# Supplementary material for: Structural basis and synergism of ATP and Na+ activation in bacterial K+ uptake system KtrAB
Source: Nat Commun. 2024 May 8;15:3850. doi: 10.1038/s41467-024-48057-y (PMC11078986; doi:10.1038/s41467-024-48057-y)
Supplement: Supplementary file 1 — Supplementary Information [file 41467_2024_48057_MOESM1_ESM.pdf]

Supplementary Information

Structural Basis and Synergism of ATP and Na^+ Activation in Bacterial K^+ Uptake System KtrAB

Wesley Tien Chiang[1,14], Yao-Kai Chang[2,14], Wei-Han Hui[3], Shu-Wei Chang[3,4], Chen-Yi Liao[1], Yi-Chuan Chang[1], Chun-Jung Chen[5], Wei-Chen Wang[6], Chien-Chen Lai[6,7], Chun-Hsiung Wang[2], Siou-Ying Luo[2], Ya-Ping Huang[2], Shan-Ho Chou[1], Tzyy-Leng Horng[8], Ming-Hon Hou[9], Stephen P. Muench[10], Ren-Shiang Chen[11], Ming-Daw Tsai[2,12*] & Nien-Jen Hu[1,13*]

[1]Graduate Institute of Biochemistry, National Chung Hsing University, Taichung City 402202, Taiwan
[2]Institute of Biological Chemistry, Academia Sinica, Taipei City 115201, Taiwan.
[3]Department of Civil Engineering, National Taiwan University, Taipei City 106319, Taiwan.
[4]Department of Biomedical Engineering, National Taiwan University, Taipei 10663, Taiwan
[5]Life Science Group, Scientific Research Division, National Synchrotron Radiation Research Center, Hsinchu, 30092, Taiwan.
[6]Institute of Molecular Biology, National Chung Hsing University, Taichung City 402202, Taiwan.
[7]Graduate Institute of Chinese Medical Science, China Medical University, Taichung City 406040, Taiwan.
[8]Department of Applied Mathematics, Feng Chia University, Taichung City 407102, Taiwan.
[9]Institute of Genomics and Bioinformatics, National Chung Hsing University, Taichung City 402202, Taiwan.
[10]School of Biomedical Sciences, Faculty of Biological Sciences and the Astbury Centre for Structural Molecular Biology, University of Leeds, Leeds LS2 9JT, UK.
[11]Department of Life Science, Tunghai University, Taichung City 407224, Taiwan.
[12]Institute of Biochemical Sciences, National Taiwan University, Taipei City 106319, Taiwan.
[13]Ph.D Program in Translational Medicine, National Chung Hsing University, Taichung, 402202, Taiwan
[14]These authors contributed equally

*e-mail: mdtsai@gate.sinica.edu.tw, njhu@nchu.edu.tw

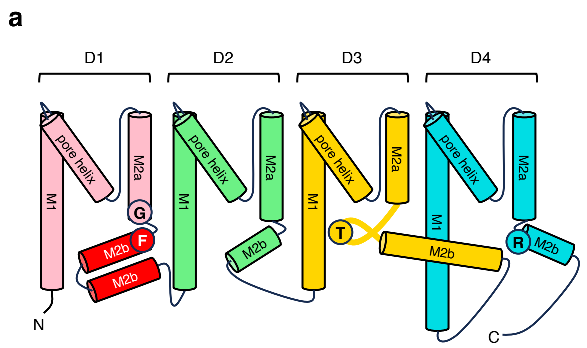

Supplementary Fig. 1 Topology maps of BsKtrB. BsKtrB models from ATP-BsKtrAB (**a**) and ADP-BsKtrAB (**b**), with the same color code as in **Fig. 1a**. D1M2b helix revealing a huge conformational change is colored as red. The intramembrane loop between the two discontinuous M2 helices in D3 domain is color as a yellow loop. The residues G87, F91, T310 and R417 are shown as circles with one-letter code of amino acids. The dotted line indicates the residues not built in the ADP-BsKtrAB model due to the poor density.

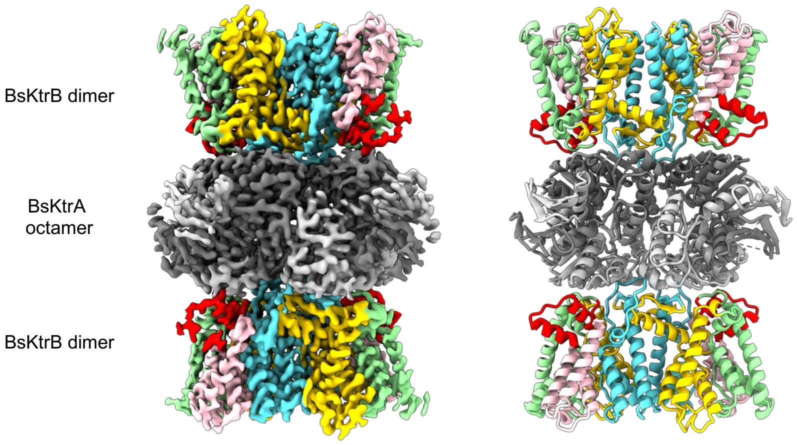

Supplementary Fig. 2 Cryo-EM density map of ATP-BsKtrAB in the presence of Mg^{2+} (Structure I). The cryo-EM map is contoured at 4.5 σ (left panel) and ribbon representation (right panel), demonstrating the $KtrB_2A_8B_2$ assembly. The map and structural model are presented with the same color code as in **Fig. 1a**.

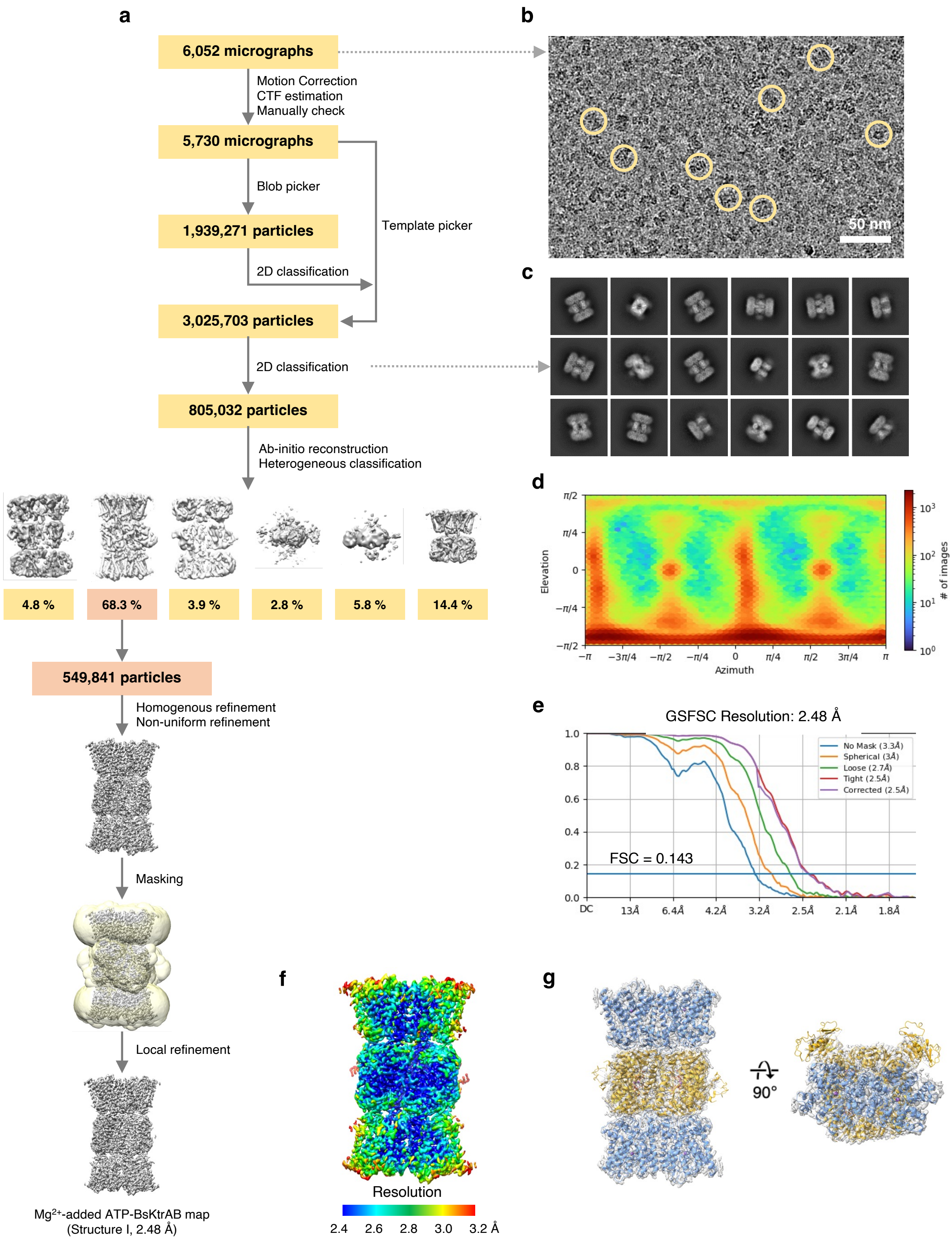

Supplementary Fig. 3 Cryo-EM data processing flowchart and map reconstruction of ATP-BsKtrAB complex in the presence of Mg^{2+} (Structure I). a, 5,730 out of 6,052 micrographs were selected for blob-based picking and 2D classification. The 2D class averages with high resolution were then used as the template for another round of template-based picking. After stack cleaning by 2D classification, 805,032 selected particles were subjected to *ab-initio* reconstruction and heterogeneous classification without imposing symmetry. 549,841 particles from a single class with $KtrB_2A_8B_2$ assembly were then used for 3D map reconstruction by homogenous, non-uniform, and local refinement with C2 symmetry to obtain a 2.48 Å map of ATP-BsKtrAB complex. The detailed processing workflow is described in Methods. **b,** A representative micrograph with the picked protein particles in yellow circles. The scale bar represents a distance of 50 nm. **c,** Representative 2D class averages after three rounds of 2D classification. Both $KtrA_8B_2$ and $KtrB_2A_8B_2$ assemblies can be observed in this dataset. **d,** Angular distribution of particles used in the final map reconstruction. The heat map represents the number of particles for different orientations. **e,** Gold standard FSC curve of the 3D reconstruction of ATP-BsKtrAB-Mg^{2+} map. The resolution was demarcated by the criterion of FSC = 0.143. **f,** The cryo-EM density map of ATP-BsKtrAB complex in the presence of Mg^{2+} (Structure I) at 2.48 Å. The local resolution is indicated as the color gradient. **g,** Two orthogonal views of model fit to the map.

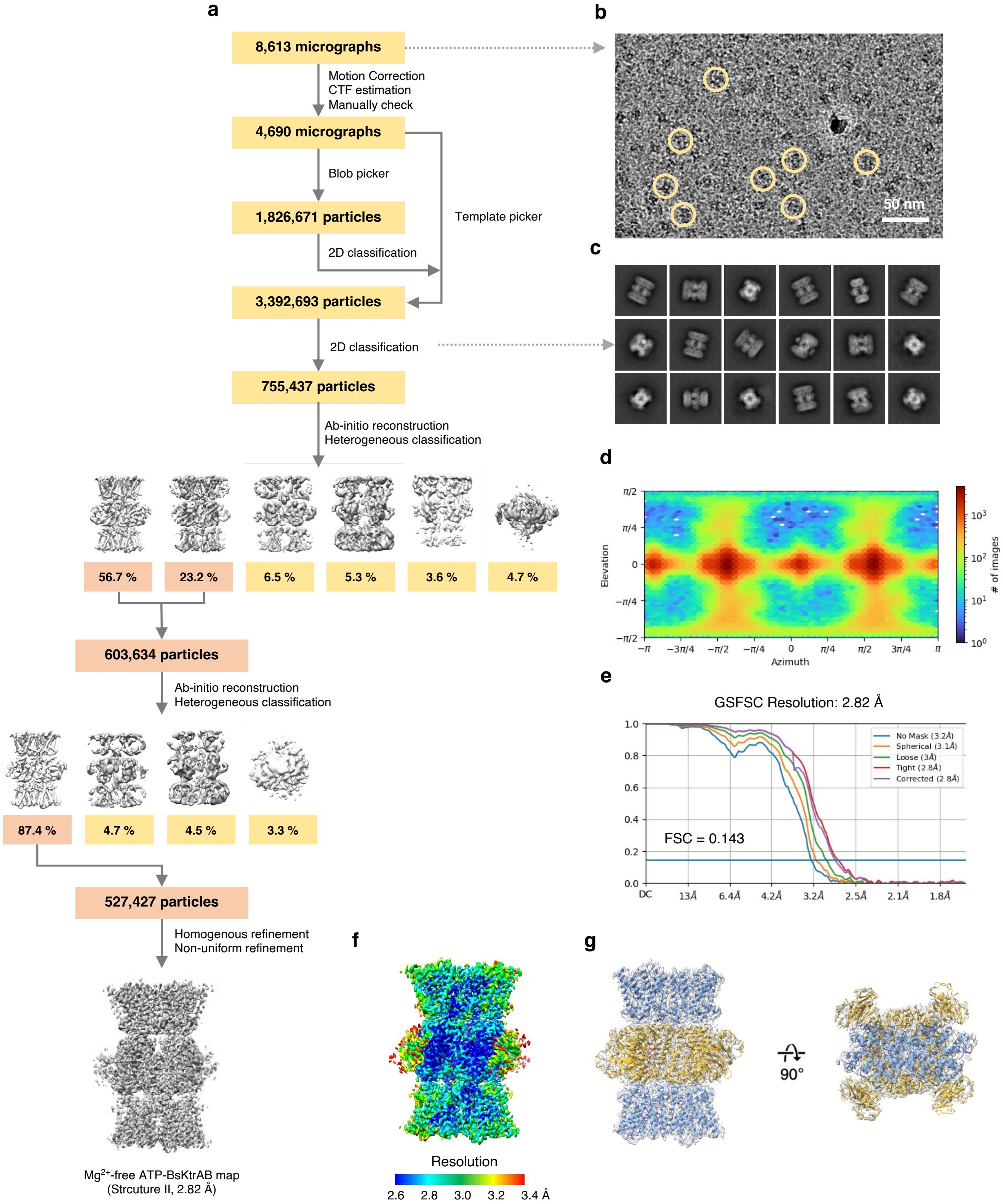

Supplementary Fig. 4 Cryo-EM data processing flowchart and map reconstruction of ATP-BsKtrAB complex in the presence of EDTA and EGTA (Structure II). a, 4,690 out of 8,613 micrographs were selected for blob-based picking and 2D classification. The 2D class averages with high resolution were then used as the template for another round of template-based picking. After stack cleaning by 2D classification, 755,437 selected particles were subjected to two rounds of *ab-initio* reconstruction and heterogeneous classification without imposing symmetry. 527,427 particles from a single class with $KtrB_2A_8B_2$ assembly were then used for 3D map reconstruction by homogenous refinement and non-uniform refinement with C2 symmetry to obtain a 2.82 Å map of ATP-BsKtrAB-EDTA-EGTA complex. The detailed processing workflow is described in Methods. **b,** A representative micrograph with the picked protein particles in yellow circles. The scale bar represents a distance of 50 nm. **c,** Representative 2D class averages after three rounds of 2D classification. Only the $KtrB_2A_8B_2$ assembly can be observed in this dataset. **d,** Angular distribution of particles used in the final map reconstruction. The heat map represents the number of particles for different orientations. **e,** Gold standard FSC curve of the 3D reconstruction of ATP-BsKtrAB-EDTA-EGTA map. The resolution was demarcated by the criterion of FSC = 0.143. **f,** The cryo-EM density map of ATP-BsKtrAB complex in the presence of EDTA and EGTA (Structure II) at 2.82 Å. The local resolution is indicated as the color gradient. **g,** Two orthogonal views of model fit to the map.

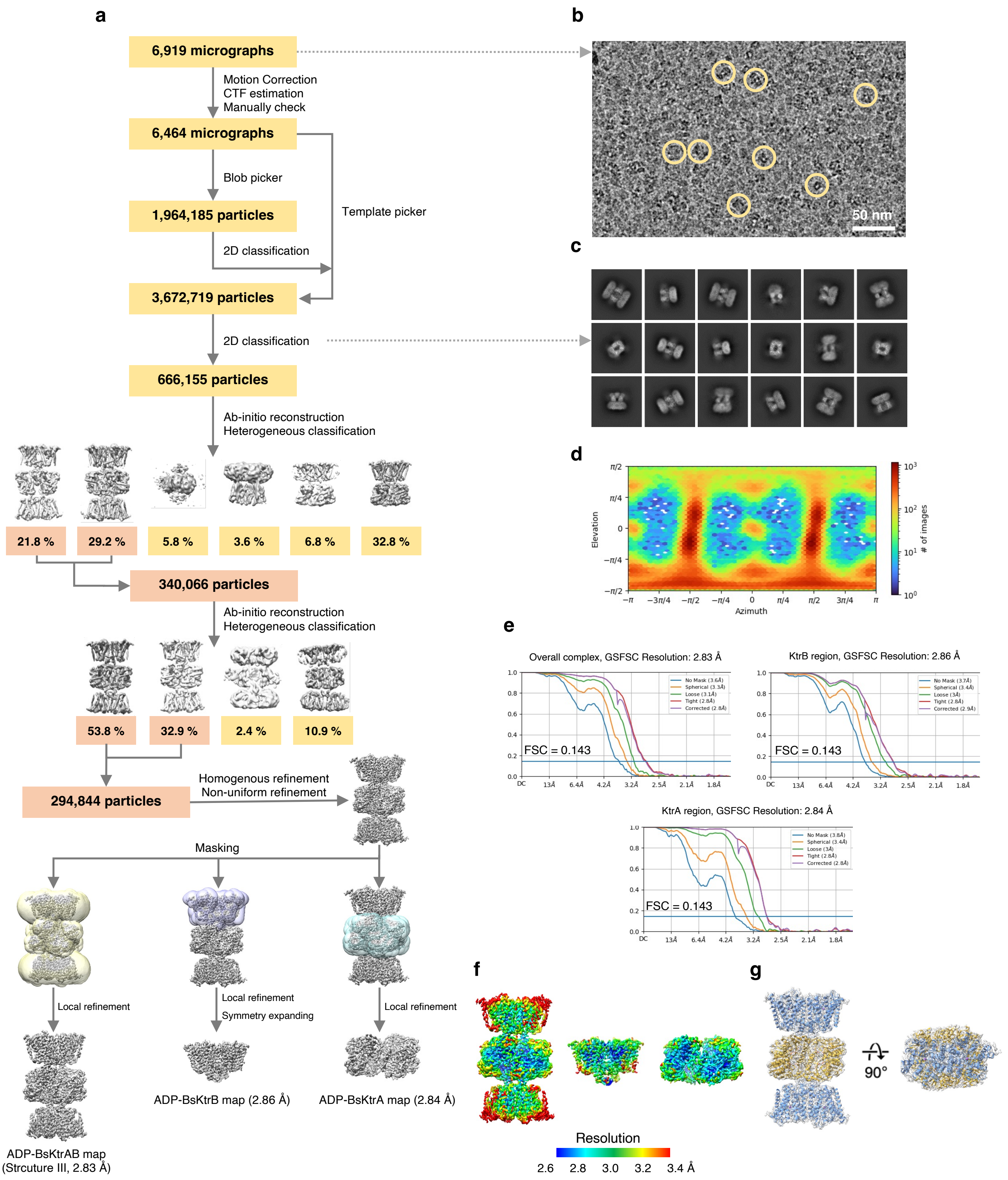

Supplementary Fig. 5 Cryo-EM data processing flowchart and map reconstruction of ADP-BsKtrAB complex (Structure III). a, 6,464 out of 6,919 micrographs were selected for blob-based picking and 2D classification. The 2D class averages with high resolution were then used as the template for another round of template-based picking. After stack cleaning by 2D classification, 666,155 selected particles were subjected to two rounds of *ab-initio* reconstruction and heterogeneous classification without imposing symmetry. 294,844 particles from two classes with $KtrB_2A_8B_2$ assembly were then used for 3D map reconstruction by homogenous, non-uniform, and local refinement with C2 symmetry to obtain a 2.83 Å map of ADP-BsKtrAB overall complex. The map quality improvement was achieved by focused refinement, resulting in a 2.84 Å BsKtrA map and a 2.86 Å BsKtrB map. The detailed processing workflow is described in Methods. **b,** A representative micrograph with the picked protein particles in yellow circles. The scale bar represents 50 nm. **c,** Representative 2D class averages after three rounds of 2D classification. Both $KtrA_8B_2$ and $KtrB_2A_8B_2$ assemblies can be observed in this dataset. **d,** Angular distribution of particles used in the final map reconstruction. The heat map represents the number of particles for different orientations. **e,** Gold standard FSC curve of the 3D reconstruction of ADP-BsKtrAB overall complex (left), BsKtrA region (middle), and BsKtrB region (right). The resolution was demarcated by the criterion of FSC = 0.143. **f,** The cryo-EM density map of ADP-BsKtrAB complex at 2.83 Å (left), BsKtrB region at 2.86 Å (middle), and BsKtrA region at 2.84 Å (right). The local resolution is indicated as the color gradient**. g,** Two orthogonal views of model fit to the map.

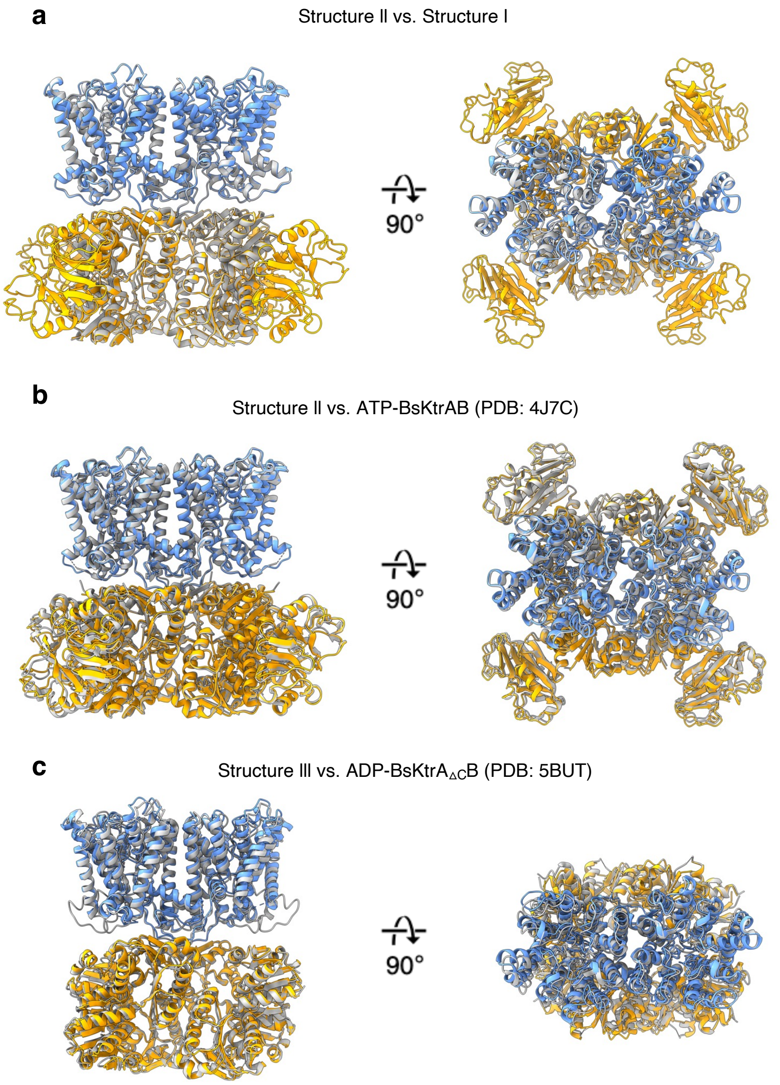

Supplementary Fig. 6 Comparison of cryo-EM structures of BsKtrAB with previously published structures. a, Superimposition of the cryo-EM structures of ATP-BsKtrAB in the presence of Mg^{2+} (Structure I) colored in gray and ATP-BsKtrAB in the presence of EDTA and EGTA (Structure II) (r.m.s.d. 0.30 Å over 2,104 C_α atoms). **b**, Superimposition of ATP-BsKtrAB crystal structure (PDB ID 4J7C) colored in gray and ATP-BsKtrAB cryo-EM structure (Structure II) (r.m.s.d. of 0.79 Å over 2,479 C_α atoms). **c**, Superimposition of ADP-BsKtrA$_{\Delta C}$B crystal structure (PDB ID 5BUT) colored in gray and ADP-BsKtrAB cryo-EM structure (Structure III) (r.m.s.d. of 1.19 Å over 1,793 C_α atoms). BsKtrB dimer and BsKtrA octamer in Structure II (**a**,**b**) and Structure III (**c**) are colored in blue and orange, respectively.

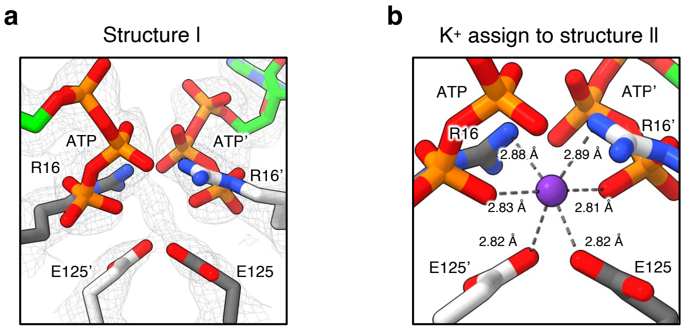

Supplementary Fig. 7 The cation binding site at the BsKtrA intra-dimer interface. a, A close-up view of the intra-dimer interface of BsKtrA from ATP-BsKtrAB in the presence of 2 mM Mg^{2+} (Structure I) with the cryo-EM map contoured at 7.0 σ. **b,** The refined model of the intra-dimer interface of BsKtrA from ATP-BsKtrAB in the presence of EDTA and EGTA (Structure II) with K^+ assigned (purple sphere) at the site. Coordination geometries of the assigned K^+ are depicted as dashed lines with distances.

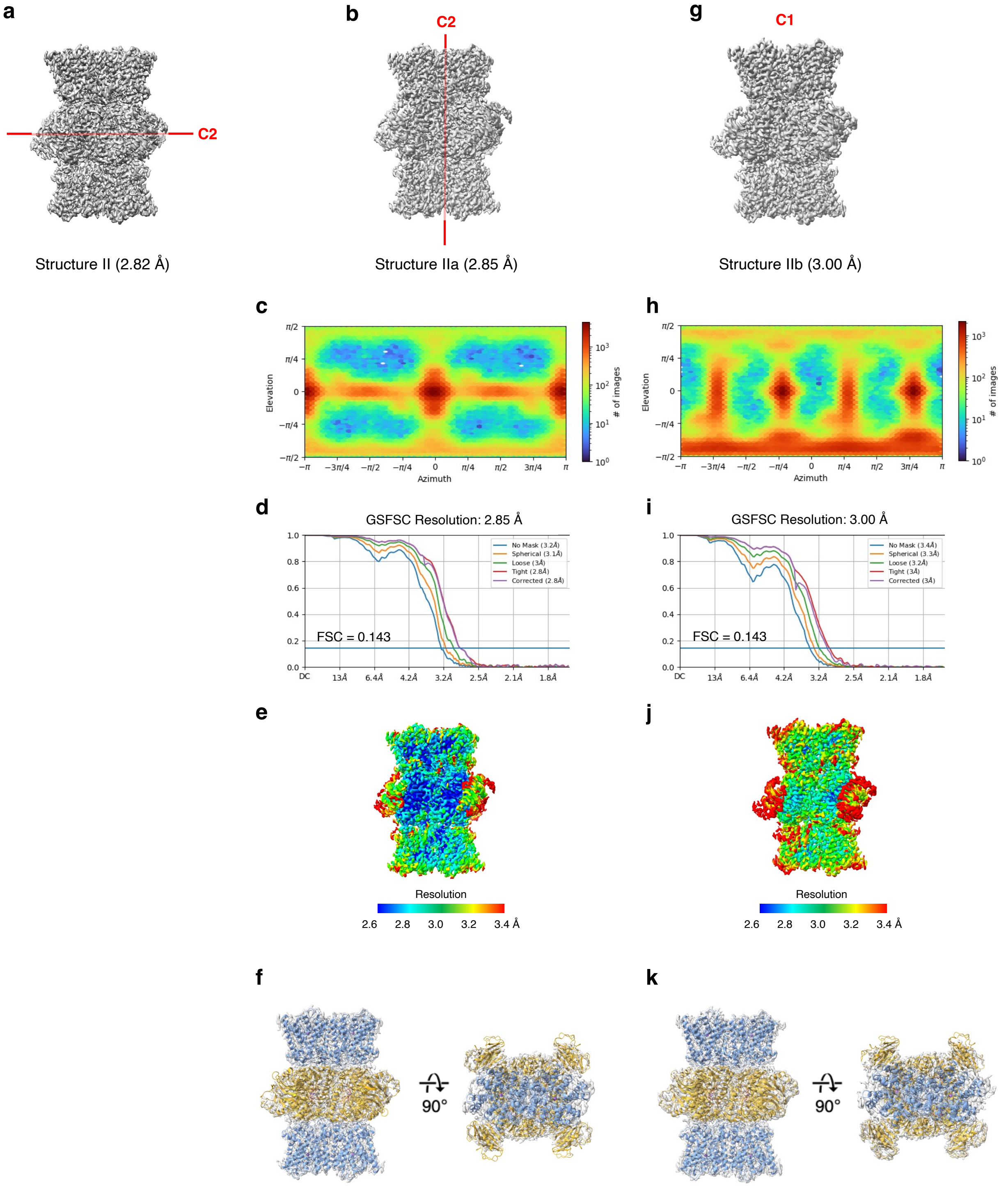

Supplementary Fig. 8 Additional map reconstructions of Mg^{2+}-free ATP-BsKtrAB with a vertical C2 symmetry axis and without imposing symmetry (C1). a, The original map reconstruction of structure II with a horizontal C2 symmetry axis. **b,** The additional map reconstruction of structure II using a vertical C2 symmetry axis resulted in a new model, structure IIa. **c,** Angular distribution of particles used in the final map reconstruction of structure IIa. The heat map represents the number of particles for different orientations. **d,** Gold standard FSC curve of the 3D reconstruction of structure IIa map. The resolution was demarcated by the criterion of FSC = 0.143. **e,** The cryo-EM density map of structure IIa was refined to 2.85 Å. The local resolution is indicated as the color gradient. **f,** Two orthogonal views of model fit to the map. **g,** The additional map reconstruction of structure II without imposing symmetry (C1) resulted in a new model, structure IIb. **h,** Angular distribution of particles used in the final map reconstruction of structure IIb. The heat map represents the number of particles for different orientations. **i,** Gold standard FSC curve of the 3D reconstruction of structure IIb map. The resolution was demarcated by the criterion of FSC = 0.143. **j,** The cryo-EM density map of structure IIb was refined to 3.00 Å. The local resolution is indicated as the color gradient. **k,** Two orthogonal views of model fit to the map.

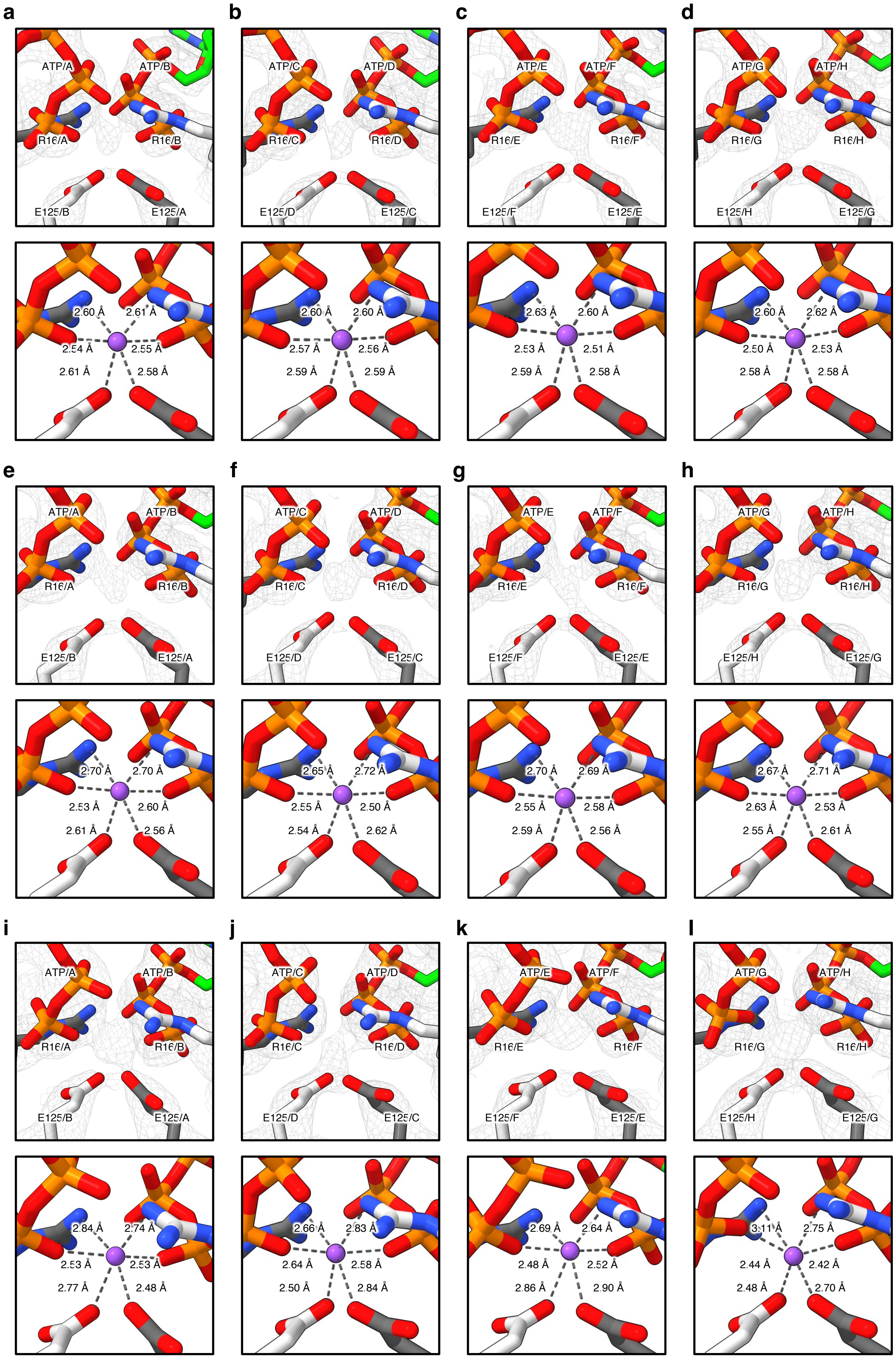

Supplementary Fig. 9 The Na+ binding sites in the ATP-BsKtrAB cryo-EM structures based on different symmetry map reconstructions. Upper panels, a close-up view of the intra-dimer interfaces of ATP-BsKtrA from ATP-BsKtrAB cryo-EM structures, Structure II based on the horizontal C2 map (**a-d**), Structure IIa based on the vertical C2 map (**e-h**) and Structure IIb based on the C1 map (**i-l**). The cryo-EM density maps (gray mesh) are contoured at 10.0 σ in (**a-h**) and 6.0 σ in (**i-l**). The coordinating amino acid side chains and ATP γ-phosphates are shown in stick from each protomer of BsKtrA dimer. Lower panels, the coordination geometries of the assigned Na^+ cations after structure refinement are depicted as dashed lines with coordination distances indicated.

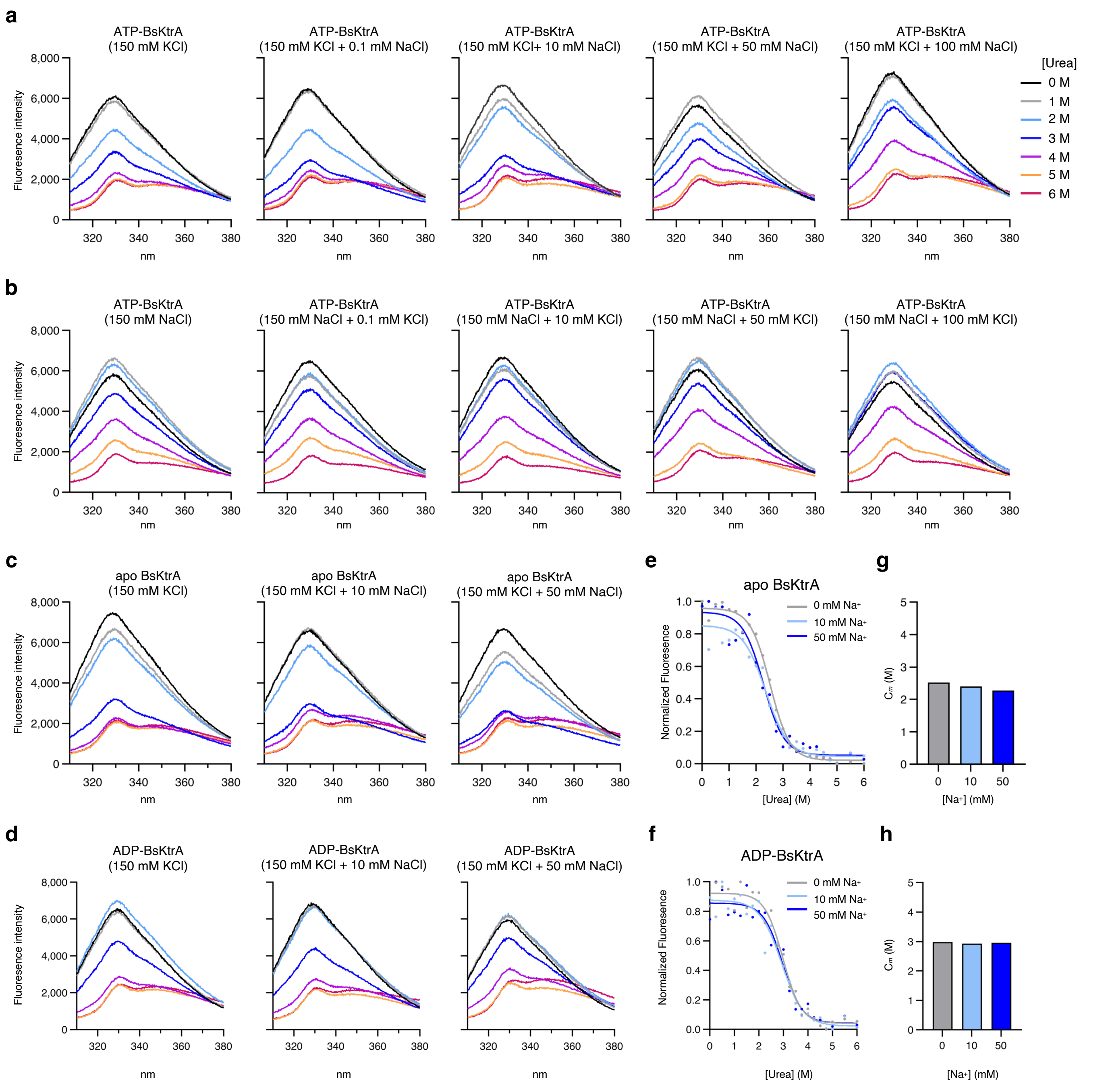

Supplementary Fig. 10 Urea unfolding assays using intrinsic tryptophan fluorescence. **a**,**b**, Fluorescence spectra of urea-unfolded ATP-BsKtrA in (**a**) K^+ Buffer titrated against NaCl, and in (**b**) Na^+ Buffer titrated against KCl. Plots of normalized fluorescence intensities of urea-unfolded ATP-BsKtrA against NaCl or KCl concentrations, and C_m values for respective experiments are shown in **Fig. 3a,b. c**,**d**, Fluorescence spectra of urea-unfolded (**c**) apo-BsKtrA and (**d**) ADP-BsKtrA in K^+ Buffer titrated against NaCl concentrations. **e**,**f**, Plots of normalized fluorescence intensities of urea-unfolded (**e**) apo-BsKtrA and (**f**) ADP-BsKtrA analyzed from the data in (**c**) and (**d**), respectively, against different NaCl concentrations. **g**,**h**, C_m values of (**g**) apo-BsKtrA and (**h**) ADP-BsKtrA, as determined in (**e**) and (**f**), plotted against Na^+ concentrations. Source data for (**a–h**) are provided as a Source Data file.

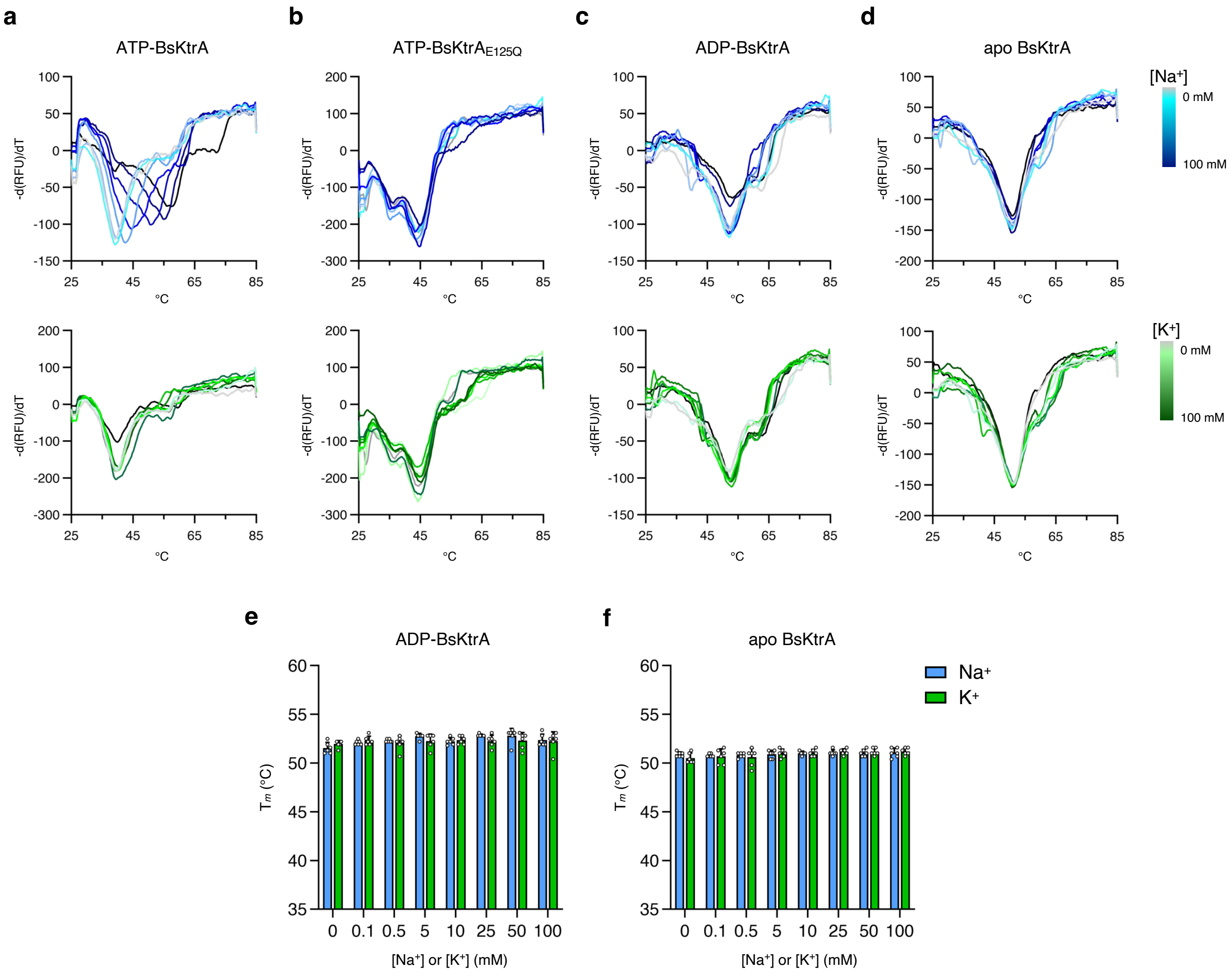

Supplementary Fig. 11 Thermostability assay using differential scanning fluorescence (DSF). **a-d,** First derivative curves of (**a**) ATP-BsKtrA, (**b**) ATP-BsKtrA$_{E125Q}$, (**c**) ADP-BsKtrA and (**d**) apo-BsKtrA in Choline Buffer titrated against NaCl (upper panel) or KCl (lower panel). NaCl and KCl concentrations are indicated as the color gradient. Plots of T_m values derived from the first derivative curves against NaCl or KCl are shown in **Fig. 3c,d**. **e** and **f**, Plots of T_m values derived from the first derivative curves in (**c**) and (**d**), respectively. Blue and green bars indicate the T_m values titrated against NaCl and KCl, respectively. Data represent the mean ± s.d.; n = 6 independent experimental replicates. Source data for (**a–f**) are provided as a Source Data file.

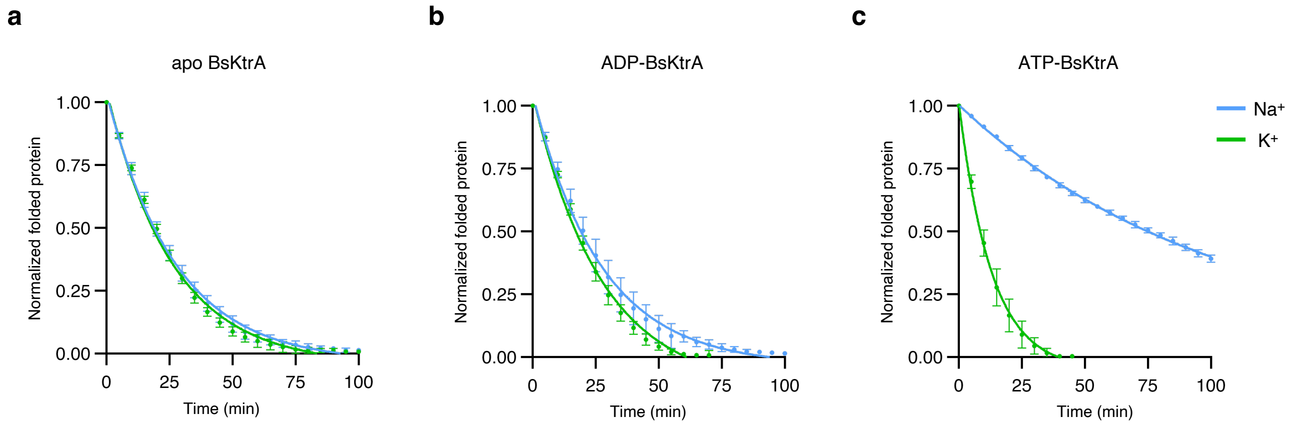

Supplementary Fig. 12 Protein stability as monitored by half-life analysis using SYPRO Orange. a-c, Unfolding curves of (**a**) apo-, (**b**) ADP-, and (**c**) ATP-BsKtrA measured at 40 °C in the presence of Na^+ (blue points) and K^+ (green points). Data represent the mean ± s.d.; n = 4 independent experimental replicates. The half-life values were calculated by fitting the data to a one-phase decay model using GraphPad Prism. Source data for (**a–c**) are provided as a Source Data file.

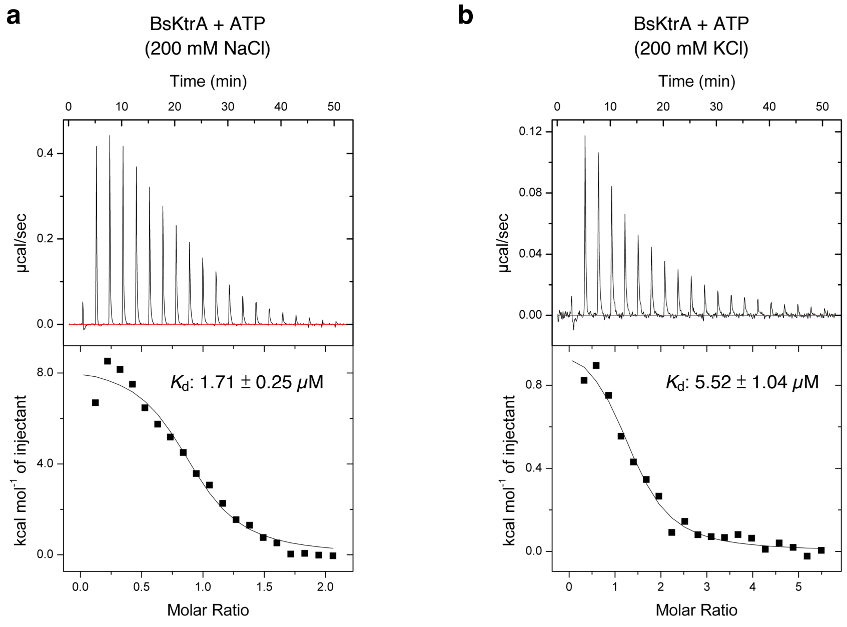

Supplementary Fig. 13 ATP binding affinity analysis using ITC. **a**,**b**, Characterization of ATP binding affinity (K_d) to BsKtrA in the presence of 200 mM (**a**) NaCl and (**b**) KCl.

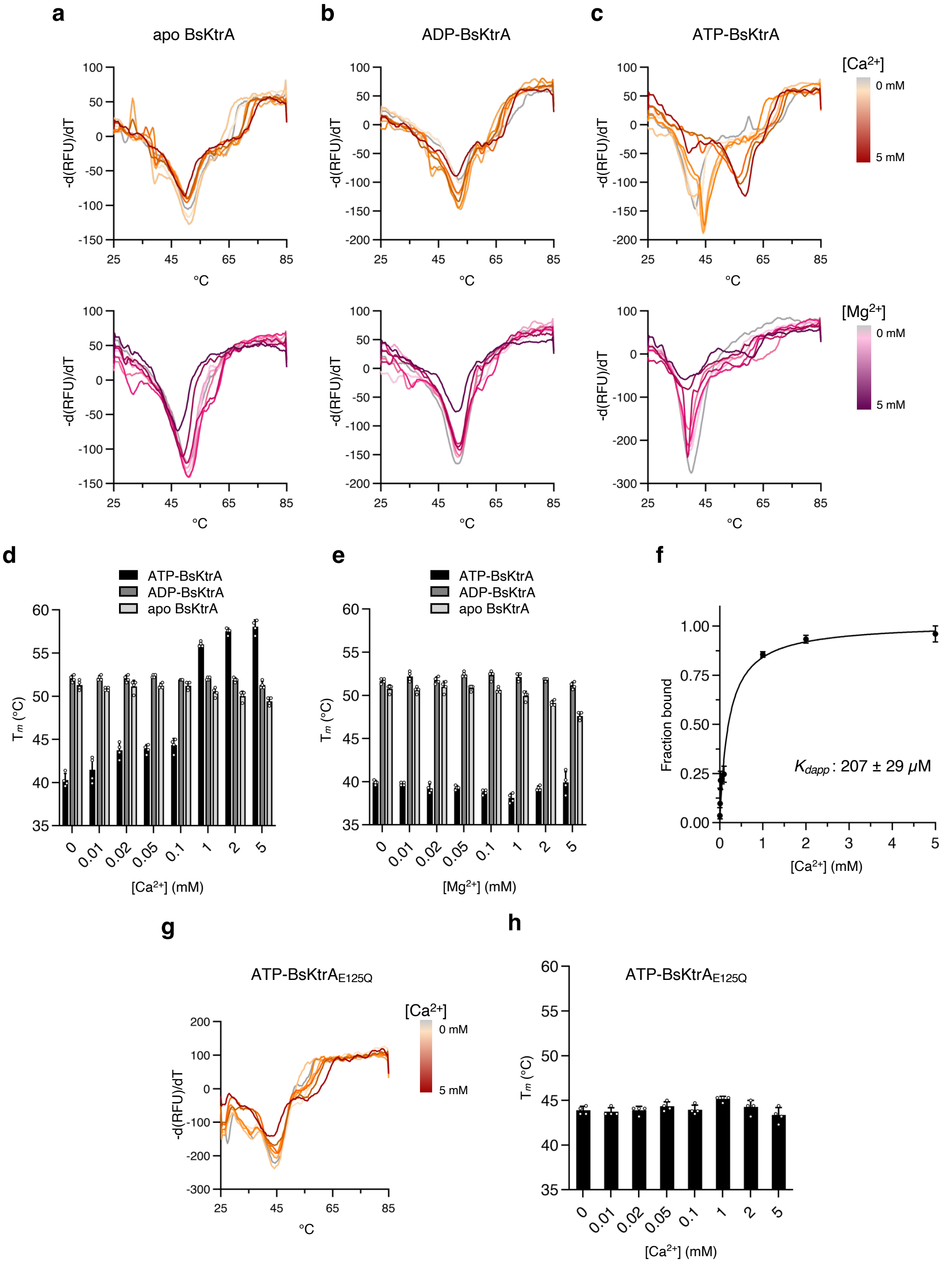

Supplementary Fig. 14 Divalent cation binding affinity to BsKtrA using DSF. **a-c,** First derivative curves of (**a**) apo BsKtrA, (**b**) ADP-BsKtrA and (**c**) ATP-BsKtrA in Choline Buffer titrated against $CaCl_2$ (upper panel) or $MgCl_2$ (lower panel) concentrations. $CaCl_2$ and $MgCl_2$ concentrations are indicated as the color gradient. **d,e,** Plots of T_m values of ATP- (black bar), ADP- (dark gray bar) and apo-BsKtrA (light gray bar) titrated with (**d**) $CaCl_2$ or (**e**) $MgCl_2$. Data represent the mean ± s.d.; *n* =4 independent experimental replicates. **f**, The apparent K_d (K_{dapp}) of Ca^{2+} to ATP-BsKtrA was analyzed by fitting the fraction bound derived from the DSF assays against the concentrations of Ca^{2+} to a one-site model using GraphPad Prism. **g**, First derivative curves of ATP-BsKtrA$_{E125Q}$ in Choline Buffer titrated against $CaCl_2$. **h**, Plots of T_m values of ATP-BsKtrA$_{E125Q}$ against $CaCl_2$ titration. Data represent the mean ± s.d.; *n* =4 independent experimental replicates. Source data for (**a–h**) are provided as a Source Data file.

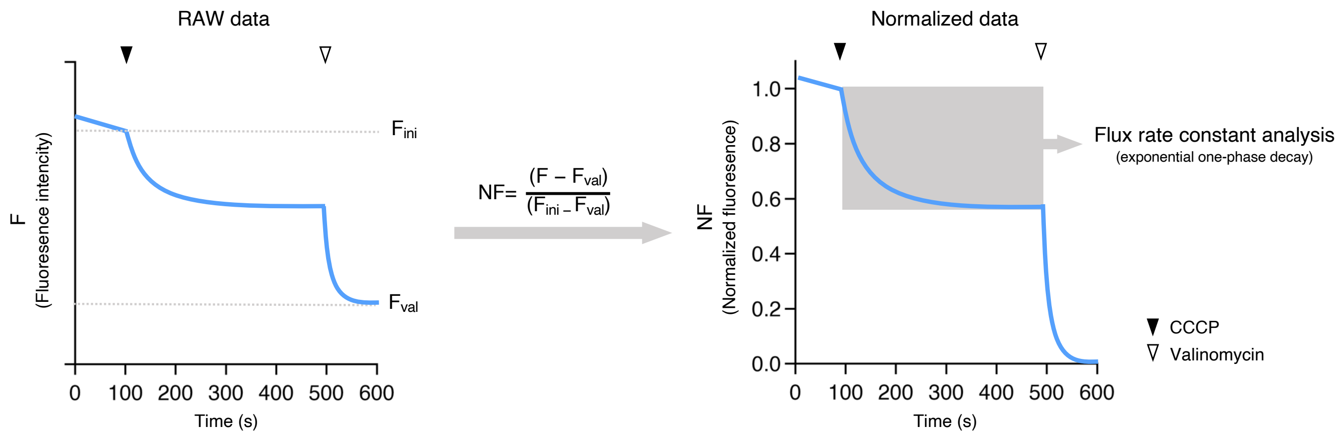

Supplementary Fig. 15 Schematic illustration of the analysis of K^+ flux assays. Left panel, the K^+ flux raw data. Right panel, the normalized K^+ flux data. The addition of H^+ ionophore CCCP and K^+ ionophore valinomycin is indicated as the black and white arrow, respectively. To normalize the fluorescence quenching curves, each dataset of individual experiment was normalized using the equation: NF = (F-F_{val})/(F_{ini}-F_{val}), where NF is the normalized fluorescence (right panel), F is the fluorescence of each time point (left panel), F_{ini} is the last baseline data point measured before CCCP addition (left panel), and F_{val} is the lowest data point measured after valinomycin addition (left panel). The K^+ flux rate constants (100-500 s, grey region) were determined using the exponential one-phase decay model in GraphPad Prism.

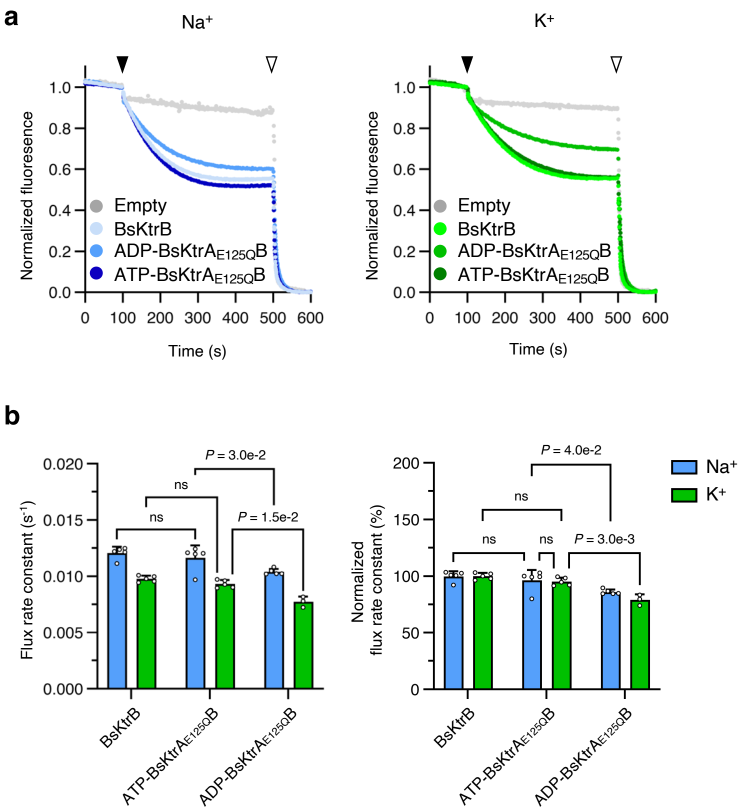

Supplementary Fig. 16 The importance of BsKtrA Glu125 in Na^+-dependent activation of BsKtrB. a, Fluorescence-based K^+ flux assays of BsKtrA$_{E125Q}$B in the presence of ATP or ADP performed in Swelling Na^+ Buffer (Na^+, left panel) or Swelling K^+ Buffer (K^+, right panel). K^+ flux activity of BsKtrB was performed as a comparison. K^+ flux rate constants were calculated by fitting the data (100-500 s) to a one-phase decay model. The addition of H^+ ionophore CCCP and K^+ ionophore valinomycin is indicated as the black and white arrow, respectively. **b**, K^+ flux rate constants (left panel) and normalized K^+ flux rate constants (right panel) of BsKtrB, ADP-BsKtrA$_{E125Q}$B and ATP-BsKtrA$_{E125Q}$B in Na^+ (blue bars) or K^+ (green bars). Normalized K^+ flux rate constants were calculated using the respective rates of KtrB in either Na^+ or K^+ as 100 %. Data represent the mean ± s.d with $n = 3$ (for ADP-BsKtrA$_{E125Q}$B in K^+) or $n = 5$ (for the others) independent experimental replicates. Statistical analyses were performed using two-way ANOVA, and ns indicates no significance ($p \geq 0.05$). Source data for (**a–b**) are provided as a Source Data file.

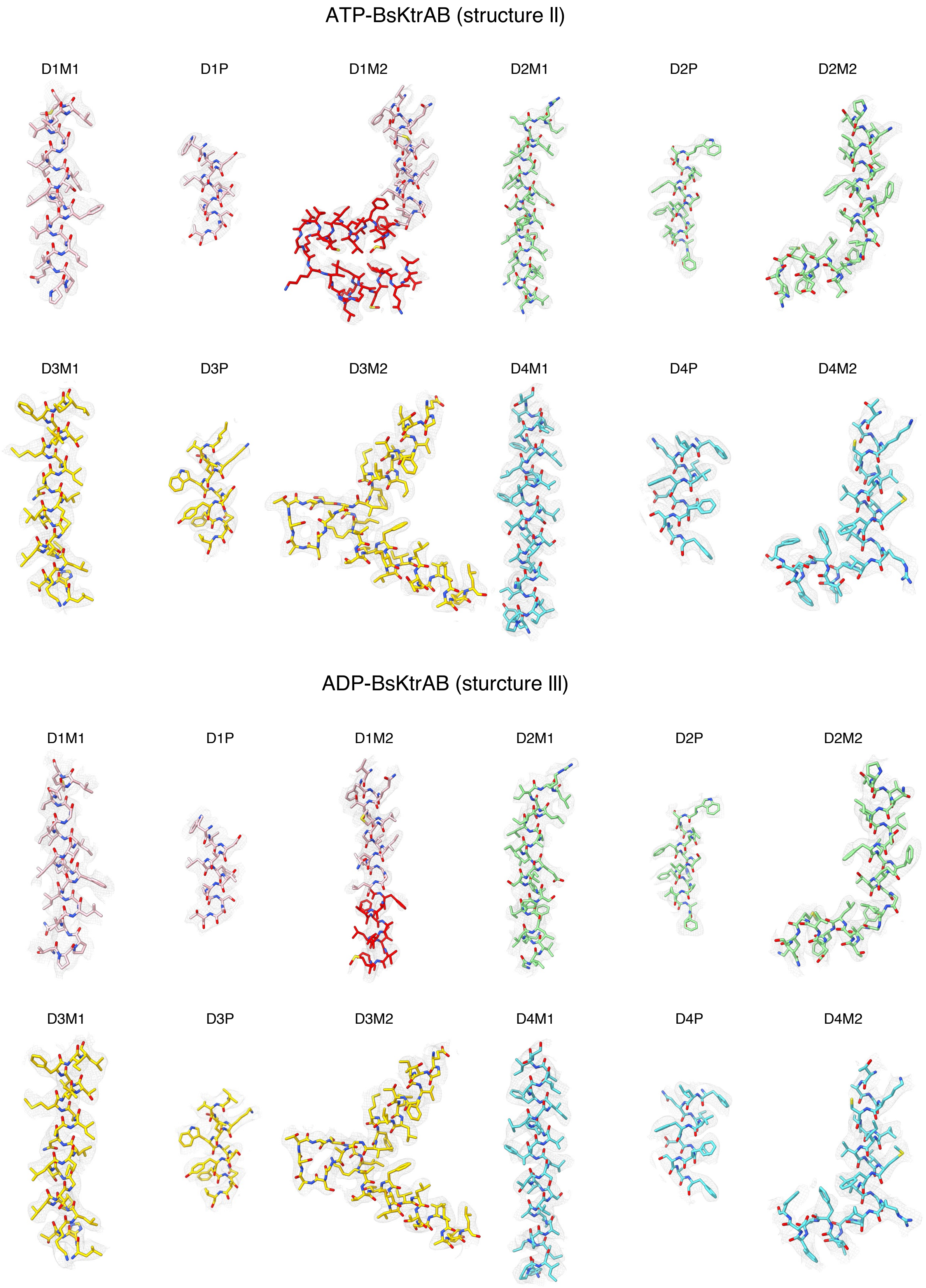

Supplementary Fig. 17 High-resolution cryo-EM maps and the built models of BsKtrB helices from ATP- (Structure II) and ADP-BsKtrAB complexes (Structure III). Cryo-EM density maps, contoured with a threshold of 5σ, of the individual helices of BsKtrB from ATP-BsKtrAB (Structure II, upper panel) and ADP-BsKtrAB (Structure III, lower panel) with the same color code as in **Fig. 1a**. D1M2b helices showing the significant conformational change in ATP- and ADP-BsKtrAB structures are colored in red.

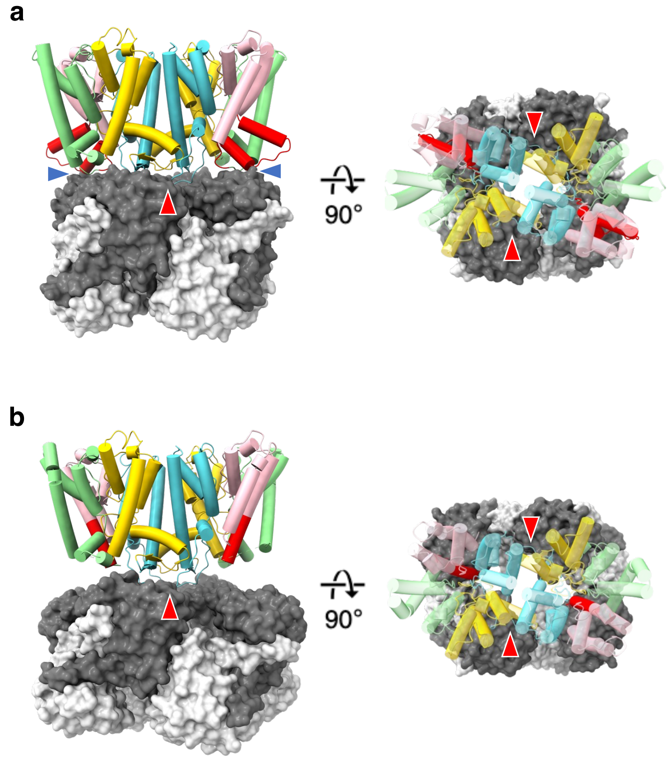

Supplementary Fig. 18 Protein contacts between BsKtrA and BsKtrB in ATP- and ADP-BsKtrAB structures. a,**b,** Two orthogonal views of (**a**) ATP-BsKtrAB (Structure II) and (**b**) ADP-BsKtrAB (Structure III) with the same color code as in **Fig. 1a**. BsKtrB dimer is shown in cylindrical representation, while BsKtrA octamer is shown in surface representation**.** The helical cylinders of BsKtrB dimer, except the D1M2b helices (red), from the top view are rendered as semi-transparent to highlight the conformational change of D1M2b helices with respect to the overall conformational change of the BsKtrA octameric ring. The red and blue arrows indicate the lateral contact and tip contact, respectively, between the BsKtrA octameric ring and the BsKtrB dimer.

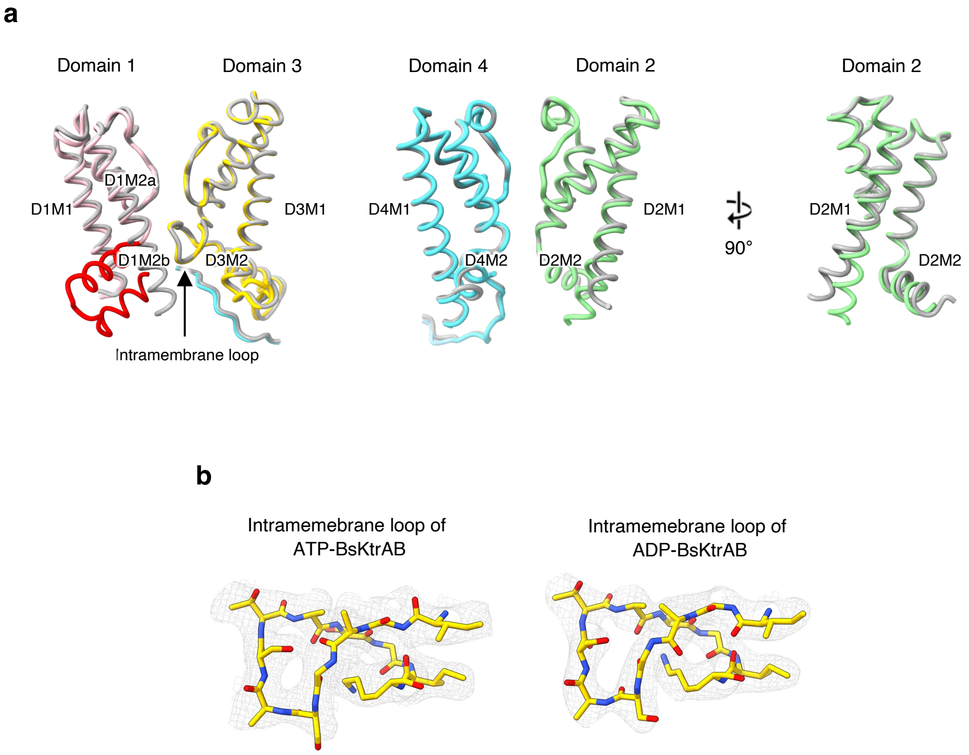

Supplementary Fig. 19 Structural comparison of the transmembrane helices and the intramembrane loop of BsKtrB from ATP- (Structure II) and ADP-BsKtrAB (Structure III). a, Superposition of the domains D1/D3 and D2/D4 of BsKtrB from ATP- and ADP-BsKtrAB structures. The BsKtrB model from ATP-BsKtrAB is colored by domains with the color code as in **Fig. 1a**. The BsKtrB model from ADP-BasKtrAB is colored in gray for comparison. The BsKtrB D1M2b helical hairpin from ATP-BsKtrAB is highlighted in red. **b**, Representative cryo-EM densities and the fitted models of BsKtrB intramembrane loop from ATP- (left panel) and ADP-BsKtrAB (right panel).

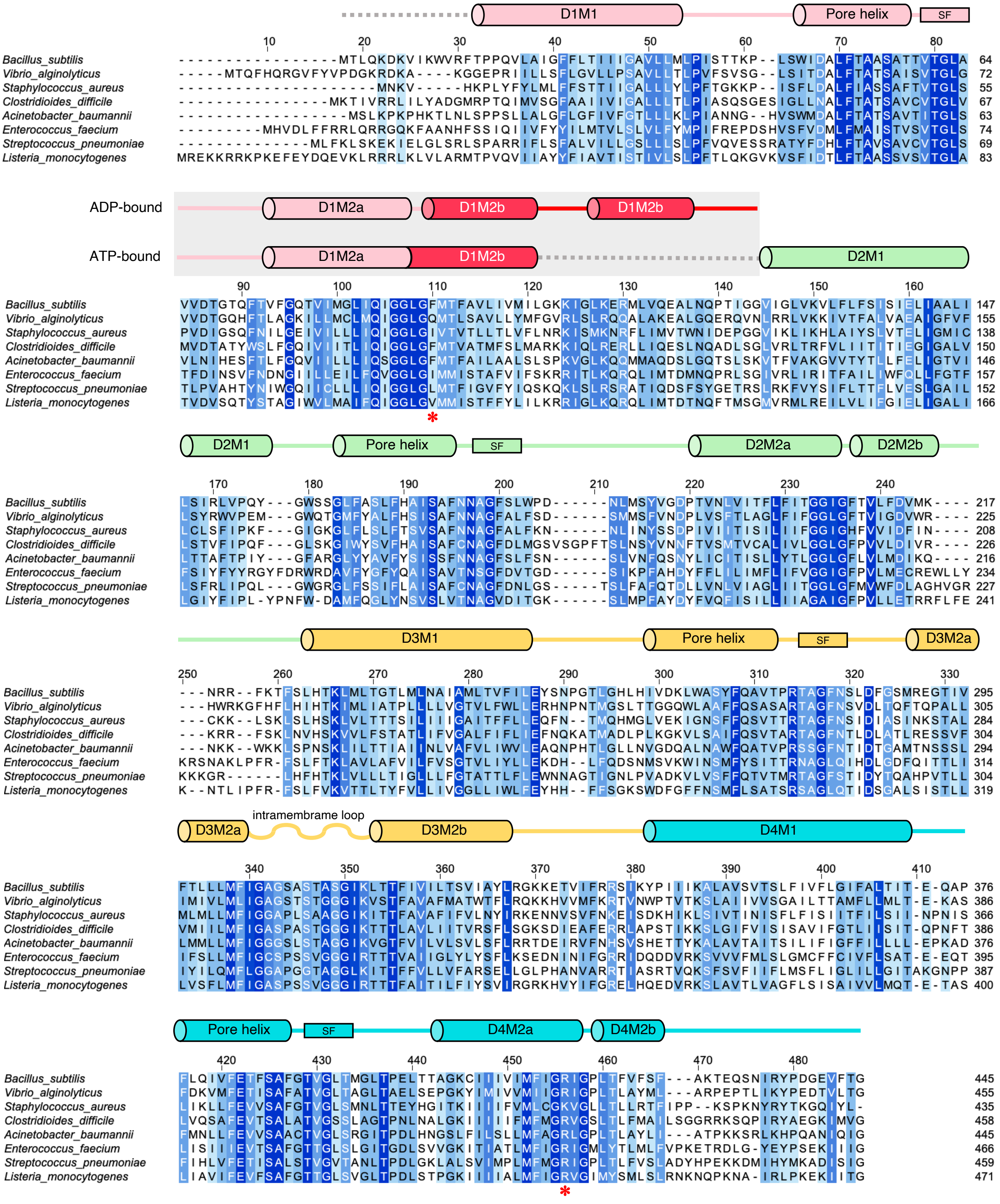

Supplementary Fig. 20 Amino acid sequence alignment of KtrB homologues. α-helices are shown as horizontal cylinders on the basis of D1-D4 domains with the color code as in **Fig. 1a**. The stretches of amino acids constituting the selectivity filter (SF) are shown as rectangles. The C-terminal region of BsKtrB D1M2b helix is shown in red to highlight the conformational change in ADP- and ATP-BsKtrAB. The stretches of amino acids that are unable to be modelled unambiguously are shown as dashed gray lines. The intramembrane loop on D3M2 helix is shown as the yellow wavy line. The red asterisks denote the residues, Phe91 and Arg417, serving as the gates of BsKtrB pore and the equivalent residues in other homologues. The sequence alignment is performed using Clustal Omega and presented using Jalview.

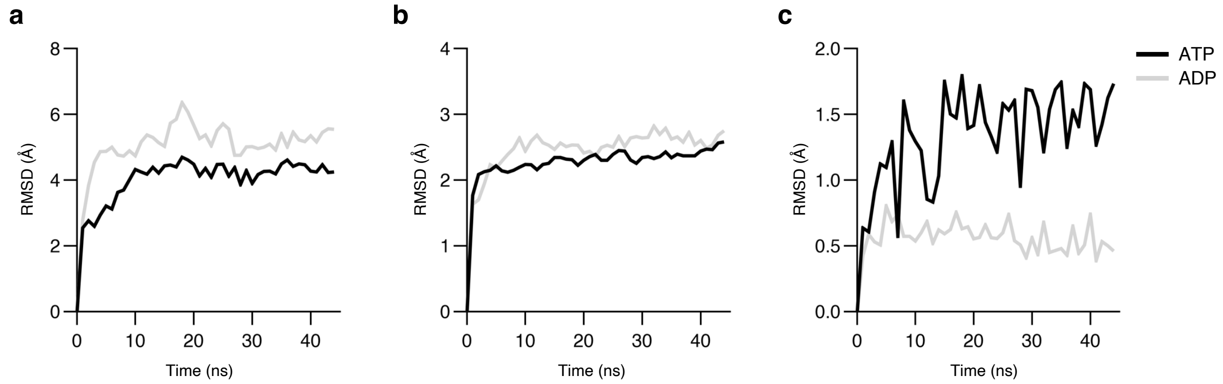

Supplementary Fig. 21 Structural stability of MD simulation. a, RMSD of the whole protein (BsKtrAB). **b**, RMSD of the BsKtrB. **c**, RMSD of the amino acids associated with loop (T310, G87 and R417) during the 45 ns MD simulations. Source data for (**a–c**) are provided as a Source Data file.

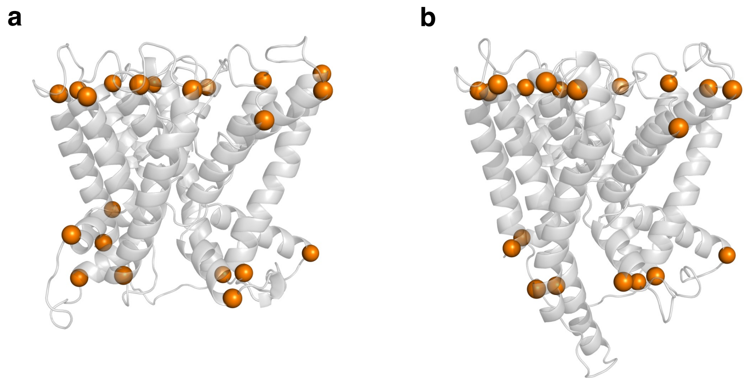

Supplementary Fig. 22 The C_α atoms of fixed amino acids of BsKtrB in SMD simulation. The selected C_α atoms in the BsKtrB models from (a) ATP-BsKtrAB (Structure II) and (b) ADP-BsKtrAB (Structure III) are shown as orange spheres.

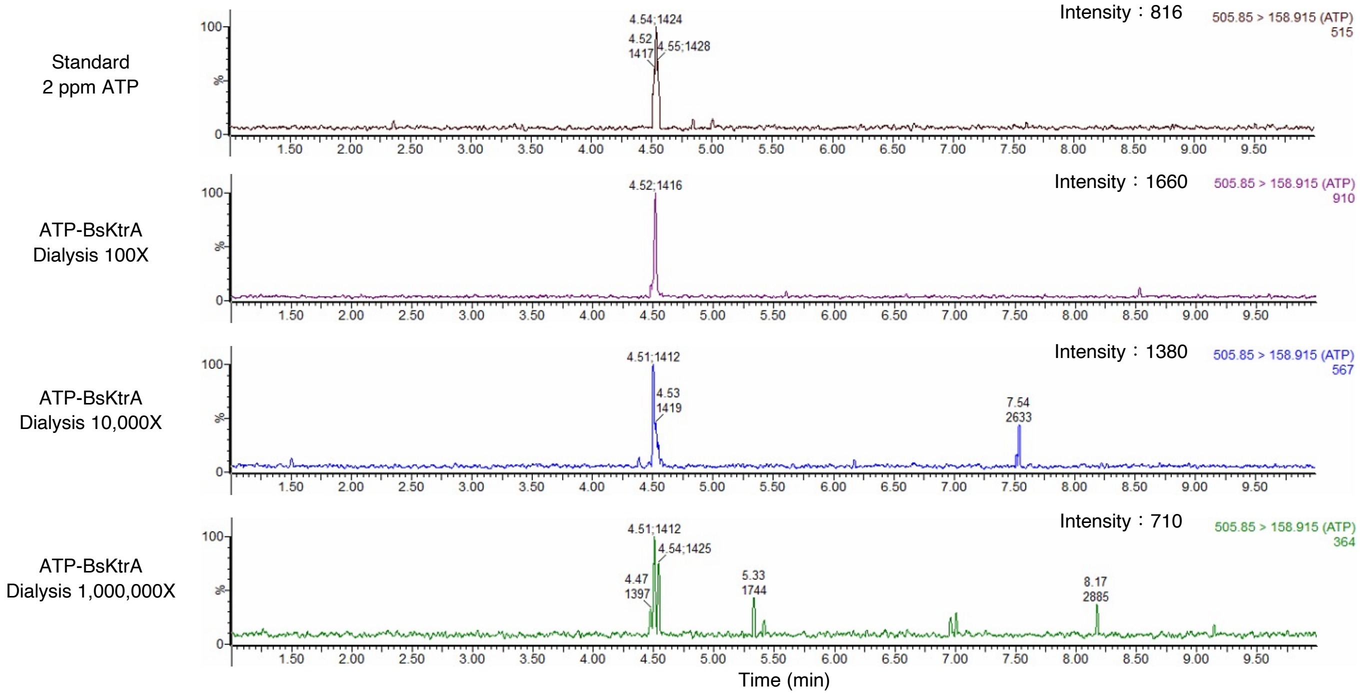

Supplementary Fig. 23 Relative quantification of residual ATP in various dialysis folds by LC/MSMS. All relative quantification data were obtained under the same multiple reaction monitoring (MRM) transition (m/z 505 to m/z 158) and retention time (approximately 4.5 min) as the ATP standard (2 ppm), with the intensity indicated. Source data are provided as a Source Data file.

Supplementary Table 1 Cryo-EM data collection and refinement statistics of BsKtrAB structures.

	Mg^{2+}-added ATP-BsKtrAB	Mg^{2+}-free ATP-BsKtrAB			ADP-BsKtrAB
	**Structure I**	**Structure II**	**Structure IIa**	**Structure IIb**	**Structure III**
**PDB code**	**8K1T**	**8K1U**	**8XMH**	**8XMI**	**8K1S**
**EMDB code**	EMD-36803	EMD-36804	EMD-38477	EMD-38478	EMD-36800/EMD-36801/EMD-36802[a]
**Data Collection**					
EM equipment	Titan Krios		Titan Krios		Titan Krios
Voltage (kV)	300		300		300
Detector	Gatan K3 Summit		Gatan K3 Summit		Gatan K3 Summit
Magnification (nominal)	105,000		105,000		105,000
Calibrated pixel size (Å)	0.83		0.83		0.83
Defocus range (µm)	-1.5 ~ -2.2		-1.4 ~ -2.2		-1.5 ~ -2.2
Electron dose (e-/Å^2)	36		54		36
Exposure time (s)	2.5		2.0		2.5
Number of frames	40		50		40
Collected micrographs	6052		8613		6919
Selected micrographs	5730		4690		6464
**3D Reconstruction**					
Number of used particles	549,841		527,427		294,844/294,844/589,688[a]
Symmetry	C2	C2	C2 (vertical)[b]	C1	C2/C1/C1[a]
Map resolution (Å, FSC=0.143)	2.48	2.82	2.85	3.00	2.83/2.84/2.86[a]
Map sharpening B factor (Å^2)	-87.7	-118.2	-120.0	-114.3	98.3/-73.0/-111.9[a]
**Refinement**					
Correlation coefficient (CC_{mask})	0.84	0.81	0.81	0.84	0.75
Model composition					
Non-hydrogen atoms	23866	26964	26948	26718	21000
Protein residues	3052	3446	3444	3415	2695
ATP/ADP	8	8	8	8	8
Na^+	4	4	4	4	0
B-factors (Å^2)					
Protein	50.59	30.12	27.63	30.33	55.94
ATP/ADP	33.28	9.45	10.57	14.80	55.37
Na^+	29.42	11.75	12.92	12.78	-
R.M.S. deviations					
Bond lengths (Å)	0.003	0.002	0.003	0.004	0.004
Bond angles (°)	0.494	0.477	0.514	0.571	0.610
**Validation**					
MolProbity score	1.35	1.36	1.50	1.68	1.43
Clashscore	2.93	2.69	2.91	3.50	5.89
Rotamer outliers	0.00	0.00	0.00	0.10	0.00
C_β deviations	0.00	0.00	0.00	0.00	0.00
EMRinger score	3.15	2.99	3.33	3.59	2.70
Ramachandran plot					
Favored (%)	97.77	97.40	97.31	97.04	97.56
Allowed (%)	2.23	2.60	2.69	2.96	2.44
Outliers (%)	0.00	0.00	0.00	0.00	0.00

[a]Apart from the overall complex of ADP-BsKtrAB map, two additional maps were created for the KtrA octamer region and KtrB dimer region, utilizing focused refinement to enhance the map quality. The three maps represent KtrAB overall complex, KtrA region and KtrB region, respectively.

[b]In contrast to the utilization of a horizontal C2 symmetry axis in map reconstructions, the map reconstruction process here employed a vertical C2 symmetry axis.

Supplementary Table 2 The stretches of amino acids of BsKtrAB built unambiguously in Structures I, II and III.

		Mg^{2+}-added ATP-BsKtrAB	Mg^{2+}-free ATP-BsKtrAB			ADP-BsKtrAB
		**Structure I**	**Structure II**	**Structure IIa**	**Structure IIb**	**Structure III**
**BsKtrA**	**chain A**	7-139	7-221	7-221	7-221	7-139
	**chain B**	7-139	7-221	7-221	7-221	7-139
	**chain C**	7-156; 160-172; 179-192; 205-217	7-221	7-221	7-221	7-140
	**chain D**	7-156; 160-201; 205-220	7-221	7-221	7-160; 165-184; 189-198; 204-221	7-139
	**chain E**	7-156; 160-201; 205-220	7-222	7-221	7-185; 202-221	7-140
	**chain F**	7-156; 160-172; 179-192; 205-217	7-221	7-221	7-221	7-139
	**chain G**	7-139	7-221	7-221	7-221	7-140
	**chain H**	7-139	7-222	7-221	7-221	7-139
**BsKtrB**	**chain I**	15-445	15-445	15-445	15-445	15-102; 127-445
	**chain J**	15-445	15-445	15-445	15-445	15-102; 127-445
	**chain K**	15-445	15-445	15-445	15-445	15-102; 127-445
	**chain L**	15-445	15-445	15-445	15-445	15-102; 127-445

Supplementary Table 3 X-ray crystallographic data collection and refinement statistics of BsKtrA structures.

	Tl^+-added ATP-BsKtrA (K^+ buffer)	Tl^+-added ATP-BsKtrA (Na^+ buffer)
**PDB code**	**8K16**	**8K1K**
**Data Collection**		
Beamline	TPS 05A, NSRRC, Taiwan	
Space group	I4	
Cell dimensions		
a, b, c (Å)	122.42, 122.42, 83.85	122.54, 122.54, 83.75
α, β, γ. (°)	90.00, 90.00, 90.00	90.00, 90.00, 90.00
Resolution (Å)	24.01 - 3.10 (3.16 - 3.10)	28.01 - 3.00 (3.06 - 3.00)
R_{merge}	3.7 (45.1)	3.9 (40.5)
$I/\sigma(I)$	55.87 (5.57)	50.39 (5.03)
Completeness (%)	99.7 (100.0)	99.6 (99.6)
Redundancy	15.2 (15.4)	15.2 (15.4)
**Refinement**		
Resolution (Å)	3.10	3.00
No. reflections	11317 (1134)	12515 (1243)
R_{work}	24.2 (41.7)	22.1 (35.5)
R_{free}	26.7 (39.9)	26.9 (36.8)
No. atoms		
Protein	3412	3412
ATP	62	62
Thallium	1	0
Sodium	0	1
B-factor ($Å^2$)		
Protein	110	99
ATP	100	86
Thallium	117	-
Sodium	-	76
r.m.s. deviations		
Bond length (Å)	0.002	0.002
Bond angles (°)	0.479	0.536
Ramachandran plot		
Favored (%)	98.37	96.28
Allowed (%)	1.63	3.72
Outliers (%)	0.00	0.00

*Values in parentheses are for highest-resolution shell.

Supplementary Table 4 ICP-MS analysis of cation abundance in apo- and ATP-BsKtrA.

Protein sample	Metal ion concentration (μg/ml)			
	Na^+	K^+	Ca^{2+}	Mg^{2+}
**Apo BsKtrA**	0.469 ± 0.069	0.627 ± 0.073	N.D.	0.113 ± 0.003
	(0.26 : 1)	(0.20 : 1)	-	(0.06 : 1)
**ATP-BsKtrA**	1.352 ± 0.097	0.717 ± 0.086	2.529 ± 0.180	0.135 ± 0.004
	(0.74 : 1)	(0.23 : 1)	(0.79 : 1)	(0.07 : 1)

NOTE: The concentrations (μg/ml) of cations in per ml of apo- or ATP-BsKtrA protein solution (80 μM). Data represent the mean ± s.d.; n = 3. N.D., not detected. Cation to protein molar ratio is indicated in the brackets. Source data are provided as a Source Data file.
