# Supplementary material for: Structural basis and synergism of ATP and Na+ activation in bacterial K+ uptake system KtrAB
Source: Nat Commun. 2024 May 8;15:3850. doi: 10.1038/s41467-024-48057-y (PMC11078986; doi:10.1038/s41467-024-48057-y)
Supplement: Supplementary file 3 — Description of Additional Supplementary Files [file 41467_2024_48057_MOESM3_ESM.pdf]

Description of Additional Supplementary Files

File Name: Supplementary Data 1

Description: MD simulation of initial ATP-KtrAB pdb model

File Name: Supplementary Data 2

Description: MD simulation of equilibrated ATP-KtrAB pdb model

File Name: Supplementary Data 3

Description: MD simulation of initial ADP-KtrAB pdb model

File Name: Supplementary Data 4

Description: MD simulation of equilibrated ADP-KtrAB pdb model

File Name: Supplementary Data 5

Description: The supplementary data contains the primer sequences used in the site-directed mutagenesis
