# Supplementary material for: Structural basis and synergism of ATP and Na+ activation in bacterial K+ uptake system KtrAB
Source: Nat Commun. 2024 May 8;15:3850. doi: 10.1038/s41467-024-48057-y (PMC11078986; doi:10.1038/s41467-024-48057-y)
Supplement: Supplementary file 9 — Reporting Summary [file 41467_2024_48057_MOESM9_ESM.pdf]

nature portfolio

Corresponding author(s): Ming-Daw Tsai, Nien-Jen Hu

Last updated by author(s): Mar 26, 2024

Reporting Summary

Nature Portfolio wishes to improve the reproducibility of the work that we publish. This form provides structure for consistency and transparency in reporting. For further information on Nature Portfolio policies, see our Editorial Policies and the Editorial Policy Checklist.

Statistics

For all statistical analyses, confirm that the following items are present in the figure legend, table legend, main text, or Methods section.

n/a	Confirmed	
☐	☒	The exact sample size (*n*) for each experimental group/condition, given as a discrete number and unit of measurement
☐	☒	A statement on whether measurements were taken from distinct samples or whether the same sample was measured repeatedly
☐	☒	The statistical test(s) used AND whether they are one- or two-sided<br>*Only common tests should be described solely by name; describe more complex techniques in the Methods section.*
☒	☐	A description of all covariates tested
☒	☐	A description of any assumptions or corrections, such as tests of normality and adjustment for multiple comparisons
☐	☒	A full description of the statistical parameters including central tendency (e.g. means) or other basic estimates (e.g. regression coefficient) AND variation (e.g. standard deviation) or associated estimates of uncertainty (e.g. confidence intervals)
☐	☒	For null hypothesis testing, the test statistic (e.g. *F*, *t*, *r*) with confidence intervals, effect sizes, degrees of freedom and *P* value noted<br>*Give P values as exact values whenever suitable.*
☒	☐	For Bayesian analysis, information on the choice of priors and Markov chain Monte Carlo settings
☒	☐	For hierarchical and complex designs, identification of the appropriate level for tests and full reporting of outcomes
☒	☐	Estimates of effect sizes (e.g. Cohen's *d*, Pearson's *r*), indicating how they were calculated

Our web collection on statistics for biologists contains articles on many of the points above.

Software and code

Policy information about availability of computer code

Data collection	cryo-EM data collection: EPU 2<br>X-ray crystallography data collection: BLU-ICE 5.1
Data analysis	Cyro-EM data analysis: MotionCor2, Relion 3.1, cryoSPARC 3.3.2<br>Cyro-EM model building: COOT 0.9.8.1, Phenix 1.19.2, chimera 1.14<br>X-ray crystallography data processing and analysis: HKL2000 v722<br>X-ray crystallography model building: COOT 0.9.8.9, Phenix 1.12-2829<br>Structural analysis: Pymol 2.5.4, Chimera X 1.6.1<br>Statistical analysis: GraphPad Prism 6<br>nitial molecular structure generation: CCBuilder 2.0 (web server), CHARMM-GUI 3.2 (web server), SWISS model (web server)<br>Molecular dynamics simulation: NAMD 2.14<br>Molecular dynamics trajectory analysis: VMD 1.9.4

For manuscripts utilizing custom algorithms or software that are central to the research but not yet described in published literature, software must be made available to editors and reviewers. We strongly encourage code deposition in a community repository (e.g. GitHub). See the Nature Portfolio guidelines for submitting code & software for further information.

Data

Policy information about availability of data

All manuscripts must include a data availability statement. This statement should provide the following information, where applicable:

- Accession codes, unique identifiers, or web links for publicly available datasets
- A description of any restrictions on data availability
- For clinical datasets or third party data, please ensure that the statement adheres to our policy

The cryo-EM maps have been deposited in the Electron Microscopy Data Bank (EMDB) under accession codes EMD-36803 [https://www.ebi.ac.uk/pdbe/entry/emdb/EMD-36803] (Structure I); EMD-36804 [https://www.ebi.ac.uk/pdbe/entry/emdb/EMD-36804] (Structure II); EMD-38477 [https://www.ebi.ac.uk/pdbe/entry/emdb/EMD-38477] (Structure IIa); EMD-38478 [https://www.ebi.ac.uk/pdbe/entry/emdb/EMD-38478] (Structure IIb); EMD-36800 [https://www.ebi.ac.uk/pdbe/entry/emdb/EMD-36800] (Structure III); EMD-36801 [https://www.ebi.ac.uk/pdbe/entry/emdb/EMD-36801] (Structure III, focused refined on KtrA octamer); and EMD-36802 [https://www.ebi.ac.uk/pdbe/entry/emdb/EMD-36802] (Structure III, focused refined on KtrB dimer). The atomic coordinates have been deposited in the Protein Data Bank (PDB) under accession codes 8KIT [https://doi.org/10.2210/pdb8K1T/pdb] (Structure I); 8K1U [https://doi.org/10.2210/pdb8K1U/pdb] (Structure II); 8XMH [https://doi.org/10.2210/pdb8XMH/pdb] (Structure IIa); 8XMI [https://doi.org/10.2210/pdb8XMI/pdb] (Structure IIb); 8K1S [https://doi.org/10.2210/pdb8K1S/pdb] (Structure III); 8K16 [https://doi.org/10.2210/pdb8K16/pdb] (Tl+-treated BsKtrA in K+ Buffer); and 8K1K [https://doi.org/10.2210/pdb8K1K/pdb] (Tl+-treated BsKtrA in Na+ Buffer).

Research involving human participants, their data, or biological material

Policy information about studies with human participants or human data. See also policy information about sex, gender (identity/presentation), and sexual orientation and race, ethnicity and racism.

Reporting on sex and gender	not applicable
Reporting on race, ethnicity, or other socially relevant groupings	not applicable
Population characteristics	not applicable
Recruitment	not applicable
Ethics oversight	not applicable

Note that full information on the approval of the study protocol must also be provided in the manuscript.

Field-specific reporting

Please select the one below that is the best fit for your research. If you are not sure, read the appropriate sections before making your selection.

☒ Life sciences ☐ Behavioural & social sciences ☐ Ecological, evolutionary & environmental sciences

For a reference copy of the document with all sections, see nature.com/documents/nr-reporting-summary-flat.pdf

Life sciences study design

All studies must disclose on these points even when the disclosure is negative.

Sample size	No statistical method was used to determine the sample size. At least three independent experiments are performed for biochemical assays to ensure reliable and repeatable results. The numbers of replication for each biochemical assay have been described in the figure legends.
Data exclusions	Particle images with poor quality were excluded in 2D and 3D classification.
Replication	Biological assays were repeated at least in triplicates independently and the replicate numbers for each assays are described in the figure legends. All the experimental data were consistently reproduced.
Randomization	The Fourier shell correlation (FSC) analysis of Cryo-EM was randomized by splitting the particle images into two. The randomization procedures were performed using cryoSPARC.
Blinding	The nature of the study does not require blinding because no clinical trials are involved.

Reporting for specific materials, systems and methods

We require information from authors about some types of materials, experimental systems and methods used in many studies. Here, indicate whether each material, system or method listed is relevant to your study. If you are not sure if a list item applies to your research, read the appropriate section before selecting a response.

Materials & experimental systems

n/a	Involved in the study
☒	☐ Antibodies
☒	☐ Eukaryotic cell lines
☒	☐ Palaeontology and archaeology
☒	☐ Animals and other organisms
☒	☐ Clinical data
☒	☐ Dual use research of concern
☒	☐ Plants

Methods

n/a	Involved in the study
☒	☐ ChIP-seq
☒	☐ Flow cytometry
☒	☐ MRI-based neuroimaging
