# Supplementary material for: Structural basis and synergism of ATP and Na+ activation in bacterial K+ uptake system KtrAB
Source: Nat Commun. 2024 May 8;15:3850. doi: 10.1038/s41467-024-48057-y (PMC11078986; doi:10.1038/s41467-024-48057-y)
Supplement: Supplementary file 4 — Supplementary Data 1 [file 41467_2024_48057_MOESM4_ESM.pdf]

```
CRYST1  199.901  200.007  200.007  90.00  90.00  90.00 P 1
1
ATOM      1  N   LYS A   7      35.099  18.110  25.277  1.00  0.00
A    N
ATOM      2  HT1 LYS A   7      36.049  18.381  25.126  1.00  0.00
A    H
ATOM      3  HT2 LYS A   7      34.528  18.922  25.399  1.00  0.00
A    H
ATOM      4  HT3 LYS A   7      34.769  17.575  24.499  1.00  0.00
A    H
ATOM      5  CA  LYS A   7      35.023  17.292  26.483  1.00  0.00
A    C
ATOM      6  HA  LYS A   7      35.603  16.395  26.303  1.00  0.00
A    H
ATOM      7  CB  LYS A   7      35.579  18.063  27.681  1.00  0.00
A    C
ATOM      8  HB1 LYS A   7      35.374  17.451  28.589  1.00  0.00
A    H
ATOM      9  HB2 LYS A   7      36.687  18.124  27.590  1.00  0.00
A    H
ATOM     10  CG  LYS A   7      34.968  19.440  27.853  1.00  0.00
A    C
ATOM     11  HG1 LYS A   7      35.107  20.021  26.913  1.00  0.00
A    H
ATOM     12  HG2 LYS A   7      33.871  19.333  28.016  1.00  0.00
A    H
ATOM     13  CD  LYS A   7      35.604  20.197  29.005  1.00  0.00
A    C
ATOM     14  HD1 LYS A   7      34.930  21.037  29.289  1.00  0.00
A    H
ATOM     15  HD2 LYS A   7      35.665  19.509  29.880  1.00  0.00
A    H
ATOM     16  CE  LYS A   7      36.984  20.716  28.644  1.00  0.00
A    C
ATOM     17  HE1 LYS A   7      37.698  19.871  28.517  1.00  0.00
A    H
ATOM     18  HE2 LYS A   7      36.948  21.292  27.693  1.00  0.00
A    H
ATOM     19  NZ  LYS A   7      37.531  21.615  29.699  1.00  0.00
A    N
ATOM     20  HZ1 LYS A   7      38.476  21.953  29.424  1.00  0.00
A    H
ATOM     21  HZ2 LYS A   7      36.897  22.429  29.824  1.00  0.00
A    H
ATOM     22  HZ3 LYS A   7      37.599  21.093  30.595  1.00  0.00
A    H
ATOM     23  C   LYS A   7      33.587  16.859  26.746  1.00  0.00
A    C
ATOM     24  O   LYS A   7      32.660  17.663  26.655  1.00  0.00
A    O
ATOM     25  N   GLN A   8      33.406  15.591  27.105  1.00  0.00
A    N
ATOM     26  HN  GLN A   8      34.147  14.982  27.384  1.00  0.00
A    H
```

ATOM 27 CA GLN A 8 32.085 14.979 27.118 1.00 0.00
A C
ATOM 28 HA GLN A 8 31.401 15.699 26.686 1.00 0.00
A H
ATOM 29 CB GLN A 8 32.082 13.710 26.279 1.00 0.00
A C
ATOM 30 HB1 GLN A 8 33.026 13.166 26.513 1.00 0.00
A H
ATOM 31 HB2 GLN A 8 31.251 13.048 26.615 1.00 0.00
A H
ATOM 32 CG GLN A 8 31.999 14.034 24.808 1.00 0.00
A C
ATOM 33 HG1 GLN A 8 30.939 14.140 24.493 1.00 0.00
A H
ATOM 34 HG2 GLN A 8 32.518 14.989 24.587 1.00 0.00
A H
ATOM 35 CD GLN A 8 32.589 12.956 23.940 1.00 0.00
A C
ATOM 36 OE1 GLN A 8 33.383 12.140 24.401 1.00 0.00
A O
ATOM 37 NE2 GLN A 8 32.212 12.949 22.667 1.00 0.00
A N
ATOM 38 HE21 GLN A 8 32.595 12.235 22.087 1.00 0.00
A H
ATOM 39 HE22 GLN A 8 31.588 13.647 22.332 1.00 0.00
A H
ATOM 40 C GLN A 8 31.616 14.697 28.537 1.00 0.00
A C
ATOM 41 O GLN A 8 32.362 14.150 29.362 1.00 0.00
A O
ATOM 42 N PHE A 9 30.366 15.064 28.796 1.00 0.00
A N
ATOM 43 HN PHE A 9 29.753 15.371 28.069 1.00 0.00
A H
ATOM 44 CA PHE A 9 29.773 15.061 30.120 1.00 0.00
A C
ATOM 45 HA PHE A 9 30.489 14.595 30.785 1.00 0.00
A H
ATOM 46 CB PHE A 9 29.392 16.469 30.533 1.00 0.00
A C
ATOM 47 HB1 PHE A 9 28.621 16.858 29.832 1.00 0.00
A H
ATOM 48 HB2 PHE A 9 28.944 16.481 31.550 1.00 0.00
A H
ATOM 49 CG PHE A 9 30.524 17.427 30.478 1.00 0.00
A C
ATOM 50 CD1 PHE A 9 31.395 17.534 31.540 1.00 0.00
A C
ATOM 51 HD1 PHE A 9 31.245 16.923 32.419 1.00 0.00
A H
ATOM 52 CE1 PHE A 9 32.447 18.410 31.500 1.00 0.00
A C
ATOM 53 HE1 PHE A 9 33.124 18.479 32.339 1.00 0.00
A H

```
ATOM     54  CZ  PHE A   9      32.640  19.197  30.386  1.00  0.00
A    C
ATOM     55  HZ  PHE A   9      33.471  19.887  30.351  1.00  0.00
A    H
ATOM     56  CD2 PHE A   9      30.730  18.213  29.360  1.00  0.00
A    C
ATOM     57  HD2 PHE A   9      30.050  18.151  28.523  1.00  0.00
A    H
ATOM     58  CE2 PHE A   9      31.782  19.097  29.310  1.00  0.00
A    C
ATOM     59  HE2 PHE A   9      31.929  19.710  28.431  1.00  0.00
A    H
ATOM     60  C   PHE A   9      28.532  14.188  30.119  1.00  0.00
A    C
ATOM     61  O   PHE A   9      27.700  14.273  29.211  1.00  0.00
A    O
ATOM     62  N   ALA A  10      28.406  13.361  31.150  1.00  0.00
A    N
ATOM     63  HN  ALA A  10      29.093  13.300  31.873  1.00  0.00
A    H
ATOM     64  CA  ALA A  10      27.256  12.482  31.304  1.00  0.00
A    C
ATOM     65  HA  ALA A  10      26.503  12.674  30.548  1.00  0.00
A    H
ATOM     66  CB  ALA A  10      27.668  11.014  31.195  1.00  0.00
A    C
ATOM     67  HB1 ALA A  10      28.100  10.821  30.190  1.00  0.00
A    H
ATOM     68  HB2 ALA A  10      28.440  10.758  31.954  1.00  0.00
A    H
ATOM     69  HB3 ALA A  10      26.799  10.333  31.331  1.00  0.00
A    H
ATOM     70  C   ALA A  10      26.599  12.762  32.644  1.00  0.00
A    C
ATOM     71  O   ALA A  10      27.168  12.452  33.695  1.00  0.00
A    O
ATOM     72  N   VAL A  11      25.416  13.349  32.613  1.00  0.00
A    N
ATOM     73  HN  VAL A  11      24.982  13.678  31.778  1.00  0.00
A    H
ATOM     74  CA  VAL A  11      24.636  13.565  33.818  1.00  0.00
A    C
ATOM     75  HA  VAL A  11      25.303  13.636  34.668  1.00  0.00
A    H
ATOM     76  CB  VAL A  11      23.783  14.829  33.740  1.00  0.00
A    C
ATOM     77  HB  VAL A  11      23.801  15.184  32.680  1.00  0.00
A    H
ATOM     78  CG1 VAL A  11      22.354  14.541  34.140  1.00  0.00
A    C
ATOM     79 HG11 VAL A  11      21.752  15.474  34.118  1.00  0.00
A    H
ATOM     80 HG12 VAL A  11      21.872  13.821  33.447  1.00  0.00
A    H
```

```
ATOM     81 HG13 VAL A  11      22.310  14.128  35.170  1.00  0.00
A    H
ATOM     82  CG2 VAL A  11      24.356  15.884  34.647  1.00  0.00
A    C
ATOM     83 HG21 VAL A  11      23.725  16.798  34.634  1.00  0.00
A    H
ATOM     84 HG22 VAL A  11      24.410  15.511  35.693  1.00  0.00
A    H
ATOM     85 HG23 VAL A  11      25.377  16.177  34.328  1.00  0.00
A    H
ATOM     86  C   VAL A  11      23.781  12.325  34.012  1.00  0.00
A    C
ATOM     87  O   VAL A  11      23.115  11.857  33.086  1.00  0.00
A    O
ATOM     88  N   ILE A  12      23.815  11.781  35.214  1.00  0.00
A    N
ATOM     89  HN  ILE A  12      24.445  12.072  35.931  1.00  0.00
A    H
ATOM     90  CA  ILE A  12      22.929  10.701  35.602  1.00  0.00
A    C
ATOM     91  HA  ILE A  12      22.184  10.582  34.825  1.00  0.00
A    H
ATOM     92  CB  ILE A  12      23.701   9.407  35.864  1.00  0.00
A    C
ATOM     93  HB  ILE A  12      24.380   9.654  36.719  1.00  0.00
A    H
ATOM     94  CG2 ILE A  12      22.733   8.290  36.078  1.00  0.00
A    C
ATOM     95 HG21 ILE A  12      23.261   7.377  36.423  1.00  0.00
A    H
ATOM     96 HG22 ILE A  12      21.984   8.559  36.852  1.00  0.00
A    H
ATOM     97 HG23 ILE A  12      22.194   8.046  35.138  1.00  0.00
A    H
ATOM     98  CG1 ILE A  12      24.589   9.073  34.677  1.00  0.00
A    C
ATOM     99 HG11 ILE A  12      25.345   9.877  34.536  1.00  0.00
A    H
ATOM    100 HG12 ILE A  12      25.144   8.133  34.896  1.00  0.00
A    H
ATOM    101  CD  ILE A  12      23.830   8.886  33.413  1.00  0.00
A    C
ATOM    102  HD1 ILE A  12      24.516   8.587  32.592  1.00  0.00
A    H
ATOM    103  HD2 ILE A  12      23.062   8.091  33.530  1.00  0.00
A    H
ATOM    104  HD3 ILE A  12      23.321   9.826  33.111  1.00  0.00
A    H
ATOM    105  C   ILE A  12      22.194  11.150  36.848  1.00  0.00
A    C
ATOM    106  O   ILE A  12      22.805  11.677  37.785  1.00  0.00
A    O
ATOM    107  N   GLY A  13      20.886  10.972  36.845  1.00  0.00
A    N
```

```
ATOM    108  HN  GLY A  13      20.393  10.555  36.082  1.00  0.00
A    H
ATOM    109  CA  GLY A  13      20.073  11.378  37.963  1.00  0.00
A    C
ATOM    110  HA1 GLY A  13      20.688  11.440  38.850  1.00  0.00
A    H
ATOM    111  HA2 GLY A  13      19.224  10.713  38.057  1.00  0.00
A    H
ATOM    112  C   GLY A  13      19.550  12.758  37.662  1.00  0.00
A    C
ATOM    113  O   GLY A  13      20.278  13.745  37.795  1.00  0.00
A    O
ATOM    114  N   LEU A  14      18.296  12.847  37.246  1.00  0.00
A    N
ATOM    115  HN  LEU A  14      17.645  12.092  37.202  1.00  0.00
A    H
ATOM    116  CA  LEU A  14      17.761  14.126  36.803  1.00  0.00
A    C
ATOM    117  HA  LEU A  14      18.598  14.807  36.720  1.00  0.00
A    H
ATOM    118  CB  LEU A  14      17.090  13.962  35.454  1.00  0.00
A    C
ATOM    119  HB1 LEU A  14      17.021  12.868  35.251  1.00  0.00
A    H
ATOM    120  HB2 LEU A  14      16.042  14.328  35.524  1.00  0.00
A    H
ATOM    121  CG  LEU A  14      17.856  14.648  34.344  1.00  0.00
A    C
ATOM    122  HG  LEU A  14      18.799  15.075  34.765  1.00  0.00
A    H
ATOM    123  CD1 LEU A  14      18.201  13.645  33.276  1.00  0.00
A    C
ATOM    124 HD11 LEU A  14      18.723  14.141  32.430  1.00  0.00
A    H
ATOM    125 HD12 LEU A  14      18.867  12.854  33.683  1.00  0.00
A    H
ATOM    126 HD13 LEU A  14      17.282  13.161  32.882  1.00  0.00
A    H
ATOM    127  CD2 LEU A  14      17.026  15.796  33.786  1.00  0.00
A    C
ATOM    128 HD21 LEU A  14      17.586  16.321  32.982  1.00  0.00
A    H
ATOM    129 HD22 LEU A  14      16.070  15.438  33.353  1.00  0.00
A    H
ATOM    130 HD23 LEU A  14      16.794  16.532  34.585  1.00  0.00
A    H
ATOM    131  C   LEU A  14      16.802  14.698  37.844  1.00  0.00
A    C
ATOM    132  O   LEU A  14      15.625  14.933  37.589  1.00  0.00
A    O
ATOM    133  N   GLY A  15      17.333  14.920  39.040  1.00  0.00
A    N
ATOM    134  HN  GLY A  15      18.246  14.609  39.301  1.00  0.00
A    H
```

```
ATOM    135  CA  GLY A  15      16.601  15.642  40.060  1.00  0.00
A    C
ATOM    136  HA1 GLY A  15      17.007  15.374  41.026  1.00  0.00
A    H
ATOM    137  HA2 GLY A  15      15.547  15.429  39.946  1.00  0.00
A    H
ATOM    138  C   GLY A  15      16.799  17.128  39.866  1.00  0.00
A    C
ATOM    139  O   GLY A  15      17.566  17.524  38.983  1.00  0.00
A    O
ATOM    140  N   ARG A  16      16.118  17.955  40.668  1.00  0.00
A    N
ATOM    141  HN  ARG A  16      15.461  17.632  41.346  1.00  0.00
A    H
ATOM    142  CA  ARG A  16      16.313  19.396  40.582  1.00  0.00
A    C
ATOM    143  HA  ARG A  16      15.834  19.751  39.677  1.00  0.00
A    H
ATOM    144  CB  ARG A  16      15.732  20.094  41.807  1.00  0.00
A    C
ATOM    145  HB1 ARG A  16      16.306  19.716  42.686  1.00  0.00
A    H
ATOM    146  HB2 ARG A  16      15.942  21.185  41.750  1.00  0.00
A    H
ATOM    147  CG  ARG A  16      14.273  19.829  42.035  1.00  0.00
A    C
ATOM    148  HG1 ARG A  16      13.683  20.541  41.416  1.00  0.00
A    H
ATOM    149  HG2 ARG A  16      14.023  18.816  41.643  1.00  0.00
A    H
ATOM    150  CD  ARG A  16      13.932  19.892  43.510  1.00  0.00
A    C
ATOM    151  HD1 ARG A  16      14.415  20.787  43.965  1.00  0.00
A    H
ATOM    152  HD2 ARG A  16      12.832  19.958  43.674  1.00  0.00
A    H
ATOM    153  NE  ARG A  16      14.380  18.704  44.233  1.00  0.00
A    N
ATOM    154  HE  ARG A  16      14.916  18.073  43.662  1.00  0.00
A    H
ATOM    155  CZ  ARG A  16      14.115  18.470  45.514  1.00  0.00
A    C
ATOM    156  NH1 ARG A  16      13.398  19.339  46.212  1.00  0.00
A    N
ATOM    157 HH11 ARG A  16      13.216  19.187  47.173  1.00  0.00
A    H
ATOM    158 HH12 ARG A  16      13.178  20.203  45.753  1.00  0.00
A    H
ATOM    159  NH2 ARG A  16      14.555  17.363  46.095  1.00  0.00
A    N
ATOM    160 HH21 ARG A  16      14.283  17.150  47.023  1.00  0.00
A    H
ATOM    161 HH22 ARG A  16      14.973  16.683  45.507  1.00  0.00
A    H
```

ATOM 162 C ARG A 16 17.781 19.745 40.460 1.00 0.00
A C
ATOM 163 O ARG A 16 18.159 20.563 39.614 1.00 0.00
A O
ATOM 164 N PHE A 17 18.615 19.099 41.263 1.00 0.00
A N
ATOM 165 HN PHE A 17 18.338 18.471 41.989 1.00 0.00
A H
ATOM 166 CA PHE A 17 20.048 19.271 41.133 1.00 0.00
A C
ATOM 167 HA PHE A 17 20.250 20.321 41.308 1.00 0.00
A H
ATOM 168 CB PHE A 17 20.761 18.379 42.139 1.00 0.00
A C
ATOM 169 HB1 PHE A 17 20.404 18.627 43.163 1.00 0.00
A H
ATOM 170 HB2 PHE A 17 20.526 17.307 41.960 1.00 0.00
A H
ATOM 171 CG PHE A 17 22.235 18.562 42.158 1.00 0.00
A C
ATOM 172 CD1 PHE A 17 22.778 19.826 42.276 1.00 0.00
A C
ATOM 173 HD1 PHE A 17 22.123 20.684 42.344 1.00 0.00
A H
ATOM 174 CE1 PHE A 17 24.137 20.006 42.300 1.00 0.00
A C
ATOM 175 HE1 PHE A 17 24.549 21.001 42.390 1.00 0.00
A H
ATOM 176 CZ PHE A 17 24.970 18.915 42.215 1.00 0.00
A C
ATOM 177 HZ PHE A 17 26.042 19.050 42.246 1.00 0.00
A H
ATOM 178 CD2 PHE A 17 23.081 17.475 42.075 1.00 0.00
A C
ATOM 179 HD2 PHE A 17 22.673 16.480 41.966 1.00 0.00
A H
ATOM 180 CE2 PHE A 17 24.439 17.647 42.103 1.00 0.00
A C
ATOM 181 HE2 PHE A 17 25.092 16.788 42.035 1.00 0.00
A H
ATOM 182 C PHE A 17 20.495 18.962 39.714 1.00 0.00
A C
ATOM 183 O PHE A 17 21.083 19.814 39.038 1.00 0.00
A O
ATOM 184 N GLY A 18 20.205 17.751 39.241 1.00 0.00
A N
ATOM 185 HN GLY A 18 19.709 17.048 39.748 1.00 0.00
A H
ATOM 186 CA GLY A 18 20.620 17.383 37.899 1.00 0.00
A C
ATOM 187 HA1 GLY A 18 20.160 16.431 37.674 1.00 0.00
A H
ATOM 188 HA2 GLY A 18 21.702 17.367 37.890 1.00 0.00
A H

```
ATOM    189  C   GLY A  18      20.159  18.384  36.860  1.00  0.00
A    C
ATOM    190  O   GLY A  18      20.974  18.982  36.148  1.00  0.00
A    O
ATOM    191  N   GLY A  19      18.848  18.616  36.801  1.00  0.00
A    N
ATOM    192  HN  GLY A  19      18.187  18.212  37.433  1.00  0.00
A    H
ATOM    193  CA  GLY A  19      18.292  19.492  35.786  1.00  0.00
A    C
ATOM    194  HA1 GLY A  19      17.248  19.628  36.033  1.00  0.00
A    H
ATOM    195  HA2 GLY A  19      18.465  19.014  34.831  1.00  0.00
A    H
ATOM    196  C   GLY A  19      18.947  20.854  35.751  1.00  0.00
A    C
ATOM    197  O   GLY A  19      19.189  21.405  34.675  1.00  0.00
A    O
ATOM    198  N   SER A  20      19.249  21.407  36.919  1.00  0.00
A    N
ATOM    199  HN  SER A  20      19.009  21.027  37.812  1.00  0.00
A    H
ATOM    200  CA  SER A  20      19.981  22.657  36.982  1.00  0.00
A    C
ATOM    201  HA  SER A  20      19.359  23.393  36.490  1.00  0.00
A    H
ATOM    202  CB  SER A  20      20.308  22.970  38.432  1.00  0.00
A    C
ATOM    203  HB1 SER A  20      20.876  22.108  38.853  1.00  0.00
A    H
ATOM    204  HB2 SER A  20      20.972  23.861  38.496  1.00  0.00
A    H
ATOM    205  OG  SER A  20      19.120  23.146  39.155  1.00  0.00
A    O
ATOM    206  HG1 SER A  20      19.351  23.119  40.092  1.00  0.00
A    H
ATOM    207  C   SER A  20      21.275  22.572  36.205  1.00  0.00
A    C
ATOM    208  O   SER A  20      21.518  23.378  35.297  1.00  0.00
A    O
ATOM    209  N   ILE A  21      22.100  21.599  36.576  1.00  0.00
A    N
ATOM    210  HN  ILE A  21      21.900  20.940  37.298  1.00  0.00
A    H
ATOM    211  CA  ILE A  21      23.393  21.413  35.942  1.00  0.00
A    C
ATOM    212  HA  ILE A  21      23.976  22.298  36.160  1.00  0.00
A    H
ATOM    213  CB  ILE A  21      24.044  20.134  36.479  1.00  0.00
A    C
ATOM    214  HB  ILE A  21      23.402  19.307  36.084  1.00  0.00
A    H
ATOM    215  CG2 ILE A  21      25.475  20.059  36.047  1.00  0.00
A    C
```

```
ATOM    216 HG21 ILE A  21      25.907  19.068  36.297  1.00  0.00
A    H
ATOM    217 HG22 ILE A  21      25.565  20.198  34.949  1.00  0.00
A    H
ATOM    218 HG23 ILE A  21      26.084  20.839  36.550  1.00  0.00
A    H
ATOM    219  CG1 ILE A  21      23.931  20.111  37.993  1.00  0.00
A    C
ATOM    220 HG11 ILE A  21      24.484  20.975  38.426  1.00  0.00
A    H
ATOM    221 HG12 ILE A  21      22.860  20.230  38.277  1.00  0.00
A    H
ATOM    222  CD  ILE A  21      24.437  18.851  38.622  1.00  0.00
A    C
ATOM    223  HD1 ILE A  21      24.273  18.875  39.721  1.00  0.00
A    H
ATOM    224  HD2 ILE A  21      23.905  17.966  38.210  1.00  0.00
A    H
ATOM    225  HD3 ILE A  21      25.524  18.723  38.440  1.00  0.00
A    H
ATOM    226  C   ILE A  21      23.238  21.349  34.438  1.00  0.00
A    C
ATOM    227  O   ILE A  21      23.904  22.077  33.693  1.00  0.00
A    O
ATOM    228  N   VAL A  22      22.367  20.462  33.969  1.00  0.00
A    N
ATOM    229  HN  VAL A  22      21.713  19.926  34.497  1.00  0.00
A    H
ATOM    230  CA  VAL A  22      22.329  20.214  32.536  1.00  0.00
A    C
ATOM    231  HA  VAL A  22      23.350  20.068  32.203  1.00  0.00
A    H
ATOM    232  CB  VAL A  22      21.521  18.949  32.221  1.00  0.00
A    C
ATOM    233  HB  VAL A  22      21.136  19.046  31.176  1.00  0.00
A    H
ATOM    234  CG1 VAL A  22      22.400  17.738  32.317  1.00  0.00
A    C
ATOM    235 HG11 VAL A  22      21.809  16.816  32.128  1.00  0.00
A    H
ATOM    236 HG12 VAL A  22      23.219  17.764  31.569  1.00  0.00
A    H
ATOM    237 HG13 VAL A  22      22.847  17.656  33.331  1.00  0.00
A    H
ATOM    238  CG2 VAL A  22      20.371  18.831  33.146  1.00  0.00
A    C
ATOM    239 HG21 VAL A  22      19.803  17.896  32.950  1.00  0.00
A    H
ATOM    240 HG22 VAL A  22      20.719  18.814  34.202  1.00  0.00
A    H
ATOM    241 HG23 VAL A  22      19.669  19.681  33.024  1.00  0.00
A    H
ATOM    242  C   VAL A  22      21.777  21.416  31.794  1.00  0.00
A    C
```

```
ATOM    243  O   VAL A  22      22.282  21.781  30.731  1.00  0.00
A    O
ATOM    244  N   LYS A  23      20.731  22.043  32.324  1.00  0.00
A    N
ATOM    245  HN  LYS A  23      20.298  21.816  33.195  1.00  0.00
A    H
ATOM    246  CA  LYS A  23      20.134  23.159  31.611  1.00  0.00
A    C
ATOM    247  HA  LYS A  23      19.940  22.831  30.596  1.00  0.00
A    H
ATOM    248  CB  LYS A  23      18.835  23.574  32.287  1.00  0.00
A    C
ATOM    249  HB1 LYS A  23      18.282  22.639  32.537  1.00  0.00
A    H
ATOM    250  HB2 LYS A  23      19.073  24.066  33.256  1.00  0.00
A    H
ATOM    251  CG  LYS A  23      17.957  24.459  31.431  1.00  0.00
A    C
ATOM    252  HG1 LYS A  23      18.527  24.772  30.527  1.00  0.00
A    H
ATOM    253  HG2 LYS A  23      17.079  23.869  31.083  1.00  0.00
A    H
ATOM    254  CD  LYS A  23      17.500  25.692  32.176  1.00  0.00
A    C
ATOM    255  HD1 LYS A  23      17.734  25.557  33.257  1.00  0.00
A    H
ATOM    256  HD2 LYS A  23      18.098  26.559  31.811  1.00  0.00
A    H
ATOM    257  CE  LYS A  23      16.008  25.916  31.948  1.00  0.00
A    C
ATOM    258  HE1 LYS A  23      15.798  26.072  30.867  1.00  0.00
A    H
ATOM    259  HE2 LYS A  23      15.424  25.037  32.298  1.00  0.00
A    H
ATOM    260  NZ  LYS A  23      15.478  27.106  32.669  1.00  0.00
A    N
ATOM    261  HZ1 LYS A  23      14.461  27.213  32.478  1.00  0.00
A    H
ATOM    262  HZ2 LYS A  23      15.627  26.989  33.691  1.00  0.00
A    H
ATOM    263  HZ3 LYS A  23      15.980  27.958  32.346  1.00  0.00
A    H
ATOM    264  C   LYS A  23      21.110  24.316  31.534  1.00  0.00
A    C
ATOM    265  O   LYS A  23      21.210  24.985  30.499  1.00  0.00
A    O
ATOM    266  N   GLU A  24      21.882  24.529  32.599  1.00  0.00
A    N
ATOM    267  HN  GLU A  24      21.834  24.051  33.471  1.00  0.00
A    H
ATOM    268  CA  GLU A  24      22.918  25.549  32.540  1.00  0.00
A    C
ATOM    269  HA  GLU A  24      22.463  26.483  32.233  1.00  0.00
A    H
```

```
ATOM    270  CB  GLU A  24      23.575  25.690  33.914  1.00  0.00
A    C
ATOM    271  HB1 GLU A  24      22.770  25.848  34.669  1.00  0.00
A    H
ATOM    272  HB2 GLU A  24      24.072  24.734  34.194  1.00  0.00
A    H
ATOM    273  CG  GLU A  24      24.584  26.821  34.072  1.00  0.00
A    C
ATOM    274  HG1 GLU A  24      24.079  27.791  33.914  1.00  0.00
A    H
ATOM    275  HG2 GLU A  24      25.040  26.807  35.081  1.00  0.00
A    H
ATOM    276  CD  GLU A  24      25.716  26.806  33.062  1.00  0.00
A    C
ATOM    277  OE1 GLU A  24      26.443  25.801  32.961  1.00  0.00
A    O
ATOM    278  OE2 GLU A  24      25.855  27.813  32.345  1.00  0.00
A    O
ATOM    279  C   GLU A  24      23.961  25.188  31.490  1.00  0.00
A    C
ATOM    280  O   GLU A  24      24.249  25.974  30.581  1.00  0.00
A    O
ATOM    281  N   LEU A  25      24.571  24.013  31.628  1.00  0.00
A    N
ATOM    282  HN  LEU A  25      24.349  23.331  32.322  1.00  0.00
A    H
ATOM    283  CA  LEU A  25      25.654  23.627  30.734  1.00  0.00
A    C
ATOM    284  HA  LEU A  25      26.434  24.367  30.850  1.00  0.00
A    H
ATOM    285  CB  LEU A  25      26.148  22.239  31.124  1.00  0.00
A    C
ATOM    286  HB1 LEU A  25      25.511  21.890  31.970  1.00  0.00
A    H
ATOM    287  HB2 LEU A  25      25.956  21.537  30.283  1.00  0.00
A    H
ATOM    288  CG  LEU A  25      27.605  22.213  31.544  1.00  0.00
A    C
ATOM    289  HG  LEU A  25      27.968  23.251  31.735  1.00  0.00
A    H
ATOM    290  CD1 LEU A  25      27.769  21.376  32.774  1.00  0.00
A    C
ATOM    291 HD11 LEU A  25      28.840  21.312  33.062  1.00  0.00
A    H
ATOM    292 HD12 LEU A  25      27.205  21.817  33.625  1.00  0.00
A    H
ATOM    293 HD13 LEU A  25      27.394  20.346  32.601  1.00  0.00
A    H
ATOM    294  CD2 LEU A  25      28.426  21.630  30.419  1.00  0.00
A    C
ATOM    295 HD21 LEU A  25      29.502  21.616  30.695  1.00  0.00
A    H
ATOM    296 HD22 LEU A  25      28.124  20.589  30.189  1.00  0.00
A    H
```

```
ATOM    297 HD23 LEU A  25      28.311  22.240  29.497  1.00  0.00
A    H
ATOM    298  C   LEU A  25      25.202  23.660  29.286  1.00  0.00
A    C
ATOM    299  O   LEU A  25      25.954  24.060  28.391  1.00  0.00
A    O
ATOM    300  N   HSE A  26      23.969  23.236  29.048  1.00  0.00
A    N
ATOM    301  HN  HSE A  26      23.403  22.785  29.738  1.00  0.00
A    H
ATOM    302  CA  HSE A  26      23.329  23.390  27.756  1.00  0.00
A    C
ATOM    303  HA  HSE A  26      23.937  22.860  27.033  1.00  0.00
A    H
ATOM    304  CB  HSE A  26      21.915  22.836  27.860  1.00  0.00
A    C
ATOM    305  HB1 HSE A  26      21.983  21.796  28.249  1.00  0.00
A    H
ATOM    306  HB2 HSE A  26      21.340  23.425  28.604  1.00  0.00
A    H
ATOM    307  ND1 HSE A  26      20.248  23.739  26.211  1.00  0.00
A    N
ATOM    308  CG  HSE A  26      21.178  22.787  26.568  1.00  0.00
A    C
ATOM    309  CE1 HSE A  26      19.742  23.430  25.032  1.00  0.00
A    C
ATOM    310  HE1 HSE A  26      18.985  24.020  24.509  1.00  0.00
A    H
ATOM    311  NE2 HSE A  26      20.299  22.307  24.619  1.00  0.00
A    N
ATOM    312  HE2 HSE A  26      20.080  21.837  23.764  1.00  0.00
A    H
ATOM    313  CD2 HSE A  26      21.197  21.880  25.564  1.00  0.00
A    C
ATOM    314  HD2 HSE A  26      21.773  20.971  25.463  1.00  0.00
A    H
ATOM    315  C   HSE A  26      23.310  24.847  27.339  1.00  0.00
A    C
ATOM    316  O   HSE A  26      23.614  25.175  26.188  1.00  0.00
A    O
ATOM    317  N   ARG A  27      22.956  25.738  28.263  1.00  0.00
A    N
ATOM    318  HN  ARG A  27      22.619  25.496  29.170  1.00  0.00
A    H
ATOM    319  CA  ARG A  27      23.055  27.156  27.961  1.00  0.00
A    C
ATOM    320  HA  ARG A  27      22.481  27.357  27.065  1.00  0.00
A    H
ATOM    321  CB  ARG A  27      22.506  27.981  29.118  1.00  0.00
A    C
ATOM    322  HB1 ARG A  27      21.590  27.453  29.477  1.00  0.00
A    H
ATOM    323  HB2 ARG A  27      23.225  27.958  29.967  1.00  0.00
A    H
```

```
ATOM    324  CG  ARG A  27      22.143  29.392  28.730  1.00  0.00
A    C
ATOM    325  HG1 ARG A  27      22.491  29.573  27.689  1.00  0.00
A    H
ATOM    326  HG2 ARG A  27      21.033  29.483  28.695  1.00  0.00
A    H
ATOM    327  CD  ARG A  27      22.690  30.394  29.725  1.00  0.00
A    C
ATOM    328  HD1 ARG A  27      22.679  31.412  29.271  1.00  0.00
A    H
ATOM    329  HD2 ARG A  27      22.085  30.414  30.661  1.00  0.00
A    H
ATOM    330  NE  ARG A  27      24.056  30.087  30.135  1.00  0.00
A    N
ATOM    331  HE  ARG A  27      24.123  29.779  31.091  1.00  0.00
A    H
ATOM    332  CZ  ARG A  27      25.120  30.195  29.346  1.00  0.00
A    C
ATOM    333  NH1 ARG A  27      24.989  30.593  28.092  1.00  0.00
A    N
ATOM    334 HH11 ARG A  27      25.778  30.650  27.496  1.00  0.00
A    H
ATOM    335 HH12 ARG A  27      24.051  30.696  27.755  1.00  0.00
A    H
ATOM    336  NH2 ARG A  27      26.321  29.903  29.813  1.00  0.00
A    N
ATOM    337 HH21 ARG A  27      27.121  30.066  29.254  1.00  0.00
A    H
ATOM    338 HH22 ARG A  27      26.393  29.727  30.786  1.00  0.00
A    H
ATOM    339  C   ARG A  27      24.492  27.547  27.655  1.00  0.00
A    C
ATOM    340  O   ARG A  27      24.731  28.466  26.867  1.00  0.00
A    O
ATOM    341  N   MET A  28      25.466  26.859  28.253  1.00  0.00
A    N
ATOM    342  HN  MET A  28      25.361  26.163  28.961  1.00  0.00
A    H
ATOM    343  CA  MET A  28      26.846  27.116  27.865  1.00  0.00
A    C
ATOM    344  HA  MET A  28      26.948  28.192  27.787  1.00  0.00
A    H
ATOM    345  CB  MET A  28      27.831  26.568  28.889  1.00  0.00
A    C
ATOM    346  HB1 MET A  28      27.583  25.490  29.032  1.00  0.00
A    H
ATOM    347  HB2 MET A  28      28.860  26.597  28.466  1.00  0.00
A    H
ATOM    348  CG  MET A  28      27.782  27.253  30.207  1.00  0.00
A    C
ATOM    349  HG1 MET A  28      27.879  28.352  30.074  1.00  0.00
A    H
ATOM    350  HG2 MET A  28      26.802  27.059  30.693  1.00  0.00
A    H
```

```
ATOM    351  SD  MET A  28      29.083  26.724  31.316  1.00  0.00
A    S
ATOM    352  CE  MET A  28      30.472  27.531  30.532  1.00  0.00
A    C
ATOM    353  HE1 MET A  28      31.416  27.314  31.077  1.00  0.00
A    H
ATOM    354  HE2 MET A  28      30.596  27.183  29.484  1.00  0.00
A    H
ATOM    355  HE3 MET A  28      30.335  28.634  30.512  1.00  0.00
A    H
ATOM    356  C   MET A  28      27.187  26.525  26.512  1.00  0.00
A    C
ATOM    357  O   MET A  28      28.322  26.686  26.055  1.00  0.00
A    O
ATOM    358  N   GLY A  29      26.249  25.838  25.872  1.00  0.00
A    N
ATOM    359  HN  GLY A  29      25.334  25.671  26.237  1.00  0.00
A    H
ATOM    360  CA  GLY A  29      26.513  25.277  24.567  1.00  0.00
A    C
ATOM    361  HA1 GLY A  29      26.934  26.065  23.958  1.00  0.00
A    H
ATOM    362  HA2 GLY A  29      25.582  24.875  24.191  1.00  0.00
A    H
ATOM    363  C   GLY A  29      27.515  24.150  24.631  1.00  0.00
A    C
ATOM    364  O   GLY A  29      28.555  24.195  23.970  1.00  0.00
A    O
ATOM    365  N   HSE A  30      27.228  23.141  25.438  1.00  0.00
A    N
ATOM    366  HN  HSE A  30      26.403  23.081  25.999  1.00  0.00
A    H
ATOM    367  CA  HSE A  30      28.123  22.010  25.570  1.00  0.00
A    C
ATOM    368  HA  HSE A  30      28.949  22.177  24.889  1.00  0.00
A    H
ATOM    369  CB  HSE A  30      28.626  21.890  27.001  1.00  0.00
A    C
ATOM    370  HB1 HSE A  30      27.754  22.006  27.681  1.00  0.00
A    H
ATOM    371  HB2 HSE A  30      29.034  20.871  27.166  1.00  0.00
A    H
ATOM    372  ND1 HSE A  30      30.899  22.601  27.815  1.00  0.00
A    N
ATOM    373  CG  HSE A  30      29.637  22.923  27.366  1.00  0.00
A    C
ATOM    374  CE1 HSE A  30      31.573  23.711  28.056  1.00  0.00
A    C
ATOM    375  HE1 HSE A  30      32.598  23.745  28.434  1.00  0.00
A    H
ATOM    376  NE2 HSE A  30      30.796  24.740  27.771  1.00  0.00
A    N
ATOM    377  HE2 HSE A  30      31.049  25.701  27.879  1.00  0.00
A    H
```

```
ATOM    378  CD2 HSE A  30      29.582  24.274  27.332  1.00  0.00
A    C
ATOM    379  HD2 HSE A  30      28.773  24.928  27.038  1.00  0.00
A    H
ATOM    380  C   HSE A  30      27.439  20.724  25.135  1.00  0.00
A    C
ATOM    381  O   HSE A  30      26.241  20.680  24.850  1.00  0.00
A    O
ATOM    382  N   GLU A  31      28.230  19.665  25.087  1.00  0.00
A    N
ATOM    383  HN  GLU A  31      29.203  19.711  25.297  1.00  0.00
A    H
ATOM    384  CA  GLU A  31      27.766  18.336  24.725  1.00  0.00
A    C
ATOM    385  HA  GLU A  31      26.845  18.412  24.161  1.00  0.00
A    H
ATOM    386  CB  GLU A  31      28.825  17.623  23.886  1.00  0.00
A    C
ATOM    387  HB1 GLU A  31      28.447  16.603  23.643  1.00  0.00
A    H
ATOM    388  HB2 GLU A  31      28.946  18.144  22.910  1.00  0.00
A    H
ATOM    389  CG  GLU A  31      30.205  17.472  24.540  1.00  0.00
A    C
ATOM    390  HG1 GLU A  31      30.082  17.143  25.588  1.00  0.00
A    H
ATOM    391  HG2 GLU A  31      30.824  16.732  23.996  1.00  0.00
A    H
ATOM    392  CD  GLU A  31      30.993  18.777  24.620  1.00  0.00
A    C
ATOM    393  OE1 GLU A  31      32.162  18.791  24.185  1.00  0.00
A    O
ATOM    394  OE2 GLU A  31      30.464  19.777  25.151  1.00  0.00
A    O
ATOM    395  C   GLU A  31      27.448  17.587  26.012  1.00  0.00
A    C
ATOM    396  O   GLU A  31      28.347  17.252  26.789  1.00  0.00
A    O
ATOM    397  N   VAL A  32      26.166  17.355  26.260  1.00  0.00
A    N
ATOM    398  HN  VAL A  32      25.431  17.589  25.629  1.00  0.00
A    H
ATOM    399  CA  VAL A  32      25.721  16.730  27.495  1.00  0.00
A    C
ATOM    400  HA  VAL A  32      26.569  16.233  27.949  1.00  0.00
A    H
ATOM    401  CB  VAL A  32      25.156  17.763  28.484  1.00  0.00
A    C
ATOM    402  HB  VAL A  32      24.748  17.202  29.360  1.00  0.00
A    H
ATOM    403  CG1 VAL A  32      26.260  18.691  28.947  1.00  0.00
A    C
ATOM    404 HG11 VAL A  32      25.852  19.465  29.633  1.00  0.00
A    H
```

```
ATOM    405 HG12 VAL A  32      27.050  18.142  29.501  1.00  0.00
A    H
ATOM    406 HG13 VAL A  32      26.725  19.209  28.082  1.00  0.00
A    H
ATOM    407  CG2 VAL A  32      24.054  18.572  27.840  1.00  0.00
A    C
ATOM    408 HG21 VAL A  32      23.673  19.345  28.542  1.00  0.00
A    H
ATOM    409 HG22 VAL A  32      24.427  19.083  26.927  1.00  0.00
A    H
ATOM    410 HG23 VAL A  32      23.197  17.928  27.554  1.00  0.00
A    H
ATOM    411  C   VAL A  32      24.690  15.665  27.168  1.00  0.00
A    C
ATOM    412  O   VAL A  32      23.679  15.942  26.511  1.00  0.00
A    O
ATOM    413  N   LEU A  33      24.953  14.447  27.613  1.00  0.00
A    N
ATOM    414  HN  LEU A  33      25.808  14.200  28.064  1.00  0.00
A    H
ATOM    415  CA  LEU A  33      24.010  13.353  27.481  1.00  0.00
A    C
ATOM    416  HA  LEU A  33      23.359  13.572  26.645  1.00  0.00
A    H
ATOM    417  CB  LEU A  33      24.727  12.023  27.284  1.00  0.00
A    C
ATOM    418  HB1 LEU A  33      25.068  11.987  26.223  1.00  0.00
A    H
ATOM    419  HB2 LEU A  33      25.650  12.014  27.904  1.00  0.00
A    H
ATOM    420  CG  LEU A  33      23.823  10.831  27.585  1.00  0.00
A    C
ATOM    421  HG  LEU A  33      23.466  10.944  28.637  1.00  0.00
A    H
ATOM    422  CD1 LEU A  33      22.625  10.809  26.642  1.00  0.00
A    C
ATOM    423 HD11 LEU A  33      21.995   9.914  26.832  1.00  0.00
A    H
ATOM    424 HD12 LEU A  33      21.998  11.714  26.786  1.00  0.00
A    H
ATOM    425 HD13 LEU A  33      22.961  10.779  25.584  1.00  0.00
A    H
ATOM    426  CD2 LEU A  33      24.609   9.539  27.493  1.00  0.00
A    C
ATOM    427 HD21 LEU A  33      23.955   8.672  27.732  1.00  0.00
A    H
ATOM    428 HD22 LEU A  33      25.021   9.381  26.476  1.00  0.00
A    H
ATOM    429 HD23 LEU A  33      25.453   9.546  28.216  1.00  0.00
A    H
ATOM    430  C   LEU A  33      23.149  13.310  28.734  1.00  0.00
A    C
ATOM    431  O   LEU A  33      23.670  13.173  29.845  1.00  0.00
A    O
```

```
ATOM    432  N   ALA A  34      21.845  13.422  28.553  1.00  0.00
A    N
ATOM    433  HN  ALA A  34      21.430  13.557  27.654  1.00  0.00
A    H
ATOM    434  CA  ALA A  34      20.913  13.354  29.665  1.00  0.00
A    C
ATOM    435  HA  ALA A  34      21.398  13.573  30.609  1.00  0.00
A    H
ATOM    436  CB  ALA A  34      19.764  14.337  29.472  1.00  0.00
A    C
ATOM    437  HB1 ALA A  34      20.162  15.374  29.445  1.00  0.00
A    H
ATOM    438  HB2 ALA A  34      19.235  14.150  28.513  1.00  0.00
A    H
ATOM    439  HB3 ALA A  34      19.027  14.274  30.301  1.00  0.00
A    H
ATOM    440  C   ALA A  34      20.391  11.933  29.772  1.00  0.00
A    C
ATOM    441  O   ALA A  34      20.007  11.333  28.765  1.00  0.00
A    O
ATOM    442  N   VAL A  35      20.398  11.392  30.985  1.00  0.00
A    N
ATOM    443  HN  VAL A  35      20.794  11.852  31.776  1.00  0.00
A    H
ATOM    444  CA  VAL A  35      19.831  10.082  31.273  1.00  0.00
A    C
ATOM    445  HA  VAL A  35      19.128   9.830  30.490  1.00  0.00
A    H
ATOM    446  CB  VAL A  35      20.911   8.989  31.381  1.00  0.00
A    C
ATOM    447  HB  VAL A  35      21.573   9.253  32.242  1.00  0.00
A    H
ATOM    448  CG1 VAL A  35      20.272   7.635  31.638  1.00  0.00
A    C
ATOM    449 HG11 VAL A  35      21.051   6.843  31.676  1.00  0.00
A    H
ATOM    450 HG12 VAL A  35      19.731   7.614  32.606  1.00  0.00
A    H
ATOM    451 HG13 VAL A  35      19.559   7.377  30.825  1.00  0.00
A    H
ATOM    452  CG2 VAL A  35      21.766   8.959  30.138  1.00  0.00
A    C
ATOM    453 HG21 VAL A  35      22.519   8.145  30.199  1.00  0.00
A    H
ATOM    454 HG22 VAL A  35      21.138   8.788  29.237  1.00  0.00
A    H
ATOM    455 HG23 VAL A  35      22.315   9.914  30.005  1.00  0.00
A    H
ATOM    456  C   VAL A  35      19.052  10.172  32.573  1.00  0.00
A    C
ATOM    457  O   VAL A  35      19.480  10.842  33.517  1.00  0.00
A    O
ATOM    458  N   ASP A  36      17.913   9.494  32.621  1.00  0.00
A    N
```

```
ATOM    459  HN  ASP A  36      17.462   9.028  31.863  1.00  0.00
A    H
ATOM    460  CA  ASP A  36      17.187   9.373  33.869  1.00  0.00
A    C
ATOM    461  HA  ASP A  36      17.884   9.042  34.631  1.00  0.00
A    H
ATOM    462  CB  ASP A  36      16.518  10.680  34.262  1.00  0.00
A    C
ATOM    463  HB1 ASP A  36      17.195  11.524  34.019  1.00  0.00
A    H
ATOM    464  HB2 ASP A  36      15.573  10.819  33.701  1.00  0.00
A    H
ATOM    465  CG  ASP A  36      16.227  10.735  35.723  1.00  0.00
A    C
ATOM    466  OD1 ASP A  36      17.171  11.019  36.485  1.00  0.00
A    O
ATOM    467  OD2 ASP A  36      15.068  10.483  36.107  1.00  0.00
A    O
ATOM    468  C   ASP A  36      16.143   8.287  33.731  1.00  0.00
A    C
ATOM    469  O   ASP A  36      15.455   8.205  32.713  1.00  0.00
A    O
ATOM    470  N   ILE A  37      16.023   7.465  34.771  1.00  0.00
A    N
ATOM    471  HN  ILE A  37      16.558   7.493  35.613  1.00  0.00
A    H
ATOM    472  CA  ILE A  37      15.030   6.401  34.730  1.00  0.00
A    C
ATOM    473  HA  ILE A  37      15.186   5.870  33.799  1.00  0.00
A    H
ATOM    474  CB  ILE A  37      15.193   5.459  35.930  1.00  0.00
A    C
ATOM    475  HB  ILE A  37      16.270   5.154  35.902  1.00  0.00
A    H
ATOM    476  CG2 ILE A  37      14.777   6.152  37.207  1.00  0.00
A    C
ATOM    477 HG21 ILE A  37      15.038   5.532  38.090  1.00  0.00
A    H
ATOM    478 HG22 ILE A  37      15.295   7.128  37.315  1.00  0.00
A    H
ATOM    479 HG23 ILE A  37      13.681   6.332  37.223  1.00  0.00
A    H
ATOM    480  CG1 ILE A  37      14.364   4.197  35.712  1.00  0.00
A    C
ATOM    481 HG11 ILE A  37      13.283   4.432  35.832  1.00  0.00
A    H
ATOM    482 HG12 ILE A  37      14.510   3.846  34.664  1.00  0.00
A    H
ATOM    483  CD  ILE A  37      14.722   3.077  36.628  1.00  0.00
A    C
ATOM    484  HD1 ILE A  37      14.126   2.172  36.382  1.00  0.00
A    H
ATOM    485  HD2 ILE A  37      15.799   2.821  36.533  1.00  0.00
A    H
```

```
ATOM    486  HD3 ILE A  37      14.516   3.346  37.686  1.00  0.00
A    H
ATOM    487  C   ILE A  37      13.628   6.977  34.676  1.00  0.00
A    C
ATOM    488  O   ILE A  37      12.705   6.332  34.170  1.00  0.00
A    O
ATOM    489  N   ASN A  38      13.433   8.186  35.178  1.00  0.00
A    N
ATOM    490  HN  ASN A  38      14.132   8.751  35.616  1.00  0.00
A    H
ATOM    491  CA  ASN A  38      12.120   8.795  35.116  1.00  0.00
A    C
ATOM    492  HA  ASN A  38      11.395   7.995  35.227  1.00  0.00
A    H
ATOM    493  CB  ASN A  38      11.950   9.857  36.187  1.00  0.00
A    C
ATOM    494  HB1 ASN A  38      11.779   9.339  37.156  1.00  0.00
A    H
ATOM    495  HB2 ASN A  38      12.879  10.454  36.288  1.00  0.00
A    H
ATOM    496  CG  ASN A  38      10.779  10.741  35.909  1.00  0.00
A    C
ATOM    497  OD1 ASN A  38      10.887  11.708  35.167  1.00  0.00
A    O
ATOM    498  ND2 ASN A  38       9.635  10.397  36.474  1.00  0.00
A    N
ATOM    499 HD21 ASN A  38       8.842  10.971  36.289  1.00  0.00
A    H
ATOM    500 HD22 ASN A  38       9.582   9.576  37.033  1.00  0.00
A    H
ATOM    501  C   ASN A  38      11.928   9.401  33.740  1.00  0.00
A    C
ATOM    502  O   ASN A  38      12.524  10.434  33.418  1.00  0.00
A    O
ATOM    503  N   GLU A  39      11.086   8.762  32.935  1.00  0.00
A    N
ATOM    504  HN  GLU A  39      10.589   7.933  33.173  1.00  0.00
A    H
ATOM    505  CA  GLU A  39      10.819   9.247  31.589  1.00  0.00
A    C
ATOM    506  HA  GLU A  39      11.756   9.272  31.046  1.00  0.00
A    H
ATOM    507  CB  GLU A  39       9.817   8.324  30.913  1.00  0.00
A    C
ATOM    508  HB1 GLU A  39       9.862   8.507  29.815  1.00  0.00
A    H
ATOM    509  HB2 GLU A  39      10.124   7.264  31.059  1.00  0.00
A    H
ATOM    510  CG  GLU A  39       8.411   8.550  31.414  1.00  0.00
A    C
ATOM    511  HG1 GLU A  39       8.221   9.635  31.502  1.00  0.00
A    H
ATOM    512  HG2 GLU A  39       7.667   8.104  30.723  1.00  0.00
A    H
```

```
ATOM    513  CD  GLU A  39       8.180   7.981  32.798  1.00  0.00
A    C
ATOM    514  OE1 GLU A  39       9.166   7.683  33.508  1.00  0.00
A    O
ATOM    515  OE2 GLU A  39       7.006   7.842  33.187  1.00  0.00
A    O
ATOM    516  C   GLU A  39      10.278  10.663  31.616  1.00  0.00
A    C
ATOM    517  O   GLU A  39      10.364  11.390  30.628  1.00  0.00
A    O
ATOM    518  N   GLU A  40       9.690  11.070  32.737  1.00  0.00
A    N
ATOM    519  HN  GLU A  40       9.624  10.564  33.593  1.00  0.00
A    H
ATOM    520  CA  GLU A  40       9.063  12.380  32.775  1.00  0.00
A    C
ATOM    521  HA  GLU A  40       8.419  12.469  31.909  1.00  0.00
A    H
ATOM    522  CB  GLU A  40       8.238  12.515  34.050  1.00  0.00
A    C
ATOM    523  HB1 GLU A  40       8.461  11.640  34.703  1.00  0.00
A    H
ATOM    524  HB2 GLU A  40       8.567  13.412  34.619  1.00  0.00
A    H
ATOM    525  CG  GLU A  40       6.716  12.586  33.880  1.00  0.00
A    C
ATOM    526  HG1 GLU A  40       6.303  11.562  33.826  1.00  0.00
A    H
ATOM    527  HG2 GLU A  40       6.246  13.124  34.726  1.00  0.00
A    H
ATOM    528  CD  GLU A  40       6.247  13.256  32.601  1.00  0.00
A    C
ATOM    529  OE1 GLU A  40       5.535  14.272  32.719  1.00  0.00
A    O
ATOM    530  OE2 GLU A  40       6.517  12.740  31.493  1.00  0.00
A    O
ATOM    531  C   GLU A  40      10.096  13.489  32.686  1.00  0.00
A    C
ATOM    532  O   GLU A  40       9.949  14.416  31.880  1.00  0.00
A    O
ATOM    533  N   LYS A  41      11.148  13.411  33.497  1.00  0.00
A    N
ATOM    534  HN  LYS A  41      11.297  12.689  34.171  1.00  0.00
A    H
ATOM    535  CA  LYS A  41      12.188  14.428  33.439  1.00  0.00
A    C
ATOM    536  HA  LYS A  41      11.700  15.389  33.552  1.00  0.00
A    H
ATOM    537  CB  LYS A  41      13.211  14.210  34.550  1.00  0.00
A    C
ATOM    538  HB1 LYS A  41      13.826  13.327  34.260  1.00  0.00
A    H
ATOM    539  HB2 LYS A  41      13.906  15.079  34.578  1.00  0.00
A    H
```

```
ATOM    540  CG  LYS A  41      12.615  13.966  35.904  1.00  0.00
A    C
ATOM    541  HG1 LYS A  41      11.592  13.542  35.781  1.00  0.00
A    H
ATOM    542  HG2 LYS A  41      13.228  13.203  36.438  1.00  0.00
A    H
ATOM    543  CD  LYS A  41      12.528  15.228  36.716  1.00  0.00
A    C
ATOM    544  HD1 LYS A  41      13.390  15.881  36.445  1.00  0.00
A    H
ATOM    545  HD2 LYS A  41      11.598  15.767  36.422  1.00  0.00
A    H
ATOM    546  CE  LYS A  41      12.525  14.878  38.188  1.00  0.00
A    C
ATOM    547  HE1 LYS A  41      12.314  15.781  38.803  1.00  0.00
A    H
ATOM    548  HE2 LYS A  41      11.748  14.113  38.406  1.00  0.00
A    H
ATOM    549  NZ  LYS A  41      13.843  14.332  38.606  1.00  0.00
A    N
ATOM    550  HZ1 LYS A  41      13.827  14.097  39.620  1.00  0.00
A    H
ATOM    551  HZ2 LYS A  41      14.054  13.475  38.056  1.00  0.00
A    H
ATOM    552  HZ3 LYS A  41      14.584  15.041  38.430  1.00  0.00
A    H
ATOM    553  C   LYS A  41      12.884  14.430  32.089  1.00  0.00
A    C
ATOM    554  O   LYS A  41      13.164  15.495  31.533  1.00  0.00
A    O
ATOM    555  N   VAL A  42      13.230  13.248  31.581  1.00  0.00
A    N
ATOM    556  HN  VAL A  42      13.113  12.370  32.038  1.00  0.00
A    H
ATOM    557  CA  VAL A  42      13.832  13.163  30.258  1.00  0.00
A    C
ATOM    558  HA  VAL A  42      14.768  13.708  30.278  1.00  0.00
A    H
ATOM    559  CB  VAL A  42      14.075  11.693  29.883  1.00  0.00
A    C
ATOM    560  HB  VAL A  42      13.079  11.230  29.671  1.00  0.00
A    H
ATOM    561  CG1 VAL A  42      14.942  11.599  28.646  1.00  0.00
A    C
ATOM    562 HG11 VAL A  42      15.149  10.535  28.401  1.00  0.00
A    H
ATOM    563 HG12 VAL A  42      14.447  12.053  27.763  1.00  0.00
A    H
ATOM    564 HG13 VAL A  42      15.916  12.108  28.810  1.00  0.00
A    H
ATOM    565  CG2 VAL A  42      14.697  10.956  31.048  1.00  0.00
A    C
ATOM    566 HG21 VAL A  42      14.914   9.902  30.776  1.00  0.00
A    H
```

```
ATOM    567 HG22 VAL A  42      15.650  11.442  31.353  1.00  0.00
A    H
ATOM    568 HG23 VAL A  42      14.017  10.942  31.925  1.00  0.00
A    H
ATOM    569  C   VAL A  42      12.931  13.843  29.249  1.00  0.00
A    C
ATOM    570  O   VAL A  42      13.370  14.665  28.436  1.00  0.00
A    O
ATOM    571  N   ASN A  43      11.646  13.515  29.311  1.00  0.00
A    N
ATOM    572  HN  ASN A  43      11.278  12.872  29.981  1.00  0.00
A    H
ATOM    573  CA  ASN A  43      10.659  14.068  28.401  1.00  0.00
A    C
ATOM    574  HA  ASN A  43      10.962  13.754  27.408  1.00  0.00
A    H
ATOM    575  CB  ASN A  43       9.280  13.542  28.787  1.00  0.00
A    C
ATOM    576  HB1 ASN A  43       9.422  12.584  29.334  1.00  0.00
A    H
ATOM    577  HB2 ASN A  43       8.778  14.248  29.477  1.00  0.00
A    H
ATOM    578  CG  ASN A  43       8.411  13.273  27.597  1.00  0.00
A    C
ATOM    579  OD1 ASN A  43       8.260  14.114  26.724  1.00  0.00
A    O
ATOM    580  ND2 ASN A  43       7.844  12.077  27.539  1.00  0.00
A    N
ATOM    581 HD21 ASN A  43       7.273  11.879  26.747  1.00  0.00
A    H
ATOM    582 HD22 ASN A  43       8.020  11.407  28.254  1.00  0.00
A    H
ATOM    583  C   ASN A  43      10.680  15.581  28.429  1.00  0.00
A    C
ATOM    584  O   ASN A  43      10.664  16.237  27.382  1.00  0.00
A    O
ATOM    585  N   ALA A  44      10.711  16.153  29.626  1.00  0.00
A    N
ATOM    586  HN  ALA A  44      10.701  15.660  30.496  1.00  0.00
A    H
ATOM    587  CA  ALA A  44      10.763  17.604  29.730  1.00  0.00
A    C
ATOM    588  HA  ALA A  44       9.967  18.055  29.148  1.00  0.00
A    H
ATOM    589  CB  ALA A  44      10.590  18.034  31.184  1.00  0.00
A    C
ATOM    590  HB1 ALA A  44       9.612  17.670  31.566  1.00  0.00
A    H
ATOM    591  HB2 ALA A  44      11.390  17.602  31.825  1.00  0.00
A    H
ATOM    592  HB3 ALA A  44      10.609  19.140  31.290  1.00  0.00
A    H
ATOM    593  C   ALA A  44      12.067  18.153  29.172  1.00  0.00
A    C
```

```
ATOM    594  O   ALA A  44      12.067  18.899  28.187  1.00  0.00
A    O
ATOM    595  N   TYR A  45      13.188  17.759  29.754  1.00  0.00
A    N
ATOM    596  HN  TYR A  45      13.256  16.985  30.381  1.00  0.00
A    H
ATOM    597  CA  TYR A  45      14.443  18.452  29.512  1.00  0.00
A    C
ATOM    598  HA  TYR A  45      14.175  19.493  29.380  1.00  0.00
A    H
ATOM    599  CB  TYR A  45      15.395  18.278  30.692  1.00  0.00
A    C
ATOM    600  HB1 TYR A  45      15.424  17.205  30.981  1.00  0.00
A    H
ATOM    601  HB2 TYR A  45      16.430  18.571  30.408  1.00  0.00
A    H
ATOM    602  CG  TYR A  45      14.980  19.045  31.914  1.00  0.00
A    C
ATOM    603  CD1 TYR A  45      13.889  19.897  31.883  1.00  0.00
A    C
ATOM    604  HD1 TYR A  45      13.328  20.019  30.967  1.00  0.00
A    H
ATOM    605  CE1 TYR A  45      13.516  20.611  32.998  1.00  0.00
A    C
ATOM    606  HE1 TYR A  45      12.654  21.260  32.972  1.00  0.00
A    H
ATOM    607  CZ  TYR A  45      14.256  20.499  34.152  1.00  0.00
A    C
ATOM    608  OH  TYR A  45      13.898  21.212  35.271  1.00  0.00
A    O
ATOM    609  HH  TYR A  45      14.485  20.947  35.981  1.00  0.00
A    H
ATOM    610  CD2 TYR A  45      15.715  18.964  33.082  1.00  0.00
A    C
ATOM    611  HD2 TYR A  45      16.612  18.361  33.116  1.00  0.00
A    H
ATOM    612  CE2 TYR A  45      15.353  19.676  34.197  1.00  0.00
A    C
ATOM    613  HE2 TYR A  45      15.946  19.602  35.096  1.00  0.00
A    H
ATOM    614  C   TYR A  45      15.153  18.010  28.248  1.00  0.00
A    C
ATOM    615  O   TYR A  45      16.301  18.414  28.037  1.00  0.00
A    O
ATOM    616  N   ALA A  46      14.520  17.190  27.412  1.00  0.00
A    N
ATOM    617  HN  ALA A  46      13.655  16.727  27.604  1.00  0.00
A    H
ATOM    618  CA  ALA A  46      15.097  16.919  26.104  1.00  0.00
A    C
ATOM    619  HA  ALA A  46      16.021  16.360  26.197  1.00  0.00
A    H
ATOM    620  CB  ALA A  46      14.123  16.100  25.262  1.00  0.00
A    C
```

```
ATOM    621  HB1 ALA A  46      13.914  15.133  25.768  1.00  0.00
A    H
ATOM    622  HB2 ALA A  46      13.156  16.636  25.136  1.00  0.00
A    H
ATOM    623  HB3 ALA A  46      14.539  15.878  24.256  1.00  0.00
A    H
ATOM    624  C   ALA A  46      15.452  18.218  25.402  1.00  0.00
A    C
ATOM    625  O   ALA A  46      16.557  18.375  24.872  1.00  0.00
A    O
ATOM    626  N   SER A  47      14.531  19.179  25.424  1.00  0.00
A    N
ATOM    627  HN  SER A  47      13.642  19.097  25.874  1.00  0.00
A    H
ATOM    628  CA  SER A  47      14.756  20.461  24.777  1.00  0.00
A    C
ATOM    629  HA  SER A  47      14.945  20.227  23.738  1.00  0.00
A    H
ATOM    630  CB  SER A  47      13.530  21.346  24.956  1.00  0.00
A    C
ATOM    631  HB1 SER A  47      13.548  22.126  24.161  1.00  0.00
A    H
ATOM    632  HB2 SER A  47      12.598  20.753  24.813  1.00  0.00
A    H
ATOM    633  OG  SER A  47      13.564  21.970  26.224  1.00  0.00
A    O
ATOM    634  HG1 SER A  47      12.908  22.677  26.210  1.00  0.00
A    H
ATOM    635  C   SER A  47      15.982  21.183  25.308  1.00  0.00
A    C
ATOM    636  O   SER A  47      16.588  21.968  24.575  1.00  0.00
A    O
ATOM    637  N   TYR A  48      16.359  20.947  26.558  1.00  0.00
A    N
ATOM    638  HN  TYR A  48      15.887  20.318  27.173  1.00  0.00
A    H
ATOM    639  CA  TYR A  48      17.517  21.604  27.140  1.00  0.00
A    C
ATOM    640  HA  TYR A  48      17.773  22.420  26.475  1.00  0.00
A    H
ATOM    641  CB  TYR A  48      17.191  22.106  28.541  1.00  0.00
A    C
ATOM    642  HB1 TYR A  48      16.967  21.236  29.197  1.00  0.00
A    H
ATOM    643  HB2 TYR A  48      18.064  22.634  28.982  1.00  0.00
A    H
ATOM    644  CG  TYR A  48      15.992  22.998  28.556  1.00  0.00
A    C
ATOM    645  CD1 TYR A  48      15.672  23.769  27.452  1.00  0.00
A    C
ATOM    646  HD1 TYR A  48      16.311  23.751  26.579  1.00  0.00
A    H
ATOM    647  CE1 TYR A  48      14.566  24.574  27.450  1.00  0.00
A    C
```

ATOM 648 HE1 TYR A 48 14.333 25.186 26.592 1.00 0.00
A H
ATOM 649 CZ TYR A 48 13.753 24.605 28.557 1.00 0.00
A C
ATOM 650 OH TYR A 48 12.642 25.409 28.562 1.00 0.00
A O
ATOM 651 HH TYR A 48 12.169 25.242 29.380 1.00 0.00
A H
ATOM 652 CD2 TYR A 48 15.152 23.037 29.651 1.00 0.00
A C
ATOM 653 HD2 TYR A 48 15.387 22.459 30.534 1.00 0.00
A H
ATOM 654 CE2 TYR A 48 14.041 23.840 29.660 1.00 0.00
A C
ATOM 655 HE2 TYR A 48 13.417 23.881 30.539 1.00 0.00
A H
ATOM 656 C TYR A 48 18.712 20.677 27.190 1.00 0.00
A C
ATOM 657 O TYR A 48 19.561 20.812 28.072 1.00 0.00
A O
ATOM 658 N ALA A 49 18.792 19.734 26.261 1.00 0.00
A N
ATOM 659 HN ALA A 49 18.195 19.685 25.461 1.00 0.00
A H
ATOM 660 CA ALA A 49 19.789 18.686 26.358 1.00 0.00
A C
ATOM 661 HA ALA A 49 20.601 18.975 27.014 1.00 0.00
A H
ATOM 662 CB ALA A 49 19.185 17.384 26.888 1.00 0.00
A C
ATOM 663 HB1 ALA A 49 18.777 17.550 27.908 1.00 0.00
A H
ATOM 664 HB2 ALA A 49 18.350 17.038 26.239 1.00 0.00
A H
ATOM 665 HB3 ALA A 49 19.945 16.576 26.949 1.00 0.00
A H
ATOM 666 C ALA A 49 20.403 18.442 24.997 1.00 0.00
A C
ATOM 667 O ALA A 49 19.700 18.324 23.992 1.00 0.00
A O
ATOM 668 N THR A 50 21.730 18.364 24.980 1.00 0.00
A N
ATOM 669 HN THR A 50 22.346 18.593 25.734 1.00 0.00
A H
ATOM 670 CA THR A 50 22.404 17.908 23.776 1.00 0.00
A C
ATOM 671 HA THR A 50 22.127 18.604 22.996 1.00 0.00
A H
ATOM 672 CB THR A 50 23.914 17.844 24.008 1.00 0.00
A C
ATOM 673 HB THR A 50 24.086 16.982 24.698 1.00 0.00
A H
ATOM 674 OG1 THR A 50 24.353 19.035 24.672 1.00 0.00
A O

```
ATOM    675  HG1 THR A  50      25.306  18.935  24.776  1.00  0.00
A    H
ATOM    676  CG2 THR A  50      24.655  17.708  22.687  1.00  0.00
A    C
ATOM    677 HG21 THR A  50      25.741  17.547  22.858  1.00  0.00
A    H
ATOM    678 HG22 THR A  50      24.268  16.839  22.113  1.00  0.00
A    H
ATOM    679 HG23 THR A  50      24.533  18.620  22.064  1.00  0.00
A    H
ATOM    680  C   THR A  50      21.881  16.550  23.349  1.00  0.00
A    C
ATOM    681  O   THR A  50      21.758  16.281  22.152  1.00  0.00
A    O
ATOM    682  N   HSE A  51      21.541  15.700  24.308  1.00  0.00
A    N
ATOM    683  HN  HSE A  51      21.602  15.893  25.286  1.00  0.00
A    H
ATOM    684  CA  HSE A  51      21.038  14.379  23.990  1.00  0.00
A    C
ATOM    685  HA  HSE A  51      20.239  14.487  23.267  1.00  0.00
A    H
ATOM    686  CB  HSE A  51      22.160  13.496  23.447  1.00  0.00
A    C
ATOM    687  HB1 HSE A  51      22.529  13.952  22.503  1.00  0.00
A    H
ATOM    688  HB2 HSE A  51      23.010  13.495  24.162  1.00  0.00
A    H
ATOM    689  ND1 HSE A  51      22.627  11.128  22.733  1.00  0.00
A    N
ATOM    690  CG  HSE A  51      21.738  12.095  23.144  1.00  0.00
A    C
ATOM    691  CE1 HSE A  51      21.982   9.991  22.541  1.00  0.00
A    C
ATOM    692  HE1 HSE A  51      22.449   9.062  22.206  1.00  0.00
A    H
ATOM    693  NE2 HSE A  51      20.706  10.188  22.815  1.00  0.00
A    N
ATOM    694  HE2 HSE A  51      19.988   9.497  22.737  1.00  0.00
A    H
ATOM    695  CD2 HSE A  51      20.526  11.494  23.194  1.00  0.00
A    C
ATOM    696  HD2 HSE A  51      19.561  11.903  23.459  1.00  0.00
A    H
ATOM    697  C   HSE A  51      20.425  13.783  25.245  1.00  0.00
A    C
ATOM    698  O   HSE A  51      21.086  13.688  26.282  1.00  0.00
A    O
ATOM    699  N   ALA A  52      19.162  13.384  25.145  1.00  0.00
A    N
ATOM    700  HN  ALA A  52      18.655  13.455  24.286  1.00  0.00
A    H
ATOM    701  CA  ALA A  52      18.424  12.819  26.261  1.00  0.00
A    C
```

```
ATOM    702  HA  ALA A  52      19.026  12.787  27.162  1.00  0.00
A    H
ATOM    703  CB  ALA A  52      17.153  13.616  26.551  1.00  0.00
A    C
ATOM    704  HB1 ALA A  52      17.422  14.656  26.837  1.00  0.00
A    H
ATOM    705  HB2 ALA A  52      16.500  13.667  25.653  1.00  0.00
A    H
ATOM    706  HB3 ALA A  52      16.571  13.170  27.387  1.00  0.00
A    H
ATOM    707  C   ALA A  52      18.063  11.385  25.937  1.00  0.00
A    C
ATOM    708  O   ALA A  52      17.887  11.035  24.769  1.00  0.00
A    O
ATOM    709  N   VAL A  53      17.942  10.565  26.972  1.00  0.00
A    N
ATOM    710  HN  VAL A  53      18.096  10.859  27.912  1.00  0.00
A    H
ATOM    711  CA  VAL A  53      17.574   9.162  26.833  1.00  0.00
A    C
ATOM    712  HA  VAL A  53      16.850   9.065  26.035  1.00  0.00
A    H
ATOM    713  CB  VAL A  53      18.797   8.260  26.571  1.00  0.00
A    C
ATOM    714  HB  VAL A  53      19.631   8.625  27.218  1.00  0.00
A    H
ATOM    715  CG1 VAL A  53      18.487   6.812  26.926  1.00  0.00
A    C
ATOM    716 HG11 VAL A  53      19.360   6.164  26.696  1.00  0.00
A    H
ATOM    717 HG12 VAL A  53      18.262   6.694  28.006  1.00  0.00
A    H
ATOM    718 HG13 VAL A  53      17.619   6.441  26.339  1.00  0.00
A    H
ATOM    719  CG2 VAL A  53      19.246   8.352  25.133  1.00  0.00
A    C
ATOM    720 HG21 VAL A  53      20.102   7.669  24.944  1.00  0.00
A    H
ATOM    721 HG22 VAL A  53      18.418   8.073  24.447  1.00  0.00
A    H
ATOM    722 HG23 VAL A  53      19.577   9.381  24.881  1.00  0.00
A    H
ATOM    723  C   VAL A  53      16.893   8.740  28.120  1.00  0.00
A    C
ATOM    724  O   VAL A  53      17.312   9.143  29.208  1.00  0.00
A    O
ATOM    725  N   ILE A  54      15.849   7.933  28.011  1.00  0.00
A    N
ATOM    726  HN  ILE A  54      15.362   7.717  27.166  1.00  0.00
A    H
ATOM    727  CA  ILE A  54      15.315   7.273  29.191  1.00  0.00
A    C
ATOM    728  HA  ILE A  54      15.539   7.894  30.050  1.00  0.00
A    H
```

```
ATOM    729  CB  ILE A  54      13.814   7.019  29.053  1.00  0.00
A    C
ATOM    730  HB  ILE A  54      13.729   6.305  28.195  1.00  0.00
A    H
ATOM    731  CG2 ILE A  54      13.248   6.536  30.362  1.00  0.00
A    C
ATOM    732 HG21 ILE A  54      12.198   6.200  30.234  1.00  0.00
A    H
ATOM    733 HG22 ILE A  54      13.831   5.674  30.752  1.00  0.00
A    H
ATOM    734 HG23 ILE A  54      13.264   7.343  31.125  1.00  0.00
A    H
ATOM    735  CG1 ILE A  54      13.113   8.303  28.639  1.00  0.00
A    C
ATOM    736 HG11 ILE A  54      12.971   8.958  29.528  1.00  0.00
A    H
ATOM    737 HG12 ILE A  54      13.764   8.855  27.923  1.00  0.00
A    H
ATOM    738  CD  ILE A  54      11.781   8.065  27.982  1.00  0.00
A    C
ATOM    739  HD1 ILE A  54      11.338   9.029  27.648  1.00  0.00
A    H
ATOM    740  HD2 ILE A  54      11.894   7.407  27.094  1.00  0.00
A    H
ATOM    741  HD3 ILE A  54      11.069   7.587  28.687  1.00  0.00
A    H
ATOM    742  C   ILE A  54      16.084   5.974  29.377  1.00  0.00
A    C
ATOM    743  O   ILE A  54      16.174   5.159  28.453  1.00  0.00
A    O
ATOM    744  N   ALA A  55      16.652   5.781  30.566  1.00  0.00
A    N
ATOM    745  HN  ALA A  55      16.566   6.427  31.324  1.00  0.00
A    H
ATOM    746  CA  ALA A  55      17.450   4.595  30.844  1.00  0.00
A    C
ATOM    747  HA  ALA A  55      16.931   3.693  30.537  1.00  0.00
A    H
ATOM    748  CB  ALA A  55      18.792   4.639  30.107  1.00  0.00
A    C
ATOM    749  HB1 ALA A  55      18.614   4.680  29.011  1.00  0.00
A    H
ATOM    750  HB2 ALA A  55      19.376   5.541  30.394  1.00  0.00
A    H
ATOM    751  HB3 ALA A  55      19.406   3.738  30.321  1.00  0.00
A    H
ATOM    752  C   ALA A  55      17.682   4.469  32.342  1.00  0.00
A    C
ATOM    753  O   ALA A  55      17.822   5.473  33.044  1.00  0.00
A    O
ATOM    754  N   ASN A  56      17.732   3.228  32.815  1.00  0.00
A    N
ATOM    755  HN  ASN A  56      17.596   2.427  32.233  1.00  0.00
A    H
```

ATOM 756 CA ASN A 56 17.987 2.922 34.218 1.00 0.00
A C
ATOM 757 HA ASN A 56 17.675 3.784 34.798 1.00 0.00
A H
ATOM 758 CB ASN A 56 17.244 1.661 34.626 1.00 0.00
A C
ATOM 759 HB1 ASN A 56 16.158 1.839 34.467 1.00 0.00
A H
ATOM 760 HB2 ASN A 56 17.540 0.812 33.977 1.00 0.00
A H
ATOM 761 CG ASN A 56 17.452 1.304 36.078 1.00 0.00
A C
ATOM 762 OD1 ASN A 56 18.338 1.836 36.749 1.00 0.00
A O
ATOM 763 ND2 ASN A 56 16.632 0.390 36.576 1.00 0.00
A N
ATOM 764 HD21 ASN A 56 16.745 0.148 37.536 1.00 0.00
A H
ATOM 765 HD22 ASN A 56 15.919 -0.005 36.004 1.00 0.00
A H
ATOM 766 C ASN A 56 19.490 2.765 34.399 1.00 0.00
A C
ATOM 767 O ASN A 56 20.098 1.837 33.866 1.00 0.00
A O
ATOM 768 N ALA A 57 20.082 3.656 35.191 1.00 0.00
A N
ATOM 769 HN ALA A 57 19.572 4.331 35.724 1.00 0.00
A H
ATOM 770 CA ALA A 57 21.530 3.708 35.339 1.00 0.00
A C
ATOM 771 HA ALA A 57 21.986 3.875 34.369 1.00 0.00
A H
ATOM 772 CB ALA A 57 21.918 4.855 36.263 1.00 0.00
A C
ATOM 773 HB1 ALA A 57 21.528 5.812 35.854 1.00 0.00
A H
ATOM 774 HB2 ALA A 57 21.485 4.715 37.277 1.00 0.00
A H
ATOM 775 HB3 ALA A 57 23.022 4.948 36.359 1.00 0.00
A H
ATOM 776 C ALA A 57 22.130 2.426 35.878 1.00 0.00
A C
ATOM 777 O ALA A 57 23.326 2.196 35.691 1.00 0.00
A O
ATOM 778 N THR A 58 21.346 1.588 36.531 1.00 0.00
A N
ATOM 779 HN THR A 58 20.347 1.630 36.560 1.00 0.00
A H
ATOM 780 CA THR A 58 21.918 0.492 37.289 1.00 0.00
A C
ATOM 781 HA THR A 58 22.814 0.904 37.733 1.00 0.00
A H
ATOM 782 CB THR A 58 20.912 0.022 38.335 1.00 0.00
A C

```
ATOM    783  HB  THR A  58      21.426  -0.780  38.920  1.00  0.00
A    H
ATOM    784  OG1 THR A  58      19.785  -0.559  37.679  1.00  0.00
A    O
ATOM    785  HG1 THR A  58      19.185  -0.824  38.386  1.00  0.00
A    H
ATOM    786  CG2 THR A  58      20.432   1.199  39.159  1.00  0.00
A    C
ATOM    787 HG21 THR A  58      19.787   0.860  39.997  1.00  0.00
A    H
ATOM    788 HG22 THR A  58      21.296   1.746  39.593  1.00  0.00
A    H
ATOM    789 HG23 THR A  58      19.847   1.910  38.537  1.00  0.00
A    H
ATOM    790  C   THR A  58      22.361  -0.685  36.432  1.00  0.00
A    C
ATOM    791  O   THR A  58      22.935  -1.634  36.973  1.00  0.00
A    O
ATOM    792  N   GLU A  59      22.117  -0.662  35.126  1.00  0.00
A    N
ATOM    793  HN  GLU A  59      21.714   0.107  34.639  1.00  0.00
A    H
ATOM    794  CA  GLU A  59      22.433  -1.803  34.274  1.00  0.00
A    C
ATOM    795  HA  GLU A  59      22.751  -2.622  34.908  1.00  0.00
A    H
ATOM    796  CB  GLU A  59      21.216  -2.216  33.456  1.00  0.00
A    C
ATOM    797  HB1 GLU A  59      20.824  -1.311  32.937  1.00  0.00
A    H
ATOM    798  HB2 GLU A  59      21.524  -2.919  32.650  1.00  0.00
A    H
ATOM    799  CG  GLU A  59      20.100  -2.829  34.254  1.00  0.00
A    C
ATOM    800  HG1 GLU A  59      19.316  -3.200  33.568  1.00  0.00
A    H
ATOM    801  HG2 GLU A  59      20.470  -3.670  34.873  1.00  0.00
A    H
ATOM    802  CD  GLU A  59      19.429  -1.825  35.149  1.00  0.00
A    C
ATOM    803  OE1 GLU A  59      19.143  -2.162  36.314  1.00  0.00
A    O
ATOM    804  OE2 GLU A  59      19.238  -0.677  34.712  1.00  0.00
A    O
ATOM    805  C   GLU A  59      23.592  -1.479  33.340  1.00  0.00
A    C
ATOM    806  O   GLU A  59      23.556  -0.478  32.618  1.00  0.00
A    O
ATOM    807  N   GLU A  60      24.589  -2.365  33.316  1.00  0.00
A    N
ATOM    808  HN  GLU A  60      24.579  -3.252  33.769  1.00  0.00
A    H
ATOM    809  CA  GLU A  60      25.821  -2.083  32.591  1.00  0.00
A    C
```

```
ATOM    810  HA  GLU A  60      26.187  -1.119  32.920  1.00  0.00
A    H
ATOM    811  CB  GLU A  60      26.879  -3.137  32.932  1.00  0.00
A    C
ATOM    812  HB1 GLU A  60      27.764  -2.965  32.278  1.00  0.00
A    H
ATOM    813  HB2 GLU A  60      27.236  -2.988  33.975  1.00  0.00
A    H
ATOM    814  CG  GLU A  60      26.478  -4.610  32.774  1.00  0.00
A    C
ATOM    815  HG1 GLU A  60      27.364  -5.252  32.925  1.00  0.00
A    H
ATOM    816  HG2 GLU A  60      25.696  -4.890  33.509  1.00  0.00
A    H
ATOM    817  CD  GLU A  60      25.963  -4.983  31.404  1.00  0.00
A    C
ATOM    818  OE1 GLU A  60      26.663  -4.709  30.410  1.00  0.00
A    O
ATOM    819  OE2 GLU A  60      24.835  -5.513  31.319  1.00  0.00
A    O
ATOM    820  C   GLU A  60      25.583  -1.986  31.091  1.00  0.00
A    C
ATOM    821  O   GLU A  60      26.285  -1.252  30.389  1.00  0.00
A    O
ATOM    822  N   ASN A  61      24.614  -2.737  30.575  1.00  0.00
A    N
ATOM    823  HN  ASN A  61      23.980  -3.311  31.091  1.00  0.00
A    H
ATOM    824  CA  ASN A  61      24.415  -2.762  29.135  1.00  0.00
A    C
ATOM    825  HA  ASN A  61      25.402  -2.866  28.697  1.00  0.00
A    H
ATOM    826  CB  ASN A  61      23.522  -3.935  28.758  1.00  0.00
A    C
ATOM    827  HB1 ASN A  61      23.140  -3.755  27.730  1.00  0.00
A    H
ATOM    828  HB2 ASN A  61      24.111  -4.875  28.734  1.00  0.00
A    H
ATOM    829  CG  ASN A  61      22.354  -4.069  29.680  1.00  0.00
A    C
ATOM    830  OD1 ASN A  61      22.428  -3.675  30.842  1.00  0.00
A    O
ATOM    831  ND2 ASN A  61      21.266  -4.632  29.180  1.00  0.00
A    N
ATOM    832 HD21 ASN A  61      20.476  -4.717  29.782  1.00  0.00
A    H
ATOM    833 HD22 ASN A  61      21.248  -4.924  28.228  1.00  0.00
A    H
ATOM    834  C   ASN A  61      23.834  -1.448  28.653  1.00  0.00
A    C
ATOM    835  O   ASN A  61      24.197  -0.956  27.581  1.00  0.00
A    O
ATOM    836  N   GLU A  62      22.928  -0.860  29.434  1.00  0.00
A    N
```

```
ATOM    837  HN  GLU A  62      22.457  -1.262  30.215  1.00  0.00
A    H
ATOM    838  CA  GLU A  62      22.551   0.517  29.161  1.00  0.00
A    C
ATOM    839  HA  GLU A  62      22.070   0.550  28.191  1.00  0.00
A    H
ATOM    840  CB  GLU A  62      21.594   1.042  30.221  1.00  0.00
A    C
ATOM    841  HB1 GLU A  62      21.933   0.663  31.213  1.00  0.00
A    H
ATOM    842  HB2 GLU A  62      21.665   2.151  30.278  1.00  0.00
A    H
ATOM    843  CG  GLU A  62      20.168   0.630  30.002  1.00  0.00
A    C
ATOM    844  HG1 GLU A  62      19.682   1.339  29.306  1.00  0.00
A    H
ATOM    845  HG2 GLU A  62      20.110  -0.392  29.580  1.00  0.00
A    H
ATOM    846  CD  GLU A  62      19.350   0.673  31.266  1.00  0.00
A    C
ATOM    847  OE1 GLU A  62      18.575   1.631  31.437  1.00  0.00
A    O
ATOM    848  OE2 GLU A  62      19.469  -0.257  32.082  1.00  0.00
A    O
ATOM    849  C   GLU A  62      23.771   1.412  29.088  1.00  0.00
A    C
ATOM    850  O   GLU A  62      23.842   2.301  28.235  1.00  0.00
A    O
ATOM    851  N   LEU A  63      24.749   1.167  29.947  1.00  0.00
A    N
ATOM    852  HN  LEU A  63      24.767   0.414  30.602  1.00  0.00
A    H
ATOM    853  CA  LEU A  63      25.919   2.024  29.991  1.00  0.00
A    C
ATOM    854  HA  LEU A  63      25.554   3.043  30.025  1.00  0.00
A    H
ATOM    855  CB  LEU A  63      26.735   1.683  31.228  1.00  0.00
A    C
ATOM    856  HB1 LEU A  63      27.266   0.725  31.018  1.00  0.00
A    H
ATOM    857  HB2 LEU A  63      27.528   2.452  31.362  1.00  0.00
A    H
ATOM    858  CG  LEU A  63      25.858   1.539  32.464  1.00  0.00
A    C
ATOM    859  HG  LEU A  63      25.142   0.710  32.247  1.00  0.00
A    H
ATOM    860  CD1 LEU A  63      26.698   1.175  33.664  1.00  0.00
A    C
ATOM    861 HD11 LEU A  63      26.068   1.117  34.577  1.00  0.00
A    H
ATOM    862 HD12 LEU A  63      27.189   0.189  33.514  1.00  0.00
A    H
ATOM    863 HD13 LEU A  63      27.487   1.937  33.836  1.00  0.00
A    H
```

ATOM 864 CD2 LEU A 63 25.056 2.797 32.722 1.00 0.00
A C
ATOM 865 HD21 LEU A 63 24.409 2.666 33.617 1.00 0.00
A H
ATOM 866 HD22 LEU A 63 25.713 3.671 32.907 1.00 0.00
A H
ATOM 867 HD23 LEU A 63 24.404 3.028 31.852 1.00 0.00
A H
ATOM 868 C LEU A 63 26.762 1.881 28.736 1.00 0.00
A C
ATOM 869 O LEU A 63 27.058 2.877 28.067 1.00 0.00
A O
ATOM 870 N LEU A 64 27.154 0.652 28.403 1.00 0.00
A N
ATOM 871 HN LEU A 64 26.978 -0.175 28.932 1.00 0.00
A H
ATOM 872 CA LEU A 64 27.906 0.428 27.177 1.00 0.00
A C
ATOM 873 HA LEU A 64 28.820 1.002 27.262 1.00 0.00
A H
ATOM 874 CB LEU A 64 28.198 -1.053 27.004 1.00 0.00
A C
ATOM 875 HB1 LEU A 64 27.290 -1.517 26.552 1.00 0.00
A H
ATOM 876 HB2 LEU A 64 29.012 -1.177 26.256 1.00 0.00
A H
ATOM 877 CG LEU A 64 28.530 -1.746 28.313 1.00 0.00
A C
ATOM 878 HG LEU A 64 27.779 -1.432 29.078 1.00 0.00
A H
ATOM 879 CD1 LEU A 64 28.464 -3.243 28.134 1.00 0.00
A C
ATOM 880 HD11 LEU A 64 28.752 -3.761 29.073 1.00 0.00
A H
ATOM 881 HD12 LEU A 64 27.434 -3.560 27.862 1.00 0.00
A H
ATOM 882 HD13 LEU A 64 29.156 -3.573 27.330 1.00 0.00
A H
ATOM 883 CD2 LEU A 64 29.895 -1.307 28.790 1.00 0.00
A C
ATOM 884 HD21 LEU A 64 30.141 -1.794 29.759 1.00 0.00
A H
ATOM 885 HD22 LEU A 64 30.689 -1.579 28.066 1.00 0.00
A H
ATOM 886 HD23 LEU A 64 29.920 -0.206 28.939 1.00 0.00
A H
ATOM 887 C LEU A 64 27.149 0.951 25.971 1.00 0.00
A C
ATOM 888 O LEU A 64 27.734 1.599 25.097 1.00 0.00
A O
ATOM 889 N SER A 65 25.844 0.686 25.913 1.00 0.00
A N
ATOM 890 HN SER A 65 25.335 0.147 26.584 1.00 0.00
A H

ATOM 891 CA SER A 65 25.035 1.187 24.813 1.00 0.00
A C
ATOM 892 HA SER A 65 25.453 0.757 23.912 1.00 0.00
A H
ATOM 893 CB SER A 65 23.578 0.796 25.022 1.00 0.00
A C
ATOM 894 HB1 SER A 65 22.979 1.234 24.191 1.00 0.00
A H
ATOM 895 HB2 SER A 65 23.461 -0.310 24.962 1.00 0.00
A H
ATOM 896 OG SER A 65 23.118 1.308 26.253 1.00 0.00
A O
ATOM 897 HG1 SER A 65 22.155 1.238 26.247 1.00 0.00
A H
ATOM 898 C SER A 65 25.153 2.694 24.691 1.00 0.00
A C
ATOM 899 O SER A 65 25.082 3.237 23.584 1.00 0.00
A O
ATOM 900 N LEU A 66 25.348 3.382 25.811 1.00 0.00
A N
ATOM 901 HN LEU A 66 25.396 2.998 26.731 1.00 0.00
A H
ATOM 902 CA LEU A 66 25.514 4.822 25.757 1.00 0.00
A C
ATOM 903 HA LEU A 66 24.790 5.182 25.037 1.00 0.00
A H
ATOM 904 CB LEU A 66 25.262 5.427 27.130 1.00 0.00
A C
ATOM 905 HB1 LEU A 66 25.879 4.854 27.859 1.00 0.00
A H
ATOM 906 HB2 LEU A 66 25.655 6.467 27.145 1.00 0.00
A H
ATOM 907 CG LEU A 66 23.796 5.366 27.524 1.00 0.00
A C
ATOM 908 HG LEU A 66 23.524 4.300 27.719 1.00 0.00
A H
ATOM 909 CD1 LEU A 66 23.577 6.174 28.772 1.00 0.00
A C
ATOM 910 HD11 LEU A 66 22.500 6.180 29.048 1.00 0.00
A H
ATOM 911 HD12 LEU A 66 24.153 5.747 29.622 1.00 0.00
A H
ATOM 912 HD13 LEU A 66 23.902 7.226 28.621 1.00 0.00
A H
ATOM 913 CD2 LEU A 66 22.929 5.871 26.390 1.00 0.00
A C
ATOM 914 HD21 LEU A 66 21.854 5.810 26.669 1.00 0.00
A H
ATOM 915 HD22 LEU A 66 23.153 6.928 26.143 1.00 0.00
A H
ATOM 916 HD23 LEU A 66 23.086 5.257 25.477 1.00 0.00
A H
ATOM 917 C LEU A 66 26.889 5.229 25.265 1.00 0.00
A C

```
ATOM    918  O   LEU A  66      27.113   6.416  25.022  1.00  0.00
A    O
ATOM    919  N   GLY A  67      27.806   4.283  25.105  1.00  0.00
A    N
ATOM    920  HN  GLY A  67      27.635   3.305  25.213  1.00  0.00
A    H
ATOM    921  CA  GLY A  67      29.161   4.646  24.748  1.00  0.00
A    C
ATOM    922  HA1 GLY A  67      29.129   5.191  23.814  1.00  0.00
A    H
ATOM    923  HA2 GLY A  67      29.759   3.745  24.717  1.00  0.00
A    H
ATOM    924  C   GLY A  67      29.744   5.558  25.802  1.00  0.00
A    C
ATOM    925  O   GLY A  67      30.373   6.576  25.499  1.00  0.00
A    O
ATOM    926  N   ILE A  68      29.510   5.192  27.060  1.00  0.00
A    N
ATOM    927  HN  ILE A  68      29.055   4.345  27.325  1.00  0.00
A    H
ATOM    928  CA  ILE A  68      29.915   6.027  28.180  1.00  0.00
A    C
ATOM    929  HA  ILE A  68      29.421   6.981  28.041  1.00  0.00
A    H
ATOM    930  CB  ILE A  68      29.493   5.348  29.485  1.00  0.00
A    C
ATOM    931  HB  ILE A  68      30.093   5.866  30.276  1.00  0.00
A    H
ATOM    932  CG2 ILE A  68      28.001   5.414  29.643  1.00  0.00
A    C
ATOM    933 HG21 ILE A  68      27.699   5.066  30.653  1.00  0.00
A    H
ATOM    934 HG22 ILE A  68      27.635   6.456  29.523  1.00  0.00
A    H
ATOM    935 HG23 ILE A  68      27.493   4.776  28.890  1.00  0.00
A    H
ATOM    936  CG1 ILE A  68      29.943   3.893  29.451  1.00  0.00
A    C
ATOM    937 HG11 ILE A  68      29.405   3.351  28.641  1.00  0.00
A    H
ATOM    938 HG12 ILE A  68      31.030   3.858  29.205  1.00  0.00
A    H
ATOM    939  CD  ILE A  68      29.736   3.185  30.726  1.00  0.00
A    C
ATOM    940  HD1 ILE A  68      30.134   2.149  30.662  1.00  0.00
A    H
ATOM    941  HD2 ILE A  68      30.260   3.708  31.555  1.00  0.00
A    H
ATOM    942  HD3 ILE A  68      28.656   3.123  30.976  1.00  0.00
A    H
ATOM    943  C   ILE A  68      31.407   6.298  28.161  1.00  0.00
A    C
ATOM    944  O   ILE A  68      31.844   7.428  28.402  1.00  0.00
A    O
```

```
ATOM    945  N   ARG A  69      32.211   5.276  27.875  1.00  0.00
A    N
ATOM    946  HN  ARG A  69      31.866   4.379  27.609  1.00  0.00
A    H
ATOM    947  CA  ARG A  69      33.654   5.426  27.939  1.00  0.00
A    C
ATOM    948  HA  ARG A  69      33.916   5.691  28.957  1.00  0.00
A    H
ATOM    949  CB  ARG A  69      34.317   4.118  27.551  1.00  0.00
A    C
ATOM    950  HB1 ARG A  69      33.951   3.871  26.526  1.00  0.00
A    H
ATOM    951  HB2 ARG A  69      35.415   4.272  27.455  1.00  0.00
A    H
ATOM    952  CG  ARG A  69      33.973   3.001  28.495  1.00  0.00
A    C
ATOM    953  HG1 ARG A  69      34.469   3.197  29.471  1.00  0.00
A    H
ATOM    954  HG2 ARG A  69      32.879   3.029  28.705  1.00  0.00
A    H
ATOM    955  CD  ARG A  69      34.332   1.671  27.898  1.00  0.00
A    C
ATOM    956  HD1 ARG A  69      35.385   1.700  27.534  1.00  0.00
A    H
ATOM    957  HD2 ARG A  69      34.233   0.848  28.643  1.00  0.00
A    H
ATOM    958  NE  ARG A  69      33.450   1.344  26.787  1.00  0.00
A    N
ATOM    959  HE  ARG A  69      32.563   0.973  27.083  1.00  0.00
A    H
ATOM    960  CZ  ARG A  69      33.771   1.512  25.511  1.00  0.00
A    C
ATOM    961  NH1 ARG A  69      34.953   2.012  25.183  1.00  0.00
A    N
ATOM    962 HH11 ARG A  69      35.190   2.165  24.234  1.00  0.00
A    H
ATOM    963 HH12 ARG A  69      35.518   2.348  25.939  1.00  0.00
A    H
ATOM    964  NH2 ARG A  69      32.910   1.186  24.560  1.00  0.00
A    N
ATOM    965 HH21 ARG A  69      33.183   1.230  23.610  1.00  0.00
A    H
ATOM    966 HH22 ARG A  69      32.089   0.709  24.844  1.00  0.00
A    H
ATOM    967  C   ARG A  69      34.153   6.549  27.058  1.00  0.00
A    C
ATOM    968  O   ARG A  69      35.182   7.152  27.374  1.00  0.00
A    O
ATOM    969  N   ASN A  70      33.443   6.852  25.975  1.00  0.00
A    N
ATOM    970  HN  ASN A  70      32.674   6.319  25.623  1.00  0.00
A    H
ATOM    971  CA  ASN A  70      33.758   8.037  25.200  1.00  0.00
A    C
```

ATOM 972 HA ASN A 70 34.777 7.901 24.854 1.00 0.00
A H
ATOM 973 CB ASN A 70 32.777 8.177 24.042 1.00 0.00
A C
ATOM 974 HB1 ASN A 70 31.801 8.506 24.460 1.00 0.00
A H
ATOM 975 HB2 ASN A 70 33.120 8.963 23.339 1.00 0.00
A H
ATOM 976 CG ASN A 70 32.563 6.875 23.314 1.00 0.00
A C
ATOM 977 OD1 ASN A 70 33.359 5.948 23.439 1.00 0.00
A O
ATOM 978 ND2 ASN A 70 31.485 6.796 22.547 1.00 0.00
A N
ATOM 979 HD21 ASN A 70 31.325 5.937 22.069 1.00 0.00
A H
ATOM 980 HD22 ASN A 70 30.856 7.565 22.490 1.00 0.00
A H
ATOM 981 C ASN A 70 33.728 9.284 26.058 1.00 0.00
A C
ATOM 982 O ASN A 70 34.543 10.190 25.854 1.00 0.00
A O
ATOM 983 N PHE A 71 32.832 9.338 27.033 1.00 0.00
A N
ATOM 984 HN PHE A 71 32.176 8.609 27.222 1.00 0.00
A H
ATOM 985 CA PHE A 71 32.732 10.482 27.919 1.00 0.00
A C
ATOM 986 HA PHE A 71 32.816 11.370 27.304 1.00 0.00
A H
ATOM 987 CB PHE A 71 31.437 10.445 28.717 1.00 0.00
A C
ATOM 988 HB1 PHE A 71 31.394 9.498 29.297 1.00 0.00
A H
ATOM 989 HB2 PHE A 71 31.395 11.279 29.449 1.00 0.00
A H
ATOM 990 CG PHE A 71 30.214 10.469 27.875 1.00 0.00
A C
ATOM 991 CD1 PHE A 71 29.822 9.344 27.174 1.00 0.00
A C
ATOM 992 HD1 PHE A 71 30.417 8.444 27.232 1.00 0.00
A H
ATOM 993 CE1 PHE A 71 28.696 9.359 26.403 1.00 0.00
A C
ATOM 994 HE1 PHE A 71 28.401 8.472 25.860 1.00 0.00
A H
ATOM 995 CZ PHE A 71 27.935 10.506 26.326 1.00 0.00
A C
ATOM 996 HZ PHE A 71 27.038 10.518 25.723 1.00 0.00
A H
ATOM 997 CD2 PHE A 71 29.445 11.613 27.795 1.00 0.00
A C
ATOM 998 HD2 PHE A 71 29.747 12.505 28.325 1.00 0.00
A H

```
ATOM    999  CE2 PHE A  71      28.311  11.636  27.026  1.00  0.00
A    C
ATOM   1000  HE2 PHE A  71      27.717  12.538  26.969  1.00  0.00
A    H
ATOM   1001  C   PHE A  71      33.917  10.483  28.868  1.00  0.00
A    C
ATOM   1002  O   PHE A  71      34.519   9.436  29.121  1.00  0.00
A    O
ATOM   1003  N   GLU A  72      34.240  11.662  29.395  1.00  0.00
A    N
ATOM   1004  HN  GLU A  72      33.791  12.529  29.195  1.00  0.00
A    H
ATOM   1005  CA  GLU A  72      35.332  11.768  30.351  1.00  0.00
A    C
ATOM   1006  HA  GLU A  72      35.768  10.784  30.475  1.00  0.00
A    H
ATOM   1007  CB  GLU A  72      36.368  12.742  29.825  1.00  0.00
A    C
ATOM   1008  HB1 GLU A  72      36.466  13.576  30.560  1.00  0.00
A    H
ATOM   1009  HB2 GLU A  72      37.366  12.250  29.789  1.00  0.00
A    H
ATOM   1010  CG  GLU A  72      36.000  13.298  28.473  1.00  0.00
A    C
ATOM   1011  HG1 GLU A  72      35.719  12.470  27.798  1.00  0.00
A    H
ATOM   1012  HG2 GLU A  72      35.153  14.008  28.553  1.00  0.00
A    H
ATOM   1013  CD  GLU A  72      37.158  13.998  27.810  1.00  0.00
A    C
ATOM   1014  OE1 GLU A  72      37.779  14.869  28.455  1.00  0.00
A    O
ATOM   1015  OE2 GLU A  72      37.446  13.674  26.637  1.00  0.00
A    O
ATOM   1016  C   GLU A  72      34.828  12.212  31.715  1.00  0.00
A    C
ATOM   1017  O   GLU A  72      35.338  11.767  32.750  1.00  0.00
A    O
ATOM   1018  N   TYR A  73      33.830  13.094  31.738  1.00  0.00
A    N
ATOM   1019  HN  TYR A  73      33.422  13.490  30.918  1.00  0.00
A    H
ATOM   1020  CA  TYR A  73      33.245  13.557  32.990  1.00  0.00
A    C
ATOM   1021  HA  TYR A  73      33.877  13.204  33.796  1.00  0.00
A    H
ATOM   1022  CB  TYR A  73      33.086  15.074  33.016  1.00  0.00
A    C
ATOM   1023  HB1 TYR A  73      32.649  15.410  32.050  1.00  0.00
A    H
ATOM   1024  HB2 TYR A  73      32.384  15.381  33.821  1.00  0.00
A    H
ATOM   1025  CG  TYR A  73      34.363  15.851  33.181  1.00  0.00
A    C
```

```
ATOM   1026  CD1 TYR A  73      35.300  15.909  32.163  1.00  0.00
A    C
ATOM   1027  HD1 TYR A  73      35.128  15.368  31.243  1.00  0.00
A    H
ATOM   1028  CE1 TYR A  73      36.462  16.628  32.311  1.00  0.00
A    C
ATOM   1029  HE1 TYR A  73      37.191  16.665  31.514  1.00  0.00
A    H
ATOM   1030  CZ  TYR A  73      36.696  17.298  33.489  1.00  0.00
A    C
ATOM   1031  OH  TYR A  73      37.852  18.017  33.655  1.00  0.00
A    O
ATOM   1032  HH  TYR A  73      37.794  18.464  34.502  1.00  0.00
A    H
ATOM   1033  CD2 TYR A  73      34.624  16.539  34.351  1.00  0.00
A    C
ATOM   1034  HD2 TYR A  73      33.927  16.486  35.176  1.00  0.00
A    H
ATOM   1035  CE2 TYR A  73      35.780  17.255  34.508  1.00  0.00
A    C
ATOM   1036  HE2 TYR A  73      35.973  17.764  35.441  1.00  0.00
A    H
ATOM   1037  C   TYR A  73      31.888  12.905  33.169  1.00  0.00
A    C
ATOM   1038  O   TYR A  73      31.074  12.896  32.240  1.00  0.00
A    O
ATOM   1039  N   VAL A  74      31.631  12.383  34.363  1.00  0.00
A    N
ATOM   1040  HN  VAL A  74      32.308  12.334  35.093  1.00  0.00
A    H
ATOM   1041  CA  VAL A  74      30.329  11.835  34.712  1.00  0.00
A    C
ATOM   1042  HA  VAL A  74      29.602  12.181  33.988  1.00  0.00
A    H
ATOM   1043  CB  VAL A  74      30.331  10.295  34.720  1.00  0.00
A    C
ATOM   1044  HB  VAL A  74      29.290   9.957  34.950  1.00  0.00
A    H
ATOM   1045  CG1 VAL A  74      30.741   9.767  33.364  1.00  0.00
A    C
ATOM   1046 HG11 VAL A  74      30.780   8.656  33.379  1.00  0.00
A    H
ATOM   1047 HG12 VAL A  74      30.020  10.061  32.573  1.00  0.00
A    H
ATOM   1048 HG13 VAL A  74      31.749  10.143  33.084  1.00  0.00
A    H
ATOM   1049  CG2 VAL A  74      31.247   9.754  35.798  1.00  0.00
A    C
ATOM   1050 HG21 VAL A  74      31.272   8.644  35.770  1.00  0.00
A    H
ATOM   1051 HG22 VAL A  74      32.282  10.132  35.656  1.00  0.00
A    H
ATOM   1052 HG23 VAL A  74      30.901  10.056  36.809  1.00  0.00
A    H
```

```
ATOM   1053  C   VAL A  74      29.923  12.388  36.066  1.00  0.00
A    C
ATOM   1054  O   VAL A  74      30.754  12.517  36.975  1.00  0.00
A    O
ATOM   1055  N   ILE A  75      28.650  12.727  36.192  1.00  0.00
A    N
ATOM   1056  HN  ILE A  75      27.979  12.594  35.464  1.00  0.00
A    H
ATOM   1057  CA  ILE A  75      28.108  13.324  37.398  1.00  0.00
A    C
ATOM   1058  HA  ILE A  75      28.829  13.181  38.193  1.00  0.00
A    H
ATOM   1059  CB  ILE A  75      27.760  14.797  37.189  1.00  0.00
A    C
ATOM   1060  HB  ILE A  75      26.737  14.908  37.631  1.00  0.00
A    H
ATOM   1061  CG2 ILE A  75      28.809  15.660  37.833  1.00  0.00
A    C
ATOM   1062 HG21 ILE A  75      28.495  16.725  37.828  1.00  0.00
A    H
ATOM   1063 HG22 ILE A  75      28.973  15.363  38.890  1.00  0.00
A    H
ATOM   1064 HG23 ILE A  75      29.775  15.580  37.292  1.00  0.00
A    H
ATOM   1065  CG1 ILE A  75      27.645  15.066  35.696  1.00  0.00
A    C
ATOM   1066 HG11 ILE A  75      28.185  14.275  35.128  1.00  0.00
A    H
ATOM   1067 HG12 ILE A  75      26.573  15.009  35.401  1.00  0.00
A    H
ATOM   1068  CD  ILE A  75      28.165  16.396  35.272  1.00  0.00
A    C
ATOM   1069  HD1 ILE A  75      27.996  16.547  34.184  1.00  0.00
A    H
ATOM   1070  HD2 ILE A  75      27.647  17.212  35.821  1.00  0.00
A    H
ATOM   1071  HD3 ILE A  75      29.255  16.477  35.465  1.00  0.00
A    H
ATOM   1072  C   ILE A  75      26.875  12.539  37.780  1.00  0.00
A    C
ATOM   1073  O   ILE A  75      25.856  12.593  37.084  1.00  0.00
A    O
ATOM   1074  N   VAL A  76      26.959  11.829  38.889  1.00  0.00
A    N
ATOM   1075  HN  VAL A  76      27.787  11.762  39.438  1.00  0.00
A    H
ATOM   1076  CA  VAL A  76      25.834  11.074  39.407  1.00  0.00
A    C
ATOM   1077  HA  VAL A  76      25.157  10.820  38.602  1.00  0.00
A    H
ATOM   1078  CB  VAL A  76      26.287   9.800  40.098  1.00  0.00
A    C
ATOM   1079  HB  VAL A  76      26.890  10.096  40.991  1.00  0.00
A    H
```

```
ATOM   1080  CG1 VAL A  76      25.103   9.009  40.539  1.00  0.00
A    C
ATOM   1081 HG11 VAL A  76      25.429   8.057  41.011  1.00  0.00
A    H
ATOM   1082 HG12 VAL A  76      24.499   9.559  41.290  1.00  0.00
A    H
ATOM   1083 HG13 VAL A  76      24.453   8.758  39.674  1.00  0.00
A    H
ATOM   1084  CG2 VAL A  76      27.126   8.992  39.144  1.00  0.00
A    C
ATOM   1085 HG21 VAL A  76      27.438   8.033  39.610  1.00  0.00
A    H
ATOM   1086 HG22 VAL A  76      26.553   8.761  38.221  1.00  0.00
A    H
ATOM   1087 HG23 VAL A  76      28.046   9.541  38.855  1.00  0.00
A    H
ATOM   1088  C   VAL A  76      25.098  12.030  40.338  1.00  0.00
A    C
ATOM   1089  O   VAL A  76      25.426  12.176  41.521  1.00  0.00
A    O
ATOM   1090  N   ALA A  77      24.120  12.731  39.774  1.00  0.00
A    N
ATOM   1091  HN  ALA A  77      23.895  12.628  38.805  1.00  0.00
A    H
ATOM   1092  CA  ALA A  77      23.304  13.693  40.507  1.00  0.00
A    C
ATOM   1093  HA  ALA A  77      23.838  14.062  41.375  1.00  0.00
A    H
ATOM   1094  CB  ALA A  77      22.976  14.891  39.627  1.00  0.00
A    C
ATOM   1095  HB1 ALA A  77      23.918  15.379  39.295  1.00  0.00
A    H
ATOM   1096  HB2 ALA A  77      22.416  14.578  38.718  1.00  0.00
A    H
ATOM   1097  HB3 ALA A  77      22.372  15.647  40.172  1.00  0.00
A    H
ATOM   1098  C   ALA A  77      22.035  13.050  41.027  1.00  0.00
A    C
ATOM   1099  O   ALA A  77      20.929  13.528  40.795  1.00  0.00
A    O
ATOM   1100  N   ILE A  78      22.185  11.943  41.729  1.00  0.00
A    N
ATOM   1101  HN  ILE A  78      23.064  11.485  41.839  1.00  0.00
A    H
ATOM   1102  CA  ILE A  78      21.083  11.288  42.407  1.00  0.00
A    C
ATOM   1103  HA  ILE A  78      20.154  11.681  42.014  1.00  0.00
A    H
ATOM   1104  CB  ILE A  78      21.177   9.768  42.264  1.00  0.00
A    C
ATOM   1105  HB  ILE A  78      20.935   9.573  41.188  1.00  0.00
A    H
ATOM   1106  CG2 ILE A  78      22.509   9.302  42.729  1.00  0.00
A    C
```

```
ATOM   1107 HG21 ILE A  78      22.653   8.228  42.488  1.00  0.00
A    H
ATOM   1108 HG22 ILE A  78      23.323   9.870  42.232  1.00  0.00
A    H
ATOM   1109 HG23 ILE A  78      22.614   9.428  43.827  1.00  0.00
A    H
ATOM   1110  CG1 ILE A  78      20.103   9.072  43.083  1.00  0.00
A    C
ATOM   1111 HG11 ILE A  78      20.203   9.355  44.154  1.00  0.00
A    H
ATOM   1112 HG12 ILE A  78      19.104   9.425  42.741  1.00  0.00
A    H
ATOM   1113  CD  ILE A  78      20.162   7.584  42.946  1.00  0.00
A    C
ATOM   1114  HD1 ILE A  78      19.329   7.113  43.510  1.00  0.00
A    H
ATOM   1115  HD2 ILE A  78      20.074   7.285  41.879  1.00  0.00
A    H
ATOM   1116  HD3 ILE A  78      21.117   7.185  43.346  1.00  0.00
A    H
ATOM   1117  C   ILE A  78      21.164  11.723  43.861  1.00  0.00
A    C
ATOM   1118  O   ILE A  78      22.256  12.005  44.363  1.00  0.00
A    O
ATOM   1119  N   GLY A  79      20.024  11.820  44.539  1.00  0.00
A    N
ATOM   1120  HN  GLY A  79      19.111  11.679  44.156  1.00  0.00
A    H
ATOM   1121  CA  GLY A  79      20.067  12.157  45.948  1.00  0.00
A    C
ATOM   1122  HA1 GLY A  79      19.630  13.142  46.028  1.00  0.00
A    H
ATOM   1123  HA2 GLY A  79      21.110  12.109  46.234  1.00  0.00
A    H
ATOM   1124  C   GLY A  79      19.293  11.226  46.853  1.00  0.00
A    C
ATOM   1125  O   GLY A  79      19.580  11.135  48.049  1.00  0.00
A    O
ATOM   1126  N   ALA A  80      18.308  10.525  46.301  1.00  0.00
A    N
ATOM   1127  HN  ALA A  80      18.097  10.495  45.325  1.00  0.00
A    H
ATOM   1128  CA  ALA A  80      17.443   9.722  47.152  1.00  0.00
A    C
ATOM   1129  HA  ALA A  80      17.457  10.117  48.162  1.00  0.00
A    H
ATOM   1130  CB  ALA A  80      16.003   9.786  46.641  1.00  0.00
A    C
ATOM   1131  HB1 ALA A  80      15.663  10.843  46.614  1.00  0.00
A    H
ATOM   1132  HB2 ALA A  80      15.927   9.376  45.610  1.00  0.00
A    H
ATOM   1133  HB3 ALA A  80      15.309   9.218  47.298  1.00  0.00
A    H
```

```
ATOM   1134  C   ALA A  80      17.883   8.269  47.253  1.00  0.00
A    C
ATOM   1135  O   ALA A  80      18.173   7.782  48.348  1.00  0.00
A    O
ATOM   1136  N   ASN A  81      17.942   7.565  46.135  1.00  0.00
A    N
ATOM   1137  HN  ASN A  81      17.881   7.969  45.223  1.00  0.00
A    H
ATOM   1138  CA  ASN A  81      18.104   6.119  46.145  1.00  0.00
A    C
ATOM   1139  HA  ASN A  81      17.606   5.753  47.037  1.00  0.00
A    H
ATOM   1140  CB  ASN A  81      17.513   5.534  44.871  1.00  0.00
A    C
ATOM   1141  HB1 ASN A  81      16.469   5.903  44.780  1.00  0.00
A    H
ATOM   1142  HB2 ASN A  81      18.073   5.896  43.985  1.00  0.00
A    H
ATOM   1143  CG  ASN A  81      17.478   4.042  44.887  1.00  0.00
A    C
ATOM   1144  OD1 ASN A  81      18.330   3.396  45.487  1.00  0.00
A    O
ATOM   1145  ND2 ASN A  81      16.486   3.473  44.220  1.00  0.00
A    N
ATOM   1146 HD21 ASN A  81      16.440   2.478  44.228  1.00  0.00
A    H
ATOM   1147 HD22 ASN A  81      15.807   4.037  43.760  1.00  0.00
A    H
ATOM   1148  C   ASN A  81      19.583   5.786  46.275  1.00  0.00
A    C
ATOM   1149  O   ASN A  81      20.265   5.581  45.267  1.00  0.00
A    O
ATOM   1150  N   ILE A  82      20.070   5.685  47.513  1.00  0.00
A    N
ATOM   1151  HN  ILE A  82      19.532   5.747  48.350  1.00  0.00
A    H
ATOM   1152  CA  ILE A  82      21.496   5.465  47.733  1.00  0.00
A    C
ATOM   1153  HA  ILE A  82      22.006   6.332  47.332  1.00  0.00
A    H
ATOM   1154  CB  ILE A  82      21.790   5.315  49.234  1.00  0.00
A    C
ATOM   1155  HB  ILE A  82      20.992   4.624  49.607  1.00  0.00
A    H
ATOM   1156  CG2 ILE A  82      23.197   4.809  49.458  1.00  0.00
A    C
ATOM   1157 HG21 ILE A  82      23.355   4.553  50.527  1.00  0.00
A    H
ATOM   1158 HG22 ILE A  82      23.388   3.893  48.862  1.00  0.00
A    H
ATOM   1159 HG23 ILE A  82      23.946   5.577  49.172  1.00  0.00
A    H
ATOM   1160  CG1 ILE A  82      21.591   6.647  49.945  1.00  0.00
A    C
```

```
ATOM   1161 HG11 ILE A  82      22.313   6.735  50.788  1.00  0.00
A    H
ATOM   1162 HG12 ILE A  82      21.815   7.473  49.232  1.00  0.00
A    H
ATOM   1163  CD  ILE A  82      20.211   6.854  50.471  1.00  0.00
A    C
ATOM   1164  HD1 ILE A  82      20.118   7.864  50.924  1.00  0.00
A    H
ATOM   1165  HD2 ILE A  82      19.464   6.770  49.653  1.00  0.00
A    H
ATOM   1166  HD3 ILE A  82      19.965   6.103  51.251  1.00  0.00
A    H
ATOM   1167  C   ILE A  82      21.987   4.263  46.944  1.00  0.00
A    C
ATOM   1168  O   ILE A  82      23.104   4.267  46.405  1.00  0.00
A    O
ATOM   1169  N   GLN A  83      21.150   3.232  46.841  1.00  0.00
A    N
ATOM   1170  HN  GLN A  83      20.248   3.192  47.268  1.00  0.00
A    H
ATOM   1171  CA  GLN A  83      21.505   2.050  46.071  1.00  0.00
A    C
ATOM   1172  HA  GLN A  83      22.290   1.541  46.616  1.00  0.00
A    H
ATOM   1173  CB  GLN A  83      20.271   1.184  45.882  1.00  0.00
A    C
ATOM   1174  HB1 GLN A  83      19.450   1.662  46.466  1.00  0.00
A    H
ATOM   1175  HB2 GLN A  83      19.956   1.220  44.814  1.00  0.00
A    H
ATOM   1176  CG  GLN A  83      20.400  -0.221  46.318  1.00  0.00
A    C
ATOM   1177  HG1 GLN A  83      21.363  -0.650  45.965  1.00  0.00
A    H
ATOM   1178  HG2 GLN A  83      20.382  -0.291  47.425  1.00  0.00
A    H
ATOM   1179  CD  GLN A  83      19.292  -1.056  45.732  1.00  0.00
A    C
ATOM   1180  OE1 GLN A  83      18.963  -0.920  44.560  1.00  0.00
A    O
ATOM   1181  NE2 GLN A  83      18.690  -1.903  46.548  1.00  0.00
A    N
ATOM   1182 HE21 GLN A  83      17.960  -2.459  46.160  1.00  0.00
A    H
ATOM   1183 HE22 GLN A  83      18.963  -1.949  47.503  1.00  0.00
A    H
ATOM   1184  C   GLN A  83      22.063   2.426  44.712  1.00  0.00
A    C
ATOM   1185  O   GLN A  83      23.237   2.173  44.414  1.00  0.00
A    O
ATOM   1186  N   ALA A  84      21.236   3.066  43.894  1.00  0.00
A    N
ATOM   1187  HN  ALA A  84      20.316   3.364  44.147  1.00  0.00
A    H
```

ATOM 1188 CA ALA A 84 21.640 3.378 42.534 1.00 0.00
A C
ATOM 1189 HA ALA A 84 21.867 2.471 41.984 1.00 0.00
A H
ATOM 1190 CB ALA A 84 20.510 4.102 41.810 1.00 0.00
A C
ATOM 1191 HB1 ALA A 84 19.609 3.455 41.785 1.00 0.00
A H
ATOM 1192 HB2 ALA A 84 20.239 5.044 42.335 1.00 0.00
A H
ATOM 1193 HB3 ALA A 84 20.786 4.347 40.762 1.00 0.00
A H
ATOM 1194 C ALA A 84 22.907 4.211 42.526 1.00 0.00
A C
ATOM 1195 O ALA A 84 23.846 3.920 41.781 1.00 0.00
A O
ATOM 1196 N SER A 85 22.949 5.254 43.352 1.00 0.00
A N
ATOM 1197 HN SER A 85 22.199 5.553 43.942 1.00 0.00
A H
ATOM 1198 CA SER A 85 24.151 6.068 43.456 1.00 0.00
A C
ATOM 1199 HA SER A 85 24.220 6.625 42.531 1.00 0.00
A H
ATOM 1200 CB SER A 85 24.059 6.964 44.685 1.00 0.00
A C
ATOM 1201 HB1 SER A 85 24.710 7.852 44.516 1.00 0.00
A H
ATOM 1202 HB2 SER A 85 23.018 7.340 44.813 1.00 0.00
A H
ATOM 1203 OG SER A 85 24.508 6.267 45.831 1.00 0.00
A O
ATOM 1204 HG1 SER A 85 24.645 6.924 46.526 1.00 0.00
A H
ATOM 1205 C SER A 85 25.387 5.192 43.550 1.00 0.00
A C
ATOM 1206 O SER A 85 26.315 5.297 42.735 1.00 0.00
A O
ATOM 1207 N THR A 86 25.396 4.286 44.518 1.00 0.00
A N
ATOM 1208 HN THR A 86 24.619 4.025 45.090 1.00 0.00
A H
ATOM 1209 CA THR A 86 26.626 3.573 44.818 1.00 0.00
A C
ATOM 1210 HA THR A 86 27.407 4.322 44.803 1.00 0.00
A H
ATOM 1211 CB THR A 86 26.504 2.901 46.175 1.00 0.00
A C
ATOM 1212 HB THR A 86 27.496 2.427 46.376 1.00 0.00
A H
ATOM 1213 OG1 THR A 86 25.521 1.865 46.099 1.00 0.00
A O
ATOM 1214 HG1 THR A 86 25.479 1.486 46.983 1.00 0.00
A H

```
ATOM   1215  CG2 THR A  86      26.098   3.917  47.217  1.00  0.00
A    C
ATOM   1216 HG21 THR A  86      26.116   3.469  48.233  1.00  0.00
A    H
ATOM   1217 HG22 THR A  86      26.799   4.778  47.212  1.00  0.00
A    H
ATOM   1218 HG23 THR A  86      25.073   4.301  47.023  1.00  0.00
A    H
ATOM   1219  C   THR A  86      26.975   2.561  43.737  1.00  0.00
A    C
ATOM   1220  O   THR A  86      28.120   2.518  43.273  1.00  0.00
A    O
ATOM   1221  N   LEU A  87      26.017   1.722  43.331  1.00  0.00
A    N
ATOM   1222  HN  LEU A  87      25.077   1.675  43.662  1.00  0.00
A    H
ATOM   1223  CA  LEU A  87      26.336   0.748  42.287  1.00  0.00
A    C
ATOM   1224  HA  LEU A  87      27.190   0.182  42.636  1.00  0.00
A    H
ATOM   1225  CB  LEU A  87      25.180  -0.217  42.020  1.00  0.00
A    C
ATOM   1226  HB1 LEU A  87      24.512   0.269  41.272  1.00  0.00
A    H
ATOM   1227  HB2 LEU A  87      25.579  -1.132  41.527  1.00  0.00
A    H
ATOM   1228  CG  LEU A  87      24.125  -0.836  42.941  1.00  0.00
A    C
ATOM   1229  HG  LEU A  87      24.268  -1.933  42.786  1.00  0.00
A    H
ATOM   1230  CD1 LEU A  87      24.225  -0.509  44.417  1.00  0.00
A    C
ATOM   1231 HD11 LEU A  87      23.384  -0.971  44.977  1.00  0.00
A    H
ATOM   1232 HD12 LEU A  87      25.178  -0.893  44.839  1.00  0.00
A    H
ATOM   1233 HD13 LEU A  87      24.187   0.590  44.578  1.00  0.00
A    H
ATOM   1234  CD2 LEU A  87      22.739  -0.460  42.409  1.00  0.00
A    C
ATOM   1235 HD21 LEU A  87      21.946  -0.909  43.045  1.00  0.00
A    H
ATOM   1236 HD22 LEU A  87      22.586   0.637  42.405  1.00  0.00
A    H
ATOM   1237 HD23 LEU A  87      22.607  -0.835  41.371  1.00  0.00
A    H
ATOM   1238  C   LEU A  87      26.744   1.449  41.004  1.00  0.00
A    C
ATOM   1239  O   LEU A  87      27.625   0.981  40.272  1.00  0.00
A    O
ATOM   1240  N   THR A  88      26.112   2.581  40.720  1.00  0.00
A    N
ATOM   1241  HN  THR A  88      25.329   2.938  41.228  1.00  0.00
A    H
```

```
ATOM   1242  CA  THR A  88      26.530   3.410  39.610  1.00  0.00
A    C
ATOM   1243  HA  THR A  88      26.395   2.810  38.720  1.00  0.00
A    H
ATOM   1244  CB  THR A  88      25.724   4.694  39.648  1.00  0.00
A    C
ATOM   1245  HB  THR A  88      25.961   5.180  40.626  1.00  0.00
A    H
ATOM   1246  OG1 THR A  88      24.332   4.366  39.636  1.00  0.00
A    O
ATOM   1247  HG1 THR A  88      23.869   5.211  39.650  1.00  0.00
A    H
ATOM   1248  CG2 THR A  88      26.071   5.564  38.481  1.00  0.00
A    C
ATOM   1249 HG21 THR A  88      25.571   6.552  38.562  1.00  0.00
A    H
ATOM   1250 HG22 THR A  88      27.167   5.738  38.439  1.00  0.00
A    H
ATOM   1251 HG23 THR A  88      25.758   5.093  37.524  1.00  0.00
A    H
ATOM   1252  C   THR A  88      28.006   3.720  39.701  1.00  0.00
A    C
ATOM   1253  O   THR A  88      28.795   3.321  38.834  1.00  0.00
A    O
ATOM   1254  N   THR A  89      28.395   4.391  40.784  1.00  0.00
A    N
ATOM   1255  HN  THR A  89      27.786   4.696  41.517  1.00  0.00
A    H
ATOM   1256  CA  THR A  89      29.794   4.743  40.982  1.00  0.00
A    C
ATOM   1257  HA  THR A  89      30.044   5.427  40.182  1.00  0.00
A    H
ATOM   1258  CB  THR A  89      29.984   5.325  42.369  1.00  0.00
A    C
ATOM   1259  HB  THR A  89      31.088   5.395  42.527  1.00  0.00
A    H
ATOM   1260  OG1 THR A  89      29.448   4.405  43.324  1.00  0.00
A    O
ATOM   1261  HG1 THR A  89      29.580   4.820  44.183  1.00  0.00
A    H
ATOM   1262  CG2 THR A  89      29.256   6.637  42.479  1.00  0.00
A    C
ATOM   1263 HG21 THR A  89      29.478   7.131  43.449  1.00  0.00
A    H
ATOM   1264 HG22 THR A  89      29.570   7.325  41.666  1.00  0.00
A    H
ATOM   1265 HG23 THR A  89      28.156   6.493  42.406  1.00  0.00
A    H
ATOM   1266  C   THR A  89      30.691   3.535  40.812  1.00  0.00
A    C
ATOM   1267  O   THR A  89      31.789   3.639  40.255  1.00  0.00
A    O
ATOM   1268  N   LEU A  90      30.246   2.383  41.305  1.00  0.00
A    N
```

```
ATOM   1269  HN  LEU A  90      29.407   2.258  41.830  1.00  0.00
A    H
ATOM   1270  CA  LEU A  90      31.006   1.162  41.095  1.00  0.00
A    C
ATOM   1271  HA  LEU A  90      31.966   1.303  41.575  1.00  0.00
A    H
ATOM   1272  CB  LEU A  90      30.265  -0.027  41.685  1.00  0.00
A    C
ATOM   1273  HB1 LEU A  90      30.453  -0.018  42.783  1.00  0.00
A    H
ATOM   1274  HB2 LEU A  90      29.171   0.129  41.559  1.00  0.00
A    H
ATOM   1275  CG  LEU A  90      30.697  -1.374  41.112  1.00  0.00
A    C
ATOM   1276  HG  LEU A  90      30.409  -1.406  40.034  1.00  0.00
A    H
ATOM   1277  CD1 LEU A  90      32.186  -1.594  41.259  1.00  0.00
A    C
ATOM   1278 HD11 LEU A  90      32.471  -2.599  40.880  1.00  0.00
A    H
ATOM   1279 HD12 LEU A  90      32.753  -0.830  40.685  1.00  0.00
A    H
ATOM   1280 HD13 LEU A  90      32.489  -1.528  42.326  1.00  0.00
A    H
ATOM   1281  CD2 LEU A  90      29.955  -2.459  41.820  1.00  0.00
A    C
ATOM   1282 HD21 LEU A  90      30.237  -3.452  41.408  1.00  0.00
A    H
ATOM   1283 HD22 LEU A  90      30.181  -2.468  42.905  1.00  0.00
A    H
ATOM   1284 HD23 LEU A  90      28.859  -2.328  41.692  1.00  0.00
A    H
ATOM   1285  C   LEU A  90      31.262   0.935  39.617  1.00  0.00
A    C
ATOM   1286  O   LEU A  90      32.407   0.753  39.196  1.00  0.00
A    O
ATOM   1287  N   LEU A  91      30.201   0.917  38.820  1.00  0.00
A    N
ATOM   1288  HN  LEU A  91      29.259   1.097  39.094  1.00  0.00
A    H
ATOM   1289  CA  LEU A  91      30.366   0.613  37.405  1.00  0.00
A    C
ATOM   1290  HA  LEU A  91      30.849  -0.353  37.346  1.00  0.00
A    H
ATOM   1291  CB  LEU A  91      29.010   0.571  36.714  1.00  0.00
A    C
ATOM   1292  HB1 LEU A  91      28.351   1.299  37.240  1.00  0.00
A    H
ATOM   1293  HB2 LEU A  91      29.121   0.953  35.675  1.00  0.00
A    H
ATOM   1294  CG  LEU A  91      28.372  -0.814  36.738  1.00  0.00
A    C
ATOM   1295  HG  LEU A  91      27.364  -0.736  36.265  1.00  0.00
A    H
```

ATOM 1296 CD1 LEU A 91 29.232 -1.786 35.953 1.00 0.00
A C
ATOM 1297 HD11 LEU A 91 28.806 -2.811 36.002 1.00 0.00
A H
ATOM 1298 HD12 LEU A 91 29.290 -1.482 34.886 1.00 0.00
A H
ATOM 1299 HD13 LEU A 91 30.263 -1.819 36.365 1.00 0.00
A H
ATOM 1300 CD2 LEU A 91 28.186 -1.301 38.157 1.00 0.00
A C
ATOM 1301 HD21 LEU A 91 27.704 -2.302 38.162 1.00 0.00
A H
ATOM 1302 HD22 LEU A 91 29.154 -1.392 38.690 1.00 0.00
A H
ATOM 1303 HD23 LEU A 91 27.538 -0.601 38.727 1.00 0.00
A H
ATOM 1304 C LEU A 91 31.279 1.622 36.736 1.00 0.00
A C
ATOM 1305 O LEU A 91 32.135 1.267 35.919 1.00 0.00
A O
ATOM 1306 N LEU A 92 31.119 2.891 37.090 1.00 0.00
A N
ATOM 1307 HN LEU A 92 30.482 3.234 37.776 1.00 0.00
A H
ATOM 1308 CA LEU A 92 31.923 3.922 36.451 1.00 0.00
A C
ATOM 1309 HA LEU A 92 31.829 3.770 35.383 1.00 0.00
A H
ATOM 1310 CB LEU A 92 31.403 5.302 36.829 1.00 0.00
A C
ATOM 1311 HB1 LEU A 92 31.090 5.255 37.897 1.00 0.00
A H
ATOM 1312 HB2 LEU A 92 32.245 6.030 36.788 1.00 0.00
A H
ATOM 1313 CG LEU A 92 30.230 5.756 35.972 1.00 0.00
A C
ATOM 1314 HG LEU A 92 30.113 6.861 36.086 1.00 0.00
A H
ATOM 1315 CD1 LEU A 92 30.519 5.416 34.528 1.00 0.00
A C
ATOM 1316 HD11 LEU A 92 29.659 5.692 33.883 1.00 0.00
A H
ATOM 1317 HD12 LEU A 92 31.416 5.966 34.169 1.00 0.00
A H
ATOM 1318 HD13 LEU A 92 30.705 4.327 34.412 1.00 0.00
A H
ATOM 1319 CD2 LEU A 92 28.946 5.106 36.407 1.00 0.00
A C
ATOM 1320 HD21 LEU A 92 28.100 5.464 35.782 1.00 0.00
A H
ATOM 1321 HD22 LEU A 92 28.992 4.003 36.310 1.00 0.00
A H
ATOM 1322 HD23 LEU A 92 28.724 5.354 37.467 1.00 0.00
A H

ATOM 1323 C LEU A 92 33.393 3.781 36.807 1.00 0.00
A C
ATOM 1324 O LEU A 92 34.252 3.725 35.919 1.00 0.00
A O
ATOM 1325 N LYS A 93 33.705 3.710 38.099 1.00 0.00
A N
ATOM 1326 HN LYS A 93 33.049 3.758 38.850 1.00 0.00
A H
ATOM 1327 CA LYS A 93 35.095 3.551 38.501 1.00 0.00
A C
ATOM 1328 HA LYS A 93 35.653 4.365 38.056 1.00 0.00
A H
ATOM 1329 CB LYS A 93 35.207 3.593 40.018 1.00 0.00
A C
ATOM 1330 HB1 LYS A 93 34.376 4.235 40.391 1.00 0.00
A H
ATOM 1331 HB2 LYS A 93 35.019 2.573 40.424 1.00 0.00
A H
ATOM 1332 CG LYS A 93 36.526 4.141 40.513 1.00 0.00
A C
ATOM 1333 HG1 LYS A 93 36.472 4.282 41.616 1.00 0.00
A H
ATOM 1334 HG2 LYS A 93 37.327 3.393 40.313 1.00 0.00
A H
ATOM 1335 CD LYS A 93 36.844 5.463 39.846 1.00 0.00
A C
ATOM 1336 HD1 LYS A 93 36.875 5.304 38.745 1.00 0.00
A H
ATOM 1337 HD2 LYS A 93 36.006 6.167 40.057 1.00 0.00
A H
ATOM 1338 CE LYS A 93 38.158 6.033 40.350 1.00 0.00
A C
ATOM 1339 HE1 LYS A 93 38.059 6.369 41.405 1.00 0.00
A H
ATOM 1340 HE2 LYS A 93 38.962 5.265 40.300 1.00 0.00
A H
ATOM 1341 NZ LYS A 93 38.606 7.205 39.545 1.00 0.00
A N
ATOM 1342 HZ1 LYS A 93 39.506 7.569 39.917 1.00 0.00
A H
ATOM 1343 HZ2 LYS A 93 38.734 6.918 38.553 1.00 0.00
A H
ATOM 1344 HZ3 LYS A 93 37.885 7.953 39.594 1.00 0.00
A H
ATOM 1345 C LYS A 93 35.669 2.256 37.955 1.00 0.00
A C
ATOM 1346 O LYS A 93 36.872 2.167 37.685 1.00 0.00
A O
ATOM 1347 N GLU A 94 34.827 1.242 37.783 1.00 0.00
A N
ATOM 1348 HN GLU A 94 33.873 1.217 38.072 1.00 0.00
A H
ATOM 1349 CA GLU A 94 35.277 0.030 37.121 1.00 0.00
A C

```
ATOM   1350  HA  GLU A  94      36.209  -0.279  37.577  1.00  0.00
A    H
ATOM   1351  CB  GLU A  94      34.238  -1.073  37.279  1.00  0.00
A    C
ATOM   1352  HB1 GLU A  94      34.293  -1.456  38.324  1.00  0.00
A    H
ATOM   1353  HB2 GLU A  94      33.215  -0.647  37.164  1.00  0.00
A    H
ATOM   1354  CG  GLU A  94      34.435  -2.222  36.318  1.00  0.00
A    C
ATOM   1355  HG1 GLU A  94      34.075  -1.930  35.314  1.00  0.00
A    H
ATOM   1356  HG2 GLU A  94      35.505  -2.506  36.253  1.00  0.00
A    H
ATOM   1357  CD  GLU A  94      33.636  -3.438  36.706  1.00  0.00
A    C
ATOM   1358  OE1 GLU A  94      33.419  -3.639  37.918  1.00  0.00
A    O
ATOM   1359  OE2 GLU A  94      33.230  -4.196  35.804  1.00  0.00
A    O
ATOM   1360  C   GLU A  94      35.556   0.286  35.652  1.00  0.00
A    C
ATOM   1361  O   GLU A  94      36.473  -0.308  35.077  1.00  0.00
A    O
ATOM   1362  N   LEU A  95      34.783   1.166  35.029  1.00  0.00
A    N
ATOM   1363  HN  LEU A  95      34.017   1.647  35.450  1.00  0.00
A    H
ATOM   1364  CA  LEU A  95      35.005   1.502  33.634  1.00  0.00
A    C
ATOM   1365  HA  LEU A  95      35.511   0.650  33.199  1.00  0.00
A    H
ATOM   1366  CB  LEU A  95      33.677   1.713  32.924  1.00  0.00
A    C
ATOM   1367  HB1 LEU A  95      32.943   2.050  33.692  1.00  0.00
A    H
ATOM   1368  HB2 LEU A  95      33.781   2.558  32.207  1.00  0.00
A    H
ATOM   1369  CG  LEU A  95      33.268   0.391  32.279  1.00  0.00
A    C
ATOM   1370  HG  LEU A  95      34.186   0.006  31.771  1.00  0.00
A    H
ATOM   1371  CD1 LEU A  95      32.808  -0.610  33.323  1.00  0.00
A    C
ATOM   1372 HD11 LEU A  95      32.468  -1.551  32.838  1.00  0.00
A    H
ATOM   1373 HD12 LEU A  95      33.636  -0.856  34.021  1.00  0.00
A    H
ATOM   1374 HD13 LEU A  95      31.961  -0.200  33.914  1.00  0.00
A    H
ATOM   1375  CD2 LEU A  95      32.207   0.597  31.229  1.00  0.00
A    C
ATOM   1376 HD21 LEU A  95      31.936  -0.373  30.758  1.00  0.00
A    H
```

ATOM 1377 HD22 LEU A 95 31.283 1.032 31.661 1.00 0.00
A H
ATOM 1378 HD23 LEU A 95 32.575 1.280 30.432 1.00 0.00
A H
ATOM 1379 C LEU A 95 35.907 2.708 33.460 1.00 0.00
A C
ATOM 1380 O LEU A 95 35.845 3.372 32.422 1.00 0.00
A O
ATOM 1381 N ASP A 96 36.722 3.016 34.465 1.00 0.00
A N
ATOM 1382 HN ASP A 96 36.581 2.736 35.411 1.00 0.00
A H
ATOM 1383 CA ASP A 96 37.930 3.811 34.282 1.00 0.00
A C
ATOM 1384 HA ASP A 96 38.397 3.891 35.256 1.00 0.00
A H
ATOM 1385 CB ASP A 96 38.863 3.133 33.287 1.00 0.00
A C
ATOM 1386 HB1 ASP A 96 38.500 3.322 32.257 1.00 0.00
A H
ATOM 1387 HB2 ASP A 96 39.890 3.540 33.375 1.00 0.00
A H
ATOM 1388 CG ASP A 96 38.915 1.644 33.486 1.00 0.00
A C
ATOM 1389 OD1 ASP A 96 38.740 1.204 34.640 1.00 0.00
A O
ATOM 1390 OD2 ASP A 96 39.101 0.914 32.493 1.00 0.00
A O
ATOM 1391 C ASP A 96 37.626 5.225 33.825 1.00 0.00
A C
ATOM 1392 O ASP A 96 38.504 5.925 33.320 1.00 0.00
A O
ATOM 1393 N ILE A 97 36.383 5.650 33.988 1.00 0.00
A N
ATOM 1394 HN ILE A 97 35.620 5.144 34.385 1.00 0.00
A H
ATOM 1395 CA ILE A 97 36.042 7.001 33.552 1.00 0.00
A C
ATOM 1396 HA ILE A 97 36.352 7.095 32.519 1.00 0.00
A H
ATOM 1397 CB ILE A 97 34.544 7.248 33.720 1.00 0.00
A C
ATOM 1398 HB ILE A 97 34.343 7.028 34.798 1.00 0.00
A H
ATOM 1399 CG2 ILE A 97 34.178 8.651 33.284 1.00 0.00
A C
ATOM 1400 HG21 ILE A 97 33.125 8.880 33.551 1.00 0.00
A H
ATOM 1401 HG22 ILE A 97 34.824 9.402 33.785 1.00 0.00
A H
ATOM 1402 HG23 ILE A 97 34.291 8.767 32.186 1.00 0.00
A H
ATOM 1403 CG1 ILE A 97 33.786 6.218 32.891 1.00 0.00
A C

```
ATOM   1404 HG11 ILE A  97      33.991   5.197  33.282  1.00  0.00
A    H
ATOM   1405 HG12 ILE A  97      32.693   6.400  33.001  1.00  0.00
A    H
ATOM   1406  CD  ILE A  97      34.135   6.271  31.425  1.00  0.00
A    C
ATOM   1407  HD1 ILE A  97      33.517   5.543  30.854  1.00  0.00
A    H
ATOM   1408  HD2 ILE A  97      33.945   7.286  31.013  1.00  0.00
A    H
ATOM   1409  HD3 ILE A  97      35.203   6.018  31.259  1.00  0.00
A    H
ATOM   1410  C   ILE A  97      36.866   7.999  34.352  1.00  0.00
A    C
ATOM   1411  O   ILE A  97      37.035   7.825  35.572  1.00  0.00
A    O
ATOM   1412  N   PRO A  98      37.429   9.023  33.719  1.00  0.00
A    N
ATOM   1413  CD  PRO A  98      37.345   9.368  32.292  1.00  0.00
A    C
ATOM   1414  HD1 PRO A  98      37.681   8.340  32.556  1.00  0.00
A    H
ATOM   1415  HD2 PRO A  98      36.719   9.349  31.370  1.00  0.00
A    H
ATOM   1416  CA  PRO A  98      38.349   9.902  34.453  1.00  0.00
A    C
ATOM   1417  HA  PRO A  98      39.207   9.330  34.789  1.00  0.00
A    H
ATOM   1418  CB  PRO A  98      38.780  10.916  33.388  1.00  0.00
A    C
ATOM   1419  HB1 PRO A  98      39.631  10.494  32.808  1.00  0.00
A    H
ATOM   1420  HB2 PRO A  98      39.087  11.889  33.821  1.00  0.00
A    H
ATOM   1421  CG  PRO A  98      38.518  10.250  32.090  1.00  0.00
A    C
ATOM   1422  HG1 PRO A  98      39.132  10.659  31.264  1.00  0.00
A    H
ATOM   1423  HG2 PRO A  98      37.439  10.353  31.835  1.00  0.00
A    H
ATOM   1424  C   PRO A  98      37.709  10.580  35.644  1.00  0.00
A    C
ATOM   1425  O   PRO A  98      38.168  10.405  36.777  1.00  0.00
A    O
ATOM   1426  N   ASN A  99      36.637  11.327  35.425  1.00  0.00
A    N
ATOM   1427  HN  ASN A  99      36.136  11.331  34.560  1.00  0.00
A    H
ATOM   1428  CA  ASN A  99      36.095  12.216  36.441  1.00  0.00
A    C
ATOM   1429  HA  ASN A  99      36.765  12.167  37.293  1.00  0.00
A    H
ATOM   1430  CB  ASN A  99      35.961  13.629  35.889  1.00  0.00
A    C
```

```
ATOM   1431  HB1 ASN A  99      35.187  13.612  35.092  1.00  0.00
A    H
ATOM   1432  HB2 ASN A  99      35.608  14.321  36.682  1.00  0.00
A    H
ATOM   1433  CG  ASN A  99      37.242  14.124  35.291  1.00  0.00
A    C
ATOM   1434  OD1 ASN A  99      37.321  14.371  34.092  1.00  0.00
A    O
ATOM   1435  ND2 ASN A  99      38.270  14.250  36.118  1.00  0.00
A    N
ATOM   1436 HD21 ASN A  99      39.125  14.588  35.737  1.00  0.00
A    H
ATOM   1437 HD22 ASN A  99      38.161  14.038  37.084  1.00  0.00
A    H
ATOM   1438  C   ASN A  99      34.746  11.688  36.887  1.00  0.00
A    C
ATOM   1439  O   ASN A  99      33.830  11.553  36.071  1.00  0.00
A    O
ATOM   1440  N   ILE A 100      34.623  11.398  38.176  1.00  0.00
A    N
ATOM   1441  HN  ILE A 100      35.363  11.520  38.833  1.00  0.00
A    H
ATOM   1442  CA  ILE A 100      33.396  10.875  38.752  1.00  0.00
A    C
ATOM   1443  HA  ILE A 100      32.616  10.946  38.004  1.00  0.00
A    H
ATOM   1444  CB  ILE A 100      33.564   9.439  39.246  1.00  0.00
A    C
ATOM   1445  HB  ILE A 100      34.540   9.445  39.795  1.00  0.00
A    H
ATOM   1446  CG2 ILE A 100      32.374   9.039  40.066  1.00  0.00
A    C
ATOM   1447 HG21 ILE A 100      32.551   8.061  40.559  1.00  0.00
A    H
ATOM   1448 HG22 ILE A 100      32.172   9.786  40.862  1.00  0.00
A    H
ATOM   1449 HG23 ILE A 100      31.467   8.949  39.431  1.00  0.00
A    H
ATOM   1450  CG1 ILE A 100      33.725   8.496  38.058  1.00  0.00
A    C
ATOM   1451 HG11 ILE A 100      32.824   8.555  37.406  1.00  0.00
A    H
ATOM   1452 HG12 ILE A 100      34.596   8.830  37.448  1.00  0.00
A    H
ATOM   1453  CD  ILE A 100      33.951   7.066  38.450  1.00  0.00
A    C
ATOM   1454  HD1 ILE A 100      34.117   6.441  37.546  1.00  0.00
A    H
ATOM   1455  HD2 ILE A 100      34.844   6.976  39.105  1.00  0.00
A    H
ATOM   1456  HD3 ILE A 100      33.073   6.658  38.992  1.00  0.00
A    H
ATOM   1457  C   ILE A 100      33.003  11.803  39.885  1.00  0.00
A    C
```

```
ATOM   1458  O   ILE A 100      33.716  11.906  40.892  1.00  0.00
A    O
ATOM   1459  N   TRP A 101      31.882  12.495  39.720  1.00  0.00
A    N
ATOM   1460  HN  TRP A 101      31.270  12.347  38.947  1.00  0.00
A    H
ATOM   1461  CA  TRP A 101      31.456  13.526  40.655  1.00  0.00
A    C
ATOM   1462  HA  TRP A 101      32.090  13.475  41.532  1.00  0.00
A    H
ATOM   1463  CB  TRP A 101      31.535  14.898  39.996  1.00  0.00
A    C
ATOM   1464  HB1 TRP A 101      31.092  14.814  38.979  1.00  0.00
A    H
ATOM   1465  HB2 TRP A 101      30.911  15.625  40.561  1.00  0.00
A    H
ATOM   1466  CG  TRP A 101      32.915  15.362  39.862  1.00  0.00
A    C
ATOM   1467  CD1 TRP A 101      33.992  14.626  39.489  1.00  0.00
A    C
ATOM   1468  HD1 TRP A 101      33.948  13.584  39.196  1.00  0.00
A    H
ATOM   1469  NE1 TRP A 101      35.117  15.398  39.499  1.00  0.00
A    N
ATOM   1470  HE1 TRP A 101      36.018  15.121  39.240  1.00  0.00
A    H
ATOM   1471  CE2 TRP A 101      34.774  16.664  39.889  1.00  0.00
A    C
ATOM   1472  CD2 TRP A 101      33.392  16.670  40.125  1.00  0.00
A    C
ATOM   1473  CE3 TRP A 101      32.785  17.852  40.538  1.00  0.00
A    C
ATOM   1474  HE3 TRP A 101      31.725  17.906  40.745  1.00  0.00
A    H
ATOM   1475  CZ3 TRP A 101      33.560  18.967  40.691  1.00  0.00
A    C
ATOM   1476  HZ3 TRP A 101      33.101  19.894  41.007  1.00  0.00
A    H
ATOM   1477  CZ2 TRP A 101      35.559  17.790  40.048  1.00  0.00
A    C
ATOM   1478  HZ2 TRP A 101      36.622  17.791  39.865  1.00  0.00
A    H
ATOM   1479  CH2 TRP A 101      34.934  18.933  40.451  1.00  0.00
A    C
ATOM   1480  HH2 TRP A 101      35.522  19.830  40.589  1.00  0.00
A    H
ATOM   1481  C   TRP A 101      30.039  13.225  41.115  1.00  0.00
A    C
ATOM   1482  O   TRP A 101      29.140  13.065  40.288  1.00  0.00
A    O
ATOM   1483  N   VAL A 102      29.834  13.156  42.426  1.00  0.00
A    N
ATOM   1484  HN  VAL A 102      30.466  13.458  43.134  1.00  0.00
A    H
```

```
ATOM   1485  CA  VAL A 102      28.588  12.598  42.930  1.00  0.00
A    C
ATOM   1486  HA  VAL A 102      27.917  12.488  42.088  1.00  0.00
A    H
ATOM   1487  CB  VAL A 102      28.831  11.223  43.579  1.00  0.00
A    C
ATOM   1488  HB  VAL A 102      29.451  11.387  44.495  1.00  0.00
A    H
ATOM   1489  CG1 VAL A 102      27.522  10.582  43.975  1.00  0.00
A    C
ATOM   1490 HG11 VAL A 102      27.701   9.574  44.407  1.00  0.00
A    H
ATOM   1491 HG12 VAL A 102      26.988  11.180  44.742  1.00  0.00
A    H
ATOM   1492 HG13 VAL A 102      26.859  10.464  43.091  1.00  0.00
A    H
ATOM   1493  CG2 VAL A 102      29.599  10.324  42.638  1.00  0.00
A    C
ATOM   1494 HG21 VAL A 102      29.742   9.317  43.087  1.00  0.00
A    H
ATOM   1495 HG22 VAL A 102      29.051  10.205  41.678  1.00  0.00
A    H
ATOM   1496 HG23 VAL A 102      30.604  10.739  42.417  1.00  0.00
A    H
ATOM   1497  C   VAL A 102      27.927  13.545  43.920  1.00  0.00
A    C
ATOM   1498  O   VAL A 102      28.595  14.288  44.647  1.00  0.00
A    O
ATOM   1499  N   LYS A 103      26.593  13.515  43.929  1.00  0.00
A    N
ATOM   1500  HN  LYS A 103      26.073  13.047  43.217  1.00  0.00
A    H
ATOM   1501  CA  LYS A 103      25.789  14.154  44.966  1.00  0.00
A    C
ATOM   1502  HA  LYS A 103      26.344  15.003  45.343  1.00  0.00
A    H
ATOM   1503  CB  LYS A 103      24.437  14.581  44.392  1.00  0.00
A    C
ATOM   1504  HB1 LYS A 103      24.633  15.406  43.670  1.00  0.00
A    H
ATOM   1505  HB2 LYS A 103      24.013  13.740  43.798  1.00  0.00
A    H
ATOM   1506  CG  LYS A 103      23.409  15.067  45.401  1.00  0.00
A    C
ATOM   1507  HG1 LYS A 103      23.395  14.372  46.271  1.00  0.00
A    H
ATOM   1508  HG2 LYS A 103      23.720  16.068  45.781  1.00  0.00
A    H
ATOM   1509  CD  LYS A 103      22.020  15.124  44.755  1.00  0.00
A    C
ATOM   1510  HD1 LYS A 103      22.062  15.827  43.892  1.00  0.00
A    H
ATOM   1511  HD2 LYS A 103      21.783  14.114  44.350  1.00  0.00
A    H
```

```
ATOM   1512  CE  LYS A 103      20.922  15.558  45.731  1.00  0.00
A    C
ATOM   1513  HE1 LYS A 103      20.990  14.975  46.676  1.00  0.00
A    H
ATOM   1514  HE2 LYS A 103      21.021  16.638  45.975  1.00  0.00
A    H
ATOM   1515  NZ  LYS A 103      19.542  15.349  45.177  1.00  0.00
A    N
ATOM   1516  HZ1 LYS A 103      18.830  15.655  45.871  1.00  0.00
A    H
ATOM   1517  HZ2 LYS A 103      19.431  15.902  44.303  1.00  0.00
A    H
ATOM   1518  HZ3 LYS A 103      19.404  14.341  44.963  1.00  0.00
A    H
ATOM   1519  C   LYS A 103      25.599  13.182  46.125  1.00  0.00
A    C
ATOM   1520  O   LYS A 103      25.102  12.070  45.931  1.00  0.00
A    O
ATOM   1521  N   ALA A 104      25.990  13.600  47.326  1.00  0.00
A    N
ATOM   1522  HN  ALA A 104      26.271  14.545  47.493  1.00  0.00
A    H
ATOM   1523  CA  ALA A 104      26.039  12.720  48.486  1.00  0.00
A    C
ATOM   1524  HA  ALA A 104      25.944  11.685  48.178  1.00  0.00
A    H
ATOM   1525  CB  ALA A 104      27.359  12.871  49.241  1.00  0.00
A    C
ATOM   1526  HB1 ALA A 104      28.205  12.627  48.564  1.00  0.00
A    H
ATOM   1527  HB2 ALA A 104      27.497  13.914  49.599  1.00  0.00
A    H
ATOM   1528  HB3 ALA A 104      27.412  12.187  50.115  1.00  0.00
A    H
ATOM   1529  C   ALA A 104      24.887  12.990  49.444  1.00  0.00
A    C
ATOM   1530  O   ALA A 104      24.388  14.113  49.547  1.00  0.00
A    O
ATOM   1531  N   GLN A 105      24.494  11.946  50.171  1.00  0.00
A    N
ATOM   1532  HN  GLN A 105      24.969  11.067  50.157  1.00  0.00
A    H
ATOM   1533  CA  GLN A 105      23.336  11.991  51.054  1.00  0.00
A    C
ATOM   1534  HA  GLN A 105      22.701  12.787  50.684  1.00  0.00
A    H
ATOM   1535  CB  GLN A 105      22.600  10.652  50.999  1.00  0.00
A    C
ATOM   1536  HB1 GLN A 105      22.432  10.421  49.921  1.00  0.00
A    H
ATOM   1537  HB2 GLN A 105      23.266   9.848  51.389  1.00  0.00
A    H
ATOM   1538  CG  GLN A 105      21.273  10.632  51.721  1.00  0.00
A    C
```

```
ATOM   1539  HG1 GLN A 105      20.846  11.656  51.776  1.00  0.00
A    H
ATOM   1540  HG2 GLN A 105      20.545   9.988  51.186  1.00  0.00
A    H
ATOM   1541  CD  GLN A 105      21.403  10.145  53.142  1.00  0.00
A    C
ATOM   1542  OE1 GLN A 105      22.372   9.473  53.491  1.00  0.00
A    O
ATOM   1543  NE2 GLN A 105      20.418  10.462  53.969  1.00  0.00
A    N
ATOM   1544 HE21 GLN A 105      20.502  10.146  54.910  1.00  0.00
A    H
ATOM   1545 HE22 GLN A 105      19.637  10.977  53.633  1.00  0.00
A    H
ATOM   1546  C   GLN A 105      23.713  12.334  52.491  1.00  0.00
A    C
ATOM   1547  O   GLN A 105      23.287  13.366  53.012  1.00  0.00
A    O
ATOM   1548  N   ASN A 106      24.514  11.495  53.142  1.00  0.00
A    N
ATOM   1549  HN  ASN A 106      24.862  10.636  52.770  1.00  0.00
A    H
ATOM   1550  CA  ASN A 106      24.955  11.787  54.497  1.00  0.00
A    C
ATOM   1551  HA  ASN A 106      24.754  12.838  54.676  1.00  0.00
A    H
ATOM   1552  CB  ASN A 106      24.263  10.911  55.541  1.00  0.00
A    C
ATOM   1553  HB1 ASN A 106      24.548  11.289  56.546  1.00  0.00
A    H
ATOM   1554  HB2 ASN A 106      23.161  11.001  55.451  1.00  0.00
A    H
ATOM   1555  CG  ASN A 106      24.676   9.450  55.456  1.00  0.00
A    C
ATOM   1556  OD1 ASN A 106      25.516   9.058  54.648  1.00  0.00
A    O
ATOM   1557  ND2 ASN A 106      24.094   8.637  56.320  1.00  0.00
A    N
ATOM   1558 HD21 ASN A 106      24.356   7.676  56.292  1.00  0.00
A    H
ATOM   1559 HD22 ASN A 106      23.445   8.996  56.983  1.00  0.00
A    H
ATOM   1560  C   ASN A 106      26.454  11.587  54.574  1.00  0.00
A    C
ATOM   1561  O   ASN A 106      27.096  11.165  53.609  1.00  0.00
A    O
ATOM   1562  N   TYR A 107      27.003  11.859  55.752  1.00  0.00
A    N
ATOM   1563  HN  TYR A 107      26.525  12.243  56.541  1.00  0.00
A    H
ATOM   1564  CA  TYR A 107      28.415  11.599  55.971  1.00  0.00
A    C
ATOM   1565  HA  TYR A 107      28.956  12.230  55.276  1.00  0.00
A    H
```

```
ATOM   1566  CB  TYR A 107      28.785  11.914  57.417  1.00  0.00
A    C
ATOM   1567  HB1 TYR A 107      29.025  12.997  57.505  1.00  0.00
A    H
ATOM   1568  HB2 TYR A 107      27.928  11.713  58.095  1.00  0.00
A    H
ATOM   1569  CG  TYR A 107      29.993  11.147  57.872  1.00  0.00
A    C
ATOM   1570  CD1 TYR A 107      31.267  11.568  57.535  1.00  0.00
A    C
ATOM   1571  HD1 TYR A 107      31.398  12.456  56.931  1.00  0.00
A    H
ATOM   1572  CE1 TYR A 107      32.373  10.866  57.936  1.00  0.00
A    C
ATOM   1573  HE1 TYR A 107      33.363  11.200  57.665  1.00  0.00
A    H
ATOM   1574  CZ  TYR A 107      32.216   9.724  58.682  1.00  0.00
A    C
ATOM   1575  OH  TYR A 107      33.318   9.013  59.090  1.00  0.00
A    O
ATOM   1576  HH  TYR A 107      33.009   8.300  59.653  1.00  0.00
A    H
ATOM   1577  CD2 TYR A 107      29.862   9.995  58.621  1.00  0.00
A    C
ATOM   1578  HD2 TYR A 107      28.878   9.617  58.865  1.00  0.00
A    H
ATOM   1579  CE2 TYR A 107      30.964   9.286  59.025  1.00  0.00
A    C
ATOM   1580  HE2 TYR A 107      30.837   8.375  59.592  1.00  0.00
A    H
ATOM   1581  C   TYR A 107      28.767  10.160  55.637  1.00  0.00
A    C
ATOM   1582  O   TYR A 107      29.798   9.891  55.009  1.00  0.00
A    O
ATOM   1583  N   TYR A 108      27.925   9.217  56.050  1.00  0.00
A    N
ATOM   1584  HN  TYR A 108      27.061   9.367  56.528  1.00  0.00
A    H
ATOM   1585  CA  TYR A 108      28.246   7.819  55.808  1.00  0.00
A    C
ATOM   1586  HA  TYR A 108      29.248   7.662  56.189  1.00  0.00
A    H
ATOM   1587  CB  TYR A 108      27.240   6.924  56.507  1.00  0.00
A    C
ATOM   1588  HB1 TYR A 108      26.216   7.188  56.161  1.00  0.00
A    H
ATOM   1589  HB2 TYR A 108      27.412   5.858  56.243  1.00  0.00
A    H
ATOM   1590  CG  TYR A 108      27.297   7.122  57.973  1.00  0.00
A    C
ATOM   1591  CD1 TYR A 108      28.498   7.003  58.641  1.00  0.00
A    C
ATOM   1592  HD1 TYR A 108      29.393   6.740  58.094  1.00  0.00
A    H
```

ATOM 1593 CE1 TYR A 108 28.581 7.189 59.985 1.00 0.00
A C
ATOM 1594 HE1 TYR A 108 29.527 7.085 60.496 1.00 0.00
A H
ATOM 1595 CZ TYR A 108 27.452 7.503 60.690 1.00 0.00
A C
ATOM 1596 OH TYR A 108 27.531 7.688 62.048 1.00 0.00
A O
ATOM 1597 HH TYR A 108 26.665 7.969 62.351 1.00 0.00
A H
ATOM 1598 CD2 TYR A 108 26.171 7.445 58.693 1.00 0.00
A C
ATOM 1599 HD2 TYR A 108 25.213 7.515 58.196 1.00 0.00
A H
ATOM 1600 CE2 TYR A 108 26.240 7.633 60.047 1.00 0.00
A C
ATOM 1601 HE2 TYR A 108 25.343 7.861 60.603 1.00 0.00
A H
ATOM 1602 C TYR A 108 28.277 7.514 54.327 1.00 0.00
A C
ATOM 1603 O TYR A 108 29.343 7.206 53.777 1.00 0.00
A O
ATOM 1604 N HSE A 109 27.126 7.652 53.668 1.00 0.00
A N
ATOM 1605 HN HSE A 109 26.250 7.855 54.104 1.00 0.00
A H
ATOM 1606 CA HSE A 109 27.060 7.513 52.223 1.00 0.00
A C
ATOM 1607 HA HSE A 109 27.093 6.453 52.002 1.00 0.00
A H
ATOM 1608 CB HSE A 109 25.781 8.162 51.714 1.00 0.00
A C
ATOM 1609 HB1 HSE A 109 24.923 7.553 52.073 1.00 0.00
A H
ATOM 1610 HB2 HSE A 109 25.674 9.172 52.163 1.00 0.00
A H
ATOM 1611 ND1 HSE A 109 25.555 9.435 49.559 1.00 0.00
A N
ATOM 1612 CG HSE A 109 25.702 8.241 50.229 1.00 0.00
A C
ATOM 1613 CE1 HSE A 109 25.511 9.205 48.260 1.00 0.00
A C
ATOM 1614 HE1 HSE A 109 25.400 9.979 47.496 1.00 0.00
A H
ATOM 1615 NE2 HSE A 109 25.615 7.903 48.064 1.00 0.00
A N
ATOM 1616 HE2 HSE A 109 25.591 7.451 47.174 1.00 0.00
A H
ATOM 1617 CD2 HSE A 109 25.732 7.277 49.282 1.00 0.00
A C
ATOM 1618 HD2 HSE A 109 25.818 6.205 49.386 1.00 0.00
A H
ATOM 1619 C HSE A 109 28.270 8.142 51.567 1.00 0.00
A C

```
ATOM   1620  O   HSE A 109      28.845   7.580  50.631  1.00  0.00
A    O
ATOM   1621  N   HSE A 110      28.706   9.283  52.092  1.00  0.00
A    N
ATOM   1622  HN  HSE A 110      28.263   9.766  52.847  1.00  0.00
A    H
ATOM   1623  CA  HSE A 110      29.902   9.934  51.585  1.00  0.00
A    C
ATOM   1624  HA  HSE A 110      29.759  10.057  50.519  1.00  0.00
A    H
ATOM   1625  CB  HSE A 110      30.072  11.268  52.286  1.00  0.00
A    C
ATOM   1626  HB1 HSE A 110      29.569  12.044  51.670  1.00  0.00
A    H
ATOM   1627  HB2 HSE A 110      29.549  11.242  53.265  1.00  0.00
A    H
ATOM   1628  ND1 HSE A 110      32.165  12.433  51.539  1.00  0.00
A    N
ATOM   1629  CG  HSE A 110      31.493  11.669  52.464  1.00  0.00
A    C
ATOM   1630  CE1 HSE A 110      33.402  12.629  51.953  1.00  0.00
A    C
ATOM   1631  HE1 HSE A 110      34.156  13.199  51.406  1.00  0.00
A    H
ATOM   1632  NE2 HSE A 110      33.555  12.019  53.114  1.00  0.00
A    N
ATOM   1633  HE2 HSE A 110      34.403  11.991  53.643  1.00  0.00
A    H
ATOM   1634  CD2 HSE A 110      32.374  11.411  53.457  1.00  0.00
A    C
ATOM   1635  HD2 HSE A 110      32.247  10.837  54.365  1.00  0.00
A    H
ATOM   1636  C   HSE A 110      31.140   9.072  51.774  1.00  0.00
A    C
ATOM   1637  O   HSE A 110      31.979   8.966  50.874  1.00  0.00
A    O
ATOM   1638  N   LYS A 111      31.307   8.484  52.951  1.00  0.00
A    N
ATOM   1639  HN  LYS A 111      30.695   8.542  53.738  1.00  0.00
A    H
ATOM   1640  CA  LYS A 111      32.492   7.662  53.151  1.00  0.00
A    C
ATOM   1641  HA  LYS A 111      33.348   8.264  52.870  1.00  0.00
A    H
ATOM   1642  CB  LYS A 111      32.612   7.231  54.603  1.00  0.00
A    C
ATOM   1643  HB1 LYS A 111      32.806   8.151  55.203  1.00  0.00
A    H
ATOM   1644  HB2 LYS A 111      31.630   6.835  54.946  1.00  0.00
A    H
ATOM   1645  CG  LYS A 111      33.730   6.227  54.823  1.00  0.00
A    C
ATOM   1646  HG1 LYS A 111      33.358   5.405  55.477  1.00  0.00
A    H
```

```
ATOM   1647  HG2 LYS A 111      34.007   5.773  53.844  1.00  0.00
A    H
ATOM   1648  CD  LYS A 111      34.977   6.840  55.480  1.00  0.00
A    C
ATOM   1649  HD1 LYS A 111      35.138   6.341  56.463  1.00  0.00
A    H
ATOM   1650  HD2 LYS A 111      35.857   6.606  54.837  1.00  0.00
A    H
ATOM   1651  CE  LYS A 111      34.844   8.329  55.659  1.00  0.00
A    C
ATOM   1652  HE1 LYS A 111      33.792   8.649  55.490  1.00  0.00
A    H
ATOM   1653  HE2 LYS A 111      35.142   8.627  56.688  1.00  0.00
A    H
ATOM   1654  NZ  LYS A 111      35.716   9.031  54.688  1.00  0.00
A    N
ATOM   1655  HZ1 LYS A 111      35.626  10.060  54.808  1.00  0.00
A    H
ATOM   1656  HZ2 LYS A 111      36.705   8.750  54.844  1.00  0.00
A    H
ATOM   1657  HZ3 LYS A 111      35.435   8.771  53.721  1.00  0.00
A    H
ATOM   1658  C   LYS A 111      32.478   6.445  52.245  1.00  0.00
A    C
ATOM   1659  O   LYS A 111      33.494   6.097  51.634  1.00  0.00
A    O
ATOM   1660  N   VAL A 112      31.330   5.788  52.142  1.00  0.00
A    N
ATOM   1661  HN  VAL A 112      30.492   5.996  52.641  1.00  0.00
A    H
ATOM   1662  CA  VAL A 112      31.225   4.650  51.235  1.00  0.00
A    C
ATOM   1663  HA  VAL A 112      31.992   3.936  51.509  1.00  0.00
A    H
ATOM   1664  CB  VAL A 112      29.855   3.972  51.369  1.00  0.00
A    C
ATOM   1665  HB  VAL A 112      30.035   2.887  51.564  1.00  0.00
A    H
ATOM   1666  CG1 VAL A 112      29.101   4.563  52.511  1.00  0.00
A    C
ATOM   1667 HG11 VAL A 112      28.093   4.101  52.587  1.00  0.00
A    H
ATOM   1668 HG12 VAL A 112      29.617   4.390  53.477  1.00  0.00
A    H
ATOM   1669 HG13 VAL A 112      28.968   5.657  52.369  1.00  0.00
A    H
ATOM   1670  CG2 VAL A 112      29.071   4.132  50.096  1.00  0.00
A    C
ATOM   1671 HG21 VAL A 112      28.062   3.678  50.198  1.00  0.00
A    H
ATOM   1672 HG22 VAL A 112      28.948   5.208  49.846  1.00  0.00
A    H
ATOM   1673 HG23 VAL A 112      29.579   3.632  49.245  1.00  0.00
A    H
```

```
ATOM   1674  C   VAL A 112      31.501   5.090  49.807  1.00  0.00
A    C
ATOM   1675  O   VAL A 112      32.045   4.326  49.000  1.00  0.00
A    O
ATOM   1676  N   LEU A 113      31.138   6.326  49.476  1.00  0.00
A    N
ATOM   1677  HN  LEU A 113      30.581   6.934  50.039  1.00  0.00
A    H
ATOM   1678  CA  LEU A 113      31.551   6.900  48.209  1.00  0.00
A    C
ATOM   1679  HA  LEU A 113      31.175   6.248  47.430  1.00  0.00
A    H
ATOM   1680  CB  LEU A 113      30.994   8.310  48.092  1.00  0.00
A    C
ATOM   1681  HB1 LEU A 113      30.026   8.328  48.644  1.00  0.00
A    H
ATOM   1682  HB2 LEU A 113      31.669   9.011  48.631  1.00  0.00
A    H
ATOM   1683  CG  LEU A 113      30.731   8.853  46.702  1.00  0.00
A    C
ATOM   1684  HG  LEU A 113      31.657   9.362  46.341  1.00  0.00
A    H
ATOM   1685  CD1 LEU A 113      30.395   7.729  45.761  1.00  0.00
A    C
ATOM   1686 HD11 LEU A 113      30.153   8.124  44.751  1.00  0.00
A    H
ATOM   1687 HD12 LEU A 113      31.253   7.028  45.665  1.00  0.00
A    H
ATOM   1688 HD13 LEU A 113      29.516   7.158  46.129  1.00  0.00
A    H
ATOM   1689  CD2 LEU A 113      29.635   9.881  46.775  1.00  0.00
A    C
ATOM   1690 HD21 LEU A 113      29.438  10.307  45.767  1.00  0.00
A    H
ATOM   1691 HD22 LEU A 113      28.687   9.444  47.147  1.00  0.00
A    H
ATOM   1692 HD23 LEU A 113      29.925  10.713  47.453  1.00  0.00
A    H
ATOM   1693  C   LEU A 113      33.061   6.920  48.099  1.00  0.00
A    C
ATOM   1694  O   LEU A 113      33.629   6.509  47.082  1.00  0.00
A    O
ATOM   1695  N   GLU A 114      33.726   7.389  49.148  1.00  0.00
A    N
ATOM   1696  HN  GLU A 114      33.319   7.708  49.999  1.00  0.00
A    H
ATOM   1697  CA  GLU A 114      35.178   7.473  49.118  1.00  0.00
A    C
ATOM   1698  HA  GLU A 114      35.462   8.118  48.296  1.00  0.00
A    H
ATOM   1699  CB  GLU A 114      35.697   8.055  50.427  1.00  0.00
A    C
ATOM   1700  HB1 GLU A 114      35.056   8.926  50.699  1.00  0.00
A    H
```

ATOM 1701 HB2 GLU A 114 35.568 7.314 51.247 1.00 0.00
A H
ATOM 1702 CG GLU A 114 37.132 8.490 50.319 1.00 0.00
A C
ATOM 1703 HG1 GLU A 114 37.369 9.186 51.144 1.00 0.00
A H
ATOM 1704 HG2 GLU A 114 37.816 7.620 50.368 1.00 0.00
A H
ATOM 1705 CD GLU A 114 37.394 9.250 49.040 1.00 0.00
A C
ATOM 1706 OE1 GLU A 114 36.687 10.247 48.792 1.00 0.00
A O
ATOM 1707 OE2 GLU A 114 38.292 8.841 48.277 1.00 0.00
A O
ATOM 1708 C GLU A 114 35.802 6.110 48.860 1.00 0.00
A C
ATOM 1709 O GLU A 114 36.740 5.980 48.066 1.00 0.00
A O
ATOM 1710 N LYS A 115 35.283 5.075 49.515 1.00 0.00
A N
ATOM 1711 HN LYS A 115 34.480 5.116 50.106 1.00 0.00
A H
ATOM 1712 CA LYS A 115 35.897 3.759 49.394 1.00 0.00
A C
ATOM 1713 HA LYS A 115 36.942 3.885 49.653 1.00 0.00
A H
ATOM 1714 CB LYS A 115 35.227 2.779 50.338 1.00 0.00
A C
ATOM 1715 HB1 LYS A 115 34.129 2.854 50.163 1.00 0.00
A H
ATOM 1716 HB2 LYS A 115 35.520 1.744 50.052 1.00 0.00
A H
ATOM 1717 CG LYS A 115 35.536 3.069 51.757 1.00 0.00
A C
ATOM 1718 HG1 LYS A 115 34.938 3.949 52.088 1.00 0.00
A H
ATOM 1719 HG2 LYS A 115 35.223 2.200 52.381 1.00 0.00
A H
ATOM 1720 CD LYS A 115 36.991 3.358 51.940 1.00 0.00
A C
ATOM 1721 HD1 LYS A 115 37.574 2.496 51.543 1.00 0.00
A H
ATOM 1722 HD2 LYS A 115 37.249 4.248 51.320 1.00 0.00
A H
ATOM 1723 CE LYS A 115 37.256 3.599 53.397 1.00 0.00
A C
ATOM 1724 HE1 LYS A 115 38.349 3.596 53.601 1.00 0.00
A H
ATOM 1725 HE2 LYS A 115 36.841 4.582 53.712 1.00 0.00
A H
ATOM 1726 NZ LYS A 115 36.614 2.523 54.199 1.00 0.00
A N
ATOM 1727 HZ1 LYS A 115 36.793 2.680 55.212 1.00 0.00
A H

```
ATOM   1728  HZ2 LYS A 115      35.589   2.525  54.025  1.00  0.00
A    H
ATOM   1729  HZ3 LYS A 115      37.006   1.602  53.919  1.00  0.00
A    H
ATOM   1730  C   LYS A 115      35.843   3.211  47.980  1.00  0.00
A    C
ATOM   1731  O   LYS A 115      36.748   2.476  47.578  1.00  0.00
A    O
ATOM   1732  N   ILE A 116      34.808   3.541  47.215  1.00  0.00
A    N
ATOM   1733  HN  ILE A 116      34.047   4.118  47.504  1.00  0.00
A    H
ATOM   1734  CA  ILE A 116      34.707   3.067  45.845  1.00  0.00
A    C
ATOM   1735  HA  ILE A 116      34.955   2.013  45.872  1.00  0.00
A    H
ATOM   1736  CB  ILE A 116      33.296   3.283  45.289  1.00  0.00
A    C
ATOM   1737  HB  ILE A 116      32.616   3.009  46.136  1.00  0.00
A    H
ATOM   1738  CG2 ILE A 116      33.169   4.678  44.738  1.00  0.00
A    C
ATOM   1739 HG21 ILE A 116      32.114   4.901  44.476  1.00  0.00
A    H
ATOM   1740 HG22 ILE A 116      33.495   5.430  45.487  1.00  0.00
A    H
ATOM   1741 HG23 ILE A 116      33.787   4.802  43.825  1.00  0.00
A    H
ATOM   1742  CG1 ILE A 116      32.999   2.303  44.157  1.00  0.00
A    C
ATOM   1743 HG11 ILE A 116      31.943   2.420  43.825  1.00  0.00
A    H
ATOM   1744 HG12 ILE A 116      33.647   2.553  43.285  1.00  0.00
A    H
ATOM   1745  CD  ILE A 116      33.231   0.879  44.497  1.00  0.00
A    C
ATOM   1746  HD1 ILE A 116      33.050   0.236  43.608  1.00  0.00
A    H
ATOM   1747  HD2 ILE A 116      34.278   0.722  44.832  1.00  0.00
A    H
ATOM   1748  HD3 ILE A 116      32.548   0.546  45.306  1.00  0.00
A    H
ATOM   1749  C   ILE A 116      35.730   3.737  44.944  1.00  0.00
A    C
ATOM   1750  O   ILE A 116      35.989   3.255  43.841  1.00  0.00
A    O
ATOM   1751  N   GLY A 117      36.332   4.828  45.394  1.00  0.00
A    N
ATOM   1752  HN  GLY A 117      36.285   5.148  46.339  1.00  0.00
A    H
ATOM   1753  CA  GLY A 117      37.118   5.641  44.497  1.00  0.00
A    C
ATOM   1754  HA1 GLY A 117      37.365   5.024  43.645  1.00  0.00
A    H
```

```
ATOM   1755  HA2 GLY A 117      37.976   5.993  45.054  1.00  0.00
A    H
ATOM   1756  C   GLY A 117      36.365   6.843  43.999  1.00  0.00
A    C
ATOM   1757  O   GLY A 117      36.461   7.183  42.817  1.00  0.00
A    O
ATOM   1758  N   ALA A 118      35.599   7.490  44.868  1.00  0.00
A    N
ATOM   1759  HN  ALA A 118      35.456   7.197  45.813  1.00  0.00
A    H
ATOM   1760  CA  ALA A 118      34.901   8.700  44.478  1.00  0.00
A    C
ATOM   1761  HA  ALA A 118      34.276   8.527  43.608  1.00  0.00
A    H
ATOM   1762  CB  ALA A 118      34.031   9.203  45.620  1.00  0.00
A    C
ATOM   1763  HB1 ALA A 118      33.270   8.435  45.875  1.00  0.00
A    H
ATOM   1764  HB2 ALA A 118      34.639   9.396  46.530  1.00  0.00
A    H
ATOM   1765  HB3 ALA A 118      33.496  10.138  45.346  1.00  0.00
A    H
ATOM   1766  C   ALA A 118      35.896   9.771  44.082  1.00  0.00
A    C
ATOM   1767  O   ALA A 118      36.607  10.320  44.926  1.00  0.00
A    O
ATOM   1768  N   ASP A 119      35.949  10.066  42.791  1.00  0.00
A    N
ATOM   1769  HN  ASP A 119      35.445   9.638  42.045  1.00  0.00
A    H
ATOM   1770  CA  ASP A 119      36.826  11.131  42.343  1.00  0.00
A    C
ATOM   1771  HA  ASP A 119      37.821  10.894  42.704  1.00  0.00
A    H
ATOM   1772  CB  ASP A 119      36.806  11.219  40.820  1.00  0.00
A    C
ATOM   1773  HB1 ASP A 119      35.755  11.278  40.472  1.00  0.00
A    H
ATOM   1774  HB2 ASP A 119      37.338  12.127  40.474  1.00  0.00
A    H
ATOM   1775  CG  ASP A 119      37.435  10.010  40.168  1.00  0.00
A    C
ATOM   1776  OD1 ASP A 119      38.633  10.082  39.819  1.00  0.00
A    O
ATOM   1777  OD2 ASP A 119      36.740   8.982  40.023  1.00  0.00
A    O
ATOM   1778  C   ASP A 119      36.439  12.459  42.962  1.00  0.00
A    C
ATOM   1779  O   ASP A 119      37.291  13.340  43.110  1.00  0.00
A    O
ATOM   1780  N   ARG A 120      35.174  12.620  43.329  1.00  0.00
A    N
ATOM   1781  HN  ARG A 120      34.476  11.924  43.180  1.00  0.00
A    H
```

```
ATOM   1782  CA  ARG A 120      34.744  13.845  43.980  1.00  0.00
A    C
ATOM   1783  HA  ARG A 120      35.398  14.044  44.821  1.00  0.00
A    H
ATOM   1784  CB  ARG A 120      34.770  15.014  43.005  1.00  0.00
A    C
ATOM   1785  HB1 ARG A 120      35.840  15.315  42.903  1.00  0.00
A    H
ATOM   1786  HB2 ARG A 120      34.453  14.665  41.997  1.00  0.00
A    H
ATOM   1787  CG  ARG A 120      33.974  16.162  43.481  1.00  0.00
A    C
ATOM   1788  HG1 ARG A 120      33.049  16.231  42.866  1.00  0.00
A    H
ATOM   1789  HG2 ARG A 120      33.627  15.957  44.520  1.00  0.00
A    H
ATOM   1790  CD  ARG A 120      34.799  17.393  43.462  1.00  0.00
A    C
ATOM   1791  HD1 ARG A 120      35.605  17.309  44.228  1.00  0.00
A    H
ATOM   1792  HD2 ARG A 120      35.270  17.554  42.465  1.00  0.00
A    H
ATOM   1793  NE  ARG A 120      33.976  18.553  43.744  1.00  0.00
A    N
ATOM   1794  HE  ARG A 120      33.006  18.325  43.885  1.00  0.00
A    H
ATOM   1795  CZ  ARG A 120      34.443  19.791  43.812  1.00  0.00
A    C
ATOM   1796  NH1 ARG A 120      35.730  20.021  43.608  1.00  0.00
A    N
ATOM   1797 HH11 ARG A 120      36.096  20.939  43.682  1.00  0.00
A    H
ATOM   1798 HH12 ARG A 120      36.317  19.213  43.518  1.00  0.00
A    H
ATOM   1799  NH2 ARG A 120      33.625  20.798  44.072  1.00  0.00
A    N
ATOM   1800 HH21 ARG A 120      33.960  21.730  44.036  1.00  0.00
A    H
ATOM   1801 HH22 ARG A 120      32.655  20.594  44.089  1.00  0.00
A    H
ATOM   1802  C   ARG A 120      33.346  13.672  44.549  1.00  0.00
A    C
ATOM   1803  O   ARG A 120      32.434  13.217  43.851  1.00  0.00
A    O
ATOM   1804  N   ILE A 121      33.182  14.069  45.805  1.00  0.00
A    N
ATOM   1805  HN  ILE A 121      33.924  14.474  46.336  1.00  0.00
A    H
ATOM   1806  CA  ILE A 121      31.924  13.958  46.528  1.00  0.00
A    C
ATOM   1807  HA  ILE A 121      31.210  13.474  45.874  1.00  0.00
A    H
ATOM   1808  CB  ILE A 121      32.121  13.189  47.843  1.00  0.00
A    C
```

```
ATOM   1809  HB  ILE A 121      32.735  13.876  48.479  1.00  0.00
A    H
ATOM   1810  CG2 ILE A 121      30.797  12.832  48.470  1.00  0.00
A    C
ATOM   1811 HG21 ILE A 121      30.947  12.418  49.488  1.00  0.00
A    H
ATOM   1812 HG22 ILE A 121      30.148  13.728  48.563  1.00  0.00
A    H
ATOM   1813 HG23 ILE A 121      30.262  12.072  47.862  1.00  0.00
A    H
ATOM   1814  CG1 ILE A 121      32.961  11.943  47.582  1.00  0.00
A    C
ATOM   1815 HG11 ILE A 121      32.532  11.371  46.729  1.00  0.00
A    H
ATOM   1816 HG12 ILE A 121      33.988  12.256  47.284  1.00  0.00
A    H
ATOM   1817  CD  ILE A 121      33.084  11.027  48.762  1.00  0.00
A    C
ATOM   1818  HD1 ILE A 121      33.753  10.173  48.521  1.00  0.00
A    H
ATOM   1819  HD2 ILE A 121      33.510  11.567  49.635  1.00  0.00
A    H
ATOM   1820  HD3 ILE A 121      32.094  10.615  49.052  1.00  0.00
A    H
ATOM   1821  C   ILE A 121      31.401  15.362  46.785  1.00  0.00
A    C
ATOM   1822  O   ILE A 121      32.188  16.309  46.892  1.00  0.00
A    O
ATOM   1823  N   ILE A 122      30.083  15.511  46.865  1.00  0.00
A    N
ATOM   1824  HN  ILE A 122      29.424  14.775  46.720  1.00  0.00
A    H
ATOM   1825  CA  ILE A 122      29.480  16.797  47.180  1.00  0.00
A    C
ATOM   1826  HA  ILE A 122      30.238  17.391  47.675  1.00  0.00
A    H
ATOM   1827  CB  ILE A 122      28.952  17.512  45.923  1.00  0.00
A    C
ATOM   1828  HB  ILE A 122      28.341  16.736  45.396  1.00  0.00
A    H
ATOM   1829  CG2 ILE A 122      28.169  18.754  46.303  1.00  0.00
A    C
ATOM   1830 HG21 ILE A 122      27.658  19.181  45.415  1.00  0.00
A    H
ATOM   1831 HG22 ILE A 122      27.390  18.516  47.057  1.00  0.00
A    H
ATOM   1832 HG23 ILE A 122      28.838  19.533  46.726  1.00  0.00
A    H
ATOM   1833  CG1 ILE A 122      30.104  17.873  44.993  1.00  0.00
A    C
ATOM   1834 HG11 ILE A 122      29.878  18.827  44.465  1.00  0.00
A    H
ATOM   1835 HG12 ILE A 122      31.020  18.044  45.604  1.00  0.00
A    H
```

ATOM 1836 CD ILE A 122 30.419 16.812 43.970 1.00 0.00
A C
ATOM 1837 HD1 ILE A 122 31.302 17.111 43.365 1.00 0.00
A H
ATOM 1838 HD2 ILE A 122 30.648 15.845 44.466 1.00 0.00
A H
ATOM 1839 HD3 ILE A 122 29.563 16.660 43.279 1.00 0.00
A H
ATOM 1840 C ILE A 122 28.357 16.597 48.179 1.00 0.00
A C
ATOM 1841 O ILE A 122 27.521 15.703 48.020 1.00 0.00
A O
ATOM 1842 N HSE A 123 28.329 17.446 49.192 1.00 0.00
A N
ATOM 1843 HN HSE A 123 29.060 18.114 49.326 1.00 0.00
A H
ATOM 1844 CA HSE A 123 27.273 17.505 50.187 1.00 0.00
A C
ATOM 1845 HA HSE A 123 26.568 16.706 49.992 1.00 0.00
A H
ATOM 1846 CB HSE A 123 27.855 17.440 51.593 1.00 0.00
A C
ATOM 1847 HB1 HSE A 123 28.572 18.281 51.706 1.00 0.00
A H
ATOM 1848 HB2 HSE A 123 27.046 17.601 52.337 1.00 0.00
A H
ATOM 1849 ND1 HSE A 123 28.083 15.206 52.737 1.00 0.00
A N
ATOM 1850 CG HSE A 123 28.586 16.172 51.893 1.00 0.00
A C
ATOM 1851 CE1 HSE A 123 28.946 14.211 52.827 1.00 0.00
A C
ATOM 1852 HE1 HSE A 123 28.802 13.319 53.442 1.00 0.00
A H
ATOM 1853 NE2 HSE A 123 29.986 14.490 52.063 1.00 0.00
A N
ATOM 1854 HE2 HSE A 123 30.793 13.911 51.955 1.00 0.00
A H
ATOM 1855 CD2 HSE A 123 29.787 15.713 51.469 1.00 0.00
A C
ATOM 1856 HD2 HSE A 123 30.507 16.168 50.804 1.00 0.00
A H
ATOM 1857 C HSE A 123 26.532 18.815 49.980 1.00 0.00
A C
ATOM 1858 O HSE A 123 26.685 19.749 50.776 1.00 0.00
A O
ATOM 1859 N PRO A 124 25.729 18.918 48.923 1.00 0.00
A N
ATOM 1860 CD PRO A 124 25.133 17.830 48.139 1.00 0.00
A C
ATOM 1861 HD1 PRO A 124 26.242 17.903 48.196 1.00 0.00
A H
ATOM 1862 HD2 PRO A 124 24.813 16.762 48.121 1.00 0.00
A H

ATOM 1863 CA PRO A 124 25.268 20.241 48.477 1.00 0.00
A C
ATOM 1864 HA PRO A 124 26.114 20.800 48.093 1.00 0.00
A H
ATOM 1865 CB PRO A 124 24.299 19.899 47.338 1.00 0.00
A C
ATOM 1866 HB1 PRO A 124 24.878 19.772 46.395 1.00 0.00
A H
ATOM 1867 HB2 PRO A 124 23.527 20.680 47.184 1.00 0.00
A H
ATOM 1868 CG PRO A 124 24.638 18.529 46.925 1.00 0.00
A C
ATOM 1869 HG1 PRO A 124 24.325 18.300 45.887 1.00 0.00
A H
ATOM 1870 HG2 PRO A 124 24.157 17.806 47.624 1.00 0.00
A H
ATOM 1871 C PRO A 124 24.582 21.055 49.550 1.00 0.00
A C
ATOM 1872 O PRO A 124 25.030 22.166 49.874 1.00 0.00
A O
ATOM 1873 N GLU A 125 23.501 20.531 50.113 1.00 0.00
A N
ATOM 1874 HN GLU A 125 23.118 19.628 49.939 1.00 0.00
A H
ATOM 1875 CA GLU A 125 22.762 21.308 51.090 1.00 0.00
A C
ATOM 1876 HA GLU A 125 22.530 22.270 50.652 1.00 0.00
A H
ATOM 1877 CB GLU A 125 21.465 20.617 51.464 1.00 0.00
A C
ATOM 1878 HB1 GLU A 125 21.678 19.532 51.609 1.00 0.00
A H
ATOM 1879 HB2 GLU A 125 21.109 20.991 52.450 1.00 0.00
A H
ATOM 1880 CG GLU A 125 20.384 20.782 50.415 1.00 0.00
A C
ATOM 1881 HG1 GLU A 125 19.417 20.988 50.910 1.00 0.00
A H
ATOM 1882 HG2 GLU A 125 20.624 21.616 49.725 1.00 0.00
A H
ATOM 1883 CD GLU A 125 20.149 19.550 49.589 1.00 0.00
A C
ATOM 1884 OE1 GLU A 125 20.945 18.592 49.720 1.00 0.00
A O
ATOM 1885 OE2 GLU A 125 19.156 19.547 48.834 1.00 0.00
A O
ATOM 1886 C GLU A 125 23.597 21.565 52.321 1.00 0.00
A C
ATOM 1887 O GLU A 125 23.433 22.595 52.965 1.00 0.00
A O
ATOM 1888 N LYS A 126 24.514 20.659 52.652 1.00 0.00
A N
ATOM 1889 HN LYS A 126 24.615 19.753 52.244 1.00 0.00
A H

ATOM 1890 CA LYS A 126 25.475 20.975 53.699 1.00 0.00
A C
ATOM 1891 HA LYS A 126 24.917 21.136 54.613 1.00 0.00
A H
ATOM 1892 CB LYS A 126 26.469 19.827 53.861 1.00 0.00
A C
ATOM 1893 HB1 LYS A 126 27.072 19.782 52.925 1.00 0.00
A H
ATOM 1894 HB2 LYS A 126 27.180 20.077 54.680 1.00 0.00
A H
ATOM 1895 CG LYS A 126 25.847 18.480 54.100 1.00 0.00
A C
ATOM 1896 HG1 LYS A 126 25.239 18.197 53.211 1.00 0.00
A H
ATOM 1897 HG2 LYS A 126 26.655 17.721 54.205 1.00 0.00
A H
ATOM 1898 CD LYS A 126 24.967 18.473 55.312 1.00 0.00
A C
ATOM 1899 HD1 LYS A 126 24.513 19.484 55.424 1.00 0.00
A H
ATOM 1900 HD2 LYS A 126 24.136 17.751 55.133 1.00 0.00
A H
ATOM 1901 CE LYS A 126 25.729 18.091 56.567 1.00 0.00
A C
ATOM 1902 HE1 LYS A 126 26.542 17.370 56.326 1.00 0.00
A H
ATOM 1903 HE2 LYS A 126 26.185 18.990 57.037 1.00 0.00
A H
ATOM 1904 NZ LYS A 126 24.824 17.452 57.564 1.00 0.00
A N
ATOM 1905 HZ1 LYS A 126 25.359 17.195 58.418 1.00 0.00
A H
ATOM 1906 HZ2 LYS A 126 24.065 18.117 57.819 1.00 0.00
A H
ATOM 1907 HZ3 LYS A 126 24.403 16.596 57.149 1.00 0.00
A H
ATOM 1908 C LYS A 126 26.208 22.271 53.380 1.00 0.00
A C
ATOM 1909 O LYS A 126 26.228 23.215 54.187 1.00 0.00
A O
ATOM 1910 N ASP A 127 26.782 22.344 52.183 1.00 0.00
A N
ATOM 1911 HN ASP A 127 26.703 21.669 51.455 1.00 0.00
A H
ATOM 1912 CA ASP A 127 27.620 23.476 51.818 1.00 0.00
A C
ATOM 1913 HA ASP A 127 28.373 23.580 52.590 1.00 0.00
A H
ATOM 1914 CB ASP A 127 28.236 23.216 50.441 1.00 0.00
A C
ATOM 1915 HB1 ASP A 127 27.476 22.749 49.783 1.00 0.00
A H
ATOM 1916 HB2 ASP A 127 28.565 24.166 49.975 1.00 0.00
A H

```
ATOM   1917  CG  ASP A 127       29.422  22.275  50.501  1.00  0.00
A    C
ATOM   1918  OD1 ASP A 127       29.191  21.065  50.726  1.00  0.00
A    O
ATOM   1919  OD2 ASP A 127       30.566  22.739  50.308  1.00  0.00
A    O
ATOM   1920  C   ASP A 127       26.811  24.763  51.816  1.00  0.00
A    C
ATOM   1921  O   ASP A 127       27.168  25.752  52.474  1.00  0.00
A    O
ATOM   1922  N   MET A 128       25.697  24.769  51.098  1.00  0.00
A    N
ATOM   1923  HN  MET A 128       25.270  24.022  50.592  1.00  0.00
A    H
ATOM   1924  CA  MET A 128       24.982  26.027  51.024  1.00  0.00
A    C
ATOM   1925  HA  MET A 128       25.721  26.810  50.916  1.00  0.00
A    H
ATOM   1926  CB  MET A 128       24.083  26.066  49.793  1.00  0.00
A    C
ATOM   1927  HB1 MET A 128       24.665  26.563  48.982  1.00  0.00
A    H
ATOM   1928  HB2 MET A 128       23.888  25.027  49.445  1.00  0.00
A    H
ATOM   1929  CG  MET A 128       22.782  26.809  49.974  1.00  0.00
A    C
ATOM   1930  HG1 MET A 128       21.961  26.098  50.207  1.00  0.00
A    H
ATOM   1931  HG2 MET A 128       22.870  27.513  50.829  1.00  0.00
A    H
ATOM   1932  SD  MET A 128       22.316  27.735  48.499  1.00  0.00
A    S
ATOM   1933  CE  MET A 128       23.721  27.462  47.413  1.00  0.00
A    C
ATOM   1934  HE1 MET A 128       23.583  27.991  46.446  1.00  0.00
A    H
ATOM   1935  HE2 MET A 128       24.661  27.834  47.874  1.00  0.00
A    H
ATOM   1936  HE3 MET A 128       23.853  26.380  47.194  1.00  0.00
A    H
ATOM   1937  C   MET A 128       24.217  26.293  52.309  1.00  0.00
A    C
ATOM   1938  O   MET A 128       23.854  27.436  52.584  1.00  0.00
A    O
ATOM   1939  N   GLY A 129       24.038  25.291  53.153  1.00  0.00
A    N
ATOM   1940  HN  GLY A 129       24.313  24.345  52.991  1.00  0.00
A    H
ATOM   1941  CA  GLY A 129       23.398  25.545  54.416  1.00  0.00
A    C
ATOM   1942  HA1 GLY A 129       23.193  24.595  54.891  1.00  0.00
A    H
ATOM   1943  HA2 GLY A 129       22.520  26.153  54.242  1.00  0.00
A    H
```

```
ATOM   1944  C   GLY A 129      24.329  26.319  55.306  1.00  0.00
A    C
ATOM   1945  O   GLY A 129      23.929  27.295  55.934  1.00  0.00
A    O
ATOM   1946  N   VAL A 130      25.578  25.876  55.390  1.00  0.00
A    N
ATOM   1947  HN  VAL A 130      25.966  25.064  54.963  1.00  0.00
A    H
ATOM   1948  CA  VAL A 130      26.522  26.642  56.193  1.00  0.00
A    C
ATOM   1949  HA  VAL A 130      26.089  26.775  57.176  1.00  0.00
A    H
ATOM   1950  CB  VAL A 130      27.833  25.856  56.388  1.00  0.00
A    C
ATOM   1951  HB  VAL A 130      28.450  26.409  57.138  1.00  0.00
A    H
ATOM   1952  CG1 VAL A 130      27.516  24.468  56.909  1.00  0.00
A    C
ATOM   1953 HG11 VAL A 130      28.449  23.874  57.018  1.00  0.00
A    H
ATOM   1954 HG12 VAL A 130      27.030  24.506  57.906  1.00  0.00
A    H
ATOM   1955 HG13 VAL A 130      26.845  23.928  56.207  1.00  0.00
A    H
ATOM   1956  CG2 VAL A 130      28.595  25.720  55.106  1.00  0.00
A    C
ATOM   1957 HG21 VAL A 130      29.515  25.117  55.259  1.00  0.00
A    H
ATOM   1958 HG22 VAL A 130      27.973  25.219  54.333  1.00  0.00
A    H
ATOM   1959 HG23 VAL A 130      28.905  26.711  54.716  1.00  0.00
A    H
ATOM   1960  C   VAL A 130      26.721  28.028  55.589  1.00  0.00
A    C
ATOM   1961  O   VAL A 130      26.821  29.028  56.317  1.00  0.00
A    O
ATOM   1962  N   LYS A 131      26.731  28.117  54.253  1.00  0.00
A    N
ATOM   1963  HN  LYS A 131      26.860  27.345  53.632  1.00  0.00
A    H
ATOM   1964  CA  LYS A 131      26.543  29.411  53.603  1.00  0.00
A    C
ATOM   1965  HA  LYS A 131      27.471  29.956  53.721  1.00  0.00
A    H
ATOM   1966  CB  LYS A 131      26.210  29.204  52.123  1.00  0.00
A    C
ATOM   1967  HB1 LYS A 131      26.895  28.412  51.741  1.00  0.00
A    H
ATOM   1968  HB2 LYS A 131      25.179  28.793  52.037  1.00  0.00
A    H
ATOM   1969  CG  LYS A 131      26.352  30.431  51.221  1.00  0.00
A    C
ATOM   1970  HG1 LYS A 131      27.339  30.911  51.410  1.00  0.00
A    H
```

ATOM 1971 HG2 LYS A 131 26.343 30.101 50.156 1.00 0.00
A H
ATOM 1972 CD LYS A 131 25.218 31.425 51.510 1.00 0.00
A C
ATOM 1973 HD1 LYS A 131 24.349 31.165 50.863 1.00 0.00
A H
ATOM 1974 HD2 LYS A 131 24.898 31.287 52.569 1.00 0.00
A H
ATOM 1975 CE LYS A 131 25.592 32.878 51.293 1.00 0.00
A C
ATOM 1976 HE1 LYS A 131 24.806 33.549 51.704 1.00 0.00
A H
ATOM 1977 HE2 LYS A 131 26.555 33.114 51.798 1.00 0.00
A H
ATOM 1978 NZ LYS A 131 25.737 33.146 49.805 1.00 0.00
A N
ATOM 1979 HZ1 LYS A 131 25.993 34.142 49.645 1.00 0.00
A H
ATOM 1980 HZ2 LYS A 131 26.479 32.531 49.413 1.00 0.00
A H
ATOM 1981 HZ3 LYS A 131 24.836 32.940 49.328 1.00 0.00
A H
ATOM 1982 C LYS A 131 25.450 30.229 54.265 1.00 0.00
A C
ATOM 1983 O LYS A 131 25.722 31.261 54.891 1.00 0.00
A O
ATOM 1984 N ILE A 132 24.211 29.790 54.117 1.00 0.00
A N
ATOM 1985 HN ILE A 132 23.948 28.887 53.784 1.00 0.00
A H
ATOM 1986 CA ILE A 132 23.089 30.649 54.451 1.00 0.00
A C
ATOM 1987 HA ILE A 132 23.256 31.594 53.949 1.00 0.00
A H
ATOM 1988 CB ILE A 132 21.776 30.013 53.978 1.00 0.00
A C
ATOM 1989 HB ILE A 132 21.888 29.932 52.867 1.00 0.00
A H
ATOM 1990 CG2 ILE A 132 21.561 28.694 54.659 1.00 0.00
A C
ATOM 1991 HG21 ILE A 132 20.698 28.159 54.209 1.00 0.00
A H
ATOM 1992 HG22 ILE A 132 22.454 28.043 54.553 1.00 0.00
A H
ATOM 1993 HG23 ILE A 132 21.355 28.834 55.741 1.00 0.00
A H
ATOM 1994 CG1 ILE A 132 20.616 30.964 54.243 1.00 0.00
A C
ATOM 1995 HG11 ILE A 132 20.139 30.714 55.217 1.00 0.00
A H
ATOM 1996 HG12 ILE A 132 21.013 32.002 54.328 1.00 0.00
A H
ATOM 1997 CD ILE A 132 19.583 30.943 53.170 1.00 0.00
A C

```
ATOM   1998  HD1 ILE A 132      18.792  31.694  53.381  1.00  0.00
A    H
ATOM   1999  HD2 ILE A 132      20.037  31.185  52.185  1.00  0.00
A    H
ATOM   2000  HD3 ILE A 132      19.101  29.946  53.101  1.00  0.00
A    H
ATOM   2001  C   ILE A 132      23.085  30.939  55.939  1.00  0.00
A    C
ATOM   2002  O   ILE A 132      22.659  32.011  56.383  1.00  0.00
A    O
ATOM   2003  N   ALA A 133      23.611  30.007  56.723  1.00  0.00
A    N
ATOM   2004  HN  ALA A 133      23.970  29.134  56.396  1.00  0.00
A    H
ATOM   2005  CA  ALA A 133      23.697  30.218  58.153  1.00  0.00
A    C
ATOM   2006  HA  ALA A 133      22.727  30.472  58.565  1.00  0.00
A    H
ATOM   2007  CB  ALA A 133      24.190  28.947  58.830  1.00  0.00
A    C
ATOM   2008  HB1 ALA A 133      23.480  28.118  58.629  1.00  0.00
A    H
ATOM   2009  HB2 ALA A 133      25.187  28.647  58.439  1.00  0.00
A    H
ATOM   2010  HB3 ALA A 133      24.267  29.074  59.931  1.00  0.00
A    H
ATOM   2011  C   ALA A 133      24.612  31.384  58.467  1.00  0.00
A    C
ATOM   2012  O   ALA A 133      24.238  32.293  59.216  1.00  0.00
A    O
ATOM   2013  N   GLN A 134      25.815  31.384  57.893  1.00  0.00
A    N
ATOM   2014  HN  GLN A 134      26.194  30.672  57.303  1.00  0.00
A    H
ATOM   2015  CA  GLN A 134      26.703  32.514  58.119  1.00  0.00
A    C
ATOM   2016  HA  GLN A 134      26.823  32.618  59.190  1.00  0.00
A    H
ATOM   2017  CB  GLN A 134      28.053  32.263  57.463  1.00  0.00
A    C
ATOM   2018  HB1 GLN A 134      27.885  32.284  56.362  1.00  0.00
A    H
ATOM   2019  HB2 GLN A 134      28.736  33.113  57.689  1.00  0.00
A    H
ATOM   2020  CG  GLN A 134      28.652  30.943  57.873  1.00  0.00
A    C
ATOM   2021  HG1 GLN A 134      27.855  30.238  58.196  1.00  0.00
A    H
ATOM   2022  HG2 GLN A 134      29.194  30.477  57.025  1.00  0.00
A    H
ATOM   2023  CD  GLN A 134      29.600  31.078  59.042  1.00  0.00
A    C
ATOM   2024  OE1 GLN A 134      29.798  32.171  59.570  1.00  0.00
A    O
```

```
ATOM   2025  NE2 GLN A 134      30.184  29.962  59.461  1.00  0.00
A    N
ATOM   2026 HE21 GLN A 134      30.808  30.043  60.233  1.00  0.00
A    H
ATOM   2027 HE22 GLN A 134      30.002  29.103  58.995  1.00  0.00
A    H
ATOM   2028  C   GLN A 134      26.080  33.798  57.601  1.00  0.00
A    C
ATOM   2029  O   GLN A 134      26.173  34.847  58.246  1.00  0.00
A    O
ATOM   2030  N   SER A 135      25.411  33.730  56.450  1.00  0.00
A    N
ATOM   2031  HN  SER A 135      25.229  32.902  55.919  1.00  0.00
A    H
ATOM   2032  CA  SER A 135      24.869  34.949  55.859  1.00  0.00
A    C
ATOM   2033  HA  SER A 135      25.662  35.683  55.920  1.00  0.00
A    H
ATOM   2034  CB  SER A 135      24.454  34.707  54.411  1.00  0.00
A    C
ATOM   2035  HB1 SER A 135      23.935  35.621  54.042  1.00  0.00
A    H
ATOM   2036  HB2 SER A 135      25.352  34.560  53.770  1.00  0.00
A    H
ATOM   2037  OG  SER A 135      23.572  33.611  54.318  1.00  0.00
A    O
ATOM   2038  HG1 SER A 135      23.173  33.641  53.439  1.00  0.00
A    H
ATOM   2039  C   SER A 135      23.691  35.489  56.656  1.00  0.00
A    C
ATOM   2040  O   SER A 135      23.378  36.680  56.567  1.00  0.00
A    O
ATOM   2041  N   LEU A 136      23.019  34.639  57.428  1.00  0.00
A    N
ATOM   2042  HN  LEU A 136      23.196  33.662  57.522  1.00  0.00
A    H
ATOM   2043  CA  LEU A 136      21.907  35.127  58.235  1.00  0.00
A    C
ATOM   2044  HA  LEU A 136      21.499  35.984  57.714  1.00  0.00
A    H
ATOM   2045  CB  LEU A 136      20.834  34.052  58.379  1.00  0.00
A    C
ATOM   2046  HB1 LEU A 136      21.303  33.081  58.100  1.00  0.00
A    H
ATOM   2047  HB2 LEU A 136      20.554  33.962  59.451  1.00  0.00
A    H
ATOM   2048  CG  LEU A 136      19.613  34.306  57.499  1.00  0.00
A    C
ATOM   2049  HG  LEU A 136      19.786  33.880  56.482  1.00  0.00
A    H
ATOM   2050  CD1 LEU A 136      18.383  33.697  58.118  1.00  0.00
A    C
ATOM   2051 HD11 LEU A 136      17.486  33.922  57.503  1.00  0.00
A    H
```

ATOM 2052 HD12 LEU A 136 18.487 32.594 58.193 1.00 0.00
A H
ATOM 2053 HD13 LEU A 136 18.215 34.104 59.139 1.00 0.00
A H
ATOM 2054 CD2 LEU A 136 19.415 35.797 57.355 1.00 0.00
A C
ATOM 2055 HD21 LEU A 136 18.540 36.009 56.701 1.00 0.00
A H
ATOM 2056 HD22 LEU A 136 19.229 36.283 58.334 1.00 0.00
A H
ATOM 2057 HD23 LEU A 136 20.312 36.267 56.897 1.00 0.00
A H
ATOM 2058 C LEU A 136 22.380 35.601 59.597 1.00 0.00
A C
ATOM 2059 O LEU A 136 21.916 36.633 60.095 1.00 0.00
A O
ATOM 2060 N SER A 137 23.297 34.858 60.216 1.00 0.00
A N
ATOM 2061 HN SER A 137 23.698 34.017 59.853 1.00 0.00
A H
ATOM 2062 CA SER A 137 23.802 35.241 61.531 1.00 0.00
A C
ATOM 2063 HA SER A 137 22.961 35.175 62.207 1.00 0.00
A H
ATOM 2064 CB SER A 137 24.941 34.310 61.932 1.00 0.00
A C
ATOM 2065 HB1 SER A 137 25.259 34.578 62.966 1.00 0.00
A H
ATOM 2066 HB2 SER A 137 24.587 33.254 61.962 1.00 0.00
A H
ATOM 2067 OG SER A 137 26.036 34.470 61.049 1.00 0.00
A O
ATOM 2068 HG1 SER A 137 26.798 34.043 61.461 1.00 0.00
A H
ATOM 2069 C SER A 137 24.273 36.688 61.535 1.00 0.00
A C
ATOM 2070 O SER A 137 23.713 37.536 62.237 1.00 0.00
A O
ATOM 2071 N ASP A 138 25.282 36.993 60.733 1.00 0.00
A N
ATOM 2072 HN ASP A 138 25.761 36.339 60.154 1.00 0.00
A H
ATOM 2073 CA ASP A 138 25.806 38.348 60.613 1.00 0.00
A C
ATOM 2074 HA ASP A 138 25.494 38.925 61.477 1.00 0.00
A H
ATOM 2075 CB ASP A 138 27.321 38.326 60.464 1.00 0.00
A C
ATOM 2076 HB1 ASP A 138 27.603 37.558 59.715 1.00 0.00
A H
ATOM 2077 HB2 ASP A 138 27.693 39.309 60.114 1.00 0.00
A H
ATOM 2078 CG ASP A 138 28.030 37.978 61.755 1.00 0.00
A C

```
ATOM   2079  OD1 ASP A 138      28.196  38.872  62.611  1.00  0.00
A    O
ATOM   2080  OD2 ASP A 138      28.416  36.803  61.914  1.00  0.00
A    O
ATOM   2081  C   ASP A 138      25.125  38.970  59.401  1.00  0.00
A    C
ATOM   2082  O   ASP A 138      25.491  38.688  58.258  1.00  0.00
A    O
ATOM   2083  N   GLU A 139      24.134  39.821  59.651  1.00  0.00
A    N
ATOM   2084  HN  GLU A 139      23.889  40.185  60.545  1.00  0.00
A    H
ATOM   2085  CA  GLU A 139      23.293  40.304  58.565  1.00  0.00
A    C
ATOM   2086  HA  GLU A 139      23.065  39.453  57.936  1.00  0.00
A    H
ATOM   2087  CB  GLU A 139      21.993  40.866  59.136  1.00  0.00
A    C
ATOM   2088  HB1 GLU A 139      21.449  41.386  58.314  1.00  0.00
A    H
ATOM   2089  HB2 GLU A 139      21.331  40.032  59.460  1.00  0.00
A    H
ATOM   2090  CG  GLU A 139      22.196  41.833  60.276  1.00  0.00
A    C
ATOM   2091  HG1 GLU A 139      22.876  41.385  61.024  1.00  0.00
A    H
ATOM   2092  HG2 GLU A 139      22.629  42.788  59.916  1.00  0.00
A    H
ATOM   2093  CD  GLU A 139      20.914  42.132  61.012  1.00  0.00
A    C
ATOM   2094  OE1 GLU A 139      20.958  42.276  62.250  1.00  0.00
A    O
ATOM   2095  OE2 GLU A 139      19.858  42.211  60.355  1.00  0.00
A    O
ATOM   2096  C   GLU A 139      23.977  41.346  57.687  1.00  0.00
A    C
ATOM   2097  O   GLU A 139      23.295  41.988  56.883  1.00  0.00
A    O
ATOM   2098  N   ASN A 140      25.293  41.533  57.811  1.00  0.00
A    N
ATOM   2099  HN  ASN A 140      25.884  41.128  58.506  1.00  0.00
A    H
ATOM   2100  CA  ASN A 140      25.981  42.403  56.865  1.00  0.00
A    C
ATOM   2101  HA  ASN A 140      25.202  42.968  56.365  1.00  0.00
A    H
ATOM   2102  CB  ASN A 140      26.951  43.343  57.581  1.00  0.00
A    C
ATOM   2103  HB1 ASN A 140      26.667  43.370  58.656  1.00  0.00
A    H
ATOM   2104  HB2 ASN A 140      27.985  42.949  57.521  1.00  0.00
A    H
ATOM   2105  CG  ASN A 140      26.890  44.762  57.037  1.00  0.00
A    C
```

```
ATOM   2106  OD1 ASN A 140      27.860  45.516  57.126  1.00  0.00
A    O
ATOM   2107  ND2 ASN A 140      25.745  45.130  56.466  1.00  0.00
A    N
ATOM   2108 HD21 ASN A 140      25.694  46.053  56.094  1.00  0.00
A    H
ATOM   2109 HD22 ASN A 140      24.993  44.482  56.395  1.00  0.00
A    H
ATOM   2110  C   ASN A 140      26.723  41.605  55.804  1.00  0.00
A    C
ATOM   2111  O   ASN A 140      26.776  42.021  54.641  1.00  0.00
A    O
ATOM   2112  N   VAL A 141      27.312  40.469  56.171  1.00  0.00
A    N
ATOM   2113  HN  VAL A 141      27.382  40.108  57.097  1.00  0.00
A    H
ATOM   2114  CA  VAL A 141      27.931  39.634  55.149  1.00  0.00
A    C
ATOM   2115  HA  VAL A 141      28.573  40.245  54.527  1.00  0.00
A    H
ATOM   2116  CB  VAL A 141      28.763  38.496  55.756  1.00  0.00
A    C
ATOM   2117  HB  VAL A 141      29.840  38.730  55.572  1.00  0.00
A    H
ATOM   2118  CG1 VAL A 141      28.534  38.386  57.241  1.00  0.00
A    C
ATOM   2119 HG11 VAL A 141      29.112  37.534  57.659  1.00  0.00
A    H
ATOM   2120 HG12 VAL A 141      28.863  39.301  57.776  1.00  0.00
A    H
ATOM   2121 HG13 VAL A 141      27.459  38.212  57.461  1.00  0.00
A    H
ATOM   2122  CG2 VAL A 141      28.546  37.164  55.027  1.00  0.00
A    C
ATOM   2123 HG21 VAL A 141      29.127  36.352  55.511  1.00  0.00
A    H
ATOM   2124 HG22 VAL A 141      27.471  36.881  55.042  1.00  0.00
A    H
ATOM   2125 HG23 VAL A 141      28.875  37.227  53.969  1.00  0.00
A    H
ATOM   2126  C   VAL A 141      26.803  39.111  54.268  1.00  0.00
A    C
ATOM   2127  O   VAL A 141      25.898  38.420  54.742  1.00  0.00
A    O
ATOM   2128  N   LEU A 142      26.796  39.529  53.007  1.00  0.00
A    N
ATOM   2129  HN  LEU A 142      27.440  40.174  52.601  1.00  0.00
A    H
ATOM   2130  CA  LEU A 142      25.782  39.036  52.087  1.00  0.00
A    C
ATOM   2131  HA  LEU A 142      24.903  38.808  52.676  1.00  0.00
A    H
ATOM   2132  CB  LEU A 142      25.450  40.095  51.032  1.00  0.00
A    C
```

```
ATOM   2133  HB1 LEU A 142      25.171  41.026  51.578  1.00  0.00
A    H
ATOM   2134  HB2 LEU A 142      26.374  40.338  50.460  1.00  0.00
A    H
ATOM   2135  CG  LEU A 142      24.311  39.694  50.098  1.00  0.00
A    C
ATOM   2136  HG  LEU A 142      24.607  38.744  49.590  1.00  0.00
A    H
ATOM   2137  CD1 LEU A 142      23.046  39.472  50.898  1.00  0.00
A    C
ATOM   2138 HD11 LEU A 142      22.197  39.226  50.225  1.00  0.00
A    H
ATOM   2139 HD12 LEU A 142      23.179  38.633  51.614  1.00  0.00
A    H
ATOM   2140 HD13 LEU A 142      22.780  40.385  51.472  1.00  0.00
A    H
ATOM   2141  CD2 LEU A 142      24.086  40.747  49.028  1.00  0.00
A    C
ATOM   2142 HD21 LEU A 142      23.267  40.434  48.345  1.00  0.00
A    H
ATOM   2143 HD22 LEU A 142      23.801  41.724  49.468  1.00  0.00
A    H
ATOM   2144 HD23 LEU A 142      25.008  40.890  48.423  1.00  0.00
A    H
ATOM   2145  C   LEU A 142      26.243  37.743  51.432  1.00  0.00
A    C
ATOM   2146  O   LEU A 142      25.588  36.705  51.557  1.00  0.00
A    O
ATOM   2147  N   ASN A 143      27.377  37.788  50.741  1.00  0.00
A    N
ATOM   2148  HN  ASN A 143      27.854  38.633  50.503  1.00  0.00
A    H
ATOM   2149  CA  ASN A 143      28.025  36.577  50.266  1.00  0.00
A    C
ATOM   2150  HA  ASN A 143      27.484  35.734  50.681  1.00  0.00
A    H
ATOM   2151  CB  ASN A 143      28.085  36.511  48.737  1.00  0.00
A    C
ATOM   2152  HB1 ASN A 143      28.726  37.348  48.384  1.00  0.00
A    H
ATOM   2153  HB2 ASN A 143      28.563  35.565  48.410  1.00  0.00
A    H
ATOM   2154  CG  ASN A 143      26.726  36.658  48.087  1.00  0.00
A    C
ATOM   2155  OD1 ASN A 143      25.861  37.372  48.585  1.00  0.00
A    O
ATOM   2156  ND2 ASN A 143      26.526  35.968  46.972  1.00  0.00
A    N
ATOM   2157 HD21 ASN A 143      25.640  36.064  46.526  1.00  0.00
A    H
ATOM   2158 HD22 ASN A 143      27.257  35.407  46.595  1.00  0.00
A    H
ATOM   2159  C   ASN A 143      29.428  36.554  50.847  1.00  0.00
A    C
```

```
ATOM   2160  O   ASN A 143      29.988  37.599  51.185  1.00  0.00
A    O
ATOM   2161  N   TYR A 144      29.999  35.360  50.954  1.00  0.00
A    N
ATOM   2162  HN  TYR A 144      29.602  34.490  50.665  1.00  0.00
A    H
ATOM   2163  CA  TYR A 144      31.327  35.254  51.537  1.00  0.00
A    C
ATOM   2164  HA  TYR A 144      31.883  36.136  51.242  1.00  0.00
A    H
ATOM   2165  CB  TYR A 144      31.260  35.136  53.061  1.00  0.00
A    C
ATOM   2166  HB1 TYR A 144      32.277  35.292  53.483  1.00  0.00
A    H
ATOM   2167  HB2 TYR A 144      30.603  35.924  53.487  1.00  0.00
A    H
ATOM   2168  CG  TYR A 144      30.796  33.794  53.565  1.00  0.00
A    C
ATOM   2169  CD1 TYR A 144      29.479  33.394  53.425  1.00  0.00
A    C
ATOM   2170  HD1 TYR A 144      28.762  34.057  52.958  1.00  0.00
A    H
ATOM   2171  CE1 TYR A 144      29.052  32.168  53.889  1.00  0.00
A    C
ATOM   2172  HE1 TYR A 144      28.021  31.867  53.780  1.00  0.00
A    H
ATOM   2173  CZ  TYR A 144      29.950  31.322  54.505  1.00  0.00
A    C
ATOM   2174  OH  TYR A 144      29.517  30.096  54.964  1.00  0.00
A    O
ATOM   2175  HH  TYR A 144      30.285  29.624  55.292  1.00  0.00
A    H
ATOM   2176  CD2 TYR A 144      31.682  32.928  54.188  1.00  0.00
A    C
ATOM   2177  HD2 TYR A 144      32.708  33.228  54.349  1.00  0.00
A    H
ATOM   2178  CE2 TYR A 144      31.270  31.700  54.657  1.00  0.00
A    C
ATOM   2179  HE2 TYR A 144      31.976  31.052  55.156  1.00  0.00
A    H
ATOM   2180  C   TYR A 144      32.038  34.056  50.938  1.00  0.00
A    C
ATOM   2181  O   TYR A 144      31.423  33.038  50.614  1.00  0.00
A    O
ATOM   2182  N   ILE A 145      33.354  34.192  50.802  1.00  0.00
A    N
ATOM   2183  HN  ILE A 145      33.836  35.039  51.021  1.00  0.00
A    H
ATOM   2184  CA  ILE A 145      34.218  33.122  50.323  1.00  0.00
A    C
ATOM   2185  HA  ILE A 145      33.713  32.183  50.516  1.00  0.00
A    H
ATOM   2186  CB  ILE A 145      34.546  33.261  48.824  1.00  0.00
A    C
```

```
ATOM   2187  HB  ILE A 145      33.959  34.155  48.491  1.00  0.00
A    H
ATOM   2188  CG2 ILE A 145      36.048  33.435  48.590  1.00  0.00
A    C
ATOM   2189 HG21 ILE A 145      36.251  33.689  47.529  1.00  0.00
A    H
ATOM   2190 HG22 ILE A 145      36.454  34.258  49.215  1.00  0.00
A    H
ATOM   2191 HG23 ILE A 145      36.600  32.503  48.835  1.00  0.00
A    H
ATOM   2192  CG1 ILE A 145      33.993  32.049  48.084  1.00  0.00
A    C
ATOM   2193 HG11 ILE A 145      33.371  31.437  48.776  1.00  0.00
A    H
ATOM   2194 HG12 ILE A 145      33.325  32.401  47.265  1.00  0.00
A    H
ATOM   2195  CD  ILE A 145      35.053  31.159  47.468  1.00  0.00
A    C
ATOM   2196  HD1 ILE A 145      34.577  30.331  46.898  1.00  0.00
A    H
ATOM   2197  HD2 ILE A 145      35.694  31.738  46.770  1.00  0.00
A    H
ATOM   2198  HD3 ILE A 145      35.699  30.710  48.251  1.00  0.00
A    H
ATOM   2199  C   ILE A 145      35.490  33.119  51.164  1.00  0.00
A    C
ATOM   2200  O   ILE A 145      36.014  34.183  51.512  1.00  0.00
A    O
ATOM   2201  N   ASP A 146      35.948  31.927  51.537  1.00  0.00
A    N
ATOM   2202  HN  ASP A 146      35.524  31.056  51.301  1.00  0.00
A    H
ATOM   2203  CA  ASP A 146      37.149  31.772  52.349  1.00  0.00
A    C
ATOM   2204  HA  ASP A 146      37.571  32.759  52.499  1.00  0.00
A    H
ATOM   2205  CB  ASP A 146      36.809  31.091  53.672  1.00  0.00
A    C
ATOM   2206  HB1 ASP A 146      37.721  30.606  54.077  1.00  0.00
A    H
ATOM   2207  HB2 ASP A 146      36.450  31.833  54.412  1.00  0.00
A    H
ATOM   2208  CG  ASP A 146      35.754  30.016  53.515  1.00  0.00
A    C
ATOM   2209  OD1 ASP A 146      36.111  28.914  53.042  1.00  0.00
A    O
ATOM   2210  OD2 ASP A 146      34.569  30.277  53.816  1.00  0.00
A    O
ATOM   2211  C   ASP A 146      38.189  30.962  51.591  1.00  0.00
A    C
ATOM   2212  O   ASP A 146      37.904  29.850  51.139  1.00  0.00
A    O
ATOM   2213  N   LEU A 147      39.390  31.515  51.462  1.00  0.00
A    N
```

```
ATOM   2214  HN  LEU A 147      39.625  32.436  51.765  1.00  0.00
A    H
ATOM   2215  CA  LEU A 147      40.492  30.788  50.846  1.00  0.00
A    C
ATOM   2216  HA  LEU A 147      40.056  30.199  50.050  1.00  0.00
A    H
ATOM   2217  CB  LEU A 147      41.501  31.784  50.283  1.00  0.00
A    C
ATOM   2218  HB1 LEU A 147      42.485  31.261  50.229  1.00  0.00
A    H
ATOM   2219  HB2 LEU A 147      41.224  32.025  49.233  1.00  0.00
A    H
ATOM   2220  CG  LEU A 147      41.592  33.021  51.171  1.00  0.00
A    C
ATOM   2221  HG  LEU A 147      40.623  33.080  51.722  1.00  0.00
A    H
ATOM   2222  CD1 LEU A 147      42.720  32.899  52.178  1.00  0.00
A    C
ATOM   2223 HD11 LEU A 147      42.804  33.825  52.784  1.00  0.00
A    H
ATOM   2224 HD12 LEU A 147      42.538  32.046  52.867  1.00  0.00
A    H
ATOM   2225 HD13 LEU A 147      43.690  32.733  51.662  1.00  0.00
A    H
ATOM   2226  CD2 LEU A 147      41.722  34.270  50.308  1.00  0.00
A    C
ATOM   2227 HD21 LEU A 147      41.770  35.178  50.947  1.00  0.00
A    H
ATOM   2228 HD22 LEU A 147      42.640  34.245  49.688  1.00  0.00
A    H
ATOM   2229 HD23 LEU A 147      40.845  34.367  49.631  1.00  0.00
A    H
ATOM   2230  C   LEU A 147      41.167  29.825  51.815  1.00  0.00
A    C
ATOM   2231  O   LEU A 147      41.757  28.831  51.379  1.00  0.00
A    O
ATOM   2232  N   SER A 148      41.082  30.087  53.112  1.00  0.00
A    N
ATOM   2233  HN  SER A 148      40.597  30.873  53.496  1.00  0.00
A    H
ATOM   2234  CA  SER A 148      41.705  29.226  54.106  1.00  0.00
A    C
ATOM   2235  HA  SER A 148      41.740  28.228  53.691  1.00  0.00
A    H
ATOM   2236  CB  SER A 148      43.120  29.733  54.430  1.00  0.00
A    C
ATOM   2237  HB1 SER A 148      43.573  29.042  55.177  1.00  0.00
A    H
ATOM   2238  HB2 SER A 148      43.762  29.699  53.520  1.00  0.00
A    H
ATOM   2239  OG  SER A 148      43.080  31.041  54.982  1.00  0.00
A    O
ATOM   2240  HG1 SER A 148      43.949  31.214  55.365  1.00  0.00
A    H
```

```
ATOM   2241  C   SER A 148      40.797  29.176  55.329  1.00  0.00
A    C
ATOM   2242  O   SER A 148      39.694  29.739  55.323  1.00  0.00
A    O
ATOM   2243  N   ASP A 149      41.257  28.504  56.385  1.00  0.00
A    N
ATOM   2244  HN  ASP A 149      42.107  27.986  56.431  1.00  0.00
A    H
ATOM   2245  CA  ASP A 149      40.504  28.473  57.633  1.00  0.00
A    C
ATOM   2246  HA  ASP A 149      39.454  28.512  57.366  1.00  0.00
A    H
ATOM   2247  CB  ASP A 149      40.824  27.195  58.404  1.00  0.00
A    C
ATOM   2248  HB1 ASP A 149      40.402  27.269  59.426  1.00  0.00
A    H
ATOM   2249  HB2 ASP A 149      40.375  26.315  57.905  1.00  0.00
A    H
ATOM   2250  CG  ASP A 149      42.312  26.963  58.544  1.00  0.00
A    C
ATOM   2251  OD1 ASP A 149      42.849  26.087  57.835  1.00  0.00
A    O
ATOM   2252  OD2 ASP A 149      42.948  27.668  59.351  1.00  0.00
A    O
ATOM   2253  C   ASP A 149      40.785  29.699  58.490  1.00  0.00
A    C
ATOM   2254  O   ASP A 149      40.255  29.815  59.601  1.00  0.00
A    O
ATOM   2255  N   GLU A 150      41.606  30.621  57.992  1.00  0.00
A    N
ATOM   2256  HN  GLU A 150      42.021  30.539  57.090  1.00  0.00
A    H
ATOM   2257  CA  GLU A 150      41.978  31.833  58.708  1.00  0.00
A    C
ATOM   2258  HA  GLU A 150      41.486  31.795  59.672  1.00  0.00
A    H
ATOM   2259  CB  GLU A 150      43.498  31.887  58.897  1.00  0.00
A    C
ATOM   2260  HB1 GLU A 150      43.814  30.963  59.434  1.00  0.00
A    H
ATOM   2261  HB2 GLU A 150      44.003  31.852  57.906  1.00  0.00
A    H
ATOM   2262  CG  GLU A 150      43.971  33.098  59.675  1.00  0.00
A    C
ATOM   2263  HG1 GLU A 150      44.718  33.652  59.078  1.00  0.00
A    H
ATOM   2264  HG2 GLU A 150      43.124  33.770  59.916  1.00  0.00
A    H
ATOM   2265  CD  GLU A 150      44.672  32.743  60.964  1.00  0.00
A    C
ATOM   2266  OE1 GLU A 150      44.441  31.632  61.480  1.00  0.00
A    O
ATOM   2267  OE2 GLU A 150      45.454  33.579  61.460  1.00  0.00
A    O
```

ATOM 2268 C GLU A 150 41.526 33.108 58.021 1.00 0.00
A C
ATOM 2269 O GLU A 150 41.169 34.074 58.698 1.00 0.00
A O
ATOM 2270 N TYR A 151 41.536 33.140 56.696 1.00 0.00
A N
ATOM 2271 HN TYR A 151 41.733 32.345 56.126 1.00 0.00
A H
ATOM 2272 CA TYR A 151 41.255 34.358 55.956 1.00 0.00
A C
ATOM 2273 HA TYR A 151 40.873 35.079 56.667 1.00 0.00
A H
ATOM 2274 CB TYR A 151 42.517 34.868 55.262 1.00 0.00
A C
ATOM 2275 HB1 TYR A 151 42.750 34.208 54.399 1.00 0.00
A H
ATOM 2276 HB2 TYR A 151 42.357 35.892 54.859 1.00 0.00
A H
ATOM 2277 CG TYR A 151 43.744 34.873 56.144 1.00 0.00
A C
ATOM 2278 CD1 TYR A 151 43.935 35.862 57.096 1.00 0.00
A C
ATOM 2279 HD1 TYR A 151 43.203 36.650 57.208 1.00 0.00
A H
ATOM 2280 CE1 TYR A 151 45.058 35.877 57.897 1.00 0.00
A C
ATOM 2281 HE1 TYR A 151 45.198 36.656 58.632 1.00 0.00
A H
ATOM 2282 CZ TYR A 151 46.009 34.893 57.750 1.00 0.00
A C
ATOM 2283 OH TYR A 151 47.128 34.899 58.546 1.00 0.00
A O
ATOM 2284 HH TYR A 151 47.628 34.104 58.346 1.00 0.00
A H
ATOM 2285 CD2 TYR A 151 44.716 33.892 56.015 1.00 0.00
A C
ATOM 2286 HD2 TYR A 151 44.617 33.129 55.256 1.00 0.00
A H
ATOM 2287 CE2 TYR A 151 45.842 33.898 56.812 1.00 0.00
A C
ATOM 2288 HE2 TYR A 151 46.595 33.136 56.681 1.00 0.00
A H
ATOM 2289 C TYR A 151 40.157 34.112 54.933 1.00 0.00
A C
ATOM 2290 O TYR A 151 39.916 32.971 54.526 1.00 0.00
A O
ATOM 2291 N SER A 152 39.497 35.190 54.518 1.00 0.00
A N
ATOM 2292 HN SER A 152 39.758 36.133 54.725 1.00 0.00
A H
ATOM 2293 CA SER A 152 38.300 35.069 53.701 1.00 0.00
A C
ATOM 2294 HA SER A 152 38.550 34.384 52.900 1.00 0.00
A H

```
ATOM   2295  CB  SER A 152      37.123  34.570  54.540  1.00  0.00
A    C
ATOM   2296  HB1 SER A 152      36.224  35.167  54.264  1.00  0.00
A    H
ATOM   2297  HB2 SER A 152      36.896  33.507  54.297  1.00  0.00
A    H
ATOM   2298  OG  SER A 152      37.397  34.747  55.921  1.00  0.00
A    O
ATOM   2299  HG1 SER A 152      36.561  34.634  56.392  1.00  0.00
A    H
ATOM   2300  C   SER A 152      37.939  36.402  53.060  1.00  0.00
A    C
ATOM   2301  O   SER A 152      38.419  37.465  53.471  1.00  0.00
A    O
ATOM   2302  N   ILE A 153      37.066  36.321  52.059  1.00  0.00
A    N
ATOM   2303  HN  ILE A 153      36.716  35.445  51.735  1.00  0.00
A    H
ATOM   2304  CA  ILE A 153      36.538  37.477  51.343  1.00  0.00
A    C
ATOM   2305  HA  ILE A 153      37.051  38.357  51.711  1.00  0.00
A    H
ATOM   2306  CB  ILE A 153      36.700  37.310  49.823  1.00  0.00
A    C
ATOM   2307  HB  ILE A 153      36.338  36.272  49.615  1.00  0.00
A    H
ATOM   2308  CG2 ILE A 153      35.931  38.383  49.070  1.00  0.00
A    C
ATOM   2309 HG21 ILE A 153      35.923  38.167  47.982  1.00  0.00
A    H
ATOM   2310 HG22 ILE A 153      34.876  38.428  49.412  1.00  0.00
A    H
ATOM   2311 HG23 ILE A 153      36.391  39.382  49.222  1.00  0.00
A    H
ATOM   2312  CG1 ILE A 153      38.177  37.348  49.431  1.00  0.00
A    C
ATOM   2313 HG11 ILE A 153      38.438  38.358  49.043  1.00  0.00
A    H
ATOM   2314 HG12 ILE A 153      38.797  37.176  50.341  1.00  0.00
A    H
ATOM   2315  CD  ILE A 153      38.559  36.302  48.391  1.00  0.00
A    C
ATOM   2316  HD1 ILE A 153      39.651  36.339  48.189  1.00  0.00
A    H
ATOM   2317  HD2 ILE A 153      38.306  35.281  48.751  1.00  0.00
A    H
ATOM   2318  HD3 ILE A 153      38.027  36.480  47.434  1.00  0.00
A    H
ATOM   2319  C   ILE A 153      35.071  37.631  51.723  1.00  0.00
A    C
ATOM   2320  O   ILE A 153      34.359  36.633  51.890  1.00  0.00
A    O
ATOM   2321  N   VAL A 154      34.612  38.874  51.872  1.00  0.00
A    N
```

```
ATOM   2322  HN  VAL A 154      35.170  39.680  51.694  1.00  0.00
A    H
ATOM   2323  CA  VAL A 154      33.251  39.168  52.310  1.00  0.00
A    C
ATOM   2324  HA  VAL A 154      32.629  38.316  52.064  1.00  0.00
A    H
ATOM   2325  CB  VAL A 154      33.185  39.435  53.828  1.00  0.00
A    C
ATOM   2326  HB  VAL A 154      33.799  40.345  54.038  1.00  0.00
A    H
ATOM   2327  CG1 VAL A 154      31.744  39.687  54.259  1.00  0.00
A    C
ATOM   2328 HG11 VAL A 154      31.691  39.841  55.358  1.00  0.00
A    H
ATOM   2329 HG12 VAL A 154      31.326  40.596  53.777  1.00  0.00
A    H
ATOM   2330 HG13 VAL A 154      31.099  38.820  54.002  1.00  0.00
A    H
ATOM   2331  CG2 VAL A 154      33.769  38.268  54.616  1.00  0.00
A    C
ATOM   2332 HG21 VAL A 154      33.682  38.449  55.709  1.00  0.00
A    H
ATOM   2333 HG22 VAL A 154      33.231  37.326  54.373  1.00  0.00
A    H
ATOM   2334 HG23 VAL A 154      34.845  38.126  54.385  1.00  0.00
A    H
ATOM   2335  C   VAL A 154      32.709  40.367  51.541  1.00  0.00
A    C
ATOM   2336  O   VAL A 154      33.431  41.341  51.293  1.00  0.00
A    O
ATOM   2337  N   GLU A 155      31.429  40.301  51.177  1.00  0.00
A    N
ATOM   2338  HN  GLU A 155      30.902  39.455  51.185  1.00  0.00
A    H
ATOM   2339  CA  GLU A 155      30.673  41.467  50.732  1.00  0.00
A    C
ATOM   2340  HA  GLU A 155      31.359  42.270  50.494  1.00  0.00
A    H
ATOM   2341  CB  GLU A 155      29.789  41.157  49.525  1.00  0.00
A    C
ATOM   2342  HB1 GLU A 155      29.373  40.131  49.654  1.00  0.00
A    H
ATOM   2343  HB2 GLU A 155      28.913  41.843  49.515  1.00  0.00
A    H
ATOM   2344  CG  GLU A 155      30.488  41.222  48.196  1.00  0.00
A    C
ATOM   2345  HG1 GLU A 155      30.102  42.082  47.618  1.00  0.00
A    H
ATOM   2346  HG2 GLU A 155      31.583  41.333  48.327  1.00  0.00
A    H
ATOM   2347  CD  GLU A 155      30.222  40.001  47.348  1.00  0.00
A    C
ATOM   2348  OE1 GLU A 155      29.618  40.147  46.263  1.00  0.00
A    O
```

```
ATOM   2349  OE2 GLU A 155      30.599  38.893  47.787  1.00  0.00
A    O
ATOM   2350  C   GLU A 155      29.801  41.942  51.883  1.00  0.00
A    C
ATOM   2351  O   GLU A 155      29.141  41.130  52.536  1.00  0.00
A    O
ATOM   2352  N   LEU A 156      29.792  43.247  52.130  1.00  0.00
A    N
ATOM   2353  HN  LEU A 156      30.392  43.909  51.684  1.00  0.00
A    H
ATOM   2354  CA  LEU A 156      28.875  43.829  53.102  1.00  0.00
A    C
ATOM   2355  HA  LEU A 156      28.111  43.089  53.306  1.00  0.00
A    H
ATOM   2356  CB  LEU A 156      29.591  44.209  54.410  1.00  0.00
A    C
ATOM   2357  HB1 LEU A 156      29.906  45.274  54.316  1.00  0.00
A    H
ATOM   2358  HB2 LEU A 156      28.858  44.178  55.246  1.00  0.00
A    H
ATOM   2359  CG  LEU A 156      30.827  43.379  54.775  1.00  0.00
A    C
ATOM   2360  HG  LEU A 156      30.775  42.471  54.127  1.00  0.00
A    H
ATOM   2361  CD1 LEU A 156      32.113  44.136  54.478  1.00  0.00
A    C
ATOM   2362 HD11 LEU A 156      32.997  43.541  54.792  1.00  0.00
A    H
ATOM   2363 HD12 LEU A 156      32.201  44.345  53.389  1.00  0.00
A    H
ATOM   2364 HD13 LEU A 156      32.134  45.103  55.023  1.00  0.00
A    H
ATOM   2365  CD2 LEU A 156      30.777  42.924  56.228  1.00  0.00
A    C
ATOM   2366 HD21 LEU A 156      31.670  42.309  56.471  1.00  0.00
A    H
ATOM   2367 HD22 LEU A 156      30.761  43.785  56.927  1.00  0.00
A    H
ATOM   2368 HD23 LEU A 156      29.870  42.308  56.411  1.00  0.00
A    H
ATOM   2369  C   LEU A 156      28.186  45.045  52.502  1.00  0.00
A    C
ATOM   2370  O   LEU A 156      28.840  45.920  51.934  1.00  0.00
A    O
ATOM   2371  N   LEU A 157      26.864  45.105  52.629  1.00  0.00
A    N
ATOM   2372  HN  LEU A 157      26.292  44.417  53.072  1.00  0.00
A    H
ATOM   2373  CA  LEU A 157      26.128  46.244  52.100  1.00  0.00
A    C
ATOM   2374  HA  LEU A 157      26.627  46.570  51.197  1.00  0.00
A    H
ATOM   2375  CB  LEU A 157      24.680  45.868  51.784  1.00  0.00
A    C
```

ATOM 2376 HB1 LEU A 157 24.600 45.768 50.677 1.00 0.00
A H
ATOM 2377 HB2 LEU A 157 24.470 44.857 52.200 1.00 0.00
A H
ATOM 2378 CG LEU A 157 23.537 46.789 52.211 1.00 0.00
A C
ATOM 2379 HG LEU A 157 23.949 47.798 52.455 1.00 0.00
A H
ATOM 2380 CD1 LEU A 157 22.534 46.904 51.080 1.00 0.00
A C
ATOM 2381 HD11 LEU A 157 21.671 47.532 51.387 1.00 0.00
A H
ATOM 2382 HD12 LEU A 157 23.003 47.369 50.186 1.00 0.00
A H
ATOM 2383 HD13 LEU A 157 22.149 45.903 50.793 1.00 0.00
A H
ATOM 2384 CD2 LEU A 157 22.863 46.240 53.462 1.00 0.00
A C
ATOM 2385 HD21 LEU A 157 22.044 46.917 53.788 1.00 0.00
A H
ATOM 2386 HD22 LEU A 157 22.423 45.239 53.285 1.00 0.00
A H
ATOM 2387 HD23 LEU A 157 23.596 46.158 54.294 1.00 0.00
A H
ATOM 2388 C LEU A 157 26.205 47.387 53.108 1.00 0.00
A C
ATOM 2389 O LEU A 157 26.085 47.167 54.318 1.00 0.00
A O
ATOM 2390 N ALA A 158 26.422 48.606 52.612 1.00 0.00
A N
ATOM 2391 HN ALA A 158 26.391 48.821 51.637 1.00 0.00
A H
ATOM 2392 CA ALA A 158 26.729 49.727 53.491 1.00 0.00
A C
ATOM 2393 HA ALA A 158 27.429 49.428 54.263 1.00 0.00
A H
ATOM 2394 CB ALA A 158 27.356 50.874 52.701 1.00 0.00
A C
ATOM 2395 HB1 ALA A 158 28.293 50.525 52.219 1.00 0.00
A H
ATOM 2396 HB2 ALA A 158 26.671 51.229 51.900 1.00 0.00
A H
ATOM 2397 HB3 ALA A 158 27.607 51.735 53.358 1.00 0.00
A H
ATOM 2398 C ALA A 158 25.480 50.213 54.211 1.00 0.00
A C
ATOM 2399 O ALA A 158 24.424 50.397 53.598 1.00 0.00
A O
ATOM 2400 N THR A 159 25.598 50.402 55.519 1.00 0.00
A N
ATOM 2401 HN THR A 159 26.411 50.173 56.054 1.00 0.00
A H
ATOM 2402 CA THR A 159 24.515 50.975 56.296 1.00 0.00
A C

ATOM 2403 HA THR A 159 23.608 50.789 55.736 1.00 0.00
A H
ATOM 2404 CB THR A 159 24.471 50.357 57.693 1.00 0.00
A C
ATOM 2405 HB THR A 159 24.446 49.250 57.542 1.00 0.00
A H
ATOM 2406 OG1 THR A 159 23.259 50.741 58.356 1.00 0.00
A O
ATOM 2407 HG1 THR A 159 23.301 50.330 59.226 1.00 0.00
A H
ATOM 2408 CG2 THR A 159 25.668 50.806 58.513 1.00 0.00
A C
ATOM 2409 HG21 THR A 159 25.703 50.273 59.487 1.00 0.00
A H
ATOM 2410 HG22 THR A 159 26.613 50.588 57.971 1.00 0.00
A H
ATOM 2411 HG23 THR A 159 25.627 51.897 58.716 1.00 0.00
A H
ATOM 2412 C THR A 159 24.669 52.492 56.382 1.00 0.00
A C
ATOM 2413 O THR A 159 25.599 53.086 55.829 1.00 0.00
A O
ATOM 2414 N ARG A 160 23.730 53.122 57.090 1.00 0.00
A N
ATOM 2415 HN ARG A 160 22.975 52.643 57.531 1.00 0.00
A H
ATOM 2416 CA ARG A 160 23.769 54.570 57.252 1.00 0.00
A C
ATOM 2417 HA ARG A 160 23.844 55.014 56.266 1.00 0.00
A H
ATOM 2418 CB ARG A 160 22.496 55.050 57.944 1.00 0.00
A C
ATOM 2419 HB1 ARG A 160 21.657 54.457 57.507 1.00 0.00
A H
ATOM 2420 HB2 ARG A 160 22.534 54.777 59.022 1.00 0.00
A H
ATOM 2421 CG ARG A 160 22.212 56.522 57.750 1.00 0.00
A C
ATOM 2422 HG1 ARG A 160 23.175 57.078 57.795 1.00 0.00
A H
ATOM 2423 HG2 ARG A 160 21.823 56.683 56.717 1.00 0.00
A H
ATOM 2424 CD ARG A 160 21.188 56.999 58.755 1.00 0.00
A C
ATOM 2425 HD1 ARG A 160 21.232 58.109 58.831 1.00 0.00
A H
ATOM 2426 HD2 ARG A 160 20.155 56.701 58.460 1.00 0.00
A H
ATOM 2427 NE ARG A 160 21.431 56.419 60.073 1.00 0.00
A N
ATOM 2428 HE ARG A 160 20.789 55.680 60.305 1.00 0.00
A H
ATOM 2429 CZ ARG A 160 22.396 56.812 60.899 1.00 0.00
A C

```
ATOM   2430  NH1 ARG A 160      23.221  57.789  60.549  1.00  0.00
A    N
ATOM   2431 HH11 ARG A 160      23.956  58.068  61.150  1.00  0.00
A    H
ATOM   2432 HH12 ARG A 160      23.141  58.121  59.607  1.00  0.00
A    H
ATOM   2433  NH2 ARG A 160      22.539  56.223  62.076  1.00  0.00
A    N
ATOM   2434 HH21 ARG A 160      23.199  56.572  62.726  1.00  0.00
A    H
ATOM   2435 HH22 ARG A 160      21.831  55.582  62.346  1.00  0.00
A    H
ATOM   2436  C   ARG A 160      24.994  55.030  58.031  1.00  0.00
A    C
ATOM   2437  O   ARG A 160      25.420  56.179  57.874  1.00  0.00
A    O
ATOM   2438  N   LYS A 161      25.574  54.159  58.860  1.00  0.00
A    N
ATOM   2439  HN  LYS A 161      25.185  53.264  59.073  1.00  0.00
A    H
ATOM   2440  CA  LYS A 161      26.836  54.458  59.525  1.00  0.00
A    C
ATOM   2441  HA  LYS A 161      26.699  55.405  60.033  1.00  0.00
A    H
ATOM   2442  CB  LYS A 161      27.183  53.349  60.520  1.00  0.00
A    C
ATOM   2443  HB1 LYS A 161      27.361  52.422  59.927  1.00  0.00
A    H
ATOM   2444  HB2 LYS A 161      28.149  53.596  61.014  1.00  0.00
A    H
ATOM   2445  CG  LYS A 161      26.110  53.076  61.551  1.00  0.00
A    C
ATOM   2446  HG1 LYS A 161      25.120  53.027  61.043  1.00  0.00
A    H
ATOM   2447  HG2 LYS A 161      26.296  52.078  62.011  1.00  0.00
A    H
ATOM   2448  CD  LYS A 161      26.091  54.164  62.603  1.00  0.00
A    C
ATOM   2449  HD1 LYS A 161      25.822  55.125  62.109  1.00  0.00
A    H
ATOM   2450  HD2 LYS A 161      25.283  53.926  63.333  1.00  0.00
A    H
ATOM   2451  CE  LYS A 161      27.438  54.257  63.296  1.00  0.00
A    C
ATOM   2452  HE1 LYS A 161      27.712  53.280  63.750  1.00  0.00
A    H
ATOM   2453  HE2 LYS A 161      28.232  54.542  62.571  1.00  0.00
A    H
ATOM   2454  NZ  LYS A 161      27.440  55.270  64.382  1.00  0.00
A    N
ATOM   2455  HZ1 LYS A 161      28.377  55.306  64.834  1.00  0.00
A    H
ATOM   2456  HZ2 LYS A 161      27.212  56.205  63.985  1.00  0.00
A    H
```

ATOM 2457 HZ3 LYS A 161 26.724 55.017 65.093 1.00 0.00
A H
ATOM 2458 C LYS A 161 27.986 54.631 58.545 1.00 0.00
A C
ATOM 2459 O LYS A 161 29.103 54.932 58.978 1.00 0.00
A O
ATOM 2460 N LEU A 162 27.744 54.446 57.247 1.00 0.00
A N
ATOM 2461 HN LEU A 162 26.831 54.293 56.874 1.00 0.00
A H
ATOM 2462 CA LEU A 162 28.802 54.451 56.252 1.00 0.00
A C
ATOM 2463 HA LEU A 162 29.691 54.769 56.781 1.00 0.00
A H
ATOM 2464 CB LEU A 162 28.971 53.044 55.661 1.00 0.00
A C
ATOM 2465 HB1 LEU A 162 27.961 52.698 55.343 1.00 0.00
A H
ATOM 2466 HB2 LEU A 162 29.580 53.112 54.733 1.00 0.00
A H
ATOM 2467 CG LEU A 162 29.562 51.959 56.565 1.00 0.00
A C
ATOM 2468 HG LEU A 162 28.749 51.658 57.270 1.00 0.00
A H
ATOM 2469 CD1 LEU A 162 29.972 50.762 55.727 1.00 0.00
A C
ATOM 2470 HD11 LEU A 162 30.447 49.985 56.363 1.00 0.00
A H
ATOM 2471 HD12 LEU A 162 29.088 50.312 55.227 1.00 0.00
A H
ATOM 2472 HD13 LEU A 162 30.702 51.062 54.944 1.00 0.00
A H
ATOM 2473 CD2 LEU A 162 30.734 52.476 57.389 1.00 0.00
A C
ATOM 2474 HD21 LEU A 162 31.127 51.671 58.047 1.00 0.00
A H
ATOM 2475 HD22 LEU A 162 31.568 52.822 56.745 1.00 0.00
A H
ATOM 2476 HD23 LEU A 162 30.413 53.323 58.033 1.00 0.00
A H
ATOM 2477 C LEU A 162 28.575 55.427 55.107 1.00 0.00
A C
ATOM 2478 O LEU A 162 29.376 55.433 54.166 1.00 0.00
A O
ATOM 2479 N ASP A 163 27.527 56.245 55.153 1.00 0.00
A N
ATOM 2480 HN ASP A 163 26.912 56.361 55.929 1.00 0.00
A H
ATOM 2481 CA ASP A 163 27.171 57.085 54.017 1.00 0.00
A C
ATOM 2482 HA ASP A 163 27.182 56.450 53.138 1.00 0.00
A H
ATOM 2483 CB ASP A 163 25.792 57.713 54.242 1.00 0.00
A C

ATOM 2484 HB1 ASP A 163 25.081 56.926 54.566 1.00 0.00
A H
ATOM 2485 HB2 ASP A 163 25.840 58.486 55.033 1.00 0.00
A H
ATOM 2486 CG ASP A 163 25.219 58.350 52.984 1.00 0.00
A C
ATOM 2487 OD1 ASP A 163 25.965 58.523 51.995 1.00 0.00
A O
ATOM 2488 OD2 ASP A 163 24.016 58.690 52.990 1.00 0.00
A O
ATOM 2489 C ASP A 163 28.214 58.171 53.788 1.00 0.00
A C
ATOM 2490 O ASP A 163 28.723 58.769 54.738 1.00 0.00
A O
ATOM 2491 N SER A 164 28.512 58.425 52.511 1.00 0.00
A N
ATOM 2492 HN SER A 164 28.148 57.876 51.759 1.00 0.00
A H
ATOM 2493 CA SER A 164 29.390 59.515 52.087 1.00 0.00
A C
ATOM 2494 HA SER A 164 29.559 59.367 51.028 1.00 0.00
A H
ATOM 2495 CB SER A 164 28.731 60.871 52.337 1.00 0.00
A C
ATOM 2496 HB1 SER A 164 29.015 61.211 53.358 1.00 0.00
A H
ATOM 2497 HB2 SER A 164 29.119 61.629 51.619 1.00 0.00
A H
ATOM 2498 OG SER A 164 27.318 60.767 52.263 1.00 0.00
A O
ATOM 2499 HG1 SER A 164 26.954 61.577 52.642 1.00 0.00
A H
ATOM 2500 C SER A 164 30.747 59.434 52.777 1.00 0.00
A C
ATOM 2501 O SER A 164 31.376 60.444 53.096 1.00 0.00
A O
ATOM 2502 N LYS A 165 31.200 58.210 53.007 1.00 0.00
A N
ATOM 2503 HN LYS A 165 30.700 57.391 52.727 1.00 0.00
A H
ATOM 2504 CA LYS A 165 32.460 57.960 53.685 1.00 0.00
A C
ATOM 2505 HA LYS A 165 32.763 58.878 54.172 1.00 0.00
A H
ATOM 2506 CB LYS A 165 32.313 56.831 54.708 1.00 0.00
A C
ATOM 2507 HB1 LYS A 165 31.904 55.947 54.165 1.00 0.00
A H
ATOM 2508 HB2 LYS A 165 33.323 56.537 55.070 1.00 0.00
A H
ATOM 2509 CG LYS A 165 31.395 57.184 55.865 1.00 0.00
A C
ATOM 2510 HG1 LYS A 165 30.353 57.288 55.485 1.00 0.00
A H

```
ATOM   2511  HG2 LYS A 165      31.399  56.346  56.600  1.00  0.00
A    H
ATOM   2512  CD  LYS A 165      31.807  58.480  56.540  1.00  0.00
A    C
ATOM   2513  HD1 LYS A 165      32.839  58.354  56.942  1.00  0.00
A    H
ATOM   2514  HD2 LYS A 165      31.847  59.278  55.763  1.00  0.00
A    H
ATOM   2515  CE  LYS A 165      30.831  58.853  57.643  1.00  0.00
A    C
ATOM   2516  HE1 LYS A 165      29.829  59.079  57.217  1.00  0.00
A    H
ATOM   2517  HE2 LYS A 165      30.724  58.017  58.369  1.00  0.00
A    H
ATOM   2518  NZ  LYS A 165      31.284  60.052  58.397  1.00  0.00
A    N
ATOM   2519  HZ1 LYS A 165      30.597  60.283  59.143  1.00  0.00
A    H
ATOM   2520  HZ2 LYS A 165      32.211  59.861  58.828  1.00  0.00
A    H
ATOM   2521  HZ3 LYS A 165      31.369  60.858  57.745  1.00  0.00
A    H
ATOM   2522  C   LYS A 165      33.530  57.616  52.658  1.00  0.00
A    C
ATOM   2523  O   LYS A 165      33.233  57.380  51.487  1.00  0.00
A    O
ATOM   2524  N   SER A 166      34.785  57.644  53.091  1.00  0.00
A    N
ATOM   2525  HN  SER A 166      35.055  57.888  54.022  1.00  0.00
A    H
ATOM   2526  CA  SER A 166      35.900  57.310  52.216  1.00  0.00
A    C
ATOM   2527  HA  SER A 166      35.458  56.986  51.282  1.00  0.00
A    H
ATOM   2528  CB  SER A 166      36.825  58.506  52.019  1.00  0.00
A    C
ATOM   2529  HB1 SER A 166      36.376  59.166  51.242  1.00  0.00
A    H
ATOM   2530  HB2 SER A 166      36.892  59.102  52.956  1.00  0.00
A    H
ATOM   2531  OG  SER A 166      38.090  58.053  51.583  1.00  0.00
A    O
ATOM   2532  HG1 SER A 166      38.571  58.824  51.257  1.00  0.00
A    H
ATOM   2533  C   SER A 166      36.706  56.143  52.763  1.00  0.00
A    C
ATOM   2534  O   SER A 166      36.626  55.806  53.948  1.00  0.00
A    O
ATOM   2535  N   ILE A 167      37.493  55.528  51.879  1.00  0.00
A    N
ATOM   2536  HN  ILE A 167      37.498  55.701  50.897  1.00  0.00
A    H
ATOM   2537  CA  ILE A 167      38.447  54.512  52.313  1.00  0.00
A    C
```

```
ATOM   2538  HA  ILE A 167      37.902  53.821  52.944  1.00  0.00
A    H
ATOM   2539  CB  ILE A 167      39.058  53.793  51.099  1.00  0.00
A    C
ATOM   2540  HB  ILE A 167      39.196  54.601  50.336  1.00  0.00
A    H
ATOM   2541  CG2 ILE A 167      40.342  53.086  51.497  1.00  0.00
A    C
ATOM   2542 HG21 ILE A 167      40.869  52.698  50.599  1.00  0.00
A    H
ATOM   2543 HG22 ILE A 167      41.033  53.782  52.017  1.00  0.00
A    H
ATOM   2544 HG23 ILE A 167      40.132  52.230  52.172  1.00  0.00
A    H
ATOM   2545  CG1 ILE A 167      38.063  52.796  50.513  1.00  0.00
A    C
ATOM   2546 HG11 ILE A 167      38.587  51.851  50.250  1.00  0.00
A    H
ATOM   2547 HG12 ILE A 167      37.303  52.547  51.288  1.00  0.00
A    H
ATOM   2548  CD  ILE A 167      37.346  53.315  49.301  1.00  0.00
A    C
ATOM   2549  HD1 ILE A 167      36.595  52.574  48.951  1.00  0.00
A    H
ATOM   2550  HD2 ILE A 167      36.816  54.263  49.534  1.00  0.00
A    H
ATOM   2551  HD3 ILE A 167      38.056  53.504  48.468  1.00  0.00
A    H
ATOM   2552  C   ILE A 167      39.526  55.141  53.180  1.00  0.00
A    C
ATOM   2553  O   ILE A 167      39.871  54.621  54.247  1.00  0.00
A    O
ATOM   2554  N   ILE A 168      40.077  56.271  52.734  1.00  0.00
A    N
ATOM   2555  HN  ILE A 168      39.900  56.701  51.851  1.00  0.00
A    H
ATOM   2556  CA  ILE A 168      41.033  56.989  53.567  1.00  0.00
A    C
ATOM   2557  HA  ILE A 168      41.743  56.256  53.929  1.00  0.00
A    H
ATOM   2558  CB  ILE A 168      41.756  58.082  52.761  1.00  0.00
A    C
ATOM   2559  HB  ILE A 168      42.088  57.566  51.825  1.00  0.00
A    H
ATOM   2560  CG2 ILE A 168      40.838  59.264  52.507  1.00  0.00
A    C
ATOM   2561 HG21 ILE A 168      41.306  59.977  51.798  1.00  0.00
A    H
ATOM   2562 HG22 ILE A 168      39.877  58.930  52.062  1.00  0.00
A    H
ATOM   2563 HG23 ILE A 168      40.618  59.807  53.451  1.00  0.00
A    H
ATOM   2564  CG1 ILE A 168      43.020  58.527  53.498  1.00  0.00
A    C
```

```
ATOM   2565 HG11 ILE A 168      43.269  59.575  53.218  1.00  0.00
A    H
ATOM   2566 HG12 ILE A 168      42.822  58.517  54.594  1.00  0.00
A    H
ATOM   2567  CD  ILE A 168      44.216  57.640  53.222  1.00  0.00
A    C
ATOM   2568  HD1 ILE A 168      45.089  57.972  53.824  1.00  0.00
A    H
ATOM   2569  HD2 ILE A 168      43.991  56.585  53.488  1.00  0.00
A    H
ATOM   2570  HD3 ILE A 168      44.502  57.680  52.150  1.00  0.00
A    H
ATOM   2571  C   ILE A 168      40.343  57.569  54.787  1.00  0.00
A    C
ATOM   2572  O   ILE A 168      40.984  57.813  55.815  1.00  0.00
A    O
ATOM   2573  N   ASP A 169      39.032  57.780  54.708  1.00  0.00
A    N
ATOM   2574  HN  ASP A 169      38.457  57.678  53.899  1.00  0.00
A    H
ATOM   2575  CA  ASP A 169      38.287  58.206  55.882  1.00  0.00
A    C
ATOM   2576  HA  ASP A 169      38.831  59.030  56.331  1.00  0.00
A    H
ATOM   2577  CB  ASP A 169      36.872  58.610  55.468  1.00  0.00
A    C
ATOM   2578  HB1 ASP A 169      36.766  58.498  54.370  1.00  0.00
A    H
ATOM   2579  HB2 ASP A 169      36.120  57.959  55.959  1.00  0.00
A    H
ATOM   2580  CG  ASP A 169      36.552  60.048  55.806  1.00  0.00
A    C
ATOM   2581  OD1 ASP A 169      37.216  60.935  55.231  1.00  0.00
A    O
ATOM   2582  OD2 ASP A 169      35.644  60.291  56.632  1.00  0.00
A    O
ATOM   2583  C   ASP A 169      38.252  57.087  56.915  1.00  0.00
A    C
ATOM   2584  O   ASP A 169      38.858  57.185  57.988  1.00  0.00
A    O
ATOM   2585  N   LEU A 170      37.567  55.997  56.586  1.00  0.00
A    N
ATOM   2586  HN  LEU A 170      37.062  55.903  55.730  1.00  0.00
A    H
ATOM   2587  CA  LEU A 170      37.493  54.829  57.457  1.00  0.00
A    C
ATOM   2588  HA  LEU A 170      37.818  55.137  58.442  1.00  0.00
A    H
ATOM   2589  CB  LEU A 170      36.060  54.312  57.519  1.00  0.00
A    C
ATOM   2590  HB1 LEU A 170      35.677  54.278  56.473  1.00  0.00
A    H
ATOM   2591  HB2 LEU A 170      36.071  53.258  57.877  1.00  0.00
A    H
```

```
ATOM   2592  CG  LEU A 170      35.090  55.147  58.363  1.00  0.00
A    C
ATOM   2593  HG  LEU A 170      35.648  55.423  59.290  1.00  0.00
A    H
ATOM   2594  CD1 LEU A 170      34.645  56.408  57.632  1.00  0.00
A    C
ATOM   2595 HD11 LEU A 170      33.907  56.972  58.241  1.00  0.00
A    H
ATOM   2596 HD12 LEU A 170      35.513  57.073  57.433  1.00  0.00
A    H
ATOM   2597 HD13 LEU A 170      34.169  56.153  56.661  1.00  0.00
A    H
ATOM   2598  CD2 LEU A 170      33.883  54.322  58.769  1.00  0.00
A    C
ATOM   2599 HD21 LEU A 170      33.194  54.928  59.396  1.00  0.00
A    H
ATOM   2600 HD22 LEU A 170      33.311  53.969  57.886  1.00  0.00
A    H
ATOM   2601 HD23 LEU A 170      34.198  53.434  59.359  1.00  0.00
A    H
ATOM   2602  C   LEU A 170      38.467  53.761  56.962  1.00  0.00
A    C
ATOM   2603  O   LEU A 170      38.099  52.789  56.301  1.00  0.00
A    O
ATOM   2604  N   ASN A 171      39.742  53.978  57.280  1.00  0.00
A    N
ATOM   2605  HN  ASN A 171      40.065  54.749  57.828  1.00  0.00
A    H
ATOM   2606  CA  ASN A 171      40.791  53.072  56.835  1.00  0.00
A    C
ATOM   2607  HA  ASN A 171      40.662  52.969  55.763  1.00  0.00
A    H
ATOM   2608  CB  ASN A 171      42.171  53.635  57.180  1.00  0.00
A    C
ATOM   2609  HB1 ASN A 171      42.927  52.860  56.929  1.00  0.00
A    H
ATOM   2610  HB2 ASN A 171      42.389  54.528  56.559  1.00  0.00
A    H
ATOM   2611  CG  ASN A 171      42.312  53.970  58.645  1.00  0.00
A    C
ATOM   2612  OD1 ASN A 171      41.359  53.871  59.413  1.00  0.00
A    O
ATOM   2613  ND2 ASN A 171      43.514  54.366  59.045  1.00  0.00
A    N
ATOM   2614 HD21 ASN A 171      43.628  54.580  60.011  1.00  0.00
A    H
ATOM   2615 HD22 ASN A 171      44.266  54.412  58.394  1.00  0.00
A    H
ATOM   2616  C   ASN A 171      40.617  51.690  57.457  1.00  0.00
A    C
ATOM   2617  O   ASN A 171      40.036  51.538  58.535  1.00  0.00
A    O
ATOM   2618  N   VAL A 172      41.107  50.675  56.749  1.00  0.00
A    N
```

ATOM 2619 HN VAL A 172 41.561 50.782 55.868 1.00 0.00
A H
ATOM 2620 CA VAL A 172 41.017 49.299 57.216 1.00 0.00
A C
ATOM 2621 HA VAL A 172 40.704 49.338 58.251 1.00 0.00
A H
ATOM 2622 CB VAL A 172 39.961 48.518 56.407 1.00 0.00
A C
ATOM 2623 HB VAL A 172 39.074 49.186 56.276 1.00 0.00
A H
ATOM 2624 CG1 VAL A 172 40.499 48.133 55.036 1.00 0.00
A C
ATOM 2625 HG11 VAL A 172 39.744 47.538 54.478 1.00 0.00
A H
ATOM 2626 HG12 VAL A 172 40.734 49.026 54.421 1.00 0.00
A H
ATOM 2627 HG13 VAL A 172 41.418 47.517 55.134 1.00 0.00
A H
ATOM 2628 CG2 VAL A 172 39.496 47.282 57.176 1.00 0.00
A C
ATOM 2629 HG21 VAL A 172 38.765 46.699 56.576 1.00 0.00
A H
ATOM 2630 HG22 VAL A 172 40.359 46.623 57.414 1.00 0.00
A H
ATOM 2631 HG23 VAL A 172 39.000 47.564 58.127 1.00 0.00
A H
ATOM 2632 C VAL A 172 42.365 48.588 57.173 1.00 0.00
A C
ATOM 2633 O VAL A 172 42.568 47.613 57.904 1.00 0.00
A O
ATOM 2634 N ARG A 173 43.295 49.048 56.333 1.00 0.00
A N
ATOM 2635 HN ARG A 173 43.158 49.840 55.744 1.00 0.00
A H
ATOM 2636 CA ARG A 173 44.590 48.383 56.245 1.00 0.00
A C
ATOM 2637 HA ARG A 173 44.420 47.312 56.223 1.00 0.00
A H
ATOM 2638 CB ARG A 173 45.324 48.817 54.980 1.00 0.00
A C
ATOM 2639 HB1 ARG A 173 44.538 49.123 54.249 1.00 0.00
A H
ATOM 2640 HB2 ARG A 173 45.920 49.732 55.191 1.00 0.00
A H
ATOM 2641 CG ARG A 173 46.166 47.712 54.381 1.00 0.00
A C
ATOM 2642 HG1 ARG A 173 47.204 47.811 54.767 1.00 0.00
A H
ATOM 2643 HG2 ARG A 173 45.797 46.730 54.760 1.00 0.00
A H
ATOM 2644 CD ARG A 173 46.095 47.711 52.863 1.00 0.00
A C
ATOM 2645 HD1 ARG A 173 46.015 46.661 52.497 1.00 0.00
A H

ATOM 2646 HD2 ARG A 173 45.214 48.286 52.494 1.00 0.00
A H
ATOM 2647 NE ARG A 173 47.268 48.327 52.246 1.00 0.00
A N
ATOM 2648 HE ARG A 173 47.071 49.222 51.831 1.00 0.00
A H
ATOM 2649 CZ ARG A 173 48.480 47.780 52.222 1.00 0.00
A C
ATOM 2650 NH1 ARG A 173 48.695 46.597 52.782 1.00 0.00
A N
ATOM 2651 HH11 ARG A 173 49.601 46.198 52.789 1.00 0.00
A H
ATOM 2652 HH12 ARG A 173 47.933 46.208 53.305 1.00 0.00
A H
ATOM 2653 NH2 ARG A 173 49.483 48.418 51.634 1.00 0.00
A N
ATOM 2654 HH21 ARG A 173 50.363 47.974 51.540 1.00 0.00
A H
ATOM 2655 HH22 ARG A 173 49.255 49.236 51.122 1.00 0.00
A H
ATOM 2656 C ARG A 173 45.444 48.663 57.472 1.00 0.00
A C
ATOM 2657 O ARG A 173 45.988 47.736 58.083 1.00 0.00
A O
ATOM 2658 N ALA A 174 45.584 49.932 57.843 1.00 0.00
A N
ATOM 2659 HN ALA A 174 45.230 50.709 57.322 1.00 0.00
A H
ATOM 2660 CA ALA A 174 46.287 50.271 59.072 1.00 0.00
A C
ATOM 2661 HA ALA A 174 47.150 49.629 59.205 1.00 0.00
A H
ATOM 2662 CB ALA A 174 46.778 51.718 59.014 1.00 0.00
A C
ATOM 2663 HB1 ALA A 174 47.461 51.846 58.147 1.00 0.00
A H
ATOM 2664 HB2 ALA A 174 45.928 52.424 58.886 1.00 0.00
A H
ATOM 2665 HB3 ALA A 174 47.335 51.999 59.934 1.00 0.00
A H
ATOM 2666 C ALA A 174 45.404 50.062 60.293 1.00 0.00
A C
ATOM 2667 O ALA A 174 45.907 49.873 61.405 1.00 0.00
A O
ATOM 2668 N LYS A 175 44.085 50.077 60.098 1.00 0.00
A N
ATOM 2669 HN LYS A 175 43.650 50.151 59.201 1.00 0.00
A H
ATOM 2670 CA LYS A 175 43.165 49.983 61.225 1.00 0.00
A C
ATOM 2671 HA LYS A 175 43.670 50.416 62.081 1.00 0.00
A H
ATOM 2672 CB LYS A 175 41.888 50.756 60.910 1.00 0.00
A C

ATOM 2673 HB1 LYS A 175 42.186 51.662 60.334 1.00 0.00
A H
ATOM 2674 HB2 LYS A 175 41.254 50.146 60.228 1.00 0.00
A H
ATOM 2675 CG LYS A 175 41.102 51.183 62.126 1.00 0.00
A C
ATOM 2676 HG1 LYS A 175 40.612 50.290 62.576 1.00 0.00
A H
ATOM 2677 HG2 LYS A 175 41.805 51.595 62.886 1.00 0.00
A H
ATOM 2678 CD LYS A 175 40.053 52.206 61.741 1.00 0.00
A C
ATOM 2679 HD1 LYS A 175 40.558 53.046 61.213 1.00 0.00
A H
ATOM 2680 HD2 LYS A 175 39.352 51.730 61.017 1.00 0.00
A H
ATOM 2681 CE LYS A 175 39.299 52.709 62.956 1.00 0.00
A C
ATOM 2682 HE1 LYS A 175 38.673 51.897 63.389 1.00 0.00
A H
ATOM 2683 HE2 LYS A 175 40.007 53.061 63.738 1.00 0.00
A H
ATOM 2684 NZ LYS A 175 38.399 53.843 62.611 1.00 0.00
A N
ATOM 2685 HZ1 LYS A 175 37.893 54.167 63.461 1.00 0.00
A H
ATOM 2686 HZ2 LYS A 175 38.962 54.627 62.224 1.00 0.00
A H
ATOM 2687 HZ3 LYS A 175 37.709 53.533 61.898 1.00 0.00
A H
ATOM 2688 C LYS A 175 42.846 48.534 61.569 1.00 0.00
A C
ATOM 2689 O LYS A 175 42.989 48.120 62.726 1.00 0.00
A O
ATOM 2690 N TYR A 176 42.411 47.745 60.585 1.00 0.00
A N
ATOM 2691 HN TYR A 176 42.329 48.043 59.635 1.00 0.00
A H
ATOM 2692 CA TYR A 176 42.016 46.365 60.822 1.00 0.00
A C
ATOM 2693 HA TYR A 176 42.198 46.194 61.876 1.00 0.00
A H
ATOM 2694 CB TYR A 176 40.531 46.168 60.479 1.00 0.00
A C
ATOM 2695 HB1 TYR A 176 40.373 46.393 59.402 1.00 0.00
A H
ATOM 2696 HB2 TYR A 176 40.227 45.112 60.642 1.00 0.00
A H
ATOM 2697 CG TYR A 176 39.609 47.080 61.256 1.00 0.00
A C
ATOM 2698 CD1 TYR A 176 39.140 48.262 60.698 1.00 0.00
A C
ATOM 2699 HD1 TYR A 176 39.446 48.544 59.700 1.00 0.00
A H

ATOM 2700 CE1 TYR A 176 38.303 49.101 61.402 1.00 0.00
A C
ATOM 2701 HE1 TYR A 176 37.949 50.021 60.960 1.00 0.00
A H
ATOM 2702 CZ TYR A 176 37.923 48.767 62.683 1.00 0.00
A C
ATOM 2703 OH TYR A 176 37.087 49.605 63.384 1.00 0.00
A O
ATOM 2704 HH TYR A 176 36.881 49.175 64.216 1.00 0.00
A H
ATOM 2705 CD2 TYR A 176 39.215 46.766 62.550 1.00 0.00
A C
ATOM 2706 HD2 TYR A 176 39.597 45.876 63.029 1.00 0.00
A H
ATOM 2707 CE2 TYR A 176 38.376 47.601 63.263 1.00 0.00
A C
ATOM 2708 HE2 TYR A 176 38.099 47.345 64.275 1.00 0.00
A H
ATOM 2709 C TYR A 176 42.841 45.345 60.053 1.00 0.00
A C
ATOM 2710 O TYR A 176 42.545 44.149 60.143 1.00 0.00
A O
ATOM 2711 N GLY A 177 43.853 45.771 59.301 1.00 0.00
A N
ATOM 2712 HN GLY A 177 44.071 46.737 59.169 1.00 0.00
A H
ATOM 2713 CA GLY A 177 44.718 44.833 58.615 1.00 0.00
A C
ATOM 2714 HA1 GLY A 177 44.990 44.093 59.356 1.00 0.00
A H
ATOM 2715 HA2 GLY A 177 45.549 45.417 58.244 1.00 0.00
A H
ATOM 2716 C GLY A 177 44.094 44.115 57.445 1.00 0.00
A C
ATOM 2717 O GLY A 177 44.630 43.095 57.001 1.00 0.00
A O
ATOM 2718 N CYS A 178 42.981 44.612 56.925 1.00 0.00
A N
ATOM 2719 HN CYS A 178 42.563 45.455 57.261 1.00 0.00
A H
ATOM 2720 CA CYS A 178 42.274 43.977 55.826 1.00 0.00
A C
ATOM 2721 HA CYS A 178 42.730 43.006 55.671 1.00 0.00
A H
ATOM 2722 CB CYS A 178 40.787 43.825 56.164 1.00 0.00
A C
ATOM 2723 HB1 CYS A 178 40.423 44.821 56.503 1.00 0.00
A H
ATOM 2724 HB2 CYS A 178 40.214 43.586 55.241 1.00 0.00
A H
ATOM 2725 SG CYS A 178 40.471 42.620 57.466 1.00 0.00
A S
ATOM 2726 HG1 CYS A 178 39.151 42.798 57.540 1.00 0.00
A H

ATOM 2727 C CYS A 178 42.469 44.790 54.552 1.00 0.00
A C
ATOM 2728 O CYS A 178 43.226 45.765 54.517 1.00 0.00
A O
ATOM 2729 N THR A 179 41.774 44.377 53.494 1.00 0.00
A N
ATOM 2730 HN THR A 179 41.128 43.615 53.531 1.00 0.00
A H
ATOM 2731 CA THR A 179 41.891 45.001 52.183 1.00 0.00
A C
ATOM 2732 HA THR A 179 42.334 45.974 52.348 1.00 0.00
A H
ATOM 2733 CB THR A 179 42.726 44.127 51.240 1.00 0.00
A C
ATOM 2734 HB THR A 179 41.995 43.556 50.618 1.00 0.00
A H
ATOM 2735 OG1 THR A 179 43.485 43.187 52.012 1.00 0.00
A O
ATOM 2736 HG1 THR A 179 43.993 42.679 51.371 1.00 0.00
A H
ATOM 2737 CG2 THR A 179 43.648 44.969 50.379 1.00 0.00
A C
ATOM 2738 HG21 THR A 179 44.164 44.341 49.622 1.00 0.00
A H
ATOM 2739 HG22 THR A 179 43.070 45.748 49.838 1.00 0.00
A H
ATOM 2740 HG23 THR A 179 44.421 45.475 50.996 1.00 0.00
A H
ATOM 2741 C THR A 179 40.509 45.247 51.594 1.00 0.00
A C
ATOM 2742 O THR A 179 39.785 44.298 51.281 1.00 0.00
A O
ATOM 2743 N ILE A 180 40.152 46.520 51.431 1.00 0.00
A N
ATOM 2744 HN ILE A 180 40.688 47.299 51.753 1.00 0.00
A H
ATOM 2745 CA ILE A 180 38.914 46.888 50.748 1.00 0.00
A C
ATOM 2746 HA ILE A 180 38.162 46.155 51.011 1.00 0.00
A H
ATOM 2747 CB ILE A 180 38.470 48.313 51.129 1.00 0.00
A C
ATOM 2748 HB ILE A 180 39.295 48.698 51.779 1.00 0.00
A H
ATOM 2749 CG2 ILE A 180 38.199 49.155 49.879 1.00 0.00
A C
ATOM 2750 HG21 ILE A 180 38.032 50.217 50.153 1.00 0.00
A H
ATOM 2751 HG22 ILE A 180 39.062 49.118 49.182 1.00 0.00
A H
ATOM 2752 HG23 ILE A 180 37.299 48.790 49.342 1.00 0.00
A H
ATOM 2753 CG1 ILE A 180 37.206 48.261 51.995 1.00 0.00
A C

ATOM 2754 HG11 ILE A 180 36.740 47.252 51.922 1.00 0.00
A H
ATOM 2755 HG12 ILE A 180 37.492 48.416 53.061 1.00 0.00
A H
ATOM 2756 CD ILE A 180 36.151 49.307 51.643 1.00 0.00
A C
ATOM 2757 HD1 ILE A 180 35.295 49.244 52.348 1.00 0.00
A H
ATOM 2758 HD2 ILE A 180 36.578 50.331 51.705 1.00 0.00
A H
ATOM 2759 HD3 ILE A 180 35.762 49.149 50.614 1.00 0.00
A H
ATOM 2760 C ILE A 180 39.151 46.757 49.250 1.00 0.00
A C
ATOM 2761 O ILE A 180 40.241 47.068 48.754 1.00 0.00
A O
ATOM 2762 N LEU A 181 38.148 46.267 48.524 1.00 0.00
A N
ATOM 2763 HN LEU A 181 37.210 46.159 48.846 1.00 0.00
A H
ATOM 2764 CA LEU A 181 38.363 45.835 47.150 1.00 0.00
A C
ATOM 2765 HA LEU A 181 39.391 46.096 46.935 1.00 0.00
A H
ATOM 2766 CB LEU A 181 38.137 44.330 47.029 1.00 0.00
A C
ATOM 2767 HB1 LEU A 181 38.176 43.908 48.059 1.00 0.00
A H
ATOM 2768 HB2 LEU A 181 37.103 44.147 46.660 1.00 0.00
A H
ATOM 2769 CG LEU A 181 39.145 43.589 46.164 1.00 0.00
A C
ATOM 2770 HG LEU A 181 38.869 43.743 45.093 1.00 0.00
A H
ATOM 2771 CD1 LEU A 181 40.563 44.100 46.385 1.00 0.00
A C
ATOM 2772 HD11 LEU A 181 41.288 43.513 45.780 1.00 0.00
A H
ATOM 2773 HD12 LEU A 181 40.645 45.168 46.089 1.00 0.00
A H
ATOM 2774 HD13 LEU A 181 40.850 44.008 47.454 1.00 0.00
A H
ATOM 2775 CD2 LEU A 181 39.014 42.133 46.470 1.00 0.00
A C
ATOM 2776 HD21 LEU A 181 39.720 41.543 45.846 1.00 0.00
A H
ATOM 2777 HD22 LEU A 181 39.242 41.916 47.533 1.00 0.00
A H
ATOM 2778 HD23 LEU A 181 37.983 41.780 46.254 1.00 0.00
A H
ATOM 2779 C LEU A 181 37.491 46.518 46.114 1.00 0.00
A C
ATOM 2780 O LEU A 181 37.999 46.872 45.051 1.00 0.00
A O

```
ATOM   2781  N   ALA A 182      36.194  46.676  46.363  1.00  0.00
A    N
ATOM   2782  HN  ALA A 182      35.750  46.447  47.229  1.00  0.00
A    H
ATOM   2783  CA  ALA A 182      35.319  47.214  45.337  1.00  0.00
A    C
ATOM   2784  HA  ALA A 182      35.799  48.023  44.797  1.00  0.00
A    H
ATOM   2785  CB  ALA A 182      34.929  46.150  44.307  1.00  0.00
A    C
ATOM   2786  HB1 ALA A 182      35.842  45.761  43.809  1.00  0.00
A    H
ATOM   2787  HB2 ALA A 182      34.413  45.293  44.795  1.00  0.00
A    H
ATOM   2788  HB3 ALA A 182      34.260  46.565  43.523  1.00  0.00
A    H
ATOM   2789  C   ALA A 182      34.078  47.806  45.978  1.00  0.00
A    C
ATOM   2790  O   ALA A 182      33.632  47.379  47.050  1.00  0.00
A    O
ATOM   2791  N   ILE A 183      33.515  48.781  45.274  1.00  0.00
A    N
ATOM   2792  HN  ILE A 183      33.832  49.012  44.357  1.00  0.00
A    H
ATOM   2793  CA  ILE A 183      32.407  49.598  45.745  1.00  0.00
A    C
ATOM   2794  HA  ILE A 183      32.035  49.151  46.659  1.00  0.00
A    H
ATOM   2795  CB  ILE A 183      32.859  51.057  45.956  1.00  0.00
A    C
ATOM   2796  HB  ILE A 183      33.265  51.371  44.960  1.00  0.00
A    H
ATOM   2797  CG2 ILE A 183      31.707  51.956  46.370  1.00  0.00
A    C
ATOM   2798 HG21 ILE A 183      32.020  53.021  46.362  1.00  0.00
A    H
ATOM   2799 HG22 ILE A 183      30.852  51.852  45.669  1.00  0.00
A    H
ATOM   2800 HG23 ILE A 183      31.355  51.705  47.393  1.00  0.00
A    H
ATOM   2801  CG1 ILE A 183      34.024  51.113  46.951  1.00  0.00
A    C
ATOM   2802 HG11 ILE A 183      33.769  50.527  47.862  1.00  0.00
A    H
ATOM   2803 HG12 ILE A 183      34.915  50.631  46.488  1.00  0.00
A    H
ATOM   2804  CD  ILE A 183      34.406  52.510  47.359  1.00  0.00
A    C
ATOM   2805  HD1 ILE A 183      35.290  52.486  48.033  1.00  0.00
A    H
ATOM   2806  HD2 ILE A 183      34.664  53.122  46.469  1.00  0.00
A    H
ATOM   2807  HD3 ILE A 183      33.574  53.008  47.900  1.00  0.00
A    H
```

ATOM 2808 C ILE A 183 31.282 49.511 44.724 1.00 0.00
A C
ATOM 2809 O ILE A 183 31.470 49.874 43.558 1.00 0.00
A O
ATOM 2810 N LYS A 184 30.112 49.043 45.160 1.00 0.00
A N
ATOM 2811 HN LYS A 184 29.973 48.796 46.118 1.00 0.00
A H
ATOM 2812 CA LYS A 184 28.948 48.853 44.294 1.00 0.00
A C
ATOM 2813 HA LYS A 184 29.191 49.197 43.296 1.00 0.00
A H
ATOM 2814 CB LYS A 184 28.509 47.392 44.298 1.00 0.00
A C
ATOM 2815 HB1 LYS A 184 29.434 46.772 44.249 1.00 0.00
A H
ATOM 2816 HB2 LYS A 184 28.028 47.161 45.275 1.00 0.00
A H
ATOM 2817 CG LYS A 184 27.584 46.921 43.170 1.00 0.00
A C
ATOM 2818 HG1 LYS A 184 27.818 45.859 42.926 1.00 0.00
A H
ATOM 2819 HG2 LYS A 184 26.529 46.954 43.528 1.00 0.00
A H
ATOM 2820 CD LYS A 184 27.705 47.730 41.896 1.00 0.00
A C
ATOM 2821 HD1 LYS A 184 27.162 48.693 42.038 1.00 0.00
A H
ATOM 2822 HD2 LYS A 184 28.781 47.974 41.741 1.00 0.00
A H
ATOM 2823 CE LYS A 184 27.140 46.924 40.730 1.00 0.00
A C
ATOM 2824 HE1 LYS A 184 27.562 45.894 40.727 1.00 0.00
A H
ATOM 2825 HE2 LYS A 184 26.033 46.850 40.807 1.00 0.00
A H
ATOM 2826 NZ LYS A 184 27.430 47.500 39.388 1.00 0.00
A N
ATOM 2827 HZ1 LYS A 184 27.017 46.899 38.646 1.00 0.00
A H
ATOM 2828 HZ2 LYS A 184 27.024 48.456 39.325 1.00 0.00
A H
ATOM 2829 HZ3 LYS A 184 28.460 47.557 39.252 1.00 0.00
A H
ATOM 2830 C LYS A 184 27.843 49.755 44.831 1.00 0.00
A C
ATOM 2831 O LYS A 184 27.169 49.402 45.802 1.00 0.00
A O
ATOM 2832 N HSE A 185 27.659 50.917 44.211 1.00 0.00
A N
ATOM 2833 HN HSE A 185 28.178 51.190 43.402 1.00 0.00
A H
ATOM 2834 CA HSE A 185 26.678 51.902 44.658 1.00 0.00
A C

ATOM 2835 HA HSE A 185 26.175 51.494 45.526 1.00 0.00
A H
ATOM 2836 CB HSE A 185 27.348 53.232 45.010 1.00 0.00
A C
ATOM 2837 HB1 HSE A 185 26.555 53.937 45.340 1.00 0.00
A H
ATOM 2838 HB2 HSE A 185 28.032 53.085 45.872 1.00 0.00
A H
ATOM 2839 ND1 HSE A 185 27.512 54.830 43.069 1.00 0.00
A N
ATOM 2840 CG HSE A 185 28.082 53.870 43.876 1.00 0.00
A C
ATOM 2841 CE1 HSE A 185 28.386 55.210 42.156 1.00 0.00
A C
ATOM 2842 HE1 HSE A 185 28.198 55.973 41.396 1.00 0.00
A H
ATOM 2843 NE2 HSE A 185 29.495 54.511 42.324 1.00 0.00
A N
ATOM 2844 HE2 HSE A 185 30.311 54.578 41.750 1.00 0.00
A H
ATOM 2845 CD2 HSE A 185 29.329 53.663 43.391 1.00 0.00
A C
ATOM 2846 HD2 HSE A 185 30.096 52.975 43.720 1.00 0.00
A H
ATOM 2847 C HSE A 185 25.623 52.097 43.582 1.00 0.00
A C
ATOM 2848 O HSE A 185 25.911 52.656 42.518 1.00 0.00
A O
ATOM 2849 N HSE A 186 24.405 51.642 43.871 1.00 0.00
A N
ATOM 2850 HN HSE A 186 24.188 51.165 44.722 1.00 0.00
A H
ATOM 2851 CA HSE A 186 23.272 51.801 42.966 1.00 0.00
A C
ATOM 2852 HA HSE A 186 22.460 51.207 43.369 1.00 0.00
A H
ATOM 2853 CB HSE A 186 22.842 53.268 42.877 1.00 0.00
A C
ATOM 2854 HB1 HSE A 186 22.909 53.706 43.896 1.00 0.00
A H
ATOM 2855 HB2 HSE A 186 23.556 53.825 42.236 1.00 0.00
A H
ATOM 2856 ND1 HSE A 186 20.354 52.887 43.008 1.00 0.00
A N
ATOM 2857 CG HSE A 186 21.443 53.455 42.384 1.00 0.00
A C
ATOM 2858 CE1 HSE A 186 19.255 53.212 42.350 1.00 0.00
A C
ATOM 2859 HE1 HSE A 186 18.248 52.893 42.631 1.00 0.00
A H
ATOM 2860 NE2 HSE A 186 19.593 53.964 41.319 1.00 0.00
A N
ATOM 2861 HE2 HSE A 186 18.958 54.334 40.642 1.00 0.00
A H

ATOM 2862 CD2 HSE A 186 20.957 54.128 41.315 1.00 0.00
A C
ATOM 2863 HD2 HSE A 186 21.479 54.695 40.558 1.00 0.00
A H
ATOM 2864 C HSE A 186 23.589 51.246 41.583 1.00 0.00
A C
ATOM 2865 O HSE A 186 23.289 51.870 40.565 1.00 0.00
A O
ATOM 2866 N GLY A 187 24.223 50.074 41.536 1.00 0.00
A N
ATOM 2867 HN GLY A 187 24.488 49.585 42.366 1.00 0.00
A H
ATOM 2868 CA GLY A 187 24.571 49.433 40.297 1.00 0.00
A C
ATOM 2869 HA1 GLY A 187 23.780 49.674 39.600 1.00 0.00
A H
ATOM 2870 HA2 GLY A 187 24.665 48.377 40.514 1.00 0.00
A H
ATOM 2871 C GLY A 187 25.881 49.907 39.703 1.00 0.00
A C
ATOM 2872 O GLY A 187 26.316 49.368 38.682 1.00 0.00
A O
ATOM 2873 N ASP A 188 26.520 50.895 40.316 1.00 0.00
A N
ATOM 2874 HN ASP A 188 26.241 51.346 41.159 1.00 0.00
A H
ATOM 2875 CA ASP A 188 27.758 51.432 39.770 1.00 0.00
A C
ATOM 2876 HA ASP A 188 27.807 51.125 38.732 1.00 0.00
A H
ATOM 2877 CB ASP A 188 27.764 52.952 39.909 1.00 0.00
A C
ATOM 2878 HB1 ASP A 188 27.656 53.221 40.979 1.00 0.00
A H
ATOM 2879 HB2 ASP A 188 28.719 53.374 39.538 1.00 0.00
A H
ATOM 2880 CG ASP A 188 26.619 53.612 39.155 1.00 0.00
A C
ATOM 2881 OD1 ASP A 188 25.678 52.888 38.766 1.00 0.00
A O
ATOM 2882 OD2 ASP A 188 26.656 54.848 38.954 1.00 0.00
A O
ATOM 2883 C ASP A 188 28.975 50.827 40.466 1.00 0.00
A C
ATOM 2884 O ASP A 188 29.050 50.786 41.697 1.00 0.00
A O
ATOM 2885 N ILE A 189 29.939 50.366 39.666 1.00 0.00
A N
ATOM 2886 HN ILE A 189 29.899 50.474 38.675 1.00 0.00
A H
ATOM 2887 CA ILE A 189 31.130 49.668 40.145 1.00 0.00
A C
ATOM 2888 HA ILE A 189 30.902 49.299 41.137 1.00 0.00
A H

```
ATOM   2889  CB  ILE A 189      31.527  48.507  39.207  1.00  0.00
A    C
ATOM   2890  HB  ILE A 189      30.681  48.435  38.478  1.00  0.00
A    H
ATOM   2891  CG2 ILE A 189      32.892  48.749  38.570  1.00  0.00
A    C
ATOM   2892 HG21 ILE A 189      33.097  47.992  37.784  1.00  0.00
A    H
ATOM   2893 HG22 ILE A 189      32.935  49.750  38.092  1.00  0.00
A    H
ATOM   2894 HG23 ILE A 189      33.701  48.689  39.328  1.00  0.00
A    H
ATOM   2895  CG1 ILE A 189      31.573  47.165  39.943  1.00  0.00
A    C
ATOM   2896 HG11 ILE A 189      31.142  46.367  39.297  1.00  0.00
A    H
ATOM   2897 HG12 ILE A 189      32.637  46.895  40.134  1.00  0.00
A    H
ATOM   2898  CD  ILE A 189      30.873  47.121  41.245  1.00  0.00
A    C
ATOM   2899  HD1 ILE A 189      31.012  46.129  41.725  1.00  0.00
A    H
ATOM   2900  HD2 ILE A 189      31.273  47.899  41.931  1.00  0.00
A    H
ATOM   2901  HD3 ILE A 189      29.784  47.291  41.118  1.00  0.00
A    H
ATOM   2902  C   ILE A 189      32.279  50.654  40.296  1.00  0.00
A    C
ATOM   2903  O   ILE A 189      32.366  51.643  39.556  1.00  0.00
A    O
ATOM   2904  N   CYS A 190      33.134  50.393  41.280  1.00  0.00
A    N
ATOM   2905  HN  CYS A 190      32.895  49.752  42.009  1.00  0.00
A    H
ATOM   2906  CA  CYS A 190      34.460  50.988  41.401  1.00  0.00
A    C
ATOM   2907  HA  CYS A 190      34.811  51.266  40.414  1.00  0.00
A    H
ATOM   2908  CB  CYS A 190      34.457  52.201  42.326  1.00  0.00
A    C
ATOM   2909  HB1 CYS A 190      33.587  52.832  42.038  1.00  0.00
A    H
ATOM   2910  HB2 CYS A 190      34.269  51.877  43.372  1.00  0.00
A    H
ATOM   2911  SG  CYS A 190      35.958  53.220  42.221  1.00  0.00
A    S
ATOM   2912  HG1 CYS A 190      35.572  54.171  43.073  1.00  0.00
A    H
ATOM   2913  C   CYS A 190      35.370  49.877  41.921  1.00  0.00
A    C
ATOM   2914  O   CYS A 190      35.295  49.497  43.092  1.00  0.00
A    O
ATOM   2915  N   LEU A 191      36.210  49.343  41.037  1.00  0.00
A    N
```

```
ATOM   2916  HN  LEU A 191      36.280  49.627  40.083  1.00  0.00
A    H
ATOM   2917  CA  LEU A 191      37.119  48.269  41.409  1.00  0.00
A    C
ATOM   2918  HA  LEU A 191      36.643  47.716  42.208  1.00  0.00
A    H
ATOM   2919  CB  LEU A 191      37.427  47.371  40.211  1.00  0.00
A    C
ATOM   2920  HB1 LEU A 191      37.593  48.038  39.335  1.00  0.00
A    H
ATOM   2921  HB2 LEU A 191      38.394  46.850  40.388  1.00  0.00
A    H
ATOM   2922  CG  LEU A 191      36.413  46.324  39.763  1.00  0.00
A    C
ATOM   2923  HG  LEU A 191      35.497  46.844  39.390  1.00  0.00
A    H
ATOM   2924  CD1 LEU A 191      37.020  45.501  38.647  1.00  0.00
A    C
ATOM   2925 HD11 LEU A 191      36.321  44.697  38.331  1.00  0.00
A    H
ATOM   2926 HD12 LEU A 191      37.239  46.140  37.764  1.00  0.00
A    H
ATOM   2927 HD13 LEU A 191      37.967  45.026  38.980  1.00  0.00
A    H
ATOM   2928  CD2 LEU A 191      36.010  45.437  40.919  1.00  0.00
A    C
ATOM   2929 HD21 LEU A 191      35.258  44.689  40.588  1.00  0.00
A    H
ATOM   2930 HD22 LEU A 191      36.877  44.883  41.332  1.00  0.00
A    H
ATOM   2931 HD23 LEU A 191      35.561  46.043  41.736  1.00  0.00
A    H
ATOM   2932  C   LEU A 191      38.423  48.835  41.955  1.00  0.00
A    C
ATOM   2933  O   LEU A 191      38.984  49.786  41.407  1.00  0.00
A    O
ATOM   2934  N   SER A 192      38.930  48.198  43.010  1.00  0.00
A    N
ATOM   2935  HN  SER A 192      38.509  47.373  43.386  1.00  0.00
A    H
ATOM   2936  CA  SER A 192      40.132  48.629  43.714  1.00  0.00
A    C
ATOM   2937  HA  SER A 192      40.106  48.155  44.687  1.00  0.00
A    H
ATOM   2938  CB  SER A 192      41.394  48.234  42.959  1.00  0.00
A    C
ATOM   2939  HB1 SER A 192      42.150  49.039  43.104  1.00  0.00
A    H
ATOM   2940  HB2 SER A 192      41.825  47.299  43.385  1.00  0.00
A    H
ATOM   2941  OG  SER A 192      41.158  48.088  41.570  1.00  0.00
A    O
ATOM   2942  HG1 SER A 192      42.020  48.062  41.137  1.00  0.00
A    H
```

ATOM 2943 C SER A 192 40.090 50.145 43.945 1.00 0.00
A C
ATOM 2944 O SER A 192 40.919 50.876 43.394 1.00 0.00
A O
ATOM 2945 N PRO A 193 39.140 50.636 44.736 1.00 0.00
A N
ATOM 2946 CD PRO A 193 38.287 49.910 45.691 1.00 0.00
A C
ATOM 2947 HD1 PRO A 193 37.470 49.354 45.178 1.00 0.00
A H
ATOM 2948 HD2 PRO A 193 38.896 49.218 46.317 1.00 0.00
A H
ATOM 2949 CA PRO A 193 38.954 52.087 44.832 1.00 0.00
A C
ATOM 2950 HA PRO A 193 38.783 52.515 43.851 1.00 0.00
A H
ATOM 2951 CB PRO A 193 37.707 52.228 45.711 1.00 0.00
A C
ATOM 2952 HB1 PRO A 193 36.800 52.148 45.070 1.00 0.00
A H
ATOM 2953 HB2 PRO A 193 37.677 53.192 46.260 1.00 0.00
A H
ATOM 2954 CG PRO A 193 37.709 51.003 46.547 1.00 0.00
A C
ATOM 2955 HG1 PRO A 193 36.703 50.741 46.931 1.00 0.00
A H
ATOM 2956 HG2 PRO A 193 38.408 51.144 47.403 1.00 0.00
A H
ATOM 2957 C PRO A 193 40.161 52.760 45.463 1.00 0.00
A C
ATOM 2958 O PRO A 193 40.656 52.344 46.513 1.00 0.00
A O
ATOM 2959 N ALA A 194 40.636 53.812 44.800 1.00 0.00
A N
ATOM 2960 HN ALA A 194 40.250 54.123 43.932 1.00 0.00
A H
ATOM 2961 CA ALA A 194 41.756 54.588 45.297 1.00 0.00
A C
ATOM 2962 HA ALA A 194 42.426 53.984 45.898 1.00 0.00
A H
ATOM 2963 CB ALA A 194 42.547 55.195 44.143 1.00 0.00
A C
ATOM 2964 HB1 ALA A 194 42.968 54.383 43.512 1.00 0.00
A H
ATOM 2965 HB2 ALA A 194 41.895 55.825 43.500 1.00 0.00
A H
ATOM 2966 HB3 ALA A 194 43.392 55.818 44.509 1.00 0.00
A H
ATOM 2967 C ALA A 194 41.231 55.681 46.208 1.00 0.00
A C
ATOM 2968 O ALA A 194 40.337 56.438 45.795 1.00 0.00
A O
ATOM 2969 N PRO A 195 41.722 55.786 47.440 1.00 0.00
A N

ATOM 2970 CD PRO A 195 42.715 54.911 48.085 1.00 0.00
A C
ATOM 2971 HD1 PRO A 195 41.785 54.474 47.657 1.00 0.00
A H
ATOM 2972 HD2 PRO A 195 43.573 54.212 47.947 1.00 0.00
A H
ATOM 2973 CA PRO A 195 41.255 56.851 48.338 1.00 0.00
A C
ATOM 2974 HA PRO A 195 40.197 56.743 48.544 1.00 0.00
A H
ATOM 2975 CB PRO A 195 42.085 56.627 49.607 1.00 0.00
A C
ATOM 2976 HB1 PRO A 195 41.582 55.863 50.239 1.00 0.00
A H
ATOM 2977 HB2 PRO A 195 42.219 57.554 50.200 1.00 0.00
A H
ATOM 2978 CG PRO A 195 42.522 55.202 49.537 1.00 0.00
A C
ATOM 2979 HG1 PRO A 195 42.767 54.777 50.531 1.00 0.00
A H
ATOM 2980 HG2 PRO A 195 43.411 55.122 48.871 1.00 0.00
A H
ATOM 2981 C PRO A 195 41.508 58.226 47.734 1.00 0.00
A C
ATOM 2982 O PRO A 195 42.572 58.487 47.169 1.00 0.00
A O
ATOM 2983 N GLU A 196 40.516 59.108 47.849 1.00 0.00
A N
ATOM 2984 HN GLU A 196 40.559 60.022 47.457 1.00 0.00
A H
ATOM 2985 CA GLU A 196 39.272 58.824 48.558 1.00 0.00
A C
ATOM 2986 HA GLU A 196 39.437 57.975 49.209 1.00 0.00
A H
ATOM 2987 CB GLU A 196 38.849 60.036 49.393 1.00 0.00
A C
ATOM 2988 HB1 GLU A 196 37.896 59.784 49.912 1.00 0.00
A H
ATOM 2989 HB2 GLU A 196 39.596 60.219 50.197 1.00 0.00
A H
ATOM 2990 CG GLU A 196 38.638 61.321 48.604 1.00 0.00
A C
ATOM 2991 HG1 GLU A 196 38.760 62.190 49.276 1.00 0.00
A H
ATOM 2992 HG2 GLU A 196 39.364 61.399 47.771 1.00 0.00
A H
ATOM 2993 CD GLU A 196 37.235 61.424 48.038 1.00 0.00
A C
ATOM 2994 OE1 GLU A 196 36.279 61.560 48.837 1.00 0.00
A O
ATOM 2995 OE2 GLU A 196 37.083 61.329 46.803 1.00 0.00
A O
ATOM 2996 C GLU A 196 38.166 58.434 47.577 1.00 0.00
A C

```
ATOM   2997  O   GLU A 196      38.346  58.533  46.367  1.00  0.00
A    O
ATOM   2998  N   ASP A 197      37.029  57.979  48.101  1.00  0.00
A    N
ATOM   2999  HN  ASP A 197      36.850  57.901  49.079  1.00  0.00
A    H
ATOM   3000  CA  ASP A 197      35.908  57.538  47.282  1.00  0.00
A    C
ATOM   3001  HA  ASP A 197      35.794  58.257  46.479  1.00  0.00
A    H
ATOM   3002  CB  ASP A 197      36.131  56.131  46.735  1.00  0.00
A    C
ATOM   3003  HB1 ASP A 197      37.207  55.995  46.501  1.00  0.00
A    H
ATOM   3004  HB2 ASP A 197      35.838  55.369  47.485  1.00  0.00
A    H
ATOM   3005  CG  ASP A 197      35.356  55.880  45.465  1.00  0.00
A    C
ATOM   3006  OD1 ASP A 197      34.952  56.863  44.808  1.00  0.00
A    O
ATOM   3007  OD2 ASP A 197      35.132  54.695  45.135  1.00  0.00
A    O
ATOM   3008  C   ASP A 197      34.619  57.570  48.085  1.00  0.00
A    C
ATOM   3009  O   ASP A 197      34.557  57.055  49.202  1.00  0.00
A    O
ATOM   3010  N   ILE A 198      33.573  58.123  47.476  1.00  0.00
A    N
ATOM   3011  HN  ILE A 198      33.584  58.446  46.531  1.00  0.00
A    H
ATOM   3012  CA  ILE A 198      32.294  58.309  48.157  1.00  0.00
A    C
ATOM   3013  HA  ILE A 198      32.508  58.748  49.123  1.00  0.00
A    H
ATOM   3014  CB  ILE A 198      31.350  59.208  47.334  1.00  0.00
A    C
ATOM   3015  HB  ILE A 198      31.656  59.036  46.272  1.00  0.00
A    H
ATOM   3016  CG2 ILE A 198      29.886  58.897  47.657  1.00  0.00
A    C
ATOM   3017 HG21 ILE A 198      29.210  59.430  46.956  1.00  0.00
A    H
ATOM   3018 HG22 ILE A 198      29.681  57.810  47.561  1.00  0.00
A    H
ATOM   3019 HG23 ILE A 198      29.631  59.212  48.691  1.00  0.00
A    H
ATOM   3020  CG1 ILE A 198      31.604  60.687  47.630  1.00  0.00
A    C
ATOM   3021 HG11 ILE A 198      31.176  60.952  48.623  1.00  0.00
A    H
ATOM   3022 HG12 ILE A 198      32.703  60.859  47.690  1.00  0.00
A    H
ATOM   3023  CD  ILE A 198      31.042  61.613  46.592  1.00  0.00
A    C
```

ATOM   3024 HD1 ILE A 198   31.307 62.666 46.830 1.00 0.00
A   H
ATOM   3025 HD2 ILE A 198   31.450 61.370 45.587 1.00 0.00
A   H
ATOM   3026 HD3 ILE A 198   29.934 61.538 46.550 1.00 0.00
A   H
ATOM   3027 C   ILE A 198   31.659 56.949 48.422 1.00 0.00
A   C
ATOM   3028 O   ILE A 198   31.369 56.185 47.493 1.00 0.00
A   O
ATOM   3029 N   ILE A 199   31.444 56.646 49.698 1.00 0.00
A   N
ATOM   3030 HN ILE A 199   31.730 57.241 50.447 1.00 0.00
A   H
ATOM   3031 CA ILE A 199   30.777 55.424 50.128 1.00 0.00
A   C
ATOM   3032 HA ILE A 199   30.659 54.781 49.264 1.00 0.00
A   H
ATOM   3033 CB ILE A 199   31.549 54.720 51.257 1.00 0.00
A   C
ATOM   3034 HB ILE A 199   31.634 55.493 52.063 1.00 0.00
A   H
ATOM   3035 CG2 ILE A 199   30.808 53.477 51.720 1.00 0.00
A   C
ATOM   3036 HG21 ILE A 199   31.280 53.058 52.632 1.00 0.00
A   H
ATOM   3037 HG22 ILE A 199   29.751 53.715 51.966 1.00 0.00
A   H
ATOM   3038 HG23 ILE A 199   30.818 52.693 50.933 1.00 0.00
A   H
ATOM   3039 CG1 ILE A 199   32.975 54.393 50.798 1.00 0.00
A   C
ATOM   3040 HG11 ILE A 199   32.950 53.955 49.775 1.00 0.00
A   H
ATOM   3041 HG12 ILE A 199   33.560 55.339 50.738 1.00 0.00
A   H
ATOM   3042 CD ILE A 199   33.721 53.452 51.703 1.00 0.00
A   C
ATOM   3043 HD1 ILE A 199   34.761 53.310 51.337 1.00 0.00
A   H
ATOM   3044 HD2 ILE A 199   33.768 53.857 52.737 1.00 0.00
A   H
ATOM   3045 HD3 ILE A 199   33.229 52.457 51.735 1.00 0.00
A   H
ATOM   3046 C   ILE A 199   29.379 55.816 50.578 1.00 0.00
A   C
ATOM   3047 O   ILE A 199   29.206 56.357 51.673 1.00 0.00
A   O
ATOM   3048 N   ARG A 200   28.389 55.571 49.729 1.00 0.00
A   N
ATOM   3049 HN ARG A 200   28.553 55.161 48.835 1.00 0.00
A   H
ATOM   3050 CA ARG A 200   27.009 55.888 50.056 1.00 0.00
A   C

```
ATOM   3051  HA  ARG A 200      26.995  56.768  50.688  1.00  0.00
A    H
ATOM   3052  CB  ARG A 200      26.210  56.141  48.782  1.00  0.00
A    C
ATOM   3053  HB1 ARG A 200      26.545  55.375  48.041  1.00  0.00
A    H
ATOM   3054  HB2 ARG A 200      25.133  55.934  48.970  1.00  0.00
A    H
ATOM   3055  CG  ARG A 200      26.427  57.519  48.203  1.00  0.00
A    C
ATOM   3056  HG1 ARG A 200      26.720  58.207  49.026  1.00  0.00
A    H
ATOM   3057  HG2 ARG A 200      27.301  57.488  47.513  1.00  0.00
A    H
ATOM   3058  CD  ARG A 200      25.210  57.995  47.436  1.00  0.00
A    C
ATOM   3059  HD1 ARG A 200      25.165  57.473  46.453  1.00  0.00
A    H
ATOM   3060  HD2 ARG A 200      24.267  57.787  47.995  1.00  0.00
A    H
ATOM   3061  NE  ARG A 200      25.243  59.436  47.205  1.00  0.00
A    N
ATOM   3062  HE  ARG A 200      26.139  59.848  47.404  1.00  0.00
A    H
ATOM   3063  CZ  ARG A 200      24.205  60.144  46.776  1.00  0.00
A    C
ATOM   3064  NH1 ARG A 200      23.049  59.544  46.527  1.00  0.00
A    N
ATOM   3065 HH11 ARG A 200      22.277  60.060  46.182  1.00  0.00
A    H
ATOM   3066 HH12 ARG A 200      23.049  58.543  46.575  1.00  0.00
A    H
ATOM   3067  NH2 ARG A 200      24.319  61.452  46.592  1.00  0.00
A    N
ATOM   3068 HH21 ARG A 200      23.522  61.989  46.356  1.00  0.00
A    H
ATOM   3069 HH22 ARG A 200      25.160  61.877  46.902  1.00  0.00
A    H
ATOM   3070  C   ARG A 200      26.381  54.758  50.868  1.00  0.00
A    C
ATOM   3071  O   ARG A 200      26.963  53.688  51.050  1.00  0.00
A    O
ATOM   3072  N   GLU A 201      25.175  55.012  51.364  1.00  0.00
A    N
ATOM   3073  HN  GLU A 201      24.708  55.891  51.336  1.00  0.00
A    H
ATOM   3074  CA  GLU A 201      24.415  53.959  52.017  1.00  0.00
A    C
ATOM   3075  HA  GLU A 201      25.034  53.503  52.780  1.00  0.00
A    H
ATOM   3076  CB  GLU A 201      23.136  54.513  52.636  1.00  0.00
A    C
ATOM   3077  HB1 GLU A 201      22.406  53.676  52.731  1.00  0.00
A    H
```

```
ATOM   3078  HB2 GLU A 201      23.339  54.861  53.674  1.00  0.00
A    H
ATOM   3079  CG  GLU A 201      22.491  55.628  51.846  1.00  0.00
A    C
ATOM   3080  HG1 GLU A 201      23.076  56.557  51.974  1.00  0.00
A    H
ATOM   3081  HG2 GLU A 201      22.442  55.374  50.769  1.00  0.00
A    H
ATOM   3082  CD  GLU A 201      21.096  55.940  52.340  1.00  0.00
A    C
ATOM   3083  OE1 GLU A 201      20.860  55.829  53.560  1.00  0.00
A    O
ATOM   3084  OE2 GLU A 201      20.233  56.296  51.509  1.00  0.00
A    O
ATOM   3085  C   GLU A 201      24.072  52.876  51.007  1.00  0.00
A    C
ATOM   3086  O   GLU A 201      24.123  53.097  49.793  1.00  0.00
A    O
ATOM   3087  N   GLN A 202      23.738  51.695  51.518  1.00  0.00
A    N
ATOM   3088  HN  GLN A 202      23.752  51.534  52.503  1.00  0.00
A    H
ATOM   3089  CA  GLN A 202      23.328  50.543  50.724  1.00  0.00
A    C
ATOM   3090  HA  GLN A 202      23.210  49.717  51.415  1.00  0.00
A    H
ATOM   3091  CB  GLN A 202      21.990  50.806  50.023  1.00  0.00
A    C
ATOM   3092  HB1 GLN A 202      22.120  51.733  49.417  1.00  0.00
A    H
ATOM   3093  HB2 GLN A 202      21.785  49.986  49.298  1.00  0.00
A    H
ATOM   3094  CG  GLN A 202      20.846  50.981  51.006  1.00  0.00
A    C
ATOM   3095  HG1 GLN A 202      21.070  50.455  51.960  1.00  0.00
A    H
ATOM   3096  HG2 GLN A 202      20.689  52.055  51.238  1.00  0.00
A    H
ATOM   3097  CD  GLN A 202      19.525  50.407  50.523  1.00  0.00
A    C
ATOM   3098  OE1 GLN A 202      19.494  49.445  49.763  1.00  0.00
A    O
ATOM   3099  NE2 GLN A 202      18.424  50.999  50.971  1.00  0.00
A    N
ATOM   3100 HE21 GLN A 202      17.554  50.625  50.662  1.00  0.00
A    H
ATOM   3101 HE22 GLN A 202      18.496  51.786  51.574  1.00  0.00
A    H
ATOM   3102  C   GLN A 202      24.389  50.144  49.709  1.00  0.00
A    C
ATOM   3103  O   GLN A 202      24.068  49.529  48.683  1.00  0.00
A    O
ATOM   3104  N   ASP A 203      25.649  50.475  49.975  1.00  0.00
A    N
```

ATOM 3105 HN ASP A 203 25.954 50.963 50.789 1.00 0.00
A H
ATOM 3106 CA ASP A 203 26.738 50.144 49.074 1.00 0.00
A C
ATOM 3107 HA ASP A 203 26.325 50.107 48.072 1.00 0.00
A H
ATOM 3108 CB ASP A 203 27.880 51.143 49.200 1.00 0.00
A C
ATOM 3109 HB1 ASP A 203 27.468 52.172 49.178 1.00 0.00
A H
ATOM 3110 HB2 ASP A 203 28.417 51.002 50.158 1.00 0.00
A H
ATOM 3111 CG ASP A 203 28.864 51.022 48.071 1.00 0.00
A C
ATOM 3112 OD1 ASP A 203 29.900 50.360 48.269 1.00 0.00
A O
ATOM 3113 OD2 ASP A 203 28.627 51.618 47.013 1.00 0.00
A O
ATOM 3114 C ASP A 203 27.283 48.760 49.374 1.00 0.00
A C
ATOM 3115 O ASP A 203 27.471 48.392 50.535 1.00 0.00
A O
ATOM 3116 N CYS A 204 27.580 48.011 48.320 1.00 0.00
A N
ATOM 3117 HN CYS A 204 27.381 48.259 47.374 1.00 0.00
A H
ATOM 3118 CA CYS A 204 28.243 46.725 48.487 1.00 0.00
A C
ATOM 3119 HA CYS A 204 27.942 46.328 49.450 1.00 0.00
A H
ATOM 3120 CB CYS A 204 27.837 45.764 47.375 1.00 0.00
A C
ATOM 3121 HB1 CYS A 204 27.880 46.330 46.417 1.00 0.00
A H
ATOM 3122 HB2 CYS A 204 28.592 44.952 47.285 1.00 0.00
A H
ATOM 3123 SG CYS A 204 26.172 45.093 47.575 1.00 0.00
A S
ATOM 3124 HG1 CYS A 204 26.139 44.389 46.441 1.00 0.00
A H
ATOM 3125 C CYS A 204 29.752 46.941 48.525 1.00 0.00
A C
ATOM 3126 O CYS A 204 30.343 47.442 47.561 1.00 0.00
A O
ATOM 3127 N LEU A 205 30.358 46.594 49.658 1.00 0.00
A N
ATOM 3128 HN LEU A 205 29.835 46.188 50.404 1.00 0.00
A H
ATOM 3129 CA LEU A 205 31.778 46.753 49.941 1.00 0.00
A C
ATOM 3130 HA LEU A 205 32.215 47.375 49.171 1.00 0.00
A H
ATOM 3131 CB LEU A 205 31.987 47.320 51.350 1.00 0.00
A C

ATOM 3132 HB1 LEU A 205 31.041 47.153 51.915 1.00 0.00
A H
ATOM 3133 HB2 LEU A 205 32.766 46.719 51.869 1.00 0.00
A H
ATOM 3134 CG LEU A 205 32.340 48.773 51.657 1.00 0.00
A C
ATOM 3135 HG LEU A 205 33.366 49.001 51.281 1.00 0.00
A H
ATOM 3136 CD1 LEU A 205 31.335 49.736 51.050 1.00 0.00
A C
ATOM 3137 HD11 LEU A 205 31.583 50.783 51.326 1.00 0.00
A H
ATOM 3138 HD12 LEU A 205 31.338 49.656 49.942 1.00 0.00
A H
ATOM 3139 HD13 LEU A 205 30.310 49.514 51.415 1.00 0.00
A H
ATOM 3140 CD2 LEU A 205 32.348 48.930 53.161 1.00 0.00
A C
ATOM 3141 HD21 LEU A 205 32.620 49.972 53.439 1.00 0.00
A H
ATOM 3142 HD22 LEU A 205 31.354 48.713 53.600 1.00 0.00
A H
ATOM 3143 HD23 LEU A 205 33.092 48.244 53.619 1.00 0.00
A H
ATOM 3144 C LEU A 205 32.410 45.373 49.834 1.00 0.00
A C
ATOM 3145 O LEU A 205 31.882 44.407 50.399 1.00 0.00
A O
ATOM 3146 N VAL A 206 33.516 45.272 49.105 1.00 0.00
A N
ATOM 3147 HN VAL A 206 33.914 46.025 48.588 1.00 0.00
A H
ATOM 3148 CA VAL A 206 34.253 44.018 49.005 1.00 0.00
A C
ATOM 3149 HA VAL A 206 33.641 43.221 49.409 1.00 0.00
A H
ATOM 3150 CB VAL A 206 34.611 43.676 47.556 1.00 0.00
A C
ATOM 3151 HB VAL A 206 35.334 44.449 47.196 1.00 0.00
A H
ATOM 3152 CG1 VAL A 206 35.259 42.310 47.486 1.00 0.00
A C
ATOM 3153 HG11 VAL A 206 35.484 42.044 46.430 1.00 0.00
A H
ATOM 3154 HG12 VAL A 206 36.217 42.282 48.045 1.00 0.00
A H
ATOM 3155 HG13 VAL A 206 34.582 41.532 47.899 1.00 0.00
A H
ATOM 3156 CG2 VAL A 206 33.354 43.713 46.694 1.00 0.00
A C
ATOM 3157 HG21 VAL A 206 33.588 43.428 45.646 1.00 0.00
A H
ATOM 3158 HG22 VAL A 206 32.591 43.006 47.086 1.00 0.00
A H

ATOM   3159 HG23 VAL A 206   32.910 44.730 46.673 1.00 0.00
A   H
ATOM   3160 C   VAL A 206   35.495 44.127 49.876 1.00 0.00
A   C
ATOM   3161 O   VAL A 206   36.370 44.970 49.637 1.00 0.00
A   O
ATOM   3162 N   ILE A 207   35.579 43.262 50.879 1.00 0.00
A   N
ATOM   3163 HN ILE A 207   34.895 42.558 51.057 1.00 0.00
A   H
ATOM   3164 CA ILE A 207   36.687 43.266 51.816 1.00 0.00
A   C
ATOM   3165 HA ILE A 207   37.452 43.918 51.414 1.00 0.00
A   H
ATOM   3166 CB ILE A 207   36.250 43.731 53.221 1.00 0.00
A   C
ATOM   3167 HB ILE A 207   35.657 44.662 53.035 1.00 0.00
A   H
ATOM   3168 CG2 ILE A 207   35.459 42.645 53.910 1.00 0.00
A   C
ATOM   3169 HG21 ILE A 207   35.011 43.026 54.852 1.00 0.00
A   H
ATOM   3170 HG22 ILE A 207   34.631 42.287 53.263 1.00 0.00
A   H
ATOM   3171 HG23 ILE A 207   36.108 41.778 54.160 1.00 0.00
A   H
ATOM   3172 CG1 ILE A 207   37.466 44.141 54.057 1.00 0.00
A   C
ATOM   3173 HG11 ILE A 207   38.170 43.284 54.144 1.00 0.00
A   H
ATOM   3174 HG12 ILE A 207   38.007 44.959 53.529 1.00 0.00
A   H
ATOM   3175 CD ILE A 207   37.115 44.620 55.421 1.00 0.00
A   C
ATOM   3176 HD1 ILE A 207   38.028 44.957 55.958 1.00 0.00
A   H
ATOM   3177 HD2 ILE A 207   36.408 45.476 55.366 1.00 0.00
A   H
ATOM   3178 HD3 ILE A 207   36.644 43.812 56.018 1.00 0.00
A   H
ATOM   3179 C   ILE A 207   37.282 41.866 51.861 1.00 0.00
A   C
ATOM   3180 O   ILE A 207   36.648 40.881 51.484 1.00 0.00
A   O
ATOM   3181 N   MET A 208   38.521 41.779 52.327 1.00 0.00
A   N
ATOM   3182 HN MET A 208   39.070 42.560 52.616 1.00 0.00
A   H
ATOM   3183 CA MET A 208   39.192 40.493 52.456 1.00 0.00
A   C
ATOM   3184 HA MET A 208   38.457 39.753 52.747 1.00 0.00
A   H
ATOM   3185 CB MET A 208   39.886 40.071 51.151 1.00 0.00
A   C

ATOM 3186 HB1 MET A 208 39.081 39.797 50.430 1.00 0.00
A H
ATOM 3187 HB2 MET A 208 40.413 40.947 50.713 1.00 0.00
A H
ATOM 3188 CG MET A 208 40.825 38.888 51.348 1.00 0.00
A C
ATOM 3189 HG1 MET A 208 41.320 38.949 52.340 1.00 0.00
A H
ATOM 3190 HG2 MET A 208 40.244 37.941 51.320 1.00 0.00
A H
ATOM 3191 SD MET A 208 42.120 38.771 50.126 1.00 0.00
A S
ATOM 3192 CE MET A 208 42.729 40.438 50.167 1.00 0.00
A C
ATOM 3193 HE1 MET A 208 43.561 40.575 49.444 1.00 0.00
A H
ATOM 3194 HE2 MET A 208 41.928 41.162 49.906 1.00 0.00
A H
ATOM 3195 HE3 MET A 208 43.111 40.700 51.177 1.00 0.00
A H
ATOM 3196 C MET A 208 40.212 40.593 53.576 1.00 0.00
A C
ATOM 3197 O MET A 208 40.835 41.641 53.762 1.00 0.00
A O
ATOM 3198 N GLY A 209 40.386 39.501 54.308 1.00 0.00
A N
ATOM 3199 HN GLY A 209 39.811 38.685 54.250 1.00 0.00
A H
ATOM 3200 CA GLY A 209 41.467 39.437 55.267 1.00 0.00
A C
ATOM 3201 HA1 GLY A 209 41.510 40.401 55.756 1.00 0.00
A H
ATOM 3202 HA2 GLY A 209 42.363 39.176 54.721 1.00 0.00
A H
ATOM 3203 C GLY A 209 41.245 38.384 56.327 1.00 0.00
A C
ATOM 3204 O GLY A 209 40.566 37.383 56.088 1.00 0.00
A O
ATOM 3205 N HSE A 210 41.815 38.607 57.507 1.00 0.00
A N
ATOM 3206 HN HSE A 210 42.414 39.378 57.716 1.00 0.00
A H
ATOM 3207 CA HSE A 210 41.595 37.701 58.619 1.00 0.00
A C
ATOM 3208 HA HSE A 210 41.751 36.691 58.260 1.00 0.00
A H
ATOM 3209 CB HSE A 210 42.521 38.033 59.784 1.00 0.00
A C
ATOM 3210 HB1 HSE A 210 43.568 37.953 59.421 1.00 0.00
A H
ATOM 3211 HB2 HSE A 210 42.363 39.087 60.095 1.00 0.00
A H
ATOM 3212 ND1 HSE A 210 42.674 35.772 60.886 1.00 0.00
A N

```
ATOM   3213  CG  HSE A 210      42.360  37.113  60.949  1.00  0.00
A    C
ATOM   3214  CE1 HSE A 210      42.424  35.209  62.055  1.00  0.00
A    C
ATOM   3215  HE1 HSE A 210      42.591  34.154  62.283  1.00  0.00
A    H
ATOM   3216  NE2 HSE A 210      41.943  36.134  62.867  1.00  0.00
A    N
ATOM   3217  HE2 HSE A 210      41.651  35.977  63.810  1.00  0.00
A    H
ATOM   3218  CD2 HSE A 210      41.885  37.330  62.197  1.00  0.00
A    C
ATOM   3219  HD2 HSE A 210      41.507  38.238  62.646  1.00  0.00
A    H
ATOM   3220  C   HSE A 210      40.148  37.798  59.062  1.00  0.00
A    C
ATOM   3221  O   HSE A 210      39.679  38.865  59.463  1.00  0.00
A    O
ATOM   3222  N   LYS A 211      39.444  36.666  58.991  1.00  0.00
A    N
ATOM   3223  HN  LYS A 211      39.842  35.772  58.790  1.00  0.00
A    H
ATOM   3224  CA  LYS A 211      38.001  36.668  59.209  1.00  0.00
A    C
ATOM   3225  HA  LYS A 211      37.565  37.282  58.431  1.00  0.00
A    H
ATOM   3226  CB  LYS A 211      37.461  35.242  59.135  1.00  0.00
A    C
ATOM   3227  HB1 LYS A 211      36.397  35.277  59.465  1.00  0.00
A    H
ATOM   3228  HB2 LYS A 211      37.448  34.913  58.072  1.00  0.00
A    H
ATOM   3229  CG  LYS A 211      38.213  34.243  59.995  1.00  0.00
A    C
ATOM   3230  HG1 LYS A 211      39.244  34.118  59.593  1.00  0.00
A    H
ATOM   3231  HG2 LYS A 211      38.306  34.651  61.028  1.00  0.00
A    H
ATOM   3232  CD  LYS A 211      37.517  32.894  60.016  1.00  0.00
A    C
ATOM   3233  HD1 LYS A 211      36.431  33.062  60.200  1.00  0.00
A    H
ATOM   3234  HD2 LYS A 211      37.618  32.436  59.005  1.00  0.00
A    H
ATOM   3235  CE  LYS A 211      38.120  31.996  61.075  1.00  0.00
A    C
ATOM   3236  HE1 LYS A 211      37.979  32.437  62.087  1.00  0.00
A    H
ATOM   3237  HE2 LYS A 211      37.638  30.994  61.059  1.00  0.00
A    H
ATOM   3238  NZ  LYS A 211      39.579  31.794  60.875  1.00  0.00
A    N
ATOM   3239  HZ1 LYS A 211      39.959  31.174  61.621  1.00  0.00
A    H
```

ATOM 3240 HZ2 LYS A 211 39.746 31.357 59.945 1.00 0.00
A H
ATOM 3241 HZ3 LYS A 211 40.064 32.714 60.913 1.00 0.00
A H
ATOM 3242 C LYS A 211 37.635 37.300 60.545 1.00 0.00
A C
ATOM 3243 O LYS A 211 36.597 37.960 60.664 1.00 0.00
A O
ATOM 3244 N LYS A 212 38.476 37.119 61.563 1.00 0.00
A N
ATOM 3245 HN LYS A 212 39.350 36.636 61.518 1.00 0.00
A H
ATOM 3246 CA LYS A 212 38.133 37.652 62.874 1.00 0.00
A C
ATOM 3247 HA LYS A 212 37.072 37.484 63.018 1.00 0.00
A H
ATOM 3248 CB LYS A 212 38.908 36.917 63.964 1.00 0.00
A C
ATOM 3249 HB1 LYS A 212 39.991 37.111 63.785 1.00 0.00
A H
ATOM 3250 HB2 LYS A 212 38.668 37.374 64.950 1.00 0.00
A H
ATOM 3251 CG LYS A 212 38.666 35.401 63.990 1.00 0.00
A C
ATOM 3252 HG1 LYS A 212 39.399 34.903 63.314 1.00 0.00
A H
ATOM 3253 HG2 LYS A 212 38.854 35.021 65.021 1.00 0.00
A H
ATOM 3254 CD LYS A 212 37.252 34.996 63.545 1.00 0.00
A C
ATOM 3255 HD1 LYS A 212 37.023 35.515 62.586 1.00 0.00
A H
ATOM 3256 HD2 LYS A 212 37.255 33.900 63.339 1.00 0.00
A H
ATOM 3257 CE LYS A 212 36.182 35.318 64.579 1.00 0.00
A C
ATOM 3258 HE1 LYS A 212 36.572 35.153 65.608 1.00 0.00
A H
ATOM 3259 HE2 LYS A 212 35.860 36.378 64.490 1.00 0.00
A H
ATOM 3260 NZ LYS A 212 34.978 34.454 64.402 1.00 0.00
A N
ATOM 3261 HZ1 LYS A 212 34.262 34.690 65.119 1.00 0.00
A H
ATOM 3262 HZ2 LYS A 212 34.580 34.604 63.453 1.00 0.00
A H
ATOM 3263 HZ3 LYS A 212 35.252 33.456 64.503 1.00 0.00
A H
ATOM 3264 C LYS A 212 38.367 39.157 62.935 1.00 0.00
A C
ATOM 3265 O LYS A 212 37.575 39.886 63.539 1.00 0.00
A O
ATOM 3266 N ASP A 213 39.424 39.650 62.287 1.00 0.00
A N

```
ATOM   3267  HN  ASP A 213      40.169  39.143  61.861  1.00  0.00
A    H
ATOM   3268  CA  ASP A 213      39.568  41.096  62.150  1.00  0.00
A    C
ATOM   3269  HA  ASP A 213      39.483  41.521  63.143  1.00  0.00
A    H
ATOM   3270  CB  ASP A 213      40.911  41.443  61.511  1.00  0.00
A    C
ATOM   3271  HB1 ASP A 213      41.070  40.801  60.622  1.00  0.00
A    H
ATOM   3272  HB2 ASP A 213      40.926  42.502  61.183  1.00  0.00
A    H
ATOM   3273  CG  ASP A 213      42.079  41.216  62.444  1.00  0.00
A    C
ATOM   3274  OD1 ASP A 213      42.379  42.118  63.256  1.00  0.00
A    O
ATOM   3275  OD2 ASP A 213      42.697  40.137  62.376  1.00  0.00
A    O
ATOM   3276  C   ASP A 213      38.430  41.680  61.323  1.00  0.00
A    C
ATOM   3277  O   ASP A 213      37.974  42.802  61.580  1.00  0.00
A    O
ATOM   3278  N   ILE A 214      37.961  40.932  60.323  1.00  0.00
A    N
ATOM   3279  HN  ILE A 214      38.326  40.045  60.048  1.00  0.00
A    H
ATOM   3280  CA  ILE A 214      36.825  41.385  59.526  1.00  0.00
A    C
ATOM   3281  HA  ILE A 214      37.060  42.386  59.185  1.00  0.00
A    H
ATOM   3282  CB  ILE A 214      36.593  40.442  58.333  1.00  0.00
A    C
ATOM   3283  HB  ILE A 214      36.430  39.437  58.799  1.00  0.00
A    H
ATOM   3284  CG2 ILE A 214      35.437  40.938  57.488  1.00  0.00
A    C
ATOM   3285 HG21 ILE A 214      35.163  40.185  56.719  1.00  0.00
A    H
ATOM   3286 HG22 ILE A 214      34.538  41.121  58.113  1.00  0.00
A    H
ATOM   3287 HG23 ILE A 214      35.701  41.884  56.969  1.00  0.00
A    H
ATOM   3288  CG1 ILE A 214      37.856  40.336  57.484  1.00  0.00
A    C
ATOM   3289 HG11 ILE A 214      38.010  41.282  56.918  1.00  0.00
A    H
ATOM   3290 HG12 ILE A 214      38.735  40.209  58.158  1.00  0.00
A    H
ATOM   3291  CD  ILE A 214      37.849  39.183  56.509  1.00  0.00
A    C
ATOM   3292  HD1 ILE A 214      38.818  39.130  55.967  1.00  0.00
A    H
ATOM   3293  HD2 ILE A 214      37.696  38.220  57.043  1.00  0.00
A    H
```

ATOM 3294 HD3 ILE A 214 37.041 39.302 55.757 1.00 0.00
A H
ATOM 3295 C ILE A 214 35.584 41.498 60.398 1.00 0.00
A C
ATOM 3296 O ILE A 214 34.831 42.474 60.313 1.00 0.00
A O
ATOM 3297 N LYS A 215 35.356 40.501 61.257 1.00 0.00
A N
ATOM 3298 HN LYS A 215 35.889 39.658 61.305 1.00 0.00
A H
ATOM 3299 CA LYS A 215 34.263 40.598 62.214 1.00 0.00
A C
ATOM 3300 HA LYS A 215 33.354 40.755 61.646 1.00 0.00
A H
ATOM 3301 CB LYS A 215 34.160 39.315 63.042 1.00 0.00
A C
ATOM 3302 HB1 LYS A 215 34.076 38.468 62.324 1.00 0.00
A H
ATOM 3303 HB2 LYS A 215 35.113 39.164 63.595 1.00 0.00
A H
ATOM 3304 CG LYS A 215 32.967 39.277 64.001 1.00 0.00
A C
ATOM 3305 HG1 LYS A 215 32.128 39.863 63.562 1.00 0.00
A H
ATOM 3306 HG2 LYS A 215 32.618 38.223 64.103 1.00 0.00
A H
ATOM 3307 CD LYS A 215 33.324 39.857 65.367 1.00 0.00
A C
ATOM 3308 HD1 LYS A 215 34.413 39.701 65.542 1.00 0.00
A H
ATOM 3309 HD2 LYS A 215 33.145 40.956 65.334 1.00 0.00
A H
ATOM 3310 CE LYS A 215 32.520 39.238 66.484 1.00 0.00
A C
ATOM 3311 HE1 LYS A 215 31.431 39.381 66.306 1.00 0.00
A H
ATOM 3312 HE2 LYS A 215 32.725 38.147 66.555 1.00 0.00
A H
ATOM 3313 NZ LYS A 215 32.868 39.866 67.783 1.00 0.00
A N
ATOM 3314 HZ1 LYS A 215 32.309 39.435 68.548 1.00 0.00
A H
ATOM 3315 HZ2 LYS A 215 33.881 39.727 67.975 1.00 0.00
A H
ATOM 3316 HZ3 LYS A 215 32.665 40.885 67.740 1.00 0.00
A H
ATOM 3317 C LYS A 215 34.437 41.797 63.130 1.00 0.00
A C
ATOM 3318 O LYS A 215 33.456 42.464 63.466 1.00 0.00
A O
ATOM 3319 N ARG A 216 35.664 42.049 63.587 1.00 0.00
A N
ATOM 3320 HN ARG A 216 36.456 41.488 63.358 1.00 0.00
A H

```
ATOM   3321  CA  ARG A 216      35.902  43.188  64.467  1.00  0.00
A    C
ATOM   3322  HA  ARG A 216      35.264  43.089  65.337  1.00  0.00
A    H
ATOM   3323  CB  ARG A 216      37.370  43.220  64.889  1.00  0.00
A    C
ATOM   3324  HB1 ARG A 216      37.970  43.186  63.949  1.00  0.00
A    H
ATOM   3325  HB2 ARG A 216      37.599  44.200  65.364  1.00  0.00
A    H
ATOM   3326  CG  ARG A 216      37.769  42.056  65.775  1.00  0.00
A    C
ATOM   3327  HG1 ARG A 216      37.408  42.257  66.808  1.00  0.00
A    H
ATOM   3328  HG2 ARG A 216      37.225  41.144  65.441  1.00  0.00
A    H
ATOM   3329  CD  ARG A 216      39.260  41.787  65.714  1.00  0.00
A    C
ATOM   3330  HD1 ARG A 216      39.638  42.025  64.693  1.00  0.00
A    H
ATOM   3331  HD2 ARG A 216      39.818  42.406  66.455  1.00  0.00
A    H
ATOM   3332  NE  ARG A 216      39.558  40.391  66.022  1.00  0.00
A    N
ATOM   3333  HE  ARG A 216      38.766  39.889  66.388  1.00  0.00
A    H
ATOM   3334  CZ  ARG A 216      40.746  39.820  65.846  1.00  0.00
A    C
ATOM   3335  NH1 ARG A 216      41.759  40.526  65.367  1.00  0.00
A    N
ATOM   3336 HH11 ARG A 216      42.640  40.102  65.209  1.00  0.00
A    H
ATOM   3337 HH12 ARG A 216      41.544  41.453  65.052  1.00  0.00
A    H
ATOM   3338  NH2 ARG A 216      40.922  38.542  66.153  1.00  0.00
A    N
ATOM   3339 HH21 ARG A 216      41.827  38.143  66.103  1.00  0.00
A    H
ATOM   3340 HH22 ARG A 216      40.178  38.087  66.622  1.00  0.00
A    H
ATOM   3341  C   ARG A 216      35.510  44.490  63.786  1.00  0.00
A    C
ATOM   3342  O   ARG A 216      34.795  45.314  64.369  1.00  0.00
A    O
ATOM   3343  N   PHE A 217      35.965  44.684  62.550  1.00  0.00
A    N
ATOM   3344  HN  PHE A 217      36.561  44.058  62.049  1.00  0.00
A    H
ATOM   3345  CA  PHE A 217      35.601  45.888  61.815  1.00  0.00
A    C
ATOM   3346  HA  PHE A 217      35.862  46.724  62.453  1.00  0.00
A    H
ATOM   3347  CB  PHE A 217      36.350  45.949  60.487  1.00  0.00
A    C
```

ATOM 3348 HB1 PHE A 217 37.345 46.418 60.652 1.00 0.00
A H
ATOM 3349 HB2 PHE A 217 36.531 44.930 60.083 1.00 0.00
A H
ATOM 3350 CG PHE A 217 35.661 46.781 59.451 1.00 0.00
A C
ATOM 3351 CD1 PHE A 217 35.613 48.157 59.570 1.00 0.00
A C
ATOM 3352 HD1 PHE A 217 36.075 48.637 60.421 1.00 0.00
A H
ATOM 3353 CE1 PHE A 217 34.978 48.926 58.619 1.00 0.00
A C
ATOM 3354 HE1 PHE A 217 34.951 50.001 58.722 1.00 0.00
A H
ATOM 3355 CZ PHE A 217 34.383 48.322 57.534 1.00 0.00
A C
ATOM 3356 HZ PHE A 217 33.892 48.922 56.782 1.00 0.00
A H
ATOM 3357 CD2 PHE A 217 35.059 46.186 58.358 1.00 0.00
A C
ATOM 3358 HD2 PHE A 217 35.065 45.110 58.257 1.00 0.00
A H
ATOM 3359 CE2 PHE A 217 34.424 46.951 57.403 1.00 0.00
A C
ATOM 3360 HE2 PHE A 217 33.957 46.473 56.554 1.00 0.00
A H
ATOM 3361 C PHE A 217 34.097 45.954 61.586 1.00 0.00
A C
ATOM 3362 O PHE A 217 33.498 47.031 61.678 1.00 0.00
A O
ATOM 3363 N GLU A 218 33.470 44.814 61.300 1.00 0.00
A N
ATOM 3364 HN GLU A 218 33.907 43.926 61.191 1.00 0.00
A H
ATOM 3365 CA GLU A 218 32.023 44.788 61.118 1.00 0.00
A C
ATOM 3366 HA GLU A 218 31.769 45.502 60.344 1.00 0.00
A H
ATOM 3367 CB GLU A 218 31.580 43.386 60.700 1.00 0.00
A C
ATOM 3368 HB1 GLU A 218 32.350 42.969 60.012 1.00 0.00
A H
ATOM 3369 HB2 GLU A 218 31.568 42.714 61.587 1.00 0.00
A H
ATOM 3370 CG GLU A 218 30.233 43.332 60.007 1.00 0.00
A C
ATOM 3371 HG1 GLU A 218 30.030 44.303 59.520 1.00 0.00
A H
ATOM 3372 HG2 GLU A 218 30.211 42.530 59.244 1.00 0.00
A H
ATOM 3373 CD GLU A 218 29.100 43.108 60.990 1.00 0.00
A C
ATOM 3374 OE1 GLU A 218 29.388 42.829 62.172 1.00 0.00
A O

```
ATOM   3375  OE2 GLU A 218      27.923  43.207  60.587  1.00  0.00
A    O
ATOM   3376  C   GLU A 218      31.296  45.216  62.383  1.00  0.00
A    C
ATOM   3377  O   GLU A 218      30.329  45.984  62.328  1.00  0.00
A    O
ATOM   3378  N   ASN A 219      31.753  44.733  63.534  1.00  0.00
A    N
ATOM   3379  HN  ASN A 219      32.571  44.169  63.630  1.00  0.00
A    H
ATOM   3380  CA  ASN A 219      31.059  45.008  64.783  1.00  0.00
A    C
ATOM   3381  HA  ASN A 219      30.003  44.879  64.572  1.00  0.00
A    H
ATOM   3382  CB  ASN A 219      31.535  44.039  65.864  1.00  0.00
A    C
ATOM   3383  HB1 ASN A 219      31.780  43.073  65.372  1.00  0.00
A    H
ATOM   3384  HB2 ASN A 219      32.464  44.416  66.338  1.00  0.00
A    H
ATOM   3385  CG  ASN A 219      30.480  43.775  66.917  1.00  0.00
A    C
ATOM   3386  OD1 ASN A 219      29.394  44.354  66.884  1.00  0.00
A    O
ATOM   3387  ND2 ASN A 219      30.792  42.895  67.860  1.00  0.00
A    N
ATOM   3388 HD21 ASN A 219      30.103  42.703  68.554  1.00  0.00
A    H
ATOM   3389 HD22 ASN A 219      31.675  42.435  67.839  1.00  0.00
A    H
ATOM   3390  C   ASN A 219      31.269  46.450  65.218  1.00  0.00
A    C
ATOM   3391  O   ASN A 219      30.345  47.101  65.717  1.00  0.00
A    O
ATOM   3392  N   GLU A 220      32.482  46.967  65.037  1.00  0.00
A    N
ATOM   3393  HN  GLU A 220      33.231  46.523  64.553  1.00  0.00
A    H
ATOM   3394  CA  GLU A 220      32.814  48.285  65.559  1.00  0.00
A    C
ATOM   3395  HA  GLU A 220      32.166  48.468  66.408  1.00  0.00
A    H
ATOM   3396  CB  GLU A 220      34.270  48.287  66.035  1.00  0.00
A    C
ATOM   3397  HB1 GLU A 220      34.928  48.217  65.138  1.00  0.00
A    H
ATOM   3398  HB2 GLU A 220      34.509  49.263  66.515  1.00  0.00
A    H
ATOM   3399  CG  GLU A 220      34.570  47.132  66.984  1.00  0.00
A    C
ATOM   3400  HG1 GLU A 220      34.486  47.482  68.029  1.00  0.00
A    H
ATOM   3401  HG2 GLU A 220      33.867  46.291  66.823  1.00  0.00
A    H
```

```
ATOM   3402  CD  GLU A 220      35.982  46.598  66.850  1.00  0.00
A    C
ATOM   3403  OE1 GLU A 220      36.593  46.782  65.777  1.00  0.00
A    O
ATOM   3404  OE2 GLU A 220      36.473  45.972  67.812  1.00  0.00
A    O
ATOM   3405  C   GLU A 220      32.568  49.405  64.550  1.00  0.00
A    C
ATOM   3406  O   GLU A 220      31.805  50.334  64.839  1.00  0.00
A    O
ATOM   3407  N   GLY A 221      33.186  49.341  63.373  1.00  0.00
A    N
ATOM   3408  HN  GLY A 221      33.790  48.585  63.120  1.00  0.00
A    H
ATOM   3409  CA  GLY A 221      33.022  50.376  62.371  1.00  0.00
A    C
ATOM   3410  HA1 GLY A 221      33.760  50.181  61.605  1.00  0.00
A    H
ATOM   3411  HA2 GLY A 221      33.148  51.323  62.878  1.00  0.00
A    H
ATOM   3412  C   GLY A 221      31.656  50.374  61.717  1.00  0.00
A    C
ATOM   3413  O   GLY A 221      31.254  51.362  61.096  1.00  0.00
A    O
ATOM   3414  N   MET A 222      30.938  49.265  61.838  1.00  0.00
A    N
ATOM   3415  HN  MET A 222      31.254  48.443  62.308  1.00  0.00
A    H
ATOM   3416  CA  MET A 222      29.597  49.160  61.281  1.00  0.00
A    C
ATOM   3417  HA  MET A 222      29.279  50.160  61.015  1.00  0.00
A    H
ATOM   3418  CB  MET A 222      29.595  48.255  60.051  1.00  0.00
A    C
ATOM   3419  HB1 MET A 222      29.875  47.234  60.399  1.00  0.00
A    H
ATOM   3420  HB2 MET A 222      28.559  48.175  59.654  1.00  0.00
A    H
ATOM   3421  CG  MET A 222      30.557  48.685  58.963  1.00  0.00
A    C
ATOM   3422  HG1 MET A 222      30.423  49.763  58.733  1.00  0.00
A    H
ATOM   3423  HG2 MET A 222      31.603  48.543  59.312  1.00  0.00
A    H
ATOM   3424  SD  MET A 222      30.310  47.749  57.442  1.00  0.00
A    S
ATOM   3425  CE  MET A 222      30.984  46.157  57.903  1.00  0.00
A    C
ATOM   3426  HE1 MET A 222      30.921  45.440  57.056  1.00  0.00
A    H
ATOM   3427  HE2 MET A 222      32.052  46.246  58.196  1.00  0.00
A    H
ATOM   3428  HE3 MET A 222      30.429  45.721  58.761  1.00  0.00
A    H
```

ATOM 3429 C MET A 222 28.621 48.630 62.321 1.00 0.00
A C
ATOM 3430 O MET A 222 27.576 49.228 62.562 1.00 0.00
A O
ATOM 3431 N ASN B 6 18.226 36.588 72.247 1.00 0.00
B N
ATOM 3432 HT1 ASN B 6 18.901 36.590 72.985 1.00 0.00
B H
ATOM 3433 HT2 ASN B 6 18.012 35.647 71.984 1.00 0.00
B H
ATOM 3434 HT3 ASN B 6 17.394 37.057 72.545 1.00 0.00
B H
ATOM 3435 CA ASN B 6 18.779 37.289 71.096 1.00 0.00
B C
ATOM 3436 HA ASN B 6 18.935 38.313 71.420 1.00 0.00
B H
ATOM 3437 CB ASN B 6 20.087 36.637 70.659 1.00 0.00
B C
ATOM 3438 HB1 ASN B 6 20.553 36.175 71.557 1.00 0.00
B H
ATOM 3439 HB2 ASN B 6 19.888 35.824 69.932 1.00 0.00
B H
ATOM 3440 CG ASN B 6 21.061 37.633 70.082 1.00 0.00
B C
ATOM 3441 OD1 ASN B 6 20.800 38.835 70.071 1.00 0.00
B O
ATOM 3442 ND2 ASN B 6 22.193 37.142 69.593 1.00 0.00
B N
ATOM 3443 HD21 ASN B 6 22.850 37.791 69.219 1.00 0.00
B H
ATOM 3444 HD22 ASN B 6 22.372 36.164 69.637 1.00 0.00
B H
ATOM 3445 C ASN B 6 17.785 37.316 69.932 1.00 0.00
B C
ATOM 3446 O ASN B 6 16.730 37.939 70.031 1.00 0.00
B O
ATOM 3447 N LYS B 7 18.125 36.647 68.829 1.00 0.00
B N
ATOM 3448 HN LYS B 7 19.008 36.190 68.733 1.00 0.00
B H
ATOM 3449 CA LYS B 7 17.238 36.527 67.677 1.00 0.00
B C
ATOM 3450 HA LYS B 7 16.352 37.114 67.887 1.00 0.00
B H
ATOM 3451 CB LYS B 7 17.937 37.034 66.416 1.00 0.00
B C
ATOM 3452 HB1 LYS B 7 17.274 36.794 65.553 1.00 0.00
B H
ATOM 3453 HB2 LYS B 7 18.003 38.145 66.461 1.00 0.00
B H
ATOM 3454 CG LYS B 7 19.303 36.414 66.189 1.00 0.00
B C
ATOM 3455 HG1 LYS B 7 19.937 36.590 67.087 1.00 0.00
B H

```
ATOM   3456  HG2 LYS B   7      19.186  35.312  66.075  1.00  0.00
B    H
ATOM   3457  CD  LYS B   7      19.991  37.004  64.970  1.00  0.00
B    C
ATOM   3458  HD1 LYS B   7      20.814  36.318  64.664  1.00  0.00
B    H
ATOM   3459  HD2 LYS B   7      19.252  37.034  64.136  1.00  0.00
B    H
ATOM   3460  CE  LYS B   7      20.530  38.396  65.247  1.00  0.00
B    C
ATOM   3461  HE1 LYS B   7      19.694  39.116  65.396  1.00  0.00
B    H
ATOM   3462  HE2 LYS B   7      21.160  38.396  66.163  1.00  0.00
B    H
ATOM   3463  NZ  LYS B   7      21.365  38.901  64.121  1.00  0.00
B    N
ATOM   3464  HZ1 LYS B   7      21.718  39.855  64.339  1.00  0.00
B    H
ATOM   3465  HZ2 LYS B   7      22.171  38.260  63.973  1.00  0.00
B    H
ATOM   3466  HZ3 LYS B   7      20.792  38.935  63.254  1.00  0.00
B    H
ATOM   3467  C   LYS B   7      16.791  35.081  67.495  1.00  0.00
B    C
ATOM   3468  O   LYS B   7      17.600  34.157  67.575  1.00  0.00
B    O
ATOM   3469  N   GLN B   8      15.506  34.887  67.218  1.00  0.00
B    N
ATOM   3470  HN  GLN B   8      14.881  35.617  66.947  1.00  0.00
B    H
ATOM   3471  CA  GLN B   8      14.895  33.569  67.291  1.00  0.00
B    C
ATOM   3472  HA  GLN B   8      15.640  32.901  67.707  1.00  0.00
B    H
ATOM   3473  CB  GLN B   8      13.675  33.599  68.201  1.00  0.00
B    C
ATOM   3474  HB1 GLN B   8      13.118  34.535  67.962  1.00  0.00
B    H
ATOM   3475  HB2 GLN B   8      12.996  32.757  67.937  1.00  0.00
B    H
ATOM   3476  CG  GLN B   8      14.084  33.575  69.655  1.00  0.00
B    C
ATOM   3477  HG1 GLN B   8      14.209  32.528  70.004  1.00  0.00
B    H
ATOM   3478  HG2 GLN B   8      15.049  34.101  69.800  1.00  0.00
B    H
ATOM   3479  CD  GLN B   8      13.059  34.199  70.560  1.00  0.00
B    C
ATOM   3480  OE1 GLN B   8      12.216  34.976  70.116  1.00  0.00
B    O
ATOM   3481  NE2 GLN B   8      13.125  33.872  71.845  1.00  0.00
B    N
ATOM   3482 HE21 GLN B   8      12.446  34.280  72.448  1.00  0.00
B    H
```

ATOM 3483 HE22 GLN B 8 13.841 33.260 72.162 1.00 0.00
B H
ATOM 3484 C GLN B 8 14.531 33.045 65.911 1.00 0.00
B C
ATOM 3485 O GLN B 8 13.936 33.760 65.091 1.00 0.00
B O
ATOM 3486 N PHE B 9 14.884 31.786 65.679 1.00 0.00
B N
ATOM 3487 HN PHE B 9 15.232 31.203 66.411 1.00 0.00
B H
ATOM 3488 CA PHE B 9 14.805 31.141 64.383 1.00 0.00
B C
ATOM 3489 HA PHE B 9 14.301 31.831 63.717 1.00 0.00
B H
ATOM 3490 CB PHE B 9 16.186 30.743 63.903 1.00 0.00
B C
ATOM 3491 HB1 PHE B 9 16.616 29.999 64.610 1.00 0.00
B H
ATOM 3492 HB2 PHE B 9 16.139 30.258 62.905 1.00 0.00
B H
ATOM 3493 CG PHE B 9 17.144 31.876 63.858 1.00 0.00
B C
ATOM 3494 CD1 PHE B 9 17.190 32.703 62.760 1.00 0.00
B C
ATOM 3495 HD1 PHE B 9 16.528 32.519 61.924 1.00 0.00
B H
ATOM 3496 CE1 PHE B 9 18.066 33.754 62.708 1.00 0.00
B C
ATOM 3497 HE1 PHE B 9 18.086 34.398 61.840 1.00 0.00
B H
ATOM 3498 CZ PHE B 9 18.915 33.991 63.766 1.00 0.00
B C
ATOM 3499 HZ PHE B 9 19.604 34.822 63.728 1.00 0.00
B H
ATOM 3500 CD2 PHE B 9 17.995 32.124 64.921 1.00 0.00
B C
ATOM 3501 HD2 PHE B 9 17.982 31.476 65.786 1.00 0.00
B H
ATOM 3502 CE2 PHE B 9 18.880 33.175 64.878 1.00 0.00
B C
ATOM 3503 HE2 PHE B 9 19.542 33.356 65.712 1.00 0.00
B H
ATOM 3504 C PHE B 9 13.933 29.903 64.482 1.00 0.00
B C
ATOM 3505 O PHE B 9 14.072 29.107 65.416 1.00 0.00
B O
ATOM 3506 N ALA B 10 13.048 29.739 63.507 1.00 0.00
B N
ATOM 3507 HN ALA B 10 12.943 30.398 62.762 1.00 0.00
B H
ATOM 3508 CA ALA B 10 12.163 28.584 63.449 1.00 0.00
B C
ATOM 3509 HA ALA B 10 12.400 27.860 64.220 1.00 0.00
B H

ATOM 3510 CB ALA B 10 10.702 29.002 63.626 1.00 0.00
B C
ATOM 3511 HB1 ALA B 10 10.568 29.473 64.623 1.00 0.00
B H
ATOM 3512 HB2 ALA B 10 10.403 29.745 62.854 1.00 0.00
B H
ATOM 3513 HB3 ALA B 10 10.016 28.131 63.563 1.00 0.00
B H
ATOM 3514 C ALA B 10 12.366 27.876 62.120 1.00 0.00
B C
ATOM 3515 O ALA B 10 11.995 28.404 61.068 1.00 0.00
B O
ATOM 3516 N VAL B 11 12.954 26.693 62.164 1.00 0.00
B N
ATOM 3517 HN VAL B 11 13.332 26.292 62.995 1.00 0.00
B H
ATOM 3518 CA VAL B 11 13.100 25.868 60.979 1.00 0.00
B C
ATOM 3519 HA VAL B 11 13.122 26.500 60.100 1.00 0.00
B H
ATOM 3520 CB VAL B 11 14.368 25.017 61.017 1.00 0.00
B C
ATOM 3521 HB VAL B 11 14.782 25.077 62.053 1.00 0.00
B H
ATOM 3522 CG1 VAL B 11 14.058 23.574 60.691 1.00 0.00
B C
ATOM 3523 HG11 VAL B 11 14.992 22.972 60.683 1.00 0.00
B H
ATOM 3524 HG12 VAL B 11 13.379 23.120 61.442 1.00 0.00
B H
ATOM 3525 HG13 VAL B 11 13.587 23.489 59.688 1.00 0.00
B H
ATOM 3526 CG2 VAL B 11 15.368 25.551 60.030 1.00 0.00
B C
ATOM 3527 HG21 VAL B 11 16.281 24.920 60.015 1.00 0.00
B H
ATOM 3528 HG22 VAL B 11 14.936 25.565 59.006 1.00 0.00
B H
ATOM 3529 HG23 VAL B 11 15.678 26.585 60.290 1.00 0.00
B H
ATOM 3530 C VAL B 11 11.853 25.008 60.891 1.00 0.00
B C
ATOM 3531 O VAL B 11 11.440 24.379 61.868 1.00 0.00
B O
ATOM 3532 N ILE B 12 11.239 24.996 59.722 1.00 0.00
B N
ATOM 3533 HN ILE B 12 11.486 25.598 58.965 1.00 0.00
B H
ATOM 3534 CA ILE B 12 10.140 24.097 59.432 1.00 0.00
B C
ATOM 3535 HA ILE B 12 10.068 23.383 60.243 1.00 0.00
B H
ATOM 3536 CB ILE B 12 8.832 24.860 59.216 1.00 0.00
B C

```
ATOM   3537  HB  ILE B  12       9.029  25.506  58.322  1.00  0.00
B    H
ATOM   3538  CG2 ILE B  12       7.705  23.886  59.104  1.00  0.00
B    C
ATOM   3539 HG21 ILE B  12       6.772  24.400  58.792  1.00  0.00
B    H
ATOM   3540 HG22 ILE B  12       7.929  23.106  58.346  1.00  0.00
B    H
ATOM   3541 HG23 ILE B  12       7.516  23.384  60.077  1.00  0.00
B    H
ATOM   3542  CG1 ILE B  12       8.566  25.792  60.385  1.00  0.00
B    C
ATOM   3543 HG11 ILE B  12       9.375  26.553  60.451  1.00  0.00
B    H
ATOM   3544 HG12 ILE B  12       7.614  26.340  60.201  1.00  0.00
B    H
ATOM   3545  CD  ILE B  12       8.453  25.085  61.686  1.00  0.00
B    C
ATOM   3546  HD1 ILE B  12       8.202  25.804  62.496  1.00  0.00
B    H
ATOM   3547  HD2 ILE B  12       7.654  24.314  61.646  1.00  0.00
B    H
ATOM   3548  HD3 ILE B  12       9.410  24.588  61.954  1.00  0.00
B    H
ATOM   3549  C   ILE B  12      10.516  23.313  58.192  1.00  0.00
B    C
ATOM   3550  O   ILE B  12      10.988  23.885  57.203  1.00  0.00
B    O
ATOM   3551  N   GLY B  13      10.341  22.006  58.255  1.00  0.00
B    N
ATOM   3552  HN  GLY B  13       9.968  21.545  59.060  1.00  0.00
B    H
ATOM   3553  CA  GLY B  13      10.681  21.149  57.149  1.00  0.00
B    C
ATOM   3554  HA1 GLY B  13      10.690  21.729  56.235  1.00  0.00
B    H
ATOM   3555  HA2 GLY B  13      10.013  20.299  57.128  1.00  0.00
B    H
ATOM   3556  C   GLY B  13      12.076  20.637  57.389  1.00  0.00
B    C
ATOM   3557  O   GLY B  13      13.053  21.358  57.171  1.00  0.00
B    O
ATOM   3558  N   LEU B  14      12.190  19.399  57.848  1.00  0.00
B    N
ATOM   3559  HN  LEU B  14      11.440  18.752  57.962  1.00  0.00
B    H
ATOM   3560  CA  LEU B  14      13.493  18.880  58.236  1.00  0.00
B    C
ATOM   3561  HA  LEU B  14      14.177  19.720  58.245  1.00  0.00
B    H
ATOM   3562  CB  LEU B  14      13.408  18.263  59.619  1.00  0.00
B    C
ATOM   3563  HB1 LEU B  14      12.329  18.203  59.887  1.00  0.00
B    H
```

```
ATOM   3564  HB2 LEU B  14      13.771  17.212  59.569  1.00  0.00
B    H
ATOM   3565  CG  LEU B  14      14.157  19.071  60.655  1.00  0.00
B    C
ATOM   3566  HG  LEU B  14      14.559  19.995  60.175  1.00  0.00
B    H
ATOM   3567  CD1 LEU B  14      13.217  19.458  61.765  1.00  0.00
B    C
ATOM   3568 HD11 LEU B  14      13.760  20.011  62.560  1.00  0.00
B    H
ATOM   3569 HD12 LEU B  14      12.403  20.110  61.379  1.00  0.00
B    H
ATOM   3570 HD13 LEU B  14      12.757  18.556  62.222  1.00  0.00
B    H
ATOM   3571  CD2 LEU B  14      15.335  18.262  61.178  1.00  0.00
B    C
ATOM   3572 HD21 LEU B  14      15.906  18.852  61.928  1.00  0.00
B    H
ATOM   3573 HD22 LEU B  14      15.004  17.324  61.669  1.00  0.00
B    H
ATOM   3574 HD23 LEU B  14      16.025  17.997  60.347  1.00  0.00
B    H
ATOM   3575  C   LEU B  14      14.005  17.880  57.203  1.00  0.00
B    C
ATOM   3576  O   LEU B  14      14.256  16.714  57.489  1.00  0.00
B    O
ATOM   3577  N   GLY B  15      14.157  18.365  55.976  1.00  0.00
B    N
ATOM   3578  HN  GLY B  15      13.833  19.268  55.698  1.00  0.00
B    H
ATOM   3579  CA  GLY B  15      14.819  17.592  54.945  1.00  0.00
B    C
ATOM   3580  HA1 GLY B  15      14.495  17.960  53.981  1.00  0.00
B    H
ATOM   3581  HA2 GLY B  15      14.614  16.544  55.113  1.00  0.00
B    H
ATOM   3582  C   GLY B  15      16.314  17.796  55.045  1.00  0.00
B    C
ATOM   3583  O   GLY B  15      16.761  18.596  55.872  1.00  0.00
B    O
ATOM   3584  N   ARG B  16      17.094  17.082  54.223  1.00  0.00
B    N
ATOM   3585  HN  ARG B  16      16.733  16.400  53.591  1.00  0.00
B    H
ATOM   3586  CA  ARG B  16      18.538  17.279  54.218  1.00  0.00
B    C
ATOM   3587  HA  ARG B  16      18.945  16.835  55.119  1.00  0.00
B    H
ATOM   3588  CB  ARG B  16      19.163  16.649  52.978  1.00  0.00
B    C
ATOM   3589  HB1 ARG B  16      18.735  17.191  52.100  1.00  0.00
B    H
ATOM   3590  HB2 ARG B  16      20.255  16.860  52.965  1.00  0.00
B    H
```

```
ATOM   3591  CG  ARG B  16      18.886  15.183  52.823  1.00  0.00
B    C
ATOM   3592  HG1 ARG B  16      19.633  14.617  53.422  1.00  0.00
B    H
ATOM   3593  HG2 ARG B  16      17.897  14.952  53.280  1.00  0.00
B    H
ATOM   3594  CD  ARG B  16      18.864  14.785  51.361  1.00  0.00
B    C
ATOM   3595  HD1 ARG B  16      19.732  15.247  50.837  1.00  0.00
B    H
ATOM   3596  HD2 ARG B  16      18.920  13.679  51.237  1.00  0.00
B    H
ATOM   3597  NE  ARG B  16      17.637  15.207  50.691  1.00  0.00
B    N
ATOM   3598  HE  ARG B  16      17.040  15.766  51.276  1.00  0.00
B    H
ATOM   3599  CZ  ARG B  16      17.328  14.892  49.438  1.00  0.00
B    C
ATOM   3600  NH1 ARG B  16      18.157  14.147  48.719  1.00  0.00
B    N
ATOM   3601 HH11 ARG B  16      17.949  13.927  47.776  1.00  0.00
B    H
ATOM   3602 HH12 ARG B  16      19.046  13.945  49.135  1.00  0.00
B    H
ATOM   3603  NH2 ARG B  16      16.190  15.312  48.904  1.00  0.00
B    N
ATOM   3604 HH21 ARG B  16      15.924  15.003  48.002  1.00  0.00
B    H
ATOM   3605 HH22 ARG B  16      15.545  15.754  49.514  1.00  0.00
B    H
ATOM   3606  C   ARG B  16      18.892  18.750  54.262  1.00  0.00
B    C
ATOM   3607  O   ARG B  16      19.757  19.159  55.045  1.00  0.00
B    O
ATOM   3608  N   PHE B  17      18.200  19.553  53.466  1.00  0.00
B    N
ATOM   3609  HN  PHE B  17      17.533  19.248  52.789  1.00  0.00
B    H
ATOM   3610  CA  PHE B  17      18.378  20.990  53.530  1.00  0.00
B    C
ATOM   3611  HA  PHE B  17      19.415  21.184  53.287  1.00  0.00
B    H
ATOM   3612  CB  PHE B  17      17.428  21.666  52.551  1.00  0.00
B    C
ATOM   3613  HB1 PHE B  17      17.617  21.269  51.529  1.00  0.00
B    H
ATOM   3614  HB2 PHE B  17      16.369  21.439  52.800  1.00  0.00
B    H
ATOM   3615  CG  PHE B  17      17.609  23.137  52.463  1.00  0.00
B    C
ATOM   3616  CD1 PHE B  17      18.863  23.673  52.253  1.00  0.00
B    C
ATOM   3617  HD1 PHE B  17      19.718  23.016  52.162  1.00  0.00
B    H
```

ATOM 3618 CE1 PHE B 17 19.040 25.029 52.164 1.00 0.00
B C
ATOM 3619 HE1 PHE B 17 20.027 25.436 51.999 1.00 0.00
B H
ATOM 3620 CZ PHE B 17 17.955 25.867 52.280 1.00 0.00
B C
ATOM 3621 HZ PHE B 17 18.088 26.937 52.201 1.00 0.00
B H
ATOM 3622 CD2 PHE B 17 16.527 23.987 52.576 1.00 0.00
B C
ATOM 3623 HD2 PHE B 17 15.541 23.586 52.759 1.00 0.00
B H
ATOM 3624 CE2 PHE B 17 16.697 25.343 52.486 1.00 0.00
B C
ATOM 3625 HE2 PHE B 17 15.843 26.000 52.579 1.00 0.00
B H
ATOM 3626 C PHE B 17 18.151 21.493 54.946 1.00 0.00
B C
ATOM 3627 O PHE B 17 19.040 22.105 55.548 1.00 0.00
B O
ATOM 3628 N GLY B 18 16.970 21.222 55.500 1.00 0.00
B N
ATOM 3629 HN GLY B 18 16.238 20.706 55.055 1.00 0.00
B H
ATOM 3630 CA GLY B 18 16.679 21.689 56.843 1.00 0.00
B C
ATOM 3631 HA1 GLY B 18 15.743 21.239 57.141 1.00 0.00
B H
ATOM 3632 HA2 GLY B 18 16.661 22.770 56.811 1.00 0.00
B H
ATOM 3633 C GLY B 18 17.739 21.268 57.839 1.00 0.00
B C
ATOM 3634 O GLY B 18 18.377 22.110 58.484 1.00 0.00
B O
ATOM 3635 N GLY B 19 17.975 19.960 57.936 1.00 0.00
B N
ATOM 3636 HN GLY B 19 17.536 19.275 57.354 1.00 0.00
B H
ATOM 3637 CA GLY B 19 18.908 19.444 58.920 1.00 0.00
B C
ATOM 3638 HA1 GLY B 19 19.031 18.390 58.707 1.00 0.00
B H
ATOM 3639 HA2 GLY B 19 18.486 19.654 59.893 1.00 0.00
B H
ATOM 3640 C GLY B 19 20.270 20.096 58.851 1.00 0.00
B C
ATOM 3641 O GLY B 19 20.882 20.379 59.883 1.00 0.00
B O
ATOM 3642 N SER B 20 20.754 20.352 57.643 1.00 0.00
B N
ATOM 3643 HN SER B 20 20.323 20.078 56.783 1.00 0.00
B H
ATOM 3644 CA SER B 20 21.998 21.080 57.478 1.00 0.00
B C

ATOM 3645 HA SER B 20 22.763 20.477 57.950 1.00 0.00
B H
ATOM 3646 CB SER B 20 22.226 21.349 56.001 1.00 0.00
B C
ATOM 3647 HB1 SER B 20 21.342 21.900 55.609 1.00 0.00
B H
ATOM 3648 HB2 SER B 20 23.112 22.008 55.860 1.00 0.00
B H
ATOM 3649 OG SER B 20 22.361 20.134 55.314 1.00 0.00
B O
ATOM 3650 HG1 SER B 20 22.279 20.329 54.372 1.00 0.00
B H
ATOM 3651 C SER B 20 21.956 22.404 58.208 1.00 0.00
B C
ATOM 3652 O SER B 20 22.814 22.680 59.058 1.00 0.00
B O
ATOM 3653 N ILE B 21 20.963 23.214 57.862 1.00 0.00
B N
ATOM 3654 HN ILE B 21 20.264 22.988 57.188 1.00 0.00
B H
ATOM 3655 CA ILE B 21 20.814 24.531 58.455 1.00 0.00
B C
ATOM 3656 HA ILE B 21 21.686 25.103 58.164 1.00 0.00
B H
ATOM 3657 CB ILE B 21 19.506 25.163 57.968 1.00 0.00
B C
ATOM 3658 HB ILE B 21 18.702 24.538 58.435 1.00 0.00
B H
ATOM 3659 CG2 ILE B 21 19.455 26.610 58.347 1.00 0.00
B C
ATOM 3660 HG21 ILE B 21 18.450 27.033 58.138 1.00 0.00
B H
ATOM 3661 HG22 ILE B 21 19.656 26.743 59.431 1.00 0.00
B H
ATOM 3662 HG23 ILE B 21 20.204 27.197 57.776 1.00 0.00
B H
ATOM 3663 CG1 ILE B 21 19.394 24.991 56.463 1.00 0.00
B C
ATOM 3664 HG11 ILE B 21 20.231 25.524 55.960 1.00 0.00
B H
ATOM 3665 HG12 ILE B 21 19.497 23.909 56.216 1.00 0.00
B H
ATOM 3666 CD ILE B 21 18.100 25.474 55.888 1.00 0.00
B C
ATOM 3667 HD1 ILE B 21 18.061 25.266 54.797 1.00 0.00
B H
ATOM 3668 HD2 ILE B 21 17.240 24.961 56.372 1.00 0.00
B H
ATOM 3669 HD3 ILE B 21 17.983 26.568 56.034 1.00 0.00
B H
ATOM 3670 C ILE B 21 20.836 24.435 59.965 1.00 0.00
B C
ATOM 3671 O ILE B 21 21.607 25.128 60.640 1.00 0.00
B O

```
ATOM   3672  N   VAL B  22      19.979  23.584  60.518  1.00  0.00
B    N
ATOM   3673  HN  VAL B  22      19.414  22.911  60.047  1.00  0.00
B    H
ATOM   3674  CA  VAL B  22      19.814  23.603  61.964  1.00  0.00
B    C
ATOM   3675  HA  VAL B  22      19.687  24.635  62.264  1.00  0.00
B    H
ATOM   3676  CB  VAL B  22      18.570  22.810  62.382  1.00  0.00
B    C
ATOM   3677  HB  VAL B  22      18.728  22.465  63.435  1.00  0.00
B    H
ATOM   3678  CG1 VAL B  22      17.355  23.685  62.322  1.00  0.00
B    C
ATOM   3679 HG11 VAL B  22      16.447  23.102  62.587  1.00  0.00
B    H
ATOM   3680 HG12 VAL B  22      17.424  24.532  63.035  1.00  0.00
B    H
ATOM   3681 HG13 VAL B  22      17.215  24.092  61.298  1.00  0.00
B    H
ATOM   3682  CG2 VAL B  22      18.400  21.623  61.512  1.00  0.00
B    C
ATOM   3683 HG21 VAL B  22      17.478  21.066  61.783  1.00  0.00
B    H
ATOM   3684 HG22 VAL B  22      18.322  21.930  60.445  1.00  0.00
B    H
ATOM   3685 HG23 VAL B  22      19.256  20.924  61.612  1.00  0.00
B    H
ATOM   3686  C   VAL B  22      21.058  23.077  62.656  1.00  0.00
B    C
ATOM   3687  O   VAL B  22      21.483  23.622  63.676  1.00  0.00
B    O
ATOM   3688  N   LYS B  23      21.654  22.010  62.130  1.00  0.00
B    N
ATOM   3689  HN  LYS B  23      21.379  21.544  61.291  1.00  0.00
B    H
ATOM   3690  CA  LYS B  23      22.809  21.440  62.801  1.00  0.00
B    C
ATOM   3691  HA  LYS B  23      22.541  21.287  63.839  1.00  0.00
B    H
ATOM   3692  CB  LYS B  23      23.186  20.115  62.153  1.00  0.00
B    C
ATOM   3693  HB1 LYS B  23      22.238  19.555  61.979  1.00  0.00
B    H
ATOM   3694  HB2 LYS B  23      23.622  20.314  61.148  1.00  0.00
B    H
ATOM   3695  CG  LYS B  23      24.119  19.270  62.990  1.00  0.00
B    C
ATOM   3696  HG1 LYS B  23      24.483  19.875  63.852  1.00  0.00
B    H
ATOM   3697  HG2 LYS B  23      23.551  18.407  63.406  1.00  0.00
B    H
ATOM   3698  CD  LYS B  23      25.308  18.783  62.193  1.00  0.00
B    C
```

ATOM 3699 HD1 LYS B 23 25.111 18.974 61.113 1.00 0.00
B H
ATOM 3700 HD2 LYS B 23 26.194 19.393 62.484 1.00 0.00
B H
ATOM 3701 CE LYS B 23 25.547 17.301 62.465 1.00 0.00
B C
ATOM 3702 HE1 LYS B 23 25.765 17.132 63.543 1.00 0.00
B H
ATOM 3703 HE2 LYS B 23 24.650 16.704 62.190 1.00 0.00
B H
ATOM 3704 NZ LYS B 23 26.693 16.740 61.699 1.00 0.00
B N
ATOM 3705 HZ1 LYS B 23 26.812 15.731 61.922 1.00 0.00
B H
ATOM 3706 HZ2 LYS B 23 26.517 16.850 60.679 1.00 0.00
B H
ATOM 3707 HZ3 LYS B 23 27.562 17.253 61.952 1.00 0.00
B H
ATOM 3708 C LYS B 23 23.969 22.417 62.773 1.00 0.00
B C
ATOM 3709 O LYS B 23 24.697 22.556 63.763 1.00 0.00
B O
ATOM 3710 N GLU B 24 24.119 23.147 61.668 1.00 0.00
B N
ATOM 3711 HN GLU B 24 23.592 23.065 60.827 1.00 0.00
B H
ATOM 3712 CA GLU B 24 25.140 24.184 61.628 1.00 0.00
B C
ATOM 3713 HA GLU B 24 26.091 23.741 61.896 1.00 0.00
B H
ATOM 3714 CB GLU B 24 25.201 24.785 60.224 1.00 0.00
B C
ATOM 3715 HB1 GLU B 24 25.317 23.952 59.493 1.00 0.00
B H
ATOM 3716 HB2 GLU B 24 24.230 25.272 59.979 1.00 0.00
B H
ATOM 3717 CG GLU B 24 26.319 25.786 59.962 1.00 0.00
B C
ATOM 3718 HG1 GLU B 24 27.298 25.287 60.083 1.00 0.00
B H
ATOM 3719 HG2 GLU B 24 26.247 26.204 58.938 1.00 0.00
B H
ATOM 3720 CD GLU B 24 26.362 26.957 60.926 1.00 0.00
B C
ATOM 3721 OE1 GLU B 24 25.364 27.689 61.057 1.00 0.00
B O
ATOM 3722 OE2 GLU B 24 27.409 27.122 61.578 1.00 0.00
B O
ATOM 3723 C GLU B 24 24.839 25.267 62.656 1.00 0.00
B C
ATOM 3724 O GLU B 24 25.677 25.588 63.506 1.00 0.00
B O
ATOM 3725 N LEU B 25 23.658 25.872 62.563 1.00 0.00
B N

```
ATOM   3726  HN  LEU B  25      22.938  25.624  61.919  1.00  0.00
B    H
ATOM   3727  CA  LEU B  25      23.323  26.989  63.434  1.00  0.00
B    C
ATOM   3728  HA  LEU B  25      24.055  27.765  63.246  1.00  0.00
B    H
ATOM   3729  CB  LEU B  25      21.915  27.470  63.107  1.00  0.00
B    C
ATOM   3730  HB1 LEU B  25      21.516  26.801  62.309  1.00  0.00
B    H
ATOM   3731  HB2 LEU B  25      21.263  27.313  63.994  1.00  0.00
B    H
ATOM   3732  CG  LEU B  25      21.863  28.909  62.632  1.00  0.00
B    C
ATOM   3733  HG  LEU B  25      22.888  29.262  62.368  1.00  0.00
B    H
ATOM   3734  CD1 LEU B  25      20.958  29.027  61.447  1.00  0.00
B    C
ATOM   3735 HD11 LEU B  25      20.876  30.086  61.122  1.00  0.00
B    H
ATOM   3736 HD12 LEU B  25      21.349  28.430  60.595  1.00  0.00
B    H
ATOM   3737 HD13 LEU B  25      19.938  28.660  61.694  1.00  0.00
B    H
ATOM   3738  CD2 LEU B  25      21.346  29.775  63.756  1.00  0.00
B    C
ATOM   3739 HD21 LEU B  25      21.315  30.840  63.440  1.00  0.00
B    H
ATOM   3740 HD22 LEU B  25      20.320  29.484  64.059  1.00  0.00
B    H
ATOM   3741 HD23 LEU B  25      22.009  29.695  64.645  1.00  0.00
B    H
ATOM   3742  C   LEU B  25      23.441  26.594  64.895  1.00  0.00
B    C
ATOM   3743  O   LEU B  25      23.891  27.380  65.735  1.00  0.00
B    O
ATOM   3744  N   HSE B  26      23.032  25.372  65.205  1.00  0.00
B    N
ATOM   3745  HN  HSE B  26      22.543  24.780  64.565  1.00  0.00
B    H
ATOM   3746  CA  HSE B  26      23.262  24.782  66.509  1.00  0.00
B    C
ATOM   3747  HA  HSE B  26      22.773  25.418  67.238  1.00  0.00
B    H
ATOM   3748  CB  HSE B  26      22.704  23.366  66.493  1.00  0.00
B    C
ATOM   3749  HB1 HSE B  26      21.644  23.419  66.161  1.00  0.00
B    H
ATOM   3750  HB2 HSE B  26      23.250  22.761  65.739  1.00  0.00
B    H
ATOM   3751  ND1 HSE B  26      23.700  21.764  68.149  1.00  0.00
B    N
ATOM   3752  CG  HSE B  26      22.730  22.680  67.813  1.00  0.00
B    C
```

ATOM 3753 CE1 HSE B 26 23.462 21.303 69.363 1.00 0.00
B C
ATOM 3754 HE1 HSE B 26 24.083 20.566 69.879 1.00 0.00
B H
ATOM 3755 NE2 HSE B 26 22.364 21.878 69.819 1.00 0.00
B N
ATOM 3756 HE2 HSE B 26 21.944 21.694 70.708 1.00 0.00
B H
ATOM 3757 CD2 HSE B 26 21.883 22.740 68.866 1.00 0.00
B C
ATOM 3758 HD2 HSE B 26 20.980 23.321 68.998 1.00 0.00
B H
ATOM 3759 C HSE B 26 24.740 24.778 66.841 1.00 0.00
B C
ATOM 3760 O HSE B 26 25.134 25.126 67.959 1.00 0.00
B O
ATOM 3761 N ARG B 27 25.576 24.387 65.882 1.00 0.00
B N
ATOM 3762 HN ARG B 27 25.281 24.015 65.005 1.00 0.00
B H
ATOM 3763 CA ARG B 27 27.009 24.496 66.097 1.00 0.00
B C
ATOM 3764 HA ARG B 27 27.262 23.956 67.002 1.00 0.00
B H
ATOM 3765 CB ARG B 27 27.766 23.901 64.917 1.00 0.00
B C
ATOM 3766 HB1 ARG B 27 27.219 22.973 64.626 1.00 0.00
B H
ATOM 3767 HB2 ARG B 27 27.692 24.586 64.043 1.00 0.00
B H
ATOM 3768 CG ARG B 27 29.197 23.551 65.236 1.00 0.00
B C
ATOM 3769 HG1 ARG B 27 29.438 23.939 66.250 1.00 0.00
B H
ATOM 3770 HG2 ARG B 27 29.291 22.443 65.309 1.00 0.00
B H
ATOM 3771 CD ARG B 27 30.140 24.058 64.164 1.00 0.00
B C
ATOM 3772 HD1 ARG B 27 31.181 24.062 64.559 1.00 0.00
B H
ATOM 3773 HD2 ARG B 27 30.106 23.417 63.252 1.00 0.00
B H
ATOM 3774 NE ARG B 27 29.808 25.408 63.720 1.00 0.00
B N
ATOM 3775 HE ARG B 27 29.445 25.437 62.782 1.00 0.00
B H
ATOM 3776 CZ ARG B 27 29.961 26.500 64.459 1.00 0.00
B C
ATOM 3777 NH1 ARG B 27 30.432 26.417 65.693 1.00 0.00
B N
ATOM 3778 HH11 ARG B 27 30.522 27.229 66.254 1.00 0.00
B H
ATOM 3779 HH12 ARG B 27 30.554 25.492 66.060 1.00 0.00
B H

```
ATOM   3780  NH2 ARG B  27      29.642  27.682  63.963  1.00  0.00
B    N
ATOM   3781 HH21 ARG B  27      29.836  28.503  64.481  1.00  0.00
B    H
ATOM   3782 HH22 ARG B  27      29.408  27.716  63.000  1.00  0.00
B    H
ATOM   3783  C   ARG B  27      27.416  25.943  66.324  1.00  0.00
B    C
ATOM   3784  O   ARG B  27      28.379  26.211  67.047  1.00  0.00
B    O
ATOM   3785  N   MET B  28      26.693  26.893  65.729  1.00  0.00
B    N
ATOM   3786  HN  MET B  28      25.958  26.761  65.069  1.00  0.00
B    H
ATOM   3787  CA  MET B  28      26.972  28.287  66.048  1.00  0.00
B    C
ATOM   3788  HA  MET B  28      28.051  28.391  66.059  1.00  0.00
B    H
ATOM   3789  CB  MET B  28      26.364  29.231  65.019  1.00  0.00
B    C
ATOM   3790  HB1 MET B  28      25.280  28.979  64.948  1.00  0.00
B    H
ATOM   3791  HB2 MET B  28      26.416  30.276  65.400  1.00  0.00
B    H
ATOM   3792  CG  MET B  28      26.973  29.132  63.667  1.00  0.00
B    C
ATOM   3793  HG1 MET B  28      28.077  29.234  63.732  1.00  0.00
B    H
ATOM   3794  HG2 MET B  28      26.752  28.134  63.230  1.00  0.00
B    H
ATOM   3795  SD  MET B  28      26.379  30.389  62.541  1.00  0.00
B    S
ATOM   3796  CE  MET B  28      27.229  31.807  63.222  1.00  0.00
B    C
ATOM   3797  HE1 MET B  28      26.981  32.729  62.654  1.00  0.00
B    H
ATOM   3798  HE2 MET B  28      26.942  31.971  64.283  1.00  0.00
B    H
ATOM   3799  HE3 MET B  28      28.331  31.668  63.184  1.00  0.00
B    H
ATOM   3800  C   MET B  28      26.460  28.681  67.418  1.00  0.00
B    C
ATOM   3801  O   MET B  28      26.646  29.833  67.820  1.00  0.00
B    O
ATOM   3802  N   GLY B  29      25.812  27.770  68.134  1.00  0.00
B    N
ATOM   3803  HN  GLY B  29      25.626  26.842  67.815  1.00  0.00
B    H
ATOM   3804  CA  GLY B  29      25.327  28.086  69.457  1.00  0.00
B    C
ATOM   3805  HA1 GLY B  29      26.147  28.530  70.002  1.00  0.00
B    H
ATOM   3806  HA2 GLY B  29      24.948  27.171  69.891  1.00  0.00
B    H
```

ATOM	3807	C	GLY	B	29	24.196	29.085	69.420	1.00	0.00	B C
ATOM	3808	O	GLY	B	29	24.279	30.151	70.036	1.00	0.00	B O
ATOM	3809	N	HSE	B	30	23.143	28.769	68.684	1.00	0.00	B N
ATOM	3810	HN	HSE	B	30	23.052	27.922	68.160	1.00	0.00	B H
ATOM	3811	CA	HSE	B	30	22.006	29.660	68.583	1.00	0.00	B C
ATOM	3812	HA	HSE	B	30	22.211	30.511	69.221	1.00	0.00	B H
ATOM	3813	CB	HSE	B	30	21.804	30.106	67.142	1.00	0.00	B C
ATOM	3814	HB1	HSE	B	30	21.883	29.208	66.492	1.00	0.00	B H
ATOM	3815	HB2	HSE	B	30	20.776	30.508	67.019	1.00	0.00	B H
ATOM	3816	ND1	HSE	B	30	22.464	32.346	66.201	1.00	0.00	B N
ATOM	3817	CG	HSE	B	30	22.813	31.102	66.680	1.00	0.00	B C
ATOM	3818	CE1	HSE	B	30	23.557	33.008	65.870	1.00	0.00	B C
ATOM	3819	HE1	HSE	B	30	23.568	34.017	65.452	1.00	0.00	B H
ATOM	3820	NE2	HSE	B	30	24.603	32.241	66.125	1.00	0.00	B N
ATOM	3821	HE2	HSE	B	30	25.555	32.489	65.952	1.00	0.00	B H
ATOM	3822	CD2	HSE	B	30	24.163	31.046	66.638	1.00	0.00	B C
ATOM	3823	HD2	HSE	B	30	24.833	30.247	66.925	1.00	0.00	B H
ATOM	3824	C	HSE	B	30	20.748	28.994	69.118	1.00	0.00	B C
ATOM	3825	O	HSE	B	30	20.721	27.808	69.451	1.00	0.00	B O
ATOM	3826	N	GLU	B	31	19.693	29.788	69.197	1.00	0.00	B N
ATOM	3827	HN	GLU	B	31	19.726	30.752	68.946	1.00	0.00	B H
ATOM	3828	CA	GLU	B	31	18.387	29.341	69.653	1.00	0.00	B C
ATOM	3829	HA	GLU	B	31	18.496	28.442	70.247	1.00	0.00	B H
ATOM	3830	CB	GLU	B	31	17.723	30.433	70.490	1.00	0.00	B C
ATOM	3831	HB1	GLU	B	31	16.718	30.066	70.805	1.00	0.00	B H
ATOM	3832	HB2	GLU	B	31	18.298	30.591	71.430	1.00	0.00	B H
ATOM	3833	CG	GLU	B	31	17.534	31.786	69.792	1.00	0.00	B C

ATOM 3834 HG1 GLU B 31 17.145 31.624 68.770 1.00 0.00
B H
ATOM 3835 HG2 GLU B 31 16.827 32.428 70.355 1.00 0.00
B H
ATOM 3836 CD GLU B 31 18.832 32.569 69.606 1.00 0.00
B C
ATOM 3837 OE1 GLU B 31 18.870 33.754 69.994 1.00 0.00
B O
ATOM 3838 OE2 GLU B 31 19.799 32.018 69.040 1.00 0.00
B O
ATOM 3839 C GLU B 31 17.565 28.975 68.425 1.00 0.00
B C
ATOM 3840 O GLU B 31 17.185 29.843 67.634 1.00 0.00
B O
ATOM 3841 N VAL B 32 17.321 27.683 68.241 1.00 0.00
B N
ATOM 3842 HN VAL B 32 17.592 26.972 68.885 1.00 0.00
B H
ATOM 3843 CA VAL B 32 16.625 27.193 67.062 1.00 0.00
B C
ATOM 3844 HA VAL B 32 16.101 28.023 66.604 1.00 0.00
B H
ATOM 3845 CB VAL B 32 17.601 26.588 66.037 1.00 0.00
B C
ATOM 3846 HB VAL B 32 16.991 26.146 65.211 1.00 0.00
B H
ATOM 3847 CG1 VAL B 32 18.499 27.672 65.479 1.00 0.00
B C
ATOM 3848 HG11 VAL B 32 19.232 27.236 64.766 1.00 0.00
B H
ATOM 3849 HG12 VAL B 32 17.919 28.441 64.927 1.00 0.00
B H
ATOM 3850 HG13 VAL B 32 19.065 28.171 66.294 1.00 0.00
B H
ATOM 3851 CG2 VAL B 32 18.446 25.510 66.675 1.00 0.00
B C
ATOM 3852 HG21 VAL B 32 19.178 25.102 65.946 1.00 0.00
B H
ATOM 3853 HG22 VAL B 32 19.008 25.918 67.543 1.00 0.00
B H
ATOM 3854 HG23 VAL B 32 17.820 24.666 67.031 1.00 0.00
B H
ATOM 3855 C VAL B 32 15.582 26.177 67.490 1.00 0.00
B C
ATOM 3856 O VAL B 32 15.897 25.192 68.168 1.00 0.00
B O
ATOM 3857 N LEU B 33 14.341 26.424 67.106 1.00 0.00
B N
ATOM 3858 HN LEU B 33 14.067 27.261 66.637 1.00 0.00
B H
ATOM 3859 CA LEU B 33 13.256 25.488 67.338 1.00 0.00
B C
ATOM 3860 HA LEU B 33 13.524 24.869 68.184 1.00 0.00
B H

ATOM 3861 CB LEU B 33 11.939 26.213 67.583 1.00 0.00
B C
ATOM 3862 HB1 LEU B 33 11.963 26.596 68.630 1.00 0.00
B H
ATOM 3863 HB2 LEU B 33 11.893 27.112 66.928 1.00 0.00
B H
ATOM 3864 CG LEU B 33 10.732 25.301 67.388 1.00 0.00
B C
ATOM 3865 HG LEU B 33 10.785 24.904 66.345 1.00 0.00
B H
ATOM 3866 CD1 LEU B 33 10.766 24.141 68.375 1.00 0.00
B C
ATOM 3867 HD11 LEU B 33 9.861 23.506 68.262 1.00 0.00
B H
ATOM 3868 HD12 LEU B 33 11.662 23.507 68.202 1.00 0.00
B H
ATOM 3869 HD13 LEU B 33 10.798 24.516 69.420 1.00 0.00
B H
ATOM 3870 CD2 LEU B 33 9.447 26.091 67.523 1.00 0.00
B C
ATOM 3871 HD21 LEU B 33 8.569 25.429 67.362 1.00 0.00
B H
ATOM 3872 HD22 LEU B 33 9.348 26.543 68.530 1.00 0.00
B H
ATOM 3873 HD23 LEU B 33 9.411 26.907 66.768 1.00 0.00
B H
ATOM 3874 C LEU B 33 13.141 24.580 66.125 1.00 0.00
B C
ATOM 3875 O LEU B 33 12.940 25.057 65.003 1.00 0.00
B O
ATOM 3876 N ALA B 34 13.265 23.283 66.349 1.00 0.00
B N
ATOM 3877 HN ALA B 34 13.452 22.903 67.254 1.00 0.00
B H
ATOM 3878 CA ALA B 34 13.134 22.310 65.279 1.00 0.00
B C
ATOM 3879 HA ALA B 34 13.299 22.757 64.306 1.00 0.00
B H
ATOM 3880 CB ALA B 34 14.127 21.166 65.460 1.00 0.00
B C
ATOM 3881 HB1 ALA B 34 15.164 21.562 65.412 1.00 0.00
B H
ATOM 3882 HB2 ALA B 34 13.995 20.675 66.449 1.00 0.00
B H
ATOM 3883 HB3 ALA B 34 14.016 20.398 64.665 1.00 0.00
B H
ATOM 3884 C ALA B 34 11.709 21.785 65.275 1.00 0.00
B C
ATOM 3885 O ALA B 34 11.168 21.441 66.330 1.00 0.00
B O
ATOM 3886 N VAL B 35 11.098 21.746 64.096 1.00 0.00
B N
ATOM 3887 HN VAL B 35 11.511 22.111 63.266 1.00 0.00
B H

```
ATOM   3888  CA  VAL B  35       9.774  21.170  63.907  1.00  0.00
B    C
ATOM   3889  HA  VAL B  35       9.569  20.498  64.730  1.00  0.00
B    H
ATOM   3890  CB  VAL B  35       8.677  22.247  63.819  1.00  0.00
B    C
ATOM   3891  HB  VAL B  35       8.890  22.874  62.920  1.00  0.00
B    H
ATOM   3892  CG1 VAL B  35       7.310  21.600  63.667  1.00  0.00
B    C
ATOM   3893 HG11 VAL B  35       6.517  22.377  63.643  1.00  0.00
B    H
ATOM   3894 HG12 VAL B  35       7.234  21.021  62.723  1.00  0.00
B    H
ATOM   3895 HG13 VAL B  35       7.100  20.920  64.520  1.00  0.00
B    H
ATOM   3896  CG2 VAL B  35       8.718  23.149  65.029  1.00  0.00
B    C
ATOM   3897 HG21 VAL B  35       7.901  23.901  64.986  1.00  0.00
B    H
ATOM   3898 HG22 VAL B  35       8.599  22.557  65.962  1.00  0.00
B    H
ATOM   3899 HG23 VAL B  35       9.679  23.701  65.086  1.00  0.00
B    H
ATOM   3900  C   VAL B  35       9.790  20.341  62.635  1.00  0.00
B    C
ATOM   3901  O   VAL B  35      10.404  20.731  61.638  1.00  0.00
B    O
ATOM   3902  N   ASP B  36       9.111  19.202  62.671  1.00  0.00
B    N
ATOM   3903  HN  ASP B  36       8.691  18.783  63.471  1.00  0.00
B    H
ATOM   3904  CA  ASP B  36       8.919  18.428  61.460  1.00  0.00
B    C
ATOM   3905  HA  ASP B  36       8.543  19.095  60.693  1.00  0.00
B    H
ATOM   3906  CB  ASP B  36      10.201  17.742  61.018  1.00  0.00
B    C
ATOM   3907  HB1 ASP B  36      11.057  18.427  61.185  1.00  0.00
B    H
ATOM   3908  HB2 ASP B  36      10.373  16.819  61.607  1.00  0.00
B    H
ATOM   3909  CG  ASP B  36      10.172  17.394  59.570  1.00  0.00
B    C
ATOM   3910  OD1 ASP B  36      10.410  18.308  58.757  1.00  0.00
B    O
ATOM   3911  OD2 ASP B  36       9.899  16.222  59.246  1.00  0.00
B    O
ATOM   3912  C   ASP B  36       7.843  17.392  61.702  1.00  0.00
B    C
ATOM   3913  O   ASP B  36       7.821  16.744  62.748  1.00  0.00
B    O
ATOM   3914  N   ILE B  37       6.962  17.232  60.718  1.00  0.00
B    N
```

```
ATOM   3915  HN  ILE B  37       6.942  17.734  59.856  1.00  0.00
B    H
ATOM   3916  CA  ILE B  37       5.904  16.244  60.859  1.00  0.00
B    C
ATOM   3917  HA  ILE B  37       5.428  16.436  61.811  1.00  0.00
B    H
ATOM   3918  CB  ILE B  37       4.894  16.361  59.709  1.00  0.00
B    C
ATOM   3919  HB  ILE B  37       4.590  17.438  59.712  1.00  0.00
B    H
ATOM   3920  CG2 ILE B  37       5.511  15.895  58.410  1.00  0.00
B    C
ATOM   3921 HG21 ILE B  37       4.842  16.124  57.555  1.00  0.00
B    H
ATOM   3922 HG22 ILE B  37       6.479  16.406  58.226  1.00  0.00
B    H
ATOM   3923 HG23 ILE B  37       5.691  14.799  58.427  1.00  0.00
B    H
ATOM   3924  CG1 ILE B  37       3.648  15.543  60.032  1.00  0.00
B    C
ATOM   3925 HG11 ILE B  37       3.875  14.457  59.939  1.00  0.00
B    H
ATOM   3926 HG12 ILE B  37       3.358  15.730  61.091  1.00  0.00
B    H
ATOM   3927  CD  ILE B  37       2.476  15.866  59.168  1.00  0.00
B    C
ATOM   3928  HD1 ILE B  37       1.587  15.282  59.489  1.00  0.00
B    H
ATOM   3929  HD2 ILE B  37       2.224  16.947  59.236  1.00  0.00
B    H
ATOM   3930  HD3 ILE B  37       2.684  15.620  58.105  1.00  0.00
B    H
ATOM   3931  C   ILE B  37       6.483  14.843  60.932  1.00  0.00
B    C
ATOM   3932  O   ILE B  37       5.869  13.942  61.511  1.00  0.00
B    O
ATOM   3933  N   ASN B  38       7.661  14.628  60.369  1.00  0.00
B    N
ATOM   3934  HN  ASN B  38       8.199  15.308  59.872  1.00  0.00
B    H
ATOM   3935  CA  ASN B  38       8.273  13.318  60.448  1.00  0.00
B    C
ATOM   3936  HA  ASN B  38       7.468  12.591  60.412  1.00  0.00
B    H
ATOM   3937  CB  ASN B  38       9.272  13.105  59.323  1.00  0.00
B    C
ATOM   3938  HB1 ASN B  38       8.698  12.897  58.394  1.00  0.00
B    H
ATOM   3939  HB2 ASN B  38       9.861  14.028  59.152  1.00  0.00
B    H
ATOM   3940  CG  ASN B  38      10.172  11.944  59.595  1.00  0.00
B    C
ATOM   3941  OD1 ASN B  38      11.179  12.079  60.275  1.00  0.00
B    O
```

```
ATOM   3942  ND2 ASN B  38       9.795  10.779  59.096  1.00  0.00
B    N
ATOM   3943 HD21 ASN B  38      10.380   9.993  59.279  1.00  0.00
B    H
ATOM   3944 HD22 ASN B  38       8.943  10.707  58.588  1.00  0.00
B    H
ATOM   3945  C   ASN B  38       8.959  13.178  61.793  1.00  0.00
B    C
ATOM   3946  O   ASN B  38      10.007  13.785  62.031  1.00  0.00
B    O
ATOM   3947  N   GLU B  39       8.367  12.369  62.664  1.00  0.00
B    N
ATOM   3948  HN  GLU B  39       7.526  11.862  62.494  1.00  0.00
B    H
ATOM   3949  CA  GLU B  39       8.930  12.154  63.990  1.00  0.00
B    C
ATOM   3950  HA  GLU B  39       8.986  13.111  64.493  1.00  0.00
B    H
ATOM   3951  CB  GLU B  39       8.048  11.179  64.756  1.00  0.00
B    C
ATOM   3952  HB1 GLU B  39       8.294  11.267  65.840  1.00  0.00
B    H
ATOM   3953  HB2 GLU B  39       6.981  11.481  64.661  1.00  0.00
B    H
ATOM   3954  CG  GLU B  39       8.246   9.755  64.300  1.00  0.00
B    C
ATOM   3955  HG1 GLU B  39       9.325   9.560  64.156  1.00  0.00
B    H
ATOM   3956  HG2 GLU B  39       7.841   9.040  65.043  1.00  0.00
B    H
ATOM   3957  CD  GLU B  39       7.598   9.472  62.960  1.00  0.00
B    C
ATOM   3958  OE1 GLU B  39       7.259  10.430  62.230  1.00  0.00
B    O
ATOM   3959  OE2 GLU B  39       7.438   8.284  62.626  1.00  0.00
B    O
ATOM   3960  C   GLU B  39      10.344  11.610  63.901  1.00  0.00
B    C
ATOM   3961  O   GLU B  39      11.126  11.733  64.843  1.00  0.00
B    O
ATOM   3962  N   GLU B  40      10.684  10.978  62.784  1.00  0.00
B    N
ATOM   3963  HN  GLU B  40      10.129  10.879  61.963  1.00  0.00
B    H
ATOM   3964  CA  GLU B  40      11.990  10.348  62.694  1.00  0.00
B    C
ATOM   3965  HA  GLU B  40      12.131   9.737  63.577  1.00  0.00
B    H
ATOM   3966  CB  GLU B  40      12.052   9.473  61.447  1.00  0.00
B    C
ATOM   3967  HB1 GLU B  40      11.140   9.672  60.838  1.00  0.00
B    H
ATOM   3968  HB2 GLU B  40      12.914   9.779  60.813  1.00  0.00
B    H
```

ATOM 3969 CG GLU B 40 12.133 7.960 61.671 1.00 0.00
B C
ATOM 3970 HG1 GLU B 40 11.114 7.550 61.799 1.00 0.00
B H
ATOM 3971 HG2 GLU B 40 12.623 7.457 60.813 1.00 0.00
B H
ATOM 3972 CD GLU B 40 12.877 7.540 62.926 1.00 0.00
B C
ATOM 3973 OE1 GLU B 40 13.885 6.823 62.777 1.00 0.00
B O
ATOM 3974 OE2 GLU B 40 12.425 7.853 64.051 1.00 0.00
B O
ATOM 3975 C GLU B 40 13.102 11.383 62.679 1.00 0.00
B C
ATOM 3976 O GLU B 40 14.074 11.265 63.435 1.00 0.00
B O
ATOM 3977 N LYS B 41 12.976 12.402 61.833 1.00 0.00
B N
ATOM 3978 HN LYS B 41 12.215 12.527 61.198 1.00 0.00
B H
ATOM 3979 CA LYS B 41 13.995 13.442 61.791 1.00 0.00
B C
ATOM 3980 HA LYS B 41 14.947 12.949 61.642 1.00 0.00
B H
ATOM 3981 CB LYS B 41 13.711 14.422 60.657 1.00 0.00
B C
ATOM 3982 HB1 LYS B 41 12.845 15.048 60.973 1.00 0.00
B H
ATOM 3983 HB2 LYS B 41 14.577 15.114 60.552 1.00 0.00
B H
ATOM 3984 CG LYS B 41 13.390 13.773 59.342 1.00 0.00
B C
ATOM 3985 HG1 LYS B 41 12.976 12.756 59.529 1.00 0.00
B H
ATOM 3986 HG2 LYS B 41 12.597 14.365 58.830 1.00 0.00
B H
ATOM 3987 CD LYS B 41 14.602 13.654 58.463 1.00 0.00
B C
ATOM 3988 HD1 LYS B 41 15.269 14.525 58.661 1.00 0.00
B H
ATOM 3989 HD2 LYS B 41 15.158 12.735 58.761 1.00 0.00
B H
ATOM 3990 CE LYS B 41 14.168 13.594 57.015 1.00 0.00
B C
ATOM 3991 HE1 LYS B 41 15.034 13.358 56.358 1.00 0.00
B H
ATOM 3992 HE2 LYS B 41 13.393 12.809 56.872 1.00 0.00
B H
ATOM 3993 NZ LYS B 41 13.597 14.896 56.579 1.00 0.00
B N
ATOM 3994 HZ1 LYS B 41 13.304 14.840 55.583 1.00 0.00
B H
ATOM 3995 HZ2 LYS B 41 12.772 15.128 57.169 1.00 0.00
B H

```
ATOM   3996  HZ3 LYS B  41       14.314  15.641  56.687  1.00  0.00
B    H
ATOM   3997  C   LYS B  41       14.073  14.190  63.111  1.00  0.00
B    C
ATOM   3998  O   LYS B  41       15.169  14.490  63.593  1.00  0.00
B    O
ATOM   3999  N   VAL B  42       12.923  14.557  63.672  1.00  0.00
B    N
ATOM   4000  HN  VAL B  42       12.020  14.424  63.272  1.00  0.00
B    H
ATOM   4001  CA  VAL B  42       12.914  15.210  64.974  1.00  0.00
B    C
ATOM   4002  HA  VAL B  42       13.456  16.143  64.886  1.00  0.00
B    H
ATOM   4003  CB  VAL B  42       11.468  15.470  65.425  1.00  0.00
B    C
ATOM   4004  HB  VAL B  42       11.019  14.485  65.701  1.00  0.00
B    H
ATOM   4005  CG1 VAL B  42       11.446  16.384  66.630  1.00  0.00
B    C
ATOM   4006 HG11 VAL B  42       10.398  16.601  66.929  1.00  0.00
B    H
ATOM   4007 HG12 VAL B  42       11.950  15.923  67.504  1.00  0.00
B    H
ATOM   4008 HG13 VAL B  42       11.945  17.351  66.399  1.00  0.00
B    H
ATOM   4009  CG2 VAL B  42       10.664  16.047  64.280  1.00  0.00
B    C
ATOM   4010 HG21 VAL B  42        9.628  16.276  64.604  1.00  0.00
B    H
ATOM   4011 HG22 VAL B  42       11.131  16.985  63.909  1.00  0.00
B    H
ATOM   4012 HG23 VAL B  42       10.600  15.331  63.434  1.00  0.00
B    H
ATOM   4013  C   VAL B  42       13.652  14.348  65.977  1.00  0.00
B    C
ATOM   4014  O   VAL B  42       14.519  14.817  66.723  1.00  0.00
B    O
ATOM   4015  N   ASN B  43       13.322  13.062  65.983  1.00  0.00
B    N
ATOM   4016  HN  ASN B  43       12.643  12.668  65.365  1.00  0.00
B    H
ATOM   4017  CA  ASN B  43       13.928  12.111  66.898  1.00  0.00
B    C
ATOM   4018  HA  ASN B  43       13.671  12.454  67.895  1.00  0.00
B    H
ATOM   4019  CB  ASN B  43       13.381  10.718  66.597  1.00  0.00
B    C
ATOM   4020  HB1 ASN B  43       12.394  10.841  66.101  1.00  0.00
B    H
ATOM   4021  HB2 ASN B  43       14.047  10.188  65.886  1.00  0.00
B    H
ATOM   4022  CG  ASN B  43       13.182   9.897  67.834  1.00  0.00
B    C
```

ATOM 4023 OD1 ASN B 43 14.072 9.779 68.661 1.00 0.00
B O
ATOM 4024 ND2 ASN B 43 11.992 9.335 67.983 1.00 0.00
B N
ATOM 4025 HD21 ASN B 43 11.840 8.795 68.807 1.00 0.00
B H
ATOM 4026 HD22 ASN B 43 11.281 9.484 67.302 1.00 0.00
B H
ATOM 4027 C ASN B 43 15.436 12.128 66.781 1.00 0.00
B C
ATOM 4028 O ASN B 43 16.152 12.152 67.788 1.00 0.00
B O
ATOM 4029 N ALA B 44 15.938 12.112 65.554 1.00 0.00
B N
ATOM 4030 HN ALA B 44 15.396 12.070 64.715 1.00 0.00
B H
ATOM 4031 CA ALA B 44 17.381 12.157 65.363 1.00 0.00
B C
ATOM 4032 HA ALA B 44 17.866 11.384 65.948 1.00 0.00
B H
ATOM 4033 CB ALA B 44 17.726 11.928 63.895 1.00 0.00
B C
ATOM 4034 HB1 ALA B 44 17.342 10.937 63.573 1.00 0.00
B H
ATOM 4035 HB2 ALA B 44 17.257 12.702 63.249 1.00 0.00
B H
ATOM 4036 HB3 ALA B 44 18.824 11.942 63.725 1.00 0.00
B H
ATOM 4037 C ALA B 44 17.959 13.481 65.838 1.00 0.00
B C
ATOM 4038 O ALA B 44 18.761 13.518 66.777 1.00 0.00
B O
ATOM 4039 N TYR B 45 17.532 14.580 65.236 1.00 0.00
B N
ATOM 4040 HN TYR B 45 16.723 14.625 64.652 1.00 0.00
B H
ATOM 4041 CA TYR B 45 18.237 15.842 65.389 1.00 0.00
B C
ATOM 4042 HA TYR B 45 19.284 15.576 65.471 1.00 0.00
B H
ATOM 4043 CB TYR B 45 17.994 16.747 64.184 1.00 0.00
B C
ATOM 4044 HB1 TYR B 45 16.906 16.767 63.956 1.00 0.00
B H
ATOM 4045 HB2 TYR B 45 18.302 17.791 64.409 1.00 0.00
B H
ATOM 4046 CG TYR B 45 18.688 16.285 62.937 1.00 0.00
B C
ATOM 4047 CD1 TYR B 45 19.543 15.195 62.961 1.00 0.00
B C
ATOM 4048 HD1 TYR B 45 19.720 14.672 63.891 1.00 0.00
B H
ATOM 4049 CE1 TYR B 45 20.190 14.778 61.821 1.00 0.00
B C

```
ATOM   4050  HE1 TYR B  45      20.841  13.916  61.843  1.00  0.00
B    H
ATOM   4051  CZ  TYR B  45      20.012  15.472  60.648  1.00  0.00
B    C
ATOM   4052  OH  TYR B  45      20.659  15.069  59.506  1.00  0.00
B    O
ATOM   4053  HH  TYR B  45      20.352  15.628  58.788  1.00  0.00
B    H
ATOM   4054  CD2 TYR B  45      18.540  16.973  61.748  1.00  0.00
B    C
ATOM   4055  HD2 TYR B  45      17.937  17.870  61.714  1.00  0.00
B    H
ATOM   4056  CE2 TYR B  45      19.187  16.568  60.609  1.00  0.00
B    C
ATOM   4057  HE2 TYR B  45      19.061  17.127  59.694  1.00  0.00
B    H
ATOM   4058  C   TYR B  45      17.869  16.601  66.647  1.00  0.00
B    C
ATOM   4059  O   TYR B  45      18.283  17.756  66.790  1.00  0.00
B    O
ATOM   4060  N   ALA B  46      17.099  16.002  67.553  1.00  0.00
B    N
ATOM   4061  HN  ALA B  46      16.625  15.132  67.423  1.00  0.00
B    H
ATOM   4062  CA  ALA B  46      16.904  16.631  68.852  1.00  0.00
B    C
ATOM   4063  HA  ALA B  46      16.339  17.550  68.755  1.00  0.00
B    H
ATOM   4064  CB  ALA B  46      16.135  15.691  69.777  1.00  0.00
B    C
ATOM   4065  HB1 ALA B  46      15.141  15.464  69.335  1.00  0.00
B    H
ATOM   4066  HB2 ALA B  46      16.678  14.729  69.909  1.00  0.00
B    H
ATOM   4067  HB3 ALA B  46      15.970  16.145  70.777  1.00  0.00
B    H
ATOM   4068  C   ALA B  46      18.241  17.011  69.464  1.00  0.00
B    C
ATOM   4069  O   ALA B  46      18.427  18.135  69.939  1.00  0.00
B    O
ATOM   4070  N   SER B  47      19.198  16.088  69.420  1.00  0.00
B    N
ATOM   4071  HN  SER B  47      19.091  15.182  69.010  1.00  0.00
B    H
ATOM   4072  CA  SER B  47      20.515  16.335  69.984  1.00  0.00
B    C
ATOM   4073  HA  SER B  47      20.342  16.565  71.027  1.00  0.00
B    H
ATOM   4074  CB  SER B  47      21.390  15.102  69.801  1.00  0.00
B    C
ATOM   4075  HB1 SER B  47      22.215  15.149  70.549  1.00  0.00
B    H
ATOM   4076  HB2 SER B  47      20.806  14.178  70.014  1.00  0.00
B    H
```

ATOM 4077 OG SER B 47 21.940 15.087 68.499 1.00 0.00
B O
ATOM 4078 HG1 SER B 47 22.648 14.431 68.497 1.00 0.00
B H
ATOM 4079 C SER B 47 21.205 17.539 69.365 1.00 0.00
B C
ATOM 4080 O SER B 47 22.030 18.172 70.026 1.00 0.00
B O
ATOM 4081 N TYR B 48 20.897 17.868 68.116 1.00 0.00
B N
ATOM 4082 HN TYR B 48 20.235 17.373 67.557 1.00 0.00
B H
ATOM 4083 CA TYR B 48 21.518 19.003 67.452 1.00 0.00
B C
ATOM 4084 HA TYR B 48 22.371 19.284 68.059 1.00 0.00
B H
ATOM 4085 CB TYR B 48 21.938 18.621 66.039 1.00 0.00
B C
ATOM 4086 HB1 TYR B 48 21.032 18.373 65.444 1.00 0.00
B H
ATOM 4087 HB2 TYR B 48 22.438 19.475 65.534 1.00 0.00
B H
ATOM 4088 CG TYR B 48 22.829 17.421 66.019 1.00 0.00
B C
ATOM 4089 CD1 TYR B 48 23.663 17.143 67.088 1.00 0.00
B C
ATOM 4090 HD1 TYR B 48 23.696 17.817 67.934 1.00 0.00
B H
ATOM 4091 CE1 TYR B 48 24.468 16.036 67.086 1.00 0.00
B C
ATOM 4092 HE1 TYR B 48 25.128 15.835 67.916 1.00 0.00
B H
ATOM 4093 CZ TYR B 48 24.435 15.180 66.013 1.00 0.00
B C
ATOM 4094 OH TYR B 48 25.239 14.070 66.004 1.00 0.00
B O
ATOM 4095 HH TYR B 48 25.026 13.565 65.217 1.00 0.00
B H
ATOM 4096 CD2 TYR B 48 22.806 16.538 64.956 1.00 0.00
B C
ATOM 4097 HD2 TYR B 48 22.178 16.741 64.100 1.00 0.00
B H
ATOM 4098 CE2 TYR B 48 23.607 15.427 64.944 1.00 0.00
B C
ATOM 4099 HE2 TYR B 48 23.598 14.768 64.089 1.00 0.00
B H
ATOM 4100 C TYR B 48 20.589 20.195 67.410 1.00 0.00
B C
ATOM 4101 O TYR B 48 20.672 21.008 66.490 1.00 0.00
B O
ATOM 4102 N ALA B 49 19.702 20.312 68.389 1.00 0.00
B N
ATOM 4103 HN ALA B 49 19.700 19.745 69.213 1.00 0.00
B H

```
ATOM   4104  CA  ALA B  49      18.648  21.306  68.314  1.00  0.00
B    C
ATOM   4105  HA  ALA B  49      18.898  22.092  67.611  1.00  0.00
B    H
ATOM   4106  CB  ALA B  49      17.318  20.683  67.884  1.00  0.00
B    C
ATOM   4107  HB1 ALA B  49      17.426  20.236  66.873  1.00  0.00
B    H
ATOM   4108  HB2 ALA B  49      17.010  19.876  68.584  1.00  0.00
B    H
ATOM   4109  HB3 ALA B  49      16.507  21.442  67.839  1.00  0.00
B    H
ATOM   4110  C   ALA B  49      18.484  21.973  69.661  1.00  0.00
B    C
ATOM   4111  O   ALA B  49      18.425  21.310  70.699  1.00  0.00
B    O
ATOM   4112  N   THR B  50      18.405  23.301  69.631  1.00  0.00
B    N
ATOM   4113  HN  THR B  50      18.589  23.886  68.842  1.00  0.00
B    H
ATOM   4114  CA  THR B  50      18.020  24.021  70.832  1.00  0.00
B    C
ATOM   4115  HA  THR B  50      18.759  23.772  71.581  1.00  0.00
B    H
ATOM   4116  CB  THR B  50      17.940  25.521  70.546  1.00  0.00
B    C
ATOM   4117  HB  THR B  50      17.039  25.667  69.901  1.00  0.00
B    H
ATOM   4118  OG1 THR B  50      19.092  25.932  69.797  1.00  0.00
B    O
ATOM   4119  HG1 THR B  50      18.985  26.880  69.662  1.00  0.00
B    H
ATOM   4120  CG2 THR B  50      17.881  26.313  71.843  1.00  0.00
B    C
ATOM   4121 HG21 THR B  50      17.709  27.391  71.640  1.00  0.00
B    H
ATOM   4122 HG22 THR B  50      17.048  25.948  72.481  1.00  0.00
B    H
ATOM   4123 HG23 THR B  50      18.828  26.214  72.416  1.00  0.00
B    H
ATOM   4124  C   THR B  50      16.688  23.517  71.357  1.00  0.00
B    C
ATOM   4125  O   THR B  50      16.490  23.441  72.572  1.00  0.00
B    O
ATOM   4126  N   HSE B  51      15.785  23.142  70.463  1.00  0.00
B    N
ATOM   4127  HN  HSE B  51      15.921  23.166  69.474  1.00  0.00
B    H
ATOM   4128  CA  HSE B  51      14.485  22.652  70.876  1.00  0.00
B    C
ATOM   4129  HA  HSE B  51      14.637  21.881  71.622  1.00  0.00
B    H
ATOM   4130  CB  HSE B  51      13.633  23.796  71.426  1.00  0.00
B    C
```

ATOM 4131 HB1 HSE B 51 14.144 24.201 72.326 1.00 0.00
B H
ATOM 4132 HB2 HSE B 51 13.590 24.618 70.681 1.00 0.00
B H
ATOM 4133 ND1 HSE B 51 11.311 24.295 72.257 1.00 0.00
B N
ATOM 4134 CG HSE B 51 12.254 23.388 71.826 1.00 0.00
B C
ATOM 4135 CE1 HSE B 51 10.189 23.659 72.539 1.00 0.00
B C
ATOM 4136 HE1 HSE B 51 9.280 24.140 72.909 1.00 0.00
B H
ATOM 4137 NE2 HSE B 51 10.369 22.374 72.304 1.00 0.00
B N
ATOM 4138 HE2 HSE B 51 9.684 21.660 72.450 1.00 0.00
B H
ATOM 4139 CD2 HSE B 51 11.653 22.177 71.858 1.00 0.00
B C
ATOM 4140 HD2 HSE B 51 12.046 21.202 71.607 1.00 0.00
B H
ATOM 4141 C HSE B 51 13.819 21.993 69.682 1.00 0.00
B C
ATOM 4142 O HSE B 51 13.661 22.614 68.628 1.00 0.00
B O
ATOM 4143 N ALA B 52 13.427 20.735 69.855 1.00 0.00
B N
ATOM 4144 HN ALA B 52 13.548 20.262 70.727 1.00 0.00
B H
ATOM 4145 CA ALA B 52 12.798 19.955 68.803 1.00 0.00
B C
ATOM 4146 HA ALA B 52 12.713 20.522 67.882 1.00 0.00
B H
ATOM 4147 CB ALA B 52 13.578 18.673 68.517 1.00 0.00
B C
ATOM 4148 HB1 ALA B 52 14.598 18.930 68.161 1.00 0.00
B H
ATOM 4149 HB2 ALA B 52 13.682 18.055 69.436 1.00 0.00
B H
ATOM 4150 HB3 ALA B 52 13.086 18.060 67.732 1.00 0.00
B H
ATOM 4151 C ALA B 52 11.385 19.609 69.223 1.00 0.00
B C
ATOM 4152 O ALA B 52 11.104 19.479 70.414 1.00 0.00
B O
ATOM 4153 N VAL B 53 10.508 19.449 68.243 1.00 0.00
B N
ATOM 4154 HN VAL B 53 10.747 19.567 67.283 1.00 0.00
B H
ATOM 4155 CA VAL B 53 9.115 19.088 68.476 1.00 0.00
B C
ATOM 4156 HA VAL B 53 9.065 18.394 69.306 1.00 0.00
B H
ATOM 4157 CB VAL B 53 8.229 20.322 68.743 1.00 0.00
B C

ATOM 4158 HB VAL B 53 8.555 21.129 68.043 1.00 0.00
B H
ATOM 4159 CG1 VAL B 53 6.763 20.001 68.485 1.00 0.00
B C
ATOM 4160 HG11 VAL B 53 6.129 20.883 68.717 1.00 0.00
B H
ATOM 4161 HG12 VAL B 53 6.584 19.734 67.423 1.00 0.00
B H
ATOM 4162 HG13 VAL B 53 6.427 19.158 69.126 1.00 0.00
B H
ATOM 4163 CG2 VAL B 53 8.404 20.827 70.155 1.00 0.00
B C
ATOM 4164 HG21 VAL B 53 7.733 21.690 70.349 1.00 0.00
B H
ATOM 4165 HG22 VAL B 53 8.165 20.027 70.888 1.00 0.00
B H
ATOM 4166 HG23 VAL B 53 9.446 21.166 70.335 1.00 0.00
B H
ATOM 4167 C VAL B 53 8.620 18.359 67.243 1.00 0.00
B C
ATOM 4168 O VAL B 53 8.959 18.734 66.119 1.00 0.00
B O
ATOM 4169 N ILE B 54 7.822 17.320 67.441 1.00 0.00
B N
ATOM 4170 HN ILE B 54 7.656 16.867 68.314 1.00 0.00
B H
ATOM 4171 CA ILE B 54 7.094 16.742 66.322 1.00 0.00
B C
ATOM 4172 HA ILE B 54 7.665 16.930 65.422 1.00 0.00
B H
ATOM 4173 CB ILE B 54 6.850 15.249 66.532 1.00 0.00
B C
ATOM 4174 HB ILE B 54 6.186 15.198 67.432 1.00 0.00
B H
ATOM 4175 CG2 ILE B 54 6.292 14.632 65.276 1.00 0.00
B C
ATOM 4176 HG21 ILE B 54 5.965 13.589 65.464 1.00 0.00
B H
ATOM 4177 HG22 ILE B 54 5.410 15.200 64.914 1.00 0.00
B H
ATOM 4178 HG23 ILE B 54 7.055 14.617 64.468 1.00 0.00
B H
ATOM 4179 CG1 ILE B 54 8.157 14.561 66.899 1.00 0.00
B C
ATOM 4180 HG11 ILE B 54 8.761 14.386 65.981 1.00 0.00
B H
ATOM 4181 HG12 ILE B 54 8.748 15.238 67.557 1.00 0.00
B H
ATOM 4182 CD ILE B 54 7.958 13.256 67.619 1.00 0.00
B C
ATOM 4183 HD1 ILE B 54 8.939 12.825 67.914 1.00 0.00
B H
ATOM 4184 HD2 ILE B 54 7.352 13.404 68.538 1.00 0.00
B H

ATOM   4185 HD3 ILE B 54   7.440 12.518 66.970 1.00 0.00
B   H
ATOM   4186 C   ILE B 54   5.785 17.506 66.181 1.00 0.00
B   C
ATOM   4187 O   ILE B 54   5.026 17.633 67.147 1.00 0.00
B   O
ATOM   4188 N   ALA B 55   5.524 18.027 64.985 1.00 0.00
B   N
ATOM   4189 HN ALA B 55   6.126 17.910 64.195 1.00 0.00
B   H
ATOM   4190 CA ALA B 55   4.323 18.815 64.744 1.00 0.00
B   C
ATOM   4191 HA ALA B 55   3.441 18.311 65.122 1.00 0.00
B   H
ATOM   4192 CB ALA B 55   4.409 20.184 65.424 1.00 0.00
B   C
ATOM   4193 HB1 ALA B 55   4.514 20.050 66.522 1.00 0.00
B   H
ATOM   4194 HB2 ALA B 55   5.292 20.754 65.063 1.00 0.00
B   H
ATOM   4195 HB3 ALA B 55   3.497 20.792 65.239 1.00 0.00
B   H
ATOM   4196 C   ALA B 55   4.111 18.989 63.248 1.00 0.00
B   C
ATOM   4197 O   ALA B 55   5.073 19.100 62.484 1.00 0.00
B   O
ATOM   4198 N   ASN B 56   2.846 19.021 62.847 1.00 0.00
B   N
ATOM   4199 HN ASN B 56   2.079 18.909 63.479 1.00 0.00
B   H
ATOM   4200 CA ASN B 56   2.458 19.224 61.455 1.00 0.00
B   C
ATOM   4201 HA ASN B 56   3.285 18.888 60.839 1.00 0.00
B   H
ATOM   4202 CB ASN B 56   1.175 18.467 61.150 1.00 0.00
B   C
ATOM   4203 HB1 ASN B 56   1.363 17.388 61.340 1.00 0.00
B   H
ATOM   4204 HB2 ASN B 56   0.364 18.789 61.835 1.00 0.00
B   H
ATOM   4205 CG ASN B 56   0.736 18.619 59.713 1.00 0.00
B   C
ATOM   4206 OD1 ASN B 56   1.226 19.477 58.980 1.00 0.00
B   O
ATOM   4207 ND2 ASN B 56   -0.206 17.782 59.302 1.00 0.00
B   N
ATOM   4208 HD21 ASN B 56   -0.503 17.856 58.354 1.00 0.00
B   H
ATOM   4209 HD22 ASN B 56   -0.566 17.094 59.924 1.00 0.00
B   H
ATOM   4210 C   ASN B 56   2.289 20.718 61.225 1.00 0.00
B   C
ATOM   4211 O   ASN B 56   1.393 21.348 61.786 1.00 0.00
B   O

ATOM 4212 N ALA B 57 3.132 21.277 60.359 1.00 0.00
B N
ATOM 4213 HN ALA B 57 3.776 20.747 59.808 1.00 0.00
B H
ATOM 4214 CA ALA B 57 3.175 22.718 60.154 1.00 0.00
B C
ATOM 4215 HA ALA B 57 3.396 23.210 61.095 1.00 0.00
B H
ATOM 4216 CB ALA B 57 4.266 23.069 59.150 1.00 0.00
B C
ATOM 4217 HB1 ALA B 57 5.245 22.693 59.518 1.00 0.00
B H
ATOM 4218 HB2 ALA B 57 4.068 22.598 58.163 1.00 0.00
B H
ATOM 4219 HB3 ALA B 57 4.353 24.168 59.006 1.00 0.00
B H
ATOM 4220 C ALA B 57 1.863 23.300 59.666 1.00 0.00
B C
ATOM 4221 O ALA B 57 1.644 24.503 59.820 1.00 0.00
B O
ATOM 4222 N THR B 58 0.990 22.492 59.093 1.00 0.00
B N
ATOM 4223 HN THR B 58 1.032 21.493 59.100 1.00 0.00
B H
ATOM 4224 CA THR B 58 −0.149 23.035 58.379 1.00 0.00
B C
ATOM 4225 HA THR B 58 0.235 23.913 57.877 1.00 0.00
B H
ATOM 4226 CB THR B 58 −0.679 21.990 57.402 1.00 0.00
B C
ATOM 4227 HB THR B 58 −1.513 22.482 56.844 1.00 0.00
B H
ATOM 4228 OG1 THR B 58 −1.220 20.890 58.133 1.00 0.00
B O
ATOM 4229 HG1 THR B 58 −1.525 20.265 57.467 1.00 0.00
B H
ATOM 4230 CG2 THR B 58 0.450 21.477 56.530 1.00 0.00
B C
ATOM 4231 HG21 THR B 58 0.063 20.801 55.739 1.00 0.00
B H
ATOM 4232 HG22 THR B 58 0.970 22.323 56.033 1.00 0.00
B H
ATOM 4233 HG23 THR B 58 1.197 20.916 57.132 1.00 0.00
B H
ATOM 4234 C THR B 58 −1.275 23.513 59.284 1.00 0.00
B C
ATOM 4235 O THR B 58 −2.254 24.067 58.778 1.00 0.00
B O
ATOM 4236 N GLU B 59 −1.175 23.320 60.595 1.00 0.00
B N
ATOM 4237 HN GLU B 59 −0.379 22.935 61.053 1.00 0.00
B H
ATOM 4238 CA GLU B 59 −2.267 23.671 61.499 1.00 0.00
B C

ATOM 4239 HA GLU B 59 -3.121 23.964 60.902 1.00 0.00
B H
ATOM 4240 CB GLU B 59 -2.629 22.487 62.388 1.00 0.00
B C
ATOM 4241 HB1 GLU B 59 -1.695 22.113 62.867 1.00 0.00
B H
ATOM 4242 HB2 GLU B 59 -3.285 22.828 63.221 1.00 0.00
B H
ATOM 4243 CG GLU B 59 -3.288 21.342 61.669 1.00 0.00
B C
ATOM 4244 HG1 GLU B 59 -3.619 20.586 62.404 1.00 0.00
B H
ATOM 4245 HG2 GLU B 59 -4.163 21.691 61.084 1.00 0.00
B H
ATOM 4246 CD GLU B 59 -2.335 20.635 60.744 1.00 0.00
B C
ATOM 4247 OE1 GLU B 59 -2.740 20.304 59.614 1.00 0.00
B O
ATOM 4248 OE2 GLU B 59 -1.165 20.460 61.122 1.00 0.00
B O
ATOM 4249 C GLU B 59 -1.890 24.865 62.367 1.00 0.00
B C
ATOM 4250 O GLU B 59 -0.848 24.855 63.030 1.00 0.00
B O
ATOM 4251 N GLU B 60 -2.774 25.862 62.404 1.00 0.00
B N
ATOM 4252 HN GLU B 60 -3.686 25.837 62.004 1.00 0.00
B H
ATOM 4253 CA GLU B 60 -2.450 27.122 63.064 1.00 0.00
B C
ATOM 4254 HA GLU B 60 -1.507 27.474 62.664 1.00 0.00
B H
ATOM 4255 CB GLU B 60 -3.523 28.167 62.742 1.00 0.00
B C
ATOM 4256 HB1 GLU B 60 -3.314 29.078 63.349 1.00 0.00
B H
ATOM 4257 HB2 GLU B 60 -3.435 28.483 61.678 1.00 0.00
B H
ATOM 4258 CG GLU B 60 -4.985 27.774 63.000 1.00 0.00
B C
ATOM 4259 HG1 GLU B 60 -5.636 28.655 62.852 1.00 0.00
B H
ATOM 4260 HG2 GLU B 60 -5.305 26.965 62.314 1.00 0.00
B H
ATOM 4261 CD GLU B 60 -5.278 27.313 64.409 1.00 0.00
B C
ATOM 4262 OE1 GLU B 60 -4.947 28.052 65.359 1.00 0.00
B O
ATOM 4263 OE2 GLU B 60 -5.801 26.191 64.568 1.00 0.00
B O
ATOM 4264 C GLU B 60 -2.267 26.942 64.563 1.00 0.00
B C
ATOM 4265 O GLU B 60 -1.494 27.670 65.194 1.00 0.00
B O

```
ATOM   4266  N   ASN B  61      -2.986  25.996  65.158  1.00  0.00
B    N
ATOM   4267  HN  ASN B  61      -3.589  25.343  64.701  1.00  0.00
B    H
ATOM   4268  CA  ASN B  61      -2.927  25.853  66.604  1.00  0.00
B    C
ATOM   4269  HA  ASN B  61      -3.006  26.855  67.009  1.00  0.00
B    H
ATOM   4270  CB  ASN B  61      -4.076  24.977  67.084  1.00  0.00
B    C
ATOM   4271  HB1 ASN B  61      -3.835  24.633  68.113  1.00  0.00
B    H
ATOM   4272  HB2 ASN B  61      -5.013  25.567  67.141  1.00  0.00
B    H
ATOM   4273  CG  ASN B  61      -4.263  23.774  66.217  1.00  0.00
B    C
ATOM   4274  OD1 ASN B  61      -3.936  23.802  65.031  1.00  0.00
B    O
ATOM   4275  ND2 ASN B  61      -4.795  22.707  66.790  1.00  0.00
B    N
ATOM   4276 HD21 ASN B  61      -4.913  21.895  66.225  1.00  0.00
B    H
ATOM   4277 HD22 ASN B  61      -5.031  22.726  67.756  1.00  0.00
B    H
ATOM   4278  C   ASN B  61      -1.588  25.288  67.031  1.00  0.00
B    C
ATOM   4279  O   ASN B  61      -1.035  25.692  68.058  1.00  0.00
B    O
ATOM   4280  N   GLU B  62      -1.045  24.352  66.253  1.00  0.00
B    N
ATOM   4281  HN  GLU B  62      -1.491  23.852  65.516  1.00  0.00
B    H
ATOM   4282  CA  GLU B  62       0.345  23.984  66.461  1.00  0.00
B    C
ATOM   4283  HA  GLU B  62       0.434  23.540  67.445  1.00  0.00
B    H
ATOM   4284  CB  GLU B  62       0.810  22.986  65.411  1.00  0.00
B    C
ATOM   4285  HB1 GLU B  62       0.376  23.286  64.430  1.00  0.00
B    H
ATOM   4286  HB2 GLU B  62       1.914  23.053  65.290  1.00  0.00
B    H
ATOM   4287  CG  GLU B  62       0.412  21.569  65.708  1.00  0.00
B    C
ATOM   4288  HG1 GLU B  62       1.160  21.109  66.380  1.00  0.00
B    H
ATOM   4289  HG2 GLU B  62      -0.585  21.530  66.191  1.00  0.00
B    H
ATOM   4290  CD  GLU B  62       0.382  20.704  64.477  1.00  0.00
B    C
ATOM   4291  OE1 GLU B  62       1.329  19.920  64.281  1.00  0.00
B    O
ATOM   4292  OE2 GLU B  62      -0.593  20.791  63.711  1.00  0.00
B    O
```

```
ATOM   4293  C   GLU B  62       1.242  25.205  66.435  1.00  0.00
B    C
ATOM   4294  O   GLU B  62       2.179  25.307  67.232  1.00  0.00
B    O
ATOM   4295  N   LEU B  63       0.948  26.149  65.553  1.00  0.00
B    N
ATOM   4296  HN  LEU B  63       0.160  26.142  64.942  1.00  0.00
B    H
ATOM   4297  CA  LEU B  63       1.800  27.315  65.414  1.00  0.00
B    C
ATOM   4298  HA  LEU B  63       2.814  26.948  65.335  1.00  0.00
B    H
ATOM   4299  CB  LEU B  63       1.387  28.082  64.169  1.00  0.00
B    C
ATOM   4300  HB1 LEU B  63       0.444  28.623  64.414  1.00  0.00
B    H
ATOM   4301  HB2 LEU B  63       2.147  28.867  63.960  1.00  0.00
B    H
ATOM   4302  CG  LEU B  63       1.172  27.158  62.979  1.00  0.00
B    C
ATOM   4303  HG  LEU B  63       0.358  26.451  63.269  1.00  0.00
B    H
ATOM   4304  CD1 LEU B  63       0.739  27.952  61.770  1.00  0.00
B    C
ATOM   4305 HD11 LEU B  63       0.627  27.286  60.887  1.00  0.00
B    H
ATOM   4306 HD12 LEU B  63      -0.237  28.450  61.957  1.00  0.00
B    H
ATOM   4307 HD13 LEU B  63       1.490  28.732  61.523  1.00  0.00
B    H
ATOM   4308  CD2 LEU B  63       2.415  26.346  62.680  1.00  0.00
B    C
ATOM   4309 HD21 LEU B  63       2.233  25.664  61.821  1.00  0.00
B    H
ATOM   4310 HD22 LEU B  63       3.275  26.994  62.418  1.00  0.00
B    H
ATOM   4311 HD23 LEU B  63       2.696  25.728  63.560  1.00  0.00
B    H
ATOM   4312  C   LEU B  63       1.728  28.206  66.643  1.00  0.00
B    C
ATOM   4313  O   LEU B  63       2.762  28.526  67.240  1.00  0.00
B    O
ATOM   4314  N   LEU B  64       0.521  28.613  67.031  1.00  0.00
B    N
ATOM   4315  HN  LEU B  64      -0.335  28.416  66.557  1.00  0.00
B    H
ATOM   4316  CA  LEU B  64       0.368  29.412  68.237  1.00  0.00
B    C
ATOM   4317  HA  LEU B  64       0.934  30.321  68.084  1.00  0.00
B    H
ATOM   4318  CB  LEU B  64      -1.102  29.712  68.484  1.00  0.00
B    C
ATOM   4319  HB1 LEU B  64      -1.537  28.823  68.998  1.00  0.00
B    H
```

ATOM 4320 HB2 LEU B 64 -1.183 30.555 69.207 1.00 0.00
B H
ATOM 4321 CG LEU B 64 -1.868 29.994 67.206 1.00 0.00
B C
ATOM 4322 HG LEU B 64 -1.600 29.214 66.454 1.00 0.00
B H
ATOM 4323 CD1 LEU B 64 -3.353 29.937 67.474 1.00 0.00
B C
ATOM 4324 HD11 LEU B 64 -3.925 30.190 66.555 1.00 0.00
B H
ATOM 4325 HD12 LEU B 64 -3.653 28.919 67.803 1.00 0.00
B H
ATOM 4326 HD13 LEU B 64 -3.636 30.660 68.268 1.00 0.00
B H
ATOM 4327 CD2 LEU B 64 -1.460 31.339 66.651 1.00 0.00
B C
ATOM 4328 HD21 LEU B 64 -2.003 31.547 65.704 1.00 0.00
B H
ATOM 4329 HD22 LEU B 64 -1.690 32.161 67.358 1.00 0.00
B H
ATOM 4330 HD23 LEU B 64 -0.369 31.357 66.438 1.00 0.00
B H
ATOM 4331 C LEU B 64 0.959 28.701 69.440 1.00 0.00
B C
ATOM 4332 O LEU B 64 1.657 29.320 70.251 1.00 0.00
B O
ATOM 4333 N SER B 65 0.700 27.400 69.564 1.00 0.00
B N
ATOM 4334 HN SER B 65 0.125 26.865 68.945 1.00 0.00
B H
ATOM 4335 CA SER B 65 1.265 26.634 70.663 1.00 0.00
B C
ATOM 4336 HA SER B 65 0.888 27.088 71.570 1.00 0.00
B H
ATOM 4337 CB SER B 65 0.864 25.171 70.534 1.00 0.00
B C
ATOM 4338 HB1 SER B 65 1.350 24.605 71.361 1.00 0.00
B H
ATOM 4339 HB2 SER B 65 -0.237 25.058 70.664 1.00 0.00
B H
ATOM 4340 OG SER B 65 1.303 24.663 69.294 1.00 0.00
B O
ATOM 4341 HG1 SER B 65 1.234 23.701 69.341 1.00 0.00
B H
ATOM 4342 C SER B 65 2.775 26.755 70.693 1.00 0.00
B C
ATOM 4343 O SER B 65 3.383 26.725 71.768 1.00 0.00
B O
ATOM 4344 N LEU B 66 3.398 26.905 69.528 1.00 0.00
B N
ATOM 4345 HN LEU B 66 2.962 26.918 68.631 1.00 0.00
B H
ATOM 4346 CA LEU B 66 4.838 27.070 69.492 1.00 0.00
B C

ATOM 4347 HA LEU B 66 5.240 26.374 70.218 1.00 0.00
B H
ATOM 4348 CB LEU B 66 5.362 26.764 68.098 1.00 0.00
B C
ATOM 4349 HB1 LEU B 66 4.748 27.353 67.379 1.00 0.00
B H
ATOM 4350 HB2 LEU B 66 6.400 27.155 68.008 1.00 0.00
B H
ATOM 4351 CG LEU B 66 5.280 25.284 67.764 1.00 0.00
B C
ATOM 4352 HG LEU B 66 4.205 25.006 67.642 1.00 0.00
B H
ATOM 4353 CD1 LEU B 66 6.016 25.016 66.482 1.00 0.00
B C
ATOM 4354 HD11 LEU B 66 6.007 23.930 66.248 1.00 0.00
B H
ATOM 4355 HD12 LEU B 66 5.541 25.560 65.637 1.00 0.00
B H
ATOM 4356 HD13 LEU B 66 7.073 25.345 66.560 1.00 0.00
B H
ATOM 4357 CD2 LEU B 66 5.851 24.462 68.901 1.00 0.00
B C
ATOM 4358 HD21 LEU B 66 5.775 23.378 68.668 1.00 0.00
B H
ATOM 4359 HD22 LEU B 66 6.920 24.694 69.078 1.00 0.00
B H
ATOM 4360 HD23 LEU B 66 5.290 24.656 69.840 1.00 0.00
B H
ATOM 4361 C LEU B 66 5.271 28.463 69.907 1.00 0.00
B C
ATOM 4362 O LEU B 66 6.472 28.695 70.071 1.00 0.00
B O
ATOM 4363 N GLY B 67 4.336 29.387 70.086 1.00 0.00
B N
ATOM 4364 HN GLY B 67 3.352 29.214 70.041 1.00 0.00
B H
ATOM 4365 CA GLY B 67 4.718 30.754 70.367 1.00 0.00
B C
ATOM 4366 HA1 GLY B 67 5.315 30.759 71.269 1.00 0.00
B H
ATOM 4367 HA2 GLY B 67 3.820 31.355 70.426 1.00 0.00
B H
ATOM 4368 C GLY B 67 5.567 31.294 69.241 1.00 0.00
B C
ATOM 4369 O GLY B 67 6.600 31.933 69.459 1.00 0.00
B O
ATOM 4370 N ILE B 68 5.129 31.012 68.016 1.00 0.00
B N
ATOM 4371 HN ILE B 68 4.269 30.546 67.819 1.00 0.00
B H
ATOM 4372 CA ILE B 68 5.898 31.372 66.835 1.00 0.00
B C
ATOM 4373 HA ILE B 68 6.858 30.884 66.938 1.00 0.00
B H

ATOM 4374 CB ILE B 68 5.144 30.901 65.588 1.00 0.00
B C
ATOM 4375 HB ILE B 68 5.614 31.468 64.745 1.00 0.00
B H
ATOM 4376 CG2 ILE B 68 5.202 29.403 65.485 1.00 0.00
B C
ATOM 4377 HG21 ILE B 68 4.796 29.063 64.510 1.00 0.00
B H
ATOM 4378 HG22 ILE B 68 6.249 29.042 65.559 1.00 0.00
B H
ATOM 4379 HG23 ILE B 68 4.609 28.926 66.293 1.00 0.00
B H
ATOM 4380 CG1 ILE B 68 3.694 31.354 65.689 1.00 0.00
B C
ATOM 4381 HG11 ILE B 68 3.200 30.849 66.549 1.00 0.00
B H
ATOM 4382 HG12 ILE B 68 3.672 32.449 65.894 1.00 0.00
B H
ATOM 4383 CD ILE B 68 2.913 31.098 64.466 1.00 0.00
B C
ATOM 4384 HD1 ILE B 68 1.882 31.500 64.575 1.00 0.00
B H
ATOM 4385 HD2 ILE B 68 3.387 31.589 63.589 1.00 0.00
B H
ATOM 4386 HD3 ILE B 68 2.837 30.009 64.263 1.00 0.00
B H
ATOM 4387 C ILE B 68 6.167 32.863 66.781 1.00 0.00
B C
ATOM 4388 O ILE B 68 7.281 33.290 66.458 1.00 0.00
B O
ATOM 4389 N ARG B 69 5.163 33.679 67.093 1.00 0.00
B N
ATOM 4390 HN ARG B 69 4.282 33.346 67.423 1.00 0.00
B H
ATOM 4391 CA ARG B 69 5.309 35.119 66.964 1.00 0.00
B C
ATOM 4392 HA ARG B 69 5.514 35.341 65.924 1.00 0.00
B H
ATOM 4393 CB ARG B 69 4.025 35.799 67.401 1.00 0.00
B C
ATOM 4394 HB1 ARG B 69 3.838 35.473 68.452 1.00 0.00
B H
ATOM 4395 HB2 ARG B 69 4.183 36.899 67.445 1.00 0.00
B H
ATOM 4396 CG ARG B 69 2.856 35.419 66.538 1.00 0.00
B C
ATOM 4397 HG1 ARG B 69 2.994 35.877 65.533 1.00 0.00
B H
ATOM 4398 HG2 ARG B 69 2.872 34.318 66.370 1.00 0.00
B H
ATOM 4399 CD ARG B 69 1.562 35.803 67.196 1.00 0.00
B C
ATOM 4400 HD1 ARG B 69 1.610 36.870 67.515 1.00 0.00
B H

```
ATOM   4401  HD2 ARG B  69       0.697  35.675  66.504  1.00  0.00
B    H
ATOM   4402  NE  ARG B  69       1.300  34.965  68.358  1.00  0.00
B    N
ATOM   4403  HE  ARG B  69       0.915  34.067  68.120  1.00  0.00
B    H
ATOM   4404  CZ  ARG B  69       1.541  35.336  69.609  1.00  0.00
B    C
ATOM   4405  NH1 ARG B  69       2.059  36.528  69.860  1.00  0.00
B    N
ATOM   4406 HH11 ARG B  69       2.265  36.802  70.789  1.00  0.00
B    H
ATOM   4407 HH12 ARG B  69       2.349  37.063  69.064  1.00  0.00
B    H
ATOM   4408  NH2 ARG B  69       1.272  34.513  70.609  1.00  0.00
B    N
ATOM   4409 HH21 ARG B  69       1.371  34.823  71.544  1.00  0.00
B    H
ATOM   4410 HH22 ARG B  69       0.780  33.682  70.385  1.00  0.00
B    H
ATOM   4411  C   ARG B  69       6.480  35.650  67.759  1.00  0.00
B    C
ATOM   4412  O   ARG B  69       7.062  36.665  67.369  1.00  0.00
B    O
ATOM   4413  N   ASN B  70       6.846  34.982  68.850  1.00  0.00
B    N
ATOM   4414  HN  ASN B  70       6.335  34.229  69.263  1.00  0.00
B    H
ATOM   4415  CA  ASN B  70       8.074  35.326  69.541  1.00  0.00
B    C
ATOM   4416  HA  ASN B  70       7.957  36.358  69.854  1.00  0.00
B    H
ATOM   4417  CB  ASN B  70       8.281  34.390  70.726  1.00  0.00
B    C
ATOM   4418  HB1 ASN B  70       8.586  33.398  70.328  1.00  0.00
B    H
ATOM   4419  HB2 ASN B  70       9.106  34.758  71.370  1.00  0.00
B    H
ATOM   4420  CG  ASN B  70       7.024  34.206  71.537  1.00  0.00
B    C
ATOM   4421  OD1 ASN B  70       6.090  34.998  71.436  1.00  0.00
B    O
ATOM   4422  ND2 ASN B  70       6.990  33.158  72.348  1.00  0.00
B    N
ATOM   4423 HD21 ASN B  70       6.160  33.018  72.881  1.00  0.00
B    H
ATOM   4424 HD22 ASN B  70       7.762  32.532  72.385  1.00  0.00
B    H
ATOM   4425  C   ASN B  70       9.269  35.261  68.615  1.00  0.00
B    C
ATOM   4426  O   ASN B  70      10.184  36.080  68.733  1.00  0.00
B    O
ATOM   4427  N   PHE B  71       9.267  34.327  67.674  1.00  0.00
B    N
```

ATOM 4428 HN PHE B 71 8.529 33.665 67.553 1.00 0.00
B H
ATOM 4429 CA PHE B 71 10.358 34.191 66.728 1.00 0.00
B C
ATOM 4430 HA PHE B 71 11.279 34.298 67.287 1.00 0.00
B H
ATOM 4431 CB PHE B 71 10.276 32.866 65.984 1.00 0.00
B C
ATOM 4432 HB1 PHE B 71 9.296 32.802 65.462 1.00 0.00
B H
ATOM 4433 HB2 PHE B 71 11.067 32.794 65.207 1.00 0.00
B H
ATOM 4434 CG PHE B 71 10.349 31.676 66.870 1.00 0.00
B C
ATOM 4435 CD1 PHE B 71 9.268 31.314 67.650 1.00 0.00
B C
ATOM 4436 HD1 PHE B 71 8.364 31.908 67.622 1.00 0.00
B H
ATOM 4437 CE1 PHE B 71 9.327 30.219 68.462 1.00 0.00
B C
ATOM 4438 HE1 PHE B 71 8.474 29.947 69.067 1.00 0.00
B H
ATOM 4439 CZ PHE B 71 10.478 29.459 68.501 1.00 0.00
B C
ATOM 4440 HZ PHE B 71 10.525 28.585 69.136 1.00 0.00
B H
ATOM 4441 CD2 PHE B 71 11.496 30.909 66.914 1.00 0.00
B C
ATOM 4442 HD2 PHE B 71 12.357 31.189 66.323 1.00 0.00
B H
ATOM 4443 CE2 PHE B 71 11.566 29.806 67.723 1.00 0.00
B C
ATOM 4444 HE2 PHE B 71 12.470 29.214 67.750 1.00 0.00
B H
ATOM 4445 C PHE B 71 10.303 35.338 65.735 1.00 0.00
B C
ATOM 4446 O PHE B 71 9.242 35.931 65.519 1.00 0.00
B O
ATOM 4447 N GLU B 72 11.450 35.639 65.129 1.00 0.00
B N
ATOM 4448 HN GLU B 72 12.327 35.197 65.295 1.00 0.00
B H
ATOM 4449 CA GLU B 72 11.499 36.693 64.125 1.00 0.00
B C
ATOM 4450 HA GLU B 72 10.510 37.125 64.042 1.00 0.00
B H
ATOM 4451 CB GLU B 72 12.502 37.748 64.554 1.00 0.00
B C
ATOM 4452 HB1 GLU B 72 13.291 37.816 63.770 1.00 0.00
B H
ATOM 4453 HB2 GLU B 72 12.011 38.746 64.580 1.00 0.00
B H
ATOM 4454 CG GLU B 72 13.135 37.431 65.885 1.00 0.00
B C

ATOM 4455 HG1 GLU B 72 12.347 37.177 66.617 1.00 0.00
B H
ATOM 4456 HG2 GLU B 72 13.839 36.580 65.797 1.00 0.00
B H
ATOM 4457 CD GLU B 72 13.871 38.612 66.461 1.00 0.00
B C
ATOM 4458 OE1 GLU B 72 14.703 39.207 65.743 1.00 0.00
B O
ATOM 4459 OE2 GLU B 72 13.616 38.947 67.638 1.00 0.00
B O
ATOM 4460 C GLU B 72 11.864 36.135 62.759 1.00 0.00
B C
ATOM 4461 O GLU B 72 11.359 36.605 61.732 1.00 0.00
B O
ATOM 4462 N TYR B 73 12.743 35.136 62.724 1.00 0.00
B N
ATOM 4463 HN TYR B 73 13.186 34.758 63.535 1.00 0.00
B H
ATOM 4464 CA TYR B 73 13.133 34.503 61.471 1.00 0.00
B C
ATOM 4465 HA TYR B 73 12.734 35.104 60.664 1.00 0.00
B H
ATOM 4466 CB TYR B 73 14.647 34.340 61.364 1.00 0.00
B C
ATOM 4467 HB1 TYR B 73 15.040 33.939 62.324 1.00 0.00
B H
ATOM 4468 HB2 TYR B 73 14.908 33.607 60.570 1.00 0.00
B H
ATOM 4469 CG TYR B 73 15.411 35.609 61.105 1.00 0.00
B C
ATOM 4470 CD1 TYR B 73 15.528 36.584 62.080 1.00 0.00
B C
ATOM 4471 HD1 TYR B 73 15.042 36.449 63.036 1.00 0.00
B H
ATOM 4472 CE1 TYR B 73 16.236 37.738 61.846 1.00 0.00
B C
ATOM 4473 HE1 TYR B 73 16.318 38.497 62.611 1.00 0.00
B H
ATOM 4474 CZ TYR B 73 16.837 37.926 60.624 1.00 0.00
B C
ATOM 4475 OH TYR B 73 17.545 39.073 60.371 1.00 0.00
B O
ATOM 4476 HH TYR B 73 17.941 38.982 59.502 1.00 0.00
B H
ATOM 4477 CD2 TYR B 73 16.031 35.823 59.888 1.00 0.00
B C
ATOM 4478 HD2 TYR B 73 15.929 35.094 59.094 1.00 0.00
B H
ATOM 4479 CE2 TYR B 73 16.736 36.971 59.645 1.00 0.00
B C
ATOM 4480 HE2 TYR B 73 17.191 37.126 58.678 1.00 0.00
B H
ATOM 4481 C TYR B 73 12.473 33.140 61.383 1.00 0.00
B C

ATOM 4482 O TYR B 73 12.519 32.363 62.342 1.00 0.00
B O
ATOM 4483 N VAL B 74 11.883 32.837 60.232 1.00 0.00
B N
ATOM 4484 HN VAL B 74 11.792 33.484 59.479 1.00 0.00
B H
ATOM 4485 CA VAL B 74 11.316 31.524 59.967 1.00 0.00
B C
ATOM 4486 HA VAL B 74 11.704 30.826 60.698 1.00 0.00
B H
ATOM 4487 CB VAL B 74 9.779 31.528 60.048 1.00 0.00
B C
ATOM 4488 HB VAL B 74 9.430 30.480 59.878 1.00 0.00
B H
ATOM 4489 CG1 VAL B 74 9.330 31.992 61.415 1.00 0.00
B C
ATOM 4490 HG11 VAL B 74 8.221 32.032 61.463 1.00 0.00
B H
ATOM 4491 HG12 VAL B 74 9.671 31.301 62.214 1.00 0.00
B H
ATOM 4492 HG13 VAL B 74 9.721 33.009 61.632 1.00 0.00
B H
ATOM 4493 CG2 VAL B 74 9.176 32.401 58.968 1.00 0.00
B C
ATOM 4494 HG21 VAL B 74 8.069 32.430 59.058 1.00 0.00
B H
ATOM 4495 HG22 VAL B 74 9.561 33.441 59.046 1.00 0.00
B H
ATOM 4496 HG23 VAL B 74 9.420 32.015 57.956 1.00 0.00
B H
ATOM 4497 C VAL B 74 11.791 31.064 58.600 1.00 0.00
B C
ATOM 4498 O VAL B 74 11.866 31.858 57.653 1.00 0.00
B O
ATOM 4499 N ILE B 75 12.124 29.787 58.505 1.00 0.00
B N
ATOM 4500 HN ILE B 75 12.034 29.146 59.264 1.00 0.00
B H
ATOM 4501 CA ILE B 75 12.650 29.197 57.287 1.00 0.00
B C
ATOM 4502 HA ILE B 75 12.461 29.887 56.474 1.00 0.00
B H
ATOM 4503 CB ILE B 75 14.133 28.856 57.423 1.00 0.00
B C
ATOM 4504 HB ILE B 75 14.220 27.817 57.016 1.00 0.00
B H
ATOM 4505 CG2 ILE B 75 14.956 29.878 56.691 1.00 0.00
B C
ATOM 4506 HG21 ILE B 75 16.020 29.563 56.646 1.00 0.00
B H
ATOM 4507 HG22 ILE B 75 14.597 30.001 55.648 1.00 0.00
B H
ATOM 4508 HG23 ILE B 75 14.908 30.865 57.198 1.00 0.00
B H

ATOM 4509 CG1 ILE B 75 14.489 28.800 58.902 1.00 0.00
B C
ATOM 4510 HG11 ILE B 75 13.732 29.362 59.493 1.00 0.00
B H
ATOM 4511 HG12 ILE B 75 14.450 27.740 59.241 1.00 0.00
B H
ATOM 4512 CD ILE B 75 15.840 29.333 59.228 1.00 0.00
B C
ATOM 4513 HD1 ILE B 75 16.054 29.207 60.312 1.00 0.00
B H
ATOM 4514 HD2 ILE B 75 16.624 28.793 58.654 1.00 0.00
B H
ATOM 4515 HD3 ILE B 75 15.910 30.415 58.989 1.00 0.00
B H
ATOM 4516 C ILE B 75 11.845 27.952 56.999 1.00 0.00
B C
ATOM 4517 O ILE B 75 11.940 26.961 57.731 1.00 0.00
B O
ATOM 4518 N VAL B 76 11.071 27.993 55.932 1.00 0.00
B N
ATOM 4519 HN VAL B 76 10.973 28.801 55.356 1.00 0.00
B H
ATOM 4520 CA VAL B 76 10.289 26.851 55.500 1.00 0.00
B C
ATOM 4521 HA VAL B 76 10.081 26.206 56.345 1.00 0.00
B H
ATOM 4522 CB VAL B 76 8.977 27.279 54.868 1.00 0.00
B C
ATOM 4523 HB VAL B 76 9.220 27.846 53.937 1.00 0.00
B H
ATOM 4524 CG1 VAL B 76 8.162 26.079 54.520 1.00 0.00
B C
ATOM 4525 HG11 VAL B 76 7.184 26.388 54.092 1.00 0.00
B H
ATOM 4526 HG12 VAL B 76 8.668 25.446 53.762 1.00 0.00
B H
ATOM 4527 HG13 VAL B 76 7.963 25.464 55.424 1.00 0.00
B H
ATOM 4528 CG2 VAL B 76 8.225 28.155 55.834 1.00 0.00
B C
ATOM 4529 HG21 VAL B 76 7.242 28.451 55.412 1.00 0.00
B H
ATOM 4530 HG22 VAL B 76 8.048 27.619 56.791 1.00 0.00
B H
ATOM 4531 HG23 VAL B 76 8.789 29.086 56.054 1.00 0.00
B H
ATOM 4532 C VAL B 76 11.191 26.077 54.546 1.00 0.00
B C
ATOM 4533 O VAL B 76 11.268 26.359 53.344 1.00 0.00
B O
ATOM 4534 N ALA B 77 11.923 25.121 55.107 1.00 0.00
B N
ATOM 4535 HN ALA B 77 11.877 24.935 56.088 1.00 0.00
B H

```
ATOM   4536  CA  ALA B  77      12.842  24.276  54.352  1.00  0.00
B    C
ATOM   4537  HA  ALA B  77      13.161  24.775  53.444  1.00  0.00
B    H
ATOM   4538  CB  ALA B  77      14.089  23.982  55.173  1.00  0.00
B    C
ATOM   4539  HB1 ALA B  77      14.594  24.935  55.439  1.00  0.00
B    H
ATOM   4540  HB2 ALA B  77      13.830  23.457  56.119  1.00  0.00
B    H
ATOM   4541  HB3 ALA B  77      14.813  23.356  54.608  1.00  0.00
B    H
ATOM   4542  C   ALA B  77      12.171  22.989  53.919  1.00  0.00
B    C
ATOM   4543  O   ALA B  77      12.664  21.891  54.166  1.00  0.00
B    O
ATOM   4544  N   ILE B  78      11.026  23.113  53.277  1.00  0.00
B    N
ATOM   4545  HN  ILE B  78      10.560  23.987  53.159  1.00  0.00
B    H
ATOM   4546  CA  ILE B  78      10.333  21.986  52.680  1.00  0.00
B    C
ATOM   4547  HA  ILE B  78      10.750  21.073  53.086  1.00  0.00
B    H
ATOM   4548  CB  ILE B  78       8.824  22.088  52.908  1.00  0.00
B    C
ATOM   4549  HB  ILE B  78       8.692  21.887  54.002  1.00  0.00
B    H
ATOM   4550  CG2 ILE B  78       8.330  23.402  52.420  1.00  0.00
B    C
ATOM   4551 HG21 ILE B  78       7.272  23.556  52.716  1.00  0.00
B    H
ATOM   4552 HG22 ILE B  78       8.926  24.234  52.851  1.00  0.00
B    H
ATOM   4553 HG23 ILE B  78       8.392  23.463  51.313  1.00  0.00
B    H
ATOM   4554  CG1 ILE B  78       8.083  20.984  52.174  1.00  0.00
B    C
ATOM   4555 HG11 ILE B  78       8.303  21.043  51.084  1.00  0.00
B    H
ATOM   4556 HG12 ILE B  78       8.456  19.998  52.533  1.00  0.00
B    H
ATOM   4557  CD  ILE B  78       6.606  21.050  52.393  1.00  0.00
B    C
ATOM   4558  HD1 ILE B  78       6.102  20.197  51.889  1.00  0.00
B    H
ATOM   4559  HD2 ILE B  78       6.368  21.004  53.478  1.00  0.00
B    H
ATOM   4560  HD3 ILE B  78       6.183  21.990  51.979  1.00  0.00
B    H
ATOM   4561  C   ILE B  78      10.684  22.010  51.202  1.00  0.00
B    C
ATOM   4562  O   ILE B  78      10.936  23.081  50.642  1.00  0.00
B    O
```

ATOM	4563	N	GLY	B	79	10.742	20.845	50.565	1.00	0.00	B N
ATOM	4564	HN	GLY	B	79	10.624	19.948	50.990	1.00	0.00	B H
ATOM	4565	CA	GLY	B	79	10.997	20.834	49.138	1.00	0.00	B C
ATOM	4566	HA1	GLY	B	79	11.977	20.392	49.017	1.00	0.00	B H
ATOM	4567	HA2	GLY	B	79	10.931	21.864	48.815	1.00	0.00	B H
ATOM	4568	C	GLY	B	79	10.015	20.025	48.320	1.00	0.00	B C
ATOM	4569	O	GLY	B	79	9.855	20.266	47.121	1.00	0.00	B O
ATOM	4570	N	ALA	B	80	9.349	19.064	48.949	1.00	0.00	B N
ATOM	4571	HN	ALA	B	80	9.377	18.891	49.934	1.00	0.00	B H
ATOM	4572	CA	ALA	B	80	8.498	18.167	48.181	1.00	0.00	B C
ATOM	4573	HA	ALA	B	80	8.834	18.141	47.150	1.00	0.00	B H
ATOM	4574	CB	ALA	B	80	8.593	16.747	48.743	1.00	0.00	B C
ATOM	4575	HB1	ALA	B	80	9.650	16.408	48.722	1.00	0.00	B H
ATOM	4576	HB2	ALA	B	80	8.243	16.711	49.797	1.00	0.00	B H
ATOM	4577	HB3	ALA	B	80	7.991	16.029	48.146	1.00	0.00	B H
ATOM	4578	C	ALA	B	80	7.041	18.606	48.147	1.00	0.00	B C
ATOM	4579	O	ALA	B	80	6.491	18.853	47.070	1.00	0.00	B O
ATOM	4580	N	ASN	B	81	6.402	18.708	49.299	1.00	0.00	B N
ATOM	4581	HN	ASN	B	81	6.859	18.680	50.187	1.00	0.00	B H
ATOM	4582	CA	ASN	B	81	4.960	18.872	49.367	1.00	0.00	B C
ATOM	4583	HA	ASN	B	81	4.543	18.339	48.518	1.00	0.00	B H
ATOM	4584	CB	ASN	B	81	4.449	18.332	50.695	1.00	0.00	B C
ATOM	4585	HB1	ASN	B	81	4.824	17.291	50.805	1.00	0.00	B H
ATOM	4586	HB2	ASN	B	81	4.861	18.926	51.537	1.00	0.00	B H
ATOM	4587	CG	ASN	B	81	2.958	18.299	50.766	1.00	0.00	B C
ATOM	4588	OD1	ASN	B	81	2.279	19.127	50.172	1.00	0.00	B O
ATOM	4589	ND2	ASN	B	81	2.430	17.334	51.503	1.00	0.00	B N

```
ATOM   4590 HD21 ASN B  81       1.436  17.289  51.554  1.00  0.00
B    H
ATOM   4591 HD22 ASN B  81       3.020  16.672  51.956  1.00  0.00
B    H
ATOM   4592  C   ASN B  81       4.618  20.346  49.199  1.00  0.00
B    C
ATOM   4593  O   ASN B  81       4.471  21.066  50.189  1.00  0.00
B    O
ATOM   4594  N   ILE B  82       4.445  20.784  47.952  1.00  0.00
B    N
ATOM   4595  HN  ILE B  82       4.458  20.214  47.134  1.00  0.00
B    H
ATOM   4596  CA  ILE B  82       4.213  22.202  47.689  1.00  0.00
B    C
ATOM   4597  HA  ILE B  82       5.100  22.726  48.020  1.00  0.00
B    H
ATOM   4598  CB  ILE B  82       3.974  22.437  46.190  1.00  0.00
B    C
ATOM   4599  HB  ILE B  82       3.263  21.626  45.889  1.00  0.00
B    H
ATOM   4600  CG2 ILE B  82       3.455  23.835  45.940  1.00  0.00
B    C
ATOM   4601 HG21 ILE B  82       3.137  23.950  44.882  1.00  0.00
B    H
ATOM   4602 HG22 ILE B  82       2.575  24.051  46.581  1.00  0.00
B    H
ATOM   4603 HG23 ILE B  82       4.238  24.593  46.152  1.00  0.00
B    H
ATOM   4604  CG1 ILE B  82       5.263  22.208  45.410  1.00  0.00
B    C
ATOM   4605 HG11 ILE B  82       5.301  22.896  44.535  1.00  0.00
B    H
ATOM   4606 HG12 ILE B  82       6.130  22.459  46.063  1.00  0.00
B    H
ATOM   4607  CD  ILE B  82       5.441  20.808  44.928  1.00  0.00
B    C
ATOM   4608  HD1 ILE B  82       6.424  20.697  44.420  1.00  0.00
B    H
ATOM   4609  HD2 ILE B  82       5.407  20.094  45.778  1.00  0.00
B    H
ATOM   4610  HD3 ILE B  82       4.646  20.532  44.203  1.00  0.00
B    H
ATOM   4611  C   ILE B  82       3.057  22.724  48.527  1.00  0.00
B    C
ATOM   4612  O   ILE B  82       3.092  23.861  49.020  1.00  0.00
B    O
ATOM   4613  N   GLN B  83       2.033  21.893  48.722  1.00  0.00
B    N
ATOM   4614  HN  GLN B  83       1.969  20.977  48.332  1.00  0.00
B    H
ATOM   4615  CA  GLN B  83       0.898  22.279  49.545  1.00  0.00
B    C
ATOM   4616  HA  GLN B  83       0.356  23.042  49.000  1.00  0.00
B    H
```

```
ATOM   4617  CB  GLN B  83       0.045  21.055  49.830  1.00  0.00
B    C
ATOM   4618  HB1 GLN B  83       0.489  20.211  49.252  1.00  0.00
B    H
ATOM   4619  HB2 GLN B  83       0.143  20.780  50.905  1.00  0.00
B    H
ATOM   4620  CG  GLN B  83      -1.382  21.168  49.473  1.00  0.00
B    C
ATOM   4621  HG1 GLN B  83      -1.791  22.144  49.812  1.00  0.00
B    H
ATOM   4622  HG2 GLN B  83      -1.518  21.106  48.374  1.00  0.00
B    H
ATOM   4623  CD  GLN B  83      -2.182  20.085  50.149  1.00  0.00
B    C
ATOM   4624  OE1 GLN B  83      -1.977  19.802  51.322  1.00  0.00
B    O
ATOM   4625  NE2 GLN B  83      -3.073  19.453  49.407  1.00  0.00
B    N
ATOM   4626 HE21 GLN B  83      -3.606  18.740  49.853  1.00  0.00
B    H
ATOM   4627 HE22 GLN B  83      -3.173  19.689  48.446  1.00  0.00
B    H
ATOM   4628  C   GLN B  83       1.352  22.889  50.857  1.00  0.00
B    C
ATOM   4629  O   GLN B  83       1.115  24.075  51.123  1.00  0.00
B    O
ATOM   4630  N   ALA B  84       2.039  22.093  51.668  1.00  0.00
B    N
ATOM   4631  HN  ALA B  84       2.322  21.164  51.434  1.00  0.00
B    H
ATOM   4632  CA  ALA B  84       2.429  22.550  52.991  1.00  0.00
B    C
ATOM   4633  HA  ALA B  84       1.556  22.799  53.584  1.00  0.00
B    H
ATOM   4634  CB  ALA B  84       3.194  21.448  53.715  1.00  0.00
B    C
ATOM   4635  HB1 ALA B  84       2.549  20.548  53.813  1.00  0.00
B    H
ATOM   4636  HB2 ALA B  84       4.105  21.156  53.148  1.00  0.00
B    H
ATOM   4637  HB3 ALA B  84       3.499  21.765  54.736  1.00  0.00
B    H
ATOM   4638  C   ALA B  84       3.260  23.815  52.901  1.00  0.00
B    C
ATOM   4639  O   ALA B  84       3.012  24.782  53.625  1.00  0.00
B    O
ATOM   4640  N   SER B  85       4.253  23.823  52.014  1.00  0.00
B    N
ATOM   4641  HN  SER B  85       4.518  23.051  51.437  1.00  0.00
B    H
ATOM   4642  CA  SER B  85       5.059  25.018  51.817  1.00  0.00
B    C
ATOM   4643  HA  SER B  85       5.668  25.122  52.705  1.00  0.00
B    H
```

```
ATOM   4644  CB  SER B  85       5.882  24.878  50.543  1.00  0.00
B    C
ATOM   4645  HB1 SER B  85       6.777  25.533  50.636  1.00  0.00
B    H
ATOM   4646  HB2 SER B  85       6.251  23.833  50.434  1.00  0.00
B    H
ATOM   4647  OG  SER B  85       5.120  25.284  49.423  1.00  0.00
B    O
ATOM   4648  HG1 SER B  85       5.735  25.392  48.686  1.00  0.00
B    H
ATOM   4649  C   SER B  85       4.179  26.252  51.727  1.00  0.00
B    C
ATOM   4650  O   SER B  85       4.328  27.209  52.498  1.00  0.00
B    O
ATOM   4651  N   THR B  86       3.217  26.224  50.814  1.00  0.00
B    N
ATOM   4652  HN  THR B  86       2.923  25.426  50.289  1.00  0.00
B    H
ATOM   4653  CA  THR B  86       2.487  27.443  50.506  1.00  0.00
B    C
ATOM   4654  HA  THR B  86       3.235  28.222  50.449  1.00  0.00
B    H
ATOM   4655  CB  THR B  86       1.738  27.269  49.197  1.00  0.00
B    C
ATOM   4656  HB  THR B  86       1.252  28.253  48.985  1.00  0.00
B    H
ATOM   4657  OG1 THR B  86       0.709  26.291  49.371  1.00  0.00
B    O
ATOM   4658  HG1 THR B  86       0.278  26.216  48.512  1.00  0.00
B    H
ATOM   4659  CG2 THR B  86       2.692  26.822  48.115  1.00  0.00
B    C
ATOM   4660 HG21 THR B  86       2.186  26.800  47.127  1.00  0.00
B    H
ATOM   4661 HG22 THR B  86       3.551  27.522  48.043  1.00  0.00
B    H
ATOM   4662 HG23 THR B  86       3.087  25.805  48.325  1.00  0.00
B    H
ATOM   4663  C   THR B  86       1.538  27.835  51.630  1.00  0.00
B    C
ATOM   4664  O   THR B  86       1.523  28.997  52.051  1.00  0.00
B    O
ATOM   4665  N   LEU B  87       0.726  26.895  52.122  1.00  0.00
B    N
ATOM   4666  HN  LEU B  87       0.660  25.942  51.831  1.00  0.00
B    H
ATOM   4667  CA  LEU B  87      -0.186  27.257  53.207  1.00  0.00
B    C
ATOM   4668  HA  LEU B  87      -0.772  28.098  52.859  1.00  0.00
B    H
ATOM   4669  CB  LEU B  87      -1.134  26.113  53.575  1.00  0.00
B    C
ATOM   4670  HB1 LEU B  87      -0.606  25.474  54.320  1.00  0.00
B    H
```

ATOM 4671 HB2 LEU B 87 -2.019 26.533 54.102 1.00 0.00
B H
ATOM 4672 CG LEU B 87 -1.804 25.022 52.731 1.00 0.00
B C
ATOM 4673 HG LEU B 87 -2.890 25.174 52.943 1.00 0.00
B H
ATOM 4674 CD1 LEU B 87 -1.562 25.065 51.238 1.00 0.00
B C
ATOM 4675 HD11 LEU B 87 -2.054 24.203 50.738 1.00 0.00
B H
ATOM 4676 HD12 LEU B 87 -1.970 26.001 50.801 1.00 0.00
B H
ATOM 4677 HD13 LEU B 87 -0.475 25.019 51.015 1.00 0.00
B H
ATOM 4678 CD2 LEU B 87 -1.397 23.658 53.295 1.00 0.00
B C
ATOM 4679 HD21 LEU B 87 -1.882 22.841 52.717 1.00 0.00
B H
ATOM 4680 HD22 LEU B 87 -0.301 23.503 53.242 1.00 0.00
B H
ATOM 4681 HD23 LEU B 87 -1.712 23.566 54.357 1.00 0.00
B H
ATOM 4682 C LEU B 87 0.587 27.712 54.430 1.00 0.00
B C
ATOM 4683 O LEU B 87 0.161 28.621 55.154 1.00 0.00
B O
ATOM 4684 N THR B 88 1.734 27.090 54.673 1.00 0.00
B N
ATOM 4685 HN THR B 88 2.062 26.288 54.175 1.00 0.00
B H
ATOM 4686 CA THR B 88 2.625 27.549 55.716 1.00 0.00
B C
ATOM 4687 HA THR B 88 2.077 27.451 56.644 1.00 0.00
B H
ATOM 4688 CB THR B 88 3.906 26.740 55.634 1.00 0.00
B C
ATOM 4689 HB THR B 88 4.333 26.939 54.621 1.00 0.00
B H
ATOM 4690 OG1 THR B 88 3.580 25.351 55.720 1.00 0.00
B O
ATOM 4691 HG1 THR B 88 4.422 24.886 55.675 1.00 0.00
B H
ATOM 4692 CG2 THR B 88 4.842 27.132 56.735 1.00 0.00
B C
ATOM 4693 HG21 THR B 88 5.824 26.628 56.616 1.00 0.00
B H
ATOM 4694 HG22 THR B 88 5.017 28.229 56.723 1.00 0.00
B H
ATOM 4695 HG23 THR B 88 4.428 26.856 57.729 1.00 0.00
B H
ATOM 4696 C THR B 88 2.928 29.021 55.549 1.00 0.00
B C
ATOM 4697 O THR B 88 2.581 29.843 56.407 1.00 0.00
B O

```
ATOM   4698  N   THR B  89       3.537  29.366  54.416  1.00  0.00
B    N
ATOM   4699  HN  THR B  89       3.800  28.730  53.691  1.00  0.00
B    H
ATOM   4700  CA  THR B  89       3.875  30.756  54.143  1.00  0.00
B    C
ATOM   4701  HA  THR B  89       4.604  31.036  54.891  1.00  0.00
B    H
ATOM   4702  CB  THR B  89       4.375  30.891  52.718  1.00  0.00
B    C
ATOM   4703  HB  THR B  89       4.436  31.988  52.514  1.00  0.00
B    H
ATOM   4704  OG1 THR B  89       3.401  30.320  51.839  1.00  0.00
B    O
ATOM   4705  HG1 THR B  89       3.766  30.419  50.953  1.00  0.00
B    H
ATOM   4706  CG2 THR B  89       5.680  30.157  52.560  1.00  0.00
B    C
ATOM   4707 HG21 THR B  89       6.117  30.340  51.556  1.00  0.00
B    H
ATOM   4708 HG22 THR B  89       6.413  30.503  53.320  1.00  0.00
B    H
ATOM   4709 HG23 THR B  89       5.540  29.061  52.685  1.00  0.00
B    H
ATOM   4710  C   THR B  89       2.678  31.660  54.348  1.00  0.00
B    C
ATOM   4711  O   THR B  89       2.812  32.779  54.854  1.00  0.00
B    O
ATOM   4712  N   LEU B  90       1.500  31.199  53.940  1.00  0.00
B    N
ATOM   4713  HN  LEU B  90       1.345  30.339  53.458  1.00  0.00
B    H
ATOM   4714  CA  LEU B  90       0.293  31.969  54.191  1.00  0.00
B    C
ATOM   4715  HA  LEU B  90       0.405  32.909  53.666  1.00  0.00
B    H
ATOM   4716  CB  LEU B  90      -0.928  31.207  53.699  1.00  0.00
B    C
ATOM   4717  HB1 LEU B  90      -0.983  31.351  52.595  1.00  0.00
B    H
ATOM   4718  HB2 LEU B  90      -0.764  30.118  53.859  1.00  0.00
B    H
ATOM   4719  CG  LEU B  90      -2.241  31.662  54.332  1.00  0.00
B    C
ATOM   4720  HG  LEU B  90      -2.210  31.417  55.421  1.00  0.00
B    H
ATOM   4721  CD1 LEU B  90      -2.471  33.144  54.140  1.00  0.00
B    C
ATOM   4722 HD11 LEU B  90      -3.452  33.445  54.565  1.00  0.00
B    H
ATOM   4723 HD12 LEU B  90      -1.676  33.732  54.646  1.00  0.00
B    H
ATOM   4724 HD13 LEU B  90      -2.466  33.406  53.060  1.00  0.00
B    H
```

ATOM 4725 CD2 LEU B 90 -3.363 30.895 53.717 1.00 0.00
B C
ATOM 4726 HD21 LEU B 90 -4.332 31.194 54.173 1.00 0.00
B H
ATOM 4727 HD22 LEU B 90 -3.435 31.078 52.625 1.00 0.00
B H
ATOM 4728 HD23 LEU B 90 -3.225 29.804 53.880 1.00 0.00
B H
ATOM 4729 C LEU B 90 0.151 32.282 55.668 1.00 0.00
B C
ATOM 4730 O LEU B 90 -0.007 33.443 56.054 1.00 0.00
B O
ATOM 4731 N LEU B 91 0.180 31.253 56.505 1.00 0.00
B N
ATOM 4732 HN LEU B 91 0.344 30.301 56.258 1.00 0.00
B H
ATOM 4733 CA LEU B 91 -0.042 31.472 57.928 1.00 0.00
B C
ATOM 4734 HA LEU B 91 -1.004 31.959 58.023 1.00 0.00
B H
ATOM 4735 CB LEU B 91 -0.042 30.144 58.672 1.00 0.00
B C
ATOM 4736 HB1 LEU B 91 0.654 29.463 58.130 1.00 0.00
B H
ATOM 4737 HB2 LEU B 91 0.399 30.295 59.682 1.00 0.00
B H
ATOM 4738 CG LEU B 91 -1.426 29.508 58.753 1.00 0.00
B C
ATOM 4739 HG LEU B 91 -1.320 28.518 59.259 1.00 0.00
B H
ATOM 4740 CD1 LEU B 91 -2.351 30.398 59.559 1.00 0.00
B C
ATOM 4741 HD11 LEU B 91 -3.377 29.972 59.587 1.00 0.00
B H
ATOM 4742 HD12 LEU B 91 -1.986 30.498 60.604 1.00 0.00
B H
ATOM 4743 HD13 LEU B 91 -2.409 31.413 59.110 1.00 0.00
B H
ATOM 4744 CD2 LEU B 91 -1.994 29.268 57.373 1.00 0.00
B C
ATOM 4745 HD21 LEU B 91 -2.994 28.787 57.445 1.00 0.00
B H
ATOM 4746 HD22 LEU B 91 -2.116 30.214 56.809 1.00 0.00
B H
ATOM 4747 HD23 LEU B 91 -1.328 28.597 56.790 1.00 0.00
B H
ATOM 4748 C LEU B 91 1.004 32.409 58.501 1.00 0.00
B C
ATOM 4749 O LEU B 91 0.696 33.297 59.304 1.00 0.00
B O
ATOM 4750 N LEU B 92 2.250 32.234 58.082 1.00 0.00
B N
ATOM 4751 HN LEU B 92 2.554 31.569 57.402 1.00 0.00
B H

```
ATOM   4752  CA  LEU B  92       3.316  33.060  58.628  1.00  0.00
B    C
ATOM   4753  HA  LEU B  92       3.225  33.008  59.705  1.00  0.00
B    H
ATOM   4754  CB  LEU B  92       4.672  32.525  58.191  1.00  0.00
B    C
ATOM   4755  HB1 LEU B  92       4.563  32.171  57.140  1.00  0.00
B    H
ATOM   4756  HB2 LEU B  92       5.399  33.366  58.157  1.00  0.00
B    H
ATOM   4757  CG  LEU B  92       5.176  31.385  59.066  1.00  0.00
B    C
ATOM   4758  HG  LEU B  92       6.273  31.263  58.893  1.00  0.00
B    H
ATOM   4759  CD1 LEU B  92       4.920  31.731  60.514  1.00  0.00
B    C
ATOM   4760 HD11 LEU B  92       5.233  30.896  61.176  1.00  0.00
B    H
ATOM   4761 HD12 LEU B  92       5.488  32.641  60.805  1.00  0.00
B    H
ATOM   4762 HD13 LEU B  92       3.839  31.923  60.686  1.00  0.00
B    H
ATOM   4763  CD2 LEU B  92       4.502  30.085  58.719  1.00  0.00
B    C
ATOM   4764 HD21 LEU B  92       4.898  29.264  59.354  1.00  0.00
B    H
ATOM   4765 HD22 LEU B  92       3.407  30.136  58.877  1.00  0.00
B    H
ATOM   4766 HD23 LEU B  92       4.689  29.822  57.656  1.00  0.00
B    H
ATOM   4767  C   LEU B  92       3.154  34.517  58.223  1.00  0.00
B    C
ATOM   4768  O   LEU B  92       3.148  35.409  59.080  1.00  0.00
B    O
ATOM   4769  N   LYS B  93       3.008  34.778  56.927  1.00  0.00
B    N
ATOM   4770  HN  LYS B  93       3.013  34.094  56.200  1.00  0.00
B    H
ATOM   4771  CA  LYS B  93       2.824  36.150  56.482  1.00  0.00
B    C
ATOM   4772  HA  LYS B  93       3.663  36.725  56.857  1.00  0.00
B    H
ATOM   4773  CB  LYS B  93       2.778  36.204  54.960  1.00  0.00
B    C
ATOM   4774  HB1 LYS B  93       3.398  35.359  54.582  1.00  0.00
B    H
ATOM   4775  HB2 LYS B  93       1.736  36.003  54.622  1.00  0.00
B    H
ATOM   4776  CG  LYS B  93       3.295  37.502  54.384  1.00  0.00
B    C
ATOM   4777  HG1 LYS B  93       3.372  37.405  53.277  1.00  0.00
B    H
ATOM   4778  HG2 LYS B  93       2.558  38.312  54.596  1.00  0.00
B    H
```

```
ATOM   4779  CD  LYS B  93       4.654  37.843  54.960  1.00  0.00
B    C
ATOM   4780  HD1 LYS B  93       4.558  37.918  56.067  1.00  0.00
B    H
ATOM   4781  HD2 LYS B  93       5.345  36.997  54.741  1.00  0.00
B    H
ATOM   4782  CE  LYS B  93       5.192  39.137  54.374  1.00  0.00
B    C
ATOM   4783  HE1 LYS B  93       5.468  38.996  53.305  1.00  0.00
B    H
ATOM   4784  HE2 LYS B  93       4.428  39.942  54.437  1.00  0.00
B    H
ATOM   4785  NZ  LYS B  93       6.408  39.614  55.091  1.00  0.00
B    N
ATOM   4786  HZ1 LYS B  93       6.750  40.499  54.663  1.00  0.00
B    H
ATOM   4787  HZ2 LYS B  93       6.178  39.780  56.092  1.00  0.00
B    H
ATOM   4788  HZ3 LYS B  93       7.153  38.891  55.028  1.00  0.00
B    H
ATOM   4789  C   LYS B  93       1.562  36.749  57.077  1.00  0.00
B    C
ATOM   4790  O   LYS B  93       1.488  37.960  57.306  1.00  0.00
B    O
ATOM   4791  N   GLU B  94       0.561  35.914  57.341  1.00  0.00
B    N
ATOM   4792  HN  GLU B  94       0.520  34.950  57.092  1.00  0.00
B    H
ATOM   4793  CA  GLU B  94      -0.612  36.391  58.054  1.00  0.00
B    C
ATOM   4794  HA  GLU B  94      -0.948  37.306  57.582  1.00  0.00
B    H
ATOM   4795  CB  GLU B  94      -1.721  35.349  58.001  1.00  0.00
B    C
ATOM   4796  HB1 GLU B  94      -2.164  35.365  56.978  1.00  0.00
B    H
ATOM   4797  HB2 GLU B  94      -1.289  34.332  58.130  1.00  0.00
B    H
ATOM   4798  CG  GLU B  94      -2.812  35.585  59.019  1.00  0.00
B    C
ATOM   4799  HG1 GLU B  94      -2.462  35.262  60.016  1.00  0.00
B    H
ATOM   4800  HG2 GLU B  94      -3.092  36.656  59.060  1.00  0.00
B    H
ATOM   4801  CD  GLU B  94      -4.047  34.773  58.732  1.00  0.00
B    C
ATOM   4802  OE1 GLU B  94      -4.319  34.508  57.544  1.00  0.00
B    O
ATOM   4803  OE2 GLU B  94      -4.752  34.402  59.692  1.00  0.00
B    O
ATOM   4804  C   GLU B  94      -0.270  36.727  59.493  1.00  0.00
B    C
ATOM   4805  O   GLU B  94      -0.831  37.667  60.066  1.00  0.00
B    O
```

```
ATOM   4806  N   LEU B  95       0.644  35.978  60.095  1.00  0.00
B    N
ATOM   4807  HN  LEU B  95       1.100  35.196  59.677  1.00  0.00
B    H
ATOM   4808  CA  LEU B  95       1.061  36.254  61.458  1.00  0.00
B    C
ATOM   4809  HA  LEU B  95       0.236  36.778  61.923  1.00  0.00
B    H
ATOM   4810  CB  LEU B  95       1.313  34.953  62.206  1.00  0.00
B    C
ATOM   4811  HB1 LEU B  95       1.604  34.189  61.448  1.00  0.00
B    H
ATOM   4812  HB2 LEU B  95       2.198  35.082  62.868  1.00  0.00
B    H
ATOM   4813  CG  LEU B  95       0.031  34.572  62.941  1.00  0.00
B    C
ATOM   4814  HG  LEU B  95      -0.326  35.509  63.434  1.00  0.00
B    H
ATOM   4815  CD1 LEU B  95      -1.028  34.073  61.975  1.00  0.00
B    C
ATOM   4816 HD11 LEU B  95      -1.939  33.755  62.526  1.00  0.00
B    H
ATOM   4817 HD12 LEU B  95      -1.314  34.873  61.260  1.00  0.00
B    H
ATOM   4818 HD13 LEU B  95      -0.651  33.203  61.395  1.00  0.00
B    H
ATOM   4819  CD2 LEU B  95       0.298  33.552  64.018  1.00  0.00
B    C
ATOM   4820 HD21 LEU B  95      -0.642  33.301  64.554  1.00  0.00
B    H
ATOM   4821 HD22 LEU B  95       0.707  32.611  63.598  1.00  0.00
B    H
ATOM   4822 HD23 LEU B  95       1.025  33.950  64.759  1.00  0.00
B    H
ATOM   4823  C   LEU B  95       2.274  37.160  61.527  1.00  0.00
B    C
ATOM   4824  O   LEU B  95       2.997  37.136  62.526  1.00  0.00
B    O
ATOM   4825  N   ASP B  96       2.524  37.934  60.474  1.00  0.00
B    N
ATOM   4826  HN  ASP B  96       2.190  37.756  59.552  1.00  0.00
B    H
ATOM   4827  CA  ASP B  96       3.326  39.147  60.564  1.00  0.00
B    C
ATOM   4828  HA  ASP B  96       3.350  39.575  59.568  1.00  0.00
B    H
ATOM   4829  CB  ASP B  96       2.705  40.119  61.560  1.00  0.00
B    C
ATOM   4830  HB1 ASP B  96       2.953  39.795  62.590  1.00  0.00
B    H
ATOM   4831  HB2 ASP B  96       3.105  41.142  61.411  1.00  0.00
B    H
ATOM   4832  CG  ASP B  96       1.207  40.165  61.445  1.00  0.00
B    C
```

ATOM 4833 OD1 ASP B 96 0.702 39.947 60.326 1.00 0.00
B O
ATOM 4834 OD2 ASP B 96 0.537 40.390 62.471 1.00 0.00
B O
ATOM 4835 C ASP B 96 4.764 38.859 60.950 1.00 0.00
B C
ATOM 4836 O ASP B 96 5.491 39.755 61.379 1.00 0.00
B O
ATOM 4837 N ILE B 97 5.179 37.610 60.811 1.00 0.00
B N
ATOM 4838 HN ILE B 97 4.652 36.833 60.472 1.00 0.00
B H
ATOM 4839 CA ILE B 97 6.554 37.285 61.181 1.00 0.00
B C
ATOM 4840 HA ILE B 97 6.708 37.637 62.193 1.00 0.00
B H
ATOM 4841 CB ILE B 97 6.793 35.781 61.058 1.00 0.00
B C
ATOM 4842 HB ILE B 97 6.510 35.539 60.002 1.00 0.00
B H
ATOM 4843 CG2 ILE B 97 8.217 35.429 61.425 1.00 0.00
B C
ATOM 4844 HG21 ILE B 97 8.431 34.366 61.186 1.00 0.00
B H
ATOM 4845 HG22 ILE B 97 8.939 36.054 60.856 1.00 0.00
B H
ATOM 4846 HG23 ILE B 97 8.398 35.584 62.509 1.00 0.00
B H
ATOM 4847 CG1 ILE B 97 5.813 35.058 61.974 1.00 0.00
B C
ATOM 4848 HG11 ILE B 97 4.770 35.248 61.635 1.00 0.00
B H
ATOM 4849 HG12 ILE B 97 5.990 33.960 61.896 1.00 0.00
B H
ATOM 4850 CD ILE B 97 5.950 35.462 63.419 1.00 0.00
B C
ATOM 4851 HD1 ILE B 97 5.258 34.867 64.053 1.00 0.00
B H
ATOM 4852 HD2 ILE B 97 6.987 35.286 63.778 1.00 0.00
B H
ATOM 4853 HD3 ILE B 97 5.706 36.536 63.557 1.00 0.00
B H
ATOM 4854 C ILE B 97 7.503 38.074 60.294 1.00 0.00
B C
ATOM 4855 O ILE B 97 7.258 38.197 59.079 1.00 0.00
B O
ATOM 4856 N PRO B 98 8.563 38.661 60.844 1.00 0.00
B N
ATOM 4857 CD PRO B 98 8.988 38.632 62.251 1.00 0.00
B C
ATOM 4858 HD1 PRO B 98 7.947 38.961 62.034 1.00 0.00
B H
ATOM 4859 HD2 PRO B 98 9.023 38.043 63.196 1.00 0.00
B H

```
ATOM   4860  CA  PRO B  98       9.396  39.550  60.025  1.00  0.00
B    C
ATOM   4861  HA  PRO B  98       8.806  40.395  59.689  1.00  0.00
B    H
ATOM   4862  CB  PRO B  98      10.470  40.021  61.011  1.00  0.00
B    C
ATOM   4863  HB1 PRO B  98      10.082  40.894  61.582  1.00  0.00
B    H
ATOM   4864  HB2 PRO B  98      11.416  40.309  60.510  1.00  0.00
B    H
ATOM   4865  CG  PRO B  98       9.880  39.811  62.355  1.00  0.00
B    C
ATOM   4866  HG1 PRO B  98      10.336  40.456  63.132  1.00  0.00
B    H
ATOM   4867  HG2 PRO B  98       9.998  38.742  62.646  1.00  0.00
B    H
ATOM   4868  C   PRO B  98      10.005  38.864  58.822  1.00  0.00
B    C
ATOM   4869  O   PRO B  98       9.763  39.279  57.685  1.00  0.00
B    O
ATOM   4870  N   ASN B  99      10.764  37.800  59.038  1.00  0.00
B    N
ATOM   4871  HN  ASN B  99      10.817  37.333  59.920  1.00  0.00
B    H
ATOM   4872  CA  ASN B  99      11.593  37.217  57.996  1.00  0.00
B    C
ATOM   4873  HA  ASN B  99      11.496  37.854  57.122  1.00  0.00
B    H
ATOM   4874  CB  ASN B  99      13.036  37.103  58.469  1.00  0.00
B    C
ATOM   4875  HB1 ASN B  99      13.064  36.360  59.297  1.00  0.00
B    H
ATOM   4876  HB2 ASN B  99      13.680  36.717  57.654  1.00  0.00
B    H
ATOM   4877  CG  ASN B  99      13.563  38.406  58.987  1.00  0.00
B    C
ATOM   4878  OD1 ASN B  99      13.879  38.531  60.165  1.00  0.00
B    O
ATOM   4879  ND2 ASN B  99      13.640  39.400  58.115  1.00  0.00
B    N
ATOM   4880 HD21 ASN B  99      14.000  40.270  58.443  1.00  0.00
B    H
ATOM   4881 HD22 ASN B  99      13.373  39.254  57.168  1.00  0.00
B    H
ATOM   4882  C   ASN B  99      11.041  35.853  57.633  1.00  0.00
B    C
ATOM   4883  O   ASN B  99      10.954  34.970  58.491  1.00  0.00
B    O
ATOM   4884  N   ILE B 100      10.678  35.681  56.369  1.00  0.00
B    N
ATOM   4885  HN  ILE B 100      10.762  36.394  55.676  1.00  0.00
B    H
ATOM   4886  CA  ILE B 100      10.123  34.433  55.873  1.00  0.00
B    C
```

```
ATOM   4887  HA  ILE B 100      10.238  33.682  56.645  1.00  0.00
B    H
ATOM   4888  CB  ILE B 100       8.660  34.584  55.457  1.00  0.00
B    C
ATOM   4889  HB  ILE B 100       8.633  35.537  54.871  1.00  0.00
B    H
ATOM   4890  CG2 ILE B 100       8.214  33.363  54.708  1.00  0.00
B    C
ATOM   4891 HG21 ILE B 100       7.209  33.522  54.265  1.00  0.00
B    H
ATOM   4892 HG22 ILE B 100       8.914  33.130  53.878  1.00  0.00
B    H
ATOM   4893 HG23 ILE B 100       8.162  32.481  55.381  1.00  0.00
B    H
ATOM   4894  CG1 ILE B 100       7.788  34.792  56.690  1.00  0.00
B    C
ATOM   4895 HG11 ILE B 100       7.886  33.918  57.372  1.00  0.00
B    H
ATOM   4896 HG12 ILE B 100       8.156  35.686  57.245  1.00  0.00
B    H
ATOM   4897  CD  ILE B 100       6.338  35.005  56.373  1.00  0.00
B    C
ATOM   4898  HD1 ILE B 100       5.765  35.208  57.303  1.00  0.00
B    H
ATOM   4899  HD2 ILE B 100       6.209  35.871  55.689  1.00  0.00
B    H
ATOM   4900  HD3 ILE B 100       5.900  34.106  55.889  1.00  0.00
B    H
ATOM   4901  C   ILE B 100      10.984  33.995  54.704  1.00  0.00
B    C
ATOM   4902  O   ILE B 100      11.027  34.668  53.667  1.00  0.00
B    O
ATOM   4903  N   TRP B 101      11.684  32.881  54.872  1.00  0.00
B    N
ATOM   4904  HN  TRP B 101      11.582  32.299  55.675  1.00  0.00
B    H
ATOM   4905  CA  TRP B 101      12.660  32.417  53.895  1.00  0.00
B    C
ATOM   4906  HA  TRP B 101      12.557  33.016  52.998  1.00  0.00
B    H
ATOM   4907  CB  TRP B 101      14.068  32.519  54.470  1.00  0.00
B    C
ATOM   4908  HB1 TRP B 101      14.042  32.116  55.507  1.00  0.00
B    H
ATOM   4909  HB2 TRP B 101      14.761  31.873  53.889  1.00  0.00
B    H
ATOM   4910  CG  TRP B 101      14.537  33.903  54.524  1.00  0.00
B    C
ATOM   4911  CD1 TRP B 101      13.824  34.994  54.897  1.00  0.00
B    C
ATOM   4912  HD1 TRP B 101      12.802  34.963  55.251  1.00  0.00
B    H
ATOM   4913  NE1 TRP B 101      14.593  36.117  54.799  1.00  0.00
B    N
```

```
ATOM   4914  HE1 TRP B 101      14.331  37.027  55.039  1.00  0.00
B    H
ATOM   4915  CE2 TRP B 101      15.834  35.757  54.350  1.00  0.00
B    C
ATOM   4916  CD2 TRP B 101      15.829  34.367  54.167  1.00  0.00
B    C
ATOM   4917  CE3 TRP B 101      16.985  33.743  53.710  1.00  0.00
B    C
ATOM   4918  HE3 TRP B 101      17.027  32.676  53.542  1.00  0.00
B    H
ATOM   4919  CZ3 TRP B 101      18.089  34.509  53.463  1.00  0.00
B    C
ATOM   4920  HZ3 TRP B 101      18.996  34.037  53.111  1.00  0.00
B    H
ATOM   4921  CZ2 TRP B 101      16.949  36.533  54.095  1.00  0.00
B    C
ATOM   4922  HZ2 TRP B 101      16.959  37.603  54.237  1.00  0.00
B    H
ATOM   4923  CH2 TRP B 101      18.068  35.892  53.651  1.00  0.00
B    C
ATOM   4924  HH2 TRP B 101      18.956  36.473  53.440  1.00  0.00
B    H
ATOM   4925  C   TRP B 101      12.334  30.984  53.509  1.00  0.00
B    C
ATOM   4926  O   TRP B 101      12.223  30.117  54.380  1.00  0.00
B    O
ATOM   4927  N   VAL B 102      12.190  30.727  52.215  1.00  0.00
B    N
ATOM   4928  HN  VAL B 102      12.450  31.331  51.466  1.00  0.00
B    H
ATOM   4929  CA  VAL B 102      11.605  29.463  51.792  1.00  0.00
B    C
ATOM   4930  HA  VAL B 102      11.545  28.824  52.664  1.00  0.00
B    H
ATOM   4931  CB  VAL B 102      10.194  29.683  51.214  1.00  0.00
B    C
ATOM   4932  HB  VAL B 102      10.304  30.266  50.267  1.00  0.00
B    H
ATOM   4933  CG1 VAL B 102       9.532  28.360  50.909  1.00  0.00
B    C
ATOM   4934 HG11 VAL B 102       8.501  28.525  50.529  1.00  0.00
B    H
ATOM   4935 HG12 VAL B 102      10.086  27.795  50.130  1.00  0.00
B    H
ATOM   4936 HG13 VAL B 102       9.467  27.734  51.824  1.00  0.00
B    H
ATOM   4937  CG2 VAL B 102       9.351  30.487  52.175  1.00  0.00
B    C
ATOM   4938 HG21 VAL B 102       8.320  30.615  51.782  1.00  0.00
B    H
ATOM   4939 HG22 VAL B 102       9.288  29.978  53.161  1.00  0.00
B    H
ATOM   4940 HG23 VAL B 102       9.776  31.500  52.333  1.00  0.00
B    H
```

ATOM 4941 C VAL B 102 12.494 28.764 50.775 1.00 0.00
B C
ATOM 4942 O VAL B 102 13.192 29.403 49.980 1.00 0.00
B O
ATOM 4943 N LYS B 103 12.464 27.430 50.820 1.00 0.00
B N
ATOM 4944 HN LYS B 103 12.040 26.938 51.579 1.00 0.00
B H
ATOM 4945 CA LYS B 103 13.042 26.585 49.780 1.00 0.00
B C
ATOM 4946 HA LYS B 103 13.868 27.124 49.332 1.00 0.00
B H
ATOM 4947 CB LYS B 103 13.503 25.256 50.380 1.00 0.00
B C
ATOM 4948 HB1 LYS B 103 14.369 25.479 51.046 1.00 0.00
B H
ATOM 4949 HB2 LYS B 103 12.698 24.856 51.038 1.00 0.00
B H
ATOM 4950 CG LYS B 103 13.932 24.189 49.385 1.00 0.00
B C
ATOM 4951 HG1 LYS B 103 13.188 24.141 48.557 1.00 0.00
B H
ATOM 4952 HG2 LYS B 103 14.909 24.482 48.937 1.00 0.00
B H
ATOM 4953 CD LYS B 103 14.026 22.826 50.081 1.00 0.00
B C
ATOM 4954 HD1 LYS B 103 14.777 22.900 50.900 1.00 0.00
B H
ATOM 4955 HD2 LYS B 103 13.040 22.607 50.552 1.00 0.00
B H
ATOM 4956 CE LYS B 103 14.403 21.690 49.125 1.00 0.00
B C
ATOM 4957 HE1 LYS B 103 13.767 21.721 48.213 1.00 0.00
B H
ATOM 4958 HE2 LYS B 103 15.468 21.778 48.814 1.00 0.00
B H
ATOM 4959 NZ LYS B 103 14.229 20.333 49.744 1.00 0.00
B N
ATOM 4960 HZ1 LYS B 103 14.494 19.595 49.061 1.00 0.00
B H
ATOM 4961 HZ2 LYS B 103 14.832 20.256 50.588 1.00 0.00
B H
ATOM 4962 HZ3 LYS B 103 13.235 20.205 50.021 1.00 0.00
B H
ATOM 4963 C LYS B 103 12.006 26.352 48.686 1.00 0.00
B C
ATOM 4964 O LYS B 103 10.908 25.864 48.964 1.00 0.00
B O
ATOM 4965 N ALA B 104 12.354 26.695 47.449 1.00 0.00
B N
ATOM 4966 HN ALA B 104 13.287 26.968 47.217 1.00 0.00
B H
ATOM 4967 CA ALA B 104 11.407 26.700 46.342 1.00 0.00
B C

```
ATOM   4968  HA  ALA B 104      10.391  26.619  46.713  1.00  0.00
B    H
ATOM   4969  CB  ALA B 104      11.513  27.990  45.528  1.00  0.00
B    C
ATOM   4970  HB1 ALA B 104      11.308  28.862  46.185  1.00  0.00
B    H
ATOM   4971  HB2 ALA B 104      12.534  28.112  45.105  1.00  0.00
B    H
ATOM   4972  HB3 ALA B 104      10.780  28.009  44.694  1.00  0.00
B    H
ATOM   4973  C   ALA B 104      11.622  25.512  45.415  1.00  0.00
B    C
ATOM   4974  O   ALA B 104      12.738  25.007  45.267  1.00  0.00
B    O
ATOM   4975  N   GLN B 105      10.538  25.092  44.765  1.00  0.00
B    N
ATOM   4976  HN  GLN B 105       9.661  25.568  44.812  1.00  0.00
B    H
ATOM   4977  CA  GLN B 105      10.533  23.901  43.928  1.00  0.00
B    C
ATOM   4978  HA  GLN B 105      11.349  23.279  44.274  1.00  0.00
B    H
ATOM   4979  CB  GLN B 105       9.199  23.170  44.087  1.00  0.00
B    C
ATOM   4980  HB1 GLN B 105       9.029  23.045  45.181  1.00  0.00
B    H
ATOM   4981  HB2 GLN B 105       8.375  23.822  43.718  1.00  0.00
B    H
ATOM   4982  CG  GLN B 105       9.139  21.815  43.421  1.00  0.00
B    C
ATOM   4983  HG1 GLN B 105      10.159  21.385  43.325  1.00  0.00
B    H
ATOM   4984  HG2 GLN B 105       8.527  21.110  44.020  1.00  0.00
B    H
ATOM   4985  CD  GLN B 105       8.571  21.891  42.025  1.00  0.00
B    C
ATOM   4986  OE1 GLN B 105       7.879  22.846  41.680  1.00  0.00
B    O
ATOM   4987  NE2 GLN B 105       8.840  20.874  41.221  1.00  0.00
B    N
ATOM   4988 HE21 GLN B 105       8.471  20.921  40.297  1.00  0.00
B    H
ATOM   4989 HE22 GLN B 105       9.373  20.105  41.557  1.00  0.00
B    H
ATOM   4990  C   GLN B 105      10.791  24.221  42.459  1.00  0.00
B    C
ATOM   4991  O   GLN B 105      11.792  23.775  41.895  1.00  0.00
B    O
ATOM   4992  N   ASN B 106       9.917  24.998  41.827  1.00  0.00
B    N
ATOM   4993  HN  ASN B 106       9.080  25.361  42.235  1.00  0.00
B    H
ATOM   4994  CA  ASN B 106      10.129  25.385  40.441  1.00  0.00
B    C
```

```
ATOM   4995  HA  ASN B 106      11.168  25.176  40.209  1.00  0.00
B    H
ATOM   4996  CB  ASN B 106       9.193  24.654  39.479  1.00  0.00
B    C
ATOM   4997  HB1 ASN B 106       9.513  24.899  38.442  1.00  0.00
B    H
ATOM   4998  HB2 ASN B 106       9.290  23.557  39.605  1.00  0.00
B    H
ATOM   4999  CG  ASN B 106       7.739  25.072  39.631  1.00  0.00
B    C
ATOM   5000  OD1 ASN B 106       7.395  25.943  40.426  1.00  0.00
B    O
ATOM   5001  ND2 ASN B 106       6.879  24.458  38.839  1.00  0.00
B    N
ATOM   5002 HD21 ASN B 106       5.921  24.722  38.911  1.00  0.00
B    H
ATOM   5003 HD22 ASN B 106       7.200  23.783  38.182  1.00  0.00
B    H
ATOM   5004  C   ASN B 106       9.923  26.881  40.318  1.00  0.00
B    C
ATOM   5005  O   ASN B 106       9.557  27.560  41.280  1.00  0.00
B    O
ATOM   5006  N   TYR B 107      10.127  27.383  39.105  1.00  0.00
B    N
ATOM   5007  HN  TYR B 107      10.464  26.874  38.315  1.00  0.00
B    H
ATOM   5008  CA  TYR B 107       9.853  28.786  38.847  1.00  0.00
B    C
ATOM   5009  HA  TYR B 107      10.522  29.353  39.483  1.00  0.00
B    H
ATOM   5010  CB  TYR B 107      10.084  29.098  37.372  1.00  0.00
B    C
ATOM   5011  HB1 TYR B 107      11.159  29.333  37.213  1.00  0.00
B    H
ATOM   5012  HB2 TYR B 107       9.844  28.215  36.742  1.00  0.00
B    H
ATOM   5013  CG  TYR B 107       9.291  30.289  36.916  1.00  0.00
B    C
ATOM   5014  CD1 TYR B 107       9.729  31.574  37.178  1.00  0.00
B    C
ATOM   5015  HD1 TYR B 107      10.650  31.727  37.723  1.00  0.00
B    H
ATOM   5016  CE1 TYR B 107       9.004  32.665  36.776  1.00  0.00
B    C
ATOM   5017  HE1 TYR B 107       9.353  33.665  36.989  1.00  0.00
B    H
ATOM   5018  CZ  TYR B 107       7.821  32.481  36.103  1.00  0.00
B    C
ATOM   5019  OH  TYR B 107       7.088  33.568  35.695  1.00  0.00
B    O
ATOM   5020  HH  TYR B 107       6.344  33.238  35.186  1.00  0.00
B    H
ATOM   5021  CD2 TYR B 107       8.097  30.131  36.239  1.00  0.00
B    C
```

```
ATOM   5022  HD2 TYR B 107       7.708  29.139  36.056  1.00  0.00
B    H
ATOM   5023  CE2 TYR B 107       7.365  31.217  35.835  1.00  0.00
B    C
ATOM   5024  HE2 TYR B 107       6.424  31.070  35.328  1.00  0.00
B    H
ATOM   5025  C   TYR B 107       8.435  29.153  39.250  1.00  0.00
B    C
ATOM   5026  O   TYR B 107       8.201  30.208  39.852  1.00  0.00
B    O
ATOM   5027  N   TYR B 108       7.471  28.296  38.925  1.00  0.00
B    N
ATOM   5028  HN  TYR B 108       7.595  27.414  38.474  1.00  0.00
B    H
ATOM   5029  CA  TYR B 108       6.089  28.628  39.235  1.00  0.00
B    C
ATOM   5030  HA  TYR B 108       5.910  29.615  38.826  1.00  0.00
B    H
ATOM   5031  CB  TYR B 108       5.156  27.597  38.629  1.00  0.00
B    C
ATOM   5032  HB1 TYR B 108       5.440  26.587  38.999  1.00  0.00
B    H
ATOM   5033  HB2 TYR B 108       4.107  27.782  38.948  1.00  0.00
B    H
ATOM   5034  CG  TYR B 108       5.269  27.597  37.153  1.00  0.00
B    C
ATOM   5035  CD1 TYR B 108       5.110  28.771  36.447  1.00  0.00
B    C
ATOM   5036  HD1 TYR B 108       4.877  29.687  36.972  1.00  0.00
B    H
ATOM   5037  CE1 TYR B 108       5.217  28.801  35.092  1.00  0.00
B    C
ATOM   5038  HE1 TYR B 108       5.083  29.726  34.551  1.00  0.00
B    H
ATOM   5039  CZ  TYR B 108       5.490  27.645  34.415  1.00  0.00
B    C
ATOM   5040  OH  TYR B 108       5.598  27.672  33.046  1.00  0.00
B    O
ATOM   5041  HH  TYR B 108       5.861  26.795  32.760  1.00  0.00
B    H
ATOM   5042  CD2 TYR B 108       5.550  26.444  36.459  1.00  0.00
B    C
ATOM   5043  HD2 TYR B 108       5.648  25.506  36.988  1.00  0.00
B    H
ATOM   5044  CE2 TYR B 108       5.659  26.460  35.095  1.00  0.00
B    C
ATOM   5045  HE2 TYR B 108       5.855  25.542  34.561  1.00  0.00
B    H
ATOM   5046  C   TYR B 108       5.871  28.717  40.729  1.00  0.00
B    C
ATOM   5047  O   TYR B 108       5.593  29.803  41.254  1.00  0.00
B    O
ATOM   5048  N   HSE B 109       6.046  27.593  41.422  1.00  0.00
B    N
```

```
ATOM   5049  HN  HSE B 109       6.224  26.700  41.010  1.00  0.00
B    H
ATOM   5050  CA  HSE B 109       5.992  27.583  42.875  1.00  0.00
B    C
ATOM   5051  HA  HSE B 109       4.947  27.625  43.156  1.00  0.00
B    H
ATOM   5052  CB  HSE B 109       6.670  26.324  43.395  1.00  0.00
B    C
ATOM   5053  HB1 HSE B 109       6.043  25.454  43.105  1.00  0.00
B    H
ATOM   5054  HB2 HSE B 109       7.652  26.198  42.894  1.00  0.00
B    H
ATOM   5055  ND1 HSE B 109       8.065  26.180  45.480  1.00  0.00
B    N
ATOM   5056  CG  HSE B 109       6.835  26.302  44.874  1.00  0.00
B    C
ATOM   5057  CE1 HSE B 109       7.911  26.186  46.791  1.00  0.00
B    C
ATOM   5058  HE1 HSE B 109       8.729  26.105  47.512  1.00  0.00
B    H
ATOM   5059  NE2 HSE B 109       6.622  26.300  47.057  1.00  0.00
B    N
ATOM   5060  HE2 HSE B 109       6.222  26.310  47.973  1.00  0.00
B    H
ATOM   5061  CD2 HSE B 109       5.928  26.370  45.875  1.00  0.00
B    C
ATOM   5062  HD2 HSE B 109       4.851  26.454  45.829  1.00  0.00
B    H
ATOM   5063  C   HSE B 109       6.657  28.817  43.447  1.00  0.00
B    C
ATOM   5064  O   HSE B 109       6.150  29.429  44.389  1.00  0.00
B    O
ATOM   5065  N   HSE B 110       7.765  29.231  42.839  1.00  0.00
B    N
ATOM   5066  HN  HSE B 110       8.201  28.758  42.074  1.00  0.00
B    H
ATOM   5067  CA  HSE B 110       8.444  30.445  43.260  1.00  0.00
B    C
ATOM   5068  HA  HSE B 110       8.628  30.344  44.323  1.00  0.00
B    H
ATOM   5069  CB  HSE B 110       9.735  30.585  42.478  1.00  0.00
B    C
ATOM   5070  HB1 HSE B 110      10.546  30.105  43.068  1.00  0.00
B    H
ATOM   5071  HB2 HSE B 110       9.652  30.024  41.523  1.00  0.00
B    H
ATOM   5072  ND1 HSE B 110      10.939  32.703  43.073  1.00  0.00
B    N
ATOM   5073  CG  HSE B 110      10.123  31.997  42.222  1.00  0.00
B    C
ATOM   5074  CE1 HSE B 110      11.109  33.924  42.602  1.00  0.00
B    C
ATOM   5075  HE1 HSE B 110      11.708  34.698  43.088  1.00  0.00
B    H
```

ATOM 5076 NE2 HSE B 110 10.433 34.032 41.473 1.00 0.00
B N
ATOM 5077 HE2 HSE B 110 10.374 34.858 40.914 1.00 0.00
B H
ATOM 5078 CD2 HSE B 110 9.807 32.839 41.213 1.00 0.00
B C
ATOM 5079 HD2 HSE B 110 9.183 32.678 40.345 1.00 0.00
B H
ATOM 5080 C HSE B 110 7.571 31.675 43.074 1.00 0.00
B C
ATOM 5081 O HSE B 110 7.518 32.548 43.946 1.00 0.00
B O
ATOM 5082 N LYS B 111 6.915 31.797 41.928 1.00 0.00
B N
ATOM 5083 HN LYS B 111 6.929 31.154 41.165 1.00 0.00
B H
ATOM 5084 CA LYS B 111 6.082 32.975 41.730 1.00 0.00
B C
ATOM 5085 HA LYS B 111 6.699 33.841 41.942 1.00 0.00
B H
ATOM 5086 CB LYS B 111 5.568 33.039 40.301 1.00 0.00
B C
ATOM 5087 HB1 LYS B 111 6.451 33.207 39.642 1.00 0.00
B H
ATOM 5088 HB2 LYS B 111 5.154 32.044 40.021 1.00 0.00
B H
ATOM 5089 CG LYS B 111 4.552 34.148 40.097 1.00 0.00
B C
ATOM 5090 HG1 LYS B 111 3.694 33.753 39.506 1.00 0.00
B H
ATOM 5091 HG2 LYS B 111 4.155 34.464 41.089 1.00 0.00
B H
ATOM 5092 CD LYS B 111 5.126 35.368 39.358 1.00 0.00
B C
ATOM 5093 HD1 LYS B 111 4.570 35.494 38.400 1.00 0.00
B H
ATOM 5094 HD2 LYS B 111 4.929 36.273 39.978 1.00 0.00
B H
ATOM 5095 CE LYS B 111 6.601 35.227 39.098 1.00 0.00
B C
ATOM 5096 HE1 LYS B 111 6.931 34.182 39.288 1.00 0.00
B H
ATOM 5097 HE2 LYS B 111 6.839 35.484 38.042 1.00 0.00
B H
ATOM 5098 NZ LYS B 111 7.357 36.134 39.992 1.00 0.00
B N
ATOM 5099 HZ1 LYS B 111 8.378 36.039 39.816 1.00 0.00
B H
ATOM 5100 HZ2 LYS B 111 7.068 37.117 39.815 1.00 0.00
B H
ATOM 5101 HZ3 LYS B 111 7.155 35.891 40.982 1.00 0.00
B H
ATOM 5102 C LYS B 111 4.920 32.997 42.704 1.00 0.00
B C

ATOM 5103 O LYS B 111 4.607 34.038 43.295 1.00 0.00
B O
ATOM 5104 N VAL B 112 4.271 31.855 42.889 1.00 0.00
B N
ATOM 5105 HN VAL B 112 4.450 30.999 42.413 1.00 0.00
B H
ATOM 5106 CA VAL B 112 3.188 31.787 43.865 1.00 0.00
B C
ATOM 5107 HA VAL B 112 2.458 32.543 43.602 1.00 0.00
B H
ATOM 5108 CB VAL B 112 2.504 30.415 43.823 1.00 0.00
B C
ATOM 5109 HB VAL B 112 1.408 30.588 43.683 1.00 0.00
B H
ATOM 5110 CG1 VAL B 112 3.029 29.615 42.680 1.00 0.00
B C
ATOM 5111 HG11 VAL B 112 2.565 28.606 42.669 1.00 0.00
B H
ATOM 5112 HG12 VAL B 112 2.801 30.094 41.705 1.00 0.00
B H
ATOM 5113 HG13 VAL B 112 4.130 29.486 42.764 1.00 0.00
B H
ATOM 5114 CG2 VAL B 112 2.738 29.679 45.114 1.00 0.00
B C
ATOM 5115 HG21 VAL B 112 2.279 28.668 45.078 1.00 0.00
B H
ATOM 5116 HG22 VAL B 112 3.826 29.564 45.306 1.00 0.00
B H
ATOM 5117 HG23 VAL B 112 2.288 30.221 45.972 1.00 0.00
B H
ATOM 5118 C VAL B 112 3.709 32.119 45.253 1.00 0.00
B C
ATOM 5119 O VAL B 112 2.993 32.694 46.080 1.00 0.00
B O
ATOM 5120 N LEU B 113 4.962 31.766 45.525 1.00 0.00
B N
ATOM 5121 HN LEU B 113 5.536 31.187 44.950 1.00 0.00
B H
ATOM 5122 CA LEU B 113 5.608 32.227 46.741 1.00 0.00
B C
ATOM 5123 HA LEU B 113 5.003 31.883 47.570 1.00 0.00
B H
ATOM 5124 CB LEU B 113 7.023 31.674 46.798 1.00 0.00
B C
ATOM 5125 HB1 LEU B 113 7.011 30.685 46.284 1.00 0.00
B H
ATOM 5126 HB2 LEU B 113 7.692 32.327 46.194 1.00 0.00
B H
ATOM 5127 CG LEU B 113 7.646 31.464 48.163 1.00 0.00
B C
ATOM 5128 HG LEU B 113 8.174 32.403 48.458 1.00 0.00
B H
ATOM 5129 CD1 LEU B 113 6.578 31.165 49.180 1.00 0.00
B C

ATOM   5130 HD11 LEU B 113   7.031 30.963 50.174 1.00 0.00
B   H
ATOM   5131 HD12 LEU B 113   5.883 32.027 49.282 1.00 0.00
B   H
ATOM   5132 HD13 LEU B 113   5.988 30.273 48.879 1.00 0.00
B   H
ATOM   5133 CD2 LEU B 113   8.669 30.364 48.072 1.00 0.00
B   C
ATOM   5134 HD21 LEU B 113   9.153 30.205 49.061 1.00 0.00
B   H
ATOM   5135 HD22 LEU B 113   8.211 29.403 47.764 1.00 0.00
B   H
ATOM   5136 HD23 LEU B 113   9.459 30.627 47.337 1.00 0.00
B   H
ATOM   5137 C   LEU B 113   5.634 33.741 46.791 1.00 0.00
B   C
ATOM   5138 O   LEU B 113   5.282 34.349 47.806 1.00 0.00
B   O
ATOM   5139 N   GLU B 114   6.041 34.363 45.691 1.00 0.00
B   N
ATOM   5140 HN GLU B 114   6.309 33.922 44.838 1.00 0.00
B   H
ATOM   5141 CA GLU B 114   6.125 35.816 45.658 1.00 0.00
B   C
ATOM   5142 HA GLU B 114   6.817 36.132 46.430 1.00 0.00
B   H
ATOM   5143 CB GLU B 114   6.630 36.283 44.299 1.00 0.00
B   C
ATOM   5144 HB1 GLU B 114   7.484 35.631 44.004 1.00 0.00
B   H
ATOM   5145 HB2 GLU B 114   5.844 36.122 43.528 1.00 0.00
B   H
ATOM   5146 CG GLU B 114   7.070 37.719 44.326 1.00 0.00
B   C
ATOM   5147 HG1 GLU B 114   7.717 37.923 43.453 1.00 0.00
B   H
ATOM   5148 HG2 GLU B 114   6.197 38.402 44.301 1.00 0.00
B   H
ATOM   5149 CD GLU B 114   7.902 38.030 45.548 1.00 0.00
B   C
ATOM   5150 OE1 GLU B 114   8.912 37.332 45.766 1.00 0.00
B   O
ATOM   5151 OE2 GLU B 114   7.537 38.958 46.298 1.00 0.00
B   O
ATOM   5152 C   GLU B 114   4.779 36.451 45.971 1.00 0.00
B   C
ATOM   5153 O   GLU B 114   4.695 37.419 46.735 1.00 0.00
B   O
ATOM   5154 N   LYS B 115   3.709 35.909 45.399 1.00 0.00
B   N
ATOM   5155 HN LYS B 115   3.716 35.083 44.838 1.00 0.00
B   H
ATOM   5156 CA LYS B 115   2.401 36.529 45.571 1.00 0.00
B   C

ATOM 5157 HA LYS B 115 2.510 37.562 45.266 1.00 0.00
B H
ATOM 5158 CB LYS B 115 1.368 35.824 44.712 1.00 0.00
B C
ATOM 5159 HB1 LYS B 115 1.453 34.734 44.925 1.00 0.00
B H
ATOM 5160 HB2 LYS B 115 0.351 36.129 45.046 1.00 0.00
B H
ATOM 5161 CG LYS B 115 1.575 36.078 43.268 1.00 0.00
B C
ATOM 5162 HG1 LYS B 115 2.435 35.466 42.911 1.00 0.00
B H
ATOM 5163 HG2 LYS B 115 0.673 35.741 42.707 1.00 0.00
B H
ATOM 5164 CD LYS B 115 1.852 37.524 43.012 1.00 0.00
B C
ATOM 5165 HD1 LYS B 115 1.013 38.123 43.435 1.00 0.00
B H
ATOM 5166 HD2 LYS B 115 2.776 37.804 43.569 1.00 0.00
B H
ATOM 5167 CE LYS B 115 2.008 37.731 41.534 1.00 0.00
B C
ATOM 5168 HE1 LYS B 115 1.993 38.817 41.288 1.00 0.00
B H
ATOM 5169 HE2 LYS B 115 2.971 37.304 41.178 1.00 0.00
B H
ATOM 5170 NZ LYS B 115 0.888 37.061 40.821 1.00 0.00
B N
ATOM 5171 HZ1 LYS B 115 0.986 37.200 39.795 1.00 0.00
B H
ATOM 5172 HZ2 LYS B 115 0.901 36.043 41.035 1.00 0.00
B H
ATOM 5173 HZ3 LYS B 115 -0.016 37.465 41.139 1.00 0.00
B H
ATOM 5174 C LYS B 115 1.935 36.531 47.015 1.00 0.00
B C
ATOM 5175 O LYS B 115 1.225 37.452 47.424 1.00 0.00
B O
ATOM 5176 N ILE B 116 2.310 35.526 47.800 1.00 0.00
B N
ATOM 5177 HN ILE B 116 2.869 34.753 47.506 1.00 0.00
B H
ATOM 5178 CA ILE B 116 1.917 35.478 49.199 1.00 0.00
B C
ATOM 5179 HA ILE B 116 0.863 35.726 49.224 1.00 0.00
B H
ATOM 5180 CB ILE B 116 2.165 34.090 49.794 1.00 0.00
B C
ATOM 5181 HB ILE B 116 1.843 33.379 48.992 1.00 0.00
B H
ATOM 5182 CG2 ILE B 116 3.590 33.983 50.268 1.00 0.00
B C
ATOM 5183 HG21 ILE B 116 3.828 32.938 50.557 1.00 0.00
B H

ATOM 5184 HG22 ILE B 116 4.298 34.279 49.466 1.00 0.00
B H
ATOM 5185 HG23 ILE B 116 3.766 34.635 51.149 1.00 0.00
B H
ATOM 5186 CG1 ILE B 116 1.253 33.838 50.992 1.00 0.00
B C
ATOM 5187 HG11 ILE B 116 1.389 32.794 51.356 1.00 0.00
B H
ATOM 5188 HG12 ILE B 116 1.552 34.518 51.823 1.00 0.00
B H
ATOM 5189 CD ILE B 116 -0.189 34.059 50.727 1.00 0.00
B C
ATOM 5190 HD1 ILE B 116 -0.779 33.913 51.657 1.00 0.00
B H
ATOM 5191 HD2 ILE B 116 -0.366 35.094 50.362 1.00 0.00
B H
ATOM 5192 HD3 ILE B 116 -0.568 33.347 49.964 1.00 0.00
B H
ATOM 5193 C ILE B 116 2.637 36.535 50.018 1.00 0.00
B C
ATOM 5194 O ILE B 116 2.219 36.838 51.136 1.00 0.00
B O
ATOM 5195 N GLY B 117 3.699 37.117 49.483 1.00 0.00
B N
ATOM 5196 HN GLY B 117 3.963 37.033 48.523 1.00 0.00
B H
ATOM 5197 CA GLY B 117 4.563 37.937 50.298 1.00 0.00
B C
ATOM 5198 HA1 GLY B 117 3.995 38.218 51.175 1.00 0.00
B H
ATOM 5199 HA2 GLY B 117 4.881 38.771 49.689 1.00 0.00
B H
ATOM 5200 C GLY B 117 5.792 37.201 50.756 1.00 0.00
B C
ATOM 5201 O GLY B 117 6.199 37.343 51.912 1.00 0.00
B O
ATOM 5202 N ALA B 118 6.387 36.400 49.881 1.00 0.00
B N
ATOM 5203 HN ALA B 118 6.042 36.221 48.961 1.00 0.00
B H
ATOM 5204 CA ALA B 118 7.618 35.717 50.229 1.00 0.00
B C
ATOM 5205 HA ALA B 118 7.495 35.129 51.131 1.00 0.00
B H
ATOM 5206 CB ALA B 118 8.056 34.803 49.094 1.00 0.00
B C
ATOM 5207 HB1 ALA B 118 7.276 34.033 48.914 1.00 0.00
B H
ATOM 5208 HB2 ALA B 118 8.195 35.376 48.151 1.00 0.00
B H
ATOM 5209 HB3 ALA B 118 9.006 34.279 49.333 1.00 0.00
B H
ATOM 5210 C ALA B 118 8.711 36.726 50.521 1.00 0.00
B C

```
ATOM   5211  O   ALA B 118       9.209  37.402  49.621  1.00  0.00
B    O
ATOM   5212  N   ASP B 119       9.079  36.828  51.791  1.00  0.00
B    N
ATOM   5213  HN  ASP B 119       8.696  36.355  52.580  1.00  0.00
B    H
ATOM   5214  CA  ASP B 119      10.167  37.721  52.143  1.00  0.00
B    C
ATOM   5215  HA  ASP B 119       9.910  38.700  51.757  1.00  0.00
B    H
ATOM   5216  CB  ASP B 119      10.343  37.760  53.657  1.00  0.00
B    C
ATOM   5217  HB1 ASP B 119      10.424  36.723  54.042  1.00  0.00
B    H
ATOM   5218  HB2 ASP B 119      11.268  38.305  53.929  1.00  0.00
B    H
ATOM   5219  CG  ASP B 119       9.173  38.415  54.352  1.00  0.00
B    C
ATOM   5220  OD1 ASP B 119       9.265  39.626  54.651  1.00  0.00
B    O
ATOM   5221  OD2 ASP B 119       8.157  37.727  54.584  1.00  0.00
B    O
ATOM   5222  C   ASP B 119      11.458  37.308  51.463  1.00  0.00
B    C
ATOM   5223  O   ASP B 119      12.328  38.152  51.231  1.00  0.00
B    O
ATOM   5224  N   ARG B 120      11.599  36.029  51.136  1.00  0.00
B    N
ATOM   5225  HN  ARG B 120      10.913  35.338  51.352  1.00  0.00
B    H
ATOM   5226  CA  ARG B 120      12.784  35.571  50.433  1.00  0.00
B    C
ATOM   5227  HA  ARG B 120      12.934  36.193  49.558  1.00  0.00
B    H
ATOM   5228  CB  ARG B 120      14.007  35.634  51.338  1.00  0.00
B    C
ATOM   5229  HB1 ARG B 120      14.313  36.707  51.381  1.00  0.00
B    H
ATOM   5230  HB2 ARG B 120      13.717  35.356  52.375  1.00  0.00
B    H
ATOM   5231  CG  ARG B 120      15.126  34.818  50.827  1.00  0.00
B    C
ATOM   5232  HG1 ARG B 120      15.232  33.919  51.472  1.00  0.00
B    H
ATOM   5233  HG2 ARG B 120      14.861  34.432  49.816  1.00  0.00
B    H
ATOM   5234  CD  ARG B 120      16.355  35.642  50.742  1.00  0.00
B    C
ATOM   5235  HD1 ARG B 120      16.227  36.418  49.952  1.00  0.00
B    H
ATOM   5236  HD2 ARG B 120      16.574  36.151  51.710  1.00  0.00
B    H
ATOM   5237  NE  ARG B 120      17.499  34.806  50.426  1.00  0.00
B    N
```

ATOM 5238 HE ARG B 120 17.264 33.832 50.337 1.00 0.00
B H
ATOM 5239 CZ ARG B 120 18.730 35.270 50.269 1.00 0.00
B C
ATOM 5240 NH1 ARG B 120 18.970 36.563 50.408 1.00 0.00
B N
ATOM 5241 HH11 ARG B 120 19.881 36.925 50.268 1.00 0.00
B H
ATOM 5242 HH12 ARG B 120 18.169 37.154 50.522 1.00 0.00
B H
ATOM 5243 NH2 ARG B 120 19.721 34.441 49.981 1.00 0.00
B N
ATOM 5244 HH21 ARG B 120 20.652 34.776 49.950 1.00 0.00
B H
ATOM 5245 HH22 ARG B 120 19.518 33.471 50.013 1.00 0.00
B H
ATOM 5246 C ARG B 120 12.580 34.153 49.930 1.00 0.00
B C
ATOM 5247 O ARG B 120 12.165 33.270 50.688 1.00 0.00
B O
ATOM 5248 N ILE B 121 12.903 33.941 48.659 1.00 0.00
B N
ATOM 5249 HN ILE B 121 13.273 34.661 48.077 1.00 0.00
B H
ATOM 5250 CA ILE B 121 12.752 32.655 47.994 1.00 0.00
B C
ATOM 5251 HA ILE B 121 12.307 31.969 48.704 1.00 0.00
B H
ATOM 5252 CB ILE B 121 11.908 32.802 46.720 1.00 0.00
B C
ATOM 5253 HB ILE B 121 12.556 33.390 46.022 1.00 0.00
B H
ATOM 5254 CG2 ILE B 121 11.516 31.456 46.166 1.00 0.00
B C
ATOM 5255 HG21 ILE B 121 11.044 31.566 45.168 1.00 0.00
B H
ATOM 5256 HG22 ILE B 121 12.406 30.803 46.047 1.00 0.00
B H
ATOM 5257 HG23 ILE B 121 10.793 30.945 46.837 1.00 0.00
B H
ATOM 5258 CG1 ILE B 121 10.678 33.654 47.020 1.00 0.00
B C
ATOM 5259 HG11 ILE B 121 10.157 33.258 47.921 1.00 0.00
B H
ATOM 5260 HG12 ILE B 121 11.006 34.691 47.258 1.00 0.00
B H
ATOM 5261 CD ILE B 121 9.694 33.731 45.890 1.00 0.00
B C
ATOM 5262 HD1 ILE B 121 8.856 34.411 46.155 1.00 0.00
B H
ATOM 5263 HD2 ILE B 121 10.183 34.122 44.972 1.00 0.00
B H
ATOM 5264 HD3 ILE B 121 9.267 32.731 45.664 1.00 0.00
B H

ATOM 5265 C ILE B 121 14.139 32.121 47.677 1.00 0.00
B C
ATOM 5266 O ILE B 121 15.076 32.901 47.485 1.00 0.00
B O
ATOM 5267 N ILE B 122 14.284 30.800 47.640 1.00 0.00
B N
ATOM 5268 HN ILE B 122 13.558 30.149 47.852 1.00 0.00
B H
ATOM 5269 CA ILE B 122 15.551 30.184 47.275 1.00 0.00
B C
ATOM 5270 HA ILE B 122 16.115 30.921 46.716 1.00 0.00
B H
ATOM 5271 CB ILE B 122 16.337 29.703 48.508 1.00 0.00
B C
ATOM 5272 HB ILE B 122 15.593 29.114 49.101 1.00 0.00
B H
ATOM 5273 CG2 ILE B 122 17.555 28.905 48.087 1.00 0.00
B C
ATOM 5274 HG21 ILE B 122 18.034 28.428 48.967 1.00 0.00
B H
ATOM 5275 HG22 ILE B 122 17.274 28.098 47.378 1.00 0.00
B H
ATOM 5276 HG23 ILE B 122 18.307 29.557 47.594 1.00 0.00
B H
ATOM 5277 CG1 ILE B 122 16.750 30.890 49.369 1.00 0.00
B C
ATOM 5278 HG11 ILE B 122 17.733 30.683 49.850 1.00 0.00
B H
ATOM 5279 HG12 ILE B 122 16.886 31.782 48.715 1.00 0.00
B H
ATOM 5280 CD ILE B 122 15.750 31.246 50.440 1.00 0.00
B C
ATOM 5281 HD1 ILE B 122 16.082 32.152 50.993 1.00 0.00
B H
ATOM 5282 HD2 ILE B 122 14.756 31.457 49.992 1.00 0.00
B H
ATOM 5283 HD3 ILE B 122 15.638 30.419 51.171 1.00 0.00
B H
ATOM 5284 C ILE B 122 15.294 29.022 46.333 1.00 0.00
B C
ATOM 5285 O ILE B 122 14.411 28.195 46.576 1.00 0.00
B O
ATOM 5286 N HSE B 123 16.083 28.955 45.275 1.00 0.00
B N
ATOM 5287 HN HSE B 123 16.741 29.679 45.074 1.00 0.00
B H
ATOM 5288 CA HSE B 123 16.084 27.861 44.319 1.00 0.00
B C
ATOM 5289 HA HSE B 123 15.299 27.165 44.587 1.00 0.00
B H
ATOM 5290 CB HSE B 123 15.938 28.387 42.898 1.00 0.00
B C
ATOM 5291 HB1 HSE B 123 16.771 29.099 42.709 1.00 0.00
B H

ATOM 5292 HB2 HSE B 123 16.057 27.550 42.178 1.00 0.00
B H
ATOM 5293 ND1 HSE B 123 13.642 28.575 41.877 1.00 0.00
B N
ATOM 5294 CG HSE B 123 14.653 29.108 42.644 1.00 0.00
B C
ATOM 5295 CE1 HSE B 123 12.641 29.435 41.813 1.00 0.00
B C
ATOM 5296 HE1 HSE B 123 11.715 29.268 41.258 1.00 0.00
B H
ATOM 5297 NE2 HSE B 123 12.964 30.503 42.518 1.00 0.00
B N
ATOM 5298 HE2 HSE B 123 12.392 31.316 42.628 1.00 0.00
B H
ATOM 5299 CD2 HSE B 123 14.219 30.325 43.046 1.00 0.00
B C
ATOM 5300 HD2 HSE B 123 14.711 31.070 43.655 1.00 0.00
B H
ATOM 5301 C HSE B 123 17.405 27.126 44.479 1.00 0.00
B C
ATOM 5302 O HSE B 123 18.292 27.246 43.625 1.00 0.00
B O
ATOM 5303 N PRO B 124 17.570 26.365 45.559 1.00 0.00
B N
ATOM 5304 CD PRO B 124 16.529 25.800 46.427 1.00 0.00
B C
ATOM 5305 HD1 PRO B 124 16.598 26.907 46.324 1.00 0.00
B H
ATOM 5306 HD2 PRO B 124 15.465 25.483 46.519 1.00 0.00
B H
ATOM 5307 CA PRO B 124 18.916 25.920 45.945 1.00 0.00
B C
ATOM 5308 HA PRO B 124 19.496 26.778 46.262 1.00 0.00
B H
ATOM 5309 CB PRO B 124 18.642 24.997 47.139 1.00 0.00
B C
ATOM 5310 HB1 PRO B 124 18.569 25.612 48.065 1.00 0.00
B H
ATOM 5311 HB2 PRO B 124 19.430 24.229 47.278 1.00 0.00
B H
ATOM 5312 CG PRO B 124 17.297 25.352 47.617 1.00 0.00
B C
ATOM 5313 HG1 PRO B 124 17.130 25.080 48.678 1.00 0.00
B H
ATOM 5314 HG2 PRO B 124 16.535 24.845 46.981 1.00 0.00
B H
ATOM 5315 C PRO B 124 19.667 25.192 44.854 1.00 0.00
B C
ATOM 5316 O PRO B 124 20.758 25.624 44.450 1.00 0.00
B O
ATOM 5317 N GLU B 125 19.112 24.090 44.365 1.00 0.00
B N
ATOM 5318 HN GLU B 125 18.221 23.715 44.604 1.00 0.00
B H

```
ATOM   5319  CA  GLU B 125      19.832  23.312  43.375  1.00  0.00
B    C
ATOM   5320  HA  GLU B 125      20.818  23.096  43.766  1.00  0.00
B    H
ATOM   5321  CB  GLU B 125      19.122  22.003  43.092  1.00  0.00
B    C
ATOM   5322  HB1 GLU B 125      18.031  22.211  43.003  1.00  0.00
B    H
ATOM   5323  HB2 GLU B 125      19.440  21.608  42.101  1.00  0.00
B    H
ATOM   5324  CG  GLU B 125      19.350  20.963  44.171  1.00  0.00
B    C
ATOM   5325  HG1 GLU B 125      19.527  19.977  43.702  1.00  0.00
B    H
ATOM   5326  HG2 GLU B 125      20.222  21.228  44.801  1.00  0.00
B    H
ATOM   5327  CD  GLU B 125      18.166  20.762  45.076  1.00  0.00
B    C
ATOM   5328  OE1 GLU B 125      17.202  21.555  44.969  1.00  0.00
B    O
ATOM   5329  OE2 GLU B 125      18.208  19.799  45.866  1.00  0.00
B    O
ATOM   5330  C   GLU B 125      20.017  24.098  42.099  1.00  0.00
B    C
ATOM   5331  O   GLU B 125      21.008  23.909  41.403  1.00  0.00
B    O
ATOM   5332  N   LYS B 126      19.093  25.003  41.785  1.00  0.00
B    N
ATOM   5333  HN  LYS B 126      18.213  25.120  42.241  1.00  0.00
B    H
ATOM   5334  CA  LYS B 126      19.347  25.923  40.686  1.00  0.00
B    C
ATOM   5335  HA  LYS B 126      19.454  25.330  39.785  1.00  0.00
B    H
ATOM   5336  CB  LYS B 126      18.192  26.910  40.551  1.00  0.00
B    C
ATOM   5337  HB1 LYS B 126      18.200  27.551  41.463  1.00  0.00
B    H
ATOM   5338  HB2 LYS B 126      18.393  27.588  39.691  1.00  0.00
B    H
ATOM   5339  CG  LYS B 126      16.833  26.282  40.416  1.00  0.00
B    C
ATOM   5340  HG1 LYS B 126      16.602  25.711  41.344  1.00  0.00
B    H
ATOM   5341  HG2 LYS B 126      16.068  27.088  40.324  1.00  0.00
B    H
ATOM   5342  CD  LYS B 126      16.756  25.356  39.240  1.00  0.00
B    C
ATOM   5343  HD1 LYS B 126      17.760  24.897  39.088  1.00  0.00
B    H
ATOM   5344  HD2 LYS B 126      16.048  24.533  39.493  1.00  0.00
B    H
ATOM   5345  CE  LYS B 126      16.301  26.069  37.982  1.00  0.00
B    C
```

ATOM 5346 HE1 LYS B 126 15.594 26.891 38.234 1.00 0.00
B H
ATOM 5347 HE2 LYS B 126 17.171 26.506 37.443 1.00 0.00
B H
ATOM 5348 NZ LYS B 126 15.608 25.127 37.059 1.00 0.00
B N
ATOM 5349 HZ1 LYS B 126 15.300 25.629 36.202 1.00 0.00
B H
ATOM 5350 HZ2 LYS B 126 16.258 24.359 36.795 1.00 0.00
B H
ATOM 5351 HZ3 LYS B 126 14.777 24.723 37.538 1.00 0.00
B H
ATOM 5352 C LYS B 126 20.660 26.665 40.899 1.00 0.00
B C
ATOM 5353 O LYS B 126 21.554 26.653 40.039 1.00 0.00
B O
ATOM 5354 N ASP B 127 20.800 27.286 42.067 1.00 0.00
B N
ATOM 5355 HN ASP B 127 20.168 27.236 42.836 1.00 0.00
B H
ATOM 5356 CA ASP B 127 21.951 28.135 42.334 1.00 0.00
B C
ATOM 5357 HA ASP B 127 22.010 28.857 41.528 1.00 0.00
B H
ATOM 5358 CB ASP B 127 21.771 28.804 43.698 1.00 0.00
B C
ATOM 5359 HB1 ASP B 127 21.344 28.071 44.411 1.00 0.00
B H
ATOM 5360 HB2 ASP B 127 22.746 29.149 44.095 1.00 0.00
B H
ATOM 5361 CG ASP B 127 20.827 29.989 43.647 1.00 0.00
B C
ATOM 5362 OD1 ASP B 127 19.605 29.751 43.501 1.00 0.00
B O
ATOM 5363 OD2 ASP B 127 21.300 31.139 43.768 1.00 0.00
B O
ATOM 5364 C ASP B 127 23.236 27.324 42.292 1.00 0.00
B C
ATOM 5365 O ASP B 127 24.186 27.655 41.565 1.00 0.00
B O
ATOM 5366 N MET B 128 23.285 26.240 43.052 1.00 0.00
B N
ATOM 5367 HN MET B 128 22.569 25.835 43.617 1.00 0.00
B H
ATOM 5368 CA MET B 128 24.546 25.526 43.080 1.00 0.00
B C
ATOM 5369 HA MET B 128 25.334 26.269 43.114 1.00 0.00
B H
ATOM 5370 CB MET B 128 24.656 24.676 44.341 1.00 0.00
B C
ATOM 5371 HB1 MET B 128 25.197 25.289 45.099 1.00 0.00
B H
ATOM 5372 HB2 MET B 128 23.638 24.496 44.755 1.00 0.00
B H

ATOM 5373 CG MET B 128 25.389 23.369 44.169 1.00 0.00
B C
ATOM 5374 HG1 MET B 128 24.668 22.539 44.009 1.00 0.00
B H
ATOM 5375 HG2 MET B 128 26.044 23.422 43.272 1.00 0.00
B H
ATOM 5376 SD MET B 128 26.399 22.958 45.604 1.00 0.00
B S
ATOM 5377 CE MET B 128 26.188 24.404 46.650 1.00 0.00
B C
ATOM 5378 HE1 MET B 128 26.772 24.303 47.590 1.00 0.00
B H
ATOM 5379 HE2 MET B 128 26.533 25.326 46.131 1.00 0.00
B H
ATOM 5380 HE3 MET B 128 25.120 24.546 46.925 1.00 0.00
B H
ATOM 5381 C MET B 128 24.737 24.711 41.813 1.00 0.00
B C
ATOM 5382 O MET B 128 25.863 24.336 41.486 1.00 0.00
B O
ATOM 5383 N GLY B 129 23.689 24.501 41.036 1.00 0.00
B N
ATOM 5384 HN GLY B 129 22.753 24.784 41.241 1.00 0.00
B H
ATOM 5385 CA GLY B 129 23.870 23.812 39.786 1.00 0.00
B C
ATOM 5386 HA1 GLY B 129 22.895 23.590 39.375 1.00 0.00
B H
ATOM 5387 HA2 GLY B 129 24.489 22.942 39.959 1.00 0.00
B H
ATOM 5388 C GLY B 129 24.589 24.707 38.818 1.00 0.00
B C
ATOM 5389 O GLY B 129 25.529 24.282 38.150 1.00 0.00
B O
ATOM 5390 N VAL B 130 24.142 25.953 38.711 1.00 0.00
B N
ATOM 5391 HN VAL B 130 23.356 26.357 39.169 1.00 0.00
B H
ATOM 5392 CA VAL B 130 24.859 26.864 37.830 1.00 0.00
B C
ATOM 5393 HA VAL B 130 24.935 26.394 36.857 1.00 0.00
B H
ATOM 5394 CB VAL B 130 24.062 28.167 37.629 1.00 0.00
B C
ATOM 5395 HB VAL B 130 24.571 28.753 36.825 1.00 0.00
B H
ATOM 5396 CG1 VAL B 130 22.646 27.832 37.202 1.00 0.00
B C
ATOM 5397 HG11 VAL B 130 22.047 28.761 37.091 1.00 0.00
B H
ATOM 5398 HG12 VAL B 130 22.627 27.307 36.224 1.00 0.00
B H
ATOM 5399 HG13 VAL B 130 22.149 27.190 37.960 1.00 0.00
B H

```
ATOM   5400  CG2 VAL B 130      24.000  28.979  38.886  1.00  0.00
B    C
ATOM   5401 HG21 VAL B 130      23.389  29.894  38.734  1.00  0.00
B    H
ATOM   5402 HG22 VAL B 130      23.545  28.388  39.710  1.00  0.00
B    H
ATOM   5403 HG23 VAL B 130      25.013  29.302  39.207  1.00  0.00
B    H
ATOM   5404  C   VAL B 130      26.277  27.084  38.343  1.00  0.00
B    C
ATOM   5405  O   VAL B 130      27.234  27.154  37.556  1.00  0.00
B    O
ATOM   5406  N   LYS B 131      26.444  27.145  39.670  1.00  0.00
B    N
ATOM   5407  HN  LYS B 131      25.710  27.299  40.329  1.00  0.00
B    H
ATOM   5408  CA  LYS B 131      27.774  26.981  40.251  1.00  0.00
B    C
ATOM   5409  HA  LYS B 131      28.311  27.903  40.066  1.00  0.00
B    H
ATOM   5410  CB  LYS B 131      27.653  26.707  41.752  1.00  0.00
B    C
ATOM   5411  HB1 LYS B 131      26.885  27.407  42.153  1.00  0.00
B    H
ATOM   5412  HB2 LYS B 131      27.248  25.680  41.902  1.00  0.00
B    H
ATOM   5413  CG  LYS B 131      28.930  26.881  42.577  1.00  0.00
B    C
ATOM   5414  HG1 LYS B 131      29.398  27.860  42.322  1.00  0.00
B    H
ATOM   5415  HG2 LYS B 131      28.662  26.913  43.658  1.00  0.00
B    H
ATOM   5416  CD  LYS B 131      29.906  25.736  42.274  1.00  0.00
B    C
ATOM   5417  HD1 LYS B 131      29.686  24.893  42.969  1.00  0.00
B    H
ATOM   5418  HD2 LYS B 131      29.708  25.374  41.238  1.00  0.00
B    H
ATOM   5419  CE  LYS B 131      31.369  26.115  42.393  1.00  0.00
B    C
ATOM   5420  HE1 LYS B 131      32.016  25.312  41.975  1.00  0.00
B    H
ATOM   5421  HE2 LYS B 131      31.575  27.057  41.838  1.00  0.00
B    H
ATOM   5422  NZ  LYS B 131      31.722  26.318  43.856  1.00  0.00
B    N
ATOM   5423  HZ1 LYS B 131      32.725  26.577  43.948  1.00  0.00
B    H
ATOM   5424  HZ2 LYS B 131      31.131  27.076  44.253  1.00  0.00
B    H
ATOM   5425  HZ3 LYS B 131      31.544  25.437  44.380  1.00  0.00
B    H
ATOM   5426  C   LYS B 131      28.554  25.862  39.586  1.00  0.00
B    C
```

ATOM 5427 O LYS B 131 29.547 26.108 38.892 1.00 0.00
B O
ATOM 5428 N ILE B 132 28.124 24.630 39.807 1.00 0.00
B N
ATOM 5429 HN ILE B 132 27.242 24.382 40.202 1.00 0.00
B H
ATOM 5430 CA ILE B 132 28.964 23.496 39.468 1.00 0.00
B C
ATOM 5431 HA ILE B 132 29.936 23.681 39.908 1.00 0.00
B H
ATOM 5432 CB ILE B 132 28.356 22.202 40.027 1.00 0.00
B C
ATOM 5433 HB ILE B 132 28.340 22.356 41.136 1.00 0.00
B H
ATOM 5434 CG2 ILE B 132 27.001 21.962 39.433 1.00 0.00
B C
ATOM 5435 HG21 ILE B 132 26.493 21.119 39.946 1.00 0.00
B H
ATOM 5436 HG22 ILE B 132 26.356 22.859 39.543 1.00 0.00
B H
ATOM 5437 HG23 ILE B 132 27.078 21.716 38.354 1.00 0.00
B H
ATOM 5438 CG1 ILE B 132 29.292 21.031 39.753 1.00 0.00
B C
ATOM 5439 HG11 ILE B 132 28.986 20.517 38.815 1.00 0.00
B H
ATOM 5440 HG12 ILE B 132 30.322 21.422 39.592 1.00 0.00
B H
ATOM 5441 CD ILE B 132 29.333 20.041 40.866 1.00 0.00
B C
ATOM 5442 HD1 ILE B 132 30.071 19.239 40.642 1.00 0.00
B H
ATOM 5443 HD2 ILE B 132 29.631 20.532 41.817 1.00 0.00
B H
ATOM 5444 HD3 ILE B 132 28.342 19.563 41.011 1.00 0.00
B H
ATOM 5445 C ILE B 132 29.166 23.432 37.968 1.00 0.00
B C
ATOM 5446 O ILE B 132 30.212 22.988 37.478 1.00 0.00
B O
ATOM 5447 N ALA B 133 28.190 23.929 37.219 1.00 0.00
B N
ATOM 5448 HN ALA B 133 27.337 24.300 37.582 1.00 0.00
B H
ATOM 5449 CA ALA B 133 28.319 23.959 35.776 1.00 0.00
B C
ATOM 5450 HA ALA B 133 28.550 22.974 35.389 1.00 0.00
B H
ATOM 5451 CB ALA B 133 27.009 24.428 35.155 1.00 0.00
B C
ATOM 5452 HB1 ALA B 133 26.194 23.726 35.432 1.00 0.00
B H
ATOM 5453 HB2 ALA B 133 26.731 25.438 35.524 1.00 0.00
B H

ATOM 5454 HB3 ALA B 133 27.072 24.462 34.046 1.00 0.00
B H
ATOM 5455 C ALA B 133 29.463 24.860 35.360 1.00 0.00
B C
ATOM 5456 O ALA B 133 30.328 24.455 34.574 1.00 0.00
B O
ATOM 5457 N GLN B 134 29.495 26.085 35.886 1.00 0.00
B N
ATOM 5458 HN GLN B 134 28.819 26.487 36.501 1.00 0.00
B H
ATOM 5459 CA GLN B 134 30.609 26.961 35.561 1.00 0.00
B C
ATOM 5460 HA GLN B 134 30.651 27.039 34.481 1.00 0.00
B H
ATOM 5461 CB GLN B 134 30.395 28.336 36.177 1.00 0.00
B C
ATOM 5462 HB1 GLN B 134 30.479 28.210 37.282 1.00 0.00
B H
ATOM 5463 HB2 GLN B 134 31.231 29.009 35.876 1.00 0.00
B H
ATOM 5464 CG GLN B 134 29.054 28.920 35.821 1.00 0.00
B C
ATOM 5465 HG1 GLN B 134 28.334 28.112 35.572 1.00 0.00
B H
ATOM 5466 HG2 GLN B 134 28.639 29.496 36.673 1.00 0.00
B H
ATOM 5467 CD GLN B 134 29.121 29.821 34.611 1.00 0.00
B C
ATOM 5468 OE1 GLN B 134 30.181 29.998 34.012 1.00 0.00
B O
ATOM 5469 NE2 GLN B 134 27.981 30.391 34.234 1.00 0.00
B N
ATOM 5470 HE21 GLN B 134 28.017 30.983 33.434 1.00 0.00
B H
ATOM 5471 HE22 GLN B 134 27.150 30.227 34.757 1.00 0.00
B H
ATOM 5472 C GLN B 134 31.923 26.357 36.027 1.00 0.00
B C
ATOM 5473 O GLN B 134 32.933 26.423 35.319 1.00 0.00
B O
ATOM 5474 N SER B 135 31.921 25.733 37.207 1.00 0.00
B N
ATOM 5475 HN SER B 135 31.126 25.574 37.792 1.00 0.00
B H
ATOM 5476 CA SER B 135 33.174 25.212 37.745 1.00 0.00
B C
ATOM 5477 HA SER B 135 33.903 26.001 37.611 1.00 0.00
B H
ATOM 5478 CB SER B 135 33.016 24.855 39.220 1.00 0.00
B C
ATOM 5479 HB1 SER B 135 33.949 24.349 39.556 1.00 0.00
B H
ATOM 5480 HB2 SER B 135 32.905 25.779 39.833 1.00 0.00
B H

ATOM 5481 OG SER B 135 31.927 23.979 39.411 1.00 0.00
B O
ATOM 5482 HG1 SER B 135 32.008 23.616 40.302 1.00 0.00
B H
ATOM 5483 C SER B 135 33.667 24.003 36.965 1.00 0.00
B C
ATOM 5484 O SER B 135 34.861 23.694 36.997 1.00 0.00
B O
ATOM 5485 N LEU B 136 32.775 23.303 36.271 1.00 0.00
B N
ATOM 5486 HN LEU B 136 31.793 23.477 36.227 1.00 0.00
B H
ATOM 5487 CA LEU B 136 33.216 22.160 35.481 1.00 0.00
B C
ATOM 5488 HA LEU B 136 34.102 21.771 35.966 1.00 0.00
B H
ATOM 5489 CB LEU B 136 32.135 21.084 35.441 1.00 0.00
B C
ATOM 5490 HB1 LEU B 136 31.181 21.565 35.757 1.00 0.00
B H
ATOM 5491 HB2 LEU B 136 31.983 20.763 34.386 1.00 0.00
B H
ATOM 5492 CG LEU B 136 32.440 19.898 36.351 1.00 0.00
B C
ATOM 5493 HG LEU B 136 32.075 20.110 37.385 1.00 0.00
B H
ATOM 5494 CD1 LEU B 136 31.798 18.644 35.817 1.00 0.00
B C
ATOM 5495 HD11 LEU B 136 32.060 17.771 36.453 1.00 0.00
B H
ATOM 5496 HD12 LEU B 136 30.692 18.747 35.803 1.00 0.00
B H
ATOM 5497 HD13 LEU B 136 32.145 18.436 34.782 1.00 0.00
B H
ATOM 5498 CD2 LEU B 136 33.938 19.703 36.416 1.00 0.00
B C
ATOM 5499 HD21 LEU B 136 34.187 18.854 37.089 1.00 0.00
B H
ATOM 5500 HD22 LEU B 136 34.365 19.478 35.418 1.00 0.00
B H
ATOM 5501 HD23 LEU B 136 34.432 20.617 36.810 1.00 0.00
B H
ATOM 5502 C LEU B 136 33.610 22.579 34.076 1.00 0.00
B C
ATOM 5503 O LEU B 136 34.612 22.095 33.537 1.00 0.00
B O
ATOM 5504 N SER B 137 32.832 23.472 33.465 1.00 0.00
B N
ATOM 5505 HN SER B 137 32.013 23.888 33.861 1.00 0.00
B H
ATOM 5506 CA SER B 137 33.137 23.925 32.113 1.00 0.00
B C
ATOM 5507 HA SER B 137 33.031 23.059 31.475 1.00 0.00
B H

ATOM 5508 CB SER B 137 32.183 25.049 31.722 1.00 0.00
B C
ATOM 5509 HB1 SER B 137 32.390 25.326 30.663 1.00 0.00
B H
ATOM 5510 HB2 SER B 137 31.128 24.695 31.767 1.00 0.00
B H
ATOM 5511 OG SER B 137 32.394 26.178 32.550 1.00 0.00
B O
ATOM 5512 HG1 SER B 137 31.942 26.922 32.134 1.00 0.00
B H
ATOM 5513 C SER B 137 34.581 24.394 32.006 1.00 0.00
B C
ATOM 5514 O SER B 137 35.388 23.806 31.278 1.00 0.00
B O
ATOM 5515 N ASP B 138 34.932 25.432 32.748 1.00 0.00
B N
ATOM 5516 HN ASP B 138 34.310 25.934 33.345 1.00 0.00
B H
ATOM 5517 CA ASP B 138 36.291 25.958 32.770 1.00 0.00
B C
ATOM 5518 HA ASP B 138 36.817 25.613 31.888 1.00 0.00
B H
ATOM 5519 CB ASP B 138 36.275 27.478 32.861 1.00 0.00
B C
ATOM 5520 HB1 ASP B 138 35.552 27.790 33.641 1.00 0.00
B H
ATOM 5521 HB2 ASP B 138 37.276 27.863 33.140 1.00 0.00
B H
ATOM 5522 CG ASP B 138 35.853 28.136 31.566 1.00 0.00
B C
ATOM 5523 OD1 ASP B 138 36.696 28.268 30.654 1.00 0.00
B O
ATOM 5524 OD2 ASP B 138 34.670 28.519 31.460 1.00 0.00
B O
ATOM 5525 C ASP B 138 36.982 25.325 33.970 1.00 0.00
B C
ATOM 5526 O ASP B 138 36.766 25.735 35.112 1.00 0.00
B O
ATOM 5527 N GLU B 139 37.819 24.323 33.709 1.00 0.00
B N
ATOM 5528 HN GLU B 139 38.130 24.043 32.806 1.00 0.00
B H
ATOM 5529 CA GLU B 139 38.364 23.526 34.797 1.00 0.00
B C
ATOM 5530 HA GLU B 139 37.550 23.324 35.482 1.00 0.00
B H
ATOM 5531 CB GLU B 139 38.893 22.203 34.246 1.00 0.00
B C
ATOM 5532 HB1 GLU B 139 39.458 21.690 35.058 1.00 0.00
B H
ATOM 5533 HB2 GLU B 139 38.041 21.531 33.996 1.00 0.00
B H
ATOM 5534 CG GLU B 139 39.793 22.360 33.045 1.00 0.00
B C

```
ATOM   5535  HG1 GLU B 139      39.303  23.012  32.299  1.00  0.00
B    H
ATOM   5536  HG2 GLU B 139      40.767  22.804  33.334  1.00  0.00
B    H
ATOM   5537  CD  GLU B 139      40.048  21.049  32.343  1.00  0.00
B    C
ATOM   5538  OE1 GLU B 139      40.121  21.045  31.099  1.00  0.00
B    O
ATOM   5539  OE2 GLU B 139      40.166  20.020  33.035  1.00  0.00
B    O
ATOM   5540  C   GLU B 139      39.454  24.240  35.586  1.00  0.00
B    C
ATOM   5541  O   GLU B 139      40.142  23.589  36.378  1.00  0.00
B    O
ATOM   5542  N   ASN B 140      39.633  25.550  35.401  1.00  0.00
B    N
ATOM   5543  HN  ASN B 140      39.188  26.114  34.706  1.00  0.00
B    H
ATOM   5544  CA  ASN B 140      40.555  26.274  36.268  1.00  0.00
B    C
ATOM   5545  HA  ASN B 140      41.148  25.512  36.763  1.00  0.00
B    H
ATOM   5546  CB  ASN B 140      41.452  27.214  35.461  1.00  0.00
B    C
ATOM   5547  HB1 ASN B 140      41.418  26.888  34.398  1.00  0.00
B    H
ATOM   5548  HB2 ASN B 140      41.061  28.251  35.505  1.00  0.00
B    H
ATOM   5549  CG  ASN B 140      42.899  27.172  35.924  1.00  0.00
B    C
ATOM   5550  OD1 ASN B 140      43.647  28.137  35.754  1.00  0.00
B    O
ATOM   5551  ND2 ASN B 140      43.301  26.049  36.517  1.00  0.00
B    N
ATOM   5552 HD21 ASN B 140      44.244  26.012  36.836  1.00  0.00
B    H
ATOM   5553 HD22 ASN B 140      42.660  25.300  36.654  1.00  0.00
B    H
ATOM   5554  C   ASN B 140      39.820  27.058  37.344  1.00  0.00
B    C
ATOM   5555  O   ASN B 140      40.302  27.155  38.477  1.00  0.00
B    O
ATOM   5556  N   VAL B 141      38.664  27.633  37.020  1.00  0.00
B    N
ATOM   5557  HN  VAL B 141      38.249  27.666  36.114  1.00  0.00
B    H
ATOM   5558  CA  VAL B 141      37.890  28.294  38.063  1.00  0.00
B    C
ATOM   5559  HA  VAL B 141      38.534  28.959  38.625  1.00  0.00
B    H
ATOM   5560  CB  VAL B 141      36.717  29.102  37.492  1.00  0.00
B    C
ATOM   5561  HB  VAL B 141      36.960  30.186  37.620  1.00  0.00
B    H
```

ATOM 5562 CG1 VAL B 141 36.521 28.816 36.026 1.00 0.00
B C
ATOM 5563 HG11 VAL B 141 35.646 29.378 35.636 1.00 0.00
B H
ATOM 5564 HG12 VAL B 141 37.404 29.123 35.426 1.00 0.00
B H
ATOM 5565 HG13 VAL B 141 36.336 27.733 35.858 1.00 0.00
B H
ATOM 5566 CG2 VAL B 141 35.429 28.916 38.305 1.00 0.00
B C
ATOM 5567 HG21 VAL B 141 34.589 29.480 37.847 1.00 0.00
B H
ATOM 5568 HG22 VAL B 141 35.147 27.842 38.349 1.00 0.00
B H
ATOM 5569 HG23 VAL B 141 35.554 29.285 39.344 1.00 0.00
B H
ATOM 5570 C VAL B 141 37.419 27.200 39.017 1.00 0.00
B C
ATOM 5571 O VAL B 141 36.702 26.278 38.619 1.00 0.00
B O
ATOM 5572 N LEU B 142 37.910 27.243 40.251 1.00 0.00
B N
ATOM 5573 HN LEU B 142 38.577 27.900 40.595 1.00 0.00
B H
ATOM 5574 CA LEU B 142 37.470 26.265 41.236 1.00 0.00
B C
ATOM 5575 HA LEU B 142 37.209 25.364 40.694 1.00 0.00
B H
ATOM 5576 CB LEU B 142 38.589 25.974 42.241 1.00 0.00
B C
ATOM 5577 HB1 LEU B 142 39.488 25.674 41.654 1.00 0.00
B H
ATOM 5578 HB2 LEU B 142 38.862 26.918 42.761 1.00 0.00
B H
ATOM 5579 CG LEU B 142 38.244 24.873 43.239 1.00 0.00
B C
ATOM 5580 HG LEU B 142 37.324 25.189 43.789 1.00 0.00
B H
ATOM 5581 CD1 LEU B 142 37.977 23.578 42.503 1.00 0.00
B C
ATOM 5582 HD11 LEU B 142 37.772 22.756 43.222 1.00 0.00
B H
ATOM 5583 HD12 LEU B 142 37.097 23.683 41.832 1.00 0.00
B H
ATOM 5584 HD13 LEU B 142 38.856 23.288 41.888 1.00 0.00
B H
ATOM 5585 CD2 LEU B 142 39.357 24.687 44.255 1.00 0.00
B C
ATOM 5586 HD21 LEU B 142 39.085 23.895 44.986 1.00 0.00
B H
ATOM 5587 HD22 LEU B 142 40.308 24.385 43.771 1.00 0.00
B H
ATOM 5588 HD23 LEU B 142 39.535 25.630 44.815 1.00 0.00
B H

ATOM 5589 C LEU B 142 36.217 26.754 41.946 1.00 0.00
B C
ATOM 5590 O LEU B 142 35.174 26.095 41.908 1.00 0.00
B O
ATOM 5591 N ASN B 143 36.301 27.913 42.589 1.00 0.00
B N
ATOM 5592 HN ASN B 143 37.158 28.398 42.759 1.00 0.00
B H
ATOM 5593 CA ASN B 143 35.119 28.581 43.108 1.00 0.00
B C
ATOM 5594 HA ASN B 143 34.252 28.027 42.765 1.00 0.00
B H
ATOM 5595 CB ASN B 143 35.142 28.700 44.635 1.00 0.00
B C
ATOM 5596 HB1 ASN B 143 35.998 29.353 44.912 1.00 0.00
B H
ATOM 5597 HB2 ASN B 143 34.217 29.193 44.998 1.00 0.00
B H
ATOM 5598 CG ASN B 143 35.328 27.367 45.327 1.00 0.00
B C
ATOM 5599 OD1 ASN B 143 36.013 26.483 44.823 1.00 0.00
B O
ATOM 5600 ND2 ASN B 143 34.704 27.213 46.487 1.00 0.00
B N
ATOM 5601 HD21 ASN B 143 34.826 26.344 46.961 1.00 0.00
B H
ATOM 5602 HD22 ASN B 143 34.165 27.958 46.866 1.00 0.00
B H
ATOM 5603 C ASN B 143 35.062 29.960 42.475 1.00 0.00
B C
ATOM 5604 O ASN B 143 36.085 30.505 42.055 1.00 0.00
B O
ATOM 5605 N TYR B 144 33.863 30.528 42.416 1.00 0.00
B N
ATOM 5606 HN TYR B 144 33.012 30.145 42.769 1.00 0.00
B H
ATOM 5607 CA TYR B 144 33.722 31.833 41.787 1.00 0.00
B C
ATOM 5608 HA TYR B 144 34.620 32.399 42.008 1.00 0.00
B H
ATOM 5609 CB TYR B 144 33.517 31.707 40.277 1.00 0.00
B C
ATOM 5610 HB1 TYR B 144 33.647 32.707 39.808 1.00 0.00
B H
ATOM 5611 HB2 TYR B 144 34.280 31.033 39.832 1.00 0.00
B H
ATOM 5612 CG TYR B 144 32.148 31.226 39.870 1.00 0.00
B C
ATOM 5613 CD1 TYR B 144 31.758 29.916 40.084 1.00 0.00
B C
ATOM 5614 HD1 TYR B 144 32.448 29.218 40.538 1.00 0.00
B H
ATOM 5615 CE1 TYR B 144 30.508 29.473 39.709 1.00 0.00
B C

ATOM 5616 HE1 TYR B 144 30.215 28.447 39.877 1.00 0.00
B H
ATOM 5617 CZ TYR B 144 29.626 30.347 39.110 1.00 0.00
B C
ATOM 5618 OH TYR B 144 28.376 29.899 38.739 1.00 0.00
B O
ATOM 5619 HH TYR B 144 27.885 30.654 38.410 1.00 0.00
B H
ATOM 5620 CD2 TYR B 144 31.247 32.088 39.264 1.00 0.00
B C
ATOM 5621 HD2 TYR B 144 31.536 33.107 39.047 1.00 0.00
B H
ATOM 5622 CE2 TYR B 144 29.994 31.660 38.884 1.00 0.00
B C
ATOM 5623 HE2 TYR B 144 29.318 32.346 38.394 1.00 0.00
B H
ATOM 5624 C TYR B 144 32.560 32.569 42.429 1.00 0.00
B C
ATOM 5625 O TYR B 144 31.564 31.968 42.834 1.00 0.00
B O
ATOM 5626 N ILE B 145 32.704 33.889 42.504 1.00 0.00
B N
ATOM 5627 HN ILE B 145 33.536 34.360 42.219 1.00 0.00
B H
ATOM 5628 CA ILE B 145 31.661 34.772 43.010 1.00 0.00
B C
ATOM 5629 HA ILE B 145 30.714 34.262 42.891 1.00 0.00
B H
ATOM 5630 CB ILE B 145 31.886 35.158 44.486 1.00 0.00
B C
ATOM 5631 HB ILE B 145 32.798 34.583 44.789 1.00 0.00
B H
ATOM 5632 CG2 ILE B 145 32.073 36.668 44.649 1.00 0.00
B C
ATOM 5633 HG21 ILE B 145 32.387 36.913 45.686 1.00 0.00
B H
ATOM 5634 HG22 ILE B 145 32.857 37.047 43.962 1.00 0.00
B H
ATOM 5635 HG23 ILE B 145 31.128 37.210 44.437 1.00 0.00
B H
ATOM 5636 CG1 ILE B 145 30.721 34.636 45.315 1.00 0.00
B C
ATOM 5637 HG11 ILE B 145 30.071 33.988 44.684 1.00 0.00
B H
ATOM 5638 HG12 ILE B 145 31.119 34.000 46.138 1.00 0.00
B H
ATOM 5639 CD ILE B 145 29.867 35.720 45.939 1.00 0.00
B C
ATOM 5640 HD1 ILE B 145 29.075 35.268 46.574 1.00 0.00
B H
ATOM 5641 HD2 ILE B 145 30.486 36.387 46.577 1.00 0.00
B H
ATOM 5642 HD3 ILE B 145 29.373 36.336 45.158 1.00 0.00
B H

```
ATOM   5643  C   ILE B 145      31.609  36.010  42.123  1.00  0.00
B    C
ATOM   5644  O   ILE B 145      32.651  36.520  41.693  1.00  0.00
B    O
ATOM   5645  N   ASP B 146      30.397  36.456  41.802  1.00  0.00
B    N
ATOM   5646  HN  ASP B 146      29.542  36.043  42.104  1.00  0.00
B    H
ATOM   5647  CA  ASP B 146      30.195  37.624  40.953  1.00  0.00
B    C
ATOM   5648  HA  ASP B 146      31.171  38.039  40.729  1.00  0.00
B    H
ATOM   5649  CB  ASP B 146      29.438  37.234  39.687  1.00  0.00
B    C
ATOM   5650  HB1 ASP B 146      28.929  38.129  39.276  1.00  0.00
B    H
ATOM   5651  HB2 ASP B 146      30.135  36.846  38.920  1.00  0.00
B    H
ATOM   5652  CG  ASP B 146      28.375  36.188  39.946  1.00  0.00
B    C
ATOM   5653  OD1 ASP B 146      27.302  36.564  40.468  1.00  0.00
B    O
ATOM   5654  OD2 ASP B 146      28.619  34.991  39.677  1.00  0.00
B    O
ATOM   5655  C   ASP B 146      29.429  38.694  41.716  1.00  0.00
B    C
ATOM   5656  O   ASP B 146      28.346  38.427  42.243  1.00  0.00
B    O
ATOM   5657  N   LEU B 147      29.988  39.898  41.766  1.00  0.00
B    N
ATOM   5658  HN  LEU B 147      30.890  40.121  41.401  1.00  0.00
B    H
ATOM   5659  CA  LEU B 147      29.296  41.025  42.380  1.00  0.00
B    C
ATOM   5660  HA  LEU B 147      28.753  40.621  43.225  1.00  0.00
B    H
ATOM   5661  CB  LEU B 147      30.322  42.053  42.845  1.00  0.00
B    C
ATOM   5662  HB1 LEU B 147      29.803  43.038  42.891  1.00  0.00
B    H
ATOM   5663  HB2 LEU B 147      30.624  41.815  43.889  1.00  0.00
B    H
ATOM   5664  CG  LEU B 147      31.506  42.108  41.884  1.00  0.00
B    C
ATOM   5665  HG  LEU B 147      31.535  41.117  41.369  1.00  0.00
B    H
ATOM   5666  CD1 LEU B 147      31.325  43.197  40.842  1.00  0.00
B    C
ATOM   5667 HD11 LEU B 147      32.216  43.256  40.181  1.00  0.00
B    H
ATOM   5668 HD12 LEU B 147      30.434  42.990  40.212  1.00  0.00
B    H
ATOM   5669 HD13 LEU B 147      31.189  44.186  41.329  1.00  0.00
B    H
```

ATOM 5670 CD2 LEU B 147 32.803 42.269 42.667 1.00 0.00
B C
ATOM 5671 HD21 LEU B 147 33.672 42.292 41.975 1.00 0.00
B H
ATOM 5672 HD22 LEU B 147 32.815 43.212 43.251 1.00 0.00
B H
ATOM 5673 HD23 LEU B 147 32.941 41.420 43.371 1.00 0.00
B H
ATOM 5674 C LEU B 147 28.279 41.662 41.443 1.00 0.00
B C
ATOM 5675 O LEU B 147 27.311 42.271 41.912 1.00 0.00
B O
ATOM 5676 N SER B 148 28.465 41.527 40.137 1.00 0.00
B N
ATOM 5677 HN SER B 148 29.227 41.027 39.727 1.00 0.00
B H
ATOM 5678 CA SER B 148 27.547 42.111 39.171 1.00 0.00
B C
ATOM 5679 HA SER B 148 26.574 42.164 39.641 1.00 0.00
B H
ATOM 5680 CB SER B 148 28.034 43.513 38.763 1.00 0.00
B C
ATOM 5681 HB1 SER B 148 27.300 43.937 38.039 1.00 0.00
B H
ATOM 5682 HB2 SER B 148 28.050 44.191 39.646 1.00 0.00
B H
ATOM 5683 OG SER B 148 29.309 43.450 38.139 1.00 0.00
B O
ATOM 5684 HG1 SER B 148 29.457 44.303 37.711 1.00 0.00
B H
ATOM 5685 C SER B 148 27.428 41.157 37.989 1.00 0.00
B C
ATOM 5686 O SER B 148 27.991 40.054 38.006 1.00 0.00
B O
ATOM 5687 N ASP B 149 26.696 41.577 36.956 1.00 0.00
B N
ATOM 5688 HN ASP B 149 26.176 42.426 36.906 1.00 0.00
B H
ATOM 5689 CA ASP B 149 26.593 40.775 35.742 1.00 0.00
B C
ATOM 5690 HA ASP B 149 26.650 39.736 36.047 1.00 0.00
B H
ATOM 5691 CB ASP B 149 25.272 41.068 35.035 1.00 0.00
B C
ATOM 5692 HB1 ASP B 149 25.288 40.605 34.027 1.00 0.00
B H
ATOM 5693 HB2 ASP B 149 24.422 40.639 35.602 1.00 0.00
B H
ATOM 5694 CG ASP B 149 25.031 42.549 34.851 1.00 0.00
B C
ATOM 5695 OD1 ASP B 149 24.197 43.116 35.588 1.00 0.00
B O
ATOM 5696 OD2 ASP B 149 25.688 43.153 33.980 1.00 0.00
B O

ATOM 5697 C ASP B 149 27.767 41.022 34.806 1.00 0.00
B C
ATOM 5698 O ASP B 149 27.818 40.449 33.711 1.00 0.00
B O
ATOM 5699 N GLU B 150 28.714 41.860 35.218 1.00 0.00
B N
ATOM 5700 HN GLU B 150 28.685 42.309 36.107 1.00 0.00
B H
ATOM 5701 CA GLU B 150 29.883 42.203 34.419 1.00 0.00
B C
ATOM 5702 HA GLU B 150 29.789 41.675 33.478 1.00 0.00
B H
ATOM 5703 CB GLU B 150 29.925 43.713 34.168 1.00 0.00
B C
ATOM 5704 HB1 GLU B 150 28.971 44.009 33.673 1.00 0.00
B H
ATOM 5705 HB2 GLU B 150 29.946 44.257 35.140 1.00 0.00
B H
ATOM 5706 CG GLU B 150 31.089 44.155 33.303 1.00 0.00
B C
ATOM 5707 HG1 GLU B 150 31.677 44.924 33.838 1.00 0.00
B H
ATOM 5708 HG2 GLU B 150 31.748 43.299 33.056 1.00 0.00
B H
ATOM 5709 CD GLU B 150 30.660 44.805 32.011 1.00 0.00
B C
ATOM 5710 OE1 GLU B 150 29.520 44.556 31.569 1.00 0.00
B O
ATOM 5711 OE2 GLU B 150 31.465 45.567 31.437 1.00 0.00
B O
ATOM 5712 C GLU B 150 31.196 41.775 35.047 1.00 0.00
B C
ATOM 5713 O GLU B 150 32.122 41.390 34.330 1.00 0.00
B O
ATOM 5714 N TYR B 151 31.305 41.837 36.368 1.00 0.00
B N
ATOM 5715 HN TYR B 151 30.543 42.057 36.975 1.00 0.00
B H
ATOM 5716 CA TYR B 151 32.564 41.584 37.046 1.00 0.00
B C
ATOM 5717 HA TYR B 151 33.243 41.173 36.310 1.00 0.00
B H
ATOM 5718 CB TYR B 151 33.112 42.869 37.659 1.00 0.00
B C
ATOM 5719 HB1 TYR B 151 32.502 43.138 38.549 1.00 0.00
B H
ATOM 5720 HB2 TYR B 151 34.157 42.724 38.009 1.00 0.00
B H
ATOM 5721 CG TYR B 151 33.065 44.062 36.732 1.00 0.00
B C
ATOM 5722 CD1 TYR B 151 33.998 44.215 35.717 1.00 0.00
B C
ATOM 5723 HD1 TYR B 151 34.777 43.476 35.587 1.00 0.00
B H

ATOM 5724 CE1 TYR B 151 33.966 45.307 34.874 1.00 0.00
B C
ATOM 5725 HE1 TYR B 151 34.701 45.418 34.091 1.00 0.00
B H
ATOM 5726 CZ TYR B 151 32.990 46.263 35.040 1.00 0.00
B C
ATOM 5727 OH TYR B 151 32.950 47.351 34.202 1.00 0.00
B O
ATOM 5728 HH TYR B 151 32.168 47.860 34.427 1.00 0.00
B H
ATOM 5729 CD2 TYR B 151 32.093 45.040 36.879 1.00 0.00
B C
ATOM 5730 HD2 TYR B 151 31.375 44.971 37.684 1.00 0.00
B H
ATOM 5731 CE2 TYR B 151 32.052 46.134 36.040 1.00 0.00
B C
ATOM 5732 HE2 TYR B 151 31.298 46.893 36.186 1.00 0.00
B H
ATOM 5733 C TYR B 151 32.378 40.526 38.124 1.00 0.00
B C
ATOM 5734 O TYR B 151 31.263 40.302 38.605 1.00 0.00
B O
ATOM 5735 N SER B 152 33.480 39.881 38.501 1.00 0.00
B N
ATOM 5736 HN SER B 152 34.410 40.132 38.229 1.00 0.00
B H
ATOM 5737 CA SER B 152 33.407 38.717 39.370 1.00 0.00
B C
ATOM 5738 HA SER B 152 32.770 39.001 40.199 1.00 0.00
B H
ATOM 5739 CB SER B 152 32.861 37.510 38.607 1.00 0.00
B C
ATOM 5740 HB1 SER B 152 33.473 36.621 38.882 1.00 0.00
B H
ATOM 5741 HB2 SER B 152 31.814 37.294 38.918 1.00 0.00
B H
ATOM 5742 OG SER B 152 32.957 37.729 37.209 1.00 0.00
B O
ATOM 5743 HG1 SER B 152 32.818 36.876 36.778 1.00 0.00
B H
ATOM 5744 C SER B 152 34.774 38.379 39.945 1.00 0.00
B C
ATOM 5745 O SER B 152 35.813 38.841 39.456 1.00 0.00
B O
ATOM 5746 N ILE B 153 34.753 37.546 40.982 1.00 0.00
B N
ATOM 5747 HN ILE B 153 33.898 37.209 41.370 1.00 0.00
B H
ATOM 5748 CA ILE B 153 35.949 37.045 41.651 1.00 0.00
B C
ATOM 5749 HA ILE B 153 36.806 37.541 41.212 1.00 0.00
B H
ATOM 5750 CB ILE B 153 35.871 37.265 43.169 1.00 0.00
B C

```
ATOM   5751  HB  ILE B 153      34.846  36.912  43.450  1.00  0.00
B    H
ATOM   5752  CG2 ILE B 153      36.985  36.525  43.889  1.00  0.00
B    C
ATOM   5753 HG21 ILE B 153      36.831  36.560  44.988  1.00  0.00
B    H
ATOM   5754 HG22 ILE B 153      37.011  35.457  43.587  1.00  0.00
B    H
ATOM   5755 HG23 ILE B 153      37.973  36.977  43.663  1.00  0.00
B    H
ATOM   5756  CG1 ILE B 153      35.930  38.757  43.502  1.00  0.00
B    C
ATOM   5757 HG11 ILE B 153      36.960  39.031  43.820  1.00  0.00
B    H
ATOM   5758 HG12 ILE B 153      35.704  39.342  42.580  1.00  0.00
B    H
ATOM   5759  CD  ILE B 153      34.945  39.179  44.585  1.00  0.00
B    C
ATOM   5760  HD1 ILE B 153      34.992  40.279  44.743  1.00  0.00
B    H
ATOM   5761  HD2 ILE B 153      33.905  38.914  44.295  1.00  0.00
B    H
ATOM   5762  HD3 ILE B 153      35.178  38.684  45.551  1.00  0.00
B    H
ATOM   5763  C   ILE B 153      36.082  35.564  41.319  1.00  0.00
B    C
ATOM   5764  O   ILE B 153      35.077  34.848  41.238  1.00  0.00
B    O
ATOM   5765  N   VAL B 154      37.314  35.097  41.117  1.00  0.00
B    N
ATOM   5766  HN  VAL B 154      38.129  35.661  41.228  1.00  0.00
B    H
ATOM   5767  CA  VAL B 154      37.584  33.720  40.716  1.00  0.00
B    C
ATOM   5768  HA  VAL B 154      36.748  33.110  41.034  1.00  0.00
B    H
ATOM   5769  CB  VAL B 154      37.762  33.594  39.188  1.00  0.00
B    C
ATOM   5770  HB  VAL B 154      38.659  34.197  38.903  1.00  0.00
B    H
ATOM   5771  CG1 VAL B 154      37.990  32.138  38.801  1.00  0.00
B    C
ATOM   5772 HG11 VAL B 154      38.079  32.043  37.697  1.00  0.00
B    H
ATOM   5773 HG12 VAL B 154      38.925  31.737  39.244  1.00  0.00
B    H
ATOM   5774 HG13 VAL B 154      37.139  31.506  39.132  1.00  0.00
B    H
ATOM   5775  CG2 VAL B 154      36.551  34.149  38.448  1.00  0.00
B    C
ATOM   5776 HG21 VAL B 154      36.667  34.019  37.351  1.00  0.00
B    H
ATOM   5777 HG22 VAL B 154      35.624  33.623  38.765  1.00  0.00
B    H
```

ATOM 5778 HG23 VAL B 154 36.421 35.234 38.645 1.00 0.00
B H
ATOM 5779 C VAL B 154 38.826 33.206 41.434 1.00 0.00
B C
ATOM 5780 O VAL B 154 39.811 33.936 41.597 1.00 0.00
B O
ATOM 5781 N GLU B 155 38.781 31.941 41.852 1.00 0.00
B N
ATOM 5782 HN GLU B 155 37.937 31.415 41.915 1.00 0.00
B H
ATOM 5783 CA GLU B 155 39.971 31.202 42.257 1.00 0.00
B C
ATOM 5784 HA GLU B 155 40.787 31.894 42.420 1.00 0.00
B H
ATOM 5785 CB GLU B 155 39.733 30.367 43.514 1.00 0.00
B C
ATOM 5786 HB1 GLU B 155 38.702 29.947 43.462 1.00 0.00
B H
ATOM 5787 HB2 GLU B 155 40.419 29.490 43.520 1.00 0.00
B H
ATOM 5788 CG GLU B 155 39.875 31.116 44.809 1.00 0.00
B C
ATOM 5789 HG1 GLU B 155 40.766 30.750 45.351 1.00 0.00
B H
ATOM 5790 HG2 GLU B 155 39.978 32.204 44.630 1.00 0.00
B H
ATOM 5791 CD GLU B 155 38.704 30.885 45.735 1.00 0.00
B C
ATOM 5792 OE1 GLU B 155 38.914 30.323 46.832 1.00 0.00
B O
ATOM 5793 OE2 GLU B 155 37.573 31.246 45.347 1.00 0.00
B O
ATOM 5794 C GLU B 155 40.380 30.286 41.115 1.00 0.00
B C
ATOM 5795 O GLU B 155 39.533 29.601 40.536 1.00 0.00
B O
ATOM 5796 N LEU B 156 41.669 30.265 40.794 1.00 0.00
B N
ATOM 5797 HN LEU B 156 42.355 30.881 41.175 1.00 0.00
B H
ATOM 5798 CA LEU B 156 42.194 29.309 39.827 1.00 0.00
B C
ATOM 5799 HA LEU B 156 41.444 28.540 39.696 1.00 0.00
B H
ATOM 5800 CB LEU B 156 42.497 29.974 38.471 1.00 0.00
B C
ATOM 5801 HB1 LEU B 156 43.566 30.292 38.492 1.00 0.00
B H
ATOM 5802 HB2 LEU B 156 42.419 29.209 37.667 1.00 0.00
B H
ATOM 5803 CG LEU B 156 41.647 31.196 38.107 1.00 0.00
B C
ATOM 5804 HG LEU B 156 40.779 31.170 38.809 1.00 0.00
B H

ATOM 5805 CD1 LEU B 156 42.419 32.490 38.311 1.00 0.00
B C
ATOM 5806 HD11 LEU B 156 41.807 33.362 37.998 1.00 0.00
B H
ATOM 5807 HD12 LEU B 156 42.691 32.620 39.380 1.00 0.00
B H
ATOM 5808 HD13 LEU B 156 43.353 32.488 37.710 1.00 0.00
B H
ATOM 5809 CD2 LEU B 156 41.108 31.089 36.686 1.00 0.00
B C
ATOM 5810 HD21 LEU B 156 40.479 31.973 36.444 1.00 0.00
B H
ATOM 5811 HD22 LEU B 156 41.927 31.045 35.940 1.00 0.00
B H
ATOM 5812 HD23 LEU B 156 40.483 30.177 36.575 1.00 0.00
B H
ATOM 5813 C LEU B 156 43.443 28.643 40.381 1.00 0.00
B C
ATOM 5814 O LEU B 156 44.349 29.316 40.872 1.00 0.00
B O
ATOM 5815 N LEU B 157 43.497 27.317 40.303 1.00 0.00
B N
ATOM 5816 HN LEU B 157 42.784 26.729 39.925 1.00 0.00
B H
ATOM 5817 CA LEU B 157 44.665 26.600 40.793 1.00 0.00
B C
ATOM 5818 HA LEU B 157 45.043 27.133 41.656 1.00 0.00
B H
ATOM 5819 CB LEU B 157 44.309 25.167 41.186 1.00 0.00
B C
ATOM 5820 HB1 LEU B 157 44.275 25.130 42.299 1.00 0.00
B H
ATOM 5821 HB2 LEU B 157 43.276 24.942 40.839 1.00 0.00
B H
ATOM 5822 CG LEU B 157 45.206 24.006 40.751 1.00 0.00
B C
ATOM 5823 HG LEU B 157 46.199 24.406 40.433 1.00 0.00
B H
ATOM 5824 CD1 LEU B 157 45.386 23.047 41.912 1.00 0.00
B C
ATOM 5825 HD11 LEU B 157 45.995 22.171 41.604 1.00 0.00
B H
ATOM 5826 HD12 LEU B 157 45.902 23.550 42.758 1.00 0.00
B H
ATOM 5827 HD13 LEU B 157 44.402 22.677 42.272 1.00 0.00
B H
ATOM 5828 CD2 LEU B 157 44.586 23.285 39.562 1.00 0.00
B C
ATOM 5829 HD21 LEU B 157 45.243 22.452 39.230 1.00 0.00
B H
ATOM 5830 HD22 LEU B 157 43.597 22.854 39.815 1.00 0.00
B H
ATOM 5831 HD23 LEU B 157 44.455 23.985 38.708 1.00 0.00
B H

ATOM 5832 C LEU B 157 45.749 26.636 39.719 1.00 0.00
B C
ATOM 5833 O LEU B 157 45.459 26.469 38.528 1.00 0.00
B O
ATOM 5834 N ALA B 158 46.993 26.871 40.134 1.00 0.00
B N
ATOM 5835 HN ALA B 158 47.265 26.876 41.096 1.00 0.00
B H
ATOM 5836 CA ALA B 158 48.062 27.141 39.180 1.00 0.00
B C
ATOM 5837 HA ALA B 158 47.718 27.811 38.400 1.00 0.00
B H
ATOM 5838 CB ALA B 158 49.253 27.796 39.877 1.00 0.00
B C
ATOM 5839 HB1 ALA B 158 48.932 28.753 40.342 1.00 0.00
B H
ATOM 5840 HB2 ALA B 158 49.653 27.142 40.682 1.00 0.00
B H
ATOM 5841 HB3 ALA B 158 50.074 28.019 39.162 1.00 0.00
B H
ATOM 5842 C ALA B 158 48.506 25.864 38.483 1.00 0.00
B C
ATOM 5843 O ALA B 158 48.726 24.834 39.125 1.00 0.00
B O
ATOM 5844 N THR B 159 48.619 25.932 37.162 1.00 0.00
B N
ATOM 5845 HN THR B 159 48.360 26.724 36.610 1.00 0.00
B H
ATOM 5846 CA THR B 159 49.147 24.818 36.396 1.00 0.00
B C
ATOM 5847 HA THR B 159 48.995 23.934 37.002 1.00 0.00
B H
ATOM 5848 CB THR B 159 48.449 24.721 35.040 1.00 0.00
B C
ATOM 5849 HB THR B 159 47.353 24.702 35.256 1.00 0.00
B H
ATOM 5850 OG1 THR B 159 48.796 23.484 34.404 1.00 0.00
B O
ATOM 5851 HG1 THR B 159 48.336 23.492 33.558 1.00 0.00
B H
ATOM 5852 CG2 THR B 159 48.849 25.884 34.149 1.00 0.00
B C
ATOM 5853 HG21 THR B 159 48.260 25.882 33.208 1.00 0.00
B H
ATOM 5854 HG22 THR B 159 48.663 26.850 34.666 1.00 0.00
B H
ATOM 5855 HG23 THR B 159 49.927 25.834 33.884 1.00 0.00
B H
ATOM 5856 C THR B 159 50.656 24.967 36.216 1.00 0.00
B C
ATOM 5857 O THR B 159 51.281 25.916 36.698 1.00 0.00
B O
ATOM 5858 N ARG B 160 51.245 24.000 35.511 1.00 0.00
B N

ATOM 5859 HN ARG B 160 50.740 23.230 35.128 1.00 0.00
B H
ATOM 5860 CA ARG B 160 52.681 24.030 35.263 1.00 0.00
B C
ATOM 5861 HA ARG B 160 53.181 24.142 36.218 1.00 0.00
B H
ATOM 5862 CB ARG B 160 53.122 22.731 34.595 1.00 0.00
B C
ATOM 5863 HB1 ARG B 160 52.558 21.910 35.100 1.00 0.00
B H
ATOM 5864 HB2 ARG B 160 52.787 22.725 33.534 1.00 0.00
B H
ATOM 5865 CG ARG B 160 54.603 22.453 34.714 1.00 0.00
B C
ATOM 5866 HG1 ARG B 160 55.153 23.412 34.596 1.00 0.00
B H
ATOM 5867 HG2 ARG B 160 54.825 22.106 35.749 1.00 0.00
B H
ATOM 5868 CD ARG B 160 55.021 21.389 33.724 1.00 0.00
B C
ATOM 5869 HD1 ARG B 160 56.125 21.426 33.583 1.00 0.00
B H
ATOM 5870 HD2 ARG B 160 54.740 20.369 34.076 1.00 0.00
B H
ATOM 5871 NE ARG B 160 54.367 21.582 32.432 1.00 0.00
B N
ATOM 5872 HE ARG B 160 53.617 20.932 32.267 1.00 0.00
B H
ATOM 5873 CZ ARG B 160 54.709 22.513 31.549 1.00 0.00
B C
ATOM 5874 NH1 ARG B 160 55.704 23.350 31.809 1.00 0.00
B N
ATOM 5875 HH11 ARG B 160 55.946 24.062 31.164 1.00 0.00
B H
ATOM 5876 HH12 ARG B 160 56.092 23.306 32.733 1.00 0.00
B H
ATOM 5877 NH2 ARG B 160 54.053 22.611 30.402 1.00 0.00
B N
ATOM 5878 HH21 ARG B 160 54.363 23.246 29.709 1.00 0.00
B H
ATOM 5879 HH22 ARG B 160 53.400 21.895 30.198 1.00 0.00
B H
ATOM 5880 C ARG B 160 53.094 25.224 34.413 1.00 0.00
B C
ATOM 5881 O ARG B 160 54.250 25.653 34.486 1.00 0.00
B O
ATOM 5882 N LYS B 161 52.176 25.772 33.613 1.00 0.00
B N
ATOM 5883 HN LYS B 161 51.271 25.376 33.468 1.00 0.00
B H
ATOM 5884 CA LYS B 161 52.435 27.007 32.883 1.00 0.00
B C
ATOM 5885 HA LYS B 161 53.350 26.849 32.326 1.00 0.00
B H

```
ATOM    5886  CB  LYS B 161      51.271  27.317  31.941  1.00  0.00
B    C
ATOM    5887  HB1 LYS B 161      50.379  27.521  32.578  1.00  0.00
B    H
ATOM    5888  HB2 LYS B 161      51.488  28.263  31.396  1.00  0.00
B    H
ATOM    5889  CG  LYS B 161      50.938  26.205  30.970  1.00  0.00
B    C
ATOM    5890  HG1 LYS B 161      50.921  25.236  31.519  1.00  0.00
B    H
ATOM    5891  HG2 LYS B 161      49.916  26.375  30.562  1.00  0.00
B    H
ATOM    5892  CD  LYS B 161      51.964  26.144  29.860  1.00  0.00
B    C
ATOM    5893  HD1 LYS B 161      52.953  25.893  30.308  1.00  0.00
B    H
ATOM    5894  HD2 LYS B 161      51.686  25.308  29.177  1.00  0.00
B    H
ATOM    5895  CE  LYS B 161      52.016  27.462  29.110  1.00  0.00
B    C
ATOM    5896  HE1 LYS B 161      51.013  27.718  28.701  1.00  0.00
B    H
ATOM    5897  HE2 LYS B 161      52.341  28.284  29.786  1.00  0.00
B    H
ATOM    5898  NZ  LYS B 161      52.965  27.420  27.968  1.00  0.00
B    N
ATOM    5899  HZ1 LYS B 161      52.972  28.338  27.478  1.00  0.00
B    H
ATOM    5900  HZ2 LYS B 161      53.920  27.207  28.319  1.00  0.00
B    H
ATOM    5901  HZ3 LYS B 161      52.671  26.677  27.303  1.00  0.00
B    H
ATOM    5902  C   LYS B 161      52.664  28.194  33.805  1.00  0.00
B    C
ATOM    5903  O   LYS B 161      52.939  29.293  33.313  1.00  0.00
B    O
ATOM    5904  N   LEU B 162      52.554  28.003  35.121  1.00  0.00
B    N
ATOM    5905  HN  LEU B 162      52.422  27.105  35.538  1.00  0.00
B    H
ATOM    5906  CA  LEU B 162      52.616  29.098  36.072  1.00  0.00
B    C
ATOM    5907  HA  LEU B 162      52.903  29.966  35.491  1.00  0.00
B    H
ATOM    5908  CB  LEU B 162      51.245  29.292  36.736  1.00  0.00
B    C
ATOM    5909  HB1 LEU B 162      50.918  28.296  37.113  1.00  0.00
B    H
ATOM    5910  HB2 LEU B 162      51.367  29.936  37.636  1.00  0.00
B    H
ATOM    5911  CG  LEU B 162      50.109  29.849  35.875  1.00  0.00
B    C
ATOM    5912  HG  LEU B 162      49.769  29.011  35.222  1.00  0.00
B    H
```

```
ATOM   5913  CD1 LEU B 162      48.963  30.295  36.765  1.00  0.00
B    C
ATOM   5914 HD11 LEU B 162      48.150  30.745  36.156  1.00  0.00
B    H
ATOM   5915 HD12 LEU B 162      48.542  29.430  37.323  1.00  0.00
B    H
ATOM   5916 HD13 LEU B 162      49.308  31.053  37.500  1.00  0.00
B    H
ATOM   5917  CD2 LEU B 162      50.577  30.987  34.976  1.00  0.00
B    C
ATOM   5918 HD21 LEU B 162      49.735  31.357  34.351  1.00  0.00
B    H
ATOM   5919 HD22 LEU B 162      50.960  31.845  35.565  1.00  0.00
B    H
ATOM   5920 HD23 LEU B 162      51.386  30.640  34.298  1.00  0.00
B    H
ATOM   5921  C   LEU B 162      53.657  28.915  37.167  1.00  0.00
B    C
ATOM   5922  O   LEU B 162      53.716  29.753  38.075  1.00  0.00
B    O
ATOM   5923  N   ASP B 163      54.473  27.865  37.114  1.00  0.00
B    N
ATOM   5924  HN  ASP B 163      54.544  27.220  36.357  1.00  0.00
B    H
ATOM   5925  CA  ASP B 163      55.377  27.552  38.213  1.00  0.00
B    C
ATOM   5926  HA  ASP B 163      54.794  27.598  39.127  1.00  0.00
B    H
ATOM   5927  CB  ASP B 163      55.992  26.165  38.006  1.00  0.00
B    C
ATOM   5928  HB1 ASP B 163      55.188  25.443  37.756  1.00  0.00
B    H
ATOM   5929  HB2 ASP B 163      56.718  26.181  37.170  1.00  0.00
B    H
ATOM   5930  CG  ASP B 163      56.701  25.640  39.247  1.00  0.00
B    C
ATOM   5931  OD1 ASP B 163      56.930  26.423  40.194  1.00  0.00
B    O
ATOM   5932  OD2 ASP B 163      57.040  24.437  39.268  1.00  0.00
B    O
ATOM   5933  C   ASP B 163      56.473  28.601  38.338  1.00  0.00
B    C
ATOM   5934  O   ASP B 163      57.015  29.073  37.336  1.00  0.00
B    O
ATOM   5935  N   SER B 164      56.800  28.948  39.585  1.00  0.00
B    N
ATOM   5936  HN  SER B 164      56.296  28.613  40.381  1.00  0.00
B    H
ATOM   5937  CA  SER B 164      57.912  29.841  39.911  1.00  0.00
B    C
ATOM   5938  HA  SER B 164      57.826  30.050  40.969  1.00  0.00
B    H
ATOM   5939  CB  SER B 164      59.252  29.171  39.610  1.00  0.00
B    C
```

ATOM 5940 HB1 SER B 164 59.532 29.415 38.559 1.00 0.00
B H
ATOM 5941 HB2 SER B 164 60.050 29.586 40.266 1.00 0.00
B H
ATOM 5942 OG SER B 164 59.154 27.763 39.744 1.00 0.00
B O
ATOM 5943 HG1 SER B 164 59.941 27.383 39.333 1.00 0.00
B H
ATOM 5944 C SER B 164 57.790 31.170 39.175 1.00 0.00
B C
ATOM 5945 O SER B 164 58.780 31.785 38.773 1.00 0.00
B O
ATOM 5946 N LYS B 165 56.555 31.616 38.998 1.00 0.00
B N
ATOM 5947 HN LYS B 165 55.755 31.128 39.344 1.00 0.00
B H
ATOM 5948 CA LYS B 165 56.264 32.849 38.287 1.00 0.00
B C
ATOM 5949 HA LYS B 165 57.152 33.131 37.735 1.00 0.00
B H
ATOM 5950 CB LYS B 165 55.079 32.663 37.338 1.00 0.00
B C
ATOM 5951 HB1 LYS B 165 54.229 32.278 37.947 1.00 0.00
B H
ATOM 5952 HB2 LYS B 165 54.763 33.659 36.954 1.00 0.00
B H
ATOM 5953 CG LYS B 165 55.365 31.700 36.199 1.00 0.00
B C
ATOM 5954 HG1 LYS B 165 55.491 30.674 36.613 1.00 0.00
B H
ATOM 5955 HG2 LYS B 165 54.486 31.677 35.515 1.00 0.00
B H
ATOM 5956 CD LYS B 165 56.619 32.085 35.435 1.00 0.00
B C
ATOM 5957 HD1 LYS B 165 56.469 33.099 35.001 1.00 0.00
B H
ATOM 5958 HD2 LYS B 165 57.461 32.154 36.162 1.00 0.00
B H
ATOM 5959 CE LYS B 165 56.928 31.067 34.351 1.00 0.00
B C
ATOM 5960 HE1 LYS B 165 57.180 30.081 34.802 1.00 0.00
B H
ATOM 5961 HE2 LYS B 165 56.051 30.932 33.679 1.00 0.00
B H
ATOM 5962 NZ LYS B 165 58.081 31.487 33.512 1.00 0.00
B N
ATOM 5963 HZ1 LYS B 165 58.269 30.770 32.780 1.00 0.00
B H
ATOM 5964 HZ2 LYS B 165 57.865 32.397 33.056 1.00 0.00
B H
ATOM 5965 HZ3 LYS B 165 58.923 31.596 34.112 1.00 0.00
B H
ATOM 5966 C LYS B 165 55.981 33.959 39.290 1.00 0.00
B C

```
ATOM   5967  O   LYS B 165      55.812  33.707  40.484  1.00  0.00
B    O
ATOM   5968  N   SER B 166      55.982  35.196  38.808  1.00  0.00
B    N
ATOM   5969  HN  SER B 166      56.172  35.429  37.855  1.00  0.00
B    H
ATOM   5970  CA  SER B 166      55.699  36.344  39.657  1.00  0.00
B    C
ATOM   5971  HA  SER B 166      55.430  35.939  40.624  1.00  0.00
B    H
ATOM   5972  CB  SER B 166      56.904  37.275  39.749  1.00  0.00
B    C
ATOM   5973  HB1 SER B 166      57.609  36.856  40.502  1.00  0.00
B    H
ATOM   5974  HB2 SER B 166      57.446  37.305  38.776  1.00  0.00
B    H
ATOM   5975  OG  SER B 166      56.475  38.557  40.160  1.00  0.00
B    O
ATOM   5976  HG1 SER B 166      57.262  39.049  40.422  1.00  0.00
B    H
ATOM   5977  C   SER B 166      54.501  37.129  39.147  1.00  0.00
B    C
ATOM   5978  O   SER B 166      54.097  37.004  37.988  1.00  0.00
B    O
ATOM   5979  N   ILE B 167      53.938  37.953  40.034  1.00  0.00
B    N
ATOM   5980  HN  ILE B 167      54.169  37.997  41.003  1.00  0.00
B    H
ATOM   5981  CA  ILE B 167      52.898  38.888  39.623  1.00  0.00
B    C
ATOM   5982  HA  ILE B 167      52.172  38.319  39.054  1.00  0.00
B    H
ATOM   5983  CB  ILE B 167      52.249  39.548  40.851  1.00  0.00
B    C
ATOM   5984  HB  ILE B 167      53.100  39.716  41.561  1.00  0.00
B    H
ATOM   5985  CG2 ILE B 167      51.519  40.817  40.447  1.00  0.00
B    C
ATOM   5986 HG21 ILE B 167      51.184  41.378  41.344  1.00  0.00
B    H
ATOM   5987 HG22 ILE B 167      52.183  41.486  39.860  1.00  0.00
B    H
ATOM   5988 HG23 ILE B 167      50.626  40.582  39.831  1.00  0.00
B    H
ATOM   5989  CG1 ILE B 167      51.289  38.578  41.534  1.00  0.00
B    C
ATOM   5990 HG11 ILE B 167      50.360  39.114  41.830  1.00  0.00
B    H
ATOM   5991 HG12 ILE B 167      50.995  37.788  40.804  1.00  0.00
B    H
ATOM   5992  CD  ILE B 167      51.877  37.908  42.740  1.00  0.00
B    C
ATOM   5993  HD1 ILE B 167      51.158  37.172  43.162  1.00  0.00
B    H
```

ATOM 5994 HD2 ILE B 167 52.811 37.368 42.473 1.00 0.00
B H
ATOM 5995 HD3 ILE B 167 52.113 38.650 43.532 1.00 0.00
B H
ATOM 5996 C ILE B 167 53.474 39.933 38.680 1.00 0.00
B C
ATOM 5997 O ILE B 167 52.894 40.237 37.632 1.00 0.00
B O
ATOM 5998 N ILE B 168 54.628 40.500 39.038 1.00 0.00
B N
ATOM 5999 HN ILE B 168 55.110 40.358 39.901 1.00 0.00
B H
ATOM 6000 CA ILE B 168 55.296 41.421 38.127 1.00 0.00
B C
ATOM 6001 HA ILE B 168 54.542 42.117 37.782 1.00 0.00
B H
ATOM 6002 CB ILE B 168 56.432 42.173 38.840 1.00 0.00
B C
ATOM 6003 HB ILE B 168 55.970 42.542 39.791 1.00 0.00
B H
ATOM 6004 CG2 ILE B 168 57.628 41.265 39.062 1.00 0.00
B C
ATOM 6005 HG21 ILE B 168 58.380 41.760 39.710 1.00 0.00
B H
ATOM 6006 HG22 ILE B 168 57.322 40.321 39.561 1.00 0.00
B H
ATOM 6007 HG23 ILE B 168 58.116 41.009 38.097 1.00 0.00
B H
ATOM 6008 CG1 ILE B 168 56.834 43.407 38.031 1.00 0.00
B C
ATOM 6009 HG11 ILE B 168 57.897 43.664 38.240 1.00 0.00
B H
ATOM 6010 HG12 ILE B 168 56.761 43.166 36.947 1.00 0.00
B H
ATOM 6011 CD ILE B 168 55.964 44.614 38.312 1.00 0.00
B C
ATOM 6012 HD1 ILE B 168 56.260 45.463 37.658 1.00 0.00
B H
ATOM 6013 HD2 ILE B 168 54.896 44.381 38.116 1.00 0.00
B H
ATOM 6014 HD3 ILE B 168 56.066 44.942 39.368 1.00 0.00
B H
ATOM 6015 C ILE B 168 55.806 40.683 36.904 1.00 0.00
B C
ATOM 6016 O ILE B 168 55.988 41.283 35.840 1.00 0.00
B O
ATOM 6017 N ASP B 169 56.021 39.376 37.022 1.00 0.00
B N
ATOM 6018 HN ASP B 169 55.966 38.833 37.856 1.00 0.00
B H
ATOM 6019 CA ASP B 169 56.379 38.586 35.856 1.00 0.00
B C
ATOM 6020 HA ASP B 169 57.175 39.110 35.339 1.00 0.00
B H

```
ATOM   6021  CB  ASP B 169      56.807  37.187  36.300  1.00  0.00
B    C
ATOM   6022  HB1 ASP B 169      56.758  37.124  37.406  1.00  0.00
B    H
ATOM   6023  HB2 ASP B 169      56.129  36.418  35.878  1.00  0.00
B    H
ATOM   6024  CG  ASP B 169      58.223  36.852  35.891  1.00  0.00
B    C
ATOM   6025  OD1 ASP B 169      59.141  37.537  36.388  1.00  0.00
B    O
ATOM   6026  OD2 ASP B 169      58.420  35.911  35.088  1.00  0.00
B    O
ATOM   6027  C   ASP B 169      55.202  38.512  34.891  1.00  0.00
B    C
ATOM   6028  O   ASP B 169      55.237  39.076  33.791  1.00  0.00
B    O
ATOM   6029  N   LEU B 170      54.134  37.842  35.309  1.00  0.00
B    N
ATOM   6030  HN  LEU B 170      54.090  37.370  36.187  1.00  0.00
B    H
ATOM   6031  CA  LEU B 170      52.917  37.735  34.510  1.00  0.00
B    C
ATOM   6032  HA  LEU B 170      53.167  38.022  33.496  1.00  0.00
B    H
ATOM   6033  CB  LEU B 170      52.399  36.302  34.534  1.00  0.00
B    C
ATOM   6034  HB1 LEU B 170      52.426  35.960  35.594  1.00  0.00
B    H
ATOM   6035  HB2 LEU B 170      51.326  36.300  34.239  1.00  0.00
B    H
ATOM   6036  CG  LEU B 170      53.183  35.300  33.682  1.00  0.00
B    C
ATOM   6037  HG  LEU B 170      53.405  35.820  32.720  1.00  0.00
B    H
ATOM   6038  CD1 LEU B 170      54.487  34.881  34.356  1.00  0.00
B    C
ATOM   6039 HD11 LEU B 170      55.014  34.119  33.743  1.00  0.00
B    H
ATOM   6040 HD12 LEU B 170      55.161  35.755  34.482  1.00  0.00
B    H
ATOM   6041 HD13 LEU B 170      54.288  34.444  35.357  1.00  0.00
B    H
ATOM   6042  CD2 LEU B 170      52.336  34.077  33.371  1.00  0.00
B    C
ATOM   6043 HD21 LEU B 170      52.907  33.364  32.738  1.00  0.00
B    H
ATOM   6044 HD22 LEU B 170      52.036  33.541  34.294  1.00  0.00
B    H
ATOM   6045 HD23 LEU B 170      51.416  34.371  32.822  1.00  0.00
B    H
ATOM   6046  C   LEU B 170      51.879  38.730  35.028  1.00  0.00
B    C
ATOM   6047  O   LEU B 170      50.948  38.389  35.758  1.00  0.00
B    O
```

```
ATOM   6048  N   ASN B 171      52.076  39.991  34.649  1.00  0.00
B    N
ATOM   6049  HN  ASN B 171      52.814  40.291  34.045  1.00  0.00
B    H
ATOM   6050  CA  ASN B 171      51.196  41.059  35.104  1.00  0.00
B    C
ATOM   6051  HA  ASN B 171      51.155  40.971  36.184  1.00  0.00
B    H
ATOM   6052  CB  ASN B 171      51.737  42.422  34.674  1.00  0.00
B    C
ATOM   6053  HB1 ASN B 171      50.977  43.188  34.940  1.00  0.00
B    H
ATOM   6054  HB2 ASN B 171      52.663  42.664  35.233  1.00  0.00
B    H
ATOM   6055  CG  ASN B 171      51.986  42.507  33.187  1.00  0.00
B    C
ATOM   6056  OD1 ASN B 171      51.844  41.525  32.464  1.00  0.00
B    O
ATOM   6057  ND2 ASN B 171      52.359  43.691  32.719  1.00  0.00
B    N
ATOM   6058 HD21 ASN B 171      52.517  43.767  31.738  1.00  0.00
B    H
ATOM   6059 HD22 ASN B 171      52.442  44.467  33.337  1.00  0.00
B    H
ATOM   6060  C   ASN B 171      49.781  40.863  34.570  1.00  0.00
B    C
ATOM   6061  O   ASN B 171      49.567  40.240  33.527  1.00  0.00
B    O
ATOM   6062  N   VAL B 172      48.808  41.381  35.316  1.00  0.00
B    N
ATOM   6063  HN  VAL B 172      48.965  41.868  36.171  1.00  0.00
B    H
ATOM   6064  CA  VAL B 172      47.408  41.274  34.934  1.00  0.00
B    C
ATOM   6065  HA  VAL B 172      47.386  40.920  33.911  1.00  0.00
B    H
ATOM   6066  CB  VAL B 172      46.676  40.252  35.826  1.00  0.00
B    C
ATOM   6067  HB  VAL B 172      47.351  39.370  35.953  1.00  0.00
B    H
ATOM   6068  CG1 VAL B 172      46.370  40.843  37.196  1.00  0.00
B    C
ATOM   6069 HG11 VAL B 172      45.808  40.111  37.816  1.00  0.00
B    H
ATOM   6070 HG12 VAL B 172      47.298  41.101  37.748  1.00  0.00
B    H
ATOM   6071 HG13 VAL B 172      45.748  41.759  37.098  1.00  0.00
B    H
ATOM   6072  CG2 VAL B 172      45.399  39.760  35.150  1.00  0.00
B    C
ATOM   6073 HG21 VAL B 172      44.852  39.054  35.811  1.00  0.00
B    H
ATOM   6074 HG22 VAL B 172      44.727  40.614  34.918  1.00  0.00
B    H
```

ATOM 6075 HG23 VAL B 172 45.625 39.227 34.203 1.00 0.00
B H
ATOM 6076 C VAL B 172 46.699 42.624 34.965 1.00 0.00
B C
ATOM 6077 O VAL B 172 45.683 42.800 34.285 1.00 0.00
B O
ATOM 6078 N ARG B 173 47.206 43.586 35.741 1.00 0.00
B N
ATOM 6079 HN ARG B 173 48.031 43.471 36.288 1.00 0.00
B H
ATOM 6080 CA ARG B 173 46.546 44.884 35.817 1.00 0.00
B C
ATOM 6081 HA ARG B 173 45.480 44.718 35.909 1.00 0.00
B H
ATOM 6082 CB ARG B 173 47.052 45.665 37.025 1.00 0.00
B C
ATOM 6083 HB1 ARG B 173 47.399 44.907 37.768 1.00 0.00
B H
ATOM 6084 HB2 ARG B 173 47.953 46.250 36.740 1.00 0.00
B H
ATOM 6085 CG ARG B 173 45.983 46.532 37.653 1.00 0.00
B C
ATOM 6086 HG1 ARG B 173 46.059 47.555 37.223 1.00 0.00
B H
ATOM 6087 HG2 ARG B 173 44.982 46.153 37.346 1.00 0.00
B H
ATOM 6088 CD ARG B 173 46.069 46.520 39.171 1.00 0.00
B C
ATOM 6089 HD1 ARG B 173 45.043 46.454 39.600 1.00 0.00
B H
ATOM 6090 HD2 ARG B 173 46.667 45.653 39.540 1.00 0.00
B H
ATOM 6091 NE ARG B 173 46.720 47.715 39.705 1.00 0.00
B N
ATOM 6092 HE ARG B 173 47.638 47.534 40.075 1.00 0.00
B H
ATOM 6093 CZ ARG B 173 46.175 48.928 39.714 1.00 0.00
B C
ATOM 6094 NH1 ARG B 173 44.961 49.122 39.215 1.00 0.00
B N
ATOM 6095 HH11 ARG B 173 44.563 50.028 39.196 1.00 0.00
B H
ATOM 6096 HH12 ARG B 173 44.544 48.341 38.745 1.00 0.00
B H
ATOM 6097 NH2 ARG B 173 46.844 49.953 40.225 1.00 0.00
B N
ATOM 6098 HH21 ARG B 173 46.405 50.836 40.309 1.00 0.00
B H
ATOM 6099 HH22 ARG B 173 47.691 49.745 40.696 1.00 0.00
B H
ATOM 6100 C ARG B 173 46.753 45.690 34.543 1.00 0.00
B C
ATOM 6101 O ARG B 173 45.794 46.210 33.966 1.00 0.00
B O

ATOM 6102 N ALA B 174 47.999 45.813 34.094 1.00 0.00
B N
ATOM 6103 HN ALA B 174 48.804 45.478 34.582 1.00 0.00
B H
ATOM 6104 CA ALA B 174 48.267 46.466 32.821 1.00 0.00
B C
ATOM 6105 HA ALA B 174 47.617 47.325 32.693 1.00 0.00
B H
ATOM 6106 CB ALA B 174 49.714 46.958 32.776 1.00 0.00
B C
ATOM 6107 HB1 ALA B 174 49.891 47.673 33.606 1.00 0.00
B H
ATOM 6108 HB2 ALA B 174 50.426 46.113 32.894 1.00 0.00
B H
ATOM 6109 HB3 ALA B 174 49.939 47.479 31.820 1.00 0.00
B H
ATOM 6110 C ALA B 174 47.988 45.538 31.650 1.00 0.00
B C
ATOM 6111 O ALA B 174 47.734 45.998 30.532 1.00 0.00
B O
ATOM 6112 N LYS B 175 48.015 44.226 31.894 1.00 0.00
B N
ATOM 6113 HN LYS B 175 48.140 43.826 32.801 1.00 0.00
B H
ATOM 6114 CA LYS B 175 47.857 43.265 30.811 1.00 0.00
B C
ATOM 6115 HA LYS B 175 48.237 43.737 29.913 1.00 0.00
B H
ATOM 6116 CB LYS B 175 48.647 41.999 31.130 1.00 0.00
B C
ATOM 6117 HB1 LYS B 175 49.584 42.318 31.642 1.00 0.00
B H
ATOM 6118 HB2 LYS B 175 48.077 41.393 31.871 1.00 0.00
B H
ATOM 6119 CG LYS B 175 49.004 41.166 29.923 1.00 0.00
B C
ATOM 6120 HG1 LYS B 175 48.087 40.661 29.545 1.00 0.00
B H
ATOM 6121 HG2 LYS B 175 49.371 41.839 29.114 1.00 0.00
B H
ATOM 6122 CD LYS B 175 50.048 40.130 30.288 1.00 0.00
B C
ATOM 6123 HD1 LYS B 175 50.918 40.654 30.749 1.00 0.00
B H
ATOM 6124 HD2 LYS B 175 49.614 39.459 31.065 1.00 0.00
B H
ATOM 6125 CE LYS B 175 50.481 39.329 29.077 1.00 0.00
B C
ATOM 6126 HE1 LYS B 175 49.646 38.687 28.716 1.00 0.00
B H
ATOM 6127 HE2 LYS B 175 50.787 40.006 28.249 1.00 0.00
B H
ATOM 6128 NZ LYS B 175 51.633 38.443 29.390 1.00 0.00
B N

```
ATOM   6129  HZ1 LYS B 175      51.909  37.904  28.543  1.00  0.00
B    H
ATOM   6130  HZ2 LYS B 175      52.438  39.019  29.709  1.00  0.00
B    H
ATOM   6131  HZ3 LYS B 175      51.365  37.782  30.147  1.00  0.00
B    H
ATOM   6132  C   LYS B 175      46.390  42.933  30.564  1.00  0.00
B    C
ATOM   6133  O   LYS B 175      45.910  43.033  29.428  1.00  0.00
B    O
ATOM   6134  N   TYR B 176      45.660  42.539  31.609  1.00  0.00
B    N
ATOM   6135  HN  TYR B 176      46.012  42.495  32.543  1.00  0.00
B    H
ATOM   6136  CA  TYR B 176      44.269  42.137  31.467  1.00  0.00
B    C
ATOM   6137  HA  TYR B 176      44.037  42.276  30.418  1.00  0.00
B    H
ATOM   6138  CB  TYR B 176      44.094  40.666  31.878  1.00  0.00
B    C
ATOM   6139  HB1 TYR B 176      44.381  40.549  32.946  1.00  0.00
B    H
ATOM   6140  HB2 TYR B 176      43.030  40.356  31.788  1.00  0.00
B    H
ATOM   6141  CG  TYR B 176      44.960  39.714  31.087  1.00  0.00
B    C
ATOM   6142  CD1 TYR B 176      46.172  39.265  31.592  1.00  0.00
B    C
ATOM   6143  HD1 TYR B 176      46.511  39.609  32.560  1.00  0.00
B    H
ATOM   6144  CE1 TYR B 176      46.970  38.400  30.874  1.00  0.00
B    C
ATOM   6145  HE1 TYR B 176      47.913  38.060  31.276  1.00  0.00
B    H
ATOM   6146  CZ  TYR B 176      46.562  37.972  29.631  1.00  0.00
B    C
ATOM   6147  OH  TYR B 176      47.358  37.108  28.915  1.00  0.00
B    O
ATOM   6148  HH  TYR B 176      46.881  36.870  28.118  1.00  0.00
B    H
ATOM   6149  CD2 TYR B 176      44.571  39.270  29.830  1.00  0.00
B    C
ATOM   6150  HD2 TYR B 176      43.653  39.634  29.389  1.00  0.00
B    H
ATOM   6151  CE2 TYR B 176      45.365  38.403  29.103  1.00  0.00
B    C
ATOM   6152  HE2 TYR B 176      45.052  38.087  28.119  1.00  0.00
B    H
ATOM   6153  C   TYR B 176      43.294  42.994  32.261  1.00  0.00
B    C
ATOM   6154  O   TYR B 176      42.095  42.695  32.252  1.00  0.00
B    O
ATOM   6155  N   GLY B 177      43.762  44.033  32.947  1.00  0.00
B    N
```

ATOM 6156 HN GLY B 177 44.734 44.255 33.015 1.00 0.00
B H
ATOM 6157 CA GLY B 177 42.866 44.925 33.653 1.00 0.00
B C
ATOM 6158 HA1 GLY B 177 42.084 45.170 32.947 1.00 0.00
B H
ATOM 6159 HA2 GLY B 177 43.470 45.769 33.958 1.00 0.00
B H
ATOM 6160 C GLY B 177 42.217 44.348 34.886 1.00 0.00
B C
ATOM 6161 O GLY B 177 41.224 44.903 35.367 1.00 0.00
B O
ATOM 6162 N CYS B 178 42.744 43.255 35.418 1.00 0.00
B N
ATOM 6163 HN CYS B 178 43.566 42.823 35.052 1.00 0.00
B H
ATOM 6164 CA CYS B 178 42.174 42.593 36.579 1.00 0.00
B C
ATOM 6165 HA CYS B 178 41.213 43.056 36.773 1.00 0.00
B H
ATOM 6166 CB CYS B 178 42.004 41.093 36.308 1.00 0.00
B C
ATOM 6167 HB1 CYS B 178 42.979 40.714 35.926 1.00 0.00
B H
ATOM 6168 HB2 CYS B 178 41.819 40.556 37.265 1.00 0.00
B H
ATOM 6169 SG CYS B 178 40.727 40.728 35.092 1.00 0.00
B S
ATOM 6170 HG1 CYS B 178 40.901 39.406 35.059 1.00 0.00
B H
ATOM 6171 C CYS B 178 43.060 42.836 37.795 1.00 0.00
B C
ATOM 6172 O CYS B 178 44.033 43.591 37.745 1.00 0.00
B O
ATOM 6173 N THR B 179 42.708 42.183 38.901 1.00 0.00
B N
ATOM 6174 HN THR B 179 41.945 41.537 38.935 1.00 0.00
B H
ATOM 6175 CA THR B 179 43.407 42.350 40.169 1.00 0.00
B C
ATOM 6176 HA THR B 179 44.369 42.784 39.931 1.00 0.00
B H
ATOM 6177 CB THR B 179 42.588 43.222 41.128 1.00 0.00
B C
ATOM 6178 HB THR B 179 42.055 42.516 41.811 1.00 0.00
B H
ATOM 6179 OG1 THR B 179 41.605 43.951 40.382 1.00 0.00
B O
ATOM 6180 HG1 THR B 179 41.133 44.484 41.031 1.00 0.00
B H
ATOM 6181 CG2 THR B 179 43.479 44.176 41.902 1.00 0.00
B C
ATOM 6182 HG21 THR B 179 42.896 44.723 42.674 1.00 0.00
B H

ATOM 6183 HG22 THR B 179 44.289 43.618 42.418 1.00 0.00
B H
ATOM 6184 HG23 THR B 179 43.948 44.924 41.227 1.00 0.00
B H
ATOM 6185 C THR B 179 43.689 40.991 40.796 1.00 0.00
B C
ATOM 6186 O THR B 179 42.759 40.281 41.191 1.00 0.00
B O
ATOM 6187 N ILE B 180 44.969 40.639 40.899 1.00 0.00
B N
ATOM 6188 HN ILE B 180 45.726 41.160 40.512 1.00 0.00
B H
ATOM 6189 CA ILE B 180 45.376 39.428 41.608 1.00 0.00
B C
ATOM 6190 HA ILE B 180 44.630 38.666 41.417 1.00 0.00
B H
ATOM 6191 CB ILE B 180 46.776 38.967 41.162 1.00 0.00
B C
ATOM 6192 HB ILE B 180 47.123 39.766 40.457 1.00 0.00
B H
ATOM 6193 CG2 ILE B 180 47.691 38.744 42.370 1.00 0.00
B C
ATOM 6194 HG21 ILE B 180 48.736 38.565 42.042 1.00 0.00
B H
ATOM 6195 HG22 ILE B 180 47.694 39.635 43.033 1.00 0.00
B H
ATOM 6196 HG23 ILE B 180 47.358 37.866 42.963 1.00 0.00
B H
ATOM 6197 CG1 ILE B 180 46.676 37.671 40.351 1.00 0.00
B C
ATOM 6198 HG11 ILE B 180 45.675 37.210 40.499 1.00 0.00
B H
ATOM 6199 HG12 ILE B 180 46.769 37.915 39.267 1.00 0.00
B H
ATOM 6200 CD ILE B 180 47.742 36.628 40.682 1.00 0.00
B C
ATOM 6201 HD1 ILE B 180 47.639 35.745 40.015 1.00 0.00
B H
ATOM 6202 HD2 ILE B 180 48.761 37.052 40.544 1.00 0.00
B H
ATOM 6203 HD3 ILE B 180 47.645 36.279 41.732 1.00 0.00
B H
ATOM 6204 C ILE B 180 45.332 39.723 43.100 1.00 0.00
B C
ATOM 6205 O ILE B 180 45.670 40.831 43.534 1.00 0.00
B O
ATOM 6206 N LEU B 181 44.886 38.751 43.891 1.00 0.00
B N
ATOM 6207 HN LEU B 181 44.760 37.801 43.613 1.00 0.00
B H
ATOM 6208 CA LEU B 181 44.535 39.019 45.279 1.00 0.00
B C
ATOM 6209 HA LEU B 181 44.808 40.054 45.438 1.00 0.00
B H

ATOM 6210 CB LEU B 181 43.039 38.800 45.497 1.00 0.00
B C
ATOM 6211 HB1 LEU B 181 42.557 38.799 44.492 1.00 0.00
B H
ATOM 6212 HB2 LEU B 181 42.879 37.782 45.916 1.00 0.00
B H
ATOM 6213 CG LEU B 181 42.348 39.841 46.362 1.00 0.00
B C
ATOM 6214 HG LEU B 181 42.565 39.606 47.432 1.00 0.00
B H
ATOM 6215 CD1 LEU B 181 42.845 41.249 46.057 1.00 0.00
B C
ATOM 6216 HD11 LEU B 181 42.293 41.997 46.666 1.00 0.00
B H
ATOM 6217 HD12 LEU B 181 43.928 41.341 46.288 1.00 0.00
B H
ATOM 6218 HD13 LEU B 181 42.692 41.496 44.984 1.00 0.00
B H
ATOM 6219 CD2 LEU B 181 40.876 39.702 46.146 1.00 0.00
B C
ATOM 6220 HD21 LEU B 181 40.323 40.432 46.775 1.00 0.00
B H
ATOM 6221 HD22 LEU B 181 40.597 39.889 45.090 1.00 0.00
B H
ATOM 6222 HD23 LEU B 181 40.537 38.680 46.422 1.00 0.00
B H
ATOM 6223 C LEU B 181 45.277 38.186 46.307 1.00 0.00
B C
ATOM 6224 O LEU B 181 45.692 38.736 47.327 1.00 0.00
B O
ATOM 6225 N ALA B 182 45.420 36.881 46.099 1.00 0.00
B N
ATOM 6226 HN ALA B 182 45.142 36.404 45.267 1.00 0.00
B H
ATOM 6227 CA ALA B 182 46.019 36.046 47.126 1.00 0.00
B C
ATOM 6228 HA ALA B 182 46.857 36.545 47.599 1.00 0.00
B H
ATOM 6229 CB ALA B 182 45.015 35.696 48.230 1.00 0.00
B C
ATOM 6230 HB1 ALA B 182 44.654 36.629 48.715 1.00 0.00
B H
ATOM 6231 HB2 ALA B 182 44.133 35.164 47.814 1.00 0.00
B H
ATOM 6232 HB3 ALA B 182 45.476 35.058 49.015 1.00 0.00
B H
ATOM 6233 C ALA B 182 46.572 34.780 46.500 1.00 0.00
B C
ATOM 6234 O ALA B 182 46.084 34.293 45.474 1.00 0.00
B O
ATOM 6235 N ILE B 183 47.588 34.243 47.168 1.00 0.00
B N
ATOM 6236 HN ILE B 183 47.871 34.594 48.058 1.00 0.00
B H

ATOM 6237 CA ILE B 183 48.376 33.117 46.694 1.00 0.00
B C
ATOM 6238 HA ILE B 183 47.877 32.712 45.823 1.00 0.00
B H
ATOM 6239 CB ILE B 183 49.821 33.558 46.383 1.00 0.00
B C
ATOM 6240 HB ILE B 183 50.191 34.002 47.341 1.00 0.00
B H
ATOM 6241 CG2 ILE B 183 50.695 32.389 45.962 1.00 0.00
B C
ATOM 6242 HG21 ILE B 183 51.759 32.700 45.895 1.00 0.00
B H
ATOM 6243 HG22 ILE B 183 50.632 31.562 46.700 1.00 0.00
B H
ATOM 6244 HG23 ILE B 183 50.386 31.998 44.969 1.00 0.00
B H
ATOM 6245 CG1 ILE B 183 49.817 34.682 45.341 1.00 0.00
B C
ATOM 6246 HG11 ILE B 183 49.180 34.392 44.476 1.00 0.00
B H
ATOM 6247 HG12 ILE B 183 49.362 35.592 45.795 1.00 0.00
B H
ATOM 6248 CD ILE B 183 51.190 35.047 44.837 1.00 0.00
B C
ATOM 6249 HD1 ILE B 183 51.126 35.903 44.131 1.00 0.00
B H
ATOM 6250 HD2 ILE B 183 51.852 35.339 45.680 1.00 0.00
B H
ATOM 6251 HD3 ILE B 183 51.657 34.194 44.301 1.00 0.00
B H
ATOM 6252 C ILE B 183 48.350 32.031 47.762 1.00 0.00
B C
ATOM 6253 O ILE B 183 48.780 32.265 48.896 1.00 0.00
B O
ATOM 6254 N LYS B 184 47.858 30.847 47.399 1.00 0.00
B N
ATOM 6255 HN LYS B 184 47.554 30.671 46.464 1.00 0.00
B H
ATOM 6256 CA LYS B 184 47.720 29.718 48.320 1.00 0.00
B C
ATOM 6257 HA LYS B 184 48.122 29.999 49.286 1.00 0.00
B H
ATOM 6258 CB LYS B 184 46.262 29.281 48.417 1.00 0.00
B C
ATOM 6259 HB1 LYS B 184 45.643 30.208 48.465 1.00 0.00
B H
ATOM 6260 HB2 LYS B 184 45.975 28.763 47.474 1.00 0.00
B H
ATOM 6261 CG LYS B 184 45.857 28.401 49.606 1.00 0.00
B C
ATOM 6262 HG1 LYS B 184 44.812 28.646 49.902 1.00 0.00
B H
ATOM 6263 HG2 LYS B 184 45.871 27.333 49.288 1.00 0.00
B H

```
ATOM   6264  CD  LYS B 184      46.739  28.570  50.825  1.00  0.00
B    C
ATOM   6265  HD1 LYS B 184      47.692  28.020  50.649  1.00  0.00
B    H
ATOM   6266  HD2 LYS B 184      46.991  29.651  50.924  1.00  0.00
B    H
ATOM   6267  CE  LYS B 184      46.002  28.052  52.057  1.00  0.00
B    C
ATOM   6268  HE1 LYS B 184      44.974  28.477  52.103  1.00  0.00
B    H
ATOM   6269  HE2 LYS B 184      45.923  26.943  52.027  1.00  0.00
B    H
ATOM   6270  NZ  LYS B 184      46.655  28.393  53.351  1.00  0.00
B    N
ATOM   6271  HZ1 LYS B 184      46.098  28.011  54.142  1.00  0.00
B    H
ATOM   6272  HZ2 LYS B 184      47.612  27.988  53.373  1.00  0.00
B    H
ATOM   6273  HZ3 LYS B 184      46.720  29.427  53.443  1.00  0.00
B    H
ATOM   6274  C   LYS B 184      48.590  28.591  47.774  1.00  0.00
B    C
ATOM   6275  O   LYS B 184      48.182  27.880  46.852  1.00  0.00
B    O
ATOM   6276  N   HSE B 185      49.786  28.429  48.333  1.00  0.00
B    N
ATOM   6277  HN  HSE B 185      50.104  28.977  49.104  1.00  0.00
B    H
ATOM   6278  CA  HSE B 185      50.744  27.431  47.868  1.00  0.00
B    C
ATOM   6279  HA  HSE B 185      50.286  26.896  47.045  1.00  0.00
B    H
ATOM   6280  CB  HSE B 185      52.051  28.086  47.414  1.00  0.00
B    C
ATOM   6281  HB1 HSE B 185      52.738  27.279  47.075  1.00  0.00
B    H
ATOM   6282  HB2 HSE B 185      51.855  28.735  46.536  1.00  0.00
B    H
ATOM   6283  ND1 HSE B 185      53.758  28.320  49.251  1.00  0.00
B    N
ATOM   6284  CG  HSE B 185      52.752  28.861  48.481  1.00  0.00
B    C
ATOM   6285  CE1 HSE B 185      54.190  29.230  50.107  1.00  0.00
B    C
ATOM   6286  HE1 HSE B 185      54.995  29.070  50.827  1.00  0.00
B    H
ATOM   6287  NE2 HSE B 185      53.481  30.332  49.936  1.00  0.00
B    N
ATOM   6288  HE2 HSE B 185      53.581  31.169  50.473  1.00  0.00
B    H
ATOM   6289  CD2 HSE B 185      52.573  30.127  48.927  1.00  0.00
B    C
ATOM   6290  HD2 HSE B 185      51.867  30.881  48.609  1.00  0.00
B    H
```

ATOM 6291 C HSE B 185 51.002 26.418 48.970 1.00 0.00
B C
ATOM 6292 O HSE B 185 51.621 26.746 49.988 1.00 0.00
B O
ATOM 6293 N HSE B 186 50.533 25.190 48.756 1.00 0.00
B N
ATOM 6294 HN HSE B 186 50.007 24.941 47.943 1.00 0.00
B H
ATOM 6295 CA HSE B 186 50.745 24.093 49.694 1.00 0.00
B C
ATOM 6296 HA HSE B 186 50.130 23.267 49.358 1.00 0.00
B H
ATOM 6297 CB HSE B 186 52.213 23.665 49.715 1.00 0.00
B C
ATOM 6298 HB1 HSE B 186 52.592 23.692 48.670 1.00 0.00
B H
ATOM 6299 HB2 HSE B 186 52.806 24.402 50.295 1.00 0.00
B H
ATOM 6300 ND1 HSE B 186 51.829 21.174 49.703 1.00 0.00
B N
ATOM 6301 CG HSE B 186 52.431 22.286 50.250 1.00 0.00
B C
ATOM 6302 CE1 HSE B 186 52.191 20.101 50.383 1.00 0.00
B C
ATOM 6303 HE1 HSE B 186 51.858 19.085 50.161 1.00 0.00
B H
ATOM 6304 NE2 HSE B 186 53.002 20.477 51.354 1.00 0.00
B N
ATOM 6305 HE2 HSE B 186 53.411 19.867 52.033 1.00 0.00
B H
ATOM 6306 CD2 HSE B 186 53.165 21.839 51.296 1.00 0.00
B C
ATOM 6307 HD2 HSE B 186 53.775 22.388 51.999 1.00 0.00
B H
ATOM 6308 C HSE B 186 50.270 24.464 51.094 1.00 0.00
B C
ATOM 6309 O HSE B 186 50.952 24.203 52.085 1.00 0.00
B O
ATOM 6310 N GLY B 187 49.103 25.102 51.184 1.00 0.00
B N
ATOM 6311 HN GLY B 187 48.566 25.337 50.374 1.00 0.00
B H
ATOM 6312 CA GLY B 187 48.533 25.498 52.444 1.00 0.00
B C
ATOM 6313 HA1 GLY B 187 48.816 24.736 53.157 1.00 0.00
B H
ATOM 6314 HA2 GLY B 187 47.466 25.585 52.286 1.00 0.00
B H
ATOM 6315 C GLY B 187 49.039 26.830 52.956 1.00 0.00
B C
ATOM 6316 O GLY B 187 48.560 27.305 53.990 1.00 0.00
B O
ATOM 6317 N ASP B 188 49.990 27.442 52.263 1.00 0.00
B N

ATOM 6318 HN ASP B 188 50.392 27.130 51.406 1.00 0.00
B H
ATOM 6319 CA ASP B 188 50.558 28.700 52.730 1.00 0.00
B C
ATOM 6320 HA ASP B 188 50.311 28.790 53.782 1.00 0.00
B H
ATOM 6321 CB ASP B 188 52.067 28.698 52.503 1.00 0.00
B C
ATOM 6322 HB1 ASP B 188 52.273 28.547 51.424 1.00 0.00
B H
ATOM 6323 HB2 ASP B 188 52.509 29.667 52.810 1.00 0.00
B H
ATOM 6324 CG ASP B 188 52.771 27.583 53.261 1.00 0.00
B C
ATOM 6325 OD1 ASP B 188 52.071 26.659 53.728 1.00 0.00
B O
ATOM 6326 OD2 ASP B 188 54.016 27.627 53.389 1.00 0.00
B O
ATOM 6327 C ASP B 188 49.911 29.891 52.023 1.00 0.00
B C
ATOM 6328 O ASP B 188 49.800 29.918 50.795 1.00 0.00
B O
ATOM 6329 N ILE B 189 49.498 30.885 52.810 1.00 0.00
B N
ATOM 6330 HN ILE B 189 49.664 30.884 53.794 1.00 0.00
B H
ATOM 6331 CA ILE B 189 48.772 32.057 52.326 1.00 0.00
B C
ATOM 6332 HA ILE B 189 48.347 31.792 51.367 1.00 0.00
B H
ATOM 6333 CB ILE B 189 47.667 32.492 53.314 1.00 0.00
B C
ATOM 6334 HB ILE B 189 47.638 31.675 54.078 1.00 0.00
B H
ATOM 6335 CG2 ILE B 189 47.945 33.882 53.882 1.00 0.00
B C
ATOM 6336 HG21 ILE B 189 47.234 34.118 54.702 1.00 0.00
B H
ATOM 6337 HG22 ILE B 189 48.972 33.941 54.300 1.00 0.00
B H
ATOM 6338 HG23 ILE B 189 47.840 34.660 53.098 1.00 0.00
B H
ATOM 6339 CG1 ILE B 189 46.284 32.512 52.656 1.00 0.00
B C
ATOM 6340 HG11 ILE B 189 45.524 32.109 53.363 1.00 0.00
B H
ATOM 6341 HG12 ILE B 189 46.003 33.569 52.439 1.00 0.00
B H
ATOM 6342 CD ILE B 189 46.166 31.763 51.385 1.00 0.00
B C
ATOM 6343 HD1 ILE B 189 45.147 31.884 50.959 1.00 0.00
B H
ATOM 6344 HD2 ILE B 189 46.902 32.135 50.640 1.00 0.00
B H

```
ATOM   6345  HD3 ILE B 189      46.344  30.678  51.545  1.00  0.00
B    H
ATOM   6346  C   ILE B 189      49.747  33.198  52.074  1.00  0.00
B    C
ATOM   6347  O   ILE B 189      50.775  33.313  52.751  1.00  0.00
B    O
ATOM   6348  N   CYS B 190      49.428  34.016  51.074  1.00  0.00
B    N
ATOM   6349  HN  CYS B 190      48.747  33.749  50.395  1.00  0.00
B    H
ATOM   6350  CA  CYS B 190      50.013  35.335  50.867  1.00  0.00
B    C
ATOM   6351  HA  CYS B 190      50.347  35.724  51.822  1.00  0.00
B    H
ATOM   6352  CB  CYS B 190      51.172  35.295  49.875  1.00  0.00
B    C
ATOM   6353  HB1 CYS B 190      51.819  34.435  50.159  1.00  0.00
B    H
ATOM   6354  HB2 CYS B 190      50.788  35.067  48.856  1.00  0.00
B    H
ATOM   6355  SG  CYS B 190      52.194  36.796  49.862  1.00  0.00
B    S
ATOM   6356  HG1 CYS B 190      53.095  36.375  48.973  1.00  0.00
B    H
ATOM   6357  C   CYS B 190      48.875  36.225  50.377  1.00  0.00
B    C
ATOM   6358  O   CYS B 190      48.427  36.104  49.234  1.00  0.00
B    O
ATOM   6359  N   LEU B 191      48.392  37.099  51.258  1.00  0.00
B    N
ATOM   6360  HN  LEU B 191      48.731  37.207  52.191  1.00  0.00
B    H
ATOM   6361  CA  LEU B 191      47.297  37.996  50.912  1.00  0.00
B    C
ATOM   6362  HA  LEU B 191      46.699  37.489  50.166  1.00  0.00
B    H
ATOM   6363  CB  LEU B 191      46.470  38.352  52.148  1.00  0.00
B    C
ATOM   6364  HB1 LEU B 191      47.187  38.551  52.977  1.00  0.00
B    H
ATOM   6365  HB2 LEU B 191      45.939  39.312  51.964  1.00  0.00
B    H
ATOM   6366  CG  LEU B 191      45.451  37.357  52.696  1.00  0.00
B    C
ATOM   6367  HG  LEU B 191      45.992  36.455  53.072  1.00  0.00
B    H
ATOM   6368  CD1 LEU B 191      44.694  38.007  53.833  1.00  0.00
B    C
ATOM   6369 HD11 LEU B 191      43.910  37.323  54.223  1.00  0.00
B    H
ATOM   6370 HD12 LEU B 191      45.383  38.259  54.668  1.00  0.00
B    H
ATOM   6371 HD13 LEU B 191      44.200  38.941  53.492  1.00  0.00
B    H
```

ATOM 6372 CD2 LEU B 191 44.500 36.910 51.609 1.00 0.00
B C
ATOM 6373 HD21 LEU B 191 43.773 36.172 52.012 1.00 0.00
B H
ATOM 6374 HD22 LEU B 191 43.922 37.761 51.196 1.00 0.00
B H
ATOM 6375 HD23 LEU B 191 45.058 36.430 50.777 1.00 0.00
B H
ATOM 6376 C LEU B 191 47.829 39.276 50.285 1.00 0.00
B C
ATOM 6377 O LEU B 191 48.811 39.856 50.755 1.00 0.00
B O
ATOM 6378 N SER B 192 47.132 39.742 49.251 1.00 0.00
B N
ATOM 6379 HN SER B 192 46.286 39.308 48.940 1.00 0.00
B H
ATOM 6380 CA SER B 192 47.521 40.916 48.476 1.00 0.00
B C
ATOM 6381 HA SER B 192 46.991 40.853 47.534 1.00 0.00
B H
ATOM 6382 CB SER B 192 47.168 42.206 49.204 1.00 0.00
B C
ATOM 6383 HB1 SER B 192 47.961 42.957 48.983 1.00 0.00
B H
ATOM 6384 HB2 SER B 192 46.210 42.620 48.817 1.00 0.00
B H
ATOM 6385 OG SER B 192 47.104 42.025 50.607 1.00 0.00
B O
ATOM 6386 HG1 SER B 192 47.101 42.904 51.007 1.00 0.00
B H
ATOM 6387 C SER B 192 49.021 40.863 48.159 1.00 0.00
B C
ATOM 6388 O SER B 192 49.782 41.712 48.634 1.00 0.00
B O
ATOM 6389 N PRO B 193 49.466 39.882 47.379 1.00 0.00
B N
ATOM 6390 CD PRO B 193 48.686 38.993 46.501 1.00 0.00
B C
ATOM 6391 HD1 PRO B 193 48.162 38.196 47.076 1.00 0.00
B H
ATOM 6392 HD2 PRO B 193 47.959 39.578 45.893 1.00 0.00
B H
ATOM 6393 CA PRO B 193 50.910 39.691 47.206 1.00 0.00
B C
ATOM 6394 HA PRO B 193 51.395 39.556 48.166 1.00 0.00
B H
ATOM 6395 CB PRO B 193 51.000 38.410 46.369 1.00 0.00
B C
ATOM 6396 HB1 PRO B 193 50.959 37.529 47.049 1.00 0.00
B H
ATOM 6397 HB2 PRO B 193 51.929 38.356 45.767 1.00 0.00
B H
ATOM 6398 CG PRO B 193 49.729 38.381 45.607 1.00 0.00
B C

ATOM 6399 HG1 PRO B 193 49.445 37.362 45.278 1.00 0.00
B H
ATOM 6400 HG2 PRO B 193 49.820 39.046 44.718 1.00 0.00
B H
ATOM 6401 C PRO B 193 51.543 40.871 46.491 1.00 0.00
B C
ATOM 6402 O PRO B 193 51.067 41.326 45.448 1.00 0.00
B O
ATOM 6403 N ALA B 194 52.632 41.370 47.073 1.00 0.00
B N
ATOM 6404 HN ALA B 194 52.992 41.017 47.937 1.00 0.00
B H
ATOM 6405 CA ALA B 194 53.377 42.469 46.490 1.00 0.00
B C
ATOM 6406 HA ALA B 194 52.738 43.116 45.899 1.00 0.00
B H
ATOM 6407 CB ALA B 194 54.050 43.303 47.574 1.00 0.00
B C
ATOM 6408 HB1 ALA B 194 53.276 43.749 48.234 1.00 0.00
B H
ATOM 6409 HB2 ALA B 194 54.718 42.676 48.203 1.00 0.00
B H
ATOM 6410 HB3 ALA B 194 54.649 44.132 47.139 1.00 0.00
B H
ATOM 6411 C ALA B 194 54.415 41.907 45.538 1.00 0.00
B C
ATOM 6412 O ALA B 194 55.196 41.029 45.940 1.00 0.00
B O
ATOM 6413 N PRO B 195 54.449 42.350 44.283 1.00 0.00
B N
ATOM 6414 CD PRO B 195 53.536 43.317 43.651 1.00 0.00
B C
ATOM 6415 HD1 PRO B 195 53.126 42.405 44.140 1.00 0.00
B H
ATOM 6416 HD2 PRO B 195 52.847 44.180 43.796 1.00 0.00
B H
ATOM 6417 CA PRO B 195 55.460 41.847 43.344 1.00 0.00
B C
ATOM 6418 HA PRO B 195 55.341 40.782 43.185 1.00 0.00
B H
ATOM 6419 CB PRO B 195 55.163 42.627 42.059 1.00 0.00
B C
ATOM 6420 HB1 PRO B 195 54.364 42.100 41.491 1.00 0.00
B H
ATOM 6421 HB2 PRO B 195 56.054 42.736 41.408 1.00 0.00
B H
ATOM 6422 CG PRO B 195 53.744 43.068 42.193 1.00 0.00
B C
ATOM 6423 HG1 PRO B 195 53.261 43.274 41.218 1.00 0.00
B H
ATOM 6424 HG2 PRO B 195 53.702 43.982 42.828 1.00 0.00
B H
ATOM 6425 C PRO B 195 56.868 42.120 43.857 1.00 0.00
B C

ATOM 6426 O PRO B 195 57.161 43.206 44.365 1.00 0.00
B O
ATOM 6427 N GLU B 196 57.742 41.124 43.730 1.00 0.00
B N
ATOM 6428 HN GLU B 196 58.678 41.181 44.067 1.00 0.00
B H
ATOM 6429 CA GLU B 196 57.419 39.854 43.087 1.00 0.00
B C
ATOM 6430 HA GLU B 196 56.533 39.995 42.479 1.00 0.00
B H
ATOM 6431 CB GLU B 196 58.580 39.397 42.201 1.00 0.00
B C
ATOM 6432 HB1 GLU B 196 58.300 38.424 41.735 1.00 0.00
B H
ATOM 6433 HB2 GLU B 196 58.716 40.111 41.358 1.00 0.00
B H
ATOM 6434 CG GLU B 196 59.909 39.216 42.921 1.00 0.00
B C
ATOM 6435 HG1 GLU B 196 60.738 39.309 42.196 1.00 0.00
B H
ATOM 6436 HG2 GLU B 196 60.035 39.974 43.720 1.00 0.00
B H
ATOM 6437 CD GLU B 196 60.047 37.834 43.535 1.00 0.00
B C
ATOM 6438 OE1 GLU B 196 60.137 36.848 42.767 1.00 0.00
B O
ATOM 6439 OE2 GLU B 196 60.023 37.731 44.777 1.00 0.00
B O
ATOM 6440 C GLU B 196 57.088 38.787 44.132 1.00 0.00
B C
ATOM 6441 O GLU B 196 57.256 39.013 45.326 1.00 0.00
B O
ATOM 6442 N ASP B 197 56.603 37.631 43.678 1.00 0.00
B N
ATOM 6443 HN ASP B 197 56.469 37.414 42.715 1.00 0.00
B H
ATOM 6444 CA ASP B 197 56.212 36.544 44.565 1.00 0.00
B C
ATOM 6445 HA ASP B 197 56.976 36.461 45.330 1.00 0.00
B H
ATOM 6446 CB ASP B 197 54.839 36.790 45.183 1.00 0.00
B C
ATOM 6447 HB1 ASP B 197 54.717 37.874 45.381 1.00 0.00
B H
ATOM 6448 HB2 ASP B 197 54.036 36.469 44.491 1.00 0.00
B H
ATOM 6449 CG ASP B 197 54.661 36.065 46.496 1.00 0.00
B C
ATOM 6450 OD1 ASP B 197 55.681 35.685 47.109 1.00 0.00
B O
ATOM 6451 OD2 ASP B 197 53.498 35.856 46.901 1.00 0.00
B O
ATOM 6452 C ASP B 197 56.198 35.224 43.813 1.00 0.00
B C

ATOM 6453 O ASP B 197 55.620 35.120 42.729 1.00 0.00
B O
ATOM 6454 N ILE B 198 56.787 34.202 44.429 1.00 0.00
B N
ATOM 6455 HN ILE B 198 57.163 34.249 45.352 1.00 0.00
B H
ATOM 6456 CA ILE B 198 56.934 32.896 43.788 1.00 0.00
B C
ATOM 6457 HA ILE B 198 57.317 33.072 42.790 1.00 0.00
B H
ATOM 6458 CB ILE B 198 57.880 31.984 44.593 1.00 0.00
B C
ATOM 6459 HB ILE B 198 57.770 32.332 45.652 1.00 0.00
B H
ATOM 6460 CG2 ILE B 198 57.552 30.510 44.347 1.00 0.00
B C
ATOM 6461 HG21 ILE B 198 58.124 29.862 45.042 1.00 0.00
B H
ATOM 6462 HG22 ILE B 198 56.472 30.311 44.515 1.00 0.00
B H
ATOM 6463 HG23 ILE B 198 57.806 30.215 43.307 1.00 0.00
B H
ATOM 6464 CG1 ILE B 198 59.338 32.226 44.203 1.00 0.00
B C
ATOM 6465 HG11 ILE B 198 59.546 31.760 43.214 1.00 0.00
B H
ATOM 6466 HG12 ILE B 198 59.505 33.321 44.093 1.00 0.00
B H
ATOM 6467 CD ILE B 198 60.324 31.702 45.206 1.00 0.00
B C
ATOM 6468 HD1 ILE B 198 61.360 31.957 44.897 1.00 0.00
B H
ATOM 6469 HD2 ILE B 198 60.139 32.148 46.207 1.00 0.00
B H
ATOM 6470 HD3 ILE B 198 60.254 30.598 45.294 1.00 0.00
B H
ATOM 6471 C ILE B 198 55.561 32.254 43.627 1.00 0.00
B C
ATOM 6472 O ILE B 198 54.852 32.001 44.610 1.00 0.00
B O
ATOM 6473 N ILE B 199 55.185 31.991 42.380 1.00 0.00
B N
ATOM 6474 HN ILE B 199 55.736 32.246 41.589 1.00 0.00
B H
ATOM 6475 CA ILE B 199 53.941 31.309 42.048 1.00 0.00
B C
ATOM 6476 HA ILE B 199 53.349 31.226 42.951 1.00 0.00
B H
ATOM 6477 CB ILE B 199 53.172 32.037 40.933 1.00 0.00
B C
ATOM 6478 HB ILE B 199 53.898 32.091 40.082 1.00 0.00
B H
ATOM 6479 CG2 ILE B 199 51.905 31.281 40.572 1.00 0.00
B C

ATOM 6480 HG21 ILE B 199 51.433 31.718 39.667 1.00 0.00
B H
ATOM 6481 HG22 ILE B 199 52.127 30.216 40.353 1.00 0.00
B H
ATOM 6482 HG23 ILE B 199 51.167 31.323 41.402 1.00 0.00
B H
ATOM 6483 CG1 ILE B 199 52.871 33.481 41.353 1.00 0.00
B C
ATOM 6484 HG11 ILE B 199 52.493 33.497 42.400 1.00 0.00
B H
ATOM 6485 HG12 ILE B 199 53.818 34.067 41.337 1.00 0.00
B H
ATOM 6486 CD ILE B 199 51.879 34.192 40.477 1.00 0.00
B C
ATOM 6487 HD1 ILE B 199 51.757 35.246 40.809 1.00 0.00
B H
ATOM 6488 HD2 ILE B 199 52.223 34.198 39.421 1.00 0.00
B H
ATOM 6489 HD3 ILE B 199 50.884 33.701 40.521 1.00 0.00
B H
ATOM 6490 C ILE B 199 54.307 29.893 41.631 1.00 0.00
B C
ATOM 6491 O ILE B 199 54.784 29.677 40.514 1.00 0.00
B O
ATOM 6492 N ARG B 200 54.112 28.937 42.531 1.00 0.00
B N
ATOM 6493 HN ARG B 200 53.753 29.137 43.441 1.00 0.00
B H
ATOM 6494 CA ARG B 200 54.410 27.547 42.239 1.00 0.00
B C
ATOM 6495 HA ARG B 200 55.252 27.508 41.558 1.00 0.00
B H
ATOM 6496 CB ARG B 200 54.737 26.796 43.527 1.00 0.00
B C
ATOM 6497 HB1 ARG B 200 54.016 27.161 44.297 1.00 0.00
B H
ATOM 6498 HB2 ARG B 200 54.521 25.714 43.393 1.00 0.00
B H
ATOM 6499 CG ARG B 200 56.147 27.034 44.016 1.00 0.00
B C
ATOM 6500 HG1 ARG B 200 56.786 27.293 43.144 1.00 0.00
B H
ATOM 6501 HG2 ARG B 200 56.156 27.935 44.671 1.00 0.00
B H
ATOM 6502 CD ARG B 200 56.667 25.846 44.801 1.00 0.00
B C
ATOM 6503 HD1 ARG B 200 56.203 25.840 45.814 1.00 0.00
B H
ATOM 6504 HD2 ARG B 200 56.427 24.883 44.292 1.00 0.00
B H
ATOM 6505 NE ARG B 200 58.119 25.886 44.947 1.00 0.00
B N
ATOM 6506 HE ARG B 200 58.517 26.774 44.689 1.00 0.00
B H

```
ATOM   6507  CZ  ARG B 200      58.852  24.864  45.375  1.00  0.00
B    C
ATOM   6508  NH1 ARG B 200      58.268  23.721  45.703  1.00  0.00
B    N
ATOM   6509 HH11 ARG B 200      58.803  22.961  46.047  1.00  0.00
B    H
ATOM   6510 HH12 ARG B 200      57.266  23.721  45.713  1.00  0.00
B    H
ATOM   6511  NH2 ARG B 200      60.168  24.985  45.478  1.00  0.00
B    N
ATOM   6512 HH21 ARG B 200      60.720  24.197  45.713  1.00  0.00
B    H
ATOM   6513 HH22 ARG B 200      60.573  25.813  45.110  1.00  0.00
B    H
ATOM   6514  C   ARG B 200      53.237  26.888  41.520  1.00  0.00
B    C
ATOM   6515  O   ARG B 200      52.157  27.465  41.378  1.00  0.00
B    O
ATOM   6516  N   GLU B 201      53.462  25.663  41.057  1.00  0.00
B    N
ATOM   6517  HN  GLU B 201      54.341  25.196  41.051  1.00  0.00
B    H
ATOM   6518  CA  GLU B 201      52.374  24.881  40.496  1.00  0.00
B    C
ATOM   6519  HA  GLU B 201      51.873  25.471  39.738  1.00  0.00
B    H
ATOM   6520  CB  GLU B 201      52.893  23.577  39.897  1.00  0.00
B    C
ATOM   6521  HB1 GLU B 201      52.052  22.845  39.878  1.00  0.00
B    H
ATOM   6522  HB2 GLU B 201      53.180  23.738  38.833  1.00  0.00
B    H
ATOM   6523  CG  GLU B 201      54.051  22.962  40.645  1.00  0.00
B    C
ATOM   6524  HG1 GLU B 201      54.970  23.541  40.441  1.00  0.00
B    H
ATOM   6525  HG2 GLU B 201      53.860  22.956  41.737  1.00  0.00
B    H
ATOM   6526  CD  GLU B 201      54.336  21.548  40.189  1.00  0.00
B    C
ATOM   6527  OE1 GLU B 201      54.155  21.266  38.986  1.00  0.00
B    O
ATOM   6528  OE2 GLU B 201      54.740  20.718  41.030  1.00  0.00
B    O
ATOM   6529  C   GLU B 201      51.352  24.578  41.580  1.00  0.00
B    C
ATOM   6530  O   GLU B 201      51.643  24.676  42.775  1.00  0.00
B    O
ATOM   6531  N   GLN B 202      50.143  24.226  41.152  1.00  0.00
B    N
ATOM   6532  HN  GLN B 202      49.925  24.203  40.178  1.00  0.00
B    H
ATOM   6533  CA  GLN B 202      49.039  23.849  42.026  1.00  0.00
B    C
```

ATOM 6534 HA GLN B 202 48.174 23.705 41.389 1.00 0.00
B H
ATOM 6535 CB GLN B 202 49.344 22.539 42.761 1.00 0.00
B C
ATOM 6536 HB1 GLN B 202 50.304 22.692 43.307 1.00 0.00
B H
ATOM 6537 HB2 GLN B 202 48.568 22.365 43.542 1.00 0.00
B H
ATOM 6538 CG GLN B 202 49.462 21.358 41.816 1.00 0.00
B C
ATOM 6539 HG1 GLN B 202 48.882 21.546 40.886 1.00 0.00
B H
ATOM 6540 HG2 GLN B 202 50.520 21.189 41.528 1.00 0.00
B H
ATOM 6541 CD GLN B 202 48.918 20.057 42.381 1.00 0.00
B C
ATOM 6542 OE1 GLN B 202 48.001 20.057 43.196 1.00 0.00
B O
ATOM 6543 NE2 GLN B 202 49.484 18.939 41.944 1.00 0.00
B N
ATOM 6544 HE21 GLN B 202 49.129 18.082 42.307 1.00 0.00
B H
ATOM 6545 HE22 GLN B 202 50.236 18.986 41.295 1.00 0.00
B H
ATOM 6546 C GLN B 202 48.699 24.950 43.019 1.00 0.00
B C
ATOM 6547 O GLN B 202 48.144 24.670 44.091 1.00 0.00
B O
ATOM 6548 N ASP B 203 49.013 26.198 42.686 1.00 0.00
B N
ATOM 6549 HN ASP B 203 49.454 26.469 41.835 1.00 0.00
B H
ATOM 6550 CA ASP B 203 48.733 27.321 43.563 1.00 0.00
B C
ATOM 6551 HA ASP B 203 48.755 26.948 44.580 1.00 0.00
B H
ATOM 6552 CB ASP B 203 49.722 28.456 43.334 1.00 0.00
B C
ATOM 6553 HB1 ASP B 203 50.752 28.044 43.311 1.00 0.00
B H
ATOM 6554 HB2 ASP B 203 49.525 28.957 42.365 1.00 0.00
B H
ATOM 6555 CG ASP B 203 49.667 29.483 44.429 1.00 0.00
B C
ATOM 6556 OD1 ASP B 203 48.994 30.512 44.230 1.00 0.00
B O
ATOM 6557 OD2 ASP B 203 50.322 29.287 45.459 1.00 0.00
B O
ATOM 6558 C ASP B 203 47.334 27.857 43.322 1.00 0.00
B C
ATOM 6559 O ASP B 203 46.900 28.000 42.178 1.00 0.00
B O
ATOM 6560 N CYS B 204 46.648 28.195 44.405 1.00 0.00
B N

```
ATOM   6561  HN  CYS B 204      46.950  28.032  45.342  1.00  0.00
B    H
ATOM   6562  CA  CYS B 204      45.353  28.853  44.286  1.00  0.00
B    C
ATOM   6563  HA  CYS B 204      44.900  28.517  43.361  1.00  0.00
B    H
ATOM   6564  CB  CYS B 204      44.458  28.493  45.467  1.00  0.00
B    C
ATOM   6565  HB1 CYS B 204      45.079  28.571  46.388  1.00  0.00
B    H
ATOM   6566  HB2 CYS B 204      43.652  29.252  45.575  1.00  0.00
B    H
ATOM   6567  SG  CYS B 204      43.777  26.821  45.372  1.00  0.00
B    S
ATOM   6568  HG1 CYS B 204      43.142  26.832  46.544  1.00  0.00
B    H
ATOM   6569  C   CYS B 204      45.566  30.359  44.178  1.00  0.00
B    C
ATOM   6570  O   CYS B 204      46.121  30.986  45.087  1.00  0.00
B    O
ATOM   6571  N   LEU B 205      45.154  30.922  43.044  1.00  0.00
B    N
ATOM   6572  HN  LEU B 205      44.706  30.370  42.343  1.00  0.00
B    H
ATOM   6573  CA  LEU B 205      45.294  32.329  42.696  1.00  0.00
B    C
ATOM   6574  HA  LEU B 205      45.959  32.796  43.412  1.00  0.00
B    H
ATOM   6575  CB  LEU B 205      45.779  32.481  41.251  1.00  0.00
B    C
ATOM   6576  HB1 LEU B 205      45.581  31.514  40.734  1.00  0.00
B    H
ATOM   6577  HB2 LEU B 205      45.149  33.240  40.737  1.00  0.00
B    H
ATOM   6578  CG  LEU B 205      47.210  32.821  40.846  1.00  0.00
B    C
ATOM   6579  HG  LEU B 205      47.458  33.861  41.169  1.00  0.00
B    H
ATOM   6580  CD1 LEU B 205      48.208  31.838  41.436  1.00  0.00
B    C
ATOM   6581 HD11 LEU B 205      49.237  32.074  41.090  1.00  0.00
B    H
ATOM   6582 HD12 LEU B 205      48.193  31.885  42.546  1.00  0.00
B    H
ATOM   6583 HD13 LEU B 205      47.967  30.800  41.124  1.00  0.00
B    H
ATOM   6584  CD2 LEU B 205      47.281  32.770  39.337  1.00  0.00
B    C
ATOM   6585 HD21 LEU B 205      48.305  33.029  38.990  1.00  0.00
B    H
ATOM   6586 HD22 LEU B 205      47.040  31.760  38.949  1.00  0.00
B    H
ATOM   6587 HD23 LEU B 205      46.568  33.496  38.890  1.00  0.00
B    H
```

ATOM 6588 C LEU B 205 43.923 32.966 42.860 1.00 0.00
B C
ATOM 6589 O LEU B 205 42.925 32.419 42.372 1.00 0.00
B O
ATOM 6590 N VAL B 206 43.863 34.100 43.550 1.00 0.00
B N
ATOM 6591 HN VAL B 206 44.645 34.515 44.008 1.00 0.00
B H
ATOM 6592 CA VAL B 206 42.616 34.843 43.693 1.00 0.00
B C
ATOM 6593 HA VAL B 206 41.798 34.217 43.360 1.00 0.00
B H
ATOM 6594 CB VAL B 206 42.358 35.256 45.145 1.00 0.00
B C
ATOM 6595 HB VAL B 206 43.150 35.991 45.432 1.00 0.00
B H
ATOM 6596 CG1 VAL B 206 40.998 35.910 45.269 1.00 0.00
B C
ATOM 6597 HG11 VAL B 206 40.793 36.175 46.329 1.00 0.00
B H
ATOM 6598 HG12 VAL B 206 40.938 36.846 44.676 1.00 0.00
B H
ATOM 6599 HG13 VAL B 206 40.198 35.219 44.927 1.00 0.00
B H
ATOM 6600 CG2 VAL B 206 42.445 34.034 46.051 1.00 0.00
B C
ATOM 6601 HG21 VAL B 206 42.221 34.309 47.104 1.00 0.00
B H
ATOM 6602 HG22 VAL B 206 41.718 33.258 45.730 1.00 0.00
B H
ATOM 6603 HG23 VAL B 206 43.462 33.589 46.030 1.00 0.00
B H
ATOM 6604 C VAL B 206 42.673 36.050 42.770 1.00 0.00
B C
ATOM 6605 O VAL B 206 43.528 36.932 42.926 1.00 0.00
B O
ATOM 6606 N ILE B 207 41.752 36.097 41.817 1.00 0.00
B N
ATOM 6607 HN ILE B 207 41.038 35.407 41.708 1.00 0.00
B H
ATOM 6608 CA ILE B 207 41.701 37.166 40.837 1.00 0.00
B C
ATOM 6609 HA ILE B 207 42.374 37.946 41.169 1.00 0.00
B H
ATOM 6610 CB ILE B 207 42.084 36.674 39.427 1.00 0.00
B C
ATOM 6611 HB ILE B 207 43.025 36.088 39.581 1.00 0.00
B H
ATOM 6612 CG2 ILE B 207 40.960 35.860 38.833 1.00 0.00
B C
ATOM 6613 HG21 ILE B 207 41.286 35.374 37.889 1.00 0.00
B H
ATOM 6614 HG22 ILE B 207 40.640 35.057 39.531 1.00 0.00
B H

```
ATOM   6615 HG23 ILE B 207      40.080  36.499  38.608  1.00  0.00
B    H
ATOM   6616  CG1 ILE B 207      42.444  37.856  38.522  1.00  0.00
B    C
ATOM   6617 HG11 ILE B 207      41.582  38.557  38.458  1.00  0.00
B    H
ATOM   6618 HG12 ILE B 207      43.291  38.416  38.979  1.00  0.00
B    H
ATOM   6619  CD  ILE B 207      42.844  37.452  37.147  1.00  0.00
B    C
ATOM   6620  HD1 ILE B 207      43.148  38.344  36.556  1.00  0.00
B    H
ATOM   6621  HD2 ILE B 207      43.702  36.748  37.179  1.00  0.00
B    H
ATOM   6622  HD3 ILE B 207      42.003  36.960  36.615  1.00  0.00
B    H
ATOM   6623  C   ILE B 207      40.300  37.760  40.851  1.00  0.00
B    C
ATOM   6624  O   ILE B 207      39.338  37.144  41.308  1.00  0.00
B    O
ATOM   6625  N   MET B 208      40.186  38.982  40.342  1.00  0.00
B    N
ATOM   6626  HN  MET B 208      40.949  39.519  39.989  1.00  0.00
B    H
ATOM   6627  CA  MET B 208      38.894  39.649  40.262  1.00  0.00
B    C
ATOM   6628  HA  MET B 208      38.139  38.904  40.042  1.00  0.00
B    H
ATOM   6629  CB  MET B 208      38.546  40.394  41.562  1.00  0.00
B    C
ATOM   6630  HB1 MET B 208      38.315  39.618  42.329  1.00  0.00
B    H
ATOM   6631  HB2 MET B 208      39.446  40.936  41.929  1.00  0.00
B    H
ATOM   6632  CG  MET B 208      37.354  41.325  41.397  1.00  0.00
B    C
ATOM   6633  HG1 MET B 208      37.358  41.781  40.385  1.00  0.00
B    H
ATOM   6634  HG2 MET B 208      36.410  40.747  41.502  1.00  0.00
B    H
ATOM   6635  SD  MET B 208      37.307  42.667  42.572  1.00  0.00
B    S
ATOM   6636  CE  MET B 208      38.968  43.273  42.411  1.00  0.00
B    C
ATOM   6637  HE1 MET B 208      39.145  44.133  43.094  1.00  0.00
B    H
ATOM   6638  HE2 MET B 208      39.707  42.481  42.661  1.00  0.00
B    H
ATOM   6639  HE3 MET B 208      39.171  43.614  41.374  1.00  0.00
B    H
ATOM   6640  C   MET B 208      38.927  40.624  39.100  1.00  0.00
B    C
ATOM   6641  O   MET B 208      39.963  41.238  38.830  1.00  0.00
B    O
```

ATOM 6642 N GLY B 209 37.795 40.771 38.427 1.00 0.00
B N
ATOM 6643 HN GLY B 209 36.985 40.199 38.553 1.00 0.00
B H
ATOM 6644 CA GLY B 209 37.675 41.815 37.431 1.00 0.00
B C
ATOM 6645 HA1 GLY B 209 38.608 41.839 36.885 1.00 0.00
B H
ATOM 6646 HA2 GLY B 209 37.444 42.732 37.957 1.00 0.00
B H
ATOM 6647 C GLY B 209 36.562 41.553 36.443 1.00 0.00
B C
ATOM 6648 O GLY B 209 35.577 40.884 36.766 1.00 0.00
B O
ATOM 6649 N HSE B 210 36.716 42.076 35.231 1.00 0.00
B N
ATOM 6650 HN HSE B 210 37.474 42.666 34.954 1.00 0.00
B H
ATOM 6651 CA HSE B 210 35.747 41.814 34.183 1.00 0.00
B C
ATOM 6652 HA HSE B 210 34.760 41.985 34.594 1.00 0.00
B H
ATOM 6653 CB HSE B 210 36.010 42.694 32.966 1.00 0.00
B C
ATOM 6654 HB1 HSE B 210 35.951 43.755 33.292 1.00 0.00
B H
ATOM 6655 HB2 HSE B 210 37.045 42.524 32.602 1.00 0.00
B H
ATOM 6656 ND1 HSE B 210 33.690 42.808 31.990 1.00 0.00
B N
ATOM 6657 CG HSE B 210 35.024 42.490 31.862 1.00 0.00
B C
ATOM 6658 CE1 HSE B 210 33.061 42.513 30.867 1.00 0.00
B C
ATOM 6659 HE1 HSE B 210 31.994 42.671 30.694 1.00 0.00
B H
ATOM 6660 NE2 HSE B 210 33.937 41.999 30.021 1.00 0.00
B N
ATOM 6661 HE2 HSE B 210 33.726 41.671 29.101 1.00 0.00
B H
ATOM 6662 CD2 HSE B 210 35.170 41.967 30.623 1.00 0.00
B C
ATOM 6663 HD2 HSE B 210 36.050 41.571 30.137 1.00 0.00
B H
ATOM 6664 C HSE B 210 35.819 40.350 33.791 1.00 0.00
B C
ATOM 6665 O HSE B 210 36.862 39.865 33.347 1.00 0.00
B O
ATOM 6666 N LYS B 211 34.695 39.652 33.956 1.00 0.00
B N
ATOM 6667 HN LYS B 211 33.813 40.058 34.193 1.00 0.00
B H
ATOM 6668 CA LYS B 211 34.685 38.203 33.793 1.00 0.00
B C

ATOM 6669 HA LYS B 211 35.344 37.797 34.550 1.00 0.00
B H
ATOM 6670 CB LYS B 211 33.265 37.668 33.970 1.00 0.00
B C
ATOM 6671 HB1 LYS B 211 33.282 36.592 33.681 1.00 0.00
B H
ATOM 6672 HB2 LYS B 211 33.000 37.697 35.050 1.00 0.00
B H
ATOM 6673 CG LYS B 211 32.218 38.387 33.141 1.00 0.00
B C
ATOM 6674 HG1 LYS B 211 32.117 39.433 33.509 1.00 0.00
B H
ATOM 6675 HG2 LYS B 211 32.566 38.440 32.083 1.00 0.00
B H
ATOM 6676 CD LYS B 211 30.871 37.693 33.226 1.00 0.00
B C
ATOM 6677 HD1 LYS B 211 31.028 36.600 33.075 1.00 0.00
B H
ATOM 6678 HD2 LYS B 211 30.473 37.834 34.257 1.00 0.00
B H
ATOM 6679 CE LYS B 211 29.911 38.255 32.198 1.00 0.00
B C
ATOM 6680 HE1 LYS B 211 30.293 38.075 31.168 1.00 0.00
B H
ATOM 6681 HE2 LYS B 211 28.912 37.776 32.292 1.00 0.00
B H
ATOM 6682 NZ LYS B 211 29.720 39.721 32.352 1.00 0.00
B N
ATOM 6683 HZ1 LYS B 211 29.058 40.071 31.631 1.00 0.00
B H
ATOM 6684 HZ2 LYS B 211 29.340 39.923 33.298 1.00 0.00
B H
ATOM 6685 HZ3 LYS B 211 30.636 40.202 32.242 1.00 0.00
B H
ATOM 6686 C LYS B 211 35.238 37.784 32.438 1.00 0.00
B C
ATOM 6687 O LYS B 211 35.893 36.741 32.322 1.00 0.00
B O
ATOM 6688 N LYS B 212 34.999 38.585 31.401 1.00 0.00
B N
ATOM 6689 HN LYS B 212 34.520 39.461 31.440 1.00 0.00
B H
ATOM 6690 CA LYS B 212 35.455 38.189 30.075 1.00 0.00
B C
ATOM 6691 HA LYS B 212 35.280 37.123 29.983 1.00 0.00
B H
ATOM 6692 CB LYS B 212 34.657 38.924 29.001 1.00 0.00
B C
ATOM 6693 HB1 LYS B 212 34.860 40.012 29.128 1.00 0.00
B H
ATOM 6694 HB2 LYS B 212 35.056 38.646 28.000 1.00 0.00
B H
ATOM 6695 CG LYS B 212 33.142 38.682 29.072 1.00 0.00
B C

ATOM 6696 HG1 LYS B 212 32.684 39.442 29.746 1.00 0.00
B H
ATOM 6697 HG2 LYS B 212 32.703 38.831 28.059 1.00 0.00
B H
ATOM 6698 CD LYS B 212 32.765 37.288 29.595 1.00 0.00
B C
ATOM 6699 HD1 LYS B 212 33.340 37.095 30.529 1.00 0.00
B H
ATOM 6700 HD2 LYS B 212 31.684 37.299 29.864 1.00 0.00
B H
ATOM 6701 CE LYS B 212 33.027 36.178 28.586 1.00 0.00
B C
ATOM 6702 HE1 LYS B 212 32.803 36.528 27.554 1.00 0.00
B H
ATOM 6703 HE2 LYS B 212 34.091 35.857 28.625 1.00 0.00
B H
ATOM 6704 NZ LYS B 212 32.176 34.983 28.859 1.00 0.00
B N
ATOM 6705 HZ1 LYS B 212 32.372 34.239 28.158 1.00 0.00
B H
ATOM 6706 HZ2 LYS B 212 32.382 34.621 29.812 1.00 0.00
B H
ATOM 6707 HZ3 LYS B 212 31.173 35.252 28.806 1.00 0.00
B H
ATOM 6708 C LYS B 212 36.953 38.419 29.918 1.00 0.00
B C
ATOM 6709 O LYS B 212 37.648 37.603 29.304 1.00 0.00
B O
ATOM 6710 N ASP B 213 37.482 39.499 30.495 1.00 0.00
B N
ATOM 6711 HN ASP B 213 37.000 40.261 30.921 1.00 0.00
B H
ATOM 6712 CA ASP B 213 38.934 39.647 30.543 1.00 0.00
B C
ATOM 6713 HA ASP B 213 39.301 39.523 29.531 1.00 0.00
B H
ATOM 6714 CB ASP B 213 39.317 41.013 31.108 1.00 0.00
B C
ATOM 6715 HB1 ASP B 213 38.727 41.208 32.026 1.00 0.00
B H
ATOM 6716 HB2 ASP B 213 40.392 41.040 31.372 1.00 0.00
B H
ATOM 6717 CG ASP B 213 39.034 42.143 30.144 1.00 0.00
B C
ATOM 6718 OD1 ASP B 213 39.887 42.410 29.272 1.00 0.00
B O
ATOM 6719 OD2 ASP B 213 37.961 42.767 30.251 1.00 0.00
B O
ATOM 6720 C ASP B 213 39.565 38.542 31.377 1.00 0.00
B C
ATOM 6721 O ASP B 213 40.672 38.073 31.075 1.00 0.00
B O
ATOM 6722 N ILE B 214 38.877 38.113 32.437 1.00 0.00
B N

ATOM 6723 HN ILE B 214 38.008 38.491 32.749 1.00 0.00
B H
ATOM 6724 CA ILE B 214 39.376 37.008 33.251 1.00 0.00
B C
ATOM 6725 HA ILE B 214 40.395 37.255 33.522 1.00 0.00
B H
ATOM 6726 CB ILE B 214 38.504 36.824 34.504 1.00 0.00
B C
ATOM 6727 HB ILE B 214 37.474 36.645 34.104 1.00 0.00
B H
ATOM 6728 CG2 ILE B 214 39.050 35.700 35.364 1.00 0.00
B C
ATOM 6729 HG21 ILE B 214 38.343 35.458 36.185 1.00 0.00
B H
ATOM 6730 HG22 ILE B 214 39.197 34.778 34.764 1.00 0.00
B H
ATOM 6731 HG23 ILE B 214 40.024 35.983 35.816 1.00 0.00
B H
ATOM 6732 CG1 ILE B 214 38.447 38.119 35.308 1.00 0.00
B C
ATOM 6733 HG11 ILE B 214 39.424 38.293 35.812 1.00 0.00
B H
ATOM 6734 HG12 ILE B 214 38.281 38.970 34.609 1.00 0.00
B H
ATOM 6735 CD ILE B 214 37.352 38.151 36.348 1.00 0.00
B C
ATOM 6736 HD1 ILE B 214 37.330 39.141 36.854 1.00 0.00
B H
ATOM 6737 HD2 ILE B 214 36.359 37.980 35.877 1.00 0.00
B H
ATOM 6738 HD3 ILE B 214 37.515 37.373 37.122 1.00 0.00
B H
ATOM 6739 C ILE B 214 39.439 35.734 32.422 1.00 0.00
B C
ATOM 6740 O ILE B 214 40.419 34.983 32.479 1.00 0.00
B O
ATOM 6741 N LYS B 215 38.395 35.474 31.633 1.00 0.00
B N
ATOM 6742 HN LYS B 215 37.550 36.006 31.613 1.00 0.00
B H
ATOM 6743 CA LYS B 215 38.438 34.343 30.714 1.00 0.00
B C
ATOM 6744 HA LYS B 215 38.629 33.458 31.308 1.00 0.00
B H
ATOM 6745 CB LYS B 215 37.109 34.211 29.966 1.00 0.00
B C
ATOM 6746 HB1 LYS B 215 36.304 34.156 30.736 1.00 0.00
B H
ATOM 6747 HB2 LYS B 215 36.925 35.143 29.387 1.00 0.00
B H
ATOM 6748 CG LYS B 215 37.015 32.981 29.057 1.00 0.00
B C
ATOM 6749 HG1 LYS B 215 37.626 32.158 29.493 1.00 0.00
B H

ATOM 6750 HG2 LYS B 215 35.958 32.632 29.029 1.00 0.00
B H
ATOM 6751 CD LYS B 215 37.516 33.284 27.648 1.00 0.00
B C
ATOM 6752 HD1 LYS B 215 37.351 34.366 27.440 1.00 0.00
B H
ATOM 6753 HD2 LYS B 215 38.616 33.104 27.625 1.00 0.00
B H
ATOM 6754 CE LYS B 215 36.834 32.438 26.601 1.00 0.00
B C
ATOM 6755 HE1 LYS B 215 36.988 31.357 26.812 1.00 0.00
B H
ATOM 6756 HE2 LYS B 215 35.741 32.641 26.586 1.00 0.00
B H
ATOM 6757 NZ LYS B 215 37.384 32.735 25.255 1.00 0.00
B N
ATOM 6758 HZ1 LYS B 215 36.910 32.148 24.539 1.00 0.00
B H
ATOM 6759 HZ2 LYS B 215 37.235 33.739 25.032 1.00 0.00
B H
ATOM 6760 HZ3 LYS B 215 38.405 32.532 25.246 1.00 0.00
B H
ATOM 6761 C LYS B 215 39.581 34.480 29.723 1.00 0.00
B C
ATOM 6762 O LYS B 215 40.228 33.487 29.389 1.00 0.00
B O
ATOM 6763 N ARG B 216 39.806 35.688 29.206 1.00 0.00
B N
ATOM 6764 HN ARG B 216 39.259 36.488 29.439 1.00 0.00
B H
ATOM 6765 CA ARG B 216 40.891 35.890 28.253 1.00 0.00
B C
ATOM 6766 HA ARG B 216 40.743 35.220 27.415 1.00 0.00
B H
ATOM 6767 CB ARG B 216 40.897 37.341 27.773 1.00 0.00
B C
ATOM 6768 HB1 ARG B 216 40.916 37.977 28.690 1.00 0.00
B H
ATOM 6769 HB2 ARG B 216 41.847 37.551 27.235 1.00 0.00
B H
ATOM 6770 CG ARG B 216 39.685 37.707 26.940 1.00 0.00
B C
ATOM 6771 HG1 ARG B 216 39.828 37.305 25.913 1.00 0.00
B H
ATOM 6772 HG2 ARG B 216 38.794 37.177 27.347 1.00 0.00
B H
ATOM 6773 CD ARG B 216 39.417 39.199 26.959 1.00 0.00
B C
ATOM 6774 HD1 ARG B 216 39.713 39.616 27.949 1.00 0.00
B H
ATOM 6775 HD2 ARG B 216 39.991 39.728 26.163 1.00 0.00
B H
ATOM 6776 NE ARG B 216 38.005 39.488 26.721 1.00 0.00
B N

```
ATOM   6777  HE  ARG B 216      37.483  38.683  26.416  1.00  0.00
B    H
ATOM   6778  CZ  ARG B 216      37.446  40.682  26.884  1.00  0.00
B    C
ATOM   6779  NH1 ARG B 216      38.177  41.712  27.282  1.00  0.00
B    N
ATOM   6780 HH11 ARG B 216      37.762  42.598  27.430  1.00  0.00
B    H
ATOM   6781 HH12 ARG B 216      39.122  41.508  27.551  1.00  0.00
B    H
ATOM   6782  NH2 ARG B 216      36.152  40.848  26.645  1.00  0.00
B    N
ATOM   6783 HH21 ARG B 216      35.755  41.754  26.683  1.00  0.00
B    H
ATOM   6784 HH22 ARG B 216      35.671  40.086  26.232  1.00  0.00
B    H
ATOM   6785  C   ARG B 216      42.231  35.524  28.872  1.00  0.00
B    C
ATOM   6786  O   ARG B 216      43.020  34.785  28.271  1.00  0.00
B    O
ATOM   6787  N   PHE B 217      42.495  36.026  30.076  1.00  0.00
B    N
ATOM   6788  HN  PHE B 217      41.899  36.642  30.589  1.00  0.00
B    H
ATOM   6789  CA  PHE B 217      43.741  35.688  30.753  1.00  0.00
B    C
ATOM   6790  HA  PHE B 217      44.538  35.923  30.057  1.00  0.00
B    H
ATOM   6791  CB  PHE B 217      43.877  36.488  32.045  1.00  0.00
B    C
ATOM   6792  HB1 PHE B 217      44.335  37.475  31.815  1.00  0.00
B    H
ATOM   6793  HB2 PHE B 217      42.883  36.686  32.500  1.00  0.00
B    H
ATOM   6794  CG  PHE B 217      44.768  35.840  33.058  1.00  0.00
B    C
ATOM   6795  CD1 PHE B 217      46.136  35.785  32.862  1.00  0.00
B    C
ATOM   6796  HD1 PHE B 217      46.566  36.213  31.967  1.00  0.00
B    H
ATOM   6797  CE1 PHE B 217      46.959  35.186  33.790  1.00  0.00
B    C
ATOM   6798  HE1 PHE B 217      48.027  35.153  33.625  1.00  0.00
B    H
ATOM   6799  CZ  PHE B 217      46.419  34.634  34.932  1.00  0.00
B    C
ATOM   6800  HZ  PHE B 217      47.061  34.171  35.668  1.00  0.00
B    H
ATOM   6801  CD2 PHE B 217      44.239  35.281  34.207  1.00  0.00
B    C
ATOM   6802  HD2 PHE B 217      43.171  35.292  34.370  1.00  0.00
B    H
ATOM   6803  CE2 PHE B 217      45.057  34.683  35.139  1.00  0.00
B    C
```

ATOM 6804 HE2 PHE B 217 44.629 34.251 36.032 1.00 0.00
B H
ATOM 6805 C PHE B 217 43.822 34.195 31.037 1.00 0.00
B C
ATOM 6806 O PHE B 217 44.891 33.592 30.906 1.00 0.00
B O
ATOM 6807 N GLU B 218 42.699 33.580 31.413 1.00 0.00
B N
ATOM 6808 HN GLU B 218 41.819 34.024 31.557 1.00 0.00
B H
ATOM 6809 CA GLU B 218 42.685 32.141 31.652 1.00 0.00
B C
ATOM 6810 HA GLU B 218 43.442 31.916 32.393 1.00 0.00
B H
ATOM 6811 CB GLU B 218 41.310 31.718 32.167 1.00 0.00
B C
ATOM 6812 HB1 GLU B 218 40.933 32.515 32.848 1.00 0.00
B H
ATOM 6813 HB2 GLU B 218 40.587 31.674 31.321 1.00 0.00
B H
ATOM 6814 CG GLU B 218 41.298 30.399 32.913 1.00 0.00
B C
ATOM 6815 HG1 GLU B 218 42.295 30.214 33.352 1.00 0.00
B H
ATOM 6816 HG2 GLU B 218 40.540 30.407 33.723 1.00 0.00
B H
ATOM 6817 CD GLU B 218 41.019 29.228 31.990 1.00 0.00
B C
ATOM 6818 OE1 GLU B 218 40.670 29.471 30.816 1.00 0.00
B O
ATOM 6819 OE2 GLU B 218 41.141 28.068 32.433 1.00 0.00
B O
ATOM 6820 C GLU B 218 43.041 31.366 30.393 1.00 0.00
B C
ATOM 6821 O GLU B 218 43.811 30.401 30.441 1.00 0.00
B O
ATOM 6822 N ASN B 219 42.491 31.778 29.256 1.00 0.00
B N
ATOM 6823 HN ASN B 219 41.920 32.593 29.161 1.00 0.00
B H
ATOM 6824 CA ASN B 219 42.694 31.036 28.021 1.00 0.00
B C
ATOM 6825 HA ASN B 219 42.579 29.989 28.281 1.00 0.00
B H
ATOM 6826 CB ASN B 219 41.664 31.471 26.979 1.00 0.00
B C
ATOM 6827 HB1 ASN B 219 40.728 31.738 27.517 1.00 0.00
B H
ATOM 6828 HB2 ASN B 219 42.013 32.381 26.449 1.00 0.00
B H
ATOM 6829 CG ASN B 219 41.340 30.375 25.986 1.00 0.00
B C
ATOM 6830 OD1 ASN B 219 41.921 29.292 26.028 1.00 0.00
B O

ATOM 6831 ND2 ASN B 219 40.406 30.653 25.083 1.00 0.00
B N
ATOM 6832 HD21 ASN B 219 40.174 29.938 24.430 1.00 0.00
B H
ATOM 6833 HD22 ASN B 219 39.948 31.536 25.096 1.00 0.00
B H
ATOM 6834 C ASN B 219 44.108 31.228 27.495 1.00 0.00
B C
ATOM 6835 O ASN B 219 44.730 30.283 26.996 1.00 0.00
B O
ATOM 6836 N GLU B 220 44.633 32.445 27.599 1.00 0.00
B N
ATOM 6837 HN GLU B 220 44.217 33.213 28.078 1.00 0.00
B H
ATOM 6838 CA GLU B 220 45.919 32.755 26.988 1.00 0.00
B C
ATOM 6839 HA GLU B 220 46.054 32.074 26.157 1.00 0.00
B H
ATOM 6840 CB GLU B 220 45.892 34.191 26.457 1.00 0.00
B C
ATOM 6841 HB1 GLU B 220 45.875 34.884 27.330 1.00 0.00
B H
ATOM 6842 HB2 GLU B 220 46.838 34.410 25.913 1.00 0.00
B H
ATOM 6843 CG GLU B 220 44.683 34.456 25.565 1.00 0.00
B C
ATOM 6844 HG1 GLU B 220 44.972 34.330 24.505 1.00 0.00
B H
ATOM 6845 HG2 GLU B 220 43.855 33.759 25.802 1.00 0.00
B H
ATOM 6846 CD GLU B 220 44.157 35.872 25.675 1.00 0.00
B C
ATOM 6847 OE1 GLU B 220 44.404 36.525 26.711 1.00 0.00
B O
ATOM 6848 OE2 GLU B 220 43.477 36.328 24.732 1.00 0.00
B O
ATOM 6849 C GLU B 220 47.096 32.547 27.939 1.00 0.00
B C
ATOM 6850 O GLU B 220 48.007 31.771 27.628 1.00 0.00
B O
ATOM 6851 N GLY B 221 47.099 33.211 29.094 1.00 0.00
B N
ATOM 6852 HN GLY B 221 46.359 33.824 29.368 1.00 0.00
B H
ATOM 6853 CA GLY B 221 48.191 33.084 30.040 1.00 0.00
B C
ATOM 6854 HA1 GLY B 221 48.041 33.852 30.787 1.00 0.00
B H
ATOM 6855 HA2 GLY B 221 49.107 33.189 29.474 1.00 0.00
B H
ATOM 6856 C GLY B 221 48.228 31.744 30.745 1.00 0.00
B C
ATOM 6857 O GLY B 221 49.250 31.365 31.323 1.00 0.00
B O

ATOM 6858 N MET B 222 47.113 31.024 30.717 1.00 0.00
B N
ATOM 6859 HN MET B 222 46.265 31.323 30.285 1.00 0.00
B H
ATOM 6860 CA MET B 222 47.042 29.706 31.331 1.00 0.00
B C
ATOM 6861 HA MET B 222 48.057 29.397 31.550 1.00 0.00
B H
ATOM 6862 CB MET B 222 46.211 29.754 32.610 1.00 0.00
B C
ATOM 6863 HB1 MET B 222 45.170 30.021 32.312 1.00 0.00
B H
ATOM 6864 HB2 MET B 222 46.155 28.733 33.052 1.00 0.00
B H
ATOM 6865 CG MET B 222 46.701 30.756 33.634 1.00 0.00
B C
ATOM 6866 HG1 MET B 222 47.791 30.630 33.807 1.00 0.00
B H
ATOM 6867 HG2 MET B 222 46.538 31.788 33.254 1.00 0.00
B H
ATOM 6868 SD MET B 222 45.855 30.569 35.214 1.00 0.00
B S
ATOM 6869 CE MET B 222 44.240 31.227 34.821 1.00 0.00
B C
ATOM 6870 HE1 MET B 222 43.572 31.197 35.709 1.00 0.00
B H
ATOM 6871 HE2 MET B 222 44.311 32.283 34.482 1.00 0.00
B H
ATOM 6872 HE3 MET B 222 43.755 30.641 34.011 1.00 0.00
B H
ATOM 6873 C MET B 222 46.453 28.691 30.362 1.00 0.00
B C
ATOM 6874 O MET B 222 47.038 27.636 30.127 1.00 0.00
B O
ATOM 6875 N LYS C 7 −18.240 35.921 23.039 1.00 0.00
C N
ATOM 6876 HT1 LYS C 7 −18.468 36.881 22.880 1.00 0.00
C H
ATOM 6877 HT2 LYS C 7 −19.074 35.397 23.214 1.00 0.00
C H
ATOM 6878 HT3 LYS C 7 −17.764 35.547 22.243 1.00 0.00
C H
ATOM 6879 CA LYS C 7 −17.366 35.825 24.204 1.00 0.00
C C
ATOM 6880 HA LYS C 7 −16.450 36.352 23.966 1.00 0.00
C H
ATOM 6881 CB LYS C 7 −18.043 36.445 25.425 1.00 0.00
C C
ATOM 6882 HB1 LYS C 7 −17.397 36.225 26.307 1.00 0.00
C H
ATOM 6883 HB2 LYS C 7 −18.050 37.553 25.313 1.00 0.00
C H
ATOM 6884 CG LYS C 7 −19.441 35.913 25.680 1.00 0.00
C C

ATOM 6885 HG1 LYS C 7 -20.061 36.066 24.767 1.00 0.00
C H
ATOM 6886 HG2 LYS C 7 -19.384 34.815 25.862 1.00 0.00
C H
ATOM 6887 CD LYS C 7 -20.103 36.612 26.855 1.00 0.00
C C
ATOM 6888 HD1 LYS C 7 -20.962 35.990 27.195 1.00 0.00
C H
ATOM 6889 HD2 LYS C 7 -19.369 36.654 27.692 1.00 0.00
C H
ATOM 6890 CE LYS C 7 -20.565 38.011 26.489 1.00 0.00
C C
ATOM 6891 HE1 LYS C 7 -19.691 38.674 26.303 1.00 0.00
C H
ATOM 6892 HE2 LYS C 7 -21.191 37.988 25.569 1.00 0.00
C H
ATOM 6893 NZ LYS C 7 -21.378 38.627 27.576 1.00 0.00
C N
ATOM 6894 HZ1 LYS C 7 -21.678 39.583 27.298 1.00 0.00
C H
ATOM 6895 HZ2 LYS C 7 -22.217 38.039 27.758 1.00 0.00
C H
ATOM 6896 HZ3 LYS C 7 -20.807 38.683 28.444 1.00 0.00
C H
ATOM 6897 C LYS C 7 -16.998 34.373 24.476 1.00 0.00
C C
ATOM 6898 O LYS C 7 -17.853 33.489 24.447 1.00 0.00
C O
ATOM 6899 N GLN C 8 -15.726 34.129 24.774 1.00 0.00
C N
ATOM 6900 HN GLN C 8 -15.065 34.841 25.005 1.00 0.00
C H
ATOM 6901 CA GLN C 8 -15.185 32.778 24.788 1.00 0.00
C C
ATOM 6902 HA GLN C 8 -15.961 32.126 24.406 1.00 0.00
C H
ATOM 6903 CB GLN C 8 -13.961 32.688 23.885 1.00 0.00
C C
ATOM 6904 HB1 GLN C 8 -13.356 33.606 24.072 1.00 0.00
C H
ATOM 6905 HB2 GLN C 8 -13.329 31.829 24.206 1.00 0.00
C H
ATOM 6906 CG GLN C 8 -14.365 32.596 22.435 1.00 0.00
C C
ATOM 6907 HG1 GLN C 8 -14.545 31.537 22.149 1.00 0.00
C H
ATOM 6908 HG2 GLN C 8 -15.300 33.161 22.250 1.00 0.00
C H
ATOM 6909 CD GLN C 8 -13.303 33.109 21.500 1.00 0.00
C C
ATOM 6910 OE1 GLN C 8 -12.423 33.865 21.902 1.00 0.00
C O
ATOM 6911 NE2 GLN C 8 -13.381 32.708 20.238 1.00 0.00
C N

```
ATOM   6912 HE21 GLN C   8      -12.679  33.041  19.615  1.00  0.00
C    H
ATOM   6913 HE22 GLN C   8      -14.128  32.117  19.952  1.00  0.00
C    H
ATOM   6914  C   GLN C   8      -14.856  32.321  26.200  1.00  0.00
C    C
ATOM   6915  O   GLN C   8      -14.228  33.052  26.980  1.00  0.00
C    O
ATOM   6916  N   PHE C   9      -15.275  31.099  26.505  1.00  0.00
C    N
ATOM   6917  HN  PHE C   9      -15.650  30.491  25.807  1.00  0.00
C    H
ATOM   6918  CA  PHE C   9      -15.237  30.531  27.839  1.00  0.00
C    C
ATOM   6919  HA  PHE C   9      -14.700  31.234  28.465  1.00  0.00
C    H
ATOM   6920  CB  PHE C   9      -16.639  30.236  28.331  1.00  0.00
C    C
ATOM   6921  HB1 PHE C   9      -17.105  29.474  27.668  1.00  0.00
C    H
ATOM   6922  HB2 PHE C   9      -16.622  29.811  29.357  1.00  0.00
C    H
ATOM   6923  CG  PHE C   9      -17.537  31.419  28.300  1.00  0.00
C    C
ATOM   6924  CD1 PHE C   9      -17.543  32.314  29.345  1.00  0.00
C    C
ATOM   6925  HD1 PHE C   9      -16.896  32.147  30.196  1.00  0.00
C    H
ATOM   6926  CE1 PHE C   9      -18.362  33.410  29.327  1.00  0.00
C    C
ATOM   6927  HE1 PHE C   9      -18.352  34.107  30.153  1.00  0.00
C    H
ATOM   6928  CZ  PHE C   9      -19.192  33.626  28.248  1.00  0.00
C    C
ATOM   6929  HZ  PHE C   9      -19.836  34.493  28.230  1.00  0.00
C    H
ATOM   6930  CD2 PHE C   9      -18.368  31.645  27.217  1.00  0.00
C    C
ATOM   6931  HD2 PHE C   9      -18.386  30.945  26.394  1.00  0.00
C    H
ATOM   6932  CE2 PHE C   9      -19.195  32.742  27.189  1.00  0.00
C    C
ATOM   6933  HE2 PHE C   9      -19.843  32.906  26.339  1.00  0.00
C    H
ATOM   6934  C   PHE C   9      -14.432  29.246  27.823  1.00  0.00
C    C
ATOM   6935  O   PHE C   9      -14.608  28.402  26.937  1.00  0.00
C    O
ATOM   6936  N   ALA C  10      -13.561  29.095  28.812  1.00  0.00
C    N
ATOM   6937  HN  ALA C  10      -13.423  29.793  29.514  1.00  0.00
C    H
ATOM   6938  CA  ALA C  10      -12.739  27.901  28.948  1.00  0.00
C    C
```

```
ATOM   6939  HA  ALA C  10     -13.010  27.145  28.220  1.00  0.00
C    H
ATOM   6940  CB  ALA C  10     -11.257  28.231  28.757  1.00  0.00
C    C
ATOM   6941  HB1 ALA C  10     -11.093  28.632  27.734  1.00  0.00
C    H
ATOM   6942  HB2 ALA C  10     -10.922  29.002  29.484  1.00  0.00
C    H
ATOM   6943  HB3 ALA C  10     -10.619  27.329  28.878  1.00  0.00
C    H
ATOM   6944  C   ALA C  10     -12.983  27.286  30.315  1.00  0.00
C    C
ATOM   6945  O   ALA C  10     -12.590  27.858  31.336  1.00  0.00
C    O
ATOM   6946  N   VAL C  11     -13.634  26.137  30.338  1.00  0.00
C    N
ATOM   6947  HN  VAL C  11     -14.029  25.705  29.531  1.00  0.00
C    H
ATOM   6948  CA  VAL C  11     -13.829  25.394  31.571  1.00  0.00
C    C
ATOM   6949  HA  VAL C  11     -13.820  26.080  32.409  1.00  0.00
C    H
ATOM   6950  CB  VAL C  11     -15.139  24.610  31.574  1.00  0.00
C    C
ATOM   6951  HB  VAL C  11     -15.546  24.627  30.533  1.00  0.00
C    H
ATOM   6952  CG1 VAL C  11     -14.908  23.175  31.992  1.00  0.00
C    C
ATOM   6953 HG11 VAL C  11     -15.872  22.624  32.029  1.00  0.00
C    H
ATOM   6954 HG12 VAL C  11     -14.249  22.641  31.276  1.00  0.00
C    H
ATOM   6955 HG13 VAL C  11     -14.447  23.130  33.002  1.00  0.00
C    H
ATOM   6956  CG2 VAL C  11     -16.114  25.256  32.520  1.00  0.00
C    C
ATOM   6957 HG21 VAL C  11     -17.060  24.677  32.567  1.00  0.00
C    H
ATOM   6958 HG22 VAL C  11     -15.687  25.310  33.545  1.00  0.00
C    H
ATOM   6959 HG23 VAL C  11     -16.368  26.286  32.195  1.00  0.00
C    H
ATOM   6960  C   VAL C  11     -12.629  24.477  31.721  1.00  0.00
C    C
ATOM   6961  O   VAL C  11     -12.245  23.769  30.788  1.00  0.00
C    O
ATOM   6962  N   ILE C  12     -12.023  24.504  32.893  1.00  0.00
C    N
ATOM   6963  HN  ILE C  12     -12.242  25.162  33.610  1.00  0.00
C    H
ATOM   6964  CA  ILE C  12     -10.973  23.568  33.246  1.00  0.00
C    C
ATOM   6965  HA  ILE C  12     -10.934  22.803  32.481  1.00  0.00
C    H
```

ATOM 6966 CB ILE C 12 -9.628 24.273 33.425 1.00 0.00
C C
ATOM 6967 HB ILE C 12 -9.795 24.981 34.276 1.00 0.00
C H
ATOM 6968 CG2 ILE C 12 -8.555 23.250 33.604 1.00 0.00
C C
ATOM 6969 HG21 ILE C 12 -7.597 23.733 33.891 1.00 0.00
C H
ATOM 6970 HG22 ILE C 12 -8.822 22.532 34.408 1.00 0.00
C H
ATOM 6971 HG23 ILE C 12 -8.388 22.680 32.666 1.00 0.00
C H
ATOM 6972 CG1 ILE C 12 -9.307 25.118 32.204 1.00 0.00
C C
ATOM 6973 HG11 ILE C 12 -10.074 25.916 32.085 1.00 0.00
C H
ATOM 6974 HG12 ILE C 12 -8.328 25.624 32.363 1.00 0.00
C H
ATOM 6975 CD ILE C 12 -9.227 24.326 30.949 1.00 0.00
C C
ATOM 6976 HD1 ILE C 12 -8.934 24.980 30.099 1.00 0.00
C H
ATOM 6977 HD2 ILE C 12 -8.471 23.518 31.043 1.00 0.00
C H
ATOM 6978 HD3 ILE C 12 -10.208 23.865 30.705 1.00 0.00
C H
ATOM 6979 C ILE C 12 -11.396 22.883 34.529 1.00 0.00
C C
ATOM 6980 O ILE C 12 -11.841 23.539 35.477 1.00 0.00
C O
ATOM 6981 N GLY C 13 -11.289 21.568 34.546 1.00 0.00
C N
ATOM 6982 HN GLY C 13 -10.938 21.040 33.775 1.00 0.00
C H
ATOM 6983 CA GLY C 13 -11.679 20.799 35.700 1.00 0.00
C C
ATOM 6984 HA1 GLY C 13 -11.662 21.434 36.576 1.00 0.00
C H
ATOM 6985 HA2 GLY C 13 -11.056 19.918 35.778 1.00 0.00
C H
ATOM 6986 C GLY C 13 -13.099 20.347 35.481 1.00 0.00
C C
ATOM 6987 O GLY C 13 -14.038 21.130 35.647 1.00 0.00
C O
ATOM 6988 N LEU C 14 -13.276 19.092 35.097 1.00 0.00
C N
ATOM 6989 HN LEU C 14 -12.561 18.399 35.028 1.00 0.00
C H
ATOM 6990 CA LEU C 14 -14.603 18.619 34.732 1.00 0.00
C C
ATOM 6991 HA LEU C 14 -15.241 19.491 34.666 1.00 0.00
C H
ATOM 6992 CB LEU C 14 -14.545 17.914 33.391 1.00 0.00
C C

```
ATOM   6993  HB1 LEU C  14      -13.469  17.782  33.135  1.00  0.00
C    H
ATOM   6994  HB2 LEU C  14      -14.964  16.890  33.502  1.00  0.00
C    H
ATOM   6995  CG  LEU C  14      -15.245  18.695  32.300  1.00  0.00
C    C
ATOM   6996  HG  LEU C  14      -15.599  19.668  32.720  1.00  0.00
C    H
ATOM   6997  CD1 LEU C  14      -14.281  18.963  31.176  1.00  0.00
C    C
ATOM   6998 HD11 LEU C  14      -14.790  19.495  30.344  1.00  0.00
C    H
ATOM   6999 HD12 LEU C  14      -13.436  19.594  31.527  1.00  0.00
C    H
ATOM   7000 HD13 LEU C  14      -13.867  18.013  30.778  1.00  0.00
C    H
ATOM   7001  CD2 LEU C  14      -16.463  17.919  31.819  1.00  0.00
C    C
ATOM   7002 HD21 LEU C  14      -16.997  18.491  31.029  1.00  0.00
C    H
ATOM   7003 HD22 LEU C  14      -16.179  16.937  31.389  1.00  0.00
C    H
ATOM   7004 HD23 LEU C  14      -17.168  17.742  32.659  1.00  0.00
C    H
ATOM   7005  C   LEU C  14      -15.173  17.712  35.821  1.00  0.00
C    C
ATOM   7006  O   LEU C  14      -15.482  16.546  35.604  1.00  0.00
C    O
ATOM   7007  N   GLY C  15      -15.304  18.279  37.014  1.00  0.00
C    N
ATOM   7008  HN  GLY C  15      -14.932  19.179  37.238  1.00  0.00
C    H
ATOM   7009  CA  GLY C  15      -16.010  17.607  38.085  1.00  0.00
C    C
ATOM   7010  HA1 GLY C  15      -15.672  18.015  39.028  1.00  0.00
C    H
ATOM   7011  HA2 GLY C  15      -15.860  16.540  37.984  1.00  0.00
C    H
ATOM   7012  C   GLY C  15      -17.492  17.882  37.961  1.00  0.00
C    C
ATOM   7013  O   GLY C  15      -17.891  18.652  37.083  1.00  0.00
C    O
ATOM   7014  N   ARG C  16      -18.312  17.262  38.819  1.00  0.00
C    N
ATOM   7015  HN  ARG C  16      -17.991  16.602  39.494  1.00  0.00
C    H
ATOM   7016  CA  ARG C  16      -19.744  17.535  38.801  1.00  0.00
C    C
ATOM   7017  HA  ARG C  16      -20.170  17.059  37.926  1.00  0.00
C    H
ATOM   7018  CB  ARG C  16      -20.407  17.016  40.072  1.00  0.00
C    C
ATOM   7019  HB1 ARG C  16      -19.955  17.586  40.919  1.00  0.00
C    H
```

ATOM 7020 HB2 ARG C 16 -21.487 17.283 40.065 1.00 0.00
C H
ATOM 7021 CG ARG C 16 -20.209 15.549 40.319 1.00 0.00
C C
ATOM 7022 HG1 ARG C 16 -20.982 14.987 39.750 1.00 0.00
C H
ATOM 7023 HG2 ARG C 16 -19.232 15.237 39.884 1.00 0.00
C H
ATOM 7024 CD ARG C 16 -20.214 15.242 41.803 1.00 0.00
C C
ATOM 7025 HD1 ARG C 16 -21.058 15.782 42.290 1.00 0.00
C H
ATOM 7026 HD2 ARG C 16 -20.331 14.150 41.994 1.00 0.00
C H
ATOM 7027 NE ARG C 16 -18.970 15.637 42.454 1.00 0.00
C N
ATOM 7028 HE ARG C 16 -18.340 16.126 41.840 1.00 0.00
C H
ATOM 7029 CZ ARG C 16 -18.683 15.385 43.728 1.00 0.00
C C
ATOM 7030 NH1 ARG C 16 -19.553 14.730 44.484 1.00 0.00
C N
ATOM 7031 HH11 ARG C 16 -19.361 14.558 45.440 1.00 0.00
C H
ATOM 7032 HH12 ARG C 16 -20.450 14.548 44.074 1.00 0.00
C H
ATOM 7033 NH2 ARG C 16 -17.526 15.775 44.243 1.00 0.00
C N
ATOM 7034 HH21 ARG C 16 -17.282 15.509 45.165 1.00 0.00
C H
ATOM 7035 HH22 ARG C 16 -16.856 16.144 43.612 1.00 0.00
C H
ATOM 7036 C ARG C 16 -20.019 19.016 38.664 1.00 0.00
C C
ATOM 7037 O ARG C 16 -20.857 19.422 37.852 1.00 0.00
C O
ATOM 7038 N PHE C 17 -19.289 19.828 39.415 1.00 0.00
C N
ATOM 7039 HN PHE C 17 -18.641 19.531 40.115 1.00 0.00
C H
ATOM 7040 CA PHE C 17 -19.391 21.267 39.262 1.00 0.00
C C
ATOM 7041 HA PHE C 17 -20.418 21.528 39.485 1.00 0.00
C H
ATOM 7042 CB PHE C 17 -18.411 21.950 40.206 1.00 0.00
C C
ATOM 7043 HB1 PHE C 17 -18.625 21.627 41.249 1.00 0.00
C H
ATOM 7044 HB2 PHE C 17 -17.364 21.653 39.981 1.00 0.00
C H
ATOM 7045 CG PHE C 17 -18.514 23.432 40.202 1.00 0.00
C C
ATOM 7046 CD1 PHE C 17 -19.739 24.044 40.371 1.00 0.00
C C

ATOM 7047 HD1 PHE C 17 −20.626 23.439 40.495 1.00 0.00
C H
ATOM 7048 CE1 PHE C 17 −19.844 25.412 40.375 1.00 0.00
C C
ATOM 7049 HE1 PHE C 17 −20.809 25.879 40.508 1.00 0.00
C H
ATOM 7050 CZ PHE C 17 −18.716 26.182 40.215 1.00 0.00
C C
ATOM 7051 HZ PHE C 17 −18.794 27.260 40.227 1.00 0.00
C H
ATOM 7052 CD2 PHE C 17 −17.388 24.215 40.045 1.00 0.00
C C
ATOM 7053 HD2 PHE C 17 −16.424 23.751 39.895 1.00 0.00
C H
ATOM 7054 CE2 PHE C 17 −17.485 25.582 40.052 1.00 0.00
C C
ATOM 7055 HE2 PHE C 17 −16.597 26.185 39.928 1.00 0.00
C H
ATOM 7056 C PHE C 17 −19.131 21.670 37.820 1.00 0.00
C C
ATOM 7057 O PHE C 17 −19.984 22.289 37.175 1.00 0.00
C O
ATOM 7058 N GLY C 18 −17.963 21.304 37.294 1.00 0.00
C N
ATOM 7059 HN GLY C 18 −17.263 20.779 37.775 1.00 0.00
C H
ATOM 7060 CA GLY C 18 −17.642 21.672 35.926 1.00 0.00
C C
ATOM 7061 HA1 GLY C 18 −16.728 21.156 35.664 1.00 0.00
C H
ATOM 7062 HA2 GLY C 18 −17.568 22.751 35.892 1.00 0.00
C H
ATOM 7063 C GLY C 18 −18.719 21.246 34.949 1.00 0.00
C C
ATOM 7064 O GLY C 18 −19.309 22.079 34.251 1.00 0.00
C O
ATOM 7065 N GLY C 19 −19.023 19.949 34.931 1.00 0.00
C N
ATOM 7066 HN GLY C 19 −18.624 19.279 35.557 1.00 0.00
C H
ATOM 7067 CA GLY C 19 −19.979 19.423 33.973 1.00 0.00
C C
ATOM 7068 HA1 GLY C 19 −20.158 18.393 34.249 1.00 0.00
C H
ATOM 7069 HA2 GLY C 19 −19.543 19.551 32.992 1.00 0.00
C H
ATOM 7070 C GLY C 19 −21.303 20.149 33.992 1.00 0.00
C C
ATOM 7071 O GLY C 19 −21.895 20.400 32.940 1.00 0.00
C O
ATOM 7072 N SER C 20 −21.779 20.503 35.179 1.00 0.00
C N
ATOM 7073 HN SER C 20 −21.368 20.259 36.058 1.00 0.00
C H

```
ATOM   7074  CA  SER C  20      -22.983  21.305  35.288  1.00  0.00
C    C
ATOM   7075  HA  SER C  20      -23.775  20.714  34.848  1.00  0.00
C    H
ATOM   7076  CB  SER C  20      -23.204  21.676  36.744  1.00  0.00
C    C
ATOM   7077  HB1 SER C  20      -22.293  22.203  37.108  1.00  0.00
C    H
ATOM   7078  HB2 SER C  20      -24.054  22.388  36.839  1.00  0.00
C    H
ATOM   7079  OG  SER C  20      -23.405  20.513  37.503  1.00  0.00
C    O
ATOM   7080  HG1 SER C  20      -23.318  20.762  38.431  1.00  0.00
C    H
ATOM   7081  C   SER C  20      -22.868  22.577  34.481  1.00  0.00
C    C
ATOM   7082  O   SER C  20      -23.706  22.846  33.607  1.00  0.00
C    O
ATOM   7083  N   ILE C  21      -21.835  23.355  34.783  1.00  0.00
C    N
ATOM   7084  HN  ILE C  21      -21.151  23.134  35.475  1.00  0.00
C    H
ATOM   7085  CA  ILE C  21      -21.614  24.622  34.113  1.00  0.00
C    C
ATOM   7086  HA  ILE C  21      -22.455  25.256  34.360  1.00  0.00
C    H
ATOM   7087  CB  ILE C  21      -20.276  25.213  34.570  1.00  0.00
C    C
ATOM   7088  HB  ILE C  21      -19.505  24.520  34.148  1.00  0.00
C    H
ATOM   7089  CG2 ILE C  21      -20.147  26.630  34.103  1.00  0.00
C    C
ATOM   7090 HG21 ILE C  21      -19.122  27.013  34.294  1.00  0.00
C    H
ATOM   7091 HG22 ILE C  21      -20.336  26.707  33.012  1.00  0.00
C    H
ATOM   7092 HG23 ILE C  21      -20.868  27.290  34.632  1.00  0.00
C    H
ATOM   7093  CG1 ILE C  21      -20.181  25.128  36.083  1.00  0.00
C    C
ATOM   7094 HG11 ILE C  21      -20.991  25.735  36.546  1.00  0.00
C    H
ATOM   7095 HG12 ILE C  21      -20.342  24.071  36.396  1.00  0.00
C    H
ATOM   7096  CD  ILE C  21      -18.865  25.576  36.637  1.00  0.00
C    C
ATOM   7097  HD1 ILE C  21      -18.841  25.435  37.739  1.00  0.00
C    H
ATOM   7098  HD2 ILE C  21      -18.032  24.989  36.193  1.00  0.00
C    H
ATOM   7099  HD3 ILE C  21      -18.689  26.653  36.425  1.00  0.00
C    H
ATOM   7100  C   ILE C  21      -21.635  24.435  32.612  1.00  0.00
C    C
```

ATOM 7101 O ILE C 21 -22.363 25.125 31.889 1.00 0.00
C O
ATOM 7102 N VAL C 22 -20.820 23.508 32.117 1.00 0.00
C N
ATOM 7103 HN VAL C 22 -20.294 22.837 32.632 1.00 0.00
C H
ATOM 7104 CA VAL C 22 -20.648 23.429 30.675 1.00 0.00
C C
ATOM 7105 HA VAL C 22 -20.465 24.433 30.313 1.00 0.00
C H
ATOM 7106 CB VAL C 22 -19.447 22.548 30.314 1.00 0.00
C C
ATOM 7107 HB VAL C 22 -19.618 22.149 29.284 1.00 0.00
C H
ATOM 7108 CG1 VAL C 22 -18.187 23.360 30.330 1.00 0.00
C C
ATOM 7109 HG11 VAL C 22 -17.309 22.716 30.107 1.00 0.00
C H
ATOM 7110 HG12 VAL C 22 -18.208 24.165 29.567 1.00 0.00
C H
ATOM 7111 HG13 VAL C 22 -18.029 23.821 31.329 1.00 0.00
C H
ATOM 7112 CG2 VAL C 22 -19.343 21.410 31.257 1.00 0.00
C C
ATOM 7113 HG21 VAL C 22 -18.451 20.789 31.026 1.00 0.00
C H
ATOM 7114 HG22 VAL C 22 -19.253 21.776 32.302 1.00 0.00
C H
ATOM 7115 HG23 VAL C 22 -20.235 20.752 31.194 1.00 0.00
C H
ATOM 7116 C VAL C 22 -21.915 22.929 30.006 1.00 0.00
C C
ATOM 7117 O VAL C 22 -22.307 23.432 28.952 1.00 0.00
C O
ATOM 7118 N LYS C 23 -22.570 21.928 30.591 1.00 0.00
C N
ATOM 7119 HN LYS C 23 -22.324 21.501 31.459 1.00 0.00
C H
ATOM 7120 CA LYS C 23 -23.750 21.380 29.947 1.00 0.00
C C
ATOM 7121 HA LYS C 23 -23.486 21.151 28.922 1.00 0.00
C H
ATOM 7122 CB LYS C 23 -24.200 20.119 30.672 1.00 0.00
C C
ATOM 7123 HB1 LYS C 23 -23.285 19.521 30.889 1.00 0.00
C H
ATOM 7124 HB2 LYS C 23 -24.629 20.402 31.659 1.00 0.00
C H
ATOM 7125 CG LYS C 23 -25.172 19.274 29.881 1.00 0.00
C C
ATOM 7126 HG1 LYS C 23 -25.500 19.842 28.980 1.00 0.00
C H
ATOM 7127 HG2 LYS C 23 -24.649 18.357 29.523 1.00 0.00
C H

ATOM 7128 CD LYS C 23 -26.389 18.900 30.698 1.00 0.00
C C
ATOM 7129 HD1 LYS C 23 -26.186 19.147 31.765 1.00 0.00
C H
ATOM 7130 HD2 LYS C 23 -27.240 19.538 30.363 1.00 0.00
C H
ATOM 7131 CE LYS C 23 -26.704 17.419 30.515 1.00 0.00
C C
ATOM 7132 HE1 LYS C 23 -26.925 17.196 29.447 1.00 0.00
C H
ATOM 7133 HE2 LYS C 23 -25.840 16.793 30.833 1.00 0.00
C H
ATOM 7134 NZ LYS C 23 -27.882 16.967 31.305 1.00 0.00
C N
ATOM 7135 HZ1 LYS C 23 -28.053 15.954 31.143 1.00 0.00
C H
ATOM 7136 HZ2 LYS C 23 -27.705 17.130 32.317 1.00 0.00
C H
ATOM 7137 HZ3 LYS C 23 -28.722 17.508 31.014 1.00 0.00
C H
ATOM 7138 C LYS C 23 -24.857 22.415 29.907 1.00 0.00
C C
ATOM 7139 O LYS C 23 -25.572 22.532 28.905 1.00 0.00
C O
ATOM 7140 N GLU C 24 -24.973 23.220 30.964 1.00 0.00
C N
ATOM 7141 HN GLU C 24 -24.455 23.162 31.812 1.00 0.00
C H
ATOM 7142 CA GLU C 24 -25.937 24.308 30.933 1.00 0.00
C C
ATOM 7143 HA GLU C 24 -26.909 23.899 30.684 1.00 0.00
C H
ATOM 7144 CB GLU C 24 -25.973 24.998 32.298 1.00 0.00
C C
ATOM 7145 HB1 GLU C 24 -26.135 24.219 33.077 1.00 0.00
C H
ATOM 7146 HB2 GLU C 24 -24.979 25.448 32.519 1.00 0.00
C H
ATOM 7147 CG GLU C 24 -27.038 26.070 32.489 1.00 0.00
C C
ATOM 7148 HG1 GLU C 24 -28.041 25.615 32.390 1.00 0.00
C H
ATOM 7149 HG2 GLU C 24 -26.949 26.544 33.486 1.00 0.00
C H
ATOM 7150 CD GLU C 24 -27.015 27.180 31.456 1.00 0.00
C C
ATOM 7151 OE1 GLU C 24 -25.979 27.850 31.288 1.00 0.00
C O
ATOM 7152 OE2 GLU C 24 -28.048 27.359 30.787 1.00 0.00
C O
ATOM 7153 C GLU C 24 -25.576 25.309 29.843 1.00 0.00
C C
ATOM 7154 O GLU C 24 -26.391 25.621 28.969 1.00 0.00
C O

```
ATOM   7155  N   LEU C  25      -24.365  25.856  29.909  1.00  0.00
C    N
ATOM   7156  HN  LEU C  25      -23.661  25.611  30.573  1.00  0.00
C    H
ATOM   7157  CA  LEU C  25      -23.967  26.900  28.973  1.00  0.00
C    C
ATOM   7158  HA  LEU C  25      -24.657  27.722  29.108  1.00  0.00
C    H
ATOM   7159  CB  LEU C  25      -22.537  27.324  29.282  1.00  0.00
C    C
ATOM   7160  HB1 LEU C  25      -22.178  26.685  30.122  1.00  0.00
C    H
ATOM   7161  HB2 LEU C  25      -21.890  27.078  28.411  1.00  0.00
C    H
ATOM   7162  CG  LEU C  25      -22.411  28.785  29.668  1.00  0.00
C    C
ATOM   7163  HG  LEU C  25      -23.417  29.209  29.903  1.00  0.00
C    H
ATOM   7164  CD1 LEU C  25      -21.505  28.927  30.851  1.00  0.00
C    C
ATOM   7165 HD11 LEU C  25      -21.368  29.998  31.111  1.00  0.00
C    H
ATOM   7166 HD12 LEU C  25      -21.930  28.405  31.734  1.00  0.00
C    H
ATOM   7167 HD13 LEU C  25      -20.506  28.494  30.634  1.00  0.00
C    H
ATOM   7168  CD2 LEU C  25      -21.843  29.551  28.497  1.00  0.00
C    C
ATOM   7169 HD21 LEU C  25      -21.758  30.631  28.746  1.00  0.00
C    H
ATOM   7170 HD22 LEU C  25      -20.833  29.189  28.221  1.00  0.00
C    H
ATOM   7171 HD23 LEU C  25      -22.505  29.451  27.610  1.00  0.00
C    H
ATOM   7172  C   LEU C  25      -24.099  26.422  27.539  1.00  0.00
C    C
ATOM   7173  O   LEU C  25      -24.504  27.177  26.648  1.00  0.00
C    O
ATOM   7174  N   HSE C  26      -23.753  25.163  27.308  1.00  0.00
C    N
ATOM   7175  HN  HSE C  26      -23.298  24.588  27.985  1.00  0.00
C    H
ATOM   7176  CA  HSE C  26      -24.008  24.508  26.040  1.00  0.00
C    C
ATOM   7177  HA  HSE C  26      -23.484  25.071  25.278  1.00  0.00
C    H
ATOM   7178  CB  HSE C  26      -23.526  23.067  26.146  1.00  0.00
C    C
ATOM   7179  HB1 HSE C  26      -22.466  23.086  26.481  1.00  0.00
C    H
ATOM   7180  HB2 HSE C  26      -24.106  22.539  26.932  1.00  0.00
C    H
ATOM   7181  ND1 HSE C  26      -24.599  21.422  24.583  1.00  0.00
C    N
```

ATOM 7182 CG HSE C 26 -23.583 22.304 24.870 1.00 0.00
C C
ATOM 7183 CE1 HSE C 26 -24.380 20.875 23.401 1.00 0.00
C C
ATOM 7184 HE1 HSE C 26 -25.035 20.141 22.925 1.00 0.00
C H
ATOM 7185 NE2 HSE C 26 -23.251 21.362 22.919 1.00 0.00
C N
ATOM 7186 HE2 HSE C 26 -22.837 21.103 22.048 1.00 0.00
C H
ATOM 7187 CD2 HSE C 26 -22.729 22.255 23.822 1.00 0.00
C C
ATOM 7188 HD2 HSE C 26 -21.797 22.778 23.662 1.00 0.00
C H
ATOM 7189 C HSE C 26 -25.483 24.560 25.696 1.00 0.00
C C
ATOM 7190 O HSE C 26 -25.853 24.860 24.557 1.00 0.00
C O
ATOM 7191 N ARG C 27 -26.343 24.274 26.672 1.00 0.00
C N
ATOM 7192 HN ARG C 27 -26.074 23.942 27.573 1.00 0.00
C H
ATOM 7193 CA ARG C 27 -27.768 24.444 26.439 1.00 0.00
C C
ATOM 7194 HA ARG C 27 -28.045 23.865 25.565 1.00 0.00
C H
ATOM 7195 CB ARG C 27 -28.560 23.964 27.648 1.00 0.00
C C
ATOM 7196 HB1 ARG C 27 -28.063 23.028 27.999 1.00 0.00
C H
ATOM 7197 HB2 ARG C 27 -28.455 24.697 28.478 1.00 0.00
C H
ATOM 7198 CG ARG C 27 -30.006 23.670 27.339 1.00 0.00
C C
ATOM 7199 HG1 ARG C 27 -30.222 24.006 26.301 1.00 0.00
C H
ATOM 7200 HG2 ARG C 27 -30.158 22.566 27.334 1.00 0.00
C H
ATOM 7201 CD ARG C 27 -30.926 24.291 28.370 1.00 0.00
C C
ATOM 7202 HD1 ARG C 27 -31.965 24.326 27.966 1.00 0.00
C H
ATOM 7203 HD2 ARG C 27 -30.931 23.705 29.319 1.00 0.00
C H
ATOM 7204 NE ARG C 27 -30.525 25.646 28.735 1.00 0.00
C N
ATOM 7205 HE ARG C 27 -30.164 25.713 29.672 1.00 0.00
C H
ATOM 7206 CZ ARG C 27 -30.617 26.698 27.929 1.00 0.00
C C
ATOM 7207 NH1 ARG C 27 -31.085 26.564 26.698 1.00 0.00
C N
ATOM 7208 HH11 ARG C 27 -31.131 27.343 26.088 1.00 0.00
C H

ATOM 7209 HH12 ARG C 27 −31.254 25.626 26.387 1.00 0.00
C H
ATOM 7210 NH2 ARG C 27 −30.237 27.890 28.354 1.00 0.00
C N
ATOM 7211 HH21 ARG C 27 −30.386 28.687 27.786 1.00 0.00
C H
ATOM 7212 HH22 ARG C 27 −30.006 27.971 29.315 1.00 0.00
C H
ATOM 7213 C ARG C 27 −28.096 25.894 26.121 1.00 0.00
C C
ATOM 7214 O ARG C 27 −29.041 26.168 25.374 1.00 0.00
C O
ATOM 7215 N MET C 28 −27.328 26.841 26.662 1.00 0.00
C N
ATOM 7216 HN MET C 28 −26.604 26.710 27.336 1.00 0.00
C H
ATOM 7217 CA MET C 28 −27.530 28.224 26.257 1.00 0.00
C C
ATOM 7218 HA MET C 28 −28.602 28.383 26.230 1.00 0.00
C H
ATOM 7219 CB MET C 28 −26.878 29.197 27.230 1.00 0.00
C C
ATOM 7220 HB1 MET C 28 −25.808 28.892 27.325 1.00 0.00
C H
ATOM 7221 HB2 MET C 28 −26.874 30.216 26.786 1.00 0.00
C H
ATOM 7222 CG MET C 28 −27.496 29.212 28.582 1.00 0.00
C C
ATOM 7223 HG1 MET C 28 −28.594 29.366 28.503 1.00 0.00
C H
ATOM 7224 HG2 MET C 28 −27.329 28.232 29.080 1.00 0.00
C H
ATOM 7225 SD MET C 28 −26.843 30.504 29.634 1.00 0.00
C S
ATOM 7226 CE MET C 28 −27.613 31.919 28.860 1.00 0.00
C C
ATOM 7227 HE1 MET C 28 −27.319 32.860 29.372 1.00 0.00
C H
ATOM 7228 HE2 MET C 28 −27.312 32.003 27.794 1.00 0.00
C H
ATOM 7229 HE3 MET C 28 −28.721 31.842 28.898 1.00 0.00
C H
ATOM 7230 C MET C 28 −26.991 28.506 24.869 1.00 0.00
C C
ATOM 7231 O MET C 28 −27.115 29.639 24.396 1.00 0.00
C O
ATOM 7232 N GLY C 29 −26.389 27.521 24.216 1.00 0.00
C N
ATOM 7233 HN GLY C 29 −26.253 26.605 24.593 1.00 0.00
C H
ATOM 7234 CA GLY C 29 −25.883 27.728 22.880 1.00 0.00
C C
ATOM 7235 HA1 GLY C 29 −26.678 28.180 22.302 1.00 0.00
C H

ATOM 7236 HA2 GLY C 29 −25.551 26.769 22.505 1.00 0.00
C H
ATOM 7237 C GLY C 29 −24.701 28.668 22.864 1.00 0.00
C C
ATOM 7238 O GLY C 29 −24.724 29.696 22.184 1.00 0.00
C O
ATOM 7239 N HSE C 30 −23.669 28.341 23.626 1.00 0.00
C N
ATOM 7240 HN HSE C 30 −23.625 27.525 24.201 1.00 0.00
C H
ATOM 7241 CA HSE C 30 −22.487 29.175 23.681 1.00 0.00
C C
ATOM 7242 HA HSE C 30 −22.644 29.996 22.991 1.00 0.00
C H
ATOM 7243 CB HSE C 30 −22.268 29.699 25.093 1.00 0.00
C C
ATOM 7244 HB1 HSE C 30 −22.395 28.848 25.797 1.00 0.00
C H
ATOM 7245 HB2 HSE C 30 −21.221 30.053 25.199 1.00 0.00
C H
ATOM 7246 ND1 HSE C 30 −22.813 32.023 25.891 1.00 0.00
C N
ATOM 7247 CG HSE C 30 −23.225 30.773 25.487 1.00 0.00
C C
ATOM 7248 CE1 HSE C 30 −23.871 32.761 26.173 1.00 0.00
C C
ATOM 7249 HE1 HSE C 30 −23.830 33.794 26.528 1.00 0.00
C H
ATOM 7250 NE2 HSE C 30 −24.954 32.036 25.956 1.00 0.00
C N
ATOM 7251 HE2 HSE C 30 −25.892 32.344 26.107 1.00 0.00
C H
ATOM 7252 CD2 HSE C 30 −24.577 30.791 25.521 1.00 0.00
C C
ATOM 7253 HD2 HSE C 30 −25.286 30.013 25.279 1.00 0.00
C H
ATOM 7254 C HSE C 30 −21.265 28.413 23.198 1.00 0.00
C C
ATOM 7255 O HSE C 30 −21.298 27.210 22.938 1.00 0.00
C O
ATOM 7256 N GLU C 31 −20.168 29.145 23.079 1.00 0.00
C N
ATOM 7257 HN GLU C 31 −20.152 30.122 23.270 1.00 0.00
C H
ATOM 7258 CA GLU C 31 −18.885 28.603 22.661 1.00 0.00
C C
ATOM 7259 HA GLU C 31 −19.039 27.676 22.122 1.00 0.00
C H
ATOM 7260 CB GLU C 31 −18.161 29.604 21.764 1.00 0.00
C C
ATOM 7261 HB1 GLU C 31 −17.176 29.167 21.480 1.00 0.00
C H
ATOM 7262 HB2 GLU C 31 −18.722 29.734 20.812 1.00 0.00
C H

```
ATOM   7263  CG  GLU C  31      -17.904  30.987  22.379  1.00  0.00
C    C
ATOM   7264  HG1 GLU C  31      -17.528  30.867  23.412  1.00  0.00
C    H
ATOM   7265  HG2 GLU C  31      -17.161  31.554  21.784  1.00  0.00
C    H
ATOM   7266  CD  GLU C  31      -19.159  31.846  22.507  1.00  0.00
C    C
ATOM   7267  OE1 GLU C  31      -19.132  33.006  22.047  1.00  0.00
C    O
ATOM   7268  OE2 GLU C  31      -20.157  31.383  23.099  1.00  0.00
C    O
ATOM   7269  C   GLU C  31      -18.089  28.269  23.916  1.00  0.00
C    C
ATOM   7270  O   GLU C  31      -17.667  29.163  24.655  1.00  0.00
C    O
ATOM   7271  N   VAL C  32      -17.915  26.981  24.180  1.00  0.00
C    N
ATOM   7272  HN  VAL C  32      -18.219  26.247  23.577  1.00  0.00
C    H
ATOM   7273  CA  VAL C  32      -17.252  26.528  25.392  1.00  0.00
C    C
ATOM   7274  HA  VAL C  32      -16.687  27.356  25.801  1.00  0.00
C    H
ATOM   7275  CB  VAL C  32      -18.262  26.039  26.444  1.00  0.00
C    C
ATOM   7276  HB  VAL C  32      -17.680  25.617  27.300  1.00  0.00
C    H
ATOM   7277  CG1 VAL C  32      -19.103  27.200  26.928  1.00  0.00
C    C
ATOM   7278 HG11 VAL C  32      -19.862  26.848  27.660  1.00  0.00
C    H
ATOM   7279 HG12 VAL C  32      -18.486  27.971  27.436  1.00  0.00
C    H
ATOM   7280 HG13 VAL C  32      -19.640  27.677  26.080  1.00  0.00
C    H
ATOM   7281  CG2 VAL C  32      -19.160  24.970  25.866  1.00  0.00
C    C
ATOM   7282 HG21 VAL C  32      -19.915  24.645  26.613  1.00  0.00
C    H
ATOM   7283 HG22 VAL C  32      -19.697  25.353  24.970  1.00  0.00
C    H
ATOM   7284 HG23 VAL C  32      -18.577  24.074  25.567  1.00  0.00
C    H
ATOM   7285  C   VAL C  32      -16.262  25.433  25.034  1.00  0.00
C    C
ATOM   7286  O   VAL C  32      -16.625  24.427  24.415  1.00  0.00
C    O
ATOM   7287  N   LEU C  33      -15.011  25.638  25.412  1.00  0.00
C    N
ATOM   7288  HN  LEU C  33      -14.695  26.487  25.833  1.00  0.00
C    H
ATOM   7289  CA  LEU C  33      -13.976  24.634  25.246  1.00  0.00
C    C
```

```
ATOM   7290  HA  LEU C  33      -14.271  23.979  24.436  1.00  0.00
C    H
ATOM   7291  CB  LEU C  33      -12.621  25.273  24.967  1.00  0.00
C    C
ATOM   7292  HB1 LEU C  33      -12.621  25.591  23.900  1.00  0.00
C    H
ATOM   7293  HB2 LEU C  33      -12.530  26.206  25.566  1.00  0.00
C    H
ATOM   7294  CG  LEU C  33      -11.466  24.312  25.228  1.00  0.00
C    C
ATOM   7295  HG  LEU C  33      -11.545  23.983  26.292  1.00  0.00
C    H
ATOM   7296  CD1 LEU C  33      -11.556  23.096  24.311  1.00  0.00
C    C
ATOM   7297 HD11 LEU C  33      -10.688  22.423  24.471  1.00  0.00
C    H
ATOM   7298 HD12 LEU C  33      -12.485  22.521  24.514  1.00  0.00
C    H
ATOM   7299 HD13 LEU C  33      -11.562  23.409  23.245  1.00  0.00
C    H
ATOM   7300  CD2 LEU C  33      -10.140  25.023  25.054  1.00  0.00
C    C
ATOM   7301 HD21 LEU C  33       -9.298  24.327  25.262  1.00  0.00
C    H
ATOM   7302 HD22 LEU C  33      -10.012  25.406  24.022  1.00  0.00
C    H
ATOM   7303 HD23 LEU C  33      -10.063  25.880  25.757  1.00  0.00
C    H
ATOM   7304  C   LEU C  33      -13.915  23.798  26.513  1.00  0.00
C    C
ATOM   7305  O   LEU C  33      -13.694  24.331  27.605  1.00  0.00
C    O
ATOM   7306  N   ALA C  34      -14.106  22.497  26.368  1.00  0.00
C    N
ATOM   7307  HN  ALA C  34      -14.310  22.073  25.486  1.00  0.00
C    H
ATOM   7308  CA  ALA C  34      -14.031  21.586  27.495  1.00  0.00
C    C
ATOM   7309  HA  ALA C  34      -14.176  22.101  28.438  1.00  0.00
C    H
ATOM   7310  CB  ALA C  34      -15.083  20.488  27.377  1.00  0.00
C    C
ATOM   7311  HB1 ALA C  34      -16.097  20.940  27.394  1.00  0.00
C    H
ATOM   7312  HB2 ALA C  34      -14.973  19.930  26.421  1.00  0.00
C    H
ATOM   7313  HB3 ALA C  34      -15.016  19.765  28.219  1.00  0.00
C    H
ATOM   7314  C   ALA C  34      -12.636  20.988  27.543  1.00  0.00
C    C
ATOM   7315  O   ALA C  34      -12.110  20.553  26.514  1.00  0.00
C    O
ATOM   7316  N   VAL C  35      -12.034  20.989  28.726  1.00  0.00
C    N
```

ATOM 7317 HN VAL C 35 -12.432 21.425 29.530 1.00 0.00
C H
ATOM 7318 CA VAL C 35 -10.743 20.357 28.962 1.00 0.00
C C
ATOM 7319 HA VAL C 35 -10.569 19.626 28.182 1.00 0.00
C H
ATOM 7320 CB VAL C 35 -9.590 21.378 28.991 1.00 0.00
C C
ATOM 7321 HB VAL C 35 -9.774 22.070 29.849 1.00 0.00
C H
ATOM 7322 CG1 VAL C 35 -8.261 20.671 29.193 1.00 0.00
C C
ATOM 7323 HG11 VAL C 35 -7.426 21.406 29.175 1.00 0.00
C H
ATOM 7324 HG12 VAL C 35 -8.219 20.148 30.171 1.00 0.00
C H
ATOM 7325 HG13 VAL C 35 -8.083 19.930 28.385 1.00 0.00
C H
ATOM 7326 CG2 VAL C 35 -9.578 22.206 27.728 1.00 0.00
C C
ATOM 7327 HG21 VAL C 35 -8.723 22.916 27.731 1.00 0.00
C H
ATOM 7328 HG22 VAL C 35 -9.487 21.552 26.834 1.00 0.00
C H
ATOM 7329 HG23 VAL C 35 -10.508 22.803 27.630 1.00 0.00
C H
ATOM 7330 C VAL C 35 -10.807 19.610 30.280 1.00 0.00
C C
ATOM 7331 O VAL C 35 -11.406 20.092 31.247 1.00 0.00
C O
ATOM 7332 N ASP C 36 -10.190 18.436 30.320 1.00 0.00
C N
ATOM 7333 HN ASP C 36 -9.789 17.946 29.550 1.00 0.00
C H
ATOM 7334 CA ASP C 36 -10.045 17.729 31.577 1.00 0.00
C C
ATOM 7335 HA ASP C 36 -9.639 18.422 32.305 1.00 0.00
C H
ATOM 7336 CB ASP C 36 -11.364 17.141 32.050 1.00 0.00
C C
ATOM 7337 HB1 ASP C 36 -12.182 17.859 31.835 1.00 0.00
C H
ATOM 7338 HB2 ASP C 36 -11.582 16.195 31.518 1.00 0.00
C H
ATOM 7339 CG ASP C 36 -11.359 16.881 33.516 1.00 0.00
C C
ATOM 7340 OD1 ASP C 36 -11.552 17.854 34.271 1.00 0.00
C O
ATOM 7341 OD2 ASP C 36 -11.150 15.718 33.913 1.00 0.00
C O
ATOM 7342 C ASP C 36 -9.024 16.626 31.407 1.00 0.00
C C
ATOM 7343 O ASP C 36 -9.032 15.914 30.402 1.00 0.00
C O

```
ATOM   7344  N   ILE C  37      -8.158  16.480  32.406  1.00  0.00
C    N
ATOM   7345  HN  ILE C  37      -8.115  17.031  33.237  1.00  0.00
C    H
ATOM   7346  CA  ILE C  37      -7.153  15.431  32.334  1.00  0.00
C    C
ATOM   7347  HA  ILE C  37      -6.663  15.539  31.375  1.00  0.00
C    H
ATOM   7348  CB  ILE C  37      -6.143  15.565  33.481  1.00  0.00
C    C
ATOM   7349  HB  ILE C  37      -5.783  16.623  33.415  1.00  0.00
C    H
ATOM   7350  CG2 ILE C  37      -6.790  15.212  34.802  1.00  0.00
C    C
ATOM   7351 HG21 ILE C  37      -6.113  15.456  35.646  1.00  0.00
C    H
ATOM   7352 HG22 ILE C  37      -7.729  15.785  34.948  1.00  0.00
C    H
ATOM   7353 HG23 ILE C  37      -7.027  14.129  34.851  1.00  0.00
C    H
ATOM   7354  CG1 ILE C  37      -4.940  14.665  33.219  1.00  0.00
C    C
ATOM   7355 HG11 ILE C  37      -5.225  13.600  33.376  1.00  0.00
C    H
ATOM   7356 HG12 ILE C  37      -4.636  14.771  32.153  1.00  0.00
C    H
ATOM   7357  CD  ILE C  37      -3.757  14.978  34.071  1.00  0.00
C    C
ATOM   7358  HD1 ILE C  37      -2.898  14.329  33.792  1.00  0.00
C    H
ATOM   7359  HD2 ILE C  37      -3.449  16.038  33.938  1.00  0.00
C    H
ATOM   7360  HD3 ILE C  37      -3.982  14.808  35.145  1.00  0.00
C    H
ATOM   7361  C   ILE C  37      -7.804  14.061  32.342  1.00  0.00
C    C
ATOM   7362  O   ILE C  37      -7.237  13.095  31.823  1.00  0.00
C    O
ATOM   7363  N   ASN C  38      -8.995  13.943  32.907  1.00  0.00
C    N
ATOM   7364  HN  ASN C  38      -9.499  14.680  33.357  1.00  0.00
C    H
ATOM   7365  CA  ASN C  38      -9.675  12.664  32.904  1.00  0.00
C    C
ATOM   7366  HA  ASN C  38      -8.910  11.900  32.990  1.00  0.00
C    H
ATOM   7367  CB  ASN C  38     -10.689  12.573  34.031  1.00  0.00
C    C
ATOM   7368  HB1 ASN C  38     -10.131  12.393  34.977  1.00  0.00
C    H
ATOM   7369  HB2 ASN C  38     -11.230  13.536  34.141  1.00  0.00
C    H
ATOM   7370  CG  ASN C  38     -11.647  11.447  33.824  1.00  0.00
C    C
```

```
ATOM   7371  OD1 ASN C  38      -12.644  11.592  33.129  1.00  0.00
C    O
ATOM   7372  ND2 ASN C  38      -11.336  10.296  34.396  1.00  0.00
C    N
ATOM   7373 HD21 ASN C  38      -11.961   9.532  34.257  1.00  0.00
C    H
ATOM   7374 HD22 ASN C  38      -10.491  10.209  34.915  1.00  0.00
C    H
ATOM   7375  C   ASN C  38      -10.361  12.478  31.565  1.00  0.00
C    C
ATOM   7376  O   ASN C  38      -11.375  13.124  31.282  1.00  0.00
C    O
ATOM   7377  N   GLU C  39       -9.810  11.587  30.749  1.00  0.00
C    N
ATOM   7378  HN  GLU C  39       -8.998  11.049  30.958  1.00  0.00
C    H
ATOM   7379  CA  GLU C  39      -10.377  11.321  29.434  1.00  0.00
C    C
ATOM   7380  HA  GLU C  39      -10.379  12.247  28.872  1.00  0.00
C    H
ATOM   7381  CB  GLU C  39       -9.544  10.258  28.735  1.00  0.00
C    C
ATOM   7382  HB1 GLU C  39       -9.780  10.291  27.646  1.00  0.00
C    H
ATOM   7383  HB2 GLU C  39       -8.463  10.508  28.820  1.00  0.00
C    H
ATOM   7384  CG  GLU C  39       -9.819   8.876  29.278  1.00  0.00
C    C
ATOM   7385  HG1 GLU C  39      -10.908   8.747  29.424  1.00  0.00
C    H
ATOM   7386  HG2 GLU C  39       -9.450   8.095  28.583  1.00  0.00
C    H
ATOM   7387  CD  GLU C  39       -9.194   8.642  30.637  1.00  0.00
C    C
ATOM   7388  OE1 GLU C  39       -8.807   9.623  31.308  1.00  0.00
C    O
ATOM   7389  OE2 GLU C  39       -9.098   7.469  31.043  1.00  0.00
C    O
ATOM   7390  C   GLU C  39      -11.817  10.858  29.545  1.00  0.00
C    C
ATOM   7391  O   GLU C  39      -12.588  10.965  28.592  1.00  0.00
C    O
ATOM   7392  N   GLU C  40      -12.196  10.315  30.696  1.00  0.00
C    N
ATOM   7393  HN  GLU C  40      -11.651  10.239  31.527  1.00  0.00
C    H
ATOM   7394  CA  GLU C  40      -13.534   9.761  30.815  1.00  0.00
C    C
ATOM   7395  HA  GLU C  40      -13.704   9.106  29.970  1.00  0.00
C    H
ATOM   7396  CB  GLU C  40      -13.648   8.969  32.113  1.00  0.00
C    C
ATOM   7397  HB1 GLU C  40      -12.729   9.157  32.716  1.00  0.00
C    H
```

ATOM 7398 HB2 GLU C 40 -14.496 9.358 32.719 1.00 0.00
C H
ATOM 7399 CG GLU C 40 -13.808 7.452 31.980 1.00 0.00
C C
ATOM 7400 HG1 GLU C 40 -12.812 6.982 31.883 1.00 0.00
C H
ATOM 7401 HG2 GLU C 40 -14.327 7.029 32.863 1.00 0.00
C H
ATOM 7402 CD GLU C 40 -14.568 6.996 30.747 1.00 0.00
C C
ATOM 7403 OE1 GLU C 40 -15.612 6.342 30.931 1.00 0.00
C O
ATOM 7404 OE2 GLU C 40 -14.094 7.215 29.608 1.00 0.00
C O
ATOM 7405 C GLU C 40 -14.590 10.853 30.757 1.00 0.00
C C
ATOM 7406 O GLU C 40 -15.563 10.740 30.003 1.00 0.00
C O
ATOM 7407 N LYS C 41 -14.413 11.914 31.541 1.00 0.00
C N
ATOM 7408 HN LYS C 41 -13.651 12.037 32.174 1.00 0.00
C H
ATOM 7409 CA LYS C 41 -15.376 13.007 31.511 1.00 0.00
C C
ATOM 7410 HA LYS C 41 -16.354 12.574 31.683 1.00 0.00
C H
ATOM 7411 CB LYS C 41 -15.046 14.037 32.586 1.00 0.00
C C
ATOM 7412 HB1 LYS C 41 -14.146 14.597 32.239 1.00 0.00
C H
ATOM 7413 HB2 LYS C 41 -15.873 14.780 32.642 1.00 0.00
C H
ATOM 7414 CG LYS C 41 -14.765 13.455 33.940 1.00 0.00
C C
ATOM 7415 HG1 LYS C 41 -14.404 12.408 33.819 1.00 0.00
C H
ATOM 7416 HG2 LYS C 41 -13.945 14.035 34.421 1.00 0.00
C H
ATOM 7417 CD LYS C 41 -15.987 13.453 34.816 1.00 0.00
C C
ATOM 7418 HD1 LYS C 41 -16.606 14.344 34.560 1.00 0.00
C H
ATOM 7419 HD2 LYS C 41 -16.589 12.548 34.570 1.00 0.00
C H
ATOM 7420 CE LYS C 41 -15.563 13.459 36.268 1.00 0.00
C C
ATOM 7421 HE1 LYS C 41 -16.443 13.309 36.932 1.00 0.00
C H
ATOM 7422 HE2 LYS C 41 -14.830 12.645 36.464 1.00 0.00
C H
ATOM 7423 NZ LYS C 41 -14.926 14.754 36.628 1.00 0.00
C N
ATOM 7424 HZ1 LYS C 41 -14.640 14.744 37.628 1.00 0.00
C H

```
ATOM   7425  HZ2 LYS C  41      -14.087  14.907  36.032  1.00  0.00
C    H
ATOM   7426  HZ3 LYS C  41      -15.603  15.528  36.471  1.00  0.00
C    H
ATOM   7427  C   LYS C  41      -15.408  13.675  30.148  1.00  0.00
C    C
ATOM   7428  O   LYS C  41      -16.486  14.002  29.640  1.00  0.00
C    O
ATOM   7429  N   VAL C  42      -14.238  13.946  29.574  1.00  0.00
C    N
ATOM   7430  HN  VAL C  42      -13.345  13.790  29.988  1.00  0.00
C    H
ATOM   7431  CA  VAL C  42      -14.189  14.517  28.235  1.00  0.00
C    C
ATOM   7432  HA  VAL C  42      -14.681  15.481  28.261  1.00  0.00
C    H
ATOM   7433  CB  VAL C  42      -12.729  14.672  27.781  1.00  0.00
C    C
ATOM   7434  HB  VAL C  42      -12.331  13.650  27.568  1.00  0.00
C    H
ATOM   7435  CG1 VAL C  42      -12.653  15.508  26.522  1.00  0.00
C    C
ATOM   7436 HG11 VAL C  42      -11.594  15.652  26.218  1.00  0.00
C    H
ATOM   7437 HG12 VAL C  42      -13.178  15.021  25.673  1.00  0.00
C    H
ATOM   7438 HG13 VAL C  42      -13.101  16.511  26.689  1.00  0.00
C    H
ATOM   7439  CG2 VAL C  42      -11.902  15.275  28.894  1.00  0.00
C    C
ATOM   7440 HG21 VAL C  42      -10.852  15.429  28.564  1.00  0.00
C    H
ATOM   7441 HG22 VAL C  42      -12.320  16.258  29.203  1.00  0.00
C    H
ATOM   7442 HG23 VAL C  42      -11.880  14.610  29.782  1.00  0.00
C    H
ATOM   7443  C   VAL C  42      -14.967  13.635  27.281  1.00  0.00
C    C
ATOM   7444  O   VAL C  42      -15.805  14.102  26.501  1.00  0.00
C    O
ATOM   7445  N   ASN C  43      -14.705  12.336  27.356  1.00  0.00
C    N
ATOM   7446  HN  ASN C  43      -14.050  11.945  28.000  1.00  0.00
C    H
ATOM   7447  CA  ASN C  43      -15.357  11.363  26.496  1.00  0.00
C    C
ATOM   7448  HA  ASN C  43      -15.078  11.629  25.481  1.00  0.00
C    H
ATOM   7449  CB  ASN C  43      -14.886   9.965  26.885  1.00  0.00
C    C
ATOM   7450  HB1 ASN C  43      -13.896  10.065  27.381  1.00  0.00
C    H
ATOM   7451  HB2 ASN C  43      -15.583   9.514  27.622  1.00  0.00
C    H
```

```
ATOM   7452  CG  ASN C  43     -14.725   9.061  25.702  1.00  0.00
C    C
ATOM   7453  OD1 ASN C  43     -15.618   8.938  24.877  1.00  0.00
C    O
ATOM   7454  ND2 ASN C  43     -13.566   8.427  25.597  1.00  0.00
C    N
ATOM   7455 HD21 ASN C  43     -13.440   7.832  24.809  1.00  0.00
C    H
ATOM   7456 HD22 ASN C  43     -12.852   8.579  26.274  1.00  0.00
C    H
ATOM   7457  C   ASN C  43     -16.863  11.467  26.600  1.00  0.00
C    C
ATOM   7458  O   ASN C  43     -17.572  11.466  25.588  1.00  0.00
C    O
ATOM   7459  N   ALA C  44     -17.371  11.552  27.823  1.00  0.00
C    N
ATOM   7460  HN  ALA C  44     -16.835  11.533  28.666  1.00  0.00
C    H
ATOM   7461  CA  ALA C  44     -18.810  11.685  27.999  1.00  0.00
C    C
ATOM   7462  HA  ALA C  44     -19.332  10.904  27.458  1.00  0.00
C    H
ATOM   7463  CB  ALA C  44     -19.172  11.564  29.475  1.00  0.00
C    C
ATOM   7464  HB1 ALA C  44     -18.843  10.576  29.860  1.00  0.00
C    H
ATOM   7465  HB2 ALA C  44     -18.667  12.351  30.076  1.00  0.00
C    H
ATOM   7466  HB3 ALA C  44     -20.269  11.647  29.635  1.00  0.00
C    H
ATOM   7467  C   ALA C  44     -19.315  13.006  27.440  1.00  0.00
C    C
ATOM   7468  O   ALA C  44     -20.110  13.027  26.494  1.00  0.00
C    O
ATOM   7469  N   TYR C  45     -18.833  14.116  27.976  1.00  0.00
C    N
ATOM   7470  HN  TYR C  45     -18.024  14.153  28.562  1.00  0.00
C    H
ATOM   7471  CA  TYR C  45     -19.469  15.401  27.741  1.00  0.00
C    C
ATOM   7472  HA  TYR C  45     -20.528  15.187  27.668  1.00  0.00
C    H
ATOM   7473  CB  TYR C  45     -19.185  16.364  28.890  1.00  0.00
C    C
ATOM   7474  HB1 TYR C  45     -18.098  16.341  29.126  1.00  0.00
C    H
ATOM   7475  HB2 TYR C  45     -19.436  17.408  28.599  1.00  0.00
C    H
ATOM   7476  CG  TYR C  45     -19.908  16.016  30.158  1.00  0.00
C    C
ATOM   7477  CD1 TYR C  45     -20.819  14.973  30.194  1.00  0.00
C    C
ATOM   7478  HD1 TYR C  45     -21.018  14.404  29.297  1.00  0.00
C    H
```

```
ATOM   7479  CE1 TYR C  45      -21.492  14.661  31.352  1.00  0.00
C    C
ATOM   7480  HE1 TYR C  45      -22.187  13.835  31.378  1.00  0.00
C    H
ATOM   7481  CZ  TYR C  45      -21.282  15.416  32.482  1.00  0.00
C    C
ATOM   7482  OH  TYR C  45      -21.955  15.118  33.641  1.00  0.00
C    O
ATOM   7483  HH  TYR C  45      -21.623  15.703  34.325  1.00  0.00
C    H
ATOM   7484  CD2 TYR C  45      -19.728  16.768  31.304  1.00  0.00
C    C
ATOM   7485  HD2 TYR C  45      -19.078  17.632  31.288  1.00  0.00
C    H
ATOM   7486  CE2 TYR C  45      -20.402  16.467  32.461  1.00  0.00
C    C
ATOM   7487  HE2 TYR C  45      -20.251  17.073  33.342  1.00  0.00
C    H
ATOM   7488  C   TYR C  45      -19.055  16.061  26.442  1.00  0.00
C    C
ATOM   7489  O   TYR C  45      -19.407  17.225  26.226  1.00  0.00
C    O
ATOM   7490  N   ALA C  46      -18.315  15.368  25.580  1.00  0.00
C    N
ATOM   7491  HN  ALA C  46      -17.889  14.483  25.767  1.00  0.00
C    H
ATOM   7492  CA  ALA C  46      -18.081  15.904  24.247  1.00  0.00
C    C
ATOM   7493  HA  ALA C  46      -17.468  16.797  24.292  1.00  0.00
C    H
ATOM   7494  CB  ALA C  46      -17.359  14.871  23.387  1.00  0.00
C    C
ATOM   7495  HB1 ALA C  46      -16.381  14.620  23.849  1.00  0.00
C    H
ATOM   7496  HB2 ALA C  46      -17.952  13.933  23.309  1.00  0.00
C    H
ATOM   7497  HB3 ALA C  46      -17.167  15.255  22.362  1.00  0.00
C    H
ATOM   7498  C   ALA C  46      -19.393  16.316  23.603  1.00  0.00
C    C
ATOM   7499  O   ALA C  46      -19.518  17.418  23.058  1.00  0.00
C    O
ATOM   7500  N   SER C  47      -20.399  15.449  23.695  1.00  0.00
C    N
ATOM   7501  HN  SER C  47      -20.341  14.566  24.159  1.00  0.00
C    H
ATOM   7502  CA  SER C  47      -21.698  15.731  23.108  1.00  0.00
C    C
ATOM   7503  HA  SER C  47      -21.508  15.886  22.054  1.00  0.00
C    H
ATOM   7504  CB  SER C  47      -22.637  14.559  23.358  1.00  0.00
C    C
ATOM   7505  HB1 SER C  47      -23.455  14.604  22.603  1.00  0.00
C    H
```

```
ATOM   7506  HB2 SER C  47      −22.102  13.594  23.206  1.00  0.00
C    H
ATOM   7507  OG  SER C  47      −23.192  14.652  24.655  1.00  0.00
C    O
ATOM   7508  HG1 SER C  47      −23.934  14.035  24.690  1.00  0.00
C    H
ATOM   7509  C   SER C  47      −22.325  17.004  23.647  1.00  0.00
C    C
ATOM   7510  O   SER C  47      −23.114  17.638  22.941  1.00  0.00
C    O
ATOM   7511  N   TYR C  48      −22.006  17.393  24.875  1.00  0.00
C    N
ATOM   7512  HN  TYR C  48      −21.373  16.899  25.468  1.00  0.00
C    H
ATOM   7513  CA  TYR C  48      −22.570  18.597  25.464  1.00  0.00
C    C
ATOM   7514  HA  TYR C  48      −23.403  18.884  24.834  1.00  0.00
C    H
ATOM   7515  CB  TYR C  48      −23.015  18.325  26.894  1.00  0.00
C    C
ATOM   7516  HB1 TYR C  48      −22.126  18.066  27.510  1.00  0.00
C    H
ATOM   7517  HB2 TYR C  48      −23.471  19.234  27.343  1.00  0.00
C    H
ATOM   7518  CG  TYR C  48      −23.969  17.178  26.980  1.00  0.00
C    C
ATOM   7519  CD1 TYR C  48      −24.811  16.878  25.924  1.00  0.00
C    C
ATOM   7520  HD1 TYR C  48      −24.804  17.498  25.038  1.00  0.00
C    H
ATOM   7521  CE1 TYR C  48      −25.673  15.818  25.987  1.00  0.00
C    C
ATOM   7522  HE1 TYR C  48      −26.341  15.603  25.166  1.00  0.00
C    H
ATOM   7523  CZ  TYR C  48      −25.690  15.029  27.112  1.00  0.00
C    C
ATOM   7524  OH  TYR C  48      −26.553  13.965  27.181  1.00  0.00
C    O
ATOM   7525  HH  TYR C  48      −26.370  13.498  27.999  1.00  0.00
C    H
ATOM   7526  CD2 TYR C  48      −23.997  16.362  28.096  1.00  0.00
C    C
ATOM   7527  HD2 TYR C  48      −23.362  16.583  28.943  1.00  0.00
C    H
ATOM   7528  CE2 TYR C  48      −24.856  15.297  28.169  1.00  0.00
C    C
ATOM   7529  HE2 TYR C  48      −24.886  14.692  29.063  1.00  0.00
C    H
ATOM   7530  C   TYR C  48      −21.579  19.739  25.440  1.00  0.00
C    C
ATOM   7531  O   TYR C  48      −21.622  20.610  26.309  1.00  0.00
C    O
ATOM   7532  N   ALA C  49      −20.681  19.750  24.463  1.00  0.00
C    N
```

ATOM   7533 HN   ALA C 49   -20.705 19.134 23.676 1.00 0.00
C   H
ATOM   7534 CA   ALA C 49   -19.577 20.689 24.485 1.00 0.00
C   C
ATOM   7535 HA   ALA C 49   -19.788 21.530 25.137 1.00 0.00
C   H
ATOM   7536 CB   ALA C 49   -18.285 20.025 24.963 1.00 0.00
C   C
ATOM   7537 HB1 ALA C 49   -18.421 19.647 25.999 1.00 0.00
C   H
ATOM   7538 HB2 ALA C 49   -18.018 19.161 24.317 1.00 0.00
C   H
ATOM   7539 HB3 ALA C 49   -17.434 20.740 24.968 1.00 0.00
C   H
ATOM   7540 C   ALA C 49   -19.372 21.264 23.101 1.00 0.00
C   C
ATOM   7541 O   ALA C 49   -19.343 20.535 22.107 1.00 0.00
C   O
ATOM   7542 N   THR C 50   -19.223 22.583 23.050 1.00 0.00
C   N
ATOM   7543 HN   THR C 50   -19.379 23.225 23.800 1.00 0.00
C   H
ATOM   7544 CA   THR C 50   -18.795 23.208 21.811 1.00 0.00
C   C
ATOM   7545 HA   THR C 50   -19.544 22.953 21.073 1.00 0.00
C   H
ATOM   7546 CB   THR C 50   -18.638 24.716 22.006 1.00 0.00
C   C
ATOM   7547 HB   THR C 50   -17.733 24.854 22.647 1.00 0.00
C   H
ATOM   7548 OG1 THR C 50   -19.768 25.233 22.719 1.00 0.00
C   O
ATOM   7549 HG1 THR C 50   -19.612 26.180 22.795 1.00 0.00
C   H
ATOM   7550 CG2 THR C 50   -18.530 25.423 20.664 1.00 0.00
C   C
ATOM   7551 HG21 THR C 50   -18.303 26.501 20.803 1.00 0.00
C   H
ATOM   7552 HG22 THR C 50   -17.714 24.977 20.056 1.00 0.00
C   H
ATOM   7553 HG23 THR C 50   -19.478 25.339 20.090 1.00 0.00
C   H
ATOM   7554 C   THR C 50   -17.490 22.604 21.328 1.00 0.00
C   C
ATOM   7555 O   THR C 50   -17.290 22.442 20.121 1.00 0.00
C   O
ATOM   7556 N   HSE C 51   -16.612 22.237 22.250 1.00 0.00
C   N
ATOM   7557 HN   HSE C 51   -16.750 22.328 23.235 1.00 0.00
C   H
ATOM   7558 CA   HSE C 51   -15.337 21.655 21.877 1.00 0.00
C   C
ATOM   7559 HA   HSE C 51   -15.526 20.849 21.179 1.00 0.00
C   H

```
ATOM   7560  CB  HSE C  51      -14.424  22.717  21.266  1.00  0.00
C    C
ATOM   7561  HB1 HSE C  51      -14.909  23.092  20.339  1.00  0.00
C    H
ATOM   7562  HB2 HSE C  51      -14.341  23.579  21.961  1.00  0.00
C    H
ATOM   7563  ND1 HSE C  51      -12.074  23.041  20.424  1.00  0.00
C    N
ATOM   7564  CG  HSE C  51      -13.066  22.214  20.903  1.00  0.00
C    C
ATOM   7565  CE1 HSE C  51      -10.986  22.331  20.190  1.00  0.00
C    C
ATOM   7566  HE1 HSE C  51      -10.051  22.740  19.798  1.00  0.00
C    H
ATOM   7567  NE2 HSE C  51      -11.236  21.073  20.502  1.00  0.00
C    N
ATOM   7568  HE2 HSE C  51      -10.589  20.317  20.404  1.00  0.00
C    H
ATOM   7569  CD2 HSE C  51      -12.529  20.972  20.949  1.00  0.00
C    C
ATOM   7570  HD2 HSE C  51      -12.974  20.035  21.256  1.00  0.00
C    H
ATOM   7571  C   HSE C  51      -14.712  21.036  23.115  1.00  0.00
C    C
ATOM   7572  O   HSE C  51      -14.528  21.711  24.131  1.00  0.00
C    O
ATOM   7573  N   ALA C  52      -14.387  19.751  23.022  1.00  0.00
C    N
ATOM   7574  HN  ALA C  52      -14.529  19.231  22.180  1.00  0.00
C    H
ATOM   7575  CA  ALA C  52      -13.806  19.005  24.125  1.00  0.00
C    C
ATOM   7576  HA  ALA C  52      -13.696  19.621  25.009  1.00  0.00
C    H
ATOM   7577  CB  ALA C  52      -14.654  17.785  24.482  1.00  0.00
C    C
ATOM   7578  HB1 ALA C  52      -15.661  18.116  24.814  1.00  0.00
C    H
ATOM   7579  HB2 ALA C  52      -14.786  17.118  23.602  1.00  0.00
C    H
ATOM   7580  HB3 ALA C  52      -14.197  17.197  25.307  1.00  0.00
C    H
ATOM   7581  C   ALA C  52      -12.411  18.560  23.738  1.00  0.00
C    C
ATOM   7582  O   ALA C  52      -12.132  18.343  22.558  1.00  0.00
C    O
ATOM   7583  N   VAL C  53      -11.547  18.415  24.732  1.00  0.00
C    N
ATOM   7584  HN  VAL C  53      -11.785  18.603  25.682  1.00  0.00
C    H
ATOM   7585  CA  VAL C  53      -10.175  17.968  24.532  1.00  0.00
C    C
ATOM   7586  HA  VAL C  53      -10.158  17.224  23.746  1.00  0.00
C    H
```

ATOM 7587 CB VAL C 53 -9.223 19.134 24.197 1.00 0.00
C C
ATOM 7588 HB VAL C 53 -9.509 20.000 24.843 1.00 0.00
C H
ATOM 7589 CG1 VAL C 53 -7.777 18.753 24.486 1.00 0.00
C C
ATOM 7590 HG11 VAL C 53 -7.095 19.584 24.205 1.00 0.00
C H
ATOM 7591 HG12 VAL C 53 -7.617 18.544 25.565 1.00 0.00
C H
ATOM 7592 HG13 VAL C 53 -7.483 17.855 23.902 1.00 0.00
C H
ATOM 7593 CG2 VAL C 53 -9.365 19.561 22.756 1.00 0.00
C C
ATOM 7594 HG21 VAL C 53 -8.648 20.374 22.515 1.00 0.00
C H
ATOM 7595 HG22 VAL C 53 -9.166 18.705 22.075 1.00 0.00
C H
ATOM 7596 HG23 VAL C 53 -10.386 19.942 22.548 1.00 0.00
C H
ATOM 7597 C VAL C 53 -9.724 17.290 25.810 1.00 0.00
C C
ATOM 7598 O VAL C 53 -10.049 17.751 26.907 1.00 0.00
C O
ATOM 7599 N ILE C 54 -8.981 16.201 25.684 1.00 0.00
C N
ATOM 7600 HN ILE C 54 -8.834 15.687 24.841 1.00 0.00
C H
ATOM 7601 CA ILE C 54 -8.291 15.655 26.841 1.00 0.00
C C
ATOM 7602 HA ILE C 54 -8.854 15.929 27.724 1.00 0.00
C H
ATOM 7603 CB ILE C 54 -8.124 14.141 26.725 1.00 0.00
C C
ATOM 7604 HB ILE C 54 -7.461 14.000 25.834 1.00 0.00
C H
ATOM 7605 CG2 ILE C 54 -7.606 13.574 28.019 1.00 0.00
C C
ATOM 7606 HG21 ILE C 54 -7.334 12.505 27.897 1.00 0.00
C H
ATOM 7607 HG22 ILE C 54 -6.696 14.116 28.353 1.00 0.00
C H
ATOM 7608 HG23 ILE C 54 -8.371 13.648 28.820 1.00 0.00
C H
ATOM 7609 CG1 ILE C 54 -9.464 13.502 26.391 1.00 0.00
C C
ATOM 7610 HG11 ILE C 54 -10.080 13.415 27.314 1.00 0.00
C H
ATOM 7611 HG12 ILE C 54 -10.016 14.168 25.688 1.00 0.00
C H
ATOM 7612 CD ILE C 54 -9.332 12.147 25.753 1.00 0.00
C C
ATOM 7613 HD1 ILE C 54 -10.333 11.752 25.477 1.00 0.00
C H

```
ATOM   7614  HD2 ILE C  54      -8.715  12.206  24.830  1.00  0.00
C    H
ATOM   7615  HD3 ILE C  54      -8.856  11.424  26.449  1.00  0.00
C    H
ATOM   7616  C   ILE C  54      -6.944  16.356  26.944  1.00  0.00
C    C
ATOM   7617  O   ILE C  54      -6.174  16.383  25.980  1.00  0.00
C    O
ATOM   7618  N   ALA C  55      -6.661  16.935  28.110  1.00  0.00
C    N
ATOM   7619  HN  ALA C  55      -7.271  16.899  28.901  1.00  0.00
C    H
ATOM   7620  CA  ALA C  55      -5.421  17.672  28.310  1.00  0.00
C    C
ATOM   7621  HA  ALA C  55      -4.565  17.099  27.971  1.00  0.00
C    H
ATOM   7622  CB  ALA C  55      -5.430  19.000  27.548  1.00  0.00
C    C
ATOM   7623  HB1 ALA C  55      -5.537  18.805  26.459  1.00  0.00
C    H
ATOM   7624  HB2 ALA C  55      -6.284  19.638  27.866  1.00  0.00
C    H
ATOM   7625  HB3 ALA C  55      -4.487  19.568  27.703  1.00  0.00
C    H
ATOM   7626  C   ALA C  55      -5.206  17.927  29.795  1.00  0.00
C    C
ATOM   7627  O   ALA C  55      -6.164  18.135  30.543  1.00  0.00
C    O
ATOM   7628  N   ASN C  56      -3.942  17.917  30.203  1.00  0.00
C    N
ATOM   7629  HN  ASN C  56      -3.181  17.725  29.585  1.00  0.00
C    H
ATOM   7630  CA  ASN C  56      -3.550  18.184  31.583  1.00  0.00
C    C
ATOM   7631  HA  ASN C  56      -4.397  17.930  32.213  1.00  0.00
C    H
ATOM   7632  CB  ASN C  56      -2.312  17.380  31.943  1.00  0.00
C    C
ATOM   7633  HB1 ASN C  56      -2.557  16.302  31.819  1.00  0.00
C    H
ATOM   7634  HB2 ASN C  56      -1.483  17.616  31.245  1.00  0.00
C    H
ATOM   7635  CG  ASN C  56      -1.871  17.597  33.372  1.00  0.00
C    C
ATOM   7636  OD1 ASN C  56      -2.319  18.523  34.048  1.00  0.00
C    O
ATOM   7637  ND2 ASN C  56      -0.978  16.738  33.841  1.00  0.00
C    N
ATOM   7638 HD21 ASN C  56      -0.681  16.856  34.785  1.00  0.00
C    H
ATOM   7639 HD22 ASN C  56      -0.651  15.994  33.266  1.00  0.00
C    H
ATOM   7640  C   ASN C  56      -3.305  19.677  31.723  1.00  0.00
C    C
```

```
ATOM   7641  O   ASN C  56      -2.374  20.224  31.131  1.00  0.00
C    O
ATOM   7642  N   ALA C  57      -4.120  20.333  32.546  1.00  0.00
C    N
ATOM   7643  HN  ALA C  57      -4.793  19.871  33.124  1.00  0.00
C    H
ATOM   7644  CA  ALA C  57      -4.088  21.785  32.663  1.00  0.00
C    C
ATOM   7645  HA  ALA C  57      -4.279  22.230  31.693  1.00  0.00
C    H
ATOM   7646  CB  ALA C  57      -5.164  22.252  33.635  1.00  0.00
C    C
ATOM   7647  HB1 ALA C  57      -6.159  21.906  33.283  1.00  0.00
C    H
ATOM   7648  HB2 ALA C  57      -4.995  21.832  34.650  1.00  0.00
C    H
ATOM   7649  HB3 ALA C  57      -5.193  23.360  33.712  1.00  0.00
C    H
ATOM   7650  C   ALA C  57      -2.748  22.324  33.125  1.00  0.00
C    C
ATOM   7651  O   ALA C  57      -2.466  23.503  32.898  1.00  0.00
C    O
ATOM   7652  N   THR C  58      -1.922  21.508  33.752  1.00  0.00
C    N
ATOM   7653  HN  THR C  58      -2.016  20.515  33.806  1.00  0.00
C    H
ATOM   7654  CA  THR C  58      -0.759  22.034  34.441  1.00  0.00
C    C
ATOM   7655  HA  THR C  58      -1.099  22.960  34.885  1.00  0.00
C    H
ATOM   7656  CB  THR C  58      -0.291  21.025  35.485  1.00  0.00
C    C
ATOM   7657  HB  THR C  58       0.566  21.505  36.017  1.00  0.00
C    H
ATOM   7658  OG1 THR C  58       0.194  19.855  34.824  1.00  0.00
C    O
ATOM   7659  HG1 THR C  58       0.462  19.255  35.529  1.00  0.00
C    H
ATOM   7660  CG2 THR C  58      -1.448  20.625  36.376  1.00  0.00
C    C
ATOM   7661 HG21 THR C  58      -1.100  19.980  37.210  1.00  0.00
C    H
ATOM   7662 HG22 THR C  58      -1.926  21.526  36.818  1.00  0.00
C    H
ATOM   7663 HG23 THR C  58      -2.220  20.067  35.803  1.00  0.00
C    H
ATOM   7664  C   THR C  58       0.394  22.395  33.517  1.00  0.00
C    C
ATOM   7665  O   THR C  58       1.399  22.928  33.996  1.00  0.00
C    O
ATOM   7666  N   GLU C  59       0.290  22.129  32.219  1.00  0.00
C    N
ATOM   7667  HN  GLU C  59      -0.524  21.759  31.780  1.00  0.00
C    H
```

```
ATOM   7668  CA  GLU C  59       1.403  22.365  31.304  1.00  0.00
C    C
ATOM   7669  HA  GLU C  59       2.269  22.648  31.888  1.00  0.00
C    H
ATOM   7670  CB  GLU C  59       1.707  21.111  30.492  1.00  0.00
C    C
ATOM   7671  HB1 GLU C  59       0.757  20.759  30.028  1.00  0.00
C    H
ATOM   7672  HB2 GLU C  59       2.384  21.365  29.646  1.00  0.00
C    H
ATOM   7673  CG  GLU C  59       2.300  19.980  31.284  1.00  0.00
C    C
ATOM   7674  HG1 GLU C  59       2.594  19.164  30.599  1.00  0.00
C    H
ATOM   7675  HG2 GLU C  59       3.189  20.317  31.853  1.00  0.00
C    H
ATOM   7676  CD  GLU C  59       1.308  19.382  32.242  1.00  0.00
C    C
ATOM   7677  OE1 GLU C  59       1.688  19.100  33.395  1.00  0.00
C    O
ATOM   7678  OE2 GLU C  59       0.131  19.245  31.867  1.00  0.00
C    O
ATOM   7679  C   GLU C  59       1.094  23.521  30.362  1.00  0.00
C    C
ATOM   7680  O   GLU C  59       0.056  23.526  29.693  1.00  0.00
C    O
ATOM   7681  N   GLU C  60       2.029  24.467  30.271  1.00  0.00
C    N
ATOM   7682  HN  GLU C  60       2.937  24.418  30.677  1.00  0.00
C    H
ATOM   7683  CA  GLU C  60       1.775  25.700  29.533  1.00  0.00
C    C
ATOM   7684  HA  GLU C  60       0.851  26.124  29.903  1.00  0.00
C    H
ATOM   7685  CB  GLU C  60       2.901  26.704  29.798  1.00  0.00
C    C
ATOM   7686  HB1 GLU C  60       2.744  27.586  29.135  1.00  0.00
C    H
ATOM   7687  HB2 GLU C  60       2.825  27.089  30.840  1.00  0.00
C    H
ATOM   7688  CG  GLU C  60       4.341  26.220  29.576  1.00  0.00
C    C
ATOM   7689  HG1 GLU C  60       5.036  27.074  29.673  1.00  0.00
C    H
ATOM   7690  HG2 GLU C  60       4.616  25.439  30.313  1.00  0.00
C    H
ATOM   7691  CD  GLU C  60       4.616  25.659  28.201  1.00  0.00
C    C
ATOM   7692  OE1 GLU C  60       4.328  26.354  27.206  1.00  0.00
C    O
ATOM   7693  OE2 GLU C  60       5.079  24.504  28.114  1.00  0.00
C    O
ATOM   7694  C   GLU C  60       1.590  25.438  28.046  1.00  0.00
C    C
```

```
ATOM   7695  O   GLU C  60       0.859  26.165  27.367  1.00  0.00
C    O
ATOM   7696  N   ASN C  61       2.259  24.420  27.516  1.00  0.00
C    N
ATOM   7697  HN  ASN C  61       2.824  23.765  28.017  1.00  0.00
C    H
ATOM   7698  CA  ASN C  61       2.201  24.192  26.081  1.00  0.00
C    C
ATOM   7699  HA  ASN C  61       2.335  25.162  25.615  1.00  0.00
C    H
ATOM   7700  CB  ASN C  61       3.304  23.229  25.664  1.00  0.00
C    C
ATOM   7701  HB1 ASN C  61       3.049  22.836  24.656  1.00  0.00
C    H
ATOM   7702  HB2 ASN C  61       4.271  23.765  25.579  1.00  0.00
C    H
ATOM   7703  CG  ASN C  61       3.422  22.074  26.605  1.00  0.00
C    C
ATOM   7704  OD1 ASN C  61       3.092  22.191  27.784  1.00  0.00
C    O
ATOM   7705  ND2 ASN C  61       3.900  20.946  26.102  1.00  0.00
C    N
ATOM   7706 HD21 ASN C  61       3.973  20.166  26.716  1.00  0.00
C    H
ATOM   7707 HD22 ASN C  61       4.143  20.894  25.138  1.00  0.00
C    H
ATOM   7708  C   ASN C  61       0.835  23.673  25.678  1.00  0.00
C    C
ATOM   7709  O   ASN C  61       0.309  24.042  24.625  1.00  0.00
C    O
ATOM   7710  N   GLU C  62       0.240  22.817  26.508  1.00  0.00
C    N
ATOM   7711  HN  GLU C  62       0.655  22.340  27.278  1.00  0.00
C    H
ATOM   7712  CA  GLU C  62      -1.167  22.510  26.312  1.00  0.00
C    C
ATOM   7713  HA  GLU C  62      -1.274  22.014  25.356  1.00  0.00
C    H
ATOM   7714  CB  GLU C  62      -1.688  21.603  27.418  1.00  0.00
C    C
ATOM   7715  HB1 GLU C  62      -1.244  21.941  28.383  1.00  0.00
C    H
ATOM   7716  HB2 GLU C  62      -2.788  21.735  27.525  1.00  0.00
C    H
ATOM   7717  CG  GLU C  62      -1.364  20.154  27.211  1.00  0.00
C    C
ATOM   7718  HG1 GLU C  62      -2.132  19.693  26.563  1.00  0.00
C    H
ATOM   7719  HG2 GLU C  62      -0.368  20.032  26.740  1.00  0.00
C    H
ATOM   7720  CD  GLU C  62      -1.386  19.364  28.492  1.00  0.00
C    C
ATOM   7721  OE1 GLU C  62      -2.375  18.645  28.729  1.00  0.00
C    O
```

ATOM 7722 OE2 GLU C 62 −0.411 19.447 29.259 1.00 0.00
C O
ATOM 7723 C GLU C 62 −1.998 23.775 26.258 1.00 0.00
C C
ATOM 7724 O GLU C 62 −2.925 23.878 25.449 1.00 0.00
C O
ATOM 7725 N LEU C 63 −1.658 24.756 27.081 1.00 0.00
C N
ATOM 7726 HN LEU C 63 −0.873 24.745 27.697 1.00 0.00
C H
ATOM 7727 CA LEU C 63 −2.448 25.971 27.143 1.00 0.00
C C
ATOM 7728 HA LEU C 63 −3.482 25.663 27.237 1.00 0.00
C H
ATOM 7729 CB LEU C 63 −2.001 26.791 28.341 1.00 0.00
C C
ATOM 7730 HB1 LEU C 63 −1.029 27.265 28.071 1.00 0.00
C H
ATOM 7731 HB2 LEU C 63 −2.719 27.627 28.497 1.00 0.00
C H
ATOM 7732 CG LEU C 63 −1.840 25.931 29.588 1.00 0.00
C C
ATOM 7733 HG LEU C 63 −1.063 25.166 29.346 1.00 0.00
C H
ATOM 7734 CD1 LEU C 63 −1.372 26.773 30.750 1.00 0.00
C C
ATOM 7735 HD11 LEU C 63 −1.299 26.158 31.673 1.00 0.00
C H
ATOM 7736 HD12 LEU C 63 −0.370 27.207 30.541 1.00 0.00
C H
ATOM 7737 HD13 LEU C 63 −2.081 27.606 30.943 1.00 0.00
C H
ATOM 7738 CD2 LEU C 63 −3.125 25.205 29.926 1.00 0.00
C C
ATOM 7739 HD21 LEU C 63 −2.983 24.569 30.827 1.00 0.00
C H
ATOM 7740 HD22 LEU C 63 −3.951 25.912 30.141 1.00 0.00
C H
ATOM 7741 HD23 LEU C 63 −3.435 24.549 29.084 1.00 0.00
C H
ATOM 7742 C LEU C 63 −2.324 26.780 25.863 1.00 0.00
C C
ATOM 7743 O LEU C 63 −3.336 27.117 25.239 1.00 0.00
C O
ATOM 7744 N LEU C 64 −1.095 27.097 25.460 1.00 0.00
C N
ATOM 7745 HN LEU C 64 −0.252 26.886 25.951 1.00 0.00
C H
ATOM 7746 CA LEU C 64 −0.895 27.812 24.208 1.00 0.00
C C
ATOM 7747 HA LEU C 64 −1.414 28.758 24.302 1.00 0.00
C H
ATOM 7748 CB LEU C 64 0.590 28.019 23.955 1.00 0.00
C C

```
ATOM   7749  HB1 LEU C  64       0.980  27.079  23.501  1.00  0.00
C    H
ATOM   7750  HB2 LEU C  64       0.718  28.810  23.183  1.00  0.00
C    H
ATOM   7751  CG  LEU C  64       1.365  28.339  25.219  1.00  0.00
C    C
ATOM   7752  HG  LEU C  64       1.050  27.622  26.015  1.00  0.00
C    H
ATOM   7753  CD1 LEU C  64       2.845  28.188  24.966  1.00  0.00
C    C
ATOM   7754 HD11 LEU C  64       3.425  28.466  25.872  1.00  0.00
C    H
ATOM   7755 HD12 LEU C  64       3.092  27.136  24.703  1.00  0.00
C    H
ATOM   7756 HD13 LEU C  64       3.171  28.844  24.132  1.00  0.00
C    H
ATOM   7757  CD2 LEU C  64       1.026  29.735  25.687  1.00  0.00
C    C
ATOM   7758 HD21 LEU C  64       1.574  29.971  26.624  1.00  0.00
C    H
ATOM   7759 HD22 LEU C  64       1.303  30.499  24.932  1.00  0.00
C    H
ATOM   7760 HD23 LEU C  64      -0.064  29.823  25.891  1.00  0.00
C    H
ATOM   7761  C   LEU C  64      -1.517  27.062  23.046  1.00  0.00
C    C
ATOM   7762  O   LEU C  64      -2.177  27.665  22.194  1.00  0.00
C    O
ATOM   7763  N   SER C  65      -1.326  25.744  23.005  1.00  0.00
C    N
ATOM   7764  HN  SER C  65      -0.783  25.219  23.659  1.00  0.00
C    H
ATOM   7765  CA  SER C  65      -1.926  24.942  21.950  1.00  0.00
C    C
ATOM   7766  HA  SER C  65      -1.522  25.319  21.019  1.00  0.00
C    H
ATOM   7767  CB  SER C  65      -1.604  23.471  22.170  1.00  0.00
C    C
ATOM   7768  HB1 SER C  65      -2.117  22.881  21.376  1.00  0.00
C    H
ATOM   7769  HB2 SER C  65      -0.510  23.292  22.056  1.00  0.00
C    H
ATOM   7770  OG  SER C  65      -2.075  23.064  23.436  1.00  0.00
C    O
ATOM   7771  HG1 SER C  65      -2.057  22.099  23.449  1.00  0.00
C    H
ATOM   7772  C   SER C  65      -3.428  25.140  21.901  1.00  0.00
C    C
ATOM   7773  O   SER C  65      -4.031  25.078  20.825  1.00  0.00
C    O
ATOM   7774  N   LEU C  66      -4.047  25.394  23.049  1.00  0.00
C    N
ATOM   7775  HN  LEU C  66      -3.614  25.440  23.948  1.00  0.00
C    H
```

ATOM 7776 CA LEU C 66 -5.477 25.637 23.065 1.00 0.00
C C
ATOM 7777 HA LEU C 66 -5.912 24.920 22.381 1.00 0.00
C H
ATOM 7778 CB LEU C 66 -6.023 25.445 24.470 1.00 0.00
C C
ATOM 7779 HB1 LEU C 66 -5.380 26.044 25.156 1.00 0.00
C H
ATOM 7780 HB2 LEU C 66 -7.039 25.894 24.528 1.00 0.00
C H
ATOM 7781 CG LEU C 66 -6.020 23.986 24.894 1.00 0.00
C C
ATOM 7782 HG LEU C 66 -4.962 23.660 25.042 1.00 0.00
C H
ATOM 7783 CD1 LEU C 66 -6.775 23.836 26.185 1.00 0.00
C C
ATOM 7784 HD11 LEU C 66 -6.824 22.767 26.485 1.00 0.00
C H
ATOM 7785 HD12 LEU C 66 -6.276 24.405 26.998 1.00 0.00
C H
ATOM 7786 HD13 LEU C 66 -7.813 24.214 26.078 1.00 0.00
C H
ATOM 7787 CD2 LEU C 66 -6.628 23.126 23.806 1.00 0.00
C C
ATOM 7788 HD21 LEU C 66 -6.611 22.055 24.106 1.00 0.00
C H
ATOM 7789 HD22 LEU C 66 -7.683 23.402 23.606 1.00 0.00
C H
ATOM 7790 HD23 LEU C 66 -6.054 23.231 22.861 1.00 0.00
C H
ATOM 7791 C LEU C 66 -5.833 27.022 22.562 1.00 0.00
C C
ATOM 7792 O LEU C 66 -7.019 27.307 22.375 1.00 0.00
C O
ATOM 7793 N GLY C 67 -4.850 27.883 22.334 1.00 0.00
C N
ATOM 7794 HN GLY C 67 -3.878 27.661 22.397 1.00 0.00
C H
ATOM 7795 CA GLY C 67 -5.157 29.248 21.966 1.00 0.00
C C
ATOM 7796 HA1 GLY C 67 -5.750 29.229 21.061 1.00 0.00
C H
ATOM 7797 HA2 GLY C 67 -4.229 29.796 21.878 1.00 0.00
C H
ATOM 7798 C GLY C 67 -5.982 29.901 23.052 1.00 0.00
C C
ATOM 7799 O GLY C 67 -6.979 30.579 22.786 1.00 0.00
C O
ATOM 7800 N ILE C 68 -5.566 29.671 24.295 1.00 0.00
C N
ATOM 7801 HN ILE C 68 -4.732 29.174 24.528 1.00 0.00
C H
ATOM 7802 CA ILE C 68 -6.319 30.142 25.446 1.00 0.00
C C

ATOM 7803 HA ILE C 68 -7.304 29.700 25.364 1.00 0.00
C H
ATOM 7804 CB ILE C 68 -5.596 29.711 26.725 1.00 0.00
C C
ATOM 7805 HB ILE C 68 -6.039 30.352 27.528 1.00 0.00
C H
ATOM 7806 CG2 ILE C 68 -5.734 28.227 26.918 1.00 0.00
C C
ATOM 7807 HG21 ILE C 68 -5.352 27.926 27.915 1.00 0.00
C H
ATOM 7808 HG22 ILE C 68 -6.799 27.918 26.859 1.00 0.00
C H
ATOM 7809 HG23 ILE C 68 -5.164 27.670 26.145 1.00 0.00
C H
ATOM 7810 CG1 ILE C 68 -4.124 30.080 26.607 1.00 0.00
C C
ATOM 7811 HG11 ILE C 68 -3.654 29.497 25.784 1.00 0.00
C H
ATOM 7812 HG12 ILE C 68 -4.043 31.157 26.335 1.00 0.00
C H
ATOM 7813 CD ILE C 68 -3.364 29.859 27.849 1.00 0.00
C C
ATOM 7814 HD1 ILE C 68 -2.313 30.199 27.724 1.00 0.00
C H
ATOM 7815 HD2 ILE C 68 -3.815 30.428 28.691 1.00 0.00
C H
ATOM 7816 HD3 ILE C 68 -3.347 28.783 28.120 1.00 0.00
C H
ATOM 7817 C ILE C 68 -6.509 31.647 25.407 1.00 0.00
C C
ATOM 7818 O ILE C 68 -7.601 32.150 25.695 1.00 0.00
C O
ATOM 7819 N ARG C 69 -5.463 32.388 25.053 1.00 0.00
C N
ATOM 7820 HN ARG C 69 -4.600 31.989 24.748 1.00 0.00
C H
ATOM 7821 CA ARG C 69 -5.531 33.838 25.093 1.00 0.00
C C
ATOM 7822 HA ARG C 69 -5.730 34.134 26.117 1.00 0.00
C H
ATOM 7823 CB ARG C 69 -4.212 34.422 24.625 1.00 0.00
C C
ATOM 7824 HB1 ARG C 69 -4.038 34.023 23.598 1.00 0.00
C H
ATOM 7825 HB2 ARG C 69 -4.312 35.524 24.513 1.00 0.00
C H
ATOM 7826 CG ARG C 69 -3.068 34.035 25.519 1.00 0.00
C C
ATOM 7827 HG1 ARG C 69 -3.186 34.560 26.493 1.00 0.00
C H
ATOM 7828 HG2 ARG C 69 -3.143 32.948 25.754 1.00 0.00
C H
ATOM 7829 CD ARG C 69 -1.753 34.310 24.848 1.00 0.00
C C

ATOM 7830 HD1 ARG C 69 -1.744 35.356 24.463 1.00 0.00
C H
ATOM 7831 HD2 ARG C 69 -0.898 34.181 25.552 1.00 0.00
C H
ATOM 7832 NE ARG C 69 -1.530 33.389 23.742 1.00 0.00
C N
ATOM 7833 HE ARG C 69 -1.194 32.489 24.040 1.00 0.00
C H
ATOM 7834 CZ ARG C 69 -1.746 33.695 22.468 1.00 0.00
C C
ATOM 7835 NH1 ARG C 69 -2.199 34.895 22.141 1.00 0.00
C N
ATOM 7836 HH11 ARG C 69 -2.386 35.122 21.195 1.00 0.00
C H
ATOM 7837 HH12 ARG C 69 -2.464 35.492 22.901 1.00 0.00
C H
ATOM 7838 NH2 ARG C 69 -1.516 32.799 21.523 1.00 0.00
C N
ATOM 7839 HH21 ARG C 69 -1.594 33.056 20.570 1.00 0.00
C H
ATOM 7840 HH22 ARG C 69 -1.069 31.958 21.800 1.00 0.00
C H
ATOM 7841 C ARG C 69 -6.670 34.381 24.258 1.00 0.00
C C
ATOM 7842 O ARG C 69 -7.200 35.447 24.581 1.00 0.00
C O
ATOM 7843 N ASN C 70 -7.065 33.668 23.207 1.00 0.00
C N
ATOM 7844 HN ASN C 70 -6.593 32.865 22.846 1.00 0.00
C H
ATOM 7845 CA ASN C 70 -8.271 34.032 22.486 1.00 0.00
C C
ATOM 7846 HA ASN C 70 -8.098 35.035 22.111 1.00 0.00
C H
ATOM 7847 CB ASN C 70 -8.522 33.037 21.358 1.00 0.00
C C
ATOM 7848 HB1 ASN C 70 -8.881 32.089 21.815 1.00 0.00
C H
ATOM 7849 HB2 ASN C 70 -9.324 33.408 20.688 1.00 0.00
C H
ATOM 7850 CG ASN C 70 -7.272 32.738 20.571 1.00 0.00
C C
ATOM 7851 OD1 ASN C 70 -6.298 33.485 20.631 1.00 0.00
C O
ATOM 7852 ND2 ASN C 70 -7.290 31.643 19.826 1.00 0.00
C N
ATOM 7853 HD21 ASN C 70 -6.466 31.427 19.309 1.00 0.00
C H
ATOM 7854 HD22 ASN C 70 -8.094 31.057 19.821 1.00 0.00
C H
ATOM 7855 C ASN C 70 -9.472 34.087 23.406 1.00 0.00
C C
ATOM 7856 O ASN C 70 -10.341 34.945 23.230 1.00 0.00
C O

```
ATOM   7857  N   PHE C  71      -9.524  33.214  24.402  1.00  0.00
C    N
ATOM   7858  HN  PHE C  71      -8.823  32.522  24.569  1.00  0.00
C    H
ATOM   7859  CA  PHE C  71     -10.624  33.194  25.346  1.00  0.00
C    C
ATOM   7860  HA  PHE C  71     -11.536  33.315  24.775  1.00  0.00
C    H
ATOM   7861  CB  PHE C  71     -10.615  31.914  26.170  1.00  0.00
C    C
ATOM   7862  HB1 PHE C  71      -9.642  31.831  26.702  1.00  0.00
C    H
ATOM   7863  HB2 PHE C  71     -11.412  31.932  26.945  1.00  0.00
C    H
ATOM   7864  CG  PHE C  71     -10.748  30.678  25.358  1.00  0.00
C    C
ATOM   7865  CD1 PHE C  71      -9.683  30.212  24.609  1.00  0.00
C    C
ATOM   7866  HD1 PHE C  71      -8.751  30.759  24.608  1.00  0.00
C    H
ATOM   7867  CE1 PHE C  71      -9.797  29.075  23.866  1.00  0.00
C    C
ATOM   7868  HE1 PHE C  71      -8.956  28.722  23.286  1.00  0.00
C    H
ATOM   7869  CZ  PHE C  71     -10.986  28.375  23.864  1.00  0.00
C    C
ATOM   7870  HZ  PHE C  71     -11.076  27.468  23.283  1.00  0.00
C    H
ATOM   7871  CD2 PHE C  71     -11.934  29.971  25.353  1.00  0.00
C    C
ATOM   7872  HD2 PHE C  71     -12.780  30.331  25.920  1.00  0.00
C    H
ATOM   7873  CE2 PHE C  71     -12.058  28.825  24.611  1.00  0.00
C    C
ATOM   7874  HE2 PHE C  71     -12.992  28.282  24.612  1.00  0.00
C    H
ATOM   7875  C   PHE C  71     -10.514  34.395  26.267  1.00  0.00
C    C
ATOM   7876  O   PHE C  71      -9.423  34.943  26.455  1.00  0.00
C    O
ATOM   7877  N   GLU C  72     -11.644  34.792  26.846  1.00  0.00
C    N
ATOM   7878  HN  GLU C  72     -12.542  34.387  26.701  1.00  0.00
C    H
ATOM   7879  CA  GLU C  72     -11.643  35.908  27.782  1.00  0.00
C    C
ATOM   7880  HA  GLU C  72     -10.632  36.293  27.847  1.00  0.00
C    H
ATOM   7881  CB  GLU C  72     -12.587  36.984  27.282  1.00  0.00
C    C
ATOM   7882  HB1 GLU C  72     -13.375  37.140  28.054  1.00  0.00
C    H
ATOM   7883  HB2 GLU C  72     -12.044  37.953  27.199  1.00  0.00
C    H
```

ATOM 7884 CG GLU C 72 −13.229 36.621 25.969 1.00 0.00
C C
ATOM 7885 HG1 GLU C 72 −12.452 36.282 25.260 1.00 0.00
C H
ATOM 7886 HG2 GLU C 72 −13.978 35.815 26.102 1.00 0.00
C H
ATOM 7887 CD GLU C 72 −13.899 37.802 25.316 1.00 0.00
C C
ATOM 7888 OE1 GLU C 72 −14.702 38.481 25.990 1.00 0.00
C O
ATOM 7889 OE2 GLU C 72 −13.621 38.050 24.123 1.00 0.00
C O
ATOM 7890 C GLU C 72 −12.042 35.454 29.177 1.00 0.00
C C
ATOM 7891 O GLU C 72 −11.518 35.960 30.178 1.00 0.00
C O
ATOM 7892 N TYR C 73 −12.973 34.507 29.267 1.00 0.00
C N
ATOM 7893 HN TYR C 73 −13.433 34.104 28.476 1.00 0.00
C H
ATOM 7894 CA TYR C 73 −13.403 33.973 30.552 1.00 0.00
C C
ATOM 7895 HA TYR C 73 −12.975 34.600 31.325 1.00 0.00
C H
ATOM 7896 CB TYR C 73 −14.923 33.897 30.658 1.00 0.00
C C
ATOM 7897 HB1 TYR C 73 −15.332 33.459 29.722 1.00 0.00
C H
ATOM 7898 HB2 TYR C 73 −15.226 33.228 31.493 1.00 0.00
C H
ATOM 7899 CG TYR C 73 −15.620 35.217 30.833 1.00 0.00
C C
ATOM 7900 CD1 TYR C 73 −15.681 36.136 29.800 1.00 0.00
C C
ATOM 7901 HD1 TYR C 73 −15.199 35.918 28.857 1.00 0.00
C H
ATOM 7902 CE1 TYR C 73 −16.329 37.338 29.957 1.00 0.00
C C
ATOM 7903 HE1 TYR C 73 −16.368 38.051 29.147 1.00 0.00
C H
ATOM 7904 CZ TYR C 73 −16.922 37.630 31.161 1.00 0.00
C C
ATOM 7905 OH TYR C 73 −17.571 38.828 31.338 1.00 0.00
C O
ATOM 7906 HH TYR C 73 −17.974 38.812 32.208 1.00 0.00
C H
ATOM 7907 CD2 TYR C 73 −16.233 35.538 32.030 1.00 0.00
C C
ATOM 7908 HD2 TYR C 73 −16.176 34.856 32.868 1.00 0.00
C H
ATOM 7909 CE2 TYR C 73 −16.878 36.735 32.198 1.00 0.00
C C
ATOM 7910 HE2 TYR C 73 −17.328 36.972 33.150 1.00 0.00
C H

ATOM 7911 C TYR C 73 -12.817 32.584 30.728 1.00 0.00
C C
ATOM 7912 O TYR C 73 -12.899 31.754 29.818 1.00 0.00
C O
ATOM 7913 N VAL C 74 -12.249 32.323 31.901 1.00 0.00
C N
ATOM 7914 HN VAL C 74 -12.126 33.010 32.613 1.00 0.00
C H
ATOM 7915 CA VAL C 74 -11.752 31.001 32.249 1.00 0.00
C C
ATOM 7916 HA VAL C 74 -12.174 30.280 31.559 1.00 0.00
C H
ATOM 7917 CB VAL C 74 -10.217 30.918 32.180 1.00 0.00
C C
ATOM 7918 HB VAL C 74 -9.925 29.866 32.417 1.00 0.00
C H
ATOM 7919 CG1 VAL C 74 -9.738 31.273 30.791 1.00 0.00
C C
ATOM 7920 HG11 VAL C 74 -8.627 31.253 30.749 1.00 0.00
C H
ATOM 7921 HG12 VAL C 74 -10.111 30.553 30.033 1.00 0.00
C H
ATOM 7922 HG13 VAL C 74 -10.074 32.294 30.508 1.00 0.00
C H
ATOM 7923 CG2 VAL C 74 -9.573 31.824 33.209 1.00 0.00
C C
ATOM 7924 HG21 VAL C 74 -8.466 31.788 33.126 1.00 0.00
C H
ATOM 7925 HG22 VAL C 74 -9.900 32.876 33.064 1.00 0.00
C H
ATOM 7926 HG23 VAL C 74 -9.841 31.514 34.241 1.00 0.00
C H
ATOM 7927 C VAL C 74 -12.258 30.651 33.639 1.00 0.00
C C
ATOM 7928 O VAL C 74 -12.294 31.505 34.535 1.00 0.00
C O
ATOM 7929 N ILE C 75 -12.657 29.401 33.809 1.00 0.00
C N
ATOM 7930 HN ILE C 75 -12.599 28.710 33.091 1.00 0.00
C H
ATOM 7931 CA ILE C 75 -13.219 28.915 35.057 1.00 0.00
C C
ATOM 7932 HA ILE C 75 -12.996 29.643 35.828 1.00 0.00
C H
ATOM 7933 CB ILE C 75 -14.718 28.645 34.930 1.00 0.00
C C
ATOM 7934 HB ILE C 75 -14.862 27.639 35.399 1.00 0.00
C H
ATOM 7935 CG2 ILE C 75 -15.489 29.752 35.592 1.00 0.00
C C
ATOM 7936 HG21 ILE C 75 -16.568 29.498 35.648 1.00 0.00
C H
ATOM 7937 HG22 ILE C 75 -15.129 29.921 36.629 1.00 0.00
C H

```
ATOM   7938 HG23 ILE C  75      -15.385  30.702  35.026  1.00  0.00
C    H
ATOM   7939  CG1 ILE C  75      -15.070  28.517  33.454  1.00  0.00
C    C
ATOM   7940 HG11 ILE C  75      -14.282  29.001  32.835  1.00  0.00
C    H
ATOM   7941 HG12 ILE C  75      -15.086  27.437  33.180  1.00  0.00
C    H
ATOM   7942  CD  ILE C  75      -16.389  29.099  33.086  1.00  0.00
C    C
ATOM   7943  HD1 ILE C  75      -16.604  28.918  32.011  1.00  0.00
C    H
ATOM   7944  HD2 ILE C  75      -17.203  28.638  33.685  1.00  0.00
C    H
ATOM   7945  HD3 ILE C  75      -16.403  30.196  33.259  1.00  0.00
C    H
ATOM   7946  C   ILE C  75      -12.483  27.649  35.426  1.00  0.00
C    C
ATOM   7947  O   ILE C  75      -12.627  26.622  34.756  1.00  0.00
C    O
ATOM   7948  N   VAL C  76      -11.713  27.717  36.495  1.00  0.00
C    N
ATOM   7949  HN  VAL C  76      -11.574  28.551  37.022  1.00  0.00
C    H
ATOM   7950  CA  VAL C  76      -10.995  26.562  37.001  1.00  0.00
C    C
ATOM   7951  HA  VAL C  76      -10.819  25.857  36.198  1.00  0.00
C    H
ATOM   7952  CB  VAL C  76       -9.663  26.959  37.616  1.00  0.00
C    C
ATOM   7953  HB  VAL C  76       -9.880  27.596  38.508  1.00  0.00
C    H
ATOM   7954  CG1 VAL C  76       -8.916  25.741  38.043  1.00  0.00
C    C
ATOM   7955 HG11 VAL C  76       -7.924  26.024  38.458  1.00  0.00
C    H
ATOM   7956 HG12 VAL C  76       -9.459  25.183  38.835  1.00  0.00
C    H
ATOM   7957 HG13 VAL C  76       -8.746  25.061  37.181  1.00  0.00
C    H
ATOM   7958  CG2 VAL C  76       -8.863  27.733  36.605  1.00  0.00
C    C
ATOM   7959 HG21 VAL C  76       -7.867  28.003  37.015  1.00  0.00
C    H
ATOM   7960 HG22 VAL C  76       -8.711  27.131  35.683  1.00  0.00
C    H
ATOM   7961 HG23 VAL C  76       -9.376  28.676  36.324  1.00  0.00
C    H
ATOM   7962  C   VAL C  76      -11.940  25.898  37.994  1.00  0.00
C    C
ATOM   7963  O   VAL C  76      -12.008  26.256  39.176  1.00  0.00
C    O
ATOM   7964  N   ALA C  77      -12.719  24.948  37.487  1.00  0.00
C    N
```

```
ATOM   7965  HN  ALA C  77      -12.678  24.701  36.520  1.00  0.00
C    H
ATOM   7966  CA  ALA C  77      -13.685  24.200  38.285  1.00  0.00
C    C
ATOM   7967  HA  ALA C  77      -13.981  24.771  39.158  1.00  0.00
C    H
ATOM   7968  CB  ALA C  77      -14.942  23.922  37.473  1.00  0.00
C    C
ATOM   7969  HB1 ALA C  77      -15.394  24.882  37.146  1.00  0.00
C    H
ATOM   7970  HB2 ALA C  77      -14.707  23.327  36.564  1.00  0.00
C    H
ATOM   7971  HB3 ALA C  77      -15.701  23.371  38.070  1.00  0.00
C    H
ATOM   7972  C   ALA C  77      -13.085  22.909  38.801  1.00  0.00
C    C
ATOM   7973  O   ALA C  77      -13.634  21.826  38.616  1.00  0.00
C    O
ATOM   7974  N   ILE C  78      -11.937  23.012  39.442  1.00  0.00
C    N
ATOM   7975  HN  ILE C  78      -11.427  23.866  39.510  1.00  0.00
C    H
ATOM   7976  CA  ILE C  78      -11.308  21.889  40.112  1.00  0.00
C    C
ATOM   7977  HA  ILE C  78      -11.770  20.975  39.759  1.00  0.00
C    H
ATOM   7978  CB  ILE C  78       -9.795  21.897  39.890  1.00  0.00
C    C
ATOM   7979  HB  ILE C  78       -9.669  21.624  38.812  1.00  0.00
C    H
ATOM   7980  CG2 ILE C  78       -9.235  23.210  40.301  1.00  0.00
C    C
ATOM   7981 HG21 ILE C  78       -8.168  23.291  40.004  1.00  0.00
C    H
ATOM   7982 HG22 ILE C  78       -9.783  24.045  39.815  1.00  0.00
C    H
ATOM   7983 HG23 ILE C  78       -9.298  23.343  41.401  1.00  0.00
C    H
ATOM   7984  CG1 ILE C  78       -9.116  20.803  40.696  1.00  0.00
C    C
ATOM   7985 HG11 ILE C  78       -9.339  20.940  41.778  1.00  0.00
C    H
ATOM   7986 HG12 ILE C  78       -9.540  19.818  40.396  1.00  0.00
C    H
ATOM   7987  CD  ILE C  78       -7.637  20.777  40.485  1.00  0.00
C    C
ATOM   7988  HD1 ILE C  78       -7.182  19.931  41.043  1.00  0.00
C    H
ATOM   7989  HD2 ILE C  78       -7.397  20.652  39.406  1.00  0.00
C    H
ATOM   7990  HD3 ILE C  78       -7.167  21.718  40.843  1.00  0.00
C    H
ATOM   7991  C   ILE C  78      -11.664  22.022  41.582  1.00  0.00
C    C
```

ATOM 7992 O ILE C 78 -11.860 23.137 42.074 1.00 0.00
C O
ATOM 7993 N GLY C 79 -11.787 20.901 42.290 1.00 0.00
C N
ATOM 7994 HN GLY C 79 -11.714 19.975 41.922 1.00 0.00
C H
ATOM 7995 CA GLY C 79 -12.048 20.992 43.712 1.00 0.00
C C
ATOM 7996 HA1 GLY C 79 -13.050 20.612 43.852 1.00 0.00
C H
ATOM 7997 HA2 GLY C 79 -11.928 22.036 43.972 1.00 0.00
C H
ATOM 7998 C GLY C 79 -11.114 20.184 44.586 1.00 0.00
C C
ATOM 7999 O GLY C 79 -10.947 20.489 45.770 1.00 0.00
C O
ATOM 8000 N ALA C 80 -10.497 19.152 44.022 1.00 0.00
C N
ATOM 8001 HN ALA C 80 -10.529 18.920 43.050 1.00 0.00
C H
ATOM 8002 CA ALA C 80 -9.698 18.261 44.850 1.00 0.00
C C
ATOM 8003 HA ALA C 80 -10.039 18.316 45.877 1.00 0.00
C H
ATOM 8004 CB ALA C 80 -9.865 16.817 44.375 1.00 0.00
C C
ATOM 8005 HB1 ALA C 80 -10.939 16.535 44.409 1.00 0.00
C H
ATOM 8006 HB2 ALA C 80 -9.513 16.698 43.328 1.00 0.00
C H
ATOM 8007 HB3 ALA C 80 -9.304 16.106 45.020 1.00 0.00
C H
ATOM 8008 C ALA C 80 -8.220 18.624 44.869 1.00 0.00
C C
ATOM 8009 O ALA C 80 -7.664 18.909 45.933 1.00 0.00
C O
ATOM 8010 N ASN C 81 -7.572 18.622 43.717 1.00 0.00
C N
ATOM 8011 HN ASN C 81 -8.025 18.565 42.828 1.00 0.00
C H
ATOM 8012 CA ASN C 81 -6.121 18.705 43.650 1.00 0.00
C C
ATOM 8013 HA ASN C 81 -5.737 18.205 44.533 1.00 0.00
C H
ATOM 8014 CB ASN C 81 -5.635 18.059 42.362 1.00 0.00
C C
ATOM 8015 HB1 ASN C 81 -6.064 17.035 42.312 1.00 0.00
C H
ATOM 8016 HB2 ASN C 81 -6.010 18.622 41.483 1.00 0.00
C H
ATOM 8017 CG ASN C 81 -4.148 17.942 42.305 1.00 0.00
C C
ATOM 8018 OD1 ASN C 81 -3.427 18.769 42.852 1.00 0.00
C O

```
ATOM   8019  ND2 ASN C  81      -3.668  16.908  41.631  1.00  0.00
C    N
ATOM   8020 HD21 ASN C  81      -2.678  16.808  41.592  1.00  0.00
C    H
ATOM   8021 HD22 ASN C  81      -4.290  16.253  41.214  1.00  0.00
C    H
ATOM   8022  C   ASN C  81      -5.703  20.167  43.730  1.00  0.00
C    C
ATOM   8023  O   ASN C  81      -5.514  20.816  42.699  1.00  0.00
C    O
ATOM   8024  N   ILE C  82      -5.513  20.670  44.950  1.00  0.00
C    N
ATOM   8025  HN  ILE C  82      -5.559  20.152  45.802  1.00  0.00
C    H
ATOM   8026  CA  ILE C  82      -5.207  22.087  45.127  1.00  0.00
C    C
ATOM   8027  HA  ILE C  82      -6.064  22.636  44.759  1.00  0.00
C    H
ATOM   8028  CB  ILE C  82      -4.963  22.401  46.612  1.00  0.00
C    C
ATOM   8029  HB  ILE C  82      -4.297  21.574  46.968  1.00  0.00
C    H
ATOM   8030  CG2 ILE C  82      -4.371  23.783  46.780  1.00  0.00
C    C
ATOM   8031 HG21 ILE C  82      -4.052  23.946  47.830  1.00  0.00
C    H
ATOM   8032 HG22 ILE C  82      -3.478  23.913  46.133  1.00  0.00
C    H
ATOM   8033 HG23 ILE C  82      -5.112  24.567  46.516  1.00  0.00
C    H
ATOM   8034  CG1 ILE C  82      -6.267  22.290  47.394  1.00  0.00
C    C
ATOM   8035 HG11 ILE C  82      -6.272  23.031  48.224  1.00  0.00
C    H
ATOM   8036 HG12 ILE C  82      -7.115  22.544  46.720  1.00  0.00
C    H
ATOM   8037  CD  ILE C  82      -6.520  20.933  47.960  1.00  0.00
C    C
ATOM   8038  HD1 ILE C  82      -7.509  20.904  48.466  1.00  0.00
C    H
ATOM   8039  HD2 ILE C  82      -6.519  20.166  47.155  1.00  0.00
C    H
ATOM   8040  HD3 ILE C  82      -5.744  20.661  48.706  1.00  0.00
C    H
ATOM   8041  C   ILE C  82      -4.021  22.496  44.268  1.00  0.00
C    C
ATOM   8042  O   ILE C  82      -3.994  23.601  43.707  1.00  0.00
C    O
ATOM   8043  N   GLN C  83      -3.043  21.603  44.132  1.00  0.00
C    N
ATOM   8044  HN  GLN C  83      -3.030  20.709  44.578  1.00  0.00
C    H
ATOM   8045  CA  GLN C  83      -1.885  21.876  43.296  1.00  0.00
C    C
```

```
ATOM   8046  HA  GLN C  83      -1.306  22.643  43.797  1.00  0.00
C    H
ATOM   8047  CB  GLN C  83      -1.098  20.595  43.092  1.00  0.00
C    C
ATOM   8048  HB1 GLN C  83      -1.589  19.813  43.717  1.00  0.00
C    H
ATOM   8049  HB2 GLN C  83      -1.205  20.260  42.034  1.00  0.00
C    H
ATOM   8050  CG  GLN C  83       0.333  20.655  43.454  1.00  0.00
C    C
ATOM   8051  HG1 GLN C  83       0.793  21.586  43.060  1.00  0.00
C    H
ATOM   8052  HG2 GLN C  83       0.460  20.653  44.557  1.00  0.00
C    H
ATOM   8053  CD  GLN C  83       1.078  19.492  42.852  1.00  0.00
C    C
ATOM   8054  OE1 GLN C  83       0.863  19.149  41.696  1.00  0.00
C    O
ATOM   8055  NE2 GLN C  83       1.930  18.860  43.637  1.00  0.00
C    N
ATOM   8056 HE21 GLN C  83       2.426  18.094  43.238  1.00  0.00
C    H
ATOM   8057 HE22 GLN C  83       2.038  19.147  44.582  1.00  0.00
C    H
ATOM   8058  C   GLN C  83      -2.300  22.428  41.946  1.00  0.00
C    C
ATOM   8059  O   GLN C  83      -2.000  23.582  41.610  1.00  0.00
C    O
ATOM   8060  N   ALA C  84      -3.024  21.622  41.180  1.00  0.00
C    N
ATOM   8061  HN  ALA C  84      -3.357  20.724  41.467  1.00  0.00
C    H
ATOM   8062  CA  ALA C  84      -3.383  22.016  39.829  1.00  0.00
C    C
ATOM   8063  HA  ALA C  84      -2.497  22.181  39.228  1.00  0.00
C    H
ATOM   8064  CB  ALA C  84      -4.203  20.913  39.167  1.00  0.00
C    C
ATOM   8065  HB1 ALA C  84      -3.607  19.977  39.129  1.00  0.00
C    H
ATOM   8066  HB2 ALA C  84      -5.130  20.705  39.744  1.00  0.00
C    H
ATOM   8067  HB3 ALA C  84      -4.486  21.182  38.127  1.00  0.00
C    H
ATOM   8068  C   ALA C  84      -4.147  23.327  39.834  1.00  0.00
C    C
ATOM   8069  O   ALA C  84      -3.845  24.233  39.055  1.00  0.00
C    O
ATOM   8070  N   SER C  85      -5.142  23.441  40.712  1.00  0.00
C    N
ATOM   8071  HN  SER C  85      -5.449  22.720  41.332  1.00  0.00
C    H
ATOM   8072  CA  SER C  85      -5.884  24.687  40.829  1.00  0.00
C    C
```

```
ATOM   8073  HA  SER C  85      -6.484  24.768  39.932  1.00  0.00
C    H
ATOM   8074  CB  SER C  85      -6.719  24.668  42.103  1.00  0.00
C    C
ATOM   8075  HB1 SER C  85      -7.579  25.363  41.963  1.00  0.00
C    H
ATOM   8076  HB2 SER C  85      -7.144  23.653  42.273  1.00  0.00
C    H
ATOM   8077  OG  SER C  85      -5.942  25.102  43.203  1.00  0.00
C    O
ATOM   8078  HG1 SER C  85      -6.554  25.287  43.927  1.00  0.00
C    H
ATOM   8079  C   SER C  85      -4.941  25.876  40.851  1.00  0.00
C    C
ATOM   8080  O   SER C  85      -5.036  26.791  40.022  1.00  0.00
C    O
ATOM   8081  N   THR C  86      -3.986  25.853  41.772  1.00  0.00
C    N
ATOM   8082  HN  THR C  86      -3.737  25.074  42.348  1.00  0.00
C    H
ATOM   8083  CA  THR C  86      -3.194  27.048  42.009  1.00  0.00
C    C
ATOM   8084  HA  THR C  86      -3.899  27.868  42.015  1.00  0.00
C    H
ATOM   8085  CB  THR C  86      -2.461  26.916  43.332  1.00  0.00
C    C
ATOM   8086  HB  THR C  86      -1.924  27.885  43.487  1.00  0.00
C    H
ATOM   8087  OG1 THR C  86      -1.484  25.877  43.227  1.00  0.00
C    O
ATOM   8088  HG1 THR C  86      -1.063  25.832  44.092  1.00  0.00
C    H
ATOM   8089  CG2 THR C  86      -3.442  26.586  44.433  1.00  0.00
C    C
ATOM   8090 HG21 THR C  86      -2.942  26.598  45.425  1.00  0.00
C    H
ATOM   8091 HG22 THR C  86      -4.264  27.333  44.456  1.00  0.00
C    H
ATOM   8092 HG23 THR C  86      -3.890  25.581  44.282  1.00  0.00
C    H
ATOM   8093  C   THR C  86      -2.221  27.320  40.870  1.00  0.00
C    C
ATOM   8094  O   THR C  86      -2.143  28.452  40.379  1.00  0.00
C    O
ATOM   8095  N   LEU C  87      -1.457  26.311  40.444  1.00  0.00
C    N
ATOM   8096  HN  LEU C  87      -1.442  25.376  40.793  1.00  0.00
C    H
ATOM   8097  CA  LEU C  87      -0.522  26.557  39.347  1.00  0.00
C    C
ATOM   8098  HA  LEU C  87       0.105  27.386  39.647  1.00  0.00
C    H
ATOM   8099  CB  LEU C  87       0.365  25.344  39.057  1.00  0.00
C    C
```

```
ATOM   8100  HB1 LEU C  87      -0.195  24.689  38.349  1.00  0.00
C    H
ATOM   8101  HB2 LEU C  87       1.273  25.682  38.510  1.00  0.00
C    H
ATOM   8102  CG  LEU C  87       0.973  24.274  39.969  1.00  0.00
C    C
ATOM   8103  HG  LEU C  87       2.066  24.354  39.756  1.00  0.00
C    H
ATOM   8104  CD1 LEU C  87       0.727  24.420  41.456  1.00  0.00
C    C
ATOM   8105 HD11 LEU C  87       1.171  23.566  42.011  1.00  0.00
C    H
ATOM   8106 HD12 LEU C  87       1.181  25.360  41.838  1.00  0.00
C    H
ATOM   8107 HD13 LEU C  87      -0.362  24.445  41.673  1.00  0.00
C    H
ATOM   8108  CD2 LEU C  87       0.497  22.901  39.486  1.00  0.00
C    C
ATOM   8109 HD21 LEU C  87       0.935  22.096  40.117  1.00  0.00
C    H
ATOM   8110 HD22 LEU C  87      -0.606  22.808  39.541  1.00  0.00
C    H
ATOM   8111 HD23 LEU C  87       0.811  22.728  38.434  1.00  0.00
C    H
ATOM   8112  C   LEU C  87      -1.265  26.976  38.092  1.00  0.00
C    C
ATOM   8113  O   LEU C  87      -0.788  27.815  37.317  1.00  0.00
C    O
ATOM   8114  N   THR C  88      -2.442  26.402  37.879  1.00  0.00
C    N
ATOM   8115  HN  THR C  88      -2.815  25.651  38.422  1.00  0.00
C    H
ATOM   8116  CA  THR C  88      -3.303  26.842  36.802  1.00  0.00
C    C
ATOM   8117  HA  THR C  88      -2.758  26.658  35.886  1.00  0.00
C    H
ATOM   8118  CB  THR C  88      -4.625  26.108  36.923  1.00  0.00
C    C
ATOM   8119  HB  THR C  88      -5.046  26.391  37.919  1.00  0.00
C    H
ATOM   8120  OG1 THR C  88      -4.373  24.701  36.925  1.00  0.00
C    O
ATOM   8121  HG1 THR C  88      -5.239  24.285  36.991  1.00  0.00
C    H
ATOM   8122  CG2 THR C  88      -5.534  26.480  35.793  1.00  0.00
C    C
ATOM   8123 HG21 THR C  88      -6.543  26.037  35.935  1.00  0.00
C    H
ATOM   8124 HG22 THR C  88      -5.652  27.584  35.737  1.00  0.00
C    H
ATOM   8125 HG23 THR C  88      -5.131  26.123  34.821  1.00  0.00
C    H
ATOM   8126  C   THR C  88      -3.529  28.335  36.876  1.00  0.00
C    C
```

```
ATOM   8127  O   THR C  88      -3.133  29.084  35.973  1.00  0.00
C    O
ATOM   8128  N   THR C  89      -4.123  28.781  37.982  1.00  0.00
C    N
ATOM   8129  HN  THR C  89      -4.423  28.205  38.742  1.00  0.00
C    H
ATOM   8130  CA  THR C  89      -4.388  30.201  38.166  1.00  0.00
C    C
ATOM   8131  HA  THR C  89      -5.097  30.472  37.396  1.00  0.00
C    H
ATOM   8132  CB  THR C  89      -4.888  30.449  39.576  1.00  0.00
C    C
ATOM   8133  HB  THR C  89      -4.892  31.558  39.713  1.00  0.00
C    H
ATOM   8134  OG1 THR C  89      -3.949  29.882  40.496  1.00  0.00
C    O
ATOM   8135  HG1 THR C  89      -4.313  30.054  41.372  1.00  0.00
C    H
ATOM   8136  CG2 THR C  89      -6.229  29.796  39.768  1.00  0.00
C    C
ATOM   8137 HG21 THR C  89      -6.661  30.063  40.756  1.00  0.00
C    H
ATOM   8138 HG22 THR C  89      -6.939  30.131  38.983  1.00  0.00
C    H
ATOM   8139 HG23 THR C  89      -6.147  28.689  39.712  1.00  0.00
C    H
ATOM   8140  C   THR C  89      -3.144  31.027  37.917  1.00  0.00
C    C
ATOM   8141  O   THR C  89      -3.217  32.119  37.342  1.00  0.00
C    O
ATOM   8142  N   LEU C  90      -1.995  30.531  38.361  1.00  0.00
C    N
ATOM   8143  HN  LEU C  90      -1.889  29.695  38.896  1.00  0.00
C    H
ATOM   8144  CA  LEU C  90      -0.747  31.218  38.073  1.00  0.00
C    C
ATOM   8145  HA  LEU C  90      -0.811  32.193  38.538  1.00  0.00
C    H
ATOM   8146  CB  LEU C  90       0.430  30.424  38.619  1.00  0.00
C    C
ATOM   8147  HB1 LEU C  90       0.488  30.632  39.713  1.00  0.00
C    H
ATOM   8148  HB2 LEU C  90       0.209  29.339  38.524  1.00  0.00
C    H
ATOM   8149  CG  LEU C  90       1.767  30.770  37.970  1.00  0.00
C    C
ATOM   8150  HG  LEU C  90       1.730  30.461  36.898  1.00  0.00
C    H
ATOM   8151  CD1 LEU C  90       2.074  32.249  38.074  1.00  0.00
C    C
ATOM   8152 HD11 LEU C  90       3.072  32.471  37.639  1.00  0.00
C    H
ATOM   8153 HD12 LEU C  90       1.314  32.846  37.526  1.00  0.00
C    H
```

```
ATOM   8154 HD13 LEU C  90       2.079  32.575  39.135  1.00  0.00
C    H
ATOM   8155  CD2 LEU C  90       2.845  29.985  38.640  1.00  0.00
C    C
ATOM   8156 HD21 LEU C  90       3.830  30.204  38.174  1.00  0.00
C    H
ATOM   8157 HD22 LEU C  90       2.922  30.230  39.719  1.00  0.00
C    H
ATOM   8158 HD23 LEU C  90       2.651  28.894  38.542  1.00  0.00
C    H
ATOM   8159  C   LEU C  90      -0.581  31.432  36.581  1.00  0.00
C    C
ATOM   8160  O   LEU C  90      -0.361  32.557  36.126  1.00  0.00
C    O
ATOM   8161  N   LEU C  91      -0.661  30.357  35.808  1.00  0.00
C    N
ATOM   8162  HN  LEU C  91      -0.878  29.431  36.112  1.00  0.00
C    H
ATOM   8163  CA  LEU C  91      -0.421  30.476  34.375  1.00  0.00
C    C
ATOM   8164  HA  LEU C  91       0.565  30.905  34.257  1.00  0.00
C    H
ATOM   8165  CB  LEU C  91      -0.488  29.108  33.713  1.00  0.00
C    C
ATOM   8166  HB1 LEU C  91      -1.223  28.499  34.290  1.00  0.00
C    H
ATOM   8167  HB2 LEU C  91      -0.916  29.221  32.692  1.00  0.00
C    H
ATOM   8168  CG  LEU C  91       0.860  28.396  33.683  1.00  0.00
C    C
ATOM   8169  HG  LEU C  91       0.703  27.384  33.236  1.00  0.00
C    H
ATOM   8170  CD1 LEU C  91       1.835  29.185  32.831  1.00  0.00
C    C
ATOM   8171 HD11 LEU C  91       2.837  28.704  32.837  1.00  0.00
C    H
ATOM   8172 HD12 LEU C  91       1.481  29.239  31.779  1.00  0.00
C    H
ATOM   8173 HD13 LEU C  91       1.945  30.220  33.218  1.00  0.00
C    H
ATOM   8174  CD2 LEU C  91       1.409  28.210  35.079  1.00  0.00
C    C
ATOM   8175 HD21 LEU C  91       2.382  27.674  35.044  1.00  0.00
C    H
ATOM   8176 HD22 LEU C  91       1.579  29.182  35.585  1.00  0.00
C    H
ATOM   8177 HD23 LEU C  91       0.706  27.612  35.698  1.00  0.00
C    H
ATOM   8178  C   LEU C  91      -1.415  31.430  33.739  1.00  0.00
C    C
ATOM   8179  O   LEU C  91      -1.056  32.250  32.886  1.00  0.00
C    O
ATOM   8180  N   LEU C  92      -2.669  31.347  34.159  1.00  0.00
C    N
```

ATOM 8181 HN LEU C 92 -3.012 30.743 34.875 1.00 0.00
C H
ATOM 8182 CA LEU C 92 -3.688 32.194 33.554 1.00 0.00
C C
ATOM 8183 HA LEU C 92 -3.595 32.072 32.483 1.00 0.00
C H
ATOM 8184 CB LEU C 92 -5.073 31.757 34.013 1.00 0.00
C C
ATOM 8185 HB1 LEU C 92 -4.986 31.463 35.084 1.00 0.00
C H
ATOM 8186 HB2 LEU C 92 -5.756 32.636 33.989 1.00 0.00
C H
ATOM 8187 CG LEU C 92 -5.632 30.594 33.205 1.00 0.00
C C
ATOM 8188 HG LEU C 92 -6.734 30.541 33.377 1.00 0.00
C H
ATOM 8189 CD1 LEU C 92 -5.351 30.836 31.741 1.00 0.00
C C
ATOM 8190 HD11 LEU C 92 -5.705 29.980 31.129 1.00 0.00
C H
ATOM 8191 HD12 LEU C 92 -5.870 31.754 31.390 1.00 0.00
C H
ATOM 8192 HD13 LEU C 92 -4.262 30.961 31.565 1.00 0.00
C H
ATOM 8193 CD2 LEU C 92 -5.030 29.285 33.636 1.00 0.00
C C
ATOM 8194 HD21 LEU C 92 -5.465 28.448 33.047 1.00 0.00
C H
ATOM 8195 HD22 LEU C 92 -3.932 29.269 33.483 1.00 0.00
C H
ATOM 8196 HD23 LEU C 92 -5.236 29.097 34.712 1.00 0.00
C H
ATOM 8197 C LEU C 92 -3.451 33.661 33.871 1.00 0.00
C C
ATOM 8198 O LEU C 92 -3.394 34.497 32.962 1.00 0.00
C O
ATOM 8199 N LYS C 93 -3.297 33.993 35.150 1.00 0.00
C N
ATOM 8200 HN LYS C 93 -3.341 33.355 35.918 1.00 0.00
C H
ATOM 8201 CA LYS C 93 -3.043 35.379 35.512 1.00 0.00
C C
ATOM 8202 HA LYS C 93 -3.847 35.973 35.097 1.00 0.00
C H
ATOM 8203 CB LYS C 93 -3.000 35.523 37.028 1.00 0.00
C C
ATOM 8204 HB1 LYS C 93 -3.667 34.736 37.451 1.00 0.00
C H
ATOM 8205 HB2 LYS C 93 -1.973 35.288 37.386 1.00 0.00
C H
ATOM 8206 CG LYS C 93 -3.451 36.880 37.519 1.00 0.00
C C
ATOM 8207 HG1 LYS C 93 -3.538 36.855 38.629 1.00 0.00
C H

ATOM 8208 HG2 LYS C 93 -2.672 37.635 37.265 1.00 0.00
C H
ATOM 8209 CD LYS C 93 -4.787 37.256 36.913 1.00 0.00
C C
ATOM 8210 HD1 LYS C 93 -4.683 37.258 35.804 1.00 0.00
C H
ATOM 8211 HD2 LYS C 93 -5.524 36.463 37.178 1.00 0.00
C H
ATOM 8212 CE LYS C 93 -5.259 38.610 37.415 1.00 0.00
C C
ATOM 8213 HE1 LYS C 93 -5.545 38.550 38.489 1.00 0.00
C H
ATOM 8214 HE2 LYS C 93 -4.453 39.368 37.308 1.00 0.00
C H
ATOM 8215 NZ LYS C 93 -6.445 39.104 36.661 1.00 0.00
C N
ATOM 8216 HZ1 LYS C 93 -6.741 40.031 37.030 1.00 0.00
C H
ATOM 8217 HZ2 LYS C 93 -6.204 39.196 35.653 1.00 0.00
C H
ATOM 8218 HZ3 LYS C 93 -7.227 38.427 36.764 1.00 0.00
C H
ATOM 8219 C LYS C 93 -1.748 35.872 34.890 1.00 0.00
C C
ATOM 8220 O LYS C 93 -1.609 37.061 34.589 1.00 0.00
C O
ATOM 8221 N GLU C 94 -0.792 34.972 34.686 1.00 0.00
C N
ATOM 8222 HN GLU C 94 -0.803 34.025 34.993 1.00 0.00
C H
ATOM 8223 CA GLU C 94 0.407 35.342 33.954 1.00 0.00
C C
ATOM 8224 HA GLU C 94 0.790 36.265 34.371 1.00 0.00
C H
ATOM 8225 CB GLU C 94 1.459 34.248 34.079 1.00 0.00
C C
ATOM 8226 HB1 GLU C 94 1.897 34.302 35.103 1.00 0.00
C H
ATOM 8227 HB2 GLU C 94 0.975 33.248 34.008 1.00 0.00
C H
ATOM 8228 CG GLU C 94 2.567 34.364 33.058 1.00 0.00
C C
ATOM 8229 HG1 GLU C 94 2.205 34.000 32.078 1.00 0.00
C H
ATOM 8230 HG2 GLU C 94 2.903 35.414 32.955 1.00 0.00
C H
ATOM 8231 CD GLU C 94 3.756 33.507 33.403 1.00 0.00
C C
ATOM 8232 OE1 GLU C 94 4.008 33.302 34.607 1.00 0.00
C O
ATOM 8233 OE2 GLU C 94 4.444 33.041 32.472 1.00 0.00
C O
ATOM 8234 C GLU C 94 0.091 35.606 32.495 1.00 0.00
C C

```
ATOM   8235  O   GLU C  94       0.704  36.478  31.871  1.00  0.00
C    O
ATOM   8236  N   LEU C  95      -0.859  34.871  31.932  1.00  0.00
C    N
ATOM   8237  HN  LEU C  95      -1.360  34.141  32.394  1.00  0.00
C    H
ATOM   8238  CA  LEU C  95      -1.254  35.084  30.553  1.00  0.00
C    C
ATOM   8239  HA  LEU C  95      -0.399  35.533  30.063  1.00  0.00
C    H
ATOM   8240  CB  LEU C  95      -1.572  33.755  29.883  1.00  0.00
C    C
ATOM   8241  HB1 LEU C  95      -1.907  33.055  30.683  1.00  0.00
C    H
ATOM   8242  HB2 LEU C  95      -2.445  33.891  29.207  1.00  0.00
C    H
ATOM   8243  CG  LEU C  95      -0.308  33.263  29.182  1.00  0.00
C    C
ATOM   8244  HG  LEU C  95       0.100  34.148  28.636  1.00  0.00
C    H
ATOM   8245  CD1 LEU C  95       0.719  32.768  30.185  1.00  0.00
C    C
ATOM   8246 HD11 LEU C  95       1.614  32.369  29.662  1.00  0.00
C    H
ATOM   8247 HD12 LEU C  95       1.045  33.595  30.852  1.00  0.00
C    H
ATOM   8248 HD13 LEU C  95       0.294  31.956  30.814  1.00  0.00
C    H
ATOM   8249  CD2 LEU C  95      -0.624  32.194  28.167  1.00  0.00
C    C
ATOM   8250 HD21 LEU C  95       0.304  31.862  27.654  1.00  0.00
C    H
ATOM   8251 HD22 LEU C  95      -1.085  31.303  28.640  1.00  0.00
C    H
ATOM   8252 HD23 LEU C  95      -1.326  32.583  27.398  1.00  0.00
C    H
ATOM   8253  C   LEU C  95      -2.417  36.047  30.419  1.00  0.00
C    C
ATOM   8254  O   LEU C  95      -3.136  36.000  29.417  1.00  0.00
C    O
ATOM   8255  N   ASP C  96      -2.630  36.896  31.420  1.00  0.00
C    N
ATOM   8256  HN  ASP C  96      -2.311  36.757  32.354  1.00  0.00
C    H
ATOM   8257  CA  ASP C  96      -3.367  38.141  31.250  1.00  0.00
C    C
ATOM   8258  HA  ASP C  96      -3.372  38.631  32.218  1.00  0.00
C    H
ATOM   8259  CB  ASP C  96      -2.691  39.017  30.202  1.00  0.00
C    C
ATOM   8260  HB1 ASP C  96      -2.952  38.644  29.191  1.00  0.00
C    H
ATOM   8261  HB2 ASP C  96      -3.038  40.067  30.286  1.00  0.00
C    H
```

```
ATOM   8262  CG  ASP C  96      -1.194  38.991  30.325  1.00  0.00
C    C
ATOM   8263  OD1 ASP C  96      -0.706  38.816  31.459  1.00  0.00
C    O
ATOM   8264  OD2 ASP C  96      -0.508  39.118  29.293  1.00  0.00
C    O
ATOM   8265  C   ASP C  96      -4.816  37.907  30.871  1.00  0.00
C    C
ATOM   8266  O   ASP C  96      -5.494  38.811  30.383  1.00  0.00
C    O
ATOM   8267  N   ILE C  97      -5.298  36.692  31.083  1.00  0.00
C    N
ATOM   8268  HN  ILE C  97      -4.814  35.911  31.472  1.00  0.00
C    H
ATOM   8269  CA  ILE C  97      -6.686  36.417  30.723  1.00  0.00
C    C
ATOM   8270  HA  ILE C  97      -6.817  36.712  29.690  1.00  0.00
C    H
ATOM   8271  CB  ILE C  97      -7.004  34.939  30.936  1.00  0.00
C    C
ATOM   8272  HB  ILE C  97      -6.739  34.747  32.007  1.00  0.00
C    H
ATOM   8273  CG2 ILE C  97      -8.445  34.641  30.581  1.00  0.00
C    C
ATOM   8274 HG21 ILE C  97      -8.717  33.607  30.882  1.00  0.00
C    H
ATOM   8275 HG22 ILE C  97      -9.134  35.336  31.105  1.00  0.00
C    H
ATOM   8276 HG23 ILE C  97      -8.613  34.738  29.488  1.00  0.00
C    H
ATOM   8277  CG1 ILE C  97      -6.061  34.110  30.074  1.00  0.00
C    C
ATOM   8278 HG11 ILE C  97      -5.011  34.265  30.409  1.00  0.00
C    H
ATOM   8279 HG12 ILE C  97      -6.295  33.031  30.217  1.00  0.00
C    H
ATOM   8280  CD  ILE C  97      -6.170  34.431  28.605  1.00  0.00
C    C
ATOM   8281  HD1 ILE C  97      -5.507  33.763  28.014  1.00  0.00
C    H
ATOM   8282  HD2 ILE C  97      -7.214  34.289  28.250  1.00  0.00
C    H
ATOM   8283  HD3 ILE C  97      -5.869  35.481  28.404  1.00  0.00
C    H
ATOM   8284  C   ILE C  97      -7.595  37.310  31.553  1.00  0.00
C    C
ATOM   8285  O   ILE C  97      -7.351  37.492  32.760  1.00  0.00
C    O
ATOM   8286  N   PRO C  98      -8.621  37.916  30.961  1.00  0.00
C    N
ATOM   8287  CD  PRO C  98      -9.041  37.823  29.555  1.00  0.00
C    C
ATOM   8288  HD1 PRO C  98      -7.985  38.109  29.759  1.00  0.00
C    H
```

```
ATOM   8289  HD2 PRO C  98      -9.102  37.179  28.648  1.00  0.00
C    H
ATOM   8290  CA  PRO C  98      -9.410  38.896  31.718  1.00  0.00
C    C
ATOM   8291  HA  PRO C  98      -8.777  39.727  32.005  1.00  0.00
C    H
ATOM   8292  CB  PRO C  98     -10.453  39.361  30.695  1.00  0.00
C    C
ATOM   8293  HB1 PRO C  98     -10.016  40.176  30.076  1.00  0.00
C    H
ATOM   8294  HB2 PRO C  98     -11.385  39.728  31.170  1.00  0.00
C    H
ATOM   8295  CG  PRO C  98      -9.869  39.039  29.371  1.00  0.00
C    C
ATOM   8296  HG1 PRO C  98     -10.286  39.659  28.553  1.00  0.00
C    H
ATOM   8297  HG2 PRO C  98     -10.043  37.962  29.146  1.00  0.00
C    H
ATOM   8298  C   PRO C  98     -10.059  38.317  32.954  1.00  0.00
C    C
ATOM   8299  O   PRO C  98      -9.801  38.789  34.066  1.00  0.00
C    O
ATOM   8300  N   ASN C  99     -10.873  37.284  32.797  1.00  0.00
C    N
ATOM   8301  HN  ASN C  99     -10.948  36.768  31.945  1.00  0.00
C    H
ATOM   8302  CA  ASN C  99     -11.736  36.811  33.868  1.00  0.00
C    C
ATOM   8303  HA  ASN C  99     -11.608  37.494  34.701  1.00  0.00
C    H
ATOM   8304  CB  ASN C  99     -13.181  36.743  33.391  1.00  0.00
C    C
ATOM   8305  HB1 ASN C  99     -13.244  35.953  32.610  1.00  0.00
C    H
ATOM   8306  HB2 ASN C  99     -13.849  36.442  34.225  1.00  0.00
C    H
ATOM   8307  CG  ASN C  99     -13.637  38.038  32.790  1.00  0.00
C    C
ATOM   8308  OD1 ASN C  99     -13.940  38.107  31.604  1.00  0.00
C    O
ATOM   8309  ND2 ASN C  99     -13.664  39.087  33.599  1.00  0.00
C    N
ATOM   8310 HD21 ASN C  99     -13.976  39.952  33.217  1.00  0.00
C    H
ATOM   8311 HD22 ASN C  99     -13.408  38.985  34.556  1.00  0.00
C    H
ATOM   8312  C   ASN C  99     -11.259  35.444  34.318  1.00  0.00
C    C
ATOM   8313  O   ASN C  99     -11.215  34.507  33.515  1.00  0.00
C    O
ATOM   8314  N   ILE C 100     -10.911  35.331  35.592  1.00  0.00
C    N
ATOM   8315  HN  ILE C 100     -10.959  36.089  36.239  1.00  0.00
C    H
```

```
ATOM   8316  CA  ILE C 100     -10.425  34.088  36.167  1.00  0.00
C    C
ATOM   8317  HA  ILE C 100     -10.575  33.299  35.441  1.00  0.00
C    H
ATOM   8318  CB  ILE C 100      -8.959  34.187  36.586  1.00  0.00
C    C
ATOM   8319  HB  ILE C 100      -8.884  35.171  37.113  1.00  0.00
C    H
ATOM   8320  CG2 ILE C 100      -8.581  32.993  37.411  1.00  0.00
C    C
ATOM   8321 HG21 ILE C 100      -7.571  33.125  37.851  1.00  0.00
C    H
ATOM   8322 HG22 ILE C 100      -9.297  32.849  38.247  1.00  0.00
C    H
ATOM   8323 HG23 ILE C 100      -8.573  32.070  36.793  1.00  0.00
C    H
ATOM   8324  CG1 ILE C 100      -8.070  34.273  35.348  1.00  0.00
C    C
ATOM   8325 HG11 ILE C 100      -8.211  33.365  34.719  1.00  0.00
C    H
ATOM   8326 HG12 ILE C 100      -8.388  35.149  34.737  1.00  0.00
C    H
ATOM   8327  CD  ILE C 100      -6.613  34.428  35.663  1.00  0.00
C    C
ATOM   8328  HD1 ILE C 100      -6.026  34.543  34.726  1.00  0.00
C    H
ATOM   8329  HD2 ILE C 100      -6.442  35.327  36.294  1.00  0.00
C    H
ATOM   8330  HD3 ILE C 100      -6.225  33.538  36.203  1.00  0.00
C    H
ATOM   8331  C   ILE C 100     -11.314  33.768  37.354  1.00  0.00
C    C
ATOM   8332  O   ILE C 100     -11.326  34.505  38.349  1.00  0.00
C    O
ATOM   8333  N   TRP C 101     -12.071  32.684  37.250  1.00  0.00
C    N
ATOM   8334  HN  TRP C 101     -11.996  32.049  36.485  1.00  0.00
C    H
ATOM   8335  CA  TRP C 101     -13.074  32.334  38.245  1.00  0.00
C    C
ATOM   8336  HA  TRP C 101     -12.945  32.981  39.104  1.00  0.00
C    H
ATOM   8337  CB  TRP C 101     -14.472  32.474  37.654  1.00  0.00
C    C
ATOM   8338  HB1 TRP C 101     -14.462  32.008  36.643  1.00  0.00
C    H
ATOM   8339  HB2 TRP C 101     -15.200  31.901  38.267  1.00  0.00
C    H
ATOM   8340  CG  TRP C 101     -14.868  33.875  37.512  1.00  0.00
C    C
ATOM   8341  CD1 TRP C 101     -14.095  34.902  37.079  1.00  0.00
C    C
ATOM   8342  HD1 TRP C 101     -13.075  34.796  36.735  1.00  0.00
C    H
```

```
ATOM   8343  NE1 TRP C 101     -14.805  36.068  37.103  1.00  0.00
C    N
ATOM   8344  HE1 TRP C 101     -14.494  36.946  36.811  1.00  0.00
C    H
ATOM   8345  CE2 TRP C 101     -16.065  35.801  37.563  1.00  0.00
C    C
ATOM   8346  CD2 TRP C 101     -16.133  34.427  37.831  1.00  0.00
C    C
ATOM   8347  CE3 TRP C 101     -17.322  33.894  38.315  1.00  0.00
C    C
ATOM   8348  HE3 TRP C 101     -17.423  32.842  38.548  1.00  0.00
C    H
ATOM   8349  CZ3 TRP C 101     -18.386  34.732  38.507  1.00  0.00
C    C
ATOM   8350  HZ3 TRP C 101     -19.318  34.330  38.879  1.00  0.00
C    H
ATOM   8351  CZ2 TRP C 101     -17.139  36.650  37.762  1.00  0.00
C    C
ATOM   8352  HZ2 TRP C 101     -17.092  37.708  37.556  1.00  0.00
C    H
ATOM   8353  CH2 TRP C 101     -18.292  36.097  38.235  1.00  0.00
C    C
ATOM   8354  HH2 TRP C 101     -19.149  36.734  38.403  1.00  0.00
C    H
ATOM   8355  C   TRP C 101     -12.827  30.911  38.721  1.00  0.00
C    C
ATOM   8356  O   TRP C 101     -12.758  29.988  37.905  1.00  0.00
C    O
ATOM   8357  N   VAL C 102     -12.702  30.727  40.029  1.00  0.00
C    N
ATOM   8358  HN  VAL C 102     -12.934  31.389  40.738  1.00  0.00
C    H
ATOM   8359  CA  VAL C 102     -12.186  29.463  40.533  1.00  0.00
C    C
ATOM   8360  HA  VAL C 102     -12.155  28.770  39.702  1.00  0.00
C    H
ATOM   8361  CB  VAL C 102     -10.768  29.643  41.107  1.00  0.00
C    C
ATOM   8362  HB  VAL C 102     -10.851  30.289  42.016  1.00  0.00
C    H
ATOM   8363  CG1 VAL C 102     -10.178  28.310  41.498  1.00  0.00
C    C
ATOM   8364 HG11 VAL C 102      -9.141  28.443  41.875  1.00  0.00
C    H
ATOM   8365 HG12 VAL C 102     -10.763  27.823  42.306  1.00  0.00
C    H
ATOM   8366 HG13 VAL C 102     -10.141  27.625  40.624  1.00  0.00
C    H
ATOM   8367  CG2 VAL C 102      -9.879  30.343  40.105  1.00  0.00
C    C
ATOM   8368 HG21 VAL C 102      -8.844  30.440  40.497  1.00  0.00
C    H
ATOM   8369 HG22 VAL C 102      -9.840  29.771  39.153  1.00  0.00
C    H
```

ATOM 8370 HG23 VAL C 102 -10.251 31.365 39.882 1.00 0.00
C H
ATOM 8371 C VAL C 102 -13.116 28.875 41.583 1.00 0.00
C C
ATOM 8372 O VAL C 102 -13.783 29.596 42.332 1.00 0.00
C O
ATOM 8373 N LYS C 103 -13.157 27.541 41.620 1.00 0.00
C N
ATOM 8374 HN LYS C 103 -12.755 26.982 40.896 1.00 0.00
C H
ATOM 8375 CA LYS C 103 -13.783 26.793 42.705 1.00 0.00
C C
ATOM 8376 HA LYS C 103 -14.581 27.401 43.113 1.00 0.00
C H
ATOM 8377 CB LYS C 103 -14.311 25.456 42.184 1.00 0.00
C C
ATOM 8378 HB1 LYS C 103 -15.161 25.683 41.499 1.00 0.00
C H
ATOM 8379 HB2 LYS C 103 -13.525 24.976 41.558 1.00 0.00
C H
ATOM 8380 CG LYS C 103 -14.799 24.475 43.238 1.00 0.00
C C
ATOM 8381 HG1 LYS C 103 -14.062 24.439 44.072 1.00 0.00
C H
ATOM 8382 HG2 LYS C 103 -15.762 24.847 43.661 1.00 0.00
C H
ATOM 8383 CD LYS C 103 -14.964 23.080 42.627 1.00 0.00
C C
ATOM 8384 HD1 LYS C 103 -15.706 23.144 41.799 1.00 0.00
C H
ATOM 8385 HD2 LYS C 103 -13.988 22.781 42.177 1.00 0.00
C H
ATOM 8386 CE LYS C 103 -15.404 22.026 43.647 1.00 0.00
C C
ATOM 8387 HE1 LYS C 103 -14.771 22.079 44.561 1.00 0.00
C H
ATOM 8388 HE2 LYS C 103 -16.464 22.187 43.943 1.00 0.00
C H
ATOM 8389 NZ LYS C 103 -15.299 20.626 43.114 1.00 0.00
C N
ATOM 8390 HZ1 LYS C 103 -15.606 19.946 43.839 1.00 0.00
C H
ATOM 8391 HZ2 LYS C 103 -15.901 20.529 42.271 1.00 0.00
C H
ATOM 8392 HZ3 LYS C 103 -14.312 20.429 42.853 1.00 0.00
C H
ATOM 8393 C LYS C 103 -12.764 26.573 43.818 1.00 0.00
C C
ATOM 8394 O LYS C 103 -11.692 26.012 43.581 1.00 0.00
C O
ATOM 8395 N ALA C 104 -13.099 27.009 45.030 1.00 0.00
C N
ATOM 8396 HN ALA C 104 -14.018 27.344 45.237 1.00 0.00
C H

```
ATOM   8397  CA  ALA C 104     -12.159  27.033  46.143  1.00  0.00
C    C
ATOM   8398  HA  ALA C 104     -11.148  26.876  45.786  1.00  0.00
C    H
ATOM   8399  CB  ALA C 104     -12.201  28.373  46.875  1.00  0.00
C    C
ATOM   8400  HB1 ALA C 104     -11.947  29.192  46.169  1.00  0.00
C    H
ATOM   8401  HB2 ALA C 104     -13.216  28.575  47.282  1.00  0.00
C    H
ATOM   8402  HB3 ALA C 104     -11.471  28.405  47.713  1.00  0.00
C    H
ATOM   8403  C   ALA C 104     -12.441  25.916  47.139  1.00  0.00
C    C
ATOM   8404  O   ALA C 104     -13.582  25.481  47.308  1.00  0.00
C    O
ATOM   8405  N   GLN C 105     -11.383  25.480  47.822  1.00  0.00
C    N
ATOM   8406  HN  GLN C 105     -10.483  25.906  47.754  1.00  0.00
C    H
ATOM   8407  CA  GLN C 105     -11.446  24.345  48.730  1.00  0.00
C    C
ATOM   8408  HA  GLN C 105     -12.292  23.747  48.415  1.00  0.00
C    H
ATOM   8409  CB  GLN C 105     -10.152  23.536  48.625  1.00  0.00
C    C
ATOM   8410  HB1 GLN C 105      -9.985  23.334  47.541  1.00  0.00
C    H
ATOM   8411  HB2 GLN C 105      -9.295  24.164  48.959  1.00  0.00
C    H
ATOM   8412  CG  GLN C 105     -10.166  22.224  49.374  1.00  0.00
C    C
ATOM   8413  HG1 GLN C 105     -11.208  21.855  49.489  1.00  0.00
C    H
ATOM   8414  HG2 GLN C 105      -9.590  21.451  48.824  1.00  0.00
C    H
ATOM   8415  CD  GLN C 105      -9.601  22.354  50.766  1.00  0.00
C    C
ATOM   8416  OE1 GLN C 105      -8.861  23.291  51.059  1.00  0.00
C    O
ATOM   8417  NE2 GLN C 105      -9.927  21.404  51.629  1.00  0.00
C    N
ATOM   8418 HE21 GLN C 105      -9.560  21.488  52.551  1.00  0.00
C    H
ATOM   8419 HE22 GLN C 105     -10.499  20.646  51.336  1.00  0.00
C    H
ATOM   8420  C   GLN C 105     -11.693  24.767  50.174  1.00  0.00
C    C
ATOM   8421  O   GLN C 105     -12.719  24.410  50.756  1.00  0.00
C    O
ATOM   8422  N   ASN C 106     -10.781  25.533  50.765  1.00  0.00
C    N
ATOM   8423  HN  ASN C 106      -9.924  25.826  50.343  1.00  0.00
C    H
```

```
ATOM   8424  CA  ASN C 106     -10.979  26.016  52.123  1.00  0.00
C    C
ATOM   8425  HA  ASN C 106     -12.028  25.877  52.359  1.00  0.00
C    H
ATOM   8426  CB  ASN C 106     -10.088  25.298  53.135  1.00  0.00
C    C
ATOM   8427  HB1 ASN C 106     -10.398  25.623  54.153  1.00  0.00
C    H
ATOM   8428  HB2 ASN C 106     -10.242  24.202  53.075  1.00  0.00
C    H
ATOM   8429  CG  ASN C 106      -8.613  25.629  52.968  1.00  0.00
C    C
ATOM   8430  OD1 ASN C 106      -8.219  26.430  52.123  1.00  0.00
C    O
ATOM   8431  ND2 ASN C 106      -7.789  25.019  53.803  1.00  0.00
C    N
ATOM   8432 HD21 ASN C 106      -6.818  25.228  53.722  1.00  0.00
C    H
ATOM   8433 HD22 ASN C 106      -8.148  24.404  54.498  1.00  0.00
C    H
ATOM   8434  C   ASN C 106     -10.695  27.503  52.155  1.00  0.00
C    C
ATOM   8435  O   ASN C 106     -10.289  28.103  51.157  1.00  0.00
C    O
ATOM   8436  N   TYR C 107     -10.877  28.090  53.334  1.00  0.00
C    N
ATOM   8437  HN  TYR C 107     -11.245  27.648  54.151  1.00  0.00
C    H
ATOM   8438  CA  TYR C 107     -10.531  29.490  53.509  1.00  0.00
C    C
ATOM   8439  HA  TYR C 107     -11.167  30.051  52.836  1.00  0.00
C    H
ATOM   8440  CB  TYR C 107     -10.751  29.904  54.959  1.00  0.00
C    C
ATOM   8441  HB1 TYR C 107     -11.813  30.205  55.095  1.00  0.00
C    H
ATOM   8442  HB2 TYR C 107     -10.561  29.050  55.645  1.00  0.00
C    H
ATOM   8443  CG  TYR C 107      -9.898  31.077  55.348  1.00  0.00
C    C
ATOM   8444  CD1 TYR C 107     -10.267  32.364  55.005  1.00  0.00
C    C
ATOM   8445  HD1 TYR C 107     -11.176  32.531  54.443  1.00  0.00
C    H
ATOM   8446  CE1 TYR C 107      -9.487  33.439  55.346  1.00  0.00
C    C
ATOM   8447  HE1 TYR C 107      -9.783  34.441  55.071  1.00  0.00
C    H
ATOM   8448  CZ  TYR C 107      -8.318  33.233  56.036  1.00  0.00
C    C
ATOM   8449  OH  TYR C 107      -7.530  34.304  56.384  1.00  0.00
C    O
ATOM   8450  HH  TYR C 107      -6.806  33.967  56.917  1.00  0.00
C    H
```

ATOM 8451 CD2 TYR C 107 -8.717 30.898 56.042 1.00 0.00
C C
ATOM 8452 HD2 TYR C 107 -8.381 29.900 56.289 1.00 0.00
C H
ATOM 8453 CE2 TYR C 107 -7.931 31.966 56.386 1.00 0.00
C C
ATOM 8454 HE2 TYR C 107 -7.002 31.801 56.911 1.00 0.00
C H
ATOM 8455 C TYR C 107 -9.093 29.755 53.094 1.00 0.00
C C
ATOM 8456 O TYR C 107 -8.802 30.758 52.431 1.00 0.00
C O
ATOM 8457 N TYR C 108 -8.177 28.871 53.479 1.00 0.00
C N
ATOM 8458 HN TYR C 108 -8.349 28.027 53.984 1.00 0.00
C H
ATOM 8459 CA TYR C 108 -6.779 29.110 53.159 1.00 0.00
C C
ATOM 8460 HA TYR C 108 -6.549 30.109 53.509 1.00 0.00
C H
ATOM 8461 CB TYR C 108 -5.904 28.071 53.834 1.00 0.00
C C
ATOM 8462 HB1 TYR C 108 -6.238 27.057 53.524 1.00 0.00
C H
ATOM 8463 HB2 TYR C 108 -4.845 28.180 53.513 1.00 0.00
C H
ATOM 8464 CG TYR C 108 -6.023 28.168 55.307 1.00 0.00
C C
ATOM 8465 CD1 TYR C 108 -5.806 29.372 55.941 1.00 0.00
C C
ATOM 8466 HD1 TYR C 108 -5.523 30.240 55.362 1.00 0.00
C H
ATOM 8467 CE1 TYR C 108 -5.918 29.491 57.291 1.00 0.00
C C
ATOM 8468 HE1 TYR C 108 -5.738 30.440 57.776 1.00 0.00
C H
ATOM 8469 CZ TYR C 108 -6.255 28.395 58.035 1.00 0.00
C C
ATOM 8470 OH TYR C 108 -6.366 28.511 59.400 1.00 0.00
C O
ATOM 8471 HH TYR C 108 -6.676 27.667 59.736 1.00 0.00
C H
ATOM 8472 CD2 TYR C 108 -6.369 27.075 56.068 1.00 0.00
C C
ATOM 8473 HD2 TYR C 108 -6.516 26.113 55.596 1.00 0.00
C H
ATOM 8474 CE2 TYR C 108 -6.482 27.180 57.427 1.00 0.00
C C
ATOM 8475 HE2 TYR C 108 -6.727 26.307 58.013 1.00 0.00
C H
ATOM 8476 C TYR C 108 -6.548 29.096 51.665 1.00 0.00
C C
ATOM 8477 O TYR C 108 -6.212 30.133 51.076 1.00 0.00
C O

```
ATOM   8478  N   HSE C 109      -6.781  27.942  51.040  1.00  0.00
C    N
ATOM   8479  HN  HSE C 109      -7.008  27.086  51.503  1.00  0.00
C    H
ATOM   8480  CA  HSE C 109      -6.721  27.840  49.591  1.00  0.00
C    C
ATOM   8481  HA  HSE C 109      -5.673  27.811  49.317  1.00  0.00
C    H
ATOM   8482  CB  HSE C 109      -7.462  26.590  49.143  1.00  0.00
C    C
ATOM   8483  HB1 HSE C 109      -6.882  25.707  49.490  1.00  0.00
C    H
ATOM   8484  HB2 HSE C 109      -8.452  26.546  49.645  1.00  0.00
C    H
ATOM   8485  ND1 HSE C 109      -8.854  26.391  47.060  1.00  0.00
C    N
ATOM   8486  CG  HSE C 109      -7.622  26.485  47.666  1.00  0.00
C    C
ATOM   8487  CE1 HSE C 109      -8.694  26.309  45.752  1.00  0.00
C    C
ATOM   8488  HE1 HSE C 109      -9.510  26.227  45.031  1.00  0.00
C    H
ATOM   8489  NE2 HSE C 109      -7.400  26.337  45.490  1.00  0.00
C    N
ATOM   8490  HE2 HSE C 109      -6.996  26.269  44.578  1.00  0.00
C    H
ATOM   8491  CD2 HSE C 109      -6.707  26.445  46.671  1.00  0.00
C    C
ATOM   8492  HD2 HSE C 109      -5.628  26.475  46.720  1.00  0.00
C    H
ATOM   8493  C   HSE C 109      -7.317  29.070  48.940  1.00  0.00
C    C
ATOM   8494  O   HSE C 109      -6.774  29.595  47.966  1.00  0.00
C    O
ATOM   8495  N   HSE C 110      -8.404  29.578  49.512  1.00  0.00
C    N
ATOM   8496  HN  HSE C 110      -8.868  29.177  50.301  1.00  0.00
C    H
ATOM   8497  CA  HSE C 110      -9.015  30.797  49.012  1.00  0.00
C    C
ATOM   8498  HA  HSE C 110      -9.200  30.641  47.956  1.00  0.00
C    H
ATOM   8499  CB  HSE C 110     -10.301  31.053  49.775  1.00  0.00
C    C
ATOM   8500  HB1 HSE C 110     -11.133  30.581  49.210  1.00  0.00
C    H
ATOM   8501  HB2 HSE C 110     -10.252  30.548  50.763  1.00  0.00
C    H
ATOM   8502  ND1 HSE C 110     -11.389  33.192  49.042  1.00  0.00
C    N
ATOM   8503  CG  HSE C 110     -10.616  32.497  49.942  1.00  0.00
C    C
ATOM   8504  CE1 HSE C 110     -11.496  34.446  49.437  1.00  0.00
C    C
```

```
ATOM   8505  HE1 HSE C 110     -12.051  35.219  48.901  1.00  0.00
C    H
ATOM   8506  NE2 HSE C 110     -10.820  34.588  50.563  1.00  0.00
C    N
ATOM   8507  HE2 HSE C 110     -10.720  35.443  51.070  1.00  0.00
C    H
ATOM   8508  CD2 HSE C 110     -10.259  33.381  50.900  1.00  0.00
C    C
ATOM   8509  HD2 HSE C 110      -9.646  33.241  51.780  1.00  0.00
C    H
ATOM   8510  C   HSE C 110      -8.079  31.990  49.130  1.00  0.00
C    C
ATOM   8511  O   HSE C 110      -7.976  32.804  48.207  1.00  0.00
C    O
ATOM   8512  N   LYS C 111      -7.423  32.147  50.272  1.00  0.00
C    N
ATOM   8513  HN  LYS C 111      -7.474  31.554  51.075  1.00  0.00
C    H
ATOM   8514  CA  LYS C 111      -6.531  33.290  50.404  1.00  0.00
C    C
ATOM   8515  HA  LYS C 111      -7.100  34.171  50.134  1.00  0.00
C    H
ATOM   8516  CB  LYS C 111      -6.020  33.415  51.830  1.00  0.00
C    C
ATOM   8517  HB1 LYS C 111      -6.895  33.670  52.470  1.00  0.00
C    H
ATOM   8518  HB2 LYS C 111      -5.660  32.419  52.174  1.00  0.00
C    H
ATOM   8519  CG  LYS C 111      -4.947  34.479  51.974  1.00  0.00
C    C
ATOM   8520  HG1 LYS C 111      -4.114  34.075  52.594  1.00  0.00
C    H
ATOM   8521  HG2 LYS C 111      -4.529  34.712  50.968  1.00  0.00
C    H
ATOM   8522  CD  LYS C 111      -5.459  35.770  52.634  1.00  0.00
C    C
ATOM   8523  HD1 LYS C 111      -4.902  35.924  53.587  1.00  0.00
C    H
ATOM   8524  HD2 LYS C 111      -5.212  36.624  51.961  1.00  0.00
C    H
ATOM   8525  CE  LYS C 111      -6.940  35.723  52.889  1.00  0.00
C    C
ATOM   8526  HE1 LYS C 111      -7.325  34.686  52.761  1.00  0.00
C    H
ATOM   8527  HE2 LYS C 111      -7.169  36.056  53.925  1.00  0.00
C    H
ATOM   8528  NZ  LYS C 111      -7.644  36.612  51.935  1.00  0.00
C    N
ATOM   8529  HZ1 LYS C 111      -8.670  36.581  52.109  1.00  0.00
C    H
ATOM   8530  HZ2 LYS C 111      -7.304  37.588  52.055  1.00  0.00
C    H
ATOM   8531  HZ3 LYS C 111      -7.450  36.299  50.963  1.00  0.00
C    H
```

```
ATOM   8532  C   LYS C 111      -5.364  33.192  49.439  1.00  0.00
C    C
ATOM   8533  O   LYS C 111      -4.994  34.176  48.788  1.00  0.00
C    O
ATOM   8534  N   VAL C 112      -4.775  32.007  49.329  1.00  0.00
C    N
ATOM   8535  HN  VAL C 112      -5.001  31.192  49.855  1.00  0.00
C    H
ATOM   8536  CA  VAL C 112      -3.694  31.823  48.367  1.00  0.00
C    C
ATOM   8537  HA  VAL C 112      -2.926  32.554  48.588  1.00  0.00
C    H
ATOM   8538  CB  VAL C 112      -3.083  30.422  48.498  1.00  0.00
C    C
ATOM   8539  HB  VAL C 112      -1.980  30.545  48.634  1.00  0.00
C    H
ATOM   8540  CG1 VAL C 112      -3.655  29.722  49.684  1.00  0.00
C    C
ATOM   8541 HG11 VAL C 112      -3.244  28.692  49.760  1.00  0.00
C    H
ATOM   8542 HG12 VAL C 112      -3.404  30.248  50.630  1.00  0.00
C    H
ATOM   8543 HG13 VAL C 112      -4.760  29.646  49.600  1.00  0.00
C    H
ATOM   8544  CG2 VAL C 112      -3.350  29.621  47.253  1.00  0.00
C    C
ATOM   8545 HG21 VAL C 112      -2.946  28.591  47.354  1.00  0.00
C    H
ATOM   8546 HG22 VAL C 112      -4.442  29.551  47.060  1.00  0.00
C    H
ATOM   8547 HG23 VAL C 112      -2.868  30.084  46.367  1.00  0.00
C    H
ATOM   8548  C   VAL C 112      -4.190  32.095  46.959  1.00  0.00
C    C
ATOM   8549  O   VAL C 112      -3.441  32.581  46.103  1.00  0.00
C    O
ATOM   8550  N   LEU C 113      -5.459  31.794  46.699  1.00  0.00
C    N
ATOM   8551  HN  LEU C 113      -6.065  31.282  47.303  1.00  0.00
C    H
ATOM   8552  CA  LEU C 113      -6.074  32.213  45.452  1.00  0.00
C    C
ATOM   8553  HA  LEU C 113      -5.485  31.788  44.650  1.00  0.00
C    H
ATOM   8554  CB  LEU C 113      -7.516  31.732  45.418  1.00  0.00
C    C
ATOM   8555  HB1 LEU C 113      -7.558  30.777  45.991  1.00  0.00
C    H
ATOM   8556  HB2 LEU C 113      -8.151  32.455  45.977  1.00  0.00
C    H
ATOM   8557  CG  LEU C 113      -8.143  31.471  44.064  1.00  0.00
C    C
ATOM   8558  HG  LEU C 113      -8.618  32.417  43.708  1.00  0.00
C    H
```

```
ATOM   8559  CD1 LEU C 113      -7.088  31.056  43.075  1.00  0.00
C    C
ATOM   8560 HD11 LEU C 113      -7.547  30.816  42.092  1.00  0.00
C    H
ATOM   8561 HD12 LEU C 113      -6.349  31.873  42.925  1.00  0.00
C    H
ATOM   8562 HD13 LEU C 113      -6.547  30.155  43.433  1.00  0.00
C    H
ATOM   8563  CD2 LEU C 113      -9.223  30.435  44.212  1.00  0.00
C    C
ATOM   8564 HD21 LEU C 113      -9.711  30.242  43.232  1.00  0.00
C    H
ATOM   8565 HD22 LEU C 113      -8.818  29.472  44.582  1.00  0.00
C    H
ATOM   8566 HD23 LEU C 113     -10.002  30.785  44.924  1.00  0.00
C    H
ATOM   8567  C   LEU C 113      -6.019  33.720  45.310  1.00  0.00
C    C
ATOM   8568  O   LEU C 113      -5.631  34.245  44.262  1.00  0.00
C    O
ATOM   8569  N   GLU C 114      -6.398  34.429  46.366  1.00  0.00
C    N
ATOM   8570  HN  GLU C 114      -6.693  34.056  47.241  1.00  0.00
C    H
ATOM   8571  CA  GLU C 114      -6.405  35.882  46.309  1.00  0.00
C    C
ATOM   8572  HA  GLU C 114      -7.075  36.186  45.514  1.00  0.00
C    H
ATOM   8573  CB  GLU C 114      -6.892  36.458  47.634  1.00  0.00
C    C
ATOM   8574  HB1 GLU C 114      -7.781  35.871  47.962  1.00  0.00
C    H
ATOM   8575  HB2 GLU C 114      -6.118  36.305  48.419  1.00  0.00
C    H
ATOM   8576  CG  GLU C 114      -7.254  37.912  47.515  1.00  0.00
C    C
ATOM   8577  HG1 GLU C 114      -7.893  38.203  48.369  1.00  0.00
C    H
ATOM   8578  HG2 GLU C 114      -6.347  38.548  47.506  1.00  0.00
C    H
ATOM   8579  CD  GLU C 114      -8.063  38.190  46.271  1.00  0.00
C    C
ATOM   8580  OE1 GLU C 114      -9.108  37.534  46.089  1.00  0.00
C    O
ATOM   8581  OE2 GLU C 114      -7.646  39.050  45.467  1.00  0.00
C    O
ATOM   8582  C   GLU C 114      -5.025  36.426  45.969  1.00  0.00
C    C
ATOM   8583  O   GLU C 114      -4.888  37.340  45.149  1.00  0.00
C    O
ATOM   8584  N   LYS C 115      -3.988  35.864  46.582  1.00  0.00
C    N
ATOM   8585  HN  LYS C 115      -4.041  35.075  47.193  1.00  0.00
C    H
```

```
ATOM   8586  CA  LYS C 115      -2.649  36.403  46.382  1.00  0.00
C    C
ATOM   8587  HA  LYS C 115      -2.705  37.457  46.623  1.00  0.00
C    H
ATOM   8588  CB  LYS C 115      -1.659  35.700  47.290  1.00  0.00
C    C
ATOM   8589  HB1 LYS C 115      -1.800  34.604  47.142  1.00  0.00
C    H
ATOM   8590  HB2 LYS C 115      -0.626  35.931  46.947  1.00  0.00
C    H
ATOM   8591  CG  LYS C 115      -1.859  36.051  48.714  1.00  0.00
C    C
ATOM   8592  HG1 LYS C 115      -2.752  35.508  49.102  1.00  0.00
C    H
ATOM   8593  HG2 LYS C 115      -0.978  35.702  49.301  1.00  0.00
C    H
ATOM   8594  CD  LYS C 115      -2.060  37.523  48.880  1.00  0.00
C    C
ATOM   8595  HD1 LYS C 115      -1.189  38.050  48.428  1.00  0.00
C    H
ATOM   8596  HD2 LYS C 115      -2.965  37.817  48.301  1.00  0.00
C    H
ATOM   8597  CE  LYS C 115      -2.211  37.829  50.341  1.00  0.00
C    C
ATOM   8598  HE1 LYS C 115      -2.139  38.924  50.519  1.00  0.00
C    H
ATOM   8599  HE2 LYS C 115      -3.197  37.475  50.715  1.00  0.00
C    H
ATOM   8600  NZ  LYS C 115      -1.131  37.145  51.102  1.00  0.00
C    N
ATOM   8601  HZ1 LYS C 115      -1.225  37.352  52.118  1.00  0.00
C    H
ATOM   8602  HZ2 LYS C 115      -1.197  36.118  50.952  1.00  0.00
C    H
ATOM   8603  HZ3 LYS C 115      -0.206  37.481  50.767  1.00  0.00
C    H
ATOM   8604  C   LYS C 115      -2.178  36.292  44.944  1.00  0.00
C    C
ATOM   8605  O   LYS C 115      -1.417  37.148  44.485  1.00  0.00
C    O
ATOM   8606  N   ILE C 116      -2.601  35.261  44.219  1.00  0.00
C    N
ATOM   8607  HN  ILE C 116      -3.201  34.537  44.555  1.00  0.00
C    H
ATOM   8608  CA  ILE C 116      -2.205  35.108  42.829  1.00  0.00
C    C
ATOM   8609  HA  ILE C 116      -1.140  35.299  42.798  1.00  0.00
C    H
ATOM   8610  CB  ILE C 116      -2.524  33.701  42.317  1.00  0.00
C    C
ATOM   8611  HB  ILE C 116      -2.244  33.024  43.164  1.00  0.00
C    H
ATOM   8612  CG2 ILE C 116      -3.951  33.640  41.840  1.00  0.00
C    C
```

```
ATOM   8613 HG21 ILE C 116      -4.242  32.593  41.613  1.00  0.00
C    H
ATOM   8614 HG22 ILE C 116      -4.646  34.021  42.618  1.00  0.00
C    H
ATOM   8615 HG23 ILE C 116      -4.089  34.246  40.920  1.00  0.00
C    H
ATOM   8616  CG1 ILE C 116      -1.620  33.328  41.144  1.00  0.00
C    C
ATOM   8617 HG11 ILE C 116      -1.809  32.274  40.843  1.00  0.00
C    H
ATOM   8618 HG12 ILE C 116      -1.879  33.971  40.272  1.00  0.00
C    H
ATOM   8619  CD  ILE C 116      -0.171  33.489  41.406  1.00  0.00
C    C
ATOM   8620  HD1 ILE C 116       0.414  33.255  40.490  1.00  0.00
C    H
ATOM   8621  HD2 ILE C 116       0.059  34.533  41.708  1.00  0.00
C    H
ATOM   8622  HD3 ILE C 116       0.166  32.805  42.213  1.00  0.00
C    H
ATOM   8623  C   ILE C 116      -2.864  36.148  41.941  1.00  0.00
C    C
ATOM   8624  O   ILE C 116      -2.427  36.360  40.810  1.00  0.00
C    O
ATOM   8625  N   GLY C 117      -3.897  36.818  42.432  1.00  0.00
C    N
ATOM   8626  HN  GLY C 117      -4.170  36.808  43.393  1.00  0.00
C    H
ATOM   8627  CA  GLY C 117      -4.712  37.630  41.560  1.00  0.00
C    C
ATOM   8628  HA1 GLY C 117      -4.127  37.825  40.672  1.00  0.00
C    H
ATOM   8629  HA2 GLY C 117      -4.989  38.517  42.114  1.00  0.00
C    H
ATOM   8630  C   GLY C 117      -5.976  36.934  41.139  1.00  0.00
C    C
ATOM   8631  O   GLY C 117      -6.371  37.026  39.974  1.00  0.00
C    O
ATOM   8632  N   ALA C 118      -6.617  36.221  42.057  1.00  0.00
C    N
ATOM   8633  HN  ALA C 118      -6.286  36.081  42.990  1.00  0.00
C    H
ATOM   8634  CA  ALA C 118      -7.882  35.583  41.742  1.00  0.00
C    C
ATOM   8635  HA  ALA C 118      -7.787  34.934  40.878  1.00  0.00
C    H
ATOM   8636  CB  ALA C 118      -8.371  34.764  42.928  1.00  0.00
C    C
ATOM   8637  HB1 ALA C 118      -7.633  33.966  43.160  1.00  0.00
C    H
ATOM   8638  HB2 ALA C 118      -8.484  35.399  43.833  1.00  0.00
C    H
ATOM   8639  HB3 ALA C 118      -9.347  34.276  42.714  1.00  0.00
C    H
```

ATOM 8640 C ALA C 118 -8.917 36.627 41.380 1.00 0.00
C C
ATOM 8641 O ALA C 118 -9.383 37.383 42.234 1.00 0.00
C O
ATOM 8642 N ASP C 119 -9.274 36.671 40.104 1.00 0.00
C N
ATOM 8643 HN ASP C 119 -8.913 36.130 39.348 1.00 0.00
C H
ATOM 8644 CA ASP C 119 -10.312 37.597 39.691 1.00 0.00
C C
ATOM 8645 HA ASP C 119 -10.005 38.584 40.020 1.00 0.00
C H
ATOM 8646 CB ASP C 119 -10.479 37.551 38.175 1.00 0.00
C C
ATOM 8647 HB1 ASP C 119 -10.611 36.498 37.854 1.00 0.00
C H
ATOM 8648 HB2 ASP C 119 -11.374 38.125 37.864 1.00 0.00
C H
ATOM 8649 CG ASP C 119 -9.273 38.101 37.450 1.00 0.00
C C
ATOM 8650 OD1 ASP C 119 -9.299 39.294 37.078 1.00 0.00
C O
ATOM 8651 OD2 ASP C 119 -8.293 37.348 37.268 1.00 0.00
C O
ATOM 8652 C ASP C 119 -11.625 37.295 40.384 1.00 0.00
C C
ATOM 8653 O ASP C 119 -12.451 38.195 40.557 1.00 0.00
C O
ATOM 8654 N ARG C 120 -11.836 36.047 40.787 1.00 0.00
C N
ATOM 8655 HN ARG C 120 -11.187 35.310 40.616 1.00 0.00
C H
ATOM 8656 CA ARG C 120 -13.046 35.696 41.508 1.00 0.00
C C
ATOM 8657 HA ARG C 120 -13.166 36.377 42.342 1.00 0.00
C H
ATOM 8658 CB ARG C 120 -14.261 35.768 40.592 1.00 0.00
C C
ATOM 8659 HB1 ARG C 120 -14.510 36.850 40.482 1.00 0.00
C H
ATOM 8660 HB2 ARG C 120 -13.982 35.413 39.575 1.00 0.00
C H
ATOM 8661 CG ARG C 120 -15.424 35.044 41.142 1.00 0.00
C C
ATOM 8662 HG1 ARG C 120 -15.573 34.113 40.552 1.00 0.00
C H
ATOM 8663 HG2 ARG C 120 -15.185 34.708 42.177 1.00 0.00
C H
ATOM 8664 CD ARG C 120 -16.607 35.936 41.167 1.00 0.00
C C
ATOM 8665 HD1 ARG C 120 -16.441 36.750 41.909 1.00 0.00
C H
ATOM 8666 HD2 ARG C 120 -16.795 36.395 40.168 1.00 0.00
C H

ATOM 8667 NE ARG C 120 -17.795 35.182 41.525 1.00 0.00
C N
ATOM 8668 HE ARG C 120 -17.612 34.204 41.675 1.00 0.00
C H
ATOM 8669 CZ ARG C 120 -19.001 35.718 41.644 1.00 0.00
C C
ATOM 8670 NH1 ARG C 120 -19.172 37.012 41.425 1.00 0.00
C N
ATOM 8671 HH11 ARG C 120 -20.063 37.428 41.535 1.00 0.00
C H
ATOM 8672 HH12 ARG C 120 -18.339 37.551 41.281 1.00 0.00
C H
ATOM 8673 NH2 ARG C 120 -20.035 34.961 41.973 1.00 0.00
C N
ATOM 8674 HH21 ARG C 120 -20.948 35.346 41.977 1.00 0.00
C H
ATOM 8675 HH22 ARG C 120 -19.883 33.982 42.002 1.00 0.00
C H
ATOM 8676 C ARG C 120 -12.920 34.303 42.098 1.00 0.00
C C
ATOM 8677 O ARG C 120 -12.549 33.354 41.398 1.00 0.00
C O
ATOM 8678 N ILE C 121 -13.260 34.186 43.376 1.00 0.00
C N
ATOM 8679 HN ILE C 121 -13.595 34.959 43.911 1.00 0.00
C H
ATOM 8680 CA ILE C 121 -13.180 32.936 44.119 1.00 0.00
C C
ATOM 8681 HA ILE C 121 -12.770 32.185 43.457 1.00 0.00
C H
ATOM 8682 CB ILE C 121 -12.335 33.117 45.389 1.00 0.00
C C
ATOM 8683 HB ILE C 121 -12.954 33.780 46.046 1.00 0.00
C H
ATOM 8684 CG2 ILE C 121 -12.018 31.789 46.026 1.00 0.00
C C
ATOM 8685 HG21 ILE C 121 -11.545 31.936 47.019 1.00 0.00
C H
ATOM 8686 HG22 ILE C 121 -12.941 31.192 46.179 1.00 0.00
C H
ATOM 8687 HG23 ILE C 121 -11.321 31.201 45.393 1.00 0.00
C H
ATOM 8688 CG1 ILE C 121 -11.060 33.884 45.048 1.00 0.00
C C
ATOM 8689 HG11 ILE C 121 -10.557 33.409 44.176 1.00 0.00
C H
ATOM 8690 HG12 ILE C 121 -11.334 34.920 44.745 1.00 0.00
C H
ATOM 8691 CD ILE C 121 -10.079 33.979 46.177 1.00 0.00
C C
ATOM 8692 HD1 ILE C 121 -9.205 34.597 45.879 1.00 0.00
C H
ATOM 8693 HD2 ILE C 121 -10.551 34.449 47.067 1.00 0.00
C H

ATOM 8694 HD3 ILE C 121 -9.707 32.973 46.466 1.00 0.00
C H
ATOM 8695 C ILE C 121 -14.595 32.498 44.458 1.00 0.00
C C
ATOM 8696 O ILE C 121 -15.490 33.337 44.595 1.00 0.00
C O
ATOM 8697 N ILE C 122 -14.809 31.191 44.575 1.00 0.00
C N
ATOM 8698 HN ILE C 122 -14.118 30.490 44.408 1.00 0.00
C H
ATOM 8699 CA ILE C 122 -16.108 30.666 44.967 1.00 0.00
C C
ATOM 8700 HA ILE C 122 -16.635 31.464 45.475 1.00 0.00
C H
ATOM 8701 CB ILE C 122 -16.913 30.152 43.760 1.00 0.00
C C
ATOM 8702 HB ILE C 122 -16.200 29.489 43.209 1.00 0.00
C H
ATOM 8703 CG2 ILE C 122 -18.175 29.446 44.219 1.00 0.00
C C
ATOM 8704 HG21 ILE C 122 -18.673 28.941 43.365 1.00 0.00
C H
ATOM 8705 HG22 ILE C 122 -17.940 28.670 44.977 1.00 0.00
C H
ATOM 8706 HG23 ILE C 122 -18.893 30.166 44.664 1.00 0.00
C H
ATOM 8707 CG1 ILE C 122 -17.260 31.305 42.823 1.00 0.00
C C
ATOM 8708 HG11 ILE C 122 -18.249 31.120 42.349 1.00 0.00
C H
ATOM 8709 HG12 ILE C 122 -17.352 32.240 43.422 1.00 0.00
C H
ATOM 8710 CD ILE C 122 -16.237 31.541 41.742 1.00 0.00
C C
ATOM 8711 HD1 ILE C 122 -16.518 32.427 41.132 1.00 0.00
C H
ATOM 8712 HD2 ILE C 122 -15.235 31.727 42.184 1.00 0.00
C H
ATOM 8713 HD3 ILE C 122 -16.166 30.666 41.062 1.00 0.00
C H
ATOM 8714 C ILE C 122 -15.918 29.552 45.979 1.00 0.00
C C
ATOM 8715 O ILE C 122 -15.079 28.666 45.795 1.00 0.00
C O
ATOM 8716 N HSE C 123 -16.714 29.590 47.033 1.00 0.00
C N
ATOM 8717 HN HSE C 123 -17.334 30.359 47.184 1.00 0.00
C H
ATOM 8718 CA HSE C 123 -16.778 28.559 48.054 1.00 0.00
C C
ATOM 8719 HA HSE C 123 -16.029 27.807 47.837 1.00 0.00
C H
ATOM 8720 CB HSE C 123 -16.610 29.163 49.442 1.00 0.00
C C

ATOM 8721 HB1 HSE C 123 −17.404 29.928 49.580 1.00 0.00
C H
ATOM 8722 HB2 HSE C 123 −16.776 28.379 50.211 1.00 0.00
C H
ATOM 8723 ND1 HSE C 123 −14.311 29.292 50.467 1.00 0.00
C N
ATOM 8724 CG HSE C 123 −15.290 29.830 49.661 1.00 0.00
C C
ATOM 8725 CE1 HSE C 123 −13.268 30.101 50.487 1.00 0.00
C C
ATOM 8726 HE1 HSE C 123 −12.354 29.920 51.059 1.00 0.00
C H
ATOM 8727 NE2 HSE C 123 −13.529 31.139 49.715 1.00 0.00
C N
ATOM 8728 HE2 HSE C 123 −12.915 31.913 49.561 1.00 0.00
C H
ATOM 8729 CD2 HSE C 123 −14.790 30.995 49.189 1.00 0.00
C C
ATOM 8730 HD2 HSE C 123 −15.239 31.727 48.532 1.00 0.00
C H
ATOM 8731 C HSE C 123 −18.135 27.886 47.930 1.00 0.00
C C
ATOM 8732 O HSE C 123 −19.018 28.106 48.768 1.00 0.00
C O
ATOM 8733 N PRO C 124 −18.335 27.070 46.897 1.00 0.00
C N
ATOM 8734 CD PRO C 124 −17.321 26.400 46.073 1.00 0.00
C C
ATOM 8735 HD1 PRO C 124 −17.332 27.512 46.109 1.00 0.00
C H
ATOM 8736 HD2 PRO C 124 −16.275 26.022 46.009 1.00 0.00
C H
ATOM 8737 CA PRO C 124 −19.701 26.674 46.528 1.00 0.00
C C
ATOM 8738 HA PRO C 124 −20.233 27.542 46.156 1.00 0.00
C H
ATOM 8739 CB PRO C 124 −19.471 25.665 45.394 1.00 0.00
C C
ATOM 8740 HB1 PRO C 124 −19.361 26.218 44.435 1.00 0.00
C H
ATOM 8741 HB2 PRO C 124 −20.299 24.934 45.297 1.00 0.00
C H
ATOM 8742 CG PRO C 124 −18.107 25.920 44.907 1.00 0.00
C C
ATOM 8743 HG1 PRO C 124 −17.949 25.576 43.866 1.00 0.00
C H
ATOM 8744 HG2 PRO C 124 −17.377 25.414 45.579 1.00 0.00
C H
ATOM 8745 C PRO C 124 −20.495 26.054 47.655 1.00 0.00
C C
ATOM 8746 O PRO C 124 −21.562 26.567 48.025 1.00 0.00
C O
ATOM 8747 N GLU C 125 −20.001 24.957 48.215 1.00 0.00
C N

ATOM 8748 HN GLU C 125 -19.130 24.522 48.005 1.00 0.00
C H
ATOM 8749 CA GLU C 125 -20.765 24.280 49.246 1.00 0.00
C C
ATOM 8750 HA GLU C 125 -21.759 24.092 48.862 1.00 0.00
C H
ATOM 8751 CB GLU C 125 -20.126 22.955 49.613 1.00 0.00
C C
ATOM 8752 HB1 GLU C 125 -19.026 23.110 49.697 1.00 0.00
C H
ATOM 8753 HB2 GLU C 125 -20.468 22.638 50.624 1.00 0.00
C H
ATOM 8754 CG GLU C 125 -20.404 21.864 48.598 1.00 0.00
C C
ATOM 8755 HG1 GLU C 125 -20.635 20.920 49.125 1.00 0.00
C H
ATOM 8756 HG2 GLU C 125 -21.257 22.136 47.946 1.00 0.00
C H
ATOM 8757 CD GLU C 125 -19.229 21.547 47.717 1.00 0.00
C C
ATOM 8758 OE1 GLU C 125 -18.225 22.293 47.782 1.00 0.00
C O
ATOM 8759 OE2 GLU C 125 -19.318 20.540 46.985 1.00 0.00
C O
ATOM 8760 C GLU C 125 -20.914 25.151 50.469 1.00 0.00
C C
ATOM 8761 O GLU C 125 -21.917 25.058 51.169 1.00 0.00
C O
ATOM 8762 N LYS C 126 -19.945 26.025 50.734 1.00 0.00
C N
ATOM 8763 HN LYS C 126 -19.058 26.069 50.277 1.00 0.00
C H
ATOM 8764 CA LYS C 126 -20.155 27.022 51.775 1.00 0.00
C C
ATOM 8765 HA LYS C 126 -20.298 26.491 52.709 1.00 0.00
C H
ATOM 8766 CB LYS C 126 -18.949 27.954 51.857 1.00 0.00
C C
ATOM 8767 HB1 LYS C 126 -18.920 28.538 50.907 1.00 0.00
C H
ATOM 8768 HB2 LYS C 126 -19.117 28.693 52.672 1.00 0.00
C H
ATOM 8769 CG LYS C 126 -17.626 27.264 52.041 1.00 0.00
C C
ATOM 8770 HG1 LYS C 126 -17.422 26.624 51.152 1.00 0.00
C H
ATOM 8771 HG2 LYS C 126 -16.820 28.032 52.090 1.00 0.00
C H
ATOM 8772 CD LYS C 126 -17.604 26.409 53.271 1.00 0.00
C C
ATOM 8773 HD1 LYS C 126 -18.631 26.013 53.443 1.00 0.00
C H
ATOM 8774 HD2 LYS C 126 -16.939 25.536 53.074 1.00 0.00
C H

ATOM 8775 CE LYS C 126 -17.117 27.173 54.488 1.00 0.00
C C
ATOM 8776 HE1 LYS C 126 -16.368 27.940 54.192 1.00 0.00
C H
ATOM 8777 HE2 LYS C 126 -17.966 27.685 54.992 1.00 0.00
C H
ATOM 8778 NZ LYS C 126 -16.479 26.254 55.471 1.00 0.00
C N
ATOM 8779 HZ1 LYS C 126 -16.149 26.791 56.299 1.00 0.00
C H
ATOM 8780 HZ2 LYS C 126 -17.169 25.539 55.776 1.00 0.00
C H
ATOM 8781 HZ3 LYS C 126 -15.669 25.779 55.024 1.00 0.00
C H
ATOM 8782 C LYS C 126 -21.425 27.817 51.505 1.00 0.00
C C
ATOM 8783 O LYS C 126 -22.323 27.905 52.358 1.00 0.00
C O
ATOM 8784 N ASP C 127 -21.528 28.372 50.300 1.00 0.00
C N
ATOM 8785 HN ASP C 127 -20.895 28.242 49.541 1.00 0.00
C H
ATOM 8786 CA ASP C 127 -22.631 29.263 49.974 1.00 0.00
C C
ATOM 8787 HA ASP C 127 -22.655 30.036 50.733 1.00 0.00
C H
ATOM 8788 CB ASP C 127 -22.409 29.837 48.574 1.00 0.00
C C
ATOM 8789 HB1 ASP C 127 -22.019 29.040 47.910 1.00 0.00
C H
ATOM 8790 HB2 ASP C 127 -23.363 30.208 48.149 1.00 0.00
C H
ATOM 8791 CG ASP C 127 -21.404 30.971 48.559 1.00 0.00
C C
ATOM 8792 OD1 ASP C 127 -20.198 30.679 48.728 1.00 0.00
C O
ATOM 8793 OD2 ASP C 127 -21.815 32.135 48.365 1.00 0.00
C O
ATOM 8794 C ASP C 127 -23.957 28.525 50.056 1.00 0.00
C C
ATOM 8795 O ASP C 127 -24.890 28.949 50.754 1.00 0.00
C O
ATOM 8796 N MET C 128 -24.060 27.400 49.362 1.00 0.00
C N
ATOM 8797 HN MET C 128 -23.365 26.924 48.828 1.00 0.00
C H
ATOM 8798 CA MET C 128 -25.356 26.753 49.368 1.00 0.00
C C
ATOM 8799 HA MET C 128 -26.103 27.532 49.284 1.00 0.00
C H
ATOM 8800 CB MET C 128 -25.506 25.834 48.161 1.00 0.00
C C
ATOM 8801 HB1 MET C 128 -26.012 26.427 47.363 1.00 0.00
C H

```
ATOM   8802  HB2 MET C 128     -24.497  25.575  47.766  1.00  0.00
C    H
ATOM   8803  CG  MET C 128     -26.308  24.580  48.407  1.00  0.00
C    C
ATOM   8804  HG1 MET C 128     -25.632  23.726  48.623  1.00  0.00
C    H
ATOM   8805  HG2 MET C 128     -26.963  24.723  49.294  1.00  0.00
C    H
ATOM   8806  SD  MET C 128     -27.332  24.136  46.992  1.00  0.00
C    S
ATOM   8807  CE  MET C 128     -27.040  25.502  45.861  1.00  0.00
C    C
ATOM   8808  HE1 MET C 128     -27.624  25.375  44.924  1.00  0.00
C    H
ATOM   8809  HE2 MET C 128     -27.337  26.470  46.320  1.00  0.00
C    H
ATOM   8810  HE3 MET C 128     -25.965  25.570  45.586  1.00  0.00
C    H
ATOM   8811  C   MET C 128     -25.596  26.028  50.681  1.00  0.00
C    C
ATOM   8812  O   MET C 128     -26.741  25.733  51.022  1.00  0.00
C    O
ATOM   8813  N   GLY C 129     -24.563  25.812  51.478  1.00  0.00
C    N
ATOM   8814  HN  GLY C 129     -23.612  26.032  51.263  1.00  0.00
C    H
ATOM   8815  CA  GLY C 129     -24.787  25.211  52.765  1.00  0.00
C    C
ATOM   8816  HA1 GLY C 129     -23.827  24.963  53.196  1.00  0.00
C    H
ATOM   8817  HA2 GLY C 129     -25.450  24.365  52.641  1.00  0.00
C    H
ATOM   8818  C   GLY C 129     -25.462  26.200  53.672  1.00  0.00
C    C
ATOM   8819  O   GLY C 129     -26.425  25.866  54.358  1.00  0.00
C    O
ATOM   8820  N   VAL C 130     -24.950  27.425  53.706  1.00  0.00
C    N
ATOM   8821  HN  VAL C 130     -24.141  27.759  53.230  1.00  0.00
C    H
ATOM   8822  CA  VAL C 130     -25.622  28.425  54.525  1.00  0.00
C    C
ATOM   8823  HA  VAL C 130     -25.727  28.021  55.524  1.00  0.00
C    H
ATOM   8824  CB  VAL C 130     -24.758  29.694  54.652  1.00  0.00
C    C
ATOM   8825  HB  VAL C 130     -25.238  30.355  55.415  1.00  0.00
C    H
ATOM   8826  CG1 VAL C 130     -23.364  29.312  55.110  1.00  0.00
C    C
ATOM   8827 HG11 VAL C 130     -22.716  30.213  55.169  1.00  0.00
C    H
ATOM   8828 HG12 VAL C 130     -23.376  28.848  56.118  1.00  0.00
C    H
```

ATOM 8829 HG13 VAL C 130 -22.897 28.600 54.396 1.00 0.00
C H
ATOM 8830 CG2 VAL C 130 -24.648 30.423 53.348 1.00 0.00
C C
ATOM 8831 HG21 VAL C 130 -23.990 31.312 53.449 1.00 0.00
C H
ATOM 8832 HG22 VAL C 130 -24.221 29.759 52.565 1.00 0.00
C H
ATOM 8833 HG23 VAL C 130 -25.640 30.778 53.001 1.00 0.00
C H
ATOM 8834 C VAL C 130 -27.024 28.687 53.988 1.00 0.00
C C
ATOM 8835 O VAL C 130 -27.979 28.856 54.763 1.00 0.00
C O
ATOM 8836 N LYS C 131 -27.181 28.675 52.659 1.00 0.00
C N
ATOM 8837 HN LYS C 131 -26.436 28.750 51.998 1.00 0.00
C H
ATOM 8838 CA LYS C 131 -28.516 28.546 52.078 1.00 0.00
C C
ATOM 8839 HA LYS C 131 -29.004 29.505 52.203 1.00 0.00
C H
ATOM 8840 CB LYS C 131 -28.403 28.174 50.598 1.00 0.00
C C
ATOM 8841 HB1 LYS C 131 -27.596 28.807 50.161 1.00 0.00
C H
ATOM 8842 HB2 LYS C 131 -28.053 27.120 50.515 1.00 0.00
C H
ATOM 8843 CG LYS C 131 -29.665 28.365 49.755 1.00 0.00
C C
ATOM 8844 HG1 LYS C 131 -30.081 29.380 49.946 1.00 0.00
C H
ATOM 8845 HG2 LYS C 131 -29.391 28.317 48.676 1.00 0.00
C H
ATOM 8846 CD LYS C 131 -30.701 27.293 50.119 1.00 0.00
C C
ATOM 8847 HD1 LYS C 131 -30.523 26.399 49.478 1.00 0.00
C H
ATOM 8848 HD2 LYS C 131 -30.527 26.987 51.176 1.00 0.00
C H
ATOM 8849 CE LYS C 131 -32.141 27.741 49.966 1.00 0.00
C C
ATOM 8850 HE1 LYS C 131 -32.831 27.000 50.428 1.00 0.00
C H
ATOM 8851 HE2 LYS C 131 -32.299 28.725 50.460 1.00 0.00
C H
ATOM 8852 NZ LYS C 131 -32.477 27.872 48.491 1.00 0.00
C N
ATOM 8853 HZ1 LYS C 131 -33.465 28.177 48.375 1.00 0.00
C H
ATOM 8854 HZ2 LYS C 131 -31.845 28.571 48.052 1.00 0.00
C H
ATOM 8855 HZ3 LYS C 131 -32.345 26.951 48.023 1.00 0.00
C H

```
ATOM   8856  C    LYS C 131     -29.356  27.513  52.804  1.00  0.00
C    C
ATOM   8857  O    LYS C 131     -30.338  27.853  53.475  1.00  0.00
C    O
ATOM   8858  N    ILE C 132     -28.992  26.248  52.663  1.00  0.00
C    N
ATOM   8859  HN   ILE C 132     -28.122  25.931  52.291  1.00  0.00
C    H
ATOM   8860  CA   ILE C 132     -29.891  25.183  53.064  1.00  0.00
C    C
ATOM   8861  HA   ILE C 132     -30.850  25.391  52.607  1.00  0.00
C    H
ATOM   8862  CB   ILE C 132     -29.351  23.827  52.589  1.00  0.00
C    C
ATOM   8863  HB   ILE C 132     -29.321  23.913  51.473  1.00  0.00
C    H
ATOM   8864  CG2  ILE C 132     -28.013  23.554  53.207  1.00  0.00
C    C
ATOM   8865 HG21 ILE C 132     -27.549  22.655  52.750  1.00  0.00
C    H
ATOM   8866 HG22 ILE C 132     -27.321  24.408  53.048  1.00  0.00
C    H
ATOM   8867 HG23 ILE C 132     -28.108  23.378  54.299  1.00  0.00
C    H
ATOM   8868  CG1  ILE C 132     -30.349  22.727  52.926  1.00  0.00
C    C
ATOM   8869 HG11 ILE C 132     -30.076  22.256  53.897  1.00  0.00
C    H
ATOM   8870 HG12 ILE C 132     -31.358  23.180  53.054  1.00  0.00
C    H
ATOM   8871  CD   ILE C 132     -30.437  21.672  51.877  1.00  0.00
C    C
ATOM   8872  HD1  ILE C 132     -31.218  20.927  52.144  1.00  0.00
C    H
ATOM   8873  HD2  ILE C 132     -30.704  22.118  50.895  1.00  0.00
C    H
ATOM   8874  HD3  ILE C 132     -29.471  21.135  51.770  1.00  0.00
C    H
ATOM   8875  C    ILE C 132     -30.104  25.222  54.563  1.00  0.00
C    C
ATOM   8876  O    ILE C 132     -31.173  24.865  55.070  1.00  0.00
C    O
ATOM   8877  N    ALA C 133     -29.107  25.712  55.289  1.00  0.00
C    N
ATOM   8878  HN   ALA C 133     -28.233  26.015  54.911  1.00  0.00
C    H
ATOM   8879  CA   ALA C 133     -29.239  25.838  56.725  1.00  0.00
C    C
ATOM   8880  HA   ALA C 133     -29.524  24.891  57.170  1.00  0.00
C    H
ATOM   8881  CB   ALA C 133     -27.910  26.274  57.327  1.00  0.00
C    C
ATOM   8882  HB1  ALA C 133     -27.131  25.515  57.101  1.00  0.00
C    H
```

ATOM 8883 HB2 ALA C 133 -27.577 27.245 56.898 1.00 0.00
C H
ATOM 8884 HB3 ALA C 133 -27.975 26.379 58.432 1.00 0.00
C H
ATOM 8885 C ALA C 133 -30.337 26.821 57.076 1.00 0.00
C C
ATOM 8886 O ALA C 133 -31.225 26.511 57.879 1.00 0.00
C O
ATOM 8887 N GLN C 134 -30.300 28.011 56.477 1.00 0.00
C N
ATOM 8888 HN GLN C 134 -29.600 28.340 55.847 1.00 0.00
C H
ATOM 8889 CA GLN C 134 -31.369 28.962 56.739 1.00 0.00
C C
ATOM 8890 HA GLN C 134 -31.412 29.109 57.812 1.00 0.00
C H
ATOM 8891 CB GLN C 134 -31.080 30.284 56.043 1.00 0.00
C C
ATOM 8892 HB1 GLN C 134 -31.165 30.097 54.947 1.00 0.00
C H
ATOM 8893 HB2 GLN C 134 -31.879 31.017 56.296 1.00 0.00
C H
ATOM 8894 CG GLN C 134 -29.711 30.817 56.373 1.00 0.00
C C
ATOM 8895 HG1 GLN C 134 -29.036 29.989 56.678 1.00 0.00
C H
ATOM 8896 HG2 GLN C 134 -29.260 31.316 55.491 1.00 0.00
C H
ATOM 8897 CD GLN C 134 -29.735 31.795 57.524 1.00 0.00
C C
ATOM 8898 OE1 GLN C 134 -30.787 32.063 58.103 1.00 0.00
C O
ATOM 8899 NE2 GLN C 134 -28.569 32.325 57.875 1.00 0.00
C N
ATOM 8900 HE21 GLN C 134 -28.577 32.967 58.636 1.00 0.00
C H
ATOM 8901 HE22 GLN C 134 -27.746 32.086 57.371 1.00 0.00
C H
ATOM 8902 C GLN C 134 -32.710 28.401 56.301 1.00 0.00
C C
ATOM 8903 O GLN C 134 -33.718 28.564 56.996 1.00 0.00
C O
ATOM 8904 N SER C 135 -32.736 27.707 55.162 1.00 0.00
C N
ATOM 8905 HN SER C 135 -31.948 27.471 54.595 1.00 0.00
C H
ATOM 8906 CA SER C 135 -34.012 27.221 54.646 1.00 0.00
C C
ATOM 8907 HA SER C 135 -34.698 28.053 54.726 1.00 0.00
C H
ATOM 8908 CB SER C 135 -33.867 26.766 53.197 1.00 0.00
C C
ATOM 8909 HB1 SER C 135 -34.825 26.290 52.886 1.00 0.00
C H

```
ATOM   8910  HB2 SER C 135     -33.705  27.643  52.529  1.00  0.00
C    H
ATOM   8911  OG  SER C 135     -32.825  25.824  53.068  1.00  0.00
C    O
ATOM   8912  HG1 SER C 135     -32.922  25.411  52.201  1.00  0.00
C    H
ATOM   8913  C   SER C 135     -34.572  26.089  55.495  1.00  0.00
C    C
ATOM   8914  O   SER C 135     -35.782  25.841  55.472  1.00  0.00
C    O
ATOM   8915  N   LEU C 136     -33.722  25.387  56.238  1.00  0.00
C    N
ATOM   8916  HN  LEU C 136     -32.733  25.512  56.279  1.00  0.00
C    H
ATOM   8917  CA  LEU C 136     -34.226  24.320  57.093  1.00  0.00
C    C
ATOM   8918  HA  LEU C 136     -35.130  23.949  56.625  1.00  0.00
C    H
ATOM   8919  CB  LEU C 136     -33.204  23.194  57.206  1.00  0.00
C    C
ATOM   8920  HB1 LEU C 136     -32.224  23.604  56.868  1.00  0.00
C    H
ATOM   8921  HB2 LEU C 136     -33.074  22.930  58.279  1.00  0.00
C    H
ATOM   8922  CG  LEU C 136     -33.567  21.971  56.367  1.00  0.00
C    C
ATOM   8923  HG  LEU C 136     -33.187  22.099  55.325  1.00  0.00
C    H
ATOM   8924  CD1 LEU C 136     -32.995  20.721  56.982  1.00  0.00
C    C
ATOM   8925 HD11 LEU C 136     -33.298  19.825  56.399  1.00  0.00
C    H
ATOM   8926 HD12 LEU C 136     -31.885  20.766  57.000  1.00  0.00
C    H
ATOM   8927 HD13 LEU C 136     -33.357  20.595  58.025  1.00  0.00
C    H
ATOM   8928  CD2 LEU C 136     -35.072  21.851  56.303  1.00  0.00
C    C
ATOM   8929 HD21 LEU C 136     -35.364  20.977  55.680  1.00  0.00
C    H
ATOM   8930 HD22 LEU C 136     -35.515  21.710  57.309  1.00  0.00
C    H
ATOM   8931 HD23 LEU C 136     -35.516  22.763  55.850  1.00  0.00
C    H
ATOM   8932  C   LEU C 136     -34.604  24.844  58.466  1.00  0.00
C    C
ATOM   8933  O   LEU C 136     -35.632  24.447  59.025  1.00  0.00
C    O
ATOM   8934  N   SER C 137     -33.782  25.731  59.027  1.00  0.00
C    N
ATOM   8935  HN  SER C 137     -32.940  26.077  58.612  1.00  0.00
C    H
ATOM   8936  CA  SER C 137     -34.069  26.282  60.346  1.00  0.00
C    C
```

ATOM 8937 HA SER C 137 -34.012 25.452 61.038 1.00 0.00
C H
ATOM 8938 CB SER C 137 -33.058 27.375 60.676 1.00 0.00
C C
ATOM 8939 HB1 SER C 137 -33.255 27.726 61.714 1.00 0.00
C H
ATOM 8940 HB2 SER C 137 -32.023 26.963 60.660 1.00 0.00
C H
ATOM 8941 OG SER C 137 -33.206 28.461 59.778 1.00 0.00
C O
ATOM 8942 HG1 SER C 137 -32.717 29.205 60.151 1.00 0.00
C H
ATOM 8943 C SER C 137 -35.486 26.831 60.413 1.00 0.00
C C
ATOM 8944 O SER C 137 -36.327 26.332 61.170 1.00 0.00
C O
ATOM 8945 N ASP C 138 -35.779 27.839 59.606 1.00 0.00
C N
ATOM 8946 HN ASP C 138 -35.129 28.270 58.985 1.00 0.00
C H
ATOM 8947 CA ASP C 138 -37.107 28.433 59.542 1.00 0.00
C C
ATOM 8948 HA ASP C 138 -37.655 28.170 60.440 1.00 0.00
C H
ATOM 8949 CB ASP C 138 -37.012 29.942 59.359 1.00 0.00
C C
ATOM 8950 HB1 ASP C 138 -36.270 30.167 58.567 1.00 0.00
C H
ATOM 8951 HB2 ASP C 138 -37.990 30.361 59.050 1.00 0.00
C H
ATOM 8952 CG ASP C 138 -36.561 30.655 60.615 1.00 0.00
C C
ATOM 8953 OD1 ASP C 138 -37.401 30.886 61.510 1.00 0.00
C O
ATOM 8954 OD2 ASP C 138 -35.360 30.980 60.707 1.00 0.00
C O
ATOM 8955 C ASP C 138 -37.826 27.765 58.379 1.00 0.00
C C
ATOM 8956 O ASP C 138 -37.584 28.091 57.215 1.00 0.00
C O
ATOM 8957 N GLU C 139 -38.716 26.827 58.692 1.00 0.00
C N
ATOM 8958 HN GLU C 139 -39.045 26.620 59.609 1.00 0.00
C H
ATOM 8959 CA GLU C 139 -39.299 25.993 57.652 1.00 0.00
C C
ATOM 8960 HA GLU C 139 -38.494 25.706 56.985 1.00 0.00
C H
ATOM 8961 CB GLU C 139 -39.898 24.736 58.278 1.00 0.00
C C
ATOM 8962 HB1 GLU C 139 -40.487 24.205 57.495 1.00 0.00
C H
ATOM 8963 HB2 GLU C 139 -39.084 24.038 58.574 1.00 0.00
C H

ATOM 8964 CG GLU C 139 -40.794 25.014 59.461 1.00 0.00
C C
ATOM 8965 HG1 GLU C 139 -40.272 25.683 60.169 1.00 0.00
C H
ATOM 8966 HG2 GLU C 139 -41.740 25.491 59.138 1.00 0.00
C H
ATOM 8967 CD GLU C 139 -41.122 23.765 60.239 1.00 0.00
C C
ATOM 8968 OE1 GLU C 139 -41.201 23.840 61.481 1.00 0.00
C O
ATOM 8969 OE2 GLU C 139 -41.292 22.702 59.610 1.00 0.00
C O
ATOM 8970 C GLU C 139 -40.345 26.715 56.812 1.00 0.00
C C
ATOM 8971 O GLU C 139 -41.064 26.053 56.055 1.00 0.00
C O
ATOM 8972 N ASN C 140 -40.455 28.041 56.915 1.00 0.00
C N
ATOM 8973 HN ASN C 140 -39.983 28.623 57.577 1.00 0.00
C H
ATOM 8974 CA ASN C 140 -41.335 28.758 55.998 1.00 0.00
C C
ATOM 8975 HA ASN C 140 -41.965 28.000 55.545 1.00 0.00
C H
ATOM 8976 CB ASN C 140 -42.183 29.790 56.741 1.00 0.00
C C
ATOM 8977 HB1 ASN C 140 -42.171 29.528 57.821 1.00 0.00
C H
ATOM 8978 HB2 ASN C 140 -41.737 30.801 56.639 1.00 0.00
C H
ATOM 8979 CG ASN C 140 -43.629 29.797 56.269 1.00 0.00
C C
ATOM 8980 OD1 ASN C 140 -44.325 30.808 56.375 1.00 0.00
C O
ATOM 8981 ND2 ASN C 140 -44.086 28.663 55.743 1.00 0.00
C N
ATOM 8982 HD21 ASN C 140 -45.028 28.656 55.419 1.00 0.00
C H
ATOM 8983 HD22 ASN C 140 -43.484 27.875 55.657 1.00 0.00
C H
ATOM 8984 C ASN C 140 -40.553 29.434 54.884 1.00 0.00
C C
ATOM 8985 O ASN C 140 -41.024 29.487 53.742 1.00 0.00
C O
ATOM 8986 N VAL C 141 -39.370 29.966 55.180 1.00 0.00
C N
ATOM 8987 HN VAL C 141 -38.958 30.034 56.085 1.00 0.00
C H
ATOM 8988 CA VAL C 141 -38.557 30.521 54.104 1.00 0.00
C C
ATOM 8989 HA VAL C 141 -39.163 31.183 53.498 1.00 0.00
C H
ATOM 8990 CB VAL C 141 -37.346 31.299 54.634 1.00 0.00
C C

ATOM 8991 HB VAL C 141 -37.530 32.384 54.438 1.00 0.00
C H
ATOM 8992 CG1 VAL C 141 -37.172 31.093 56.117 1.00 0.00
C C
ATOM 8993 HG11 VAL C 141 -36.269 31.632 56.478 1.00 0.00
C H
ATOM 8994 HG12 VAL C 141 -38.040 31.482 56.689 1.00 0.00
C H
ATOM 8995 HG13 VAL C 141 -37.046 30.015 56.352 1.00 0.00
C H
ATOM 8996 CG2 VAL C 141 -36.066 30.996 53.844 1.00 0.00
C C
ATOM 8997 HG21 VAL C 141 -35.200 31.543 54.274 1.00 0.00
C H
ATOM 8998 HG22 VAL C 141 -35.841 29.909 53.868 1.00 0.00
C H
ATOM 8999 HG23 VAL C 141 -36.166 31.308 52.783 1.00 0.00
C H
ATOM 9000 C VAL C 141 -38.140 29.348 53.223 1.00 0.00
C C
ATOM 9001 O VAL C 141 -37.476 28.416 53.681 1.00 0.00
C O
ATOM 9002 N LEU C 142 -38.623 29.340 51.986 1.00 0.00
C N
ATOM 9003 HN LEU C 142 -39.253 30.009 51.598 1.00 0.00
C H
ATOM 9004 CA LEU C 142 -38.232 28.281 51.064 1.00 0.00
C C
ATOM 9005 HA LEU C 142 -38.021 27.403 51.659 1.00 0.00
C H
ATOM 9006 CB LEU C 142 -39.359 27.989 50.071 1.00 0.00
C C
ATOM 9007 HB1 LEU C 142 -40.274 27.772 50.669 1.00 0.00
C H
ATOM 9008 HB2 LEU C 142 -39.581 28.913 49.492 1.00 0.00
C H
ATOM 9009 CG LEU C 142 -39.068 26.812 49.143 1.00 0.00
C C
ATOM 9010 HG LEU C 142 -38.132 27.045 48.583 1.00 0.00
C H
ATOM 9011 CD1 LEU C 142 -38.874 25.552 49.960 1.00 0.00
C C
ATOM 9012 HD11 LEU C 142 -38.709 24.677 49.295 1.00 0.00
C H
ATOM 9013 HD12 LEU C 142 -37.993 25.652 50.630 1.00 0.00
C H
ATOM 9014 HD13 LEU C 142 -39.769 25.348 50.586 1.00 0.00
C H
ATOM 9015 CD2 LEU C 142 -40.186 26.623 48.133 1.00 0.00
C C
ATOM 9016 HD21 LEU C 142 -39.953 25.775 47.453 1.00 0.00
C H
ATOM 9017 HD22 LEU C 142 -41.152 26.400 48.629 1.00 0.00
C H

ATOM 9018 HD23 LEU C 142 -40.312 27.539 47.515 1.00 0.00
C H
ATOM 9019 C LEU C 142 -36.951 28.660 50.335 1.00 0.00
C C
ATOM 9020 O LEU C 142 -35.945 27.950 50.422 1.00 0.00
C O
ATOM 9021 N ASN C 143 -36.971 29.781 49.622 1.00 0.00
C N
ATOM 9022 HN ASN C 143 -37.800 30.298 49.417 1.00 0.00
C H
ATOM 9023 CA ASN C 143 -35.753 30.352 49.073 1.00 0.00
C C
ATOM 9024 HA ASN C 143 -34.919 29.774 49.455 1.00 0.00
C H
ATOM 9025 CB ASN C 143 -35.763 30.378 47.541 1.00 0.00
C C
ATOM 9026 HB1 ASN C 143 -36.583 31.057 47.218 1.00 0.00
C H
ATOM 9027 HB2 ASN C 143 -34.811 30.799 47.156 1.00 0.00
C H
ATOM 9028 CG ASN C 143 -36.016 29.017 46.930 1.00 0.00
C C
ATOM 9029 OD1 ASN C 143 -36.749 28.203 47.483 1.00 0.00
C O
ATOM 9030 ND2 ASN C 143 -35.396 28.758 45.786 1.00 0.00
C N
ATOM 9031 HD21 ASN C 143 -35.561 27.870 45.366 1.00 0.00
C H
ATOM 9032 HD22 ASN C 143 -34.817 29.450 45.365 1.00 0.00
C H
ATOM 9033 C ASN C 143 -35.626 31.762 49.621 1.00 0.00
C C
ATOM 9034 O ASN C 143 -36.620 32.385 49.999 1.00 0.00
C O
ATOM 9035 N TYR C 144 -34.398 32.270 49.654 1.00 0.00
C N
ATOM 9036 HN TYR C 144 -33.566 31.821 49.331 1.00 0.00
C H
ATOM 9037 CA TYR C 144 -34.191 33.600 50.203 1.00 0.00
C C
ATOM 9038 HA TYR C 144 -35.057 34.198 49.942 1.00 0.00
C H
ATOM 9039 CB TYR C 144 -33.999 33.557 51.720 1.00 0.00
C C
ATOM 9040 HB1 TYR C 144 -34.078 34.589 52.126 1.00 0.00
C H
ATOM 9041 HB2 TYR C 144 -34.799 32.953 52.200 1.00 0.00
C H
ATOM 9042 CG TYR C 144 -32.660 33.031 52.166 1.00 0.00
C C
ATOM 9043 CD1 TYR C 144 -32.340 31.691 52.035 1.00 0.00
C C
ATOM 9044 HD1 TYR C 144 -33.064 31.004 51.619 1.00 0.00
C H

ATOM 9045 CE1 TYR C 144 −31.116 31.207 52.447 1.00 0.00
C C
ATOM 9046 HE1 TYR C 144 −30.877 30.159 52.345 1.00 0.00
C H
ATOM 9047 CZ TYR C 144 −30.192 32.069 52.999 1.00 0.00
C C
ATOM 9048 OH TYR C 144 −28.969 31.579 53.406 1.00 0.00
C O
ATOM 9049 HH TYR C 144 −28.441 32.326 53.693 1.00 0.00
C H
ATOM 9050 CD2 TYR C 144 −31.718 33.879 52.725 1.00 0.00
C C
ATOM 9051 HD2 TYR C 144 −31.953 34.924 52.879 1.00 0.00
C H
ATOM 9052 CE2 TYR C 144 −30.490 33.410 53.140 1.00 0.00
C C
ATOM 9053 HE2 TYR C 144 −29.781 34.089 53.591 1.00 0.00
C H
ATOM 9054 C TYR C 144 −32.990 34.233 49.527 1.00 0.00
C C
ATOM 9055 O TYR C 144 −32.024 33.558 49.168 1.00 0.00
C O
ATOM 9056 N ILE C 145 −33.063 35.552 49.371 1.00 0.00
C N
ATOM 9057 HN ILE C 145 −33.870 36.082 49.621 1.00 0.00
C H
ATOM 9058 CA ILE C 145 −31.972 36.348 48.820 1.00 0.00
C C
ATOM 9059 HA ILE C 145 −31.054 35.797 48.976 1.00 0.00
C H
ATOM 9060 CB ILE C 145 −32.171 36.653 47.322 1.00 0.00
C C
ATOM 9061 HB ILE C 145 −33.110 36.110 47.047 1.00 0.00
C H
ATOM 9062 CG2 ILE C 145 −32.276 38.159 47.064 1.00 0.00
C C
ATOM 9063 HG21 ILE C 145 −32.573 38.355 46.013 1.00 0.00
C H
ATOM 9064 HG22 ILE C 145 −33.041 38.621 47.723 1.00 0.00
C H
ATOM 9065 HG23 ILE C 145 −31.304 38.663 47.250 1.00 0.00
C H
ATOM 9066 CG1 ILE C 145 −31.030 36.021 46.535 1.00 0.00
C C
ATOM 9067 HG11 ILE C 145 −30.418 35.380 47.208 1.00 0.00
C H
ATOM 9068 HG12 ILE C 145 −31.459 35.357 45.749 1.00 0.00
C H
ATOM 9069 CD ILE C 145 −30.118 37.018 45.852 1.00 0.00
C C
ATOM 9070 HD1 ILE C 145 −29.348 36.487 45.252 1.00 0.00
C H
ATOM 9071 HD2 ILE C 145 −30.697 37.676 45.169 1.00 0.00
C H

```
ATOM   9072  HD3 ILE C 145      -29.596  37.654  46.596  1.00  0.00
C    H
ATOM   9073  C   ILE C 145      -31.860  37.632  49.630  1.00  0.00
C    C
ATOM   9074  O   ILE C 145      -32.875  38.221  50.020  1.00  0.00
C    O
ATOM   9075  N   ASP C 146      -30.626  38.033  49.933  1.00  0.00
C    N
ATOM   9076  HN  ASP C 146      -29.792  37.558  49.662  1.00  0.00
C    H
ATOM   9077  CA  ASP C 146      -30.366  39.239  50.710  1.00  0.00
C    C
ATOM   9078  HA  ASP C 146      -31.320  39.717  50.902  1.00  0.00
C    H
ATOM   9079  CB  ASP C 146      -29.637  38.888  52.003  1.00  0.00
C    C
ATOM   9080  HB1 ASP C 146      -29.085  39.779  52.364  1.00  0.00
C    H
ATOM   9081  HB2 ASP C 146      -30.359  38.585  52.788  1.00  0.00
C    H
ATOM   9082  CG  ASP C 146      -28.629  37.773  51.816  1.00  0.00
C    C
ATOM   9083  OD1 ASP C 146      -27.536  38.060  51.282  1.00  0.00
C    O
ATOM   9084  OD2 ASP C 146      -28.938  36.611  52.156  1.00  0.00
C    O
ATOM   9085  C   ASP C 146      -29.542  40.218  49.890  1.00  0.00
C    C
ATOM   9086  O   ASP C 146      -28.471  39.864  49.388  1.00  0.00
C    O
ATOM   9087  N   LEU C 147      -30.036  41.445  49.761  1.00  0.00
C    N
ATOM   9088  HN  LEU C 147      -30.926  41.736  50.104  1.00  0.00
C    H
ATOM   9089  CA  LEU C 147      -29.283  42.494  49.085  1.00  0.00
C    C
ATOM   9090  HA  LEU C 147      -28.759  42.011  48.271  1.00  0.00
C    H
ATOM   9091  CB  LEU C 147      -30.250  43.545  48.550  1.00  0.00
C    C
ATOM   9092  HB1 LEU C 147      -29.679  44.496  48.448  1.00  0.00
C    H
ATOM   9093  HB2 LEU C 147      -30.560  43.260  47.520  1.00  0.00
C    H
ATOM   9094  CG  LEU C 147      -31.435  43.721  49.498  1.00  0.00
C    C
ATOM   9095  HG  LEU C 147      -31.518  42.767  50.072  1.00  0.00
C    H
ATOM   9096  CD1 LEU C 147      -31.201  44.859  50.472  1.00  0.00
C    C
ATOM   9097 HD11 LEU C 147      -32.090  45.006  51.122  1.00  0.00
C    H
ATOM   9098 HD12 LEU C 147      -30.325  44.644  51.122  1.00  0.00
C    H
```

ATOM 9099 HD13 LEU C 147 -31.009 45.809 49.928 1.00 0.00
C H
ATOM 9100 CD2 LEU C 147 -32.717 43.901 48.696 1.00 0.00
C C
ATOM 9101 HD21 LEU C 147 -33.588 44.012 49.378 1.00 0.00
C H
ATOM 9102 HD22 LEU C 147 -32.676 44.805 48.056 1.00 0.00
C H
ATOM 9103 HD23 LEU C 147 -32.896 43.019 48.044 1.00 0.00
C H
ATOM 9104 C LEU C 147 -28.237 43.134 49.990 1.00 0.00
C C
ATOM 9105 O LEU C 147 -27.236 43.660 49.492 1.00 0.00
C O
ATOM 9106 N SER C 148 -28.436 43.088 51.300 1.00 0.00
C N
ATOM 9107 HN SER C 148 -29.226 42.655 51.735 1.00 0.00
C H
ATOM 9108 CA SER C 148 -27.494 43.682 52.236 1.00 0.00
C C
ATOM 9109 HA SER C 148 -26.517 43.655 51.771 1.00 0.00
C H
ATOM 9110 CB SER C 148 -27.907 45.129 52.554 1.00 0.00
C C
ATOM 9111 HB1 SER C 148 -27.156 45.557 53.255 1.00 0.00
C H
ATOM 9112 HB2 SER C 148 -27.884 45.750 51.630 1.00 0.00
C H
ATOM 9113 OG SER C 148 -29.186 45.172 53.170 1.00 0.00
C O
ATOM 9114 HG1 SER C 148 -29.291 46.056 53.543 1.00 0.00
C H
ATOM 9115 C SER C 148 -27.430 42.796 53.474 1.00 0.00
C C
ATOM 9116 O SER C 148 -28.050 41.726 53.520 1.00 0.00
C O
ATOM 9117 N ASP C 149 -26.680 43.240 54.485 1.00 0.00
C N
ATOM 9118 HN ASP C 149 -26.116 44.061 54.487 1.00 0.00
C H
ATOM 9119 CA ASP C 149 -26.626 42.510 55.747 1.00 0.00
C C
ATOM 9120 HA ASP C 149 -26.736 41.458 55.506 1.00 0.00
C H
ATOM 9121 CB ASP C 149 -25.295 42.775 56.446 1.00 0.00
C C
ATOM 9122 HB1 ASP C 149 -25.340 42.377 57.479 1.00 0.00
C H
ATOM 9123 HB2 ASP C 149 -24.466 42.269 55.913 1.00 0.00
C H
ATOM 9124 CG ASP C 149 -24.977 44.251 56.540 1.00 0.00
C C
ATOM 9125 OD1 ASP C 149 -24.111 44.726 55.775 1.00 0.00
C O

```
ATOM   9126  OD2 ASP C 149     -25.604  44.940  57.367  1.00  0.00
C    O
ATOM   9127  C   ASP C 149     -27.790  42.875  56.657  1.00  0.00
C    C
ATOM   9128  O   ASP C 149     -27.877  42.374  57.784  1.00  0.00
C    O
ATOM   9129  N   GLU C 150     -28.690  43.735  56.187  1.00  0.00
C    N
ATOM   9130  HN  GLU C 150     -28.632  44.128  55.274  1.00  0.00
C    H
ATOM   9131  CA  GLU C 150     -29.843  44.187  56.955  1.00  0.00
C    C
ATOM   9132  HA  GLU C 150     -29.782  43.712  57.926  1.00  0.00
C    H
ATOM   9133  CB  GLU C 150     -29.805  45.711  57.113  1.00  0.00
C    C
ATOM   9134  HB1 GLU C 150     -28.839  45.987  57.596  1.00  0.00
C    H
ATOM   9135  HB2 GLU C 150     -29.794  46.194  56.110  1.00  0.00
C    H
ATOM   9136  CG  GLU C 150     -30.947  46.264  57.941  1.00  0.00
C    C
ATOM   9137  HG1 GLU C 150     -31.490  47.028  57.356  1.00  0.00
C    H
ATOM   9138  HG2 GLU C 150     -31.651  45.460  58.233  1.00  0.00
C    H
ATOM   9139  CD  GLU C 150     -30.491  46.969  59.194  1.00  0.00
C    C
ATOM   9140  OE1 GLU C 150     -29.368  46.688  59.658  1.00  0.00
C    O
ATOM   9141  OE2 GLU C 150     -31.256  47.805  59.714  1.00  0.00
C    O
ATOM   9142  C   GLU C 150     -31.173  43.792  56.343  1.00  0.00
C    C
ATOM   9143  O   GLU C 150     -32.121  43.500  57.076  1.00  0.00
C    O
ATOM   9144  N   TYR C 151     -31.273  43.777  55.022  1.00  0.00
C    N
ATOM   9145  HN  TYR C 151     -30.498  43.919  54.409  1.00  0.00
C    H
ATOM   9146  CA  TYR C 151     -32.541  43.550  54.349  1.00  0.00
C    C
ATOM   9147  HA  TYR C 151     -33.245  43.221  55.104  1.00  0.00
C    H
ATOM   9148  CB  TYR C 151     -33.017  44.823  53.655  1.00  0.00
C    C
ATOM   9149  HB1 TYR C 151     -32.389  45.004  52.755  1.00  0.00
C    H
ATOM   9150  HB2 TYR C 151     -34.067  44.711  53.306  1.00  0.00
C    H
ATOM   9151  CG  TYR C 151     -32.911  46.065  54.508  1.00  0.00
C    C
ATOM   9152  CD1 TYR C 151     -33.839  46.329  55.505  1.00  0.00
C    C
```

```
ATOM   9153  HD1 TYR C 151      -34.658  45.644  55.672  1.00  0.00
C    H
ATOM   9154  CE1 TYR C 151      -33.752  47.467  56.281  1.00  0.00
C    C
ATOM   9155  HE1 TYR C 151      -34.484  47.664  57.050  1.00  0.00
C    H
ATOM   9156  CZ  TYR C 151      -32.727  48.359  56.063  1.00  0.00
C    C
ATOM   9157  OH  TYR C 151      -32.633  49.492  56.834  1.00  0.00
C    O
ATOM   9158  HH  TYR C 151      -31.824  49.944  56.585  1.00  0.00
C    H
ATOM   9159  CD2 TYR C 151      -31.888  46.980  54.309  1.00  0.00
C    C
ATOM   9160  HD2 TYR C 151      -31.171  46.824  53.516  1.00  0.00
C    H
ATOM   9161  CE2 TYR C 151      -31.793  48.120  55.080  1.00  0.00
C    C
ATOM   9162  HE2 TYR C 151      -30.999  48.828  54.894  1.00  0.00
C    H
ATOM   9163  C   TYR C 151      -32.406  42.420  53.340  1.00  0.00
C    C
ATOM   9164  O   TYR C 151      -31.302  42.108  52.883  1.00  0.00
C    O
ATOM   9165  N   SER C 152      -33.538  41.811  52.994  1.00  0.00
C    N
ATOM   9166  HN  SER C 152      -34.454  42.127  53.243  1.00  0.00
C    H
ATOM   9167  CA  SER C 152      -33.524  40.594  52.199  1.00  0.00
C    C
ATOM   9168  HA  SER C 152      -32.869  40.791  51.360  1.00  0.00
C    H
ATOM   9169  CB  SER C 152      -33.045  39.409  53.038  1.00  0.00
C    C
ATOM   9170  HB1 SER C 152      -33.702  38.537  52.813  1.00  0.00
C    H
ATOM   9171  HB2 SER C 152      -32.009  39.121  52.747  1.00  0.00
C    H
ATOM   9172  OG  SER C 152      -33.136  39.718  54.419  1.00  0.00
C    O
ATOM   9173  HG1 SER C 152      -33.043  38.888  54.903  1.00  0.00
C    H
ATOM   9174  C   SER C 152      -34.904  40.294  51.634  1.00  0.00
C    C
ATOM   9175  O   SER C 152      -35.918  40.838  52.088  1.00  0.00
C    O
ATOM   9176  N   ILE C 153      -34.923  39.399  50.651  1.00  0.00
C    N
ATOM   9177  HN  ILE C 153      -34.083  38.996  50.291  1.00  0.00
C    H
ATOM   9178  CA  ILE C 153      -36.140  38.921  50.005  1.00  0.00
C    C
ATOM   9179  HA  ILE C 153      -36.971  39.488  50.405  1.00  0.00
C    H
```

```
ATOM   9180  CB  ILE C 153     -36.043  39.044  48.475  1.00  0.00
C    C
ATOM   9181  HB  ILE C 153     -35.038  38.621  48.224  1.00  0.00
C    H
ATOM   9182  CG2 ILE C 153     -37.192  38.320  47.795  1.00  0.00
C    C
ATOM   9183 HG21 ILE C 153     -37.032  38.279  46.697  1.00  0.00
C    H
ATOM   9184 HG22 ILE C 153     -37.275  37.275  48.161  1.00  0.00
C    H
ATOM   9185 HG23 ILE C 153     -38.157  38.836  47.986  1.00  0.00
C    H
ATOM   9186  CG1 ILE C 153     -36.021  40.513  48.054  1.00  0.00
C    C
ATOM   9187 HG11 ILE C 153     -37.034  40.822  47.711  1.00  0.00
C    H
ATOM   9188 HG12 ILE C 153     -35.768  41.139  48.940  1.00  0.00
C    H
ATOM   9189  CD  ILE C 153     -35.012  40.817  46.953  1.00  0.00
C    C
ATOM   9190  HD1 ILE C 153     -35.000  41.905  46.731  1.00  0.00
C    H
ATOM   9191  HD2 ILE C 153     -33.989  40.515  47.266  1.00  0.00
C    H
ATOM   9192  HD3 ILE C 153     -35.268  40.277  46.017  1.00  0.00
C    H
ATOM   9193  C   ILE C 153     -36.353  37.472  50.425  1.00  0.00
C    C
ATOM   9194  O   ILE C 153     -35.386  36.710  50.557  1.00  0.00
C    O
ATOM   9195  N   VAL C 154     -37.609  37.085  50.646  1.00  0.00
C    N
ATOM   9196  HN  VAL C 154     -38.392  37.682  50.495  1.00  0.00
C    H
ATOM   9197  CA  VAL C 154     -37.952  35.751  51.128  1.00  0.00
C    C
ATOM   9198  HA  VAL C 154     -37.148  35.078  50.855  1.00  0.00
C    H
ATOM   9199  CB  VAL C 154     -38.144  35.728  52.659  1.00  0.00
C    C
ATOM   9200  HB  VAL C 154     -39.008  36.395  52.900  1.00  0.00
C    H
ATOM   9201  CG1 VAL C 154     -38.451  34.312  53.133  1.00  0.00
C    C
ATOM   9202 HG11 VAL C 154     -38.551  34.289  54.239  1.00  0.00
C    H
ATOM   9203 HG12 VAL C 154     -39.404  33.935  52.708  1.00  0.00
C    H
ATOM   9204 HG13 VAL C 154     -37.634  33.616  52.848  1.00  0.00
C    H
ATOM   9205  CG2 VAL C 154     -36.909  36.262  53.374  1.00  0.00
C    C
ATOM   9206 HG21 VAL C 154     -37.039  36.206  54.476  1.00  0.00
C    H
```

```
ATOM   9207 HG22 VAL C 154     -36.011  35.670  53.096  1.00  0.00
C    H
ATOM   9208 HG23 VAL C 154     -36.722  37.324  53.112  1.00  0.00
C    H
ATOM   9209  C   VAL C 154     -39.217  35.259  50.432  1.00  0.00
C    C
ATOM   9210  O   VAL C 154     -40.162  36.028  50.217  1.00  0.00
C    O
ATOM   9211  N   GLU C 155     -39.238  33.970  50.094  1.00  0.00
C    N
ATOM   9212  HN  GLU C 155     -38.424  33.398  50.071  1.00  0.00
C    H
ATOM   9213  CA  GLU C 155     -40.464  33.272  49.725  1.00  0.00
C    C
ATOM   9214  HA  GLU C 155     -41.241  33.996  49.513  1.00  0.00
C    H
ATOM   9215  CB  GLU C 155     -40.263  32.350  48.524  1.00  0.00
C    C
ATOM   9216  HB1 GLU C 155     -39.256  31.882  48.610  1.00  0.00
C    H
ATOM   9217  HB2 GLU C 155     -40.994  31.511  48.566  1.00  0.00
C    H
ATOM   9218  CG  GLU C 155     -40.360  33.024  47.184  1.00  0.00
C    C
ATOM   9219  HG1 GLU C 155     -41.268  32.673  46.659  1.00  0.00
C    H
ATOM   9220  HG2 GLU C 155     -40.406  34.126  47.296  1.00  0.00
C    H
ATOM   9221  CD  GLU C 155     -39.199  32.675  46.283  1.00  0.00
C    C
ATOM   9222  OE1 GLU C 155     -39.433  32.060  45.220  1.00  0.00
C    O
ATOM   9223  OE2 GLU C 155     -38.053  33.000  46.658  1.00  0.00
C    O
ATOM   9224  C   GLU C 155     -40.926  32.449  50.917  1.00  0.00
C    C
ATOM   9225  O   GLU C 155     -40.117  31.759  51.543  1.00  0.00
C    O
ATOM   9226  N   LEU C 156     -42.215  32.517  51.230  1.00  0.00
C    N
ATOM   9227  HN  LEU C 156     -42.865  33.144  50.806  1.00  0.00
C    H
ATOM   9228  CA  LEU C 156     -42.794  31.650  52.249  1.00  0.00
C    C
ATOM   9229  HA  LEU C 156     -42.087  30.852  52.433  1.00  0.00
C    H
ATOM   9230  CB  LEU C 156     -43.068  32.412  53.559  1.00  0.00
C    C
ATOM   9231  HB1 LEU C 156     -44.118  32.783  53.511  1.00  0.00
C    H
ATOM   9232  HB2 LEU C 156     -43.033  31.695  54.409  1.00  0.00
C    H
ATOM   9233  CG  LEU C 156     -42.155  33.609  53.855  1.00  0.00
C    C
```

ATOM 9234 HG LEU C 156 -41.286 33.495 53.163 1.00 0.00
C H
ATOM 9235 CD1 LEU C 156 -42.856 34.927 53.567 1.00 0.00
C C
ATOM 9236 HD11 LEU C 156 -42.200 35.784 53.830 1.00 0.00
C H
ATOM 9237 HD12 LEU C 156 -43.115 35.005 52.489 1.00 0.00
C H
ATOM 9238 HD13 LEU C 156 -43.791 35.012 54.160 1.00 0.00
C H
ATOM 9239 CD2 LEU C 156 -41.630 33.561 55.284 1.00 0.00
C C
ATOM 9240 HD21 LEU C 156 -40.956 34.423 55.478 1.00 0.00
C H
ATOM 9241 HD22 LEU C 156 -42.453 33.605 56.025 1.00 0.00
C H
ATOM 9242 HD23 LEU C 156 -41.055 32.625 55.456 1.00 0.00
C H
ATOM 9243 C LEU C 156 -44.074 31.018 51.727 1.00 0.00
C C
ATOM 9244 O LEU C 156 -44.942 31.707 51.189 1.00 0.00
C O
ATOM 9245 N LEU C 157 -44.198 29.705 51.886 1.00 0.00
C N
ATOM 9246 HN LEU C 157 -43.519 29.106 52.305 1.00 0.00
C H
ATOM 9247 CA LEU C 157 -45.400 29.020 51.431 1.00 0.00
C C
ATOM 9248 HA LEU C 157 -45.745 29.518 50.533 1.00 0.00
C H
ATOM 9249 CB LEU C 157 -45.120 27.550 51.129 1.00 0.00
C C
ATOM 9250 HB1 LEU C 157 -45.082 27.444 50.020 1.00 0.00
C H
ATOM 9251 HB2 LEU C 157 -44.101 27.293 51.497 1.00 0.00
C H
ATOM 9252 CG LEU C 157 -46.077 26.467 51.627 1.00 0.00
C C
ATOM 9253 HG LEU C 157 -47.049 26.938 51.912 1.00 0.00
C H
ATOM 9254 CD1 LEU C 157 -46.304 25.450 50.525 1.00 0.00
C C
ATOM 9255 HD11 LEU C 157 -46.960 24.628 50.882 1.00 0.00
C H
ATOM 9256 HD12 LEU C 157 -46.789 25.926 49.646 1.00 0.00
C H
ATOM 9257 HD13 LEU C 157 -45.340 25.006 50.196 1.00 0.00
C H
ATOM 9258 CD2 LEU C 157 -45.502 25.789 52.863 1.00 0.00
C C
ATOM 9259 HD21 LEU C 157 -46.204 25.013 53.240 1.00 0.00
C H
ATOM 9260 HD22 LEU C 157 -44.536 25.290 52.645 1.00 0.00
C H

```
ATOM   9261 HD23 LEU C 157     -45.339  26.531  53.674  1.00  0.00
C    H
ATOM   9262  C   LEU C 157     -46.486  29.180  52.492  1.00  0.00
C    C
ATOM   9263  O   LEU C 157     -46.210  29.071  53.693  1.00  0.00
C    O
ATOM   9264  N   ALA C 158     -47.714  29.454  52.054  1.00  0.00
C    N
ATOM   9265  HN  ALA C 158     -47.981  29.416  51.091  1.00  0.00
C    H
ATOM   9266  CA  ALA C 158     -48.771  29.839  52.982  1.00  0.00
C    C
ATOM   9267  HA  ALA C 158     -48.396  30.537  53.722  1.00  0.00
C    H
ATOM   9268  CB  ALA C 158     -49.923  30.511  52.236  1.00  0.00
C    C
ATOM   9269  HB1 ALA C 158     -49.550  31.420  51.717  1.00  0.00
C    H
ATOM   9270  HB2 ALA C 158     -50.353  29.831  51.469  1.00  0.00
C    H
ATOM   9271  HB3 ALA C 158     -50.734  30.821  52.930  1.00  0.00
C    H
ATOM   9272  C   ALA C 158     -49.285  28.631  53.752  1.00  0.00
C    C
ATOM   9273  O   ALA C 158     -49.556  27.576  53.173  1.00  0.00
C    O
ATOM   9274  N   THR C 159     -49.400  28.785  55.065  1.00  0.00
C    N
ATOM   9275  HN  THR C 159     -49.102  29.595  55.569  1.00  0.00
C    H
ATOM   9276  CA  THR C 159     -49.990  27.750  55.893  1.00  0.00
C    C
ATOM   9277  HA  THR C 159     -49.883  26.824  55.344  1.00  0.00
C    H
ATOM   9278  CB  THR C 159     -49.304  27.700  57.258  1.00  0.00
C    C
ATOM   9279  HB  THR C 159     -48.209  27.610  57.052  1.00  0.00
C    H
ATOM   9280  OG1 THR C 159     -49.719  26.524  57.967  1.00  0.00
C    O
ATOM   9281  HG1 THR C 159     -49.262  26.561  58.814  1.00  0.00
C    H
ATOM   9282  CG2 THR C 159     -49.646  28.935  58.074  1.00  0.00
C    C
ATOM   9283 HG21 THR C 159     -49.061  28.960  59.018  1.00  0.00
C    H
ATOM   9284 HG22 THR C 159     -49.406  29.857  57.500  1.00  0.00
C    H
ATOM   9285 HG23 THR C 159     -50.726  28.957  58.333  1.00  0.00
C    H
ATOM   9286  C   THR C 159     -51.490  27.990  56.052  1.00  0.00
C    C
ATOM   9287  O   THR C 159     -52.061  28.939  55.509  1.00  0.00
C    O
```

```
ATOM   9288  N   ARG C 160     -52.132  27.100  56.811  1.00  0.00
C    N
ATOM   9289  HN  ARG C 160     -51.671  26.328  57.244  1.00  0.00
C    H
ATOM   9290  CA  ARG C 160     -53.566  27.221  57.045  1.00  0.00
C    C
ATOM   9291  HA  ARG C 160     -54.054  27.301  56.081  1.00  0.00
C    H
ATOM   9292  CB  ARG C 160     -54.078  25.989  57.788  1.00  0.00
C    C
ATOM   9293  HB1 ARG C 160     -53.556  25.110  57.339  1.00  0.00
C    H
ATOM   9294  HB2 ARG C 160     -53.749  26.031  58.850  1.00  0.00
C    H
ATOM   9295  CG  ARG C 160     -55.571  25.783  57.674  1.00  0.00
C    C
ATOM   9296  HG1 ARG C 160     -56.070  26.775  57.728  1.00  0.00
C    H
ATOM   9297  HG2 ARG C 160     -55.805  25.385  56.660  1.00  0.00
C    H
ATOM   9298  CD  ARG C 160     -56.048  24.805  58.726  1.00  0.00
C    C
ATOM   9299  HD1 ARG C 160     -57.151  24.909  58.856  1.00  0.00
C    H
ATOM   9300  HD2 ARG C 160     -55.821  23.752  58.439  1.00  0.00
C    H
ATOM   9301  NE  ARG C 160     -55.391  25.042  60.007  1.00  0.00
C    N
ATOM   9302  HE  ARG C 160     -54.677  24.364  60.217  1.00  0.00
C    H
ATOM   9303  CZ  ARG C 160     -55.688  26.043  60.829  1.00  0.00
C    C
ATOM   9304  NH1 ARG C 160     -56.636  26.913  60.511  1.00  0.00
C    N
ATOM   9305 HH11 ARG C 160     -56.843  27.675  61.109  1.00  0.00
C    H
ATOM   9306 HH12 ARG C 160     -57.021  26.833  59.589  1.00  0.00
C    H
ATOM   9307  NH2 ARG C 160     -55.033  26.176  61.973  1.00  0.00
C    N
ATOM   9308 HH21 ARG C 160     -55.312  26.869  62.624  1.00  0.00
C    H
ATOM   9309 HH22 ARG C 160     -54.420  25.440  62.227  1.00  0.00
C    H
ATOM   9310  C   ARG C 160     -53.919  28.483  57.819  1.00  0.00
C    C
ATOM   9311  O   ARG C 160     -55.050  28.969  57.710  1.00  0.00
C    O
ATOM   9312  N   LYS C 161     -52.977  29.031  58.589  1.00  0.00
C    N
ATOM   9313  HN  LYS C 161     -52.094  28.598  58.764  1.00  0.00
C    H
ATOM   9314  CA  LYS C 161     -53.173  30.320  59.241  1.00  0.00
C    C
```

```
ATOM   9315  HA  LYS C 161     -54.098  30.245  59.799  1.00  0.00
C    H
ATOM   9316  CB  LYS C 161     -51.998  30.626  60.171  1.00  0.00
C    C
ATOM   9317  HB1 LYS C 161     -51.094  30.743  59.529  1.00  0.00
C    H
ATOM   9318  HB2 LYS C 161     -52.169  31.614  60.655  1.00  0.00
C    H
ATOM   9319  CG  LYS C 161     -51.729  29.560  61.211  1.00  0.00
C    C
ATOM   9320  HG1 LYS C 161     -51.761  28.559  60.723  1.00  0.00
C    H
ATOM   9321  HG2 LYS C 161     -50.701  29.700  61.616  1.00  0.00
C    H
ATOM   9322  CD  LYS C 161     -52.763  29.621  62.316  1.00  0.00
C    C
ATOM   9323  HD1 LYS C 161     -53.762  29.396  61.876  1.00  0.00
C    H
ATOM   9324  HD2 LYS C 161     -52.533  28.815  63.051  1.00  0.00
C    H
ATOM   9325  CE  LYS C 161     -52.748  30.983  62.983  1.00  0.00
C    C
ATOM   9326  HE1 LYS C 161     -51.735  31.211  63.383  1.00  0.00
C    H
ATOM   9327  HE2 LYS C 161     -53.026  31.778  62.255  1.00  0.00
C    H
ATOM   9328  NZ  LYS C 161     -53.703  31.062  64.117  1.00  0.00
C    N
ATOM   9329  HZ1 LYS C 161     -53.664  32.007  64.550  1.00  0.00
C    H
ATOM   9330  HZ2 LYS C 161     -54.667  30.878  63.773  1.00  0.00
C    H
ATOM   9331  HZ3 LYS C 161     -53.452  30.347  64.830  1.00  0.00
C    H
ATOM   9332  C   LYS C 161     -53.336  31.459  58.246  1.00  0.00
C    C
ATOM   9333  O   LYS C 161     -53.553  32.599  58.669  1.00  0.00
C    O
ATOM   9334  N   LEU C 162     -53.229  31.181  56.945  1.00  0.00
C    N
ATOM   9335  HN  LEU C 162     -53.143  30.254  56.585  1.00  0.00
C    H
ATOM   9336  CA  LEU C 162     -53.230  32.219  55.929  1.00  0.00
C    C
ATOM   9337  HA  LEU C 162     -53.473  33.134  56.454  1.00  0.00
C    H
ATOM   9338  CB  LEU C 162     -51.848  32.300  55.265  1.00  0.00
C    C
ATOM   9339  HB1 LEU C 162     -51.572  31.267  54.952  1.00  0.00
C    H
ATOM   9340  HB2 LEU C 162     -51.930  32.894  54.328  1.00  0.00
C    H
ATOM   9341  CG  LEU C 162     -50.687  32.847  56.100  1.00  0.00
C    C
```

ATOM 9342 HG LEU C 162 -50.394 32.033 56.807 1.00 0.00
C H
ATOM 9343 CD1 LEU C 162 -49.514 33.177 55.193 1.00 0.00
C C
ATOM 9344 HD11 LEU C 162 -48.681 33.620 55.779 1.00 0.00
C H
ATOM 9345 HD12 LEU C 162 -49.138 32.260 54.691 1.00 0.00
C H
ATOM 9346 HD13 LEU C 162 -49.816 33.907 54.412 1.00 0.00
C H
ATOM 9347 CD2 LEU C 162 -51.098 34.062 56.923 1.00 0.00
C C
ATOM 9348 HD21 LEU C 162 -50.241 34.424 57.532 1.00 0.00
C H
ATOM 9349 HD22 LEU C 162 -51.432 34.900 56.279 1.00 0.00
C H
ATOM 9350 HD23 LEU C 162 -51.928 33.801 57.614 1.00 0.00
C H
ATOM 9351 C LEU C 162 -54.273 32.024 54.839 1.00 0.00
C C
ATOM 9352 O LEU C 162 -54.284 32.805 53.882 1.00 0.00
C O
ATOM 9353 N ASP C 163 -55.143 31.023 54.951 1.00 0.00
C N
ATOM 9354 HN ASP C 163 -55.252 30.431 55.745 1.00 0.00
C H
ATOM 9355 CA ASP C 163 -56.057 30.691 53.865 1.00 0.00
C C
ATOM 9356 HA ASP C 163 -55.469 30.650 52.955 1.00 0.00
C H
ATOM 9357 CB ASP C 163 -56.747 29.354 54.152 1.00 0.00
C C
ATOM 9358 HB1 ASP C 163 -55.985 28.607 54.452 1.00 0.00
C H
ATOM 9359 HB2 ASP C 163 -57.475 29.461 54.980 1.00 0.00
C H
ATOM 9360 CG ASP C 163 -57.477 28.791 52.940 1.00 0.00
C C
ATOM 9361 OD1 ASP C 163 -57.660 29.526 51.946 1.00 0.00
C O
ATOM 9362 OD2 ASP C 163 -57.880 27.610 52.990 1.00 0.00
C O
ATOM 9363 C ASP C 163 -57.097 31.787 53.668 1.00 0.00
C C
ATOM 9364 O ASP C 163 -57.618 32.347 54.636 1.00 0.00
C O
ATOM 9365 N SER C 164 -57.399 32.074 52.400 1.00 0.00
C N
ATOM 9366 HN SER C 164 -56.909 31.666 51.630 1.00 0.00
C H
ATOM 9367 CA SER C 164 -58.461 33.002 52.011 1.00 0.00
C C
ATOM 9368 HA SER C 164 -58.359 33.142 50.943 1.00 0.00
C H

```
ATOM   9369  CB  SER C 164      -59.836  32.423  52.343  1.00  0.00
C    C
ATOM   9370  HB1 SER C 164      -60.108  32.745  53.374  1.00  0.00
C    H
ATOM   9371  HB2 SER C 164      -60.609  32.839  51.657  1.00  0.00
C    H
ATOM   9372  OG  SER C 164      -59.812  31.006  52.296  1.00  0.00
C    O
ATOM   9373  HG1 SER C 164      -60.620  30.695  52.722  1.00  0.00
C    H
ATOM   9374  C   SER C 164      -58.273  34.366  52.666  1.00  0.00
C    C
ATOM   9375  O   SER C 164      -59.230  35.055  53.022  1.00  0.00
C    O
ATOM   9376  N   LYS C 165      -57.016  34.756  52.824  1.00  0.00
C    N
ATOM   9377  HN  LYS C 165      -56.242  34.206  52.514  1.00  0.00
C    H
ATOM   9378  CA  LYS C 165      -56.664  36.013  53.461  1.00  0.00
C    C
ATOM   9379  HA  LYS C 165      -57.538  36.376  53.988  1.00  0.00
C    H
ATOM   9380  CB  LYS C 165      -55.495  35.824  54.429  1.00  0.00
C    C
ATOM   9381  HB1 LYS C 165      -54.663  35.358  53.851  1.00  0.00
C    H
ATOM   9382  HB2 LYS C 165      -55.128  36.823  54.754  1.00  0.00
C    H
ATOM   9383  CG  LYS C 165      -55.836  34.948  55.622  1.00  0.00
C    C
ATOM   9384  HG1 LYS C 165      -56.016  33.907  55.271  1.00  0.00
C    H
ATOM   9385  HG2 LYS C 165      -54.963  34.919  56.313  1.00  0.00
C    H
ATOM   9386  CD  LYS C 165      -57.072  35.445  56.352  1.00  0.00
C    C
ATOM   9387  HD1 LYS C 165      -56.871  36.476  56.725  1.00  0.00
C    H
ATOM   9388  HD2 LYS C 165      -57.906  35.513  55.616  1.00  0.00
C    H
ATOM   9389  CE  LYS C 165      -57.438  34.513  57.495  1.00  0.00
C    C
ATOM   9390  HE1 LYS C 165      -57.739  33.516  57.103  1.00  0.00
C    H
ATOM   9391  HE2 LYS C 165      -56.573  34.375  58.179  1.00  0.00
C    H
ATOM   9392  NZ  LYS C 165      -58.571  35.044  58.297  1.00  0.00
C    N
ATOM   9393  HZ1 LYS C 165      -58.800  34.386  59.070  1.00  0.00
C    H
ATOM   9394  HZ2 LYS C 165      -58.310  35.969  58.697  1.00  0.00
C    H
ATOM   9395  HZ3 LYS C 165      -59.404  35.160  57.685  1.00  0.00
C    H
```

```
ATOM   9396  C   LYS C 165     -56.317  37.042  52.395  1.00  0.00
C    C
ATOM   9397  O   LYS C 165     -56.157  36.710  51.220  1.00  0.00
C    O
ATOM   9398  N   SER C 166     -56.256  38.306  52.800  1.00  0.00
C    N
ATOM   9399  HN  SER C 166     -56.437  38.607  53.736  1.00  0.00
C    H
ATOM   9400  CA  SER C 166     -55.909  39.382  51.885  1.00  0.00
C    C
ATOM   9401  HA  SER C 166     -55.657  38.905  50.947  1.00  0.00
C    H
ATOM   9402  CB  SER C 166     -57.062  40.368  51.728  1.00  0.00
C    C
ATOM   9403  HB1 SER C 166     -57.784  39.941  50.995  1.00  0.00
C    H
ATOM   9404  HB2 SER C 166     -57.605  40.486  52.693  1.00  0.00
C    H
ATOM   9405  OG  SER C 166     -56.563  41.598  51.242  1.00  0.00
C    O
ATOM   9406  HG1 SER C 166     -57.323  42.114  50.944  1.00  0.00
C    H
ATOM   9407  C   SER C 166     -54.672  40.135  52.356  1.00  0.00
C    C
ATOM   9408  O   SER C 166     -54.280  40.059  53.524  1.00  0.00
C    O
ATOM   9409  N   ILE C 167     -54.063  40.871  51.424  1.00  0.00
C    N
ATOM   9410  HN  ILE C 167     -54.286  40.867  50.452  1.00  0.00
C    H
ATOM   9411  CA  ILE C 167     -52.975  41.774  51.785  1.00  0.00
C    C
ATOM   9412  HA  ILE C 167     -52.284  41.205  52.393  1.00  0.00
C    H
ATOM   9413  CB  ILE C 167     -52.288  42.322  50.524  1.00  0.00
C    C
ATOM   9414  HB  ILE C 167     -53.125  42.489  49.799  1.00  0.00
C    H
ATOM   9415  CG2 ILE C 167     -51.494  43.573  50.857  1.00  0.00
C    C
ATOM   9416 HG21 ILE C 167     -51.126  44.060  49.929  1.00  0.00
C    H
ATOM   9417 HG22 ILE C 167     -52.124  44.310  51.396  1.00  0.00
C    H
ATOM   9418 HG23 ILE C 167     -50.617  43.330  51.492  1.00  0.00
C    H
ATOM   9419  CG1 ILE C 167     -51.378  41.263  49.910  1.00  0.00
C    C
ATOM   9420 HG11 ILE C 167     -50.420  41.730  49.589  1.00  0.00
C    H
ATOM   9421 HG12 ILE C 167     -51.130  40.506  50.689  1.00  0.00
C    H
ATOM   9422  CD  ILE C 167     -51.996  40.552  48.742  1.00  0.00
C    C
```

ATOM 9423 HD1 ILE C 167 -51.315 39.754 48.372 1.00 0.00
C H
ATOM 9424 HD2 ILE C 167 -52.958 40.079 49.035 1.00 0.00
C H
ATOM 9425 HD3 ILE C 167 -52.188 41.254 47.905 1.00 0.00
C H
ATOM 9426 C ILE C 167 -53.501 42.903 52.659 1.00 0.00
C C
ATOM 9427 O ILE C 167 -52.910 43.240 53.691 1.00 0.00
C O
ATOM 9428 N ILE C 168 -54.622 43.507 52.258 1.00 0.00
C N
ATOM 9429 HN ILE C 168 -55.106 43.338 51.403 1.00 0.00
C H
ATOM 9430 CA ILE C 168 -55.243 44.516 53.105 1.00 0.00
C C
ATOM 9431 HA ILE C 168 -54.454 45.191 53.413 1.00 0.00
C H
ATOM 9432 CB ILE C 168 -56.335 45.282 52.339 1.00 0.00
C C
ATOM 9433 HB ILE C 168 -55.850 45.568 51.371 1.00 0.00
C H
ATOM 9434 CG2 ILE C 168 -57.576 44.425 52.165 1.00 0.00
C C
ATOM 9435 HG21 ILE C 168 -58.299 44.918 51.482 1.00 0.00
C H
ATOM 9436 HG22 ILE C 168 -57.317 43.439 51.726 1.00 0.00
C H
ATOM 9437 HG23 ILE C 168 -58.082 44.254 53.139 1.00 0.00
C H
ATOM 9438 CG1 ILE C 168 -56.675 46.582 53.069 1.00 0.00
C C
ATOM 9439 HG11 ILE C 168 -57.721 46.882 52.836 1.00 0.00
C H
ATOM 9440 HG12 ILE C 168 -56.621 46.405 54.167 1.00 0.00
C H
ATOM 9441 CD ILE C 168 -55.741 47.722 52.722 1.00 0.00
C C
ATOM 9442 HD1 ILE C 168 -55.994 48.624 53.320 1.00 0.00
C H
ATOM 9443 HD2 ILE C 168 -54.687 47.444 52.941 1.00 0.00
C H
ATOM 9444 HD3 ILE C 168 -55.820 47.989 51.648 1.00 0.00
C H
ATOM 9445 C ILE C 168 -55.797 43.883 54.367 1.00 0.00
C C
ATOM 9446 O ILE C 168 -55.953 44.555 55.392 1.00 0.00
C O
ATOM 9447 N ASP C 169 -56.081 42.583 54.327 1.00 0.00
C N
ATOM 9448 HN ASP C 169 -56.051 41.988 53.528 1.00 0.00
C H
ATOM 9449 CA ASP C 169 -56.485 41.886 55.538 1.00 0.00
C C

```
ATOM   9450  HA  ASP C 169     -57.254  42.483  56.016  1.00  0.00
C    H
ATOM   9451  CB  ASP C 169     -56.985  40.487  55.176  1.00  0.00
C    C
ATOM   9452  HB1 ASP C 169     -56.936  40.354  54.077  1.00  0.00
C    H
ATOM   9453  HB2 ASP C 169     -56.351  39.712  55.650  1.00  0.00
C    H
ATOM   9454  CG  ASP C 169     -58.419  40.252  55.593  1.00  0.00
C    C
ATOM   9455  OD1 ASP C 169     -59.297  40.954  55.049  1.00  0.00
C    O
ATOM   9456  OD2 ASP C 169     -58.669  39.376  56.450  1.00  0.00
C    O
ATOM   9457  C   ASP C 169     -55.318  41.810  56.513  1.00  0.00
C    C
ATOM   9458  O   ASP C 169     -55.328  42.441  57.578  1.00  0.00
C    O
ATOM   9459  N   LEU C 170     -54.286  41.061  56.146  1.00  0.00
C    N
ATOM   9460  HN  LEU C 170     -54.263  40.535  55.299  1.00  0.00
C    H
ATOM   9461  CA  LEU C 170     -53.080  40.939  56.960  1.00  0.00
C    C
ATOM   9462  HA  LEU C 170     -53.319  41.300  57.952  1.00  0.00
C    H
ATOM   9463  CB  LEU C 170     -52.638  39.481  57.026  1.00  0.00
C    C
ATOM   9464  HB1 LEU C 170     -52.680  39.078  55.988  1.00  0.00
C    H
ATOM   9465  HB2 LEU C 170     -51.568  39.441  57.329  1.00  0.00
C    H
ATOM   9466  CG  LEU C 170     -53.478  38.576  57.932  1.00  0.00
C    C
ATOM   9467  HG  LEU C 170     -53.676  39.166  58.860  1.00  0.00
C    H
ATOM   9468  CD1 LEU C 170     -54.798  38.186  57.276  1.00  0.00
C    C
ATOM   9469 HD11 LEU C 170     -55.369  37.492  57.929  1.00  0.00
C    H
ATOM   9470 HD12 LEU C 170     -55.424  39.086  57.091  1.00  0.00
C    H
ATOM   9471 HD13 LEU C 170     -54.618  37.677  56.304  1.00  0.00
C    H
ATOM   9472  CD2 LEU C 170     -52.699  37.332  58.323  1.00  0.00
C    C
ATOM   9473 HD21 LEU C 170     -53.309  36.690  58.996  1.00  0.00
C    H
ATOM   9474 HD22 LEU C 170     -52.424  36.726  57.438  1.00  0.00
C    H
ATOM   9475 HD23 LEU C 170     -51.766  37.610  58.860  1.00  0.00
C    H
ATOM   9476  C   LEU C 170     -51.988  41.844  56.390  1.00  0.00
C    C
```

```
ATOM   9477  O   LEU C 170     -51.072  41.411  55.689  1.00  0.00
C    O
ATOM   9478  N   ASN C 171     -52.120  43.134  56.690  1.00  0.00
C    N
ATOM   9479  HN  ASN C 171     -52.844  43.509  57.267  1.00  0.00
C    H
ATOM   9480  CA  ASN C 171     -51.182  44.124  56.176  1.00  0.00
C    C
ATOM   9481  HA  ASN C 171     -51.141  43.969  55.104  1.00  0.00
C    H
ATOM   9482  CB  ASN C 171     -51.652  45.538  56.519  1.00  0.00
C    C
ATOM   9483  HB1 ASN C 171     -50.852  46.247  56.213  1.00  0.00
C    H
ATOM   9484  HB2 ASN C 171     -52.563  45.793  55.939  1.00  0.00
C    H
ATOM   9485  CG  ASN C 171     -51.904  45.727  57.996  1.00  0.00
C    C
ATOM   9486  OD1 ASN C 171     -51.817  44.785  58.779  1.00  0.00
C    O
ATOM   9487  ND2 ASN C 171     -52.215  46.956  58.388  1.00  0.00
C    N
ATOM   9488 HD21 ASN C 171     -52.373  47.099  59.362  1.00  0.00
C    H
ATOM   9489 HD22 ASN C 171     -52.254  47.696  57.724  1.00  0.00
C    H
ATOM   9490  C   ASN C 171     -49.782  43.887  56.732  1.00  0.00
C    C
ATOM   9491  O   ASN C 171     -49.606  43.320  57.814  1.00  0.00
C    O
ATOM   9492  N   VAL C 172     -48.780  44.307  55.964  1.00  0.00
C    N
ATOM   9493  HN  VAL C 172     -48.907  44.750  55.079  1.00  0.00
C    H
ATOM   9494  CA  VAL C 172     -47.388  44.152  56.364  1.00  0.00
C    C
ATOM   9495  HA  VAL C 172     -47.390  43.860  57.407  1.00  0.00
C    H
ATOM   9496  CB  VAL C 172     -46.708  43.038  55.540  1.00  0.00
C    C
ATOM   9497  HB  VAL C 172     -47.429  42.187  55.462  1.00  0.00
C    H
ATOM   9498  CG1 VAL C 172     -46.365  43.528  54.139  1.00  0.00
C    C
ATOM   9499 HG11 VAL C 172     -45.842  42.732  53.569  1.00  0.00
C    H
ATOM   9500 HG12 VAL C 172     -47.276  43.800  53.566  1.00  0.00
C    H
ATOM   9501 HG13 VAL C 172     -45.696  44.414  54.186  1.00  0.00
C    H
ATOM   9502  CG2 VAL C 172     -45.461  42.523  56.256  1.00  0.00
C    C
ATOM   9503 HG21 VAL C 172     -44.949  41.749  55.645  1.00  0.00
C    H
```

```
ATOM   9504 HG22 VAL C 172     -44.746  43.353  56.441  1.00  0.00
C    H
ATOM   9505 HG23 VAL C 172     -45.720  42.062  57.232  1.00  0.00
C    H
ATOM   9506  C   VAL C 172     -46.610  45.458  56.254  1.00  0.00
C    C
ATOM   9507  O   VAL C 172     -45.588  45.621  56.931  1.00  0.00
C    O
ATOM   9508  N   ARG C 173     -47.062  46.395  55.418  1.00  0.00
C    N
ATOM   9509  HN  ARG C 173     -47.890  46.290  54.872  1.00  0.00
C    H
ATOM   9510  CA  ARG C 173     -46.333  47.649  55.268  1.00  0.00
C    C
ATOM   9511  HA  ARG C 173     -45.277  47.421  55.195  1.00  0.00
C    H
ATOM   9512  CB  ARG C 173     -46.792  48.381  54.010  1.00  0.00
C    C
ATOM   9513  HB1 ARG C 173     -47.176  47.599  53.313  1.00  0.00
C    H
ATOM   9514  HB2 ARG C 173     -47.662  49.029  54.253  1.00  0.00
C    H
ATOM   9515  CG  ARG C 173     -45.675  49.150  53.339  1.00  0.00
C    C
ATOM   9516  HG1 ARG C 173     -45.698  50.199  53.706  1.00  0.00
C    H
ATOM   9517  HG2 ARG C 173     -44.698  48.737  53.676  1.00  0.00
C    H
ATOM   9518  CD  ARG C 173     -45.755  49.049  51.824  1.00  0.00
C    C
ATOM   9519  HD1 ARG C 173     -44.731  48.905  51.407  1.00  0.00
C    H
ATOM   9520  HD2 ARG C 173     -46.396  48.195  51.503  1.00  0.00
C    H
ATOM   9521  NE  ARG C 173     -46.339  50.241  51.213  1.00  0.00
C    N
ATOM   9522  HE  ARG C 173     -47.264  50.086  50.847  1.00  0.00
C    H
ATOM   9523  CZ  ARG C 173     -45.730  51.423  51.135  1.00  0.00
C    C
ATOM   9524  NH1 ARG C 173     -44.511  51.582  51.630  1.00  0.00
C    N
ATOM   9525 HH11 ARG C 173     -44.064  52.465  51.596  1.00  0.00
C    H
ATOM   9526 HH12 ARG C 173     -44.139  50.810  52.149  1.00  0.00
C    H
ATOM   9527  NH2 ARG C 173     -46.342  52.447  50.558  1.00  0.00
C    N
ATOM   9528 HH21 ARG C 173     -45.857  53.300  50.422  1.00  0.00
C    H
ATOM   9529 HH22 ARG C 173     -47.197  52.256  50.093  1.00  0.00
C    H
ATOM   9530  C   ARG C 173     -46.503  48.541  56.488  1.00  0.00
C    C
```

```
ATOM   9531  O   ARG C 173      -45.520  49.046  57.040  1.00  0.00
C    O
ATOM   9532  N   ALA C 174      -47.743  48.757  56.919  1.00  0.00
C    N
ATOM   9533  HN  ALA C 174      -48.563  48.436  56.445  1.00  0.00
C    H
ATOM   9534  CA  ALA C 174      -47.981  49.501  58.148  1.00  0.00
C    C
ATOM   9535  HA  ALA C 174      -47.288  50.330  58.230  1.00  0.00
C    H
ATOM   9536  CB  ALA C 174      -49.401  50.069  58.152  1.00  0.00
C    C
ATOM   9537  HB1 ALA C 174      -49.535  50.741  57.278  1.00  0.00
C    H
ATOM   9538  HB2 ALA C 174      -50.156  49.257  58.078  1.00  0.00
C    H
ATOM   9539  HB3 ALA C 174      -49.604  50.659  59.072  1.00  0.00
C    H
ATOM   9540  C   ALA C 174      -47.757  48.632  59.376  1.00  0.00
C    C
ATOM   9541  O   ALA C 174      -47.484  49.146  60.466  1.00  0.00
C    O
ATOM   9542  N   LYS C 175      -47.852  47.312  59.211  1.00  0.00
C    N
ATOM   9543  HN  LYS C 175      -47.993  46.864  58.329  1.00  0.00
C    H
ATOM   9544  CA  LYS C 175      -47.750  46.411  60.352  1.00  0.00
C    C
ATOM   9545  HA  LYS C 175      -48.110  46.956  61.217  1.00  0.00
C    H
ATOM   9546  CB  LYS C 175      -48.606  45.172  60.106  1.00  0.00
C    C
ATOM   9547  HB1 LYS C 175      -49.524  45.509  59.571  1.00  0.00
C    H
ATOM   9548  HB2 LYS C 175      -48.067  44.493  59.407  1.00  0.00
C    H
ATOM   9549  CG  LYS C 175      -49.011  44.434  61.359  1.00  0.00
C    C
ATOM   9550  HG1 LYS C 175      -48.123  43.904  61.773  1.00  0.00
C    H
ATOM   9551  HG2 LYS C 175      -49.344  45.173  62.123  1.00  0.00
C    H
ATOM   9552  CD  LYS C 175      -50.107  43.435  61.049  1.00  0.00
C    C
ATOM   9553  HD1 LYS C 175      -50.946  43.975  60.552  1.00  0.00
C    H
ATOM   9554  HD2 LYS C 175      -49.706  42.696  60.317  1.00  0.00
C    H
ATOM   9555  CE  LYS C 175      -50.586  42.733  62.303  1.00  0.00
C    C
ATOM   9556  HE1 LYS C 175      -49.788  42.071  62.708  1.00  0.00
C    H
ATOM   9557  HE2 LYS C 175      -50.859  43.475  63.086  1.00  0.00
C    H
```

ATOM 9558 NZ LYS C 175 -51.784 41.890 62.036 1.00 0.00
C N
ATOM 9559 HZ1 LYS C 175 -52.091 41.420 62.912 1.00 0.00
C H
ATOM 9560 HZ2 LYS C 175 -52.556 42.488 61.677 1.00 0.00
C H
ATOM 9561 HZ3 LYS C 175 -51.548 41.171 61.323 1.00 0.00
C H
ATOM 9562 C LYS C 175 -46.304 46.019 60.631 1.00 0.00
C C
ATOM 9563 O LYS C 175 -45.825 46.163 61.762 1.00 0.00
C O
ATOM 9564 N TYR C 176 -45.591 45.524 59.618 1.00 0.00
C N
ATOM 9565 HN TYR C 176 -45.942 45.440 58.687 1.00 0.00
C H
ATOM 9566 CA TYR C 176 -44.224 45.058 59.794 1.00 0.00
C C
ATOM 9567 HA TYR C 176 -43.989 45.250 60.834 1.00 0.00
C H
ATOM 9568 CB TYR C 176 -44.125 43.558 59.475 1.00 0.00
C C
ATOM 9569 HB1 TYR C 176 -44.413 43.392 58.414 1.00 0.00
C H
ATOM 9570 HB2 TYR C 176 -43.079 43.200 59.590 1.00 0.00
C H
ATOM 9571 CG TYR C 176 -45.044 42.702 60.317 1.00 0.00
C C
ATOM 9572 CD1 TYR C 176 -46.275 42.288 59.829 1.00 0.00
C C
ATOM 9573 HD1 TYR C 176 -46.590 42.590 58.840 1.00 0.00
C H
ATOM 9574 CE1 TYR C 176 -47.121 41.512 60.593 1.00 0.00
C C
ATOM 9575 HE1 TYR C 176 -48.079 41.199 60.206 1.00 0.00
C H
ATOM 9576 CZ TYR C 176 -46.743 41.139 61.864 1.00 0.00
C C
ATOM 9577 OH TYR C 176 -47.587 40.365 62.624 1.00 0.00
C O
ATOM 9578 HH TYR C 176 -47.127 40.151 63.438 1.00 0.00
C H
ATOM 9579 CD2 TYR C 176 -44.686 42.318 61.601 1.00 0.00
C C
ATOM 9580 HD2 TYR C 176 -43.753 42.660 62.026 1.00 0.00
C H
ATOM 9581 CE2 TYR C 176 -45.527 41.540 62.374 1.00 0.00
C C
ATOM 9582 HE2 TYR C 176 -45.235 41.269 63.378 1.00 0.00
C H
ATOM 9583 C TYR C 176 -43.202 45.811 58.957 1.00 0.00
C C
ATOM 9584 O TYR C 176 -42.021 45.452 58.993 1.00 0.00
C O

ATOM 9585 N GLY C 177 −43.612 46.830 58.206 1.00 0.00
C N
ATOM 9586 HN GLY C 177 −44.570 47.099 58.117 1.00 0.00
C H
ATOM 9587 CA GLY C 177 −42.665 47.630 57.454 1.00 0.00
C C
ATOM 9588 HA1 GLY C 177 −41.875 47.876 58.150 1.00 0.00
C H
ATOM 9589 HA2 GLY C 177 −43.221 48.485 57.094 1.00 0.00
C H
ATOM 9590 C GLY C 177 −42.042 46.945 56.264 1.00 0.00
C C
ATOM 9591 O GLY C 177 −41.020 47.416 55.757 1.00 0.00
C O
ATOM 9592 N CYS C 178 −42.625 45.850 55.795 1.00 0.00
C N
ATOM 9593 HN CYS C 178 −43.470 45.485 56.181 1.00 0.00
C H
ATOM 9594 CA CYS C 178 −42.086 45.089 54.680 1.00 0.00
C C
ATOM 9595 HA CYS C 178 −41.101 45.488 54.467 1.00 0.00
C H
ATOM 9596 CB CYS C 178 −41.996 43.603 55.042 1.00 0.00
C C
ATOM 9597 HB1 CYS C 178 −42.991 43.299 55.440 1.00 0.00
C H
ATOM 9598 HB2 CYS C 178 −41.835 42.999 54.121 1.00 0.00
C H
ATOM 9599 SG CYS C 178 −40.744 43.246 56.290 1.00 0.00
C S
ATOM 9600 HG1 CYS C 178 −40.988 41.940 56.402 1.00 0.00
C H
ATOM 9601 C CYS C 178 −42.951 45.303 53.445 1.00 0.00
C C
ATOM 9602 O CYS C 178 −43.884 46.111 53.442 1.00 0.00
C O
ATOM 9603 N THR C 179 −42.630 44.566 52.384 1.00 0.00
C N
ATOM 9604 HN THR C 179 −41.903 43.882 52.396 1.00 0.00
C H
ATOM 9605 CA THR C 179 −43.313 44.692 51.103 1.00 0.00
C C
ATOM 9606 HA THR C 179 −44.252 45.190 51.307 1.00 0.00
C H
ATOM 9607 CB THR C 179 −42.445 45.459 50.099 1.00 0.00
C C
ATOM 9608 HB THR C 179 −41.946 44.686 49.465 1.00 0.00
C H
ATOM 9609 OG1 THR C 179 −41.428 46.180 50.806 1.00 0.00
C O
ATOM 9610 HG1 THR C 179 −40.926 46.647 50.128 1.00 0.00
C H
ATOM 9611 CG2 THR C 179 −43.280 46.409 49.261 1.00 0.00
C C

ATOM   9612 HG21 THR C 179   -42.666 46.877 48.462 1.00 0.00
C   H
ATOM   9613 HG22 THR C 179   -44.116 45.863 48.773 1.00 0.00
C   H
ATOM   9614 HG23 THR C 179   -43.712 47.220 49.885 1.00 0.00
C   H
ATOM   9615 C   THR C 179   -43.663 43.314 50.558 1.00 0.00
C   C
ATOM   9616 O   THR C 179   -42.772 42.532 50.213 1.00 0.00
C   O
ATOM   9617 N   ILE C 180   -44.960 43.024 50.466 1.00 0.00
C   N
ATOM   9618 HN ILE C 180   -45.690 43.607 50.816 1.00 0.00
C   H
ATOM   9619 CA ILE C 180   -45.428 41.794 49.830 1.00 0.00
C   C
ATOM   9620 HA ILE C 180   -44.725 41.008 50.073 1.00 0.00
C   H
ATOM   9621 CB ILE C 180   -46.853 41.436 50.292 1.00 0.00
C   C
ATOM   9622 HB ILE C 180   -47.160 42.293 50.944 1.00 0.00
C   H
ATOM   9623 CG2 ILE C 180   -47.771 41.189 49.092 1.00 0.00
C   C
ATOM   9624 HG21 ILE C 180   -48.826 41.085 49.423 1.00 0.00
C   H
ATOM   9625 HG22 ILE C 180   -47.724 42.036 48.376 1.00 0.00
C   H
ATOM   9626 HG23 ILE C 180   -47.484 40.260 48.556 1.00 0.00
C   H
ATOM   9627 CG1 ILE C 180   -46.825 40.189 51.181 1.00 0.00
C   C
ATOM   9628 HG11 ILE C 180   -45.848 39.668 51.068 1.00 0.00
C   H
ATOM   9629 HG12 ILE C 180   -46.908 40.503 52.246 1.00 0.00
C   H
ATOM   9630 CD ILE C 180   -47.942 39.186 50.906 1.00 0.00
C   C
ATOM   9631 HD1 ILE C 180   -47.887 38.341 51.626 1.00 0.00
C   H
ATOM   9632 HD2 ILE C 180   -48.938 39.668 51.011 1.00 0.00
C   H
ATOM   9633 HD3 ILE C 180   -47.859 38.769 49.880 1.00 0.00
C   H
ATOM   9634 C   ILE C 180   -45.361 41.995 48.322 1.00 0.00
C   C
ATOM   9635 O   ILE C 180   -45.638 43.091 47.819 1.00 0.00
C   O
ATOM   9636 N   LEU C 181   -44.964 40.953 47.596 1.00 0.00
C   N
ATOM   9637 HN LEU C 181   -44.890 40.017 47.933 1.00 0.00
C   H
ATOM   9638 CA LEU C 181   -44.592 41.117 46.197 1.00 0.00
C   C

```
ATOM   9639  HA  LEU C 181      -44.809  42.154  45.973  1.00  0.00
C    H
ATOM   9640  CB  LEU C 181      -43.109  40.808  46.005  1.00  0.00
C    C
ATOM   9641  HB1 LEU C 181      -42.633  40.843  47.012  1.00  0.00
C    H
ATOM   9642  HB2 LEU C 181      -43.001  39.759  45.650  1.00  0.00
C    H
ATOM   9643  CG  LEU C 181      -42.361  41.756  45.083  1.00  0.00
C    C
ATOM   9644  HG  LEU C 181      -42.585  41.467  44.027  1.00  0.00
C    H
ATOM   9645  CD1 LEU C 181      -42.783  43.204  45.297  1.00  0.00
C    C
ATOM   9646 HD11 LEU C 181      -42.190  43.884  44.647  1.00  0.00
C    H
ATOM   9647 HD12 LEU C 181      -43.859  43.339  45.053  1.00  0.00
C    H
ATOM   9648 HD13 LEU C 181      -42.623  43.508  46.354  1.00  0.00
C    H
ATOM   9649  CD2 LEU C 181      -40.900  41.553  45.318  1.00  0.00
C    C
ATOM   9650 HD21 LEU C 181      -40.306  42.214  44.650  1.00  0.00
C    H
ATOM   9651 HD22 LEU C 181      -40.616  41.789  46.363  1.00  0.00
C    H
ATOM   9652 HD23 LEU C 181      -40.614  40.499  45.107  1.00  0.00
C    H
ATOM   9653  C   LEU C 181      -45.373  40.264  45.216  1.00  0.00
C    C
ATOM   9654  O   LEU C 181      -45.754  40.771  44.161  1.00  0.00
C    O
ATOM   9655  N   ALA C 182      -45.586  38.983  45.501  1.00  0.00
C    N
ATOM   9656  HN  ALA C 182      -45.337  38.544  46.364  1.00  0.00
C    H
ATOM   9657  CA  ALA C 182      -46.223  38.119  44.523  1.00  0.00
C    C
ATOM   9658  HA  ALA C 182      -47.031  38.632  44.014  1.00  0.00
C    H
ATOM   9659  CB  ALA C 182      -45.235  37.650  43.451  1.00  0.00
C    C
ATOM   9660  HB1 ALA C 182      -44.823  38.532  42.914  1.00  0.00
C    H
ATOM   9661  HB2 ALA C 182      -44.384  37.098  43.906  1.00  0.00
C    H
ATOM   9662  HB3 ALA C 182      -45.726  36.991  42.702  1.00  0.00
C    H
ATOM   9663  C   ALA C 182      -46.847  36.924  45.220  1.00  0.00
C    C
ATOM   9664  O   ALA C 182      -46.389  36.477  46.279  1.00  0.00
C    O
ATOM   9665  N   ILE C 183      -47.886  36.402  44.579  1.00  0.00
C    N
```

```
ATOM   9666  HN  ILE C 183     -48.147  36.713  43.667  1.00  0.00
C    H
ATOM   9667  CA  ILE C 183     -48.735  35.349  45.115  1.00  0.00
C    C
ATOM   9668  HA  ILE C 183     -48.262  34.972  46.012  1.00  0.00
C    H
ATOM   9669  CB  ILE C 183     -50.156  35.884  45.388  1.00  0.00
C    C
ATOM   9670  HB  ILE C 183     -50.498  36.287  44.400  1.00  0.00
C    H
ATOM   9671  CG2 ILE C 183     -51.093  34.791  45.872  1.00  0.00
C    C
ATOM   9672 HG21 ILE C 183     -52.139  35.161  45.911  1.00  0.00
C    H
ATOM   9673 HG22 ILE C 183     -51.071  33.918  45.186  1.00  0.00
C    H
ATOM   9674 HG23 ILE C 183     -50.809  34.446  46.889  1.00  0.00
C    H
ATOM   9675  CG1 ILE C 183     -50.098  37.070  46.358  1.00  0.00
C    C
ATOM   9676 HG11 ILE C 183     -49.481  36.801  47.244  1.00  0.00
C    H
ATOM   9677 HG12 ILE C 183     -49.595  37.926  45.853  1.00  0.00
C    H
ATOM   9678  CD  ILE C 183     -51.450  37.536  46.828  1.00  0.00
C    C
ATOM   9679  HD1 ILE C 183     -51.345  38.430  47.480  1.00  0.00
C    H
ATOM   9680  HD2 ILE C 183     -52.093  37.809  45.964  1.00  0.00
C    H
ATOM   9681  HD3 ILE C 183     -51.963  36.743  47.412  1.00  0.00
C    H
ATOM   9682  C   ILE C 183     -48.762  34.201  44.115  1.00  0.00
C    C
ATOM   9683  O   ILE C 183     -49.173  34.387  42.966  1.00  0.00
C    O
ATOM   9684  N   LYS C 184     -48.335  33.017  44.554  1.00  0.00
C    N
ATOM   9685  HN  LYS C 184     -48.046  32.884  45.500  1.00  0.00
C    H
ATOM   9686  CA  LYS C 184     -48.252  31.828  43.705  1.00  0.00
C    C
ATOM   9687  HA  LYS C 184     -48.633  32.070  42.720  1.00  0.00
C    H
ATOM   9688  CB  LYS C 184     -46.819  31.309  43.646  1.00  0.00
C    C
ATOM   9689  HB1 LYS C 184     -46.152  32.197  43.546  1.00  0.00
C    H
ATOM   9690  HB2 LYS C 184     -46.564  30.836  44.620  1.00  0.00
C    H
ATOM   9691  CG  LYS C 184     -46.455  30.338  42.516  1.00  0.00
C    C
ATOM   9692  HG1 LYS C 184     -45.397  30.510  42.214  1.00  0.00
C    H
```

ATOM 9693 HG2 LYS C 184 -46.526 29.294 42.898 1.00 0.00
C H
ATOM 9694 CD LYS C 184 -47.322 30.479 41.281 1.00 0.00
C C
ATOM 9695 HD1 LYS C 184 -48.304 29.992 41.483 1.00 0.00
C H
ATOM 9696 HD2 LYS C 184 -47.516 31.564 41.114 1.00 0.00
C H
ATOM 9697 CE LYS C 184 -46.608 29.848 40.090 1.00 0.00
C C
ATOM 9698 HE1 LYS C 184 -45.559 30.215 40.025 1.00 0.00
C H
ATOM 9699 HE2 LYS C 184 -46.588 28.740 40.188 1.00 0.00
C H
ATOM 9700 NZ LYS C 184 -47.236 30.142 38.772 1.00 0.00
C N
ATOM 9701 HZ1 LYS C 184 -46.696 29.683 38.010 1.00 0.00
C H
ATOM 9702 HZ2 LYS C 184 -48.214 29.787 38.767 1.00 0.00
C H
ATOM 9703 HZ3 LYS C 184 -47.245 31.171 38.616 1.00 0.00
C H
ATOM 9704 C LYS C 184 -49.184 30.785 44.311 1.00 0.00
C C
ATOM 9705 O LYS C 184 -48.817 30.110 45.278 1.00 0.00
C O
ATOM 9706 N HSE C 185 -50.384 30.651 43.754 1.00 0.00
C N
ATOM 9707 HN HSE C 185 -50.670 31.169 42.948 1.00 0.00
C H
ATOM 9708 CA HSE C 185 -51.396 29.735 44.271 1.00 0.00
C C
ATOM 9709 HA HSE C 185 -50.971 29.228 45.129 1.00 0.00
C H
ATOM 9710 CB HSE C 185 -52.668 30.484 44.675 1.00 0.00
C C
ATOM 9711 HB1 HSE C 185 -53.398 29.738 45.057 1.00 0.00
C H
ATOM 9712 HB2 HSE C 185 -52.441 31.176 45.514 1.00 0.00
C H
ATOM 9713 ND1 HSE C 185 -54.352 30.696 42.814 1.00 0.00
C N
ATOM 9714 CG HSE C 185 -53.322 31.229 43.558 1.00 0.00
C C
ATOM 9715 CE1 HSE C 185 -54.731 31.572 41.901 1.00 0.00
C C
ATOM 9716 HE1 HSE C 185 -55.541 31.411 41.185 1.00 0.00
C H
ATOM 9717 NE2 HSE C 185 -53.966 32.644 42.009 1.00 0.00
C N
ATOM 9718 HE2 HSE C 185 -54.018 33.450 41.420 1.00 0.00
C H
ATOM 9719 CD2 HSE C 185 -53.074 32.454 43.036 1.00 0.00
C C

```
ATOM   9720  HD2 HSE C 185      -52.331  33.188  43.314  1.00  0.00
C    H
ATOM   9721  C   HSE C 185      -51.701  28.672  43.231  1.00  0.00
C    C
ATOM   9722  O   HSE C 185      -52.298  28.968  42.190  1.00  0.00
C    O
ATOM   9723  N   HSE C 186      -51.298  27.436  43.524  1.00  0.00
C    N
ATOM   9724  HN  HSE C 186      -50.790  27.210  44.354  1.00  0.00
C    H
ATOM   9725  CA  HSE C 186      -51.564  26.296  42.653  1.00  0.00
C    C
ATOM   9726  HA  HSE C 186      -50.994  25.461  43.043  1.00  0.00
C    H
ATOM   9727  CB  HSE C 186      -53.054  25.946  42.646  1.00  0.00
C    C
ATOM   9728  HB1 HSE C 186      -53.436  26.058  43.683  1.00  0.00
C    H
ATOM   9729  HB2 HSE C 186      -53.603  26.677  42.017  1.00  0.00
C    H
ATOM   9730  ND1 HSE C 186      -52.801  23.443  42.813  1.00  0.00
C    N
ATOM   9731  CG  HSE C 186      -53.341  24.549  42.195  1.00  0.00
C    C
ATOM   9732  CE1 HSE C 186      -53.217  22.351  42.198  1.00  0.00
C    C
ATOM   9733  HE1 HSE C 186      -52.940  21.334  42.484  1.00  0.00
C    H
ATOM   9734  NE2 HSE C 186      -54.002  22.710  41.198  1.00  0.00
C    N
ATOM   9735  HE2 HSE C 186      -54.438  22.082  40.554  1.00  0.00
C    H
ATOM   9736  CD2 HSE C 186      -54.093  24.079  41.173  1.00  0.00
C    C
ATOM   9737  HD2 HSE C 186      -54.671  24.616  40.434  1.00  0.00
C    H
ATOM   9738  C   HSE C 186      -51.065  26.556  41.237  1.00  0.00
C    C
ATOM   9739  O   HSE C 186      -51.755  26.271  40.258  1.00  0.00
C    O
ATOM   9740  N   GLY C 187      -49.865  27.124  41.116  1.00  0.00
C    N
ATOM   9741  HN  GLY C 187      -49.319  27.378  41.914  1.00  0.00
C    H
ATOM   9742  CA  GLY C 187      -49.270  27.412  39.839  1.00  0.00
C    C
ATOM   9743  HA1 GLY C 187      -49.590  26.623  39.172  1.00  0.00
C    H
ATOM   9744  HA2 GLY C 187      -48.202  27.452  40.000  1.00  0.00
C    H
ATOM   9745  C   GLY C 187      -49.702  28.734  39.242  1.00  0.00
C    C
ATOM   9746  O   GLY C 187      -49.194  29.119  38.185  1.00  0.00
C    O
```

```
ATOM   9747  N   ASP C 188      -50.623  29.438  39.890  1.00  0.00
C    N
ATOM   9748  HN  ASP C 188      -51.044  29.200  40.761  1.00  0.00
C    H
ATOM   9749  CA  ASP C 188      -51.120  30.692  39.343  1.00  0.00
C    C
ATOM   9750  HA  ASP C 188      -50.864  30.703  38.289  1.00  0.00
C    H
ATOM   9751  CB  ASP C 188      -52.628  30.784  39.558  1.00  0.00
C    C
ATOM   9752  HB1 ASP C 188      -52.846  30.713  40.642  1.00  0.00
C    H
ATOM   9753  HB2 ASP C 188      -53.016  31.754  39.188  1.00  0.00
C    H
ATOM   9754  CG  ASP C 188      -53.387  29.662  38.865  1.00  0.00
C    C
ATOM   9755  OD1 ASP C 188      -52.735  28.676  38.460  1.00  0.00
C    O
ATOM   9756  OD2 ASP C 188      -54.628  29.764  38.724  1.00  0.00
C    O
ATOM   9757  C   ASP C 188      -50.415  31.889  39.981  1.00  0.00
C    C
ATOM   9758  O   ASP C 188      -50.308  31.984  41.207  1.00  0.00
C    O
ATOM   9759  N   ILE C 189      -49.946  32.809  39.138  1.00  0.00
C    N
ATOM   9760  HN  ILE C 189      -50.107  32.756  38.154  1.00  0.00
C    H
ATOM   9761  CA  ILE C 189      -49.161  33.970  39.555  1.00  0.00
C    C
ATOM   9762  HA  ILE C 189      -48.754  33.741  40.532  1.00  0.00
C    H
ATOM   9763  CB  ILE C 189      -48.030  34.284  38.551  1.00  0.00
C    C
ATOM   9764  HB  ILE C 189      -48.041  33.420  37.839  1.00  0.00
C    H
ATOM   9765  CG2 ILE C 189      -48.231  35.649  37.898  1.00  0.00
C    C
ATOM   9766 HG21 ILE C 189      -47.505  35.797  37.071  1.00  0.00
C    H
ATOM   9767 HG22 ILE C 189      -49.251  35.737  37.470  1.00  0.00
C    H
ATOM   9768 HG23 ILE C 189      -48.087  36.468  38.635  1.00  0.00
C    H
ATOM   9769  CG1 ILE C 189      -46.651  34.272  39.217  1.00  0.00
C    C
ATOM   9770 HG11 ILE C 189      -45.911  33.786  38.543  1.00  0.00
C    H
ATOM   9771 HG12 ILE C 189      -46.316  35.323  39.371  1.00  0.00
C    H
ATOM   9772  CD  ILE C 189      -46.579  33.596  40.532  1.00  0.00
C    C
ATOM   9773  HD1 ILE C 189      -45.557  33.690  40.958  1.00  0.00
C    H
```

ATOM 9774 HD2 ILE C 189 -47.297 34.052 41.247 1.00 0.00
C H
ATOM 9775 HD3 ILE C 189 -46.813 32.514 40.437 1.00 0.00
C H
ATOM 9776 C ILE C 189 -50.075 35.174 39.730 1.00 0.00
C C
ATOM 9777 O ILE C 189 -51.094 35.300 39.038 1.00 0.00
C O
ATOM 9778 N CYS C 190 -49.718 36.033 40.680 1.00 0.00
C N
ATOM 9779 HN CYS C 190 -49.055 35.773 41.380 1.00 0.00
C H
ATOM 9780 CA CYS C 190 -50.234 37.391 40.801 1.00 0.00
C C
ATOM 9781 HA CYS C 190 -50.542 37.738 39.822 1.00 0.00
C H
ATOM 9782 CB CYS C 190 -51.397 37.473 41.786 1.00 0.00
C C
ATOM 9783 HB1 CYS C 190 -52.087 36.631 41.551 1.00 0.00
C H
ATOM 9784 HB2 CYS C 190 -51.029 37.288 42.819 1.00 0.00
C H
ATOM 9785 SG CYS C 190 -52.339 39.023 41.699 1.00 0.00
C S
ATOM 9786 HG1 CYS C 190 -53.263 38.707 42.607 1.00 0.00
C H
ATOM 9787 C CYS C 190 -49.051 38.248 41.245 1.00 0.00
C C
ATOM 9788 O CYS C 190 -48.616 38.175 42.397 1.00 0.00
C O
ATOM 9789 N LEU C 191 -48.519 39.040 40.316 1.00 0.00
C N
ATOM 9790 HN LEU C 191 -48.847 39.106 39.376 1.00 0.00
C H
ATOM 9791 CA LEU C 191 -47.381 39.898 40.615 1.00 0.00
C C
ATOM 9792 HA LEU C 191 -46.812 39.408 41.396 1.00 0.00
C H
ATOM 9793 CB LEU C 191 -46.530 40.133 39.367 1.00 0.00
C C
ATOM 9794 HB1 LEU C 191 -47.231 40.317 38.520 1.00 0.00
C H
ATOM 9795 HB2 LEU C 191 -45.949 41.073 39.497 1.00 0.00
C H
ATOM 9796 CG LEU C 191 -45.562 39.053 38.889 1.00 0.00
C C
ATOM 9797 HG LEU C 191 -46.148 38.160 38.563 1.00 0.00
C H
ATOM 9798 CD1 LEU C 191 -44.767 39.594 37.719 1.00 0.00
C C
ATOM 9799 HD11 LEU C 191 -44.018 38.846 37.378 1.00 0.00
C H
ATOM 9800 HD12 LEU C 191 -45.438 39.831 36.865 1.00 0.00
C H

```
ATOM   9801 HD13 LEU C 191      -44.226  40.520  38.007  1.00  0.00
C    H
ATOM   9802  CD2 LEU C 191      -44.641  38.626  40.008  1.00  0.00
C    C
ATOM   9803 HD21 LEU C 191      -43.952  37.827  39.657  1.00  0.00
C    H
ATOM   9804 HD22 LEU C 191      -44.020  39.469  40.373  1.00  0.00
C    H
ATOM   9805 HD23 LEU C 191      -45.227  38.228  40.865  1.00  0.00
C    H
ATOM   9806  C   LEU C 191      -47.847  41.239  41.159  1.00  0.00
C    C
ATOM   9807  O   LEU C 191      -48.793  41.841  40.647  1.00  0.00
C    O
ATOM   9808  N   SER C 192      -47.130  41.732  42.169  1.00  0.00
C    N
ATOM   9809  HN  SER C 192      -46.311  41.274  42.512  1.00  0.00
C    H
ATOM   9810  CA  SER C 192      -47.460  42.969  42.868  1.00  0.00
C    C
ATOM   9811  HA  SER C 192      -46.938  42.936  43.815  1.00  0.00
C    H
ATOM   9812  CB  SER C 192      -47.036  44.193  42.065  1.00  0.00
C    C
ATOM   9813  HB1 SER C 192      -47.791  44.995  42.233  1.00  0.00
C    H
ATOM   9814  HB2 SER C 192      -46.060  44.580  42.434  1.00  0.00
C    H
ATOM   9815  OG  SER C 192      -46.975  43.922  40.676  1.00  0.00
C    O
ATOM   9816  HG1 SER C 192      -46.924  44.774  40.223  1.00  0.00
C    H
ATOM   9817  C   SER C 192      -48.962  43.015  43.174  1.00  0.00
C    C
ATOM   9818  O   SER C 192      -49.675  43.871  42.643  1.00  0.00
C    O
ATOM   9819  N   PRO C 193      -49.462  42.109  44.010  1.00  0.00
C    N
ATOM   9820  CD  PRO C 193      -48.735  41.235  44.947  1.00  0.00
C    C
ATOM   9821  HD1 PRO C 193      -48.251  40.379  44.426  1.00  0.00
C    H
ATOM   9822  HD2 PRO C 193      -47.980  41.817  45.524  1.00  0.00
C    H
ATOM   9823  CA  PRO C 193      -50.915  42.004  44.183  1.00  0.00
C    C
ATOM   9824  HA  PRO C 193      -51.403  41.836  43.229  1.00  0.00
C    H
ATOM   9825  CB  PRO C 193      -51.077  40.783  45.096  1.00  0.00
C    C
ATOM   9826  HB1 PRO C 193      -51.078  39.861  44.473  1.00  0.00
C    H
ATOM   9827  HB2 PRO C 193      -52.011  40.816  45.692  1.00  0.00
C    H
```

ATOM 9828 CG PRO C 193 -49.812 40.735 45.868 1.00 0.00
C C
ATOM 9829 HG1 PRO C 193 -49.584 39.724 46.262 1.00 0.00
C H
ATOM 9830 HG2 PRO C 193 -49.871 41.457 46.714 1.00 0.00
C H
ATOM 9831 C PRO C 193 -51.488 43.259 44.820 1.00 0.00
C C
ATOM 9832 O PRO C 193 -50.993 43.750 45.837 1.00 0.00
C O
ATOM 9833 N ALA C 194 -52.546 43.776 44.200 1.00 0.00
C N
ATOM 9834 HN ALA C 194 -52.922 43.391 43.358 1.00 0.00
C H
ATOM 9835 CA ALA C 194 -53.234 44.947 44.710 1.00 0.00
C C
ATOM 9836 HA ALA C 194 -52.564 45.594 45.265 1.00 0.00
C H
ATOM 9837 CB ALA C 194 -53.857 45.747 43.572 1.00 0.00
C C
ATOM 9838 HB1 ALA C 194 -53.058 46.110 42.890 1.00 0.00
C H
ATOM 9839 HB2 ALA C 194 -54.554 45.117 42.976 1.00 0.00
C H
ATOM 9840 HB3 ALA C 194 -54.414 46.631 43.950 1.00 0.00
C H
ATOM 9841 C ALA C 194 -54.305 44.500 45.687 1.00 0.00
C C
ATOM 9842 O ALA C 194 -55.129 43.641 45.332 1.00 0.00
C O
ATOM 9843 N PRO C 195 -54.321 45.020 46.912 1.00 0.00
C N
ATOM 9844 CD PRO C 195 -53.362 45.976 47.489 1.00 0.00
C C
ATOM 9845 HD1 PRO C 195 -52.997 45.014 47.060 1.00 0.00
C H
ATOM 9846 HD2 PRO C 195 -52.628 46.792 47.298 1.00 0.00
C H
ATOM 9847 CA PRO C 195 -55.362 44.629 47.871 1.00 0.00
C C
ATOM 9848 HA PRO C 195 -55.300 43.571 48.096 1.00 0.00
C H
ATOM 9849 CB PRO C 195 -55.030 45.470 49.109 1.00 0.00
C C
ATOM 9850 HB1 PRO C 195 -54.262 44.937 49.713 1.00 0.00
C H
ATOM 9851 HB2 PRO C 195 -55.917 45.665 49.744 1.00 0.00
C H
ATOM 9852 CG PRO C 195 -53.589 45.827 48.959 1.00 0.00
C C
ATOM 9853 HG1 PRO C 195 -53.101 46.067 49.924 1.00 0.00
C H
ATOM 9854 HG2 PRO C 195 -53.496 46.697 48.270 1.00 0.00
C H

ATOM 9855 C PRO C 195 -56.751 44.944 47.332 1.00 0.00
C C
ATOM 9856 O PRO C 195 -56.984 46.010 46.757 1.00 0.00
C O
ATOM 9857 N GLU C 196 -57.678 44.005 47.513 1.00 0.00
C N
ATOM 9858 HN GLU C 196 -58.607 44.090 47.167 1.00 0.00
C H
ATOM 9859 CA GLU C 196 -57.424 42.762 48.234 1.00 0.00
C C
ATOM 9860 HA GLU C 196 -56.534 42.893 48.838 1.00 0.00
C H
ATOM 9861 CB GLU C 196 -58.613 42.420 49.138 1.00 0.00
C C
ATOM 9862 HB1 GLU C 196 -58.386 41.464 49.663 1.00 0.00
C H
ATOM 9863 HB2 GLU C 196 -58.714 43.190 49.935 1.00 0.00
C H
ATOM 9864 CG GLU C 196 -59.946 42.266 48.420 1.00 0.00
C C
ATOM 9865 HG1 GLU C 196 -60.771 42.448 49.132 1.00 0.00
C H
ATOM 9866 HG2 GLU C 196 -60.028 42.981 47.577 1.00 0.00
C H
ATOM 9867 CD GLU C 196 -60.153 40.859 47.891 1.00 0.00
C C
ATOM 9868 OE1 GLU C 196 -60.299 39.928 48.717 1.00 0.00
C O
ATOM 9869 OE2 GLU C 196 -60.130 40.679 46.657 1.00 0.00
C O
ATOM 9870 C GLU C 196 -57.145 41.617 47.260 1.00 0.00
C C
ATOM 9871 O GLU C 196 -57.296 41.778 46.052 1.00 0.00
C O
ATOM 9872 N ASP C 197 -56.725 40.467 47.786 1.00 0.00
C N
ATOM 9873 HN ASP C 197 -56.606 40.303 48.762 1.00 0.00
C H
ATOM 9874 CA ASP C 197 -56.388 39.308 46.971 1.00 0.00
C C
ATOM 9875 HA ASP C 197 -57.152 39.218 46.208 1.00 0.00
C H
ATOM 9876 CB ASP C 197 -55.000 39.443 46.348 1.00 0.00
C C
ATOM 9877 HB1 ASP C 197 -54.821 40.505 46.085 1.00 0.00
C H
ATOM 9878 HB2 ASP C 197 -54.218 39.124 47.065 1.00 0.00
C H
ATOM 9879 CG ASP C 197 -54.857 38.631 45.085 1.00 0.00
C C
ATOM 9880 OD1 ASP C 197 -55.891 38.268 44.487 1.00 0.00
C O
ATOM 9881 OD2 ASP C 197 -53.703 38.337 44.701 1.00 0.00
C O

```
ATOM   9882  C   ASP C 197     -56.447  38.039  47.802  1.00  0.00
C    C
ATOM   9883  O   ASP C 197     -55.880  37.970  48.894  1.00  0.00
C    O
ATOM   9884  N   ILE C 198     -57.086  37.012  47.245  1.00  0.00
C    N
ATOM   9885  HN  ILE C 198     -57.455  37.022  46.317  1.00  0.00
C    H
ATOM   9886  CA  ILE C 198     -57.305  35.759  47.963  1.00  0.00
C    C
ATOM   9887  HA  ILE C 198     -57.682  36.015  48.945  1.00  0.00
C    H
ATOM   9888  CB  ILE C 198     -58.294  34.850  47.208  1.00  0.00
C    C
ATOM   9889  HB  ILE C 198     -58.162  35.125  46.131  1.00  0.00
C    H
ATOM   9890  CG2 ILE C 198     -58.046  33.378  47.546  1.00  0.00
C    C
ATOM   9891 HG21 ILE C 198     -58.650  32.718  46.888  1.00  0.00
C    H
ATOM   9892 HG22 ILE C 198     -56.978  33.112  47.399  1.00  0.00
C    H
ATOM   9893 HG23 ILE C 198     -58.320  33.160  48.599  1.00  0.00
C    H
ATOM   9894  CG1 ILE C 198     -59.740  35.191  47.571  1.00  0.00
C    C
ATOM   9895 HG11 ILE C 198     -59.976  34.798  48.585  1.00  0.00
C    H
ATOM   9896 HG12 ILE C 198     -59.849  36.298  47.613  1.00  0.00
C    H
ATOM   9897  CD  ILE C 198     -60.747  34.659  46.594  1.00  0.00
C    C
ATOM   9898  HD1 ILE C 198     -61.770  34.986  46.878  1.00  0.00
C    H
ATOM   9899  HD2 ILE C 198     -60.534  35.032  45.570  1.00  0.00
C    H
ATOM   9900  HD3 ILE C 198     -60.735  33.548  46.575  1.00  0.00
C    H
ATOM   9901  C   ILE C 198     -55.969  35.057  48.174  1.00  0.00
C    C
ATOM   9902  O   ILE C 198     -55.270  34.706  47.213  1.00  0.00
C    O
ATOM   9903  N   ILE C 199     -55.612  34.850  49.437  1.00  0.00
C    N
ATOM   9904  HN  ILE C 199     -56.152  35.181  50.207  1.00  0.00
C    H
ATOM   9905  CA  ILE C 199     -54.408  34.124  49.820  1.00  0.00
C    C
ATOM   9906  HA  ILE C 199     -53.818  33.955  48.927  1.00  0.00
C    H
ATOM   9907  CB  ILE C 199     -53.606  34.879  50.894  1.00  0.00
C    C
ATOM   9908  HB  ILE C 199     -54.331  35.022  51.735  1.00  0.00
C    H
```

```
ATOM   9909  CG2 ILE C 199     -52.384  34.081  51.310  1.00  0.00
C    C
ATOM   9910 HG21 ILE C 199     -51.893  34.548  52.189  1.00  0.00
C    H
ATOM   9911 HG22 ILE C 199     -52.664  33.045  51.591  1.00  0.00
C    H
ATOM   9912 HG23 ILE C 199     -51.642  34.031  50.486  1.00  0.00
C    H
ATOM   9913  CG1 ILE C 199     -53.228  36.276  50.389  1.00  0.00
C    C
ATOM   9914 HG11 ILE C 199     -52.845  36.208  49.346  1.00  0.00
C    H
ATOM   9915 HG12 ILE C 199     -54.143  36.911  50.363  1.00  0.00
C    H
ATOM   9916  CD  ILE C 199     -52.204  36.987  51.227  1.00  0.00
C    C
ATOM   9917  HD1 ILE C 199     -52.025  38.010  50.833  1.00  0.00
C    H
ATOM   9918  HD2 ILE C 199     -52.551  37.075  52.279  1.00  0.00
C    H
ATOM   9919  HD3 ILE C 199     -51.236  36.442  51.221  1.00  0.00
C    H
ATOM   9920  C   ILE C 199     -54.851  32.760  50.319  1.00  0.00
C    C
ATOM   9921  O   ILE C 199     -55.344  32.638  51.444  1.00  0.00
C    O
ATOM   9922  N   ARG C 200     -54.703  31.741  49.481  1.00  0.00
C    N
ATOM   9923  HN  ARG C 200     -54.332  31.866  48.564  1.00  0.00
C    H
ATOM   9924  CA  ARG C 200     -55.075  30.388  49.855  1.00  0.00
C    C
ATOM   9925  HA  ARG C 200     -55.921  30.434  50.531  1.00  0.00
C    H
ATOM   9926  CB  ARG C 200     -55.437  29.580  48.612  1.00  0.00
C    C
ATOM   9927  HB1 ARG C 200     -54.694  29.858  47.828  1.00  0.00
C    H
ATOM   9928  HB2 ARG C 200     -55.279  28.497  48.813  1.00  0.00
C    H
ATOM   9929  CG  ARG C 200     -56.829  29.860  48.099  1.00  0.00
C    C
ATOM   9930  HG1 ARG C 200     -57.457  30.206  48.949  1.00  0.00
C    H
ATOM   9931  HG2 ARG C 200     -56.788  30.717  47.389  1.00  0.00
C    H
ATOM   9932  CD  ARG C 200     -57.408  28.656  47.384  1.00  0.00
C    C
ATOM   9933  HD1 ARG C 200     -56.940  28.563  46.377  1.00  0.00
C    H
ATOM   9934  HD2 ARG C 200     -57.223  27.714  47.953  1.00  0.00
C    H
ATOM   9935  NE  ARG C 200     -58.855  28.763  47.224  1.00  0.00
C    N
```

```
ATOM   9936  HE  ARG C 200     -59.208  29.684  47.423  1.00  0.00
C    H
ATOM   9937  CZ  ARG C 200     -59.639  27.756  46.854  1.00  0.00
C    C
ATOM   9938  NH1 ARG C 200     -59.116  26.565  46.601  1.00  0.00
C    N
ATOM   9939 HH11 ARG C 200     -59.689  25.817  46.300  1.00  0.00
C    H
ATOM   9940 HH12 ARG C 200     -58.115  26.513  46.598  1.00  0.00
C    H
ATOM   9941  NH2 ARG C 200     -60.946  27.939  46.733  1.00  0.00
C    N
ATOM   9942 HH21 ARG C 200     -61.537  27.168  46.542  1.00  0.00
C    H
ATOM   9943 HH22 ARG C 200     -61.308  28.807  47.046  1.00  0.00
C    H
ATOM   9944  C   ARG C 200     -53.942  29.715  50.622  1.00  0.00
C    C
ATOM   9945  O   ARG C 200     -52.833  30.241  50.737  1.00  0.00
C    O
ATOM   9946  N   GLU C 201     -54.234  28.534  51.157  1.00  0.00
C    N
ATOM   9947  HN  GLU C 201     -55.137  28.115  51.183  1.00  0.00
C    H
ATOM   9948  CA  GLU C 201     -53.191  27.731  51.773  1.00  0.00
C    C
ATOM   9949  HA  GLU C 201     -52.663  28.339  52.498  1.00  0.00
C    H
ATOM   9950  CB  GLU C 201     -53.780  26.495  52.447  1.00  0.00
C    C
ATOM   9951  HB1 GLU C 201     -52.980  25.723  52.517  1.00  0.00
C    H
ATOM   9952  HB2 GLU C 201     -54.063  26.736  53.496  1.00  0.00
C    H
ATOM   9953  CG  GLU C 201     -54.966  25.897  51.728  1.00  0.00
C    C
ATOM   9954  HG1 GLU C 201     -55.855  26.534  51.889  1.00  0.00
C    H
ATOM   9955  HG2 GLU C 201     -54.771  25.813  50.641  1.00  0.00
C    H
ATOM   9956  CD  GLU C 201     -55.327  24.531  52.269  1.00  0.00
C    C
ATOM   9957  OE1 GLU C 201     -55.168  24.314  53.486  1.00  0.00
C    O
ATOM   9958  OE2 GLU C 201     -55.772  23.674  51.476  1.00  0.00
C    O
ATOM   9959  C   GLU C 201     -52.182  27.309  50.718  1.00  0.00
C    C
ATOM   9960  O   GLU C 201     -52.461  27.349  49.516  1.00  0.00
C    O
ATOM   9961  N   GLN C 202     -50.995  26.922  51.175  1.00  0.00
C    N
ATOM   9962  HN  GLN C 202     -50.782  26.946  52.151  1.00  0.00
C    H
```

```
ATOM   9963  CA  GLN C 202     -49.908  26.434  50.334  1.00  0.00
C    C
ATOM   9964  HA  GLN C 202     -49.056  26.285  50.986  1.00  0.00
C    H
ATOM   9965  CB  GLN C 202     -50.278  25.098  49.678  1.00  0.00
C    C
ATOM   9966  HB1 GLN C 202     -51.226  25.267  49.117  1.00  0.00
C    H
ATOM   9967  HB2 GLN C 202     -49.509  24.836  48.917  1.00  0.00
C    H
ATOM   9968  CG  GLN C 202     -50.463  23.985  50.694  1.00  0.00
C    C
ATOM   9969  HG1 GLN C 202     -49.878  24.199  51.614  1.00  0.00
C    H
ATOM   9970  HG2 GLN C 202     -51.530  23.890  50.983  1.00  0.00
C    H
ATOM   9971  CD  GLN C 202     -49.987  22.625  50.212  1.00  0.00
C    C
ATOM   9972  OE1 GLN C 202     -49.068  22.528  49.405  1.00  0.00
C    O
ATOM   9973  NE2 GLN C 202     -50.612  21.567  50.712  1.00  0.00
C    N
ATOM   9974 HE21 GLN C 202     -50.301  20.673  50.404  1.00  0.00
C    H
ATOM   9975 HE22 GLN C 202     -51.363  21.692  51.351  1.00  0.00
C    H
ATOM   9976  C   GLN C 202     -49.505  27.452  49.277  1.00  0.00
C    C
ATOM   9977  O   GLN C 202     -48.962  27.078  48.230  1.00  0.00
C    O
ATOM   9978  N   ASP C 203     -49.755  28.733  49.532  1.00  0.00
C    N
ATOM   9979  HN  ASP C 203     -50.185  29.079  50.362  1.00  0.00
C    H
ATOM   9980  CA  ASP C 203     -49.412  29.785  48.591  1.00  0.00
C    C
ATOM   9981  HA  ASP C 203     -49.449  29.351  47.598  1.00  0.00
C    H
ATOM   9982  CB  ASP C 203     -50.341  30.981  48.742  1.00  0.00
C    C
ATOM   9983  HB1 ASP C 203     -51.390  30.626  48.782  1.00  0.00
C    H
ATOM   9984  HB2 ASP C 203     -50.123  31.529  49.680  1.00  0.00
C    H
ATOM   9985  CG  ASP C 203     -50.227  31.935  47.587  1.00  0.00
C    C
ATOM   9986  OD1 ASP C 203     -49.500  32.937  47.728  1.00  0.00
C    O
ATOM   9987  OD2 ASP C 203     -50.887  31.710  46.566  1.00  0.00
C    O
ATOM   9988  C   ASP C 203     -47.989  30.258  48.809  1.00  0.00
C    C
ATOM   9989  O   ASP C 203     -47.552  30.450  49.946  1.00  0.00
C    O
```

```
ATOM   9990  N   CYS C 204     -47.279  30.494  47.713  1.00  0.00
C    N
ATOM   9991  HN  CYS C 204     -47.586  30.290  46.784  1.00  0.00
C    H
ATOM   9992  CA  CYS C 204     -45.953  31.089  47.801  1.00  0.00
C    C
ATOM   9993  HA  CYS C 204     -45.525  30.787  48.750  1.00  0.00
C    H
ATOM   9994  CB  CYS C 204     -45.072  30.610  46.652  1.00  0.00
C    C
ATOM   9995  HB1 CYS C 204     -45.683  30.663  45.723  1.00  0.00
C    H
ATOM   9996  HB2 CYS C 204     -44.226  31.318  46.504  1.00  0.00
C    H
ATOM   9997  SG  CYS C 204     -44.482  28.915  46.854  1.00  0.00
C    S
ATOM   9998  HG1 CYS C 204     -43.841  28.822  45.689  1.00  0.00
C    H
ATOM   9999  C   CYS C 204     -46.087  32.609  47.815  1.00  0.00
C    C
ATOM  10000  O   CYS C 204     -46.602  33.206  46.865  1.00  0.00
C    O
ATOM  10001  N   LEU C 205     -45.650  33.217  48.916  1.00  0.00
C    N
ATOM  10002  HN  LEU C 205     -45.234  32.686  49.652  1.00  0.00
C    H
ATOM  10003  CA  LEU C 205     -45.717  34.648  49.176  1.00  0.00
C    C
ATOM  10004  HA  LEU C 205     -46.352  35.105  48.429  1.00  0.00
C    H
ATOM  10005  CB  LEU C 205     -46.199  34.914  50.606  1.00  0.00
C    C
ATOM  10006  HB1 LEU C 205     -46.054  33.970  51.183  1.00  0.00
C    H
ATOM  10007  HB2 LEU C 205     -45.532  35.668  51.079  1.00  0.00
C    H
ATOM  10008  CG  LEU C 205     -47.613  35.352  50.979  1.00  0.00
C    C
ATOM  10009  HG  LEU C 205     -47.805  36.381  50.591  1.00  0.00
C    H
ATOM  10010  CD1 LEU C 205     -48.658  34.390  50.443  1.00  0.00
C    C
ATOM  10011 HD11 LEU C 205     -49.675  34.700  50.765  1.00  0.00
C    H
ATOM  10012 HD12 LEU C 205     -48.635  34.366  49.333  1.00  0.00
C    H
ATOM  10013 HD13 LEU C 205     -48.474  33.361  50.819  1.00  0.00
C    H
ATOM  10014  CD2 LEU C 205     -47.692  35.398  52.487  1.00  0.00
C    C
ATOM  10015 HD21 LEU C 205     -48.701  35.732  52.811  1.00  0.00
C    H
ATOM  10016 HD22 LEU C 205     -47.505  34.403  52.938  1.00  0.00
C    H
```

```
ATOM  10017 HD23 LEU C 205      -46.943  36.112  52.893  1.00  0.00
C    H
ATOM  10018  C   LEU C 205      -44.313  35.201  48.985  1.00  0.00
C    C
ATOM  10019  O   LEU C 205      -43.348  34.633  49.513  1.00  0.00
C    O
ATOM  10020  N   VAL C 206      -44.190  36.286  48.228  1.00  0.00
C    N
ATOM  10021  HN  VAL C 206      -44.947  36.714  47.741  1.00  0.00
C    H
ATOM  10022  CA  VAL C 206      -42.905  36.952  48.049  1.00  0.00
C    C
ATOM  10023  HA  VAL C 206      -42.123  36.305  48.427  1.00  0.00
C    H
ATOM  10024  CB  VAL C 206      -42.619  37.261  46.577  1.00  0.00
C    C
ATOM  10025  HB  VAL C 206      -43.370  38.017  46.239  1.00  0.00
C    H
ATOM  10026  CG1 VAL C 206      -41.226  37.833  46.423  1.00  0.00
C    C
ATOM  10027 HG11 VAL C 206      -41.002  38.021  45.351  1.00  0.00
C    H
ATOM  10028 HG12 VAL C 206      -41.118  38.799  46.958  1.00  0.00
C    H
ATOM  10029 HG13 VAL C 206      -40.465  37.124  46.813  1.00  0.00
C    H
ATOM  10030  CG2 VAL C 206      -42.767  35.992  45.746  1.00  0.00
C    C
ATOM  10031 HG21 VAL C 206      -42.524  36.189  44.680  1.00  0.00
C    H
ATOM  10032 HG22 VAL C 206      -42.084  35.199  46.119  1.00  0.00
C    H
ATOM  10033 HG23 VAL C 206      -43.806  35.605  45.787  1.00  0.00
C    H
ATOM  10034  C   VAL C 206      -42.902  38.215  48.896  1.00  0.00
C    C
ATOM  10035  O   VAL C 206      -43.709  39.129  48.680  1.00  0.00
C    O
ATOM  10036  N   ILE C 207      -41.985  38.270  49.853  1.00  0.00
C    N
ATOM  10037  HN  ILE C 207      -41.310  37.552  50.011  1.00  0.00
C    H
ATOM  10038  CA  ILE C 207      -41.881  39.395  50.765  1.00  0.00
C    C
ATOM  10039  HA  ILE C 207      -42.511  40.187  50.381  1.00  0.00
C    H
ATOM  10040  CB  ILE C 207      -42.296  39.010  52.200  1.00  0.00
C    C
ATOM  10041  HB  ILE C 207      -43.265  38.465  52.075  1.00  0.00
C    H
ATOM  10042  CG2 ILE C 207      -41.219  38.176  52.852  1.00  0.00
C    C
ATOM  10043 HG21 ILE C 207      -41.574  37.767  53.820  1.00  0.00
C    H
```

ATOM 10044 HG22 ILE C 207 -40.939 37.316 52.206 1.00 0.00
C H
ATOM 10045 HG23 ILE C 207 -40.309 38.781 53.044 1.00 0.00
C H
ATOM 10046 CG1 ILE C 207 -42.597 40.263 53.028 1.00 0.00
C C
ATOM 10047 HG11 ILE C 207 -41.700 40.922 53.055 1.00 0.00
C H
ATOM 10048 HG12 ILE C 207 -43.411 40.837 52.530 1.00 0.00
C H
ATOM 10049 CD ILE C 207 -43.023 39.966 54.422 1.00 0.00
C C
ATOM 10050 HD1 ILE C 207 -43.283 40.906 54.955 1.00 0.00
C H
ATOM 10051 HD2 ILE C 207 -43.917 39.306 54.425 1.00 0.00
C H
ATOM 10052 HD3 ILE C 207 -42.211 39.463 54.988 1.00 0.00
C H
ATOM 10053 C ILE C 207 -40.450 39.912 50.726 1.00 0.00
C C
ATOM 10054 O ILE C 207 -39.521 39.219 50.315 1.00 0.00
C O
ATOM 10055 N MET C 208 -40.273 41.156 51.160 1.00 0.00
C N
ATOM 10056 HN MET C 208 -41.008 41.752 51.474 1.00 0.00
C H
ATOM 10057 CA MET C 208 -38.949 41.756 51.209 1.00 0.00
C C
ATOM 10058 HA MET C 208 -38.236 40.988 51.479 1.00 0.00
C H
ATOM 10059 CB MET C 208 -38.557 42.401 49.870 1.00 0.00
C C
ATOM 10060 HB1 MET C 208 -38.364 41.568 49.153 1.00 0.00
C H
ATOM 10061 HB2 MET C 208 -39.424 42.967 49.464 1.00 0.00
C H
ATOM 10062 CG MET C 208 -37.317 43.277 49.986 1.00 0.00
C C
ATOM 10063 HG1 MET C 208 -37.300 43.794 50.968 1.00 0.00
C H
ATOM 10064 HG2 MET C 208 -36.405 42.644 49.923 1.00 0.00
C H
ATOM 10065 SD MET C 208 -37.194 44.540 48.731 1.00 0.00
C S
ATOM 10066 CE MET C 208 -38.821 45.241 48.842 1.00 0.00
C C
ATOM 10067 HE1 MET C 208 -38.949 46.065 48.107 1.00 0.00
C H
ATOM 10068 HE2 MET C 208 -39.600 44.475 48.636 1.00 0.00
C H
ATOM 10069 HE3 MET C 208 -39.010 45.655 49.855 1.00 0.00
C H
ATOM 10070 C MET C 208 -38.936 42.802 52.310 1.00 0.00
C C

ATOM 10071 O   MET C 208   -39.939 43.484 52.533 1.00 0.00
C   O
ATOM 10072 N   GLY C 209   -37.800 42.930 52.981 1.00 0.00
C   N
ATOM 10073 HN GLY C 209   -37.020 42.310 52.896 1.00 0.00
C   H
ATOM 10074 CA GLY C 209   -37.629 44.025 53.912 1.00 0.00
C   C
ATOM 10075 HA1 GLY C 209   -38.563 44.131 54.447 1.00 0.00
C   H
ATOM 10076 HA2 GLY C 209   -37.348 44.894 53.334 1.00 0.00
C   H
ATOM 10077 C   GLY C 209   -36.537 43.765 54.923 1.00 0.00
C   C
ATOM 10078 O   GLY C 209   -35.587 43.029 54.649 1.00 0.00
C   O
ATOM 10079 N   HSE C 210   -36.669 44.370 56.099 1.00 0.00
C   N
ATOM 10080 HN HSE C 210   -37.396 45.014 56.332 1.00 0.00
C   H
ATOM 10081 CA HSE C 210   -35.720 44.123 57.169 1.00 0.00
C   C
ATOM 10082 HA HSE C 210   -34.723 44.217 56.757 1.00 0.00
C   H
ATOM 10083 CB HSE C 210   -35.941 45.087 58.328 1.00 0.00
C   C
ATOM 10084 HB1 HSE C 210   -35.825 46.121 57.939 1.00 0.00
C   H
ATOM 10085 HB2 HSE C 210   -36.984 44.993 58.694 1.00 0.00
C   H
ATOM 10086 ND1 HSE C 210   -33.623 45.139 59.313 1.00 0.00
C   N
ATOM 10087 CG HSE C 210   -34.972 44.900 59.449 1.00 0.00
C   C
ATOM 10088 CE1 HSE C 210   -33.015 44.880 60.457 1.00 0.00
C   C
ATOM 10089 HE1 HSE C 210   -31.941 44.993 60.627 1.00 0.00
C   H
ATOM 10090 NE2 HSE C 210   -33.920 44.467 61.325 1.00 0.00
C   N
ATOM 10091 HE2 HSE C 210   -33.730 44.184 62.265 1.00 0.00
C   H
ATOM 10092 CD2 HSE C 210   -35.152 44.462 60.717 1.00 0.00
C   C
ATOM 10093 HD2 HSE C 210   -36.054 44.143 61.219 1.00 0.00
C   H
ATOM 10094 C   HSE C 210   -35.870 42.692 57.649 1.00 0.00
C   C
ATOM 10095 O   HSE C 210   -36.939 42.289 58.114 1.00 0.00
C   O
ATOM 10096 N   LYS C 211   -34.784 41.926 57.536 1.00 0.00
C   N
ATOM 10097 HN LYS C 211   -33.881 42.269 57.281 1.00 0.00
C   H

ATOM 10098 CA LYS C 211 -34.852 40.490 57.787 1.00 0.00
C C
ATOM 10099 HA LYS C 211 -35.528 40.073 57.051 1.00 0.00
C H
ATOM 10100 CB LYS C 211 -33.462 39.872 57.653 1.00 0.00
C C
ATOM 10101 HB1 LYS C 211 -33.536 38.817 58.008 1.00 0.00
C H
ATOM 10102 HB2 LYS C 211 -33.190 39.821 56.575 1.00 0.00
C H
ATOM 10103 CG LYS C 211 -32.381 40.584 58.445 1.00 0.00
C C
ATOM 10104 HG1 LYS C 211 -32.222 41.599 58.015 1.00 0.00
C H
ATOM 10105 HG2 LYS C 211 -32.730 40.719 59.494 1.00 0.00
C H
ATOM 10106 CD LYS C 211 -31.073 39.817 58.413 1.00 0.00
C C
ATOM 10107 HD1 LYS C 211 -31.288 38.745 58.630 1.00 0.00
C H
ATOM 10108 HD2 LYS C 211 -30.663 39.873 57.378 1.00 0.00
C H
ATOM 10109 CE LYS C 211 -30.089 40.390 59.413 1.00 0.00
C C
ATOM 10110 HE1 LYS C 211 -30.485 40.293 60.449 1.00 0.00
C H
ATOM 10111 HE2 LYS C 211 -29.117 39.854 59.356 1.00 0.00
C H
ATOM 10112 NZ LYS C 211 -29.821 41.831 59.171 1.00 0.00
C N
ATOM 10113 HZ1 LYS C 211 -29.144 42.190 59.875 1.00 0.00
C H
ATOM 10114 HZ2 LYS C 211 -29.425 41.955 58.217 1.00 0.00
C H
ATOM 10115 HZ3 LYS C 211 -30.710 42.366 59.244 1.00 0.00
C H
ATOM 10116 C LYS C 211 -35.433 40.185 59.160 1.00 0.00
C C
ATOM 10117 O LYS C 211 -36.141 39.187 59.335 1.00 0.00
C O
ATOM 10118 N LYS C 212 -35.155 41.035 60.149 1.00 0.00
C N
ATOM 10119 HN LYS C 212 -34.631 41.881 60.060 1.00 0.00
C H
ATOM 10120 CA LYS C 212 -35.638 40.745 61.492 1.00 0.00
C C
ATOM 10121 HA LYS C 212 -35.520 39.680 61.651 1.00 0.00
C H
ATOM 10122 CB LYS C 212 -34.806 41.501 62.526 1.00 0.00
C C
ATOM 10123 HB1 LYS C 212 -34.951 42.589 62.333 1.00 0.00
C H
ATOM 10124 HB2 LYS C 212 -35.223 41.305 63.539 1.00 0.00
C H

ATOM 10125 CG LYS C 212 -33.307 41.178 62.481 1.00 0.00
C C
ATOM 10126 HG1 LYS C 212 -32.806 41.869 61.765 1.00 0.00
C H
ATOM 10127 HG2 LYS C 212 -32.865 41.364 63.487 1.00 0.00
C H
ATOM 10128 CD LYS C 212 -33.001 39.736 62.048 1.00 0.00
C C
ATOM 10129 HD1 LYS C 212 -33.580 39.515 61.122 1.00 0.00
C H
ATOM 10130 HD2 LYS C 212 -31.919 39.674 61.788 1.00 0.00
C H
ATOM 10131 CE LYS C 212 -33.326 38.705 63.120 1.00 0.00
C C
ATOM 10132 HE1 LYS C 212 -33.088 39.105 64.130 1.00 0.00
C H
ATOM 10133 HE2 LYS C 212 -34.405 38.439 63.092 1.00 0.00
C H
ATOM 10134 NZ LYS C 212 -32.539 37.452 62.928 1.00 0.00
C N
ATOM 10135 HZ1 LYS C 212 -32.777 36.764 63.671 1.00 0.00
C H
ATOM 10136 HZ2 LYS C 212 -32.759 37.044 61.997 1.00 0.00
C H
ATOM 10137 HZ3 LYS C 212 -31.523 37.671 62.973 1.00 0.00
C H
ATOM 10138 C LYS C 212 -37.123 41.063 61.623 1.00 0.00
C C
ATOM 10139 O LYS C 212 -37.862 40.324 62.281 1.00 0.00
C O
ATOM 10140 N ASP C 213 -37.591 42.132 60.978 1.00 0.00
C N
ATOM 10141 HN ASP C 213 -37.067 42.841 60.510 1.00 0.00
C H
ATOM 10142 CA ASP C 213 -39.032 42.352 60.909 1.00 0.00
C C
ATOM 10143 HA ASP C 213 -39.410 42.310 61.924 1.00 0.00
C H
ATOM 10144 CB ASP C 213 -39.340 43.700 60.260 1.00 0.00
C C
ATOM 10145 HB1 ASP C 213 -38.737 43.806 59.336 1.00 0.00
C H
ATOM 10146 HB2 ASP C 213 -40.412 43.766 59.986 1.00 0.00
C H
ATOM 10147 CG ASP C 213 -39.003 44.871 61.154 1.00 0.00
C C
ATOM 10148 OD1 ASP C 213 -39.844 45.236 62.003 1.00 0.00
C O
ATOM 10149 OD2 ASP C 213 -37.897 45.428 61.018 1.00 0.00
C O
ATOM 10150 C ASP C 213 -39.718 41.233 60.139 1.00 0.00
C C
ATOM 10151 O ASP C 213 -40.848 40.843 60.461 1.00 0.00
C O

ATOM 10152 N ILE C 214 −39.049 40.704 59.113 1.00 0.00
C N
ATOM 10153 HN ILE C 214 −38.160 41.015 58.785 1.00 0.00
C H
ATOM 10154 CA ILE C 214 −39.602 39.579 58.365 1.00 0.00
C C
ATOM 10155 HA ILE C 214 −40.604 39.863 58.071 1.00 0.00
C H
ATOM 10156 CB ILE C 214 −38.735 39.273 57.132 1.00 0.00
C C
ATOM 10157 HB ILE C 214 −37.718 39.065 57.549 1.00 0.00
C H
ATOM 10158 CG2 ILE C 214 −39.336 38.130 56.338 1.00 0.00
C C
ATOM 10159 HG21 ILE C 214 −38.639 37.801 55.539 1.00 0.00
C H
ATOM 10160 HG22 ILE C 214 −39.534 37.255 56.993 1.00 0.00
C H
ATOM 10161 HG23 ILE C 214 −40.291 38.436 55.862 1.00 0.00
C H
ATOM 10162 CG1 ILE C 214 −38.606 40.513 56.250 1.00 0.00
C C
ATOM 10163 HG11 ILE C 214 −39.569 40.707 55.729 1.00 0.00
C H
ATOM 10164 HG12 ILE C 214 −38.398 41.396 56.898 1.00 0.00
C H
ATOM 10165 CD ILE C 214 −37.506 40.423 55.220 1.00 0.00
C C
ATOM 10166 HD1 ILE C 214 −37.428 41.376 54.654 1.00 0.00
C H
ATOM 10167 HD2 ILE C 214 −36.527 40.228 55.708 1.00 0.00
C H
ATOM 10168 HD3 ILE C 214 −37.706 39.608 54.493 1.00 0.00
C H
ATOM 10169 C ILE C 214 −39.736 38.363 59.269 1.00 0.00
C C
ATOM 10170 O ILE C 214 −40.755 37.664 59.249 1.00 0.00
C O
ATOM 10171 N LYS C 215 −38.711 38.098 60.081 1.00 0.00
C N
ATOM 10172 HN LYS C 215 −37.838 38.584 60.076 1.00 0.00
C H
ATOM 10173 CA LYS C 215 −38.817 37.030 61.067 1.00 0.00
C C
ATOM 10174 HA LYS C 215 −39.053 36.121 60.527 1.00 0.00
C H
ATOM 10175 CB LYS C 215 −37.500 36.874 61.831 1.00 0.00
C C
ATOM 10176 HB1 LYS C 215 −36.696 36.731 61.074 1.00 0.00
C H
ATOM 10177 HB2 LYS C 215 −37.270 37.829 62.354 1.00 0.00
C H
ATOM 10178 CG LYS C 215 −37.477 35.699 62.814 1.00 0.00
C C

ATOM 10179 HG1 LYS C 215   -38.130 34.885 62.424 1.00 0.00
C   H
ATOM 10180 HG2 LYS C 215   -36.440 35.295 62.871 1.00 0.00
C   H
ATOM 10181 CD LYS C 215   -37.966 36.113 64.198 1.00 0.00
C   C
ATOM 10182 HD1 LYS C 215   -37.742 37.195 64.340 1.00 0.00
C   H
ATOM 10183 HD2 LYS C 215   -39.073 35.995 64.224 1.00 0.00
C   H
ATOM 10184 CE LYS C 215   -37.335 35.299 65.300 1.00 0.00
C   C
ATOM 10185 HE1 LYS C 215   -37.545 34.216 65.154 1.00 0.00
C   H
ATOM 10186 HE2 LYS C 215   -36.232 35.444 65.311 1.00 0.00
C   H
ATOM 10187 NZ LYS C 215   -37.876 35.706 66.621 1.00 0.00
C   N
ATOM 10188 HZ1 LYS C 215   -37.437 35.140 67.376 1.00 0.00
C   H
ATOM 10189 HZ2 LYS C 215   -37.673 36.713 66.784 1.00 0.00
C   H
ATOM 10190 HZ3 LYS C 215   -38.905 35.559 66.634 1.00 0.00
C   H
ATOM 10191 C   LYS C 215   -39.956 37.288 62.038 1.00 0.00
C   C
ATOM 10192 O   LYS C 215   -40.656 36.352 62.428 1.00 0.00
C   O
ATOM 10193 N   ARG C 216   -40.119 38.535 62.480 1.00 0.00
C   N
ATOM 10194 HN ARG C 216   -39.529 39.290 62.203 1.00 0.00
C   H
ATOM 10195 CA ARG C 216   -41.195 38.852 63.410 1.00 0.00
C   C
ATOM 10196 HA ARG C 216   -41.087 38.228 64.288 1.00 0.00
C   H
ATOM 10197 CB ARG C 216   -41.127 40.328 63.800 1.00 0.00
C   C
ATOM 10198 HB1 ARG C 216   -41.109 40.906 62.845 1.00 0.00
C   H
ATOM 10199 HB2 ARG C 216   -42.069 40.619 64.316 1.00 0.00
C   H
ATOM 10200 CG ARG C 216   -39.901 40.679 64.619 1.00 0.00
C   C
ATOM 10201 HG1 ARG C 216   -40.069 40.350 65.668 1.00 0.00
C   H
ATOM 10202 HG2 ARG C 216   -39.037 40.079 64.252 1.00 0.00
C   H
ATOM 10203 CD ARG C 216   -39.555 42.152 64.510 1.00 0.00
C   C
ATOM 10204 HD1 ARG C 216   -39.823 42.522 63.494 1.00 0.00
C   H
ATOM 10205 HD2 ARG C 216   -40.104 42.759 65.267 1.00 0.00
C   H

ATOM 10206 NE ARG C 216   -38.131 42.379 64.742 1.00 0.00
C   N
ATOM 10207 HE ARG C 216   -37.654 41.568 65.100 1.00 0.00
C   H
ATOM 10208 CZ ARG C 216   -37.508 43.531 64.511 1.00 0.00
C   C
ATOM 10209 NH1 ARG C 216   -38.182 44.571 64.045 1.00 0.00
C   N
ATOM 10210 HH11 ARG C 216   -37.719 45.424 63.847 1.00 0.00
C   H
ATOM 10211 HH12 ARG C 216   -39.135 44.402 63.782 1.00 0.00
C   H
ATOM 10212 NH2 ARG C 216   -36.208 43.643 64.749 1.00 0.00
C   N
ATOM 10213 HH21 ARG C 216   -35.765 44.523 64.658 1.00 0.00
C   H
ATOM 10214 HH22 ARG C 216   -35.770 42.884 65.211 1.00 0.00
C   H
ATOM 10215 C   ARG C 216   -42.550 38.518 62.804 1.00 0.00
C   C
ATOM 10216 O   ARG C 216   -43.381 37.861 63.443 1.00 0.00
C   O
ATOM 10217 N   PHE C 217   -42.782 38.960 61.569 1.00 0.00
C   N
ATOM 10218 HN PHE C 217   -42.153 39.511 61.024 1.00 0.00
C   H
ATOM 10219 CA PHE C 217   -44.041 38.646 60.904 1.00 0.00
C   C
ATOM 10220 HA PHE C 217   -44.827 38.965 61.577 1.00 0.00
C   H
ATOM 10221 CB PHE C 217   -44.130 39.373 59.565 1.00 0.00
C   C
ATOM 10222 HB1 PHE C 217   -44.537 40.394 59.732 1.00 0.00
C   H
ATOM 10223 HB2 PHE C 217   -43.125 39.491 59.106 1.00 0.00
C   H
ATOM 10224 CG PHE C 217   -45.049 38.710 58.586 1.00 0.00
C   C
ATOM 10225 CD1 PHE C 217   -46.418 38.740 58.776 1.00 0.00
C   C
ATOM 10226 HD1 PHE C 217   -46.828 39.244 59.640 1.00 0.00
C   H
ATOM 10227 CE1 PHE C 217   -47.268 38.129 57.878 1.00 0.00
C   C
ATOM 10228 HE1 PHE C 217   -48.336 38.164 58.036 1.00 0.00
C   H
ATOM 10229 CZ PHE C 217   -46.752 37.480 56.778 1.00 0.00
C   C
ATOM 10230 HZ PHE C 217   -47.415 37.008 56.066 1.00 0.00
C   H
ATOM 10231 CD2 PHE C 217   -44.545 38.055 57.478 1.00 0.00
C   C
ATOM 10232 HD2 PHE C 217   -43.477 38.001 57.322 1.00 0.00
C   H

ATOM 10233 CE2 PHE C 217 -45.390 37.444 56.578 1.00 0.00
C C
ATOM 10234 HE2 PHE C 217 -44.982 36.936 55.716 1.00 0.00
C H
ATOM 10235 C PHE C 217 -44.200 37.145 60.712 1.00 0.00
C C
ATOM 10236 O PHE C 217 -45.300 36.608 60.871 1.00 0.00
C O
ATOM 10237 N GLU C 218 -43.110 36.451 60.382 1.00 0.00
C N
ATOM 10238 HN GLU C 218 -42.206 36.838 60.219 1.00 0.00
C H
ATOM 10239 CA GLU C 218 -43.170 35.002 60.232 1.00 0.00
C C
ATOM 10240 HA GLU C 218 -43.935 34.772 59.500 1.00 0.00
C H
ATOM 10241 CB GLU C 218 -41.818 34.475 59.755 1.00 0.00
C C
ATOM 10242 HB1 GLU C 218 -41.395 35.209 59.029 1.00 0.00
C H
ATOM 10243 HB2 GLU C 218 -41.103 34.444 60.607 1.00 0.00
C H
ATOM 10244 CG GLU C 218 -41.872 33.114 59.089 1.00 0.00
C C
ATOM 10245 HG1 GLU C 218 -42.876 32.955 58.656 1.00 0.00
C H
ATOM 10246 HG2 GLU C 218 -41.112 33.033 58.287 1.00 0.00
C H
ATOM 10247 CD GLU C 218 -41.660 31.989 60.084 1.00 0.00
C C
ATOM 10248 OE1 GLU C 218 -41.303 32.285 61.244 1.00 0.00
C O
ATOM 10249 OE2 GLU C 218 -41.841 30.812 59.713 1.00 0.00
C O
ATOM 10250 C GLU C 218 -43.572 34.325 61.533 1.00 0.00
C C
ATOM 10251 O GLU C 218 -44.392 33.400 61.537 1.00 0.00
C O
ATOM 10252 N ASN C 219 -43.007 34.776 62.647 1.00 0.00
C N
ATOM 10253 HN ASN C 219 -42.395 35.565 62.697 1.00 0.00
C H
ATOM 10254 CA ASN C 219 -43.254 34.122 63.924 1.00 0.00
C C
ATOM 10255 HA ASN C 219 -43.194 33.056 63.730 1.00 0.00
C H
ATOM 10256 CB ASN C 219 -42.207 34.566 64.944 1.00 0.00
C C
ATOM 10257 HB1 ASN C 219 -41.256 34.750 64.399 1.00 0.00
C H
ATOM 10258 HB2 ASN C 219 -42.510 35.523 65.416 1.00 0.00
C H
ATOM 10259 CG ASN C 219 -41.946 33.518 66.005 1.00 0.00
C C

ATOM 10260 OD1 ASN C 219 −42.584 32.466 66.025 1.00 0.00
C O
ATOM 10261 ND2 ASN C 219 −41.003 33.801 66.896 1.00 0.00
C N
ATOM 10262 HD21 ASN C 219 −40.811 33.115 67.594 1.00 0.00
C H
ATOM 10263 HD22 ASN C 219 −40.498 34.656 66.833 1.00 0.00
C H
ATOM 10264 C ASN C 219 −44.658 34.420 64.425 1.00 0.00
C C
ATOM 10265 O ASN C 219 −45.332 33.542 64.976 1.00 0.00
C O
ATOM 10266 N GLU C 220 −45.118 35.655 64.244 1.00 0.00
C N
ATOM 10267 HN GLU C 220 −44.660 36.369 63.722 1.00 0.00
C H
ATOM 10268 CA GLU C 220 −46.389 36.069 64.824 1.00 0.00
C C
ATOM 10269 HA GLU C 220 −46.563 35.448 65.694 1.00 0.00
C H
ATOM 10270 CB GLU C 220 −46.288 37.531 65.267 1.00 0.00
C C
ATOM 10271 HB1 GLU C 220 −46.230 38.167 64.354 1.00 0.00
C H
ATOM 10272 HB2 GLU C 220 −47.224 37.832 65.791 1.00 0.00
C H
ATOM 10273 CG GLU C 220 −45.071 37.786 66.150 1.00 0.00
C C
ATOM 10274 HG1 GLU C 220 −45.371 37.741 67.213 1.00 0.00
C H
ATOM 10275 HG2 GLU C 220 −44.279 37.035 65.962 1.00 0.00
C H
ATOM 10276 CD GLU C 220 −44.470 39.164 65.959 1.00 0.00
C C
ATOM 10277 OE1 GLU C 220 −44.677 39.763 64.883 1.00 0.00
C O
ATOM 10278 OE2 GLU C 220 −43.771 39.639 66.877 1.00 0.00
C O
ATOM 10279 C GLU C 220 −47.571 35.865 63.879 1.00 0.00
C C
ATOM 10280 O GLU C 220 −48.524 35.159 64.231 1.00 0.00
C O
ATOM 10281 N GLY C 221 −47.534 36.456 62.686 1.00 0.00
C N
ATOM 10282 HN GLY C 221 −46.760 37.011 62.382 1.00 0.00
C H
ATOM 10283 CA GLY C 221 −48.626 36.329 61.741 1.00 0.00
C C
ATOM 10284 HA1 GLY C 221 −48.431 37.040 60.950 1.00 0.00
C H
ATOM 10285 HA2 GLY C 221 −49.537 36.516 62.291 1.00 0.00
C H
ATOM 10286 C GLY C 221 −48.731 34.952 61.118 1.00 0.00
C C

ATOM 10287 O   GLY C 221   -49.769 34.593 60.556 1.00 0.00
C   O
ATOM 10288 N   MET C 222   -47.656 34.177 61.200 1.00 0.00
C   N
ATOM 10289 HN MET C 222   -46.796 34.457 61.620 1.00 0.00
C   H
ATOM 10290 CA MET C 222   -47.652 32.822 60.667 1.00 0.00
C   C
ATOM 10291 HA MET C 222   -48.681 32.554 60.459 1.00 0.00
C   H
ATOM 10292 CB MET C 222   -46.813 32.747 59.394 1.00 0.00
C   C
ATOM 10293 HB1 MET C 222   -45.761 32.978 59.685 1.00 0.00
C   H
ATOM 10294 HB2 MET C 222   -46.809 31.700 59.017 1.00 0.00
C   H
ATOM 10295 CG MET C 222   -47.246 33.709 58.307 1.00 0.00
C   C
ATOM 10296 HG1 MET C 222   -48.341 33.629 58.134 1.00 0.00
C   H
ATOM 10297 HG2 MET C 222   -47.031 34.753 58.625 1.00 0.00
C   H
ATOM 10298 SD MET C 222   -46.404 33.382 56.749 1.00 0.00
C   S
ATOM 10299 CE MET C 222   -44.758 33.977 57.113 1.00 0.00
C   C
ATOM 10300 HE1 MET C 222   -44.090 33.858 56.234 1.00 0.00
C   H
ATOM 10301 HE2 MET C 222   -44.774 35.054 57.386 1.00 0.00
C   H
ATOM 10302 HE3 MET C 222   -44.308 33.416 57.961 1.00 0.00
C   H
ATOM 10303 C   MET C 222   -47.122 31.838 61.701 1.00 0.00
C   C
ATOM 10304 O   MET C 222   -47.763 30.832 61.995 1.00 0.00
C   O
ATOM 10305 N   ASN D   6   -37.128 22.031 71.321 1.00 0.00
D   N
ATOM 10306 HT1 ASN D   6   -37.119 22.803 71.956 1.00 0.00
D   H
ATOM 10307 HT2 ASN D   6   -36.744 21.221 71.763 1.00 0.00
D   H
ATOM 10308 HT3 ASN D   6   -38.063 21.844 71.022 1.00 0.00
D   H
ATOM 10309 CA ASN D   6   -36.317 22.365 70.158 1.00 0.00
D   C
ATOM 10310 HA ASN D   6   -36.811 23.207 69.686 1.00 0.00
D   H
ATOM 10311 CB ASN D   6   -34.900 22.726 70.587 1.00 0.00
D   C
ATOM 10312 HB1 ASN D   6   -34.691 22.203 71.545 1.00 0.00
D   H
ATOM 10313 HB2 ASN D   6   -34.167 22.360 69.840 1.00 0.00
D   H

ATOM	10314	CG	ASN	D	6	−34.725	24.211	70.812	1.00	0.00	D	C
ATOM	10315	OD1	ASN	D	6	−35.322	25.029	70.114	1.00	0.00	D	O
ATOM	10316	ND2	ASN	D	6	−33.896	24.569	71.786	1.00	0.00	D	N
ATOM	10317	HD21	ASN	D	6	−33.781	25.545	71.952	1.00	0.00	D	H
ATOM	10318	HD22	ASN	D	6	−33.443	23.874	72.335	1.00	0.00	D	H
ATOM	10319	C	ASN	D	6	−36.294	21.218	69.151	1.00	0.00	D	C
ATOM	10320	O	ASN	D	6	−37.216	20.410	69.113	1.00	0.00	D	O
ATOM	10321	N	LYS	D	7	−35.237	21.143	68.347	1.00	0.00	D	N
ATOM	10322	HN	LYS	D	7	−34.411	21.692	68.467	1.00	0.00	D	H
ATOM	10323	CA	LYS	D	7	−35.214	20.231	67.209	1.00	0.00	D	C
ATOM	10324	HA	LYS	D	7	−35.849	19.389	67.455	1.00	0.00	D	H
ATOM	10325	CB	LYS	D	7	−35.719	20.940	65.954	1.00	0.00	D	C
ATOM	10326	HB1	LYS	D	7	−35.552	20.247	65.096	1.00	0.00	D	H
ATOM	10327	HB2	LYS	D	7	−36.820	21.077	66.034	1.00	0.00	D	H
ATOM	10328	CG	LYS	D	7	−35.023	22.259	65.679	1.00	0.00	D	C
ATOM	10329	HG1	LYS	D	7	−35.126	22.919	66.570	1.00	0.00	D	H
ATOM	10330	HG2	LYS	D	7	−33.934	22.070	65.530	1.00	0.00	D	H
ATOM	10331	CD	LYS	D	7	−35.610	22.964	64.468	1.00	0.00	D	C
ATOM	10332	HD1	LYS	D	7	−34.884	23.735	64.124	1.00	0.00	D	H
ATOM	10333	HD2	LYS	D	7	−35.715	22.215	63.648	1.00	0.00	D	H
ATOM	10334	CE	LYS	D	7	−36.955	23.594	64.782	1.00	0.00	D	C
ATOM	10335	HE1	LYS	D	7	−37.720	22.808	64.971	1.00	0.00	D	H
ATOM	10336	HE2	LYS	D	7	−36.883	24.239	65.686	1.00	0.00	D	H
ATOM	10337	NZ	LYS	D	7	−37.444	24.441	63.657	1.00	0.00	D	N
ATOM	10338	HZ1	LYS	D	7	−38.366	24.858	63.899	1.00	0.00	D	H
ATOM	10339	HZ2	LYS	D	7	−36.759	25.202	63.472	1.00	0.00	D	H
ATOM	10340	HZ3	LYS	D	7	−37.544	23.856	62.802	1.00	0.00	D	H

```
ATOM  10341  C   LYS D   7      -33.807  19.690  66.987  1.00  0.00
D    C
ATOM  10342  O   LYS D   7      -32.831  20.439  67.020  1.00  0.00
D    O
ATOM  10343  N   GLN D   8      -33.705  18.390  66.728  1.00  0.00
D    N
ATOM  10344  HN  GLN D   8      -34.482  17.808  66.493  1.00  0.00
D    H
ATOM  10345  CA  GLN D   8      -32.426  17.697  66.768  1.00  0.00
D    C
ATOM  10346  HA  GLN D   8      -31.698  18.405  67.146  1.00  0.00
D    H
ATOM  10347  CB  GLN D   8      -32.502  16.498  67.702  1.00  0.00
D    C
ATOM  10348  HB1 GLN D   8      -33.479  15.998  67.505  1.00  0.00
D    H
ATOM  10349  HB2 GLN D   8      -31.715  15.761  67.422  1.00  0.00
D    H
ATOM  10350  CG  GLN D   8      -32.400  16.928  69.145  1.00  0.00
D    C
ATOM  10351  HG1 GLN D   8      -31.335  16.992  69.455  1.00  0.00
D    H
ATOM  10352  HG2 GLN D   8      -32.857  17.929  69.290  1.00  0.00
D    H
ATOM  10353  CD  GLN D   8      -33.056  15.961  70.091  1.00  0.00
D    C
ATOM  10354  OE1 GLN D   8      -33.899  15.162  69.690  1.00  0.00
D    O
ATOM  10355  NE2 GLN D   8      -32.680  16.029  71.362  1.00  0.00
D    N
ATOM  10356 HE21 GLN D   8      -33.107  15.387  71.993  1.00  0.00
D    H
ATOM  10357 HE22 GLN D   8      -32.013  16.710  71.644  1.00  0.00
D    H
ATOM  10358  C   GLN D   8      -31.975  17.277  65.378  1.00  0.00
D    C
ATOM  10359  O   GLN D   8      -32.753  16.715  64.593  1.00  0.00
D    O
ATOM  10360  N   PHE D   9      -30.705  17.544  65.097  1.00  0.00
D    N
ATOM  10361  HN  PHE D   9      -30.075  17.866  65.802  1.00  0.00
D    H
ATOM  10362  CA  PHE D   9      -30.112  17.402  63.781  1.00  0.00
D    C
ATOM  10363  HA  PHE D   9      -30.856  16.932  63.148  1.00  0.00
D    H
ATOM  10364  CB  PHE D   9      -29.643  18.748  63.262  1.00  0.00
D    C
ATOM  10365  HB1 PHE D   9      -28.850  19.141  63.935  1.00  0.00
D    H
ATOM  10366  HB2 PHE D   9      -29.196  18.653  62.249  1.00  0.00
D    H
ATOM  10367  CG  PHE D   9      -30.714  19.776  63.237  1.00  0.00
D    C
```

ATOM 10368 CD1 PHE D 9 -31.576 19.855 62.166 1.00 0.00
D C
ATOM 10369 HD1 PHE D 9 -31.463 19.170 61.337 1.00 0.00
D H
ATOM 10370 CE1 PHE D 9 -32.571 20.795 62.133 1.00 0.00
D C
ATOM 10371 HE1 PHE D 9 -33.243 20.842 61.289 1.00 0.00
D H
ATOM 10372 CZ PHE D 9 -32.715 21.676 63.183 1.00 0.00
D C
ATOM 10373 HZ PHE D 9 -33.502 22.417 63.161 1.00 0.00
D H
ATOM 10374 CD2 PHE D 9 -30.871 20.658 64.291 1.00 0.00
D C
ATOM 10375 HD2 PHE D 9 -30.196 20.619 65.134 1.00 0.00
D H
ATOM 10376 CE2 PHE D 9 -31.864 21.606 64.267 1.00 0.00
D C
ATOM 10377 HE2 PHE D 9 -31.974 22.293 65.095 1.00 0.00
D H
ATOM 10378 C PHE D 9 -28.929 16.456 63.855 1.00 0.00
D C
ATOM 10379 O PHE D 9 -28.093 16.559 64.758 1.00 0.00
D O
ATOM 10380 N ALA D 10 -28.855 15.546 62.891 1.00 0.00
D N
ATOM 10381 HN ALA D 10 -29.545 15.471 62.171 1.00 0.00
D H
ATOM 10382 CA ALA D 10 -27.762 14.587 62.812 1.00 0.00
D C
ATOM 10383 HA ALA D 10 -26.998 14.789 63.554 1.00 0.00
D H
ATOM 10384 CB ALA D 10 -28.266 13.160 63.030 1.00 0.00
D C
ATOM 10385 HB1 ALA D 10 -28.710 13.073 64.045 1.00 0.00
D H
ATOM 10386 HB2 ALA D 10 -29.053 12.896 62.290 1.00 0.00
D H
ATOM 10387 HB3 ALA D 10 -27.443 12.419 62.950 1.00 0.00
D H
ATOM 10388 C ALA D 10 -27.090 14.721 61.456 1.00 0.00
D C
ATOM 10389 O ALA D 10 -27.677 14.367 60.429 1.00 0.00
D O
ATOM 10390 N VAL D 11 -25.871 15.234 61.448 1.00 0.00
D N
ATOM 10391 HN VAL D 11 -25.419 15.600 62.257 1.00 0.00
D H
ATOM 10392 CA VAL D 11 -25.080 15.306 60.234 1.00 0.00
D C
ATOM 10393 HA VAL D 11 -25.740 15.353 59.377 1.00 0.00
D H
ATOM 10394 CB VAL D 11 -24.150 16.518 60.218 1.00 0.00
D C

```
ATOM  10395  HB  VAL D  11     -24.147  16.954  61.248  1.00  0.00
D    H
ATOM  10396  CG1 VAL D  11     -22.741  16.110  59.848  1.00  0.00
D    C
ATOM  10397 HG11 VAL D  11     -22.081  17.003  59.801  1.00  0.00
D    H
ATOM  10398 HG12 VAL D  11     -22.305  15.417  60.597  1.00  0.00
D    H
ATOM  10399 HG13 VAL D  11     -22.723  15.617  58.853  1.00  0.00
D    H
ATOM  10400  CG2 VAL D  11     -24.654  17.532  59.229  1.00  0.00
D    C
ATOM  10401 HG21 VAL D  11     -23.967  18.404  59.176  1.00  0.00
D    H
ATOM  10402 HG22 VAL D  11     -24.731  17.084  58.215  1.00  0.00
D    H
ATOM  10403 HG23 VAL D  11     -25.656  17.912  59.519  1.00  0.00
D    H
ATOM  10404  C   VAL D  11     -24.304  14.005  60.140  1.00  0.00
D    C
ATOM  10405  O   VAL D  11     -23.670  13.569  61.102  1.00  0.00
D    O
ATOM  10406  N   ILE D  12     -24.372  13.372  58.983  1.00  0.00
D    N
ATOM  10407  HN  ILE D  12     -24.983  13.644  58.243  1.00  0.00
D    H
ATOM  10408  CA  ILE D  12     -23.556  12.212  58.684  1.00  0.00
D    C
ATOM  10409  HA  ILE D  12     -22.819  12.109  59.471  1.00  0.00
D    H
ATOM  10410  CB  ILE D  12     -24.407  10.953  58.519  1.00  0.00
D    C
ATOM  10411  HB  ILE D  12     -25.070  11.176  57.645  1.00  0.00
D    H
ATOM  10412  CG2 ILE D  12     -23.511   9.764  58.396  1.00  0.00
D    C
ATOM  10413 HG21 ILE D  12     -24.095   8.862  58.120  1.00  0.00
D    H
ATOM  10414 HG22 ILE D  12     -22.746   9.924  57.607  1.00  0.00
D    H
ATOM  10415 HG23 ILE D  12     -22.989   9.559  59.354  1.00  0.00
D    H
ATOM  10416  CG1 ILE D  12     -25.314  10.768  59.724  1.00  0.00
D    C
ATOM  10417 HG11 ILE D  12     -26.019  11.625  59.800  1.00  0.00
D    H
ATOM  10418 HG12 ILE D  12     -25.926   9.849  59.576  1.00  0.00
D    H
ATOM  10419  CD  ILE D  12     -24.569  10.632  61.002  1.00  0.00
D    C
ATOM  10420  HD1 ILE D  12     -25.274  10.441  61.840  1.00  0.00
D    H
ATOM  10421  HD2 ILE D  12     -23.853   9.785  60.951  1.00  0.00
D    H
```

```
ATOM  10422  HD3 ILE D  12     -24.003  11.559  61.234  1.00  0.00
D    H
ATOM  10423  C   ILE D  12     -22.795  12.516  57.410  1.00  0.00
D    C
ATOM  10424  O   ILE D  12     -23.371  13.007  56.434  1.00  0.00
D    O
ATOM  10425  N   GLY D  13     -21.500  12.259  57.434  1.00  0.00
D    N
ATOM  10426  HN  GLY D  13     -21.035  11.871  58.229  1.00  0.00
D    H
ATOM  10427  CA  GLY D  13     -20.662  12.525  56.292  1.00  0.00
D    C
ATOM  10428  HA1 GLY D  13     -21.272  12.556  55.400  1.00  0.00
D    H
ATOM  10429  HA2 GLY D  13     -19.858  11.803  56.255  1.00  0.00
D    H
ATOM  10430  C   GLY D  13     -20.053  13.889  56.488  1.00  0.00
D    C
ATOM  10431  O   GLY D  13     -20.718  14.906  56.276  1.00  0.00
D    O
ATOM  10432  N   LEU D  14     -18.797  13.931  56.902  1.00  0.00
D    N
ATOM  10433  HN  LEU D  14     -18.195  13.143  57.008  1.00  0.00
D    H
ATOM  10434  CA  LEU D  14     -18.183  15.204  57.248  1.00  0.00
D    C
ATOM  10435  HA  LEU D  14     -18.975  15.941  57.273  1.00  0.00
D    H
ATOM  10436  CB  LEU D  14     -17.523  15.104  58.609  1.00  0.00
D    C
ATOM  10437  HB1 LEU D  14     -17.523  14.027  58.897  1.00  0.00
D    H
ATOM  10438  HB2 LEU D  14     -16.455  15.397  58.516  1.00  0.00
D    H
ATOM  10439  CG  LEU D  14     -18.245  15.919  59.659  1.00  0.00
D    C
ATOM  10440  HG  LEU D  14     -19.159  16.371  59.202  1.00  0.00
D    H
ATOM  10441  CD1 LEU D  14     -18.652  15.025  60.799  1.00  0.00
D    C
ATOM  10442 HD11 LEU D  14     -19.142  15.616  61.602  1.00  0.00
D    H
ATOM  10443 HD12 LEU D  14     -19.367  14.249  60.451  1.00  0.00
D    H
ATOM  10444 HD13 LEU D  14     -17.766  14.517  61.235  1.00  0.00
D    H
ATOM  10445  CD2 LEU D  14     -17.346  17.053  60.130  1.00  0.00
D    C
ATOM  10446 HD21 LEU D  14     -17.871  17.673  60.889  1.00  0.00
D    H
ATOM  10447 HD22 LEU D  14     -16.414  16.671  60.595  1.00  0.00
D    H
ATOM  10448 HD23 LEU D  14     -17.068  17.709  59.278  1.00  0.00
D    H
```

ATOM 10449 C LEU D 14 -17.189 15.633 56.171 1.00 0.00
D C
ATOM 10450 O LEU D 14 -16.000 15.813 56.413 1.00 0.00
D O
ATOM 10451 N GLY D 15 -17.706 15.795 54.958 1.00 0.00
D N
ATOM 10452 HN GLY D 15 -18.637 15.523 54.717 1.00 0.00
D H
ATOM 10453 CA GLY D 15 -16.930 16.389 53.890 1.00 0.00
D C
ATOM 10454 HA1 GLY D 15 -17.351 16.073 52.946 1.00 0.00
D H
ATOM 10455 HA2 GLY D 15 -15.891 16.119 54.025 1.00 0.00
D H
ATOM 10456 C GLY D 15 -17.034 17.895 53.967 1.00 0.00
D C
ATOM 10457 O GLY D 15 -17.775 18.406 54.813 1.00 0.00
D O
ATOM 10458 N ARG D 16 -16.302 18.614 53.107 1.00 0.00
D N
ATOM 10459 HN ARG D 16 -15.667 18.199 52.460 1.00 0.00
D H
ATOM 10460 CA ARG D 16 -16.406 20.067 53.082 1.00 0.00
D C
ATOM 10461 HA ARG D 16 -15.906 20.460 53.959 1.00 0.00
D H
ATOM 10462 CB ARG D 16 -15.783 20.630 51.809 1.00 0.00
D C
ATOM 10463 HB1 ARG D 16 -16.381 20.222 50.959 1.00 0.00
D H
ATOM 10464 HB2 ARG D 16 -15.924 21.733 51.782 1.00 0.00
D H
ATOM 10465 CG ARG D 16 -14.343 20.257 51.610 1.00 0.00
D C
ATOM 10466 HG1 ARG D 16 -13.710 20.976 52.175 1.00 0.00
D H
ATOM 10467 HG2 ARG D 16 -14.159 19.263 52.078 1.00 0.00
D H
ATOM 10468 CD ARG D 16 -13.999 20.184 50.135 1.00 0.00
D C
ATOM 10469 HD1 ARG D 16 -14.425 21.071 49.612 1.00 0.00
D H
ATOM 10470 HD2 ARG D 16 -12.896 20.169 49.973 1.00 0.00
D H
ATOM 10471 NE ARG D 16 -14.520 18.976 49.504 1.00 0.00
D N
ATOM 10472 HE ARG D 16 -15.095 18.424 50.119 1.00 0.00
D H
ATOM 10473 CZ ARG D 16 -14.271 18.626 48.246 1.00 0.00
D C
ATOM 10474 NH1 ARG D 16 -13.501 19.393 47.488 1.00 0.00
D N
ATOM 10475 HH11 ARG D 16 -13.329 19.156 46.542 1.00 0.00
D H

ATOM 10476 HH12 ARG D 16 −13.228 20.274 47.880 1.00 0.00
D H
ATOM 10477 NH2 ARG D 16 −14.780 17.508 47.749 1.00 0.00
D N
ATOM 10478 HH21 ARG D 16 −14.521 17.208 46.842 1.00 0.00
D H
ATOM 10479 HH22 ARG D 16 −15.240 16.903 48.386 1.00 0.00
D H
ATOM 10480 C ARG D 16 −17.849 20.515 53.168 1.00 0.00
D C
ATOM 10481 O ARG D 16 −18.175 21.418 53.948 1.00 0.00
D O
ATOM 10482 N PHE D 17 −18.723 19.862 52.414 1.00 0.00
D N
ATOM 10483 HN PHE D 17 −18.485 19.164 51.741 1.00 0.00
D H
ATOM 10484 CA PHE D 17 −20.143 20.134 52.524 1.00 0.00
D C
ATOM 10485 HA PHE D 17 −20.278 21.178 52.268 1.00 0.00
D H
ATOM 10486 CB PHE D 17 −20.910 19.212 51.585 1.00 0.00
D C
ATOM 10487 HB1 PHE D 17 −20.539 19.359 50.547 1.00 0.00
D H
ATOM 10488 HB2 PHE D 17 −20.742 18.145 51.846 1.00 0.00
D H
ATOM 10489 CG PHE D 17 −22.370 19.485 51.545 1.00 0.00
D C
ATOM 10490 CD1 PHE D 17 −22.832 20.768 51.329 1.00 0.00
D C
ATOM 10491 HD1 PHE D 17 −22.126 21.577 51.199 1.00 0.00
D H
ATOM 10492 CE1 PHE D 17 −24.176 21.030 51.283 1.00 0.00
D C
ATOM 10493 HE1 PHE D 17 −24.525 22.038 51.111 1.00 0.00
D H
ATOM 10494 CZ PHE D 17 −25.077 20.003 51.448 1.00 0.00
D C
ATOM 10495 HZ PHE D 17 −26.137 20.203 51.404 1.00 0.00
D H
ATOM 10496 CD2 PHE D 17 −23.282 18.462 51.707 1.00 0.00
D C
ATOM 10497 HD2 PHE D 17 −22.938 17.455 51.895 1.00 0.00
D H
ATOM 10498 CE2 PHE D 17 −24.627 18.717 51.661 1.00 0.00
D C
ATOM 10499 HE2 PHE D 17 −25.333 17.909 51.791 1.00 0.00
D H
ATOM 10500 C PHE D 17 −20.608 19.963 53.960 1.00 0.00
D C
ATOM 10501 O PHE D 17 −21.141 20.900 54.565 1.00 0.00
D O
ATOM 10502 N GLY D 18 −20.395 18.776 54.526 1.00 0.00
D N

ATOM 10503 HN GLY D 18 −19.943 18.006 54.078 1.00 0.00
D H
ATOM 10504 CA GLY D 18 −20.832 18.540 55.890 1.00 0.00
D C
ATOM 10505 HA1 GLY D 18 −20.432 17.581 56.191 1.00 0.00
D H
ATOM 10506 HA2 GLY D 18 −21.912 18.592 55.896 1.00 0.00
D H
ATOM 10507 C GLY D 18 −20.309 19.587 56.851 1.00 0.00
D C
ATOM 10508 O GLY D 18 −21.085 20.289 57.511 1.00 0.00
D O
ATOM 10509 N GLY D 19 −18.986 19.741 56.898 1.00 0.00
D N
ATOM 10510 HN GLY D 19 −18.352 19.250 56.302 1.00 0.00
D H
ATOM 10511 CA GLY D 19 −18.377 20.655 57.846 1.00 0.00
D C
ATOM 10512 HA1 GLY D 19 −17.326 20.707 57.596 1.00 0.00
D H
ATOM 10513 HA2 GLY D 19 −18.580 20.265 58.834 1.00 0.00
D H
ATOM 10514 C GLY D 19 −18.944 22.054 57.773 1.00 0.00
D C
ATOM 10515 O GLY D 19 −19.151 22.701 58.802 1.00 0.00
D O
ATOM 10516 N SER D 20 −19.211 22.534 56.565 1.00 0.00
D N
ATOM 10517 HN SER D 20 −18.994 22.073 55.705 1.00 0.00
D H
ATOM 10518 CA SER D 20 −19.864 23.818 56.402 1.00 0.00
D C
ATOM 10519 HA SER D 20 −19.196 24.551 56.839 1.00 0.00
D H
ATOM 10520 CB SER D 20 −20.169 24.039 54.931 1.00 0.00
D C
ATOM 10521 HB1 SER D 20 −20.790 23.185 54.575 1.00 0.00
D H
ATOM 10522 HB2 SER D 20 −20.775 24.963 54.795 1.00 0.00
D H
ATOM 10523 OG SER D 20 −18.973 24.083 54.201 1.00 0.00
D O
ATOM 10524 HG1 SER D 20 −19.208 23.998 53.268 1.00 0.00
D H
ATOM 10525 C SER D 20 −21.161 23.875 57.177 1.00 0.00
D C
ATOM 10526 O SER D 20 −21.352 24.762 58.019 1.00 0.00
D O
ATOM 10527 N ILE D 21 −22.045 22.930 56.878 1.00 0.00
D N
ATOM 10528 HN ILE D 21 −21.887 22.205 56.209 1.00 0.00
D H
ATOM 10529 CA ILE D 21 −23.346 22.875 57.517 1.00 0.00
D C

ATOM 10530 HA ILE D 21   -23.872 23.777 57.230 1.00 0.00
D   H
ATOM 10531 CB ILE D 21   -24.077 21.602 57.079 1.00 0.00
D   C
ATOM 10532 HB ILE D 21   -23.489 20.769 57.540 1.00 0.00
D   H
ATOM 10533 CG2 ILE D 21   -25.510 21.649 57.507 1.00 0.00
D   C
ATOM 10534 HG21 ILE D 21   -26.003 20.670 57.332 1.00 0.00
D   H
ATOM 10535 HG22 ILE D 21   -25.593 21.878 58.590 1.00 0.00
D   H
ATOM 10536 HG23 ILE D 21   -26.069 22.424 56.942 1.00 0.00
D   H
ATOM 10537 CG1 ILE D 21   -23.964 21.454 55.570 1.00 0.00
D   C
ATOM 10538 HG11 ILE D 21   -24.460 22.315 55.069 1.00 0.00
D   H
ATOM 10539 HG12 ILE D 21   -22.888 21.482 55.285 1.00 0.00
D   H
ATOM 10540 CD ILE D 21   -24.549 20.184 55.037 1.00 0.00
D   C
ATOM 10541 HD1 ILE D 21   -24.381 20.112 53.941 1.00 0.00
D   H
ATOM 10542 HD2 ILE D 21   -24.075 19.301 55.518 1.00 0.00
D   H
ATOM 10543 HD3 ILE D 21   -25.643 20.140 55.221 1.00 0.00
D   H
ATOM 10544 C   ILE D 21   -23.195 22.917 59.022 1.00 0.00
D   C
ATOM 10545 O   ILE D 21   -23.815 23.741 59.706 1.00 0.00
D   O
ATOM 10546 N   VAL D 22   -22.382 22.015 59.563 1.00 0.00
D   N
ATOM 10547 HN VAL D 22   -21.764 21.400 59.082 1.00 0.00
D   H
ATOM 10548 CA VAL D 22   -22.361 21.877 61.011 1.00 0.00
D   C
ATOM 10549 HA VAL D 22   -23.388 21.822 61.349 1.00 0.00
D   H
ATOM 10550 CB VAL D 22   -21.633 20.592 61.426 1.00 0.00
D   C
ATOM 10551 HB VAL D 22   -21.243 20.744 62.462 1.00 0.00
D   H
ATOM 10552 CG1 VAL D 22   -22.586 19.434 61.420 1.00 0.00
D   C
ATOM 10553 HG11 VAL D 22   -22.053 18.495 61.682 1.00 0.00
D   H
ATOM 10554 HG12 VAL D 22   -23.401 19.570 62.160 1.00 0.00
D   H
ATOM 10555 HG13 VAL D 22   -23.037 19.304 60.413 1.00 0.00
D   H
ATOM 10556 CG2 VAL D 22   -20.492 20.331 60.520 1.00 0.00
D   C

```
ATOM  10557 HG21 VAL D  22      -19.985  19.381  60.790  1.00  0.00
D    H
ATOM  10558 HG22 VAL D  22      -20.840  20.255  59.467  1.00  0.00
D    H
ATOM  10559 HG23 VAL D  22      -19.738  21.142  60.580  1.00  0.00
D    H
ATOM  10560  C   VAL D  22      -21.734  23.096  61.661  1.00  0.00
D    C
ATOM  10561  O   VAL D  22      -22.214  23.573  62.690  1.00  0.00
D    O
ATOM  10562  N   LYS D  23      -20.650  23.614  61.089  1.00  0.00
D    N
ATOM  10563  HN  LYS D  23      -20.232  23.295  60.240  1.00  0.00
D    H
ATOM  10564  CA  LYS D  23      -19.985  24.742  61.718  1.00  0.00
D    C
ATOM  10565  HA  LYS D  23      -19.813  24.481  62.755  1.00  0.00
D    H
ATOM  10566  CB  LYS D  23      -18.661  25.022  61.018  1.00  0.00
D    C
ATOM  10567  HB1 LYS D  23      -18.168  24.037  60.842  1.00  0.00
D    H
ATOM  10568  HB2 LYS D  23      -18.868  25.452  60.013  1.00  0.00
D    H
ATOM  10569  CG  LYS D  23      -17.730  25.912  61.807  1.00  0.00
D    C
ATOM  10570  HG1 LYS D  23      -18.280  26.329  62.683  1.00  0.00
D    H
ATOM  10571  HG2 LYS D  23      -16.891  25.297  62.204  1.00  0.00
D    H
ATOM  10572  CD  LYS D  23      -17.196  27.055  60.971  1.00  0.00
D    C
ATOM  10573  HD1 LYS D  23      -17.438  26.852  59.903  1.00  0.00
D    H
ATOM  10574  HD2 LYS D  23      -17.738  27.983  61.266  1.00  0.00
D    H
ATOM  10575  CE  LYS D  23      -15.693  27.201  61.188  1.00  0.00
D    C
ATOM  10576  HE1 LYS D  23      -15.474  27.425  62.256  1.00  0.00
D    H
ATOM  10577  HE2 LYS D  23      -15.165  26.263  60.909  1.00  0.00
D    H
ATOM  10578  NZ  LYS D  23      -15.089  28.297  60.382  1.00  0.00
D    N
ATOM  10579  HZ1 LYS D  23      -14.067  28.354  60.569  1.00  0.00
D    H
ATOM  10580  HZ2 LYS D  23      -15.247  28.112  59.371  1.00  0.00
D    H
ATOM  10581  HZ3 LYS D  23      -15.537  29.201  60.637  1.00  0.00
D    H
ATOM  10582  C   LYS D  23      -20.886  25.960  61.700  1.00  0.00
D    C
ATOM  10583  O   LYS D  23      -20.943  26.712  62.680  1.00  0.00
D    O
```

ATOM 10584 N   GLU D 24   -21.643 26.139 60.618 1.00 0.00
D   N
ATOM 10585 HN GLU D 24   -21.625 25.594 59.785 1.00 0.00
D   H
ATOM 10586 CA GLU D 24   -22.614 27.223 60.594 1.00 0.00
D   C
ATOM 10587 HA GLU D 24   -22.102 28.148 60.830 1.00 0.00
D   H
ATOM 10588 CB GLU D 24   -23.259 27.297 59.210 1.00 0.00
D   C
ATOM 10589 HB1 GLU D 24   -22.446 27.347 58.449 1.00 0.00
D   H
ATOM 10590 HB2 GLU D 24   -23.815 26.355 59.002 1.00 0.00
D   H
ATOM 10591 CG GLU D 24   -24.195 28.474 58.961 1.00 0.00
D   C
ATOM 10592 HG1 GLU D 24   -23.630 29.419 59.046 1.00 0.00
D   H
ATOM 10593 HG2 GLU D 24   -24.652 28.411 57.953 1.00 0.00
D   H
ATOM 10594 CD GLU D 24   -25.326 28.608 59.963 1.00 0.00
D   C
ATOM 10595 OE1 GLU D 24   -26.116 27.661 60.137 1.00 0.00
D   O
ATOM 10596 OE2 GLU D 24   -25.401 29.674 60.600 1.00 0.00
D   O
ATOM 10597 C   GLU D 24   -23.677 27.009 61.664 1.00 0.00
D   C
ATOM 10598 O   GLU D 24   -23.915 27.880 62.507 1.00 0.00
D   O
ATOM 10599 N   LEU D 25   -24.360 25.868 61.613 1.00 0.00
D   N
ATOM 10600 HN LEU D 25   -24.182 25.123 60.974 1.00 0.00
D   H
ATOM 10601 CA LEU D 25   -25.464 25.620 62.530 1.00 0.00
D   C
ATOM 10602 HA LEU D 25   -26.197 26.397 62.353 1.00 0.00
D   H
ATOM 10603 CB LEU D 25   -26.044 24.241 62.244 1.00 0.00
D   C
ATOM 10604 HB1 LEU D 25   -25.431 23.788 61.431 1.00 0.00
D   H
ATOM 10605 HB2 LEU D 25   -25.897 23.594 63.137 1.00 0.00
D   H
ATOM 10606 CG LEU D 25   -27.500 24.272 61.821 1.00 0.00
D   C
ATOM 10607 HG LEU D 25   -27.798 25.314 61.550 1.00 0.00
D   H
ATOM 10608 CD1 LEU D 25   -27.716 23.356 60.657 1.00 0.00
D   C
ATOM 10609 HD11 LEU D 25   -28.790 23.337 60.370 1.00 0.00
D   H
ATOM 10610 HD12 LEU D 25   -27.126 23.694 59.779 1.00 0.00
D   H

```
ATOM  10611 HD13 LEU D  25     -27.405  22.320  60.910  1.00  0.00
D    H
ATOM  10612  CD2 LEU D  25     -28.356  23.831  62.983  1.00  0.00
D    C
ATOM  10613 HD21 LEU D  25     -29.431  23.863  62.705  1.00  0.00
D    H
ATOM  10614 HD22 LEU D  25     -28.120  22.793  63.294  1.00  0.00
D    H
ATOM  10615 HD23 LEU D  25     -28.203  24.502  63.855  1.00  0.00
D    H
ATOM  10616  C   LEU D  25     -25.011  25.738  63.972  1.00  0.00
D    C
ATOM  10617  O   LEU D  25     -25.737  26.251  64.830  1.00  0.00
D    O
ATOM  10618  N   HSE D  26     -23.808  25.256  64.248  1.00  0.00
D    N
ATOM  10619  HN  HSE D  26     -23.272  24.718  63.599  1.00  0.00
D    H
ATOM  10620  CA  HSE D  26     -23.159  25.469  65.527  1.00  0.00
D    C
ATOM  10621  HA  HSE D  26     -23.798  25.035  66.286  1.00  0.00
D    H
ATOM  10622  CB  HSE D  26     -21.783  24.822  65.474  1.00  0.00
D    C
ATOM  10623  HB1 HSE D  26     -21.915  23.762  65.165  1.00  0.00
D    H
ATOM  10624  HB2 HSE D  26     -21.171  25.315  64.690  1.00  0.00
D    H
ATOM  10625  ND1 HSE D  26     -20.063  25.742  67.055  1.00  0.00
D    N
ATOM  10626  CG  HSE D  26     -21.049  24.826  66.770  1.00  0.00
D    C
ATOM  10627  CE1 HSE D  26     -19.576  25.495  68.257  1.00  0.00
D    C
ATOM  10628  HE1 HSE D  26     -18.784  26.075  68.737  1.00  0.00
D    H
ATOM  10629  NE2 HSE D  26     -20.203  24.443  68.753  1.00  0.00
D    N
ATOM  10630  HE2 HSE D  26     -20.015  24.027  69.643  1.00  0.00
D    H
ATOM  10631  CD2 HSE D  26     -21.126  24.003  67.839  1.00  0.00
D    C
ATOM  10632  HD2 HSE D  26     -21.758  23.141  68.007  1.00  0.00
D    H
ATOM  10633  C   HSE D  26     -23.049  26.949  65.831  1.00  0.00
D    C
ATOM  10634  O   HSE D  26     -23.332  27.384  66.953  1.00  0.00
D    O
ATOM  10635  N   ARG D  27     -22.640  27.742  64.843  1.00  0.00
D    N
ATOM  10636  HN  ARG D  27     -22.319  27.410  63.958  1.00  0.00
D    H
ATOM  10637  CA  ARG D  27     -22.649  29.183  65.034  1.00  0.00
D    C
```

```
ATOM  10638  HA  ARG D  27      -22.064  29.416  65.916  1.00  0.00
D    H
ATOM  10639  CB  ARG D  27      -22.050  29.881  63.820  1.00  0.00
D    C
ATOM  10640  HB1 ARG D  27      -21.169  29.270  63.509  1.00  0.00
D    H
ATOM  10641  HB2 ARG D  27      -22.769  29.836  62.973  1.00  0.00
D    H
ATOM  10642  CG  ARG D  27      -21.598  31.291  64.100  1.00  0.00
D    C
ATOM  10643  HG1 ARG D  27      -21.933  31.574  65.122  1.00  0.00
D    H
ATOM  10644  HG2 ARG D  27      -20.485  31.315  64.132  1.00  0.00
D    H
ATOM  10645  CD  ARG D  27      -22.082  32.246  63.029  1.00  0.00
D    C
ATOM  10646  HD1 ARG D  27      -22.007  33.293  63.404  1.00  0.00
D    H
ATOM  10647  HD2 ARG D  27      -21.477  32.156  62.096  1.00  0.00
D    H
ATOM  10648  NE  ARG D  27      -23.465  31.995  62.636  1.00  0.00
D    N
ATOM  10649  HE  ARG D  27      -23.550  31.620  61.706  1.00  0.00
D    H
ATOM  10650  CZ  ARG D  27      -24.520  32.229  63.409  1.00  0.00
D    C
ATOM  10651  NH1 ARG D  27      -24.363  32.714  64.630  1.00  0.00
D    N
ATOM  10652 HH11 ARG D  27      -25.146  32.866  65.217  1.00  0.00
D    H
ATOM  10653 HH12 ARG D  27      -23.419  32.783  64.963  1.00  0.00
D    H
ATOM  10654  NH2 ARG D  27      -25.736  31.978  62.961  1.00  0.00
D    N
ATOM  10655 HH21 ARG D  27      -26.524  32.233  63.503  1.00  0.00
D    H
ATOM  10656 HH22 ARG D  27      -25.820  31.732  62.004  1.00  0.00
D    H
ATOM  10657  C   ARG D  27      -24.059  29.685  65.303  1.00  0.00
D    C
ATOM  10658  O   ARG D  27      -24.241  30.676  66.016  1.00  0.00
D    O
ATOM  10659  N   MET D  28      -25.074  29.016  64.755  1.00  0.00
D    N
ATOM  10660  HN  MET D  28      -25.012  28.263  64.104  1.00  0.00
D    H
ATOM  10661  CA  MET D  28      -26.435  29.389  65.115  1.00  0.00
D    C
ATOM  10662  HA  MET D  28      -26.469  30.471  65.109  1.00  0.00
D    H
ATOM  10663  CB  MET D  28      -27.452  28.825  64.131  1.00  0.00
D    C
ATOM  10664  HB1 MET D  28      -27.273  27.726  64.073  1.00  0.00
D    H
```

ATOM 10665 HB2 MET D 28 −28.476 28.953 64.546 1.00 0.00
D H
ATOM 10666 CG MET D 28 −27.361 29.402 62.765 1.00 0.00
D C
ATOM 10667 HG1 MET D 28 −27.387 30.512 62.811 1.00 0.00
D H
ATOM 10668 HG2 MET D 28 −26.394 29.109 62.299 1.00 0.00
D H
ATOM 10669 SD MET D 28 −28.692 28.873 61.694 1.00 0.00
D S
ATOM 10670 CE MET D 28 −30.029 29.823 62.406 1.00 0.00
D C
ATOM 10671 HE1 MET D 28 −30.984 29.624 61.874 1.00 0.00
D H
ATOM 10672 HE2 MET D 28 −30.175 29.564 63.477 1.00 0.00
D H
ATOM 10673 HE3 MET D 28 −29.823 30.913 62.342 1.00 0.00
D H
ATOM 10674 C MET D 28 −26.813 28.925 66.508 1.00 0.00
D C
ATOM 10675 O MET D 28 −27.935 29.192 66.944 1.00 0.00
D O
ATOM 10676 N GLY D 29 −25.920 28.233 67.203 1.00 0.00
D N
ATOM 10677 HN GLY D 29 −25.017 27.980 66.857 1.00 0.00
D H
ATOM 10678 CA GLY D 29 −26.219 27.792 68.546 1.00 0.00
D C
ATOM 10679 HA1 GLY D 29 −26.590 28.649 69.090 1.00 0.00
D H
ATOM 10680 HA2 GLY D 29 −25.314 27.363 68.957 1.00 0.00
D H
ATOM 10681 C GLY D 29 −27.289 26.728 68.564 1.00 0.00
D C
ATOM 10682 O GLY D 29 −28.324 26.888 69.215 1.00 0.00
D O
ATOM 10683 N HSE D 30 −27.065 25.644 67.839 1.00 0.00
D N
ATOM 10684 HN HSE D 30 −26.245 25.489 67.289 1.00 0.00
D H
ATOM 10685 CA HSE D 30 −28.030 24.565 67.790 1.00 0.00
D C
ATOM 10686 HA HSE D 30 −28.845 24.834 68.453 1.00 0.00
D H
ATOM 10687 CB HSE D 30 −28.538 24.367 66.370 1.00 0.00
D C
ATOM 10688 HB1 HSE D 30 −27.660 24.376 65.688 1.00 0.00
D H
ATOM 10689 HB2 HSE D 30 −29.008 23.365 66.281 1.00 0.00
D H
ATOM 10690 ND1 HSE D 30 −30.764 25.153 65.492 1.00 0.00
D N
ATOM 10691 CG HSE D 30 −29.484 25.430 65.921 1.00 0.00
D C

```
ATOM  10692  CE1 HSE D  30     -31.366  26.281  65.163  1.00  0.00
D    C
ATOM  10693  HE1 HSE D  30     -32.387  26.351  64.779  1.00  0.00
D    H
ATOM  10694  NE2 HSE D  30     -30.526  27.279  65.372  1.00  0.00
D    N
ATOM  10695  HE2 HSE D  30     -30.719  28.242  65.188  1.00  0.00
D    H
ATOM  10696  CD2 HSE D  30     -29.344  26.773  65.852  1.00  0.00
D    C
ATOM  10697  HD2 HSE D  30     -28.495  27.395  66.099  1.00  0.00
D    H
ATOM  10698  C   HSE D  30     -27.428  23.276  68.326  1.00  0.00
D    C
ATOM  10699  O   HSE D  30     -26.235  23.178  68.619  1.00  0.00
D    O
ATOM  10700  N   GLU D  31     -28.284  22.275  68.452  1.00  0.00
D    N
ATOM  10701  HN  GLU D  31     -29.252  22.366  68.234  1.00  0.00
D    H
ATOM  10702  CA  GLU D  31     -27.905  20.951  68.917  1.00  0.00
D    C
ATOM  10703  HA  GLU D  31     -26.980  21.012  69.478  1.00  0.00
D    H
ATOM  10704  CB  GLU D  31     -29.007  20.373  69.803  1.00  0.00
D    C
ATOM  10705  HB1 GLU D  31     -28.694  19.353  70.126  1.00  0.00
D    H
ATOM  10706  HB2 GLU D  31     -29.095  20.974  70.735  1.00  0.00
D    H
ATOM  10707  CG  GLU D  31     -30.393  20.258  69.156  1.00  0.00
D    C
ATOM  10708  HG1 GLU D  31     -30.291  19.842  68.137  1.00  0.00
D    H
ATOM  10709  HG2 GLU D  31     -31.057  19.602  69.753  1.00  0.00
D    H
ATOM  10710  CD  GLU D  31     -31.097  21.600  68.973  1.00  0.00
D    C
ATOM  10711  OE1 GLU D  31     -32.264  21.721  69.398  1.00  0.00
D    O
ATOM  10712  OE2 GLU D  31     -30.506  22.522  68.368  1.00  0.00
D    O
ATOM  10713  C   GLU D  31     -27.634  20.086  67.694  1.00  0.00
D    C
ATOM  10714  O   GLU D  31     -28.552  19.750  66.940  1.00  0.00
D    O
ATOM  10715  N   VAL D  32     -26.368  19.757  67.470  1.00  0.00
D    N
ATOM  10716  HN  VAL D  32     -25.620  19.993  68.084  1.00  0.00
D    H
ATOM  10717  CA  VAL D  32     -25.964  19.012  66.289  1.00  0.00
D    C
ATOM  10718  HA  VAL D  32     -26.841  18.535  65.871  1.00  0.00
D    H
```

```
ATOM  10719  CB  VAL D  32     -25.336  19.928  65.225  1.00  0.00
D    C
ATOM  10720  HB  VAL D  32     -24.963  19.276  64.396  1.00  0.00
D    H
ATOM  10721  CG1 VAL D  32     -26.379  20.884  64.687  1.00  0.00
D    C
ATOM  10722 HG11 VAL D  32     -25.923  21.576  63.946  1.00  0.00
D    H
ATOM  10723 HG12 VAL D  32     -27.203  20.345  64.174  1.00  0.00
D    H
ATOM  10724 HG13 VAL D  32     -26.811  21.496  65.507  1.00  0.00
D    H
ATOM  10725  CG2 VAL D  32     -24.185  20.714  65.811  1.00  0.00
D    C
ATOM  10726 HG21 VAL D  32     -23.755  21.405  65.054  1.00  0.00
D    H
ATOM  10727 HG22 VAL D  32     -24.526  21.316  66.680  1.00  0.00
D    H
ATOM  10728 HG23 VAL D  32     -23.370  20.041  66.151  1.00  0.00
D    H
ATOM  10729  C   VAL D  32     -25.002  17.912  66.702  1.00  0.00
D    C
ATOM  10730  O   VAL D  32     -23.976  18.175  67.341  1.00  0.00
D    O
ATOM  10731  N   LEU D  33     -25.341  16.683  66.350  1.00  0.00
D    N
ATOM  10732  HN  LEU D  33     -26.210  16.456  65.915  1.00  0.00
D    H
ATOM  10733  CA  LEU D  33     -24.468  15.545  66.571  1.00  0.00
D    C
ATOM  10734  HA  LEU D  33     -23.805  15.786  67.391  1.00  0.00
D    H
ATOM  10735  CB  LEU D  33     -25.267  14.282  66.866  1.00  0.00
D    C
ATOM  10736  HB1 LEU D  33     -25.609  14.350  67.925  1.00  0.00
D    H
ATOM  10737  HB2 LEU D  33     -26.189  14.283  66.244  1.00  0.00
D    H
ATOM  10738  CG  LEU D  33     -24.440  13.016  66.663  1.00  0.00
D    C
ATOM  10739  HG  LEU D  33     -24.077  13.025  65.607  1.00  0.00
D    H
ATOM  10740  CD1 LEU D  33     -23.246  12.992  67.610  1.00  0.00
D    C
ATOM  10741 HD11 LEU D  33     -22.674  12.047  67.491  1.00  0.00
D    H
ATOM  10742 HD12 LEU D  33     -22.562  13.842  67.399  1.00  0.00
D    H
ATOM  10743 HD13 LEU D  33     -23.582  13.065  68.666  1.00  0.00
D    H
ATOM  10744  CD2 LEU D  33     -25.305  11.786  66.849  1.00  0.00
D    C
ATOM  10745 HD21 LEU D  33     -24.706  10.864  66.683  1.00  0.00
D    H
```

```
ATOM  10746 HD22 LEU D  33      -25.726  11.733  67.874  1.00  0.00
D    H
ATOM  10747 HD23 LEU D  33      -26.147  11.789  66.125  1.00  0.00
D    H
ATOM  10748  C   LEU D  33      -23.612  15.351  65.331  1.00  0.00
D    C
ATOM  10749  O   LEU D  33      -24.140  15.163  64.230  1.00  0.00
D    O
ATOM  10750  N   ALA D  34      -22.302  15.395  65.508  1.00  0.00
D    N
ATOM  10751  HN  ALA D  34      -21.880  15.574  66.395  1.00  0.00
D    H
ATOM  10752  CA  ALA D  34      -21.377  15.184  64.409  1.00  0.00
D    C
ATOM  10753  HA  ALA D  34      -21.847  15.360  63.448  1.00  0.00
D    H
ATOM  10754  CB  ALA D  34      -20.169  16.106  64.532  1.00  0.00
D    C
ATOM  10755  HB1 ALA D  34      -20.501  17.165  64.476  1.00  0.00
D    H
ATOM  10756  HB2 ALA D  34      -19.652  15.960  65.506  1.00  0.00
D    H
ATOM  10757  HB3 ALA D  34      -19.437  15.933  63.713  1.00  0.00
D    H
ATOM  10758  C   ALA D  34      -20.946  13.729  64.414  1.00  0.00
D    C
ATOM  10759  O   ALA D  34      -20.600  13.185  65.467  1.00  0.00
D    O
ATOM  10760  N   VAL D  35      -20.986  13.097  63.246  1.00  0.00
D    N
ATOM  10761  HN  VAL D  35      -21.352  13.519  62.420  1.00  0.00
D    H
ATOM  10762  CA  VAL D  35      -20.503  11.736  63.063  1.00  0.00
D    C
ATOM  10763  HA  VAL D  35      -19.817  11.502  63.867  1.00  0.00
D    H
ATOM  10764  CB  VAL D  35      -21.650  10.709  63.033  1.00  0.00
D    C
ATOM  10765  HB  VAL D  35      -22.293  10.947  62.150  1.00  0.00
D    H
ATOM  10766  CG1 VAL D  35      -21.096   9.300  62.885  1.00  0.00
D    C
ATOM  10767 HG11 VAL D  35      -21.923   8.557  62.903  1.00  0.00
D    H
ATOM  10768 HG12 VAL D  35      -20.556   9.171  61.925  1.00  0.00
D    H
ATOM  10769 HG13 VAL D  35      -20.401   9.062  63.719  1.00  0.00
D    H
ATOM  10770  CG2 VAL D  35      -22.505  10.827  64.271  1.00  0.00
D    C
ATOM  10771 HG21 VAL D  35      -23.308  10.060  64.270  1.00  0.00
D    H
ATOM  10772 HG22 VAL D  35      -21.889  10.686  65.186  1.00  0.00
D    H
```

```
ATOM  10773 HG23 VAL D  35     -22.992  11.822  64.330  1.00  0.00
D    H
ATOM  10774  C   VAL D  35     -19.720  11.676  61.764  1.00  0.00
D    C
ATOM  10775  O   VAL D  35     -20.105  12.298  60.769  1.00  0.00
D    O
ATOM  10776  N   ASP D  36     -18.626  10.927  61.773  1.00  0.00
D    N
ATOM  10777  HN  ASP D  36     -18.207  10.492  62.567  1.00  0.00
D    H
ATOM  10778  CA  ASP D  36     -17.908  10.666  60.541  1.00  0.00
D    C
ATOM  10779  HA  ASP D  36     -18.624  10.322  59.804  1.00  0.00
D    H
ATOM  10780  CB  ASP D  36     -17.159  11.894  60.053  1.00  0.00
D    C
ATOM  10781  HB1 ASP D  36     -17.781  12.794  60.227  1.00  0.00
D    H
ATOM  10782  HB2 ASP D  36     -16.206  12.016  60.605  1.00  0.00
D    H
ATOM  10783  CG  ASP D  36     -16.865  11.818  58.593  1.00  0.00
D    C
ATOM  10784  OD1 ASP D  36     -17.789  12.100  57.808  1.00  0.00
D    O
ATOM  10785  OD2 ASP D  36     -15.724  11.464  58.236  1.00  0.00
D    O
ATOM  10786  C   ASP D  36     -16.935   9.530  60.768  1.00  0.00
D    C
ATOM  10787  O   ASP D  36     -16.252   9.485  61.792  1.00  0.00
D    O
ATOM  10788  N   ILE D  37     -16.866   8.625  59.796  1.00  0.00
D    N
ATOM  10789  HN  ILE D  37     -17.397   8.623  58.951  1.00  0.00
D    H
ATOM  10790  CA  ILE D  37     -15.943   7.508  59.924  1.00  0.00
D    C
ATOM  10791  HA  ILE D  37     -16.131   7.061  60.891  1.00  0.00
D    H
ATOM  10792  CB  ILE D  37     -16.164   6.487  58.799  1.00  0.00
D    C
ATOM  10793  HB  ILE D  37     -17.257   6.253  58.844  1.00  0.00
D    H
ATOM  10794  CG2 ILE D  37     -15.705   7.051  57.473  1.00  0.00
D    C
ATOM  10795 HG21 ILE D  37     -16.005   6.382  56.640  1.00  0.00
D    H
ATOM  10796 HG22 ILE D  37     -16.161   8.046  57.287  1.00  0.00
D    H
ATOM  10797 HG23 ILE D  37     -14.600   7.162  57.449  1.00  0.00
D    H
ATOM  10798  CG1 ILE D  37     -15.416   5.197  59.117  1.00  0.00
D    C
ATOM  10799 HG11 ILE D  37     -14.322   5.353  58.983  1.00  0.00
D    H
```

```
ATOM  10800 HG12 ILE D  37      -15.583   4.938  60.187  1.00  0.00
D    H
ATOM  10801  CD  ILE D  37      -15.843   4.033  58.287  1.00  0.00
D    C
ATOM  10802  HD1 ILE D  37      -15.304   3.115  58.605  1.00  0.00
D    H
ATOM  10803  HD2 ILE D  37      -16.934   3.853  58.397  1.00  0.00
D    H
ATOM  10804  HD3 ILE D  37      -15.620   4.208  57.214  1.00  0.00
D    H
ATOM  10805  C   ILE D  37      -14.506   7.996  59.938  1.00  0.00
D    C
ATOM  10806  O   ILE D  37      -13.626   7.336  60.498  1.00  0.00
D    O
ATOM  10807  N   ASN D  38      -14.237   9.148  59.347  1.00  0.00
D    N
ATOM  10808  HN  ASN D  38      -14.899   9.720  58.863  1.00  0.00
D    H
ATOM  10809  CA  ASN D  38      -12.888   9.677  59.368  1.00  0.00
D    C
ATOM  10810  HA  ASN D  38      -12.215   8.827  59.323  1.00  0.00
D    H
ATOM  10811  CB  ASN D  38      -12.652  10.640  58.219  1.00  0.00
D    C
ATOM  10812  HB1 ASN D  38      -12.514  10.039  57.294  1.00  0.00
D    H
ATOM  10813  HB2 ASN D  38      -13.542  11.285  58.068  1.00  0.00
D    H
ATOM  10814  CG  ASN D  38      -11.428  11.468  58.434  1.00  0.00
D    C
ATOM  10815  OD1 ASN D  38      -11.474  12.494  59.098  1.00  0.00
D    O
ATOM  10816  ND2 ASN D  38      -10.306  11.011  57.903  1.00  0.00
D    N
ATOM  10817 HD21 ASN D  38       -9.480  11.547  58.048  1.00  0.00
D    H
ATOM  10818 HD22 ASN D  38      -10.305  10.147  57.409  1.00  0.00
D    H
ATOM  10819  C   ASN D  38      -12.658  10.374  60.694  1.00  0.00
D    C
ATOM  10820  O   ASN D  38      -13.189  11.464  60.933  1.00  0.00
D    O
ATOM  10821  N   GLU D  39      -11.858   9.748  61.549  1.00  0.00
D    N
ATOM  10822  HN  GLU D  39      -11.412   8.873  61.378  1.00  0.00
D    H
ATOM  10823  CA  GLU D  39      -11.561  10.317  62.856  1.00  0.00
D    C
ATOM  10824  HA  GLU D  39      -12.495  10.442  63.390  1.00  0.00
D    H
ATOM  10825  CB  GLU D  39      -10.619   9.389  63.606  1.00  0.00
D    C
ATOM  10826  HB1 GLU D  39      -10.651   9.659  64.686  1.00  0.00
D    H
```

ATOM 10827 HB2 GLU D 39   -10.991   8.342 63.542 1.00 0.00
D   H
ATOM 10828 CG GLU D 39   -9.202   9.487 63.096 1.00 0.00
D   C
ATOM 10829 HG1 GLU D 39   -8.943 10.547 62.926 1.00 0.00
D   H
ATOM 10830 HG2 GLU D 39   -8.488   9.049 63.822 1.00 0.00
D   H
ATOM 10831 CD GLU D 39   -9.007   8.799 61.761 1.00 0.00
D   C
ATOM 10832 OE1 GLU D 39   -10.009   8.510 61.071 1.00 0.00
D   O
ATOM 10833 OE2 GLU D 39   -7.843   8.558 61.391 1.00 0.00
D   O
ATOM 10834 C   GLU D 39   -10.932 11.691 62.722 1.00 0.00
D   C
ATOM 10835 O   GLU D 39   -10.972 12.496 63.652 1.00 0.00
D   O
ATOM 10836 N   GLU D 40   -10.320 11.972 61.577 1.00 0.00
D   N
ATOM 10837 HN GLU D 40   -10.286 11.399 60.763 1.00 0.00
D   H
ATOM 10838 CA GLU D 40   -9.612 13.233 61.440 1.00 0.00
D   C
ATOM 10839 HA GLU D 40   -8.964 13.349 62.300 1.00 0.00
D   H
ATOM 10840 CB GLU D 40   -8.779 13.218 60.163 1.00 0.00
D   C
ATOM 10841 HB1 GLU D 40   -9.056 12.311 59.578 1.00 0.00
D   H
ATOM 10842 HB2 GLU D 40   -9.051 14.087 59.525 1.00 0.00
D   H
ATOM 10843 CG GLU D 40   -7.256 13.206 60.335 1.00 0.00
D   C
ATOM 10844 HG1 GLU D 40   -6.908 12.165 60.470 1.00 0.00
D   H
ATOM 10845 HG2 GLU D 40   -6.754 13.647 59.452 1.00 0.00
D   H
ATOM 10846 CD GLU D 40   -6.746 13.943 61.561 1.00 0.00
D   C
ATOM 10847 OE1 GLU D 40   -5.972 14.899 61.368 1.00 0.00
D   O
ATOM 10848 OE2 GLU D 40   -7.048 13.531 62.705 1.00 0.00
D   O
ATOM 10849 C   GLU D 40   -10.573 14.409 61.439 1.00 0.00
D   C
ATOM 10850 O   GLU D 40   -10.367 15.384 62.172 1.00 0.00
D   O
ATOM 10851 N   LYS D 41   -11.628 14.334 60.632 1.00 0.00
D   N
ATOM 10852 HN LYS D 41   -11.823 13.573 60.015 1.00 0.00
D   H
ATOM 10853 CA LYS D 41   -12.602 15.417 60.605 1.00 0.00
D   C

```
ATOM  10854  HA  LYS D  41     -12.055  16.333  60.421  1.00  0.00
D    H
ATOM  10855  CB  LYS D  41     -13.637  15.177  59.510  1.00  0.00
D    C
ATOM  10856  HB1 LYS D  41     -14.306  14.359  59.864  1.00  0.00
D    H
ATOM  10857  HB2 LYS D  41     -14.276  16.083  59.412  1.00  0.00
D    H
ATOM  10858  CG  LYS D  41     -13.057  14.792  58.181  1.00  0.00
D    C
ATOM  10859  HG1 LYS D  41     -12.062  14.317  58.341  1.00  0.00
D    H
ATOM  10860  HG2 LYS D  41     -13.715  14.030  57.704  1.00  0.00
D    H
ATOM  10861  CD  LYS D  41     -12.892  15.981  57.275  1.00  0.00
D    C
ATOM  10862  HD1 LYS D  41     -13.712  16.704  57.490  1.00  0.00
D    H
ATOM  10863  HD2 LYS D  41     -11.929  16.481  57.532  1.00  0.00
D    H
ATOM  10864  CE  LYS D  41     -12.910  15.518  55.835  1.00  0.00
D    C
ATOM  10865  HE1 LYS D  41     -12.643  16.355  55.153  1.00  0.00
D    H
ATOM  10866  HE2 LYS D  41     -12.182  14.692  55.681  1.00  0.00
D    H
ATOM  10867  NZ  LYS D  41     -14.261  15.024  55.454  1.00  0.00
D    N
ATOM  10868  HZ1 LYS D  41     -14.258  14.711  54.461  1.00  0.00
D    H
ATOM  10869  HZ2 LYS D  41     -14.525  14.227  56.067  1.00  0.00
D    H
ATOM  10870  HZ3 LYS D  41     -14.955  15.790  55.571  1.00  0.00
D    H
ATOM  10871  C   LYS D  41     -13.297  15.565  61.948  1.00  0.00
D    C
ATOM  10872  O   LYS D  41     -13.509  16.686  62.419  1.00  0.00
D    O
ATOM  10873  N   VAL D  42     -13.716  14.450  62.544  1.00  0.00
D    N
ATOM  10874  HN  VAL D  42     -13.654  13.534  62.157  1.00  0.00
D    H
ATOM  10875  CA  VAL D  42     -14.323  14.505  63.867  1.00  0.00
D    C
ATOM  10876  HA  VAL D  42     -15.222  15.104  63.800  1.00  0.00
D    H
ATOM  10877  CB  VAL D  42     -14.657  13.087  64.353  1.00  0.00
D    C
ATOM  10878  HB  VAL D  42     -13.693  12.580  64.605  1.00  0.00
D    H
ATOM  10879  CG1 VAL D  42     -15.528  13.143  65.590  1.00  0.00
D    C
ATOM  10880 HG11 VAL D  42     -15.801  12.117  65.916  1.00  0.00
D    H
```

ATOM 10881 HG12 VAL D 42 -15.006 13.632 66.438 1.00 0.00
D H
ATOM 10882 HG13 VAL D 42 -16.468 13.699 65.383 1.00 0.00
D H
ATOM 10883 CG2 VAL D 42 -15.324 12.303 63.245 1.00 0.00
D C
ATOM 10884 HG21 VAL D 42 -15.607 11.288 63.597 1.00 0.00
D H
ATOM 10885 HG22 VAL D 42 -16.244 12.822 62.898 1.00 0.00
D H
ATOM 10886 HG23 VAL D 42 -14.645 12.178 62.375 1.00 0.00
D H
ATOM 10887 C VAL D 42 -13.381 15.204 64.825 1.00 0.00
D C
ATOM 10888 O VAL D 42 -13.767 16.112 65.570 1.00 0.00
D O
ATOM 10889 N ASN D 43 -12.119 14.792 64.794 1.00 0.00
D N
ATOM 10890 HN ASN D 43 -11.791 14.078 64.177 1.00 0.00
D H
ATOM 10891 CA ASN D 43 -11.099 15.350 65.665 1.00 0.00
D C
ATOM 10892 HA ASN D 43 -11.421 15.134 66.678 1.00 0.00
D H
ATOM 10893 CB ASN D 43 -9.756 14.710 65.327 1.00 0.00
D C
ATOM 10894 HB1 ASN D 43 -9.959 13.724 64.855 1.00 0.00
D H
ATOM 10895 HB2 ASN D 43 -9.210 15.329 64.586 1.00 0.00
D H
ATOM 10896 CG ASN D 43 -8.906 14.480 66.539 1.00 0.00
D C
ATOM 10897 OD1 ASN D 43 -8.702 15.376 67.344 1.00 0.00
D O
ATOM 10898 ND2 ASN D 43 -8.415 13.260 66.691 1.00 0.00
D N
ATOM 10899 HD21 ASN D 43 -7.857 13.089 67.499 1.00 0.00
D H
ATOM 10900 HD22 ASN D 43 -8.633 12.549 66.030 1.00 0.00
D H
ATOM 10901 C ASN D 43 -11.025 16.856 65.519 1.00 0.00
D C
ATOM 10902 O ASN D 43 -10.968 17.588 66.512 1.00 0.00
D O
ATOM 10903 N ALA D 44 -11.020 17.334 64.283 1.00 0.00
D N
ATOM 10904 HN ALA D 44 -11.041 16.776 63.453 1.00 0.00
D H
ATOM 10905 CA ALA D 44 -10.981 18.774 64.067 1.00 0.00
D C
ATOM 10906 HA ALA D 44 -10.159 19.218 64.616 1.00 0.00
D H
ATOM 10907 CB ALA D 44 -10.780 19.077 62.584 1.00 0.00
D C

```
ATOM  10908  HB1 ALA D  44      -9.827  18.625  62.236  1.00  0.00
D    H
ATOM  10909  HB2 ALA D  44     -11.605  18.649  61.974  1.00  0.00
D    H
ATOM  10910  HB3 ALA D  44     -10.731  20.172  62.394  1.00  0.00
D    H
ATOM  10911  C   ALA D  44     -12.248  19.443  64.574  1.00  0.00
D    C
ATOM  10912  O   ALA D  44     -12.201  20.262  65.498  1.00  0.00
D    O
ATOM  10913  N   TYR D  45     -13.392  19.077  64.019  1.00  0.00
D    N
ATOM  10914  HN  TYR D  45     -13.508  18.262  63.453  1.00  0.00
D    H
ATOM  10915  CA  TYR D  45     -14.601  19.864  64.200  1.00  0.00
D    C
ATOM  10916  HA  TYR D  45     -14.268  20.893  64.252  1.00  0.00
D    H
ATOM  10917  CB  TYR D  45     -15.561  19.660  63.032  1.00  0.00
D    C
ATOM  10918  HB1 TYR D  45     -15.658  18.572  62.826  1.00  0.00
D    H
ATOM  10919  HB2 TYR D  45     -16.575  20.038  63.287  1.00  0.00
D    H
ATOM  10920  CG  TYR D  45     -15.099  20.301  61.758  1.00  0.00
D    C
ATOM  10921  CD1 TYR D  45     -13.957  21.084  61.728  1.00  0.00
D    C
ATOM  10922  HD1 TYR D  45     -13.390  21.242  62.635  1.00  0.00
D    H
ATOM  10923  CE1 TYR D  45     -13.540  21.684  60.563  1.00  0.00
D    C
ATOM  10924  HE1 TYR D  45     -12.637  22.278  60.544  1.00  0.00
D    H
ATOM  10925  CZ  TYR D  45     -14.285  21.531  59.417  1.00  0.00
D    C
ATOM  10926  OH  TYR D  45     -13.883  22.131  58.249  1.00  0.00
D    O
ATOM  10927  HH  TYR D  45     -14.486  21.849  57.558  1.00  0.00
D    H
ATOM  10928  CD2 TYR D  45     -15.837  20.177  60.595  1.00  0.00
D    C
ATOM  10929  HD2 TYR D  45     -16.771  19.632  60.604  1.00  0.00
D    H
ATOM  10930  CE2 TYR D  45     -15.432  20.778  59.431  1.00  0.00
D    C
ATOM  10931  HE2 TYR D  45     -16.029  20.673  58.538  1.00  0.00
D    H
ATOM  10932  C   TYR D  45     -15.336  19.567  65.491  1.00  0.00
D    C
ATOM  10933  O   TYR D  45     -16.457  20.056  65.664  1.00  0.00
D    O
ATOM  10934  N   ALA D  46     -14.756  18.776  66.390  1.00  0.00
D    N
```

ATOM 10935 HN ALA D 46 -13.921 18.247 66.239 1.00 0.00
D H
ATOM 10936 CA ALA D 46 -15.349 18.642 67.713 1.00 0.00
D C
ATOM 10937 HA ALA D 46 -16.307 18.136 67.658 1.00 0.00
D H
ATOM 10938 CB ALA D 46 -14.429 17.831 68.621 1.00 0.00
D C
ATOM 10939 HB1 ALA D 46 -14.281 16.817 68.191 1.00 0.00
D H
ATOM 10940 HB2 ALA D 46 -13.431 18.313 68.711 1.00 0.00
D H
ATOM 10941 HB3 ALA D 46 -14.858 17.713 69.639 1.00 0.00
D H
ATOM 10942 C ALA D 46 -15.623 20.012 68.312 1.00 0.00
D C
ATOM 10943 O ALA D 46 -16.715 20.278 68.822 1.00 0.00
D O
ATOM 10944 N SER D 47 -14.643 20.908 68.219 1.00 0.00
D N
ATOM 10945 HN SER D 47 -13.760 20.736 67.780 1.00 0.00
D H
ATOM 10946 CA SER D 47 -14.787 22.248 68.765 1.00 0.00
D C
ATOM 10947 HA SER D 47 -14.991 22.107 69.818 1.00 0.00
D H
ATOM 10948 CB SER D 47 -13.507 23.037 68.524 1.00 0.00
D C
ATOM 10949 HB1 SER D 47 -13.474 23.875 69.256 1.00 0.00
D H
ATOM 10950 HB2 SER D 47 -12.616 22.399 68.717 1.00 0.00
D H
ATOM 10951 OG SER D 47 -13.502 23.563 67.212 1.00 0.00
D O
ATOM 10952 HG1 SER D 47 -12.802 24.227 67.175 1.00 0.00
D H
ATOM 10953 C SER D 47 -15.964 23.002 68.174 1.00 0.00
D C
ATOM 10954 O SER D 47 -16.520 23.877 68.841 1.00 0.00
D O
ATOM 10955 N TYR D 48 -16.356 22.694 66.944 1.00 0.00
D N
ATOM 10956 HN TYR D 48 -15.925 21.991 66.381 1.00 0.00
D H
ATOM 10957 CA TYR D 48 -17.471 23.376 66.307 1.00 0.00
D C
ATOM 10958 HA TYR D 48 -17.675 24.255 66.906 1.00 0.00
D H
ATOM 10959 CB TYR D 48 -17.114 23.747 64.874 1.00 0.00
D C
ATOM 10960 HB1 TYR D 48 -16.943 22.817 64.289 1.00 0.00
D H
ATOM 10961 HB2 TYR D 48 -17.951 24.292 64.388 1.00 0.00
D H

ATOM 10962 CG TYR D 48 −15.862 24.558 64.797 1.00 0.00
D C
ATOM 10963 CD1 TYR D 48 −15.493 25.391 65.839 1.00 0.00
D C
ATOM 10964 HD1 TYR D 48 −16.132 25.480 66.707 1.00 0.00
D H
ATOM 10965 CE1 TYR D 48 −14.339 26.123 65.784 1.00 0.00
D C
ATOM 10966 HE1 TYR D 48 −14.069 26.784 66.594 1.00 0.00
D H
ATOM 10967 CZ TYR D 48 −13.525 26.017 64.682 1.00 0.00
D C
ATOM 10968 OH TYR D 48 −12.367 26.748 64.622 1.00 0.00
D O
ATOM 10969 HH TYR D 48 −11.905 26.489 63.822 1.00 0.00
D H
ATOM 10970 CD2 TYR D 48 −15.020 24.461 63.704 1.00 0.00
D C
ATOM 10971 HD2 TYR D 48 −15.292 23.833 62.867 1.00 0.00
D H
ATOM 10972 CE2 TYR D 48 −13.861 25.188 63.640 1.00 0.00
D C
ATOM 10973 HE2 TYR D 48 −13.233 25.120 62.763 1.00 0.00
D H
ATOM 10974 C TYR D 48 −18.722 22.525 66.324 1.00 0.00
D C
ATOM 10975 O TYR D 48 −19.560 22.643 65.429 1.00 0.00
D O
ATOM 10976 N ALA D 49 −18.860 21.662 67.323 1.00 0.00
D N
ATOM 10977 HN ALA D 49 −18.266 21.637 68.127 1.00 0.00
D H
ATOM 10978 CA ALA D 49 −19.920 20.674 67.301 1.00 0.00
D C
ATOM 10979 HA ALA D 49 −20.713 20.961 66.621 1.00 0.00
D H
ATOM 10980 CB ALA D 49 −19.400 19.300 66.875 1.00 0.00
D C
ATOM 10981 HB1 ALA D 49 −18.982 19.362 65.847 1.00 0.00
D H
ATOM 10982 HB2 ALA D 49 −18.591 18.952 67.553 1.00 0.00
D H
ATOM 10983 HB3 ALA D 49 −20.210 18.539 66.871 1.00 0.00
D H
ATOM 10984 C ALA D 49 −20.550 20.575 68.673 1.00 0.00
D C
ATOM 10985 O ALA D 49 −19.855 20.491 69.688 1.00 0.00
D O
ATOM 10986 N THR D 50 −21.879 20.582 68.690 1.00 0.00
D N
ATOM 10987 HN THR D 50 −22.479 20.790 67.918 1.00 0.00
D H
ATOM 10988 CA THR D 50 −22.580 20.263 69.922 1.00 0.00
D C

```
ATOM  10989  HA  THR D  50     -22.258  20.998  70.648  1.00  0.00
D    H
ATOM  10990  CB  THR D  50     -24.091  20.276  69.688  1.00  0.00
D    C
ATOM  10991  HB  THR D  50     -24.316  19.376  69.067  1.00  0.00
D    H
ATOM  10992  OG1 THR D  50     -24.454  21.437  68.932  1.00  0.00
D    O
ATOM  10993  HG1 THR D  50     -25.411  21.389  68.832  1.00  0.00
D    H
ATOM  10994  CG2 THR D  50     -24.840  20.288  71.012  1.00  0.00
D    C
ATOM  10995 HG21 THR D  50     -25.933  20.182  70.849  1.00  0.00
D    H
ATOM  10996 HG22 THR D  50     -24.507  19.444  71.653  1.00  0.00
D    H
ATOM  10997 HG23 THR D  50     -24.660  21.235  71.564  1.00  0.00
D    H
ATOM  10998  C   THR D  50     -22.144  18.912  70.454  1.00  0.00
D    C
ATOM  10999  O   THR D  50     -22.037  18.730  71.670  1.00  0.00
D    O
ATOM  11000  N   HSE D  51     -21.858  17.972  69.566  1.00  0.00
D    N
ATOM  11001  HN  HSE D  51     -21.907  18.093  68.576  1.00  0.00
D    H
ATOM  11002  CA  HSE D  51     -21.438  16.650  69.987  1.00  0.00
D    C
ATOM  11003  HA  HSE D  51     -20.634  16.763  70.704  1.00  0.00
D    H
ATOM  11004  CB  HSE D  51     -22.614  15.882  70.591  1.00  0.00
D    C
ATOM  11005  HB1 HSE D  51     -22.954  16.432  71.495  1.00  0.00
D    H
ATOM  11006  HB2 HSE D  51     -23.462  15.878  69.874  1.00  0.00
D    H
ATOM  11007  ND1 HSE D  51     -23.229  13.611  71.483  1.00  0.00
D    N
ATOM  11008  CG  HSE D  51     -22.280  14.486  71.003  1.00  0.00
D    C
ATOM  11009  CE1 HSE D  51     -22.656  12.455  71.766  1.00  0.00
D    C
ATOM  11010  HE1 HSE D  51     -23.180  11.585  72.169  1.00  0.00
D    H
ATOM  11011  NE2 HSE D  51     -21.370  12.548  71.483  1.00  0.00
D    N
ATOM  11012  HE2 HSE D  51     -20.697  11.822  71.617  1.00  0.00
D    H
ATOM  11013  CD2 HSE D  51     -21.109  13.809  71.006  1.00  0.00
D    C
ATOM  11014  HD2 HSE D  51     -20.119  14.134  70.716  1.00  0.00
D    H
ATOM  11015  C   HSE D  51     -20.864  15.922  68.784  1.00  0.00
D    C
```

```
ATOM  11016  O   HSE D  51      -21.530  15.788  67.755  1.00  0.00
D    O
ATOM  11017  N   ALA D  52      -19.628  15.454  68.922  1.00  0.00
D    N
ATOM  11018  HN  ALA D  52      -19.117  15.559  69.774  1.00  0.00
D    H
ATOM  11019  CA  ALA D  52      -18.927  14.759  67.856  1.00  0.00
D    C
ATOM  11020  HA  ALA D  52      -19.529  14.696  66.958  1.00  0.00
D    H
ATOM  11021  CB  ALA D  52      -17.609  15.450  67.512  1.00  0.00
D    C
ATOM  11022  HB1 ALA D  52      -17.813  16.478  67.146  1.00  0.00
D    H
ATOM  11023  HB2 ALA D  52      -16.954  15.529  68.406  1.00  0.00
D    H
ATOM  11024  HB3 ALA D  52      -17.057  14.905  66.716  1.00  0.00
D    H
ATOM  11025  C   ALA D  52      -18.657  13.334  68.291  1.00  0.00
D    C
ATOM  11026  O   ALA D  52      -18.504  13.065  69.483  1.00  0.00
D    O
ATOM  11027  N   VAL D  53      -18.588  12.431  67.322  1.00  0.00
D    N
ATOM  11028  HN  VAL D  53      -18.723  12.661  66.362  1.00  0.00
D    H
ATOM  11029  CA  VAL D  53      -18.308  11.023  67.571  1.00  0.00
D    C
ATOM  11030  HA  VAL D  53      -17.590  10.942  68.378  1.00  0.00
D    H
ATOM  11031  CB  VAL D  53      -19.585  10.221  67.896  1.00  0.00
D    C
ATOM  11032  HB  VAL D  53      -20.395  10.587  67.217  1.00  0.00
D    H
ATOM  11033  CG1 VAL D  53      -19.367   8.735  67.655  1.00  0.00
D    C
ATOM  11034 HG11 VAL D  53      -20.279   8.162  67.928  1.00  0.00
D    H
ATOM  11035 HG12 VAL D  53      -19.150   8.521  66.588  1.00  0.00
D    H
ATOM  11036 HG13 VAL D  53      -18.525   8.356  68.273  1.00  0.00
D    H
ATOM  11037  CG2 VAL D  53      -20.029  10.452  69.321  1.00  0.00
D    C
ATOM  11038 HG21 VAL D  53      -20.925   9.841  69.558  1.00  0.00
D    H
ATOM  11039 HG22 VAL D  53      -19.219  10.176  70.031  1.00  0.00
D    H
ATOM  11040 HG23 VAL D  53      -20.294  11.516  69.491  1.00  0.00
D    H
ATOM  11041  C   VAL D  53      -17.655  10.461  66.323  1.00  0.00
D    C
ATOM  11042  O   VAL D  53      -18.047  10.804  65.207  1.00  0.00
D    O
```

ATOM 11043 N ILE D 54 −16.663 9.601 66.501 1.00 0.00
D N
ATOM 11044 HN ILE D 54 −16.191 9.422 67.362 1.00 0.00
D H
ATOM 11045 CA ILE D 54 −16.172 8.819 65.378 1.00 0.00
D C
ATOM 11046 HA ILE D 54 −16.355 9.385 64.473 1.00 0.00
D H
ATOM 11047 CB ILE D 54 −14.690 8.483 65.541 1.00 0.00
D C
ATOM 11048 HB ILE D 54 −14.649 7.833 66.451 1.00 0.00
D H
ATOM 11049 CG2 ILE D 54 −14.156 7.866 64.276 1.00 0.00
D C
ATOM 11050 HG21 ILE D 54 −13.128 7.477 64.435 1.00 0.00
D H
ATOM 11051 HG22 ILE D 54 −14.791 7.015 63.951 1.00 0.00
D H
ATOM 11052 HG23 ILE D 54 −14.121 8.612 63.454 1.00 0.00
D H
ATOM 11053 CG1 ILE D 54 −13.909 9.749 65.859 1.00 0.00
D C
ATOM 11054 HG11 ILE D 54 −13.728 10.325 64.924 1.00 0.00
D H
ATOM 11055 HG12 ILE D 54 −14.524 10.393 66.529 1.00 0.00
D H
ATOM 11056 CD ILE D 54 −12.595 9.480 66.537 1.00 0.00
D C
ATOM 11057 HD1 ILE D 54 −12.094 10.437 66.800 1.00 0.00
D H
ATOM 11058 HD2 ILE D 54 −12.749 8.901 67.473 1.00 0.00
D H
ATOM 11059 HD3 ILE D 54 −11.915 8.906 65.874 1.00 0.00
D H
ATOM 11060 C ILE D 54 −17.022 7.560 65.288 1.00 0.00
D C
ATOM 11061 O ILE D 54 −17.163 6.827 66.271 1.00 0.00
D O
ATOM 11062 N ALA D 55 −17.600 7.312 64.114 1.00 0.00
D N
ATOM 11063 HN ALA D 55 −17.472 7.891 63.308 1.00 0.00
D H
ATOM 11064 CA ALA D 55 −18.470 6.160 63.925 1.00 0.00
D C
ATOM 11065 HA ALA D 55 −18.010 5.254 64.301 1.00 0.00
D H
ATOM 11066 CB ALA D 55 −19.807 6.345 64.649 1.00 0.00
D C
ATOM 11067 HB1 ALA D 55 −19.628 6.460 65.739 1.00 0.00
D H
ATOM 11068 HB2 ALA D 55 −20.332 7.258 64.290 1.00 0.00
D H
ATOM 11069 HB3 ALA D 55 −20.477 5.470 64.501 1.00 0.00
D H

ATOM 11070 C ALA D 55 -18.710 5.935 62.439 1.00 0.00
D C
ATOM 11071 O ALA D 55 -18.786 6.888 61.662 1.00 0.00
D O
ATOM 11072 N ASN D 56 -18.837 4.667 62.063 1.00 0.00
D N
ATOM 11073 HN ASN D 56 -18.751 3.906 62.705 1.00 0.00
D H
ATOM 11074 CA ASN D 56 -19.113 4.269 60.687 1.00 0.00
D C
ATOM 11075 HA ASN D 56 -18.747 5.063 60.043 1.00 0.00
D H
ATOM 11076 CB ASN D 56 -18.449 2.936 60.381 1.00 0.00
D C
ATOM 11077 HB1 ASN D 56 -17.354 3.058 60.530 1.00 0.00
D H
ATOM 11078 HB2 ASN D 56 -18.797 2.160 61.092 1.00 0.00
D H
ATOM 11079 CG ASN D 56 -18.679 2.483 58.959 1.00 0.00
D C
ATOM 11080 OD1 ASN D 56 -19.530 3.015 58.246 1.00 0.00
D O
ATOM 11081 ND2 ASN D 56 -17.919 1.483 58.537 1.00 0.00
D N
ATOM 11082 HD21 ASN D 56 -18.046 1.176 57.597 1.00 0.00
D H
ATOM 11083 HD22 ASN D 56 -17.232 1.090 59.140 1.00 0.00
D H
ATOM 11084 C ASN D 56 -20.621 4.193 60.511 1.00 0.00
D C
ATOM 11085 O ASN D 56 -21.286 3.349 61.111 1.00 0.00
D O
ATOM 11086 N ALA D 57 -21.156 5.055 59.649 1.00 0.00
D N
ATOM 11087 HN ALA D 57 -20.605 5.653 59.067 1.00 0.00
D H
ATOM 11088 CA ALA D 57 -22.598 5.187 59.492 1.00 0.00
D C
ATOM 11089 HA ALA D 57 -23.042 5.455 60.444 1.00 0.00
D H
ATOM 11090 CB ALA D 57 -22.914 6.281 58.479 1.00 0.00
D C
ATOM 11091 HB1 ALA D 57 -22.464 7.239 58.815 1.00 0.00
D H
ATOM 11092 HB2 ALA D 57 -22.490 6.036 57.481 1.00 0.00
D H
ATOM 11093 HB3 ALA D 57 -24.009 6.435 58.371 1.00 0.00
D H
ATOM 11094 C ALA D 57 -23.278 3.907 59.049 1.00 0.00
D C
ATOM 11095 O ALA D 57 -24.486 3.767 59.248 1.00 0.00
D O
ATOM 11096 N THR D 58 -22.548 2.973 58.467 1.00 0.00
D N

```
ATOM  11097  HN  THR D  58     -21.549   2.950  58.440  1.00  0.00
D    H
ATOM  11098  CA  THR D  58     -23.188   1.861  57.792  1.00  0.00
D    C
ATOM  11099  HA  THR D  58     -24.056   2.292  57.313  1.00  0.00
D    H
ATOM  11100  CB  THR D  58     -22.212   1.248  56.791  1.00  0.00
D    C
ATOM  11101  HB  THR D  58     -22.775   0.437  56.267  1.00  0.00
D    H
ATOM  11102  OG1 THR D  58     -21.125   0.651  57.495  1.00  0.00
D    O
ATOM  11103  HG1 THR D  58     -20.543   0.295  56.815  1.00  0.00
D    H
ATOM  11104  CG2 THR D  58     -21.660   2.327  55.881  1.00  0.00
D    C
ATOM  11105 HG21 THR D  58     -21.038   1.884  55.075  1.00  0.00
D    H
ATOM  11106 HG22 THR D  58     -22.488   2.891  55.402  1.00  0.00
D    H
ATOM  11107 HG23 THR D  58     -21.032   3.047  56.449  1.00  0.00
D    H
ATOM  11108  C   THR D  58     -23.704   0.783  58.735  1.00  0.00
D    C
ATOM  11109  O   THR D  58     -24.336  -0.167  58.266  1.00  0.00
D    O
ATOM  11110  N   GLU D  59     -23.459   0.891  60.037  1.00  0.00
D    N
ATOM  11111  HN  GLU D  59     -23.008   1.670  60.465  1.00  0.00
D    H
ATOM  11112  CA  GLU D  59     -23.846  -0.159  60.973  1.00  0.00
D    C
ATOM  11113  HA  GLU D  59     -24.213  -1.004  60.403  1.00  0.00
D    H
ATOM  11114  CB  GLU D  59     -22.657  -0.582  61.827  1.00  0.00
D    C
ATOM  11115  HB1 GLU D  59     -22.208   0.334  62.276  1.00  0.00
D    H
ATOM  11116  HB2 GLU D  59     -23.009  -1.201  62.682  1.00  0.00
D    H
ATOM  11117  CG  GLU D  59     -21.582  -1.324  61.083  1.00  0.00
D    C
ATOM  11118  HG1 GLU D  59     -20.823  -1.691  61.798  1.00  0.00
D    H
ATOM  11119  HG2 GLU D  59     -22.005  -2.185  60.528  1.00  0.00
D    H
ATOM  11120  CD  GLU D  59     -20.849  -0.435  60.117  1.00  0.00
D    C
ATOM  11121  OE1 GLU D  59     -20.585  -0.879  58.983  1.00  0.00
D    O
ATOM  11122  OE2 GLU D  59     -20.586   0.728  60.466  1.00  0.00
D    O
ATOM  11123  C   GLU D  59     -24.982   0.307  61.873  1.00  0.00
D    C
```

ATOM 11124 O GLU D 59 -24.883 1.358 62.515 1.00 0.00
D O
ATOM 11125 N GLU D 60 -26.032 -0.509 61.961 1.00 0.00
D N
ATOM 11126 HN GLU D 60 -26.079 -1.428 61.579 1.00 0.00
D H
ATOM 11127 CA GLU D 60 -27.244 -0.094 62.656 1.00 0.00
D C
ATOM 11128 HA GLU D 60 -27.549 0.863 62.251 1.00 0.00
D H
ATOM 11129 CB GLU D 60 -28.366 -1.104 62.391 1.00 0.00
D C
ATOM 11130 HB1 GLU D 60 -29.240 -0.827 63.025 1.00 0.00
D H
ATOM 11131 HB2 GLU D 60 -28.714 -1.014 61.337 1.00 0.00
D H
ATOM 11132 CG GLU D 60 -28.058 -2.583 62.665 1.00 0.00
D C
ATOM 11133 HG1 GLU D 60 -28.984 -3.177 62.560 1.00 0.00
D H
ATOM 11134 HG2 GLU D 60 -27.296 -2.967 61.958 1.00 0.00
D H
ATOM 11135 CD GLU D 60 -27.568 -2.881 64.061 1.00 0.00
D C
ATOM 11136 OE1 GLU D 60 -28.249 -2.487 65.029 1.00 0.00
D O
ATOM 11137 OE2 GLU D 60 -26.476 -3.472 64.193 1.00 0.00
D O
ATOM 11138 C GLU D 60 -27.001 0.102 64.145 1.00 0.00
D C
ATOM 11139 O GLU D 60 -27.655 0.931 64.785 1.00 0.00
D O
ATOM 11140 N ASN D 61 -26.082 -0.665 64.721 1.00 0.00
D N
ATOM 11141 HN ASN D 61 -25.485 -1.315 64.253 1.00 0.00
D H
ATOM 11142 CA ASN D 61 -25.884 -0.593 66.161 1.00 0.00
D C
ATOM 11143 HA ASN D 61 -26.876 -0.600 66.602 1.00 0.00
D H
ATOM 11144 CB ASN D 61 -25.066 -1.786 66.632 1.00 0.00
D C
ATOM 11145 HB1 ASN D 61 -24.672 -1.551 67.645 1.00 0.00
D H
ATOM 11146 HB2 ASN D 61 -25.713 -2.683 66.727 1.00 0.00
D H
ATOM 11147 CG ASN D 61 -23.910 -2.064 65.729 1.00 0.00
D C
ATOM 11148 OD1 ASN D 61 -23.959 -1.756 64.539 1.00 0.00
D O
ATOM 11149 ND2 ASN D 61 -22.858 -2.654 66.275 1.00 0.00
D N
ATOM 11150 HD21 ASN D 61 -22.077 -2.834 65.684 1.00 0.00
D H

ATOM 11151 HD22 ASN D 61 −22.859 −2.872 67.246 1.00 0.00
D H
ATOM 11152 C ASN D 61 −25.222 0.716 66.542 1.00 0.00
D C
ATOM 11153 O ASN D 61 −25.553 1.311 67.572 1.00 0.00
D O
ATOM 11154 N GLU D 62 −24.281 1.184 65.723 1.00 0.00
D N
ATOM 11155 HN GLU D 62 −23.836 0.695 64.978 1.00 0.00
D H
ATOM 11156 CA GLU D 62 −23.818 2.551 65.891 1.00 0.00
D C
ATOM 11157 HA GLU D 62 −23.336 2.629 66.858 1.00 0.00
D H
ATOM 11158 CB GLU D 62 −22.830 2.933 64.799 1.00 0.00
D C
ATOM 11159 HB1 GLU D 62 −23.191 2.501 63.837 1.00 0.00
D H
ATOM 11160 HB2 GLU D 62 −22.831 4.037 64.657 1.00 0.00
D H
ATOM 11161 CG GLU D 62 −21.432 2.450 65.056 1.00 0.00
D C
ATOM 11162 HG1 GLU D 62 −20.903 3.178 65.696 1.00 0.00
D H
ATOM 11163 HG2 GLU D 62 −21.439 1.461 65.556 1.00 0.00
D H
ATOM 11164 CD GLU D 62 −20.613 2.344 63.797 1.00 0.00
D C
ATOM 11165 OE1 GLU D 62 −19.779 3.236 63.556 1.00 0.00
D O
ATOM 11166 OE2 GLU D 62 −20.790 1.363 63.053 1.00 0.00
D O
ATOM 11167 C GLU D 62 −24.980 3.524 65.889 1.00 0.00
D C
ATOM 11168 O GLU D 62 −24.994 4.479 66.671 1.00 0.00
D O
ATOM 11169 N LEU D 63 −25.971 3.276 65.047 1.00 0.00
D N
ATOM 11170 HN LEU D 63 −26.037 2.477 64.452 1.00 0.00
D H
ATOM 11171 CA LEU D 63 −27.084 4.198 64.931 1.00 0.00
D C
ATOM 11172 HA LEU D 63 −26.656 5.187 64.820 1.00 0.00
D H
ATOM 11173 CB LEU D 63 −27.921 3.814 63.720 1.00 0.00
D C
ATOM 11174 HB1 LEU D 63 −28.511 2.912 64.001 1.00 0.00
D H
ATOM 11175 HB2 LEU D 63 −28.663 4.620 63.524 1.00 0.00
D H
ATOM 11176 CG LEU D 63 −27.055 3.521 62.504 1.00 0.00
D C
ATOM 11177 HG LEU D 63 −26.391 2.669 62.788 1.00 0.00
D H

```
ATOM  11178  CD1 LEU D  63      -27.915   3.119  61.331  1.00  0.00
D    C
ATOM  11179 HD11 LEU D  63      -27.290   2.951  60.428  1.00  0.00
D    H
ATOM  11180 HD12 LEU D  63      -28.467   2.181  61.553  1.00  0.00
D    H
ATOM  11181 HD13 LEU D  63      -28.655   3.914  61.096  1.00  0.00
D    H
ATOM  11182  CD2 LEU D  63      -26.176   4.703  62.153  1.00  0.00
D    C
ATOM  11183 HD21 LEU D  63      -25.538   4.463  61.275  1.00  0.00
D    H
ATOM  11184 HD22 LEU D  63      -26.777   5.598  61.897  1.00  0.00
D    H
ATOM  11185 HD23 LEU D  63      -25.510   4.960  63.005  1.00  0.00
D    H
ATOM  11186  C   LEU D  63      -27.934   4.205  66.190  1.00  0.00
D    C
ATOM  11187  O   LEU D  63      -28.167   5.266  66.779  1.00  0.00
D    O
ATOM  11188  N   LEU D  64      -28.403   3.033  66.614  1.00  0.00
D    N
ATOM  11189  HN  LEU D  64      -28.279   2.158  66.151  1.00  0.00
D    H
ATOM  11190  CA  LEU D  64      -29.167   2.952  67.850  1.00  0.00
D    C
ATOM  11191  HA  LEU D  64      -30.043   3.574  67.716  1.00  0.00
D    H
ATOM  11192  CB  LEU D  64      -29.552   1.509  68.135  1.00  0.00
D    C
ATOM  11193  HB1 LEU D  64      -28.675   1.026  68.625  1.00  0.00
D    H
ATOM  11194  HB2 LEU D  64      -30.372   1.495  68.887  1.00  0.00
D    H
ATOM  11195  CG  LEU D  64      -29.927   0.741  66.883  1.00  0.00
D    C
ATOM  11196  HG  LEU D  64      -29.158   0.947  66.100  1.00  0.00
D    H
ATOM  11197  CD1 LEU D  64      -29.955  -0.740  67.176  1.00  0.00
D    C
ATOM  11198 HD11 LEU D  64      -30.276  -1.309  66.278  1.00  0.00
D    H
ATOM  11199 HD12 LEU D  64      -28.947  -1.098  67.475  1.00  0.00
D    H
ATOM  11200 HD13 LEU D  64      -30.666  -0.963  68.000  1.00  0.00
D    H
ATOM  11201  CD2 LEU D  64      -31.262   1.225  66.366  1.00  0.00
D    C
ATOM  11202 HD21 LEU D  64      -31.537   0.681  65.437  1.00  0.00
D    H
ATOM  11203 HD22 LEU D  64      -32.071   1.060  67.105  1.00  0.00
D    H
ATOM  11204 HD23 LEU D  64      -31.218   2.310  66.132  1.00  0.00
D    H
```

ATOM 11205 C LEU D 64 -28.379 3.517 69.017 1.00 0.00
D C
ATOM 11206 O LEU D 64 -28.923 4.267 69.835 1.00 0.00
D O
ATOM 11207 N SER D 65 -27.094 3.177 69.101 1.00 0.00
D N
ATOM 11208 HN SER D 65 -26.619 2.558 68.475 1.00 0.00
D H
ATOM 11209 CA SER D 65 -26.255 3.710 70.163 1.00 0.00
D C
ATOM 11210 HA SER D 65 -26.700 3.379 71.092 1.00 0.00
D H
ATOM 11211 CB SER D 65 -24.827 3.214 69.991 1.00 0.00
D C
ATOM 11212 HB1 SER D 65 -24.201 3.676 70.789 1.00 0.00
D H
ATOM 11213 HB2 SER D 65 -24.779 2.111 70.137 1.00 0.00
D H
ATOM 11214 OG SER D 65 -24.335 3.599 68.728 1.00 0.00
D O
ATOM 11215 HG1 SER D 65 -23.378 3.469 68.743 1.00 0.00
D H
ATOM 11216 C SER D 65 -26.278 5.226 70.168 1.00 0.00
D C
ATOM 11217 O SER D 65 -26.174 5.847 71.230 1.00 0.00
D O
ATOM 11218 N LEU D 66 -26.430 5.837 68.997 1.00 0.00
D N
ATOM 11219 HN LEU D 66 -26.503 5.386 68.110 1.00 0.00
D H
ATOM 11220 CA LEU D 66 -26.505 7.284 68.938 1.00 0.00
D C
ATOM 11221 HA LEU D 66 -25.759 7.652 69.631 1.00 0.00
D H
ATOM 11222 CB LEU D 66 -26.216 7.764 67.526 1.00 0.00
D C
ATOM 11223 HB1 LEU D 66 -26.868 7.177 66.840 1.00 0.00
D H
ATOM 11224 HB2 LEU D 66 -26.543 8.823 67.430 1.00 0.00
D H
ATOM 11225 CG LEU D 66 -24.756 7.581 67.144 1.00 0.00
D C
ATOM 11226 HG LEU D 66 -24.552 6.489 67.033 1.00 0.00
D H
ATOM 11227 CD1 LEU D 66 -24.486 8.276 65.838 1.00 0.00
D C
ATOM 11228 HD11 LEU D 66 -23.412 8.193 65.567 1.00 0.00
D H
ATOM 11229 HD12 LEU D 66 -25.088 7.822 65.021 1.00 0.00
D H
ATOM 11230 HD13 LEU D 66 -24.744 9.354 65.907 1.00 0.00
D H
ATOM 11231 CD2 LEU D 66 -23.860 8.117 68.240 1.00 0.00
D C

ATOM 11232 HD21 LEU D 66   −22.791   7.968 67.973 1.00 0.00
D   H
ATOM 11233 HD22 LEU D 66   −24.017   9.201 68.405 1.00 0.00
D   H
ATOM 11234 HD23 LEU D 66   −24.056   7.586 69.197 1.00 0.00
D   H
ATOM 11235 C   LEU D 66   −27.852   7.812 69.392 1.00 0.00
D   C
ATOM 11236 O   LEU D 66   −28.002   9.027 69.541 1.00 0.00
D   O
ATOM 11237 N   GLY D 67   −28.827   6.941 69.620 1.00 0.00
D   N
ATOM 11238 HN GLY D 67   −28.718   5.947 69.588 1.00 0.00
D   H
ATOM 11239 CA GLY D 67   −30.155   7.414 69.942 1.00 0.00
D   C
ATOM 11240 HA1 GLY D 67   −30.090   8.026 70.831 1.00 0.00
D   H
ATOM 11241 HA2 GLY D 67   −30.810   6.559 70.038 1.00 0.00
D   H
ATOM 11242 C   GLY D 67   −30.680   8.278 68.817 1.00 0.00
D   C
ATOM 11243 O   GLY D 67   −31.244   9.353 69.037 1.00 0.00
D   O
ATOM 11244 N   ILE D 68   −30.469   7.802 67.592 1.00 0.00
D   N
ATOM 11245 HN ILE D 68   −30.067   6.910 67.395 1.00 0.00
D   H
ATOM 11246 CA ILE D 68   −30.821   8.572 66.409 1.00 0.00
D   C
ATOM 11247 HA ILE D 68   −30.269   9.501 66.477 1.00 0.00
D   H
ATOM 11248 CB ILE D 68   −30.444   7.768 65.162 1.00 0.00
D   C
ATOM 11249 HB ILE D 68   −31.009   8.259 64.330 1.00 0.00
D   H
ATOM 11250 CG2 ILE D 68   −28.950   7.729 65.007 1.00 0.00
D   C
ATOM 11251 HG21 ILE D 68   −28.670   7.285 64.029 1.00 0.00
D   H
ATOM 11252 HG22 ILE D 68   −28.521   8.752 65.048 1.00 0.00
D   H
ATOM 11253 HG23 ILE D 68   −28.482   7.119 65.810 1.00 0.00
D   H
ATOM 11254 CG1 ILE D 68   −30.984   6.352 65.306 1.00 0.00
D   C
ATOM 11255 HG11 ILE D 68   −30.479   5.842 66.158 1.00 0.00
D   H
ATOM 11256 HG12 ILE D 68   −32.070   6.403 65.549 1.00 0.00
D   H
ATOM 11257 CD ILE D 68   −30.821   5.536 64.090 1.00 0.00
D   C
ATOM 11258 HD1 ILE D 68   −31.283   4.536 64.232 1.00 0.00
D   H

ATOM 11259 HD2 ILE D 68 −31.311 6.026 63.221 1.00 0.00
D H
ATOM 11260 HD3 ILE D 68 −29.747 5.388 63.851 1.00 0.00
D H
ATOM 11261 C ILE D 68 −32.294 8.935 66.400 1.00 0.00
D C
ATOM 11262 O ILE D 68 −32.659 10.068 66.071 1.00 0.00
D O
ATOM 11263 N ARG D 69 −33.160 7.990 66.761 1.00 0.00
D N
ATOM 11264 HN ARG D 69 −32.872 7.097 67.097 1.00 0.00
D H
ATOM 11265 CA ARG D 69 −34.592 8.226 66.677 1.00 0.00
D C
ATOM 11266 HA ARG D 69 −34.836 8.428 65.641 1.00 0.00
D H
ATOM 11267 CB ARG D 69 −35.335 6.996 67.162 1.00 0.00
D C
ATOM 11268 HB1 ARG D 69 −34.985 6.807 68.204 1.00 0.00
D H
ATOM 11269 HB2 ARG D 69 −36.421 7.225 67.240 1.00 0.00
D H
ATOM 11270 CG ARG D 69 −35.061 5.791 66.308 1.00 0.00
D C
ATOM 11271 HG1 ARG D 69 −35.545 5.941 65.317 1.00 0.00
D H
ATOM 11272 HG2 ARG D 69 −33.967 5.734 66.102 1.00 0.00
D H
ATOM 11273 CD ARG D 69 −35.502 4.535 67.004 1.00 0.00
D C
ATOM 11274 HD1 ARG D 69 −36.552 4.657 67.358 1.00 0.00
D H
ATOM 11275 HD2 ARG D 69 −35.455 3.652 66.325 1.00 0.00
D H
ATOM 11276 NE ARG D 69 −34.643 4.240 68.142 1.00 0.00
D N
ATOM 11277 HE ARG D 69 −33.781 3.792 67.880 1.00 0.00
D H
ATOM 11278 CZ ARG D 69 −34.954 4.525 69.399 1.00 0.00
D C
ATOM 11279 NH1 ARG D 69 −36.102 5.122 69.682 1.00 0.00
D N
ATOM 11280 HH11 ARG D 69 −36.329 5.362 70.615 1.00 0.00
D H
ATOM 11281 HH12 ARG D 69 −36.645 5.432 68.898 1.00 0.00
D H
ATOM 11282 NH2 ARG D 69 −34.115 4.221 70.376 1.00 0.00
D N
ATOM 11283 HH21 ARG D 69 −34.385 4.355 71.319 1.00 0.00
D H
ATOM 11284 HH22 ARG D 69 −33.325 3.674 70.133 1.00 0.00
D H
ATOM 11285 C ARG D 69 −35.018 9.442 67.468 1.00 0.00
D C

ATOM 11286 O ARG D 69 −36.008 10.082 67.101 1.00 0.00
D O
ATOM 11287 N ASN D 70 −34.291 9.783 68.527 1.00 0.00
D N
ATOM 11288 HN ASN D 70 −33.557 9.232 68.923 1.00 0.00
D H
ATOM 11289 CA ASN D 70 −34.531 11.042 69.207 1.00 0.00
D C
ATOM 11290 HA ASN D 70 −35.557 10.998 69.557 1.00 0.00
D H
ATOM 11291 CB ASN D 70 −33.543 11.209 70.355 1.00 0.00
D C
ATOM 11292 HB1 ASN D 70 −32.548 11.442 69.917 1.00 0.00
D H
ATOM 11293 HB2 ASN D 70 −33.835 12.067 70.994 1.00 0.00
D H
ATOM 11294 CG ASN D 70 −33.411 9.957 71.182 1.00 0.00
D C
ATOM 11295 OD1 ASN D 70 −34.264 9.074 71.126 1.00 0.00
D O
ATOM 11296 ND2 ASN D 70 −32.340 9.869 71.958 1.00 0.00
D N
ATOM 11297 HD21 ASN D 70 −32.236 9.041 72.501 1.00 0.00
D H
ATOM 11298 HD22 ASN D 70 −31.665 10.599 71.959 1.00 0.00
D H
ATOM 11299 C ASN D 70 −34.423 12.215 68.255 1.00 0.00
D C
ATOM 11300 O ASN D 70 −35.179 13.182 68.385 1.00 0.00
D O
ATOM 11301 N PHE D 71 −33.526 12.137 67.284 1.00 0.00
D N
ATOM 11302 HN PHE D 71 −32.917 11.356 67.154 1.00 0.00
D H
ATOM 11303 CA PHE D 71 −33.354 13.201 66.313 1.00 0.00
D C
ATOM 11304 HA PHE D 71 −33.383 14.137 66.857 1.00 0.00
D H
ATOM 11305 CB PHE D 71 −32.064 13.021 65.526 1.00 0.00
D C
ATOM 11306 HB1 PHE D 71 −32.080 12.030 65.021 1.00 0.00
D H
ATOM 11307 HB2 PHE D 71 −31.969 13.792 64.731 1.00 0.00
D H
ATOM 11308 CG PHE D 71 −30.842 13.033 66.370 1.00 0.00
D C
ATOM 11309 CD1 PHE D 71 −30.521 11.944 67.158 1.00 0.00
D C
ATOM 11310 HD1 PHE D 71 −31.171 11.080 67.166 1.00 0.00
D H
ATOM 11311 CE1 PHE D 71 −29.397 11.947 67.930 1.00 0.00
D C
ATOM 11312 HE1 PHE D 71 −29.159 11.088 68.541 1.00 0.00
D H

ATOM 11313 CZ PHE D 71 −28.563 13.047 67.922 1.00 0.00
D C
ATOM 11314 HZ PHE D 71 −27.666 13.049 68.525 1.00 0.00
D H
ATOM 11315 CD2 PHE D 71 −30.002 14.130 66.365 1.00 0.00
D C
ATOM 11316 HD2 PHE D 71 −30.249 14.996 65.766 1.00 0.00
D H
ATOM 11317 CE2 PHE D 71 −28.869 14.141 67.136 1.00 0.00
D C
ATOM 11318 HE2 PHE D 71 −28.220 15.006 67.126 1.00 0.00
D H
ATOM 11319 C PHE D 71 −34.536 13.203 65.360 1.00 0.00
D C
ATOM 11320 O PHE D 71 −35.202 12.178 65.186 1.00 0.00
D O
ATOM 11321 N GLU D 72 −34.784 14.356 64.743 1.00 0.00
D N
ATOM 11322 HN GLU D 72 −34.282 15.205 64.877 1.00 0.00
D H
ATOM 11323 CA GLU D 72 −35.868 14.455 63.776 1.00 0.00
D C
ATOM 11324 HA GLU D 72 −36.366 13.495 63.725 1.00 0.00
D H
ATOM 11325 CB GLU D 72 −36.840 15.531 64.220 1.00 0.00
D C
ATOM 11326 HB1 GLU D 72 −36.885 16.310 63.425 1.00 0.00
D H
ATOM 11327 HB2 GLU D 72 −37.867 15.106 64.290 1.00 0.00
D H
ATOM 11328 CG GLU D 72 −36.439 16.165 65.528 1.00 0.00
D C
ATOM 11329 HG1 GLU D 72 −36.211 15.374 66.267 1.00 0.00
D H
ATOM 11330 HG2 GLU D 72 −35.548 16.811 65.399 1.00 0.00
D H
ATOM 11331 CD GLU D 72 −37.550 16.985 66.129 1.00 0.00
D C
ATOM 11332 OE1 GLU D 72 −38.115 17.841 65.416 1.00 0.00
D O
ATOM 11333 OE2 GLU D 72 −37.858 16.771 67.322 1.00 0.00
D O
ATOM 11334 C GLU D 72 −35.337 14.761 62.384 1.00 0.00
D C
ATOM 11335 O GLU D 72 −35.874 14.269 61.384 1.00 0.00
D O
ATOM 11336 N TYR D 73 −34.286 15.573 62.299 1.00 0.00
D N
ATOM 11337 HN TYR D 73 −33.854 16.006 63.089 1.00 0.00
D H
ATOM 11338 CA TYR D 73 −33.672 15.900 61.018 1.00 0.00
D C
ATOM 11339 HA TYR D 73 −34.325 15.526 60.239 1.00 0.00
D H

```
ATOM  11340  CB  TYR D  73      -33.419  17.399  60.876  1.00  0.00
D    C
ATOM  11341  HB1 TYR D  73      -32.962  17.782  61.815  1.00  0.00
D    H
ATOM  11342  HB2 TYR D  73      -32.699  17.599  60.054  1.00  0.00
D    H
ATOM  11343  CG  TYR D  73      -34.644  18.239  60.645  1.00  0.00
D    C
ATOM  11344  CD1 TYR D  73      -35.576  18.434  61.650  1.00  0.00
D    C
ATOM  11345  HD1 TYR D  73      -35.439  17.957  62.611  1.00  0.00
D    H
ATOM  11346  CE1 TYR D  73      -36.690  19.211  61.443  1.00  0.00
D    C
ATOM  11347  HE1 TYR D  73      -37.415  19.355  62.231  1.00  0.00
D    H
ATOM  11348  CZ  TYR D  73      -36.881  19.800  60.216  1.00  0.00
D    C
ATOM  11349  OH  TYR D  73      -37.990  20.577  59.990  1.00  0.00
D    O
ATOM  11350  HH  TYR D  73      -37.904  20.951  59.111  1.00  0.00
D    H
ATOM  11351  CD2 TYR D  73      -34.862  18.850  59.425  1.00  0.00
D    C
ATOM  11352  HD2 TYR D  73      -34.170  18.690  58.610  1.00  0.00
D    H
ATOM  11353  CE2 TYR D  73      -35.970  19.623  59.207  1.00  0.00
D    C
ATOM  11354  HE2 TYR D  73      -36.130  20.070  58.238  1.00  0.00
D    H
ATOM  11355  C   TYR D  73      -32.359  15.154  60.896  1.00  0.00
D    C
ATOM  11356  O   TYR D  73      -31.547  15.166  61.827  1.00  0.00
D    O
ATOM  11357  N   VAL D  74      -32.135  14.526  59.746  1.00  0.00
D    N
ATOM  11358  HN  VAL D  74      -32.813  14.464  59.019  1.00  0.00
D    H
ATOM  11359  CA  VAL D  74      -30.870  13.872  59.448  1.00  0.00
D    C
ATOM  11360  HA  VAL D  74      -30.122  14.227  60.147  1.00  0.00
D    H
ATOM  11361  CB  VAL D  74      -30.968  12.339  59.558  1.00  0.00
D    C
ATOM  11362  HB  VAL D  74      -29.951  11.920  59.359  1.00  0.00
D    H
ATOM  11363  CG1 VAL D  74      -31.411  11.944  60.950  1.00  0.00
D    C
ATOM  11364 HG11 VAL D  74      -31.521  10.841  61.021  1.00  0.00
D    H
ATOM  11365 HG12 VAL D  74      -30.672  12.253  61.718  1.00  0.00
D    H
ATOM  11366 HG13 VAL D  74      -32.393  12.404  61.195  1.00  0.00
D    H
```

ATOM 11367 CG2 VAL D 74 −31.917 11.775 58.521 1.00 0.00
D C
ATOM 11368 HG21 VAL D 74 −32.012 10.674 58.634 1.00 0.00
D H
ATOM 11369 HG22 VAL D 74 −32.927 12.227 58.629 1.00 0.00
D H
ATOM 11370 HG23 VAL D 74 −31.553 11.976 57.492 1.00 0.00
D H
ATOM 11371 C VAL D 74 −30.429 14.293 58.057 1.00 0.00
D C
ATOM 11372 O VAL D 74 −31.251 14.402 57.137 1.00 0.00
D O
ATOM 11373 N ILE D 75 −29.137 14.541 57.912 1.00 0.00
D N
ATOM 11374 HN ILE D 75 −28.477 14.422 58.650 1.00 0.00
D H
ATOM 11375 CA ILE D 75 −28.559 15.008 56.665 1.00 0.00
D C
ATOM 11376 HA ILE D 75 −29.287 14.849 55.879 1.00 0.00
D H
ATOM 11377 CB ILE D 75 −28.120 16.468 56.762 1.00 0.00
D C
ATOM 11378 HB ILE D 75 −27.092 16.481 56.319 1.00 0.00
D H
ATOM 11379 CG2 ILE D 75 −29.112 17.342 56.049 1.00 0.00
D C
ATOM 11380 HG21 ILE D 75 −28.733 18.383 55.974 1.00 0.00
D H
ATOM 11381 HG22 ILE D 75 −29.294 16.975 55.017 1.00 0.00
D H
ATOM 11382 HG23 ILE D 75 −30.083 17.365 56.590 1.00 0.00
D H
ATOM 11383 CG1 ILE D 75 −27.989 16.845 58.231 1.00 0.00
D C
ATOM 11384 HG11 ILE D 75 −28.577 16.135 58.856 1.00 0.00
D H
ATOM 11385 HG12 ILE D 75 −26.922 16.745 58.535 1.00 0.00
D H
ATOM 11386 CD ILE D 75 −28.424 18.233 58.549 1.00 0.00
D C
ATOM 11387 HD1 ILE D 75 −28.246 18.456 59.624 1.00 0.00
D H
ATOM 11388 HD2 ILE D 75 −27.856 18.970 57.943 1.00 0.00
D H
ATOM 11389 HD3 ILE D 75 −29.507 18.367 58.345 1.00 0.00
D H
ATOM 11390 C ILE D 75 −27.378 14.120 56.351 1.00 0.00
D C
ATOM 11391 O ILE D 75 −26.359 14.164 57.046 1.00 0.00
D O
ATOM 11392 N VAL D 76 −27.507 13.333 55.300 1.00 0.00
D N
ATOM 11393 HN VAL D 76 −28.338 13.277 54.753 1.00 0.00
D H

ATOM 11394 CA VAL D 76   -26.431 12.472 54.847 1.00 0.00
D   C
ATOM 11395 HA VAL D 76   -25.772 12.238 55.673 1.00 0.00
D   H
ATOM 11396 CB VAL D 76   -26.963 11.179 54.254 1.00 0.00
D   C
ATOM 11397 HB VAL D 76   -27.546 11.443 53.338 1.00 0.00
D   H
ATOM 11398 CG1 VAL D 76   -25.831 10.283 53.881 1.00 0.00
D   C
ATOM 11399 HG11 VAL D 76   -26.216   9.320 53.483 1.00 0.00
D   H
ATOM 11400 HG12 VAL D 76   -25.193 10.735 53.093 1.00 0.00
D   H
ATOM 11401 HG13 VAL D 76   -25.198 10.060 54.766 1.00 0.00
D   H
ATOM 11402 CG2 VAL D 76   -27.851 10.502 55.263 1.00 0.00
D   C
ATOM 11403 HG21 VAL D 76   -28.224   9.532 54.870 1.00 0.00
D   H
ATOM 11404 HG22 VAL D 76   -27.294 10.308 56.204 1.00 0.00
D   H
ATOM 11405 HG23 VAL D 76   -28.735 11.128 55.504 1.00 0.00
D   H
ATOM 11406 C   VAL D 76   -25.637 13.306 53.849 1.00 0.00
D   C
ATOM 11407 O   VAL D 76   -25.955 13.380 52.656 1.00 0.00
D   O
ATOM 11408 N   ALA D 77   -24.616 13.985 54.362 1.00 0.00
D   N
ATOM 11409 HN ALA D 77   -24.397 13.942 55.337 1.00 0.00
D   H
ATOM 11410 CA ALA D 77   -23.742 14.835 53.561 1.00 0.00
D   C
ATOM 11411 HA ALA D 77   -24.252 15.170 52.665 1.00 0.00
D   H
ATOM 11412 CB ALA D 77   -23.340 16.074 54.349 1.00 0.00
D   C
ATOM 11413 HB1 ALA D 77   -24.249 16.644 54.637 1.00 0.00
D   H
ATOM 11414 HB2 ALA D 77   -22.800 15.798 55.281 1.00 0.00
D   H
ATOM 11415 HB3 ALA D 77   -22.689 16.747 53.750 1.00 0.00
D   H
ATOM 11416 C   ALA D 77   -22.516 14.075 53.098 1.00 0.00
D   C
ATOM 11417 O   ALA D 77   -21.382 14.501 53.299 1.00 0.00
D   O
ATOM 11418 N   ILE D 78   -22.735 12.930 52.483 1.00 0.00
D   N
ATOM 11419 HN ILE D 78   -23.641 12.519 52.405 1.00 0.00
D   H
ATOM 11420 CA ILE D 78   -21.676 12.156 51.862 1.00 0.00
D   C

ATOM 11421 HA ILE D 78   -20.725 12.519 52.229 1.00 0.00
D   H
ATOM 11422 CB ILE D 78   -21.865 10.661 52.122 1.00 0.00
D   C
ATOM 11423 HB ILE D 78   -21.635 10.534 53.210 1.00 0.00
D   H
ATOM 11424 CG2 ILE D 78   -23.224 10.244 51.689 1.00 0.00
D   C
ATOM 11425 HG21 ILE D 78   -23.435   9.203 52.011 1.00 0.00
D   H
ATOM 11426 HG22 ILE D 78   -24.001 10.898 52.138 1.00 0.00
D   H
ATOM 11427 HG23 ILE D 78   -23.321 10.292 50.584 1.00 0.00
D   H
ATOM 11428 CG1 ILE D 78   -20.837   9.838 51.364 1.00 0.00
D   C
ATOM 11429 HG11 ILE D 78   -20.921 10.043 50.273 1.00 0.00
D   H
ATOM 11430 HG12 ILE D 78   -19.818 10.154 51.681 1.00 0.00
D   H
ATOM 11431 CD ILE D 78   -20.989   8.372 51.615 1.00 0.00
D   C
ATOM 11432 HD1 ILE D 78   -20.187   7.807 51.092 1.00 0.00
D   H
ATOM 11433 HD2 ILE D 78   -20.918   8.152 52.702 1.00 0.00
D   H
ATOM 11434 HD3 ILE D 78   -21.968   8.003 51.243 1.00 0.00
D   H
ATOM 11435 C   ILE D 78   -21.729 12.482 50.380 1.00 0.00
D   C
ATOM 11436 O   ILE D 78   -22.801 12.792 49.852 1.00 0.00
D   O
ATOM 11437 N   GLY D 79   -20.585 12.456 49.702 1.00 0.00
D   N
ATOM 11438 HN GLY D 79   -19.683 12.288 50.098 1.00 0.00
D   H
ATOM 11439 CA GLY D 79   -20.608 12.684 48.270 1.00 0.00
D   C
ATOM 11440 HA1 GLY D 79   -20.110 13.632 48.116 1.00 0.00
D   H
ATOM 11441 HA2 GLY D 79   -21.651 12.678 47.983 1.00 0.00
D   H
ATOM 11442 C   GLY D 79   -19.893 11.640 47.444 1.00 0.00
D   C
ATOM 11443 O   GLY D 79   -20.185 11.475 46.257 1.00 0.00
D   O
ATOM 11444 N   ALA D 80   -18.954 10.924 48.053 1.00 0.00
D   N
ATOM 11445 HN ALA D 80   -18.743 10.957 49.030 1.00 0.00
D   H
ATOM 11446 CA ALA D 80   -18.142 10.004 47.270 1.00 0.00
D   C
ATOM 11447 HA ALA D 80   -18.132 10.320 46.233 1.00 0.00
D   H

```
ATOM  11448  CB  ALA D  80     -16.700  10.017  47.783  1.00  0.00
D    C
ATOM  11449  HB1 ALA D  80     -16.295  11.051  47.729  1.00  0.00
D    H
ATOM  11450  HB2 ALA D  80     -16.649   9.684  48.842  1.00  0.00
D    H
ATOM  11451  HB3 ALA D  80     -16.043   9.360  47.173  1.00  0.00
D    H
ATOM  11452  C   ALA D  80     -18.672   8.578  47.279  1.00  0.00
D    C
ATOM  11453  O   ALA D  80     -18.992   8.027  46.223  1.00  0.00
D    O
ATOM  11454  N   ASN D  81     -18.775   7.967  48.447  1.00  0.00
D    N
ATOM  11455  HN  ASN D  81     -18.689   8.435  49.325  1.00  0.00
D    H
ATOM  11456  CA  ASN D  81     -19.027   6.539  48.548  1.00  0.00
D    C
ATOM  11457  HA  ASN D  81     -18.553   6.074  47.690  1.00  0.00
D    H
ATOM  11458  CB  ASN D  81     -18.475   6.017  49.867  1.00  0.00
D    C
ATOM  11459  HB1 ASN D  81     -17.408   6.327  49.935  1.00  0.00
D    H
ATOM  11460  HB2 ASN D  81     -19.011   6.481  50.720  1.00  0.00
D    H
ATOM  11461  CG  ASN D  81     -18.533   4.529  49.965  1.00  0.00
D    C
ATOM  11462  OD1 ASN D  81     -19.423   3.894  49.413  1.00  0.00
D    O
ATOM  11463  ND2 ASN D  81     -17.579   3.952  50.679  1.00  0.00
D    N
ATOM  11464 HD21 ASN D  81     -17.595   2.958  50.747  1.00  0.00
D    H
ATOM  11465 HD22 ASN D  81     -16.866   4.506  51.098  1.00  0.00
D    H
ATOM  11466  C   ASN D  81     -20.524   6.289  48.437  1.00  0.00
D    C
ATOM  11467  O   ASN D  81     -21.217   6.205  49.454  1.00  0.00
D    O
ATOM  11468  N   ILE D  82     -21.016   6.123  47.208  1.00  0.00
D    N
ATOM  11469  HN  ILE D  82     -20.475   6.084  46.371  1.00  0.00
D    H
ATOM  11470  CA  ILE D  82     -22.454   5.978  46.999  1.00  0.00
D    C
ATOM  11471  HA  ILE D  82     -22.908   6.904  47.331  1.00  0.00
D    H
ATOM  11472  CB  ILE D  82     -22.757   5.729  45.513  1.00  0.00
D    C
ATOM  11473  HB  ILE D  82     -22.004   4.963  45.198  1.00  0.00
D    H
ATOM  11474  CG2 ILE D  82     -24.192   5.296  45.321  1.00  0.00
D    C
```

```
ATOM  11475 HG21 ILE D  82      -24.365   4.968  44.275  1.00  0.00
D    H
ATOM  11476 HG22 ILE D  82      -24.441   4.443  45.986  1.00  0.00
D    H
ATOM  11477 HG23 ILE D  82      -24.892   6.130  45.544  1.00  0.00
D    H
ATOM  11478  CG1 ILE D  82      -22.475   6.988  44.702  1.00  0.00
D    C
ATOM  11479 HG11 ILE D  82      -23.189   7.056  43.851  1.00  0.00
D    H
ATOM  11480 HG12 ILE D  82      -22.647   7.880  45.348  1.00  0.00
D    H
ATOM  11481  CD  ILE D  82      -21.083   7.068  44.169  1.00  0.00
D    C
ATOM  11482  HD1 ILE D  82      -20.927   8.033  43.638  1.00  0.00
D    H
ATOM  11483  HD2 ILE D  82      -20.343   7.002  44.995  1.00  0.00
D    H
ATOM  11484  HD3 ILE D  82      -20.884   6.245  43.450  1.00  0.00
D    H
ATOM  11485  C   ILE D  82      -23.019   4.872  47.876  1.00  0.00
D    C
ATOM  11486  O   ILE D  82      -24.134   4.988  48.407  1.00  0.00
D    O
ATOM  11487  N   GLN D  83      -22.248   3.802  48.062  1.00  0.00
D    N
ATOM  11488  HN  GLN D  83      -21.351   3.672  47.645  1.00  0.00
D    H
ATOM  11489  CA  GLN D  83      -22.677   2.706  48.920  1.00  0.00
D    C
ATOM  11490  HA  GLN D  83      -23.492   2.207  48.411  1.00  0.00
D    H
ATOM  11491  CB  GLN D  83      -21.500   1.783  49.180  1.00  0.00
D    C
ATOM  11492  HB1 GLN D  83      -20.651   2.162  48.565  1.00  0.00
D    H
ATOM  11493  HB2 GLN D  83      -21.182   1.881  50.243  1.00  0.00
D    H
ATOM  11494  CG  GLN D  83      -21.716   0.360  48.853  1.00  0.00
D    C
ATOM  11495  HG1 GLN D  83      -22.704   0.021  49.233  1.00  0.00
D    H
ATOM  11496  HG2 GLN D  83      -21.701   0.202  47.755  1.00  0.00
D    H
ATOM  11497  CD  GLN D  83      -20.663  -0.496  49.506  1.00  0.00
D    C
ATOM  11498  OE1 GLN D  83      -20.326  -0.289  50.666  1.00  0.00
D    O
ATOM  11499  NE2 GLN D  83      -20.115  -1.438  48.762  1.00  0.00
D    N
ATOM  11500 HE21 GLN D  83      -19.422  -2.007  49.195  1.00  0.00
D    H
ATOM  11501 HE22 GLN D  83      -20.390  -1.540  47.812  1.00  0.00
D    H
```

ATOM 11502 C GLN D 83 −23.210 3.222 50.242 1.00 0.00
D C
ATOM 11503 O GLN D 83 −24.398 3.066 50.553 1.00 0.00
D O
ATOM 11504 N ALA D 84 −22.345 3.869 51.013 1.00 0.00
D N
ATOM 11505 HN ALA D 84 −21.408 4.089 50.742 1.00 0.00
D H
ATOM 11506 CA ALA D 84 −22.728 4.310 52.342 1.00 0.00
D C
ATOM 11507 HA ALA D 84 −23.011 3.465 52.960 1.00 0.00
D H
ATOM 11508 CB ALA D 84 −21.555 5.015 53.014 1.00 0.00
D C
ATOM 11509 HB1 ALA D 84 −20.696 4.317 53.093 1.00 0.00
D H
ATOM 11510 HB2 ALA D 84 −21.226 5.896 52.422 1.00 0.00
D H
ATOM 11511 HB3 ALA D 84 −21.815 5.356 54.040 1.00 0.00
D H
ATOM 11512 C ALA D 84 −23.941 5.219 52.281 1.00 0.00
D C
ATOM 11513 O ALA D 84 −24.896 5.045 53.041 1.00 0.00
D O
ATOM 11514 N SER D 85 −23.917 6.195 51.376 1.00 0.00
D N
ATOM 11515 HN SER D 85 −23.150 6.400 50.768 1.00 0.00
D H
ATOM 11516 CA SER D 85 −25.066 7.073 51.204 1.00 0.00
D C
ATOM 11517 HA SER D 85 −25.099 7.702 52.083 1.00 0.00
D H
ATOM 11518 CB SER D 85 −24.918 7.863 49.909 1.00 0.00
D C
ATOM 11519 HB1 SER D 85 −25.513 8.801 50.007 1.00 0.00
D H
ATOM 11520 HB2 SER D 85 −23.856 8.163 49.757 1.00 0.00
D H
ATOM 11521 OG SER D 85 −25.410 7.111 48.819 1.00 0.00
D O
ATOM 11522 HG1 SER D 85 −25.505 7.719 48.075 1.00 0.00
D H
ATOM 11523 C SER D 85 −26.355 6.272 51.171 1.00 0.00
D C
ATOM 11524 O SER D 85 −27.273 6.496 51.972 1.00 0.00
D O
ATOM 11525 N THR D 86 −26.420 5.296 50.277 1.00 0.00
D N
ATOM 11526 HN THR D 86 −25.661 4.944 49.730 1.00 0.00
D H
ATOM 11527 CA THR D 86 −27.692 4.640 50.025 1.00 0.00
D C
ATOM 11528 HA THR D 86 −28.425 5.434 49.979 1.00 0.00
D H

ATOM 11529 CB THR D 86 -27.613 3.858 48.725 1.00 0.00
D C
ATOM 11530 HB THR D 86 -28.633 3.433 48.557 1.00 0.00
D H
ATOM 11531 OG1 THR D 86 -26.697 2.773 48.886 1.00 0.00
D O
ATOM 11532 HG1 THR D 86 -26.679 2.324 48.034 1.00 0.00
D H
ATOM 11533 CG2 THR D 86 -27.143 4.763 47.610 1.00 0.00
D C
ATOM 11534 HG21 THR D 86 -27.189 4.241 46.632 1.00 0.00
D H
ATOM 11535 HG22 THR D 86 -27.789 5.666 47.546 1.00 0.00
D H
ATOM 11536 HG23 THR D 86 -26.096 5.096 47.779 1.00 0.00
D H
ATOM 11537 C THR D 86 -28.104 3.737 51.180 1.00 0.00
D C
ATOM 11538 O THR D 86 -29.250 3.803 51.640 1.00 0.00
D O
ATOM 11539 N LEU D 87 -27.201 2.875 51.654 1.00 0.00
D N
ATOM 11540 HN LEU D 87 -26.265 2.744 51.333 1.00 0.00
D H
ATOM 11541 CA LEU D 87 -27.581 2.006 52.768 1.00 0.00
D C
ATOM 11542 HA LEU D 87 -28.469 1.470 52.460 1.00 0.00
D H
ATOM 11543 CB LEU D 87 -26.487 0.993 53.115 1.00 0.00
D C
ATOM 11544 HB1 LEU D 87 -25.791 1.493 53.826 1.00 0.00
D H
ATOM 11545 HB2 LEU D 87 -26.943 0.146 53.673 1.00 0.00
D H
ATOM 11546 CG LEU D 87 -25.472 0.241 52.249 1.00 0.00
D C
ATOM 11547 HG LEU D 87 -25.685 -0.829 52.487 1.00 0.00
D H
ATOM 11548 CD1 LEU D 87 -25.551 0.460 50.752 1.00 0.00
D C
ATOM 11549 HD11 LEU D 87 -24.740 -0.096 50.234 1.00 0.00
D H
ATOM 11550 HD12 LEU D 87 -26.526 0.105 50.356 1.00 0.00
D H
ATOM 11551 HD13 LEU D 87 -25.444 1.538 50.507 1.00 0.00
D H
ATOM 11552 CD2 LEU D 87 -24.066 0.569 52.757 1.00 0.00
D C
ATOM 11553 HD21 LEU D 87 -23.303 0.024 52.161 1.00 0.00
D H
ATOM 11554 HD22 LEU D 87 -23.844 1.652 52.677 1.00 0.00
D H
ATOM 11555 HD23 LEU D 87 -23.957 0.267 53.821 1.00 0.00
D H

ATOM 11556 C LEU D 87 -27.943 2.827 53.991 1.00 0.00
D C
ATOM 11557 O LEU D 87 -28.852 2.473 54.753 1.00 0.00
D O
ATOM 11558 N THR D 88 -27.242 3.936 54.190 1.00 0.00
D N
ATOM 11559 HN THR D 88 -26.438 4.204 53.659 1.00 0.00
D H
ATOM 11560 CA THR D 88 -27.606 4.873 55.232 1.00 0.00
D C
ATOM 11561 HA THR D 88 -27.510 4.336 56.166 1.00 0.00
D H
ATOM 11562 CB THR D 88 -26.722 6.098 55.098 1.00 0.00
D C
ATOM 11563 HB THR D 88 -26.929 6.519 54.084 1.00 0.00
D H
ATOM 11564 OG1 THR D 88 -25.353 5.684 55.143 1.00 0.00
D O
ATOM 11565 HG1 THR D 88 -24.838 6.495 55.065 1.00 0.00
D H
ATOM 11566 CG2 THR D 88 -27.013 7.075 56.194 1.00 0.00
D C
ATOM 11567 HG21 THR D 88 -26.453 8.021 56.039 1.00 0.00
D H
ATOM 11568 HG22 THR D 88 -28.097 7.319 56.217 1.00 0.00
D H
ATOM 11569 HG23 THR D 88 -26.729 6.661 57.186 1.00 0.00
D H
ATOM 11570 C THR D 88 -29.061 5.267 55.110 1.00 0.00
D C
ATOM 11571 O THR D 88 -29.873 4.986 56.000 1.00 0.00
D O
ATOM 11572 N THR D 89 -29.407 5.876 53.977 1.00 0.00
D N
ATOM 11573 HN THR D 89 -28.780 6.085 53.226 1.00 0.00
D H
ATOM 11574 CA THR D 89 -30.781 6.298 53.745 1.00 0.00
D C
ATOM 11575 HA THR D 89 -30.987 7.057 54.488 1.00 0.00
D H
ATOM 11576 CB THR D 89 -30.933 6.782 52.317 1.00 0.00
D C
ATOM 11577 HB THR D 89 -32.030 6.911 52.149 1.00 0.00
D H
ATOM 11578 OG1 THR D 89 -30.456 5.760 51.437 1.00 0.00
D O
ATOM 11579 HG1 THR D 89 -30.563 6.115 50.549 1.00 0.00
D H
ATOM 11580 CG2 THR D 89 -30.124 8.034 52.109 1.00 0.00
D C
ATOM 11581 HG21 THR D 89 -30.314 8.465 51.104 1.00 0.00
D H
ATOM 11582 HG22 THR D 89 -30.395 8.801 52.866 1.00 0.00
D H

ATOM 11583 HG23 THR D 89 -29.036 7.826 52.199 1.00 0.00
D H
ATOM 11584 C THR D 89 -31.752 5.166 54.004 1.00 0.00
D C
ATOM 11585 O THR D 89 -32.841 5.381 54.545 1.00 0.00
D O
ATOM 11586 N LEU D 90 -31.380 3.954 53.603 1.00 0.00
D N
ATOM 11587 HN LEU D 90 -30.551 3.737 53.093 1.00 0.00
D H
ATOM 11588 CA LEU D 90 -32.216 2.803 53.903 1.00 0.00
D C
ATOM 11589 HA LEU D 90 -33.166 2.967 53.409 1.00 0.00
D H
ATOM 11590 CB LEU D 90 -31.550 1.528 53.409 1.00 0.00
D C
ATOM 11591 HB1 LEU D 90 -31.735 1.463 52.312 1.00 0.00
D H
ATOM 11592 HB2 LEU D 90 -30.449 1.625 53.531 1.00 0.00
D H
ATOM 11593 CG LEU D 90 -32.066 0.258 54.082 1.00 0.00
D C
ATOM 11594 HG LEU D 90 -31.781 0.291 55.161 1.00 0.00
D H
ATOM 11595 CD1 LEU D 90 -33.566 0.120 53.945 1.00 0.00
D C
ATOM 11596 HD11 LEU D 90 -33.913 -0.832 54.398 1.00 0.00
D H
ATOM 11597 HD12 LEU D 90 -34.084 0.960 54.456 1.00 0.00
D H
ATOM 11598 HD13 LEU D 90 -33.864 0.124 52.875 1.00 0.00
D H
ATOM 11599 CD2 LEU D 90 -31.394 -0.923 53.463 1.00 0.00
D C
ATOM 11600 HD21 LEU D 90 -31.739 -1.861 53.948 1.00 0.00
D H
ATOM 11601 HD22 LEU D 90 -31.619 -1.001 52.380 1.00 0.00
D H
ATOM 11602 HD23 LEU D 90 -30.292 -0.852 53.588 1.00 0.00
D H
ATOM 11603 C LEU D 90 -32.485 2.706 55.392 1.00 0.00
D C
ATOM 11604 O LEU D 90 -33.640 2.630 55.820 1.00 0.00
D O
ATOM 11605 N LEU D 91 -31.428 2.684 56.193 1.00 0.00
D N
ATOM 11606 HN LEU D 91 -30.476 2.784 55.910 1.00 0.00
D H
ATOM 11607 CA LEU D 91 -31.611 2.501 57.626 1.00 0.00
D C
ATOM 11608 HA LEU D 91 -32.154 1.574 57.758 1.00 0.00
D H
ATOM 11609 CB LEU D 91 -30.260 2.427 58.325 1.00 0.00
D C

ATOM 11610 HB1 LEU D 91   -29.556   3.070 57.748 1.00 0.00
D   H
ATOM 11611 HB2 LEU D 91   -30.348   2.894 59.331 1.00 0.00
D   H
ATOM 11612 CG LEU D 91   -29.710   1.008 58.410 1.00 0.00
D   C
ATOM 11613 HG LEU D 91   -28.698   1.059 58.881 1.00 0.00
D   H
ATOM 11614 CD1 LEU D 91   -30.629   0.155 59.264 1.00 0.00
D   C
ATOM 11615 HD11 LEU D 91   -30.268 -0.895 59.297 1.00 0.00
D   H
ATOM 11616 HD12 LEU D 91   -30.668   0.544 60.304 1.00 0.00
D   H
ATOM 11617 HD13 LEU D 91   -31.661   0.155 58.850 1.00 0.00
D   H
ATOM 11618 CD2 LEU D 91   -29.555   0.401 57.034 1.00 0.00
D   C
ATOM 11619 HD21 LEU D 91   -29.136 -0.625 57.110 1.00 0.00
D   H
ATOM 11620 HD22 LEU D 91   -30.527   0.330 56.506 1.00 0.00
D   H
ATOM 11621 HD23 LEU D 91   -28.865   1.013 56.414 1.00 0.00
D   H
ATOM 11622 C   LEU D 91   -32.459   3.614 58.211 1.00 0.00
D   C
ATOM 11623 O   LEU D 91   -33.336   3.377 59.049 1.00 0.00
D   O
ATOM 11624 N   LEU D 92   -32.221   4.838 57.761 1.00 0.00
D   N
ATOM 11625 HN LEU D 92   -31.563   5.088 57.053 1.00 0.00
D   H
ATOM 11626 CA LEU D 92   -32.957   5.964 58.316 1.00 0.00
D   C
ATOM 11627 HA LEU D 92   -32.874   5.889 59.392 1.00 0.00
D   H
ATOM 11628 CB LEU D 92   -32.352   7.276 57.835 1.00 0.00
D   C
ATOM 11629 HB1 LEU D 92   -32.042   7.126 56.774 1.00 0.00
D   H
ATOM 11630 HB2 LEU D 92   -33.146   8.056 57.816 1.00 0.00
D   H
ATOM 11631 CG LEU D 92   -31.153   7.721 58.660 1.00 0.00
D   C
ATOM 11632 HG LEU D 92   -30.968   8.804 58.462 1.00 0.00
D   H
ATOM 11633 CD1 LEU D 92   -31.463   7.512 60.123 1.00 0.00
D   C
ATOM 11634 HD11 LEU D 92   -30.586   7.782 60.750 1.00 0.00
D   H
ATOM 11635 HD12 LEU D 92   -32.323   8.142 60.434 1.00 0.00
D   H
ATOM 11636 HD13 LEU D 92   -31.716   6.449 60.323 1.00 0.00
D   H

ATOM 11637 CD2 LEU D 92 −29.912 6.960 58.283 1.00 0.00
D C
ATOM 11638 HD21 LEU D 92 −29.045 7.313 58.884 1.00 0.00
D H
ATOM 11639 HD22 LEU D 92 −30.026 5.872 58.464 1.00 0.00
D H
ATOM 11640 HD23 LEU D 92 −29.674 7.112 57.208 1.00 0.00
D H
ATOM 11641 C LEU D 92 −34.434 5.889 57.964 1.00 0.00
D C
ATOM 11642 O LEU D 92 −35.294 5.955 58.850 1.00 0.00
D O
ATOM 11643 N LYS D 93 −34.749 5.738 56.680 1.00 0.00
D N
ATOM 11644 HN LYS D 93 −34.092 5.685 55.931 1.00 0.00
D H
ATOM 11645 CA LYS D 93 −36.145 5.635 56.285 1.00 0.00
D C
ATOM 11646 HA LYS D 93 −36.653 6.516 56.662 1.00 0.00
D H
ATOM 11647 CB LYS D 93 −36.255 5.566 54.767 1.00 0.00
D C
ATOM 11648 HB1 LYS D 93 −35.386 6.125 54.350 1.00 0.00
D H
ATOM 11649 HB2 LYS D 93 −36.131 4.509 54.443 1.00 0.00
D H
ATOM 11650 CG LYS D 93 −37.538 6.156 54.226 1.00 0.00
D C
ATOM 11651 HG1 LYS D 93 −37.474 6.208 53.115 1.00 0.00
D H
ATOM 11652 HG2 LYS D 93 −38.384 5.476 54.478 1.00 0.00
D H
ATOM 11653 CD LYS D 93 −37.772 7.543 54.787 1.00 0.00
D C
ATOM 11654 HD1 LYS D 93 −37.813 7.471 55.897 1.00 0.00
D H
ATOM 11655 HD2 LYS D 93 −36.892 8.176 54.526 1.00 0.00
D H
ATOM 11656 CE LYS D 93 −39.048 8.154 54.235 1.00 0.00
D C
ATOM 11657 HE1 LYS D 93 −38.927 8.403 53.157 1.00 0.00
D H
ATOM 11658 HE2 LYS D 93 −39.898 7.444 54.340 1.00 0.00
D H
ATOM 11659 NZ LYS D 93 −39.422 9.410 54.945 1.00 0.00
D N
ATOM 11660 HZ1 LYS D 93 −40.297 9.800 54.541 1.00 0.00
D H
ATOM 11661 HZ2 LYS D 93 −39.567 9.208 55.954 1.00 0.00
D H
ATOM 11662 HZ3 LYS D 93 −38.655 10.106 54.843 1.00 0.00
D H
ATOM 11663 C LYS D 93 −36.801 4.425 56.926 1.00 0.00
D C

```
ATOM  11664  O   LYS D  93     -38.006   4.432  57.195  1.00  0.00
D    O
ATOM  11665  N   GLU D  94     -36.023   3.377  57.179  1.00  0.00
D    N
ATOM  11666  HN  GLU D  94     -35.073   3.270  56.899  1.00  0.00
D    H
ATOM  11667  CA  GLU D  94     -36.548   2.249  57.931  1.00  0.00
D    C
ATOM  11668  HA  GLU D  94     -37.498   1.964  57.496  1.00  0.00
D    H
ATOM  11669  CB  GLU D  94     -35.581   1.075  57.863  1.00  0.00
D    C
ATOM  11670  HB1 GLU D  94     -35.661   0.617  56.850  1.00  0.00
D    H
ATOM  11671  HB2 GLU D  94     -34.534   1.443  57.949  1.00  0.00
D    H
ATOM  11672  CG  GLU D  94     -35.850   0.018  58.908  1.00  0.00
D    C
ATOM  11673  HG1 GLU D  94     -35.471   0.364  59.888  1.00  0.00
D    H
ATOM  11674  HG2 GLU D  94     -36.935  -0.192  58.990  1.00  0.00
D    H
ATOM  11675  CD  GLU D  94     -35.129  -1.271  58.619  1.00  0.00
D    C
ATOM  11676  OE1 GLU D  94     -34.924  -1.579  57.428  1.00  0.00
D    O
ATOM  11677  OE2 GLU D  94     -34.770  -1.982  59.580  1.00  0.00
D    O
ATOM  11678  C   GLU D  94     -36.811   2.636  59.373  1.00  0.00
D    C
ATOM  11679  O   GLU D  94     -37.764   2.146  59.988  1.00  0.00
D    O
ATOM  11680  N   LEU D  95     -35.985   3.511  59.932  1.00  0.00
D    N
ATOM  11681  HN  LEU D  95     -35.191   3.911  59.478  1.00  0.00
D    H
ATOM  11682  CA  LEU D  95     -36.186   3.966  61.295  1.00  0.00
D    C
ATOM  11683  HA  LEU D  95     -36.745   3.184  61.793  1.00  0.00
D    H
ATOM  11684  CB  LEU D  95     -34.847   4.149  61.993  1.00  0.00
D    C
ATOM  11685  HB1 LEU D  95     -34.093   4.378  61.204  1.00  0.00
D    H
ATOM  11686  HB2 LEU D  95     -34.897   5.051  62.642  1.00  0.00
D    H
ATOM  11687  CG  LEU D  95     -34.522   2.856  62.740  1.00  0.00
D    C
ATOM  11688  HG  LEU D  95     -35.462   2.570  63.271  1.00  0.00
D    H
ATOM  11689  CD1 LEU D  95     -34.124   1.751  61.779  1.00  0.00
D    C
ATOM  11690 HD11 LEU D  95     -33.844   0.832  62.336  1.00  0.00
D    H
```

```
ATOM  11691 HD12 LEU D  95      -34.966   1.504  61.097  1.00  0.00
D    H
ATOM  11692 HD13 LEU D  95      -33.254   2.061  61.162  1.00  0.00
D    H
ATOM  11693  CD2 LEU D  95      -33.449   3.077  63.776  1.00  0.00
D    C
ATOM  11694 HD21 LEU D  95      -33.240   2.131  64.321  1.00  0.00
D    H
ATOM  11695 HD22 LEU D  95      -32.499   3.417  63.316  1.00  0.00
D    H
ATOM  11696 HD23 LEU D  95      -33.773   3.839  64.516  1.00  0.00
D    H
ATOM  11697  C   LEU D  95      -37.010   5.237  61.371  1.00  0.00
D    C
ATOM  11698  O   LEU D  95      -36.906   5.974  62.355  1.00  0.00
D    O
ATOM  11699  N   ASP D  96      -37.804   5.517  60.341  1.00  0.00
D    N
ATOM  11700  HN  ASP D  96      -37.680   5.157  59.421  1.00  0.00
D    H
ATOM  11701  CA  ASP D  96      -38.959   6.397  60.456  1.00  0.00
D    C
ATOM  11702  HA  ASP D  96      -39.420   6.431  59.475  1.00  0.00
D    H
ATOM  11703  CB  ASP D  96      -39.933   5.857  61.497  1.00  0.00
D    C
ATOM  11704  HB1 ASP D  96      -39.557   6.103  62.511  1.00  0.00
D    H
ATOM  11705  HB2 ASP D  96      -40.932   6.320  61.374  1.00  0.00
D    H
ATOM  11706  CG  ASP D  96      -40.078   4.363  61.413  1.00  0.00
D    C
ATOM  11707  OD1 ASP D  96      -39.932   3.826  60.297  1.00  0.00
D    O
ATOM  11708  OD2 ASP D  96      -40.310   3.727  62.458  1.00  0.00
D    O
ATOM  11709  C   ASP D  96      -38.567   7.820  60.805  1.00  0.00
D    C
ATOM  11710  O   ASP D  96      -39.399   8.610  61.250  1.00  0.00
D    O
ATOM  11711  N   ILE D  97      -37.300   8.152  60.616  1.00  0.00
D    N
ATOM  11712  HN  ILE D  97      -36.570   7.571  60.261  1.00  0.00
D    H
ATOM  11713  CA  ILE D  97      -36.876   9.509  60.948  1.00  0.00
D    C
ATOM  11714  HA  ILE D  97      -37.180   9.701  61.969  1.00  0.00
D    H
ATOM  11715  CB  ILE D  97      -35.365   9.649  60.769  1.00  0.00
D    C
ATOM  11716  HB  ILE D  97      -35.178   9.332  59.711  1.00  0.00
D    H
ATOM  11717  CG2 ILE D  97      -34.911  11.055  61.097  1.00  0.00
D    C
```

ATOM 11718 HG21 ILE D 97   -33.846 11.197 60.818 1.00 0.00
D   H
ATOM 11719 HG22 ILE D 97   -35.508 11.805 60.537 1.00 0.00
D   H
ATOM 11720 HG23 ILE D 97   -35.015 11.264 62.183 1.00 0.00
D   H
ATOM 11721 CG1 ILE D 97   -34.673   8.641 61.678 1.00 0.00
D   C
ATOM 11722 HG11 ILE D 97   -34.941   7.607 61.366 1.00 0.00
D   H
ATOM 11723 HG12 ILE D 97   -33.571   8.746 61.560 1.00 0.00
D   H
ATOM 11724 CD ILE D 97   -35.016   8.828 63.134 1.00 0.00
D   C
ATOM 11725 HD1 ILE D 97   -34.445   8.109 63.761 1.00 0.00
D   H
ATOM 11726 HD2 ILE D 97   -34.764   9.857 63.467 1.00 0.00
D   H
ATOM 11727 HD3 ILE D 97   -36.098   8.654 63.314 1.00 0.00
D   H
ATOM 11728 C   ILE D 97   -37.634 10.491 60.071 1.00 0.00
D   C
ATOM 11729 O   ILE D 97   -37.814 10.234 58.865 1.00 0.00
D   O
ATOM 11730 N   PRO D 98   -38.133 11.596 60.620 1.00 0.00
D   N
ATOM 11731 CD PRO D 98   -38.028 12.043 62.016 1.00 0.00
D   C
ATOM 11732 HD1 PRO D 98   -38.428 11.020 61.831 1.00 0.00
D   H
ATOM 11733 HD2 PRO D 98   -37.404 12.056 62.940 1.00 0.00
D   H
ATOM 11734 CA PRO D 98   -38.996 12.471 59.815 1.00 0.00
D   C
ATOM 11735 HA PRO D 98   -39.887 11.929 59.519 1.00 0.00
D   H
ATOM 11736 CB PRO D 98   -39.362 13.589 60.797 1.00 0.00
D   C
ATOM 11737 HB1 PRO D 98   -40.239 13.267 61.403 1.00 0.00
D   H
ATOM 11738 HB2 PRO D 98   -39.608 14.543 60.287 1.00 0.00
D   H
ATOM 11739 CG PRO D 98   -39.143 13.010 62.143 1.00 0.00
D   C
ATOM 11740 HG1 PRO D 98   -39.731 13.519 62.932 1.00 0.00
D   H
ATOM 11741 HG2 PRO D 98   -38.059 13.065 62.394 1.00 0.00
D   H
ATOM 11742 C   PRO D 98   -38.314 13.014 58.580 1.00 0.00
D   C
ATOM 11743 O   PRO D 98   -38.784 12.781 57.461 1.00 0.00
D   O
ATOM 11744 N   ASN D 99   -37.198 13.707 58.745 1.00 0.00
D   N

```
ATOM  11745  HN  ASN D  99     -36.698  13.747  59.609  1.00  0.00
D    H
ATOM  11746  CA  ASN D  99     -36.600  14.479  57.667  1.00  0.00
D    C
ATOM  11747  HA  ASN D  99     -37.273  14.407  56.818  1.00  0.00
D    H
ATOM  11748  CB  ASN D  99     -36.377  15.920  58.108  1.00  0.00
D    C
ATOM  11749  HB1 ASN D  99     -35.605  15.915  58.908  1.00  0.00
D    H
ATOM  11750  HB2 ASN D  99     -35.981  16.524  57.267  1.00  0.00
D    H
ATOM  11751  CG  ASN D  99     -37.626  16.538  58.660  1.00  0.00
D    C
ATOM  11752  OD1 ASN D  99     -37.689  16.881  59.836  1.00  0.00
D    O
ATOM  11753  ND2 ASN D  99     -38.644  16.663  57.821  1.00  0.00
D    N
ATOM  11754 HD21 ASN D  99     -39.476  17.083  58.171  1.00  0.00
D    H
ATOM  11755 HD22 ASN D  99     -38.548  16.370  56.875  1.00  0.00
D    H
ATOM  11756  C   ASN D  99     -35.288  13.834  57.268  1.00  0.00
D    C
ATOM  11757  O   ASN D  99     -34.383  13.707  58.098  1.00  0.00
D    O
ATOM  11758  N   ILE D 100     -35.183  13.439  56.006  1.00  0.00
D    N
ATOM  11759  HN  ILE D 100     -35.914  13.557  55.337  1.00  0.00
D    H
ATOM  11760  CA  ILE D 100     -33.991  12.798  55.479  1.00  0.00
D    C
ATOM  11761  HA  ILE D 100     -33.209  12.878  56.223  1.00  0.00
D    H
ATOM  11762  CB  ILE D 100     -34.249  11.341  55.096  1.00  0.00
D    C
ATOM  11763  HB  ILE D 100     -35.222  11.365  54.543  1.00  0.00
D    H
ATOM  11764  CG2 ILE D 100     -33.086  10.805  54.315  1.00  0.00
D    C
ATOM  11765 HG21 ILE D 100     -33.324   9.804  53.898  1.00  0.00
D    H
ATOM  11766 HG22 ILE D 100     -32.839  11.474  53.463  1.00  0.00
D    H
ATOM  11767 HG23 ILE D 100     -32.186  10.709  54.958  1.00  0.00
D    H
ATOM  11768  CG1 ILE D 100     -34.469  10.505  56.352  1.00  0.00
D    C
ATOM  11769 HG11 ILE D 100     -33.567  10.558  57.003  1.00  0.00
D    H
ATOM  11770 HG12 ILE D 100     -35.318  10.939  56.929  1.00  0.00
D    H
ATOM  11771  CD  ILE D 100     -34.784   9.066  56.070  1.00  0.00
D    C
```

```
ATOM  11772  HD1 ILE D 100     -34.990   8.524  57.019  1.00  0.00
D    H
ATOM  11773  HD2 ILE D 100     -35.680   8.981  55.419  1.00  0.00
D    H
ATOM  11774  HD3 ILE D 100     -33.932   8.563  55.566  1.00  0.00
D    H
ATOM  11775  C   ILE D 100     -33.540  13.610  54.280  1.00  0.00
D    C
ATOM  11776  O   ILE D 100     -34.246  13.678  53.265  1.00  0.00
D    O
ATOM  11777  N   TRP D 101     -32.379  14.239  54.396  1.00  0.00
D    N
ATOM  11778  HN  TRP D 101     -31.776  14.114  55.181  1.00  0.00
D    H
ATOM  11779  CA  TRP D 101     -31.890  15.167  53.386  1.00  0.00
D    C
ATOM  11780  HA  TRP D 101     -32.525  15.088  52.511  1.00  0.00
D    H
ATOM  11781  CB  TRP D 101     -31.882  16.588  53.937  1.00  0.00
D    C
ATOM  11782  HB1 TRP D 101     -31.445  16.554  54.960  1.00  0.00
D    H
ATOM  11783  HB2 TRP D 101     -31.213  17.228  53.322  1.00  0.00
D    H
ATOM  11784  CG  TRP D 101     -33.230  17.146  54.029  1.00  0.00
D    C
ATOM  11785  CD1 TRP D 101     -34.351  16.510  54.451  1.00  0.00
D    C
ATOM  11786  HD1 TRP D 101     -34.372  15.495  54.823  1.00  0.00
D    H
ATOM  11787  NE1 TRP D 101     -35.425  17.348  54.377  1.00  0.00
D    N
ATOM  11788  HE1 TRP D 101     -36.341  17.149  54.652  1.00  0.00
D    H
ATOM  11789  CE2 TRP D 101     -35.003  18.556  53.893  1.00  0.00
D    C
ATOM  11790  CD2 TRP D 101     -33.624  18.458  53.663  1.00  0.00
D    C
ATOM  11791  CE3 TRP D 101     -32.944  19.563  53.163  1.00  0.00
D    C
ATOM  11792  HE3 TRP D 101     -31.883  19.536  52.959  1.00  0.00
D    H
ATOM  11793  CZ3 TRP D 101     -33.647  20.710  52.921  1.00  0.00
D    C
ATOM  11794  HZ3 TRP D 101     -33.131  21.579  52.536  1.00  0.00
D    H
ATOM  11795  CZ2 TRP D 101     -35.715  19.714  53.644  1.00  0.00
D    C
ATOM  11796  HZ2 TRP D 101     -36.776  19.795  53.821  1.00  0.00
D    H
ATOM  11797  CH2 TRP D 101     -35.020  20.781  53.156  1.00  0.00
D    C
ATOM  11798  HH2 TRP D 101     -35.551  21.701  52.948  1.00  0.00
D    H
```

```
ATOM  11799  C   TRP D 101     -30.494  14.743  52.958  1.00  0.00
D    C
ATOM  11800  O   TRP D 101     -29.606  14.592  53.800  1.00  0.00
D    O
ATOM  11801  N   VAL D 102     -30.292  14.561  51.657  1.00  0.00
D    N
ATOM  11802  HN  VAL D 102     -30.904  14.847  50.925  1.00  0.00
D    H
ATOM  11803  CA  VAL D 102     -29.084  13.889  51.204  1.00  0.00
D    C
ATOM  11804  HA  VAL D 102     -28.421  13.803  52.055  1.00  0.00
D    H
ATOM  11805  CB  VAL D 102     -29.413  12.486  50.661  1.00  0.00
D    C
ATOM  11806  HB  VAL D 102     -30.021  12.617  49.733  1.00  0.00
D    H
ATOM  11807  CG1 VAL D 102     -28.147  11.735  50.323  1.00  0.00
D    C
ATOM  11808 HG11 VAL D 102     -28.389  10.710  49.968  1.00  0.00
D    H
ATOM  11809 HG12 VAL D 102     -27.576  12.238  49.515  1.00  0.00
D    H
ATOM  11810 HG13 VAL D 102     -27.493  11.645  51.217  1.00  0.00
D    H
ATOM  11811  CG2 VAL D 102     -30.236  11.711  51.665  1.00  0.00
D    C
ATOM  11812 HG21 VAL D 102     -30.442  10.685  51.296  1.00  0.00
D    H
ATOM  11813 HG22 VAL D 102     -29.696  11.633  52.634  1.00  0.00
D    H
ATOM  11814 HG23 VAL D 102     -31.213  12.204  51.849  1.00  0.00
D    H
ATOM  11815  C   VAL D 102     -28.366  14.713  50.148  1.00  0.00
D    C
ATOM  11816  O   VAL D 102     -28.986  15.438  49.361  1.00  0.00
D    O
ATOM  11817  N   LYS D 103     -27.035  14.599  50.147  1.00  0.00
D    N
ATOM  11818  HN  LYS D 103     -26.545  14.156  50.896  1.00  0.00
D    H
ATOM  11819  CA  LYS D 103     -26.193  15.105  49.068  1.00  0.00
D    C
ATOM  11820  HA  LYS D 103     -26.694  15.956  48.623  1.00  0.00
D    H
ATOM  11821  CB  LYS D 103     -24.816  15.489  49.614  1.00  0.00
D    C
ATOM  11822  HB1 LYS D 103     -24.960  16.378  50.271  1.00  0.00
D    H
ATOM  11823  HB2 LYS D 103     -24.446  14.671  50.272  1.00  0.00
D    H
ATOM  11824  CG  LYS D 103     -23.760  15.832  48.576  1.00  0.00
D    C
ATOM  11825  HG1 LYS D 103     -23.789  15.072  47.762  1.00  0.00
D    H
```

ATOM 11826 HG2 LYS D 103 −24.008 16.817 48.119 1.00 0.00
D H
ATOM 11827 CD LYS D 103 −22.370 15.852 49.222 1.00 0.00
D C
ATOM 11828 HD1 LYS D 103 −22.368 16.621 50.028 1.00 0.00
D H
ATOM 11829 HD2 LYS D 103 −22.199 14.862 49.705 1.00 0.00
D H
ATOM 11830 CE LYS D 103 −21.247 16.138 48.222 1.00 0.00
D C
ATOM 11831 HE1 LYS D 103 −21.350 15.489 47.324 1.00 0.00
D H
ATOM 11832 HE2 LYS D 103 −21.277 17.200 47.894 1.00 0.00
D H
ATOM 11833 NZ LYS D 103 −19.883 15.888 48.797 1.00 0.00
D N
ATOM 11834 HZ1 LYS D 103 −19.153 16.094 48.085 1.00 0.00
D H
ATOM 11835 HZ2 LYS D 103 −19.738 16.499 49.626 1.00 0.00
D H
ATOM 11836 HZ3 LYS D 103 −19.808 14.892 49.089 1.00 0.00
D H
ATOM 11837 C LYS D 103 −26.064 14.037 47.988 1.00 0.00
D C
ATOM 11838 O LYS D 103 −25.638 12.915 48.269 1.00 0.00
D O
ATOM 11839 N ALA D 104 −26.429 14.384 46.756 1.00 0.00
D N
ATOM 11840 HN ALA D 104 −26.650 15.330 46.516 1.00 0.00
D H
ATOM 11841 CA ALA D 104 −26.532 13.422 45.667 1.00 0.00
D C
ATOM 11842 HA ALA D 104 −26.503 12.410 46.055 1.00 0.00
D H
ATOM 11843 CB ALA D 104 −27.840 13.597 44.896 1.00 0.00
D C
ATOM 11844 HB1 ALA D 104 −28.701 13.461 45.585 1.00 0.00
D H
ATOM 11845 HB2 ALA D 104 −27.912 14.617 44.458 1.00 0.00
D H
ATOM 11846 HB3 ALA D 104 −27.935 12.853 44.077 1.00 0.00
D H
ATOM 11847 C ALA D 104 −25.366 13.545 44.698 1.00 0.00
D C
ATOM 11848 O ALA D 104 −24.797 14.624 44.511 1.00 0.00
D O
ATOM 11849 N GLN D 105 −25.039 12.424 44.054 1.00 0.00
D N
ATOM 11850 HN GLN D 105 −25.567 11.581 44.133 1.00 0.00
D H
ATOM 11851 CA GLN D 105 −23.880 12.330 43.177 1.00 0.00
D C
ATOM 11852 HA GLN D 105 −23.197 13.111 43.486 1.00 0.00
D H

```
ATOM  11853  CB  GLN D 105     -23.230  10.955  43.338  1.00  0.00
D    C
ATOM  11854  HB1 GLN D 105     -23.078  10.797  44.431  1.00  0.00
D    H
ATOM  11855  HB2 GLN D 105     -23.946  10.167  43.008  1.00  0.00
D    H
ATOM  11856  CG  GLN D 105     -21.907  10.797  42.627  1.00  0.00
D    C
ATOM  11857  HG1 GLN D 105     -21.418  11.786  42.495  1.00  0.00
D    H
ATOM  11858  HG2 GLN D 105     -21.220  10.153  43.213  1.00  0.00
D    H
ATOM  11859  CD  GLN D 105     -22.068  10.210  41.246  1.00  0.00
D    C
ATOM  11860  OE1 GLN D 105     -23.076   9.577  40.946  1.00  0.00
D    O
ATOM  11861  NE2 GLN D 105     -21.064  10.401  40.402  1.00  0.00
D    N
ATOM  11862 HE21 GLN D 105     -21.166  10.020  39.488  1.00  0.00
D    H
ATOM  11863 HE22 GLN D 105     -20.252  10.892  40.701  1.00  0.00
D    H
ATOM  11864  C   GLN D 105     -24.235  12.583  41.716  1.00  0.00
D    C
ATOM  11865  O   GLN D 105     -23.745  13.544  41.118  1.00  0.00
D    O
ATOM  11866  N   ASN D 106     -25.087  11.749  41.126  1.00  0.00
D    N
ATOM  11867  HN  ASN D 106     -25.488  10.944  41.561  1.00  0.00
D    H
ATOM  11868  CA  ASN D 106     -25.508  11.963  39.751  1.00  0.00
D    C
ATOM  11869  HA  ASN D 106     -25.242  12.983  39.494  1.00  0.00
D    H
ATOM  11870  CB  ASN D 106     -24.873  10.966  38.782  1.00  0.00
D    C
ATOM  11871  HB1 ASN D 106     -25.134  11.283  37.749  1.00  0.00
D    H
ATOM  11872  HB2 ASN D 106     -23.768  10.993  38.870  1.00  0.00
D    H
ATOM  11873  CG  ASN D 106     -25.377   9.545  38.977  1.00  0.00
D    C
ATOM  11874  OD1 ASN D 106     -26.239   9.270  39.808  1.00  0.00
D    O
ATOM  11875  ND2 ASN D 106     -24.846   8.633  38.180  1.00  0.00
D    N
ATOM  11876 HD21 ASN D 106     -25.168   7.695  38.280  1.00  0.00
D    H
ATOM  11877 HD22 ASN D 106     -24.176   8.899  37.495  1.00  0.00
D    H
ATOM  11878  C   ASN D 106     -27.018  11.851  39.684  1.00  0.00
D    C
ATOM  11879  O   ASN D 106     -27.685  11.546  40.674  1.00  0.00
D    O
```

```
ATOM  11880  N   TYR D 107     -27.548  12.065  38.484  1.00  0.00
D    N
ATOM  11881  HN  TYR D 107     -27.047  12.355  37.671  1.00  0.00
D    H
ATOM  11882  CA  TYR D 107     -28.974  11.879  38.279  1.00  0.00
D    C
ATOM  11883  HA  TYR D 107     -29.475  12.594  38.920  1.00  0.00
D    H
ATOM  11884  CB  TYR D 107     -29.324  12.103  36.812  1.00  0.00
D    C
ATOM  11885  HB1 TYR D 107     -29.496  13.189  36.640  1.00  0.00
D    H
ATOM  11886  HB2 TYR D 107     -28.480  11.796  36.156  1.00  0.00
D    H
ATOM  11887  CG  TYR D 107     -30.577  11.381  36.411  1.00  0.00
D    C
ATOM  11888  CD1 TYR D 107     -31.822  11.905  36.709  1.00  0.00
D    C
ATOM  11889  HD1 TYR D 107     -31.897  12.843  37.241  1.00  0.00
D    H
ATOM  11890  CE1 TYR D 107     -32.969  11.245  36.358  1.00  0.00
D    C
ATOM  11891  HE1 TYR D 107     -33.937  11.660  36.598  1.00  0.00
D    H
ATOM  11892  CZ  TYR D 107     -32.884  10.041  35.702  1.00  0.00
D    C
ATOM  11893  OH  TYR D 107     -34.030   9.372  35.345  1.00  0.00
D    O
ATOM  11894  HH  TYR D 107     -33.766   8.599  34.839  1.00  0.00
D    H
ATOM  11895  CD2 TYR D 107     -30.519  10.167  35.753  1.00  0.00
D    C
ATOM  11896  HD2 TYR D 107     -29.561   9.711  35.545  1.00  0.00
D    H
ATOM  11897  CE2 TYR D 107     -31.662   9.500  35.400  1.00  0.00
D    C
ATOM  11898  HE2 TYR D 107     -31.593   8.543  34.905  1.00  0.00
D    H
ATOM  11899  C   TYR D 107     -29.415  10.494  38.723  1.00  0.00
D    C
ATOM  11900  O   TYR D 107     -30.461  10.339  39.363  1.00  0.00
D    O
ATOM  11901  N   TYR D 108     -28.634   9.472  38.386  1.00  0.00
D    N
ATOM  11902  HN  TYR D 108     -27.763   9.531  37.902  1.00  0.00
D    H
ATOM  11903  CA  TYR D 108     -29.041   8.119  38.734  1.00  0.00
D    C
ATOM  11904  HA  TYR D 108     -30.051   7.996  38.362  1.00  0.00
D    H
ATOM  11905  CB  TYR D 108     -28.094   7.112  38.111  1.00  0.00
D    C
ATOM  11906  HB1 TYR D 108     -27.056   7.336  38.440  1.00  0.00
D    H
```

ATOM 11907 HB2 TYR D 108 -28.333 6.082 38.455 1.00 0.00
D H
ATOM 11908 CG TYR D 108 -28.139 7.199 36.634 1.00 0.00
D C
ATOM 11909 CD1 TYR D 108 -29.344 7.104 35.971 1.00 0.00
D C
ATOM 11910 HD1 TYR D 108 -30.253 6.939 36.531 1.00 0.00
D H
ATOM 11911 CE1 TYR D 108 -29.416 7.190 34.616 1.00 0.00
D C
ATOM 11912 HE1 TYR D 108 -30.366 7.107 34.110 1.00 0.00
D H
ATOM 11913 CZ TYR D 108 -28.269 7.377 33.894 1.00 0.00
D C
ATOM 11914 OH TYR D 108 -28.337 7.463 32.526 1.00 0.00
D O
ATOM 11915 HH TYR D 108 -27.456 7.663 32.205 1.00 0.00
D H
ATOM 11916 CD2 TYR D 108 -26.995 7.395 35.895 1.00 0.00
D C
ATOM 11917 HD2 TYR D 108 -26.035 7.442 36.389 1.00 0.00
D H
ATOM 11918 CE2 TYR D 108 -27.052 7.480 34.531 1.00 0.00
D C
ATOM 11919 HE2 TYR D 108 -26.142 7.607 33.964 1.00 0.00
D H
ATOM 11920 C TYR D 108 -29.093 7.931 40.233 1.00 0.00
D C
ATOM 11921 O TYR D 108 -30.174 7.734 40.801 1.00 0.00
D O
ATOM 11922 N HSE D 109 -27.935 8.047 40.885 1.00 0.00
D N
ATOM 11923 HN HSE D 109 -27.048 8.161 40.439 1.00 0.00
D H
ATOM 11924 CA HSE D 109 -27.877 8.016 42.337 1.00 0.00
D C
ATOM 11925 HA HSE D 109 -27.977 6.981 42.639 1.00 0.00
D H
ATOM 11926 CB HSE D 109 -26.561 8.621 42.801 1.00 0.00
D C
ATOM 11927 HB1 HSE D 109 -25.742 7.934 42.494 1.00 0.00
D H
ATOM 11928 HB2 HSE D 109 -26.390 9.585 42.276 1.00 0.00
D H
ATOM 11929 ND1 HSE D 109 -26.256 10.040 44.853 1.00 0.00
D N
ATOM 11930 CG HSE D 109 -26.476 8.810 44.276 1.00 0.00
D C
ATOM 11931 CE1 HSE D 109 -26.226 9.909 46.166 1.00 0.00
D C
ATOM 11932 HE1 HSE D 109 -26.068 10.732 46.868 1.00 0.00
D H
ATOM 11933 NE2 HSE D 109 -26.410 8.635 46.461 1.00 0.00
D N

ATOM 11934 HE2 HSE D 109 −26.413 8.252 47.384 1.00 0.00
D H
ATOM 11935 CD2 HSE D 109 −26.567 7.926 45.295 1.00 0.00
D C
ATOM 11936 HD2 HSE D 109 −26.721 6.857 45.273 1.00 0.00
D H
ATOM 11937 C HSE D 109 −29.047 8.769 42.937 1.00 0.00
D C
ATOM 11938 O HSE D 109 −29.655 8.318 43.910 1.00 0.00
D O
ATOM 11939 N HSE D 110 −29.410 9.891 42.324 1.00 0.00
D N
ATOM 11940 HN HSE D 110 −28.939 10.284 41.536 1.00 0.00
D H
ATOM 11941 CA HSE D 110 −30.562 10.652 42.773 1.00 0.00
D C
ATOM 11942 HA HSE D 110 −30.412 10.848 43.828 1.00 0.00
D H
ATOM 11943 CB HSE D 110 −30.648 11.936 41.971 1.00 0.00
D C
ATOM 11944 HB1 HSE D 110 −30.097 12.725 42.529 1.00 0.00
D H
ATOM 11945 HB2 HSE D 110 −30.127 11.802 40.999 1.00 0.00
D H
ATOM 11946 ND1 HSE D 110 −32.663 13.284 42.615 1.00 0.00
D N
ATOM 11947 CG HSE D 110 −32.041 12.410 41.755 1.00 0.00
D C
ATOM 11948 CE1 HSE D 110 −33.887 13.524 42.181 1.00 0.00
D C
ATOM 11949 HE1 HSE D 110 −34.604 14.181 42.680 1.00 0.00
D H
ATOM 11950 NE2 HSE D 110 −34.077 12.837 41.070 1.00 0.00
D N
ATOM 11951 HE2 HSE D 110 −34.925 12.822 40.541 1.00 0.00
D H
ATOM 11952 CD2 HSE D 110 −32.936 12.132 40.781 1.00 0.00
D C
ATOM 11953 HD2 HSE D 110 −32.847 11.484 39.920 1.00 0.00
D H
ATOM 11954 C HSE D 110 −31.852 9.857 42.645 1.00 0.00
D C
ATOM 11955 O HSE D 110 −32.695 9.875 43.547 1.00 0.00
D O
ATOM 11956 N LYS D 111 −32.055 9.192 41.516 1.00 0.00
D N
ATOM 11957 HN LYS D 111 −31.441 9.151 40.730 1.00 0.00
D H
ATOM 11958 CA LYS D 111 −33.291 8.434 41.375 1.00 0.00
D C
ATOM 11959 HA LYS D 111 −34.106 9.108 41.605 1.00 0.00
D H
ATOM 11960 CB LYS D 111 −33.437 7.900 39.959 1.00 0.00
D C

ATOM 11961 HB1 LYS D 111 −33.572 8.780 39.289 1.00 0.00
D H
ATOM 11962 HB2 LYS D 111 −32.482 7.418 39.653 1.00 0.00
D H
ATOM 11963 CG LYS D 111 −34.615 6.953 39.812 1.00 0.00
D C
ATOM 11964 HG1 LYS D 111 −34.295 6.062 39.225 1.00 0.00
D H
ATOM 11965 HG2 LYS D 111 −34.919 6.594 40.822 1.00 0.00
D H
ATOM 11966 CD LYS D 111 −35.821 7.591 39.104 1.00 0.00
D C
ATOM 11967 HD1 LYS D 111 −36.014 7.028 38.162 1.00 0.00
D H
ATOM 11968 HD2 LYS D 111 −36.714 7.465 39.758 1.00 0.00
D H
ATOM 11969 CE LYS D 111 −35.595 9.050 38.811 1.00 0.00
D C
ATOM 11970 HE1 LYS D 111 −34.525 9.315 38.958 1.00 0.00
D H
ATOM 11971 HE2 LYS D 111 −35.875 9.285 37.760 1.00 0.00
D H
ATOM 11972 NZ LYS D 111 −36.421 9.878 39.721 1.00 0.00
D N
ATOM 11973 HZ1 LYS D 111 −36.267 10.888 39.523 1.00 0.00
D H
ATOM 11974 HZ2 LYS D 111 −37.426 9.649 39.584 1.00 0.00
D H
ATOM 11975 HZ3 LYS D 111 −36.156 9.676 40.707 1.00 0.00
D H
ATOM 11976 C LYS D 111 −33.352 7.291 42.371 1.00 0.00
D C
ATOM 11977 O LYS D 111 −34.389 7.056 43.003 1.00 0.00
D O
ATOM 11978 N VAL D 112 −32.247 6.573 42.530 1.00 0.00
D N
ATOM 11979 HN VAL D 112 −31.398 6.689 42.021 1.00 0.00
D H
ATOM 11980 CA VAL D 112 −32.214 5.505 43.523 1.00 0.00
D C
ATOM 11981 HA VAL D 112 −33.024 4.822 43.301 1.00 0.00
D H
ATOM 11982 CB VAL D 112 −30.891 4.733 43.448 1.00 0.00
D C
ATOM 11983 HB VAL D 112 −31.138 3.649 43.336 1.00 0.00
D H
ATOM 11984 CG1 VAL D 112 −30.100 5.187 42.269 1.00 0.00
D C
ATOM 11985 HG11 VAL D 112 −29.122 4.659 42.233 1.00 0.00
D H
ATOM 11986 HG12 VAL D 112 −30.626 4.974 41.316 1.00 0.00
D H
ATOM 11987 HG13 VAL D 112 −29.898 6.278 42.327 1.00 0.00
D H

ATOM 11988 CG2 VAL D 112   -30.096   4.942 44.708 1.00 0.00
D   C
ATOM 11989 HG21 VAL D 112   -29.119   4.419 44.647 1.00 0.00
D   H
ATOM 11990 HG22 VAL D 112   -29.904   6.024 44.874 1.00 0.00
D   H
ATOM 11991 HG23 VAL D 112   -30.635   4.543 45.593 1.00 0.00
D   H
ATOM 11992 C   VAL D 112   -32.463   6.069 44.911 1.00 0.00
D   C
ATOM 11993 O   VAL D 112   -33.053   5.406 45.771 1.00 0.00
D   O
ATOM 11994 N   LEU D 113   -32.023   7.303 45.148 1.00 0.00
D   N
ATOM 11995 HN LEU D 113   -31.430   7.830 44.543 1.00 0.00
D   H
ATOM 11996 CA LEU D 113   -32.399   7.996 46.365 1.00 0.00
D   C
ATOM 11997 HA LEU D 113   -32.065   7.384 47.193 1.00 0.00
D   H
ATOM 11998 CB LEU D 113   -31.755   9.374 46.377 1.00 0.00
D   C
ATOM 11999 HB1 LEU D 113   -30.787   9.290 45.830 1.00 0.00
D   H
ATOM 12000 HB2 LEU D 113   -32.385 10.073 45.782 1.00 0.00
D   H
ATOM 12001 CG LEU D 113   -31.457 10.006 47.721 1.00 0.00
D   C
ATOM 12002 HG LEU D 113   -32.351 10.598 48.037 1.00 0.00
D   H
ATOM 12003 CD1 LEU D 113   -31.193   8.938 48.748 1.00 0.00
D   C
ATOM 12004 HD11 LEU D 113   -30.927   9.395 49.725 1.00 0.00
D   H
ATOM 12005 HD12 LEU D 113   -32.092   8.303 48.893 1.00 0.00
D   H
ATOM 12006 HD13 LEU D 113   -30.351   8.287 48.428 1.00 0.00
D   H
ATOM 12007 CD2 LEU D 113   -30.300 10.954 47.574 1.00 0.00
D   C
ATOM 12008 HD21 LEU D 113   -30.076 11.445 48.547 1.00 0.00
D   H
ATOM 12009 HD22 LEU D 113   -29.381 10.431 47.243 1.00 0.00
D   H
ATOM 12010 HD23 LEU D 113   -30.538 11.747 46.833 1.00 0.00
D   H
ATOM 12011 C   LEU D 113   -33.905   8.119 46.466 1.00 0.00
D   C
ATOM 12012 O   LEU D 113   -34.498   7.825 47.508 1.00 0.00
D   O
ATOM 12013 N   GLU D 114   -34.539   8.547 45.381 1.00 0.00
D   N
ATOM 12014 HN GLU D 114   -34.112   8.772 44.509 1.00 0.00
D   H

```
ATOM  12015  CA  GLU D 114     -35.982   8.725  45.396  1.00  0.00
D    C
ATOM  12016  HA  GLU D 114     -36.225   9.447  46.165  1.00  0.00
D    H
ATOM  12017  CB  GLU D 114     -36.465   9.235  44.044  1.00  0.00
D    C
ATOM  12018  HB1 GLU D 114     -35.771  10.040  43.708  1.00  0.00
D    H
ATOM  12019  HB2 GLU D 114     -36.382   8.426  43.284  1.00  0.00
D    H
ATOM  12020  CG  GLU D 114     -37.868   9.767  44.111  1.00  0.00
D    C
ATOM  12021  HG1 GLU D 114     -38.061  10.412  43.234  1.00  0.00
D    H
ATOM  12022  HG2 GLU D 114     -38.606   8.940  44.125  1.00  0.00
D    H
ATOM  12023  CD  GLU D 114     -38.082  10.637  45.326  1.00  0.00
D    C
ATOM  12024  OE1 GLU D 114     -37.314  11.604  45.501  1.00  0.00
D    O
ATOM  12025  OE2 GLU D 114     -39.004  10.345  46.115  1.00  0.00
D    O
ATOM  12026  C   GLU D 114     -36.691   7.427  45.756  1.00  0.00
D    C
ATOM  12027  O   GLU D 114     -37.635   7.418  46.553  1.00  0.00
D    O
ATOM  12028  N   LYS D 115     -36.238   6.314  45.185  1.00  0.00
D    N
ATOM  12029  HN  LYS D 115     -35.433   6.259  44.597  1.00  0.00
D    H
ATOM  12030  CA  LYS D 115     -36.933   5.052  45.404  1.00  0.00
D    C
ATOM  12031  HA  LYS D 115     -37.967   5.222  45.130  1.00  0.00
D    H
ATOM  12032  CB  LYS D 115     -36.326   3.962  44.542  1.00  0.00
D    C
ATOM  12033  HB1 LYS D 115     -35.226   3.979  44.717  1.00  0.00
D    H
ATOM  12034  HB2 LYS D 115     -36.685   2.972  44.904  1.00  0.00
D    H
ATOM  12035  CG  LYS D 115     -36.615   4.161  43.103  1.00  0.00
D    C
ATOM  12036  HG1 LYS D 115     -35.963   4.974  42.710  1.00  0.00
D    H
ATOM  12037  HG2 LYS D 115     -36.357   3.230  42.549  1.00  0.00
D    H
ATOM  12038  CD  LYS D 115     -38.050   4.525  42.891  1.00  0.00
D    C
ATOM  12039  HD1 LYS D 115     -38.686   3.733  43.351  1.00  0.00
D    H
ATOM  12040  HD2 LYS D 115     -38.252   5.474  43.439  1.00  0.00
D    H
ATOM  12041  CE  LYS D 115     -38.300   4.669  41.418  1.00  0.00
D    C
```

```
ATOM  12042  HE1 LYS D 115     -39.391   4.719  41.210  1.00  0.00
D    H
ATOM  12043  HE2 LYS D 115     -37.824   5.596  41.030  1.00  0.00
D    H
ATOM  12044  NZ  LYS D 115     -37.726   3.496  40.705  1.00  0.00
D    N
ATOM  12045  HZ1 LYS D 115     -37.894   3.585  39.681  1.00  0.00
D    H
ATOM  12046  HZ2 LYS D 115     -36.703   3.448  40.884  1.00  0.00
D    H
ATOM  12047  HZ3 LYS D 115     -38.175   2.626  41.054  1.00  0.00
D    H
ATOM  12048  C   LYS D 115     -36.914   4.613  46.856  1.00  0.00
D    C
ATOM  12049  O   LYS D 115     -37.863   3.969  47.309  1.00  0.00
D    O
ATOM  12050  N   ILE D 116     -35.860   4.935  47.599  1.00  0.00
D    N
ATOM  12051  HN  ILE D 116     -35.063   5.438  47.269  1.00  0.00
D    H
ATOM  12052  CA  ILE D 116     -35.789   4.563  49.002  1.00  0.00
D    C
ATOM  12053  HA  ILE D 116     -36.103   3.528  49.055  1.00  0.00
D    H
ATOM  12054  CB  ILE D 116     -34.367   4.733  49.546  1.00  0.00
D    C
ATOM  12055  HB  ILE D 116     -33.706   4.353  48.726  1.00  0.00
D    H
ATOM  12056  CG2 ILE D 116     -34.152   6.156  49.988  1.00  0.00
D    C
ATOM  12057 HG21 ILE D 116     -33.085   6.331  50.237  1.00  0.00
D    H
ATOM  12058 HG22 ILE D 116     -34.430   6.867  49.182  1.00  0.00
D    H
ATOM  12059 HG23 ILE D 116     -34.762   6.389  50.886  1.00  0.00
D    H
ATOM  12060  CG1 ILE D 116     -34.132   3.826  50.751  1.00  0.00
D    C
ATOM  12061 HG11 ILE D 116     -33.071   3.900  51.077  1.00  0.00
D    H
ATOM  12062 HG12 ILE D 116     -34.762   4.182  51.598  1.00  0.00
D    H
ATOM  12063  CD  ILE D 116     -34.453   2.398  50.522  1.00  0.00
D    C
ATOM  12064  HD1 ILE D 116     -34.312   1.815  51.458  1.00  0.00
D    H
ATOM  12065  HD2 ILE D 116     -35.509   2.281  50.195  1.00  0.00
D    H
ATOM  12066  HD3 ILE D 116     -33.793   1.961  49.743  1.00  0.00
D    H
ATOM  12067  C   ILE D 116     -36.768   5.363  49.843  1.00  0.00
D    C
ATOM  12068  O   ILE D 116     -37.057   4.985  50.979  1.00  0.00
D    O
```

ATOM 12069 N GLY D 117 -37.301 6.452 49.308 1.00 0.00
D N
ATOM 12070 HN GLY D 117 -37.234 6.695 48.340 1.00 0.00
D H
ATOM 12071 CA GLY D 117 -38.034 7.380 50.135 1.00 0.00
D C
ATOM 12072 HA1 GLY D 117 -38.319 6.846 51.031 1.00 0.00
D H
ATOM 12073 HA2 GLY D 117 -38.868 7.741 49.548 1.00 0.00
D H
ATOM 12074 C GLY D 117 -37.207 8.567 50.543 1.00 0.00
D C
ATOM 12075 O GLY D 117 -37.282 9.002 51.695 1.00 0.00
D O
ATOM 12076 N ALA D 118 -36.401 9.095 49.631 1.00 0.00
D N
ATOM 12077 HN ALA D 118 -36.276 8.724 48.712 1.00 0.00
D H
ATOM 12078 CA ALA D 118 -35.629 10.285 49.931 1.00 0.00
D C
ATOM 12079 HA ALA D 118 -35.019 10.141 50.815 1.00 0.00
D H
ATOM 12080 CB ALA D 118 -34.730 10.643 48.757 1.00 0.00
D C
ATOM 12081 HB1 ALA D 118 -34.017 9.812 48.566 1.00 0.00
D H
ATOM 12082 HB2 ALA D 118 -35.326 10.803 47.832 1.00 0.00
D H
ATOM 12083 HB3 ALA D 118 -34.138 11.562 48.961 1.00 0.00
D H
ATOM 12084 C ALA D 118 -36.555 11.444 50.236 1.00 0.00
D C
ATOM 12085 O ALA D 118 -37.231 11.970 49.350 1.00 0.00
D O
ATOM 12086 N ASP D 119 -36.589 11.841 51.502 1.00 0.00
D N
ATOM 12087 HN ASP D 119 -36.113 11.443 52.282 1.00 0.00
D H
ATOM 12088 CA ASP D 119 -37.399 12.989 51.861 1.00 0.00
D C
ATOM 12089 HA ASP D 119 -38.406 12.789 51.512 1.00 0.00
D H
ATOM 12090 CB ASP D 119 -37.373 13.194 53.373 1.00 0.00
D C
ATOM 12091 HB1 ASP D 119 -36.321 13.213 53.721 1.00 0.00
D H
ATOM 12092 HB2 ASP D 119 -37.848 14.157 53.645 1.00 0.00
D H
ATOM 12093 CG ASP D 119 -38.077 12.080 54.114 1.00 0.00
D C
ATOM 12094 OD1 ASP D 119 -39.267 12.253 54.450 1.00 0.00
D O
ATOM 12095 OD2 ASP D 119 -37.447 11.026 54.341 1.00 0.00
D O

ATOM 12096 C ASP D 119 -36.928 14.239 51.144 1.00 0.00
D C
ATOM 12097 O ASP D 119 -37.723 15.157 50.925 1.00 0.00
D O
ATOM 12098 N ARG D 120 -35.655 14.292 50.772 1.00 0.00
D N
ATOM 12099 HN ARG D 120 -35.002 13.567 50.979 1.00 0.00
D H
ATOM 12100 CA ARG D 120 -35.149 15.433 50.031 1.00 0.00
D C
ATOM 12101 HA ARG D 120 -35.790 15.605 49.174 1.00 0.00
D H
ATOM 12102 CB ARG D 120 -35.102 16.674 50.914 1.00 0.00
D C
ATOM 12103 HB1 ARG D 120 -36.151 17.048 50.988 1.00 0.00
D H
ATOM 12104 HB2 ARG D 120 -34.807 16.385 51.947 1.00 0.00
D H
ATOM 12105 CG ARG D 120 -34.235 17.730 50.354 1.00 0.00
D C
ATOM 12106 HG1 ARG D 120 -33.308 17.788 50.965 1.00 0.00
D H
ATOM 12107 HG2 ARG D 120 -33.903 17.424 49.336 1.00 0.00
D H
ATOM 12108 CD ARG D 120 -34.982 19.007 50.274 1.00 0.00
D C
ATOM 12109 HD1 ARG D 120 -35.792 18.916 49.513 1.00 0.00
D H
ATOM 12110 HD2 ARG D 120 -35.442 19.274 51.254 1.00 0.00
D H
ATOM 12111 NE ARG D 120 -34.087 20.089 49.908 1.00 0.00
D N
ATOM 12112 HE ARG D 120 -33.134 19.791 49.789 1.00 0.00
D H
ATOM 12113 CZ ARG D 120 -34.476 21.344 49.743 1.00 0.00
D C
ATOM 12114 NH1 ARG D 120 -35.746 21.671 49.921 1.00 0.00
D N
ATOM 12115 HH11 ARG D 120 -36.053 22.601 49.776 1.00 0.00
D H
ATOM 12116 HH12 ARG D 120 -36.384 20.911 50.070 1.00 0.00
D H
ATOM 12117 NH2 ARG D 120 -33.596 22.275 49.408 1.00 0.00
D N
ATOM 12118 HH21 ARG D 120 -33.872 23.225 49.371 1.00 0.00
D H
ATOM 12119 HH22 ARG D 120 -32.640 22.010 49.411 1.00 0.00
D H
ATOM 12120 C ARG D 120 -33.765 15.130 49.483 1.00 0.00
D C
ATOM 12121 O ARG D 120 -32.883 14.674 50.219 1.00 0.00
D O
ATOM 12122 N ILE D 121 -33.578 15.418 48.202 1.00 0.00
D N

ATOM 12123 HN ILE D 121 -34.293 15.825 47.637 1.00 0.00
D H
ATOM 12124 CA ILE D 121 -32.327 15.174 47.495 1.00 0.00
D C
ATOM 12125 HA ILE D 121 -31.645 14.698 48.189 1.00 0.00
D H
ATOM 12126 CB ILE D 121 -32.572 14.319 46.243 1.00 0.00
D C
ATOM 12127 HB ILE D 121 -33.142 14.992 45.552 1.00 0.00
D H
ATOM 12128 CG2 ILE D 121 -31.274 13.833 45.652 1.00 0.00
D C
ATOM 12129 HG21 ILE D 121 -31.449 13.351 44.668 1.00 0.00
D H
ATOM 12130 HG22 ILE D 121 -30.570 14.677 45.494 1.00 0.00
D H
ATOM 12131 HG23 ILE D 121 -30.787 13.090 46.319 1.00 0.00
D H
ATOM 12132 CG1 ILE D 121 -33.490 13.152 46.595 1.00 0.00
D C
ATOM 12133 HG11 ILE D 121 -33.097 12.622 47.492 1.00 0.00
D H
ATOM 12134 HG12 ILE D 121 -34.495 13.551 46.863 1.00 0.00
D H
ATOM 12135 CD ILE D 121 -33.670 12.157 45.488 1.00 0.00
D C
ATOM 12136 HD1 ILE D 121 -34.392 11.368 45.792 1.00 0.00
D H
ATOM 12137 HD2 ILE D 121 -34.061 12.654 44.574 1.00 0.00
D H
ATOM 12138 HD3 ILE D 121 -32.707 11.663 45.236 1.00 0.00
D H
ATOM 12139 C ILE D 121 -31.718 16.519 47.133 1.00 0.00
D C
ATOM 12140 O ILE D 121 -32.444 17.500 46.950 1.00 0.00
D O
ATOM 12141 N ILE D 122 -30.393 16.577 47.049 1.00 0.00
D N
ATOM 12142 HN ILE D 122 -29.781 15.815 47.254 1.00 0.00
D H
ATOM 12143 CA ILE D 122 -29.712 17.796 46.639 1.00 0.00
D C
ATOM 12144 HA ILE D 122 -30.430 18.395 46.095 1.00 0.00
D H
ATOM 12145 CB ILE D 122 -29.138 18.571 47.840 1.00 0.00
D C
ATOM 12146 HB ILE D 122 -28.577 17.800 48.427 1.00 0.00
D H
ATOM 12147 CG2 ILE D 122 -28.280 19.728 47.368 1.00 0.00
D C
ATOM 12148 HG21 ILE D 122 -27.743 20.190 48.223 1.00 0.00
D H
ATOM 12149 HG22 ILE D 122 -27.518 19.384 46.638 1.00 0.00
D H

ATOM 12150 HG23 ILE D 122   -28.900 20.512 46.885 1.00 0.00
D   H
ATOM 12151 CG1 ILE D 122   -30.266 19.074 48.734 1.00 0.00
D   C
ATOM 12152 HG11 ILE D 122   -29.980 20.049 49.188 1.00 0.00
D   H
ATOM 12153 HG12 ILE D 122   -31.169 19.255 48.107 1.00 0.00
D   H
ATOM 12154 CD ILE D 122   -30.647 18.116 49.833 1.00 0.00
D   C
ATOM 12155 HD1 ILE D 122   -31.509 18.514 50.410 1.00 0.00
D   H
ATOM 12156 HD2 ILE D 122   -30.937 17.130 49.412 1.00 0.00
D   H
ATOM 12157 HD3 ILE D 122   -29.802 17.964 50.539 1.00 0.00
D   H
ATOM 12158 C   ILE D 122   -28.602 17.449 45.664 1.00 0.00
D   C
ATOM 12159 O   ILE D 122   -27.824 16.519 45.895 1.00 0.00
D   O
ATOM 12160 N   HSE D 123   -28.521 18.214 44.590 1.00 0.00
D   N
ATOM 12161 HN HSE D 123   -29.208 18.914 44.402 1.00 0.00
D   H
ATOM 12162 CA HSE D 123   -27.464 18.129 43.596 1.00 0.00
D   C
ATOM 12163 HA HSE D 123   -26.810 17.305 43.855 1.00 0.00
D   H
ATOM 12164 CB HSE D 123   -28.048 17.993 42.197 1.00 0.00
D   C
ATOM 12165 HB1 HSE D 123   -28.712 18.867 42.018 1.00 0.00
D   H
ATOM 12166 HB2 HSE D 123   -27.231 18.047 41.447 1.00 0.00
D   H
ATOM 12167 ND1 HSE D 123   -28.416 15.696 41.227 1.00 0.00
D   N
ATOM 12168 CG HSE D 123   -28.857 16.753 41.993 1.00 0.00
D   C
ATOM 12169 CE1 HSE D 123   -29.340 14.753 41.211 1.00 0.00
D   C
ATOM 12170 HE1 HSE D 123   -29.252 13.809 40.667 1.00 0.00
D   H
ATOM 12171 NE2 HSE D 123   -30.361 15.154 41.946 1.00 0.00
D   N
ATOM 12172 HE2 HSE D 123   -31.203 14.637 42.096 1.00 0.00
D   H
ATOM 12173 CD2 HSE D 123   -30.085 16.405 42.444 1.00 0.00
D   C
ATOM 12174 HD2 HSE D 123   -30.776 16.954 43.068 1.00 0.00
D   H
ATOM 12175 C   HSE D 123   -26.642 19.403 43.706 1.00 0.00
D   C
ATOM 12176 O   HSE D 123   -26.736 20.281 42.840 1.00 0.00
D   O

ATOM 12177 N PRO D 124 −25.834 19.537 44.757 1.00 0.00
D N
ATOM 12178 CD PRO D 124 −25.307 18.477 45.625 1.00 0.00
D C
ATOM 12179 HD1 PRO D 124 −26.410 18.615 45.559 1.00 0.00
D H
ATOM 12180 HD2 PRO D 124 −25.055 17.396 45.725 1.00 0.00
D H
ATOM 12181 CA PRO D 124 −25.291 20.858 45.101 1.00 0.00
D C
ATOM 12182 HA PRO D 124 −26.099 21.497 45.437 1.00 0.00
D H
ATOM 12183 CB PRO D 124 −24.345 20.546 46.269 1.00 0.00
D C
ATOM 12184 HB1 PRO D 124 −24.931 20.529 47.216 1.00 0.00
D H
ATOM 12185 HB2 PRO D 124 −23.525 21.286 46.366 1.00 0.00
D H
ATOM 12186 CG PRO D 124 −24.768 19.234 46.784 1.00 0.00
D C
ATOM 12187 HG1 PRO D 124 −24.472 19.068 47.838 1.00 0.00
D H
ATOM 12188 HG2 PRO D 124 −24.333 18.432 46.145 1.00 0.00
D H
ATOM 12189 C PRO D 124 −24.556 21.543 43.972 1.00 0.00
D C
ATOM 12190 O PRO D 124 −24.932 22.651 43.562 1.00 0.00
D O
ATOM 12191 N GLU D 125 −23.510 20.909 43.457 1.00 0.00
D N
ATOM 12192 HN GLU D 125 −23.184 20.000 43.701 1.00 0.00
D H
ATOM 12193 CA GLU D 125 −22.722 21.561 42.426 1.00 0.00
D C
ATOM 12194 HA GLU D 125 −22.430 22.538 42.791 1.00 0.00
D H
ATOM 12195 CB GLU D 125 −21.471 20.764 42.113 1.00 0.00
D C
ATOM 12196 HB1 GLU D 125 −21.752 19.686 42.053 1.00 0.00
D H
ATOM 12197 HB2 GLU D 125 −21.093 21.037 41.103 1.00 0.00
D H
ATOM 12198 CG GLU D 125 −20.382 20.943 43.152 1.00 0.00
D C
ATOM 12199 HG1 GLU D 125 −19.405 21.049 42.647 1.00 0.00
D H
ATOM 12200 HG2 GLU D 125 −20.571 21.840 43.775 1.00 0.00
D H
ATOM 12201 CD GLU D 125 −20.225 19.765 44.071 1.00 0.00
D C
ATOM 12202 OE1 GLU D 125 −21.080 18.852 44.010 1.00 0.00
D O
ATOM 12203 OE2 GLU D 125 −19.234 19.758 44.828 1.00 0.00
D O

ATOM 12204 C GLU D 125 -23.539 21.775 41.176 1.00 0.00
D C
ATOM 12205 O GLU D 125 -23.312 22.739 40.454 1.00 0.00
D O
ATOM 12206 N LYS D 126 -24.511 20.905 40.911 1.00 0.00
D N
ATOM 12207 HN LYS D 126 -24.669 20.041 41.387 1.00 0.00
D H
ATOM 12208 CA LYS D 126 -25.452 21.199 39.837 1.00 0.00
D C
ATOM 12209 HA LYS D 126 -24.886 21.253 38.916 1.00 0.00
D H
ATOM 12210 CB LYS D 126 -26.515 20.107 39.759 1.00 0.00
D C
ATOM 12211 HB1 LYS D 126 -27.120 20.172 40.693 1.00 0.00
D H
ATOM 12212 HB2 LYS D 126 -27.209 20.337 38.920 1.00 0.00
D H
ATOM 12213 CG LYS D 126 -25.979 18.709 39.628 1.00 0.00
D C
ATOM 12214 HG1 LYS D 126 -25.391 18.457 40.540 1.00 0.00
D H
ATOM 12215 HG2 LYS D 126 -26.834 17.996 39.577 1.00 0.00
D H
ATOM 12216 CD LYS D 126 -25.101 18.553 38.424 1.00 0.00
D C
ATOM 12217 HD1 LYS D 126 -24.585 19.523 38.237 1.00 0.00
D H
ATOM 12218 HD2 LYS D 126 -24.317 17.798 38.663 1.00 0.00
D H
ATOM 12219 CE LYS D 126 -25.885 18.123 37.198 1.00 0.00
D C
ATOM 12220 HE1 LYS D 126 -26.741 17.474 37.491 1.00 0.00
D H
ATOM 12221 HE2 LYS D 126 -26.284 19.010 36.659 1.00 0.00
D H
ATOM 12222 NZ LYS D 126 -25.022 17.356 36.259 1.00 0.00
D N
ATOM 12223 HZ1 LYS D 126 -25.573 17.067 35.425 1.00 0.00
D H
ATOM 12224 HZ2 LYS D 126 -24.224 17.950 35.956 1.00 0.00
D H
ATOM 12225 HZ3 LYS D 126 -24.655 16.510 36.740 1.00 0.00
D H
ATOM 12226 C LYS D 126 -26.101 22.560 40.052 1.00 0.00
D C
ATOM 12227 O LYS D 126 -26.062 23.437 39.174 1.00 0.00
D O
ATOM 12228 N ASP D 127 -26.670 22.760 41.237 1.00 0.00
D N
ATOM 12229 HN ASP D 127 -26.633 22.140 42.016 1.00 0.00
D H
ATOM 12230 CA ASP D 127 -27.435 23.967 41.509 1.00 0.00
D C

ATOM 12231 HA ASP D 127 −28.181 24.059 40.728 1.00 0.00
D H
ATOM 12232 CB ASP D 127 −28.066 23.853 42.899 1.00 0.00
D C
ATOM 12233 HB1 ASP D 127 −27.337 23.391 43.595 1.00 0.00
D H
ATOM 12234 HB2 ASP D 127 −28.334 24.855 43.290 1.00 0.00
D H
ATOM 12235 CG ASP D 127 −29.309 22.986 42.906 1.00 0.00
D C
ATOM 12236 OD1 ASP D 127 −29.154 21.750 42.777 1.00 0.00
D O
ATOM 12237 OD2 ASP D 127 −30.422 23.534 43.057 1.00 0.00
D O
ATOM 12238 C ASP D 127 −26.547 25.197 41.415 1.00 0.00
D C
ATOM 12239 O ASP D 127 −26.842 26.152 40.682 1.00 0.00
D O
ATOM 12240 N MET D 128 −25.434 25.189 42.138 1.00 0.00
D N
ATOM 12241 HN MET D 128 −25.055 24.459 42.702 1.00 0.00
D H
ATOM 12242 CA MET D 128 −24.642 26.402 42.117 1.00 0.00
D C
ATOM 12243 HA MET D 128 −25.332 27.236 42.160 1.00 0.00
D H
ATOM 12244 CB MET D 128 −23.743 26.478 43.346 1.00 0.00
D C
ATOM 12245 HB1 MET D 128 −24.293 27.072 44.114 1.00 0.00
D H
ATOM 12246 HB2 MET D 128 −23.615 25.459 43.774 1.00 0.00
D H
ATOM 12247 CG MET D 128 −22.398 27.123 43.116 1.00 0.00
D C
ATOM 12248 HG1 MET D 128 −21.623 26.346 42.943 1.00 0.00
D H
ATOM 12249 HG2 MET D 128 −22.442 27.764 42.208 1.00 0.00
D H
ATOM 12250 SD MET D 128 −21.875 28.129 44.517 1.00 0.00
D S
ATOM 12251 CE MET D 128 −23.294 28.029 45.614 1.00 0.00
D C
ATOM 12252 HE1 MET D 128 −23.122 28.622 46.539 1.00 0.00
D H
ATOM 12253 HE2 MET D 128 −24.208 28.422 45.121 1.00 0.00
D H
ATOM 12254 HE3 MET D 128 −23.493 26.978 45.915 1.00 0.00
D H
ATOM 12255 C MET D 128 −23.861 26.519 40.819 1.00 0.00
D C
ATOM 12256 O MET D 128 −23.427 27.611 40.458 1.00 0.00
D O
ATOM 12257 N GLY D 129 −23.746 25.446 40.056 1.00 0.00
D N

ATOM 12258 HN GLY D 129 -24.081 24.533 40.289 1.00 0.00
D H
ATOM 12259 CA GLY D 129 -23.092 25.561 38.781 1.00 0.00
D C
ATOM 12260 HA1 GLY D 129 -22.946 24.566 38.381 1.00 0.00
D H
ATOM 12261 HA2 GLY D 129 -22.178 26.125 38.912 1.00 0.00
D H
ATOM 12262 C GLY D 129 -23.972 26.321 37.829 1.00 0.00
D C
ATOM 12263 O GLY D 129 -23.511 27.219 37.129 1.00 0.00
D O
ATOM 12264 N VAL D 130 -25.247 25.952 37.773 1.00 0.00
D N
ATOM 12265 HN VAL D 130 -25.685 25.201 38.261 1.00 0.00
D H
ATOM 12266 CA VAL D 130 -26.140 26.710 36.909 1.00 0.00
D C
ATOM 12267 HA VAL D 130 -25.700 26.741 35.920 1.00 0.00
D H
ATOM 12268 CB VAL D 130 -27.498 25.995 36.769 1.00 0.00
D C
ATOM 12269 HB VAL D 130 -28.079 26.527 35.976 1.00 0.00
D H
ATOM 12270 CG1 VAL D 130 -27.268 24.554 36.358 1.00 0.00
D C
ATOM 12271 HG11 VAL D 130 -28.236 24.013 36.290 1.00 0.00
D H
ATOM 12272 HG12 VAL D 130 -26.781 24.484 35.364 1.00 0.00
D H
ATOM 12273 HG13 VAL D 130 -26.632 24.030 37.104 1.00 0.00
D H
ATOM 12274 CG2 VAL D 130 -28.267 26.007 38.054 1.00 0.00
D C
ATOM 12275 HG21 VAL D 130 -29.224 25.453 37.944 1.00 0.00
D H
ATOM 12276 HG22 VAL D 130 -27.677 25.529 38.866 1.00 0.00
D H
ATOM 12277 HG23 VAL D 130 -28.513 27.043 38.366 1.00 0.00
D H
ATOM 12278 C VAL D 130 -26.253 28.149 37.404 1.00 0.00
D C
ATOM 12279 O VAL D 130 -26.289 29.094 36.602 1.00 0.00
D O
ATOM 12280 N LYS D 131 -26.256 28.341 38.729 1.00 0.00
D N
ATOM 12281 HN LYS D 131 -26.434 27.629 39.407 1.00 0.00
D H
ATOM 12282 CA LYS D 131 -25.988 29.668 39.278 1.00 0.00
D C
ATOM 12283 HA LYS D 131 -26.880 30.259 39.113 1.00 0.00
D H
ATOM 12284 CB LYS D 131 -25.668 29.555 40.771 1.00 0.00
D C

ATOM 12285 HB1 LYS D 131 -26.400 28.839 41.210 1.00 0.00
D H
ATOM 12286 HB2 LYS D 131 -24.664 29.089 40.892 1.00 0.00
D H
ATOM 12287 CG LYS D 131 -25.733 30.855 41.576 1.00 0.00
D C
ATOM 12288 HG1 LYS D 131 -26.688 31.379 41.346 1.00 0.00
D H
ATOM 12289 HG2 LYS D 131 -25.744 30.608 42.663 1.00 0.00
D H
ATOM 12290 CD LYS D 131 -24.540 31.750 41.217 1.00 0.00
D C
ATOM 12291 HD1 LYS D 131 -23.689 31.488 41.886 1.00 0.00
D H
ATOM 12292 HD2 LYS D 131 -24.229 31.511 40.173 1.00 0.00
D H
ATOM 12293 CE LYS D 131 -24.821 33.236 41.319 1.00 0.00
D C
ATOM 12294 HE1 LYS D 131 -23.993 33.821 40.861 1.00 0.00
D H
ATOM 12295 HE2 LYS D 131 -25.766 33.492 40.793 1.00 0.00
D H
ATOM 12296 NZ LYS D 131 -24.949 33.625 42.782 1.00 0.00
D N
ATOM 12297 HZ1 LYS D 131 -25.141 34.645 42.863 1.00 0.00
D H
ATOM 12298 HZ2 LYS D 131 -25.729 33.091 43.216 1.00 0.00
D H
ATOM 12299 HZ3 LYS D 131 -24.064 33.401 43.278 1.00 0.00
D H
ATOM 12300 C LYS D 131 -24.846 30.362 38.561 1.00 0.00
D C
ATOM 12301 O LYS D 131 -25.052 31.358 37.856 1.00 0.00
D O
ATOM 12302 N ILE D 132 -23.636 29.859 38.748 1.00 0.00
D N
ATOM 12303 HN ILE D 132 -23.431 28.969 39.151 1.00 0.00
D H
ATOM 12304 CA ILE D 132 -22.463 30.618 38.354 1.00 0.00
D C
ATOM 12305 HA ILE D 132 -22.569 31.607 38.781 1.00 0.00
D H
ATOM 12306 CB ILE D 132 -21.192 29.938 38.881 1.00 0.00
D C
ATOM 12307 HB ILE D 132 -21.309 29.950 39.994 1.00 0.00
D H
ATOM 12308 CG2 ILE D 132 -21.060 28.561 38.305 1.00 0.00
D C
ATOM 12309 HG21 ILE D 132 -20.233 28.008 38.800 1.00 0.00
D H
ATOM 12310 HG22 ILE D 132 -21.992 27.976 38.457 1.00 0.00
D H
ATOM 12311 HG23 ILE D 132 -20.846 28.604 37.216 1.00 0.00
D H

ATOM 12312 CG1 ILE D 132 -19.975 30.792 38.549 1.00 0.00
D C
ATOM 12313 HG11 ILE D 132 -19.514 30.439 37.600 1.00 0.00
D H
ATOM 12314 HG12 ILE D 132 -20.306 31.844 38.383 1.00 0.00
D H
ATOM 12315 CD ILE D 132 -18.944 30.790 39.626 1.00 0.00
D C
ATOM 12316 HD1 ILE D 132 -18.108 31.471 39.361 1.00 0.00
D H
ATOM 12317 HD2 ILE D 132 -19.381 31.134 40.588 1.00 0.00
D H
ATOM 12318 HD3 ILE D 132 -18.525 29.772 39.773 1.00 0.00
D H
ATOM 12319 C ILE D 132 -22.440 30.791 36.849 1.00 0.00
D C
ATOM 12320 O ILE D 132 -21.949 31.797 36.325 1.00 0.00
D O
ATOM 12321 N ALA D 133 -23.024 29.837 36.136 1.00 0.00
D N
ATOM 12322 HN ALA D 133 -23.435 29.015 36.529 1.00 0.00
D H
ATOM 12323 CA ALA D 133 -23.097 29.942 34.692 1.00 0.00
D C
ATOM 12324 HA ALA D 133 -22.112 30.102 34.267 1.00 0.00
D H
ATOM 12325 CB ALA D 133 -23.669 28.655 34.113 1.00 0.00
D C
ATOM 12326 HB1 ALA D 133 -23.011 27.802 34.382 1.00 0.00
D H
ATOM 12327 HB2 ALA D 133 -24.681 28.449 34.523 1.00 0.00
D H
ATOM 12328 HB3 ALA D 133 -23.737 28.701 33.005 1.00 0.00
D H
ATOM 12329 C ALA D 133 -23.937 31.135 34.286 1.00 0.00
D C
ATOM 12330 O ALA D 133 -23.505 31.958 33.471 1.00 0.00
D O
ATOM 12331 N GLN D 134 -25.138 31.253 34.852 1.00 0.00
D N
ATOM 12332 HN GLN D 134 -25.560 30.614 35.493 1.00 0.00
D H
ATOM 12333 CA GLN D 134 -25.952 32.417 34.536 1.00 0.00
D C
ATOM 12334 HA GLN D 134 -26.065 32.446 33.459 1.00 0.00
D H
ATOM 12335 CB GLN D 134 -27.315 32.301 35.202 1.00 0.00
D C
ATOM 12336 HB1 GLN D 134 -27.145 32.395 36.300 1.00 0.00
D H
ATOM 12337 HB2 GLN D 134 -27.944 33.172 34.909 1.00 0.00
D H
ATOM 12338 CG GLN D 134 -27.996 30.994 34.892 1.00 0.00
D C

ATOM 12339 HG1 GLN D 134   -27.245 30.219 34.628 1.00 0.00
D   H
ATOM 12340 HG2 GLN D 134   -28.567 30.630 35.771 1.00 0.00
D   H
ATOM 12341 CD GLN D 134   -28.933 31.098 33.713 1.00 0.00
D   C
ATOM 12342 OE1 GLN D 134   -29.062 32.158 33.101 1.00 0.00
D   O
ATOM 12343 NE2 GLN D 134   -29.587 29.991 33.377 1.00 0.00
D   N
ATOM 12344 HE21 GLN D 134   -30.204 30.053 32.598 1.00 0.00
D   H
ATOM 12345 HE22 GLN D 134   -29.460 29.161 33.909 1.00 0.00
D   H
ATOM 12346 C   GLN D 134   -25.250 33.696 34.957 1.00 0.00
D   C
ATOM 12347 O   GLN D 134   -25.277 34.695 34.232 1.00 0.00
D   O
ATOM 12348 N   SER D 135   -24.586 33.675 36.114 1.00 0.00
D   N
ATOM 12349 HN SER D 135   -24.457 32.881 36.707 1.00 0.00
D   H
ATOM 12350 CA SER D 135   -23.969 34.900 36.611 1.00 0.00
D   C
ATOM 12351 HA SER D 135   -24.715 35.675 36.489 1.00 0.00
D   H
ATOM 12352 CB SER D 135   -23.571 34.744 38.075 1.00 0.00
D   C
ATOM 12353 HB1 SER D 135   -22.995 35.649 38.375 1.00 0.00
D   H
ATOM 12354 HB2 SER D 135   -24.476 34.703 38.721 1.00 0.00
D   H
ATOM 12355 OG SER D 135   -22.760 33.605 38.257 1.00 0.00
D   O
ATOM 12356 HG1 SER D 135   -22.360 33.678 39.133 1.00 0.00
D   H
ATOM 12357 C   SER D 135   -22.758 35.301 35.781 1.00 0.00
D   C
ATOM 12358 O   SER D 135   -22.373 36.474 35.780 1.00 0.00
D   O
ATOM 12359 N   LEU D 136   -22.142 34.355 35.080 1.00 0.00
D   N
ATOM 12360 HN LEU D 136   -22.380 33.386 35.060 1.00 0.00
D   H
ATOM 12361 CA LEU D 136   -21.002 34.708 34.243 1.00 0.00
D   C
ATOM 12362 HA LEU D 136   -20.540 35.575 34.698 1.00 0.00
D   H
ATOM 12363 CB LEU D 136   -19.999 33.560 34.188 1.00 0.00
D   C
ATOM 12364 HB1 LEU D 136   -20.528 32.643 34.539 1.00 0.00
D   H
ATOM 12365 HB2 LEU D 136   -19.725 33.369 33.126 1.00 0.00
D   H

ATOM 12366 CG LEU D 136 −18.764 33.804 35.051 1.00 0.00
D C
ATOM 12367 HG LEU D 136 −18.962 33.470 36.098 1.00 0.00
D H
ATOM 12368 CD1 LEU D 136 −17.574 33.074 34.487 1.00 0.00
D C
ATOM 12369 HD11 LEU D 136 −16.664 33.289 35.087 1.00 0.00
D H
ATOM 12370 HD12 LEU D 136 −17.747 31.976 34.497 1.00 0.00
D H
ATOM 12371 HD13 LEU D 136 −17.381 33.389 33.440 1.00 0.00
D H
ATOM 12372 CD2 LEU D 136 −18.472 35.286 35.081 1.00 0.00
D C
ATOM 12373 HD21 LEU D 136 −17.587 35.493 35.720 1.00 0.00
D H
ATOM 12374 HD22 LEU D 136 −18.255 35.681 34.067 1.00 0.00
D H
ATOM 12375 HD23 LEU D 136 −19.339 35.845 35.496 1.00 0.00
D H
ATOM 12376 C LEU D 136 −21.444 35.105 32.845 1.00 0.00
D C
ATOM 12377 O LEU D 136 −20.916 36.064 32.272 1.00 0.00
D O
ATOM 12378 N SER D 137 −22.405 34.374 32.281 1.00 0.00
D N
ATOM 12379 HN SER D 137 −22.856 33.590 32.706 1.00 0.00
D H
ATOM 12380 CA SER D 137 −22.886 34.686 30.940 1.00 0.00
D C
ATOM 12381 HA SER D 137 −22.051 34.515 30.274 1.00 0.00
D H
ATOM 12382 CB SER D 137 −24.080 33.799 30.604 1.00 0.00
D C
ATOM 12383 HB1 SER D 137 −24.381 34.007 29.552 1.00 0.00
D H
ATOM 12384 HB2 SER D 137 −23.792 32.725 30.656 1.00 0.00
D H
ATOM 12385 OG SER D 137 −25.163 34.095 31.468 1.00 0.00
D O
ATOM 12386 HG1 SER D 137 −25.949 33.686 31.086 1.00 0.00
D H
ATOM 12387 C SER D 137 −23.265 36.154 30.821 1.00 0.00
D C
ATOM 12388 O SER D 137 −22.653 36.909 30.058 1.00 0.00
D O
ATOM 12389 N ASP D 138 −24.253 36.583 31.592 1.00 0.00
D N
ATOM 12390 HN ASP D 138 −24.773 36.007 32.216 1.00 0.00
D H
ATOM 12391 CA ASP D 138 −24.690 37.974 31.604 1.00 0.00
D C
ATOM 12392 HA ASP D 138 −24.342 38.462 30.701 1.00 0.00
D H

ATOM 12393 CB ASP D 138 -26.204 38.058 31.747 1.00 0.00
D C
ATOM 12394 HB1 ASP D 138 -26.533 37.370 32.552 1.00 0.00
D H
ATOM 12395 HB2 ASP D 138 -26.514 39.087 32.019 1.00 0.00
D H
ATOM 12396 CG ASP D 138 -26.933 37.656 30.484 1.00 0.00
D C
ATOM 12397 OD1 ASP D 138 -27.042 38.491 29.560 1.00 0.00
D O
ATOM 12398 OD2 ASP D 138 -27.393 36.498 30.414 1.00 0.00
D O
ATOM 12399 C ASP D 138 -23.972 38.643 32.769 1.00 0.00
D C
ATOM 12400 O ASP D 138 -24.354 38.474 33.929 1.00 0.00
D O
ATOM 12401 N GLU D 139 -22.930 39.408 32.459 1.00 0.00
D N
ATOM 12402 HN GLU D 139 -22.662 39.686 31.540 1.00 0.00
D H
ATOM 12403 CA GLU D 139 -22.061 39.921 33.508 1.00 0.00
D C
ATOM 12404 HA GLU D 139 -21.887 39.108 34.202 1.00 0.00
D H
ATOM 12405 CB GLU D 139 -20.727 40.354 32.903 1.00 0.00
D C
ATOM 12406 HB1 GLU D 139 -20.152 40.899 33.687 1.00 0.00
D H
ATOM 12407 HB2 GLU D 139 -20.120 39.457 32.647 1.00 0.00
D H
ATOM 12408 CG GLU D 139 -20.869 41.242 31.690 1.00 0.00
D C
ATOM 12409 HG1 GLU D 139 -21.576 40.780 30.976 1.00 0.00
D H
ATOM 12410 HG2 GLU D 139 -21.241 42.246 31.973 1.00 0.00
D H
ATOM 12411 CD GLU D 139 -19.571 41.401 30.940 1.00 0.00
D C
ATOM 12412 OE1 GLU D 139 -19.605 41.452 29.695 1.00 0.00
D O
ATOM 12413 OE2 GLU D 139 -18.512 41.465 31.594 1.00 0.00
D O
ATOM 12414 C GLU D 139 -22.677 41.068 34.299 1.00 0.00
D C
ATOM 12415 O GLU D 139 -21.956 41.726 35.056 1.00 0.00
D O
ATOM 12416 N ASN D 140 -23.979 41.327 34.155 1.00 0.00
D N
ATOM 12417 HN ASN D 140 -24.594 40.907 33.489 1.00 0.00
D H
ATOM 12418 CA ASN D 140 -24.612 42.308 35.029 1.00 0.00
D C
ATOM 12419 HA ASN D 140 -23.797 42.859 35.488 1.00 0.00
D H

ATOM 12420 CB ASN D 140   -25.520 43.250 34.238 1.00 0.00
D   C
ATOM 12421 HB1 ASN D 140   -25.233 43.177 33.166 1.00 0.00
D   H
ATOM 12422 HB2 ASN D 140   -26.577 42.927 34.323 1.00 0.00
D   H
ATOM 12423 CG ASN D 140   -25.370 44.699 34.671 1.00 0.00
D   C
ATOM 12424 OD1 ASN D 140   -26.291 45.503 34.519 1.00 0.00
D   O
ATOM 12425 ND2 ASN D 140   -24.204 45.038 35.218 1.00 0.00
D   N
ATOM 12426 HD21 ASN D 140   -24.096 45.982 35.517 1.00 0.00
D   H
ATOM 12427 HD22 ASN D 140   -23.494 44.353 35.342 1.00 0.00
D   H
ATOM 12428 C   ASN D 140   -25.402 41.643 36.144 1.00 0.00
D   C
ATOM 12429 O   ASN D 140   -25.429 42.149 37.271 1.00 0.00
D   O
ATOM 12430 N   VAL D 141   -26.061 40.522 35.863 1.00 0.00
D   N
ATOM 12431 HN VAL D 141   -26.153 40.095 34.967 1.00 0.00
D   H
ATOM 12432 CA VAL D 141   -26.731 39.808 36.942 1.00 0.00
D   C
ATOM 12433 HA VAL D 141   -27.334 40.503 37.513 1.00 0.00
D   H
ATOM 12434 CB VAL D 141   -27.632 38.680 36.421 1.00 0.00
D   C
ATOM 12435 HB VAL D 141   -28.693 38.993 36.582 1.00 0.00
D   H
ATOM 12436 CG1 VAL D 141   -27.411 38.442 34.950 1.00 0.00
D   C
ATOM 12437 HG11 VAL D 141   -28.041 37.597 34.597 1.00 0.00
D   H
ATOM 12438 HG12 VAL D 141   -27.683 39.332 34.346 1.00 0.00
D   H
ATOM 12439 HG13 VAL D 141   -26.349 38.186 34.749 1.00 0.00
D   H
ATOM 12440 CG2 VAL D 141   -27.500 37.397 37.253 1.00 0.00
D   C
ATOM 12441 HG21 VAL D 141   -28.132 36.588 36.829 1.00 0.00
D   H
ATOM 12442 HG22 VAL D 141   -26.445 37.048 37.265 1.00 0.00
D   H
ATOM 12443 HG23 VAL D 141   -27.825 37.563 38.301 1.00 0.00
D   H
ATOM 12444 C   VAL D 141   -25.638 39.284 37.867 1.00 0.00
D   C
ATOM 12445 O   VAL D 141   -24.777 38.504 37.453 1.00 0.00
D   O
ATOM 12446 N   LEU D 142   -25.606 39.797 39.092 1.00 0.00
D   N

ATOM 12447 HN LEU D 142 -26.208 40.511 39.444 1.00 0.00
D H
ATOM 12448 CA LEU D 142 -24.623 39.314 40.053 1.00 0.00
D C
ATOM 12449 HA LEU D 142 -23.760 38.987 39.488 1.00 0.00
D H
ATOM 12450 CB LEU D 142 -24.227 40.428 41.025 1.00 0.00
D C
ATOM 12451 HB1 LEU D 142 -23.891 41.296 40.412 1.00 0.00
D H
ATOM 12452 HB2 LEU D 142 -25.133 40.770 41.572 1.00 0.00
D H
ATOM 12453 CG LEU D 142 -23.115 40.030 41.992 1.00 0.00
D C
ATOM 12454 HG LEU D 142 -23.469 39.142 42.569 1.00 0.00
D H
ATOM 12455 CD1 LEU D 142 -21.867 39.668 41.217 1.00 0.00
D C
ATOM 12456 HD11 LEU D 142 -21.034 39.423 41.910 1.00 0.00
D H
ATOM 12457 HD12 LEU D 142 -22.052 38.786 40.567 1.00 0.00
D H
ATOM 12458 HD13 LEU D 142 -21.544 40.515 40.575 1.00 0.00
D H
ATOM 12459 CD2 LEU D 142 -22.823 41.146 42.980 1.00 0.00
D C
ATOM 12460 HD21 LEU D 142 -22.025 40.836 43.689 1.00 0.00
D H
ATOM 12461 HD22 LEU D 142 -22.479 42.067 42.467 1.00 0.00
D H
ATOM 12462 HD23 LEU D 142 -23.734 41.393 43.568 1.00 0.00
D H
ATOM 12463 C LEU D 142 -25.166 38.107 40.803 1.00 0.00
D C
ATOM 12464 O LEU D 142 -24.576 37.023 40.761 1.00 0.00
D O
ATOM 12465 N ASN D 143 -26.294 38.275 41.483 1.00 0.00
D N
ATOM 12466 HN ASN D 143 -26.716 39.165 41.653 1.00 0.00
D H
ATOM 12467 CA ASN D 143 -27.017 37.148 42.047 1.00 0.00
D C
ATOM 12468 HA ASN D 143 -26.530 36.242 41.702 1.00 0.00
D H
ATOM 12469 CB ASN D 143 -27.080 37.204 43.576 1.00 0.00
D C
ATOM 12470 HB1 ASN D 143 -27.667 38.106 43.859 1.00 0.00
D H
ATOM 12471 HB2 ASN D 143 -27.617 36.319 43.972 1.00 0.00
D H
ATOM 12472 CG ASN D 143 -25.715 37.316 44.219 1.00 0.00
D C
ATOM 12473 OD1 ASN D 143 -24.808 37.934 43.671 1.00 0.00
D O

```
ATOM  12474  ND2 ASN D 143     -25.560  36.703  45.385  1.00  0.00
D    N
ATOM  12475 HD21 ASN D 143     -24.669  36.777  45.826  1.00  0.00
D    H
ATOM  12476 HD22 ASN D 143     -26.325  36.220  45.799  1.00  0.00
D    H
ATOM  12477  C   ASN D 143     -28.418  37.169  41.462  1.00  0.00
D    C
ATOM  12478  O   ASN D 143     -28.912  38.217  41.041  1.00  0.00
D    O
ATOM  12479  N   TYR D 144     -29.064  36.008  41.445  1.00  0.00
D    N
ATOM  12480  HN  TYR D 144     -28.722  35.140  41.801  1.00  0.00
D    H
ATOM  12481  CA  TYR D 144     -30.395  35.940  40.865  1.00  0.00
D    C
ATOM  12482  HA  TYR D 144     -30.894  36.875  41.087  1.00  0.00
D    H
ATOM  12483  CB  TYR D 144     -30.336  35.701  39.355  1.00  0.00
D    C
ATOM  12484  HB1 TYR D 144     -31.342  35.887  38.917  1.00  0.00
D    H
ATOM  12485  HB2 TYR D 144     -29.630  36.412  38.872  1.00  0.00
D    H
ATOM  12486  CG  TYR D 144     -29.957  34.298  38.958  1.00  0.00
D    C
ATOM  12487  CD1 TYR D 144     -28.667  33.828  39.134  1.00  0.00
D    C
ATOM  12488  HD1 TYR D 144     -27.910  34.480  39.550  1.00  0.00
D    H
ATOM  12489  CE1 TYR D 144     -28.319  32.546  38.769  1.00  0.00
D    C
ATOM  12490  HE1 TYR D 144     -27.309  32.190  38.906  1.00  0.00
D    H
ATOM  12491  CZ  TYR D 144     -29.268  31.712  38.216  1.00  0.00
D    C
ATOM  12492  OH  TYR D 144     -28.912  30.430  37.854  1.00  0.00
D    O
ATOM  12493  HH  TYR D 144     -29.708  29.983  37.560  1.00  0.00
D    H
ATOM  12494  CD2 TYR D 144     -30.896  33.443  38.398  1.00  0.00
D    C
ATOM  12495  HD2 TYR D 144     -31.901  33.794  38.209  1.00  0.00
D    H
ATOM  12496  CE2 TYR D 144     -30.561  32.160  38.029  1.00  0.00
D    C
ATOM  12497  HE2 TYR D 144     -31.304  31.520  37.576  1.00  0.00
D    H
ATOM  12498  C   TYR D 144     -31.180  34.839  41.552  1.00  0.00
D    C
ATOM  12499  O   TYR D 144     -30.630  33.813  41.955  1.00  0.00
D    O
ATOM  12500  N   ILE D 145     -32.485  35.068  41.670  1.00  0.00
D    N
```

```
ATOM  12501  HN  ILE D 145     -32.911  35.924  41.386  1.00  0.00
D    H
ATOM  12502  CA  ILE D 145     -33.415  34.093  42.225  1.00  0.00
D    C
ATOM  12503  HA  ILE D 145     -32.970  33.112  42.106  1.00  0.00
D    H
ATOM  12504  CB  ILE D 145     -33.734  34.367  43.708  1.00  0.00
D    C
ATOM  12505  HB  ILE D 145     -33.092  35.245  43.974  1.00  0.00
D    H
ATOM  12506  CG2 ILE D 145     -35.222  34.653  43.920  1.00  0.00
D    C
ATOM  12507 HG21 ILE D 145     -35.409  35.000  44.957  1.00  0.00
D    H
ATOM  12508 HG22 ILE D 145     -35.574  35.449  43.230  1.00  0.00
D    H
ATOM  12509 HG23 ILE D 145     -35.830  33.741  43.744  1.00  0.00
D    H
ATOM  12510  CG1 ILE D 145     -33.258  33.184  44.542  1.00  0.00
D    C
ATOM  12511 HG11 ILE D 145     -32.676  32.483  43.903  1.00  0.00
D    H
ATOM  12512 HG12 ILE D 145     -32.569  33.556  45.335  1.00  0.00
D    H
ATOM  12513  CD  ILE D 145     -34.370  32.413  45.219  1.00  0.00
D    C
ATOM  12514  HD1 ILE D 145     -33.948  31.604  45.854  1.00  0.00
D    H
ATOM  12515  HD2 ILE D 145     -34.974  33.083  45.867  1.00  0.00
D    H
ATOM  12516  HD3 ILE D 145     -35.044  31.945  44.470  1.00  0.00
D    H
ATOM  12517  C   ILE D 145     -34.683  34.105  41.382  1.00  0.00
D    C
ATOM  12518  O   ILE D 145     -35.141  35.170  40.950  1.00  0.00
D    O
ATOM  12519  N   ASP D 146     -35.217  32.918  41.099  1.00  0.00
D    N
ATOM  12520  HN  ASP D 146     -34.849  32.043  41.403  1.00  0.00
D    H
ATOM  12521  CA  ASP D 146     -36.426  32.777  40.295  1.00  0.00
D    C
ATOM  12522  HA  ASP D 146     -36.784  33.774  40.066  1.00  0.00
D    H
ATOM  12523  CB  ASP D 146     -36.128  31.976  39.031  1.00  0.00
D    C
ATOM  12524  HB1 ASP D 146     -37.067  31.519  38.658  1.00  0.00
D    H
ATOM  12525  HB2 ASP D 146     -35.722  32.634  38.238  1.00  0.00
D    H
ATOM  12526  CG  ASP D 146     -35.143  30.852  39.276  1.00  0.00
D    C
ATOM  12527  OD1 ASP D 146     -35.568  29.814  39.829  1.00  0.00
D    O
```

ATOM 12528 OD2 ASP D 146 -33.944 31.014 38.960 1.00 0.00
D O
ATOM 12529 C ASP D 146 -37.513 32.095 41.108 1.00 0.00
D C
ATOM 12530 O ASP D 146 -37.298 31.006 41.646 1.00 0.00
D O
ATOM 12531 N LEU D 147 -38.678 32.729 41.188 1.00 0.00
D N
ATOM 12532 HN LEU D 147 -38.855 33.638 40.814 1.00 0.00
D H
ATOM 12533 CA LEU D 147 -39.823 32.123 41.853 1.00 0.00
D C
ATOM 12534 HA LEU D 147 -39.426 31.570 42.694 1.00 0.00
D H
ATOM 12535 CB LEU D 147 -40.768 33.221 42.333 1.00 0.00
D C
ATOM 12536 HB1 LEU D 147 -41.781 32.767 42.422 1.00 0.00
D H
ATOM 12537 HB2 LEU D 147 -40.476 33.525 43.363 1.00 0.00
D H
ATOM 12538 CG LEU D 147 -40.780 34.389 41.352 1.00 0.00
D C
ATOM 12539 HG LEU D 147 -39.809 34.344 40.803 1.00 0.00
D H
ATOM 12540 CD1 LEU D 147 -41.914 34.260 40.352 1.00 0.00
D C
ATOM 12541 HD11 LEU D 147 -41.939 35.141 39.675 1.00 0.00
D H
ATOM 12542 HD12 LEU D 147 -41.787 33.347 39.731 1.00 0.00
D H
ATOM 12543 HD13 LEU D 147 -42.893 34.196 40.874 1.00 0.00
D H
ATOM 12544 CD2 LEU D 147 -40.832 35.707 42.116 1.00 0.00
D C
ATOM 12545 HD21 LEU D 147 -40.822 36.564 41.408 1.00 0.00
D H
ATOM 12546 HD22 LEU D 147 -41.750 35.788 42.731 1.00 0.00
D H
ATOM 12547 HD23 LEU D 147 -39.951 35.802 42.788 1.00 0.00
D H
ATOM 12548 C LEU D 147 -40.557 31.133 40.958 1.00 0.00
D C
ATOM 12549 O LEU D 147 -41.207 30.213 41.465 1.00 0.00
D O
ATOM 12550 N SER D 148 -40.456 31.287 39.645 1.00 0.00
D N
ATOM 12551 HN SER D 148 -39.923 32.009 39.203 1.00 0.00
D H
ATOM 12552 CA SER D 148 -41.130 30.393 38.717 1.00 0.00
D C
ATOM 12553 HA SER D 148 -41.227 29.433 39.207 1.00 0.00
D H
ATOM 12554 CB SER D 148 -42.512 30.961 38.348 1.00 0.00
D C

ATOM 12555 HB1 SER D 148   -43.007 30.244 37.653 1.00 0.00
D   H
ATOM 12556 HB2 SER D 148   -43.154 31.037 39.253 1.00 0.00
D   H
ATOM 12557 OG SER D 148   -42.389 32.218 37.697 1.00 0.00
D   O
ATOM 12558 HG1 SER D 148   -43.246 32.415 37.297 1.00 0.00
D   H
ATOM 12559 C   SER D 148   -40.227 30.193 37.506 1.00 0.00
D   C
ATOM 12560 O   SER D 148   -39.092 30.684 37.475 1.00 0.00
D   O
ATOM 12561 N   ASP D 149   -40.730 29.472 36.503 1.00 0.00
D   N
ATOM 12562 HN ASP D 149   -41.611 29.006 36.491 1.00 0.00
D   H
ATOM 12563 CA ASP D 149   -39.979 29.297 35.264 1.00 0.00
D   C
ATOM 12564 HA ASP D 149   -38.929 29.291 35.532 1.00 0.00
D   H
ATOM 12565 CB ASP D 149   -40.379 27.987 34.591 1.00 0.00
D   C
ATOM 12566 HB1 ASP D 149   -39.954 27.957 33.568 1.00 0.00
D   H
ATOM 12567 HB2 ASP D 149   -39.985 27.120 35.158 1.00 0.00
D   H
ATOM 12568 CG ASP D 149   -41.879 27.837 34.464 1.00 0.00
D   C
ATOM 12569 OD1 ASP D 149   -42.471 27.055 35.236 1.00 0.00
D   O
ATOM 12570 OD2 ASP D 149   -42.470 28.516 33.601 1.00 0.00
D   O
ATOM 12571 C   ASP D 149   -40.183 30.468 34.313 1.00 0.00
D   C
ATOM 12572 O   ASP D 149   -39.648 30.464 33.199 1.00 0.00
D   O
ATOM 12573 N   GLU D 150   -40.945 31.475 34.736 1.00 0.00
D   N
ATOM 12574 HN GLU D 150   -41.365 31.488 35.639 1.00 0.00
D   H
ATOM 12575 CA GLU D 150   -41.240 32.649 33.927 1.00 0.00
D   C
ATOM 12576 HA GLU D 150   -40.752 32.507 32.971 1.00 0.00
D   H
ATOM 12577 CB GLU D 150   -42.754 32.784 33.726 1.00 0.00
D   C
ATOM 12578 HB1 GLU D 150   -43.127 31.843 33.259 1.00 0.00
D   H
ATOM 12579 HB2 GLU D 150   -43.261 32.856 34.714 1.00 0.00
D   H
ATOM 12580 CG GLU D 150   -43.151 33.959 32.855 1.00 0.00
D   C
ATOM 12581 HG1 GLU D 150   -43.862 34.602 33.405 1.00 0.00
D   H

ATOM 12582 HG2 GLU D 150 −42.264 34.558 32.568 1.00 0.00
D H
ATOM 12583 CD GLU D 150 −43.871 33.550 31.594 1.00 0.00
D C
ATOM 12584 OE1 GLU D 150 −43.711 32.390 31.167 1.00 0.00
D O
ATOM 12585 OE2 GLU D 150 −44.600 34.392 31.032 1.00 0.00
D O
ATOM 12586 C GLU D 150 −40.708 33.943 34.516 1.00 0.00
D C
ATOM 12587 O GLU D 150 −40.291 34.829 33.767 1.00 0.00
D O
ATOM 12588 N TYR D 151 −40.717 34.078 35.835 1.00 0.00
D N
ATOM 12589 HN TYR D 151 −40.964 33.343 36.463 1.00 0.00
D H
ATOM 12590 CA TYR D 151 −40.361 35.329 36.480 1.00 0.00
D C
ATOM 12591 HA TYR D 151 −39.934 35.968 35.717 1.00 0.00
D H
ATOM 12592 CB TYR D 151 −41.587 35.969 37.126 1.00 0.00
D C
ATOM 12593 HB1 TYR D 151 −41.862 35.392 38.036 1.00 0.00
D H
ATOM 12594 HB2 TYR D 151 −41.362 37.008 37.451 1.00 0.00
D H
ATOM 12595 CG TYR D 151 −42.812 35.983 36.240 1.00 0.00
D C
ATOM 12596 CD1 TYR D 151 −42.940 36.906 35.213 1.00 0.00
D C
ATOM 12597 HD1 TYR D 151 −42.159 37.635 35.044 1.00 0.00
D H
ATOM 12598 CE1 TYR D 151 −44.061 36.929 34.409 1.00 0.00
D C
ATOM 12599 HE1 TYR D 151 −44.152 37.657 33.616 1.00 0.00
D H
ATOM 12600 CZ TYR D 151 −45.071 36.020 34.626 1.00 0.00
D C
ATOM 12601 OH TYR D 151 −46.188 36.035 33.826 1.00 0.00
D O
ATOM 12602 HH TYR D 151 −46.736 35.291 34.084 1.00 0.00
D H
ATOM 12603 CD2 TYR D 151 −43.843 35.078 36.439 1.00 0.00
D C
ATOM 12604 HD2 TYR D 151 −43.792 34.371 37.255 1.00 0.00
D H
ATOM 12605 CE2 TYR D 151 −44.967 35.092 35.639 1.00 0.00
D C
ATOM 12606 HE2 TYR D 151 −45.767 34.390 35.826 1.00 0.00
D H
ATOM 12607 C TYR D 151 −39.280 35.094 37.524 1.00 0.00
D C
ATOM 12608 O TYR D 151 −39.110 33.975 38.018 1.00 0.00
D O

```
ATOM  12609  N   SER D 152     -38.553  36.158  37.858  1.00  0.00
D    N
ATOM  12610  HN  SER D 152     -38.755  37.096  37.577  1.00  0.00
D    H
ATOM  12611  CA  SER D 152     -37.366  36.026  38.688  1.00  0.00
D    C
ATOM  12612  HA  SER D 152     -37.658  35.423  39.537  1.00  0.00
D    H
ATOM  12613  CB  SER D 152     -36.223  35.390  37.896  1.00  0.00
D    C
ATOM  12614  HB1 SER D 152     -35.288  35.948  38.129  1.00  0.00
D    H
ATOM  12615  HB2 SER D 152     -36.065  34.336  38.220  1.00  0.00
D    H
ATOM  12616  OG  SER D 152     -36.485  35.477  36.504  1.00  0.00
D    O
ATOM  12617  HG1 SER D 152     -35.659  35.275  36.046  1.00  0.00
D    H
ATOM  12618  C   SER D 152     -36.922  37.379  39.225  1.00  0.00
D    C
ATOM  12619  O   SER D 152     -37.335  38.435  38.732  1.00  0.00
D    O
ATOM  12620  N   ILE D 153     -36.055  37.321  40.234  1.00  0.00
D    N
ATOM  12621  HN  ILE D 153     -35.761  36.452  40.626  1.00  0.00
D    H
ATOM  12622  CA  ILE D 153     -35.457  38.494  40.862  1.00  0.00
D    C
ATOM  12623  HA  ILE D 153     -35.913  39.373  40.425  1.00  0.00
D    H
ATOM  12624  CB  ILE D 153     -35.629  38.455  42.389  1.00  0.00
D    C
ATOM  12625  HB  ILE D 153     -35.332  37.415  42.678  1.00  0.00
D    H
ATOM  12626  CG2 ILE D 153     -34.795  39.531  43.060  1.00  0.00
D    C
ATOM  12627 HG21 ILE D 153     -34.801  39.399  44.163  1.00  0.00
D    H
ATOM  12628 HG22 ILE D 153     -33.739  39.483  42.722  1.00  0.00
D    H
ATOM  12629 HG23 ILE D 153     -35.192  40.543  42.830  1.00  0.00
D    H
ATOM  12630  CG1 ILE D 153     -37.101  38.615  42.769  1.00  0.00
D    C
ATOM  12631 HG11 ILE D 153     -37.299  39.667  43.077  1.00  0.00
D    H
ATOM  12632 HG12 ILE D 153     -37.730  38.411  41.873  1.00  0.00
D    H
ATOM  12633  CD  ILE D 153     -37.547  37.678  43.886  1.00  0.00
D    C
ATOM  12634  HD1 ILE D 153     -38.635  37.797  44.079  1.00  0.00
D    H
ATOM  12635  HD2 ILE D 153     -37.358  36.618  43.607  1.00  0.00
D    H
```

ATOM 12636 HD3 ILE D 153 −37.004 37.896 44.829 1.00 0.00
D H
ATOM 12637 C ILE D 153 −33.983 38.526 40.478 1.00 0.00
D C
ATOM 12638 O ILE D 153 −33.336 37.475 40.392 1.00 0.00
D O
ATOM 12639 N VAL D 154 −33.446 39.721 40.236 1.00 0.00
D N
ATOM 12640 HN VAL D 154 −33.953 40.572 40.350 1.00 0.00
D H
ATOM 12641 CA VAL D 154 −32.070 39.895 39.783 1.00 0.00
D C
ATOM 12642 HA VAL D 154 −31.503 39.027 40.097 1.00 0.00
D H
ATOM 12643 CB VAL D 154 −31.987 40.040 38.250 1.00 0.00
D C
ATOM 12644 HB VAL D 154 −32.541 40.969 37.969 1.00 0.00
D H
ATOM 12645 CG1 VAL D 154 −30.533 40.167 37.808 1.00 0.00
D C
ATOM 12646 HG11 VAL D 154 −30.470 40.232 36.700 1.00 0.00
D H
ATOM 12647 HG12 VAL D 154 −30.058 41.082 38.220 1.00 0.00
D H
ATOM 12648 HG13 VAL D 154 −29.945 39.282 38.134 1.00 0.00
D H
ATOM 12649 CG2 VAL D 154 −32.642 38.855 37.552 1.00 0.00
D C
ATOM 12650 HG21 VAL D 154 −32.543 38.946 36.449 1.00 0.00
D H
ATOM 12651 HG22 VAL D 154 −32.165 37.902 37.869 1.00 0.00
D H
ATOM 12652 HG23 VAL D 154 −33.725 38.798 37.788 1.00 0.00
D H
ATOM 12653 C VAL D 154 −31.453 41.115 40.460 1.00 0.00
D C
ATOM 12654 O VAL D 154 −32.114 42.147 40.629 1.00 0.00
D O
ATOM 12655 N GLU D 155 −30.179 40.997 40.835 1.00 0.00
D N
ATOM 12656 HN GLU D 155 −29.707 40.122 40.897 1.00 0.00
D H
ATOM 12657 CA GLU D 155 −29.353 42.143 41.192 1.00 0.00
D C
ATOM 12658 HA GLU D 155 −29.986 43.005 41.363 1.00 0.00
D H
ATOM 12659 CB GLU D 155 −28.490 41.873 42.424 1.00 0.00
D C
ATOM 12660 HB1 GLU D 155 −28.140 40.816 42.377 1.00 0.00
D H
ATOM 12661 HB2 GLU D 155 −27.572 42.500 42.387 1.00 0.00
D H
ATOM 12662 CG GLU D 155 −29.183 42.085 43.741 1.00 0.00
D C

ATOM 12663 HG1 GLU D 155 -28.745 42.961 44.252 1.00 0.00
D H
ATOM 12664 HG2 GLU D 155 -30.269 42.254 43.595 1.00 0.00
D H
ATOM 12665 CD GLU D 155 -28.995 40.917 44.681 1.00 0.00
D C
ATOM 12666 OE1 GLU D 155 -28.384 41.109 45.754 1.00 0.00
D O
ATOM 12667 OE2 GLU D 155 -29.440 39.805 44.326 1.00 0.00
D O
ATOM 12668 C GLU D 155 -28.452 42.473 40.013 1.00 0.00
D C
ATOM 12669 O GLU D 155 -27.845 41.573 39.426 1.00 0.00
D O
ATOM 12670 N LEU D 156 -28.362 43.752 39.666 1.00 0.00
D N
ATOM 12671 HN LEU D 156 -28.919 44.483 40.055 1.00 0.00
D H
ATOM 12672 CA LEU D 156 -27.410 44.198 38.657 1.00 0.00
D C
ATOM 12673 HA LEU D 156 -26.695 43.399 38.515 1.00 0.00
D H
ATOM 12674 CB LEU D 156 -28.101 44.520 37.320 1.00 0.00
D C
ATOM 12675 HB1 LEU D 156 -28.350 45.607 37.331 1.00 0.00
D H
ATOM 12676 HB2 LEU D 156 -27.372 44.380 36.492 1.00 0.00
D H
ATOM 12677 CG LEU D 156 -29.386 43.744 37.013 1.00 0.00
D C
ATOM 12678 HG LEU D 156 -29.390 42.888 37.729 1.00 0.00
D H
ATOM 12679 CD1 LEU D 156 -30.621 44.600 37.246 1.00 0.00
D C
ATOM 12680 HD11 LEU D 156 -31.540 44.039 36.973 1.00 0.00
D H
ATOM 12681 HD12 LEU D 156 -30.696 44.896 38.315 1.00 0.00
D H
ATOM 12682 HD13 LEU D 156 -30.582 45.523 36.629 1.00 0.00
D H
ATOM 12683 CD2 LEU D 156 -29.365 43.176 35.600 1.00 0.00
D C
ATOM 12684 HD21 LEU D 156 -30.295 42.602 35.401 1.00 0.00
D H
ATOM 12685 HD22 LEU D 156 -29.295 43.978 34.837 1.00 0.00
D H
ATOM 12686 HD23 LEU D 156 -28.499 42.492 35.469 1.00 0.00
D H
ATOM 12687 C LEU D 156 -26.646 45.412 39.164 1.00 0.00
D C
ATOM 12688 O LEU D 156 -27.243 46.367 39.661 1.00 0.00
D O
ATOM 12689 N LEU D 157 -25.323 45.379 39.040 1.00 0.00
D N

ATOM 12690 HN LEU D 157 −24.796 44.624 38.655 1.00 0.00
D H
ATOM 12691 CA LEU D 157 −24.517 46.507 39.483 1.00 0.00
D C
ATOM 12692 HA LEU D 157 −24.995 46.933 40.356 1.00 0.00
D H
ATOM 12693 CB LEU D 157 −23.096 46.067 39.834 1.00 0.00
D C
ATOM 12694 HB1 LEU D 157 −23.022 46.049 40.946 1.00 0.00
D H
ATOM 12695 HB2 LEU D 157 −22.950 45.016 39.498 1.00 0.00
D H
ATOM 12696 CG LEU D 157 −21.897 46.879 39.343 1.00 0.00
D C
ATOM 12697 HG LEU D 157 −22.244 47.890 39.020 1.00 0.00
D H
ATOM 12698 CD1 LEU D 157 −20.888 47.018 40.468 1.00 0.00
D C
ATOM 12699 HD11 LEU D 157 −19.986 47.565 40.118 1.00 0.00
D H
ATOM 12700 HD12 LEU D 157 −21.327 47.580 41.321 1.00 0.00
D H
ATOM 12701 HD13 LEU D 157 −20.568 46.019 40.833 1.00 0.00
D H
ATOM 12702 CD2 LEU D 157 −21.259 46.194 38.142 1.00 0.00
D C
ATOM 12703 HD21 LEU D 157 −20.398 46.791 37.769 1.00 0.00
D H
ATOM 12704 HD22 LEU D 157 −20.881 45.185 38.399 1.00 0.00
D H
ATOM 12705 HD23 LEU D 157 −21.995 46.093 37.315 1.00 0.00
D H
ATOM 12706 C LEU D 157 −24.521 47.572 38.390 1.00 0.00
D C
ATOM 12707 O LEU D 157 −24.415 47.252 37.200 1.00 0.00
D O
ATOM 12708 N ALA D 158 −24.663 48.836 38.789 1.00 0.00
D N
ATOM 12709 HN ALA D 158 −24.619 49.124 39.745 1.00 0.00
D H
ATOM 12710 CA ALA D 158 −24.898 49.903 37.824 1.00 0.00
D C
ATOM 12711 HA ALA D 158 −25.615 49.590 37.074 1.00 0.00
D H
ATOM 12712 CB ALA D 158 −25.452 51.145 38.520 1.00 0.00
D C
ATOM 12713 HB1 ALA D 158 −26.411 50.895 39.023 1.00 0.00
D H
ATOM 12714 HB2 ALA D 158 −24.746 51.516 39.295 1.00 0.00
D H
ATOM 12715 HB3 ALA D 158 −25.648 51.967 37.798 1.00 0.00
D H
ATOM 12716 C ALA D 158 −23.620 50.253 37.076 1.00 0.00
D C

ATOM 12717 O   ALA D 158   -22.556 50.417 37.678 1.00 0.00
D   O
ATOM 12718 N   THR D 159   -23.726 50.348 35.757 1.00 0.00
D   N
ATOM 12719 HN THR D 159   -24.551 50.130 35.237 1.00 0.00
D   H
ATOM 12720 CA THR D 159   -22.610 50.790 34.944 1.00 0.00
D   C
ATOM 12721 HA THR D 159   -21.717 50.593 35.521 1.00 0.00
D   H
ATOM 12722 CB THR D 159   -22.605 50.064 33.598 1.00 0.00
D   C
ATOM 12723 HB THR D 159   -22.647 48.973 33.834 1.00 0.00
D   H
ATOM 12724 OG1 THR D 159   -21.371 50.320 32.913 1.00 0.00
D   O
ATOM 12725 HG1 THR D 159   -21.438 49.847 32.077 1.00 0.00
D   H
ATOM 12726 CG2 THR D 159   -23.771 50.523 32.739 1.00 0.00
D   C
ATOM 12727 HG21 THR D 159   -23.839 49.919 31.810 1.00 0.00
D   H
ATOM 12728 HG22 THR D 159   -24.728 50.408 33.292 1.00 0.00
D   H
ATOM 12729 HG23 THR D 159   -23.661 51.591 32.452 1.00 0.00
D   H
ATOM 12730 C   THR D 159   -22.669 52.302 34.740 1.00 0.00
D   C
ATOM 12731 O   THR D 159   -23.559 52.994 35.242 1.00 0.00
D   O
ATOM 12732 N   ARG D 160   -21.692 52.816 33.990 1.00 0.00
D   N
ATOM 12733 HN ARG D 160   -20.969 52.257 33.590 1.00 0.00
D   H
ATOM 12734 CA ARG D 160   -21.639 54.246 33.717 1.00 0.00
D   C
ATOM 12735 HA ARG D 160   -21.687 54.769 34.666 1.00 0.00
D   H
ATOM 12736 CB ARG D 160   -20.339 54.592 32.996 1.00 0.00
D   C
ATOM 12737 HB1 ARG D 160   -19.538 53.985 33.482 1.00 0.00
D   H
ATOM 12738 HB2 ARG D 160   -20.393 54.241 31.942 1.00 0.00
D   H
ATOM 12739 CG ARG D 160   -19.964 56.054 33.078 1.00 0.00
D   C
ATOM 12740 HG1 ARG D 160   -20.890 56.662 32.985 1.00 0.00
D   H
ATOM 12741 HG2 ARG D 160   -19.566 56.269 34.096 1.00 0.00
D   H
ATOM 12742 CD ARG D 160   -18.911 56.386 32.043 1.00 0.00
D   C
ATOM 12743 HD1 ARG D 160   -18.885 57.489 31.882 1.00 0.00
D   H

ATOM 12744 HD2 ARG D 160 -17.900 56.048 32.365 1.00 0.00
D H
ATOM 12745 NE ARG D 160 -19.190 55.724 30.773 1.00 0.00
D N
ATOM 12746 HE ARG D 160 -18.596 54.930 30.602 1.00 0.00
D H
ATOM 12747 CZ ARG D 160 -20.128 56.111 29.915 1.00 0.00
D C
ATOM 12748 NH1 ARG D 160 -20.891 57.161 30.184 1.00 0.00
D N
ATOM 12749 HH11 ARG D 160 -21.608 57.436 29.559 1.00 0.00
D H
ATOM 12750 HH12 ARG D 160 -20.791 57.559 31.098 1.00 0.00
D H
ATOM 12751 NH2 ARG D 160 -20.308 55.443 28.785 1.00 0.00
D N
ATOM 12752 HH21 ARG D 160 -20.946 55.781 28.108 1.00 0.00
D H
ATOM 12753 HH22 ARG D 160 -19.642 54.741 28.569 1.00 0.00
D H
ATOM 12754 C ARG D 160 -22.833 54.720 32.899 1.00 0.00
D C
ATOM 12755 O ARG D 160 -23.186 55.903 32.965 1.00 0.00
D O
ATOM 12756 N LYS D 161 -23.467 53.826 32.137 1.00 0.00
D N
ATOM 12757 HN LYS D 161 -23.135 52.895 31.996 1.00 0.00
D H
ATOM 12758 CA LYS D 161 -24.707 54.151 31.445 1.00 0.00
D C
ATOM 12759 HA LYS D 161 -24.511 55.045 30.865 1.00 0.00
D H
ATOM 12760 CB LYS D 161 -25.123 52.993 30.536 1.00 0.00
D C
ATOM 12761 HB1 LYS D 161 -25.360 52.128 31.196 1.00 0.00
D H
ATOM 12762 HB2 LYS D 161 -26.073 53.261 30.020 1.00 0.00
D H
ATOM 12763 CG LYS D 161 -24.071 52.574 29.534 1.00 0.00
D C
ATOM 12764 HG1 LYS D 161 -23.086 52.503 30.049 1.00 0.00
D H
ATOM 12765 HG2 LYS D 161 -24.319 51.558 29.151 1.00 0.00
D H
ATOM 12766 CD LYS D 161 -23.983 53.576 28.402 1.00 0.00
D C
ATOM 12767 HD1 LYS D 161 -23.655 54.554 28.822 1.00 0.00
D H
ATOM 12768 HD2 LYS D 161 -23.191 53.233 27.697 1.00 0.00
D H
ATOM 12769 CE LYS D 161 -25.320 53.699 27.697 1.00 0.00
D C
ATOM 12770 HE1 LYS D 161 -25.654 52.708 27.317 1.00 0.00
D H

ATOM 12771 HE2 LYS D 161 −26.095 54.087 28.394 1.00 0.00
D H
ATOM 12772 NZ LYS D 161 −25.259 54.624 26.537 1.00 0.00
D N
ATOM 12773 HZ1 LYS D 161 −26.191 54.683 26.078 1.00 0.00
D H
ATOM 12774 HZ2 LYS D 161 −24.974 55.571 26.861 1.00 0.00
D H
ATOM 12775 HZ3 LYS D 161 −24.560 54.273 25.852 1.00 0.00
D H
ATOM 12776 C LYS D 161 −25.844 54.472 32.402 1.00 0.00
D C
ATOM 12777 O LYS D 161 −26.941 54.808 31.942 1.00 0.00
D O
ATOM 12778 N LEU D 162 −25.615 54.372 33.712 1.00 0.00
D N
ATOM 12779 HN LEU D 162 −24.714 54.190 34.100 1.00 0.00
D H
ATOM 12780 CA LEU D 162 −26.670 54.521 34.699 1.00 0.00
D C
ATOM 12781 HA LEU D 162 −27.537 54.852 34.142 1.00 0.00
D H
ATOM 12782 CB LEU D 162 −26.927 53.176 35.395 1.00 0.00
D C
ATOM 12783 HB1 LEU D 162 −25.941 52.793 35.744 1.00 0.00
D H
ATOM 12784 HB2 LEU D 162 −27.531 53.354 36.312 1.00 0.00
D H
ATOM 12785 CG LEU D 162 −27.584 52.064 34.575 1.00 0.00
D C
ATOM 12786 HG LEU D 162 −26.792 51.660 33.899 1.00 0.00
D H
ATOM 12787 CD1 LEU D 162 −28.071 50.963 35.501 1.00 0.00
D C
ATOM 12788 HD11 LEU D 162 −28.593 50.170 34.924 1.00 0.00
D H
ATOM 12789 HD12 LEU D 162 −27.216 50.498 36.038 1.00 0.00
D H
ATOM 12790 HD13 LEU D 162 −28.780 51.368 36.253 1.00 0.00
D H
ATOM 12791 CD2 LEU D 162 −28.722 52.588 33.706 1.00 0.00
D C
ATOM 12792 HD21 LEU D 162 −29.165 51.760 33.111 1.00 0.00
D H
ATOM 12793 HD22 LEU D 162 −29.532 53.034 34.318 1.00 0.00
D H
ATOM 12794 HD23 LEU D 162 −28.349 53.363 33.001 1.00 0.00
D H
ATOM 12795 C LEU D 162 −26.383 55.566 35.766 1.00 0.00
D C
ATOM 12796 O LEU D 162 −27.182 55.694 36.700 1.00 0.00
D O
ATOM 12797 N ASP D 163 −25.285 56.311 35.662 1.00 0.00
D N

ATOM 12798 HN ASP D 163 −24.665 56.329 34.882 1.00 0.00
D H
ATOM 12799 CA ASP D 163 −24.877 57.211 36.732 1.00 0.00
D C
ATOM 12800 HA ASP D 163 −24.927 56.649 37.658 1.00 0.00
D H
ATOM 12801 CB ASP D 163 −23.462 57.733 36.466 1.00 0.00
D C
ATOM 12802 HB1 ASP D 163 −22.802 56.881 36.207 1.00 0.00
D H
ATOM 12803 HB2 ASP D 163 −23.462 58.445 35.616 1.00 0.00
D H
ATOM 12804 CG ASP D 163 −22.850 58.428 37.675 1.00 0.00
D C
ATOM 12805 OD1 ASP D 163 −23.582 58.723 38.643 1.00 0.00
D O
ATOM 12806 OD2 ASP D 163 −21.628 58.690 37.648 1.00 0.00
D O
ATOM 12807 C ASP D 163 −25.850 58.375 36.872 1.00 0.00
D C
ATOM 12808 O ASP D 163 −26.321 58.930 35.877 1.00 0.00
D O
ATOM 12809 N SER D 164 −26.132 58.745 38.123 1.00 0.00
D N
ATOM 12810 HN SER D 164 −25.802 58.234 38.916 1.00 0.00
D H
ATOM 12811 CA SER D 164 −26.940 59.918 38.459 1.00 0.00
D C
ATOM 12812 HA SER D 164 −27.117 59.862 39.525 1.00 0.00
D H
ATOM 12813 CB SER D 164 −26.197 61.206 38.108 1.00 0.00
D C
ATOM 12814 HB1 SER D 164 −26.459 61.482 37.062 1.00 0.00
D H
ATOM 12815 HB2 SER D 164 −26.538 62.041 38.762 1.00 0.00
D H
ATOM 12816 OG SER D 164 −24.794 61.020 38.197 1.00 0.00
D O
ATOM 12817 HG1 SER D 164 −24.380 61.774 37.758 1.00 0.00
D H
ATOM 12818 C SER D 164 −28.299 59.868 37.770 1.00 0.00
D C
ATOM 12819 O SER D 164 −28.864 60.888 37.371 1.00 0.00
D O
ATOM 12820 N LYS D 165 −28.828 58.661 37.632 1.00 0.00
D N
ATOM 12821 HN LYS D 165 −28.380 57.838 37.977 1.00 0.00
D H
ATOM 12822 CA LYS D 165 −30.101 58.438 36.970 1.00 0.00
D C
ATOM 12823 HA LYS D 165 −30.346 59.333 36.411 1.00 0.00
D H
ATOM 12824 CB LYS D 165 −30.025 57.228 36.038 1.00 0.00
D C

ATOM 12825 HB1 LYS D 165 -29.674 56.365 36.649 1.00 0.00
D H
ATOM 12826 HB2 LYS D 165 -31.052 56.970 35.695 1.00 0.00
D H
ATOM 12827 CG LYS D 165 -29.086 57.432 34.861 1.00 0.00
D C
ATOM 12828 HG1 LYS D 165 -28.039 57.501 35.238 1.00 0.00
D H
ATOM 12829 HG2 LYS D 165 -29.141 56.541 34.194 1.00 0.00
D H
ATOM 12830 CD LYS D 165 -29.417 58.695 34.086 1.00 0.00
D C
ATOM 12831 HD1 LYS D 165 -30.454 58.602 33.689 1.00 0.00
D H
ATOM 12832 HD2 LYS D 165 -29.408 59.552 34.798 1.00 0.00
D H
ATOM 12833 CE LYS D 165 -28.419 58.920 32.962 1.00 0.00
D C
ATOM 12834 HE1 LYS D 165 -27.404 59.116 33.376 1.00 0.00
D H
ATOM 12835 HE2 LYS D 165 -28.364 58.026 32.304 1.00 0.00
D H
ATOM 12836 NZ LYS D 165 -28.796 60.083 32.116 1.00 0.00
D N
ATOM 12837 HZ1 LYS D 165 -28.095 60.214 31.358 1.00 0.00
D H
ATOM 12838 HZ2 LYS D 165 -29.733 59.919 31.696 1.00 0.00
D H
ATOM 12839 HZ3 LYS D 165 -28.831 60.941 32.704 1.00 0.00
D H
ATOM 12840 C LYS D 165 -31.191 58.243 38.014 1.00 0.00
D C
ATOM 12841 O LYS D 165 -30.908 58.080 39.202 1.00 0.00
D O
ATOM 12842 N SER D 166 -32.442 58.317 37.575 1.00 0.00
D N
ATOM 12843 HN SER D 166 -32.696 58.507 36.627 1.00 0.00
D H
ATOM 12844 CA SER D 166 -33.574 58.121 38.468 1.00 0.00
D C
ATOM 12845 HA SER D 166 -33.154 57.843 39.425 1.00 0.00
D H
ATOM 12846 CB SER D 166 -34.423 59.385 38.568 1.00 0.00
D C
ATOM 12847 HB1 SER D 166 -33.934 60.074 39.294 1.00 0.00
D H
ATOM 12848 HB2 SER D 166 -34.452 59.912 37.587 1.00 0.00
D H
ATOM 12849 OG SER D 166 -35.715 59.045 39.030 1.00 0.00
D O
ATOM 12850 HG1 SER D 166 -36.146 59.867 39.295 1.00 0.00
D H
ATOM 12851 C SER D 166 -34.453 56.968 38.008 1.00 0.00
D C

ATOM 12852 O SER D 166 -34.394 56.536 36.853 1.00 0.00
D O
ATOM 12853 N ILE D 167 -35.277 56.474 38.933 1.00 0.00
D N
ATOM 12854 HN ILE D 167 -35.273 56.723 39.899 1.00 0.00
D H
ATOM 12855 CA ILE D 167 -36.292 55.488 38.573 1.00 0.00
D C
ATOM 12856 HA ILE D 167 -35.790 54.718 38.000 1.00 0.00
D H
ATOM 12857 CB ILE D 167 -36.948 54.904 39.836 1.00 0.00
D C
ATOM 12858 HB ILE D 167 -37.036 55.775 40.534 1.00 0.00
D H
ATOM 12859 CG2 ILE D 167 -38.274 54.251 39.489 1.00 0.00
D C
ATOM 12860 HG21 ILE D 167 -38.823 53.968 40.410 1.00 0.00
D H
ATOM 12861 HG22 ILE D 167 -38.919 54.947 38.913 1.00 0.00
D H
ATOM 12862 HG23 ILE D 167 -38.118 53.335 38.882 1.00 0.00
D H
ATOM 12863 CG1 ILE D 167 -36.018 53.896 40.503 1.00 0.00
D C
ATOM 12864 HG11 ILE D 167 -36.600 53.009 40.836 1.00 0.00
D H
ATOM 12865 HG12 ILE D 167 -35.275 53.540 39.753 1.00 0.00
D H
ATOM 12866 CD ILE D 167 -35.269 54.461 41.675 1.00 0.00
D C
ATOM 12867 HD1 ILE D 167 -34.566 53.704 42.084 1.00 0.00
D H
ATOM 12868 HD2 ILE D 167 -34.681 55.353 41.371 1.00 0.00
D H
ATOM 12869 HD3 ILE D 167 -35.966 54.757 42.486 1.00 0.00
D H
ATOM 12870 C ILE D 167 -37.330 56.115 37.655 1.00 0.00
D C
ATOM 12871 O ILE D 167 -37.707 55.538 36.630 1.00 0.00
D O
ATOM 12872 N ILE D 168 -37.809 57.309 38.010 1.00 0.00
D N
ATOM 12873 HN ILE D 168 -37.605 57.795 38.858 1.00 0.00
D H
ATOM 12874 CA ILE D 168 -38.718 58.018 37.119 1.00 0.00
D C
ATOM 12875 HA ILE D 168 -39.471 57.304 36.811 1.00 0.00
D H
ATOM 12876 CB ILE D 168 -39.372 59.212 37.836 1.00 0.00
D C
ATOM 12877 HB ILE D 168 -39.736 58.792 38.807 1.00 0.00
D H
ATOM 12878 CG2 ILE D 168 -38.381 60.351 38.003 1.00 0.00
D C

```
ATOM  12879 HG21 ILE D 168     -38.804  61.145  38.653  1.00  0.00
D    H
ATOM  12880 HG22 ILE D 168     -37.443  59.993  38.475  1.00  0.00
D    H
ATOM  12881 HG23 ILE D 168     -38.128  60.805  37.021  1.00  0.00
D    H
ATOM  12882  CG1 ILE D 168     -40.605  59.679  37.062  1.00  0.00
D    C
ATOM  12883 HG11 ILE D 168     -40.787  60.759  37.259  1.00  0.00
D    H
ATOM  12884 HG12 ILE D 168     -40.407  59.572  35.970  1.00  0.00
D    H
ATOM  12885  CD  ILE D 168     -41.853  58.893  37.399  1.00  0.00
D    C
ATOM  12886  HD1 ILE D 168     -42.704  59.233  36.769  1.00  0.00
D    H
ATOM  12887  HD2 ILE D 168     -41.696  57.809  37.215  1.00  0.00
D    H
ATOM  12888  HD3 ILE D 168     -42.137  59.033  38.463  1.00  0.00
D    H
ATOM  12889  C   ILE D 168     -37.992  58.459  35.862  1.00  0.00
D    C
ATOM  12890  O   ILE D 168     -38.617  58.662  34.816  1.00  0.00
D    O
ATOM  12891  N   ASP D 169     -36.671  58.593  35.932  1.00  0.00
D    N
ATOM  12892  HN  ASP D 169     -36.104  58.518  36.748  1.00  0.00
D    H
ATOM  12893  CA  ASP D 169     -35.902  58.879  34.732  1.00  0.00
D    C
ATOM  12894  HA  ASP D 169     -36.393  59.697  34.218  1.00  0.00
D    H
ATOM  12895  CB  ASP D 169     -34.464  59.224  35.121  1.00  0.00
D    C
ATOM  12896  HB1 ASP D 169     -34.365  59.191  36.224  1.00  0.00
D    H
ATOM  12897  HB2 ASP D 169     -33.755  58.492  34.686  1.00  0.00
D    H
ATOM  12898  CG  ASP D 169     -34.053  60.609  34.674  1.00  0.00
D    C
ATOM  12899  OD1 ASP D 169     -34.661  61.577  35.176  1.00  0.00
D    O
ATOM  12900  OD2 ASP D 169     -33.131  60.731  33.836  1.00  0.00
D    O
ATOM  12901  C   ASP D 169     -35.936  57.683  33.789  1.00  0.00
D    C
ATOM  12902  O   ASP D 169     -36.535  57.736  32.707  1.00  0.00
D    O
ATOM  12903  N   LEU D 170     -35.321  56.582  34.203  1.00  0.00
D    N
ATOM  12904  HN  LEU D 170     -34.823  56.524  35.067  1.00  0.00
D    H
ATOM  12905  CA  LEU D 170     -35.320  55.348  33.425  1.00  0.00
D    C
```

ATOM 12906 HA LEU D 170   -35.626 55.599 32.417 1.00 0.00
D   H
ATOM 12907 CB LEU D 170   -33.922 54.739 33.410 1.00 0.00
D   C
ATOM 12908 HB1 LEU D 170   -33.543 54.761 34.458 1.00 0.00
D   H
ATOM 12909 HB2 LEU D 170   -33.999 53.664 33.134 1.00 0.00
D   H
ATOM 12910 CG LEU D 170   -32.903 55.443 32.510 1.00 0.00
D   C
ATOM 12911 HG LEU D 170   -33.441 55.681 31.562 1.00 0.00
D   H
ATOM 12912 CD1 LEU D 170   -32.379 56.728 33.144 1.00 0.00
D   C
ATOM 12913 HD11 LEU D 170   -31.606 57.195 32.496 1.00 0.00
D   H
ATOM 12914 HD12 LEU D 170   -33.204 57.459 33.287 1.00 0.00
D   H
ATOM 12915 HD13 LEU D 170   -31.920 56.518 34.134 1.00 0.00
D   H
ATOM 12916 CD2 LEU D 170   -31.748 54.515 32.175 1.00 0.00
D   C
ATOM 12917 HD21 LEU D 170   -31.022 55.027 31.506 1.00 0.00
D   H
ATOM 12918 HD22 LEU D 170   -31.201 54.196 33.085 1.00 0.00
D   H
ATOM 12919 HD23 LEU D 170   -32.119 53.606 31.653 1.00 0.00
D   H
ATOM 12920 C   LEU D 170   -36.359 54.384 33.997 1.00 0.00
D   C
ATOM 12921 O   LEU D 170   -36.053 53.445 34.732 1.00 0.00
D   O
ATOM 12922 N   ASN D 171   -37.618 54.655 33.656 1.00 0.00
D   N
ATOM 12923 HN ASN D 171   -37.892 55.400 33.048 1.00 0.00
D   H
ATOM 12924 CA ASN D 171   -38.722 53.853 34.165 1.00 0.00
D   C
ATOM 12925 HA ASN D 171   -38.599 53.825 35.242 1.00 0.00
D   H
ATOM 12926 CB ASN D 171   -40.064 54.473 33.770 1.00 0.00
D   C
ATOM 12927 HB1 ASN D 171   -40.868 53.768 34.077 1.00 0.00
D   H
ATOM 12928 HB2 ASN D 171   -40.226 55.423 34.319 1.00 0.00
D   H
ATOM 12929 CG ASN D 171   -40.185 54.702 32.283 1.00 0.00
D   C
ATOM 12930 OD1 ASN D 171   -39.239 54.484 31.530 1.00 0.00
D   O
ATOM 12931 ND2 ASN D 171   -41.358 55.141 31.848 1.00 0.00
D   N
ATOM 12932 HD21 ASN D 171   -41.458 55.286 30.867 1.00 0.00
D   H

```
ATOM  12933 HD22 ASN D 171     -42.106  55.284  32.490  1.00  0.00
D    H
ATOM  12934  C   ASN D 171     -38.636  52.420  33.652  1.00  0.00
D    C
ATOM  12935  O   ASN D 171     -38.065  52.149  32.591  1.00  0.00
D    O
ATOM  12936  N   VAL D 172     -39.188  51.495  34.434  1.00  0.00
D    N
ATOM  12937  HN  VAL D 172     -39.634  51.698  35.302  1.00  0.00
D    H
ATOM  12938  CA  VAL D 172     -39.185  50.084  34.074  1.00  0.00
D    C
ATOM  12939  HA  VAL D 172     -38.869  50.022  33.040  1.00  0.00
D    H
ATOM  12940  CB  VAL D 172     -38.180  49.303  34.945  1.00  0.00
D    C
ATOM  12941  HB  VAL D 172     -37.253  49.922  35.028  1.00  0.00
D    H
ATOM  12942  CG1 VAL D 172     -38.741  49.059  36.340  1.00  0.00
D    C
ATOM  12943 HG11 VAL D 172     -38.024  48.462  36.945  1.00  0.00
D    H
ATOM  12944 HG12 VAL D 172     -38.919  50.010  36.882  1.00  0.00
D    H
ATOM  12945 HG13 VAL D 172     -39.697  48.496  36.286  1.00  0.00
D    H
ATOM  12946  CG2 VAL D 172     -37.794  47.985  34.277  1.00  0.00
D    C
ATOM  12947 HG21 VAL D 172     -37.101  47.406  34.923  1.00  0.00
D    H
ATOM  12948 HG22 VAL D 172     -38.696  47.365  34.087  1.00  0.00
D    H
ATOM  12949 HG23 VAL D 172     -37.281  48.160  33.308  1.00  0.00
D    H
ATOM  12950  C   VAL D 172     -40.575  49.464  34.166  1.00  0.00
D    C
ATOM  12951  O   VAL D 172     -40.839  48.450  33.511  1.00  0.00
D    O
ATOM  12952  N   ARG D 173     -41.474  50.044  34.962  1.00  0.00
D    N
ATOM  12953  HN  ARG D 173     -41.287  50.870  35.489  1.00  0.00
D    H
ATOM  12954  CA  ARG D 173     -42.809  49.471  35.096  1.00  0.00
D    C
ATOM  12955  HA  ARG D 173     -42.708  48.397  35.202  1.00  0.00
D    H
ATOM  12956  CB  ARG D 173     -43.513  50.045  36.320  1.00  0.00
D    C
ATOM  12957  HB1 ARG D 173     -42.709  50.356  37.030  1.00  0.00
D    H
ATOM  12958  HB2 ARG D 173     -44.049  50.979  36.038  1.00  0.00
D    H
ATOM  12959  CG  ARG D 173     -44.423  49.046  36.997  1.00  0.00
D    C
```

ATOM 12960 HG1 ARG D 173   -45.454 49.180 36.601 1.00 0.00
D   H
ATOM 12961 HG2 ARG D 173   -44.118 48.017 36.697 1.00 0.00
D   H
ATOM 12962 CD ARG D 173   -44.352 49.157 38.512 1.00 0.00
D   C
ATOM 12963 HD1 ARG D 173   -44.338 48.136 38.958 1.00 0.00
D   H
ATOM 12964 HD2 ARG D 173   -43.437 49.703 38.839 1.00 0.00
D   H
ATOM 12965 NE ARG D 173   -45.484 49.892 39.073 1.00 0.00
D   N
ATOM 12966 HE ARG D 173   -45.232 50.803 39.418 1.00 0.00
D   H
ATOM 12967 CZ ARG D 173   -46.729 49.426 39.134 1.00 0.00
D   C
ATOM 12968 NH1 ARG D 173   -47.016 48.218 38.666 1.00 0.00
D   N
ATOM 12969 HH11 ARG D 173   -47.946 47.877 38.686 1.00 0.00
D   H
ATOM 12970 HH12 ARG D 173   -46.280 47.743 38.178 1.00 0.00
D   H
ATOM 12971 NH2 ARG D 173   -47.690 50.168 39.666 1.00 0.00
D   N
ATOM 12972 HH21 ARG D 173   -48.596 49.788 39.790 1.00 0.00
D   H
ATOM 12973 HH22 ARG D 173   -47.412 51.007 40.114 1.00 0.00
D   H
ATOM 12974 C   ARG D 173   -43.642 49.708 33.846 1.00 0.00
D   C
ATOM 12975 O   ARG D 173   -44.244 48.773 33.306 1.00 0.00
D   O
ATOM 12976 N   ALA D 174   -43.703 50.951 33.378 1.00 0.00
D   N
ATOM 12977 HN ALA D 174   -43.300 51.741 33.839 1.00 0.00
D   H
ATOM 12978 CA ALA D 174   -44.382 51.238 32.123 1.00 0.00
D   C
ATOM 12979 HA ALA D 174   -45.284 50.643 32.036 1.00 0.00
D   H
ATOM 12980 CB ALA D 174   -44.782 52.713 32.067 1.00 0.00
D   C
ATOM 12981 HB1 ALA D 174   -45.456 52.950 32.918 1.00 0.00
D   H
ATOM 12982 HB2 ALA D 174   -43.890 53.372 32.143 1.00 0.00
D   H
ATOM 12983 HB3 ALA D 174   -45.321 52.955 31.125 1.00 0.00
D   H
ATOM 12984 C   ALA D 174   -43.514 50.880 30.926 1.00 0.00
D   C
ATOM 12985 O   ALA D 174   -44.029 50.638 29.830 1.00 0.00
D   O
ATOM 12986 N   LYS D 175   -42.197 50.828 31.126 1.00 0.00
D   N

ATOM 12987 HN LYS D 175 −41.758 50.943 32.016 1.00 0.00
D H
ATOM 12988 CA LYS D 175 −41.286 50.590 30.014 1.00 0.00
D C
ATOM 12989 HA LYS D 175 −41.762 50.985 29.125 1.00 0.00
D H
ATOM 12990 CB LYS D 175 −39.962 51.303 30.275 1.00 0.00
D C
ATOM 12991 HB1 LYS D 175 −40.203 52.268 30.779 1.00 0.00
D H
ATOM 12992 HB2 LYS D 175 −39.368 50.708 31.005 1.00 0.00
D H
ATOM 12993 CG LYS D 175 −39.151 51.586 29.033 1.00 0.00
D C
ATOM 12994 HG1 LYS D 175 −38.719 50.631 28.655 1.00 0.00
D H
ATOM 12995 HG2 LYS D 175 −39.827 51.980 28.240 1.00 0.00
D H
ATOM 12996 CD LYS D 175 −38.039 52.567 29.343 1.00 0.00
D C
ATOM 12997 HD1 LYS D 175 −38.490 53.476 29.804 1.00 0.00
D H
ATOM 12998 HD2 LYS D 175 −37.370 52.104 30.105 1.00 0.00
D H
ATOM 12999 CE LYS D 175 −37.256 52.926 28.097 1.00 0.00
D C
ATOM 13000 HE1 LYS D 175 −36.682 52.047 27.731 1.00 0.00
D H
ATOM 13001 HE2 LYS D 175 −37.942 53.262 27.288 1.00 0.00
D H
ATOM 13002 NZ LYS D 175 −36.286 54.026 28.358 1.00 0.00
D N
ATOM 13003 HZ1 LYS D 175 −35.762 54.252 27.489 1.00 0.00
D H
ATOM 13004 HZ2 LYS D 175 −36.799 54.871 28.682 1.00 0.00
D H
ATOM 13005 HZ3 LYS D 175 −35.617 53.728 29.096 1.00 0.00
D H
ATOM 13006 C LYS D 175 −41.057 49.101 29.784 1.00 0.00
D C
ATOM 13007 O LYS D 175 −41.226 48.610 28.662 1.00 0.00
D O
ATOM 13008 N TYR D 176 −40.673 48.365 30.828 1.00 0.00
D N
ATOM 13009 HN TYR D 176 −40.573 48.729 31.752 1.00 0.00
D H
ATOM 13010 CA TYR D 176 −40.365 46.949 30.699 1.00 0.00
D C
ATOM 13011 HA TYR D 176 −40.557 46.709 29.660 1.00 0.00
D H
ATOM 13012 CB TYR D 176 −38.895 46.686 31.064 1.00 0.00
D C
ATOM 13013 HB1 TYR D 176 −38.724 46.984 32.122 1.00 0.00
D H

ATOM 13014 HB2 TYR D 176   -38.658 45.603 30.984 1.00 0.00
D   H
ATOM 13015 CG TYR D 176   -37.918 47.476 30.224 1.00 0.00
D   C
ATOM 13016 CD1 TYR D 176   -37.376 48.665 30.691 1.00 0.00
D   C
ATOM 13017 HD1 TYR D 176   -37.664 49.042 31.662 1.00 0.00
D   H
ATOM 13018 CE1 TYR D 176   -36.488 49.394 29.928 1.00 0.00
D   C
ATOM 13019 HE1 TYR D 176   -36.076 50.320 30.300 1.00 0.00
D   H
ATOM 13020 CZ TYR D 176   -36.130 48.940 28.679 1.00 0.00
D   C
ATOM 13021 OH TYR D 176   -35.243 49.667 27.920 1.00 0.00
D   O
ATOM 13022 HH TYR D 176   -35.064 49.162 27.124 1.00 0.00
D   H
ATOM 13023 CD2 TYR D 176   -37.545 47.039 28.960 1.00 0.00
D   C
ATOM 13024 HD2 TYR D 176   -37.982 46.139 28.549 1.00 0.00
D   H
ATOM 13025 CE2 TYR D 176   -36.655 47.763 28.189 1.00 0.00
D   C
ATOM 13026 HE2 TYR D 176   -36.395 47.414 27.201 1.00 0.00
D   H
ATOM 13027 C   TYR D 176   -41.253 46.044 31.540 1.00 0.00
D   C
ATOM 13028 O   TYR D 176   -41.032 44.829 31.544 1.00 0.00
D   O
ATOM 13029 N   GLY D 177   -42.236 46.590 32.252 1.00 0.00
D   N
ATOM 13030 HN GLY D 177   -42.394 47.575 32.310 1.00 0.00
D   H
ATOM 13031 CA GLY D 177   -43.159 45.764 33.006 1.00 0.00
D   C
ATOM 13032 HA1 GLY D 177   -43.477 44.987 32.323 1.00 0.00
D   H
ATOM 13033 HA2 GLY D 177   -43.952 46.425 33.327 1.00 0.00
D   H
ATOM 13034 C   GLY D 177   -42.581 45.101 34.231 1.00 0.00
D   C
ATOM 13035 O   GLY D 177   -43.180 44.154 34.749 1.00 0.00
D   O
ATOM 13036 N   CYS D 178   -41.439 45.566 34.715 1.00 0.00
D   N
ATOM 13037 HN CYS D 178   -40.968 46.353 34.318 1.00 0.00
D   H
ATOM 13038 CA CYS D 178   -40.773 44.975 35.863 1.00 0.00
D   C
ATOM 13039 HA CYS D 178   -41.289 44.050 36.090 1.00 0.00
D   H
ATOM 13040 CB CYS D 178   -39.299 44.705 35.546 1.00 0.00
D   C

```
ATOM  13041  HB1 CYS D 178     -38.872  45.646  35.132  1.00  0.00
D    H
ATOM  13042  HB2 CYS D 178     -38.741  44.502  36.487  1.00  0.00
D    H
ATOM  13043  SG  CYS D 178     -39.058  43.385  34.342  1.00  0.00
D    S
ATOM  13044  HG1 CYS D 178     -37.730  43.474  34.261  1.00  0.00
D    H
ATOM  13045  C   CYS D 178     -40.917  45.894  37.070  1.00  0.00
D    C
ATOM  13046  O   CYS D 178     -41.610  46.914  37.027  1.00  0.00
D    O
ATOM  13047  N   THR D 179     -40.249  45.521  38.159  1.00  0.00
D    N
ATOM  13048  HN  THR D 179     -39.652  44.720  38.186  1.00  0.00
D    H
ATOM  13049  CA  THR D 179     -40.327  46.250  39.419  1.00  0.00
D    C
ATOM  13050  HA  THR D 179     -40.707  47.234  39.178  1.00  0.00
D    H
ATOM  13051  CB  THR D 179     -41.215  45.505  40.422  1.00  0.00
D    C
ATOM  13052  HB  THR D 179     -40.521  44.939  41.091  1.00  0.00
D    H
ATOM  13053  OG1 THR D 179     -42.031  44.558  39.721  1.00  0.00
D    O
ATOM  13054  HG1 THR D 179     -42.570  44.133  40.397  1.00  0.00
D    H
ATOM  13055  CG2 THR D 179     -42.083  46.467  41.211  1.00  0.00
D    C
ATOM  13056 HG21 THR D 179     -42.638  45.933  42.011  1.00  0.00
D    H
ATOM  13057 HG22 THR D 179     -41.457  47.248  41.693  1.00  0.00
D    H
ATOM  13058 HG23 THR D 179     -42.822  46.972  40.553  1.00  0.00
D    H
ATOM  13059  C   THR D 179     -38.932  46.454  39.994  1.00  0.00
D    C
ATOM  13060  O   THR D 179     -38.269  45.489  40.382  1.00  0.00
D    O
ATOM  13061  N   ILE D 180     -38.496  47.711  40.060  1.00  0.00
D    N
ATOM  13062  HN  ILE D 180     -38.981  48.492  39.674  1.00  0.00
D    H
ATOM  13063  CA  ILE D 180     -37.237  48.053  40.719  1.00  0.00
D    C
ATOM  13064  HA  ILE D 180     -36.531  47.258  40.516  1.00  0.00
D    H
ATOM  13065  CB  ILE D 180     -36.704  49.413  40.231  1.00  0.00
D    C
ATOM  13066  HB  ILE D 180     -37.504  49.797  39.548  1.00  0.00
D    H
ATOM  13067  CG2 ILE D 180     -36.382  50.331  41.414  1.00  0.00
D    C
```

ATOM 13068 HG21 ILE D 180   -36.149 51.357 41.060 1.00 0.00
D   H
ATOM 13069 HG22 ILE D 180   -37.247 50.402 42.106 1.00 0.00
D   H
ATOM 13070 HG23 ILE D 180   -35.507 49.953 41.983 1.00 0.00
D   H
ATOM 13071 CG1 ILE D 180   -35.446 49.216 39.379 1.00 0.00
D   C
ATOM 13072 HG11 ILE D 180   -35.045 48.189 39.531 1.00 0.00
D   H
ATOM 13073 HG12 ILE D 180   -35.722 49.305 38.303 1.00 0.00
D   H
ATOM 13074 CD ILE D 180   -34.328 50.217 39.654 1.00 0.00
D   C
ATOM 13075 HD1 ILE D 180   -33.478 50.045 38.960 1.00 0.00
D   H
ATOM 13076 HD2 ILE D 180   -34.690 51.257 39.510 1.00 0.00
D   H
ATOM 13077 HD3 ILE D 180   -33.950 50.116 40.693 1.00 0.00
D   H
ATOM 13078 C   ILE D 180   -37.482 48.052 42.222 1.00 0.00
D   C
ATOM 13079 O   ILE D 180   -38.550 48.467 42.687 1.00 0.00
D   O
ATOM 13080 N   LEU D 181   -36.513 47.558 42.988 1.00 0.00
D   N
ATOM 13081 HN LEU D 181   -35.583 47.368 42.679 1.00 0.00
D   H
ATOM 13082 CA LEU D 181   -36.753 47.248 44.390 1.00 0.00
D   C
ATOM 13083 HA LEU D 181   -37.763 47.588 44.580 1.00 0.00
D   H
ATOM 13084 CB LEU D 181   -36.623 45.745 44.628 1.00 0.00
D   C
ATOM 13085 HB1 LEU D 181   -36.690 45.247 43.633 1.00 0.00
D   H
ATOM 13086 HB2 LEU D 181   -35.604 45.526 45.014 1.00 0.00
D   H
ATOM 13087 CG LEU D 181   -37.674 45.137 45.542 1.00 0.00
D   C
ATOM 13088 HG LEU D 181   -37.389 45.356 46.599 1.00 0.00
D   H
ATOM 13089 CD1 LEU D 181   -39.058 45.718 45.275 1.00 0.00
D   C
ATOM 13090 HD11 LEU D 181   -39.818 45.227 45.920 1.00 0.00
D   H
ATOM 13091 HD12 LEU D 181   -39.073 46.809 45.488 1.00 0.00
D   H
ATOM 13092 HD13 LEU D 181   -39.351 45.564 44.215 1.00 0.00
D   H
ATOM 13093 CD2 LEU D 181   -37.636 43.656 45.350 1.00 0.00
D   C
ATOM 13094 HD21 LEU D 181   -38.378 43.161 46.014 1.00 0.00
D   H

ATOM 13095 HD22 LEU D 181 -37.877 43.372 44.306 1.00 0.00
D H
ATOM 13096 HD23 LEU D 181 -36.628 43.257 45.598 1.00 0.00
D H
ATOM 13097 C LEU D 181 -35.841 47.953 45.375 1.00 0.00
D C
ATOM 13098 O LEU D 181 -36.326 48.420 46.404 1.00 0.00
D O
ATOM 13099 N ALA D 182 -34.536 48.009 45.120 1.00 0.00
D N
ATOM 13100 HN ALA D 182 -34.108 47.686 44.277 1.00 0.00
D H
ATOM 13101 CA ALA D 182 -33.629 48.569 46.106 1.00 0.00
D C
ATOM 13102 HA ALA D 182 -34.058 49.446 46.579 1.00 0.00
D H
ATOM 13103 CB ALA D 182 -33.306 47.565 47.216 1.00 0.00
D C
ATOM 13104 HB1 ALA D 182 -34.242 47.273 47.739 1.00 0.00
D H
ATOM 13105 HB2 ALA D 182 -32.845 46.643 46.799 1.00 0.00
D H
ATOM 13106 HB3 ALA D 182 -32.613 47.998 47.970 1.00 0.00
D H
ATOM 13107 C ALA D 182 -32.353 49.030 45.427 1.00 0.00
D C
ATOM 13108 O ALA D 182 -31.936 48.495 44.394 1.00 0.00
D O
ATOM 13109 N ILE D 183 -31.730 50.021 46.057 1.00 0.00
D N
ATOM 13110 HN ILE D 183 -32.033 50.342 46.952 1.00 0.00
D H
ATOM 13111 CA ILE D 183 -30.574 50.727 45.530 1.00 0.00
D C
ATOM 13112 HA ILE D 183 -30.231 50.189 44.656 1.00 0.00
D H
ATOM 13113 CB ILE D 183 -30.933 52.192 45.206 1.00 0.00
D C
ATOM 13114 HB ILE D 183 -31.319 52.606 46.171 1.00 0.00
D H
ATOM 13115 CG2 ILE D 183 -29.726 52.983 44.729 1.00 0.00
D C
ATOM 13116 HG21 ILE D 183 -29.971 54.062 44.652 1.00 0.00
D H
ATOM 13117 HG22 ILE D 183 -28.879 52.880 45.440 1.00 0.00
D H
ATOM 13118 HG23 ILE D 183 -29.389 52.632 43.731 1.00 0.00
D H
ATOM 13119 CG1 ILE D 183 -32.091 52.243 44.203 1.00 0.00
D C
ATOM 13120 HG11 ILE D 183 -31.872 51.573 43.342 1.00 0.00
D H
ATOM 13121 HG12 ILE D 183 -33.012 51.855 44.696 1.00 0.00
D H

```
ATOM  13122  CD  ILE D 183     -32.385  53.626  43.687  1.00  0.00
D    C
ATOM  13123  HD1 ILE D 183     -33.268  53.606  43.012  1.00  0.00
D    H
ATOM  13124  HD2 ILE D 183     -32.604  54.320  44.526  1.00  0.00
D    H
ATOM  13125  HD3 ILE D 183     -31.523  54.028  43.113  1.00  0.00
D    H
ATOM  13126  C   ILE D 183     -29.455  50.650  46.560  1.00  0.00
D    C
ATOM  13127  O   ILE D 183     -29.621  51.113  47.694  1.00  0.00
D    O
ATOM  13128  N   LYS D 184     -28.318  50.077  46.167  1.00  0.00
D    N
ATOM  13129  HN  LYS D 184     -28.195  49.747  45.232  1.00  0.00
D    H
ATOM  13130  CA  LYS D 184     -27.169  49.882  47.051  1.00  0.00
D    C
ATOM  13131  HA  LYS D 184     -27.389  50.316  48.019  1.00  0.00
D    H
ATOM  13132  CB  LYS D 184     -26.821  48.400  47.161  1.00  0.00
D    C
ATOM  13133  HB1 LYS D 184     -27.782  47.844  47.253  1.00  0.00
D    H
ATOM  13134  HB2 LYS D 184     -26.355  48.065  46.208  1.00  0.00
D    H
ATOM  13135  CG  LYS D 184     -25.927  47.960  48.327  1.00  0.00
D    C
ATOM  13136  HG1 LYS D 184     -26.228  46.938  48.651  1.00  0.00
D    H
ATOM  13137  HG2 LYS D 184     -24.872  47.900  47.973  1.00  0.00
D    H
ATOM  13138  CD  LYS D 184     -25.997  48.872  49.535  1.00  0.00
D    C
ATOM  13139  HD1 LYS D 184     -25.394  49.785  49.322  1.00  0.00
D    H
ATOM  13140  HD2 LYS D 184     -27.056  49.193  49.666  1.00  0.00
D    H
ATOM  13141  CE  LYS D 184     -25.484  48.124  50.761  1.00  0.00
D    C
ATOM  13142  HE1 LYS D 184     -25.971  47.126  50.841  1.00  0.00
D    H
ATOM  13143  HE2 LYS D 184     -24.384  47.974  50.696  1.00  0.00
D    H
ATOM  13144  NZ  LYS D 184     -25.738  48.819  52.054  1.00  0.00
D    N
ATOM  13145  HZ1 LYS D 184     -25.363  48.252  52.842  1.00  0.00
D    H
ATOM  13146  HZ2 LYS D 184     -25.272  49.749  52.046  1.00  0.00
D    H
ATOM  13147  HZ3 LYS D 184     -26.762  48.950  52.180  1.00  0.00
D    H
ATOM  13148  C   LYS D 184     -26.009  50.669  46.452  1.00  0.00
D    C
```

ATOM 13149 O LYS D 184 -25.358 50.200 45.514 1.00 0.00
D O
ATOM 13150 N HSE D 185 -25.751 51.861 46.981 1.00 0.00
D N
ATOM 13151 HN HSE D 185 -26.252 52.227 47.764 1.00 0.00
D H
ATOM 13152 CA HSE D 185 -24.712 52.745 46.465 1.00 0.00
D C
ATOM 13153 HA HSE D 185 -24.236 52.241 45.633 1.00 0.00
D H
ATOM 13154 CB HSE D 185 -25.297 54.084 46.008 1.00 0.00
D C
ATOM 13155 HB1 HSE D 185 -24.461 54.711 45.630 1.00 0.00
D H
ATOM 13156 HB2 HSE D 185 -25.988 53.915 45.156 1.00 0.00
D H
ATOM 13157 ND1 HSE D 185 -25.359 55.834 47.819 1.00 0.00
D N
ATOM 13158 CG HSE D 185 -25.990 54.852 47.087 1.00 0.00
D C
ATOM 13159 CE1 HSE D 185 -26.208 56.337 48.697 1.00 0.00
D C
ATOM 13160 HE1 HSE D 185 -25.971 57.142 49.396 1.00 0.00
D H
ATOM 13161 NE2 HSE D 185 -27.359 55.698 48.577 1.00 0.00
D N
ATOM 13162 HE2 HSE D 185 -28.169 55.860 49.140 1.00 0.00
D H
ATOM 13163 CD2 HSE D 185 -27.247 54.761 47.579 1.00 0.00
D C
ATOM 13164 HD2 HSE D 185 -28.056 54.099 47.300 1.00 0.00
D H
ATOM 13165 C HSE D 185 -23.646 52.957 47.527 1.00 0.00
D C
ATOM 13166 O HSE D 185 -23.899 53.613 48.543 1.00 0.00
D O
ATOM 13167 N HSE D 186 -22.459 52.406 47.280 1.00 0.00
D N
ATOM 13168 HN HSE D 186 -22.273 51.852 46.469 1.00 0.00
D H
ATOM 13169 CA HSE D 186 -21.318 52.563 48.176 1.00 0.00
D C
ATOM 13170 HA HSE D 186 -20.545 51.890 47.824 1.00 0.00
D H
ATOM 13171 CB HSE D 186 -20.798 54.002 48.154 1.00 0.00
D C
ATOM 13172 HB1 HSE D 186 -20.838 54.365 47.104 1.00 0.00
D H
ATOM 13173 HB2 HSE D 186 -21.476 54.650 48.748 1.00 0.00
D H
ATOM 13174 ND1 HSE D 186 -18.338 53.458 48.065 1.00 0.00
D N
ATOM 13175 CG HSE D 186 -19.389 54.139 48.637 1.00 0.00
D C

ATOM 13176 CE1 HSE D 186 -17.221 53.763 48.700 1.00 0.00
D C
ATOM 13177 HE1 HSE D 186 -16.235 53.362 48.450 1.00 0.00
D H
ATOM 13178 NE2 HSE D 186 -17.511 54.611 49.670 1.00 0.00
D N
ATOM 13179 HE2 HSE D 186 -16.853 54.991 50.319 1.00 0.00
D H
ATOM 13180 CD2 HSE D 186 -18.861 54.860 49.654 1.00 0.00
D C
ATOM 13181 HD2 HSE D 186 -19.346 55.516 50.363 1.00 0.00
D H
ATOM 13182 C HSE D 186 -21.670 52.136 49.595 1.00 0.00
D C
ATOM 13183 O HSE D 186 -21.331 52.818 50.565 1.00 0.00
D O
ATOM 13184 N GLY D 187 -22.376 51.014 49.731 1.00 0.00
D N
ATOM 13185 HN GLY D 187 -22.671 50.479 48.940 1.00 0.00
D H
ATOM 13186 CA GLY D 187 -22.763 50.493 51.013 1.00 0.00
D C
ATOM 13187 HA1 GLY D 187 -21.959 50.738 51.694 1.00 0.00
D H
ATOM 13188 HA2 GLY D 187 -22.923 49.432 50.878 1.00 0.00
D H
ATOM 13189 C GLY D 187 -24.041 51.092 51.562 1.00 0.00
D C
ATOM 13190 O GLY D 187 -24.509 50.663 52.621 1.00 0.00
D O
ATOM 13191 N ASP D 188 -24.617 52.069 50.872 1.00 0.00
D N
ATOM 13192 HN ASP D 188 -24.308 52.435 49.998 1.00 0.00
D H
ATOM 13193 CA ASP D 188 -25.819 52.723 51.369 1.00 0.00
D C
ATOM 13194 HA ASP D 188 -25.887 52.501 52.428 1.00 0.00
D H
ATOM 13195 CB ASP D 188 -25.730 54.224 51.114 1.00 0.00
D C
ATOM 13196 HB1 ASP D 188 -25.605 54.404 50.027 1.00 0.00
D H
ATOM 13197 HB2 ASP D 188 -26.656 54.733 51.447 1.00 0.00
D H
ATOM 13198 CG ASP D 188 -24.545 54.868 51.819 1.00 0.00
D C
ATOM 13199 OD1 ASP D 188 -23.651 54.119 52.268 1.00 0.00
D O
ATOM 13200 OD2 ASP D 188 -24.505 56.116 51.925 1.00 0.00
D O
ATOM 13201 C ASP D 188 -27.072 52.142 50.715 1.00 0.00
D C
ATOM 13202 O ASP D 188 -27.149 52.011 49.491 1.00 0.00
D O

ATOM 13203 N ILE D 189 -28.062 51.806 51.544 1.00 0.00
D N
ATOM 13204 HN ILE D 189 -28.016 51.988 52.523 1.00 0.00
D H
ATOM 13205 CA ILE D 189 -29.294 51.150 51.114 1.00 0.00
D C
ATOM 13206 HA ILE D 189 -29.091 50.691 50.155 1.00 0.00
D H
ATOM 13207 CB ILE D 189 -29.764 50.092 52.137 1.00 0.00
D C
ATOM 13208 HB ILE D 189 -28.924 50.024 52.874 1.00 0.00
D H
ATOM 13209 CG2 ILE D 189 -31.111 50.467 52.746 1.00 0.00
D C
ATOM 13210 HG21 ILE D 189 -31.363 49.788 53.587 1.00 0.00
D H
ATOM 13211 HG22 ILE D 189 -31.091 51.503 53.146 1.00 0.00
D H
ATOM 13212 HG23 ILE D 189 -31.922 50.398 51.990 1.00 0.00
D H
ATOM 13213 CG1 ILE D 189 -29.894 48.702 51.507 1.00 0.00
D C
ATOM 13214 HG11 ILE D 189 -29.513 47.931 52.214 1.00 0.00
D H
ATOM 13215 HG12 ILE D 189 -30.972 48.486 51.332 1.00 0.00
D H
ATOM 13216 CD ILE D 189 -29.198 48.514 50.214 1.00 0.00
D C
ATOM 13217 HD1 ILE D 189 -29.400 47.498 49.812 1.00 0.00
D H
ATOM 13218 HD2 ILE D 189 -29.550 49.259 49.468 1.00 0.00
D H
ATOM 13219 HD3 ILE D 189 -28.100 48.625 50.333 1.00 0.00
D H
ATOM 13220 C ILE D 189 -30.380 52.191 50.883 1.00 0.00
D C
ATOM 13221 O ILE D 189 -30.404 53.237 51.543 1.00 0.00
D O
ATOM 13222 N CYS D 190 -31.250 51.908 49.917 1.00 0.00
D N
ATOM 13223 HN CYS D 190 -31.052 51.200 49.242 1.00 0.00
D H
ATOM 13224 CA CYS D 190 -32.537 52.574 49.744 1.00 0.00
D C
ATOM 13225 HA CYS D 190 -32.870 52.948 50.705 1.00 0.00
D H
ATOM 13226 CB CYS D 190 -32.456 53.710 48.729 1.00 0.00
D C
ATOM 13227 HB1 CYS D 190 -31.547 54.306 48.972 1.00 0.00
D H
ATOM 13228 HB2 CYS D 190 -32.289 53.295 47.711 1.00 0.00
D H
ATOM 13229 SG CYS D 190 -33.890 54.826 48.748 1.00 0.00
D S

ATOM 13230 HG1 CYS D 190 -33.445 55.683 47.827 1.00 0.00
D H
ATOM 13231 C CYS D 190 -33.512 51.485 49.306 1.00 0.00
D C
ATOM 13232 O CYS D 190 -33.461 51.012 48.168 1.00 0.00
D O
ATOM 13233 N LEU D 191 -34.384 51.075 50.225 1.00 0.00
D N
ATOM 13234 HN LEU D 191 -34.437 51.436 51.154 1.00 0.00
D H
ATOM 13235 CA LEU D 191 -35.360 50.035 49.932 1.00 0.00
D C
ATOM 13236 HA LEU D 191 -34.920 49.393 49.180 1.00 0.00
D H
ATOM 13237 CB LEU D 191 -35.724 49.253 51.194 1.00 0.00
D C
ATOM 13238 HB1 LEU D 191 -35.849 49.994 52.016 1.00 0.00
D H
ATOM 13239 HB2 LEU D 191 -36.722 48.781 51.052 1.00 0.00
D H
ATOM 13240 CG LEU D 191 -34.777 48.182 51.727 1.00 0.00
D C
ATOM 13241 HG LEU D 191 -33.830 48.671 52.062 1.00 0.00
D H
ATOM 13242 CD1 LEU D 191 -35.434 47.487 52.900 1.00 0.00
D C
ATOM 13243 HD11 LEU D 191 -34.788 46.667 53.280 1.00 0.00
D H
ATOM 13244 HD12 LEU D 191 -35.613 48.205 53.730 1.00 0.00
D H
ATOM 13245 HD13 LEU D 191 -36.410 47.048 52.600 1.00 0.00
D H
ATOM 13246 CD2 LEU D 191 -34.431 47.185 50.644 1.00 0.00
D C
ATOM 13247 HD21 LEU D 191 -33.726 46.420 51.036 1.00 0.00
D H
ATOM 13248 HD22 LEU D 191 -35.330 46.655 50.272 1.00 0.00
D H
ATOM 13249 HD23 LEU D 191 -33.946 47.697 49.786 1.00 0.00
D H
ATOM 13250 C LEU D 191 -36.625 50.636 49.338 1.00 0.00
D C
ATOM 13251 O LEU D 191 -37.125 51.661 49.809 1.00 0.00
D O
ATOM 13252 N SER D 192 -37.171 49.953 48.333 1.00 0.00
D N
ATOM 13253 HN SER D 192 -36.802 49.077 48.024 1.00 0.00
D H
ATOM 13254 CA SER D 192 -38.344 50.403 47.591 1.00 0.00
D C
ATOM 13255 HA SER D 192 -38.348 49.855 46.658 1.00 0.00
D H
ATOM 13256 CB SER D 192 -39.628 50.147 48.369 1.00 0.00
D C

```
ATOM  13257  HB1 SER D 192     -40.333  50.984  48.159  1.00  0.00
D    H
ATOM  13258  HB2 SER D 192     -40.116  49.211  48.013  1.00  0.00
D    H
ATOM  13259  OG  SER D 192     -39.402  50.094  49.767  1.00  0.00
D    O
ATOM  13260  HG1 SER D 192     -40.264  50.155  50.196  1.00  0.00
D    H
ATOM  13261  C   SER D 192     -38.207  51.891  47.245  1.00  0.00
D    C
ATOM  13262  O   SER D 192     -38.988  52.713  47.734  1.00  0.00
D    O
ATOM  13263  N   PRO D 193     -37.228  52.260  46.423  1.00  0.00
D    N
ATOM  13264  CD  PRO D 193     -36.421  51.410  45.530  1.00  0.00
D    C
ATOM  13265  HD1 PRO D 193     -35.640  50.845  46.089  1.00  0.00
D    H
ATOM  13266  HD2 PRO D 193     -37.072  50.710  44.955  1.00  0.00
D    H
ATOM  13267  CA  PRO D 193     -36.952  53.685  46.217  1.00  0.00
D    C
ATOM  13268  HA  PRO D 193     -36.753  54.177  47.162  1.00  0.00
D    H
ATOM  13269  CB  PRO D 193     -35.698  53.679  45.335  1.00  0.00
D    C
ATOM  13270  HB1 PRO D 193     -34.798  53.591  45.984  1.00  0.00
D    H
ATOM  13271  HB2 PRO D 193     -35.606  54.593  44.714  1.00  0.00
D    H
ATOM  13272  CG  PRO D 193     -35.776  52.396  44.597  1.00  0.00
D    C
ATOM  13273  HG1 PRO D 193     -34.789  52.044  44.239  1.00  0.00
D    H
ATOM  13274  HG2 PRO D 193     -36.465  52.514  43.729  1.00  0.00
D    H
ATOM  13275  C   PRO D 193     -38.114  54.381  45.530  1.00  0.00
D    C
ATOM  13276  O   PRO D 193     -38.634  53.917  44.512  1.00  0.00
D    O
ATOM  13277  N   ALA D 194     -38.521  55.508  46.108  1.00  0.00
D    N
ATOM  13278  HN  ALA D 194     -38.118  55.860  46.952  1.00  0.00
D    H
ATOM  13279  CA  ALA D 194     -39.591  56.313  45.547  1.00  0.00
D    C
ATOM  13280  HA  ALA D 194     -40.297  55.707  44.991  1.00  0.00
D    H
ATOM  13281  CB  ALA D 194     -40.342  57.055  46.646  1.00  0.00
D    C
ATOM  13282  HB1 ALA D 194     -40.813  56.323  47.336  1.00  0.00
D    H
ATOM  13283  HB2 ALA D 194     -39.652  57.691  47.242  1.00  0.00
D    H
```

ATOM 13284 HB3 ALA D 194 −41.147 57.699 46.229 1.00 0.00
D H
ATOM 13285 C ALA D 194 −38.998 57.297 44.557 1.00 0.00
D C
ATOM 13286 O ALA D 194 −38.059 58.026 44.915 1.00 0.00
D O
ATOM 13287 N PRO D 195 −39.482 57.338 43.317 1.00 0.00
D N
ATOM 13288 CD PRO D 195 −40.527 56.478 42.737 1.00 0.00
D C
ATOM 13289 HD1 PRO D 195 −39.626 56.018 43.203 1.00 0.00
D H
ATOM 13290 HD2 PRO D 195 −41.427 55.848 42.924 1.00 0.00
D H
ATOM 13291 CA PRO D 195 −38.950 58.298 42.343 1.00 0.00
D C
ATOM 13292 HA PRO D 195 −37.900 58.108 42.150 1.00 0.00
D H
ATOM 13293 CB PRO D 195 −39.791 58.030 41.091 1.00 0.00
D C
ATOM 13294 HB1 PRO D 195 −39.336 57.189 40.522 1.00 0.00
D H
ATOM 13295 HB2 PRO D 195 −39.867 58.915 40.428 1.00 0.00
D H
ATOM 13296 CG PRO D 195 −40.315 56.644 41.268 1.00 0.00
D C
ATOM 13297 HG1 PRO D 195 −40.587 56.160 40.309 1.00 0.00
D H
ATOM 13298 HG2 PRO D 195 −41.207 56.671 41.935 1.00 0.00
D H
ATOM 13299 C PRO D 195 −39.115 59.730 42.838 1.00 0.00
D C
ATOM 13300 O PRO D 195 −40.160 60.099 43.377 1.00 0.00
D O
ATOM 13301 N GLU D 196 −38.070 60.535 42.661 1.00 0.00
D N
ATOM 13302 HN GLU D 196 −38.055 61.478 42.982 1.00 0.00
D H
ATOM 13303 CA GLU D 196 −36.846 60.120 41.982 1.00 0.00
D C
ATOM 13304 HA GLU D 196 −37.063 59.235 41.397 1.00 0.00
D H
ATOM 13305 CB GLU D 196 −36.347 61.235 41.058 1.00 0.00
D C
ATOM 13306 HB1 GLU D 196 −35.411 60.885 40.564 1.00 0.00
D H
ATOM 13307 HB2 GLU D 196 −37.081 61.402 40.238 1.00 0.00
D H
ATOM 13308 CG GLU D 196 −36.057 62.562 41.747 1.00 0.00
D C
ATOM 13309 HG1 GLU D 196 −36.123 63.383 41.009 1.00 0.00
D H
ATOM 13310 HG2 GLU D 196 −36.778 62.750 42.567 1.00 0.00
D H

```
ATOM  13311  CD  GLU D 196     -34.649  62.620  42.310  1.00  0.00
D    C
ATOM  13312  OE1 GLU D 196     -33.687  62.635  41.507  1.00  0.00
D    O
ATOM  13313  OE2 GLU D 196     -34.504  62.612  43.548  1.00  0.00
D    O
ATOM  13314  C   GLU D 196     -35.766  59.739  42.996  1.00  0.00
D    C
ATOM  13315  O   GLU D 196     -35.940  59.942  44.193  1.00  0.00
D    O
ATOM  13316  N   ASP D 197     -34.660  59.173  42.512  1.00  0.00
D    N
ATOM  13317  HN  ASP D 197     -34.486  59.008  41.545  1.00  0.00
D    H
ATOM  13318  CA  ASP D 197     -33.570  58.730  43.369  1.00  0.00
D    C
ATOM  13319  HA  ASP D 197     -33.411  59.500  44.115  1.00  0.00
D    H
ATOM  13320  CB  ASP D 197     -33.880  57.385  44.021  1.00  0.00
D    C
ATOM  13321  HB1 ASP D 197     -34.962  57.334  44.257  1.00  0.00
D    H
ATOM  13322  HB2 ASP D 197     -33.634  56.551  43.333  1.00  0.00
D    H
ATOM  13323  CG  ASP D 197     -33.123  57.184  45.311  1.00  0.00
D    C
ATOM  13324  OD1 ASP D 197     -32.657  58.187  45.892  1.00  0.00
D    O
ATOM  13325  OD2 ASP D 197     -32.974  56.017  45.732  1.00  0.00
D    O
ATOM  13326  C   ASP D 197     -32.281  58.618  42.572  1.00  0.00
D    C
ATOM  13327  O   ASP D 197     -32.251  58.016  41.498  1.00  0.00
D    O
ATOM  13328  N   ILE D 198     -31.203  59.150  43.143  1.00  0.00
D    N
ATOM  13329  HN  ILE D 198     -31.194  59.543  44.060  1.00  0.00
D    H
ATOM  13330  CA  ILE D 198     -29.914  59.203  42.455  1.00  0.00
D    C
ATOM  13331  HA  ILE D 198     -30.100  59.579  41.457  1.00  0.00
D    H
ATOM  13332  CB  ILE D 198     -28.915  60.102  43.210  1.00  0.00
D    C
ATOM  13333  HB  ILE D 198     -29.231  60.033  44.282  1.00  0.00
D    H
ATOM  13334  CG2 ILE D 198     -27.474  59.675  42.920  1.00  0.00
D    C
ATOM  13335 HG21 ILE D 198     -26.765  60.217  43.581  1.00  0.00
D    H
ATOM  13336 HG22 ILE D 198     -27.339  58.589  43.102  1.00  0.00
D    H
ATOM  13337 HG23 ILE D 198     -27.200  59.891  41.866  1.00  0.00
D    H
```

```
ATOM  13338  CG1 ILE D 198     -29.076  61.565  42.800  1.00  0.00
D    C
ATOM  13339 HG11 ILE D 198     -28.632  61.725  41.793  1.00  0.00
D    H
ATOM  13340 HG12 ILE D 198     -30.163  61.800  42.723  1.00  0.00
D    H
ATOM  13341  CD  ILE D 198     -28.456  62.532  43.767  1.00  0.00
D    C
ATOM  13342  HD1 ILE D 198     -28.655  63.578  43.448  1.00  0.00
D    H
ATOM  13343  HD2 ILE D 198     -28.879  62.393  44.785  1.00  0.00
D    H
ATOM  13344  HD3 ILE D 198     -27.356  62.393  43.820  1.00  0.00
D    H
ATOM  13345  C   ILE D 198     -29.366  57.789  42.298  1.00  0.00
D    C
ATOM  13346  O   ILE D 198     -29.123  57.082  43.286  1.00  0.00
D    O
ATOM  13347  N   ILE D 199     -29.170  57.375  41.051  1.00  0.00
D    N
ATOM  13348  HN  ILE D 199     -29.416  57.929  40.258  1.00  0.00
D    H
ATOM  13349  CA  ILE D 199     -28.580  56.084  40.719  1.00  0.00
D    C
ATOM  13350  HA  ILE D 199     -28.503  55.505  41.630  1.00  0.00
D    H
ATOM  13351  CB  ILE D 199     -29.396  55.346  39.645  1.00  0.00
D    C
ATOM  13352  HB  ILE D 199     -29.433  56.059  38.783  1.00  0.00
D    H
ATOM  13353  CG2 ILE D 199     -28.734  54.026  39.283  1.00  0.00
D    C
ATOM  13354 HG21 ILE D 199     -29.231  53.569  38.402  1.00  0.00
D    H
ATOM  13355 HG22 ILE D 199     -27.665  54.177  39.024  1.00  0.00
D    H
ATOM  13356 HG23 ILE D 199     -28.792  53.307  40.127  1.00  0.00
D    H
ATOM  13357  CG1 ILE D 199     -30.839  55.144  40.119  1.00  0.00
D    C
ATOM  13358 HG11 ILE D 199     -30.843  54.785  41.172  1.00  0.00
D    H
ATOM  13359 HG12 ILE D 199     -31.364  56.127  40.105  1.00  0.00
D    H
ATOM  13360  CD  ILE D 199     -31.643  54.186  39.287  1.00  0.00
D    C
ATOM  13361  HD1 ILE D 199     -32.690  54.137  39.657  1.00  0.00
D    H
ATOM  13362  HD2 ILE D 199     -31.664  54.511  38.225  1.00  0.00
D    H
ATOM  13363  HD3 ILE D 199     -31.214  53.163  39.333  1.00  0.00
D    H
ATOM  13364  C   ILE D 199     -27.160  56.353  40.248  1.00  0.00
D    C
```

```
ATOM  13365  O   ILE D 199      -26.954  56.796  39.115  1.00  0.00
D    O
ATOM  13366  N   ARG D 200      -26.188  56.112  41.118  1.00  0.00
D    N
ATOM  13367  HN  ARG D 200      -26.378  55.782  42.040  1.00  0.00
D    H
ATOM  13368  CA  ARG D 200      -24.791  56.316  40.774  1.00  0.00
D    C
ATOM  13369  HA  ARG D 200      -24.723  57.143  40.076  1.00  0.00
D    H
ATOM  13370  CB  ARG D 200      -23.978  56.616  42.029  1.00  0.00
D    C
ATOM  13371  HB1 ARG D 200      -24.360  55.932  42.824  1.00  0.00
D    H
ATOM  13372  HB2 ARG D 200      -22.916  56.328  41.862  1.00  0.00
D    H
ATOM  13373  CG  ARG D 200      -24.108  58.046  42.499  1.00  0.00
D    C
ATOM  13374  HG1 ARG D 200      -24.358  58.684  41.624  1.00  0.00
D    H
ATOM  13375  HG2 ARG D 200      -24.982  58.123  43.186  1.00  0.00
D    H
ATOM  13376  CD  ARG D 200      -22.863  58.502  43.232  1.00  0.00
D    C
ATOM  13377  HD1 ARG D 200      -22.850  58.056  44.253  1.00  0.00
D    H
ATOM  13378  HD2 ARG D 200      -21.936  58.193  42.695  1.00  0.00
D    H
ATOM  13379  NE  ARG D 200      -22.805  59.956  43.352  1.00  0.00
D    N
ATOM  13380  HE  ARG D 200      -23.674  60.407  43.118  1.00  0.00
D    H
ATOM  13381  CZ  ARG D 200      -21.725  60.629  43.731  1.00  0.00
D    C
ATOM  13382  NH1 ARG D 200      -20.610  59.979  44.031  1.00  0.00
D    N
ATOM  13383 HH11 ARG D 200      -19.807  60.470  44.338  1.00  0.00
D    H
ATOM  13384 HH12 ARG D 200      -20.673  58.979  44.059  1.00  0.00
D    H
ATOM  13385  NH2 ARG D 200      -21.757  61.952  43.813  1.00  0.00
D    N
ATOM  13386 HH21 ARG D 200      -20.928  62.455  44.011  1.00  0.00
D    H
ATOM  13387 HH22 ARG D 200      -22.570  62.402  43.466  1.00  0.00
D    H
ATOM  13388  C   ARG D 200      -24.235  55.091  40.055  1.00  0.00
D    C
ATOM  13389  O   ARG D 200      -24.883  54.048  39.953  1.00  0.00
D    O
ATOM  13390  N   GLU D 201      -23.015  55.230  39.546  1.00  0.00
D    N
ATOM  13391  HN  GLU D 201      -22.493  56.078  39.508  1.00  0.00
D    H
```

ATOM 13392 CA GLU D 201   -22.324 54.084 38.980 1.00 0.00
D   C
ATOM 13393 HA GLU D 201   -22.970 53.609 38.253 1.00 0.00
D   H
ATOM 13394 CB GLU D 201   -21.011 54.507 38.327 1.00 0.00
D   C
ATOM 13395 HB1 GLU D 201   -20.336 53.622 38.300 1.00 0.00
D   H
ATOM 13396 HB2 GLU D 201   -21.191 54.786 37.264 1.00 0.00
D   H
ATOM 13397 CG GLU D 201   -20.298 55.637 39.032 1.00 0.00
D   C
ATOM 13398 HG1 GLU D 201   -20.823 56.588 38.829 1.00 0.00
D   H
ATOM 13399 HG2 GLU D 201   -20.264 55.464 40.126 1.00 0.00
D   H
ATOM 13400 CD GLU D 201   -18.886 55.822 38.521 1.00 0.00
D   C
ATOM 13401 OE1 GLU D 201   -18.658 55.604 37.315 1.00 0.00
D   O
ATOM 13402 OE2 GLU D 201   -18.003 56.187 39.327 1.00 0.00
D   O
ATOM 13403 C   GLU D 201   -22.048 53.063 40.073 1.00 0.00
D   C
ATOM 13404 O   GLU D 201   -22.086 53.379 41.264 1.00 0.00
D   O
ATOM 13405 N   GLN D 202   -21.790 51.826 39.656 1.00 0.00
D   N
ATOM 13406 HN GLN D 202   -21.815 51.590 38.686 1.00 0.00
D   H
ATOM 13407 CA GLN D 202   -21.453 50.716 40.538 1.00 0.00
D   C
ATOM 13408 HA GLN D 202   -21.386 49.833 39.914 1.00 0.00
D   H
ATOM 13409 CB GLN D 202   -20.101 50.949 41.222 1.00 0.00
D   C
ATOM 13410 HB1 GLN D 202   -20.172 51.926 41.755 1.00 0.00
D   H
ATOM 13411 HB2 GLN D 202   -19.948 50.176 42.010 1.00 0.00
D   H
ATOM 13412 CG GLN D 202   -18.948 50.975 40.234 1.00 0.00
D   C
ATOM 13413 HG1 GLN D 202   -19.205 50.392 39.324 1.00 0.00
D   H
ATOM 13414 HG2 GLN D 202   -18.723 52.015 39.920 1.00 0.00
D   H
ATOM 13415 CD GLN D 202   -17.665 50.358 40.767 1.00 0.00
D   C
ATOM 13416 OE1 GLN D 202   -17.695 49.457 41.599 1.00 0.00
D   O
ATOM 13417 NE2 GLN D 202   -16.529 50.843 40.281 1.00 0.00
D   N
ATOM 13418 HE21 GLN D 202   -15.684 50.440 40.622 1.00 0.00
D   H

ATOM 13419 HE22 GLN D 202 −16.550 51.586 39.620 1.00 0.00
D H
ATOM 13420 C GLN D 202 −22.537 50.463 41.575 1.00 0.00
D C
ATOM 13421 O GLN D 202 −22.255 49.910 42.647 1.00 0.00
D O
ATOM 13422 N ASP D 203 −23.773 50.851 41.278 1.00 0.00
D N
ATOM 13423 HN ASP D 203 −24.046 51.294 40.428 1.00 0.00
D H
ATOM 13424 CA ASP D 203 −24.881 50.659 42.197 1.00 0.00
D C
ATOM 13425 HA ASP D 203 −24.471 50.674 43.201 1.00 0.00
D H
ATOM 13426 CB ASP D 203 −25.958 51.714 41.989 1.00 0.00
D C
ATOM 13427 HB1 ASP D 203 −25.482 52.714 41.933 1.00 0.00
D H
ATOM 13428 HB2 ASP D 203 −26.503 51.534 41.041 1.00 0.00
D H
ATOM 13429 CG ASP D 203 −26.947 51.744 43.118 1.00 0.00
D C
ATOM 13430 OD1 ASP D 203 −28.023 51.135 42.968 1.00 0.00
D O
ATOM 13431 OD2 ASP D 203 −26.673 52.403 44.128 1.00 0.00
D O
ATOM 13432 C ASP D 203 −25.511 49.294 42.002 1.00 0.00
D C
ATOM 13433 O ASP D 203 −25.723 48.850 40.872 1.00 0.00
D O
ATOM 13434 N CYS D 204 −25.855 48.648 43.108 1.00 0.00
D N
ATOM 13435 HN CYS D 204 −25.640 48.956 44.033 1.00 0.00
D H
ATOM 13436 CA CYS D 204 −26.597 47.397 43.038 1.00 0.00
D C
ATOM 13437 HA CYS D 204 −26.322 46.909 42.109 1.00 0.00
D H
ATOM 13438 CB CYS D 204 −26.253 46.501 44.222 1.00 0.00
D C
ATOM 13439 HB1 CYS D 204 −26.259 47.141 45.133 1.00 0.00
D H
ATOM 13440 HB2 CYS D 204 −27.058 45.747 44.371 1.00 0.00
D H
ATOM 13441 SG CYS D 204 −24.633 45.713 44.083 1.00 0.00
D S
ATOM 13442 HG1 CYS D 204 −24.645 45.098 45.267 1.00 0.00
D H
ATOM 13443 C CYS D 204 −28.091 47.704 42.976 1.00 0.00
D C
ATOM 13444 O CYS D 204 −28.648 48.313 43.895 1.00 0.00
D O
ATOM 13445 N LEU D 205 −28.718 47.309 41.870 1.00 0.00
D N

ATOM 13446 HN LEU D 205 -28.219 46.814 41.161 1.00 0.00
D H
ATOM 13447 CA LEU D 205 -30.124 47.533 41.567 1.00 0.00
D C
ATOM 13448 HA LEU D 205 -30.522 48.239 42.285 1.00 0.00
D H
ATOM 13449 CB LEU D 205 -30.297 48.002 40.119 1.00 0.00
D C
ATOM 13450 HB1 LEU D 205 -29.363 47.733 39.572 1.00 0.00
D H
ATOM 13451 HB2 LEU D 205 -31.112 47.414 39.644 1.00 0.00
D H
ATOM 13452 CG LEU D 205 -30.558 49.446 39.699 1.00 0.00
D C
ATOM 13453 HG LEU D 205 -31.568 49.766 40.052 1.00 0.00
D H
ATOM 13454 CD1 LEU D 205 -29.494 50.389 40.235 1.00 0.00
D C
ATOM 13455 HD11 LEU D 205 -29.677 51.425 39.879 1.00 0.00
D H
ATOM 13456 HD12 LEU D 205 -29.502 50.396 41.346 1.00 0.00
D H
ATOM 13457 HD13 LEU D 205 -28.485 50.077 39.892 1.00 0.00
D H
ATOM 13458 CD2 LEU D 205 -30.556 49.487 38.188 1.00 0.00
D C
ATOM 13459 HD21 LEU D 205 -30.761 50.519 37.830 1.00 0.00
D H
ATOM 13460 HD22 LEU D 205 -29.577 49.174 37.771 1.00 0.00
D H
ATOM 13461 HD23 LEU D 205 -31.341 48.815 37.780 1.00 0.00
D H
ATOM 13462 C LEU D 205 -30.841 46.208 41.779 1.00 0.00
D C
ATOM 13463 O LEU D 205 -30.375 45.170 41.293 1.00 0.00
D O
ATOM 13464 N VAL D 206 -31.952 46.234 42.508 1.00 0.00
D N
ATOM 13465 HN VAL D 206 -32.301 47.048 42.963 1.00 0.00
D H
ATOM 13466 CA VAL D 206 -32.766 45.040 42.701 1.00 0.00
D C
ATOM 13467 HA VAL D 206 -32.206 44.177 42.362 1.00 0.00
D H
ATOM 13468 CB VAL D 206 -33.144 44.833 44.170 1.00 0.00
D C
ATOM 13469 HB VAL D 206 -33.816 45.674 44.467 1.00 0.00
D H
ATOM 13470 CG1 VAL D 206 -33.877 43.520 44.342 1.00 0.00
D C
ATOM 13471 HG11 VAL D 206 -34.117 43.350 45.415 1.00 0.00
D H
ATOM 13472 HG12 VAL D 206 -34.835 43.509 43.784 1.00 0.00
D H

ATOM 13473 HG13 VAL D 206   -33.250 42.672 43.992 1.00 0.00
D   H
ATOM 13474 CG2 VAL D 206   -31.887 44.857 45.033 1.00 0.00
D   C
ATOM 13475 HG21 VAL D 206   -32.138 44.669 46.098 1.00 0.00
D   H
ATOM 13476 HG22 VAL D 206   -31.169 44.077 44.700 1.00 0.00
D   H
ATOM 13477 HG23 VAL D 206   -31.381 45.843 44.977 1.00 0.00
D   H
ATOM 13478 C   VAL D 206   -33.999 45.158 41.818 1.00 0.00
D   C
ATOM 13479 O   VAL D 206   -34.819 46.070 41.986 1.00 0.00
D   O
ATOM 13480 N   ILE D 207   -34.136 44.226 40.883 1.00 0.00
D   N
ATOM 13481 HN ILE D 207   -33.498 43.469 40.763 1.00 0.00
D   H
ATOM 13482 CA ILE D 207   -35.242 44.226 39.943 1.00 0.00
D   C
ATOM 13483 HA ILE D 207   -35.965 44.955 40.289 1.00 0.00
D   H
ATOM 13484 CB ILE D 207   -34.776 44.552 38.509 1.00 0.00
D   C
ATOM 13485 HB ILE D 207   -34.125 45.456 38.624 1.00 0.00
D   H
ATOM 13486 CG2 ILE D 207   -34.056 43.370 37.909 1.00 0.00
D   C
ATOM 13487 HG21 ILE D 207   -33.584 43.648 36.944 1.00 0.00
D   H
ATOM 13488 HG22 ILE D 207   -33.251 43.012 38.585 1.00 0.00
D   H
ATOM 13489 HG23 ILE D 207   -34.757 42.530 37.723 1.00 0.00
D   H
ATOM 13490 CG1 ILE D 207   -35.964 44.973 37.638 1.00 0.00
D   C
ATOM 13491 HG11 ILE D 207   -36.720 44.158 37.615 1.00 0.00
D   H
ATOM 13492 HG12 ILE D 207   -36.452 45.862 38.099 1.00 0.00
D   H
ATOM 13493 CD ILE D 207   -35.584 45.323 36.244 1.00 0.00
D   C
ATOM 13494 HD1 ILE D 207   -36.474 45.674 35.679 1.00 0.00
D   H
ATOM 13495 HD2 ILE D 207   -34.825 46.134 36.236 1.00 0.00
D   H
ATOM 13496 HD3 ILE D 207   -35.166 44.442 35.713 1.00 0.00
D   H
ATOM 13497 C   ILE D 207   -35.923 42.867 40.003 1.00 0.00
D   C
ATOM 13498 O   ILE D 207   -35.352 41.876 40.458 1.00 0.00
D   O
ATOM 13499 N   MET D 208   -37.167 42.823 39.540 1.00 0.00
D   N

ATOM 13500 HN MET D 208 -37.666 43.613 39.190 1.00 0.00
D H
ATOM 13501 CA MET D 208 -37.916 41.575 39.507 1.00 0.00
D C
ATOM 13502 HA MET D 208 -37.228 40.770 39.276 1.00 0.00
D H
ATOM 13503 CB MET D 208 -38.635 41.298 40.837 1.00 0.00
D C
ATOM 13504 HB1 MET D 208 -37.849 41.031 41.581 1.00 0.00
D H
ATOM 13505 HB2 MET D 208 -39.106 42.236 41.204 1.00 0.00
D H
ATOM 13506 CG MET D 208 -39.646 40.165 40.727 1.00 0.00
D C
ATOM 13507 HG1 MET D 208 -40.136 40.180 39.731 1.00 0.00
D H
ATOM 13508 HG2 MET D 208 -39.125 39.188 40.830 1.00 0.00
D H
ATOM 13509 SD MET D 208 -40.946 40.224 41.948 1.00 0.00
D S
ATOM 13510 CE MET D 208 -41.450 41.918 41.775 1.00 0.00
D C
ATOM 13511 HE1 MET D 208 -42.272 42.162 42.482 1.00 0.00
D H
ATOM 13512 HE2 MET D 208 -40.604 42.609 41.983 1.00 0.00
D H
ATOM 13513 HE3 MET D 208 -41.814 42.124 40.745 1.00 0.00
D H
ATOM 13514 C MET D 208 -38.928 41.651 38.377 1.00 0.00
D C
ATOM 13515 O MET D 208 -39.484 42.719 38.109 1.00 0.00
D O
ATOM 13516 N GLY D 209 -39.169 40.520 37.731 1.00 0.00
D N
ATOM 13517 HN GLY D 209 -38.646 39.678 37.854 1.00 0.00
D H
ATOM 13518 CA GLY D 209 -40.254 40.449 36.775 1.00 0.00
D C
ATOM 13519 HA1 GLY D 209 -40.237 41.374 36.213 1.00 0.00
D H
ATOM 13520 HA2 GLY D 209 -41.163 40.287 37.336 1.00 0.00
D H
ATOM 13521 C GLY D 209 -40.097 39.306 35.799 1.00 0.00
D C
ATOM 13522 O GLY D 209 -39.482 38.286 36.118 1.00 0.00
D O
ATOM 13523 N HSE D 210 -40.652 39.473 34.604 1.00 0.00
D N
ATOM 13524 HN HSE D 210 -41.200 40.262 34.334 1.00 0.00
D H
ATOM 13525 CA HSE D 210 -40.489 38.472 33.566 1.00 0.00
D C
ATOM 13526 HA HSE D 210 -40.709 37.504 34.001 1.00 0.00
D H

ATOM 13527 CB HSE D 210 −41.392 38.770 32.374 1.00 0.00
D C
ATOM 13528 HB1 HSE D 210 −42.442 38.784 32.738 1.00 0.00
D H
ATOM 13529 HB2 HSE D 210 −41.168 39.785 31.985 1.00 0.00
D H
ATOM 13530 ND1 HSE D 210 −41.687 36.445 31.447 1.00 0.00
D N
ATOM 13531 CG HSE D 210 −41.290 37.754 31.284 1.00 0.00
D C
ATOM 13532 CE1 HSE D 210 −41.472 35.780 30.327 1.00 0.00
D C
ATOM 13533 HE1 HSE D 210 −41.704 34.722 30.180 1.00 0.00
D H
ATOM 13534 NE2 HSE D 210 −40.935 36.606 29.448 1.00 0.00
D N
ATOM 13535 HE2 HSE D 210 −40.653 36.358 28.522 1.00 0.00
D H
ATOM 13536 CD2 HSE D 210 −40.802 37.845 30.025 1.00 0.00
D C
ATOM 13537 HD2 HSE D 210 −40.367 38.690 29.510 1.00 0.00
D H
ATOM 13538 C HSE D 210 −39.038 38.442 33.123 1.00 0.00
D C
ATOM 13539 O HSE D 210 −38.503 39.445 32.643 1.00 0.00
D O
ATOM 13540 N LYS D 211 −38.408 37.279 33.285 1.00 0.00
D N
ATOM 13541 HN LYS D 211 −38.860 36.430 33.553 1.00 0.00
D H
ATOM 13542 CA LYS D 211 −36.967 37.173 33.074 1.00 0.00
D C
ATOM 13543 HA LYS D 211 −36.493 37.817 33.805 1.00 0.00
D H
ATOM 13544 CB LYS D 211 −36.518 35.725 33.260 1.00 0.00
D C
ATOM 13545 HB1 LYS D 211 −35.454 35.667 32.935 1.00 0.00
D H
ATOM 13546 HB2 LYS D 211 −36.526 35.480 34.346 1.00 0.00
D H
ATOM 13547 CG LYS D 211 −37.331 34.712 32.476 1.00 0.00
D C
ATOM 13548 HG1 LYS D 211 −38.368 34.684 32.882 1.00 0.00
D H
ATOM 13549 HG2 LYS D 211 −37.398 35.044 31.415 1.00 0.00
D H
ATOM 13550 CD LYS D 211 −36.721 33.326 32.562 1.00 0.00
D C
ATOM 13551 HD1 LYS D 211 −35.627 33.410 32.371 1.00 0.00
D H
ATOM 13552 HD2 LYS D 211 −36.850 32.954 33.605 1.00 0.00
D H
ATOM 13553 CE LYS D 211 −37.380 32.387 31.572 1.00 0.00
D C

ATOM 13554 HE1 LYS D 211 -37.212 32.740 30.530 1.00 0.00
D H
ATOM 13555 HE2 LYS D 211 -36.961 31.362 31.668 1.00 0.00
D H
ATOM 13556 NZ LYS D 211 -38.847 32.292 31.780 1.00 0.00
D N
ATOM 13557 HZ1 LYS D 211 -39.264 31.642 31.083 1.00 0.00
D H
ATOM 13558 HZ2 LYS D 211 -39.040 31.940 32.740 1.00 0.00
D H
ATOM 13559 HZ3 LYS D 211 -39.273 33.234 31.670 1.00 0.00
D H
ATOM 13560 C LYS D 211 -36.563 37.676 31.695 1.00 0.00
D C
ATOM 13561 O LYS D 211 -35.485 38.260 31.531 1.00 0.00
D O
ATOM 13562 N LYS D 212 -37.414 37.470 30.690 1.00 0.00
D N
ATOM 13563 HN LYS D 212 -38.316 37.049 30.768 1.00 0.00
D H
ATOM 13564 CA LYS D 212 -37.036 37.878 29.343 1.00 0.00
D C
ATOM 13565 HA LYS D 212 -35.988 37.634 29.218 1.00 0.00
D H
ATOM 13566 CB LYS D 212 -37.857 37.110 28.310 1.00 0.00
D C
ATOM 13567 HB1 LYS D 212 -38.926 37.385 28.469 1.00 0.00
D H
ATOM 13568 HB2 LYS D 212 -37.590 37.473 27.293 1.00 0.00
D H
ATOM 13569 CG LYS D 212 -37.710 35.585 28.402 1.00 0.00
D C
ATOM 13570 HG1 LYS D 212 -38.473 35.188 29.111 1.00 0.00
D H
ATOM 13571 HG2 LYS D 212 -37.921 35.140 27.403 1.00 0.00
D H
ATOM 13572 CD LYS D 212 -36.325 35.127 28.884 1.00 0.00
D C
ATOM 13573 HD1 LYS D 212 -36.064 35.703 29.801 1.00 0.00
D H
ATOM 13574 HD2 LYS D 212 -36.397 34.053 29.175 1.00 0.00
D H
ATOM 13575 CE LYS D 212 -35.237 35.300 27.833 1.00 0.00
D C
ATOM 13576 HE1 LYS D 212 -35.637 35.082 26.818 1.00 0.00
D H
ATOM 13577 HE2 LYS D 212 -34.849 36.343 27.841 1.00 0.00
D H
ATOM 13578 NZ LYS D 212 -34.089 34.380 28.082 1.00 0.00
D N
ATOM 13579 HZ1 LYS D 212 -33.360 34.515 27.352 1.00 0.00
D H
ATOM 13580 HZ2 LYS D 212 -33.682 34.578 29.018 1.00 0.00
D H

ATOM 13581 HZ3 LYS D 212 -34.423 33.395 28.057 1.00 0.00
D H
ATOM 13582 C LYS D 212 -37.176 39.385 29.166 1.00 0.00
D C
ATOM 13583 O LYS D 212 -36.340 40.015 28.511 1.00 0.00
D O
ATOM 13584 N ASP D 213 -38.199 39.992 29.769 1.00 0.00
D N
ATOM 13585 HN ASP D 213 -38.974 39.567 30.230 1.00 0.00
D H
ATOM 13586 CA ASP D 213 -38.253 41.451 29.794 1.00 0.00
D C
ATOM 13587 HA ASP D 213 -38.142 41.792 28.771 1.00 0.00
D H
ATOM 13588 CB ASP D 213 -39.571 41.929 30.397 1.00 0.00
D C
ATOM 13589 HB1 ASP D 213 -39.770 41.368 31.333 1.00 0.00
D H
ATOM 13590 HB2 ASP D 213 -39.520 43.008 30.642 1.00 0.00
D H
ATOM 13591 CG ASP D 213 -40.751 41.704 29.478 1.00 0.00
D C
ATOM 13592 OD1 ASP D 213 -40.993 42.557 28.599 1.00 0.00
D O
ATOM 13593 OD2 ASP D 213 -41.437 40.675 29.627 1.00 0.00
D O
ATOM 13594 C ASP D 213 -37.081 42.024 30.578 1.00 0.00
D C
ATOM 13595 O ASP D 213 -36.555 43.093 30.238 1.00 0.00
D O
ATOM 13596 N ILE D 214 -36.660 41.327 31.636 1.00 0.00
D N
ATOM 13597 HN ILE D 214 -37.080 40.489 31.977 1.00 0.00
D H
ATOM 13598 CA ILE D 214 -35.498 41.768 32.401 1.00 0.00
D C
ATOM 13599 HA ILE D 214 -35.670 42.804 32.662 1.00 0.00
D H
ATOM 13600 CB ILE D 214 -35.325 40.907 33.664 1.00 0.00
D C
ATOM 13601 HB ILE D 214 -35.226 39.861 33.279 1.00 0.00
D H
ATOM 13602 CG2 ILE D 214 -34.140 41.394 34.475 1.00 0.00
D C
ATOM 13603 HG21 ILE D 214 -33.914 40.688 35.300 1.00 0.00
D H
ATOM 13604 HG22 ILE D 214 -33.232 41.471 33.841 1.00 0.00
D H
ATOM 13605 HG23 ILE D 214 -34.344 42.392 34.918 1.00 0.00
D H
ATOM 13606 CG1 ILE D 214 -36.593 40.947 34.513 1.00 0.00
D C
ATOM 13607 HG11 ILE D 214 -36.688 41.942 35.003 1.00 0.00
D H

```
ATOM  13608 HG12 ILE D 214     -37.478  40.824  33.847  1.00  0.00
D    H
ATOM  13609  CD  ILE D 214     -36.658  39.874  35.573  1.00  0.00
D    C
ATOM  13610  HD1 ILE D 214     -37.628  39.924  36.114  1.00  0.00
D    H
ATOM  13611  HD2 ILE D 214     -36.566  38.866  35.117  1.00  0.00
D    H
ATOM  13612  HD3 ILE D 214     -35.843  39.999  36.317  1.00  0.00
D    H
ATOM  13613  C   ILE D 214     -34.252  41.736  31.529  1.00  0.00
D    C
ATOM  13614  O   ILE D 214     -33.439  42.666  31.542  1.00  0.00
D    O
ATOM  13615  N   LYS D 215     -34.086  40.663  30.751  1.00  0.00
D    N
ATOM  13616  HN  LYS D 215     -34.669  39.853  30.764  1.00  0.00
D    H
ATOM  13617  CA  LYS D 215     -32.989  40.618  29.794  1.00  0.00
D    C
ATOM  13618  HA  LYS D 215     -32.073  40.762  30.354  1.00  0.00
D    H
ATOM  13619  CB  LYS D 215     -32.967  39.271  29.068  1.00  0.00
D    C
ATOM  13620  HB1 LYS D 215     -32.937  38.478  29.850  1.00  0.00
D    H
ATOM  13621  HB2 LYS D 215     -33.929  39.137  28.524  1.00  0.00
D    H
ATOM  13622  CG  LYS D 215     -31.779  39.084  28.120  1.00  0.00
D    C
ATOM  13623  HG1 LYS D 215     -30.904  39.649  28.516  1.00  0.00
D    H
ATOM  13624  HG2 LYS D 215     -31.497  38.007  28.100  1.00  0.00
D    H
ATOM  13625  CD  LYS D 215     -32.098  39.579  26.712  1.00  0.00
D    C
ATOM  13626  HD1 LYS D 215     -33.195  39.480  26.544  1.00  0.00
D    H
ATOM  13627  HD2 LYS D 215     -31.849  40.664  26.661  1.00  0.00
D    H
ATOM  13628  CE  LYS D 215     -31.336  38.826  25.651  1.00  0.00
D    C
ATOM  13629  HE1 LYS D 215     -30.240  38.913  25.823  1.00  0.00
D    H
ATOM  13630  HE2 LYS D 215     -31.610  37.748  25.664  1.00  0.00
D    H
ATOM  13631  NZ  LYS D 215     -31.644  39.372  24.304  1.00  0.00
D    N
ATOM  13632  HZ1 LYS D 215     -31.114  38.849  23.578  1.00  0.00
D    H
ATOM  13633  HZ2 LYS D 215     -32.663  39.284  24.118  1.00  0.00
D    H
ATOM  13634  HZ3 LYS D 215     -31.376  40.376  24.269  1.00  0.00
D    H
```

ATOM 13635 C LYS D 215 −33.088 41.752 28.787 1.00 0.00
D C
ATOM 13636 O LYS D 215 −32.067 42.328 28.406 1.00 0.00
D O
ATOM 13637 N ARG D 216 −34.296 42.044 28.306 1.00 0.00
D N
ATOM 13638 HN ARG D 216 −35.121 41.552 28.575 1.00 0.00
D H
ATOM 13639 CA ARG D 216 −34.463 43.123 27.341 1.00 0.00
D C
ATOM 13640 HA ARG D 216 −33.833 42.918 26.484 1.00 0.00
D H
ATOM 13641 CB ARG D 216 −35.926 43.215 26.910 1.00 0.00
D C
ATOM 13642 HB1 ARG D 216 −36.526 43.290 27.848 1.00 0.00
D H
ATOM 13643 HB2 ARG D 216 −36.094 44.168 26.362 1.00 0.00
D H
ATOM 13644 CG ARG D 216 −36.397 42.014 26.113 1.00 0.00
D C
ATOM 13645 HG1 ARG D 216 −36.025 42.114 25.070 1.00 0.00
D H
ATOM 13646 HG2 ARG D 216 −35.910 41.097 26.518 1.00 0.00
D H
ATOM 13647 CD ARG D 216 −37.902 41.843 26.189 1.00 0.00
D C
ATOM 13648 HD1 ARG D 216 −38.264 42.182 27.187 1.00 0.00
D H
ATOM 13649 HD2 ARG D 216 −38.422 42.437 25.401 1.00 0.00
D H
ATOM 13650 NE ARG D 216 −38.287 40.449 25.986 1.00 0.00
D N
ATOM 13651 HE ARG D 216 −37.528 39.872 25.662 1.00 0.00
D H
ATOM 13652 CZ ARG D 216 −39.508 39.969 26.201 1.00 0.00
D C
ATOM 13653 NH1 ARG D 216 −40.475 40.772 26.619 1.00 0.00
D N
ATOM 13654 HH11 ARG D 216 −41.380 40.417 26.805 1.00 0.00
D H
ATOM 13655 HH12 ARG D 216 −40.203 41.707 26.861 1.00 0.00
D H
ATOM 13656 NH2 ARG D 216 −39.765 38.685 25.993 1.00 0.00
D N
ATOM 13657 HH21 ARG D 216 −40.693 38.348 26.069 1.00 0.00
D H
ATOM 13658 HH22 ARG D 216 −39.051 38.148 25.562 1.00 0.00
D H
ATOM 13659 C ARG D 216 −33.989 44.447 27.921 1.00 0.00
D C
ATOM 13660 O ARG D 216 −33.225 45.178 27.280 1.00 0.00
D O
ATOM 13661 N PHE D 217 −34.432 44.764 29.137 1.00 0.00
D N

ATOM 13662 HN PHE D 217 -35.066 44.218 29.682 1.00 0.00
D H
ATOM 13663 CA PHE D 217 -33.993 45.996 29.777 1.00 0.00
D C
ATOM 13664 HA PHE D 217 -34.201 46.795 29.075 1.00 0.00
D H
ATOM 13665 CB PHE D 217 -34.737 46.206 31.093 1.00 0.00
D C
ATOM 13666 HB1 PHE D 217 -35.700 46.723 30.887 1.00 0.00
D H
ATOM 13667 HB2 PHE D 217 -34.982 45.235 31.573 1.00 0.00
D H
ATOM 13668 CG PHE D 217 -33.997 47.071 32.067 1.00 0.00
D C
ATOM 13669 CD1 PHE D 217 -33.863 48.428 31.842 1.00 0.00
D C
ATOM 13670 HD1 PHE D 217 -34.294 48.868 30.953 1.00 0.00
D H
ATOM 13671 CE1 PHE D 217 -33.180 49.227 32.734 1.00 0.00
D C
ATOM 13672 HE1 PHE D 217 -33.085 50.287 32.548 1.00 0.00
D H
ATOM 13673 CZ PHE D 217 -32.624 48.672 33.865 1.00 0.00
D C
ATOM 13674 HZ PHE D 217 -32.096 49.295 34.571 1.00 0.00
D H
ATOM 13675 CD2 PHE D 217 -33.433 46.526 33.205 1.00 0.00
D C
ATOM 13676 HD2 PHE D 217 -33.506 45.463 33.389 1.00 0.00
D H
ATOM 13677 CE2 PHE D 217 -32.752 47.320 34.100 1.00 0.00
D C
ATOM 13678 HE2 PHE D 217 -32.316 46.880 34.987 1.00 0.00
D H
ATOM 13679 C PHE D 217 -32.488 45.986 30.008 1.00 0.00
D C
ATOM 13680 O PHE D 217 -31.823 47.013 29.837 1.00 0.00
D O
ATOM 13681 N GLU D 218 -31.933 44.834 30.385 1.00 0.00
D N
ATOM 13682 HN GLU D 218 -32.425 43.986 30.559 1.00 0.00
D H
ATOM 13683 CA GLU D 218 -30.491 44.731 30.575 1.00 0.00
D C
ATOM 13684 HA GLU D 218 -30.193 45.485 31.292 1.00 0.00
D H
ATOM 13685 CB GLU D 218 -30.137 43.341 31.102 1.00 0.00
D C
ATOM 13686 HB1 GLU D 218 -30.932 43.027 31.817 1.00 0.00
D H
ATOM 13687 HB2 GLU D 218 -30.167 42.603 30.269 1.00 0.00
D H
ATOM 13688 CG GLU D 218 -28.796 43.256 31.803 1.00 0.00
D C

ATOM 13689 HG1 GLU D 218 −28.532 44.247 32.216 1.00 0.00
D H
ATOM 13690 HG2 GLU D 218 −28.824 42.514 32.626 1.00 0.00
D H
ATOM 13691 CD GLU D 218 −27.678 42.887 30.846 1.00 0.00
D C
ATOM 13692 OE1 GLU D 218 −27.984 42.536 29.688 1.00 0.00
D O
ATOM 13693 OE2 GLU D 218 −26.498 42.943 31.246 1.00 0.00
D O
ATOM 13694 C GLU D 218 −29.740 45.015 29.284 1.00 0.00
D C
ATOM 13695 O GLU D 218 −28.726 45.722 29.285 1.00 0.00
D O
ATOM 13696 N ASN D 219 −30.225 44.474 28.172 1.00 0.00
D N
ATOM 13697 HN ASN D 219 −31.077 43.955 28.115 1.00 0.00
D H
ATOM 13698 CA ASN D 219 −29.515 44.608 26.909 1.00 0.00
D C
ATOM 13699 HA ASN D 219 −28.469 44.431 27.135 1.00 0.00
D H
ATOM 13700 CB ASN D 219 −30.051 43.590 25.903 1.00 0.00
D C
ATOM 13701 HB1 ASN D 219 −30.356 42.682 26.467 1.00 0.00
D H
ATOM 13702 HB2 ASN D 219 −30.955 43.986 25.397 1.00 0.00
D H
ATOM 13703 CG ASN D 219 −29.013 43.180 24.879 1.00 0.00
D C
ATOM 13704 OD1 ASN D 219 −27.894 43.691 24.873 1.00 0.00
D O
ATOM 13705 ND2 ASN D 219 −29.381 42.250 24.005 1.00 0.00
D N
ATOM 13706 HD21 ASN D 219 −28.706 41.962 23.332 1.00 0.00
D H
ATOM 13707 HD22 ASN D 219 −30.290 41.849 24.057 1.00 0.00
D H
ATOM 13708 C ASN D 219 −29.635 46.022 26.363 1.00 0.00
D C
ATOM 13709 O ASN D 219 −28.671 46.573 25.820 1.00 0.00
D O
ATOM 13710 N GLU D 220 −30.812 46.626 26.498 1.00 0.00
D N
ATOM 13711 HN GLU D 220 −31.587 46.267 27.011 1.00 0.00
D H
ATOM 13712 CA GLU D 220 −31.061 47.919 25.874 1.00 0.00
D C
ATOM 13713 HA GLU D 220 −30.404 47.996 25.018 1.00 0.00
D H
ATOM 13714 CB GLU D 220 −32.514 47.974 25.392 1.00 0.00
D C
ATOM 13715 HB1 GLU D 220 −33.175 48.015 26.288 1.00 0.00
D H

ATOM 13716 HB2 GLU D 220 −32.691 48.923 24.838 1.00 0.00
D H
ATOM 13717 CG GLU D 220 −32.886 46.770 24.533 1.00 0.00
D C
ATOM 13718 HG1 GLU D 220 −32.780 47.033 23.465 1.00 0.00
D H
ATOM 13719 HG2 GLU D 220 −32.236 45.903 24.763 1.00 0.00
D H
ATOM 13720 CD GLU D 220 −34.328 46.338 24.701 1.00 0.00
D C
ATOM 13721 OE1 GLU D 220 −34.927 46.642 25.753 1.00 0.00
D O
ATOM 13722 OE2 GLU D 220 −34.858 45.672 23.788 1.00 0.00
D O
ATOM 13723 C GLU D 220 −30.745 49.096 26.794 1.00 0.00
D C
ATOM 13724 O GLU D 220 −29.925 49.950 26.439 1.00 0.00
D O
ATOM 13725 N GLY D 221 −31.367 49.161 27.970 1.00 0.00
D N
ATOM 13726 HN GLY D 221 −32.016 48.467 28.278 1.00 0.00
D H
ATOM 13727 CA GLY D 221 −31.138 50.258 28.891 1.00 0.00
D C
ATOM 13728 HA1 GLY D 221 −31.887 50.170 29.666 1.00 0.00
D H
ATOM 13729 HA2 GLY D 221 −31.204 51.169 28.312 1.00 0.00
D H
ATOM 13730 C GLY D 221 −29.775 50.221 29.550 1.00 0.00
D C
ATOM 13731 O GLY D 221 −29.311 51.227 30.094 1.00 0.00
D O
ATOM 13732 N MET D 222 −29.127 49.062 29.517 1.00 0.00
D N
ATOM 13733 HN MET D 222 −29.494 48.229 29.111 1.00 0.00
D H
ATOM 13734 CA MET D 222 −27.796 48.917 30.088 1.00 0.00
D C
ATOM 13735 HA MET D 222 −27.417 49.913 30.278 1.00 0.00
D H
ATOM 13736 CB MET D 222 −27.851 48.112 31.383 1.00 0.00
D C
ATOM 13737 HB1 MET D 222 −28.195 47.086 31.114 1.00 0.00
D H
ATOM 13738 HB2 MET D 222 −26.822 47.998 31.791 1.00 0.00
D H
ATOM 13739 CG MET D 222 −28.784 48.684 32.431 1.00 0.00
D C
ATOM 13740 HG1 MET D 222 −28.583 49.766 32.578 1.00 0.00
D H
ATOM 13741 HG2 MET D 222 −29.838 48.581 32.090 1.00 0.00
D H
ATOM 13742 SD MET D 222 −28.596 47.855 34.020 1.00 0.00
D S

```
ATOM  13743  CE  MET D 222     -29.369  46.277  33.680  1.00  0.00
D    C
ATOM  13744  HE1 MET D 222     -29.350  45.625  34.579  1.00  0.00
D    H
ATOM  13745  HE2 MET D 222     -30.429  46.410  33.376  1.00  0.00
D    H
ATOM  13746  HE3 MET D 222     -28.843  45.743  32.861  1.00  0.00
D    H
ATOM  13747  C   MET D 222     -26.855  48.248  29.097  1.00  0.00
D    C
ATOM  13748  O   MET D 222     -25.774  48.761  28.815  1.00  0.00
D    O
ATOM  13749  N   LYS E   7     -34.167 -18.336  23.272  1.00  0.00
E    N
ATOM  13750  HT1 LYS E   7     -35.104 -18.607  23.053  1.00  0.00
E    H
ATOM  13751  HT2 LYS E   7     -33.626 -19.142  23.511  1.00  0.00
E    H
ATOM  13752  HT3 LYS E   7     -33.755 -17.869  22.490  1.00  0.00
E    H
ATOM  13753  CA  LYS E   7     -34.191 -17.425  24.410  1.00  0.00
E    C
ATOM  13754  HA  LYS E   7     -34.735 -16.539  24.106  1.00  0.00
E    H
ATOM  13755  CB  LYS E   7     -34.873 -18.091  25.606  1.00  0.00
E    C
ATOM  13756  HB1 LYS E   7     -34.742 -17.409  26.478  1.00  0.00
E    H
ATOM  13757  HB2 LYS E   7     -35.968 -18.147  25.414  1.00  0.00
E    H
ATOM  13758  CG  LYS E   7     -34.306 -19.456  25.944  1.00  0.00
E    C
ATOM  13759  HG1 LYS E   7     -34.368 -20.109  25.045  1.00  0.00
E    H
ATOM  13760  HG2 LYS E   7     -33.228 -19.348  26.203  1.00  0.00
E    H
ATOM  13761  CD  LYS E   7     -35.063 -20.111  27.087  1.00  0.00
E    C
ATOM  13762  HD1 LYS E   7     -34.434 -20.933  27.499  1.00  0.00
E    H
ATOM  13763  HD2 LYS E   7     -35.194 -19.354  27.894  1.00  0.00
E    H
ATOM  13764  CE  LYS E   7     -36.413 -20.641  26.636  1.00  0.00
E    C
ATOM  13765  HE1 LYS E   7     -37.094 -19.801  26.375  1.00  0.00
E    H
ATOM  13766  HE2 LYS E   7     -36.298 -21.293  25.742  1.00  0.00
E    H
ATOM  13767  NZ  LYS E   7     -37.075 -21.447  27.702  1.00  0.00
E    N
ATOM  13768  HZ1 LYS E   7     -37.996 -21.795  27.366  1.00  0.00
E    H
ATOM  13769  HZ2 LYS E   7     -36.471 -22.255  27.953  1.00  0.00
E    H
```

```
ATOM  13770  HZ3 LYS E   7      -37.218 -20.853  28.544  1.00  0.00
E    H
ATOM  13771  C   LYS E   7      -32.778 -16.990  24.775  1.00  0.00
E    C
ATOM  13772  O   LYS E   7      -31.862 -17.808  24.838  1.00  0.00
E    O
ATOM  13773  N   GLN E   8      -32.606 -15.700  25.048  1.00  0.00
E    N
ATOM  13774  HN  GLN E   8      -33.358 -15.063  25.205  1.00  0.00
E    H
ATOM  13775  CA  GLN E   8      -31.282 -15.103  25.141  1.00  0.00
E    C
ATOM  13776  HA  GLN E   8      -30.573 -15.863  24.833  1.00  0.00
E    H
ATOM  13777  CB  GLN E   8      -31.174 -13.905  24.208  1.00  0.00
E    C
ATOM  13778  HB1 GLN E   8      -32.126 -13.333  24.307  1.00  0.00
E    H
ATOM  13779  HB2 GLN E   8      -30.366 -13.228  24.569  1.00  0.00
E    H
ATOM  13780  CG  GLN E   8      -30.959 -14.348  22.781  1.00  0.00
E    C
ATOM  13781  HG1 GLN E   8      -29.876 -14.492  22.579  1.00  0.00
E    H
ATOM  13782  HG2 GLN E   8      -31.472 -15.312  22.586  1.00  0.00
E    H
ATOM  13783  CD  GLN E   8      -31.443 -13.337  21.778  1.00  0.00
E    C
ATOM  13784  OE1 GLN E   8      -32.261 -12.476  22.095  1.00  0.00
E    O
ATOM  13785  NE2 GLN E   8      -30.948 -13.437  20.551  1.00  0.00
E    N
ATOM  13786 HE21 GLN E   8      -31.261 -12.767  19.883  1.00  0.00
E    H
ATOM  13787 HE22 GLN E   8      -30.308 -14.167  20.334  1.00  0.00
E    H
ATOM  13788  C   GLN E   8      -30.943 -14.713  26.571  1.00  0.00
E    C
ATOM  13789  O   GLN E   8      -31.753 -14.093  27.274  1.00  0.00
E    O
ATOM  13790  N   PHE E   9      -29.731 -15.073  26.977  1.00  0.00
E    N
ATOM  13791  HN  PHE E   9      -29.059 -15.444  26.338  1.00  0.00
E    H
ATOM  13792  CA  PHE E   9      -29.264 -14.971  28.347  1.00  0.00
E    C
ATOM  13793  HA  PHE E   9      -30.032 -14.445  28.903  1.00  0.00
E    H
ATOM  13794  CB  PHE E   9      -28.953 -16.346  28.904  1.00  0.00
E    C
ATOM  13795  HB1 PHE E   9      -28.127 -16.799  28.314  1.00  0.00
E    H
ATOM  13796  HB2 PHE E   9      -28.605 -16.280  29.957  1.00  0.00
E    H
```

ATOM 13797 CG PHE E 9 -30.093 -17.291 28.818 1.00 0.00
E C
ATOM 13798 CD1 PHE E 9 -31.062 -17.303 29.795 1.00 0.00
E C
ATOM 13799 HD1 PHE E 9 -30.983 -16.625 30.635 1.00 0.00
E H
ATOM 13800 CE1 PHE E 9 -32.122 -18.166 29.724 1.00 0.00
E C
ATOM 13801 HE1 PHE E 9 -32.878 -18.159 30.496 1.00 0.00
E H
ATOM 13802 CZ PHE E 9 -32.225 -19.037 28.661 1.00 0.00
E C
ATOM 13803 HZ PHE E 9 -33.062 -19.718 28.602 1.00 0.00
E H
ATOM 13804 CD2 PHE E 9 -30.208 -18.163 27.749 1.00 0.00
E C
ATOM 13805 HD2 PHE E 9 -29.449 -18.175 26.979 1.00 0.00
E H
ATOM 13806 CE2 PHE E 9 -31.267 -19.035 27.669 1.00 0.00
E C
ATOM 13807 HE2 PHE E 9 -31.343 -19.714 26.831 1.00 0.00
E H
ATOM 13808 C PHE E 9 -28.014 -14.115 28.396 1.00 0.00
E C
ATOM 13809 O PHE E 9 -27.102 -14.283 27.582 1.00 0.00
E O
ATOM 13810 N ALA E 10 -27.970 -13.210 29.365 1.00 0.00
E N
ATOM 13811 HN ALA E 10 -28.721 -13.082 30.012 1.00 0.00
E H
ATOM 13812 CA ALA E 10 -26.822 -12.335 29.559 1.00 0.00
E C
ATOM 13813 HA ALA E 10 -26.005 -12.594 28.897 1.00 0.00
E H
ATOM 13814 CB ALA E 10 -27.194 -10.875 29.295 1.00 0.00
E C
ATOM 13815 HB1 ALA E 10 -27.525 -10.759 28.241 1.00 0.00
E H
ATOM 13816 HB2 ALA E 10 -28.030 -10.550 29.953 1.00 0.00
E H
ATOM 13817 HB3 ALA E 10 -26.330 -10.195 29.460 1.00 0.00
E H
ATOM 13818 C ALA E 10 -26.301 -12.514 30.974 1.00 0.00
E C
ATOM 13819 O ALA E 10 -26.961 -12.113 31.939 1.00 0.00
E O
ATOM 13820 N VAL E 11 -25.131 -13.115 31.102 1.00 0.00
E N
ATOM 13821 HN VAL E 11 -24.627 -13.516 30.342 1.00 0.00
E H
ATOM 13822 CA VAL E 11 -24.474 -13.242 32.390 1.00 0.00
E C
ATOM 13823 HA VAL E 11 -25.220 -13.237 33.176 1.00 0.00
E H

```
ATOM  13824  CB  VAL E  11     -23.643 -14.519  32.495  1.00  0.00
E    C
ATOM  13825  HB  VAL E  11     -23.568 -14.958  31.470  1.00  0.00
E    H
ATOM  13826  CG1 VAL E  11     -22.252 -14.216  33.005  1.00  0.00
E    C
ATOM  13827 HG11 VAL E  11     -21.668 -15.155  33.115  1.00  0.00
E    H
ATOM  13828 HG12 VAL E  11     -21.693 -13.559  32.308  1.00  0.00
E    H
ATOM  13829 HG13 VAL E  11     -22.298 -13.721  34.000  1.00  0.00
E    H
ATOM  13830  CG2 VAL E  11     -24.318 -15.490  33.423  1.00  0.00
E    C
ATOM  13831 HG21 VAL E  11     -23.706 -16.410  33.542  1.00  0.00
E    H
ATOM  13832 HG22 VAL E  11     -24.464 -15.034  34.426  1.00  0.00
E    H
ATOM  13833 HG23 VAL E  11     -25.311 -15.797  33.032  1.00  0.00
E    H
ATOM  13834  C   VAL E  11     -23.617 -12.002  32.567  1.00  0.00
E    C
ATOM  13835  O   VAL E  11     -22.858 -11.617  31.675  1.00  0.00
E    O
ATOM  13836  N   ILE E  12     -23.754 -11.362  33.713  1.00  0.00
E    N
ATOM  13837  HN  ILE E  12     -24.455 -11.586  34.388  1.00  0.00
E    H
ATOM  13838  CA  ILE E  12     -22.888 -10.264  34.098  1.00  0.00
E    C
ATOM  13839  HA  ILE E  12     -22.071 -10.218  33.390  1.00  0.00
E    H
ATOM  13840  CB  ILE E  12     -23.656  -8.945  34.182  1.00  0.00
E    C
ATOM  13841  HB  ILE E  12     -24.417  -9.115  34.984  1.00  0.00
E    H
ATOM  13842  CG2 ILE E  12     -22.691  -7.826  34.399  1.00  0.00
E    C
ATOM  13843 HG21 ILE E  12     -23.231  -6.881  34.619  1.00  0.00
E    H
ATOM  13844 HG22 ILE E  12     -22.023  -8.039  35.260  1.00  0.00
E    H
ATOM  13845 HG23 ILE E  12     -22.061  -7.663  33.498  1.00  0.00
E    H
ATOM  13846  CG1 ILE E  12     -24.420  -8.696  32.892  1.00  0.00
E    C
ATOM  13847 HG11 ILE E  12     -25.176  -9.499  32.742  1.00  0.00
E    H
ATOM  13848 HG12 ILE E  12     -24.975  -7.734  32.982  1.00  0.00
E    H
ATOM  13849  CD  ILE E  12     -23.543  -8.621  31.696  1.00  0.00
E    C
ATOM  13850  HD1 ILE E  12     -24.145  -8.381  30.792  1.00  0.00
E    H
```

ATOM 13851 HD2 ILE E 12   -22.774 -7.829 31.823 1.00 0.00
E   H
ATOM 13852 HD3 ILE E 12   -23.027 -9.588 31.519 1.00 0.00
E   H
ATOM 13853 C   ILE E 12   -22.284 -10.620 35.441 1.00 0.00
E   C
ATOM 13854 O   ILE E 12   -22.990 -11.063 36.353 1.00 0.00
E   O
ATOM 13855 N   GLY E 13   -20.978 -10.459 35.549 1.00 0.00
E   N
ATOM 13856 HN GLY E 13   -20.408 -10.109 34.806 1.00 0.00
E   H
ATOM 13857 CA GLY E 13   -20.282 -10.783 36.768 1.00 0.00
E   C
ATOM 13858 HA1 GLY E 13   -20.979 -10.765 37.594 1.00 0.00
E   H
ATOM 13859 HA2 GLY E 13   -19.434 -10.123 36.888 1.00 0.00
E   H
ATOM 13860 C   GLY E 13   -19.759 -12.188 36.629 1.00 0.00
E   C
ATOM 13861 O   GLY E 13   -20.516 -13.153 36.769 1.00 0.00
E   O
ATOM 13862 N   LEU E 14   -18.474 -12.326 36.343 1.00 0.00
E   N
ATOM 13863 HN LEU E 14   -17.806 -11.584 36.301 1.00 0.00
E   H
ATOM 13864 CA LEU E 14   -17.924 -13.642 36.057 1.00 0.00
E   C
ATOM 13865 HA LEU E 14   -18.764 -14.317 35.950 1.00 0.00
E   H
ATOM 13866 CB LEU E 14   -17.126 -13.595 34.768 1.00 0.00
E   C
ATOM 13867 HB1 LEU E 14   -17.019 -12.521 34.487 1.00 0.00
E   H
ATOM 13868 HB2 LEU E 14   -16.096 -13.965 34.967 1.00 0.00
E   H
ATOM 13869 CG LEU E 14   -17.796 -14.359 33.648 1.00 0.00
E   C
ATOM 13870 HG LEU E 14   -18.782 -14.741 34.009 1.00 0.00
E   H
ATOM 13871 CD1 LEU E 14   -18.019 -13.441 32.476 1.00 0.00
E   C
ATOM 13872 HD11 LEU E 14   -18.467 -13.996 31.625 1.00 0.00
E   H
ATOM 13873 HD12 LEU E 14   -18.707 -12.613 32.753 1.00 0.00
E   H
ATOM 13874 HD13 LEU E 14   -17.058 -13.000 32.135 1.00 0.00
E   H
ATOM 13875 CD2 LEU E 14   -16.940 -15.558 33.265 1.00 0.00
E   C
ATOM 13876 HD21 LEU E 14   -17.431 -16.140 32.454 1.00 0.00
E   H
ATOM 13877 HD22 LEU E 14   -15.941 -15.247 32.898 1.00 0.00
E   H

ATOM 13878 HD23 LEU E 14 −16.798 −16.230 34.138 1.00 0.00
E H
ATOM 13879 C LEU E 14 −17.079 −14.141 37.225 1.00 0.00
E C
ATOM 13880 O LEU E 14 −15.888 −14.408 37.104 1.00 0.00
E O
ATOM 13881 N GLY E 15 −17.725 −14.258 38.379 1.00 0.00
E N
ATOM 13882 HN GLY E 15 −18.653 −13.917 38.527 1.00 0.00
E H
ATOM 13883 CA GLY E 15 −17.107 −14.906 39.518 1.00 0.00
E C
ATOM 13884 HA1 GLY E 15 −17.597 −14.556 40.417 1.00 0.00
E H
ATOM 13885 HA2 GLY E 15 −16.043 −14.714 39.489 1.00 0.00
E H
ATOM 13886 C GLY E 15 −17.315 −16.399 39.425 1.00 0.00
E C
ATOM 13887 O GLY E 15 −18.002 −16.856 38.506 1.00 0.00
E O
ATOM 13888 N ARG E 16 −16.728 −17.167 40.351 1.00 0.00
E N
ATOM 13889 HN ARG E 16 −16.132 −16.798 41.060 1.00 0.00
E H
ATOM 13890 CA ARG E 16 −16.942 −18.609 40.360 1.00 0.00
E C
ATOM 13891 HA ARG E 16 −16.387 −19.041 39.536 1.00 0.00
E H
ATOM 13892 CB ARG E 16 −16.493 −19.212 41.687 1.00 0.00
E C
ATOM 13893 HB1 ARG E 16 −17.141 −18.759 42.474 1.00 0.00
E H
ATOM 13894 HB2 ARG E 16 −16.718 −20.301 41.695 1.00 0.00
E H
ATOM 13895 CG ARG E 16 −15.058 −18.946 42.032 1.00 0.00
E C
ATOM 13896 HG1 ARG E 16 −14.426 −19.713 41.532 1.00 0.00
E H
ATOM 13897 HG2 ARG E 16 −14.753 −17.970 41.588 1.00 0.00
E H
ATOM 13898 CD ARG E 16 −14.860 −18.893 43.534 1.00 0.00
E C
ATOM 13899 HD1 ARG E 16 −15.400 −19.744 44.009 1.00 0.00
E H
ATOM 13900 HD2 ARG E 16 −13.781 −18.959 43.807 1.00 0.00
E H
ATOM 13901 NE ARG E 16 −15.351 −17.648 44.113 1.00 0.00
E N
ATOM 13902 HE ARG E 16 −15.819 −17.058 43.445 1.00 0.00
E H
ATOM 13903 CZ ARG E 16 −15.203 −17.314 45.392 1.00 0.00
E C
ATOM 13904 NH1 ARG E 16 −14.573 −18.133 46.221 1.00 0.00
E N

```
ATOM  13905 HH11 ARG E  16      -14.479 -17.905  47.181  1.00  0.00
E    H
ATOM  13906 HH12 ARG E  16      -14.328 -19.033  45.855  1.00  0.00
E    H
ATOM  13907  NH2 ARG E  16      -15.676 -16.159  45.840  1.00  0.00
E    N
ATOM  13908 HH21 ARG E  16      -15.488 -15.875  46.769  1.00  0.00
E    H
ATOM  13909 HH22 ARG E  16      -16.025 -15.524  45.162  1.00  0.00
E    H
ATOM  13910  C   ARG E  16      -18.398 -18.949  40.126  1.00  0.00
E    C
ATOM  13911  O   ARG E  16      -18.710 -19.827  39.314  1.00  0.00
E    O
ATOM  13912  N   PHE E  17      -19.292 -18.230  40.793  1.00  0.00
E    N
ATOM  13913  HN  PHE E  17      -19.073 -17.549  41.490  1.00  0.00
E    H
ATOM  13914  CA  PHE E  17      -20.710 -18.396  40.539  1.00  0.00
E    C
ATOM  13915  HA  PHE E  17      -20.947 -19.426  40.775  1.00  0.00
E    H
ATOM  13916  CB  PHE E  17      -21.498 -17.416  41.399  1.00  0.00
E    C
ATOM  13917  HB1 PHE E  17      -21.245 -17.585  42.468  1.00  0.00
E    H
ATOM  13918  HB2 PHE E  17      -21.227 -16.366  41.159  1.00  0.00
E    H
ATOM  13919  CG  PHE E  17      -22.970 -17.581  41.291  1.00  0.00
E    C
ATOM  13920  CD1 PHE E  17      -23.546 -18.825  41.455  1.00  0.00
E    C
ATOM  13921  HD1 PHE E  17      -22.919 -19.683  41.653  1.00  0.00
E    H
ATOM  13922  CE1 PHE E  17      -24.905 -18.986  41.364  1.00  0.00
E    C
ATOM  13923  HE1 PHE E  17      -25.343 -19.965  41.494  1.00  0.00
E    H
ATOM  13924  CZ  PHE E  17      -25.705 -17.896  41.112  1.00  0.00
E    C
ATOM  13925  HZ  PHE E  17      -26.776 -18.017  41.049  1.00  0.00
E    H
ATOM  13926  CD2 PHE E  17      -23.783 -16.494  41.042  1.00  0.00
E    C
ATOM  13927  HD2 PHE E  17      -23.347 -15.515  40.894  1.00  0.00
E    H
ATOM  13928  CE2 PHE E  17      -25.141 -16.647  40.952  1.00  0.00
E    C
ATOM  13929  HE2 PHE E  17      -25.768 -15.789  40.754  1.00  0.00
E    H
ATOM  13930  C   PHE E  17      -21.015 -18.196  39.063  1.00  0.00
E    C
ATOM  13931  O   PHE E  17      -21.553 -19.093  38.405  1.00  0.00
E    O
```

ATOM 13932 N GLY E 18 -20.657 -17.031 38.527 1.00 0.00
E N
ATOM 13933 HN GLY E 18 -20.196 -16.295 39.021 1.00 0.00
E H
ATOM 13934 CA GLY E 18 -20.937 -16.767 37.127 1.00 0.00
E C
ATOM 13935 HA1 GLY E 18 -20.439 -15.842 36.872 1.00 0.00
E H
ATOM 13936 HA2 GLY E 18 -22.012 -16.739 37.012 1.00 0.00
E H
ATOM 13937 C GLY E 18 -20.398 -17.854 36.219 1.00 0.00
E C
ATOM 13938 O GLY E 18 -21.155 -18.497 35.482 1.00 0.00
E O
ATOM 13939 N GLY E 19 -19.092 -18.105 36.305 1.00 0.00
E N
ATOM 13940 HN GLY E 19 -18.486 -17.661 36.963 1.00 0.00
E H
ATOM 13941 CA GLY E 19 -18.461 -19.066 35.419 1.00 0.00
E C
ATOM 13942 HA1 GLY E 19 -17.447 -19.195 35.773 1.00 0.00
E H
ATOM 13943 HA2 GLY E 19 -18.533 -18.665 34.417 1.00 0.00
E H
ATOM 13944 C GLY E 19 -19.134 -20.419 35.429 1.00 0.00
E C
ATOM 13945 O GLY E 19 -19.284 -21.051 34.382 1.00 0.00
E O
ATOM 13946 N SER E 20 -19.556 -20.873 36.602 1.00 0.00
E N
ATOM 13947 HN SER E 20 -19.394 -20.426 37.482 1.00 0.00
E H
ATOM 13948 CA SER E 20 -20.317 -22.105 36.693 1.00 0.00
E C
ATOM 13949 HA SER E 20 -19.666 -22.886 36.323 1.00 0.00
E H
ATOM 13950 CB SER E 20 -20.785 -22.297 38.125 1.00 0.00
E C
ATOM 13951 HB1 SER E 20 -21.373 -21.399 38.420 1.00 0.00
E H
ATOM 13952 HB2 SER E 20 -21.467 -23.174 38.196 1.00 0.00
E H
ATOM 13953 OG SER E 20 -19.674 -22.427 38.972 1.00 0.00
E O
ATOM 13954 HG1 SER E 20 -19.994 -22.321 39.876 1.00 0.00
E H
ATOM 13955 C SER E 20 -21.529 -22.067 35.793 1.00 0.00
E C
ATOM 13956 O SER E 20 -21.701 -22.940 34.930 1.00 0.00
E O
ATOM 13957 N ILE E 21 -22.366 -21.058 36.004 1.00 0.00
E N
ATOM 13958 HN ILE E 21 -22.223 -20.345 36.688 1.00 0.00
E H

```
ATOM  13959  CA  ILE E  21     -23.589 -20.909  35.237  1.00  0.00
E    C
ATOM  13960  HA  ILE E  21     -24.207 -21.768  35.466  1.00  0.00
E    H
ATOM  13961  CB  ILE E  21     -24.264 -19.584  35.605  1.00  0.00
E    C
ATOM  13962  HB  ILE E  21     -23.572 -18.798  35.210  1.00  0.00
E    H
ATOM  13963  CG2 ILE E  21     -25.646 -19.527  35.034  1.00  0.00
E    C
ATOM  13964 HG21 ILE E  21     -26.080 -18.513  35.162  1.00  0.00
E    H
ATOM  13965 HG22 ILE E  21     -25.635 -19.752  33.947  1.00  0.00
E    H
ATOM  13966 HG23 ILE E  21     -26.314 -20.257  35.537  1.00  0.00
E    H
ATOM  13967  CG1 ILE E  21     -24.293 -19.440  37.117  1.00  0.00
E    C
ATOM  13968 HG11 ILE E  21     -24.900 -20.260  37.562  1.00  0.00
E    H
ATOM  13969 HG12 ILE E  21     -23.256 -19.548  37.510  1.00  0.00
E    H
ATOM  13970  CD  ILE E  21     -24.832 -18.127  37.594  1.00  0.00
E    C
ATOM  13971  HD1 ILE E  21     -24.772 -18.065  38.702  1.00  0.00
E    H
ATOM  13972  HD2 ILE E  21     -24.247 -17.285  37.166  1.00  0.00
E    H
ATOM  13973  HD3 ILE E  21     -25.895 -18.003  37.300  1.00  0.00
E    H
ATOM  13974  C   ILE E  21     -23.291 -20.967  33.755  1.00  0.00
E    C
ATOM  13975  O   ILE E  21     -23.897 -21.744  33.009  1.00  0.00
E    O
ATOM  13976  N   VAL E  22     -22.363 -20.130  33.302  1.00  0.00
E    N
ATOM  13977  HN  VAL E  22     -21.752 -19.562  33.846  1.00  0.00
E    H
ATOM  13978  CA  VAL E  22     -22.186 -19.999  31.864  1.00  0.00
E    C
ATOM  13979  HA  VAL E  22     -23.167 -19.869  31.425  1.00  0.00
E    H
ATOM  13980  CB  VAL E  22     -21.327 -18.773  31.529  1.00  0.00
E    C
ATOM  13981  HB  VAL E  22     -20.846 -18.959  30.537  1.00  0.00
E    H
ATOM  13982  CG1 VAL E  22     -22.187 -17.548  31.444  1.00  0.00
E    C
ATOM  13983 HG11 VAL E  22     -21.562 -16.652  31.240  1.00  0.00
E    H
ATOM  13984 HG12 VAL E  22     -22.931 -17.626  30.625  1.00  0.00
E    H
ATOM  13985 HG13 VAL E  22     -22.727 -17.380  32.400  1.00  0.00
E    H
```

ATOM 13986 CG2 VAL E 22   -20.267 -18.594 32.547 1.00 0.00
E   C
ATOM 13987 HG21 VAL E 22   -19.665 -17.685 32.332 1.00 0.00
E   H
ATOM 13988 HG22 VAL E 22   -20.711 -18.488 33.560 1.00 0.00
E   H
ATOM 13989 HG23 VAL E 22   -19.572 -19.460 32.560 1.00 0.00
E   H
ATOM 13990 C   VAL E 22   -21.589 -21.263 31.276 1.00 0.00
E   C
ATOM 13991 O   VAL E 22   -21.997 -21.707 30.202 1.00 0.00
E   O
ATOM 13992 N   LYS E 23   -20.609 -21.858 31.952 1.00 0.00
E   N
ATOM 13993 HN LYS E 23   -20.255 -21.568 32.840 1.00 0.00
E   H
ATOM 13994 CA LYS E 23   -19.970 -23.035 31.389 1.00 0.00
E   C
ATOM 13995 HA LYS E 23   -19.675 -22.792 30.375 1.00 0.00
E   H
ATOM 13996 CB LYS E 23   -18.748 -23.410 32.217 1.00 0.00
E   C
ATOM 13997 HB1 LYS E 23   -18.204 -22.465 32.446 1.00 0.00
E   H
ATOM 13998 HB2 LYS E 23   -19.086 -23.821 33.196 1.00 0.00
E   H
ATOM 13999 CG LYS E 23   -17.810 -24.370 31.522 1.00 0.00
E   C
ATOM 14000 HG1 LYS E 23   -18.297 -24.748 30.594 1.00 0.00
E   H
ATOM 14001 HG2 LYS E 23   -16.891 -23.820 31.215 1.00 0.00
E   H
ATOM 14002 CD LYS E 23   -17.450 -25.546 32.403 1.00 0.00
E   C
ATOM 14003 HD1 LYS E 23   -17.783 -25.321 33.443 1.00 0.00
E   H
ATOM 14004 HD2 LYS E 23   -18.028 -26.432 32.053 1.00 0.00
E   H
ATOM 14005 CE LYS E 23   -15.949 -25.805 32.337 1.00 0.00
E   C
ATOM 14006 HE1 LYS E 23   -15.641 -26.049 31.296 1.00 0.00
E   H
ATOM 14007 HE2 LYS E 23   -15.383 -24.907 32.671 1.00 0.00
E   H
ATOM 14008 NZ LYS E 23   -15.511 -26.939 33.197 1.00 0.00
E   N
ATOM 14009 HZ1 LYS E 23   -14.482 -27.073 33.113 1.00 0.00
E   H
ATOM 14010 HZ2 LYS E 23   -15.754 -26.738 34.188 1.00 0.00
E   H
ATOM 14011 HZ3 LYS E 23   -15.996 -27.809 32.895 1.00 0.00
E   H
ATOM 14012 C   LYS E 23   -20.955 -24.183 31.311 1.00 0.00
E   C

```
ATOM  14013  O   LYS E  23      -20.971 -24.932  30.327  1.00  0.00
E    O
ATOM  14014  N   GLU E  24      -21.830 -24.301  32.310  1.00  0.00
E    N
ATOM  14015  HN  GLU E  24      -21.855 -23.755  33.143  1.00  0.00
E    H
ATOM  14016  CA  GLU E  24      -22.875 -25.310  32.233  1.00  0.00
E    C
ATOM  14017  HA  GLU E  24      -22.412 -26.270  32.046  1.00  0.00
E    H
ATOM  14018  CB  GLU E  24      -23.661 -25.332  33.544  1.00  0.00
E    C
ATOM  14019  HB1 GLU E  24      -22.935 -25.439  34.382  1.00  0.00
E    H
ATOM  14020  HB2 GLU E  24      -24.164 -24.351  33.699  1.00  0.00
E    H
ATOM  14021  CG  GLU E  24      -24.702 -26.434  33.693  1.00  0.00
E    C
ATOM  14022  HG1 GLU E  24      -24.202 -27.419  33.660  1.00  0.00
E    H
ATOM  14023  HG2 GLU E  24      -25.251 -26.336  34.650  1.00  0.00
E    H
ATOM  14024  CD  GLU E  24      -25.733 -26.487  32.581  1.00  0.00
E    C
ATOM  14025  OE1 GLU E  24      -26.428 -25.486  32.333  1.00  0.00
E    O
ATOM  14026  OE2 GLU E  24      -25.823 -27.548  31.936  1.00  0.00
E    O
ATOM  14027  C   GLU E  24      -23.807 -25.022  31.062  1.00  0.00
E    C
ATOM  14028  O   GLU E  24      -24.022 -25.876  30.195  1.00  0.00
E    O
ATOM  14029  N   LEU E  25      -24.404 -23.834  31.047  1.00  0.00
E    N
ATOM  14030  HN  LEU E  25      -24.235 -23.101  31.704  1.00  0.00
E    H
ATOM  14031  CA  LEU E  25      -25.390 -23.507  30.026  1.00  0.00
E    C
ATOM  14032  HA  LEU E  25      -26.192 -24.227  30.124  1.00  0.00
E    H
ATOM  14033  CB  LEU E  25      -25.891 -22.087  30.256  1.00  0.00
E    C
ATOM  14034  HB1 LEU E  25      -25.331 -21.678  31.128  1.00  0.00
E    H
ATOM  14035  HB2 LEU E  25      -25.607 -21.457  29.385  1.00  0.00
E    H
ATOM  14036  CG  LEU E  25      -27.380 -22.010  30.531  1.00  0.00
E    C
ATOM  14037  HG  LEU E  25      -27.780 -23.025  30.768  1.00  0.00
E    H
ATOM  14038  CD1 LEU E  25      -27.644 -21.075  31.671  1.00  0.00
E    C
ATOM  14039 HD11 LEU E  25      -28.736 -20.975  31.848  1.00  0.00
E    H
```

```
ATOM  14040 HD12 LEU E  25      -27.173 -21.452  32.604  1.00  0.00
E    H
ATOM  14041 HD13 LEU E  25      -27.234 -20.066  31.454  1.00  0.00
E    H
ATOM  14042  CD2 LEU E  25      -28.080 -21.509  29.291  1.00  0.00
E    C
ATOM  14043 HD21 LEU E  25      -29.177 -21.460  29.460  1.00  0.00
E    H
ATOM  14044 HD22 LEU E  25      -27.737 -20.493  29.010  1.00  0.00
E    H
ATOM  14045 HD23 LEU E  25      -27.889 -22.192  28.435  1.00  0.00
E    H
ATOM  14046  C   LEU E  25      -24.803 -23.663  28.636  1.00  0.00
E    C
ATOM  14047  O   LEU E  25      -25.475 -24.124  27.707  1.00  0.00
E    O
ATOM  14048  N   HSE E  26      -23.546 -23.273  28.484  1.00  0.00
E    N
ATOM  14049  HN  HSE E  26      -23.039 -22.774  29.186  1.00  0.00
E    H
ATOM  14050  CA  HSE E  26      -22.789 -23.538  27.276  1.00  0.00
E    C
ATOM  14051  HA  HSE E  26      -23.315 -23.061  26.458  1.00  0.00
E    H
ATOM  14052  CB  HSE E  26      -21.380 -22.993  27.471  1.00  0.00
E    C
ATOM  14053  HB1 HSE E  26      -21.465 -21.926  27.768  1.00  0.00
E    H
ATOM  14054  HB2 HSE E  26      -20.889 -23.527  28.311  1.00  0.00
E    H
ATOM  14055  ND1 HSE E  26      -19.584 -24.045  26.066  1.00  0.00
E    N
ATOM  14056  CG  HSE E  26      -20.523 -23.058  26.255  1.00  0.00
E    C
ATOM  14057  CE1 HSE E  26      -18.962 -23.839  24.920  1.00  0.00
E    C
ATOM  14058  HE1 HSE E  26      -18.171 -24.479  24.521  1.00  0.00
E    H
ATOM  14059  NE2 HSE E  26      -19.455 -22.746  24.369  1.00  0.00
E    N
ATOM  14060  HE2 HSE E  26      -19.148 -22.349  23.505  1.00  0.00
E    H
ATOM  14061  CD2 HSE E  26      -20.431 -22.235  25.186  1.00  0.00
E    C
ATOM  14062  HD2 HSE E  26      -20.976 -21.330  24.959  1.00  0.00
E    H
ATOM  14063  C   HSE E  26      -22.759 -25.024  26.979  1.00  0.00
E    C
ATOM  14064  O   HSE E  26      -22.960 -25.439  25.834  1.00  0.00
E    O
ATOM  14065  N   ARG E  27      -22.512 -25.841  28.000  1.00  0.00
E    N
ATOM  14066  HN  ARG E  27      -22.258 -25.530  28.913  1.00  0.00
E    H
```

```
ATOM  14067  CA  ARG E  27      -22.609 -27.278  27.803  1.00  0.00
E    C
ATOM  14068  HA  ARG E  27      -21.956 -27.558  26.984  1.00  0.00
E    H
ATOM  14069  CB  ARG E  27      -22.188 -28.014  29.069  1.00  0.00
E    C
ATOM  14070  HB1 ARG E  27      -21.301 -27.469  29.470  1.00  0.00
E    H
ATOM  14071  HB2 ARG E  27      -22.984 -27.913  29.840  1.00  0.00
E    H
ATOM  14072  CG  ARG E  27      -21.817 -29.455  28.830  1.00  0.00
E    C
ATOM  14073  HG1 ARG E  27      -22.068 -29.714  27.778  1.00  0.00
E    H
ATOM  14074  HG2 ARG E  27      -20.711 -29.562  28.908  1.00  0.00
E    H
ATOM  14075  CD  ARG E  27      -22.476 -30.368  29.843  1.00  0.00
E    C
ATOM  14076  HD1 ARG E  27      -22.441 -31.419  29.473  1.00  0.00
E    H
ATOM  14077  HD2 ARG E  27      -21.962 -30.320  30.832  1.00  0.00
E    H
ATOM  14078  NE  ARG E  27      -23.868 -30.013  30.095  1.00  0.00
E    N
ATOM  14079  HE  ARG E  27      -24.018 -29.628  31.013  1.00  0.00
E    H
ATOM  14080  CZ  ARG E  27      -24.854 -30.171  29.220  1.00  0.00
E    C
ATOM  14081  NH1 ARG E  27      -24.612 -30.671  28.018  1.00  0.00
E    N
ATOM  14082 HH11 ARG E  27      -25.342 -30.766  27.354  1.00  0.00
E    H
ATOM  14083 HH12 ARG E  27      -23.648 -30.810  27.781  1.00  0.00
E    H
ATOM  14084  NH2 ARG E  27      -26.087 -29.828  29.545  1.00  0.00
E    N
ATOM  14085 HH21 ARG E  27      -26.834 -30.027  28.926  1.00  0.00
E    H
ATOM  14086 HH22 ARG E  27      -26.247 -29.573  30.489  1.00  0.00
E    H
ATOM  14087  C   ARG E  27      -24.018 -27.675  27.392  1.00  0.00
E    C
ATOM  14088  O   ARG E  27      -24.199 -28.652  26.659  1.00  0.00
E    O
ATOM  14089  N   MET E  28      -25.031 -26.930  27.838  1.00  0.00
E    N
ATOM  14090  HN  MET E  28      -24.979 -26.182  28.495  1.00  0.00
E    H
ATOM  14091  CA  MET E  28      -26.372 -27.202  27.341  1.00  0.00
E    C
ATOM  14092  HA  MET E  28      -26.487 -28.279  27.339  1.00  0.00
E    H
ATOM  14093  CB  MET E  28      -27.438 -26.561  28.220  1.00  0.00
E    C
```

ATOM 14094 HB1 MET E 28 -27.184 -25.478 28.300 1.00 0.00
E H
ATOM 14095 HB2 MET E 28 -28.422 -26.613 27.702 1.00 0.00
E H
ATOM 14096 CG MET E 28 -27.528 -27.139 29.586 1.00 0.00
E C
ATOM 14097 HG1 MET E 28 -27.634 -28.243 29.532 1.00 0.00
E H
ATOM 14098 HG2 MET E 28 -26.595 -26.917 30.149 1.00 0.00
E H
ATOM 14099 SD MET E 28 -28.918 -26.507 30.520 1.00 0.00
E S
ATOM 14100 CE MET E 28 -30.242 -27.359 29.672 1.00 0.00
E C
ATOM 14101 HE1 MET E 28 -31.229 -27.088 30.105 1.00 0.00
E H
ATOM 14102 HE2 MET E 28 -30.260 -27.095 28.593 1.00 0.00
E H
ATOM 14103 HE3 MET E 28 -30.125 -28.461 29.753 1.00 0.00
E H
ATOM 14104 C MET E 28 -26.572 -26.716 25.919 1.00 0.00
E C
ATOM 14105 O MET E 28 -27.661 -26.901 25.370 1.00 0.00
E O
ATOM 14106 N GLY E 29 -25.565 -26.095 25.320 1.00 0.00
E N
ATOM 14107 HN GLY E 29 -24.685 -25.909 25.756 1.00 0.00
E H
ATOM 14108 CA GLY E 29 -25.693 -25.637 23.955 1.00 0.00
E C
ATOM 14109 HA1 GLY E 29 -26.070 -26.466 23.373 1.00 0.00
E H
ATOM 14110 HA2 GLY E 29 -24.723 -25.277 23.640 1.00 0.00
E H
ATOM 14111 C GLY E 29 -26.675 -24.497 23.834 1.00 0.00
E C
ATOM 14112 O GLY E 29 -27.648 -24.582 23.082 1.00 0.00
E O
ATOM 14113 N HSE E 30 -26.445 -23.430 24.583 1.00 0.00
E N
ATOM 14114 HN HSE E 30 -25.677 -23.335 25.214 1.00 0.00
E H
ATOM 14115 CA HSE E 30 -27.327 -22.282 24.538 1.00 0.00
E C
ATOM 14116 HA HSE E 30 -28.088 -22.493 23.796 1.00 0.00
E H
ATOM 14117 CB HSE E 30 -27.960 -22.042 25.901 1.00 0.00
E C
ATOM 14118 HB1 HSE E 30 -27.159 -22.115 26.668 1.00 0.00
E H
ATOM 14119 HB2 HSE E 30 -28.361 -21.008 25.947 1.00 0.00
E H
ATOM 14120 ND1 HSE E 30 -30.314 -22.658 26.546 1.00 0.00
E N

```
ATOM  14121  CG  HSE E  30      -29.023 -23.030  26.248  1.00  0.00
E    C
ATOM  14122  CE1 HSE E  30      -31.029 -23.736  26.809  1.00  0.00
E    C
ATOM  14123  HE1 HSE E  30      -32.085 -23.728  27.088  1.00  0.00
E    H
ATOM  14124  NE2 HSE E  30      -30.249 -24.795  26.682  1.00  0.00
E    N
ATOM  14125  HE2 HSE E  30      -30.530 -25.741  26.841  1.00  0.00
E    H
ATOM  14126  CD2 HSE E  30      -28.990 -24.380  26.325  1.00  0.00
E    C
ATOM  14127  HD2 HSE E  30      -28.169 -25.064  26.164  1.00  0.00
E    H
ATOM  14128  C   HSE E  30      -26.580 -21.043  24.071  1.00  0.00
E    C
ATOM  14129  O   HSE E  30      -25.360 -21.035  23.899  1.00  0.00
E    O
ATOM  14130  N   GLU E  31      -27.343 -19.982  23.863  1.00  0.00
E    N
ATOM  14131  HN  GLU E  31      -28.332 -20.000  23.982  1.00  0.00
E    H
ATOM  14132  CA  GLU E  31      -26.821 -18.691  23.444  1.00  0.00
E    C
ATOM  14133  HA  GLU E  31      -25.852 -18.823  22.979  1.00  0.00
E    H
ATOM  14134  CB  GLU E  31      -27.782 -18.036  22.454  1.00  0.00
E    C
ATOM  14135  HB1 GLU E  31      -27.364 -17.043  22.168  1.00  0.00
E    H
ATOM  14136  HB2 GLU E  31      -27.821 -18.633  21.515  1.00  0.00
E    H
ATOM  14137  CG  GLU E  31      -29.215 -17.817  22.959  1.00  0.00
E    C
ATOM  14138  HG1 GLU E  31      -29.185 -17.406  23.985  1.00  0.00
E    H
ATOM  14139  HG2 GLU E  31      -29.766 -17.117  22.300  1.00  0.00
E    H
ATOM  14140  CD  GLU E  31      -30.031 -19.102  23.065  1.00  0.00
E    C
ATOM  14141  OE1 GLU E  31      -31.154 -19.138  22.523  1.00  0.00
E    O
ATOM  14142  OE2 GLU E  31      -29.574 -20.063  23.723  1.00  0.00
E    O
ATOM  14143  C   GLU E  31      -26.611 -17.846  24.693  1.00  0.00
E    C
ATOM  14144  O   GLU E  31      -27.574 -17.438  25.350  1.00  0.00
E    O
ATOM  14145  N   VAL E  32      -25.354 -17.610  25.043  1.00  0.00
E    N
ATOM  14146  HN  VAL E  32      -24.567 -17.902  24.506  1.00  0.00
E    H
ATOM  14147  CA  VAL E  32      -25.017 -16.893  26.262  1.00  0.00
E    C
```

```
ATOM  14148  HA  VAL E  32     -25.894 -16.351  26.594  1.00  0.00
E    H
ATOM  14149  CB  VAL E  32     -24.568 -17.849  27.380  1.00  0.00
E    C
ATOM  14150  HB  VAL E  32     -24.233 -17.225  28.244  1.00  0.00
E    H
ATOM  14151  CG1 VAL E  32     -25.729 -18.724  27.807  1.00  0.00
E    C
ATOM  14152 HG11 VAL E  32     -25.402 -19.445  28.587  1.00  0.00
E    H
ATOM  14153 HG12 VAL E  32     -26.557 -18.123  28.237  1.00  0.00
E    H
ATOM  14154 HG13 VAL E  32     -26.121 -19.304  26.945  1.00  0.00
E    H
ATOM  14155  CG2 VAL E  32     -23.426 -18.721  26.910  1.00  0.00
E    C
ATOM  14156 HG21 VAL E  32     -23.128 -19.439  27.704  1.00  0.00
E    H
ATOM  14157 HG22 VAL E  32     -23.721 -19.299  26.008  1.00  0.00
E    H
ATOM  14158 HG23 VAL E  32     -22.533 -18.111  26.657  1.00  0.00
E    H
ATOM  14159  C   VAL E  32     -23.938 -15.869  25.952  1.00  0.00
E    C
ATOM  14160  O   VAL E  32     -22.877 -16.209  25.419  1.00  0.00
E    O
ATOM  14161  N   LEU E  33     -24.219 -14.617  26.272  1.00  0.00
E    N
ATOM  14162  HN  LEU E  33     -25.108 -14.323  26.619  1.00  0.00
E    H
ATOM  14163  CA  LEU E  33     -23.247 -13.547  26.147  1.00  0.00
E    C
ATOM  14164  HA  LEU E  33     -22.523 -13.840  25.396  1.00  0.00
E    H
ATOM  14165  CB  LEU E  33     -23.916 -12.229  25.777  1.00  0.00
E    C
ATOM  14166  HB1 LEU E  33     -24.154 -12.275  24.690  1.00  0.00
E    H
ATOM  14167  HB2 LEU E  33     -24.894 -12.160  26.304  1.00  0.00
E    H
ATOM  14168  CG  LEU E  33     -23.021 -11.027  26.068  1.00  0.00
E    C
ATOM  14169  HG  LEU E  33     -22.768 -11.060  27.156  1.00  0.00
E    H
ATOM  14170  CD1 LEU E  33     -21.740 -11.095  25.246  1.00  0.00
E    C
ATOM  14171 HD11 LEU E  33     -21.113 -10.196  25.425  1.00  0.00
E    H
ATOM  14172 HD12 LEU E  33     -21.146 -11.994  25.519  1.00  0.00
E    H
ATOM  14173 HD13 LEU E  33     -21.972 -11.146  24.161  1.00  0.00
E    H
ATOM  14174  CD2 LEU E  33     -23.769  -9.738  25.800  1.00  0.00
E    C
```

```
ATOM  14175 HD21 LEU E  33      -23.124  -8.862  26.030  1.00  0.00
E    H
ATOM  14176 HD22 LEU E  33      -24.080  -9.658  24.739  1.00  0.00
E    H
ATOM  14177 HD23 LEU E  33      -24.678  -9.676  26.437  1.00  0.00
E    H
ATOM  14178  C   LEU E  33      -22.509 -13.414  27.467  1.00  0.00
E    C
ATOM  14179  O   LEU E  33      -23.129 -13.182  28.510  1.00  0.00
E    O
ATOM  14180  N   ALA E  34      -21.194 -13.555  27.422  1.00  0.00
E    N
ATOM  14181  HN  ALA E  34      -20.699 -13.768  26.581  1.00  0.00
E    H
ATOM  14182  CA  ALA E  34      -20.372 -13.410  28.609  1.00  0.00
E    C
ATOM  14183  HA  ALA E  34      -20.949 -13.548  29.517  1.00  0.00
E    H
ATOM  14184  CB  ALA E  34      -19.229 -14.419  28.606  1.00  0.00
E    C
ATOM  14185  HB1 ALA E  34      -19.642 -15.450  28.622  1.00  0.00
E    H
ATOM  14186  HB2 ALA E  34      -18.607 -14.315  27.690  1.00  0.00
E    H
ATOM  14187  HB3 ALA E  34      -18.573 -14.298  29.494  1.00  0.00
E    H
ATOM  14188  C   ALA E  34      -19.834 -11.991  28.655  1.00  0.00
E    C
ATOM  14189  O   ALA E  34      -19.345 -11.478  27.644  1.00  0.00
E    O
ATOM  14190  N   VAL E  35      -19.945 -11.353  29.814  1.00  0.00
E    N
ATOM  14191  HN  VAL E  35      -20.424 -11.744  30.597  1.00  0.00
E    H
ATOM  14192  CA  VAL E  35      -19.383 -10.031  30.051  1.00  0.00
E    C
ATOM  14193  HA  VAL E  35      -18.605  -9.851  29.321  1.00  0.00
E    H
ATOM  14194  CB  VAL E  35      -20.448  -8.921  29.969  1.00  0.00
E    C
ATOM  14195  HB  VAL E  35      -21.194  -9.107  30.780  1.00  0.00
E    H
ATOM  14196  CG1 VAL E  35      -19.810  -7.558  30.178  1.00  0.00
E    C
ATOM  14197 HG11 VAL E  35      -20.573  -6.756  30.079  1.00  0.00
E    H
ATOM  14198 HG12 VAL E  35      -19.362  -7.466  31.189  1.00  0.00
E    H
ATOM  14199 HG13 VAL E  35      -19.018  -7.374  29.420  1.00  0.00
E    H
ATOM  14200  CG2 VAL E  35      -21.180  -8.981  28.650  1.00  0.00
E    C
ATOM  14201 HG21 VAL E  35      -21.920  -8.155  28.574  1.00  0.00
E    H
```

```
ATOM  14202 HG22 VAL E  35      -20.466  -8.889  27.803  1.00  0.00
E    H
ATOM  14203 HG23 VAL E  35      -21.731  -9.937  28.540  1.00  0.00
E    H
ATOM  14204  C   VAL E  35      -18.733 -10.025  31.422  1.00  0.00
E    C
ATOM  14205  O   VAL E  35      -19.261 -10.614  32.371  1.00  0.00
E    O
ATOM  14206  N   ASP E  36      -17.590  -9.359  31.526  1.00  0.00
E    N
ATOM  14207  HN  ASP E  36      -17.062  -8.960  30.781  1.00  0.00
E    H
ATOM  14208  CA  ASP E  36      -16.983  -9.148  32.825  1.00  0.00
E    C
ATOM  14209  HA  ASP E  36      -17.743  -8.749  33.487  1.00  0.00
E    H
ATOM  14210  CB  ASP E  36      -16.381 -10.426  33.383  1.00  0.00
E    C
ATOM  14211  HB1 ASP E  36      -17.047 -11.279  33.144  1.00  0.00
E    H
ATOM  14212  HB2 ASP E  36      -15.389 -10.622  32.928  1.00  0.00
E    H
ATOM  14213  CG  ASP E  36      -16.229 -10.367  34.864  1.00  0.00
E    C
ATOM  14214  OD1 ASP E  36      -17.247 -10.577  35.551  1.00  0.00
E    O
ATOM  14215  OD2 ASP E  36      -15.107 -10.098  35.337  1.00  0.00
E    O
ATOM  14216  C   ASP E  36      -15.911  -8.088  32.702  1.00  0.00
E    C
ATOM  14217  O   ASP E  36      -15.127  -8.097  31.752  1.00  0.00
E    O
ATOM  14218  N   ILE E  37      -15.873  -7.187  33.679  1.00  0.00
E    N
ATOM  14219  HN  ILE E  37      -16.486  -7.141  34.466  1.00  0.00
E    H
ATOM  14220  CA  ILE E  37      -14.861  -6.142  33.650  1.00  0.00
E    C
ATOM  14221  HA  ILE E  37      -14.917  -5.686  32.670  1.00  0.00
E    H
ATOM  14222  CB  ILE E  37      -15.119  -5.104  34.751  1.00  0.00
E    C
ATOM  14223  HB  ILE E  37      -16.182  -4.790  34.597  1.00  0.00
E    H
ATOM  14224  CG2 ILE E  37      -14.838  -5.696  36.114  1.00  0.00
E    C
ATOM  14225 HG21 ILE E  37      -15.171  -5.004  36.916  1.00  0.00
E    H
ATOM  14226 HG22 ILE E  37      -15.382  -6.655  36.248  1.00  0.00
E    H
ATOM  14227 HG23 ILE E  37      -13.753  -5.888  36.248  1.00  0.00
E    H
ATOM  14228  CG1 ILE E  37      -14.248  -3.874  34.516  1.00  0.00
E    C
```

```
ATOM  14229 HG11 ILE E  37      -13.188  -4.110  34.758  1.00  0.00
E    H
ATOM  14230 HG12 ILE E  37      -14.286  -3.607  33.435  1.00  0.00
E    H
ATOM  14231  CD  ILE E  37      -14.669  -2.680  35.303  1.00  0.00
E    C
ATOM  14232  HD1 ILE E  37      -14.034  -1.804  35.044  1.00  0.00
E    H
ATOM  14233  HD2 ILE E  37      -15.727  -2.419  35.083  1.00  0.00
E    H
ATOM  14234  HD3 ILE E  37      -14.570  -2.866  36.393  1.00  0.00
E    H
ATOM  14235  C   ILE E  37      -13.471  -6.736  33.779  1.00  0.00
E    C
ATOM  14236  O   ILE E  37      -12.492  -6.145  33.312  1.00  0.00
E    O
ATOM  14237  N   ASN E  38      -13.349  -7.903  34.390  1.00  0.00
E    N
ATOM  14238  HN  ASN E  38      -14.096  -8.423  34.802  1.00  0.00
E    H
ATOM  14239  CA  ASN E  38      -12.048  -8.530  34.502  1.00  0.00
E    C
ATOM  14240  HA  ASN E  38      -11.321  -7.733  34.618  1.00  0.00
E    H
ATOM  14241  CB  ASN E  38      -12.000  -9.505  35.664  1.00  0.00
E    C
ATOM  14242  HB1 ASN E  38      -11.912  -8.913  36.601  1.00  0.00
E    H
ATOM  14243  HB2 ASN E  38      -12.946 -10.081  35.723  1.00  0.00
E    H
ATOM  14244  CG  ASN E  38      -10.826 -10.422  35.571  1.00  0.00
E    C
ATOM  14245  OD1 ASN E  38      -10.881 -11.444  34.902  1.00  0.00
E    O
ATOM  14246  ND2 ASN E  38       -9.733 -10.046  36.214  1.00  0.00
E    N
ATOM  14247 HD21 ASN E  38       -8.937 -10.642  36.152  1.00  0.00
E    H
ATOM  14248 HD22 ASN E  38       -9.718  -9.183  36.709  1.00  0.00
E    H
ATOM  14249  C   ASN E  38      -11.738  -9.248  33.203  1.00  0.00
E    C
ATOM  14250  O   ASN E  38      -12.321 -10.296  32.908  1.00  0.00
E    O
ATOM  14251  N   GLU E  39      -10.811  -8.685  32.435  1.00  0.00
E    N
ATOM  14252  HN  GLU E  39      -10.322  -7.846  32.654  1.00  0.00
E    H
ATOM  14253  CA  GLU E  39      -10.427  -9.279  31.162  1.00  0.00
E    C
ATOM  14254  HA  GLU E  39      -11.309  -9.336  30.536  1.00  0.00
E    H
ATOM  14255  CB  GLU E  39       -9.347  -8.426  30.516  1.00  0.00
E    C
```

ATOM 14256 HB1 GLU E 39 -9.291 -8.697 29.436 1.00 0.00
E H
ATOM 14257 HB2 GLU E 39 -9.645 -7.354 30.546 1.00 0.00
E H
ATOM 14258 CG GLU E 39 -8.000 -8.627 31.165 1.00 0.00
E C
ATOM 14259 HG1 GLU E 39 -7.840 -9.703 31.356 1.00 0.00
E H
ATOM 14260 HG2 GLU E 39 -7.187 -8.246 30.516 1.00 0.00
E H
ATOM 14261 CD GLU E 39 -7.890 -7.951 32.515 1.00 0.00
E C
ATOM 14262 OE1 GLU E 39 -8.933 -7.586 33.102 1.00 0.00
E O
ATOM 14263 OE2 GLU E 39 -6.756 -7.796 33.003 1.00 0.00
E O
ATOM 14264 C GLU E 39 -9.918 -10.696 31.354 1.00 0.00
E C
ATOM 14265 O GLU E 39 -9.925 -11.498 30.422 1.00 0.00
E O
ATOM 14266 N GLU E 40 -9.447 -11.017 32.553 1.00 0.00
E N
ATOM 14267 HN GLU E 40 -9.452 -10.444 33.369 1.00 0.00
E H
ATOM 14268 CA GLU E 40 -8.852 -12.327 32.755 1.00 0.00
E C
ATOM 14269 HA GLU E 40 -8.130 -12.494 31.965 1.00 0.00
E H
ATOM 14270 CB GLU E 40 -8.153 -12.369 34.111 1.00 0.00
E C
ATOM 14271 HB1 GLU E 40 -8.420 -11.441 34.668 1.00 0.00
E H
ATOM 14272 HB2 GLU E 40 -8.553 -13.213 34.715 1.00 0.00
E H
ATOM 14273 CG GLU E 40 -6.624 -12.471 34.092 1.00 0.00
E C
ATOM 14274 HG1 GLU E 40 -6.188 -11.460 33.998 1.00 0.00
E H
ATOM 14275 HG2 GLU E 40 -6.247 -12.945 35.020 1.00 0.00
E H
ATOM 14276 CD GLU E 40 -6.050 -13.248 32.921 1.00 0.00
E C
ATOM 14277 OE1 GLU E 40 -5.372 -14.259 33.187 1.00 0.00
E O
ATOM 14278 OE2 GLU E 40 -6.203 -12.819 31.756 1.00 0.00
E O
ATOM 14279 C GLU E 40 -9.894 -13.428 32.654 1.00 0.00
E C
ATOM 14280 O GLU E 40 -9.689 -14.418 31.943 1.00 0.00
E O
ATOM 14281 N LYS E 41 -11.016 -13.272 33.352 1.00 0.00
E N
ATOM 14282 HN LYS E 41 -11.215 -12.496 33.949 1.00 0.00
E H

```
ATOM  14283  CA  LYS E  41     -12.065 -14.279  33.277  1.00  0.00
E    C
ATOM  14284  HA  LYS E  41     -11.609 -15.233  33.511  1.00  0.00
E    H
ATOM  14285  CB  LYS E  41     -13.184 -13.960  34.263  1.00  0.00
E    C
ATOM  14286  HB1 LYS E  41     -13.750 -13.095  33.846  1.00  0.00
E    H
ATOM  14287  HB2 LYS E  41     -13.895 -14.816  34.292  1.00  0.00
E    H
ATOM  14288  CG  LYS E  41     -12.714 -13.615  35.646  1.00  0.00
E    C
ATOM  14289  HG1 LYS E  41     -11.676 -13.216  35.589  1.00  0.00
E    H
ATOM  14290  HG2 LYS E  41     -13.359 -12.806  36.057  1.00  0.00
E    H
ATOM  14291  CD  LYS E  41     -12.729 -14.809  36.559  1.00  0.00
E    C
ATOM  14292  HD1 LYS E  41     -13.574 -15.470  36.259  1.00  0.00
E    H
ATOM  14293  HD2 LYS E  41     -11.786 -15.380  36.399  1.00  0.00
E    H
ATOM  14294  CE  LYS E  41     -12.858 -14.341  37.992  1.00  0.00
E    C
ATOM  14295  HE1 LYS E  41     -12.722 -15.194  38.694  1.00  0.00
E    H
ATOM  14296  HE2 LYS E  41     -12.090 -13.571  38.222  1.00  0.00
E    H
ATOM  14297  NZ  LYS E  41     -14.200 -13.748  38.237  1.00  0.00
E    N
ATOM  14298  HZ1 LYS E  41     -14.275 -13.433  39.225  1.00  0.00
E    H
ATOM  14299  HZ2 LYS E  41     -14.341 -12.935  37.603  1.00  0.00
E    H
ATOM  14300  HZ3 LYS E  41     -14.933 -14.461  38.047  1.00  0.00
E    H
ATOM  14301  C   LYS E  41     -12.631 -14.380  31.870  1.00  0.00
E    C
ATOM  14302  O   LYS E  41     -12.876 -15.485  31.375  1.00  0.00
E    O
ATOM  14303  N   VAL E  42     -12.904 -13.240  31.239  1.00  0.00
E    N
ATOM  14304  HN  VAL E  42     -12.814 -12.329  31.634  1.00  0.00
E    H
ATOM  14305  CA  VAL E  42     -13.376 -13.254  29.863  1.00  0.00
E    C
ATOM  14306  HA  VAL E  42     -14.320 -13.784  29.835  1.00  0.00
E    H
ATOM  14307  CB  VAL E  42     -13.554 -11.817  29.351  1.00  0.00
E    C
ATOM  14308  HB  VAL E  42     -12.534 -11.383  29.199  1.00  0.00
E    H
ATOM  14309  CG1 VAL E  42     -14.298 -11.812  28.033  1.00  0.00
E    C
```

```
ATOM  14310 HG11 VAL E  42     -14.459 -10.769  27.684  1.00  0.00
E    H
ATOM  14311 HG12 VAL E  42     -13.730 -12.341  27.239  1.00  0.00
E    H
ATOM  14312 HG13 VAL E  42     -15.292 -12.296  28.141  1.00  0.00
E    H
ATOM  14313  CG2 VAL E  42     -14.269 -10.982  30.389  1.00  0.00
E    C
ATOM  14314 HG21 VAL E  42     -14.439  -9.950  30.015  1.00  0.00
E    H
ATOM  14315 HG22 VAL E  42     -15.255 -11.430  30.640  1.00  0.00
E    H
ATOM  14316 HG23 VAL E  42     -13.673 -10.905  31.323  1.00  0.00
E    H
ATOM  14317  C   VAL E  42     -12.397 -14.024  29.001  1.00  0.00
E    C
ATOM  14318  O   VAL E  42     -12.772 -14.904  28.217  1.00  0.00
E    O
ATOM  14319  N   ASN E  43     -11.117 -13.707  29.160  1.00  0.00
E    N
ATOM  14320  HN  ASN E  43     -10.801 -13.017  29.810  1.00  0.00
E    H
ATOM  14321  CA  ASN E  43     -10.060 -14.342  28.395  1.00  0.00
E    C
ATOM  14322  HA  ASN E  43     -10.262 -14.106  27.355  1.00  0.00
E    H
ATOM  14323  CB  ASN E  43      -8.713 -13.803  28.869  1.00  0.00
E    C
ATOM  14324  HB1 ASN E  43      -8.887 -12.804  29.322  1.00  0.00
E    H
ATOM  14325  HB2 ASN E  43      -8.292 -14.459  29.658  1.00  0.00
E    H
ATOM  14326  CG  ASN E  43      -7.731 -13.641  27.749  1.00  0.00
E    C
ATOM  14327  OD1 ASN E  43      -7.514 -14.552  26.965  1.00  0.00
E    O
ATOM  14328  ND2 ASN E  43      -7.138 -12.461  27.652  1.00  0.00
E    N
ATOM  14329 HD21 ASN E  43      -6.490 -12.333  26.905  1.00  0.00
E    H
ATOM  14330 HD22 ASN E  43      -7.368 -11.733  28.291  1.00  0.00
E    H
ATOM  14331  C   ASN E  43     -10.112 -15.849  28.541  1.00  0.00
E    C
ATOM  14332  O   ASN E  43     -10.010 -16.587  27.555  1.00  0.00
E    O
ATOM  14333  N   ALA E  44     -10.267 -16.322  29.770  1.00  0.00
E    N
ATOM  14334  HN  ALA E  44     -10.330 -15.761  30.595  1.00  0.00
E    H
ATOM  14335  CA  ALA E  44     -10.357 -17.759  29.983  1.00  0.00
E    C
ATOM  14336  HA  ALA E  44      -9.519 -18.265  29.518  1.00  0.00
E    H
```

ATOM 14337 CB ALA E 44 -10.331 -18.072 31.476 1.00 0.00
E C
ATOM 14338 HB1 ALA E 44 -9.387 -17.691 31.920 1.00 0.00
E H
ATOM 14339 HB2 ALA E 44 -11.179 -17.583 32.002 1.00 0.00
E H
ATOM 14340 HB3 ALA E 44 -10.382 -19.167 31.666 1.00 0.00
E H
ATOM 14341 C ALA E 44 -11.612 -18.336 29.347 1.00 0.00
E C
ATOM 14342 O ALA E 44 -11.533 -19.159 28.430 1.00 0.00
E O
ATOM 14343 N TYR E 45 -12.776 -17.884 29.787 1.00 0.00
E N
ATOM 14344 HN TYR E 45 -12.888 -17.061 30.342 1.00 0.00
E H
ATOM 14345 CA TYR E 45 -14.016 -18.578 29.481 1.00 0.00
E C
ATOM 14346 HA TYR E 45 -13.756 -19.630 29.459 1.00 0.00
E H
ATOM 14347 CB TYR E 45 -15.071 -18.300 30.547 1.00 0.00
E C
ATOM 14348 HB1 TYR E 45 -15.107 -17.207 30.747 1.00 0.00
E H
ATOM 14349 HB2 TYR E 45 -16.079 -18.603 30.190 1.00 0.00
E H
ATOM 14350 CG TYR E 45 -14.789 -18.969 31.859 1.00 0.00
E C
ATOM 14351 CD1 TYR E 45 -13.717 -19.835 32.001 1.00 0.00
E C
ATOM 14352 HD1 TYR E 45 -13.075 -20.038 31.155 1.00 0.00
E H
ATOM 14353 CE1 TYR E 45 -13.465 -20.461 33.200 1.00 0.00
E C
ATOM 14354 HE1 TYR E 45 -12.617 -21.120 33.308 1.00 0.00
E H
ATOM 14355 CZ TYR E 45 -14.308 -20.248 34.265 1.00 0.00
E C
ATOM 14356 OH TYR E 45 -14.071 -20.873 35.464 1.00 0.00
E O
ATOM 14357 HH TYR E 45 -14.717 -20.545 36.093 1.00 0.00
E H
ATOM 14358 CD2 TYR E 45 -15.629 -18.787 32.942 1.00 0.00
E C
ATOM 14359 HD2 TYR E 45 -16.514 -18.174 32.842 1.00 0.00
E H
ATOM 14360 CE2 TYR E 45 -15.389 -19.413 34.138 1.00 0.00
E C
ATOM 14361 HE2 TYR E 45 -16.063 -19.261 34.968 1.00 0.00
E H
ATOM 14362 C TYR E 45 -14.594 -18.232 28.123 1.00 0.00
E C
ATOM 14363 O TYR E 45 -15.725 -18.637 27.836 1.00 0.00
E O

ATOM 14364 N   ALA E 46   -13.868 -17.489 27.290 1.00 0.00
E   N
ATOM 14365 HN ALA E 46   -13.016 -17.022 27.526 1.00 0.00
E   H
ATOM 14366 CA ALA E 46   -14.314 -17.317 25.916 1.00 0.00
E   C
ATOM 14367 HA ALA E 46   -15.232 -16.741 25.875 1.00 0.00
E   H
ATOM 14368 CB ALA E 46   -13.249 -16.580 25.109 1.00 0.00
E   C
ATOM 14369 HB1 ALA E 46   -13.070 -15.579 25.555 1.00 0.00
E   H
ATOM 14370 HB2 ALA E 46   -12.286 -17.136 25.118 1.00 0.00
E   H
ATOM 14371 HB3 ALA E 46   -13.564 -16.435 24.053 1.00 0.00
E   H
ATOM 14372 C   ALA E 46   -14.627 -18.665 25.287 1.00 0.00
E   C
ATOM 14373 O   ALA E 46   -15.680 -18.851 24.669 1.00 0.00
E   O
ATOM 14374 N   SER E 47   -13.731 -19.630 25.474 1.00 0.00
E   N
ATOM 14375 HN SER E 47   -12.886 -19.524 26.000 1.00 0.00
E   H
ATOM 14376 CA SER E 47   -13.918 -20.958 24.911 1.00 0.00
E   C
ATOM 14377 HA SER E 47   -14.003 -20.806 23.843 1.00 0.00
E   H
ATOM 14378 CB SER E 47   -12.731 -21.840 25.277 1.00 0.00
E   C
ATOM 14379 HB1 SER E 47   -12.687 -22.681 24.547 1.00 0.00
E   H
ATOM 14380 HB2 SER E 47   -11.780 -21.271 25.177 1.00 0.00
E   H
ATOM 14381 OG SER E 47   -12.898 -22.359 26.580 1.00 0.00
E   O
ATOM 14382 HG1 SER E 47   -12.256 -23.074 26.685 1.00 0.00
E   H
ATOM 14383 C   SER E 47   -15.202 -21.620 25.377 1.00 0.00
E   C
ATOM 14384 O   SER E 47   -15.751 -22.455 24.654 1.00 0.00
E   O
ATOM 14385 N   TYR E 48   -15.692 -21.281 26.563 1.00 0.00
E   N
ATOM 14386 HN TYR E 48   -15.266 -20.610 27.168 1.00 0.00
E   H
ATOM 14387 CA TYR E 48   -16.912 -21.875 27.082 1.00 0.00
E   C
ATOM 14388 HA TYR E 48   -17.120 -22.739 26.463 1.00 0.00
E   H
ATOM 14389 CB TYR E 48   -16.730 -22.266 28.542 1.00 0.00
E   C
ATOM 14390 HB1 TYR E 48   -16.551 -21.349 29.145 1.00 0.00
E   H

ATOM 14391 HB2 TYR E 48 -17.651 -22.746 28.939 1.00 0.00
E H
ATOM 14392 CG TYR E 48 -15.555 -23.169 28.742 1.00 0.00
E C
ATOM 14393 CD1 TYR E 48 -15.147 -24.030 27.738 1.00 0.00
E C
ATOM 14394 HD1 TYR E 48 -15.701 -24.074 26.810 1.00 0.00
E H
ATOM 14395 CE1 TYR E 48 -14.061 -24.845 27.907 1.00 0.00
E C
ATOM 14396 HE1 TYR E 48 -13.760 -25.529 27.126 1.00 0.00
E H
ATOM 14397 CZ TYR E 48 -13.356 -24.796 29.085 1.00 0.00
E C
ATOM 14398 OH TYR E 48 -12.268 -25.611 29.259 1.00 0.00
E O
ATOM 14399 HH TYR E 48 -11.870 -25.384 30.102 1.00 0.00
E H
ATOM 14400 CD2 TYR E 48 -14.822 -23.129 29.913 1.00 0.00
E C
ATOM 14401 HD2 TYR E 48 -15.129 -22.479 30.721 1.00 0.00
E H
ATOM 14402 CE2 TYR E 48 -13.733 -23.942 30.092 1.00 0.00
E C
ATOM 14403 HE2 TYR E 48 -13.195 -23.919 31.027 1.00 0.00
E H
ATOM 14404 C TYR E 48 -18.088 -20.933 26.943 1.00 0.00
E C
ATOM 14405 O TYR E 48 -19.018 -20.987 27.747 1.00 0.00
E O
ATOM 14406 N ALA E 49 -18.061 -20.067 25.939 1.00 0.00
E N
ATOM 14407 HN ALA E 49 -17.389 -20.090 25.200 1.00 0.00
E H
ATOM 14408 CA ALA E 49 -19.042 -19.002 25.856 1.00 0.00
E C
ATOM 14409 HA ALA E 49 -19.918 -19.228 26.452 1.00 0.00
E H
ATOM 14410 CB ALA E 49 -18.465 -17.670 26.338 1.00 0.00
E C
ATOM 14411 HB1 ALA E 49 -18.159 -17.760 27.402 1.00 0.00
E H
ATOM 14412 HB2 ALA E 49 -17.567 -17.386 25.747 1.00 0.00
E H
ATOM 14413 HB3 ALA E 49 -19.211 -16.849 26.263 1.00 0.00
E H
ATOM 14414 C ALA E 49 -19.521 -18.862 24.428 1.00 0.00
E C
ATOM 14415 O ALA E 49 -18.723 -18.833 23.489 1.00 0.00
E O
ATOM 14416 N THR E 50 -20.839 -18.770 24.278 1.00 0.00
E N
ATOM 14417 HN THR E 50 -21.527 -18.929 24.985 1.00 0.00
E H

```
ATOM  14418  CA  THR E  50     -21.386 -18.405  22.983  1.00  0.00
E    C
ATOM  14419  HA  THR E  50     -21.049 -19.165  22.291  1.00  0.00
E    H
ATOM  14420  CB  THR E  50     -22.909 -18.304  23.063  1.00  0.00
E    C
ATOM  14421  HB  THR E  50     -23.128 -17.388  23.662  1.00  0.00
E    H
ATOM  14422  OG1 THR E  50     -23.432 -19.433  23.775  1.00  0.00
E    O
ATOM  14423  HG1 THR E  50     -24.389 -19.314  23.778  1.00  0.00
E    H
ATOM  14424  CG2 THR E  50     -23.520 -18.266  21.671  1.00  0.00
E    C
ATOM  14425 HG21 THR E  50     -24.613 -18.079  21.723  1.00  0.00
E    H
ATOM  14426 HG22 THR E  50     -23.063 -17.451  21.070  1.00  0.00
E    H
ATOM  14427 HG23 THR E  50     -23.357 -19.227  21.135  1.00  0.00
E    H
ATOM  14428  C   THR E  50     -20.798 -17.091  22.503  1.00  0.00
E    C
ATOM  14429  O   THR E  50     -20.557 -16.921  21.305  1.00  0.00
E    O
ATOM  14430  N   HSE E  51     -20.535 -16.171  23.419  1.00  0.00
E    N
ATOM  14431  HN  HSE E  51     -20.692 -16.283  24.399  1.00  0.00
E    H
ATOM  14432  CA  HSE E  51     -19.978 -14.887  23.047  1.00  0.00
E    C
ATOM  14433  HA  HSE E  51     -19.116 -15.063  22.415  1.00  0.00
E    H
ATOM  14434  CB  HSE E  51     -21.026 -14.036  22.331  1.00  0.00
E    C
ATOM  14435  HB1 HSE E  51     -21.313 -14.563  21.395  1.00  0.00
E    H
ATOM  14436  HB2 HSE E  51     -21.940 -13.968  22.958  1.00  0.00
E    H
ATOM  14437  ND1 HSE E  51     -21.379 -11.728  21.390  1.00  0.00
E    N
ATOM  14438  CG  HSE E  51     -20.551 -12.669  21.960  1.00  0.00
E    C
ATOM  14439  CE1 HSE E  51     -20.696 -10.619  21.172  1.00  0.00
E    C
ATOM  14440  HE1 HSE E  51     -21.111  -9.714  20.721  1.00  0.00
E    H
ATOM  14441  NE2 HSE E  51     -19.456 -10.807  21.583  1.00  0.00
E    N
ATOM  14442  HE2 HSE E  51     -18.721 -10.133  21.519  1.00  0.00
E    H
ATOM  14443  CD2 HSE E  51     -19.338 -12.082  22.079  1.00  0.00
E    C
ATOM  14444  HD2 HSE E  51     -18.411 -12.479  22.468  1.00  0.00
E    H
```

```
ATOM  14445  C   HSE E  51     -19.475 -14.198  24.306  1.00  0.00
E    C
ATOM  14446  O   HSE E  51     -20.231 -14.012  25.263  1.00  0.00
E    O
ATOM  14447  N   ALA E  52     -18.201 -13.823  24.295  1.00  0.00
E    N
ATOM  14448  HN  ALA E  52     -17.616 -13.968  23.497  1.00  0.00
E    H
ATOM  14449  CA  ALA E  52     -17.561 -13.180  25.429  1.00  0.00
E    C
ATOM  14450  HA  ALA E  52     -18.245 -13.069  26.263  1.00  0.00
E    H
ATOM  14451  CB  ALA E  52     -16.339 -13.966  25.901  1.00  0.00
E    C
ATOM  14452  HB1 ALA E  52     -16.654 -14.975  26.242  1.00  0.00
E    H
ATOM  14453  HB2 ALA E  52     -15.606 -14.096  25.076  1.00  0.00
E    H
ATOM  14454  HB3 ALA E  52     -15.830 -13.460  26.750  1.00  0.00
E    H
ATOM  14455  C   ALA E  52     -17.144 -11.780  25.029  1.00  0.00
E    C
ATOM  14456  O   ALA E  52     -16.851 -11.527  23.859  1.00  0.00
E    O
ATOM  14457  N   VAL E  53     -17.105 -10.881  26.002  1.00  0.00
E    N
ATOM  14458  HN  VAL E  53     -17.353 -11.097  26.943  1.00  0.00
E    H
ATOM  14459  CA  VAL E  53     -16.699  -9.499  25.790  1.00  0.00
E    C
ATOM  14460  HA  VAL E  53     -15.900  -9.474  25.059  1.00  0.00
E    H
ATOM  14461  CB  VAL E  53     -17.873  -8.606  25.341  1.00  0.00
E    C
ATOM  14462  HB  VAL E  53     -18.772  -8.907  25.933  1.00  0.00
E    H
ATOM  14463  CG1 VAL E  53     -17.571  -7.138  25.609  1.00  0.00
E    C
ATOM  14464 HG11 VAL E  53     -18.405  -6.500  25.246  1.00  0.00
E    H
ATOM  14465 HG12 VAL E  53     -17.447  -6.937  26.693  1.00  0.00
E    H
ATOM  14466 HG13 VAL E  53     -16.644  -6.826  25.080  1.00  0.00
E    H
ATOM  14467  CG2 VAL E  53     -18.187  -8.808  23.877  1.00  0.00
E    C
ATOM  14468 HG21 VAL E  53     -19.007  -8.133  23.554  1.00  0.00
E    H
ATOM  14469 HG22 VAL E  53     -17.292  -8.595  23.253  1.00  0.00
E    H
ATOM  14470 HG23 VAL E  53     -18.513  -9.850  23.677  1.00  0.00
E    H
ATOM  14471  C   VAL E  53     -16.135  -8.983  27.098  1.00  0.00
E    C
```

ATOM 14472 O VAL E 53 −16.662 −9.292 28.169 1.00 0.00
E O
ATOM 14473 N ILE E 54 −15.069 −8.199 27.026 1.00 0.00
E N
ATOM 14474 HN ILE E 54 −14.501 −8.057 26.218 1.00 0.00
E H
ATOM 14475 CA ILE E 54 −14.638 −7.452 28.196 1.00 0.00
E C
ATOM 14476 HA ILE E 54 −14.953 −8.000 29.075 1.00 0.00
E H
ATOM 14477 CB ILE E 54 −13.126 −7.227 28.184 1.00 0.00
E C
ATOM 14478 HB ILE E 54 −12.945 −6.585 27.285 1.00 0.00
E H
ATOM 14479 CG2 ILE E 54 −12.676 −6.648 29.499 1.00 0.00
E C
ATOM 14480 HG21 ILE E 54 −11.611 −6.337 29.446 1.00 0.00
E H
ATOM 14481 HG22 ILE E 54 −13.275 −5.751 29.762 1.00 0.00
E H
ATOM 14482 HG23 ILE E 54 −12.779 −7.392 30.317 1.00 0.00
E H
ATOM 14483 CG1 ILE E 54 −12.412 −8.549 27.941 1.00 0.00
E C
ATOM 14484 HG11 ILE E 54 −12.369 −9.134 28.887 1.00 0.00
E H
ATOM 14485 HG12 ILE E 54 −13.003 −9.148 27.210 1.00 0.00
E H
ATOM 14486 CD ILE E 54 −11.020 −8.381 27.399 1.00 0.00
E C
ATOM 14487 HD1 ILE E 54 −10.566 −9.372 27.186 1.00 0.00
E H
ATOM 14488 HD2 ILE E 54 −11.035 −7.795 26.455 1.00 0.00
E H
ATOM 14489 HD3 ILE E 54 −10.369 −7.856 28.130 1.00 0.00
E H
ATOM 14490 C ILE E 54 −15.395 −6.133 28.204 1.00 0.00
E C
ATOM 14491 O ILE E 54 −15.381 −5.395 27.215 1.00 0.00
E O
ATOM 14492 N ALA E 55 −16.069 −5.839 29.315 1.00 0.00
E N
ATOM 14493 HN ALA E 55 −16.068 −6.424 30.126 1.00 0.00
E H
ATOM 14494 CA ALA E 55 −16.866 −4.625 29.421 1.00 0.00
E C
ATOM 14495 HA ALA E 55 −16.303 −3.757 29.096 1.00 0.00
E H
ATOM 14496 CB ALA E 55 −18.133 −4.712 28.565 1.00 0.00
E C
ATOM 14497 HB1 ALA E 55 −17.854 −4.844 27.498 1.00 0.00
E H
ATOM 14498 HB2 ALA E 55 −18.758 −5.581 28.865 1.00 0.00
E H

ATOM 14499 HB3 ALA E 55   -18.747 -3.790 28.648 1.00 0.00
E   H
ATOM 14500 C   ALA E 55   -17.237 -4.377 30.875 1.00 0.00
E   C
ATOM 14501 O   ALA E 55   -17.461 -5.319 31.638 1.00 0.00
E   O
ATOM 14502 N   ASN E 56   -17.307 -3.102 31.241 1.00 0.00
E   N
ATOM 14503 HN ASN E 56   -17.101 -2.352 30.615 1.00 0.00
E   H
ATOM 14504 CA ASN E 56   -17.688 -2.681 32.585 1.00 0.00
E   C
ATOM 14505 HA ASN E 56   -17.449 -3.496 33.258 1.00 0.00
E   H
ATOM 14506 CB ASN E 56   -16.962 -1.400 32.962 1.00 0.00
E   C
ATOM 14507 HB1 ASN E 56   -15.870 -1.604 32.923 1.00 0.00
E   H
ATOM 14508 HB2 ASN E 56   -17.178 -0.602 32.222 1.00 0.00
E   H
ATOM 14509 CG ASN E 56   -17.300 -0.925 34.355 1.00 0.00
E   C
ATOM 14510 OD1 ASN E 56   -18.256 -1.390 34.976 1.00 0.00
E   O
ATOM 14511 ND2 ASN E 56   -16.513   0.017 34.856 1.00 0.00
E   N
ATOM 14512 HD21 ASN E 56   -16.712   0.336 35.778 1.00 0.00
E   H
ATOM 14513 HD22 ASN E 56   -15.742   0.356 34.325 1.00 0.00
E   H
ATOM 14514 C   ASN E 56   -19.197 -2.492 32.608 1.00 0.00
E   C
ATOM 14515 O   ASN E 56   -19.735 -1.603 31.949 1.00 0.00
E   O
ATOM 14516 N   ALA E 57   -19.879 -3.309 33.409 1.00 0.00
E   N
ATOM 14517 HN ALA E 57   -19.435 -3.944 34.040 1.00 0.00
E   H
ATOM 14518 CA ALA E 57   -21.336 -3.333 33.419 1.00 0.00
E   C
ATOM 14519 HA ALA E 57   -21.700 -3.571 32.427 1.00 0.00
E   H
ATOM 14520 CB ALA E 57   -21.832 -4.397 34.390 1.00 0.00
E   C
ATOM 14521 HB1 ALA E 57   -21.424 -5.388 34.098 1.00 0.00
E   H
ATOM 14522 HB2 ALA E 57   -21.494 -4.180 35.427 1.00 0.00
E   H
ATOM 14523 HB3 ALA E 57   -22.941 -4.468 34.387 1.00 0.00
E   H
ATOM 14524 C   ALA E 57   -21.959 -2.003 33.796 1.00 0.00
E   C
ATOM 14525 O   ALA E 57   -23.128 -1.776 33.477 1.00 0.00
E   O

```
ATOM  14526  N   THR E  58     -21.224  -1.125  34.453  1.00  0.00
E    N
ATOM  14527  HN  THR E  58     -20.234  -1.177  34.582  1.00  0.00
E    H
ATOM  14528  CA  THR E  58     -21.845   0.034  35.064  1.00  0.00
E    C
ATOM  14529  HA  THR E  58     -22.787  -0.329  35.451  1.00  0.00
E    H
ATOM  14530  CB  THR E  58     -20.933   0.576  36.161  1.00  0.00
E    C
ATOM  14531  HB  THR E  58     -21.483   1.429  36.629  1.00  0.00
E    H
ATOM  14532  OG1 THR E  58     -19.737   1.089  35.572  1.00  0.00
E    O
ATOM  14533  HG1 THR E  58     -19.203   1.403  36.310  1.00  0.00
E    H
ATOM  14534  CG2 THR E  58     -20.556  -0.538  37.117  1.00  0.00
E    C
ATOM  14535 HG21 THR E  58     -19.987  -0.139  37.984  1.00  0.00
E    H
ATOM  14536 HG22 THR E  58     -21.467  -1.038  37.509  1.00  0.00
E    H
ATOM  14537 HG23 THR E  58     -19.929  -1.303  36.612  1.00  0.00
E    H
ATOM  14538  C   THR E  58     -22.181   1.144  34.079  1.00  0.00
E    C
ATOM  14539  O   THR E  58     -22.785   2.140  34.484  1.00  0.00
E    O
ATOM  14540  N   GLU E  59     -21.816   1.013  32.808  1.00  0.00
E    N
ATOM  14541  HN  GLU E  59     -21.383   0.202  32.424  1.00  0.00
E    H
ATOM  14542  CA  GLU E  59     -22.028   2.086  31.842  1.00  0.00
E    C
ATOM  14543  HA  GLU E  59     -22.387   2.957  32.376  1.00  0.00
E    H
ATOM  14544  CB  GLU E  59     -20.730   2.417  31.114  1.00  0.00
E    C
ATOM  14545  HB1 GLU E  59     -20.307   1.469  30.708  1.00  0.00
E    H
ATOM  14546  HB2 GLU E  59     -20.947   3.057  30.229  1.00  0.00
E    H
ATOM  14547  CG  GLU E  59     -19.683   3.080  31.965  1.00  0.00
E    C
ATOM  14548  HG1 GLU E  59     -18.832   3.387  31.331  1.00  0.00
E    H
ATOM  14549  HG2 GLU E  59     -20.096   3.972  32.478  1.00  0.00
E    H
ATOM  14550  CD  GLU E  59     -19.120   2.142  32.996  1.00  0.00
E    C
ATOM  14551  OE1 GLU E  59     -18.939   2.569  34.152  1.00  0.00
E    O
ATOM  14552  OE2 GLU E  59     -18.910   0.961  32.670  1.00  0.00
E    O
```

ATOM 14553 C GLU E 59 -23.099 1.702 30.829 1.00 0.00
E C
ATOM 14554 O GLU E 59 -23.014 0.646 30.196 1.00 0.00
E O
ATOM 14555 N GLU E 60 -24.071 2.595 30.640 1.00 0.00
E N
ATOM 14556 HN GLU E 60 -24.088 3.515 31.019 1.00 0.00
E H
ATOM 14557 CA GLU E 60 -25.235 2.268 29.824 1.00 0.00
E C
ATOM 14558 HA GLU E 60 -25.650 1.339 30.193 1.00 0.00
E H
ATOM 14559 CB GLU E 60 -26.300 3.359 29.979 1.00 0.00
E C
ATOM 14560 HB1 GLU E 60 -27.123 3.145 29.259 1.00 0.00
E H
ATOM 14561 HB2 GLU E 60 -26.757 3.299 30.992 1.00 0.00
E H
ATOM 14562 CG GLU E 60 -25.857 4.810 29.744 1.00 0.00
E C
ATOM 14563 HG1 GLU E 60 -26.742 5.473 29.757 1.00 0.00
E H
ATOM 14564 HG2 GLU E 60 -25.144 5.139 30.525 1.00 0.00
E H
ATOM 14565 CD GLU E 60 -25.206 5.066 28.405 1.00 0.00
E C
ATOM 14566 OE1 GLU E 60 -25.815 4.721 27.372 1.00 0.00
E O
ATOM 14567 OE2 GLU E 60 -24.067 5.573 28.386 1.00 0.00
E O
ATOM 14568 C GLU E 60 -24.858 2.048 28.367 1.00 0.00
E C
ATOM 14569 O GLU E 60 -25.504 1.269 27.661 1.00 0.00
E O
ATOM 14570 N ASN E 61 -23.831 2.744 27.889 1.00 0.00
E N
ATOM 14571 HN ASN E 61 -23.237 3.350 28.418 1.00 0.00
E H
ATOM 14572 CA ASN E 61 -23.496 2.651 26.477 1.00 0.00
E C
ATOM 14573 HA ASN E 61 -24.434 2.731 25.939 1.00 0.00
E H
ATOM 14574 CB ASN E 61 -22.548 3.780 26.095 1.00 0.00
E C
ATOM 14575 HB1 ASN E 61 -22.073 3.512 25.125 1.00 0.00
E H
ATOM 14576 HB2 ASN E 61 -23.113 4.721 25.939 1.00 0.00
E H
ATOM 14577 CG ASN E 61 -21.471 3.974 27.111 1.00 0.00
E C
ATOM 14578 OD1 ASN E 61 -21.662 3.675 28.290 1.00 0.00
E O
ATOM 14579 ND2 ASN E 61 -20.329 4.483 26.675 1.00 0.00
E N

```
ATOM  14580 HD21 ASN E  61      −19.599   4.606  27.342  1.00  0.00
E    H
ATOM  14581 HD22 ASN E  61      −20.215   4.697  25.710  1.00  0.00
E    H
ATOM  14582  C   ASN E  61      −22.897   1.297  26.158  1.00  0.00
E    C
ATOM  14583  O   ASN E  61      −23.166   0.724  25.098  1.00  0.00
E    O
ATOM  14584  N   GLU E  62      −22.081   0.762  27.067  1.00  0.00
E    N
ATOM  14585  HN  GLU E  62      −21.678   1.220  27.855  1.00  0.00
E    H
ATOM  14586  CA  GLU E  62      −21.706  −0.636  26.940  1.00  0.00
E    C
ATOM  14587  HA  GLU E  62      −21.136  −0.753  26.028  1.00  0.00
E    H
ATOM  14588  CB  GLU E  62      −20.864  −1.086  28.125  1.00  0.00
E    C
ATOM  14589  HB1 GLU E  62      −21.288  −0.626  29.047  1.00  0.00
E    H
ATOM  14590  HB2 GLU E  62      −20.961  −2.187  28.260  1.00  0.00
E    H
ATOM  14591  CG  GLU E  62      −19.415  −0.709  28.012  1.00  0.00
E    C
ATOM  14592  HG1 GLU E  62      −18.879  −1.477  27.425  1.00  0.00
E    H
ATOM  14593  HG2 GLU E  62      −19.298   0.275  27.517  1.00  0.00
E    H
ATOM  14594  CD  GLU E  62      −18.722  −0.659  29.348  1.00  0.00
E    C
ATOM  14595  OE1 GLU E  62      −17.985  −1.610  29.668  1.00  0.00
E    O
ATOM  14596  OE2 GLU E  62      −18.899   0.334  30.073  1.00  0.00
E    O
ATOM  14597  C   GLU E  62      −22.930  −1.519  26.821  1.00  0.00
E    C
ATOM  14598  O   GLU E  62      −22.937  −2.474  26.038  1.00  0.00
E    O
ATOM  14599  N   LEU E  63      −23.980  −1.195  27.560  1.00  0.00
E    N
ATOM  14600  HN  LEU E  63      −24.045  −0.393  28.150  1.00  0.00
E    H
ATOM  14601  CA  LEU E  63      −25.166  −2.032  27.560  1.00  0.00
E    C
ATOM  14602  HA  LEU E  63      −24.826  −3.049  27.710  1.00  0.00
E    H
ATOM  14603  CB  LEU E  63      −26.088  −1.584  28.682  1.00  0.00
E    C
ATOM  14604  HB1 LEU E  63      −26.579  −0.642  28.347  1.00  0.00
E    H
ATOM  14605  HB2 LEU E  63      −26.904  −2.331  28.800  1.00  0.00
E    H
ATOM  14606  CG  LEU E  63      −25.330  −1.350  29.980  1.00  0.00
E    C
```

```
ATOM  14607  HG  LEU E  63     -24.580  -0.550  29.769  1.00  0.00
E    H
ATOM  14608  CD1 LEU E  63     -26.272  -0.882  31.063  1.00  0.00
E    C
ATOM  14609 HD11 LEU E  63     -25.730  -0.757  32.025  1.00  0.00
E    H
ATOM  14610 HD12 LEU E  63     -26.728   0.093  30.790  1.00  0.00
E    H
ATOM  14611 HD13 LEU E  63     -27.089  -1.619  31.218  1.00  0.00
E    H
ATOM  14612  CD2 LEU E  63     -24.581  -2.594  30.413  1.00  0.00
E    C
ATOM  14613 HD21 LEU E  63     -24.019  -2.399  31.351  1.00  0.00
E    H
ATOM  14614 HD22 LEU E  63     -25.269  -3.441  30.603  1.00  0.00
E    H
ATOM  14615 HD23 LEU E  63     -23.854  -2.902  29.630  1.00  0.00
E    H
ATOM  14616  C   LEU E  63     -25.883  -1.980  26.222  1.00  0.00
E    C
ATOM  14617  O   LEU E  63     -26.133  -3.024  25.609  1.00  0.00
E    O
ATOM  14618  N   LEU E  64     -26.217  -0.778  25.758  1.00  0.00
E    N
ATOM  14619  HN  LEU E  64     -26.076   0.087  26.235  1.00  0.00
E    H
ATOM  14620  CA  LEU E  64     -26.846  -0.645  24.450  1.00  0.00
E    C
ATOM  14621  HA  LEU E  64     -27.775  -1.199  24.492  1.00  0.00
E    H
ATOM  14622  CB  LEU E  64     -27.092   0.822  24.135  1.00  0.00
E    C
ATOM  14623  HB1 LEU E  64     -26.137   1.237  23.737  1.00  0.00
E    H
ATOM  14624  HB2 LEU E  64     -27.828   0.895  23.304  1.00  0.00
E    H
ATOM  14625  CG  LEU E  64     -27.533   1.620  25.346  1.00  0.00
E    C
ATOM  14626  HG  LEU E  64     -26.864   1.360  26.202  1.00  0.00
E    H
ATOM  14627  CD1 LEU E  64     -27.420   3.097  25.057  1.00  0.00
E    C
ATOM  14628 HD11 LEU E  64     -27.786   3.692  25.922  1.00  0.00
E    H
ATOM  14629 HD12 LEU E  64     -26.363   3.378  24.863  1.00  0.00
E    H
ATOM  14630 HD13 LEU E  64     -28.025   3.371  24.167  1.00  0.00
E    H
ATOM  14631  CD2 LEU E  64     -28.945   1.238  25.724  1.00  0.00
E    C
ATOM  14632 HD21 LEU E  64     -29.272   1.805  26.622  1.00  0.00
E    H
ATOM  14633 HD22 LEU E  64     -29.662   1.461  24.908  1.00  0.00
E    H
```

```
ATOM  14634 HD23 LEU E  64      -29.007   0.153  25.957  1.00  0.00
E    H
ATOM  14635  C   LEU E  64      -25.989  -1.271  23.368  1.00  0.00
E    C
ATOM  14636  O   LEU E  64      -26.501  -1.981  22.496  1.00  0.00
E    O
ATOM  14637  N   SER E  65      -24.679  -1.028  23.414  1.00  0.00
E    N
ATOM  14638  HN  SER E  65      -24.226  -0.443  24.087  1.00  0.00
E    H
ATOM  14639  CA  SER E  65      -23.779  -1.625  22.441  1.00  0.00
E    C
ATOM  14640  HA  SER E  65      -24.102  -1.264  21.473  1.00  0.00
E    H
ATOM  14641  CB  SER E  65      -22.343  -1.235  22.756  1.00  0.00
E    C
ATOM  14642  HB1 SER E  65      -21.677  -1.747  22.023  1.00  0.00
E    H
ATOM  14643  HB2 SER E  65      -22.199  -0.139  22.619  1.00  0.00
E    H
ATOM  14644  OG  SER E  65      -22.010  -1.651  24.063  1.00  0.00
E    O
ATOM  14645  HG1 SER E  65      -21.050  -1.593  24.144  1.00  0.00
E    H
ATOM  14646  C   SER E  65      -23.914  -3.135  22.427  1.00  0.00
E    C
ATOM  14647  O   SER E  65      -23.750  -3.766  21.378  1.00  0.00
E    O
ATOM  14648  N   LEU E  66      -24.228  -3.729  23.574  1.00  0.00
E    N
ATOM  14649  HN  LEU E  66      -24.356  -3.271  24.453  1.00  0.00
E    H
ATOM  14650  CA  LEU E  66      -24.416  -5.166  23.619  1.00  0.00
E    C
ATOM  14651  HA  LEU E  66      -23.634  -5.592  23.004  1.00  0.00
E    H
ATOM  14652  CB  LEU E  66      -24.307  -5.662  25.051  1.00  0.00
E    C
ATOM  14653  HB1 LEU E  66      -24.980  -5.025  25.672  1.00  0.00
E    H
ATOM  14654  HB2 LEU E  66      -24.719  -6.694  25.110  1.00  0.00
E    H
ATOM  14655  CG  LEU E  66      -22.884  -5.586  25.580  1.00  0.00
E    C
ATOM  14656  HG  LEU E  66      -22.612  -4.511  25.715  1.00  0.00
E    H
ATOM  14657  CD1 LEU E  66      -22.799  -6.295  26.903  1.00  0.00
E    C
ATOM  14658 HD11 LEU E  66      -21.754  -6.291  27.281  1.00  0.00
E    H
ATOM  14659 HD12 LEU E  66      -23.445  -5.795  27.656  1.00  0.00
E    H
ATOM  14660 HD13 LEU E  66      -23.127  -7.351  26.803  1.00  0.00
E    H
```

ATOM 14661 CD2 LEU E 66 -21.923 -6.190 24.577 1.00 0.00
E C
ATOM 14662 HD21 LEU E 66 -20.879 -6.119 24.952 1.00 0.00
E H
ATOM 14663 HD22 LEU E 66 -22.143 -7.261 24.393 1.00 0.00
E H
ATOM 14664 HD23 LEU E 66 -21.981 -5.650 23.608 1.00 0.00
E H
ATOM 14665 C LEU E 66 -25.746 -5.594 23.030 1.00 0.00
E C
ATOM 14666 O LEU E 66 -25.970 -6.795 22.862 1.00 0.00
E O
ATOM 14667 N GLY E 67 -26.625 -4.654 22.708 1.00 0.00
E N
ATOM 14668 HN GLY E 67 -26.446 -3.672 22.754 1.00 0.00
E H
ATOM 14669 CA GLY E 67 -27.946 -5.028 22.253 1.00 0.00
E C
ATOM 14670 HA1 GLY E 67 -27.838 -5.646 21.372 1.00 0.00
E H
ATOM 14671 HA2 GLY E 67 -28.523 -4.126 22.095 1.00 0.00
E H
ATOM 14672 C GLY E 67 -28.645 -5.847 23.315 1.00 0.00
E C
ATOM 14673 O GLY E 67 -29.261 -6.879 23.034 1.00 0.00
E O
ATOM 14674 N ILE E 68 -28.523 -5.383 24.557 1.00 0.00
E N
ATOM 14675 HN ILE E 68 -28.079 -4.523 24.798 1.00 0.00
E H
ATOM 14676 CA ILE E 68 -29.048 -6.121 25.696 1.00 0.00
E C
ATOM 14677 HA ILE E 68 -28.564 -7.089 25.678 1.00 0.00
E H
ATOM 14678 CB ILE E 68 -28.740 -5.344 26.977 1.00 0.00
E C
ATOM 14679 HB ILE E 68 -29.422 -5.789 27.745 1.00 0.00
E H
ATOM 14680 CG2 ILE E 68 -27.270 -5.415 27.282 1.00 0.00
E C
ATOM 14681 HG21 ILE E 68 -27.058 -4.990 28.286 1.00 0.00
E H
ATOM 14682 HG22 ILE E 68 -26.916 -6.467 27.280 1.00 0.00
E H
ATOM 14683 HG23 ILE E 68 -26.681 -4.845 26.533 1.00 0.00
E H
ATOM 14684 CG1 ILE E 68 -29.156 -3.892 26.785 1.00 0.00
E C
ATOM 14685 HG11 ILE E 68 -28.532 -3.423 25.992 1.00 0.00
E H
ATOM 14686 HG12 ILE E 68 -30.212 -3.862 26.434 1.00 0.00
E H
ATOM 14687 CD ILE E 68 -29.057 -3.086 28.015 1.00 0.00
E C

ATOM 14688 HD1 ILE E 68 -29.427 -2.054 27.830 1.00 0.00
E H
ATOM 14689 HD2 ILE E 68 -29.668 -3.535 28.827 1.00 0.00
E H
ATOM 14690 HD3 ILE E 68 -28.005 -3.018 28.363 1.00 0.00
E H
ATOM 14691 C ILE E 68 -30.537 -6.374 25.554 1.00 0.00
E C
ATOM 14692 O ILE E 68 -31.017 -7.476 25.840 1.00 0.00
E O
ATOM 14693 N ARG E 69 -31.291 -5.370 25.113 1.00 0.00
E N
ATOM 14694 HN ARG E 69 -30.905 -4.501 24.810 1.00 0.00
E H
ATOM 14695 CA ARG E 69 -32.737 -5.497 25.050 1.00 0.00
E C
ATOM 14696 HA ARG E 69 -33.099 -5.676 26.055 1.00 0.00
E H
ATOM 14697 CB ARG E 69 -33.334 -4.217 24.499 1.00 0.00
E C
ATOM 14698 HB1 ARG E 69 -32.867 -4.058 23.497 1.00 0.00
E H
ATOM 14699 HB2 ARG E 69 -34.420 -4.364 24.310 1.00 0.00
E H
ATOM 14700 CG ARG E 69 -33.059 -3.032 25.380 1.00 0.00
E C
ATOM 14701 HG1 ARG E 69 -33.649 -3.143 26.316 1.00 0.00
E H
ATOM 14702 HG2 ARG E 69 -31.990 -3.055 25.696 1.00 0.00
E H
ATOM 14703 CD ARG E 69 -33.334 -1.750 24.648 1.00 0.00
E C
ATOM 14704 HD1 ARG E 69 -34.348 -1.795 24.190 1.00 0.00
E H
ATOM 14705 HD2 ARG E 69 -33.289 -0.871 25.333 1.00 0.00
E H
ATOM 14706 NE ARG E 69 -32.345 -1.524 23.604 1.00 0.00
E N
ATOM 14707 HE ARG E 69 -31.481 -1.142 23.953 1.00 0.00
E H
ATOM 14708 CZ ARG E 69 -32.548 -1.789 22.320 1.00 0.00
E C
ATOM 14709 NH1 ARG E 69 -33.702 -2.301 21.922 1.00 0.00
E N
ATOM 14710 HH11 ARG E 69 -33.852 -2.525 20.969 1.00 0.00
E H
ATOM 14711 HH12 ARG E 69 -34.342 -2.569 22.645 1.00 0.00
E H
ATOM 14712 NH2 ARG E 69 -31.594 -1.552 21.434 1.00 0.00
E N
ATOM 14713 HH21 ARG E 69 -31.777 -1.669 20.469 1.00 0.00
E H
ATOM 14714 HH22 ARG E 69 -30.794 -1.064 21.757 1.00 0.00
E H

```
ATOM  14715  C   ARG E  69      -33.172  -6.682  24.216  1.00  0.00
E    C
ATOM  14716  O   ARG E  69      -34.237  -7.245  24.479  1.00  0.00
E    O
ATOM  14717  N   ASN E  70      -32.368  -7.078  23.234  1.00  0.00
E    N
ATOM  14718  HN  ASN E  70      -31.560  -6.584  22.917  1.00  0.00
E    H
ATOM  14719  CA  ASN E  70      -32.631  -8.319  22.528  1.00  0.00
E    C
ATOM  14720  HA  ASN E  70      -33.610  -8.199  22.077  1.00  0.00
E    H
ATOM  14721  CB  ASN E  70      -31.547  -8.563  21.484  1.00  0.00
E    C
ATOM  14722  HB1 ASN E  70      -30.622  -8.869  22.019  1.00  0.00
E    H
ATOM  14723  HB2 ASN E  70      -31.837  -9.399  20.815  1.00  0.00
E    H
ATOM  14724  CG  ASN E  70      -31.240  -7.327  20.680  1.00  0.00
E    C
ATOM  14725  OD1 ASN E  70      -32.027  -6.383  20.654  1.00  0.00
E    O
ATOM  14726  ND2 ASN E  70      -30.093  -7.322  20.017  1.00  0.00
E    N
ATOM  14727 HD21 ASN E  70      -29.873  -6.505  19.491  1.00  0.00
E    H
ATOM  14728 HD22 ASN E  70      -29.477  -8.101  20.082  1.00  0.00
E    H
ATOM  14729  C   ASN E  70      -32.706  -9.494  23.481  1.00  0.00
E    C
ATOM  14730  O   ASN E  70      -33.515 -10.402  23.272  1.00  0.00
E    O
ATOM  14731  N   PHE E  71      -31.909  -9.479  24.539  1.00  0.00
E    N
ATOM  14732  HN  PHE E  71      -31.260  -8.745  24.732  1.00  0.00
E    H
ATOM  14733  CA  PHE E  71      -31.915 -10.549  25.518  1.00  0.00
E    C
ATOM  14734  HA  PHE E  71      -31.958 -11.482  24.969  1.00  0.00
E    H
ATOM  14735  CB  PHE E  71      -30.701 -10.463  26.431  1.00  0.00
E    C
ATOM  14736  HB1 PHE E  71      -30.695  -9.472  26.935  1.00  0.00
E    H
ATOM  14737  HB2 PHE E  71      -30.744 -11.235  27.229  1.00  0.00
E    H
ATOM  14738  CG  PHE E  71      -29.404 -10.569  25.714  1.00  0.00
E    C
ATOM  14739  CD1 PHE E  71      -28.925  -9.509  24.967  1.00  0.00
E    C
ATOM  14740  HD1 PHE E  71      -29.505  -8.599  24.897  1.00  0.00
E    H
ATOM  14741  CE1 PHE E  71      -27.733  -9.599  24.312  1.00  0.00
E    C
```

ATOM 14742 HE1 PHE E 71 -27.370 -8.762 23.731 1.00 0.00
E H
ATOM 14743 CZ PHE E 71 -26.989 -10.758 24.399 1.00 0.00
E C
ATOM 14744 HZ PHE E 71 -26.039 -10.828 23.889 1.00 0.00
E H
ATOM 14745 CD2 PHE E 71 -28.653 -11.725 25.799 1.00 0.00
E C
ATOM 14746 HD2 PHE E 71 -29.021 -12.568 26.366 1.00 0.00
E H
ATOM 14747 CE2 PHE E 71 -27.451 -11.823 25.147 1.00 0.00
E C
ATOM 14748 HE2 PHE E 71 -26.872 -12.733 25.219 1.00 0.00
E H
ATOM 14749 C PHE E 71 -33.183 -10.460 26.346 1.00 0.00
E C
ATOM 14750 O PHE E 71 -33.786 -9.389 26.457 1.00 0.00
E O
ATOM 14751 N GLU E 72 -33.577 -11.589 26.931 1.00 0.00
E N
ATOM 14752 HN GLU E 72 -33.129 -12.474 26.844 1.00 0.00
E H
ATOM 14753 CA GLU E 72 -34.757 -11.604 27.784 1.00 0.00
E C
ATOM 14754 HA GLU E 72 -35.184 -10.609 27.787 1.00 0.00
E H
ATOM 14755 CB GLU E 72 -35.758 -12.607 27.238 1.00 0.00
E C
ATOM 14756 HB1 GLU E 72 -35.940 -13.377 28.022 1.00 0.00
E H
ATOM 14757 HB2 GLU E 72 -36.738 -12.107 27.068 1.00 0.00
E H
ATOM 14758 CG GLU E 72 -35.274 -13.273 25.976 1.00 0.00
E C
ATOM 14759 HG1 GLU E 72 -34.914 -12.505 25.268 1.00 0.00
E H
ATOM 14760 HG2 GLU E 72 -34.452 -13.983 26.193 1.00 0.00
E H
ATOM 14761 CD GLU E 72 -36.378 -14.010 25.263 1.00 0.00
E C
ATOM 14762 OE1 GLU E 72 -37.072 -14.820 25.912 1.00 0.00
E O
ATOM 14763 OE2 GLU E 72 -36.547 -13.779 24.045 1.00 0.00
E O
ATOM 14764 C GLU E 72 -34.394 -11.944 29.220 1.00 0.00
E C
ATOM 14765 O GLU E 72 -34.990 -11.411 30.163 1.00 0.00
E O
ATOM 14766 N TYR E 73 -33.419 -12.831 29.408 1.00 0.00
E N
ATOM 14767 HN TYR E 73 -32.942 -13.297 28.664 1.00 0.00
E H
ATOM 14768 CA TYR E 73 -32.964 -13.199 30.742 1.00 0.00
E C

```
ATOM  14769  HA  TYR E  73     -33.663 -12.775  31.453  1.00  0.00
E    H
ATOM  14770  CB  TYR E  73     -32.838 -14.712  30.903  1.00  0.00
E    C
ATOM  14771  HB1 TYR E  73     -32.318 -15.130  30.014  1.00  0.00
E    H
ATOM  14772  HB2 TYR E  73     -32.221 -14.962  31.793  1.00  0.00
E    H
ATOM  14773  CG  TYR E  73     -34.140 -15.458  31.006  1.00  0.00
E    C
ATOM  14774  CD1 TYR E  73     -34.978 -15.587  29.912  1.00  0.00
E    C
ATOM  14775  HD1 TYR E  73     -34.710 -15.124  28.972  1.00  0.00
E    H
ATOM  14776  CE1 TYR E  73     -36.161 -16.278  30.003  1.00  0.00
E    C
ATOM  14777  HE1 TYR E  73     -36.811 -16.371  29.146  1.00  0.00
E    H
ATOM  14778  CZ  TYR E  73     -36.519 -16.848  31.201  1.00  0.00
E    C
ATOM  14779  OH  TYR E  73     -37.699 -17.538  31.312  1.00  0.00
E    O
ATOM  14780  HH  TYR E  73     -37.730 -17.915  32.194  1.00  0.00
E    H
ATOM  14781  CD2 TYR E  73     -34.523 -16.047  32.197  1.00  0.00
E    C
ATOM  14782  HD2 TYR E  73     -33.907 -15.934  33.078  1.00  0.00
E    H
ATOM  14783  CE2 TYR E  73     -35.703 -16.735  32.297  1.00  0.00
E    C
ATOM  14784  HE2 TYR E  73     -35.992 -17.166  33.244  1.00  0.00
E    H
ATOM  14785  C   TYR E  73     -31.617 -12.553  30.999  1.00  0.00
E    C
ATOM  14786  O   TYR E  73     -30.719 -12.627  30.154  1.00  0.00
E    O
ATOM  14787  N   VAL E  74     -31.465 -11.939  32.168  1.00  0.00
E    N
ATOM  14788  HN  VAL E  74     -32.207 -11.822  32.824  1.00  0.00
E    H
ATOM  14789  CA  VAL E  74     -30.191 -11.379  32.595  1.00  0.00
E    C
ATOM  14790  HA  VAL E  74     -29.406 -11.791  31.974  1.00  0.00
E    H
ATOM  14791  CB  VAL E  74     -30.164  -9.845  32.481  1.00  0.00
E    C
ATOM  14792  HB  VAL E  74     -29.144  -9.501  32.782  1.00  0.00
E    H
ATOM  14793  CG1 VAL E  74     -30.434  -9.422  31.054  1.00  0.00
E    C
ATOM  14794 HG11 VAL E  74     -30.454  -8.313  30.978  1.00  0.00
E    H
ATOM  14795 HG12 VAL E  74     -29.647  -9.786  30.363  1.00  0.00
E    H
```

```
ATOM  14796 HG13 VAL E  74      -31.417  -9.807  30.710  1.00  0.00
E    H
ATOM  14797  CG2 VAL E  74      -31.168  -9.207  33.420  1.00  0.00
E    C
ATOM  14798 HG21 VAL E  74      -31.170  -8.103  33.302  1.00  0.00
E    H
ATOM  14799 HG22 VAL E  74      -32.192  -9.584  33.211  1.00  0.00
E    H
ATOM  14800 HG23 VAL E  74      -30.925  -9.431  34.480  1.00  0.00
E    H
ATOM  14801  C   VAL E  74      -29.926 -11.827  34.021  1.00  0.00
E    C
ATOM  14802  O   VAL E  74      -30.841 -11.872  34.854  1.00  0.00
E    O
ATOM  14803  N   ILE E  75      -28.677 -12.170  34.295  1.00  0.00
E    N
ATOM  14804  HN  ILE E  75      -27.939 -12.106  33.626  1.00  0.00
E    H
ATOM  14805  CA  ILE E  75      -28.263 -12.673  35.592  1.00  0.00
E    C
ATOM  14806  HA  ILE E  75      -29.053 -12.457  36.300  1.00  0.00
E    H
ATOM  14807  CB  ILE E  75      -27.926 -14.162  35.534  1.00  0.00
E    C
ATOM  14808  HB  ILE E  75      -26.952 -14.250  36.079  1.00  0.00
E    H
ATOM  14809  CG2 ILE E  75      -29.047 -14.958  36.139  1.00  0.00
E    C
ATOM  14810 HG21 ILE E  75      -28.755 -16.023  36.248  1.00  0.00
E    H
ATOM  14811 HG22 ILE E  75      -29.305 -14.575  37.149  1.00  0.00
E    H
ATOM  14812 HG23 ILE E  75      -29.957 -14.911  35.503  1.00  0.00
E    H
ATOM  14813  CG1 ILE E  75      -27.676 -14.552  34.083  1.00  0.00
E    C
ATOM  14814 HG11 ILE E  75      -28.144 -13.804  33.406  1.00  0.00
E    H
ATOM  14815 HG12 ILE E  75      -26.580 -14.532  33.888  1.00  0.00
E    H
ATOM  14816  CD  ILE E  75      -28.179 -15.906  33.717  1.00  0.00
E    C
ATOM  14817  HD1 ILE E  75      -27.911 -16.145  32.666  1.00  0.00
E    H
ATOM  14818  HD2 ILE E  75      -27.731 -16.681  34.375  1.00  0.00
E    H
ATOM  14819  HD3 ILE E  75      -29.284 -15.959  33.811  1.00  0.00
E    H
ATOM  14820  C   ILE E  75      -27.058 -11.874  36.027  1.00  0.00
E    C
ATOM  14821  O   ILE E  75      -25.979 -11.996  35.437  1.00  0.00
E    O
ATOM  14822  N   VAL E  76      -27.232 -11.077  37.062  1.00  0.00
E    N
```

```
ATOM  14823  HN  VAL E  76      -28.108 -10.957  37.522  1.00  0.00
E    H
ATOM  14824  CA  VAL E  76      -26.147 -10.297  37.626  1.00  0.00
E    C
ATOM  14825  HA  VAL E  76      -25.393 -10.115  36.871  1.00  0.00
E    H
ATOM  14826  CB  VAL E  76      -26.639  -8.965  38.167  1.00  0.00
E    C
ATOM  14827  HB  VAL E  76      -27.330  -9.182  39.019  1.00  0.00
E    H
ATOM  14828  CG1 VAL E  76      -25.486  -8.155  38.656  1.00  0.00
E    C
ATOM  14829 HG11 VAL E  76      -25.836  -7.164  39.017  1.00  0.00
E    H
ATOM  14830 HG12 VAL E  76      -24.966  -8.650  39.502  1.00  0.00
E    H
ATOM  14831 HG13 VAL E  76      -24.752  -7.984  37.839  1.00  0.00
E    H
ATOM  14832  CG2 VAL E  76      -27.368  -8.228  37.076  1.00  0.00
E    C
ATOM  14833 HG21 VAL E  76      -27.705  -7.231  37.433  1.00  0.00
E    H
ATOM  14834 HG22 VAL E  76      -26.705  -8.079  36.196  1.00  0.00
E    H
ATOM  14835 HG23 VAL E  76      -28.267  -8.787  36.745  1.00  0.00
E    H
ATOM  14836  C   VAL E  76      -25.521 -11.183  38.694  1.00  0.00
E    C
ATOM  14837  O   VAL E  76      -25.962 -11.230  39.849  1.00  0.00
E    O
ATOM  14838  N   ALA E  77      -24.507 -11.938  38.284  1.00  0.00
E    N
ATOM  14839  HN  ALA E  77      -24.190 -11.916  37.336  1.00  0.00
E    H
ATOM  14840  CA  ALA E  77      -23.783 -12.848  39.165  1.00  0.00
E    C
ATOM  14841  HA  ALA E  77      -24.403 -13.140  40.004  1.00  0.00
E    H
ATOM  14842  CB  ALA E  77      -23.397 -14.116  38.418  1.00  0.00
E    C
ATOM  14843  HB1 ALA E  77      -24.313 -14.617  38.038  1.00  0.00
E    H
ATOM  14844  HB2 ALA E  77      -22.747 -13.884  37.546  1.00  0.00
E    H
ATOM  14845  HB3 ALA E  77      -22.861 -14.833  39.078  1.00  0.00
E    H
ATOM  14846  C   ALA E  77      -22.558 -12.179  39.753  1.00  0.00
E    C
ATOM  14847  O   ALA E  77      -21.443 -12.689  39.665  1.00  0.00
E    O
ATOM  14848  N   ILE E  78      -22.751 -11.018  40.347  1.00  0.00
E    N
ATOM  14849  HN  ILE E  78      -23.628 -10.541  40.336  1.00  0.00
E    H
```

```
ATOM  14850  CA  ILE E  78     -21.706 -10.324  41.075  1.00  0.00
E    C
ATOM  14851  HA  ILE E  78     -20.752 -10.758  40.804  1.00  0.00
E    H
ATOM  14852  CB  ILE E  78     -21.757  -8.820  40.804  1.00  0.00
E    C
ATOM  14853  HB  ILE E  78     -21.410  -8.715  39.745  1.00  0.00
E    H
ATOM  14854  CG2 ILE E  78     -23.118  -8.302  41.099  1.00  0.00
E    C
ATOM  14855 HG21 ILE E  78     -23.217  -7.249  40.761  1.00  0.00
E    H
ATOM  14856 HG22 ILE E  78     -23.892  -8.899  40.572  1.00  0.00
E    H
ATOM  14857 HG23 ILE E  78     -23.328  -8.338  42.189  1.00  0.00
E    H
ATOM  14858  CG1 ILE E  78     -20.752  -8.073  41.664  1.00  0.00
E    C
ATOM  14859 HG11 ILE E  78     -20.959  -8.268  42.740  1.00  0.00
E    H
ATOM  14860 HG12 ILE E  78     -19.732  -8.465  41.449  1.00  0.00
E    H
ATOM  14861  CD  ILE E  78     -20.769  -6.601  41.406  1.00  0.00
E    C
ATOM  14862  HD1 ILE E  78     -19.984  -6.095  42.009  1.00  0.00
E    H
ATOM  14863  HD2 ILE E  78     -20.574  -6.389  40.332  1.00  0.00
E    H
ATOM  14864  HD3 ILE E  78     -21.750  -6.159  41.680  1.00  0.00
E    H
ATOM  14865  C   ILE E  78     -21.932 -10.640  42.543  1.00  0.00
E    C
ATOM  14866  O   ILE E  78     -23.072 -10.868  42.959  1.00  0.00
E    O
ATOM  14867  N   GLY E  79     -20.864 -10.696  43.333  1.00  0.00
E    N
ATOM  14868  HN  GLY E  79     -19.917 -10.595  43.030  1.00  0.00
E    H
ATOM  14869  CA  GLY E  79     -21.047 -10.917  44.753  1.00  0.00
E    C
ATOM  14870  HA1 GLY E  79     -20.639 -11.899  44.953  1.00  0.00
E    H
ATOM  14871  HA2 GLY E  79     -22.111 -10.834  44.933  1.00  0.00
E    H
ATOM  14872  C   GLY E  79     -20.343  -9.926  45.652  1.00  0.00
E    C
ATOM  14873  O   GLY E  79     -20.741  -9.736  46.804  1.00  0.00
E    O
ATOM  14874  N   ALA E  80     -19.298  -9.283  45.143  1.00  0.00
E    N
ATOM  14875  HN  ALA E  80     -18.995  -9.334  44.192  1.00  0.00
E    H
ATOM  14876  CA  ALA E  80     -18.501  -8.425  46.008  1.00  0.00
E    C
```

```
ATOM  14877  HA  ALA E  80      -18.618  -8.736  47.039  1.00  0.00
E    H
ATOM  14878  CB  ALA E  80      -17.021  -8.546  45.644  1.00  0.00
E    C
ATOM  14879  HB1 ALA E  80      -16.701  -9.606  45.734  1.00  0.00
E    H
ATOM  14880  HB2 ALA E  80      -16.839  -8.222  44.596  1.00  0.00
E    H
ATOM  14881  HB3 ALA E  80      -16.382  -7.936  46.319  1.00  0.00
E    H
ATOM  14882  C   ALA E  80      -18.922  -6.963  45.951  1.00  0.00
E    C
ATOM  14883  O   ALA E  80      -19.305  -6.386  46.972  1.00  0.00
E    O
ATOM  14884  N   ASN E  81      -18.861  -6.350  44.781  1.00  0.00
E    N
ATOM  14885  HN  ASN E  81      -18.721  -6.826  43.915  1.00  0.00
E    H
ATOM  14886  CA  ASN E  81      -18.995  -4.907  44.661  1.00  0.00
E    C
ATOM  14887  HA  ASN E  81      -18.577  -4.477  45.565  1.00  0.00
E    H
ATOM  14888  CB  ASN E  81      -18.275  -4.433  43.407  1.00  0.00
E    C
ATOM  14889  HB1 ASN E  81      -17.234  -4.820  43.446  1.00  0.00
E    H
ATOM  14890  HB2 ASN E  81      -18.755  -4.858  42.503  1.00  0.00
E    H
ATOM  14891  CG  ASN E  81      -18.213  -2.945  43.309  1.00  0.00
E    C
ATOM  14892  OD1 ASN E  81      -19.105  -2.243  43.772  1.00  0.00
E    O
ATOM  14893  ND2 ASN E  81      -17.152  -2.443  42.697  1.00  0.00
E    N
ATOM  14894 HD21 ASN E  81      -17.088  -1.451  42.631  1.00  0.00
E    H
ATOM  14895 HD22 ASN E  81      -16.443  -3.050  42.349  1.00  0.00
E    H
ATOM  14896  C   ASN E  81      -20.474  -4.546  44.621  1.00  0.00
E    C
ATOM  14897  O   ASN E  81      -21.052  -4.416  43.540  1.00  0.00
E    O
ATOM  14898  N   ILE E  82      -21.072  -4.341  45.795  1.00  0.00
E    N
ATOM  14899  HN  ILE E  82      -20.617  -4.342  46.683  1.00  0.00
E    H
ATOM  14900  CA  ILE E  82      -22.509  -4.088  45.860  1.00  0.00
E    C
ATOM  14901  HA  ILE E  82      -22.996  -4.979  45.481  1.00  0.00
E    H
ATOM  14902  CB  ILE E  82      -22.940  -3.813  47.308  1.00  0.00
E    C
ATOM  14903  HB  ILE E  82      -22.168  -3.104  47.700  1.00  0.00
E    H
```

ATOM 14904 CG2 ILE E 82   -24.353 -3.274 47.357 1.00 0.00
E   C
ATOM 14905 HG21 ILE E 82   -24.606 -2.931 48.381 1.00 0.00
E   H
ATOM 14906 HG22 ILE E 82   -24.469 -2.407 46.673 1.00 0.00
E   H
ATOM 14907 HG23 ILE E 82   -25.086 -4.054 47.061 1.00 0.00
E   H
ATOM 14908 CG1 ILE E 82   -22.836 -5.086 48.139 1.00 0.00
E   C
ATOM 14909 HG11 ILE E 82   -23.636 -5.098 48.913 1.00 0.00
E   H
ATOM 14910 HG12 ILE E 82   -23.007 -5.965 47.476 1.00 0.00
E   H
ATOM 14911 CD ILE E 82   -21.515 -5.267 48.810 1.00 0.00
E   C
ATOM 14912 HD1 ILE E 82   -21.487 -6.238 49.349 1.00 0.00
E   H
ATOM 14913 HD2 ILE E 82   -20.692 -5.260 48.064 1.00 0.00
E   H
ATOM 14914 HD3 ILE E 82   -21.330 -4.459 49.548 1.00 0.00
E   H
ATOM 14915 C   ILE E 82   -22.900 -2.947 44.935 1.00 0.00
E   C
ATOM 14916 O   ILE E 82   -23.960 -2.982 44.293 1.00 0.00
E   O
ATOM 14917 N   GLN E 83   -22.037 -1.937 44.832 1.00 0.00
E   N
ATOM 14918 HN GLN E 83   -21.180 -1.873 45.340 1.00 0.00
E   H
ATOM 14919 CA GLN E 83   -22.293 -0.816 43.940 1.00 0.00
E   C
ATOM 14920 HA GLN E 83   -23.116 -0.255 44.365 1.00 0.00
E   H
ATOM 14921 CB GLN E 83   -21.032   0.016 43.803 1.00 0.00
E   C
ATOM 14922 HB1 GLN E 83   -20.279 -0.423 44.499 1.00 0.00
E   H
ATOM 14923 HB2 GLN E 83   -20.617 -0.110 42.777 1.00 0.00
E   H
ATOM 14924 CG GLN E 83   -21.173   1.453 44.111 1.00 0.00
E   C
ATOM 14925 HG1 GLN E 83   -22.090   1.863 43.636 1.00 0.00
E   H
ATOM 14926 HG2 GLN E 83   -21.257   1.613 45.206 1.00 0.00
E   H
ATOM 14927 CD GLN E 83   -19.999   2.226 43.571 1.00 0.00
E   C
ATOM 14928 OE1 GLN E 83   -19.564   1.991 42.450 1.00 0.00
E   O
ATOM 14929 NE2 GLN E 83   -19.460   3.128 44.371 1.00 0.00
E   N
ATOM 14930 HE21 GLN E 83   -18.687   3.643 44.013 1.00 0.00
E   H

```
ATOM  14931 HE22 GLN E  83      -19.822   3.253  45.290  1.00  0.00
E    H
ATOM  14932  C   GLN E  83      -22.728  -1.294  42.568  1.00  0.00
E    C
ATOM  14933  O   GLN E  83      -23.864  -1.053  42.139  1.00  0.00
E    O
ATOM  14934  N   ALA E  84      -21.840  -2.008  41.885  1.00  0.00
E    N
ATOM  14935  HN  ALA E  84      -20.954  -2.295  42.249  1.00  0.00
E    H
ATOM  14936  CA  ALA E  84      -22.120  -2.424  40.522  1.00  0.00
E    C
ATOM  14937  HA  ALA E  84      -22.275  -1.562  39.883  1.00  0.00
E    H
ATOM  14938  CB  ALA E  84      -20.940  -3.216  39.970  1.00  0.00
E    C
ATOM  14939  HB1 ALA E  84      -20.028  -2.582  39.980  1.00  0.00
E    H
ATOM  14940  HB2 ALA E  84      -20.738  -4.116  40.590  1.00  0.00
E    H
ATOM  14941  HB3 ALA E  84      -21.120  -3.541  38.922  1.00  0.00
E    H
ATOM  14942  C   ALA E  84      -23.396  -3.240  40.458  1.00  0.00
E    C
ATOM  14943  O   ALA E  84      -24.254  -2.998  39.606  1.00  0.00
E    O
ATOM  14944  N   SER E  85      -23.536  -4.211  41.357  1.00  0.00
E    N
ATOM  14945  HN  SER E  85      -22.852  -4.470  42.038  1.00  0.00
E    H
ATOM  14946  CA  SER E  85      -24.757  -5.000  41.409  1.00  0.00
E    C
ATOM  14947  HA  SER E  85      -24.749  -5.629  40.528  1.00  0.00
E    H
ATOM  14948  CB  SER E  85      -24.800  -5.796  42.708  1.00  0.00
E    C
ATOM  14949  HB1 SER E  85      -25.449  -6.687  42.546  1.00  0.00
E    H
ATOM  14950  HB2 SER E  85      -23.785  -6.173  42.965  1.00  0.00
E    H
ATOM  14951  OG  SER E  85      -25.343  -5.005  43.747  1.00  0.00
E    O
ATOM  14952  HG1 SER E  85      -25.558  -5.601  44.475  1.00  0.00
E    H
ATOM  14953  C   SER E  85      -25.981  -4.106  41.314  1.00  0.00
E    C
ATOM  14954  O   SER E  85      -26.828  -4.264  40.424  1.00  0.00
E    O
ATOM  14955  N   THR E  86      -26.063  -3.125  42.203  1.00  0.00
E    N
ATOM  14956  HN  THR E  86      -25.339  -2.828  42.825  1.00  0.00
E    H
ATOM  14957  CA  THR E  86      -27.301  -2.375  42.327  1.00  0.00
E    C
```

```
ATOM  14958  HA  THR E  86      -28.092  -3.113  42.296  1.00  0.00
E    H
ATOM  14959  CB  THR E  86      -27.297  -1.598  43.631  1.00  0.00
E    C
ATOM  14960  HB  THR E  86      -28.294  -1.098  43.697  1.00  0.00
E    H
ATOM  14961  OG1 THR E  86      -26.291  -0.583  43.569  1.00  0.00
E    O
ATOM  14962  HG1 THR E  86      -26.326  -0.134  44.420  1.00  0.00
E    H
ATOM  14963  CG2 THR E  86      -27.010  -2.532  44.784  1.00  0.00
E    C
ATOM  14964 HG21 THR E  86      -27.116  -2.004  45.756  1.00  0.00
E    H
ATOM  14965 HG22 THR E  86      -27.724  -3.384  44.780  1.00  0.00
E    H
ATOM  14966 HG23 THR E  86      -25.979  -2.941  44.721  1.00  0.00
E    H
ATOM  14967  C   THR E  86      -27.527  -1.449  41.140  1.00  0.00
E    C
ATOM  14968  O   THR E  86      -28.622  -1.432  40.566  1.00  0.00
E    O
ATOM  14969  N   LEU E  87      -26.520  -0.658  40.763  1.00  0.00
E    N
ATOM  14970  HN  LEU E  87      -25.614  -0.595  41.178  1.00  0.00
E    H
ATOM  14971  CA  LEU E  87      -26.721   0.233  39.619  1.00  0.00
E    C
ATOM  14972  HA  LEU E  87      -27.594   0.835  39.838  1.00  0.00
E    H
ATOM  14973  CB  LEU E  87      -25.526   1.160  39.389  1.00  0.00
E    C
ATOM  14974  HB1 LEU E  87      -24.800   0.608  38.748  1.00  0.00
E    H
ATOM  14975  HB2 LEU E  87      -25.858   2.037  38.791  1.00  0.00
E    H
ATOM  14976  CG  LEU E  87      -24.549   1.838  40.356  1.00  0.00
E    C
ATOM  14977  HG  LEU E  87      -24.657   2.920  40.101  1.00  0.00
E    H
ATOM  14978  CD1 LEU E  87      -24.795   1.632  41.836  1.00  0.00
E    C
ATOM  14979 HD11 LEU E  87      -24.001   2.127  42.435  1.00  0.00
E    H
ATOM  14980 HD12 LEU E  87      -25.776   2.061  42.133  1.00  0.00
E    H
ATOM  14981 HD13 LEU E  87      -24.795   0.550  42.086  1.00  0.00
E    H
ATOM  14982  CD2 LEU E  87      -23.127   1.405  39.990  1.00  0.00
E    C
ATOM  14983 HD21 LEU E  87      -22.390   1.895  40.662  1.00  0.00
E    H
ATOM  14984 HD22 LEU E  87      -22.995   0.309  40.086  1.00  0.00
E    H
```

```
ATOM  14985 HD23 LEU E  87      -22.890   1.696  38.943  1.00  0.00
E    H
ATOM  14986  C   LEU E  87      -27.017  -0.564  38.363  1.00  0.00
E    C
ATOM  14987  O   LEU E  87      -27.816  -0.145  37.515  1.00  0.00
E    O
ATOM  14988  N   THR E  88      -26.385  -1.722  38.231  1.00  0.00
E    N
ATOM  14989  HN  THR E  88      -25.661  -2.046  38.839  1.00  0.00
E    H
ATOM  14990  CA  THR E  88      -26.711  -2.633  37.155  1.00  0.00
E    C
ATOM  14991  HA  THR E  88      -26.482  -2.108  36.236  1.00  0.00
E    H
ATOM  14992  CB  THR E  88      -25.937  -3.918  37.372  1.00  0.00
E    C
ATOM  14993  HB  THR E  88      -26.275  -4.321  38.358  1.00  0.00
E    H
ATOM  14994  OG1 THR E  88      -24.544  -3.609  37.467  1.00  0.00
E    O
ATOM  14995  HG1 THR E  88      -24.102  -4.455  37.592  1.00  0.00
E    H
ATOM  14996  CG2 THR E  88      -26.188  -4.876  36.248  1.00  0.00
E    C
ATOM  14997 HG21 THR E  88      -25.718  -5.860  36.455  1.00  0.00
E    H
ATOM  14998 HG22 THR E  88      -27.279  -5.041  36.115  1.00  0.00
E    H
ATOM  14999 HG23 THR E  88      -25.776  -4.488  35.292  1.00  0.00
E    H
ATOM  15000  C   THR E  88      -28.194  -2.917  37.128  1.00  0.00
E    C
ATOM  15001  O   THR E  88      -28.890  -2.581  36.161  1.00  0.00
E    O
ATOM  15002  N   THR E  89      -28.697  -3.496  38.217  1.00  0.00
E    N
ATOM  15003  HN  THR E  89      -28.168  -3.748  39.027  1.00  0.00
E    H
ATOM  15004  CA  THR E  89      -30.115  -3.814  38.308  1.00  0.00
E    C
ATOM  15005  HA  THR E  89      -30.301  -4.556  37.544  1.00  0.00
E    H
ATOM  15006  CB  THR E  89      -30.447  -4.280  39.712  1.00  0.00
E    C
ATOM  15007  HB  THR E  89      -31.562  -4.325  39.768  1.00  0.00
E    H
ATOM  15008  OG1 THR E  89      -29.986  -3.292  40.639  1.00  0.00
E    O
ATOM  15009  HG1 THR E  89      -30.207  -3.635  41.511  1.00  0.00
E    H
ATOM  15010  CG2 THR E  89      -29.758  -5.587  39.995  1.00  0.00
E    C
ATOM  15011 HG21 THR E  89      -30.080  -6.000  40.975  1.00  0.00
E    H
```

```
ATOM  15012 HG22 THR E  89      -30.007  -6.334  39.212  1.00  0.00
E    H
ATOM  15013 HG23 THR E  89      -28.654  -5.460  40.018  1.00  0.00
E    H
ATOM  15014  C   THR E  89      -30.968  -2.612  37.958  1.00  0.00
E    C
ATOM  15015  O   THR E  89      -32.011  -2.748  37.309  1.00  0.00
E    O
ATOM  15016  N   LEU E  90      -30.551  -1.430  38.399  1.00  0.00
E    N
ATOM  15017  HN  LEU E  90      -29.764  -1.274  38.992  1.00  0.00
E    H
ATOM  15018  CA  LEU E  90      -31.264  -0.221  38.021  1.00  0.00
E    C
ATOM  15019  HA  LEU E  90      -32.268  -0.311  38.417  1.00  0.00
E    H
ATOM  15020  CB  LEU E  90      -30.558   1.003  38.584  1.00  0.00
E    C
ATOM  15021  HB1 LEU E  90      -30.848   1.085  39.657  1.00  0.00
E    H
ATOM  15022  HB2 LEU E  90      -29.461   0.824  38.574  1.00  0.00
E    H
ATOM  15023  CG  LEU E  90      -30.908   2.304  37.868  1.00  0.00
E    C
ATOM  15024  HG  LEU E  90      -30.520   2.247  36.823  1.00  0.00
E    H
ATOM  15025  CD1 LEU E  90      -32.400   2.554  37.855  1.00  0.00
E    C
ATOM  15026 HD11 LEU E  90      -32.628   3.528  37.373  1.00  0.00
E    H
ATOM  15027 HD12 LEU E  90      -32.925   1.754  37.291  1.00  0.00
E    H
ATOM  15028 HD13 LEU E  90      -32.803   2.577  38.890  1.00  0.00
E    H
ATOM  15029  CD2 LEU E  90      -30.215   3.435  38.557  1.00  0.00
E    C
ATOM  15030 HD21 LEU E  90      -30.437   4.395  38.043  1.00  0.00
E    H
ATOM  15031 HD22 LEU E  90      -30.541   3.533  39.612  1.00  0.00
E    H
ATOM  15032 HD23 LEU E  90      -29.113   3.281  38.544  1.00  0.00
E    H
ATOM  15033  C   LEU E  90      -31.374  -0.109  36.513  1.00  0.00
E    C
ATOM  15034  O   LEU E  90      -32.471   0.051  35.971  1.00  0.00
E    O
ATOM  15035  N   LEU E  91      -30.242  -0.168  35.823  1.00  0.00
E    N
ATOM  15036  HN  LEU E  91      -29.333  -0.337  36.199  1.00  0.00
E    H
ATOM  15037  CA  LEU E  91      -30.266   0.023  34.379  1.00  0.00
E    C
ATOM  15038  HA  LEU E  91      -30.723   0.987  34.197  1.00  0.00
E    H
```

```
ATOM  15039  CB  LEU E  91      -28.850  -0.008  33.820  1.00  0.00
E    C
ATOM  15040  HB1 LEU E  91      -28.257  -0.698  34.463  1.00  0.00
E    H
ATOM  15041  HB2 LEU E  91      -28.870  -0.471  32.809  1.00  0.00
E    H
ATOM  15042  CG  LEU E  91      -28.191   1.367  33.795  1.00  0.00
E    C
ATOM  15043  HG  LEU E  91      -27.144   1.239  33.429  1.00  0.00
E    H
ATOM  15044  CD1 LEU E  91      -28.953   2.283  32.857  1.00  0.00
E    C
ATOM  15045 HD11 LEU E  91      -28.513   3.303  32.866  1.00  0.00
E    H
ATOM  15046 HD12 LEU E  91      -28.917   1.894  31.817  1.00  0.00
E    H
ATOM  15047 HD13 LEU E  91      -30.018   2.362  33.165  1.00  0.00
E    H
ATOM  15048  CD2 LEU E  91      -28.131   1.965  35.183  1.00  0.00
E    C
ATOM  15049 HD21 LEU E  91      -27.632   2.958  35.155  1.00  0.00
E    H
ATOM  15050 HD22 LEU E  91      -29.143   2.109  35.612  1.00  0.00
E    H
ATOM  15051 HD23 LEU E  91      -27.552   1.306  35.866  1.00  0.00
E    H
ATOM  15052  C   LEU E  91      -31.131  -1.028  33.707  1.00  0.00
E    C
ATOM  15053  O   LEU E  91      -31.900  -0.728  32.786  1.00  0.00
E    O
ATOM  15054  N   LEU E  92      -31.031  -2.264  34.173  1.00  0.00
E    N
ATOM  15055  HN  LEU E  92      -30.468  -2.559  34.943  1.00  0.00
E    H
ATOM  15056  CA  LEU E  92      -31.789  -3.335  33.543  1.00  0.00
E    C
ATOM  15057  HA  LEU E  92      -31.592  -3.270  32.481  1.00  0.00
E    H
ATOM  15058  CB  LEU E  92      -31.335  -4.686  34.078  1.00  0.00
E    C
ATOM  15059  HB1 LEU E  92      -31.123  -4.556  35.164  1.00  0.00
E    H
ATOM  15060  HB2 LEU E  92      -32.182  -5.405  34.013  1.00  0.00
E    H
ATOM  15061  CG  LEU E  92      -30.094  -5.221  33.375  1.00  0.00
E    C
ATOM  15062  HG  LEU E  92      -30.010  -6.314  33.586  1.00  0.00
E    H
ATOM  15063  CD1 LEU E  92      -30.239  -4.994  31.888  1.00  0.00
E    C
ATOM  15064 HD11 LEU E  92      -29.326  -5.331  31.351  1.00  0.00
E    H
ATOM  15065 HD12 LEU E  92      -31.108  -5.560  31.489  1.00  0.00
E    H
```

```
ATOM  15066 HD13 LEU E  92      -30.392  -3.916  31.669  1.00  0.00
E    H
ATOM  15067  CD2 LEU E  92      -28.844  -4.552  33.879  1.00  0.00
E    C
ATOM  15068 HD21 LEU E  92      -27.950  -4.969  33.368  1.00  0.00
E    H
ATOM  15069 HD22 LEU E  92      -28.861  -3.460  33.691  1.00  0.00
E    H
ATOM  15070 HD23 LEU E  92      -28.728  -4.718  34.972  1.00  0.00
E    H
ATOM  15071  C   LEU E  92      -33.284  -3.148  33.744  1.00  0.00
E    C
ATOM  15072  O   LEU E  92      -34.053  -3.153  32.776  1.00  0.00
E    O
ATOM  15073  N   LYS E  93      -33.715  -2.969  34.991  1.00  0.00
E    N
ATOM  15074  HN  LYS E  93      -33.134  -2.963  35.803  1.00  0.00
E    H
ATOM  15075  CA  LYS E  93      -35.132  -2.763  35.244  1.00  0.00
E    C
ATOM  15076  HA  LYS E  93      -35.662  -3.604  34.814  1.00  0.00
E    H
ATOM  15077  CB  LYS E  93      -35.389  -2.681  36.743  1.00  0.00
E    C
ATOM  15078  HB1 LYS E  93      -34.611  -3.302  37.244  1.00  0.00
E    H
ATOM  15079  HB2 LYS E  93      -35.220  -1.635  37.083  1.00  0.00
E    H
ATOM  15080  CG  LYS E  93      -36.760  -3.171  37.150  1.00  0.00
E    C
ATOM  15081  HG1 LYS E  93      -36.814  -3.223  38.261  1.00  0.00
E    H
ATOM  15082  HG2 LYS E  93      -37.523  -2.432  36.815  1.00  0.00
E    H
ATOM  15083  CD  LYS E  93      -37.038  -4.539  36.563  1.00  0.00
E    C
ATOM  15084  HD1 LYS E  93      -36.961  -4.469  35.453  1.00  0.00
E    H
ATOM  15085  HD2 LYS E  93      -36.238  -5.234  36.909  1.00  0.00
E    H
ATOM  15086  CE  LYS E  93      -38.406  -5.051  36.981  1.00  0.00
E    C
ATOM  15087  HE1 LYS E  93      -38.414  -5.303  38.065  1.00  0.00
E    H
ATOM  15088  HE2 LYS E  93      -39.185  -4.280  36.793  1.00  0.00
E    H
ATOM  15089  NZ  LYS E  93      -38.798  -6.279  36.232  1.00  0.00
E    N
ATOM  15090  HZ1 LYS E  93      -39.736  -6.602  36.544  1.00  0.00
E    H
ATOM  15091  HZ2 LYS E  93      -38.825  -6.070  35.213  1.00  0.00
E    H
ATOM  15092  HZ3 LYS E  93      -38.099  -7.029  36.409  1.00  0.00
E    H
```

```
ATOM  15093  C   LYS E  93     -35.629  -1.509  34.545  1.00  0.00
E    C
ATOM  15094  O   LYS E  93     -36.797  -1.427  34.156  1.00  0.00
E    O
ATOM  15095  N   GLU E  94     -34.754  -0.522  34.376  1.00  0.00
E    N
ATOM  15096  HN  GLU E  94     -33.832  -0.484  34.751  1.00  0.00
E    H
ATOM  15097  CA  GLU E  94     -35.116   0.638  33.580  1.00  0.00
E    C
ATOM  15098  HA  GLU E  94     -36.082   0.994  33.916  1.00  0.00
E    H
ATOM  15099  CB  GLU E  94     -34.076   1.738  33.749  1.00  0.00
E    C
ATOM  15100  HB1 GLU E  94     -34.223   2.204  34.751  1.00  0.00
E    H
ATOM  15101  HB2 GLU E  94     -33.056   1.292  33.767  1.00  0.00
E    H
ATOM  15102  CG  GLU E  94     -34.158   2.808  32.686  1.00  0.00
E    C
ATOM  15103  HG1 GLU E  94     -33.709   2.432  31.748  1.00  0.00
E    H
ATOM  15104  HG2 GLU E  94     -35.211   3.099  32.495  1.00  0.00
E    H
ATOM  15105  CD  GLU E  94     -33.376   4.042  33.052  1.00  0.00
E    C
ATOM  15106  OE1 GLU E  94     -33.271   4.338  34.259  1.00  0.00
E    O
ATOM  15107  OE2 GLU E  94     -32.872   4.720  32.136  1.00  0.00
E    O
ATOM  15108  C   GLU E  94     -35.259   0.267  32.116  1.00  0.00
E    C
ATOM  15109  O   GLU E  94     -36.106   0.824  31.411  1.00  0.00
E    O
ATOM  15110  N   LEU E  95     -34.448  -0.668  31.640  1.00  0.00
E    N
ATOM  15111  HN  LEU E  95     -33.735  -1.124  32.169  1.00  0.00
E    H
ATOM  15112  CA  LEU E  95     -34.544  -1.113  30.262  1.00  0.00
E    C
ATOM  15113  HA  LEU E  95     -34.990  -0.293  29.714  1.00  0.00
E    H
ATOM  15114  CB  LEU E  95     -33.159  -1.397  29.701  1.00  0.00
E    C
ATOM  15115  HB1 LEU E  95     -32.507  -1.678  30.560  1.00  0.00
E    H
ATOM  15116  HB2 LEU E  95     -33.210  -2.295  29.046  1.00  0.00
E    H
ATOM  15117  CG  LEU E  95     -32.666  -0.135  28.996  1.00  0.00
E    C
ATOM  15118  HG  LEU E  95     -33.523   0.219  28.374  1.00  0.00
E    H
ATOM  15119  CD1 LEU E  95     -32.287   0.941  29.997  1.00  0.00
E    C
```

```
ATOM  15120 HD11 LEU E  95      -31.885   1.836  29.475  1.00  0.00
E    H
ATOM  15121 HD12 LEU E  95      -33.173   1.252  30.592  1.00  0.00
E    H
ATOM  15122 HD13 LEU E  95      -31.508   0.570  30.697  1.00  0.00
E    H
ATOM  15123  CD2 LEU E  95      -31.513  -0.437  28.073  1.00  0.00
E    C
ATOM  15124 HD21 LEU E  95      -31.181   0.488  27.554  1.00  0.00
E    H
ATOM  15125 HD22 LEU E  95      -30.643  -0.845  28.624  1.00  0.00
E    H
ATOM  15126 HD23 LEU E  95      -31.817  -1.177  27.301  1.00  0.00
E    H
ATOM  15127  C   LEU E  95      -35.449  -2.319  30.098  1.00  0.00
E    C
ATOM  15128  O   LEU E  95      -35.300  -3.064  29.126  1.00  0.00
E    O
ATOM  15129  N   ASP E  96      -36.361  -2.535  31.041  1.00  0.00
E    N
ATOM  15130  HN  ASP E  96      -36.305  -2.181  31.971  1.00  0.00
E    H
ATOM  15131  CA  ASP E  96      -37.561  -3.328  30.807  1.00  0.00
E    C
ATOM  15132  HA  ASP E  96      -38.119  -3.324  31.736  1.00  0.00
E    H
ATOM  15133  CB  ASP E  96      -38.382  -2.720  29.677  1.00  0.00
E    C
ATOM  15134  HB1 ASP E  96      -37.926  -2.996  28.705  1.00  0.00
E    H
ATOM  15135  HB2 ASP E  96      -39.420  -3.108  29.697  1.00  0.00
E    H
ATOM  15136  CG  ASP E  96      -38.424  -1.220  29.751  1.00  0.00
E    C
ATOM  15137  OD1 ASP E  96      -38.351  -0.691  30.879  1.00  0.00
E    O
ATOM  15138  OD2 ASP E  96      -38.500  -0.571  28.690  1.00  0.00
E    O
ATOM  15139  C   ASP E  96      -37.242  -4.777  30.494  1.00  0.00
E    C
ATOM  15140  O   ASP E  96      -38.082  -5.506  29.964  1.00  0.00
E    O
ATOM  15141  N   ILE E  97      -36.028  -5.202  30.808  1.00  0.00
E    N
ATOM  15142  HN  ILE E  97      -35.297  -4.675  31.236  1.00  0.00
E    H
ATOM  15143  CA  ILE E  97      -35.674  -6.587  30.515  1.00  0.00
E    C
ATOM  15144  HA  ILE E  97      -35.887  -6.760  29.468  1.00  0.00
E    H
ATOM  15145  CB  ILE E  97      -34.204  -6.838  30.844  1.00  0.00
E    C
ATOM  15146  HB  ILE E  97      -34.101  -6.534  31.917  1.00  0.00
E    H
```

ATOM 15147 CG2 ILE E 97   -33.825 -8.275 30.559 1.00 0.00
E   C
ATOM 15148 HG21 ILE E 97   -32.807 -8.495 30.943 1.00 0.00
E   H
ATOM 15149 HG22 ILE E 97   -34.530 -8.976 31.054 1.00 0.00
E   H
ATOM 15150 HG23 ILE E 97   -33.836 -8.480 29.468 1.00 0.00
E   H
ATOM 15151 CG1 ILE E 97   -33.352 -5.888 30.014 1.00 0.00
E   C
ATOM 15152 HG11 ILE E 97   -33.573 -4.836 30.301 1.00 0.00
E   H
ATOM 15153 HG12 ILE E 97   -32.277 -6.073 30.243 1.00 0.00
E   H
ATOM 15154 CD ILE E 97   -33.560 -6.054 28.530 1.00 0.00
E   C
ATOM 15155 HD1 ILE E 97   -32.876 -5.381 27.968 1.00 0.00
E   H
ATOM 15156 HD2 ILE E 97   -33.352 -7.101 28.220 1.00 0.00
E   H
ATOM 15157 HD3 ILE E 97   -34.603 -5.803 28.243 1.00 0.00
E   H
ATOM 15158 C   ILE E 97   -36.589 -7.508 31.308 1.00 0.00
E   C
ATOM 15159 O   ILE E 97   -36.869 -7.234 32.490 1.00 0.00
E   O
ATOM 15160 N   PRO E 98   -37.110 -8.574 30.708 1.00 0.00
E   N
ATOM 15161 CD PRO E 98   -36.898 -9.033 29.327 1.00 0.00
E   C
ATOM 15162 HD1 PRO E 98   -37.238 -7.983 29.475 1.00 0.00
E   H
ATOM 15163 HD2 PRO E 98   -36.188 -9.095 28.471 1.00 0.00
E   H
ATOM 15164 CA PRO E 98   -38.112 -9.379 31.418 1.00 0.00
E   C
ATOM 15165 HA PRO E 98   -38.985 -8.772 31.624 1.00 0.00
E   H
ATOM 15166 CB PRO E 98   -38.460 -10.471 30.399 1.00 0.00
E   C
ATOM 15167 HB1 PRO E 98   -39.243 -10.087 29.709 1.00 0.00
E   H
ATOM 15168 HB2 PRO E 98   -38.825 -11.403 30.877 1.00 0.00
E   H
ATOM 15169 CG PRO E 98   -38.064 -9.915 29.083 1.00 0.00
E   C
ATOM 15170 HG1 PRO E 98   -38.604 -10.381 28.237 1.00 0.00
E   H
ATOM 15171 HG2 PRO E 98   -36.966 -10.050 28.942 1.00 0.00
E   H
ATOM 15172 C   PRO E 98   -37.601 -9.967 32.713 1.00 0.00
E   C
ATOM 15173 O   PRO E 98   -38.162 -9.696 33.780 1.00 0.00
E   O

```
ATOM  15174  N   ASN E  99     -36.528 -10.742  32.658  1.00  0.00
E    N
ATOM  15175  HN  ASN E  99     -35.946 -10.822  31.849  1.00  0.00
E    H
ATOM  15176  CA  ASN E  99     -36.102 -11.553  33.787  1.00  0.00
E    C
ATOM  15177  HA  ASN E  99     -36.849 -11.428  34.564  1.00  0.00
E    H
ATOM  15178  CB  ASN E  99     -35.942 -13.007  33.365  1.00  0.00
E    C
ATOM  15179  HB1 ASN E  99     -35.095 -13.062  32.647  1.00  0.00
E    H
ATOM  15180  HB2 ASN E  99     -35.679 -13.636  34.240  1.00  0.00
E    H
ATOM  15181  CG  ASN E  99     -37.171 -13.534  32.688  1.00  0.00
E    C
ATOM  15182  OD1 ASN E  99     -37.141 -13.875  31.510  1.00  0.00
E    O
ATOM  15183  ND2 ASN E  99     -38.275 -13.581  33.419  1.00  0.00
E    N
ATOM  15184 HD21 ASN E  99     -39.096 -13.939  32.985  1.00  0.00
E    H
ATOM  15185 HD22 ASN E  99     -38.254 -13.292  34.372  1.00  0.00
E    H
ATOM  15186  C   ASN E  99     -34.791 -11.006  34.319  1.00  0.00
E    C
ATOM  15187  O   ASN E  99     -33.799 -10.948  33.586  1.00  0.00
E    O
ATOM  15188  N   ILE E 100     -34.785 -10.616  35.586  1.00  0.00
E    N
ATOM  15189  HN  ILE E 100     -35.587 -10.676  36.178  1.00  0.00
E    H
ATOM  15190  CA  ILE E 100     -33.608 -10.063  36.234  1.00  0.00
E    C
ATOM  15191  HA  ILE E 100     -32.763 -10.204  35.573  1.00  0.00
E    H
ATOM  15192  CB  ILE E 100     -33.795  -8.589  36.595  1.00  0.00
E    C
ATOM  15193  HB  ILE E 100     -34.818  -8.539  37.046  1.00  0.00
E    H
ATOM  15194  CG2 ILE E 100     -32.680  -8.138  37.492  1.00  0.00
E    C
ATOM  15195 HG21 ILE E 100     -32.884  -7.121  37.887  1.00  0.00
E    H
ATOM  15196 HG22 ILE E 100     -32.570  -8.821  38.361  1.00  0.00
E    H
ATOM  15197 HG23 ILE E 100     -31.715  -8.110  36.942  1.00  0.00
E    H
ATOM  15198  CG1 ILE E 100     -33.824  -7.743  35.326  1.00  0.00
E    C
ATOM  15199 HG11 ILE E 100     -32.868  -7.866  34.770  1.00  0.00
E    H
ATOM  15200 HG12 ILE E 100     -34.640  -8.114  34.664  1.00  0.00
E    H
```

ATOM 15201 CD ILE E 100 -34.057 -6.284 35.581 1.00 0.00
E C
ATOM 15202 HD1 ILE E 100 -34.126 -5.731 34.620 1.00 0.00
E H
ATOM 15203 HD2 ILE E 100 -35.006 -6.131 36.139 1.00 0.00
E H
ATOM 15204 HD3 ILE E 100 -33.226 -5.845 36.171 1.00 0.00
E H
ATOM 15205 C ILE E 100 -33.342 -10.901 37.469 1.00 0.00
E C
ATOM 15206 O ILE E 100 -34.148 -10.913 38.407 1.00 0.00
E O
ATOM 15207 N TRP E 101 -32.224 -11.616 37.468 1.00 0.00
E N
ATOM 15208 HN TRP E 101 -31.537 -11.538 36.749 1.00 0.00
E H
ATOM 15209 CA TRP E 101 -31.909 -12.573 38.519 1.00 0.00
E C
ATOM 15210 HA TRP E 101 -32.622 -12.444 39.324 1.00 0.00
E H
ATOM 15211 CB TRP E 101 -31.951 -13.993 37.966 1.00 0.00
E C
ATOM 15212 HB1 TRP E 101 -31.412 -13.996 36.992 1.00 0.00
E H
ATOM 15213 HB2 TRP E 101 -31.397 -14.679 38.643 1.00 0.00
E H
ATOM 15214 CG TRP E 101 -33.321 -14.450 37.736 1.00 0.00
E C
ATOM 15215 CD1 TRP E 101 -34.344 -13.734 37.205 1.00 0.00
E C
ATOM 15216 HD1 TRP E 101 -34.252 -12.720 36.837 1.00 0.00
E H
ATOM 15217 NE1 TRP E 101 -35.479 -14.489 37.167 1.00 0.00
E N
ATOM 15218 HE1 TRP E 101 -36.345 -14.224 36.801 1.00 0.00
E H
ATOM 15219 CE2 TRP E 101 -35.199 -15.724 37.686 1.00 0.00
E C
ATOM 15220 CD2 TRP E 101 -33.846 -15.727 38.054 1.00 0.00
E C
ATOM 15221 CE3 TRP E 101 -33.304 -16.879 38.615 1.00 0.00
E C
ATOM 15222 HE3 TRP E 101 -32.270 -16.930 38.926 1.00 0.00
E H
ATOM 15223 CZ3 TRP E 101 -34.111 -17.970 38.782 1.00 0.00
E C
ATOM 15224 HZ3 TRP E 101 -33.702 -18.873 39.214 1.00 0.00
E H
ATOM 15225 CZ2 TRP E 101 -36.017 -16.826 37.859 1.00 0.00
E C
ATOM 15226 HZ2 TRP E 101 -37.058 -16.828 37.576 1.00 0.00
E H
ATOM 15227 CH2 TRP E 101 -35.456 -17.939 38.408 1.00 0.00
E C

ATOM 15228 HH2 TRP E 101 -36.071 -18.816 38.556 1.00 0.00
E H
ATOM 15229 C TRP E 101 -30.536 -12.253 39.087 1.00 0.00
E C
ATOM 15230 O TRP E 101 -29.559 -12.171 38.338 1.00 0.00
E O
ATOM 15231 N VAL E 102 -30.453 -12.082 40.401 1.00 0.00
E N
ATOM 15232 HN VAL E 102 -31.155 -12.320 41.067 1.00 0.00
E H
ATOM 15233 CA VAL E 102 -29.251 -11.500 40.977 1.00 0.00
E C
ATOM 15234 HA VAL E 102 -28.500 -11.466 40.197 1.00 0.00
E H
ATOM 15235 CB VAL E 102 -29.527 -10.074 41.489 1.00 0.00
E C
ATOM 15236 HB VAL E 102 -30.234 -10.156 42.351 1.00 0.00
E H
ATOM 15237 CG1 VAL E 102 -28.250 -9.418 41.956 1.00 0.00
E C
ATOM 15238 HG11 VAL E 102 -28.450 -8.376 42.288 1.00 0.00
E H
ATOM 15239 HG12 VAL E 102 -27.803 -9.958 42.816 1.00 0.00
E H
ATOM 15240 HG13 VAL E 102 -27.505 -9.379 41.133 1.00 0.00
E H
ATOM 15241 CG2 VAL E 102 -30.185 -9.245 40.411 1.00 0.00
E C
ATOM 15242 HG21 VAL E 102 -30.351 -8.204 40.762 1.00 0.00
E H
ATOM 15243 HG22 VAL E 102 -29.547 -9.211 39.501 1.00 0.00
E H
ATOM 15244 HG23 VAL E 102 -31.173 -9.665 40.128 1.00 0.00
E H
ATOM 15245 C VAL E 102 -28.706 -12.372 42.097 1.00 0.00
E C
ATOM 15246 O VAL E 102 -29.454 -13.046 42.813 1.00 0.00
E O
ATOM 15247 N LYS E 103 -27.378 -12.357 42.231 1.00 0.00
E N
ATOM 15248 HN LYS E 103 -26.783 -11.955 41.537 1.00 0.00
E H
ATOM 15249 CA LYS E 103 -26.689 -12.919 43.387 1.00 0.00
E C
ATOM 15250 HA LYS E 103 -27.294 -13.730 43.776 1.00 0.00
E H
ATOM 15251 CB LYS E 103 -25.296 -13.407 42.982 1.00 0.00
E C
ATOM 15252 HB1 LYS E 103 -25.439 -14.285 42.310 1.00 0.00
E H
ATOM 15253 HB2 LYS E 103 -24.802 -12.620 42.368 1.00 0.00
E H
ATOM 15254 CG LYS E 103 -24.378 -13.823 44.120 1.00 0.00
E C

```
ATOM  15255  HG1 LYS E 103     -24.432 -13.060  44.930  1.00  0.00
E    H
ATOM  15256  HG2 LYS E 103     -24.742 -14.787  44.545  1.00  0.00
E    H
ATOM  15257  CD  LYS E 103     -22.935 -13.948  43.617  1.00  0.00
E    C
ATOM  15258  HD1 LYS E 103     -22.908 -14.717  42.811  1.00  0.00
E    H
ATOM  15259  HD2 LYS E 103     -22.642 -12.975  43.158  1.00  0.00
E    H
ATOM  15260  CE  LYS E 103     -21.943 -14.314  44.724  1.00  0.00
E    C
ATOM  15261  HE1 LYS E 103     -22.088 -13.657  45.611  1.00  0.00
E    H
ATOM  15262  HE2 LYS E 103     -22.086 -15.370  45.043  1.00  0.00
E    H
ATOM  15263  NZ  LYS E 103     -20.513 -14.167  44.291  1.00  0.00
E    N
ATOM  15264  HZ1 LYS E 103     -19.876 -14.424  45.072  1.00  0.00
E    H
ATOM  15265  HZ2 LYS E 103     -20.332 -14.790  43.478  1.00  0.00
E    H
ATOM  15266  HZ3 LYS E 103     -20.337 -13.181  44.012  1.00  0.00
E    H
ATOM  15267  C   LYS E 103     -26.590 -11.861  44.480  1.00  0.00
E    C
ATOM  15268  O   LYS E 103     -26.055 -10.774  44.247  1.00  0.00
E    O
ATOM  15269  N   ALA E 104     -27.101 -12.176  45.668  1.00  0.00
E    N
ATOM  15270  HN  ALA E 104     -27.414 -13.101  45.882  1.00  0.00
E    H
ATOM  15271  CA  ALA E 104     -27.242 -11.204  46.744  1.00  0.00
E    C
ATOM  15272  HA  ALA E 104     -27.099 -10.199  46.365  1.00  0.00
E    H
ATOM  15273  CB  ALA E 104     -28.630 -11.279  47.378  1.00  0.00
E    C
ATOM  15274  HB1 ALA E 104     -29.404 -11.080  46.605  1.00  0.00
E    H
ATOM  15275  HB2 ALA E 104     -28.822 -12.289  47.802  1.00  0.00
E    H
ATOM  15276  HB3 ALA E 104     -28.752 -10.526  48.187  1.00  0.00
E    H
ATOM  15277  C   ALA E 104     -26.190 -11.410  47.826  1.00  0.00
E    C
ATOM  15278  O   ALA E 104     -25.726 -12.528  48.064  1.00  0.00
E    O
ATOM  15279  N   GLN E 105     -25.849 -10.315  48.503  1.00  0.00
E    N
ATOM  15280  HN  GLN E 105     -26.303  -9.436  48.375  1.00  0.00
E    H
ATOM  15281  CA  GLN E 105     -24.780 -10.303  49.493  1.00  0.00
E    C
```

ATOM 15282 HA GLN E 105   -24.128 -11.134 49.251 1.00 0.00
E   H
ATOM 15283 CB GLN E 105   -24.017 -8.982 49.404 1.00 0.00
E   C
ATOM 15284 HB1 GLN E 105   -23.744 -8.838 48.333 1.00 0.00
E   H
ATOM 15285 HB2 GLN E 105   -24.702 -8.143 49.664 1.00 0.00
E   H
ATOM 15286 CG GLN E 105   -22.764 -8.919 50.245 1.00 0.00
E   C
ATOM 15287 HG1 GLN E 105   -22.363 -9.941 50.421 1.00 0.00
E   H
ATOM 15288 HG2 GLN E 105   -21.977 -8.328 49.733 1.00 0.00
E   H
ATOM 15289 CD GLN E 105   -23.019 -8.319 51.605 1.00 0.00
E   C
ATOM 15290 OE1 GLN E 105   -24.002 -7.610 51.805 1.00 0.00
E   O
ATOM 15291 NE2 GLN E 105   -22.122 -8.579 52.546 1.00 0.00
E   N
ATOM 15292 HE21 GLN E 105   -22.289 -8.189 53.447 1.00 0.00
E   H
ATOM 15293 HE22 GLN E 105   -21.323 -9.128 52.328 1.00 0.00
E   H
ATOM 15294 C   GLN E 105   -25.298 -10.524 50.910 1.00 0.00
E   C
ATOM 15295 O   GLN E 105   -24.944 -11.517 51.550 1.00 0.00
E   O
ATOM 15296 N   ASN E 106   -26.142 -9.628 51.414 1.00 0.00
E   N
ATOM 15297 HN ASN E 106   -26.436 -8.798 50.943 1.00 0.00
E   H
ATOM 15298 CA ASN E 106   -26.713 -9.805 52.739 1.00 0.00
E   C
ATOM 15299 HA ASN E 106   -26.551 -10.840 53.019 1.00 0.00
E   H
ATOM 15300 CB ASN E 106   -26.107 -8.855 53.771 1.00 0.00
E   C
ATOM 15301 HB1 ASN E 106   -26.493 -9.147 54.771 1.00 0.00
E   H
ATOM 15302 HB2 ASN E 106   -25.004 -8.964 53.794 1.00 0.00
E   H
ATOM 15303 CG ASN E 106   -26.482 -7.400 53.532 1.00 0.00
E   C
ATOM 15304 OD1 ASN E 106   -27.234 -7.066 52.619 1.00 0.00
E   O
ATOM 15305 ND2 ASN E 106   -25.968 -6.528 54.382 1.00 0.00
E   N
ATOM 15306 HD21 ASN E 106   -26.208 -5.570 54.255 1.00 0.00
E   H
ATOM 15307 HD22 ASN E 106   -25.392 -6.841 55.130 1.00 0.00
E   H
ATOM 15308 C   ASN E 106   -28.210 -9.581 52.655 1.00 0.00
E   C

```
ATOM  15309  O   ASN E 106     -28.749  -9.231  51.604  1.00  0.00
E    O
ATOM  15310  N   TYR E 107     -28.872  -9.752  53.794  1.00  0.00
E    N
ATOM  15311  HN  TYR E 107     -28.479 -10.075  54.653  1.00  0.00
E    H
ATOM  15312  CA  TYR E 107     -30.294  -9.458  53.855  1.00  0.00
E    C
ATOM  15313  HA  TYR E 107     -30.779 -10.136  53.163  1.00  0.00
E    H
ATOM  15314  CB  TYR E 107     -30.805  -9.651  55.278  1.00  0.00
E    C
ATOM  15315  HB1 TYR E 107     -31.073 -10.719  55.428  1.00  0.00
E    H
ATOM  15316  HB2 TYR E 107     -30.012  -9.404  56.018  1.00  0.00
E    H
ATOM  15317  CG  TYR E 107     -32.036  -8.837  55.554  1.00  0.00
E    C
ATOM  15318  CD1 TYR E 107     -33.280  -9.268  55.131  1.00  0.00
E    C
ATOM  15319  HD1 TYR E 107     -33.370 -10.199  54.589  1.00  0.00
E    H
ATOM  15320  CE1 TYR E 107     -34.405  -8.523  55.367  1.00  0.00
E    C
ATOM  15321  HE1 TYR E 107     -35.372  -8.867  55.030  1.00  0.00
E    H
ATOM  15322  CZ  TYR E 107     -34.297  -7.326  56.033  1.00  0.00
E    C
ATOM  15323  OH  TYR E 107     -35.419  -6.572  56.276  1.00  0.00
E    O
ATOM  15324  HH  TYR E 107     -35.151  -5.820  56.807  1.00  0.00
E    H
ATOM  15325  CD2 TYR E 107     -31.954  -7.629  56.219  1.00  0.00
E    C
ATOM  15326  HD2 TYR E 107     -30.991  -7.244  56.526  1.00  0.00
E    H
ATOM  15327  CE2 TYR E 107     -33.075  -6.877  56.458  1.00  0.00
E    C
ATOM  15328  HE2 TYR E 107     -32.985  -5.926  56.961  1.00  0.00
E    H
ATOM  15329  C   TYR E 107     -30.585  -8.046  53.375  1.00  0.00
E    C
ATOM  15330  O   TYR E 107     -31.546  -7.816  52.633  1.00  0.00
E    O
ATOM  15331  N   TYR E 108     -29.768  -7.084  53.793  1.00  0.00
E    N
ATOM  15332  HN  TYR E 108     -28.957  -7.206  54.364  1.00  0.00
E    H
ATOM  15333  CA  TYR E 108     -30.036  -5.706  53.411  1.00  0.00
E    C
ATOM  15334  HA  TYR E 108     -31.067  -5.507  53.680  1.00  0.00
E    H
ATOM  15335  CB  TYR E 108     -29.084  -4.770  54.130  1.00  0.00
E    C
```

ATOM 15336 HB1 TYR E 108   -28.037 -5.072 53.906 1.00 0.00
E   H
ATOM 15337 HB2 TYR E 108   -29.209 -3.726 53.767 1.00 0.00
E   H
ATOM 15338 CG TYR E 108   -29.284 -4.848 55.595 1.00 0.00
E   C
ATOM 15339 CD1 TYR E 108   -30.539 -4.661 56.133 1.00 0.00
E   C
ATOM 15340 HD1 TYR E 108   -31.373 -4.431 55.484 1.00 0.00
E   H
ATOM 15341 CE1 TYR E 108   -30.753 -4.737 57.474 1.00 0.00
E   C
ATOM 15342 HE1 TYR E 108   -31.740 -4.582 57.882 1.00 0.00
E   H
ATOM 15343 CZ TYR E 108   -29.702 -5.006 58.307 1.00 0.00
E   C
ATOM 15344 OH TYR E 108   -29.913 -5.082 59.661 1.00 0.00
E   O
ATOM 15345 HH TYR E 108   -29.085 -5.347 60.068 1.00 0.00
E   H
ATOM 15346 CD2 TYR E 108   -28.238 -5.125 56.443 1.00 0.00
E   C
ATOM 15347 HD2 TYR E 108   -27.239 -5.245 56.048 1.00 0.00
E   H
ATOM 15348 CE2 TYR E 108   -28.438 -5.203 57.795 1.00 0.00
E   C
ATOM 15349 HE2 TYR E 108   -27.602 -5.396 58.451 1.00 0.00
E   H
ATOM 15350 C   TYR E 108   -29.922 -5.520 51.914 1.00 0.00
E   C
ATOM 15351 O   TYR E 108   -30.924 -5.245 51.242 1.00 0.00
E   O
ATOM 15352 N   HSE E 109   -28.716 -5.724 51.382 1.00 0.00
E   N
ATOM 15353 HN HSE E 109   -27.889 -5.903 51.913 1.00 0.00
E   H
ATOM 15354 CA HSE E 109   -28.511 -5.702 49.944 1.00 0.00
E   C
ATOM 15355 HA HSE E 109   -28.505 -4.662 49.638 1.00 0.00
E   H
ATOM 15356 CB HSE E 109   -27.203 -6.405 49.612 1.00 0.00
E   C
ATOM 15357 HB1 HSE E 109   -26.371 -5.779 50.002 1.00 0.00
E   H
ATOM 15358 HB2 HSE E 109   -27.158 -7.376 50.148 1.00 0.00
E   H
ATOM 15359 ND1 HSE E 109   -26.798 -7.849 47.596 1.00 0.00
E   N
ATOM 15360 CG HSE E 109   -26.984 -6.605 48.152 1.00 0.00
E   C
ATOM 15361 CE1 HSE E 109   -26.627 -7.726 46.292 1.00 0.00
E   C
ATOM 15362 HE1 HSE E 109   -26.461 -8.561 45.607 1.00 0.00
E   H

ATOM 15363 NE2 HSE E 109 −26.686 −6.442 45.986 1.00 0.00
E N
ATOM 15364 HE2 HSE E 109 −26.568 −6.062 45.069 1.00 0.00
E H
ATOM 15365 CD2 HSE E 109 −26.907 −5.720 47.133 1.00 0.00
E C
ATOM 15366 HD2 HSE E 109 −26.982 −4.642 47.144 1.00 0.00
E H
ATOM 15367 C HSE E 109 −29.666 −6.369 49.226 1.00 0.00
E C
ATOM 15368 O HSE E 109 −30.139 −5.876 48.199 1.00 0.00
E O
ATOM 15369 N HSE E 110 −30.171 −7.458 49.795 1.00 0.00
E N
ATOM 15370 HN HSE E 110 −29.811 −7.883 50.625 1.00 0.00
E H
ATOM 15371 CA HSE E 110 −31.327 −8.133 49.229 1.00 0.00
E C
ATOM 15372 HA HSE E 110 −31.086 −8.343 48.194 1.00 0.00
E H
ATOM 15373 CB HSE E 110 −31.587 −9.404 50.013 1.00 0.00
E C
ATOM 15374 HB1 HSE E 110 −31.044 −10.233 49.512 1.00 0.00
E H
ATOM 15375 HB2 HSE E 110 −31.159 −9.305 51.033 1.00 0.00
E H
ATOM 15376 ND1 HSE E 110 −33.622 −10.601 49.164 1.00 0.00
E N
ATOM 15377 CG HSE E 110 −33.026 −9.774 50.086 1.00 0.00
E C
ATOM 15378 CE1 HSE E 110 −34.897 −10.748 49.471 1.00 0.00
E C
ATOM 15379 HE1 HSE E 110 −35.608 −11.351 48.900 1.00 0.00
E H
ATOM 15380 NE2 HSE E 110 −35.147 −10.046 50.561 1.00 0.00
E N
ATOM 15381 HE2 HSE E 110 −36.040 −9.966 51.002 1.00 0.00
E H
ATOM 15382 CD2 HSE E 110 −33.992 −9.426 50.966 1.00 0.00
E C
ATOM 15383 HD2 HSE E 110 −33.941 −8.783 51.833 1.00 0.00
E H
ATOM 15384 C HSE E 110 −32.560 −7.245 49.230 1.00 0.00
E C
ATOM 15385 O HSE E 110 −33.307 −7.202 48.248 1.00 0.00
E O
ATOM 15386 N LYS E 111 −32.825 −6.562 50.335 1.00 0.00
E N
ATOM 15387 HN LYS E 111 −32.291 −6.564 51.178 1.00 0.00
E H
ATOM 15388 CA LYS E 111 −34.009 −5.712 50.355 1.00 0.00
E C
ATOM 15389 HA LYS E 111 −34.846 −6.325 50.041 1.00 0.00
E H

ATOM 15390 CB LYS E 111 -34.257 -5.165 51.751 1.00 0.00
E C
ATOM 15391 HB1 LYS E 111 -34.524 -6.031 52.399 1.00 0.00
E H
ATOM 15392 HB2 LYS E 111 -33.304 -4.754 52.153 1.00 0.00
E H
ATOM 15393 CG LYS E 111 -35.370 -4.133 51.782 1.00 0.00
E C
ATOM 15394 HG1 LYS E 111 -35.047 -3.267 52.401 1.00 0.00
E H
ATOM 15395 HG2 LYS E 111 -35.545 -3.756 50.748 1.00 0.00
E H
ATOM 15396 CD LYS E 111 -36.686 -4.678 52.362 1.00 0.00
E C
ATOM 15397 HD1 LYS E 111 -36.931 -4.099 53.282 1.00 0.00
E H
ATOM 15398 HD2 LYS E 111 -37.497 -4.487 51.622 1.00 0.00
E H
ATOM 15399 CE LYS E 111 -36.599 -6.147 52.671 1.00 0.00
E C
ATOM 15400 HE1 LYS E 111 -35.542 -6.492 52.630 1.00 0.00
E H
ATOM 15401 HE2 LYS E 111 -36.999 -6.358 53.687 1.00 0.00
E H
ATOM 15402 NZ LYS E 111 -37.390 -6.916 51.679 1.00 0.00
E N
ATOM 15403 HZ1 LYS E 111 -37.330 -7.934 51.889 1.00 0.00
E H
ATOM 15404 HZ2 LYS E 111 -38.384 -6.612 51.717 1.00 0.00
E H
ATOM 15405 HZ3 LYS E 111 -37.014 -6.738 50.726 1.00 0.00
E H
ATOM 15406 C LYS E 111 -33.885 -4.573 49.361 1.00 0.00
E C
ATOM 15407 O LYS E 111 -34.833 -4.263 48.630 1.00 0.00
E O
ATOM 15408 N VAL E 112 -32.719 -3.940 49.318 1.00 0.00
E N
ATOM 15409 HN VAL E 112 -31.936 -4.116 49.908 1.00 0.00
E H
ATOM 15410 CA VAL E 112 -32.508 -2.880 48.337 1.00 0.00
E C
ATOM 15411 HA VAL E 112 -33.283 -2.138 48.480 1.00 0.00
E H
ATOM 15412 CB VAL E 112 -31.144 -2.209 48.548 1.00 0.00
E C
ATOM 15413 HB VAL E 112 -31.320 -1.109 48.640 1.00 0.00
E H
ATOM 15414 CG1 VAL E 112 -30.512 -2.715 49.800 1.00 0.00
E C
ATOM 15415 HG11 VAL E 112 -29.507 -2.261 49.936 1.00 0.00
E H
ATOM 15416 HG12 VAL E 112 -31.115 -2.459 50.696 1.00 0.00
E H

```
ATOM  15417 HG13 VAL E 112      -30.388  -3.818  49.758  1.00  0.00
E    H
ATOM  15418  CG2 VAL E 112      -30.246  -2.479  47.373  1.00  0.00
E    C
ATOM  15419 HG21 VAL E 112      -29.242  -2.031  47.534  1.00  0.00
E    H
ATOM  15420 HG22 VAL E 112      -30.120  -3.573  47.222  1.00  0.00
E    H
ATOM  15421 HG23 VAL E 112      -30.662  -2.044  46.441  1.00  0.00
E    H
ATOM  15422  C   VAL E 112      -32.657  -3.429  46.929  1.00  0.00
E    C
ATOM  15423  O   VAL E 112      -33.107  -2.726  46.017  1.00  0.00
E    O
ATOM  15424  N   LEU E 113      -32.287  -4.692  46.734  1.00  0.00
E    N
ATOM  15425  HN  LEU E 113      -31.799  -5.259  47.393  1.00  0.00
E    H
ATOM  15426  CA  LEU E 113      -32.590  -5.360  45.481  1.00  0.00
E    C
ATOM  15427  HA  LEU E 113      -32.130  -4.778  44.694  1.00  0.00
E    H
ATOM  15428  CB  LEU E 113      -32.051  -6.782  45.529  1.00  0.00
E    C
ATOM  15429  HB1 LEU E 113      -31.140  -6.768  46.171  1.00  0.00
E    H
ATOM  15430  HB2 LEU E 113      -32.788  -7.430  46.055  1.00  0.00
E    H
ATOM  15431  CG  LEU E 113      -31.668  -7.439  44.219  1.00  0.00
E    C
ATOM  15432  HG  LEU E 113      -32.567  -7.963  43.812  1.00  0.00
E    H
ATOM  15433  CD1 LEU E 113      -31.223  -6.398  43.228  1.00  0.00
E    C
ATOM  15434 HD11 LEU E 113      -30.895  -6.876  42.280  1.00  0.00
E    H
ATOM  15435 HD12 LEU E 113      -32.055  -5.698  42.996  1.00  0.00
E    H
ATOM  15436 HD13 LEU E 113      -30.372  -5.810  43.632  1.00  0.00
E    H
ATOM  15437  CD2 LEU E 113      -30.604  -8.471  44.477  1.00  0.00
E    C
ATOM  15438 HD21 LEU E 113      -30.321  -8.979  43.530  1.00  0.00
E    H
ATOM  15439 HD22 LEU E 113      -29.687  -8.015  44.902  1.00  0.00
E    H
ATOM  15440 HD23 LEU E 113      -30.975  -9.241  45.187  1.00  0.00
E    H
ATOM  15441  C   LEU E 113      -34.082  -5.372  45.228  1.00  0.00
E    C
ATOM  15442  O   LEU E 113      -34.545  -5.038  44.132  1.00  0.00
E    O
ATOM  15443  N   GLU E 114      -34.852  -5.748  46.243  1.00  0.00
E    N
```

ATOM 15444 HN GLU E 114 -34.533 -6.002 47.152 1.00 0.00
E H
ATOM 15445 CA GLU E 114 -36.297 -5.817 46.081 1.00 0.00
E C
ATOM 15446 HA GLU E 114 -36.515 -6.523 45.289 1.00 0.00
E H
ATOM 15447 CB GLU E 114 -36.949 -6.286 47.376 1.00 0.00
E C
ATOM 15448 HB1 GLU E 114 -36.353 -7.139 47.776 1.00 0.00
E H
ATOM 15449 HB2 GLU E 114 -36.884 -5.483 48.144 1.00 0.00
E H
ATOM 15450 CG GLU E 114 -38.374 -6.712 47.166 1.00 0.00
E C
ATOM 15451 HG1 GLU E 114 -38.702 -7.338 48.016 1.00 0.00
E H
ATOM 15452 HG2 GLU E 114 -39.043 -5.833 47.080 1.00 0.00
E H
ATOM 15453 CD GLU E 114 -38.529 -7.568 45.931 1.00 0.00
E C
ATOM 15454 OE1 GLU E 114 -37.822 -8.591 45.831 1.00 0.00
E O
ATOM 15455 OE2 GLU E 114 -39.343 -7.212 45.055 1.00 0.00
E O
ATOM 15456 C GLU E 114 -36.868 -4.472 45.658 1.00 0.00
E C
ATOM 15457 O GLU E 114 -37.722 -4.396 44.770 1.00 0.00
E O
ATOM 15458 N LYS E 115 -36.393 -3.394 46.275 1.00 0.00
E N
ATOM 15459 HN LYS E 115 -35.648 -3.396 46.941 1.00 0.00
E H
ATOM 15460 CA LYS E 115 -36.967 -2.085 45.993 1.00 0.00
E C
ATOM 15461 HA LYS E 115 -38.034 -2.177 46.160 1.00 0.00
E H
ATOM 15462 CB LYS E 115 -36.370 -1.040 46.916 1.00 0.00
E C
ATOM 15463 HB1 LYS E 115 -35.263 -1.141 46.853 1.00 0.00
E H
ATOM 15464 HB2 LYS E 115 -36.615 -0.028 46.523 1.00 0.00
E H
ATOM 15465 CG LYS E 115 -36.818 -1.211 48.317 1.00 0.00
E C
ATOM 15466 HG1 LYS E 115 -36.271 -2.068 48.772 1.00 0.00
E H
ATOM 15467 HG2 LYS E 115 -36.548 -0.299 48.897 1.00 0.00
E H
ATOM 15468 CD LYS E 115 -38.289 -1.467 48.382 1.00 0.00
E C
ATOM 15469 HD1 LYS E 115 -38.815 -0.633 47.864 1.00 0.00
E H
ATOM 15470 HD2 LYS E 115 -38.504 -2.402 47.812 1.00 0.00
E H

```
ATOM  15471  CE  LYS E 115      -38.695  -1.587  49.822  1.00  0.00
E    C
ATOM  15472  HE1 LYS E 115      -39.803  -1.555  49.919  1.00  0.00
E    H
ATOM  15473  HE2 LYS E 115      -38.331  -2.546  50.253  1.00  0.00
E    H
ATOM  15474  NZ  LYS E 115      -38.111  -0.458  50.594  1.00  0.00
E    N
ATOM  15475  HZ1 LYS E 115      -38.388  -0.531  51.595  1.00  0.00
E    H
ATOM  15476  HZ2 LYS E 115      -37.074  -0.486  50.520  1.00  0.00
E    H
ATOM  15477  HZ3 LYS E 115      -38.458   0.442  50.205  1.00  0.00
E    H
ATOM  15478  C   LYS E 115      -36.769  -1.653  44.551  1.00  0.00
E    C
ATOM  15479  O   LYS E 115      -37.617  -0.942  44.007  1.00  0.00
E    O
ATOM  15480  N   ILE E 116      -35.672  -2.056  43.917  1.00  0.00
E    N
ATOM  15481  HN  ILE E 116      -34.953  -2.615  44.323  1.00  0.00
E    H
ATOM  15482  CA  ILE E 116      -35.433  -1.695  42.530  1.00  0.00
E    C
ATOM  15483  HA  ILE E 116      -35.661  -0.640  42.449  1.00  0.00
E    H
ATOM  15484  CB  ILE E 116      -33.980  -1.971  42.132  1.00  0.00
E    C
ATOM  15485  HB  ILE E 116      -33.379  -1.638  43.016  1.00  0.00
E    H
ATOM  15486  CG2 ILE E 116      -33.828  -3.407  41.708  1.00  0.00
E    C
ATOM  15487 HG21 ILE E 116      -32.757  -3.661  41.567  1.00  0.00
E    H
ATOM  15488 HG22 ILE E 116      -34.238  -4.093  42.479  1.00  0.00
E    H
ATOM  15489 HG23 ILE E 116      -34.359  -3.597  40.751  1.00  0.00
E    H
ATOM  15490  CG1 ILE E 116      -33.558  -1.088  40.959  1.00  0.00
E    C
ATOM  15491 HG11 ILE E 116      -32.478  -1.243  40.741  1.00  0.00
E    H
ATOM  15492 HG12 ILE E 116      -34.125  -1.399  40.052  1.00  0.00
E    H
ATOM  15493  CD  ILE E 116      -33.794   0.361  41.161  1.00  0.00
E    C
ATOM  15494  HD1 ILE E 116      -33.516   0.928  40.247  1.00  0.00
E    H
ATOM  15495  HD2 ILE E 116      -34.866   0.556  41.380  1.00  0.00
E    H
ATOM  15496  HD3 ILE E 116      -33.185   0.750  42.005  1.00  0.00
E    H
ATOM  15497  C   ILE E 116      -36.379  -2.423  41.592  1.00  0.00
E    C
```

```
ATOM  15498  O   ILE E 116     -36.523  -2.028  40.434  1.00  0.00
E    O
ATOM  15499  N   GLY E 117     -37.041  -3.467  42.066  1.00  0.00
E    N
ATOM  15500  HN  GLY E 117     -37.090  -3.710  43.034  1.00  0.00
E    H
ATOM  15501  CA  GLY E 117     -37.755  -4.341  41.166  1.00  0.00
E    C
ATOM  15502  HA1 GLY E 117     -37.909  -3.790  40.248  1.00  0.00
E    H
ATOM  15503  HA2 GLY E 117     -38.669  -4.637  41.665  1.00  0.00
E    H
ATOM  15504  C   GLY E 117     -36.981  -5.587  40.837  1.00  0.00
E    C
ATOM  15505  O   GLY E 117     -36.972  -6.019  39.683  1.00  0.00
E    O
ATOM  15506  N   ALA E 118     -36.313  -6.171  41.825  1.00  0.00
E    N
ATOM  15507  HN  ALA E 118     -36.255  -5.807  42.754  1.00  0.00
E    H
ATOM  15508  CA  ALA E 118     -35.605  -7.416  41.599  1.00  0.00
E    C
ATOM  15509  HA  ALA E 118     -34.900  -7.320  40.781  1.00  0.00
E    H
ATOM  15510  CB  ALA E 118     -34.858  -7.836  42.856  1.00  0.00
E    C
ATOM  15511  HB1 ALA E 118     -34.109  -7.059  43.121  1.00  0.00
E    H
ATOM  15512  HB2 ALA E 118     -35.554  -7.947  43.716  1.00  0.00
E    H
ATOM  15513  HB3 ALA E 118     -34.319  -8.797  42.711  1.00  0.00
E    H
ATOM  15514  C   ALA E 118     -36.579  -8.504  41.197  1.00  0.00
E    C
ATOM  15515  O   ALA E 118     -37.378  -8.975  42.009  1.00  0.00
E    O
ATOM  15516  N   ASP E 119     -36.515  -8.901  39.933  1.00  0.00
E    N
ATOM  15517  HN  ASP E 119     -35.934  -8.542  39.207  1.00  0.00
E    H
ATOM  15518  CA  ASP E 119     -37.368  -9.987  39.488  1.00  0.00
E    C
ATOM  15519  HA  ASP E 119     -38.387  -9.711  39.735  1.00  0.00
E    H
ATOM  15520  CB  ASP E 119     -37.204 -10.198  37.987  1.00  0.00
E    C
ATOM  15521  HB1 ASP E 119     -36.126 -10.298  37.747  1.00  0.00
E    H
ATOM  15522  HB2 ASP E 119     -37.720 -11.124  37.664  1.00  0.00
E    H
ATOM  15523  CG  ASP E 119     -37.745  -9.038  37.184  1.00  0.00
E    C
ATOM  15524  OD1 ASP E 119     -38.907  -9.125  36.728  1.00  0.00
E    O
```

```
ATOM  15525  OD2 ASP E 119     -37.020  -8.034  37.025  1.00  0.00
E    O
ATOM  15526  C   ASP E 119     -37.066 -11.267  40.245  1.00  0.00
E    C
ATOM  15527  O   ASP E 119     -37.944 -12.123  40.379  1.00  0.00
E    O
ATOM  15528  N   ARG E 120     -35.844 -11.413  40.743  1.00  0.00
E    N
ATOM  15529  HN  ARG E 120     -35.121 -10.738  40.607  1.00  0.00
E    H
ATOM  15530  CA  ARG E 120     -35.500 -12.586  41.526  1.00  0.00
E    C
ATOM  15531  HA  ARG E 120     -36.236 -12.710  42.312  1.00  0.00
E    H
ATOM  15532  CB  ARG E 120     -35.458 -13.829  40.648  1.00  0.00
E    C
ATOM  15533  HB1 ARG E 120     -36.519 -14.125  40.468  1.00  0.00
E    H
ATOM  15534  HB2 ARG E 120     -35.040 -13.567  39.652  1.00  0.00
E    H
ATOM  15535  CG  ARG E 120     -34.732 -14.945  41.289  1.00  0.00
E    C
ATOM  15536  HG1 ARG E 120     -33.755 -15.073  40.774  1.00  0.00
E    H
ATOM  15537  HG2 ARG E 120     -34.483 -14.661  42.337  1.00  0.00
E    H
ATOM  15538  CD  ARG E 120     -35.576 -16.163  41.287  1.00  0.00
E    C
ATOM  15539  HD1 ARG E 120     -36.449 -16.009  41.962  1.00  0.00
E    H
ATOM  15540  HD2 ARG E 120     -35.952 -16.399  40.264  1.00  0.00
E    H
ATOM  15541  NE  ARG E 120     -34.805 -17.307  41.738  1.00  0.00
E    N
ATOM  15542  HE  ARG E 120     -33.848 -17.080  41.952  1.00  0.00
E    H
ATOM  15543  CZ  ARG E 120     -35.301 -18.529  41.858  1.00  0.00
E    C
ATOM  15544  NH1 ARG E 120     -36.568 -18.761  41.552  1.00  0.00
E    N
ATOM  15545 HH11 ARG E 120     -36.955 -19.665  41.661  1.00  0.00
E    H
ATOM  15546 HH12 ARG E 120     -37.127 -17.956  41.342  1.00  0.00
E    H
ATOM  15547  NH2 ARG E 120     -34.531 -19.522  42.274  1.00  0.00
E    N
ATOM  15548 HH21 ARG E 120     -34.879 -20.448  42.280  1.00  0.00
E    H
ATOM  15549 HH22 ARG E 120     -33.563 -19.329  42.368  1.00  0.00
E    H
ATOM  15550  C   ARG E 120     -34.161 -12.385  42.212  1.00  0.00
E    C
ATOM  15551  O   ARG E 120     -33.177 -11.997  41.571  1.00  0.00
E    O
```

```
ATOM  15552  N   ILE E 121     -34.124 -12.680  43.505  1.00  0.00
E    N
ATOM  15553  HN  ILE E 121     -34.921 -13.031  43.993  1.00  0.00
E    H
ATOM  15554  CA  ILE E 121     -32.937 -12.527  44.335  1.00  0.00
E    C
ATOM  15555  HA  ILE E 121     -32.156 -12.106  43.715  1.00  0.00
E    H
ATOM  15556  CB  ILE E 121     -33.244 -11.653  45.559  1.00  0.00
E    C
ATOM  15557  HB  ILE E 121     -33.929 -12.279  46.185  1.00  0.00
E    H
ATOM  15558  CG2 ILE E 121     -31.979 -11.263  46.281  1.00  0.00
E    C
ATOM  15559 HG21 ILE E 121     -32.217 -10.767  47.244  1.00  0.00
E    H
ATOM  15560 HG22 ILE E 121     -31.359 -12.156  46.505  1.00  0.00
E    H
ATOM  15561 HG23 ILE E 121     -31.374 -10.561  45.669  1.00  0.00
E    H
ATOM  15562  CG1 ILE E 121     -34.032 -10.422  45.122  1.00  0.00
E    C
ATOM  15563 HG11 ILE E 121     -33.513  -9.925  44.271  1.00  0.00
E    H
ATOM  15564 HG12 ILE E 121     -35.032 -10.745  44.753  1.00  0.00
E    H
ATOM  15565  CD  ILE E 121     -34.247  -9.412  46.209  1.00  0.00
E    C
ATOM  15566  HD1 ILE E 121     -34.875  -8.573  45.838  1.00  0.00
E    H
ATOM  15567  HD2 ILE E 121     -34.764  -9.875  47.077  1.00  0.00
E    H
ATOM  15568  HD3 ILE E 121     -33.280  -8.991  46.558  1.00  0.00
E    H
ATOM  15569  C   ILE E 121     -32.469 -13.912  44.751  1.00  0.00
E    C
ATOM  15570  O   ILE E 121     -33.282 -14.837  44.855  1.00  0.00
E    O
ATOM  15571  N   ILE E 122     -31.167 -14.069  44.967  1.00  0.00
E    N
ATOM  15572  HN  ILE E 122     -30.483 -13.356  44.828  1.00  0.00
E    H
ATOM  15573  CA  ILE E 122     -30.623 -15.334  45.439  1.00  0.00
E    C
ATOM  15574  HA  ILE E 122     -31.435 -15.876  45.905  1.00  0.00
E    H
ATOM  15575  CB  ILE E 122     -29.990 -16.152  44.299  1.00  0.00
E    C
ATOM  15576  HB  ILE E 122     -29.318 -15.428  43.774  1.00  0.00
E    H
ATOM  15577  CG2 ILE E 122     -29.272 -17.369  44.850  1.00  0.00
E    C
ATOM  15578 HG21 ILE E 122     -28.687 -17.872  44.053  1.00  0.00
E    H
```

```
ATOM  15579 HG22 ILE E 122     -28.565 -17.080  45.655  1.00  0.00
E    H
ATOM  15580 HG23 ILE E 122     -29.995 -18.102  45.265  1.00  0.00
E    H
ATOM  15581  CG1 ILE E 122     -31.058 -16.574  43.295  1.00  0.00
E    C
ATOM  15582 HG11 ILE E 122     -30.801 -17.570  42.868  1.00  0.00
E    H
ATOM  15583 HG12 ILE E 122     -32.030 -16.685  43.827  1.00  0.00
E    H
ATOM  15584  CD  ILE E 122     -31.253 -15.595  42.165  1.00  0.00
E    C
ATOM  15585  HD1 ILE E 122     -32.080 -15.930  41.503  1.00  0.00
E    H
ATOM  15586  HD2 ILE E 122     -31.509 -14.588  42.559  1.00  0.00
E    H
ATOM  15587  HD3 ILE E 122     -30.333 -15.509  41.549  1.00  0.00
E    H
ATOM  15588  C   ILE E 122     -29.595 -15.067  46.523  1.00  0.00
E    C
ATOM  15589  O   ILE E 122     -28.731 -14.198  46.375  1.00  0.00
E    O
ATOM  15590  N   HSE E 123     -29.679 -15.832  47.597  1.00  0.00
E    N
ATOM  15591  HN  HSE E 123     -30.432 -16.479  47.712  1.00  0.00
E    H
ATOM  15592  CA  HSE E 123     -28.724 -15.823  48.692  1.00  0.00
E    C
ATOM  15593  HA  HSE E 123     -27.988 -15.051  48.504  1.00  0.00
E    H
ATOM  15594  CB  HSE E 123     -29.435 -15.638  50.026  1.00  0.00
E    C
ATOM  15595  HB1 HSE E 123     -30.176 -16.459  50.135  1.00  0.00
E    H
ATOM  15596  HB2 HSE E 123     -28.704 -15.749  50.854  1.00  0.00
E    H
ATOM  15597  ND1 HSE E 123     -29.727 -13.317  50.962  1.00  0.00
E    N
ATOM  15598  CG  HSE E 123     -30.166 -14.341  50.153  1.00  0.00
E    C
ATOM  15599  CE1 HSE E 123     -30.575 -12.307  50.889  1.00  0.00
E    C
ATOM  15600  HE1 HSE E 123     -30.473 -11.370  51.443  1.00  0.00
E    H
ATOM  15601  NE2 HSE E 123     -31.543 -12.635  50.054  1.00  0.00
E    N
ATOM  15602  HE2 HSE E 123     -32.326 -12.057  49.825  1.00  0.00
E    H
ATOM  15603  CD2 HSE E 123     -31.312 -13.903  49.580  1.00  0.00
E    C
ATOM  15604  HD2 HSE E 123     -31.975 -14.401  48.886  1.00  0.00
E    H
ATOM  15605  C   HSE E 123     -27.992 -17.154  48.660  1.00  0.00
E    C
```

```
ATOM  15606  O   HSE E 123     -28.237 -18.019  49.509  1.00  0.00
E    O
ATOM  15607  N   PRO E 124     -27.095 -17.350  47.696  1.00  0.00
E    N
ATOM  15608  CD  PRO E 124     -26.406 -16.336  46.890  1.00  0.00
E    C
ATOM  15609  HD1 PRO E 124     -27.517 -16.391  46.845  1.00  0.00
E    H
ATOM  15610  HD2 PRO E 124     -26.065 -15.277  46.819  1.00  0.00
E    H
ATOM  15611  CA  PRO E 124     -26.620 -18.710  47.403  1.00  0.00
E    C
ATOM  15612  HA  PRO E 124     -27.436 -19.288  46.986  1.00  0.00
E    H
ATOM  15613  CB  PRO E 124     -25.540 -18.473  46.337  1.00  0.00
E    C
ATOM  15614  HB1 PRO E 124     -26.025 -18.415  45.336  1.00  0.00
E    H
ATOM  15615  HB2 PRO E 124     -24.772 -19.271  46.320  1.00  0.00
E    H
ATOM  15616  CG  PRO E 124     -25.811 -17.136  45.787  1.00  0.00
E    C
ATOM  15617  HG1 PRO E 124     -25.398 -16.995  44.769  1.00  0.00
E    H
ATOM  15618  HG2 PRO E 124     -25.385 -16.365  46.470  1.00  0.00
E    H
ATOM  15619  C   PRO E 124     -26.054 -19.443  48.597  1.00  0.00
E    C
ATOM  15620  O   PRO E 124     -26.552 -20.519  48.963  1.00  0.00
E    O
ATOM  15621  N   GLU E 125     -25.021 -18.888  49.218  1.00  0.00
E    N
ATOM  15622  HN  GLU E 125     -24.606 -18.006  49.012  1.00  0.00
E    H
ATOM  15623  CA  GLU E 125     -24.393 -19.593  50.319  1.00  0.00
E    C
ATOM  15624  HA  GLU E 125     -24.140 -20.590  49.983  1.00  0.00
E    H
ATOM  15625  CB  GLU E 125     -23.123 -18.890  50.760  1.00  0.00
E    C
ATOM  15626  HB1 GLU E 125     -23.327 -17.795  50.797  1.00  0.00
E    H
ATOM  15627  HB2 GLU E 125     -22.869 -19.188  51.802  1.00  0.00
E    H
ATOM  15628  CG  GLU E 125     -21.951 -19.152  49.836  1.00  0.00
E    C
ATOM  15629  HG1 GLU E 125     -21.041 -19.327  50.437  1.00  0.00
E    H
ATOM  15630  HG2 GLU E 125     -22.141 -20.034  49.195  1.00  0.00
E    H
ATOM  15631  CD  GLU E 125     -21.615 -17.993  48.941  1.00  0.00
E    C
ATOM  15632  OE1 GLU E 125     -22.403 -17.018  48.920  1.00  0.00
E    O
```

ATOM 15633 OE2 GLU E 125   -20.556 -18.062 48.288 1.00 0.00
E   O
ATOM 15634 C   GLU E 125   -25.345 -19.740 51.481 1.00 0.00
E   C
ATOM 15635 O   GLU E 125   -25.263 -20.717 52.218 1.00 0.00
E   O
ATOM 15636 N   LYS E 126   -26.272 -18.800 51.650 1.00 0.00
E   N
ATOM 15637 HN LYS E 126   -26.316 -17.930 51.163 1.00 0.00
E   H
ATOM 15638 CA LYS E 126   -27.334 -19.020 52.622 1.00 0.00
E   C
ATOM 15639 HA LYS E 126   -26.868 -19.113 53.595 1.00 0.00
E   H
ATOM 15640 CB LYS E 126   -28.316 -17.852 52.597 1.00 0.00
E   C
ATOM 15641 HB1 LYS E 126   -28.828 -17.876 51.608 1.00 0.00
E   H
ATOM 15642 HB2 LYS E 126   -29.106 -18.027 53.362 1.00 0.00
E   H
ATOM 15643 CG LYS E 126   -27.693 -16.497 52.787 1.00 0.00
E   C
ATOM 15644 HG1 LYS E 126   -26.999 -16.294 51.941 1.00 0.00
E   H
ATOM 15645 HG2 LYS E 126   -28.493 -15.722 52.754 1.00 0.00
E   H
ATOM 15646 CD LYS E 126   -26.932 -16.402 54.074 1.00 0.00
E   C
ATOM 15647 HD1 LYS E 126   -26.511 -17.407 54.309 1.00 0.00
E   H
ATOM 15648 HD2 LYS E 126   -26.074 -15.709 53.919 1.00 0.00
E   H
ATOM 15649 CE LYS E 126   -27.802 -15.911 55.216 1.00 0.00
E   C
ATOM 15650 HE1 LYS E 126   -28.574 -15.202 54.841 1.00 0.00
E   H
ATOM 15651 HE2 LYS E 126   -28.318 -16.763 55.709 1.00 0.00
E   H
ATOM 15652 NZ LYS E 126   -26.984 -15.206 56.241 1.00 0.00
E   N
ATOM 15653 HZ1 LYS E 126   -27.593 -14.874 57.016 1.00 0.00
E   H
ATOM 15654 HZ2 LYS E 126   -26.267 -15.858 56.620 1.00 0.00
E   H
ATOM 15655 HZ3 LYS E 126   -26.508 -14.392 55.803 1.00 0.00
E   H
ATOM 15656 C   LYS E 126   -28.058 -20.329 52.337 1.00 0.00
E   C
ATOM 15657 O   LYS E 126   -28.172 -21.204 53.211 1.00 0.00
E   O
ATOM 15658 N   ASP E 127   -28.519 -20.491 51.100 1.00 0.00
E   N
ATOM 15659 HN ASP E 127   -28.359 -19.877 50.332 1.00 0.00
E   H

ATOM 15660 CA ASP E 127   -29.338 -21.639 50.747 1.00 0.00
E   C
ATOM 15661 HA ASP E 127   -30.164 -21.673 51.450 1.00 0.00
E   H
ATOM 15662 CB ASP E 127   -29.816 -21.484 49.302 1.00 0.00
E   C
ATOM 15663 HB1 ASP E 127   -28.988 -21.080 48.684 1.00 0.00
E   H
ATOM 15664 HB2 ASP E 127   -30.117 -22.464 48.883 1.00 0.00
E   H
ATOM 15665 CG ASP E 127   -30.985 -20.526 49.173 1.00 0.00
E   C
ATOM 15666 OD1 ASP E 127   -30.752 -19.305 49.323 1.00 0.00
E   O
ATOM 15667 OD2 ASP E 127   -32.114 -20.991 48.909 1.00 0.00
E   O
ATOM 15668 C   ASP E 127   -28.558 -22.931 50.925 1.00 0.00
E   C
ATOM 15669 O   ASP E 127   -28.996 -23.860 51.622 1.00 0.00
E   O
ATOM 15670 N   MET E 128   -27.382 -23.008 50.319 1.00 0.00
E   N
ATOM 15671 HN MET E 128   -26.894 -22.311 49.798 1.00 0.00
E   H
ATOM 15672 CA MET E 128   -26.688 -24.276 50.414 1.00 0.00
E   C
ATOM 15673 HA MET E 128   -27.429 -25.057 50.298 1.00 0.00
E   H
ATOM 15674 CB MET E 128   -25.677 -24.423 49.281 1.00 0.00
E   C
ATOM 15675 HB1 MET E 128   -26.189 -24.976 48.459 1.00 0.00
E   H
ATOM 15676 HB2 MET E 128   -25.431 -23.418 48.873 1.00 0.00
E   H
ATOM 15677 CG MET E 128   -24.414 -25.165 49.644 1.00 0.00
E   C
ATOM 15678 HG1 MET E 128   -23.605 -24.448 49.898 1.00 0.00
E   H
ATOM 15679 HG2 MET E 128   -24.596 -25.798 50.540 1.00 0.00
E   H
ATOM 15680 SD MET E 128   -23.829 -26.213 48.298 1.00 0.00
E   S
ATOM 15681 CE MET E 128   -25.118 -26.011 47.064 1.00 0.00
E   C
ATOM 15682 HE1 MET E 128   -24.900 -26.617 46.159 1.00 0.00
E   H
ATOM 15683 HE2 MET E 128   -26.105 -26.333 47.461 1.00 0.00
E   H
ATOM 15684 HE3 MET E 128   -25.207 -24.949 46.748 1.00 0.00
E   H
ATOM 15685 C   MET E 128   -26.052 -24.446 51.783 1.00 0.00
E   C
ATOM 15686 O   MET E 128   -25.739 -25.567 52.181 1.00 0.00
E   O

```
ATOM  15687  N   GLY E 129     -25.935 -23.382  52.558  1.00  0.00
E    N
ATOM  15688  HN  GLY E 129     -26.177 -22.449  52.297  1.00  0.00
E    H
ATOM  15689  CA  GLY E 129     -25.422 -23.542  53.893  1.00  0.00
E    C
ATOM  15690  HA1 GLY E 129     -25.244 -22.559  54.309  1.00  0.00
E    H
ATOM  15691  HA2 GLY E 129     -24.544 -24.173  53.852  1.00  0.00
E    H
ATOM  15692  C   GLY E 129     -26.448 -24.230  54.748  1.00  0.00
E    C
ATOM  15693  O   GLY E 129     -26.128 -25.157  55.486  1.00  0.00
E    O
ATOM  15694  N   VAL E 130     -27.691 -23.768  54.677  1.00  0.00
E    N
ATOM  15695  HN  VAL E 130     -28.021 -22.988  54.152  1.00  0.00
E    H
ATOM  15696  CA  VAL E 130     -28.721 -24.455  55.443  1.00  0.00
E    C
ATOM  15697  HA  VAL E 130     -28.387 -24.514  56.471  1.00  0.00
E    H
ATOM  15698  CB  VAL E 130     -30.029 -23.641  55.449  1.00  0.00
E    C
ATOM  15699  HB  VAL E 130     -30.725 -24.125  56.178  1.00  0.00
E    H
ATOM  15700  CG1 VAL E 130     -29.735 -22.219  55.887  1.00  0.00
E    C
ATOM  15701 HG11 VAL E 130     -30.663 -21.607  55.859  1.00  0.00
E    H
ATOM  15702 HG12 VAL E 130     -29.347 -22.182  56.925  1.00  0.00
E    H
ATOM  15703 HG13 VAL E 130     -28.992 -21.745  55.211  1.00  0.00
E    H
ATOM  15704  CG2 VAL E 130     -30.664 -23.599  54.093  1.00  0.00
E    C
ATOM  15705 HG21 VAL E 130     -31.583 -22.975  54.109  1.00  0.00
E    H
ATOM  15706 HG22 VAL E 130     -29.962 -23.169  53.347  1.00  0.00
E    H
ATOM  15707 HG23 VAL E 130     -30.954 -24.615  53.754  1.00  0.00
E    H
ATOM  15708  C   VAL E 130     -28.889 -25.882  54.934  1.00  0.00
E    C
ATOM  15709  O   VAL E 130     -29.077 -26.820  55.726  1.00  0.00
E    O
ATOM  15710  N   LYS E 131     -28.774 -26.078  53.615  1.00  0.00
E    N
ATOM  15711  HN  LYS E 131     -28.829 -25.358  52.926  1.00  0.00
E    H
ATOM  15712  CA  LYS E 131     -28.550 -27.423  53.090  1.00  0.00
E    C
ATOM  15713  HA  LYS E 131     -29.496 -27.947  53.161  1.00  0.00
E    H
```

ATOM 15714 CB LYS E 131   -28.075 -27.339 51.638 1.00 0.00
E   C
ATOM 15715 HB1 LYS E 131   -28.705 -26.573 51.130 1.00 0.00
E   H
ATOM 15716 HB2 LYS E 131   -27.032 -26.949 51.620 1.00 0.00
E   H
ATOM 15717 CG LYS E 131   -28.155 -28.634 50.825 1.00 0.00
E   C
ATOM 15718 HG1 LYS E 131   -29.165 -29.086 50.957 1.00 0.00
E   H
ATOM 15719 HG2 LYS E 131   -28.039 -28.390 49.744 1.00 0.00
E   H
ATOM 15720 CD LYS E 131   -27.073 -29.613 51.299 1.00 0.00
E   C
ATOM 15721 HD1 LYS E 131   -26.143 -29.417 50.718 1.00 0.00
E   H
ATOM 15722 HD2 LYS E 131   -26.851 -29.394 52.368 1.00 0.00
E   H
ATOM 15723 CE LYS E 131   -27.453 -31.075 51.162 1.00 0.00
E   C
ATOM 15724 HE1 LYS E 131   -26.722 -31.720 51.699 1.00 0.00
E   H
ATOM 15725 HE2 LYS E 131   -28.464 -31.258 51.588 1.00 0.00
E   H
ATOM 15726 NZ LYS E 131   -27.461 -31.460 49.693 1.00 0.00
E   N
ATOM 15727 HZ1 LYS E 131   -27.719 -32.462 49.589 1.00 0.00
E   H
ATOM 15728 HZ2 LYS E 131   -28.151 -30.870 49.183 1.00 0.00
E   H
ATOM 15729 HZ3 LYS E 131   -26.515 -31.304 49.289 1.00 0.00
E   H
ATOM 15730 C   LYS E 131   -27.541 -28.198 53.916 1.00 0.00
E   C
ATOM 15731 O   LYS E 131   -27.892 -29.173 54.593 1.00 0.00
E   O
ATOM 15732 N   ILE E 132   -26.286 -27.787 53.854 1.00 0.00
E   N
ATOM 15733 HN ILE E 132   -25.976 -26.917 53.478 1.00 0.00
E   H
ATOM 15734 CA ILE E 132   -25.218 -28.629 54.360 1.00 0.00
E   C
ATOM 15735 HA ILE E 132   -25.354 -29.609 53.920 1.00 0.00
E   H
ATOM 15736 CB ILE E 132   -23.854 -28.048 53.967 1.00 0.00
E   C
ATOM 15737 HB ILE E 132   -23.858 -28.056 52.848 1.00 0.00
E   H
ATOM 15738 CG2 ILE E 132   -23.677 -26.682 54.559 1.00 0.00
E   C
ATOM 15739 HG21 ILE E 132   -22.765 -26.195 54.153 1.00 0.00
E   H
ATOM 15740 HG22 ILE E 132   -24.542 -26.030 54.317 1.00 0.00
E   H

```
ATOM  15741 HG23 ILE E 132      -23.578 -26.737  55.663  1.00  0.00
E    H
ATOM  15742  CG1 ILE E 132      -22.742 -28.989  54.416  1.00  0.00
E    C
ATOM  15743 HG11 ILE E 132      -22.354 -28.668  55.409  1.00  0.00
E    H
ATOM  15744 HG12 ILE E 132      -23.164 -30.012  54.544  1.00  0.00
E    H
ATOM  15745  CD  ILE E 132      -21.611 -29.066  53.448  1.00  0.00
E    C
ATOM  15746  HD1 ILE E 132      -20.857 -29.807  53.794  1.00  0.00
E    H
ATOM  15747  HD2 ILE E 132      -21.974 -29.380  52.445  1.00  0.00
E    H
ATOM  15748  HD3 ILE E 132      -21.105 -28.082  53.348  1.00  0.00
E    H
ATOM  15749  C   ILE E 132      -25.359 -28.798  55.859  1.00  0.00
E    C
ATOM  15750  O   ILE E 132      -24.999 -29.836  56.426  1.00  0.00
E    O
ATOM  15751  N   ALA E 133      -25.939 -27.801  56.514  1.00  0.00
E    N
ATOM  15752  HN  ALA E 133      -26.248 -26.951  56.086  1.00  0.00
E    H
ATOM  15753  CA  ALA E 133      -26.164 -27.895  57.942  1.00  0.00
E    C
ATOM  15754  HA  ALA E 133      -25.242 -28.128  58.463  1.00  0.00
E    H
ATOM  15755  CB  ALA E 133      -26.695 -26.568  58.467  1.00  0.00
E    C
ATOM  15756  HB1 ALA E 133      -25.953 -25.765  58.270  1.00  0.00
E    H
ATOM  15757  HB2 ALA E 133      -27.643 -26.289  57.958  1.00  0.00
E    H
ATOM  15758  HB3 ALA E 133      -26.879 -26.604  59.562  1.00  0.00
E    H
ATOM  15759  C   ALA E 133      -27.128 -29.021  58.257  1.00  0.00
E    C
ATOM  15760  O   ALA E 133      -26.843 -29.872  59.109  1.00  0.00
E    O
ATOM  15761  N   GLN E 134      -28.271 -29.052  57.572  1.00  0.00
E    N
ATOM  15762  HN  GLN E 134      -28.579 -28.386  56.895  1.00  0.00
E    H
ATOM  15763  CA  GLN E 134      -29.197 -30.151  57.801  1.00  0.00
E    C
ATOM  15764  HA  GLN E 134      -29.419 -30.167  58.861  1.00  0.00
E    H
ATOM  15765  CB  GLN E 134      -30.473 -29.937  57.001  1.00  0.00
E    C
ATOM  15766  HB1 GLN E 134      -30.202 -30.047  55.925  1.00  0.00
E    H
ATOM  15767  HB2 GLN E 134      -31.192 -30.759  57.225  1.00  0.00
E    H
```

ATOM 15768 CG GLN E 134   -31.083 -28.582 57.246 1.00 0.00
E   C
ATOM 15769 HG1 GLN E 134   -30.307 -27.864 57.587 1.00 0.00
E   H
ATOM 15770 HG2 GLN E 134   -31.535 -28.180 56.316 1.00 0.00
E   H
ATOM 15771 CD GLN E 134   -32.139 -28.612 58.325 1.00 0.00
E   C
ATOM 15772 OE1 GLN E 134   -32.409 -29.656 58.917 1.00 0.00
E   O
ATOM 15773 NE2 GLN E 134   -32.740 -27.458 58.597 1.00 0.00
E   N
ATOM 15774 HE21 GLN E 134   -33.435 -27.471 59.310 1.00 0.00
E   H
ATOM 15775 HE22 GLN E 134   -32.498 -26.642 58.083 1.00 0.00
E   H
ATOM 15776 C   GLN E 134   -28.552 -31.481 57.447 1.00 0.00
E   C
ATOM 15777 O   GLN E 134   -28.727 -32.472 58.162 1.00 0.00
E   O
ATOM 15778 N   SER E 135   -27.776 -31.512 56.363 1.00 0.00
E   N
ATOM 15779 HN SER E 135   -27.528 -30.732 55.789 1.00 0.00
E   H
ATOM 15780 CA SER E 135   -27.205 -32.781 55.925 1.00 0.00
E   C
ATOM 15781 HA SER E 135   -28.014 -33.498 55.968 1.00 0.00
E   H
ATOM 15782 CB SER E 135   -26.651 -32.661 54.510 1.00 0.00
E   C
ATOM 15783 HB1 SER E 135   -26.117 -33.607 54.266 1.00 0.00
E   H
ATOM 15784 HB2 SER E 135   -27.483 -32.556 53.777 1.00 0.00
E   H
ATOM 15785 OG SER E 135   -25.743 -31.586 54.415 1.00 0.00
E   O
ATOM 15786 HG1 SER E 135   -25.264 -31.691 53.583 1.00 0.00
E   H
ATOM 15787 C   SER E 135   -26.118 -33.269 56.872 1.00 0.00
E   C
ATOM 15788 O   SER E 135   -25.822 -34.467 56.908 1.00 0.00
E   O
ATOM 15789 N   LEU E 136   -25.506 -32.369 57.636 1.00 0.00
E   N
ATOM 15790 HN LEU E 136   -25.673 -31.385 57.635 1.00 0.00
E   H
ATOM 15791 CA LEU E 136   -24.486 -32.802 58.581 1.00 0.00
E   C
ATOM 15792 HA LEU E 136   -24.046 -33.703 58.171 1.00 0.00
E   H
ATOM 15793 CB LEU E 136   -23.410 -31.732 58.742 1.00 0.00
E   C
ATOM 15794 HB1 LEU E 136   -23.831 -30.781 58.342 1.00 0.00
E   H

ATOM 15795 HB2 LEU E 136 -23.231 -31.559 59.826 1.00 0.00
E H
ATOM 15796 CG LEU E 136 -22.116 -32.070 58.007 1.00 0.00
E C
ATOM 15797 HG LEU E 136 -22.183 -31.725 56.946 1.00 0.00
E H
ATOM 15798 CD1 LEU E 136 -20.938 -31.428 58.691 1.00 0.00
E C
ATOM 15799 HD11 LEU E 136 -19.991 -31.712 58.184 1.00 0.00
E H
ATOM 15800 HD12 LEU E 136 -21.027 -30.321 58.667 1.00 0.00
E H
ATOM 15801 HD13 LEU E 136 -20.875 -31.753 59.751 1.00 0.00
E H
ATOM 15802 CD2 LEU E 136 -21.934 -33.570 58.001 1.00 0.00
E C
ATOM 15803 HD21 LEU E 136 -21.006 -33.843 57.454 1.00 0.00
E H
ATOM 15804 HD22 LEU E 136 -21.851 -33.977 59.028 1.00 0.00
E H
ATOM 15805 HD23 LEU E 136 -22.793 -34.064 57.496 1.00 0.00
E H
ATOM 15806 C LEU E 136 -25.094 -33.160 59.925 1.00 0.00
E C
ATOM 15807 O LEU E 136 -24.699 -34.153 60.545 1.00 0.00
E O
ATOM 15808 N SER E 137 -26.051 -32.359 60.394 1.00 0.00
E N
ATOM 15809 HN SER E 137 -26.398 -31.544 59.928 1.00 0.00
E H
ATOM 15810 CA SER E 137 -26.686 -32.628 61.679 1.00 0.00
E C
ATOM 15811 HA SER E 137 -25.911 -32.517 62.426 1.00 0.00
E H
ATOM 15812 CB SER E 137 -27.838 -31.655 61.895 1.00 0.00
E C
ATOM 15813 HB1 SER E 137 -28.258 -31.835 62.911 1.00 0.00
E H
ATOM 15814 HB2 SER E 137 -27.467 -30.605 61.877 1.00 0.00
E H
ATOM 15815 OG SER E 137 -28.848 -31.873 60.926 1.00 0.00
E O
ATOM 15816 HG1 SER E 137 -29.637 -31.405 61.228 1.00 0.00
E H
ATOM 15817 C SER E 137 -27.183 -34.065 61.752 1.00 0.00
E C
ATOM 15818 O SER E 137 -26.708 -34.861 62.570 1.00 0.00
E O
ATOM 15819 N ASP E 138 -28.116 -34.422 60.884 1.00 0.00
E N
ATOM 15820 HN ASP E 138 -28.526 -33.811 60.212 1.00 0.00
E H
ATOM 15821 CA ASP E 138 -28.652 -35.776 60.822 1.00 0.00
E C

ATOM 15822 HA ASP E 138   -28.435 -36.285 61.754 1.00 0.00
E   H
ATOM 15823 CB ASP E 138   -30.146 -35.748 60.527 1.00 0.00
E   C
ATOM 15824 HB1 ASP E 138   -30.341 -35.040 59.696 1.00 0.00
E   H
ATOM 15825 HB2 ASP E 138   -30.503 -36.751 60.220 1.00 0.00
E   H
ATOM 15826 CG ASP E 138   -30.967 -35.289 61.713 1.00 0.00
E   C
ATOM 15827 OD1 ASP E 138   -31.230 -36.110 62.616 1.00 0.00
E   O
ATOM 15828 OD2 ASP E 138   -31.344 -34.099 61.740 1.00 0.00
E   O
ATOM 15829 C   ASP E 138   -27.873 -36.501 59.733 1.00 0.00
E   C
ATOM 15830 O   ASP E 138   -28.123 -36.308 58.542 1.00 0.00
E   O
ATOM 15831 N   GLU E 139   -26.926 -37.341 60.143 1.00 0.00
E   N
ATOM 15832 HN GLU E 139   -26.773 -37.634 61.083 1.00 0.00
E   H
ATOM 15833 CA GLU E 139   -25.997 -37.920 59.185 1.00 0.00
E   C
ATOM 15834 HA GLU E 139   -25.693 -37.125 58.516 1.00 0.00
E   H
ATOM 15835 CB GLU E 139   -24.767 -38.448 59.920 1.00 0.00
E   C
ATOM 15836 HB1 GLU E 139   -24.158 -39.037 59.196 1.00 0.00
E   H
ATOM 15837 HB2 GLU E 139   -24.124 -37.597 60.239 1.00 0.00
E   H
ATOM 15838 CG GLU E 139   -25.096 -39.319 61.108 1.00 0.00
E   C
ATOM 15839 HG1 GLU E 139   -25.835 -38.805 61.749 1.00 0.00
E   H
ATOM 15840 HG2 GLU E 139   -25.509 -40.295 60.784 1.00 0.00
E   H
ATOM 15841 CD GLU E 139   -23.894 -39.571 61.985 1.00 0.00
E   C
ATOM 15842 OE1 GLU E 139   -24.059 -39.616 63.221 1.00 0.00
E   O
ATOM 15843 OE2 GLU E 139   -22.783 -39.717 61.441 1.00 0.00
E   O
ATOM 15844 C   GLU E 139   -26.613 -39.020 58.331 1.00 0.00
E   C
ATOM 15845 O   GLU E 139   -25.870 -39.733 57.648 1.00 0.00
E   O
ATOM 15846 N   ASN E 140   -27.938 -39.182 58.341 1.00 0.00
E   N
ATOM 15847 HN ASN E 140   -28.584 -38.715 58.944 1.00 0.00
E   H
ATOM 15848 CA ASN E 140   -28.551 -40.117 57.406 1.00 0.00
E   C

ATOM 15849 HA ASN E 140   -27.739 -40.730 57.030 1.00 0.00
E   H
ATOM 15850 CB ASN E 140   -29.602 -40.985 58.098 1.00 0.00
E   C
ATOM 15851 HB1 ASN E 140   -29.421 -40.929 59.194 1.00 0.00
E   H
ATOM 15852 HB2 ASN E 140   -30.619 -40.585 57.908 1.00 0.00
E   H
ATOM 15853 CG ASN E 140   -29.518 -42.443 57.676 1.00 0.00
E   C
ATOM 15854 OD1 ASN E 140   -30.506 -43.176 57.730 1.00 0.00
E   O
ATOM 15855 ND2 ASN E 140   -28.331 -42.869 57.248 1.00 0.00
E   N
ATOM 15856 HD21 ASN E 140   -28.263 -43.820 56.956 1.00 0.00
E   H
ATOM 15857 HD22 ASN E 140   -27.562 -42.238 57.199 1.00 0.00
E   H
ATOM 15858 C   ASN E 140   -29.174 -39.398 56.218 1.00 0.00
E   C
ATOM 15859 O   ASN E 140   -29.125 -39.905 55.093 1.00 0.00
E   O
ATOM 15860 N   VAL E 141   -29.772 -38.229 56.436 1.00 0.00
E   N
ATOM 15861 HN VAL E 141   -29.922 -37.793 57.320 1.00 0.00
E   H
ATOM 15862 CA VAL E 141   -30.277 -37.472 55.297 1.00 0.00
E   C
ATOM 15863 HA VAL E 141   -30.869 -38.123 54.666 1.00 0.00
E   H
ATOM 15864 CB VAL E 141   -31.140 -36.278 55.730 1.00 0.00
E   C
ATOM 15865 HB VAL E 141   -32.199 -36.513 55.462 1.00 0.00
E   H
ATOM 15866 CG1 VAL E 141   -31.049 -36.053 57.217 1.00 0.00
E   C
ATOM 15867 HG11 VAL E 141   -31.647 -35.163 57.509 1.00 0.00
E   H
ATOM 15868 HG12 VAL E 141   -31.445 -36.919 57.788 1.00 0.00
E   H
ATOM 15869 HG13 VAL E 141   -29.997 -35.875 57.524 1.00 0.00
E   H
ATOM 15870 CG2 VAL E 141   -30.829 -35.012 54.923 1.00 0.00
E   C
ATOM 15871 HG21 VAL E 141   -31.438 -34.157 55.283 1.00 0.00
E   H
ATOM 15872 HG22 VAL E 141   -29.754 -34.741 55.019 1.00 0.00
E   H
ATOM 15873 HG23 VAL E 141   -31.057 -35.155 53.846 1.00 0.00
E   H
ATOM 15874 C   VAL E 141   -29.060 -37.035 54.491 1.00 0.00
E   C
ATOM 15875 O   VAL E 141   -28.190 -36.319 54.993 1.00 0.00
E   O

```
ATOM  15876  N   LEU E 142     -28.942 -37.554  53.273  1.00  0.00
E    N
ATOM  15877  HN  LEU E 142     -29.556 -38.223  52.859  1.00  0.00
E    H
ATOM  15878  CA  LEU E 142     -27.835 -37.147  52.418  1.00  0.00
E    C
ATOM  15879  HA  LEU E 142     -27.011 -36.882  53.069  1.00  0.00
E    H
ATOM  15880  CB  LEU E 142     -27.426 -38.291  51.486  1.00  0.00
E    C
ATOM  15881  HB1 LEU E 142     -27.220 -39.179  52.127  1.00  0.00
E    H
ATOM  15882  HB2 LEU E 142     -28.296 -38.567  50.849  1.00  0.00
E    H
ATOM  15883  CG  LEU E 142     -26.197 -37.980  50.638  1.00  0.00
E    C
ATOM  15884  HG  LEU E 142     -26.425 -37.071  50.031  1.00  0.00
E    H
ATOM  15885  CD1 LEU E 142     -25.009 -37.709  51.536  1.00  0.00
E    C
ATOM  15886 HD11 LEU E 142     -24.095 -37.529  50.930  1.00  0.00
E    H
ATOM  15887 HD12 LEU E 142     -25.192 -36.814  52.167  1.00  0.00
E    H
ATOM  15888 HD13 LEU E 142     -24.816 -38.577  52.203  1.00  0.00
E    H
ATOM  15889  CD2 LEU E 142     -25.891 -39.118  49.680  1.00  0.00
E    C
ATOM  15890 HD21 LEU E 142     -25.005 -38.870  49.056  1.00  0.00
E    H
ATOM  15891 HD22 LEU E 142     -25.668 -40.060  50.220  1.00  0.00
E    H
ATOM  15892 HD23 LEU E 142     -26.753 -39.299  49.002  1.00  0.00
E    H
ATOM  15893  C   LEU E 142     -28.208 -35.906  51.623  1.00  0.00
E    C
ATOM  15894  O   LEU E 142     -27.546 -34.868  51.727  1.00  0.00
E    O
ATOM  15895  N   ASN E 143     -29.271 -35.993  50.831  1.00  0.00
E    N
ATOM  15896  HN  ASN E 143     -29.739 -36.849  50.615  1.00  0.00
E    H
ATOM  15897  CA  ASN E 143     -29.848 -34.816  50.202  1.00  0.00
E    C
ATOM  15898  HA  ASN E 143     -29.333 -33.948  50.598  1.00  0.00
E    H
ATOM  15899  CB  ASN E 143     -29.762 -34.873  48.673  1.00  0.00
E    C
ATOM  15900  HB1 ASN E 143     -30.382 -35.730  48.329  1.00  0.00
E    H
ATOM  15901  HB2 ASN E 143     -30.190 -33.952  48.228  1.00  0.00
E    H
ATOM  15902  CG  ASN E 143     -28.350 -35.088  48.172  1.00  0.00
E    C
```

ATOM 15903 OD1 ASN E 143   -27.551 -35.769 48.805 1.00 0.00
E   O
ATOM 15904 ND2 ASN E 143   -28.033 -34.492 47.029 1.00 0.00
E   N
ATOM 15905 HD21 ASN E 143   -27.111 -34.633 46.680 1.00 0.00
E   H
ATOM 15906 HD22 ASN E 143   -28.715 -33.956 46.541 1.00 0.00
E   H
ATOM 15907 C   ASN E 143   -31.299 -34.730 50.643 1.00 0.00
E   C
ATOM 15908 O   ASN E 143   -31.909 -35.738 51.007 1.00 0.00
E   O
ATOM 15909 N   TYR E 144   -31.854 -33.525 50.599 1.00 0.00
E   N
ATOM 15910 HN TYR E 144   -31.415 -32.686 50.282 1.00 0.00
E   H
ATOM 15911 CA TYR E 144   -33.229 -33.357 51.044 1.00 0.00
E   C
ATOM 15912 HA TYR E 144   -33.772 -34.253 50.768 1.00 0.00
E   H
ATOM 15913 CB TYR E 144   -33.305 -33.117 52.552 1.00 0.00
E   C
ATOM 15914 HB1 TYR E 144   -34.360 -33.226 52.885 1.00 0.00
E   H
ATOM 15915 HB2 TYR E 144   -32.707 -33.876 53.101 1.00 0.00
E   H
ATOM 15916 CG TYR E 144   -32.866 -31.745 52.991 1.00 0.00
E   C
ATOM 15917 CD1 TYR E 144   -31.532 -31.373 52.946 1.00 0.00
E   C
ATOM 15918 HD1 TYR E 144   -30.788 -32.081 52.605 1.00 0.00
E   H
ATOM 15919 CE1 TYR E 144   -31.128 -30.119 53.350 1.00 0.00
E   C
ATOM 15920 HE1 TYR E 144   -30.086 -29.840 53.316 1.00 0.00
E   H
ATOM 15921 CZ TYR E 144   -32.063 -29.215 53.809 1.00 0.00
E   C
ATOM 15922 OH TYR E 144   -31.652 -27.961 54.209 1.00 0.00
E   O
ATOM 15923 HH TYR E 144   -32.438 -27.456 54.425 1.00 0.00
E   H
ATOM 15924 CD2 TYR E 144   -33.789 -30.821 53.456 1.00 0.00
E   C
ATOM 15925 HD2 TYR E 144   -34.832 -31.095 53.541 1.00 0.00
E   H
ATOM 15926 CE2 TYR E 144   -33.399 -29.564 53.863 1.00 0.00
E   C
ATOM 15927 HE2 TYR E 144   -34.136 -28.871 54.240 1.00 0.00
E   H
ATOM 15928 C   TYR E 144   -33.857 -32.202 50.285 1.00 0.00
E   C
ATOM 15929 O   TYR E 144   -33.195 -31.221 49.943 1.00 0.00
E   O

ATOM 15930 N   ILE E 145   -35.157 -32.334 50.035 1.00 0.00
E   N
ATOM 15931 HN ILE E 145   -35.672 -33.156 50.271 1.00 0.00
E   H
ATOM 15932 CA ILE E 145   -35.952 -31.295 49.393 1.00 0.00
E   C
ATOM 15933 HA ILE E 145   -35.449 -30.351 49.559 1.00 0.00
E   H
ATOM 15934 CB ILE E 145   -36.139 -31.550 47.884 1.00 0.00
E   C
ATOM 15935 HB ILE E 145   -35.542 -32.474 47.680 1.00 0.00
E   H
ATOM 15936 CG2 ILE E 145   -37.615 -31.726 47.522 1.00 0.00
E   C
ATOM 15937 HG21 ILE E 145   -37.722 -32.061 46.470 1.00 0.00
E   H
ATOM 15938 HG22 ILE E 145   -38.093 -32.491 48.169 1.00 0.00
E   H
ATOM 15939 HG23 ILE E 145   -38.170 -30.771 47.640 1.00 0.00
E   H
ATOM 15940 CG1 ILE E 145   -35.495 -30.409 47.108 1.00 0.00
E   C
ATOM 15941 HG11 ILE E 145   -34.931 -29.751 47.805 1.00 0.00
E   H
ATOM 15942 HG12 ILE E 145   -34.759 -30.833 46.387 1.00 0.00
E   H
ATOM 15943 CD ILE E 145   -36.474 -29.559 46.324 1.00 0.00
E   C
ATOM 15944 HD1 ILE E 145   -35.931 -28.785 45.740 1.00 0.00
E   H
ATOM 15945 HD2 ILE E 145   -37.057 -30.186 45.615 1.00 0.00
E   H
ATOM 15946 HD3 ILE E 145   -37.184 -29.041 47.003 1.00 0.00
E   H
ATOM 15947 C   ILE E 145   -37.296 -31.210 50.104 1.00 0.00
E   C
ATOM 15948 O   ILE E 145   -37.872 -32.237 50.483 1.00 0.00
E   O
ATOM 15949 N   ASP E 146   -37.766 -29.986 50.336 1.00 0.00
E   N
ATOM 15950 HN ASP E 146   -37.304 -29.143 50.074 1.00 0.00
E   H
ATOM 15951 CA ASP E 146   -39.035 -29.753 51.015 1.00 0.00
E   C
ATOM 15952 HA ASP E 146   -39.489 -30.719 51.202 1.00 0.00
E   H
ATOM 15953 CB ASP E 146   -38.808 -28.972 52.307 1.00 0.00
E   C
ATOM 15954 HB1 ASP E 146   -39.743 -28.446 52.584 1.00 0.00
E   H
ATOM 15955 HB2 ASP E 146   -38.533 -29.656 53.133 1.00 0.00
E   H
ATOM 15956 CG ASP E 146   -37.722 -27.925 52.167 1.00 0.00
E   C

ATOM 15957 OD1 ASP E 146 -38.011 -26.861 51.577 1.00 0.00
E O
ATOM 15958 OD2 ASP E 146 -36.576 -28.174 52.600 1.00 0.00
E O
ATOM 15959 C ASP E 146 -39.981 -28.995 50.100 1.00 0.00
E C
ATOM 15960 O ASP E 146 -39.634 -27.926 49.590 1.00 0.00
E O
ATOM 15961 N LEU E 147 -41.176 -29.542 49.900 1.00 0.00
E N
ATOM 15962 HN LEU E 147 -41.458 -30.433 50.250 1.00 0.00
E H
ATOM 15963 CA LEU E 147 -42.200 -28.854 49.125 1.00 0.00
E C
ATOM 15964 HA LEU E 147 -41.678 -28.335 48.331 1.00 0.00
E H
ATOM 15965 CB LEU E 147 -43.170 -29.879 48.549 1.00 0.00
E C
ATOM 15966 HB1 LEU E 147 -44.133 -29.352 48.360 1.00 0.00
E H
ATOM 15967 HB2 LEU E 147 -42.799 -30.208 47.552 1.00 0.00
E H
ATOM 15968 CG LEU E 147 -43.369 -31.040 49.519 1.00 0.00
E C
ATOM 15969 HG LEU E 147 -42.457 -31.067 50.162 1.00 0.00
E H
ATOM 15970 CD1 LEU E 147 -44.585 -30.825 50.400 1.00 0.00
E C
ATOM 15971 HD11 LEU E 147 -44.744 -31.699 51.066 1.00 0.00
E H
ATOM 15972 HD12 LEU E 147 -44.453 -29.922 51.035 1.00 0.00
E H
ATOM 15973 HD13 LEU E 147 -45.498 -30.689 49.783 1.00 0.00
E H
ATOM 15974 CD2 LEU E 147 -43.441 -32.353 48.748 1.00 0.00
E C
ATOM 15975 HD21 LEU E 147 -43.567 -33.207 49.449 1.00 0.00
E H
ATOM 15976 HD22 LEU E 147 -44.295 -32.368 48.042 1.00 0.00
E H
ATOM 15977 HD23 LEU E 147 -42.506 -32.515 48.168 1.00 0.00
E H
ATOM 15978 C LEU E 147 -42.945 -27.808 49.947 1.00 0.00
E C
ATOM 15979 O LEU E 147 -43.472 -26.845 49.379 1.00 0.00
E O
ATOM 15980 N SER E 148 -42.988 -27.966 51.262 1.00 0.00
E N
ATOM 15981 HN SER E 148 -42.558 -28.724 51.752 1.00 0.00
E H
ATOM 15982 CA SER E 148 -43.685 -27.020 52.121 1.00 0.00
E C
ATOM 15983 HA SER E 148 -43.661 -26.058 51.626 1.00 0.00
E H

ATOM 15984 CB SER E 148   -45.135 -27.483 52.348 1.00 0.00
E   C
ATOM 15985 HB1 SER E 148   -45.642 -26.729 52.992 1.00 0.00
E   H
ATOM 15986 HB2 SER E 148   -45.688 -27.513 51.381 1.00 0.00
E   H
ATOM 15987 OG SER E 148   -45.173 -28.744 53.001 1.00 0.00
E   O
ATOM 15988 HG1 SER E 148   -46.077 -28.874 53.312 1.00 0.00
E   H
ATOM 15989 C   SER E 148   -42.897 -26.884 53.418 1.00 0.00
E   C
ATOM 15990 O   SER E 148   -41.809 -27.458 53.561 1.00 0.00
E   O
ATOM 15991 N   ASP E 149   -43.441 -26.123 54.368 1.00 0.00
E   N
ATOM 15992 HN ASP E 149   -44.281 -25.592 54.292 1.00 0.00
E   H
ATOM 15993 CA ASP E 149   -42.809 -26.001 55.677 1.00 0.00
E   C
ATOM 15994 HA ASP E 149   -41.740 -26.074 55.517 1.00 0.00
E   H
ATOM 15995 CB ASP E 149   -43.177 -24.661 56.311 1.00 0.00
E   C
ATOM 15996 HB1 ASP E 149   -42.854 -24.659 57.371 1.00 0.00
E   H
ATOM 15997 HB2 ASP E 149   -42.665 -23.829 55.789 1.00 0.00
E   H
ATOM 15998 CG ASP E 149   -44.666 -24.402 56.288 1.00 0.00
E   C
ATOM 15999 OD1 ASP E 149   -45.117 -23.579 55.463 1.00 0.00
E   O
ATOM 16000 OD2 ASP E 149   -45.389 -25.031 57.083 1.00 0.00
E   O
ATOM 16001 C   ASP E 149   -43.195 -27.150 56.596 1.00 0.00
E   C
ATOM 16002 O   ASP E 149   -42.774 -27.183 57.759 1.00 0.00
E   O
ATOM 16003 N   GLU E 150   -43.982 -28.099 56.096 1.00 0.00
E   N
ATOM 16004 HN GLU E 150   -44.308 -28.085 55.155 1.00 0.00
E   H
ATOM 16005 CA GLU E 150   -44.443 -29.245 56.867 1.00 0.00
E   C
ATOM 16006 HA GLU E 150   -44.044 -29.136 57.868 1.00 0.00
E   H
ATOM 16007 CB GLU E 150   -45.975 -29.266 56.912 1.00 0.00
E   C
ATOM 16008 HB1 GLU E 150   -46.322 -28.299 57.342 1.00 0.00
E   H
ATOM 16009 HB2 GLU E 150   -46.382 -29.305 55.877 1.00 0.00
E   H
ATOM 16010 CG GLU E 150   -46.544 -30.406 57.735 1.00 0.00
E   C

ATOM 16011 HG1 GLU E 150 -47.242 -30.996 57.114 1.00 0.00
E H
ATOM 16012 HG2 GLU E 150 -45.738 -31.068 58.108 1.00 0.00
E H
ATOM 16013 CD GLU E 150 -47.356 -29.940 58.918 1.00 0.00
E C
ATOM 16014 OE1 GLU E 150 -47.153 -28.794 59.366 1.00 0.00
E O
ATOM 16015 OE2 GLU E 150 -48.198 -30.725 59.402 1.00 0.00
E O
ATOM 16016 C GLU E 150 -43.953 -30.577 56.329 1.00 0.00
E C
ATOM 16017 O GLU E 150 -43.680 -31.489 57.112 1.00 0.00
E O
ATOM 16018 N TYR E 151 -43.838 -30.716 55.015 1.00 0.00
E N
ATOM 16019 HN TYR E 151 -43.964 -29.966 54.368 1.00 0.00
E H
ATOM 16020 CA TYR E 151 -43.512 -31.993 54.404 1.00 0.00
E C
ATOM 16021 HA TYR E 151 -43.213 -32.659 55.204 1.00 0.00
E H
ATOM 16022 CB TYR E 151 -44.711 -32.541 53.636 1.00 0.00
E C
ATOM 16023 HB1 TYR E 151 -44.851 -31.949 52.705 1.00 0.00
E H
ATOM 16024 HB2 TYR E 151 -44.534 -33.596 53.331 1.00 0.00
E H
ATOM 16025 CG TYR E 151 -46.017 -32.462 54.393 1.00 0.00
E C
ATOM 16026 CD1 TYR E 151 -46.317 -33.369 55.398 1.00 0.00
E C
ATOM 16027 HD1 TYR E 151 -45.613 -34.153 55.642 1.00 0.00
E H
ATOM 16028 CE1 TYR E 151 -47.511 -33.306 56.086 1.00 0.00
E C
ATOM 16029 HE1 TYR E 151 -47.736 -34.021 56.862 1.00 0.00
E H
ATOM 16030 CZ TYR E 151 -48.424 -32.325 55.771 1.00 0.00
E C
ATOM 16031 OH TYR E 151 -49.614 -32.255 56.454 1.00 0.00
E O
ATOM 16032 HH TYR E 151 -50.077 -31.473 56.146 1.00 0.00
E H
ATOM 16033 CD2 TYR E 151 -46.953 -31.483 54.095 1.00 0.00
E C
ATOM 16034 HD2 TYR E 151 -46.767 -30.785 53.291 1.00 0.00
E H
ATOM 16035 CE2 TYR E 151 -48.150 -31.411 54.778 1.00 0.00
E C
ATOM 16036 HE2 TYR E 151 -48.873 -30.654 54.516 1.00 0.00
E H
ATOM 16037 C TYR E 151 -42.318 -31.842 53.474 1.00 0.00
E C

ATOM 16038 O TYR E 151 −42.016 −30.740 53.006 1.00 0.00
E O
ATOM 16039 N SER E 152 −41.642 −32.958 53.212 1.00 0.00
E N
ATOM 16040 HN SER E 152 −41.940 −33.878 53.466 1.00 0.00
E H
ATOM 16041 CA SER E 152 −40.371 −32.916 52.506 1.00 0.00
E C
ATOM 16042 HA SER E 152 −40.531 −32.296 51.634 1.00 0.00
E H
ATOM 16043 CB SER E 152 −39.270 −32.365 53.412 1.00 0.00
E C
ATOM 16044 HB1 SER E 152 −38.359 −32.992 53.272 1.00 0.00
E H
ATOM 16045 HB2 SER E 152 −39.002 −31.327 53.110 1.00 0.00
E H
ATOM 16046 OG SER E 152 −39.676 −32.428 54.771 1.00 0.00
E O
ATOM 16047 HG1 SER E 152 −38.888 −32.287 55.310 1.00 0.00
E H
ATOM 16048 C SER E 152 −39.977 −34.301 52.011 1.00 0.00
E C
ATOM 16049 O SER E 152 −40.514 −35.322 52.456 1.00 0.00
E O
ATOM 16050 N ILE E 153 −39.012 −34.312 51.094 1.00 0.00
E N
ATOM 16051 HN ILE E 153 −38.615 −33.468 50.737 1.00 0.00
E H
ATOM 16052 CA ILE E 153 −38.441 −35.527 50.526 1.00 0.00
E C
ATOM 16053 HA ILE E 153 −39.003 −36.369 50.912 1.00 0.00
E H
ATOM 16054 CB ILE E 153 −38.455 −35.482 48.990 1.00 0.00
E C
ATOM 16055 HB ILE E 153 −38.054 −34.468 48.737 1.00 0.00
E H
ATOM 16056 CG2 ILE E 153 −37.640 −36.619 48.401 1.00 0.00
E C
ATOM 16057 HG21 ILE E 153 −37.525 −36.491 47.304 1.00 0.00
E H
ATOM 16058 HG22 ILE E 153 −36.622 −36.648 48.844 1.00 0.00
E H
ATOM 16059 HG23 ILE E 153 −38.131 −37.598 48.585 1.00 0.00
E H
ATOM 16060 CG1 ILE E 153 −39.890 −35.533 48.461 1.00 0.00
E C
ATOM 16061 HG11 ILE E 153 −40.132 −36.568 48.131 1.00 0.00
E H
ATOM 16062 HG12 ILE E 153 −40.589 −35.280 49.291 1.00 0.00
E H
ATOM 16063 CD ILE E 153 −40.150 −34.570 47.310 1.00 0.00
E C
ATOM 16064 HD1 ILE E 153 −41.219 −34.610 47.008 1.00 0.00
E H

ATOM 16065 HD2 ILE E 153 -39.912 -33.526 47.611 1.00 0.00
E H
ATOM 16066 HD3 ILE E 153 -39.533 -34.831 46.424 1.00 0.00
E H
ATOM 16067 C ILE E 153 -37.020 -35.667 51.056 1.00 0.00
E C
ATOM 16068 O ILE E 153 -36.308 -34.668 51.211 1.00 0.00
E O
ATOM 16069 N VAL E 154 -36.601 -36.899 51.345 1.00 0.00
E N
ATOM 16070 HN VAL E 154 -37.156 -37.710 51.177 1.00 0.00
E H
ATOM 16071 CA VAL E 154 -35.294 -37.173 51.934 1.00 0.00
E C
ATOM 16072 HA VAL E 154 -34.635 -36.351 51.683 1.00 0.00
E H
ATOM 16073 CB VAL E 154 -35.376 -37.317 53.468 1.00 0.00
E C
ATOM 16074 HB VAL E 154 -36.025 -38.201 53.689 1.00 0.00
E H
ATOM 16075 CG1 VAL E 154 -33.988 -37.551 54.052 1.00 0.00
E C
ATOM 16076 HG11 VAL E 154 -34.042 -37.616 55.161 1.00 0.00
E H
ATOM 16077 HG12 VAL E 154 -33.544 -38.500 53.687 1.00 0.00
E H
ATOM 16078 HG13 VAL E 154 -33.305 -36.715 53.790 1.00 0.00
E H
ATOM 16079 CG2 VAL E 154 -36.009 -36.085 54.101 1.00 0.00
E C
ATOM 16080 HG21 VAL E 154 -36.029 -36.178 55.208 1.00 0.00
E H
ATOM 16081 HG22 VAL E 154 -35.432 -35.171 53.838 1.00 0.00
E H
ATOM 16082 HG23 VAL E 154 -37.055 -35.948 53.758 1.00 0.00
E H
ATOM 16083 C VAL E 154 -34.704 -38.437 51.318 1.00 0.00
E C
ATOM 16084 O VAL E 154 -35.418 -39.418 51.080 1.00 0.00
E O
ATOM 16085 N GLU E 155 -33.394 -38.415 51.074 1.00 0.00
E N
ATOM 16086 HN GLU E 155 -32.854 -37.578 51.064 1.00 0.00
E H
ATOM 16087 CA GLU E 155 -32.622 -39.621 50.797 1.00 0.00
E C
ATOM 16088 HA GLU E 155 -33.297 -40.434 50.558 1.00 0.00
E H
ATOM 16089 CB GLU E 155 -31.623 -39.420 49.659 1.00 0.00
E C
ATOM 16090 HB1 GLU E 155 -31.201 -38.392 49.746 1.00 0.00
E H
ATOM 16091 HB2 GLU E 155 -30.763 -40.115 49.786 1.00 0.00
E H

```
ATOM  16092  CG  GLU E 155     -32.194 -39.584  48.278  1.00  0.00
E    C
ATOM  16093  HG1 GLU E 155     -31.772 -40.492  47.809  1.00  0.00
E    H
ATOM  16094  HG2 GLU E 155     -33.297 -39.671  48.312  1.00  0.00
E    H
ATOM  16095  CD  GLU E 155     -31.824 -38.437  47.367  1.00  0.00
E    C
ATOM  16096  OE1 GLU E 155     -31.124 -38.678  46.359  1.00  0.00
E    O
ATOM  16097  OE2 GLU E 155     -32.220 -37.294  47.679  1.00  0.00
E    O
ATOM  16098  C   GLU E 155     -31.873 -40.013  52.060  1.00  0.00
E    C
ATOM  16099  O   GLU E 155     -31.263 -39.158  52.707  1.00  0.00
E    O
ATOM  16100  N   LEU E 156     -31.912 -41.293  52.409  1.00  0.00
E    N
ATOM  16101  HN  LEU E 156     -32.481 -41.981  51.962  1.00  0.00
E    H
ATOM  16102  CA  LEU E 156     -31.102 -41.806  53.507  1.00  0.00
E    C
ATOM  16103  HA  LEU E 156     -30.348 -41.062  53.725  1.00  0.00
E    H
ATOM  16104  CB  LEU E 156     -31.947 -42.071  54.766  1.00  0.00
E    C
ATOM  16105  HB1 LEU E 156     -32.273 -43.136  54.726  1.00  0.00
E    H
ATOM  16106  HB2 LEU E 156     -31.296 -41.983  55.664  1.00  0.00
E    H
ATOM  16107  CG  LEU E 156     -33.196 -41.201  54.945  1.00  0.00
E    C
ATOM  16108  HG  LEU E 156     -33.064 -40.349  54.234  1.00  0.00
E    H
ATOM  16109  CD1 LEU E 156     -34.462 -41.963  54.585  1.00  0.00
E    C
ATOM  16110 HD11 LEU E 156     -35.360 -41.335  54.766  1.00  0.00
E    H
ATOM  16111 HD12 LEU E 156     -34.451 -42.257  53.513  1.00  0.00
E    H
ATOM  16112 HD13 LEU E 156     -34.553 -42.884  55.200  1.00  0.00
E    H
ATOM  16113  CD2 LEU E 156     -33.274 -40.631  56.356  1.00  0.00
E    C
ATOM  16114 HD21 LEU E 156     -34.175 -39.988  56.463  1.00  0.00
E    H
ATOM  16115 HD22 LEU E 156     -33.341 -41.433  57.118  1.00  0.00
E    H
ATOM  16116 HD23 LEU E 156     -32.377 -40.012  56.576  1.00  0.00
E    H
ATOM  16117  C   LEU E 156     -30.384 -43.075  53.073  1.00  0.00
E    C
ATOM  16118  O   LEU E 156     -30.997 -43.985  52.517  1.00  0.00
E    O
```

ATOM 16119 N LEU E 157 -29.082 -43.139 53.331 1.00 0.00
E N
ATOM 16120 HN LEU E 157 -28.542 -42.425 53.770 1.00 0.00
E H
ATOM 16121 CA LEU E 157 -28.320 -44.326 52.966 1.00 0.00
E C
ATOM 16122 HA LEU E 157 -28.737 -44.718 52.047 1.00 0.00
E H
ATOM 16123 CB LEU E 157 -26.843 -43.994 52.763 1.00 0.00
E C
ATOM 16124 HB1 LEU E 157 -26.657 -43.985 51.663 1.00 0.00
E H
ATOM 16125 HB2 LEU E 157 -26.654 -42.956 53.116 1.00 0.00
E H
ATOM 16126 CG LEU E 157 -25.763 -44.891 53.368 1.00 0.00
E C
ATOM 16127 HG LEU E 157 -26.216 -45.871 53.650 1.00 0.00
E H
ATOM 16128 CD1 LEU E 157 -24.660 -45.109 52.351 1.00 0.00
E C
ATOM 16129 HD11 LEU E 157 -23.841 -45.719 52.788 1.00 0.00
E H
ATOM 16130 HD12 LEU E 157 -25.051 -45.639 51.456 1.00 0.00
E H
ATOM 16131 HD13 LEU E 157 -24.230 -44.137 52.023 1.00 0.00
E H
ATOM 16132 CD2 LEU E 157 -25.200 -44.251 54.631 1.00 0.00
E C
ATOM 16133 HD21 LEU E 157 -24.427 -44.909 55.085 1.00 0.00
E H
ATOM 16134 HD22 LEU E 157 -24.725 -43.272 54.417 1.00 0.00
E H
ATOM 16135 HD23 LEU E 157 -26.005 -44.094 55.380 1.00 0.00
E H
ATOM 16136 C LEU E 157 -28.515 -45.384 54.049 1.00 0.00
E C
ATOM 16137 O LEU E 157 -28.505 -45.068 55.244 1.00 0.00
E O
ATOM 16138 N ALA E 158 -28.708 -46.636 53.633 1.00 0.00
E N
ATOM 16139 HN ALA E 158 -28.589 -46.930 52.685 1.00 0.00
E H
ATOM 16140 CA ALA E 158 -29.118 -47.679 54.565 1.00 0.00
E C
ATOM 16141 HA ALA E 158 -29.882 -47.310 55.240 1.00 0.00
E H
ATOM 16142 CB ALA E 158 -29.689 -48.879 53.811 1.00 0.00
E C
ATOM 16143 HB1 ALA E 158 -30.571 -48.559 53.216 1.00 0.00
E H
ATOM 16144 HB2 ALA E 158 -28.938 -49.303 53.109 1.00 0.00
E H
ATOM 16145 HB3 ALA E 158 -30.016 -49.681 54.507 1.00 0.00
E H

ATOM 16146 C ALA E 158 -27.951 -48.119 55.437 1.00 0.00
E C
ATOM 16147 O ALA E 158 -26.846 -48.364 54.944 1.00 0.00
E O
ATOM 16148 N THR E 159 -28.197 -48.202 56.738 1.00 0.00
E N
ATOM 16149 HN THR E 159 -29.053 -47.923 57.172 1.00 0.00
E H
ATOM 16150 CA THR E 159 -27.204 -48.722 57.658 1.00 0.00
E C
ATOM 16151 HA THR E 159 -26.245 -48.592 57.174 1.00 0.00
E H
ATOM 16152 CB THR E 159 -27.280 -47.995 59.000 1.00 0.00
E C
ATOM 16153 HB THR E 159 -27.219 -46.904 58.765 1.00 0.00
E H
ATOM 16154 OG1 THR E 159 -26.144 -48.339 59.804 1.00 0.00
E O
ATOM 16155 HG1 THR E 159 -26.261 -47.859 60.632 1.00 0.00
E H
ATOM 16156 CG2 THR E 159 -28.558 -48.363 59.735 1.00 0.00
E C
ATOM 16157 HG21 THR E 159 -28.673 -47.752 60.655 1.00 0.00
E H
ATOM 16158 HG22 THR E 159 -29.443 -48.177 59.089 1.00 0.00
E H
ATOM 16159 HG23 THR E 159 -28.558 -49.435 60.026 1.00 0.00
E H
ATOM 16160 C THR E 159 -27.395 -50.226 57.848 1.00 0.00
E C
ATOM 16161 O THR E 159 -28.280 -50.852 57.256 1.00 0.00
E O
ATOM 16162 N ARG E 160 -26.540 -50.808 58.690 1.00 0.00
E N
ATOM 16163 HN ARG E 160 -25.821 -50.303 59.162 1.00 0.00
E H
ATOM 16164 CA ARG E 160 -26.621 -52.238 58.962 1.00 0.00
E C
ATOM 16165 HA ARG E 160 -26.610 -52.759 58.012 1.00 0.00
E H
ATOM 16166 CB ARG E 160 -25.428 -52.675 59.808 1.00 0.00
E C
ATOM 16167 HB1 ARG E 160 -24.540 -52.131 59.408 1.00 0.00
E H
ATOM 16168 HB2 ARG E 160 -25.562 -52.317 60.853 1.00 0.00
E H
ATOM 16169 CG ARG E 160 -25.157 -54.162 59.759 1.00 0.00
E C
ATOM 16170 HG1 ARG E 160 -26.131 -54.700 59.757 1.00 0.00
E H
ATOM 16171 HG2 ARG E 160 -24.676 -54.410 58.786 1.00 0.00
E H
ATOM 16172 CD ARG E 160 -24.241 -54.567 60.894 1.00 0.00
E C

```
ATOM  16173  HD1 ARG E 160     -24.312 -55.668  61.052  1.00  0.00      E    H
ATOM  16174  HD2 ARG E 160     -23.179 -54.305  60.675  1.00  0.00      E    H
ATOM  16175  NE  ARG E 160     -24.596 -53.882  62.131  1.00  0.00      E    N
ATOM  16176  HE  ARG E 160     -23.964 -53.135  62.366  1.00  0.00      E    H
ATOM  16177  CZ  ARG E 160     -25.643 -54.195  62.889  1.00  0.00      E    C
ATOM  16178  NH1 ARG E 160     -26.450 -55.188  62.540  1.00  0.00      E    N
ATOM  16179 HH11 ARG E 160     -27.244 -55.408  63.088  1.00  0.00      E    H
ATOM  16180 HH12 ARG E 160     -26.287 -55.597  61.640  1.00  0.00      E    H
ATOM  16181  NH2 ARG E 160     -25.886 -53.513  63.998  1.00  0.00      E    N
ATOM  16182 HH21 ARG E 160     -26.612 -53.800  64.606  1.00  0.00      E    H
ATOM  16183 HH22 ARG E 160     -25.195 -52.861  64.284  1.00  0.00      E    H
ATOM  16184  C   ARG E 160     -27.923 -52.620  59.654  1.00  0.00      E    C
ATOM  16185  O   ARG E 160     -28.354 -53.773  59.548  1.00  0.00      E    O
ATOM  16186  N   LYS E 161     -28.562 -51.678  60.352  1.00  0.00      E    N
ATOM  16187  HN  LYS E 161     -28.177 -50.774  60.529  1.00  0.00      E    H
ATOM  16188  CA  LYS E 161     -29.886 -51.907  60.914  1.00  0.00      E    C
ATOM  16189  HA  LYS E 161     -29.816 -52.811  61.507  1.00  0.00      E    H
ATOM  16190  CB  LYS E 161     -30.304 -50.719  61.780  1.00  0.00      E    C
ATOM  16191  HB1 LYS E 161     -30.409 -49.840  61.104  1.00  0.00      E    H
ATOM  16192  HB2 LYS E 161     -31.318 -50.914  62.198  1.00  0.00      E    H
ATOM  16193  CG  LYS E 161     -29.330 -50.375  62.885  1.00  0.00      E    C
ATOM  16194  HG1 LYS E 161     -28.295 -50.379  62.472  1.00  0.00      E    H
ATOM  16195  HG2 LYS E 161     -29.540 -49.342  63.245  1.00  0.00      E    H
ATOM  16196  CD  LYS E 161     -29.431 -51.376  64.016  1.00  0.00      E    C
ATOM  16197  HD1 LYS E 161     -29.135 -52.377  63.628  1.00  0.00      E    H
ATOM  16198  HD2 LYS E 161     -28.692 -51.090  64.800  1.00  0.00      E    H
ATOM  16199  CE  LYS E 161     -30.838 -51.398  64.583  1.00  0.00      E    C
```

ATOM 16200 HE1 LYS E 161 -31.135 -50.383 64.931 1.00 0.00
E H
ATOM 16201 HE2 LYS E 161 -31.565 -51.730 63.810 1.00 0.00
E H
ATOM 16202 NZ LYS E 161 -30.963 -52.320 65.740 1.00 0.00
E N
ATOM 16203 HZ1 LYS E 161 -31.939 -52.308 66.101 1.00 0.00
E H
ATOM 16204 HZ2 LYS E 161 -30.717 -53.285 65.441 1.00 0.00
E H
ATOM 16205 HZ3 LYS E 161 -30.313 -52.018 66.493 1.00 0.00
E H
ATOM 16206 C LYS E 161 -30.942 -52.146 59.846 1.00 0.00
E C
ATOM 16207 O LYS E 161 -32.100 -52.398 60.192 1.00 0.00
E O
ATOM 16208 N LEU E 162 -30.574 -52.068 58.566 1.00 0.00
E N
ATOM 16209 HN LEU E 162 -29.627 -51.956 58.271 1.00 0.00
E H
ATOM 16210 CA LEU E 162 -31.534 -52.141 57.478 1.00 0.00
E C
ATOM 16211 HA LEU E 162 -32.475 -52.405 57.943 1.00 0.00
E H
ATOM 16212 CB LEU E 162 -31.618 -50.784 56.765 1.00 0.00
E C
ATOM 16213 HB1 LEU E 162 -30.576 -50.477 56.518 1.00 0.00
E H
ATOM 16214 HB2 LEU E 162 -32.137 -50.920 55.790 1.00 0.00
E H
ATOM 16215 CG LEU E 162 -32.270 -49.622 57.518 1.00 0.00
E C
ATOM 16216 HG LEU E 162 -31.523 -49.275 58.273 1.00 0.00
E H
ATOM 16217 CD1 LEU E 162 -32.578 -48.492 56.553 1.00 0.00
E C
ATOM 16218 HD11 LEU E 162 -33.094 -47.660 57.078 1.00 0.00
E H
ATOM 16219 HD12 LEU E 162 -31.642 -48.094 56.106 1.00 0.00
E H
ATOM 16220 HD13 LEU E 162 -33.236 -48.845 55.731 1.00 0.00
E H
ATOM 16221 CD2 LEU E 162 -33.526 -50.058 58.266 1.00 0.00
E C
ATOM 16222 HD21 LEU E 162 -33.965 -49.199 58.817 1.00 0.00
E H
ATOM 16223 HD22 LEU E 162 -34.301 -50.446 57.574 1.00 0.00
E H
ATOM 16224 HD23 LEU E 162 -33.284 -50.856 59.001 1.00 0.00
E H
ATOM 16225 C LEU E 162 -31.219 -53.208 56.440 1.00 0.00
E C
ATOM 16226 O LEU E 162 -31.927 -53.280 55.430 1.00 0.00
E O

```
ATOM  16227  N   ASP E 163     -30.195 -54.032  56.651  1.00  0.00
E    N
ATOM  16228  HN  ASP E 163     -29.658 -54.092  57.488  1.00  0.00
E    H
ATOM  16229  CA  ASP E 163     -29.749 -54.965  55.624  1.00  0.00
E    C
ATOM  16230  HA  ASP E 163     -29.665 -54.404  54.700  1.00  0.00
E    H
ATOM  16231  CB  ASP E 163     -28.411 -55.589  56.029  1.00  0.00
E    C
ATOM  16232  HB1 ASP E 163     -27.718 -54.787  56.356  1.00  0.00
E    H
ATOM  16233  HB2 ASP E 163     -28.549 -56.296  56.871  1.00  0.00
E    H
ATOM  16234  CG  ASP E 163     -27.734 -56.332  54.885  1.00  0.00
E    C
ATOM  16235  OD1 ASP E 163     -28.385 -56.574  53.847  1.00  0.00
E    O
ATOM  16236  OD2 ASP E 163     -26.543 -56.684  55.033  1.00  0.00
E    O
ATOM  16237  C   ASP E 163     -30.787 -56.053  55.383  1.00  0.00
E    C
ATOM  16238  O   ASP E 163     -31.396 -56.567  56.323  1.00  0.00
E    O
ATOM  16239  N   SER E 164     -30.968 -56.406  54.108  1.00  0.00
E    N
ATOM  16240  HN  SER E 164     -30.523 -55.924  53.353  1.00  0.00
E    H
ATOM  16241  CA  SER E 164     -31.823 -57.517  53.688  1.00  0.00
E    C
ATOM  16242  HA  SER E 164     -31.887 -57.453  52.609  1.00  0.00
E    H
ATOM  16243  CB  SER E 164     -31.216 -58.855  54.106  1.00  0.00
E    C
ATOM  16244  HB1 SER E 164     -31.603 -59.109  55.120  1.00  0.00
E    H
ATOM  16245  HB2 SER E 164     -31.550 -59.665  53.418  1.00  0.00
E    H
ATOM  16246  OG  SER E 164     -29.802 -58.774  54.160  1.00  0.00
E    O
ATOM  16247  HG1 SER E 164     -29.491 -59.556  54.635  1.00  0.00
E    H
ATOM  16248  C   SER E 164     -33.237 -57.364  54.237  1.00  0.00
E    C
ATOM  16249  O   SER E 164     -33.914 -58.337  54.572  1.00  0.00
E    O
ATOM  16250  N   LYS E 165     -33.686 -56.120  54.325  1.00  0.00
E    N
ATOM  16251  HN  LYS E 165     -33.145 -55.333  54.031  1.00  0.00
E    H
ATOM  16252  CA  LYS E 165     -34.999 -55.801  54.856  1.00  0.00
E    C
ATOM  16253  HA  LYS E 165     -35.365 -56.673  55.385  1.00  0.00
E    H
```

ATOM 16254 CB LYS E 165   -34.928 -54.596 55.796 1.00 0.00
E   C
ATOM 16255 HB1 LYS E 165   -34.452 -53.764 55.228 1.00 0.00
E   H
ATOM 16256 HB2 LYS E 165   -35.962 -54.261 56.035 1.00 0.00
E   H
ATOM 16257 CG LYS E 165   -34.130 -54.866 57.062 1.00 0.00
E   C
ATOM 16258 HG1 LYS E 165   -33.059 -55.013 56.793 1.00 0.00
E   H
ATOM 16259 HG2 LYS E 165   -34.187 -53.972 57.724 1.00 0.00
E   H
ATOM 16260 CD LYS E 165   -34.630 -56.098 57.793 1.00 0.00
E   C
ATOM 16261 HD1 LYS E 165   -35.692 -55.928 58.083 1.00 0.00
E   H
ATOM 16262 HD2 LYS E 165   -34.611 -56.956 57.081 1.00 0.00
E   H
ATOM 16263 CE LYS E 165   -33.771 -56.392 59.012 1.00 0.00
E   C
ATOM 16264 HE1 LYS E 165   -32.737 -56.664 58.704 1.00 0.00
E   H
ATOM 16265 HE2 LYS E 165   -33.718 -55.502 59.676 1.00 0.00
E   H
ATOM 16266 NZ LYS E 165   -34.315 -57.521 59.810 1.00 0.00
E   N
ATOM 16267 HZ1 LYS E 165   -33.707 -57.699 60.636 1.00 0.00
E   H
ATOM 16268 HZ2 LYS E 165   -35.276 -57.287 60.134 1.00 0.00
E   H
ATOM 16269 HZ3 LYS E 165   -34.353 -58.376 59.219 1.00 0.00
E   H
ATOM 16270 C   LYS E 165   -35.960 -55.529 53.708 1.00 0.00
E   C
ATOM 16271 O   LYS E 165   -35.550 -55.391 52.556 1.00 0.00
E   O
ATOM 16272 N   SER E 166   -37.252 -55.507 54.020 1.00 0.00
E   N
ATOM 16273 HN SER E 166   -37.613 -55.673 54.937 1.00 0.00
E   H
ATOM 16274 CA SER E 166   -38.271 -55.232 53.018 1.00 0.00
E   C
ATOM 16275 HA SER E 166   -37.737 -54.990 52.108 1.00 0.00
E   H
ATOM 16276 CB SER E 166   -39.197 -56.429 52.828 1.00 0.00
E   C
ATOM 16277 HB1 SER E 166   -38.690 -57.155 52.152 1.00 0.00
E   H
ATOM 16278 HB2 SER E 166   -39.365 -56.948 53.799 1.00 0.00
E   H
ATOM 16279 OG SER E 166   -40.406 -55.997 52.239 1.00 0.00
E   O
ATOM 16280 HG1 SER E 166   -40.869 -56.786 51.931 1.00 0.00
E   H

ATOM 16281 C SER E 166 -39.103 -54.015 53.392 1.00 0.00
E C
ATOM 16282 O SER E 166 -39.129 -53.586 54.548 1.00 0.00
E O
ATOM 16283 N ILE E 167 -39.792 -53.464 52.390 1.00 0.00
E N
ATOM 16284 HN ILE E 167 -39.708 -53.716 51.429 1.00 0.00
E H
ATOM 16285 CA ILE E 167 -40.761 -52.405 52.650 1.00 0.00
E C
ATOM 16286 HA ILE E 167 -40.265 -51.673 53.274 1.00 0.00
E H
ATOM 16287 CB ILE E 167 -41.241 -51.779 51.330 1.00 0.00
E C
ATOM 16288 HB ILE E 167 -41.322 -52.643 50.623 1.00 0.00
E H
ATOM 16289 CG2 ILE E 167 -42.543 -51.027 51.545 1.00 0.00
E C
ATOM 16290 HG21 ILE E 167 -42.975 -50.707 50.573 1.00 0.00
E H
ATOM 16291 HG22 ILE E 167 -43.294 -51.671 52.050 1.00 0.00
E H
ATOM 16292 HG23 ILE E 167 -42.382 -50.122 52.167 1.00 0.00
E H
ATOM 16293 CG1 ILE E 167 -40.176 -50.845 50.765 1.00 0.00
E C
ATOM 16294 HG11 ILE E 167 -40.655 -49.917 50.379 1.00 0.00
E H
ATOM 16295 HG12 ILE E 167 -39.488 -50.543 51.587 1.00 0.00
E H
ATOM 16296 CD ILE E 167 -39.358 -51.467 49.671 1.00 0.00
E C
ATOM 16297 HD1 ILE E 167 -38.563 -50.765 49.338 1.00 0.00
E H
ATOM 16298 HD2 ILE E 167 -38.871 -52.400 50.028 1.00 0.00
E H
ATOM 16299 HD3 ILE E 167 -39.990 -51.713 48.791 1.00 0.00
E H
ATOM 16300 C ILE E 167 -41.930 -52.950 53.456 1.00 0.00
E C
ATOM 16301 O ILE E 167 -42.365 -52.343 54.441 1.00 0.00
E O
ATOM 16302 N ILE E 168 -42.459 -54.106 53.050 1.00 0.00
E N
ATOM 16303 HN ILE E 168 -42.207 -54.609 52.226 1.00 0.00
E H
ATOM 16304 CA ILE E 168 -43.503 -54.743 53.842 1.00 0.00
E C
ATOM 16305 HA ILE E 168 -44.229 -53.975 54.074 1.00 0.00
E H
ATOM 16306 CB ILE E 168 -44.167 -55.888 53.059 1.00 0.00
E C
ATOM 16307 HB ILE E 168 -44.399 -55.444 52.058 1.00 0.00
E H

```
ATOM  16308  CG2 ILE E 168     -43.252 -57.098  52.988  1.00  0.00
E    C
ATOM  16309 HG21 ILE E 168     -43.666 -57.860  52.295  1.00  0.00
E    H
ATOM  16310 HG22 ILE E 168     -42.246 -56.813  52.613  1.00  0.00
E    H
ATOM  16311 HG23 ILE E 168     -43.134 -57.566  53.988  1.00  0.00
E    H
ATOM  16312  CG1 ILE E 168     -45.503 -56.259  53.703  1.00  0.00
E    C
ATOM  16313 HG11 ILE E 168     -45.744 -57.323  53.484  1.00  0.00
E    H
ATOM  16314 HG12 ILE E 168     -45.409 -56.165  54.810  1.00  0.00
E    H
ATOM  16315  CD  ILE E 168     -46.650 -55.383  53.245  1.00  0.00
E    C
ATOM  16316  HD1 ILE E 168     -47.583 -55.656  53.784  1.00  0.00
E    H
ATOM  16317  HD2 ILE E 168     -46.431 -54.312  53.449  1.00  0.00
E    H
ATOM  16318  HD3 ILE E 168     -46.834 -55.504  52.157  1.00  0.00
E    H
ATOM  16319  C   ILE E 168     -42.943 -55.231  55.164  1.00  0.00
E    C
ATOM  16320  O   ILE E 168     -43.681 -55.385  56.141  1.00  0.00
E    O
ATOM  16321  N   ASP E 169     -41.634 -55.464  55.227  1.00  0.00
E    N
ATOM  16322  HN  ASP E 169     -40.984 -55.435  54.472  1.00  0.00
E    H
ATOM  16323  CA  ASP E 169     -41.012 -55.802  56.497  1.00  0.00
E    C
ATOM  16324  HA  ASP E 169     -41.611 -56.580  56.956  1.00  0.00
E    H
ATOM  16325  CB  ASP E 169     -39.572 -56.255  56.253  1.00  0.00
E    C
ATOM  16326  HB1 ASP E 169     -39.362 -56.233  55.165  1.00  0.00
E    H
ATOM  16327  HB2 ASP E 169     -38.858 -55.575  56.760  1.00  0.00
E    H
ATOM  16328  CG  ASP E 169     -39.313 -57.664  56.735  1.00  0.00
E    C
ATOM  16329  OD1 ASP E 169     -39.937 -58.587  56.170  1.00  0.00
E    O
ATOM  16330  OD2 ASP E 169     -38.491 -57.851  57.661  1.00  0.00
E    O
ATOM  16331  C   ASP E 169     -41.054 -54.604  57.438  1.00  0.00
E    C
ATOM  16332  O   ASP E 169     -41.760 -54.608  58.453  1.00  0.00
E    O
ATOM  16333  N   LEU E 170     -40.320 -53.553  57.091  1.00  0.00
E    N
ATOM  16334  HN  LEU E 170     -39.735 -53.533  56.282  1.00  0.00
E    H
```

```
ATOM  16335  CA  LEU E 170     -40.305 -52.320  57.871  1.00  0.00
E    C
ATOM  16336  HA  LEU E 170     -40.729 -52.544  58.841  1.00  0.00
E    H
ATOM  16337  CB  LEU E 170     -38.874 -51.816  58.028  1.00  0.00
E    C
ATOM  16338  HB1 LEU E 170     -38.394 -51.871  57.024  1.00  0.00
E    H
ATOM  16339  HB2 LEU E 170     -38.899 -50.737  58.299  1.00  0.00
E    H
ATOM  16340  CG  LEU E 170     -38.006 -52.591  59.024  1.00  0.00
E    C
ATOM  16341  HG  LEU E 170     -38.654 -52.786  59.913  1.00  0.00
E    H
ATOM  16342  CD1 LEU E 170     -37.518 -53.913  58.441  1.00  0.00
E    C
ATOM  16343 HD11 LEU E 170     -36.853 -54.435  59.161  1.00  0.00
E    H
ATOM  16344 HD12 LEU E 170     -38.376 -54.581  58.212  1.00  0.00
E    H
ATOM  16345 HD13 LEU E 170     -36.947 -53.741  57.503  1.00  0.00
E    H
ATOM  16346  CD2 LEU E 170     -36.825 -51.750  59.478  1.00  0.00
E    C
ATOM  16347 HD21 LEU E 170     -36.211 -52.312  60.214  1.00  0.00
E    H
ATOM  16348 HD22 LEU E 170     -36.167 -51.475  58.628  1.00  0.00
E    H
ATOM  16349 HD23 LEU E 170     -37.178 -50.814  59.963  1.00  0.00
E    H
ATOM  16350  C   LEU E 170     -41.207 -51.283  57.201  1.00  0.00
E    C
ATOM  16351  O   LEU E 170     -40.760 -50.373  56.503  1.00  0.00
E    O
ATOM  16352  N   ASN E 171     -42.510 -51.459  57.411  1.00  0.00
E    N
ATOM  16353  HN  ASN E 171     -42.898 -52.180  57.984  1.00  0.00
E    H
ATOM  16354  CA  ASN E 171     -43.496 -50.579  56.798  1.00  0.00
E    C
ATOM  16355  HA  ASN E 171     -43.264 -50.563  55.738  1.00  0.00
E    H
ATOM  16356  CB  ASN E 171     -44.912 -51.096  57.053  1.00  0.00
E    C
ATOM  16357  HB1 ASN E 171     -45.626 -50.336  56.669  1.00  0.00
E    H
ATOM  16358  HB2 ASN E 171     -45.088 -52.033  56.486  1.00  0.00
E    H
ATOM  16359  CG  ASN E 171     -45.199 -51.310  58.520  1.00  0.00
E    C
ATOM  16360  OD1 ASN E 171     -44.320 -51.161  59.365  1.00  0.00
E    O
ATOM  16361  ND2 ASN E 171     -46.439 -51.659  58.832  1.00  0.00
E    N
```

ATOM 16362 HD21 ASN E 171   -46.648 -51.793 59.797 1.00 0.00
E   H
ATOM 16363 HD22 ASN E 171   -47.126 -51.749 58.117 1.00 0.00
E   H
ATOM 16364 C   ASN E 171   -43.355 -49.155 57.323 1.00 0.00
E   C
ATOM 16365 O   ASN E 171   -42.876 -48.922 58.437 1.00 0.00
E   O
ATOM 16366 N   VAL E 172   -43.756 -48.193 56.493 1.00 0.00
E   N
ATOM 16367 HN VAL E 172   -44.125 -48.365 55.584 1.00 0.00
E   H
ATOM 16368 CA VAL E 172   -43.683 -46.785 56.857 1.00 0.00
E   C
ATOM 16369 HA VAL E 172   -43.469 -46.744 57.917 1.00 0.00
E   H
ATOM 16370 CB VAL E 172   -42.540 -46.084 56.095 1.00 0.00
E   C
ATOM 16371 HB VAL E 172   -41.658 -46.770 56.103 1.00 0.00
E   H
ATOM 16372 CG1 VAL E 172   -42.939 -45.805 54.651 1.00 0.00
E   C
ATOM 16373 HG11 VAL E 172   -42.124 -45.265 54.123 1.00 0.00
E   H
ATOM 16374 HG12 VAL E 172   -43.131 -46.743 54.091 1.00 0.00
E   H
ATOM 16375 HG13 VAL E 172   -43.852 -45.172 54.611 1.00 0.00
E   H
ATOM 16376 CG2 VAL E 172   -42.127 -44.797 56.804 1.00 0.00
E   C
ATOM 16377 HG21 VAL E 172   -41.331 -44.272 56.233 1.00 0.00
E   H
ATOM 16378 HG22 VAL E 172   -42.996 -44.111 56.904 1.00 0.00
E   H
ATOM 16379 HG23 VAL E 172   -41.729 -45.008 57.819 1.00 0.00
E   H
ATOM 16380 C   VAL E 172   -45.007 -46.065 56.629 1.00 0.00
E   C
ATOM 16381 O   VAL E 172   -45.259 -45.031 57.257 1.00 0.00
E   O
ATOM 16382 N   ARG E 173   -45.863 -46.579 55.742 1.00 0.00
E   N
ATOM 16383 HN ARG E 173   -45.685 -47.418 55.232 1.00 0.00
E   H
ATOM 16384 CA ARG E 173   -47.130 -45.909 55.478 1.00 0.00
E   C
ATOM 16385 HA ARG E 173   -46.940 -44.845 55.387 1.00 0.00
E   H
ATOM 16386 CB ARG E 173   -47.749 -46.434 54.187 1.00 0.00
E   C
ATOM 16387 HB1 ARG E 173   -46.903 -46.807 53.560 1.00 0.00
E   H
ATOM 16388 HB2 ARG E 173   -48.379 -47.324 54.411 1.00 0.00
E   H

```
ATOM  16389  CG  ARG E 173     -48.509 -45.371  53.425  1.00  0.00
E    C
ATOM  16390  HG1 ARG E 173     -49.581 -45.426  53.716  1.00  0.00
E    H
ATOM  16391  HG2 ARG E 173     -48.159 -44.367  53.760  1.00  0.00
E    H
ATOM  16392  CD  ARG E 173     -48.294 -45.493  51.925  1.00  0.00
E    C
ATOM  16393  HD1 ARG E 173     -48.159 -44.477  51.487  1.00  0.00
E    H
ATOM  16394  HD2 ARG E 173     -47.394 -46.107  51.688  1.00  0.00
E    H
ATOM  16395  NE  ARG E 173     -49.415 -46.143  51.249  1.00  0.00
E    N
ATOM  16396  HE  ARG E 173     -49.198 -47.071  50.927  1.00  0.00
E    H
ATOM  16397  CZ  ARG E 173     -50.610 -45.586  51.066  1.00  0.00
E    C
ATOM  16398  NH1 ARG E 173     -50.852 -44.359  51.508  1.00  0.00
E    N
ATOM  16399 HH11 ARG E 173     -51.747 -43.950  51.396  1.00  0.00
E    H
ATOM  16400 HH12 ARG E 173     -50.136 -43.940  52.068  1.00  0.00
E    H
ATOM  16401  NH2 ARG E 173     -51.564 -46.256  50.436  1.00  0.00
E    N
ATOM  16402 HH21 ARG E 173     -52.422 -45.810  50.223  1.00  0.00
E    H
ATOM  16403 HH22 ARG E 173     -51.306 -47.116  50.014  1.00  0.00
E    H
ATOM  16404  C   ARG E 173     -48.101 -46.078  56.636  1.00  0.00
E    C
ATOM  16405  O   ARG E 173     -48.683 -45.100  57.117  1.00  0.00
E    O
ATOM  16406  N   ALA E 174     -48.300 -47.312  57.091  1.00  0.00
E    N
ATOM  16407  HN  ALA E 174     -47.913 -48.132  56.671  1.00  0.00
E    H
ATOM  16408  CA  ALA E 174     -49.123 -47.543  58.270  1.00  0.00
E    C
ATOM  16409  HA  ALA E 174     -49.982 -46.882  58.268  1.00  0.00
E    H
ATOM  16410  CB  ALA E 174     -49.633 -48.985  58.280  1.00  0.00
E    C
ATOM  16411  HB1 ALA E 174     -50.234 -49.173  57.364  1.00  0.00
E    H
ATOM  16412  HB2 ALA E 174     -48.788 -49.708  58.289  1.00  0.00
E    H
ATOM  16413  HB3 ALA E 174     -50.281 -49.185  59.161  1.00  0.00
E    H
ATOM  16414  C   ALA E 174     -48.355 -47.246  59.549  1.00  0.00
E    C
ATOM  16415  O   ALA E 174     -48.958 -46.963  60.589  1.00  0.00
E    O
```

ATOM 16416 N   LYS E 175   -47.024 -47.293 59.484 1.00 0.00
E   N
ATOM 16417 HN LYS E 175   -46.508 -47.444 58.642 1.00 0.00
E   H
ATOM 16418 CA LYS E 175   -46.214 -47.120 60.683 1.00 0.00
E   C
ATOM 16419 HA LYS E 175   -46.807 -47.476 61.517 1.00 0.00
E   H
ATOM 16420 CB LYS E 175   -44.927 -47.931 60.554 1.00 0.00
E   C
ATOM 16421 HB1 LYS E 175   -45.187 -48.877 60.025 1.00 0.00
E   H
ATOM 16422 HB2 LYS E 175   -44.221 -47.385 59.889 1.00 0.00
E   H
ATOM 16423 CG LYS E 175   -44.269 -48.268 61.870 1.00 0.00
E   C
ATOM 16424 HG1 LYS E 175   -43.806 -47.347 62.293 1.00 0.00
E   H
ATOM 16425 HG2 LYS E 175   -45.049 -48.607 62.589 1.00 0.00
E   H
ATOM 16426 CD LYS E 175   -43.208 -49.330 61.670 1.00 0.00
E   C
ATOM 16427 HD1 LYS E 175   -43.677 -50.205 61.162 1.00 0.00
E   H
ATOM 16428 HD2 LYS E 175   -42.433 -48.921 60.981 1.00 0.00
E   H
ATOM 16429 CE LYS E 175   -42.582 -49.742 62.986 1.00 0.00
E   C
ATOM 16430 HE1 LYS E 175   -41.984 -48.907 63.412 1.00 0.00
E   H
ATOM 16431 HE2 LYS E 175   -43.369 -50.023 63.722 1.00 0.00
E   H
ATOM 16432 NZ LYS E 175   -41.676 -50.911 62.821 1.00 0.00
E   N
ATOM 16433 HZ1 LYS E 175   -41.260 -51.172 63.737 1.00 0.00
E   H
ATOM 16434 HZ2 LYS E 175   -42.214 -51.718 62.444 1.00 0.00
E   H
ATOM 16435 HZ3 LYS E 175   -40.915 -50.667 62.155 1.00 0.00
E   H
ATOM 16436 C   LYS E 175   -45.900 -45.651 60.941 1.00 0.00
E   C
ATOM 16437 O   LYS E 175   -46.145 -45.145 62.042 1.00 0.00
E   O
ATOM 16438 N   TYR E 176   -45.360 -44.949 59.943 1.00 0.00
E   N
ATOM 16439 HN TYR E 176   -45.196 -45.322 59.031 1.00 0.00
E   H
ATOM 16440 CA TYR E 176   -44.962 -43.560 60.108 1.00 0.00
E   C
ATOM 16441 HA TYR E 176   -45.238 -43.302 61.124 1.00 0.00
E   H
ATOM 16442 CB TYR E 176   -43.448 -43.408 59.895 1.00 0.00
E   C

ATOM 16443 HB1 TYR E 176 -43.193 -43.721 58.858 1.00 0.00
E H
ATOM 16444 HB2 TYR E 176 -43.141 -42.345 60.002 1.00 0.00
E H
ATOM 16445 CG TYR E 176 -42.621 -44.266 60.826 1.00 0.00
E C
ATOM 16446 CD1 TYR E 176 -42.125 -45.493 60.410 1.00 0.00
E C
ATOM 16447 HD1 TYR E 176 -42.340 -45.850 59.413 1.00 0.00
E H
ATOM 16448 CE1 TYR E 176 -41.374 -46.283 61.256 1.00 0.00
E C
ATOM 16449 HE1 TYR E 176 -40.995 -47.238 60.923 1.00 0.00
E H
ATOM 16450 CZ TYR E 176 -41.111 -45.852 62.536 1.00 0.00
E C
ATOM 16451 OH TYR E 176 -40.362 -46.640 63.378 1.00 0.00
E O
ATOM 16452 HH TYR E 176 -40.227 -46.147 64.190 1.00 0.00
E H
ATOM 16453 CD2 TYR E 176 -42.345 -43.853 62.122 1.00 0.00
E C
ATOM 16454 HD2 TYR E 176 -42.752 -42.922 62.491 1.00 0.00
E H
ATOM 16455 CE2 TYR E 176 -41.594 -44.638 62.976 1.00 0.00
E C
ATOM 16456 HE2 TYR E 176 -41.410 -44.306 63.986 1.00 0.00
E H
ATOM 16457 C TYR E 176 -45.691 -42.595 59.186 1.00 0.00
E C
ATOM 16458 O TYR E 176 -45.381 -41.399 59.208 1.00 0.00
E O
ATOM 16459 N GLY E 177 -46.635 -43.068 58.375 1.00 0.00
E N
ATOM 16460 HN GLY E 177 -46.858 -44.039 58.298 1.00 0.00
E H
ATOM 16461 CA GLY E 177 -47.413 -42.179 57.538 1.00 0.00
E C
ATOM 16462 HA1 GLY E 177 -47.740 -41.379 58.188 1.00 0.00
E H
ATOM 16463 HA2 GLY E 177 -48.217 -42.780 57.136 1.00 0.00
E H
ATOM 16464 C GLY E 177 -46.668 -41.564 56.380 1.00 0.00
E C
ATOM 16465 O GLY E 177 -47.139 -40.578 55.808 1.00 0.00
E O
ATOM 16466 N CYS E 178 -45.519 -42.115 56.011 1.00 0.00
E N
ATOM 16467 HN CYS E 178 -45.151 -42.932 56.451 1.00 0.00
E H
ATOM 16468 CA CYS E 178 -44.700 -41.578 54.937 1.00 0.00
E C
ATOM 16469 HA CYS E 178 -45.121 -40.618 54.663 1.00 0.00
E H

ATOM 16470 CB CYS E 178 -43.249 -41.417 55.403 1.00 0.00
E C
ATOM 16471 HB1 CYS E 178 -42.938 -42.387 55.853 1.00 0.00
E H
ATOM 16472 HB2 CYS E 178 -42.586 -41.261 54.523 1.00 0.00
E H
ATOM 16473 SG CYS E 178 -43.034 -40.115 56.631 1.00 0.00
E S
ATOM 16474 HG1 CYS E 178 -41.730 -40.302 56.845 1.00 0.00
E H
ATOM 16475 C CYS E 178 -44.790 -42.489 53.718 1.00 0.00
E C
ATOM 16476 O CYS E 178 -45.557 -43.454 53.688 1.00 0.00
E O
ATOM 16477 N THR E 179 -43.989 -42.170 52.703 1.00 0.00
E N
ATOM 16478 HN THR E 179 -43.335 -41.414 52.741 1.00 0.00
E H
ATOM 16479 CA THR E 179 -43.994 -42.896 51.440 1.00 0.00
E C
ATOM 16480 HA THR E 179 -44.469 -43.848 51.638 1.00 0.00
E H
ATOM 16481 CB THR E 179 -44.718 -42.091 50.355 1.00 0.00
E C
ATOM 16482 HB THR E 179 -43.920 -41.579 49.763 1.00 0.00
E H
ATOM 16483 OG1 THR E 179 -45.528 -41.083 50.975 1.00 0.00
E O
ATOM 16484 HG1 THR E 179 -45.964 -40.622 50.250 1.00 0.00
E H
ATOM 16485 CG2 THR E 179 -45.572 -42.988 49.478 1.00 0.00
E C
ATOM 16486 HG21 THR E 179 -46.002 -42.417 48.628 1.00 0.00
E H
ATOM 16487 HG22 THR E 179 -44.960 -43.815 49.059 1.00 0.00
E H
ATOM 16488 HG23 THR E 179 -46.409 -43.434 50.057 1.00 0.00
E H
ATOM 16489 C THR E 179 -42.567 -43.206 51.007 1.00 0.00
E C
ATOM 16490 O THR E 179 -41.798 -42.292 50.691 1.00 0.00
E O
ATOM 16491 N ILE E 180 -42.221 -44.492 50.980 1.00 0.00
E N
ATOM 16492 HN ILE E 180 -42.799 -45.235 51.311 1.00 0.00
E H
ATOM 16493 CA ILE E 180 -40.931 -44.927 50.450 1.00 0.00
E C
ATOM 16494 HA ILE E 180 -40.193 -44.185 50.725 1.00 0.00
E H
ATOM 16495 CB ILE E 180 -40.553 -46.321 50.983 1.00 0.00
E C
ATOM 16496 HB ILE E 180 -41.443 -46.643 51.580 1.00 0.00
E H

ATOM 16497 CG2 ILE E 180 -40.182 -47.264 49.835 1.00 0.00
E C
ATOM 16498 HG21 ILE E 180 -40.063 -48.303 50.207 1.00 0.00
E H
ATOM 16499 HG22 ILE E 180 -40.976 -47.274 49.059 1.00 0.00
E H
ATOM 16500 HG23 ILE E 180 -39.228 -46.955 49.358 1.00 0.00
E H
ATOM 16501 CG1 ILE E 180 -39.376 -46.215 51.959 1.00 0.00
E C
ATOM 16502 HG11 ILE E 180 -38.886 -45.221 51.853 1.00 0.00
E H
ATOM 16503 HG12 ILE E 180 -39.765 -46.281 53.001 1.00 0.00
E H
ATOM 16504 CD ILE E 180 -38.312 -47.298 51.794 1.00 0.00
E C
ATOM 16505 HD1 ILE E 180 -37.525 -47.188 52.572 1.00 0.00
E H
ATOM 16506 HD2 ILE E 180 -38.763 -48.309 51.896 1.00 0.00
E H
ATOM 16507 HD3 ILE E 180 -37.824 -47.229 50.799 1.00 0.00
E H
ATOM 16508 C ILE E 180 -41.023 -44.914 48.930 1.00 0.00
E C
ATOM 16509 O ILE E 180 -42.066 -45.250 48.359 1.00 0.00
E O
ATOM 16510 N LEU E 181 -39.947 -44.495 48.268 1.00 0.00
E N
ATOM 16511 HN LEU E 181 -39.042 -44.372 48.670 1.00 0.00
E H
ATOM 16512 CA LEU E 181 -40.021 -44.174 46.849 1.00 0.00
E C
ATOM 16513 HA LEU E 181 -41.030 -44.440 46.559 1.00 0.00
E H
ATOM 16514 CB LEU E 181 -39.757 -42.686 46.631 1.00 0.00
E C
ATOM 16515 HB1 LEU E 181 -39.886 -42.181 47.617 1.00 0.00
E H
ATOM 16516 HB2 LEU E 181 -38.690 -42.545 46.350 1.00 0.00
E H
ATOM 16517 CG LEU E 181 -40.663 -42.005 45.619 1.00 0.00
E C
ATOM 16518 HG LEU E 181 -40.291 -42.247 44.595 1.00 0.00
E H
ATOM 16519 CD1 LEU E 181 -42.105 -42.480 45.743 1.00 0.00
E C
ATOM 16520 HD11 LEU E 181 -42.759 -41.936 45.028 1.00 0.00
E H
ATOM 16521 HD12 LEU E 181 -42.179 -43.567 45.526 1.00 0.00
E H
ATOM 16522 HD13 LEU E 181 -42.491 -42.300 46.769 1.00 0.00
E H
ATOM 16523 CD2 LEU E 181 -40.535 -40.529 45.820 1.00 0.00
E C

```
ATOM  16524 HD21 LEU E 181     -41.167 -39.984  45.086  1.00  0.00
E    H
ATOM  16525 HD22 LEU E 181     -40.858 -40.225  46.836  1.00  0.00
E    H
ATOM  16526 HD23 LEU E 181     -39.480 -40.207  45.677  1.00  0.00
E    H
ATOM  16527  C   LEU E 181     -39.070 -44.947  45.959  1.00  0.00
E    C
ATOM  16528  O   LEU E 181     -39.481 -45.381  44.884  1.00  0.00
E    O
ATOM  16529  N   ALA E 182     -37.805 -45.100  46.343  1.00  0.00
E    N
ATOM  16530  HN  ALA E 182     -37.441 -44.808  47.226  1.00  0.00
E    H
ATOM  16531  CA  ALA E 182     -36.847 -45.729  45.451  1.00  0.00
E    C
ATOM  16532  HA  ALA E 182     -37.289 -46.573  44.933  1.00  0.00
E    H
ATOM  16533  CB  ALA E 182     -36.341 -44.756  44.383  1.00  0.00
E    C
ATOM  16534  HB1 ALA E 182     -37.195 -44.396  43.771  1.00  0.00
E    H
ATOM  16535  HB2 ALA E 182     -35.857 -43.869  44.848  1.00  0.00
E    H
ATOM  16536  HB3 ALA E 182     -35.610 -45.240  43.701  1.00  0.00
E    H
ATOM  16537  C   ALA E 182     -35.684 -46.282  46.254  1.00  0.00
E    C
ATOM  16538  O   ALA E 182     -35.333 -45.775  47.325  1.00  0.00
E    O
ATOM  16539  N   ILE E 183     -35.077 -47.317  45.685  1.00  0.00
E    N
ATOM  16540  HN  ILE E 183     -35.309 -47.617  44.763  1.00  0.00
E    H
ATOM  16541  CA  ILE E 183     -34.034 -48.107  46.324  1.00  0.00
E    C
ATOM  16542  HA  ILE E 183     -33.742 -47.592  47.231  1.00  0.00
E    H
ATOM  16543  CB  ILE E 183     -34.532 -49.539  46.604  1.00  0.00
E    C
ATOM  16544  HB  ILE E 183     -34.849 -49.927  45.603  1.00  0.00
E    H
ATOM  16545  CG2 ILE E 183     -33.441 -50.416  47.196  1.00  0.00
E    C
ATOM  16546 HG21 ILE E 183     -33.772 -51.473  47.244  1.00  0.00
E    H
ATOM  16547 HG22 ILE E 183     -32.522 -50.378  46.574  1.00  0.00
E    H
ATOM  16548 HG23 ILE E 183     -33.182 -50.087  48.225  1.00  0.00
E    H
ATOM  16549  CG1 ILE E 183     -35.786 -49.500  47.484  1.00  0.00
E    C
ATOM  16550 HG11 ILE E 183     -35.606 -48.846  48.367  1.00  0.00
E    H
```

ATOM 16551 HG12 ILE E 183 -36.621 -49.047 46.902 1.00 0.00
E H
ATOM 16552 CD ILE E 183 -36.233 -50.856 47.963 1.00 0.00
E C
ATOM 16553 HD1 ILE E 183 -37.175 -50.767 48.546 1.00 0.00
E H
ATOM 16554 HD2 ILE E 183 -36.418 -51.534 47.102 1.00 0.00
E H
ATOM 16555 HD3 ILE E 183 -35.466 -51.319 48.619 1.00 0.00
E H
ATOM 16556 C ILE E 183 -32.815 -48.115 45.411 1.00 0.00
E C
ATOM 16557 O ILE E 183 -32.900 -48.568 44.265 1.00 0.00
E O
ATOM 16558 N LYS E 184 -31.684 -47.627 45.920 1.00 0.00
E N
ATOM 16559 HN LYS E 184 -31.631 -47.303 46.864 1.00 0.00
E H
ATOM 16560 CA LYS E 184 -30.440 -47.521 45.157 1.00 0.00
E C
ATOM 16561 HA LYS E 184 -30.594 -47.942 44.171 1.00 0.00
E H
ATOM 16562 CB LYS E 184 -29.975 -46.070 45.087 1.00 0.00
E C
ATOM 16563 HB1 LYS E 184 -30.879 -45.445 44.901 1.00 0.00
E H
ATOM 16564 HB2 LYS E 184 -29.585 -45.767 46.085 1.00 0.00
E H
ATOM 16565 CG LYS E 184 -28.938 -45.701 44.020 1.00 0.00
E C
ATOM 16566 HG1 LYS E 184 -29.127 -44.660 43.672 1.00 0.00
E H
ATOM 16567 HG2 LYS E 184 -27.923 -45.718 44.479 1.00 0.00
E H
ATOM 16568 CD LYS E 184 -28.953 -46.610 42.807 1.00 0.00
E C
ATOM 16569 HD1 LYS E 184 -28.444 -47.564 43.076 1.00 0.00
E H
ATOM 16570 HD2 LYS E 184 -30.014 -46.852 42.569 1.00 0.00
E H
ATOM 16571 CE LYS E 184 -28.264 -45.906 41.642 1.00 0.00
E C
ATOM 16572 HE1 LYS E 184 -28.664 -44.875 41.518 1.00 0.00
E H
ATOM 16573 HE2 LYS E 184 -27.168 -45.839 41.818 1.00 0.00
E H
ATOM 16574 NZ LYS E 184 -28.438 -46.585 40.327 1.00 0.00
E N
ATOM 16575 HZ1 LYS E 184 -27.945 -46.051 39.583 1.00 0.00
E H
ATOM 16576 HZ2 LYS E 184 -28.046 -47.547 40.379 1.00 0.00
E H
ATOM 16577 HZ3 LYS E 184 -29.451 -46.641 40.098 1.00 0.00
E H

ATOM 16578 C LYS E 184 -29.408 -48.391 45.866 1.00 0.00
E C
ATOM 16579 O LYS E 184 -28.821 -47.967 46.867 1.00 0.00
E O
ATOM 16580 N HSE E 185 -29.188 -49.600 45.360 1.00 0.00
E N
ATOM 16581 HN HSE E 185 -29.632 -49.932 44.529 1.00 0.00
E H
ATOM 16582 CA HSE E 185 -28.273 -50.557 45.976 1.00 0.00
E C
ATOM 16583 HA HSE E 185 -27.848 -50.086 46.854 1.00 0.00
E H
ATOM 16584 CB HSE E 185 -29.000 -51.847 46.366 1.00 0.00
E C
ATOM 16585 HB1 HSE E 185 -28.256 -52.534 46.825 1.00 0.00
E H
ATOM 16586 HB2 HSE E 185 -29.759 -51.624 47.144 1.00 0.00
E H
ATOM 16587 ND1 HSE E 185 -29.009 -53.593 44.551 1.00 0.00
E N
ATOM 16588 CG HSE E 185 -29.635 -52.565 45.220 1.00 0.00
E C
ATOM 16589 CE1 HSE E 185 -29.800 -54.035 43.590 1.00 0.00
E C
ATOM 16590 HE1 HSE E 185 -29.555 -54.859 42.915 1.00 0.00
E H
ATOM 16591 NE2 HSE E 185 -30.905 -53.312 43.595 1.00 0.00
E N
ATOM 16592 HE2 HSE E 185 -31.663 -53.416 42.952 1.00 0.00
E H
ATOM 16593 CD2 HSE E 185 -30.827 -52.382 44.603 1.00 0.00
E C
ATOM 16594 HD2 HSE E 185 -31.607 -51.662 44.802 1.00 0.00
E H
ATOM 16595 C HSE E 185 -27.125 -50.851 45.025 1.00 0.00
E C
ATOM 16596 O HSE E 185 -27.321 -51.489 43.987 1.00 0.00
E O
ATOM 16597 N HSE E 186 -25.931 -50.388 45.393 1.00 0.00
E N
ATOM 16598 HN HSE E 186 -25.787 -49.847 46.220 1.00 0.00
E H
ATOM 16599 CA HSE E 186 -24.721 -50.633 44.616 1.00 0.00
E C
ATOM 16600 HA HSE E 186 -23.939 -50.019 45.046 1.00 0.00
E H
ATOM 16601 CB HSE E 186 -24.314 -52.106 44.684 1.00 0.00
E C
ATOM 16602 HB1 HSE E 186 -24.486 -52.460 45.723 1.00 0.00
E H
ATOM 16603 HB2 HSE E 186 -24.975 -52.705 44.023 1.00 0.00
E H
ATOM 16604 ND1 HSE E 186 -21.842 -51.747 45.023 1.00 0.00
E N

ATOM 16605 CG HSE E 186 -22.877 -52.349 44.345 1.00 0.00
E C
ATOM 16606 CE1 HSE E 186 -20.692 -52.136 44.502 1.00 0.00
E C
ATOM 16607 HE1 HSE E 186 -19.710 -51.808 44.853 1.00 0.00
E H
ATOM 16608 NE2 HSE E 186 -20.944 -52.964 43.505 1.00 0.00
E N
ATOM 16609 HE2 HSE E 186 -20.255 -53.393 42.923 1.00 0.00
E H
ATOM 16610 CD2 HSE E 186 -22.305 -53.112 43.384 1.00 0.00
E C
ATOM 16611 HD2 HSE E 186 -22.763 -53.733 42.627 1.00 0.00
E H
ATOM 16612 C HSE E 186 -24.895 -50.187 43.169 1.00 0.00
E C
ATOM 16613 O HSE E 186 -24.512 -50.894 42.237 1.00 0.00
E O
ATOM 16614 N GLY E 187 -25.499 -49.016 42.969 1.00 0.00
E N
ATOM 16615 HN GLY E 187 -25.832 -48.458 43.729 1.00 0.00
E H
ATOM 16616 CA GLY E 187 -25.716 -48.472 41.655 1.00 0.00
E C
ATOM 16617 HA1 GLY E 187 -24.868 -48.778 41.058 1.00 0.00
E H
ATOM 16618 HA2 GLY E 187 -25.809 -47.401 41.778 1.00 0.00
E H
ATOM 16619 C GLY E 187 -26.973 -48.976 40.979 1.00 0.00
E C
ATOM 16620 O GLY E 187 -27.298 -48.517 39.880 1.00 0.00
E O
ATOM 16621 N ASP E 188 -27.685 -49.905 41.604 1.00 0.00
E N
ATOM 16622 HN ASP E 188 -27.495 -50.291 42.503 1.00 0.00
E H
ATOM 16623 CA ASP E 188 -28.876 -50.470 40.985 1.00 0.00
E C
ATOM 16624 HA ASP E 188 -28.820 -50.246 39.925 1.00 0.00
E H
ATOM 16625 CB ASP E 188 -28.925 -51.972 41.242 1.00 0.00
E C
ATOM 16626 HB1 ASP E 188 -28.923 -52.156 42.336 1.00 0.00
E H
ATOM 16627 HB2 ASP E 188 -29.848 -52.413 40.816 1.00 0.00
E H
ATOM 16628 CG ASP E 188 -27.726 -52.706 40.657 1.00 0.00
E C
ATOM 16629 OD1 ASP E 188 -26.739 -52.027 40.303 1.00 0.00
E O
ATOM 16630 OD2 ASP E 188 -27.769 -53.953 40.550 1.00 0.00
E O
ATOM 16631 C ASP E 188 -30.143 -49.795 41.511 1.00 0.00
E C

```
ATOM  16632  O   ASP E 188     -30.332 -49.655  42.723  1.00  0.00
E    O
ATOM  16633  N   ILE E 189     -31.016 -49.390  40.588  1.00  0.00
E    N
ATOM  16634  HN  ILE E 189     -30.885 -49.578  39.617  1.00  0.00
E    H
ATOM  16635  CA  ILE E 189     -32.234 -48.642  40.895  1.00  0.00
E    C
ATOM  16636  HA  ILE E 189     -32.094 -48.197  41.873  1.00  0.00
E    H
ATOM  16637  CB  ILE E 189     -32.518 -47.555  39.835  1.00  0.00
E    C
ATOM  16638  HB  ILE E 189     -31.606 -47.551  39.187  1.00  0.00
E    H
ATOM  16639  CG2 ILE E 189     -33.822 -47.832  39.091  1.00  0.00
E    C
ATOM  16640 HG21 ILE E 189     -33.936 -47.138  38.232  1.00  0.00
E    H
ATOM  16641 HG22 ILE E 189     -33.837 -48.867  38.691  1.00  0.00
E    H
ATOM  16642 HG23 ILE E 189     -34.697 -47.701  39.761  1.00  0.00
E    H
ATOM  16643  CG1 ILE E 189     -32.608 -46.158  40.454  1.00  0.00
E    C
ATOM  16644 HG11 ILE E 189     -32.102 -45.420  39.792  1.00  0.00
E    H
ATOM  16645 HG12 ILE E 189     -33.680 -45.861  40.521  1.00  0.00
E    H
ATOM  16646  CD  ILE E 189     -32.034 -46.017  41.810  1.00  0.00
E    C
ATOM  16647  HD1 ILE E 189     -32.199 -44.987  42.194  1.00  0.00
E    H
ATOM  16648  HD2 ILE E 189     -32.512 -46.732  42.514  1.00  0.00
E    H
ATOM  16649  HD3 ILE E 189     -30.940 -46.209  41.802  1.00  0.00
E    H
ATOM  16650  C   ILE E 189     -33.411 -49.599  41.012  1.00  0.00
E    C
ATOM  16651  O   ILE E 189     -33.447 -50.642  40.348  1.00  0.00
E    O
ATOM  16652  N   CYS E 190     -34.350 -49.249  41.886  1.00  0.00
E    N
ATOM  16653  HN  CYS E 190     -34.169 -48.555  42.580  1.00  0.00
E    H
ATOM  16654  CA  CYS E 190     -35.694 -49.817  41.926  1.00  0.00
E    C
ATOM  16655  HA  CYS E 190     -35.956 -50.169  40.935  1.00  0.00
E    H
ATOM  16656  CB  CYS E 190     -35.802 -50.951  42.939  1.00  0.00
E    C
ATOM  16657  HB1 CYS E 190     -34.920 -51.613  42.788  1.00  0.00
E    H
ATOM  16658  HB2 CYS E 190     -35.708 -50.546  43.971  1.00  0.00
E    H
```

```
ATOM  16659  SG  CYS E 190     -37.304 -51.958  42.772  1.00  0.00
E    S
ATOM  16660  HG1 CYS E 190     -37.019 -52.842  43.731  1.00  0.00
E    H
ATOM  16661  C   CYS E 190     -36.626 -48.657  42.268  1.00  0.00
E    C
ATOM  16662  O   CYS E 190     -36.654 -48.184  43.407  1.00  0.00
E    O
ATOM  16663  N   LEU E 191     -37.368 -48.186  41.267  1.00  0.00
E    N
ATOM  16664  HN  LEU E 191     -37.353 -48.544  40.335  1.00  0.00
E    H
ATOM  16665  CA  LEU E 191     -38.288 -47.076  41.466  1.00  0.00
E    C
ATOM  16666  HA  LEU E 191     -37.879 -46.466  42.261  1.00  0.00
E    H
ATOM  16667  CB  LEU E 191     -38.464 -46.273  40.176  1.00  0.00
E    C
ATOM  16668  HB1 LEU E 191     -38.559 -47.006  39.342  1.00  0.00
E    H
ATOM  16669  HB2 LEU E 191     -39.434 -45.728  40.218  1.00  0.00
E    H
ATOM  16670  CG  LEU E 191     -37.391 -45.277  39.746  1.00  0.00
E    C
ATOM  16671  HG  LEU E 191     -36.454 -45.835  39.506  1.00  0.00
E    H
ATOM  16672  CD1 LEU E 191     -37.874 -44.540  38.515  1.00  0.00
E    C
ATOM  16673 HD11 LEU E 191     -37.134 -43.773  38.205  1.00  0.00
E    H
ATOM  16674 HD12 LEU E 191     -38.022 -45.245  37.669  1.00  0.00
E    H
ATOM  16675 HD13 LEU E 191     -38.840 -44.029  38.717  1.00  0.00
E    H
ATOM  16676  CD2 LEU E 191     -37.083 -44.306  40.861  1.00  0.00
E    C
ATOM  16677 HD21 LEU E 191     -36.288 -43.596  40.546  1.00  0.00
E    H
ATOM  16678 HD22 LEU E 191     -37.975 -43.709  41.142  1.00  0.00
E    H
ATOM  16679 HD23 LEU E 191     -36.728 -44.849  41.763  1.00  0.00
E    H
ATOM  16680  C   LEU E 191     -39.648 -47.580  41.926  1.00  0.00
E    C
ATOM  16681  O   LEU E 191     -40.173 -48.566  41.404  1.00  0.00
E    O
ATOM  16682  N   SER E 192     -40.240 -46.853  42.873  1.00  0.00
E    N
ATOM  16683  HN  SER E 192     -39.841 -46.006  43.222  1.00  0.00
E    H
ATOM  16684  CA  SER E 192     -41.512 -47.213  43.491  1.00  0.00
E    C
ATOM  16685  HA  SER E 192     -41.571 -46.662  44.421  1.00  0.00
E    H
```

ATOM 16686 CB SER E 192 -42.689 -46.865 42.589 1.00 0.00
E C
ATOM 16687 HB1 SER E 192 -43.470 -47.647 42.723 1.00 0.00
E H
ATOM 16688 HB2 SER E 192 -43.140 -45.895 42.897 1.00 0.00
E H
ATOM 16689 OG SER E 192 -42.320 -46.834 41.222 1.00 0.00
E O
ATOM 16690 HG1 SER E 192 -43.138 -46.832 40.708 1.00 0.00
E H
ATOM 16691 C SER E 192 -41.521 -48.706 43.844 1.00 0.00
E C
ATOM 16692 O SER E 192 -42.308 -49.469 43.276 1.00 0.00
E O
ATOM 16693 N PRO E 193 -40.660 -49.144 44.759 1.00 0.00
E N
ATOM 16694 CD PRO E 193 -39.886 -48.353 45.732 1.00 0.00
E C
ATOM 16695 HD1 PRO E 193 -39.014 -47.850 45.257 1.00 0.00
E H
ATOM 16696 HD2 PRO E 193 -40.538 -47.605 46.241 1.00 0.00
E H
ATOM 16697 CA PRO E 193 -40.512 -50.585 44.986 1.00 0.00
E C
ATOM 16698 HA PRO E 193 -40.256 -51.093 44.064 1.00 0.00
E H
ATOM 16699 CB PRO E 193 -39.357 -50.669 45.990 1.00 0.00
E C
ATOM 16700 HB1 PRO E 193 -38.392 -50.651 45.435 1.00 0.00
E H
ATOM 16701 HB2 PRO E 193 -39.397 -51.584 46.614 1.00 0.00
E H
ATOM 16702 CG PRO E 193 -39.413 -49.381 46.722 1.00 0.00
E C
ATOM 16703 HG1 PRO E 193 -38.443 -49.101 47.178 1.00 0.00
E H
ATOM 16704 HG2 PRO E 193 -40.193 -49.444 47.516 1.00 0.00
E H
ATOM 16705 C PRO E 193 -41.787 -51.190 45.550 1.00 0.00
E C
ATOM 16706 O PRO E 193 -42.371 -50.684 46.512 1.00 0.00
E O
ATOM 16707 N ALA E 194 -42.216 -52.286 44.930 1.00 0.00
E N
ATOM 16708 HN ALA E 194 -41.755 -52.670 44.130 1.00 0.00
E H
ATOM 16709 CA ALA E 194 -43.393 -53.007 45.376 1.00 0.00
E C
ATOM 16710 HA ALA E 194 -44.106 -52.349 45.861 1.00 0.00
E H
ATOM 16711 CB ALA E 194 -44.083 -53.695 44.204 1.00 0.00
E C
ATOM 16712 HB1 ALA E 194 -44.427 -52.933 43.473 1.00 0.00
E H

ATOM 16713 HB2 ALA E 194 −43.384 −54.383 43.678 1.00 0.00
E H
ATOM 16714 HB3 ALA E 194 −44.970 −54.278 44.535 1.00 0.00
E H
ATOM 16715 C ALA E 194 −42.978 −54.028 46.417 1.00 0.00
E C
ATOM 16716 O ALA E 194 −42.064 −54.827 46.153 1.00 0.00
E O
ATOM 16717 N PRO E 195 −43.585 −54.028 47.601 1.00 0.00
E N
ATOM 16718 CD PRO E 195 −44.618 −53.093 48.076 1.00 0.00
E C
ATOM 16719 HD1 PRO E 195 −43.643 −52.703 47.705 1.00 0.00
E H
ATOM 16720 HD2 PRO E 195 −45.445 −52.398 47.802 1.00 0.00
E H
ATOM 16721 CA PRO E 195 −43.227 −55.023 48.620 1.00 0.00
E C
ATOM 16722 HA PRO E 195 −42.191 −54.912 48.919 1.00 0.00
E H
ATOM 16723 CB PRO E 195 −44.168 −54.688 49.782 1.00 0.00
E C
ATOM 16724 HB1 PRO E 195 −43.712 −53.881 50.399 1.00 0.00
E H
ATOM 16725 HB2 PRO E 195 −44.376 −55.563 50.431 1.00 0.00
E H
ATOM 16726 CG PRO E 195 −44.568 −53.268 49.560 1.00 0.00
E C
ATOM 16727 HG1 PRO E 195 −44.898 −52.761 50.488 1.00 0.00
E H
ATOM 16728 HG2 PRO E 195 −45.388 −53.232 48.807 1.00 0.00
E H
ATOM 16729 C PRO E 195 −43.448 −56.439 48.105 1.00 0.00
E C
ATOM 16730 O PRO E 195 −44.458 −56.733 47.463 1.00 0.00
E O
ATOM 16731 N GLU E 196 −42.488 −57.320 48.384 1.00 0.00
E N
ATOM 16732 HN GLU E 196 −42.511 −58.263 48.062 1.00 0.00
E H
ATOM 16733 CA GLU E 196 −41.311 −56.995 49.185 1.00 0.00
E C
ATOM 16734 HA GLU E 196 −41.520 −56.094 49.748 1.00 0.00
E H
ATOM 16735 CB GLU E 196 −40.992 −58.141 50.148 1.00 0.00
E C
ATOM 16736 HB1 GLU E 196 −40.087 −57.859 50.735 1.00 0.00
E H
ATOM 16737 HB2 GLU E 196 −41.814 −58.250 50.890 1.00 0.00
E H
ATOM 16738 CG GLU E 196 −40.733 −59.488 49.488 1.00 0.00
E C
ATOM 16739 HG1 GLU E 196 −40.934 −60.298 50.212 1.00 0.00
E H

```
ATOM  16740  HG2 GLU E 196     -41.379 -59.624  48.599  1.00  0.00
E    H
ATOM  16741  CD  GLU E 196     -39.284 -59.653  49.069  1.00  0.00
E    C
ATOM  16742  OE1 GLU E 196     -38.411 -59.736  49.965  1.00  0.00
E    O
ATOM  16743  OE2 GLU E 196     -39.015 -59.659  47.851  1.00  0.00
E    O
ATOM  16744  C   GLU E 196     -40.110 -56.699  48.286  1.00  0.00
E    C
ATOM  16745  O   GLU E 196     -40.176 -56.892  47.075  1.00  0.00
E    O
ATOM  16746  N   ASP E 197     -39.018 -56.216  48.881  1.00  0.00
E    N
ATOM  16747  HN  ASP E 197     -38.930 -56.061  49.861  1.00  0.00
E    H
ATOM  16748  CA  ASP E 197     -37.818 -55.856  48.140  1.00  0.00
E    C
ATOM  16749  HA  ASP E 197     -37.642 -56.638  47.410  1.00  0.00
E    H
ATOM  16750  CB  ASP E 197     -37.960 -54.495  47.465  1.00  0.00
E    C
ATOM  16751  HB1 ASP E 197     -39.006 -54.366  47.119  1.00  0.00
E    H
ATOM  16752  HB2 ASP E 197     -37.725 -53.679  48.177  1.00  0.00
E    H
ATOM  16753  CG  ASP E 197     -37.064 -54.355  46.258  1.00  0.00
E    C
ATOM  16754  OD1 ASP E 197     -36.618 -55.392  45.723  1.00  0.00
E    O
ATOM  16755  OD2 ASP E 197     -36.787 -53.203  45.860  1.00  0.00
E    O
ATOM  16756  C   ASP E 197     -36.611 -55.837  49.062  1.00  0.00
E    C
ATOM  16757  O   ASP E 197     -36.645 -55.236  50.137  1.00  0.00
E    O
ATOM  16758  N   ILE E 198     -35.523 -56.450  48.602  1.00  0.00
E    N
ATOM  16759  HN  ILE E 198     -35.451 -56.846  47.689  1.00  0.00
E    H
ATOM  16760  CA  ILE E 198     -34.317 -56.596  49.416  1.00  0.00
E    C
ATOM  16761  HA  ILE E 198     -34.630 -56.954  50.388  1.00  0.00
E    H
ATOM  16762  CB  ILE E 198     -33.318 -57.569  48.760  1.00  0.00
E    C
ATOM  16763  HB  ILE E 198     -33.521 -57.481  47.663  1.00  0.00
E    H
ATOM  16764  CG2 ILE E 198     -31.885 -57.251  49.196  1.00  0.00
E    C
ATOM  16765 HG21 ILE E 198     -31.157 -57.846  48.608  1.00  0.00
E    H
ATOM  16766 HG22 ILE E 198     -31.652 -56.177  49.035  1.00  0.00
E    H
```

ATOM 16767 HG23 ILE E 198   -31.736 -57.485 50.271 1.00 0.00
E   H
ATOM 16768 CG1 ILE E 198   -33.628 -59.016 49.146 1.00 0.00
E   C
ATOM 16769 HG11 ILE E 198   -33.301 -59.205 50.194 1.00 0.00
E   H
ATOM 16770 HG12 ILE E 198   -34.731 -59.170 49.113 1.00 0.00
E   H
ATOM 16771 CD ILE E 198   -32.987 -60.030 48.243 1.00 0.00
E   C
ATOM 16772 HD1 ILE E 198   -33.294 -61.057 48.537 1.00 0.00
E   H
ATOM 16773 HD2 ILE E 198   -33.294 -59.863 47.188 1.00 0.00
E   H
ATOM 16774 HD3 ILE E 198   -31.880 -59.972 48.301 1.00 0.00
E   H
ATOM 16775 C   ILE E 198   -33.685 -55.226 49.632 1.00 0.00
E   C
ATOM 16776 O   ILE E 198   -33.292 -54.543 48.677 1.00 0.00
E   O
ATOM 16777 N   ILE E 199   -33.586 -54.824 50.894 1.00 0.00
E   N
ATOM 16778 HN ILE E 199   -33.951 -55.355 51.656 1.00 0.00
E   H
ATOM 16779 CA ILE E 199   -32.938 -53.580 51.289 1.00 0.00
E   C
ATOM 16780 HA ILE E 199   -32.728 -53.009 50.393 1.00 0.00
E   H
ATOM 16781 CB ILE E 199   -33.800 -52.778 52.279 1.00 0.00
E   C
ATOM 16782 HB ILE E 199   -33.977 -53.483 53.130 1.00 0.00
E   H
ATOM 16783 CG2 ILE E 199   -33.082 -51.511 52.711 1.00 0.00
E   C
ATOM 16784 HG21 ILE E 199   -33.631 -51.014 53.538 1.00 0.00
E   H
ATOM 16785 HG22 ILE E 199   -32.059 -51.739 53.075 1.00 0.00
E   H
ATOM 16786 HG23 ILE E 199   -33.001 -50.793 51.868 1.00 0.00
E   H
ATOM 16787 CG1 ILE E 199   -35.170 -52.472 51.662 1.00 0.00
E   C
ATOM 16788 HG11 ILE E 199   -35.041 -52.118 50.615 1.00 0.00
E   H
ATOM 16789 HG12 ILE E 199   -35.765 -53.412 51.620 1.00 0.00
E   H
ATOM 16790 CD ILE E 199   -35.980 -51.454 52.414 1.00 0.00
E   C
ATOM 16791 HD1 ILE E 199   -36.978 -51.328 51.940 1.00 0.00
E   H
ATOM 16792 HD2 ILE E 199   -36.132 -51.774 53.467 1.00 0.00
E   H
ATOM 16793 HD3 ILE E 199   -35.475 -50.465 52.415 1.00 0.00
E   H

```
ATOM  16794  C   ILE E 199      -31.597 -53.951  51.900  1.00  0.00
E    C
ATOM  16795  O   ILE E 199      -31.539 -54.405  53.046  1.00  0.00
E    O
ATOM  16796  N   ARG E 200      -30.527 -53.787  51.132  1.00  0.00
E    N
ATOM  16797  HN  ARG E 200      -30.599 -53.449  50.197  1.00  0.00
E    H
ATOM  16798  CA  ARG E 200      -29.191 -54.091  51.615  1.00  0.00
E    C
ATOM  16799  HA  ARG E 200      -29.254 -54.918  52.313  1.00  0.00
E    H
ATOM  16800  CB  ARG E 200      -28.280 -54.456  50.447  1.00  0.00
E    C
ATOM  16801  HB1 ARG E 200      -28.529 -53.748  49.620  1.00  0.00
E    H
ATOM  16802  HB2 ARG E 200      -27.222 -54.246  50.721  1.00  0.00
E    H
ATOM  16803  CG  ARG E 200      -28.468 -55.874  49.961  1.00  0.00
E    C
ATOM  16804  HG1 ARG E 200      -28.851 -56.489  50.805  1.00  0.00
E    H
ATOM  16805  HG2 ARG E 200      -29.273 -55.889  49.190  1.00  0.00
E    H
ATOM  16806  CD  ARG E 200      -27.193 -56.423  49.355  1.00  0.00
E    C
ATOM  16807  HD1 ARG E 200      -27.045 -55.983  48.342  1.00  0.00
E    H
ATOM  16808  HD2 ARG E 200      -26.304 -56.182  49.983  1.00  0.00
E    H
ATOM  16809  NE  ARG E 200      -27.231 -57.878  49.236  1.00  0.00
E    N
ATOM  16810  HE  ARG E 200      -28.149 -58.263  49.380  1.00  0.00
E    H
ATOM  16811  CZ  ARG E 200      -26.171 -58.632  48.965  1.00  0.00
E    C
ATOM  16812  NH1 ARG E 200      -24.985 -58.067  48.781  1.00  0.00
E    N
ATOM  16813 HH11 ARG E 200      -24.194 -58.617  48.554  1.00  0.00
E    H
ATOM  16814 HH12 ARG E 200      -24.971 -57.065  48.751  1.00  0.00
E    H
ATOM  16815  NH2 ARG E 200      -26.293 -59.948  48.874  1.00  0.00
E    N
ATOM  16816 HH21 ARG E 200      -25.487 -60.511  48.758  1.00  0.00
E    H
ATOM  16817 HH22 ARG E 200      -27.166 -60.336  49.135  1.00  0.00
E    H
ATOM  16818  C   ARG E 200      -28.621 -52.908  52.391  1.00  0.00
E    C
ATOM  16819  O   ARG E 200      -29.196 -51.820  52.432  1.00  0.00
E    O
ATOM  16820  N   GLU E 201      -27.472 -53.136  53.020  1.00  0.00
E    N
```

```
ATOM  16821  HN  GLU E 201      -27.022 -54.020  53.107  1.00  0.00
E    H
ATOM  16822  CA  GLU E 201      -26.758 -52.043  53.657  1.00  0.00
E    C
ATOM  16823  HA  GLU E 201      -27.436 -51.519  54.319  1.00  0.00
E    H
ATOM  16824  CB  GLU E 201      -25.553 -52.560  54.438  1.00  0.00
E    C
ATOM  16825  HB1 GLU E 201      -24.820 -51.726  54.537  1.00  0.00
E    H
ATOM  16826  HB2 GLU E 201      -25.860 -52.821  55.476  1.00  0.00
E    H
ATOM  16827  CG  GLU E 201      -24.857 -53.742  53.803  1.00  0.00
E    C
ATOM  16828  HG1 GLU E 201      -25.469 -54.651  53.947  1.00  0.00
E    H
ATOM  16829  HG2 GLU E 201      -24.700 -53.575  52.719  1.00  0.00
E    H
ATOM  16830  CD  GLU E 201      -23.522 -54.030  54.453  1.00  0.00
E    C
ATOM  16831  OE1 GLU E 201      -23.401 -53.825  55.677  1.00  0.00
E    O
ATOM  16832  OE2 GLU E 201      -22.592 -54.462  53.739  1.00  0.00
E    O
ATOM  16833  C   GLU E 201      -26.299 -51.049  52.602  1.00  0.00
E    C
ATOM  16834  O   GLU E 201      -26.239 -51.366  51.412  1.00  0.00
E    O
ATOM  16835  N   GLN E 202      -25.992 -49.833  53.048  1.00  0.00
E    N
ATOM  16836  HN  GLN E 202      -26.097 -49.593  54.011  1.00  0.00
E    H
ATOM  16837  CA  GLN E 202      -25.486 -48.754  52.208  1.00  0.00
E    C
ATOM  16838  HA  GLN E 202      -25.417 -47.877  52.840  1.00  0.00
E    H
ATOM  16839  CB  GLN E 202      -24.093 -49.089  51.663  1.00  0.00
E    C
ATOM  16840  HB1 GLN E 202      -24.183 -50.059  51.122  1.00  0.00
E    H
ATOM  16841  HB2 GLN E 202      -23.805 -48.331  50.898  1.00  0.00
E    H
ATOM  16842  CG  GLN E 202      -23.051 -49.197  52.762  1.00  0.00
E    C
ATOM  16843  HG1 GLN E 202      -23.355 -48.595  53.645  1.00  0.00
E    H
ATOM  16844  HG2 GLN E 202      -22.936 -50.250  53.091  1.00  0.00
E    H
ATOM  16845  CD  GLN E 202      -21.679 -48.679  52.364  1.00  0.00
E    C
ATOM  16846  OE1 GLN E 202      -21.559 -47.782  51.537  1.00  0.00
E    O
ATOM  16847  NE2 GLN E 202      -20.637 -49.246  52.961  1.00  0.00
E    N
```

ATOM 16848 HE21 GLN E 202   -19.734 -48.908 52.708 1.00 0.00
E   H
ATOM 16849 HE22 GLN E 202   -20.781 -49.983 53.612 1.00 0.00
E   H
ATOM 16850 C   GLN E 202   -26.439 -48.425 51.068 1.00 0.00
E   C
ATOM 16851 O   GLN E 202   -26.010 -47.899 50.033 1.00 0.00
E   O
ATOM 16852 N   ASP E 203   -27.725 -48.719 51.238 1.00 0.00
E   N
ATOM 16853 HN ASP E 203   -28.114 -49.136 52.055 1.00 0.00
E   H
ATOM 16854 CA ASP E 203   -28.717 -48.450 50.213 1.00 0.00
E   C
ATOM 16855 HA ASP E 203   -28.210 -48.498 49.256 1.00 0.00
E   H
ATOM 16856 CB ASP E 203   -29.884 -49.421 50.308 1.00 0.00
E   C
ATOM 16857 HB1 ASP E 203   -29.492 -50.453 50.408 1.00 0.00
E   H
ATOM 16858 HB2 ASP E 203   -30.508 -49.197 51.196 1.00 0.00
E   H
ATOM 16859 CG ASP E 203   -30.755 -49.380 49.083 1.00 0.00
E   C
ATOM 16860 OD1 ASP E 203   -31.791 -48.692 49.128 1.00 0.00
E   O
ATOM 16861 OD2 ASP E 203   -30.431 -50.061 48.104 1.00 0.00
E   O
ATOM 16862 C   ASP E 203   -29.261 -47.040 50.349 1.00 0.00
E   C
ATOM 16863 O   ASP E 203   -29.551 -46.578 51.454 1.00 0.00
E   O
ATOM 16864 N   CYS E 204   -29.442 -46.374 49.217 1.00 0.00
E   N
ATOM 16865 HN CYS E 204   -29.160 -46.700 48.317 1.00 0.00
E   H
ATOM 16866 CA CYS E 204   -30.093 -45.071 49.217 1.00 0.00
E   C
ATOM 16867 HA CYS E 204   -29.877 -44.601 50.169 1.00 0.00
E   H
ATOM 16868 CB CYS E 204   -29.566 -44.207 48.077 1.00 0.00
E   C
ATOM 16869 HB1 CYS E 204   -29.527 -44.848 47.167 1.00 0.00
E   H
ATOM 16870 HB2 CYS E 204   -30.293 -43.397 47.851 1.00 0.00
E   H
ATOM 16871 SG CYS E 204   -27.914 -43.541 48.381 1.00 0.00
E   S
ATOM 16872 HG1 CYS E 204   -27.761 -42.931 47.205 1.00 0.00
E   H
ATOM 16873 C   CYS E 204   -31.603 -45.267 49.127 1.00 0.00
E   C
ATOM 16874 O   CYS E 204   -32.109 -45.835 48.154 1.00 0.00
E   O

```
ATOM  16875  N   LEU E 205     -32.307 -44.823  50.166  1.00  0.00
E    N
ATOM  16876  HN  LEU E 205     -31.848 -44.364  50.924  1.00  0.00
E    H
ATOM  16877  CA  LEU E 205     -33.750 -44.941  50.324  1.00  0.00
E    C
ATOM  16878  HA  LEU E 205     -34.126 -45.617  49.567  1.00  0.00
E    H
ATOM  16879  CB  LEU E 205     -34.101 -45.391  51.746  1.00  0.00
E    C
ATOM  16880  HB1 LEU E 205     -33.210 -45.190  52.384  1.00  0.00
E    H
ATOM  16881  HB2 LEU E 205     -34.914 -44.741  52.140  1.00  0.00
E    H
ATOM  16882  CG  LEU E 205     -34.510 -46.809  52.131  1.00  0.00
E    C
ATOM  16883  HG  LEU E 205     -35.500 -47.052  51.676  1.00  0.00
E    H
ATOM  16884  CD1 LEU E 205     -33.472 -47.830  51.702  1.00  0.00
E    C
ATOM  16885 HD11 LEU E 205     -33.766 -48.849  52.035  1.00  0.00
E    H
ATOM  16886 HD12 LEU E 205     -33.368 -47.841  50.595  1.00  0.00
E    H
ATOM  16887 HD13 LEU E 205     -32.481 -47.593  52.145  1.00  0.00
E    H
ATOM  16888  CD2 LEU E 205     -34.664 -46.846  53.634  1.00  0.00
E    C
ATOM  16889 HD21 LEU E 205     -34.981 -47.858  53.965  1.00  0.00
E    H
ATOM  16890 HD22 LEU E 205     -33.713 -46.605  54.150  1.00  0.00
E    H
ATOM  16891 HD23 LEU E 205     -35.436 -46.115  53.964  1.00  0.00
E    H
ATOM  16892  C   LEU E 205     -34.342 -43.568  50.047  1.00  0.00
E    C
ATOM  16893  O   LEU E 205     -33.851 -42.564  50.582  1.00  0.00
E    O
ATOM  16894  N   VAL E 206     -35.372 -43.511  49.210  1.00  0.00
E    N
ATOM  16895  HN  VAL E 206     -35.733 -44.299  48.717  1.00  0.00
E    H
ATOM  16896  CA  VAL E 206     -36.072 -42.261  48.941  1.00  0.00
E    C
ATOM  16897  HA  VAL E 206     -35.487 -41.442  49.339  1.00  0.00
E    H
ATOM  16898  CB  VAL E 206     -36.284 -42.033  47.442  1.00  0.00
E    C
ATOM  16899  HB  VAL E 206     -36.984 -42.824  47.076  1.00  0.00
E    H
ATOM  16900  CG1 VAL E 206     -36.896 -40.669  47.202  1.00  0.00
E    C
ATOM  16901 HG11 VAL E 206     -37.014 -40.486  46.112  1.00  0.00
E    H
```

```
ATOM  16902 HG12 VAL E 206     -37.903 -40.585  47.662  1.00  0.00
E    H
ATOM  16903 HG13 VAL E 206     -36.247 -39.869  47.616  1.00  0.00
E    H
ATOM  16904  CG2 VAL E 206     -34.952 -42.153  46.710  1.00  0.00
E    C
ATOM  16905 HG21 VAL E 206     -35.081 -41.951  45.625  1.00  0.00
E    H
ATOM  16906 HG22 VAL E 206     -34.216 -41.426  47.116  1.00  0.00
E    H
ATOM  16907 HG23 VAL E 206     -34.528 -43.173  46.812  1.00  0.00
E    H
ATOM  16908  C   VAL E 206     -37.393 -42.285  49.695  1.00  0.00
E    C
ATOM  16909  O   VAL E 206     -38.257 -43.133  49.441  1.00  0.00
E    O
ATOM  16910  N   ILE E 207     -37.554 -41.342  50.614  1.00  0.00
E    N
ATOM  16911  HN  ILE E 207     -36.877 -40.634  50.801  1.00  0.00
E    H
ATOM  16912  CA  ILE E 207     -38.746 -41.257  51.438  1.00  0.00
E    C
ATOM  16913  HA  ILE E 207     -39.483 -41.931  51.017  1.00  0.00
E    H
ATOM  16914  CB  ILE E 207     -38.453 -41.612  52.910  1.00  0.00
E    C
ATOM  16915  HB  ILE E 207     -37.863 -42.562  52.856  1.00  0.00
E    H
ATOM  16916  CG2 ILE E 207     -37.710 -40.484  53.585  1.00  0.00
E    C
ATOM  16917 HG21 ILE E 207     -37.360 -40.793  54.591  1.00  0.00
E    H
ATOM  16918 HG22 ILE E 207     -36.817 -40.189  52.995  1.00  0.00
E    H
ATOM  16919 HG23 ILE E 207     -38.362 -39.593  53.703  1.00  0.00
E    H
ATOM  16920  CG1 ILE E 207     -39.749 -41.940  53.656  1.00  0.00
E    C
ATOM  16921 HG11 ILE E 207     -40.442 -41.070  53.606  1.00  0.00
E    H
ATOM  16922 HG12 ILE E 207     -40.253 -42.792  53.145  1.00  0.00
E    H
ATOM  16923  CD  ILE E 207     -39.539 -42.312  55.081  1.00  0.00
E    C
ATOM  16924  HD1 ILE E 207     -40.504 -42.595  55.553  1.00  0.00
E    H
ATOM  16925  HD2 ILE E 207     -38.847 -43.177  55.162  1.00  0.00
E    H
ATOM  16926  HD3 ILE E 207     -39.112 -41.463  55.654  1.00  0.00
E    H
ATOM  16927  C   ILE E 207     -39.315 -39.851  51.315  1.00  0.00
E    C
ATOM  16928  O   ILE E 207     -38.630 -38.907  50.924  1.00  0.00
E    O
```

```
ATOM  16929  N   MET E 208      -40.592 -39.713  51.652  1.00  0.00
E    N
ATOM  16930  HN  MET E 208      -41.181 -40.463  51.946  1.00  0.00
E    H
ATOM  16931  CA  MET E 208      -41.246 -38.413  51.614  1.00  0.00
E    C
ATOM  16932  HA  MET E 208      -40.527 -37.661  51.915  1.00  0.00
E    H
ATOM  16933  CB  MET E 208      -41.806 -38.088  50.219  1.00  0.00
E    C
ATOM  16934  HB1 MET E 208      -40.931 -37.884  49.558  1.00  0.00
E    H
ATOM  16935  HB2 MET E 208      -42.306 -38.991  49.802  1.00  0.00
E    H
ATOM  16936  CG  MET E 208      -42.736 -36.883  50.232  1.00  0.00
E    C
ATOM  16937  HG1 MET E 208      -43.324 -36.859  51.173  1.00  0.00
E    H
ATOM  16938  HG2 MET E 208      -42.136 -35.947  50.185  1.00  0.00
E    H
ATOM  16939  SD  MET E 208      -43.906 -36.849  48.886  1.00  0.00
E    S
ATOM  16940  CE  MET E 208      -44.550 -38.501  48.999  1.00  0.00
E    C
ATOM  16941  HE1 MET E 208      -45.312 -38.685  48.212  1.00  0.00
E    H
ATOM  16942  HE2 MET E 208      -43.741 -39.253  48.874  1.00  0.00
E    H
ATOM  16943  HE3 MET E 208      -45.030 -38.676  49.986  1.00  0.00
E    H
ATOM  16944  C   MET E 208      -42.369 -38.410  52.635  1.00  0.00
E    C
ATOM  16945  O   MET E 208      -43.027 -39.433  52.842  1.00  0.00
E    O
ATOM  16946  N   GLY E 209      -42.590 -37.261  53.258  1.00  0.00
E    N
ATOM  16947  HN  GLY E 209      -41.996 -36.461  53.192  1.00  0.00
E    H
ATOM  16948  CA  GLY E 209      -43.756 -37.107  54.101  1.00  0.00
E    C
ATOM  16949  HA1 GLY E 209      -43.865 -38.028  54.659  1.00  0.00
E    H
ATOM  16950  HA2 GLY E 209      -44.591 -36.879  53.452  1.00  0.00
E    H
ATOM  16951  C   GLY E 209      -43.614 -35.975  55.092  1.00  0.00
E    C
ATOM  16952  O   GLY E 209      -42.897 -35.005  54.841  1.00  0.00
E    O
ATOM  16953  N   HSE E 210      -44.298 -36.096  56.225  1.00  0.00
E    N
ATOM  16954  HN  HSE E 210      -44.928 -36.841  56.436  1.00  0.00
E    H
ATOM  16955  CA  HSE E 210      -44.167 -35.107  57.278  1.00  0.00
E    C
```

```
ATOM  16956  HA  HSE E 210     -44.270 -34.128  56.827  1.00  0.00
E    H
ATOM  16957  CB  HSE E 210     -45.205 -35.333  58.371  1.00  0.00
E    C
ATOM  16958  HB1 HSE E 210     -46.211 -35.271  57.905  1.00  0.00
E    H
ATOM  16959  HB2 HSE E 210     -45.097 -36.360  58.777  1.00  0.00
E    H
ATOM  16960  ND1 HSE E 210     -45.418 -32.989  59.273  1.00  0.00
E    N
ATOM  16961  CG  HSE E 210     -45.138 -34.324  59.471  1.00  0.00
E    C
ATOM  16962  CE1 HSE E 210     -45.269 -32.337  60.412  1.00  0.00
E    C
ATOM  16963  HE1 HSE E 210     -45.437 -31.265  60.539  1.00  0.00
E    H
ATOM  16964  NE2 HSE E 210     -44.885 -33.199  61.337  1.00  0.00
E    N
ATOM  16965  HE2 HSE E 210     -44.680 -32.970  62.288  1.00  0.00
E    H
ATOM  16966  CD2 HSE E 210     -44.788 -34.446  60.772  1.00  0.00
E    C
ATOM  16967  HD2 HSE E 210     -44.472 -35.319  61.326  1.00  0.00
E    H
ATOM  16968  C   HSE E 210     -42.770 -35.183  57.865  1.00  0.00
E    C
ATOM  16969  O   HSE E 210     -42.362 -36.221  58.392  1.00  0.00
E    O
ATOM  16970  N   LYS E 211     -42.041 -34.070  57.771  1.00  0.00
E    N
ATOM  16971  HN  LYS E 211     -42.401 -33.191  57.463  1.00  0.00
E    H
ATOM  16972  CA  LYS E 211     -40.626 -34.071  58.128  1.00  0.00
E    C
ATOM  16973  HA  LYS E 211     -40.130 -34.751  57.445  1.00  0.00
E    H
ATOM  16974  CB  LYS E 211     -40.053 -32.662  57.993  1.00  0.00
E    C
ATOM  16975  HB1 LYS E 211     -39.026 -32.682  58.424  1.00  0.00
E    H
ATOM  16976  HB2 LYS E 211     -39.934 -32.421  56.913  1.00  0.00
E    H
ATOM  16977  CG  LYS E 211     -40.864 -31.588  58.696  1.00  0.00
E    C
ATOM  16978  HG1 LYS E 211     -41.850 -31.484  58.189  1.00  0.00
E    H
ATOM  16979  HG2 LYS E 211     -41.062 -31.912  59.744  1.00  0.00
E    H
ATOM  16980  CD  LYS E 211     -40.148 -30.251  58.676  1.00  0.00
E    C
ATOM  16981  HD1 LYS E 211     -39.088 -30.415  58.976  1.00  0.00
E    H
ATOM  16982  HD2 LYS E 211     -40.143 -29.876  57.626  1.00  0.00
E    H
```

ATOM 16983 CE LYS E 211 -40.831 -29.262 59.598 1.00 0.00
E C
ATOM 16984 HE1 LYS E 211 -40.795 -29.621 60.651 1.00 0.00
E H
ATOM 16985 HE2 LYS E 211 -40.330 -28.270 59.548 1.00 0.00
E H
ATOM 16986 NZ LYS E 211 -42.260 -29.061 59.245 1.00 0.00
E N
ATOM 16987 HZ1 LYS E 211 -42.695 -28.379 59.898 1.00 0.00
E H
ATOM 16988 HZ2 LYS E 211 -42.328 -28.699 58.272 1.00 0.00
E H
ATOM 16989 HZ3 LYS E 211 -42.763 -29.969 59.309 1.00 0.00
E H
ATOM 16990 C LYS E 211 -40.401 -34.598 59.538 1.00 0.00
E C
ATOM 16991 O LYS E 211 -39.392 -35.259 59.807 1.00 0.00
E O
ATOM 16992 N LYS E 212 -41.330 -34.326 60.453 1.00 0.00
E N
ATOM 16993 HN LYS E 212 -42.186 -33.839 60.287 1.00 0.00
E H
ATOM 16994 CA LYS E 212 -41.122 -34.757 61.828 1.00 0.00
E C
ATOM 16995 HA LYS E 212 -40.076 -34.591 62.059 1.00 0.00
E H
ATOM 16996 CB LYS E 212 -41.984 -33.926 62.777 1.00 0.00
E C
ATOM 16997 HB1 LYS E 212 -43.048 -34.121 62.510 1.00 0.00
E H
ATOM 16998 HB2 LYS E 212 -41.849 -34.305 63.815 1.00 0.00
E H
ATOM 16999 CG LYS E 212 -41.715 -32.417 62.707 1.00 0.00
E C
ATOM 17000 HG1 LYS E 212 -42.370 -31.966 61.927 1.00 0.00
E H
ATOM 17001 HG2 LYS E 212 -41.992 -31.954 63.682 1.00 0.00
E H
ATOM 17002 CD LYS E 212 -40.259 -32.064 62.369 1.00 0.00
E C
ATOM 17003 HD1 LYS E 212 -39.950 -32.661 61.480 1.00 0.00
E H
ATOM 17004 HD2 LYS E 212 -40.221 -30.989 62.078 1.00 0.00
E H
ATOM 17005 CE LYS E 212 -39.298 -32.315 63.523 1.00 0.00
E C
ATOM 17006 HE1 LYS E 212 -39.781 -32.064 64.493 1.00 0.00
E H
ATOM 17007 HE2 LYS E 212 -38.990 -33.382 63.549 1.00 0.00
E H
ATOM 17008 NZ LYS E 212 -38.066 -31.483 63.394 1.00 0.00
E N
ATOM 17009 HZ1 LYS E 212 -37.426 -31.670 64.193 1.00 0.00
E H

ATOM 17010 HZ2 LYS E 212   -37.583 -31.713 62.503 1.00 0.00
E   H
ATOM 17011 HZ3 LYS E 212   -38.328 -30.476 63.390 1.00 0.00
E   H
ATOM 17012 C   LYS E 212   -41.391 -36.248 61.985 1.00 0.00
E   C
ATOM 17013 O   LYS E 212   -40.674 -36.937 62.718 1.00 0.00
E   O
ATOM 17014 N   ASP E 213   -42.390 -36.779 61.280 1.00 0.00
E   N
ATOM 17015 HN ASP E 213   -43.081 -36.298 60.746 1.00 0.00
E   H
ATOM 17016 CA ASP E 213   -42.549 -38.230 61.243 1.00 0.00
E   C
ATOM 17017 HA ASP E 213   -42.567 -38.576 62.271 1.00 0.00
E   H
ATOM 17018 CB ASP E 213   -43.832 -38.611 60.509 1.00 0.00
E   C
ATOM 17019 HB1 ASP E 213   -43.893 -38.040 59.561 1.00 0.00
E   H
ATOM 17020 HB2 ASP E 213   -43.836 -39.692 60.266 1.00 0.00
E   H
ATOM 17021 CG ASP E 213   -45.078 -38.297 61.305 1.00 0.00
E   C
ATOM 17022 OD1 ASP E 213   -45.471 -39.126 62.152 1.00 0.00
E   O
ATOM 17023 OD2 ASP E 213   -45.666 -37.219 61.093 1.00 0.00
E   O
ATOM 17024 C   ASP E 213   -41.349 -38.892 60.579 1.00 0.00
E   C
ATOM 17025 O   ASP E 213   -40.942 -39.995 60.966 1.00 0.00
E   O
ATOM 17026 N   ILE E 214   -40.773 -38.232 59.572 1.00 0.00
E   N
ATOM 17027 HN ILE E 214   -41.094 -37.366 59.194 1.00 0.00
E   H
ATOM 17028 CA ILE E 214   -39.577 -38.760 58.926 1.00 0.00
E   C
ATOM 17029 HA ILE E 214   -39.799 -39.782 58.645 1.00 0.00
E   H
ATOM 17030 CB ILE E 214   -39.215 -37.919 57.690 1.00 0.00
E   C
ATOM 17031 HB ILE E 214   -39.077 -36.882 58.088 1.00 0.00
E   H
ATOM 17032 CG2 ILE E 214   -37.993 -38.495 57.001 1.00 0.00
E   C
ATOM 17033 HG21 ILE E 214   -37.635 -37.810 56.205 1.00 0.00
E   H
ATOM 17034 HG22 ILE E 214   -37.162 -38.638 57.722 1.00 0.00
E   H
ATOM 17035 HG23 ILE E 214   -38.226 -39.476 56.535 1.00 0.00
E   H
ATOM 17036 CG1 ILE E 214   -40.390 -37.868 56.718 1.00 0.00
E   C

ATOM 17037 HG11 ILE E 214   -40.508 -38.855 56.217 1.00 0.00
E   H
ATOM 17038 HG12 ILE E 214   -41.325 -37.677 57.293 1.00 0.00
E   H
ATOM 17039 CD ILE E 214   -40.268 -36.797 55.661 1.00 0.00
E   C
ATOM 17040 HD1 ILE E 214   -41.180 -36.776 55.025 1.00 0.00
E   H
ATOM 17041 HD2 ILE E 214   -40.147 -35.796 56.129 1.00 0.00
E   H
ATOM 17042 HD3 ILE E 214   -39.395 -36.985 55.002 1.00 0.00
E   H
ATOM 17043 C   ILE E 214   -38.425 -38.817 59.919 1.00 0.00
E   C
ATOM 17044 O   ILE E 214   -37.687 -39.806 59.984 1.00 0.00
E   O
ATOM 17045 N   LYS E 215   -38.261 -37.758 60.714 1.00 0.00
E   N
ATOM 17046 HN LYS E 215   -38.780 -36.908 60.645 1.00 0.00
E   H
ATOM 17047 CA LYS E 215   -37.265 -37.790 61.777 1.00 0.00
E   C
ATOM 17048 HA LYS E 215   -36.309 -38.003 61.312 1.00 0.00
E   H
ATOM 17049 CB LYS E 215   -37.217 -36.447 62.507 1.00 0.00
E   C
ATOM 17050 HB1 LYS E 215   -37.050 -35.660 61.736 1.00 0.00
E   H
ATOM 17051 HB2 LYS E 215   -38.217 -36.241 62.952 1.00 0.00
E   H
ATOM 17052 CG LYS E 215   -36.119 -36.345 63.571 1.00 0.00
E   C
ATOM 17053 HG1 LYS E 215   -35.254 -36.974 63.262 1.00 0.00
E   H
ATOM 17054 HG2 LYS E 215   -35.761 -35.291 63.623 1.00 0.00
E   H
ATOM 17055 CD LYS E 215   -36.614 -36.810 64.937 1.00 0.00
E   C
ATOM 17056 HD1 LYS E 215   -37.711 -36.629 64.994 1.00 0.00
E   H
ATOM 17057 HD2 LYS E 215   -36.453 -37.910 65.008 1.00 0.00
E   H
ATOM 17058 CE LYS E 215   -35.908 -36.113 66.074 1.00 0.00
E   C
ATOM 17059 HE1 LYS E 215   -34.810 -36.283 66.013 1.00 0.00
E   H
ATOM 17060 HE2 LYS E 215   -36.097 -35.017 66.038 1.00 0.00
E   H
ATOM 17061 NZ LYS E 215   -36.390 -36.629 67.379 1.00 0.00
E   N
ATOM 17062 HZ1 LYS E 215   -35.898 -36.145 68.158 1.00 0.00
E   H
ATOM 17063 HZ2 LYS E 215   -37.414 -36.464 67.461 1.00 0.00
E   H

ATOM 17064 HZ3 LYS E 215 −36.204 −37.651 67.436 1.00 0.00
E H
ATOM 17065 C LYS E 215 −37.548 −38.910 62.764 1.00 0.00
E C
ATOM 17066 O LYS E 215 −36.617 −39.560 63.244 1.00 0.00
E O
ATOM 17067 N ARG E 216 −38.817 −39.110 63.120 1.00 0.00
E N
ATOM 17068 HN ARG E 216 −39.572 −38.559 62.772 1.00 0.00
E H
ATOM 17069 CA ARG E 216 −39.160 −40.171 64.060 1.00 0.00
E C
ATOM 17070 HA ARG E 216 −38.605 −40.011 64.977 1.00 0.00
E H
ATOM 17071 CB ARG E 216 −40.662 −40.152 64.341 1.00 0.00
E C
ATOM 17072 HB1 ARG E 216 −41.169 −40.187 63.347 1.00 0.00
E H
ATOM 17073 HB2 ARG E 216 −40.954 −41.088 64.867 1.00 0.00
E H
ATOM 17074 CG ARG E 216 −41.120 −38.917 65.090 1.00 0.00
E C
ATOM 17075 HG1 ARG E 216 −40.862 −39.039 66.164 1.00 0.00
E H
ATOM 17076 HG2 ARG E 216 −40.530 −38.041 64.739 1.00 0.00
E H
ATOM 17077 CD ARG E 216 −42.594 −38.635 64.864 1.00 0.00
E C
ATOM 17078 HD1 ARG E 216 −42.878 −38.950 63.834 1.00 0.00
E H
ATOM 17079 HD2 ARG E 216 −43.231 −39.186 65.594 1.00 0.00
E H
ATOM 17080 NE ARG E 216 −42.892 −37.216 65.032 1.00 0.00
E N
ATOM 17081 HE ARG E 216 −42.128 −36.696 65.433 1.00 0.00
E H
ATOM 17082 CZ ARG E 216 −44.046 −36.647 64.698 1.00 0.00
E C
ATOM 17083 NH1 ARG E 216 −45.023 −37.378 64.181 1.00 0.00
E N
ATOM 17084 HH11 ARG E 216 −45.876 −36.957 63.906 1.00 0.00
E H
ATOM 17085 HH12 ARG E 216 −44.797 −38.330 63.964 1.00 0.00
E H
ATOM 17086 NH2 ARG E 216 −44.227 −35.347 64.885 1.00 0.00
E N
ATOM 17087 HH21 ARG E 216 −45.115 −34.943 64.716 1.00 0.00
E H
ATOM 17088 HH22 ARG E 216 −43.521 −34.864 65.386 1.00 0.00
E H
ATOM 17089 C ARG E 216 −38.731 −41.528 63.524 1.00 0.00
E C
ATOM 17090 O ARG E 216 −38.090 −42.311 64.237 1.00 0.00
E O

ATOM 17091 N PHE E 217 -39.070 -41.816 62.269 1.00 0.00
E N
ATOM 17092 HN PHE E 217 -39.603 -41.225 61.665 1.00 0.00
E H
ATOM 17093 CA PHE E 217 -38.662 -43.079 61.671 1.00 0.00
E C
ATOM 17094 HA PHE E 217 -38.998 -43.858 62.345 1.00 0.00
E H
ATOM 17095 CB PHE E 217 -39.282 -43.238 60.287 1.00 0.00
E C
ATOM 17096 HB1 PHE E 217 -40.297 -43.679 60.391 1.00 0.00
E H
ATOM 17097 HB2 PHE E 217 -39.404 -42.252 59.787 1.00 0.00
E H
ATOM 17098 CG PHE E 217 -38.515 -44.159 59.389 1.00 0.00
E C
ATOM 17099 CD1 PHE E 217 -38.506 -45.522 59.620 1.00 0.00
E C
ATOM 17100 HD1 PHE E 217 -39.056 -45.927 60.459 1.00 0.00
E H
ATOM 17101 CE1 PHE E 217 -37.798 -46.372 58.798 1.00 0.00
E C
ATOM 17102 HE1 PHE E 217 -37.802 -47.436 58.988 1.00 0.00
E H
ATOM 17103 CZ PHE E 217 -37.091 -45.864 57.731 1.00 0.00
E C
ATOM 17104 HZ PHE E 217 -36.541 -46.527 57.079 1.00 0.00
E H
ATOM 17105 CD2 PHE E 217 -37.800 -43.662 58.315 1.00 0.00
E C
ATOM 17106 HD2 PHE E 217 -37.775 -42.597 58.129 1.00 0.00
E H
ATOM 17107 CE2 PHE E 217 -37.093 -44.507 57.489 1.00 0.00
E C
ATOM 17108 HE2 PHE E 217 -36.540 -44.103 56.653 1.00 0.00
E H
ATOM 17109 C PHE E 217 -37.144 -43.182 61.592 1.00 0.00
E C
ATOM 17110 O PHE E 217 -36.578 -44.254 61.826 1.00 0.00
E O
ATOM 17111 N GLU E 218 -36.470 -42.075 61.278 1.00 0.00
E N
ATOM 17112 HN GLU E 218 -36.879 -41.194 61.057 1.00 0.00
E H
ATOM 17113 CA GLU E 218 -35.013 -42.080 61.235 1.00 0.00
E C
ATOM 17114 HA GLU E 218 -34.700 -42.855 60.547 1.00 0.00
E H
ATOM 17115 CB GLU E 218 -34.506 -40.722 60.752 1.00 0.00
E C
ATOM 17116 HB1 GLU E 218 -35.199 -40.354 59.961 1.00 0.00
E H
ATOM 17117 HB2 GLU E 218 -34.567 -39.981 61.580 1.00 0.00
E H

```
ATOM  17118  CG  GLU E 218      -33.098 -40.740  60.190  1.00  0.00
E    C
ATOM  17119  HG1 GLU E 218      -32.868 -41.749  59.803  1.00  0.00
E    H
ATOM  17120  HG2 GLU E 218      -32.988 -40.001  59.371  1.00  0.00
E    H
ATOM  17121  CD  GLU E 218      -32.059 -40.452  61.256  1.00  0.00
E    C
ATOM  17122  OE1 GLU E 218      -32.453 -40.074  62.379  1.00  0.00
E    O
ATOM  17123  OE2 GLU E 218      -30.852 -40.596  60.976  1.00  0.00
E    O
ATOM  17124  C   GLU E 218      -34.419 -42.415  62.593  1.00  0.00
E    C
ATOM  17125  O   GLU E 218      -33.465 -43.195  62.692  1.00  0.00
E    O
ATOM  17126  N   ASN E 219      -34.972 -41.835  63.653  1.00  0.00
E    N
ATOM  17127  HN  ASN E 219      -35.785 -41.255  63.625  1.00  0.00
E    H
ATOM  17128  CA  ASN E 219      -34.405 -42.017  64.981  1.00  0.00
E    C
ATOM  17129  HA  ASN E 219      -33.331 -41.918  64.863  1.00  0.00
E    H
ATOM  17130  CB  ASN E 219      -34.963 -40.959  65.932  1.00  0.00
E    C
ATOM  17131  HB1 ASN E 219      -35.141 -40.033  65.344  1.00  0.00
E    H
ATOM  17132  HB2 ASN E 219      -35.940 -41.286  66.343  1.00  0.00
E    H
ATOM  17133  CG  ASN E 219      -34.006 -40.624  67.057  1.00  0.00
E    C
ATOM  17134  OD1 ASN E 219      -32.934 -41.217  67.174  1.00  0.00
E    O
ATOM  17135  ND2 ASN E 219      -34.390 -39.667  67.893  1.00  0.00
E    N
ATOM  17136 HD21 ASN E 219      -33.766 -39.427  68.632  1.00  0.00
E    H
ATOM  17137 HD22 ASN E 219      -35.256 -39.199  67.751  1.00  0.00
E    H
ATOM  17138  C   ASN E 219      -34.683 -43.416  65.506  1.00  0.00
E    C
ATOM  17139  O   ASN E 219      -33.823 -44.036  66.141  1.00  0.00
E    O
ATOM  17140  N   GLU E 220      -35.883 -43.932  65.252  1.00  0.00
E    N
ATOM  17141  HN  GLU E 220      -36.573 -43.519  64.665  1.00  0.00
E    H
ATOM  17142  CA  GLU E 220      -36.289 -45.200  65.843  1.00  0.00
E    C
ATOM  17143  HA  GLU E 220      -35.729 -45.323  66.761  1.00  0.00
E    H
ATOM  17144  CB  GLU E 220      -37.782 -45.146  66.175  1.00  0.00
E    C
```

ATOM 17145 HB1 GLU E 220 -38.352 -45.141 65.216 1.00 0.00
E H
ATOM 17146 HB2 GLU E 220 -38.085 -46.078 66.704 1.00 0.00
E H
ATOM 17147 CG GLU E 220 -38.149 -43.916 66.997 1.00 0.00
E C
ATOM 17148 HG1 GLU E 220 -38.171 -44.182 68.069 1.00 0.00
E H
ATOM 17149 HG2 GLU E 220 -37.416 -43.099 66.838 1.00 0.00
E H
ATOM 17150 CD GLU E 220 -39.531 -43.378 66.686 1.00 0.00
E C
ATOM 17151 OE1 GLU E 220 -40.042 -43.641 65.578 1.00 0.00
E O
ATOM 17152 OE2 GLU E 220 -40.099 -42.672 67.543 1.00 0.00
E O
ATOM 17153 C GLU E 220 -35.970 -46.401 64.953 1.00 0.00
E C
ATOM 17154 O GLU E 220 -35.255 -47.313 65.386 1.00 0.00
E O
ATOM 17155 N GLY E 221 -36.473 -46.424 63.721 1.00 0.00
E N
ATOM 17156 HN GLY E 221 -37.034 -45.684 63.351 1.00 0.00
E H
ATOM 17157 CA GLY E 221 -36.235 -47.539 62.823 1.00 0.00
E C
ATOM 17158 HA1 GLY E 221 -36.893 -47.398 61.977 1.00 0.00
E H
ATOM 17159 HA2 GLY E 221 -36.426 -48.440 63.389 1.00 0.00
E H
ATOM 17160 C GLY E 221 -34.813 -47.605 62.305 1.00 0.00
E C
ATOM 17161 O GLY E 221 -34.373 -48.644 61.806 1.00 0.00
E O
ATOM 17162 N MET E 222 -34.088 -46.497 62.407 1.00 0.00
E N
ATOM 17163 HN MET E 222 -34.432 -45.637 62.777 1.00 0.00
E H
ATOM 17164 CA MET E 222 -32.699 -46.453 61.974 1.00 0.00
E C
ATOM 17165 HA MET E 222 -32.377 -47.476 61.819 1.00 0.00
E H
ATOM 17166 CB MET E 222 -32.564 -45.651 60.682 1.00 0.00
E C
ATOM 17167 HB1 MET E 222 -32.857 -44.601 60.920 1.00 0.00
E H
ATOM 17168 HB2 MET E 222 -31.493 -45.615 60.381 1.00 0.00
E H
ATOM 17169 CG MET E 222 -33.425 -46.155 59.544 1.00 0.00
E C
ATOM 17170 HG1 MET E 222 -33.290 -47.250 59.413 1.00 0.00
E H
ATOM 17171 HG2 MET E 222 -34.497 -45.975 59.779 1.00 0.00
E H

ATOM 17172 SD MET E 222   -33.018 -45.348 57.986 1.00 0.00
E   S
ATOM 17173 CE MET E 222   -33.701 -43.717 58.252 1.00 0.00
E   C
ATOM 17174 HE1 MET E 222   -33.545 -43.071 57.361 1.00 0.00
E   H
ATOM 17175 HE2 MET E 222   -34.794 -43.769 58.447 1.00 0.00
E   H
ATOM 17176 HE3 MET E 222   -33.222 -43.219 59.122 1.00 0.00
E   H
ATOM 17177 C   MET E 222   -31.815 -45.852 63.058 1.00 0.00
E   C
ATOM 17178 O   MET E 222   -30.809 -46.442 63.445 1.00 0.00
E   O
ATOM 17179 N   ASN F   6   -23.916 -35.353 70.791 1.00 0.00
F   N
ATOM 17180 HT1 ASN F   6   -24.782 -35.314 71.289 1.00 0.00
F   H
ATOM 17181 HT2 ASN F   6   -23.326 -36.055 71.190 1.00 0.00
F   H
ATOM 17182 HT3 ASN F   6   -24.085 -35.557 69.827 1.00 0.00
F   H
ATOM 17183 CA ASN F   6   -23.251 -34.061 70.894 1.00 0.00
F   C
ATOM 17184 HA ASN F   6   -23.894 -33.346 70.393 1.00 0.00
F   H
ATOM 17185 CB ASN F   6   -23.046 -33.690 72.354 1.00 0.00
F   C
ATOM 17186 HB1 ASN F   6   -23.786 -34.259 72.957 1.00 0.00
F   H
ATOM 17187 HB2 ASN F   6   -22.035 -33.996 72.692 1.00 0.00
F   H
ATOM 17188 CG ASN F   6   -23.262 -32.228 72.602 1.00 0.00
F   C
ATOM 17189 OD1 ASN F   6   -24.008 -31.573 71.875 1.00 0.00
F   O
ATOM 17190 ND2 ASN F   6   -22.622 -31.701 73.634 1.00 0.00
F   N
ATOM 17191 HD21 ASN F   6   -22.766 -30.732 73.816 1.00 0.00
F   H
ATOM 17192 HD22 ASN F   6   -22.047 -32.274 74.210 1.00 0.00
F   H
ATOM 17193 C   ASN F   6   -21.919 -34.093 70.140 1.00 0.00
F   C
ATOM 17194 O   ASN F   6   -21.318 -35.155 70.003 1.00 0.00
F   O
ATOM 17195 N   LYS F   7   -21.454 -32.937 69.657 1.00 0.00
F   N
ATOM 17196 HN LYS F   7   -21.756 -32.042 69.982 1.00 0.00
F   H
ATOM 17197 CA LYS F   7   -20.452 -32.905 68.597 1.00 0.00
F   C
ATOM 17198 HA LYS F   7   -19.602 -33.484 68.938 1.00 0.00
F   H

ATOM 17199 CB LYS F 7 -21.026 -33.497 67.310 1.00 0.00
F C
ATOM 17200 HB1 LYS F 7 -20.273 -33.323 66.506 1.00 0.00
F H
ATOM 17201 HB2 LYS F 7 -21.112 -34.601 67.425 1.00 0.00
F H
ATOM 17202 CG LYS F 7 -22.352 -32.885 66.899 1.00 0.00
F C
ATOM 17203 HG1 LYS F 7 -23.077 -32.992 67.738 1.00 0.00
F H
ATOM 17204 HG2 LYS F 7 -22.208 -31.795 66.722 1.00 0.00
F H
ATOM 17205 CD LYS F 7 -22.920 -33.555 65.660 1.00 0.00
F C
ATOM 17206 HD1 LYS F 7 -23.696 -32.887 65.223 1.00 0.00
F H
ATOM 17207 HD2 LYS F 7 -22.100 -33.649 64.910 1.00 0.00
F H
ATOM 17208 CE LYS F 7 -23.503 -34.921 65.978 1.00 0.00
F C
ATOM 17209 HE1 LYS F 7 -22.697 -35.633 66.262 1.00 0.00
F H
ATOM 17210 HE2 LYS F 7 -24.225 -34.851 66.821 1.00 0.00
F H
ATOM 17211 NZ LYS F 7 -24.226 -35.499 64.809 1.00 0.00
F N
ATOM 17212 HZ1 LYS F 7 -24.613 -36.433 65.055 1.00 0.00
F H
ATOM 17213 HZ2 LYS F 7 -25.003 -34.865 64.533 1.00 0.00
F H
ATOM 17214 HZ3 LYS F 7 -23.567 -35.599 64.010 1.00 0.00
F H
ATOM 17215 C LYS F 7 -19.969 -31.481 68.362 1.00 0.00
F C
ATOM 17216 O LYS F 7 -20.768 -30.547 68.292 1.00 0.00
F O
ATOM 17217 N GLN F 8 -18.659 -31.316 68.207 1.00 0.00
F N
ATOM 17218 HN GLN F 8 -18.021 -32.069 68.053 1.00 0.00
F H
ATOM 17219 CA GLN F 8 -18.040 -30.000 68.250 1.00 0.00
F C
ATOM 17220 HA GLN F 8 -18.813 -29.298 68.538 1.00 0.00
F H
ATOM 17221 CB GLN F 8 -16.920 -29.975 69.282 1.00 0.00
F C
ATOM 17222 HB1 GLN F 8 -16.356 -30.929 69.167 1.00 0.00
F H
ATOM 17223 HB2 GLN F 8 -16.205 -29.159 69.030 1.00 0.00
F H
ATOM 17224 CG GLN F 8 -17.476 -29.842 70.679 1.00 0.00
F C
ATOM 17225 HG1 GLN F 8 -17.622 -28.771 70.938 1.00 0.00
F H

ATOM 17226 HG2 GLN F 8 −18.460 −30.349 70.760 1.00 0.00
F H
ATOM 17227 CD GLN F 8 −16.558 −30.408 71.728 1.00 0.00
F C
ATOM 17228 OE1 GLN F 8 −15.687 −31.220 71.429 1.00 0.00
F O
ATOM 17229 NE2 GLN F 8 −16.753 −29.989 72.972 1.00 0.00
F N
ATOM 17230 HE21 GLN F 8 −16.145 −30.357 73.670 1.00 0.00
F H
ATOM 17231 HE22 GLN F 8 −17.489 −29.351 73.170 1.00 0.00
F H
ATOM 17232 C GLN F 8 −17.528 −29.578 66.881 1.00 0.00
F C
ATOM 17233 O GLN F 8 −16.862 −30.353 66.179 1.00 0.00
F O
ATOM 17234 N PHE F 9 −17.836 −28.337 66.526 1.00 0.00
F N
ATOM 17235 HN PHE F 9 −18.249 −27.699 67.175 1.00 0.00
F H
ATOM 17236 CA PHE F 9 −17.615 −27.787 65.202 1.00 0.00
F C
ATOM 17237 HA PHE F 9 −17.055 −28.527 64.644 1.00 0.00
F H
ATOM 17238 CB PHE F 9 −18.934 −27.414 64.556 1.00 0.00
F C
ATOM 17239 HB1 PHE F 9 −19.423 −26.618 65.161 1.00 0.00
F H
ATOM 17240 HB2 PHE F 9 −18.778 −27.001 63.536 1.00 0.00
F H
ATOM 17241 CG PHE F 9 −19.900 −28.539 64.491 1.00 0.00
F C
ATOM 17242 CD1 PHE F 9 −19.843 −29.444 63.455 1.00 0.00
F C
ATOM 17243 HD1 PHE F 9 −19.097 −29.325 62.681 1.00 0.00
F H
ATOM 17244 CE1 PHE F 9 −20.724 −30.489 63.387 1.00 0.00
F C
ATOM 17245 HE1 PHE F 9 −20.664 −31.194 62.569 1.00 0.00
F H
ATOM 17246 CZ PHE F 9 −21.681 −30.644 64.365 1.00 0.00
F C
ATOM 17247 HZ PHE F 9 −22.374 −31.471 64.315 1.00 0.00
F H
ATOM 17248 CD2 PHE F 9 −20.858 −28.706 65.475 1.00 0.00
F C
ATOM 17249 HD2 PHE F 9 −20.926 −27.998 66.289 1.00 0.00
F H
ATOM 17250 CE2 PHE F 9 −21.748 −29.750 65.415 1.00 0.00
F C
ATOM 17251 HE2 PHE F 9 −22.496 −29.866 66.187 1.00 0.00
F H
ATOM 17252 C PHE F 9 −16.742 −26.551 65.304 1.00 0.00
F C

ATOM 17253 O   PHE F   9   -16.963 -25.689 66.160 1.00 0.00
F   O
ATOM 17254 N   ALA F 10   -15.758 -26.463 64.417 1.00 0.00
F   N
ATOM 17255 HN ALA F 10   -15.587 -27.174 63.736 1.00 0.00
F   H
ATOM 17256 CA ALA F 10   -14.855 -25.322 64.371 1.00 0.00
F   C
ATOM 17257 HA ALA F 10   -15.159 -24.543 65.061 1.00 0.00
F   H
ATOM 17258 CB ALA F 10   -13.427 -25.738 64.727 1.00 0.00
F   C
ATOM 17259 HB1 ALA F 10   -13.402 -26.136 65.763 1.00 0.00
F   H
ATOM 17260 HB2 ALA F 10   -13.061 -26.536 64.045 1.00 0.00
F   H
ATOM 17261 HB3 ALA F 10   -12.725 -24.877 64.674 1.00 0.00
F   H
ATOM 17262 C   ALA F 10   -14.909 -24.709 62.981 1.00 0.00
F   C
ATOM 17263 O   ALA F 10   -14.439 -25.314 62.013 1.00 0.00
F   O
ATOM 17264 N   VAL F 11   -15.483 -23.522 62.881 1.00 0.00
F   N
ATOM 17265 HN VAL F 11   -15.938 -23.059 63.637 1.00 0.00
F   H
ATOM 17266 CA VAL F 11   -15.493 -22.782 61.632 1.00 0.00
F   C
ATOM 17267 HA VAL F 11   -15.433 -23.475 60.803 1.00 0.00
F   H
ATOM 17268 CB VAL F 11   -16.745 -21.922 61.478 1.00 0.00
F   C
ATOM 17269 HB VAL F 11   -17.266 -21.904 62.467 1.00 0.00
F   H
ATOM 17270 CG1 VAL F 11   -16.383 -20.507 61.085 1.00 0.00
F   C
ATOM 17271 HG11 VAL F 11   -17.302 -19.900 60.938 1.00 0.00
F   H
ATOM 17272 HG12 VAL F 11   -15.778 -20.006 61.869 1.00 0.00
F   H
ATOM 17273 HG13 VAL F 11   -15.809 -20.498 60.134 1.00 0.00
F   H
ATOM 17274 CG2 VAL F 11   -17.646 -22.518 60.432 1.00 0.00
F   C
ATOM 17275 HG21 VAL F 11   -18.545 -21.883 60.279 1.00 0.00
F   H
ATOM 17276 HG22 VAL F 11   -17.112 -22.609 59.462 1.00 0.00
F   H
ATOM 17277 HG23 VAL F 11   -17.997 -23.528 60.732 1.00 0.00
F   H
ATOM 17278 C   VAL F 11   -14.231 -21.939 61.613 1.00 0.00
F   C
ATOM 17279 O   VAL F 11   -13.911 -21.245 62.581 1.00 0.00
F   O

```
ATOM  17280  N   ILE F  12     -13.501 -22.015  60.517  1.00  0.00
F    N
ATOM  17281  HN  ILE F  12     -13.677 -22.666  59.782  1.00  0.00
F    H
ATOM  17282  CA  ILE F  12     -12.364 -21.147  60.279  1.00  0.00
F    C
ATOM  17283  HA  ILE F  12     -12.363 -20.378  61.041  1.00  0.00
F    H
ATOM  17284  CB  ILE F  12     -11.052 -21.933  60.254  1.00  0.00
F    C
ATOM  17285  HB  ILE F  12     -11.165 -22.639  59.393  1.00  0.00
F    H
ATOM  17286  CG2 ILE F  12      -9.906 -20.978  60.192  1.00  0.00
F    C
ATOM  17287 HG21 ILE F  12      -8.954 -21.520  60.015  1.00  0.00
F    H
ATOM  17288 HG22 ILE F  12     -10.039 -20.253  59.362  1.00  0.00
F    H
ATOM  17289 HG23 ILE F  12      -9.810 -20.410  61.142  1.00  0.00
F    H
ATOM  17290  CG1 ILE F  12     -10.922 -22.782  61.507  1.00  0.00
F    C
ATOM  17291 HG11 ILE F  12     -11.745 -23.531  61.541  1.00  0.00
F    H
ATOM  17292 HG12 ILE F  12      -9.963 -23.348  61.461  1.00  0.00
F    H
ATOM  17293  CD  ILE F  12     -10.933 -21.984  62.760  1.00  0.00
F    C
ATOM  17294  HD1 ILE F  12     -10.777 -22.645  63.640  1.00  0.00
F    H
ATOM  17295  HD2 ILE F  12     -10.123 -21.224  62.748  1.00  0.00
F    H
ATOM  17296  HD3 ILE F  12     -11.905 -21.462  62.891  1.00  0.00
F    H
ATOM  17297  C   ILE F  12     -12.599 -20.452  58.955  1.00  0.00
F    C
ATOM  17298  O   ILE F  12     -12.974 -21.090  57.965  1.00  0.00
F    O
ATOM  17299  N   GLY F  13     -12.412 -19.145  58.944  1.00  0.00
F    N
ATOM  17300  HN  GLY F  13     -12.117 -18.631  59.749  1.00  0.00
F    H
ATOM  17301  CA  GLY F  13     -12.624 -18.367  57.751  1.00  0.00
F    C
ATOM  17302  HA1 GLY F  13     -12.547 -19.010  56.885  1.00  0.00
F    H
ATOM  17303  HA2 GLY F  13     -11.946 -17.525  57.741  1.00  0.00
F    H
ATOM  17304  C   GLY F  13     -14.030 -17.828  57.808  1.00  0.00
F    C
ATOM  17305  O   GLY F  13     -14.989 -18.556  57.541  1.00  0.00
F    O
ATOM  17306  N   LEU F  14     -14.173 -16.561  58.165  1.00  0.00
F    N
```

```
ATOM  17307  HN  LEU F  14      -13.429 -15.912  58.310  1.00  0.00
F    H
ATOM  17308  CA  LEU F  14      -15.501 -16.006  58.378  1.00  0.00
F    C
ATOM  17309  HA  LEU F  14      -16.194 -16.837  58.376  1.00  0.00
F    H
ATOM  17310  CB  LEU F  14      -15.549 -15.292  59.715  1.00  0.00
F    C
ATOM  17311  HB1 LEU F  14      -14.502 -15.222  60.089  1.00  0.00
F    H
ATOM  17312  HB2 LEU F  14      -15.889 -14.244  59.554  1.00  0.00
F    H
ATOM  17313  CG  LEU F  14      -16.413 -16.018  60.723  1.00  0.00
F    C
ATOM  17314  HG  LEU F  14      -16.776 -16.972  60.269  1.00  0.00
F    H
ATOM  17315  CD1 LEU F  14      -15.598 -16.332  61.949  1.00  0.00
F    C
ATOM  17316 HD11 LEU F  14      -16.228 -16.824  62.721  1.00  0.00
F    H
ATOM  17317 HD12 LEU F  14      -14.758 -17.014  61.697  1.00  0.00
F    H
ATOM  17318 HD13 LEU F  14      -15.175 -15.402  62.386  1.00  0.00
F    H
ATOM  17319  CD2 LEU F  14      -17.627 -15.166  61.062  1.00  0.00
F    C
ATOM  17320 HD21 LEU F  14      -18.281 -15.696  61.789  1.00  0.00
F    H
ATOM  17321 HD22 LEU F  14      -17.334 -14.198  61.517  1.00  0.00
F    H
ATOM  17322 HD23 LEU F  14      -18.223 -14.956  60.148  1.00  0.00
F    H
ATOM  17323  C   LEU F  14      -15.889 -15.079  57.229  1.00  0.00
F    C
ATOM  17324  O   LEU F  14      -16.150 -13.893  57.405  1.00  0.00
F    O
ATOM  17325  N   GLY F  15      -15.920 -15.648  56.030  1.00  0.00
F    N
ATOM  17326  HN  GLY F  15      -15.581 -16.570  55.852  1.00  0.00
F    H
ATOM  17327  CA  GLY F  15      -16.462 -14.947  54.885  1.00  0.00
F    C
ATOM  17328  HA1 GLY F  15      -16.046 -15.386  53.988  1.00  0.00
F    H
ATOM  17329  HA2 GLY F  15      -16.259 -13.890  54.999  1.00  0.00
F    H
ATOM  17330  C   GLY F  15      -17.962 -15.132  54.843  1.00  0.00
F    C
ATOM  17331  O   GLY F  15      -18.503 -15.868  55.675  1.00  0.00
F    O
ATOM  17332  N   ARG F  16      -18.643 -14.474  53.898  1.00  0.00
F    N
ATOM  17333  HN  ARG F  16      -18.208 -13.841  53.261  1.00  0.00
F    H
```

ATOM 17334 CA ARG F 16   -20.081 -14.660 53.756 1.00 0.00
F   C
ATOM 17335 HA ARG F 16   -20.573 -14.150 54.575 1.00 0.00
F   H
ATOM 17336 CB ARG F 16   -20.566 -14.116 52.417 1.00 0.00
F   C
ATOM 17337 HB1 ARG F 16   -20.058 -14.721 51.629 1.00 0.00
F   H
ATOM 17338 HB2 ARG F 16   -21.655 -14.319 52.305 1.00 0.00
F   H
ATOM 17339 CG ARG F 16   -20.254 -12.667 52.188 1.00 0.00
F   C
ATOM 17340 HG1 ARG F 16   -21.050 -12.054 52.665 1.00 0.00
F   H
ATOM 17341 HG2 ARG F 16   -19.314 -12.409 52.729 1.00 0.00
F   H
ATOM 17342 CD ARG F 16   -20.075 -12.374 50.711 1.00 0.00
F   C
ATOM 17343 HD1 ARG F 16   -20.891 -12.867 50.134 1.00 0.00
F   H
ATOM 17344 HD2 ARG F 16   -20.104 -11.279 50.505 1.00 0.00
F   H
ATOM 17345 NE ARG F 16   -18.792 -12.851 50.204 1.00 0.00
F   N
ATOM 17346 HE ARG F 16   -18.266 -13.371 50.887 1.00 0.00
F   H
ATOM 17347 CZ ARG F 16   -18.351 -12.629 48.971 1.00 0.00
F   C
ATOM 17348 NH1 ARG F 16   -19.091 -11.932 48.120 1.00 0.00
F   N
ATOM 17349 HH11 ARG F 16   -18.785 -11.781 47.190 1.00 0.00
F   H
ATOM 17350 HH12 ARG F 16   -20.015 -11.693 48.427 1.00 0.00
F   H
ATOM 17351 NH2 ARG F 16   -17.170 -13.094 48.589 1.00 0.00
F   N
ATOM 17352 HH21 ARG F 16   -16.810 -12.852 47.699 1.00 0.00
F   H
ATOM 17353 HH22 ARG F 16   -16.598 -13.496 49.291 1.00 0.00
F   H
ATOM 17354 C   ARG F 16   -20.459 -16.120 53.867 1.00 0.00
F   C
ATOM 17355 O   ARG F 16   -21.406 -16.468 54.582 1.00 0.00
F   O
ATOM 17356 N   PHE F 17   -19.701 -16.984 53.205 1.00 0.00
F   N
ATOM 17357 HN PHE F 17   -18.963 -16.734 52.580 1.00 0.00
F   H
ATOM 17358 CA PHE F 17   -19.906 -18.412 53.352 1.00 0.00
F   C
ATOM 17359 HA PHE F 17   -20.916 -18.614 53.017 1.00 0.00
F   H
ATOM 17360 CB PHE F 17   -18.870 -19.162 52.525 1.00 0.00
F   C

```
ATOM  17361  HB1 PHE F  17      -18.947 -18.838  51.464  1.00  0.00
F    H
ATOM  17362  HB2 PHE F  17      -17.838 -18.925  52.865  1.00  0.00
F    H
ATOM  17363  CG  PHE F  17      -19.062 -20.635  52.524  1.00  0.00
F    C
ATOM  17364  CD1 PHE F  17      -20.296 -21.176  52.222  1.00  0.00
F    C
ATOM  17365  HD1 PHE F  17      -21.126 -20.521  51.997  1.00  0.00
F    H
ATOM  17366  CE1 PHE F  17      -20.482 -22.534  52.211  1.00  0.00
F    C
ATOM  17367  HE1 PHE F  17      -21.453 -22.944  51.973  1.00  0.00
F    H
ATOM  17368  CZ  PHE F  17      -19.427 -23.368  52.498  1.00  0.00
F    C
ATOM  17369  HZ  PHE F  17      -19.566 -24.439  52.482  1.00  0.00
F    H
ATOM  17370  CD2 PHE F  17      -18.010 -21.482  52.808  1.00  0.00
F    C
ATOM  17371  HD2 PHE F  17      -17.041 -21.075  53.062  1.00  0.00
F    H
ATOM  17372  CE2 PHE F  17      -18.189 -22.840  52.796  1.00  0.00
F    C
ATOM  17373  HE2 PHE F  17      -17.358 -23.494  53.022  1.00  0.00
F    H
ATOM  17374  C   PHE F  17      -19.833 -18.814  54.815  1.00  0.00
F    C
ATOM  17375  O   PHE F  17      -20.787 -19.375  55.363  1.00  0.00
F    O
ATOM  17376  N   GLY F  18      -18.710 -18.512  55.468  1.00  0.00
F    N
ATOM  17377  HN  GLY F  18      -17.930 -18.035  55.067  1.00  0.00
F    H
ATOM  17378  CA  GLY F  18      -18.567 -18.885  56.863  1.00  0.00
F    C
ATOM  17379  HA1 GLY F  18      -17.660 -18.421  57.224  1.00  0.00
F    H
ATOM  17380  HA2 GLY F  18      -18.562 -19.965  56.909  1.00  0.00
F    H
ATOM  17381  C   GLY F  18      -19.718 -18.386  57.712  1.00  0.00
F    C
ATOM  17382  O   GLY F  18      -20.430 -19.174  58.345  1.00  0.00
F    O
ATOM  17383  N   GLY F  19      -19.943 -17.072  57.692  1.00  0.00
F    N
ATOM  17384  HN  GLY F  19      -19.438 -16.433  57.114  1.00  0.00
F    H
ATOM  17385  CA  GLY F  19      -20.965 -16.481  58.535  1.00  0.00
F    C
ATOM  17386  HA1 GLY F  19      -21.049 -15.444  58.238  1.00  0.00
F    H
ATOM  17387  HA2 GLY F  19      -20.649 -16.624  59.559  1.00  0.00
F    H
```

```
ATOM  17388  C   GLY F  19      -22.321 -17.126  58.371  1.00  0.00
F    C
ATOM  17389  O   GLY F  19      -23.041 -17.330  59.351  1.00  0.00
F    O
ATOM  17390  N   SER F  20      -22.683 -17.464  57.140  1.00  0.00
F    N
ATOM  17391  HN  SER F  20      -22.163 -17.254  56.313  1.00  0.00
F    H
ATOM  17392  CA  SER F  20      -23.913 -18.194  56.900  1.00  0.00
F    C
ATOM  17393  HA  SER F  20      -24.713 -17.553  57.246  1.00  0.00
F    H
ATOM  17394  CB  SER F  20      -23.993 -18.566  55.429  1.00  0.00
F    C
ATOM  17395  HB1 SER F  20      -23.081 -19.151  55.170  1.00  0.00
F    H
ATOM  17396  HB2 SER F  20      -24.870 -19.226  55.243  1.00  0.00
F    H
ATOM  17397  OG  SER F  20      -24.038 -17.401  54.649  1.00  0.00
F    O
ATOM  17398  HG1 SER F  20      -23.863 -17.665  53.736  1.00  0.00
F    H
ATOM  17399  C   SER F  20      -23.966 -19.462  57.720  1.00  0.00
F    C
ATOM  17400  O   SER F  20      -24.910 -19.670  58.495  1.00  0.00
F    O
ATOM  17401  N   ILE F  21      -22.955 -20.302  57.537  1.00  0.00
F    N
ATOM  17402  HN  ILE F  21      -22.186 -20.130  56.924  1.00  0.00
F    H
ATOM  17403  CA  ILE F  21      -22.885 -21.574  58.234  1.00  0.00
F    C
ATOM  17404  HA  ILE F  21      -23.730 -22.161  57.896  1.00  0.00
F    H
ATOM  17405  CB  ILE F  21      -21.544 -22.249  57.931  1.00  0.00
F    C
ATOM  17406  HB  ILE F  21      -20.784 -21.598  58.434  1.00  0.00
F    H
ATOM  17407  CG2 ILE F  21      -21.553 -23.665  58.414  1.00  0.00
F    C
ATOM  17408 HG21 ILE F  21      -20.539 -24.110  58.340  1.00  0.00
F    H
ATOM  17409 HG22 ILE F  21      -21.868 -23.719  59.477  1.00  0.00
F    H
ATOM  17410 HG23 ILE F  21      -22.249 -24.286  57.811  1.00  0.00
F    H
ATOM  17411  CG1 ILE F  21      -21.277 -22.185  56.437  1.00  0.00
F    C
ATOM  17412 HG11 ILE F  21      -22.065 -22.748  55.888  1.00  0.00
F    H
ATOM  17413 HG12 ILE F  21      -21.338 -21.123  56.105  1.00  0.00
F    H
ATOM  17414  CD  ILE F  21      -19.936 -22.717  56.036  1.00  0.00
F    C
```

```
ATOM  17415  HD1 ILE F  21      -19.781 -22.587  54.942  1.00  0.00
F    H
ATOM  17416  HD2 ILE F  21      -19.124 -22.176  56.568  1.00  0.00
F    H
ATOM  17417  HD3 ILE F  21      -19.851 -23.799  56.269  1.00  0.00
F    H
ATOM  17418  C   ILE F  21      -23.062 -21.370  59.722  1.00  0.00
F    C
ATOM  17419  O   ILE F  21      -23.907 -22.008  60.361  1.00  0.00
F    O
ATOM  17420  N   VAL F  22      -22.254 -20.488  60.300  1.00  0.00
F    N
ATOM  17421  HN  VAL F  22      -21.635 -19.854  59.845  1.00  0.00
F    H
ATOM  17422  CA  VAL F  22      -22.239 -20.405  61.753  1.00  0.00
F    C
ATOM  17423  HA  VAL F  22      -22.159 -21.415  62.136  1.00  0.00
F    H
ATOM  17424  CB  VAL F  22      -21.034 -19.592  62.242  1.00  0.00
F    C
ATOM  17425  HB  VAL F  22      -21.294 -19.173  63.245  1.00  0.00
F    H
ATOM  17426  CG1 VAL F  22      -19.832 -20.479  62.370  1.00  0.00
F    C
ATOM  17427 HG11 VAL F  22      -18.947 -19.885  62.686  1.00  0.00
F    H
ATOM  17428 HG12 VAL F  22      -19.987 -21.272  63.129  1.00  0.00
F    H
ATOM  17429 HG13 VAL F  22      -19.593 -20.959  61.396  1.00  0.00
F    H
ATOM  17430  CG2 VAL F  22      -20.757 -18.473  61.312  1.00  0.00
F    C
ATOM  17431 HG21 VAL F  22      -19.861 -17.903  61.639  1.00  0.00
F    H
ATOM  17432 HG22 VAL F  22      -20.574 -18.855  60.284  1.00  0.00
F    H
ATOM  17433 HG23 VAL F  22      -21.610 -17.763  61.274  1.00  0.00
F    H
ATOM  17434  C   VAL F  22      -23.539 -19.823  62.273  1.00  0.00
F    C
ATOM  17435  O   VAL F  22      -24.076 -20.290  63.280  1.00  0.00
F    O
ATOM  17436  N   LYS F  23      -24.064 -18.792  61.616  1.00  0.00
F    N
ATOM  17437  HN  LYS F  23      -23.697 -18.388  60.779  1.00  0.00
F    H
ATOM  17438  CA  LYS F  23      -25.274 -18.167  62.122  1.00  0.00
F    C
ATOM  17439  HA  LYS F  23      -25.112 -17.942  63.169  1.00  0.00
F    H
ATOM  17440  CB  LYS F  23      -25.562 -16.888  61.346  1.00  0.00
F    C
ATOM  17441  HB1 LYS F  23      -24.593 -16.348  61.232  1.00  0.00
F    H
```

ATOM 17442 HB2 LYS F 23 -25.894 -17.154 60.318 1.00 0.00
F H
ATOM 17443 CG LYS F 23 -26.564 -15.980 62.020 1.00 0.00
F C
ATOM 17444 HG1 LYS F 23 -27.024 -16.519 62.880 1.00 0.00
F H
ATOM 17445 HG2 LYS F 23 -26.029 -15.092 62.432 1.00 0.00
F H
ATOM 17446 CD LYS F 23 -27.657 -15.541 61.071 1.00 0.00
F C
ATOM 17447 HD1 LYS F 23 -27.353 -15.810 60.034 1.00 0.00
F H
ATOM 17448 HD2 LYS F 23 -28.577 -16.123 61.310 1.00 0.00
F H
ATOM 17449 CE LYS F 23 -27.901 -14.042 61.213 1.00 0.00
F C
ATOM 17450 HE1 LYS F 23 -28.227 -13.796 62.248 1.00 0.00
F H
ATOM 17451 HE2 LYS F 23 -26.972 -13.473 60.992 1.00 0.00
F H
ATOM 17452 NZ LYS F 23 -28.954 -13.530 60.294 1.00 0.00
F N
ATOM 17453 HZ1 LYS F 23 -29.080 -12.507 60.432 1.00 0.00
F H
ATOM 17454 HZ2 LYS F 23 -28.675 -13.713 59.309 1.00 0.00
F H
ATOM 17455 HZ3 LYS F 23 -29.852 -14.018 60.491 1.00 0.00
F H
ATOM 17456 C LYS F 23 -26.438 -19.134 62.041 1.00 0.00
F C
ATOM 17457 O LYS F 23 -27.266 -19.197 62.958 1.00 0.00
F O
ATOM 17458 N GLU F 24 -26.485 -19.941 60.982 1.00 0.00
F N
ATOM 17459 HN GLU F 24 -25.873 -19.924 60.197 1.00 0.00
F H
ATOM 17460 CA GLU F 24 -27.510 -20.969 60.908 1.00 0.00
F C
ATOM 17461 HA GLU F 24 -28.477 -20.501 61.046 1.00 0.00
F H
ATOM 17462 CB GLU F 24 -27.435 -21.670 59.551 1.00 0.00
F C
ATOM 17463 HB1 GLU F 24 -27.462 -20.890 58.755 1.00 0.00
F H
ATOM 17464 HB2 GLU F 24 -26.451 -22.180 59.443 1.00 0.00
F H
ATOM 17465 CG GLU F 24 -28.536 -22.679 59.246 1.00 0.00
F C
ATOM 17466 HG1 GLU F 24 -29.513 -22.164 59.231 1.00 0.00
F H
ATOM 17467 HG2 GLU F 24 -28.366 -23.167 58.266 1.00 0.00
F H
ATOM 17468 CD GLU F 24 -28.693 -23.778 60.280 1.00 0.00
F C

ATOM 17469 OE1 GLU F 24 -27.726 -24.505 60.565 1.00 0.00
F O
ATOM 17470 OE2 GLU F 24 -29.804 -23.887 60.830 1.00 0.00
F O
ATOM 17471 C GLU F 24 -27.333 -21.979 62.036 1.00 0.00
F C
ATOM 17472 O GLU F 24 -28.258 -22.233 62.814 1.00 0.00
F O
ATOM 17473 N LEU F 25 -26.158 -22.598 62.108 1.00 0.00
F N
ATOM 17474 HN LEU F 25 -25.372 -22.403 61.526 1.00 0.00
F H
ATOM 17475 CA LEU F 25 -25.929 -23.653 63.087 1.00 0.00
F C
ATOM 17476 HA LEU F 25 -26.649 -24.434 62.879 1.00 0.00
F H
ATOM 17477 CB LEU F 25 -24.503 -24.165 62.941 1.00 0.00
F C
ATOM 17478 HB1 LEU F 25 -24.015 -23.558 62.144 1.00 0.00
F H
ATOM 17479 HB2 LEU F 25 -23.944 -23.949 63.878 1.00 0.00
F H
ATOM 17480 CG LEU F 25 -24.423 -25.635 62.577 1.00 0.00
F C
ATOM 17481 HG LEU F 25 -25.421 -25.999 62.233 1.00 0.00
F H
ATOM 17482 CD1 LEU F 25 -23.402 -25.844 61.503 1.00 0.00
F C
ATOM 17483 HD11 LEU F 25 -23.302 -26.925 61.265 1.00 0.00
F H
ATOM 17484 HD12 LEU F 25 -23.694 -25.306 60.576 1.00 0.00
F H
ATOM 17485 HD13 LEU F 25 -22.409 -25.468 61.828 1.00 0.00
F H
ATOM 17486 CD2 LEU F 25 -24.037 -26.421 63.806 1.00 0.00
F C
ATOM 17487 HD21 LEU F 25 -23.989 -27.506 63.571 1.00 0.00
F H
ATOM 17488 HD22 LEU F 25 -23.043 -26.117 64.192 1.00 0.00
F H
ATOM 17489 HD23 LEU F 25 -24.785 -26.273 64.614 1.00 0.00
F H
ATOM 17490 C LEU F 25 -26.192 -23.153 64.495 1.00 0.00
F C
ATOM 17491 O LEU F 25 -26.738 -23.874 65.337 1.00 0.00
F O
ATOM 17492 N HSE F 26 -25.799 -21.915 64.759 1.00 0.00
F N
ATOM 17493 HN HSE F 26 -25.237 -21.375 64.135 1.00 0.00
F H
ATOM 17494 CA HSE F 26 -26.153 -21.232 65.988 1.00 0.00
F C
ATOM 17495 HA HSE F 26 -25.752 -21.817 66.806 1.00 0.00
F H

ATOM 17496 CB HSE F 26 -25.575 -19.824 65.930 1.00 0.00
F C
ATOM 17497 HB1 HSE F 26 -24.488 -19.910 65.716 1.00 0.00
F H
ATOM 17498 HB2 HSE F 26 -26.031 -19.271 65.082 1.00 0.00
F H
ATOM 17499 ND1 HSE F 26 -26.715 -18.100 67.357 1.00 0.00
F N
ATOM 17500 CG HSE F 26 -25.729 -19.045 67.189 1.00 0.00
F C
ATOM 17501 CE1 HSE F 26 -26.596 -17.557 68.555 1.00 0.00
F C
ATOM 17502 HE1 HSE F 26 -27.255 -16.780 68.952 1.00 0.00
F H
ATOM 17503 NE2 HSE F 26 -25.559 -18.105 69.160 1.00 0.00
F N
ATOM 17504 HE2 HSE F 26 -25.230 -17.860 70.072 1.00 0.00
F H
ATOM 17505 CD2 HSE F 26 -24.995 -19.036 68.326 1.00 0.00
F C
ATOM 17506 HD2 HSE F 26 -24.118 -19.611 68.590 1.00 0.00
F H
ATOM 17507 C HSE F 26 -27.658 -21.193 66.165 1.00 0.00
F C
ATOM 17508 O HSE F 26 -28.169 -21.457 67.257 1.00 0.00
F O
ATOM 17509 N ARG F 27 -28.384 -20.866 65.098 1.00 0.00
F N
ATOM 17510 HN ARG F 27 -27.996 -20.560 64.232 1.00 0.00
F H
ATOM 17511 CA ARG F 27 -29.833 -20.949 65.171 1.00 0.00
F C
ATOM 17512 HA ARG F 27 -30.171 -20.345 66.005 1.00 0.00
F H
ATOM 17513 CB ARG F 27 -30.457 -20.434 63.879 1.00 0.00
F C
ATOM 17514 HB1 ARG F 27 -29.868 -19.534 63.582 1.00 0.00
F H
ATOM 17515 HB2 ARG F 27 -30.304 -21.181 63.069 1.00 0.00
F H
ATOM 17516 CG ARG F 27 -31.908 -20.052 64.023 1.00 0.00
F C
ATOM 17517 HG1 ARG F 27 -32.257 -20.364 65.032 1.00 0.00
F H
ATOM 17518 HG2 ARG F 27 -31.992 -18.941 64.008 1.00 0.00
F H
ATOM 17519 CD ARG F 27 -32.743 -20.628 62.898 1.00 0.00
F C
ATOM 17520 HD1 ARG F 27 -33.820 -20.597 63.183 1.00 0.00
F H
ATOM 17521 HD2 ARG F 27 -32.607 -20.053 61.952 1.00 0.00
F H
ATOM 17522 NE ARG F 27 -32.386 -22.007 62.586 1.00 0.00
F N

ATOM 17523 HE ARG F 27   -31.928 -22.105 61.695 1.00 0.00
F   H
ATOM 17524 CZ ARG F 27   -32.630 -23.044 63.380 1.00 0.00
F   C
ATOM 17525 NH1 ARG F 27   -33.224 -22.869 64.550 1.00 0.00
F   N
ATOM 17526 HH11 ARG F 27   -33.384 -23.637 65.155 1.00 0.00
F   H
ATOM 17527 HH12 ARG F 27   -33.370 -21.919 64.836 1.00 0.00
F   H
ATOM 17528 NH2 ARG F 27   -32.278 -24.260 63.005 1.00 0.00
F   N
ATOM 17529 HH21 ARG F 27   -32.537 -25.041 63.556 1.00 0.00
F   H
ATOM 17530 HH22 ARG F 27   -31.948 -24.365 62.076 1.00 0.00
F   H
ATOM 17531 C   ARG F 27   -30.282 -22.373 65.456 1.00 0.00
F   C
ATOM 17532 O   ARG F 27   -31.319 -22.582 66.092 1.00 0.00
F   O
ATOM 17533 N   MET F 28   -29.517 -23.368 65.007 1.00 0.00
F   N
ATOM 17534 HN MET F 28   -28.715 -23.290 64.418 1.00 0.00
F   H
ATOM 17535 CA MET F 28   -29.847 -24.734 65.393 1.00 0.00
F   C
ATOM 17536 HA MET F 28   -30.922 -24.828 65.300 1.00 0.00
F   H
ATOM 17537 CB MET F 28   -29.151 -25.754 64.502 1.00 0.00
F   C
ATOM 17538 HB1 MET F 28   -28.061 -25.516 64.526 1.00 0.00
F   H
ATOM 17539 HB2 MET F 28   -29.258 -26.768 64.948 1.00 0.00
F   H
ATOM 17540 CG MET F 28   -29.614 -25.747 63.089 1.00 0.00
F   C
ATOM 17541 HG1 MET F 28   -30.721 -25.834 63.045 1.00 0.00
F   H
ATOM 17542 HG2 MET F 28   -29.334 -24.784 62.608 1.00 0.00
F   H
ATOM 17543 SD MET F 28   -28.926 -27.086 62.123 1.00 0.00
F   S
ATOM 17544 CE MET F 28   -29.862 -28.445 62.810 1.00 0.00
F   C
ATOM 17545 HE1 MET F 28   -29.570 -29.407 62.337 1.00 0.00
F   H
ATOM 17546 HE2 MET F 28   -29.688 -28.535 63.904 1.00 0.00
F   H
ATOM 17547 HE3 MET F 28   -30.952 -28.302 62.648 1.00 0.00
F   H
ATOM 17548 C   MET F 28   -29.484 -25.033 66.833 1.00 0.00
F   C
ATOM 17549 O   MET F 28   -29.727 -26.152 67.293 1.00 0.00
F   O

ATOM 17550 N GLY F 29 -28.900 -24.078 67.545 1.00 0.00
F N
ATOM 17551 HN GLY F 29 -28.667 -23.176 67.182 1.00 0.00
F H
ATOM 17552 CA GLY F 29 -28.559 -24.301 68.932 1.00 0.00
F C
ATOM 17553 HA1 GLY F 29 -29.439 -24.698 69.419 1.00 0.00
F H
ATOM 17554 HA2 GLY F 29 -28.214 -23.359 69.337 1.00 0.00
F H
ATOM 17555 C GLY F 29 -27.445 -25.310 69.082 1.00 0.00
F C
ATOM 17556 O GLY F 29 -27.606 -26.327 69.760 1.00 0.00
F O
ATOM 17557 N HSE F 30 -26.317 -25.053 68.439 1.00 0.00
F N
ATOM 17558 HN HSE F 30 -26.159 -24.248 67.869 1.00 0.00
F H
ATOM 17559 CA HSE F 30 -25.189 -25.958 68.519 1.00 0.00
F C
ATOM 17560 HA HSE F 30 -25.471 -26.760 69.190 1.00 0.00
F H
ATOM 17561 CB HSE F 30 -24.846 -26.507 67.142 1.00 0.00
F C
ATOM 17562 HB1 HSE F 30 -24.843 -25.657 66.426 1.00 0.00
F H
ATOM 17563 HB2 HSE F 30 -23.817 -26.924 67.155 1.00 0.00
F H
ATOM 17564 ND1 HSE F 30 -25.438 -28.803 66.298 1.00 0.00
F N
ATOM 17565 CG HSE F 30 -25.816 -27.526 66.648 1.00 0.00
F C
ATOM 17566 CE1 HSE F 30 -26.501 -29.480 65.902 1.00 0.00
F C
ATOM 17567 HE1 HSE F 30 -26.484 -30.516 65.557 1.00 0.00
F H
ATOM 17568 NE2 HSE F 30 -27.555 -28.689 65.993 1.00 0.00
F N
ATOM 17569 HE2 HSE F 30 -28.488 -28.943 65.739 1.00 0.00
F H
ATOM 17570 CD2 HSE F 30 -27.154 -27.464 66.463 1.00 0.00
F C
ATOM 17571 HD2 HSE F 30 -27.839 -26.643 66.621 1.00 0.00
F H
ATOM 17572 C HSE F 30 -23.983 -25.264 69.134 1.00 0.00
F C
ATOM 17573 O HSE F 30 -23.973 -24.058 69.384 1.00 0.00
F O
ATOM 17574 N GLU F 31 -22.953 -26.059 69.377 1.00 0.00
F N
ATOM 17575 HN GLU F 31 -22.975 -27.037 69.194 1.00 0.00
F H
ATOM 17576 CA GLU F 31 -21.695 -25.590 69.933 1.00 0.00
F C

ATOM 17577 HA GLU F 31   -21.851 -24.650 70.448 1.00 0.00
F   H
ATOM 17578 CB GLU F 31   -21.136 -26.623 70.909 1.00 0.00
F   C
ATOM 17579 HB1 GLU F 31   -20.165 -26.241 71.301 1.00 0.00
F   H
ATOM 17580 HB2 GLU F 31   -21.808 -26.709 71.793 1.00 0.00
F   H
ATOM 17581 CG GLU F 31   -20.896 -28.025 70.332 1.00 0.00
F   C
ATOM 17582 HG1 GLU F 31   -20.401 -27.938 69.347 1.00 0.00
F   H
ATOM 17583 HG2 GLU F 31   -20.259 -28.630 71.008 1.00 0.00
F   H
ATOM 17584 CD GLU F 31   -22.179 -28.808 70.068 1.00 0.00
F   C
ATOM 17585 OE1 GLU F 31   -22.274 -29.964 70.531 1.00 0.00
F   O
ATOM 17586 OE2 GLU F 31   -23.076 -28.293 69.366 1.00 0.00
F   O
ATOM 17587 C   GLU F 31   -20.745 -25.317 68.775 1.00 0.00
F   C
ATOM 17588 O   GLU F 31   -20.299 -26.243 68.091 1.00 0.00
F   O
ATOM 17589 N   VAL F 32   -20.465 -24.045 68.526 1.00 0.00
F   N
ATOM 17590 HN VAL F 32   -20.792 -23.288 69.086 1.00 0.00
F   H
ATOM 17591 CA VAL F 32   -19.645 -23.644 67.394 1.00 0.00
F   C
ATOM 17592 HA VAL F 32   -19.088 -24.509 67.053 1.00 0.00
F   H
ATOM 17593 CB VAL F 32   -20.500 -23.107 66.233 1.00 0.00
F   C
ATOM 17594 HB VAL F 32   -19.802 -22.729 65.446 1.00 0.00
F   H
ATOM 17595 CG1 VAL F 32   -21.352 -24.222 65.663 1.00 0.00
F   C
ATOM 17596 HG11 VAL F 32   -22.002 -23.834 64.849 1.00 0.00
F   H
ATOM 17597 HG12 VAL F 32   -20.729 -25.033 65.229 1.00 0.00
F   H
ATOM 17598 HG13 VAL F 32   -22.007 -24.657 66.448 1.00 0.00
F   H
ATOM 17599 CG2 VAL F 32   -21.392 -21.981 66.703 1.00 0.00
F   C
ATOM 17600 HG21 VAL F 32   -22.037 -21.619 65.875 1.00 0.00
F   H
ATOM 17601 HG22 VAL F 32   -22.046 -22.322 67.534 1.00 0.00
F   H
ATOM 17602 HG23 VAL F 32   -20.793 -21.118 67.063 1.00 0.00
F   H
ATOM 17603 C   VAL F 32   -18.637 -22.607 67.856 1.00 0.00
F   C

```
ATOM  17604  O   VAL F  32     -19.005 -21.575  68.427  1.00  0.00
F    O
ATOM  17605  N   LEU F  33     -17.365 -22.890  67.621  1.00  0.00
F    N
ATOM  17606  HN  LEU F  33     -17.057 -23.760  67.241  1.00  0.00
F    H
ATOM  17607  CA  LEU F  33     -16.297 -21.948  67.898  1.00  0.00
F    C
ATOM  17608  HA  LEU F  33     -16.640 -21.268  68.666  1.00  0.00
F    H
ATOM  17609  CB  LEU F  33     -15.024 -22.664  68.329  1.00  0.00
F    C
ATOM  17610  HB1 LEU F  33     -15.162 -22.970  69.392  1.00  0.00
F    H
ATOM  17611  HB2 LEU F  33     -14.924 -23.608  67.747  1.00  0.00
F    H
ATOM  17612  CG  LEU F  33     -13.789 -21.775  68.195  1.00  0.00
F    C
ATOM  17613  HG  LEU F  33     -13.729 -21.452  67.127  1.00  0.00
F    H
ATOM  17614  CD1 LEU F  33     -13.908 -20.547  69.091  1.00  0.00
F    C
ATOM  17615 HD11 LEU F  33     -12.988 -19.928  69.027  1.00  0.00
F    H
ATOM  17616 HD12 LEU F  33     -14.773 -19.920  68.783  1.00  0.00
F    H
ATOM  17617 HD13 LEU F  33     -14.052 -20.848  70.151  1.00  0.00
F    H
ATOM  17618  CD2 LEU F  33     -12.537 -22.564  68.518  1.00  0.00
F    C
ATOM  17619 HD21 LEU F  33     -11.636 -21.923  68.403  1.00  0.00
F    H
ATOM  17620 HD22 LEU F  33     -12.549 -22.943  69.560  1.00  0.00
F    H
ATOM  17621 HD23 LEU F  33     -12.435 -23.432  67.831  1.00  0.00
F    H
ATOM  17622  C   LEU F  33     -16.045 -21.130  66.642  1.00  0.00
F    C
ATOM  17623  O   LEU F  33     -15.737 -21.687  65.584  1.00  0.00
F    O
ATOM  17624  N   ALA F  34     -16.172 -19.819  66.760  1.00  0.00
F    N
ATOM  17625  HN  ALA F  34     -16.449 -19.374  67.611  1.00  0.00
F    H
ATOM  17626  CA  ALA F  34     -15.917 -18.925  65.644  1.00  0.00
F    C
ATOM  17627  HA  ALA F  34     -15.987 -19.440  64.693  1.00  0.00
F    H
ATOM  17628  CB  ALA F  34     -16.908 -17.765  65.640  1.00  0.00
F    C
ATOM  17629  HB1 ALA F  34     -17.940 -18.157  65.511  1.00  0.00
F    H
ATOM  17630  HB2 ALA F  34     -16.872 -17.206  66.601  1.00  0.00
F    H
```

ATOM 17631 HB3 ALA F 34 -16.704 -17.056 64.810 1.00 0.00
F H
ATOM 17632 C ALA F 34 -14.493 -18.414 65.751 1.00 0.00
F C
ATOM 17633 O ALA F 34 -14.059 -17.998 66.830 1.00 0.00
F O
ATOM 17634 N VAL F 35 -13.763 -18.462 64.642 1.00 0.00
F N
ATOM 17635 HN VAL F 35 -14.094 -18.882 63.801 1.00 0.00
F H
ATOM 17636 CA VAL F 35 -12.419 -17.911 64.551 1.00 0.00
F C
ATOM 17637 HA VAL F 35 -12.290 -17.183 65.341 1.00 0.00
F H
ATOM 17638 CB VAL F 35 -11.334 -19.000 64.654 1.00 0.00
F C
ATOM 17639 HB VAL F 35 -11.462 -19.688 63.783 1.00 0.00
F H
ATOM 17640 CG1 VAL F 35 -9.949 -18.374 64.599 1.00 0.00
F C
ATOM 17641 HG11 VAL F 35 -9.168 -19.157 64.712 1.00 0.00
F H
ATOM 17642 HG12 VAL F 35 -9.768 -17.865 63.630 1.00 0.00
F H
ATOM 17643 HG13 VAL F 35 -9.818 -17.637 65.419 1.00 0.00
F H
ATOM 17644 CG2 VAL F 35 -11.512 -19.812 65.913 1.00 0.00
F C
ATOM 17645 HG21 VAL F 35 -10.706 -20.572 66.008 1.00 0.00
F H
ATOM 17646 HG22 VAL F 35 -11.481 -19.155 66.809 1.00 0.00
F H
ATOM 17647 HG23 VAL F 35 -12.481 -20.352 65.909 1.00 0.00
F H
ATOM 17648 C VAL F 35 -12.291 -17.175 63.230 1.00 0.00
F C
ATOM 17649 O VAL F 35 -12.806 -17.631 62.203 1.00 0.00
F O
ATOM 17650 N ASP F 36 -11.603 -16.041 63.255 1.00 0.00
F N
ATOM 17651 HN ASP F 36 -11.259 -15.569 64.063 1.00 0.00
F H
ATOM 17652 CA ASP F 36 -11.277 -15.357 62.020 1.00 0.00
F C
ATOM 17653 HA ASP F 36 -10.834 -16.080 61.344 1.00 0.00
F H
ATOM 17654 CB ASP F 36 -12.496 -14.696 61.400 1.00 0.00
F C
ATOM 17655 HB1 ASP F 36 -13.375 -15.360 61.525 1.00 0.00
F H
ATOM 17656 HB2 ASP F 36 -12.715 -13.731 61.900 1.00 0.00
F H
ATOM 17657 CG ASP F 36 -12.313 -14.452 59.942 1.00 0.00
F C

```
ATOM  17658  OD1 ASP F  36     -12.479 -15.419  59.174  1.00  0.00
F    O
ATOM  17659  OD2 ASP F  36     -11.991 -13.308  59.566  1.00  0.00
F    O
ATOM  17660  C   ASP F  36     -10.216 -14.314  62.298  1.00  0.00
F    C
ATOM  17661  O   ASP F  36     -10.293 -13.593  63.294  1.00  0.00
F    O
ATOM  17662  N   ILE F  37      -9.237 -14.232  61.401  1.00  0.00
F    N
ATOM  17663  HN  ILE F  37      -9.135 -14.793  60.583  1.00  0.00
F    H
ATOM  17664  CA  ILE F  37      -8.184 -13.243  61.581  1.00  0.00
F    C
ATOM  17665  HA  ILE F  37      -7.810 -13.371  62.589  1.00  0.00
F    H
ATOM  17666  CB  ILE F  37      -7.063 -13.450  60.554  1.00  0.00
F    C
ATOM  17667  HB  ILE F  37      -6.776 -14.527  60.665  1.00  0.00
F    H
ATOM  17668  CG2 ILE F  37      -7.537 -13.073  59.169  1.00  0.00
F    C
ATOM  17669 HG21 ILE F  37      -6.786 -13.366  58.406  1.00  0.00
F    H
ATOM  17670 HG22 ILE F  37      -8.488 -13.589  58.922  1.00  0.00
F    H
ATOM  17671 HG23 ILE F  37      -7.702 -11.978  59.090  1.00  0.00
F    H
ATOM  17672  CG1 ILE F  37      -5.845 -12.619  60.946  1.00  0.00
F    C
ATOM  17673 HG11 ILE F  37      -6.046 -11.541  60.754  1.00  0.00
F    H
ATOM  17674 HG12 ILE F  37      -5.668 -12.731  62.040  1.00  0.00
F    H
ATOM  17675  CD  ILE F  37      -4.595 -13.011  60.234  1.00  0.00
F    C
ATOM  17676  HD1 ILE F  37      -3.735 -12.410  60.603  1.00  0.00
F    H
ATOM  17677  HD2 ILE F  37      -4.367 -14.086  60.405  1.00  0.00
F    H
ATOM  17678  HD3 ILE F  37      -4.689 -12.840  59.140  1.00  0.00
F    H
ATOM  17679  C   ILE F  37      -8.747 -11.837  61.496  1.00  0.00
F    C
ATOM  17680  O   ILE F  37      -8.183 -10.901  62.071  1.00  0.00
F    O
ATOM  17681  N   ASN F  38      -9.858 -11.654  60.800  1.00  0.00
F    N
ATOM  17682  HN  ASN F  38     -10.351 -12.364  60.299  1.00  0.00
F    H
ATOM  17683  CA  ASN F  38     -10.456 -10.337  60.722  1.00  0.00
F    C
ATOM  17684  HA  ASN F  38      -9.642  -9.620  60.719  1.00  0.00
F    H
```

```
ATOM  17685  CB  ASN F  38     -11.330 -10.198  59.490  1.00  0.00
F    C
ATOM  17686  HB1 ASN F  38     -10.661 -10.061  58.612  1.00  0.00
F    H
ATOM  17687  HB2 ASN F  38     -11.913 -11.127  59.323  1.00  0.00
F    H
ATOM  17688  CG  ASN F  38     -12.237  -9.014  59.583  1.00  0.00
F    C
ATOM  17689  OD1 ASN F  38     -13.310  -9.092  60.163  1.00  0.00
F    O
ATOM  17690  ND2 ASN F  38     -11.794  -7.890  59.046  1.00  0.00
F    N
ATOM  17691 HD21 ASN F  38     -12.382  -7.089  59.111  1.00  0.00
F    H
ATOM  17692 HD22 ASN F  38     -10.892  -7.861  58.626  1.00  0.00
F    H
ATOM  17693  C   ASN F  38     -11.274 -10.096  61.976  1.00  0.00
F    C
ATOM  17694  O   ASN F  38     -12.350 -10.677  62.146  1.00  0.00
F    O
ATOM  17695  N   GLU F  39     -10.764  -9.232  62.845  1.00  0.00
F    N
ATOM  17696  HN  GLU F  39      -9.903  -8.745  62.728  1.00  0.00
F    H
ATOM  17697  CA  GLU F  39     -11.457  -8.918  64.087  1.00  0.00
F    C
ATOM  17698  HA  GLU F  39     -11.577  -9.837  64.648  1.00  0.00
F    H
ATOM  17699  CB  GLU F  39     -10.645  -7.898  64.870  1.00  0.00
F    C
ATOM  17700  HB1 GLU F  39     -11.003  -7.906  65.926  1.00  0.00
F    H
ATOM  17701  HB2 GLU F  39      -9.578  -8.214  64.908  1.00  0.00
F    H
ATOM  17702  CG  GLU F  39     -10.773  -6.509  64.296  1.00  0.00
F    C
ATOM  17703  HG1 GLU F  39     -11.828  -6.316  64.029  1.00  0.00
F    H
ATOM  17704  HG2 GLU F  39     -10.437  -5.745  65.025  1.00  0.00
F    H
ATOM  17705  CD  GLU F  39      -9.988  -6.326  63.015  1.00  0.00
F    C
ATOM  17706  OE1 GLU F  39      -9.588  -7.336  62.393  1.00  0.00
F    O
ATOM  17707  OE2 GLU F  39      -9.776  -5.166  62.617  1.00  0.00
F    O
ATOM  17708  C   GLU F  39     -12.846  -8.371  63.815  1.00  0.00
F    C
ATOM  17709  O   GLU F  39     -13.722  -8.422  64.675  1.00  0.00
F    O
ATOM  17710  N   GLU F  40     -13.061  -7.819  62.626  1.00  0.00
F    N
ATOM  17711  HN  GLU F  40     -12.423  -7.783  61.862  1.00  0.00
F    H
```

ATOM 17712 CA GLU F 40 -14.340 -7.188 62.357 1.00 0.00
F C
ATOM 17713 HA GLU F 40 -14.563 -6.514 63.175 1.00 0.00
F H
ATOM 17714 CB GLU F 40 -14.261 -6.404 61.051 1.00 0.00
F C
ATOM 17715 HB1 GLU F 40 -13.294 -6.653 60.555 1.00 0.00
F H
ATOM 17716 HB2 GLU F 40 -15.058 -6.748 60.355 1.00 0.00
F H
ATOM 17717 CG GLU F 40 -14.343 -4.878 61.160 1.00 0.00
F C
ATOM 17718 HG1 GLU F 40 -13.337 -4.468 61.365 1.00 0.00
F H
ATOM 17719 HG2 GLU F 40 -14.734 -4.434 60.223 1.00 0.00
F H
ATOM 17720 CD GLU F 40 -15.207 -4.365 62.298 1.00 0.00
F C
ATOM 17721 OE1 GLU F 40 -16.183 -3.652 61.996 1.00 0.00
F O
ATOM 17722 OE2 GLU F 40 -14.877 -4.599 63.484 1.00 0.00
F O
ATOM 17723 C GLU F 40 -15.460 -8.213 62.300 1.00 0.00
F C
ATOM 17724 O GLU F 40 -16.503 -8.034 62.940 1.00 0.00
F O
ATOM 17725 N LYS F 41 -15.263 -9.291 61.545 1.00 0.00
F N
ATOM 17726 HN LYS F 41 -14.443 -9.466 61.001 1.00 0.00
F H
ATOM 17727 CA LYS F 41 -16.286 -10.324 61.471 1.00 0.00
F C
ATOM 17728 HA LYS F 41 -17.211 -9.836 61.190 1.00 0.00
F H
ATOM 17729 CB LYS F 41 -15.902 -11.384 60.444 1.00 0.00
F C
ATOM 17730 HB1 LYS F 41 -15.083 -11.993 60.891 1.00 0.00
F H
ATOM 17731 HB2 LYS F 41 -16.762 -12.075 60.298 1.00 0.00
F H
ATOM 17732 CG LYS F 41 -15.437 -10.834 59.127 1.00 0.00
F C
ATOM 17733 HG1 LYS F 41 -15.028 -9.810 59.283 1.00 0.00
F H
ATOM 17734 HG2 LYS F 41 -14.606 -11.469 58.744 1.00 0.00
F H
ATOM 17735 CD LYS F 41 -16.552 -10.768 58.121 1.00 0.00
F C
ATOM 17736 HD1 LYS F 41 -17.248 -11.618 58.310 1.00 0.00
F H
ATOM 17737 HD2 LYS F 41 -17.122 -9.826 58.295 1.00 0.00
F H
ATOM 17738 CE LYS F 41 -15.970 -10.815 56.725 1.00 0.00
F C

ATOM 17739 HE1 LYS F 41   -16.759 -10.620 55.966 1.00 0.00
F   H
ATOM 17740 HE2 LYS F 41   -15.173 -10.048 56.609 1.00 0.00
F   H
ATOM 17741 NZ LYS F 41   -15.376 -12.149 56.443 1.00 0.00
F   N
ATOM 17742 HZ1 LYS F 41   -14.980 -12.167 55.481 1.00 0.00
F   H
ATOM 17743 HZ2 LYS F 41   -14.620 -12.345 57.131 1.00 0.00
F   H
ATOM 17744 HZ3 LYS F 41   -16.111 -12.880 56.527 1.00 0.00
F   H
ATOM 17745 C   LYS F 41   -16.511 -10.975 62.824 1.00 0.00
F   C
ATOM 17746 O   LYS F 41   -17.655 -11.232 63.211 1.00 0.00
F   O
ATOM 17747 N   VAL F 42   -15.431 -11.309 63.527 1.00 0.00
F   N
ATOM 17748 HN VAL F 42   -14.490 -11.211 63.214 1.00 0.00
F   H
ATOM 17749 CA VAL F 42   -15.564 -11.868 64.865 1.00 0.00
F   C
ATOM 17750 HA VAL F 42   -16.108 -12.802 64.788 1.00 0.00
F   H
ATOM 17751 CB VAL F 42   -14.177 -12.106 65.480 1.00 0.00
F   C
ATOM 17752 HB VAL F 42   -13.743 -11.106 65.731 1.00 0.00
F   H
ATOM 17753 CG1 VAL F 42   -14.292 -12.932 66.743 1.00 0.00
F   C
ATOM 17754 HG11 VAL F 42   -13.284 -13.135 67.164 1.00 0.00
F   H
ATOM 17755 HG12 VAL F 42   -14.877 -12.405 67.525 1.00 0.00
F   H
ATOM 17756 HG13 VAL F 42   -14.778 -13.907 66.531 1.00 0.00
F   H
ATOM 17757 CG2 VAL F 42   -13.268 -12.768 64.469 1.00 0.00
F   C
ATOM 17758 HG21 VAL F 42   -12.273 -12.981 64.915 1.00 0.00
F   H
ATOM 17759 HG22 VAL F 42   -13.706 -13.728 64.119 1.00 0.00
F   H
ATOM 17760 HG23 VAL F 42   -13.106 -12.117 63.585 1.00 0.00
F   H
ATOM 17761 C   VAL F 42   -16.389 -10.932 65.723 1.00 0.00
F   C
ATOM 17762 O   VAL F 42   -17.336 -11.340 66.406 1.00 0.00
F   O
ATOM 17763 N   ASN F 43   -16.043 -9.651 65.674 1.00 0.00
F   N
ATOM 17764 HN ASN F 43   -15.297 -9.307 65.105 1.00 0.00
F   H
ATOM 17765 CA ASN F 43   -16.726 -8.632 66.451 1.00 0.00
F   C

```
ATOM  17766  HA  ASN F  43      -16.578  -8.904  67.491  1.00  0.00
F    H
ATOM  17767  CB  ASN F  43      -16.131  -7.269  66.111  1.00  0.00
F    C
ATOM  17768  HB1 ASN F  43      -15.100  -7.433  65.729  1.00  0.00
F    H
ATOM  17769  HB2 ASN F  43      -16.713  -6.787  65.300  1.00  0.00
F    H
ATOM  17770  CG  ASN F  43      -16.048  -6.363  67.301  1.00  0.00
F    C
ATOM  17771  OD1 ASN F  43      -17.018  -6.179  68.021  1.00  0.00
F    O
ATOM  17772  ND2 ASN F  43      -14.872  -5.800  67.532  1.00  0.00
F    N
ATOM  17773 HD21 ASN F  43      -14.799  -5.204  68.327  1.00  0.00
F    H
ATOM  17774 HD22 ASN F  43      -14.098  -6.002  66.941  1.00  0.00
F    H
ATOM  17775  C   ASN F  43      -18.215  -8.647  66.180  1.00  0.00
F    C
ATOM  17776  O   ASN F  43      -19.030  -8.594  67.106  1.00  0.00
F    O
ATOM  17777  N   ALA F  44      -18.588  -8.715  64.908  1.00  0.00
F    N
ATOM  17778  HN  ALA F  44      -17.961  -8.738  64.130  1.00  0.00
F    H
ATOM  17779  CA  ALA F  44      -20.003  -8.763  64.573  1.00  0.00
F    C
ATOM  17780  HA  ALA F  44      -20.533  -7.946  65.049  1.00  0.00
F    H
ATOM  17781  CB  ALA F  44      -20.192  -8.636  63.065  1.00  0.00
F    C
ATOM  17782  HB1 ALA F  44      -19.762  -7.673  62.714  1.00  0.00
F    H
ATOM  17783  HB2 ALA F  44      -19.672  -9.459  62.526  1.00  0.00
F    H
ATOM  17784  HB3 ALA F  44      -21.268  -8.653  62.785  1.00  0.00
F    H
ATOM  17785  C   ALA F  44      -20.647 -10.046  65.077  1.00  0.00
F    C
ATOM  17786  O   ALA F  44      -21.542 -10.010  65.928  1.00  0.00
F    O
ATOM  17787  N   TYR F  45      -20.175 -11.188  64.602  1.00  0.00
F    N
ATOM  17788  HN  TYR F  45      -19.311 -11.280  64.111  1.00  0.00
F    H
ATOM  17789  CA  TYR F  45      -20.910 -12.430  64.769  1.00  0.00
F    C
ATOM  17790  HA  TYR F  45      -21.956 -12.152  64.722  1.00  0.00
F    H
ATOM  17791  CB  TYR F  45      -20.559 -13.422  63.663  1.00  0.00
F    C
ATOM  17792  HB1 TYR F  45      -19.454 -13.466  63.551  1.00  0.00
F    H
```

ATOM 17793 HB2 TYR F 45 -20.905 -14.445 63.928 1.00 0.00
F H
ATOM 17794 CG TYR F 45 -21.114 -13.044 62.321 1.00 0.00
F C
ATOM 17795 CD1 TYR F 45 -21.950 -11.949 62.179 1.00 0.00
F C
ATOM 17796 HD1 TYR F 45 -22.214 -11.359 63.046 1.00 0.00
F H
ATOM 17797 CE1 TYR F 45 -22.471 -11.610 60.953 1.00 0.00
F C
ATOM 17798 HE1 TYR F 45 -23.107 -10.744 60.846 1.00 0.00
F H
ATOM 17799 CZ TYR F 45 -22.183 -12.388 59.856 1.00 0.00
F C
ATOM 17800 OH TYR F 45 -22.703 -12.062 58.627 1.00 0.00
F O
ATOM 17801 HH TYR F 45 -22.333 -12.674 57.987 1.00 0.00
F H
ATOM 17802 CD2 TYR F 45 -20.854 -13.817 61.205 1.00 0.00
F C
ATOM 17803 HD2 TYR F 45 -20.263 -14.717 61.297 1.00 0.00
F H
ATOM 17804 CE2 TYR F 45 -21.375 -13.489 59.980 1.00 0.00
F C
ATOM 17805 HE2 TYR F 45 -21.164 -14.113 59.123 1.00 0.00
F H
ATOM 17806 C TYR F 45 -20.685 -13.100 66.110 1.00 0.00
F C
ATOM 17807 O TYR F 45 -21.129 -14.239 66.288 1.00 0.00
F O
ATOM 17808 N ALA F 46 -20.004 -12.443 67.045 1.00 0.00
F N
ATOM 17809 HN ALA F 46 -19.508 -11.587 66.903 1.00 0.00
F H
ATOM 17810 CA ALA F 46 -19.952 -12.979 68.398 1.00 0.00
F C
ATOM 17811 HA ALA F 46 -19.394 -13.907 68.426 1.00 0.00
F H
ATOM 17812 CB ALA F 46 -19.270 -11.982 69.330 1.00 0.00
F C
ATOM 17813 HB1 ALA F 46 -18.233 -11.794 68.980 1.00 0.00
F H
ATOM 17814 HB2 ALA F 46 -19.809 -11.010 69.340 1.00 0.00
F H
ATOM 17815 HB3 ALA F 46 -19.216 -12.366 70.372 1.00 0.00
F H
ATOM 17816 C ALA F 46 -21.352 -13.306 68.893 1.00 0.00
F C
ATOM 17817 O ALA F 46 -21.602 -14.392 69.425 1.00 0.00
F O
ATOM 17818 N SER F 47 -22.285 -12.381 68.686 1.00 0.00
F N
ATOM 17819 HN SER F 47 -22.124 -11.507 68.228 1.00 0.00
F H

ATOM 17820 CA SER F 47   -23.658 -12.578 69.126 1.00 0.00
F   C
ATOM 17821 HA SER F 47   -23.596 -12.734 70.195 1.00 0.00
F   H
ATOM 17822 CB SER F 47   -24.490 -11.355 68.768 1.00 0.00
F   C
ATOM 17823 HB1 SER F 47   -25.388 -11.343 69.427 1.00 0.00
F   H
ATOM 17824 HB2 SER F 47   -23.919 -10.422 68.975 1.00 0.00
F   H
ATOM 17825 OG SER F 47   -24.902 -11.428 67.418 1.00 0.00
F   O
ATOM 17826 HG1 SER F 47   -25.597 -10.769 67.298 1.00 0.00
F   H
ATOM 17827 C   SER F 47   -24.296 -13.817 68.525 1.00 0.00
F   C
ATOM 17828 O   SER F 47   -25.196 -14.396 69.141 1.00 0.00
F   O
ATOM 17829 N   TYR F 48   -23.866 -14.237 67.342 1.00 0.00
F   N
ATOM 17830 HN TYR F 48   -23.142 -13.787 66.822 1.00 0.00
F   H
ATOM 17831 CA TYR F 48   -24.432 -15.411 66.698 1.00 0.00
F   C
ATOM 17832 HA TYR F 48   -25.348 -15.641 67.230 1.00 0.00
F   H
ATOM 17833 CB TYR F 48   -24.699 -15.129 65.226 1.00 0.00
F   C
ATOM 17834 HB1 TYR F 48   -23.733 -14.930 64.712 1.00 0.00
F   H
ATOM 17835 HB2 TYR F 48   -25.156 -16.014 64.733 1.00 0.00
F   H
ATOM 17836 CG TYR F 48   -25.566 -13.928 65.029 1.00 0.00
F   C
ATOM 17837 CD1 TYR F 48   -26.501 -13.567 65.984 1.00 0.00
F   C
ATOM 17838 HD1 TYR F 48   -26.630 -14.178 66.867 1.00 0.00
F   H
ATOM 17839 CE1 TYR F 48   -27.286 -12.459 65.821 1.00 0.00
F   C
ATOM 17840 HE1 TYR F 48   -28.026 -12.196 66.561 1.00 0.00
F   H
ATOM 17841 CZ TYR F 48   -27.130 -11.681 64.698 1.00 0.00
F   C
ATOM 17842 OH TYR F 48   -27.913 -10.569 64.529 1.00 0.00
F   O
ATOM 17843 HH TYR F 48   -27.612 -10.123 63.734 1.00 0.00
F   H
ATOM 17844 CD2 TYR F 48   -25.420 -13.124 63.915 1.00 0.00
F   C
ATOM 17845 HD2 TYR F 48   -24.710 -13.392 63.144 1.00 0.00
F   H
ATOM 17846 CE2 TYR F 48   -26.200 -12.010 63.743 1.00 0.00
F   C

```
ATOM  17847  HE2 TYR F  48      -26.093 -11.412  62.849  1.00  0.00
F    H
ATOM  17848  C   TYR F  48      -23.522 -16.611  66.836  1.00  0.00
F    C
ATOM  17849  O   TYR F  48      -23.521 -17.487  65.972  1.00  0.00
F    O
ATOM  17850  N   ALA F  49      -22.741 -16.665  67.908  1.00  0.00
F    N
ATOM  17851  HN  ALA F  49      -22.816 -16.041  68.685  1.00  0.00
F    H
ATOM  17852  CA  ALA F  49      -21.700 -17.668  68.013  1.00  0.00
F    C
ATOM  17853  HA  ALA F  49      -21.888 -18.501  67.345  1.00  0.00
F    H
ATOM  17854  CB  ALA F  49      -20.325 -17.087  67.679  1.00  0.00
F    C
ATOM  17855  HB1 ALA F  49      -20.321 -16.712  66.634  1.00  0.00
F    H
ATOM  17856  HB2 ALA F  49      -20.078 -16.234  68.349  1.00  0.00
F    H
ATOM  17857  HB3 ALA F  49      -19.524 -17.853  67.772  1.00  0.00
F    H
ATOM  17858  C   ALA F  49      -21.685 -18.239  69.413  1.00  0.00
F    C
ATOM  17859  O   ALA F  49      -21.723 -17.504  70.402  1.00  0.00
F    O
ATOM  17860  N   THR F  50      -21.623 -19.565  69.485  1.00  0.00
F    N
ATOM  17861  HN  THR F  50      -21.734 -20.204  68.724  1.00  0.00
F    H
ATOM  17862  CA  THR F  50      -21.373 -20.200  70.767  1.00  0.00
F    C
ATOM  17863  HA  THR F  50      -22.182 -19.894  71.416  1.00  0.00
F    H
ATOM  17864  CB  THR F  50      -21.288 -21.718  70.597  1.00  0.00
F    C
ATOM  17865  HB  THR F  50      -20.328 -21.916  70.061  1.00  0.00
F    H
ATOM  17866  OG1 THR F  50      -22.361 -22.173  69.764  1.00  0.00
F    O
ATOM  17867  HG1 THR F  50      -22.254 -23.129  69.709  1.00  0.00
F    H
ATOM  17868  CG2 THR F  50      -21.373 -22.416  71.945  1.00  0.00
F    C
ATOM  17869 HG21 THR F  50      -21.197 -23.506  71.836  1.00  0.00
F    H
ATOM  17870 HG22 THR F  50      -20.604 -22.013  72.639  1.00  0.00
F    H
ATOM  17871 HG23 THR F  50      -22.371 -22.269  72.409  1.00  0.00
F    H
ATOM  17872  C   THR F  50      -20.097 -19.670  71.390  1.00  0.00
F    C
ATOM  17873  O   THR F  50      -20.022 -19.508  72.611  1.00  0.00
F    O
```

```
ATOM  17874  N   HSE F  51      -19.101 -19.367  70.571  1.00  0.00
F    N
ATOM  17875  HN  HSE F  51      -19.135 -19.461  69.577  1.00  0.00
F    H
ATOM  17876  CA  HSE F  51      -17.843 -18.858  71.082  1.00  0.00
F    C
ATOM  17877  HA  HSE F  51      -18.058 -18.035  71.752  1.00  0.00
F    H
ATOM  17878  CB  HSE F  51      -17.069 -19.966  71.795  1.00  0.00
F    C
ATOM  17879  HB1 HSE F  51      -17.675 -20.301  72.665  1.00  0.00
F    H
ATOM  17880  HB2 HSE F  51      -16.962 -20.839  71.118  1.00  0.00
F    H
ATOM  17881  ND1 HSE F  51      -14.852 -20.421  72.896  1.00  0.00
F    N
ATOM  17882  CG  HSE F  51      -15.732 -19.540  72.306  1.00  0.00
F    C
ATOM  17883  CE1 HSE F  51      -13.755 -19.774  73.248  1.00  0.00
F    C
ATOM  17884  HE1 HSE F  51      -12.896 -20.234  73.743  1.00  0.00
F    H
ATOM  17885  NE2 HSE F  51      -13.892 -18.507  72.905  1.00  0.00
F    N
ATOM  17886  HE2 HSE F  51      -13.215 -17.790  73.070  1.00  0.00
F    H
ATOM  17887  CD2 HSE F  51      -15.120 -18.334  72.315  1.00  0.00
F    C
ATOM  17888  HD2 HSE F  51      -15.470 -17.376  71.958  1.00  0.00
F    H
ATOM  17889  C   HSE F  51      -17.047 -18.290  69.919  1.00  0.00
F    C
ATOM  17890  O   HSE F  51      -16.792 -18.986  68.933  1.00  0.00
F    O
ATOM  17891  N   ALA F  52      -16.657 -17.026  70.043  1.00  0.00
F    N
ATOM  17892  HN  ALA F  52      -16.860 -16.490  70.863  1.00  0.00
F    H
ATOM  17893  CA  ALA F  52      -15.913 -16.328  69.010  1.00  0.00
F    C
ATOM  17894  HA  ALA F  52      -15.743 -16.959  68.146  1.00  0.00
F    H
ATOM  17895  CB  ALA F  52      -16.641 -15.064  68.554  1.00  0.00
F    C
ATOM  17896  HB1 ALA F  52      -17.623 -15.337  68.113  1.00  0.00
F    H
ATOM  17897  HB2 ALA F  52      -16.829 -14.381  69.411  1.00  0.00
F    H
ATOM  17898  HB3 ALA F  52      -16.060 -14.513  67.783  1.00  0.00
F    H
ATOM  17899  C   ALA F  52      -14.546 -15.963  69.549  1.00  0.00
F    C
ATOM  17900  O   ALA F  52      -14.387 -15.750  70.751  1.00  0.00
F    O
```

```
ATOM  17901  N   VAL F  53     -13.570 -15.880  68.656  1.00  0.00
F    N
ATOM  17902  HN  VAL F  53     -13.711 -16.064  67.686  1.00  0.00
F    H
ATOM  17903  CA  VAL F  53     -12.203 -15.514  69.006  1.00  0.00
F    C
ATOM  17904  HA  VAL F  53     -12.228 -14.764  69.787  1.00  0.00
F    H
ATOM  17905  CB  VAL F  53     -11.367 -16.731  69.450  1.00  0.00
F    C
ATOM  17906  HB  VAL F  53     -11.632 -17.585  68.779  1.00  0.00
F    H
ATOM  17907  CG1 VAL F  53      -9.878 -16.440  69.323  1.00  0.00
F    C
ATOM  17908 HG11 VAL F  53      -9.283 -17.308  69.681  1.00  0.00
F    H
ATOM  17909 HG12 VAL F  53      -9.587 -16.252  68.268  1.00  0.00
F    H
ATOM  17910 HG13 VAL F  53      -9.598 -15.555  69.933  1.00  0.00
F    H
ATOM  17911  CG2 VAL F  53     -11.694 -17.133  70.868  1.00  0.00
F    C
ATOM  17912 HG21 VAL F  53     -11.058 -17.985  71.191  1.00  0.00
F    H
ATOM  17913 HG22 VAL F  53     -11.520 -16.283  71.565  1.00  0.00
F    H
ATOM  17914 HG23 VAL F  53     -12.753 -17.450  70.962  1.00  0.00
F    H
ATOM  17915  C   VAL F  53     -11.573 -14.877  67.784  1.00  0.00
F    C
ATOM  17916  O   VAL F  53     -11.801 -15.329  66.659  1.00  0.00
F    O
ATOM  17917  N   ILE F  54     -10.785 -13.832  67.989  1.00  0.00
F    N
ATOM  17918  HN  ILE F  54     -10.704 -13.319  68.841  1.00  0.00
F    H
ATOM  17919  CA  ILE F  54      -9.937 -13.342  66.914  1.00  0.00
F    C
ATOM  17920  HA  ILE F  54     -10.414 -13.590  65.974  1.00  0.00
F    H
ATOM  17921  CB  ILE F  54      -9.694 -11.839  67.042  1.00  0.00
F    C
ATOM  17922  HB  ILE F  54      -9.126 -11.727  68.001  1.00  0.00
F    H
ATOM  17923  CG2 ILE F  54      -9.002 -11.317  65.811  1.00  0.00
F    C
ATOM  17924 HG21 ILE F  54      -8.682 -10.265  65.959  1.00  0.00
F    H
ATOM  17925 HG22 ILE F  54      -8.094 -11.915  65.584  1.00  0.00
F    H
ATOM  17926 HG23 ILE F  54      -9.677 -11.355  64.929  1.00  0.00
F    H
ATOM  17927  CG1 ILE F  54     -11.021 -11.117  67.222  1.00  0.00
F    C
```

ATOM 17928 HG11 ILE F 54   -11.524 -11.003 66.236 1.00 0.00
F   H
ATOM 17929 HG12 ILE F 54   -11.687 -11.741 67.861 1.00 0.00
F   H
ATOM 17930 CD ILE F 54   -10.879 -9.766 67.866 1.00 0.00
F   C
ATOM 17931 HD1 ILE F 54   -11.880 -9.309 68.027 1.00 0.00
F   H
ATOM 17932 HD2 ILE F 54   -10.374 -9.852 68.852 1.00 0.00
F   H
ATOM 17933 HD3 ILE F 54   -10.287 -9.079 67.224 1.00 0.00
F   H
ATOM 17934 C   ILE F 54   -8.632 -14.122 66.964 1.00 0.00
F   C
ATOM 17935 O   ILE F 54   -7.978 -14.185 68.009 1.00 0.00
F   O
ATOM 17936 N   ALA F 55   -8.257 -14.729 65.840 1.00 0.00
F   N
ATOM 17937 HN ALA F 55   -8.773 -14.665 64.986 1.00 0.00
F   H
ATOM 17938 CA ALA F 55   -7.049 -15.541 65.782 1.00 0.00
F   C
ATOM 17939 HA ALA F 55   -6.204 -15.016 66.212 1.00 0.00
F   H
ATOM 17940 CB ALA F 55   -7.224 -16.858 66.544 1.00 0.00
F   C
ATOM 17941 HB1 ALA F 55   -7.440 -16.646 67.612 1.00 0.00
F   H
ATOM 17942 HB2 ALA F 55   -8.074 -17.446 66.134 1.00 0.00
F   H
ATOM 17943 HB3 ALA F 55   -6.307 -17.484 66.497 1.00 0.00
F   H
ATOM 17944 C   ALA F 55   -6.686 -15.824 64.331 1.00 0.00
F   C
ATOM 17945 O   ALA F 55   -7.566 -15.982 63.482 1.00 0.00
F   O
ATOM 17946 N   ASN F 56   -5.387 -15.894 64.067 1.00 0.00
F   N
ATOM 17947 HN ASN F 56   -4.688 -15.741 64.765 1.00 0.00
F   H
ATOM 17948 CA ASN F 56   -4.861 -16.198 62.741 1.00 0.00
F   C
ATOM 17949 HA ASN F 56   -5.615 -15.900 62.020 1.00 0.00
F   H
ATOM 17950 CB ASN F 56   -3.543 -15.473 62.518 1.00 0.00
F   C
ATOM 17951 HB1 ASN F 56   -3.736 -14.381 62.610 1.00 0.00
F   H
ATOM 17952 HB2 ASN F 56   -2.812 -15.749 63.305 1.00 0.00
F   H
ATOM 17953 CG ASN F 56   -2.960 -15.730 61.149 1.00 0.00
F   C
ATOM 17954 OD1 ASN F 56   -3.385 -16.636 60.430 1.00 0.00
F   O

```
ATOM  17955  ND2 ASN F  56       -1.970 -14.931  60.779  1.00  0.00
F    N
ATOM  17956 HD21 ASN F  56       -1.578 -15.077  59.875  1.00  0.00
F    H
ATOM  17957 HD22 ASN F  56       -1.666 -14.202  61.385  1.00  0.00
F    H
ATOM  17958  C   ASN F  56       -4.691 -17.705  62.634  1.00  0.00
F    C
ATOM  17959  O   ASN F  56       -3.867 -18.299  63.329  1.00  0.00
F    O
ATOM  17960  N   ALA F  57       -5.449 -18.319  61.728  1.00  0.00
F    N
ATOM  17961  HN  ALA F  57       -6.024 -17.824  61.077  1.00  0.00
F    H
ATOM  17962  CA  ALA F  57       -5.491 -19.771  61.621  1.00  0.00
F    C
ATOM  17963  HA  ALA F  57       -5.815 -20.194  62.566  1.00  0.00
F    H
ATOM  17964  CB  ALA F  57       -6.478 -20.184  60.536  1.00  0.00
F    C
ATOM  17965  HB1 ALA F  57       -7.484 -19.776  60.774  1.00  0.00
F    H
ATOM  17966  HB2 ALA F  57       -6.172 -19.784  59.545  1.00  0.00
F    H
ATOM  17967  HB3 ALA F  57       -6.565 -21.289  60.461  1.00  0.00
F    H
ATOM  17968  C   ALA F  57       -4.145 -20.394  61.314  1.00  0.00
F    C
ATOM  17969  O   ALA F  57       -3.959 -21.585  61.574  1.00  0.00
F    O
ATOM  17970  N   THR F  58       -3.205 -19.636  60.780  1.00  0.00
F    N
ATOM  17971  HN  THR F  58       -3.233 -18.639  60.712  1.00  0.00
F    H
ATOM  17972  CA  THR F  58       -2.007 -20.237  60.227  1.00  0.00
F    C
ATOM  17973  HA  THR F  58       -2.350 -21.145  59.751  1.00  0.00
F    H
ATOM  17974  CB  THR F  58       -1.364 -19.269  59.239  1.00  0.00
F    C
ATOM  17975  HB  THR F  58       -0.484 -19.805  58.808  1.00  0.00
F    H
ATOM  17976  OG1 THR F  58       -0.886 -18.123  59.944  1.00  0.00
F    O
ATOM  17977  HG1 THR F  58       -0.506 -17.548  59.271  1.00  0.00
F    H
ATOM  17978  CG2 THR F  58       -2.390 -18.811  58.222  1.00  0.00
F    C
ATOM  17979 HG21 THR F  58       -1.913 -18.197  57.429  1.00  0.00
F    H
ATOM  17980 HG22 THR F  58       -2.868 -19.687  57.734  1.00  0.00
F    H
ATOM  17981 HG23 THR F  58       -3.186 -18.203  58.702  1.00  0.00
F    H
```

```
ATOM  17982  C   THR F  58      -0.987 -20.657  61.276  1.00  0.00
F    C
ATOM  17983  O   THR F  58       0.031 -21.253  60.914  1.00  0.00
F    O
ATOM  17984  N   GLU F  59      -1.218 -20.371  62.553  1.00  0.00
F    N
ATOM  17985  HN  GLU F  59      -2.053 -19.949  62.897  1.00  0.00
F    H
ATOM  17986  CA  GLU F  59      -0.232 -20.663  63.587  1.00  0.00
F    C
ATOM  17987  HA  GLU F  59       0.676 -21.004  63.104  1.00  0.00
F    H
ATOM  17988  CB  GLU F  59       0.056 -19.421  64.423  1.00  0.00
F    C
ATOM  17989  HB1 GLU F  59      -0.917 -19.007  64.775  1.00  0.00
F    H
ATOM  17990  HB2 GLU F  59       0.617 -19.706  65.340  1.00  0.00
F    H
ATOM  17991  CG  GLU F  59       0.800 -18.336  63.698  1.00  0.00
F    C
ATOM  17992  HG1 GLU F  59       1.064 -17.532  64.408  1.00  0.00
F    H
ATOM  17993  HG2 GLU F  59       1.726 -18.731  63.234  1.00  0.00
F    H
ATOM  17994  CD  GLU F  59      -0.041 -17.690  62.632  1.00  0.00
F    C
ATOM  17995  OE1 GLU F  59       0.482 -17.444  61.528  1.00  0.00
F    O
ATOM  17996  OE2 GLU F  59      -1.242 -17.480  62.873  1.00  0.00
F    O
ATOM  17997  C   GLU F  59      -0.712 -21.790  64.492  1.00  0.00
F    C
ATOM  17998  O   GLU F  59      -1.817 -21.725  65.042  1.00  0.00
F    O
ATOM  17999  N   GLU F  60       0.148 -22.789  64.692  1.00  0.00
F    N
ATOM  18000  HN  GLU F  60       1.097 -22.798  64.389  1.00  0.00
F    H
ATOM  18001  CA  GLU F  60      -0.260 -23.996  65.401  1.00  0.00
F    C
ATOM  18002  HA  GLU F  60      -1.162 -24.368  64.932  1.00  0.00
F    H
ATOM  18003  CB  GLU F  60       0.825 -25.069  65.267  1.00  0.00
F    C
ATOM  18004  HB1 GLU F  60       0.542 -25.932  65.912  1.00  0.00
F    H
ATOM  18005  HB2 GLU F  60       0.842 -25.460  64.224  1.00  0.00
F    H
ATOM  18006  CG  GLU F  60       2.258 -24.669  65.647  1.00  0.00
F    C
ATOM  18007  HG1 GLU F  60       2.908 -25.562  65.631  1.00  0.00
F    H
ATOM  18008  HG2 GLU F  60       2.660 -23.914  64.943  1.00  0.00
F    H
```

```
ATOM  18009  CD  GLU F  60       2.411 -24.110  67.042  1.00  0.00
F    C
ATOM  18010  OE1 GLU F  60       1.974 -24.776  68.001  1.00  0.00
F    O
ATOM  18011  OE2 GLU F  60       2.931 -22.984  67.175  1.00  0.00
F    O
ATOM  18012  C   GLU F  60      -0.594 -23.708  66.856  1.00  0.00
F    C
ATOM  18013  O   GLU F  60      -1.439 -24.383  67.453  1.00  0.00
F    O
ATOM  18014  N   ASN F  61       0.073 -22.726  67.455  1.00  0.00
F    N
ATOM  18015  HN  ASN F  61       0.728 -22.110  67.017  1.00  0.00
F    H
ATOM  18016  CA  ASN F  61      -0.130 -22.480  68.873  1.00  0.00
F    C
ATOM  18017  HA  ASN F  61      -0.108 -23.452  69.354  1.00  0.00
F    H
ATOM  18018  CB  ASN F  61       0.975 -21.580  69.407  1.00  0.00
F    C
ATOM  18019  HB1 ASN F  61       0.633 -21.162  70.379  1.00  0.00
F    H
ATOM  18020  HB2 ASN F  61       1.893 -22.171  69.601  1.00  0.00
F    H
ATOM  18021  CG  ASN F  61       1.266 -20.444  68.482  1.00  0.00
F    C
ATOM  18022  OD1 ASN F  61       1.064 -20.554  67.273  1.00  0.00
F    O
ATOM  18023  ND2 ASN F  61       1.752 -19.342  69.030  1.00  0.00
F    N
ATOM  18024 HD21 ASN F  61       1.940 -18.574  68.425  1.00  0.00
F    H
ATOM  18025 HD22 ASN F  61       1.889 -19.295  70.015  1.00  0.00
F    H
ATOM  18026  C   ASN F  61      -1.500 -21.877  69.118  1.00  0.00
F    C
ATOM  18027  O   ASN F  61      -2.161 -22.202  70.109  1.00  0.00
F    O
ATOM  18028  N   GLU F  62      -1.945 -20.995  68.225  1.00  0.00
F    N
ATOM  18029  HN  GLU F  62      -1.419 -20.551  67.504  1.00  0.00
F    H
ATOM  18030  CA  GLU F  62      -3.345 -20.602  68.261  1.00  0.00
F    C
ATOM  18031  HA  GLU F  62      -3.529 -20.090  69.197  1.00  0.00
F    H
ATOM  18032  CB  GLU F  62      -3.684 -19.678  67.101  1.00  0.00
F    C
ATOM  18033  HB1 GLU F  62      -3.155 -20.051  66.193  1.00  0.00
F    H
ATOM  18034  HB2 GLU F  62      -4.771 -19.746  66.869  1.00  0.00
F    H
ATOM  18035  CG  GLU F  62      -3.299 -18.248  67.336  1.00  0.00
F    C
```

```
ATOM  18036  HG1 GLU F  62      -4.105 -17.735  67.892  1.00  0.00
F    H
ATOM  18037  HG2 GLU F  62      -2.356 -18.180  67.916  1.00  0.00
F    H
ATOM  18038  CD  GLU F  62      -3.129 -17.472  66.058  1.00  0.00
F    C
ATOM  18039  OE1 GLU F  62      -4.040 -16.699  65.710  1.00  0.00
F    O
ATOM  18040  OE2 GLU F  62      -2.081 -17.621  65.406  1.00  0.00
F    O
ATOM  18041  C   GLU F  62      -4.251 -21.816  68.227  1.00  0.00
F    C
ATOM  18042  O   GLU F  62      -5.267 -21.854  68.929  1.00  0.00
F    O
ATOM  18043  N   LEU F  63      -3.881 -22.822  67.450  1.00  0.00
F    N
ATOM  18044  HN  LEU F  63      -3.033 -22.865  66.926  1.00  0.00
F    H
ATOM  18045  CA  LEU F  63      -4.731 -23.989  67.305  1.00  0.00
F    C
ATOM  18046  HA  LEU F  63      -5.728 -23.622  67.096  1.00  0.00
F    H
ATOM  18047  CB  LEU F  63      -4.204 -24.847  66.168  1.00  0.00
F    C
ATOM  18048  HB1 LEU F  63      -3.299 -25.376  66.546  1.00  0.00
F    H
ATOM  18049  HB2 LEU F  63      -4.949 -25.640  65.936  1.00  0.00
F    H
ATOM  18050  CG  LEU F  63      -3.854 -24.012  64.943  1.00  0.00
F    C
ATOM  18051  HG  LEU F  63      -3.065 -23.291  65.266  1.00  0.00
F    H
ATOM  18052  CD1 LEU F  63      -3.311 -24.893  63.844  1.00  0.00
F    C
ATOM  18053 HD11 LEU F  63      -3.100 -24.294  62.933  1.00  0.00
F    H
ATOM  18054 HD12 LEU F  63      -2.368 -25.383  64.166  1.00  0.00
F    H
ATOM  18055 HD13 LEU F  63      -4.044 -25.684  63.578  1.00  0.00
F    H
ATOM  18056  CD2 LEU F  63      -5.047 -23.214  64.461  1.00  0.00
F    C
ATOM  18057 HD21 LEU F  63      -4.768 -22.597  63.579  1.00  0.00
F    H
ATOM  18058 HD22 LEU F  63      -5.885 -23.873  64.158  1.00  0.00
F    H
ATOM  18059 HD23 LEU F  63      -5.409 -22.533  65.260  1.00  0.00
F    H
ATOM  18060  C   LEU F  63      -4.800 -24.790  68.595  1.00  0.00
F    C
ATOM  18061  O   LEU F  63      -5.893 -25.060  69.103  1.00  0.00
F    O
ATOM  18062  N   LEU F  64      -3.645 -25.177  69.133  1.00  0.00
F    N
```

ATOM 18063 HN LEU F 64 -2.741 -25.022 68.738 1.00 0.00
F H
ATOM 18064 CA LEU F 64 -3.629 -25.890 70.402 1.00 0.00
F C
ATOM 18065 HA LEU F 64 -4.190 -26.803 70.254 1.00 0.00
F H
ATOM 18066 CB LEU F 64 -2.196 -26.182 70.821 1.00 0.00
F C
ATOM 18067 HB1 LEU F 64 -1.803 -25.262 71.312 1.00 0.00
F H
ATOM 18068 HB2 LEU F 64 -2.202 -26.970 71.607 1.00 0.00
F H
ATOM 18069 CG LEU F 64 -1.307 -26.561 69.652 1.00 0.00
F C
ATOM 18070 HG LEU F 64 -1.486 -25.834 68.823 1.00 0.00
F H
ATOM 18071 CD1 LEU F 64 0.143 -26.495 70.068 1.00 0.00
F C
ATOM 18072 HD11 LEU F 64 0.802 -26.816 69.234 1.00 0.00
F H
ATOM 18073 HD12 LEU F 64 0.422 -25.458 70.352 1.00 0.00
F H
ATOM 18074 HD13 LEU F 64 0.333 -27.160 70.936 1.00 0.00
F H
ATOM 18075 CD2 LEU F 64 -1.675 -27.939 69.154 1.00 0.00
F C
ATOM 18076 HD21 LEU F 64 -1.041 -28.218 68.285 1.00 0.00
F H
ATOM 18077 HD22 LEU F 64 -1.531 -28.710 69.938 1.00 0.00
F H
ATOM 18078 HD23 LEU F 64 -2.738 -27.964 68.831 1.00 0.00
F H
ATOM 18079 C LEU F 64 -4.331 -25.091 71.484 1.00 0.00
F C
ATOM 18080 O LEU F 64 -5.117 -25.645 72.260 1.00 0.00
F O
ATOM 18081 N SER F 65 -4.066 -23.786 71.543 1.00 0.00
F N
ATOM 18082 HN SER F 65 -3.421 -23.302 70.951 1.00 0.00
F H
ATOM 18083 CA SER F 65 -4.730 -22.939 72.521 1.00 0.00
F C
ATOM 18084 HA SER F 65 -4.454 -23.328 73.492 1.00 0.00
F H
ATOM 18085 CB SER F 65 -4.297 -21.492 72.331 1.00 0.00
F C
ATOM 18086 HB1 SER F 65 -4.857 -20.864 73.061 1.00 0.00
F H
ATOM 18087 HB2 SER F 65 -3.214 -21.378 72.566 1.00 0.00
F H
ATOM 18088 OG SER F 65 -4.599 -21.071 71.020 1.00 0.00
F O
ATOM 18089 HG1 SER F 65 -4.521 -20.108 71.006 1.00 0.00
F H

```
ATOM  18090  C   SER F  65      -6.237 -23.046  72.403  1.00  0.00
F    C
ATOM  18091  O   SER F  65      -6.951 -22.936  73.404  1.00  0.00
F    O
ATOM  18092  N   LEU F  66      -6.739 -23.276  71.192  1.00  0.00
F    N
ATOM  18093  HN  LEU F  66      -6.213 -23.356  70.349  1.00  0.00
F    H
ATOM  18094  CA  LEU F  66      -8.170 -23.433  71.019  1.00  0.00
F    C
ATOM  18095  HA  LEU F  66      -8.634 -22.683  71.648  1.00  0.00
F    H
ATOM  18096  CB  LEU F  66      -8.543 -23.224  69.560  1.00  0.00
F    C
ATOM  18097  HB1 LEU F  66      -7.866 -23.867  68.952  1.00  0.00
F    H
ATOM  18098  HB2 LEU F  66      -9.572 -23.612  69.390  1.00  0.00
F    H
ATOM  18099  CG  LEU F  66      -8.406 -21.772  69.134  1.00  0.00
F    C
ATOM  18100  HG  LEU F  66      -7.320 -21.511  69.104  1.00  0.00
F    H
ATOM  18101  CD1 LEU F  66      -9.001 -21.589  67.766  1.00  0.00
F    C
ATOM  18102 HD11 LEU F  66      -8.952 -20.522  67.457  1.00  0.00
F    H
ATOM  18103 HD12 LEU F  66      -8.450 -22.195  67.015  1.00  0.00
F    H
ATOM  18104 HD13 LEU F  66     -10.067 -21.904  67.757  1.00  0.00
F    H
ATOM  18105  CD2 LEU F  66      -9.078 -20.865  70.144  1.00  0.00
F    C
ATOM  18106 HD21 LEU F  66      -8.963 -19.801  69.845  1.00  0.00
F    H
ATOM  18107 HD22 LEU F  66     -10.163 -21.077  70.226  1.00  0.00
F    H
ATOM  18108 HD23 LEU F  66      -8.620 -20.995  71.149  1.00  0.00
F    H
ATOM  18109  C   LEU F  66      -8.663 -24.789  71.483  1.00  0.00
F    C
ATOM  18110  O   LEU F  66      -9.877 -25.001  71.537  1.00  0.00
F    O
ATOM  18111  N   GLY F  67      -7.765 -25.705  71.822  1.00  0.00
F    N
ATOM  18112  HN  GLY F  67      -6.781 -25.541  71.867  1.00  0.00
F    H
ATOM  18113  CA  GLY F  67      -8.193 -27.045  72.160  1.00  0.00
F    C
ATOM  18114  HA1 GLY F  67      -8.880 -26.981  72.992  1.00  0.00
F    H
ATOM  18115  HA2 GLY F  67      -7.315 -27.647  72.353  1.00  0.00
F    H
ATOM  18116  C   GLY F  67      -8.931 -27.658  70.991  1.00  0.00
F    C
```

```
ATOM  18117  O   GLY F  67      -9.990 -28.273  71.146  1.00  0.00
F    O
ATOM  18118  N   ILE F  68      -8.364 -27.468  69.801  1.00  0.00
F    N
ATOM  18119  HN  ILE F  68      -7.481 -27.025  69.662  1.00  0.00
F    H
ATOM  18120  CA  ILE F  68      -9.012 -27.905  68.575  1.00  0.00
F    C
ATOM  18121  HA  ILE F  68      -9.971 -27.404  68.544  1.00  0.00
F    H
ATOM  18122  CB  ILE F  68      -8.128 -27.530  67.384  1.00  0.00
F    C
ATOM  18123  HB  ILE F  68      -8.517 -28.152  66.539  1.00  0.00
F    H
ATOM  18124  CG2 ILE F  68      -8.154 -26.043  67.171  1.00  0.00
F    C
ATOM  18125 HG21 ILE F  68      -7.646 -25.776  66.221  1.00  0.00
F    H
ATOM  18126 HG22 ILE F  68      -9.198 -25.670  67.109  1.00  0.00
F    H
ATOM  18127 HG23 ILE F  68      -7.640 -25.514  68.001  1.00  0.00
F    H
ATOM  18128  CG1 ILE F  68      -6.703 -27.985  67.667  1.00  0.00
F    C
ATOM  18129 HG11 ILE F  68      -6.293 -27.422  68.535  1.00  0.00
F    H
ATOM  18130 HG12 ILE F  68      -6.718 -29.063  67.950  1.00  0.00
F    H
ATOM  18131  CD  ILE F  68      -5.797 -27.823  66.516  1.00  0.00
F    C
ATOM  18132  HD1 ILE F  68      -4.789 -28.223  66.759  1.00  0.00
F    H
ATOM  18133  HD2 ILE F  68      -6.186 -28.373  65.631  1.00  0.00
F    H
ATOM  18134  HD3 ILE F  68      -5.685 -26.752  66.245  1.00  0.00
F    H
ATOM  18135  C   ILE F  68      -9.297 -29.395  68.598  1.00  0.00
F    C
ATOM  18136  O   ILE F  68     -10.378 -29.835  68.193  1.00  0.00
F    O
ATOM  18137  N   ARG F  69      -8.343 -30.194  69.071  1.00  0.00
F    N
ATOM  18138  HN  ARG F  69      -7.497 -29.843  69.469  1.00  0.00
F    H
ATOM  18139  CA  ARG F  69      -8.494 -31.637  69.029  1.00  0.00
F    C
ATOM  18140  HA  ARG F  69      -8.595 -31.931  67.991  1.00  0.00
F    H
ATOM  18141  CB  ARG F  69      -7.272 -32.293  69.643  1.00  0.00
F    C
ATOM  18142  HB1 ARG F  69      -7.188 -31.894  70.682  1.00  0.00
F    H
ATOM  18143  HB2 ARG F  69      -7.450 -33.386  69.749  1.00  0.00
F    H
```

ATOM 18144 CG ARG F 69   -6.015 -31.985 68.880 1.00 0.00
F   C
ATOM 18145 HG1 ARG F 69   -6.056 -32.513 67.902 1.00 0.00
F   H
ATOM 18146 HG2 ARG F 69   -5.998 -30.899 68.634 1.00 0.00
F   H
ATOM 18147 CD ARG F 69   -4.802 -32.330 69.695 1.00 0.00
F   C
ATOM 18148 HD1 ARG F 69   -4.897 -33.371 70.081 1.00 0.00
F   H
ATOM 18149 HD2 ARG F 69   -3.868 -32.259 69.089 1.00 0.00
F   H
ATOM 18150 NE ARG F 69   -4.648 -31.414 70.816 1.00 0.00
F   N
ATOM 18151 HE ARG F 69   -4.226 -30.538 70.556 1.00 0.00
F   H
ATOM 18152 CZ ARG F 69   -5.022 -31.692 72.058 1.00 0.00
F   C
ATOM 18153 NH1 ARG F 69   -5.581 -32.860 72.338 1.00 0.00
F   N
ATOM 18154 HH11 ARG F 69   -5.886 -33.065 73.258 1.00 0.00
F   H
ATOM 18155 HH12 ARG F 69   -5.796 -33.448 71.556 1.00 0.00
F   H
ATOM 18156 NH2 ARG F 69   -4.845 -30.801 73.020 1.00 0.00
F   N
ATOM 18157 HH21 ARG F 69   -5.044 -31.043 73.960 1.00 0.00
F   H
ATOM 18158 HH22 ARG F 69   -4.320 -29.992 72.791 1.00 0.00
F   H
ATOM 18159 C   ARG F 69   -9.748 -32.103 69.733 1.00 0.00
F   C
ATOM 18160 O   ARG F 69   -10.303 -33.138 69.356 1.00 0.00
F   O
ATOM 18161 N   ASN F 70   -10.214 -31.355 70.730 1.00 0.00
F   N
ATOM 18162 HN ASN F 70   -9.738 -30.578 71.139 1.00 0.00
F   H
ATOM 18163 CA ASN F 70   -11.513 -31.640 71.314 1.00 0.00
F   C
ATOM 18164 HA ASN F 70   -11.443 -32.648 71.709 1.00 0.00
F   H
ATOM 18165 CB ASN F 70   -11.827 -30.621 72.401 1.00 0.00
F   C
ATOM 18166 HB1 ASN F 70   -12.076 -29.657 71.904 1.00 0.00
F   H
ATOM 18167 HB2 ASN F 70   -12.720 -30.937 72.979 1.00 0.00
F   H
ATOM 18168 CG ASN F 70   -10.658 -30.388 73.323 1.00 0.00
F   C
ATOM 18169 OD1 ASN F 70   -9.730 -31.192 73.375 1.00 0.00
F   O
ATOM 18170 ND2 ASN F 70   -10.691 -29.285 74.057 1.00 0.00
F   N

```
ATOM  18171 HD21 ASN F  70      -9.918 -29.114  74.662  1.00  0.00
F    H
ATOM  18172 HD22 ASN F  70     -11.453 -28.652  73.969  1.00  0.00
F    H
ATOM  18173  C   ASN F  70     -12.605 -31.633  70.265  1.00  0.00
F    C
ATOM  18174  O   ASN F  70     -13.539 -32.435  70.347  1.00  0.00
F    O
ATOM  18175  N   PHE F  71     -12.492 -30.769  69.266  1.00  0.00
F    N
ATOM  18176  HN  PHE F  71     -11.737 -30.122  69.176  1.00  0.00
F    H
ATOM  18177  CA  PHE F  71     -13.479 -30.693  68.205  1.00  0.00
F    C
ATOM  18178  HA  PHE F  71     -14.454 -30.754  68.672  1.00  0.00
F    H
ATOM  18179  CB  PHE F  71     -13.300 -29.425  67.382  1.00  0.00
F    C
ATOM  18180  HB1 PHE F  71     -12.271 -29.406  66.962  1.00  0.00
F    H
ATOM  18181  HB2 PHE F  71     -14.005 -29.404  66.523  1.00  0.00
F    H
ATOM  18182  CG  PHE F  71     -13.448 -28.175  68.170  1.00  0.00
F    C
ATOM  18183  CD1 PHE F  71     -12.447 -27.765  69.032  1.00  0.00
F    C
ATOM  18184  HD1 PHE F  71     -11.555 -28.366  69.139  1.00  0.00
F    H
ATOM  18185  CE1 PHE F  71     -12.574 -26.614  69.753  1.00  0.00
F    C
ATOM  18186  HE1 PHE F  71     -11.782 -26.306  70.421  1.00  0.00
F    H
ATOM  18187  CZ  PHE F  71     -13.712 -25.845  69.620  1.00  0.00
F    C
ATOM  18188  HZ  PHE F  71     -13.812 -24.929  70.185  1.00  0.00
F    H
ATOM  18189  CD2 PHE F  71     -14.582 -27.399  68.040  1.00  0.00
F    C
ATOM  18190  HD2 PHE F  71     -15.381 -27.715  67.384  1.00  0.00
F    H
ATOM  18191  CE2 PHE F  71     -14.718 -26.239  68.759  1.00  0.00
F    C
ATOM  18192  HE2 PHE F  71     -15.612 -25.640  68.650  1.00  0.00
F    H
ATOM  18193  C   PHE F  71     -13.339 -31.908  67.306  1.00  0.00
F    C
ATOM  18194  O   PHE F  71     -12.270 -32.522  67.243  1.00  0.00
F    O
ATOM  18195  N   GLU F  72     -14.420 -32.243  66.607  1.00  0.00
F    N
ATOM  18196  HN  GLU F  72     -15.304 -31.785  66.651  1.00  0.00
F    H
ATOM  18197  CA  GLU F  72     -14.381 -33.366  65.681  1.00  0.00
F    C
```

ATOM 18198 HA GLU F 72 -13.395 -33.810 65.731 1.00 0.00
F H
ATOM 18199 CB GLU F 72 -15.439 -34.379 66.076 1.00 0.00
F C
ATOM 18200 HB1 GLU F 72 -16.144 -34.497 65.221 1.00 0.00
F H
ATOM 18201 HB2 GLU F 72 -14.968 -35.378 66.223 1.00 0.00
F H
ATOM 18202 CG GLU F 72 -16.201 -33.965 67.308 1.00 0.00
F C
ATOM 18203 HG1 GLU F 72 -15.489 -33.664 68.099 1.00 0.00
F H
ATOM 18204 HG2 GLU F 72 -16.880 -33.117 67.088 1.00 0.00
F H
ATOM 18205 CD GLU F 72 -17.009 -35.097 67.887 1.00 0.00
F C
ATOM 18206 OE1 GLU F 72 -17.771 -35.734 67.130 1.00 0.00
F O
ATOM 18207 OE2 GLU F 72 -16.881 -35.348 69.105 1.00 0.00
F O
ATOM 18208 C GLU F 72 -14.596 -32.905 64.248 1.00 0.00
F C
ATOM 18209 O GLU F 72 -13.994 -33.451 63.314 1.00 0.00
F O
ATOM 18210 N TYR F 73 -15.452 -31.904 64.052 1.00 0.00
F N
ATOM 18211 HN TYR F 73 -15.972 -31.467 64.784 1.00 0.00
F H
ATOM 18212 CA TYR F 73 -15.702 -31.359 62.725 1.00 0.00
F C
ATOM 18213 HA TYR F 73 -15.230 -32.019 62.007 1.00 0.00
F H
ATOM 18214 CB TYR F 73 -17.194 -31.194 62.450 1.00 0.00
F C
ATOM 18215 HB1 TYR F 73 -17.678 -30.724 63.333 1.00 0.00
F H
ATOM 18216 HB2 TYR F 73 -17.362 -30.518 61.584 1.00 0.00
F H
ATOM 18217 CG TYR F 73 -17.946 -32.473 62.202 1.00 0.00
F C
ATOM 18218 CD1 TYR F 73 -18.177 -33.376 63.225 1.00 0.00
F C
ATOM 18219 HD1 TYR F 73 -17.790 -33.177 64.215 1.00 0.00
F H
ATOM 18220 CE1 TYR F 73 -18.874 -34.538 63.001 1.00 0.00
F C
ATOM 18221 HE1 TYR F 73 -19.046 -35.239 63.804 1.00 0.00
F H
ATOM 18222 CZ TYR F 73 -19.347 -34.808 61.740 1.00 0.00
F C
ATOM 18223 OH TYR F 73 -20.043 -35.965 61.496 1.00 0.00
F O
ATOM 18224 HH TYR F 73 -20.345 -35.933 60.586 1.00 0.00
F H

ATOM 18225 CD2 TYR F 73 −18.440 −32.769 60.945 1.00 0.00
F C
ATOM 18226 HD2 TYR F 73 −18.248 −32.100 60.118 1.00 0.00
F H
ATOM 18227 CE2 TYR F 73 −19.133 −33.926 60.712 1.00 0.00
F C
ATOM 18228 HE2 TYR F 73 −19.488 −34.147 59.716 1.00 0.00
F H
ATOM 18229 C TYR F 73 −15.018 −30.011 62.609 1.00 0.00
F C
ATOM 18230 O TYR F 73 −15.150 −29.167 63.501 1.00 0.00
F O
ATOM 18231 N VAL F 74 −14.307 −29.795 61.507 1.00 0.00
F N
ATOM 18232 HN VAL F 74 −14.147 −30.497 60.817 1.00 0.00
F H
ATOM 18233 CA VAL F 74 −13.697 −28.509 61.210 1.00 0.00
F C
ATOM 18234 HA VAL F 74 −14.148 −27.757 61.846 1.00 0.00
F H
ATOM 18235 CB VAL F 74 −12.177 −28.518 61.450 1.00 0.00
F C
ATOM 18236 HB VAL F 74 −11.796 −27.488 61.243 1.00 0.00
F H
ATOM 18237 CG1 VAL F 74 −11.878 −28.886 62.887 1.00 0.00
F C
ATOM 18238 HG11 VAL F 74 −10.779 −28.932 63.052 1.00 0.00
F H
ATOM 18239 HG12 VAL F 74 −12.287 −28.137 63.595 1.00 0.00
F H
ATOM 18240 HG13 VAL F 74 −12.304 −29.882 63.134 1.00 0.00
F H
ATOM 18241 CG2 VAL F 74 −11.478 −29.471 60.503 1.00 0.00
F C
ATOM 18242 HG21 VAL F 74 −10.387 −29.502 60.710 1.00 0.00
F H
ATOM 18243 HG22 VAL F 74 −11.884 −30.500 60.615 1.00 0.00
F H
ATOM 18244 HG23 VAL F 74 −11.611 −29.158 59.447 1.00 0.00
F H
ATOM 18245 C VAL F 74 −14.022 −28.144 59.772 1.00 0.00
F C
ATOM 18246 O VAL F 74 −14.011 −29.004 58.881 1.00 0.00
F O
ATOM 18247 N ILE F 75 −14.324 −26.875 59.553 1.00 0.00
F N
ATOM 18248 HN ILE F 75 −14.303 −26.181 60.270 1.00 0.00
F H
ATOM 18249 CA ILE F 75 −14.713 −26.370 58.249 1.00 0.00
F C
ATOM 18250 HA ILE F 75 −14.451 −27.117 57.511 1.00 0.00
F H
ATOM 18251 CB ILE F 75 −16.198 −26.009 58.207 1.00 0.00
F C

```
ATOM  18252  HB  ILE F  75      -16.227 -25.001  57.722  1.00  0.00
F    H
ATOM  18253  CG2 ILE F  75      -16.956 -27.076  57.468  1.00  0.00
F    C
ATOM  18254 HG21 ILE F  75      -18.005 -26.757  57.291  1.00  0.00
F    H
ATOM  18255 HG22 ILE F  75      -16.494 -27.277  56.479  1.00  0.00
F    H
ATOM  18256 HG23 ILE F  75      -16.974 -28.023  58.046  1.00  0.00
F    H
ATOM  18257  CG1 ILE F  75      -16.703 -25.844  59.634  1.00  0.00
F    C
ATOM  18258 HG11 ILE F  75      -16.019 -26.368  60.338  1.00  0.00
F    H
ATOM  18259 HG12 ILE F  75      -16.684 -24.763  59.901  1.00  0.00
F    H
ATOM  18260  CD  ILE F  75      -18.089 -26.343  59.855  1.00  0.00
F    C
ATOM  18261  HD1 ILE F  75      -18.411 -26.139  60.899  1.00  0.00
F    H
ATOM  18262  HD2 ILE F  75      -18.801 -25.840  59.166  1.00  0.00
F    H
ATOM  18263  HD3 ILE F  75      -18.149 -27.439  59.686  1.00  0.00
F    H
ATOM  18264  C   ILE F  75      -13.865 -25.154  57.960  1.00  0.00
F    C
ATOM  18265  O   ILE F  75      -14.021 -24.112  58.606  1.00  0.00
F    O
ATOM  18266  N   VAL F  76      -12.987 -25.277  56.983  1.00  0.00
F    N
ATOM  18267  HN  VAL F  76      -12.841 -26.124  56.478  1.00  0.00
F    H
ATOM  18268  CA  VAL F  76      -12.148 -24.174  56.557  1.00  0.00
F    C
ATOM  18269  HA  VAL F  76      -12.019 -23.472  57.371  1.00  0.00
F    H
ATOM  18270  CB  VAL F  76      -10.783 -24.655  56.096  1.00  0.00
F    C
ATOM  18271  HB  VAL F  76      -10.938 -25.287  55.186  1.00  0.00
F    H
ATOM  18272  CG1 VAL F  76       -9.921 -23.489  55.751  1.00  0.00
F    C
ATOM  18273 HG11 VAL F  76       -8.909 -23.835  55.450  1.00  0.00
F    H
ATOM  18274 HG12 VAL F  76      -10.338 -22.908  54.902  1.00  0.00
F    H
ATOM  18275 HG13 VAL F  76       -9.807 -22.813  56.624  1.00  0.00
F    H
ATOM  18276  CG2 VAL F  76      -10.148 -25.465  57.193  1.00  0.00
F    C
ATOM  18277 HG21 VAL F  76       -9.130 -25.798  56.897  1.00  0.00
F    H
ATOM  18278 HG22 VAL F  76      -10.063 -24.864  58.123  1.00  0.00
F    H
```

ATOM 18279 HG23 VAL F 76 -10.745 -26.374 57.419 1.00 0.00
F H
ATOM 18280 C VAL F 76 -12.935 -23.465 55.463 1.00 0.00
F C
ATOM 18281 O VAL F 76 -12.893 -23.831 54.281 1.00 0.00
F O
ATOM 18282 N ALA F 77 -13.707 -22.465 55.874 1.00 0.00
F N
ATOM 18283 HN ALA F 77 -13.758 -22.209 56.840 1.00 0.00
F H
ATOM 18284 CA ALA F 77 -14.532 -21.669 54.971 1.00 0.00
F C
ATOM 18285 HA ALA F 77 -14.764 -22.229 54.072 1.00 0.00
F H
ATOM 18286 CB ALA F 77 -15.852 -21.308 55.637 1.00 0.00
F C
ATOM 18287 HB1 ALA F 77 -16.396 -22.237 55.914 1.00 0.00
F H
ATOM 18288 HB2 ALA F 77 -15.684 -20.720 56.565 1.00 0.00
F H
ATOM 18289 HB3 ALA F 77 -16.505 -20.719 54.957 1.00 0.00
F H
ATOM 18290 C ALA F 77 -13.801 -20.422 54.521 1.00 0.00
F C
ATOM 18291 O ALA F 77 -14.300 -19.306 54.639 1.00 0.00
F O
ATOM 18292 N ILE F 78 -12.597 -20.599 54.012 1.00 0.00
F N
ATOM 18293 HN ILE F 78 -12.135 -21.484 54.005 1.00 0.00
F H
ATOM 18294 CA ILE F 78 -11.830 -19.523 53.413 1.00 0.00
F C
ATOM 18295 HA ILE F 78 -12.272 -18.580 53.708 1.00 0.00
F H
ATOM 18296 CB ILE F 78 -10.355 -19.620 53.802 1.00 0.00
F C
ATOM 18297 HB ILE F 78 -10.334 -19.343 54.887 1.00 0.00
F H
ATOM 18298 CG2 ILE F 78 -9.833 -20.968 53.462 1.00 0.00
F C
ATOM 18299 HG21 ILE F 78 -8.814 -21.108 53.877 1.00 0.00
F H
ATOM 18300 HG22 ILE F 78 -10.482 -21.762 53.886 1.00 0.00
F H
ATOM 18301 HG23 ILE F 78 -9.782 -21.108 52.361 1.00 0.00
F H
ATOM 18302 CG1 ILE F 78 -9.526 -18.576 53.073 1.00 0.00
F C
ATOM 18303 HG11 ILE F 78 -9.634 -18.710 51.973 1.00 0.00
F H
ATOM 18304 HG12 ILE F 78 -9.919 -17.564 53.321 1.00 0.00
F H
ATOM 18305 CD ILE F 78 -8.081 -18.637 53.448 1.00 0.00
F C

```
ATOM  18306  HD1 ILE F  78      -7.515 -17.825  52.941  1.00  0.00
F    H
ATOM  18307  HD2 ILE F  78      -7.955 -18.515  54.546  1.00  0.00
F    H
ATOM  18308  HD3 ILE F  78      -7.632 -19.607  53.148  1.00  0.00
F    H
ATOM  18309  C   ILE F  78     -12.028 -19.650  51.912  1.00  0.00
F    C
ATOM  18310  O   ILE F  78     -12.236 -20.757  51.406  1.00  0.00
F    O
ATOM  18311  N   GLY F  79     -12.004 -18.533  51.191  1.00  0.00
F    N
ATOM  18312  HN  GLY F  79     -11.917 -17.609  51.562  1.00  0.00
F    H
ATOM  18313  CA  GLY F  79     -12.109 -18.622  49.748  1.00  0.00
F    C
ATOM  18314  HA1 GLY F  79     -13.065 -18.183  49.496  1.00  0.00
F    H
ATOM  18315  HA2 GLY F  79     -12.025 -19.672  49.507  1.00  0.00
F    H
ATOM  18316  C   GLY F  79     -11.037 -17.881  48.982  1.00  0.00
F    C
ATOM  18317  O   GLY F  79     -10.758 -18.208  47.824  1.00  0.00
F    O
ATOM  18318  N   ALA F  80     -10.426 -16.881  49.607  1.00  0.00
F    N
ATOM  18319  HN  ALA F  80     -10.552 -16.638  50.568  1.00  0.00
F    H
ATOM  18320  CA  ALA F  80      -9.487 -16.048  48.869  1.00  0.00
F    C
ATOM  18321  HA  ALA F  80      -9.715 -16.094  47.810  1.00  0.00
F    H
ATOM  18322  CB  ALA F  80      -9.619 -14.592  49.317  1.00  0.00
F    C
ATOM  18323  HB1 ALA F  80     -10.664 -14.248  49.162  1.00  0.00
F    H
ATOM  18324  HB2 ALA F  80      -9.379 -14.483  50.398  1.00  0.00
F    H
ATOM  18325  HB3 ALA F  80      -8.948 -13.923  48.737  1.00  0.00
F    H
ATOM  18326  C   ALA F  80      -8.041 -16.499  49.017  1.00  0.00
F    C
ATOM  18327  O   ALA F  80      -7.387 -16.827  48.024  1.00  0.00
F    O
ATOM  18328  N   ASN F  81      -7.526 -16.524  50.235  1.00  0.00
F    N
ATOM  18329  HN  ASN F  81      -8.071 -16.430  51.067  1.00  0.00
F    H
ATOM  18330  CA  ASN F  81      -6.100 -16.693  50.464  1.00  0.00
F    C
ATOM  18331  HA  ASN F  81      -5.591 -16.225  49.628  1.00  0.00
F    H
ATOM  18332  CB  ASN F  81      -5.721 -16.062  51.796  1.00  0.00
F    C
```

```
ATOM  18333  HB1 ASN F  81      -6.091 -15.013  51.793  1.00  0.00
F    H
ATOM  18334  HB2 ASN F  81      -6.226 -16.591  52.630  1.00  0.00
F    H
ATOM  18335  CG  ASN F  81      -4.246 -16.034  52.019  1.00  0.00
F    C
ATOM  18336  OD1 ASN F  81      -3.520 -16.908  51.558  1.00  0.00
F    O
ATOM  18337  ND2 ASN F  81      -3.782 -15.024  52.738  1.00  0.00
F    N
ATOM  18338 HD21 ASN F  81      -2.798 -14.982  52.888  1.00  0.00
F    H
ATOM  18339 HD22 ASN F  81      -4.406 -14.328  53.080  1.00  0.00
F    H
ATOM  18340  C   ASN F  81      -5.765 -18.177  50.436  1.00  0.00
F    C
ATOM  18341  O   ASN F  81      -5.731 -18.825  51.485  1.00  0.00
F    O
ATOM  18342  N   ILE F  82      -5.470 -18.703  49.247  1.00  0.00
F    N
ATOM  18343  HN  ILE F  82      -5.390 -18.193  48.394  1.00  0.00
F    H
ATOM  18344  CA  ILE F  82      -5.233 -20.138  49.110  1.00  0.00
F    C
ATOM  18345  HA  ILE F  82      -6.158 -20.631  49.384  1.00  0.00
F    H
ATOM  18346  CB  ILE F  82      -4.844 -20.482  47.664  1.00  0.00
F    C
ATOM  18347  HB  ILE F  82      -4.094 -19.699  47.382  1.00  0.00
F    H
ATOM  18348  CG2 ILE F  82      -4.322 -21.898  47.569  1.00  0.00
F    C
ATOM  18349 HG21 ILE F  82      -3.898 -22.091  46.561  1.00  0.00
F    H
ATOM  18350 HG22 ILE F  82      -3.516 -22.073  48.312  1.00  0.00
F    H
ATOM  18351 HG23 ILE F  82      -5.134 -22.634  47.750  1.00  0.00
F    H
ATOM  18352  CG1 ILE F  82      -6.043 -20.300  46.741  1.00  0.00
F    C
ATOM  18353 HG11 ILE F  82      -6.001 -21.049  45.918  1.00  0.00
F    H
ATOM  18354 HG12 ILE F  82      -6.975 -20.497  47.318  1.00  0.00
F    H
ATOM  18355  CD  ILE F  82      -6.150 -18.936  46.144  1.00  0.00
F    C
ATOM  18356  HD1 ILE F  82      -7.074 -18.855  45.531  1.00  0.00
F    H
ATOM  18357  HD2 ILE F  82      -6.193 -18.162  46.941  1.00  0.00
F    H
ATOM  18358  HD3 ILE F  82      -5.282 -18.719  45.488  1.00  0.00
F    H
ATOM  18359  C   ILE F  82      -4.177 -20.608  50.098  1.00  0.00
F    C
```

ATOM 18360 O   ILE F 82   -4.278 -21.706 50.664 1.00 0.00
F   O
ATOM 18361 N   GLN F 83   -3.168 -19.773 50.339 1.00 0.00
F   N
ATOM 18362 HN GLN F 83   -3.052 -18.886 49.896 1.00 0.00
F   H
ATOM 18363 CA GLN F 83   -2.128 -20.107 51.300 1.00 0.00
F   C
ATOM 18364 HA GLN F 83   -1.545 -20.911 50.869 1.00 0.00
F   H
ATOM 18365 CB GLN F 83   -1.292 -18.872 51.586 1.00 0.00
F   C
ATOM 18366 HB1 GLN F 83   -1.661 -18.069 50.907 1.00 0.00
F   H
ATOM 18367 HB2 GLN F 83   -1.496 -18.521 52.623 1.00 0.00
F   H
ATOM 18368 CG GLN F 83   0.163 -19.021 51.388 1.00 0.00
F   C
ATOM 18369 HG1 GLN F 83   0.520 -19.973 51.836 1.00 0.00
F   H
ATOM 18370 HG2 GLN F 83   0.412 -19.040 50.307 1.00 0.00
F   H
ATOM 18371 CD GLN F 83   0.905 -17.897 52.064 1.00 0.00
F   C
ATOM 18372 OE1 GLN F 83   0.584 -17.530 53.188 1.00 0.00
F   O
ATOM 18373 NE2 GLN F 83   1.877 -17.326 51.377 1.00 0.00
F   N
ATOM 18374 HE21 GLN F 83   2.371 -16.586 51.826 1.00 0.00
F   H
ATOM 18375 HE22 GLN F 83   2.073 -17.630 50.451 1.00 0.00
F   H
ATOM 18376 C   GLN F 83   -2.723 -20.618 52.597 1.00 0.00
F   C
ATOM 18377 O   GLN F 83   -2.533 -21.784 52.970 1.00 0.00
F   O
ATOM 18378 N   ALA F 84   -3.478 -19.761 53.276 1.00 0.00
F   N
ATOM 18379 HN ALA F 84   -3.723 -18.849 52.949 1.00 0.00
F   H
ATOM 18380 CA ALA F 84   -4.009 -20.120 54.580 1.00 0.00
F   C
ATOM 18381 HA ALA F 84   -3.206 -20.332 55.275 1.00 0.00
F   H
ATOM 18382 CB ALA F 84   -4.829 -18.963 55.141 1.00 0.00
F   C
ATOM 18383 HB1 ALA F 84   -4.184 -18.064 55.242 1.00 0.00
F   H
ATOM 18384 HB2 ALA F 84   -5.672 -18.706 54.465 1.00 0.00
F   H
ATOM 18385 HB3 ALA F 84   -5.242 -19.204 56.145 1.00 0.00
F   H
ATOM 18386 C   ALA F 84   -4.845 -21.382 54.494 1.00 0.00
F   C

```
ATOM  18387  O   ALA F  84      -4.687 -22.297  55.305  1.00  0.00
F    O
ATOM  18388  N   SER F  85      -5.741 -21.446  53.512  1.00  0.00
F    N
ATOM  18389  HN  SER F  85      -5.934 -20.716  52.857  1.00  0.00
F    H
ATOM  18390  CA  SER F  85      -6.540 -22.647  53.317  1.00  0.00
F    C
ATOM  18391  HA  SER F  85      -7.239 -22.684  54.142  1.00  0.00
F    H
ATOM  18392  CB  SER F  85      -7.225 -22.592  51.957  1.00  0.00
F    C
ATOM  18393  HB1 SER F  85      -8.135 -23.234  52.002  1.00  0.00
F    H
ATOM  18394  HB2 SER F  85      -7.567 -21.555  51.737  1.00  0.00
F    H
ATOM  18395  OG  SER F  85      -6.358 -23.082  50.953  1.00  0.00
F    O
ATOM  18396  HG1 SER F  85      -6.896 -23.238  50.166  1.00  0.00
F    H
ATOM  18397  C   SER F  85      -5.673 -23.891  53.405  1.00  0.00
F    C
ATOM  18398  O   SER F  85      -5.916 -24.789  54.221  1.00  0.00
F    O
ATOM  18399  N   THR F  86      -4.623 -23.934  52.596  1.00  0.00
F    N
ATOM  18400  HN  THR F  86      -4.265 -23.178  52.048  1.00  0.00
F    H
ATOM  18401  CA  THR F  86      -3.883 -25.176  52.453  1.00  0.00
F    C
ATOM  18402  HA  THR F  86      -4.632 -25.953  52.374  1.00  0.00
F    H
ATOM  18403  CB  THR F  86      -3.000 -25.104  51.219  1.00  0.00
F    C
ATOM  18404  HB  THR F  86      -2.510 -26.103  51.129  1.00  0.00
F    H
ATOM  18405  OG1 THR F  86      -1.980 -24.123  51.430  1.00  0.00
F    O
ATOM  18406  HG1 THR F  86      -1.463 -24.112  50.618  1.00  0.00
F    H
ATOM  18407  CG2 THR F  86      -3.831 -24.727  50.015  1.00  0.00
F    C
ATOM  18408 HG21 THR F  86      -3.227 -24.780  49.086  1.00  0.00
F    H
ATOM  18409 HG22 THR F  86      -4.690 -25.423  49.904  1.00  0.00
F    H
ATOM  18410 HG23 THR F  86      -4.231 -23.695  50.112  1.00  0.00
F    H
ATOM  18411  C   THR F  86      -3.060 -25.494  53.694  1.00  0.00
F    C
ATOM  18412  O   THR F  86      -3.106 -26.624  54.196  1.00  0.00
F    O
ATOM  18413  N   LEU F  87      -2.289 -24.527  54.200  1.00  0.00
F    N
```

```
ATOM  18414  HN  LEU F  87      -2.181 -23.598  53.852  1.00  0.00
F    H
ATOM  18415  CA  LEU F  87      -1.499 -24.817  55.396  1.00  0.00
F    C
ATOM  18416  HA  LEU F  87      -0.893 -25.685  55.171  1.00  0.00
F    H
ATOM  18417  CB  LEU F  87      -0.578 -23.657  55.778  1.00  0.00
F    C
ATOM  18418  HB1 LEU F  87      -1.172 -22.962  56.416  1.00  0.00
F    H
ATOM  18419  HB2 LEU F  87       0.242 -24.042  56.423  1.00  0.00
F    H
ATOM  18420  CG  LEU F  87       0.191 -22.635  54.935  1.00  0.00
F    C
ATOM  18421  HG  LEU F  87       1.247 -22.778  55.269  1.00  0.00
F    H
ATOM  18422  CD1 LEU F  87       0.104 -22.782  53.431  1.00  0.00
F    C
ATOM  18423 HD11 LEU F  87       0.658 -21.961  52.926  1.00  0.00
F    H
ATOM  18424 HD12 LEU F  87       0.542 -23.750  53.106  1.00  0.00
F    H
ATOM  18425 HD13 LEU F  87      -0.954 -22.743  53.093  1.00  0.00
F    H
ATOM  18426  CD2 LEU F  87      -0.252 -21.231  55.356  1.00  0.00
F    C
ATOM  18427 HD21 LEU F  87       0.301 -20.461  54.777  1.00  0.00
F    H
ATOM  18428 HD22 LEU F  87      -1.335 -21.072  55.178  1.00  0.00
F    H
ATOM  18429 HD23 LEU F  87      -0.047 -21.066  56.437  1.00  0.00
F    H
ATOM  18430  C   LEU F  87      -2.400 -25.178  56.562  1.00  0.00
F    C
ATOM  18431  O   LEU F  87      -2.065 -26.037  57.388  1.00  0.00
F    O
ATOM  18432  N   THR F  88      -3.557 -24.534  56.639  1.00  0.00
F    N
ATOM  18433  HN  THR F  88      -3.820 -23.767  56.055  1.00  0.00
F    H
ATOM  18434  CA  THR F  88      -4.558 -24.910  57.615  1.00  0.00
F    C
ATOM  18435  HA  THR F  88      -4.107 -24.750  58.585  1.00  0.00
F    H
ATOM  18436  CB  THR F  88      -5.811 -24.100  57.343  1.00  0.00
F    C
ATOM  18437  HB  THR F  88      -6.135 -24.367  56.307  1.00  0.00
F    H
ATOM  18438  OG1 THR F  88      -5.476 -22.710  57.366  1.00  0.00
F    O
ATOM  18439  HG1 THR F  88      -6.303 -22.243  57.200  1.00  0.00
F    H
ATOM  18440  CG2 THR F  88      -6.861 -24.404  58.366  1.00  0.00
F    C
```

ATOM 18441 HG21 THR F 88   -7.819 -23.904 58.110 1.00 0.00
F   H
ATOM 18442 HG22 THR F 88   -7.050 -25.499 58.414 1.00 0.00
F   H
ATOM 18443 HG23 THR F 88   -6.547 -24.061 59.375 1.00 0.00
F   H
ATOM 18444 C   THR F 88   -4.863 -26.388 57.522 1.00 0.00
F   C
ATOM 18445 O   THR F 88   -4.617 -27.149 58.466 1.00 0.00
F   O
ATOM 18446 N   THR F 89   -5.357 -26.809 56.358 1.00 0.00
F   N
ATOM 18447 HN THR F 89   -5.535 -26.224 55.568 1.00 0.00
F   H
ATOM 18448 CA THR F 89   -5.685 -28.212 56.150 1.00 0.00
F   C
ATOM 18449 HA THR F 89   -6.491 -28.433 56.837 1.00 0.00
F   H
ATOM 18450 CB THR F 89   -6.039 -28.445 54.694 1.00 0.00
F   C
ATOM 18451 HB THR F 89   -6.094 -29.553 54.562 1.00 0.00
F   H
ATOM 18452 OG1 THR F 89   -4.971 -27.944 53.883 1.00 0.00
F   O
ATOM 18453 HG1 THR F 89   -5.245 -28.102 52.973 1.00 0.00
F   H
ATOM 18454 CG2 THR F 89   -7.308 -27.715 54.351 1.00 0.00
F   C
ATOM 18455 HG21 THR F 89   -7.643 -27.965 53.321 1.00 0.00
F   H
ATOM 18456 HG22 THR F 89   -8.121 -28.000 55.053 1.00 0.00
F   H
ATOM 18457 HG23 THR F 89   -7.166 -26.614 54.411 1.00 0.00
F   H
ATOM 18458 C   THR F 89   -4.529 -29.108 56.541 1.00 0.00
F   C
ATOM 18459 O   THR F 89   -4.731 -30.187 57.107 1.00 0.00
F   O
ATOM 18460 N   LEU F 90   -3.309 -28.686 56.226 1.00 0.00
F   N
ATOM 18461 HN LEU F 90   -3.093 -27.865 55.701 1.00 0.00
F   H
ATOM 18462 CA LEU F 90   -2.145 -29.444 56.654 1.00 0.00
F   C
ATOM 18463 HA LEU F 90   -2.215 -30.419 56.188 1.00 0.00
F   H
ATOM 18464 CB LEU F 90   -0.870 -28.727 56.239 1.00 0.00
F   C
ATOM 18465 HB1 LEU F 90   -0.703 -28.949 55.160 1.00 0.00
F   H
ATOM 18466 HB2 LEU F 90   -1.034 -27.628 56.305 1.00 0.00
F   H
ATOM 18467 CG LEU F 90   0.364 -29.146 57.035 1.00 0.00
F   C

```
ATOM  18468  HG  LEU F  90       0.224 -28.824  58.095  1.00  0.00
F    H
ATOM  18469  CD1 LEU F  90       0.591 -30.640  56.973  1.00  0.00
F    C
ATOM  18470 HD11 LEU F  90       1.520 -30.917  57.518  1.00  0.00
F    H
ATOM  18471 HD12 LEU F  90      -0.260 -31.185  57.435  1.00  0.00
F    H
ATOM  18472 HD13 LEU F  90       0.695 -30.978  55.920  1.00  0.00
F    H
ATOM  18473  CD2 LEU F  90       1.556 -28.432  56.488  1.00  0.00
F    C
ATOM  18474 HD21 LEU F  90       2.468 -28.706  57.063  1.00  0.00
F    H
ATOM  18475 HD22 LEU F  90       1.737 -28.692  55.425  1.00  0.00
F    H
ATOM  18476 HD23 LEU F  90       1.416 -27.332  56.560  1.00  0.00
F    H
ATOM  18477  C   LEU F  90      -2.160 -29.651  58.156  1.00  0.00
F    C
ATOM  18478  O   LEU F  90      -2.060 -30.783  58.637  1.00  0.00
F    O
ATOM  18479  N   LEU F  91      -2.260 -28.565  58.911  1.00  0.00
F    N
ATOM  18480  HN  LEU F  91      -2.385 -27.631  58.582  1.00  0.00
F    H
ATOM  18481  CA  LEU F  91      -2.189 -28.683  60.362  1.00  0.00
F    C
ATOM  18482  HA  LEU F  91      -1.249 -29.169  60.592  1.00  0.00
F    H
ATOM  18483  CB  LEU F  91      -2.246 -27.306  61.007  1.00  0.00
F    C
ATOM  18484  HB1 LEU F  91      -2.873 -26.661  60.349  1.00  0.00
F    H
ATOM  18485  HB2 LEU F  91      -2.791 -27.382  61.973  1.00  0.00
F    H
ATOM  18486  CG  LEU F  91      -0.869 -26.675  61.186  1.00  0.00
F    C
ATOM  18487  HG  LEU F  91      -1.013 -25.652  61.608  1.00  0.00
F    H
ATOM  18488  CD1 LEU F  91      -0.045 -27.513  62.144  1.00  0.00
F    C
ATOM  18489 HD11 LEU F  91       0.979 -27.093  62.248  1.00  0.00
F    H
ATOM  18490 HD12 LEU F  91      -0.517 -27.535  63.150  1.00  0.00
F    H
ATOM  18491 HD13 LEU F  91       0.045 -28.556  61.775  1.00  0.00
F    H
ATOM  18492  CD2 LEU F  91      -0.159 -26.538  59.858  1.00  0.00
F    C
ATOM  18493 HD21 LEU F  91       0.835 -26.060  59.999  1.00  0.00
F    H
ATOM  18494 HD22 LEU F  91       0.007 -27.523  59.378  1.00  0.00
F    H
```

ATOM 18495 HD23 LEU F 91 -0.752 -25.906 59.163 1.00 0.00
F H
ATOM 18496 C LEU F 91 -3.303 -29.570 60.887 1.00 0.00
F C
ATOM 18497 O LEU F 91 -3.092 -30.401 61.778 1.00 0.00
F O
ATOM 18498 N LEU F 92 -4.496 -29.417 60.330 1.00 0.00
F N
ATOM 18499 HN LEU F 92 -4.718 -28.802 59.577 1.00 0.00
F H
ATOM 18500 CA LEU F 92 -5.624 -30.194 60.820 1.00 0.00
F C
ATOM 18501 HA LEU F 92 -5.644 -30.066 61.895 1.00 0.00
F H
ATOM 18502 CB LEU F 92 -6.920 -29.681 60.208 1.00 0.00
F C
ATOM 18503 HB1 LEU F 92 -6.698 -29.403 59.152 1.00 0.00
F H
ATOM 18504 HB2 LEU F 92 -7.653 -30.518 60.159 1.00 0.00
F H
ATOM 18505 CG LEU F 92 -7.496 -28.478 60.943 1.00 0.00
F C
ATOM 18506 HG LEU F 92 -8.567 -28.360 60.649 1.00 0.00
F H
ATOM 18507 CD1 LEU F 92 -7.394 -28.722 62.430 1.00 0.00
F C
ATOM 18508 HD11 LEU F 92 -7.762 -27.839 62.996 1.00 0.00
F H
ATOM 18509 HD12 LEU F 92 -8.001 -29.604 62.724 1.00 0.00
F H
ATOM 18510 HD13 LEU F 92 -6.340 -28.908 62.726 1.00 0.00
F H
ATOM 18511 CD2 LEU F 92 -6.771 -27.212 60.578 1.00 0.00
F C
ATOM 18512 HD21 LEU F 92 -7.217 -26.344 61.110 1.00 0.00
F H
ATOM 18513 HD22 LEU F 92 -5.698 -27.260 60.854 1.00 0.00
F H
ATOM 18514 HD23 LEU F 92 -6.844 -27.024 59.485 1.00 0.00
F H
ATOM 18515 C LEU F 92 -5.443 -31.677 60.537 1.00 0.00
F C
ATOM 18516 O LEU F 92 -5.538 -32.505 61.450 1.00 0.00
F O
ATOM 18517 N LYS F 93 -5.168 -32.031 59.285 1.00 0.00
F N
ATOM 18518 HN LYS F 93 -5.088 -31.399 58.515 1.00 0.00
F H
ATOM 18519 CA LYS F 93 -4.959 -33.432 58.958 1.00 0.00
F C
ATOM 18520 HA LYS F 93 -5.840 -33.973 59.282 1.00 0.00
F H
ATOM 18521 CB LYS F 93 -4.757 -33.595 57.457 1.00 0.00
F C

ATOM 18522 HB1 LYS F 93 −5.324 −32.775 56.960 1.00 0.00
F H
ATOM 18523 HB2 LYS F 93 −3.684 −33.425 57.215 1.00 0.00
F H
ATOM 18524 CG LYS F 93 −5.231 −34.928 56.923 1.00 0.00
F C
ATOM 18525 HG1 LYS F 93 −5.192 −34.910 55.810 1.00 0.00
F H
ATOM 18526 HG2 LYS F 93 −4.532 −35.725 57.266 1.00 0.00
F H
ATOM 18527 CD LYS F 93 −6.647 −35.217 57.378 1.00 0.00
F C
ATOM 18528 HD1 LYS F 93 −6.667 −35.213 58.492 1.00 0.00
F H
ATOM 18529 HD2 LYS F 93 −7.300 −34.384 57.030 1.00 0.00
F H
ATOM 18530 CE LYS F 93 −7.141 −36.545 56.832 1.00 0.00
F C
ATOM 18531 HE1 LYS F 93 −7.304 −36.479 55.733 1.00 0.00
F H
ATOM 18532 HE2 LYS F 93 −6.400 −37.350 57.030 1.00 0.00
F H
ATOM 18533 NZ LYS F 93 −8.431 −36.961 57.451 1.00 0.00
F N
ATOM 18534 HZ1 LYS F 93 −8.741 −37.871 57.054 1.00 0.00
F H
ATOM 18535 HZ2 LYS F 93 −8.308 −37.056 58.479 1.00 0.00
F H
ATOM 18536 HZ3 LYS F 93 −9.154 −36.239 57.259 1.00 0.00
F H
ATOM 18537 C LYS F 93 −3.775 −33.996 59.722 1.00 0.00
F C
ATOM 18538 O LYS F 93 −3.741 −35.188 60.041 1.00 0.00
F O
ATOM 18539 N GLU F 94 −2.793 −33.152 60.029 1.00 0.00
F N
ATOM 18540 HN GLU F 94 −2.713 −32.209 59.720 1.00 0.00
F H
ATOM 18541 CA GLU F 94 −1.708 −33.585 60.891 1.00 0.00
F C
ATOM 18542 HA GLU F 94 −1.338 −34.533 60.522 1.00 0.00
F H
ATOM 18543 CB GLU F 94 −0.584 −32.557 60.880 1.00 0.00
F C
ATOM 18544 HB1 GLU F 94 −0.038 −32.649 59.913 1.00 0.00
F H
ATOM 18545 HB2 GLU F 94 −1.012 −31.530 60.892 1.00 0.00
F H
ATOM 18546 CG GLU F 94 0.393 −32.728 62.020 1.00 0.00
F C
ATOM 18547 HG1 GLU F 94 −0.053 −32.332 62.951 1.00 0.00
F H
ATOM 18548 HG2 GLU F 94 0.653 −33.796 62.164 1.00 0.00
F H

```
ATOM  18549  CD  GLU F  94       1.663 -31.947  61.807  1.00  0.00
F    C
ATOM  18550  OE1 GLU F  94       2.060 -31.771  60.638  1.00  0.00
F    O
ATOM  18551  OE2 GLU F  94       2.271 -31.514  62.807  1.00  0.00
F    O
ATOM  18552  C   GLU F  94      -2.200 -33.815  62.307  1.00  0.00
F    C
ATOM  18553  O   GLU F  94      -1.714 -34.715  62.999  1.00  0.00
F    O
ATOM  18554  N   LEU F  95      -3.161 -33.018  62.757  1.00  0.00
F    N
ATOM  18555  HN  LEU F  95      -3.561 -32.264  62.240  1.00  0.00
F    H
ATOM  18556  CA  LEU F  95      -3.718 -33.193  64.085  1.00  0.00
F    C
ATOM  18557  HA  LEU F  95      -2.952 -33.688  64.668  1.00  0.00
F    H
ATOM  18558  CB  LEU F  95      -4.029 -31.840  64.710  1.00  0.00
F    C
ATOM  18559  HB1 LEU F  95      -4.230 -31.131  63.874  1.00  0.00
F    H
ATOM  18560  HB2 LEU F  95      -4.980 -31.917  65.284  1.00  0.00
F    H
ATOM  18561  CG  LEU F  95      -2.823 -31.417  65.546  1.00  0.00
F    C
ATOM  18562  HG  LEU F  95      -2.533 -32.319  66.138  1.00  0.00
F    H
ATOM  18563  CD1 LEU F  95      -1.663 -30.996  64.662  1.00  0.00
F    C
ATOM  18564 HD11 LEU F  95      -0.809 -30.645  65.281  1.00  0.00
F    H
ATOM  18565 HD12 LEU F  95      -1.316 -31.848  64.040  1.00  0.00
F    H
ATOM  18566 HD13 LEU F  95      -1.965 -30.168  63.986  1.00  0.00
F    H
ATOM  18567  CD2 LEU F  95      -3.185 -30.321  66.515  1.00  0.00
F    C
ATOM  18568 HD21 LEU F  95      -2.301 -30.039  67.127  1.00  0.00
F    H
ATOM  18569 HD22 LEU F  95      -3.535 -29.409  65.990  1.00  0.00
F    H
ATOM  18570 HD23 LEU F  95      -3.990 -30.660  67.203  1.00  0.00
F    H
ATOM  18571  C   LEU F  95      -4.946 -34.083  64.091  1.00  0.00
F    C
ATOM  18572  O   LEU F  95      -5.768 -33.983  65.006  1.00  0.00
F    O
ATOM  18573  N   ASP F  96      -5.097 -34.929  63.076  1.00  0.00
F    N
ATOM  18574  HN  ASP F  96      -4.667 -34.821  62.183  1.00  0.00
F    H
ATOM  18575  CA  ASP F  96      -5.922 -36.127  63.167  1.00  0.00
F    C
```

```
ATOM  18576  HA  ASP F  96      -5.850 -36.625  62.207  1.00  0.00
F    H
ATOM  18577  CB  ASP F  96      -5.421 -37.030  64.288  1.00  0.00
F    C
ATOM  18578  HB1 ASP F  96      -5.769 -36.631  65.263  1.00  0.00
F    H
ATOM  18579  HB2 ASP F  96      -5.819 -38.057  64.169  1.00  0.00
F    H
ATOM  18580  CG  ASP F  96      -3.921 -37.094  64.333  1.00  0.00
F    C
ATOM  18581  OD1 ASP F  96      -3.299 -36.960  63.260  1.00  0.00
F    O
ATOM  18582  OD2 ASP F  96      -3.362 -37.251  65.436  1.00  0.00
F    O
ATOM  18583  C   ASP F  96      -7.387 -35.801  63.382  1.00  0.00
F    C
ATOM  18584  O   ASP F  96      -8.168 -36.659  63.794  1.00  0.00
F    O
ATOM  18585  N   ILE F  97      -7.769 -34.562  63.112  1.00  0.00
F    N
ATOM  18586  HN  ILE F  97      -7.199 -33.815  62.776  1.00  0.00
F    H
ATOM  18587  CA  ILE F  97      -9.169 -34.202  63.314  1.00  0.00
F    C
ATOM  18588  HA  ILE F  97      -9.431 -34.479  64.328  1.00  0.00
F    H
ATOM  18589  CB  ILE F  97      -9.372 -32.709  63.061  1.00  0.00
F    C
ATOM  18590  HB  ILE F  97      -8.978 -32.545  62.026  1.00  0.00
F    H
ATOM  18591  CG2 ILE F  97     -10.822 -32.322  63.252  1.00  0.00
F    C
ATOM  18592 HG21 ILE F  97     -10.994 -31.277  62.918  1.00  0.00
F    H
ATOM  18593 HG22 ILE F  97     -11.490 -32.979  62.657  1.00  0.00
F    H
ATOM  18594 HG23 ILE F  97     -11.116 -32.397  64.320  1.00  0.00
F    H
ATOM  18595  CG1 ILE F  97      -8.481 -31.929  64.020  1.00  0.00
F    C
ATOM  18596 HG11 ILE F  97      -7.412 -32.150  63.805  1.00  0.00
F    H
ATOM  18597 HG12 ILE F  97      -8.634 -30.839  63.848  1.00  0.00
F    H
ATOM  18598  CD  ILE F  97      -8.773 -32.228  65.469  1.00  0.00
F    C
ATOM  18599  HD1 ILE F  97      -8.141 -31.595  66.129  1.00  0.00
F    H
ATOM  18600  HD2 ILE F  97      -9.838 -32.021  65.705  1.00  0.00
F    H
ATOM  18601  HD3 ILE F  97      -8.560 -33.291  65.707  1.00  0.00
F    H
ATOM  18602  C   ILE F  97     -10.033 -35.046  62.391  1.00  0.00
F    C
```

```
ATOM  18603  O   ILE F  97      -9.666 -35.257  61.220  1.00  0.00
F    O
ATOM  18604  N   PRO F  98     -11.152 -35.585  62.868  1.00  0.00
F    N
ATOM  18605  CD  PRO F  98     -11.720 -35.452  64.217  1.00  0.00
F    C
ATOM  18606  HD1 PRO F  98     -10.666 -35.802  64.132  1.00  0.00
F    H
ATOM  18607  HD2 PRO F  98     -11.844 -34.797  65.109  1.00  0.00
F    H
ATOM  18608  CA  PRO F  98     -11.909 -36.524  62.031  1.00  0.00
F    C
ATOM  18609  HA  PRO F  98     -11.300 -37.394  61.819  1.00  0.00
F    H
ATOM  18610  CB  PRO F  98     -13.086 -36.916  62.932  1.00  0.00
F    C
ATOM  18611  HB1 PRO F  98     -12.771 -37.750  63.599  1.00  0.00
F    H
ATOM  18612  HB2 PRO F  98     -13.980 -37.233  62.358  1.00  0.00
F    H
ATOM  18613  CG  PRO F  98     -12.635 -36.615  64.312  1.00  0.00
F    C
ATOM  18614  HG1 PRO F  98     -13.176 -37.199  65.080  1.00  0.00
F    H
ATOM  18615  HG2 PRO F  98     -12.767 -35.527  64.512  1.00  0.00
F    H
ATOM  18616  C   PRO F  98     -12.381 -35.921  60.727  1.00  0.00
F    C
ATOM  18617  O   PRO F  98     -12.030 -36.418  59.653  1.00  0.00
F    O
ATOM  18618  N   ASN F  99     -13.143 -34.839  60.788  1.00  0.00
F    N
ATOM  18619  HN  ASN F  99     -13.280 -34.309  61.624  1.00  0.00
F    H
ATOM  18620  CA  ASN F  99     -13.851 -34.327  59.627  1.00  0.00
F    C
ATOM  18621  HA  ASN F  99     -13.673 -35.025  58.816  1.00  0.00
F    H
ATOM  18622  CB  ASN F  99     -15.334 -34.168  59.939  1.00  0.00
F    C
ATOM  18623  HB1 ASN F  99     -15.437 -33.367  60.704  1.00  0.00
F    H
ATOM  18624  HB2 ASN F  99     -15.885 -33.838  59.035  1.00  0.00
F    H
ATOM  18625  CG  ASN F  99     -15.931 -35.427  60.490  1.00  0.00
F    C
ATOM  18626  OD1 ASN F  99     -16.368 -35.465  61.635  1.00  0.00
F    O
ATOM  18627  ND2 ASN F  99     -15.932 -36.481  59.686  1.00  0.00
F    N
ATOM  18628 HD21 ASN F  99     -16.336 -37.322  60.036  1.00  0.00
F    H
ATOM  18629 HD22 ASN F  99     -15.567 -36.404  58.764  1.00  0.00
F    H
```

```
ATOM  18630  C   ASN F  99     -13.246 -32.995  59.228  1.00  0.00
F    C
ATOM  18631  O   ASN F  99     -13.235 -32.054  60.026  1.00  0.00
F    O
ATOM  18632  N   ILE F 100     -12.752 -32.916  57.999  1.00  0.00
F    N
ATOM  18633  HN  ILE F 100     -12.775 -33.678  57.354  1.00  0.00
F    H
ATOM  18634  CA  ILE F 100     -12.131 -31.711  57.477  1.00  0.00
F    C
ATOM  18635  HA  ILE F 100     -12.313 -30.907  58.178  1.00  0.00
F    H
ATOM  18636  CB  ILE F 100     -10.636 -31.902  57.226  1.00  0.00
F    C
ATOM  18637  HB  ILE F 100     -10.562 -32.895  56.715  1.00  0.00
F    H
ATOM  18638  CG2 ILE F 100     -10.097 -30.741  56.444  1.00  0.00
F    C
ATOM  18639 HG21 ILE F 100      -9.054 -30.938  56.120  1.00  0.00
F    H
ATOM  18640 HG22 ILE F 100     -10.703 -30.563  55.531  1.00  0.00
F    H
ATOM  18641 HG23 ILE F 100     -10.101 -29.814  57.055  1.00  0.00
F    H
ATOM  18642  CG1 ILE F 100      -9.898 -32.027  58.555  1.00  0.00
F    C
ATOM  18643 HG11 ILE F 100     -10.052 -31.106  59.160  1.00  0.00
F    H
ATOM  18644 HG12 ILE F 100     -10.334 -32.875  59.131  1.00  0.00
F    H
ATOM  18645  CD  ILE F 100      -8.426 -32.272  58.406  1.00  0.00
F    C
ATOM  18646  HD1 ILE F 100      -7.954 -32.412  59.403  1.00  0.00
F    H
ATOM  18647  HD2 ILE F 100      -8.240 -33.186  57.803  1.00  0.00
F    H
ATOM  18648  HD3 ILE F 100      -7.927 -31.414  57.908  1.00  0.00
F    H
ATOM  18649  C   ILE F 100     -12.861 -31.352  56.198  1.00  0.00
F    C
ATOM  18650  O   ILE F 100     -12.807 -32.096  55.210  1.00  0.00
F    O
ATOM  18651  N   TRP F 101     -13.559 -30.223  56.212  1.00  0.00
F    N
ATOM  18652  HN  TRP F 101     -13.531 -29.586  56.978  1.00  0.00
F    H
ATOM  18653  CA  TRP F 101     -14.421 -29.824  55.110  1.00  0.00
F    C
ATOM  18654  HA  TRP F 101     -14.236 -30.487  54.273  1.00  0.00
F    H
ATOM  18655  CB  TRP F 101     -15.883 -29.875  55.542  1.00  0.00
F    C
ATOM  18656  HB1 TRP F 101     -15.958 -29.399  56.544  1.00  0.00
F    H
```

```
ATOM  18657  HB2 TRP F 101     -16.503 -29.266  54.848  1.00  0.00
F    H
ATOM  18658  CG  TRP F 101     -16.375 -31.247  55.644  1.00  0.00
F    C
ATOM  18659  CD1 TRP F 101     -15.720 -32.315  56.164  1.00  0.00
F    C
ATOM  18660  HD1 TRP F 101     -14.739 -32.266  56.619  1.00  0.00
F    H
ATOM  18661  NE1 TRP F 101     -16.491 -33.436  56.067  1.00  0.00
F    N
ATOM  18662  HE1 TRP F 101     -16.268 -34.329  56.396  1.00  0.00
F    H
ATOM  18663  CE2 TRP F 101     -17.674 -33.100  55.467  1.00  0.00
F    C
ATOM  18664  CD2 TRP F 101     -17.629 -31.728  55.189  1.00  0.00
F    C
ATOM  18665  CE3 TRP F 101     -18.723 -31.128  54.572  1.00  0.00
F    C
ATOM  18666  HE3 TRP F 101     -18.732 -30.076  54.325  1.00  0.00
F    H
ATOM  18667  CZ3 TRP F 101     -19.807 -31.903  54.265  1.00  0.00
F    C
ATOM  18668  HZ3 TRP F 101     -20.667 -31.451  53.790  1.00  0.00
F    H
ATOM  18669  CZ2 TRP F 101     -18.768 -33.884  55.153  1.00  0.00
F    C
ATOM  18670  HZ2 TRP F 101     -18.808 -34.942  55.367  1.00  0.00
F    H
ATOM  18671  CH2 TRP F 101     -19.825 -33.269  54.551  1.00  0.00
F    C
ATOM  18672  HH2 TRP F 101     -20.693 -33.857  54.290  1.00  0.00
F    H
ATOM  18673  C   TRP F 101     -14.037 -28.424  54.660  1.00  0.00
F    C
ATOM  18674  O   TRP F 101     -14.004 -27.498  55.475  1.00  0.00
F    O
ATOM  18675  N   VAL F 102     -13.757 -28.262  53.372  1.00  0.00
F    N
ATOM  18676  HN  VAL F 102     -13.948 -28.916  52.645  1.00  0.00
F    H
ATOM  18677  CA  VAL F 102     -13.112 -27.035  52.924  1.00  0.00
F    C
ATOM  18678  HA  VAL F 102     -13.132 -26.336  53.752  1.00  0.00
F    H
ATOM  18679  CB  VAL F 102     -11.653 -27.306  52.513  1.00  0.00
F    C
ATOM  18680  HB  VAL F 102     -11.674 -27.954  51.602  1.00  0.00
F    H
ATOM  18681  CG1 VAL F 102     -10.944 -26.014  52.186  1.00  0.00
F    C
ATOM  18682 HG11 VAL F 102      -9.881 -26.212  51.928  1.00  0.00
F    H
ATOM  18683 HG12 VAL F 102     -11.406 -25.502  51.317  1.00  0.00
F    H
```

```
ATOM  18684 HG13 VAL F 102     -10.963 -25.324  53.057  1.00  0.00
F    H
ATOM  18685  CG2 VAL F 102     -10.924 -28.046  53.611  1.00  0.00
F    C
ATOM  18686 HG21 VAL F 102      -9.861 -28.209  53.336  1.00  0.00
F    H
ATOM  18687 HG22 VAL F 102     -10.956 -27.467  54.559  1.00  0.00
F    H
ATOM  18688 HG23 VAL F 102     -11.379 -29.042  53.795  1.00  0.00
F    H
ATOM  18689  C   VAL F 102     -13.882 -26.404  51.775  1.00  0.00
F    C
ATOM  18690  O   VAL F 102     -14.504 -27.092  50.959  1.00  0.00
F    O
ATOM  18691  N   LYS F 103     -13.837 -25.070  51.728  1.00  0.00
F    N
ATOM  18692  HN  LYS F 103     -13.486 -24.528  52.490  1.00  0.00
F    H
ATOM  18693  CA  LYS F 103     -14.293 -24.299  50.577  1.00  0.00
F    C
ATOM  18694  HA  LYS F 103     -15.076 -24.864  50.085  1.00  0.00
F    H
ATOM  18695  CB  LYS F 103     -14.794 -22.926  51.031  1.00  0.00
F    C
ATOM  18696  HB1 LYS F 103     -15.726 -23.095  51.618  1.00  0.00
F    H
ATOM  18697  HB2 LYS F 103     -14.055 -22.487  51.739  1.00  0.00
F    H
ATOM  18698  CG  LYS F 103     -15.102 -21.930  49.925  1.00  0.00
F    C
ATOM  18699  HG1 LYS F 103     -14.276 -21.947  49.178  1.00  0.00
F    H
ATOM  18700  HG2 LYS F 103     -16.031 -22.248  49.400  1.00  0.00
F    H
ATOM  18701  CD  LYS F 103     -15.249 -20.520  50.510  1.00  0.00
F    C
ATOM  18702  HD1 LYS F 103     -16.082 -20.531  51.250  1.00  0.00
F    H
ATOM  18703  HD2 LYS F 103     -14.313 -20.275  51.064  1.00  0.00
F    H
ATOM  18704  CE  LYS F 103     -15.509 -19.453  49.442  1.00  0.00
F    C
ATOM  18705  HE1 LYS F 103     -14.783 -19.554  48.604  1.00  0.00
F    H
ATOM  18706  HE2 LYS F 103     -16.538 -19.555  49.031  1.00  0.00
F    H
ATOM  18707  NZ  LYS F 103     -15.379 -18.056  49.978  1.00  0.00
F    N
ATOM  18708  HZ1 LYS F 103     -15.562 -17.366  49.221  1.00  0.00
F    H
ATOM  18709  HZ2 LYS F 103     -16.064 -17.915  50.748  1.00  0.00
F    H
ATOM  18710  HZ3 LYS F 103     -14.417 -17.916  50.347  1.00  0.00
F    H
```

ATOM 18711 C   LYS F 103   -13.147 -24.151 49.584 1.00 0.00
F   C
ATOM 18712 O   LYS F 103   -12.075 -23.653 49.938 1.00 0.00
F   O
ATOM 18713 N   ALA F 104   -13.370 -24.580 48.344 1.00 0.00
F   N
ATOM 18714 HN ALA F 104   -14.278 -24.863 48.036 1.00 0.00
F   H
ATOM 18715 CA ALA F 104   -12.315 -24.670 47.343 1.00 0.00
F   C
ATOM 18716 HA ALA F 104   -11.342 -24.569 47.811 1.00 0.00
F   H
ATOM 18717 CB ALA F 104   -12.356 -26.014 46.617 1.00 0.00
F   C
ATOM 18718 HB1 ALA F 104   -12.232 -26.838 47.351 1.00 0.00
F   H
ATOM 18719 HB2 ALA F 104   -13.329 -26.159 46.100 1.00 0.00
F   H
ATOM 18720 HB3 ALA F 104   -11.541 -26.099 45.866 1.00 0.00
F   H
ATOM 18721 C   ALA F 104   -12.417 -23.549 46.319 1.00 0.00
F   C
ATOM 18722 O   ALA F 104   -13.503 -23.049 46.021 1.00 0.00
F   O
ATOM 18723 N   GLN F 105   -11.265 -23.185 45.757 1.00 0.00
F   N
ATOM 18724 HN GLN F 105   -10.405 -23.662 45.928 1.00 0.00
F   H
ATOM 18725 CA GLN F 105   -11.156 -22.057 44.843 1.00 0.00
F   C
ATOM 18726 HA GLN F 105   -11.995 -21.406 45.058 1.00 0.00
F   H
ATOM 18727 CB GLN F 105   -9.836 -21.325 45.088 1.00 0.00
F   C
ATOM 18728 HB1 GLN F 105   -9.779 -21.123 46.183 1.00 0.00
F   H
ATOM 18729 HB2 GLN F 105   -8.988 -22.007 44.853 1.00 0.00
F   H
ATOM 18730 CG GLN F 105   -9.688 -20.023 44.338 1.00 0.00
F   C
ATOM 18731 HG1 GLN F 105   -10.686 -19.593 44.105 1.00 0.00
F   H
ATOM 18732 HG2 GLN F 105   -9.132 -19.280 44.946 1.00 0.00
F   H
ATOM 18733 CD GLN F 105   -8.979 -20.202 43.017 1.00 0.00
F   C
ATOM 18734 OE1 GLN F 105   -8.271 -21.184 42.813 1.00 0.00
F   O
ATOM 18735 NE2 GLN F 105   -9.150 -19.243 42.120 1.00 0.00
F   N
ATOM 18736 HE21 GLN F 105   -8.689 -19.359 41.245 1.00 0.00
F   H
ATOM 18737 HE22 GLN F 105   -9.706 -18.450 42.344 1.00 0.00
F   H

```
ATOM  18738  C   GLN F 105     -11.266 -22.480  43.381  1.00  0.00
F    C
ATOM  18739  O   GLN F 105     -12.198 -22.067  42.688  1.00  0.00
F    O
ATOM  18740  N   ASN F 106     -10.343 -23.305  42.899  1.00  0.00
F    N
ATOM  18741  HN  ASN F 106      -9.558 -23.644  43.416  1.00  0.00
F    H
ATOM  18742  CA  ASN F 106     -10.417 -23.789  41.529  1.00  0.00
F    C
ATOM  18743  HA  ASN F 106     -11.423 -23.589  41.177  1.00  0.00
F    H
ATOM  18744  CB  ASN F 106      -9.377 -23.136  40.620  1.00  0.00
F    C
ATOM  18745  HB1 ASN F 106      -9.591 -23.452  39.576  1.00  0.00
F    H
ATOM  18746  HB2 ASN F 106      -9.469 -22.032  40.658  1.00  0.00
F    H
ATOM  18747  CG  ASN F 106      -7.953 -23.552  40.952  1.00  0.00
F    C
ATOM  18748  OD1 ASN F 106      -7.705 -24.367  41.838  1.00  0.00
F    O
ATOM  18749  ND2 ASN F 106      -7.006 -23.002  40.212  1.00  0.00
F    N
ATOM  18750 HD21 ASN F 106      -6.064 -23.267  40.402  1.00  0.00
F    H
ATOM  18751 HD22 ASN F 106      -7.249 -22.373  39.480  1.00  0.00
F    H
ATOM  18752  C   ASN F 106     -10.222 -25.291  41.534  1.00  0.00
F    C
ATOM  18753  O   ASN F 106      -9.967 -25.903  42.574  1.00  0.00
F    O
ATOM  18754  N   TYR F 107     -10.306 -25.877  40.345  1.00  0.00
F    N
ATOM  18755  HN  TYR F 107     -10.551 -25.424  39.490  1.00  0.00
F    H
ATOM  18756  CA  TYR F 107     -10.028 -27.298  40.216  1.00  0.00
F    C
ATOM  18757  HA  TYR F 107     -10.767 -27.812  40.818  1.00  0.00
F    H
ATOM  18758  CB  TYR F 107     -10.110 -27.713  38.751  1.00  0.00
F    C
ATOM  18759  HB1 TYR F 107     -11.166 -27.952  38.499  1.00  0.00
F    H
ATOM  18760  HB2 TYR F 107      -9.793 -26.879  38.088  1.00  0.00
F    H
ATOM  18761  CG  TYR F 107      -9.292 -28.939  38.464  1.00  0.00
F    C
ATOM  18762  CD1 TYR F 107      -9.774 -30.198  38.769  1.00  0.00
F    C
ATOM  18763  HD1 TYR F 107     -10.748 -30.305  39.226  1.00  0.00
F    H
ATOM  18764  CE1 TYR F 107      -9.027 -31.320  38.521  1.00  0.00
F    C
```

ATOM 18765 HE1 TYR F 107 −9.410 −32.299 38.766 1.00 0.00
F H
ATOM 18766 CZ TYR F 107 −7.779 −31.193 37.965 1.00 0.00
F C
ATOM 18767 OH TYR F 107 −7.022 −32.312 37.712 1.00 0.00
F O
ATOM 18768 HH TYR F 107 −6.225 −32.025 37.262 1.00 0.00
F H
ATOM 18769 CD2 TYR F 107 −8.032 −28.838 37.906 1.00 0.00
F C
ATOM 18770 HD2 TYR F 107 −7.611 −27.864 37.695 1.00 0.00
F H
ATOM 18771 CE2 TYR F 107 −7.279 −29.955 37.657 1.00 0.00
F C
ATOM 18772 HE2 TYR F 107 −6.288 −29.851 37.240 1.00 0.00
F H
ATOM 18773 C TYR F 107 −8.664 −27.644 40.789 1.00 0.00
F C
ATOM 18774 O TYR F 107 −8.510 −28.655 41.484 1.00 0.00
F O
ATOM 18775 N TYR F 108 −7.659 −26.820 40.505 1.00 0.00
F N
ATOM 18776 HN TYR F 108 −7.722 −25.972 39.981 1.00 0.00
F H
ATOM 18777 CA TYR F 108 −6.322 −27.139 40.981 1.00 0.00
F C
ATOM 18778 HA TYR F 108 −6.116 −28.154 40.664 1.00 0.00
F H
ATOM 18779 CB TYR F 108 −5.316 −26.160 40.403 1.00 0.00
F C
ATOM 18780 HB1 TYR F 108 −5.621 −25.124 40.668 1.00 0.00
F H
ATOM 18781 HB2 TYR F 108 −4.309 −26.328 40.840 1.00 0.00
F H
ATOM 18782 CG TYR F 108 −5.277 −26.265 38.927 1.00 0.00
F C
ATOM 18783 CD1 TYR F 108 −5.063 −27.487 38.326 1.00 0.00
F C
ATOM 18784 HD1 TYR F 108 −4.898 −28.365 38.936 1.00 0.00
F H
ATOM 18785 CE1 TYR F 108 −5.032 −27.613 36.973 1.00 0.00
F C
ATOM 18786 HE1 TYR F 108 −4.858 −28.576 36.514 1.00 0.00
F H
ATOM 18787 CZ TYR F 108 −5.216 −26.507 36.190 1.00 0.00
F C
ATOM 18788 OH TYR F 108 −5.182 −26.631 34.823 1.00 0.00
F O
ATOM 18789 HH TYR F 108 −5.401 −25.774 34.451 1.00 0.00
F H
ATOM 18790 CD2 TYR F 108 −5.469 −25.163 38.128 1.00 0.00
F C
ATOM 18791 HD2 TYR F 108 −5.608 −24.188 38.576 1.00 0.00
F H

```
ATOM  18792  CE2 TYR F 108      -5.437 -25.274  36.765  1.00  0.00
F    C
ATOM  18793  HE2 TYR F 108      -5.563 -24.394  36.152  1.00  0.00
F    H
ATOM  18794  C   TYR F 108      -6.259 -27.122  42.491  1.00  0.00
F    C
ATOM  18795  O   TYR F 108      -6.054 -28.171  43.118  1.00  0.00
F    O
ATOM  18796  N   HSE F 109      -6.491 -25.950  43.082  1.00  0.00
F    N
ATOM  18797  HN  HSE F 109      -6.613 -25.087  42.593  1.00  0.00
F    H
ATOM  18798  CA  HSE F 109      -6.585 -25.837  44.529  1.00  0.00
F    C
ATOM  18799  HA  HSE F 109      -5.575 -25.868  44.918  1.00  0.00
F    H
ATOM  18800  CB  HSE F 109      -7.295 -24.540  44.885  1.00  0.00
F    C
ATOM  18801  HB1 HSE F 109      -6.628 -23.696  44.601  1.00  0.00
F    H
ATOM  18802  HB2 HSE F 109      -8.218 -24.443  44.276  1.00  0.00
F    H
ATOM  18803  ND1 HSE F 109      -8.895 -24.237  46.799  1.00  0.00
F    N
ATOM  18804  CG  HSE F 109      -7.612 -24.411  46.335  1.00  0.00
F    C
ATOM  18805  CE1 HSE F 109      -8.877 -24.151  48.116  1.00  0.00
F    C
ATOM  18806  HE1 HSE F 109      -9.763 -24.013  48.741  1.00  0.00
F    H
ATOM  18807  NE2 HSE F 109      -7.624 -24.254  48.521  1.00  0.00
F    N
ATOM  18808  HE2 HSE F 109      -7.321 -24.200  49.472  1.00  0.00
F    H
ATOM  18809  CD2 HSE F 109      -6.812 -24.414  47.426  1.00  0.00
F    C
ATOM  18810  HD2 HSE F 109      -5.738 -24.508  47.497  1.00  0.00
F    H
ATOM  18811  C   HSE F 109      -7.324 -27.023  45.112  1.00  0.00
F    C
ATOM  18812  O   HSE F 109      -6.924 -27.569  46.144  1.00  0.00
F    O
ATOM  18813  N   HSE F 110      -8.368 -27.471  44.425  1.00  0.00
F    N
ATOM  18814  HN  HSE F 110      -8.717 -27.051  43.588  1.00  0.00
F    H
ATOM  18815  CA  HSE F 110      -9.104 -28.646  44.857  1.00  0.00
F    C
ATOM  18816  HA  HSE F 110      -9.397 -28.467  45.885  1.00  0.00
F    H
ATOM  18817  CB  HSE F 110     -10.309 -28.833  43.957  1.00  0.00
F    C
ATOM  18818  HB1 HSE F 110     -11.170 -28.306  44.425  1.00  0.00
F    H
```

```
ATOM  18819  HB2 HSE F 110     -10.121 -28.342  42.979  1.00  0.00
F    H
ATOM  18820  ND1 HSE F 110     -11.600 -30.895  44.572  1.00  0.00
F    N
ATOM  18821  CG  HSE F 110     -10.691 -30.257  43.761  1.00  0.00
F    C
ATOM  18822  CE1 HSE F 110     -11.738 -32.144  44.172  1.00  0.00
F    C
ATOM  18823  HE1 HSE F 110     -12.395 -32.878  44.645  1.00  0.00
F    H
ATOM  18824  NE2 HSE F 110     -10.951 -32.338  43.130  1.00  0.00
F    N
ATOM  18825  HE2 HSE F 110     -10.846 -33.203  42.640  1.00  0.00
F    H
ATOM  18826  CD2 HSE F 110     -10.284 -31.171  42.852  1.00  0.00
F    C
ATOM  18827  HD2 HSE F 110      -9.572 -31.078  42.045  1.00  0.00
F    H
ATOM  18828  C   HSE F 110      -8.234 -29.893  44.850  1.00  0.00
F    C
ATOM  18829  O   HSE F 110      -8.285 -30.702  45.782  1.00  0.00
F    O
ATOM  18830  N   LYS F 111      -7.467 -30.101  43.789  1.00  0.00
F    N
ATOM  18831  HN  LYS F 111      -7.393 -29.515  42.984  1.00  0.00
F    H
ATOM  18832  CA  LYS F 111      -6.635 -31.297  43.762  1.00  0.00
F    C
ATOM  18833  HA  LYS F 111      -7.283 -32.140  43.970  1.00  0.00
F    H
ATOM  18834  CB  LYS F 111      -5.977 -31.466  42.402  1.00  0.00
F    C
ATOM  18835  HB1 LYS F 111      -6.790 -31.674  41.668  1.00  0.00
F    H
ATOM  18836  HB2 LYS F 111      -5.522 -30.498  42.097  1.00  0.00
F    H
ATOM  18837  CG  LYS F 111      -4.962 -32.594  42.383  1.00  0.00
F    C
ATOM  18838  HG1 LYS F 111      -4.042 -32.248  41.859  1.00  0.00
F    H
ATOM  18839  HG2 LYS F 111      -4.674 -32.841  43.431  1.00  0.00
F    H
ATOM  18840  CD  LYS F 111      -5.474 -33.859  41.676  1.00  0.00
F    C
ATOM  18841  HD1 LYS F 111      -4.825 -34.055  40.792  1.00  0.00
F    H
ATOM  18842  HD2 LYS F 111      -5.355 -34.720  42.375  1.00  0.00
F    H
ATOM  18843  CE  LYS F 111      -6.913 -33.727  41.255  1.00  0.00
F    C
ATOM  18844  HE1 LYS F 111      -7.246 -32.668  41.336  1.00  0.00
F    H
ATOM  18845  HE2 LYS F 111      -7.044 -34.058  40.202  1.00  0.00
F    H
```

ATOM 18846 NZ LYS F 111   −7.770 −34.563 42.128 1.00 0.00
F   N
ATOM 18847 HZ1 LYS F 111   −8.766 −34.473 41.841 1.00 0.00
F   H
ATOM 18848 HZ2 LYS F 111   −7.478 −35.558 42.051 1.00 0.00
F   H
ATOM 18849 HZ3 LYS F 111   −7.667 −34.252 43.115 1.00 0.00
F   H
ATOM 18850 C   LYS F 111   −5.580 −31.257 44.851 1.00 0.00
F   C
ATOM 18851 O   LYS F 111   −5.344 −32.255 45.542 1.00 0.00
F   O
ATOM 18852 N   VAL F 112   −4.937 −30.109 45.021 1.00 0.00
F   N
ATOM 18853 HN VAL F 112   −5.053 −29.287 44.469 1.00 0.00
F   H
ATOM 18854 CA VAL F 112   −3.959 −29.981 46.097 1.00 0.00
F   C
ATOM 18855 HA VAL F 112   −3.218 −30.760 45.965 1.00 0.00
F   H
ATOM 18856 CB VAL F 112   −3.254 −28.619 46.030 1.00 0.00
F   C
ATOM 18857 HB VAL F 112   −2.152 −28.809 46.018 1.00 0.00
F   H
ATOM 18858 CG1 VAL F 112   −3.647 −27.899 44.785 1.00 0.00
F   C
ATOM 18859 HG11 VAL F 112   −3.169 −26.896 44.752 1.00 0.00
F   H
ATOM 18860 HG12 VAL F 112   −3.326 −28.448 43.876 1.00 0.00
F   H
ATOM 18861 HG13 VAL F 112   −4.748 −27.756 44.745 1.00 0.00
F   H
ATOM 18862 CG2 VAL F 112   −3.609 −27.792 47.235 1.00 0.00
F   C
ATOM 18863 HG21 VAL F 112   −3.135 −26.789 47.176 1.00 0.00
F   H
ATOM 18864 HG22 VAL F 112   −4.710 −27.655 47.303 1.00 0.00
F   H
ATOM 18865 HG23 VAL F 112   −3.259 −28.273 48.171 1.00 0.00
F   H
ATOM 18866 C   VAL F 112   −4.625 −30.207 47.443 1.00 0.00
F   C
ATOM 18867 O   VAL F 112   −4.006 −30.728 48.379 1.00 0.00
F   O
ATOM 18868 N   LEU F 113   −5.895 −29.828 47.559 1.00 0.00
F   N
ATOM 18869 HN LEU F 113   −6.399 −29.287 46.888 1.00 0.00
F   H
ATOM 18870 CA LEU F 113   −6.668 −30.196 48.731 1.00 0.00
F   C
ATOM 18871 HA LEU F 113   −6.146 −29.799 49.591 1.00 0.00
F   H
ATOM 18872 CB LEU F 113   −8.074 −29.630 48.601 1.00 0.00
F   C

ATOM 18873 HB1 LEU F 113   -7.994 -28.680 48.023 1.00 0.00
F   H
ATOM 18874 HB2 LEU F 113   -8.685 -30.319 47.977 1.00 0.00
F   H
ATOM 18875 CG LEU F 113   -8.831 -29.318 49.876 1.00 0.00
F   C
ATOM 18876 HG LEU F 113   -9.400 -30.230 50.180 1.00 0.00
F   H
ATOM 18877 CD1 LEU F 113   -7.869 -28.956 50.974 1.00 0.00
F   C
ATOM 18878 HD11 LEU F 113   -8.419 -28.680 51.899 1.00 0.00
F   H
ATOM 18879 HD12 LEU F 113   -7.202 -29.814 51.208 1.00 0.00
F   H
ATOM 18880 HD13 LEU F 113   -7.238 -28.092 50.675 1.00 0.00
F   H
ATOM 18881 CD2 LEU F 113   -9.823 -28.221 49.603 1.00 0.00
F   C
ATOM 18882 HD21 LEU F 113   -10.405 -27.989 50.522 1.00 0.00
F   H
ATOM 18883 HD22 LEU F 113   -9.322 -27.287 49.276 1.00 0.00
F   H
ATOM 18884 HD23 LEU F 113   -10.537 -28.531 48.810 1.00 0.00
F   H
ATOM 18885 C   LEU F 113   -6.721 -31.702 48.884 1.00 0.00
F   C
ATOM 18886 O   LEU F 113   -6.484 -32.238 49.970 1.00 0.00
F   O
ATOM 18887 N   GLU F 114   -7.022 -32.398 47.794 1.00 0.00
F   N
ATOM 18888 HN GLU F 114   -7.196 -32.017 46.889 1.00 0.00
F   H
ATOM 18889 CA GLU F 114   -7.123 -33.849 47.854 1.00 0.00
F   C
ATOM 18890 HA GLU F 114   -7.895 -34.104 48.569 1.00 0.00
F   H
ATOM 18891 CB GLU F 114   -7.492 -34.409 46.486 1.00 0.00
F   C
ATOM 18892 HB1 GLU F 114   -8.301 -33.773 46.058 1.00 0.00
F   H
ATOM 18893 HB2 GLU F 114   -6.627 -34.310 45.792 1.00 0.00
F   H
ATOM 18894 CG GLU F 114   -7.954 -35.836 46.569 1.00 0.00
F   C
ATOM 18895 HG1 GLU F 114   -8.511 -36.097 45.650 1.00 0.00
F   H
ATOM 18896 HG2 GLU F 114   -7.094 -36.526 46.682 1.00 0.00
F   H
ATOM 18897 CD GLU F 114   -8.911 -36.053 47.716 1.00 0.00
F   C
ATOM 18898 OE1 GLU F 114   -9.928 -35.334 47.779 1.00 0.00
F   O
ATOM 18899 OE2 GLU F 114   -8.639 -36.927 48.564 1.00 0.00
F   O

ATOM 18900 C   GLU F 114   -5.826 -34.470 48.350 1.00 0.00
F   C
ATOM 18901 O   GLU F 114   -5.835 -35.381 49.184 1.00 0.00
F   O
ATOM 18902 N   LYS F 115   -4.694 -33.977 47.854 1.00 0.00
F   N
ATOM 18903 HN LYS F 115   -4.630 -33.192 47.240 1.00 0.00
F   H
ATOM 18904 CA LYS F 115   -3.421 -34.593 48.204 1.00 0.00
F   C
ATOM 18905 HA LYS F 115   -3.513 -35.645 47.962 1.00 0.00
F   H
ATOM 18906 CB LYS F 115   -2.295 -33.959 47.410 1.00 0.00
F   C
ATOM 18907 HB1 LYS F 115   -2.385 -32.855 47.537 1.00 0.00
F   H
ATOM 18908 HB2 LYS F 115   -1.322 -34.248 47.868 1.00 0.00
F   H
ATOM 18909 CG LYS F 115   -2.356 -34.313 45.973 1.00 0.00
F   C
ATOM 18910 HG1 LYS F 115   -3.166 -33.723 45.487 1.00 0.00
F   H
ATOM 18911 HG2 LYS F 115   -1.396 -34.024 45.487 1.00 0.00
F   H
ATOM 18912 CD LYS F 115   -2.626 -35.772 45.793 1.00 0.00
F   C
ATOM 18913 HD1 LYS F 115   -1.844 -36.345 46.342 1.00 0.00
F   H
ATOM 18914 HD2 LYS F 115   -3.606 -36.005 46.269 1.00 0.00
F   H
ATOM 18915 CE LYS F 115   -2.631 -36.083 44.324 1.00 0.00
F   C
ATOM 18916 HE1 LYS F 115   -2.607 -37.183 44.159 1.00 0.00
F   H
ATOM 18917 HE2 LYS F 115   -3.546 -35.674 43.842 1.00 0.00
F   H
ATOM 18918 NZ LYS F 115   -1.435 -35.474 43.686 1.00 0.00
F   N
ATOM 18919 HZ1 LYS F 115   -1.429 -35.685 42.667 1.00 0.00
F   H
ATOM 18920 HZ2 LYS F 115   -1.453 -34.443 43.825 1.00 0.00
F   H
ATOM 18921 HZ3 LYS F 115   -0.574 -35.862 44.124 1.00 0.00
F   H
ATOM 18922 C   LYS F 115   -3.107 -34.494 49.686 1.00 0.00
F   C
ATOM 18923 O   LYS F 115   -2.454 -35.389 50.229 1.00 0.00
F   O
ATOM 18924 N   ILE F 116   -3.545 -33.433 50.355 1.00 0.00
F   N
ATOM 18925 HN ILE F 116   -4.059 -32.680 49.952 1.00 0.00
F   H
ATOM 18926 CA ILE F 116   -3.298 -33.289 51.780 1.00 0.00
F   C

ATOM 18927 HA ILE F 116   -2.256 -33.543 51.932 1.00 0.00
F   H
ATOM 18928 CB ILE F 116   -3.586 -31.860 52.247 1.00 0.00
F   C
ATOM 18929 HB ILE F 116   -3.173 -31.211 51.434 1.00 0.00
F   H
ATOM 18930 CG2 ILE F 116   -5.050 -31.709 52.562 1.00 0.00
F   C
ATOM 18931 HG21 ILE F 116   -5.301 -30.645 52.751 1.00 0.00
F   H
ATOM 18932 HG22 ILE F 116   -5.675 -32.056 51.713 1.00 0.00
F   H
ATOM 18933 HG23 ILE F 116   -5.326 -32.295 53.463 1.00 0.00
F   H
ATOM 18934 CG1 ILE F 116   -2.798 -31.530 53.513 1.00 0.00
F   C
ATOM 18935 HG11 ILE F 116   -2.955 -30.463 53.787 1.00 0.00
F   H
ATOM 18936 HG12 ILE F 116   -3.190 -32.147 54.353 1.00 0.00
F   H
ATOM 18937 CD ILE F 116   -1.340 -31.780 53.415 1.00 0.00
F   C
ATOM 18938 HD1 ILE F 116   -0.847 -31.572 54.389 1.00 0.00
F   H
ATOM 18939 HD2 ILE F 116   -1.142 -32.839 53.144 1.00 0.00
F   H
ATOM 18940 HD3 ILE F 116   -0.875 -31.126 52.648 1.00 0.00
F   H
ATOM 18941 C   ILE F 116   -4.113 -34.279 52.593 1.00 0.00
F   C
ATOM 18942 O   ILE F 116   -3.817 -34.505 53.766 1.00 0.00
F   O
ATOM 18943 N   GLY F 117   -5.123 -34.891 51.992 1.00 0.00
F   N
ATOM 18944 HN GLY F 117   -5.286 -34.874 51.006 1.00 0.00
F   H
ATOM 18945 CA GLY F 117   -6.078 -35.644 52.770 1.00 0.00
F   C
ATOM 18946 HA1 GLY F 117   -5.607 -35.866 53.718 1.00 0.00
F   H
ATOM 18947 HA2 GLY F 117   -6.345 -36.518 52.191 1.00 0.00
F   H
ATOM 18948 C   GLY F 117   -7.336 -34.869 53.044 1.00 0.00
F   C
ATOM 18949 O   GLY F 117   -7.863 -34.925 54.159 1.00 0.00
F   O
ATOM 18950 N   ALA F 118   -7.827 -34.129 52.058 1.00 0.00
F   N
ATOM 18951 HN ALA F 118   -7.387 -34.018 51.168 1.00 0.00
F   H
ATOM 18952 CA ALA F 118   -9.077 -33.413 52.228 1.00 0.00
F   C
ATOM 18953 HA ALA F 118   -9.040 -32.762 53.094 1.00 0.00
F   H

```
ATOM  18954  CB  ALA F 118      -9.382 -32.580  50.992  1.00  0.00
F    C
ATOM  18955  HB1 ALA F 118      -8.577 -31.830  50.840  1.00  0.00
F    H
ATOM  18956  HB2 ALA F 118      -9.432 -33.218  50.082  1.00  0.00
F    H
ATOM  18957  HB3 ALA F 118     -10.345 -32.033  51.094  1.00  0.00
F    H
ATOM  18958  C   ALA F 118     -10.209 -34.390  52.475  1.00  0.00
F    C
ATOM  18959  O   ALA F 118     -10.621 -35.126  51.578  1.00  0.00
F    O
ATOM  18960  N   ASP F 119     -10.708 -34.399  53.704  1.00  0.00
F    N
ATOM  18961  HN  ASP F 119     -10.401 -33.873  54.493  1.00  0.00
F    H
ATOM  18962  CA  ASP F 119     -11.838 -35.257  54.002  1.00  0.00
F    C
ATOM  18963  HA  ASP F 119     -11.557 -36.263  53.714  1.00  0.00
F    H
ATOM  18964  CB  ASP F 119     -12.171 -35.186  55.489  1.00  0.00
F    C
ATOM  18965  HB1 ASP F 119     -12.275 -34.124  55.789  1.00  0.00
F    H
ATOM  18966  HB2 ASP F 119     -13.127 -35.703  55.701  1.00  0.00
F    H
ATOM  18967  CG  ASP F 119     -11.087 -35.799  56.347  1.00  0.00
F    C
ATOM  18968  OD1 ASP F 119     -11.227 -36.984  56.719  1.00  0.00
F    O
ATOM  18969  OD2 ASP F 119     -10.091 -35.104  56.634  1.00  0.00
F    O
ATOM  18970  C   ASP F 119     -13.047 -34.884  53.166  1.00  0.00
F    C
ATOM  18971  O   ASP F 119     -13.900 -35.737  52.905  1.00  0.00
F    O
ATOM  18972  N   ARG F 120     -13.134 -33.631  52.737  1.00  0.00
F    N
ATOM  18973  HN  ARG F 120     -12.464 -32.931  52.974  1.00  0.00
F    H
ATOM  18974  CA  ARG F 120     -14.234 -33.217  51.884  1.00  0.00
F    C
ATOM  18975  HA  ARG F 120     -14.301 -33.898  51.043  1.00  0.00
F    H
ATOM  18976  CB  ARG F 120     -15.545 -33.206  52.659  1.00  0.00
F    C
ATOM  18977  HB1 ARG F 120     -15.869 -34.270  52.744  1.00  0.00
F    H
ATOM  18978  HB2 ARG F 120     -15.360 -32.857  53.699  1.00  0.00
F    H
ATOM  18979  CG  ARG F 120     -16.594 -32.420  51.978  1.00  0.00
F    C
ATOM  18980  HG1 ARG F 120     -16.751 -31.476  52.545  1.00  0.00
F    H
```

```
ATOM  18981  HG2 ARG F 120     -16.221 -32.109  50.975  1.00  0.00
F    H
ATOM  18982  CD  ARG F 120     -17.819 -33.240  51.825  1.00  0.00
F    C
ATOM  18983  HD1 ARG F 120     -17.621 -34.071  51.110  1.00  0.00
F    H
ATOM  18984  HD2 ARG F 120     -18.144 -33.676  52.798  1.00  0.00
F    H
ATOM  18985  NE  ARG F 120     -18.911 -32.421  51.334  1.00  0.00
F    N
ATOM  18986  HE  ARG F 120     -18.654 -31.457  51.201  1.00  0.00
F    H
ATOM  18987  CZ  ARG F 120     -20.127 -32.885  51.083  1.00  0.00
F    C
ATOM  18988  NH1 ARG F 120     -20.399 -34.163  51.287  1.00  0.00
F    N
ATOM  18989 HH11 ARG F 120     -21.296 -34.527  51.079  1.00  0.00
F    H
ATOM  18990 HH12 ARG F 120     -19.623 -34.750  51.525  1.00  0.00
F    H
ATOM  18991  NH2 ARG F 120     -21.070 -32.071  50.638  1.00  0.00
F    N
ATOM  18992 HH21 ARG F 120     -21.997 -32.400  50.534  1.00  0.00
F    H
ATOM  18993 HH22 ARG F 120     -20.856 -31.103  50.623  1.00  0.00
F    H
ATOM  18994  C   ARG F 120     -13.958 -31.840  51.307  1.00  0.00
F    C
ATOM  18995  O   ARG F 120     -13.612 -30.907  52.040  1.00  0.00
F    O
ATOM  18996  N   ILE F 121     -14.146 -31.715  49.998  1.00  0.00
F    N
ATOM  18997  HN  ILE F 121     -14.467 -32.473  49.433  1.00  0.00
F    H
ATOM  18998  CA  ILE F 121     -13.909 -30.482  49.262  1.00  0.00
F    C
ATOM  18999  HA  ILE F 121     -13.529 -29.749  49.963  1.00  0.00
F    H
ATOM  19000  CB  ILE F 121     -12.941 -30.726  48.096  1.00  0.00
F    C
ATOM  19001  HB  ILE F 121     -13.521 -31.358  47.377  1.00  0.00
F    H
ATOM  19002  CG2 ILE F 121     -12.475 -29.425  47.493  1.00  0.00
F    C
ATOM  19003 HG21 ILE F 121     -11.904 -29.610  46.559  1.00  0.00
F    H
ATOM  19004 HG22 ILE F 121     -13.339 -28.776  47.237  1.00  0.00
F    H
ATOM  19005 HG23 ILE F 121     -11.817 -28.873  48.197  1.00  0.00
F    H
ATOM  19006  CG1 ILE F 121     -11.760 -31.562  48.581  1.00  0.00
F    C
ATOM  19007 HG11 ILE F 121     -11.329 -31.108  49.501  1.00  0.00
F    H
```

ATOM 19008 HG12 ILE F 121   -12.126 -32.578 48.857 1.00 0.00
F   H
ATOM 19009 CD ILE F 121   -10.667 -31.728 47.568 1.00 0.00
F   C
ATOM 19010 HD1 ILE F 121   -9.870 -32.393 47.965 1.00 0.00
F   H
ATOM 19011 HD2 ILE F 121   -11.064 -32.180 46.633 1.00 0.00
F   H
ATOM 19012 HD3 ILE F 121   -10.205 -30.750 47.318 1.00 0.00
F   H
ATOM 19013 C   ILE F 121   -15.248 -29.962 48.766 1.00 0.00
F   C
ATOM 19014 O   ILE F 121   -16.172 -30.748 48.533 1.00 0.00
F   O
ATOM 19015 N   ILE F 122   -15.369 -28.646 48.622 1.00 0.00
F   N
ATOM 19016 HN ILE F 122   -14.660 -27.986 48.863 1.00 0.00
F   H
ATOM 19017 CA ILE F 122   -16.583 -28.049 48.085 1.00 0.00
F   C
ATOM 19018 HA ILE F 122   -17.097 -28.820 47.524 1.00 0.00
F   H
ATOM 19019 CB ILE F 122   -17.484 -27.475 49.193 1.00 0.00
F   C
ATOM 19020 HB ILE F 122   -16.796 -26.851 49.819 1.00 0.00
F   H
ATOM 19021 CG2 ILE F 122   -18.641 -26.701 48.592 1.00 0.00
F   C
ATOM 19022 HG21 ILE F 122   -19.200 -26.158 49.383 1.00 0.00
F   H
ATOM 19023 HG22 ILE F 122   -18.277 -25.949 47.861 1.00 0.00
F   H
ATOM 19024 HG23 ILE F 122   -19.347 -27.382 48.073 1.00 0.00
F   H
ATOM 19025 CG1 ILE F 122   -18.002 -28.595 50.088 1.00 0.00
F   C
ATOM 19026 HG11 ILE F 122   -19.026 -28.347 50.449 1.00 0.00
F   H
ATOM 19027 HG12 ILE F 122   -18.082 -29.529 49.487 1.00 0.00
F   H
ATOM 19028 CD ILE F 122   -17.122 -28.881 51.279 1.00 0.00
F   C
ATOM 19029 HD1 ILE F 122   -17.522 -29.742 51.857 1.00 0.00
F   H
ATOM 19030 HD2 ILE F 122   -16.090 -29.131 50.952 1.00 0.00
F   H
ATOM 19031 HD3 ILE F 122   -17.073 -28.004 51.958 1.00 0.00
F   H
ATOM 19032 C   ILE F 122   -16.213 -26.959 47.095 1.00 0.00
F   C
ATOM 19033 O   ILE F 122   -15.348 -26.122 47.370 1.00 0.00
F   O
ATOM 19034 N   HSE F 123   -16.888 -26.961 45.960 1.00 0.00
F   N

```
ATOM  19035  HN  HSE F 123     -17.532 -27.693  45.743  1.00  0.00
F    H
ATOM  19036  CA  HSE F 123     -16.776 -25.939  44.934  1.00  0.00
F    C
ATOM  19037  HA  HSE F 123     -16.012 -25.231  45.232  1.00  0.00
F    H
ATOM  19038  CB  HSE F 123     -16.491 -26.567  43.576  1.00  0.00
F    C
ATOM  19039  HB1 HSE F 123     -17.310 -27.285  43.352  1.00  0.00
F    H
ATOM  19040  HB2 HSE F 123     -16.522 -25.783  42.791  1.00  0.00
F    H
ATOM  19041  ND1 HSE F 123     -14.105 -26.843  42.815  1.00  0.00
F    N
ATOM  19042  CG  HSE F 123     -15.198 -27.313  43.508  1.00  0.00
F    C
ATOM  19043  CE1 HSE F 123     -13.115 -27.712  42.914  1.00  0.00
F    C
ATOM  19044  HE1 HSE F 123     -12.135 -27.592  42.447  1.00  0.00
F    H
ATOM  19045  NE2 HSE F 123     -13.525 -28.726  43.656  1.00  0.00
F    N
ATOM  19046  HE2 HSE F 123     -12.979 -29.532  43.882  1.00  0.00
F    H
ATOM  19047  CD2 HSE F 123     -14.825 -28.501  44.038  1.00  0.00
F    C
ATOM  19048  HD2 HSE F 123     -15.388 -29.198  44.643  1.00  0.00
F    H
ATOM  19049  C   HSE F 123     -18.095 -25.186  44.904  1.00  0.00
F    C
ATOM  19050  O   HSE F 123     -18.891 -25.361  43.973  1.00  0.00
F    O
ATOM  19051  N   PRO F 124     -18.358 -24.347  45.905  1.00  0.00
F    N
ATOM  19052  CD  PRO F 124     -17.405 -23.730  46.834  1.00  0.00
F    C
ATOM  19053  HD1 PRO F 124     -17.480 -24.841  46.801  1.00  0.00
F    H
ATOM  19054  HD2 PRO F 124     -16.351 -23.415  47.012  1.00  0.00
F    H
ATOM  19055  CA  PRO F 124     -19.731 -23.867  46.116  1.00  0.00
F    C
ATOM  19056  HA  PRO F 124     -20.352 -24.697  46.430  1.00  0.00
F    H
ATOM  19057  CB  PRO F 124     -19.568 -22.862  47.266  1.00  0.00
F    C
ATOM  19058  HB1 PRO F 124     -19.601 -23.410  48.234  1.00  0.00
F    H
ATOM  19059  HB2 PRO F 124     -20.356 -22.081  47.267  1.00  0.00
F    H
ATOM  19060  CG  PRO F 124     -18.285 -23.193  47.904  1.00  0.00
F    C
ATOM  19061  HG1 PRO F 124     -18.224 -22.848  48.954  1.00  0.00
F    H
```

ATOM 19062 HG2 PRO F 124 -17.455 -22.739 47.316 1.00 0.00
F H
ATOM 19063 C PRO F 124 -20.355 -23.213 44.906 1.00 0.00
F C
ATOM 19064 O PRO F 124 -21.404 -23.666 44.422 1.00 0.00
F O
ATOM 19065 N GLU F 125 -19.737 -22.153 44.400 1.00 0.00
F N
ATOM 19066 HN GLU F 125 -18.870 -21.768 44.705 1.00 0.00
F H
ATOM 19067 CA GLU F 125 -20.339 -21.442 43.288 1.00 0.00
F C
ATOM 19068 HA GLU F 125 -21.358 -21.191 43.558 1.00 0.00
F H
ATOM 19069 CB GLU F 125 -19.586 -20.162 42.989 1.00 0.00
F C
ATOM 19070 HB1 GLU F 125 -18.494 -20.383 43.028 1.00 0.00
F H
ATOM 19071 HB2 GLU F 125 -19.794 -19.836 41.945 1.00 0.00
F H
ATOM 19072 CG GLU F 125 -19.908 -19.046 43.963 1.00 0.00
F C
ATOM 19073 HG1 GLU F 125 -20.022 -18.095 43.411 1.00 0.00
F H
ATOM 19074 HG2 GLU F 125 -20.843 -19.261 44.517 1.00 0.00
F H
ATOM 19075 CD GLU F 125 -18.822 -18.790 44.969 1.00 0.00
F C
ATOM 19076 OE1 GLU F 125 -17.863 -19.594 45.019 1.00 0.00
F O
ATOM 19077 OE2 GLU F 125 -18.930 -17.773 45.682 1.00 0.00
F O
ATOM 19078 C GLU F 125 -20.404 -22.316 42.059 1.00 0.00
F C
ATOM 19079 O GLU F 125 -21.314 -22.170 41.252 1.00 0.00
F O
ATOM 19080 N LYS F 126 -19.466 -23.248 41.907 1.00 0.00
F N
ATOM 19081 HN LYS F 126 -18.640 -23.339 42.461 1.00 0.00
F H
ATOM 19082 CA LYS F 126 -19.618 -24.242 40.853 1.00 0.00
F C
ATOM 19083 HA LYS F 126 -19.624 -23.714 39.908 1.00 0.00
F H
ATOM 19084 CB LYS F 126 -18.470 -25.246 40.911 1.00 0.00
F C
ATOM 19085 HB1 LYS F 126 -18.582 -25.818 41.860 1.00 0.00
F H
ATOM 19086 HB2 LYS F 126 -18.591 -25.982 40.085 1.00 0.00
F H
ATOM 19087 CG LYS F 126 -17.095 -24.637 40.873 1.00 0.00
F C
ATOM 19088 HG1 LYS F 126 -16.953 -24.002 41.777 1.00 0.00
F H

```
ATOM  19089  HG2 LYS F 126     -16.337 -25.452  40.919  1.00  0.00
F    H
ATOM  19090  CD  LYS F 126     -16.885 -23.799  39.649  1.00  0.00
F    C
ATOM  19091  HD1 LYS F 126     -17.861 -23.345  39.362  1.00  0.00
F    H
ATOM  19092  HD2 LYS F 126     -16.195 -22.964  39.915  1.00  0.00
F    H
ATOM  19093  CE  LYS F 126     -16.313 -24.603  38.498  1.00  0.00
F    C
ATOM  19094  HE1 LYS F 126     -15.648 -25.411  38.877  1.00  0.00
F    H
ATOM  19095  HE2 LYS F 126     -17.129 -25.069  37.903  1.00  0.00
F    H
ATOM  19096  NZ  LYS F 126     -15.514 -23.734  37.589  1.00  0.00
F    N
ATOM  19097  HZ1 LYS F 126     -15.127 -24.298  36.805  1.00  0.00
F    H
ATOM  19098  HZ2 LYS F 126     -16.121 -22.981  37.205  1.00  0.00
F    H
ATOM  19099  HZ3 LYS F 126     -14.732 -23.303  38.121  1.00  0.00
F    H
ATOM  19100  C   LYS F 126     -20.956 -24.958  40.983  1.00  0.00
F    C
ATOM  19101  O   LYS F 126     -21.757 -25.000  40.035  1.00  0.00
F    O
ATOM  19102  N   ASP F 127     -21.226 -25.493  42.170  1.00  0.00
F    N
ATOM  19103  HN  ASP F 127     -20.675 -25.393  42.995  1.00  0.00
F    H
ATOM  19104  CA  ASP F 127     -22.410 -26.312  42.375  1.00  0.00
F    C
ATOM  19105  HA  ASP F 127     -22.397 -27.090  41.620  1.00  0.00
F    H
ATOM  19106  CB  ASP F 127     -22.381 -26.884  43.794  1.00  0.00
F    C
ATOM  19107  HB1 ASP F 127     -22.019 -26.104  44.495  1.00  0.00
F    H
ATOM  19108  HB2 ASP F 127     -23.397 -27.192  44.111  1.00  0.00
F    H
ATOM  19109  CG  ASP F 127     -21.454 -28.076  43.925  1.00  0.00
F    C
ATOM  19110  OD1 ASP F 127     -20.221 -27.858  43.891  1.00  0.00
F    O
ATOM  19111  OD2 ASP F 127     -21.954 -29.211  44.077  1.00  0.00
F    O
ATOM  19112  C   ASP F 127     -23.672 -25.498  42.143  1.00  0.00
F    C
ATOM  19113  O   ASP F 127     -24.546 -25.872  41.346  1.00  0.00
F    O
ATOM  19114  N   MET F 128     -23.783 -24.361  42.816  1.00  0.00
F    N
ATOM  19115  HN  MET F 128     -23.124 -23.922  43.422  1.00  0.00
F    H
```

ATOM 19116 CA MET F 128 -25.029 -23.638 42.663 1.00 0.00
F C
ATOM 19117 HA MET F 128 -25.828 -24.370 42.667 1.00 0.00
F H
ATOM 19118 CB MET F 128 -25.257 -22.700 43.843 1.00 0.00
F C
ATOM 19119 HB1 MET F 128 -25.883 -23.252 44.581 1.00 0.00
F H
ATOM 19120 HB2 MET F 128 -24.286 -22.499 44.347 1.00 0.00
F H
ATOM 19121 CG MET F 128 -25.949 -21.402 43.504 1.00 0.00
F C
ATOM 19122 HG1 MET F 128 -25.202 -20.592 43.362 1.00 0.00
F H
ATOM 19123 HG2 MET F 128 -26.508 -21.515 42.549 1.00 0.00
F H
ATOM 19124 SD MET F 128 -27.096 -20.884 44.794 1.00 0.00
F S
ATOM 19125 CE MET F 128 -27.014 -22.253 45.956 1.00 0.00
F C
ATOM 19126 HE1 MET F 128 -27.690 -22.081 46.820 1.00 0.00
F H
ATOM 19127 HE2 MET F 128 -27.316 -23.206 45.470 1.00 0.00
F H
ATOM 19128 HE3 MET F 128 -25.983 -22.381 46.350 1.00 0.00
F H
ATOM 19129 C MET F 128 -25.078 -22.915 41.329 1.00 0.00
F C
ATOM 19130 O MET F 128 -26.158 -22.555 40.861 1.00 0.00
F O
ATOM 19131 N GLY F 129 -23.952 -22.768 40.651 1.00 0.00
F N
ATOM 19132 HN GLY F 129 -23.046 -23.042 40.972 1.00 0.00
F H
ATOM 19133 CA GLY F 129 -23.993 -22.169 39.345 1.00 0.00
F C
ATOM 19134 HA1 GLY F 129 -22.978 -21.984 39.023 1.00 0.00
F H
ATOM 19135 HA2 GLY F 129 -24.614 -21.284 39.390 1.00 0.00
F H
ATOM 19136 C GLY F 129 -24.622 -23.125 38.372 1.00 0.00
F C
ATOM 19137 O GLY F 129 -25.482 -22.741 37.583 1.00 0.00
F O
ATOM 19138 N VAL F 130 -24.185 -24.379 38.400 1.00 0.00
F N
ATOM 19139 HN VAL F 130 -23.456 -24.756 38.965 1.00 0.00
F H
ATOM 19140 CA VAL F 130 -24.820 -25.346 37.514 1.00 0.00
F C
ATOM 19141 HA VAL F 130 -24.789 -24.945 36.509 1.00 0.00
F H
ATOM 19142 CB VAL F 130 -24.025 -26.666 37.491 1.00 0.00
F C

```
ATOM  19143  HB  VAL F 130     -24.456 -27.304  36.681  1.00  0.00
F    H
ATOM  19144  CG1 VAL F 130     -22.569 -26.371  37.191  1.00  0.00
F    C
ATOM  19145 HG11 VAL F 130     -21.975 -27.310  37.209  1.00  0.00
F    H
ATOM  19146 HG12 VAL F 130     -22.441 -25.918  36.186  1.00  0.00
F    H
ATOM  19147 HG13 VAL F 130     -22.144 -25.680  37.950  1.00  0.00
F    H
ATOM  19148  CG2 VAL F 130     -24.106 -27.385  38.801  1.00  0.00
F    C
ATOM  19149 HG21 VAL F 130     -23.495 -28.313  38.778  1.00  0.00
F    H
ATOM  19150 HG22 VAL F 130     -23.731 -26.740  39.625  1.00  0.00
F    H
ATOM  19151 HG23 VAL F 130     -25.150 -27.677  39.036  1.00  0.00
F    H
ATOM  19152  C   VAL F 130     -26.287 -25.518  37.894  1.00  0.00
F    C
ATOM  19153  O   VAL F 130     -27.158 -25.637  37.018  1.00  0.00
F    O
ATOM  19154  N   LYS F 131     -26.590 -25.483  39.197  1.00  0.00
F    N
ATOM  19155  HN  LYS F 131     -25.931 -25.594  39.939  1.00  0.00
F    H
ATOM  19156  CA  LYS F 131     -27.970 -25.268  39.623  1.00  0.00
F    C
ATOM  19157  HA  LYS F 131     -28.498 -26.198  39.448  1.00  0.00
F    H
ATOM  19158  CB  LYS F 131     -28.000 -24.888  41.107  1.00  0.00
F    C
ATOM  19159  HB1 LYS F 131     -27.286 -25.564  41.633  1.00  0.00
F    H
ATOM  19160  HB2 LYS F 131     -27.598 -23.857  41.225  1.00  0.00
F    H
ATOM  19161  CG  LYS F 131     -29.358 -24.995  41.804  1.00  0.00
F    C
ATOM  19162  HG1 LYS F 131     -29.812 -25.985  41.570  1.00  0.00
F    H
ATOM  19163  HG2 LYS F 131     -29.204 -24.952  42.907  1.00  0.00
F    H
ATOM  19164  CD  LYS F 131     -30.280 -23.867  41.323  1.00  0.00
F    C
ATOM  19165  HD1 LYS F 131     -30.120 -22.979  41.976  1.00  0.00
F    H
ATOM  19166  HD2 LYS F 131     -29.971 -23.582  40.290  1.00  0.00
F    H
ATOM  19167  CE  LYS F 131     -31.754 -24.226  41.315  1.00  0.00
F    C
ATOM  19168  HE1 LYS F 131     -32.342 -23.449  40.777  1.00  0.00
F    H
ATOM  19169  HE2 LYS F 131     -31.915 -25.203  40.810  1.00  0.00
F    H
```

ATOM 19170 NZ LYS F 131   -32.258 -24.321 42.744 1.00 0.00
F   N
ATOM 19171 HZ1 LYS F 131   -33.269 -24.566 42.749 1.00 0.00
F   H
ATOM 19172 HZ2 LYS F 131   -31.723 -25.054 43.253 1.00 0.00
F   H
ATOM 19173 HZ3 LYS F 131   -32.123 -23.406 43.220 1.00 0.00
F   H
ATOM 19174 C   LYS F 131   -28.661 -24.195 38.805 1.00 0.00
F   C
ATOM 19175 O   LYS F 131   -29.581 -24.482 38.029 1.00 0.00
F   O
ATOM 19176 N   ILE F 132   -28.239 -22.952 38.982 1.00 0.00
F   N
ATOM 19177 HN ILE F 132   -27.398 -22.683 39.446 1.00 0.00
F   H
ATOM 19178 CA ILE F 132   -29.022 -21.840 38.479 1.00 0.00
F   C
ATOM 19179 HA ILE F 132   -30.036 -21.986 38.827 1.00 0.00
F   H
ATOM 19180 CB ILE F 132   -28.457 -20.513 39.005 1.00 0.00
F   C
ATOM 19181 HB ILE F 132   -28.557 -20.589 40.118 1.00 0.00
F   H
ATOM 19182 CG2 ILE F 132   -27.044 -20.327 38.539 1.00 0.00
F   C
ATOM 19183 HG21 ILE F 132   -26.579 -19.452 39.041 1.00 0.00
F   H
ATOM 19184 HG22 ILE F 132   -26.427 -21.218 38.778 1.00 0.00
F   H
ATOM 19185 HG23 ILE F 132   -27.007 -20.158 37.442 1.00 0.00
F   H
ATOM 19186 CG1 ILE F 132   -29.342 -19.359 38.554 1.00 0.00
F   C
ATOM 19187 HG11 ILE F 132   -28.934 -18.914 37.619 1.00 0.00
F   H
ATOM 19188 HG12 ILE F 132   -30.356 -19.753 38.316 1.00 0.00
F   H
ATOM 19189 CD ILE F 132   -29.484 -18.291 39.583 1.00 0.00
F   C
ATOM 19190 HD1 ILE F 132   -30.184 -17.503 39.227 1.00 0.00
F   H
ATOM 19191 HD2 ILE F 132   -29.886 -18.711 40.531 1.00 0.00
F   H
ATOM 19192 HD3 ILE F 132   -28.506 -17.810 39.797 1.00 0.00
F   H
ATOM 19193 C   ILE F 132   -29.068 -21.882 36.964 1.00 0.00
F   C
ATOM 19194 O   ILE F 132   -30.051 -21.467 36.339 1.00 0.00
F   O
ATOM 19195 N   ALA F 133   -28.028 -22.438 36.358 1.00 0.00
F   N
ATOM 19196 HN ALA F 133   -27.221 -22.788 36.832 1.00 0.00
F   H

```
ATOM  19197  CA  ALA F 133     -28.007 -22.571  34.914  1.00  0.00
F    C
ATOM  19198  HA  ALA F 133     -28.182 -21.614  34.437  1.00  0.00
F    H
ATOM  19199  CB  ALA F 133     -26.648 -23.091  34.467  1.00  0.00
F    C
ATOM  19200  HB1 ALA F 133     -25.855 -22.378  34.778  1.00  0.00
F    H
ATOM  19201  HB2 ALA F 133     -26.424 -24.075  34.935  1.00  0.00
F    H
ATOM  19202  HB3 ALA F 133     -26.596 -23.204  33.363  1.00  0.00
F    H
ATOM  19203  C   ALA F 133     -29.116 -23.490  34.447  1.00  0.00
F    C
ATOM  19204  O   ALA F 133     -29.889 -23.136  33.549  1.00  0.00
F    O
ATOM  19205  N   GLN F 134     -29.219 -24.674  35.051  1.00  0.00
F    N
ATOM  19206  HN  GLN F 134     -28.615 -25.037  35.758  1.00  0.00
F    H
ATOM  19207  CA  GLN F 134     -30.307 -25.563  34.674  1.00  0.00
F    C
ATOM  19208  HA  GLN F 134     -30.239 -25.717  33.604  1.00  0.00
F    H
ATOM  19209  CB  GLN F 134     -30.178 -26.892  35.405  1.00  0.00
F    C
ATOM  19210  HB1 GLN F 134     -30.375 -26.688  36.483  1.00  0.00
F    H
ATOM  19211  HB2 GLN F 134     -30.988 -27.578  35.066  1.00  0.00
F    H
ATOM  19212  CG  GLN F 134     -28.815 -27.510  35.232  1.00  0.00
F    C
ATOM  19213  HG1 GLN F 134     -28.061 -26.727  35.002  1.00  0.00
F    H
ATOM  19214  HG2 GLN F 134     -28.498 -28.026  36.161  1.00  0.00
F    H
ATOM  19215  CD  GLN F 134     -28.770 -28.495  34.088  1.00  0.00
F    C
ATOM  19216  OE1 GLN F 134     -29.766 -28.705  33.397  1.00  0.00
F    O
ATOM  19217  NE2 GLN F 134     -27.607 -29.098  33.873  1.00  0.00
F    N
ATOM  19218 HE21 GLN F 134     -27.569 -29.746  33.118  1.00  0.00
F    H
ATOM  19219 HE22 GLN F 134     -26.832 -28.905  34.466  1.00  0.00
F    H
ATOM  19220  C   GLN F 134     -31.653 -24.918  34.959  1.00  0.00
F    C
ATOM  19221  O   GLN F 134     -32.585 -25.028  34.156  1.00  0.00
F    O
ATOM  19222  N   SER F 135     -31.763 -24.211  36.085  1.00  0.00
F    N
ATOM  19223  HN  SER F 135     -31.030 -24.016  36.736  1.00  0.00
F    H
```

ATOM 19224 CA SER F 135   -33.057 -23.645 36.454 1.00 0.00
F   C
ATOM 19225 HA SER F 135   -33.779 -24.437 36.300 1.00 0.00
F   H
ATOM 19226 CB SER F 135   -33.047 -23.184 37.907 1.00 0.00
F   C
ATOM 19227 HB1 SER F 135   -34.003 -22.649 38.107 1.00 0.00
F   H
ATOM 19228 HB2 SER F 135   -33.014 -24.062 38.593 1.00 0.00
F   H
ATOM 19229 OG SER F 135   -31.971 -22.305 38.148 1.00 0.00
F   O
ATOM 19230 HG1 SER F 135   -32.139 -21.877 38.998 1.00 0.00
F   H
ATOM 19231 C   SER F 135   -33.450 -22.490 35.544 1.00 0.00
F   C
ATOM 19232 O   SER F 135   -34.637 -22.171 35.429 1.00 0.00
F   O
ATOM 19233 N   LEU F 136   -32.481 -21.848 34.897 1.00 0.00
F   N
ATOM 19234 HN LEU F 136   -31.503 -22.033 34.968 1.00 0.00
F   H
ATOM 19235 CA LEU F 136   -32.822 -20.763 33.987 1.00 0.00
F   C
ATOM 19236 HA LEU F 136   -33.747 -20.334 34.350 1.00 0.00
F   H
ATOM 19237 CB LEU F 136   -31.727 -19.700 33.985 1.00 0.00
F   C
ATOM 19238 HB1 LEU F 136   -30.817 -20.164 34.432 1.00 0.00
F   H
ATOM 19239 HB2 LEU F 136   -31.463 -19.456 32.932 1.00 0.00
F   H
ATOM 19240 CG LEU F 136   -32.107 -18.449 34.773 1.00 0.00
F   C
ATOM 19241 HG LEU F 136   -31.852 -18.589 35.851 1.00 0.00
F   H
ATOM 19242 CD1 LEU F 136   -31.396 -17.242 34.221 1.00 0.00
F   C
ATOM 19243 HD11 LEU F 136   -31.708 -16.323 34.764 1.00 0.00
F   H
ATOM 19244 HD12 LEU F 136   -30.295 -17.352 34.328 1.00 0.00
F   H
ATOM 19245 HD13 LEU F 136   -31.632 -17.105 33.144 1.00 0.00
F   H
ATOM 19246 CD2 LEU F 136   -33.600 -18.238 34.668 1.00 0.00
F   C
ATOM 19247 HD21 LEU F 136   -33.906 -17.342 35.251 1.00 0.00
F   H
ATOM 19248 HD22 LEU F 136   -33.919 -18.083 33.618 1.00 0.00
F   H
ATOM 19249 HD23 LEU F 136   -34.147 -19.118 35.073 1.00 0.00
F   H
ATOM 19250 C   LEU F 136   -33.075 -21.277 32.582 1.00 0.00
F   C

ATOM 19251 O   LEU F 136   -34.008 -20.826 31.910 1.00 0.00
F   O
ATOM 19252 N   SER F 137   -32.251 -22.217 32.119 1.00 0.00
F   N
ATOM 19253 HN SER F 137   -31.483 -22.609 32.626 1.00 0.00
F   H
ATOM 19254 CA SER F 137   -32.422 -22.764 30.778 1.00 0.00
F   C
ATOM 19255 HA SER F 137   -32.240 -21.946 30.094 1.00 0.00
F   H
ATOM 19256 CB SER F 137   -31.449 -23.919 30.568 1.00 0.00
F   C
ATOM 19257 HB1 SER F 137   -31.549 -24.269 29.516 1.00 0.00
F   H
ATOM 19258 HB2 SER F 137   -30.400 -23.570 30.698 1.00 0.00
F   H
ATOM 19259 OG SER F 137   -31.761 -24.984 31.448 1.00 0.00
F   O
ATOM 19260 HG1 SER F 137   -31.280 -25.760 31.135 1.00 0.00
F   H
ATOM 19261 C   SER F 137   -33.854 -23.229 30.556 1.00 0.00
F   C
ATOM 19262 O   SER F 137   -34.573 -22.688 29.709 1.00 0.00
F   O
ATOM 19263 N   ASP F 138   -34.294 -24.209 31.330 1.00 0.00
F   N
ATOM 19264 HN ASP F 138   -33.746 -24.673 32.021 1.00 0.00
F   H
ATOM 19265 CA ASP F 138   -35.656 -24.722 31.247 1.00 0.00
F   C
ATOM 19266 HA ASP F 138   -36.083 -24.436 30.292 1.00 0.00
F   H
ATOM 19267 CB ASP F 138   -35.673 -26.232 31.445 1.00 0.00
F   C
ATOM 19268 HB1 ASP F 138   -35.039 -26.493 32.316 1.00 0.00
F   H
ATOM 19269 HB2 ASP F 138   -36.703 -26.588 31.644 1.00 0.00
F   H
ATOM 19270 CG ASP F 138   -35.129 -26.984 30.250 1.00 0.00
F   C
ATOM 19271 OD1 ASP F 138   -35.876 -27.174 29.267 1.00 0.00
F   O
ATOM 19272 OD2 ASP F 138   -33.946 -27.381 30.295 1.00 0.00
F   O
ATOM 19273 C   ASP F 138   -36.457 -24.000 32.321 1.00 0.00
F   C
ATOM 19274 O   ASP F 138   -36.367 -24.329 33.505 1.00 0.00
F   O
ATOM 19275 N   GLU F 139   -37.249 -23.013 31.905 1.00 0.00
F   N
ATOM 19276 HN GLU F 139   -37.462 -22.797 30.957 1.00 0.00
F   H
ATOM 19277 CA GLU F 139   -37.892 -22.136 32.871 1.00 0.00
F   C

```
ATOM  19278  HA  GLU F 139     -37.151 -21.890  33.622  1.00  0.00
F    H
ATOM  19279  CB  GLU F 139     -38.341 -20.852  32.177  1.00  0.00
F    C
ATOM  19280  HB1 GLU F 139     -38.979 -20.279  32.888  1.00  0.00
F    H
ATOM  19281  HB2 GLU F 139     -37.458 -20.206  31.970  1.00  0.00
F    H
ATOM  19282  CG  GLU F 139     -39.115 -21.088  30.903  1.00  0.00
F    C
ATOM  19283  HG1 GLU F 139     -38.559 -21.795  30.259  1.00  0.00
F    H
ATOM  19284  HG2 GLU F 139     -40.118 -21.505  31.119  1.00  0.00
F    H
ATOM  19285  CD  GLU F 139     -39.278 -19.830  30.088  1.00  0.00
F    C
ATOM  19286  OE1 GLU F 139     -39.222 -19.913  28.846  1.00  0.00
F    O
ATOM  19287  OE2 GLU F 139     -39.452 -18.752  30.690  1.00  0.00
F    O
ATOM  19288  C   GLU F 139     -39.067 -22.785  33.591  1.00  0.00
F    C
ATOM  19289  O   GLU F 139     -39.824 -22.074  34.260  1.00  0.00
F    O
ATOM  19290  N   ASN F 140     -39.244 -24.103  33.482  1.00  0.00
F    N
ATOM  19291  HN  ASN F 140     -38.737 -24.719  32.879  1.00  0.00
F    H
ATOM  19292  CA  ASN F 140     -40.263 -24.757  34.296  1.00  0.00
F    C
ATOM  19293  HA  ASN F 140     -40.893 -23.959  34.674  1.00  0.00
F    H
ATOM  19294  CB  ASN F 140     -41.085 -25.745  33.469  1.00  0.00
F    C
ATOM  19295  HB1 ASN F 140     -40.936 -25.496  32.396  1.00  0.00
F    H
ATOM  19296  HB2 ASN F 140     -40.715 -26.779  33.625  1.00  0.00
F    H
ATOM  19297  CG  ASN F 140     -42.571 -25.660  33.776  1.00  0.00
F    C
ATOM  19298  OD1 ASN F 140     -43.311 -26.629  33.597  1.00  0.00
F    O
ATOM  19299  ND2 ASN F 140     -43.015 -24.495  34.243  1.00  0.00
F    N
ATOM  19300 HD21 ASN F 140     -43.986 -24.428  34.459  1.00  0.00
F    H
ATOM  19301 HD22 ASN F 140     -42.380 -23.742  34.391  1.00  0.00
F    H
ATOM  19302  C   ASN F 140     -39.652 -25.467  35.495  1.00  0.00
F    C
ATOM  19303  O   ASN F 140     -40.250 -25.479  36.576  1.00  0.00
F    O
ATOM  19304  N   VAL F 141     -38.478 -26.072  35.335  1.00  0.00
F    N
```

ATOM 19305 HN VAL F 141   -37.973 -26.174 34.482 1.00 0.00
F   H
ATOM 19306 CA VAL F 141   -37.825 -26.661 36.496 1.00 0.00
F   C
ATOM 19307 HA VAL F 141   -38.534 -27.280 37.033 1.00 0.00
F   H
ATOM 19308 CB VAL F 141   -36.612 -27.517 36.108 1.00 0.00
F   C
ATOM 19309 HB VAL F 141   -36.882 -28.587 36.286 1.00 0.00
F   H
ATOM 19310 CG1 VAL F 141   -36.262 -27.338 34.653 1.00 0.00
F   C
ATOM 19311 HG11 VAL F 141   -35.359 -27.933 34.396 1.00 0.00
F   H
ATOM 19312 HG12 VAL F 141   -37.083 -27.680 33.989 1.00 0.00
F   H
ATOM 19313 HG13 VAL F 141   -36.046 -26.272 34.430 1.00 0.00
F   H
ATOM 19314 CG2 VAL F 141   -35.412 -27.282 37.035 1.00 0.00
F   C
ATOM 19315 HG21 VAL F 141   -34.538 -27.884 36.707 1.00 0.00
F   H
ATOM 19316 HG22 VAL F 141   -35.119 -26.210 37.032 1.00 0.00
F   H
ATOM 19317 HG23 VAL F 141   -35.647 -27.575 38.079 1.00 0.00
F   H
ATOM 19318 C   VAL F 141   -37.438 -25.507 37.414 1.00 0.00
F   C
ATOM 19319 O   VAL F 141   -36.672 -24.620 37.030 1.00 0.00
F   O
ATOM 19320 N   LEU F 142   -38.055 -25.457 38.590 1.00 0.00
F   N
ATOM 19321 HN LEU F 142   -38.764 -26.082 38.908 1.00 0.00
F   H
ATOM 19322 CA LEU F 142   -37.705 -24.415 39.546 1.00 0.00
F   C
ATOM 19323 HA LEU F 142   -37.376 -23.557 38.973 1.00 0.00
F   H
ATOM 19324 CB LEU F 142   -38.917 -24.044 40.405 1.00 0.00
F   C
ATOM 19325 HB1 LEU F 142   -39.745 -23.779 39.709 1.00 0.00
F   H
ATOM 19326 HB2 LEU F 142   -39.257 -24.947 40.959 1.00 0.00
F   H
ATOM 19327 CG LEU F 142   -38.660 -22.877 41.355 1.00 0.00
F   C
ATOM 19328 HG LEU F 142   -37.807 -23.159 42.018 1.00 0.00
F   H
ATOM 19329 CD1 LEU F 142   -38.301 -21.640 40.560 1.00 0.00
F   C
ATOM 19330 HD11 LEU F 142   -38.158 -20.770 41.237 1.00 0.00
F   H
ATOM 19331 HD12 LEU F 142   -37.359 -21.798 39.993 1.00 0.00
F   H

```
ATOM  19332 HD13 LEU F 142     -39.107 -21.389  39.839  1.00  0.00
F    H
ATOM  19333  CD2 LEU F 142     -39.870 -22.611  42.234  1.00  0.00
F    C
ATOM  19334 HD21 LEU F 142     -39.663 -21.772  42.932  1.00  0.00
F    H
ATOM  19335 HD22 LEU F 142     -40.761 -22.337  41.633  1.00  0.00
F    H
ATOM  19336 HD23 LEU F 142     -40.118 -23.511  42.837  1.00  0.00
F    H
ATOM  19337  C   LEU F 142     -36.540 -24.860  40.415  1.00  0.00
F    C
ATOM  19338  O   LEU F 142     -35.487 -24.213  40.437  1.00  0.00
F    O
ATOM  19339  N   ASN F 143     -36.705 -25.970  41.125  1.00  0.00
F    N
ATOM  19340  HN  ASN F 143     -37.583 -26.435  41.238  1.00  0.00
F    H
ATOM  19341  CA  ASN F 143     -35.593 -26.609  41.809  1.00  0.00
F    C
ATOM  19342  HA  ASN F 143     -34.688 -26.085  41.521  1.00  0.00
F    H
ATOM  19343  CB  ASN F 143     -35.774 -26.617  43.330  1.00  0.00
F    C
ATOM  19344  HB1 ASN F 143     -36.663 -27.242  43.563  1.00  0.00
F    H
ATOM  19345  HB2 ASN F 143     -34.897 -27.088  43.820  1.00  0.00
F    H
ATOM  19346  CG  ASN F 143     -36.012 -25.237  43.904  1.00  0.00
F    C
ATOM  19347  OD1 ASN F 143     -36.628 -24.386  43.271  1.00  0.00
F    O
ATOM  19348  ND2 ASN F 143     -35.508 -25.004  45.109  1.00  0.00
F    N
ATOM  19349 HD21 ASN F 143     -35.665 -24.104  45.505  1.00  0.00
F    H
ATOM  19350 HD22 ASN F 143     -35.021 -25.725  45.593  1.00  0.00
F    H
ATOM  19351  C   ASN F 143     -35.490 -28.028  41.283  1.00  0.00
F    C
ATOM  19352  O   ASN F 143     -36.473 -28.595  40.800  1.00  0.00
F    O
ATOM  19353  N   TYR F 144     -34.301 -28.608  41.389  1.00  0.00
F    N
ATOM  19354  HN  TYR F 144     -33.486 -28.206  41.802  1.00  0.00
F    H
ATOM  19355  CA  TYR F 144     -34.115 -29.955  40.872  1.00  0.00
F    C
ATOM  19356  HA  TYR F 144     -35.038 -30.497  41.038  1.00  0.00
F    H
ATOM  19357  CB  TYR F 144     -33.753 -29.940  39.386  1.00  0.00
F    C
ATOM  19358  HB1 TYR F 144     -33.849 -30.969  38.978  1.00  0.00
F    H
```

```
ATOM  19359  HB2 TYR F 144     -34.456 -29.294  38.817  1.00  0.00
F    H
ATOM  19360  CG  TYR F 144     -32.342 -29.499  39.090  1.00  0.00
F    C
ATOM  19361  CD1 TYR F 144     -31.959 -28.178  39.252  1.00  0.00
F    C
ATOM  19362  HD1 TYR F 144     -32.682 -27.445  39.582  1.00  0.00
F    H
ATOM  19363  CE1 TYR F 144     -30.669 -27.773  38.978  1.00  0.00
F    C
ATOM  19364  HE1 TYR F 144     -30.381 -26.740  39.101  1.00  0.00
F    H
ATOM  19365  CZ  TYR F 144     -29.744 -28.694  38.536  1.00  0.00
F    C
ATOM  19366  OH  TYR F 144     -28.456 -28.283  38.266  1.00  0.00
F    O
ATOM  19367  HH  TYR F 144     -27.944 -29.063  38.043  1.00  0.00
F    H
ATOM  19368  CD2 TYR F 144     -31.396 -30.408  38.644  1.00  0.00
F    C
ATOM  19369  HD2 TYR F 144     -31.676 -31.438  38.470  1.00  0.00
F    H
ATOM  19370  CE2 TYR F 144     -30.105 -30.017  38.366  1.00  0.00
F    C
ATOM  19371  HE2 TYR F 144     -29.393 -30.741  37.999  1.00  0.00
F    H
ATOM  19372  C   TYR F 144     -33.035 -30.652  41.680  1.00  0.00
F    C
ATOM  19373  O   TYR F 144     -32.077 -30.031  42.144  1.00  0.00
F    O
ATOM  19374  N   ILE F 145     -33.205 -31.962  41.833  1.00  0.00
F    N
ATOM  19375  HN  ILE F 145     -34.011 -32.445  41.497  1.00  0.00
F    H
ATOM  19376  CA  ILE F 145     -32.234 -32.815  42.505  1.00  0.00
F    C
ATOM  19377  HA  ILE F 145     -31.272 -32.321  42.451  1.00  0.00
F    H
ATOM  19378  CB  ILE F 145     -32.615 -33.093  43.973  1.00  0.00
F    C
ATOM  19379  HB  ILE F 145     -33.544 -32.491  44.139  1.00  0.00
F    H
ATOM  19380  CG2 ILE F 145     -32.839 -34.586  44.223  1.00  0.00
F    C
ATOM  19381 HG21 ILE F 145     -33.262 -34.754  45.236  1.00  0.00
F    H
ATOM  19382 HG22 ILE F 145     -33.554 -35.008  43.486  1.00  0.00
F    H
ATOM  19383 HG23 ILE F 145     -31.885 -35.149  44.149  1.00  0.00
F    H
ATOM  19384  CG1 ILE F 145     -31.533 -32.521  44.880  1.00  0.00
F    C
ATOM  19385 HG11 ILE F 145     -30.813 -31.925  44.278  1.00  0.00
F    H
```

ATOM 19386 HG12 ILE F 145   -32.006 -31.825 45.611 1.00 0.00
F   H
ATOM 19387 CD ILE F 145   -30.764 -33.563 45.664 1.00 0.00
F   C
ATOM 19388 HD1 ILE F 145   -30.035 -33.074 46.345 1.00 0.00
F   H
ATOM 19389 HD2 ILE F 145   -31.455 -34.179 46.279 1.00 0.00
F   H
ATOM 19390 HD3 ILE F 145   -30.201 -34.237 44.985 1.00 0.00
F   H
ATOM 19391 C   ILE F 145   -32.109 -34.113 41.717 1.00 0.00
F   C
ATOM 19392 O   ILE F 145   -33.108 -34.644 41.219 1.00 0.00
F   O
ATOM 19393 N   ASP F 146   -30.875 -34.590 41.557 1.00 0.00
F   N
ATOM 19394 HN ASP F 146   -30.050 -34.163 41.917 1.00 0.00
F   H
ATOM 19395 CA ASP F 146   -30.605 -35.817 40.818 1.00 0.00
F   C
ATOM 19396 HA ASP F 146   -31.559 -36.239 40.524 1.00 0.00
F   H
ATOM 19397 CB ASP F 146   -29.716 -35.524 39.613 1.00 0.00
F   C
ATOM 19398 HB1 ASP F 146   -29.180 -36.450 39.320 1.00 0.00
F   H
ATOM 19399 HB2 ASP F 146   -30.325 -35.186 38.752 1.00 0.00
F   H
ATOM 19400 CG ASP F 146   -28.670 -34.469 39.907 1.00 0.00
F   C
ATOM 19401 OD1 ASP F 146   -27.662 -34.815 40.561 1.00 0.00
F   O
ATOM 19402 OD2 ASP F 146   -28.868 -33.294 39.529 1.00 0.00
F   O
ATOM 19403 C   ASP F 146   -29.937 -36.834 41.729 1.00 0.00
F   C
ATOM 19404 O   ASP F 146   -28.910 -36.540 42.345 1.00 0.00
F   O
ATOM 19405 N   LEU F 147   -30.515 -38.029 41.806 1.00 0.00
F   N
ATOM 19406 HN LEU F 147   -31.377 -38.270 41.367 1.00 0.00
F   H
ATOM 19407 CA LEU F 147   -29.907 -39.113 42.565 1.00 0.00
F   C
ATOM 19408 HA LEU F 147   -29.449 -38.653 43.431 1.00 0.00
F   H
ATOM 19409 CB LEU F 147   -30.990 -40.098 42.993 1.00 0.00
F   C
ATOM 19410 HB1 LEU F 147   -30.493 -41.081 43.161 1.00 0.00
F   H
ATOM 19411 HB2 LEU F 147   -31.395 -39.785 43.981 1.00 0.00
F   H
ATOM 19412 CG LEU F 147   -32.070 -40.213 41.920 1.00 0.00
F   C

```
ATOM  19413  HG  LEU F 147     -32.031 -39.262  41.337  1.00  0.00
F    H
ATOM  19414  CD1 LEU F 147     -31.798 -41.375  40.982  1.00  0.00
F    C
ATOM  19415 HD11 LEU F 147     -32.617 -41.474  40.237  1.00  0.00
F    H
ATOM  19416 HD12 LEU F 147     -30.844 -41.221  40.434  1.00  0.00
F    H
ATOM  19417 HD13 LEU F 147     -31.727 -42.328  41.548  1.00  0.00
F    H
ATOM  19418  CD2 LEU F 147     -33.442 -40.309  42.575  1.00  0.00
F    C
ATOM  19419 HD21 LEU F 147     -34.237 -40.374  41.799  1.00  0.00
F    H
ATOM  19420 HD22 LEU F 147     -33.527 -41.207  43.219  1.00  0.00
F    H
ATOM  19421 HD23 LEU F 147     -33.639 -39.411  43.199  1.00  0.00
F    H
ATOM  19422  C   LEU F 147     -28.808 -39.824  41.786  1.00  0.00
F    C
ATOM  19423  O   LEU F 147     -27.902 -40.403  42.395  1.00  0.00
F    O
ATOM  19424  N   SER F 148     -28.856 -39.780  40.461  1.00  0.00
F    N
ATOM  19425  HN  SER F 148     -29.565 -39.305  39.940  1.00  0.00
F    H
ATOM  19426  CA  SER F 148     -27.853 -40.439  39.640  1.00  0.00
F    C
ATOM  19427  HA  SER F 148     -26.935 -40.464  40.211  1.00  0.00
F    H
ATOM  19428  CB  SER F 148     -28.316 -41.862  39.283  1.00  0.00
F    C
ATOM  19429  HB1 SER F 148     -27.518 -42.343  38.672  1.00  0.00
F    H
ATOM  19430  HB2 SER F 148     -28.434 -42.474  40.206  1.00  0.00
F    H
ATOM  19431  OG  SER F 148     -29.518 -41.835  38.527  1.00  0.00
F    O
ATOM  19432  HG1 SER F 148     -29.634 -42.715  38.149  1.00  0.00
F    H
ATOM  19433  C   SER F 148     -27.599 -39.572  38.412  1.00  0.00
F    C
ATOM  19434  O   SER F 148     -28.145 -38.467  38.293  1.00  0.00
F    O
ATOM  19435  N   ASP F 149     -26.770 -40.070  37.493  1.00  0.00
F    N
ATOM  19436  HN  ASP F 149     -26.259 -40.924  37.557  1.00  0.00
F    H
ATOM  19437  CA  ASP F 149     -26.532 -39.357  36.243  1.00  0.00
F    C
ATOM  19438  HA  ASP F 149     -26.604 -38.299  36.467  1.00  0.00
F    H
ATOM  19439  CB  ASP F 149     -25.149 -39.709  35.698  1.00  0.00
F    C
```

ATOM 19440 HB1 ASP F 149 -25.054 -39.320 34.664 1.00 0.00
F H
ATOM 19441 HB2 ASP F 149 -24.356 -39.247 36.318 1.00 0.00
F H
ATOM 19442 CG ASP F 149 -24.912 -41.202 35.645 1.00 0.00
F C
ATOM 19443 OD1 ASP F 149 -24.167 -41.720 36.504 1.00 0.00
F O
ATOM 19444 OD2 ASP F 149 -25.485 -41.861 34.756 1.00 0.00
F O
ATOM 19445 C ASP F 149 -27.607 -39.662 35.210 1.00 0.00
F C
ATOM 19446 O ASP F 149 -27.536 -39.169 34.078 1.00 0.00
F O
ATOM 19447 N GLU F 150 -28.603 -40.462 35.579 1.00 0.00
F N
ATOM 19448 HN GLU F 150 -28.672 -40.847 36.495 1.00 0.00
F H
ATOM 19449 CA GLU F 150 -29.688 -40.852 34.689 1.00 0.00
F C
ATOM 19450 HA GLU F 150 -29.491 -40.393 33.729 1.00 0.00
F H
ATOM 19451 CB GLU F 150 -29.726 -42.377 34.542 1.00 0.00
F C
ATOM 19452 HB1 GLU F 150 -28.731 -42.715 34.170 1.00 0.00
F H
ATOM 19453 HB2 GLU F 150 -29.854 -42.849 35.542 1.00 0.00
F H
ATOM 19454 CG GLU F 150 -30.801 -42.871 33.595 1.00 0.00
F C
ATOM 19455 HG1 GLU F 150 -31.450 -43.596 34.119 1.00 0.00
F H
ATOM 19456 HG2 GLU F 150 -31.420 -42.031 33.223 1.00 0.00
F H
ATOM 19457 CD GLU F 150 -30.250 -43.614 32.403 1.00 0.00
F C
ATOM 19458 OE1 GLU F 150 -29.068 -43.406 32.065 1.00 0.00
F O
ATOM 19459 OE2 GLU F 150 -31.004 -44.409 31.803 1.00 0.00
F O
ATOM 19460 C GLU F 150 -31.052 -40.373 35.147 1.00 0.00
F C
ATOM 19461 O GLU F 150 -31.894 -40.033 34.312 1.00 0.00
F O
ATOM 19462 N TYR F 151 -31.299 -40.338 36.449 1.00 0.00
F N
ATOM 19463 HN TYR F 151 -30.606 -40.520 37.146 1.00 0.00
F H
ATOM 19464 CA TYR F 151 -32.617 -40.028 36.975 1.00 0.00
F C
ATOM 19465 HA TYR F 151 -33.211 -39.666 36.144 1.00 0.00
F H
ATOM 19466 CB TYR F 151 -33.244 -41.263 37.616 1.00 0.00
F C

ATOM 19467 HB1 TYR F 151 -32.733 -41.471 38.581 1.00 0.00
F H
ATOM 19468 HB2 TYR F 151 -34.317 -41.085 37.845 1.00 0.00
F H
ATOM 19469 CG TYR F 151 -33.119 -42.519 36.785 1.00 0.00
F C
ATOM 19470 CD1 TYR F 151 -33.943 -42.738 35.692 1.00 0.00
F C
ATOM 19471 HD1 TYR F 151 -34.695 -42.005 35.431 1.00 0.00
F H
ATOM 19472 CE1 TYR F 151 -33.841 -43.886 34.935 1.00 0.00
F C
ATOM 19473 HE1 TYR F 151 -34.493 -44.048 34.089 1.00 0.00
F H
ATOM 19474 CZ TYR F 151 -32.902 -44.836 35.269 1.00 0.00
F C
ATOM 19475 OH TYR F 151 -32.791 -45.981 34.518 1.00 0.00
F O
ATOM 19476 HH TYR F 151 -32.044 -46.478 34.859 1.00 0.00
F H
ATOM 19477 CD2 TYR F 151 -32.180 -43.490 37.100 1.00 0.00
F C
ATOM 19478 HD2 TYR F 151 -31.549 -43.369 37.968 1.00 0.00
F H
ATOM 19479 CE2 TYR F 151 -32.069 -44.643 36.349 1.00 0.00
F C
ATOM 19480 HE2 TYR F 151 -31.345 -45.395 36.625 1.00 0.00
F H
ATOM 19481 C TYR F 151 -32.527 -38.898 37.988 1.00 0.00
F C
ATOM 19482 O TYR F 151 -31.464 -38.648 38.565 1.00 0.00
F O
ATOM 19483 N SER F 152 -33.652 -38.220 38.203 1.00 0.00
F N
ATOM 19484 HN SER F 152 -34.552 -38.483 37.856 1.00 0.00
F H
ATOM 19485 CA SER F 152 -33.652 -36.996 38.991 1.00 0.00
F C
ATOM 19486 HA SER F 152 -33.108 -37.223 39.899 1.00 0.00
F H
ATOM 19487 CB SER F 152 -33.013 -35.850 38.205 1.00 0.00
F C
ATOM 19488 HB1 SER F 152 -33.638 -34.940 38.352 1.00 0.00
F H
ATOM 19489 HB2 SER F 152 -32.001 -35.620 38.609 1.00 0.00
F H
ATOM 19490 OG SER F 152 -32.968 -36.169 36.824 1.00 0.00
F O
ATOM 19491 HG1 SER F 152 -32.773 -35.351 36.350 1.00 0.00
F H
ATOM 19492 C SER F 152 -35.067 -36.609 39.396 1.00 0.00
F C
ATOM 19493 O SER F 152 -36.055 -37.097 38.835 1.00 0.00
F O

```
ATOM  19494  N   ILE F 153     -35.140 -35.704  40.369  1.00  0.00
F    N
ATOM  19495  HN  ILE F 153     -34.324 -35.347  40.819  1.00  0.00
F    H
ATOM  19496  CA  ILE F 153     -36.392 -35.148  40.872  1.00  0.00
F    C
ATOM  19497  HA  ILE F 153     -37.206 -35.668  40.384  1.00  0.00
F    H
ATOM  19498  CB  ILE F 153     -36.473 -35.260  42.403  1.00  0.00
F    C
ATOM  19499  HB  ILE F 153     -35.478 -34.896  42.764  1.00  0.00
F    H
ATOM  19500  CG2 ILE F 153     -37.644 -34.462  42.948  1.00  0.00
F    C
ATOM  19501 HG21 ILE F 153     -37.606 -34.419  44.057  1.00  0.00
F    H
ATOM  19502 HG22 ILE F 153     -37.623 -33.419  42.570  1.00  0.00
F    H
ATOM  19503 HG23 ILE F 153     -38.611 -34.922  42.652  1.00  0.00
F    H
ATOM  19504  CG1 ILE F 153     -36.588 -36.723  42.831  1.00  0.00
F    C
ATOM  19505 HG11 ILE F 153     -37.649 -36.967  43.060  1.00  0.00
F    H
ATOM  19506 HG12 ILE F 153     -36.277 -37.373  41.981  1.00  0.00
F    H
ATOM  19507  CD  ILE F 153     -35.725 -37.075  44.037  1.00  0.00
F    C
ATOM  19508  HD1 ILE F 153     -35.805 -38.160  44.265  1.00  0.00
F    H
ATOM  19509  HD2 ILE F 153     -34.657 -36.838  43.838  1.00  0.00
F    H
ATOM  19510  HD3 ILE F 153     -36.049 -36.510  44.936  1.00  0.00
F    H
ATOM  19511  C   ILE F 153     -36.467 -33.693  40.425  1.00  0.00
F    C
ATOM  19512  O   ILE F 153     -35.449 -32.992  40.399  1.00  0.00
F    O
ATOM  19513  N   VAL F 154     -37.665 -33.233  40.064  1.00  0.00
F    N
ATOM  19514  HN  VAL F 154     -38.494 -33.782  40.129  1.00  0.00
F    H
ATOM  19515  CA  VAL F 154     -37.872 -31.887  39.541  1.00  0.00
F    C
ATOM  19516  HA  VAL F 154     -37.065 -31.261  39.901  1.00  0.00
F    H
ATOM  19517  CB  VAL F 154     -37.891 -31.869  37.999  1.00  0.00
F    C
ATOM  19518  HB  VAL F 154     -38.762 -32.484  37.665  1.00  0.00
F    H
ATOM  19519  CG1 VAL F 154     -38.057 -30.443  37.488  1.00  0.00
F    C
ATOM  19520 HG11 VAL F 154     -38.030 -30.426  36.377  1.00  0.00
F    H
```

ATOM 19521 HG12 VAL F 154   -39.026 -30.004 37.803 1.00 0.00
F   H
ATOM 19522 HG13 VAL F 154   -37.235 -29.794 37.861 1.00 0.00
F   H
ATOM 19523 CG2 VAL F 154   -36.619 -32.483 37.428 1.00 0.00
F   C
ATOM 19524 HG21 VAL F 154   -36.620 -32.431 36.318 1.00 0.00
F   H
ATOM 19525 HG22 VAL F 154   -35.722 -31.943 37.803 1.00 0.00
F   H
ATOM 19526 HG23 VAL F 154   -36.525 -33.552 37.713 1.00 0.00
F   H
ATOM 19527 C   VAL F 154   -39.174 -31.314 40.089 1.00 0.00
F   C
ATOM 19528 O   VAL F 154   -40.182 -32.023 40.200 1.00 0.00
F   O
ATOM 19529 N   GLU F 155   -39.155 -30.022 40.419 1.00 0.00
F   N
ATOM 19530 HN GLU F 155   -38.315 -29.500 40.532 1.00 0.00
F   H
ATOM 19531 CA GLU F 155   -40.369 -29.248 40.646 1.00 0.00
F   C
ATOM 19532 HA GLU F 155   -41.207 -29.922 40.772 1.00 0.00
F   H
ATOM 19533 CB GLU F 155   -40.249 -28.327 41.859 1.00 0.00
F   C
ATOM 19534 HB1 GLU F 155   -39.211 -27.920 41.885 1.00 0.00
F   H
ATOM 19535 HB2 GLU F 155   -40.918 -27.447 41.731 1.00 0.00
F   H
ATOM 19536 CG GLU F 155   -40.535 -28.980 43.181 1.00 0.00
F   C
ATOM 19537 HG1 GLU F 155   -41.473 -28.571 43.599 1.00 0.00
F   H
ATOM 19538 HG2 GLU F 155   -40.635 -30.078 43.068 1.00 0.00
F   H
ATOM 19539 CD GLU F 155   -39.462 -28.692 44.205 1.00 0.00
F   C
ATOM 19540 OE1 GLU F 155   -39.776 -28.053 45.233 1.00 0.00
F   O
ATOM 19541 OE2 GLU F 155   -38.302 -29.088 43.963 1.00 0.00
F   O
ATOM 19542 C   GLU F 155   -40.645 -28.413 39.406 1.00 0.00
F   C
ATOM 19543 O   GLU F 155   -39.732 -27.778 38.872 1.00 0.00
F   O
ATOM 19544 N   LEU F 156   -41.893 -28.406 38.953 1.00 0.00
F   N
ATOM 19545 HN LEU F 156   -42.624 -28.987 39.304 1.00 0.00
F   H
ATOM 19546 CA LEU F 156   -42.302 -27.518 37.871 1.00 0.00
F   C
ATOM 19547 HA LEU F 156   -41.532 -26.765 37.765 1.00 0.00
F   H

ATOM 19548 CB LEU F 156 -42.473 -28.276 36.542 1.00 0.00
F C
ATOM 19549 HB1 LEU F 156 -43.542 -28.583 36.474 1.00 0.00
F H
ATOM 19550 HB2 LEU F 156 -42.301 -27.570 35.699 1.00 0.00
F H
ATOM 19551 CG LEU F 156 -41.609 -29.527 36.354 1.00 0.00
F C
ATOM 19552 HG LEU F 156 -40.817 -29.456 37.138 1.00 0.00
F H
ATOM 19553 CD1 LEU F 156 -42.416 -30.798 36.568 1.00 0.00
F C
ATOM 19554 HD11 LEU F 156 -41.787 -31.695 36.380 1.00 0.00
F H
ATOM 19555 HD12 LEU F 156 -42.796 -30.850 37.610 1.00 0.00
F H
ATOM 19556 HD13 LEU F 156 -43.284 -30.833 35.875 1.00 0.00
F H
ATOM 19557 CD2 LEU F 156 -40.925 -29.526 34.993 1.00 0.00
F C
ATOM 19558 HD21 LEU F 156 -40.288 -30.429 34.880 1.00 0.00
F H
ATOM 19559 HD22 LEU F 156 -41.662 -29.528 34.165 1.00 0.00
F H
ATOM 19560 HD23 LEU F 156 -40.278 -28.629 34.884 1.00 0.00
F H
ATOM 19561 C LEU F 156 -43.593 -26.805 38.245 1.00 0.00
F C
ATOM 19562 O LEU F 156 -44.553 -27.435 38.685 1.00 0.00
F O
ATOM 19563 N LEU F 157 -43.619 -25.487 38.068 1.00 0.00
F N
ATOM 19564 HN LEU F 157 -42.862 -24.934 37.724 1.00 0.00
F H
ATOM 19565 CA LEU F 157 -44.820 -24.729 38.383 1.00 0.00
F C
ATOM 19566 HA LEU F 157 -45.292 -25.196 39.238 1.00 0.00
F H
ATOM 19567 CB LEU F 157 -44.487 -23.274 38.709 1.00 0.00
F C
ATOM 19568 HB1 LEU F 157 -44.566 -23.157 39.814 1.00 0.00
F H
ATOM 19569 HB2 LEU F 157 -43.421 -23.081 38.455 1.00 0.00
F H
ATOM 19570 CG LEU F 157 -45.316 -22.141 38.103 1.00 0.00
F C
ATOM 19571 HG LEU F 157 -46.277 -22.556 37.714 1.00 0.00
F H
ATOM 19572 CD1 LEU F 157 -45.602 -21.100 39.169 1.00 0.00
F C
ATOM 19573 HD11 LEU F 157 -46.163 -20.244 38.737 1.00 0.00
F H
ATOM 19574 HD12 LEU F 157 -46.210 -21.537 39.990 1.00 0.00
F H

ATOM 19575 HD13 LEU F 157   -44.655 -20.711 39.601 1.00 0.00
F   H
ATOM 19576 CD2 LEU F 157   -44.567 -21.511 36.937 1.00 0.00
F   C
ATOM 19577 HD21 LEU F 157   -45.174 -20.699 36.481 1.00 0.00
F   H
ATOM 19578 HD22 LEU F 157   -43.603 -21.070 37.259 1.00 0.00
F   H
ATOM 19579 HD23 LEU F 157   -44.358 -22.271 36.152 1.00 0.00
F   H
ATOM 19580 C   LEU F 157   -45.788 -24.834 37.208 1.00 0.00
F   C
ATOM 19581 O   LEU F 157   -45.374 -24.755 36.045 1.00 0.00
F   O
ATOM 19582 N   ALA F 158   -47.072 -25.030 37.507 1.00 0.00
F   N
ATOM 19583 HN ALA F 158   -47.441 -24.965 38.434 1.00 0.00
F   H
ATOM 19584 CA ALA F 158   -48.041 -25.360 36.468 1.00 0.00
F   C
ATOM 19585 HA ALA F 158   -47.629 -26.087 35.778 1.00 0.00
F   H
ATOM 19586 CB ALA F 158   -49.306 -25.955 37.083 1.00 0.00
F   C
ATOM 19587 HB1 ALA F 158   -49.048 -26.878 37.645 1.00 0.00
F   H
ATOM 19588 HB2 ALA F 158   -49.777 -25.242 37.794 1.00 0.00
F   H
ATOM 19589 HB3 ALA F 158   -50.051 -26.223 36.304 1.00 0.00
F   H
ATOM 19590 C   ALA F 158   -48.391 -24.133 35.641 1.00 0.00
F   C
ATOM 19591 O   ALA F 158   -48.661 -23.057 36.182 1.00 0.00
F   O
ATOM 19592 N   THR F 159   -48.369 -24.293 34.324 1.00 0.00
F   N
ATOM 19593 HN THR F 159   -48.067 -25.125 33.858 1.00 0.00
F   H
ATOM 19594 CA THR F 159   -48.799 -23.234 33.431 1.00 0.00
F   C
ATOM 19595 HA THR F 159   -48.697 -22.311 33.985 1.00 0.00
F   H
ATOM 19596 CB THR F 159   -47.964 -23.239 32.151 1.00 0.00
F   C
ATOM 19597 HB THR F 159   -46.896 -23.212 32.476 1.00 0.00
F   H
ATOM 19598 OG1 THR F 159   -48.225 -22.048 31.396 1.00 0.00
F   O
ATOM 19599 HG1 THR F 159   -47.681 -22.121 30.605 1.00 0.00
F   H
ATOM 19600 CG2 THR F 159   -48.287 -24.460 31.307 1.00 0.00
F   C
ATOM 19601 HG21 THR F 159   -47.605 -24.529 30.434 1.00 0.00
F   H

```
ATOM  19602 HG22 THR F 159     -48.168 -25.387  31.907  1.00  0.00
F    H
ATOM  19603 HG23 THR F 159     -49.332 -24.422  30.930  1.00  0.00
F    H
ATOM  19604  C   THR F 159     -50.283 -23.385  33.107  1.00  0.00
F    C
ATOM  19605  O   THR F 159     -50.969 -24.293  33.587  1.00  0.00
F    O
ATOM  19606  N   ARG F 160     -50.783 -22.467  32.277  1.00  0.00
F    N
ATOM  19607  HN  ARG F 160     -50.231 -21.729  31.895  1.00  0.00
F    H
ATOM  19608  CA  ARG F 160     -52.186 -22.504  31.885  1.00  0.00
F    C
ATOM  19609  HA  ARG F 160     -52.783 -22.544  32.788  1.00  0.00
F    H
ATOM  19610  CB  ARG F 160     -52.536 -21.252  31.085  1.00  0.00
F    C
ATOM  19611  HB1 ARG F 160     -52.015 -20.402  31.586  1.00  0.00
F    H
ATOM  19612  HB2 ARG F 160     -52.095 -21.326  30.067  1.00  0.00
F    H
ATOM  19613  CG  ARG F 160     -54.018 -20.956  31.030  1.00  0.00
F    C
ATOM  19614  HG1 ARG F 160     -54.567 -21.917  30.925  1.00  0.00
F    H
ATOM  19615  HG2 ARG F 160     -54.339 -20.533  32.010  1.00  0.00
F    H
ATOM  19616  CD  ARG F 160     -54.316 -19.963  29.929  1.00  0.00
F    C
ATOM  19617  HD1 ARG F 160     -55.401 -20.004  29.677  1.00  0.00
F    H
ATOM  19618  HD2 ARG F 160     -54.060 -18.923  30.236  1.00  0.00
F    H
ATOM  19619  NE  ARG F 160     -53.536 -20.252  28.729  1.00  0.00
F    N
ATOM  19620  HE  ARG F 160     -52.763 -19.620  28.599  1.00  0.00
F    H
ATOM  19621  CZ  ARG F 160     -53.800 -21.241  27.883  1.00  0.00
F    C
ATOM  19622  NH1 ARG F 160     -54.828 -22.050  28.096  1.00  0.00
F    N
ATOM  19623 HH11 ARG F 160     -55.012 -22.804  27.481  1.00  0.00
F    H
ATOM  19624 HH12 ARG F 160     -55.307 -21.939  28.970  1.00  0.00
F    H
ATOM  19625  NH2 ARG F 160     -53.030 -21.425  26.820  1.00  0.00
F    N
ATOM  19626 HH21 ARG F 160     -53.276 -22.106  26.145  1.00  0.00
F    H
ATOM  19627 HH22 ARG F 160     -52.348 -20.731  26.636  1.00  0.00
F    H
ATOM  19628  C   ARG F 160     -52.527 -23.751  31.082  1.00  0.00
F    C
```

ATOM 19629 O ARG F 160 -53.690 -24.167 31.065 1.00 0.00
F O
ATOM 19630 N LYS F 161 -51.540 -24.362 30.423 1.00 0.00
F N
ATOM 19631 HN LYS F 161 -50.619 -23.984 30.345 1.00 0.00
F H
ATOM 19632 CA LYS F 161 -51.740 -25.644 29.759 1.00 0.00
F C
ATOM 19633 HA LYS F 161 -52.591 -25.520 29.101 1.00 0.00
F H
ATOM 19634 CB LYS F 161 -50.490 -26.029 28.967 1.00 0.00
F C
ATOM 19635 HB1 LYS F 161 -49.671 -26.193 29.705 1.00 0.00
F H
ATOM 19636 HB2 LYS F 161 -50.663 -27.009 28.470 1.00 0.00
F H
ATOM 19637 CG LYS F 161 -50.043 -24.992 27.959 1.00 0.00
F C
ATOM 19638 HG1 LYS F 161 -50.066 -23.986 28.437 1.00 0.00
F H
ATOM 19639 HG2 LYS F 161 -48.986 -25.199 27.671 1.00 0.00
F H
ATOM 19640 CD LYS F 161 -50.948 -25.002 26.747 1.00 0.00
F C
ATOM 19641 HD1 LYS F 161 -51.974 -24.713 27.071 1.00 0.00
F H
ATOM 19642 HD2 LYS F 161 -50.589 -24.220 26.040 1.00 0.00
F H
ATOM 19643 CE LYS F 161 -50.942 -26.371 26.090 1.00 0.00
F C
ATOM 19644 HE1 LYS F 161 -49.906 -26.663 25.807 1.00 0.00
F H
ATOM 19645 HE2 LYS F 161 -51.347 -27.140 26.785 1.00 0.00
F H
ATOM 19646 NZ LYS F 161 -51.767 -26.405 24.856 1.00 0.00
F N
ATOM 19647 HZ1 LYS F 161 -51.738 -27.356 24.433 1.00 0.00
F H
ATOM 19648 HZ2 LYS F 161 -52.751 -26.160 25.089 1.00 0.00
F H
ATOM 19649 HZ3 LYS F 161 -51.396 -25.714 24.173 1.00 0.00
F H
ATOM 19650 C LYS F 161 -52.080 -26.761 30.734 1.00 0.00
F C
ATOM 19651 O LYS F 161 -52.318 -27.890 30.293 1.00 0.00
F O
ATOM 19652 N LEU F 162 -52.103 -26.477 32.036 1.00 0.00
F N
ATOM 19653 HN LEU F 162 -52.003 -25.552 32.399 1.00 0.00
F H
ATOM 19654 CA LEU F 162 -52.279 -27.502 33.051 1.00 0.00
F C
ATOM 19655 HA LEU F 162 -52.516 -28.407 32.506 1.00 0.00
F H

ATOM 19656 CB LEU F 162 -50.987 -27.657 33.865 1.00 0.00
F C
ATOM 19657 HB1 LEU F 162 -50.686 -26.638 34.203 1.00 0.00
F H
ATOM 19658 HB2 LEU F 162 -51.209 -28.234 34.790 1.00 0.00
F H
ATOM 19659 CG LEU F 162 -49.776 -28.282 33.167 1.00 0.00
F C
ATOM 19660 HG LEU F 162 -49.357 -27.495 32.494 1.00 0.00
F H
ATOM 19661 CD1 LEU F 162 -48.733 -28.670 34.200 1.00 0.00
F C
ATOM 19662 HD11 LEU F 162 -47.868 -29.170 33.713 1.00 0.00
F H
ATOM 19663 HD12 LEU F 162 -48.360 -27.772 34.737 1.00 0.00
F H
ATOM 19664 HD13 LEU F 162 -49.162 -29.372 34.947 1.00 0.00
F H
ATOM 19665 CD2 LEU F 162 -50.165 -29.479 32.308 1.00 0.00
F C
ATOM 19666 HD21 LEU F 162 -49.269 -29.898 31.801 1.00 0.00
F H
ATOM 19667 HD22 LEU F 162 -50.619 -30.288 32.915 1.00 0.00
F H
ATOM 19668 HD23 LEU F 162 -50.896 -29.176 31.527 1.00 0.00
F H
ATOM 19669 C LEU F 162 -53.424 -27.233 34.016 1.00 0.00
F C
ATOM 19670 O LEU F 162 -53.588 -28.003 34.968 1.00 0.00
F O
ATOM 19671 N ASP F 163 -54.214 -26.183 33.805 1.00 0.00
F N
ATOM 19672 HN ASP F 163 -54.198 -25.594 33.002 1.00 0.00
F H
ATOM 19673 CA ASP F 163 -55.222 -25.787 34.780 1.00 0.00
F C
ATOM 19674 HA ASP F 163 -54.737 -25.772 35.750 1.00 0.00
F H
ATOM 19675 CB ASP F 163 -55.792 -24.413 34.412 1.00 0.00
F C
ATOM 19676 HB1 ASP F 163 -54.957 -23.717 34.197 1.00 0.00
F H
ATOM 19677 HB2 ASP F 163 -56.428 -24.484 33.509 1.00 0.00
F H
ATOM 19678 CG ASP F 163 -56.617 -23.796 35.533 1.00 0.00
F C
ATOM 19679 OD1 ASP F 163 -56.954 -24.507 36.504 1.00 0.00
F O
ATOM 19680 OD2 ASP F 163 -56.940 -22.592 35.434 1.00 0.00
F O
ATOM 19681 C ASP F 163 -56.341 -26.816 34.864 1.00 0.00
F C
ATOM 19682 O ASP F 163 -56.784 -27.354 33.846 1.00 0.00
F O

```
ATOM  19683  N   SER F 164     -56.799 -27.071  36.091  1.00  0.00
F    N
ATOM  19684  HN  SER F 164     -56.375 -26.684  36.910  1.00  0.00
F    H
ATOM  19685  CA  SER F 164     -57.952 -27.930  36.362  1.00  0.00
F    C
ATOM  19686  HA  SER F 164     -57.977 -28.065  37.435  1.00  0.00
F    H
ATOM  19687  CB  SER F 164     -59.244 -27.273  35.876  1.00  0.00
F    C
ATOM  19688  HB1 SER F 164     -59.418 -27.588  34.823  1.00  0.00
F    H
ATOM  19689  HB2 SER F 164     -60.111 -27.635  36.474  1.00  0.00
F    H
ATOM  19690  OG  SER F 164     -59.140 -25.860  35.922  1.00  0.00
F    O
ATOM  19691  HG1 SER F 164     -59.875 -25.506  35.406  1.00  0.00
F    H
ATOM  19692  C   SER F 164     -57.774 -29.310  35.737  1.00  0.00
F    C
ATOM  19693  O   SER F 164     -58.726 -29.944  35.280  1.00  0.00
F    O
ATOM  19694  N   LYS F 165     -56.533 -29.775  35.723  1.00  0.00
F    N
ATOM  19695  HN  LYS F 165     -55.766 -29.270  36.116  1.00  0.00
F    H
ATOM  19696  CA  LYS F 165     -56.189 -31.058  35.134  1.00  0.00
F    C
ATOM  19697  HA  LYS F 165     -57.019 -31.373  34.514  1.00  0.00
F    H
ATOM  19698  CB  LYS F 165     -54.910 -30.949  34.302  1.00  0.00
F    C
ATOM  19699  HB1 LYS F 165     -54.121 -30.527  34.967  1.00  0.00
F    H
ATOM  19700  HB2 LYS F 165     -54.570 -31.972  34.025  1.00  0.00
F    H
ATOM  19701  CG  LYS F 165     -55.063 -30.068  33.074  1.00  0.00
F    C
ATOM  19702  HG1 LYS F 165     -55.217 -29.013  33.399  1.00  0.00
F    H
ATOM  19703  HG2 LYS F 165     -54.117 -30.099  32.486  1.00  0.00
F    H
ATOM  19704  CD  LYS F 165     -56.237 -30.497  32.213  1.00  0.00
F    C
ATOM  19705  HD1 LYS F 165     -56.059 -31.542  31.869  1.00  0.00
F    H
ATOM  19706  HD2 LYS F 165     -57.150 -30.508  32.852  1.00  0.00
F    H
ATOM  19707  CE  LYS F 165     -56.418 -29.557  31.034  1.00  0.00
F    C
ATOM  19708  HE1 LYS F 165     -56.701 -28.540  31.386  1.00  0.00
F    H
ATOM  19709  HE2 LYS F 165     -55.475 -29.477  30.449  1.00  0.00
F    H
```

```
ATOM  19710  NZ  LYS F 165     -57.485 -30.028  30.112  1.00  0.00
F    N
ATOM  19711  HZ1 LYS F 165     -57.586 -29.365  29.316  1.00  0.00
F    H
ATOM  19712  HZ2 LYS F 165     -57.237 -30.969  29.746  1.00  0.00
F    H
ATOM  19713  HZ3 LYS F 165     -58.386 -30.087  30.628  1.00  0.00
F    H
ATOM  19714  C   LYS F 165     -56.026 -32.095  36.236  1.00  0.00
F    C
ATOM  19715  O   LYS F 165     -55.978 -31.760  37.420  1.00  0.00
F    O
ATOM  19716  N   SER F 166     -55.996 -33.363  35.845  1.00  0.00
F    N
ATOM  19717  HN  SER F 166     -56.090 -33.662  34.896  1.00  0.00
F    H
ATOM  19718  CA  SER F 166     -55.819 -34.449  36.797  1.00  0.00
F    C
ATOM  19719  HA  SER F 166     -55.646 -33.979  37.756  1.00  0.00
F    H
ATOM  19720  CB  SER F 166     -57.040 -35.362  36.829  1.00  0.00
F    C
ATOM  19721  HB1 SER F 166     -57.812 -34.886  37.474  1.00  0.00
F    H
ATOM  19722  HB2 SER F 166     -57.479 -35.459  35.810  1.00  0.00
F    H
ATOM  19723  OG  SER F 166     -56.674 -36.615  37.372  1.00  0.00
F    O
ATOM  19724  HG1 SER F 166     -57.492 -37.081  37.584  1.00  0.00
F    H
ATOM  19725  C   SER F 166     -54.585 -35.279  36.471  1.00  0.00
F    C
ATOM  19726  O   SER F 166     -54.063 -35.239  35.354  1.00  0.00
F    O
ATOM  19727  N   ILE F 167     -54.130 -36.040  37.466  1.00  0.00
F    N
ATOM  19728  HN  ILE F 167     -54.459 -36.013  38.407  1.00  0.00
F    H
ATOM  19729  CA  ILE F 167     -53.065 -37.011  37.233  1.00  0.00
F    C
ATOM  19730  HA  ILE F 167     -52.277 -36.490  36.704  1.00  0.00
F    H
ATOM  19731  CB  ILE F 167     -52.557 -37.585  38.565  1.00  0.00
F    C
ATOM  19732  HB  ILE F 167     -53.478 -37.695  39.193  1.00  0.00
F    H
ATOM  19733  CG2 ILE F 167     -51.808 -38.885  38.328  1.00  0.00
F    C
ATOM  19734 HG21 ILE F 167     -51.575 -39.383  39.292  1.00  0.00
F    H
ATOM  19735 HG22 ILE F 167     -52.418 -39.589  37.724  1.00  0.00
F    H
ATOM  19736 HG23 ILE F 167     -50.852 -38.701  37.793  1.00  0.00
F    H
```

ATOM 19737 CG1 ILE F 167   -51.658 -36.576 39.273 1.00 0.00
F   C
ATOM 19738 HG11 ILE F 167   -50.772 -37.096 39.701 1.00 0.00
F   H
ATOM 19739 HG12 ILE F 167   -51.279 -35.843 38.524 1.00 0.00
F   H
ATOM 19740 CD ILE F 167   -52.358 -35.817 40.362 1.00 0.00
F   C
ATOM 19741 HD1 ILE F 167   -51.675 -35.058 40.803 1.00 0.00
F   H
ATOM 19742 HD2 ILE F 167   -53.250 -35.291 39.962 1.00 0.00
F   H
ATOM 19743 HD3 ILE F 167   -52.685 -36.498 41.176 1.00 0.00
F   H
ATOM 19744 C   ILE F 167   -53.557 -38.116 36.311 1.00 0.00
F   C
ATOM 19745 O   ILE F 167   -52.877 -38.498 35.352 1.00 0.00
F   O
ATOM 19746 N   ILE F 168   -54.750 -38.648 36.586 1.00 0.00
F   N
ATOM 19747 HN ILE F 168   -55.315 -38.440 37.381 1.00 0.00
F   H
ATOM 19748 CA ILE F 168   -55.333 -39.627 35.679 1.00 0.00
F   C
ATOM 19749 HA ILE F 168   -54.557 -40.350 35.462 1.00 0.00
F   H
ATOM 19750 CB ILE F 168   -56.548 -40.317 36.320 1.00 0.00
F   C
ATOM 19751 HB ILE F 168   -56.193 -40.620 37.338 1.00 0.00
F   H
ATOM 19752 CG2 ILE F 168   -57.747 -39.386 36.352 1.00 0.00
F   C
ATOM 19753 HG21 ILE F 168   -58.570 -39.829 36.952 1.00 0.00
F   H
ATOM 19754 HG22 ILE F 168   -57.481 -38.412 36.815 1.00 0.00
F   H
ATOM 19755 HG23 ILE F 168   -58.130 -39.195 35.327 1.00 0.00
F   H
ATOM 19756 CG1 ILE F 168   -56.882 -41.602 35.563 1.00 0.00
F   C
ATOM 19757 HG11 ILE F 168   -57.964 -41.837 35.678 1.00 0.00
F   H
ATOM 19758 HG12 ILE F 168   -56.694 -41.440 34.477 1.00 0.00
F   H
ATOM 19759 CD ILE F 168   -56.063 -42.793 36.015 1.00 0.00
F   C
ATOM 19760 HD1 ILE F 168   -56.303 -43.684 35.396 1.00 0.00
F   H
ATOM 19761 HD2 ILE F 168   -54.976 -42.582 35.915 1.00 0.00
F   H
ATOM 19762 HD3 ILE F 168   -56.277 -43.044 37.076 1.00 0.00
F   H
ATOM 19763 C   ILE F 168   -55.704 -38.974 34.360 1.00 0.00
F   C

```
ATOM  19764  O   ILE F 168     -55.786 -39.647  33.327  1.00  0.00
F    O
ATOM  19765  N   ASP F 169     -55.912 -37.660  34.363  1.00  0.00
F    N
ATOM  19766  HN  ASP F 169     -55.936 -37.060  35.158  1.00  0.00
F    H
ATOM  19767  CA  ASP F 169     -56.136 -36.953  33.112  1.00  0.00
F    C
ATOM  19768  HA  ASP F 169     -56.881 -37.508  32.553  1.00  0.00
F    H
ATOM  19769  CB  ASP F 169     -56.587 -35.523  33.411  1.00  0.00
F    C
ATOM  19770  HB1 ASP F 169     -56.652 -35.382  34.509  1.00  0.00
F    H
ATOM  19771  HB2 ASP F 169     -55.858 -34.791  33.008  1.00  0.00
F    H
ATOM  19772  CG  ASP F 169     -57.949 -35.208  32.835  1.00  0.00
F    C
ATOM  19773  OD1 ASP F 169     -58.923 -35.849  33.280  1.00  0.00
F    O
ATOM  19774  OD2 ASP F 169     -58.049 -34.326  31.952  1.00  0.00
F    O
ATOM  19775  C   ASP F 169     -54.864 -36.957  32.273  1.00  0.00
F    C
ATOM  19776  O   ASP F 169     -54.795 -37.597  31.217  1.00  0.00
F    O
ATOM  19777  N   LEU F 170     -53.836 -36.266  32.750  1.00  0.00
F    N
ATOM  19778  HN  LEU F 170     -53.876 -35.732  33.592  1.00  0.00
F    H
ATOM  19779  CA  LEU F 170     -52.542 -36.225  32.077  1.00  0.00
F    C
ATOM  19780  HA  LEU F 170     -52.691 -36.582  31.066  1.00  0.00
F    H
ATOM  19781  CB  LEU F 170     -52.008 -34.798  32.054  1.00  0.00
F    C
ATOM  19782  HB1 LEU F 170     -52.139 -34.381  33.079  1.00  0.00
F    H
ATOM  19783  HB2 LEU F 170     -50.911 -34.825  31.871  1.00  0.00
F    H
ATOM  19784  CG  LEU F 170     -52.687 -33.854  31.057  1.00  0.00
F    C
ATOM  19785  HG  LEU F 170     -52.815 -34.441  30.116  1.00  0.00
F    H
ATOM  19786  CD1 LEU F 170     -54.046 -33.379  31.560  1.00  0.00
F    C
ATOM  19787 HD11 LEU F 170     -54.497 -32.659  30.843  1.00  0.00
F    H
ATOM  19788 HD12 LEU F 170     -54.742 -34.238  31.677  1.00  0.00
F    H
ATOM  19789 HD13 LEU F 170     -53.945 -32.872  32.543  1.00  0.00
F    H
ATOM  19790  CD2 LEU F 170     -51.795 -32.663  30.750  1.00  0.00
F    C
```

ATOM 19791 HD21 LEU F 170   -52.286 -31.992 30.012 1.00 0.00
F   H
ATOM 19792 HD22 LEU F 170   -51.583 -32.065 31.659 1.00 0.00
F   H
ATOM 19793 HD23 LEU F 170   -50.827 -33.002 30.320 1.00 0.00
F   H
ATOM 19794 C   LEU F 170   -51.577 -37.188 32.768 1.00 0.00
F   C
ATOM 19795 O   LEU F 170   -50.722 -36.803 33.565 1.00 0.00
F   O
ATOM 19796 N   ASN F 171   -51.752 -38.472 32.460 1.00 0.00
F   N
ATOM 19797 HN ASN F 171   -52.427 -38.810 31.805 1.00 0.00
F   H
ATOM 19798 CA ASN F 171   -50.940 -39.510 33.079 1.00 0.00
F   C
ATOM 19799 HA ASN F 171   -51.009 -39.345 34.149 1.00 0.00
F   H
ATOM 19800 CB ASN F 171   -51.453 -40.897 32.691 1.00 0.00
F   C
ATOM 19801 HB1 ASN F 171   -50.735 -41.648 33.088 1.00 0.00
F   H
ATOM 19802 HB2 ASN F 171   -52.436 -41.092 33.166 1.00 0.00
F   H
ATOM 19803 CG ASN F 171   -51.549 -41.086 31.196 1.00 0.00
F   C
ATOM 19804 OD1 ASN F 171   -51.319 -40.158 30.424 1.00 0.00
F   O
ATOM 19805 ND2 ASN F 171   -51.888 -42.297 30.777 1.00 0.00
F   N
ATOM 19806 HD21 ASN F 171   -51.945 -42.442 29.793 1.00 0.00
F   H
ATOM 19807 HD22 ASN F 171   -52.046 -43.026 31.435 1.00 0.00
F   H
ATOM 19808 C   ASN F 171   -49.476 -39.363 32.682 1.00 0.00
F   C
ATOM 19809 O   ASN F 171   -49.146 -38.818 31.624 1.00 0.00
F   O
ATOM 19810 N   VAL F 172   -48.591 -39.833 33.559 1.00 0.00
F   N
ATOM 19811 HN VAL F 172   -48.843 -40.257 34.426 1.00 0.00
F   H
ATOM 19812 CA VAL F 172   -47.158 -39.764 33.317 1.00 0.00
F   C
ATOM 19813 HA VAL F 172   -47.026 -39.485 32.279 1.00 0.00
F   H
ATOM 19814 CB VAL F 172   -46.506 -38.686 34.207 1.00 0.00
F   C
ATOM 19815 HB VAL F 172   -47.178 -37.793 34.201 1.00 0.00
F   H
ATOM 19816 CG1 VAL F 172   -46.352 -39.181 35.640 1.00 0.00
F   C
ATOM 19817 HG11 VAL F 172   -45.846 -38.411 36.261 1.00 0.00
F   H

ATOM 19818 HG12 VAL F 172   -47.335 -39.391 36.109 1.00 0.00
F   H
ATOM 19819 HG13 VAL F 172   -45.737 -40.106 35.671 1.00 0.00
F   H
ATOM 19820 CG2 VAL F 172   -45.159 -38.253 33.633 1.00 0.00
F   C
ATOM 19821 HG21 VAL F 172   -44.672 -37.506 34.296 1.00 0.00
F   H
ATOM 19822 HG22 VAL F 172   -44.479 -39.126 33.532 1.00 0.00
F   H
ATOM 19823 HG23 VAL F 172   -45.279 -37.787 32.634 1.00 0.00
F   H
ATOM 19824 C   VAL F 172   -46.476 -41.114 33.517 1.00 0.00
F   C
ATOM 19825 O   VAL F 172   -45.397 -41.345 32.960 1.00 0.00
F   O
ATOM 19826 N   ARG F 173   -47.074 -42.014 34.301 1.00 0.00
F   N
ATOM 19827 HN ARG F 173   -47.950 -41.854 34.750 1.00 0.00
F   H
ATOM 19828 CA ARG F 173   -46.444 -43.308 34.536 1.00 0.00
F   C
ATOM 19829 HA ARG F 173   -45.390 -43.143 34.725 1.00 0.00
F   H
ATOM 19830 CB ARG F 173   -47.083 -43.997 35.737 1.00 0.00
F   C
ATOM 19831 HB1 ARG F 173   -47.494 -43.186 36.384 1.00 0.00
F   H
ATOM 19832 HB2 ARG F 173   -47.958 -44.596 35.401 1.00 0.00
F   H
ATOM 19833 CG ARG F 173   -46.097 -44.824 36.533 1.00 0.00
F   C
ATOM 19834 HG1 ARG F 173   -46.143 -45.874 36.170 1.00 0.00
F   H
ATOM 19835 HG2 ARG F 173   -45.064 -44.474 36.306 1.00 0.00
F   H
ATOM 19836 CD ARG F 173   -46.339 -44.704 38.029 1.00 0.00
F   C
ATOM 19837 HD1 ARG F 173   -45.360 -44.615 38.556 1.00 0.00
F   H
ATOM 19838 HD2 ARG F 173   -46.958 -43.808 38.272 1.00 0.00
F   H
ATOM 19839 NE ARG F 173   -47.058 -45.853 38.575 1.00 0.00
F   N
ATOM 19840 HE ARG F 173   -48.007 -45.639 38.834 1.00 0.00
F   H
ATOM 19841 CZ ARG F 173   -46.534 -47.066 38.726 1.00 0.00
F   C
ATOM 19842 NH1 ARG F 173   -45.278 -47.303 38.370 1.00 0.00
F   N
ATOM 19843 HH11 ARG F 173   -44.893 -48.212 38.457 1.00 0.00
F   H
ATOM 19844 HH12 ARG F 173   -44.804 -46.561 37.893 1.00 0.00
F   H

```
ATOM  19845  NH2 ARG F 173      -47.267 -48.046  39.236  1.00  0.00
F    N
ATOM  19846 HH21 ARG F 173      -46.851 -48.924  39.428  1.00  0.00
F    H
ATOM  19847 HH22 ARG F 173      -48.155 -47.799  39.600  1.00  0.00
F    H
ATOM  19848  C   ARG F 173      -46.531 -44.200  33.308  1.00  0.00
F    C
ATOM  19849  O   ARG F 173      -45.524 -44.769  32.872  1.00  0.00
F    O
ATOM  19850  N   ALA F 174      -47.725 -44.347  32.743  1.00  0.00
F    N
ATOM  19851  HN  ALA F 174      -48.572 -43.973  33.120  1.00  0.00
F    H
ATOM  19852  CA  ALA F 174      -47.869 -45.088  31.498  1.00  0.00
F    C
ATOM  19853  HA  ALA F 174      -47.223 -45.958  31.497  1.00  0.00
F    H
ATOM  19854  CB  ALA F 174      -49.312 -45.570  31.337  1.00  0.00
F    C
ATOM  19855  HB1 ALA F 174      -49.584 -46.224  32.193  1.00  0.00
F    H
ATOM  19856  HB2 ALA F 174      -50.020 -44.714  31.321  1.00  0.00
F    H
ATOM  19857  HB3 ALA F 174      -49.445 -46.157  30.402  1.00  0.00
F    H
ATOM  19858  C   ALA F 174      -47.458 -44.247  30.298  1.00  0.00
F    C
ATOM  19859  O   ALA F 174      -47.097 -44.788  29.249  1.00  0.00
F    O
ATOM  19860  N   LYS F 175      -47.491 -42.922  30.447  1.00  0.00
F    N
ATOM  19861  HN  LYS F 175      -47.703 -42.458  31.305  1.00  0.00
F    H
ATOM  19862  CA  LYS F 175      -47.209 -42.041  29.321  1.00  0.00
F    C
ATOM  19863  HA  LYS F 175      -47.503 -42.572  28.424  1.00  0.00
F    H
ATOM  19864  CB  LYS F 175      -48.010 -40.750  29.466  1.00  0.00
F    C
ATOM  19865  HB1 LYS F 175      -49.000 -41.025  29.897  1.00  0.00
F    H
ATOM  19866  HB2 LYS F 175      -47.512 -40.097  30.217  1.00  0.00
F    H
ATOM  19867  CG  LYS F 175      -48.228 -40.003  28.173  1.00  0.00
F    C
ATOM  19868  HG1 LYS F 175      -47.269 -39.531  27.859  1.00  0.00
F    H
ATOM  19869  HG2 LYS F 175      -48.517 -40.728  27.379  1.00  0.00
F    H
ATOM  19870  CD  LYS F 175      -49.290 -38.937  28.354  1.00  0.00
F    C
ATOM  19871  HD1 LYS F 175      -50.209 -39.421  28.756  1.00  0.00
F    H
```

```
ATOM  19872  HD2 LYS F 175     -48.930 -38.215  29.123  1.00  0.00
F    H
ATOM  19873  CE  LYS F 175     -49.582 -38.221  27.052  1.00  0.00
F    C
ATOM  19874  HE1 LYS F 175     -48.706 -37.613  26.736  1.00  0.00
F    H
ATOM  19875  HE2 LYS F 175     -49.811 -38.953  26.246  1.00  0.00
F    H
ATOM  19876  NZ  LYS F 175     -50.749 -37.305  27.180  1.00  0.00
F    N
ATOM  19877  HZ1 LYS F 175     -50.927 -36.826  26.273  1.00  0.00
F    H
ATOM  19878  HZ2 LYS F 175     -51.591 -37.851  27.453  1.00  0.00
F    H
ATOM  19879  HZ3 LYS F 175     -50.550 -36.594  27.912  1.00  0.00
F    H
ATOM  19880  C   LYS F 175     -45.720 -41.738  29.204  1.00  0.00
F    C
ATOM  19881  O   LYS F 175     -45.128 -41.922  28.135  1.00  0.00
F    O
ATOM  19882  N   TYR F 176     -45.096 -41.276  30.289  1.00  0.00
F    N
ATOM  19883  HN  TYR F 176     -45.541 -41.161  31.175  1.00  0.00
F    H
ATOM  19884  CA  TYR F 176     -43.691 -40.895  30.264  1.00  0.00
F    C
ATOM  19885  HA  TYR F 176     -43.354 -41.112  29.257  1.00  0.00
F    H
ATOM  19886  CB  TYR F 176     -43.538 -39.400  30.587  1.00  0.00
F    C
ATOM  19887  HB1 TYR F 176     -43.933 -39.205  31.608  1.00  0.00
F    H
ATOM  19888  HB2 TYR F 176     -42.467 -39.105  30.587  1.00  0.00
F    H
ATOM  19889  CG  TYR F 176     -44.304 -38.500  29.644  1.00  0.00
F    C
ATOM  19890  CD1 TYR F 176     -45.554 -38.008  29.990  1.00  0.00
F    C
ATOM  19891  HD1 TYR F 176     -45.995 -38.279  30.940  1.00  0.00
F    H
ATOM  19892  CE1 TYR F 176     -46.262 -37.191  29.133  1.00  0.00
F    C
ATOM  19893  HE1 TYR F 176     -47.238 -36.818  29.410  1.00  0.00
F    H
ATOM  19894  CZ  TYR F 176     -45.723 -36.855  27.912  1.00  0.00
F    C
ATOM  19895  OH  TYR F 176     -46.428 -36.039  27.059  1.00  0.00
F    O
ATOM  19896  HH  TYR F 176     -45.867 -35.862  26.301  1.00  0.00
F    H
ATOM  19897  CD2 TYR F 176     -43.782 -38.151  28.407  1.00  0.00
F    C
ATOM  19898  HD2 TYR F 176     -42.829 -38.552  28.089  1.00  0.00
F    H
```

ATOM 19899 CE2 TYR F 176   -44.484 -37.332 27.542 1.00 0.00
F   C
ATOM 19900 HE2 TYR F 176   -44.067 -37.089 26.576 1.00 0.00
F   H
ATOM 19901 C   TYR F 176   -42.816 -41.699 31.213 1.00 0.00
F   C
ATOM 19902 O   TYR F 176   -41.618 -41.411 31.307 1.00 0.00
F   O
ATOM 19903 N   GLY F 177   -43.368 -42.684 31.918 1.00 0.00
F   N
ATOM 19904 HN GLY F 177   -44.344 -42.894 31.900 1.00 0.00
F   H
ATOM 19905 CA GLY F 177   -42.561 -43.530 32.774 1.00 0.00
F   C
ATOM 19906 HA1 GLY F 177   -41.715 -43.829 32.171 1.00 0.00
F   H
ATOM 19907 HA2 GLY F 177   -43.205 -44.345 33.074 1.00 0.00
F   H
ATOM 19908 C   GLY F 177   -42.034 -42.871 34.024 1.00 0.00
F   C
ATOM 19909 O   GLY F 177   -41.105 -43.397 34.643 1.00 0.00
F   O
ATOM 19910 N   CYS F 178   -42.598 -41.739 34.420 1.00 0.00
F   N
ATOM 19911 HN CYS F 178   -43.370 -41.328 33.941 1.00 0.00
F   H
ATOM 19912 CA CYS F 178   -42.141 -40.999 35.585 1.00 0.00
F   C
ATOM 19913 HA CYS F 178   -41.212 -41.453 35.909 1.00 0.00
F   H
ATOM 19914 CB CYS F 178   -41.922 -39.524 35.229 1.00 0.00
F   C
ATOM 19915 HB1 CYS F 178   -42.847 -39.166 34.723 1.00 0.00
F   H
ATOM 19916 HB2 CYS F 178   -41.829 -38.922 36.160 1.00 0.00
F   H
ATOM 19917 SG CYS F 178   -40.520 -39.257 34.129 1.00 0.00
F   S
ATOM 19918 HG1 CYS F 178   -40.671 -37.939 33.985 1.00 0.00
F   H
ATOM 19919 C   CYS F 178   -43.150 -41.149 36.717 1.00 0.00
F   C
ATOM 19920 O   CYS F 178   -44.124 -41.899 36.619 1.00 0.00
F   O
ATOM 19921 N   THR F 179   -42.905 -40.420 37.805 1.00 0.00
F   N
ATOM 19922 HN THR F 179   -42.142 -39.779 37.871 1.00 0.00
F   H
ATOM 19923 CA THR F 179   -43.733 -40.492 39.002 1.00 0.00
F   C
ATOM 19924 HA THR F 179   -44.671 -40.935 38.697 1.00 0.00
F   H
ATOM 19925 CB THR F 179   -43.030 -41.299 40.099 1.00 0.00
F   C

ATOM 19926 HB THR F 179   -42.559 -40.549 40.781 1.00 0.00
F   H
ATOM 19927 OG1 THR F 179   -41.985 -42.086 39.512 1.00 0.00
F   O
ATOM 19928 HG1 THR F 179   -41.592 -42.576 40.243 1.00 0.00
F   H
ATOM 19929 CG2 THR F 179   -44.009 -42.189 40.842 1.00 0.00
F   C
ATOM 19930 HG21 THR F 179   -43.516 -42.683 41.706 1.00 0.00
F   H
ATOM 19931 HG22 THR F 179   -44.860 -41.590 41.231 1.00 0.00
F   H
ATOM 19932 HG23 THR F 179   -44.416 -42.979 40.176 1.00 0.00
F   H
ATOM 19933 C   THR F 179   -44.058 -39.090 39.499 1.00 0.00
F   C
ATOM 19934 O   THR F 179   -43.164 -38.359 39.938 1.00 0.00
F   O
ATOM 19935 N   ILE F 180   -45.337 -38.722 39.444 1.00 0.00
F   N
ATOM 19936 HN ILE F 180   -46.057 -39.263 39.016 1.00 0.00
F   H
ATOM 19937 CA ILE F 180   -45.797 -37.460 40.020 1.00 0.00
F   C
ATOM 19938 HA ILE F 180   -45.025 -36.720 39.855 1.00 0.00
F   H
ATOM 19939 CB ILE F 180   -47.137 -37.022 39.400 1.00 0.00
F   C
ATOM 19940 HB ILE F 180   -47.420 -37.867 38.721 1.00 0.00
F   H
ATOM 19941 CG2 ILE F 180   -48.168 -36.708 40.489 1.00 0.00
F   C
ATOM 19942 HG21 ILE F 180   -49.171 -36.545 40.042 1.00 0.00
F   H
ATOM 19943 HG22 ILE F 180   -48.252 -37.548 41.209 1.00 0.00
F   H
ATOM 19944 HG23 ILE F 180   -47.886 -35.792 41.050 1.00 0.00
F   H
ATOM 19945 CG1 ILE F 180   -46.935 -35.789 38.514 1.00 0.00
F   C
ATOM 19946 HG11 ILE F 180   -45.947 -35.325 38.733 1.00 0.00
F   H
ATOM 19947 HG12 ILE F 180   -46.919 -36.109 37.447 1.00 0.00
F   H
ATOM 19948 CD ILE F 180   -48.014 -34.717 38.659 1.00 0.00
F   C
ATOM 19949 HD1 ILE F 180   -47.830 -33.885 37.945 1.00 0.00
F   H
ATOM 19950 HD2 ILE F 180   -49.019 -35.142 38.445 1.00 0.00
F   H
ATOM 19951 HD3 ILE F 180   -48.021 -34.294 39.685 1.00 0.00
F   H
ATOM 19952 C   ILE F 180   -45.911 -37.649 41.526 1.00 0.00
F   C

ATOM 19953 O ILE F 180 -46.308 -38.721 42.000 1.00 0.00
F O
ATOM 19954 N LEU F 181 -45.535 -36.625 42.288 1.00 0.00
F N
ATOM 19955 HN LEU F 181 -45.368 -35.698 41.958 1.00 0.00
F H
ATOM 19956 CA LEU F 181 -45.332 -36.796 43.721 1.00 0.00
F C
ATOM 19957 HA LEU F 181 -45.634 -37.815 43.924 1.00 0.00
F H
ATOM 19958 CB LEU F 181 -43.864 -36.574 44.075 1.00 0.00
F C
ATOM 19959 HB1 LEU F 181 -43.282 -36.650 43.128 1.00 0.00
F H
ATOM 19960 HB2 LEU F 181 -43.732 -35.530 44.435 1.00 0.00
F H
ATOM 19961 CG LEU F 181 -43.281 -37.555 45.080 1.00 0.00
F C
ATOM 19962 HG LEU F 181 -43.603 -37.243 46.103 1.00 0.00
F H
ATOM 19963 CD1 LEU F 181 -43.763 -38.978 44.824 1.00 0.00
F C
ATOM 19964 HD11 LEU F 181 -43.288 -39.684 45.538 1.00 0.00
F H
ATOM 19965 HD12 LEU F 181 -44.865 -39.045 44.947 1.00 0.00
F H
ATOM 19966 HD13 LEU F 181 -43.505 -39.301 43.794 1.00 0.00
F H
ATOM 19967 CD2 LEU F 181 -41.792 -37.441 45.008 1.00 0.00
F C
ATOM 19968 HD21 LEU F 181 -41.317 -38.129 45.741 1.00 0.00
F H
ATOM 19969 HD22 LEU F 181 -41.409 -37.705 44.002 1.00 0.00
F H
ATOM 19970 HD23 LEU F 181 -41.470 -36.404 45.244 1.00 0.00
F H
ATOM 19971 C LEU F 181 -46.164 -35.887 44.605 1.00 0.00
F C
ATOM 19972 O LEU F 181 -46.690 -36.359 45.612 1.00 0.00
F O
ATOM 19973 N ALA F 182 -46.266 -34.599 44.292 1.00 0.00
F N
ATOM 19974 HN ALA F 182 -45.898 -34.184 43.460 1.00 0.00
F H
ATOM 19975 CA ALA F 182 -46.955 -33.688 45.190 1.00 0.00
F C
ATOM 19976 HA ALA F 182 -47.844 -34.146 45.608 1.00 0.00
F H
ATOM 19977 CB ALA F 182 -46.066 -33.269 46.365 1.00 0.00
F C
ATOM 19978 HB1 ALA F 182 -45.770 -34.167 46.948 1.00 0.00
F H
ATOM 19979 HB2 ALA F 182 -45.137 -32.772 46.006 1.00 0.00
F H

ATOM 19980 HB3 ALA F 182   -46.595 -32.574 47.052 1.00 0.00
F   H
ATOM 19981 C   ALA F 182   -47.424 -32.466 44.423 1.00 0.00
F   C
ATOM 19982 O   ALA F 182   -46.825 -32.058 43.421 1.00 0.00
F   O
ATOM 19983 N   ILE F 183   -48.494 -31.876 44.943 1.00 0.00
F   N
ATOM 19984 HN ILE F 183   -48.874 -32.163 45.820 1.00 0.00
F   H
ATOM 19985 CA ILE F 183   -49.214 -30.780 44.311 1.00 0.00
F   C
ATOM 19986 HA ILE F 183   -48.622 -30.442 43.470 1.00 0.00
F   H
ATOM 19987 CB ILE F 183   -50.625 -31.233 43.883 1.00 0.00
F   C
ATOM 19988 HB ILE F 183   -51.097 -31.605 44.826 1.00 0.00
F   H
ATOM 19989 CG2 ILE F 183   -51.434 -30.091 43.293 1.00 0.00
F   C
ATOM 19990 HG21 ILE F 183   -52.489 -30.398 43.138 1.00 0.00
F   H
ATOM 19991 HG22 ILE F 183   -51.436 -29.214 43.974 1.00 0.00
F   H
ATOM 19992 HG23 ILE F 183   -51.018 -29.773 42.314 1.00 0.00
F   H
ATOM 19993 CG1 ILE F 183   -50.530 -32.429 42.929 1.00 0.00
F   C
ATOM 19994 HG11 ILE F 183   -49.803 -32.206 42.117 1.00 0.00
F   H
ATOM 19995 HG12 ILE F 183   -50.138 -33.308 43.490 1.00 0.00
F   H
ATOM 19996 CD ILE F 183   -51.848 -32.818 42.313 1.00 0.00
F   C
ATOM 19997 HD1 ILE F 183   -51.724 -33.724 41.680 1.00 0.00
F   H
ATOM 19998 HD2 ILE F 183   -52.597 -33.045 43.101 1.00 0.00
F   H
ATOM 19999 HD3 ILE F 183   -52.244 -32.003 41.672 1.00 0.00
F   H
ATOM 20000 C   ILE F 183   -49.281 -29.622 45.296 1.00 0.00
F   C
ATOM 20001 O   ILE F 183   -49.829 -29.771 46.395 1.00 0.00
F   O
ATOM 20002 N   LYS F 184   -48.738 -28.471 44.904 1.00 0.00
F   N
ATOM 20003 HN LYS F 184   -48.338 -28.364 43.995 1.00 0.00
F   H
ATOM 20004 CA LYS F 184   -48.678 -27.280 45.752 1.00 0.00
F   C
ATOM 20005 HA LYS F 184   -49.181 -27.487 46.689 1.00 0.00
F   H
ATOM 20006 CB LYS F 184   -47.232 -26.847 45.970 1.00 0.00
F   C

ATOM 20007 HB1 LYS F 184 −46.636 −27.771 46.148 1.00 0.00
F H
ATOM 20008 HB2 LYS F 184 −46.842 −26.400 45.027 1.00 0.00
F H
ATOM 20009 CG LYS F 184 −46.939 −25.887 47.129 1.00 0.00
F C
ATOM 20010 HG1 LYS F 184 −45.933 −26.118 47.547 1.00 0.00
F H
ATOM 20011 HG2 LYS F 184 −46.905 −24.844 46.737 1.00 0.00
F H
ATOM 20012 CD LYS F 184 −47.943 −25.962 48.259 1.00 0.00
F C
ATOM 20013 HD1 LYS F 184 −48.866 −25.421 47.948 1.00 0.00
F H
ATOM 20014 HD2 LYS F 184 −48.219 −27.032 48.407 1.00 0.00
F H
ATOM 20015 CE LYS F 184 −47.330 −25.363 49.522 1.00 0.00
F C
ATOM 20016 HE1 LYS F 184 −46.319 −25.789 49.705 1.00 0.00
F H
ATOM 20017 HE2 LYS F 184 −47.234 −24.259 49.423 1.00 0.00
F H
ATOM 20018 NZ LYS F 184 −48.117 −25.606 50.761 1.00 0.00
F N
ATOM 20019 HZ1 LYS F 184 −47.640 −25.171 51.578 1.00 0.00
F H
ATOM 20020 HZ2 LYS F 184 −49.067 −25.194 50.656 1.00 0.00
F H
ATOM 20021 HZ3 LYS F 184 −48.206 −26.630 50.919 1.00 0.00
F H
ATOM 20022 C LYS F 184 −49.472 −26.189 45.042 1.00 0.00
F C
ATOM 20023 O LYS F 184 −48.959 −25.548 44.120 1.00 0.00
F O
ATOM 20024 N HSE F 185 −50.716 −25.978 45.461 1.00 0.00
F N
ATOM 20025 HN HSE F 185 −51.120 −26.468 46.232 1.00 0.00
F H
ATOM 20026 CA HSE F 185 −51.607 −25.009 44.831 1.00 0.00
F C
ATOM 20027 HA HSE F 185 −51.059 −24.537 44.024 1.00 0.00
F H
ATOM 20028 CB HSE F 185 −52.869 −25.685 44.290 1.00 0.00
F C
ATOM 20029 HB1 HSE F 185 −53.505 −24.900 43.825 1.00 0.00
F H
ATOM 20030 HB2 HSE F 185 −52.592 −26.397 43.485 1.00 0.00
F H
ATOM 20031 ND1 HSE F 185 −54.759 −25.776 45.952 1.00 0.00
F N
ATOM 20032 CG HSE F 185 −53.688 −26.378 45.330 1.00 0.00
F C
ATOM 20033 CE1 HSE F 185 −55.290 −26.618 46.820 1.00 0.00
F C

ATOM 20034 HE1 HSE F 185 -56.163 -26.401 47.441 1.00 0.00
F H
ATOM 20035 NE2 HSE F 185 -54.584 -27.735 46.803 1.00 0.00
F N
ATOM 20036 HE2 HSE F 185 -54.750 -28.531 47.383 1.00 0.00
F H
ATOM 20037 CD2 HSE F 185 -53.573 -27.609 45.881 1.00 0.00
F C
ATOM 20038 HD2 HSE F 185 -52.848 -28.388 45.691 1.00 0.00
F H
ATOM 20039 C HSE F 185 -51.962 -23.918 45.827 1.00 0.00
F C
ATOM 20040 O HSE F 185 -52.688 -24.168 46.794 1.00 0.00
F O
ATOM 20041 N HSE F 186 -51.454 -22.711 45.576 1.00 0.00
F N
ATOM 20042 HN HSE F 186 -50.845 -22.525 44.807 1.00 0.00
F H
ATOM 20043 CA HSE F 186 -51.747 -21.550 46.407 1.00 0.00
F C
ATOM 20044 HA HSE F 186 -51.089 -20.753 46.079 1.00 0.00
F H
ATOM 20045 CB HSE F 186 -53.204 -21.110 46.246 1.00 0.00
F C
ATOM 20046 HB1 HSE F 186 -53.474 -21.208 45.172 1.00 0.00
F H
ATOM 20047 HB2 HSE F 186 -53.863 -21.800 46.811 1.00 0.00
F H
ATOM 20048 ND1 HSE F 186 -52.784 -18.629 46.098 1.00 0.00
F N
ATOM 20049 CG HSE F 186 -53.454 -19.695 46.657 1.00 0.00
F C
ATOM 20050 CE1 HSE F 186 -53.198 -17.508 46.659 1.00 0.00
F C
ATOM 20051 HE1 HSE F 186 -52.829 -16.512 46.401 1.00 0.00
F H
ATOM 20052 NE2 HSE F 186 -54.110 -17.808 47.566 1.00 0.00
F N
ATOM 20053 HE2 HSE F 186 -54.576 -17.147 48.155 1.00 0.00
F H
ATOM 20054 CD2 HSE F 186 -54.286 -19.170 47.587 1.00 0.00
F C
ATOM 20055 HD2 HSE F 186 -54.975 -19.663 48.259 1.00 0.00
F H
ATOM 20056 C HSE F 186 -51.424 -21.824 47.871 1.00 0.00
F C
ATOM 20057 O HSE F 186 -52.201 -21.487 48.765 1.00 0.00
F O
ATOM 20058 N GLY F 187 -50.282 -22.460 48.126 1.00 0.00
F N
ATOM 20059 HN GLY F 187 -49.667 -22.754 47.395 1.00 0.00
F H
ATOM 20060 CA GLY F 187 -49.851 -22.770 49.463 1.00 0.00
F C

ATOM 20061 HA1 GLY F 187 -50.194 -21.956 50.087 1.00 0.00
F H
ATOM 20062 HA2 GLY F 187 -48.776 -22.875 49.423 1.00 0.00
F H
ATOM 20063 C GLY F 187 -50.426 -24.058 50.013 1.00 0.00
F C
ATOM 20064 O GLY F 187 -50.063 -24.461 51.122 1.00 0.00
F O
ATOM 20065 N ASP F 188 -51.310 -24.712 49.271 1.00 0.00
F N
ATOM 20066 HN ASP F 188 -51.617 -24.457 48.358 1.00 0.00
F H
ATOM 20067 CA ASP F 188 -51.940 -25.929 49.762 1.00 0.00
F C
ATOM 20068 HA ASP F 188 -51.805 -25.944 50.837 1.00 0.00
F H
ATOM 20069 CB ASP F 188 -53.417 -25.933 49.381 1.00 0.00
F C
ATOM 20070 HB1 ASP F 188 -53.510 -25.859 48.279 1.00 0.00
F H
ATOM 20071 HB2 ASP F 188 -53.902 -26.873 49.709 1.00 0.00
F H
ATOM 20072 CG ASP F 188 -54.179 -24.761 49.981 1.00 0.00
F C
ATOM 20073 OD1 ASP F 188 -53.519 -23.811 50.452 1.00 0.00
F O
ATOM 20074 OD2 ASP F 188 -55.432 -24.786 49.982 1.00 0.00
F O
ATOM 20075 C ASP F 188 -51.242 -27.172 49.211 1.00 0.00
F C
ATOM 20076 O ASP F 188 -51.004 -27.287 48.006 1.00 0.00
F O
ATOM 20077 N ILE F 189 -50.925 -28.109 50.106 1.00 0.00
F N
ATOM 20078 HN ILE F 189 -51.190 -28.037 51.065 1.00 0.00
F H
ATOM 20079 CA ILE F 189 -50.171 -29.320 49.783 1.00 0.00
F C
ATOM 20080 HA ILE F 189 -49.646 -29.126 48.856 1.00 0.00
F H
ATOM 20081 CB ILE F 189 -49.180 -29.690 50.908 1.00 0.00
F C
ATOM 20082 HB ILE F 189 -49.218 -28.820 51.612 1.00 0.00
F H
ATOM 20083 CG2 ILE F 189 -49.535 -31.033 51.541 1.00 0.00
F C
ATOM 20084 HG21 ILE F 189 -48.917 -31.215 52.445 1.00 0.00
F H
ATOM 20085 HG22 ILE F 189 -50.600 -31.055 51.852 1.00 0.00
F H
ATOM 20086 HG23 ILE F 189 -49.360 -31.867 50.828 1.00 0.00
F H
ATOM 20087 CG1 ILE F 189 -47.737 -29.767 50.400 1.00 0.00
F C

```
ATOM  20088 HG11 ILE F 189     -47.049 -29.319  51.152  1.00  0.00
F    H
ATOM  20089 HG12 ILE F 189     -47.451 -30.837  50.288  1.00  0.00
F    H
ATOM  20090  CD  ILE F 189     -47.478 -29.111  49.099  1.00  0.00
F    C
ATOM  20091  HD1 ILE F 189     -46.422 -29.270  48.790  1.00  0.00
F    H
ATOM  20092  HD2 ILE F 189     -48.138 -29.530  48.310  1.00  0.00
F    H
ATOM  20093  HD3 ILE F 189     -47.656 -28.017  49.162  1.00  0.00
F    H
ATOM  20094  C   ILE F 189     -51.131 -30.468  49.512  1.00  0.00
F    C
ATOM  20095  O   ILE F 189     -52.226 -30.526  50.085  1.00  0.00
F    O
ATOM  20096  N   CYS F 190     -50.723 -31.357  48.610  1.00  0.00
F    N
ATOM  20097  HN  CYS F 190     -49.973 -31.144  47.988  1.00  0.00
F    H
ATOM  20098  CA  CYS F 190     -51.303 -32.684  48.437  1.00  0.00
F    C
ATOM  20099  HA  CYS F 190     -51.739 -33.000  49.377  1.00  0.00
F    H
ATOM  20100  CB  CYS F 190     -52.352 -32.706  47.330  1.00  0.00
F    C
ATOM  20101  HB1 CYS F 190     -53.013 -31.823  47.484  1.00  0.00
F    H
ATOM  20102  HB2 CYS F 190     -51.862 -32.554  46.343  1.00  0.00
F    H
ATOM  20103  SG  CYS F 190     -53.389 -34.197  47.317  1.00  0.00
F    S
ATOM  20104  HG1 CYS F 190     -54.187 -33.835  46.311  1.00  0.00
F    H
ATOM  20105  C   CYS F 190     -50.133 -33.614  48.132  1.00  0.00
F    C
ATOM  20106  O   CYS F 190     -49.569 -33.580  47.036  1.00  0.00
F    O
ATOM  20107  N   LEU F 191     -49.756 -34.427  49.117  1.00  0.00
F    N
ATOM  20108  HN  LEU F 191     -50.191 -34.465  50.015  1.00  0.00
F    H
ATOM  20109  CA  LEU F 191     -48.645 -35.353  48.951  1.00  0.00
F    C
ATOM  20110  HA  LEU F 191     -47.967 -34.906  48.235  1.00  0.00
F    H
ATOM  20111  CB  LEU F 191     -47.955 -35.626  50.288  1.00  0.00
F    C
ATOM  20112  HB1 LEU F 191     -48.756 -35.760  51.051  1.00  0.00
F    H
ATOM  20113  HB2 LEU F 191     -47.422 -36.602  50.227  1.00  0.00
F    H
ATOM  20114  CG  LEU F 191     -46.983 -34.602  50.867  1.00  0.00
F    C
```

ATOM 20115 HG LEU F 191   -47.547 -33.672 51.121 1.00 0.00
F   H
ATOM 20116 CD1 LEU F 191   -46.357 -35.176 52.119 1.00 0.00
F   C
ATOM 20117 HD11 LEU F 191   -45.607 -34.471 52.539 1.00 0.00
F   H
ATOM 20118 HD12 LEU F 191   -47.131 -35.362 52.893 1.00 0.00
F   H
ATOM 20119 HD13 LEU F 191   -45.844 -36.136 51.897 1.00 0.00
F   H
ATOM 20120 CD2 LEU F 191   -45.919 -34.241 49.856 1.00 0.00
F   C
ATOM 20121 HD21 LEU F 191   -45.226 -33.481 50.278 1.00 0.00
F   H
ATOM 20122 HD22 LEU F 191   -45.313 -35.123 49.566 1.00 0.00
F   H
ATOM 20123 HD23 LEU F 191   -46.381 -33.817 48.938 1.00 0.00
F   H
ATOM 20124 C   LEU F 191   -49.128 -36.671 48.364 1.00 0.00
F   C
ATOM 20125 O   LEU F 191   -50.161 -37.210 48.769 1.00 0.00
F   O
ATOM 20126 N   SER F 192   -48.334 -37.215 47.442 1.00 0.00
F   N
ATOM 20127 HN SER F 192   -47.456 -36.810 47.190 1.00 0.00
F   H
ATOM 20128 CA SER F 192   -48.658 -38.438 46.716 1.00 0.00
F   C
ATOM 20129 HA SER F 192   -48.033 -38.447 45.832 1.00 0.00
F   H
ATOM 20130 CB SER F 192   -48.401 -39.677 47.565 1.00 0.00
F   C
ATOM 20131 HB1 SER F 192   -49.179 -40.434 47.316 1.00 0.00
F   H
ATOM 20132 HB2 SER F 192   -47.414 -40.125 47.309 1.00 0.00
F   H
ATOM 20133 OG SER F 192   -48.479 -39.396 48.952 1.00 0.00
F   O
ATOM 20134 HG1 SER F 192   -48.531 -40.244 49.411 1.00 0.00
F   H
ATOM 20135 C   SER F 192   -50.117 -38.397 46.242 1.00 0.00
F   C
ATOM 20136 O   SER F 192   -50.935 -39.205 46.695 1.00 0.00
F   O
ATOM 20137 N   PRO F 193   -50.466 -37.472 45.353 1.00 0.00
F   N
ATOM 20138 CD PRO F 193   -49.586 -36.653 44.500 1.00 0.00
F   C
ATOM 20139 HD1 PRO F 193   -49.112 -35.821 45.069 1.00 0.00
F   H
ATOM 20140 HD2 PRO F 193   -48.809 -37.285 44.013 1.00 0.00
F   H
ATOM 20141 CA PRO F 193   -51.880 -37.283 45.018 1.00 0.00
F   C

```
ATOM  20142  HA  PRO F 193      -52.460 -37.076  45.911  1.00  0.00
F    H
ATOM  20143  CB  PRO F 193      -51.866 -36.065  44.089  1.00  0.00
F    C
ATOM  20144  HB1 PRO F 193      -51.882 -35.138  44.706  1.00  0.00
F    H
ATOM  20145  HB2 PRO F 193      -52.727 -36.047  43.392  1.00  0.00
F    H
ATOM  20146  CG  PRO F 193      -50.523 -36.099  43.462  1.00  0.00
F    C
ATOM  20147  HG1 PRO F 193      -50.192 -35.108  43.094  1.00  0.00
F    H
ATOM  20148  HG2 PRO F 193      -50.531 -36.824  42.616  1.00  0.00
F    H
ATOM  20149  C   PRO F 193      -52.455 -38.508  44.326  1.00  0.00
F    C
ATOM  20150  O   PRO F 193      -51.880 -39.038  43.373  1.00  0.00
F    O
ATOM  20151  N   ALA F 194      -53.604 -38.955  44.826  1.00  0.00
F    N
ATOM  20152  HN  ALA F 194      -54.046 -38.539  45.620  1.00  0.00
F    H
ATOM  20153  CA  ALA F 194      -54.301 -40.088  44.247  1.00  0.00
F    C
ATOM  20154  HA  ALA F 194      -53.615 -40.781  43.772  1.00  0.00
F    H
ATOM  20155  CB  ALA F 194      -55.094 -40.838  45.312  1.00  0.00
F    C
ATOM  20156  HB1 ALA F 194      -54.399 -41.242  46.078  1.00  0.00
F    H
ATOM  20157  HB2 ALA F 194      -55.813 -40.161  45.824  1.00  0.00
F    H
ATOM  20158  HB3 ALA F 194      -55.659 -41.690  44.877  1.00  0.00
F    H
ATOM  20159  C   ALA F 194      -55.228 -39.588  43.155  1.00  0.00
F    C
ATOM  20160  O   ALA F 194      -56.033 -38.678  43.412  1.00  0.00
F    O
ATOM  20161  N   PRO F 195      -55.139 -40.119  41.938  1.00  0.00
F    N
ATOM  20162  CD  PRO F 195      -54.180 -41.136  41.474  1.00  0.00
F    C
ATOM  20163  HD1 PRO F 195      -53.809 -40.193  41.938  1.00  0.00
F    H
ATOM  20164  HD2 PRO F 195      -53.521 -41.991  41.750  1.00  0.00
F    H
ATOM  20165  CA  PRO F 195      -56.041 -39.678  40.867  1.00  0.00
F    C
ATOM  20166  HA  PRO F 195      -55.890 -38.628  40.647  1.00  0.00
F    H
ATOM  20167  CB  PRO F 195      -55.624 -40.549  39.677  1.00  0.00
F    C
ATOM  20168  HB1 PRO F 195      -54.763 -40.069  39.161  1.00  0.00
F    H
```

ATOM 20169 HB2 PRO F 195   -56.445 -40.699 38.947 1.00 0.00
F   H
ATOM 20170 CG PRO F 195   -54.233 -40.989 39.989 1.00 0.00
F   C
ATOM 20171 HG1 PRO F 195   -53.656 -41.267 39.085 1.00 0.00
F   H
ATOM 20172 HG2 PRO F 195   -54.271 -41.856 40.687 1.00 0.00
F   H
ATOM 20173 C   PRO F 195   -57.497 -39.904 41.249 1.00 0.00
F   C
ATOM 20174 O   PRO F 195   -57.857 -40.947 41.798 1.00 0.00
F   O
ATOM 20175 N   GLU F 196   -58.339 -38.913 40.962 1.00 0.00
F   N
ATOM 20176 HN GLU F 196   -59.305 -38.939 41.202 1.00 0.00
F   H
ATOM 20177 CA GLU F 196   -57.933 -37.694 40.269 1.00 0.00
F   C
ATOM 20178 HA GLU F 196   -56.991 -37.884 39.769 1.00 0.00
F   H
ATOM 20179 CB GLU F 196   -58.991 -37.292 39.237 1.00 0.00
F   C
ATOM 20180 HB1 GLU F 196   -58.650 -36.357 38.734 1.00 0.00
F   H
ATOM 20181 HB2 GLU F 196   -59.050 -38.064 38.437 1.00 0.00
F   H
ATOM 20182 CG GLU F 196   -60.385 -37.051 39.801 1.00 0.00
F   C
ATOM 20183 HG1 GLU F 196   -61.136 -37.190 39.003 1.00 0.00
F   H
ATOM 20184 HG2 GLU F 196   -60.603 -37.749 40.633 1.00 0.00
F   H
ATOM 20185 CD GLU F 196   -60.563 -35.629 40.298 1.00 0.00
F   C
ATOM 20186 OE1 GLU F 196   -60.561 -34.699 39.457 1.00 0.00
F   O
ATOM 20187 OE2 GLU F 196   -60.667 -35.437 41.526 1.00 0.00
F   O
ATOM 20188 C   GLU F 196   -57.696 -36.557 41.264 1.00 0.00
F   C
ATOM 20189 O   GLU F 196   -57.989 -36.696 42.447 1.00 0.00
F   O
ATOM 20190 N   ASP F 197   -57.151 -35.439 40.784 1.00 0.00
F   N
ATOM 20191 HN ASP F 197   -56.915 -35.292 39.826 1.00 0.00
F   H
ATOM 20192 CA ASP F 197   -56.837 -34.296 41.627 1.00 0.00
F   C
ATOM 20193 HA ASP F 197   -57.675 -34.152 42.301 1.00 0.00
F   H
ATOM 20194 CB ASP F 197   -55.538 -34.506 42.400 1.00 0.00
F   C
ATOM 20195 HB1 ASP F 197   -55.451 -35.574 42.685 1.00 0.00
F   H

ATOM 20196 HB2 ASP F 197   -54.663 -34.241 41.773 1.00 0.00
F   H
ATOM 20197 CG ASP F 197   -55.487 -33.691 43.670 1.00 0.00
F   C
ATOM 20198 OD1 ASP F 197   -56.558 -33.261 44.146 1.00 0.00
F   O
ATOM 20199 OD2 ASP F 197   -54.368 -33.461 44.179 1.00 0.00
F   O
ATOM 20200 C   ASP F 197   -56.726 -33.032 40.789 1.00 0.00
F   C
ATOM 20201 O   ASP F 197   -56.039 -33.010 39.767 1.00 0.00
F   O
ATOM 20202 N   ILE F 198   -57.360 -31.966 41.267 1.00 0.00
F   N
ATOM 20203 HN ILE F 198   -57.830 -31.945 42.148 1.00 0.00
F   H
ATOM 20204 CA ILE F 198   -57.422 -30.707 40.525 1.00 0.00
F   C
ATOM 20205 HA ILE F 198   -57.702 -30.950 39.508 1.00 0.00
F   H
ATOM 20206 CB ILE F 198   -58.432 -29.734 41.162 1.00 0.00
F   C
ATOM 20207 HB ILE F 198   -58.437 -30.006 42.248 1.00 0.00
F   H
ATOM 20208 CG2 ILE F 198   -58.059 -28.283 40.848 1.00 0.00
F   C
ATOM 20209 HG21 ILE F 198   -58.691 -27.582 41.432 1.00 0.00
F   H
ATOM 20210 HG22 ILE F 198   -57.000 -28.080 41.112 1.00 0.00
F   H
ATOM 20211 HG23 ILE F 198   -58.200 -28.060 39.769 1.00 0.00
F   H
ATOM 20212 CG1 ILE F 198   -59.846 -29.991 40.641 1.00 0.00
F   C
ATOM 20213 HG11 ILE F 198   -59.944 -29.595 39.605 1.00 0.00
F   H
ATOM 20214 HG12 ILE F 198   -60.017 -31.091 40.590 1.00 0.00
F   H
ATOM 20215 CD ILE F 198   -60.921 -29.391 41.497 1.00 0.00
F   C
ATOM 20216 HD1 ILE F 198   -61.924 -29.659 41.102 1.00 0.00
F   H
ATOM 20217 HD2 ILE F 198   -60.848 -29.766 42.540 1.00 0.00
F   H
ATOM 20218 HD3 ILE F 198   -60.844 -28.283 41.514 1.00 0.00
F   H
ATOM 20219 C   ILE F 198   -56.031 -30.088 40.462 1.00 0.00
F   C
ATOM 20220 O   ILE F 198   -55.423 -29.771 41.493 1.00 0.00
F   O
ATOM 20221 N   ILE F 199   -55.524 -29.917 39.246 1.00 0.00
F   N
ATOM 20222 HN ILE F 199   -55.994 -30.223 38.421 1.00 0.00
F   H

ATOM 20223 CA ILE F 199   -54.243 -29.269 38.997 1.00 0.00
F   C
ATOM 20224 HA ILE F 199   -53.746 -29.127 39.948 1.00 0.00
F   H
ATOM 20225 CB ILE F 199   -53.374 -30.082 38.022 1.00 0.00
F   C
ATOM 20226 HB ILE F 199   -54.008 -30.191 37.106 1.00 0.00
F   H
ATOM 20227 CG2 ILE F 199   -52.065 -29.362 37.742 1.00 0.00
F   C
ATOM 20228 HG21 ILE F 199   -51.509 -29.866 36.924 1.00 0.00
F   H
ATOM 20229 HG22 ILE F 199   -52.249 -28.314 37.427 1.00 0.00
F   H
ATOM 20230 HG23 ILE F 199   -51.419 -29.349 38.644 1.00 0.00
F   H
ATOM 20231 CG1 ILE F 199   -53.138 -31.494 38.572 1.00 0.00
F   C
ATOM 20232 HG11 ILE F 199   -52.870 -31.437 39.650 1.00 0.00
F   H
ATOM 20233 HG12 ILE F 199   -54.088 -32.072 38.499 1.00 0.00
F   H
ATOM 20234 CD ILE F 199   -52.073 -32.272 37.855 1.00 0.00
F   C
ATOM 20235 HD1 ILE F 199   -52.001 -33.300 38.271 1.00 0.00
F   H
ATOM 20236 HD2 ILE F 199   -52.307 -32.352 36.772 1.00 0.00
F   H
ATOM 20237 HD3 ILE F 199   -51.080 -31.786 37.967 1.00 0.00
F   H
ATOM 20238 C   ILE F 199   -54.544 -27.886 38.447 1.00 0.00
F   C
ATOM 20239 O   ILE F 199   -54.901 -27.746 37.273 1.00 0.00
F   O
ATOM 20240 N   ARG F 200   -54.429 -26.869 39.292 1.00 0.00
F   N
ATOM 20241 HN ARG F 200   -54.169 -27.006 40.245 1.00 0.00
F   H
ATOM 20242 CA ARG F 200   -54.675 -25.500 38.873 1.00 0.00
F   C
ATOM 20243 HA ARG F 200   -55.442 -25.504 38.108 1.00 0.00
F   H
ATOM 20244 CB ARG F 200   -55.123 -24.658 40.064 1.00 0.00
F   C
ATOM 20245 HB1 ARG F 200   -54.489 -24.972 40.928 1.00 0.00
F   H
ATOM 20246 HB2 ARG F 200   -54.878 -23.589 39.878 1.00 0.00
F   H
ATOM 20247 CG ARG F 200   -56.578 -24.849 40.420 1.00 0.00
F   C
ATOM 20248 HG1 ARG F 200   -57.127 -25.166 39.507 1.00 0.00
F   H
ATOM 20249 HG2 ARG F 200   -56.668 -25.701 41.134 1.00 0.00
F   H

```
ATOM  20250  CD  ARG F 200      -57.159 -23.606  41.061  1.00  0.00
F    C
ATOM  20251  HD1 ARG F 200      -56.802 -23.530  42.114  1.00  0.00
F    H
ATOM  20252  HD2 ARG F 200      -56.856 -22.683  40.513  1.00  0.00
F    H
ATOM  20253  NE  ARG F 200      -58.619 -23.624  41.059  1.00  0.00
F    N
ATOM  20254  HE  ARG F 200      -59.003 -24.524  40.826  1.00  0.00
F    H
ATOM  20255  CZ  ARG F 200      -59.377 -22.570  41.335  1.00  0.00
F    C
ATOM  20256  NH1 ARG F 200      -58.813 -21.409  41.641  1.00  0.00
F    N
ATOM  20257 HH11 ARG F 200      -59.370 -20.623  41.873  1.00  0.00
F    H
ATOM  20258 HH12 ARG F 200      -57.818 -21.416  41.755  1.00  0.00
F    H
ATOM  20259  NH2 ARG F 200      -60.699 -22.672  41.310  1.00  0.00
F    N
ATOM  20260 HH21 ARG F 200      -61.258 -21.865  41.431  1.00  0.00
F    H
ATOM  20261 HH22 ARG F 200      -61.076 -23.520  40.963  1.00  0.00
F    H
ATOM  20262  C   ARG F 200      -53.425 -24.904  38.235  1.00  0.00
F    C
ATOM  20263  O   ARG F 200      -52.344 -25.496  38.247  1.00  0.00
F    O
ATOM  20264  N   GLU F 201      -53.583 -23.713  37.667  1.00  0.00
F    N
ATOM  20265  HN  GLU F 201      -54.451 -23.242  37.538  1.00  0.00
F    H
ATOM  20266  CA  GLU F 201      -52.432 -22.980  37.168  1.00  0.00
F    C
ATOM  20267  HA  GLU F 201      -51.864 -23.626  36.509  1.00  0.00
F    H
ATOM  20268  CB  GLU F 201      -52.867 -21.719  36.427  1.00  0.00
F    C
ATOM  20269  HB1 GLU F 201      -52.019 -20.996  36.444  1.00  0.00
F    H
ATOM  20270  HB2 GLU F 201      -53.045 -21.954  35.354  1.00  0.00
F    H
ATOM  20271  CG  GLU F 201      -54.088 -21.044  37.007  1.00  0.00
F    C
ATOM  20272  HG1 GLU F 201      -54.990 -21.629  36.749  1.00  0.00
F    H
ATOM  20273  HG2 GLU F 201      -54.010 -20.961  38.109  1.00  0.00
F    H
ATOM  20274  CD  GLU F 201      -54.303 -19.664  36.425  1.00  0.00
F    C
ATOM  20275  OE1 GLU F 201      -53.996 -19.470  35.232  1.00  0.00
F    O
ATOM  20276  OE2 GLU F 201      -54.779 -18.773  37.159  1.00  0.00
F    O
```

```
ATOM  20277  C   GLU F 201     -51.522 -22.608  38.327  1.00  0.00
F    C
ATOM  20278  O   GLU F 201     -51.935 -22.618  39.491  1.00  0.00
F    O
ATOM  20279  N   GLN F 202     -50.270 -22.297  38.004  1.00  0.00
F    N
ATOM  20280  HN  GLN F 202     -49.953 -22.345  37.059  1.00  0.00
F    H
ATOM  20281  CA  GLN F 202     -49.258 -21.866  38.960  1.00  0.00
F    C
ATOM  20282  HA  GLN F 202     -48.330 -21.774  38.408  1.00  0.00
F    H
ATOM  20283  CB  GLN F 202     -49.617 -20.504  39.565  1.00  0.00
F    C
ATOM  20284  HB1 GLN F 202     -50.631 -20.611  40.017  1.00  0.00
F    H
ATOM  20285  HB2 GLN F 202     -48.924 -20.281  40.407  1.00  0.00
F    H
ATOM  20286  CG  GLN F 202     -49.620 -19.393  38.531  1.00  0.00
F    C
ATOM  20287  HG1 GLN F 202     -48.949 -19.651  37.683  1.00  0.00
F    H
ATOM  20288  HG2 GLN F 202     -50.640 -19.237  38.124  1.00  0.00
F    H
ATOM  20289  CD  GLN F 202     -49.118 -18.059  39.058  1.00  0.00
F    C
ATOM  20290  OE1 GLN F 202     -48.291 -18.008  39.961  1.00  0.00
F    O
ATOM  20291  NE2 GLN F 202     -49.620 -16.970  38.486  1.00  0.00
F    N
ATOM  20292 HE21 GLN F 202     -49.291 -16.093  38.824  1.00  0.00
F    H
ATOM  20293 HE22 GLN F 202     -50.301 -17.059  37.768  1.00  0.00
F    H
ATOM  20294  C   GLN F 202     -49.037 -22.895  40.058  1.00  0.00
F    C
ATOM  20295  O   GLN F 202     -48.592 -22.543  41.159  1.00  0.00
F    O
ATOM  20296  N   ASP F 203     -49.333 -24.161  39.782  1.00  0.00
F    N
ATOM  20297  HN  ASP F 203     -49.686 -24.490  38.909  1.00  0.00
F    H
ATOM  20298  CA  ASP F 203     -49.162 -25.222  40.760  1.00  0.00
F    C
ATOM  20299  HA  ASP F 203     -49.282 -24.776  41.741  1.00  0.00
F    H
ATOM  20300  CB  ASP F 203     -50.138 -26.363  40.510  1.00  0.00
F    C
ATOM  20301  HB1 ASP F 203     -51.153 -25.947  40.351  1.00  0.00
F    H
ATOM  20302  HB2 ASP F 203     -49.849 -26.932  39.604  1.00  0.00
F    H
ATOM  20303  CG  ASP F 203     -50.210 -27.310  41.674  1.00  0.00
F    C
```

```
ATOM  20304  OD1 ASP F 203     -49.536 -28.354  41.619  1.00  0.00
F    O
ATOM  20305  OD2 ASP F 203     -50.966 -27.035  42.614  1.00  0.00
F    O
ATOM  20306  C   ASP F 203     -47.754 -25.782  40.703  1.00  0.00
F    C
ATOM  20307  O   ASP F 203     -47.206 -26.011  39.624  1.00  0.00
F    O
ATOM  20308  N   CYS F 204     -47.186 -26.048  41.873  1.00  0.00
F    N
ATOM  20309  HN  CYS F 204     -47.582 -25.817  42.760  1.00  0.00
F    H
ATOM  20310  CA  CYS F 204     -45.897 -26.722  41.937  1.00  0.00
F    C
ATOM  20311  HA  CYS F 204     -45.347 -26.455  41.042  1.00  0.00
F    H
ATOM  20312  CB  CYS F 204     -45.123 -26.284  43.175  1.00  0.00
F    C
ATOM  20313  HB1 CYS F 204     -45.836 -26.292  44.030  1.00  0.00
F    H
ATOM  20314  HB2 CYS F 204     -44.343 -27.039  43.420  1.00  0.00
F    H
ATOM  20315  SG  CYS F 204     -44.413 -24.629  43.033  1.00  0.00
F    S
ATOM  20316  HG1 CYS F 204     -43.900 -24.562  44.263  1.00  0.00
F    H
ATOM  20317  C   CYS F 204     -46.119 -28.230  41.912  1.00  0.00
F    C
ATOM  20318  O   CYS F 204     -46.774 -28.786  42.801  1.00  0.00
F    O
ATOM  20319  N   LEU F 205     -45.600 -28.875  40.870  1.00  0.00
F    N
ATOM  20320  HN  LEU F 205     -45.074 -28.378  40.183  1.00  0.00
F    H
ATOM  20321  CA  LEU F 205     -45.725 -30.302  40.609  1.00  0.00
F    C
ATOM  20322  HA  LEU F 205     -46.466 -30.712  41.283  1.00  0.00
F    H
ATOM  20323  CB  LEU F 205     -46.060 -30.555  39.135  1.00  0.00
F    C
ATOM  20324  HB1 LEU F 205     -45.795 -29.630  38.574  1.00  0.00
F    H
ATOM  20325  HB2 LEU F 205     -45.392 -31.353  38.744  1.00  0.00
F    H
ATOM  20326  CG  LEU F 205     -47.448 -30.911  38.609  1.00  0.00
F    C
ATOM  20327  HG  LEU F 205     -47.743 -31.924  38.977  1.00  0.00
F    H
ATOM  20328  CD1 LEU F 205     -48.486 -29.883  39.021  1.00  0.00
F    C
ATOM  20329 HD11 LEU F 205     -49.477 -30.135  38.589  1.00  0.00
F    H
ATOM  20330 HD12 LEU F 205     -48.586 -29.849  40.127  1.00  0.00
F    H
```

ATOM 20331 HD13 LEU F 205   -48.198 -28.871 38.664 1.00 0.00
F   H
ATOM 20332 CD2 LEU F 205   -47.361 -30.969 37.101 1.00 0.00
F   C
ATOM 20333 HD21 LEU F 205   -48.347 -31.244 36.668 1.00 0.00
F   H
ATOM 20334 HD22 LEU F 205   -47.066 -29.991 36.670 1.00 0.00
F   H
ATOM 20335 HD23 LEU F 205   -46.617 -31.730 36.783 1.00 0.00
F   H
ATOM 20336 C   LEU F 205   -44.387 -30.937 40.958 1.00 0.00
F   C
ATOM 20337 O   LEU F 205   -43.337 -30.432 40.540 1.00 0.00
F   O
ATOM 20338 N   VAL F 206   -44.415 -32.019 41.728 1.00 0.00
F   N
ATOM 20339 HN VAL F 206   -45.246 -32.395 42.131 1.00 0.00
F   H
ATOM 20340 CA VAL F 206   -43.200 -32.758 42.052 1.00 0.00
F   C
ATOM 20341 HA VAL F 206   -42.343 -32.164 41.762 1.00 0.00
F   H
ATOM 20342 CB VAL F 206   -43.099 -33.068 43.548 1.00 0.00
F   C
ATOM 20343 HB VAL F 206   -43.926 -33.775 43.803 1.00 0.00
F   H
ATOM 20344 CG1 VAL F 206   -41.768 -33.721 43.858 1.00 0.00
F   C
ATOM 20345 HG11 VAL F 206   -41.678 -33.911 44.949 1.00 0.00
F   H
ATOM 20346 HG12 VAL F 206   -41.661 -34.698 43.343 1.00 0.00
F   H
ATOM 20347 HG13 VAL F 206   -40.928 -33.062 43.553 1.00 0.00
F   H
ATOM 20348 CG2 VAL F 206   -43.261 -31.784 44.353 1.00 0.00
F   C
ATOM 20349 HG21 VAL F 206   -43.150 -31.984 45.440 1.00 0.00
F   H
ATOM 20350 HG22 VAL F 206   -42.494 -31.037 44.055 1.00 0.00
F   H
ATOM 20351 HG23 VAL F 206   -44.264 -31.335 44.195 1.00 0.00
F   H
ATOM 20352 C   VAL F 206   -43.179 -34.028 41.216 1.00 0.00
F   C
ATOM 20353 O   VAL F 206   -44.058 -34.891 41.343 1.00 0.00
F   O
ATOM 20354 N   ILE F 207   -42.166 -34.148 40.368 1.00 0.00
F   N
ATOM 20355 HN ILE F 207   -41.435 -33.474 40.284 1.00 0.00
F   H
ATOM 20356 CA ILE F 207   -42.030 -35.286 39.478 1.00 0.00
F   C
ATOM 20357 HA ILE F 207   -42.745 -36.035 39.794 1.00 0.00
F   H

```
ATOM  20358  CB  ILE F 207     -42.258 -34.894  38.003  1.00  0.00
F    C
ATOM  20359  HB  ILE F 207     -43.200 -34.290  38.016  1.00  0.00
F    H
ATOM  20360  CG2 ILE F 207     -41.068 -34.131  37.473  1.00  0.00
F    C
ATOM  20361 HG21 ILE F 207     -41.288 -33.712  36.469  1.00  0.00
F    H
ATOM  20362 HG22 ILE F 207     -40.812 -33.283  38.142  1.00  0.00
F    H
ATOM  20363 HG23 ILE F 207     -40.179 -34.790  37.388  1.00  0.00
F    H
ATOM  20364  CG1 ILE F 207     -42.541 -36.134  37.152  1.00  0.00
F    C
ATOM  20365 HG11 ILE F 207     -41.688 -36.845  37.228  1.00  0.00
F    H
ATOM  20366 HG12 ILE F 207     -43.439 -36.652  37.558  1.00  0.00
F    H
ATOM  20367  CD  ILE F 207     -42.791 -35.826  35.717  1.00  0.00
F    C
ATOM  20368  HD1 ILE F 207     -43.047 -36.755  35.162  1.00  0.00
F    H
ATOM  20369  HD2 ILE F 207     -43.637 -35.114  35.611  1.00  0.00
F    H
ATOM  20370  HD3 ILE F 207     -41.892 -35.380  35.243  1.00  0.00
F    H
ATOM  20371  C   ILE F 207     -40.647 -35.888  39.678  1.00  0.00
F    C
ATOM  20372  O   ILE F 207     -39.728 -35.247  40.187  1.00  0.00
F    O
ATOM  20373  N   MET F 208     -40.498 -37.143  39.271  1.00  0.00
F    N
ATOM  20374  HN  MET F 208     -41.228 -37.697  38.878  1.00  0.00
F    H
ATOM  20375  CA  MET F 208     -39.214 -37.824  39.372  1.00  0.00
F    C
ATOM  20376  HA  MET F 208     -38.430 -37.102  39.181  1.00  0.00
F    H
ATOM  20377  CB  MET F 208     -39.014 -38.476  40.750  1.00  0.00
F    C
ATOM  20378  HB1 MET F 208     -38.853 -37.648  41.480  1.00  0.00
F    H
ATOM  20379  HB2 MET F 208     -39.955 -38.984  41.058  1.00  0.00
F    H
ATOM  20380  CG  MET F 208     -37.824 -39.426  40.776  1.00  0.00
F    C
ATOM  20381  HG1 MET F 208     -37.730 -39.953  39.803  1.00  0.00
F    H
ATOM  20382  HG2 MET F 208     -36.888 -38.848  40.938  1.00  0.00
F    H
ATOM  20383  SD  MET F 208     -37.917 -40.681  42.042  1.00  0.00
F    S
ATOM  20384  CE  MET F 208     -39.562 -41.284  41.752  1.00  0.00
F    C
```

ATOM 20385 HE1 MET F 208 −39.821 −42.090 42.471 1.00 0.00
F H
ATOM 20386 HE2 MET F 208 −40.312 −40.471 41.868 1.00 0.00
F H
ATOM 20387 HE3 MET F 208 −39.662 −41.697 40.726 1.00 0.00
F H
ATOM 20388 C MET F 208 −39.142 −38.879 38.284 1.00 0.00
F C
ATOM 20389 O MET F 208 −40.154 −39.503 37.952 1.00 0.00
F O
ATOM 20390 N GLY F 209 −37.949 −39.082 37.744 1.00 0.00
F N
ATOM 20391 HN GLY F 209 −37.148 −38.508 37.914 1.00 0.00
F H
ATOM 20392 CA GLY F 209 −37.742 −40.195 36.842 1.00 0.00
F C
ATOM 20393 HA1 GLY F 209 −38.615 −40.251 36.207 1.00 0.00
F H
ATOM 20394 HA2 GLY F 209 −37.579 −41.073 37.452 1.00 0.00
F H
ATOM 20395 C GLY F 209 −36.531 −40.011 35.958 1.00 0.00
F C
ATOM 20396 O GLY F 209 −35.574 −39.329 36.334 1.00 0.00
F O
ATOM 20397 N HSE F 210 −36.566 −40.620 34.779 1.00 0.00
F N
ATOM 20398 HN HSE F 210 −37.300 −41.222 34.467 1.00 0.00
F H
ATOM 20399 CA HSE F 210 −35.492 −40.440 33.820 1.00 0.00
F C
ATOM 20400 HA HSE F 210 −34.554 −40.589 34.341 1.00 0.00
F H
ATOM 20401 CB HSE F 210 −35.640 −41.402 32.646 1.00 0.00
F C
ATOM 20402 HB1 HSE F 210 −35.630 −42.438 33.050 1.00 0.00
F H
ATOM 20403 HB2 HSE F 210 −36.628 −41.251 32.166 1.00 0.00
F H
ATOM 20404 ND1 HSE F 210 −33.233 −41.602 31.927 1.00 0.00
F N
ATOM 20405 CG HSE F 210 −34.543 −41.284 31.640 1.00 0.00
F C
ATOM 20406 CE1 HSE F 210 −32.488 −41.393 30.857 1.00 0.00
F C
ATOM 20407 HE1 HSE F 210 −31.411 −41.571 30.807 1.00 0.00
F H
ATOM 20408 NE2 HSE F 210 −33.265 −40.935 29.892 1.00 0.00
F N
ATOM 20409 HE2 HSE F 210 −32.954 −40.674 28.979 1.00 0.00
F H
ATOM 20410 CD2 HSE F 210 −34.553 −40.850 30.358 1.00 0.00
F C
ATOM 20411 HD2 HSE F 210 −35.372 −40.483 29.756 1.00 0.00
F H

ATOM 20412 C HSE F 210 -35.501 -39.008 33.321 1.00 0.00
F C
ATOM 20413 O HSE F 210 -36.485 -38.547 32.738 1.00 0.00
F O
ATOM 20414 N LYS F 211 -34.389 -38.308 33.552 1.00 0.00
F N
ATOM 20415 HN LYS F 211 -33.543 -38.702 33.907 1.00 0.00
F H
ATOM 20416 CA LYS F 211 -34.341 -36.873 33.289 1.00 0.00
F C
ATOM 20417 HA LYS F 211 -35.069 -36.409 33.945 1.00 0.00
F H
ATOM 20418 CB LYS F 211 -32.940 -36.336 33.575 1.00 0.00
F C
ATOM 20419 HB1 LYS F 211 -32.912 -35.284 33.210 1.00 0.00
F H
ATOM 20420 HB2 LYS F 211 -32.788 -36.289 34.676 1.00 0.00
F H
ATOM 20421 CG LYS F 211 -31.824 -37.121 32.911 1.00 0.00
F C
ATOM 20422 HG1 LYS F 211 -31.775 -38.138 33.362 1.00 0.00
F H
ATOM 20423 HG2 LYS F 211 -32.061 -37.247 31.829 1.00 0.00
F H
ATOM 20424 CD LYS F 211 -30.483 -36.433 33.086 1.00 0.00
F C
ATOM 20425 HD1 LYS F 211 -30.607 -35.352 32.843 1.00 0.00
F H
ATOM 20426 HD2 LYS F 211 -30.195 -36.502 34.160 1.00 0.00
F H
ATOM 20427 CE LYS F 211 -29.431 -37.074 32.205 1.00 0.00
F C
ATOM 20428 HE1 LYS F 211 -29.702 -36.964 31.131 1.00 0.00
F H
ATOM 20429 HE2 LYS F 211 -28.440 -36.596 32.368 1.00 0.00
F H
ATOM 20430 NZ LYS F 211 -29.278 -38.526 32.480 1.00 0.00
F N
ATOM 20431 HZ1 LYS F 211 -28.551 -38.932 31.858 1.00 0.00
F H
ATOM 20432 HZ2 LYS F 211 -28.999 -38.664 33.473 1.00 0.00
F H
ATOM 20433 HZ3 LYS F 211 -30.185 -39.008 32.311 1.00 0.00
F H
ATOM 20434 C LYS F 211 -34.747 -36.548 31.858 1.00 0.00
F C
ATOM 20435 O LYS F 211 -35.370 -35.512 31.601 1.00 0.00
F O
ATOM 20436 N LYS F 212 -34.413 -37.423 30.910 1.00 0.00
F N
ATOM 20437 HN LYS F 212 -33.953 -38.296 31.060 1.00 0.00
F H
ATOM 20438 CA LYS F 212 -34.725 -37.120 29.520 1.00 0.00
F C

```
ATOM  20439  HA  LYS F 212     -34.526 -36.065  29.370  1.00  0.00
F    H
ATOM  20440  CB  LYS F 212     -33.831 -37.934  28.588  1.00  0.00
F    C
ATOM  20441  HB1 LYS F 212     -34.062 -39.010  28.768  1.00  0.00
F    H
ATOM  20442  HB2 LYS F 212     -34.120 -37.725  27.534  1.00  0.00
F    H
ATOM  20443  CG  LYS F 212     -32.329 -37.700  28.799  1.00  0.00
F    C
ATOM  20444  HG1 LYS F 212     -31.954 -38.413  29.569  1.00  0.00
F    H
ATOM  20445  HG2 LYS F 212     -31.790 -37.924  27.849  1.00  0.00
F    H
ATOM  20446  CD  LYS F 212     -31.986 -36.274  29.258  1.00  0.00
F    C
ATOM  20447  HD1 LYS F 212     -32.649 -36.011  30.114  1.00  0.00
F    H
ATOM  20448  HD2 LYS F 212     -30.938 -36.275  29.638  1.00  0.00
F    H
ATOM  20449  CE  LYS F 212     -32.127 -35.238  28.153  1.00  0.00
F    C
ATOM  20450  HE1 LYS F 212     -31.803 -35.663  27.176  1.00  0.00
F    H
ATOM  20451  HE2 LYS F 212     -33.186 -34.908  28.059  1.00  0.00
F    H
ATOM  20452  NZ  LYS F 212     -31.293 -34.032  28.427  1.00  0.00
F    N
ATOM  20453  HZ1 LYS F 212     -31.404 -33.339  27.659  1.00  0.00
F    H
ATOM  20454  HZ2 LYS F 212     -31.590 -33.602  29.327  1.00  0.00
F    H
ATOM  20455  HZ3 LYS F 212     -30.294 -34.312  28.496  1.00  0.00
F    H
ATOM  20456  C   LYS F 212     -36.202 -37.349  29.225  1.00  0.00
F    C
ATOM  20457  O   LYS F 212     -36.818 -36.575  28.486  1.00  0.00
F    O
ATOM  20458  N   ASP F 213     -36.804 -38.382  29.818  1.00  0.00
F    N
ATOM  20459  HN  ASP F 213     -36.379 -39.115  30.344  1.00  0.00
F    H
ATOM  20460  CA  ASP F 213     -38.254 -38.515  29.726  1.00  0.00
F    C
ATOM  20461  HA  ASP F 213     -38.513 -38.462  28.674  1.00  0.00
F    H
ATOM  20462  CB  ASP F 213     -38.712 -39.835  30.342  1.00  0.00
F    C
ATOM  20463  HB1 ASP F 213     -38.223 -39.967  31.328  1.00  0.00
F    H
ATOM  20464  HB2 ASP F 213     -39.809 -39.834  30.495  1.00  0.00
F    H
ATOM  20465  CG  ASP F 213     -38.349 -41.034  29.495  1.00  0.00
F    C
```

ATOM 20466 OD1 ASP F 213   −39.112 −41.356 28.560 1.00 0.00
F   O
ATOM 20467 OD2 ASP F 213   −37.301 −41.655 29.756 1.00 0.00
F   O
ATOM 20468 C   ASP F 213   −38.952 −37.348 30.411 1.00 0.00
F   C
ATOM 20469 O   ASP F 213   −40.014 −36.896 29.962 1.00 0.00
F   O
ATOM 20470 N   ILE F 214   −38.370 −36.850 31.504 1.00 0.00
F   N
ATOM 20471 HN ILE F 214   −37.543 −37.210 31.929 1.00 0.00
F   H
ATOM 20472 CA ILE F 214   −38.934 −35.686 32.181 1.00 0.00
F   C
ATOM 20473 HA ILE F 214   −39.978 −35.906 32.363 1.00 0.00
F   H
ATOM 20474 CB ILE F 214   −38.193 −35.420 33.502 1.00 0.00
F   C
ATOM 20475 HB ILE F 214   −37.124 −35.276 33.200 1.00 0.00
F   H
ATOM 20476 CG2 ILE F 214   −38.808 −34.234 34.218 1.00 0.00
F   C
ATOM 20477 HG21 ILE F 214   −38.186 −33.938 35.090 1.00 0.00
F   H
ATOM 20478 HG22 ILE F 214   −38.879 −33.356 33.542 1.00 0.00
F   H
ATOM 20479 HG23 ILE F 214   −39.827 −34.476 34.587 1.00 0.00
F   H
ATOM 20480 CG1 ILE F 214   −38.238 −36.655 34.396 1.00 0.00
F   C
ATOM 20481 HG11 ILE F 214   −39.265 −36.787 34.806 1.00 0.00
F   H
ATOM 20482 HG12 ILE F 214   −38.013 −37.556 33.779 1.00 0.00
F   H
ATOM 20483 CD ILE F 214   −37.256 −36.620 35.543 1.00 0.00
F   C
ATOM 20484 HD1 ILE F 214   −37.300 −37.571 36.118 1.00 0.00
F   H
ATOM 20485 HD2 ILE F 214   −36.218 −36.489 35.167 1.00 0.00
F   H
ATOM 20486 HD3 ILE F 214   −37.486 −35.787 36.240 1.00 0.00
F   H
ATOM 20487 C   ILE F 214   −38.893 −34.474 31.262 1.00 0.00
F   C
ATOM 20488 O   ILE F 214   −39.863 −33.715 31.165 1.00 0.00
F   O
ATOM 20489 N   LYS F 215   −37.769 −34.279 30.569 1.00 0.00
F   N
ATOM 20490 HN LYS F 215   −36.934 −34.816 30.673 1.00 0.00
F   H
ATOM 20491 CA LYS F 215   −37.701 −33.217 29.573 1.00 0.00
F   C
ATOM 20492 HA LYS F 215   −37.940 −32.290 30.081 1.00 0.00
F   H

```
ATOM  20493  CB  LYS F 215     -36.300 -33.148  28.960  1.00  0.00
F    C
ATOM  20494  HB1 LYS F 215     -35.578 -33.044  29.803  1.00  0.00
F    H
ATOM  20495  HB2 LYS F 215     -36.071 -34.121  28.470  1.00  0.00
F    H
ATOM  20496  CG  LYS F 215     -36.097 -31.987  27.983  1.00  0.00
F    C
ATOM  20497  HG1 LYS F 215     -36.738 -31.132  28.295  1.00  0.00
F    H
ATOM  20498  HG2 LYS F 215     -35.038 -31.646  28.040  1.00  0.00
F    H
ATOM  20499  CD  LYS F 215     -36.454 -32.386  26.553  1.00  0.00
F    C
ATOM  20500  HD1 LYS F 215     -36.283 -33.481  26.440  1.00  0.00
F    H
ATOM  20501  HD2 LYS F 215     -37.543 -32.201  26.402  1.00  0.00
F    H
ATOM  20502  CE  LYS F 215     -35.655 -31.622  25.526  1.00  0.00
F    C
ATOM  20503  HE1 LYS F 215     -35.814 -30.527  25.644  1.00  0.00
F    H
ATOM  20504  HE2 LYS F 215     -34.569 -31.834  25.640  1.00  0.00
F    H
ATOM  20505  NZ  LYS F 215     -36.069 -32.010  24.154  1.00  0.00
F    N
ATOM  20506  HZ1 LYS F 215     -35.515 -31.479  23.451  1.00  0.00
F    H
ATOM  20507  HZ2 LYS F 215     -35.911 -33.030  24.019  1.00  0.00
F    H
ATOM  20508  HZ3 LYS F 215     -37.080 -31.801  24.026  1.00  0.00
F    H
ATOM  20509  C   LYS F 215     -38.739 -33.416  28.482  1.00  0.00
F    C
ATOM  20510  O   LYS F 215     -39.333 -32.444  28.013  1.00  0.00
F    O
ATOM  20511  N   ARG F 216     -38.927 -34.656  28.030  1.00  0.00
F    N
ATOM  20512  HN  ARG F 216     -38.418 -35.442  28.373  1.00  0.00
F    H
ATOM  20513  CA  ARG F 216     -39.910 -34.918  26.987  1.00  0.00
F    C
ATOM  20514  HA  ARG F 216     -39.667 -34.310  26.124  1.00  0.00
F    H
ATOM  20515  CB  ARG F 216     -39.888 -36.399  26.611  1.00  0.00
F    C
ATOM  20516  HB1 ARG F 216     -40.011 -36.968  27.564  1.00  0.00
F    H
ATOM  20517  HB2 ARG F 216     -40.781 -36.640  25.993  1.00  0.00
F    H
ATOM  20518  CG  ARG F 216     -38.602 -36.832  25.937  1.00  0.00
F    C
ATOM  20519  HG1 ARG F 216     -38.631 -36.504  24.875  1.00  0.00
F    H
```

ATOM 20520 HG2 ARG F 216   -37.749 -36.281 26.397 1.00 0.00
F   H
ATOM 20521 CD ARG F 216   -38.359 -38.322 26.088 1.00 0.00
F   C
ATOM 20522 HD1 ARG F 216   -38.763 -38.666 27.069 1.00 0.00
F   H
ATOM 20523 HD2 ARG F 216   -38.856 -38.902 25.275 1.00 0.00
F   H
ATOM 20524 NE ARG F 216   -36.935 -38.636 26.019 1.00 0.00
F   N
ATOM 20525 HE ARG F 216   -36.373 -37.858 25.713 1.00 0.00
F   H
ATOM 20526 CZ ARG F 216   -36.412 -39.819 26.322 1.00 0.00
F   C
ATOM 20527 NH1 ARG F 216   -37.196 -40.813 26.713 1.00 0.00
F   N
ATOM 20528 HH11 ARG F 216   -36.811 -41.690 26.965 1.00 0.00
F   H
ATOM 20529 HH12 ARG F 216   -38.160 -40.584 26.867 1.00 0.00
F   H
ATOM 20530 NH2 ARG F 216   -35.103 -40.012 26.231 1.00 0.00
F   N
ATOM 20531 HH21 ARG F 216   -34.726 -40.916 26.373 1.00 0.00
F   H
ATOM 20532 HH22 ARG F 216   -34.570 -39.286 25.818 1.00 0.00
F   H
ATOM 20533 C   ARG F 216   -41.301 -34.498 27.436 1.00 0.00
F   C
ATOM 20534 O   ARG F 216   -42.014 -33.800 26.706 1.00 0.00
F   O
ATOM 20535 N   PHE F 217   -41.696 -34.912 28.638 1.00 0.00
F   N
ATOM 20536 HN PHE F 217   -41.165 -35.495 29.253 1.00 0.00
F   H
ATOM 20537 CA PHE F 217   -42.999 -34.518 29.157 1.00 0.00
F   C
ATOM 20538 HA PHE F 217   -43.723 -34.795 28.401 1.00 0.00
F   H
ATOM 20539 CB PHE F 217   -43.280 -35.223 30.481 1.00 0.00
F   C
ATOM 20540 HB1 PHE F 217   -43.726 -36.220 30.275 1.00 0.00
F   H
ATOM 20541 HB2 PHE F 217   -42.341 -35.395 31.050 1.00 0.00
F   H
ATOM 20542 CG PHE F 217   -44.260 -34.497 31.349 1.00 0.00
F   C
ATOM 20543 CD1 PHE F 217   -45.600 -34.446 31.008 1.00 0.00
F   C
ATOM 20544 HD1 PHE F 217   -45.941 -34.935 30.105 1.00 0.00
F   H
ATOM 20545 CE1 PHE F 217   -46.505 -33.776 31.802 1.00 0.00
F   C
ATOM 20546 HE1 PHE F 217   -47.550 -33.747 31.526 1.00 0.00
F   H

```
ATOM  20547  CZ  PHE F 217     -46.077 -33.148  32.951  1.00  0.00
F    C
ATOM  20548  HZ  PHE F 217     -46.785 -32.629  33.582  1.00  0.00
F    H
ATOM  20549  CD2 PHE F 217     -43.844 -33.861  32.503  1.00  0.00
F    C
ATOM  20550  HD2 PHE F 217     -42.798 -33.868  32.777  1.00  0.00
F    H
ATOM  20551  CE2 PHE F 217     -44.745 -33.191  33.301  1.00  0.00
F    C
ATOM  20552  HE2 PHE F 217     -44.405 -32.699  34.202  1.00  0.00
F    H
ATOM  20553  C   PHE F 217     -43.086 -33.007  29.326  1.00  0.00
F    C
ATOM  20554  O   PHE F 217     -44.128 -32.407  29.043  1.00  0.00
F    O
ATOM  20555  N   GLU F 218     -42.000 -32.376  29.771  1.00  0.00
F    N
ATOM  20556  HN  GLU F 218     -41.145 -32.813  30.034  1.00  0.00
F    H
ATOM  20557  CA  GLU F 218     -41.990 -30.923  29.909  1.00  0.00
F    C
ATOM  20558  HA  GLU F 218     -42.817 -30.641  30.549  1.00  0.00
F    H
ATOM  20559  CB  GLU F 218     -40.670 -30.474  30.533  1.00  0.00
F    C
ATOM  20560  HB1 GLU F 218     -40.377 -31.223  31.304  1.00  0.00
F    H
ATOM  20561  HB2 GLU F 218     -39.863 -30.495  29.766  1.00  0.00
F    H
ATOM  20562  CG  GLU F 218     -40.715 -29.104  31.182  1.00  0.00
F    C
ATOM  20563  HG1 GLU F 218     -41.750 -28.881  31.499  1.00  0.00
F    H
ATOM  20564  HG2 GLU F 218     -40.046 -29.061  32.063  1.00  0.00
F    H
ATOM  20565  CD  GLU F 218     -40.326 -28.005  30.212  1.00  0.00
F    C
ATOM  20566  OE1 GLU F 218     -39.861 -28.334  29.101  1.00  0.00
F    O
ATOM  20567  OE2 GLU F 218     -40.475 -26.816  30.557  1.00  0.00
F    O
ATOM  20568  C   GLU F 218     -42.203 -30.238  28.568  1.00  0.00
F    C
ATOM  20569  O   GLU F 218     -42.960 -29.266  28.469  1.00  0.00
F    O
ATOM  20570  N   ASN F 219     -41.546 -30.734  27.527  1.00  0.00
F    N
ATOM  20571  HN  ASN F 219     -40.981 -31.558  27.548  1.00  0.00
F    H
ATOM  20572  CA  ASN F 219     -41.609 -30.080  26.227  1.00  0.00
F    C
ATOM  20573  HA  ASN F 219     -41.506 -29.018  26.422  1.00  0.00
F    H
```

ATOM 20574 CB ASN F 219 -40.484 -30.596 25.333 1.00 0.00
F C
ATOM 20575 HB1 ASN F 219 -39.612 -30.829 25.983 1.00 0.00
F H
ATOM 20576 HB2 ASN F 219 -40.789 -31.539 24.835 1.00 0.00
F H
ATOM 20577 CG ASN F 219 -40.044 -29.577 24.303 1.00 0.00
F C
ATOM 20578 OD1 ASN F 219 -40.610 -28.489 24.207 1.00 0.00
F O
ATOM 20579 ND2 ASN F 219 -39.025 -29.925 23.524 1.00 0.00
F N
ATOM 20580 HD21 ASN F 219 -38.717 -29.260 22.849 1.00 0.00
F H
ATOM 20581 HD22 ASN F 219 -38.582 -30.807 23.648 1.00 0.00
F H
ATOM 20582 C ASN F 219 -42.964 -30.299 25.573 1.00 0.00
F C
ATOM 20583 O ASN F 219 -43.518 -29.387 24.947 1.00 0.00
F O
ATOM 20584 N GLU F 220 -43.515 -31.502 25.707 1.00 0.00
F N
ATOM 20585 HN GLU F 220 -43.161 -32.236 26.280 1.00 0.00
F H
ATOM 20586 CA GLU F 220 -44.736 -31.845 24.990 1.00 0.00
F C
ATOM 20587 HA GLU F 220 -44.774 -31.224 24.103 1.00 0.00
F H
ATOM 20588 CB GLU F 220 -44.675 -33.316 24.567 1.00 0.00
F C
ATOM 20589 HB1 GLU F 220 -44.757 -33.945 25.483 1.00 0.00
F H
ATOM 20590 HB2 GLU F 220 -45.563 -33.567 23.944 1.00 0.00
F H
ATOM 20591 CG GLU F 220 -43.385 -33.653 23.826 1.00 0.00
F C
ATOM 20592 HG1 GLU F 220 -43.561 -33.601 22.735 1.00 0.00
F H
ATOM 20593 HG2 GLU F 220 -42.574 -32.947 24.097 1.00 0.00
F H
ATOM 20594 CD GLU F 220 -42.894 -35.062 24.089 1.00 0.00
F C
ATOM 20595 OE1 GLU F 220 -43.255 -35.636 25.137 1.00 0.00
F O
ATOM 20596 OE2 GLU F 220 -42.126 -35.587 23.256 1.00 0.00
F O
ATOM 20597 C GLU F 220 -46.001 -31.562 25.796 1.00 0.00
F C
ATOM 20598 O GLU F 220 -46.864 -30.804 25.338 1.00 0.00
F O
ATOM 20599 N GLY F 221 -46.133 -32.142 26.988 1.00 0.00
F N
ATOM 20600 HN GLY F 221 -45.434 -32.739 27.380 1.00 0.00
F H

```
ATOM  20601  CA  GLY F 221     -47.314 -31.939  27.805  1.00  0.00
F    C
ATOM  20602  HA1 GLY F 221     -47.252 -32.652  28.616  1.00  0.00
F    H
ATOM  20603  HA2 GLY F 221     -48.168 -32.077  27.157  1.00  0.00
F    H
ATOM  20604  C   GLY F 221     -47.404 -30.552  28.407  1.00  0.00
F    C
ATOM  20605  O   GLY F 221     -48.475 -30.126  28.847  1.00  0.00
F    O
ATOM  20606  N   MET F 222     -46.282 -29.844  28.443  1.00  0.00
F    N
ATOM  20607  HN  MET F 222     -45.399 -30.180  28.124  1.00  0.00
F    H
ATOM  20608  CA  MET F 222     -46.255 -28.486  28.967  1.00  0.00
F    C
ATOM  20609  HA  MET F 222     -47.282 -28.156  29.059  1.00  0.00
F    H
ATOM  20610  CB  MET F 222     -45.561 -28.447  30.326  1.00  0.00
F    C
ATOM  20611  HB1 MET F 222     -44.499 -28.744  30.156  1.00  0.00
F    H
ATOM  20612  HB2 MET F 222     -45.536 -27.398  30.698  1.00  0.00
F    H
ATOM  20613  CG  MET F 222     -46.169 -29.371  31.361  1.00  0.00
F    C
ATOM  20614  HG1 MET F 222     -47.268 -29.224  31.410  1.00  0.00
F    H
ATOM  20615  HG2 MET F 222     -45.983 -30.428  31.072  1.00  0.00
F    H
ATOM  20616  SD  MET F 222     -45.487 -29.078  33.003  1.00  0.00
F    S
ATOM  20617  CE  MET F 222     -43.850 -29.774  32.827  1.00  0.00
F    C
ATOM  20618  HE1 MET F 222     -43.278 -29.686  33.776  1.00  0.00
F    H
ATOM  20619  HE2 MET F 222     -43.901 -30.851  32.558  1.00  0.00
F    H
ATOM  20620  HE3 MET F 222     -43.275 -29.250  32.033  1.00  0.00
F    H
ATOM  20621  C   MET F 222     -45.555 -27.546  27.996  1.00  0.00
F    C
ATOM  20622  O   MET F 222     -46.098 -26.508  27.627  1.00  0.00
F    O
ATOM  20623  N   LYS G   7      19.012 -35.381  27.901  1.00  0.00
G    N
ATOM  20624  HT1 LYS G   7      19.290 -36.340  27.853  1.00  0.00
G    H
ATOM  20625  HT2 LYS G   7      19.805 -34.806  28.106  1.00  0.00
G    H
ATOM  20626  HT3 LYS G   7      18.602 -35.100  27.033  1.00  0.00
G    H
ATOM  20627  CA  LYS G   7      18.026 -35.225  28.965  1.00  0.00
G    C
```

```
ATOM  20628  HA  LYS G   7       17.158 -35.812  28.691  1.00  0.00
G    H
ATOM  20629  CB  LYS G   7       18.603 -35.703  30.297  1.00  0.00
G    C
ATOM  20630  HB1 LYS G   7       17.866 -35.436  31.088  1.00  0.00
G    H
ATOM  20631  HB2 LYS G   7       18.663 -36.814  30.288  1.00  0.00
G    H
ATOM  20632  CG  LYS G   7       19.948 -35.086  30.632  1.00  0.00
G    C
ATOM  20633  HG1 LYS G   7       20.661 -35.289  29.800  1.00  0.00
G    H
ATOM  20634  HG2 LYS G   7       19.832 -33.981  30.706  1.00  0.00
G    H
ATOM  20635  CD  LYS G   7       20.517 -35.649  31.923  1.00  0.00
G    C
ATOM  20636  HD1 LYS G   7       21.315 -34.961  32.285  1.00  0.00
G    H
ATOM  20637  HD2 LYS G   7       19.706 -35.651  32.688  1.00  0.00
G    H
ATOM  20638  CE  LYS G   7       21.065 -37.052  31.733  1.00  0.00
G    C
ATOM  20639  HE1 LYS G   7       20.239 -37.769  31.528  1.00  0.00
G    H
ATOM  20640  HE2 LYS G   7       21.776 -37.081  30.878  1.00  0.00
G    H
ATOM  20641  NZ  LYS G   7       21.791 -37.531  32.943  1.00  0.00
G    N
ATOM  20642  HZ1 LYS G   7       22.153 -38.494  32.783  1.00  0.00
G    H
ATOM  20643  HZ2 LYS G   7       22.586 -36.893  33.148  1.00  0.00
G    H
ATOM  20644  HZ3 LYS G   7       21.141 -37.538  33.755  1.00  0.00
G    H
ATOM  20645  C   LYS G   7       17.579 -33.772  29.068  1.00  0.00
G    C
ATOM  20646  O   LYS G   7       18.399 -32.855  29.038  1.00  0.00
G    O
ATOM  20647  N   GLN G   8       16.274 -33.562  29.221  1.00  0.00
G    N
ATOM  20648  HN  GLN G   8       15.621 -34.281  29.452  1.00  0.00
G    H
ATOM  20649  CA  GLN G   8       15.685 -32.242  29.059  1.00  0.00
G    C
ATOM  20650  HA  GLN G   8       16.470 -31.590  28.695  1.00  0.00
G    H
ATOM  20651  CB  GLN G   8       14.553 -32.288  28.042  1.00  0.00
G    C
ATOM  20652  HB1 GLN G   8       13.967 -33.212  28.253  1.00  0.00
G    H
ATOM  20653  HB2 GLN G   8       13.861 -31.434  28.222  1.00  0.00
G    H
ATOM  20654  CG  GLN G   8       15.093 -32.304  26.632  1.00  0.00
G    C
```

```
ATOM  20655  HG1 GLN G   8      15.259 -31.268  26.269  1.00  0.00
G    H
ATOM  20656  HG2 GLN G   8      16.063 -32.840  26.589  1.00  0.00
G    H
ATOM  20657  CD  GLN G   8      14.148 -32.946  25.653  1.00  0.00
G    C
ATOM  20658  OE1 GLN G   8      13.261 -33.705  26.038  1.00  0.00
G    O
ATOM  20659  NE2 GLN G   8      14.334 -32.654  24.372  1.00  0.00
G    N
ATOM  20660 HE21 GLN G   8      13.709 -33.072  23.720  1.00  0.00
G    H
ATOM  20661 HE22 GLN G   8      15.082 -32.055  24.105  1.00  0.00
G    H
ATOM  20662  C   GLN G   8      15.201 -31.679  30.386  1.00  0.00
G    C
ATOM  20663  O   GLN G   8      14.529 -32.366  31.167  1.00  0.00
G    O
ATOM  20664  N   PHE G   9      15.543 -30.416  30.617  1.00  0.00
G    N
ATOM  20665  HN  PHE G   9      15.962 -29.855  29.904  1.00  0.00
G    H
ATOM  20666  CA  PHE G   9      15.353 -29.736  31.883  1.00  0.00
G    C
ATOM  20667  HA  PHE G   9      14.785 -30.406  32.518  1.00  0.00
G    H
ATOM  20668  CB  PHE G   9      16.689 -29.335  32.476  1.00  0.00
G    C
ATOM  20669  HB1 PHE G   9      17.188 -28.613  31.793  1.00  0.00
G    H
ATOM  20670  HB2 PHE G   9      16.556 -28.823  33.454  1.00  0.00
G    H
ATOM  20671  CG  PHE G   9      17.629 -30.472  32.638  1.00  0.00
G    C
ATOM  20672  CD1 PHE G   9      17.566 -31.272  33.757  1.00  0.00
G    C
ATOM  20673  HD1 PHE G   9      16.833 -31.062  34.524  1.00  0.00
G    H
ATOM  20674  CE1 PHE G   9      18.423 -32.327  33.916  1.00  0.00
G    C
ATOM  20675  HE1 PHE G   9      18.358 -32.948  34.798  1.00  0.00
G    H
ATOM  20676  CZ  PHE G   9      19.363 -32.597  32.945  1.00  0.00
G    C
ATOM  20677  HZ  PHE G   9      20.039 -33.431  33.067  1.00  0.00
G    H
ATOM  20678  CD2 PHE G   9      18.569 -30.755  31.664  1.00  0.00
G    C
ATOM  20679  HD2 PHE G   9      18.640 -30.130  30.784  1.00  0.00
G    H
ATOM  20680  CE2 PHE G   9      19.437 -31.810  31.814  1.00  0.00
G    C
ATOM  20681  HE2 PHE G   9      20.171 -32.017  31.047  1.00  0.00
G    H
```

```
ATOM  20682  C   PHE G   9      14.506 -28.496  31.673  1.00  0.00
G    C
ATOM  20683  O   PHE G   9      14.736 -27.725  30.735  1.00  0.00
G    O
ATOM  20684  N   ALA G  10      13.537 -28.299  32.559  1.00  0.00
G    N
ATOM  20685  HN  ALA G  10      13.359 -28.936  33.308  1.00  0.00
G    H
ATOM  20686  CA  ALA G  10      12.661 -27.138  32.505  1.00  0.00
G    C
ATOM  20687  HA  ALA G  10      12.973 -26.436  31.740  1.00  0.00
G    H
ATOM  20688  CB  ALA G  10      11.219 -27.551  32.207  1.00  0.00
G    C
ATOM  20689  HB1 ALA G  10      11.170 -28.047  31.214  1.00  0.00
G    H
ATOM  20690  HB2 ALA G  10      10.843 -28.269  32.967  1.00  0.00
G    H
ATOM  20691  HB3 ALA G  10      10.538 -26.672  32.185  1.00  0.00
G    H
ATOM  20692  C   ALA G  10      12.748 -26.395  33.828  1.00  0.00
G    C
ATOM  20693  O   ALA G  10      12.278 -26.893  34.856  1.00  0.00
G    O
ATOM  20694  N   VAL G  11      13.349 -25.218  33.807  1.00  0.00
G    N
ATOM  20695  HN  VAL G  11      13.804 -24.842  33.004  1.00  0.00
G    H
ATOM  20696  CA  VAL G  11      13.395 -24.363  34.979  1.00  0.00
G    C
ATOM  20697  HA  VAL G  11      13.330 -24.972  35.871  1.00  0.00
G    H
ATOM  20698  CB  VAL G  11      14.668 -23.522  35.035  1.00  0.00
G    C
ATOM  20699  HB  VAL G  11      15.175 -23.611  34.042  1.00  0.00
G    H
ATOM  20700  CG1 VAL G  11      14.343 -22.068  35.295  1.00  0.00
G    C
ATOM  20701 HG11 VAL G  11      15.278 -21.472  35.372  1.00  0.00
G    H
ATOM  20702 HG12 VAL G  11      13.739 -21.630  34.473  1.00  0.00
G    H
ATOM  20703 HG13 VAL G  11      13.783 -21.955  36.248  1.00  0.00
G    H
ATOM  20704  CG2 VAL G  11      15.569 -24.037  36.124  1.00  0.00
G    C
ATOM  20705 HG21 VAL G  11      16.483 -23.412  36.205  1.00  0.00
G    H
ATOM  20706 HG22 VAL G  11      15.045 -24.020  37.105  1.00  0.00
G    H
ATOM  20707 HG23 VAL G  11      15.892 -25.079  35.919  1.00  0.00
G    H
ATOM  20708  C   VAL G  11      12.152 -23.492  34.931  1.00  0.00
G    C
```

ATOM 20709 O   VAL G 11   11.835 -22.887 33.905 1.00 0.00
G   O
ATOM 20710 N   ILE G 12   11.435 -23.445 36.039 1.00 0.00
G   N
ATOM 20711 HN ILE G 12   11.606 -24.028 36.830 1.00 0.00
G   H
ATOM 20712 CA ILE G 12   10.322 -22.530 36.203 1.00 0.00
G   C
ATOM 20713 HA ILE G 12   10.331 -21.837 35.371 1.00 0.00
G   H
ATOM 20714 CB ILE G 12   8.993 -23.279 36.319 1.00 0.00
G   C
ATOM 20715 HB ILE G 12   9.102 -23.902 37.243 1.00 0.00
G   H
ATOM 20716 CG2 ILE G 12   7.870 -22.294 36.302 1.00 0.00
G   C
ATOM 20717 HG21 ILE G 12   6.908 -22.794 36.540 1.00 0.00
G   H
ATOM 20718 HG22 ILE G 12   8.031 -21.497 37.057 1.00 0.00
G   H
ATOM 20719 HG23 ILE G 12   7.775 -21.817 35.303 1.00 0.00
G   H
ATOM 20720 CG1 ILE G 12   8.826 -24.241 35.156 1.00 0.00
G   C
ATOM 20721 HG11 ILE G 12   9.630 -25.010 35.184 1.00 0.00
G   H
ATOM 20722 HG12 ILE G 12   7.855 -24.776 35.267 1.00 0.00
G   H
ATOM 20723 CD ILE G 12   8.839 -23.567 33.832 1.00 0.00
G   C
ATOM 20724 HD1 ILE G 12   8.656 -24.306 33.021 1.00 0.00
G   H
ATOM 20725 HD2 ILE G 12   8.047 -22.789 33.779 1.00 0.00
G   H
ATOM 20726 HD3 ILE G 12   9.820 -23.085 33.640 1.00 0.00
G   H
ATOM 20727 C   ILE G 12   10.591 -21.717 37.453 1.00 0.00
G   C
ATOM 20728 O   ILE G 12   10.965 -22.267 38.494 1.00 0.00
G   O
ATOM 20729 N   GLY G 13   10.434 -20.411 37.339 1.00 0.00
G   N
ATOM 20730 HN GLY G 13   10.141 -19.970 36.492 1.00 0.00
G   H
ATOM 20731 CA GLY G 13   10.681 -19.527 38.449 1.00 0.00
G   C
ATOM 20732 HA1 GLY G 13   10.600 -20.082 39.374 1.00 0.00
G   H
ATOM 20733 HA2 GLY G 13   10.022 -18.673 38.387 1.00 0.00
G   H
ATOM 20734 C   GLY G 13   12.096 -19.031 38.325 1.00 0.00
G   C
ATOM 20735 O   GLY G 13   13.043 -19.753 38.650 1.00 0.00
G   O

ATOM 20736 N LEU G 14 12.263 -17.808 37.846 1.00 0.00
G N
ATOM 20737 HN LEU G 14 11.532 -17.157 37.647 1.00 0.00
G H
ATOM 20738 CA LEU G 14 13.601 -17.308 37.566 1.00 0.00
G C
ATOM 20739 HA LEU G 14 14.274 -18.153 37.641 1.00 0.00
G H
ATOM 20740 CB LEU G 14 13.648 -16.727 36.165 1.00 0.00
G C
ATOM 20741 HB1 LEU G 14 12.597 -16.667 35.798 1.00 0.00
G H
ATOM 20742 HB2 LEU G 14 14.015 -15.678 36.221 1.00 0.00
G H
ATOM 20743 CG LEU G 14 14.480 -17.567 35.222 1.00 0.00
G C
ATOM 20744 HG LEU G 14 14.827 -18.481 35.761 1.00 0.00
G H
ATOM 20745 CD1 LEU G 14 13.642 -17.977 34.042 1.00 0.00
G C
ATOM 20746 HD11 LEU G 14 14.250 -18.556 33.314 1.00 0.00
G H
ATOM 20747 HD12 LEU G 14 12.790 -18.613 34.369 1.00 0.00
G H
ATOM 20748 HD13 LEU G 14 13.234 -17.085 33.522 1.00 0.00
G H
ATOM 20749 CD2 LEU G 14 15.709 -16.780 34.789 1.00 0.00
G C
ATOM 20750 HD21 LEU G 14 16.340 -17.393 34.109 1.00 0.00
G H
ATOM 20751 HD22 LEU G 14 15.432 -15.853 34.247 1.00 0.00
G H
ATOM 20752 HD23 LEU G 14 16.322 -16.498 35.672 1.00 0.00
G H
ATOM 20753 C LEU G 14 14.026 -16.284 38.615 1.00 0.00
G C
ATOM 20754 O LEU G 14 14.311 -15.127 38.323 1.00 0.00
G O
ATOM 20755 N GLY G 15 14.061 -16.736 39.863 1.00 0.00
G N
ATOM 20756 HN GLY G 15 13.702 -17.629 40.133 1.00 0.00
G H
ATOM 20757 CA GLY G 15 14.634 -15.942 40.930 1.00 0.00
G C
ATOM 20758 HA1 GLY G 15 14.220 -16.284 41.869 1.00 0.00
G H
ATOM 20759 HA2 GLY G 15 14.454 -14.897 40.717 1.00 0.00
G H
ATOM 20760 C GLY G 15 16.129 -16.159 40.972 1.00 0.00
G C
ATOM 20761 O GLY G 15 16.642 -16.984 40.209 1.00 0.00
G O
ATOM 20762 N ARG G 16 16.838 -15.429 41.842 1.00 0.00
G N

ATOM 20763 HN ARG G 16 16.427 -14.728 42.420 1.00 0.00
G H
ATOM 20764 CA ARG G 16 18.273 -15.635 41.984 1.00 0.00
G C
ATOM 20765 HA ARG G 16 18.765 -15.218 41.114 1.00 0.00
G H
ATOM 20766 CB ARG G 16 18.789 -14.977 43.260 1.00 0.00
G C
ATOM 20767 HB1 ARG G 16 18.277 -15.491 44.108 1.00 0.00
G H
ATOM 20768 HB2 ARG G 16 19.873 -15.195 43.378 1.00 0.00
G H
ATOM 20769 CG ARG G 16 18.513 -13.505 43.350 1.00 0.00
G C
ATOM 20770 HG1 ARG G 16 19.317 -12.961 42.809 1.00 0.00
G H
ATOM 20771 HG2 ARG G 16 17.573 -13.278 42.797 1.00 0.00
G H
ATOM 20772 CD ARG G 16 18.360 -13.068 44.794 1.00 0.00
G C
ATOM 20773 HD1 ARG G 16 19.172 -13.522 45.407 1.00 0.00
G H
ATOM 20774 HD2 ARG G 16 18.415 -11.958 44.893 1.00 0.00
G H
ATOM 20775 NE ARG G 16 17.074 -13.464 45.360 1.00 0.00
G N
ATOM 20776 HE ARG G 16 16.527 -14.034 44.737 1.00 0.00
G H
ATOM 20777 CZ ARG G 16 16.656 -13.113 46.571 1.00 0.00
G C
ATOM 20778 NH1 ARG G 16 17.422 -12.356 47.343 1.00 0.00
G N
ATOM 20779 HH11 ARG G 16 17.131 -12.111 48.257 1.00 0.00
G H
ATOM 20780 HH12 ARG G 16 18.347 -12.171 47.005 1.00 0.00
G H
ATOM 20781 NH2 ARG G 16 15.468 -13.511 47.009 1.00 0.00
G N
ATOM 20782 HH21 ARG G 16 15.125 -13.176 47.875 1.00 0.00
G H
ATOM 20783 HH22 ARG G 16 14.878 -13.964 46.354 1.00 0.00
G H
ATOM 20784 C ARG G 16 18.616 -17.109 42.010 1.00 0.00
G C
ATOM 20785 O ARG G 16 19.545 -17.545 41.321 1.00 0.00
G O
ATOM 20786 N PHE G 17 17.847 -17.885 42.761 1.00 0.00
G N
ATOM 20787 HN PHE G 17 17.123 -17.559 43.365 1.00 0.00
G H
ATOM 20788 CA PHE G 17 18.017 -19.326 42.750 1.00 0.00
G C
ATOM 20789 HA PHE G 17 19.027 -19.519 43.091 1.00 0.00
G H

```
ATOM  20790  CB  PHE G  17      16.975 -19.968  43.656  1.00  0.00
G    C
ATOM  20791  HB1 PHE G  17      17.073 -19.545  44.680  1.00  0.00
G    H
ATOM  20792  HB2 PHE G  17      15.946 -19.740  43.306  1.00  0.00
G    H
ATOM  20793  CG  PHE G  17      17.134 -21.438  43.798  1.00  0.00
G    C
ATOM  20794  CD1 PHE G  17      18.360 -21.976  44.135  1.00  0.00
G    C
ATOM  20795  HD1 PHE G  17      19.208 -21.322  44.287  1.00  0.00
G    H
ATOM  20796  CE1 PHE G  17      18.514 -23.331  44.275  1.00  0.00
G    C
ATOM  20797  HE1 PHE G  17      19.479 -23.740  44.540  1.00  0.00
G    H
ATOM  20798  CZ  PHE G  17      17.437 -24.164  44.082  1.00  0.00
G    C
ATOM  20799  HZ  PHE G  17      17.553 -25.232  44.199  1.00  0.00
G    H
ATOM  20800  CD2 PHE G  17      16.059 -22.283  43.608  1.00  0.00
G    C
ATOM  20801  HD2 PHE G  17      15.097 -21.878  43.327  1.00  0.00
G    H
ATOM  20802  CE2 PHE G  17      16.207 -23.637  43.748  1.00  0.00
G    C
ATOM  20803  HE2 PHE G  17      15.359 -24.290  43.596  1.00  0.00
G    H
ATOM  20804  C   PHE G  17      17.915 -19.864  41.333  1.00  0.00
G    C
ATOM  20805  O   PHE G  17      18.849 -20.498  40.830  1.00  0.00
G    O
ATOM  20806  N   GLY G  18      16.792 -19.600  40.667  1.00  0.00
G    N
ATOM  20807  HN  GLY G  18      16.028 -19.068  41.030  1.00  0.00
G    H
ATOM  20808  CA  GLY G  18      16.621 -20.101  39.315  1.00  0.00
G    C
ATOM  20809  HA1 GLY G  18      15.719 -19.653  38.921  1.00  0.00
G    H
ATOM  20810  HA2 GLY G  18      16.591 -21.181  39.372  1.00  0.00
G    H
ATOM  20811  C   GLY G  18      17.770 -19.713  38.410  1.00  0.00
G    C
ATOM  20812  O   GLY G  18      18.457 -20.576  37.848  1.00  0.00
G    O
ATOM  20813  N   GLY G  19      18.026 -18.410  38.301  1.00  0.00
G    N
ATOM  20814  HN  GLY G  19      17.543 -17.707  38.821  1.00  0.00
G    H
ATOM  20815  CA  GLY G  19      19.050 -17.927  37.394  1.00  0.00
G    C
ATOM  20816  HA1 GLY G  19      19.162 -16.869  37.588  1.00  0.00
G    H
```

ATOM 20817 HA2 GLY G 19   18.717 -18.161 36.392 1.00 0.00
G   H
ATOM 20818 C   GLY G 19   20.394 -18.585 37.604 1.00 0.00
G   C
ATOM 20819 O   GLY G 19   21.095 -18.901 36.639 1.00 0.00
G   O
ATOM 20820 N   SER G 20   20.763 -18.812 38.856 1.00 0.00
G   N
ATOM 20821 HN SER G 20   20.259 -18.512 39.666 1.00 0.00
G   H
ATOM 20822 CA SER G 20   21.980 -19.545 39.152 1.00 0.00
G   C
ATOM 20823 HA SER G 20   22.790 -18.960 38.737 1.00 0.00
G   H
ATOM 20824 CB SER G 20   22.071 -19.776 40.651 1.00 0.00
G   C
ATOM 20825 HB1 SER G 20   21.148 -20.311 40.975 1.00 0.00
G   H
ATOM 20826 HB2 SER G 20   22.934 -20.437 40.890 1.00 0.00
G   H
ATOM 20827 OG SER G 20   22.153 -18.544 41.315 1.00 0.00
G   O
ATOM 20828 HG1 SER G 20   21.985 -18.714 42.250 1.00 0.00
G   H
ATOM 20829 C   SER G 20   21.994 -20.887 38.457 1.00 0.00
G   C
ATOM 20830 O   SER G 20   22.922 -21.192 37.695 1.00 0.00
G   O
ATOM 20831 N   ILE G 21   20.965 -21.682 38.731 1.00 0.00
G   N
ATOM 20832 HN ILE G 21   20.209 -21.433 39.332 1.00 0.00
G   H
ATOM 20833 CA ILE G 21   20.858 -23.012 38.162 1.00 0.00
G   C
ATOM 20834 HA ILE G 21   21.694 -23.583 38.545 1.00 0.00
G   H
ATOM 20835 CB ILE G 21   19.505 -23.622 38.543 1.00 0.00
G   C
ATOM 20836 HB ILE G 21   18.754 -23.004 37.989 1.00 0.00
G   H
ATOM 20837 CG2 ILE G 21   19.475 -25.078 38.198 1.00 0.00
G   C
ATOM 20838 HG21 ILE G 21   18.452 -25.489 38.325 1.00 0.00
G   H
ATOM 20839 HG22 ILE G 21   19.774 -25.242 37.142 1.00 0.00
G   H
ATOM 20840 HG23 ILE G 21   20.164 -25.655 38.851 1.00 0.00
G   H
ATOM 20841 CG1 ILE G 21   19.260 -23.409 40.027 1.00 0.00
G   C
ATOM 20842 HG11 ILE G 21   20.043 -23.935 40.617 1.00 0.00
G   H
ATOM 20843 HG12 ILE G 21   19.350 -22.322 40.255 1.00 0.00
G   H

ATOM 20844 CD ILE G 21 17.914 -23.868 40.492 1.00 0.00
G C
ATOM 20845 HD1 ILE G 21 17.776 -23.631 41.570 1.00 0.00
G H
ATOM 20846 HD2 ILE G 21 17.107 -23.361 39.920 1.00 0.00
G H
ATOM 20847 HD3 ILE G 21 17.800 -24.965 40.364 1.00 0.00
G H
ATOM 20848 C ILE G 21 21.019 -22.956 36.658 1.00 0.00
G C
ATOM 20849 O ILE G 21 21.841 -23.673 36.074 1.00 0.00
G O
ATOM 20850 N VAL G 22 20.223 -22.115 36.007 1.00 0.00
G N
ATOM 20851 HN VAL G 22 19.624 -21.425 36.406 1.00 0.00
G H
ATOM 20852 CA VAL G 22 20.191 -22.171 34.554 1.00 0.00
G C
ATOM 20853 HA VAL G 22 20.082 -23.211 34.270 1.00 0.00
G H
ATOM 20854 CB VAL G 22 18.998 -21.380 34.003 1.00 0.00
G C
ATOM 20855 HB VAL G 22 19.255 -21.064 32.962 1.00 0.00
G H
ATOM 20856 CG1 VAL G 22 17.774 -22.246 33.974 1.00 0.00
G C
ATOM 20857 HG11 VAL G 22 16.899 -21.664 33.612 1.00 0.00
G H
ATOM 20858 HG12 VAL G 22 17.900 -23.112 33.291 1.00 0.00
G H
ATOM 20859 HG13 VAL G 22 17.538 -22.626 34.991 1.00 0.00
G H
ATOM 20860 CG2 VAL G 22 18.759 -20.171 34.823 1.00 0.00
G C
ATOM 20861 HG21 VAL G 22 17.871 -19.614 34.453 1.00 0.00
G H
ATOM 20862 HG22 VAL G 22 18.581 -20.447 35.884 1.00 0.00
G H
ATOM 20863 HG23 VAL G 22 19.627 -19.480 34.783 1.00 0.00
G H
ATOM 20864 C VAL G 22 21.497 -21.673 33.965 1.00 0.00
G C
ATOM 20865 O VAL G 22 22.009 -22.247 33.002 1.00 0.00
G O
ATOM 20866 N LYS G 23 22.053 -20.597 34.514 1.00 0.00
G N
ATOM 20867 HN LYS G 23 21.706 -20.106 35.312 1.00 0.00
G H
ATOM 20868 CA LYS G 23 23.270 -20.052 33.936 1.00 0.00
G C
ATOM 20869 HA LYS G 23 23.100 -19.925 32.874 1.00 0.00
G H
ATOM 20870 CB LYS G 23 23.598 -18.712 34.582 1.00 0.00
G C

ATOM 20871 HB1 LYS G 23   22.643 -18.140 34.655 1.00 0.00
G   H
ATOM 20872 HB2 LYS G 23   23.937 -18.887 35.628 1.00 0.00
G   H
ATOM 20873 CG LYS G 23   24.612 -17.897 33.812 1.00 0.00
G   C
ATOM 20874 HG1 LYS G 23   25.048 -18.526 33.003 1.00 0.00
G   H
ATOM 20875 HG2 LYS G 23   24.093 -17.041 33.324 1.00 0.00
G   H
ATOM 20876 CD LYS G 23   25.727 -17.396 34.702 1.00 0.00
G   C
ATOM 20877 HD1 LYS G 23   25.431 -17.558 35.764 1.00 0.00
G   H
ATOM 20878 HD2 LYS G 23   26.631 -18.019 34.509 1.00 0.00
G   H
ATOM 20879 CE LYS G 23   26.003 -15.923 34.415 1.00 0.00
G   C
ATOM 20880 HE1 LYS G 23   26.319 -15.785 33.357 1.00 0.00
G   H
ATOM 20881 HE2 LYS G 23   25.090 -15.313 34.592 1.00 0.00
G   H
ATOM 20882 NZ LYS G 23   27.081 -15.351 35.267 1.00 0.00
G   N
ATOM 20883 HZ1 LYS G 23   27.228 -14.348 35.030 1.00 0.00
G   H
ATOM 20884 HZ2 LYS G 23   26.812 -15.433 36.269 1.00 0.00
G   H
ATOM 20885 HZ3 LYS G 23   27.964 -15.876 35.107 1.00 0.00
G   H
ATOM 20886 C   LYS G 23   24.413 -21.035 34.097 1.00 0.00
G   C
ATOM 20887 O   LYS G 23   25.226 -21.206 33.181 1.00 0.00
G   O
ATOM 20888 N   GLU G 24   24.456 -21.737 35.229 1.00 0.00
G   N
ATOM 20889 HN GLU G 24   23.855 -21.628 36.016 1.00 0.00
G   H
ATOM 20890 CA GLU G 24   25.459 -22.779 35.389 1.00 0.00
G   C
ATOM 20891 HA GLU G 24   26.434 -22.349 35.196 1.00 0.00
G   H
ATOM 20892 CB GLU G 24   25.385 -23.344 36.808 1.00 0.00
G   C
ATOM 20893 HB1 GLU G 24   25.441 -22.492 37.524 1.00 0.00
G   H
ATOM 20894 HB2 GLU G 24   24.392 -23.817 36.974 1.00 0.00
G   H
ATOM 20895 CG GLU G 24   26.467 -24.344 37.197 1.00 0.00
G   C
ATOM 20896 HG1 GLU G 24   27.456 -23.854 37.150 1.00 0.00
G   H
ATOM 20897 HG2 GLU G 24   26.299 -24.733 38.221 1.00 0.00
G   H

ATOM 20898 CD GLU G 24   26.586 -25.540 36.271 1.00 0.00
G   C
ATOM 20899 OE1 GLU G 24   25.597 -26.269 36.068 1.00 0.00
G   O
ATOM 20900 OE2 GLU G 24   27.686 -25.730 35.722 1.00 0.00
G   O
ATOM 20901 C   GLU G 24   25.242 -23.887 34.365 1.00 0.00
G   C
ATOM 20902 O   GLU G 24   26.151 -24.237 33.605 1.00 0.00
G   O
ATOM 20903 N   LEU G 25   24.052 -24.482 34.367 1.00 0.00
G   N
ATOM 20904 HN LEU G 25   23.278 -24.212 34.937 1.00 0.00
G   H
ATOM 20905 CA LEU G 25   23.788 -25.619 33.496 1.00 0.00
G   C
ATOM 20906 HA LEU G 25   24.491 -26.394 33.772 1.00 0.00
G   H
ATOM 20907 CB LEU G 25   22.352 -26.081 33.708 1.00 0.00
G   C
ATOM 20908 HB1 LEU G 25   21.890 -25.389 34.449 1.00 0.00
G   H
ATOM 20909 HB2 LEU G 25   21.784 -25.942 32.761 1.00 0.00
G   H
ATOM 20910 CG LEU G 25   22.243 -27.507 34.212 1.00 0.00
G   C
ATOM 20911 HG LEU G 25   23.237 -27.861 34.579 1.00 0.00
G   H
ATOM 20912 CD1 LEU G 25   21.231 -27.586 35.313 1.00 0.00
G   C
ATOM 20913 HD11 LEU G 25   21.111 -28.636 35.655 1.00 0.00
G   H
ATOM 20914 HD12 LEU G 25   21.548 -26.970 36.181 1.00 0.00
G   H
ATOM 20915 HD13 LEU G 25   20.243 -27.220 34.964 1.00 0.00
G   H
ATOM 20916 CD2 LEU G 25   21.822 -28.398 33.068 1.00 0.00
G   C
ATOM 20917 HD21 LEU G 25   21.753 -29.454 33.407 1.00 0.00
G   H
ATOM 20918 HD22 LEU G 25   20.830 -28.107 32.667 1.00 0.00
G   H
ATOM 20919 HD23 LEU G 25   22.564 -28.346 32.242 1.00 0.00
G   H
ATOM 20920 C   LEU G 25   24.042 -25.264 32.045 1.00 0.00
G   C
ATOM 20921 O   LEU G 25   24.560 -26.075 31.269 1.00 0.00
G   O
ATOM 20922 N   HSE G 26   23.674 -24.048 31.667 1.00 0.00
G   N
ATOM 20923 HN HSE G 26   23.133 -23.437 32.243 1.00 0.00
G   H
ATOM 20924 CA HSE G 26   24.027 -23.494 30.374 1.00 0.00
G   C

```
ATOM  20925  HA  HSE G  26      23.601 -24.145  29.620  1.00  0.00
G    H
ATOM  20926  CB  HSE G  26      23.482 -22.074  30.302  1.00  0.00
G    C
ATOM  20927  HB1 HSE G  26      22.396 -22.113  30.536  1.00  0.00
G    H
ATOM  20928  HB2 HSE G  26      23.962 -21.453  31.087  1.00  0.00
G    H
ATOM  20929  ND1 HSE G  26      24.642 -20.524  28.703  1.00  0.00
G    N
ATOM  20930  CG  HSE G  26      23.635 -21.423  28.972  1.00  0.00
G    C
ATOM  20931  CE1 HSE G  26      24.518 -20.094  27.461  1.00  0.00
G    C
ATOM  20932  HE1 HSE G  26      25.188 -19.375  26.984  1.00  0.00
G    H
ATOM  20933  NE2 HSE G  26      23.461 -20.673  26.921  1.00  0.00
G    N
ATOM  20934  HE2 HSE G  26      23.125 -20.510  25.994  1.00  0.00
G    H
ATOM  20935  CD2 HSE G  26      22.888 -21.505  27.849  1.00  0.00
G    C
ATOM  20936  HD2 HSE G  26      21.995 -22.083  27.650  1.00  0.00
G    H
ATOM  20937  C   HSE G  26      25.529 -23.509  30.177  1.00  0.00
G    C
ATOM  20938  O   HSE G  26      26.019 -23.890  29.109  1.00  0.00
G    O
ATOM  20939  N   ARG G  27      26.277 -23.099  31.199  1.00  0.00
G    N
ATOM  20940  HN  ARG G  27      25.908 -22.702  32.036  1.00  0.00
G    H
ATOM  20941  CA  ARG G  27      27.724 -23.223  31.118  1.00  0.00
G    C
ATOM  20942  HA  ARG G  27      28.063 -22.711  30.226  1.00  0.00
G    H
ATOM  20943  CB  ARG G  27      28.375 -22.602  32.347  1.00  0.00
G    C
ATOM  20944  HB1 ARG G  27      27.811 -21.662  32.561  1.00  0.00
G    H
ATOM  20945  HB2 ARG G  27      28.215 -23.262  33.227  1.00  0.00
G    H
ATOM  20946  CG  ARG G  27      29.832 -22.271  32.151  1.00  0.00
G    C
ATOM  20947  HG1 ARG G  27      30.162 -22.687  31.174  1.00  0.00
G    H
ATOM  20948  HG2 ARG G  27      29.943 -21.166  32.058  1.00  0.00
G    H
ATOM  20949  CD  ARG G  27      30.669 -22.755  33.317  1.00  0.00
G    C
ATOM  20950  HD1 ARG G  27      31.743 -22.777  33.018  1.00  0.00
G    H
ATOM  20951  HD2 ARG G  27      30.558 -22.089  34.205  1.00  0.00
G    H
```

ATOM 20952 NE ARG G 27   30.286 -24.090 33.763 1.00 0.00
G   N
ATOM 20953 HE ARG G 27   29.838 -24.092 34.665 1.00 0.00
G   H
ATOM 20954 CZ ARG G 27   30.495 -25.203 33.071 1.00 0.00
G   C
ATOM 20955 NH1 ARG G 27   31.077 -25.156 31.883 1.00 0.00
G   N
ATOM 20956 HH11 ARG G 27   31.211 -25.982 31.354 1.00 0.00
G   H
ATOM 20957 HH12 ARG G 27   31.240 -24.242 31.504 1.00 0.00
G   H
ATOM 20958 NH2 ARG G 27   30.122 -26.369 33.565 1.00 0.00
G   N
ATOM 20959 HH21 ARG G 27   30.355 -27.205 33.089 1.00 0.00
G   H
ATOM 20960 HH22 ARG G 27   29.801 -26.377 34.503 1.00 0.00
G   H
ATOM 20961 C   ARG G 27   28.136 -24.679 30.967 1.00 0.00
G   C
ATOM 20962 O   ARG G 27   29.158 -24.972 30.341 1.00 0.00
G   O
ATOM 20963 N   MET G 28   27.354 -25.607 31.518 1.00 0.00
G   N
ATOM 20964 HN MET G 28   26.563 -25.452 32.105 1.00 0.00
G   H
ATOM 20965 CA MET G 28   27.647 -27.011 31.262 1.00 0.00
G   C
ATOM 20966 HA MET G 28   28.720 -27.122 31.351 1.00 0.00
G   H
ATOM 20967 CB MET G 28   26.940 -27.924 32.255 1.00 0.00
G   C
ATOM 20968 HB1 MET G 28   25.856 -27.665 32.220 1.00 0.00
G   H
ATOM 20969 HB2 MET G 28   27.019 -28.979 31.908 1.00 0.00
G   H
ATOM 20970 CG MET G 28   27.423 -27.792 33.654 1.00 0.00
G   C
ATOM 20971 HG1 MET G 28   28.528 -27.902 33.693 1.00 0.00
G   H
ATOM 20972 HG2 MET G 28   27.173 -26.782 34.042 1.00 0.00
G   H
ATOM 20973 SD MET G 28   26.718 -29.015 34.754 1.00 0.00
G   S
ATOM 20974 CE MET G 28   27.613 -30.456 34.189 1.00 0.00
G   C
ATOM 20975 HE1 MET G 28   27.306 -31.362 34.756 1.00 0.00
G   H
ATOM 20976 HE2 MET G 28   27.422 -30.647 33.111 1.00 0.00
G   H
ATOM 20977 HE3 MET G 28   28.708 -30.324 34.324 1.00 0.00
G   H
ATOM 20978 C   MET G 28   27.258 -27.439 29.861 1.00 0.00
G   C

```
ATOM  20979  O   MET G  28      27.470 -28.602  29.507  1.00  0.00
G    O
ATOM  20980  N   GLY G  29      26.687 -26.542  29.067  1.00  0.00
G    N
ATOM  20981  HN  GLY G  29      26.481 -25.604  29.343  1.00  0.00
G    H
ATOM  20982  CA  GLY G  29      26.321 -26.889  27.712  1.00  0.00
G    C
ATOM  20983  HA1 GLY G  29      27.184 -27.353  27.256  1.00  0.00
G    H
ATOM  20984  HA2 GLY G  29      25.991 -25.983  27.222  1.00  0.00
G    H
ATOM  20985  C   GLY G  29      25.183 -27.880  27.672  1.00  0.00
G    C
ATOM  20986  O   GLY G  29      25.312 -28.961  27.095  1.00  0.00
G    O
ATOM  20987  N   HSE G  30      24.070 -27.536  28.301  1.00  0.00
G    N
ATOM  20988  HN  HSE G  30      23.939 -26.676  28.792  1.00  0.00
G    H
ATOM  20989  CA  HSE G  30      22.920 -28.417  28.320  1.00  0.00
G    C
ATOM  20990  HA  HSE G  30      23.175 -29.287  27.727  1.00  0.00
G    H
ATOM  20991  CB  HSE G  30      22.583 -28.823  29.748  1.00  0.00
G    C
ATOM  20992  HB1 HSE G  30      22.611 -27.908  30.378  1.00  0.00
G    H
ATOM  20993  HB2 HSE G  30      21.545 -29.214  29.787  1.00  0.00
G    H
ATOM  20994  ND1 HSE G  30      23.135 -31.042  30.802  1.00  0.00
G    N
ATOM  20995  CG  HSE G  30      23.536 -29.813  30.326  1.00  0.00
G    C
ATOM  20996  CE1 HSE G  30      24.187 -31.702  31.249  1.00  0.00
G    C
ATOM  20997  HE1 HSE G  30      24.151 -32.700  31.694  1.00  0.00
G    H
ATOM  20998  NE2 HSE G  30      25.258 -30.949  31.071  1.00  0.00
G    N
ATOM  20999  HE2 HSE G  30      26.188 -31.199  31.338  1.00  0.00
G    H
ATOM  21000  CD2 HSE G  30      24.878 -29.765  30.489  1.00  0.00
G    C
ATOM  21001  HD2 HSE G  30      25.579 -28.979  30.244  1.00  0.00
G    H
ATOM  21002  C   HSE G  30      21.723 -27.757  27.656  1.00  0.00
G    C
ATOM  21003  O   HSE G  30      21.737 -26.581  27.291  1.00  0.00
G    O
ATOM  21004  N   GLU G  31      20.672 -28.546  27.501  1.00  0.00
G    N
ATOM  21005  HN  GLU G  31      20.674 -29.503  27.778  1.00  0.00
G    H
```

```
ATOM  21006  CA  GLU G  31      19.417 -28.103  26.917  1.00  0.00
G    C
ATOM  21007  HA  GLU G  31      19.587 -27.222  26.311  1.00  0.00
G    H
ATOM  21008  CB  GLU G  31      18.821 -29.211  26.052  1.00  0.00
G    C
ATOM  21009  HB1 GLU G  31      17.853 -28.846  25.637  1.00  0.00
G    H
ATOM  21010  HB2 GLU G  31      19.478 -29.398  25.173  1.00  0.00
G    H
ATOM  21011  CG  GLU G  31      18.558 -30.545  26.764  1.00  0.00
G    C
ATOM  21012  HG1 GLU G  31      18.079 -30.352  27.742  1.00  0.00
G    H
ATOM  21013  HG2 GLU G  31      17.898 -31.196  26.157  1.00  0.00
G    H
ATOM  21014  CD  GLU G  31      19.826 -31.329  27.087  1.00  0.00
G    C
ATOM  21015  OE1 GLU G  31      19.888 -32.526  26.735  1.00  0.00
G    O
ATOM  21016  OE2 GLU G  31      20.742 -30.771  27.726  1.00  0.00
G    O
ATOM  21017  C   GLU G  31      18.489 -27.697  28.055  1.00  0.00
G    C
ATOM  21018  O   GLU G  31      18.032 -28.542  28.830  1.00  0.00
G    O
ATOM  21019  N   VAL G  32      18.242 -26.400  28.183  1.00  0.00
G    N
ATOM  21020  HN  VAL G  32      18.577 -25.709  27.548  1.00  0.00
G    H
ATOM  21021  CA  VAL G  32      17.446 -25.873  29.280  1.00  0.00
G    C
ATOM  21022  HA  VAL G  32      16.875 -26.687  29.709  1.00  0.00
G    H
ATOM  21023  CB  VAL G  32      18.329 -25.248  30.374  1.00  0.00
G    C
ATOM  21024  HB  VAL G  32      17.651 -24.780  31.129  1.00  0.00
G    H
ATOM  21025  CG1 VAL G  32      19.163 -26.323  31.040  1.00  0.00
G    C
ATOM  21026 HG11 VAL G  32      19.832 -25.874  31.805  1.00  0.00
G    H
ATOM  21027 HG12 VAL G  32      18.528 -27.074  31.555  1.00  0.00
G    H
ATOM  21028 HG13 VAL G  32      19.797 -26.846  30.293  1.00  0.00
G    H
ATOM  21029  CG2 VAL G  32      19.240 -24.194  29.788  1.00  0.00
G    C
ATOM  21030 HG21 VAL G  32      19.906 -23.770  30.570  1.00  0.00
G    H
ATOM  21031 HG22 VAL G  32      19.876 -24.629  28.986  1.00  0.00
G    H
ATOM  21032 HG23 VAL G  32      18.657 -23.355  29.354  1.00  0.00
G    H
```

ATOM 21033 C   VAL G 32   16.456 -24.861 28.733 1.00 0.00
G   C
ATOM 21034 O   VAL G 32   16.841 -23.898 28.060 1.00 0.00
G   O
ATOM 21035 N   LEU G 33   15.182 -25.090 29.008 1.00 0.00
G   N
ATOM 21036 HN LEU G 33   14.859 -25.913 29.471 1.00 0.00
G   H
ATOM 21037 CA LEU G 33   14.132 -24.153 28.655 1.00 0.00
G   C
ATOM 21038 HA LEU G 33   14.482 -23.559 27.820 1.00 0.00
G   H
ATOM 21039 CB LEU G 33   12.836 -24.876 28.308 1.00 0.00
G   C
ATOM 21040 HB1 LEU G 33   12.953 -25.285 27.278 1.00 0.00
G   H
ATOM 21041 HB2 LEU G 33   12.723 -25.757 28.978 1.00 0.00
G   H
ATOM 21042 CG LEU G 33   11.625 -23.950 28.370 1.00 0.00
G   C
ATOM 21043 HG LEU G 33   11.587 -23.526 29.402 1.00 0.00
G   H
ATOM 21044 CD1 LEU G 33   11.759 -22.817 27.358 1.00 0.00
G   C
ATOM 21045 HD11 LEU G 33   10.854 -22.172 27.373 1.00 0.00
G   H
ATOM 21046 HD12 LEU G 33   12.642 -22.184 27.594 1.00 0.00
G   H
ATOM 21047 HD13 LEU G 33   11.881 -23.221 26.330 1.00 0.00
G   H
ATOM 21048 CD2 LEU G 33   10.350 -24.735 28.138 1.00 0.00
G   C
ATOM 21049 HD21 LEU G 33   9.467 -24.064 28.201 1.00 0.00
G   H
ATOM 21050 HD22 LEU G 33   10.339 -25.212 27.137 1.00 0.00
G   H
ATOM 21051 HD23 LEU G 33   10.238 -25.531 28.906 1.00 0.00
G   H
ATOM 21052 C   LEU G 33   13.915 -23.213 29.827 1.00 0.00
G   C
ATOM 21053 O   LEU G 33   13.609 -23.658 30.937 1.00 0.00
G   O
ATOM 21054 N   ALA G 34   14.071 -21.922 29.582 1.00 0.00
G   N
ATOM 21055 HN ALA G 34   14.344 -21.568 28.689 1.00 0.00
G   H
ATOM 21056 CA ALA G 34   13.852 -20.920 30.610 1.00 0.00
G   C
ATOM 21057 HA ALA G 34   13.923 -21.343 31.606 1.00 0.00
G   H
ATOM 21058 CB ALA G 34   14.869 -19.789 30.491 1.00 0.00
G   C
ATOM 21059 HB1 ALA G 34   15.893 -20.191 30.644 1.00 0.00
G   H

```
ATOM  21060  HB2 ALA G  34      14.833 -19.324  29.482  1.00  0.00
G    H
ATOM  21061  HB3 ALA G  34      14.694 -18.999  31.251  1.00  0.00
G    H
ATOM  21062  C   ALA G  34      12.437 -20.386  30.471  1.00  0.00
G    C
ATOM  21063  O   ALA G  34      11.998 -20.067  29.362  1.00  0.00
G    O
ATOM  21064  N   VAL G  35      11.722 -20.312  31.587  1.00  0.00
G    N
ATOM  21065  HN  VAL G  35      12.055 -20.657  32.461  1.00  0.00
G    H
ATOM  21066  CA  VAL G  35      10.392 -19.722  31.640  1.00  0.00
G    C
ATOM  21067  HA  VAL G  35      10.268 -19.070  30.784  1.00  0.00
G    H
ATOM  21068  CB  VAL G  35       9.281 -20.789  31.654  1.00  0.00
G    C
ATOM  21069  HB  VAL G  35       9.406 -21.394  32.586  1.00  0.00
G    H
ATOM  21070  CG1 VAL G  35       7.913 -20.128  31.664  1.00  0.00
G    C
ATOM  21071 HG11 VAL G  35       7.113 -20.898  31.635  1.00  0.00
G    H
ATOM  21072 HG12 VAL G  35       7.756 -19.524  32.582  1.00  0.00
G    H
ATOM  21073 HG13 VAL G  35       7.788 -19.470  30.779  1.00  0.00
G    H
ATOM  21074  CG2 VAL G  35       9.423 -21.723  30.477  1.00  0.00
G    C
ATOM  21075 HG21 VAL G  35       8.600 -22.468  30.465  1.00  0.00
G    H
ATOM  21076 HG22 VAL G  35       9.395 -21.155  29.523  1.00  0.00
G    H
ATOM  21077 HG23 VAL G  35      10.381 -22.283  30.522  1.00  0.00
G    H
ATOM  21078  C   VAL G  35      10.300 -18.859  32.885  1.00  0.00
G    C
ATOM  21079  O   VAL G  35      10.818 -19.227  33.945  1.00  0.00
G    O
ATOM  21080  N   ASP G  36       9.638 -17.717  32.760  1.00  0.00
G    N
ATOM  21081  HN  ASP G  36       9.295 -17.317  31.914  1.00  0.00
G    H
ATOM  21082  CA  ASP G  36       9.344 -16.910  33.926  1.00  0.00
G    C
ATOM  21083  HA  ASP G  36       8.894 -17.554  34.673  1.00  0.00
G    H
ATOM  21084  CB  ASP G  36      10.587 -16.222  34.465  1.00  0.00
G    C
ATOM  21085  HB1 ASP G  36      11.448 -16.916  34.395  1.00  0.00
G    H
ATOM  21086  HB2 ASP G  36      10.820 -15.316  33.871  1.00  0.00
G    H
```

```
ATOM  21087  CG  ASP G  36      10.429 -15.835  35.895  1.00  0.00
G    C
ATOM  21088  OD1 ASP G  36      10.583 -16.728  36.751  1.00  0.00
G    O
ATOM  21089  OD2 ASP G  36      10.138 -14.652  36.163  1.00  0.00
G    O
ATOM  21090  C   ASP G  36       8.304 -15.873  33.561  1.00  0.00
G    C
ATOM  21091  O   ASP G  36       8.383 -15.253  32.501  1.00  0.00
G    O
ATOM  21092  N   ILE G  37       7.338 -15.681  34.457  1.00  0.00
G    N
ATOM  21093  HN  ILE G  37       7.235 -16.159  35.326  1.00  0.00
G    H
ATOM  21094  CA  ILE G  37       6.306 -14.690  34.194  1.00  0.00
G    C
ATOM  21095  HA  ILE G  37       5.917 -14.904  33.208  1.00  0.00
G    H
ATOM  21096  CB  ILE G  37       5.195 -14.770  35.249  1.00  0.00
G    C
ATOM  21097  HB  ILE G  37       4.883 -15.845  35.246  1.00  0.00
G    H
ATOM  21098  CG2 ILE G  37       5.696 -14.273  36.586  1.00  0.00
G    C
ATOM  21099 HG21 ILE G  37       4.950 -14.474  37.382  1.00  0.00
G    H
ATOM  21100 HG22 ILE G  37       6.638 -14.787  36.872  1.00  0.00
G    H
ATOM  21101 HG23 ILE G  37       5.888 -13.180  36.558  1.00  0.00
G    H
ATOM  21102  CG1 ILE G  37       3.990 -13.952  34.793  1.00  0.00
G    C
ATOM  21103 HG11 ILE G  37       4.219 -12.866  34.877  1.00  0.00
G    H
ATOM  21104 HG12 ILE G  37       3.797 -14.165  33.717  1.00  0.00
G    H
ATOM  21105  CD  ILE G  37       2.742 -14.244  35.554  1.00  0.00
G    C
ATOM  21106  HD1 ILE G  37       1.891 -13.662  35.138  1.00  0.00
G    H
ATOM  21107  HD2 ILE G  37       2.488 -15.324  35.491  1.00  0.00
G    H
ATOM  21108  HD3 ILE G  37       2.854 -13.972  36.625  1.00  0.00
G    H
ATOM  21109  C   ILE G  37       6.903 -13.296  34.137  1.00  0.00
G    C
ATOM  21110  O   ILE G  37       6.353 -12.406  33.481  1.00  0.00
G    O
ATOM  21111  N   ASN G  38       8.026 -13.074  34.799  1.00  0.00
G    N
ATOM  21112  HN  ASN G  38       8.511 -13.744  35.361  1.00  0.00
G    H
ATOM  21113  CA  ASN G  38       8.655 -11.770  34.744  1.00  0.00
G    C
```

```
ATOM  21114  HA  ASN G  38       7.857 -11.037  34.687  1.00  0.00
G    H
ATOM  21115  CB  ASN G  38       9.549 -11.534  35.948  1.00  0.00
G    C
ATOM  21116  HB1 ASN G  38       8.896 -11.298  36.816  1.00  0.00
G    H
ATOM  21117  HB2 ASN G  38      10.113 -12.456  36.196  1.00  0.00
G    H
ATOM  21118  CG  ASN G  38      10.481 -10.387  35.729  1.00  0.00
G    C
ATOM  21119  OD1 ASN G  38      11.546 -10.547  35.149  1.00  0.00
G    O
ATOM  21120  ND2 ASN G  38      10.072  -9.207  36.162  1.00  0.00
G    N
ATOM  21121 HD21 ASN G  38      10.677  -8.430  36.013  1.00  0.00
G    H
ATOM  21122 HD22 ASN G  38       9.178  -9.115  36.589  1.00  0.00
G    H
ATOM  21123  C   ASN G  38       9.462 -11.672  33.463  1.00  0.00
G    C
ATOM  21124  O   ASN G  38      10.522 -12.291  33.338  1.00  0.00
G    O
ATOM  21125  N   GLU G  39       8.960 -10.882  32.521  1.00  0.00
G    N
ATOM  21126  HN  GLU G  39       8.111 -10.365  32.600  1.00  0.00
G    H
ATOM  21127  CA  GLU G  39       9.643 -10.705  31.246  1.00  0.00
G    C
ATOM  21128  HA  GLU G  39       9.735 -11.675  30.775  1.00  0.00
G    H
ATOM  21129  CB  GLU G  39       8.843  -9.746  30.378  1.00  0.00
G    C
ATOM  21130  HB1 GLU G  39       9.186  -9.863  29.324  1.00  0.00
G    H
ATOM  21131  HB2 GLU G  39       7.769 -10.038  30.384  1.00  0.00
G    H
ATOM  21132  CG  GLU G  39       9.012  -8.312  30.815  1.00  0.00
G    C
ATOM  21133  HG1 GLU G  39      10.075  -8.121  31.052  1.00  0.00
G    H
ATOM  21134  HG2 GLU G  39       8.685  -7.613  30.020  1.00  0.00
G    H
ATOM  21135  CD  GLU G  39       8.247  -7.988  32.081  1.00  0.00
G    C
ATOM  21136  OE1 GLU G  39       7.834  -8.924  32.801  1.00  0.00
G    O
ATOM  21137  OE2 GLU G  39       8.069  -6.790  32.368  1.00  0.00
G    O
ATOM  21138  C   GLU G  39      11.047 -10.168  31.450  1.00  0.00
G    C
ATOM  21139  O   GLU G  39      11.911 -10.322  30.587  1.00  0.00
G    O
ATOM  21140  N   GLU G  40      11.291  -9.511  32.577  1.00  0.00
G    N
```

ATOM 21141 HN GLU G 40   10.664 -9.386 33.342 1.00 0.00
G   H
ATOM 21142 CA GLU G 40   12.589 -8.887 32.769 1.00 0.00
G   C
ATOM 21143 HA GLU G 40   12.816 -8.302 31.886 1.00 0.00
G   H
ATOM 21144 CB GLU G 40   12.544 -7.979 33.995 1.00 0.00
G   C
ATOM 21145 HB1 GLU G 40   11.579 -8.155 34.522 1.00 0.00
G   H
ATOM 21146 HB2 GLU G 40   13.343 -8.274 34.712 1.00 0.00
G   H
ATOM 21147 CG GLU G 40   12.660 -6.474 33.739 1.00 0.00
G   C
ATOM 21148 HG1 GLU G 40   11.662 -6.060 33.507 1.00 0.00
G   H
ATOM 21149 HG2 GLU G 40   13.074 -5.951 34.624 1.00 0.00
G   H
ATOM 21150 CD GLU G 40   13.520 -6.092 32.547 1.00 0.00
G   C
ATOM 21151 OE1 GLU G 40   14.516 -5.378 32.768 1.00 0.00
G   O
ATOM 21152 OE2 GLU G 40   13.169 -6.432 31.394 1.00 0.00
G   O
ATOM 21153 C   GLU G 40   13.685 -9.928 32.913 1.00 0.00
G   C
ATOM 21154 O   GLU G 40   14.723 -9.837 32.246 1.00 0.00
G   O
ATOM 21155 N   LYS G 41   13.473 -10.924 33.769 1.00 0.00
G   N
ATOM 21156 HN LYS G 41   12.657 -11.027 34.337 1.00 0.00
G   H
ATOM 21157 CA LYS G 41   14.474 -11.970 33.931 1.00 0.00
G   C
ATOM 21158 HA LYS G 41   15.414 -11.480 34.154 1.00 0.00
G   H
ATOM 21159 CB LYS G 41   14.080 -12.917 35.061 1.00 0.00
G   C
ATOM 21160 HB1 LYS G 41   13.241 -13.546 34.683 1.00 0.00
G   H
ATOM 21161 HB2 LYS G 41   14.926 -13.611 35.263 1.00 0.00
G   H
ATOM 21162 CG LYS G 41   13.645 -12.231 36.322 1.00 0.00
G   C
ATOM 21163 HG1 LYS G 41   13.259 -11.217 36.071 1.00 0.00
G   H
ATOM 21164 HG2 LYS G 41   12.804 -12.805 36.775 1.00 0.00
G   H
ATOM 21165 CD LYS G 41   14.775 -12.097 37.306 1.00 0.00
G   C
ATOM 21166 HD1 LYS G 41   15.448 -12.977 37.192 1.00 0.00
G   H
ATOM 21167 HD2 LYS G 41   15.363 -11.190 37.036 1.00 0.00
G   H

ATOM 21168 CE LYS G 41 14.210 -11.995 38.706 1.00 0.00
G C
ATOM 21169 HE1 LYS G 41 15.014 -11.746 39.433 1.00 0.00
G H
ATOM 21170 HE2 LYS G 41 13.432 -11.202 38.756 1.00 0.00
G H
ATOM 21171 NZ LYS G 41 13.590 -13.281 39.121 1.00 0.00
G N
ATOM 21172 HZ1 LYS G 41 13.208 -13.198 40.086 1.00 0.00
G H
ATOM 21173 HZ2 LYS G 41 12.821 -13.525 38.465 1.00 0.00
G H
ATOM 21174 HZ3 LYS G 41 14.308 -14.034 39.100 1.00 0.00
G H
ATOM 21175 C LYS G 41 14.665 -12.753 32.644 1.00 0.00
G C
ATOM 21176 O LYS G 41 15.798 -13.073 32.271 1.00 0.00
G O
ATOM 21177 N VAL G 42 13.567 -13.127 31.989 1.00 0.00
G N
ATOM 21178 HN VAL G 42 12.633 -12.976 32.302 1.00 0.00
G H
ATOM 21179 CA VAL G 42 13.671 -13.815 30.710 1.00 0.00
G C
ATOM 21180 HA VAL G 42 14.195 -14.749 30.871 1.00 0.00
G H
ATOM 21181 CB VAL G 42 12.270 -14.076 30.137 1.00 0.00
G C
ATOM 21182 HB VAL G 42 11.857 -13.095 29.795 1.00 0.00
G H
ATOM 21183 CG1 VAL G 42 12.349 -15.022 28.958 1.00 0.00
G C
ATOM 21184 HG11 VAL G 42 11.330 -15.240 28.569 1.00 0.00
G H
ATOM 21185 HG12 VAL G 42 12.936 -14.587 28.122 1.00 0.00
G H
ATOM 21186 HG13 VAL G 42 12.816 -15.984 29.258 1.00 0.00
G H
ATOM 21187 CG2 VAL G 42 11.360 -14.617 31.217 1.00 0.00
G C
ATOM 21188 HG21 VAL G 42 10.355 -14.847 30.804 1.00 0.00
G H
ATOM 21189 HG22 VAL G 42 11.782 -15.549 31.651 1.00 0.00
G H
ATOM 21190 HG23 VAL G 42 11.226 -13.881 32.036 1.00 0.00
G H
ATOM 21191 C VAL G 42 14.505 -12.984 29.758 1.00 0.00
G C
ATOM 21192 O VAL G 42 15.433 -13.479 29.105 1.00 0.00
G O
ATOM 21193 N ASN G 43 14.188 -11.697 29.687 1.00 0.00
G N
ATOM 21194 HN ASN G 43 13.459 -11.282 30.228 1.00 0.00
G H

ATOM 21195 CA ASN G 43   14.884 -10.774 28.807 1.00 0.00
G   C
ATOM 21196 HA ASN G 43   14.716 -11.140 27.800 1.00 0.00
G   H
ATOM 21197 CB ASN G 43   14.326 -9.370 29.021 1.00 0.00
G   C
ATOM 21198 HB1 ASN G 43   13.296 -9.473 29.428 1.00 0.00
G   H
ATOM 21199 HB2 ASN G 43   14.929 -8.826 29.776 1.00 0.00
G   H
ATOM 21200 CG ASN G 43   14.248 -8.581 27.750 1.00 0.00
G   C
ATOM 21201 OD1 ASN G 43   15.212 -8.492 27.004 1.00 0.00
G   O
ATOM 21202 ND2 ASN G 43   13.081 -8.015 27.478 1.00 0.00
G   N
ATOM 21203 HD21 ASN G 43   13.011 -7.496 26.631 1.00 0.00
G   H
ATOM 21204 HD22 ASN G 43   12.310 -8.141 28.095 1.00 0.00
G   H
ATOM 21205 C   ASN G 43   16.377 -10.799 29.061 1.00 0.00
G   C
ATOM 21206 O   ASN G 43   17.180 -10.854 28.125 1.00 0.00
G   O
ATOM 21207 N   ALA G 44   16.765 -10.754 30.329 1.00 0.00
G   N
ATOM 21208 HN ALA G 44   16.148 -10.687 31.113 1.00 0.00
G   H
ATOM 21209 CA ALA G 44   18.183 -10.803 30.651 1.00 0.00
G   C
ATOM 21210 HA ALA G 44   18.726 -10.049 30.092 1.00 0.00
G   H
ATOM 21211 CB ALA G 44   18.395 -10.537 32.138 1.00 0.00
G   C
ATOM 21212 HB1 ALA G 44   17.991 -9.535 32.398 1.00 0.00
G   H
ATOM 21213 HB2 ALA G 44   17.863 -11.290 32.759 1.00 0.00
G   H
ATOM 21214 HB3 ALA G 44   19.472 -10.554 32.408 1.00 0.00
G   H
ATOM 21215 C   ALA G 44   18.790 -12.144 30.266 1.00 0.00
G   C
ATOM 21216 O   ALA G 44   19.674 -12.211 29.406 1.00 0.00
G   O
ATOM 21217 N   TYR G 45   18.299 -13.223 30.855 1.00 0.00
G   N
ATOM 21218 HN TYR G 45   17.440 -13.246 31.363 1.00 0.00
G   H
ATOM 21219 CA TYR G 45   19.003 -14.494 30.799 1.00 0.00
G   C
ATOM 21220 HA TYR G 45   20.056 -14.239 30.807 1.00 0.00
G   H
ATOM 21221 CB TYR G 45   18.644 -15.365 31.999 1.00 0.00
G   C

```
ATOM  21222  HB1 TYR G  45      17.540 -15.371  32.127  1.00  0.00
G    H
ATOM  21223  HB2 TYR G  45      18.962 -16.417  31.830  1.00  0.00
G    H
ATOM  21224  CG  TYR G  45      19.226 -14.874  33.292  1.00  0.00
G    C
ATOM  21225  CD1 TYR G  45      20.089 -13.791  33.319  1.00  0.00
G    C
ATOM  21226  HD1 TYR G  45      20.355 -13.293  32.397  1.00  0.00
G    H
ATOM  21227  CE1 TYR G  45      20.634 -13.348  34.501  1.00  0.00
G    C
ATOM  21228  HE1 TYR G  45      21.291 -12.491  34.516  1.00  0.00
G    H
ATOM  21229  CZ  TYR G  45      20.344 -14.009  35.670  1.00  0.00
G    C
ATOM  21230  OH  TYR G  45      20.887 -13.581  36.856  1.00  0.00
G    O
ATOM  21231  HH  TYR G  45      20.513 -14.120  37.556  1.00  0.00
G    H
ATOM  21232  CD2 TYR G  45      18.964 -15.530  34.480  1.00  0.00
G    C
ATOM  21233  HD2 TYR G  45      18.352 -16.421  34.482  1.00  0.00
G    H
ATOM  21234  CE2 TYR G  45      19.509 -15.099  35.663  1.00  0.00
G    C
ATOM  21235  HE2 TYR G  45      19.295 -15.631  36.577  1.00  0.00
G    H
ATOM  21236  C   TYR G  45      18.744 -15.283  29.532  1.00  0.00
G    C
ATOM  21237  O   TYR G  45      19.159 -16.444  29.459  1.00  0.00
G    O
ATOM  21238  N   ALA G  46      18.066 -14.703  28.545  1.00  0.00
G    N
ATOM  21239  HN  ALA G  46      17.592 -13.826  28.609  1.00  0.00
G    H
ATOM  21240  CA  ALA G  46      17.984 -15.364  27.251  1.00  0.00
G    C
ATOM  21241  HA  ALA G  46      17.404 -16.277  27.319  1.00  0.00
G    H
ATOM  21242  CB  ALA G  46      17.312 -14.445  26.235  1.00  0.00
G    C
ATOM  21243  HB1 ALA G  46      16.284 -14.199  26.577  1.00  0.00
G    H
ATOM  21244  HB2 ALA G  46      17.874 -13.491  26.127  1.00  0.00
G    H
ATOM  21245  HB3 ALA G  46      17.235 -14.925  25.236  1.00  0.00
G    H
ATOM  21246  C   ALA G  46      19.368 -15.770  26.773  1.00  0.00
G    C
ATOM  21247  O   ALA G  46      19.586 -16.908  26.346  1.00  0.00
G    O
ATOM  21248  N   SER G  47      20.326 -14.853  26.880  1.00  0.00
G    N
```

```
ATOM  21249  HN  SER G  47      20.191 -13.935  27.254  1.00  0.00
G    H
ATOM  21250  CA  SER G  47      21.687 -15.124  26.446  1.00  0.00
G    C
ATOM  21251  HA  SER G  47      21.607 -15.381  25.398  1.00  0.00
G    H
ATOM  21252  CB  SER G  47      22.552 -13.893  26.675  1.00  0.00
G    C
ATOM  21253  HB1 SER G  47      23.440 -13.965  26.007  1.00  0.00
G    H
ATOM  21254  HB2 SER G  47      21.998 -12.971  26.386  1.00  0.00
G    H
ATOM  21255  OG  SER G  47      22.981 -13.846  28.021  1.00  0.00
G    O
ATOM  21256  HG1 SER G  47      23.692 -13.195  28.070  1.00  0.00
G    H
ATOM  21257  C   SER G  47      22.306 -16.316  27.156  1.00  0.00
G    C
ATOM  21258  O   SER G  47      23.182 -16.972  26.588  1.00  0.00
G    O
ATOM  21259  N   TYR G  48      21.882 -16.609  28.380  1.00  0.00
G    N
ATOM  21260  HN  TYR G  48      21.175 -16.095  28.862  1.00  0.00
G    H
ATOM  21261  CA  TYR G  48      22.430 -17.730  29.126  1.00  0.00
G    C
ATOM  21262  HA  TYR G  48      23.331 -18.032  28.607  1.00  0.00
G    H
ATOM  21263  CB  TYR G  48      22.723 -17.314  30.562  1.00  0.00
G    C
ATOM  21264  HB1 TYR G  48      21.769 -17.043  31.064  1.00  0.00
G    H
ATOM  21265  HB2 TYR G  48      23.167 -18.158  31.133  1.00  0.00
G    H
ATOM  21266  CG  TYR G  48      23.619 -16.120  30.632  1.00  0.00
G    C
ATOM  21267  CD1 TYR G  48      24.550 -15.876  29.636  1.00  0.00
G    C
ATOM  21268  HD1 TYR G  48      24.653 -16.572  28.815  1.00  0.00
G    H
ATOM  21269  CE1 TYR G  48      25.361 -14.776  29.683  1.00  0.00
G    C
ATOM  21270  HE1 TYR G  48      26.097 -14.603  28.912  1.00  0.00
G    H
ATOM  21271  CZ  TYR G  48      25.239 -13.891  30.727  1.00  0.00
G    C
ATOM  21272  OH  TYR G  48      26.049 -12.786  30.779  1.00  0.00
G    O
ATOM  21273  HH  TYR G  48      25.769 -12.259  31.530  1.00  0.00
G    H
ATOM  21274  CD2 TYR G  48      23.508 -15.209  31.665  1.00  0.00
G    C
ATOM  21275  HD2 TYR G  48      22.803 -15.385  32.466  1.00  0.00
G    H
```

ATOM 21276 CE2 TYR G 48   24.315 -14.103 31.720 1.00 0.00
G   C
ATOM 21277 HE2 TYR G 48   24.233 -13.421 32.553 1.00 0.00
G   H
ATOM 21278 C   TYR G 48   21.490 -18.915 29.114 1.00 0.00
G   C
ATOM 21279 O   TYR G 48   21.481 -19.704 30.060 1.00 0.00
G   O
ATOM 21280 N   ALA G 49   20.694 -19.052 28.061 1.00 0.00
G   N
ATOM 21281 HN ALA G 49   20.772 -18.508 27.226 1.00 0.00
G   H
ATOM 21282 CA ALA G 49   19.629 -20.035 28.066 1.00 0.00
G   C
ATOM 21283 HA ALA G 49   19.806 -20.804 28.809 1.00 0.00
G   H
ATOM 21284 CB ALA G 49   18.272 -19.393 28.357 1.00 0.00
G   C
ATOM 21285 HB1 ALA G 49   18.291 -18.919 29.362 1.00 0.00
G   H
ATOM 21286 HB2 ALA G 49   18.035 -18.603 27.612 1.00 0.00
G   H
ATOM 21287 HB3 ALA G 49   17.453 -20.144 28.348 1.00 0.00
G   H
ATOM 21288 C   ALA G 49   19.582 -20.738 26.727 1.00 0.00
G   C
ATOM 21289 O   ALA G 49   19.624 -20.102 25.672 1.00 0.00
G   O
ATOM 21290 N   THR G 50   19.488 -22.063 26.784 1.00 0.00
G   N
ATOM 21291 HN THR G 50   19.594 -22.629 27.601 1.00 0.00
G   H
ATOM 21292 CA THR G 50   19.207 -22.812 25.572 1.00 0.00
G   C
ATOM 21293 HA THR G 50   20.015 -22.589 24.888 1.00 0.00
G   H
ATOM 21294 CB THR G 50   19.089 -24.303 25.888 1.00 0.00
G   C
ATOM 21295 HB THR G 50   18.132 -24.426 26.452 1.00 0.00
G   H
ATOM 21296 OG1 THR G 50   20.163 -24.702 26.749 1.00 0.00
G   O
ATOM 21297 HG1 THR G 50   20.036 -25.646 26.897 1.00 0.00
G   H
ATOM 21298 CG2 THR G 50   19.139 -25.129 24.612 1.00 0.00
G   C
ATOM 21299 HG21 THR G 50   18.940 -26.200 24.826 1.00 0.00
G   H
ATOM 21300 HG22 THR G 50   18.371 -24.777 23.892 1.00 0.00
G   H
ATOM 21301 HG23 THR G 50   20.135 -25.052 24.125 1.00 0.00
G   H
ATOM 21302 C   THR G 50   17.935 -22.313 24.915 1.00 0.00
G   C

ATOM 21303 O   THR G 50   17.848 -22.268 23.685 1.00 0.00
G   O
ATOM 21304 N   HSE G 51   16.957 -21.909 25.713 1.00 0.00
G   N
ATOM 21305 HN HSE G 51   17.001 -21.907 26.711 1.00 0.00
G   H
ATOM 21306 CA HSE G 51   15.705 -21.421 25.169 1.00 0.00
G   C
ATOM 21307 HA HSE G 51   15.931 -20.671 24.422 1.00 0.00
G   H
ATOM 21308 CB HSE G 51   14.895 -22.574 24.575 1.00 0.00
G   C
ATOM 21309 HB1 HSE G 51   15.481 -23.006 23.735 1.00 0.00
G   H
ATOM 21310 HB2 HSE G 51   14.777 -23.374 25.335 1.00 0.00
G   H
ATOM 21311 ND1 HSE G 51   12.654 -23.079 23.548 1.00 0.00
G   N
ATOM 21312 CG HSE G 51   13.561 -22.167 24.041 1.00 0.00
G   C
ATOM 21313 CE1 HSE G 51   11.568 -22.443 23.149 1.00 0.00
G   C
ATOM 21314 HE1 HSE G 51   10.691 -22.928 22.709 1.00 0.00
G   H
ATOM 21315 NE2 HSE G 51   11.738 -21.152 23.366 1.00 0.00
G   N
ATOM 21316 HE2 HSE G 51   11.075 -20.438 23.140 1.00 0.00
G   H
ATOM 21317 CD2 HSE G 51   12.977 -20.952 23.923 1.00 0.00
G   C
ATOM 21318 HD2 HSE G 51   13.354 -19.973 24.183 1.00 0.00
G   H
ATOM 21319 C   HSE G 51   14.938 -20.726 26.281 1.00 0.00
G   C
ATOM 21320 O   HSE G 51   14.680 -21.317 27.333 1.00 0.00
G   O
ATOM 21321 N   ALA G 52   14.576 -19.470 26.040 1.00 0.00
G   N
ATOM 21322 HN ALA G 52   14.780 -19.021 25.171 1.00 0.00
G   H
ATOM 21323 CA ALA G 52   13.861 -18.658 27.011 1.00 0.00
G   C
ATOM 21324 HA ALA G 52   13.688 -19.199 27.934 1.00 0.00
G   H
ATOM 21325 CB ALA G 52   14.623 -17.374 27.334 1.00 0.00
G   C
ATOM 21326 HB1 ALA G 52   15.605 -17.627 27.788 1.00 0.00
G   H
ATOM 21327 HB2 ALA G 52   14.816 -16.781 26.413 1.00 0.00
G   H
ATOM 21328 HB3 ALA G 52   14.065 -16.737 28.054 1.00 0.00
G   H
ATOM 21329 C   ALA G 52   12.496 -18.314 26.454 1.00 0.00
G   C

```
ATOM  21330  O   ALA G  52      12.325 -18.214  25.239  1.00  0.00
G    O
ATOM  21331  N   VAL G  53      11.533 -18.122  27.346  1.00  0.00
G    N
ATOM  21332  HN  VAL G  53      11.682 -18.215  28.327  1.00  0.00
G    H
ATOM  21333  CA  VAL G  53      10.171 -17.758  26.977  1.00  0.00
G    C
ATOM  21334  HA  VAL G  53      10.203 -17.087  26.128  1.00  0.00
G    H
ATOM  21335  CB  VAL G  53       9.301 -18.992  26.662  1.00  0.00
G    C
ATOM  21336  HB  VAL G  53       9.555 -19.784  27.409  1.00  0.00
G    H
ATOM  21337  CG1 VAL G  53       7.821 -18.655  26.778  1.00  0.00
G    C
ATOM  21338 HG11 VAL G  53       7.202 -19.538  26.511  1.00  0.00
G    H
ATOM  21339 HG12 VAL G  53       7.548 -18.359  27.812  1.00  0.00
G    H
ATOM  21340 HG13 VAL G  53       7.552 -17.826  26.088  1.00  0.00
G    H
ATOM  21341  CG2 VAL G  53       9.600 -19.536  25.286  1.00  0.00
G    C
ATOM  21342 HG21 VAL G  53       8.941 -20.400  25.053  1.00  0.00
G    H
ATOM  21343 HG22 VAL G  53       9.437 -18.754  24.513  1.00  0.00
G    H
ATOM  21344 HG23 VAL G  53      10.650 -19.886  25.211  1.00  0.00
G    H
ATOM  21345  C   VAL G  53       9.573 -16.992  28.140  1.00  0.00
G    C
ATOM  21346  O   VAL G  53       9.804 -17.340  29.300  1.00  0.00
G    O
ATOM  21347  N   ILE G  54       8.805 -15.954  27.844  1.00  0.00
G    N
ATOM  21348  HN  ILE G  54       8.722 -15.524  26.947  1.00  0.00
G    H
ATOM  21349  CA  ILE G  54       7.984 -15.342  28.877  1.00  0.00
G    C
ATOM  21350  HA  ILE G  54       8.469 -15.510  29.830  1.00  0.00
G    H
ATOM  21351  CB  ILE G  54       7.774 -13.852  28.607  1.00  0.00
G    C
ATOM  21352  HB  ILE G  54       7.196 -13.821  27.649  1.00  0.00
G    H
ATOM  21353  CG2 ILE G  54       7.110 -13.199  29.790  1.00  0.00
G    C
ATOM  21354 HG21 ILE G  54       6.813 -12.158  29.544  1.00  0.00
G    H
ATOM  21355 HG22 ILE G  54       6.192 -13.751  30.084  1.00  0.00
G    H
ATOM  21356 HG23 ILE G  54       7.795 -13.167  30.663  1.00  0.00
G    H
```

```
ATOM  21357  CG1 ILE G  54       9.115 -13.184  28.343  1.00  0.00
G    C
ATOM  21358 HG11 ILE G  54       9.634 -12.989  29.308  1.00  0.00
G    H
ATOM  21359 HG12 ILE G  54       9.758 -13.884  27.760  1.00  0.00
G    H
ATOM  21360  CD  ILE G  54       8.995 -11.897  27.574  1.00  0.00
G    C
ATOM  21361  HD1 ILE G  54      10.003 -11.482  27.359  1.00  0.00
G    H
ATOM  21362  HD2 ILE G  54       8.474 -12.065  26.608  1.00  0.00
G    H
ATOM  21363  HD3 ILE G  54       8.428 -11.139  28.153  1.00  0.00
G    H
ATOM  21364  C   ILE G  54       6.661 -16.092  28.916  1.00  0.00
G    C
ATOM  21365  O   ILE G  54       5.992 -16.239  27.890  1.00  0.00
G    O
ATOM  21366  N   ALA G  55       6.287 -16.579  30.098  1.00  0.00
G    N
ATOM  21367  HN  ALA G  55       6.816 -16.446  30.936  1.00  0.00
G    H
ATOM  21368  CA  ALA G  55       5.062 -17.352  30.247  1.00  0.00
G    C
ATOM  21369  HA  ALA G  55       4.223 -16.851  29.778  1.00  0.00
G    H
ATOM  21370  CB  ALA G  55       5.197 -18.740  29.614  1.00  0.00
G    C
ATOM  21371  HB1 ALA G  55       5.402 -18.635  28.528  1.00  0.00
G    H
ATOM  21372  HB2 ALA G  55       6.039 -19.307  30.068  1.00  0.00
G    H
ATOM  21373  HB3 ALA G  55       4.266 -19.336  29.732  1.00  0.00
G    H
ATOM  21374  C   ALA G  55       4.713 -17.486  31.722  1.00  0.00
G    C
ATOM  21375  O   ALA G  55       5.600 -17.582  32.573  1.00  0.00
G    O
ATOM  21376  N   ASN G  56       3.416 -17.499  32.007  1.00  0.00
G    N
ATOM  21377  HN  ASN G  56       2.712 -17.398  31.306  1.00  0.00
G    H
ATOM  21378  CA  ASN G  56       2.901 -17.662  33.362  1.00  0.00
G    C
ATOM  21379  HA  ASN G  56       3.671 -17.314  34.042  1.00  0.00
G    H
ATOM  21380  CB  ASN G  56       1.603 -16.887  33.529  1.00  0.00
G    C
ATOM  21381  HB1 ASN G  56       1.819 -15.815  33.329  1.00  0.00
G    H
ATOM  21382  HB2 ASN G  56       0.856 -17.221  32.782  1.00  0.00
G    H
ATOM  21383  CG  ASN G  56       1.034 -16.998  34.923  1.00  0.00
G    C
```

```
ATOM  21384  OD1 ASN G  56       1.447 -17.841  35.721  1.00  0.00
G    O
ATOM  21385  ND2 ASN G  56       0.067 -16.143  35.226  1.00  0.00
G    N
ATOM  21386 HD21 ASN G  56      -0.316 -16.192  36.145  1.00  0.00
G    H
ATOM  21387 HD22 ASN G  56      -0.230 -15.469  34.557  1.00  0.00
G    H
ATOM  21388  C   ASN G  56       2.699 -19.147  33.615  1.00  0.00
G    C
ATOM  21389  O   ASN G  56       1.852 -19.785  32.991  1.00  0.00
G    O
ATOM  21390  N   ALA G  57       3.454 -19.689  34.568  1.00  0.00
G    N
ATOM  21391  HN  ALA G  57       4.050 -19.148  35.161  1.00  0.00
G    H
ATOM  21392  CA  ALA G  57       3.465 -21.125  34.813  1.00  0.00
G    C
ATOM  21393  HA  ALA G  57       3.766 -21.644  33.910  1.00  0.00
G    H
ATOM  21394  CB  ALA G  57       4.456 -21.456  35.921  1.00  0.00
G    C
ATOM  21395  HB1 ALA G  57       5.468 -21.098  35.636  1.00  0.00
G    H
ATOM  21396  HB2 ALA G  57       4.173 -20.956  36.874  1.00  0.00
G    H
ATOM  21397  HB3 ALA G  57       4.518 -22.552  36.101  1.00  0.00
G    H
ATOM  21398  C   ALA G  57       2.108 -21.683  35.195  1.00  0.00
G    C
ATOM  21399  O   ALA G  57       1.893 -22.889  35.052  1.00  0.00
G    O
ATOM  21400  N   THR G  58       1.193 -20.855  35.664  1.00  0.00
G    N
ATOM  21401  HN  THR G  58       1.244 -19.857  35.634  1.00  0.00
G    H
ATOM  21402  CA  THR G  58      -0.010 -21.372  36.285  1.00  0.00
G    C
ATOM  21403  HA  THR G  58       0.319 -22.239  36.842  1.00  0.00
G    H
ATOM  21404  CB  THR G  58      -0.616 -20.297  37.183  1.00  0.00
G    C
ATOM  21405  HB  THR G  58      -1.502 -20.767  37.675  1.00  0.00
G    H
ATOM  21406  OG1 THR G  58      -1.078 -19.213  36.376  1.00  0.00
G    O
ATOM  21407  HG1 THR G  58      -1.437 -18.567  36.995  1.00  0.00
G    H
ATOM  21408  CG2 THR G  58       0.432 -19.769  38.140  1.00  0.00
G    C
ATOM  21409 HG21 THR G  58      -0.019 -19.070  38.875  1.00  0.00
G    H
ATOM  21410 HG22 THR G  58       0.897 -20.605  38.705  1.00  0.00
G    H
```

```
ATOM  21411 HG23 THR G  58       1.236 -19.229  37.595  1.00  0.00
G    H
ATOM  21412  C   THR G  58      -1.053 -21.865  35.294  1.00  0.00
G    C
ATOM  21413  O   THR G  58      -2.079 -22.399  35.723  1.00  0.00
G    O
ATOM  21414  N   GLU G  59      -0.833 -21.708  33.993  1.00  0.00
G    N
ATOM  21415  HN  GLU G  59       0.006 -21.342  33.600  1.00  0.00
G    H
ATOM  21416  CA  GLU G  59      -1.841 -22.075  33.003  1.00  0.00
G    C
ATOM  21417  HA  GLU G  59      -2.749 -22.346  33.526  1.00  0.00
G    H
ATOM  21418  CB  GLU G  59      -2.110 -20.912  32.054  1.00  0.00
G    C
ATOM  21419  HB1 GLU G  59      -1.133 -20.559  31.651  1.00  0.00
G    H
ATOM  21420  HB2 GLU G  59      -2.691 -21.271  31.174  1.00  0.00
G    H
ATOM  21421  CG  GLU G  59      -2.820 -19.745  32.680  1.00  0.00
G    C
ATOM  21422  HG1 GLU G  59      -3.076 -19.006  31.899  1.00  0.00
G    H
ATOM  21423  HG2 GLU G  59      -3.748 -20.071  33.190  1.00  0.00
G    H
ATOM  21424  CD  GLU G  59      -1.949 -19.020  33.669  1.00  0.00
G    C
ATOM  21425  OE1 GLU G  59      -2.451 -18.657  34.749  1.00  0.00
G    O
ATOM  21426  OE2 GLU G  59      -0.748 -18.863  33.395  1.00  0.00
G    O
ATOM  21427  C   GLU G  59      -1.397 -23.294  32.204  1.00  0.00
G    C
ATOM  21428  O   GLU G  59      -0.300 -23.310  31.638  1.00  0.00
G    O
ATOM  21429  N   GLU G  60      -2.283 -24.287  32.113  1.00  0.00
G    N
ATOM  21430  HN  GLU G  60      -3.228 -24.244  32.426  1.00  0.00
G    H
ATOM  21431  CA  GLU G  60      -1.913 -25.566  31.517  1.00  0.00
G    C
ATOM  21432  HA  GLU G  60      -1.014 -25.914  32.010  1.00  0.00
G    H
ATOM  21433  CB  GLU G  60      -3.021 -26.594  31.766  1.00  0.00
G    C
ATOM  21434  HB1 GLU G  60      -2.766 -27.522  31.203  1.00  0.00
G    H
ATOM  21435  HB2 GLU G  60      -3.032 -26.883  32.841  1.00  0.00
G    H
ATOM  21436  CG  GLU G  60      -4.448 -26.199  31.366  1.00  0.00
G    C
ATOM  21437  HG1 GLU G  60      -5.118 -27.071  31.476  1.00  0.00
G    H
```

```
ATOM  21438  HG2 GLU G  60      -4.823 -25.370  31.999  1.00  0.00
G    H
ATOM  21439  CD  GLU G  60      -4.608 -25.773  29.925  1.00  0.00
G    C
ATOM  21440  OE1 GLU G  60      -4.198 -26.538  29.030  1.00  0.00
G    O
ATOM  21441  OE2 GLU G  60      -5.103 -24.652  29.690  1.00  0.00
G    O
ATOM  21442  C   GLU G  60      -1.592 -25.427  30.038  1.00  0.00
G    C
ATOM  21443  O   GLU G  60      -0.772 -26.176  29.499  1.00  0.00
G    O
ATOM  21444  N   ASN G  61      -2.244 -24.491  29.355  1.00  0.00
G    N
ATOM  21445  HN  ASN G  61      -2.879 -23.822  29.739  1.00  0.00
G    H
ATOM  21446  CA  ASN G  61      -2.054 -24.387  27.916  1.00  0.00
G    C
ATOM  21447  HA  ASN G  61      -2.105 -25.400  27.531  1.00  0.00
G    H
ATOM  21448  CB  ASN G  61      -3.146 -23.517  27.312  1.00  0.00
G    C
ATOM  21449  HB1 ASN G  61      -2.809 -23.203  26.300  1.00  0.00
G    H
ATOM  21450  HB2 ASN G  61      -4.079 -24.102  27.186  1.00  0.00
G    H
ATOM  21451  CG  ASN G  61      -3.399 -22.291  28.127  1.00  0.00
G    C
ATOM  21452  OD1 ASN G  61      -3.182 -22.289  29.337  1.00  0.00
G    O
ATOM  21453  ND2 ASN G  61      -3.867 -21.235  27.480  1.00  0.00
G    N
ATOM  21454 HD21 ASN G  61      -4.030 -20.408  28.011  1.00  0.00
G    H
ATOM  21455 HD22 ASN G  61      -4.015 -21.278  26.496  1.00  0.00
G    H
ATOM  21456  C   ASN G  61      -0.676 -23.844  27.599  1.00  0.00
G    C
ATOM  21457  O   ASN G  61      -0.035 -24.278  26.638  1.00  0.00
G    O
ATOM  21458  N   GLU G  62      -0.197 -22.891  28.399  1.00  0.00
G    N
ATOM  21459  HN  GLU G  62      -0.704 -22.368  29.080  1.00  0.00
G    H
ATOM  21460  CA  GLU G  62       1.210 -22.538  28.309  1.00  0.00
G    C
ATOM  21461  HA  GLU G  62       1.392 -22.122  27.326  1.00  0.00
G    H
ATOM  21462  CB  GLU G  62       1.586 -21.515  29.372  1.00  0.00
G    C
ATOM  21463  HB1 GLU G  62       1.061 -21.787  30.317  1.00  0.00
G    H
ATOM  21464  HB2 GLU G  62       2.673 -21.586  29.595  1.00  0.00
G    H
```

ATOM 21465 CG GLU G 62   1.230 -20.105 29.003 1.00 0.00
G   C
ATOM 21466 HG1 GLU G 62   2.040 -19.668 28.391 1.00 0.00
G   H
ATOM 21467 HG2 GLU G 62   0.282 -20.072 28.430 1.00 0.00
G   H
ATOM 21468 CD GLU G 62   1.095 -19.206 30.203 1.00 0.00
G   C
ATOM 21469 OE1 GLU G 62   2.029 -18.425 30.465 1.00 0.00
G   O
ATOM 21470 OE2 GLU G 62   0.053 -19.267 30.879 1.00 0.00
G   O
ATOM 21471 C   GLU G 62   2.089 -23.764 28.449 1.00 0.00
G   C
ATOM 21472 O   GLU G 62   3.094 -23.894 27.744 1.00 0.00
G   O
ATOM 21473 N   LEU G 63   1.707 -24.682 29.324 1.00 0.00
G   N
ATOM 21474 HN LEU G 63   0.865 -24.653 29.860 1.00 0.00
G   H
ATOM 21475 CA LEU G 63   2.531 -25.850 29.571 1.00 0.00
G   C
ATOM 21476 HA LEU G 63   3.538 -25.488 29.733 1.00 0.00
G   H
ATOM 21477 CB LEU G 63   2.001 -26.581 30.792 1.00 0.00
G   C
ATOM 21478 HB1 LEU G 63   1.079 -27.123 30.478 1.00 0.00
G   H
ATOM 21479 HB2 LEU G 63   2.731 -27.366 31.091 1.00 0.00
G   H
ATOM 21480 CG LEU G 63   1.687 -25.625 31.933 1.00 0.00
G   C
ATOM 21481 HG LEU G 63   0.910 -24.920 31.550 1.00 0.00
G   H
ATOM 21482 CD1 LEU G 63   1.138 -26.382 33.118 1.00 0.00
G   C
ATOM 21483 HD11 LEU G 63   0.953 -25.693 33.970 1.00 0.00
G   H
ATOM 21484 HD12 LEU G 63   0.179 -26.878 32.856 1.00 0.00
G   H
ATOM 21485 HD13 LEU G 63   1.855 -27.161 33.453 1.00 0.00
G   H
ATOM 21486 CD2 LEU G 63   2.903 -24.812 32.323 1.00 0.00
G   C
ATOM 21487 HD21 LEU G 63   2.650 -24.107 33.145 1.00 0.00
G   H
ATOM 21488 HD22 LEU G 63   3.731 -25.459 32.679 1.00 0.00
G   H
ATOM 21489 HD23 LEU G 63   3.270 -24.220 31.457 1.00 0.00
G   H
ATOM 21490 C   LEU G 63   2.565 -26.772 28.364 1.00 0.00
G   C
ATOM 21491 O   LEU G 63   3.645 -27.116 27.872 1.00 0.00
G   O

ATOM 21492 N LEU G 64 1.393 -27.181 27.879 1.00 0.00
G N
ATOM 21493 HN LEU G 64 0.499 -26.968 28.268 1.00 0.00
G H
ATOM 21494 CA LEU G 64 1.344 -28.011 26.684 1.00 0.00
G C
ATOM 21495 HA LEU G 64 1.885 -28.920 26.912 1.00 0.00
G H
ATOM 21496 CB LEU G 64 -0.100 -28.308 26.311 1.00 0.00
G C
ATOM 21497 HB1 LEU G 64 -0.479 -27.431 25.738 1.00 0.00
G H
ATOM 21498 HB2 LEU G 64 -0.122 -29.169 25.606 1.00 0.00
G H
ATOM 21499 CG LEU G 64 -0.982 -28.551 27.521 1.00 0.00
G C
ATOM 21500 HG LEU G 64 -0.776 -27.752 28.274 1.00 0.00
G H
ATOM 21501 CD1 LEU G 64 -2.436 -28.491 27.118 1.00 0.00
G C
ATOM 21502 HD11 LEU G 64 -3.091 -28.715 27.987 1.00 0.00
G H
ATOM 21503 HD12 LEU G 64 -2.695 -27.480 26.737 1.00 0.00
G H
ATOM 21504 HD13 LEU G 64 -2.653 -29.233 26.320 1.00 0.00
G H
ATOM 21505 CD2 LEU G 64 -0.638 -29.883 28.145 1.00 0.00
G C
ATOM 21506 HD21 LEU G 64 -1.267 -30.063 29.044 1.00 0.00
G H
ATOM 21507 HD22 LEU G 64 -0.811 -30.722 27.441 1.00 0.00
G H
ATOM 21508 HD23 LEU G 64 0.428 -29.903 28.459 1.00 0.00
G H
ATOM 21509 C LEU G 64 2.049 -27.337 25.522 1.00 0.00
G C
ATOM 21510 O LEU G 64 2.812 -27.982 24.794 1.00 0.00
G O
ATOM 21511 N SER G 65 1.813 -26.038 25.342 1.00 0.00
G N
ATOM 21512 HN SER G 65 1.188 -25.483 25.891 1.00 0.00
G H
ATOM 21513 CA SER G 65 2.483 -25.304 24.279 1.00 0.00
G C
ATOM 21514 HA SER G 65 2.185 -25.779 23.353 1.00 0.00
G H
ATOM 21515 CB SER G 65 2.085 -23.837 24.333 1.00 0.00
G C
ATOM 21516 HB1 SER G 65 2.650 -23.297 23.539 1.00 0.00
G H
ATOM 21517 HB2 SER G 65 1.003 -23.720 24.100 1.00 0.00
G H
ATOM 21518 OG SER G 65 2.415 -23.298 25.594 1.00 0.00
G O

ATOM 21519 HG1 SER G 65   2.359 -22.338 25.516 1.00 0.00
G   H
ATOM 21520 C   SER G 65   3.990 -25.437 24.390 1.00 0.00
G   C
ATOM 21521 O   SER G 65   4.691 -25.441 23.375 1.00 0.00
G   O
ATOM 21522 N   LEU G 66   4.502 -25.561 25.610 1.00 0.00
G   N
ATOM 21523 HN LEU G 66   3.986 -25.547 26.463 1.00 0.00
G   H
ATOM 21524 CA LEU G 66   5.932 -25.735 25.782 1.00 0.00
G   C
ATOM 21525 HA LEU G 66   6.404 -25.060 25.080 1.00 0.00
G   H
ATOM 21526 CB LEU G 66   6.329 -25.395 27.209 1.00 0.00
G   C
ATOM 21527 HB1 LEU G 66   5.647 -25.961 27.884 1.00 0.00
G   H
ATOM 21528 HB2 LEU G 66   7.351 -25.790 27.403 1.00 0.00
G   H
ATOM 21529 CG LEU G 66   6.230 -23.907 27.496 1.00 0.00
G   C
ATOM 21530 HG LEU G 66   5.151 -23.618 27.512 1.00 0.00
G   H
ATOM 21531 CD1 LEU G 66   6.849 -23.608 28.833 1.00 0.00
G   C
ATOM 21532 HD11 LEU G 66   6.827 -22.516 29.037 1.00 0.00
G   H
ATOM 21533 HD12 LEU G 66   6.295 -24.126 29.645 1.00 0.00
G   H
ATOM 21534 HD13 LEU G 66   7.906 -23.945 28.859 1.00 0.00
G   H
ATOM 21535 CD2 LEU G 66   6.910 -23.118 26.395 1.00 0.00
G   C
ATOM 21536 HD21 LEU G 66   6.822 -22.027 26.592 1.00 0.00
G   H
ATOM 21537 HD22 LEU G 66   7.989 -23.363 26.323 1.00 0.00
G   H
ATOM 21538 HD23 LEU G 66   6.436 -23.334 25.413 1.00 0.00
G   H
ATOM 21539 C   LEU G 66   6.388 -27.141 25.445 1.00 0.00
G   C
ATOM 21540 O   LEU G 66   7.595 -27.386 25.398 1.00 0.00
G   O
ATOM 21541 N   GLY G 67   5.464 -28.062 25.205 1.00 0.00
G   N
ATOM 21542 HN GLY G 67   4.482 -27.881 25.154 1.00 0.00
G   H
ATOM 21543 CA GLY G 67   5.857 -29.439 24.995 1.00 0.00
G   C
ATOM 21544 HA1 GLY G 67   6.534 -29.472 24.152 1.00 0.00
G   H
ATOM 21545 HA2 GLY G 67   4.963 -30.035 24.870 1.00 0.00
G   H

ATOM 21546 C   GLY G 67   6.595 -29.955 26.208 1.00 0.00
G   C
ATOM 21547 O   GLY G 67   7.638 -30.608 26.102 1.00 0.00
G   O
ATOM 21548 N   ILE G 68   6.049 -29.637 27.379 1.00 0.00
G   N
ATOM 21549 HN ILE G 68   5.179 -29.162 27.486 1.00 0.00
G   H
ATOM 21550 CA ILE G 68   6.704 -29.970 28.635 1.00 0.00
G   C
ATOM 21551 HA ILE G 68   7.675 -29.492 28.606 1.00 0.00
G   H
ATOM 21552 CB ILE G 68   5.844 -29.463 29.795 1.00 0.00
G   C
ATOM 21553 HB ILE G 68   6.230 -30.012 30.690 1.00 0.00
G   H
ATOM 21554 CG2 ILE G 68   5.908 -27.964 29.864 1.00 0.00
G   C
ATOM 21555 HG21 ILE G 68   5.418 -27.594 30.789 1.00 0.00
G   H
ATOM 21556 HG22 ILE G 68   6.961 -27.612 29.878 1.00 0.00
G   H
ATOM 21557 HG23 ILE G 68   5.395 -27.504 28.993 1.00 0.00
G   H
ATOM 21558 CG1 ILE G 68   4.406 -29.907 29.573 1.00 0.00
G   C
ATOM 21559 HG11 ILE G 68   3.998 -29.420 28.660 1.00 0.00
G   H
ATOM 21560 HG12 ILE G 68   4.392 -31.007 29.393 1.00 0.00
G   H
ATOM 21561 CD ILE G 68   3.519 -29.615 30.714 1.00 0.00
G   C
ATOM 21562 HD1 ILE G 68   2.499 -30.011 30.521 1.00 0.00
G   H
ATOM 21563 HD2 ILE G 68   3.907 -30.087 31.642 1.00 0.00
G   H
ATOM 21564 HD3 ILE G 68   3.436 -28.521 30.883 1.00 0.00
G   H
ATOM 21565 C   ILE G 68   6.954 -31.462 28.752 1.00 0.00
G   C
ATOM 21566 O   ILE G 68   8.030 -31.888 29.186 1.00 0.00
G   O
ATOM 21567 N   ARG G 69   5.975 -32.280 28.370 1.00 0.00
G   N
ATOM 21568 HN ARG G 69   5.132 -31.949 27.952 1.00 0.00
G   H
ATOM 21569 CA ARG G 69   6.095 -33.717 28.550 1.00 0.00
G   C
ATOM 21570 HA ARG G 69   6.203 -33.912 29.609 1.00 0.00
G   H
ATOM 21571 CB ARG G 69   4.850 -34.399 28.015 1.00 0.00
G   C
ATOM 21572 HB1 ARG G 69   4.763 -34.099 26.943 1.00 0.00
G   H

```
ATOM  21573  HB2 ARG G  69       5.002 -35.501  28.015  1.00  0.00
G    H
ATOM  21574  CG  ARG G  69       3.611 -33.989  28.758  1.00  0.00
G    C
ATOM  21575  HG1 ARG G  69       3.653 -34.420  29.782  1.00  0.00
G    H
ATOM  21576  HG2 ARG G  69       3.622 -32.883  28.898  1.00  0.00
G    H
ATOM  21577  CD  ARG G  69       2.379 -34.381  27.994  1.00  0.00
G    C
ATOM  21578  HD1 ARG G  69       2.447 -35.455  27.708  1.00  0.00
G    H
ATOM  21579  HD2 ARG G  69       1.456 -34.230  28.601  1.00  0.00
G    H
ATOM  21580  NE  ARG G  69       2.231 -33.572  26.792  1.00  0.00
G    N
ATOM  21581  HE  ARG G  69       1.833 -32.665  26.972  1.00  0.00
G    H
ATOM  21582  CZ  ARG G  69       2.582 -33.978  25.579  1.00  0.00
G    C
ATOM  21583  NH1 ARG G  69       3.109 -35.180  25.406  1.00  0.00
G    N
ATOM  21584 HH11 ARG G  69       3.396 -35.480  24.507  1.00  0.00
G    H
ATOM  21585 HH12 ARG G  69       3.322 -35.696  26.239  1.00  0.00
G    H
ATOM  21586  NH2 ARG G  69       2.412 -33.180  24.537  1.00  0.00
G    N
ATOM  21587 HH21 ARG G  69       2.593 -33.515  23.624  1.00  0.00
G    H
ATOM  21588 HH22 ARG G  69       1.910 -32.339  24.694  1.00  0.00
G    H
ATOM  21589  C   ARG G  69       7.329 -34.277  27.878  1.00  0.00
G    C
ATOM  21590  O   ARG G  69       7.864 -35.285  28.346  1.00  0.00
G    O
ATOM  21591  N   ASN G  70       7.798 -33.641  26.810  1.00  0.00
G    N
ATOM  21592  HN  ASN G  70       7.333 -32.896  26.333  1.00  0.00
G    H
ATOM  21593  CA  ASN G  70       9.081 -34.010  26.241  1.00  0.00
G    C
ATOM  21594  HA  ASN G  70       8.984 -35.049  25.945  1.00  0.00
G    H
ATOM  21595  CB  ASN G  70       9.404 -33.107  25.056  1.00  0.00
G    C
ATOM  21596  HB1 ASN G  70       9.680 -32.108  25.456  1.00  0.00
G    H
ATOM  21597  HB2 ASN G  70      10.281 -33.499  24.501  1.00  0.00
G    H
ATOM  21598  CG  ASN G  70       8.228 -32.937  24.131  1.00  0.00
G    C
ATOM  21599  OD1 ASN G  70       7.281 -33.720  24.166  1.00  0.00
G    O
```

```
ATOM  21600  ND2 ASN G  70       8.277 -31.912  23.293  1.00  0.00
G    N
ATOM  21601 HD21 ASN G  70       7.501 -31.782  22.684  1.00  0.00
G    H
ATOM  21602 HD22 ASN G  70       9.055 -31.292  23.311  1.00  0.00
G    H
ATOM  21603  C   ASN G  70      10.188 -33.929  27.273  1.00  0.00
G    C
ATOM  21604  O   ASN G  70      11.102 -34.759  27.258  1.00  0.00
G    O
ATOM  21605  N   PHE G  71      10.108 -32.971  28.183  1.00  0.00
G    N
ATOM  21606  HN  PHE G  71       9.368 -32.301  28.219  1.00  0.00
G    H
ATOM  21607  CA  PHE G  71      11.110 -32.817  29.221  1.00  0.00
G    C
ATOM  21608  HA  PHE G  71      12.078 -32.945  28.751  1.00  0.00
G    H
ATOM  21609  CB  PHE G  71      10.972 -31.473  29.920  1.00  0.00
G    C
ATOM  21610  HB1 PHE G  71       9.950 -31.389  30.349  1.00  0.00
G    H
ATOM  21611  HB2 PHE G  71      11.689 -31.386  30.765  1.00  0.00
G    H
ATOM  21612  CG  PHE G  71      11.137 -30.308  29.014  1.00  0.00
G    C
ATOM  21613  CD1 PHE G  71      10.135 -29.958  28.129  1.00  0.00
G    C
ATOM  21614  HD1 PHE G  71       9.227 -30.544  28.090  1.00  0.00
G    H
ATOM  21615  CE1 PHE G  71      10.278 -28.886  27.299  1.00  0.00
G    C
ATOM  21616  HE1 PHE G  71       9.486 -28.624  26.613  1.00  0.00
G    H
ATOM  21617  CZ  PHE G  71      11.435 -28.134  27.345  1.00  0.00
G    C
ATOM  21618  HZ  PHE G  71      11.548 -27.278  26.694  1.00  0.00
G    H
ATOM  21619  CD2 PHE G  71      12.291 -29.549  29.056  1.00  0.00
G    C
ATOM  21620  HD2 PHE G  71      13.091 -29.819  29.731  1.00  0.00
G    H
ATOM  21621  CE2 PHE G  71      12.444 -28.468  28.227  1.00  0.00
G    C
ATOM  21622  HE2 PHE G  71      13.354 -27.884  28.267  1.00  0.00
G    H
ATOM  21623  C   PHE G  71      10.955 -33.937  30.235  1.00  0.00
G    C
ATOM  21624  O   PHE G  71       9.873 -34.516  30.368  1.00  0.00
G    O
ATOM  21625  N   GLU G  72      12.038 -34.229  30.951  1.00  0.00
G    N
ATOM  21626  HN  GLU G  72      12.930 -33.797  30.854  1.00  0.00
G    H
```

ATOM 21627 CA GLU G 72   11.986 -35.257 31.981 1.00 0.00
G   C
ATOM 21628 HA GLU G 72   10.990 -35.681 31.985 1.00 0.00
G   H
ATOM 21629 CB GLU G 72   13.014 -36.328 31.673 1.00 0.00
G   C
ATOM 21630 HB1 GLU G 72   13.728 -36.381 32.528 1.00 0.00
G   H
ATOM 21631 HB2 GLU G 72   12.518 -37.325 31.629 1.00 0.00
G   H
ATOM 21632 CG GLU G 72   13.768 -36.052 30.398 1.00 0.00
G   C
ATOM 21633 HG1 GLU G 72   13.053 -35.813 29.591 1.00 0.00
G   H
ATOM 21634 HG2 GLU G 72   14.470 -35.204 30.527 1.00 0.00
G   H
ATOM 21635 CD GLU G 72   14.543 -37.254 29.922 1.00 0.00
G   C
ATOM 21636 OE1 GLU G 72   15.301 -37.834 30.728 1.00 0.00
G   O
ATOM 21637 OE2 GLU G 72   14.393 -37.618 28.735 1.00 0.00
G   O
ATOM 21638 C   GLU G 72   12.230 -34.665 33.360 1.00 0.00
G   C
ATOM 21639 O   GLU G 72   11.629 -35.104 34.349 1.00 0.00
G   O
ATOM 21640 N   TYR G 73   13.112 -33.671 33.450 1.00 0.00
G   N
ATOM 21641 HN TYR G 73   13.632 -33.320 32.673 1.00 0.00
G   H
ATOM 21642 CA TYR G 73   13.392 -33.007 34.715 1.00 0.00
G   C
ATOM 21643 HA TYR G 73   12.916 -33.584 35.498 1.00 0.00
G   H
ATOM 21644 CB TYR G 73   14.891 -32.853 34.957 1.00 0.00
G   C
ATOM 21645 HB1 TYR G 73   15.374 -32.481 34.027 1.00 0.00
G   H
ATOM 21646 HB2 TYR G 73   15.085 -32.101 35.752 1.00 0.00
G   H
ATOM 21647 CG TYR G 73   15.618 -34.119 35.317 1.00 0.00
G   C
ATOM 21648 CD1 TYR G 73   15.814 -35.122 34.382 1.00 0.00
G   C
ATOM 21649 HD1 TYR G 73   15.418 -35.009 33.382 1.00 0.00
G   H
ATOM 21650 CE1 TYR G 73   16.486 -36.274 34.710 1.00 0.00
G   C
ATOM 21651 HE1 TYR G 73   16.632 -37.053 33.976 1.00 0.00
G   H
ATOM 21652 CZ TYR G 73   16.970 -36.432 35.987 1.00 0.00
G   C
ATOM 21653 OH TYR G 73   17.642 -37.577 36.332 1.00 0.00
G   O

```
ATOM  21654  HH  TYR G  73      17.958 -37.466  37.231  1.00  0.00
G    H
ATOM  21655  CD2 TYR G  73      16.121 -34.305  36.591  1.00  0.00
G    C
ATOM  21656  HD2 TYR G  73      15.955 -33.556  37.353  1.00  0.00
G    H
ATOM  21657  CE2 TYR G  73      16.791 -35.451  36.927  1.00  0.00
G    C
ATOM  21658  HE2 TYR G  73      17.154 -35.583  37.936  1.00  0.00
G    H
ATOM  21659  C   TYR G  73      12.740 -31.639  34.708  1.00  0.00
G    C
ATOM  21660  O   TYR G  73      12.880 -30.888  33.737  1.00  0.00
G    O
ATOM  21661  N   VAL G  74      12.050 -31.301  35.792  1.00  0.00
G    N
ATOM  21662  HN  VAL G  74      11.884 -31.929  36.549  1.00  0.00
G    H
ATOM  21663  CA  VAL G  74      11.474 -29.977  35.970  1.00  0.00
G    C
ATOM  21664  HA  VAL G  74      11.933 -29.300  35.260  1.00  0.00
G    H
ATOM  21665  CB  VAL G  74       9.950 -29.973  35.750  1.00  0.00
G    C
ATOM  21666  HB  VAL G  74       9.597 -28.918  35.859  1.00  0.00
G    H
ATOM  21667  CG1 VAL G  74       9.623 -30.470  34.360  1.00  0.00
G    C
ATOM  21668 HG11 VAL G  74       8.522 -30.504  34.212  1.00  0.00
G    H
ATOM  21669 HG12 VAL G  74      10.041 -29.804  33.577  1.00  0.00
G    H
ATOM  21670 HG13 VAL G  74      10.023 -31.495  34.205  1.00  0.00
G    H
ATOM  21671  CG2 VAL G  74       9.243 -30.814  36.793  1.00  0.00
G    C
ATOM  21672 HG21 VAL G  74       8.149 -30.837  36.602  1.00  0.00
G    H
ATOM  21673 HG22 VAL G  74       9.623 -31.858  36.776  1.00  0.00
G    H
ATOM  21674 HG23 VAL G  74       9.397 -30.404  37.812  1.00  0.00
G    H
ATOM  21675  C   VAL G  74      11.827 -29.485  37.363  1.00  0.00
G    C
ATOM  21676  O   VAL G  74      11.807 -30.254  38.333  1.00  0.00
G    O
ATOM  21677  N   ILE G  75      12.161 -28.208  37.456  1.00  0.00
G    N
ATOM  21678  HN  ILE G  75      12.147 -27.587  36.676  1.00  0.00
G    H
ATOM  21679  CA  ILE G  75      12.579 -27.590  38.701  1.00  0.00
G    C
ATOM  21680  HA  ILE G  75      12.310 -28.256  39.510  1.00  0.00
G    H
```

```
ATOM  21681  CB  ILE G  75      14.072 -27.263  38.691  1.00  0.00
G    C
ATOM  21682  HB  ILE G  75      14.131 -26.214  39.076  1.00  0.00
G    H
ATOM  21683  CG2 ILE G  75      14.815 -28.270  39.521  1.00  0.00
G    C
ATOM  21684 HG21 ILE G  75      15.873 -27.963  39.654  1.00  0.00
G    H
ATOM  21685 HG22 ILE G  75      14.361 -28.363  40.531  1.00  0.00
G    H
ATOM  21686 HG23 ILE G  75      14.802 -29.270  39.039  1.00  0.00
G    H
ATOM  21687  CG1 ILE G  75      14.560 -27.248  37.249  1.00  0.00
G    C
ATOM  21688 HG11 ILE G  75      13.855 -27.821  36.607  1.00  0.00
G    H
ATOM  21689 HG12 ILE G  75      14.562 -26.197  36.880  1.00  0.00
G    H
ATOM  21690  CD  ILE G  75      15.931 -27.799  37.062  1.00  0.00
G    C
ATOM  21691  HD1 ILE G  75      16.243 -27.704  36.000  1.00  0.00
G    H
ATOM  21692  HD2 ILE G  75      16.664 -27.249  37.691  1.00  0.00
G    H
ATOM  21693  HD3 ILE G  75      15.969 -28.874  37.336  1.00  0.00
G    H
ATOM  21694  C   ILE G  75      11.763 -26.332  38.881  1.00  0.00
G    C
ATOM  21695  O   ILE G  75      11.933 -25.362  38.136  1.00  0.00
G    O
ATOM  21696  N   VAL G  76      10.896 -26.340  39.875  1.00  0.00
G    N
ATOM  21697  HN  VAL G  76      10.737 -27.131  40.461  1.00  0.00
G    H
ATOM  21698  CA  VAL G  76      10.087 -25.180  40.202  1.00  0.00
G    C
ATOM  21699  HA  VAL G  76       9.964 -24.556  39.325  1.00  0.00
G    H
ATOM  21700  CB  VAL G  76       8.719 -25.582  40.723  1.00  0.00
G    C
ATOM  21701  HB  VAL G  76       8.871 -26.127  41.686  1.00  0.00
G    H
ATOM  21702  CG1 VAL G  76       7.888 -24.368  40.964  1.00  0.00
G    C
ATOM  21703 HG11 VAL G  76       6.872 -24.659  41.308  1.00  0.00
G    H
ATOM  21704 HG12 VAL G  76       8.329 -23.720  41.749  1.00  0.00
G    H
ATOM  21705 HG13 VAL G  76       7.779 -23.775  40.031  1.00  0.00
G    H
ATOM  21706  CG2 VAL G  76       8.051 -26.479  39.716  1.00  0.00
G    C
ATOM  21707 HG21 VAL G  76       7.029 -26.757  40.054  1.00  0.00
G    H
```

```
ATOM  21708 HG22 VAL G  76       7.967 -25.968  38.733  1.00  0.00
G    H
ATOM  21709 HG23 VAL G  76       8.624 -27.418  39.573  1.00  0.00
G    H
ATOM  21710  C   VAL G  76      10.906 -24.388  41.214  1.00  0.00
G    C
ATOM  21711  O   VAL G  76      10.870 -24.638  42.425  1.00  0.00
G    O
ATOM  21712  N   ALA G  77      11.694 -23.452  40.699  1.00  0.00
G    N
ATOM  21713  HN  ALA G  77      11.738 -23.291  39.714  1.00  0.00
G    H
ATOM  21714  CA  ALA G  77      12.550 -22.593  41.512  1.00  0.00
G    C
ATOM  21715  HA  ALA G  77      12.780 -23.071  42.458  1.00  0.00
G    H
ATOM  21716  CB  ALA G  77      13.868 -22.330  40.801  1.00  0.00
G    C
ATOM  21717  HB1 ALA G  77      14.387 -23.293  40.608  1.00  0.00
G    H
ATOM  21718  HB2 ALA G  77      13.702 -21.829  39.823  1.00  0.00
G    H
ATOM  21719  HB3 ALA G  77      14.544 -21.694  41.414  1.00  0.00
G    H
ATOM  21720  C   ALA G  77      11.853 -21.291  41.849  1.00  0.00
G    C
ATOM  21721  O   ALA G  77      12.377 -20.204  41.619  1.00  0.00
G    O
ATOM  21722  N   ILE G  78      10.653 -21.390  42.387  1.00  0.00
G    N
ATOM  21723  HN  ILE G  78      10.170 -22.258  42.484  1.00  0.00
G    H
ATOM  21724  CA  ILE G  78       9.919 -20.243  42.888  1.00  0.00
G    C
ATOM  21725  HA  ILE G  78      10.379 -19.344  42.499  1.00  0.00
G    H
ATOM  21726  CB  ILE G  78       8.437 -20.340  42.526  1.00  0.00
G    C
ATOM  21727  HB  ILE G  78       8.407 -20.167  41.420  1.00  0.00
G    H
ATOM  21728  CG2 ILE G  78       7.888 -21.637  43.001  1.00  0.00
G    C
ATOM  21729 HG21 ILE G  78       6.860 -21.792  42.612  1.00  0.00
G    H
ATOM  21730 HG22 ILE G  78       8.513 -22.484  42.649  1.00  0.00
G    H
ATOM  21731 HG23 ILE G  78       7.848 -21.670  44.110  1.00  0.00
G    H
ATOM  21732  CG1 ILE G  78       7.642 -19.213  43.161  1.00  0.00
G    C
ATOM  21733 HG11 ILE G  78       7.762 -19.244  44.268  1.00  0.00
G    H
ATOM  21734 HG12 ILE G  78       8.055 -18.238  42.813  1.00  0.00
G    H
```

```
ATOM  21735  CD  ILE G  78       6.191 -19.274  42.809  1.00  0.00
G    C
ATOM  21736  HD1 ILE G  78       5.651 -18.404  43.242  1.00  0.00
G    H
ATOM  21737  HD2 ILE G  78       6.054 -19.255  41.706  1.00  0.00
G    H
ATOM  21738  HD3 ILE G  78       5.723 -20.200  43.206  1.00  0.00
G    H
ATOM  21739  C   ILE G  78      10.134 -20.230  44.392  1.00  0.00
G    C
ATOM  21740  O   ILE G  78      10.323 -21.288  45.000  1.00  0.00
G    O
ATOM  21741  N   GLY G  79      10.145 -19.048  45.001  1.00  0.00
G    N
ATOM  21742  HN  GLY G  79      10.074 -18.163  44.545  1.00  0.00
G    H
ATOM  21743  CA  GLY G  79      10.268 -19.001  46.444  1.00  0.00
G    C
ATOM  21744  HA1 GLY G  79      11.237 -18.563  46.642  1.00  0.00
G    H
ATOM  21745  HA2 GLY G  79      10.163 -20.021  46.787  1.00  0.00
G    H
ATOM  21746  C   GLY G  79       9.225 -18.164  47.149  1.00  0.00
G    C
ATOM  21747  O   GLY G  79       8.954 -18.371  48.335  1.00  0.00
G    O
ATOM  21748  N   ALA G  80       8.627 -17.215  46.437  1.00  0.00
G    N
ATOM  21749  HN  ALA G  80       8.745 -17.069  45.456  1.00  0.00
G    H
ATOM  21750  CA  ALA G  80       7.718 -16.293  47.102  1.00  0.00
G    C
ATOM  21751  HA  ALA G  80       7.959 -16.242  48.158  1.00  0.00
G    H
ATOM  21752  CB  ALA G  80       7.876 -14.890  46.514  1.00  0.00
G    C
ATOM  21753  HB1 ALA G  80       8.930 -14.556  46.623  1.00  0.00
G    H
ATOM  21754  HB2 ALA G  80       7.624 -14.880  45.432  1.00  0.00
G    H
ATOM  21755  HB3 ALA G  80       7.228 -14.152  47.035  1.00  0.00
G    H
ATOM  21756  C   ALA G  80       6.260 -16.720  47.014  1.00  0.00
G    C
ATOM  21757  O   ALA G  80       5.613 -16.936  48.042  1.00  0.00
G    O
ATOM  21758  N   ASN G  81       5.728 -16.849  45.811  1.00  0.00
G    N
ATOM  21759  HN  ASN G  81       6.263 -16.848  44.967  1.00  0.00
G    H
ATOM  21760  CA  ASN G  81       4.295 -17.005  45.616  1.00  0.00
G    C
ATOM  21761  HA  ASN G  81       3.808 -16.448  46.409  1.00  0.00
G    H
```

```
ATOM  21762  CB  ASN G  81       3.913 -16.497  44.233  1.00  0.00
G    C
ATOM  21763  HB1 ASN G  81       4.306 -15.462  44.130  1.00  0.00
G    H
ATOM  21764  HB2 ASN G  81       4.395 -17.117  43.448  1.00  0.00
G    H
ATOM  21765  CG  ASN G  81       2.435 -16.455  44.025  1.00  0.00
G    C
ATOM  21766  OD1 ASN G  81       1.697 -17.263  44.575  1.00  0.00
G    O
ATOM  21767  ND2 ASN G  81       1.986 -15.507  43.218  1.00  0.00
G    N
ATOM  21768 HD21 ASN G  81       1.001 -15.455  43.076  1.00  0.00
G    H
ATOM  21769 HD22 ASN G  81       2.621 -14.862  42.804  1.00  0.00
G    H
ATOM  21770  C   ASN G  81       3.927 -18.471  45.789  1.00  0.00
G    C
ATOM  21771  O   ASN G  81       3.863 -19.217  44.808  1.00  0.00
G    O
ATOM  21772  N   ILE G  82       3.636 -18.875  47.026  1.00  0.00
G    N
ATOM  21773  HN  ILE G  82       3.580 -18.282  47.827  1.00  0.00
G    H
ATOM  21774  CA  ILE G  82       3.368 -20.283  47.303  1.00  0.00
G    C
ATOM  21775  HA  ILE G  82       4.278 -20.821  47.067  1.00  0.00
G    H
ATOM  21776  CB  ILE G  82       2.992 -20.477  48.781  1.00  0.00
G    C
ATOM  21777  HB  ILE G  82       2.263 -19.654  48.996  1.00  0.00
G    H
ATOM  21778  CG2 ILE G  82       2.439 -21.865  49.018  1.00  0.00
G    C
ATOM  21779 HG21 ILE G  82       2.026 -21.950  50.045  1.00  0.00
G    H
ATOM  21780 HG22 ILE G  82       1.619 -22.091  48.305  1.00  0.00
G    H
ATOM  21781 HG23 ILE G  82       3.230 -22.634  48.898  1.00  0.00
G    H
ATOM  21782  CG1 ILE G  82       4.206 -20.237  49.669  1.00  0.00
G    C
ATOM  21783 HG11 ILE G  82       4.158 -20.901  50.561  1.00  0.00
G    H
ATOM  21784 HG12 ILE G  82       5.126 -20.511  49.104  1.00  0.00
G    H
ATOM  21785  CD  ILE G  82       4.353 -18.826  50.129  1.00  0.00
G    C
ATOM  21786  HD1 ILE G  82       5.286 -18.708  50.722  1.00  0.00
G    H
ATOM  21787  HD2 ILE G  82       4.403 -18.133  49.261  1.00  0.00
G    H
ATOM  21788  HD3 ILE G  82       3.498 -18.525  50.771  1.00  0.00
G    H
```

```
ATOM  21789  C   ILE G  82       2.288 -20.819  46.378  1.00  0.00
G    C
ATOM  21790  O   ILE G  82       2.357 -21.970  45.919  1.00  0.00
G    O
ATOM  21791  N   GLN G  83       1.295 -19.987  46.069  1.00  0.00
G    N
ATOM  21792  HN  GLN G  83       1.204 -19.059  46.426  1.00  0.00
G    H
ATOM  21793  CA  GLN G  83       0.235 -20.387  45.156  1.00  0.00
G    C
ATOM  21794  HA  GLN G  83      -0.359 -21.134  45.669  1.00  0.00
G    H
ATOM  21795  CB  GLN G  83      -0.575 -19.166  44.762  1.00  0.00
G    C
ATOM  21796  HB1 GLN G  83      -0.178 -18.311  45.356  1.00  0.00
G    H
ATOM  21797  HB2 GLN G  83      -0.377 -18.922  43.693  1.00  0.00
G    H
ATOM  21798  CG  GLN G  83      -2.030 -19.261  44.991  1.00  0.00
G    C
ATOM  21799  HG1 GLN G  83      -2.415 -20.243  44.641  1.00  0.00
G    H
ATOM  21800  HG2 GLN G  83      -2.265 -19.169  46.071  1.00  0.00
G    H
ATOM  21801  CD  GLN G  83      -2.756 -18.189  44.217  1.00  0.00
G    C
ATOM  21802  OE1 GLN G  83      -2.442 -17.939  43.060  1.00  0.00
G    O
ATOM  21803  NE2 GLN G  83      -3.705 -17.532  44.857  1.00  0.00
G    N
ATOM  21804 HE21 GLN G  83      -4.188 -16.828  44.345  1.00  0.00
G    H
ATOM  21805 HE22 GLN G  83      -3.896 -17.741  45.810  1.00  0.00
G    H
ATOM  21806  C   GLN G  83       0.801 -21.035  43.907  1.00  0.00
G    C
ATOM  21807  O   GLN G  83       0.579 -22.226  43.651  1.00  0.00
G    O
ATOM  21808  N   ALA G  84       1.566 -20.267  43.141  1.00  0.00
G    N
ATOM  21809  HN  ALA G  84       1.837 -19.334  43.376  1.00  0.00
G    H
ATOM  21810  CA  ALA G  84       2.071 -20.761  41.872  1.00  0.00
G    C
ATOM  21811  HA  ALA G  84       1.254 -21.020  41.209  1.00  0.00
G    H
ATOM  21812  CB  ALA G  84       2.909 -19.683  41.192  1.00  0.00
G    C
ATOM  21813  HB1 ALA G  84       2.284 -18.783  41.013  1.00  0.00
G    H
ATOM  21814  HB2 ALA G  84       3.767 -19.381  41.832  1.00  0.00
G    H
ATOM  21815  HB3 ALA G  84       3.303 -20.028  40.212  1.00  0.00
G    H
```

ATOM 21816 C   ALA G 84   2.879 -22.029 42.069 1.00 0.00
G   C
ATOM 21817 O   ALA G 84   2.688 -23.013 41.351 1.00 0.00
G   O
ATOM 21818 N   SER G 85   3.786 -22.020 43.044 1.00 0.00
G   N
ATOM 21819 HN SER G 85   4.004 -21.234 43.623 1.00 0.00
G   H
ATOM 21820 CA SER G 85   4.560 -23.215 43.344 1.00 0.00
G   C
ATOM 21821 HA SER G 85   5.247 -23.347 42.519 1.00 0.00
G   H
ATOM 21822 CB SER G 85   5.265 -23.046 44.684 1.00 0.00
G   C
ATOM 21823 HB1 SER G 85   6.159 -23.711 44.691 1.00 0.00
G   H
ATOM 21824 HB2 SER G 85   5.633 -22.001 44.800 1.00 0.00
G   H
ATOM 21825 OG SER G 85   4.401 -23.417 45.740 1.00 0.00
G   O
ATOM 21826 HG1 SER G 85   4.945 -23.511 46.532 1.00 0.00
G   H
ATOM 21827 C   SER G 85   3.664 -24.440 43.387 1.00 0.00
G   C
ATOM 21828 O   SER G 85   3.875 -25.419 42.656 1.00 0.00
G   O
ATOM 21829 N   THR G 86   2.624 -24.381 44.207 1.00 0.00
G   N
ATOM 21830 HN THR G 86   2.292 -23.567 44.682 1.00 0.00
G   H
ATOM 21831 CA THR G 86   1.857 -25.585 44.477 1.00 0.00
G   C
ATOM 21832 HA THR G 86   2.589 -26.368 44.623 1.00 0.00
G   H
ATOM 21833 CB THR G 86   0.993 -25.373 45.707 1.00 0.00
G   C
ATOM 21834 HB THR G 86   0.481 -26.348 45.898 1.00 0.00
G   H
ATOM 21835 OG1 THR G 86   -0.006 -24.393 45.415 1.00 0.00
G   O
ATOM 21836 HG1 THR G 86   -0.512 -24.292 46.229 1.00 0.00
G   H
ATOM 21837 CG2 THR G 86   1.849 -24.903 46.861 1.00 0.00
G   C
ATOM 21838 HG21 THR G 86   1.255 -24.852 47.798 1.00 0.00
G   H
ATOM 21839 HG22 THR G 86   2.693 -25.606 47.029 1.00 0.00
G   H
ATOM 21840 HG23 THR G 86   2.271 -23.894 46.661 1.00 0.00
G   H
ATOM 21841 C   THR G 86   1.012 -26.001 43.281 1.00 0.00
G   C
ATOM 21842 O   THR G 86   1.024 -27.174 42.891 1.00 0.00
G   O

ATOM 21843 N   LEU G 87   0.256 -25.068 42.694 1.00 0.00
G   N
ATOM 21844 HN LEU G 87   0.171 -24.108 42.953 1.00 0.00
G   H
ATOM 21845 CA LEU G 87   -0.557 -25.453 41.540 1.00 0.00
G   C
ATOM 21846 HA LEU G 87   -1.179 -26.280 41.856 1.00 0.00
G   H
ATOM 21847 CB LEU G 87   -1.457 -24.313 41.058 1.00 0.00
G   C
ATOM 21848 HB1 LEU G 87   -0.857 -23.699 40.348 1.00 0.00
G   H
ATOM 21849 HB2 LEU G 87   -2.295 -24.740 40.464 1.00 0.00
G   H
ATOM 21850 CG LEU G 87   -2.190 -23.196 41.808 1.00 0.00
G   C
ATOM 21851 HG LEU G 87   -3.253 -23.346 41.501 1.00 0.00
G   H
ATOM 21852 CD1 LEU G 87   -2.086 -23.200 43.318 1.00 0.00
G   C
ATOM 21853 HD11 LEU G 87   -2.615 -22.322 43.747 1.00 0.00
G   H
ATOM 21854 HD12 LEU G 87   -2.542 -24.122 43.739 1.00 0.00
G   H
ATOM 21855 HD13 LEU G 87   -1.023 -23.157 43.638 1.00 0.00
G   H
ATOM 21856 CD2 LEU G 87   -1.721 -21.850 41.249 1.00 0.00
G   C
ATOM 21857 HD21 LEU G 87   -2.249 -21.016 41.759 1.00 0.00
G   H
ATOM 21858 HD22 LEU G 87   -0.633 -21.702 41.399 1.00 0.00
G   H
ATOM 21859 HD23 LEU G 87   -1.937 -21.784 40.161 1.00 0.00
G   H
ATOM 21860 C   LEU G 87   0.320 -25.945 40.405 1.00 0.00
G   C
ATOM 21861 O   LEU G 87   -0.046 -26.872 39.670 1.00 0.00
G   O
ATOM 21862 N   THR G 88   1.490 -25.339 40.252 1.00 0.00
G   N
ATOM 21863 HN THR G 88   1.780 -24.526 40.757 1.00 0.00
G   H
ATOM 21864 CA THR G 88   2.469 -25.832 39.307 1.00 0.00
G   C
ATOM 21865 HA THR G 88   2.009 -25.754 38.330 1.00 0.00
G   H
ATOM 21866 CB THR G 88   3.743 -25.031 39.485 1.00 0.00
G   C
ATOM 21867 HB THR G 88   4.075 -25.205 40.537 1.00 0.00
G   H
ATOM 21868 OG1 THR G 88   3.441 -23.641 39.333 1.00 0.00
G   O
ATOM 21869 HG1 THR G 88   4.280 -23.182 39.443 1.00 0.00
G   H

```
ATOM  21870  CG2 THR G  88       4.773 -25.457  38.484  1.00  0.00
G    C
ATOM  21871 HG21 THR G  88       5.745 -24.957  38.679  1.00  0.00
G    H
ATOM  21872 HG22 THR G  88       4.936 -26.554  38.539  1.00  0.00
G    H
ATOM  21873 HG23 THR G  88       4.455 -25.204  37.449  1.00  0.00
G    H
ATOM  21874  C   THR G  88       2.742 -27.301  39.538  1.00  0.00
G    C
ATOM  21875  O   THR G  88       2.465 -28.143  38.674  1.00  0.00
G    O
ATOM  21876  N   THR G  89       3.241 -27.619  40.731  1.00  0.00
G    N
ATOM  21877  HN  THR G  89       3.441 -26.966  41.461  1.00  0.00
G    H
ATOM  21878  CA  THR G  89       3.540 -29.004  41.069  1.00  0.00
G    C
ATOM  21879  HA  THR G  89       4.331 -29.308  40.397  1.00  0.00
G    H
ATOM  21880  CB  THR G  89       3.907 -29.104  42.536  1.00  0.00
G    C
ATOM  21881  HB  THR G  89       3.940 -30.196  42.774  1.00  0.00
G    H
ATOM  21882  OG1 THR G  89       2.863 -28.503  43.308  1.00  0.00
G    O
ATOM  21883  HG1 THR G  89       3.145 -28.580  44.226  1.00  0.00
G    H
ATOM  21884  CG2 THR G  89       5.198 -28.376  42.793  1.00  0.00
G    C
ATOM  21885 HG21 THR G  89       5.540 -28.535  43.838  1.00  0.00
G    H
ATOM  21886 HG22 THR G  89       5.994 -28.746  42.113  1.00  0.00
G    H
ATOM  21887 HG23 THR G  89       5.081 -27.283  42.629  1.00  0.00
G    H
ATOM  21888  C   THR G  89       2.359 -29.906  40.779  1.00  0.00
G    C
ATOM  21889  O   THR G  89       2.529 -31.039  40.317  1.00  0.00
G    O
ATOM  21890  N   LEU G  90       1.153 -29.426  41.066  1.00  0.00
G    N
ATOM  21891  HN  LEU G  90       0.963 -28.553  41.512  1.00  0.00
G    H
ATOM  21892  CA  LEU G  90      -0.034 -30.194  40.726  1.00  0.00
G    C
ATOM  21893  HA  LEU G  90       0.022 -31.120  41.282  1.00  0.00
G    H
ATOM  21894  CB  LEU G  90      -1.288 -29.409  41.084  1.00  0.00
G    C
ATOM  21895  HB1 LEU G  90      -1.444 -29.522  42.181  1.00  0.00
G    H
ATOM  21896  HB2 LEU G  90      -1.099 -28.327  40.910  1.00  0.00
G    H
```

ATOM 21897 CG LEU G 90   -2.540 -29.873 40.347 1.00 0.00
G   C
ATOM 21898 HG LEU G 90   -2.407 -29.657 39.259 1.00 0.00
G   H
ATOM 21899 CD1 LEU G 90   -2.801 -31.348 40.556 1.00 0.00
G   C
ATOM 21900 HD11 LEU G 90   -3.742 -31.654 40.051 1.00 0.00
G   H
ATOM 21901 HD12 LEU G 90   -1.968 -31.955 40.139 1.00 0.00
G   H
ATOM 21902 HD13 LEU G 90   -2.897 -31.580 41.637 1.00 0.00
G   H
ATOM 21903 CD2 LEU G 90   -3.709 -29.081 40.836 1.00 0.00
G   C
ATOM 21904 HD21 LEU G 90   -4.633 -29.387 40.300 1.00 0.00
G   H
ATOM 21905 HD22 LEU G 90   -3.881 -29.234 41.920 1.00 0.00
G   H
ATOM 21906 HD23 LEU G 90   -3.546 -27.997 40.656 1.00 0.00
G   H
ATOM 21907 C   LEU G 90   -0.044 -30.545 39.251 1.00 0.00
G   C
ATOM 21908 O   LEU G 90   -0.176 -31.714 38.882 1.00 0.00
G   O
ATOM 21909 N   LEU G 91   0.071 -29.537 38.393 1.00 0.00
G   N
ATOM 21910 HN LEU G 91   0.221 -28.580 38.630 1.00 0.00
G   H
ATOM 21911 CA LEU G 91   -0.023 -29.793 36.962 1.00 0.00
G   C
ATOM 21912 HA LEU G 91   -0.977 -30.276 36.791 1.00 0.00
G   H
ATOM 21913 CB LEU G 91   0.058 -28.487 36.187 1.00 0.00
G   C
ATOM 21914 HB1 LEU G 91   0.708 -27.797 36.772 1.00 0.00
G   H
ATOM 21915 HB2 LEU G 91   0.587 -28.668 35.226 1.00 0.00
G   H
ATOM 21916 CG LEU G 91   -1.307 -27.843 35.965 1.00 0.00
G   C
ATOM 21917 HG LEU G 91   -1.145 -26.868 35.444 1.00 0.00
G   H
ATOM 21918 CD1 LEU G 91   -2.164 -28.748 35.101 1.00 0.00
G   C
ATOM 21919 HD11 LEU G 91   -3.179 -28.315 34.969 1.00 0.00
G   H
ATOM 21920 HD12 LEU G 91   -1.707 -28.878 34.096 1.00 0.00
G   H
ATOM 21921 HD13 LEU G 91   -2.273 -29.749 35.569 1.00 0.00
G   H
ATOM 21922 CD2 LEU G 91   -1.997 -27.562 37.280 1.00 0.00
G   C
ATOM 21923 HD21 LEU G 91   -2.981 -27.076 37.103 1.00 0.00
G   H

```
ATOM  21924 HD22 LEU G  91      -2.179 -28.492  37.854  1.00  0.00
G    H
ATOM  21925 HD23 LEU G  91      -1.380 -26.880  37.905  1.00  0.00
G    H
ATOM  21926  C   LEU G  91       1.063 -30.753  36.511  1.00  0.00
G    C
ATOM  21927  O   LEU G  91       0.821 -31.660  35.707  1.00  0.00
G    O
ATOM  21928  N   LEU G  92       2.267 -30.576  37.039  1.00  0.00
G    N
ATOM  21929  HN  LEU G  92       2.513 -29.896  37.727  1.00  0.00
G    H
ATOM  21930  CA  LEU G  92       3.371 -31.424  36.613  1.00  0.00
G    C
ATOM  21931  HA  LEU G  92       3.380 -31.400  35.531  1.00  0.00
G    H
ATOM  21932  CB  LEU G  92       4.687 -30.885  37.158  1.00  0.00
G    C
ATOM  21933  HB1 LEU G  92       4.486 -30.502  38.185  1.00  0.00
G    H
ATOM  21934  HB2 LEU G  92       5.401 -31.730  37.280  1.00  0.00
G    H
ATOM  21935  CG  LEU G  92       5.278 -29.773  36.304  1.00  0.00
G    C
ATOM  21936  HG  LEU G  92       6.356 -29.654  36.573  1.00  0.00
G    H
ATOM  21937  CD1 LEU G  92       5.151 -30.155  34.848  1.00  0.00
G    C
ATOM  21938 HD11 LEU G  92       5.531 -29.339  34.196  1.00  0.00
G    H
ATOM  21939 HD12 LEU G  92       5.736 -31.076  34.634  1.00  0.00
G    H
ATOM  21940 HD13 LEU G  92       4.089 -30.344  34.584  1.00  0.00
G    H
ATOM  21941  CD2 LEU G  92       4.588 -28.459  36.554  1.00  0.00
G    C
ATOM  21942 HD21 LEU G  92       5.047 -27.659  35.934  1.00  0.00
G    H
ATOM  21943 HD22 LEU G  92       3.511 -28.508  36.298  1.00  0.00
G    H
ATOM  21944 HD23 LEU G  92       4.681 -28.168  37.622  1.00  0.00
G    H
ATOM  21945  C   LEU G  92       3.159 -32.867  37.038  1.00  0.00
G    C
ATOM  21946  O   LEU G  92       3.223 -33.781  36.209  1.00  0.00
G    O
ATOM  21947  N   LYS G  93       2.893 -33.092  38.323  1.00  0.00
G    N
ATOM  21948  HN  LYS G  93       2.838 -32.389  39.030  1.00  0.00
G    H
ATOM  21949  CA  LYS G  93       2.656 -34.452  38.785  1.00  0.00
G    C
ATOM  21950  HA  LYS G  93       3.520 -35.042  38.505  1.00  0.00
G    H
```

```
ATOM  21951  CB  LYS G  93       2.472 -34.464  40.297  1.00  0.00
G    C
ATOM  21952  HB1 LYS G  93       3.064 -33.614  40.707  1.00  0.00
G    H
ATOM  21953  HB2 LYS G  93       1.406 -34.246  40.533  1.00  0.00
G    H
ATOM  21954  CG  LYS G  93       2.922 -35.750  40.951  1.00  0.00
G    C
ATOM  21955  HG1 LYS G  93       2.899 -35.624  42.058  1.00  0.00
G    H
ATOM  21956  HG2 LYS G  93       2.200 -36.560  40.694  1.00  0.00
G    H
ATOM  21957  CD  LYS G  93       4.325 -36.116  40.511  1.00  0.00
G    C
ATOM  21958  HD1 LYS G  93       4.330 -36.219  39.402  1.00  0.00
G    H
ATOM  21959  HD2 LYS G  93       5.001 -35.270  40.770  1.00  0.00
G    H
ATOM  21960  CE  LYS G  93       4.795 -37.397  41.176  1.00  0.00
G    C
ATOM  21961  HE1 LYS G  93       4.973 -37.230  42.262  1.00  0.00
G    H
ATOM  21962  HE2 LYS G  93       4.032 -38.199  41.063  1.00  0.00
G    H
ATOM  21963  NZ  LYS G  93       6.067 -37.901  40.585  1.00  0.00
G    N
ATOM  21964  HZ1 LYS G  93       6.360 -38.777  41.065  1.00  0.00
G    H
ATOM  21965  HZ2 LYS G  93       5.928 -38.092  39.572  1.00  0.00
G    H
ATOM  21966  HZ3 LYS G  93       6.810 -37.181  40.698  1.00  0.00
G    H
ATOM  21967  C   LYS G  93       1.450 -35.056  38.091  1.00  0.00
G    C
ATOM  21968  O   LYS G  93       1.384 -36.272  37.890  1.00  0.00
G    O
ATOM  21969  N   GLU G  94       0.484 -34.222  37.716  1.00  0.00
G    N
ATOM  21970  HN  GLU G  94       0.429 -33.252  37.934  1.00  0.00
G    H
ATOM  21971  CA  GLU G  94      -0.623 -34.710  36.912  1.00  0.00
G    C
ATOM  21972  HA  GLU G  94      -1.009 -35.610  37.375  1.00  0.00
G    H
ATOM  21973  CB  GLU G  94      -1.723 -33.659  36.837  1.00  0.00
G    C
ATOM  21974  HB1 GLU G  94      -2.256 -33.644  37.815  1.00  0.00
G    H
ATOM  21975  HB2 GLU G  94      -1.271 -32.648  36.720  1.00  0.00
G    H
ATOM  21976  CG  GLU G  94      -2.719 -33.915  35.731  1.00  0.00
G    C
ATOM  21977  HG1 GLU G  94      -2.277 -33.623  34.761  1.00  0.00
G    H
```

```
ATOM  21978  HG2 GLU G  94      -3.005 -34.986  35.694  1.00  0.00
G    H
ATOM  21979  CD  GLU G  94      -3.968 -33.087  35.881  1.00  0.00
G    C
ATOM  21980  OE1 GLU G  94      -4.345 -32.790  37.033  1.00  0.00
G    O
ATOM  21981  OE2 GLU G  94      -4.579 -32.738  34.852  1.00  0.00
G    O
ATOM  21982  C   GLU G  94      -0.156 -35.086  35.519  1.00  0.00
G    C
ATOM  21983  O   GLU G  94      -0.671 -36.037  34.922  1.00  0.00
G    O
ATOM  21984  N   LEU G  95       0.816 -34.360  34.985  1.00  0.00
G    N
ATOM  21985  HN  LEU G  95       1.241 -33.570  35.422  1.00  0.00
G    H
ATOM  21986  CA  LEU G  95       1.353 -34.675  33.673  1.00  0.00
G    C
ATOM  21987  HA  LEU G  95       0.568 -35.205  33.148  1.00  0.00
G    H
ATOM  21988  CB  LEU G  95       1.685 -33.396  32.917  1.00  0.00
G    C
ATOM  21989  HB1 LEU G  95       1.914 -32.614  33.678  1.00  0.00
G    H
ATOM  21990  HB2 LEU G  95       2.624 -33.550  32.342  1.00  0.00
G    H
ATOM  21991  CG  LEU G  95       0.477 -33.026  32.059  1.00  0.00
G    C
ATOM  21992  HG  LEU G  95       0.159 -33.974  31.560  1.00  0.00
G    H
ATOM  21993  CD1 LEU G  95      -0.660 -32.494  32.910  1.00  0.00
G    C
ATOM  21994 HD11 LEU G  95      -1.514 -32.183  32.270  1.00  0.00
G    H
ATOM  21995 HD12 LEU G  95      -1.018 -33.273  33.616  1.00  0.00
G    H
ATOM  21996 HD13 LEU G  95      -0.330 -31.611  33.499  1.00  0.00
G    H
ATOM  21997  CD2 LEU G  95       0.852 -32.037  30.984  1.00  0.00
G    C
ATOM  21998 HD21 LEU G  95      -0.033 -31.793  30.358  1.00  0.00
G    H
ATOM  21999 HD22 LEU G  95       1.231 -31.089  31.415  1.00  0.00
G    H
ATOM  22000 HD23 LEU G  95       1.639 -32.461  30.323  1.00  0.00
G    H
ATOM  22001  C   LEU G  95       2.560 -35.591  33.739  1.00  0.00
G    C
ATOM  22002  O   LEU G  95       3.371 -35.599  32.809  1.00  0.00
G    O
ATOM  22003  N   ASP G  96       2.704 -36.339  34.829  1.00  0.00
G    N
ATOM  22004  HN  ASP G  96       2.289 -36.135  35.712  1.00  0.00
G    H
```

ATOM 22005 CA ASP G 96   3.501 -37.559 34.844 1.00 0.00
G   C
ATOM 22006 HA ASP G 96   3.429 -37.961 35.848 1.00 0.00
G   H
ATOM 22007 CB ASP G 96   2.964 -38.553 33.821 1.00 0.00
G   C
ATOM 22008 HB1 ASP G 96   3.309 -38.258 32.810 1.00 0.00
G   H
ATOM 22009 HB2 ASP G 96   3.339 -39.574 34.033 1.00 0.00
G   H
ATOM 22010 CG ASP G 96   1.462 -38.585 33.799 1.00 0.00
G   C
ATOM 22011 OD1 ASP G 96   0.858 -38.334 34.862 1.00 0.00
G   O
ATOM 22012 OD2 ASP G 96   0.885 -38.834 32.723 1.00 0.00
G   O
ATOM 22013 C   ASP G 96   4.970 -37.291 34.584 1.00 0.00
G   C
ATOM 22014 O   ASP G 96   5.725 -38.203 34.246 1.00 0.00
G   O
ATOM 22015 N   ILE G 97   5.383 -36.041 34.727 1.00 0.00
G   N
ATOM 22016 HN ILE G 97   4.834 -35.252 34.995 1.00 0.00
G   H
ATOM 22017 CA ILE G 97   6.788 -35.736 34.477 1.00 0.00
G   C
ATOM 22018 HA ILE G 97   7.030 -36.115 33.492 1.00 0.00
G   H
ATOM 22019 CB ILE G 97   7.028 -34.231 34.581 1.00 0.00
G   C
ATOM 22020 HB ILE G 97   6.654 -33.957 35.601 1.00 0.00
G   H
ATOM 22021 CG2 ILE G 97   8.484 -33.899 34.337 1.00 0.00
G   C
ATOM 22022 HG21 ILE G 97   8.686 -32.832 34.567 1.00 0.00
G   H
ATOM 22023 HG22 ILE G 97   9.144 -34.512 34.986 1.00 0.00
G   H
ATOM 22024 HG23 ILE G 97   8.762 -34.084 33.279 1.00 0.00
G   H
ATOM 22025 CG1 ILE G 97   6.143 -33.526 33.563 1.00 0.00
G   C
ATOM 22026 HG11 ILE G 97   5.072 -33.701 33.810 1.00 0.00
G   H
ATOM 22027 HG12 ILE G 97   6.322 -32.428 33.628 1.00 0.00
G   H
ATOM 22028 CD ILE G 97   6.408 -33.969 32.146 1.00 0.00
G   C
ATOM 22029 HD1 ILE G 97   5.781 -33.387 31.436 1.00 0.00
G   H
ATOM 22030 HD2 ILE G 97   7.475 -33.810 31.878 1.00 0.00
G   H
ATOM 22031 HD3 ILE G 97   6.168 -35.045 32.013 1.00 0.00
G   H

ATOM 22032 C   ILE G 97   7.645 -36.508 35.467 1.00 0.00
G   C
ATOM 22033 O   ILE G 97   7.290 -36.596 36.656 1.00 0.00
G   O
ATOM 22034 N   PRO G 98   8.745 -37.116 35.031 1.00 0.00
G   N
ATOM 22035 CD PRO G 98   9.298 -37.128 33.669 1.00 0.00
G   C
ATOM 22036 HD1 PRO G 98   8.237 -37.443 33.797 1.00 0.00
G   H
ATOM 22037 HD2 PRO G 98   9.424 -36.564 32.716 1.00 0.00
G   H
ATOM 22038 CA PRO G 98   9.492 -37.989 35.945 1.00 0.00
G   C
ATOM 22039 HA PRO G 98   8.866 -38.820 36.247 1.00 0.00
G   H
ATOM 22040 CB PRO G 98   10.647 -38.493 35.073 1.00 0.00
G   C
ATOM 22041 HB1 PRO G 98   10.304 -39.379 34.492 1.00 0.00
G   H
ATOM 22042 HB2 PRO G 98   11.541 -38.775 35.665 1.00 0.00
G   H
ATOM 22043 CG PRO G 98   10.185 -38.315 33.676 1.00 0.00
G   C
ATOM 22044 HG1 PRO G 98   10.703 -38.984 32.961 1.00 0.00
G   H
ATOM 22045 HG2 PRO G 98   10.340 -37.255 33.371 1.00 0.00
G   H
ATOM 22046 C   PRO G 98   9.995 -37.275 37.180 1.00 0.00
G   C
ATOM 22047 O   PRO G 98   9.648 -37.659 38.301 1.00 0.00
G   O
ATOM 22048 N   ASN G 99   10.781 -36.223 37.007 1.00 0.00
G   N
ATOM 22049 HN ASN G 99   10.920 -35.780 36.122 1.00 0.00
G   H
ATOM 22050 CA ASN G 99   11.517 -35.618 38.106 1.00 0.00
G   C
ATOM 22051 HA ASN G 99   11.333 -36.231 38.982 1.00 0.00
G   H
ATOM 22052 CB ASN G 99   12.998 -35.527 37.763 1.00 0.00
G   C
ATOM 22053 HB1 ASN G 99   13.109 -34.806 36.923 1.00 0.00
G   H
ATOM 22054 HB2 ASN G 99   13.569 -35.124 38.624 1.00 0.00
G   H
ATOM 22055 CG ASN G 99   13.558 -36.847 37.329 1.00 0.00
G   C
ATOM 22056 OD1 ASN G 99   13.979 -37.005 36.187 1.00 0.00
G   O
ATOM 22057 ND2 ASN G 99   13.547 -37.819 38.229 1.00 0.00
G   N
ATOM 22058 HD21 ASN G 99   13.927 -38.698 37.957 1.00 0.00
G   H

```
ATOM  22059 HD22 ASN G  99      13.195 -37.646  39.144  1.00  0.00
G    H
ATOM  22060  C   ASN G  99      10.947 -34.241  38.380  1.00  0.00
G    C
ATOM  22061  O   ASN G  99      10.947 -33.381  37.495  1.00  0.00
G    O
ATOM  22062  N   ILE G 100      10.472 -34.032  39.602  1.00  0.00
G    N
ATOM  22063  HN  ILE G 100      10.486 -34.728  40.317  1.00  0.00
G    H
ATOM  22064  CA  ILE G 100       9.885 -32.768  40.013  1.00  0.00
G    C
ATOM  22065  HA  ILE G 100      10.076 -32.040  39.235  1.00  0.00
G    H
ATOM  22066  CB  ILE G 100       8.390 -32.898  40.298  1.00  0.00
G    C
ATOM  22067  HB  ILE G 100       8.300 -33.836  40.903  1.00  0.00
G    H
ATOM  22068  CG2 ILE G 100       7.888 -31.656  40.971  1.00  0.00
G    C
ATOM  22069 HG21 ILE G 100       6.845 -31.796  41.323  1.00  0.00
G    H
ATOM  22070 HG22 ILE G 100       8.511 -31.405  41.855  1.00  0.00
G    H
ATOM  22071 HG23 ILE G 100       7.906 -30.791  40.274  1.00  0.00
G    H
ATOM  22072  CG1 ILE G 100       7.631 -33.133  38.995  1.00  0.00
G    C
ATOM  22073 HG11 ILE G 100       7.798 -32.278  38.303  1.00  0.00
G    H
ATOM  22074 HG12 ILE G 100       8.041 -34.043  38.499  1.00  0.00
G    H
ATOM  22075  CD  ILE G 100       6.156 -33.327  39.184  1.00  0.00
G    C
ATOM  22076  HD1 ILE G 100       5.669 -33.551  38.211  1.00  0.00
G    H
ATOM  22077  HD2 ILE G 100       5.959 -34.175  39.875  1.00  0.00
G    H
ATOM  22078  HD3 ILE G 100       5.684 -32.414  39.603  1.00  0.00
G    H
ATOM  22079  C   ILE G 100      10.641 -32.305  41.242  1.00  0.00
G    C
ATOM  22080  O   ILE G 100      10.583 -32.950  42.297  1.00  0.00
G    O
ATOM  22081  N   TRP G 101      11.364 -31.201  41.111  1.00  0.00
G    N
ATOM  22082  HN  TRP G 101      11.341 -30.640  40.287  1.00  0.00
G    H
ATOM  22083  CA  TRP G 101      12.251 -30.718  42.160  1.00  0.00
G    C
ATOM  22084  HA  TRP G 101      12.061 -31.293  43.059  1.00  0.00
G    H
ATOM  22085  CB  TRP G 101      13.704 -30.845  41.719  1.00  0.00
G    C
```

```
ATOM  22086  HB1 TRP G 101      13.777 -30.469  40.674  1.00  0.00
G    H
ATOM  22087  HB2 TRP G 101      14.347 -30.188  42.344  1.00  0.00
G    H
ATOM  22088  CG  TRP G 101      14.164 -32.233  41.744  1.00  0.00
G    C
ATOM  22089  CD1 TRP G 101      13.477 -33.329  41.337  1.00  0.00
G    C
ATOM  22090  HD1 TRP G 101      12.492 -33.300  40.891  1.00  0.00
G    H
ATOM  22091  NE1 TRP G 101      14.223 -34.455  41.533  1.00  0.00
G    N
ATOM  22092  HE1 TRP G 101      13.975 -35.369  41.295  1.00  0.00
G    H
ATOM  22093  CE2 TRP G 101      15.422 -34.091  42.084  1.00  0.00
G    C
ATOM  22094  CD2 TRP G 101      15.412 -32.697  42.230  1.00  0.00
G    C
ATOM  22095  CE3 TRP G 101      16.527 -32.069  42.773  1.00  0.00
G    C
ATOM  22096  HE3 TRP G 101      16.565 -30.997  42.916  1.00  0.00
G    H
ATOM  22097  CZ3 TRP G 101      17.598 -32.836  43.140  1.00  0.00
G    C
ATOM  22098  HZ3 TRP G 101      18.473 -32.360  43.561  1.00  0.00
G    H
ATOM  22099  CZ2 TRP G 101      16.502 -34.868  42.459  1.00  0.00
G    C
ATOM  22100  HZ2 TRP G 101      16.516 -35.941  42.348  1.00  0.00
G    H
ATOM  22101  CH2 TRP G 101      17.581 -34.222  42.986  1.00  0.00
G    C
ATOM  22102  HH2 TRP G 101      18.440 -34.803  43.291  1.00  0.00
G    H
ATOM  22103  C   TRP G 101      11.905 -29.272  42.478  1.00  0.00
G    C
ATOM  22104  O   TRP G 101      11.881 -28.429  41.578  1.00  0.00
G    O
ATOM  22105  N   VAL G 102      11.646 -28.981  43.747  1.00  0.00
G    N
ATOM  22106  HN  VAL G 102      11.831 -29.567  44.532  1.00  0.00
G    H
ATOM  22107  CA  VAL G 102      11.035 -27.703  44.081  1.00  0.00
G    C
ATOM  22108  HA  VAL G 102      11.060 -27.087  43.190  1.00  0.00
G    H
ATOM  22109  CB  VAL G 102       9.576 -27.897  44.533  1.00  0.00
G    C
ATOM  22110  HB  VAL G 102       9.594 -28.456  45.501  1.00  0.00
G    H
ATOM  22111  CG1 VAL G 102       8.901 -26.563  44.744  1.00  0.00
G    C
ATOM  22112 HG11 VAL G 102       7.838 -26.710  45.032  1.00  0.00
G    H
```

ATOM 22113 HG12 VAL G 102   9.386 -25.982 45.556 1.00 0.00
G   H
ATOM 22114 HG13 VAL G 102   8.925 -25.959 43.811 1.00 0.00
G   H
ATOM 22115 CG2 VAL G 102   8.816 -28.722 43.521 1.00 0.00
G   C
ATOM 22116 HG21 VAL G 102   7.753 -28.832 43.822 1.00 0.00
G   H
ATOM 22117 HG22 VAL G 102   8.848 -28.238 42.521 1.00 0.00
G   H
ATOM 22118 HG23 VAL G 102   9.245 -29.741 43.429 1.00 0.00
G   H
ATOM 22119 C   VAL G 102   11.834 -26.983 45.157 1.00 0.00
G   C
ATOM 22120 O   VAL G 102   12.452 -27.604 46.028 1.00 0.00
G   O
ATOM 22121 N   LYS G 103   11.821 -25.650 45.075 1.00 0.00
G   N
ATOM 22122 HN LYS G 103   11.472 -25.175 44.268 1.00 0.00
G   H
ATOM 22123 CA LYS G 103   12.310 -24.782 46.141 1.00 0.00
G   C
ATOM 22124 HA LYS G 103   13.087 -25.315 46.676 1.00 0.00
G   H
ATOM 22125 CB LYS G 103   12.836 -23.472 45.551 1.00 0.00
G   C
ATOM 22126 HB1 LYS G 103   13.756 -23.719 44.972 1.00 0.00
G   H
ATOM 22127 HB2 LYS G 103   12.098 -23.085 44.813 1.00 0.00
G   H
ATOM 22128 CG LYS G 103   13.182 -22.382 46.554 1.00 0.00
G   C
ATOM 22129 HG1 LYS G 103   12.365 -22.307 47.308 1.00 0.00
G   H
ATOM 22130 HG2 LYS G 103   14.111 -22.671 47.097 1.00 0.00
G   H
ATOM 22131 CD LYS G 103   13.353 -21.038 45.834 1.00 0.00
G   C
ATOM 22132 HD1 LYS G 103   14.176 -21.141 45.089 1.00 0.00
G   H
ATOM 22133 HD2 LYS G 103   12.416 -20.826 45.268 1.00 0.00
G   H
ATOM 22134 CE LYS G 103   13.652 -19.880 46.791 1.00 0.00
G   C
ATOM 22135 HE1 LYS G 103   12.935 -19.883 47.642 1.00 0.00
G   H
ATOM 22136 HE2 LYS G 103   14.683 -19.967 47.200 1.00 0.00
G   H
ATOM 22137 NZ LYS G 103   13.546 -18.539 46.125 1.00 0.00
G   N
ATOM 22138 HZ1 LYS G 103   13.755 -17.784 46.809 1.00 0.00
G   H
ATOM 22139 HZ2 LYS G 103   14.224 -18.489 45.338 1.00 0.00
G   H

ATOM 22140 HZ3 LYS G 103   12.583 -18.412 45.755 1.00 0.00
G   H
ATOM 22141 C   LYS G 103   11.180 -24.512 47.129 1.00 0.00
G   C
ATOM 22142 O   LYS G 103   10.117 -24.025 46.741 1.00 0.00
G   O
ATOM 22143 N   ALA G 104   11.411 -24.825 48.402 1.00 0.00
G   N
ATOM 22144 HN ALA G 104   12.316 -25.099 48.725 1.00 0.00
G   H
ATOM 22145 CA ALA G 104   10.368 -24.794 49.418 1.00 0.00
G   C
ATOM 22146 HA ALA G 104   9.391 -24.716 48.955 1.00 0.00
G   H
ATOM 22147 CB ALA G 104   10.388 -26.063 50.270 1.00 0.00
G   C
ATOM 22148 HB1 ALA G 104   10.236 -26.950 49.620 1.00 0.00
G   H
ATOM 22149 HB2 ALA G 104   11.365 -26.181 50.788 1.00 0.00
G   H
ATOM 22150 HB3 ALA G 104   9.582 -26.055 51.035 1.00 0.00
G   H
ATOM 22151 C   ALA G 104   10.508 -23.582 50.328 1.00 0.00
G   C
ATOM 22152 O   ALA G 104   11.611 -23.082 50.565 1.00 0.00
G   O
ATOM 22153 N   GLN G 105   9.374 -23.139 50.867 1.00 0.00
G   N
ATOM 22154 HN GLN G 105   8.501 -23.610 50.754 1.00 0.00
G   H
ATOM 22155 CA GLN G 105   9.304 -21.925 51.669 1.00 0.00
G   C
ATOM 22156 HA GLN G 105   10.155 -21.320 51.381 1.00 0.00
G   H
ATOM 22157 CB GLN G 105   7.997 -21.190 51.369 1.00 0.00
G   C
ATOM 22158 HB1 GLN G 105   7.929 -21.093 50.260 1.00 0.00
G   H
ATOM 22159 HB2 GLN G 105   7.136 -21.826 51.679 1.00 0.00
G   H
ATOM 22160 CG GLN G 105   7.889 -19.818 51.992 1.00 0.00
G   C
ATOM 22161 HG1 GLN G 105   8.899 -19.393 52.171 1.00 0.00
G   H
ATOM 22162 HG2 GLN G 105   7.341 -19.124 51.322 1.00 0.00
G   H
ATOM 22163 CD GLN G 105   7.195 -19.853 53.331 1.00 0.00
G   C
ATOM 22164 OE1 GLN G 105   6.466 -20.793 53.637 1.00 0.00
G   O
ATOM 22165 NE2 GLN G 105   7.399 -18.816 54.131 1.00 0.00
G   N
ATOM 22166 HE21 GLN G 105   6.946 -18.837 55.018 1.00 0.00
G   H

ATOM 22167 HE22 GLN G 105   7.969 -18.061 53.826 1.00 0.00
G   H
ATOM 22168 C   GLN G 105   9.424 -22.208 53.163 1.00 0.00
G   C
ATOM 22169 O   GLN G 105   10.374 -21.754 53.803 1.00 0.00
G   O
ATOM 22170 N   ASN G 106   8.488 -22.961 53.733 1.00 0.00
G   N
ATOM 22171 HN ASN G 106   7.690 -23.330 53.259 1.00 0.00
G   H
ATOM 22172 CA ASN G 106   8.570 -23.313 55.141 1.00 0.00
G   C
ATOM 22173 HA ASN G 106   9.585 -23.105 55.461 1.00 0.00
G   H
ATOM 22174 CB ASN G 106   7.558 -22.551 55.996 1.00 0.00
G   C
ATOM 22175 HB1 ASN G 106   7.779 -22.772 57.063 1.00 0.00
G   H
ATOM 22176 HB2 ASN G 106   7.675 -21.458 55.850 1.00 0.00
G   H
ATOM 22177 CG ASN G 106   6.120 -22.963 55.722 1.00 0.00
G   C
ATOM 22178 OD1 ASN G 106   5.841 -23.852 54.920 1.00 0.00
G   O
ATOM 22179 ND2 ASN G 106   5.197 -22.321 56.416 1.00 0.00
G   N
ATOM 22180 HD21 ASN G 106   4.247 -22.580 56.263 1.00 0.00
G   H
ATOM 22181 HD22 ASN G 106   5.463 -21.632 57.082 1.00 0.00
G   H
ATOM 22182 C   ASN G 106   8.340 -24.804 55.284 1.00 0.00
G   C
ATOM 22183 O   ASN G 106   8.057 -25.506 54.310 1.00 0.00
G   O
ATOM 22184 N   TYR G 107   8.428 -25.275 56.522 1.00 0.00
G   N
ATOM 22185 HN TYR G 107   8.697 -24.747 57.326 1.00 0.00
G   H
ATOM 22186 CA TYR G 107   8.119 -26.669 56.791 1.00 0.00
G   C
ATOM 22187 HA TYR G 107   8.838 -27.257 56.234 1.00 0.00
G   H
ATOM 22188 CB TYR G 107   8.211 -26.944 58.289 1.00 0.00
G   C
ATOM 22189 HB1 TYR G 107   9.265 -27.182 58.551 1.00 0.00
G   H
ATOM 22190 HB2 TYR G 107   7.922 -26.043 58.872 1.00 0.00
G   H
ATOM 22191 CG TYR G 107   7.369 -28.116 58.700 1.00 0.00
G   C
ATOM 22192 CD1 TYR G 107   7.818 -29.410 58.513 1.00 0.00
G   C
ATOM 22193 HD1 TYR G 107   8.784 -29.584 58.058 1.00 0.00
G   H

ATOM 22194 CE1 TYR G 107 7.049 -30.486 58.876 1.00 0.00
G C
ATOM 22195 HE1 TYR G 107 7.406 -31.493 58.723 1.00 0.00
G H
ATOM 22196 CZ TYR G 107 5.811 -30.275 59.432 1.00 0.00
G C
ATOM 22197 OH TYR G 107 5.034 -31.346 59.800 1.00 0.00
G O
ATOM 22198 HH TYR G 107 4.249 -30.998 60.229 1.00 0.00
G H
ATOM 22199 CD2 TYR G 107 6.120 -27.933 59.261 1.00 0.00
G C
ATOM 22200 HD2 TYR G 107 5.724 -26.934 59.383 1.00 0.00
G H
ATOM 22201 CE2 TYR G 107 5.344 -29.002 59.625 1.00 0.00
G C
ATOM 22202 HE2 TYR G 107 4.361 -28.834 60.041 1.00 0.00
G H
ATOM 22203 C TYR G 107 6.740 -27.036 56.270 1.00 0.00
G C
ATOM 22204 O TYR G 107 6.553 -28.105 55.676 1.00 0.00
G O
ATOM 22205 N TYR G 108 5.758 -26.165 56.483 1.00 0.00
G N
ATOM 22206 HN TYR G 108 5.848 -25.273 56.922 1.00 0.00
G H
ATOM 22207 CA TYR G 108 4.407 -26.496 56.057 1.00 0.00
G C
ATOM 22208 HA TYR G 108 4.183 -27.470 56.473 1.00 0.00
G H
ATOM 22209 CB TYR G 108 3.433 -25.442 56.549 1.00 0.00
G C
ATOM 22210 HB1 TYR G 108 3.758 -24.445 56.181 1.00 0.00
G H
ATOM 22211 HB2 TYR G 108 2.416 -25.627 56.141 1.00 0.00
G H
ATOM 22212 CG TYR G 108 3.410 -25.405 58.029 1.00 0.00
G C
ATOM 22213 CD1 TYR G 108 3.177 -26.557 58.747 1.00 0.00
G C
ATOM 22214 HD1 TYR G 108 2.985 -27.485 58.226 1.00 0.00
G H
ATOM 22215 CE1 TYR G 108 3.161 -26.553 60.106 1.00 0.00
G C
ATOM 22216 HE1 TYR G 108 2.970 -27.462 60.656 1.00 0.00
G H
ATOM 22217 CZ TYR G 108 3.382 -25.380 60.776 1.00 0.00
G C
ATOM 22218 OH TYR G 108 3.363 -25.372 62.149 1.00 0.00
G O
ATOM 22219 HH TYR G 108 3.607 -24.490 62.435 1.00 0.00
G H
ATOM 22220 CD2 TYR G 108 3.639 -24.234 58.715 1.00 0.00
G C

ATOM 22221 HD2 TYR G 108 3.794 -23.311 58.173 1.00 0.00
G H
ATOM 22222 CE2 TYR G 108 3.622 -24.215 60.083 1.00 0.00
G C
ATOM 22223 HE2 TYR G 108 3.777 -23.283 60.608 1.00 0.00
G H
ATOM 22224 C TYR G 108 4.325 -26.623 54.553 1.00 0.00
G C
ATOM 22225 O TYR G 108 4.086 -27.721 54.032 1.00 0.00
G O
ATOM 22226 N HSE G 109 4.574 -25.519 53.849 1.00 0.00
G N
ATOM 22227 HN HSE G 109 4.721 -24.617 54.253 1.00 0.00
G H
ATOM 22228 CA HSE G 109 4.652 -25.546 52.397 1.00 0.00
G C
ATOM 22229 HA HSE G 109 3.636 -25.589 52.024 1.00 0.00
G H
ATOM 22230 CB HSE G 109 5.386 -24.308 51.910 1.00 0.00
G C
ATOM 22231 HB1 HSE G 109 4.743 -23.425 52.118 1.00 0.00
G H
ATOM 22232 HB2 HSE G 109 6.319 -24.176 52.496 1.00 0.00
G H
ATOM 22233 ND1 HSE G 109 6.966 -24.228 49.958 1.00 0.00
G N
ATOM 22234 CG HSE G 109 5.685 -24.326 50.451 1.00 0.00
G C
ATOM 22235 CE1 HSE G 109 6.933 -24.269 48.638 1.00 0.00
G C
ATOM 22236 HE1 HSE G 109 7.814 -24.212 47.993 1.00 0.00
G H
ATOM 22237 NE2 HSE G 109 5.673 -24.379 48.258 1.00 0.00
G N
ATOM 22238 HE2 HSE G 109 5.357 -24.410 47.311 1.00 0.00
G H
ATOM 22239 CD2 HSE G 109 4.873 -24.414 49.374 1.00 0.00
G C
ATOM 22240 HD2 HSE G 109 3.796 -24.490 49.323 1.00 0.00
G H
ATOM 22241 C HSE G 109 5.354 -26.801 51.921 1.00 0.00
G C
ATOM 22242 O HSE G 109 4.929 -27.433 50.952 1.00 0.00
G O
ATOM 22243 N HSE G 110 6.399 -27.205 52.637 1.00 0.00
G N
ATOM 22244 HN HSE G 110 6.770 -26.715 53.425 1.00 0.00
G H
ATOM 22245 CA HSE G 110 7.101 -28.435 52.311 1.00 0.00
G C
ATOM 22246 HA HSE G 110 7.383 -28.363 51.267 1.00 0.00
G H
ATOM 22247 CB HSE G 110 8.315 -28.563 53.212 1.00 0.00
G C

ATOM 22248 HB1 HSE G 110 9.181 -28.105 52.687 1.00 0.00
G H
ATOM 22249 HB2 HSE G 110 8.150 -27.978 54.141 1.00 0.00
G H
ATOM 22250 ND1 HSE G 110 9.549 -30.704 52.783 1.00 0.00
G N
ATOM 22251 CG HSE G 110 8.666 -29.970 53.540 1.00 0.00
G C
ATOM 22252 CE1 HSE G 110 9.664 -31.913 53.300 1.00 0.00
G C
ATOM 22253 HE1 HSE G 110 10.298 -32.705 52.892 1.00 0.00
G H
ATOM 22254 NE2 HSE G 110 8.887 -31.987 54.365 1.00 0.00
G N
ATOM 22255 HE2 HSE G 110 8.770 -32.798 54.938 1.00 0.00
G H
ATOM 22256 CD2 HSE G 110 8.250 -30.783 54.537 1.00 0.00
G C
ATOM 22257 HD2 HSE G 110 7.550 -30.595 55.338 1.00 0.00
G H
ATOM 22258 C HSE G 110 6.204 -29.654 52.449 1.00 0.00
G C
ATOM 22259 O HSE G 110 6.223 -30.549 51.599 1.00 0.00
G O
ATOM 22260 N LYS G 111 5.446 -29.741 53.533 1.00 0.00
G N
ATOM 22261 HN LYS G 111 5.396 -29.078 54.278 1.00 0.00
G H
ATOM 22262 CA LYS G 111 4.588 -30.907 53.686 1.00 0.00
G C
ATOM 22263 HA LYS G 111 5.213 -31.782 53.554 1.00 0.00
G H
ATOM 22264 CB LYS G 111 3.945 -30.929 55.062 1.00 0.00
G C
ATOM 22265 HB1 LYS G 111 4.762 -31.085 55.803 1.00 0.00
G H
ATOM 22266 HB2 LYS G 111 3.515 -29.925 55.277 1.00 0.00
G H
ATOM 22267 CG LYS G 111 2.904 -32.026 55.202 1.00 0.00
G C
ATOM 22268 HG1 LYS G 111 2.000 -31.609 55.702 1.00 0.00
G H
ATOM 22269 HG2 LYS G 111 2.595 -32.365 54.186 1.00 0.00
G H
ATOM 22270 CD LYS G 111 3.397 -33.230 56.021 1.00 0.00
G C
ATOM 22271 HD1 LYS G 111 2.755 -33.325 56.926 1.00 0.00
G H
ATOM 22272 HD2 LYS G 111 3.250 -34.150 55.410 1.00 0.00
G H
ATOM 22273 CE LYS G 111 4.843 -33.091 56.411 1.00 0.00
G C
ATOM 22274 HE1 LYS G 111 5.200 -32.054 56.225 1.00 0.00
G H

ATOM 22275 HE2 LYS G 111 4.981 -33.323 57.490 1.00 0.00
G H
ATOM 22276 NZ LYS G 111 5.670 -34.028 55.613 1.00 0.00
G N
ATOM 22277 HZ1 LYS G 111 6.671 -33.934 55.879 1.00 0.00
G H
ATOM 22278 HZ2 LYS G 111 5.356 -35.004 55.789 1.00 0.00
G H
ATOM 22279 HZ3 LYS G 111 5.560 -33.811 54.602 1.00 0.00
G H
ATOM 22280 C LYS G 111 3.519 -30.948 52.609 1.00 0.00
G C
ATOM 22281 O LYS G 111 3.251 -32.001 52.020 1.00 0.00
G O
ATOM 22282 N VAL G 112 2.900 -29.806 52.337 1.00 0.00
G N
ATOM 22283 HN VAL G 112 3.042 -28.938 52.805 1.00 0.00
G H
ATOM 22284 CA VAL G 112 1.911 -29.757 51.265 1.00 0.00
G C
ATOM 22285 HA VAL G 112 1.154 -30.502 51.479 1.00 0.00
G H
ATOM 22286 CB VAL G 112 1.238 -28.379 51.208 1.00 0.00
G C
ATOM 22287 HB VAL G 112 0.133 -28.541 51.250 1.00 0.00
G H
ATOM 22288 CG1 VAL G 112 1.664 -27.553 52.374 1.00 0.00
G C
ATOM 22289 HG11 VAL G 112 1.210 -26.540 52.316 1.00 0.00
G H
ATOM 22290 HG12 VAL G 112 1.343 -28.005 53.335 1.00 0.00
G H
ATOM 22291 HG13 VAL G 112 2.769 -27.434 52.389 1.00 0.00
G H
ATOM 22292 CG2 VAL G 112 1.595 -27.680 49.925 1.00 0.00
G C
ATOM 22293 HG21 VAL G 112 1.146 -26.665 49.892 1.00 0.00
G H
ATOM 22294 HG22 VAL G 112 2.698 -27.578 49.830 1.00 0.00
G H
ATOM 22295 HG23 VAL G 112 1.221 -28.241 49.044 1.00 0.00
G H
ATOM 22296 C VAL G 112 2.552 -30.128 49.939 1.00 0.00
G C
ATOM 22297 O VAL G 112 1.910 -30.721 49.065 1.00 0.00
G O
ATOM 22298 N LEU G 113 3.829 -29.792 49.774 1.00 0.00
G N
ATOM 22299 HN LEU G 113 4.355 -29.203 50.383 1.00 0.00
G H
ATOM 22300 CA LEU G 113 4.578 -30.290 48.634 1.00 0.00
G C
ATOM 22301 HA LEU G 113 4.054 -29.965 47.744 1.00 0.00
G H

ATOM 22302 CB LEU G 113   5.998 -29.748 48.693 1.00 0.00
G   C
ATOM 22303 HB1 LEU G 113   5.948 -28.746 49.179 1.00 0.00
G   H
ATOM 22304 HB2 LEU G 113   6.602 -30.390 49.372 1.00 0.00
G   H
ATOM 22305 CG LEU G 113   6.744 -29.578 47.385 1.00 0.00
G   C
ATOM 22306 HG LEU G 113   7.287 -30.529 47.164 1.00 0.00
G   H
ATOM 22307 CD1 LEU G 113   5.777 -29.300 46.267 1.00 0.00
G   C
ATOM 22308 HD11 LEU G 113   6.321 -29.127 45.314 1.00 0.00
G   H
ATOM 22309 HD12 LEU G 113   5.088 -30.161 46.124 1.00 0.00
G   H
ATOM 22310 HD13 LEU G 113   5.169 -28.397 46.489 1.00 0.00
G   H
ATOM 22311 CD2 LEU G 113   7.765 -28.484 47.539 1.00 0.00
G   C
ATOM 22312 HD21 LEU G 113   8.339 -28.355 46.596 1.00 0.00
G   H
ATOM 22313 HD22 LEU G 113   7.291 -27.512 47.781 1.00 0.00
G   H
ATOM 22314 HD23 LEU G 113   8.483 -28.733 48.350 1.00 0.00
G   H
ATOM 22315 C   LEU G 113   4.594 -31.804 48.626 1.00 0.00
G   C
ATOM 22316 O   LEU G 113   4.331 -32.437 47.599 1.00 0.00
G   O
ATOM 22317 N   GLU G 114   4.894 -32.400 49.774 1.00 0.00
G   N
ATOM 22318 HN GLU G 114   5.088 -31.939 50.635 1.00 0.00
G   H
ATOM 22319 CA GLU G 114   4.961 -33.852 49.852 1.00 0.00
G   C
ATOM 22320 HA GLU G 114   5.717 -34.193 49.156 1.00 0.00
G   H
ATOM 22321 CB GLU G 114   5.337 -34.286 51.264 1.00 0.00
G   C
ATOM 22322 HB1 GLU G 114   6.167 -33.632 51.619 1.00 0.00
G   H
ATOM 22323 HB2 GLU G 114   4.485 -34.101 51.955 1.00 0.00
G   H
ATOM 22324 CG GLU G 114   5.764 -35.725 51.314 1.00 0.00
G   C
ATOM 22325 HG1 GLU G 114   6.327 -35.911 52.246 1.00 0.00
G   H
ATOM 22326 HG2 GLU G 114   4.886 -36.401 51.277 1.00 0.00
G   H
ATOM 22327 CD GLU G 114   6.700 -36.075 50.181 1.00 0.00
G   C
ATOM 22328 OE1 GLU G 114   7.733 -35.390 50.039 1.00 0.00
G   O

ATOM 22329 OE2 GLU G 114   6.397 -37.020 49.424 1.00 0.00
G   O
ATOM 22330 C   GLU G 114   3.643 -34.487 49.434 1.00 0.00
G   C
ATOM 22331 O   GLU G 114   3.621 -35.473 48.692 1.00 0.00
G   O
ATOM 22332 N   LYS G 115   2.530 -33.921 49.893 1.00 0.00
G   N
ATOM 22333 HN LYS G 115   2.493 -33.080 50.431 1.00 0.00
G   H
ATOM 22334 CA LYS G 115   1.239 -34.536 49.617 1.00 0.00
G   C
ATOM 22335 HA LYS G 115   1.311 -35.562 49.959 1.00 0.00
G   H
ATOM 22336 CB LYS G 115   0.138 -33.803 50.360 1.00 0.00
G   C
ATOM 22337 HB1 LYS G 115   0.250 -32.719 50.126 1.00 0.00
G   H
ATOM 22338 HB2 LYS G 115   -0.847 -34.112 49.944 1.00 0.00
G   H
ATOM 22339 CG LYS G 115   0.212 -34.018 51.823 1.00 0.00
G   C
ATOM 22340 HG1 LYS G 115   1.041 -33.402 52.241 1.00 0.00
G   H
ATOM 22341 HG2 LYS G 115   -0.735 -33.660 52.290 1.00 0.00
G   H
ATOM 22342 CD LYS G 115   0.450 -35.459 52.141 1.00 0.00
G   C
ATOM 22343 HD1 LYS G 115   -0.352 -36.063 51.657 1.00 0.00
G   H
ATOM 22344 HD2 LYS G 115   1.419 -35.760 51.678 1.00 0.00
G   H
ATOM 22345 CE LYS G 115   0.468 -35.628 53.631 1.00 0.00
G   C
ATOM 22346 HE1 LYS G 115   0.421 -36.707 53.902 1.00 0.00
G   H
ATOM 22347 HE2 LYS G 115   1.399 -35.198 54.063 1.00 0.00
G   H
ATOM 22348 NZ LYS G 115   -0.705 -34.931 54.222 1.00 0.00
G   N
ATOM 22349 HZ1 LYS G 115   -0.703 -35.044 55.256 1.00 0.00
G   H
ATOM 22350 HZ2 LYS G 115   -0.664 -33.919 53.984 1.00 0.00
G   H
ATOM 22351 HZ3 LYS G 115   -1.580 -35.338 53.833 1.00 0.00
G   H
ATOM 22352 C   LYS G 115   0.907 -34.574 48.137 1.00 0.00
G   C
ATOM 22353 O   LYS G 115   0.227 -35.501 47.689 1.00 0.00
G   O
ATOM 22354 N   ILE G 116   1.360 -33.593 47.364 1.00 0.00
G   N
ATOM 22355 HN ILE G 116   1.896 -32.816 47.688 1.00 0.00
G   H

```
ATOM  22356  CA  ILE G 116       1.097 -33.580  45.935  1.00  0.00
G    C
ATOM  22357  HA  ILE G 116       0.048 -33.822  45.821  1.00  0.00
G    H
ATOM  22358  CB  ILE G 116       1.411 -32.209  45.328  1.00  0.00
G    C
ATOM  22359  HB  ILE G 116       1.025 -31.475  46.080  1.00  0.00
G    H
ATOM  22360  CG2 ILE G 116       2.874 -32.124  44.984  1.00  0.00
G    C
ATOM  22361 HG21 ILE G 116       3.147 -31.089  44.690  1.00  0.00
G    H
ATOM  22362 HG22 ILE G 116       3.503 -32.404  45.856  1.00  0.00
G    H
ATOM  22363 HG23 ILE G 116       3.124 -32.801  44.140  1.00  0.00
G    H
ATOM  22364  CG1 ILE G 116       0.613 -31.984  44.047  1.00  0.00
G    C
ATOM  22365 HG11 ILE G 116       0.792 -30.952  43.669  1.00  0.00
G    H
ATOM  22366 HG12 ILE G 116       0.979 -32.688  43.265  1.00  0.00
G    H
ATOM  22367  CD  ILE G 116      -0.848 -32.187  44.184  1.00  0.00
G    C
ATOM  22368  HD1 ILE G 116      -1.349 -32.062  43.200  1.00  0.00
G    H
ATOM  22369  HD2 ILE G 116      -1.068 -33.209  44.558  1.00  0.00
G    H
ATOM  22370  HD3 ILE G 116      -1.288 -31.451  44.890  1.00  0.00
G    H
ATOM  22371  C   ILE G 116       1.878 -34.663  45.212  1.00  0.00
G    C
ATOM  22372  O   ILE G 116       1.561 -34.992  44.069  1.00  0.00
G    O
ATOM  22373  N   GLY G 117       2.882 -35.238  45.857  1.00  0.00
G    N
ATOM  22374  HN  GLY G 117       3.060 -35.130  46.834  1.00  0.00
G    H
ATOM  22375  CA  GLY G 117       3.809 -36.084  45.144  1.00  0.00
G    C
ATOM  22376  HA1 GLY G 117       3.320 -36.384  44.227  1.00  0.00
G    H
ATOM  22377  HA2 GLY G 117       4.063 -36.906  45.800  1.00  0.00
G    H
ATOM  22378  C   GLY G 117       5.081 -35.370  44.782  1.00  0.00
G    C
ATOM  22379  O   GLY G 117       5.590 -35.545  43.673  1.00  0.00
G    O
ATOM  22380  N   ALA G 118       5.602 -34.550  45.687  1.00  0.00
G    N
ATOM  22381  HN  ALA G 118       5.177 -34.344  46.567  1.00  0.00
G    H
ATOM  22382  CA  ALA G 118       6.865 -33.885  45.435  1.00  0.00
G    C
```

ATOM 22383 HA ALA G 118   6.831 -33.319 44.510 1.00 0.00
G   H
ATOM 22384 CB ALA G 118   7.206 -32.944 46.582 1.00 0.00
G   C
ATOM 22385 HB1 ALA G 118   6.420 -32.164 46.670 1.00 0.00
G   H
ATOM 22386 HB2 ALA G 118   7.254 -33.493 47.548 1.00 0.00
G   H
ATOM 22387 HB3 ALA G 118   8.179 -32.433 46.417 1.00 0.00
G   H
ATOM 22388 C   ALA G 118   7.970 -34.908 45.270 1.00 0.00
G   C
ATOM 22389 O   ALA G 118   8.378 -35.564 46.230 1.00 0.00
G   O
ATOM 22390 N   ASP G 119   8.452 -35.046 44.043 1.00 0.00
G   N
ATOM 22391 HN ASP G 119   8.147 -34.590 43.210 1.00 0.00
G   H
ATOM 22392 CA ASP G 119   9.559 -35.957 43.815 1.00 0.00
G   C
ATOM 22393 HA ASP G 119   9.259 -36.924 44.202 1.00 0.00
G   H
ATOM 22394 CB ASP G 119   9.873 -36.036 42.325 1.00 0.00
G   C
ATOM 22395 HB1 ASP G 119   9.997 -35.010 41.922 1.00 0.00
G   H
ATOM 22396 HB2 ASP G 119   10.814 -36.595 42.153 1.00 0.00
G   H
ATOM 22397 CG ASP G 119   8.764 -36.702 41.542 1.00 0.00
G   C
ATOM 22398 OD1 ASP G 119   8.872 -37.921 41.285 1.00 0.00
G   O
ATOM 22399 OD2 ASP G 119   7.780 -36.015 41.201 1.00 0.00
G   O
ATOM 22400 C   ASP G 119   10.786 -35.535 44.599 1.00 0.00
G   C
ATOM 22401 O   ASP G 119   11.624 -36.377 44.930 1.00 0.00
G   O
ATOM 22402 N   ARG G 120   10.909 -34.248 44.903 1.00 0.00
G   N
ATOM 22403 HN ARG G 120   10.254 -33.559 44.605 1.00 0.00
G   H
ATOM 22404 CA ARG G 120   12.030 -33.781 45.700 1.00 0.00
G   C
ATOM 22405 HA ARG G 120   12.092 -34.379 46.601 1.00 0.00
G   H
ATOM 22406 CB ARG G 120   13.330 -33.875 44.912 1.00 0.00
G   C
ATOM 22407 HB1 ARG G 120   13.628 -34.951 44.925 1.00 0.00
G   H
ATOM 22408 HB2 ARG G 120   13.138 -33.623 43.846 1.00 0.00
G   H
ATOM 22409 CG ARG G 120   14.406 -33.054 45.502 1.00 0.00
G   C

```
ATOM  22410  HG1 ARG G 120      14.577 -32.172  44.847  1.00  0.00
G    H
ATOM  22411  HG2 ARG G 120      14.053 -32.640  46.474  1.00  0.00
G    H
ATOM  22412  CD  ARG G 120      15.614 -33.884  45.720  1.00  0.00
G    C
ATOM  22413  HD1 ARG G 120      15.407 -34.637  46.515  1.00  0.00
G    H
ATOM  22414  HD2 ARG G 120      15.916 -34.419  44.790  1.00  0.00
G    H
ATOM  22415  NE  ARG G 120      16.731 -33.048  46.118  1.00  0.00
G    N
ATOM  22416  HE  ARG G 120      16.499 -32.070  46.160  1.00  0.00
G    H
ATOM  22417  CZ  ARG G 120      17.939 -33.515  46.398  1.00  0.00
G    C
ATOM  22418  NH1 ARG G 120      18.179 -34.813  46.316  1.00  0.00
G    N
ATOM  22419 HH11 ARG G 120      19.071 -35.177  46.548  1.00  0.00
G    H
ATOM  22420 HH12 ARG G 120      17.386 -35.402  46.145  1.00  0.00
G    H
ATOM  22421  NH2 ARG G 120      18.907 -32.685  46.753  1.00  0.00
G    N
ATOM  22422 HH21 ARG G 120      19.828 -33.025  46.877  1.00  0.00
G    H
ATOM  22423 HH22 ARG G 120      18.716 -31.715  46.677  1.00  0.00
G    H
ATOM  22424  C   ARG G 120      11.793 -32.348  46.146  1.00  0.00
G    C
ATOM  22425  O   ARG G 120      11.459 -31.481  45.330  1.00  0.00
G    O
ATOM  22426  N   ILE G 121      12.002 -32.103  47.434  1.00  0.00
G    N
ATOM  22427  HN  ILE G 121      12.312 -32.811  48.066  1.00  0.00
G    H
ATOM  22428  CA  ILE G 121      11.803 -30.799  48.049  1.00  0.00
G    C
ATOM  22429  HA  ILE G 121      11.431 -30.129  47.285  1.00  0.00
G    H
ATOM  22430  CB  ILE G 121      10.845 -30.907  49.245  1.00  0.00
G    C
ATOM  22431  HB  ILE G 121      11.420 -31.482  50.015  1.00  0.00
G    H
ATOM  22432  CG2 ILE G 121      10.417 -29.543  49.725  1.00  0.00
G    C
ATOM  22433 HG21 ILE G 121       9.854 -29.623  50.679  1.00  0.00
G    H
ATOM  22434 HG22 ILE G 121      11.298 -28.894  49.908  1.00  0.00
G    H
ATOM  22435 HG23 ILE G 121       9.763 -29.046  48.978  1.00  0.00
G    H
ATOM  22436  CG1 ILE G 121       9.639 -31.758  48.856  1.00  0.00
G    C
```

```
ATOM  22437 HG11 ILE G 121       9.206 -31.384  47.901  1.00  0.00
G    H
ATOM  22438 HG12 ILE G 121       9.978 -32.803  48.676  1.00  0.00
G    H
ATOM  22439  CD  ILE G 121       8.556 -31.799  49.893  1.00  0.00
G    C
ATOM  22440  HD1 ILE G 121       7.739 -32.480  49.571  1.00  0.00
G    H
ATOM  22441  HD2 ILE G 121       8.955 -32.169  50.862  1.00  0.00
G    H
ATOM  22442  HD3 ILE G 121       8.119 -30.791  50.054  1.00  0.00
G    H
ATOM  22443  C   ILE G 121      13.160 -30.267  48.478  1.00  0.00
G    C
ATOM  22444  O   ILE G 121      14.069 -31.049  48.775  1.00  0.00
G    O
ATOM  22445  N   ILE G 122      13.312 -28.947  48.494  1.00  0.00
G    N
ATOM  22446  HN  ILE G 122      12.616 -28.296  48.199  1.00  0.00
G    H
ATOM  22447  CA  ILE G 122      14.547 -28.330  48.957  1.00  0.00
G    C
ATOM  22448  HA  ILE G 122      15.050 -29.055  49.584  1.00  0.00
G    H
ATOM  22449  CB  ILE G 122      15.447 -27.887  47.789  1.00  0.00
G    C
ATOM  22450  HB  ILE G 122      14.766 -27.309  47.115  1.00  0.00
G    H
ATOM  22451  CG2 ILE G 122      16.629 -27.086  48.299  1.00  0.00
G    C
ATOM  22452 HG21 ILE G 122      17.190 -26.636  47.453  1.00  0.00
G    H
ATOM  22453 HG22 ILE G 122      16.292 -26.258  48.957  1.00  0.00
G    H
ATOM  22454 HG23 ILE G 122      17.327 -27.729  48.875  1.00  0.00
G    H
ATOM  22455  CG1 ILE G 122      15.926 -29.100  46.999  1.00  0.00
G    C
ATOM  22456 HG11 ILE G 122      16.949 -28.912  46.605  1.00  0.00
G    H
ATOM  22457 HG12 ILE G 122      15.993 -29.974  47.686  1.00  0.00
G    H
ATOM  22458  CD  ILE G 122      15.024 -29.477  45.853  1.00  0.00
G    C
ATOM  22459  HD1 ILE G 122      15.396 -30.399  45.356  1.00  0.00
G    H
ATOM  22460  HD2 ILE G 122      13.990 -29.669  46.214  1.00  0.00
G    H
ATOM  22461  HD3 ILE G 122      14.987 -28.668  45.092  1.00  0.00
G    H
ATOM  22462  C   ILE G 122      14.216 -27.142  49.841  1.00  0.00
G    C
ATOM  22463  O   ILE G 122      13.367 -26.314  49.497  1.00  0.00
G    O
```

ATOM 22464 N   HSE G 123   14.906 -27.051 50.964 1.00 0.00
G   N
ATOM 22465 HN HSE G 123   15.538 -27.774 51.243 1.00 0.00
G   H
ATOM 22466 CA HSE G 123   14.831 -25.933 51.888 1.00 0.00
G   C
ATOM 22467 HA HSE G 123   14.079 -25.238 51.531 1.00 0.00
G   H
ATOM 22468 CB HSE G 123   14.550 -26.420 53.303 1.00 0.00
G   C
ATOM 22469 HB1 HSE G 123   15.356 -27.133 53.585 1.00 0.00
G   H
ATOM 22470 HB2 HSE G 123   14.611 -25.566 54.008 1.00 0.00
G   H
ATOM 22471 ND1 HSE G 123   12.169 -26.565 54.114 1.00 0.00
G   N
ATOM 22472 CG HSE G 123   13.242 -27.125 53.458 1.00 0.00
G   C
ATOM 22473 CE1 HSE G 123   11.159 -27.415 54.110 1.00 0.00
G   C
ATOM 22474 HE1 HSE G 123   10.188 -27.226 54.574 1.00 0.00
G   H
ATOM 22475 NE2 HSE G 123   11.535 -28.505 53.465 1.00 0.00
G   N
ATOM 22476 HE2 HSE G 123   10.968 -29.316 53.324 1.00 0.00
G   H
ATOM 22477 CD2 HSE G 123   12.834 -28.349 53.048 1.00 0.00
G   C
ATOM 22478 HD2 HSE G 123   13.373 -29.113 52.505 1.00 0.00
G   H
ATOM 22479 C   HSE G 123   16.167 -25.211 51.831 1.00 0.00
G   C
ATOM 22480 O   HSE G 123   16.972 -25.316 52.765 1.00 0.00
G   O
ATOM 22481 N   PRO G 124   16.437 -24.480 50.750 1.00 0.00
G   N
ATOM 22482 CD PRO G 124   15.485 -23.932 49.777 1.00 0.00
G   C
ATOM 22483 HD1 PRO G 124   15.535 -25.036 49.915 1.00 0.00
G   H
ATOM 22484 HD2 PRO G 124   14.436 -23.611 49.581 1.00 0.00
G   H
ATOM 22485 CA PRO G 124   17.816 -24.055 50.477 1.00 0.00
G   C
ATOM 22486 HA PRO G 124   18.414 -24.927 50.238 1.00 0.00
G   H
ATOM 22487 CB PRO G 124   17.661 -23.161 49.240 1.00 0.00
G   C
ATOM 22488 HB1 PRO G 124   17.667 -23.799 48.328 1.00 0.00
G   H
ATOM 22489 HB2 PRO G 124   18.466 -22.404 49.154 1.00 0.00
G   H
ATOM 22490 CG PRO G 124   16.363 -23.520 48.651 1.00 0.00
G   C

ATOM 22491 HG1 PRO G 124 16.295 -23.276 47.573 1.00 0.00
G H
ATOM 22492 HG2 PRO G 124 15.551 -22.992 49.203 1.00 0.00
G H
ATOM 22493 C PRO G 124 18.472 -23.302 51.613 1.00 0.00
G C
ATOM 22494 O PRO G 124 19.517 -23.732 52.127 1.00 0.00
G O
ATOM 22495 N GLU G 125 17.885 -22.185 52.021 1.00 0.00
G N
ATOM 22496 HN GLU G 125 17.022 -21.811 51.692 1.00 0.00
G H
ATOM 22497 CA GLU G 125 18.519 -21.386 53.052 1.00 0.00
G C
ATOM 22498 HA GLU G 125 19.539 -21.188 52.748 1.00 0.00
G H
ATOM 22499 CB GLU G 125 17.798 -20.064 53.235 1.00 0.00
G C
ATOM 22500 HB1 GLU G 125 16.701 -20.263 53.229 1.00 0.00
G H
ATOM 22501 HB2 GLU G 125 18.026 -19.646 54.241 1.00 0.00
G H
ATOM 22502 CG GLU G 125 18.132 -19.055 52.156 1.00 0.00
G C
ATOM 22503 HG1 GLU G 125 18.274 -18.059 52.613 1.00 0.00
G H
ATOM 22504 HG2 GLU G 125 19.055 -19.343 51.615 1.00 0.00
G H
ATOM 22505 CD GLU G 125 17.039 -18.870 51.141 1.00 0.00
G C
ATOM 22506 OE1 GLU G 125 16.061 -19.654 51.180 1.00 0.00
G O
ATOM 22507 OE2 GLU G 125 17.161 -17.929 50.333 1.00 0.00
G O
ATOM 22508 C GLU G 125 18.579 -22.139 54.360 1.00 0.00
G C
ATOM 22509 O GLU G 125 19.505 -21.937 55.138 1.00 0.00
G O
ATOM 22510 N LYS G 126 17.622 -23.029 54.611 1.00 0.00
G N
ATOM 22511 HN LYS G 126 16.786 -23.154 54.078 1.00 0.00
G H
ATOM 22512 CA LYS G 126 17.767 -23.920 55.753 1.00 0.00
G C
ATOM 22513 HA LYS G 126 17.799 -23.304 56.643 1.00 0.00
G H
ATOM 22514 CB LYS G 126 16.595 -24.897 55.806 1.00 0.00
G C
ATOM 22515 HB1 LYS G 126 16.681 -25.561 54.915 1.00 0.00
G H
ATOM 22516 HB2 LYS G 126 16.711 -25.554 56.698 1.00 0.00
G H
ATOM 22517 CG LYS G 126 15.235 -24.256 55.801 1.00 0.00
G C

ATOM 22518 HG1 LYS G 126   15.095 -23.707 54.842 1.00 0.00
G   H
ATOM 22519 HG2 LYS G 126   14.458 -25.053 55.844 1.00 0.00
G   H
ATOM 22520 CD LYS G 126   15.060 -23.298 56.940 1.00 0.00
G   C
ATOM 22521 HD1 LYS G 126   16.051 -22.842 57.171 1.00 0.00
G   H
ATOM 22522 HD2 LYS G 126   14.386 -22.478 56.603 1.00 0.00
G   H
ATOM 22523 CE LYS G 126   14.486 -23.974 58.171 1.00 0.00
G   C
ATOM 22524 HE1 LYS G 126   13.798 -24.798 57.878 1.00 0.00
G   H
ATOM 22525 HE2 LYS G 126   15.300 -24.401 58.798 1.00 0.00
G   H
ATOM 22526 NZ LYS G 126   13.720 -23.003 59.000 1.00 0.00
G   N
ATOM 22527 HZ1 LYS G 126   13.331 -23.480 59.840 1.00 0.00
G   H
ATOM 22528 HZ2 LYS G 126   14.350 -22.232 59.302 1.00 0.00
G   H
ATOM 22529 HZ3 LYS G 126   12.941 -22.607 58.437 1.00 0.00
G   H
ATOM 22530 C   LYS G 126   19.086 -24.677 55.679 1.00 0.00
G   C
ATOM 22531 O   LYS G 126   19.899 -24.649 56.617 1.00 0.00
G   O
ATOM 22532 N   ASP G 127   19.328 -25.330 54.545 1.00 0.00
G   N
ATOM 22533 HN ASP G 127   18.770 -25.296 53.720 1.00 0.00
G   H
ATOM 22534 CA ASP G 127   20.489 -26.194 54.407 1.00 0.00
G   C
ATOM 22535 HA ASP G 127   20.468 -26.896 55.234 1.00 0.00
G   H
ATOM 22536 CB ASP G 127   20.429 -26.898 53.050 1.00 0.00
G   C
ATOM 22537 HB1 ASP G 127   20.075 -26.181 52.283 1.00 0.00
G   H
ATOM 22538 HB2 ASP G 127   21.432 -27.260 52.752 1.00 0.00
G   H
ATOM 22539 CG ASP G 127   19.473 -28.074 53.045 1.00 0.00
G   C
ATOM 22540 OD1 ASP G 127   18.246 -27.824 53.073 1.00 0.00
G   O
ATOM 22541 OD2 ASP G 127   19.944 -29.230 52.998 1.00 0.00
G   O
ATOM 22542 C   ASP G 127   21.773 -25.392 54.546 1.00 0.00
G   C
ATOM 22543 O   ASP G 127   22.649 -25.709 55.364 1.00 0.00
G   O
ATOM 22544 N   MET G 128   21.901 -24.327 53.765 1.00 0.00
G   N

ATOM 22545 HN MET G 128 21.244 -23.932 53.126 1.00 0.00
G H
ATOM 22546 CA MET G 128 23.166 -23.624 53.833 1.00 0.00
G C
ATOM 22547 HA MET G 128 23.947 -24.372 53.891 1.00 0.00
G H
ATOM 22548 CB MET G 128 23.397 -22.808 52.566 1.00 0.00
G C
ATOM 22549 HB1 MET G 128 24.001 -23.444 51.877 1.00 0.00
G H
ATOM 22550 HB2 MET G 128 22.424 -22.633 52.057 1.00 0.00
G H
ATOM 22551 CG MET G 128 24.124 -21.502 52.771 1.00 0.00
G C
ATOM 22552 HG1 MET G 128 23.398 -20.663 52.842 1.00 0.00
G H
ATOM 22553 HG2 MET G 128 24.693 -21.536 53.725 1.00 0.00
G H
ATOM 22554 SD MET G 128 25.265 -21.137 51.423 1.00 0.00
G S
ATOM 22555 CE MET G 128 25.136 -22.608 50.401 1.00 0.00
G C
ATOM 22556 HE1 MET G 128 25.804 -22.536 49.516 1.00 0.00
G H
ATOM 22557 HE2 MET G 128 25.424 -23.517 50.972 1.00 0.00
G H
ATOM 22558 HE3 MET G 128 24.098 -22.750 50.033 1.00 0.00
G H
ATOM 22559 C MET G 128 23.249 -22.777 55.091 1.00 0.00
G C
ATOM 22560 O MET G 128 24.342 -22.400 55.510 1.00 0.00
G O
ATOM 22561 N GLY G 129 22.135 -22.539 55.764 1.00 0.00
G N
ATOM 22562 HN GLY G 129 21.219 -22.821 55.482 1.00 0.00
G H
ATOM 22563 CA GLY G 129 22.209 -21.818 57.006 1.00 0.00
G C
ATOM 22564 HA1 GLY G 129 21.203 -21.577 57.321 1.00 0.00
G H
ATOM 22565 HA2 GLY G 129 22.849 -20.957 56.868 1.00 0.00
G H
ATOM 22566 C GLY G 129 22.828 -22.692 58.059 1.00 0.00
G C
ATOM 22567 O GLY G 129 23.707 -22.255 58.799 1.00 0.00
G O
ATOM 22568 N VAL G 130 22.362 -23.931 58.157 1.00 0.00
G N
ATOM 22569 HN VAL G 130 21.616 -24.342 57.639 1.00 0.00
G H
ATOM 22570 CA VAL G 130 22.986 -24.823 59.124 1.00 0.00
G C
ATOM 22571 HA VAL G 130 22.978 -24.328 60.087 1.00 0.00
G H

```
ATOM  22572  CB  VAL G 130      22.163 -26.115  59.284  1.00  0.00
G    C
ATOM  22573  HB  VAL G 130      22.591 -26.683  60.145  1.00  0.00
G    H
ATOM  22574  CG1 VAL G 130      20.717 -25.759  59.572  1.00  0.00
G    C
ATOM  22575 HG11 VAL G 130      20.102 -26.681  59.651  1.00  0.00
G    H
ATOM  22576 HG12 VAL G 130      20.614 -25.209  60.530  1.00  0.00
G    H
ATOM  22577 HG13 VAL G 130      20.297 -25.134  58.755  1.00  0.00
G    H
ATOM  22578  CG2 VAL G 130      22.208 -26.960  58.048  1.00  0.00
G    C
ATOM  22579 HG21 VAL G 130      21.578 -27.866  58.169  1.00  0.00
G    H
ATOM  22580 HG22 VAL G 130      21.836 -26.388  57.171  1.00  0.00
G    H
ATOM  22581 HG23 VAL G 130      23.243 -27.298  57.830  1.00  0.00
G    H
ATOM  22582  C   VAL G 130      24.444 -25.067  58.747  1.00  0.00
G    C
ATOM  22583  O   VAL G 130      25.324 -25.123  59.620  1.00  0.00
G    O
ATOM  22584  N   LYS G 131      24.730 -25.165  57.443  1.00  0.00
G    N
ATOM  22585  HN  LYS G 131      24.057 -25.332  56.724  1.00  0.00
G    H
ATOM  22586  CA  LYS G 131      26.109 -25.024  56.982  1.00  0.00
G    C
ATOM  22587  HA  LYS G 131      26.618 -25.945  57.239  1.00  0.00
G    H
ATOM  22588  CB  LYS G 131      26.128 -24.789  55.469  1.00  0.00
G    C
ATOM  22589  HB1 LYS G 131      25.392 -25.495  55.018  1.00  0.00
G    H
ATOM  22590  HB2 LYS G 131      25.748 -23.764  55.257  1.00  0.00
G    H
ATOM  22591  CG  LYS G 131      27.473 -24.995  54.770  1.00  0.00
G    C
ATOM  22592  HG1 LYS G 131      27.907 -25.969  55.093  1.00  0.00
G    H
ATOM  22593  HG2 LYS G 131      27.306 -25.054  53.670  1.00  0.00
G    H
ATOM  22594  CD  LYS G 131      28.428 -23.849  55.130  1.00  0.00
G    C
ATOM  22595  HD1 LYS G 131      28.279 -23.023  54.397  1.00  0.00
G    H
ATOM  22596  HD2 LYS G 131      28.139 -23.460  56.134  1.00  0.00
G    H
ATOM  22597  CE  LYS G 131      29.892 -24.241  55.155  1.00  0.00
G    C
ATOM  22598  HE1 LYS G 131      30.505 -23.432  55.610  1.00  0.00
G    H
```

ATOM 22599 HE2 LYS G 131 30.037 -25.170 55.750 1.00 0.00
G H
ATOM 22600 NZ LYS G 131 30.374 -24.485 53.736 1.00 0.00
G N
ATOM 22601 HZ1 LYS G 131 31.379 -24.754 53.743 1.00 0.00
G H
ATOM 22602 HZ2 LYS G 131 29.816 -25.248 53.305 1.00 0.00
G H
ATOM 22603 HZ3 LYS G 131 30.254 -23.616 53.176 1.00 0.00
G H
ATOM 22604 C LYS G 131 26.834 -23.894 57.685 1.00 0.00
G C
ATOM 22605 O LYS G 131 27.759 -24.128 58.475 1.00 0.00
G O
ATOM 22606 N ILE G 132 26.439 -22.665 57.395 1.00 0.00
G N
ATOM 22607 HN ILE G 132 25.598 -22.421 56.917 1.00 0.00
G H
ATOM 22608 CA ILE G 132 27.255 -21.528 57.779 1.00 0.00
G C
ATOM 22609 HA ILE G 132 28.261 -21.732 57.436 1.00 0.00
G H
ATOM 22610 CB ILE G 132 26.712 -20.246 57.134 1.00 0.00
G C
ATOM 22611 HB ILE G 132 26.795 -20.430 56.032 1.00 0.00
G H
ATOM 22612 CG2 ILE G 132 25.311 -19.981 57.595 1.00 0.00
G C
ATOM 22613 HG21 ILE G 132 24.859 -19.148 57.016 1.00 0.00
G H
ATOM 22614 HG22 ILE G 132 24.670 -20.876 57.451 1.00 0.00
G H
ATOM 22615 HG23 ILE G 132 25.292 -19.705 58.671 1.00 0.00
G H
ATOM 22616 CG1 ILE G 132 27.631 -19.074 57.462 1.00 0.00
G C
ATOM 22617 HG11 ILE G 132 27.245 -18.533 58.355 1.00 0.00
G H
ATOM 22618 HG12 ILE G 132 28.639 -19.469 57.726 1.00 0.00
G H
ATOM 22619 CD ILE G 132 27.782 -18.114 56.334 1.00 0.00
G C
ATOM 22620 HD1 ILE G 132 28.504 -17.312 56.603 1.00 0.00
G H
ATOM 22621 HD2 ILE G 132 28.161 -18.631 55.426 1.00 0.00
G H
ATOM 22622 HD3 ILE G 132 26.813 -17.633 56.086 1.00 0.00
G H
ATOM 22623 C ILE G 132 27.320 -21.427 59.290 1.00 0.00
G C
ATOM 22624 O ILE G 132 28.321 -20.977 59.861 1.00 0.00
G O
ATOM 22625 N ALA G 133 26.276 -21.897 59.960 1.00 0.00
G N

ATOM 22626 HN ALA G 133   25.455 -22.273 59.531 1.00 0.00
G   H
ATOM 22627 CA ALA G 133   26.272 -21.889 61.409 1.00 0.00
G   C
ATOM 22628 HA ALA G 133   26.476 -20.895 61.790 1.00 0.00
G   H
ATOM 22629 CB ALA G 133   24.907 -22.332 61.920 1.00 0.00
G   C
ATOM 22630 HB1 ALA G 133   24.126 -21.631 61.553 1.00 0.00
G   H
ATOM 22631 HB2 ALA G 133   24.654 -23.350 61.552 1.00 0.00
G   H
ATOM 22632 HB3 ALA G 133   24.869 -22.337 63.031 1.00 0.00
G   H
ATOM 22633 C   ALA G 133   27.366 -22.787 61.950 1.00 0.00
G   C
ATOM 22634 O   ALA G 133   28.159 -22.366 62.800 1.00 0.00
G   O
ATOM 22635 N   GLN G 134   27.433 -24.024 61.462 1.00 0.00
G   N
ATOM 22636 HN GLN G 134   26.811 -24.438 60.799 1.00 0.00
G   H
ATOM 22637 CA GLN G 134   28.506 -24.900 61.910 1.00 0.00
G   C
ATOM 22638 HA GLN G 134   28.449 -24.949 62.990 1.00 0.00
G   H
ATOM 22639 CB GLN G 134   28.336 -26.288 61.312 1.00 0.00
G   C
ATOM 22640 HB1 GLN G 134   28.523 -26.194 60.217 1.00 0.00
G   H
ATOM 22641 HB2 GLN G 134   29.134 -26.960 61.705 1.00 0.00
G   H
ATOM 22642 CG GLN G 134   26.962 -26.854 61.559 1.00 0.00
G   C
ATOM 22643 HG1 GLN G 134   26.229 -26.034 61.721 1.00 0.00
G   H
ATOM 22644 HG2 GLN G 134   26.621 -27.448 60.688 1.00 0.00
G   H
ATOM 22645 CD GLN G 134   26.910 -27.723 62.792 1.00 0.00
G   C
ATOM 22646 OE1 GLN G 134   27.910 -27.891 63.491 1.00 0.00
G   O
ATOM 22647 NE2 GLN G 134   25.736 -28.275 63.079 1.00 0.00
G   N
ATOM 22648 HE21 GLN G 134   25.695 -28.846 63.894 1.00 0.00
G   H
ATOM 22649 HE22 GLN G 134   24.957 -28.120 62.480 1.00 0.00
G   H
ATOM 22650 C   GLN G 134   29.861 -24.317 61.549 1.00 0.00
G   C
ATOM 22651 O   GLN G 134   30.802 -24.371 62.348 1.00 0.00
G   O
ATOM 22652 N   SER G 135   29.974 -23.725 60.359 1.00 0.00
G   N

ATOM 22653 HN SER G 135   29.236 -23.574 59.701 1.00 0.00
G   H
ATOM 22654 CA SER G 135   31.275 -23.228 59.923 1.00 0.00
G   C
ATOM 22655 HA SER G 135   31.980 -24.017 60.145 1.00 0.00
G   H
ATOM 22656 CB SER G 135   31.254 -22.908 58.432 1.00 0.00
G   C
ATOM 22657 HB1 SER G 135   32.219 -22.417 58.170 1.00 0.00
G   H
ATOM 22658 HB2 SER G 135   31.192 -23.846 57.834 1.00 0.00
G   H
ATOM 22659 OG SER G 135   30.196 -22.030 58.120 1.00 0.00
G   O
ATOM 22660 HG1 SER G 135   30.362 -21.690 57.232 1.00 0.00
G   H
ATOM 22661 C   SER G 135   31.705 -22.001 60.713 1.00 0.00
G   C
ATOM 22662 O   SER G 135   32.902 -21.701 60.783 1.00 0.00
G   O
ATOM 22663 N   LEU G 136   30.761 -21.277 61.306 1.00 0.00
G   N
ATOM 22664 HN LEU G 136   29.778 -21.444 61.266 1.00 0.00
G   H
ATOM 22665 CA LEU G 136   31.138 -20.117 62.103 1.00 0.00
G   C
ATOM 22666 HA LEU G 136   32.068 -19.746 61.690 1.00 0.00
G   H
ATOM 22667 CB LEU G 136   30.069 -19.033 62.016 1.00 0.00
G   C
ATOM 22668 HB1 LEU G 136   29.143 -19.514 61.627 1.00 0.00
G   H
ATOM 22669 HB2 LEU G 136   29.825 -18.682 63.043 1.00 0.00
G   H
ATOM 22670 CG LEU G 136   30.466 -17.873 61.107 1.00 0.00
G   C
ATOM 22671 HG LEU G 136   30.193 -18.110 60.050 1.00 0.00
G   H
ATOM 22672 CD1 LEU G 136   29.789 -16.602 61.548 1.00 0.00
G   C
ATOM 22673 HD11 LEU G 136   30.115 -15.747 60.916 1.00 0.00
G   H
ATOM 22674 HD12 LEU G 136   28.686 -16.697 61.464 1.00 0.00
G   H
ATOM 22675 HD13 LEU G 136   30.042 -16.369 62.604 1.00 0.00
G   H
ATOM 22676 CD2 LEU G 136   31.965 -17.690 61.174 1.00 0.00
G   C
ATOM 22677 HD21 LEU G 136   32.283 -16.861 60.505 1.00 0.00
G   H
ATOM 22678 HD22 LEU G 136   32.302 -17.441 62.200 1.00 0.00
G   H
ATOM 22679 HD23 LEU G 136   32.485 -18.617 60.850 1.00 0.00
G   H

ATOM 22680 C LEU G 136 31.399 -20.500 63.548 1.00 0.00
G C
ATOM 22681 O LEU G 136 32.351 -20.009 64.164 1.00 0.00
G O
ATOM 22682 N SER G 137 30.559 -21.371 64.108 1.00 0.00
G N
ATOM 22683 HN SER G 137 29.775 -21.790 63.650 1.00 0.00
G H
ATOM 22684 CA SER G 137 30.736 -21.791 65.494 1.00 0.00
G C
ATOM 22685 HA SER G 137 30.581 -20.907 66.098 1.00 0.00
G H
ATOM 22686 CB SER G 137 29.741 -22.897 65.825 1.00 0.00
G C
ATOM 22687 HB1 SER G 137 29.849 -23.147 66.906 1.00 0.00
G H
ATOM 22688 HB2 SER G 137 28.698 -22.537 65.676 1.00 0.00
G H
ATOM 22689 OG SER G 137 30.016 -24.049 65.049 1.00 0.00
G O
ATOM 22690 HG1 SER G 137 29.521 -24.780 65.441 1.00 0.00
G H
ATOM 22691 C SER G 137 32.161 -22.267 65.744 1.00 0.00
G C
ATOM 22692 O SER G 137 32.903 -21.664 66.527 1.00 0.00
G O
ATOM 22693 N ASP G 138 32.568 -23.327 65.064 1.00 0.00
G N
ATOM 22694 HN ASP G 138 31.999 -23.841 64.427 1.00 0.00
G H
ATOM 22695 CA ASP G 138 33.918 -23.862 65.179 1.00 0.00
G C
ATOM 22696 HA ASP G 138 34.365 -23.496 66.097 1.00 0.00
G H
ATOM 22697 CB ASP G 138 33.898 -25.384 65.127 1.00 0.00
G C
ATOM 22698 HB1 ASP G 138 33.246 -25.712 64.292 1.00 0.00
G H
ATOM 22699 HB2 ASP G 138 34.916 -25.783 64.952 1.00 0.00
G H
ATOM 22700 CG ASP G 138 33.353 -26.004 66.395 1.00 0.00
G C
ATOM 22701 OD1 ASP G 138 34.109 -26.118 67.383 1.00 0.00
G O
ATOM 22702 OD2 ASP G 138 32.161 -26.375 66.403 1.00 0.00
G O
ATOM 22703 C ASP G 138 34.721 -23.265 64.032 1.00 0.00
G C
ATOM 22704 O ASP G 138 34.608 -23.704 62.887 1.00 0.00
G O
ATOM 22705 N GLU G 139 35.540 -22.263 64.342 1.00 0.00
G N
ATOM 22706 HN GLU G 139 35.771 -21.961 65.263 1.00 0.00
G H

ATOM 22707 CA GLU G 139   36.190 -21.498 63.289 1.00 0.00
G   C
ATOM 22708 HA GLU G 139   35.445 -21.309 62.527 1.00 0.00
G   H
ATOM 22709 CB GLU G 139   36.678 -20.165 63.850 1.00 0.00
G   C
ATOM 22710 HB1 GLU G 139   37.320 -19.678 63.080 1.00 0.00
G   H
ATOM 22711 HB2 GLU G 139   35.813 -19.481 64.003 1.00 0.00
G   H
ATOM 22712 CG GLU G 139   37.463 -20.297 65.133 1.00 0.00
G   C
ATOM 22713 HG1 GLU G 139   36.901 -20.925 65.847 1.00 0.00
G   H
ATOM 22714 HG2 GLU G 139   38.455 -20.756 64.946 1.00 0.00
G   H
ATOM 22715 CD GLU G 139   37.666 -18.970 65.821 1.00 0.00
G   C
ATOM 22716 OE1 GLU G 139   37.626 -18.932 67.066 1.00 0.00
G   O
ATOM 22717 OE2 GLU G 139   37.857 -17.959 65.117 1.00 0.00
G   O
ATOM 22718 C   GLU G 139   37.341 -22.242 62.621 1.00 0.00
G   C
ATOM 22719 O   GLU G 139   38.105 -21.616 61.878 1.00 0.00
G   O
ATOM 22720 N   ASN G 140   37.489 -23.547 62.855 1.00 0.00
G   N
ATOM 22721 HN ASN G 140   36.976 -24.091 63.518 1.00 0.00
G   H
ATOM 22722 CA ASN G 140   38.481 -24.300 62.095 1.00 0.00
G   C
ATOM 22723 HA ASN G 140   39.124 -23.556 61.635 1.00 0.00
G   H
ATOM 22724 CB ASN G 140   39.291 -25.224 63.005 1.00 0.00
G   C
ATOM 22725 HB1 ASN G 140   39.163 -24.869 64.051 1.00 0.00
G   H
ATOM 22726 HB2 ASN G 140   38.896 -26.259 62.954 1.00 0.00
G   H
ATOM 22727 CG ASN G 140   40.776 -25.204 62.674 1.00 0.00
G   C
ATOM 22728 OD1 ASN G 140   41.495 -26.169 62.938 1.00 0.00
G   O
ATOM 22729 ND2 ASN G 140   41.240 -24.100 62.092 1.00 0.00
G   N
ATOM 22730 HD21 ASN G 140   42.208 -24.078 61.860 1.00 0.00
G   H
ATOM 22731 HD22 ASN G 140   40.620 -23.351 61.879 1.00 0.00
G   H
ATOM 22732 C   ASN G 140   37.839 -25.107 60.978 1.00 0.00
G   C
ATOM 22733 O   ASN G 140   38.421 -25.238 59.896 1.00 0.00
G   O

ATOM 22734 N VAL G 141 36.654 -25.666 61.209 1.00 0.00
G N
ATOM 22735 HN VAL G 141 36.159 -25.673 62.074 1.00 0.00
G H
ATOM 22736 CA VAL G 141 35.970 -26.348 60.117 1.00 0.00
G C
ATOM 22737 HA VAL G 141 36.657 -27.032 59.635 1.00 0.00
G H
ATOM 22738 CB VAL G 141 34.743 -27.133 60.600 1.00 0.00
G C
ATOM 22739 HB VAL G 141 34.987 -28.222 60.521 1.00 0.00
G H
ATOM 22740 CG1 VAL G 141 34.418 -26.807 62.034 1.00 0.00
G C
ATOM 22741 HG11 VAL G 141 33.505 -27.353 62.357 1.00 0.00
G H
ATOM 22742 HG12 VAL G 141 35.239 -27.103 62.719 1.00 0.00
G H
ATOM 22743 HG13 VAL G 141 34.228 -25.718 62.157 1.00 0.00
G H
ATOM 22744 CG2 VAL G 141 33.537 -26.960 59.668 1.00 0.00
G C
ATOM 22745 HG21 VAL G 141 32.654 -27.506 60.062 1.00 0.00
G H
ATOM 22746 HG22 VAL G 141 33.269 -25.885 59.572 1.00 0.00
G H
ATOM 22747 HG23 VAL G 141 33.751 -27.357 58.654 1.00 0.00
G H
ATOM 22748 C VAL G 141 35.598 -25.278 59.097 1.00 0.00
G C
ATOM 22749 O VAL G 141 34.857 -24.341 59.403 1.00 0.00
G O
ATOM 22750 N LEU G 142 36.200 -25.356 57.914 1.00 0.00
G N
ATOM 22751 HN LEU G 142 36.890 -26.026 57.650 1.00 0.00
G H
ATOM 22752 CA LEU G 142 35.860 -24.402 56.868 1.00 0.00
G C
ATOM 22753 HA LEU G 142 35.559 -23.486 57.360 1.00 0.00
G H
ATOM 22754 CB LEU G 142 37.069 -24.145 55.963 1.00 0.00
G C
ATOM 22755 HB1 LEU G 142 37.913 -23.834 56.620 1.00 0.00
G H
ATOM 22756 HB2 LEU G 142 37.380 -25.105 55.494 1.00 0.00
G H
ATOM 22757 CG LEU G 142 36.826 -23.069 54.908 1.00 0.00
G C
ATOM 22758 HG LEU G 142 35.958 -23.394 54.285 1.00 0.00
G H
ATOM 22759 CD1 LEU G 142 36.505 -21.753 55.583 1.00 0.00
G C
ATOM 22760 HD11 LEU G 142 36.374 -20.949 54.828 1.00 0.00
G H

ATOM 22761 HD12 LEU G 142   35.567 -21.835 56.173 1.00 0.00
G   H
ATOM 22762 HD13 LEU G 142   37.326 -21.453 56.269 1.00 0.00
G   H
ATOM 22763 CD2 LEU G 142   38.030 -22.917 53.994 1.00 0.00
G   C
ATOM 22764 HD21 LEU G 142   37.831 -22.144 53.220 1.00 0.00
G   H
ATOM 22765 HD22 LEU G 142   38.934 -22.608 54.556 1.00 0.00
G   H
ATOM 22766 HD23 LEU G 142   38.250 -23.877 53.478 1.00 0.00
G   H
ATOM 22767 C   LEU G 142   34.673 -24.901 56.059 1.00 0.00
G   C
ATOM 22768 O   LEU G 142   33.637 -24.234 55.985 1.00 0.00
G   O
ATOM 22769 N   ASN G 143   34.805 -26.077 55.456 1.00 0.00
G   N
ATOM 22770 HN ASN G 143   35.670 -26.572 55.377 1.00 0.00
G   H
ATOM 22771 CA ASN G 143   33.668 -26.751 54.850 1.00 0.00
G   C
ATOM 22772 HA ASN G 143   32.780 -26.181 55.099 1.00 0.00
G   H
ATOM 22773 CB ASN G 143   33.830 -26.910 53.335 1.00 0.00
G   C
ATOM 22774 HB1 ASN G 143   34.702 -27.576 53.154 1.00 0.00
G   H
ATOM 22775 HB2 ASN G 143   32.937 -27.407 52.902 1.00 0.00
G   H
ATOM 22776 CG ASN G 143   34.090 -25.598 52.628 1.00 0.00
G   C
ATOM 22777 OD1 ASN G 143   34.734 -24.705 53.169 1.00 0.00
G   O
ATOM 22778 ND2 ASN G 143   33.575 -25.469 51.411 1.00 0.00
G   N
ATOM 22779 HD21 ASN G 143   33.748 -24.615 50.930 1.00 0.00
G   H
ATOM 22780 HD22 ASN G 143   33.066 -26.221 51.003 1.00 0.00
G   H
ATOM 22781 C   ASN G 143   33.541 -28.112 55.511 1.00 0.00
G   C
ATOM 22782 O   ASN G 143   34.517 -28.653 56.035 1.00 0.00
G   O
ATOM 22783 N   TYR G 144   32.337 -28.670 55.474 1.00 0.00
G   N
ATOM 22784 HN TYR G 144   31.525 -28.290 55.034 1.00 0.00
G   H
ATOM 22785 CA TYR G 144   32.127 -29.956 56.120 1.00 0.00
G   C
ATOM 22786 HA TYR G 144   33.035 -30.533 55.997 1.00 0.00
G   H
ATOM 22787 CB TYR G 144   31.786 -29.790 57.602 1.00 0.00
G   C

ATOM 22788 HB1 TYR G 144   31.863 -30.779 58.106 1.00 0.00
G   H
ATOM 22789 HB2 TYR G 144   32.512 -29.111 58.099 1.00 0.00
G   H
ATOM 22790 CG TYR G 144   30.390 -29.288 57.871 1.00 0.00
G   C
ATOM 22791 CD1 TYR G 144   30.035 -27.981 57.587 1.00 0.00
G   C
ATOM 22792 HD1 TYR G 144   30.770 -27.301 57.181 1.00 0.00
G   H
ATOM 22793 CE1 TYR G 144   28.759 -27.521 57.835 1.00 0.00
G   C
ATOM 22794 HE1 TYR G 144   28.493 -26.498 57.615 1.00 0.00
G   H
ATOM 22795 CZ TYR G 144   27.818 -28.373 58.375 1.00 0.00
G   C
ATOM 22796 OH TYR G 144   26.544 -27.905 58.618 1.00 0.00
G   O
ATOM 22797 HH TYR G 144   26.018 -28.649 58.921 1.00 0.00
G   H
ATOM 22798 CD2 TYR G 144   29.430 -30.127 58.414 1.00 0.00
G   C
ATOM 22799 HD2 TYR G 144   29.689 -31.142 58.684 1.00 0.00
G   H
ATOM 22800 CE2 TYR G 144   28.152 -29.681 58.667 1.00 0.00
G   C
ATOM 22801 HE2 TYR G 144   27.429 -30.349 59.110 1.00 0.00
G   H
ATOM 22802 C   TYR G 144   31.021 -30.701 55.396 1.00 0.00
G   C
ATOM 22803 O   TYR G 144   30.071 -30.104 54.886 1.00 0.00
G   O
ATOM 22804 N   ILE G 145   31.159 -32.024 55.367 1.00 0.00
G   N
ATOM 22805 HN ILE G 145   31.958 -32.492 55.739 1.00 0.00
G   H
ATOM 22806 CA ILE G 145   30.160 -32.914 54.792 1.00 0.00
G   C
ATOM 22807 HA ILE G 145   29.210 -32.393 54.809 1.00 0.00
G   H
ATOM 22808 CB ILE G 145   30.514 -33.340 53.354 1.00 0.00
G   C
ATOM 22809 HB ILE G 145   31.455 -32.780 53.120 1.00 0.00
G   H
ATOM 22810 CG2 ILE G 145   30.701 -34.855 53.246 1.00 0.00
G   C
ATOM 22811 HG21 ILE G 145   31.105 -35.129 52.249 1.00 0.00
G   H
ATOM 22812 HG22 ILE G 145   31.416 -35.222 54.012 1.00 0.00
G   H
ATOM 22813 HG23 ILE G 145   29.736 -35.385 53.385 1.00 0.00
G   H
ATOM 22814 CG1 ILE G 145   29.434 -32.832 52.408 1.00 0.00
G   C

ATOM 22815 HG11 ILE G 145   28.735 -32.164 52.959 1.00 0.00
G   H
ATOM 22816 HG12 ILE G 145   29.913 -32.222 51.609 1.00 0.00
G   H
ATOM 22817 CD ILE G 145   28.630 -33.926 51.736 1.00 0.00
G   C
ATOM 22818 HD1 ILE G 145   27.904 -33.486 51.019 1.00 0.00
G   H
ATOM 22819 HD2 ILE G 145   29.298 -34.614 51.174 1.00 0.00
G   H
ATOM 22820 HD3 ILE G 145   28.060 -34.518 52.482 1.00 0.00
G   H
ATOM 22821 C   ILE G 145   30.015 -34.126 55.703 1.00 0.00
G   C
ATOM 22822 O   ILE G 145   31.009 -34.631 56.238 1.00 0.00
G   O
ATOM 22823 N   ASP G 146   28.775 -34.555 55.923 1.00 0.00
G   N
ATOM 22824 HN ASP G 146   27.955 -34.144 55.534 1.00 0.00
G   H
ATOM 22825 CA ASP G 146   28.485 -35.700 56.778 1.00 0.00
G   C
ATOM 22826 HA ASP G 146   29.433 -36.115 57.101 1.00 0.00
G   H
ATOM 22827 CB ASP G 146   27.620 -35.271 57.960 1.00 0.00
G   C
ATOM 22828 HB1 ASP G 146   27.067 -36.151 58.348 1.00 0.00
G   H
ATOM 22829 HB2 ASP G 146   28.248 -34.867 58.778 1.00 0.00
G   H
ATOM 22830 CG ASP G 146   26.595 -34.224 57.578 1.00 0.00
G   C
ATOM 22831 OD1 ASP G 146   25.571 -34.607 56.971 1.00 0.00
G   O
ATOM 22832 OD2 ASP G 146   26.824 -33.023 57.838 1.00 0.00
G   O
ATOM 22833 C   ASP G 146   27.782 -36.784 55.978 1.00 0.00
G   C
ATOM 22834 O   ASP G 146   26.754 -36.525 55.347 1.00 0.00
G   O
ATOM 22835 N   LEU G 147   28.332 -37.993 56.010 1.00 0.00
G   N
ATOM 22836 HN LEU G 147   29.195 -38.212 56.459 1.00 0.00
G   H
ATOM 22837 CA LEU G 147   27.688 -39.130 55.365 1.00 0.00
G   C
ATOM 22838 HA LEU G 147   27.228 -38.745 54.464 1.00 0.00
G   H
ATOM 22839 CB LEU G 147   28.743 -40.178 55.021 1.00 0.00
G   C
ATOM 22840 HB1 LEU G 147   28.222 -41.161 54.954 1.00 0.00
G   H
ATOM 22841 HB2 LEU G 147   29.142 -39.970 54.004 1.00 0.00
G   H

```
ATOM  22842  CG  LEU G 147      29.834 -40.215  56.089  1.00  0.00
G    C
ATOM  22843  HG  LEU G 147      29.824 -39.212  56.578  1.00  0.00
G    H
ATOM  22844  CD1 LEU G 147      29.549 -41.274  57.137  1.00  0.00
G    C
ATOM  22845 HD11 LEU G 147      30.375 -41.321  57.878  1.00  0.00
G    H
ATOM  22846 HD12 LEU G 147      28.606 -41.046  57.678  1.00  0.00
G    H
ATOM  22847 HD13 LEU G 147      29.449 -42.275  56.665  1.00  0.00
G    H
ATOM  22848  CD2 LEU G 147      31.196 -40.406  55.431  1.00  0.00
G    C
ATOM  22849 HD21 LEU G 147      31.999 -40.416  56.200  1.00  0.00
G    H
ATOM  22850 HD22 LEU G 147      31.252 -41.364  54.875  1.00  0.00
G    H
ATOM  22851 HD23 LEU G 147      31.405 -39.577  54.721  1.00  0.00
G    H
ATOM  22852  C   LEU G 147      26.584 -39.736  56.221  1.00  0.00
G    C
ATOM  22853  O   LEU G 147      25.658 -40.350  55.681  1.00  0.00
G    O
ATOM  22854  N   SER G 148      26.652 -39.567  57.535  1.00  0.00
G    N
ATOM  22855  HN  SER G 148      27.379 -39.062  58.000  1.00  0.00
G    H
ATOM  22856  CA  SER G 148      25.645 -40.119  58.428  1.00  0.00
G    C
ATOM  22857  HA  SER G 148      24.719 -40.178  57.872  1.00  0.00
G    H
ATOM  22858  CB  SER G 148      26.080 -41.513  58.915  1.00  0.00
G    C
ATOM  22859  HB1 SER G 148      25.279 -41.913  59.580  1.00  0.00
G    H
ATOM  22860  HB2 SER G 148      26.171 -42.213  58.055  1.00  0.00
G    H
ATOM  22861  OG  SER G 148      27.292 -41.442  59.650  1.00  0.00
G    O
ATOM  22862  HG1 SER G 148      27.394 -42.285  60.111  1.00  0.00
G    H
ATOM  22863  C   SER G 148      25.428 -39.133  59.569  1.00  0.00
G    C
ATOM  22864  O   SER G 148      26.000 -38.035  59.575  1.00  0.00
G    O
ATOM  22865  N   ASP G 149      24.600 -39.521  60.541  1.00  0.00
G    N
ATOM  22866  HN  ASP G 149      24.069 -40.364  60.565  1.00  0.00
G    H
ATOM  22867  CA  ASP G 149      24.395 -38.687  61.719  1.00  0.00
G    C
ATOM  22868  HA  ASP G 149      24.489 -37.657  61.394  1.00  0.00
G    H
```

ATOM 22869 CB ASP G 149 23.013 -38.950 62.311 1.00 0.00
G C
ATOM 22870 HB1 ASP G 149 22.941 -38.462 63.304 1.00 0.00
G H
ATOM 22871 HB2 ASP G 149 22.222 -38.531 61.658 1.00 0.00
G H
ATOM 22872 CG ASP G 149 22.742 -40.425 62.510 1.00 0.00
G C
ATOM 22873 OD1 ASP G 149 21.974 -41.005 61.714 1.00 0.00
G O
ATOM 22874 OD2 ASP G 149 23.311 -41.010 63.452 1.00 0.00
G O
ATOM 22875 C ASP G 149 25.476 -38.917 62.766 1.00 0.00
G C
ATOM 22876 O ASP G 149 25.433 -38.315 63.844 1.00 0.00
G O
ATOM 22877 N GLU G 150 26.450 -39.771 62.463 1.00 0.00
G N
ATOM 22878 HN GLU G 150 26.497 -40.244 61.588 1.00 0.00
G H
ATOM 22879 CA GLU G 150 27.538 -40.100 63.374 1.00 0.00
G C
ATOM 22880 HA GLU G 150 27.365 -39.546 64.287 1.00 0.00
G H
ATOM 22881 CB GLU G 150 27.543 -41.604 63.666 1.00 0.00
G C
ATOM 22882 HB1 GLU G 150 26.545 -41.881 64.079 1.00 0.00
G H
ATOM 22883 HB2 GLU G 150 27.649 -42.173 62.716 1.00 0.00
G H
ATOM 22884 CG GLU G 150 28.618 -42.030 64.645 1.00 0.00
G C
ATOM 22885 HG1 GLU G 150 29.244 -42.817 64.186 1.00 0.00
G H
ATOM 22886 HG2 GLU G 150 29.259 -41.172 64.928 1.00 0.00
G H
ATOM 22887 CD GLU G 150 28.067 -42.642 65.909 1.00 0.00
G C
ATOM 22888 OE1 GLU G 150 26.895 -42.374 66.239 1.00 0.00
G O
ATOM 22889 OE2 GLU G 150 28.810 -43.394 66.573 1.00 0.00
G O
ATOM 22890 C GLU G 150 28.906 -39.699 62.857 1.00 0.00
G C
ATOM 22891 O GLU G 150 29.766 -39.302 63.646 1.00 0.00
G O
ATOM 22892 N TYR G 151 29.134 -39.796 61.555 1.00 0.00
G N
ATOM 22893 HN TYR G 151 28.429 -40.027 60.887 1.00 0.00
G H
ATOM 22894 CA TYR G 151 30.453 -39.570 60.987 1.00 0.00
G C
ATOM 22895 HA TYR G 151 31.066 -39.144 61.772 1.00 0.00
G H

ATOM 22896 CB TYR G 151 31.043 -40.876 60.460 1.00 0.00
G C
ATOM 22897 HB1 TYR G 151 30.513 -41.164 59.526 1.00 0.00
G H
ATOM 22898 HB2 TYR G 151 32.117 -40.747 60.204 1.00 0.00
G H
ATOM 22899 CG TYR G 151 30.901 -42.042 61.410 1.00 0.00
G C
ATOM 22900 CD1 TYR G 151 31.734 -42.174 62.509 1.00 0.00
G C
ATOM 22901 HD1 TYR G 151 32.505 -41.438 62.690 1.00 0.00
G H
ATOM 22902 CE1 TYR G 151 31.616 -43.243 63.374 1.00 0.00
G C
ATOM 22903 HE1 TYR G 151 32.276 -43.338 64.224 1.00 0.00
G H
ATOM 22904 CZ TYR G 151 30.651 -44.197 63.143 1.00 0.00
G C
ATOM 22905 OH TYR G 151 30.524 -45.262 64.003 1.00 0.00
G O
ATOM 22906 HH TYR G 151 29.761 -45.770 63.720 1.00 0.00
G H
ATOM 22907 CD2 TYR G 151 29.936 -43.016 61.200 1.00 0.00
G C
ATOM 22908 HD2 TYR G 151 29.296 -42.964 60.330 1.00 0.00
G H
ATOM 22909 CE2 TYR G 151 29.808 -44.088 62.059 1.00 0.00
G C
ATOM 22910 HE2 TYR G 151 29.064 -44.846 61.865 1.00 0.00
G H
ATOM 22911 C TYR G 151 30.375 -38.540 59.870 1.00 0.00
G C
ATOM 22912 O TYR G 151 29.311 -38.321 59.284 1.00 0.00
G O
ATOM 22913 N SER G 152 31.513 -37.913 59.579 1.00 0.00
G N
ATOM 22914 HN SER G 152 32.411 -38.163 59.939 1.00 0.00
G H
ATOM 22915 CA SER G 152 31.530 -36.771 58.677 1.00 0.00
G C
ATOM 22916 HA SER G 152 30.968 -37.070 57.801 1.00 0.00
G H
ATOM 22917 CB SER G 152 30.928 -35.540 59.355 1.00 0.00
G C
ATOM 22918 HB1 SER G 152 31.572 -34.663 59.115 1.00 0.00
G H
ATOM 22919 HB2 SER G 152 29.915 -35.326 58.943 1.00 0.00
G H
ATOM 22920 OG SER G 152 30.894 -35.724 60.761 1.00 0.00
G O
ATOM 22921 HG1 SER G 152 30.724 -34.859 61.156 1.00 0.00
G H
ATOM 22922 C SER G 152 32.947 -36.459 58.221 1.00 0.00
G C

ATOM 22923 O   SER G 152   33.931 -36.914 58.815 1.00 0.00
G   O
ATOM 22924 N   ILE G 153   33.028 -35.653 57.164 1.00 0.00
G   N
ATOM 22925 HN ILE G 153   32.214 -35.321 56.691 1.00 0.00
G   H
ATOM 22926 CA ILE G 153   34.285 -35.178 56.595 1.00 0.00
G   C
ATOM 22927 HA ILE G 153   35.093 -35.669 57.123 1.00 0.00
G   H
ATOM 22928 CB ILE G 153   34.343 -35.439 55.081 1.00 0.00
G   C
ATOM 22929 HB ILE G 153   33.351 -35.086 54.698 1.00 0.00
G   H
ATOM 22930 CG2 ILE G 153   35.524 -34.725 54.448 1.00 0.00
G   C
ATOM 22931 HG21 ILE G 153   35.471 -34.788 53.342 1.00 0.00
G   H
ATOM 22932 HG22 ILE G 153   35.532 -33.650 54.724 1.00 0.00
G   H
ATOM 22933 HG23 ILE G 153   36.484 -35.178 54.776 1.00 0.00
G   H
ATOM 22934 CG1 ILE G 153   34.418 -36.939 54.795 1.00 0.00
G   C
ATOM 22935 HG11 ILE G 153   35.470 -37.228 54.578 1.00 0.00
G   H
ATOM 22936 HG12 ILE G 153   34.104 -37.496 55.707 1.00 0.00
G   H
ATOM 22937 CD ILE G 153   33.532 -37.384 53.638 1.00 0.00
G   C
ATOM 22938 HD1 ILE G 153   33.584 -38.487 53.514 1.00 0.00
G   H
ATOM 22939 HD2 ILE G 153   32.473 -37.104 53.824 1.00 0.00
G   H
ATOM 22940 HD3 ILE G 153   33.857 -36.916 52.685 1.00 0.00
G   H
ATOM 22941 C   ILE G 153   34.399 -33.689 56.899 1.00 0.00
G   C
ATOM 22942 O   ILE G 153   33.398 -32.965 56.869 1.00 0.00
G   O
ATOM 22943 N   VAL G 154   35.612 -33.227 57.200 1.00 0.00
G   N
ATOM 22944 HN VAL G 154   36.428 -33.799 57.180 1.00 0.00
G   H
ATOM 22945 CA VAL G 154   35.857 -31.841 57.588 1.00 0.00
G   C
ATOM 22946 HA VAL G 154   35.060 -31.234 57.179 1.00 0.00
G   H
ATOM 22947 CB VAL G 154   35.897 -31.676 59.122 1.00 0.00
G   C
ATOM 22948 HB VAL G 154   36.758 -32.277 59.504 1.00 0.00
G   H
ATOM 22949 CG1 VAL G 154   36.102 -30.211 59.491 1.00 0.00
G   C

ATOM 22950 HG11 VAL G 154   36.092 -30.087 60.596 1.00 0.00
G   H
ATOM 22951 HG12 VAL G 154   37.078 -29.829 59.125 1.00 0.00
G   H
ATOM 22952 HG13 VAL G 154   35.291 -29.582 59.067 1.00 0.00
G   H
ATOM 22953 CG2 VAL G 154   34.619 -32.202 59.764 1.00 0.00
G   C
ATOM 22954 HG21 VAL G 154   34.637 -32.044 60.863 1.00 0.00
G   H
ATOM 22955 HG22 VAL G 154   33.730 -31.679 59.349 1.00 0.00
G   H
ATOM 22956 HG23 VAL G 154   34.497 -33.290 59.584 1.00 0.00
G   H
ATOM 22957 C   VAL G 154   37.165 -31.355 56.973 1.00 0.00
G   C
ATOM 22958 O   VAL G 154   38.155 -32.096 56.920 1.00 0.00
G   O
ATOM 22959 N   GLU G 155   37.170 -30.101 56.522 1.00 0.00
G   N
ATOM 22960 HN GLU G 155   36.340 -29.571 56.370 1.00 0.00
G   H
ATOM 22961 CA GLU G 155   38.399 -29.381 56.207 1.00 0.00
G   C
ATOM 22962 HA GLU G 155   39.219 -30.084 56.136 1.00 0.00
G   H
ATOM 22963 CB GLU G 155   38.283 -28.578 54.913 1.00 0.00
G   C
ATOM 22964 HB1 GLU G 155   37.255 -28.151 54.860 1.00 0.00
G   H
ATOM 22965 HB2 GLU G 155   38.974 -27.707 54.947 1.00 0.00
G   H
ATOM 22966 CG GLU G 155   38.536 -29.362 53.656 1.00 0.00
G   C
ATOM 22967 HG1 GLU G 155   39.477 -29.018 53.190 1.00 0.00
G   H
ATOM 22968 HG2 GLU G 155   38.611 -30.446 53.872 1.00 0.00
G   H
ATOM 22969 CD GLU G 155   37.457 -29.148 52.621 1.00 0.00
G   C
ATOM 22970 OE1 GLU G 155   37.771 -28.618 51.534 1.00 0.00
G   O
ATOM 22971 OE2 GLU G 155   36.291 -29.491 52.915 1.00 0.00
G   O
ATOM 22972 C   GLU G 155   38.710 -28.437 57.357 1.00 0.00
G   C
ATOM 22973 O   GLU G 155   37.819 -27.733 57.839 1.00 0.00
G   O
ATOM 22974 N   LEU G 156   39.965 -28.418 57.794 1.00 0.00
G   N
ATOM 22975 HN LEU G 156   40.677 -29.048 57.492 1.00 0.00
G   H
ATOM 22976 CA LEU G 156   40.409 -27.440 58.781 1.00 0.00
G   C

ATOM 22977 HA LEU G 156   39.657 -26.663 58.824 1.00 0.00
G   H
ATOM 22978 CB LEU G 156   40.580 -28.070 60.174 1.00 0.00
G   C
ATOM 22979 HB1 LEU G 156   41.643 -28.395 60.261 1.00 0.00
G   H
ATOM 22980 HB2 LEU G 156   40.436 -27.284 60.948 1.00 0.00
G   H
ATOM 22981 CG LEU G 156   39.690 -29.277 60.492 1.00 0.00
G   C
ATOM 22982 HG LEU G 156   38.888 -29.263 59.714 1.00 0.00
G   H
ATOM 22983 CD1 LEU G 156   40.465 -30.582 60.393 1.00 0.00
G   C
ATOM 22984 HD11 LEU G 156   39.818 -31.441 60.672 1.00 0.00
G   H
ATOM 22985 HD12 LEU G 156   40.830 -30.741 59.356 1.00 0.00
G   H
ATOM 22986 HD13 LEU G 156   41.341 -30.571 61.075 1.00 0.00
G   H
ATOM 22987 CD2 LEU G 156   39.025 -29.129 61.854 1.00 0.00
G   C
ATOM 22988 HD21 LEU G 156   38.369 -30.003 62.061 1.00 0.00
G   H
ATOM 22989 HD22 LEU G 156   39.773 -29.069 62.671 1.00 0.00
G   H
ATOM 22990 HD23 LEU G 156   38.400 -28.210 61.885 1.00 0.00
G   H
ATOM 22991 C   LEU G 156   41.710 -26.797 58.325 1.00 0.00
G   C
ATOM 22992 O   LEU G 156   42.650 -27.489 57.937 1.00 0.00
G   O
ATOM 22993 N   LEU G 157   41.768 -25.470 58.375 1.00 0.00
G   N
ATOM 22994 HN LEU G 157   41.030 -24.868 58.673 1.00 0.00
G   H
ATOM 22995 CA LEU G 157   42.983 -24.774 57.974 1.00 0.00
G   C
ATOM 22996 HA LEU G 157   43.433 -25.332 57.162 1.00 0.00
G   H
ATOM 22997 CB LEU G 157   42.677 -23.350 57.513 1.00 0.00
G   C
ATOM 22998 HB1 LEU G 157   42.744 -23.342 56.401 1.00 0.00
G   H
ATOM 22999 HB2 LEU G 157   41.620 -23.109 57.759 1.00 0.00
G   H
ATOM 23000 CG LEU G 157   43.540 -22.184 57.998 1.00 0.00
G   C
ATOM 23001 HG LEU G 157   44.496 -22.583 58.416 1.00 0.00
G   H
ATOM 23002 CD1 LEU G 157   43.836 -21.257 56.833 1.00 0.00
G   C
ATOM 23003 HD11 LEU G 157   44.422 -20.377 57.174 1.00 0.00
G   H

ATOM 23004 HD12 LEU G 157   44.423 -21.786 56.052 1.00 0.00
G   H
ATOM 23005 HD13 LEU G 157   42.893 -20.889 56.376 1.00 0.00
G   H
ATOM 23006 CD2 LEU G 157   42.822 -21.427 59.107 1.00 0.00
G   C
ATOM 23007 HD21 LEU G 157   43.454 -20.590 59.476 1.00 0.00
G   H
ATOM 23008 HD22 LEU G 157   41.864 -20.994 58.754 1.00 0.00
G   H
ATOM 23009 HD23 LEU G 157   42.606 -22.102 59.964 1.00 0.00
G   H
ATOM 23010 C   LEU G 157   43.963 -24.790 59.143 1.00 0.00
G   C
ATOM 23011 O   LEU G 157   43.567 -24.588 60.297 1.00 0.00
G   O
ATOM 23012 N   ALA G 158   45.239 -25.044 58.850 1.00 0.00
G   N
ATOM 23013 HN ALA G 158   45.597 -25.077 57.917 1.00 0.00
G   H
ATOM 23014 CA ALA G 158   46.213 -25.296 59.904 1.00 0.00
G   C
ATOM 23015 HA ALA G 158   45.794 -25.943 60.666 1.00 0.00
G   H
ATOM 23016 CB ALA G 158   47.456 -25.977 59.335 1.00 0.00
G   C
ATOM 23017 HB1 ALA G 158   47.171 -26.944 58.868 1.00 0.00
G   H
ATOM 23018 HB2 ALA G 158   47.934 -25.348 58.553 1.00 0.00
G   H
ATOM 23019 HB3 ALA G 158   48.207 -26.187 60.127 1.00 0.00
G   H
ATOM 23020 C   ALA G 158   46.603 -24.003 60.606 1.00 0.00
G   C
ATOM 23021 O   ALA G 158   46.890 -22.992 59.961 1.00 0.00
G   O
ATOM 23022 N   THR G 159   46.595 -24.036 61.932 1.00 0.00
G   N
ATOM 23023 HN THR G 159   46.280 -24.811 62.477 1.00 0.00
G   H
ATOM 23024 CA THR G 159   47.061 -22.907 62.714 1.00 0.00
G   C
ATOM 23025 HA THR G 159   46.973 -22.038 62.075 1.00 0.00
G   H
ATOM 23026 CB THR G 159   46.244 -22.769 63.998 1.00 0.00
G   C
ATOM 23027 HB THR G 159   45.172 -22.749 63.682 1.00 0.00
G   H
ATOM 23028 OG1 THR G 159   46.542 -21.517 64.631 1.00 0.00
G   O
ATOM 23029 HG1 THR G 159   46.007 -21.501 65.432 1.00 0.00
G   H
ATOM 23030 CG2 THR G 159   46.549 -23.911 64.951 1.00 0.00
G   C

ATOM 23031 HG21 THR G 159   45.877 -23.879 65.834 1.00 0.00
G   H
ATOM 23032 HG22 THR G 159   46.401 -24.889 64.444 1.00 0.00
G   H
ATOM 23033 HG23 THR G 159   47.599 -23.861 65.312 1.00 0.00
G   H
ATOM 23034 C   THR G 159   48.546 -23.062 63.034 1.00 0.00
G   C
ATOM 23035 O   THR G 159   49.203 -24.028 62.637 1.00 0.00
G   O
ATOM 23036 N   ARG G 160   49.078 -22.081 63.766 1.00 0.00
G   N
ATOM 23037 HN ARG G 160   48.548 -21.297 64.081 1.00 0.00
G   H
ATOM 23038 CA ARG G 160   50.485 -22.113 64.144 1.00 0.00
G   C
ATOM 23039 HA ARG G 160   51.070 -22.255 63.242 1.00 0.00
G   H
ATOM 23040 CB ARG G 160   50.874 -20.799 64.816 1.00 0.00
G   C
ATOM 23041 HB1 ARG G 160   50.365 -19.989 64.240 1.00 0.00
G   H
ATOM 23042 HB2 ARG G 160   50.445 -20.763 65.841 1.00 0.00
G   H
ATOM 23043 CG ARG G 160   52.363 -20.535 64.825 1.00 0.00
G   C
ATOM 23044 HG1 ARG G 160   52.891 -21.495 65.016 1.00 0.00
G   H
ATOM 23045 HG2 ARG G 160   52.680 -20.216 63.805 1.00 0.00
G   H
ATOM 23046 CD ARG G 160   52.698 -19.448 65.822 1.00 0.00
G   C
ATOM 23047 HD1 ARG G 160   53.785 -19.490 66.065 1.00 0.00
G   H
ATOM 23048 HD2 ARG G 160   52.461 -18.436 65.419 1.00 0.00
G   H
ATOM 23049 NE ARG G 160   51.928 -19.602 67.052 1.00 0.00
G   N
ATOM 23050 HE ARG G 160   51.171 -18.943 67.130 1.00 0.00
G   H
ATOM 23051 CZ ARG G 160   52.180 -20.512 67.988 1.00 0.00
G   C
ATOM 23052 NH1 ARG G 160   53.186 -21.362 67.841 1.00 0.00
G   N
ATOM 23053 HH11 ARG G 160   53.363 -22.058 68.523 1.00 0.00
G   H
ATOM 23054 HH12 ARG G 160   53.655 -21.346 66.955 1.00 0.00
G   H
ATOM 23055 NH2 ARG G 160   51.422 -20.575 69.071 1.00 0.00
G   N
ATOM 23056 HH21 ARG G 160   51.662 -21.194 69.806 1.00 0.00
G   H
ATOM 23057 HH22 ARG G 160   50.758 -19.850 69.196 1.00 0.00
G   H

ATOM 23058 C   ARG G 160   50.808 -23.286 65.059 1.00 0.00
G   C
ATOM 23059 O   ARG G 160   51.962 -23.726 65.103 1.00 0.00
G   O
ATOM 23060 N   LYS G 161   49.816 -23.808 65.785 1.00 0.00
G   N
ATOM 23061 HN LYS G 161   48.904 -23.403 65.836 1.00 0.00
G   H
ATOM 23062 CA LYS G 161   49.996 -25.024 66.567 1.00 0.00
G   C
ATOM 23063 HA LYS G 161   50.858 -24.857 67.201 1.00 0.00
G   H
ATOM 23064 CB LYS G 161   48.748 -25.301 67.407 1.00 0.00
G   C
ATOM 23065 HB1 LYS G 161   47.916 -25.516 66.698 1.00 0.00
G   H
ATOM 23066 HB2 LYS G 161   48.906 -26.233 67.994 1.00 0.00
G   H
ATOM 23067 CG LYS G 161   48.339 -24.161 68.314 1.00 0.00
G   C
ATOM 23068 HG1 LYS G 161   48.380 -23.207 67.742 1.00 0.00
G   H
ATOM 23069 HG2 LYS G 161   47.281 -24.312 68.631 1.00 0.00
G   H
ATOM 23070 CD LYS G 161   49.259 -24.078 69.512 1.00 0.00
G   C
ATOM 23071 HD1 LYS G 161   50.287 -23.845 69.151 1.00 0.00
G   H
ATOM 23072 HD2 LYS G 161   48.927 -23.222 70.145 1.00 0.00
G   H
ATOM 23073 CE LYS G 161   49.231 -25.376 70.298 1.00 0.00
G   C
ATOM 23074 HE1 LYS G 161   48.193 -25.614 70.620 1.00 0.00
G   H
ATOM 23075 HE2 LYS G 161   49.609 -26.217 69.676 1.00 0.00
G   H
ATOM 23076 NZ LYS G 161   50.072 -25.311 71.520 1.00 0.00
G   N
ATOM 23077 HZ1 LYS G 161   50.028 -26.216 72.032 1.00 0.00
G   H
ATOM 23078 HZ2 LYS G 161   51.058 -25.114 71.253 1.00 0.00
G   H
ATOM 23079 HZ3 LYS G 161   49.727 -24.549 72.137 1.00 0.00
G   H
ATOM 23080 C   LYS G 161   50.297 -26.236 65.701 1.00 0.00
G   C
ATOM 23081 O   LYS G 161   50.515 -27.323 66.245 1.00 0.00
G   O
ATOM 23082 N   LEU G 162   50.309 -26.080 64.376 1.00 0.00
G   N
ATOM 23083 HN LEU G 162   50.224 -25.193 63.927 1.00 0.00
G   H
ATOM 23084 CA LEU G 162   50.447 -27.201 63.464 1.00 0.00
G   C

ATOM 23085 HA LEU G 162   50.671 -28.054 64.091 1.00 0.00
G   H
ATOM 23086 CB LEU G 162   49.140 -27.403 62.682 1.00 0.00
G   C
ATOM 23087 HB1 LEU G 162   48.859 -26.415 62.251 1.00 0.00
G   H
ATOM 23088 HB2 LEU G 162   49.337 -28.073 61.815 1.00 0.00
G   H
ATOM 23089 CG LEU G 162   47.926 -27.928 63.451 1.00 0.00
G   C
ATOM 23090 HG LEU G 162   47.536 -27.070 64.050 1.00 0.00
G   H
ATOM 23091 CD1 LEU G 162   46.860 -28.389 62.472 1.00 0.00
G   C
ATOM 23092 HD11 LEU G 162   45.991 -28.817 63.016 1.00 0.00
G   H
ATOM 23093 HD12 LEU G 162   46.500 -27.537 61.855 1.00 0.00
G   H
ATOM 23094 HD13 LEU G 162   47.263 -29.170 61.792 1.00 0.00
G   H
ATOM 23095 CD2 LEU G 162   48.300 -29.046 64.417 1.00 0.00
G   C
ATOM 23096 HD21 LEU G 162   47.401 -29.393 64.972 1.00 0.00
G   H
ATOM 23097 HD22 LEU G 162   48.725 -29.922 63.886 1.00 0.00
G   H
ATOM 23098 HD23 LEU G 162   49.048 -28.688 65.157 1.00 0.00
G   H
ATOM 23099 C   LEU G 162   51.585 -27.054 62.464 1.00 0.00
G   C
ATOM 23100 O   LEU G 162   51.718 -27.915 61.588 1.00 0.00
G   O
ATOM 23101 N   ASP G 163   52.402 -26.007 62.564 1.00 0.00
G   N
ATOM 23102 HN ASP G 163   52.409 -25.343 63.307 1.00 0.00
G   H
ATOM 23103 CA ASP G 163   53.405 -25.731 61.545 1.00 0.00
G   C
ATOM 23104 HA ASP G 163   52.907 -25.797 60.584 1.00 0.00
G   H
ATOM 23105 CB ASP G 163   54.011 -24.342 61.771 1.00 0.00
G   C
ATOM 23106 HB1 ASP G 163   53.196 -23.609 61.927 1.00 0.00
G   H
ATOM 23107 HB2 ASP G 163   54.658 -24.341 62.671 1.00 0.00
G   H
ATOM 23108 CG ASP G 163   54.836 -23.856 60.587 1.00 0.00
G   C
ATOM 23109 OD1 ASP G 163   55.142 -24.665 59.686 1.00 0.00
G   O
ATOM 23110 OD2 ASP G 163   55.187 -22.656 60.566 1.00 0.00
G   O
ATOM 23111 C   ASP G 163   54.498 -26.790 61.547 1.00 0.00
G   C

ATOM 23112 O   ASP G 163   54.943 -27.239 62.607 1.00 0.00
G   O
ATOM 23113 N   SER G 164   54.934 -27.173 60.345 1.00 0.00
G   N
ATOM 23114 HN SER G 164   54.509 -26.855 59.497 1.00 0.00
G   H
ATOM 23115 CA SER G 164   56.064 -28.081 60.144 1.00 0.00
G   C
ATOM 23116 HA SER G 164   56.072 -28.318 59.089 1.00 0.00
G   H
ATOM 23117 CB SER G 164   57.377 -27.413 60.550 1.00 0.00
G   C
ATOM 23118 HB1 SER G 164   57.558 -27.631 61.627 1.00 0.00
G   H
ATOM 23119 HB2 SER G 164   58.227 -27.851 59.980 1.00 0.00
G   H
ATOM 23120 OG SER G 164   57.304 -26.008 60.371 1.00 0.00
G   O
ATOM 23121 HG1 SER G 164   58.054 -25.623 60.842 1.00 0.00
G   H
ATOM 23122 C   SER G 164   55.863 -29.390 60.901 1.00 0.00
G   C
ATOM 23123 O   SER G 164   56.806 -30.000 61.407 1.00 0.00
G   O
ATOM 23124 N   LYS G 165   54.612 -29.822 60.975 1.00 0.00
G   N
ATOM 23125 HN LYS G 165   53.852 -29.338 60.544 1.00 0.00
G   H
ATOM 23126 CA LYS G 165   54.247 -31.034 61.689 1.00 0.00
G   C
ATOM 23127 HA LYS G 165   55.078 -31.308 62.327 1.00 0.00
G   H
ATOM 23128 CB LYS G 165   52.982 -30.815 62.520 1.00 0.00
G   C
ATOM 23129 HB1 LYS G 165   52.194 -30.439 61.826 1.00 0.00
G   H
ATOM 23130 HB2 LYS G 165   52.622 -31.798 62.898 1.00 0.00
G   H
ATOM 23131 CG LYS G 165   53.172 -29.823 63.656 1.00 0.00
G   C
ATOM 23132 HG1 LYS G 165   53.345 -28.809 63.229 1.00 0.00
G   H
ATOM 23133 HG2 LYS G 165   52.234 -29.775 64.255 1.00 0.00
G   H
ATOM 23134 CD LYS G 165   54.348 -30.197 64.541 1.00 0.00
G   C
ATOM 23135 HD1 LYS G 165   54.149 -31.199 64.986 1.00 0.00
G   H
ATOM 23136 HD2 LYS G 165   55.252 -30.291 63.896 1.00 0.00
G   H
ATOM 23137 CE LYS G 165   54.566 -29.151 65.622 1.00 0.00
G   C
ATOM 23138 HE1 LYS G 165   54.867 -28.180 65.170 1.00 0.00
G   H

ATOM 23139 HE2 LYS G 165   53.633 -28.993 66.207 1.00 0.00
G   H
ATOM 23140 NZ LYS G 165   55.634 -29.558 66.573 1.00 0.00
G   N
ATOM 23141 HZ1 LYS G 165   55.762 -28.823 67.299 1.00 0.00
G   H
ATOM 23142 HZ2 LYS G 165   55.370 -30.454 67.030 1.00 0.00
G   H
ATOM 23143 HZ3 LYS G 165   56.527 -29.688 66.055 1.00 0.00
G   H
ATOM 23144 C   LYS G 165   54.045 -32.168 60.694 1.00 0.00
G   C
ATOM 23145 O   LYS G 165   53.989 -31.947 59.483 1.00 0.00
G   O
ATOM 23146 N   SER G 166   53.992 -33.392 61.205 1.00 0.00
G   N
ATOM 23147 HN SER G 166   54.093 -33.601 62.178 1.00 0.00
G   H
ATOM 23148 CA SER G 166   53.776 -34.559 60.364 1.00 0.00
G   C
ATOM 23149 HA SER G 166   53.601 -34.179 59.366 1.00 0.00
G   H
ATOM 23150 CB SER G 166   54.976 -35.501 60.406 1.00 0.00
G   C
ATOM 23151 HB1 SER G 166   55.750 -35.107 59.709 1.00 0.00
G   H
ATOM 23152 HB2 SER G 166   55.427 -35.509 61.424 1.00 0.00
G   H
ATOM 23153 OG SER G 166   54.573 -36.791 59.991 1.00 0.00
G   O
ATOM 23154 HG1 SER G 166   55.377 -37.296 59.814 1.00 0.00
G   H
ATOM 23155 C   SER G 166   52.529 -35.323 60.782 1.00 0.00
G   C
ATOM 23156 O   SER G 166   52.023 -35.165 61.896 1.00 0.00
G   O
ATOM 23157 N   ILE G 167   52.042 -36.165 59.869 1.00 0.00
G   N
ATOM 23158 HN ILE G 167   52.359 -36.237 58.926 1.00 0.00
G   H
ATOM 23159 CA ILE G 167   50.959 -37.083 60.208 1.00 0.00
G   C
ATOM 23160 HA ILE G 167   50.191 -36.496 60.693 1.00 0.00
G   H
ATOM 23161 CB ILE G 167   50.420 -37.771 58.943 1.00 0.00
G   C
ATOM 23162 HB ILE G 167   51.329 -37.963 58.319 1.00 0.00
G   H
ATOM 23163 CG2 ILE G 167   49.644 -39.023 59.312 1.00 0.00
G   C
ATOM 23164 HG21 ILE G 167   49.387 -39.606 58.403 1.00 0.00
G   H
ATOM 23165 HG22 ILE G 167   50.246 -39.681 59.975 1.00 0.00
G   H

```
ATOM  23166 HG23 ILE G 167      48.700 -38.765  59.837  1.00  0.00
G    H
ATOM  23167  CG1 ILE G 167      49.535 -36.813  58.151  1.00  0.00
G    C
ATOM  23168 HG11 ILE G 167      48.631 -37.349  57.786  1.00  0.00
G    H
ATOM  23169 HG12 ILE G 167      49.184 -36.002  58.831  1.00  0.00
G    H
ATOM  23170  CD  ILE G 167      50.237 -36.180  56.986  1.00  0.00
G    C
ATOM  23171  HD1 ILE G 167      49.567 -35.450  56.482  1.00  0.00
G    H
ATOM  23172  HD2 ILE G 167      51.148 -35.640  57.324  1.00  0.00
G    H
ATOM  23173  HD3 ILE G 167      50.536 -36.943  56.238  1.00  0.00
G    H
ATOM  23174  C   ILE G 167      51.439 -38.107  61.226  1.00  0.00
G    C
ATOM  23175  O   ILE G 167      50.763 -38.378  62.225  1.00  0.00
G    O
ATOM  23176  N   ILE G 168      52.616 -38.690  60.991  1.00  0.00
G    N
ATOM  23177  HN  ILE G 168      53.175 -38.572  60.174  1.00  0.00
G    H
ATOM  23178  CA  ILE G 168      53.188 -39.593  61.980  1.00  0.00
G    C
ATOM  23179  HA  ILE G 168      52.399 -40.273  62.273  1.00  0.00
G    H
ATOM  23180  CB  ILE G 168      54.378 -40.371  61.395  1.00  0.00
G    C
ATOM  23181  HB  ILE G 168      54.002 -40.761  60.416  1.00  0.00
G    H
ATOM  23182  CG2 ILE G 168      55.597 -39.476  61.260  1.00  0.00
G    C
ATOM  23183 HG21 ILE G 168      56.401 -39.993  60.696  1.00  0.00
G    H
ATOM  23184 HG22 ILE G 168      55.346 -38.545  60.710  1.00  0.00
G    H
ATOM  23185 HG23 ILE G 168      55.998 -39.197  62.257  1.00  0.00
G    H
ATOM  23186  CG1 ILE G 168      54.693 -41.585  62.268  1.00  0.00
G    C
ATOM  23187 HG11 ILE G 168      55.768 -41.856  62.163  1.00  0.00
G    H
ATOM  23188 HG12 ILE G 168      54.524 -41.315  63.336  1.00  0.00
G    H
ATOM  23189  CD  ILE G 168      53.841 -42.793  61.942  1.00  0.00
G    C
ATOM  23190  HD1 ILE G 168      54.070 -43.626  62.642  1.00  0.00
G    H
ATOM  23191  HD2 ILE G 168      52.761 -42.548  62.034  1.00  0.00
G    H
ATOM  23192  HD3 ILE G 168      54.034 -43.149  60.909  1.00  0.00
G    H
```

```
ATOM  23193  C   ILE G 168      53.591 -38.825  63.226  1.00  0.00
G    C
ATOM  23194  O   ILE G 168      53.672 -39.398  64.318  1.00  0.00
G    O
ATOM  23195  N   ASP G 169      53.829 -37.523  63.094  1.00  0.00
G    N
ATOM  23196  HN  ASP G 169      53.856 -37.003  62.244  1.00  0.00
G    H
ATOM  23197  CA  ASP G 169      54.086 -36.705  64.268  1.00  0.00
G    C
ATOM  23198  HA  ASP G 169      54.826 -37.221  64.869  1.00  0.00
G    H
ATOM  23199  CB  ASP G 169      54.567 -35.321  63.828  1.00  0.00
G    C
ATOM  23200  HB1 ASP G 169      54.621 -35.289  62.721  1.00  0.00
G    H
ATOM  23201  HB2 ASP G 169      53.861 -34.536  64.166  1.00  0.00
G    H
ATOM  23202  CG  ASP G 169      55.943 -34.984  64.355  1.00  0.00
G    C
ATOM  23203  OD1 ASP G 169      56.896 -35.689  63.963  1.00  0.00
G    O
ATOM  23204  OD2 ASP G 169      56.074 -34.025  65.149  1.00  0.00
G    O
ATOM  23205  C   ASP G 169      52.827 -36.597  65.119  1.00  0.00
G    C
ATOM  23206  O   ASP G 169      52.757 -37.132  66.232  1.00  0.00
G    O
ATOM  23207  N   LEU G 170      51.808 -35.931  64.589  1.00  0.00
G    N
ATOM  23208  HN  LEU G 170      51.849 -35.483  63.698  1.00  0.00
G    H
ATOM  23209  CA  LEU G 170      50.525 -35.795  65.270  1.00  0.00
G    C
ATOM  23210  HA  LEU G 170      50.679 -36.057  66.309  1.00  0.00
G    H
ATOM  23211  CB  LEU G 170      50.024 -34.359  65.161  1.00  0.00
G    C
ATOM  23212  HB1 LEU G 170      50.150 -34.046  64.098  1.00  0.00
G    H
ATOM  23213  HB2 LEU G 170      48.929 -34.342  65.358  1.00  0.00
G    H
ATOM  23214  CG  LEU G 170      50.737 -33.340  66.055  1.00  0.00
G    C
ATOM  23215  HG  LEU G 170      50.865 -33.836  67.047  1.00  0.00
G    H
ATOM  23216  CD1 LEU G 170      52.099 -32.948  65.493  1.00  0.00
G    C
ATOM  23217 HD11 LEU G 170      52.576 -32.174  66.131  1.00  0.00
G    H
ATOM  23218 HD12 LEU G 170      52.773 -33.831  65.451  1.00  0.00
G    H
ATOM  23219 HD13 LEU G 170      51.996 -32.536  64.467  1.00  0.00
G    H
```

ATOM 23220 CD2 LEU G 170   49.877 -32.104 66.255 1.00 0.00
G   C
ATOM 23221 HD21 LEU G 170   50.393 -31.378 66.919 1.00 0.00
G   H
ATOM 23222 HD22 LEU G 170   49.666 -31.591 65.295 1.00 0.00
G   H
ATOM 23223 HD23 LEU G 170   48.907 -32.377 66.726 1.00 0.00
G   H
ATOM 23224 C   LEU G 170   49.529 -36.796 64.685 1.00 0.00
G   C
ATOM 23225 O   LEU G 170   48.671 -36.468 63.865 1.00 0.00
G   O
ATOM 23226 N   ASN G 171   49.678 -38.048 65.113 1.00 0.00
G   N
ATOM 23227 HN ASN G 171   50.355 -38.336 65.789 1.00 0.00
G   H
ATOM 23228 CA ASN G 171   48.835 -39.120 64.607 1.00 0.00
G   C
ATOM 23229 HA ASN G 171   48.893 -39.061 63.525 1.00 0.00
G   H
ATOM 23230 CB ASN G 171   49.320 -40.476 65.120 1.00 0.00
G   C
ATOM 23231 HB1 ASN G 171   48.580 -41.244 64.806 1.00 0.00
G   H
ATOM 23232 HB2 ASN G 171   50.292 -40.739 64.653 1.00 0.00
G   H
ATOM 23233 CG ASN G 171   49.433 -40.523 66.626 1.00 0.00
G   C
ATOM 23234 OD1 ASN G 171   49.234 -39.520 67.307 1.00 0.00
G   O
ATOM 23235 ND2 ASN G 171   49.750 -41.696 67.156 1.00 0.00
G   N
ATOM 23236 HD21 ASN G 171   49.817 -41.747 68.149 1.00 0.00
G   H
ATOM 23237 HD22 ASN G 171   49.881 -42.489 66.568 1.00 0.00
G   H
ATOM 23238 C   ASN G 171   47.379 -38.901 65.004 1.00 0.00
G   C
ATOM 23239 O   ASN G 171   47.076 -38.250 66.008 1.00 0.00
G   O
ATOM 23240 N   VAL G 172   46.472 -39.433 64.186 1.00 0.00
G   N
ATOM 23241 HN VAL G 172   46.702 -39.944 63.362 1.00 0.00
G   H
ATOM 23242 CA VAL G 172   45.043 -39.306 64.437 1.00 0.00
G   C
ATOM 23243 HA VAL G 172   44.931 -38.924 65.443 1.00 0.00
G   H
ATOM 23244 CB VAL G 172   44.406 -38.302 63.455 1.00 0.00
G   C
ATOM 23245 HB VAL G 172   45.098 -37.429 63.367 1.00 0.00
G   H
ATOM 23246 CG1 VAL G 172   44.220 -38.929 62.078 1.00 0.00
G   C

```
ATOM  23247 HG11 VAL G 172      43.725 -38.210  61.391  1.00  0.00
G    H
ATOM  23248 HG12 VAL G 172      45.192 -39.207  61.620  1.00  0.00
G    H
ATOM  23249 HG13 VAL G 172      43.583 -39.837  62.143  1.00  0.00
G    H
ATOM  23250  CG2 VAL G 172      43.076 -37.784  64.000  1.00  0.00
G    C
ATOM  23251 HG21 VAL G 172      42.598 -37.091  63.275  1.00  0.00
G    H
ATOM  23252 HG22 VAL G 172      42.378 -38.628  64.191  1.00  0.00
G    H
ATOM  23253 HG23 VAL G 172      43.221 -37.228  64.949  1.00  0.00
G    H
ATOM  23254  C   VAL G 172      44.329 -40.652  64.376  1.00  0.00
G    C
ATOM  23255  O   VAL G 172      43.253 -40.802  64.965  1.00  0.00
G    O
ATOM  23256  N   ARG G 173      44.895 -41.636  63.675  1.00  0.00
G    N
ATOM  23257  HN  ARG G 173      45.768 -41.540  63.202  1.00  0.00
G    H
ATOM  23258  CA  ARG G 173      44.233 -42.932  63.572  1.00  0.00
G    C
ATOM  23259  HA  ARG G 173      43.180 -42.760  63.380  1.00  0.00
G    H
ATOM  23260  CB  ARG G 173      44.840 -43.748  62.436  1.00  0.00
G    C
ATOM  23261  HB1 ARG G 173      45.261 -43.013  61.709  1.00  0.00
G    H
ATOM  23262  HB2 ARG G 173      45.707 -44.331  62.818  1.00  0.00
G    H
ATOM  23263  CG  ARG G 173      43.824 -44.624  61.736  1.00  0.00
G    C
ATOM  23264  HG1 ARG G 173      43.851 -45.636  62.198  1.00  0.00
G    H
ATOM  23265  HG2 ARG G 173      42.803 -44.229  61.940  1.00  0.00
G    H
ATOM  23266  CD  ARG G 173      44.049 -44.653  60.232  1.00  0.00
G    C
ATOM  23267  HD1 ARG G 173      43.067 -44.591  59.710  1.00  0.00
G    H
ATOM  23268  HD2 ARG G 173      44.686 -43.801  59.898  1.00  0.00
G    H
ATOM  23269  NE  ARG G 173      44.734 -45.866  59.790  1.00  0.00
G    N
ATOM  23270  HE  ARG G 173      45.683 -45.702  59.500  1.00  0.00
G    H
ATOM  23271  CZ  ARG G 173      44.181 -47.076  59.764  1.00  0.00
G    C
ATOM  23272  NH1 ARG G 173      42.925 -47.247  60.155  1.00  0.00
G    N
ATOM  23273 HH11 ARG G 173      42.518 -48.150  60.161  1.00  0.00
G    H
```

ATOM 23274 HH12 ARG G 173   42.474 -46.451 60.564 1.00 0.00
G   H
ATOM 23275 NH2 ARG G 173   44.884 -48.119 59.342 1.00 0.00
G   N
ATOM 23276 HH21 ARG G 173   44.446 -49.001 59.242 1.00 0.00
G   H
ATOM 23277 HH22 ARG G 173   45.771 -47.929 58.944 1.00 0.00
G   H
ATOM 23278 C   ARG G 173   44.316 -43.704 64.880 1.00 0.00
G   C
ATOM 23279 O   ARG G 173   43.303 -44.204 65.380 1.00 0.00
G   O
ATOM 23280 N   ALA G 174   45.514 -43.825 65.444 1.00 0.00
G   N
ATOM 23281 HN ALA G 174   46.363 -43.508 65.023 1.00 0.00
G   H
ATOM 23282 CA ALA G 174   45.658 -44.446 66.752 1.00 0.00
G   C
ATOM 23283 HA ALA G 174   44.992 -45.296 66.844 1.00 0.00
G   H
ATOM 23284 CB ALA G 174   47.092 -44.946 66.942 1.00 0.00
G   C
ATOM 23285 HB1 ALA G 174   47.337 -45.685 66.150 1.00 0.00
G   H
ATOM 23286 HB2 ALA G 174   47.820 -44.109 66.866 1.00 0.00
G   H
ATOM 23287 HB3 ALA G 174   47.225 -45.443 67.927 1.00 0.00
G   H
ATOM 23288 C   ALA G 174   45.283 -43.484 67.869 1.00 0.00
G   C
ATOM 23289 O   ALA G 174   44.925 -43.913 68.970 1.00 0.00
G   O
ATOM 23290 N   LYS G 175   45.344 -42.180 67.594 1.00 0.00
G   N
ATOM 23291 HN LYS G 175   45.555 -41.805 66.692 1.00 0.00
G   H
ATOM 23292 CA LYS G 175   45.097 -41.189 68.633 1.00 0.00
G   C
ATOM 23293 HA LYS G 175   45.390 -41.640 69.574 1.00 0.00
G   H
ATOM 23294 CB LYS G 175   45.925 -39.938 68.355 1.00 0.00
G   C
ATOM 23295 HB1 LYS G 175   46.903 -40.278 67.941 1.00 0.00
G   H
ATOM 23296 HB2 LYS G 175   45.432 -39.349 67.549 1.00 0.00
G   H
ATOM 23297 CG LYS G 175   46.178 -39.076 69.568 1.00 0.00
G   C
ATOM 23298 HG1 LYS G 175   45.235 -38.554 69.847 1.00 0.00
G   H
ATOM 23299 HG2 LYS G 175   46.462 -39.729 70.425 1.00 0.00
G   H
ATOM 23300 CD LYS G 175   47.261 -38.057 69.273 1.00 0.00
G   C

ATOM 23301 HD1 LYS G 175 48.164 -38.599 68.909 1.00 0.00
G H
ATOM 23302 HD2 LYS G 175 46.906 -37.404 68.442 1.00 0.00
G H
ATOM 23303 CE LYS G 175 47.588 -37.228 70.497 1.00 0.00
G C
ATOM 23304 HE1 LYS G 175 46.730 -36.571 70.763 1.00 0.00
G H
ATOM 23305 HE2 LYS G 175 47.811 -37.884 71.367 1.00 0.00
G H
ATOM 23306 NZ LYS G 175 48.774 -36.356 70.268 1.00 0.00
G N
ATOM 23307 HZ1 LYS G 175 48.976 -35.797 71.122 1.00 0.00
G H
ATOM 23308 HZ2 LYS G 175 49.600 -36.947 70.038 1.00 0.00
G H
ATOM 23309 HZ3 LYS G 175 48.582 -35.714 69.472 1.00 0.00
G H
ATOM 23310 C LYS G 175 43.617 -40.841 68.737 1.00 0.00
G C
ATOM 23311 O LYS G 175 43.036 -40.908 69.826 1.00 0.00
G O
ATOM 23312 N TYR G 176 42.989 -40.470 67.620 1.00 0.00
G N
ATOM 23313 HN TYR G 176 43.424 -40.453 66.721 1.00 0.00
G H
ATOM 23314 CA TYR G 176 41.595 -40.054 67.624 1.00 0.00
G C
ATOM 23315 HA TYR G 176 41.267 -40.165 68.651 1.00 0.00
G H
ATOM 23316 CB TYR G 176 41.471 -38.594 67.161 1.00 0.00
G C
ATOM 23317 HB1 TYR G 176 41.855 -38.508 66.121 1.00 0.00
G H
ATOM 23318 HB2 TYR G 176 40.406 -38.276 67.146 1.00 0.00
G H
ATOM 23319 CG TYR G 176 42.271 -37.627 68.002 1.00 0.00
G C
ATOM 23320 CD1 TYR G 176 43.527 -37.201 67.598 1.00 0.00
G C
ATOM 23321 HD1 TYR G 176 43.950 -37.574 66.674 1.00 0.00
G H
ATOM 23322 CE1 TYR G 176 44.265 -36.321 68.364 1.00 0.00
G C
ATOM 23323 HE1 TYR G 176 45.244 -36.000 68.041 1.00 0.00
G H
ATOM 23324 CZ TYR G 176 43.750 -35.857 69.553 1.00 0.00
G C
ATOM 23325 OH TYR G 176 44.485 -34.981 70.315 1.00 0.00
G O
ATOM 23326 HH TYR G 176 43.939 -34.718 71.059 1.00 0.00
G H
ATOM 23327 CD2 TYR G 176 41.774 -37.147 69.207 1.00 0.00
G C

ATOM 23328 HD2 TYR G 176   40.816 -37.493 69.572 1.00 0.00
G   H
ATOM 23329 CE2 TYR G 176   42.505 -36.268 69.981 1.00 0.00
G   C
ATOM 23330 HE2 TYR G 176   42.106 -35.924 70.924 1.00 0.00
G   H
ATOM 23331 C   TYR G 176   40.688 -40.925 66.767 1.00 0.00
G   C
ATOM 23332 O   TYR G 176   39.497 -40.619 66.658 1.00 0.00
G   O
ATOM 23333 N   GLY G 177   41.208 -41.986 66.153 1.00 0.00
G   N
ATOM 23334 HN GLY G 177   42.180 -42.216 66.180 1.00 0.00
G   H
ATOM 23335 CA GLY G 177   40.371 -42.890 65.391 1.00 0.00
G   C
ATOM 23336 HA1 GLY G 177   39.526 -43.110 66.030 1.00 0.00
G   H
ATOM 23337 HA2 GLY G 177   40.991 -43.746 65.165 1.00 0.00
G   H
ATOM 23338 C   GLY G 177   39.842 -42.342 64.090 1.00 0.00
G   C
ATOM 23339 O   GLY G 177   38.892 -42.902 63.536 1.00 0.00
G   O
ATOM 23340 N   CYS G 178   40.426 -41.267 63.580 1.00 0.00
G   N
ATOM 23341 HN CYS G 178   41.215 -40.831 64.009 1.00 0.00
G   H
ATOM 23342 CA CYS G 178   39.970 -40.631 62.355 1.00 0.00
G   C
ATOM 23343 HA CYS G 178   39.027 -41.093 62.087 1.00 0.00
G   H
ATOM 23344 CB CYS G 178   39.790 -39.125 62.570 1.00 0.00
G   C
ATOM 23345 HB1 CYS G 178   40.730 -38.742 63.029 1.00 0.00
G   H
ATOM 23346 HB2 CYS G 178   39.699 -38.612 61.587 1.00 0.00
G   H
ATOM 23347 SG CYS G 178   38.410 -38.719 63.655 1.00 0.00
G   S
ATOM 23348 HG1 CYS G 178   38.592 -37.398 63.669 1.00 0.00
G   H
ATOM 23349 C   CYS G 178   40.960 -40.913 61.231 1.00 0.00
G   C
ATOM 23350 O   CYS G 178   41.919 -41.674 61.390 1.00 0.00
G   O
ATOM 23351 N   THR G 179   40.717 -40.287 60.082 1.00 0.00
G   N
ATOM 23352 HN THR G 179   39.966 -39.638 59.962 1.00 0.00
G   H
ATOM 23353 CA THR G 179   41.528 -40.493 58.888 1.00 0.00
G   C
ATOM 23354 HA THR G 179   42.460 -40.927 59.223 1.00 0.00
G   H

```
ATOM  23355  CB  THR G 179      40.791 -41.385  57.881  1.00  0.00
G    C
ATOM  23356  HB  THR G 179      40.328 -40.694  57.135  1.00  0.00
G    H
ATOM  23357  OG1 THR G 179      39.736 -42.087  58.553  1.00  0.00
G    O
ATOM  23358  HG1 THR G 179      39.322 -42.634  57.877  1.00  0.00
G    H
ATOM  23359  CG2 THR G 179      41.739 -42.365  57.215  1.00  0.00
G    C
ATOM  23360 HG21 THR G 179      41.223 -42.928  56.409  1.00  0.00
G    H
ATOM  23361 HG22 THR G 179      42.597 -41.826  56.760  1.00  0.00
G    H
ATOM  23362 HG23 THR G 179      42.138 -43.097  57.950  1.00  0.00
G    H
ATOM  23363  C   THR G 179      41.876 -39.154  58.255  1.00  0.00
G    C
ATOM  23364  O   THR G 179      40.994 -38.447  57.757  1.00  0.00
G    O
ATOM  23365  N   ILE G 180      43.165 -38.812  58.259  1.00  0.00
G    N
ATOM  23366  HN  ILE G 180      43.879 -39.328  58.728  1.00  0.00
G    H
ATOM  23367  CA  ILE G 180      43.646 -37.624  57.560  1.00  0.00
G    C
ATOM  23368  HA  ILE G 180      42.894 -36.853  57.662  1.00  0.00
G    H
ATOM  23369  CB  ILE G 180      45.004 -37.161  58.119  1.00  0.00
G    C
ATOM  23370  HB  ILE G 180      45.279 -37.942  58.872  1.00  0.00
G    H
ATOM  23371  CG2 ILE G 180      46.026 -36.976  56.994  1.00  0.00
G    C
ATOM  23372 HG21 ILE G 180      47.038 -36.796  57.412  1.00  0.00
G    H
ATOM  23373 HG22 ILE G 180      46.082 -37.884  56.357  1.00  0.00
G    H
ATOM  23374 HG23 ILE G 180      45.757 -36.112  56.350  1.00  0.00
G    H
ATOM  23375  CG1 ILE G 180      44.843 -35.843  58.884  1.00  0.00
G    C
ATOM  23376 HG11 ILE G 180      43.863 -35.380  58.633  1.00  0.00
G    H
ATOM  23377 HG12 ILE G 180      44.834 -36.058  59.977  1.00  0.00
G    H
ATOM  23378  CD  ILE G 180      45.943 -34.817  58.625  1.00  0.00
G    C
ATOM  23379  HD1 ILE G 180      45.787 -33.916  59.256  1.00  0.00
G    H
ATOM  23380  HD2 ILE G 180      46.941 -35.243  58.866  1.00  0.00
G    H
ATOM  23381  HD3 ILE G 180      45.946 -34.496  57.562  1.00  0.00
G    H
```

ATOM 23382 C ILE G 180 43.735 -37.959 56.077 1.00 0.00
G C
ATOM 23383 O ILE G 180 44.101 -39.080 55.705 1.00 0.00
G O
ATOM 23384 N LEU G 181 43.372 -37.004 55.224 1.00 0.00
G N
ATOM 23385 HN LEU G 181 43.230 -36.046 55.466 1.00 0.00
G H
ATOM 23386 CA LEU G 181 43.146 -37.306 53.817 1.00 0.00
G C
ATOM 23387 HA LEU G 181 43.421 -38.347 53.710 1.00 0.00
G H
ATOM 23388 CB LEU G 181 41.678 -37.084 53.459 1.00 0.00
G C
ATOM 23389 HB1 LEU G 181 41.107 -37.053 54.416 1.00 0.00
G H
ATOM 23390 HB2 LEU G 181 41.565 -36.076 53.002 1.00 0.00
G H
ATOM 23391 CG LEU G 181 41.059 -38.143 52.561 1.00 0.00
G C
ATOM 23392 HG LEU G 181 41.374 -37.939 51.509 1.00 0.00
G H
ATOM 23393 CD1 LEU G 181 41.513 -39.545 52.947 1.00 0.00
G C
ATOM 23394 HD11 LEU G 181 41.013 -40.306 52.310 1.00 0.00
G H
ATOM 23395 HD12 LEU G 181 42.612 -39.650 52.819 1.00 0.00
G H
ATOM 23396 HD13 LEU G 181 41.262 -39.761 54.007 1.00 0.00
G H
ATOM 23397 CD2 LEU G 181 39.576 -37.987 52.638 1.00 0.00
G C
ATOM 23398 HD21 LEU G 181 39.074 -38.731 51.981 1.00 0.00
G H
ATOM 23399 HD22 LEU G 181 39.201 -38.143 53.670 1.00 0.00
G H
ATOM 23400 HD23 LEU G 181 39.274 -36.971 52.307 1.00 0.00
G H
ATOM 23401 C LEU G 181 43.986 -36.508 52.840 1.00 0.00
G C
ATOM 23402 O LEU G 181 44.488 -37.086 51.878 1.00 0.00
G O
ATOM 23403 N ALA G 182 44.123 -35.197 53.026 1.00 0.00
G N
ATOM 23404 HN ALA G 182 43.775 -34.696 53.818 1.00 0.00
G H
ATOM 23405 CA ALA G 182 44.819 -34.394 52.037 1.00 0.00
G C
ATOM 23406 HA ALA G 182 45.692 -34.911 51.656 1.00 0.00
G H
ATOM 23407 CB ALA G 182 43.924 -34.068 50.838 1.00 0.00
G C
ATOM 23408 HB1 ALA G 182 43.601 -35.010 50.347 1.00 0.00
G H

ATOM 23409 HB2 ALA G 182   43.012 -33.518 51.157 1.00 0.00
G   H
ATOM 23410 HB3 ALA G 182   44.461 -33.454 50.082 1.00 0.00
G   H
ATOM 23411 C   ALA G 182   45.325 -33.115 52.678 1.00 0.00
G   C
ATOM 23412 O   ALA G 182   44.750 -32.599 53.642 1.00 0.00
G   O
ATOM 23413 N   ILE G 183   46.402 -32.604 52.092 1.00 0.00
G   N
ATOM 23414 HN ILE G 183   46.763 -32.982 51.242 1.00 0.00
G   H
ATOM 23415 CA ILE G 183   47.155 -31.470 52.607 1.00 0.00
G   C
ATOM 23416 HA ILE G 183   46.582 -31.039 53.417 1.00 0.00
G   H
ATOM 23417 CB ILE G 183   48.562 -31.914 53.060 1.00 0.00
G   C
ATOM 23418 HB ILE G 183   49.013 -32.386 52.151 1.00 0.00
G   H
ATOM 23419 CG2 ILE G 183   49.405 -30.739 53.529 1.00 0.00
G   C
ATOM 23420 HG21 ILE G 183   50.455 -31.056 53.700 1.00 0.00
G   H
ATOM 23421 HG22 ILE G 183   49.417 -29.932 52.767 1.00 0.00
G   H
ATOM 23422 HG23 ILE G 183   49.010 -30.320 54.477 1.00 0.00
G   H
ATOM 23423 CG1 ILE G 183   48.453 -33.010 54.126 1.00 0.00
G   C
ATOM 23424 HG11 ILE G 183   47.742 -32.693 54.921 1.00 0.00
G   H
ATOM 23425 HG12 ILE G 183   48.033 -33.929 53.656 1.00 0.00
G   H
ATOM 23426 CD ILE G 183   49.769 -33.370 54.762 1.00 0.00
G   C
ATOM 23427 HD1 ILE G 183   49.634 -34.207 55.480 1.00 0.00
G   H
ATOM 23428 HD2 ILE G 183   50.503 -33.690 53.990 1.00 0.00
G   H
ATOM 23429 HD3 ILE G 183   50.194 -32.507 55.317 1.00 0.00
G   H
ATOM 23430 C   ILE G 183   47.236 -30.414 51.513 1.00 0.00
G   C
ATOM 23431 O   ILE G 183   47.765 -30.681 50.429 1.00 0.00
G   O
ATOM 23432 N   LYS G 184   46.724 -29.217 51.800 1.00 0.00
G   N
ATOM 23433 HN LYS G 184   46.339 -29.014 52.699 1.00 0.00
G   H
ATOM 23434 CA LYS G 184   46.681 -28.112 50.840 1.00 0.00
G   C
ATOM 23435 HA LYS G 184   47.167 -28.421 49.923 1.00 0.00
G   H

ATOM 23436 CB LYS G 184   45.241 -27.668 50.600 1.00 0.00
G   C
ATOM 23437 HB1 LYS G 184   44.622 -28.592 50.519 1.00 0.00
G   H
ATOM 23438 HB2 LYS G 184   44.875 -27.124 51.499 1.00 0.00
G   H
ATOM 23439 CG LYS G 184   44.954 -26.817 49.356 1.00 0.00
G   C
ATOM 23440 HG1 LYS G 184   43.938 -27.062 48.973 1.00 0.00
G   H
ATOM 23441 HG2 LYS G 184   44.949 -25.741 49.647 1.00 0.00
G   H
ATOM 23442 CD LYS G 184   45.942 -27.024 48.227 1.00 0.00
G   C
ATOM 23443 HD1 LYS G 184   46.880 -26.477 48.475 1.00 0.00
G   H
ATOM 23444 HD2 LYS G 184   46.191 -28.109 48.180 1.00 0.00
G   H
ATOM 23445 CE LYS G 184   45.326 -26.535 46.922 1.00 0.00
G   C
ATOM 23446 HE1 LYS G 184   44.302 -26.952 46.792 1.00 0.00
G   H
ATOM 23447 HE2 LYS G 184   45.256 -25.425 46.915 1.00 0.00
G   H
ATOM 23448 NZ LYS G 184   46.090 -26.914 45.701 1.00 0.00
G   N
ATOM 23449 HZ1 LYS G 184   45.610 -26.548 44.853 1.00 0.00
G   H
ATOM 23450 HZ2 LYS G 184   47.049 -26.516 45.755 1.00 0.00
G   H
ATOM 23451 HZ3 LYS G 184   46.152 -27.951 45.641 1.00 0.00
G   H
ATOM 23452 C   LYS G 184   47.508 -26.977 51.434 1.00 0.00
G   C
ATOM 23453 O   LYS G 184   47.024 -26.239 52.296 1.00 0.00
G   O
ATOM 23454 N   HSE G 185   48.751 -26.838 50.983 1.00 0.00
G   N
ATOM 23455 HN HSE G 185   49.133 -27.410 50.258 1.00 0.00
G   H
ATOM 23456 CA HSE G 185   49.673 -25.835 51.507 1.00 0.00
G   C
ATOM 23457 HA HSE G 185   49.149 -25.275 52.271 1.00 0.00
G   H
ATOM 23458 CB HSE G 185   50.926 -26.486 52.096 1.00 0.00
G   C
ATOM 23459 HB1 HSE G 185   51.586 -25.676 52.475 1.00 0.00
G   H
ATOM 23460 HB2 HSE G 185   50.645 -27.110 52.969 1.00 0.00
G   H
ATOM 23461 ND1 HSE G 185   52.791 -26.782 50.429 1.00 0.00
G   N
ATOM 23462 CG HSE G 185   51.714 -27.296 51.118 1.00 0.00
G   C

```
ATOM  23463  CE1 HSE G 185      53.291 -27.716  49.640  1.00  0.00
G    C
ATOM  23464  HE1 HSE G 185      54.161 -27.581  48.992  1.00  0.00
G    H
ATOM  23465  NE2 HSE G 185      52.560 -28.808  49.772  1.00  0.00
G    N
ATOM  23466  HE2 HSE G 185      52.700 -29.660  49.268  1.00  0.00
G    H
ATOM  23467  CD2 HSE G 185      51.565 -28.570  50.690  1.00  0.00
G    C
ATOM  23468  HD2 HSE G 185      50.826 -29.310  50.963  1.00  0.00
G    H
ATOM  23469  C   HSE G 185      50.039 -24.853  50.406  1.00  0.00
G    C
ATOM  23470  O   HSE G 185      50.745 -25.213  49.459  1.00  0.00
G    O
ATOM  23471  N   HSE G 186      49.563 -23.616  50.546  1.00  0.00
G    N
ATOM  23472  HN  HSE G 186      48.970 -23.342  51.301  1.00  0.00
G    H
ATOM  23473  CA  HSE G 186      49.870 -22.547  49.603  1.00  0.00
G    C
ATOM  23474  HA  HSE G 186      49.235 -21.708  49.860  1.00  0.00
G    H
ATOM  23475  CB  HSE G 186      51.338 -22.129  49.706  1.00  0.00
G    C
ATOM  23476  HB1 HSE G 186      51.620 -22.131  50.781  1.00  0.00
G    H
ATOM  23477  HB2 HSE G 186      51.975 -22.886  49.202  1.00  0.00
G    H
ATOM  23478  ND1 HSE G 186      50.977 -19.637  49.617  1.00  0.00
G    N
ATOM  23479  CG  HSE G 186      51.616 -20.766  49.157  1.00  0.00
G    C
ATOM  23480  CE1 HSE G 186      51.410 -18.585  48.946  1.00  0.00
G    C
ATOM  23481  HE1 HSE G 186      51.068 -17.560  49.111  1.00  0.00
G    H
ATOM  23482  NE2 HSE G 186      52.302 -18.992  48.063  1.00  0.00
G    N
ATOM  23483  HE2 HSE G 186      52.775 -18.403  47.409  1.00  0.00
G    H
ATOM  23484  CD2 HSE G 186      52.447 -20.353  48.171  1.00  0.00
G    C
ATOM  23485  HD2 HSE G 186      53.114 -20.926  47.542  1.00  0.00
G    H
ATOM  23486  C   HSE G 186      49.521 -22.952  48.177  1.00  0.00
G    C
ATOM  23487  O   HSE G 186      50.293 -22.722  47.245  1.00  0.00
G    O
ATOM  23488  N   GLY G 187      48.360 -23.584  47.996  1.00  0.00
G    N
ATOM  23489  HN  GLY G 187      47.749 -23.792  48.759  1.00  0.00
G    H
```

```
ATOM  23490  CA  GLY G 187      47.906 -24.009  46.701  1.00  0.00
G    C
ATOM  23491  HA1 GLY G 187      48.259 -23.267  45.997  1.00  0.00
G    H
ATOM  23492  HA2 GLY G 187      46.829 -24.085  46.763  1.00  0.00
G    H
ATOM  23493  C   GLY G 187      48.443 -25.358  46.270  1.00  0.00
G    C
ATOM  23494  O   GLY G 187      48.056 -25.856  45.210  1.00  0.00
G    O
ATOM  23495  N   ASP G 188      49.322 -25.958  47.063  1.00  0.00
G    N
ATOM  23496  HN  ASP G 188      49.648 -25.625  47.945  1.00  0.00
G    H
ATOM  23497  CA  ASP G 188      49.917 -27.231  46.683  1.00  0.00
G    C
ATOM  23498  HA  ASP G 188      49.767 -27.346  45.615  1.00  0.00
G    H
ATOM  23499  CB  ASP G 188      51.399 -27.234  47.047  1.00  0.00
G    C
ATOM  23500  HB1 ASP G 188      51.508 -27.056  48.135  1.00  0.00
G    H
ATOM  23501  HB2 ASP G 188      51.858 -28.213  46.806  1.00  0.00
G    H
ATOM  23502  CG  ASP G 188      52.180 -26.143  46.327  1.00  0.00
G    C
ATOM  23503  OD1 ASP G 188      51.535 -25.228  45.775  1.00  0.00
G    O
ATOM  23504  OD2 ASP G 188      53.431 -26.200  46.315  1.00  0.00
G    O
ATOM  23505  C   ASP G 188      49.198 -28.399  47.359  1.00  0.00
G    C
ATOM  23506  O   ASP G 188      48.974 -28.392  48.572  1.00  0.00
G    O
ATOM  23507  N   ILE G 189      48.849 -29.410  46.563  1.00  0.00
G    N
ATOM  23508  HN  ILE G 189      49.103 -29.437  45.598  1.00  0.00
G    H
ATOM  23509  CA  ILE G 189      48.072 -30.564  47.009  1.00  0.00
G    C
ATOM  23510  HA  ILE G 189      47.564 -30.270  47.919  1.00  0.00
G    H
ATOM  23511  CB  ILE G 189      47.057 -31.017  45.936  1.00  0.00
G    C
ATOM  23512  HB  ILE G 189      47.105 -30.220  45.151  1.00  0.00
G    H
ATOM  23513  CG2 ILE G 189      47.372 -32.424  45.431  1.00  0.00
G    C
ATOM  23514 HG21 ILE G 189      46.736 -32.676  44.557  1.00  0.00
G    H
ATOM  23515 HG22 ILE G 189      48.432 -32.501  45.112  1.00  0.00
G    H
ATOM  23516 HG23 ILE G 189      47.188 -33.180  46.223  1.00  0.00
G    H
```

```
ATOM  23517  CG1 ILE G 189      45.620 -31.011  46.465  1.00  0.00
G    C
ATOM  23518 HG11 ILE G 189      44.932 -30.621  45.683  1.00  0.00
G    H
ATOM  23519 HG12 ILE G 189      45.311 -32.059  46.683  1.00  0.00
G    H
ATOM  23520  CD  ILE G 189      45.393 -30.227  47.700  1.00  0.00
G    C
ATOM  23521  HD1 ILE G 189      44.338 -30.331  48.034  1.00  0.00
G    H
ATOM  23522  HD2 ILE G 189      46.054 -30.584  48.519  1.00  0.00
G    H
ATOM  23523  HD3 ILE G 189      45.594 -29.148  47.529  1.00  0.00
G    H
ATOM  23524  C   ILE G 189      49.008 -31.705  47.378  1.00  0.00
G    C
ATOM  23525  O   ILE G 189      50.094 -31.845  46.801  1.00  0.00
G    O
ATOM  23526  N   CYS G 190      48.593 -32.493  48.365  1.00  0.00
G    N
ATOM  23527  HN  CYS G 190      47.856 -32.204  48.973  1.00  0.00
G    H
ATOM  23528  CA  CYS G 190      49.144 -33.811  48.659  1.00  0.00
G    C
ATOM  23529  HA  CYS G 190      49.560 -34.228  47.749  1.00  0.00
G    H
ATOM  23530  CB  CYS G 190      50.208 -33.752  49.752  1.00  0.00
G    C
ATOM  23531  HB1 CYS G 190      50.886 -32.903  49.507  1.00  0.00
G    H
ATOM  23532  HB2 CYS G 190      49.734 -33.495  50.725  1.00  0.00
G    H
ATOM  23533  SG  CYS G 190      51.211 -35.259  49.896  1.00  0.00
G    S
ATOM  23534  HG1 CYS G 190      52.031 -34.822  50.854  1.00  0.00
G    H
ATOM  23535  C   CYS G 190      47.958 -34.679  49.066  1.00  0.00
G    C
ATOM  23536  O   CYS G 190      47.409 -34.526  50.160  1.00  0.00
G    O
ATOM  23537  N   LEU G 191      47.549 -35.573  48.168  1.00  0.00
G    N
ATOM  23538  HN  LEU G 191      47.970 -35.707  47.273  1.00  0.00
G    H
ATOM  23539  CA  LEU G 191      46.419 -36.453  48.435  1.00  0.00
G    C
ATOM  23540  HA  LEU G 191      45.760 -35.924  49.110  1.00  0.00
G    H
ATOM  23541  CB  LEU G 191      45.705 -36.836  47.139  1.00  0.00
G    C
ATOM  23542  HB1 LEU G 191      46.493 -37.061  46.383  1.00  0.00
G    H
ATOM  23543  HB2 LEU G 191      45.151 -37.787  47.299  1.00  0.00
G    H
```

```
ATOM  23544  CG  LEU G 191      44.748 -35.849  46.475  1.00  0.00
G    C
ATOM  23545  HG  LEU G 191      45.330 -34.962  46.126  1.00  0.00
G    H
ATOM  23546  CD1 LEU G 191      44.093 -36.524  45.291  1.00  0.00
G    C
ATOM  23547 HD11 LEU G 191      43.354 -35.846  44.814  1.00  0.00
G    H
ATOM  23548 HD12 LEU G 191      44.853 -36.803  44.529  1.00  0.00
G    H
ATOM  23549 HD13 LEU G 191      43.562 -37.446  45.609  1.00  0.00
G    H
ATOM  23550  CD2 LEU G 191      43.707 -35.367  47.458  1.00  0.00
G    C
ATOM  23551 HD21 LEU G 191      43.027 -34.635  46.972  1.00  0.00
G    H
ATOM  23552 HD22 LEU G 191      43.086 -36.203  47.839  1.00  0.00
G    H
ATOM  23553 HD23 LEU G 191      44.192 -34.869  48.325  1.00  0.00
G    H
ATOM  23554  C   LEU G 191      46.880 -37.719  49.141  1.00  0.00
G    C
ATOM  23555  O   LEU G 191      47.895 -38.319  48.778  1.00  0.00
G    O
ATOM  23556  N   SER G 192      46.086 -38.153  50.120  1.00  0.00
G    N
ATOM  23557  HN  SER G 192      45.220 -37.706  50.342  1.00  0.00
G    H
ATOM  23558  CA  SER G 192      46.393 -39.308  50.957  1.00  0.00
G    C
ATOM  23559  HA  SER G 192      45.781 -39.217  51.845  1.00  0.00
G    H
ATOM  23560  CB  SER G 192      46.096 -40.616  50.233  1.00  0.00
G    C
ATOM  23561  HB1 SER G 192      46.859 -41.365  50.545  1.00  0.00
G    H
ATOM  23562  HB2 SER G 192      45.102 -41.014  50.543  1.00  0.00
G    H
ATOM  23563  OG  SER G 192      46.161 -40.471  48.826  1.00  0.00
G    O
ATOM  23564  HG1 SER G 192      46.187 -41.360  48.450  1.00  0.00
G    H
ATOM  23565  C   SER G 192      47.858 -39.258  51.407  1.00  0.00
G    C
ATOM  23566  O   SER G 192      48.651 -40.124  51.025  1.00  0.00
G    O
ATOM  23567  N   PRO G 193      48.240 -38.260  52.199  1.00  0.00
G    N
ATOM  23568  CD  PRO G 193      47.391 -37.342  52.979  1.00  0.00
G    C
ATOM  23569  HD1 PRO G 193      46.929 -36.558  52.338  1.00  0.00
G    H
ATOM  23570  HD2 PRO G 193      46.606 -37.905  53.534  1.00  0.00
G    H
```

ATOM 23571 CA PRO G 193 49.664 -38.073 52.498 1.00 0.00
G C
ATOM 23572 HA PRO G 193 50.235 -37.966 51.584 1.00 0.00
G H
ATOM 23573 CB PRO G 193 49.689 -36.772 53.306 1.00 0.00
G C
ATOM 23574 HB1 PRO G 193 49.717 -35.909 52.604 1.00 0.00
G H
ATOM 23575 HB2 PRO G 193 50.560 -36.708 53.989 1.00 0.00
G H
ATOM 23576 CG PRO G 193 48.354 -36.713 53.949 1.00 0.00
G C
ATOM 23577 HG1 PRO G 193 48.053 -35.683 54.224 1.00 0.00
G H
ATOM 23578 HG2 PRO G 193 48.357 -37.354 54.861 1.00 0.00
G H
ATOM 23579 C PRO G 193 50.219 -39.239 53.299 1.00 0.00
G C
ATOM 23580 O PRO G 193 49.645 -39.662 54.305 1.00 0.00
G O
ATOM 23581 N ALA G 194 51.351 -39.760 52.831 1.00 0.00
G N
ATOM 23582 HN ALA G 194 51.792 -39.434 51.996 1.00 0.00
G H
ATOM 23583 CA ALA G 194 52.029 -40.848 53.509 1.00 0.00
G C
ATOM 23584 HA ALA G 194 51.333 -41.474 54.056 1.00 0.00
G H
ATOM 23585 CB ALA G 194 52.790 -41.716 52.512 1.00 0.00
G C
ATOM 23586 HB1 ALA G 194 52.075 -42.174 51.796 1.00 0.00
G H
ATOM 23587 HB2 ALA G 194 53.517 -41.110 51.930 1.00 0.00
G H
ATOM 23588 HB3 ALA G 194 53.340 -42.536 53.021 1.00 0.00
G H
ATOM 23589 C ALA G 194 52.982 -40.268 54.537 1.00 0.00
G C
ATOM 23590 O ALA G 194 53.804 -39.407 54.184 1.00 0.00
G O
ATOM 23591 N PRO G 195 52.897 -40.678 55.800 1.00 0.00
G N
ATOM 23592 CD PRO G 195 51.922 -41.623 56.370 1.00 0.00
G C
ATOM 23593 HD1 PRO G 195 51.566 -40.721 55.822 1.00 0.00
G H
ATOM 23594 HD2 PRO G 195 51.240 -42.485 56.186 1.00 0.00
G H
ATOM 23595 CA PRO G 195 53.824 -40.158 56.814 1.00 0.00
G C
ATOM 23596 HA PRO G 195 53.702 -39.088 56.934 1.00 0.00
G H
ATOM 23597 CB PRO G 195 53.403 -40.901 58.086 1.00 0.00
G C

```
ATOM  23598  HB1 PRO G 195      52.560 -40.352  58.564  1.00  0.00
G    H
ATOM  23599  HB2 PRO G 195      54.229 -41.000  58.818  1.00  0.00
G    H
ATOM  23600  CG  PRO G 195      51.998 -41.335  57.835  1.00  0.00
G    C
ATOM  23601  HG1 PRO G 195      51.428 -41.512  58.768  1.00  0.00
G    H
ATOM  23602  HG2 PRO G 195      52.006 -42.266  57.223  1.00  0.00
G    H
ATOM  23603  C   PRO G 195      55.269 -40.454  56.439  1.00  0.00
G    C
ATOM  23604  O   PRO G 195      55.597 -41.554  55.989  1.00  0.00
G    O
ATOM  23605  N   GLU G 196      56.137 -39.460  56.620  1.00  0.00
G    N
ATOM  23606  HN  GLU G 196      57.099 -39.532  56.371  1.00  0.00
G    H
ATOM  23607  CA  GLU G 196      55.768 -38.171  57.197  1.00  0.00
G    C
ATOM  23608  HA  GLU G 196      54.830 -38.289  57.723  1.00  0.00
G    H
ATOM  23609  CB  GLU G 196      56.849 -37.698  58.173  1.00  0.00
G    C
ATOM  23610  HB1 GLU G 196      56.537 -36.711  58.585  1.00  0.00
G    H
ATOM  23611  HB2 GLU G 196      56.901 -38.390  59.043  1.00  0.00
G    H
ATOM  23612  CG  GLU G 196      58.240 -37.546  57.572  1.00  0.00
G    C
ATOM  23613  HG1 GLU G 196      58.998 -37.627  58.372  1.00  0.00
G    H
ATOM  23614  HG2 GLU G 196      58.431 -38.327  56.809  1.00  0.00
G    H
ATOM  23615  CD  GLU G 196      58.445 -36.182  56.939  1.00  0.00
G    C
ATOM  23616  OE1 GLU G 196      58.474 -35.177  57.687  1.00  0.00
G    O
ATOM  23617  OE2 GLU G 196      58.536 -36.112  55.697  1.00  0.00
G    O
ATOM  23618  C   GLU G 196      55.544 -37.131  56.099  1.00  0.00
G    C
ATOM  23619  O   GLU G 196      55.818 -37.390  54.931  1.00  0.00
G    O
ATOM  23620  N   ASP G 197      55.030 -35.959  56.476  1.00  0.00
G    N
ATOM  23621  HN  ASP G 197      54.811 -35.716  57.417  1.00  0.00
G    H
ATOM  23622  CA  ASP G 197      54.732 -34.894  55.530  1.00  0.00
G    C
ATOM  23623  HA  ASP G 197      55.564 -34.836  54.836  1.00  0.00
G    H
ATOM  23624  CB  ASP G 197      53.418 -35.147  54.796  1.00  0.00
G    C
```

```
ATOM  23625  HB1 ASP G 197      53.304 -36.235  54.615  1.00  0.00
G    H
ATOM  23626  HB2 ASP G 197      52.559 -34.801  55.403  1.00  0.00
G    H
ATOM  23627  CG  ASP G 197      53.369 -34.456  53.454  1.00  0.00
G    C
ATOM  23628  OD1 ASP G 197      54.443 -34.100  52.926  1.00  0.00
G    O
ATOM  23629  OD2 ASP G 197      52.249 -34.250  52.938  1.00  0.00
G    O
ATOM  23630  C   ASP G 197      54.662 -33.555  56.243  1.00  0.00
G    C
ATOM  23631  O   ASP G 197      53.989 -33.418  57.266  1.00  0.00
G    O
ATOM  23632  N   ILE G 198      55.313 -32.554  55.657  1.00  0.00
G    N
ATOM  23633  HN  ILE G 198      55.770 -32.628  54.774  1.00  0.00
G    H
ATOM  23634  CA  ILE G 198      55.414 -31.232  56.275  1.00  0.00
G    C
ATOM  23635  HA  ILE G 198      55.702 -31.383  57.307  1.00  0.00
G    H
ATOM  23636  CB  ILE G 198      56.438 -30.348  55.536  1.00  0.00
G    C
ATOM  23637  HB  ILE G 198      56.421 -30.723  54.481  1.00  0.00
G    H
ATOM  23638  CG2 ILE G 198      56.102 -28.866  55.713  1.00  0.00
G    C
ATOM  23639 HG21 ILE G 198      56.742 -28.238  55.058  1.00  0.00
G    H
ATOM  23640 HG22 ILE G 198      55.044 -28.664  55.443  1.00  0.00
G    H
ATOM  23641 HG23 ILE G 198      56.262 -28.544  56.764  1.00  0.00
G    H
ATOM  23642  CG1 ILE G 198      57.852 -30.589  56.063  1.00  0.00
G    C
ATOM  23643 HG11 ILE G 198      57.974 -30.098  57.056  1.00  0.00
G    H
ATOM  23644 HG12 ILE G 198      57.999 -31.683  56.217  1.00  0.00
G    H
ATOM  23645  CD  ILE G 198      58.929 -30.098  55.141  1.00  0.00
G    C
ATOM  23646  HD1 ILE G 198      59.931 -30.352  55.548  1.00  0.00
G    H
ATOM  23647  HD2 ILE G 198      58.832 -30.570  54.139  1.00  0.00
G    H
ATOM  23648  HD3 ILE G 198      58.878 -28.996  55.018  1.00  0.00
G    H
ATOM  23649  C   ILE G 198      54.038 -30.577  56.294  1.00  0.00
G    C
ATOM  23650  O   ILE G 198      53.424 -30.344  55.243  1.00  0.00
G    O
ATOM  23651  N   ILE G 199      53.552 -30.278  57.493  1.00  0.00
G    N
```

ATOM 23652 HN ILE G 199   54.026 -30.516 58.339 1.00 0.00
G   H
ATOM 23653 CA ILE G 199   52.290 -29.578 57.693 1.00 0.00
G   C
ATOM 23654 HA ILE G 199   51.784 -29.516 56.737 1.00 0.00
G   H
ATOM 23655 CB ILE G 199   51.416 -30.271 58.752 1.00 0.00
G   C
ATOM 23656 HB ILE G 199   52.060 -30.307 59.667 1.00 0.00
G   H
ATOM 23657 CG2 ILE G 199   50.128 -29.498 58.975 1.00 0.00
G   C
ATOM 23658 HG21 ILE G 199   49.572 -29.908 59.845 1.00 0.00
G   H
ATOM 23659 HG22 ILE G 199   50.341 -28.429 59.185 1.00 0.00
G   H
ATOM 23660 HG23 ILE G 199   49.470 -29.556 58.084 1.00 0.00
G   H
ATOM 23661 CG1 ILE G 199   51.140 -31.723 58.343 1.00 0.00
G   C
ATOM 23662 HG11 ILE G 199   50.859 -31.764 57.267 1.00 0.00
G   H
ATOM 23663 HG12 ILE G 199   52.076 -32.316 58.461 1.00 0.00
G   H
ATOM 23664 CD ILE G 199   50.066 -32.404 59.143 1.00 0.00
G   C
ATOM 23665 HD1 ILE G 199   49.965 -33.466 58.828 1.00 0.00
G   H
ATOM 23666 HD2 ILE G 199   50.312 -32.385 60.227 1.00 0.00
G   H
ATOM 23667 HD3 ILE G 199   49.084 -31.908 58.995 1.00 0.00
G   H
ATOM 23668 C   ILE G 199   52.629 -28.155 58.104 1.00 0.00
G   C
ATOM 23669 O   ILE G 199   53.005 -27.912 59.255 1.00 0.00
G   O
ATOM 23670 N   ARG G 200   52.526 -27.221 57.166 1.00 0.00
G   N
ATOM 23671 HN ARG G 200   52.251 -27.443 56.233 1.00 0.00
G   H
ATOM 23672 CA ARG G 200   52.809 -25.825 57.448 1.00 0.00
G   C
ATOM 23673 HA ARG G 200   53.586 -25.774 58.202 1.00 0.00
G   H
ATOM 23674 CB ARG G 200   53.260 -25.113 56.177 1.00 0.00
G   C
ATOM 23675 HB1 ARG G 200   52.608 -25.492 55.355 1.00 0.00
G   H
ATOM 23676 HB2 ARG G 200   53.043 -24.025 56.263 1.00 0.00
G   H
ATOM 23677 CG ARG G 200   54.706 -25.372 55.824 1.00 0.00
G   C
ATOM 23678 HG1 ARG G 200   55.260 -25.614 56.758 1.00 0.00
G   H

```
ATOM  23679  HG2 ARG G 200      54.766 -26.290  55.196  1.00  0.00
G    H
ATOM  23680  CD  ARG G 200      55.306 -24.210  55.060  1.00  0.00
G    C
ATOM  23681  HD1 ARG G 200      54.937 -24.228  54.009  1.00  0.00
G    H
ATOM  23682  HD2 ARG G 200      55.030 -23.232  55.520  1.00  0.00
G    H
ATOM  23683  NE  ARG G 200      56.764 -24.263  55.048  1.00  0.00
G    N
ATOM  23684  HE  ARG G 200      57.131 -25.145  55.364  1.00  0.00
G    H
ATOM  23685  CZ  ARG G 200      57.543 -23.259  54.662  1.00  0.00
G    C
ATOM  23686  NH1 ARG G 200      57.003 -22.120  54.253  1.00  0.00
G    N
ATOM  23687 HH11 ARG G 200      57.574 -21.375  53.940  1.00  0.00
G    H
ATOM  23688 HH12 ARG G 200      56.005 -22.113  54.153  1.00  0.00
G    H
ATOM  23689  NH2 ARG G 200      58.862 -23.390  54.682  1.00  0.00
G    N
ATOM  23690 HH21 ARG G 200      59.439 -22.612  54.477  1.00  0.00
G    H
ATOM  23691 HH22 ARG G 200      59.225 -24.209  55.107  1.00  0.00
G    H
ATOM  23692  C   ARG G 200      51.582 -25.140  58.040  1.00  0.00
G    C
ATOM  23693  O   ARG G 200      50.488 -25.705  58.098  1.00  0.00
G    O
ATOM  23694  N   GLU G 201      51.775 -23.906  58.490  1.00  0.00
G    N
ATOM  23695  HN  GLU G 201      52.655 -23.445  58.565  1.00  0.00
G    H
ATOM  23696  CA  GLU G 201      50.648 -23.101  58.929  1.00  0.00
G    C
ATOM  23697  HA  GLU G 201      50.075 -23.666  59.653  1.00  0.00
G    H
ATOM  23698  CB  GLU G 201      51.121 -21.785  59.539  1.00  0.00
G    C
ATOM  23699  HB1 GLU G 201      50.289 -21.047  59.463  1.00  0.00
G    H
ATOM  23700  HB2 GLU G 201      51.309 -21.920  60.628  1.00  0.00
G    H
ATOM  23701  CG  GLU G 201      52.349 -21.198  58.884  1.00  0.00
G    C
ATOM  23702  HG1 GLU G 201      53.241 -21.776  59.187  1.00  0.00
G    H
ATOM  23703  HG2 GLU G 201      52.258 -21.218  57.780  1.00  0.00
G    H
ATOM  23704  CD  GLU G 201      52.604 -19.774  59.328  1.00  0.00
G    C
ATOM  23705  OE1 GLU G 201      52.317 -19.458  60.501  1.00  0.00
G    O
```

```
ATOM  23706  OE2 GLU G 201      53.091 -18.970  58.506  1.00  0.00
G    O
ATOM  23707  C   GLU G 201      49.731 -22.820  57.749  1.00  0.00
G    C
ATOM  23708  O   GLU G 201      50.128 -22.952  56.588  1.00  0.00
G    O
ATOM  23709  N   GLN G 202      48.491 -22.449  58.055  1.00  0.00
G    N
ATOM  23710  HN  GLN G 202      48.186 -22.398  59.004  1.00  0.00
G    H
ATOM  23711  CA  GLN G 202      47.475 -22.088  57.075  1.00  0.00
G    C
ATOM  23712  HA  GLN G 202      46.558 -21.921  57.626  1.00  0.00
G    H
ATOM  23713  CB  GLN G 202      47.858 -20.800  56.337  1.00  0.00
G    C
ATOM  23714  HB1 GLN G 202      48.863 -20.973  55.885  1.00  0.00
G    H
ATOM  23715  HB2 GLN G 202      47.158 -20.640  55.486  1.00  0.00
G    H
ATOM  23716  CG  GLN G 202      47.901 -19.594  57.259  1.00  0.00
G    C
ATOM  23717  HG1 GLN G 202      47.237 -19.754  58.136  1.00  0.00
G    H
ATOM  23718  HG2 GLN G 202      48.930 -19.425  57.637  1.00  0.00
G    H
ATOM  23719  CD  GLN G 202      47.422 -18.305  56.611  1.00  0.00
G    C
ATOM  23720  OE1 GLN G 202      46.584 -18.321  55.716  1.00  0.00
G    O
ATOM  23721  NE2 GLN G 202      47.955 -17.179  57.070  1.00  0.00
G    N
ATOM  23722 HE21 GLN G 202      47.643 -16.330  56.653  1.00  0.00
G    H
ATOM  23723 HE22 GLN G 202      48.645 -17.214  57.786  1.00  0.00
G    H
ATOM  23724  C   GLN G 202      47.216 -23.212  56.082  1.00  0.00
G    C
ATOM  23725  O   GLN G 202      46.765 -22.956  54.958  1.00  0.00
G    O
ATOM  23726  N   ASP G 203      47.488 -24.453  56.476  1.00  0.00
G    N
ATOM  23727  HN  ASP G 203      47.847 -24.705  57.370  1.00  0.00
G    H
ATOM  23728  CA  ASP G 203      47.279 -25.597  55.607  1.00  0.00
G    C
ATOM  23729  HA  ASP G 203      47.397 -25.251  54.586  1.00  0.00
G    H
ATOM  23730  CB  ASP G 203      48.232 -26.731  55.954  1.00  0.00
G    C
ATOM  23731  HB1 ASP G 203      49.259 -26.326  56.060  1.00  0.00
G    H
ATOM  23732  HB2 ASP G 203      47.943 -27.205  56.914  1.00  0.00
G    H
```

```
ATOM  23733  CG  ASP G 203      48.268 -27.787  54.885  1.00  0.00
G    C
ATOM  23734  OD1 ASP G 203      47.569 -28.805  55.048  1.00  0.00
G    O
ATOM  23735  OD2 ASP G 203      49.015 -27.623  53.914  1.00  0.00
G    O
ATOM  23736  C   ASP G 203      45.859 -26.116  55.732  1.00  0.00
G    C
ATOM  23737  O   ASP G 203      45.321 -26.226  56.835  1.00  0.00
G    O
ATOM  23738  N   CYS G 204      45.270 -26.478  54.600  1.00  0.00
G    N
ATOM  23739  HN  CYS G 204      45.657 -26.342  53.691  1.00  0.00
G    H
ATOM  23740  CA  CYS G 204      43.964 -27.124  54.617  1.00  0.00
G    C
ATOM  23741  HA  CYS G 204      43.434 -26.761  55.489  1.00  0.00
G    H
ATOM  23742  CB  CYS G 204      43.184 -26.790  53.350  1.00  0.00
G    C
ATOM  23743  HB1 CYS G 204      43.886 -26.897  52.492  1.00  0.00
G    H
ATOM  23744  HB2 CYS G 204      42.384 -27.546  53.189  1.00  0.00
G    H
ATOM  23745  SG  CYS G 204      42.513 -25.111  53.340  1.00  0.00
G    S
ATOM  23746  HG1 CYS G 204      41.986 -25.149  52.115  1.00  0.00
G    H
ATOM  23747  C   CYS G 204      44.153 -28.629  54.783  1.00  0.00
G    C
ATOM  23748  O   CYS G 204      44.782 -29.283  53.946  1.00  0.00
G    O
ATOM  23749  N   LEU G 205      43.634 -29.158  55.890  1.00  0.00
G    N
ATOM  23750  HN  LEU G 205      43.129 -28.585  56.531  1.00  0.00
G    H
ATOM  23751  CA  LEU G 205      43.729 -30.556  56.284  1.00  0.00
G    C
ATOM  23752  HA  LEU G 205      44.451 -31.047  55.645  1.00  0.00
G    H
ATOM  23753  CB  LEU G 205      44.078 -30.673  57.772  1.00  0.00
G    C
ATOM  23754  HB1 LEU G 205      43.842 -29.691  58.243  1.00  0.00
G    H
ATOM  23755  HB2 LEU G 205      43.396 -31.414  58.246  1.00  0.00
G    H
ATOM  23756  CG  LEU G 205      45.464 -31.011  58.314  1.00  0.00
G    C
ATOM  23757  HG  LEU G 205      45.731 -32.061  58.042  1.00  0.00
G    H
ATOM  23758  CD1 LEU G 205      46.520 -30.052  57.793  1.00  0.00
G    C
ATOM  23759 HD11 LEU G 205      47.511 -30.285  58.237  1.00  0.00
G    H
```

ATOM 23760 HD12 LEU G 205   46.606 -30.128 56.687 1.00 0.00
G   H
ATOM 23761 HD13 LEU G 205   46.262 -29.004 58.054 1.00 0.00
G   H
ATOM 23762 CD2 LEU G 205   45.397 -30.922 59.822 1.00 0.00
G   C
ATOM 23763 HD21 LEU G 205   46.382 -31.178 60.267 1.00 0.00
G   H
ATOM 23764 HD22 LEU G 205   45.130 -29.900 60.160 1.00 0.00
G   H
ATOM 23765 HD23 LEU G 205   44.640 -31.631 60.221 1.00 0.00
G   H
ATOM 23766 C   LEU G 205   42.372 -31.188 56.013 1.00 0.00
G   C
ATOM 23767 O   LEU G 205   41.340 -30.621 56.393 1.00 0.00
G   O
ATOM 23768 N   VAL G 206   42.365 -32.340 55.350 1.00 0.00
G   N
ATOM 23769 HN VAL G 206   43.181 -32.772 54.976 1.00 0.00
G   H
ATOM 23770 CA VAL G 206   41.129 -33.077 55.113 1.00 0.00
G   C
ATOM 23771 HA VAL G 206   40.290 -32.437 55.355 1.00 0.00
G   H
ATOM 23772 CB VAL G 206   41.001 -33.527 53.655 1.00 0.00
G   C
ATOM 23773 HB VAL G 206   41.810 -34.274 53.460 1.00 0.00
G   H
ATOM 23774 CG1 VAL G 206   39.652 -34.174 53.424 1.00 0.00
G   C
ATOM 23775 HG11 VAL G 206   39.541 -34.466 52.358 1.00 0.00
G   H
ATOM 23776 HG12 VAL G 206   39.530 -35.094 54.033 1.00 0.00
G   H
ATOM 23777 HG13 VAL G 206   38.831 -33.470 53.674 1.00 0.00
G   H
ATOM 23778 CG2 VAL G 206   41.182 -32.330 52.728 1.00 0.00
G   C
ATOM 23779 HG21 VAL G 206   41.053 -32.631 51.667 1.00 0.00
G   H
ATOM 23780 HG22 VAL G 206   40.435 -31.540 52.961 1.00 0.00
G   H
ATOM 23781 HG23 VAL G 206   42.196 -31.891 52.831 1.00 0.00
G   H
ATOM 23782 C   VAL G 206   41.091 -34.260 56.069 1.00 0.00
G   C
ATOM 23783 O   VAL G 206   41.948 -35.151 56.015 1.00 0.00
G   O
ATOM 23784 N   ILE G 207   40.086 -34.274 56.935 1.00 0.00
G   N
ATOM 23785 HN ILE G 207   39.372 -33.578 56.961 1.00 0.00
G   H
ATOM 23786 CA ILE G 207   39.936 -35.318 57.932 1.00 0.00
G   C

ATOM 23787 HA ILE G 207 40.630 -36.110 57.683 1.00 0.00
G H
ATOM 23788 CB ILE G 207 40.193 -34.790 59.359 1.00 0.00
G C
ATOM 23789 HB ILE G 207 41.149 -34.213 59.276 1.00 0.00
G H
ATOM 23790 CG2 ILE G 207 39.028 -33.953 59.827 1.00 0.00
G C
ATOM 23791 HG21 ILE G 207 39.272 -33.445 60.784 1.00 0.00
G H
ATOM 23792 HG22 ILE G 207 38.781 -33.167 59.083 1.00 0.00
G H
ATOM 23793 HG23 ILE G 207 38.127 -34.580 59.989 1.00 0.00
G H
ATOM 23794 CG1 ILE G 207 40.460 -35.950 60.323 1.00 0.00
G C
ATOM 23795 HG11 ILE G 207 39.591 -36.645 60.325 1.00 0.00
G H
ATOM 23796 HG12 ILE G 207 41.341 -36.527 59.958 1.00 0.00
G H
ATOM 23797 CD ILE G 207 40.736 -35.513 61.718 1.00 0.00
G C
ATOM 23798 HD1 ILE G 207 40.978 -36.390 62.356 1.00 0.00
G H
ATOM 23799 HD2 ILE G 207 41.600 -34.813 61.745 1.00 0.00
G H
ATOM 23800 HD3 ILE G 207 39.854 -35.001 62.157 1.00 0.00
G H
ATOM 23801 C ILE G 207 38.537 -35.903 57.807 1.00 0.00
G C
ATOM 23802 O ILE G 207 37.627 -35.291 57.248 1.00 0.00
G O
ATOM 23803 N MET G 208 38.365 -37.109 58.334 1.00 0.00
G N
ATOM 23804 HN MET G 208 39.088 -37.642 58.770 1.00 0.00
G H
ATOM 23805 CA MET G 208 37.067 -37.765 58.313 1.00 0.00
G C
ATOM 23806 HA MET G 208 36.302 -37.009 58.443 1.00 0.00
G H
ATOM 23807 CB MET G 208 36.831 -38.541 57.007 1.00 0.00
G C
ATOM 23808 HB1 MET G 208 36.677 -37.784 56.203 1.00 0.00
G H
ATOM 23809 HB2 MET G 208 37.755 -39.099 56.737 1.00 0.00
G H
ATOM 23810 CG MET G 208 35.620 -39.461 57.087 1.00 0.00
G C
ATOM 23811 HG1 MET G 208 35.528 -39.890 58.107 1.00 0.00
G H
ATOM 23812 HG2 MET G 208 34.695 -38.879 56.881 1.00 0.00
G H
ATOM 23813 SD MET G 208 35.668 -40.832 55.947 1.00 0.00
G S

ATOM 23814 CE MET G 208 37.302 -41.444 56.275 1.00 0.00
G C
ATOM 23815 HE1 MET G 208 37.532 -42.323 55.634 1.00 0.00
G H
ATOM 23816 HE2 MET G 208 38.069 -40.664 56.073 1.00 0.00
G H
ATOM 23817 HE3 MET G 208 37.406 -41.760 57.334 1.00 0.00
G H
ATOM 23818 C MET G 208 36.984 -38.709 59.499 1.00 0.00
G C
ATOM 23819 O MET G 208 37.986 -39.322 59.878 1.00 0.00
G O
ATOM 23820 N GLY G 209 35.794 -38.830 60.069 1.00 0.00
G N
ATOM 23821 HN GLY G 209 35.004 -38.257 59.853 1.00 0.00
G H
ATOM 23822 CA GLY G 209 35.573 -39.846 61.077 1.00 0.00
G C
ATOM 23823 HA1 GLY G 209 36.454 -39.862 61.705 1.00 0.00
G H
ATOM 23824 HA2 GLY G 209 35.383 -40.775 60.556 1.00 0.00
G H
ATOM 23825 C GLY G 209 34.379 -39.550 61.952 1.00 0.00
G C
ATOM 23826 O GLY G 209 33.433 -38.884 61.523 1.00 0.00
G O
ATOM 23827 N HSE G 210 34.417 -40.042 63.185 1.00 0.00
G N
ATOM 23828 HN HSE G 210 35.140 -40.629 63.544 1.00 0.00
G H
ATOM 23829 CA HSE G 210 33.360 -39.746 64.134 1.00 0.00
G C
ATOM 23830 HA HSE G 210 32.412 -39.921 63.640 1.00 0.00
G H
ATOM 23831 CB HSE G 210 33.502 -40.595 65.393 1.00 0.00
G C
ATOM 23832 HB1 HSE G 210 33.463 -41.663 65.091 1.00 0.00
G H
ATOM 23833 HB2 HSE G 210 34.500 -40.422 65.845 1.00 0.00
G H
ATOM 23834 ND1 HSE G 210 31.101 -40.666 66.155 1.00 0.00
G N
ATOM 23835 CG HSE G 210 32.422 -40.354 66.395 1.00 0.00
G C
ATOM 23836 CE1 HSE G 210 30.376 -40.337 67.208 1.00 0.00
G C
ATOM 23837 HE1 HSE G 210 29.296 -40.484 67.288 1.00 0.00
G H
ATOM 23838 NE2 HSE G 210 31.175 -39.808 68.116 1.00 0.00
G N
ATOM 23839 HE2 HSE G 210 30.884 -39.454 69.004 1.00 0.00
G H
ATOM 23840 CD2 HSE G 210 32.460 -39.799 67.629 1.00 0.00
G C

ATOM 23841 HD2 HSE G 210 33.295 -39.396 68.183 1.00 0.00
G H
ATOM 23842 C HSE G 210 33.408 -38.273 64.493 1.00 0.00
G C
ATOM 23843 O HSE G 210 34.411 -37.782 65.017 1.00 0.00
G O
ATOM 23844 N LYS G 211 32.310 -37.571 64.208 1.00 0.00
G N
ATOM 23845 HN LYS G 211 31.451 -37.977 63.902 1.00 0.00
G H
ATOM 23846 CA LYS G 211 32.299 -36.118 64.332 1.00 0.00
G C
ATOM 23847 HA LYS G 211 33.029 -35.736 63.627 1.00 0.00
G H
ATOM 23848 CB LYS G 211 30.907 -35.578 64.012 1.00 0.00
G C
ATOM 23849 HB1 LYS G 211 30.907 -34.494 64.274 1.00 0.00
G H
ATOM 23850 HB2 LYS G 211 30.740 -35.634 62.913 1.00 0.00
G H
ATOM 23851 CG LYS G 211 29.781 -36.267 64.760 1.00 0.00
G C
ATOM 23852 HG1 LYS G 211 29.704 -37.323 64.411 1.00 0.00
G H
ATOM 23853 HG2 LYS G 211 30.031 -36.294 65.847 1.00 0.00
G H
ATOM 23854 CD LYS G 211 28.455 -35.568 64.536 1.00 0.00
G C
ATOM 23855 HD1 LYS G 211 28.608 -34.472 64.672 1.00 0.00
G H
ATOM 23856 HD2 LYS G 211 28.151 -35.733 63.476 1.00 0.00
G H
ATOM 23857 CE LYS G 211 27.400 -36.095 65.486 1.00 0.00
G C
ATOM 23858 HE1 LYS G 211 27.688 -35.890 66.541 1.00 0.00
G H
ATOM 23859 HE2 LYS G 211 26.419 -35.611 65.289 1.00 0.00
G H
ATOM 23860 NZ LYS G 211 27.210 -37.564 65.352 1.00 0.00
G N
ATOM 23861 HZ1 LYS G 211 26.482 -37.891 66.019 1.00 0.00
G H
ATOM 23862 HZ2 LYS G 211 26.915 -37.788 64.380 1.00 0.00
G H
ATOM 23863 HZ3 LYS G 211 28.107 -38.048 65.558 1.00 0.00
G H
ATOM 23864 C LYS G 211 32.731 -35.667 65.720 1.00 0.00
G C
ATOM 23865 O LYS G 211 33.381 -34.626 65.868 1.00 0.00
G O
ATOM 23866 N LYS G 212 32.390 -36.439 66.752 1.00 0.00
G N
ATOM 23867 HN LYS G 212 31.909 -37.312 66.691 1.00 0.00
G H

ATOM 23868 CA LYS G 212   32.728 -36.011 68.103 1.00 0.00
G   C
ATOM 23869 HA LYS G 212   32.555 -34.943 68.153 1.00 0.00
G   H
ATOM 23870 CB LYS G 212   31.828 -36.711 69.118 1.00 0.00
G   C
ATOM 23871 HB1 LYS G 212   32.032 -37.803 69.039 1.00 0.00
G   H
ATOM 23872 HB2 LYS G 212   32.136 -36.409 70.145 1.00 0.00
G   H
ATOM 23873 CG LYS G 212   30.330 -36.461 68.903 1.00 0.00
G   C
ATOM 23874 HG1 LYS G 212   29.927 -37.234 68.209 1.00 0.00
G   H
ATOM 23875 HG2 LYS G 212   29.799 -36.579 69.875 1.00 0.00
G   H
ATOM 23876 CD LYS G 212   30.012 -35.079 68.313 1.00 0.00
G   C
ATOM 23877 HD1 LYS G 212   30.670 -34.915 67.428 1.00 0.00
G   H
ATOM 23878 HD2 LYS G 212   28.959 -35.091 67.947 1.00 0.00
G   H
ATOM 23879 CE LYS G 212   30.192 -33.944 69.312 1.00 0.00
G   C
ATOM 23880 HE1 LYS G 212   29.872 -34.266 70.328 1.00 0.00
G   H
ATOM 23881 HE2 LYS G 212   31.258 -33.633 69.361 1.00 0.00
G   H
ATOM 23882 NZ LYS G 212   29.382 -32.751 68.931 1.00 0.00
G   N
ATOM 23883 HZ1 LYS G 212   29.519 -31.990 69.628 1.00 0.00
G   H
ATOM 23884 HZ2 LYS G 212   29.676 -32.416 67.992 1.00 0.00
G   H
ATOM 23885 HZ3 LYS G 212   28.375 -33.012 68.900 1.00 0.00
G   H
ATOM 23886 C   LYS G 212   34.203 -36.247 68.401 1.00 0.00
G   C
ATOM 23887 O   LYS G 212   34.846 -35.420 69.056 1.00 0.00
G   O
ATOM 23888 N   ASP G 213   34.772 -37.345 67.903 1.00 0.00
G   N
ATOM 23889 HN ASP G 213   34.323 -38.115 67.455 1.00 0.00
G   H
ATOM 23890 CA ASP G 213   36.222 -37.505 67.991 1.00 0.00
G   C
ATOM 23891 HA ASP G 213   36.497 -37.357 69.030 1.00 0.00
G   H
ATOM 23892 CB ASP G 213   36.640 -38.888 67.500 1.00 0.00
G   C
ATOM 23893 HB1 ASP G 213   36.133 -39.103 66.538 1.00 0.00
G   H
ATOM 23894 HB2 ASP G 213   37.735 -38.930 67.335 1.00 0.00
G   H

```
ATOM  23895  CG  ASP G 213      36.262 -39.991  68.462  1.00  0.00
G    C
ATOM  23896  OD1 ASP G 213      37.028 -40.240  69.416  1.00  0.00
G    O
ATOM  23897  OD2 ASP G 213      35.197 -40.609  68.275  1.00  0.00
G    O
ATOM  23898  C   ASP G 213      36.936 -36.426  67.190  1.00  0.00
G    C
ATOM  23899  O   ASP G 213      38.014 -35.958  67.580  1.00  0.00
G    O
ATOM  23900  N   ILE G 214      36.351 -36.021  66.061  1.00  0.00
G    N
ATOM  23901  HN  ILE G 214      35.510 -36.400  65.681  1.00  0.00
G    H
ATOM  23902  CA  ILE G 214      36.932 -34.941  65.269  1.00  0.00
G    C
ATOM  23903  HA  ILE G 214      37.968 -35.202  65.097  1.00  0.00
G    H
ATOM  23904  CB  ILE G 214      36.180 -34.785  63.936  1.00  0.00
G    C
ATOM  23905  HB  ILE G 214      35.118 -34.588  64.235  1.00  0.00
G    H
ATOM  23906  CG2 ILE G 214      36.811 -33.688  63.102  1.00  0.00
G    C
ATOM  23907 HG21 ILE G 214      36.185 -33.463  62.213  1.00  0.00
G    H
ATOM  23908 HG22 ILE G 214      36.911 -32.751  63.689  1.00  0.00
G    H
ATOM  23909 HG23 ILE G 214      37.820 -33.988  62.748  1.00  0.00
G    H
ATOM  23910  CG1 ILE G 214      36.184 -36.101  63.165  1.00  0.00
G    C
ATOM  23911 HG11 ILE G 214      37.202 -36.295  62.758  1.00  0.00
G    H
ATOM  23912 HG12 ILE G 214      35.947 -36.931  63.868  1.00  0.00
G    H
ATOM  23913  CD  ILE G 214      35.188 -36.153  62.031  1.00  0.00
G    C
ATOM  23914  HD1 ILE G 214      35.204 -37.155  61.549  1.00  0.00
G    H
ATOM  23915  HD2 ILE G 214      34.159 -35.961  62.403  1.00  0.00
G    H
ATOM  23916  HD3 ILE G 214      35.429 -35.397  61.254  1.00  0.00
G    H
ATOM  23917  C   ILE G 214      36.931 -33.646  66.066  1.00  0.00
G    C
ATOM  23918  O   ILE G 214      37.920 -32.903  66.079  1.00  0.00
G    O
ATOM  23919  N   LYS G 215      35.822 -33.358  66.750  1.00  0.00
G    N
ATOM  23920  HN  LYS G 215      34.973 -33.883  66.707  1.00  0.00
G    H
ATOM  23921  CA  LYS G 215      35.791 -32.203  67.640  1.00  0.00
G    C
```

ATOM 23922 HA LYS G 215 36.045 -31.335 67.044 1.00 0.00
G H
ATOM 23923 CB LYS G 215 34.401 -32.042 68.259 1.00 0.00
G C
ATOM 23924 HB1 LYS G 215 33.670 -32.002 67.419 1.00 0.00
G H
ATOM 23925 HB2 LYS G 215 34.156 -32.958 68.843 1.00 0.00
G H
ATOM 23926 CG LYS G 215 34.237 -30.789 69.123 1.00 0.00
G C
ATOM 23927 HG1 LYS G 215 34.894 -29.983 68.724 1.00 0.00
G H
ATOM 23928 HG2 LYS G 215 33.186 -30.431 69.045 1.00 0.00
G H
ATOM 23929 CD LYS G 215 34.604 -31.056 70.580 1.00 0.00
G C
ATOM 23930 HD1 LYS G 215 34.410 -32.131 70.801 1.00 0.00
G H
ATOM 23931 HD2 LYS G 215 35.698 -30.883 70.700 1.00 0.00
G H
ATOM 23932 CE LYS G 215 33.838 -30.180 71.538 1.00 0.00
G C
ATOM 23933 HE1 LYS G 215 34.021 -29.105 71.314 1.00 0.00
G H
ATOM 23934 HE2 LYS G 215 32.746 -30.375 71.458 1.00 0.00
G H
ATOM 23935 NZ LYS G 215 34.261 -30.444 72.936 1.00 0.00
G N
ATOM 23936 HZ1 LYS G 215 33.729 -29.835 73.591 1.00 0.00
G H
ATOM 23937 HZ2 LYS G 215 34.081 -31.441 73.170 1.00 0.00
G H
ATOM 23938 HZ3 LYS G 215 35.278 -30.248 73.033 1.00 0.00
G H
ATOM 23939 C LYS G 215 36.839 -32.323 68.733 1.00 0.00
G C
ATOM 23940 O LYS G 215 37.462 -31.324 69.100 1.00 0.00
G O
ATOM 23941 N ARG G 216 37.004 -33.517 69.301 1.00 0.00
G N
ATOM 23942 HN ARG G 216 36.472 -34.320 69.042 1.00 0.00
G H
ATOM 23943 CA ARG G 216 37.996 -33.701 70.354 1.00 0.00
G C
ATOM 23944 HA ARG G 216 37.779 -33.007 71.156 1.00 0.00
G H
ATOM 23945 CB ARG G 216 37.945 -35.139 70.870 1.00 0.00
G C
ATOM 23946 HB1 ARG G 216 38.043 -35.799 69.975 1.00 0.00
G H
ATOM 23947 HB2 ARG G 216 38.841 -35.341 71.498 1.00 0.00
G H
ATOM 23948 CG ARG G 216 36.659 -35.474 71.597 1.00 0.00
G C

ATOM 23949 HG1 ARG G 216   36.710 -35.047 72.622 1.00 0.00
G   H
ATOM 23950 HG2 ARG G 216   35.812 -34.950 71.096 1.00 0.00
G   H
ATOM 23951 CD ARG G 216   36.380 -36.965 71.593 1.00 0.00
G   C
ATOM 23952 HD1 ARG G 216   36.761 -37.410 70.645 1.00 0.00
G   H
ATOM 23953 HD2 ARG G 216   36.875 -37.476 72.451 1.00 0.00
G   H
ATOM 23954 NE ARG G 216   34.950 -37.237 71.709 1.00 0.00
G   N
ATOM 23955 HE ARG G 216   34.410 -36.422 71.946 1.00 0.00
G   H
ATOM 23956 CZ ARG G 216   34.396 -38.431 71.526 1.00 0.00
G   C
ATOM 23957 NH1 ARG G 216   35.152 -39.476 71.223 1.00 0.00
G   N
ATOM 23958 HH11 ARG G 216   34.743 -40.363 71.060 1.00 0.00
G   H
ATOM 23959 HH12 ARG G 216   36.119 -39.286 71.038 1.00 0.00
G   H
ATOM 23960 NH2 ARG G 216   33.084 -38.583 71.650 1.00 0.00
G   N
ATOM 23961 HH21 ARG G 216   32.685 -39.488 71.598 1.00 0.00
G   H
ATOM 23962 HH22 ARG G 216   32.574 -37.808 71.998 1.00 0.00
G   H
ATOM 23963 C   ARG G 216   39.389 -33.361 69.850 1.00 0.00
G   C
ATOM 23964 O   ARG G 216   40.128 -32.613 70.501 1.00 0.00
G   O
ATOM 23965 N   PHE G 217   39.758 -33.897 68.688 1.00 0.00
G   N
ATOM 23966 HN PHE G 217   39.206 -34.521 68.138 1.00 0.00
G   H
ATOM 23967 CA PHE G 217   41.063 -33.586 68.119 1.00 0.00
G   C
ATOM 23968 HA PHE G 217   41.791 -33.807 68.890 1.00 0.00
G   H
ATOM 23969 CB PHE G 217   41.310 -34.421 66.866 1.00 0.00
G   C
ATOM 23970 HB1 PHE G 217   41.735 -35.405 67.162 1.00 0.00
G   H
ATOM 23971 HB2 PHE G 217   40.359 -34.624 66.327 1.00 0.00
G   H
ATOM 23972 CG PHE G 217   42.295 -33.805 65.921 1.00 0.00
G   C
ATOM 23973 CD1 PHE G 217   43.640 -33.755 66.241 1.00 0.00
G   C
ATOM 23974 HD1 PHE G 217   43.981 -34.162 67.184 1.00 0.00
G   H
ATOM 23975 CE1 PHE G 217   44.550 -33.186 65.376 1.00 0.00
G   C

ATOM 23976 HE1 PHE G 217 45.599 -33.156 65.636 1.00 0.00
G H
ATOM 23977 CZ PHE G 217 44.120 -32.662 64.177 1.00 0.00
G C
ATOM 23978 HZ PHE G 217 44.831 -32.223 63.491 1.00 0.00
G H
ATOM 23979 CD2 PHE G 217 41.878 -33.273 64.717 1.00 0.00
G C
ATOM 23980 HD2 PHE G 217 40.828 -33.281 64.458 1.00 0.00
G H
ATOM 23981 CE2 PHE G 217 42.783 -32.706 63.848 1.00 0.00
G C
ATOM 23982 HE2 PHE G 217 42.443 -32.295 62.908 1.00 0.00
G H
ATOM 23983 C PHE G 217 41.183 -32.101 67.806 1.00 0.00
G C
ATOM 23984 O PHE G 217 42.241 -31.501 68.018 1.00 0.00
G O
ATOM 23985 N GLU G 218 40.106 -31.489 67.313 1.00 0.00
G N
ATOM 23986 HN GLU G 218 39.238 -31.930 67.102 1.00 0.00
G H
ATOM 23987 CA GLU G 218 40.126 -30.057 67.036 1.00 0.00
G C
ATOM 23988 HA GLU G 218 40.950 -29.857 66.362 1.00 0.00
G H
ATOM 23989 CB GLU G 218 38.808 -29.638 66.387 1.00 0.00
G C
ATOM 23990 HB1 GLU G 218 38.488 -30.450 65.695 1.00 0.00
G H
ATOM 23991 HB2 GLU G 218 38.012 -29.565 67.162 1.00 0.00
G H
ATOM 23992 CG GLU G 218 38.877 -28.339 65.609 1.00 0.00
G C
ATOM 23993 HG1 GLU G 218 39.912 -28.172 65.260 1.00 0.00
G H
ATOM 23994 HG2 GLU G 218 38.196 -28.364 64.735 1.00 0.00
G H
ATOM 23995 CD GLU G 218 38.526 -27.142 66.472 1.00 0.00
G C
ATOM 23996 OE1 GLU G 218 38.069 -27.351 67.616 1.00 0.00
G O
ATOM 23997 OE2 GLU G 218 38.698 -25.995 66.013 1.00 0.00
G O
ATOM 23998 C GLU G 218 40.374 -29.251 68.302 1.00 0.00
G C
ATOM 23999 O GLU G 218 41.154 -28.293 68.299 1.00 0.00
G O
ATOM 24000 N ASN G 219 39.719 -29.629 69.394 1.00 0.00
G N
ATOM 24001 HN ASN G 219 39.134 -30.437 69.458 1.00 0.00
G H
ATOM 24002 CA ASN G 219 39.815 -28.855 70.623 1.00 0.00
G C

ATOM 24003 HA ASN G 219   39.734 -27.815 70.327 1.00 0.00
G   H
ATOM 24004 CB ASN G 219   38.691 -29.256 71.577 1.00 0.00
G   C
ATOM 24005 HB1 ASN G 219   37.805 -29.529 70.964 1.00 0.00
G   H
ATOM 24006 HB2 ASN G 219   38.982 -30.153 72.161 1.00 0.00
G   H
ATOM 24007 CG ASN G 219   38.288 -28.132 72.509 1.00 0.00
G   C
ATOM 24008 OD1 ASN G 219   38.880 -27.054 72.493 1.00 0.00
G   O
ATOM 24009 ND2 ASN G 219   37.273 -28.378 73.329 1.00 0.00
G   N
ATOM 24010 HD21 ASN G 219   36.990 -27.645 73.940 1.00 0.00
G   H
ATOM 24011 HD22 ASN G 219   36.809 -29.258 73.297 1.00 0.00
G   H
ATOM 24012 C   ASN G 219   41.173 -29.043 71.280 1.00 0.00
G   C
ATOM 24013 O   ASN G 219   41.756 -28.090 71.810 1.00 0.00
G   O
ATOM 24014 N   GLU G 220   41.695 -30.266 71.257 1.00 0.00
G   N
ATOM 24015 HN GLU G 220   41.316 -31.043 70.763 1.00 0.00
G   H
ATOM 24016 CA GLU G 220   42.917 -30.568 71.990 1.00 0.00
G   C
ATOM 24017 HA GLU G 220   42.982 -29.866 72.812 1.00 0.00
G   H
ATOM 24018 CB GLU G 220   42.829 -31.989 72.553 1.00 0.00
G   C
ATOM 24019 HB1 GLU G 220   42.884 -32.704 71.701 1.00 0.00
G   H
ATOM 24020 HB2 GLU G 220   43.719 -32.200 73.187 1.00 0.00
G   H
ATOM 24021 CG GLU G 220   41.541 -32.223 73.338 1.00 0.00
G   C
ATOM 24022 HG1 GLU G 220   41.734 -32.071 74.416 1.00 0.00
G   H
ATOM 24023 HG2 GLU G 220   40.743 -31.528 73.009 1.00 0.00
G   H
ATOM 24024 CD GLU G 220   41.015 -33.638 73.217 1.00 0.00
G   C
ATOM 24025 OE1 GLU G 220   41.348 -34.319 72.226 1.00 0.00
G   O
ATOM 24026 OE2 GLU G 220   40.247 -34.063 74.105 1.00 0.00
G   O
ATOM 24027 C   GLU G 220   44.178 -30.394 71.146 1.00 0.00
G   C
ATOM 24028 O   GLU G 220   45.064 -29.616 71.519 1.00 0.00
G   O
ATOM 24029 N   GLY G 221   44.280 -31.088 70.014 1.00 0.00
G   N

```
ATOM  24030  HN  GLY G 221      43.562 -31.702  69.690  1.00  0.00
G    H
ATOM  24031  CA  GLY G 221      45.454 -30.994  69.168  1.00  0.00
G    C
ATOM  24032  HA1 GLY G 221      45.365 -31.780  68.431  1.00  0.00
G    H
ATOM  24033  HA2 GLY G 221      46.314 -31.089  69.818  1.00  0.00
G    H
ATOM  24034  C   GLY G 221      45.568 -29.674  68.434  1.00  0.00
G    C
ATOM  24035  O   GLY G 221      46.642 -29.317  67.942  1.00  0.00
G    O
ATOM  24036  N   MET G 222      44.462 -28.945  68.343  1.00  0.00
G    N
ATOM  24037  HN  MET G 222      43.575 -29.226  68.704  1.00  0.00
G    H
ATOM  24038  CA  MET G 222      44.458 -27.643  67.691  1.00  0.00
G    C
ATOM  24039  HA  MET G 222      45.492 -27.348  67.556  1.00  0.00
G    H
ATOM  24040  CB  MET G 222      43.747 -27.719  66.343  1.00  0.00
G    C
ATOM  24041  HB1 MET G 222      42.681 -27.972  66.553  1.00  0.00
G    H
ATOM  24042  HB2 MET G 222      43.741 -26.711  65.872  1.00  0.00
G    H
ATOM  24043  CG  MET G 222      44.320 -28.752  65.395  1.00  0.00
G    C
ATOM  24044  HG1 MET G 222      45.422 -28.637  65.319  1.00  0.00
G    H
ATOM  24045  HG2 MET G 222      44.114 -29.773  65.786  1.00  0.00
G    H
ATOM  24046  SD  MET G 222      43.623 -28.601  63.740  1.00  0.00
G    S
ATOM  24047  CE  MET G 222      41.972 -29.238  64.000  1.00  0.00
G    C
ATOM  24048  HE1 MET G 222      41.389 -29.227  63.054  1.00  0.00
G    H
ATOM  24049  HE2 MET G 222      42.002 -30.285  64.371  1.00  0.00
G    H
ATOM  24050  HE3 MET G 222      41.421 -28.626  64.747  1.00  0.00
G    H
ATOM  24051  C   MET G 222      43.794 -26.599  68.576  1.00  0.00
G    C
ATOM  24052  O   MET G 222      44.365 -25.542  68.835  1.00  0.00
G    O
ATOM  24053  N   ASN H   6      33.160 -17.920  75.969  1.00  0.00
H    N
ATOM  24054  HT1 ASN H   6      33.157 -18.493  76.788  1.00  0.00
H    H
ATOM  24055  HT2 ASN H   6      33.615 -17.051  76.163  1.00  0.00
H    H
ATOM  24056  HT3 ASN H   6      33.632 -18.393  75.224  1.00  0.00
H    H
```

```
ATOM  24057  CA  ASN H   6       31.786 -17.656  75.568  1.00  0.00
H    C
ATOM  24058  HA  ASN H   6       31.364 -18.624  75.316  1.00  0.00
H    H
ATOM  24059  CB  ASN H   6       31.023 -16.984  76.713  1.00  0.00
H    C
ATOM  24060  HB1 ASN H   6       30.020 -16.694  76.329  1.00  0.00
H    H
ATOM  24061  HB2 ASN H   6       30.867 -17.700  77.545  1.00  0.00
H    H
ATOM  24062  CG  ASN H   6       31.714 -15.727  77.221  1.00  0.00
H    C
ATOM  24063  OD1 ASN H   6       32.886 -15.488  76.929  1.00  0.00
H    O
ATOM  24064  ND2 ASN H   6       30.980 -14.903  77.964  1.00  0.00
H    N
ATOM  24065 HD21 ASN H   6       31.418 -14.071  78.293  1.00  0.00
H    H
ATOM  24066 HD22 ASN H   6       30.025 -15.113  78.146  1.00  0.00
H    H
ATOM  24067  C   ASN H   6       31.721 -16.781  74.315  1.00  0.00
H    C
ATOM  24068  O   ASN H   6       31.370 -15.606  74.398  1.00  0.00
H    O
ATOM  24069  N   LYS H   7       32.039 -17.341  73.151  1.00  0.00
H    N
ATOM  24070  HN  LYS H   7       32.251 -18.308  73.018  1.00  0.00
H    H
ATOM  24071  CA  LYS H   7       32.097 -16.523  71.945  1.00  0.00
H    C
ATOM  24072  HA  LYS H   7       32.679 -15.640  72.180  1.00  0.00
H    H
ATOM  24073  CB  LYS H   7       32.743 -17.308  70.804  1.00  0.00
H    C
ATOM  24074  HB1 LYS H   7       32.638 -16.692  69.881  1.00  0.00
H    H
ATOM  24075  HB2 LYS H   7       33.835 -17.396  70.999  1.00  0.00
H    H
ATOM  24076  CG  LYS H   7       32.117 -18.671  70.575  1.00  0.00
H    C
ATOM  24077  HG1 LYS H   7       32.154 -19.254  71.523  1.00  0.00
H    H
ATOM  24078  HG2 LYS H   7       31.043 -18.536  70.310  1.00  0.00
H    H
ATOM  24079  CD  LYS H   7       32.840 -19.444  69.486  1.00  0.00
H    C
ATOM  24080  HD1 LYS H   7       32.176 -20.269  69.139  1.00  0.00
H    H
ATOM  24081  HD2 LYS H   7       33.000 -18.759  68.621  1.00  0.00
H    H
ATOM  24082  CE  LYS H   7       34.169 -19.995  69.975  1.00  0.00
H    C
ATOM  24083  HE1 LYS H   7       34.886 -19.167  70.168  1.00  0.00
H    H
```

```
ATOM  24084  HE2 LYS H   7       34.031 -20.568  70.918  1.00  0.00
H    H
ATOM  24085  NZ  LYS H   7       34.790 -20.909  68.974  1.00  0.00
H    N
ATOM  24086  HZ1 LYS H   7       35.697 -21.269  69.335  1.00  0.00
H    H
ATOM  24087  HZ2 LYS H   7       34.150 -21.708  68.789  1.00  0.00
H    H
ATOM  24088  HZ3 LYS H   7       34.953 -20.390  68.087  1.00  0.00
H    H
ATOM  24089  C   LYS H   7       30.702 -16.056  71.549  1.00  0.00
H    C
ATOM  24090  O   LYS H   7       29.750 -16.838  71.552  1.00  0.00
H    O
ATOM  24091  N   GLN H   8       30.584 -14.786  71.176  1.00  0.00
H    N
ATOM  24092  HN  GLN H   8       31.361 -14.196  70.967  1.00  0.00
H    H
ATOM  24093  CA  GLN H   8       29.285 -14.143  71.040  1.00  0.00
H    C
ATOM  24094  HA  GLN H   8       28.546 -14.846  71.407  1.00  0.00
H    H
ATOM  24095  CB  GLN H   8       29.234 -12.872  71.876  1.00  0.00
H    C
ATOM  24096  HB1 GLN H   8       30.209 -12.352  71.730  1.00  0.00
H    H
ATOM  24097  HB2 GLN H   8       28.454 -12.191  71.464  1.00  0.00
H    H
ATOM  24098  CG  GLN H   8       29.008 -13.191  73.333  1.00  0.00
H    C
ATOM  24099  HG1 GLN H   8       27.920 -13.273  73.548  1.00  0.00
H    H
ATOM  24100  HG2 GLN H   8       29.481 -14.158  73.602  1.00  0.00
H    H
ATOM  24101  CD  GLN H   8       29.539 -12.127  74.254  1.00  0.00
H    C
ATOM  24102  OE1 GLN H   8       30.391 -11.330  73.869  1.00  0.00
H    O
ATOM  24103  NE2 GLN H   8       29.045 -12.110  75.486  1.00  0.00
H    N
ATOM  24104 HE21 GLN H   8       29.390 -11.405  76.098  1.00  0.00
H    H
ATOM  24105 HE22 GLN H   8       28.376 -12.792  75.760  1.00  0.00
H    H
ATOM  24106  C   GLN H   8       28.956 -13.852  69.585  1.00  0.00
H    C
ATOM  24107  O   GLN H   8       29.788 -13.324  68.833  1.00  0.00
H    O
ATOM  24108  N   PHE H   9       27.728 -14.190  69.209  1.00  0.00
H    N
ATOM  24109  HN  PHE H   9       27.044 -14.480  69.876  1.00  0.00
H    H
ATOM  24110  CA  PHE H   9       27.260 -14.175  67.837  1.00  0.00
H    C
```

```
ATOM  24111  HA  PHE H   9      28.046 -13.727  67.240  1.00  0.00
H    H
ATOM  24112  CB  PHE H   9      26.887 -15.574  67.389  1.00  0.00
H    C
ATOM  24113  HB1 PHE H   9      26.045 -15.944  68.015  1.00  0.00
H    H
ATOM  24114  HB2 PHE H   9      26.536 -15.577  66.334  1.00  0.00
H    H
ATOM  24115  CG  PHE H   9      27.987 -16.558  67.548  1.00  0.00
H    C
ATOM  24116  CD1 PHE H   9      28.949 -16.688  66.572  1.00  0.00
H    C
ATOM  24117  HD1 PHE H   9      28.895 -16.075  65.683  1.00  0.00
H    H
ATOM  24118  CE1 PHE H   9      29.971 -17.588  66.710  1.00  0.00
H    C
ATOM  24119  HE1 PHE H   9      30.722 -17.676  65.938  1.00  0.00
H    H
ATOM  24120  CZ  PHE H   9      30.043 -18.376  67.837  1.00  0.00
H    C
ATOM  24121  HZ  PHE H   9      30.852 -19.084  67.949  1.00  0.00
H    H
ATOM  24122  CD2 PHE H   9      28.070 -17.348  68.681  1.00  0.00
H    C
ATOM  24123  HD2 PHE H   9      27.315 -17.267  69.451  1.00  0.00
H    H
ATOM  24124  CE2 PHE H   9      29.090 -18.256  68.827  1.00  0.00
H    C
ATOM  24125  HE2 PHE H   9      29.140 -18.871  69.715  1.00  0.00
H    H
ATOM  24126  C   PHE H   9      26.046 -13.273  67.722  1.00  0.00
H    C
ATOM  24127  O   PHE H   9      25.131 -13.337  68.549  1.00  0.00
H    O
ATOM  24128  N   ALA H  10      26.037 -12.445  66.685  1.00  0.00
H    N
ATOM  24129  HN  ALA H  10      26.790 -12.398  66.030  1.00  0.00
H    H
ATOM  24130  CA  ALA H  10      24.926 -11.540  66.426  1.00  0.00
H    C
ATOM  24131  HA  ALA H  10      24.102 -11.714  67.108  1.00  0.00
H    H
ATOM  24132  CB  ALA H  10      25.361 -10.081  66.573  1.00  0.00
H    C
ATOM  24133  HB1 ALA H  10      25.703  -9.897  67.614  1.00  0.00
H    H
ATOM  24134  HB2 ALA H  10      26.205  -9.845  65.889  1.00  0.00
H    H
ATOM  24135  HB3 ALA H  10      24.524  -9.382  66.357  1.00  0.00
H    H
ATOM  24136  C   ALA H  10      24.390 -11.806  65.030  1.00  0.00
H    C
ATOM  24137  O   ALA H  10      25.062 -11.511  64.036  1.00  0.00
H    O
```

```
ATOM  24138  N   VAL H  11      23.196 -12.366  64.951  1.00  0.00
H    N
ATOM  24139  HN  VAL H  11      22.679 -12.685  65.741  1.00  0.00
H    H
ATOM  24140  CA  VAL H  11      22.528 -12.564  63.678  1.00  0.00
H    C
ATOM  24141  HA  VAL H  11      23.269 -12.653  62.893  1.00  0.00
H    H
ATOM  24142  CB  VAL H  11      21.642 -13.809  63.676  1.00  0.00
H    C
ATOM  24143  HB  VAL H  11      21.552 -14.161  64.733  1.00  0.00
H    H
ATOM  24144  CG1 VAL H  11      20.263 -13.488  63.144  1.00  0.00
H    C
ATOM  24145 HG11 VAL H  11      19.639 -14.407  63.110  1.00  0.00
H    H
ATOM  24146 HG12 VAL H  11      19.735 -12.755  63.789  1.00  0.00
H    H
ATOM  24147 HG13 VAL H  11      20.325 -13.076  62.113  1.00  0.00
H    H
ATOM  24148  CG2 VAL H  11      22.270 -14.878  62.824  1.00  0.00
H    C
ATOM  24149 HG21 VAL H  11      21.619 -15.776  62.779  1.00  0.00
H    H
ATOM  24150 HG22 VAL H  11      22.429 -14.508  61.788  1.00  0.00
H    H
ATOM  24151 HG23 VAL H  11      23.251 -15.195  63.235  1.00  0.00
H    H
ATOM  24152  C   VAL H  11      21.723 -11.306  63.405  1.00  0.00
H    C
ATOM  24153  O   VAL H  11      20.985 -10.821  64.265  1.00  0.00
H    O
ATOM  24154  N   ILE H  12      21.882 -10.764  62.213  1.00  0.00
H    N
ATOM  24155  HN  ILE H  12      22.570 -11.068  61.557  1.00  0.00
H    H
ATOM  24156  CA  ILE H  12      21.061  -9.664  61.744  1.00  0.00
H    C
ATOM  24157  HA  ILE H  12      20.249  -9.526  62.449  1.00  0.00
H    H
ATOM  24158  CB  ILE H  12      21.884  -8.390  61.556  1.00  0.00
H    C
ATOM  24159  HB  ILE H  12      22.634  -8.655  60.769  1.00  0.00
H    H
ATOM  24160  CG2 ILE H  12      20.966  -7.250  61.254  1.00  0.00
H    C
ATOM  24161 HG21 ILE H  12      21.545  -6.350  60.960  1.00  0.00
H    H
ATOM  24162 HG22 ILE H  12      20.286  -7.502  60.413  1.00  0.00
H    H
ATOM  24163 HG23 ILE H  12      20.348  -6.992  62.140  1.00  0.00
H    H
ATOM  24164  CG1 ILE H  12      22.665  -8.074  62.821  1.00  0.00
H    C
```

```
ATOM  24165 HG11 ILE H  12      23.386  -8.894  63.033  1.00  0.00
H    H
ATOM  24166 HG12 ILE H  12      23.259  -7.147  62.655  1.00  0.00
H    H
ATOM  24167  CD  ILE H  12      21.797  -7.868  64.009  1.00  0.00
H    C
ATOM  24168  HD1 ILE H  12      22.411  -7.584  64.890  1.00  0.00
H    H
ATOM  24169  HD2 ILE H  12      21.062  -7.055  63.820  1.00  0.00
H    H
ATOM  24170  HD3 ILE H  12      21.241  -8.795  64.262  1.00  0.00
H    H
ATOM  24171  C   ILE H  12      20.435 -10.098  60.435  1.00  0.00
H    C
ATOM  24172  O   ILE H  12      21.117 -10.640  59.559  1.00  0.00
H    O
ATOM  24173  N   GLY H  13      19.138  -9.890  60.316  1.00  0.00
H    N
ATOM  24174  HN  GLY H  13      18.588  -9.459  61.030  1.00  0.00
H    H
ATOM  24175  CA  GLY H  13      18.421 -10.278  59.127  1.00  0.00
H    C
ATOM  24176  HA1 GLY H  13      19.114 -10.355  58.300  1.00  0.00
H    H
ATOM  24177  HA2 GLY H  13      17.601  -9.594  58.957  1.00  0.00
H    H
ATOM  24178  C   GLY H  13      17.840 -11.645  59.377  1.00  0.00
H    C
ATOM  24179  O   GLY H  13      18.555 -12.649  59.311  1.00  0.00
H    O
ATOM  24180  N   LEU H  14      16.552 -11.703  59.674  1.00  0.00
H    N
ATOM  24181  HN  LEU H  14      15.917 -10.934  59.660  1.00  0.00
H    H
ATOM  24182  CA  LEU H  14      15.949 -12.969  60.064  1.00  0.00
H    C
ATOM  24183  HA  LEU H  14      16.759 -13.669  60.222  1.00  0.00
H    H
ATOM  24184  CB  LEU H  14      15.159 -12.787  61.346  1.00  0.00
H    C
ATOM  24185  HB1 LEU H  14      15.098 -11.692  61.542  1.00  0.00
H    H
ATOM  24186  HB2 LEU H  14      14.114 -13.128  61.178  1.00  0.00
H    H
ATOM  24187  CG  LEU H  14      15.802 -13.489  62.521  1.00  0.00
H    C
ATOM  24188  HG  LEU H  14      16.769 -13.939  62.190  1.00  0.00
H    H
ATOM  24189  CD1 LEU H  14      16.069 -12.493  63.618  1.00  0.00
H    C
ATOM  24190 HD11 LEU H  14      16.498 -13.000  64.509  1.00  0.00
H    H
ATOM  24191 HD12 LEU H  14      16.789 -11.718  63.276  1.00  0.00
H    H
```

ATOM 24192 HD13 LEU H 14   15.129 -11.987 63.925 1.00 0.00
H   H
ATOM 24193 CD2 LEU H 14   14.898 -14.616 63.000 1.00 0.00
H   C
ATOM 24194 HD21 LEU H 14   15.368 -15.153 63.853 1.00 0.00
H   H
ATOM 24195 HD22 LEU H 14   13.915 -14.236 63.344 1.00 0.00
H   H
ATOM 24196 HD23 LEU H 14   14.723 -15.349 62.182 1.00 0.00
H   H
ATOM 24197 C   LEU H 14   15.076 -13.521 58.939 1.00 0.00
H   C
ATOM 24198 O   LEU H 14   13.876 -13.727 59.083 1.00 0.00
H   O
ATOM 24199 N   GLY H 15   15.712 -13.757 57.798 1.00 0.00
H   N
ATOM 24200 HN GLY H 15   16.652 -13.469 57.623 1.00 0.00
H   H
ATOM 24201 CA GLY H 15   15.061 -14.463 56.714 1.00 0.00
H   C
ATOM 24202 HA1 GLY H 15   15.560 -14.205 55.790 1.00 0.00
H   H
ATOM 24203 HA2 GLY H 15   14.005 -14.225 56.730 1.00 0.00
H   H
ATOM 24204 C   GLY H 15   15.205 -15.953 56.924 1.00 0.00
H   C
ATOM 24205 O   GLY H 15   15.878 -16.366 57.874 1.00 0.00
H   O
ATOM 24206 N   ARG H 16   14.582 -16.765 56.061 1.00 0.00
H   N
ATOM 24207 HN ARG H 16   13.998 -16.428 55.326 1.00 0.00
H   H
ATOM 24208 CA ARG H 16   14.735 -18.211 56.165 1.00 0.00
H   C
ATOM 24209 HA ARG H 16   14.166 -18.553 57.022 1.00 0.00
H   H
ATOM 24210 CB ARG H 16   14.254 -18.896 54.890 1.00 0.00
H   C
ATOM 24211 HB1 ARG H 16   14.917 -18.534 54.068 1.00 0.00
H   H
ATOM 24212 HB2 ARG H 16   14.431 -19.992 54.966 1.00 0.00
H   H
ATOM 24213 CG ARG H 16   12.829 -18.597 54.527 1.00 0.00
H   C
ATOM 24214 HG1 ARG H 16   12.168 -19.294 55.087 1.00 0.00
H   H
ATOM 24215 HG2 ARG H 16   12.569 -17.577 54.893 1.00 0.00
H   H
ATOM 24216 CD ARG H 16   12.626 -18.654 53.026 1.00 0.00
H   C
ATOM 24217 HD1 ARG H 16   13.127 -19.561 52.619 1.00 0.00
H   H
ATOM 24218 HD2 ARG H 16   11.544 -18.694 52.762 1.00 0.00
H   H

```
ATOM  24219  NE  ARG H  16      13.167 -17.479  52.350  1.00  0.00
H    N
ATOM  24220  HE  ARG H  16      13.662 -16.860  52.969  1.00  0.00
H    H
ATOM  24221  CZ  ARG H  16      13.028 -17.240  51.051  1.00  0.00
H    C
ATOM  24222  NH1 ARG H  16      12.358 -18.093  50.289  1.00  0.00
H    N
ATOM  24223 HH11 ARG H  16      12.270 -17.938  49.315  1.00  0.00
H    H
ATOM  24224 HH12 ARG H  16      12.076 -18.951  50.725  1.00  0.00
H    H
ATOM  24225  NH2 ARG H  16      13.546 -16.144  50.513  1.00  0.00
H    N
ATOM  24226 HH21 ARG H  16      13.366 -15.927  49.563  1.00  0.00
H    H
ATOM  24227 HH22 ARG H  16      13.923 -15.474  51.138  1.00  0.00
H    H
ATOM  24228  C   ARG H  16      16.176 -18.594  56.423  1.00  0.00
H    C
ATOM  24229  O   ARG H  16      16.454 -19.418  57.300  1.00  0.00
H    O
ATOM  24230  N   PHE H  17      17.096 -17.969  55.700  1.00  0.00
H    N
ATOM  24231  HN  PHE H  17      16.903 -17.337  54.951  1.00  0.00
H    H
ATOM  24232  CA  PHE H  17      18.507 -18.174  55.964  1.00  0.00
H    C
ATOM  24233  HA  PHE H  17      18.700 -19.229  55.808  1.00  0.00
H    H
ATOM  24234  CB  PHE H  17      19.332 -17.300  55.029  1.00  0.00
H    C
ATOM  24235  HB1 PHE H  17      19.066 -17.540  53.977  1.00  0.00
H    H
ATOM  24236  HB2 PHE H  17      19.106 -16.223  55.185  1.00  0.00
H    H
ATOM  24237  CG  PHE H  17      20.796 -17.518  55.147  1.00  0.00
H    C
ATOM  24238  CD1 PHE H  17      21.318 -18.794  55.080  1.00  0.00
H    C
ATOM  24239  HD1 PHE H  17      20.653 -19.638  54.950  1.00  0.00
H    H
ATOM  24240  CE1 PHE H  17      22.668 -19.006  55.181  1.00  0.00
H    C
ATOM  24241  HE1 PHE H  17      23.064 -20.010  55.127  1.00  0.00
H    H
ATOM  24242  CZ  PHE H  17      23.516 -17.934  55.344  1.00  0.00
H    C
ATOM  24243  HZ  PHE H  17      24.582 -18.095  55.414  1.00  0.00
H    H
ATOM  24244  CD2 PHE H  17      21.656 -16.450  55.309  1.00  0.00
H    C
ATOM  24245  HD2 PHE H  17      21.263 -15.446  55.380  1.00  0.00
H    H
```

ATOM 24246 CE2 PHE H 17 23.007 -16.654 55.408 1.00 0.00
H C
ATOM 24247 HE2 PHE H 17 23.670 -15.810 55.537 1.00 0.00
H H
ATOM 24248 C PHE H 17 18.828 -17.873 57.418 1.00 0.00
H C
ATOM 24249 O PHE H 17 19.330 -18.737 58.145 1.00 0.00
H O
ATOM 24250 N GLY H 18 18.523 -16.654 57.863 1.00 0.00
H N
ATOM 24251 HN GLY H 18 18.091 -15.941 57.313 1.00 0.00
H H
ATOM 24252 CA GLY H 18 18.819 -16.294 59.237 1.00 0.00
H C
ATOM 24253 HA1 GLY H 18 18.361 -15.331 59.419 1.00 0.00
H H
ATOM 24254 HA2 GLY H 18 19.894 -16.302 59.347 1.00 0.00
H H
ATOM 24255 C GLY H 18 18.240 -17.282 60.229 1.00 0.00
H C
ATOM 24256 O GLY H 18 18.972 -17.898 61.013 1.00 0.00
H O
ATOM 24257 N GLY H 19 16.924 -17.483 60.165 1.00 0.00
H N
ATOM 24258 HN GLY H 19 16.334 -17.066 59.475 1.00 0.00
H H
ATOM 24259 CA GLY H 19 16.257 -18.344 61.124 1.00 0.00
H C
ATOM 24260 HA1 GLY H 19 15.237 -18.456 60.782 1.00 0.00
H H
ATOM 24261 HA2 GLY H 19 16.351 -17.869 62.091 1.00 0.00
H H
ATOM 24262 C GLY H 19 16.872 -19.721 61.219 1.00 0.00
H C
ATOM 24263 O GLY H 19 17.000 -20.276 62.313 1.00 0.00
H O
ATOM 24264 N SER H 20 17.268 -20.283 60.085 1.00 0.00
H N
ATOM 24265 HN SER H 20 17.120 -19.899 59.174 1.00 0.00
H H
ATOM 24266 CA SER H 20 17.975 -21.550 60.088 1.00 0.00
H C
ATOM 24267 HA SER H 20 17.293 -22.273 60.519 1.00 0.00
H H
ATOM 24268 CB SER H 20 18.427 -21.873 58.675 1.00 0.00
H C
ATOM 24269 HB1 SER H 20 19.052 -21.026 58.310 1.00 0.00
H H
ATOM 24270 HB2 SER H 20 19.072 -22.780 58.673 1.00 0.00
H H
ATOM 24271 OG SER H 20 17.308 -22.022 57.843 1.00 0.00
H O
ATOM 24272 HG1 SER H 20 17.627 -22.001 56.932 1.00 0.00
H H

```
ATOM  24273  C   SER H  20      19.192 -21.494  60.982  1.00  0.00
H    C
ATOM  24274  O   SER H  20      19.331 -22.304  61.909  1.00  0.00
H    O
ATOM  24275  N   ILE H  21      20.070 -20.541  60.691  1.00  0.00
H    N
ATOM  24276  HN  ILE H  21      19.953 -19.879  59.955  1.00  0.00
H    H
ATOM  24277  CA  ILE H  21      21.303 -20.385  61.441  1.00  0.00
H    C
ATOM  24278  HA  ILE H  21      21.882 -21.285  61.279  1.00  0.00
H    H
ATOM  24279  CB  ILE H  21      22.031 -19.123  60.969  1.00  0.00
H    C
ATOM  24280  HB  ILE H  21      21.376 -18.279  61.303  1.00  0.00
H    H
ATOM  24281  CG2 ILE H  21      23.418 -19.080  61.532  1.00  0.00
H    C
ATOM  24282 HG21 ILE H  21      23.894 -18.100  61.324  1.00  0.00
H    H
ATOM  24283 HG22 ILE H  21      23.403 -19.220  62.633  1.00  0.00
H    H
ATOM  24284 HG23 ILE H  21      24.052 -19.876  61.087  1.00  0.00
H    H
ATOM  24285  CG1 ILE H  21      22.060 -19.099  59.451  1.00  0.00
H    C
ATOM  24286 HG11 ILE H  21      22.629 -19.976  59.071  1.00  0.00
H    H
ATOM  24287 HG12 ILE H  21      21.017 -19.193  59.070  1.00  0.00
H    H
ATOM  24288  CD  ILE H  21      22.651 -17.852  58.872  1.00  0.00
H    C
ATOM  24289  HD1 ILE H  21      22.589 -17.874  57.763  1.00  0.00
H    H
ATOM  24290  HD2 ILE H  21      22.105 -16.955  59.233  1.00  0.00
H    H
ATOM  24291  HD3 ILE H  21      23.720 -17.751  59.153  1.00  0.00
H    H
ATOM  24292  C   ILE H  21      21.010 -20.314  62.924  1.00  0.00
H    C
ATOM  24293  O   ILE H  21      21.586 -21.057  63.728  1.00  0.00
H    O
ATOM  24294  N   VAL H  22      20.121 -19.407  63.312  1.00  0.00
H    N
ATOM  24295  HN  VAL H  22      19.532 -18.856  62.726  1.00  0.00
H    H
ATOM  24296  CA  VAL H  22      19.956 -19.156  64.735  1.00  0.00
H    C
ATOM  24297  HA  VAL H  22      20.944 -19.034  65.161  1.00  0.00
H    H
ATOM  24298  CB  VAL H  22      19.152 -17.872  64.974  1.00  0.00
H    C
ATOM  24299  HB  VAL H  22      18.669 -17.958  65.979  1.00  0.00
H    H
```

ATOM 24300 CG1 VAL H 22   20.063 -16.681 64.961 1.00 0.00
H   C
ATOM 24301 HG11 VAL H 22   19.478 -15.746 65.095 1.00 0.00
H   H
ATOM 24302 HG12 VAL H 22   20.808 -16.726 65.783 1.00 0.00
H   H
ATOM 24303 HG13 VAL H 22   20.605 -16.612 63.994 1.00 0.00
H   H
ATOM 24304 CG2 VAL H 22   18.095 -17.727 63.946 1.00 0.00
H   C
ATOM 24305 HG21 VAL H 22   17.534 -16.779 64.090 1.00 0.00
H   H
ATOM 24306 HG22 VAL H 22   18.540 -17.720 62.927 1.00 0.00
H   H
ATOM 24307 HG23 VAL H 22   17.364 -18.560 64.002 1.00 0.00
H   H
ATOM 24308 C   VAL H 22   19.308 -20.343 65.422 1.00 0.00
H   C
ATOM 24309 O   VAL H 22   19.703 -20.718 66.527 1.00 0.00
H   O
ATOM 24310 N   LYS H 23   18.301 -20.947 64.795 1.00 0.00
H   N
ATOM 24311 HN LYS H 23   17.957 -20.713 63.888 1.00 0.00
H   H
ATOM 24312 CA LYS H 23   17.615 -22.047 65.451 1.00 0.00
H   C
ATOM 24313 HA LYS H 23   17.338 -21.713 66.444 1.00 0.00
H   H
ATOM 24314 CB LYS H 23   16.375 -22.432 64.656 1.00 0.00
H   C
ATOM 24315 HB1 LYS H 23   15.870 -21.484 64.356 1.00 0.00
H   H
ATOM 24316 HB2 LYS H 23   16.690 -22.931 63.713 1.00 0.00
H   H
ATOM 24317 CG LYS H 23   15.401 -23.294 65.426 1.00 0.00
H   C
ATOM 24318 HG1 LYS H 23   15.877 -23.619 66.379 1.00 0.00
H   H
ATOM 24319 HG2 LYS H 23   14.508 -22.684 65.692 1.00 0.00
H   H
ATOM 24320 CD LYS H 23   14.986 -24.519 64.640 1.00 0.00
H   C
ATOM 24321 HD1 LYS H 23   15.322 -24.391 63.586 1.00 0.00
H   H
ATOM 24322 HD2 LYS H 23   15.528 -25.400 65.059 1.00 0.00
H   H
ATOM 24323 CE LYS H 23   13.475 -24.708 64.728 1.00 0.00
H   C
ATOM 24324 HE1 LYS H 23   13.161 -24.856 65.786 1.00 0.00
H   H
ATOM 24325 HE2 LYS H 23   12.946 -23.816 64.326 1.00 0.00
H   H
ATOM 24326 NZ LYS H 23   12.985 -25.886 63.961 1.00 0.00
H   N

ATOM 24327 HZ1 LYS H 23   11.953 -25.969 64.056 1.00 0.00
H   H
ATOM 24328 HZ2 LYS H 23   13.232 -25.775 62.957 1.00 0.00
H   H
ATOM 24329 HZ3 LYS H 23   13.436 -26.749 64.329 1.00 0.00
H   H
ATOM 24330 C   LYS H 23   18.552 -23.227 65.616 1.00 0.00
H   C
ATOM 24331 O   LYS H 23   18.540 -23.896 66.657 1.00 0.00
H   O
ATOM 24332 N   GLU H 24   19.415 -23.460 64.628 1.00 0.00
H   N
ATOM 24333 HN GLU H 24   19.459 -22.983 63.755 1.00 0.00
H   H
ATOM 24334 CA GLU H 24   20.417 -24.503 64.783 1.00 0.00
H   C
ATOM 24335 HA GLU H 24   19.915 -25.426 65.044 1.00 0.00
H   H
ATOM 24336 CB GLU H 24   21.195 -24.662 63.476 1.00 0.00
H   C
ATOM 24337 HB1 GLU H 24   20.460 -24.804 62.650 1.00 0.00
H   H
ATOM 24338 HB2 GLU H 24   21.738 -23.719 63.243 1.00 0.00
H   H
ATOM 24339 CG GLU H 24   22.187 -25.816 63.412 1.00 0.00
H   C
ATOM 24340 HG1 GLU H 24   21.646 -26.773 63.523 1.00 0.00
H   H
ATOM 24341 HG2 GLU H 24   22.736 -25.815 62.449 1.00 0.00
H   H
ATOM 24342 CD GLU H 24   23.221 -25.826 64.523 1.00 0.00
H   C
ATOM 24343 OE1 GLU H 24   23.959 -24.838 64.692 1.00 0.00
H   O
ATOM 24344 OE2 GLU H 24   23.268 -26.835 65.249 1.00 0.00
H   O
ATOM 24345 C   GLU H 24   21.366 -24.165 65.926 1.00 0.00
H   C
ATOM 24346 O   GLU H 24   21.549 -24.956 66.857 1.00 0.00
H   O
ATOM 24347 N   LEU H 25   22.013 -23.006 65.846 1.00 0.00
H   N
ATOM 24348 HN LEU H 25   21.871 -22.320 65.135 1.00 0.00
H   H
ATOM 24349 CA LEU H 25   23.017 -22.642 66.837 1.00 0.00
H   C
ATOM 24350 HA LEU H 25   23.788 -23.401 66.794 1.00 0.00
H   H
ATOM 24351 CB LEU H 25   23.577 -21.268 66.495 1.00 0.00
H   C
ATOM 24352 HB1 LEU H 25   23.030 -20.904 65.595 1.00 0.00
H   H
ATOM 24353 HB2 LEU H 25   23.325 -20.560 67.316 1.00 0.00
H   H

```
ATOM  24354  CG  LEU H  25      25.067 -21.276  66.212  1.00  0.00
H    C
ATOM  24355  HG  LEU H  25      25.422 -22.324  66.055  1.00  0.00
H    H
ATOM  24356  CD1 LEU H  25      25.365 -20.446  65.003  1.00  0.00
H    C
ATOM  24357 HD11 LEU H  25      26.459 -20.408  64.818  1.00  0.00
H    H
ATOM  24358 HD12 LEU H  25      24.872 -20.874  64.104  1.00  0.00
H    H
ATOM  24359 HD13 LEU H  25      25.000 -19.406  65.142  1.00  0.00
H    H
ATOM  24360  CD2 LEU H  25      25.793 -20.711  67.410  1.00  0.00
H    C
ATOM  24361 HD21 LEU H  25      26.891 -20.723  67.236  1.00  0.00
H    H
ATOM  24362 HD22 LEU H  25      25.496 -19.663  67.612  1.00  0.00
H    H
ATOM  24363 HD23 LEU H  25      25.579 -21.317  68.316  1.00  0.00
H    H
ATOM  24364  C   LEU H  25      22.431 -22.663  68.236  1.00  0.00
H    C
ATOM  24365  O   LEU H  25      23.087 -23.079  69.197  1.00  0.00
H    O
ATOM  24366  N   HSE H  26      21.192 -22.210  68.359  1.00  0.00
H    N
ATOM  24367  HN  HSE H  26      20.704 -21.747  67.621  1.00  0.00
H    H
ATOM  24368  CA  HSE H  26      20.431 -22.347  69.585  1.00  0.00
H    C
ATOM  24369  HA  HSE H  26      20.980 -21.829  70.362  1.00  0.00
H    H
ATOM  24370  CB  HSE H  26      19.046 -21.760  69.351  1.00  0.00
H    C
ATOM  24371  HB1 HSE H  26      19.174 -20.724  68.970  1.00  0.00
H    H
ATOM  24372  HB2 HSE H  26      18.528 -22.337  68.556  1.00  0.00
H    H
ATOM  24373  ND1 HSE H  26      17.213 -22.620  70.837  1.00  0.00
H    N
ATOM  24374  CG  HSE H  26      18.193 -21.692  70.569  1.00  0.00
H    C
ATOM  24375  CE1 HSE H  26      16.605 -22.299  71.963  1.00  0.00
H    C
ATOM  24376  HE1 HSE H  26      15.789 -22.871  72.412  1.00  0.00
H    H
ATOM  24377  NE2 HSE H  26      17.148 -21.188  72.428  1.00  0.00
H    N
ATOM  24378  HE2 HSE H  26      16.864 -20.711  73.259  1.00  0.00
H    H
ATOM  24379  CD2 HSE H  26      18.140 -20.785  71.570  1.00  0.00
H    C
ATOM  24380  HD2 HSE H  26      18.725 -19.889  71.725  1.00  0.00
H    H
```

```
ATOM  24381  C   HSE H  26      20.340 -23.802  69.998  1.00  0.00
H    C
ATOM  24382  O   HSE H  26      20.527 -24.136  71.173  1.00  0.00
H    O
ATOM  24383  N   ARG H  27      20.052 -24.686  69.044  1.00  0.00
H    N
ATOM  24384  HN  ARG H  27      19.806 -24.437  68.110  1.00  0.00
H    H
ATOM  24385  CA  ARG H  27      20.089 -26.106  69.353  1.00  0.00
H    C
ATOM  24386  HA  ARG H  27      19.429 -26.292  70.192  1.00  0.00
H    H
ATOM  24387  CB  ARG H  27      19.631 -26.919  68.150  1.00  0.00
H    C
ATOM  24388  HB1 ARG H  27      18.765 -26.370  67.709  1.00  0.00
H    H
ATOM  24389  HB2 ARG H  27      20.426 -26.912  67.373  1.00  0.00
H    H
ATOM  24390  CG  ARG H  27      19.201 -28.321  68.501  1.00  0.00
H    C
ATOM  24391  HG1 ARG H  27      19.445 -28.510  69.569  1.00  0.00
H    H
ATOM  24392  HG2 ARG H  27      18.091 -28.386  68.433  1.00  0.00
H    H
ATOM  24393  CD  ARG H  27      19.815 -29.337  67.561  1.00  0.00
H    C
ATOM  24394  HD1 ARG H  27      19.737 -30.354  68.012  1.00  0.00
H    H
ATOM  24395  HD2 ARG H  27      19.298 -29.345  66.573  1.00  0.00
H    H
ATOM  24396  NE  ARG H  27      21.220 -29.062  67.280  1.00  0.00
H    N
ATOM  24397  HE  ARG H  27      21.382 -28.756  66.335  1.00  0.00
H    H
ATOM  24398  CZ  ARG H  27      22.203 -29.195  68.164  1.00  0.00
H    C
ATOM  24399  NH1 ARG H  27      21.944 -29.587  69.401  1.00  0.00
H    N
ATOM  24400 HH11 ARG H  27      22.673 -29.662  70.069  1.00  0.00
H    H
ATOM  24401 HH12 ARG H  27      20.977 -29.666  69.649  1.00  0.00
H    H
ATOM  24402  NH2 ARG H  27      23.447 -28.931  67.811  1.00  0.00
H    N
ATOM  24403 HH21 ARG H  27      24.188 -29.113  68.442  1.00  0.00
H    H
ATOM  24404 HH22 ARG H  27      23.614 -28.758  66.849  1.00  0.00
H    H
ATOM  24405  C   ARG H  27      21.482 -26.530  69.792  1.00  0.00
H    C
ATOM  24406  O   ARG H  27      21.625 -27.453  70.598  1.00  0.00
H    O
ATOM  24407  N   MET H  28      22.523 -25.866  69.288  1.00  0.00
H    N
```

```
ATOM  24408  HN  MET H  28      22.498 -25.168  68.574  1.00  0.00
H    H
ATOM  24409  CA  MET H  28      23.854 -26.155  69.801  1.00  0.00
H    C
ATOM  24410  HA  MET H  28      23.924 -27.232  69.888  1.00  0.00
H    H
ATOM  24411  CB  MET H  28      24.943 -25.632  68.874  1.00  0.00
H    C
ATOM  24412  HB1 MET H  28      24.735 -24.548  68.710  1.00  0.00
H    H
ATOM  24413  HB2 MET H  28      25.927 -25.685  69.392  1.00  0.00
H    H
ATOM  24414  CG  MET H  28      25.001 -26.317  67.557  1.00  0.00
H    C
ATOM  24415  HG1 MET H  28      25.060 -27.417  67.697  1.00  0.00
H    H
ATOM  24416  HG2 MET H  28      24.076 -26.100  66.981  1.00  0.00
H    H
ATOM  24417  SD  MET H  28      26.412 -25.821  66.574  1.00  0.00
H    S
ATOM  24418  CE  MET H  28      27.703 -26.658  67.484  1.00  0.00
H    C
ATOM  24419  HE1 MET H  28      28.699 -26.466  67.029  1.00  0.00
H    H
ATOM  24420  HE2 MET H  28      27.737 -26.311  68.539  1.00  0.00
H    H
ATOM  24421  HE3 MET H  28      27.539 -27.758  67.490  1.00  0.00
H    H
ATOM  24422  C   MET H  28      24.082 -25.569  71.181  1.00  0.00
H    C
ATOM  24423  O   MET H  28      25.165 -25.757  71.741  1.00  0.00
H    O
ATOM  24424  N   GLY H  29      23.105 -24.860  71.732  1.00  0.00
H    N
ATOM  24425  HN  GLY H  29      22.233 -24.672  71.283  1.00  0.00
H    H
ATOM  24426  CA  GLY H  29      23.260 -24.302  73.056  1.00  0.00
H    C
ATOM  24427  HA1 GLY H  29      23.604 -25.099  73.701  1.00  0.00
H    H
ATOM  24428  HA2 GLY H  29      22.308 -23.877  73.343  1.00  0.00
H    H
ATOM  24429  C   GLY H  29      24.289 -23.199  73.086  1.00  0.00
H    C
ATOM  24430  O   GLY H  29      25.262 -23.268  73.841  1.00  0.00
H    O
ATOM  24431  N   HSE H  30      24.102 -22.185  72.256  1.00  0.00
H    N
ATOM  24432  HN  HSE H  30      23.334 -22.106  71.621  1.00  0.00
H    H
ATOM  24433  CA  HSE H  30      25.032 -21.077  72.209  1.00  0.00
H    C
ATOM  24434  HA  HSE H  30      25.787 -21.262  72.963  1.00  0.00
H    H
```

```
ATOM  24435  CB  HSE H  30      25.668 -20.971  70.831  1.00  0.00
H    C
ATOM  24436  HB1 HSE H  30      24.861 -21.068  70.073  1.00  0.00
H    H
ATOM  24437  HB2 HSE H  30      26.113 -19.962  70.705  1.00  0.00
H    H
ATOM  24438  ND1 HSE H  30      27.990 -21.736  70.231  1.00  0.00
H    N
ATOM  24439  CG  HSE H  30      26.685 -22.027  70.561  1.00  0.00
H    C
ATOM  24440  CE1 HSE H  30      28.657 -22.861  70.053  1.00  0.00
H    C
ATOM  24441  HE1 HSE H  30      29.712 -22.919  69.773  1.00  0.00
H    H
ATOM  24442  NE2 HSE H  30      27.833 -23.872  70.264  1.00  0.00
H    N
ATOM  24443  HE2 HSE H  30      28.073 -24.839  70.179  1.00  0.00
H    H
ATOM  24444  CD2 HSE H  30      26.595 -23.377  70.590  1.00  0.00
H    C
ATOM  24445  HD2 HSE H  30      25.747 -24.011  70.806  1.00  0.00
H    H
ATOM  24446  C   HSE H  30      24.340 -19.774  72.579  1.00  0.00
H    C
ATOM  24447  O   HSE H  30      23.122 -19.701  72.751  1.00  0.00
H    O
ATOM  24448  N   GLU H  31      25.148 -18.734  72.702  1.00  0.00
H    N
ATOM  24449  HN  GLU H  31      26.134 -18.803  72.582  1.00  0.00
H    H
ATOM  24450  CA  GLU H  31      24.685 -17.393  73.020  1.00  0.00
H    C
ATOM  24451  HA  GLU H  31      23.714 -17.446  73.497  1.00  0.00
H    H
ATOM  24452  CB  GLU H  31      25.677 -16.704  73.953  1.00  0.00
H    C
ATOM  24453  HB1 GLU H  31      25.302 -15.675  74.160  1.00  0.00
H    H
ATOM  24454  HB2 GLU H  31      25.694 -17.225  74.937  1.00  0.00
H    H
ATOM  24455  CG  GLU H  31      27.115 -16.587  73.431  1.00  0.00
H    C
ATOM  24456  HG1 GLU H  31      27.098 -16.257  72.376  1.00  0.00
H    H
ATOM  24457  HG2 GLU H  31      27.698 -15.860  74.031  1.00  0.00
H    H
ATOM  24458  CD  GLU H  31      27.875 -17.911  73.424  1.00  0.00
H    C
ATOM  24459  OE1 GLU H  31      28.999 -17.951  73.965  1.00  0.00
H    O
ATOM  24460  OE2 GLU H  31      27.375 -18.899  72.845  1.00  0.00
H    O
ATOM  24461  C   GLU H  31      24.505 -16.638  71.709  1.00  0.00
H    C
```

```
ATOM  24462  O   GLU H  31      25.480 -16.326  71.019  1.00  0.00
H    O
ATOM  24463  N   VAL H  32      23.257 -16.378  71.343  1.00  0.00
H    N
ATOM  24464  HN  VAL H  32      22.461 -16.594  71.903  1.00  0.00
H    H
ATOM  24465  CA  VAL H  32      22.944 -15.744  70.073  1.00  0.00
H    C
ATOM  24466  HA  VAL H  32      23.842 -15.268  69.699  1.00  0.00
H    H
ATOM  24467  CB  VAL H  32      22.450 -16.766  69.034  1.00  0.00
H    C
ATOM  24468  HB  VAL H  32      22.138 -16.197  68.124  1.00  0.00
H    H
ATOM  24469  CG1 VAL H  32      23.570 -17.720  68.675  1.00  0.00
H    C
ATOM  24470 HG11 VAL H  32      23.209 -18.485  67.953  1.00  0.00
H    H
ATOM  24471 HG12 VAL H  32      24.421 -17.192  68.197  1.00  0.00
H    H
ATOM  24472 HG13 VAL H  32      23.941 -18.248  69.579  1.00  0.00
H    H
ATOM  24473  CG2 VAL H  32      21.273 -17.548  69.572  1.00  0.00
H    C
ATOM  24474 HG21 VAL H  32      20.941 -18.312  68.838  1.00  0.00
H    H
ATOM  24475 HG22 VAL H  32      21.548 -18.066  70.517  1.00  0.00
H    H
ATOM  24476 HG23 VAL H  32      20.409 -16.882  69.777  1.00  0.00
H    H
ATOM  24477  C   VAL H  32      21.912 -14.655  70.302  1.00  0.00
H    C
ATOM  24478  O   VAL H  32      20.839 -14.907  70.863  1.00  0.00
H    O
ATOM  24479  N   LEU H  33      22.244 -13.444  69.884  1.00  0.00
H    N
ATOM  24480  HN  LEU H  33      23.144 -13.217  69.515  1.00  0.00
H    H
ATOM  24481  CA  LEU H  33      21.319 -12.327  69.928  1.00  0.00
H    C
ATOM  24482  HA  LEU H  33      20.587 -12.530  70.700  1.00  0.00
H    H
ATOM  24483  CB  LEU H  33      22.045 -11.014  70.192  1.00  0.00
H    C
ATOM  24484  HB1 LEU H  33      22.286 -10.985  71.281  1.00  0.00
H    H
ATOM  24485  HB2 LEU H  33      23.022 -11.028  69.661  1.00  0.00
H    H
ATOM  24486  CG  LEU H  33      21.202  -9.802  69.809  1.00  0.00
H    C
ATOM  24487  HG  LEU H  33      20.942  -9.909  68.729  1.00  0.00
H    H
ATOM  24488  CD1 LEU H  33      19.922  -9.751  70.637  1.00  0.00
H    C
```

```
ATOM  24489 HD11 LEU H  33      19.334  -8.841  70.389  1.00  0.00
H    H
ATOM  24490 HD12 LEU H  33      19.289 -10.642  70.435  1.00  0.00
H    H
ATOM  24491 HD13 LEU H  33      20.157  -9.727  71.722  1.00  0.00
H    H
ATOM  24492  CD2 LEU H  33      22.005  -8.529  69.974  1.00  0.00
H    C
ATOM  24493 HD21 LEU H  33      21.397  -7.648  69.677  1.00  0.00
H    H
ATOM  24494 HD22 LEU H  33      22.324  -8.380  71.025  1.00  0.00
H    H
ATOM  24495 HD23 LEU H  33      22.913  -8.556  69.333  1.00  0.00
H    H
ATOM  24496  C   LEU H  33      20.581 -12.266  68.602  1.00  0.00
H    C
ATOM  24497  O   LEU H  33      21.204 -12.144  67.544  1.00  0.00
H    O
ATOM  24498  N   ALA H  34      19.261 -12.348  68.659  1.00  0.00
H    N
ATOM  24499  HN  ALA H  34      18.762 -12.473  69.516  1.00  0.00
H    H
ATOM  24500  CA  ALA H  34      18.440 -12.260  67.466  1.00  0.00
H    C
ATOM  24501  HA  ALA H  34      19.005 -12.493  66.570  1.00  0.00
H    H
ATOM  24502  CB  ALA H  34      17.255 -13.216  67.551  1.00  0.00
H    C
ATOM  24503  HB1 ALA H  34      17.624 -14.262  67.614  1.00  0.00
H    H
ATOM  24504  HB2 ALA H  34      16.643 -13.014  68.458  1.00  0.00
H    H
ATOM  24505  HB3 ALA H  34      16.600 -13.136  66.658  1.00  0.00
H    H
ATOM  24506  C   ALA H  34      17.964 -10.827  67.311  1.00  0.00
H    C
ATOM  24507  O   ALA H  34      17.502 -10.216  68.280  1.00  0.00
H    O
ATOM  24508  N   VAL H  35      18.096 -10.288  66.105  1.00  0.00
H    N
ATOM  24509  HN  VAL H  35      18.553 -10.758  65.354  1.00  0.00
H    H
ATOM  24510  CA  VAL H  35      17.589  -8.966  65.767  1.00  0.00
H    C
ATOM  24511  HA  VAL H  35      16.822  -8.697  66.482  1.00  0.00
H    H
ATOM  24512  CB  VAL H  35      18.701  -7.899  65.760  1.00  0.00
H    C
ATOM  24513  HB  VAL H  35      19.434  -8.180  64.964  1.00  0.00
H    H
ATOM  24514  CG1 VAL H  35      18.119  -6.530  65.446  1.00  0.00
H    C
ATOM  24515 HG11 VAL H  35      18.916  -5.755  65.480  1.00  0.00
H    H
```

```
ATOM  24516 HG12 VAL H  35      17.670  -6.498  64.431  1.00  0.00
H    H
ATOM  24517 HG13 VAL H  35      17.341  -6.254  66.189  1.00  0.00
H    H
ATOM  24518  CG2 VAL H  35      19.436  -7.886  67.077  1.00  0.00
H    C
ATOM  24519 HG21 VAL H  35      20.210  -7.090  67.087  1.00  0.00
H    H
ATOM  24520 HG22 VAL H  35      18.731  -7.699  67.916  1.00  0.00
H    H
ATOM  24521 HG23 VAL H  35      19.947  -8.855  67.262  1.00  0.00
H    H
ATOM  24522  C   VAL H  35      16.933  -9.039  64.400  1.00  0.00
H    C
ATOM  24523  O   VAL H  35      17.431  -9.722  63.499  1.00  0.00
H    O
ATOM  24524  N   ASP H  36      15.819  -8.335  64.246  1.00  0.00
H    N
ATOM  24525  HN  ASP H  36      15.311  -7.858  64.959  1.00  0.00
H    H
ATOM  24526  CA  ASP H  36      15.215  -8.199  62.936  1.00  0.00
H    C
ATOM  24527  HA  ASP H  36      15.988  -7.886  62.243  1.00  0.00
H    H
ATOM  24528  CB  ASP H  36      14.556  -9.491  62.481  1.00  0.00
H    C
ATOM  24529  HB1 ASP H  36      15.188 -10.350  62.785  1.00  0.00
H    H
ATOM  24530  HB2 ASP H  36      13.560  -9.608  62.952  1.00  0.00
H    H
ATOM  24531  CG  ASP H  36      14.400  -9.542  61.000  1.00  0.00
H    C
ATOM  24532  OD1 ASP H  36      15.404  -9.848  60.329  1.00  0.00
H    O
ATOM  24533  OD2 ASP H  36      13.289  -9.262  60.509  1.00  0.00
H    O
ATOM  24534  C   ASP H  36      14.189  -7.089  62.976  1.00  0.00
H    C
ATOM  24535  O   ASP H  36      13.411  -6.990  63.926  1.00  0.00
H    O
ATOM  24536  N   ILE H  37      14.185  -6.266  61.931  1.00  0.00
H    N
ATOM  24537  HN  ILE H  37      14.795  -6.307  61.143  1.00  0.00
H    H
ATOM  24538  CA  ILE H  37      13.219  -5.179  61.881  1.00  0.00
H    C
ATOM  24539  HA  ILE H  37      13.299  -4.650  62.822  1.00  0.00
H    H
ATOM  24540  CB  ILE H  37      13.514  -4.243  60.701  1.00  0.00
H    C
ATOM  24541  HB  ILE H  37      14.591  -3.963  60.829  1.00  0.00
H    H
ATOM  24542  CG2 ILE H  37      13.203  -4.928  59.390  1.00  0.00
H    C
```

```
ATOM  24543 HG21 ILE H  37      13.560  -4.316  58.536  1.00  0.00
H    H
ATOM  24544 HG22 ILE H  37      13.706  -5.915  59.330  1.00  0.00
H    H
ATOM  24545 HG23 ILE H  37      12.110  -5.083  59.272  1.00  0.00
H    H
ATOM  24546  CG1 ILE H  37      12.699  -2.962  60.842  1.00  0.00
H    C
ATOM  24547 HG11 ILE H  37      11.628  -3.170  60.622  1.00  0.00
H    H
ATOM  24548 HG12 ILE H  37      12.755  -2.612  61.898  1.00  0.00
H    H
ATOM  24549  CD  ILE H  37      13.166  -1.852  59.963  1.00  0.00
H    C
ATOM  24550  HD1 ILE H  37      12.571  -0.932  60.153  1.00  0.00
H    H
ATOM  24551  HD2 ILE H  37      14.235  -1.621  60.158  1.00  0.00
H    H
ATOM  24552  HD3 ILE H  37      13.053  -2.119  58.891  1.00  0.00
H    H
ATOM  24553  C   ILE H  37      11.804  -5.722  61.802  1.00  0.00
H    C
ATOM  24554  O   ILE H  37      10.853  -5.055  62.221  1.00  0.00
H    O
ATOM  24555  N   ASN H  38      11.630  -6.927  61.284  1.00  0.00
H    N
ATOM  24556  HN  ASN H  38      12.352  -7.509  60.912  1.00  0.00
H    H
ATOM  24557  CA  ASN H  38      10.302  -7.504  61.224  1.00  0.00
H    C
ATOM  24558  HA  ASN H  38       9.611  -6.687  61.046  1.00  0.00
H    H
ATOM  24559  CB  ASN H  38      10.208  -8.564  60.141  1.00  0.00
H    C
ATOM  24560  HB1 ASN H  38      10.141  -8.043  59.161  1.00  0.00
H    H
ATOM  24561  HB2 ASN H  38      11.128  -9.182  60.126  1.00  0.00
H    H
ATOM  24562  CG  ASN H  38       8.996  -9.420  60.307  1.00  0.00
H    C
ATOM  24563  OD1 ASN H  38       9.010 -10.388  61.056  1.00  0.00
H    O
ATOM  24564  ND2 ASN H  38       7.917  -9.050  59.639  1.00  0.00
H    N
ATOM  24565 HD21 ASN H  38       7.097  -9.604  59.749  1.00  0.00
H    H
ATOM  24566 HD22 ASN H  38       7.936  -8.228  59.078  1.00  0.00
H    H
ATOM  24567  C   ASN H  38       9.968  -8.104  62.574  1.00  0.00
H    C
ATOM  24568  O   ASN H  38      10.507  -9.149  62.950  1.00  0.00
H    O
ATOM  24569  N   GLU H  39       9.070  -7.444  63.297  1.00  0.00
H    N
```

ATOM 24570 HN GLU H 39 8.615 -6.604 63.014 1.00 0.00
H H
ATOM 24571 CA GLU H 39 8.669 -7.920 64.614 1.00 0.00
H C
ATOM 24572 HA GLU H 39 9.551 -7.967 65.242 1.00 0.00
H H
ATOM 24573 CB GLU H 39 7.630 -6.973 65.193 1.00 0.00
H C
ATOM 24574 HB1 GLU H 39 7.567 -7.156 66.291 1.00 0.00
H H
ATOM 24575 HB2 GLU H 39 7.972 -5.921 65.079 1.00 0.00
H H
ATOM 24576 CG GLU H 39 6.271 -7.166 64.564 1.00 0.00
H C
ATOM 24577 HG1 GLU H 39 6.064 -8.247 64.459 1.00 0.00
H H
ATOM 24578 HG2 GLU H 39 5.477 -6.701 65.182 1.00 0.00
H H
ATOM 24579 CD GLU H 39 6.184 -6.595 63.165 1.00 0.00
H C
ATOM 24580 OE1 GLU H 39 7.238 -6.321 62.550 1.00 0.00
H O
ATOM 24581 OE2 GLU H 39 5.054 -6.429 62.668 1.00 0.00
H O
ATOM 24582 C GLU H 39 8.098 -9.324 64.534 1.00 0.00
H C
ATOM 24583 O GLU H 39 8.075 -10.051 65.527 1.00 0.00
H O
ATOM 24584 N GLU H 40 7.608 -9.718 63.365 1.00 0.00
H N
ATOM 24585 HN GLU H 40 7.633 -9.212 62.508 1.00 0.00
H H
ATOM 24586 CA GLU H 40 6.957 -11.012 63.267 1.00 0.00
H C
ATOM 24587 HA GLU H 40 6.234 -11.086 64.069 1.00 0.00
H H
ATOM 24588 CB GLU H 40 6.250 -11.131 61.921 1.00 0.00
H C
ATOM 24589 HB1 GLU H 40 6.553 -10.263 61.291 1.00 0.00
H H
ATOM 24590 HB2 GLU H 40 6.610 -12.037 61.383 1.00 0.00
H H
ATOM 24591 CG GLU H 40 4.718 -11.165 61.949 1.00 0.00
H C
ATOM 24592 HG1 GLU H 40 4.326 -10.132 61.964 1.00 0.00
H H
ATOM 24593 HG2 GLU H 40 4.316 -11.694 61.062 1.00 0.00
H H
ATOM 24594 CD GLU H 40 4.117 -11.823 63.178 1.00 0.00
H C
ATOM 24595 OE1 GLU H 40 3.396 -12.822 62.994 1.00 0.00
H O
ATOM 24596 OE2 GLU H 40 4.295 -11.311 64.307 1.00 0.00
H O

```
ATOM  24597  C   GLU H  40       7.951 -12.146  63.452  1.00  0.00
H    C
ATOM  24598  O   GLU H  40       7.707 -13.068  64.240  1.00  0.00
H    O
ATOM  24599  N   LYS H  41       9.075 -12.094  62.742  1.00  0.00
H    N
ATOM  24600  HN  LYS H  41       9.303 -11.376  62.086  1.00  0.00
H    H
ATOM  24601  CA  LYS H  41      10.081 -13.135  62.895  1.00  0.00
H    C
ATOM  24602  HA  LYS H  41       9.584 -14.084  62.737  1.00  0.00
H    H
ATOM  24603  CB  LYS H  41      11.208 -12.943  61.885  1.00  0.00
H    C
ATOM  24604  HB1 LYS H  41      11.814 -12.073  62.231  1.00  0.00
H    H
ATOM  24605  HB2 LYS H  41      11.882 -13.828  61.921  1.00  0.00
H    H
ATOM  24606  CG  LYS H  41      10.746 -12.687  60.481  1.00  0.00
H    C
ATOM  24607  HG1 LYS H  41       9.726 -12.240  60.508  1.00  0.00
H    H
ATOM  24608  HG2 LYS H  41      11.424 -11.939  60.007  1.00  0.00
H    H
ATOM  24609  CD  LYS H  41      10.706 -13.948  59.663  1.00  0.00
H    C
ATOM  24610  HD1 LYS H  41      11.523 -14.619  60.012  1.00  0.00
H    H
ATOM  24611  HD2 LYS H  41       9.739 -14.464  59.869  1.00  0.00
H    H
ATOM  24612  CE  LYS H  41      10.848 -13.600  58.198  1.00  0.00
H    C
ATOM  24613  HE1 LYS H  41      10.672 -14.499  57.566  1.00  0.00
H    H
ATOM  24614  HE2 LYS H  41      10.112 -12.818  57.909  1.00  0.00
H    H
ATOM  24615  NZ  LYS H  41      12.212 -13.086  57.905  1.00  0.00
H    N
ATOM  24616  HZ1 LYS H  41      12.295 -12.853  56.895  1.00  0.00
H    H
ATOM  24617  HZ2 LYS H  41      12.390 -12.232  58.473  1.00  0.00
H    H
ATOM  24618  HZ3 LYS H  41      12.915 -13.812  58.150  1.00  0.00
H    H
ATOM  24619  C   LYS H  41      10.649 -13.150  64.304  1.00  0.00
H    C
ATOM  24620  O   LYS H  41      10.851 -14.222  64.884  1.00  0.00
H    O
ATOM  24621  N   VAL H  42      10.973 -11.977  64.843  1.00  0.00
H    N
ATOM  24622  HN  VAL H  42      10.920 -11.097  64.378  1.00  0.00
H    H
ATOM  24623  CA  VAL H  42      11.451 -11.904  66.217  1.00  0.00
H    C
```

```
ATOM  24624  HA  VAL H  42      12.371 -12.470  66.284  1.00  0.00
H    H
ATOM  24625  CB  VAL H  42      11.693 -10.440  66.614  1.00  0.00
H    C
ATOM  24626  HB  VAL H  42      10.693  -9.952  66.733  1.00  0.00
H    H
ATOM  24627  CG1 VAL H  42      12.443 -10.364  67.927  1.00  0.00
H    C
ATOM  24628 HG11 VAL H  42      12.650  -9.304  68.192  1.00  0.00
H    H
ATOM  24629 HG12 VAL H  42      11.857 -10.804  68.760  1.00  0.00
H    H
ATOM  24630 HG13 VAL H  42      13.415 -10.897  67.855  1.00  0.00
H    H
ATOM  24631  CG2 VAL H  42      12.437  -9.719  65.512  1.00  0.00
H    C
ATOM  24632 HG21 VAL H  42      12.652  -8.670  65.805  1.00  0.00
H    H
ATOM  24633 HG22 VAL H  42      13.401 -10.228  65.295  1.00  0.00
H    H
ATOM  24634 HG23 VAL H  42      11.841  -9.690  64.577  1.00  0.00
H    H
ATOM  24635  C   VAL H  42      10.445 -12.561  67.137  1.00  0.00
H    C
ATOM  24636  O   VAL H  42      10.786 -13.392  67.987  1.00  0.00
H    O
ATOM  24637  N   ASN H  43       9.179 -12.203  66.956  1.00  0.00
H    N
ATOM  24638  HN  ASN H  43       8.889 -11.554  66.254  1.00  0.00
H    H
ATOM  24639  CA  ASN H  43       8.099 -12.731  67.771  1.00  0.00
H    C
ATOM  24640  HA  ASN H  43       8.316 -12.423  68.788  1.00  0.00
H    H
ATOM  24641  CB  ASN H  43       6.775 -12.174  67.258  1.00  0.00
H    C
ATOM  24642  HB1 ASN H  43       6.990 -11.221  66.727  1.00  0.00
H    H
ATOM  24643  HB2 ASN H  43       6.323 -12.870  66.523  1.00  0.00
H    H
ATOM  24644  CG  ASN H  43       5.806 -11.882  68.363  1.00  0.00
H    C
ATOM  24645  OD1 ASN H  43       5.554 -12.718  69.216  1.00  0.00
H    O
ATOM  24646  ND2 ASN H  43       5.264 -10.673  68.367  1.00  0.00
H    N
ATOM  24647 HD21 ASN H  43       4.626 -10.460  69.103  1.00  0.00
H    H
ATOM  24648 HD22 ASN H  43       5.522 -10.009  67.672  1.00  0.00
H    H
ATOM  24649  C   ASN H  43       8.087 -14.245  67.743  1.00  0.00
H    C
ATOM  24650  O   ASN H  43       7.958 -14.898  68.783  1.00  0.00
H    O
```

```
ATOM  24651  N   ALA H  44       8.216 -14.819  66.554  1.00  0.00
H    N
ATOM  24652  HN  ALA H  44       8.299 -14.327  65.688  1.00  0.00
H    H
ATOM  24653  CA  ALA H  44       8.242 -16.271  66.454  1.00  0.00
H    C
ATOM  24654  HA  ALA H  44       7.386 -16.702  66.960  1.00  0.00
H    H
ATOM  24655  CB  ALA H  44       8.195 -16.699  64.990  1.00  0.00
H    C
ATOM  24656  HB1 ALA H  44       7.266 -16.314  64.518  1.00  0.00
H    H
ATOM  24657  HB2 ALA H  44       9.061 -16.288  64.426  1.00  0.00
H    H
ATOM  24658  HB3 ALA H  44       8.199 -17.806  64.887  1.00  0.00
H    H
ATOM  24659  C   ALA H  44       9.476 -16.849  67.131  1.00  0.00
H    C
ATOM  24660  O   ALA H  44       9.366 -17.594  68.110  1.00  0.00
H    O
ATOM  24661  N   TYR H  45      10.655 -16.483  66.655  1.00  0.00
H    N
ATOM  24662  HN  TYR H  45      10.799 -15.712  66.037  1.00  0.00
H    H
ATOM  24663  CA  TYR H  45      11.865 -17.204  67.013  1.00  0.00
H    C
ATOM  24664  HA  TYR H  45      11.561 -18.239  67.119  1.00  0.00
H    H
ATOM  24665  CB  TYR H  45      12.926 -17.055  65.927  1.00  0.00
H    C
ATOM  24666  HB1 TYR H  45      13.007 -15.984  65.641  1.00  0.00
H    H
ATOM  24667  HB2 TYR H  45      13.923 -17.371  66.304  1.00  0.00
H    H
ATOM  24668  CG  TYR H  45      12.610 -17.813  64.671  1.00  0.00
H    C
ATOM  24669  CD1 TYR H  45      11.501 -18.640  64.599  1.00  0.00
H    C
ATOM  24670  HD1 TYR H  45      10.856 -18.747  65.460  1.00  0.00
H    H
ATOM  24671  CE1 TYR H  45      11.217 -19.347  63.454  1.00  0.00
H    C
ATOM  24672  HE1 TYR H  45      10.340 -19.975  63.399  1.00  0.00
H    H
ATOM  24673  CZ  TYR H  45      12.064 -19.255  62.374  1.00  0.00
H    C
ATOM  24674  OH  TYR H  45      11.794 -19.960  61.227  1.00  0.00
H    O
ATOM  24675  HH  TYR H  45      12.451 -19.710  60.575  1.00  0.00
H    H
ATOM  24676  CD2 TYR H  45      13.451 -17.752  63.577  1.00  0.00
H    C
ATOM  24677  HD2 TYR H  45      14.362 -17.170  63.626  1.00  0.00
H    H
```

```
ATOM  24678  CE2 TYR H  45      13.179 -18.458  62.432  1.00  0.00
H    C
ATOM  24679  HE2 TYR H  45      13.855 -18.399  61.593  1.00  0.00
H    H
ATOM  24680  C   TYR H  45      12.465 -16.778  68.338  1.00  0.00
H    C
ATOM  24681  O   TYR H  45      13.579 -17.208  68.654  1.00  0.00
H    O
ATOM  24682  N   ALA H  46      11.775 -15.942  69.112  1.00  0.00
H    N
ATOM  24683  HN  ALA H  46      10.943 -15.458  68.842  1.00  0.00
H    H
ATOM  24684  CA  ALA H  46      12.235 -15.683  70.468  1.00  0.00
H    C
ATOM  24685  HA  ALA H  46      13.176 -15.145  70.461  1.00  0.00
H    H
ATOM  24686  CB  ALA H  46      11.207 -14.839  71.216  1.00  0.00
H    C
ATOM  24687  HB1 ALA H  46      11.069 -13.869  70.693  1.00  0.00
H    H
ATOM  24688  HB2 ALA H  46      10.221 -15.352  71.252  1.00  0.00
H    H
ATOM  24689  HB3 ALA H  46      11.532 -14.624  72.256  1.00  0.00
H    H
ATOM  24690  C   ALA H  46      12.494 -16.989  71.200  1.00  0.00
H    C
ATOM  24691  O   ALA H  46      13.540 -17.170  71.829  1.00  0.00
H    O
ATOM  24692  N   SER H  47      11.556 -17.927  71.090  1.00  0.00
H    N
ATOM  24693  HN  SER H  47      10.714 -17.825  70.560  1.00  0.00
H    H
ATOM  24694  CA  SER H  47      11.689 -19.212  71.756  1.00  0.00
H    C
ATOM  24695  HA  SER H  47      11.786 -18.981  72.808  1.00  0.00
H    H
ATOM  24696  CB  SER H  47      10.465 -20.069  71.462  1.00  0.00
H    C
ATOM  24697  HB1 SER H  47      10.389 -20.848  72.255  1.00  0.00
H    H
ATOM  24698  HB2 SER H  47       9.539 -19.453  71.517  1.00  0.00
H    H
ATOM  24699  OG  SER H  47      10.602 -20.696  70.203  1.00  0.00
H    O
ATOM  24700  HG1 SER H  47       9.930 -21.388  70.156  1.00  0.00
H    H
ATOM  24701  C   SER H  47      12.942 -19.964  71.341  1.00  0.00
H    C
ATOM  24702  O   SER H  47      13.459 -20.762  72.126  1.00  0.00
H    O
ATOM  24703  N   TYR H  48      13.440 -19.739  70.131  1.00  0.00
H    N
ATOM  24704  HN  TYR H  48      13.042 -19.099  69.475  1.00  0.00
H    H
```

```
ATOM  24705  CA  TYR H  48      14.632 -20.424  69.659  1.00  0.00
H    C
ATOM  24706  HA  TYR H  48      14.806 -21.244  70.345  1.00  0.00
H    H
ATOM  24707  CB  TYR H  48      14.426 -20.920  68.233  1.00  0.00
H    C
ATOM  24708  HB1 TYR H  48      14.283 -20.046  67.561  1.00  0.00
H    H
ATOM  24709  HB2 TYR H  48      15.323 -21.468  67.874  1.00  0.00
H    H
ATOM  24710  CG  TYR H  48      13.212 -21.784  68.106  1.00  0.00
H    C
ATOM  24711  CD1 TYR H  48      12.772 -22.546  69.175  1.00  0.00
H    C
ATOM  24712  HD1 TYR H  48      13.328 -22.541  70.103  1.00  0.00
H    H
ATOM  24713  CE1 TYR H  48      11.653 -23.325  69.074  1.00  0.00
H    C
ATOM  24714  HE1 TYR H  48      11.327 -23.931  69.907  1.00  0.00
H    H
ATOM  24715  CZ  TYR H  48      10.945 -23.338  67.896  1.00  0.00
H    C
ATOM  24716  OH  TYR H  48       9.821 -24.117  67.787  1.00  0.00
H    O
ATOM  24717  HH  TYR H  48       9.430 -23.940  66.930  1.00  0.00
H    H
ATOM  24718  CD2 TYR H  48      12.476 -21.806  66.937  1.00  0.00
H    C
ATOM  24719  HD2 TYR H  48      12.808 -21.236  66.079  1.00  0.00
H    H
ATOM  24720  CE2 TYR H  48      11.352 -22.582  66.825  1.00  0.00
H    C
ATOM  24721  HE2 TYR H  48      10.810 -22.609  65.891  1.00  0.00
H    H
ATOM  24722  C   TYR H  48      15.846 -19.525  69.721  1.00  0.00
H    C
ATOM  24723  O   TYR H  48      16.770 -19.681  68.922  1.00  0.00
H    O
ATOM  24724  N   ALA H  49      15.862 -18.583  70.654  1.00  0.00
H    N
ATOM  24725  HN  ALA H  49      15.194 -18.519  71.395  1.00  0.00
H    H
ATOM  24726  CA  ALA H  49      16.887 -17.559  70.651  1.00  0.00
H    C
ATOM  24727  HA  ALA H  49      17.752 -17.868  70.073  1.00  0.00
H    H
ATOM  24728  CB  ALA H  49      16.366 -16.243  70.068  1.00  0.00
H    C
ATOM  24729  HB1 ALA H  49      16.052 -16.401  69.014  1.00  0.00
H    H
ATOM  24730  HB2 ALA H  49      15.484 -15.876  70.636  1.00  0.00
H    H
ATOM  24731  HB3 ALA H  49      17.147 -15.452  70.077  1.00  0.00
H    H
```

```
ATOM  24732  C   ALA H  49      17.378 -17.328  72.063  1.00  0.00
H    C
ATOM  24733  O   ALA H  49      16.588 -17.192  72.998  1.00  0.00
H    O
ATOM  24734  N   THR H  50      18.700 -17.280  72.204  1.00  0.00
H    N
ATOM  24735  HN  THR H  50      19.378 -17.523  71.511  1.00  0.00
H    H
ATOM  24736  CA  THR H  50      19.268 -16.839  73.464  1.00  0.00
H    C
ATOM  24737  HA  THR H  50      18.902 -17.526  74.215  1.00  0.00
H    H
ATOM  24738  CB  THR H  50      20.795 -16.810  73.375  1.00  0.00
H    C
ATOM  24739  HB  THR H  50      21.050 -15.953  72.705  1.00  0.00
H    H
ATOM  24740  OG1 THR H  50      21.266 -18.012  72.754  1.00  0.00
H    O
ATOM  24741  HG1 THR H  50      22.226 -17.935  72.739  1.00  0.00
H    H
ATOM  24742  CG2 THR H  50      21.413 -16.689  74.759  1.00  0.00
H    C
ATOM  24743 HG21 THR H  50      22.513 -16.554  74.690  1.00  0.00
H    H
ATOM  24744 HG22 THR H  50      20.993 -15.811  75.295  1.00  0.00
H    H
ATOM  24745 HG23 THR H  50      21.212 -17.598  75.368  1.00  0.00
H    H
ATOM  24746  C   THR H  50      18.741 -15.467  73.842  1.00  0.00
H    C
ATOM  24747  O   THR H  50      18.512 -15.194  75.023  1.00  0.00
H    O
ATOM  24748  N   HSE H  51      18.512 -14.612  72.856  1.00  0.00
H    N
ATOM  24749  HN  HSE H  51      18.660 -14.807  71.888  1.00  0.00
H    H
ATOM  24750  CA  HSE H  51      18.011 -13.279  73.128  1.00  0.00
H    C
ATOM  24751  HA  HSE H  51      17.145 -13.367  73.773  1.00  0.00
H    H
ATOM  24752  CB  HSE H  51      19.099 -12.421  73.773  1.00  0.00
H    C
ATOM  24753  HB1 HSE H  51      19.368 -12.885  74.746  1.00  0.00
H    H
ATOM  24754  HB2 HSE H  51      20.011 -12.442  73.141  1.00  0.00
H    H
ATOM  24755  ND1 HSE H  51      19.554 -10.064  74.529  1.00  0.00
H    N
ATOM  24756  CG  HSE H  51      18.683 -11.010  74.037  1.00  0.00
H    C
ATOM  24757  CE1 HSE H  51      18.920  -8.913  74.661  1.00  0.00
H    C
ATOM  24758  HE1 HSE H  51      19.375  -7.994  75.039  1.00  0.00
H    H
```

```
ATOM  24759  NE2 HSE H  51      17.671  -9.078  74.269  1.00  0.00
H    N
ATOM  24760  HE2 HSE H  51      16.966  -8.370  74.280  1.00  0.00
H    H
ATOM  24761  CD2 HSE H  51      17.497 -10.382  73.874  1.00  0.00
H    C
ATOM  24762  HD2 HSE H  51      16.551 -10.767  73.520  1.00  0.00
H    H
ATOM  24763  C   HSE H  51      17.533 -12.671  71.820  1.00  0.00
H    C
ATOM  24764  O   HSE H  51      18.290 -12.592  70.850  1.00  0.00
H    O
ATOM  24765  N   ALA H  52      16.276 -12.242  71.805  1.00  0.00
H    N
ATOM  24766  HN  ALA H  52      15.690 -12.299  72.612  1.00  0.00
H    H
ATOM  24767  CA  ALA H  52      15.657 -11.662  70.624  1.00  0.00
H    C
ATOM  24768  HA  ALA H  52      16.340 -11.646  69.783  1.00  0.00
H    H
ATOM  24769  CB  ALA H  52      14.401 -12.430  70.216  1.00  0.00
H    C
ATOM  24770  HB1 ALA H  52      14.671 -13.475  69.957  1.00  0.00
H    H
ATOM  24771  HB2 ALA H  52      13.666 -12.463  71.050  1.00  0.00
H    H
ATOM  24772  HB3 ALA H  52      13.911 -11.972  69.330  1.00  0.00
H    H
ATOM  24773  C   ALA H  52      15.303 -10.218  70.913  1.00  0.00
H    C
ATOM  24774  O   ALA H  52      15.027  -9.863  72.060  1.00  0.00
H    O
ATOM  24775  N   VAL H  53      15.298  -9.397  69.872  1.00  0.00
H    N
ATOM  24776  HN  VAL H  53      15.532  -9.696  68.951  1.00  0.00
H    H
ATOM  24777  CA  VAL H  53      14.952  -7.987  69.977  1.00  0.00
H    C
ATOM  24778  HA  VAL H  53      14.158  -7.871  70.704  1.00  0.00
H    H
ATOM  24779  CB  VAL H  53      16.165  -7.113  70.353  1.00  0.00
H    C
ATOM  24780  HB  VAL H  53      17.048  -7.499  69.786  1.00  0.00
H    H
ATOM  24781  CG1 VAL H  53      15.924  -5.659  69.971  1.00  0.00
H    C
ATOM  24782 HG11 VAL H  53      16.787  -5.031  70.281  1.00  0.00
H    H
ATOM  24783 HG12 VAL H  53      15.803  -5.538  68.875  1.00  0.00
H    H
ATOM  24784 HG13 VAL H  53      15.015  -5.267  70.475  1.00  0.00
H    H
ATOM  24785  CG2 VAL H  53      16.477  -7.213  71.827  1.00  0.00
H    C
```

ATOM 24786 HG21 VAL H 53   17.327 -6.550 72.094 1.00 0.00
H   H
ATOM 24787 HG22 VAL H 53   15.595 -6.914 72.433 1.00 0.00
H   H
ATOM 24788 HG23 VAL H 53   16.758 -8.249 72.108 1.00 0.00
H   H
ATOM 24789 C   VAL H 53   14.403 -7.551 68.632 1.00 0.00
H   C
ATOM 24790 O   VAL H 53   14.911 -7.966 67.589 1.00 0.00
H   O
ATOM 24791 N   ILE H 54   13.372 -6.719 68.646 1.00 0.00
H   N
ATOM 24792 HN ILE H 54   12.814 -6.490 69.441 1.00 0.00
H   H
ATOM 24793 CA ILE H 54   12.966 -6.048 67.421 1.00 0.00
H   C
ATOM 24794 HA ILE H 54   13.253 -6.676 66.587 1.00 0.00
H   H
ATOM 24795 CB ILE H 54   11.466 -5.759 67.418 1.00 0.00
H   C
ATOM 24796 HB ILE H 54   11.318 -5.042 68.265 1.00 0.00
H   H
ATOM 24797 CG2 ILE H 54   11.035 -5.265 66.062 1.00 0.00
H   C
ATOM 24798 HG21 ILE H 54   9.986 -4.905 66.093 1.00 0.00
H   H
ATOM 24799 HG22 ILE H 54   11.670 -4.417 65.729 1.00 0.00
H   H
ATOM 24800 HG23 ILE H 54   11.103 -6.073 65.305 1.00 0.00
H   H
ATOM 24801 CG1 ILE H 54   10.698 -7.025 67.765 1.00 0.00
H   C
ATOM 24802 HG11 ILE H 54   10.625 -7.680 66.868 1.00 0.00
H   H
ATOM 24803 HG12 ILE H 54   11.266 -7.591 68.539 1.00 0.00
H   H
ATOM 24804 CD ILE H 54   9.317 -6.755 68.294 1.00 0.00
H   C
ATOM 24805 HD1 ILE H 54   8.823 -7.707 68.585 1.00 0.00
H   H
ATOM 24806 HD2 ILE H 54   9.362 -6.099 69.190 1.00 0.00
H   H
ATOM 24807 HD3 ILE H 54   8.686 -6.262 67.526 1.00 0.00
H   H
ATOM 24808 C   ILE H 54   13.780 -4.768 67.308 1.00 0.00
H   C
ATOM 24809 O   ILE H 54   13.803 -3.955 68.237 1.00 0.00
H   O
ATOM 24810 N   ALA H 55   14.460 -4.590 66.177 1.00 0.00
H   N
ATOM 24811 HN ALA H 55   14.430 -5.235 65.414 1.00 0.00
H   H
ATOM 24812 CA ALA H 55   15.307 -3.424 65.975 1.00 0.00
H   C

```
ATOM  24813  HA  ALA H  55      14.784  -2.511  66.232  1.00  0.00
H    H
ATOM  24814  CB  ALA H  55      16.574  -3.498  66.833  1.00  0.00
H    C
ATOM  24815  HB1 ALA H  55      16.295  -3.534  67.907  1.00  0.00
H    H
ATOM  24816  HB2 ALA H  55      17.160  -4.413  66.601  1.00  0.00
H    H
ATOM  24817  HB3 ALA H  55      17.226  -2.611  66.677  1.00  0.00
H    H
ATOM  24818  C   ALA H  55      15.681  -3.306  64.505  1.00  0.00
H    C
ATOM  24819  O   ALA H  55      15.862  -4.314  63.819  1.00  0.00
H    O
ATOM  24820  N   ASN H  56      15.803  -2.068  64.040  1.00  0.00
H    N
ATOM  24821  HN  ASN H  56      15.631  -1.263  64.606  1.00  0.00
H    H
ATOM  24822  CA  ASN H  56      16.197  -1.770  62.667  1.00  0.00
H    C
ATOM  24823  HA  ASN H  56      15.920  -2.625  62.059  1.00  0.00
H    H
ATOM  24824  CB  ASN H  56      15.524  -0.491  62.192  1.00  0.00
H    C
ATOM  24825  HB1 ASN H  56      14.424  -0.645  62.249  1.00  0.00
H    H
ATOM  24826  HB2 ASN H  56      15.777   0.352  62.867  1.00  0.00
H    H
ATOM  24827  CG  ASN H  56      15.875  -0.143  60.766  1.00  0.00
H    C
ATOM  24828  OD1 ASN H  56      16.806  -0.696  60.181  1.00  0.00
H    O
ATOM  24829  ND2 ASN H  56      15.126   0.790  60.194  1.00  0.00
H    N
ATOM  24830 HD21 ASN H  56      15.333   1.027  59.248  1.00  0.00
H    H
ATOM  24831 HD22 ASN H  56      14.372   1.202  60.697  1.00  0.00
H    H
ATOM  24832  C   ASN H  56      17.712  -1.648  62.627  1.00  0.00
H    C
ATOM  24833  O   ASN H  56      18.290  -0.734  63.214  1.00  0.00
H    O
ATOM  24834  N   ALA H  57      18.354  -2.553  61.891  1.00  0.00
H    N
ATOM  24835  HN  ALA H  57      17.880  -3.216  61.312  1.00  0.00
H    H
ATOM  24836  CA  ALA H  57      19.808  -2.641  61.880  1.00  0.00
H    C
ATOM  24837  HA  ALA H  57      20.167  -2.816  62.888  1.00  0.00
H    H
ATOM  24838  CB  ALA H  57      20.254  -3.797  60.995  1.00  0.00
H    C
ATOM  24839  HB1 ALA H  57      19.804  -4.744  61.365  1.00  0.00
H    H
```

ATOM 24840 HB2 ALA H 57 19.921 -3.647 59.945 1.00 0.00
H H
ATOM 24841 HB3 ALA H 57 21.359 -3.916 61.002 1.00 0.00
H H
ATOM 24842 C ALA H 57 20.486 -1.373 61.399 1.00 0.00
H C
ATOM 24843 O ALA H 57 21.665 -1.171 61.698 1.00 0.00
H O
ATOM 24844 N THR H 58 19.786 -0.518 60.677 1.00 0.00
H N
ATOM 24845 HN THR H 58 18.794 -0.538 60.555 1.00 0.00
H H
ATOM 24846 CA THR H 58 20.452 0.563 59.977 1.00 0.00
H C
ATOM 24847 HA THR H 58 21.376 0.129 59.617 1.00 0.00
H H
ATOM 24848 CB THR H 58 19.559 1.055 58.841 1.00 0.00
H C
ATOM 24849 HB THR H 58 20.143 1.844 58.307 1.00 0.00
H H
ATOM 24850 OG1 THR H 58 18.389 1.663 59.390 1.00 0.00
H O
ATOM 24851 HG1 THR H 58 17.864 1.941 58.632 1.00 0.00
H H
ATOM 24852 CG2 THR H 58 19.130 -0.112 57.976 1.00 0.00
H C
ATOM 24853 HG21 THR H 58 18.575 0.241 57.082 1.00 0.00
H H
ATOM 24854 HG22 THR H 58 20.018 -0.681 57.625 1.00 0.00
H H
ATOM 24855 HG23 THR H 58 18.472 -0.808 58.542 1.00 0.00
H H
ATOM 24856 C THR H 58 20.841 1.731 60.871 1.00 0.00
H C
ATOM 24857 O THR H 58 21.484 2.665 60.388 1.00 0.00
H O
ATOM 24858 N GLU H 59 20.475 1.715 62.149 1.00 0.00
H N
ATOM 24859 HN GLU H 59 20.010 0.956 62.596 1.00 0.00
H H
ATOM 24860 CA GLU H 59 20.738 2.850 63.027 1.00 0.00
H C
ATOM 24861 HA GLU H 59 21.132 3.661 62.427 1.00 0.00
H H
ATOM 24862 CB GLU H 59 19.460 3.292 63.730 1.00 0.00
H C
ATOM 24863 HB1 GLU H 59 18.999 2.397 64.209 1.00 0.00
H H
ATOM 24864 HB2 GLU H 59 19.708 3.989 64.561 1.00 0.00
H H
ATOM 24865 CG GLU H 59 18.438 3.931 62.831 1.00 0.00
H C
ATOM 24866 HG1 GLU H 59 17.603 4.322 63.441 1.00 0.00
H H

ATOM 24867 HG2 GLU H 59 18.885 4.762 62.250 1.00 0.00
H H
ATOM 24868 CD GLU H 59 17.829 2.940 61.877 1.00 0.00
H C
ATOM 24869 OE1 GLU H 59 17.661 3.282 60.691 1.00 0.00
H O
ATOM 24870 OE2 GLU H 59 17.571 1.799 62.295 1.00 0.00
H O
ATOM 24871 C GLU H 59 21.797 2.501 64.065 1.00 0.00
H C
ATOM 24872 O GLU H 59 21.671 1.502 64.780 1.00 0.00
H O
ATOM 24873 N GLU H 60 22.808 3.363 64.183 1.00 0.00
H N
ATOM 24874 HN GLU H 60 22.861 4.250 63.732 1.00 0.00
H H
ATOM 24875 CA GLU H 60 23.960 3.053 65.020 1.00 0.00
H C
ATOM 24876 HA GLU H 60 24.333 2.080 64.725 1.00 0.00
H H
ATOM 24877 CB GLU H 60 25.070 4.081 64.779 1.00 0.00
H C
ATOM 24878 HB1 GLU H 60 25.886 3.889 65.513 1.00 0.00
H H
ATOM 24879 HB2 GLU H 60 25.520 3.921 63.773 1.00 0.00
H H
ATOM 24880 CG GLU H 60 24.690 5.563 64.898 1.00 0.00
H C
ATOM 24881 HG1 GLU H 60 25.601 6.185 64.831 1.00 0.00
H H
ATOM 24882 HG2 GLU H 60 23.987 5.859 64.095 1.00 0.00
H H
ATOM 24883 CD GLU H 60 24.058 5.952 66.216 1.00 0.00
H C
ATOM 24884 OE1 GLU H 60 24.657 5.662 67.271 1.00 0.00
H O
ATOM 24885 OE2 GLU H 60 22.942 6.506 66.196 1.00 0.00
H O
ATOM 24886 C GLU H 60 23.581 2.963 66.490 1.00 0.00
H C
ATOM 24887 O GLU H 60 24.197 2.215 67.254 1.00 0.00
H O
ATOM 24888 N ASN H 61 22.587 3.738 66.914 1.00 0.00
H N
ATOM 24889 HN ASN H 61 22.018 4.327 66.341 1.00 0.00
H H
ATOM 24890 CA ASN H 61 22.256 3.771 68.329 1.00 0.00
H C
ATOM 24891 HA ASN H 61 23.200 3.851 68.858 1.00 0.00
H H
ATOM 24892 CB ASN H 61 21.359 4.965 68.623 1.00 0.00
H C
ATOM 24893 HB1 ASN H 61 20.878 4.794 69.610 1.00 0.00
H H

ATOM 24894 HB2 ASN H 61   21.964   5.891 68.703 1.00 0.00
H   H
ATOM 24895 CG ASN H 61   20.286   5.125 67.597 1.00 0.00
H   C
ATOM 24896 OD1 ASN H 61   20.458   4.727 66.445 1.00 0.00
H   O
ATOM 24897 ND2 ASN H 61   19.169   5.714 67.993 1.00 0.00
H   N
ATOM 24898 HD21 ASN H 61   18.441   5.815 67.319 1.00 0.00
H   H
ATOM 24899 HD22 ASN H 61   19.068   6.009 68.937 1.00 0.00
H   H
ATOM 24900 C   ASN H 61   21.601   2.471 68.754 1.00 0.00
H   C
ATOM 24901 O   ASN H 61   21.851   1.972 69.856 1.00 0.00
H   O
ATOM 24902 N   GLU H 62   20.758   1.903 67.892 1.00 0.00
H   N
ATOM 24903 HN GLU H 62   20.371   2.314 67.071 1.00 0.00
H   H
ATOM 24904 CA GLU H 62   20.324   0.536 68.129 1.00 0.00
H   C
ATOM 24905 HA GLU H 62   19.755   0.516 69.049 1.00 0.00
H   H
ATOM 24906 CB GLU H 62   19.459   0.032 66.983 1.00 0.00
H   C
ATOM 24907 HB1 GLU H 62   19.898   0.401 66.028 1.00 0.00
H   H
ATOM 24908 HB2 GLU H 62   19.509 -1.080 66.934 1.00 0.00
H   H
ATOM 24909 CG GLU H 62   18.028   0.477 67.069 1.00 0.00
H   C
ATOM 24910 HG1 GLU H 62   17.463 -0.218 67.716 1.00 0.00
H   H
ATOM 24911 HG2 GLU H 62   17.955   1.503 67.483 1.00 0.00
H   H
ATOM 24912 CD GLU H 62   17.331   0.452 65.734 1.00 0.00
H   C
ATOM 24913 OE1 GLU H 62   16.553 -0.489 65.492 1.00 0.00
H   O
ATOM 24914 OE2 GLU H 62   17.546   1.378 64.933 1.00 0.00
H   O
ATOM 24915 C   GLU H 62   21.512 -0.387 68.314 1.00 0.00
H   C
ATOM 24916 O   GLU H 62   21.482 -1.276 69.170 1.00 0.00
H   O
ATOM 24917 N   LEU H 63   22.570 -0.167 67.550 1.00 0.00
H   N
ATOM 24918 HN LEU H 63   22.666   0.584 66.899 1.00 0.00
H   H
ATOM 24919 CA LEU H 63   23.719 -1.051 67.613 1.00 0.00
H   C
ATOM 24920 HA LEU H 63   23.336 -2.061 67.543 1.00 0.00
H   H

ATOM 24921 CB LEU H 63   24.655 -0.731 66.459 1.00 0.00
H   C
ATOM 24922 HB1 LEU H 63   25.187   0.213 66.719 1.00 0.00
H   H
ATOM 24923 HB2 LEU H 63   25.438 -1.519 66.399 1.00 0.00
H   H
ATOM 24924 CG LEU H 63   23.900 -0.568 65.147 1.00 0.00
H   C
ATOM 24925 HG LEU H 63   23.186   0.278 65.296 1.00 0.00
H   H
ATOM 24926 CD1 LEU H 63   24.857 -0.227 64.030 1.00 0.00
H   C
ATOM 24927 HD11 LEU H 63   24.315 -0.154 63.062 1.00 0.00
H   H
ATOM 24928 HD12 LEU H 63   25.355   0.747 64.225 1.00 0.00
H   H
ATOM 24929 HD13 LEU H 63   25.640 -1.008 63.930 1.00 0.00
H   H
ATOM 24930 CD2 LEU H 63   23.096 -1.808 64.815 1.00 0.00
H   C
ATOM 24931 HD21 LEU H 63   22.539 -1.663 63.864 1.00 0.00
H   H
ATOM 24932 HD22 LEU H 63   23.747 -2.696 64.690 1.00 0.00
H   H
ATOM 24933 HD23 LEU H 63   22.361 -2.023 65.620 1.00 0.00
H   H
ATOM 24934 C   LEU H 63   24.444 -0.925 68.943 1.00 0.00
H   C
ATOM 24935 O   LEU H 63   24.654 -1.928 69.635 1.00 0.00
H   O
ATOM 24936 N   LEU H 64   24.832   0.294 69.311 1.00 0.00
H   N
ATOM 24937 HN LEU H 64   24.726   1.124 68.767 1.00 0.00
H   H
ATOM 24938 CA LEU H 64   25.473   0.502 70.602 1.00 0.00
H   C
ATOM 24939 HA LEU H 64   26.377 -0.093 70.602 1.00 0.00
H   H
ATOM 24940 CB LEU H 64   25.781   1.977 70.802 1.00 0.00
H   C
ATOM 24941 HB1 LEU H 64   24.847   2.462 71.167 1.00 0.00
H   H
ATOM 24942 HB2 LEU H 64   26.523   2.083 71.624 1.00 0.00
H   H
ATOM 24943 CG LEU H 64   26.250   2.658 69.531 1.00 0.00
H   C
ATOM 24944 HG LEU H 64   25.567   2.362 68.698 1.00 0.00
H   H
ATOM 24945 CD1 LEU H 64   26.202   4.157 69.703 1.00 0.00
H   C
ATOM 24946 HD11 LEU H 64   26.590   4.667 68.795 1.00 0.00
H   H
ATOM 24947 HD12 LEU H 64   25.159   4.498 69.877 1.00 0.00
H   H

```
ATOM  24948 HD13 LEU H  64      26.823   4.472  70.569  1.00  0.00
H    H
ATOM  24949  CD2 LEU H  64      27.643   2.188  69.183  1.00  0.00
H    C
ATOM  24950 HD21 LEU H  64      27.989   2.668  68.242  1.00  0.00
H    H
ATOM  24951 HD22 LEU H  64      28.373   2.443  69.978  1.00  0.00
H    H
ATOM  24952 HD23 LEU H  64      27.656   1.086  69.036  1.00  0.00
H    H
ATOM  24953  C   LEU H  64      24.595   0.000  71.732  1.00  0.00
H    C
ATOM  24954  O   LEU H  64      25.081  -0.661  72.656  1.00  0.00
H    O
ATOM  24955  N   SER H  65      23.296   0.295  71.669  1.00  0.00
H    N
ATOM  24956  HN  SER H  65      22.864   0.844  70.953  1.00  0.00
H    H
ATOM  24957  CA  SER H  65      22.376  -0.186  72.688  1.00  0.00
H    C
ATOM  24958  HA  SER H  65      22.719   0.235  73.623  1.00  0.00
H    H
ATOM  24959  CB  SER H  65      20.956   0.239  72.344  1.00  0.00
H    C
ATOM  24960  HB1 SER H  65      20.273  -0.183  73.116  1.00  0.00
H    H
ATOM  24961  HB2 SER H  65      20.860   1.348  72.395  1.00  0.00
H    H
ATOM  24962  OG  SER H  65      20.600  -0.264  71.076  1.00  0.00
H    O
ATOM  24963  HG1 SER H  65      19.643  -0.170  70.992  1.00  0.00
H    H
ATOM  24964  C   SER H  65      22.447  -1.695  72.819  1.00  0.00
H    C
ATOM  24965  O   SER H  65      22.261  -2.235  73.915  1.00  0.00
H    O
ATOM  24966  N   LEU H  66      22.730  -2.389  71.722  1.00  0.00
H    N
ATOM  24967  HN  LEU H  66      22.872  -2.008  70.812  1.00  0.00
H    H
ATOM  24968  CA  LEU H  66      22.856  -3.832  71.790  1.00  0.00
H    C
ATOM  24969  HA  LEU H  66      22.059  -4.174  72.438  1.00  0.00
H    H
ATOM  24970  CB  LEU H  66      22.719  -4.433  70.401  1.00  0.00
H    C
ATOM  24971  HB1 LEU H  66      23.414  -3.876  69.731  1.00  0.00
H    H
ATOM  24972  HB2 LEU H  66      23.086  -5.483  70.422  1.00  0.00
H    H
ATOM  24973  CG  LEU H  66      21.298  -4.338  69.871  1.00  0.00
H    C
ATOM  24974  HG  LEU H  66      21.070  -3.267  69.652  1.00  0.00
H    H
```

ATOM 24975 CD1 LEU H 66   21.177 -5.144 68.608 1.00 0.00
H   C
ATOM 24976 HD11 LEU H 66   20.132 -5.124 68.232 1.00 0.00
H   H
ATOM 24977 HD12 LEU H 66   21.840 -4.733 67.816 1.00 0.00
H   H
ATOM 24978 HD13 LEU H 66   21.462 -6.202 68.789 1.00 0.00
H   H
ATOM 24979 CD2 LEU H 66   20.318 -4.821 70.919 1.00 0.00
H   C
ATOM 24980 HD21 LEU H 66   19.276 -4.735 70.542 1.00 0.00
H   H
ATOM 24981 HD22 LEU H 66   20.493 -5.882 71.186 1.00 0.00
H   H
ATOM 24982 HD23 LEU H 66   20.403 -4.208 71.843 1.00 0.00
H   H
ATOM 24983 C   LEU H 66   24.169 -4.269 72.408 1.00 0.00
H   C
ATOM 24984 O   LEU H 66   24.343 -5.462 72.669 1.00 0.00
H   O
ATOM 24985 N   GLY H 67   25.090 -3.346 72.654 1.00 0.00
H   N
ATOM 24986 HN GLY H 67   24.952 -2.365 72.530 1.00 0.00
H   H
ATOM 24987 CA GLY H 67   26.397 -3.740 73.135 1.00 0.00
H   C
ATOM 24988 HA1 GLY H 67   26.266 -4.282 74.061 1.00 0.00
H   H
ATOM 24989 HA2 GLY H 67   27.011 -2.854 73.221 1.00 0.00
H   H
ATOM 24990 C   GLY H 67   27.054 -4.667 72.139 1.00 0.00
H   C
ATOM 24991 O   GLY H 67   27.628 -5.700 72.499 1.00 0.00
H   O
ATOM 24992 N   ILE H 68   26.946 -4.298 70.865 1.00 0.00
H   N
ATOM 24993 HN ILE H 68   26.536 -3.441 70.558 1.00 0.00
H   H
ATOM 24994 CA ILE H 68   27.433 -5.145 69.786 1.00 0.00
H   C
ATOM 24995 HA ILE H 68   26.908 -6.086 69.879 1.00 0.00
H   H
ATOM 24996 CB ILE H 68   27.152 -4.456 68.449 1.00 0.00
H   C
ATOM 24997 HB ILE H 68   27.811 -4.989 67.716 1.00 0.00
H   H
ATOM 24998 CG2 ILE H 68   25.679 -4.489 68.153 1.00 0.00
H   C
ATOM 24999 HG21 ILE H 68   25.481 -4.135 67.120 1.00 0.00
H   H
ATOM 25000 HG22 ILE H 68   25.281 -5.522 68.238 1.00 0.00
H   H
ATOM 25001 HG23 ILE H 68   25.119 -3.838 68.856 1.00 0.00
H   H

```
ATOM  25002  CG1 ILE H  68      27.630  -3.013  68.526  1.00  0.00
H    C
ATOM  25003 HG11 ILE H  68      27.031  -2.457  69.281  1.00  0.00
H    H
ATOM  25004 HG12 ILE H  68      28.689  -3.002  68.872  1.00  0.00
H    H
ATOM  25005  CD  ILE H  68      27.559  -2.303  67.237  1.00  0.00
H    C
ATOM  25006  HD1 ILE H  68      27.973  -1.276  67.339  1.00  0.00
H    H
ATOM  25007  HD2 ILE H  68      28.147  -2.839  66.461  1.00  0.00
H    H
ATOM  25008  HD3 ILE H  68      26.509  -2.216  66.887  1.00  0.00
H    H
ATOM  25009  C   ILE H  68      28.911  -5.449  69.945  1.00  0.00
H    C
ATOM  25010  O   ILE H  68      29.342  -6.589  69.745  1.00  0.00
H    O
ATOM  25011  N   ARG H  69      29.708  -4.446  70.306  1.00  0.00
H    N
ATOM  25012  HN  ARG H  69      29.361  -3.542  70.541  1.00  0.00
H    H
ATOM  25013  CA  ARG H  69      31.147  -4.630  70.375  1.00  0.00
H    C
ATOM  25014  HA  ARG H  69      31.495  -4.904  69.386  1.00  0.00
H    H
ATOM  25015  CB  ARG H  69      31.803  -3.338  70.823  1.00  0.00
H    C
ATOM  25016  HB1 ARG H  69      31.348  -3.081  71.810  1.00  0.00
H    H
ATOM  25017  HB2 ARG H  69      32.882  -3.517  71.020  1.00  0.00
H    H
ATOM  25018  CG  ARG H  69      31.574  -2.215  69.852  1.00  0.00
H    C
ATOM  25019  HG1 ARG H  69      32.154  -2.424  68.927  1.00  0.00
H    H
ATOM  25020  HG2 ARG H  69      30.504  -2.218  69.542  1.00  0.00
H    H
ATOM  25021  CD  ARG H  69      31.906  -0.893  70.481  1.00  0.00
H    C
ATOM  25022  HD1 ARG H  69      32.920  -0.945  70.940  1.00  0.00
H    H
ATOM  25023  HD2 ARG H  69      31.896  -0.068  69.731  1.00  0.00
H    H
ATOM  25024  NE  ARG H  69      30.933  -0.543  71.506  1.00  0.00
H    N
ATOM  25025  HE  ARG H  69      30.085  -0.152  71.129  1.00  0.00
H    H
ATOM  25026  CZ  ARG H  69      31.130  -0.716  72.806  1.00  0.00
H    C
ATOM  25027  NH1 ARG H  69      32.264  -1.243  73.241  1.00  0.00
H    N
ATOM  25028 HH11 ARG H  69      32.410  -1.399  74.208  1.00  0.00
H    H
```

```
ATOM  25029 HH12 ARG H  69      32.889  -1.593  72.540  1.00  0.00
H    H
ATOM  25030  NH2 ARG H  69      30.193  -0.369  73.672  1.00  0.00
H    N
ATOM  25031 HH21 ARG H  69      30.376  -0.418  74.643  1.00  0.00
H    H
ATOM  25032 HH22 ARG H  69      29.413   0.126  73.314  1.00  0.00
H    H
ATOM  25033  C   ARG H  69      31.536  -5.763  71.298  1.00  0.00
H    C
ATOM  25034  O   ARG H  69      32.576  -6.390  71.079  1.00  0.00
H    O
ATOM  25035  N   ASN H  70      30.721  -6.047  72.310  1.00  0.00
H    N
ATOM  25036  HN  ASN H  70      29.937  -5.495  72.590  1.00  0.00
H    H
ATOM  25037  CA  ASN H  70      30.935  -7.239  73.111  1.00  0.00
H    C
ATOM  25038  HA  ASN H  70      31.920  -7.127  73.550  1.00  0.00
H    H
ATOM  25039  CB  ASN H  70      29.847  -7.354  74.172  1.00  0.00
H    C
ATOM  25040  HB1 ASN H  70      28.907  -7.662  73.663  1.00  0.00
H    H
ATOM  25041  HB2 ASN H  70      30.105  -8.147  74.903  1.00  0.00
H    H
ATOM  25042  CG  ASN H  70      29.597  -6.046  74.877  1.00  0.00
H    C
ATOM  25043  OD1 ASN H  70      30.423  -5.137  74.828  1.00  0.00
H    O
ATOM  25044  ND2 ASN H  70      28.455  -5.940  75.540  1.00  0.00
H    N
ATOM  25045 HD21 ASN H  70      28.272  -5.077  76.002  1.00  0.00
H    H
ATOM  25046 HD22 ASN H  70      27.805  -6.695  75.539  1.00  0.00
H    H
ATOM  25047  C   ASN H  70      30.956  -8.486  72.252  1.00  0.00
H    C
ATOM  25048  O   ASN H  70      31.726  -9.410  72.530  1.00  0.00
H    O
ATOM  25049  N   PHE H  71      30.154  -8.521  71.198  1.00  0.00
H    N
ATOM  25050  HN  PHE H  71      29.536  -7.777  70.949  1.00  0.00
H    H
ATOM  25051  CA  PHE H  71      30.110  -9.663  70.306  1.00  0.00
H    C
ATOM  25052  HA  PHE H  71      30.115 -10.551  70.926  1.00  0.00
H    H
ATOM  25053  CB  PHE H  71      28.896  -9.597  69.391  1.00  0.00
H    C
ATOM  25054  HB1 PHE H  71      28.929  -8.649  68.811  1.00  0.00
H    H
ATOM  25055  HB2 PHE H  71      28.902 -10.430  68.657  1.00  0.00
H    H
```

```
ATOM  25056  CG  PHE H  71      27.599  -9.591  70.116  1.00  0.00
H    C
ATOM  25057  CD1 PHE H  71      27.170  -8.456  70.779  1.00  0.00
H    C
ATOM  25058  HD1 PHE H  71      27.789  -7.569  70.776  1.00  0.00
H    H
ATOM  25059  CE1 PHE H  71      25.978  -8.443  71.440  1.00  0.00
H    C
ATOM  25060  HE1 PHE H  71      25.654  -7.549  71.954  1.00  0.00
H    H
ATOM  25061  CZ  PHE H  71      25.185  -9.573  71.446  1.00  0.00
H    C
ATOM  25062  HZ  PHE H  71      24.236  -9.563  71.963  1.00  0.00
H    H
ATOM  25063  CD2 PHE H  71      26.799 -10.717  70.123  1.00  0.00
H    C
ATOM  25064  HD2 PHE H  71      27.129 -11.618  69.624  1.00  0.00
H    H
ATOM  25065  CE2 PHE H  71      25.598 -10.712  70.782  1.00  0.00
H    C
ATOM  25066  HE2 PHE H  71      24.980 -11.600  70.782  1.00  0.00
H    H
ATOM  25067  C   PHE H  71      31.376  -9.694  69.472  1.00  0.00
H    C
ATOM  25068  O   PHE H  71      32.024  -8.661  69.276  1.00  0.00
H    O
ATOM  25069  N   GLU H  72      31.719 -10.881  68.976  1.00  0.00
H    N
ATOM  25070  HN  GLU H  72      31.234 -11.736  69.132  1.00  0.00
H    H
ATOM  25071  CA  GLU H  72      32.894 -11.013  68.125  1.00  0.00
H    C
ATOM  25072  HA  GLU H  72      33.362 -10.040  68.043  1.00  0.00
H    H
ATOM  25073  CB  GLU H  72      33.853 -12.012  68.745  1.00  0.00
H    C
ATOM  25074  HB1 GLU H  72      33.998 -12.848  68.023  1.00  0.00
H    H
ATOM  25075  HB2 GLU H  72      34.854 -11.543  68.874  1.00  0.00
H    H
ATOM  25076  CG  GLU H  72      33.348 -12.556  70.057  1.00  0.00
H    C
ATOM  25077  HG1 GLU H  72      33.025 -11.720  70.704  1.00  0.00
H    H
ATOM  25078  HG2 GLU H  72      32.495 -13.245  69.899  1.00  0.00
H    H
ATOM  25079  CD  GLU H  72      34.422 -13.282  70.824  1.00  0.00
H    C
ATOM  25080  OE1 GLU H  72      35.079 -14.168  70.239  1.00  0.00
H    O
ATOM  25081  OE2 GLU H  72      34.607 -12.964  72.019  1.00  0.00
H    O
ATOM  25082  C   GLU H  72      32.508 -11.448  66.721  1.00  0.00
H    C
```

```
ATOM  25083  O   GLU H  72      33.123 -11.016  65.737  1.00  0.00
H    O
ATOM  25084  N   TYR H  73      31.496 -12.305  66.604  1.00  0.00
H    N
ATOM  25085  HN  TYR H  73      31.003 -12.690  67.383  1.00  0.00
H    H
ATOM  25086  CA  TYR H  73      31.020 -12.757  65.304  1.00  0.00
H    C
ATOM  25087  HA  TYR H  73      31.732 -12.420  64.561  1.00  0.00
H    H
ATOM  25088  CB  TYR H  73      30.828 -14.270  65.262  1.00  0.00
H    C
ATOM  25089  HB1 TYR H  73      30.296 -14.595  66.183  1.00  0.00
H    H
ATOM  25090  HB2 TYR H  73      30.197 -14.563  64.396  1.00  0.00
H    H
ATOM  25091  CG  TYR H  73      32.096 -15.077  65.216  1.00  0.00
H    C
ATOM  25092  CD1 TYR H  73      32.934 -15.155  66.316  1.00  0.00
H    C
ATOM  25093  HD1 TYR H  73      32.691 -14.609  67.217  1.00  0.00
H    H
ATOM  25094  CE1 TYR H  73      34.086 -15.902  66.277  1.00  0.00
H    C
ATOM  25095  HE1 TYR H  73      34.735 -15.956  67.139  1.00  0.00
H    H
ATOM  25096  CZ  TYR H  73      34.413 -16.578  65.126  1.00  0.00
H    C
ATOM  25097  OH  TYR H  73      35.562 -17.325  65.067  1.00  0.00
H    O
ATOM  25098  HH  TYR H  73      35.574 -17.770  64.218  1.00  0.00
H    H
ATOM  25099  CD2 TYR H  73      32.449 -15.773  64.074  1.00  0.00
H    C
ATOM  25100  HD2 TYR H  73      31.833 -15.702  63.187  1.00  0.00
H    H
ATOM  25101  CE2 TYR H  73      33.597 -16.517  64.026  1.00  0.00
H    C
ATOM  25102  HE2 TYR H  73      33.863 -17.033  63.116  1.00  0.00
H    H
ATOM  25103  C   TYR H  73      29.700 -12.074  64.999  1.00  0.00
H    C
ATOM  25104  O   TYR H  73      28.805 -12.045  65.849  1.00  0.00
H    O
ATOM  25105  N   VAL H  74      29.567 -11.548  63.786  1.00  0.00
H    N
ATOM  25106  HN  VAL H  74      30.310 -11.516  63.121  1.00  0.00
H    H
ATOM  25107  CA  VAL H  74      28.317 -10.970  63.319  1.00  0.00
H    C
ATOM  25108  HA  VAL H  74      27.519 -11.298  63.972  1.00  0.00
H    H
ATOM  25109  CB  VAL H  74      28.357  -9.431  63.312  1.00  0.00
H    C
```

ATOM 25110 HB VAL H 74   27.351 -9.069 62.986 1.00 0.00
H   H
ATOM 25111 CG1 VAL H 74   28.651 -8.910 64.701 1.00 0.00
H   C
ATOM 25112 HG11 VAL H 74   28.719 -7.800 64.691 1.00 0.00
H   H
ATOM 25113 HG12 VAL H 74   27.853 -9.186 65.420 1.00 0.00
H   H
ATOM 25114 HG13 VAL H 74   29.619 -9.310 65.073 1.00 0.00
H   H
ATOM 25115 CG2 VAL H 74   29.381 -8.913 62.324 1.00 0.00
H   C
ATOM 25116 HG21 VAL H 74   29.431 -7.803 62.355 1.00 0.00
H   H
ATOM 25117 HG22 VAL H 74   30.391 -9.315 62.561 1.00 0.00
H   H
ATOM 25118 HG23 VAL H 74   29.124 -9.208 61.286 1.00 0.00
H   H
ATOM 25119 C   VAL H 74   28.025 -11.516 61.932 1.00 0.00
H   C
ATOM 25120 O   VAL H 74   28.934 -11.665 61.104 1.00 0.00
H   O
ATOM 25121 N   ILE H 75   26.762 -11.825 61.688 1.00 0.00
H   N
ATOM 25122 HN ILE H 75   26.031 -11.675 62.349 1.00 0.00
H   H
ATOM 25123 CA ILE H 75   26.321 -12.411 60.435 1.00 0.00
H   C
ATOM 25124 HA ILE H 75   27.116 -12.286 59.710 1.00 0.00
H   H
ATOM 25125 CB ILE H 75   25.921 -13.875 60.610 1.00 0.00
H   C
ATOM 25126 HB ILE H 75   24.942 -13.964 60.075 1.00 0.00
H   H
ATOM 25127 CG2 ILE H 75   27.005 -14.763 60.067 1.00 0.00
H   C
ATOM 25128 HG21 ILE H 75   26.669 -15.820 60.042 1.00 0.00
H   H
ATOM 25129 HG22 ILE H 75   27.275 -14.471 59.030 1.00 0.00
H   H
ATOM 25130 HG23 ILE H 75   27.919 -14.705 60.697 1.00 0.00
H   H
ATOM 25131 CG1 ILE H 75   25.662 -14.140 62.087 1.00 0.00
H   C
ATOM 25132 HG11 ILE H 75   26.165 -13.361 62.703 1.00 0.00
H   H
ATOM 25133 HG12 ILE H 75   24.568 -14.058 62.282 1.00 0.00
H   H
ATOM 25134 CD ILE H 75   26.108 -15.480 62.557 1.00 0.00
H   C
ATOM 25135 HD1 ILE H 75   25.837 -15.624 63.625 1.00 0.00
H   H
ATOM 25136 HD2 ILE H 75   25.625 -16.285 61.963 1.00 0.00
H   H

```
ATOM  25137  HD3 ILE H  75      27.209 -15.587  62.466  1.00  0.00
H    H
ATOM  25138  C   ILE H  75      25.148 -11.598  59.942  1.00  0.00
H    C
ATOM  25139  O   ILE H  75      24.069 -11.627  60.540  1.00  0.00
H    O
ATOM  25140  N   VAL H  76      25.352 -10.892  58.846  1.00  0.00
H    N
ATOM  25141  HN  VAL H  76      26.229 -10.846  58.376  1.00  0.00
H    H
ATOM  25142  CA  VAL H  76      24.298 -10.111  58.225  1.00  0.00
H    C
ATOM  25143  HA  VAL H  76      23.555  -9.839  58.964  1.00  0.00
H    H
ATOM  25144  CB  VAL H  76      24.844  -8.849  57.581  1.00  0.00
H    C
ATOM  25145  HB  VAL H  76      25.520  -9.161  56.747  1.00  0.00
H    H
ATOM  25146  CG1 VAL H  76      23.723  -8.031  57.032  1.00  0.00
H    C
ATOM  25147 HG11 VAL H  76      24.113  -7.088  56.594  1.00  0.00
H    H
ATOM  25148 HG12 VAL H  76      23.180  -8.569  56.228  1.00  0.00
H    H
ATOM  25149 HG13 VAL H  76      23.002  -7.765  57.834  1.00  0.00
H    H
ATOM  25150  CG2 VAL H  76      25.608  -8.060  58.608  1.00  0.00
H    C
ATOM  25151 HG21 VAL H  76      25.985  -7.110  58.173  1.00  0.00
H    H
ATOM  25152 HG22 VAL H  76      24.957  -7.814  59.475  1.00  0.00
H    H
ATOM  25153 HG23 VAL H  76      26.485  -8.629  58.982  1.00  0.00
H    H
ATOM  25154  C   VAL H  76      23.630 -11.052  57.229  1.00  0.00
H    C
ATOM  25155  O   VAL H  76      24.062 -11.207  56.082  1.00  0.00
H    O
ATOM  25156  N   ALA H  77      22.587 -11.728  57.700  1.00  0.00
H    N
ATOM  25157  HN  ALA H  77      22.276 -11.619  58.644  1.00  0.00
H    H
ATOM  25158  CA  ALA H  77      21.820 -12.672  56.894  1.00  0.00
H    C
ATOM  25159  HA  ALA H  77      22.424 -13.055  56.079  1.00  0.00
H    H
ATOM  25160  CB  ALA H  77      21.384 -13.860  57.739  1.00  0.00
H    C
ATOM  25161  HB1 ALA H  77      22.279 -14.370  58.156  1.00  0.00
H    H
ATOM  25162  HB2 ALA H  77      20.749 -13.533  58.591  1.00  0.00
H    H
ATOM  25163  HB3 ALA H  77      20.816 -14.603  57.139  1.00  0.00
H    H
```

```
ATOM  25164  C   ALA H  77      20.622 -12.000  56.258  1.00  0.00
H    C
ATOM  25165  O   ALA H  77      19.487 -12.453  56.387  1.00  0.00
H    O
ATOM  25166  N   ILE H  78      20.862 -10.898  55.573  1.00  0.00
H    N
ATOM  25167  HN  ILE H  78      21.758 -10.460  55.546  1.00  0.00
H    H
ATOM  25168  CA  ILE H  78      19.843 -10.218  54.795  1.00  0.00
H    C
ATOM  25169  HA  ILE H  78      18.873 -10.589  55.101  1.00  0.00
H    H
ATOM  25170  CB  ILE H  78      19.959  -8.702  54.948  1.00  0.00
H    C
ATOM  25171  HB  ILE H  78      19.622  -8.500  55.996  1.00  0.00
H    H
ATOM  25172  CG2 ILE H  78      21.339  -8.267  54.610  1.00  0.00
H    C
ATOM  25173 HG21 ILE H  78      21.484  -7.196  54.865  1.00  0.00
H    H
ATOM  25174 HG22 ILE H  78      22.089  -8.853  55.181  1.00  0.00
H    H
ATOM  25175 HG23 ILE H  78      21.543  -8.397  53.526  1.00  0.00
H    H
ATOM  25176  CG1 ILE H  78      18.983  -7.981  54.033  1.00  0.00
H    C
ATOM  25177 HG11 ILE H  78      19.178  -8.267  52.976  1.00  0.00
H    H
ATOM  25178 HG12 ILE H  78      17.948  -8.311  54.278  1.00  0.00
H    H
ATOM  25179  CD  ILE H  78      19.063  -6.496  54.176  1.00  0.00
H    C
ATOM  25180  HD1 ILE H  78      18.298  -6.005  53.536  1.00  0.00
H    H
ATOM  25181  HD2 ILE H  78      18.882  -6.193  55.230  1.00  0.00
H    H
ATOM  25182  HD3 ILE H  78      20.061  -6.118  53.868  1.00  0.00
H    H
ATOM  25183  C   ILE H  78      20.048 -10.657  53.356  1.00  0.00
H    C
ATOM  25184  O   ILE H  78      21.175 -10.967  52.957  1.00  0.00
H    O
ATOM  25185  N   GLY H  79      18.975 -10.729  52.575  1.00  0.00
H    N
ATOM  25186  HN  GLY H  79      18.035 -10.565  52.871  1.00  0.00
H    H
ATOM  25187  CA  GLY H  79      19.142 -11.070  51.176  1.00  0.00
H    C
ATOM  25188  HA1 GLY H  79      18.690 -12.045  51.055  1.00  0.00
H    H
ATOM  25189  HA2 GLY H  79      20.207 -11.047  50.988  1.00  0.00
H    H
ATOM  25190  C   GLY H  79      18.476 -10.122  50.203  1.00  0.00
H    C
```

```
ATOM  25191  O   GLY H  79      18.876 -10.039  49.039  1.00  0.00
H    O
ATOM  25192  N   ALA H  80      17.461  -9.398  50.662  1.00  0.00
H    N
ATOM  25193  HN  ALA H  80      17.161  -9.362  51.614  1.00  0.00
H    H
ATOM  25194  CA  ALA H  80      16.698  -8.575  49.735  1.00  0.00
H    C
ATOM  25195  HA  ALA H  80      16.796  -8.971  48.731  1.00  0.00
H    H
ATOM  25196  CB  ALA H  80      15.215  -8.604  50.109  1.00  0.00
H    C
ATOM  25197  HB1 ALA H  80      14.850  -9.654  50.104  1.00  0.00
H    H
ATOM  25198  HB2 ALA H  80      15.053  -8.191  51.128  1.00  0.00
H    H
ATOM  25199  HB3 ALA H  80      14.600  -8.023  49.389  1.00  0.00
H    H
ATOM  25200  C   ALA H  80      17.180  -7.133  49.675  1.00  0.00
H    C
ATOM  25201  O   ALA H  80      17.583  -6.655  48.612  1.00  0.00
H    O
ATOM  25202  N   ASN H  81      17.151  -6.428  50.793  1.00  0.00
H    N
ATOM  25203  HN  ASN H  81      16.995  -6.829  51.695  1.00  0.00
H    H
ATOM  25204  CA  ASN H  81      17.347  -4.987  50.800  1.00  0.00
H    C
ATOM  25205  HA  ASN H  81      16.942  -4.611  49.867  1.00  0.00
H    H
ATOM  25206  CB  ASN H  81      16.655  -4.386  52.015  1.00  0.00
H    C
ATOM  25207  HB1 ASN H  81      15.598  -4.731  52.010  1.00  0.00
H    H
ATOM  25208  HB2 ASN H  81      17.122  -4.759  52.950  1.00  0.00
H    H
ATOM  25209  CG  ASN H  81      16.656  -2.894  51.995  1.00  0.00
H    C
ATOM  25210  OD1 ASN H  81      17.575  -2.270  51.478  1.00  0.00
H    O
ATOM  25211  ND2 ASN H  81      15.619  -2.301  52.568  1.00  0.00
H    N
ATOM  25212 HD21 ASN H  81      15.597  -1.306  52.555  1.00  0.00
H    H
ATOM  25213 HD22 ASN H  81      14.889  -2.848  52.964  1.00  0.00
H    H
ATOM  25214  C   ASN H  81      18.840  -4.689  50.809  1.00  0.00
H    C
ATOM  25215  O   ASN H  81      19.429  -4.499  51.875  1.00  0.00
H    O
ATOM  25216  N   ILE H  82      19.441  -4.601  49.622  1.00  0.00
H    N
ATOM  25217  HN  ILE H  82      18.981  -4.650  48.738  1.00  0.00
H    H
```

```
ATOM  25218  CA  ILE H  82      20.887  -4.416  49.536  1.00  0.00
H    C
ATOM  25219  HA  ILE H  82      21.337  -5.294  49.982  1.00  0.00
H    H
ATOM  25220  CB  ILE H  82      21.323  -4.274  48.069  1.00  0.00
H    C
ATOM  25221  HB  ILE H  82      20.579  -3.564  47.624  1.00  0.00
H    H
ATOM  25222  CG2 ILE H  82      22.756  -3.801  47.976  1.00  0.00
H    C
ATOM  25223 HG21 ILE H  82      23.018  -3.550  46.927  1.00  0.00
H    H
ATOM  25224 HG22 ILE H  82      22.913  -2.889  48.590  1.00  0.00
H    H
ATOM  25225 HG23 ILE H  82      23.458  -4.586  48.330  1.00  0.00
H    H
ATOM  25226  CG1 ILE H  82      21.160  -5.603  47.341  1.00  0.00
H    C
ATOM  25227 HG11 ILE H  82      21.955  -5.710  46.569  1.00  0.00
H    H
ATOM  25228 HG12 ILE H  82      21.297  -6.433  48.072  1.00  0.00
H    H
ATOM  25229  CD  ILE H  82      19.830  -5.778  46.689  1.00  0.00
H    C
ATOM  25230  HD1 ILE H  82      19.756  -6.787  46.228  1.00  0.00
H    H
ATOM  25231  HD2 ILE H  82      19.012  -5.676  47.433  1.00  0.00
H    H
ATOM  25232  HD3 ILE H  82      19.676  -5.024  45.889  1.00  0.00
H    H
ATOM  25233  C   ILE H  82      21.330  -3.224  50.368  1.00  0.00
H    C
ATOM  25234  O   ILE H  82      22.392  -3.253  51.008  1.00  0.00
H    O
ATOM  25235  N   GLN H  83      20.512  -2.173  50.393  1.00  0.00
H    N
ATOM  25236  HN  GLN H  83      19.655  -2.112  49.883  1.00  0.00
H    H
ATOM  25237  CA  GLN H  83      20.821  -0.998  51.193  1.00  0.00
H    C
ATOM  25238  HA  GLN H  83      21.665  -0.508  50.723  1.00  0.00
H    H
ATOM  25239  CB  GLN H  83      19.596  -0.104  51.267  1.00  0.00
H    C
ATOM  25240  HB1 GLN H  83      18.822  -0.564  50.609  1.00  0.00
H    H
ATOM  25241  HB2 GLN H  83      19.182  -0.131  52.300  1.00  0.00
H    H
ATOM  25242  CG  GLN H  83      19.797   1.297  50.846  1.00  0.00
H    C
ATOM  25243  HG1 GLN H  83      20.732   1.703  51.287  1.00  0.00
H    H
ATOM  25244  HG2 GLN H  83      19.883   1.367  49.742  1.00  0.00
H    H
```

ATOM 25245 CD GLN H 83 18.660 2.159 51.326 1.00 0.00
H C
ATOM 25246 OE1 GLN H 83 18.220 2.031 52.462 1.00 0.00
H O
ATOM 25247 NE2 GLN H 83 18.156 3.019 50.460 1.00 0.00
H N
ATOM 25248 HE21 GLN H 83 17.407 3.592 50.778 1.00 0.00
H H
ATOM 25249 HE22 GLN H 83 18.516 3.057 49.534 1.00 0.00
H H
ATOM 25250 C GLN H 83 21.241 −1.386 52.598 1.00 0.00
H C
ATOM 25251 O GLN H 83 22.389 −1.159 53.004 1.00 0.00
H O
ATOM 25252 N ALA H 84 20.327 −2.004 53.335 1.00 0.00
H N
ATOM 25253 HN ALA H 84 19.428 −2.281 52.997 1.00 0.00
H H
ATOM 25254 CA ALA H 84 20.596 −2.324 54.726 1.00 0.00
H C
ATOM 25255 HA ALA H 84 20.790 −1.422 55.296 1.00 0.00
H H
ATOM 25256 CB ALA H 84 19.386 −3.020 55.342 1.00 0.00
H C
ATOM 25257 HB1 ALA H 84 18.501 −2.350 55.283 1.00 0.00
H H
ATOM 25258 HB2 ALA H 84 19.144 −3.957 54.795 1.00 0.00
H H
ATOM 25259 HB3 ALA H 84 19.558 −3.268 56.412 1.00 0.00
H H
ATOM 25260 C ALA H 84 21.836 −3.187 54.852 1.00 0.00
H C
ATOM 25261 O ALA H 84 22.707 −2.916 55.682 1.00 0.00
H O
ATOM 25262 N SER H 85 21.930 −4.231 54.033 1.00 0.00
H N
ATOM 25263 HN SER H 85 21.232 −4.513 53.375 1.00 0.00
H H
ATOM 25264 CA SER H 85 23.116 −5.073 54.040 1.00 0.00
H C
ATOM 25265 HA SER H 85 23.086 −5.631 54.967 1.00 0.00
H H
ATOM 25266 CB SER H 85 23.119 −5.969 52.807 1.00 0.00
H C
ATOM 25267 HB1 SER H 85 23.730 −6.871 53.036 1.00 0.00
H H
ATOM 25268 HB2 SER H 85 22.086 −6.320 52.583 1.00 0.00
H H
ATOM 25269 OG SER H 85 23.690 −5.285 51.709 1.00 0.00
H O
ATOM 25270 HG1 SER H 85 23.875 −5.946 51.029 1.00 0.00
H H
ATOM 25271 C SER H 85 24.378 −4.227 54.063 1.00 0.00
H C

```
ATOM  25272  O   SER H  85      25.222  -4.352  54.961  1.00  0.00
H    O
ATOM  25273  N   THR H  86      24.497  -3.323  53.101  1.00  0.00
H    N
ATOM  25274  HN  THR H  86      23.783  -3.044  52.459  1.00  0.00
H    H
ATOM  25275  CA  THR H  86      25.766  -2.639  52.916  1.00  0.00
H    C
ATOM  25276  HA  THR H  86      26.524  -3.405  53.004  1.00  0.00
H    H
ATOM  25277  CB  THR H  86      25.787  -1.967  51.554  1.00  0.00
H    C
ATOM  25278  HB  THR H  86      26.805  -1.516  51.446  1.00  0.00
H    H
ATOM  25279  OG1 THR H  86      24.826  -0.908  51.539  1.00  0.00
H    O
ATOM  25280  HG1 THR H  86      24.876  -0.529  50.655  1.00  0.00
H    H
ATOM  25281  CG2 THR H  86      25.456  -2.974  50.478  1.00  0.00
H    C
ATOM  25282 HG21 THR H  86      25.578  -2.528  49.469  1.00  0.00
H    H
ATOM  25283 HG22 THR H  86      26.133  -3.852  50.548  1.00  0.00
H    H
ATOM  25284 HG23 THR H  86      24.409  -3.334  50.576  1.00  0.00
H    H
ATOM  25285  C   THR H  86      26.036  -1.633  54.025  1.00  0.00
H    C
ATOM  25286  O   THR H  86      27.134  -1.618  54.595  1.00  0.00
H    O
ATOM  25287  N   LEU H  87      25.065  -0.772  54.342  1.00  0.00
H    N
ATOM  25288  HN  LEU H  87      24.161  -0.704  53.924  1.00  0.00
H    H
ATOM  25289  CA  LEU H  87      25.309   0.196  55.411  1.00  0.00
H    C
ATOM  25290  HA  LEU H  87      26.205   0.741  55.144  1.00  0.00
H    H
ATOM  25291  CB  LEU H  87      24.156   1.188  55.571  1.00  0.00
H    C
ATOM  25292  HB1 LEU H  87      23.410   0.719  56.253  1.00  0.00
H    H
ATOM  25293  HB2 LEU H  87      24.528   2.094  56.098  1.00  0.00
H    H
ATOM  25294  CG  LEU H  87      23.205   1.830  54.554  1.00  0.00
H    C
ATOM  25295  HG  LEU H  87      23.360   2.923  54.723  1.00  0.00
H    H
ATOM  25296  CD1 LEU H  87      23.434   1.498  53.096  1.00  0.00
H    C
ATOM  25297 HD11 LEU H  87      22.660   1.978  52.460  1.00  0.00
H    H
ATOM  25298 HD12 LEU H  87      24.432   1.859  52.764  1.00  0.00
H    H
```

```
ATOM  25299 HD13 LEU H  87      23.386   0.400  52.931  1.00  0.00
H    H
ATOM  25300  CD2 LEU H  87      21.767   1.488  54.956  1.00  0.00
H    C
ATOM  25301 HD21 LEU H  87      21.048   1.954  54.248  1.00  0.00
H    H
ATOM  25302 HD22 LEU H  87      21.589   0.394  54.946  1.00  0.00
H    H
ATOM  25303 HD23 LEU H  87      21.547   1.868  55.977  1.00  0.00
H    H
ATOM  25304  C   LEU H  87      25.578  -0.513  56.726  1.00  0.00
H    C
ATOM  25305  O   LEU H  87      26.398  -0.064  57.538  1.00  0.00
H    O
ATOM  25306  N   THR H  88      24.897  -1.629  56.950  1.00  0.00
H    N
ATOM  25307  HN  THR H  88      24.156  -1.968  56.371  1.00  0.00
H    H
ATOM  25308  CA  THR H  88      25.189  -2.466  58.094  1.00  0.00
H    C
ATOM  25309  HA  THR H  88      24.988  -1.861  58.968  1.00  0.00
H    H
ATOM  25310  CB  THR H  88      24.360  -3.730  57.978  1.00  0.00
H    C
ATOM  25311  HB  THR H  88      24.676  -4.222  57.025  1.00  0.00
H    H
ATOM  25312  OG1 THR H  88      22.981  -3.370  57.863  1.00  0.00
H    O
ATOM  25313  HG1 THR H  88      22.503  -4.204  57.805  1.00  0.00
H    H
ATOM  25314  CG2 THR H  88      24.577  -4.606  59.173  1.00  0.00
H    C
ATOM  25315 HG21 THR H  88      24.064  -5.583  59.045  1.00  0.00
H    H
ATOM  25316 HG22 THR H  88      25.660  -4.807  59.316  1.00  0.00
H    H
ATOM  25317 HG23 THR H  88      24.187  -4.128  60.097  1.00  0.00
H    H
ATOM  25318  C   THR H  88      26.661  -2.810  58.139  1.00  0.00
H    C
ATOM  25319  O   THR H  88      27.375  -2.429  59.076  1.00  0.00
H    O
ATOM  25320  N   THR H  89      27.132  -3.493  57.098  1.00  0.00
H    N
ATOM  25321  HN  THR H  89      26.588  -3.783  56.312  1.00  0.00
H    H
ATOM  25322  CA  THR H  89      28.535  -3.878  57.031  1.00  0.00
H    C
ATOM  25323  HA  THR H  89      28.693  -4.566  57.850  1.00  0.00
H    H
ATOM  25324  CB  THR H  89      28.840  -4.467  55.666  1.00  0.00
H    C
ATOM  25325  HB  THR H  89      29.951  -4.565  55.613  1.00  0.00
H    H
```

```
ATOM  25326  OG1 THR H  89      28.417  -3.534  54.665  1.00  0.00
H    O
ATOM  25327  HG1 THR H  89      28.619  -3.953  53.822  1.00  0.00
H    H
ATOM  25328  CG2 THR H  89      28.094  -5.761  55.488  1.00  0.00
H    C
ATOM  25329 HG21 THR H  89      28.392  -6.262  54.544  1.00  0.00
H    H
ATOM  25330 HG22 THR H  89      28.315  -6.454  56.328  1.00  0.00
H    H
ATOM  25331 HG23 THR H  89      26.996  -5.590  55.459  1.00  0.00
H    H
ATOM  25332  C   THR H  89      29.441  -2.690  57.284  1.00  0.00
H    C
ATOM  25333  O   THR H  89      30.479  -2.819  57.939  1.00  0.00
H    O
ATOM  25334  N   LEU H  90      29.071  -1.530  56.753  1.00  0.00
H    N
ATOM  25335  HN  LEU H  90      28.287  -1.387  56.152  1.00  0.00
H    H
ATOM  25336  CA  LEU H  90      29.837  -0.327  57.033  1.00  0.00
H    C
ATOM  25337  HA  LEU H  90      30.834  -0.491  56.644  1.00  0.00
H    H
ATOM  25338  CB  LEU H  90      29.181   0.878  56.377  1.00  0.00
H    C
ATOM  25339  HB1 LEU H  90      29.468   0.863  55.300  1.00  0.00
H    H
ATOM  25340  HB2 LEU H  90      28.076   0.748  56.402  1.00  0.00
H    H
ATOM  25341  CG  LEU H  90      29.590   2.216  56.988  1.00  0.00
H    C
ATOM  25342  HG  LEU H  90      29.203   2.255  58.035  1.00  0.00
H    H
ATOM  25343  CD1 LEU H  90      31.091   2.402  56.980  1.00  0.00
H    C
ATOM  25344 HD11 LEU H  90      31.362   3.400  57.385  1.00  0.00
H    H
ATOM  25345 HD12 LEU H  90      31.585   1.626  57.604  1.00  0.00
H    H
ATOM  25346 HD13 LEU H  90      31.490   2.327  55.947  1.00  0.00
H    H
ATOM  25347  CD2 LEU H  90      28.942   3.318  56.214  1.00  0.00
H    C
ATOM  25348 HD21 LEU H  90      29.209   4.304  56.651  1.00  0.00
H    H
ATOM  25349 HD22 LEU H  90      29.267   3.320  55.154  1.00  0.00
H    H
ATOM  25350 HD23 LEU H  90      27.836   3.213  56.241  1.00  0.00
H    H
ATOM  25351  C   LEU H  90      29.959  -0.102  58.528  1.00  0.00
H    C
ATOM  25352  O   LEU H  90      31.064   0.053  59.055  1.00  0.00
H    O
```

ATOM 25353 N LEU H 91 28.830 -0.059 59.222 1.00 0.00
H N
ATOM 25354 HN LEU H 91 27.913 -0.218 58.861 1.00 0.00
H H
ATOM 25355 CA LEU H 91 28.868 0.244 60.647 1.00 0.00
H C
ATOM 25356 HA LEU H 91 29.366 1.199 60.752 1.00 0.00
H H
ATOM 25357 CB LEU H 91 27.456 0.318 61.209 1.00 0.00
H C
ATOM 25358 HB1 LEU H 91 26.831 -0.395 60.624 1.00 0.00
H H
ATOM 25359 HB2 LEU H 91 27.461 -0.064 62.254 1.00 0.00
H H
ATOM 25360 CG LEU H 91 26.855 1.718 61.126 1.00 0.00
H C
ATOM 25361 HG LEU H 91 25.806 1.664 61.504 1.00 0.00
H H
ATOM 25362 CD1 LEU H 91 27.661 2.670 61.988 1.00 0.00
H C
ATOM 25363 HD11 LEU H 91 27.265 3.705 61.901 1.00 0.00
H H
ATOM 25364 HD12 LEU H 91 27.613 2.367 63.057 1.00 0.00
H H
ATOM 25365 HD13 LEU H 91 28.726 2.679 61.673 1.00 0.00
H H
ATOM 25366 CD2 LEU H 91 26.814 2.207 59.696 1.00 0.00
H C
ATOM 25367 HD21 LEU H 91 26.357 3.219 59.647 1.00 0.00
H H
ATOM 25368 HD22 LEU H 91 27.829 2.274 59.256 1.00 0.00
H H
ATOM 25369 HD23 LEU H 91 26.205 1.521 59.067 1.00 0.00
H H
ATOM 25370 C LEU H 91 29.691 -0.786 61.397 1.00 0.00
H C
ATOM 25371 O LEU H 91 30.476 -0.449 62.291 1.00 0.00
H O
ATOM 25372 N LEU H 92 29.536 -2.051 61.030 1.00 0.00
H N
ATOM 25373 HN LEU H 92 28.956 -2.381 60.287 1.00 0.00
H H
ATOM 25374 CA LEU H 92 30.252 -3.099 61.741 1.00 0.00
H C
ATOM 25375 HA LEU H 92 30.063 -2.943 62.795 1.00 0.00
H H
ATOM 25376 CB LEU H 92 29.737 -4.468 61.315 1.00 0.00
H C
ATOM 25377 HB1 LEU H 92 29.526 -4.415 60.222 1.00 0.00
H H
ATOM 25378 HB2 LEU H 92 30.553 -5.216 61.434 1.00 0.00
H H
ATOM 25379 CG LEU H 92 28.478 -4.893 62.058 1.00 0.00
H C

ATOM 25380 HG LEU H 92 28.347 -5.994 61.933 1.00 0.00
H H
ATOM 25381 CD1 LEU H 92 28.641 -4.557 63.523 1.00 0.00
H C
ATOM 25382 HD11 LEU H 92 27.718 -4.812 64.086 1.00 0.00
H H
ATOM 25383 HD12 LEU H 92 29.487 -5.128 63.963 1.00 0.00
H H
ATOM 25384 HD13 LEU H 92 28.842 -3.474 63.657 1.00 0.00
H H
ATOM 25385 CD2 LEU H 92 27.256 -4.213 61.506 1.00 0.00
H C
ATOM 25386 HD21 LEU H 92 26.348 -4.551 62.050 1.00 0.00
H H
ATOM 25387 HD22 LEU H 92 27.319 -3.111 61.608 1.00 0.00
H H
ATOM 25388 HD23 LEU H 92 27.128 -4.459 60.429 1.00 0.00
H H
ATOM 25389 C LEU H 92 31.752 -2.994 61.522 1.00 0.00
H C
ATOM 25390 O LEU H 92 32.526 -2.957 62.487 1.00 0.00
H O
ATOM 25391 N LYS H 93 32.184 -2.932 60.266 1.00 0.00
H N
ATOM 25392 HN LYS H 93 31.600 -2.965 59.456 1.00 0.00
H H
ATOM 25393 CA LYS H 93 33.608 -2.806 59.995 1.00 0.00
H C
ATOM 25394 HA LYS H 93 34.104 -3.632 60.489 1.00 0.00
H H
ATOM 25395 CB LYS H 93 33.860 -2.853 58.493 1.00 0.00
H C
ATOM 25396 HB1 LYS H 93 33.054 -3.477 58.045 1.00 0.00
H H
ATOM 25397 HB2 LYS H 93 33.734 -1.830 58.072 1.00 0.00
H H
ATOM 25398 CG LYS H 93 35.207 -3.432 58.124 1.00 0.00
H C
ATOM 25399 HG1 LYS H 93 35.254 -3.572 57.020 1.00 0.00
H H
ATOM 25400 HG2 LYS H 93 36.003 -2.702 58.400 1.00 0.00
H H
ATOM 25401 CD LYS H 93 35.430 -4.761 58.815 1.00 0.00
H C
ATOM 25402 HD1 LYS H 93 35.361 -4.601 59.916 1.00 0.00
H H
ATOM 25403 HD2 LYS H 93 34.600 -5.446 58.526 1.00 0.00
H H
ATOM 25404 CE LYS H 93 36.772 -5.362 58.437 1.00 0.00
H C
ATOM 25405 HE1 LYS H 93 36.763 -5.698 57.376 1.00 0.00
H H
ATOM 25406 HE2 LYS H 93 37.584 -4.614 58.563 1.00 0.00
H H

ATOM 25407 NZ LYS H 93 37.115 -6.543 59.279 1.00 0.00
H N
ATOM 25408 HZ1 LYS H 93 38.038 -6.929 58.992 1.00 0.00
H H
ATOM 25409 HZ2 LYS H 93 37.156 -6.257 60.278 1.00 0.00
H H
ATOM 25410 HZ3 LYS H 93 36.384 -7.275 59.162 1.00 0.00
H H
ATOM 25411 C LYS H 93 34.161 -1.524 60.591 1.00 0.00
H C
ATOM 25412 O LYS H 93 35.334 -1.462 60.972 1.00 0.00
H O
ATOM 25413 N GLU H 94 33.329 -0.491 60.686 1.00 0.00
H N
ATOM 25414 HN GLU H 94 32.407 -0.444 60.312 1.00 0.00
H H
ATOM 25415 CA GLU H 94 33.744 0.712 61.388 1.00 0.00
H C
ATOM 25416 HA GLU H 94 34.722 0.998 61.022 1.00 0.00
H H
ATOM 25417 CB GLU H 94 32.750 1.838 61.134 1.00 0.00
H C
ATOM 25418 HB1 GLU H 94 32.912 2.217 60.099 1.00 0.00
H H
ATOM 25419 HB2 GLU H 94 31.712 1.437 61.154 1.00 0.00
H H
ATOM 25420 CG GLU H 94 32.885 2.983 62.110 1.00 0.00
H C
ATOM 25421 HG1 GLU H 94 32.425 2.702 63.076 1.00 0.00
H H
ATOM 25422 HG2 GLU H 94 33.950 3.242 62.275 1.00 0.00
H H
ATOM 25423 CD GLU H 94 32.154 4.217 61.650 1.00 0.00
H C
ATOM 25424 OE1 GLU H 94 32.054 4.422 60.423 1.00 0.00
H O
ATOM 25425 OE2 GLU H 94 31.682 4.987 62.511 1.00 0.00
H O
ATOM 25426 C GLU H 94 33.879 0.451 62.875 1.00 0.00
H C
ATOM 25427 O GLU H 94 34.752 1.024 63.534 1.00 0.00
H O
ATOM 25428 N LEU H 95 33.031 -0.409 63.424 1.00 0.00
H N
ATOM 25429 HN LEU H 95 32.297 -0.873 62.933 1.00 0.00
H H
ATOM 25430 CA LEU H 95 33.115 -0.748 64.833 1.00 0.00
H C
ATOM 25431 HA LEU H 95 33.598 0.092 65.315 1.00 0.00
H H
ATOM 25432 CB LEU H 95 31.721 -0.927 65.417 1.00 0.00
H C
ATOM 25433 HB1 LEU H 95 31.054 -1.247 64.584 1.00 0.00
H H

ATOM 25434 HB2 LEU H 95 31.738 -1.773 66.140 1.00 0.00
H H
ATOM 25435 CG LEU H 95 31.286 0.406 66.021 1.00 0.00
H C
ATOM 25436 HG LEU H 95 32.160 0.772 66.612 1.00 0.00
H H
ATOM 25437 CD1 LEU H 95 30.948 1.415 64.939 1.00 0.00
H C
ATOM 25438 HD11 LEU H 95 30.587 2.364 65.390 1.00 0.00
H H
ATOM 25439 HD12 LEU H 95 31.844 1.640 64.322 1.00 0.00
H H
ATOM 25440 HD13 LEU H 95 30.151 1.023 64.273 1.00 0.00
H H
ATOM 25441 CD2 LEU H 95 30.127 0.226 66.968 1.00 0.00
H C
ATOM 25442 HD21 LEU H 95 29.835 1.203 67.412 1.00 0.00
H H
ATOM 25443 HD22 LEU H 95 29.236 -0.187 66.452 1.00 0.00
H H
ATOM 25444 HD23 LEU H 95 30.403 -0.463 67.795 1.00 0.00
H H
ATOM 25445 C LEU H 95 33.968 -1.975 65.090 1.00 0.00
H C
ATOM 25446 O LEU H 95 33.794 -2.635 66.117 1.00 0.00
H O
ATOM 25447 N ASP H 96 34.865 -2.305 64.165 1.00 0.00
H N
ATOM 25448 HN ASP H 96 34.817 -2.024 63.210 1.00 0.00
H H
ATOM 25449 CA ASP H 96 36.032 -3.126 64.458 1.00 0.00
H C
ATOM 25450 HA ASP H 96 36.584 -3.219 63.530 1.00 0.00
H H
ATOM 25451 CB ASP H 96 36.884 -2.468 65.537 1.00 0.00
H C
ATOM 25452 HB1 ASP H 96 36.420 -2.647 66.527 1.00 0.00
H H
ATOM 25453 HB2 ASP H 96 37.904 -2.899 65.546 1.00 0.00
H H
ATOM 25454 CG ASP H 96 36.988 -0.982 65.344 1.00 0.00
H C
ATOM 25455 OD1 ASP H 96 36.933 -0.540 64.179 1.00 0.00
H O
ATOM 25456 OD2 ASP H 96 37.098 -0.255 66.352 1.00 0.00
H O
ATOM 25457 C ASP H 96 35.653 -4.531 64.885 1.00 0.00
H C
ATOM 25458 O ASP H 96 36.463 -5.252 65.468 1.00 0.00
H O
ATOM 25459 N ILE H 97 34.421 -4.927 64.606 1.00 0.00
H N
ATOM 25460 HN ILE H 97 33.711 -4.405 64.139 1.00 0.00
H H

ATOM 25461 CA ILE H 97   34.010 -6.270 65.007 1.00 0.00
H   C
ATOM 25462 HA ILE H 97   34.221 -6.369 66.064 1.00 0.00
H   H
ATOM 25463 CB ILE H 97   32.528 -6.482 64.701 1.00 0.00
H   C
ATOM 25464 HB ILE H 97   32.433 -6.258 63.609 1.00 0.00
H   H
ATOM 25465 CG2 ILE H 97   32.090 -7.875 65.099 1.00 0.00
H   C
ATOM 25466 HG21 ILE H 97   31.061 -8.080 64.736 1.00 0.00
H   H
ATOM 25467 HG22 ILE H 97   32.762 -8.642 64.660 1.00 0.00
H   H
ATOM 25468 HG23 ILE H 97   32.098 -7.994 66.203 1.00 0.00
H   H
ATOM 25469 CG1 ILE H 97   31.721 -5.433 65.457 1.00 0.00
H   C
ATOM 25470 HG11 ILE H 97   31.984 -4.417 65.087 1.00 0.00
H   H
ATOM 25471 HG12 ILE H 97   30.639 -5.590 65.246 1.00 0.00
H   H
ATOM 25472 CD ILE H 97   31.930 -5.492 66.948 1.00 0.00
H   C
ATOM 25473 HD1 ILE H 97   31.280 -4.750 67.459 1.00 0.00
H   H
ATOM 25474 HD2 ILE H 97   31.680 -6.501 67.339 1.00 0.00
H   H
ATOM 25475 HD3 ILE H 97   32.985 -5.263 67.212 1.00 0.00
H   H
ATOM 25476 C   ILE H 97   34.880 -7.287 64.288 1.00 0.00
H   C
ATOM 25477 O   ILE H 97   35.166 -7.119 63.088 1.00 0.00
H   O
ATOM 25478 N   PRO H 98   35.358 -8.325 64.969 1.00 0.00
H   N
ATOM 25479 CD PRO H 98   35.134 -8.665 66.382 1.00 0.00
H   C
ATOM 25480 HD1 PRO H 98   35.518 -7.644 66.152 1.00 0.00
H   H
ATOM 25481 HD2 PRO H 98   34.426 -8.629 67.242 1.00 0.00
H   H
ATOM 25482 CA PRO H 98   36.322 -9.225 64.323 1.00 0.00
H   C
ATOM 25483 HA PRO H 98   37.220 -8.674 64.068 1.00 0.00
H   H
ATOM 25484 CB PRO H 98   36.628 -10.247 65.424 1.00 0.00
H   C
ATOM 25485 HB1 PRO H 98   37.431 -9.844 66.081 1.00 0.00
H   H
ATOM 25486 HB2 PRO H 98   36.952 -11.228 65.020 1.00 0.00
H   H
ATOM 25487 CG PRO H 98   36.263 -9.574 66.692 1.00 0.00
H   C

```
ATOM  25488  HG1 PRO H  98      36.788  -9.996  67.571  1.00  0.00
H    H
ATOM  25489  HG2 PRO H  98      35.162  -9.651  66.845  1.00  0.00
H    H
ATOM  25490  C   PRO H  98      35.779  -9.890  63.078  1.00  0.00
H    C
ATOM  25491  O   PRO H  98      36.347  -9.727  61.993  1.00  0.00
H    O
ATOM  25492  N   ASN H  99      34.675 -10.611  63.195  1.00  0.00
H    N
ATOM  25493  HN  ASN H  99      34.094 -10.602  64.008  1.00  0.00
H    H
ATOM  25494  CA  ASN H  99      34.209 -11.489  62.133  1.00  0.00
H    C
ATOM  25495  HA  ASN H  99      34.956 -11.457  61.348  1.00  0.00
H    H
ATOM  25496  CB  ASN H  99      33.990 -12.898  62.669  1.00  0.00
H    C
ATOM  25497  HB1 ASN H  99      33.146 -12.860  63.391  1.00  0.00
H    H
ATOM  25498  HB2 ASN H  99      33.695 -13.581  61.846  1.00  0.00
H    H
ATOM  25499  CG  ASN H  99      35.200 -13.422  63.383  1.00  0.00
H    C
ATOM  25500  OD1 ASN H  99      35.160 -13.668  64.584  1.00  0.00
H    O
ATOM  25501  ND2 ASN H  99      36.296 -13.572  62.656  1.00  0.00
H    N
ATOM  25502 HD21 ASN H  99      37.104 -13.931  63.116  1.00  0.00
H    H
ATOM  25503 HD22 ASN H  99      36.284 -13.359  61.684  1.00  0.00
H    H
ATOM  25504  C   ASN H  99      32.920 -10.930  61.563  1.00  0.00
H    C
ATOM  25505  O   ASN H  99      31.936 -10.772  62.291  1.00  0.00
H    O
ATOM  25506  N   ILE H 100      32.924 -10.640  60.269  1.00  0.00
H    N
ATOM  25507  HN  ILE H 100      33.719 -10.782  59.683  1.00  0.00
H    H
ATOM  25508  CA  ILE H 100      31.769 -10.089  59.582  1.00  0.00
H    C
ATOM  25509  HA  ILE H 100      30.922 -10.142  60.254  1.00  0.00
H    H
ATOM  25510  CB  ILE H 100      32.016  -8.657  59.106  1.00  0.00
H    C
ATOM  25511  HB  ILE H 100      33.038  -8.686  58.651  1.00  0.00
H    H
ATOM  25512  CG2 ILE H 100      30.917  -8.232  58.180  1.00  0.00
H    C
ATOM  25513 HG21 ILE H 100      31.161  -7.258  57.705  1.00  0.00
H    H
ATOM  25514 HG22 ILE H 100      30.773  -8.975  57.368  1.00  0.00
H    H
```

```
ATOM  25515 HG23 ILE H 100      29.958  -8.120  58.727  1.00  0.00
H    H
ATOM  25516  CG1 ILE H 100      32.087  -7.718  60.305  1.00  0.00
H    C
ATOM  25517 HG11 ILE H 100      31.129  -7.754  60.870  1.00  0.00
H    H
ATOM  25518 HG12 ILE H 100      32.889  -8.071  60.993  1.00  0.00
H    H
ATOM  25519  CD  ILE H 100      32.383  -6.294  59.937  1.00  0.00
H    C
ATOM  25520  HD1 ILE H 100      32.479  -5.671  60.852  1.00  0.00
H    H
ATOM  25521  HD2 ILE H 100      33.335  -6.225  59.367  1.00  0.00
H    H
ATOM  25522  HD3 ILE H 100      31.569  -5.866  59.315  1.00  0.00
H    H
ATOM  25523  C   ILE H 100      31.462 -11.010  58.417  1.00  0.00
H    C
ATOM  25524  O   ILE H 100      32.262 -11.129  57.481  1.00  0.00
H    O
ATOM  25525  N   TRP H 101      30.315 -11.674  58.475  1.00  0.00
H    N
ATOM  25526  HN  TRP H 101      29.636 -11.510  59.188  1.00  0.00
H    H
ATOM  25527  CA  TRP H 101      29.954 -12.696  57.504  1.00  0.00
H    C
ATOM  25528  HA  TRP H 101      30.667 -12.662  56.689  1.00  0.00
H    H
ATOM  25529  CB  TRP H 101      29.939 -14.068  58.166  1.00  0.00
H    C
ATOM  25530  HB1 TRP H 101      29.405 -13.971  59.138  1.00  0.00
H    H
ATOM  25531  HB2 TRP H 101      29.353 -14.780  57.546  1.00  0.00
H    H
ATOM  25532  CG  TRP H 101      31.289 -14.564  58.429  1.00  0.00
H    C
ATOM  25533  CD1 TRP H 101      32.343 -13.854  58.900  1.00  0.00
H    C
ATOM  25534  HD1 TRP H 101      32.296 -12.812  59.189  1.00  0.00
H    H
ATOM  25535  NE1 TRP H 101      33.446 -14.652  58.995  1.00  0.00
H    N
ATOM  25536  HE1 TRP H 101      34.324 -14.396  59.338  1.00  0.00
H    H
ATOM  25537  CE2 TRP H 101      33.111 -15.909  58.575  1.00  0.00
H    C
ATOM  25538  CD2 TRP H 101      31.758 -15.884  58.210  1.00  0.00
H    C
ATOM  25539  CE3 TRP H 101      31.163 -17.052  57.742  1.00  0.00
H    C
ATOM  25540  HE3 TRP H 101      30.126 -17.082  57.438  1.00  0.00
H    H
ATOM  25541  CZ3 TRP H 101      31.922 -18.186  57.660  1.00  0.00
H    C
```

```
ATOM  25542  HZ3 TRP H 101      31.474 -19.101  57.301  1.00  0.00
H    H
ATOM  25543  CZ2 TRP H 101      33.879 -17.055  58.488  1.00  0.00
H    C
ATOM  25544  HZ2 TRP H 101      34.920 -17.080  58.769  1.00  0.00
H    H
ATOM  25545  CH2 TRP H 101      33.269 -18.184  58.029  1.00  0.00
H    C
ATOM  25546  HH2 TRP H 101      33.845 -19.096  57.948  1.00  0.00
H    H
ATOM  25547  C   TRP H 101      28.593 -12.363  56.914  1.00  0.00
H    C
ATOM  25548  O   TRP H 101      27.624 -12.180  57.656  1.00  0.00
H    O
ATOM  25549  N   VAL H 102      28.511 -12.292  55.591  1.00  0.00
H    N
ATOM  25550  HN  VAL H 102      29.199 -12.611  54.944  1.00  0.00
H    H
ATOM  25551  CA  VAL H 102      27.331 -11.704  54.974  1.00  0.00
H    C
ATOM  25552  HA  VAL H 102      26.587 -11.578  55.749  1.00  0.00
H    H
ATOM  25553  CB  VAL H 102      27.666 -10.337  54.350  1.00  0.00
H    C
ATOM  25554  HB  VAL H 102      28.364 -10.519  53.495  1.00  0.00
H    H
ATOM  25555  CG1 VAL H 102      26.415  -9.666  53.835  1.00  0.00
H    C
ATOM  25556 HG11 VAL H 102      26.657  -8.664  53.422  1.00  0.00
H    H
ATOM  25557 HG12 VAL H 102      25.940 -10.253  53.022  1.00  0.00
H    H
ATOM  25558 HG13 VAL H 102      25.676  -9.531  54.655  1.00  0.00
H    H
ATOM  25559  CG2 VAL H 102      28.363  -9.455  55.359  1.00  0.00
H    C
ATOM  25560 HG21 VAL H 102      28.572  -8.453  54.927  1.00  0.00
H    H
ATOM  25561 HG22 VAL H 102      27.732  -9.321  56.264  1.00  0.00
H    H
ATOM  25562 HG23 VAL H 102      29.333  -9.893  55.673  1.00  0.00
H    H
ATOM  25563  C   VAL H 102      26.744 -12.638  53.926  1.00  0.00
H    C
ATOM  25564  O   VAL H 102      27.459 -13.397  53.263  1.00  0.00
H    O
ATOM  25565  N   LYS H 103      25.417 -12.576  53.792  1.00  0.00
H    N
ATOM  25566  HN  LYS H 103      24.843 -12.095  54.453  1.00  0.00
H    H
ATOM  25567  CA  LYS H 103      24.698 -13.198  52.685  1.00  0.00
H    C
ATOM  25568  HA  LYS H 103      25.266 -14.062  52.361  1.00  0.00
H    H
```

ATOM 25569 CB LYS H 103 23.289 −13.592 53.130 1.00 0.00
H C
ATOM 25570 HB1 LYS H 103 23.398 −14.421 53.868 1.00 0.00
H H
ATOM 25571 HB2 LYS H 103 22.832 −12.741 53.683 1.00 0.00
H H
ATOM 25572 CG LYS H 103 22.348 −14.057 52.029 1.00 0.00
H C
ATOM 25573 HG1 LYS H 103 22.432 −13.363 51.162 1.00 0.00
H H
ATOM 25574 HG2 LYS H 103 22.669 −15.065 51.680 1.00 0.00
H H
ATOM 25575 CD LYS H 103 20.904 −14.080 52.544 1.00 0.00
H C
ATOM 25576 HD1 LYS H 103 20.849 −14.782 53.407 1.00 0.00
H H
ATOM 25577 HD2 LYS H 103 20.656 −13.062 52.926 1.00 0.00
H H
ATOM 25578 CE LYS H 103 19.892 −14.489 51.470 1.00 0.00
H C
ATOM 25579 HE1 LYS H 103 20.060 −13.909 50.536 1.00 0.00
H H
ATOM 25580 HE2 LYS H 103 19.987 −15.571 51.236 1.00 0.00
H H
ATOM 25581 NZ LYS H 103 18.471 −14.248 51.893 1.00 0.00
H N
ATOM 25582 HZ1 LYS H 103 17.820 −14.537 51.135 1.00 0.00
H H
ATOM 25583 HZ2 LYS H 103 18.267 −14.797 52.753 1.00 0.00
H H
ATOM 25584 HZ3 LYS H 103 18.338 −13.236 52.094 1.00 0.00
H H
ATOM 25585 C LYS H 103 24.640 −12.224 51.513 1.00 0.00
H C
ATOM 25586 O LYS H 103 24.153 −11.101 51.661 1.00 0.00
H O
ATOM 25587 N ALA H 104 25.131 −12.654 50.353 1.00 0.00
H N
ATOM 25588 HN ALA H 104 25.403 −13.605 50.213 1.00 0.00
H H
ATOM 25589 CA ALA H 104 25.308 −11.775 49.204 1.00 0.00
H C
ATOM 25590 HA ALA H 104 25.209 −10.738 49.503 1.00 0.00
H H
ATOM 25591 CB ALA H 104 26.689 −11.960 48.575 1.00 0.00
H C
ATOM 25592 HB1 ALA H 104 27.475 −11.734 49.328 1.00 0.00
H H
ATOM 25593 HB2 ALA H 104 26.835 −13.007 48.232 1.00 0.00
H H
ATOM 25594 HB3 ALA H 104 26.839 −11.279 47.709 1.00 0.00
H H
ATOM 25595 C ALA H 104 24.244 −12.021 48.143 1.00 0.00
H C

ATOM 25596 O   ALA H 104   23.730 -13.131 47.993 1.00 0.00
H   O
ATOM 25597 N   GLN H 105   23.946 -10.968 47.383 1.00 0.00
H   N
ATOM 25598 HN GLN H 105   24.437 -10.101 47.442 1.00 0.00
H   H
ATOM 25599 CA GLN H 105   22.874 -10.988 46.396 1.00 0.00
H   C
ATOM 25600 HA GLN H 105   22.189 -11.767 46.704 1.00 0.00
H   H
ATOM 25601 CB GLN H 105   22.168 -9.632 46.383 1.00 0.00
H   C
ATOM 25602 HB1 GLN H 105   21.906 -9.395 47.440 1.00 0.00
H   H
ATOM 25603 HB2 GLN H 105   22.886 -8.845 46.057 1.00 0.00
H   H
ATOM 25604 CG GLN H 105   20.914 -9.582 45.541 1.00 0.00
H   C
ATOM 25605 HG1 GLN H 105   20.471 -10.596 45.447 1.00 0.00
H   H
ATOM 25606 HG2 GLN H 105   20.155 -8.920 46.006 1.00 0.00
H   H
ATOM 25607 CD GLN H 105   21.188 -9.101 44.138 1.00 0.00
H   C
ATOM 25608 OE1 GLN H 105   22.199 -8.452 43.882 1.00 0.00
H   O
ATOM 25609 NE2 GLN H 105   20.276 -9.395 43.223 1.00 0.00
H   N
ATOM 25610 HE21 GLN H 105   20.454 -9.084 42.294 1.00 0.00
H   H
ATOM 25611 HE22 GLN H 105   19.455 -9.891 43.485 1.00 0.00
H   H
ATOM 25612 C   GLN H 105   23.375 -11.341 45.000 1.00 0.00
H   C
ATOM 25613 O   GLN H 105   22.976 -12.364 44.441 1.00 0.00
H   O
ATOM 25614 N   ASN H 106   24.252 -10.523 44.427 1.00 0.00
H   N
ATOM 25615 HN ASN H 106   24.585 -9.671 44.830 1.00 0.00
H   H
ATOM 25616 CA ASN H 106   24.809 -10.828 43.119 1.00 0.00
H   C
ATOM 25617 HA ASN H 106   24.602 -11.874 42.921 1.00 0.00
H   H
ATOM 25618 CB ASN H 106   24.239 -9.936 42.016 1.00 0.00
H   C
ATOM 25619 HB1 ASN H 106   24.608 -10.322 41.041 1.00 0.00
H   H
ATOM 25620 HB2 ASN H 106   23.132 -10.000 42.003 1.00 0.00
H   H
ATOM 25621 CG ASN H 106   24.676 -8.486 42.140 1.00 0.00
H   C
ATOM 25622 OD1 ASN H 106   25.446 -8.112 43.022 1.00 0.00
H   O

```
ATOM  25623  ND2 ASN H 106      24.197  -7.660  41.226  1.00  0.00
H    N
ATOM  25624 HD21 ASN H 106      24.478  -6.706  41.279  1.00  0.00
H    H
ATOM  25625 HD22 ASN H 106      23.604  -8.004  40.505  1.00  0.00
H    H
ATOM  25626  C   ASN H 106      26.315 -10.663  43.182  1.00  0.00
H    C
ATOM  25627  O   ASN H 106      26.874 -10.254  44.203  1.00  0.00
H    O
ATOM  25628  N   TYR H 107      26.964 -10.950  42.059  1.00  0.00
H    N
ATOM  25629  HN  TYR H 107      26.552 -11.323  41.229  1.00  0.00
H    H
ATOM  25630  CA  TYR H 107      28.397 -10.723  41.973  1.00  0.00
H    C
ATOM  25631  HA  TYR H 107      28.856 -11.365  42.715  1.00  0.00
H    H
ATOM  25632  CB  TYR H 107      28.892 -11.049  40.568  1.00  0.00
H    C
ATOM  25633  HB1 TYR H 107      29.114 -12.137  40.504  1.00  0.00
H    H
ATOM  25634  HB2 TYR H 107      28.106 -10.829  39.814  1.00  0.00
H    H
ATOM  25635  CG  TYR H 107      30.154 -10.311  40.228  1.00  0.00
H    C
ATOM  25636  CD1 TYR H 107      31.381 -10.762  40.681  1.00  0.00
H    C
ATOM  25637  HD1 TYR H 107      31.434 -11.651  41.294  1.00  0.00
H    H
ATOM  25638  CE1 TYR H 107      32.536 -10.086  40.385  1.00  0.00
H    C
ATOM  25639  HE1 TYR H 107      33.490 -10.443  40.747  1.00  0.00
H    H
ATOM  25640  CZ  TYR H 107      32.476  -8.941  39.629  1.00  0.00
H    C
ATOM  25641  OH  TYR H 107      33.628  -8.258  39.326  1.00  0.00
H    O
ATOM  25642  HH  TYR H 107      33.390  -7.538  38.738  1.00  0.00
H    H
ATOM  25643  CD2 TYR H 107      30.122  -9.157  39.470  1.00  0.00
H    C
ATOM  25644  HD2 TYR H 107      29.174  -8.757  39.135  1.00  0.00
H    H
ATOM  25645  CE2 TYR H 107      31.272  -8.475  39.171  1.00  0.00
H    C
ATOM  25646  HE2 TYR H 107      31.220  -7.563  38.596  1.00  0.00
H    H
ATOM  25647  C   TYR H 107      28.749  -9.291  42.341  1.00  0.00
H    C
ATOM  25648  O   TYR H 107      29.723  -9.045  43.061  1.00  0.00
H    O
ATOM  25649  N   TYR H 108      27.972  -8.330  41.850  1.00  0.00
H    N
```

ATOM 25650 HN TYR H 108 27.154 -8.462 41.292 1.00 0.00
H H
ATOM 25651 CA TYR H 108 28.301 -6.940 42.122 1.00 0.00
H C
ATOM 25652 HA TYR H 108 29.338 -6.808 41.837 1.00 0.00
H H
ATOM 25653 CB TYR H 108 27.386 -6.023 41.333 1.00 0.00
H C
ATOM 25654 HB1 TYR H 108 26.329 -6.261 41.582 1.00 0.00
H H
ATOM 25655 HB2 TYR H 108 27.559 -4.962 41.613 1.00 0.00
H H
ATOM 25656 CG TYR H 108 27.575 -6.224 39.879 1.00 0.00
H C
ATOM 25657 CD1 TYR H 108 28.835 -6.134 39.326 1.00 0.00
H C
ATOM 25658 HD1 TYR H 108 29.682 -5.891 39.953 1.00 0.00
H H
ATOM 25659 CE1 TYR H 108 29.038 -6.324 37.995 1.00 0.00
H C
ATOM 25660 HE1 TYR H 108 30.030 -6.243 37.574 1.00 0.00
H H
ATOM 25661 CZ TYR H 108 27.972 -6.613 37.188 1.00 0.00
H C
ATOM 25662 OH TYR H 108 28.174 -6.802 35.843 1.00 0.00
H O
ATOM 25663 HH TYR H 108 27.333 -7.063 35.459 1.00 0.00
H H
ATOM 25664 CD2 TYR H 108 26.514 -6.522 39.057 1.00 0.00
H C
ATOM 25665 HD2 TYR H 108 25.513 -6.570 39.462 1.00 0.00
H H
ATOM 25666 CE2 TYR H 108 26.704 -6.713 37.715 1.00 0.00
H C
ATOM 25667 HE2 TYR H 108 25.857 -6.918 37.077 1.00 0.00
H H
ATOM 25668 C TYR H 108 28.202 -6.633 43.599 1.00 0.00
H C
ATOM 25669 O TYR H 108 29.218 -6.349 44.247 1.00 0.00
H O
ATOM 25670 N HSE H 109 26.991 -6.744 44.149 1.00 0.00
H N
ATOM 25671 HN HSE H 109 26.155 -6.927 43.634 1.00 0.00
H H
ATOM 25672 CA HSE H 109 26.794 -6.600 45.582 1.00 0.00
H C
ATOM 25673 HA HSE H 109 26.832 -5.541 45.804 1.00 0.00
H H
ATOM 25674 CB HSE H 109 25.460 -7.218 45.969 1.00 0.00
H C
ATOM 25675 HB1 HSE H 109 24.653 -6.591 45.532 1.00 0.00
H H
ATOM 25676 HB2 HSE H 109 25.372 -8.226 45.511 1.00 0.00
H H

ATOM 25677 ND1 HSE H 109 25.004 -8.482 48.092 1.00 0.00
H N
ATOM 25678 CG HSE H 109 25.240 -7.294 47.440 1.00 0.00
H C
ATOM 25679 CE1 HSE H 109 24.844 -8.250 49.383 1.00 0.00
H C
ATOM 25680 HE1 HSE H 109 24.645 -9.020 50.132 1.00 0.00
H H
ATOM 25681 NE2 HSE H 109 24.960 -6.950 49.587 1.00 0.00
H N
ATOM 25682 HE2 HSE H 109 24.863 -6.496 50.472 1.00 0.00
H H
ATOM 25683 CD2 HSE H 109 25.204 -6.329 48.387 1.00 0.00
H C
ATOM 25684 HD2 HSE H 109 25.324 -5.259 48.291 1.00 0.00
H H
ATOM 25685 C HSE H 109 27.924 -7.257 46.347 1.00 0.00
H C
ATOM 25686 O HSE H 109 28.422 -6.706 47.332 1.00 0.00
H O
ATOM 25687 N HSE H 110 28.380 -8.408 45.864 1.00 0.00
H N
ATOM 25688 HN HSE H 110 27.998 -8.880 45.071 1.00 0.00
H H
ATOM 25689 CA HSE H 110 29.507 -9.086 46.480 1.00 0.00
H C
ATOM 25690 HA HSE H 110 29.264 -9.204 47.528 1.00 0.00
H H
ATOM 25691 CB HSE H 110 29.710 -10.425 45.797 1.00 0.00
H C
ATOM 25692 HB1 HSE H 110 29.134 -11.188 46.363 1.00 0.00
H H
ATOM 25693 HB2 HSE H 110 29.282 -10.388 44.773 1.00 0.00
H H
ATOM 25694 ND1 HSE H 110 31.696 -11.637 46.734 1.00 0.00
H N
ATOM 25695 CG HSE H 110 31.131 -10.859 45.751 1.00 0.00
H C
ATOM 25696 CE1 HSE H 110 32.962 -11.862 46.438 1.00 0.00
H C
ATOM 25697 HE1 HSE H 110 33.648 -12.449 47.054 1.00 0.00
H H
ATOM 25698 NE2 HSE H 110 33.236 -11.259 45.296 1.00 0.00
H N
ATOM 25699 HE2 HSE H 110 34.130 -11.252 44.849 1.00 0.00
H H
ATOM 25700 CD2 HSE H 110 32.107 -10.624 44.845 1.00 0.00
H C
ATOM 25701 HD2 HSE H 110 32.079 -10.049 43.930 1.00 0.00
H H
ATOM 25702 C HSE H 110 30.777 -8.253 46.407 1.00 0.00
H C
ATOM 25703 O HSE H 110 31.530 -8.167 47.382 1.00 0.00
H O

ATOM 25704 N   LYS H 111   31.067 -7.671 45.251 1.00 0.00
H   N
ATOM 25705 HN LYS H 111   30.530 -7.717 44.411 1.00 0.00
H   H
ATOM 25706 CA LYS H 111   32.285 -6.878 45.164 1.00 0.00
H   C
ATOM 25707 HA LYS H 111   33.097 -7.499 45.523 1.00 0.00
H   H
ATOM 25708 CB LYS H 111   32.549 -6.452 43.729 1.00 0.00
H   C
ATOM 25709 HB1 LYS H 111   32.776 -7.377 43.149 1.00 0.00
H   H
ATOM 25710 HB2 LYS H 111   31.613 -6.034 43.296 1.00 0.00
H   H
ATOM 25711 CG LYS H 111   33.706 -5.475 43.615 1.00 0.00
H   C
ATOM 25712 HG1 LYS H 111   33.415 -4.645 42.930 1.00 0.00
H   H
ATOM 25713 HG2 LYS H 111   33.901 -5.026 44.616 1.00 0.00
H   H
ATOM 25714 CD LYS H 111   34.994 -6.119 43.076 1.00 0.00
H   C
ATOM 25715 HD1 LYS H 111   35.258 -5.625 42.112 1.00 0.00
H   H
ATOM 25716 HD2 LYS H 111   35.816 -5.906 43.797 1.00 0.00
H   H
ATOM 25717 CE LYS H 111   34.843 -7.603 42.885 1.00 0.00
H   C
ATOM 25718 HE1 LYS H 111   33.773 -7.899 42.954 1.00 0.00
H   H
ATOM 25719 HE2 LYS H 111   35.228 -7.910 41.887 1.00 0.00
H   H
ATOM 25720 NZ LYS H 111   35.604 -8.324 43.932 1.00 0.00
H   N
ATOM 25721 HZ1 LYS H 111   35.501 -9.351 43.805 1.00 0.00
H   H
ATOM 25722 HZ2 LYS H 111   36.610 -8.067 43.869 1.00 0.00
H   H
ATOM 25723 HZ3 LYS H 111   35.241 -8.055 44.869 1.00 0.00
H   H
ATOM 25724 C   LYS H 111   32.215 -5.660 46.065 1.00 0.00
H   C
ATOM 25725 O   LYS H 111   33.178 -5.335 46.768 1.00 0.00
H   O
ATOM 25726 N   VAL H 112   31.078 -4.976 46.060 1.00 0.00
H   N
ATOM 25727 HN VAL H 112   30.286 -5.164 45.486 1.00 0.00
H   H
ATOM 25728 CA VAL H 112   30.915 -3.835 46.955 1.00 0.00
H   C
ATOM 25729 HA VAL H 112   31.720 -3.140 46.754 1.00 0.00
H   H
ATOM 25730 CB VAL H 112   29.581 -3.125 46.694 1.00 0.00
H   C

```
ATOM  25731  HB  VAL H 112      29.804  -2.044  46.517  1.00  0.00
H    H
ATOM  25732  CG1 VAL H 112      28.922  -3.700  45.488  1.00  0.00
H    C
ATOM  25733 HG11 VAL H 112      27.937  -3.214  45.317  1.00  0.00
H    H
ATOM  25734 HG12 VAL H 112      29.531  -3.541  44.574  1.00  0.00
H    H
ATOM  25735 HG13 VAL H 112      28.750  -4.790  45.616  1.00  0.00
H    H
ATOM  25736  CG2 VAL H 112      28.678  -3.264  47.888  1.00  0.00
H    C
ATOM  25737 HG21 VAL H 112      27.694  -2.786  47.693  1.00  0.00
H    H
ATOM  25738 HG22 VAL H 112      28.506  -4.336  48.125  1.00  0.00
H    H
ATOM  25739 HG23 VAL H 112      29.116  -2.775  48.783  1.00  0.00
H    H
ATOM  25740  C   VAL H 112      31.048  -4.278  48.402  1.00  0.00
H    C
ATOM  25741  O   VAL H 112      31.532  -3.526  49.256  1.00  0.00
H    O
ATOM  25742  N   LEU H 113      30.627  -5.505  48.697  1.00  0.00
H    N
ATOM  25743  HN  LEU H 113      30.111  -6.100  48.084  1.00  0.00
H    H
ATOM  25744  CA  LEU H 113      30.905  -6.086  49.997  1.00  0.00
H    C
ATOM  25745  HA  LEU H 113      30.475  -5.424  50.738  1.00  0.00
H    H
ATOM  25746  CB  LEU H 113      30.308  -7.483  50.061  1.00  0.00
H    C
ATOM  25747  HB1 LEU H 113      29.396  -7.480  49.421  1.00  0.00
H    H
ATOM  25748  HB2 LEU H 113      31.014  -8.200  49.587  1.00  0.00
H    H
ATOM  25749  CG  LEU H 113      29.903  -8.017  51.419  1.00  0.00
H    C
ATOM  25750  HG  LEU H 113      30.781  -8.546  51.865  1.00  0.00
H    H
ATOM  25751  CD1 LEU H 113      29.507  -6.883  52.325  1.00  0.00
H    C
ATOM  25752 HD11 LEU H 113      29.164  -7.272  53.308  1.00  0.00
H    H
ATOM  25753 HD12 LEU H 113      30.368  -6.204  52.501  1.00  0.00
H    H
ATOM  25754 HD13 LEU H 113      28.680  -6.293  51.877  1.00  0.00
H    H
ATOM  25755  CD2 LEU H 113      28.796  -9.019  51.244  1.00  0.00
H    C
ATOM  25756 HD21 LEU H 113      28.495  -9.438  52.229  1.00  0.00
H    H
ATOM  25757 HD22 LEU H 113      27.897  -8.560  50.786  1.00  0.00
H    H
```

ATOM 25758 HD23 LEU H 113 29.129 -9.859 50.596 1.00 0.00
H H
ATOM 25759 C LEU H 113 32.398 -6.141 50.248 1.00 0.00
H C
ATOM 25760 O LEU H 113 32.879 -5.742 51.312 1.00 0.00
H O
ATOM 25761 N GLU H 114 33.146 -6.627 49.263 1.00 0.00
H N
ATOM 25762 HN GLU H 114 32.813 -6.937 48.377 1.00 0.00
H H
ATOM 25763 CA GLU H 114 34.588 -6.746 49.428 1.00 0.00
H C
ATOM 25764 HA GLU H 114 34.779 -7.396 50.273 1.00 0.00
H H
ATOM 25765 CB GLU H 114 35.212 -7.342 48.172 1.00 0.00
H C
ATOM 25766 HB1 GLU H 114 34.579 -8.197 47.841 1.00 0.00
H H
ATOM 25767 HB2 GLU H 114 35.178 -6.599 47.345 1.00 0.00
H H
ATOM 25768 CG GLU H 114 36.620 -7.811 48.413 1.00 0.00
H C
ATOM 25769 HG1 GLU H 114 36.916 -8.514 47.614 1.00 0.00
H H
ATOM 25770 HG2 GLU H 114 37.326 -6.958 48.428 1.00 0.00
H H
ATOM 25771 CD GLU H 114 36.744 -8.574 49.711 1.00 0.00
H C
ATOM 25772 OE1 GLU H 114 35.994 -9.554 49.893 1.00 0.00
H O
ATOM 25773 OE2 GLU H 114 37.576 -8.185 50.556 1.00 0.00
H O
ATOM 25774 C GLU H 114 35.217 -5.397 49.744 1.00 0.00
H C
ATOM 25775 O GLU H 114 36.078 -5.288 50.623 1.00 0.00
H O
ATOM 25776 N LYS H 115 34.785 -4.352 49.045 1.00 0.00
H N
ATOM 25777 HN LYS H 115 34.039 -4.374 48.381 1.00 0.00
H H
ATOM 25778 CA LYS H 115 35.416 -3.050 49.223 1.00 0.00
H C
ATOM 25779 HA LYS H 115 36.477 -3.200 49.063 1.00 0.00
H H
ATOM 25780 CB LYS H 115 34.860 -2.056 48.222 1.00 0.00
H C
ATOM 25781 HB1 LYS H 115 33.749 -2.103 48.295 1.00 0.00
H H
ATOM 25782 HB2 LYS H 115 35.151 -1.028 48.533 1.00 0.00
H H
ATOM 25783 CG LYS H 115 35.292 -2.356 46.837 1.00 0.00
H C
ATOM 25784 HG1 LYS H 115 34.707 -3.221 46.453 1.00 0.00
H H

ATOM 25785 HG2 LYS H 115 35.059 -1.481 46.187 1.00 0.00
H H
ATOM 25786 CD LYS H 115 36.751 -2.679 46.790 1.00 0.00
H C
ATOM 25787 HD1 LYS H 115 37.314 -1.830 47.241 1.00 0.00
H H
ATOM 25788 HD2 LYS H 115 36.929 -3.574 47.430 1.00 0.00
H H
ATOM 25789 CE LYS H 115 37.145 -2.928 45.364 1.00 0.00
H C
ATOM 25790 HE1 LYS H 115 38.253 -2.950 45.263 1.00 0.00
H H
ATOM 25791 HE2 LYS H 115 36.739 -3.901 45.010 1.00 0.00
H H
ATOM 25792 NZ LYS H 115 36.606 -1.839 44.506 1.00 0.00
H N
ATOM 25793 HZ1 LYS H 115 36.875 -2.001 43.514 1.00 0.00
H H
ATOM 25794 HZ2 LYS H 115 35.568 -1.817 44.584 1.00 0.00
H H
ATOM 25795 HZ3 LYS H 115 36.992 -0.926 44.823 1.00 0.00
H H
ATOM 25796 C LYS H 115 35.244 -2.499 50.626 1.00 0.00
H C
ATOM 25797 O LYS H 115 36.124 -1.784 51.112 1.00 0.00
H O
ATOM 25798 N ILE H 116 34.134 -2.803 51.292 1.00 0.00
H N
ATOM 25799 HN ILE H 116 33.390 -3.361 50.931 1.00 0.00
H H
ATOM 25800 CA ILE H 116 33.917 -2.325 52.647 1.00 0.00
H C
ATOM 25801 HA ILE H 116 34.192 -1.278 52.644 1.00 0.00
H H
ATOM 25802 CB ILE H 116 32.456 -2.507 53.068 1.00 0.00
H C
ATOM 25803 HB ILE H 116 31.865 -2.219 52.162 1.00 0.00
H H
ATOM 25804 CG2 ILE H 116 32.246 -3.898 53.604 1.00 0.00
H C
ATOM 25805 HG21 ILE H 116 31.166 -4.095 53.766 1.00 0.00
H H
ATOM 25806 HG22 ILE H 116 32.623 -4.659 52.888 1.00 0.00
H H
ATOM 25807 HG23 ILE H 116 32.773 -4.035 54.572 1.00 0.00
H H
ATOM 25808 CG1 ILE H 116 32.078 -1.519 54.168 1.00 0.00
H C
ATOM 25809 HG11 ILE H 116 30.994 -1.609 54.400 1.00 0.00
H H
ATOM 25810 HG12 ILE H 116 32.636 -1.782 55.096 1.00 0.00
H H
ATOM 25811 CD ILE H 116 32.374 -0.101 53.854 1.00 0.00
H C

ATOM 25812 HD1 ILE H 116 32.125 0.547 54.722 1.00 0.00
H H
ATOM 25813 HD2 ILE H 116 33.452 0.031 53.617 1.00 0.00
H H
ATOM 25814 HD3 ILE H 116 31.777 0.247 52.984 1.00 0.00
H H
ATOM 25815 C ILE H 116 34.836 −3.018 53.639 1.00 0.00
H C
ATOM 25816 O ILE H 116 35.003 −2.540 54.761 1.00 0.00
H O
ATOM 25817 N GLY H 117 35.451 −4.123 53.245 1.00 0.00
H N
ATOM 25818 HN GLY H 117 35.485 −4.443 52.299 1.00 0.00
H H
ATOM 25819 CA GLY H 117 36.132 −4.954 54.210 1.00 0.00
H C
ATOM 25820 HA1 GLY H 117 36.314 −4.340 55.082 1.00 0.00
H H
ATOM 25821 HA2 GLY H 117 37.029 −5.327 53.735 1.00 0.00
H H
ATOM 25822 C GLY H 117 35.307 −6.136 54.637 1.00 0.00
H C
ATOM 25823 O GLY H 117 35.285 −6.476 55.821 1.00 0.00
H O
ATOM 25824 N ALA H 118 34.610 −6.766 53.699 1.00 0.00
H N
ATOM 25825 HN ALA H 118 34.562 −6.473 52.744 1.00 0.00
H H
ATOM 25826 CA ALA H 118 33.850 −7.959 54.023 1.00 0.00
H C
ATOM 25827 HA ALA H 118 33.153 −7.769 54.831 1.00 0.00
H H
ATOM 25828 CB ALA H 118 33.080 −8.443 52.804 1.00 0.00
H C
ATOM 25829 HB1 ALA H 118 32.364 −7.658 52.481 1.00 0.00
H H
ATOM 25830 HB2 ALA H 118 33.766 −8.651 51.955 1.00 0.00
H H
ATOM 25831 HB3 ALA H 118 32.502 −9.366 53.026 1.00 0.00
H H
ATOM 25832 C ALA H 118 34.780 −9.053 54.508 1.00 0.00
H C
ATOM 25833 O ALA H 118 35.553 −9.619 53.733 1.00 0.00
H O
ATOM 25834 N ASP H 119 34.704 −9.346 55.799 1.00 0.00
H N
ATOM 25835 HN ASP H 119 34.144 −8.906 56.495 1.00 0.00
H H
ATOM 25836 CA ASP H 119 35.512 −10.430 56.325 1.00 0.00
H C
ATOM 25837 HA ASP H 119 36.540 −10.218 56.056 1.00 0.00
H H
ATOM 25838 CB ASP H 119 35.347 −10.516 57.839 1.00 0.00
H C

```
ATOM  25839  HB1 ASP H 119      34.268 -10.549  58.088  1.00  0.00
H    H
ATOM  25840  HB2 ASP H 119      35.825 -11.435  58.232  1.00  0.00
H    H
ATOM  25841  CG  ASP H 119      35.942  -9.320  58.548  1.00  0.00
H    C
ATOM  25842  OD1 ASP H 119      37.100  -9.420  59.007  1.00  0.00
H    O
ATOM  25843  OD2 ASP H 119      35.260  -8.277  58.629  1.00  0.00
H    O
ATOM  25844  C   ASP H 119      35.152 -11.750  55.672  1.00  0.00
H    C
ATOM  25845  O   ASP H 119      35.993 -12.651  55.604  1.00  0.00
H    O
ATOM  25846  N   ARG H 120      33.923 -11.883  55.189  1.00  0.00
H    N
ATOM  25847  HN  ARG H 120      33.231 -11.169  55.274  1.00  0.00
H    H
ATOM  25848  CA  ARG H 120      33.527 -13.097  54.501  1.00  0.00
H    C
ATOM  25849  HA  ARG H 120      34.253 -13.313  53.725  1.00  0.00
H    H
ATOM  25850  CB  ARG H 120      33.435 -14.265  55.473  1.00  0.00
H    C
ATOM  25851  HB1 ARG H 120      34.484 -14.591  55.674  1.00  0.00
H    H
ATOM  25852  HB2 ARG H 120      33.034 -13.907  56.448  1.00  0.00
H    H
ATOM  25853  CG  ARG H 120      32.659 -15.396  54.924  1.00  0.00
H    C
ATOM  25854  HG1 ARG H 120      31.680 -15.442  55.449  1.00  0.00
H    H
ATOM  25855  HG2 ARG H 120      32.417 -15.184  53.857  1.00  0.00
H    H
ATOM  25856  CD  ARG H 120      33.451 -16.645  55.019  1.00  0.00
H    C
ATOM  25857  HD1 ARG H 120      34.327 -16.581  54.333  1.00  0.00
H    H
ATOM  25858  HD2 ARG H 120      33.821 -16.816  56.057  1.00  0.00
H    H
ATOM  25859  NE  ARG H 120      32.630 -17.786  54.661  1.00  0.00
H    N
ATOM  25860  HE  ARG H 120      31.683 -17.536  54.431  1.00  0.00
H    H
ATOM  25861  CZ  ARG H 120      33.073 -19.035  54.636  1.00  0.00
H    C
ATOM  25862  NH1 ARG H 120      34.330 -19.295  54.958  1.00  0.00
H    N
ATOM  25863 HH11 ARG H 120      34.679 -20.221  54.919  1.00  0.00
H    H
ATOM  25864 HH12 ARG H 120      34.925 -18.501  55.103  1.00  0.00
H    H
ATOM  25865  NH2 ARG H 120      32.259 -20.022  54.299  1.00  0.00
H    N
```

ATOM 25866 HH21 ARG H 120   32.567 -20.960 54.366 1.00 0.00
H   H
ATOM 25867 HH22 ARG H 120   31.300 -19.797 54.191 1.00 0.00
H   H
ATOM 25868 C   ARG H 120   32.193 -12.893 53.803 1.00 0.00
H   C
ATOM 25869 O   ARG H 120   31.230 -12.415 54.414 1.00 0.00
H   O
ATOM 25870 N   ILE H 121   32.138 -13.288 52.537 1.00 0.00
H   N
ATOM 25871 HN ILE H 121   32.917 -13.709 52.077 1.00 0.00
H   H
ATOM 25872 CA ILE H 121   30.954 -13.149 51.700 1.00 0.00
H   C
ATOM 25873 HA ILE H 121   30.194 -12.647 52.286 1.00 0.00
H   H
ATOM 25874 CB ILE H 121   31.291 -12.386 50.410 1.00 0.00
H   C
ATOM 25875 HB ILE H 121   31.945 -13.088 49.834 1.00 0.00
H   H
ATOM 25876 CG2 ILE H 121   30.040 -12.000 49.663 1.00 0.00
H   C
ATOM 25877 HG21 ILE H 121   30.294 -11.591 48.663 1.00 0.00
H   H
ATOM 25878 HG22 ILE H 121   29.383 -12.881 49.510 1.00 0.00
H   H
ATOM 25879 HG23 ILE H 121   29.468 -11.227 50.219 1.00 0.00
H   H
ATOM 25880 CG1 ILE H 121   32.132 -11.160 50.749 1.00 0.00
H   C
ATOM 25881 HG11 ILE H 121   31.640 -10.576 51.559 1.00 0.00
H   H
ATOM 25882 HG12 ILE H 121   33.119 -11.497 51.140 1.00 0.00
H   H
ATOM 25883 CD ILE H 121   32.386 -10.248 49.585 1.00 0.00
H   C
ATOM 25884 HD1 ILE H 121   33.050 -9.411 49.890 1.00 0.00
H   H
ATOM 25885 HD2 ILE H 121   32.879 -10.800 48.756 1.00 0.00
H   H
ATOM 25886 HD3 ILE H 121   31.437 -9.814 49.206 1.00 0.00
H   H
ATOM 25887 C   ILE H 121   30.426 -14.540 51.395 1.00 0.00
H   C
ATOM 25888 O   ILE H 121   31.197 -15.505 51.361 1.00 0.00
H   O
ATOM 25889 N   ILE H 122   29.117 -14.659 51.193 1.00 0.00
H   N
ATOM 25890 HN ILE H 122   28.464 -13.908 51.277 1.00 0.00
H   H
ATOM 25891 CA ILE H 122   28.517 -15.932 50.822 1.00 0.00
H   C
ATOM 25892 HA ILE H 122   29.303 -16.543 50.398 1.00 0.00
H   H

```
ATOM  25893  CB  ILE H 122      27.856 -16.631  52.025  1.00  0.00
H    C
ATOM  25894  HB  ILE H 122      27.217 -15.839  52.493  1.00  0.00
H    H
ATOM  25895  CG2 ILE H 122      27.083 -17.854  51.572  1.00  0.00
H    C
ATOM  25896 HG21 ILE H 122      26.481 -18.268  52.408  1.00  0.00
H    H
ATOM  25897 HG22 ILE H 122      26.384 -17.599  50.748  1.00  0.00
H    H
ATOM  25898 HG23 ILE H 122      27.772 -18.649  51.213  1.00  0.00
H    H
ATOM  25899  CG1 ILE H 122      28.908 -17.018  53.057  1.00  0.00
H    C
ATOM  25900 HG11 ILE H 122      28.611 -17.965  53.561  1.00  0.00
H    H
ATOM  25901 HG12 ILE H 122      29.872 -17.213  52.534  1.00  0.00
H    H
ATOM  25902  CD  ILE H 122      29.152 -15.962  54.105  1.00  0.00
H    C
ATOM  25903  HD1 ILE H 122      29.967 -16.280  54.790  1.00  0.00
H    H
ATOM  25904  HD2 ILE H 122      29.448 -15.002  53.632  1.00  0.00
H    H
ATOM  25905  HD3 ILE H 122      28.240 -15.788  54.714  1.00  0.00
H    H
ATOM  25906  C   ILE H 122      27.495 -15.707  49.723  1.00  0.00
H    C
ATOM  25907  O   ILE H 122      26.670 -14.793  49.804  1.00  0.00
H    O
ATOM  25908  N   HSE H 123      27.542 -16.556  48.712  1.00  0.00
H    N
ATOM  25909  HN  HSE H 123      28.267 -17.241  48.645  1.00  0.00
H    H
ATOM  25910  CA  HSE H 123      26.583 -16.591  47.622  1.00  0.00
H    C
ATOM  25911  HA  HSE H 123      25.882 -15.776  47.750  1.00  0.00
H    H
ATOM  25912  CB  HSE H 123      27.294 -16.543  46.276  1.00  0.00
H    C
ATOM  25913  HB1 HSE H 123      27.999 -17.401  46.231  1.00  0.00
H    H
ATOM  25914  HB2 HSE H 123      26.555 -16.686  45.461  1.00  0.00
H    H
ATOM  25915  ND1 HSE H 123      27.681 -14.316  45.160  1.00  0.00
H    N
ATOM  25916  CG  HSE H 123      28.080 -15.292  46.047  1.00  0.00
H    C
ATOM  25917  CE1 HSE H 123      28.571 -13.341  45.152  1.00  0.00
H    C
ATOM  25918  HE1 HSE H 123      28.505 -12.447  44.528  1.00  0.00
H    H
ATOM  25919  NE2 HSE H 123      29.528 -13.644  46.010  1.00  0.00
H    N
```

```
ATOM  25920  HE2 HSE H 123      30.336 -13.084  46.193  1.00  0.00
H    H
ATOM  25921  CD2 HSE H 123      29.246 -14.861  46.581  1.00  0.00
H    C
ATOM  25922  HD2 HSE H 123      29.891 -15.332  47.309  1.00  0.00
H    H
ATOM  25923  C   HSE H 123      25.795 -17.883  47.758  1.00  0.00
H    C
ATOM  25924  O   HSE H 123      26.000 -18.822  46.980  1.00  0.00
H    O
ATOM  25925  N   PRO H 124      24.895 -17.966  48.736  1.00  0.00
H    N
ATOM  25926  CD  PRO H 124      24.254 -16.863  49.462  1.00  0.00
H    C
ATOM  25927  HD1 PRO H 124      25.362 -16.963  49.510  1.00  0.00
H    H
ATOM  25928  HD2 PRO H 124      23.958 -15.789  49.450  1.00  0.00
H    H
ATOM  25929  CA  PRO H 124      24.364 -19.276  49.137  1.00  0.00
H    C
ATOM  25930  HA  PRO H 124      25.157 -19.855  49.596  1.00  0.00
H    H
ATOM  25931  CB  PRO H 124      23.301 -18.911  50.182  1.00  0.00
H    C
ATOM  25932  HB1 PRO H 124      23.792 -18.797  51.174  1.00  0.00
H    H
ATOM  25933  HB2 PRO H 124      22.499 -19.673  50.263  1.00  0.00
H    H
ATOM  25934  CG  PRO H 124      23.631 -17.548  50.624  1.00  0.00
H    C
ATOM  25935  HG1 PRO H 124      23.230 -17.310  51.629  1.00  0.00
H    H
ATOM  25936  HG2 PRO H 124      23.234 -16.816  49.884  1.00  0.00
H    H
ATOM  25937  C   PRO H 124      23.761 -20.076  48.004  1.00  0.00
H    C
ATOM  25938  O   PRO H 124      24.211 -21.197  47.723  1.00  0.00
H    O
ATOM  25939  N   GLU H 125      22.751 -19.527  47.343  1.00  0.00
H    N
ATOM  25940  HN  GLU H 125      22.375 -18.614  47.480  1.00  0.00
H    H
ATOM  25941  CA  GLU H 125      22.086 -20.288  46.301  1.00  0.00
H    C
ATOM  25942  HA  GLU H 125      21.793 -21.245  46.716  1.00  0.00
H    H
ATOM  25943  CB  GLU H 125      20.847 -19.568  45.809  1.00  0.00
H    C
ATOM  25944  HB1 GLU H 125      21.097 -18.489  45.685  1.00  0.00
H    H
ATOM  25945  HB2 GLU H 125      20.575 -19.936  44.794  1.00  0.00
H    H
ATOM  25946  CG  GLU H 125      19.669 -19.707  46.752  1.00  0.00
H    C
```

```
ATOM  25947  HG1 GLU H 125      18.749 -19.891  46.169  1.00  0.00
H    H
ATOM  25948  HG2 GLU H 125      19.825 -20.545  47.461  1.00  0.00
H    H
ATOM  25949  CD  GLU H 125      19.387 -18.467  47.555  1.00  0.00
H    C
ATOM  25950  OE1 GLU H 125      20.215 -17.529  47.498  1.00  0.00
H    O
ATOM  25951  OE2 GLU H 125      18.330 -18.440  48.213  1.00  0.00
H    O
ATOM  25952  C   GLU H 125      23.026 -20.567  45.153  1.00  0.00
H    C
ATOM  25953  O   GLU H 125      22.899 -21.594  44.496  1.00  0.00
H    O
ATOM  25954  N   LYS H 126      23.991 -19.684  44.910  1.00  0.00
H    N
ATOM  25955  HN  LYS H 126      24.075 -18.780  45.327  1.00  0.00
H    H
ATOM  25956  CA  LYS H 126      25.039 -20.024  43.956  1.00  0.00
H    C
ATOM  25957  HA  LYS H 126      24.565 -20.173  42.993  1.00  0.00
H    H
ATOM  25958  CB  LYS H 126      26.069 -18.900  43.888  1.00  0.00
H    C
ATOM  25959  HB1 LYS H 126      26.584 -18.868  44.877  1.00  0.00
H    H
ATOM  25960  HB2 LYS H 126      26.847 -19.167  43.139  1.00  0.00
H    H
ATOM  25961  CG  LYS H 126      25.505 -17.539  43.594  1.00  0.00
H    C
ATOM  25962  HG1 LYS H 126      24.824 -17.239  44.423  1.00  0.00
H    H
ATOM  25963  HG2 LYS H 126      26.337 -16.799  43.564  1.00  0.00
H    H
ATOM  25964  CD  LYS H 126      24.741 -17.513  42.304  1.00  0.00
H    C
ATOM  25965  HD1 LYS H 126      24.277 -18.515  42.150  1.00  0.00
H    H
ATOM  25966  HD2 LYS H 126      23.913 -16.774  42.405  1.00  0.00
H    H
ATOM  25967  CE  LYS H 126      25.626 -17.150  41.126  1.00  0.00
H    C
ATOM  25968  HE1 LYS H 126      26.428 -16.447  41.443  1.00  0.00
H    H
ATOM  25969  HE2 LYS H 126      26.103 -18.060  40.700  1.00  0.00
H    H
ATOM  25970  NZ  LYS H 126      24.832 -16.493  40.050  1.00  0.00
H    N
ATOM  25971  HZ1 LYS H 126      25.451 -16.249  39.250  1.00  0.00
H    H
ATOM  25972  HZ2 LYS H 126      24.086 -17.141  39.724  1.00  0.00
H    H
ATOM  25973  HZ3 LYS H 126      24.394 -15.627  40.424  1.00  0.00
H    H
```

ATOM 25974 C LYS H 126 25.707 -21.337 44.341 1.00 0.00
H C
ATOM 25975 O LYS H 126 25.781 -22.281 43.538 1.00 0.00
H O
ATOM 25976 N ASP H 127 26.167 -21.421 45.587 1.00 0.00
H N
ATOM 25977 HN ASP H 127 26.036 -20.742 46.305 1.00 0.00
H H
ATOM 25978 CA ASP H 127 26.939 -22.571 46.028 1.00 0.00
H C
ATOM 25979 HA ASP H 127 27.759 -22.695 45.329 1.00 0.00
H H
ATOM 25980 CB ASP H 127 27.430 -22.324 47.455 1.00 0.00
H C
ATOM 25981 HB1 ASP H 127 26.623 -21.838 48.040 1.00 0.00
H H
ATOM 25982 HB2 ASP H 127 27.691 -23.281 47.949 1.00 0.00
H H
ATOM 25983 CG ASP H 127 28.639 -21.411 47.506 1.00 0.00
H C
ATOM 25984 OD1 ASP H 127 28.458 -20.195 47.263 1.00 0.00
H O
ATOM 25985 OD2 ASP H 127 29.749 -21.901 47.805 1.00 0.00
H O
ATOM 25986 C ASP H 127 26.104 -23.840 45.952 1.00 0.00
H C
ATOM 25987 O ASP H 127 26.497 -24.837 45.329 1.00 0.00
H O
ATOM 25988 N MET H 128 24.928 -23.818 46.564 1.00 0.00
H N
ATOM 25989 HN MET H 128 24.474 -23.060 47.029 1.00 0.00
H H
ATOM 25990 CA MET H 128 24.180 -25.059 46.570 1.00 0.00
H C
ATOM 25991 HA MET H 128 24.888 -25.859 46.745 1.00 0.00
H H
ATOM 25992 CB MET H 128 23.170 -25.073 47.712 1.00 0.00
H C
ATOM 25993 HB1 MET H 128 23.662 -25.582 48.574 1.00 0.00
H H
ATOM 25994 HB2 MET H 128 22.969 -24.029 48.040 1.00 0.00
H H
ATOM 25995 CG MET H 128 21.874 -25.787 47.411 1.00 0.00
H C
ATOM 25996 HG1 MET H 128 21.095 -25.058 47.103 1.00 0.00
H H
ATOM 25997 HG2 MET H 128 22.025 -26.494 46.568 1.00 0.00
H H
ATOM 25998 SD MET H 128 21.251 -26.700 48.836 1.00 0.00
H S
ATOM 25999 CE MET H 128 22.555 -26.458 50.048 1.00 0.00
H C
ATOM 26000 HE1 MET H 128 22.316 -26.982 50.998 1.00 0.00
H H

```
ATOM  26001  HE2 MET H 128      23.525 -26.853  49.675  1.00  0.00
H    H
ATOM  26002  HE3 MET H 128      22.692 -25.380  50.279  1.00  0.00
H    H
ATOM  26003  C   MET H 128      23.531 -25.308  45.219  1.00  0.00
H    C
ATOM  26004  O   MET H 128      23.168 -26.442  44.911  1.00  0.00
H    O
ATOM  26005  N   GLY H 129      23.455 -24.304  44.363  1.00  0.00
H    N
ATOM  26006  HN  GLY H 129      23.736 -23.363  44.551  1.00  0.00
H    H
ATOM  26007  CA  GLY H 129      22.931 -24.545  43.046  1.00  0.00
H    C
ATOM  26008  HA1 GLY H 129      22.793 -23.591  42.556  1.00  0.00
H    H
ATOM  26009  HA2 GLY H 129      22.026 -25.133  43.137  1.00  0.00
H    H
ATOM  26010  C   GLY H 129      23.922 -25.341  42.246  1.00  0.00
H    C
ATOM  26011  O   GLY H 129      23.559 -26.309  41.583  1.00  0.00
H    O
ATOM  26012  N   VAL H 130      25.183 -24.929  42.279  1.00  0.00
H    N
ATOM  26013  HN  VAL H 130      25.548 -24.126  42.742  1.00  0.00
H    H
ATOM  26014  CA  VAL H 130      26.179 -25.718  41.567  1.00  0.00
H    C
ATOM  26015  HA  VAL H 130      25.838 -25.843  40.547  1.00  0.00
H    H
ATOM  26016  CB  VAL H 130      27.521 -24.962  41.496  1.00  0.00
H    C
ATOM  26017  HB  VAL H 130      28.192 -25.531  40.807  1.00  0.00
H    H
ATOM  26018  CG1 VAL H 130      27.285 -23.569  40.948  1.00  0.00
H    C
ATOM  26019 HG11 VAL H 130      28.238 -22.997  40.927  1.00  0.00
H    H
ATOM  26020 HG12 VAL H 130      26.894 -23.598  39.910  1.00  0.00
H    H
ATOM  26021 HG13 VAL H 130      26.565 -23.012  41.584  1.00  0.00
H    H
ATOM  26022  CG2 VAL H 130      28.163 -24.843  42.843  1.00  0.00
H    C
ATOM  26023 HG21 VAL H 130      29.107 -24.262  42.778  1.00  0.00
H    H
ATOM  26024 HG22 VAL H 130      27.483 -24.327  43.555  1.00  0.00
H    H
ATOM  26025 HG23 VAL H 130      28.411 -25.841  43.259  1.00  0.00
H    H
ATOM  26026  C   VAL H 130      26.289 -27.106  42.186  1.00  0.00
H    C
ATOM  26027  O   VAL H 130      26.433 -28.111  41.470  1.00  0.00
H    O
```

ATOM 26028 N LYS H 131 26.172 -27.194 43.517 1.00 0.00
H N
ATOM 26029 HN LYS H 131 26.260 -26.425 44.148 1.00 0.00
H H
ATOM 26030 CA LYS H 131 25.894 -28.483 44.145 1.00 0.00
H C
ATOM 26031 HA LYS H 131 26.817 -29.049 44.113 1.00 0.00
H H
ATOM 26032 CB LYS H 131 25.430 -28.266 45.588 1.00 0.00
H C
ATOM 26033 HB1 LYS H 131 26.094 -27.489 46.033 1.00 0.00
H H
ATOM 26034 HB2 LYS H 131 24.404 -27.831 45.578 1.00 0.00
H H
ATOM 26035 CG LYS H 131 25.458 -29.494 46.500 1.00 0.00
H C
ATOM 26036 HG1 LYS H 131 26.447 -29.997 46.404 1.00 0.00
H H
ATOM 26037 HG2 LYS H 131 25.356 -29.162 47.559 1.00 0.00
H H
ATOM 26038 CD LYS H 131 24.333 -30.461 46.105 1.00 0.00
H C
ATOM 26039 HD1 LYS H 131 23.415 -30.181 46.668 1.00 0.00
H H
ATOM 26040 HD2 LYS H 131 24.117 -30.318 45.021 1.00 0.00
H H
ATOM 26041 CE LYS H 131 24.651 -31.922 46.356 1.00 0.00
H C
ATOM 26042 HE1 LYS H 131 23.890 -32.574 45.873 1.00 0.00
H H
ATOM 26043 HE2 LYS H 131 25.650 -32.181 45.943 1.00 0.00
H H
ATOM 26044 NZ LYS H 131 24.651 -32.190 47.850 1.00 0.00
H N
ATOM 26045 HZ1 LYS H 131 24.867 -33.192 48.032 1.00 0.00
H H
ATOM 26046 HZ2 LYS H 131 25.367 -31.593 48.310 1.00 0.00
H H
ATOM 26047 HZ3 LYS H 131 23.714 -31.963 48.242 1.00 0.00
H H
ATOM 26048 C LYS H 131 24.849 -29.276 43.385 1.00 0.00
H C
ATOM 26049 O LYS H 131 25.153 -30.315 42.786 1.00 0.00
H O
ATOM 26050 N ILE H 132 23.611 -28.807 43.416 1.00 0.00
H N
ATOM 26051 HN ILE H 132 23.341 -27.898 43.724 1.00 0.00
H H
ATOM 26052 CA ILE H 132 22.506 -29.641 42.979 1.00 0.00
H C
ATOM 26053 HA ILE H 132 22.604 -30.588 43.494 1.00 0.00
H H
ATOM 26054 CB ILE H 132 21.170 -28.972 43.329 1.00 0.00
H C

```
ATOM  26055  HB  ILE H 132      21.181 -28.892  44.445  1.00  0.00
H    H
ATOM  26056  CG2 ILE H 132      21.049 -27.651  42.631  1.00  0.00
H    C
ATOM  26057 HG21 ILE H 132      20.163 -27.094  42.999  1.00  0.00
H    H
ATOM  26058 HG22 ILE H 132      21.944 -27.020  42.819  1.00  0.00
H    H
ATOM  26059 HG23 ILE H 132      20.941 -27.789  41.535  1.00  0.00
H    H
ATOM  26060  CG1 ILE H 132      20.017 -29.898  42.956  1.00  0.00
H    C
ATOM  26061 HG11 ILE H 132      19.638 -29.638  41.942  1.00  0.00
H    H
ATOM  26062 HG12 ILE H 132      20.396 -30.945  42.908  1.00  0.00
H    H
ATOM  26063  CD  ILE H 132      18.889 -29.850  43.928  1.00  0.00
H    C
ATOM  26064  HD1 ILE H 132      18.104 -30.582  43.644  1.00  0.00
H    H
ATOM  26065  HD2 ILE H 132      19.244 -30.099  44.952  1.00  0.00
H    H
ATOM  26066  HD3 ILE H 132      18.426 -28.840  43.952  1.00  0.00
H    H
ATOM  26067  C   ILE H 132      22.633 -29.932  41.498  1.00  0.00
H    C
ATOM  26068  O   ILE H 132      22.226 -30.995  41.014  1.00  0.00
H    O
ATOM  26069  N   ALA H 133      23.252 -29.015  40.766  1.00  0.00
H    N
ATOM  26070  HN  ALA H 133      23.599 -28.149  41.125  1.00  0.00
H    H
ATOM  26071  CA  ALA H 133      23.465 -29.231  39.350  1.00  0.00
H    C
ATOM  26072  HA  ALA H 133      22.532 -29.463  38.850  1.00  0.00
H    H
ATOM  26073  CB  ALA H 133      24.050 -27.972  38.723  1.00  0.00
H    C
ATOM  26074  HB1 ALA H 133      23.343 -27.126  38.857  1.00  0.00
H    H
ATOM  26075  HB2 ALA H 133      25.012 -27.694  39.207  1.00  0.00
H    H
ATOM  26076  HB3 ALA H 133      24.227 -28.101  37.633  1.00  0.00
H    H
ATOM  26077  C   ALA H 133      24.379 -30.417  39.122  1.00  0.00
H    C
ATOM  26078  O   ALA H 133      24.053 -31.319  38.340  1.00  0.00
H    O
ATOM  26079  N   GLN H 134      25.523 -30.444  39.806  1.00  0.00
H    N
ATOM  26080  HN  GLN H 134      25.862 -29.740  40.428  1.00  0.00
H    H
ATOM  26081  CA  GLN H 134      26.400 -31.595  39.663  1.00  0.00
H    C
```

ATOM 26082 HA GLN H 134   26.616 -31.703 38.606 1.00 0.00
H   H
ATOM 26083 CB GLN H 134   27.689 -31.374 40.441 1.00 0.00
H   C
ATOM 26084 HB1 GLN H 134   27.419 -31.389 41.522 1.00 0.00
H   H
ATOM 26085 HB2 GLN H 134   28.370 -32.240 40.279 1.00 0.00
H   H
ATOM 26086 CG GLN H 134   28.354 -30.070 40.089 1.00 0.00
H   C
ATOM 26087 HG1 GLN H 134   27.607 -29.348 39.695 1.00 0.00
H   H
ATOM 26088 HG2 GLN H 134   28.825 -29.615 40.984 1.00 0.00
H   H
ATOM 26089 CD GLN H 134   29.403 -30.229 39.015 1.00 0.00
H   C
ATOM 26090 OE1 GLN H 134   29.624 -31.328 38.506 1.00 0.00
H   O
ATOM 26091 NE2 GLN H 134   30.050 -29.127 38.652 1.00 0.00
H   N
ATOM 26092 HE21 GLN H 134   30.741 -29.224 37.940 1.00 0.00
H   H
ATOM 26093 HE22 GLN H 134   29.846 -28.263 39.099 1.00 0.00
H   H
ATOM 26094 C   GLN H 134   25.702 -32.865 40.120 1.00 0.00
H   C
ATOM 26095 O   GLN H 134   25.831 -33.916 39.485 1.00 0.00
H   O
ATOM 26096 N   SER H 135   24.931 -32.778 41.204 1.00 0.00
H   N
ATOM 26097 HN SER H 135   24.720 -31.946 41.717 1.00 0.00
H   H
ATOM 26098 CA SER H 135   24.308 -33.983 41.741 1.00 0.00
H   C
ATOM 26099 HA SER H 135   25.085 -34.736 41.753 1.00 0.00
H   H
ATOM 26100 CB SER H 135   23.766 -33.729 43.144 1.00 0.00
H   C
ATOM 26101 HB1 SER H 135   23.193 -34.630 43.462 1.00 0.00
H   H
ATOM 26102 HB2 SER H 135   24.604 -33.602 43.867 1.00 0.00
H   H
ATOM 26103 OG SER H 135   22.905 -32.612 43.156 1.00 0.00
H   O
ATOM 26104 HG1 SER H 135   22.426 -32.632 43.994 1.00 0.00
H   H
ATOM 26105 C   SER H 135   23.196 -34.497 40.837 1.00 0.00
H   C
ATOM 26106 O   SER H 135   22.849 -35.680 40.896 1.00 0.00
H   O
ATOM 26107 N   LEU H 136   22.619 -33.633 40.006 1.00 0.00
H   N
ATOM 26108 HN LEU H 136   22.828 -32.660 39.929 1.00 0.00
H   H

```
ATOM  26109  CA  LEU H 136      21.576 -34.096  39.099  1.00  0.00
H    C
ATOM  26110  HA  LEU H 136      21.101 -34.941  39.579  1.00  0.00
H    H
ATOM  26111  CB  LEU H 136      20.548 -32.996  38.857  1.00  0.00
H    C
ATOM  26112  HB1 LEU H 136      21.011 -32.035  39.179  1.00  0.00
H    H
ATOM  26113  HB2 LEU H 136      20.371 -32.900  37.763  1.00  0.00
H    H
ATOM  26114  CG  LEU H 136      19.244 -33.219  39.619  1.00  0.00
H    C
ATOM  26115  HG  LEU H 136      19.329 -32.797  40.650  1.00  0.00
H    H
ATOM  26116  CD1 LEU H 136      18.091 -32.583  38.888  1.00  0.00
H    C
ATOM  26117 HD11 LEU H 136      17.134 -32.786  39.417  1.00  0.00
H    H
ATOM  26118 HD12 LEU H 136      18.227 -31.482  38.824  1.00  0.00
H    H
ATOM  26119 HD13 LEU H 136      18.010 -32.987  37.856  1.00  0.00
H    H
ATOM  26120  CD2 LEU H 136      18.998 -34.706  39.743  1.00  0.00
H    C
ATOM  26121 HD21 LEU H 136      18.062 -34.897  40.312  1.00  0.00
H    H
ATOM  26122 HD22 LEU H 136      18.892 -35.188  38.750  1.00  0.00
H    H
ATOM  26123 HD23 LEU H 136      19.837 -35.195  40.283  1.00  0.00
H    H
ATOM  26124  C   LEU H 136      22.162 -34.583  37.786  1.00  0.00
H    C
ATOM  26125  O   LEU H 136      21.723 -35.604  37.247  1.00  0.00
H    O
ATOM  26126  N   SER H 137      23.150 -33.862  37.255  1.00  0.00
H    N
ATOM  26127  HN  SER H 137      23.534 -33.030  37.655  1.00  0.00
H    H
ATOM  26128  CA  SER H 137      23.767 -34.259  35.994  1.00  0.00
H    C
ATOM  26129  HA  SER H 137      22.993 -34.174  35.242  1.00  0.00
H    H
ATOM  26130  CB  SER H 137      24.959 -33.355  35.701  1.00  0.00
H    C
ATOM  26131  HB1 SER H 137      25.365 -33.631  34.701  1.00  0.00
H    H
ATOM  26132  HB2 SER H 137      24.633 -32.292  35.640  1.00  0.00
H    H
ATOM  26133  OG  SER H 137      25.964 -33.540  36.682  1.00  0.00
H    O
ATOM  26134  HG1 SER H 137      26.770 -33.130  36.343  1.00  0.00
H    H
ATOM  26135  C   SER H 137      24.202 -35.716  36.033  1.00  0.00
H    C
```

ATOM 26136 O SER H 137 23.689 -36.552 35.281 1.00 0.00
H O
ATOM 26137 N ASP H 138 25.125 -36.044 36.924 1.00 0.00
H N
ATOM 26138 HN ASP H 138 25.565 -35.401 37.545 1.00 0.00
H H
ATOM 26139 CA ASP H 138 25.602 -37.411 37.092 1.00 0.00
H C
ATOM 26140 HA ASP H 138 25.359 -37.981 36.204 1.00 0.00
H H
ATOM 26141 CB ASP H 138 27.097 -37.424 37.381 1.00 0.00
H C
ATOM 26142 HB1 ASP H 138 27.326 -36.662 38.154 1.00 0.00
H H
ATOM 26143 HB2 ASP H 138 27.412 -38.415 37.764 1.00 0.00
H H
ATOM 26144 CG ASP H 138 27.931 -37.095 36.162 1.00 0.00
H C
ATOM 26145 OD1 ASP H 138 28.154 -37.994 35.325 1.00 0.00
H O
ATOM 26146 OD2 ASP H 138 28.358 -35.928 36.040 1.00 0.00
H O
ATOM 26147 C ASP H 138 24.798 -38.014 38.235 1.00 0.00
H C
ATOM 26148 O ASP H 138 25.061 -37.739 39.408 1.00 0.00
H O
ATOM 26149 N GLU H 139 23.814 -38.842 37.893 1.00 0.00
H N
ATOM 26150 HN GLU H 139 23.644 -39.201 36.980 1.00 0.00
H H
ATOM 26151 CA GLU H 139 22.865 -39.305 38.896 1.00 0.00
H C
ATOM 26152 HA GLU H 139 22.600 -38.447 39.501 1.00 0.00
H H
ATOM 26153 CB GLU H 139 21.611 -39.836 38.207 1.00 0.00
H C
ATOM 26154 HB1 GLU H 139 20.980 -40.340 38.974 1.00 0.00
H H
ATOM 26155 HB2 GLU H 139 21.003 -38.986 37.823 1.00 0.00
H H
ATOM 26156 CG GLU H 139 21.897 -40.810 37.089 1.00 0.00
H C
ATOM 26157 HG1 GLU H 139 22.653 -40.379 36.409 1.00 0.00
H H
ATOM 26158 HG2 GLU H 139 22.271 -41.774 37.489 1.00 0.00
H H
ATOM 26159 CD GLU H 139 20.681 -41.079 36.237 1.00 0.00
H C
ATOM 26160 OE1 GLU H 139 20.837 -41.227 35.009 1.00 0.00
H O
ATOM 26161 OE2 GLU H 139 19.568 -41.133 36.793 1.00 0.00
H O
ATOM 26162 C GLU H 139 23.439 -40.360 39.833 1.00 0.00
H C

ATOM 26163 O GLU H 139 22.670 -40.986 40.570 1.00 0.00
H O
ATOM 26164 N ASN H 140 24.756 -40.578 39.833 1.00 0.00
H N
ATOM 26165 HN ASN H 140 25.419 -40.188 39.195 1.00 0.00
H H
ATOM 26166 CA ASN H 140 25.333 -41.463 40.838 1.00 0.00
H C
ATOM 26167 HA ASN H 140 24.497 -42.009 41.261 1.00 0.00
H H
ATOM 26168 CB ASN H 140 26.342 -42.427 40.214 1.00 0.00
H C
ATOM 26169 HB1 ASN H 140 26.159 -42.449 39.118 1.00 0.00
H H
ATOM 26170 HB2 ASN H 140 27.377 -42.057 40.372 1.00 0.00
H H
ATOM 26171 CG ASN H 140 26.198 -43.843 40.750 1.00 0.00
H C
ATOM 26172 OD1 ASN H 140 27.154 -44.620 40.751 1.00 0.00
H O
ATOM 26173 ND2 ASN H 140 24.996 -44.183 41.212 1.00 0.00
H N
ATOM 26174 HD21 ASN H 140 24.890 -45.105 41.576 1.00 0.00
H H
ATOM 26175 HD22 ASN H 140 24.256 -43.519 41.212 1.00 0.00
H H
ATOM 26176 C ASN H 140 25.991 -40.681 41.964 1.00 0.00
H C
ATOM 26177 O ASN H 140 25.927 -41.096 43.126 1.00 0.00
H O
ATOM 26178 N VAL H 141 26.638 -39.560 41.654 1.00 0.00
H N
ATOM 26179 HN VAL H 141 26.802 -39.202 40.739 1.00 0.00
H H
ATOM 26180 CA VAL H 141 27.180 -38.738 42.729 1.00 0.00
H C
ATOM 26181 HA VAL H 141 27.746 -39.362 43.409 1.00 0.00
H H
ATOM 26182 CB VAL H 141 28.090 -37.620 42.203 1.00 0.00
H C
ATOM 26183 HB VAL H 141 29.140 -37.878 42.487 1.00 0.00
H H
ATOM 26184 CG1 VAL H 141 28.003 -37.508 40.703 1.00 0.00
H C
ATOM 26185 HG11 VAL H 141 28.637 -36.669 40.342 1.00 0.00
H H
ATOM 26186 HG12 VAL H 141 28.359 -38.431 40.202 1.00 0.00
H H
ATOM 26187 HG13 VAL H 141 26.957 -37.310 40.384 1.00 0.00
H H
ATOM 26188 CG2 VAL H 141 27.837 -36.282 42.910 1.00 0.00
H C
ATOM 26189 HG21 VAL H 141 28.481 -35.484 42.483 1.00 0.00
H H

```
ATOM  26190 HG22 VAL H 141      26.775 -35.974  42.796  1.00  0.00
H    H
ATOM  26191 HG23 VAL H 141      28.065 -36.351  43.994  1.00  0.00
H    H
ATOM  26192  C   VAL H 141      25.987 -38.186  43.501  1.00  0.00
H    C
ATOM  26193  O   VAL H 141      25.146 -37.475  42.947  1.00  0.00
H    O
ATOM  26194  N   LEU H 142      25.853 -38.603  44.757  1.00  0.00
H    N
ATOM  26195  HN  LEU H 142      26.439 -39.263  45.221  1.00  0.00
H    H
ATOM  26196  CA  LEU H 142      24.769 -38.084  45.578  1.00  0.00
H    C
ATOM  26197  HA  LEU H 142      23.955 -37.836  44.909  1.00  0.00
H    H
ATOM  26198  CB  LEU H 142      24.316 -39.133  46.598  1.00  0.00
H    C
ATOM  26199  HB1 LEU H 142      24.068 -40.058  46.029  1.00  0.00
H    H
ATOM  26200  HB2 LEU H 142      25.176 -39.395  47.253  1.00  0.00
H    H
ATOM  26201  CG  LEU H 142      23.105 -38.704  47.421  1.00  0.00
H    C
ATOM  26202  HG  LEU H 142      23.374 -37.761  47.954  1.00  0.00
H    H
ATOM  26203  CD1 LEU H 142      21.925 -38.454  46.506  1.00  0.00
H    C
ATOM  26204 HD11 LEU H 142      21.023 -38.188  47.097  1.00  0.00
H    H
ATOM  26205 HD12 LEU H 142      22.144 -37.619  45.807  1.00  0.00
H    H
ATOM  26206 HD13 LEU H 142      21.693 -39.361  45.909  1.00  0.00
H    H
ATOM  26207  CD2 LEU H 142      22.756 -39.751  48.465  1.00  0.00
H    C
ATOM  26208 HD21 LEU H 142      21.884 -39.418  49.070  1.00  0.00
H    H
ATOM  26209 HD22 LEU H 142      22.491 -40.722  48.000  1.00  0.00
H    H
ATOM  26210 HD23 LEU H 142      23.613 -39.915  49.154  1.00  0.00
H    H
ATOM  26211  C   LEU H 142      25.198 -36.802  46.274  1.00  0.00
H    C
ATOM  26212  O   LEU H 142      24.581 -35.748  46.089  1.00  0.00
H    O
ATOM  26213  N   ASN H 143      26.261 -36.872  47.069  1.00  0.00
H    N
ATOM  26214  HN  ASN H 143      26.693 -37.727  47.350  1.00  0.00
H    H
ATOM  26215  CA  ASN H 143      26.891 -35.675  47.602  1.00  0.00
H    C
ATOM  26216  HA  ASN H 143      26.410 -34.820  47.139  1.00  0.00
H    H
```

ATOM 26217 CB ASN H 143   26.809 -35.609 49.130 1.00 0.00
H   C
ATOM 26218 HB1 ASN H 143   27.394 -36.461 49.540 1.00 0.00
H   H
ATOM 26219 HB2 ASN H 143   27.277 -34.674 49.501 1.00 0.00
H   H
ATOM 26220 CG ASN H 143   25.392 -35.723 49.650 1.00 0.00
H   C
ATOM 26221 OD1 ASN H 143   24.562 -36.417 49.074 1.00 0.00
H   O
ATOM 26222 ND2 ASN H 143   25.106 -35.027 50.743 1.00 0.00
H   N
ATOM 26223 HD21 ASN H 143   24.181 -35.101 51.104 1.00 0.00
H   H
ATOM 26224 HD22 ASN H 143   25.812 -34.484 51.186 1.00 0.00
H   H
ATOM 26225 C   ASN H 143   28.341 -35.687 47.153 1.00 0.00
H   C
ATOM 26226 O   ASN H 143   28.906 -36.745 46.869 1.00 0.00
H   O
ATOM 26227 N   TYR H 144   28.947 -34.506 47.101 1.00 0.00
H   N
ATOM 26228 HN TYR H 144   28.546 -33.627 47.352 1.00 0.00
H   H
ATOM 26229 CA TYR H 144   30.326 -34.433 46.643 1.00 0.00
H   C
ATOM 26230 HA TYR H 144   30.831 -35.327 46.987 1.00 0.00
H   H
ATOM 26231 CB TYR H 144   30.405 -34.316 45.121 1.00 0.00
H   C
ATOM 26232 HB1 TYR H 144   31.453 -34.496 44.796 1.00 0.00
H   H
ATOM 26233 HB2 TYR H 144   29.772 -35.089 44.634 1.00 0.00
H   H
ATOM 26234 CG TYR H 144   30.022 -32.964 44.576 1.00 0.00
H   C
ATOM 26235 CD1 TYR H 144   28.706 -32.533 44.594 1.00 0.00
H   C
ATOM 26236 HD1 TYR H 144   27.934 -33.178 44.992 1.00 0.00
H   H
ATOM 26237 CE1 TYR H 144   28.354 -31.299 44.093 1.00 0.00
H   C
ATOM 26238 HE1 TYR H 144   27.324 -30.974 44.106 1.00 0.00
H   H
ATOM 26239 CZ TYR H 144   29.324 -30.474 43.564 1.00 0.00
H   C
ATOM 26240 OH TYR H 144   28.965 -29.239 43.067 1.00 0.00
H   O
ATOM 26241 HH TYR H 144   29.771 -28.785 42.813 1.00 0.00
H   H
ATOM 26242 CD2 TYR H 144   30.982 -32.119 44.039 1.00 0.00
H   C
ATOM 26243 HD2 TYR H 144   32.011 -32.444 43.974 1.00 0.00
H   H

```
ATOM  26244  CE2 TYR H 144      30.643 -30.883  43.535  1.00  0.00
H    C
ATOM  26245  HE2 TYR H 144      31.406 -30.253  43.103  1.00  0.00
H    H
ATOM  26246  C   TYR H 144      31.006 -33.250  47.309  1.00  0.00
H    C
ATOM  26247  O   TYR H 144      30.388 -32.218  47.574  1.00  0.00
H    O
ATOM  26248  N   ILE H 145      32.300 -33.418  47.566  1.00  0.00
H    N
ATOM  26249  HN  ILE H 145      32.779 -34.276  47.393  1.00  0.00
H    H
ATOM  26250  CA  ILE H 145      33.142 -32.367  48.123  1.00  0.00
H    C
ATOM  26251  HA  ILE H 145      32.680 -31.417  47.885  1.00  0.00
H    H
ATOM  26252  CB  ILE H 145      33.325 -32.511  49.647  1.00  0.00
H    C
ATOM  26253  HB  ILE H 145      32.688 -33.390  49.924  1.00  0.00
H    H
ATOM  26254  CG2 ILE H 145      34.794 -32.720  50.020  1.00  0.00
H    C
ATOM  26255 HG21 ILE H 145      34.892 -32.977  51.095  1.00  0.00
H    H
ATOM  26256 HG22 ILE H 145      35.236 -33.553  49.433  1.00  0.00
H    H
ATOM  26257 HG23 ILE H 145      35.388 -31.802  49.827  1.00  0.00
H    H
ATOM  26258  CG1 ILE H 145      32.735 -31.286  50.332  1.00  0.00
H    C
ATOM  26259 HG11 ILE H 145      32.195 -30.660  49.586  1.00  0.00
H    H
ATOM  26260 HG12 ILE H 145      31.985 -31.620  51.085  1.00  0.00
H    H
ATOM  26261  CD  ILE H 145      33.753 -30.420  51.045  1.00  0.00
H    C
ATOM  26262  HD1 ILE H 145      33.246 -29.581  51.568  1.00  0.00
H    H
ATOM  26263  HD2 ILE H 145      34.312 -31.014  51.800  1.00  0.00
H    H
ATOM  26264  HD3 ILE H 145      34.480 -29.988  50.327  1.00  0.00
H    H
ATOM  26265  C   ILE H 145      34.485 -32.395  47.405  1.00  0.00
H    C
ATOM  26266  O   ILE H 145      35.014 -33.472  47.106  1.00  0.00
H    O
ATOM  26267  N   ASP H 146      35.005 -31.215  47.077  1.00  0.00
H    N
ATOM  26268  HN  ASP H 146      34.582 -30.333  47.273  1.00  0.00
H    H
ATOM  26269  CA  ASP H 146      36.280 -31.090  46.380  1.00  0.00
H    C
ATOM  26270  HA  ASP H 146      36.691 -32.087  46.268  1.00  0.00
H    H
```

ATOM 26271 CB ASP H 146   36.080 -30.402 45.033 1.00 0.00
H   C
ATOM 26272 HB1 ASP H 146   37.036 -29.940 44.714 1.00 0.00
H   H
ATOM 26273 HB2 ASP H 146   35.774 -31.137 44.261 1.00 0.00
H   H
ATOM 26274 CG ASP H 146   35.040 -29.302 45.091 1.00 0.00
H   C
ATOM 26275 OD1 ASP H 146   35.377 -28.208 45.595 1.00 0.00
H   O
ATOM 26276 OD2 ASP H 146   33.882 -29.536 44.681 1.00 0.00
H   O
ATOM 26277 C   ASP H 146   37.262 -30.303 47.231 1.00 0.00
H   C
ATOM 26278 O   ASP H 146   36.963 -29.184 47.656 1.00 0.00
H   O
ATOM 26279 N   LEU H 147   38.434 -30.883 47.472 1.00 0.00
H   N
ATOM 26280 HN LEU H 147   38.674 -31.810 47.192 1.00 0.00
H   H
ATOM 26281 CA LEU H 147   39.490 -30.181 48.188 1.00 0.00
H   C
ATOM 26282 HA LEU H 147   38.996 -29.580 48.940 1.00 0.00
H   H
ATOM 26283 CB LEU H 147   40.419 -31.200 48.843 1.00 0.00
H   C
ATOM 26284 HB1 LEU H 147   41.404 -30.700 48.988 1.00 0.00
H   H
ATOM 26285 HB2 LEU H 147   40.039 -31.433 49.862 1.00 0.00
H   H
ATOM 26286 CG LEU H 147   40.563 -32.440 47.966 1.00 0.00
H   C
ATOM 26287 HG LEU H 147   39.647 -32.477 47.328 1.00 0.00
H   H
ATOM 26288 CD1 LEU H 147   41.782 -32.346 47.068 1.00 0.00
H   C
ATOM 26289 HD11 LEU H 147   41.900 -33.276 46.471 1.00 0.00
H   H
ATOM 26290 HD12 LEU H 147   41.685 -31.491 46.365 1.00 0.00
H   H
ATOM 26291 HD13 LEU H 147   42.704 -32.203 47.671 1.00 0.00
H   H
ATOM 26292 CD2 LEU H 147   40.582 -33.691 48.836 1.00 0.00
H   C
ATOM 26293 HD21 LEU H 147   40.668 -34.601 48.204 1.00 0.00
H   H
ATOM 26294 HD22 LEU H 147   41.440 -33.688 49.538 1.00 0.00
H   H
ATOM 26295 HD23 LEU H 147   39.645 -33.768 49.429 1.00 0.00
H   H
ATOM 26296 C   LEU H 147   40.274 -29.237 47.287 1.00 0.00
H   C
ATOM 26297 O   LEU H 147   40.845 -28.255 47.776 1.00 0.00
H   O

ATOM 26298 N SER H 148 40.304 −29.498 45.988 1.00 0.00
H N
ATOM 26299 HN SER H 148 39.840 −30.273 45.560 1.00 0.00
H H
ATOM 26300 CA SER H 148 41.036 −28.654 45.056 1.00 0.00
H C
ATOM 26301 HA SER H 148 41.056 −27.656 45.473 1.00 0.00
H H
ATOM 26302 CB SER H 148 42.464 −29.194 44.865 1.00 0.00
H C
ATOM 26303 HB1 SER H 148 43.000 −28.514 44.164 1.00 0.00
H H
ATOM 26304 HB2 SER H 148 43.019 −29.172 45.830 1.00 0.00
H H
ATOM 26305 OG SER H 148 42.445 −30.502 44.311 1.00 0.00
H O
ATOM 26306 HG1 SER H 148 43.342 −30.696 44.010 1.00 0.00
H H
ATOM 26307 C SER H 148 40.248 −28.585 43.754 1.00 0.00
H C
ATOM 26308 O SER H 148 39.136 −29.122 43.658 1.00 0.00
H O
ATOM 26309 N ASP H 149 40.821 −27.926 42.745 1.00 0.00
H N
ATOM 26310 HN ASP H 149 41.684 −27.429 42.777 1.00 0.00
H H
ATOM 26311 CA ASP H 149 40.187 −27.878 41.433 1.00 0.00
H C
ATOM 26312 HA ASP H 149 39.116 −27.893 41.601 1.00 0.00
H H
ATOM 26313 CB ASP H 149 40.608 −26.610 40.696 1.00 0.00
H C
ATOM 26314 HB1 ASP H 149 40.280 −26.676 39.639 1.00 0.00
H H
ATOM 26315 HB2 ASP H 149 40.134 −25.717 41.153 1.00 0.00
H H
ATOM 26316 CG ASP H 149 42.108 −26.412 40.695 1.00 0.00
H C
ATOM 26317 OD1 ASP H 149 42.597 −25.548 41.453 1.00 0.00
H O
ATOM 26318 OD2 ASP H 149 42.799 −27.134 39.951 1.00 0.00
H O
ATOM 26319 C ASP H 149 40.519 −29.113 40.605 1.00 0.00
H C
ATOM 26320 O ASP H 149 40.092 −29.217 39.450 1.00 0.00
H O
ATOM 26321 N GLU H 150 41.267 −30.051 41.178 1.00 0.00
H N
ATOM 26322 HN GLU H 150 41.599 −29.978 42.115 1.00 0.00
H H
ATOM 26323 CA GLU H 150 41.676 −31.273 40.498 1.00 0.00
H C
ATOM 26324 HA GLU H 150 41.277 −31.225 39.492 1.00 0.00
H H

ATOM 26325 CB GLU H 150   43.205 −31.363 40.451 1.00 0.00
H   C
ATOM 26326 HB1 GLU H 150   43.592 −30.448 39.946 1.00 0.00
H   H
ATOM 26327 HB2 GLU H 150   43.616 −31.338 41.485 1.00 0.00
H   H
ATOM 26328 CG GLU H 150   43.720 −32.586 39.720 1.00 0.00
H   C
ATOM 26329 HG1 GLU H 150   44.395 −33.156 40.384 1.00 0.00
H   H
ATOM 26330 HG2 GLU H 150   42.884 −33.241 39.401 1.00 0.00
H   H
ATOM 26331 CD GLU H 150   44.546 −32.250 38.502 1.00 0.00
H   C
ATOM 26332 OE1 GLU H 150   44.390 −31.134 37.967 1.00 0.00
H   O
ATOM 26333 OE2 GLU H 150   45.351 −33.105 38.080 1.00 0.00
H   O
ATOM 26334 C   GLU H 150   41.132 −32.536 41.139 1.00 0.00
H   C
ATOM 26335 O   GLU H 150   40.816 −33.495 40.431 1.00 0.00
H   O
ATOM 26336 N   TYR H 151   41.018 −32.567 42.460 1.00 0.00
H   N
ATOM 26337 HN TYR H 151   41.180 −31.775 43.046 1.00 0.00
H   H
ATOM 26338 CA TYR H 151   40.641 −33.777 43.170 1.00 0.00
H   C
ATOM 26339 HA TYR H 151   40.309 −34.489 42.425 1.00 0.00
H   H
ATOM 26340 CB TYR H 151   41.820 −34.315 43.977 1.00 0.00
H   C
ATOM 26341 HB1 TYR H 151   41.989 −33.658 44.858 1.00 0.00
H   H
ATOM 26342 HB2 TYR H 151   41.599 −35.333 44.364 1.00 0.00
H   H
ATOM 26343 CG TYR H 151   43.124 −34.351 43.214 1.00 0.00
H   C
ATOM 26344 CD1 TYR H 151   43.380 −35.345 42.281 1.00 0.00
H   C
ATOM 26345 HD1 TYR H 151   42.642 −36.115 42.100 1.00 0.00
H   H
ATOM 26346 CE1 TYR H 151   44.571 −35.388 41.590 1.00 0.00
H   C
ATOM 26347 HE1 TYR H 151   44.762 −36.172 40.872 1.00 0.00
H   H
ATOM 26348 CZ TYR H 151   45.527 −34.426 41.825 1.00 0.00
H   C
ATOM 26349 OH TYR H 151   46.716 −34.459 41.136 1.00 0.00
H   O
ATOM 26350 HH TYR H 151   47.214 −33.677 41.382 1.00 0.00
H   H
ATOM 26351 CD2 TYR H 151   44.102 −33.393 43.432 1.00 0.00
H   C

ATOM 26352 HD2 TYR H 151   43.951 -32.626 44.180 1.00 0.00
H   H
ATOM 26353 CE2 TYR H 151   45.297 -33.426 42.744 1.00 0.00
H   C
ATOM 26354 HE2 TYR H 151   46.052 -32.682 42.945 1.00 0.00
H   H
ATOM 26355 C   TYR H 151   39.458 -33.503 44.087 1.00 0.00
H   C
ATOM 26356 O   TYR H 151   39.207 -32.356 44.469 1.00 0.00
H   O
ATOM 26357 N   SER H 152   38.737 -34.565 44.437 1.00 0.00
H   N
ATOM 26358 HN SER H 152   38.994 -35.514 44.254 1.00 0.00
H   H
ATOM 26359 CA SER H 152   37.472 -34.415 45.140 1.00 0.00
H   C
ATOM 26360 HA SER H 152   37.664 -33.736 45.961 1.00 0.00
H   H
ATOM 26361 CB SER H 152   36.392 -33.889 44.195 1.00 0.00
H   C
ATOM 26362 HB1 SER H 152   35.456 -34.464 44.385 1.00 0.00
H   H
ATOM 26363 HB2 SER H 152   36.170 -32.821 44.417 1.00 0.00
H   H
ATOM 26364 OG SER H 152   36.788 -34.074 42.845 1.00 0.00
H   O
ATOM 26365 HG1 SER H 152   36.003 -33.941 42.298 1.00 0.00
H   H
ATOM 26366 C   SER H 152   37.022 -35.737 45.742 1.00 0.00
H   C
ATOM 26367 O   SER H 152   37.513 -36.813 45.377 1.00 0.00
H   O
ATOM 26368 N   ILE H 153   36.062 -35.635 46.658 1.00 0.00
H   N
ATOM 26369 HN ILE H 153   35.705 -34.750 46.949 1.00 0.00
H   H
ATOM 26370 CA ILE H 153   35.443 -36.777 47.321 1.00 0.00
H   C
ATOM 26371 HA ILE H 153   35.967 -37.669 47.003 1.00 0.00
H   H
ATOM 26372 CB ILE H 153   35.467 -36.612 48.849 1.00 0.00
H   C
ATOM 26373 HB ILE H 153   35.111 -35.565 49.023 1.00 0.00
H   H
ATOM 26374 CG2 ILE H 153   34.606 -37.664 49.527 1.00 0.00
H   C
ATOM 26375 HG21 ILE H 153   34.503 -37.445 50.611 1.00 0.00
H   H
ATOM 26376 HG22 ILE H 153   33.586 -37.684 49.089 1.00 0.00
H   H
ATOM 26377 HG23 ILE H 153   35.054 -38.675 49.418 1.00 0.00
H   H
ATOM 26378 CG1 ILE H 153   36.900 -36.684 49.378 1.00 0.00
H   C

```
ATOM  26379 HG11 ILE H 153      37.099 -37.699  49.788  1.00  0.00
H    H
ATOM  26380 HG12 ILE H 153      37.606 -36.527  48.529  1.00  0.00
H    H
ATOM  26381  CD  ILE H 153      37.208 -35.645  50.449  1.00  0.00
H    C
ATOM  26382  HD1 ILE H 153      38.275 -35.708  50.751  1.00  0.00
H    H
ATOM  26383  HD2 ILE H 153      37.014 -34.619  50.067  1.00  0.00
H    H
ATOM  26384  HD3 ILE H 153      36.584 -35.808  51.353  1.00  0.00
H    H
ATOM  26385  C   ILE H 153      34.015 -36.897  46.805  1.00  0.00
H    C
ATOM  26386  O   ILE H 153      33.345 -35.883  46.575  1.00  0.00
H    O
ATOM  26387  N   VAL H 154      33.542 -38.129  46.614  1.00  0.00
H    N
ATOM  26388  HN  VAL H 154      34.062 -38.947  46.845  1.00  0.00
H    H
ATOM  26389  CA  VAL H 154      32.222 -38.392  46.051  1.00  0.00
H    C
ATOM  26390  HA  VAL H 154      31.599 -37.526  46.238  1.00  0.00
H    H
ATOM  26391  CB  VAL H 154      32.290 -38.660  44.533  1.00  0.00
H    C
ATOM  26392  HB  VAL H 154      32.899 -39.585  44.381  1.00  0.00
H    H
ATOM  26393  CG1 VAL H 154      30.891 -38.878  43.971  1.00  0.00
H    C
ATOM  26394 HG11 VAL H 154      30.937 -39.033  42.872  1.00  0.00
H    H
ATOM  26395 HG12 VAL H 154      30.409 -39.775  44.411  1.00  0.00
H    H
ATOM  26396 HG13 VAL H 154      30.246 -37.995  44.167  1.00  0.00
H    H
ATOM  26397  CG2 VAL H 154      32.972 -37.508  43.804  1.00  0.00
H    C
ATOM  26398 HG21 VAL H 154      32.982 -37.688  42.708  1.00  0.00
H    H
ATOM  26399 HG22 VAL H 154      32.436 -36.553  43.996  1.00  0.00
H    H
ATOM  26400 HG23 VAL H 154      34.025 -37.390  44.134  1.00  0.00
H    H
ATOM  26401  C   VAL H 154      31.582 -39.577  46.766  1.00  0.00
H    C
ATOM  26402  O   VAL H 154      32.255 -40.567  47.079  1.00  0.00
H    O
ATOM  26403  N   GLU H 155      30.274 -39.480  47.009  1.00  0.00
H    N
ATOM  26404  HN  GLU H 155      29.770 -38.622  46.953  1.00  0.00
H    H
ATOM  26405  CA  GLU H 155      29.455 -40.627  47.381  1.00  0.00
H    C
```

ATOM 26406 HA GLU H 155   30.097 -41.446 47.680 1.00 0.00
H   H
ATOM 26407 CB GLU H 155   28.470 -40.294 48.501 1.00 0.00
H   C
ATOM 26408 HB1 GLU H 155   28.093 -39.259 48.336 1.00 0.00
H   H
ATOM 26409 HB2 GLU H 155   27.580 -40.959 48.431 1.00 0.00
H   H
ATOM 26410 CG GLU H 155   29.039 -40.374 49.890 1.00 0.00
H   C
ATOM 26411 HG1 GLU H 155   28.580 -41.223 50.428 1.00 0.00
H   H
ATOM 26412 HG2 GLU H 155   30.139 -40.511 49.861 1.00 0.00
H   H
ATOM 26413 CD GLU H 155   28.725 -39.145 50.710 1.00 0.00
H   C
ATOM 26414 OE1 GLU H 155   28.020 -39.276 51.733 1.00 0.00
H   O
ATOM 26415 OE2 GLU H 155   29.167 -38.048 50.308 1.00 0.00
H   O
ATOM 26416 C   GLU H 155   28.682 -41.083 46.154 1.00 0.00
H   C
ATOM 26417 O   GLU H 155   28.106 -40.257 45.442 1.00 0.00
H   O
ATOM 26418 N   LEU H 156   28.666 -42.388 45.907 1.00 0.00
H   N
ATOM 26419 HN LEU H 156   29.206 -43.063 46.406 1.00 0.00
H   H
ATOM 26420 CA LEU H 156   27.829 -42.950 44.853 1.00 0.00
H   C
ATOM 26421 HA LEU H 156   27.105 -42.194 44.579 1.00 0.00
H   H
ATOM 26422 CB LEU H 156   28.655 -43.348 43.617 1.00 0.00
H   C
ATOM 26423 HB1 LEU H 156   28.935 -44.420 43.740 1.00 0.00
H   H
ATOM 26424 HB2 LEU H 156   28.005 -43.301 42.716 1.00 0.00
H   H
ATOM 26425 CG LEU H 156   29.940 -42.549 43.369 1.00 0.00
H   C
ATOM 26426 HG LEU H 156   29.847 -41.639 44.010 1.00 0.00
H   H
ATOM 26427 CD1 LEU H 156   31.173 -43.334 43.785 1.00 0.00
H   C
ATOM 26428 HD11 LEU H 156   32.097 -42.761 43.554 1.00 0.00
H   H
ATOM 26429 HD12 LEU H 156   31.156 -43.542 44.877 1.00 0.00
H   H
ATOM 26430 HD13 LEU H 156   31.223 -44.304 43.244 1.00 0.00
H   H
ATOM 26431 CD2 LEU H 156   30.035 -42.095 41.917 1.00 0.00
H   C
ATOM 26432 HD21 LEU H 156   30.962 -41.502 41.758 1.00 0.00
H   H

ATOM 26433 HD22 LEU H 156 30.063 -42.956 41.219 1.00 0.00
H H
ATOM 26434 HD23 LEU H 156 29.165 -41.457 41.651 1.00 0.00
H H
ATOM 26435 C LEU H 156 27.060 -44.149 45.386 1.00 0.00
H C
ATOM 26436 O LEU H 156 27.636 -45.038 46.011 1.00 0.00
H O
ATOM 26437 N LEU H 157 25.755 -44.178 45.136 1.00 0.00
H N
ATOM 26438 HN LEU H 157 25.244 -43.478 44.643 1.00 0.00
H H
ATOM 26439 CA LEU H 157 24.945 -45.299 45.594 1.00 0.00
H C
ATOM 26440 HA LEU H 157 25.349 -45.636 46.539 1.00 0.00
H H
ATOM 26441 CB LEU H 157 23.485 -44.888 45.773 1.00 0.00
H C
ATOM 26442 HB1 LEU H 157 23.304 -44.786 46.868 1.00 0.00
H H
ATOM 26443 HB2 LEU H 157 23.338 -43.874 45.341 1.00 0.00
H H
ATOM 26444 CG LEU H 157 22.363 -45.783 45.242 1.00 0.00
H C
ATOM 26445 HG LEU H 157 22.773 -46.801 45.037 1.00 0.00
H H
ATOM 26446 CD1 LEU H 157 21.257 -45.873 46.274 1.00 0.00
H C
ATOM 26447 HD11 LEU H 157 20.410 -46.480 45.887 1.00 0.00
H H
ATOM 26448 HD12 LEU H 157 21.630 -46.348 47.207 1.00 0.00
H H
ATOM 26449 HD13 LEU H 157 20.871 -44.861 46.526 1.00 0.00
H H
ATOM 26450 CD2 LEU H 157 21.823 -45.221 43.934 1.00 0.00
H C
ATOM 26451 HD21 LEU H 157 21.020 -45.878 43.535 1.00 0.00
H H
ATOM 26452 HD22 LEU H 157 21.392 -44.208 44.070 1.00 0.00
H H
ATOM 26453 HD23 LEU H 157 22.631 -45.159 43.174 1.00 0.00
H H
ATOM 26454 C LEU H 157 25.090 -46.445 44.597 1.00 0.00
H C
ATOM 26455 O LEU H 157 25.087 -46.224 43.381 1.00 0.00
H O
ATOM 26456 N ALA H 158 25.232 -47.667 45.109 1.00 0.00
H N
ATOM 26457 HN ALA H 158 25.105 -47.880 46.077 1.00 0.00
H H
ATOM 26458 CA ALA H 158 25.592 -48.797 44.261 1.00 0.00
H C
ATOM 26459 HA ALA H 158 26.367 -48.517 43.558 1.00 0.00
H H

ATOM 26460 CB ALA H 158 26.114 -49.957 45.106 1.00 0.00
H C
ATOM 26461 HB1 ALA H 158 27.013 -49.631 45.673 1.00 0.00
H H
ATOM 26462 HB2 ALA H 158 25.350 -50.293 45.840 1.00 0.00
H H
ATOM 26463 HB3 ALA H 158 26.404 -50.825 44.475 1.00 0.00
H H
ATOM 26464 C ALA H 158 24.402 -49.254 43.429 1.00 0.00
H C
ATOM 26465 O ALA H 158 23.292 -49.413 43.940 1.00 0.00
H O
ATOM 26466 N THR H 159 24.638 -49.448 42.137 1.00 0.00
H N
ATOM 26467 HN THR H 159 25.502 -49.240 41.681 1.00 0.00
H H
ATOM 26468 CA THR H 159 23.619 -49.996 41.263 1.00 0.00
H C
ATOM 26469 HA THR H 159 22.669 -49.789 41.737 1.00 0.00
H H
ATOM 26470 CB THR H 159 23.720 -49.380 39.868 1.00 0.00
H C
ATOM 26471 HB THR H 159 23.706 -48.272 40.017 1.00 0.00
H H
ATOM 26472 OG1 THR H 159 22.566 -49.737 39.095 1.00 0.00
H O
ATOM 26473 HG1 THR H 159 22.699 -49.330 38.232 1.00 0.00
H H
ATOM 26474 CG2 THR H 159 24.978 -49.858 39.162 1.00 0.00
H C
ATOM 26475 HG21 THR H 159 25.115 -49.327 38.197 1.00 0.00
H H
ATOM 26476 HG22 THR H 159 25.873 -49.661 39.791 1.00 0.00
H H
ATOM 26477 HG23 THR H 159 24.930 -50.948 38.955 1.00 0.00
H H
ATOM 26478 C THR H 159 23.745 -51.517 41.191 1.00 0.00
H C
ATOM 26479 O THR H 159 24.605 -52.132 41.828 1.00 0.00
H O
ATOM 26480 N ARG H 160 22.862 -52.126 40.398 1.00 0.00
H N
ATOM 26481 HN ARG H 160 22.162 -51.629 39.890 1.00 0.00
H H
ATOM 26482 CA ARG H 160 22.882 -53.575 40.239 1.00 0.00
H C
ATOM 26483 HA ARG H 160 22.854 -54.019 41.228 1.00 0.00
H H
ATOM 26484 CB ARG H 160 21.667 -54.026 39.431 1.00 0.00
H C
ATOM 26485 HB1 ARG H 160 20.805 -53.413 39.789 1.00 0.00
H H
ATOM 26486 HB2 ARG H 160 21.811 -53.756 38.362 1.00 0.00
H H

ATOM 26487 CG ARG H 160   21.332 -55.491 39.598 1.00 0.00
H   C
ATOM 26488 HG1 ARG H 160   22.281 -56.069 39.641 1.00 0.00
H   H
ATOM 26489 HG2 ARG H 160   20.845 -55.641 40.589 1.00 0.00
H   H
ATOM 26490 CD ARG H 160   20.395 -55.944 38.500 1.00 0.00
H   C
ATOM 26491 HD1 ARG H 160   20.418 -57.056 38.428 1.00 0.00
H   H
ATOM 26492 HD2 ARG H 160   19.346 -55.621 38.698 1.00 0.00
H   H
ATOM 26493 NE ARG H 160   20.773 -55.374 37.211 1.00 0.00
H   N
ATOM 26494 HE ARG H 160   20.173 -54.620 36.920 1.00 0.00
H   H
ATOM 26495 CZ ARG H 160   21.802 -55.790 36.478 1.00 0.00
H   C
ATOM 26496 NH1 ARG H 160   22.566 -56.785 36.903 1.00 0.00
H   N
ATOM 26497 HH11 ARG H 160   23.348 -57.081 36.372 1.00 0.00
H   H
ATOM 26498 HH12 ARG H 160   22.391 -57.114 37.833 1.00 0.00
H   H
ATOM 26499 NH2 ARG H 160   22.067 -55.207 35.320 1.00 0.00
H   N
ATOM 26500 HH21 ARG H 160   22.777 -55.572 34.734 1.00 0.00
H   H
ATOM 26501 HH22 ARG H 160   21.403 -54.551 34.986 1.00 0.00
H   H
ATOM 26502 C   ARG H 160   24.163 -54.064 39.577 1.00 0.00
H   C
ATOM 26503 O   ARG H 160   24.545 -55.223 39.773 1.00 0.00
H   O
ATOM 26504 N   LYS H 161   24.837 -53.209 38.806 1.00 0.00
H   N
ATOM 26505 HN LYS H 161   24.490 -52.306 38.560 1.00 0.00
H   H
ATOM 26506 CA LYS H 161   26.148 -53.538 38.261 1.00 0.00
H   C
ATOM 26507 HA LYS H 161   26.037 -54.482 37.741 1.00 0.00
H   H
ATOM 26508 CB LYS H 161   26.613 -52.440 37.304 1.00 0.00
H   C
ATOM 26509 HB1 LYS H 161   26.758 -51.516 37.911 1.00 0.00
H   H
ATOM 26510 HB2 LYS H 161   27.614 -52.710 36.902 1.00 0.00
H   H
ATOM 26511 CG LYS H 161   25.647 -52.142 36.177 1.00 0.00
H   C
ATOM 26512 HG1 LYS H 161   24.615 -52.069 36.591 1.00 0.00
H   H
ATOM 26513 HG2 LYS H 161   25.898 -51.150 35.737 1.00 0.00
H   H

ATOM 26514 CD LYS H 161   25.700 -53.231 35.129 1.00 0.00
H   C
ATOM 26515 HD1 LYS H 161   25.363 -54.186 35.595 1.00 0.00
H   H
ATOM 26516 HD2 LYS H 161   24.969 -52.976 34.327 1.00 0.00
H   H
ATOM 26517 CE LYS H 161   27.103 -53.358 34.563 1.00 0.00
H   C
ATOM 26518 HE1 LYS H 161   27.440 -52.388 34.137 1.00 0.00
H   H
ATOM 26519 HE2 LYS H 161   27.819 -53.660 35.359 1.00 0.00
H   H
ATOM 26520 NZ LYS H 161   27.182 -54.372 33.482 1.00 0.00
H   N
ATOM 26521 HZ1 LYS H 161   28.156 -54.430 33.119 1.00 0.00
H   H
ATOM 26522 HZ2 LYS H 161   26.896 -55.301 33.854 1.00 0.00
H   H
ATOM 26523 HZ3 LYS H 161   26.542 -54.103 32.708 1.00 0.00
H   H
ATOM 26524 C   LYS H 161   27.197 -53.738 39.344 1.00 0.00
H   C
ATOM 26525 O   LYS H 161   28.342 -54.065 39.016 1.00 0.00
H   O
ATOM 26526 N   LEU H 162   26.840 -53.544 40.614 1.00 0.00
H   N
ATOM 26527 HN LEU H 162   25.900 -53.369 40.900 1.00 0.00
H   H
ATOM 26528 CA LEU H 162   27.800 -53.572 41.704 1.00 0.00
H   C
ATOM 26529 HA LEU H 162   28.727 -53.910 41.260 1.00 0.00
H   H
ATOM 26530 CB LEU H 162   27.947 -52.169 42.307 1.00 0.00
H   C
ATOM 26531 HB1 LEU H 162   26.920 -51.798 42.531 1.00 0.00
H   H
ATOM 26532 HB2 LEU H 162   28.465 -52.250 43.289 1.00 0.00
H   H
ATOM 26533 CG LEU H 162   28.644 -51.099 41.464 1.00 0.00
H   C
ATOM 26534 HG LEU H 162   27.908 -50.781 40.687 1.00 0.00
H   H
ATOM 26535 CD1 LEU H 162   29.003 -49.911 42.338 1.00 0.00
H   C
ATOM 26536 HD11 LEU H 162   29.553 -49.146 41.749 1.00 0.00
H   H
ATOM 26537 HD12 LEU H 162   28.087 -49.439 42.754 1.00 0.00
H   H
ATOM 26538 HD13 LEU H 162   29.650 -50.227 43.184 1.00 0.00
H   H
ATOM 26539 CD2 LEU H 162   29.876 -51.645 40.751 1.00 0.00
H   C
ATOM 26540 HD21 LEU H 162   30.349 -50.851 40.134 1.00 0.00
H   H

```
ATOM  26541 HD22 LEU H 162      30.637 -52.009  41.469  1.00  0.00
H    H
ATOM  26542 HD23 LEU H 162      29.596 -52.487  40.081  1.00  0.00
H    H
ATOM  26543  C   LEU H 162      27.446 -54.541  42.822  1.00  0.00
H    C
ATOM  26544  O   LEU H 162      28.155 -54.564  43.834  1.00  0.00
H    O
ATOM  26545  N   ASP H 163      26.387 -55.335  42.677  1.00  0.00
H    N
ATOM  26546  HN  ASP H 163      25.845 -55.436  41.848  1.00  0.00
H    H
ATOM  26547  CA  ASP H 163      25.907 -56.164  43.776  1.00  0.00
H    C
ATOM  26548  HA  ASP H 163      25.850 -55.529  44.653  1.00  0.00
H    H
ATOM  26549  CB  ASP H 163      24.541 -56.760  43.422  1.00  0.00
H    C
ATOM  26550  HB1 ASP H 163      23.882 -55.957  43.035  1.00  0.00
H    H
ATOM  26551  HB2 ASP H 163      24.644 -57.535  42.638  1.00  0.00
H    H
ATOM  26552  CG  ASP H 163      23.839 -57.381  44.622  1.00  0.00
H    C
ATOM  26553  OD1 ASP H 163      24.485 -57.570  45.675  1.00  0.00
H    O
ATOM  26554  OD2 ASP H 163      22.633 -57.693  44.504  1.00  0.00
H    O
ATOM  26555  C   ASP H 163      26.898 -57.273  44.100  1.00  0.00
H    C
ATOM  26556  O   ASP H 163      27.480 -57.886  43.201  1.00  0.00
H    O
ATOM  26557  N   SER H 164      27.071 -57.532  45.399  1.00  0.00
H    N
ATOM  26558  HN  SER H 164      26.651 -56.974  46.113  1.00  0.00
H    H
ATOM  26559  CA  SER H 164      27.880 -58.642  45.903  1.00  0.00
H    C
ATOM  26560  HA  SER H 164      27.954 -58.497  46.973  1.00  0.00
H    H
ATOM  26561  CB  SER H 164      27.215 -59.983  45.591  1.00  0.00
H    C
ATOM  26562  HB1 SER H 164      27.584 -60.331  44.599  1.00  0.00
H    H
ATOM  26563  HB2 SER H 164      27.518 -60.750  46.340  1.00  0.00
H    H
ATOM  26564  OG  SER H 164      25.805 -59.846  45.534  1.00  0.00
H    O
ATOM  26565  HG1 SER H 164      25.458 -60.648  45.122  1.00  0.00
H    H
ATOM  26566  C   SER H 164      29.296 -58.594  45.341  1.00  0.00
H    C
ATOM  26567  O   SER H 164      29.928 -59.619  45.081  1.00  0.00
H    O
```

ATOM 26568 N LYS H 165 29.797 -57.381 45.154 1.00 0.00
H N
ATOM 26569 HN LYS H 165 29.292 -56.551 45.387 1.00 0.00
H H
ATOM 26570 CA LYS H 165 31.120 -57.162 44.598 1.00 0.00
H C
ATOM 26571 HA LYS H 165 31.446 -58.088 44.140 1.00 0.00
H H
ATOM 26572 CB LYS H 165 31.096 -56.032 43.566 1.00 0.00
H C
ATOM 26573 HB1 LYS H 165 30.660 -55.139 44.070 1.00 0.00
H H
ATOM 26574 HB2 LYS H 165 32.143 -55.762 43.300 1.00 0.00
H H
ATOM 26575 CG LYS H 165 30.281 -56.365 42.328 1.00 0.00
H C
ATOM 26576 HG1 LYS H 165 29.206 -56.444 42.609 1.00 0.00
H H
ATOM 26577 HG2 LYS H 165 30.373 -55.528 41.598 1.00 0.00
H H
ATOM 26578 CD LYS H 165 30.724 -57.671 41.693 1.00 0.00
H C
ATOM 26579 HD1 LYS H 165 31.791 -57.570 41.389 1.00 0.00
H H
ATOM 26580 HD2 LYS H 165 30.674 -58.469 42.470 1.00 0.00
H H
ATOM 26581 CE LYS H 165 29.847 -58.022 40.504 1.00 0.00
H C
ATOM 26582 HE1 LYS H 165 28.804 -58.225 40.836 1.00 0.00
H H
ATOM 26583 HE2 LYS H 165 29.828 -57.185 39.773 1.00 0.00
H H
ATOM 26584 NZ LYS H 165 30.339 -59.233 39.795 1.00 0.00
H N
ATOM 26585 HZ1 LYS H 165 29.719 -59.449 38.988 1.00 0.00
H H
ATOM 26586 HZ2 LYS H 165 31.307 -59.066 39.452 1.00 0.00
H H
ATOM 26587 HZ3 LYS H 165 30.344 -60.039 40.451 1.00 0.00
H H
ATOM 26588 C LYS H 165 32.099 -56.842 45.719 1.00 0.00
H C
ATOM 26589 O LYS H 165 31.699 -56.597 46.858 1.00 0.00
H O
ATOM 26590 N SER H 166 33.388 -56.901 45.406 1.00 0.00
H N
ATOM 26591 HN SER H 166 33.738 -57.155 44.505 1.00 0.00
H H
ATOM 26592 CA SER H 166 34.422 -56.591 46.381 1.00 0.00
H C
ATOM 26593 HA SER H 166 33.903 -56.255 47.268 1.00 0.00
H H
ATOM 26594 CB SER H 166 35.298 -57.808 46.662 1.00 0.00
H C

```
ATOM  26595  HB1 SER H 166      34.764 -58.457  47.393  1.00  0.00
H    H
ATOM  26596  HB2 SER H 166      35.439 -58.407  45.735  1.00  0.00
H    H
ATOM  26597  OG  SER H 166      36.527 -57.384  47.214  1.00  0.00
H    O
ATOM  26598  HG1 SER H 166      36.957 -58.165  47.584  1.00  0.00
H    H
ATOM  26599  C   SER H 166      35.304 -55.444  45.911  1.00  0.00
H    C
ATOM  26600  O   SER H 166      35.343 -55.107  44.725  1.00  0.00
H    O
ATOM  26601  N   ILE H 167      36.020 -54.847  46.865  1.00  0.00
H    N
ATOM  26602  HN  ILE H 167      35.930 -55.019  47.844  1.00  0.00
H    H
ATOM  26603  CA  ILE H 167      37.032 -53.853  46.523  1.00  0.00
H    C
ATOM  26604  HA  ILE H 167      36.565 -53.150  45.845  1.00  0.00
H    H
ATOM  26605  CB  ILE H 167      37.546 -53.146  47.788  1.00  0.00
H    C
ATOM  26606  HB  ILE H 167      37.594 -53.955  48.560  1.00  0.00
H    H
ATOM  26607  CG2 ILE H 167      38.878 -52.471  47.513  1.00  0.00
H    C
ATOM  26608 HG21 ILE H 167      39.327 -52.094  48.455  1.00  0.00
H    H
ATOM  26609 HG22 ILE H 167      39.597 -53.183  47.058  1.00  0.00
H    H
ATOM  26610 HG23 ILE H 167      38.751 -51.611  46.821  1.00  0.00
H    H
ATOM  26611  CG1 ILE H 167      36.524 -52.127  48.281  1.00  0.00
H    C
ATOM  26612 HG11 ILE H 167      37.044 -51.193  48.593  1.00  0.00
H    H
ATOM  26613 HG12 ILE H 167      35.846 -51.860  47.439  1.00  0.00
H    H
ATOM  26614  CD  ILE H 167      35.685 -52.626  49.420  1.00  0.00
H    C
ATOM  26615  HD1 ILE H 167      34.923 -51.867  49.699  1.00  0.00
H    H
ATOM  26616  HD2 ILE H 167      35.157 -53.562  49.138  1.00  0.00
H    H
ATOM  26617  HD3 ILE H 167      36.309 -52.830  50.315  1.00  0.00
H    H
ATOM  26618  C   ILE H 167      38.173 -54.508  45.760  1.00  0.00
H    C
ATOM  26619  O   ILE H 167      38.628 -54.000  44.729  1.00  0.00
H    O
ATOM  26620  N   ILE H 168      38.654 -55.652  46.254  1.00  0.00
H    N
ATOM  26621  HN  ILE H 168      38.386 -56.078  47.116  1.00  0.00
H    H
```

```
ATOM  26622  CA  ILE H 168      39.667 -56.392  45.513  1.00  0.00
H    C
ATOM  26623  HA  ILE H 168      40.422 -55.675  45.219  1.00  0.00
H    H
ATOM  26624  CB  ILE H 168      40.285 -57.500  46.381  1.00  0.00
H    C
ATOM  26625  HB  ILE H 168      40.540 -56.991  47.345  1.00  0.00
H    H
ATOM  26626  CG2 ILE H 168      39.320 -58.660  46.549  1.00  0.00
H    C
ATOM  26627 HG21 ILE H 168      39.704 -59.383  47.299  1.00  0.00
H    H
ATOM  26628 HG22 ILE H 168      38.329 -58.304  46.902  1.00  0.00
H    H
ATOM  26629 HG23 ILE H 168      39.176 -59.199  45.589  1.00  0.00
H    H
ATOM  26630  CG1 ILE H 168      41.601 -57.977  45.766  1.00  0.00
H    C
ATOM  26631 HG11 ILE H 168      41.797 -59.030  46.067  1.00  0.00
H    H
ATOM  26632 HG12 ILE H 168      41.505 -57.964  44.656  1.00  0.00
H    H
ATOM  26633  CD  ILE H 168      42.786 -57.118  46.153  1.00  0.00
H    C
ATOM  26634  HD1 ILE H 168      43.704 -57.471  45.635  1.00  0.00
H    H
ATOM  26635  HD2 ILE H 168      42.612 -56.059  45.868  1.00  0.00
H    H
ATOM  26636  HD3 ILE H 168      42.970 -57.163  47.248  1.00  0.00
H    H
ATOM  26637  C   ILE H 168      39.079 -56.958  44.234  1.00  0.00
H    C
ATOM  26638  O   ILE H 168      39.806 -57.219  43.270  1.00  0.00
H    O
ATOM  26639  N   ASP H 169      37.761 -57.138  44.191  1.00  0.00
H    N
ATOM  26640  HN  ASP H 169      37.116 -57.020  44.941  1.00  0.00
H    H
ATOM  26641  CA  ASP H 169      37.120 -57.549  42.952  1.00  0.00
H    C
ATOM  26642  HA  ASP H 169      37.685 -58.385  42.555  1.00  0.00
H    H
ATOM  26643  CB  ASP H 169      35.663 -57.918  43.233  1.00  0.00
H    C
ATOM  26644  HB1 ASP H 169      35.459 -57.802  44.316  1.00  0.00
H    H
ATOM  26645  HB2 ASP H 169      34.977 -57.250  42.675  1.00  0.00
H    H
ATOM  26646  CG  ASP H 169      35.342 -59.348  42.865  1.00  0.00
H    C
ATOM  26647  OD1 ASP H 169      35.929 -60.250  43.499  1.00  0.00
H    O
ATOM  26648  OD2 ASP H 169      34.508 -59.573  41.958  1.00  0.00
H    O
```

ATOM 26649 C ASP H 169 37.207 -56.430 41.922 1.00 0.00
H C
ATOM 26650 O ASP H 169 37.908 -56.544 40.909 1.00 0.00
H O
ATOM 26651 N LEU H 170 36.520 -55.325 42.185 1.00 0.00
H N
ATOM 26652 HN LEU H 170 35.940 -55.219 42.991 1.00 0.00
H H
ATOM 26653 CA LEU H 170 36.554 -54.156 41.311 1.00 0.00
H C
ATOM 26654 HA LEU H 170 36.963 -54.473 40.361 1.00 0.00
H H
ATOM 26655 CB LEU H 170 35.146 -53.606 41.118 1.00 0.00
H C
ATOM 26656 HB1 LEU H 170 34.669 -53.562 42.124 1.00 0.00
H H
ATOM 26657 HB2 LEU H 170 35.214 -52.553 40.763 1.00 0.00
H H
ATOM 26658 CG LEU H 170 34.241 -54.419 40.187 1.00 0.00
H C
ATOM 26659 HG LEU H 170 34.876 -54.709 39.316 1.00 0.00
H H
ATOM 26660 CD1 LEU H 170 33.699 -55.669 40.872 1.00 0.00
H C
ATOM 26661 HD11 LEU H 170 33.008 -56.216 40.195 1.00 0.00
H H
ATOM 26662 HD12 LEU H 170 34.529 -56.352 41.151 1.00 0.00
H H
ATOM 26663 HD13 LEU H 170 33.140 -55.401 41.794 1.00 0.00
H H
ATOM 26664 CD2 LEU H 170 33.095 -53.566 39.671 1.00 0.00
H C
ATOM 26665 HD21 LEU H 170 32.454 -54.157 38.982 1.00 0.00
H H
ATOM 26666 HD22 LEU H 170 32.453 -53.199 40.496 1.00 0.00
H H
ATOM 26667 HD23 LEU H 170 33.486 -52.687 39.113 1.00 0.00
H H
ATOM 26668 C LEU H 170 37.503 -53.111 41.896 1.00 0.00
H C
ATOM 26669 O LEU H 170 37.098 -52.129 42.521 1.00 0.00
H O
ATOM 26670 N ASN H 171 38.797 -53.358 41.697 1.00 0.00
H N
ATOM 26671 HN ASN H 171 39.150 -54.137 41.181 1.00 0.00
H H
ATOM 26672 CA ASN H 171 39.822 -52.476 42.239 1.00 0.00
H C
ATOM 26673 HA ASN H 171 39.596 -52.368 43.295 1.00 0.00
H H
ATOM 26674 CB ASN H 171 41.213 -53.071 42.023 1.00 0.00
H C
ATOM 26675 HB1 ASN H 171 41.961 -52.313 42.344 1.00 0.00
H H

ATOM 26676 HB2 ASN H 171 41.352 -53.967 42.662 1.00 0.00
H H
ATOM 26677 CG ASN H 171 41.483 -53.412 40.577 1.00 0.00
H C
ATOM 26678 OD1 ASN H 171 40.608 -53.291 39.724 1.00 0.00
H O
ATOM 26679 ND2 ASN H 171 42.707 -53.837 40.292 1.00 0.00
H N
ATOM 26680 HD21 ASN H 171 42.905 -54.056 39.341 1.00 0.00
H H
ATOM 26681 HD22 ASN H 171 43.392 -53.899 41.010 1.00 0.00
H H
ATOM 26682 C ASN H 171 39.739 -51.092 41.605 1.00 0.00
H C
ATOM 26683 O ASN H 171 39.264 -50.928 40.477 1.00 0.00
H O
ATOM 26684 N VAL H 172 40.184 -50.087 42.356 1.00 0.00
H N
ATOM 26685 HN VAL H 172 40.552 -50.203 43.275 1.00 0.00
H H
ATOM 26686 CA VAL H 172 40.170 -48.710 41.883 1.00 0.00
H C
ATOM 26687 HA VAL H 172 39.954 -48.742 40.822 1.00 0.00
H H
ATOM 26688 CB VAL H 172 39.061 -47.903 42.590 1.00 0.00
H C
ATOM 26689 HB VAL H 172 38.152 -48.550 42.637 1.00 0.00
H H
ATOM 26690 CG1 VAL H 172 39.480 -47.529 44.006 1.00 0.00
H C
ATOM 26691 HG11 VAL H 172 38.691 -46.914 44.492 1.00 0.00
H H
ATOM 26692 HG12 VAL H 172 39.636 -48.428 44.639 1.00 0.00
H H
ATOM 26693 HG13 VAL H 172 40.419 -46.934 43.994 1.00 0.00
H H
ATOM 26694 CG2 VAL H 172 38.700 -46.659 41.783 1.00 0.00
H C
ATOM 26695 HG21 VAL H 172 37.931 -46.057 42.312 1.00 0.00
H H
ATOM 26696 HG22 VAL H 172 39.596 -46.020 41.627 1.00 0.00
H H
ATOM 26697 HG23 VAL H 172 38.288 -46.931 40.789 1.00 0.00
H H
ATOM 26698 C VAL H 172 41.524 -48.031 42.053 1.00 0.00
H C
ATOM 26699 O VAL H 172 41.818 -47.061 41.344 1.00 0.00
H O
ATOM 26700 N ARG H 173 42.362 -48.511 42.974 1.00 0.00
H N
ATOM 26701 HN ARG H 173 42.152 -49.300 43.547 1.00 0.00
H H
ATOM 26702 CA ARG H 173 43.658 -47.876 43.184 1.00 0.00
H C

ATOM 26703 HA ARG H 173 43.513 −46.802 43.191 1.00 0.00
H H
ATOM 26704 CB ARG H 173 44.260 −48.325 44.511 1.00 0.00
H C
ATOM 26705 HB1 ARG H 173 43.403 −48.612 45.166 1.00 0.00
H H
ATOM 26706 HB2 ARG H 173 44.851 −49.255 44.356 1.00 0.00
H H
ATOM 26707 CG ARG H 173 45.069 −47.240 45.186 1.00 0.00
H C
ATOM 26708 HG1 ARG H 173 46.136 −47.363 44.898 1.00 0.00
H H
ATOM 26709 HG2 ARG H 173 44.761 −46.251 44.775 1.00 0.00
H H
ATOM 26710 CD ARG H 173 44.857 −47.234 46.692 1.00 0.00
H C
ATOM 26711 HD1 ARG H 173 44.766 −46.182 47.049 1.00 0.00
H H
ATOM 26712 HD2 ARG H 173 43.932 −47.789 46.977 1.00 0.00
H H
ATOM 26713 NE ARG H 173 45.952 −47.877 47.414 1.00 0.00
H N
ATOM 26714 HE ARG H 173 45.697 −48.768 47.808 1.00 0.00
H H
ATOM 26715 CZ ARG H 173 47.170 −47.359 47.551 1.00 0.00
H C
ATOM 26716 NH1 ARG H 173 47.462 −46.182 47.015 1.00 0.00
H N
ATOM 26717 HH11 ARG H 173 48.375 −45.805 47.093 1.00 0.00
H H
ATOM 26718 HH12 ARG H 173 46.762 −45.777 46.423 1.00 0.00
H H
ATOM 26719 NH2 ARG H 173 48.099 −48.019 48.230 1.00 0.00
H N
ATOM 26720 HH21 ARG H 173 48.977 −47.595 48.406 1.00 0.00
H H
ATOM 26721 HH22 ARG H 173 47.807 −48.831 48.718 1.00 0.00
H H
ATOM 26722 C ARG H 173 44.616 −48.178 42.040 1.00 0.00
H C
ATOM 26723 O ARG H 173 45.235 −47.266 41.484 1.00 0.00
H O
ATOM 26724 N ALA H 174 44.759 −49.450 41.684 1.00 0.00
H N
ATOM 26725 HN ALA H 174 44.340 −50.217 42.169 1.00 0.00
H H
ATOM 26726 CA ALA H 174 45.565 −49.808 40.525 1.00 0.00
H C
ATOM 26727 HA ALA H 174 46.451 −49.187 40.474 1.00 0.00
H H
ATOM 26728 CB ALA H 174 46.014 −51.266 40.628 1.00 0.00
H C
ATOM 26729 HB1 ALA H 174 46.611 −51.409 41.555 1.00 0.00
H H

ATOM 26730 HB2 ALA H 174 45.140 -51.951 40.675 1.00 0.00
H H
ATOM 26731 HB3 ALA H 174 46.648 -51.561 39.763 1.00 0.00
H H
ATOM 26732 C ALA H 174 44.805 -49.580 39.228 1.00 0.00
H C
ATOM 26733 O ALA H 174 45.414 -49.405 38.168 1.00 0.00
H O
ATOM 26734 N LYS H 175 43.473 -49.564 39.299 1.00 0.00
H N
ATOM 26735 HN LYS H 175 42.955 -49.626 40.151 1.00 0.00
H H
ATOM 26736 CA LYS H 175 42.665 -49.450 38.091 1.00 0.00
H C
ATOM 26737 HA LYS H 175 43.238 -49.896 37.286 1.00 0.00
H H
ATOM 26738 CB LYS H 175 41.347 -50.193 38.285 1.00 0.00
H C
ATOM 26739 HB1 LYS H 175 41.570 -51.105 38.886 1.00 0.00
H H
ATOM 26740 HB2 LYS H 175 40.668 -49.567 38.907 1.00 0.00
H H
ATOM 26741 CG LYS H 175 40.667 -50.603 37.001 1.00 0.00
H C
ATOM 26742 HG1 LYS H 175 40.242 -49.699 36.509 1.00 0.00
H H
ATOM 26743 HG2 LYS H 175 41.428 -51.032 36.309 1.00 0.00
H H
ATOM 26744 CD LYS H 175 39.563 -51.600 37.286 1.00 0.00
H C
ATOM 26745 HD1 LYS H 175 39.996 -52.451 37.860 1.00 0.00
H H
ATOM 26746 HD2 LYS H 175 38.809 -51.106 37.942 1.00 0.00
H H
ATOM 26747 CE LYS H 175 38.914 -52.087 36.006 1.00 0.00
H C
ATOM 26748 HE1 LYS H 175 38.351 -51.261 35.517 1.00 0.00
H H
ATOM 26749 HE2 LYS H 175 39.685 -52.457 35.294 1.00 0.00
H H
ATOM 26750 NZ LYS H 175 37.959 -53.200 36.265 1.00 0.00
H N
ATOM 26751 HZ1 LYS H 175 37.527 -53.514 35.372 1.00 0.00
H H
ATOM 26752 HZ2 LYS H 175 38.464 -53.997 36.703 1.00 0.00
H H
ATOM 26753 HZ3 LYS H 175 37.213 -52.872 36.911 1.00 0.00
H H
ATOM 26754 C LYS H 175 42.413 -47.995 37.719 1.00 0.00
H C
ATOM 26755 O LYS H 175 42.674 -47.587 36.581 1.00 0.00
H O
ATOM 26756 N TYR H 176 41.908 -47.194 38.660 1.00 0.00
H N

ATOM 26757 HN TYR H 176 41.732 -47.488 39.598 1.00 0.00
H H
ATOM 26758 CA TYR H 176 41.569 -45.806 38.388 1.00 0.00
H C
ATOM 26759 HA TYR H 176 41.850 -45.641 37.355 1.00 0.00
H H
ATOM 26760 CB TYR H 176 40.064 -45.573 38.591 1.00 0.00
H C
ATOM 26761 HB1 TYR H 176 39.800 -45.794 39.649 1.00 0.00
H H
ATOM 26762 HB2 TYR H 176 39.802 -44.510 38.401 1.00 0.00
H H
ATOM 26763 CG TYR H 176 39.197 -46.465 37.731 1.00 0.00
H C
ATOM 26764 CD1 TYR H 176 38.650 -47.634 38.242 1.00 0.00
H C
ATOM 26765 HD1 TYR H 176 38.857 -47.923 39.264 1.00 0.00
H H
ATOM 26766 CE1 TYR H 176 37.862 -48.454 37.462 1.00 0.00
H C
ATOM 26767 HE1 TYR H 176 37.445 -49.364 37.869 1.00 0.00
H H
ATOM 26768 CZ TYR H 176 37.612 -48.114 36.153 1.00 0.00
H C
ATOM 26769 OH TYR H 176 36.825 -48.933 35.376 1.00 0.00
H O
ATOM 26770 HH TYR H 176 36.707 -48.499 34.529 1.00 0.00
H H
ATOM 26771 CD2 TYR H 176 38.932 -46.144 36.407 1.00 0.00
H C
ATOM 26772 HD2 TYR H 176 39.376 -45.263 35.966 1.00 0.00
H H
ATOM 26773 CE2 TYR H 176 38.144 -46.960 35.618 1.00 0.00
H C
ATOM 26774 HE2 TYR H 176 37.969 -46.700 34.585 1.00 0.00
H H
ATOM 26775 C TYR H 176 42.343 -44.804 39.230 1.00 0.00
H C
ATOM 26776 O TYR H 176 42.084 -43.602 39.114 1.00 0.00
H O
ATOM 26777 N GLY H 177 43.270 -45.253 40.073 1.00 0.00
H N
ATOM 26778 HN GLY H 177 43.452 -46.223 40.225 1.00 0.00
H H
ATOM 26779 CA GLY H 177 44.090 -44.334 40.838 1.00 0.00
H C
ATOM 26780 HA1 GLY H 177 44.447 -43.602 40.127 1.00 0.00
H H
ATOM 26781 HA2 GLY H 177 44.869 -44.937 41.286 1.00 0.00
H H
ATOM 26782 C GLY H 177 43.376 -43.599 41.945 1.00 0.00
H C
ATOM 26783 O GLY H 177 43.893 -42.592 42.438 1.00 0.00
H O

ATOM 26784 N CYS H 178 42.208 -44.071 42.358 1.00 0.00
H N
ATOM 26785 HN CYS H 178 41.804 -44.903 41.985 1.00 0.00
H H
ATOM 26786 CA CYS H 178 41.418 -43.417 43.388 1.00 0.00
H C
ATOM 26787 HA CYS H 178 41.881 -42.457 43.585 1.00 0.00
H H
ATOM 26788 CB CYS H 178 39.971 -43.231 42.913 1.00 0.00
H C
ATOM 26789 HB1 CYS H 178 39.617 -44.219 42.541 1.00 0.00
H H
ATOM 26790 HB2 CYS H 178 39.320 -42.978 43.779 1.00 0.00
H H
ATOM 26791 SG CYS H 178 39.807 -42.021 41.588 1.00 0.00
H S
ATOM 26792 HG1 CYS H 178 38.495 -42.168 41.391 1.00 0.00
H H
ATOM 26793 C CYS H 178 41.473 -44.233 44.674 1.00 0.00
H C
ATOM 26794 O CYS H 178 42.201 -45.224 44.778 1.00 0.00
H O
ATOM 26795 N THR H 179 40.693 -43.801 45.661 1.00 0.00
H N
ATOM 26796 HN THR H 179 40.072 -43.024 45.566 1.00 0.00
H H
ATOM 26797 CA THR H 179 40.672 -44.425 46.979 1.00 0.00
H C
ATOM 26798 HA THR H 179 41.107 -45.409 46.856 1.00 0.00
H H
ATOM 26799 CB THR H 179 41.436 -43.570 47.995 1.00 0.00
H C
ATOM 26800 HB THR H 179 40.665 -42.980 48.547 1.00 0.00
H H
ATOM 26801 OG1 THR H 179 42.286 -42.649 47.297 1.00 0.00
H O
ATOM 26802 HG1 THR H 179 42.744 -42.152 47.983 1.00 0.00
H H
ATOM 26803 CG2 THR H 179 42.255 -44.432 48.938 1.00 0.00
H C
ATOM 26804 HG21 THR H 179 42.714 -43.815 49.739 1.00 0.00
H H
ATOM 26805 HG22 THR H 179 41.611 -45.196 49.422 1.00 0.00
H H
ATOM 26806 HG23 THR H 179 43.070 -44.957 48.394 1.00 0.00
H H
ATOM 26807 C THR H 179 39.237 -44.639 47.436 1.00 0.00
H C
ATOM 26808 O THR H 179 38.508 -43.672 47.681 1.00 0.00
H O
ATOM 26809 N ILE H 180 38.836 -45.903 47.565 1.00 0.00
H N
ATOM 26810 HN ILE H 180 39.381 -46.693 47.293 1.00 0.00
H H

```
ATOM  26811  CA  ILE H 180      37.531 -46.240  48.130  1.00  0.00
H    C
ATOM  26812  HA  ILE H 180      36.823 -45.490  47.798  1.00  0.00
H    H
ATOM  26813  CB  ILE H 180      37.091 -47.654  47.708  1.00  0.00
H    C
ATOM  26814  HB  ILE H 180      37.964 -48.060  47.136  1.00  0.00
H    H
ATOM  26815  CG2 ILE H 180      36.686 -48.488  48.926  1.00  0.00
H    C
ATOM  26816 HG21 ILE H 180      36.521 -49.548  48.636  1.00  0.00
H    H
ATOM  26817 HG22 ILE H 180      37.482 -48.471  49.700  1.00  0.00
H    H
ATOM  26818 HG23 ILE H 180      35.748 -48.102  49.378  1.00  0.00
H    H
ATOM  26819  CG1 ILE H 180      35.915 -47.574  46.728  1.00  0.00
H    C
ATOM  26820 HG11 ILE H 180      35.469 -46.555  46.758  1.00  0.00
H    H
ATOM  26821 HG12 ILE H 180      36.295 -47.737  45.694  1.00  0.00
H    H
ATOM  26822  CD  ILE H 180      34.807 -48.595  46.980  1.00  0.00
H    C
ATOM  26823  HD1 ILE H 180      34.022 -48.513  46.198  1.00  0.00
H    H
ATOM  26824  HD2 ILE H 180      35.214 -49.629  46.957  1.00  0.00
H    H
ATOM  26825  HD3 ILE H 180      34.328 -48.427  47.967  1.00  0.00
H    H
ATOM  26826  C   ILE H 180      37.632 -46.112  49.643  1.00  0.00
H    C
ATOM  26827  O   ILE H 180      38.663 -46.448  50.238  1.00  0.00
H    O
ATOM  26828  N   LEU H 181      36.577 -45.597  50.273  1.00  0.00
H    N
ATOM  26829  HN  LEU H 181      35.675 -45.469  49.865  1.00  0.00
H    H
ATOM  26830  CA  LEU H 181      36.672 -45.169  51.661  1.00  0.00
H    C
ATOM  26831  HA  LEU H 181      37.669 -45.454  51.971  1.00  0.00
H    H
ATOM  26832  CB  LEU H 181      36.473 -43.658  51.762  1.00  0.00
H    C
ATOM  26833  HB1 LEU H 181      36.618 -43.238  50.740  1.00  0.00
H    H
ATOM  26834  HB2 LEU H 181      35.414 -43.450  52.033  1.00  0.00
H    H
ATOM  26835  CG  LEU H 181      37.412 -42.940  52.717  1.00  0.00
H    C
ATOM  26836  HG  LEU H 181      37.033 -43.086  53.758  1.00  0.00
H    H
ATOM  26837  CD1 LEU H 181      38.832 -43.485  52.628  1.00  0.00
H    C
```

ATOM 26838 HD11 LEU H 181   39.511 -42.915 53.298 1.00 0.00
H   H
ATOM 26839 HD12 LEU H 181   38.861 -44.554 52.931 1.00 0.00
H   H
ATOM 26840 HD13 LEU H 181   39.220 -43.403 51.591 1.00 0.00
H   H
ATOM 26841 CD2 LEU H 181   37.345 -41.481 52.401 1.00 0.00
H   C
ATOM 26842 HD21 LEU H 181   38.004 -40.907 53.089 1.00 0.00
H   H
ATOM 26843 HD22 LEU H 181   37.676 -41.271 51.364 1.00 0.00
H   H
ATOM 26844 HD23 LEU H 181   36.306 -41.104 52.521 1.00 0.00
H   H
ATOM 26845 C   LEU H 181   35.693 -45.829 52.611 1.00 0.00
H   C
ATOM 26846 O   LEU H 181   36.091 -46.194 53.715 1.00 0.00
H   O
ATOM 26847 N   ALA H 182   34.421 -45.956 52.242 1.00 0.00
H   N
ATOM 26848 HN ALA H 182   34.064 -45.719 51.339 1.00 0.00
H   H
ATOM 26849 CA ALA H 182   33.442 -46.474 53.183 1.00 0.00
H   C
ATOM 26850 HA ALA H 182   33.850 -47.293 53.764 1.00 0.00
H   H
ATOM 26851 CB ALA H 182   32.982 -45.398 54.171 1.00 0.00
H   C
ATOM 26852 HB1 ALA H 182   33.854 -45.030 54.753 1.00 0.00
H   H
ATOM 26853 HB2 ALA H 182   32.535 -44.530 53.639 1.00 0.00
H   H
ATOM 26854 HB3 ALA H 182   32.234 -45.796 54.890 1.00 0.00
H   H
ATOM 26855 C   ALA H 182   32.252 -47.036 52.427 1.00 0.00
H   C
ATOM 26856 O   ALA H 182   31.918 -46.600 51.320 1.00 0.00
H   O
ATOM 26857 N   ILE H 183   31.604 -47.998 53.076 1.00 0.00
H   N
ATOM 26858 HN ILE H 183   31.830 -48.236 54.018 1.00 0.00
H   H
ATOM 26859 CA ILE H 183   30.526 -48.788 52.503 1.00 0.00
H   C
ATOM 26860 HA ILE H 183   30.252 -48.333 51.560 1.00 0.00
H   H
ATOM 26861 CB ILE H 183   30.961 -50.259 52.335 1.00 0.00
H   C
ATOM 26862 HB ILE H 183   31.265 -50.580 53.364 1.00 0.00
H   H
ATOM 26863 CG2 ILE H 183   29.831 -51.132 51.815 1.00 0.00
H   C
ATOM 26864 HG21 ILE H 183   30.117 -52.204 51.850 1.00 0.00
H   H

```
ATOM  26865 HG22 ILE H 183      28.917 -51.006  52.432  1.00  0.00
H    H
ATOM  26866 HG23 ILE H 183      29.583 -50.874  50.764  1.00  0.00
H    H
ATOM  26867  CG1 ILE H 183      32.211 -50.343  51.453  1.00  0.00
H    C
ATOM  26868 HG11 ILE H 183      32.056 -49.752  50.522  1.00  0.00
H    H
ATOM  26869 HG12 ILE H 183      33.067 -49.882  51.995  1.00  0.00
H    H
ATOM  26870  CD  ILE H 183      32.597 -51.750  51.080  1.00  0.00
H    C
ATOM  26871  HD1 ILE H 183      33.539 -51.748  50.491  1.00  0.00
H    H
ATOM  26872  HD2 ILE H 183      32.756 -52.367  51.991  1.00  0.00
H    H
ATOM  26873  HD3 ILE H 183      31.807 -52.230  50.464  1.00  0.00
H    H
ATOM  26874  C   ILE H 183      29.312 -48.675  53.415  1.00  0.00
H    C
ATOM  26875  O   ILE H 183      29.383 -49.039  54.593  1.00  0.00
H    O
ATOM  26876  N   LYS H 184      28.200 -48.179  52.872  1.00  0.00
H    N
ATOM  26877  HN  LYS H 184      28.157 -47.929  51.907  1.00  0.00
H    H
ATOM  26878  CA  LYS H 184      26.965 -47.961  53.627  1.00  0.00
H    C
ATOM  26879  HA  LYS H 184      27.106 -48.309  54.643  1.00  0.00
H    H
ATOM  26880  CB  LYS H 184      26.562 -46.490  53.582  1.00  0.00
H    C
ATOM  26881  HB1 LYS H 184      27.493 -45.891  53.718  1.00  0.00
H    H
ATOM  26882  HB2 LYS H 184      26.181 -46.249  52.565  1.00  0.00
H    H
ATOM  26883  CG  LYS H 184      25.548 -45.995  54.620  1.00  0.00
H    C
ATOM  26884  HG1 LYS H 184      25.783 -44.939  54.886  1.00  0.00
H    H
ATOM  26885  HG2 LYS H 184      24.530 -46.005  54.165  1.00  0.00
H    H
ATOM  26886  CD  LYS H 184      25.530 -46.805  55.900  1.00  0.00
H    C
ATOM  26887  HD1 LYS H 184      24.980 -47.755  55.708  1.00  0.00
H    H
ATOM  26888  HD2 LYS H 184      26.581 -47.074  56.154  1.00  0.00
H    H
ATOM  26889  CE  LYS H 184      24.878 -45.984  57.008  1.00  0.00
H    C
ATOM  26890  HE1 LYS H 184      25.323 -44.965  57.050  1.00  0.00
H    H
ATOM  26891  HE2 LYS H 184      23.786 -45.883  56.829  1.00  0.00
H    H
```

ATOM 26892 NZ LYS H 184 25.029 -46.565 58.370 1.00 0.00
H N
ATOM 26893 HZ1 LYS H 184 24.564 -45.954 59.071 1.00 0.00
H H
ATOM 26894 HZ2 LYS H 184 24.597 -47.510 58.395 1.00 0.00
H H
ATOM 26895 HZ3 LYS H 184 26.040 -46.646 58.601 1.00 0.00
H H
ATOM 26896 C LYS H 184 25.894 -48.838 52.989 1.00 0.00
H C
ATOM 26897 O LYS H 184 25.321 -48.469 51.959 1.00 0.00
H O
ATOM 26898 N HSE H 185 25.625 -49.993 53.588 1.00 0.00
H N
ATOM 26899 HN HSE H 185 26.058 -50.275 54.442 1.00 0.00
H H
ATOM 26900 CA HSE H 185 24.668 -50.956 53.051 1.00 0.00
H C
ATOM 26901 HA HSE H 185 24.258 -50.538 52.140 1.00 0.00
H H
ATOM 26902 CB HSE H 185 25.336 -52.302 52.762 1.00 0.00
H C
ATOM 26903 HB1 HSE H 185 24.561 -52.990 52.360 1.00 0.00
H H
ATOM 26904 HB2 HSE H 185 26.100 -52.173 51.967 1.00 0.00
H H
ATOM 26905 ND1 HSE H 185 25.280 -53.899 54.708 1.00 0.00
H N
ATOM 26906 CG HSE H 185 25.947 -52.954 53.959 1.00 0.00
H C
ATOM 26907 CE1 HSE H 185 26.058 -54.299 55.699 1.00 0.00
H C
ATOM 26908 HE1 HSE H 185 25.782 -55.057 56.436 1.00 0.00
H H
ATOM 26909 NE2 HSE H 185 27.193 -53.626 55.635 1.00 0.00
H N
ATOM 26910 HE2 HSE H 185 27.950 -53.711 56.283 1.00 0.00
H H
ATOM 26911 CD2 HSE H 185 27.148 -52.776 54.558 1.00 0.00
H C
ATOM 26912 HD2 HSE H 185 27.958 -52.107 54.302 1.00 0.00
H H
ATOM 26913 C HSE H 185 23.514 -51.125 54.024 1.00 0.00
H C
ATOM 26914 O HSE H 185 23.687 -51.689 55.109 1.00 0.00
H O
ATOM 26915 N HSE H 186 22.338 -50.642 53.623 1.00 0.00
H N
ATOM 26916 HN HSE H 186 22.213 -50.162 52.756 1.00 0.00
H H
ATOM 26917 CA HSE H 186 21.122 -50.773 54.419 1.00 0.00
H C
ATOM 26918 HA HSE H 186 20.366 -50.161 53.942 1.00 0.00
H H

ATOM 26919 CB HSE H 186   20.651 -52.229 54.466 1.00 0.00
H   C
ATOM 26920 HB1 HSE H 186   20.802 -52.669 53.457 1.00 0.00
H   H
ATOM 26921 HB2 HSE H 186   21.290 -52.802 55.170 1.00 0.00
H   H
ATOM 26922 ND1 HSE H 186   18.196 -51.790 54.105 1.00 0.00
H   N
ATOM 26923 CG HSE H 186   19.209 -52.383 54.827 1.00 0.00
H   C
ATOM 26924 CE1 HSE H 186   17.034 -52.088 54.657 1.00 0.00
H   C
ATOM 26925 HE1 HSE H 186   16.065 -51.748 54.283 1.00 0.00
H   H
ATOM 26926 NE2 HSE H 186   17.256 -52.846 55.715 1.00 0.00
H   N
ATOM 26927 HE2 HSE H 186   16.551 -53.198 56.329 1.00 0.00
H   H
ATOM 26928 CD2 HSE H 186   18.609 -53.043 55.846 1.00 0.00
H   C
ATOM 26929 HD2 HSE H 186   19.044 -53.623 56.647 1.00 0.00
H   H
ATOM 26930 C   HSE H 186   21.322 -50.223 55.826 1.00 0.00
H   C
ATOM 26931 O   HSE H 186   20.914 -50.838 56.810 1.00 0.00
H   O
ATOM 26932 N   GLY H 187   21.976 -49.066 55.933 1.00 0.00
H   N
ATOM 26933 HN GLY H 187   22.328 -48.583 55.132 1.00 0.00
H   H
ATOM 26934 CA GLY H 187   22.222 -48.431 57.199 1.00 0.00
H   C
ATOM 26935 HA1 GLY H 187   21.365 -48.654 57.820 1.00 0.00
H   H
ATOM 26936 HA2 GLY H 187   22.360 -47.378 56.993 1.00 0.00
H   H
ATOM 26937 C   GLY H 187   23.461 -48.935 57.910 1.00 0.00
H   C
ATOM 26938 O   GLY H 187   23.810 -48.405 58.969 1.00 0.00
H   O
ATOM 26939 N   ASP H 188   24.130 -49.939 57.360 1.00 0.00
H   N
ATOM 26940 HN ASP H 188   23.918 -50.385 56.494 1.00 0.00
H   H
ATOM 26941 CA ASP H 188   25.299 -50.505 58.019 1.00 0.00
H   C
ATOM 26942 HA ASP H 188   25.258 -50.198 59.057 1.00 0.00
H   H
ATOM 26943 CB ASP H 188   25.282 -52.023 57.880 1.00 0.00
H   C
ATOM 26944 HB1 ASP H 188   25.267 -52.292 56.805 1.00 0.00
H   H
ATOM 26945 HB2 ASP H 188   26.187 -52.467 58.338 1.00 0.00
H   H

```
ATOM  26946  CG  ASP H 188      24.057 -52.655  58.524  1.00  0.00
H    C
ATOM  26947  OD1 ASP H 188      23.101 -51.910  58.824  1.00  0.00
H    O
ATOM  26948  OD2 ASP H 188      24.046 -53.892  58.726  1.00  0.00
H    O
ATOM  26949  C   ASP H 188      26.590 -49.929  57.440  1.00  0.00
H    C
ATOM  26950  O   ASP H 188      26.780 -49.891  56.221  1.00  0.00
H    O
ATOM  26951  N   ILE H 189      27.484 -49.490  58.326  1.00  0.00
H    N
ATOM  26952  HN  ILE H 189      27.351 -49.595  59.309  1.00  0.00
H    H
ATOM  26953  CA  ILE H 189      28.731 -48.821  57.960  1.00  0.00
H    C
ATOM  26954  HA  ILE H 189      28.604 -48.448  56.951  1.00  0.00
H    H
ATOM  26955  CB  ILE H 189      29.066 -47.668  58.931  1.00  0.00
H    C
ATOM  26956  HB  ILE H 189      28.158 -47.574  59.579  1.00  0.00
H    H
ATOM  26957  CG2 ILE H 189      30.361 -47.941  59.693  1.00  0.00
H    C
ATOM  26958 HG21 ILE H 189      30.509 -47.186  60.494  1.00  0.00
H    H
ATOM  26959 HG22 ILE H 189      30.334 -48.941  60.173  1.00  0.00
H    H
ATOM  26960 HG23 ILE H 189      31.238 -47.901  59.013  1.00  0.00
H    H
ATOM  26961  CG1 ILE H 189      29.213 -46.328  58.204  1.00  0.00
H    C
ATOM  26962 HG11 ILE H 189      28.744 -45.519  58.807  1.00  0.00
H    H
ATOM  26963 HG12 ILE H 189      30.296 -46.084  58.112  1.00  0.00
H    H
ATOM  26964  CD  ILE H 189      28.638 -46.270  56.842  1.00  0.00
H    C
ATOM  26965  HD1 ILE H 189      28.845 -45.281  56.378  1.00  0.00
H    H
ATOM  26966  HD2 ILE H 189      29.082 -47.058  56.196  1.00  0.00
H    H
ATOM  26967  HD3 ILE H 189      27.537 -46.414  56.868  1.00  0.00
H    H
ATOM  26968  C   ILE H 189      29.866 -49.833  57.916  1.00  0.00
H    C
ATOM  26969  O   ILE H 189      29.861 -50.821  58.660  1.00  0.00
H    O
ATOM  26970  N   CYS H 190      30.815 -49.593  57.016  1.00  0.00
H    N
ATOM  26971  HN  CYS H 190      30.660 -48.950  56.269  1.00  0.00
H    H
ATOM  26972  CA  CYS H 190      32.133 -50.219  57.019  1.00  0.00
H    C
```

ATOM 26973 HA CYS H 190 32.383 -50.503 58.034 1.00 0.00
H H
ATOM 26974 CB CYS H 190 32.187 -51.434 56.096 1.00 0.00
H C
ATOM 26975 HB1 CYS H 190 31.279 -52.044 56.301 1.00 0.00
H H
ATOM 26976 HB2 CYS H 190 32.106 -51.106 55.036 1.00 0.00
H H
ATOM 26977 SG CYS H 190 33.646 -52.488 56.340 1.00 0.00
H S
ATOM 26978 HG1 CYS H 190 33.320 -53.431 55.455 1.00 0.00
H H
ATOM 26979 C CYS H 190 33.112 -49.131 56.586 1.00 0.00
H C
ATOM 26980 O CYS H 190 33.155 -48.751 55.413 1.00 0.00
H O
ATOM 26981 N LEU H 191 33.878 -48.616 57.545 1.00 0.00
H N
ATOM 26982 HN LEU H 191 33.851 -48.899 58.501 1.00 0.00
H H
ATOM 26983 CA LEU H 191 34.844 -47.564 57.259 1.00 0.00
H C
ATOM 26984 HA LEU H 191 34.458 -47.001 56.420 1.00 0.00
H H
ATOM 26985 CB LEU H 191 35.060 -46.672 58.481 1.00 0.00
H C
ATOM 26986 HB1 LEU H 191 35.129 -47.341 59.369 1.00 0.00
H H
ATOM 26987 HB2 LEU H 191 36.051 -46.174 58.395 1.00 0.00
H H
ATOM 26988 CG LEU H 191 34.032 -45.600 58.834 1.00 0.00
H C
ATOM 26989 HG LEU H 191 33.074 -46.096 59.120 1.00 0.00
H H
ATOM 26990 CD1 LEU H 191 34.553 -44.790 60.003 1.00 0.00
H C
ATOM 26991 HD11 LEU H 191 33.848 -43.969 60.254 1.00 0.00
H H
ATOM 26992 HD12 LEU H 191 34.674 -45.433 60.901 1.00 0.00
H H
ATOM 26993 HD13 LEU H 191 35.538 -44.338 59.759 1.00 0.00
H H
ATOM 26994 CD2 LEU H 191 33.761 -44.707 57.645 1.00 0.00
H C
ATOM 26995 HD21 LEU H 191 32.998 -43.940 57.905 1.00 0.00
H H
ATOM 26996 HD22 LEU H 191 34.675 -44.173 57.317 1.00 0.00
H H
ATOM 26997 HD23 LEU H 191 33.377 -45.303 56.789 1.00 0.00
H H
ATOM 26998 C LEU H 191 36.179 -48.160 56.837 1.00 0.00
H C
ATOM 26999 O LEU H 191 36.663 -49.124 57.434 1.00 0.00
H O

ATOM 27000 N   SER H 192   36.797 -47.537 55.834 1.00 0.00
H   N
ATOM 27001 HN SER H 192   36.432 -46.703 55.421 1.00 0.00
H   H
ATOM 27002 CA SER H 192   38.048 -47.998 55.245 1.00 0.00
H   C
ATOM 27003 HA SER H 192   38.125 -47.525 54.275 1.00 0.00
H   H
ATOM 27004 CB SER H 192   39.243 -47.631 56.114 1.00 0.00
H   C
ATOM 27005 HB1 SER H 192   39.991 -48.453 56.039 1.00 0.00
H   H
ATOM 27006 HB2 SER H 192   39.733 -46.707 55.731 1.00 0.00
H   H
ATOM 27007 OG SER H 192   38.882 -47.478 57.476 1.00 0.00
H   O
ATOM 27008 HG1 SER H 192   39.701 -47.471 57.988 1.00 0.00
H   H
ATOM 27009 C   SER H 192   37.993 -49.513 55.010 1.00 0.00
H   C
ATOM 27010 O   SER H 192   38.749 -50.261 55.636 1.00 0.00
H   O
ATOM 27011 N   PRO H 193   37.110 -49.983 54.134 1.00 0.00
H   N
ATOM 27012 CD PRO H 193   36.365 -49.238 53.103 1.00 0.00
H   C
ATOM 27013 HD1 PRO H 193   35.517 -48.663 53.538 1.00 0.00
H   H
ATOM 27014 HD2 PRO H 193   37.045 -48.562 52.536 1.00 0.00
H   H
ATOM 27015 CA PRO H 193   36.899 -51.429 54.020 1.00 0.00
H   C
ATOM 27016 HA PRO H 193   36.627 -51.852 54.979 1.00 0.00
H   H
ATOM 27017 CB PRO H 193   35.736 -51.542 53.028 1.00 0.00
H   C
ATOM 27018 HB1 PRO H 193   34.776 -51.440 53.581 1.00 0.00
H   H
ATOM 27019 HB2 PRO H 193   35.734 -52.505 52.478 1.00 0.00
H   H
ATOM 27020 CG PRO H 193   35.844 -50.319 52.198 1.00 0.00
H   C
ATOM 27021 HG1 PRO H 193   34.885 -50.035 51.722 1.00 0.00
H   H
ATOM 27022 HG2 PRO H 193   36.616 -50.478 51.410 1.00 0.00
H   H
ATOM 27023 C   PRO H 193   38.144 -52.131 53.503 1.00 0.00
H   C
ATOM 27024 O   PRO H 193   38.744 -51.728 52.505 1.00 0.00
H   O
ATOM 27025 N   ALA H 194   38.529 -53.193 54.208 1.00 0.00
H   N
ATOM 27026 HN ALA H 194   38.057 -53.493 55.036 1.00 0.00
H   H

ATOM 27027 CA ALA H 194   39.673 -53.996 53.817 1.00 0.00
H   C
ATOM 27028 HA ALA H 194   40.409 -53.408 53.281 1.00 0.00
H   H
ATOM 27029 CB ALA H 194   40.338 -54.620 55.038 1.00 0.00
H   C
ATOM 27030 HB1 ALA H 194   40.717 -53.818 55.706 1.00 0.00
H   H
ATOM 27031 HB2 ALA H 194   39.614 -55.234 55.617 1.00 0.00
H   H
ATOM 27032 HB3 ALA H 194   41.198 -55.263 54.751 1.00 0.00
H   H
ATOM 27033 C   ALA H 194   39.209 -55.078 52.860 1.00 0.00
H   C
ATOM 27034 O   ALA H 194   38.263 -55.813 53.187 1.00 0.00
H   O
ATOM 27035 N   PRO H 195   39.810 -55.197 51.678 1.00 0.00
H   N
ATOM 27036 CD PRO H 195   40.879 -54.346 51.129 1.00 0.00
H   C
ATOM 27037 HD1 PRO H 195   39.923 -53.886 51.470 1.00 0.00
H   H
ATOM 27038 HD2 PRO H 195   41.736 -53.668 51.348 1.00 0.00
H   H
ATOM 27039 CA PRO H 195   39.405 -56.251 50.741 1.00 0.00
H   C
ATOM 27040 HA PRO H 195   38.373 -56.119 50.437 1.00 0.00
H   H
ATOM 27041 CB PRO H 195   40.353 -56.049 49.555 1.00 0.00
H   C
ATOM 27042 HB1 PRO H 195   39.928 -55.274 48.878 1.00 0.00
H   H
ATOM 27043 HB2 PRO H 195   40.520 -56.980 48.976 1.00 0.00
H   H
ATOM 27044 CG PRO H 195   40.815 -54.634 49.664 1.00 0.00
H   C
ATOM 27045 HG1 PRO H 195   41.160 -54.217 48.698 1.00 0.00
H   H
ATOM 27046 HG2 PRO H 195   41.641 -54.575 50.410 1.00 0.00
H   H
ATOM 27047 C   PRO H 195   39.567 -57.631 51.364 1.00 0.00
H   C
ATOM 27048 O   PRO H 195   40.568 -57.917 52.026 1.00 0.00
H   O
ATOM 27049 N   GLU H 196   38.570 -58.489 51.157 1.00 0.00
H   N
ATOM 27050 HN GLU H 196   38.554 -59.404 51.552 1.00 0.00
H   H
ATOM 27051 CA GLU H 196   37.404 -58.177 50.336 1.00 0.00
H   C
ATOM 27052 HA GLU H 196   37.648 -57.332 49.703 1.00 0.00
H   H
ATOM 27053 CB GLU H 196   37.032 -59.380 49.465 1.00 0.00
H   C

ATOM 27054 HB1 GLU H 196 36.137 -59.107 48.859 1.00 0.00
H H
ATOM 27055 HB2 GLU H 196 37.846 -59.582 48.732 1.00 0.00
H H
ATOM 27056 CG GLU H 196 36.719 -60.659 50.229 1.00 0.00
H C
ATOM 27057 HG1 GLU H 196 36.882 -61.532 49.571 1.00 0.00
H H
ATOM 27058 HG2 GLU H 196 37.363 -60.754 51.126 1.00 0.00
H H
ATOM 27059 CD GLU H 196 35.266 -60.729 50.662 1.00 0.00
H C
ATOM 27060 OE1 GLU H 196 34.387 -60.843 49.777 1.00 0.00
H O
ATOM 27061 OE2 GLU H 196 35.003 -60.628 51.877 1.00 0.00
H O
ATOM 27062 C GLU H 196 36.220 -57.761 51.210 1.00 0.00
H C
ATOM 27063 O GLU H 196 36.284 -57.861 52.431 1.00 0.00
H O
ATOM 27064 N ASP H 197 35.148 -57.279 50.582 1.00 0.00
H N
ATOM 27065 HN ASP H 197 35.062 -57.197 49.592 1.00 0.00
H H
ATOM 27066 CA ASP H 197 33.967 -56.812 51.294 1.00 0.00
H C
ATOM 27067 HA ASP H 197 33.762 -57.527 52.082 1.00 0.00
H H
ATOM 27068 CB ASP H 197 34.171 -55.409 51.860 1.00 0.00
H C
ATOM 27069 HB1 ASP H 197 35.223 -55.298 52.192 1.00 0.00
H H
ATOM 27070 HB2 ASP H 197 33.967 -54.642 51.087 1.00 0.00
H H
ATOM 27071 CG ASP H 197 33.287 -55.137 53.053 1.00 0.00
H C
ATOM 27072 OD1 ASP H 197 32.801 -56.110 53.669 1.00 0.00
H O
ATOM 27073 OD2 ASP H 197 33.062 -53.947 53.360 1.00 0.00
H O
ATOM 27074 C ASP H 197 32.758 -56.813 50.374 1.00 0.00
H C
ATOM 27075 O ASP H 197 32.812 -56.300 49.256 1.00 0.00
H O
ATOM 27076 N ILE H 198 31.647 -57.341 50.883 1.00 0.00
H N
ATOM 27077 HN ILE H 198 31.563 -57.662 51.824 1.00 0.00
H H
ATOM 27078 CA ILE H 198 30.432 -57.499 50.085 1.00 0.00
H C
ATOM 27079 HA ILE H 198 30.725 -57.945 49.143 1.00 0.00
H H
ATOM 27080 CB ILE H 198 29.396 -58.374 50.816 1.00 0.00
H C

ATOM 27081 HB ILE H 198   29.607 -58.208 51.903 1.00 0.00
H   H
ATOM 27082 CG2 ILE H 198   27.976 -58.029 50.360 1.00 0.00
H   C
ATOM 27083 HG21 ILE H 198   27.225 -58.544 50.994 1.00 0.00
H   H
ATOM 27084 HG22 ILE H 198   27.789 -56.937 50.437 1.00 0.00
H   H
ATOM 27085 HG23 ILE H 198   27.811 -58.339 49.306 1.00 0.00
H   H
ATOM 27086 CG1 ILE H 198   29.642 -59.858 50.544 1.00 0.00
H   C
ATOM 27087 HG11 ILE H 198   29.302 -60.113 49.516 1.00 0.00
H   H
ATOM 27088 HG12 ILE H 198   30.737 -60.056 50.587 1.00 0.00
H   H
ATOM 27089 CD ILE H 198   28.963 -60.770 51.525 1.00 0.00
H   C
ATOM 27090 HD1 ILE H 198   29.224 -61.829 51.312 1.00 0.00
H   H
ATOM 27091 HD2 ILE H 198   29.280 -60.535 52.564 1.00 0.00
H   H
ATOM 27092 HD3 ILE H 198   27.859 -60.670 51.463 1.00 0.00
H   H
ATOM 27093 C   ILE H 198   29.857 -56.124 49.763 1.00 0.00
H   C
ATOM 27094 O   ILE H 198   29.500 -55.352 50.662 1.00 0.00
H   O
ATOM 27095 N   ILE H 199   29.770 -55.818 48.473 1.00 0.00
H   N
ATOM 27096 HN ILE H 199   30.110 -56.420 47.754 1.00 0.00
H   H
ATOM 27097 CA ILE H 199   29.174 -54.582 47.984 1.00 0.00
H   C
ATOM 27098 HA ILE H 199   28.992 -53.936 48.833 1.00 0.00
H   H
ATOM 27099 CB ILE H 199   30.064 -53.898 46.932 1.00 0.00
H   C
ATOM 27100 HB ILE H 199   30.207 -54.674 46.138 1.00 0.00
H   H
ATOM 27101 CG2 ILE H 199   29.398 -52.639 46.403 1.00 0.00
H   C
ATOM 27102 HG21 ILE H 199   29.963 -52.232 45.538 1.00 0.00
H   H
ATOM 27103 HG22 ILE H 199   28.364 -52.852 46.060 1.00 0.00
H   H
ATOM 27104 HG23 ILE H 199   29.353 -51.854 47.187 1.00 0.00
H   H
ATOM 27105 CG1 ILE H 199   31.448 -53.603 47.521 1.00 0.00
H   C
ATOM 27106 HG11 ILE H 199   31.340 -53.163 48.538 1.00 0.00
H   H
ATOM 27107 HG12 ILE H 199   32.003 -54.562 47.636 1.00 0.00
H   H

ATOM 27108 CD ILE H 199 32.297 -52.682 46.691 1.00 0.00
H C
ATOM 27109 HD1 ILE H 199 33.301 -52.563 47.151 1.00 0.00
H H
ATOM 27110 HD2 ILE H 199 32.430 -53.090 45.665 1.00 0.00
H H
ATOM 27111 HD3 ILE H 199 31.834 -51.676 46.613 1.00 0.00
H H
ATOM 27112 C ILE H 199 27.815 -54.942 47.406 1.00 0.00
H C
ATOM 27113 O ILE H 199 27.732 -55.481 46.299 1.00 0.00
H O
ATOM 27114 N ARG H 200 26.755 -54.672 48.159 1.00 0.00
H N
ATOM 27115 HN ARG H 200 26.845 -54.265 49.065 1.00 0.00
H H
ATOM 27116 CA ARG H 200 25.406 -54.957 47.704 1.00 0.00
H C
ATOM 27117 HA ARG H 200 25.431 -55.838 47.073 1.00 0.00
H H
ATOM 27118 CB ARG H 200 24.486 -55.190 48.898 1.00 0.00
H C
ATOM 27119 HB1 ARG H 200 24.769 -54.430 49.667 1.00 0.00
H H
ATOM 27120 HB2 ARG H 200 23.437 -54.958 48.611 1.00 0.00
H H
ATOM 27121 CG ARG H 200 24.616 -56.572 49.495 1.00 0.00
H C
ATOM 27122 HG1 ARG H 200 24.969 -57.267 48.702 1.00 0.00
H H
ATOM 27123 HG2 ARG H 200 25.422 -56.560 50.264 1.00 0.00
H H
ATOM 27124 CD ARG H 200 23.322 -57.017 50.144 1.00 0.00
H C
ATOM 27125 HD1 ARG H 200 23.197 -56.494 51.120 1.00 0.00
H H
ATOM 27126 HD2 ARG H 200 22.441 -56.788 49.500 1.00 0.00
H H
ATOM 27127 NE ARG H 200 23.299 -58.459 50.377 1.00 0.00
H N
ATOM 27128 HE ARG H 200 24.200 -58.892 50.262 1.00 0.00
H H
ATOM 27129 CZ ARG H 200 22.209 -59.142 50.708 1.00 0.00
H C
ATOM 27130 NH1 ARG H 200 21.050 -58.515 50.848 1.00 0.00
H N
ATOM 27131 HH11 ARG H 200 20.238 -59.012 51.119 1.00 0.00
H H
ATOM 27132 HH12 ARG H 200 21.078 -57.514 50.800 1.00 0.00
H H
ATOM 27133 NH2 ARG H 200 22.275 -60.452 50.900 1.00 0.00
H N
ATOM 27134 HH21 ARG H 200 21.447 -60.969 51.062 1.00 0.00
H H

ATOM 27135 HH22 ARG H 200 23.130 -60.897 50.668 1.00 0.00
H H
ATOM 27136 C ARG H 200 24.883 -53.815 46.839 1.00 0.00
H C
ATOM 27137 O ARG H 200 25.504 -52.758 46.712 1.00 0.00
H O
ATOM 27138 N GLU H 201 23.722 -54.041 46.233 1.00 0.00
H N
ATOM 27139 HN GLU H 201 23.233 -54.909 46.216 1.00 0.00
H H
ATOM 27140 CA GLU H 201 23.052 -52.971 45.512 1.00 0.00
H C
ATOM 27141 HA GLU H 201 23.750 -52.530 44.811 1.00 0.00
H H
ATOM 27142 CB GLU H 201 21.822 -53.496 44.776 1.00 0.00
H C
ATOM 27143 HB1 GLU H 201 21.124 -52.643 44.614 1.00 0.00
H H
ATOM 27144 HB2 GLU H 201 22.112 -53.850 43.762 1.00 0.00
H H
ATOM 27145 CG GLU H 201 21.081 -54.594 45.502 1.00 0.00
H C
ATOM 27146 HG1 GLU H 201 21.653 -55.537 45.428 1.00 0.00
H H
ATOM 27147 HG2 GLU H 201 20.938 -54.338 46.570 1.00 0.00
H H
ATOM 27148 CD GLU H 201 19.730 -54.874 44.880 1.00 0.00
H C
ATOM 27149 OE1 GLU H 201 19.612 -54.761 43.644 1.00 0.00
H O
ATOM 27150 OE2 GLU H 201 18.787 -55.208 45.627 1.00 0.00
H O
ATOM 27151 C GLU H 201 22.641 -51.880 46.486 1.00 0.00
H C
ATOM 27152 O GLU H 201 22.574 -52.099 47.699 1.00 0.00
H O
ATOM 27153 N GLN H 202 22.384 -50.691 45.948 1.00 0.00
H N
ATOM 27154 HN GLN H 202 22.494 -50.532 44.969 1.00 0.00
H H
ATOM 27155 CA GLN H 202 21.930 -49.529 46.700 1.00 0.00
H C
ATOM 27156 HA GLN H 202 21.896 -48.701 46.002 1.00 0.00
H H
ATOM 27157 CB GLN H 202 20.526 -49.760 47.273 1.00 0.00
H C
ATOM 27158 HB1 GLN H 202 20.577 -50.688 47.887 1.00 0.00
H H
ATOM 27159 HB2 GLN H 202 20.275 -48.934 47.978 1.00 0.00
H H
ATOM 27160 CG GLN H 202 19.475 -49.908 46.187 1.00 0.00
H C
ATOM 27161 HG1 GLN H 202 19.799 -49.389 45.260 1.00 0.00
H H

```
ATOM  27162  HG2 GLN H 202      19.313 -50.978  45.940  1.00  0.00
H    H
ATOM  27163  CD  GLN H 202      18.128 -49.303  46.546  1.00  0.00
H    C
ATOM  27164  OE1 GLN H 202      18.050 -48.339  47.301  1.00  0.00
H    O
ATOM  27165  NE2 GLN H 202      17.060 -49.869  45.998  1.00  0.00
H    N
ATOM  27166 HE21 GLN H 202      16.174 -49.474  46.226  1.00  0.00
H    H
ATOM  27167 HE22 GLN H 202      17.168 -50.661  45.405  1.00  0.00
H    H
ATOM  27168  C   GLN H 202      22.901 -49.153  47.809  1.00  0.00
H    C
ATOM  27169  O   GLN H 202      22.500 -48.529  48.802  1.00  0.00
H    O
ATOM  27170  N   ASP H 203      24.172 -49.513  47.661  1.00  0.00
H    N
ATOM  27171  HN  ASP H 203      24.539 -50.011  46.879  1.00  0.00
H    H
ATOM  27172  CA  ASP H 203      25.180 -49.207  48.661  1.00  0.00
H    C
ATOM  27173  HA  ASP H 203      24.676 -49.159  49.620  1.00  0.00
H    H
ATOM  27174  CB  ASP H 203      26.304 -50.232  48.641  1.00  0.00
H    C
ATOM  27175  HB1 ASP H 203      25.867 -51.251  48.623  1.00  0.00
H    H
ATOM  27176  HB2 ASP H 203      26.931 -50.106  47.736  1.00  0.00
H    H
ATOM  27177  CG  ASP H 203      27.182 -50.134  49.857  1.00  0.00
H    C
ATOM  27178  OD1 ASP H 203      28.247 -49.496  49.756  1.00  0.00
H    O
ATOM  27179  OD2 ASP H 203      26.833 -50.721  50.887  1.00  0.00
H    O
ATOM  27180  C   ASP H 203      25.782 -47.837  48.414  1.00  0.00
H    C
ATOM  27181  O   ASP H 203      26.087 -47.475  47.276  1.00  0.00
H    O
ATOM  27182  N   CYS H 204      25.998 -47.093  49.491  1.00  0.00
H    N
ATOM  27183  HN  CYS H 204      25.706 -47.336  50.414  1.00  0.00
H    H
ATOM  27184  CA  CYS H 204      26.703 -45.824  49.387  1.00  0.00
H    C
ATOM  27185  HA  CYS H 204      26.504 -45.421  48.402  1.00  0.00
H    H
ATOM  27186  CB  CYS H 204      26.219 -44.851  50.457  1.00  0.00
H    C
ATOM  27187  HB1 CYS H 204      26.159 -45.416  51.414  1.00  0.00
H    H
ATOM  27188  HB2 CYS H 204      26.981 -44.057  50.618  1.00  0.00
H    H
```

```
ATOM  27189  SG  CYS H 204      24.594 -44.141  50.104  1.00  0.00
H    S
ATOM  27190  HG1 CYS H 204      24.473 -43.434  51.229  1.00  0.00
H    H
ATOM  27191  C   CYS H 204      28.204 -46.075  49.491  1.00  0.00
H    C
ATOM  27192  O   CYS H 204      28.690 -46.588  50.504  1.00  0.00
H    O
ATOM  27193  N   LEU H 205      28.921 -45.745  48.418  1.00  0.00
H    N
ATOM  27194  HN  LEU H 205      28.479 -45.328  47.627  1.00  0.00
H    H
ATOM  27195  CA  LEU H 205      30.357 -45.937  48.268  1.00  0.00
H    C
ATOM  27196  HA  LEU H 205      30.706 -46.567  49.076  1.00  0.00
H    H
ATOM  27197  CB  LEU H 205      30.682 -46.511  46.884  1.00  0.00
H    C
ATOM  27198  HB1 LEU H 205      29.797 -46.323  46.233  1.00  0.00
H    H
ATOM  27199  HB2 LEU H 205      31.520 -45.930  46.439  1.00  0.00
H    H
ATOM  27200  CG  LEU H 205      31.028 -47.972  46.610  1.00  0.00
H    C
ATOM  27201  HG  LEU H 205      32.008 -48.222  47.081  1.00  0.00
H    H
ATOM  27202  CD1 LEU H 205      29.949 -48.910  47.121  1.00  0.00
H    C
ATOM  27203 HD11 LEU H 205      30.197 -49.963  46.869  1.00  0.00
H    H
ATOM  27204 HD12 LEU H 205      29.850 -48.828  48.224  1.00  0.00
H    H
ATOM  27205 HD13 LEU H 205      28.967 -48.666  46.662  1.00  0.00
H    H
ATOM  27206  CD2 LEU H 205      31.172 -48.131  45.115  1.00  0.00
H    C
ATOM  27207 HD21 LEU H 205      31.444 -49.179  44.863  1.00  0.00
H    H
ATOM  27208 HD22 LEU H 205      30.229 -47.891  44.584  1.00  0.00
H    H
ATOM  27209 HD23 LEU H 205      31.971 -47.463  44.728  1.00  0.00
H    H
ATOM  27210  C   LEU H 205      31.008 -44.572  48.435  1.00  0.00
H    C
ATOM  27211  O   LEU H 205      30.559 -43.594  47.824  1.00  0.00
H    O
ATOM  27212  N   VAL H 206      32.044 -44.495  49.264  1.00  0.00
H    N
ATOM  27213  HN  VAL H 206      32.374 -45.257  49.816  1.00  0.00
H    H
ATOM  27214  CA  VAL H 206      32.798 -43.258  49.433  1.00  0.00
H    C
ATOM  27215  HA  VAL H 206      32.246 -42.449  48.973  1.00  0.00
H    H
```

```
ATOM  27216  CB  VAL H 206      33.026 -42.923  50.908  1.00  0.00
H    C
ATOM  27217  HB  VAL H 206      33.694 -43.712  51.335  1.00  0.00
H    H
ATOM  27218  CG1 VAL H 206      33.698 -41.572  51.040  1.00  0.00
H    C
ATOM  27219 HG11 VAL H 206      33.829 -41.310  52.112  1.00  0.00
H    H
ATOM  27220 HG12 VAL H 206      34.704 -41.567  50.573  1.00  0.00
H    H
ATOM  27221 HG13 VAL H 206      33.081 -40.779  50.565  1.00  0.00
H    H
ATOM  27222  CG2 VAL H 206      31.694 -42.929  51.651  1.00  0.00
H    C
ATOM  27223 HG21 VAL H 206      31.837 -42.647  52.715  1.00  0.00
H    H
ATOM  27224 HG22 VAL H 206      30.988 -42.206  51.190  1.00  0.00
H    H
ATOM  27225 HG23 VAL H 206      31.227 -43.935  51.629  1.00  0.00
H    H
ATOM  27226  C   VAL H 206      34.113 -43.398  48.681  1.00  0.00
H    C
ATOM  27227  O   VAL H 206      34.941 -44.261  49.000  1.00  0.00
H    O
ATOM  27228  N   ILE H 207      34.310 -42.537  47.691  1.00  0.00
H    N
ATOM  27229  HN  ILE H 207      33.662 -41.816  47.450  1.00  0.00
H    H
ATOM  27230  CA  ILE H 207      35.500 -42.569  46.861  1.00  0.00
H    C
ATOM  27231  HA  ILE H 207      36.210 -43.237  47.331  1.00  0.00
H    H
ATOM  27232  CB  ILE H 207      35.184 -43.025  45.421  1.00  0.00
H    C
ATOM  27233  HB  ILE H 207      34.554 -43.941  45.549  1.00  0.00
H    H
ATOM  27234  CG2 ILE H 207      34.487 -41.922  44.661  1.00  0.00
H    C
ATOM  27235 HG21 ILE H 207      34.120 -42.294  43.682  1.00  0.00
H    H
ATOM  27236 HG22 ILE H 207      33.611 -41.544  45.228  1.00  0.00
H    H
ATOM  27237 HG23 ILE H 207      35.177 -41.073  44.472  1.00  0.00
H    H
ATOM  27238  CG1 ILE H 207      36.463 -43.466  44.701  1.00  0.00
H    C
ATOM  27239 HG11 ILE H 207      37.192 -42.625  44.682  1.00  0.00
H    H
ATOM  27240 HG12 ILE H 207      36.932 -44.296  45.277  1.00  0.00
H    H
ATOM  27241  CD  ILE H 207      36.229 -43.939  43.310  1.00  0.00
H    C
ATOM  27242  HD1 ILE H 207      37.179 -44.298  42.861  1.00  0.00
H    H
```

ATOM 27243 HD2 ILE H 207 35.499 -44.776 43.299 1.00 0.00
H H
ATOM 27244 HD3 ILE H 207 35.834 -43.120 42.672 1.00 0.00
H H
ATOM 27245 C ILE H 207 36.128 -41.183 46.872 1.00 0.00
H C
ATOM 27246 O ILE H 207 35.485 -40.183 47.189 1.00 0.00
H O
ATOM 27247 N MET H 208 37.408 -41.126 46.523 1.00 0.00
H N
ATOM 27248 HN MET H 208 37.963 -41.921 46.286 1.00 0.00
H H
ATOM 27249 CA MET H 208 38.118 -39.857 46.458 1.00 0.00
H C
ATOM 27250 HA MET H 208 37.431 -39.100 46.101 1.00 0.00
H H
ATOM 27251 CB MET H 208 38.697 -39.447 47.822 1.00 0.00
H C
ATOM 27252 HB1 MET H 208 37.835 -39.155 48.466 1.00 0.00
H H
ATOM 27253 HB2 MET H 208 39.161 -40.335 48.308 1.00 0.00
H H
ATOM 27254 CG MET H 208 39.677 -38.287 47.714 1.00 0.00
H C
ATOM 27255 HG1 MET H 208 40.261 -38.361 46.772 1.00 0.00
H H
ATOM 27256 HG2 MET H 208 39.118 -37.327 47.688 1.00 0.00
H H
ATOM 27257 SD MET H 208 40.855 -38.200 49.051 1.00 0.00
H S
ATOM 27258 CE MET H 208 41.427 -39.881 49.067 1.00 0.00
H C
ATOM 27259 HE1 MET H 208 42.185 -40.035 49.865 1.00 0.00
H H
ATOM 27260 HE2 MET H 208 40.589 -40.587 49.252 1.00 0.00
H H
ATOM 27261 HE3 MET H 208 41.896 -40.153 48.096 1.00 0.00
H H
ATOM 27262 C MET H 208 39.235 -39.981 45.438 1.00 0.00
H C
ATOM 27263 O MET H 208 39.848 -41.045 45.311 1.00 0.00
H O
ATOM 27264 N GLY H 209 39.501 -38.896 44.727 1.00 0.00
H N
ATOM 27265 HN GLY H 209 38.942 -38.068 44.730 1.00 0.00
H H
ATOM 27266 CA GLY H 209 40.669 -38.858 43.872 1.00 0.00
H C
ATOM 27267 HA1 GLY H 209 40.735 -39.823 43.389 1.00 0.00
H H
ATOM 27268 HA2 GLY H 209 41.516 -38.616 44.500 1.00 0.00
H H
ATOM 27269 C GLY H 209 40.571 -37.802 42.796 1.00 0.00
H C

ATOM 27270 O   GLY H 209   39.896 -36.785 42.971 1.00 0.00
H   O
ATOM 27271 N   HSE H 210   41.242 -38.040 41.675 1.00 0.00
H   N
ATOM 27272 HN HSE H 210   41.838 -38.827 41.522 1.00 0.00
H   H
ATOM 27273 CA HSE H 210   41.149 -37.132 40.547 1.00 0.00
H   C
ATOM 27274 HA HSE H 210   41.295 -36.125 40.920 1.00 0.00
H   H
ATOM 27275 CB HSE H 210   42.170 -37.486 39.474 1.00 0.00
H   C
ATOM 27276 HB1 HSE H 210   43.181 -37.430 39.932 1.00 0.00
H   H
ATOM 27277 HB2 HSE H 210   42.018 -38.537 39.149 1.00 0.00
H   H
ATOM 27278 ND1 HSE H 210   42.478 -35.231 38.391 1.00 0.00
H   N
ATOM 27279 CG HSE H 210   42.141 -36.564 38.299 1.00 0.00
H   C
ATOM 27280 CE1 HSE H 210   42.352 -34.665 37.205 1.00 0.00
H   C
ATOM 27281 HE1 HSE H 210   42.564 -33.613 36.993 1.00 0.00
H   H
ATOM 27282 NE2 HSE H 210   41.928 -35.579 36.350 1.00 0.00
H   N
ATOM 27283 HE2 HSE H 210   41.729 -35.416 35.384 1.00 0.00
H   H
ATOM 27284 CD2 HSE H 210   41.780 -36.774 37.012 1.00 0.00
H   C
ATOM 27285 HD2 HSE H 210   41.424 -37.673 36.529 1.00 0.00
H   H
ATOM 27286 C   HSE H 210   39.747 -37.194 39.971 1.00 0.00
H   C
ATOM 27287 O   HSE H 210   39.292 -38.251 39.527 1.00 0.00
H   O
ATOM 27288 N   LYS H 211   39.066 -36.046 39.978 1.00 0.00
H   N
ATOM 27289 HN LYS H 211   39.462 -35.161 40.217 1.00 0.00
H   H
ATOM 27290 CA LYS H 211   37.651 -36.015 39.626 1.00 0.00
H   C
ATOM 27291 HA LYS H 211   37.131 -36.619 40.360 1.00 0.00
H   H
ATOM 27292 CB LYS H 211   37.139 -34.576 39.650 1.00 0.00
H   C
ATOM 27293 HB1 LYS H 211   36.110 -34.586 39.223 1.00 0.00
H   H
ATOM 27294 HB2 LYS H 211   37.035 -34.246 40.709 1.00 0.00
H   H
ATOM 27295 CG LYS H 211   37.991 -33.596 38.864 1.00 0.00
H   C
ATOM 27296 HG1 LYS H 211   38.983 -33.495 39.361 1.00 0.00
H   H

ATOM 27297 HG2 LYS H 211 38.171 -34.008 37.845 1.00 0.00
H H
ATOM 27298 CD LYS H 211 37.333 -32.232 38.780 1.00 0.00
H C
ATOM 27299 HD1 LYS H 211 36.265 -32.373 38.496 1.00 0.00
H H
ATOM 27300 HD2 LYS H 211 37.349 -31.776 39.796 1.00 0.00
H H
ATOM 27301 CE LYS H 211 38.053 -31.349 37.782 1.00 0.00
H C
ATOM 27302 HE1 LYS H 211 37.997 -31.789 36.761 1.00 0.00
H H
ATOM 27303 HE2 LYS H 211 37.594 -30.336 37.755 1.00 0.00
H H
ATOM 27304 NZ LYS H 211 39.491 -31.182 38.117 1.00 0.00
H N
ATOM 27305 HZ1 LYS H 211 39.952 -30.573 37.411 1.00 0.00
H H
ATOM 27306 HZ2 LYS H 211 39.580 -30.748 39.059 1.00 0.00
H H
ATOM 27307 HZ3 LYS H 211 39.955 -32.113 38.125 1.00 0.00
H H
ATOM 27308 C LYS H 211 37.397 -36.640 38.261 1.00 0.00
H C
ATOM 27309 O LYS H 211 36.358 -37.277 38.046 1.00 0.00
H O
ATOM 27310 N LYS H 212 38.333 -36.480 37.326 1.00 0.00
H N
ATOM 27311 HN LYS H 212 39.209 -36.019 37.453 1.00 0.00
H H
ATOM 27312 CA LYS H 212 38.099 -37.008 35.989 1.00 0.00
H C
ATOM 27313 HA LYS H 212 37.061 -36.815 35.746 1.00 0.00
H H
ATOM 27314 CB LYS H 212 38.990 -36.292 34.976 1.00 0.00
H C
ATOM 27315 HB1 LYS H 212 40.046 -36.512 35.256 1.00 0.00
H H
ATOM 27316 HB2 LYS H 212 38.831 -36.744 33.971 1.00 0.00
H H
ATOM 27317 CG LYS H 212 38.787 -34.771 34.929 1.00 0.00
H C
ATOM 27318 HG1 LYS H 212 39.465 -34.290 35.671 1.00 0.00
H H
ATOM 27319 HG2 LYS H 212 39.079 -34.397 33.921 1.00 0.00
H H
ATOM 27320 CD LYS H 212 37.348 -34.332 35.241 1.00 0.00
H C
ATOM 27321 HD1 LYS H 212 37.019 -34.843 36.174 1.00 0.00
H H
ATOM 27322 HD2 LYS H 212 37.357 -33.237 35.447 1.00 0.00
H H
ATOM 27323 CE LYS H 212 36.371 -34.630 34.112 1.00 0.00
H C

ATOM 27324 HE1 LYS H 212 36.859 -34.478 33.123 1.00 0.00
H H
ATOM 27325 HE2 LYS H 212 36.017 -35.683 34.169 1.00 0.00
H H
ATOM 27326 NZ LYS H 212 35.177 -33.739 34.176 1.00 0.00
H N
ATOM 27327 HZ1 LYS H 212 34.525 -33.959 33.395 1.00 0.00
H H
ATOM 27328 HZ2 LYS H 212 34.689 -33.878 35.083 1.00 0.00
H H
ATOM 27329 HZ3 LYS H 212 35.482 -32.747 34.100 1.00 0.00
H H
ATOM 27330 C LYS H 212 38.302 -38.517 35.949 1.00 0.00
H C
ATOM 27331 O LYS H 212 37.554 -39.230 35.273 1.00 0.00
H O
ATOM 27332 N ASP H 213 39.283 -39.034 36.692 1.00 0.00
H N
ATOM 27333 HN ASP H 213 39.997 -38.544 37.184 1.00 0.00
H H
ATOM 27334 CA ASP H 213 39.379 -40.483 36.842 1.00 0.00
H C
ATOM 27335 HA ASP H 213 39.378 -40.908 35.844 1.00 0.00
H H
ATOM 27336 CB ASP H 213 40.649 -40.860 37.602 1.00 0.00
H C
ATOM 27337 HB1 ASP H 213 40.740 -40.220 38.503 1.00 0.00
H H
ATOM 27338 HB2 ASP H 213 40.608 -41.919 37.929 1.00 0.00
H H
ATOM 27339 CG ASP H 213 41.903 -40.663 36.782 1.00 0.00
H C
ATOM 27340 OD1 ASP H 213 42.255 -41.571 36.001 1.00 0.00
H O
ATOM 27341 OD2 ASP H 213 42.538 -39.598 36.907 1.00 0.00
H O
ATOM 27342 C ASP H 213 38.156 -41.039 37.558 1.00 0.00
H C
ATOM 27343 O ASP H 213 37.700 -42.150 37.259 1.00 0.00
H O
ATOM 27344 N ILE H 214 37.614 -40.278 38.511 1.00 0.00
H N
ATOM 27345 HN ILE H 214 37.973 -39.401 38.820 1.00 0.00
H H
ATOM 27346 CA ILE H 214 36.399 -40.703 39.199 1.00 0.00
H C
ATOM 27347 HA ILE H 214 36.578 -41.708 39.560 1.00 0.00
H H
ATOM 27348 CB ILE H 214 36.078 -39.753 40.365 1.00 0.00
H C
ATOM 27349 HB ILE H 214 35.983 -38.745 39.887 1.00 0.00
H H
ATOM 27350 CG2 ILE H 214 34.838 -40.220 41.099 1.00 0.00
H C

ATOM 27351 HG21 ILE H 214   34.512 -39.459 41.839 1.00 0.00
H   H
ATOM 27352 HG22 ILE H 214   33.997 -40.384 40.393 1.00 0.00
H   H
ATOM 27353 HG23 ILE H 214   35.030 -41.171 41.641 1.00 0.00
H   H
ATOM 27354 CG1 ILE H 214   37.260 -39.676 41.329 1.00 0.00
H   C
ATOM 27355 HG11 ILE H 214   37.339 -40.624 41.906 1.00 0.00
H   H
ATOM 27356 HG12 ILE H 214   38.200 -39.571 40.740 1.00 0.00
H   H
ATOM 27357 CD ILE H 214   37.190 -38.521 42.299 1.00 0.00
H   C
ATOM 27358 HD1 ILE H 214   38.104 -38.490 42.930 1.00 0.00
H   H
ATOM 27359 HD2 ILE H 214   37.110 -37.555 41.754 1.00 0.00
H   H
ATOM 27360 HD3 ILE H 214   36.312 -38.619 42.972 1.00 0.00
H   H
ATOM 27361 C   ILE H 214   35.242 -40.788 38.215 1.00 0.00
H   C
ATOM 27362 O   ILE H 214   34.461 -41.746 38.229 1.00 0.00
H   O
ATOM 27363 N   LYS H 215   35.117 -39.788 37.339 1.00 0.00
H   N
ATOM 27364 HN LYS H 215   35.672 -38.957 37.340 1.00 0.00
H   H
ATOM 27365 CA LYS H 215   34.116 -39.861 36.283 1.00 0.00
H   C
ATOM 27366 HA LYS H 215   33.154 -39.997 36.765 1.00 0.00
H   H
ATOM 27367 CB LYS H 215   34.122 -38.577 35.450 1.00 0.00
H   C
ATOM 27368 HB1 LYS H 215   33.992 -37.726 36.158 1.00 0.00
H   H
ATOM 27369 HB2 LYS H 215   35.127 -38.449 34.989 1.00 0.00
H   H
ATOM 27370 CG LYS H 215   33.024 -38.513 34.384 1.00 0.00
H   C
ATOM 27371 HG1 LYS H 215   32.135 -39.079 34.743 1.00 0.00
H   H
ATOM 27372 HG2 LYS H 215   32.711 -37.451 34.250 1.00 0.00
H   H
ATOM 27373 CD LYS H 215   33.492 -39.103 33.057 1.00 0.00
H   C
ATOM 27374 HD1 LYS H 215   34.596 -38.975 32.984 1.00 0.00
H   H
ATOM 27375 HD2 LYS H 215   33.284 -40.198 33.072 1.00 0.00
H   H
ATOM 27376 CE LYS H 215   32.811 -38.467 31.872 1.00 0.00
H   C
ATOM 27377 HE1 LYS H 215   31.707 -38.585 31.948 1.00 0.00
H   H

ATOM 27378 HE2 LYS H 215   33.047 -37.381 31.823 1.00 0.00
H   H
ATOM 27379 NZ LYS H 215   33.264 -39.105 30.609 1.00 0.00
H   N
ATOM 27380 HZ1 LYS H 215   32.789 -38.662 29.796 1.00 0.00
H   H
ATOM 27381 HZ2 LYS H 215   34.293 -38.991 30.513 1.00 0.00
H   H
ATOM 27382 HZ3 LYS H 215   33.034 -40.119 30.632 1.00 0.00
H   H
ATOM 27383 C   LYS H 215   34.347 -41.065 35.387 1.00 0.00
H   C
ATOM 27384 O   LYS H 215   33.386 -41.710 34.961 1.00 0.00
H   O
ATOM 27385 N   ARG H 216   35.605 -41.347 35.046 1.00 0.00
H   N
ATOM 27386 HN ARG H 216   36.385 -40.805 35.349 1.00 0.00
H   H
ATOM 27387 CA ARG H 216   35.897 -42.492 34.192 1.00 0.00
H   C
ATOM 27388 HA ARG H 216   35.345 -42.379 33.266 1.00 0.00
H   H
ATOM 27389 CB ARG H 216   37.397 -42.559 33.908 1.00 0.00
H   C
ATOM 27390 HB1 ARG H 216   37.907 -42.537 34.901 1.00 0.00
H   H
ATOM 27391 HB2 ARG H 216   37.648 -43.544 33.458 1.00 0.00
H   H
ATOM 27392 CG ARG H 216   37.903 -41.407 33.063 1.00 0.00
H   C
ATOM 27393 HG1 ARG H 216   37.635 -41.602 32.002 1.00 0.00
H   H
ATOM 27394 HG2 ARG H 216   37.351 -40.481 33.346 1.00 0.00
H   H
ATOM 27395 CD ARG H 216   39.389 -41.172 33.263 1.00 0.00
H   C
ATOM 27396 HD1 ARG H 216   39.664 -41.416 34.315 1.00 0.00
H   H
ATOM 27397 HD2 ARG H 216   39.999 -41.805 32.578 1.00 0.00
H   H
ATOM 27398 NE ARG H 216   39.747 -39.784 32.985 1.00 0.00
H   N
ATOM 27399 HE ARG H 216   39.004 -39.265 32.546 1.00 0.00
H   H
ATOM 27400 CZ ARG H 216   40.926 -39.241 33.271 1.00 0.00
H   C
ATOM 27401 NH1 ARG H 216   41.872 -39.970 33.843 1.00 0.00
H   N
ATOM 27402 HH11 ARG H 216   42.744 -39.566 34.082 1.00 0.00
H   H
ATOM 27403 HH12 ARG H 216   41.608 -40.891 34.136 1.00 0.00
H   H
ATOM 27404 NH2 ARG H 216   41.161 -37.969 32.983 1.00 0.00
H   N

ATOM 27405 HH21 ARG H 216   42.066 -37.591 33.118 1.00 0.00
H   H
ATOM 27406 HH22 ARG H 216   40.474 -37.496 32.445 1.00 0.00
H   H
ATOM 27407 C   ARG H 216   35.413 -43.783 34.832 1.00 0.00
H   C
ATOM 27408 O   ARG H 216   34.737 -44.592 34.185 1.00 0.00
H   O
ATOM 27409 N   PHE H 217   35.746 -43.986 36.106 1.00 0.00
H   N
ATOM 27410 HN PHE H 217   36.306 -43.373 36.661 1.00 0.00
H   H
ATOM 27411 CA PHE H 217   35.287 -45.180 36.801 1.00 0.00
H   C
ATOM 27412 HA PHE H 217   35.587 -46.022 36.190 1.00 0.00
H   H
ATOM 27413 CB PHE H 217   35.908 -45.257 38.193 1.00 0.00
H   C
ATOM 27414 HB1 PHE H 217   36.902 -45.749 38.122 1.00 0.00
H   H
ATOM 27415 HB2 PHE H 217   36.075 -44.241 38.613 1.00 0.00
H   H
ATOM 27416 CG PHE H 217   35.107 -46.070 39.162 1.00 0.00
H   C
ATOM 27417 CD1 PHE H 217   35.038 -47.445 39.038 1.00 0.00
H   C
ATOM 27418 HD1 PHE H 217   35.566 -47.937 38.233 1.00 0.00
H   H
ATOM 27419 CE1 PHE H 217   34.298 -48.197 39.925 1.00 0.00
H   C
ATOM 27420 HE1 PHE H 217   34.255 -49.272 39.819 1.00 0.00
H   H
ATOM 27421 CZ PHE H 217   33.619 -47.577 40.950 1.00 0.00
H   C
ATOM 27422 HZ PHE H 217   33.045 -48.163 41.653 1.00 0.00
H   H
ATOM 27423 CD2 PHE H 217   34.419 -45.461 40.194 1.00 0.00
H   C
ATOM 27424 HD2 PHE H 217   34.440 -44.384 40.297 1.00 0.00
H   H
ATOM 27425 CE2 PHE H 217   33.680 -46.208 41.085 1.00 0.00
H   C
ATOM 27426 HE2 PHE H 217   33.148 -45.717 41.888 1.00 0.00
H   H
ATOM 27427 C   PHE H 217   33.767 -45.211 36.890 1.00 0.00
H   C
ATOM 27428 O   PHE H 217   33.155 -46.273 36.742 1.00 0.00
H   O
ATOM 27429 N   GLU H 218   33.143 -44.055 37.118 1.00 0.00
H   N
ATOM 27430 HN GLU H 218   33.589 -43.178 37.267 1.00 0.00
H   H
ATOM 27431 CA GLU H 218   31.686 -43.995 37.165 1.00 0.00
H   C

```
ATOM  27432  HA  GLU H 218      31.344 -44.701  37.910  1.00  0.00
H    H
ATOM  27433  CB  GLU H 218      31.240 -42.582  37.539  1.00  0.00
H    C
ATOM  27434  HB1 GLU H 218      31.954 -42.182  38.297  1.00  0.00
H    H
ATOM  27435  HB2 GLU H 218      31.327 -41.912  36.655  1.00  0.00
H    H
ATOM  27436  CG  GLU H 218      29.835 -42.496  38.104  1.00  0.00
H    C
ATOM  27437  HG1 GLU H 218      29.565 -43.462  38.570  1.00  0.00
H    H
ATOM  27438  HG2 GLU H 218      29.761 -41.693  38.863  1.00  0.00
H    H
ATOM  27439  CD  GLU H 218      28.804 -42.248  37.020  1.00  0.00
H    C
ATOM  27440  OE1 GLU H 218      29.208 -41.976  35.871  1.00  0.00
H    O
ATOM  27441  OE2 GLU H 218      27.593 -42.318  37.312  1.00  0.00
H    O
ATOM  27442  C   GLU H 218      31.072 -44.409  35.836  1.00  0.00
H    C
ATOM  27443  O   GLU H 218      30.085 -45.153  35.801  1.00  0.00
H    O
ATOM  27444  N   ASN H 219      31.644 -43.939  34.734  1.00  0.00
H    N
ATOM  27445  HN  ASN H 219      32.482 -43.393  34.715  1.00  0.00
H    H
ATOM  27446  CA  ASN H 219      31.062 -44.198  33.425  1.00  0.00
H    C
ATOM  27447  HA  ASN H 219      29.995 -44.045  33.538  1.00  0.00
H    H
ATOM  27448  CB  ASN H 219      31.661 -43.243  32.394  1.00  0.00
H    C
ATOM  27449  HB1 ASN H 219      31.882 -42.283  32.907  1.00  0.00
H    H
ATOM  27450  HB2 ASN H 219      32.620 -43.644  32.008  1.00  0.00
H    H
ATOM  27451  CG  ASN H 219      30.714 -42.956  31.247  1.00  0.00
H    C
ATOM  27452  OD1 ASN H 219      29.616 -43.510  31.179  1.00  0.00
H    O
ATOM  27453  ND2 ASN H 219      31.133 -42.084  30.338  1.00  0.00
H    N
ATOM  27454 HD21 ASN H 219      30.516 -41.877  29.584  1.00  0.00
H    H
ATOM  27455 HD22 ASN H 219      32.020 -41.644  30.441  1.00  0.00
H    H
ATOM  27456  C   ASN H 219      31.280 -45.645  33.011  1.00  0.00
H    C
ATOM  27457  O   ASN H 219      30.390 -46.275  32.428  1.00  0.00
H    O
ATOM  27458  N   GLU H 220      32.457 -46.191  33.303  1.00  0.00
H    N
```

ATOM 27459 HN GLU H 220 33.168 -45.763 33.854 1.00 0.00
H H
ATOM 27460 CA GLU H 220 32.805 -47.518 32.813 1.00 0.00
H C
ATOM 27461 HA GLU H 220 32.235 -47.687 31.909 1.00 0.00
H H
ATOM 27462 CB GLU H 220 34.298 -47.554 32.475 1.00 0.00
H C
ATOM 27463 HB1 GLU H 220 34.872 -47.499 33.430 1.00 0.00
H H
ATOM 27464 HB2 GLU H 220 34.558 -48.536 32.019 1.00 0.00
H H
ATOM 27465 CG GLU H 220 34.713 -46.407 31.559 1.00 0.00
H C
ATOM 27466 HG1 GLU H 220 34.717 -46.756 30.510 1.00 0.00
H H
ATOM 27467 HG2 GLU H 220 34.016 -45.551 31.655 1.00 0.00
H H
ATOM 27468 CD GLU H 220 36.118 -45.906 31.824 1.00 0.00
H C
ATOM 27469 OE1 GLU H 220 36.622 -46.104 32.949 1.00 0.00
H O
ATOM 27470 OE2 GLU H 220 36.712 -45.295 30.912 1.00 0.00
H O
ATOM 27471 C GLU H 220 32.441 -48.630 33.794 1.00 0.00
H C
ATOM 27472 O GLU H 220 31.686 -49.541 33.436 1.00 0.00
H O
ATOM 27473 N GLY H 221 32.948 -48.578 35.025 1.00 0.00
H N
ATOM 27474 HN GLY H 221 33.542 -47.836 35.334 1.00 0.00
H H
ATOM 27475 CA GLY H 221 32.667 -49.608 36.006 1.00 0.00
H C
ATOM 27476 HA1 GLY H 221 33.335 -49.429 36.838 1.00 0.00
H H
ATOM 27477 HA2 GLY H 221 32.817 -50.558 35.512 1.00 0.00
H H
ATOM 27478 C GLY H 221 31.246 -49.572 36.531 1.00 0.00
H C
ATOM 27479 O GLY H 221 30.765 -50.550 37.110 1.00 0.00
H O
ATOM 27480 N MET H 222 30.568 -48.447 36.343 1.00 0.00
H N
ATOM 27481 HN MET H 222 30.947 -47.634 35.907 1.00 0.00
H H
ATOM 27482 CA MET H 222 29.185 -48.309 36.773 1.00 0.00
H C
ATOM 27483 HA MET H 222 28.820 -49.301 37.008 1.00 0.00
H H
ATOM 27484 CB MET H 222 29.090 -47.403 37.999 1.00 0.00
H C
ATOM 27485 HB1 MET H 222 29.426 -46.390 37.679 1.00 0.00
H H

ATOM 27486 HB2 MET H 222   28.024 -47.299 38.298 1.00 0.00
H   H
ATOM 27487 CG MET H 222   29.935 -47.853 39.171 1.00 0.00
H   C
ATOM 27488 HG1 MET H 222   29.754 -48.927 39.388 1.00 0.00
H   H
ATOM 27489 HG2 MET H 222   31.013 -47.738 38.922 1.00 0.00
H   H
ATOM 27490 SD MET H 222   29.570 -46.909 40.662 1.00 0.00
H   S
ATOM 27491 CE MET H 222   30.322 -45.335 40.268 1.00 0.00
H   C
ATOM 27492 HE1 MET H 222   30.197 -44.615 41.104 1.00 0.00
H   H
ATOM 27493 HE2 MET H 222   31.410 -45.450 40.075 1.00 0.00
H   H
ATOM 27494 HE3 MET H 222   29.860 -44.888 39.361 1.00 0.00
H   H
ATOM 27495 C   MET H 222   28.323 -47.758 35.647 1.00 0.00
H   C
ATOM 27496 O   MET H 222   27.290 -48.332 35.310 1.00 0.00
H   O
ATOM 27497 N   THR I 35   -19.128 19.269   9.291 1.00 0.00
I   N
ATOM 27498 HT1 THR I 35   -19.958 19.031   9.796 1.00 0.00
I   H
ATOM 27499 HT2 THR I 35   -19.143 18.837   8.389 1.00 0.00
I   H
ATOM 27500 HT3 THR I 35   -19.060 20.261   9.192 1.00 0.00
I   H
ATOM 27501 CA THR I 35   -17.928 18.768 10.054 1.00 0.00
I   C
ATOM 27502 HA THR I 35   -17.918 19.326 10.981 1.00 0.00
I   H
ATOM 27503 CB THR I 35   -18.034 17.245 10.202 1.00 0.00
I   C
ATOM 27504 HB THR I 35   -17.888 16.822   9.178 1.00 0.00
I   H
ATOM 27505 OG1 THR I 35   -19.343 16.890 10.637 1.00 0.00
I   O
ATOM 27506 HG1 THR I 35   -19.334 15.930 10.719 1.00 0.00
I   H
ATOM 27507 CG2 THR I 35   -17.084 16.735 11.282 1.00 0.00
I   C
ATOM 27508 HG21 THR I 35   -17.077 15.625 11.310 1.00 0.00
I   H
ATOM 27509 HG22 THR I 35   -16.047 17.077 11.078 1.00 0.00
I   H
ATOM 27510 HG23 THR I 35   -17.382 17.106 12.286 1.00 0.00
I   H
ATOM 27511 C   THR I 35   -16.645 19.093   9.347 1.00 0.00
I   C
ATOM 27512 O   THR I 35   -16.703 19.020   8.129 1.00 0.00
I   O

```
ATOM  27513  N   PRO I  36     -15.510  19.456   9.951  1.00  0.00
I    N
ATOM  27514  CD  PRO I  36     -15.400  19.859  11.351  1.00  0.00
I    C
ATOM  27515  HD1 PRO I  36     -15.738  20.912  11.480  1.00  0.00
I    H
ATOM  27516  HD2 PRO I  36     -15.990  19.177  12.006  1.00  0.00
I    H
ATOM  27517  CA  PRO I  36     -14.283  19.824   9.248  1.00  0.00
I    C
ATOM  27518  HA  PRO I  36     -14.368  20.879   9.017  1.00  0.00
I    H
ATOM  27519  CB  PRO I  36     -13.196  19.904  10.331  1.00  0.00
I    C
ATOM  27520  HB1 PRO I  36     -13.223  20.915  10.798  1.00  0.00
I    H
ATOM  27521  HB2 PRO I  36     -12.179  19.718   9.933  1.00  0.00
I    H
ATOM  27522  CG  PRO I  36     -13.911  19.743  11.681  1.00  0.00
I    C
ATOM  27523  HG1 PRO I  36     -13.342  20.178  12.526  1.00  0.00
I    H
ATOM  27524  HG2 PRO I  36     -14.091  18.660  11.873  1.00  0.00
I    H
ATOM  27525  C   PRO I  36     -13.890  19.068   7.985  1.00  0.00
I    C
ATOM  27526  O   PRO I  36     -13.805  19.771   6.976  1.00  0.00
I    O
ATOM  27527  N   PRO I  37     -13.729  17.745   7.922  1.00  0.00
I    N
ATOM  27528  CD  PRO I  37     -13.712  16.831   9.068  1.00  0.00
I    C
ATOM  27529  HD1 PRO I  37     -12.840  16.583   9.714  1.00  0.00
I    H
ATOM  27530  HD2 PRO I  37     -14.493  17.367   9.655  1.00  0.00
I    H
ATOM  27531  CA  PRO I  37     -13.501  17.008   6.679  1.00  0.00
I    C
ATOM  27532  HA  PRO I  37     -12.508  17.240   6.314  1.00  0.00
I    H
ATOM  27533  CB  PRO I  37     -13.537  15.539   7.138  1.00  0.00
I    C
ATOM  27534  HB1 PRO I  37     -12.527  15.250   7.507  1.00  0.00
I    H
ATOM  27535  HB2 PRO I  37     -13.838  14.844   6.328  1.00  0.00
I    H
ATOM  27536  CG  PRO I  37     -14.292  15.565   8.470  1.00  0.00
I    C
ATOM  27537  HG1 PRO I  37     -14.058  14.696   9.116  1.00  0.00
I    H
ATOM  27538  HG2 PRO I  37     -15.388  15.592   8.270  1.00  0.00
I    H
ATOM  27539  C   PRO I  37     -14.509  17.282   5.582  1.00  0.00
I    C
```

```
ATOM  27540  O   PRO I  37      -14.133  17.439   4.423  1.00  0.00
I    O
ATOM  27541  N   GLN I  38      -15.809  17.362   5.912  1.00  0.00
I    N
ATOM  27542  HN  GLN I  38      -16.145  17.176   6.834  1.00  0.00
I    H
ATOM  27543  CA  GLN I  38      -16.836  17.725   4.961  1.00  0.00
I    C
ATOM  27544  HA  GLN I  38      -16.705  17.071   4.108  1.00  0.00
I    H
ATOM  27545  CB  GLN I  38      -18.247  17.610   5.578  1.00  0.00
I    C
ATOM  27546  HB1 GLN I  38      -18.349  18.447   6.306  1.00  0.00
I    H
ATOM  27547  HB2 GLN I  38      -19.011  17.791   4.787  1.00  0.00
I    H
ATOM  27548  CG  GLN I  38      -18.537  16.281   6.303  1.00  0.00
I    C
ATOM  27549  HG1 GLN I  38      -18.708  15.467   5.566  1.00  0.00
I    H
ATOM  27550  HG2 GLN I  38      -17.680  15.986   6.943  1.00  0.00
I    H
ATOM  27551  CD  GLN I  38      -19.798  16.402   7.149  1.00  0.00
I    C
ATOM  27552  OE1 GLN I  38      -20.163  17.500   7.578  1.00  0.00
I    O
ATOM  27553  NE2 GLN I  38      -20.433  15.259   7.470  1.00  0.00
I    N
ATOM  27554 HE21 GLN I  38      -21.259  15.342   8.019  1.00  0.00
I    H
ATOM  27555 HE22 GLN I  38      -20.061  14.386   7.171  1.00  0.00
I    H
ATOM  27556  C   GLN I  38      -16.701  19.153   4.467  1.00  0.00
I    C
ATOM  27557  O   GLN I  38      -16.822  19.432   3.280  1.00  0.00
I    O
ATOM  27558  N   VAL I  39      -16.421  20.097   5.392  1.00  0.00
I    N
ATOM  27559  HN  VAL I  39      -16.289  19.848   6.348  1.00  0.00
I    H
ATOM  27560  CA  VAL I  39      -16.283  21.523   5.115  1.00  0.00
I    C
ATOM  27561  HA  VAL I  39      -17.195  21.860   4.638  1.00  0.00
I    H
ATOM  27562  CB  VAL I  39      -16.091  22.336   6.411  1.00  0.00
I    C
ATOM  27563  HB  VAL I  39      -15.143  21.988   6.890  1.00  0.00
I    H
ATOM  27564  CG1 VAL I  39      -15.972  23.847   6.142  1.00  0.00
I    C
ATOM  27565 HG11 VAL I  39      -15.874  24.403   7.100  1.00  0.00
I    H
ATOM  27566 HG12 VAL I  39      -15.078  24.085   5.529  1.00  0.00
I    H
```

ATOM 27567 HG13 VAL I 39   -16.875 24.225   5.617 1.00 0.00
I   H
ATOM 27568 CG2 VAL I 39   -17.290 22.141   7.361 1.00 0.00
I   C
ATOM 27569 HG21 VAL I 39   -17.170 22.757   8.277 1.00 0.00
I   H
ATOM 27570 HG22 VAL I 39   -18.237 22.439   6.860 1.00 0.00
I   H
ATOM 27571 HG23 VAL I 39   -17.382 21.082   7.680 1.00 0.00
I   H
ATOM 27572 C   VAL I 39   -15.140 21.755   4.133 1.00 0.00
I   C
ATOM 27573 O   VAL I 39   -15.276 22.476   3.146 1.00 0.00
I   O
ATOM 27574 N   LEU I 40   -13.995 21.072   4.336 1.00 0.00
I   N
ATOM 27575 HN LEU I 40   -13.836 20.523   5.155 1.00 0.00
I   H
ATOM 27576 CA LEU I 40   -12.893 21.067   3.394 1.00 0.00
I   C
ATOM 27577 HA LEU I 40   -12.679 22.111   3.199 1.00 0.00
I   H
ATOM 27578 CB LEU I 40   -11.661 20.442   4.097 1.00 0.00
I   C
ATOM 27579 HB1 LEU I 40   -11.343 21.151   4.895 1.00 0.00
I   H
ATOM 27580 HB2 LEU I 40   -11.976 19.511   4.619 1.00 0.00
I   H
ATOM 27581 CG LEU I 40   -10.434 20.146   3.209 1.00 0.00
I   C
ATOM 27582 HG LEU I 40   -10.541 20.776   2.293 1.00 0.00
I   H
ATOM 27583 CD1 LEU I 40   -9.125 20.467   3.939 1.00 0.00
I   C
ATOM 27584 HD11 LEU I 40   -8.251 20.203   3.306 1.00 0.00
I   H
ATOM 27585 HD12 LEU I 40   -9.068 21.550   4.181 1.00 0.00
I   H
ATOM 27586 HD13 LEU I 40   -9.053 19.891   4.886 1.00 0.00
I   H
ATOM 27587 CD2 LEU I 40   -10.397 18.675   2.770 1.00 0.00
I   C
ATOM 27588 HD21 LEU I 40   -9.517 18.489   2.118 1.00 0.00
I   H
ATOM 27589 HD22 LEU I 40   -10.323 17.991   3.639 1.00 0.00
I   H
ATOM 27590 HD23 LEU I 40   -11.314 18.417   2.199 1.00 0.00
I   H
ATOM 27591 C   LEU I 40   -13.215 20.406   2.060 1.00 0.00
I   C
ATOM 27592 O   LEU I 40   -12.976 20.989   1.002 1.00 0.00
I   O
ATOM 27593 N   ALA I 41   -13.818 19.201   2.058 1.00 0.00
I   N

```
ATOM  27594  HN  ALA I  41      -14.073  18.746   2.910  1.00  0.00
I    H
ATOM  27595  CA  ALA I  41      -14.143  18.476   0.842  1.00  0.00
I    C
ATOM  27596  HA  ALA I  41      -13.239  18.344   0.260  1.00  0.00
I    H
ATOM  27597  CB  ALA I  41      -14.676  17.077   1.211  1.00  0.00
I    C
ATOM  27598  HB1 ALA I  41      -13.925  16.544   1.834  1.00  0.00
I    H
ATOM  27599  HB2 ALA I  41      -15.618  17.149   1.797  1.00  0.00
I    H
ATOM  27600  HB3 ALA I  41      -14.868  16.461   0.305  1.00  0.00
I    H
ATOM  27601  C   ALA I  41      -15.141  19.176  -0.067  1.00  0.00
I    C
ATOM  27602  O   ALA I  41      -14.924  19.301  -1.272  1.00  0.00
I    O
ATOM  27603  N   ILE I  42      -16.249  19.699   0.495  1.00  0.00
I    N
ATOM  27604  HN  ILE I  42      -16.472  19.605   1.462  1.00  0.00
I    H
ATOM  27605  CA  ILE I  42      -17.225  20.452  -0.270  1.00  0.00
I    C
ATOM  27606  HA  ILE I  42      -17.398  19.892  -1.181  1.00  0.00
I    H
ATOM  27607  CB  ILE I  42      -18.578  20.539   0.424  1.00  0.00
I    C
ATOM  27608  HB  ILE I  42      -18.377  21.131   1.353  1.00  0.00
I    H
ATOM  27609  CG2 ILE I  42      -19.619  21.185  -0.517  1.00  0.00
I    C
ATOM  27610 HG21 ILE I  42      -20.562  21.394   0.030  1.00  0.00
I    H
ATOM  27611 HG22 ILE I  42      -19.243  22.147  -0.922  1.00  0.00
I    H
ATOM  27612 HG23 ILE I  42      -19.854  20.513  -1.368  1.00  0.00
I    H
ATOM  27613  CG1 ILE I  42      -19.083  19.147   0.896  1.00  0.00
I    C
ATOM  27614 HG11 ILE I  42      -18.454  18.784   1.739  1.00  0.00
I    H
ATOM  27615 HG12 ILE I  42      -20.123  19.253   1.284  1.00  0.00
I    H
ATOM  27616  CD  ILE I  42      -19.117  18.041  -0.171  1.00  0.00
I    C
ATOM  27617  HD1 ILE I  42      -19.546  17.108   0.253  1.00  0.00
I    H
ATOM  27618  HD2 ILE I  42      -19.743  18.350  -1.036  1.00  0.00
I    H
ATOM  27619  HD3 ILE I  42      -18.095  17.812  -0.540  1.00  0.00
I    H
ATOM  27620  C   ILE I  42      -16.669  21.806  -0.684  1.00  0.00
I    C
```

```
ATOM  27621  O   ILE I  42     -16.893  22.262  -1.803  1.00  0.00
I    O
ATOM  27622  N   GLY I  43     -15.869  22.464   0.187  1.00  0.00
I    N
ATOM  27623  HN  GLY I  43     -15.789  22.148   1.132  1.00  0.00
I    H
ATOM  27624  CA  GLY I  43     -15.079  23.647  -0.153  1.00  0.00
I    C
ATOM  27625  HA1 GLY I  43     -14.426  23.806   0.694  1.00  0.00
I    H
ATOM  27626  HA2 GLY I  43     -15.799  24.438  -0.313  1.00  0.00
I    H
ATOM  27627  C   GLY I  43     -14.225  23.541  -1.386  1.00  0.00
I    C
ATOM  27628  O   GLY I  43     -14.377  24.329  -2.312  1.00  0.00
I    O
ATOM  27629  N   PHE I  44     -13.327  22.539  -1.462  1.00  0.00
I    N
ATOM  27630  HN  PHE I  44     -13.140  21.919  -0.701  1.00  0.00
I    H
ATOM  27631  CA  PHE I  44     -12.548  22.270  -2.664  1.00  0.00
I    C
ATOM  27632  HA  PHE I  44     -12.068  23.209  -2.912  1.00  0.00
I    H
ATOM  27633  CB  PHE I  44     -11.476  21.171  -2.442  1.00  0.00
I    C
ATOM  27634  HB1 PHE I  44     -11.886  20.396  -1.759  1.00  0.00
I    H
ATOM  27635  HB2 PHE I  44     -11.219  20.664  -3.398  1.00  0.00
I    H
ATOM  27636  CG  PHE I  44     -10.236  21.728  -1.790  1.00  0.00
I    C
ATOM  27637  CD1 PHE I  44      -9.226  22.324  -2.565  1.00  0.00
I    C
ATOM  27638  HD1 PHE I  44      -9.367  22.437  -3.631  1.00  0.00
I    H
ATOM  27639  CE1 PHE I  44      -8.035  22.768  -1.971  1.00  0.00
I    C
ATOM  27640  HE1 PHE I  44      -7.267  23.228  -2.577  1.00  0.00
I    H
ATOM  27641  CZ  PHE I  44      -7.847  22.620  -0.590  1.00  0.00
I    C
ATOM  27642  HZ  PHE I  44      -6.931  22.960  -0.128  1.00  0.00
I    H
ATOM  27643  CD2 PHE I  44     -10.034  21.600  -0.410  1.00  0.00
I    C
ATOM  27644  HD2 PHE I  44     -10.791  21.135   0.207  1.00  0.00
I    H
ATOM  27645  CE2 PHE I  44      -8.851  22.041   0.193  1.00  0.00
I    C
ATOM  27646  HE2 PHE I  44      -8.717  21.931   1.261  1.00  0.00
I    H
ATOM  27647  C   PHE I  44     -13.411  21.878  -3.862  1.00  0.00
I    C
```

ATOM 27648 O PHE I 44 -13.148 22.301 -4.981 1.00 0.00
I O
ATOM 27649 N PHE I 45 -14.493 21.093 -3.669 1.00 0.00
I N
ATOM 27650 HN PHE I 45 -14.709 20.690 -2.781 1.00 0.00
I H
ATOM 27651 CA PHE I 45 -15.441 20.761 -4.731 1.00 0.00
I C
ATOM 27652 HA PHE I 45 -14.861 20.270 -5.503 1.00 0.00
I H
ATOM 27653 CB PHE I 45 -16.527 19.789 -4.159 1.00 0.00
I C
ATOM 27654 HB1 PHE I 45 -16.119 18.755 -4.146 1.00 0.00
I H
ATOM 27655 HB2 PHE I 45 -16.787 20.052 -3.111 1.00 0.00
I H
ATOM 27656 CG PHE I 45 -17.800 19.703 -4.954 1.00 0.00
I C
ATOM 27657 CD1 PHE I 45 -17.843 19.014 -6.172 1.00 0.00
I C
ATOM 27658 HD1 PHE I 45 -16.976 18.458 -6.502 1.00 0.00
I H
ATOM 27659 CE1 PHE I 45 -18.998 19.048 -6.964 1.00 0.00
I C
ATOM 27660 HE1 PHE I 45 -19.025 18.513 -7.903 1.00 0.00
I H
ATOM 27661 CZ PHE I 45 -20.120 19.771 -6.534 1.00 0.00
I C
ATOM 27662 HZ PHE I 45 -21.002 19.820 -7.156 1.00 0.00
I H
ATOM 27663 CD2 PHE I 45 -18.946 20.378 -4.507 1.00 0.00
I C
ATOM 27664 HD2 PHE I 45 -18.934 20.889 -3.554 1.00 0.00
I H
ATOM 27665 CE2 PHE I 45 -20.101 20.422 -5.294 1.00 0.00
I C
ATOM 27666 HE2 PHE I 45 -20.972 20.959 -4.943 1.00 0.00
I H
ATOM 27667 C PHE I 45 -16.058 22.009 -5.357 1.00 0.00
I C
ATOM 27668 O PHE I 45 -16.098 22.158 -6.575 1.00 0.00
I O
ATOM 27669 N LEU I 46 -16.487 22.966 -4.523 1.00 0.00
I N
ATOM 27670 HN LEU I 46 -16.484 22.845 -3.532 1.00 0.00
I H
ATOM 27671 CA LEU I 46 -16.990 24.250 -4.963 1.00 0.00
I C
ATOM 27672 HA LEU I 46 -17.808 24.027 -5.637 1.00 0.00
I H
ATOM 27673 CB LEU I 46 -17.628 24.993 -3.759 1.00 0.00
I C
ATOM 27674 HB1 LEU I 46 -18.397 24.314 -3.323 1.00 0.00
I H

ATOM 27675 HB2 LEU I 46   -16.856 25.131 -2.969 1.00 0.00
I   H
ATOM 27676 CG LEU I 46   -18.313 26.351 -4.042 1.00 0.00
I   C
ATOM 27677 HG LEU I 46   -18.602 26.346 -5.121 1.00 0.00
I   H
ATOM 27678 CD1 LEU I 46   -19.556 26.542 -3.156 1.00 0.00
I   C
ATOM 27679 HD11 LEU I 46   -20.012 27.540 -3.333 1.00 0.00
I   H
ATOM 27680 HD12 LEU I 46   -20.319 25.766 -3.379 1.00 0.00
I   H
ATOM 27681 HD13 LEU I 46   -19.287 26.470 -2.081 1.00 0.00
I   H
ATOM 27682 CD2 LEU I 46   -17.348 27.524 -3.814 1.00 0.00
I   C
ATOM 27683 HD21 LEU I 46   -17.853 28.488 -4.040 1.00 0.00
I   H
ATOM 27684 HD22 LEU I 46   -16.997 27.567 -2.764 1.00 0.00
I   H
ATOM 27685 HD23 LEU I 46   -16.461 27.432 -4.477 1.00 0.00
I   H
ATOM 27686 C   LEU I 46   -15.966 25.072 -5.740 1.00 0.00
I   C
ATOM 27687 O   LEU I 46   -16.307 25.662 -6.763 1.00 0.00
I   O
ATOM 27688 N   THR I 47   -14.668 25.095 -5.355 1.00 0.00
I   N
ATOM 27689 HN THR I 47   -14.321 24.661 -4.524 1.00 0.00
I   H
ATOM 27690 CA THR I 47   -13.642 25.778 -6.152 1.00 0.00
I   C
ATOM 27691 HA THR I 47   -14.006 26.791 -6.254 1.00 0.00
I   H
ATOM 27692 CB THR I 47   -12.245 25.855 -5.510 1.00 0.00
I   C
ATOM 27693 HB THR I 47   -11.663 26.587 -6.122 1.00 0.00
I   H
ATOM 27694 OG1 THR I 47   -11.535 24.623 -5.570 1.00 0.00
I   O
ATOM 27695 HG1 THR I 47   -10.693 24.790 -5.134 1.00 0.00
I   H
ATOM 27696 CG2 THR I 47   -12.289 26.256 -4.029 1.00 0.00
I   C
ATOM 27697 HG21 THR I 47   -11.264 26.412 -3.630 1.00 0.00
I   H
ATOM 27698 HG22 THR I 47   -12.853 27.204 -3.901 1.00 0.00
I   H
ATOM 27699 HG23 THR I 47   -12.784 25.472 -3.416 1.00 0.00
I   H
ATOM 27700 C   THR I 47   -13.485 25.225 -7.573 1.00 0.00
I   C
ATOM 27701 O   THR I 47   -13.322 25.981 -8.532 1.00 0.00
I   O

```
ATOM  27702  N   ILE I  48     -13.549  23.886  -7.750  1.00  0.00
I    N
ATOM  27703  HN  ILE I  48     -13.630  23.260  -6.977  1.00  0.00
I    H
ATOM  27704  CA  ILE I  48     -13.507  23.226  -9.051  1.00  0.00
I    C
ATOM  27705  HA  ILE I  48     -12.627  23.610  -9.551  1.00  0.00
I    H
ATOM  27706  CB  ILE I  48     -13.414  21.702  -8.933  1.00  0.00
I    C
ATOM  27707  HB  ILE I  48     -14.358  21.404  -8.411  1.00  0.00
I    H
ATOM  27708  CG2 ILE I  48     -13.211  21.075 -10.332  1.00  0.00
I    C
ATOM  27709 HG21 ILE I  48     -13.298  19.969 -10.281  1.00  0.00
I    H
ATOM  27710 HG22 ILE I  48     -13.979  21.441 -11.046  1.00  0.00
I    H
ATOM  27711 HG23 ILE I  48     -12.208  21.326 -10.738  1.00  0.00
I    H
ATOM  27712  CG1 ILE I  48     -12.254  21.268  -8.013  1.00  0.00
I    C
ATOM  27713 HG11 ILE I  48     -11.304  21.241  -8.592  1.00  0.00
I    H
ATOM  27714 HG12 ILE I  48     -12.128  22.027  -7.208  1.00  0.00
I    H
ATOM  27715  CD  ILE I  48     -12.474  19.914  -7.358  1.00  0.00
I    C
ATOM  27716  HD1 ILE I  48     -11.637  19.680  -6.665  1.00  0.00
I    H
ATOM  27717  HD2 ILE I  48     -13.420  19.910  -6.775  1.00  0.00
I    H
ATOM  27718  HD3 ILE I  48     -12.525  19.107  -8.119  1.00  0.00
I    H
ATOM  27719  C   ILE I  48     -14.700  23.593  -9.918  1.00  0.00
I    C
ATOM  27720  O   ILE I  48     -14.540  23.950 -11.085  1.00  0.00
I    O
ATOM  27721  N   ILE I  49     -15.929  23.580  -9.354  1.00  0.00
I    N
ATOM  27722  HN  ILE I  49     -16.091  23.299  -8.410  1.00  0.00
I    H
ATOM  27723  CA  ILE I  49     -17.134  23.975 -10.075  1.00  0.00
I    C
ATOM  27724  HA  ILE I  49     -17.159  23.368 -10.971  1.00  0.00
I    H
ATOM  27725  CB  ILE I  49     -18.397  23.760  -9.240  1.00  0.00
I    C
ATOM  27726  HB  ILE I  49     -18.208  24.332  -8.296  1.00  0.00
I    H
ATOM  27727  CG2 ILE I  49     -19.656  24.248  -9.991  1.00  0.00
I    C
ATOM  27728 HG21 ILE I  49     -20.540  24.233  -9.321  1.00  0.00
I    H
```

```
ATOM  27729 HG22 ILE I  49     -19.523  25.291 -10.351  1.00  0.00
I    H
ATOM  27730 HG23 ILE I  49     -19.873  23.600 -10.866  1.00  0.00
I    H
ATOM  27731  CG1 ILE I  49     -18.587  22.284  -8.812  1.00  0.00
I    C
ATOM  27732 HG11 ILE I  49     -17.710  21.950  -8.213  1.00  0.00
I    H
ATOM  27733 HG12 ILE I  49     -19.483  22.212  -8.153  1.00  0.00
I    H
ATOM  27734  CD  ILE I  49     -18.787  21.277  -9.952  1.00  0.00
I    C
ATOM  27735  HD1 ILE I  49     -18.971  20.261  -9.541  1.00  0.00
I    H
ATOM  27736  HD2 ILE I  49     -19.660  21.563 -10.578  1.00  0.00
I    H
ATOM  27737  HD3 ILE I  49     -17.889  21.226 -10.601  1.00  0.00
I    H
ATOM  27738  C   ILE I  49     -17.073  25.426 -10.528  1.00  0.00
I    C
ATOM  27739  O   ILE I  49     -17.343  25.740 -11.686  1.00  0.00
I    O
ATOM  27740  N   ILE I  50     -16.642  26.348  -9.641  1.00  0.00
I    N
ATOM  27741  HN  ILE I  50     -16.428  26.130  -8.690  1.00  0.00
I    H
ATOM  27742  CA  ILE I  50     -16.448  27.747  -9.991  1.00  0.00
I    C
ATOM  27743  HA  ILE I  50     -17.389  28.082 -10.407  1.00  0.00
I    H
ATOM  27744  CB  ILE I  50     -16.161  28.608  -8.760  1.00  0.00
I    C
ATOM  27745  HB  ILE I  50     -15.348  28.060  -8.218  1.00  0.00
I    H
ATOM  27746  CG2 ILE I  50     -15.756  30.041  -9.171  1.00  0.00
I    C
ATOM  27747 HG21 ILE I  50     -15.399  30.614  -8.290  1.00  0.00
I    H
ATOM  27748 HG22 ILE I  50     -14.933  30.022  -9.915  1.00  0.00
I    H
ATOM  27749 HG23 ILE I  50     -16.616  30.585  -9.614  1.00  0.00
I    H
ATOM  27750  CG1 ILE I  50     -17.376  28.654  -7.792  1.00  0.00
I    C
ATOM  27751 HG11 ILE I  50     -17.509  27.661  -7.307  1.00  0.00
I    H
ATOM  27752 HG12 ILE I  50     -17.165  29.389  -6.982  1.00  0.00
I    H
ATOM  27753  CD  ILE I  50     -18.713  29.054  -8.433  1.00  0.00
I    C
ATOM  27754  HD1 ILE I  50     -19.509  29.116  -7.659  1.00  0.00
I    H
ATOM  27755  HD2 ILE I  50     -18.630  30.046  -8.928  1.00  0.00
I    H
```

ATOM 27756 HD3 ILE I 50 -19.030 28.307 -9.192 1.00 0.00
I H
ATOM 27757 C ILE I 50 -15.397 27.929 -11.079 1.00 0.00
I C
ATOM 27758 O ILE I 50 -15.647 28.594 -12.078 1.00 0.00
I O
ATOM 27759 N GLY I 51 -14.223 27.272 -10.993 1.00 0.00
I N
ATOM 27760 HN GLY I 51 -13.971 26.708 -10.207 1.00 0.00
I H
ATOM 27761 CA GLY I 51 -13.227 27.345 -12.063 1.00 0.00
I C
ATOM 27762 HA1 GLY I 51 -12.395 26.739 -11.733 1.00 0.00
I H
ATOM 27763 HA2 GLY I 51 -12.993 28.394 -12.184 1.00 0.00
I H
ATOM 27764 C GLY I 51 -13.690 26.816 -13.396 1.00 0.00
I C
ATOM 27765 O GLY I 51 -13.378 27.372 -14.443 1.00 0.00
I O
ATOM 27766 N ALA I 52 -14.490 25.740 -13.398 1.00 0.00
I N
ATOM 27767 HN ALA I 52 -14.732 25.262 -12.554 1.00 0.00
I H
ATOM 27768 CA ALA I 52 -15.059 25.192 -14.606 1.00 0.00
I C
ATOM 27769 HA ALA I 52 -14.269 25.025 -15.330 1.00 0.00
I H
ATOM 27770 CB ALA I 52 -15.669 23.820 -14.275 1.00 0.00
I C
ATOM 27771 HB1 ALA I 52 -14.890 23.164 -13.830 1.00 0.00
I H
ATOM 27772 HB2 ALA I 52 -16.496 23.918 -13.539 1.00 0.00
I H
ATOM 27773 HB3 ALA I 52 -16.062 23.316 -15.185 1.00 0.00
I H
ATOM 27774 C ALA I 52 -16.069 26.098 -15.279 1.00 0.00
I C
ATOM 27775 O ALA I 52 -16.005 26.328 -16.484 1.00 0.00
I O
ATOM 27776 N VAL I 53 -17.008 26.695 -14.518 1.00 0.00
I N
ATOM 27777 HN VAL I 53 -17.104 26.568 -13.534 1.00 0.00
I H
ATOM 27778 CA VAL I 53 -17.983 27.600 -15.104 1.00 0.00
I C
ATOM 27779 HA VAL I 53 -18.407 27.091 -15.960 1.00 0.00
I H
ATOM 27780 CB VAL I 53 -19.216 27.836 -14.239 1.00 0.00
I C
ATOM 27781 HB VAL I 53 -19.942 28.434 -14.843 1.00 0.00
I H
ATOM 27782 CG1 VAL I 53 -19.844 26.477 -13.862 1.00 0.00
I C

ATOM 27783 HG11 VAL I 53   -20.722 26.630 -13.199 1.00 0.00
I   H
ATOM 27784 HG12 VAL I 53   -20.199 25.926 -14.758 1.00 0.00
I   H
ATOM 27785 HG13 VAL I 53   -19.111 25.842 -13.320 1.00 0.00
I   H
ATOM 27786 CG2 VAL I 53   -18.886 28.663 -12.987 1.00 0.00
I   C
ATOM 27787 HG21 VAL I 53   -19.788 28.790 -12.351 1.00 0.00
I   H
ATOM 27788 HG22 VAL I 53   -18.100 28.160 -12.384 1.00 0.00
I   H
ATOM 27789 HG23 VAL I 53   -18.522 29.675 -13.259 1.00 0.00
I   H
ATOM 27790 C   VAL I 53   -17.349 28.892 -15.608 1.00 0.00
I   C
ATOM 27791 O   VAL I 53   -17.715 29.411 -16.660 1.00 0.00
I   O
ATOM 27792 N   LEU I 54   -16.325 29.431 -14.911 1.00 0.00
I   N
ATOM 27793 HN LEU I 54   -16.004 29.078 -14.035 1.00 0.00
I   H
ATOM 27794 CA LEU I 54   -15.593 30.589 -15.393 1.00 0.00
I   C
ATOM 27795 HA LEU I 54   -16.349 31.309 -15.675 1.00 0.00
I   H
ATOM 27796 CB LEU I 54   -14.764 31.256 -14.265 1.00 0.00
I   C
ATOM 27797 HB1 LEU I 54   -13.981 30.525 -13.956 1.00 0.00
I   H
ATOM 27798 HB2 LEU I 54   -14.218 32.130 -14.686 1.00 0.00
I   H
ATOM 27799 CG LEU I 54   -15.550 31.687 -12.997 1.00 0.00
I   C
ATOM 27800 HG LEU I 54   -15.757 30.712 -12.492 1.00 0.00
I   H
ATOM 27801 CD1 LEU I 54   -14.674 32.569 -12.096 1.00 0.00
I   C
ATOM 27802 HD11 LEU I 54   -15.251 32.918 -11.212 1.00 0.00
I   H
ATOM 27803 HD12 LEU I 54   -13.790 32.002 -11.734 1.00 0.00
I   H
ATOM 27804 HD13 LEU I 54   -14.316 33.462 -12.651 1.00 0.00
I   H
ATOM 27805 CD2 LEU I 54   -16.896 32.381 -13.260 1.00 0.00
I   C
ATOM 27806 HD21 LEU I 54   -17.393 32.634 -12.299 1.00 0.00
I   H
ATOM 27807 HD22 LEU I 54   -16.767 33.322 -13.831 1.00 0.00
I   H
ATOM 27808 HD23 LEU I 54   -17.572 31.712 -13.835 1.00 0.00
I   H
ATOM 27809 C   LEU I 54   -14.730 30.312 -16.633 1.00 0.00
I   C

```
ATOM  27810  O   LEU I  54      -14.559  31.184 -17.482  1.00  0.00
I    O
ATOM  27811  N   LEU I  55      -14.205  29.079 -16.825  1.00  0.00
I    N
ATOM  27812  HN  LEU I  55      -14.264  28.346 -16.149  1.00  0.00
I    H
ATOM  27813  CA  LEU I  55      -13.501  28.710 -18.050  1.00  0.00
I    C
ATOM  27814  HA  LEU I  55      -12.959  29.593 -18.365  1.00  0.00
I    H
ATOM  27815  CB  LEU I  55      -12.418  27.612 -17.810  1.00  0.00
I    C
ATOM  27816  HB1 LEU I  55      -12.795  26.951 -16.995  1.00  0.00
I    H
ATOM  27817  HB2 LEU I  55      -12.346  26.971 -18.717  1.00  0.00
I    H
ATOM  27818  CG  LEU I  55      -11.008  28.131 -17.408  1.00  0.00
I    C
ATOM  27819  HG  LEU I  55      -10.411  27.190 -17.339  1.00  0.00
I    H
ATOM  27820  CD1 LEU I  55      -10.418  29.082 -18.452  1.00  0.00
I    C
ATOM  27821 HD11 LEU I  55       -9.434  29.471 -18.113  1.00  0.00
I    H
ATOM  27822 HD12 LEU I  55      -10.270  28.557 -19.421  1.00  0.00
I    H
ATOM  27823 HD13 LEU I  55      -11.093  29.947 -18.622  1.00  0.00
I    H
ATOM  27824  CD2 LEU I  55      -10.938  28.837 -16.051  1.00  0.00
I    C
ATOM  27825 HD21 LEU I  55       -9.896  29.158 -15.836  1.00  0.00
I    H
ATOM  27826 HD22 LEU I  55      -11.580  29.740 -16.026  1.00  0.00
I    H
ATOM  27827 HD23 LEU I  55      -11.266  28.151 -15.240  1.00  0.00
I    H
ATOM  27828  C   LEU I  55      -14.468  28.329 -19.184  1.00  0.00
I    C
ATOM  27829  O   LEU I  55      -14.075  28.212 -20.341  1.00  0.00
I    O
ATOM  27830  N   MET I  56      -15.781  28.182 -18.902  1.00  0.00
I    N
ATOM  27831  HN  MET I  56      -16.121  28.179 -17.964  1.00  0.00
I    H
ATOM  27832  CA  MET I  56      -16.815  28.015 -19.918  1.00  0.00
I    C
ATOM  27833  HA  MET I  56      -16.384  27.387 -20.688  1.00  0.00
I    H
ATOM  27834  CB  MET I  56      -18.046  27.303 -19.299  1.00  0.00
I    C
ATOM  27835  HB1 MET I  56      -17.664  26.402 -18.764  1.00  0.00
I    H
ATOM  27836  HB2 MET I  56      -18.499  27.957 -18.521  1.00  0.00
I    H
```

ATOM 27837 CG MET I 56 -19.115 26.857 -20.321 1.00 0.00
I C
ATOM 27838 HG1 MET I 56 -19.816 27.692 -20.536 1.00 0.00
I H
ATOM 27839 HG2 MET I 56 -18.622 26.577 -21.278 1.00 0.00
I H
ATOM 27840 SD MET I 56 -20.118 25.446 -19.776 1.00 0.00
I S
ATOM 27841 CE MET I 56 -18.787 24.260 -20.113 1.00 0.00
I C
ATOM 27842 HE1 MET I 56 -19.109 23.223 -19.876 1.00 0.00
I H
ATOM 27843 HE2 MET I 56 -18.490 24.288 -21.184 1.00 0.00
I H
ATOM 27844 HE3 MET I 56 -17.887 24.485 -19.501 1.00 0.00
I H
ATOM 27845 C MET I 56 -17.229 29.334 -20.575 1.00 0.00
I C
ATOM 27846 O MET I 56 -17.805 29.373 -21.661 1.00 0.00
I O
ATOM 27847 N LEU I 57 -16.905 30.476 -19.939 1.00 0.00
I N
ATOM 27848 HN LEU I 57 -16.500 30.462 -19.028 1.00 0.00
I H
ATOM 27849 CA LEU I 57 -17.101 31.806 -20.485 1.00 0.00
I C
ATOM 27850 HA LEU I 57 -18.157 31.873 -20.712 1.00 0.00
I H
ATOM 27851 CB LEU I 57 -16.617 32.827 -19.425 1.00 0.00
I C
ATOM 27852 HB1 LEU I 57 -17.219 32.654 -18.504 1.00 0.00
I H
ATOM 27853 HB2 LEU I 57 -15.564 32.594 -19.153 1.00 0.00
I H
ATOM 27854 CG LEU I 57 -16.729 34.323 -19.778 1.00 0.00
I C
ATOM 27855 HG LEU I 57 -16.179 34.452 -20.741 1.00 0.00
I H
ATOM 27856 CD1 LEU I 57 -18.174 34.805 -19.927 1.00 0.00
I C
ATOM 27857 HD11 LEU I 57 -18.201 35.896 -20.133 1.00 0.00
I H
ATOM 27858 HD12 LEU I 57 -18.678 34.278 -20.765 1.00 0.00
I H
ATOM 27859 HD13 LEU I 57 -18.750 34.613 -18.996 1.00 0.00
I H
ATOM 27860 CD2 LEU I 57 -16.020 35.161 -18.710 1.00 0.00
I C
ATOM 27861 HD21 LEU I 57 -16.078 36.241 -18.964 1.00 0.00
I H
ATOM 27862 HD22 LEU I 57 -16.480 35.025 -17.710 1.00 0.00
I H
ATOM 27863 HD23 LEU I 57 -14.946 34.877 -18.642 1.00 0.00
I H

ATOM 27864 C LEU I 57 -16.331 32.061 -21.783 1.00 0.00
I C
ATOM 27865 O LEU I 57 -15.111 31.897 -21.760 1.00 0.00
I O
ATOM 27866 N PRO I 58 -16.916 32.516 -22.911 1.00 0.00
I N
ATOM 27867 CD PRO I 58 -18.365 32.667 -23.108 1.00 0.00
I C
ATOM 27868 HD1 PRO I 58 -17.978 31.696 -22.723 1.00 0.00
I H
ATOM 27869 HD2 PRO I 58 -19.329 32.927 -22.613 1.00 0.00
I H
ATOM 27870 CA PRO I 58 -16.194 32.649 -24.178 1.00 0.00
I C
ATOM 27871 HA PRO I 58 -15.844 31.674 -24.498 1.00 0.00
I H
ATOM 27872 CB PRO I 58 -17.247 33.128 -25.185 1.00 0.00
I C
ATOM 27873 HB1 PRO I 58 -17.790 32.245 -25.591 1.00 0.00
I H
ATOM 27874 HB2 PRO I 58 -16.807 33.699 -26.028 1.00 0.00
I H
ATOM 27875 CG PRO I 58 -18.561 32.588 -24.623 1.00 0.00
I C
ATOM 27876 HG1 PRO I 58 -19.348 32.479 -25.394 1.00 0.00
I H
ATOM 27877 HG2 PRO I 58 -19.220 31.857 -24.447 1.00 0.00
I H
ATOM 27878 C PRO I 58 -15.025 33.594 -24.110 1.00 0.00
I C
ATOM 27879 O PRO I 58 -14.076 33.449 -24.862 1.00 0.00
I O
ATOM 27880 N ILE I 59 -15.069 34.573 -23.196 1.00 0.00
I N
ATOM 27881 HN ILE I 59 -15.857 34.682 -22.594 1.00 0.00
I H
ATOM 27882 CA ILE I 59 -14.019 35.546 -22.983 1.00 0.00
I C
ATOM 27883 HA ILE I 59 -13.818 35.988 -23.951 1.00 0.00
I H
ATOM 27884 CB ILE I 59 -14.484 36.642 -22.021 1.00 0.00
I C
ATOM 27885 HB ILE I 59 -14.526 36.139 -21.022 1.00 0.00
I H
ATOM 27886 CG2 ILE I 59 -13.523 37.849 -22.097 1.00 0.00
I C
ATOM 27887 HG21 ILE I 59 -13.755 38.583 -21.297 1.00 0.00
I H
ATOM 27888 HG22 ILE I 59 -12.470 37.526 -21.961 1.00 0.00
I H
ATOM 27889 HG23 ILE I 59 -13.609 38.365 -23.076 1.00 0.00
I H
ATOM 27890 CG1 ILE I 59 -15.933 37.079 -22.370 1.00 0.00
I C

ATOM 27891 HG11 ILE I 59   -15.975 37.437 -23.422 1.00 0.00
I   H
ATOM 27892 HG12 ILE I 59   -16.604 36.193 -22.297 1.00 0.00
I   H
ATOM 27893 CD ILE I 59   -16.493 38.170 -21.453 1.00 0.00
I   C
ATOM 27894 HD1 ILE I 59   -17.548 38.394 -21.720 1.00 0.00
I   H
ATOM 27895 HD2 ILE I 59   -16.464 37.844 -20.392 1.00 0.00
I   H
ATOM 27896 HD3 ILE I 59   -15.909 39.110 -21.550 1.00 0.00
I   H
ATOM 27897 C   ILE I 59   -12.722 34.889 -22.520 1.00 0.00
I   C
ATOM 27898 O   ILE I 59   -11.627 35.354 -22.821 1.00 0.00
I   O
ATOM 27899 N   SER I 60   -12.797 33.758 -21.786 1.00 0.00
I   N
ATOM 27900 HN SER I 60   -13.665 33.343 -21.511 1.00 0.00
I   H
ATOM 27901 CA SER I 60   -11.621 33.037 -21.327 1.00 0.00
I   C
ATOM 27902 HA SER I 60   -10.961 33.808 -20.950 1.00 0.00
I   H
ATOM 27903 CB SER I 60   -11.977 32.068 -20.172 1.00 0.00
I   C
ATOM 27904 HB1 SER I 60   -11.031 31.773 -19.663 1.00 0.00
I   H
ATOM 27905 HB2 SER I 60   -12.609 32.584 -19.414 1.00 0.00
I   H
ATOM 27906 OG SER I 60   -12.624 30.876 -20.615 1.00 0.00
I   O
ATOM 27907 HG1 SER I 60   -12.603 30.252 -19.878 1.00 0.00
I   H
ATOM 27908 C   SER I 60   -10.872 32.297 -22.412 1.00 0.00
I   C
ATOM 27909 O   SER I 60   -9.661 32.112 -22.331 1.00 0.00
I   O
ATOM 27910 N   THR I 61   -11.582 31.868 -23.460 1.00 0.00
I   N
ATOM 27911 HN THR I 61   -12.497 32.226 -23.645 1.00 0.00
I   H
ATOM 27912 CA THR I 61   -11.126 30.871 -24.416 1.00 0.00
I   C
ATOM 27913 HA THR I 61   -10.116 30.614 -24.122 1.00 0.00
I   H
ATOM 27914 CB THR I 61   -12.013 29.626 -24.414 1.00 0.00
I   C
ATOM 27915 HB THR I 61   -12.033 29.261 -23.359 1.00 0.00
I   H
ATOM 27916 OG1 THR I 61   -11.479 28.574 -25.199 1.00 0.00
I   O
ATOM 27917 HG1 THR I 61   -12.118 27.856 -25.135 1.00 0.00
I   H

ATOM 27918 CG2 THR I 61   -13.442 29.902 -24.905 1.00 0.00
I   C
ATOM 27919 HG21 THR I 61   -14.083 29.003 -24.781 1.00 0.00
I   H
ATOM 27920 HG22 THR I 61   -13.899 30.729 -24.321 1.00 0.00
I   H
ATOM 27921 HG23 THR I 61   -13.448 30.189 -25.978 1.00 0.00
I   H
ATOM 27922 C   THR I 61   -11.033 31.465 -25.810 1.00 0.00
I   C
ATOM 27923 O   THR I 61   -11.640 32.482 -26.117 1.00 0.00
I   O
ATOM 27924 N   THR I 62   -10.230 30.878 -26.710 1.00 0.00
I   N
ATOM 27925 HN THR I 62   -9.663 30.086 -26.487 1.00 0.00
I   H
ATOM 27926 CA THR I 62   -10.107 31.333 -28.088 1.00 0.00
I   C
ATOM 27927 HA THR I 62   -10.576 32.308 -28.126 1.00 0.00
I   H
ATOM 27928 CB THR I 62   -8.665 31.382 -28.601 1.00 0.00
I   C
ATOM 27929 HB THR I 62   -8.719 31.778 -29.643 1.00 0.00
I   H
ATOM 27930 OG1 THR I 62   -8.057 30.099 -28.689 1.00 0.00
I   O
ATOM 27931 HG1 THR I 62   -7.163 30.260 -29.007 1.00 0.00
I   H
ATOM 27932 CG2 THR I 62   -7.783 32.232 -27.677 1.00 0.00
I   C
ATOM 27933 HG21 THR I 62   -6.767 32.360 -28.109 1.00 0.00
I   H
ATOM 27934 HG22 THR I 62   -8.226 33.241 -27.540 1.00 0.00
I   H
ATOM 27935 HG23 THR I 62   -7.678 31.759 -26.677 1.00 0.00
I   H
ATOM 27936 C   THR I 62   -10.900 30.438 -29.015 1.00 0.00
I   C
ATOM 27937 O   THR I 62   -11.192 30.797 -30.153 1.00 0.00
I   O
ATOM 27938 N   LYS I 63   -11.283 29.239 -28.534 1.00 0.00
I   N
ATOM 27939 HN LYS I 63   -11.060 28.975 -27.597 1.00 0.00
I   H
ATOM 27940 CA LYS I 63   -12.024 28.254 -29.290 1.00 0.00
I   C
ATOM 27941 HA LYS I 63   -12.496 28.760 -30.123 1.00 0.00
I   H
ATOM 27942 CB LYS I 63   -11.071 27.132 -29.793 1.00 0.00
I   C
ATOM 27943 HB1 LYS I 63   -10.792 26.516 -28.906 1.00 0.00
I   H
ATOM 27944 HB2 LYS I 63   -11.634 26.457 -30.477 1.00 0.00
I   H

ATOM 27945 CG LYS I 63   -9.801 27.684 -30.463 1.00 0.00
I   C
ATOM 27946 HG1 LYS I 63   -10.087 28.488 -31.180 1.00 0.00
I   H
ATOM 27947 HG2 LYS I 63   -9.153 28.149 -29.686 1.00 0.00
I   H
ATOM 27948 CD LYS I 63   -8.991 26.635 -31.232 1.00 0.00
I   C
ATOM 27949 HD1 LYS I 63   -8.573 25.906 -30.499 1.00 0.00
I   H
ATOM 27950 HD2 LYS I 63   -9.688 26.077 -31.897 1.00 0.00
I   H
ATOM 27951 CE LYS I 63   -7.876 27.293 -32.049 1.00 0.00
I   C
ATOM 27952 HE1 LYS I 63   -8.277 28.146 -32.639 1.00 0.00
I   H
ATOM 27953 HE2 LYS I 63   -7.074 27.673 -31.379 1.00 0.00
I   H
ATOM 27954 NZ LYS I 63   -7.276 26.322 -32.988 1.00 0.00
I   N
ATOM 27955 HZ1 LYS I 63   -6.521 26.779 -33.538 1.00 0.00
I   H
ATOM 27956 HZ2 LYS I 63   -6.878 25.523 -32.454 1.00 0.00
I   H
ATOM 27957 HZ3 LYS I 63   -8.009 25.969 -33.636 1.00 0.00
I   H
ATOM 27958 C   LYS I 63   -13.141 27.674 -28.416 1.00 0.00
I   C
ATOM 27959 O   LYS I 63   -13.091 27.831 -27.195 1.00 0.00
I   O
ATOM 27960 N   PRO I 64   -14.182 27.019 -28.928 1.00 0.00
I   N
ATOM 27961 CD PRO I 64   -14.504 26.966 -30.353 1.00 0.00
I   C
ATOM 27962 HD1 PRO I 64   -14.407 27.917 -29.781 1.00 0.00
I   H
ATOM 27963 HD2 PRO I 64   -14.126 27.096 -31.393 1.00 0.00
I   H
ATOM 27964 CA PRO I 64   -15.107 26.209 -28.127 1.00 0.00
I   C
ATOM 27965 HA PRO I 64   -15.699 26.866 -27.501 1.00 0.00
I   H
ATOM 27966 CB PRO I 64   -16.035 25.571 -29.179 1.00 0.00
I   C
ATOM 27967 HB1 PRO I 64   -16.857 26.283 -29.416 1.00 0.00
I   H
ATOM 27968 HB2 PRO I 64   -16.476 24.613 -28.837 1.00 0.00
I   H
ATOM 27969 CG PRO I 64   -15.960 26.519 -30.379 1.00 0.00
I   C
ATOM 27970 HG1 PRO I 64   -16.844 26.447 -31.043 1.00 0.00
I   H
ATOM 27971 HG2 PRO I 64   -16.198 27.380 -30.827 1.00 0.00
I   H

ATOM 27972 C PRO I 64 -14.432 25.156 -27.250 1.00 0.00
I C
ATOM 27973 O PRO I 64 -13.326 24.719 -27.565 1.00 0.00
I O
ATOM 27974 N LEU I 65 -15.081 24.725 -26.151 1.00 0.00
I N
ATOM 27975 HN LEU I 65 -15.951 25.105 -25.843 1.00 0.00
I H
ATOM 27976 CA LEU I 65 -14.566 23.662 -25.312 1.00 0.00
I C
ATOM 27977 HA LEU I 65 -14.002 22.990 -25.946 1.00 0.00
I H
ATOM 27978 CB LEU I 65 -13.678 24.234 -24.173 1.00 0.00
I C
ATOM 27979 HB1 LEU I 65 -13.024 25.015 -24.625 1.00 0.00
I H
ATOM 27980 HB2 LEU I 65 -14.326 24.763 -23.440 1.00 0.00
I H
ATOM 27981 CG LEU I 65 -12.783 23.207 -23.448 1.00 0.00
I C
ATOM 27982 HG LEU I 65 -13.465 22.486 -22.936 1.00 0.00
I H
ATOM 27983 CD1 LEU I 65 -11.866 22.472 -24.432 1.00 0.00
I C
ATOM 27984 HD11 LEU I 65 -11.195 21.772 -23.890 1.00 0.00
I H
ATOM 27985 HD12 LEU I 65 -12.466 21.886 -25.162 1.00 0.00
I H
ATOM 27986 HD13 LEU I 65 -11.234 23.192 -24.995 1.00 0.00
I H
ATOM 27987 CD2 LEU I 65 -11.920 23.888 -22.375 1.00 0.00
I C
ATOM 27988 HD21 LEU I 65 -11.294 23.135 -21.849 1.00 0.00
I H
ATOM 27989 HD22 LEU I 65 -11.239 24.643 -22.817 1.00 0.00
I H
ATOM 27990 HD23 LEU I 65 -12.563 24.393 -21.622 1.00 0.00
I H
ATOM 27991 C LEU I 65 -15.730 22.876 -24.730 1.00 0.00
I C
ATOM 27992 O LEU I 65 -16.755 23.446 -24.368 1.00 0.00
I O
ATOM 27993 N SER I 66 -15.624 21.533 -24.648 1.00 0.00
I N
ATOM 27994 HN SER I 66 -14.839 21.037 -25.021 1.00 0.00
I H
ATOM 27995 CA SER I 66 -16.625 20.680 -24.018 1.00 0.00
I C
ATOM 27996 HA SER I 66 -17.572 20.995 -24.436 1.00 0.00
I H
ATOM 27997 CB SER I 66 -16.327 19.169 -24.175 1.00 0.00
I C
ATOM 27998 HB1 SER I 66 -15.349 18.956 -23.687 1.00 0.00
I H

ATOM 27999 HB2 SER I 66   -17.097 18.564 -23.643 1.00 0.00
I   H
ATOM 28000 OG SER I 66   -16.242 18.791 -25.545 1.00 0.00
I   O
ATOM 28001 HG1 SER I 66   -15.841 17.912 -25.572 1.00 0.00
I   H
ATOM 28002 C   SER I 66   -16.707 20.875 -22.522 1.00 0.00
I   C
ATOM 28003 O   SER I 66   -15.726 21.224 -21.872 1.00 0.00
I   O
ATOM 28004 N   TRP I 67   -17.871 20.558 -21.912 1.00 0.00
I   N
ATOM 28005 HN TRP I 67   -18.685 20.339 -22.444 1.00 0.00
I   H
ATOM 28006 CA TRP I 67   -18.047 20.504 -20.468 1.00 0.00
I   C
ATOM 28007 HA TRP I 67   -17.900 21.514 -20.102 1.00 0.00
I   H
ATOM 28008 CB TRP I 67   -19.463 19.951 -20.131 1.00 0.00
I   C
ATOM 28009 HB1 TRP I 67   -19.580 18.974 -20.649 1.00 0.00
I   H
ATOM 28010 HB2 TRP I 67   -19.541 19.750 -19.040 1.00 0.00
I   H
ATOM 28011 CG TRP I 67   -20.627 20.821 -20.575 1.00 0.00
I   C
ATOM 28012 CD1 TRP I 67   -21.275 21.796 -19.864 1.00 0.00
I   C
ATOM 28013 HD1 TRP I 67   -21.004 22.099 -18.860 1.00 0.00
I   H
ATOM 28014 NE1 TRP I 67   -22.279 22.354 -20.622 1.00 0.00
I   N
ATOM 28015 HE1 TRP I 67   -22.849 23.107 -20.371 1.00 0.00
I   H
ATOM 28016 CE2 TRP I 67   -22.325 21.710 -21.833 1.00 0.00
I   C
ATOM 28017 CD2 TRP I 67   -21.307 20.731 -21.843 1.00 0.00
I   C
ATOM 28018 CE3 TRP I 67   -21.123 19.898 -22.948 1.00 0.00
I   C
ATOM 28019 HE3 TRP I 67   -20.357 19.135 -22.966 1.00 0.00
I   H
ATOM 28020 CZ3 TRP I 67   -21.974 20.074 -24.052 1.00 0.00
I   C
ATOM 28021 HZ3 TRP I 67   -21.855 19.443 -24.922 1.00 0.00
I   H
ATOM 28022 CZ2 TRP I 67   -23.166 21.880 -22.922 1.00 0.00
I   C
ATOM 28023 HZ2 TRP I 67   -23.946 22.626 -22.925 1.00 0.00
I   H
ATOM 28024 CH2 TRP I 67   -22.979 21.054 -24.041 1.00 0.00
I   C
ATOM 28025 HH2 TRP I 67   -23.619 21.172 -24.904 1.00 0.00
I   H

ATOM 28026 C   TRP I 67   -17.017 19.624 -19.764 1.00 0.00
I   C
ATOM 28027 O   TRP I 67   -16.421 20.017 -18.766 1.00 0.00
I   O
ATOM 28028 N   ILE I 68   -16.740 18.427 -20.318 1.00 0.00
I   N
ATOM 28029 HN ILE I 68   -17.226 18.114 -21.131 1.00 0.00
I   H
ATOM 28030 CA ILE I 68   -15.748 17.491 -19.822 1.00 0.00
I   C
ATOM 28031 HA ILE I 68   -16.019 17.302 -18.791 1.00 0.00
I   H
ATOM 28032 CB ILE I 68   -15.773 16.238 -20.702 1.00 0.00
I   C
ATOM 28033 HB ILE I 68   -15.618 16.623 -21.741 1.00 0.00
I   H
ATOM 28034 CG2 ILE I 68   -14.697 15.230 -20.250 1.00 0.00
I   C
ATOM 28035 HG21 ILE I 68   -14.611 14.397 -20.980 1.00 0.00
I   H
ATOM 28036 HG22 ILE I 68   -13.703 15.718 -20.178 1.00 0.00
I   H
ATOM 28037 HG23 ILE I 68   -14.949 14.797 -19.259 1.00 0.00
I   H
ATOM 28038 CG1 ILE I 68   -17.169 15.571 -20.683 1.00 0.00
I   C
ATOM 28039 HG11 ILE I 68   -17.058 14.469 -20.582 1.00 0.00
I   H
ATOM 28040 HG12 ILE I 68   -17.727 15.929 -19.787 1.00 0.00
I   H
ATOM 28041 CD ILE I 68   -18.015 15.876 -21.925 1.00 0.00
I   C
ATOM 28042 HD1 ILE I 68   -19.023 15.419 -21.827 1.00 0.00
I   H
ATOM 28043 HD2 ILE I 68   -18.141 16.973 -22.055 1.00 0.00
I   H
ATOM 28044 HD3 ILE I 68   -17.541 15.466 -22.841 1.00 0.00
I   H
ATOM 28045 C   ILE I 68   -14.343 18.066 -19.809 1.00 0.00
I   C
ATOM 28046 O   ILE I 68   -13.653 18.047 -18.792 1.00 0.00
I   O
ATOM 28047 N   ASP I 69   -13.901 18.642 -20.939 1.00 0.00
I   N
ATOM 28048 HN ASP I 69   -14.454 18.734 -21.763 1.00 0.00
I   H
ATOM 28049 CA ASP I 69   -12.576 19.190 -21.088 1.00 0.00
I   C
ATOM 28050 HA ASP I 69   -11.887 18.460 -20.679 1.00 0.00
I   H
ATOM 28051 CB ASP I 69   -12.223 19.318 -22.594 1.00 0.00
I   C
ATOM 28052 HB1 ASP I 69   -12.819 20.140 -23.040 1.00 0.00
I   H

```
ATOM  28053  HB2 ASP I  69      -11.149  19.556 -22.723  1.00  0.00
I    H
ATOM  28054  CG  ASP I  69      -12.544  18.036 -23.364  1.00  0.00
I    C
ATOM  28055  OD1 ASP I  69      -12.052  16.931 -23.007  1.00  0.00
I    O
ATOM  28056  OD2 ASP I  69      -13.339  18.130 -24.333  1.00  0.00
I    O
ATOM  28057  C   ASP I  69      -12.397  20.478 -20.266  1.00  0.00
I    C
ATOM  28058  O   ASP I  69      -11.370  20.686 -19.624  1.00  0.00
I    O
ATOM  28059  N   ALA I  70      -13.429  21.350 -20.191  1.00  0.00
I    N
ATOM  28060  HN  ALA I  70      -14.239  21.237 -20.766  1.00  0.00
I    H
ATOM  28061  CA  ALA I  70      -13.455  22.495 -19.294  1.00  0.00
I    C
ATOM  28062  HA  ALA I  70      -12.603  23.131 -19.504  1.00  0.00
I    H
ATOM  28063  CB  ALA I  70      -14.734  23.320 -19.538  1.00  0.00
I    C
ATOM  28064  HB1 ALA I  70      -14.778  23.633 -20.603  1.00  0.00
I    H
ATOM  28065  HB2 ALA I  70      -15.644  22.719 -19.320  1.00  0.00
I    H
ATOM  28066  HB3 ALA I  70      -14.755  24.236 -18.910  1.00  0.00
I    H
ATOM  28067  C   ALA I  70      -13.366  22.128 -17.819  1.00  0.00
I    C
ATOM  28068  O   ALA I  70      -12.587  22.712 -17.070  1.00  0.00
I    O
ATOM  28069  N   LEU I  71      -14.123  21.110 -17.367  1.00  0.00
I    N
ATOM  28070  HN  LEU I  71      -14.801  20.651 -17.939  1.00  0.00
I    H
ATOM  28071  CA  LEU I  71      -14.029  20.585 -16.020  1.00  0.00
I    C
ATOM  28072  HA  LEU I  71      -14.131  21.437 -15.360  1.00  0.00
I    H
ATOM  28073  CB  LEU I  71      -15.218  19.641 -15.744  1.00  0.00
I    C
ATOM  28074  HB1 LEU I  71      -16.131  20.135 -16.149  1.00  0.00
I    H
ATOM  28075  HB2 LEU I  71      -15.085  18.708 -16.335  1.00  0.00
I    H
ATOM  28076  CG  LEU I  71      -15.465  19.300 -14.259  1.00  0.00
I    C
ATOM  28077  HG  LEU I  71      -15.051  20.152 -13.667  1.00  0.00
I    H
ATOM  28078  CD1 LEU I  71      -16.963  19.126 -13.981  1.00  0.00
I    C
ATOM  28079 HD11 LEU I  71      -17.131  18.833 -12.922  1.00  0.00
I    H
```

ATOM 28080 HD12 LEU I 71   -17.508 20.075 -14.172 1.00 0.00
I   H
ATOM 28081 HD13 LEU I 71   -17.394 18.336 -14.631 1.00 0.00
I   H
ATOM 28082 CD2 LEU I 71   -14.739 18.026 -13.826 1.00 0.00
I   C
ATOM 28083 HD21 LEU I 71   -14.925 17.823 -12.750 1.00 0.00
I   H
ATOM 28084 HD22 LEU I 71   -15.085 17.142 -14.400 1.00 0.00
I   H
ATOM 28085 HD23 LEU I 71   -13.643 18.132 -13.976 1.00 0.00
I   H
ATOM 28086 C   LEU I 71   -12.683 19.943 -15.734 1.00 0.00
I   C
ATOM 28087 O   LEU I 71   -12.106 20.161 -14.672 1.00 0.00
I   O
ATOM 28088 N   PHE I 72   -12.104 19.191 -16.697 1.00 0.00
I   N
ATOM 28089 HN PHE I 72   -12.572 18.943 -17.544 1.00 0.00
I   H
ATOM 28090 CA PHE I 72   -10.751 18.677 -16.584 1.00 0.00
I   C
ATOM 28091 HA PHE I 72   -10.748 18.056 -15.696 1.00 0.00
I   H
ATOM 28092 CB PHE I 72   -10.394 17.833 -17.851 1.00 0.00
I   C
ATOM 28093 HB1 PHE I 72   -11.125 17.003 -17.954 1.00 0.00
I   H
ATOM 28094 HB2 PHE I 72   -10.465 18.448 -18.775 1.00 0.00
I   H
ATOM 28095 CG PHE I 72   -9.031 17.180 -17.800 1.00 0.00
I   C
ATOM 28096 CD1 PHE I 72   -7.896 17.955 -18.084 1.00 0.00
I   C
ATOM 28097 HD1 PHE I 72   -8.010 19.000 -18.335 1.00 0.00
I   H
ATOM 28098 CE1 PHE I 72   -6.615 17.402 -18.054 1.00 0.00
I   C
ATOM 28099 HE1 PHE I 72   -5.754 18.020 -18.269 1.00 0.00
I   H
ATOM 28100 CZ PHE I 72   -6.450 16.051 -17.739 1.00 0.00
I   C
ATOM 28101 HZ PHE I 72   -5.459 15.623 -17.683 1.00 0.00
I   H
ATOM 28102 CD2 PHE I 72   -8.858 15.813 -17.522 1.00 0.00
I   C
ATOM 28103 HD2 PHE I 72   -9.720 15.182 -17.356 1.00 0.00
I   H
ATOM 28104 CE2 PHE I 72   -7.573 15.251 -17.485 1.00 0.00
I   C
ATOM 28105 HE2 PHE I 72   -7.451 14.201 -17.261 1.00 0.00
I   H
ATOM 28106 C   PHE I 72   -9.726 19.775 -16.362 1.00 0.00
I   C

ATOM 28107 O   PHE I 72   -8.961 19.713 -15.403 1.00 0.00
I   O
ATOM 28108 N   THR I 73   -9.703 20.836 -17.197 1.00 0.00
I   N
ATOM 28109 HN THR I 73   -10.361 21.017 -17.927 1.00 0.00
I   H
ATOM 28110 CA THR I 73   -8.654 21.841 -17.073 1.00 0.00
I   C
ATOM 28111 HA THR I 73   -7.742 21.292 -16.875 1.00 0.00
I   H
ATOM 28112 CB THR I 73   -8.425 22.691 -18.318 1.00 0.00
I   C
ATOM 28113 HB THR I 73   -9.194 23.502 -18.286 1.00 0.00
I   H
ATOM 28114 OG1 THR I 73   -8.652 21.942 -19.503 1.00 0.00
I   O
ATOM 28115 HG1 THR I 73   -8.474 22.549 -20.229 1.00 0.00
I   H
ATOM 28116 CG2 THR I 73   -6.945 23.082 -18.370 1.00 0.00
I   C
ATOM 28117 HG21 THR I 73   -6.753 23.796 -19.198 1.00 0.00
I   H
ATOM 28118 HG22 THR I 73   -6.640 23.571 -17.420 1.00 0.00
I   H
ATOM 28119 HG23 THR I 73   -6.300 22.190 -18.523 1.00 0.00
I   H
ATOM 28120 C   THR I 73   -8.888 22.736 -15.875 1.00 0.00
I   C
ATOM 28121 O   THR I 73   -7.969 23.277 -15.268 1.00 0.00
I   O
ATOM 28122 N   ALA I 74   -10.158 22.876 -15.453 1.00 0.00
I   N
ATOM 28123 HN ALA I 74   -10.938 22.532 -15.975 1.00 0.00
I   H
ATOM 28124 CA ALA I 74   -10.486 23.533 -14.213 1.00 0.00
I   C
ATOM 28125 HA ALA I 74   -10.007 24.505 -14.178 1.00 0.00
I   H
ATOM 28126 CB ALA I 74   -12.003 23.691 -14.107 1.00 0.00
I   C
ATOM 28127 HB1 ALA I 74   -12.376 24.288 -14.967 1.00 0.00
I   H
ATOM 28128 HB2 ALA I 74   -12.509 22.702 -14.132 1.00 0.00
I   H
ATOM 28129 HB3 ALA I 74   -12.295 24.216 -13.173 1.00 0.00
I   H
ATOM 28130 C   ALA I 74   -10.006 22.779 -12.997 1.00 0.00
I   C
ATOM 28131 O   ALA I 74   -9.333 23.340 -12.135 1.00 0.00
I   O
ATOM 28132 N   ALA I 75   -10.280 21.459 -12.937 1.00 0.00
I   N
ATOM 28133 HN ALA I 75   -10.840 21.009 -13.632 1.00 0.00
I   H

ATOM 28134 CA ALA I 75   -9.798 20.605 -11.883 1.00 0.00
I   C
ATOM 28135 HA ALA I 75   -10.119 20.988 -10.920 1.00 0.00
I   H
ATOM 28136 CB ALA I 75   -10.319 19.170 -12.027 1.00 0.00
I   C
ATOM 28137 HB1 ALA I 75   -11.430 19.174 -12.015 1.00 0.00
I   H
ATOM 28138 HB2 ALA I 75   -9.989 18.720 -12.988 1.00 0.00
I   H
ATOM 28139 HB3 ALA I 75   -9.969 18.522 -11.194 1.00 0.00
I   H
ATOM 28140 C   ALA I 75   -8.307 20.545 -11.836 1.00 0.00
I   C
ATOM 28141 O   ALA I 75   -7.742 20.645 -10.758 1.00 0.00
I   O
ATOM 28142 N   SER I 76   -7.625 20.439 -12.983 1.00 0.00
I   N
ATOM 28143 HN SER I 76   -8.033 20.439 -13.896 1.00 0.00
I   H
ATOM 28144 CA SER I 76   -6.188 20.313 -12.978 1.00 0.00
I   C
ATOM 28145 HA SER I 76   -5.976 19.583 -12.208 1.00 0.00
I   H
ATOM 28146 CB SER I 76   -5.663 19.820 -14.344 1.00 0.00
I   C
ATOM 28147 HB1 SER I 76   -4.562 19.676 -14.260 1.00 0.00
I   H
ATOM 28148 HB2 SER I 76   -6.105 18.829 -14.594 1.00 0.00
I   H
ATOM 28149 OG SER I 76   -5.912 20.756 -15.391 1.00 0.00
I   O
ATOM 28150 HG1 SER I 76   -5.371 20.489 -16.144 1.00 0.00
I   H
ATOM 28151 C   SER I 76   -5.464 21.589 -12.602 1.00 0.00
I   C
ATOM 28152 O   SER I 76   -4.273 21.551 -12.321 1.00 0.00
I   O
ATOM 28153 N   ALA I 77   -6.158 22.746 -12.606 1.00 0.00
I   N
ATOM 28154 HN ALA I 77   -7.064 22.795 -13.026 1.00 0.00
I   H
ATOM 28155 CA ALA I 77   -5.674 23.980 -12.026 1.00 0.00
I   C
ATOM 28156 HA ALA I 77   -4.602 24.037 -12.180 1.00 0.00
I   H
ATOM 28157 CB ALA I 77   -6.293 25.161 -12.791 1.00 0.00
I   C
ATOM 28158 HB1 ALA I 77   -6.074 25.059 -13.876 1.00 0.00
I   H
ATOM 28159 HB2 ALA I 77   -7.397 25.184 -12.666 1.00 0.00
I   H
ATOM 28160 HB3 ALA I 77   -5.878 26.134 -12.448 1.00 0.00
I   H

```
ATOM  28161  C   ALA I  77      -5.902  24.130 -10.514  1.00  0.00
I    C
ATOM  28162  O   ALA I  77      -5.004  24.511  -9.771  1.00  0.00
I    O
ATOM  28163  N   THR I  78      -7.104  23.846  -9.979  1.00  0.00
I    N
ATOM  28164  HN  THR I  78      -7.914  23.583 -10.503  1.00  0.00
I    H
ATOM  28165  CA  THR I  78      -7.320  23.906  -8.516  1.00  0.00
I    C
ATOM  28166  HA  THR I  78      -6.769  24.776  -8.186  1.00  0.00
I    H
ATOM  28167  CB  THR I  78      -8.769  24.109  -8.106  1.00  0.00
I    C
ATOM  28168  HB  THR I  78      -9.038  25.147  -8.421  1.00  0.00
I    H
ATOM  28169  OG1 THR I  78      -9.004  24.060  -6.700  1.00  0.00
I    O
ATOM  28170  HG1 THR I  78      -9.952  24.192  -6.593  1.00  0.00
I    H
ATOM  28171  CG2 THR I  78      -9.622  23.056  -8.787  1.00  0.00
I    C
ATOM  28172 HG21 THR I  78     -10.701  23.242  -8.602  1.00  0.00
I    H
ATOM  28173 HG22 THR I  78      -9.455  23.074  -9.886  1.00  0.00
I    H
ATOM  28174 HG23 THR I  78      -9.375  22.038  -8.414  1.00  0.00
I    H
ATOM  28175  C   THR I  78      -6.730  22.716  -7.781  1.00  0.00
I    C
ATOM  28176  O   THR I  78      -6.205  22.829  -6.678  1.00  0.00
I    O
ATOM  28177  N   THR I  79      -6.736  21.511  -8.372  1.00  0.00
I    N
ATOM  28178  HN  THR I  79      -7.292  21.259  -9.164  1.00  0.00
I    H
ATOM  28179  CA  THR I  79      -5.891  20.445  -7.876  1.00  0.00
I    C
ATOM  28180  HA  THR I  79      -5.737  20.591  -6.815  1.00  0.00
I    H
ATOM  28181  CB  THR I  79      -6.434  19.023  -8.096  1.00  0.00
I    C
ATOM  28182  HB  THR I  79      -5.798  18.349  -7.471  1.00  0.00
I    H
ATOM  28183  OG1 THR I  79      -6.324  18.524  -9.419  1.00  0.00
I    O
ATOM  28184  HG1 THR I  79      -6.712  17.642  -9.394  1.00  0.00
I    H
ATOM  28185  CG2 THR I  79      -7.927  18.952  -7.753  1.00  0.00
I    C
ATOM  28186 HG21 THR I  79      -8.294  17.906  -7.801  1.00  0.00
I    H
ATOM  28187 HG22 THR I  79      -8.107  19.330  -6.724  1.00  0.00
I    H
```

ATOM 28188 HG23 THR I 79 -8.529 19.565 -8.459 1.00 0.00
I H
ATOM 28189 C THR I 79 -4.558 20.680 -8.539 1.00 0.00
I C
ATOM 28190 O THR I 79 -4.498 20.866 -9.737 1.00 0.00
I O
ATOM 28191 N VAL I 80 -3.446 20.748 -7.785 1.00 0.00
I N
ATOM 28192 HN VAL I 80 -3.442 20.415 -6.846 1.00 0.00
I H
ATOM 28193 CA VAL I 80 -2.167 21.296 -8.240 1.00 0.00
I C
ATOM 28194 HA VAL I 80 -2.357 22.344 -8.436 1.00 0.00
I H
ATOM 28195 CB VAL I 80 -1.187 21.175 -7.078 1.00 0.00
I C
ATOM 28196 HB VAL I 80 -1.333 20.167 -6.619 1.00 0.00
I H
ATOM 28197 CG1 VAL I 80 0.279 21.289 -7.501 1.00 0.00
I C
ATOM 28198 HG11 VAL I 80 0.943 21.233 -6.612 1.00 0.00
I H
ATOM 28199 HG12 VAL I 80 0.573 20.465 -8.185 1.00 0.00
I H
ATOM 28200 HG13 VAL I 80 0.467 22.259 -8.009 1.00 0.00
I H
ATOM 28201 CG2 VAL I 80 -1.509 22.279 -6.055 1.00 0.00
I C
ATOM 28202 HG21 VAL I 80 -0.791 22.251 -5.208 1.00 0.00
I H
ATOM 28203 HG22 VAL I 80 -1.451 23.281 -6.530 1.00 0.00
I H
ATOM 28204 HG23 VAL I 80 -2.528 22.152 -5.633 1.00 0.00
I H
ATOM 28205 C VAL I 80 -1.608 20.685 -9.519 1.00 0.00
I C
ATOM 28206 O VAL I 80 -1.065 21.396 -10.349 1.00 0.00
I O
ATOM 28207 N THR I 81 -1.825 19.380 -9.713 1.00 0.00
I N
ATOM 28208 HN THR I 81 -2.362 18.917 -9.009 1.00 0.00
I H
ATOM 28209 CA THR I 81 -1.394 18.509 -10.811 1.00 0.00
I C
ATOM 28210 HA THR I 81 -0.599 17.963 -10.319 1.00 0.00
I H
ATOM 28211 CB THR I 81 -2.495 17.476 -11.095 1.00 0.00
I C
ATOM 28212 HB THR I 81 -2.525 16.801 -10.205 1.00 0.00
I H
ATOM 28213 OG1 THR I 81 -2.261 16.620 -12.212 1.00 0.00
I O
ATOM 28214 HG1 THR I 81 -3.034 16.048 -12.263 1.00 0.00
I H

```
ATOM  28215  CG2 THR I  81      -3.792  18.241 -11.322  1.00  0.00
I    C
ATOM  28216 HG21 THR I  81      -4.651  17.544 -11.420  1.00  0.00
I    H
ATOM  28217 HG22 THR I  81      -3.998  18.916 -10.464  1.00  0.00
I    H
ATOM  28218 HG23 THR I  81      -3.737  18.858 -12.245  1.00  0.00
I    H
ATOM  28219  C   THR I  81      -0.813  19.024 -12.143  1.00  0.00
I    C
ATOM  28220  O   THR I  81       0.120  18.398 -12.604  1.00  0.00
I    O
ATOM  28221  N   GLY I  82      -1.268  20.069 -12.881  1.00  0.00
I    N
ATOM  28222  HN  GLY I  82      -2.104  20.593 -12.727  1.00  0.00
I    H
ATOM  28223  CA  GLY I  82      -0.453  20.491 -14.042  1.00  0.00
I    C
ATOM  28224  HA1 GLY I  82       0.556  20.200 -13.781  1.00  0.00
I    H
ATOM  28225  HA2 GLY I  82      -0.604  21.560 -14.106  1.00  0.00
I    H
ATOM  28226  C   GLY I  82      -0.771  19.903 -15.369  1.00  0.00
I    C
ATOM  28227  O   GLY I  82      -0.056  20.111 -16.344  1.00  0.00
I    O
ATOM  28228  N   LEU I  83      -1.858  19.133 -15.470  1.00  0.00
I    N
ATOM  28229  HN  LEU I  83      -2.495  18.956 -14.722  1.00  0.00
I    H
ATOM  28230  CA  LEU I  83      -2.203  18.479 -16.710  1.00  0.00
I    C
ATOM  28231  HA  LEU I  83      -1.276  18.365 -17.259  1.00  0.00
I    H
ATOM  28232  CB  LEU I  83      -2.792  17.068 -16.476  1.00  0.00
I    C
ATOM  28233  HB1 LEU I  83      -3.703  17.192 -15.845  1.00  0.00
I    H
ATOM  28234  HB2 LEU I  83      -3.144  16.658 -17.448  1.00  0.00
I    H
ATOM  28235  CG  LEU I  83      -1.803  16.114 -15.781  1.00  0.00
I    C
ATOM  28236  HG  LEU I  83      -1.684  16.470 -14.729  1.00  0.00
I    H
ATOM  28237  CD1 LEU I  83      -2.331  14.673 -15.777  1.00  0.00
I    C
ATOM  28238 HD11 LEU I  83      -1.589  13.987 -15.316  1.00  0.00
I    H
ATOM  28239 HD12 LEU I  83      -3.277  14.604 -15.198  1.00  0.00
I    H
ATOM  28240 HD13 LEU I  83      -2.529  14.325 -16.814  1.00  0.00
I    H
ATOM  28241  CD2 LEU I  83      -0.437  16.192 -16.464  1.00  0.00
I    C
```

ATOM 28242 HD21 LEU I 83   0.288 15.520 -15.956 1.00 0.00
I   H
ATOM 28243 HD22 LEU I 83   -0.493 15.884 -17.528 1.00 0.00
I   H
ATOM 28244 HD23 LEU I 83   -0.039 17.229 -16.421 1.00 0.00
I   H
ATOM 28245 C   LEU I 83   -3.132 19.307 -17.553 1.00 0.00
I   C
ATOM 28246 O   LEU I 83   -4.033 19.986 -17.072 1.00 0.00
I   O
ATOM 28247 N   ALA I 84   -2.938 19.268 -18.877 1.00 0.00
I   N
ATOM 28248 HN ALA I 84   -2.203 18.730 -19.290 1.00 0.00
I   H
ATOM 28249 CA ALA I 84   -3.765 19.992 -19.800 1.00 0.00
I   C
ATOM 28250 HA ALA I 84   -4.626 20.427 -19.304 1.00 0.00
I   H
ATOM 28251 CB ALA I 84   -2.914 21.110 -20.421 1.00 0.00
I   C
ATOM 28252 HB1 ALA I 84   -2.578 21.810 -19.625 1.00 0.00
I   H
ATOM 28253 HB2 ALA I 84   -2.008 20.695 -20.915 1.00 0.00
I   H
ATOM 28254 HB3 ALA I 84   -3.489 21.695 -21.171 1.00 0.00
I   H
ATOM 28255 C   ALA I 84   -4.313 19.035 -20.847 1.00 0.00
I   C
ATOM 28256 O   ALA I 84   -3.568 18.337 -21.529 1.00 0.00
I   O
ATOM 28257 N   VAL I 85   -5.654 18.947 -20.975 1.00 0.00
I   N
ATOM 28258 HN VAL I 85   -6.271 19.372 -20.317 1.00 0.00
I   H
ATOM 28259 CA VAL I 85   -6.325 18.240 -22.062 1.00 0.00
I   C
ATOM 28260 HA VAL I 85   -5.839 17.279 -22.174 1.00 0.00
I   H
ATOM 28261 CB VAL I 85   -7.791 17.975 -21.713 1.00 0.00
I   C
ATOM 28262 HB VAL I 85   -7.809 17.269 -20.846 1.00 0.00
I   H
ATOM 28263 CG1 VAL I 85   -8.528 19.262 -21.309 1.00 0.00
I   C
ATOM 28264 HG11 VAL I 85   -9.598 19.043 -21.099 1.00 0.00
I   H
ATOM 28265 HG12 VAL I 85   -8.096 19.711 -20.391 1.00 0.00
I   H
ATOM 28266 HG13 VAL I 85   -8.485 20.012 -22.127 1.00 0.00
I   H
ATOM 28267 CG2 VAL I 85   -8.550 17.292 -22.864 1.00 0.00
I   C
ATOM 28268 HG21 VAL I 85   -9.619 17.148 -22.598 1.00 0.00
I   H

ATOM 28269 HG22 VAL I 85   -8.499 17.909 -23.786 1.00 0.00
I   H
ATOM 28270 HG23 VAL I 85   -8.124 16.292 -23.089 1.00 0.00
I   H
ATOM 28271 C   VAL I 85   -6.165 18.989 -23.380 1.00 0.00
I   C
ATOM 28272 O   VAL I 85   -5.969 18.408 -24.444 1.00 0.00
I   O
ATOM 28273 N   VAL I 86   -6.211 20.330 -23.314 1.00 0.00
I   N
ATOM 28274 HN VAL I 86   -6.418 20.791 -22.455 1.00 0.00
I   H
ATOM 28275 CA VAL I 86   -5.979 21.221 -24.431 1.00 0.00
I   C
ATOM 28276 HA VAL I 86   -5.653 20.646 -25.288 1.00 0.00
I   H
ATOM 28277 CB VAL I 86   -7.193 22.091 -24.734 1.00 0.00
I   C
ATOM 28278 HB VAL I 86   -7.361 22.756 -23.852 1.00 0.00
I   H
ATOM 28279 CG1 VAL I 86   -6.927 22.953 -25.981 1.00 0.00
I   C
ATOM 28280 HG11 VAL I 86   -7.823 23.560 -26.229 1.00 0.00
I   H
ATOM 28281 HG12 VAL I 86   -6.084 23.657 -25.820 1.00 0.00
I   H
ATOM 28282 HG13 VAL I 86   -6.693 22.313 -26.859 1.00 0.00
I   H
ATOM 28283 CG2 VAL I 86   -8.428 21.204 -24.960 1.00 0.00
I   C
ATOM 28284 HG21 VAL I 86   -9.313 21.822 -25.222 1.00 0.00
I   H
ATOM 28285 HG22 VAL I 86   -8.247 20.485 -25.787 1.00 0.00
I   H
ATOM 28286 HG23 VAL I 86   -8.681 20.627 -24.045 1.00 0.00
I   H
ATOM 28287 C   VAL I 86   -4.835 22.127 -24.031 1.00 0.00
I   C
ATOM 28288 O   VAL I 86   -4.801 22.591 -22.892 1.00 0.00
I   O
ATOM 28289 N   ASP I 87   -3.867 22.412 -24.933 1.00 0.00
I   N
ATOM 28290 HN ASP I 87   -3.813 22.014 -25.845 1.00 0.00
I   H
ATOM 28291 CA ASP I 87   -2.785 23.348 -24.657 1.00 0.00
I   C
ATOM 28292 HA ASP I 87   -2.248 22.940 -23.809 1.00 0.00
I   H
ATOM 28293 CB ASP I 87   -1.836 23.454 -25.875 1.00 0.00
I   C
ATOM 28294 HB1 ASP I 87   -1.472 22.441 -26.144 1.00 0.00
I   H
ATOM 28295 HB2 ASP I 87   -2.369 23.875 -26.750 1.00 0.00
I   H

ATOM 28296 CG ASP I 87   -0.635 24.322 -25.542 1.00 0.00
I   C
ATOM 28297 OD1 ASP I 87   0.267 23.824 -24.831 1.00 0.00
I   O
ATOM 28298 OD2 ASP I 87   -0.659 25.515 -25.941 1.00 0.00
I   O
ATOM 28299 C   ASP I 87   -3.271 24.745 -24.244 1.00 0.00
I   C
ATOM 28300 O   ASP I 87   -4.221 25.302 -24.793 1.00 0.00
I   O
ATOM 28301 N   THR I 88   -2.631 25.332 -23.216 1.00 0.00
I   N
ATOM 28302 HN THR I 88   -1.782 24.993 -22.814 1.00 0.00
I   H
ATOM 28303 CA THR I 88   -3.144 26.544 -22.595 1.00 0.00
I   C
ATOM 28304 HA THR I 88   -4.219 26.435 -22.646 1.00 0.00
I   H
ATOM 28305 CB THR I 88   -2.867 26.613 -21.094 1.00 0.00
I   C
ATOM 28306 HB THR I 88   -3.343 25.705 -20.648 1.00 0.00
I   H
ATOM 28307 OG1 THR I 88   -3.473 27.735 -20.465 1.00 0.00
I   O
ATOM 28308 HG1 THR I 88   -3.220 27.676 -19.537 1.00 0.00
I   H
ATOM 28309 CG2 THR I 88   -1.374 26.663 -20.797 1.00 0.00
I   C
ATOM 28310 HG21 THR I 88   -1.187 26.598 -19.705 1.00 0.00
I   H
ATOM 28311 HG22 THR I 88   -0.854 25.810 -21.284 1.00 0.00
I   H
ATOM 28312 HG23 THR I 88   -0.923 27.606 -21.173 1.00 0.00
I   H
ATOM 28313 C   THR I 88   -2.803 27.834 -23.331 1.00 0.00
I   C
ATOM 28314 O   THR I 88   -3.619 28.753 -23.416 1.00 0.00
I   O
ATOM 28315 N   GLY I 89   -1.604 27.945 -23.939 1.00 0.00
I   N
ATOM 28316 HN GLY I 89   -0.947 27.194 -23.973 1.00 0.00
I   H
ATOM 28317 CA GLY I 89   -1.184 29.181 -24.593 1.00 0.00
I   C
ATOM 28318 HA1 GLY I 89   -0.138 29.048 -24.833 1.00 0.00
I   H
ATOM 28319 HA2 GLY I 89   -1.379 29.976 -23.887 1.00 0.00
I   H
ATOM 28320 C   GLY I 89   -1.916 29.500 -25.865 1.00 0.00
I   C
ATOM 28321 O   GLY I 89   -1.991 30.657 -26.269 1.00 0.00
I   O
ATOM 28322 N   THR I 90   -2.479 28.481 -26.541 1.00 0.00
I   N

ATOM 28323 HN THR I 90 −2.392 27.525 −26.263 1.00 0.00
I H
ATOM 28324 CA THR I 90 −3.272 28.685 −27.747 1.00 0.00
I C
ATOM 28325 HA THR I 90 −2.959 29.645 −28.134 1.00 0.00
I H
ATOM 28326 CB THR I 90 −3.014 27.607 −28.812 1.00 0.00
I C
ATOM 28327 HB THR I 90 −3.446 28.003 −29.764 1.00 0.00
I H
ATOM 28328 OG1 THR I 90 −3.679 26.371 −28.563 1.00 0.00
I O
ATOM 28329 HG1 THR I 90 −3.425 25.793 −29.290 1.00 0.00
I H
ATOM 28330 CG2 THR I 90 −1.512 27.289 −28.908 1.00 0.00
I C
ATOM 28331 HG21 THR I 90 −1.308 26.597 −29.752 1.00 0.00
I H
ATOM 28332 HG22 THR I 90 −0.930 28.219 −29.081 1.00 0.00
I H
ATOM 28333 HG23 THR I 90 −1.142 26.816 −27.974 1.00 0.00
I H
ATOM 28334 C THR I 90 −4.761 28.799 −27.456 1.00 0.00
I C
ATOM 28335 O THR I 90 −5.463 29.635 −28.024 1.00 0.00
I O
ATOM 28336 N GLN I 91 −5.292 27.946 −26.558 1.00 0.00
I N
ATOM 28337 HN GLN I 91 −4.716 27.340 −26.011 1.00 0.00
I H
ATOM 28338 CA GLN I 91 −6.708 27.822 −26.298 1.00 0.00
I C
ATOM 28339 HA GLN I 91 −7.192 27.871 −27.265 1.00 0.00
I H
ATOM 28340 CB GLN I 91 −6.978 26.493 −25.569 1.00 0.00
I C
ATOM 28341 HB1 GLN I 91 −6.604 25.680 −26.233 1.00 0.00
I H
ATOM 28342 HB2 GLN I 91 −6.361 26.448 −24.643 1.00 0.00
I H
ATOM 28343 CG GLN I 91 −8.453 26.171 −25.208 1.00 0.00
I C
ATOM 28344 HG1 GLN I 91 −8.505 25.277 −24.549 1.00 0.00
I H
ATOM 28345 HG2 GLN I 91 −8.921 27.020 −24.668 1.00 0.00
I H
ATOM 28346 CD GLN I 91 −9.341 25.840 −26.412 1.00 0.00
I C
ATOM 28347 OE1 GLN I 91 −8.897 25.618 −27.539 1.00 0.00
I O
ATOM 28348 NE2 GLN I 91 −10.663 25.732 −26.141 1.00 0.00
I N
ATOM 28349 HE21 GLN I 91 −11.263 25.513 −26.905 1.00 0.00
I H

```
ATOM  28350 HE22 GLN I  91      -10.993  25.886 -25.215  1.00  0.00
I    H
ATOM  28351  C   GLN I  91       -7.265  28.934 -25.457  1.00  0.00
I    C
ATOM  28352  O   GLN I  91       -8.426  29.281 -25.599  1.00  0.00
I    O
ATOM  28353  N   PHE I  92       -6.486  29.537 -24.549  1.00  0.00
I    N
ATOM  28354  HN  PHE I  92       -5.503  29.391 -24.447  1.00  0.00
I    H
ATOM  28355  CA  PHE I  92       -7.052  30.486 -23.611  1.00  0.00
I    C
ATOM  28356  HA  PHE I  92       -8.122  30.493 -23.779  1.00  0.00
I    H
ATOM  28357  CB  PHE I  92       -6.772  30.133 -22.128  1.00  0.00
I    C
ATOM  28358  HB1 PHE I  92       -5.673  30.094 -21.965  1.00  0.00
I    H
ATOM  28359  HB2 PHE I  92       -7.176  30.914 -21.449  1.00  0.00
I    H
ATOM  28360  CG  PHE I  92       -7.343  28.779 -21.821  1.00  0.00
I    C
ATOM  28361  CD1 PHE I  92       -8.686  28.587 -21.471  1.00  0.00
I    C
ATOM  28362  HD1 PHE I  92       -9.339  29.443 -21.363  1.00  0.00
I    H
ATOM  28363  CE1 PHE I  92       -9.185  27.292 -21.256  1.00  0.00
I    C
ATOM  28364  HE1 PHE I  92      -10.222  27.150 -20.988  1.00  0.00
I    H
ATOM  28365  CZ  PHE I  92       -8.342  26.180 -21.396  1.00  0.00
I    C
ATOM  28366  HZ  PHE I  92       -8.730  25.183 -21.248  1.00  0.00
I    H
ATOM  28367  CD2 PHE I  92       -6.516  27.663 -21.932  1.00  0.00
I    C
ATOM  28368  HD2 PHE I  92       -5.466  27.788 -22.156  1.00  0.00
I    H
ATOM  28369  CE2 PHE I  92       -6.997  26.364 -21.737  1.00  0.00
I    C
ATOM  28370  HE2 PHE I  92       -6.330  25.521 -21.850  1.00  0.00
I    H
ATOM  28371  C   PHE I  92       -6.547  31.867 -23.921  1.00  0.00
I    C
ATOM  28372  O   PHE I  92       -5.377  32.093 -24.216  1.00  0.00
I    O
ATOM  28373  N   THR I  93       -7.443  32.864 -23.853  1.00  0.00
I    N
ATOM  28374  HN  THR I  93       -8.426  32.692 -23.786  1.00  0.00
I    H
ATOM  28375  CA  THR I  93       -7.083  34.270 -23.869  1.00  0.00
I    C
ATOM  28376  HA  THR I  93       -6.467  34.418 -24.744  1.00  0.00
I    H
```

```
ATOM  28377  CB  THR I  93      -8.296  35.210 -23.902  1.00  0.00
I    C
ATOM  28378  HB  THR I  93      -7.889  36.233 -24.089  1.00  0.00
I    H
ATOM  28379  OG1 THR I  93      -8.974  35.258 -22.651  1.00  0.00
I    O
ATOM  28380  HG1 THR I  93      -9.715  35.859 -22.784  1.00  0.00
I    H
ATOM  28381  CG2 THR I  93      -9.296  34.758 -24.978  1.00  0.00
I    C
ATOM  28382 HG21 THR I  93     -10.120  35.495 -25.087  1.00  0.00
I    H
ATOM  28383 HG22 THR I  93      -8.790  34.665 -25.963  1.00  0.00
I    H
ATOM  28384 HG23 THR I  93      -9.743  33.773 -24.722  1.00  0.00
I    H
ATOM  28385  C   THR I  93      -6.209  34.593 -22.664  1.00  0.00
I    C
ATOM  28386  O   THR I  93      -6.179  33.847 -21.689  1.00  0.00
I    O
ATOM  28387  N   VAL I  94      -5.489  35.729 -22.651  1.00  0.00
I    N
ATOM  28388  HN  VAL I  94      -5.398  36.344 -23.430  1.00  0.00
I    H
ATOM  28389  CA  VAL I  94      -4.763  36.157 -21.457  1.00  0.00
I    C
ATOM  28390  HA  VAL I  94      -4.044  35.379 -21.232  1.00  0.00
I    H
ATOM  28391  CB  VAL I  94      -3.986  37.439 -21.714  1.00  0.00
I    C
ATOM  28392  HB  VAL I  94      -4.701  38.190 -22.131  1.00  0.00
I    H
ATOM  28393  CG1 VAL I  94      -3.377  38.003 -20.417  1.00  0.00
I    C
ATOM  28394 HG11 VAL I  94      -2.782  38.916 -20.635  1.00  0.00
I    H
ATOM  28395 HG12 VAL I  94      -4.162  38.286 -19.685  1.00  0.00
I    H
ATOM  28396 HG13 VAL I  94      -2.701  37.258 -19.944  1.00  0.00
I    H
ATOM  28397  CG2 VAL I  94      -2.848  37.118 -22.702  1.00  0.00
I    C
ATOM  28398 HG21 VAL I  94      -2.226  38.019 -22.890  1.00  0.00
I    H
ATOM  28399 HG22 VAL I  94      -2.192  36.319 -22.296  1.00  0.00
I    H
ATOM  28400 HG23 VAL I  94      -3.249  36.777 -23.680  1.00  0.00
I    H
ATOM  28401  C   VAL I  94      -5.677  36.302 -20.242  1.00  0.00
I    C
ATOM  28402  O   VAL I  94      -5.313  35.935 -19.133  1.00  0.00
I    O
ATOM  28403  N   PHE I  95      -6.937  36.748 -20.429  1.00  0.00
I    N
```

```
ATOM  28404  HN  PHE I  95      -7.246  37.129 -21.298  1.00  0.00
I    H
ATOM  28405  CA  PHE I  95      -7.955  36.716 -19.387  1.00  0.00
I    C
ATOM  28406  HA  PHE I  95      -7.541  37.290 -18.566  1.00  0.00
I    H
ATOM  28407  CB  PHE I  95      -9.239  37.442 -19.903  1.00  0.00
I    C
ATOM  28408  HB1 PHE I  95      -9.062  38.540 -19.900  1.00  0.00
I    H
ATOM  28409  HB2 PHE I  95      -9.471  37.150 -20.950  1.00  0.00
I    H
ATOM  28410  CG  PHE I  95     -10.470  37.224 -19.053  1.00  0.00
I    C
ATOM  28411  CD1 PHE I  95     -10.761  38.014 -17.935  1.00  0.00
I    C
ATOM  28412  HD1 PHE I  95     -10.158  38.887 -17.725  1.00  0.00
I    H
ATOM  28413  CE1 PHE I  95     -11.824  37.677 -17.083  1.00  0.00
I    C
ATOM  28414  HE1 PHE I  95     -12.027  38.280 -16.209  1.00  0.00
I    H
ATOM  28415  CZ  PHE I  95     -12.627  36.564 -17.367  1.00  0.00
I    C
ATOM  28416  HZ  PHE I  95     -13.431  36.286 -16.701  1.00  0.00
I    H
ATOM  28417  CD2 PHE I  95     -11.316  36.151 -19.353  1.00  0.00
I    C
ATOM  28418  HD2 PHE I  95     -11.134  35.548 -20.231  1.00  0.00
I    H
ATOM  28419  CE2 PHE I  95     -12.389  35.816 -18.526  1.00  0.00
I    C
ATOM  28420  HE2 PHE I  95     -13.027  34.983 -18.784  1.00  0.00
I    H
ATOM  28421  C   PHE I  95      -8.238  35.307 -18.853  1.00  0.00
I    C
ATOM  28422  O   PHE I  95      -8.348  35.111 -17.644  1.00  0.00
I    O
ATOM  28423  N   GLY I  96      -8.324  34.269 -19.712  1.00  0.00
I    N
ATOM  28424  HN  GLY I  96      -8.264  34.369 -20.704  1.00  0.00
I    H
ATOM  28425  CA  GLY I  96      -8.514  32.904 -19.225  1.00  0.00
I    C
ATOM  28426  HA1 GLY I  96      -8.683  32.286 -20.096  1.00  0.00
I    H
ATOM  28427  HA2 GLY I  96      -9.343  32.930 -18.531  1.00  0.00
I    H
ATOM  28428  C   GLY I  96      -7.320  32.364 -18.499  1.00  0.00
I    C
ATOM  28429  O   GLY I  96      -7.442  31.695 -17.480  1.00  0.00
I    O
ATOM  28430  N   GLN I  97      -6.118  32.710 -18.976  1.00  0.00
I    N
```

```
ATOM  28431  HN  GLN I  97      -6.024  33.190 -19.846  1.00  0.00
I    H
ATOM  28432  CA  GLN I  97      -4.868  32.436 -18.306  1.00  0.00
I    C
ATOM  28433  HA  GLN I  97      -4.848  31.367 -18.131  1.00  0.00
I    H
ATOM  28434  CB  GLN I  97      -3.692  32.780 -19.244  1.00  0.00
I    C
ATOM  28435  HB1 GLN I  97      -3.729  33.880 -19.417  1.00  0.00
I    H
ATOM  28436  HB2 GLN I  97      -2.730  32.580 -18.719  1.00  0.00
I    H
ATOM  28437  CG  GLN I  97      -3.715  32.054 -20.617  1.00  0.00
I    C
ATOM  28438  HG1 GLN I  97      -3.409  30.992 -20.499  1.00  0.00
I    H
ATOM  28439  HG2 GLN I  97      -4.735  32.068 -21.052  1.00  0.00
I    H
ATOM  28440  CD  GLN I  97      -2.729  32.709 -21.584  1.00  0.00
I    C
ATOM  28441  OE1 GLN I  97      -1.765  33.354 -21.179  1.00  0.00
I    O
ATOM  28442  NE2 GLN I  97      -2.939  32.566 -22.907  1.00  0.00
I    N
ATOM  28443 HE21 GLN I  97      -2.281  32.999 -23.516  1.00  0.00
I    H
ATOM  28444 HE22 GLN I  97      -3.733  32.061 -23.229  1.00  0.00
I    H
ATOM  28445  C   GLN I  97      -4.752  33.130 -16.939  1.00  0.00
I    C
ATOM  28446  O   GLN I  97      -4.238  32.542 -15.991  1.00  0.00
I    O
ATOM  28447  N   THR I  98      -5.274  34.368 -16.767  1.00  0.00
I    N
ATOM  28448  HN  THR I  98      -5.606  34.893 -17.550  1.00  0.00
I    H
ATOM  28449  CA  THR I  98      -5.413  35.059 -15.473  1.00  0.00
I    C
ATOM  28450  HA  THR I  98      -4.416  35.081 -15.055  1.00  0.00
I    H
ATOM  28451  CB  THR I  98      -6.003  36.464 -15.598  1.00  0.00
I    C
ATOM  28452  HB  THR I  98      -7.045  36.330 -15.977  1.00  0.00
I    H
ATOM  28453  OG1 THR I  98      -5.277  37.202 -16.565  1.00  0.00
I    O
ATOM  28454  HG1 THR I  98      -5.689  38.072 -16.587  1.00  0.00
I    H
ATOM  28455  CG2 THR I  98      -5.875  37.272 -14.300  1.00  0.00
I    C
ATOM  28456 HG21 THR I  98      -6.410  38.241 -14.383  1.00  0.00
I    H
ATOM  28457 HG22 THR I  98      -6.316  36.711 -13.448  1.00  0.00
I    H
```

ATOM 28458 HG23 THR I 98   -4.810 37.483 -14.063 1.00 0.00
I   H
ATOM 28459 C   THR I 98   -6.275 34.294 -14.489 1.00 0.00
I   C
ATOM 28460 O   THR I 98   -5.931 34.156 -13.316 1.00 0.00
I   O
ATOM 28461 N   VAL I 99   -7.410 33.715 -14.941 1.00 0.00
I   N
ATOM 28462 HN VAL I 99   -7.758 33.850 -15.865 1.00 0.00
I   H
ATOM 28463 CA VAL I 99   -8.229 32.836 -14.110 1.00 0.00
I   C
ATOM 28464 HA VAL I 99   -8.492 33.393 -13.220 1.00 0.00
I   H
ATOM 28465 CB VAL I 99   -9.521 32.427 -14.815 1.00 0.00
I   C
ATOM 28466 HB VAL I 99   -9.238 31.858 -15.735 1.00 0.00
I   H
ATOM 28467 CG1 VAL I 99   -10.381 31.522 -13.912 1.00 0.00
I   C
ATOM 28468 HG11 VAL I 99   -11.335 31.264 -14.422 1.00 0.00
I   H
ATOM 28469 HG12 VAL I 99   -9.864 30.569 -13.675 1.00 0.00
I   H
ATOM 28470 HG13 VAL I 99   -10.629 32.039 -12.961 1.00 0.00
I   H
ATOM 28471 CG2 VAL I 99   -10.347 33.674 -15.186 1.00 0.00
I   C
ATOM 28472 HG21 VAL I 99   -11.306 33.382 -15.663 1.00 0.00
I   H
ATOM 28473 HG22 VAL I 99   -10.576 34.274 -14.280 1.00 0.00
I   H
ATOM 28474 HG23 VAL I 99   -9.798 34.320 -15.903 1.00 0.00
I   H
ATOM 28475 C   VAL I 99   -7.453 31.598 -13.662 1.00 0.00
I   C
ATOM 28476 O   VAL I 99   -7.469 31.223 -12.490 1.00 0.00
I   O
ATOM 28477 N   ILE I 100   -6.679 30.974 -14.578 1.00 0.00
I   N
ATOM 28478 HN ILE I 100   -6.684 31.235 -15.541 1.00 0.00
I   H
ATOM 28479 CA ILE I 100   -5.774 29.878 -14.260 1.00 0.00
I   C
ATOM 28480 HA ILE I 100   -6.387 29.118 -13.793 1.00 0.00
I   H
ATOM 28481 CB ILE I 100   -5.156 29.276 -15.529 1.00 0.00
I   C
ATOM 28482 HB ILE I 100   -4.685 30.147 -16.050 1.00 0.00
I   H
ATOM 28483 CG2 ILE I 100   -4.125 28.176 -15.202 1.00 0.00
I   C
ATOM 28484 HG21 ILE I 100   -3.580 27.866 -16.118 1.00 0.00
I   H

```
ATOM  28485 HG22 ILE I 100      -3.373  28.542 -14.471  1.00  0.00
I    H
ATOM  28486 HG23 ILE I 100      -4.622  27.279 -14.774  1.00  0.00
I    H
ATOM  28487  CG1 ILE I 100      -6.214  28.715 -16.519  1.00  0.00
I    C
ATOM  28488 HG11 ILE I 100      -6.862  29.541 -16.887  1.00  0.00
I    H
ATOM  28489 HG12 ILE I 100      -5.690  28.289 -17.405  1.00  0.00
I    H
ATOM  28490  CD  ILE I 100      -7.135  27.610 -15.979  1.00  0.00
I    C
ATOM  28491  HD1 ILE I 100      -7.809  27.243 -16.782  1.00  0.00
I    H
ATOM  28492  HD2 ILE I 100      -6.537  26.750 -15.605  1.00  0.00
I    H
ATOM  28493  HD3 ILE I 100      -7.765  27.989 -15.147  1.00  0.00
I    H
ATOM  28494  C   ILE I 100      -4.701  30.272 -13.237  1.00  0.00
I    C
ATOM  28495  O   ILE I 100      -4.488  29.543 -12.276  1.00  0.00
I    O
ATOM  28496  N   MET I 101      -4.050  31.457 -13.332  1.00  0.00
I    N
ATOM  28497  HN  MET I 101      -4.170  32.076 -14.105  1.00  0.00
I    H
ATOM  28498  CA  MET I 101      -3.112  31.937 -12.312  1.00  0.00
I    C
ATOM  28499  HA  MET I 101      -2.315  31.204 -12.270  1.00  0.00
I    H
ATOM  28500  CB  MET I 101      -2.647  33.383 -12.617  1.00  0.00
I    C
ATOM  28501  HB1 MET I 101      -3.567  34.005 -12.719  1.00  0.00
I    H
ATOM  28502  HB2 MET I 101      -2.095  33.787 -11.738  1.00  0.00
I    H
ATOM  28503  CG  MET I 101      -1.812  33.553 -13.880  1.00  0.00
I    C
ATOM  28504  HG1 MET I 101      -0.844  33.020 -13.777  1.00  0.00
I    H
ATOM  28505  HG2 MET I 101      -2.351  33.110 -14.746  1.00  0.00
I    H
ATOM  28506  SD  MET I 101      -1.450  35.274 -14.253  1.00  0.00
I    S
ATOM  28507  CE  MET I 101       0.013  35.359 -13.189  1.00  0.00
I    C
ATOM  28508  HE1 MET I 101       0.464  36.374 -13.216  1.00  0.00
I    H
ATOM  28509  HE2 MET I 101      -0.249  35.131 -12.133  1.00  0.00
I    H
ATOM  28510  HE3 MET I 101       0.787  34.632 -13.514  1.00  0.00
I    H
ATOM  28511  C   MET I 101      -3.721  32.033 -10.922  1.00  0.00
I    C
```

```
ATOM  28512  O   MET I 101      -3.167  31.534  -9.942  1.00  0.00
I    O
ATOM  28513  N   GLY I 102      -4.916  32.657 -10.832  1.00  0.00
I    N
ATOM  28514  HN  GLY I 102      -5.289  33.131 -11.629  1.00  0.00
I    H
ATOM  28515  CA  GLY I 102      -5.741  32.699  -9.632  1.00  0.00
I    C
ATOM  28516  HA1 GLY I 102      -6.687  33.129  -9.930  1.00  0.00
I    H
ATOM  28517  HA2 GLY I 102      -5.195  33.285  -8.905  1.00  0.00
I    H
ATOM  28518  C   GLY I 102      -6.012  31.352  -9.030  1.00  0.00
I    C
ATOM  28519  O   GLY I 102      -5.735  31.117  -7.862  1.00  0.00
I    O
ATOM  28520  N   LEU I 103      -6.544  30.420  -9.838  1.00  0.00
I    N
ATOM  28521  HN  LEU I 103      -6.733  30.603 -10.800  1.00  0.00
I    H
ATOM  28522  CA  LEU I 103      -6.894  29.081  -9.407  1.00  0.00
I    C
ATOM  28523  HA  LEU I 103      -7.548  29.215  -8.556  1.00  0.00
I    H
ATOM  28524  CB  LEU I 103      -7.753  28.398 -10.503  1.00  0.00
I    C
ATOM  28525  HB1 LEU I 103      -8.403  29.185 -10.950  1.00  0.00
I    H
ATOM  28526  HB2 LEU I 103      -7.084  28.051 -11.322  1.00  0.00
I    H
ATOM  28527  CG  LEU I 103      -8.641  27.244 -10.007  1.00  0.00
I    C
ATOM  28528  HG  LEU I 103      -7.939  26.461  -9.635  1.00  0.00
I    H
ATOM  28529  CD1 LEU I 103      -9.583  27.692  -8.876  1.00  0.00
I    C
ATOM  28530 HD11 LEU I 103     -10.247  26.856  -8.567  1.00  0.00
I    H
ATOM  28531 HD12 LEU I 103      -9.000  28.020  -7.988  1.00  0.00
I    H
ATOM  28532 HD13 LEU I 103     -10.222  28.537  -9.209  1.00  0.00
I    H
ATOM  28533  CD2 LEU I 103      -9.465  26.639 -11.152  1.00  0.00
I    C
ATOM  28534 HD21 LEU I 103     -10.086  25.796 -10.778  1.00  0.00
I    H
ATOM  28535 HD22 LEU I 103     -10.149  27.386 -11.605  1.00  0.00
I    H
ATOM  28536 HD23 LEU I 103      -8.795  26.251 -11.950  1.00  0.00
I    H
ATOM  28537  C   LEU I 103      -5.702  28.242  -8.946  1.00  0.00
I    C
ATOM  28538  O   LEU I 103      -5.773  27.566  -7.919  1.00  0.00
I    O
```

```
ATOM  28539  N   ILE I 104      -4.544  28.350  -9.636  1.00  0.00
I    N
ATOM  28540  HN  ILE I 104      -4.506  28.851 -10.499  1.00  0.00
I    H
ATOM  28541  CA  ILE I 104      -3.267  27.771  -9.223  1.00  0.00
I    C
ATOM  28542  HA  ILE I 104      -3.442  26.705  -9.144  1.00  0.00
I    H
ATOM  28543  CB  ILE I 104      -2.187  28.057 -10.277  1.00  0.00
I    C
ATOM  28544  HB  ILE I 104      -2.315  29.141 -10.522  1.00  0.00
I    H
ATOM  28545  CG2 ILE I 104      -0.767  27.727  -9.775  1.00  0.00
I    C
ATOM  28546 HG21 ILE I 104      -0.004  28.090 -10.497  1.00  0.00
I    H
ATOM  28547 HG22 ILE I 104      -0.568  28.219  -8.800  1.00  0.00
I    H
ATOM  28548 HG23 ILE I 104      -0.634  26.633  -9.650  1.00  0.00
I    H
ATOM  28549  CG1 ILE I 104      -2.430  27.288 -11.597  1.00  0.00
I    C
ATOM  28550 HG11 ILE I 104      -3.420  27.570 -12.020  1.00  0.00
I    H
ATOM  28551 HG12 ILE I 104      -1.656  27.593 -12.339  1.00  0.00
I    H
ATOM  28552  CD  ILE I 104      -2.374  25.771 -11.473  1.00  0.00
I    C
ATOM  28553  HD1 ILE I 104      -2.500  25.298 -12.470  1.00  0.00
I    H
ATOM  28554  HD2 ILE I 104      -1.396  25.448 -11.055  1.00  0.00
I    H
ATOM  28555  HD3 ILE I 104      -3.182  25.396 -10.810  1.00  0.00
I    H
ATOM  28556  C   ILE I 104      -2.818  28.250  -7.837  1.00  0.00
I    C
ATOM  28557  O   ILE I 104      -2.409  27.444  -7.001  1.00  0.00
I    O
ATOM  28558  N   GLN I 105      -2.938  29.562  -7.520  1.00  0.00
I    N
ATOM  28559  HN  GLN I 105      -3.204  30.251  -8.193  1.00  0.00
I    H
ATOM  28560  CA  GLN I 105      -2.692  30.089  -6.177  1.00  0.00
I    C
ATOM  28561  HA  GLN I 105      -1.681  29.789  -5.930  1.00  0.00
I    H
ATOM  28562  CB  GLN I 105      -2.778  31.640  -6.150  1.00  0.00
I    C
ATOM  28563  HB1 GLN I 105      -3.699  31.922  -6.711  1.00  0.00
I    H
ATOM  28564  HB2 GLN I 105      -2.936  31.983  -5.102  1.00  0.00
I    H
ATOM  28565  CG  GLN I 105      -1.563  32.364  -6.777  1.00  0.00
I    C
```

ATOM 28566 HG1 GLN I 105   -1.368 31.978 -7.801 1.00 0.00
I   H
ATOM 28567 HG2 GLN I 105   -1.753 33.455 -6.854 1.00 0.00
I   H
ATOM 28568 CD GLN I 105   -0.270 32.153 -5.991 1.00 0.00
I   C
ATOM 28569 OE1 GLN I 105   0.668 31.518 -6.469 1.00 0.00
I   O
ATOM 28570 NE2 GLN I 105   -0.208 32.728 -4.779 1.00 0.00
I   N
ATOM 28571 HE21 GLN I 105   0.634 32.599 -4.263 1.00 0.00
I   H
ATOM 28572 HE22 GLN I 105   -0.988 33.239 -4.433 1.00 0.00
I   H
ATOM 28573 C   GLN I 105   -3.614 29.529 -5.100 1.00 0.00
I   C
ATOM 28574 O   GLN I 105   -3.167 29.156 -4.017 1.00 0.00
I   O
ATOM 28575 N   ILE I 106   -4.931 29.410 -5.368 1.00 0.00
I   N
ATOM 28576 HN ILE I 106   -5.346 29.717 -6.221 1.00 0.00
I   H
ATOM 28577 CA ILE I 106   -5.875 28.816 -4.423 1.00 0.00
I   C
ATOM 28578 HA ILE I 106   -5.736 29.341 -3.487 1.00 0.00
I   H
ATOM 28579 CB ILE I 106   -7.326 28.973 -4.894 1.00 0.00
I   C
ATOM 28580 HB ILE I 106   -7.372 28.399 -5.854 1.00 0.00
I   H
ATOM 28581 CG2 ILE I 106   -8.311 28.447 -3.826 1.00 0.00
I   C
ATOM 28582 HG21 ILE I 106   -9.345 28.417 -4.230 1.00 0.00
I   H
ATOM 28583 HG22 ILE I 106   -8.043 27.416 -3.513 1.00 0.00
I   H
ATOM 28584 HG23 ILE I 106   -8.309 29.100 -2.928 1.00 0.00
I   H
ATOM 28585 CG1 ILE I 106   -7.696 30.436 -5.258 1.00 0.00
I   C
ATOM 28586 HG11 ILE I 106   -7.209 30.719 -6.218 1.00 0.00
I   H
ATOM 28587 HG12 ILE I 106   -8.797 30.501 -5.413 1.00 0.00
I   H
ATOM 28588 CD ILE I 106   -7.326 31.493 -4.214 1.00 0.00
I   C
ATOM 28589 HD1 ILE I 106   -7.681 32.496 -4.538 1.00 0.00
I   H
ATOM 28590 HD2 ILE I 106   -7.795 31.258 -3.234 1.00 0.00
I   H
ATOM 28591 HD3 ILE I 106   -6.226 31.547 -4.075 1.00 0.00
I   H
ATOM 28592 C   ILE I 106   -5.552 27.345 -4.158 1.00 0.00
I   C

```
ATOM  28593  O   ILE I 106      -5.566  26.874  -3.020  1.00  0.00
I    O
ATOM  28594  N   GLY I 107      -5.204  26.580  -5.211  1.00  0.00
I    N
ATOM  28595  HN  GLY I 107      -5.121  26.976  -6.124  1.00  0.00
I    H
ATOM  28596  CA  GLY I 107      -4.928  25.153  -5.115  1.00  0.00
I    C
ATOM  28597  HA1 GLY I 107      -4.913  24.793  -6.135  1.00  0.00
I    H
ATOM  28598  HA2 GLY I 107      -5.724  24.740  -4.510  1.00  0.00
I    H
ATOM  28599  C   GLY I 107      -3.622  24.789  -4.474  1.00  0.00
I    C
ATOM  28600  O   GLY I 107      -3.517  23.804  -3.737  1.00  0.00
I    O
ATOM  28601  N   GLY I 108      -2.571  25.581  -4.754  1.00  0.00
I    N
ATOM  28602  HN  GLY I 108      -2.637  26.331  -5.411  1.00  0.00
I    H
ATOM  28603  CA  GLY I 108      -1.273  25.409  -4.127  1.00  0.00
I    C
ATOM  28604  HA1 GLY I 108      -0.585  26.029  -4.684  1.00  0.00
I    H
ATOM  28605  HA2 GLY I 108      -1.052  24.350  -4.168  1.00  0.00
I    H
ATOM  28606  C   GLY I 108      -1.225  25.833  -2.696  1.00  0.00
I    C
ATOM  28607  O   GLY I 108      -0.462  25.286  -1.919  1.00  0.00
I    O
ATOM  28608  N   LEU I 109      -2.038  26.809  -2.269  1.00  0.00
I    N
ATOM  28609  HN  LEU I 109      -2.704  27.296  -2.829  1.00  0.00
I    H
ATOM  28610  CA  LEU I 109      -1.994  27.238  -0.885  1.00  0.00
I    C
ATOM  28611  HA  LEU I 109      -1.018  26.957  -0.512  1.00  0.00
I    H
ATOM  28612  CB  LEU I 109      -2.225  28.764  -0.842  1.00  0.00
I    C
ATOM  28613  HB1 LEU I 109      -1.486  29.227  -1.535  1.00  0.00
I    H
ATOM  28614  HB2 LEU I 109      -3.229  28.989  -1.267  1.00  0.00
I    H
ATOM  28615  CG  LEU I 109      -2.073  29.412   0.543  1.00  0.00
I    C
ATOM  28616  HG  LEU I 109      -2.872  28.940   1.163  1.00  0.00
I    H
ATOM  28617  CD1 LEU I 109      -0.709  29.144   1.168  1.00  0.00
I    C
ATOM  28618 HD11 LEU I 109      -0.619  29.666   2.144  1.00  0.00
I    H
ATOM  28619 HD12 LEU I 109      -0.565  28.056   1.340  1.00  0.00
I    H
```

```
ATOM  28620 HD13 LEU I 109       0.105  29.506   0.505  1.00  0.00
I    H
ATOM  28621  CD2 LEU I 109      -2.348  30.920   0.482  1.00  0.00
I    C
ATOM  28622 HD21 LEU I 109      -2.252  31.371   1.494  1.00  0.00
I    H
ATOM  28623 HD22 LEU I 109      -1.634  31.440  -0.187  1.00  0.00
I    H
ATOM  28624 HD23 LEU I 109      -3.378  31.114   0.112  1.00  0.00
I    H
ATOM  28625  C   LEU I 109      -3.030  26.536  -0.032  1.00  0.00
I    C
ATOM  28626  O   LEU I 109      -2.827  26.301   1.159  1.00  0.00
I    O
ATOM  28627  N   GLY I 110      -4.168  26.139  -0.628  1.00  0.00
I    N
ATOM  28628  HN  GLY I 110      -4.318  26.273  -1.608  1.00  0.00
I    H
ATOM  28629  CA  GLY I 110      -5.254  25.493   0.091  1.00  0.00
I    C
ATOM  28630  HA1 GLY I 110      -4.856  25.186   1.049  1.00  0.00
I    H
ATOM  28631  HA2 GLY I 110      -5.604  24.677  -0.527  1.00  0.00
I    H
ATOM  28632  C   GLY I 110      -6.417  26.416   0.343  1.00  0.00
I    C
ATOM  28633  O   GLY I 110      -6.263  27.572   0.709  1.00  0.00
I    O
ATOM  28634  N   PHE I 111      -7.659  25.907   0.208  1.00  0.00
I    N
ATOM  28635  HN  PHE I 111      -7.808  24.937   0.020  1.00  0.00
I    H
ATOM  28636  CA  PHE I 111      -8.882  26.696   0.320  1.00  0.00
I    C
ATOM  28637  HA  PHE I 111      -8.780  27.469  -0.433  1.00  0.00
I    H
ATOM  28638  CB  PHE I 111     -10.106  25.785  -0.019  1.00  0.00
I    C
ATOM  28639  HB1 PHE I 111     -10.045  25.474  -1.085  1.00  0.00
I    H
ATOM  28640  HB2 PHE I 111     -10.096  24.857   0.594  1.00  0.00
I    H
ATOM  28641  CG  PHE I 111     -11.445  26.463   0.137  1.00  0.00
I    C
ATOM  28642  CD1 PHE I 111     -11.740  27.661  -0.536  1.00  0.00
I    C
ATOM  28643  HD1 PHE I 111     -11.016  28.083  -1.220  1.00  0.00
I    H
ATOM  28644  CE1 PHE I 111     -12.962  28.314  -0.323  1.00  0.00
I    C
ATOM  28645  HE1 PHE I 111     -13.176  29.241  -0.837  1.00  0.00
I    H
ATOM  28646  CZ  PHE I 111     -13.908  27.762   0.550  1.00  0.00
I    C
```

ATOM 28647 HZ PHE I 111   -14.850 28.265   0.716 1.00 0.00
I   H
ATOM 28648 CD2 PHE I 111   -12.410 25.909   0.991 1.00 0.00
I   C
ATOM 28649 HD2 PHE I 111   -12.203 24.985   1.513 1.00 0.00
I   H
ATOM 28650 CE2 PHE I 111   -13.636 26.553   1.200 1.00 0.00
I   C
ATOM 28651 HE2 PHE I 111   -14.367 26.115   1.864 1.00 0.00
I   H
ATOM 28652 C   PHE I 111   -9.067 27.401   1.665 1.00 0.00
I   C
ATOM 28653 O   PHE I 111   -9.441 28.571   1.727 1.00 0.00
I   O
ATOM 28654 N   MET I 112   -8.783 26.731   2.797 1.00 0.00
I   N
ATOM 28655 HN MET I 112   -8.364 25.826   2.840 1.00 0.00
I   H
ATOM 28656 CA MET I 112   -9.083 27.312   4.089 1.00 0.00
I   C
ATOM 28657 HA MET I 112   -10.067 27.754   3.987 1.00 0.00
I   H
ATOM 28658 CB MET I 112   -9.132 26.254   5.202 1.00 0.00
I   C
ATOM 28659 HB1 MET I 112   -8.150 25.725   5.191 1.00 0.00
I   H
ATOM 28660 HB2 MET I 112   -9.202 26.761   6.191 1.00 0.00
I   H
ATOM 28661 CG MET I 112   -10.267 25.238   5.007 1.00 0.00
I   C
ATOM 28662 HG1 MET I 112   -10.064 24.598   4.123 1.00 0.00
I   H
ATOM 28663 HG2 MET I 112   -10.331 24.574   5.896 1.00 0.00
I   H
ATOM 28664 SD MET I 112   -11.914 25.967   4.752 1.00 0.00
I   S
ATOM 28665 CE MET I 112   -12.640 24.318   4.717 1.00 0.00
I   C
ATOM 28666 HE1 MET I 112   -13.738 24.372   4.553 1.00 0.00
I   H
ATOM 28667 HE2 MET I 112   -12.201 23.707   3.898 1.00 0.00
I   H
ATOM 28668 HE3 MET I 112   -12.463 23.783   5.675 1.00 0.00
I   H
ATOM 28669 C   MET I 112   -8.139 28.402   4.519 1.00 0.00
I   C
ATOM 28670 O   MET I 112   -8.450 29.159   5.429 1.00 0.00
I   O
ATOM 28671 N   THR I 113   -6.976 28.544   3.862 1.00 0.00
I   N
ATOM 28672 HN THR I 113   -6.637 27.891   3.185 1.00 0.00
I   H
ATOM 28673 CA THR I 113   -6.099 29.681   4.086 1.00 0.00
I   C

ATOM 28674 HA THR I 113   -5.996 29.776   5.159 1.00 0.00
I   H
ATOM 28675 CB THR I 113   -4.744 29.498   3.417 1.00 0.00
I   C
ATOM 28676 HB THR I 113   -4.098 30.328   3.791 1.00 0.00
I   H
ATOM 28677 OG1 THR I 113   -4.848 29.643   2.012 1.00 0.00
I   O
ATOM 28678 HG1 THR I 113   -3.958 29.501   1.673 1.00 0.00
I   H
ATOM 28679 CG2 THR I 113   -4.232 28.070   3.669 1.00 0.00
I   C
ATOM 28680 HG21 THR I 113   -3.196 27.948   3.288 1.00 0.00
I   H
ATOM 28681 HG22 THR I 113   -4.223 27.849   4.758 1.00 0.00
I   H
ATOM 28682 HG23 THR I 113   -4.878 27.319   3.167 1.00 0.00
I   H
ATOM 28683 C   THR I 113   -6.759 30.964   3.600 1.00 0.00
I   C
ATOM 28684 O   THR I 113   -6.831 31.960   4.305 1.00 0.00
I   O
ATOM 28685 N   PHE I 114   -7.338 30.935   2.386 1.00 0.00
I   N
ATOM 28686 HN PHE I 114   -7.263 30.131   1.799 1.00 0.00
I   H
ATOM 28687 CA PHE I 114   -8.101 32.020   1.818 1.00 0.00
I   C
ATOM 28688 HA PHE I 114   -7.496 32.909   1.948 1.00 0.00
I   H
ATOM 28689 CB PHE I 114   -8.344 31.811   0.302 1.00 0.00
I   C
ATOM 28690 HB1 PHE I 114   -8.244 30.730   0.063 1.00 0.00
I   H
ATOM 28691 HB2 PHE I 114   -9.374 32.115   0.016 1.00 0.00
I   H
ATOM 28692 CG PHE I 114   -7.314 32.557 -0.509 1.00 0.00
I   C
ATOM 28693 CD1 PHE I 114   -7.718 33.469 -1.502 1.00 0.00
I   C
ATOM 28694 HD1 PHE I 114   -8.771 33.609 -1.702 1.00 0.00
I   H
ATOM 28695 CE1 PHE I 114   -6.771 34.194 -2.238 1.00 0.00
I   C
ATOM 28696 HE1 PHE I 114   -7.096 34.898 -2.991 1.00 0.00
I   H
ATOM 28697 CZ PHE I 114   -5.406 34.010 -1.993 1.00 0.00
I   C
ATOM 28698 HZ PHE I 114   -4.673 34.583 -2.542 1.00 0.00
I   H
ATOM 28699 CD2 PHE I 114   -5.938 32.366 -0.293 1.00 0.00
I   C
ATOM 28700 HD2 PHE I 114   -5.601 31.638   0.431 1.00 0.00
I   H

ATOM 28701 CE2 PHE I 114   -4.989 33.089 -1.026 1.00 0.00
I   C
ATOM 28702 HE2 PHE I 114   -3.936 32.933 -0.845 1.00 0.00
I   H
ATOM 28703 C   PHE I 114   -9.391 32.250   2.564 1.00 0.00
I   C
ATOM 28704 O   PHE I 114   -9.769 33.394   2.780 1.00 0.00
I   O
ATOM 28705 N   ALA I 115   -10.077 31.194   3.048 1.00 0.00
I   N
ATOM 28706 HN ALA I 115   -9.856 30.245   2.825 1.00 0.00
I   H
ATOM 28707 CA ALA I 115   -11.206 31.376   3.943 1.00 0.00
I   C
ATOM 28708 HA ALA I 115   -11.983 31.936   3.437 1.00 0.00
I   H
ATOM 28709 CB ALA I 115   -11.838 30.006   4.269 1.00 0.00
I   C
ATOM 28710 HB1 ALA I 115   -12.118 29.491   3.325 1.00 0.00
I   H
ATOM 28711 HB2 ALA I 115   -11.120 29.355   4.813 1.00 0.00
I   H
ATOM 28712 HB3 ALA I 115   -12.756 30.116   4.887 1.00 0.00
I   H
ATOM 28713 C   ALA I 115   -10.856 32.170   5.214 1.00 0.00
I   C
ATOM 28714 O   ALA I 115   -11.523 33.147   5.538 1.00 0.00
I   O
ATOM 28715 N   VAL I 116   -9.743 31.856   5.911 1.00 0.00
I   N
ATOM 28716 HN VAL I 116   -9.134 31.096   5.698 1.00 0.00
I   H
ATOM 28717 CA VAL I 116   -9.341 32.645   7.072 1.00 0.00
I   C
ATOM 28718 HA VAL I 116   -10.245 32.822   7.641 1.00 0.00
I   H
ATOM 28719 CB VAL I 116   -8.475 31.822   8.023 1.00 0.00
I   C
ATOM 28720 HB VAL I 116   -9.029 30.878   8.252 1.00 0.00
I   H
ATOM 28721 CG1 VAL I 116   -7.133 31.460   7.390 1.00 0.00
I   C
ATOM 28722 HG11 VAL I 116   -6.505 30.895   8.113 1.00 0.00
I   H
ATOM 28723 HG12 VAL I 116   -7.261 30.818   6.494 1.00 0.00
I   H
ATOM 28724 HG13 VAL I 116   -6.578 32.376   7.096 1.00 0.00
I   H
ATOM 28725 CG2 VAL I 116   -8.250 32.541   9.364 1.00 0.00
I   C
ATOM 28726 HG21 VAL I 116   -7.586 31.943 10.024 1.00 0.00
I   H
ATOM 28727 HG22 VAL I 116   -7.778 33.534   9.200 1.00 0.00
I   H

ATOM 28728 HG23 VAL I 116   -9.208 32.695   9.904 1.00 0.00
I   H
ATOM 28729 C   VAL I 116   -8.736 34.028   6.717 1.00 0.00
I   C
ATOM 28730 O   VAL I 116   -8.797 34.975   7.491 1.00 0.00
I   O
ATOM 28731 N   LEU I 117   -8.163 34.223   5.509 1.00 0.00
I   N
ATOM 28732 HN LEU I 117   -8.020 33.478   4.861 1.00 0.00
I   H
ATOM 28733 CA LEU I 117   -7.700 35.531   5.035 1.00 0.00
I   C
ATOM 28734 HA LEU I 117   -7.212 35.995   5.883 1.00 0.00
I   H
ATOM 28735 CB LEU I 117   -6.708 35.385   3.844 1.00 0.00
I   C
ATOM 28736 HB1 LEU I 117   -7.199 34.733   3.085 1.00 0.00
I   H
ATOM 28737 HB2 LEU I 117   -6.579 36.377   3.358 1.00 0.00
I   H
ATOM 28738 CG LEU I 117   -5.333 34.778   4.202 1.00 0.00
I   C
ATOM 28739 HG LEU I 117   -5.561 33.928   4.891 1.00 0.00
I   H
ATOM 28740 CD1 LEU I 117   -4.617 34.253   2.946 1.00 0.00
I   C
ATOM 28741 HD11 LEU I 117   -3.610 33.862   3.206 1.00 0.00
I   H
ATOM 28742 HD12 LEU I 117   -5.201 33.431   2.478 1.00 0.00
I   H
ATOM 28743 HD13 LEU I 117   -4.492 35.065   2.199 1.00 0.00
I   H
ATOM 28744 CD2 LEU I 117   -4.430 35.770   4.953 1.00 0.00
I   C
ATOM 28745 HD21 LEU I 117   -3.460 35.292   5.210 1.00 0.00
I   H
ATOM 28746 HD22 LEU I 117   -4.212 36.669   4.342 1.00 0.00
I   H
ATOM 28747 HD23 LEU I 117   -4.916 36.099   5.898 1.00 0.00
I   H
ATOM 28748 C   LEU I 117   -8.836 36.450   4.598 1.00 0.00
I   C
ATOM 28749 O   LEU I 117   -8.727 37.670   4.658 1.00 0.00
I   O
ATOM 28750 N   ILE I 118   -9.975 35.888   4.153 1.00 0.00
I   N
ATOM 28751 HN ILE I 118   -10.029 34.918   3.927 1.00 0.00
I   H
ATOM 28752 CA ILE I 118   -11.220 36.616   3.959 1.00 0.00
I   C
ATOM 28753 HA ILE I 118   -10.973 37.485   3.364 1.00 0.00
I   H
ATOM 28754 CB ILE I 118   -12.268 35.708   3.316 1.00 0.00
I   C

ATOM 28755 HB ILE I 118   -12.125 34.717   3.818 1.00 0.00
I   H
ATOM 28756 CG2 ILE I 118   -13.721 36.220   3.492 1.00 0.00
I   C
ATOM 28757 HG21 ILE I 118   -14.449 35.456   3.149 1.00 0.00
I   H
ATOM 28758 HG22 ILE I 118   -13.939 36.437   4.559 1.00 0.00
I   H
ATOM 28759 HG23 ILE I 118   -13.889 37.147   2.905 1.00 0.00
I   H
ATOM 28760 CG1 ILE I 118   -11.935 35.517   1.825 1.00 0.00
I   C
ATOM 28761 HG11 ILE I 118   -12.175 36.447   1.263 1.00 0.00
I   H
ATOM 28762 HG12 ILE I 118   -10.840 35.342   1.720 1.00 0.00
I   H
ATOM 28763 CD ILE I 118   -12.680 34.335   1.198 1.00 0.00
I   C
ATOM 28764 HD1 ILE I 118   -12.367 34.199   0.140 1.00 0.00
I   H
ATOM 28765 HD2 ILE I 118   -12.460 33.396   1.749 1.00 0.00
I   H
ATOM 28766 HD3 ILE I 118   -13.777 34.503   1.212 1.00 0.00
I   H
ATOM 28767 C   ILE I 118   -11.770 37.126   5.267 1.00 0.00
I   C
ATOM 28768 O   ILE I 118   -12.331 38.216   5.353 1.00 0.00
I   O
ATOM 28769 N   VAL I 119   -11.685 36.318   6.331 1.00 0.00
I   N
ATOM 28770 HN VAL I 119   -11.340 35.383   6.305 1.00 0.00
I   H
ATOM 28771 CA VAL I 119   -12.106 36.765   7.641 1.00 0.00
I   C
ATOM 28772 HA VAL I 119   -13.057 37.255   7.475 1.00 0.00
I   H
ATOM 28773 CB VAL I 119   -12.603 35.665   8.575 1.00 0.00
I   C
ATOM 28774 HB VAL I 119   -13.193 36.154   9.388 1.00 0.00
I   H
ATOM 28775 CG1 VAL I 119   -13.521 34.635   7.877 1.00 0.00
I   C
ATOM 28776 HG11 VAL I 119   -13.821 33.839   8.592 1.00 0.00
I   H
ATOM 28777 HG12 VAL I 119   -14.452 35.106   7.498 1.00 0.00
I   H
ATOM 28778 HG13 VAL I 119   -12.994 34.152   7.026 1.00 0.00
I   H
ATOM 28779 CG2 VAL I 119   -11.448 34.994   9.323 1.00 0.00
I   C
ATOM 28780 HG21 VAL I 119   -11.822 34.171   9.968 1.00 0.00
I   H
ATOM 28781 HG22 VAL I 119   -10.713 34.570   8.605 1.00 0.00
I   H

```
ATOM  28782 HG23 VAL I 119     -10.919  35.718   9.977  1.00  0.00
I    H
ATOM  28783  C   VAL I 119     -11.179  37.778   8.355  1.00  0.00
I    C
ATOM  28784  O   VAL I 119      -9.978  37.921   8.131  1.00  0.00
I    O
ATOM  28785  N   MET I 120     -11.768  38.570   9.275  1.00  0.00
I    N
ATOM  28786  HN  MET I 120     -12.756  38.544   9.409  1.00  0.00
I    H
ATOM  28787  CA  MET I 120     -11.067  39.511  10.141  1.00  0.00
I    C
ATOM  28788  HA  MET I 120     -10.326  40.022   9.538  1.00  0.00
I    H
ATOM  28789  CB  MET I 120     -12.090  40.516  10.723  1.00  0.00
I    C
ATOM  28790  HB1 MET I 120     -12.867  39.917  11.254  1.00  0.00
I    H
ATOM  28791  HB2 MET I 120     -11.594  41.139  11.500  1.00  0.00
I    H
ATOM  28792  CG  MET I 120     -12.763  41.388   9.659  1.00  0.00
I    C
ATOM  28793  HG1 MET I 120     -13.148  40.758   8.830  1.00  0.00
I    H
ATOM  28794  HG2 MET I 120     -13.629  41.922  10.106  1.00  0.00
I    H
ATOM  28795  SD  MET I 120     -11.649  42.617   8.933  1.00  0.00
I    S
ATOM  28796  CE  MET I 120     -11.339  43.577  10.443  1.00  0.00
I    C
ATOM  28797  HE1 MET I 120     -10.659  44.431  10.237  1.00  0.00
I    H
ATOM  28798  HE2 MET I 120     -12.287  43.987  10.855  1.00  0.00
I    H
ATOM  28799  HE3 MET I 120     -10.868  42.947  11.227  1.00  0.00
I    H
ATOM  28800  C   MET I 120     -10.330  38.762  11.236  1.00  0.00
I    C
ATOM  28801  O   MET I 120     -10.738  37.684  11.618  1.00  0.00
I    O
ATOM  28802  N   ILE I 121      -9.204  39.246  11.798  1.00  0.00
I    N
ATOM  28803  HN  ILE I 121      -8.783  40.144  11.687  1.00  0.00
I    H
ATOM  28804  CA  ILE I 121      -8.475  38.346  12.709  1.00  0.00
I    C
ATOM  28805  HA  ILE I 121      -8.785  37.345  12.435  1.00  0.00
I    H
ATOM  28806  CB  ILE I 121      -6.958  38.357  12.474  1.00  0.00
I    C
ATOM  28807  HB  ILE I 121      -6.687  39.443  12.447  1.00  0.00
I    H
ATOM  28808  CG2 ILE I 121      -6.221  37.573  13.589  1.00  0.00
I    C
```

ATOM 28809 HG21 ILE I 121   -5.123 37.713 13.505 1.00 0.00
I   H
ATOM 28810 HG22 ILE I 121   -6.530 37.930 14.594 1.00 0.00
I   H
ATOM 28811 HG23 ILE I 121   -6.438 36.486 13.521 1.00 0.00
I   H
ATOM 28812 CG1 ILE I 121   -6.614 37.774 11.071 1.00 0.00
I   C
ATOM 28813 HG11 ILE I 121   -7.236 38.271 10.294 1.00 0.00
I   H
ATOM 28814 HG12 ILE I 121   -5.550 38.004 10.836 1.00 0.00
I   H
ATOM 28815 CD ILE I 121   -6.793 36.253 10.919 1.00 0.00
I   C
ATOM 28816 HD1 ILE I 121   -6.469 35.926   9.907 1.00 0.00
I   H
ATOM 28817 HD2 ILE I 121   -6.183 35.709 11.672 1.00 0.00
I   H
ATOM 28818 HD3 ILE I 121   -7.856 35.962 11.049 1.00 0.00
I   H
ATOM 28819 C   ILE I 121   -8.858 38.542 14.186 1.00 0.00
I   C
ATOM 28820 O   ILE I 121   -8.688 37.674 15.037 1.00 0.00
I   O
ATOM 28821 N   LEU I 122   -9.427 39.694 14.554 1.00 0.00
I   N
ATOM 28822 HN LEU I 122   -9.729 40.374 13.889 1.00 0.00
I   H
ATOM 28823 CA LEU I 122   -9.661 40.069 15.938 1.00 0.00
I   C
ATOM 28824 HA LEU I 122   -8.683 39.960 16.390 1.00 0.00
I   H
ATOM 28825 CB LEU I 122   -10.103 41.545 15.862 1.00 0.00
I   C
ATOM 28826 HB1 LEU I 122   -9.344 42.084 15.249 1.00 0.00
I   H
ATOM 28827 HB2 LEU I 122   -11.058 41.609 15.294 1.00 0.00
I   H
ATOM 28828 CG LEU I 122   -10.253 42.307 17.186 1.00 0.00
I   C
ATOM 28829 HG LEU I 122   -10.939 41.712 17.837 1.00 0.00
I   H
ATOM 28830 CD1 LEU I 122   -8.907 42.491 17.897 1.00 0.00
I   C
ATOM 28831 HD11 LEU I 122   -9.035 43.086 18.827 1.00 0.00
I   H
ATOM 28832 HD12 LEU I 122   -8.470 41.506 18.170 1.00 0.00
I   H
ATOM 28833 HD13 LEU I 122   -8.186 43.025 17.241 1.00 0.00
I   H
ATOM 28834 CD2 LEU I 122   -10.920 43.662 16.906 1.00 0.00
I   C
ATOM 28835 HD21 LEU I 122   -11.057 44.227 17.853 1.00 0.00
I   H

ATOM 28836 HD22 LEU I 122   -10.307 44.288 16.227 1.00 0.00
I   H
ATOM 28837 HD23 LEU I 122   -11.918 43.516 16.440 1.00 0.00
I   H
ATOM 28838 C   LEU I 122   -10.655 39.245 16.776 1.00 0.00
I   C
ATOM 28839 O   LEU I 122   -11.860 39.468 16.764 1.00 0.00
I   O
ATOM 28840 N   GLY I 123   -10.181 38.303 17.615 1.00 0.00
I   N
ATOM 28841 HN GLY I 123   -9.222 38.022 17.609 1.00 0.00
I   H
ATOM 28842 CA GLY I 123   -11.034 37.624 18.594 1.00 0.00
I   C
ATOM 28843 HA1 GLY I 123   -11.508 38.412 19.163 1.00 0.00
I   H
ATOM 28844 HA2 GLY I 123   -10.375 36.991 19.173 1.00 0.00
I   H
ATOM 28845 C   GLY I 123   -12.116 36.756 18.011 1.00 0.00
I   C
ATOM 28846 O   GLY I 123   -11.869 35.661 17.527 1.00 0.00
I   O
ATOM 28847 N   LYS I 124   -13.382 37.223 18.036 1.00 0.00
I   N
ATOM 28848 HN LYS I 124   -13.629 38.108 18.426 1.00 0.00
I   H
ATOM 28849 CA LYS I 124   -14.489 36.459 17.491 1.00 0.00
I   C
ATOM 28850 HA LYS I 124   -14.394 35.457 17.893 1.00 0.00
I   H
ATOM 28851 CB LYS I 124   -15.834 37.156 17.771 1.00 0.00
I   C
ATOM 28852 HB1 LYS I 124   -15.714 38.227 17.487 1.00 0.00
I   H
ATOM 28853 HB2 LYS I 124   -16.609 36.736 17.090 1.00 0.00
I   H
ATOM 28854 CG LYS I 124   -16.308 37.085 19.220 1.00 0.00
I   C
ATOM 28855 HG1 LYS I 124   -16.397 36.016 19.522 1.00 0.00
I   H
ATOM 28856 HG2 LYS I 124   -15.539 37.553 19.876 1.00 0.00
I   H
ATOM 28857 CD LYS I 124   -17.664 37.786 19.356 1.00 0.00
I   C
ATOM 28858 HD1 LYS I 124   -17.540 38.852 19.059 1.00 0.00
I   H
ATOM 28859 HD2 LYS I 124   -18.370 37.318 18.632 1.00 0.00
I   H
ATOM 28860 CE LYS I 124   -18.212 37.681 20.775 1.00 0.00
I   C
ATOM 28861 HE1 LYS I 124   -18.387 36.616 21.047 1.00 0.00
I   H
ATOM 28862 HE2 LYS I 124   -17.494 38.118 21.504 1.00 0.00
I   H

ATOM 28863 NZ LYS I 124 −19.492 38.410 20.880 1.00 0.00
I N
ATOM 28864 HZ1 LYS I 124 −19.866 38.336 21.848 1.00 0.00
I H
ATOM 28865 HZ2 LYS I 124 −19.343 39.411 20.642 1.00 0.00
I H
ATOM 28866 HZ3 LYS I 124 −20.178 38.000 20.214 1.00 0.00
I H
ATOM 28867 C LYS I 124 −14.444 36.347 15.996 1.00 0.00
I C
ATOM 28868 O LYS I 124 −15.069 35.467 15.424 1.00 0.00
I O
ATOM 28869 N LYS I 125 −13.701 37.242 15.339 1.00 0.00
I N
ATOM 28870 HN LYS I 125 −13.226 37.985 15.808 1.00 0.00
I H
ATOM 28871 CA LYS I 125 −13.525 37.209 13.923 1.00 0.00
I C
ATOM 28872 HA LYS I 125 −14.521 37.248 13.500 1.00 0.00
I H
ATOM 28873 CB LYS I 125 −12.716 38.440 13.522 1.00 0.00
I C
ATOM 28874 HB1 LYS I 125 −11.670 38.270 13.865 1.00 0.00
I H
ATOM 28875 HB2 LYS I 125 −12.676 38.501 12.411 1.00 0.00
I H
ATOM 28876 CG LYS I 125 −13.111 39.835 14.043 1.00 0.00
I C
ATOM 28877 HG1 LYS I 125 −13.320 39.771 15.135 1.00 0.00
I H
ATOM 28878 HG2 LYS I 125 −12.250 40.528 13.911 1.00 0.00
I H
ATOM 28879 CD LYS I 125 −14.339 40.515 13.419 1.00 0.00
I C
ATOM 28880 HD1 LYS I 125 −14.360 40.275 12.331 1.00 0.00
I H
ATOM 28881 HD2 LYS I 125 −15.253 40.069 13.876 1.00 0.00
I H
ATOM 28882 CE LYS I 125 −14.285 42.035 13.661 1.00 0.00
I C
ATOM 28883 HE1 LYS I 125 −14.372 42.261 14.747 1.00 0.00
I H
ATOM 28884 HE2 LYS I 125 −13.324 42.456 13.290 1.00 0.00
I H
ATOM 28885 NZ LYS I 125 −15.352 42.827 12.998 1.00 0.00
I N
[truncated: 449,111 more chars]
