# Supplementary material for: Structural basis and synergism of ATP and Na+ activation in bacterial K+ uptake system KtrAB
Source: Nat Commun. 2024 May 8;15:3850. doi: 10.1038/s41467-024-48057-y (PMC11078986; doi:10.1038/s41467-024-48057-y)
Supplement: Supplementary file 5 — Supplementary Data 2 [file 41467_2024_48057_MOESM5_ESM.pdf]

```
CRYST1    0.000    0.000    0.000  90.00  90.00  90.00 P 1
1
ATOM      1  N   LYS A   7      29.767  20.210  24.478  0.00  0.00
A
ATOM      2  HT1 LYS A   7      30.570  20.839  24.278  0.00  0.00
A
ATOM      3  HT2 LYS A   7      29.224  20.636  25.256  0.00  0.00
A
ATOM      4  HT3 LYS A   7      29.199  20.214  23.606  0.00  0.00
A
ATOM      5  CA  LYS A   7      30.283  18.878  24.971  0.00  0.00
A
ATOM      6  HA  LYS A   7      31.099  18.477  24.388  0.00  0.00
A
ATOM      7  CB  LYS A   7      30.846  18.987  26.353  0.00  0.00
A
ATOM      8  HB1 LYS A   7      30.049  19.348  27.038  0.00  0.00
A
ATOM      9  HB2 LYS A   7      31.083  17.958  26.699  0.00  0.00
A
ATOM     10  CG  LYS A   7      32.102  19.857  26.659  0.00  0.00
A
ATOM     11  HG1 LYS A   7      32.895  19.612  25.920  0.00  0.00
A
ATOM     12  HG2 LYS A   7      31.895  20.942  26.539  0.00  0.00
A
ATOM     13  CD  LYS A   7      32.592  19.829  28.179  0.00  0.00
A
ATOM     14  HD1 LYS A   7      31.794  20.181  28.868  0.00  0.00
A
ATOM     15  HD2 LYS A   7      32.718  18.760  28.453  0.00  0.00
A
ATOM     16  CE  LYS A   7      33.778  20.758  28.341  0.00  0.00
A
ATOM     17  HE1 LYS A   7      34.455  20.730  27.460  0.00  0.00
A
ATOM     18  HE2 LYS A   7      33.555  21.788  28.692  0.00  0.00
A
ATOM     19  NZ  LYS A   7      34.588  20.234  29.443  0.00  0.00
A
ATOM     20  HZ1 LYS A   7      33.957  20.183  30.269  0.00  0.00
A
ATOM     21  HZ2 LYS A   7      34.981  19.302  29.202  0.00  0.00
A
ATOM     22  HZ3 LYS A   7      35.422  20.842  29.567  0.00  0.00
A
ATOM     23  C   LYS A   7      29.180  17.838  24.972  0.00  0.00
A
ATOM     24  O   LYS A   7      28.023  18.179  24.729  0.00  0.00
A
ATOM     25  N   GLN A   8      29.545  16.605  25.325  0.00  0.00
A
ATOM     26  HN  GLN A   8      30.488  16.340  25.510  0.00  0.00
A
```

ATOM	27	CA	GLN	A	8	28.475	15.550	25.253	0.00	0.00	A
ATOM	28	HA	GLN	A	8	27.556	16.025	24.943	0.00	0.00	A
ATOM	29	CB	GLN	A	8	28.949	14.576	24.178	0.00	0.00	A
ATOM	30	HB1	GLN	A	8	29.965	14.309	24.540	0.00	0.00	A
ATOM	31	HB2	GLN	A	8	28.296	13.678	24.226	0.00	0.00	A
ATOM	32	CG	GLN	A	8	28.784	15.207	22.770	0.00	0.00	A
ATOM	33	HG1	GLN	A	8	27.705	15.471	22.753	0.00	0.00	A
ATOM	34	HG2	GLN	A	8	29.332	16.172	22.732	0.00	0.00	A
ATOM	35	CD	GLN	A	8	29.265	14.253	21.608	0.00	0.00	A
ATOM	36	OE1	GLN	A	8	28.335	13.850	20.898	0.00	0.00	A
ATOM	37	NE2	GLN	A	8	30.546	13.858	21.552	0.00	0.00	A
ATOM	38	HE21	GLN	A	8	30.903	13.311	20.795	0.00	0.00	A
ATOM	39	HE22	GLN	A	8	31.253	14.237	22.149	0.00	0.00	A
ATOM	40	C	GLN	A	8	28.347	14.845	26.537	0.00	0.00	A
ATOM	41	O	GLN	A	8	29.291	14.138	27.011	0.00	0.00	A
ATOM	42	N	PHE	A	9	27.162	14.960	27.114	0.00	0.00	A
ATOM	43	HN	PHE	A	9	26.410	15.452	26.683	0.00	0.00	A
ATOM	44	CA	PHE	A	9	26.886	14.439	28.506	0.00	0.00	A
ATOM	45	HA	PHE	A	9	27.732	13.919	28.932	0.00	0.00	A
ATOM	46	CB	PHE	A	9	26.500	15.627	29.461	0.00	0.00	A
ATOM	47	HB1	PHE	A	9	25.631	16.172	29.036	0.00	0.00	A
ATOM	48	HB2	PHE	A	9	26.144	15.194	30.420	0.00	0.00	A
ATOM	49	CG	PHE	A	9	27.611	16.599	29.789	0.00	0.00	A
ATOM	50	CD1	PHE	A	9	28.924	16.093	29.964	0.00	0.00	A
ATOM	51	HD1	PHE	A	9	29.086	15.054	29.716	0.00	0.00	A
ATOM	52	CE1	PHE	A	9	30.013	16.928	30.207	0.00	0.00	A
ATOM	53	HE1	PHE	A	9	30.995	16.506	30.361	0.00	0.00	A

```
ATOM     54  CZ  PHE A   9      29.759  18.341  30.308  0.00  0.00
A
ATOM     55  HZ  PHE A   9      30.620  18.959  30.513  0.00  0.00
A
ATOM     56  CD2 PHE A   9      27.389  17.962  29.899  0.00  0.00
A
ATOM     57  HD2 PHE A   9      26.443  18.414  29.638  0.00  0.00
A
ATOM     58  CE2 PHE A   9      28.424  18.824  30.202  0.00  0.00
A
ATOM     59  HE2 PHE A   9      28.275  19.886  30.326  0.00  0.00
A
ATOM     60  C   PHE A   9      25.751  13.378  28.527  0.00  0.00
A
ATOM     61  O   PHE A   9      24.898  13.264  27.642  0.00  0.00
A
ATOM     62  N   ALA A  10      25.699  12.542  29.555  0.00  0.00
A
ATOM     63  HN  ALA A  10      26.324  12.624  30.327  0.00  0.00
A
ATOM     64  CA  ALA A  10      24.736  11.464  29.719  0.00  0.00
A
ATOM     65  HA  ALA A  10      23.889  11.663  29.079  0.00  0.00
A
ATOM     66  CB  ALA A  10      25.405  10.079  29.307  0.00  0.00
A
ATOM     67  HB1 ALA A  10      25.605  10.065  28.215  0.00  0.00
A
ATOM     68  HB2 ALA A  10      26.341   9.857  29.863  0.00  0.00
A
ATOM     69  HB3 ALA A  10      24.677   9.245  29.405  0.00  0.00
A
ATOM     70  C   ALA A  10      24.227  11.298  31.166  0.00  0.00
A
ATOM     71  O   ALA A  10      25.013  11.303  32.068  0.00  0.00
A
ATOM     72  N   VAL A  11      22.917  11.261  31.306  0.00  0.00
A
ATOM     73  HN  VAL A  11      22.504  11.564  30.451  0.00  0.00
A
ATOM     74  CA  VAL A  11      22.204  11.356  32.492  0.00  0.00
A
ATOM     75  HA  VAL A  11      22.972  11.458  33.245  0.00  0.00
A
ATOM     76  CB  VAL A  11      21.311  12.675  32.560  0.00  0.00
A
ATOM     77  HB  VAL A  11      20.582  12.713  31.722  0.00  0.00
A
ATOM     78  CG1 VAL A  11      20.605  12.710  33.898  0.00  0.00
A
ATOM     79 HG11 VAL A  11      21.292  12.555  34.757  0.00  0.00
A
ATOM     80 HG12 VAL A  11      20.118  13.695  34.061  0.00  0.00
A
```

```
ATOM     81 HG13 VAL A  11      19.794  11.957  33.996  0.00  0.00
A
ATOM     82  CG2 VAL A  11      22.191  13.879  32.390  0.00  0.00
A
ATOM     83 HG21 VAL A  11      21.615  14.766  32.729  0.00  0.00
A
ATOM     84 HG22 VAL A  11      23.071  13.787  33.062  0.00  0.00
A
ATOM     85 HG23 VAL A  11      22.580  13.909  31.350  0.00  0.00
A
ATOM     86  C   VAL A  11      21.413  10.101  32.714  0.00  0.00
A
ATOM     87  O   VAL A  11      20.544   9.618  31.888  0.00  0.00
A
ATOM     88  N   ILE A  12      21.628   9.482  33.901  0.00  0.00
A
ATOM     89  HN  ILE A  12      22.292   9.855  34.544  0.00  0.00
A
ATOM     90  CA  ILE A  12      20.841   8.376  34.335  0.00  0.00
A
ATOM     91  HA  ILE A  12      20.257   8.063  33.482  0.00  0.00
A
ATOM     92  CB  ILE A  12      21.673   7.194  34.716  0.00  0.00
A
ATOM     93  HB  ILE A  12      22.251   7.577  35.584  0.00  0.00
A
ATOM     94  CG2 ILE A  12      20.691   6.032  35.100  0.00  0.00
A
ATOM     95 HG21 ILE A  12      20.144   5.649  34.212  0.00  0.00
A
ATOM     96 HG22 ILE A  12      21.341   5.290  35.611  0.00  0.00
A
ATOM     97 HG23 ILE A  12      19.955   6.379  35.856  0.00  0.00
A
ATOM     98  CG1 ILE A  12      22.712   6.723  33.630  0.00  0.00
A
ATOM     99 HG11 ILE A  12      22.292   6.504  32.624  0.00  0.00
A
ATOM    100 HG12 ILE A  12      23.330   7.623  33.424  0.00  0.00
A
ATOM    101  CD  ILE A  12      23.654   5.630  34.133  0.00  0.00
A
ATOM    102  HD1 ILE A  12      23.166   4.635  34.214  0.00  0.00
A
ATOM    103  HD2 ILE A  12      24.540   5.562  33.467  0.00  0.00
A
ATOM    104  HD3 ILE A  12      24.024   5.739  35.175  0.00  0.00
A
ATOM    105  C   ILE A  12      19.890   8.943  35.427  0.00  0.00
A
ATOM    106  O   ILE A  12      20.368   9.430  36.401  0.00  0.00
A
ATOM    107  N   GLY A  13      18.589   9.040  35.132  0.00  0.00
A
```

```
ATOM    108  HN  GLY A  13      18.209   8.618  34.312  0.00  0.00
A
ATOM    109  CA  GLY A  13      17.518   9.476  35.999  0.00  0.00
A
ATOM    110  HA1 GLY A  13      17.872   9.381  37.015  0.00  0.00
A
ATOM    111  HA2 GLY A  13      16.602   8.927  35.834  0.00  0.00
A
ATOM    112  C   GLY A  13      17.082  10.914  35.886  0.00  0.00
A
ATOM    113  O   GLY A  13      17.739  11.908  36.239  0.00  0.00
A
ATOM    114  N   LEU A  14      15.853  11.203  35.318  0.00  0.00
A
ATOM    115  HN  LEU A  14      15.324  10.394  35.072  0.00  0.00
A
ATOM    116  CA  LEU A  14      15.345  12.471  34.924  0.00  0.00
A
ATOM    117  HA  LEU A  14      16.154  13.154  34.711  0.00  0.00
A
ATOM    118  CB  LEU A  14      14.493  12.290  33.705  0.00  0.00
A
ATOM    119  HB1 LEU A  14      13.806  11.464  33.986  0.00  0.00
A
ATOM    120  HB2 LEU A  14      13.897  13.212  33.536  0.00  0.00
A
ATOM    121  CG  LEU A  14      15.212  12.011  32.391  0.00  0.00
A
ATOM    122  HG  LEU A  14      15.846  11.099  32.406  0.00  0.00
A
ATOM    123  CD1 LEU A  14      14.088  11.752  31.303  0.00  0.00
A
ATOM    124 HD11 LEU A  14      14.548  11.797  30.293  0.00  0.00
A
ATOM    125 HD12 LEU A  14      13.582  10.775  31.461  0.00  0.00
A
ATOM    126 HD13 LEU A  14      13.337  12.561  31.426  0.00  0.00
A
ATOM    127  CD2 LEU A  14      16.045  13.328  32.085  0.00  0.00
A
ATOM    128 HD21 LEU A  14      16.644  13.137  31.170  0.00  0.00
A
ATOM    129 HD22 LEU A  14      15.337  14.184  32.082  0.00  0.00
A
ATOM    130 HD23 LEU A  14      16.766  13.525  32.907  0.00  0.00
A
ATOM    131  C   LEU A  14      14.494  13.107  36.109  0.00  0.00
A
ATOM    132  O   LEU A  14      13.532  13.830  35.835  0.00  0.00
A
ATOM    133  N   GLY A  15      14.779  12.791  37.329  0.00  0.00
A
ATOM    134  HN  GLY A  15      15.612  12.276  37.518  0.00  0.00
A
```

ATOM 135 CA GLY A 15 14.087 13.458 38.467 0.00 0.00
A
ATOM 136 HA1 GLY A 15 14.439 12.787 39.237 0.00 0.00
A
ATOM 137 HA2 GLY A 15 13.025 13.374 38.291 0.00 0.00
A
ATOM 138 C GLY A 15 14.343 14.934 38.806 0.00 0.00
A
ATOM 139 O GLY A 15 14.915 15.681 37.997 0.00 0.00
A
ATOM 140 N ARG A 16 13.898 15.459 39.971 0.00 0.00
A
ATOM 141 HN ARG A 16 13.521 14.801 40.618 0.00 0.00
A
ATOM 142 CA ARG A 16 13.957 16.893 40.473 0.00 0.00
A
ATOM 143 HA ARG A 16 13.499 17.375 39.622 0.00 0.00
A
ATOM 144 CB ARG A 16 13.183 17.016 41.744 0.00 0.00
A
ATOM 145 HB1 ARG A 16 12.144 16.655 41.591 0.00 0.00
A
ATOM 146 HB2 ARG A 16 13.789 16.405 42.447 0.00 0.00
A
ATOM 147 CG ARG A 16 13.135 18.393 42.382 0.00 0.00
A
ATOM 148 HG1 ARG A 16 14.209 18.659 42.479 0.00 0.00
A
ATOM 149 HG2 ARG A 16 12.677 19.074 41.633 0.00 0.00
A
ATOM 150 CD ARG A 16 12.400 18.433 43.730 0.00 0.00
A
ATOM 151 HD1 ARG A 16 12.510 19.480 44.084 0.00 0.00
A
ATOM 152 HD2 ARG A 16 11.333 18.128 43.680 0.00 0.00
A
ATOM 153 NE ARG A 16 13.046 17.532 44.672 0.00 0.00
A
ATOM 154 HE ARG A 16 13.827 16.959 44.423 0.00 0.00
A
ATOM 155 CZ ARG A 16 12.687 17.477 45.938 0.00 0.00
A
ATOM 156 NH1 ARG A 16 11.739 18.269 46.464 0.00 0.00
A
ATOM 157 HH11 ARG A 16 11.380 17.927 47.332 0.00 0.00
A
ATOM 158 HH12 ARG A 16 11.497 19.210 46.227 0.00 0.00
A
ATOM 159 NH2 ARG A 16 13.069 16.415 46.666 0.00 0.00
A
ATOM 160 HH21 ARG A 16 12.503 16.240 47.471 0.00 0.00
A
ATOM 161 HH22 ARG A 16 13.523 15.695 46.142 0.00 0.00
A

ATOM 162 C ARG A 16 15.437 17.446 40.469 0.00 0.00
A
ATOM 163 O ARG A 16 15.732 18.590 40.041 0.00 0.00
A
ATOM 164 N PHE A 17 16.428 16.633 40.876 0.00 0.00
A
ATOM 165 HN PHE A 17 16.236 15.781 41.357 0.00 0.00
A
ATOM 166 CA PHE A 17 17.867 16.928 40.684 0.00 0.00
A
ATOM 167 HA PHE A 17 18.197 17.846 41.147 0.00 0.00
A
ATOM 168 CB PHE A 17 18.616 15.894 41.552 0.00 0.00
A
ATOM 169 HB1 PHE A 17 18.362 16.045 42.623 0.00 0.00
A
ATOM 170 HB2 PHE A 17 18.387 14.846 41.264 0.00 0.00
A
ATOM 171 CG PHE A 17 20.184 15.951 41.474 0.00 0.00
A
ATOM 172 CD1 PHE A 17 20.987 15.018 40.804 0.00 0.00
A
ATOM 173 HD1 PHE A 17 20.576 14.282 40.130 0.00 0.00
A
ATOM 174 CE1 PHE A 17 22.389 14.986 40.959 0.00 0.00
A
ATOM 175 HE1 PHE A 17 22.946 14.267 40.377 0.00 0.00
A
ATOM 176 CZ PHE A 17 22.992 15.965 41.755 0.00 0.00
A
ATOM 177 HZ PHE A 17 24.068 16.060 41.760 0.00 0.00
A
ATOM 178 CD2 PHE A 17 20.812 16.888 42.291 0.00 0.00
A
ATOM 179 HD2 PHE A 17 20.150 17.555 42.823 0.00 0.00
A
ATOM 180 CE2 PHE A 17 22.194 16.911 42.419 0.00 0.00
A
ATOM 181 HE2 PHE A 17 22.669 17.697 42.988 0.00 0.00
A
ATOM 182 C PHE A 17 18.330 16.919 39.218 0.00 0.00
A
ATOM 183 O PHE A 17 18.821 17.894 38.624 0.00 0.00
A
ATOM 184 N GLY A 18 18.055 15.761 38.625 0.00 0.00
A
ATOM 185 HN GLY A 18 17.611 15.049 39.163 0.00 0.00
A
ATOM 186 CA GLY A 18 18.737 15.433 37.347 0.00 0.00
A
ATOM 187 HA1 GLY A 18 18.649 14.358 37.283 0.00 0.00
A
ATOM 188 HA2 GLY A 18 19.742 15.827 37.371 0.00 0.00
A

ATOM A	189	C	GLY	A	18	18.191	15.958	36.143	0.00	0.00
ATOM A	190	O	GLY	A	18	18.913	16.189	35.170	0.00	0.00
ATOM A	191	N	GLY	A	19	16.841	16.275	36.095	0.00	0.00
ATOM A	192	HN	GLY	A	19	16.275	16.205	36.913	0.00	0.00
ATOM A	193	CA	GLY	A	19	16.218	16.982	34.948	0.00	0.00
ATOM A	194	HA1	GLY	A	19	15.139	16.982	34.986	0.00	0.00
ATOM A	195	HA2	GLY	A	19	16.676	16.603	34.047	0.00	0.00
ATOM A	196	C	GLY	A	19	16.588	18.427	34.865	0.00	0.00
ATOM A	197	O	GLY	A	19	16.616	19.127	33.857	0.00	0.00
ATOM A	198	N	SER	A	20	16.825	19.066	36.085	0.00	0.00
ATOM A	199	HN	SER	A	20	16.867	18.563	36.945	0.00	0.00
ATOM A	200	CA	SER	A	20	17.205	20.461	36.200	0.00	0.00
ATOM A	201	HA	SER	A	20	16.583	21.040	35.533	0.00	0.00
ATOM A	202	CB	SER	A	20	17.156	20.959	37.658	0.00	0.00
ATOM A	203	HB1	SER	A	20	16.222	20.668	38.185	0.00	0.00
ATOM A	204	HB2	SER	A	20	17.994	20.527	38.246	0.00	0.00
ATOM A	205	OG	SER	A	20	17.267	22.392	37.793	0.00	0.00
ATOM A	206	HG1	SER	A	20	17.021	22.652	38.684	0.00	0.00
ATOM A	207	C	SER	A	20	18.566	20.636	35.733	0.00	0.00
ATOM A	208	O	SER	A	20	18.984	21.672	35.135	0.00	0.00
ATOM A	209	N	ILE	A	21	19.399	19.617	35.921	0.00	0.00
ATOM A	210	HN	ILE	A	21	19.132	18.824	36.465	0.00	0.00
ATOM A	211	CA	ILE	A	21	20.745	19.533	35.353	0.00	0.00
ATOM A	212	HA	ILE	A	21	21.239	20.480	35.514	0.00	0.00
ATOM A	213	CB	ILE	A	21	21.524	18.390	35.887	0.00	0.00
ATOM A	214	HB	ILE	A	21	20.806	17.543	35.886	0.00	0.00
ATOM A	215	CG2	ILE	A	21	22.800	17.814	35.072	0.00	0.00

```
ATOM    216 HG21 ILE A  21      22.455  17.295  34.152  0.00  0.00
A
ATOM    217 HG22 ILE A  21      23.435  18.655  34.719  0.00  0.00
A
ATOM    218 HG23 ILE A  21      23.521  17.310  35.751  0.00  0.00
A
ATOM    219  CG1 ILE A  21      21.840  18.749  37.418  0.00  0.00
A
ATOM    220 HG11 ILE A  21      22.641  19.516  37.486  0.00  0.00
A
ATOM    221 HG12 ILE A  21      20.937  19.197  37.885  0.00  0.00
A
ATOM    222  CD  ILE A  21      22.212  17.516  38.253  0.00  0.00
A
ATOM    223  HD1 ILE A  21      22.496  17.831  39.280  0.00  0.00
A
ATOM    224  HD2 ILE A  21      21.311  16.866  38.237  0.00  0.00
A
ATOM    225  HD3 ILE A  21      23.039  16.919  37.813  0.00  0.00
A
ATOM    226  C   ILE A  21      20.716  19.467  33.841  0.00  0.00
A
ATOM    227  O   ILE A  21      21.537  20.212  33.278  0.00  0.00
A
ATOM    228  N   VAL A  22      19.879  18.624  33.132  0.00  0.00
A
ATOM    229  HN  VAL A  22      19.379  17.887  33.579  0.00  0.00
A
ATOM    230  CA  VAL A  22      19.688  18.622  31.741  0.00  0.00
A
ATOM    231  HA  VAL A  22      20.644  18.257  31.397  0.00  0.00
A
ATOM    232  CB  VAL A  22      18.637  17.611  31.335  0.00  0.00
A
ATOM    233  HB  VAL A  22      17.687  17.812  31.875  0.00  0.00
A
ATOM    234  CG1 VAL A  22      18.231  17.544  29.852  0.00  0.00
A
ATOM    235 HG11 VAL A  22      19.007  17.782  29.093  0.00  0.00
A
ATOM    236 HG12 VAL A  22      17.847  16.528  29.616  0.00  0.00
A
ATOM    237 HG13 VAL A  22      17.386  18.255  29.729  0.00  0.00
A
ATOM    238  CG2 VAL A  22      19.130  16.196  31.773  0.00  0.00
A
ATOM    239 HG21 VAL A  22      19.257  16.118  32.874  0.00  0.00
A
ATOM    240 HG22 VAL A  22      18.457  15.354  31.506  0.00  0.00
A
ATOM    241 HG23 VAL A  22      20.144  15.997  31.364  0.00  0.00
A
ATOM    242  C   VAL A  22      19.363  19.957  31.118  0.00  0.00
A
```

ATOM	243	O	VAL	A	22	19.967	20.395	30.160	0.00	0.00	A
ATOM	244	N	LYS	A	23	18.403	20.679	31.788	0.00	0.00	A
ATOM	245	HN	LYS	A	23	17.913	20.202	32.514	0.00	0.00	A
ATOM	246	CA	LYS	A	23	17.924	22.018	31.503	0.00	0.00	A
ATOM	247	HA	LYS	A	23	17.647	22.036	30.459	0.00	0.00	A
ATOM	248	CB	LYS	A	23	16.739	22.454	32.469	0.00	0.00	A
ATOM	249	HB1	LYS	A	23	17.145	22.270	33.486	0.00	0.00	A
ATOM	250	HB2	LYS	A	23	16.586	23.552	32.392	0.00	0.00	A
ATOM	251	CG	LYS	A	23	15.541	21.654	32.248	0.00	0.00	A
ATOM	252	HG1	LYS	A	23	15.103	22.000	31.287	0.00	0.00	A
ATOM	253	HG2	LYS	A	23	15.849	20.586	32.238	0.00	0.00	A
ATOM	254	CD	LYS	A	23	14.436	21.818	33.373	0.00	0.00	A
ATOM	255	HD1	LYS	A	23	14.878	21.398	34.302	0.00	0.00	A
ATOM	256	HD2	LYS	A	23	14.303	22.903	33.570	0.00	0.00	A
ATOM	257	CE	LYS	A	23	13.067	21.287	33.104	0.00	0.00	A
ATOM	258	HE1	LYS	A	23	12.527	21.794	32.276	0.00	0.00	A
ATOM	259	HE2	LYS	A	23	13.089	20.228	32.767	0.00	0.00	A
ATOM	260	NZ	LYS	A	23	12.288	21.306	34.322	0.00	0.00	A
ATOM	261	HZ1	LYS	A	23	12.659	20.619	35.009	0.00	0.00	A
ATOM	262	HZ2	LYS	A	23	12.263	22.254	34.748	0.00	0.00	A
ATOM	263	HZ3	LYS	A	23	11.330	20.974	34.090	0.00	0.00	A
ATOM	264	C	LYS	A	23	18.948	23.138	31.547	0.00	0.00	A
ATOM	265	O	LYS	A	23	19.000	23.881	30.566	0.00	0.00	A
ATOM	266	N	GLU	A	24	19.773	23.215	32.608	0.00	0.00	A
ATOM	267	HN	GLU	A	24	19.728	22.520	33.322	0.00	0.00	A
ATOM	268	CA	GLU	A	24	20.930	24.079	32.730	0.00	0.00	A
ATOM	269	HA	GLU	A	24	20.680	25.115	32.553	0.00	0.00	A

ATOM 270 CB GLU A 24 21.542 23.862 34.135 0.00 0.00
A
ATOM 271 HB1 GLU A 24 21.841 22.812 34.342 0.00 0.00
A
ATOM 272 HB2 GLU A 24 22.501 24.420 34.196 0.00 0.00
A
ATOM 273 CG GLU A 24 20.650 24.470 35.244 0.00 0.00
A
ATOM 274 HG1 GLU A 24 19.636 24.059 35.054 0.00 0.00
A
ATOM 275 HG2 GLU A 24 21.055 24.207 36.245 0.00 0.00
A
ATOM 276 CD GLU A 24 20.572 25.926 35.043 0.00 0.00
A
ATOM 277 OE1 GLU A 24 21.602 26.644 34.935 0.00 0.00
A
ATOM 278 OE2 GLU A 24 19.425 26.415 35.125 0.00 0.00
A
ATOM 279 C GLU A 24 22.021 23.755 31.638 0.00 0.00
A
ATOM 280 O GLU A 24 22.392 24.616 30.889 0.00 0.00
A
ATOM 281 N LEU A 25 22.443 22.476 31.444 0.00 0.00
A
ATOM 282 HN LEU A 25 22.114 21.703 31.981 0.00 0.00
A
ATOM 283 CA LEU A 25 23.292 22.143 30.411 0.00 0.00
A
ATOM 284 HA LEU A 25 24.141 22.785 30.594 0.00 0.00
A
ATOM 285 CB LEU A 25 23.675 20.655 30.521 0.00 0.00
A
ATOM 286 HB1 LEU A 25 22.771 20.009 30.544 0.00 0.00
A
ATOM 287 HB2 LEU A 25 24.250 20.340 29.625 0.00 0.00
A
ATOM 288 CG LEU A 25 24.453 20.224 31.770 0.00 0.00
A
ATOM 289 HG LEU A 25 24.015 20.604 32.718 0.00 0.00
A
ATOM 290 CD1 LEU A 25 24.320 18.732 31.797 0.00 0.00
A
ATOM 291 HD11 LEU A 25 24.590 18.307 32.788 0.00 0.00
A
ATOM 292 HD12 LEU A 25 23.272 18.426 31.595 0.00 0.00
A
ATOM 293 HD13 LEU A 25 24.947 18.236 31.026 0.00 0.00
A
ATOM 294 CD2 LEU A 25 25.862 20.937 31.699 0.00 0.00
A
ATOM 295 HD21 LEU A 25 25.839 22.046 31.757 0.00 0.00
A
ATOM 296 HD22 LEU A 25 26.559 20.609 32.500 0.00 0.00
A

ATOM	297	HD23	LEU	A	25	26.332	20.709	30.719	0.00	0.00	A
ATOM	298	C	LEU	A	25	22.804	22.455	29.016	0.00	0.00	A
ATOM	299	O	LEU	A	25	23.604	22.679	28.172	0.00	0.00	A
ATOM	300	N	HSE	A	26	21.499	22.375	28.701	0.00	0.00	A
ATOM	301	HN	HSE	A	26	20.833	21.910	29.280	0.00	0.00	A
ATOM	302	CA	HSE	A	26	20.817	22.713	27.436	0.00	0.00	A
ATOM	303	HA	HSE	A	26	21.300	22.085	26.702	0.00	0.00	A
ATOM	304	CB	HSE	A	26	19.375	22.296	27.516	0.00	0.00	A
ATOM	305	HB1	HSE	A	26	19.401	21.186	27.560	0.00	0.00	A
ATOM	306	HB2	HSE	A	26	18.885	22.867	28.334	0.00	0.00	A
ATOM	307	ND1	HSE	A	26	19.398	22.008	25.048	0.00	0.00	A
ATOM	308	CG	HSE	A	26	18.752	22.594	26.161	0.00	0.00	A
ATOM	309	CE1	HSE	A	26	18.519	22.227	24.098	0.00	0.00	A
ATOM	310	HE1	HSE	A	26	18.682	21.963	23.053	0.00	0.00	A
ATOM	311	NE2	HSE	A	26	17.371	22.749	24.532	0.00	0.00	A
ATOM	312	HE2	HSE	A	26	16.560	22.877	23.962	0.00	0.00	A
ATOM	313	CD2	HSE	A	26	17.532	23.075	25.901	0.00	0.00	A
ATOM	314	HD2	HSE	A	26	16.808	23.585	26.524	0.00	0.00	A
ATOM	315	C	HSE	A	26	21.112	24.152	27.085	0.00	0.00	A
ATOM	316	O	HSE	A	26	21.578	24.482	26.002	0.00	0.00	A
ATOM	317	N	ARG	A	27	20.861	25.012	28.073	0.00	0.00	A
ATOM	318	HN	ARG	A	27	20.457	24.640	28.906	0.00	0.00	A
ATOM	319	CA	ARG	A	27	21.201	26.383	28.034	0.00	0.00	A
ATOM	320	HA	ARG	A	27	20.747	26.778	27.136	0.00	0.00	A
ATOM	321	CB	ARG	A	27	20.604	27.036	29.304	0.00	0.00	A
ATOM	322	HB1	ARG	A	27	19.620	26.582	29.547	0.00	0.00	A
ATOM	323	HB2	ARG	A	27	21.206	26.952	30.235	0.00	0.00	A

ATOM 324 CG ARG A 27 20.289 28.564 29.086 0.00 0.00
A
ATOM 325 HG1 ARG A 27 21.247 29.076 28.853 0.00 0.00
A
ATOM 326 HG2 ARG A 27 19.608 28.736 28.226 0.00 0.00
A
ATOM 327 CD ARG A 27 19.622 29.302 30.324 0.00 0.00
A
ATOM 328 HD1 ARG A 27 19.321 30.347 30.100 0.00 0.00
A
ATOM 329 HD2 ARG A 27 18.796 28.627 30.634 0.00 0.00
A
ATOM 330 NE ARG A 27 20.679 29.366 31.402 0.00 0.00
A
ATOM 331 HE ARG A 27 21.508 29.873 31.164 0.00 0.00
A
ATOM 332 CZ ARG A 27 20.841 28.472 32.392 0.00 0.00
A
ATOM 333 NH1 ARG A 27 19.825 27.627 32.740 0.00 0.00
A
ATOM 334 HH11 ARG A 27 20.013 27.062 33.543 0.00 0.00
A
ATOM 335 HH12 ARG A 27 18.888 27.861 32.479 0.00 0.00
A
ATOM 336 NH2 ARG A 27 22.086 28.219 32.865 0.00 0.00
A
ATOM 337 HH21 ARG A 27 21.979 27.608 33.650 0.00 0.00
A
ATOM 338 HH22 ARG A 27 22.801 28.884 32.652 0.00 0.00
A
ATOM 339 C ARG A 27 22.688 26.852 27.949 0.00 0.00
A
ATOM 340 O ARG A 27 23.076 27.827 27.271 0.00 0.00
A
ATOM 341 N MET A 28 23.555 26.129 28.669 0.00 0.00
A
ATOM 342 HN MET A 28 23.286 25.436 29.333 0.00 0.00
A
ATOM 343 CA MET A 28 24.984 26.150 28.490 0.00 0.00
A
ATOM 344 HA MET A 28 25.408 27.143 28.463 0.00 0.00
A
ATOM 345 CB MET A 28 25.665 25.334 29.595 0.00 0.00
A
ATOM 346 HB1 MET A 28 25.353 24.270 29.660 0.00 0.00
A
ATOM 347 HB2 MET A 28 26.765 25.271 29.456 0.00 0.00
A
ATOM 348 CG MET A 28 25.458 26.040 30.961 0.00 0.00
A
ATOM 349 HG1 MET A 28 25.862 27.072 30.883 0.00 0.00
A
ATOM 350 HG2 MET A 28 24.373 26.114 31.187 0.00 0.00
A

ATOM 351 SD MET A 28 26.415 25.290 32.285 0.00 0.00
A
ATOM 352 CE MET A 28 25.288 25.994 33.533 0.00 0.00
A
ATOM 353 HE1 MET A 28 25.483 27.063 33.764 0.00 0.00
A
ATOM 354 HE2 MET A 28 24.227 25.817 33.258 0.00 0.00
A
ATOM 355 HE3 MET A 28 25.599 25.494 34.475 0.00 0.00
A
ATOM 356 C MET A 28 25.571 25.557 27.172 0.00 0.00
A
ATOM 357 O MET A 28 26.746 25.742 26.869 0.00 0.00
A
ATOM 358 N GLY A 29 24.699 24.781 26.446 0.00 0.00
A
ATOM 359 HN GLY A 29 23.843 24.541 26.897 0.00 0.00
A
ATOM 360 CA GLY A 29 24.990 24.396 25.056 0.00 0.00
A
ATOM 361 HA1 GLY A 29 25.972 24.764 24.798 0.00 0.00
A
ATOM 362 HA2 GLY A 29 24.258 24.759 24.349 0.00 0.00
A
ATOM 363 C GLY A 29 25.228 23.003 24.783 0.00 0.00
A
ATOM 364 O GLY A 29 25.258 22.579 23.638 0.00 0.00
A
ATOM 365 N HSE A 30 25.332 22.149 25.818 0.00 0.00
A
ATOM 366 HN HSE A 30 25.137 22.476 26.739 0.00 0.00
A
ATOM 367 CA HSE A 30 25.813 20.763 25.705 0.00 0.00
A
ATOM 368 HA HSE A 30 26.573 20.763 24.938 0.00 0.00
A
ATOM 369 CB HSE A 30 26.383 20.303 27.054 0.00 0.00
A
ATOM 370 HB1 HSE A 30 25.686 20.174 27.910 0.00 0.00
A
ATOM 371 HB2 HSE A 30 26.971 19.365 26.967 0.00 0.00
A
ATOM 372 ND1 HSE A 30 28.603 21.562 26.771 0.00 0.00
A
ATOM 373 CG HSE A 30 27.438 21.319 27.509 0.00 0.00
A
ATOM 374 CE1 HSE A 30 29.141 22.562 27.419 0.00 0.00
A
ATOM 375 HE1 HSE A 30 30.091 22.990 27.101 0.00 0.00
A
ATOM 376 NE2 HSE A 30 28.379 22.964 28.468 0.00 0.00
A
ATOM 377 HE2 HSE A 30 28.581 23.665 29.151 0.00 0.00
A

```
ATOM    378  CD2 HSE A  30      27.270  22.215  28.480  0.00  0.00
A
ATOM    379  HD2 HSE A  30      26.401  22.278  29.124  0.00  0.00
A
ATOM    380  C   HSE A  30      24.703  19.881  25.263  0.00  0.00
A
ATOM    381  O   HSE A  30      23.571  20.047  25.697  0.00  0.00
A
ATOM    382  N   GLU A  31      25.108  18.872  24.569  0.00  0.00
A
ATOM    383  HN  GLU A  31      26.082  18.683  24.471  0.00  0.00
A
ATOM    384  CA  GLU A  31      24.207  17.834  24.081  0.00  0.00
A
ATOM    385  HA  GLU A  31      23.218  18.194  23.841  0.00  0.00
A
ATOM    386  CB  GLU A  31      24.749  17.242  22.712  0.00  0.00
A
ATOM    387  HB1 GLU A  31      25.724  16.713  22.773  0.00  0.00
A
ATOM    388  HB2 GLU A  31      24.039  16.489  22.308  0.00  0.00
A
ATOM    389  CG  GLU A  31      24.999  18.406  21.642  0.00  0.00
A
ATOM    390  HG1 GLU A  31      24.146  19.110  21.546  0.00  0.00
A
ATOM    391  HG2 GLU A  31      25.852  18.985  22.056  0.00  0.00
A
ATOM    392  CD  GLU A  31      25.477  17.977  20.322  0.00  0.00
A
ATOM    393  OE1 GLU A  31      24.737  17.239  19.617  0.00  0.00
A
ATOM    394  OE2 GLU A  31      26.545  18.516  19.876  0.00  0.00
A
ATOM    395  C   GLU A  31      24.140  16.693  25.019  0.00  0.00
A
ATOM    396  O   GLU A  31      25.160  16.088  25.400  0.00  0.00
A
ATOM    397  N   VAL A  32      22.921  16.403  25.582  0.00  0.00
A
ATOM    398  HN  VAL A  32      22.160  16.996  25.328  0.00  0.00
A
ATOM    399  CA  VAL A  32      22.615  15.433  26.601  0.00  0.00
A
ATOM    400  HA  VAL A  32      23.600  15.096  26.889  0.00  0.00
A
ATOM    401  CB  VAL A  32      21.847  16.132  27.805  0.00  0.00
A
ATOM    402  HB  VAL A  32      20.825  16.347  27.427  0.00  0.00
A
ATOM    403  CG1 VAL A  32      21.788  15.248  29.002  0.00  0.00
A
ATOM    404 HG11 VAL A  32      21.357  15.785  29.874  0.00  0.00
A
```

```
ATOM    405 HG12 VAL A  32      21.132  14.365  28.843  0.00  0.00
A
ATOM    406 HG13 VAL A  32      22.860  15.038  29.207  0.00  0.00
A
ATOM    407  CG2 VAL A  32      22.475  17.494  28.155  0.00  0.00
A
ATOM    408 HG21 VAL A  32      22.220  17.654  29.225  0.00  0.00
A
ATOM    409 HG22 VAL A  32      23.568  17.400  27.977  0.00  0.00
A
ATOM    410 HG23 VAL A  32      22.034  18.377  27.646  0.00  0.00
A
ATOM    411  C   VAL A  32      21.814  14.269  26.173  0.00  0.00
A
ATOM    412  O   VAL A  32      20.717  14.378  25.596  0.00  0.00
A
ATOM    413  N   LEU A  33      22.293  13.051  26.572  0.00  0.00
A
ATOM    414  HN  LEU A  33      23.210  12.989  26.958  0.00  0.00
A
ATOM    415  CA  LEU A  33      21.632  11.762  26.452  0.00  0.00
A
ATOM    416  HA  LEU A  33      21.009  11.752  25.569  0.00  0.00
A
ATOM    417  CB  LEU A  33      22.669  10.616  26.207  0.00  0.00
A
ATOM    418  HB1 LEU A  33      23.065  10.812  25.188  0.00  0.00
A
ATOM    419  HB2 LEU A  33      23.548  10.653  26.886  0.00  0.00
A
ATOM    420  CG  LEU A  33      22.285   9.220  26.243  0.00  0.00
A
ATOM    421  HG  LEU A  33      21.902   9.104  27.279  0.00  0.00
A
ATOM    422  CD1 LEU A  33      21.171   8.976  25.257  0.00  0.00
A
ATOM    423 HD11 LEU A  33      21.396   9.431  24.268  0.00  0.00
A
ATOM    424 HD12 LEU A  33      20.980   7.885  25.173  0.00  0.00
A
ATOM    425 HD13 LEU A  33      20.215   9.445  25.574  0.00  0.00
A
ATOM    426  CD2 LEU A  33      23.443   8.317  26.105  0.00  0.00
A
ATOM    427 HD21 LEU A  33      23.027   7.328  26.391  0.00  0.00
A
ATOM    428 HD22 LEU A  33      23.880   8.274  25.085  0.00  0.00
A
ATOM    429 HD23 LEU A  33      24.317   8.547  26.752  0.00  0.00
A
ATOM    430  C   LEU A  33      20.885  11.541  27.737  0.00  0.00
A
ATOM    431  O   LEU A  33      21.524  11.603  28.800  0.00  0.00
A
```

```
ATOM    432  N   ALA A  34      19.571  11.164  27.709  0.00  0.00
A
ATOM    433  HN  ALA A  34      19.117  11.170  26.822  0.00  0.00
A
ATOM    434  CA  ALA A  34      18.757  10.839  28.811  0.00  0.00
A
ATOM    435  HA  ALA A  34      19.346  11.020  29.698  0.00  0.00
A
ATOM    436  CB  ALA A  34      17.519  11.753  28.896  0.00  0.00
A
ATOM    437  HB1 ALA A  34      17.802  12.817  29.043  0.00  0.00
A
ATOM    438  HB2 ALA A  34      17.056  11.639  27.893  0.00  0.00
A
ATOM    439  HB3 ALA A  34      16.781  11.483  29.682  0.00  0.00
A
ATOM    440  C   ALA A  34      18.296   9.381  28.810  0.00  0.00
A
ATOM    441  O   ALA A  34      17.555   8.864  27.971  0.00  0.00
A
ATOM    442  N   VAL A  35      18.653   8.599  29.888  0.00  0.00
A
ATOM    443  HN  VAL A  35      19.252   8.957  30.600  0.00  0.00
A
ATOM    444  CA  VAL A  35      18.204   7.237  30.262  0.00  0.00
A
ATOM    445  HA  VAL A  35      17.549   6.959  29.450  0.00  0.00
A
ATOM    446  CB  VAL A  35      19.334   6.301  30.266  0.00  0.00
A
ATOM    447  HB  VAL A  35      20.014   6.583  31.099  0.00  0.00
A
ATOM    448  CG1 VAL A  35      18.852   4.859  30.541  0.00  0.00
A
ATOM    449 HG11 VAL A  35      19.725   4.174  30.597  0.00  0.00
A
ATOM    450 HG12 VAL A  35      18.378   4.782  31.543  0.00  0.00
A
ATOM    451 HG13 VAL A  35      18.276   4.578  29.633  0.00  0.00
A
ATOM    452  CG2 VAL A  35      20.157   6.258  28.999  0.00  0.00
A
ATOM    453 HG21 VAL A  35      19.488   5.968  28.160  0.00  0.00
A
ATOM    454 HG22 VAL A  35      20.559   7.239  28.665  0.00  0.00
A
ATOM    455 HG23 VAL A  35      20.962   5.531  29.239  0.00  0.00
A
ATOM    456  C   VAL A  35      17.505   7.312  31.631  0.00  0.00
A
ATOM    457  O   VAL A  35      18.068   7.762  32.684  0.00  0.00
A
ATOM    458  N   ASP A  36      16.177   6.955  31.608  0.00  0.00
A
```

ATOM 459 HN ASP A 36 15.767 6.547 30.796 0.00 0.00
A
ATOM 460 CA ASP A 36 15.386 6.781 32.789 0.00 0.00
A
ATOM 461 HA ASP A 36 16.017 6.465 33.607 0.00 0.00
A
ATOM 462 CB ASP A 36 14.495 8.018 33.077 0.00 0.00
A
ATOM 463 HB1 ASP A 36 15.189 8.883 33.014 0.00 0.00
A
ATOM 464 HB2 ASP A 36 13.804 8.066 32.208 0.00 0.00
A
ATOM 465 CG ASP A 36 13.823 8.160 34.466 0.00 0.00
A
ATOM 466 OD1 ASP A 36 14.180 9.119 35.152 0.00 0.00
A
ATOM 467 OD2 ASP A 36 13.122 7.256 34.877 0.00 0.00
A
ATOM 468 C ASP A 36 14.578 5.473 32.389 0.00 0.00
A
ATOM 469 O ASP A 36 14.761 4.850 31.376 0.00 0.00
A
ATOM 470 N ILE A 37 13.720 4.910 33.289 0.00 0.00
A
ATOM 471 HN ILE A 37 13.726 5.255 34.224 0.00 0.00
A
ATOM 472 CA ILE A 37 12.842 3.742 33.032 0.00 0.00
A
ATOM 473 HA ILE A 37 13.098 3.289 32.086 0.00 0.00
A
ATOM 474 CB ILE A 37 13.212 2.660 33.999 0.00 0.00
A
ATOM 475 HB ILE A 37 14.290 2.462 33.820 0.00 0.00
A
ATOM 476 CG2 ILE A 37 13.036 3.144 35.460 0.00 0.00
A
ATOM 477 HG21 ILE A 37 13.282 2.349 36.196 0.00 0.00
A
ATOM 478 HG22 ILE A 37 13.662 4.037 35.671 0.00 0.00
A
ATOM 479 HG23 ILE A 37 12.032 3.584 35.636 0.00 0.00
A
ATOM 480 CG1 ILE A 37 12.360 1.322 33.807 0.00 0.00
A
ATOM 481 HG11 ILE A 37 12.763 0.729 34.655 0.00 0.00
A
ATOM 482 HG12 ILE A 37 11.289 1.600 33.906 0.00 0.00
A
ATOM 483 CD ILE A 37 12.595 0.756 32.393 0.00 0.00
A
ATOM 484 HD1 ILE A 37 12.299 -0.309 32.506 0.00 0.00
A
ATOM 485 HD2 ILE A 37 12.051 1.273 31.574 0.00 0.00
A

ATOM	486	HD3	ILE	A	37	13.655	0.730	32.060	0.00	0.00	A
ATOM	487	C	ILE	A	37	11.448	4.214	33.077	0.00	0.00	A
ATOM	488	O	ILE	A	37	10.463	3.522	32.787	0.00	0.00	A
ATOM	489	N	ASN	A	38	11.222	5.463	33.611	0.00	0.00	A
ATOM	490	HN	ASN	A	38	11.965	5.989	34.018	0.00	0.00	A
ATOM	491	CA	ASN	A	38	9.916	6.007	33.782	0.00	0.00	A
ATOM	492	HA	ASN	A	38	9.178	5.257	34.028	0.00	0.00	A
ATOM	493	CB	ASN	A	38	9.952	6.939	35.066	0.00	0.00	A
ATOM	494	HB1	ASN	A	38	10.583	6.412	35.814	0.00	0.00	A
ATOM	495	HB2	ASN	A	38	10.439	7.925	34.904	0.00	0.00	A
ATOM	496	CG	ASN	A	38	8.596	7.390	35.578	0.00	0.00	A
ATOM	497	OD1	ASN	A	38	8.043	8.430	35.196	0.00	0.00	A
ATOM	498	ND2	ASN	A	38	7.907	6.558	36.381	0.00	0.00	A
ATOM	499	HD21	ASN	A	38	7.095	6.852	36.885	0.00	0.00	A
ATOM	500	HD22	ASN	A	38	8.186	5.610	36.534	0.00	0.00	A
ATOM	501	C	ASN	A	38	9.413	6.668	32.523	0.00	0.00	A
ATOM	502	O	ASN	A	38	9.953	7.699	32.113	0.00	0.00	A
ATOM	503	N	GLU	A	39	8.365	6.205	31.872	0.00	0.00	A
ATOM	504	HN	GLU	A	39	7.840	5.452	32.260	0.00	0.00	A
ATOM	505	CA	GLU	A	39	7.894	6.629	30.579	0.00	0.00	A
ATOM	506	HA	GLU	A	39	8.708	6.190	30.021	0.00	0.00	A
ATOM	507	CB	GLU	A	39	6.641	5.938	30.231	0.00	0.00	A
ATOM	508	HB1	GLU	A	39	6.801	4.922	30.651	0.00	0.00	A
ATOM	509	HB2	GLU	A	39	5.777	6.341	30.803	0.00	0.00	A
ATOM	510	CG	GLU	A	39	6.462	5.851	28.707	0.00	0.00	A
ATOM	511	HG1	GLU	A	39	5.897	6.730	28.330	0.00	0.00	A
ATOM	512	HG2	GLU	A	39	7.512	5.802	28.349	0.00	0.00	A

```
ATOM    513  CD  GLU A  39       5.724   4.637  28.254  0.00  0.00
A
ATOM    514  OE1 GLU A  39       5.320   3.762  29.011  0.00  0.00
A
ATOM    515  OE2 GLU A  39       5.646   4.533  27.004  0.00  0.00
A
ATOM    516  C   GLU A  39       7.762   8.125  30.416  0.00  0.00
A
ATOM    517  O   GLU A  39       8.270   8.867  29.561  0.00  0.00
A
ATOM    518  N   GLU A  40       7.013   8.645  31.322  0.00  0.00
A
ATOM    519  HN  GLU A  40       6.657   8.096  32.073  0.00  0.00
A
ATOM    520  CA  GLU A  40       6.609  10.006  31.450  0.00  0.00
A
ATOM    521  HA  GLU A  40       5.911  10.173  30.643  0.00  0.00
A
ATOM    522  CB  GLU A  40       5.733  10.177  32.699  0.00  0.00
A
ATOM    523  HB1 GLU A  40       4.959   9.382  32.639  0.00  0.00
A
ATOM    524  HB2 GLU A  40       6.272   9.928  33.638  0.00  0.00
A
ATOM    525  CG  GLU A  40       4.878  11.477  32.733  0.00  0.00
A
ATOM    526  HG1 GLU A  40       5.576  12.341  32.702  0.00  0.00
A
ATOM    527  HG2 GLU A  40       4.214  11.571  31.847  0.00  0.00
A
ATOM    528  CD  GLU A  40       4.000  11.659  33.966  0.00  0.00
A
ATOM    529  OE1 GLU A  40       2.774  11.946  33.883  0.00  0.00
A
ATOM    530  OE2 GLU A  40       4.518  11.360  35.095  0.00  0.00
A
ATOM    531  C   GLU A  40       7.823  10.971  31.411  0.00  0.00
A
ATOM    532  O   GLU A  40       7.855  11.999  30.724  0.00  0.00
A
ATOM    533  N   LYS A  41       8.901  10.624  32.148  0.00  0.00
A
ATOM    534  HN  LYS A  41       8.954   9.789  32.691  0.00  0.00
A
ATOM    535  CA  LYS A  41      10.108  11.439  32.187  0.00  0.00
A
ATOM    536  HA  LYS A  41       9.814  12.469  32.324  0.00  0.00
A
ATOM    537  CB  LYS A  41      10.900  11.111  33.417  0.00  0.00
A
ATOM    538  HB1 LYS A  41      11.234  10.053  33.470  0.00  0.00
A
ATOM    539  HB2 LYS A  41      11.746  11.817  33.560  0.00  0.00
A
```

```
ATOM    540  CG  LYS A  41       9.887  11.291  34.639  0.00  0.00
A
ATOM    541  HG1 LYS A  41       9.486  12.326  34.674  0.00  0.00
A
ATOM    542  HG2 LYS A  41       9.089  10.525  34.534  0.00  0.00
A
ATOM    543  CD  LYS A  41      10.660  11.202  36.006  0.00  0.00
A
ATOM    544  HD1 LYS A  41      11.203  10.234  36.037  0.00  0.00
A
ATOM    545  HD2 LYS A  41      11.476  11.956  35.974  0.00  0.00
A
ATOM    546  CE  LYS A  41       9.730  11.480  37.180  0.00  0.00
A
ATOM    547  HE1 LYS A  41       9.618  12.566  37.387  0.00  0.00
A
ATOM    548  HE2 LYS A  41       8.758  11.064  36.839  0.00  0.00
A
ATOM    549  NZ  LYS A  41      10.166  10.823  38.405  0.00  0.00
A
ATOM    550  HZ1 LYS A  41       9.390  10.898  39.093  0.00  0.00
A
ATOM    551  HZ2 LYS A  41      10.399   9.820  38.255  0.00  0.00
A
ATOM    552  HZ3 LYS A  41      11.074  11.187  38.757  0.00  0.00
A
ATOM    553  C   LYS A  41      10.880  11.487  30.901  0.00  0.00
A
ATOM    554  O   LYS A  41      11.196  12.570  30.394  0.00  0.00
A
ATOM    555  N   VAL A  42      11.031  10.355  30.213  0.00  0.00
A
ATOM    556  HN  VAL A  42      10.947   9.495  30.710  0.00  0.00
A
ATOM    557  CA  VAL A  42      11.687  10.135  28.873  0.00  0.00
A
ATOM    558  HA  VAL A  42      12.722  10.404  29.027  0.00  0.00
A
ATOM    559  CB  VAL A  42      11.662   8.659  28.375  0.00  0.00
A
ATOM    560  HB  VAL A  42      10.638   8.227  28.344  0.00  0.00
A
ATOM    561  CG1 VAL A  42      12.321   8.475  26.994  0.00  0.00
A
ATOM    562 HG11 VAL A  42      11.957   9.073  26.132  0.00  0.00
A
ATOM    563 HG12 VAL A  42      13.389   8.565  27.287  0.00  0.00
A
ATOM    564 HG13 VAL A  42      12.182   7.436  26.628  0.00  0.00
A
ATOM    565  CG2 VAL A  42      12.480   7.879  29.469  0.00  0.00
A
ATOM    566 HG21 VAL A  42      12.822   6.901  29.068  0.00  0.00
A
```

```
ATOM    567 HG22 VAL A  42      13.357   8.488  29.777  0.00  0.00
A
ATOM    568 HG23 VAL A  42      11.925   7.702  30.414  0.00  0.00
A
ATOM    569  C   VAL A  42      11.005  11.118  27.847  0.00  0.00
A
ATOM    570  O   VAL A  42      11.768  11.744  27.118  0.00  0.00
A
ATOM    571  N   ASN A  43       9.658  11.188  27.795  0.00  0.00
A
ATOM    572  HN  ASN A  43       9.222  10.732  28.567  0.00  0.00
A
ATOM    573  CA  ASN A  43       8.884  12.003  26.901  0.00  0.00
A
ATOM    574  HA  ASN A  43       9.356  11.978  25.930  0.00  0.00
A
ATOM    575  CB  ASN A  43       7.440  11.526  27.056  0.00  0.00
A
ATOM    576  HB1 ASN A  43       7.162  11.436  28.127  0.00  0.00
A
ATOM    577  HB2 ASN A  43       6.880  12.308  26.499  0.00  0.00
A
ATOM    578  CG  ASN A  43       7.284  10.272  26.252  0.00  0.00
A
ATOM    579  OD1 ASN A  43       6.785  10.321  25.127  0.00  0.00
A
ATOM    580  ND2 ASN A  43       7.579   9.076  26.817  0.00  0.00
A
ATOM    581 HD21 ASN A  43       7.532   8.350  26.132  0.00  0.00
A
ATOM    582 HD22 ASN A  43       7.697   8.885  27.792  0.00  0.00
A
ATOM    583  C   ASN A  43       8.944  13.557  27.293  0.00  0.00
A
ATOM    584  O   ASN A  43       9.144  14.387  26.379  0.00  0.00
A
ATOM    585  N   ALA A  44       8.909  13.907  28.619  0.00  0.00
A
ATOM    586  HN  ALA A  44       8.794  13.231  29.342  0.00  0.00
A
ATOM    587  CA  ALA A  44       8.986  15.238  29.127  0.00  0.00
A
ATOM    588  HA  ALA A  44       8.174  15.695  28.581  0.00  0.00
A
ATOM    589  CB  ALA A  44       8.731  15.279  30.662  0.00  0.00
A
ATOM    590  HB1 ALA A  44       9.557  14.800  31.229  0.00  0.00
A
ATOM    591  HB2 ALA A  44       8.521  16.315  31.006  0.00  0.00
A
ATOM    592  HB3 ALA A  44       7.833  14.727  31.014  0.00  0.00
A
ATOM    593  C   ALA A  44      10.280  15.898  28.721  0.00  0.00
A
```

ATOM 594 O ALA A 44 10.292 17.012 28.212 0.00 0.00
A
ATOM 595 N TYR A 45 11.347 15.199 28.790 0.00 0.00
A
ATOM 596 HN TYR A 45 11.349 14.244 29.077 0.00 0.00
A
ATOM 597 CA TYR A 45 12.662 15.675 28.626 0.00 0.00
A
ATOM 598 HA TYR A 45 12.700 16.750 28.723 0.00 0.00
A
ATOM 599 CB TYR A 45 13.661 15.155 29.790 0.00 0.00
A
ATOM 600 HB1 TYR A 45 13.463 14.065 29.873 0.00 0.00
A
ATOM 601 HB2 TYR A 45 14.721 15.339 29.513 0.00 0.00
A
ATOM 602 CG TYR A 45 13.372 15.855 31.023 0.00 0.00
A
ATOM 603 CD1 TYR A 45 12.363 15.454 31.832 0.00 0.00
A
ATOM 604 HD1 TYR A 45 11.770 14.624 31.475 0.00 0.00
A
ATOM 605 CE1 TYR A 45 12.174 16.008 33.081 0.00 0.00
A
ATOM 606 HE1 TYR A 45 11.294 15.713 33.633 0.00 0.00
A
ATOM 607 CZ TYR A 45 12.935 17.093 33.420 0.00 0.00
A
ATOM 608 OH TYR A 45 12.790 17.775 34.600 0.00 0.00
A
ATOM 609 HH TYR A 45 12.099 17.339 35.104 0.00 0.00
A
ATOM 610 CD2 TYR A 45 14.243 16.888 31.465 0.00 0.00
A
ATOM 611 HD2 TYR A 45 15.028 17.233 30.808 0.00 0.00
A
ATOM 612 CE2 TYR A 45 13.981 17.532 32.608 0.00 0.00
A
ATOM 613 HE2 TYR A 45 14.593 18.405 32.782 0.00 0.00
A
ATOM 614 C TYR A 45 13.274 15.411 27.243 0.00 0.00
A
ATOM 615 O TYR A 45 14.384 15.978 27.035 0.00 0.00
A
ATOM 616 N ALA A 46 12.637 14.811 26.254 0.00 0.00
A
ATOM 617 HN ALA A 46 11.799 14.330 26.499 0.00 0.00
A
ATOM 618 CA ALA A 46 13.053 14.486 24.885 0.00 0.00
A
ATOM 619 HA ALA A 46 13.935 13.862 24.899 0.00 0.00
A
ATOM 620 CB ALA A 46 11.969 13.699 24.225 0.00 0.00
A

ATOM 621 HB1 ALA A 46 11.995 12.671 24.646 0.00 0.00
A
ATOM 622 HB2 ALA A 46 10.958 14.109 24.431 0.00 0.00
A
ATOM 623 HB3 ALA A 46 12.036 13.716 23.116 0.00 0.00
A
ATOM 624 C ALA A 46 13.364 15.737 24.051 0.00 0.00
A
ATOM 625 O ALA A 46 14.372 15.786 23.340 0.00 0.00
A
ATOM 626 N SER A 47 12.532 16.795 24.130 0.00 0.00
A
ATOM 627 HN SER A 47 11.700 16.760 24.677 0.00 0.00
A
ATOM 628 CA SER A 47 12.878 18.129 23.629 0.00 0.00
A
ATOM 629 HA SER A 47 13.032 17.997 22.568 0.00 0.00
A
ATOM 630 CB SER A 47 11.635 19.104 23.813 0.00 0.00
A
ATOM 631 HB1 SER A 47 11.943 20.166 23.700 0.00 0.00
A
ATOM 632 HB2 SER A 47 10.847 18.878 23.063 0.00 0.00
A
ATOM 633 OG SER A 47 10.926 18.857 25.041 0.00 0.00
A
ATOM 634 HG1 SER A 47 10.336 19.611 25.113 0.00 0.00
A
ATOM 635 C SER A 47 14.170 18.837 24.151 0.00 0.00
A
ATOM 636 O SER A 47 14.767 19.507 23.379 0.00 0.00
A
ATOM 637 N TYR A 48 14.550 18.570 25.376 0.00 0.00
A
ATOM 638 HN TYR A 48 13.916 18.147 26.020 0.00 0.00
A
ATOM 639 CA TYR A 48 15.803 18.986 25.977 0.00 0.00
A
ATOM 640 HA TYR A 48 16.082 19.920 25.510 0.00 0.00
A
ATOM 641 CB TYR A 48 15.853 19.083 27.531 0.00 0.00
A
ATOM 642 HB1 TYR A 48 15.755 18.078 27.995 0.00 0.00
A
ATOM 643 HB2 TYR A 48 16.872 19.356 27.880 0.00 0.00
A
ATOM 644 CG TYR A 48 14.843 20.126 28.069 0.00 0.00
A
ATOM 645 CD1 TYR A 48 13.611 19.591 28.411 0.00 0.00
A
ATOM 646 HD1 TYR A 48 13.413 18.530 28.369 0.00 0.00
A
ATOM 647 CE1 TYR A 48 12.611 20.396 29.001 0.00 0.00
A

ATOM 648 HE1 TYR A 48 11.621 19.993 29.162 0.00 0.00
A
ATOM 649 CZ TYR A 48 12.858 21.738 29.273 0.00 0.00
A
ATOM 650 OH TYR A 48 11.961 22.662 29.763 0.00 0.00
A
ATOM 651 HH TYR A 48 12.171 23.413 29.203 0.00 0.00
A
ATOM 652 CD2 TYR A 48 15.120 21.455 28.341 0.00 0.00
A
ATOM 653 HD2 TYR A 48 16.001 22.004 28.043 0.00 0.00
A
ATOM 654 CE2 TYR A 48 14.139 22.254 28.991 0.00 0.00
A
ATOM 655 HE2 TYR A 48 14.397 23.268 29.260 0.00 0.00
A
ATOM 656 C TYR A 48 16.884 18.079 25.602 0.00 0.00
A
ATOM 657 O TYR A 48 17.929 18.570 25.137 0.00 0.00
A
ATOM 658 N ALA A 49 16.662 16.776 25.718 0.00 0.00
A
ATOM 659 HN ALA A 49 15.827 16.439 26.145 0.00 0.00
A
ATOM 660 CA ALA A 49 17.640 15.817 25.344 0.00 0.00
A
ATOM 661 HA ALA A 49 18.494 16.182 25.896 0.00 0.00
A
ATOM 662 CB ALA A 49 17.331 14.437 25.983 0.00 0.00
A
ATOM 663 HB1 ALA A 49 16.685 14.679 26.854 0.00 0.00
A
ATOM 664 HB2 ALA A 49 16.800 13.697 25.346 0.00 0.00
A
ATOM 665 HB3 ALA A 49 18.301 13.999 26.302 0.00 0.00
A
ATOM 666 C ALA A 49 17.978 15.842 23.898 0.00 0.00
A
ATOM 667 O ALA A 49 17.215 16.241 22.996 0.00 0.00
A
ATOM 668 N THR A 50 19.241 15.500 23.570 0.00 0.00
A
ATOM 669 HN THR A 50 19.927 15.359 24.280 0.00 0.00
A
ATOM 670 CA THR A 50 19.588 15.143 22.126 0.00 0.00
A
ATOM 671 HA THR A 50 19.131 15.924 21.537 0.00 0.00
A
ATOM 672 CB THR A 50 21.108 15.241 21.717 0.00 0.00
A
ATOM 673 HB THR A 50 21.543 16.124 22.231 0.00 0.00
A
ATOM 674 OG1 THR A 50 21.277 15.296 20.305 0.00 0.00
A

```
ATOM    675  HG1 THR A  50      22.235  15.319  20.257  0.00  0.00
A
ATOM    676  CG2 THR A  50      21.947  14.111  22.266  0.00  0.00
A
ATOM    677 HG21 THR A  50      21.627  13.152  21.806  0.00  0.00
A
ATOM    678 HG22 THR A  50      23.040  14.229  22.103  0.00  0.00
A
ATOM    679 HG23 THR A  50      21.705  13.904  23.330  0.00  0.00
A
ATOM    680  C   THR A  50      19.041  13.765  21.799  0.00  0.00
A
ATOM    681  O   THR A  50      18.621  13.418  20.673  0.00  0.00
A
ATOM    682  N   HSE A  51      19.039  12.871  22.762  0.00  0.00
A
ATOM    683  HN  HSE A  51      19.322  13.049  23.701  0.00  0.00
A
ATOM    684  CA  HSE A  51      18.355  11.596  22.582  0.00  0.00
A
ATOM    685  HA  HSE A  51      17.559  11.752  21.869  0.00  0.00
A
ATOM    686  CB  HSE A  51      19.329  10.462  22.268  0.00  0.00
A
ATOM    687  HB1 HSE A  51      20.179  10.430  22.982  0.00  0.00
A
ATOM    688  HB2 HSE A  51      18.819   9.482  22.386  0.00  0.00
A
ATOM    689  ND1 HSE A  51      19.235  10.288  19.746  0.00  0.00
A
ATOM    690  CG  HSE A  51      19.879  10.537  20.877  0.00  0.00
A
ATOM    691  CE1 HSE A  51      20.138  10.297  18.779  0.00  0.00
A
ATOM    692  HE1 HSE A  51      19.916  10.220  17.715  0.00  0.00
A
ATOM    693  NE2 HSE A  51      21.396  10.586  19.286  0.00  0.00
A
ATOM    694  HE2 HSE A  51      22.234  10.564  18.741  0.00  0.00
A
ATOM    695  CD2 HSE A  51      21.243  10.682  20.699  0.00  0.00
A
ATOM    696  HD2 HSE A  51      22.035  10.827  21.423  0.00  0.00
A
ATOM    697  C   HSE A  51      17.761  11.255  23.957  0.00  0.00
A
ATOM    698  O   HSE A  51      18.401  11.487  24.959  0.00  0.00
A
ATOM    699  N   ALA A  52      16.599  10.628  23.925  0.00  0.00
A
ATOM    700  HN  ALA A  52      16.159  10.421  23.055  0.00  0.00
A
ATOM    701  CA  ALA A  52      15.934  10.079  25.124  0.00  0.00
A
```

ATOM	702	HA	ALA	A	52	16.556	10.042	26.006	0.00	0.00	A
ATOM	703	CB	ALA	A	52	14.739	10.991	25.565	0.00	0.00	A
ATOM	704	HB1	ALA	A	52	15.172	11.985	25.807	0.00	0.00	A
ATOM	705	HB2	ALA	A	52	14.126	11.218	24.666	0.00	0.00	A
ATOM	706	HB3	ALA	A	52	14.129	10.667	26.436	0.00	0.00	A
ATOM	707	C	ALA	A	52	15.509	8.584	24.958	0.00	0.00	A
ATOM	708	O	ALA	A	52	15.111	8.201	23.868	0.00	0.00	A
ATOM	709	N	VAL	A	53	15.728	7.701	25.913	0.00	0.00	A
ATOM	710	HN	VAL	A	53	16.251	7.991	26.711	0.00	0.00	A
ATOM	711	CA	VAL	A	53	15.468	6.312	25.746	0.00	0.00	A
ATOM	712	HA	VAL	A	53	14.651	6.190	25.051	0.00	0.00	A
ATOM	713	CB	VAL	A	53	16.692	5.744	25.002	0.00	0.00	A
ATOM	714	HB	VAL	A	53	16.952	6.205	24.026	0.00	0.00	A
ATOM	715	CG1	VAL	A	53	17.933	5.898	25.871	0.00	0.00	A
ATOM	716	HG11	VAL	A	53	18.251	6.944	26.067	0.00	0.00	A
ATOM	717	HG12	VAL	A	53	17.738	5.409	26.850	0.00	0.00	A
ATOM	718	HG13	VAL	A	53	18.903	5.492	25.512	0.00	0.00	A
ATOM	719	CG2	VAL	A	53	16.441	4.220	24.755	0.00	0.00	A
ATOM	720	HG21	VAL	A	53	17.311	3.901	24.142	0.00	0.00	A
ATOM	721	HG22	VAL	A	53	16.499	3.639	25.701	0.00	0.00	A
ATOM	722	HG23	VAL	A	53	15.539	4.040	24.134	0.00	0.00	A
ATOM	723	C	VAL	A	53	15.070	5.702	27.074	0.00	0.00	A
ATOM	724	O	VAL	A	53	15.433	6.139	28.148	0.00	0.00	A
ATOM	725	N	ILE	A	54	14.387	4.548	27.057	0.00	0.00	A
ATOM	726	HN	ILE	A	54	14.233	4.114	26.172	0.00	0.00	A
ATOM	727	CA	ILE	A	54	13.906	3.910	28.219	0.00	0.00	A
ATOM	728	HA	ILE	A	54	13.943	4.496	29.126	0.00	0.00	A

ATOM	729	CB	ILE	A	54	12.391	3.566	27.873	0.00	0.00	A
ATOM	730	HB	ILE	A	54	12.401	2.927	26.964	0.00	0.00	A
ATOM	731	CG2	ILE	A	54	11.737	2.870	29.124	0.00	0.00	A
ATOM	732	HG21	ILE	A	54	10.645	2.813	28.926	0.00	0.00	A
ATOM	733	HG22	ILE	A	54	12.223	1.876	29.215	0.00	0.00	A
ATOM	734	HG23	ILE	A	54	11.885	3.480	30.041	0.00	0.00	A
ATOM	735	CG1	ILE	A	54	11.629	4.802	27.447	0.00	0.00	A
ATOM	736	HG11	ILE	A	54	11.276	5.339	28.353	0.00	0.00	A
ATOM	737	HG12	ILE	A	54	12.224	5.514	26.836	0.00	0.00	A
ATOM	738	CD	ILE	A	54	10.399	4.427	26.618	0.00	0.00	A
ATOM	739	HD1	ILE	A	54	9.849	3.590	27.098	0.00	0.00	A
ATOM	740	HD2	ILE	A	54	9.671	5.232	26.383	0.00	0.00	A
ATOM	741	HD3	ILE	A	54	10.697	4.060	25.612	0.00	0.00	A
ATOM	742	C	ILE	A	54	14.652	2.611	28.445	0.00	0.00	A
ATOM	743	O	ILE	A	54	14.778	1.652	27.666	0.00	0.00	A
ATOM	744	N	ALA	A	55	15.137	2.531	29.636	0.00	0.00	A
ATOM	745	HN	ALA	A	55	15.099	3.287	30.285	0.00	0.00	A
ATOM	746	CA	ALA	A	55	15.956	1.442	30.134	0.00	0.00	A
ATOM	747	HA	ALA	A	55	15.495	0.525	29.796	0.00	0.00	A
ATOM	748	CB	ALA	A	55	17.382	1.571	29.613	0.00	0.00	A
ATOM	749	HB1	ALA	A	55	18.009	0.779	30.076	0.00	0.00	A
ATOM	750	HB2	ALA	A	55	17.604	1.580	28.525	0.00	0.00	A
ATOM	751	HB3	ALA	A	55	17.824	2.485	30.064	0.00	0.00	A
ATOM	752	C	ALA	A	55	16.122	1.373	31.675	0.00	0.00	A
ATOM	753	O	ALA	A	55	16.148	2.358	32.384	0.00	0.00	A
ATOM	754	N	ASN	A	56	16.345	0.097	32.156	0.00	0.00	A
ATOM	755	HN	ASN	A	56	16.269	-0.656	31.507	0.00	0.00	A

```
ATOM    756  CA  ASN A  56      16.912  -0.138  33.466  0.00  0.00
A
ATOM    757  HA  ASN A  56      16.520   0.539  34.210  0.00  0.00
A
ATOM    758  CB  ASN A  56      16.656  -1.568  33.981  0.00  0.00
A
ATOM    759  HB1 ASN A  56      16.959  -2.254  33.161  0.00  0.00
A
ATOM    760  HB2 ASN A  56      17.100  -1.912  34.939  0.00  0.00
A
ATOM    761  CG  ASN A  56      15.176  -1.876  34.112  0.00  0.00
A
ATOM    762  OD1 ASN A  56      14.528  -1.579  35.140  0.00  0.00
A
ATOM    763  ND2 ASN A  56      14.563  -2.497  33.094  0.00  0.00
A
ATOM    764 HD21 ASN A  56      13.623  -2.808  33.228  0.00  0.00
A
ATOM    765 HD22 ASN A  56      15.045  -2.748  32.254  0.00  0.00
A
ATOM    766  C   ASN A  56      18.462   0.029  33.421  0.00  0.00
A
ATOM    767  O   ASN A  56      19.220  -0.614  32.651  0.00  0.00
A
ATOM    768  N   ALA A  57      18.967   1.025  34.279  0.00  0.00
A
ATOM    769  HN  ALA A  57      18.351   1.474  34.922  0.00  0.00
A
ATOM    770  CA  ALA A  57      20.369   1.216  34.428  0.00  0.00
A
ATOM    771  HA  ALA A  57      20.850   1.095  33.469  0.00  0.00
A
ATOM    772  CB  ALA A  57      20.598   2.726  34.675  0.00  0.00
A
ATOM    773  HB1 ALA A  57      20.428   3.286  33.731  0.00  0.00
A
ATOM    774  HB2 ALA A  57      19.987   3.155  35.498  0.00  0.00
A
ATOM    775  HB3 ALA A  57      21.666   2.918  34.915  0.00  0.00
A
ATOM    776  C   ALA A  57      21.206   0.303  35.370  0.00  0.00
A
ATOM    777  O   ALA A  57      22.365   0.629  35.784  0.00  0.00
A
ATOM    778  N   THR A  58      20.494  -0.809  35.827  0.00  0.00
A
ATOM    779  HN  THR A  58      19.570  -0.893  35.464  0.00  0.00
A
ATOM    780  CA  THR A  58      21.037  -1.855  36.571  0.00  0.00
A
ATOM    781  HA  THR A  58      21.977  -1.669  37.071  0.00  0.00
A
ATOM    782  CB  THR A  58      19.985  -2.211  37.611  0.00  0.00
A
```

ATOM	783	HB	THR	A	58	20.318	-3.173	38.055	0.00	0.00	A
ATOM	784	OG1	THR	A	58	18.685	-2.344	37.064	0.00	0.00	A
ATOM	785	HG1	THR	A	58	18.519	-3.289	37.050	0.00	0.00	A
ATOM	786	CG2	THR	A	58	19.907	-1.115	38.695	0.00	0.00	A
ATOM	787	HG21	THR	A	58	20.821	-1.005	39.317	0.00	0.00	A
ATOM	788	HG22	THR	A	58	19.709	-0.169	38.148	0.00	0.00	A
ATOM	789	HG23	THR	A	58	19.076	-1.392	39.380	0.00	0.00	A
ATOM	790	C	THR	A	58	21.345	-3.063	35.664	0.00	0.00	A
ATOM	791	O	THR	A	58	21.905	-4.087	36.143	0.00	0.00	A
ATOM	792	N	GLU	A	59	21.035	-3.028	34.331	0.00	0.00	A
ATOM	793	HN	GLU	A	59	20.821	-2.106	34.017	0.00	0.00	A
ATOM	794	CA	GLU	A	59	21.071	-4.069	33.361	0.00	0.00	A
ATOM	795	HA	GLU	A	59	21.230	-5.023	33.841	0.00	0.00	A
ATOM	796	CB	GLU	A	59	19.703	-4.458	32.713	0.00	0.00	A
ATOM	797	HB1	GLU	A	59	19.220	-3.495	32.445	0.00	0.00	A
ATOM	798	HB2	GLU	A	59	19.799	-5.120	31.826	0.00	0.00	A
ATOM	799	CG	GLU	A	59	18.638	-5.063	33.708	0.00	0.00	A
ATOM	800	HG1	GLU	A	59	18.393	-4.358	34.531	0.00	0.00	A
ATOM	801	HG2	GLU	A	59	17.623	-5.160	33.268	0.00	0.00	A
ATOM	802	CD	GLU	A	59	19.115	-6.397	34.271	0.00	0.00	A
ATOM	803	OE1	GLU	A	59	19.404	-7.363	33.509	0.00	0.00	A
ATOM	804	OE2	GLU	A	59	18.789	-6.575	35.474	0.00	0.00	A
ATOM	805	C	GLU	A	59	22.103	-3.762	32.371	0.00	0.00	A
ATOM	806	O	GLU	A	59	22.039	-2.808	31.644	0.00	0.00	A
ATOM	807	N	GLU	A	60	23.100	-4.724	32.213	0.00	0.00	A
ATOM	808	HN	GLU	A	60	23.161	-5.497	32.839	0.00	0.00	A
ATOM	809	CA	GLU	A	60	24.117	-4.685	31.253	0.00	0.00	A

```
ATOM    810  HA  GLU A  60      24.681  -3.798  31.504  0.00  0.00
A
ATOM    811  CB  GLU A  60      25.036  -5.998  31.431  0.00  0.00
A
ATOM    812  HB1 GLU A  60      25.565  -5.932  32.405  0.00  0.00
A
ATOM    813  HB2 GLU A  60      24.330  -6.853  31.499  0.00  0.00
A
ATOM    814  CG  GLU A  60      26.183  -6.058  30.388  0.00  0.00
A
ATOM    815  HG1 GLU A  60      25.757  -6.147  29.366  0.00  0.00
A
ATOM    816  HG2 GLU A  60      26.760  -5.112  30.308  0.00  0.00
A
ATOM    817  CD  GLU A  60      27.294  -7.098  30.485  0.00  0.00
A
ATOM    818  OE1 GLU A  60      28.457  -6.713  30.778  0.00  0.00
A
ATOM    819  OE2 GLU A  60      27.071  -8.308  30.232  0.00  0.00
A
ATOM    820  C   GLU A  60      23.586  -4.670  29.840  0.00  0.00
A
ATOM    821  O   GLU A  60      24.177  -3.938  28.975  0.00  0.00
A
ATOM    822  N   ASN A  61      22.558  -5.362  29.437  0.00  0.00
A
ATOM    823  HN  ASN A  61      22.128  -6.029  30.042  0.00  0.00
A
ATOM    824  CA  ASN A  61      22.170  -5.396  28.015  0.00  0.00
A
ATOM    825  HA  ASN A  61      23.022  -5.456  27.354  0.00  0.00
A
ATOM    826  CB  ASN A  61      21.174  -6.690  27.757  0.00  0.00
A
ATOM    827  HB1 ASN A  61      20.184  -6.540  28.238  0.00  0.00
A
ATOM    828  HB2 ASN A  61      20.989  -6.725  26.662  0.00  0.00
A
ATOM    829  CG  ASN A  61      21.837  -8.033  28.139  0.00  0.00
A
ATOM    830  OD1 ASN A  61      23.014  -8.223  28.155  0.00  0.00
A
ATOM    831  ND2 ASN A  61      20.956  -9.017  28.583  0.00  0.00
A
ATOM    832 HD21 ASN A  61      21.460  -9.779  28.990  0.00  0.00
A
ATOM    833 HD22 ASN A  61      20.019  -8.765  28.824  0.00  0.00
A
ATOM    834  C   ASN A  61      21.374  -4.157  27.699  0.00  0.00
A
ATOM    835  O   ASN A  61      21.353  -3.745  26.550  0.00  0.00
A
ATOM    836  N   GLU A  62      20.836  -3.540  28.765  0.00  0.00
A
```

ATOM 837 HN GLU A 62 21.002 -3.785 29.717 0.00 0.00
A
ATOM 838 CA GLU A 62 20.002 -2.353 28.570 0.00 0.00
A
ATOM 839 HA GLU A 62 19.445 -2.634 27.688 0.00 0.00
A
ATOM 840 CB GLU A 62 18.904 -2.094 29.643 0.00 0.00
A
ATOM 841 HB1 GLU A 62 19.372 -2.122 30.651 0.00 0.00
A
ATOM 842 HB2 GLU A 62 18.313 -1.194 29.369 0.00 0.00
A
ATOM 843 CG GLU A 62 17.862 -3.271 29.620 0.00 0.00
A
ATOM 844 HG1 GLU A 62 17.412 -3.207 28.606 0.00 0.00
A
ATOM 845 HG2 GLU A 62 18.238 -4.316 29.648 0.00 0.00
A
ATOM 846 CD GLU A 62 16.579 -3.165 30.463 0.00 0.00
A
ATOM 847 OE1 GLU A 62 16.144 -4.196 31.107 0.00 0.00
A
ATOM 848 OE2 GLU A 62 16.044 -2.024 30.520 0.00 0.00
A
ATOM 849 C GLU A 62 20.923 -1.123 28.523 0.00 0.00
A
ATOM 850 O GLU A 62 20.887 -0.373 27.510 0.00 0.00
A
ATOM 851 N LEU A 63 21.840 -1.034 29.488 0.00 0.00
A
ATOM 852 HN LEU A 63 21.781 -1.631 30.284 0.00 0.00
A
ATOM 853 CA LEU A 63 22.693 0.185 29.544 0.00 0.00
A
ATOM 854 HA LEU A 63 22.002 1.012 29.474 0.00 0.00
A
ATOM 855 CB LEU A 63 23.332 0.193 30.955 0.00 0.00
A
ATOM 856 HB1 LEU A 63 22.602 -0.148 31.720 0.00 0.00
A
ATOM 857 HB2 LEU A 63 24.109 -0.597 30.867 0.00 0.00
A
ATOM 858 CG LEU A 63 24.122 1.451 31.359 0.00 0.00
A
ATOM 859 HG LEU A 63 24.831 1.757 30.560 0.00 0.00
A
ATOM 860 CD1 LEU A 63 23.322 2.720 31.558 0.00 0.00
A
ATOM 861 HD11 LEU A 63 22.882 2.673 32.577 0.00 0.00
A
ATOM 862 HD12 LEU A 63 23.972 3.618 31.486 0.00 0.00
A
ATOM 863 HD13 LEU A 63 22.462 2.669 30.857 0.00 0.00
A

```
ATOM    864  CD2 LEU A  63      24.897   1.177  32.695  0.00  0.00
A
ATOM    865 HD21 LEU A  63      24.161   0.915  33.486  0.00  0.00
A
ATOM    866 HD22 LEU A  63      25.684   0.395  32.654  0.00  0.00
A
ATOM    867 HD23 LEU A  63      25.406   2.122  32.985  0.00  0.00
A
ATOM    868  C   LEU A  63      23.771   0.334  28.421  0.00  0.00
A
ATOM    869  O   LEU A  63      23.975   1.432  27.893  0.00  0.00
A
ATOM    870  N   LEU A  64      24.382  -0.777  28.114  0.00  0.00
A
ATOM    871  HN  LEU A  64      23.994  -1.605  28.511  0.00  0.00
A
ATOM    872  CA  LEU A  64      25.410  -0.867  27.109  0.00  0.00
A
ATOM    873  HA  LEU A  64      26.276  -0.284  27.383  0.00  0.00
A
ATOM    874  CB  LEU A  64      25.936  -2.319  27.028  0.00  0.00
A
ATOM    875  HB1 LEU A  64      26.247  -2.717  28.017  0.00  0.00
A
ATOM    876  HB2 LEU A  64      25.031  -2.930  26.822  0.00  0.00
A
ATOM    877  CG  LEU A  64      27.048  -2.683  26.014  0.00  0.00
A
ATOM    878  HG  LEU A  64      26.886  -2.330  24.973  0.00  0.00
A
ATOM    879  CD1 LEU A  64      28.327  -2.077  26.456  0.00  0.00
A
ATOM    880 HD11 LEU A  64      29.162  -2.270  25.748  0.00  0.00
A
ATOM    881 HD12 LEU A  64      28.427  -0.985  26.638  0.00  0.00
A
ATOM    882 HD13 LEU A  64      28.575  -2.471  27.464  0.00  0.00
A
ATOM    883  CD2 LEU A  64      27.215  -4.164  25.928  0.00  0.00
A
ATOM    884 HD21 LEU A  64      27.417  -4.681  26.891  0.00  0.00
A
ATOM    885 HD22 LEU A  64      26.270  -4.698  25.694  0.00  0.00
A
ATOM    886 HD23 LEU A  64      27.984  -4.385  25.158  0.00  0.00
A
ATOM    887  C   LEU A  64      25.010  -0.424  25.731  0.00  0.00
A
ATOM    888  O   LEU A  64      25.747   0.096  24.901  0.00  0.00
A
ATOM    889  N   SER A  65      23.758  -0.669  25.334  0.00  0.00
A
ATOM    890  HN  SER A  65      23.182  -1.152  25.989  0.00  0.00
A
```

ATOM 891 CA SER A 65 23.278 −0.581 23.982 0.00 0.00
A
ATOM 892 HA SER A 65 24.039 −1.018 23.353 0.00 0.00
A
ATOM 893 CB SER A 65 21.997 −1.331 23.790 0.00 0.00
A
ATOM 894 HB1 SER A 65 21.184 −0.955 24.446 0.00 0.00
A
ATOM 895 HB2 SER A 65 21.760 −1.242 22.708 0.00 0.00
A
ATOM 896 OG SER A 65 22.310 −2.682 24.113 0.00 0.00
A
ATOM 897 HG1 SER A 65 21.652 −2.984 24.744 0.00 0.00
A
ATOM 898 C SER A 65 22.850 0.805 23.633 0.00 0.00
A
ATOM 899 O SER A 65 22.203 0.894 22.598 0.00 0.00
A
ATOM 900 N LEU A 66 23.145 1.794 24.435 0.00 0.00
A
ATOM 901 HN LEU A 66 23.471 1.562 25.348 0.00 0.00
A
ATOM 902 CA LEU A 66 22.742 3.124 24.192 0.00 0.00
A
ATOM 903 HA LEU A 66 22.050 3.195 23.365 0.00 0.00
A
ATOM 904 CB LEU A 66 21.855 3.550 25.337 0.00 0.00
A
ATOM 905 HB1 LEU A 66 22.511 3.570 26.234 0.00 0.00
A
ATOM 906 HB2 LEU A 66 21.468 4.577 25.164 0.00 0.00
A
ATOM 907 CG LEU A 66 20.689 2.626 25.738 0.00 0.00
A
ATOM 908 HG LEU A 66 21.052 1.593 25.927 0.00 0.00
A
ATOM 909 CD1 LEU A 66 20.012 3.116 27.017 0.00 0.00
A
ATOM 910 HD11 LEU A 66 19.096 2.575 27.338 0.00 0.00
A
ATOM 911 HD12 LEU A 66 20.695 3.093 27.893 0.00 0.00
A
ATOM 912 HD13 LEU A 66 19.598 4.140 26.897 0.00 0.00
A
ATOM 913 CD2 LEU A 66 19.592 2.460 24.643 0.00 0.00
A
ATOM 914 HD21 LEU A 66 18.789 1.948 25.215 0.00 0.00
A
ATOM 915 HD22 LEU A 66 19.254 3.432 24.226 0.00 0.00
A
ATOM 916 HD23 LEU A 66 19.988 1.800 23.841 0.00 0.00
A
ATOM 917 C LEU A 66 23.984 4.009 24.032 0.00 0.00
A

ATOM 918 O LEU A 66 23.802 5.209 24.238 0.00 0.00
A
ATOM 919 N GLY A 67 25.151 3.401 23.773 0.00 0.00
A
ATOM 920 HN GLY A 67 25.280 2.422 23.638 0.00 0.00
A
ATOM 921 CA GLY A 67 26.397 4.216 23.659 0.00 0.00
A
ATOM 922 HA1 GLY A 67 25.979 5.149 23.310 0.00 0.00
A
ATOM 923 HA2 GLY A 67 27.119 3.653 23.087 0.00 0.00
A
ATOM 924 C GLY A 67 27.024 4.597 24.992 0.00 0.00
A
ATOM 925 O GLY A 67 28.027 5.328 24.931 0.00 0.00
A
ATOM 926 N ILE A 68 26.625 4.103 26.187 0.00 0.00
A
ATOM 927 HN ILE A 68 26.022 3.309 26.193 0.00 0.00
A
ATOM 928 CA ILE A 68 27.123 4.564 27.478 0.00 0.00
A
ATOM 929 HA ILE A 68 27.005 5.633 27.577 0.00 0.00
A
ATOM 930 CB ILE A 68 26.087 3.886 28.558 0.00 0.00
A
ATOM 931 HB ILE A 68 26.047 2.786 28.409 0.00 0.00
A
ATOM 932 CG2 ILE A 68 26.671 4.229 29.908 0.00 0.00
A
ATOM 933 HG21 ILE A 68 27.663 3.754 30.065 0.00 0.00
A
ATOM 934 HG22 ILE A 68 26.776 5.325 29.761 0.00 0.00
A
ATOM 935 HG23 ILE A 68 25.995 3.984 30.756 0.00 0.00
A
ATOM 936 CG1 ILE A 68 24.664 4.457 28.396 0.00 0.00
A
ATOM 937 HG11 ILE A 68 24.277 4.537 27.357 0.00 0.00
A
ATOM 938 HG12 ILE A 68 24.003 3.736 28.922 0.00 0.00
A
ATOM 939 CD ILE A 68 24.393 5.941 28.926 0.00 0.00
A
ATOM 940 HD1 ILE A 68 24.380 6.132 30.021 0.00 0.00
A
ATOM 941 HD2 ILE A 68 25.028 6.623 28.322 0.00 0.00
A
ATOM 942 HD3 ILE A 68 23.367 6.166 28.562 0.00 0.00
A
ATOM 943 C ILE A 68 28.603 4.323 27.782 0.00 0.00
A
ATOM 944 O ILE A 68 29.196 5.143 28.368 0.00 0.00
A

ATOM	945	N	ARG	A	69	29.176	3.284	27.223	0.00	0.00
A										
ATOM	946	HN	ARG	A	69	28.710	2.607	26.658	0.00	0.00
A										
ATOM	947	CA	ARG	A	69	30.610	2.969	27.480	0.00	0.00
A										
ATOM	948	HA	ARG	A	69	30.869	3.122	28.517	0.00	0.00
A										
ATOM	949	CB	ARG	A	69	31.009	1.525	26.965	0.00	0.00
A										
ATOM	950	HB1	ARG	A	69	30.394	0.711	27.406	0.00	0.00
A										
ATOM	951	HB2	ARG	A	69	30.795	1.488	25.876	0.00	0.00
A										
ATOM	952	CG	ARG	A	69	32.452	1.005	27.198	0.00	0.00
A										
ATOM	953	HG1	ARG	A	69	33.190	1.606	26.624	0.00	0.00
A										
ATOM	954	HG2	ARG	A	69	32.783	1.084	28.255	0.00	0.00
A										
ATOM	955	CD	ARG	A	69	32.525	−0.553	26.873	0.00	0.00
A										
ATOM	956	HD1	ARG	A	69	33.511	−1.060	26.947	0.00	0.00
A										
ATOM	957	HD2	ARG	A	69	31.740	−1.108	27.429	0.00	0.00
A										
ATOM	958	NE	ARG	A	69	32.128	−0.607	25.443	0.00	0.00
A										
ATOM	959	HE	ARG	A	69	32.357	0.133	24.809	0.00	0.00
A										
ATOM	960	CZ	ARG	A	69	31.891	−1.759	24.819	0.00	0.00
A										
ATOM	961	NH1	ARG	A	69	31.655	−2.830	25.531	0.00	0.00
A										
ATOM	962	HH11	ARG	A	69	31.598	−3.683	25.012	0.00	0.00
A										
ATOM	963	HH12	ARG	A	69	31.608	−2.880	26.529	0.00	0.00
A										
ATOM	964	NH2	ARG	A	69	31.863	−1.852	23.476	0.00	0.00
A										
ATOM	965	HH21	ARG	A	69	31.758	−2.741	23.030	0.00	0.00
A										
ATOM	966	HH22	ARG	A	69	32.099	−1.119	22.837	0.00	0.00
A										
ATOM	967	C	ARG	A	69	31.533	3.945	26.772	0.00	0.00
A										
ATOM	968	O	ARG	A	69	32.688	4.036	27.061	0.00	0.00
A										
ATOM	969	N	ASN	A	70	31.008	4.731	25.821	0.00	0.00
A										
ATOM	970	HN	ASN	A	70	30.089	4.549	25.481	0.00	0.00
A										
ATOM	971	CA	ASN	A	70	31.743	5.794	25.094	0.00	0.00
A										

ATOM 972 HA ASN A 70 32.746 5.397 25.056 0.00 0.00
A
ATOM 973 CB ASN A 70 31.167 6.178 23.748 0.00 0.00
A
ATOM 974 HB1 ASN A 70 30.122 6.540 23.854 0.00 0.00
A
ATOM 975 HB2 ASN A 70 31.837 6.904 23.242 0.00 0.00
A
ATOM 976 CG ASN A 70 31.171 4.876 23.006 0.00 0.00
A
ATOM 977 OD1 ASN A 70 32.219 4.247 22.748 0.00 0.00
A
ATOM 978 ND2 ASN A 70 29.938 4.389 22.771 0.00 0.00
A
ATOM 979 HD21 ASN A 70 29.766 3.598 22.185 0.00 0.00
A
ATOM 980 HD22 ASN A 70 29.108 4.908 22.974 0.00 0.00
A
ATOM 981 C ASN A 70 31.810 7.147 25.881 0.00 0.00
A
ATOM 982 O ASN A 70 32.536 8.074 25.402 0.00 0.00
A
ATOM 983 N PHE A 71 31.004 7.245 26.895 0.00 0.00
A
ATOM 984 HN PHE A 71 30.453 6.529 27.316 0.00 0.00
A
ATOM 985 CA PHE A 71 30.885 8.493 27.606 0.00 0.00
A
ATOM 986 HA PHE A 71 30.974 9.315 26.911 0.00 0.00
A
ATOM 987 CB PHE A 71 29.527 8.605 28.341 0.00 0.00
A
ATOM 988 HB1 PHE A 71 29.261 7.589 28.704 0.00 0.00
A
ATOM 989 HB2 PHE A 71 29.588 9.420 29.093 0.00 0.00
A
ATOM 990 CG PHE A 71 28.582 9.139 27.219 0.00 0.00
A
ATOM 991 CD1 PHE A 71 27.819 8.203 26.446 0.00 0.00
A
ATOM 992 HD1 PHE A 71 28.050 7.149 26.468 0.00 0.00
A
ATOM 993 CE1 PHE A 71 26.866 8.657 25.491 0.00 0.00
A
ATOM 994 HE1 PHE A 71 26.344 7.934 24.881 0.00 0.00
A
ATOM 995 CZ PHE A 71 26.658 9.997 25.330 0.00 0.00
A
ATOM 996 HZ PHE A 71 26.022 10.405 24.559 0.00 0.00
A
ATOM 997 CD2 PHE A 71 28.220 10.478 27.027 0.00 0.00
A
ATOM 998 HD2 PHE A 71 28.667 11.178 27.716 0.00 0.00
A

ATOM 999 CE2 PHE A 71 27.316 10.930 26.102 0.00 0.00
A
ATOM 1000 HE2 PHE A 71 27.134 11.993 26.053 0.00 0.00
A
ATOM 1001 C PHE A 71 32.116 8.725 28.533 0.00 0.00
A
ATOM 1002 O PHE A 71 32.634 7.845 29.206 0.00 0.00
A
ATOM 1003 N GLU A 72 32.708 9.966 28.525 0.00 0.00
A
ATOM 1004 HN GLU A 72 32.346 10.806 28.129 0.00 0.00
A
ATOM 1005 CA GLU A 72 33.773 10.321 29.422 0.00 0.00
A
ATOM 1006 HA GLU A 72 34.491 9.515 29.459 0.00 0.00
A
ATOM 1007 CB GLU A 72 34.409 11.557 28.740 0.00 0.00
A
ATOM 1008 HB1 GLU A 72 34.798 11.242 27.749 0.00 0.00
A
ATOM 1009 HB2 GLU A 72 33.711 12.410 28.601 0.00 0.00
A
ATOM 1010 CG GLU A 72 35.523 12.242 29.667 0.00 0.00
A
ATOM 1011 HG1 GLU A 72 35.200 12.404 30.717 0.00 0.00
A
ATOM 1012 HG2 GLU A 72 36.357 11.509 29.622 0.00 0.00
A
ATOM 1013 CD GLU A 72 35.963 13.576 29.091 0.00 0.00
A
ATOM 1014 OE1 GLU A 72 35.118 14.504 29.208 0.00 0.00
A
ATOM 1015 OE2 GLU A 72 37.081 13.678 28.572 0.00 0.00
A
ATOM 1016 C GLU A 72 33.208 10.682 30.803 0.00 0.00
A
ATOM 1017 O GLU A 72 33.756 10.419 31.921 0.00 0.00
A
ATOM 1018 N TYR A 73 31.977 11.340 30.791 0.00 0.00
A
ATOM 1019 HN TYR A 73 31.377 11.497 30.010 0.00 0.00
A
ATOM 1020 CA TYR A 73 31.186 11.494 32.022 0.00 0.00
A
ATOM 1021 HA TYR A 73 31.716 11.159 32.902 0.00 0.00
A
ATOM 1022 CB TYR A 73 30.776 12.997 32.090 0.00 0.00
A
ATOM 1023 HB1 TYR A 73 30.595 13.397 31.069 0.00 0.00
A
ATOM 1024 HB2 TYR A 73 29.821 13.282 32.582 0.00 0.00
A
ATOM 1025 CG TYR A 73 31.903 13.866 32.672 0.00 0.00
A

```
ATOM   1026  CD1 TYR A  73      33.082  14.187  31.995  0.00  0.00
A
ATOM   1027  HD1 TYR A  73      33.093  13.926  30.948  0.00  0.00
A
ATOM   1028  CE1 TYR A  73      34.074  15.000  32.493  0.00  0.00
A
ATOM   1029  HE1 TYR A  73      34.873  15.390  31.880  0.00  0.00
A
ATOM   1030  CZ  TYR A  73      33.922  15.411  33.850  0.00  0.00
A
ATOM   1031  OH  TYR A  73      34.871  16.229  34.593  0.00  0.00
A
ATOM   1032  HH  TYR A  73      34.373  16.756  35.221  0.00  0.00
A
ATOM   1033  CD2 TYR A  73      31.757  14.372  33.999  0.00  0.00
A
ATOM   1034  HD2 TYR A  73      30.812  14.252  34.508  0.00  0.00
A
ATOM   1035  CE2 TYR A  73      32.796  15.017  34.599  0.00  0.00
A
ATOM   1036  HE2 TYR A  73      32.617  15.431  35.580  0.00  0.00
A
ATOM   1037  C   TYR A  73      29.914  10.804  31.913  0.00  0.00
A
ATOM   1038  O   TYR A  73      29.261  10.784  30.833  0.00  0.00
A
ATOM   1039  N   VAL A  74      29.566  10.055  32.935  0.00  0.00
A
ATOM   1040  HN  VAL A  74      30.186  10.022  33.715  0.00  0.00
A
ATOM   1041  CA  VAL A  74      28.253   9.540  33.102  0.00  0.00
A
ATOM   1042  HA  VAL A  74      27.592   9.945  32.350  0.00  0.00
A
ATOM   1043  CB  VAL A  74      28.195   8.007  33.127  0.00  0.00
A
ATOM   1044  HB  VAL A  74      28.984   7.622  33.808  0.00  0.00
A
ATOM   1045  CG1 VAL A  74      26.833   7.580  33.580  0.00  0.00
A
ATOM   1046 HG11 VAL A  74      26.052   8.293  33.239  0.00  0.00
A
ATOM   1047 HG12 VAL A  74      26.440   6.552  33.428  0.00  0.00
A
ATOM   1048 HG13 VAL A  74      26.824   7.510  34.689  0.00  0.00
A
ATOM   1049  CG2 VAL A  74      28.439   7.566  31.643  0.00  0.00
A
ATOM   1050 HG21 VAL A  74      29.366   8.041  31.258  0.00  0.00
A
ATOM   1051 HG22 VAL A  74      28.662   6.480  31.572  0.00  0.00
A
ATOM   1052 HG23 VAL A  74      27.632   8.001  31.016  0.00  0.00
A
```

ATOM 1053 C VAL A 74 27.887 10.181 34.454 0.00 0.00
A
ATOM 1054 O VAL A 74 28.692 10.256 35.421 0.00 0.00
A
ATOM 1055 N ILE A 75 26.587 10.636 34.604 0.00 0.00
A
ATOM 1056 HN ILE A 75 25.994 10.654 33.803 0.00 0.00
A
ATOM 1057 CA ILE A 75 26.056 11.173 35.841 0.00 0.00
A
ATOM 1058 HA ILE A 75 26.658 10.852 36.677 0.00 0.00
A
ATOM 1059 CB ILE A 75 25.839 12.668 35.849 0.00 0.00
A
ATOM 1060 HB ILE A 75 26.810 13.089 35.508 0.00 0.00
A
ATOM 1061 CG2 ILE A 75 24.644 13.065 34.872 0.00 0.00
A
ATOM 1062 HG21 ILE A 75 23.660 12.839 35.335 0.00 0.00
A
ATOM 1063 HG22 ILE A 75 24.582 14.134 34.578 0.00 0.00
A
ATOM 1064 HG23 ILE A 75 24.671 12.459 33.942 0.00 0.00
A
ATOM 1065 CG1 ILE A 75 25.549 13.247 37.248 0.00 0.00
A
ATOM 1066 HG11 ILE A 75 24.452 13.122 37.371 0.00 0.00
A
ATOM 1067 HG12 ILE A 75 26.028 12.520 37.938 0.00 0.00
A
ATOM 1068 CD ILE A 75 25.961 14.706 37.392 0.00 0.00
A
ATOM 1069 HD1 ILE A 75 25.842 15.113 38.419 0.00 0.00
A
ATOM 1070 HD2 ILE A 75 27.024 14.743 37.071 0.00 0.00
A
ATOM 1071 HD3 ILE A 75 25.287 15.329 36.766 0.00 0.00
A
ATOM 1072 C ILE A 75 24.772 10.426 36.274 0.00 0.00
A
ATOM 1073 O ILE A 75 23.889 10.283 35.480 0.00 0.00
A
ATOM 1074 N VAL A 76 24.709 9.897 37.498 0.00 0.00
A
ATOM 1075 HN VAL A 76 25.442 9.795 38.166 0.00 0.00
A
ATOM 1076 CA VAL A 76 23.632 9.183 38.117 0.00 0.00
A
ATOM 1077 HA VAL A 76 22.937 8.908 37.337 0.00 0.00
A
ATOM 1078 CB VAL A 76 24.121 7.944 38.861 0.00 0.00
A
ATOM 1079 HB VAL A 76 24.760 8.313 39.692 0.00 0.00
A

```
ATOM   1080  CG1 VAL A  76      22.952   7.190  39.487  0.00  0.00
A
ATOM   1081 HG11 VAL A  76      22.608   7.661  40.433  0.00  0.00
A
ATOM   1082 HG12 VAL A  76      22.131   6.957  38.776  0.00  0.00
A
ATOM   1083 HG13 VAL A  76      23.376   6.205  39.777  0.00  0.00
A
ATOM   1084  CG2 VAL A  76      24.988   7.172  37.843  0.00  0.00
A
ATOM   1085 HG21 VAL A  76      24.613   7.192  36.797  0.00  0.00
A
ATOM   1086 HG22 VAL A  76      26.019   7.585  37.872  0.00  0.00
A
ATOM   1087 HG23 VAL A  76      25.031   6.087  38.076  0.00  0.00
A
ATOM   1088  C   VAL A  76      22.883  10.160  39.036  0.00  0.00
A
ATOM   1089  O   VAL A  76      23.337  10.538  40.080  0.00  0.00
A
ATOM   1090  N   ALA A  77      21.629  10.340  38.726  0.00  0.00
A
ATOM   1091  HN  ALA A  77      21.240   9.969  37.887  0.00  0.00
A
ATOM   1092  CA  ALA A  77      20.742  11.203  39.498  0.00  0.00
A
ATOM   1093  HA  ALA A  77      21.343  11.799  40.169  0.00  0.00
A
ATOM   1094  CB  ALA A  77      20.140  12.103  38.452  0.00  0.00
A
ATOM   1095  HB1 ALA A  77      19.622  11.486  37.688  0.00  0.00
A
ATOM   1096  HB2 ALA A  77      19.487  12.876  38.911  0.00  0.00
A
ATOM   1097  HB3 ALA A  77      20.956  12.454  37.784  0.00  0.00
A
ATOM   1098  C   ALA A  77      19.696  10.468  40.341  0.00  0.00
A
ATOM   1099  O   ALA A  77      18.685  10.994  40.890  0.00  0.00
A
ATOM   1100  N   ILE A  78      19.944   9.119  40.349  0.00  0.00
A
ATOM   1101  HN  ILE A  78      20.528   8.762  39.624  0.00  0.00
A
ATOM   1102  CA  ILE A  78      19.244   8.145  41.167  0.00  0.00
A
ATOM   1103  HA  ILE A  78      18.179   8.323  41.193  0.00  0.00
A
ATOM   1104  CB  ILE A  78      19.510   6.800  40.521  0.00  0.00
A
ATOM   1105  HB  ILE A  78      20.549   6.440  40.676  0.00  0.00
A
ATOM   1106  CG2 ILE A  78      18.648   5.754  41.346  0.00  0.00
A
```

ATOM 1107 HG21 ILE A 78 17.668 6.230 41.563 0.00 0.00
A
ATOM 1108 HG22 ILE A 78 18.644 4.688 41.032 0.00 0.00
A
ATOM 1109 HG23 ILE A 78 19.239 5.849 42.282 0.00 0.00
A
ATOM 1110 CG1 ILE A 78 19.108 6.733 38.951 0.00 0.00
A
ATOM 1111 HG11 ILE A 78 18.184 7.303 38.715 0.00 0.00
A
ATOM 1112 HG12 ILE A 78 19.961 7.196 38.411 0.00 0.00
A
ATOM 1113 CD ILE A 78 18.941 5.287 38.455 0.00 0.00
A
ATOM 1114 HD1 ILE A 78 18.027 4.793 38.847 0.00 0.00
A
ATOM 1115 HD2 ILE A 78 18.863 5.406 37.353 0.00 0.00
A
ATOM 1116 HD3 ILE A 78 19.825 4.627 38.583 0.00 0.00
A
ATOM 1117 C ILE A 78 19.860 8.145 42.498 0.00 0.00
A
ATOM 1118 O ILE A 78 21.061 7.890 42.584 0.00 0.00
A
ATOM 1119 N GLY A 79 19.029 8.356 43.522 0.00 0.00
A
ATOM 1120 HN GLY A 79 18.113 8.690 43.316 0.00 0.00
A
ATOM 1121 CA GLY A 79 19.425 8.250 44.924 0.00 0.00
A
ATOM 1122 HA1 GLY A 79 19.983 9.132 45.204 0.00 0.00
A
ATOM 1123 HA2 GLY A 79 19.978 7.338 45.096 0.00 0.00
A
ATOM 1124 C GLY A 79 18.229 8.209 45.843 0.00 0.00
A
ATOM 1125 O GLY A 79 18.340 7.864 46.997 0.00 0.00
A
ATOM 1126 N ALA A 80 17.002 8.545 45.443 0.00 0.00
A
ATOM 1127 HN ALA A 80 16.756 8.827 44.519 0.00 0.00
A
ATOM 1128 CA ALA A 80 15.793 8.186 46.159 0.00 0.00
A
ATOM 1129 HA ALA A 80 15.803 8.580 47.165 0.00 0.00
A
ATOM 1130 CB ALA A 80 14.519 8.576 45.545 0.00 0.00
A
ATOM 1131 HB1 ALA A 80 13.618 8.134 46.023 0.00 0.00
A
ATOM 1132 HB2 ALA A 80 14.364 9.673 45.624 0.00 0.00
A
ATOM 1133 HB3 ALA A 80 14.546 8.268 44.478 0.00 0.00
A

ATOM 1134 C ALA A 80 15.674 6.594 46.238 0.00 0.00
A
ATOM 1135 O ALA A 80 15.225 5.990 47.247 0.00 0.00
A
ATOM 1136 N ASN A 81 15.941 5.926 45.122 0.00 0.00
A
ATOM 1137 HN ASN A 81 16.056 6.477 44.299 0.00 0.00
A
ATOM 1138 CA ASN A 81 16.314 4.513 45.049 0.00 0.00
A
ATOM 1139 HA ASN A 81 15.751 4.039 45.840 0.00 0.00
A
ATOM 1140 CB ASN A 81 15.748 3.973 43.774 0.00 0.00
A
ATOM 1141 HB1 ASN A 81 14.783 4.475 43.548 0.00 0.00
A
ATOM 1142 HB2 ASN A 81 16.432 4.107 42.908 0.00 0.00
A
ATOM 1143 CG ASN A 81 15.360 2.426 44.050 0.00 0.00
A
ATOM 1144 OD1 ASN A 81 16.174 1.715 44.603 0.00 0.00
A
ATOM 1145 ND2 ASN A 81 14.191 2.028 43.661 0.00 0.00
A
ATOM 1146 HD21 ASN A 81 13.925 1.073 43.531 0.00 0.00
A
ATOM 1147 HD22 ASN A 81 13.611 2.731 43.248 0.00 0.00
A
ATOM 1148 C ASN A 81 17.795 4.212 45.338 0.00 0.00
A
ATOM 1149 O ASN A 81 18.635 4.256 44.420 0.00 0.00
A
ATOM 1150 N ILE A 82 18.149 3.827 46.573 0.00 0.00
A
ATOM 1151 HN ILE A 82 17.406 3.601 47.199 0.00 0.00
A
ATOM 1152 CA ILE A 82 19.553 3.448 46.891 0.00 0.00
A
ATOM 1153 HA ILE A 82 20.116 4.211 46.374 0.00 0.00
A
ATOM 1154 CB ILE A 82 19.747 3.380 48.434 0.00 0.00
A
ATOM 1155 HB ILE A 82 19.131 2.520 48.773 0.00 0.00
A
ATOM 1156 CG2 ILE A 82 21.235 3.143 48.778 0.00 0.00
A
ATOM 1157 HG21 ILE A 82 21.535 2.205 48.264 0.00 0.00
A
ATOM 1158 HG22 ILE A 82 21.891 3.998 48.507 0.00 0.00
A
ATOM 1159 HG23 ILE A 82 21.335 2.995 49.874 0.00 0.00
A
ATOM 1160 CG1 ILE A 82 19.154 4.615 49.143 0.00 0.00
A

ATOM 1161 HG11 ILE A 82 19.660 5.514 48.732 0.00 0.00
A
ATOM 1162 HG12 ILE A 82 18.079 4.600 48.862 0.00 0.00
A
ATOM 1163 CD ILE A 82 19.284 4.747 50.681 0.00 0.00
A
ATOM 1164 HD1 ILE A 82 18.914 5.725 51.056 0.00 0.00
A
ATOM 1165 HD2 ILE A 82 18.556 4.058 51.161 0.00 0.00
A
ATOM 1166 HD3 ILE A 82 20.316 4.504 51.016 0.00 0.00
A
ATOM 1167 C ILE A 82 19.975 2.182 46.156 0.00 0.00
A
ATOM 1168 O ILE A 82 21.040 2.048 45.592 0.00 0.00
A
ATOM 1169 N GLN A 83 19.093 1.176 46.288 0.00 0.00
A
ATOM 1170 HN GLN A 83 18.261 1.384 46.795 0.00 0.00
A
ATOM 1171 CA GLN A 83 19.207 -0.145 45.613 0.00 0.00
A
ATOM 1172 HA GLN A 83 20.074 -0.661 45.999 0.00 0.00
A
ATOM 1173 CB GLN A 83 17.919 -1.040 45.851 0.00 0.00
A
ATOM 1174 HB1 GLN A 83 17.014 -0.491 45.515 0.00 0.00
A
ATOM 1175 HB2 GLN A 83 17.937 -1.995 45.284 0.00 0.00
A
ATOM 1176 CG GLN A 83 17.691 -1.240 47.370 0.00 0.00
A
ATOM 1177 HG1 GLN A 83 18.297 -2.148 47.577 0.00 0.00
A
ATOM 1178 HG2 GLN A 83 18.031 -0.395 48.007 0.00 0.00
A
ATOM 1179 CD GLN A 83 16.285 -1.642 47.731 0.00 0.00
A
ATOM 1180 OE1 GLN A 83 16.037 -2.755 48.237 0.00 0.00
A
ATOM 1181 NE2 GLN A 83 15.353 -0.737 47.532 0.00 0.00
A
ATOM 1182 HE21 GLN A 83 14.443 -1.150 47.502 0.00 0.00
A
ATOM 1183 HE22 GLN A 83 15.591 0.025 46.930 0.00 0.00
A
ATOM 1184 C GLN A 83 19.470 -0.166 44.099 0.00 0.00
A
ATOM 1185 O GLN A 83 20.317 -0.911 43.635 0.00 0.00
A
ATOM 1186 N ALA A 84 18.644 0.672 43.409 0.00 0.00
A
ATOM 1187 HN ALA A 84 17.911 1.149 43.886 0.00 0.00
A

ATOM 1188 CA ALA A 84 18.908 0.905 42.009 0.00 0.00
A
ATOM 1189 HA ALA A 84 18.993 -0.078 41.569 0.00 0.00
A
ATOM 1190 CB ALA A 84 17.695 1.698 41.545 0.00 0.00
A
ATOM 1191 HB1 ALA A 84 16.807 1.059 41.744 0.00 0.00
A
ATOM 1192 HB2 ALA A 84 17.582 2.716 41.975 0.00 0.00
A
ATOM 1193 HB3 ALA A 84 17.681 1.784 40.437 0.00 0.00
A
ATOM 1194 C ALA A 84 20.198 1.630 41.724 0.00 0.00
A
ATOM 1195 O ALA A 84 20.923 1.346 40.734 0.00 0.00
A
ATOM 1196 N SER A 85 20.524 2.658 42.519 0.00 0.00
A
ATOM 1197 HN SER A 85 19.943 2.918 43.287 0.00 0.00
A
ATOM 1198 CA SER A 85 21.697 3.566 42.426 0.00 0.00
A
ATOM 1199 HA SER A 85 21.690 4.026 41.449 0.00 0.00
A
ATOM 1200 CB SER A 85 21.610 4.658 43.512 0.00 0.00
A
ATOM 1201 HB1 SER A 85 20.605 5.129 43.474 0.00 0.00
A
ATOM 1202 HB2 SER A 85 21.731 4.059 44.440 0.00 0.00
A
ATOM 1203 OG SER A 85 22.657 5.689 43.469 0.00 0.00
A
ATOM 1204 HG1 SER A 85 22.363 6.530 43.112 0.00 0.00
A
ATOM 1205 C SER A 85 23.073 2.918 42.593 0.00 0.00
A
ATOM 1206 O SER A 85 24.042 3.264 41.890 0.00 0.00
A
ATOM 1207 N THR A 86 23.216 2.027 43.561 0.00 0.00
A
ATOM 1208 HN THR A 86 22.479 1.869 44.213 0.00 0.00
A
ATOM 1209 CA THR A 86 24.518 1.450 43.940 0.00 0.00
A
ATOM 1210 HA THR A 86 25.292 2.203 43.970 0.00 0.00
A
ATOM 1211 CB THR A 86 24.551 0.862 45.363 0.00 0.00
A
ATOM 1212 HB THR A 86 25.439 0.216 45.532 0.00 0.00
A
ATOM 1213 OG1 THR A 86 23.347 0.161 45.710 0.00 0.00
A
ATOM 1214 HG1 THR A 86 23.189 0.388 46.629 0.00 0.00
A

```
ATOM   1215  CG2 THR A  86      24.630   1.972  46.354  0.00  0.00
A
ATOM   1216 HG21 THR A  86      24.771   1.479  47.339  0.00  0.00
A
ATOM   1217 HG22 THR A  86      25.480   2.662  46.166  0.00  0.00
A
ATOM   1218 HG23 THR A  86      23.671   2.533  46.352  0.00  0.00
A
ATOM   1219  C   THR A  86      24.955   0.378  42.912  0.00  0.00
A
ATOM   1220  O   THR A  86      26.116   0.206  42.574  0.00  0.00
A
ATOM   1221  N   LEU A  87      23.982  -0.321  42.345  0.00  0.00
A
ATOM   1222  HN  LEU A  87      23.052  -0.173  42.671  0.00  0.00
A
ATOM   1223  CA  LEU A  87      24.061  -1.331  41.248  0.00  0.00
A
ATOM   1224  HA  LEU A  87      24.870  -1.974  41.563  0.00  0.00
A
ATOM   1225  CB  LEU A  87      22.794  -2.232  41.298  0.00  0.00
A
ATOM   1226  HB1 LEU A  87      22.769  -2.709  42.301  0.00  0.00
A
ATOM   1227  HB2 LEU A  87      21.872  -1.620  41.198  0.00  0.00
A
ATOM   1228  CG  LEU A  87      22.711  -3.371  40.239  0.00  0.00
A
ATOM   1229  HG  LEU A  87      22.922  -2.956  39.230  0.00  0.00
A
ATOM   1230  CD1 LEU A  87      23.796  -4.522  40.404  0.00  0.00
A
ATOM   1231 HD11 LEU A  87      23.490  -5.268  39.640  0.00  0.00
A
ATOM   1232 HD12 LEU A  87      24.827  -4.238  40.103  0.00  0.00
A
ATOM   1233 HD13 LEU A  87      23.899  -4.945  41.427  0.00  0.00
A
ATOM   1234  CD2 LEU A  87      21.284  -3.999  40.361  0.00  0.00
A
ATOM   1235 HD21 LEU A  87      21.198  -4.307  41.425  0.00  0.00
A
ATOM   1236 HD22 LEU A  87      20.392  -3.349  40.228  0.00  0.00
A
ATOM   1237 HD23 LEU A  87      21.264  -4.880  39.685  0.00  0.00
A
ATOM   1238  C   LEU A  87      24.362  -0.662  39.938  0.00  0.00
A
ATOM   1239  O   LEU A  87      25.263  -1.073  39.157  0.00  0.00
A
ATOM   1240  N   THR A  88      23.703   0.556  39.719  0.00  0.00
A
ATOM   1241  HN  THR A  88      22.919   0.731  40.309  0.00  0.00
A
```

ATOM 1242 CA THR A 88 23.906 1.412 38.558 0.00 0.00
A
ATOM 1243 HA THR A 88 23.828 0.940 37.590 0.00 0.00
A
ATOM 1244 CB THR A 88 23.012 2.682 38.598 0.00 0.00
A
ATOM 1245 HB THR A 88 23.189 3.289 39.512 0.00 0.00
A
ATOM 1246 OG1 THR A 88 21.674 2.326 38.393 0.00 0.00
A
ATOM 1247 HG1 THR A 88 21.573 1.841 39.216 0.00 0.00
A
ATOM 1248 CG2 THR A 88 23.321 3.601 37.411 0.00 0.00
A
ATOM 1249 HG21 THR A 88 24.254 4.204 37.404 0.00 0.00
A
ATOM 1250 HG22 THR A 88 23.197 3.095 36.430 0.00 0.00
A
ATOM 1251 HG23 THR A 88 22.544 4.394 37.454 0.00 0.00
A
ATOM 1252 C THR A 88 25.363 1.878 38.581 0.00 0.00
A
ATOM 1253 O THR A 88 26.145 1.643 37.626 0.00 0.00
A
ATOM 1254 N THR A 89 25.754 2.344 39.812 0.00 0.00
A
ATOM 1255 HN THR A 89 25.044 2.365 40.511 0.00 0.00
A
ATOM 1256 CA THR A 89 27.108 2.746 40.055 0.00 0.00
A
ATOM 1257 HA THR A 89 27.316 3.535 39.348 0.00 0.00
A
ATOM 1258 CB THR A 89 27.309 3.389 41.500 0.00 0.00
A
ATOM 1259 HB THR A 89 27.004 2.611 42.233 0.00 0.00
A
ATOM 1260 OG1 THR A 89 26.495 4.503 41.634 0.00 0.00
A
ATOM 1261 HG1 THR A 89 25.604 4.147 41.591 0.00 0.00
A
ATOM 1262 CG2 THR A 89 28.712 3.987 41.606 0.00 0.00
A
ATOM 1263 HG21 THR A 89 29.593 3.316 41.522 0.00 0.00
A
ATOM 1264 HG22 THR A 89 28.873 4.870 40.952 0.00 0.00
A
ATOM 1265 HG23 THR A 89 28.802 4.343 42.655 0.00 0.00
A
ATOM 1266 C THR A 89 28.245 1.680 39.932 0.00 0.00
A
ATOM 1267 O THR A 89 29.350 1.946 39.336 0.00 0.00
A
ATOM 1268 N LEU A 90 27.981 0.456 40.427 0.00 0.00
A

ATOM 1269 HN LEU A 90 27.236 0.216 41.044 0.00 0.00
A
ATOM 1270 CA LEU A 90 28.835 −0.709 40.223 0.00 0.00
A
ATOM 1271 HA LEU A 90 29.825 −0.474 40.584 0.00 0.00
A
ATOM 1272 CB LEU A 90 28.116 −1.837 40.995 0.00 0.00
A
ATOM 1273 HB1 LEU A 90 27.874 −1.564 42.045 0.00 0.00
A
ATOM 1274 HB2 LEU A 90 27.165 −1.965 40.436 0.00 0.00
A
ATOM 1275 CG LEU A 90 29.104 −3.021 41.155 0.00 0.00
A
ATOM 1276 HG LEU A 90 29.351 −3.456 40.163 0.00 0.00
A
ATOM 1277 CD1 LEU A 90 30.408 −2.641 41.993 0.00 0.00
A
ATOM 1278 HD11 LEU A 90 30.086 −2.090 42.902 0.00 0.00
A
ATOM 1279 HD12 LEU A 90 31.067 −3.499 42.243 0.00 0.00
A
ATOM 1280 HD13 LEU A 90 31.123 −1.932 41.523 0.00 0.00
A
ATOM 1281 CD2 LEU A 90 28.385 −4.093 41.923 0.00 0.00
A
ATOM 1282 HD21 LEU A 90 29.017 −4.986 42.116 0.00 0.00
A
ATOM 1283 HD22 LEU A 90 27.970 −3.647 42.853 0.00 0.00
A
ATOM 1284 HD23 LEU A 90 27.559 −4.351 41.226 0.00 0.00
A
ATOM 1285 C LEU A 90 29.052 −1.097 38.737 0.00 0.00
A
ATOM 1286 O LEU A 90 30.078 −1.214 38.128 0.00 0.00
A
ATOM 1287 N LEU A 91 27.906 −1.180 37.978 0.00 0.00
A
ATOM 1288 HN LEU A 91 27.054 −1.006 38.465 0.00 0.00
A
ATOM 1289 CA LEU A 91 27.930 −1.420 36.513 0.00 0.00
A
ATOM 1290 HA LEU A 91 28.413 −2.342 36.225 0.00 0.00
A
ATOM 1291 CB LEU A 91 26.423 −1.588 36.099 0.00 0.00
A
ATOM 1292 HB1 LEU A 91 26.070 −2.383 36.790 0.00 0.00
A
ATOM 1293 HB2 LEU A 91 25.840 −0.671 36.329 0.00 0.00
A
ATOM 1294 CG LEU A 91 26.208 −2.064 34.662 0.00 0.00
A
ATOM 1295 HG LEU A 91 26.409 −1.124 34.105 0.00 0.00
A

ATOM 1296 CD1 LEU A 91 27.145 -3.163 34.169 0.00 0.00
A
ATOM 1297 HD11 LEU A 91 28.155 -2.804 33.878 0.00 0.00
A
ATOM 1298 HD12 LEU A 91 27.154 -3.842 35.048 0.00 0.00
A
ATOM 1299 HD13 LEU A 91 26.679 -3.595 33.258 0.00 0.00
A
ATOM 1300 CD2 LEU A 91 24.806 -2.450 34.556 0.00 0.00
A
ATOM 1301 HD21 LEU A 91 24.601 -3.423 35.052 0.00 0.00
A
ATOM 1302 HD22 LEU A 91 24.059 -1.688 34.864 0.00 0.00
A
ATOM 1303 HD23 LEU A 91 24.568 -2.617 33.483 0.00 0.00
A
ATOM 1304 C LEU A 91 28.616 -0.393 35.667 0.00 0.00
A
ATOM 1305 O LEU A 91 29.417 -0.870 34.791 0.00 0.00
A
ATOM 1306 N LEU A 92 28.470 0.940 35.907 0.00 0.00
A
ATOM 1307 HN LEU A 92 27.858 1.229 36.639 0.00 0.00
A
ATOM 1308 CA LEU A 92 29.312 1.921 35.268 0.00 0.00
A
ATOM 1309 HA LEU A 92 29.082 1.821 34.218 0.00 0.00
A
ATOM 1310 CB LEU A 92 28.863 3.335 35.751 0.00 0.00
A
ATOM 1311 HB1 LEU A 92 28.913 3.390 36.860 0.00 0.00
A
ATOM 1312 HB2 LEU A 92 29.509 4.142 35.343 0.00 0.00
A
ATOM 1313 CG LEU A 92 27.499 3.730 35.331 0.00 0.00
A
ATOM 1314 HG LEU A 92 26.728 2.953 35.522 0.00 0.00
A
ATOM 1315 CD1 LEU A 92 27.173 4.955 36.217 0.00 0.00
A
ATOM 1316 HD11 LEU A 92 26.122 5.246 36.006 0.00 0.00
A
ATOM 1317 HD12 LEU A 92 27.201 4.409 37.184 0.00 0.00
A
ATOM 1318 HD13 LEU A 92 27.950 5.739 36.092 0.00 0.00
A
ATOM 1319 CD2 LEU A 92 27.438 4.056 33.819 0.00 0.00
A
ATOM 1320 HD21 LEU A 92 26.365 4.232 33.589 0.00 0.00
A
ATOM 1321 HD22 LEU A 92 28.058 4.955 33.616 0.00 0.00
A
ATOM 1322 HD23 LEU A 92 27.880 3.243 33.203 0.00 0.00
A

```
ATOM   1323  C   LEU A  92      30.835   1.740  35.428  0.00  0.00
A
ATOM   1324  O   LEU A  92      31.665   1.865  34.524  0.00  0.00
A
ATOM   1325  N   LYS A  93      31.202   1.346  36.668  0.00  0.00
A
ATOM   1326  HN  LYS A  93      30.538   1.238  37.404  0.00  0.00
A
ATOM   1327  CA  LYS A  93      32.631   1.030  37.071  0.00  0.00
A
ATOM   1328  HA  LYS A  93      33.382   1.753  36.792  0.00  0.00
A
ATOM   1329  CB  LYS A  93      32.794   0.879  38.594  0.00  0.00
A
ATOM   1330  HB1 LYS A  93      32.324   1.717  39.151  0.00  0.00
A
ATOM   1331  HB2 LYS A  93      32.298  -0.052  38.942  0.00  0.00
A
ATOM   1332  CG  LYS A  93      34.266   0.772  39.097  0.00  0.00
A
ATOM   1333  HG1 LYS A  93      34.378   0.500  40.169  0.00  0.00
A
ATOM   1334  HG2 LYS A  93      34.724   0.014  38.426  0.00  0.00
A
ATOM   1335  CD  LYS A  93      35.063   2.089  38.960  0.00  0.00
A
ATOM   1336  HD1 LYS A  93      35.421   2.171  37.911  0.00  0.00
A
ATOM   1337  HD2 LYS A  93      34.404   2.983  38.943  0.00  0.00
A
ATOM   1338  CE  LYS A  93      36.205   2.310  39.953  0.00  0.00
A
ATOM   1339  HE1 LYS A  93      36.665   3.297  39.731  0.00  0.00
A
ATOM   1340  HE2 LYS A  93      35.934   2.226  41.028  0.00  0.00
A
ATOM   1341  NZ  LYS A  93      37.252   1.255  39.674  0.00  0.00
A
ATOM   1342  HZ1 LYS A  93      37.273   1.057  38.653  0.00  0.00
A
ATOM   1343  HZ2 LYS A  93      38.231   1.405  39.990  0.00  0.00
A
ATOM   1344  HZ3 LYS A  93      36.873   0.391  40.111  0.00  0.00
A
ATOM   1345  C   LYS A  93      33.145  -0.228  36.314  0.00  0.00
A
ATOM   1346  O   LYS A  93      34.308  -0.381  36.002  0.00  0.00
A
ATOM   1347  N   GLU A  94      32.213  -1.112  35.981  0.00  0.00
A
ATOM   1348  HN  GLU A  94      31.278  -0.912  36.264  0.00  0.00
A
ATOM   1349  CA  GLU A  94      32.461  -2.286  35.130  0.00  0.00
A
```

ATOM 1350 HA GLU A 94 33.375 -2.729 35.497 0.00 0.00
A
ATOM 1351 CB GLU A 94 31.263 -3.238 35.282 0.00 0.00
A
ATOM 1352 HB1 GLU A 94 30.929 -3.259 36.341 0.00 0.00
A
ATOM 1353 HB2 GLU A 94 30.397 -2.874 34.689 0.00 0.00
A
ATOM 1354 CG GLU A 94 31.554 -4.752 34.882 0.00 0.00
A
ATOM 1355 HG1 GLU A 94 30.643 -5.287 35.226 0.00 0.00
A
ATOM 1356 HG2 GLU A 94 31.739 -4.919 33.799 0.00 0.00
A
ATOM 1357 CD GLU A 94 32.665 -5.580 35.546 0.00 0.00
A
ATOM 1358 OE1 GLU A 94 32.876 -6.762 35.115 0.00 0.00
A
ATOM 1359 OE2 GLU A 94 33.277 -5.154 36.511 0.00 0.00
A
ATOM 1360 C GLU A 94 32.781 -1.930 33.614 0.00 0.00
A
ATOM 1361 O GLU A 94 33.674 -2.544 33.019 0.00 0.00
A
ATOM 1362 N LEU A 95 32.030 -0.877 33.173 0.00 0.00
A
ATOM 1363 HN LEU A 95 31.268 -0.473 33.673 0.00 0.00
A
ATOM 1364 CA LEU A 95 32.285 -0.304 31.830 0.00 0.00
A
ATOM 1365 HA LEU A 95 32.362 -1.081 31.084 0.00 0.00
A
ATOM 1366 CB LEU A 95 31.030 0.519 31.274 0.00 0.00
A
ATOM 1367 HB1 LEU A 95 30.899 1.462 31.846 0.00 0.00
A
ATOM 1368 HB2 LEU A 95 31.304 0.704 30.213 0.00 0.00
A
ATOM 1369 CG LEU A 95 29.667 -0.179 31.352 0.00 0.00
A
ATOM 1370 HG LEU A 95 29.421 -0.602 32.349 0.00 0.00
A
ATOM 1371 CD1 LEU A 95 28.665 0.920 31.056 0.00 0.00
A
ATOM 1372 HD11 LEU A 95 27.650 0.497 31.218 0.00 0.00
A
ATOM 1373 HD12 LEU A 95 28.778 1.871 31.618 0.00 0.00
A
ATOM 1374 HD13 LEU A 95 28.607 1.153 29.972 0.00 0.00
A
ATOM 1375 CD2 LEU A 95 29.604 -1.366 30.305 0.00 0.00
A
ATOM 1376 HD21 LEU A 95 29.585 -1.050 29.240 0.00 0.00
A

ATOM 1377 HD22 LEU A 95 30.372 -2.167 30.377 0.00 0.00
A
ATOM 1378 HD23 LEU A 95 28.667 -1.962 30.259 0.00 0.00
A
ATOM 1379 C LEU A 95 33.532 0.562 31.762 0.00 0.00
A
ATOM 1380 O LEU A 95 34.075 0.788 30.713 0.00 0.00
A
ATOM 1381 N ASP A 96 33.930 1.057 32.971 0.00 0.00
A
ATOM 1382 HN ASP A 96 33.353 0.848 33.757 0.00 0.00
A
ATOM 1383 CA ASP A 96 35.139 1.736 33.177 0.00 0.00
A
ATOM 1384 HA ASP A 96 35.031 1.873 34.243 0.00 0.00
A
ATOM 1385 CB ASP A 96 36.362 0.819 32.852 0.00 0.00
A
ATOM 1386 HB1 ASP A 96 36.214 -0.225 33.201 0.00 0.00
A
ATOM 1387 HB2 ASP A 96 36.633 0.811 31.775 0.00 0.00
A
ATOM 1388 CG ASP A 96 37.503 1.354 33.715 0.00 0.00
A
ATOM 1389 OD1 ASP A 96 38.631 1.740 33.322 0.00 0.00
A
ATOM 1390 OD2 ASP A 96 37.176 1.344 34.984 0.00 0.00
A
ATOM 1391 C ASP A 96 35.093 3.140 32.662 0.00 0.00
A
ATOM 1392 O ASP A 96 35.980 3.598 31.946 0.00 0.00
A
ATOM 1393 N ILE A 97 34.024 3.783 33.053 0.00 0.00
A
ATOM 1394 HN ILE A 97 33.393 3.327 33.674 0.00 0.00
A
ATOM 1395 CA ILE A 97 33.725 5.120 32.648 0.00 0.00
A
ATOM 1396 HA ILE A 97 33.841 5.255 31.583 0.00 0.00
A
ATOM 1397 CB ILE A 97 32.322 5.511 33.056 0.00 0.00
A
ATOM 1398 HB ILE A 97 32.275 5.499 34.166 0.00 0.00
A
ATOM 1399 CG2 ILE A 97 32.131 6.963 32.651 0.00 0.00
A
ATOM 1400 HG21 ILE A 97 32.700 7.704 33.252 0.00 0.00
A
ATOM 1401 HG22 ILE A 97 32.351 7.136 31.576 0.00 0.00
A
ATOM 1402 HG23 ILE A 97 31.083 7.327 32.712 0.00 0.00
A
ATOM 1403 CG1 ILE A 97 31.201 4.614 32.668 0.00 0.00
A

ATOM 1404 HG11 ILE A 97 31.327 3.571 33.029 0.00 0.00
A
ATOM 1405 HG12 ILE A 97 30.312 5.084 33.141 0.00 0.00
A
ATOM 1406 CD ILE A 97 30.949 4.592 31.163 0.00 0.00
A
ATOM 1407 HD1 ILE A 97 31.739 4.071 30.580 0.00 0.00
A
ATOM 1408 HD2 ILE A 97 30.040 3.978 30.988 0.00 0.00
A
ATOM 1409 HD3 ILE A 97 30.727 5.571 30.687 0.00 0.00
A
ATOM 1410 C ILE A 97 34.698 6.125 33.462 0.00 0.00
A
ATOM 1411 O ILE A 97 34.839 5.894 34.675 0.00 0.00
A
ATOM 1412 N PRO A 98 35.377 7.087 32.761 0.00 0.00
A
ATOM 1413 CD PRO A 98 35.526 7.127 31.316 0.00 0.00
A
ATOM 1414 HD1 PRO A 98 35.622 6.135 30.825 0.00 0.00
A
ATOM 1415 HD2 PRO A 98 34.557 7.544 30.969 0.00 0.00
A
ATOM 1416 CA PRO A 98 36.347 8.051 33.479 0.00 0.00
A
ATOM 1417 HA PRO A 98 37.132 7.374 33.780 0.00 0.00
A
ATOM 1418 CB PRO A 98 36.787 8.937 32.301 0.00 0.00
A
ATOM 1419 HB1 PRO A 98 37.847 9.232 32.456 0.00 0.00
A
ATOM 1420 HB2 PRO A 98 36.130 9.833 32.314 0.00 0.00
A
ATOM 1421 CG PRO A 98 36.704 8.069 31.018 0.00 0.00
A
ATOM 1422 HG1 PRO A 98 37.610 7.455 30.825 0.00 0.00
A
ATOM 1423 HG2 PRO A 98 36.624 8.748 30.143 0.00 0.00
A
ATOM 1424 C PRO A 98 35.714 8.776 34.625 0.00 0.00
A
ATOM 1425 O PRO A 98 36.125 8.546 35.748 0.00 0.00
A
ATOM 1426 N ASN A 99 34.558 9.498 34.434 0.00 0.00
A
ATOM 1427 HN ASN A 99 34.253 9.615 33.492 0.00 0.00
A
ATOM 1428 CA ASN A 99 33.951 10.202 35.556 0.00 0.00
A
ATOM 1429 HA ASN A 99 34.488 10.011 36.474 0.00 0.00
A
ATOM 1430 CB ASN A 99 33.861 11.775 35.361 0.00 0.00
A

```
ATOM   1431  HB1 ASN A  99      33.346  11.906  34.385  0.00  0.00
A
ATOM   1432  HB2 ASN A  99      33.295  12.255  36.188  0.00  0.00
A
ATOM   1433  CG  ASN A  99      35.254  12.353  35.294  0.00  0.00
A
ATOM   1434  OD1 ASN A  99      35.932  12.581  36.277  0.00  0.00
A
ATOM   1435  ND2 ASN A  99      35.747  12.642  34.057  0.00  0.00
A
ATOM   1436 HD21 ASN A  99      36.703  12.934  34.079  0.00  0.00
A
ATOM   1437 HD22 ASN A  99      35.306  12.333  33.214  0.00  0.00
A
ATOM   1438  C   ASN A  99      32.513   9.621  35.769  0.00  0.00
A
ATOM   1439  O   ASN A  99      31.612   9.834  34.975  0.00  0.00
A
ATOM   1440  N   ILE A 100      32.313   8.943  36.915  0.00  0.00
A
ATOM   1441  HN  ILE A 100      33.119   8.706  37.453  0.00  0.00
A
ATOM   1442  CA  ILE A 100      30.997   8.648  37.436  0.00  0.00
A
ATOM   1443  HA  ILE A 100      30.218   8.803  36.703  0.00  0.00
A
ATOM   1444  CB  ILE A 100      30.874   7.175  37.899  0.00  0.00
A
ATOM   1445  HB  ILE A 100      31.577   7.066  38.752  0.00  0.00
A
ATOM   1446  CG2 ILE A 100      29.422   6.898  38.408  0.00  0.00
A
ATOM   1447 HG21 ILE A 100      28.587   7.382  37.857  0.00  0.00
A
ATOM   1448 HG22 ILE A 100      29.155   5.826  38.290  0.00  0.00
A
ATOM   1449 HG23 ILE A 100      29.223   7.063  39.489  0.00  0.00
A
ATOM   1450  CG1 ILE A 100      31.299   6.238  36.694  0.00  0.00
A
ATOM   1451 HG11 ILE A 100      30.589   6.302  35.842  0.00  0.00
A
ATOM   1452 HG12 ILE A 100      32.280   6.436  36.210  0.00  0.00
A
ATOM   1453  CD  ILE A 100      31.536   4.842  37.197  0.00  0.00
A
ATOM   1454  HD1 ILE A 100      31.762   4.238  36.292  0.00  0.00
A
ATOM   1455  HD2 ILE A 100      32.472   4.732  37.786  0.00  0.00
A
ATOM   1456  HD3 ILE A 100      30.679   4.568  37.848  0.00  0.00
A
ATOM   1457  C   ILE A 100      30.597   9.475  38.670  0.00  0.00
A
```

ATOM	1458	O	ILE	A	100	31.154	9.455	39.729	0.00	0.00	A
ATOM	1459	N	TRP	A	101	29.531	10.290	38.495	0.00	0.00	A
ATOM	1460	HN	TRP	A	101	29.266	10.532	37.564	0.00	0.00	A
ATOM	1461	CA	TRP	A	101	29.109	11.120	39.588	0.00	0.00	A
ATOM	1462	HA	TRP	A	101	29.762	10.957	40.433	0.00	0.00	A
ATOM	1463	CB	TRP	A	101	29.104	12.639	39.216	0.00	0.00	A
ATOM	1464	HB1	TRP	A	101	28.533	12.806	38.277	0.00	0.00	A
ATOM	1465	HB2	TRP	A	101	28.696	13.216	40.073	0.00	0.00	A
ATOM	1466	CG	TRP	A	101	30.469	13.400	38.916	0.00	0.00	A
ATOM	1467	CD1	TRP	A	101	31.690	12.903	38.833	0.00	0.00	A
ATOM	1468	HD1	TRP	A	101	32.059	11.888	38.807	0.00	0.00	A
ATOM	1469	NE1	TRP	A	101	32.648	14.006	38.558	0.00	0.00	A
ATOM	1470	HE1	TRP	A	101	33.624	13.992	38.546	0.00	0.00	A
ATOM	1471	CE2	TRP	A	101	31.911	15.138	38.491	0.00	0.00	A
ATOM	1472	CD2	TRP	A	101	30.580	14.812	38.788	0.00	0.00	A
ATOM	1473	CE3	TRP	A	101	29.610	15.868	38.840	0.00	0.00	A
ATOM	1474	HE3	TRP	A	101	28.595	15.502	38.896	0.00	0.00	A
ATOM	1475	CZ3	TRP	A	101	29.988	17.190	38.855	0.00	0.00	A
ATOM	1476	HZ3	TRP	A	101	29.229	17.955	38.776	0.00	0.00	A
ATOM	1477	CZ2	TRP	A	101	32.316	16.473	38.396	0.00	0.00	A
ATOM	1478	HZ2	TRP	A	101	33.357	16.721	38.256	0.00	0.00	A
ATOM	1479	CH2	TRP	A	101	31.324	17.446	38.641	0.00	0.00	A
ATOM	1480	HH2	TRP	A	101	31.654	18.474	38.636	0.00	0.00	A
ATOM	1481	C	TRP	A	101	27.760	10.703	40.049	0.00	0.00	A
ATOM	1482	O	TRP	A	101	26.875	10.518	39.199	0.00	0.00	A
ATOM	1483	N	VAL	A	102	27.516	10.416	41.382	0.00	0.00	A
ATOM	1484	HN	VAL	A	102	28.281	10.511	42.014	0.00	0.00	A

ATOM 1485 CA VAL A 102 26.280 9.813 41.806 0.00 0.00
A
ATOM 1486 HA VAL A 102 25.564 9.677 41.009 0.00 0.00
A
ATOM 1487 CB VAL A 102 26.608 8.387 42.360 0.00 0.00
A
ATOM 1488 HB VAL A 102 27.303 8.598 43.200 0.00 0.00
A
ATOM 1489 CG1 VAL A 102 25.317 7.754 42.784 0.00 0.00
A
ATOM 1490 HG11 VAL A 102 25.365 6.652 42.914 0.00 0.00
A
ATOM 1491 HG12 VAL A 102 24.931 8.236 43.708 0.00 0.00
A
ATOM 1492 HG13 VAL A 102 24.575 7.842 41.963 0.00 0.00
A
ATOM 1493 CG2 VAL A 102 27.343 7.544 41.332 0.00 0.00
A
ATOM 1494 HG21 VAL A 102 28.351 7.960 41.119 0.00 0.00
A
ATOM 1495 HG22 VAL A 102 27.512 6.534 41.762 0.00 0.00
A
ATOM 1496 HG23 VAL A 102 26.861 7.589 40.332 0.00 0.00
A
ATOM 1497 C VAL A 102 25.580 10.640 42.914 0.00 0.00
A
ATOM 1498 O VAL A 102 26.174 10.973 43.915 0.00 0.00
A
ATOM 1499 N LYS A 103 24.248 11.005 42.767 0.00 0.00
A
ATOM 1500 HN LYS A 103 23.811 10.762 41.904 0.00 0.00
A
ATOM 1501 CA LYS A 103 23.441 11.585 43.893 0.00 0.00
A
ATOM 1502 HA LYS A 103 24.028 12.446 44.178 0.00 0.00
A
ATOM 1503 CB LYS A 103 22.015 11.990 43.468 0.00 0.00
A
ATOM 1504 HB1 LYS A 103 22.109 12.562 42.521 0.00 0.00
A
ATOM 1505 HB2 LYS A 103 21.473 11.061 43.190 0.00 0.00
A
ATOM 1506 CG LYS A 103 21.270 12.841 44.478 0.00 0.00
A
ATOM 1507 HG1 LYS A 103 21.289 12.463 45.522 0.00 0.00
A
ATOM 1508 HG2 LYS A 103 21.802 13.806 44.332 0.00 0.00
A
ATOM 1509 CD LYS A 103 19.774 13.015 44.117 0.00 0.00
A
ATOM 1510 HD1 LYS A 103 19.264 13.804 44.710 0.00 0.00
A
ATOM 1511 HD2 LYS A 103 19.747 13.369 43.064 0.00 0.00
A

```
ATOM   1512  CE  LYS A 103      18.927  11.693  44.209  0.00  0.00
A
ATOM   1513  HE1 LYS A 103      19.325  11.031  43.411  0.00  0.00
A
ATOM   1514  HE2 LYS A 103      19.281  11.263  45.170  0.00  0.00
A
ATOM   1515  NZ  LYS A 103      17.512  11.922  43.963  0.00  0.00
A
ATOM   1516  HZ1 LYS A 103      16.866  12.228  44.719  0.00  0.00
A
ATOM   1517  HZ2 LYS A 103      17.320  12.649  43.244  0.00  0.00
A
ATOM   1518  HZ3 LYS A 103      17.144  10.997  43.662  0.00  0.00
A
ATOM   1519  C   LYS A 103      23.389  10.656  45.144  0.00  0.00
A
ATOM   1520  O   LYS A 103      23.266   9.471  44.965  0.00  0.00
A
ATOM   1521  N   ALA A 104      23.529  11.146  46.409  0.00  0.00
A
ATOM   1522  HN  ALA A 104      23.899  12.060  46.554  0.00  0.00
A
ATOM   1523  CA  ALA A 104      23.330  10.320  47.520  0.00  0.00
A
ATOM   1524  HA  ALA A 104      22.827   9.405  47.243  0.00  0.00
A
ATOM   1525  CB  ALA A 104      24.680   9.839  48.318  0.00  0.00
A
ATOM   1526  HB1 ALA A 104      24.311   9.217  49.161  0.00  0.00
A
ATOM   1527  HB2 ALA A 104      25.383   9.364  47.600  0.00  0.00
A
ATOM   1528  HB3 ALA A 104      25.353  10.628  48.715  0.00  0.00
A
ATOM   1529  C   ALA A 104      22.368  10.937  48.537  0.00  0.00
A
ATOM   1530  O   ALA A 104      22.410  12.117  48.886  0.00  0.00
A
ATOM   1531  N   GLN A 105      21.444  10.141  48.965  0.00  0.00
A
ATOM   1532  HN  GLN A 105      21.488   9.211  48.608  0.00  0.00
A
ATOM   1533  CA  GLN A 105      20.431  10.510  49.873  0.00  0.00
A
ATOM   1534  HA  GLN A 105      19.978  11.469  49.668  0.00  0.00
A
ATOM   1535  CB  GLN A 105      19.381   9.416  49.896  0.00  0.00
A
ATOM   1536  HB1 GLN A 105      19.085   9.145  48.860  0.00  0.00
A
ATOM   1537  HB2 GLN A 105      19.877   8.524  50.336  0.00  0.00
A
ATOM   1538  CG  GLN A 105      18.131   9.805  50.657  0.00  0.00
A
```

ATOM 1539 HG1 GLN A 105 18.407 9.698 51.728 0.00 0.00
A
ATOM 1540 HG2 GLN A 105 18.000 10.906 50.592 0.00 0.00
A
ATOM 1541 CD GLN A 105 16.828 9.012 50.431 0.00 0.00
A
ATOM 1542 OE1 GLN A 105 15.774 9.454 50.934 0.00 0.00
A
ATOM 1543 NE2 GLN A 105 16.952 7.850 49.662 0.00 0.00
A
ATOM 1544 HE21 GLN A 105 16.127 7.291 49.585 0.00 0.00
A
ATOM 1545 HE22 GLN A 105 17.757 7.731 49.081 0.00 0.00
A
ATOM 1546 C GLN A 105 20.803 10.612 51.318 0.00 0.00
A
ATOM 1547 O GLN A 105 20.320 11.524 52.018 0.00 0.00
A
ATOM 1548 N ASN A 106 21.719 9.692 51.854 0.00 0.00
A
ATOM 1549 HN ASN A 106 22.149 8.977 51.308 0.00 0.00
A
ATOM 1550 CA ASN A 106 21.989 9.546 53.245 0.00 0.00
A
ATOM 1551 HA ASN A 106 21.943 10.536 53.674 0.00 0.00
A
ATOM 1552 CB ASN A 106 20.792 8.818 53.890 0.00 0.00
A
ATOM 1553 HB1 ASN A 106 20.908 8.843 54.994 0.00 0.00
A
ATOM 1554 HB2 ASN A 106 19.822 9.220 53.527 0.00 0.00
A
ATOM 1555 CG ASN A 106 20.689 7.284 53.560 0.00 0.00
A
ATOM 1556 OD1 ASN A 106 21.356 6.743 52.689 0.00 0.00
A
ATOM 1557 ND2 ASN A 106 19.730 6.609 54.218 0.00 0.00
A
ATOM 1558 HD21 ASN A 106 19.548 5.626 54.179 0.00 0.00
A
ATOM 1559 HD22 ASN A 106 19.261 7.099 54.953 0.00 0.00
A
ATOM 1560 C ASN A 106 23.356 8.810 53.511 0.00 0.00
A
ATOM 1561 O ASN A 106 24.023 8.370 52.570 0.00 0.00
A
ATOM 1562 N TYR A 107 23.903 8.724 54.753 0.00 0.00
A
ATOM 1563 HN TYR A 107 23.417 9.181 55.494 0.00 0.00
A
ATOM 1564 CA TYR A 107 25.129 8.036 55.052 0.00 0.00
A
ATOM 1565 HA TYR A 107 25.864 8.534 54.438 0.00 0.00
A

ATOM 1566 CB TYR A 107 25.282 8.136 56.608 0.00 0.00
A
ATOM 1567 HB1 TYR A 107 25.323 9.197 56.935 0.00 0.00
A
ATOM 1568 HB2 TYR A 107 24.381 7.640 57.029 0.00 0.00
A
ATOM 1569 CG TYR A 107 26.454 7.466 57.262 0.00 0.00
A
ATOM 1570 CD1 TYR A 107 26.265 6.405 58.127 0.00 0.00
A
ATOM 1571 HD1 TYR A 107 25.252 6.032 58.154 0.00 0.00
A
ATOM 1572 CE1 TYR A 107 27.323 5.745 58.739 0.00 0.00
A
ATOM 1573 HE1 TYR A 107 27.158 4.902 59.394 0.00 0.00
A
ATOM 1574 CZ TYR A 107 28.631 6.194 58.449 0.00 0.00
A
ATOM 1575 OH TYR A 107 29.708 5.435 59.056 0.00 0.00
A
ATOM 1576 HH TYR A 107 29.291 4.902 59.737 0.00 0.00
A
ATOM 1577 CD2 TYR A 107 27.747 7.872 56.996 0.00 0.00
A
ATOM 1578 HD2 TYR A 107 27.820 8.764 56.391 0.00 0.00
A
ATOM 1579 CE2 TYR A 107 28.835 7.264 57.614 0.00 0.00
A
ATOM 1580 HE2 TYR A 107 29.862 7.594 57.560 0.00 0.00
A
ATOM 1581 C TYR A 107 25.263 6.564 54.625 0.00 0.00
A
ATOM 1582 O TYR A 107 26.297 6.102 54.117 0.00 0.00
A
ATOM 1583 N TYR A 108 24.168 5.798 54.780 0.00 0.00
A
ATOM 1584 HN TYR A 108 23.406 6.166 55.307 0.00 0.00
A
ATOM 1585 CA TYR A 108 24.225 4.377 54.388 0.00 0.00
A
ATOM 1586 HA TYR A 108 25.129 3.900 54.736 0.00 0.00
A
ATOM 1587 CB TYR A 108 22.964 3.591 54.746 0.00 0.00
A
ATOM 1588 HB1 TYR A 108 22.007 3.952 54.311 0.00 0.00
A
ATOM 1589 HB2 TYR A 108 23.162 2.550 54.410 0.00 0.00
A
ATOM 1590 CG TYR A 108 22.856 3.683 56.213 0.00 0.00
A
ATOM 1591 CD1 TYR A 108 21.800 4.410 56.846 0.00 0.00
A
ATOM 1592 HD1 TYR A 108 21.090 4.861 56.168 0.00 0.00
A

ATOM 1593 CE1 TYR A 108 21.602 4.523 58.199 0.00 0.00
A
ATOM 1594 HE1 TYR A 108 20.701 5.021 58.528 0.00 0.00
A
ATOM 1595 CZ TYR A 108 22.587 4.094 58.993 0.00 0.00
A
ATOM 1596 OH TYR A 108 22.538 4.339 60.405 0.00 0.00
A
ATOM 1597 HH TYR A 108 21.621 4.492 60.645 0.00 0.00
A
ATOM 1598 CD2 TYR A 108 23.903 3.218 57.024 0.00 0.00
A
ATOM 1599 HD2 TYR A 108 24.732 2.671 56.601 0.00 0.00
A
ATOM 1600 CE2 TYR A 108 23.794 3.471 58.443 0.00 0.00
A
ATOM 1601 HE2 TYR A 108 24.548 3.091 59.116 0.00 0.00
A
ATOM 1602 C TYR A 108 24.357 4.118 52.851 0.00 0.00
A
ATOM 1603 O TYR A 108 25.075 3.195 52.476 0.00 0.00
A
ATOM 1604 N HSE A 109 23.767 4.984 52.039 0.00 0.00
A
ATOM 1605 HN HSE A 109 23.179 5.711 52.385 0.00 0.00
A
ATOM 1606 CA HSE A 109 24.012 5.098 50.574 0.00 0.00
A
ATOM 1607 HA HSE A 109 23.958 4.131 50.095 0.00 0.00
A
ATOM 1608 CB HSE A 109 23.001 6.096 49.954 0.00 0.00
A
ATOM 1609 HB1 HSE A 109 21.941 5.857 50.186 0.00 0.00
A
ATOM 1610 HB2 HSE A 109 23.167 7.113 50.370 0.00 0.00
A
ATOM 1611 ND1 HSE A 109 22.202 7.082 47.917 0.00 0.00
A
ATOM 1612 CG HSE A 109 23.143 6.263 48.463 0.00 0.00
A
ATOM 1613 CE1 HSE A 109 22.306 6.919 46.621 0.00 0.00
A
ATOM 1614 HE1 HSE A 109 21.953 7.673 45.918 0.00 0.00
A
ATOM 1615 NE2 HSE A 109 23.257 6.012 46.270 0.00 0.00
A
ATOM 1616 HE2 HSE A 109 23.487 5.794 45.322 0.00 0.00
A
ATOM 1617 CD2 HSE A 109 23.813 5.539 47.491 0.00 0.00
A
ATOM 1618 HD2 HSE A 109 24.559 4.754 47.513 0.00 0.00
A
ATOM 1619 C HSE A 109 25.440 5.674 50.376 0.00 0.00
A

ATOM 1620 O HSE A 109 26.118 5.028 49.614 0.00 0.00
A
ATOM 1621 N HSE A 110 25.963 6.674 51.107 0.00 0.00
A
ATOM 1622 HN HSE A 110 25.310 7.294 51.534 0.00 0.00
A
ATOM 1623 CA HSE A 110 27.162 7.345 50.771 0.00 0.00
A
ATOM 1624 HA HSE A 110 27.273 7.438 49.701 0.00 0.00
A
ATOM 1625 CB HSE A 110 27.310 8.691 51.577 0.00 0.00
A
ATOM 1626 HB1 HSE A 110 26.602 9.356 51.038 0.00 0.00
A
ATOM 1627 HB2 HSE A 110 26.907 8.559 52.604 0.00 0.00
A
ATOM 1628 ND1 HSE A 110 29.870 9.022 51.950 0.00 0.00
A
ATOM 1629 CG HSE A 110 28.648 9.496 51.517 0.00 0.00
A
ATOM 1630 CE1 HSE A 110 30.660 9.966 51.530 0.00 0.00
A
ATOM 1631 HE1 HSE A 110 31.726 9.957 51.756 0.00 0.00
A
ATOM 1632 NE2 HSE A 110 30.035 10.959 50.865 0.00 0.00
A
ATOM 1633 HE2 HSE A 110 30.419 11.876 50.757 0.00 0.00
A
ATOM 1634 CD2 HSE A 110 28.713 10.687 50.842 0.00 0.00
A
ATOM 1635 HD2 HSE A 110 27.892 11.210 50.367 0.00 0.00
A
ATOM 1636 C HSE A 110 28.306 6.446 51.067 0.00 0.00
A
ATOM 1637 O HSE A 110 29.235 6.345 50.304 0.00 0.00
A
ATOM 1638 N LYS A 111 28.153 5.629 52.120 0.00 0.00
A
ATOM 1639 HN LYS A 111 27.387 5.787 52.738 0.00 0.00
A
ATOM 1640 CA LYS A 111 29.148 4.722 52.548 0.00 0.00
A
ATOM 1641 HA LYS A 111 30.090 5.224 52.715 0.00 0.00
A
ATOM 1642 CB LYS A 111 28.762 4.189 54.000 0.00 0.00
A
ATOM 1643 HB1 LYS A 111 28.561 5.155 54.510 0.00 0.00
A
ATOM 1644 HB2 LYS A 111 27.783 3.672 53.907 0.00 0.00
A
ATOM 1645 CG LYS A 111 29.821 3.381 54.823 0.00 0.00
A
ATOM 1646 HG1 LYS A 111 29.980 2.431 54.270 0.00 0.00
A

ATOM 1647 HG2 LYS A 111 30.813 3.877 54.876 0.00 0.00
A
ATOM 1648 CD LYS A 111 29.433 2.939 56.206 0.00 0.00
A
ATOM 1649 HD1 LYS A 111 28.997 3.793 56.768 0.00 0.00
A
ATOM 1650 HD2 LYS A 111 28.695 2.114 56.107 0.00 0.00
A
ATOM 1651 CE LYS A 111 30.472 2.473 57.193 0.00 0.00
A
ATOM 1652 HE1 LYS A 111 30.066 2.012 58.120 0.00 0.00
A
ATOM 1653 HE2 LYS A 111 31.143 1.736 56.703 0.00 0.00
A
ATOM 1654 NZ LYS A 111 31.229 3.725 57.504 0.00 0.00
A
ATOM 1655 HZ1 LYS A 111 31.484 4.363 56.722 0.00 0.00
A
ATOM 1656 HZ2 LYS A 111 30.617 4.297 58.121 0.00 0.00
A
ATOM 1657 HZ3 LYS A 111 32.118 3.443 57.964 0.00 0.00
A
ATOM 1658 C LYS A 111 29.509 3.633 51.570 0.00 0.00
A
ATOM 1659 O LYS A 111 30.702 3.444 51.364 0.00 0.00
A
ATOM 1660 N VAL A 112 28.496 3.041 50.885 0.00 0.00
A
ATOM 1661 HN VAL A 112 27.550 3.301 51.064 0.00 0.00
A
ATOM 1662 CA VAL A 112 28.709 2.038 49.803 0.00 0.00
A
ATOM 1663 HA VAL A 112 29.430 1.317 50.160 0.00 0.00
A
ATOM 1664 CB VAL A 112 27.346 1.501 49.393 0.00 0.00
A
ATOM 1665 HB VAL A 112 26.668 2.345 49.145 0.00 0.00
A
ATOM 1666 CG1 VAL A 112 27.369 0.536 48.211 0.00 0.00
A
ATOM 1667 HG11 VAL A 112 26.384 0.025 48.166 0.00 0.00
A
ATOM 1668 HG12 VAL A 112 27.451 0.969 47.191 0.00 0.00
A
ATOM 1669 HG13 VAL A 112 28.119 -0.245 48.461 0.00 0.00
A
ATOM 1670 CG2 VAL A 112 26.805 0.677 50.497 0.00 0.00
A
ATOM 1671 HG21 VAL A 112 25.746 0.383 50.336 0.00 0.00
A
ATOM 1672 HG22 VAL A 112 27.455 -0.224 50.516 0.00 0.00
A
ATOM 1673 HG23 VAL A 112 26.752 1.098 51.524 0.00 0.00
A

ATOM 1674 C VAL A 112 29.397 2.635 48.614 0.00 0.00
A
ATOM 1675 O VAL A 112 30.239 2.060 47.919 0.00 0.00
A
ATOM 1676 N LEU A 113 29.070 3.879 48.243 0.00 0.00
A
ATOM 1677 HN LEU A 113 28.389 4.398 48.754 0.00 0.00
A
ATOM 1678 CA LEU A 113 29.538 4.423 46.974 0.00 0.00
A
ATOM 1679 HA LEU A 113 29.308 3.798 46.124 0.00 0.00
A
ATOM 1680 CB LEU A 113 28.721 5.727 46.722 0.00 0.00
A
ATOM 1681 HB1 LEU A 113 28.952 6.530 47.454 0.00 0.00
A
ATOM 1682 HB2 LEU A 113 29.100 6.000 45.714 0.00 0.00
A
ATOM 1683 CG LEU A 113 27.189 5.605 46.412 0.00 0.00
A
ATOM 1684 HG LEU A 113 26.694 5.135 47.288 0.00 0.00
A
ATOM 1685 CD1 LEU A 113 26.571 7.063 46.291 0.00 0.00
A
ATOM 1686 HD11 LEU A 113 27.114 7.699 47.022 0.00 0.00
A
ATOM 1687 HD12 LEU A 113 26.891 7.424 45.290 0.00 0.00
A
ATOM 1688 HD13 LEU A 113 25.485 7.015 46.519 0.00 0.00
A
ATOM 1689 CD2 LEU A 113 26.897 4.725 45.168 0.00 0.00
A
ATOM 1690 HD21 LEU A 113 25.824 4.447 45.230 0.00 0.00
A
ATOM 1691 HD22 LEU A 113 27.125 5.225 44.202 0.00 0.00
A
ATOM 1692 HD23 LEU A 113 27.487 3.785 45.240 0.00 0.00
A
ATOM 1693 C LEU A 113 31.007 4.853 46.922 0.00 0.00
A
ATOM 1694 O LEU A 113 31.695 4.700 45.938 0.00 0.00
A
ATOM 1695 N GLU A 114 31.460 5.428 48.026 0.00 0.00
A
ATOM 1696 HN GLU A 114 30.835 5.581 48.788 0.00 0.00
A
ATOM 1697 CA GLU A 114 32.784 5.513 48.470 0.00 0.00
A
ATOM 1698 HA GLU A 114 33.182 6.158 47.700 0.00 0.00
A
ATOM 1699 CB GLU A 114 32.795 6.106 49.891 0.00 0.00
A
ATOM 1700 HB1 GLU A 114 32.156 7.002 49.740 0.00 0.00
A

ATOM 1701 HB2 GLU A 114 32.394 5.511 50.738 0.00 0.00
A
ATOM 1702 CG GLU A 114 34.213 6.579 50.295 0.00 0.00
A
ATOM 1703 HG1 GLU A 114 34.756 5.610 50.288 0.00 0.00
A
ATOM 1704 HG2 GLU A 114 34.558 7.384 49.612 0.00 0.00
A
ATOM 1705 CD GLU A 114 34.229 7.146 51.763 0.00 0.00
A
ATOM 1706 OE1 GLU A 114 33.897 6.398 52.709 0.00 0.00
A
ATOM 1707 OE2 GLU A 114 34.628 8.347 51.950 0.00 0.00
A
ATOM 1708 C GLU A 114 33.642 4.260 48.488 0.00 0.00
A
ATOM 1709 O GLU A 114 34.773 4.175 48.029 0.00 0.00
A
ATOM 1710 N LYS A 115 32.982 3.174 48.931 0.00 0.00
A
ATOM 1711 HN LYS A 115 32.068 3.323 49.298 0.00 0.00
A
ATOM 1712 CA LYS A 115 33.583 1.879 48.910 0.00 0.00
A
ATOM 1713 HA LYS A 115 34.598 2.080 49.221 0.00 0.00
A
ATOM 1714 CB LYS A 115 32.952 0.924 49.865 0.00 0.00
A
ATOM 1715 HB1 LYS A 115 32.861 1.439 50.845 0.00 0.00
A
ATOM 1716 HB2 LYS A 115 31.919 0.814 49.473 0.00 0.00
A
ATOM 1717 CG LYS A 115 33.523 -0.485 50.106 0.00 0.00
A
ATOM 1718 HG1 LYS A 115 32.655 -1.009 50.561 0.00 0.00
A
ATOM 1719 HG2 LYS A 115 33.887 -1.023 49.204 0.00 0.00
A
ATOM 1720 CD LYS A 115 34.650 -0.440 51.150 0.00 0.00
A
ATOM 1721 HD1 LYS A 115 34.575 0.345 51.932 0.00 0.00
A
ATOM 1722 HD2 LYS A 115 34.603 -1.361 51.770 0.00 0.00
A
ATOM 1723 CE LYS A 115 36.130 -0.311 50.557 0.00 0.00
A
ATOM 1724 HE1 LYS A 115 36.097 -1.116 49.792 0.00 0.00
A
ATOM 1725 HE2 LYS A 115 36.339 0.669 50.077 0.00 0.00
A
ATOM 1726 NZ LYS A 115 37.195 -0.625 51.523 0.00 0.00
A
ATOM 1727 HZ1 LYS A 115 38.027 -1.089 51.105 0.00 0.00
A

ATOM   1728 HZ2 LYS A 115   37.584   0.222 51.984 0.00 0.00
A
ATOM   1729 HZ3 LYS A 115   36.863 -1.151 52.356 0.00 0.00
A
ATOM   1730 C   LYS A 115   33.749   1.155 47.491 0.00 0.00
A
ATOM   1731 O   LYS A 115   34.605   0.266 47.258 0.00 0.00
A
ATOM   1732 N   ILE A 116   32.886   1.563 46.515 0.00 0.00
A
ATOM   1733 HN ILE A 116   32.184   2.241 46.719 0.00 0.00
A
ATOM   1734 CA ILE A 116   32.859   1.104 45.129 0.00 0.00
A
ATOM   1735 HA ILE A 116   33.265   0.110 45.244 0.00 0.00
A
ATOM   1736 CB ILE A 116   31.441   1.142 44.488 0.00 0.00
A
ATOM   1737 HB ILE A 116   31.059   2.161 44.710 0.00 0.00
A
ATOM   1738 CG2 ILE A 116   31.514   1.028 42.854 0.00 0.00
A
ATOM   1739 HG21 ILE A 116   32.165   1.774 42.348 0.00 0.00
A
ATOM   1740 HG22 ILE A 116   31.749 -0.038 42.647 0.00 0.00
A
ATOM   1741 HG23 ILE A 116   30.525   1.339 42.455 0.00 0.00
A
ATOM   1742 CG1 ILE A 116   30.429   0.153 45.085 0.00 0.00
A
ATOM   1743 HG11 ILE A 116   30.816 -0.861 44.847 0.00 0.00
A
ATOM   1744 HG12 ILE A 116   30.394   0.195 46.195 0.00 0.00
A
ATOM   1745 CD ILE A 116   28.961   0.423 44.738 0.00 0.00
A
ATOM   1746 HD1 ILE A 116   28.270 -0.045 45.471 0.00 0.00
A
ATOM   1747 HD2 ILE A 116   28.758   1.515 44.707 0.00 0.00
A
ATOM   1748 HD3 ILE A 116   28.722 -0.109 43.792 0.00 0.00
A
ATOM   1749 C   ILE A 116   33.917   1.888 44.330 0.00 0.00
A
ATOM   1750 O   ILE A 116   34.630   1.483 43.381 0.00 0.00
A
ATOM   1751 N   GLY A 117   34.033   3.173 44.707 0.00 0.00
A
ATOM   1752 HN GLY A 117   33.688   3.394 45.616 0.00 0.00
A
ATOM   1753 CA GLY A 117   35.088   4.040 44.140 0.00 0.00
A
ATOM   1754 HA1 GLY A 117   35.772   3.477 43.522 0.00 0.00
A

ATOM 1755 HA2 GLY A 117 35.522 4.595 44.958 0.00 0.00
A
ATOM 1756 C GLY A 117 34.538 5.001 43.181 0.00 0.00
A
ATOM 1757 O GLY A 117 35.124 5.340 42.160 0.00 0.00
A
ATOM 1758 N ALA A 118 33.255 5.549 43.500 0.00 0.00
A
ATOM 1759 HN ALA A 118 32.852 5.404 44.400 0.00 0.00
A
ATOM 1760 CA ALA A 118 32.638 6.637 42.693 0.00 0.00
A
ATOM 1761 HA ALA A 118 32.529 6.214 41.705 0.00 0.00
A
ATOM 1762 CB ALA A 118 31.208 6.851 43.290 0.00 0.00
A
ATOM 1763 HB1 ALA A 118 30.619 5.914 43.186 0.00 0.00
A
ATOM 1764 HB2 ALA A 118 31.252 7.044 44.383 0.00 0.00
A
ATOM 1765 HB3 ALA A 118 30.642 7.689 42.830 0.00 0.00
A
ATOM 1766 C ALA A 118 33.414 7.875 42.589 0.00 0.00
A
ATOM 1767 O ALA A 118 34.001 8.385 43.534 0.00 0.00
A
ATOM 1768 N ASP A 119 33.511 8.499 41.374 0.00 0.00
A
ATOM 1769 HN ASP A 119 33.035 8.136 40.577 0.00 0.00
A
ATOM 1770 CA ASP A 119 34.369 9.652 41.078 0.00 0.00
A
ATOM 1771 HA ASP A 119 35.382 9.433 41.385 0.00 0.00
A
ATOM 1772 CB ASP A 119 34.465 9.865 39.521 0.00 0.00
A
ATOM 1773 HB1 ASP A 119 33.425 10.025 39.167 0.00 0.00
A
ATOM 1774 HB2 ASP A 119 35.045 10.790 39.312 0.00 0.00
A
ATOM 1775 CG ASP A 119 35.190 8.737 38.891 0.00 0.00
A
ATOM 1776 OD1 ASP A 119 34.419 7.849 38.389 0.00 0.00
A
ATOM 1777 OD2 ASP A 119 36.423 8.585 38.952 0.00 0.00
A
ATOM 1778 C ASP A 119 33.968 10.843 41.902 0.00 0.00
A
ATOM 1779 O ASP A 119 34.805 11.591 42.424 0.00 0.00
A
ATOM 1780 N ARG A 120 32.624 11.001 42.061 0.00 0.00
A
ATOM 1781 HN ARG A 120 31.954 10.416 41.611 0.00 0.00
A

ATOM 1782 CA ARG A 120 32.168 12.080 42.924 0.00 0.00
A
ATOM 1783 HA ARG A 120 32.875 11.967 43.733 0.00 0.00
A
ATOM 1784 CB ARG A 120 32.178 13.499 42.249 0.00 0.00
A
ATOM 1785 HB1 ARG A 120 32.992 13.643 41.506 0.00 0.00
A
ATOM 1786 HB2 ARG A 120 31.272 13.660 41.626 0.00 0.00
A
ATOM 1787 CG ARG A 120 32.291 14.628 43.200 0.00 0.00
A
ATOM 1788 HG1 ARG A 120 32.119 15.618 42.725 0.00 0.00
A
ATOM 1789 HG2 ARG A 120 31.397 14.531 43.852 0.00 0.00
A
ATOM 1790 CD ARG A 120 33.556 14.687 44.054 0.00 0.00
A
ATOM 1791 HD1 ARG A 120 33.650 13.757 44.654 0.00 0.00
A
ATOM 1792 HD2 ARG A 120 34.465 14.719 43.416 0.00 0.00
A
ATOM 1793 NE ARG A 120 33.413 15.974 44.817 0.00 0.00
A
ATOM 1794 HE ARG A 120 32.580 16.510 44.682 0.00 0.00
A
ATOM 1795 CZ ARG A 120 34.216 16.438 45.812 0.00 0.00
A
ATOM 1796 NH1 ARG A 120 35.098 15.623 46.323 0.00 0.00
A
ATOM 1797 HH11 ARG A 120 35.741 16.016 46.980 0.00 0.00
A
ATOM 1798 HH12 ARG A 120 35.346 14.795 45.821 0.00 0.00
A
ATOM 1799 NH2 ARG A 120 34.032 17.615 46.314 0.00 0.00
A
ATOM 1800 HH21 ARG A 120 34.671 18.001 46.980 0.00 0.00
A
ATOM 1801 HH22 ARG A 120 33.374 18.243 45.899 0.00 0.00
A
ATOM 1802 C ARG A 120 30.814 11.736 43.527 0.00 0.00
A
ATOM 1803 O ARG A 120 29.980 11.115 42.846 0.00 0.00
A
ATOM 1804 N ILE A 121 30.456 12.074 44.779 0.00 0.00
A
ATOM 1805 HN ILE A 121 31.087 12.578 45.363 0.00 0.00
A
ATOM 1806 CA ILE A 121 29.153 11.788 45.381 0.00 0.00
A
ATOM 1807 HA ILE A 121 28.491 11.210 44.754 0.00 0.00
A
ATOM 1808 CB ILE A 121 29.248 11.078 46.711 0.00 0.00
A

```
ATOM   1809  HB  ILE A 121      29.835  11.682  47.435  0.00  0.00
A
ATOM   1810  CG2 ILE A 121      27.835  10.762  47.241  0.00  0.00
A
ATOM   1811 HG21 ILE A 121      27.219  10.089  46.608  0.00  0.00
A
ATOM   1812 HG22 ILE A 121      27.925  10.190  48.189  0.00  0.00
A
ATOM   1813 HG23 ILE A 121      27.290  11.668  47.584  0.00  0.00
A
ATOM   1814  CG1 ILE A 121      30.023   9.695  46.593  0.00  0.00
A
ATOM   1815 HG11 ILE A 121      29.401   9.113  45.881  0.00  0.00
A
ATOM   1816 HG12 ILE A 121      30.955   9.926  46.034  0.00  0.00
A
ATOM   1817  CD  ILE A 121      30.388   8.947  47.884  0.00  0.00
A
ATOM   1818  HD1 ILE A 121      30.920   9.615  48.595  0.00  0.00
A
ATOM   1819  HD2 ILE A 121      29.486   8.611  48.439  0.00  0.00
A
ATOM   1820  HD3 ILE A 121      31.112   8.132  47.670  0.00  0.00
A
ATOM   1821  C   ILE A 121      28.516  13.165  45.509  0.00  0.00
A
ATOM   1822  O   ILE A 121      29.269  14.045  45.777  0.00  0.00
A
ATOM   1823  N   ILE A 122      27.149  13.355  45.355  0.00  0.00
A
ATOM   1824  HN  ILE A 122      26.623  12.549  45.094  0.00  0.00
A
ATOM   1825  CA  ILE A 122      26.472  14.645  45.570  0.00  0.00
A
ATOM   1826  HA  ILE A 122      27.127  15.359  46.046  0.00  0.00
A
ATOM   1827  CB  ILE A 122      25.907  15.224  44.170  0.00  0.00
A
ATOM   1828  HB  ILE A 122      25.182  14.525  43.701  0.00  0.00
A
ATOM   1829  CG2 ILE A 122      25.135  16.574  44.495  0.00  0.00
A
ATOM   1830 HG21 ILE A 122      24.861  17.186  43.609  0.00  0.00
A
ATOM   1831 HG22 ILE A 122      24.275  16.349  45.161  0.00  0.00
A
ATOM   1832 HG23 ILE A 122      25.687  17.205  45.223  0.00  0.00
A
ATOM   1833  CG1 ILE A 122      27.024  15.425  43.158  0.00  0.00
A
ATOM   1834 HG11 ILE A 122      26.779  16.280  42.491  0.00  0.00
A
ATOM   1835 HG12 ILE A 122      27.997  15.658  43.642  0.00  0.00
A
```

ATOM 1836 CD ILE A 122 27.169 14.209 42.244 0.00 0.00
A
ATOM 1837 HD1 ILE A 122 27.452 13.303 42.822 0.00 0.00
A
ATOM 1838 HD2 ILE A 122 26.138 14.026 41.874 0.00 0.00
A
ATOM 1839 HD3 ILE A 122 27.925 14.470 41.473 0.00 0.00
A
ATOM 1840 C ILE A 122 25.436 14.352 46.555 0.00 0.00
A
ATOM 1841 O ILE A 122 24.444 13.673 46.288 0.00 0.00
A
ATOM 1842 N HSE A 123 25.471 14.984 47.736 0.00 0.00
A
ATOM 1843 HN HSE A 123 26.215 15.540 48.099 0.00 0.00
A
ATOM 1844 CA HSE A 123 24.325 15.094 48.650 0.00 0.00
A
ATOM 1845 HA HSE A 123 23.513 14.446 48.355 0.00 0.00
A
ATOM 1846 CB HSE A 123 24.861 14.644 49.986 0.00 0.00
A
ATOM 1847 HB1 HSE A 123 25.344 13.658 49.814 0.00 0.00
A
ATOM 1848 HB2 HSE A 123 25.735 15.266 50.271 0.00 0.00
A
ATOM 1849 ND1 HSE A 123 23.296 15.408 51.780 0.00 0.00
A
ATOM 1850 CG HSE A 123 23.993 14.374 51.124 0.00 0.00
A
ATOM 1851 CE1 HSE A 123 22.822 14.852 52.841 0.00 0.00
A
ATOM 1852 HE1 HSE A 123 22.219 15.371 53.586 0.00 0.00
A
ATOM 1853 NE2 HSE A 123 23.096 13.519 52.913 0.00 0.00
A
ATOM 1854 HE2 HSE A 123 22.655 12.925 53.586 0.00 0.00
A
ATOM 1855 CD2 HSE A 123 23.729 13.228 51.772 0.00 0.00
A
ATOM 1856 HD2 HSE A 123 24.134 12.228 51.679 0.00 0.00
A
ATOM 1857 C HSE A 123 23.849 16.575 48.645 0.00 0.00
A
ATOM 1858 O HSE A 123 24.604 17.513 48.865 0.00 0.00
A
ATOM 1859 N PRO A 124 22.538 16.749 48.268 0.00 0.00
A
ATOM 1860 CD PRO A 124 21.849 15.827 47.315 0.00 0.00
A
ATOM 1861 HD1 PRO A 124 22.450 15.282 46.556 0.00 0.00
A
ATOM 1862 HD2 PRO A 124 21.362 15.060 47.954 0.00 0.00
A

ATOM 1863 CA PRO A 124 22.087 18.049 48.018 0.00 0.00
A
ATOM 1864 HA PRO A 124 22.891 18.762 48.119 0.00 0.00
A
ATOM 1865 CB PRO A 124 21.554 18.051 46.587 0.00 0.00
A
ATOM 1866 HB1 PRO A 124 22.391 18.159 45.864 0.00 0.00
A
ATOM 1867 HB2 PRO A 124 20.891 18.939 46.521 0.00 0.00
A
ATOM 1868 CG PRO A 124 20.877 16.677 46.464 0.00 0.00
A
ATOM 1869 HG1 PRO A 124 20.801 16.453 45.378 0.00 0.00
A
ATOM 1870 HG2 PRO A 124 19.859 16.677 46.910 0.00 0.00
A
ATOM 1871 C PRO A 124 20.982 18.443 49.011 0.00 0.00
A
ATOM 1872 O PRO A 124 20.370 19.506 48.900 0.00 0.00
A
ATOM 1873 N GLU A 125 20.825 17.610 50.041 0.00 0.00
A
ATOM 1874 HN GLU A 125 21.411 16.816 50.176 0.00 0.00
A
ATOM 1875 CA GLU A 125 19.683 17.572 50.952 0.00 0.00
A
ATOM 1876 HA GLU A 125 18.826 17.436 50.309 0.00 0.00
A
ATOM 1877 CB GLU A 125 19.939 16.503 51.963 0.00 0.00
A
ATOM 1878 HB1 GLU A 125 20.335 15.599 51.452 0.00 0.00
A
ATOM 1879 HB2 GLU A 125 20.791 16.819 52.603 0.00 0.00
A
ATOM 1880 CG GLU A 125 18.745 16.208 52.936 0.00 0.00
A
ATOM 1881 HG1 GLU A 125 18.480 17.006 53.662 0.00 0.00
A
ATOM 1882 HG2 GLU A 125 17.837 15.850 52.405 0.00 0.00
A
ATOM 1883 CD GLU A 125 19.184 15.134 53.908 0.00 0.00
A
ATOM 1884 OE1 GLU A 125 18.344 14.253 54.124 0.00 0.00
A
ATOM 1885 OE2 GLU A 125 20.282 15.182 54.516 0.00 0.00
A
ATOM 1886 C GLU A 125 19.319 18.895 51.652 0.00 0.00
A
ATOM 1887 O GLU A 125 18.152 19.313 51.708 0.00 0.00
A
ATOM 1888 N LYS A 126 20.293 19.498 52.217 0.00 0.00
A
ATOM 1889 HN LYS A 126 21.236 19.183 52.136 0.00 0.00
A

ATOM 1890 CA LYS A 126 20.237 20.746 52.887 0.00 0.00
A
ATOM 1891 HA LYS A 126 19.222 21.039 53.113 0.00 0.00
A
ATOM 1892 CB LYS A 126 21.066 20.692 54.213 0.00 0.00
A
ATOM 1893 HB1 LYS A 126 21.070 21.591 54.866 0.00 0.00
A
ATOM 1894 HB2 LYS A 126 20.861 19.793 54.832 0.00 0.00
A
ATOM 1895 CG LYS A 126 22.580 20.649 53.839 0.00 0.00
A
ATOM 1896 HG1 LYS A 126 22.772 19.666 53.358 0.00 0.00
A
ATOM 1897 HG2 LYS A 126 22.803 21.517 53.181 0.00 0.00
A
ATOM 1898 CD LYS A 126 23.455 20.737 55.052 0.00 0.00
A
ATOM 1899 HD1 LYS A 126 23.079 21.571 55.682 0.00 0.00
A
ATOM 1900 HD2 LYS A 126 23.344 19.730 55.507 0.00 0.00
A
ATOM 1901 CE LYS A 126 24.971 20.758 54.817 0.00 0.00
A
ATOM 1902 HE1 LYS A 126 25.337 19.981 54.113 0.00 0.00
A
ATOM 1903 HE2 LYS A 126 25.200 21.765 54.406 0.00 0.00
A
ATOM 1904 NZ LYS A 126 25.789 20.650 56.061 0.00 0.00
A
ATOM 1905 HZ1 LYS A 126 25.495 19.745 56.480 0.00 0.00
A
ATOM 1906 HZ2 LYS A 126 26.815 20.655 55.893 0.00 0.00
A
ATOM 1907 HZ3 LYS A 126 25.567 21.429 56.714 0.00 0.00
A
ATOM 1908 C LYS A 126 20.614 22.034 52.071 0.00 0.00
A
ATOM 1909 O LYS A 126 20.491 23.139 52.608 0.00 0.00
A
ATOM 1910 N ASP A 127 21.088 21.864 50.790 0.00 0.00
A
ATOM 1911 HN ASP A 127 21.067 20.963 50.364 0.00 0.00
A
ATOM 1912 CA ASP A 127 21.687 22.951 50.108 0.00 0.00
A
ATOM 1913 HA ASP A 127 22.524 23.236 50.728 0.00 0.00
A
ATOM 1914 CB ASP A 127 22.197 22.373 48.863 0.00 0.00
A
ATOM 1915 HB1 ASP A 127 22.618 21.362 49.049 0.00 0.00
A
ATOM 1916 HB2 ASP A 127 21.534 22.242 47.981 0.00 0.00
A

ATOM 1917 CG ASP A 127 23.435 23.122 48.307 0.00 0.00
A
ATOM 1918 OD1 ASP A 127 24.502 23.016 48.801 0.00 0.00
A
ATOM 1919 OD2 ASP A 127 23.100 23.905 47.370 0.00 0.00
A
ATOM 1920 C ASP A 127 20.741 24.192 49.851 0.00 0.00
A
ATOM 1921 O ASP A 127 21.224 25.305 50.009 0.00 0.00
A
ATOM 1922 N MET A 128 19.449 24.012 49.539 0.00 0.00
A
ATOM 1923 HN MET A 128 19.063 23.094 49.497 0.00 0.00
A
ATOM 1924 CA MET A 128 18.474 25.096 49.572 0.00 0.00
A
ATOM 1925 HA MET A 128 18.920 25.827 48.914 0.00 0.00
A
ATOM 1926 CB MET A 128 17.162 24.576 48.942 0.00 0.00
A
ATOM 1927 HB1 MET A 128 17.177 23.805 48.142 0.00 0.00
A
ATOM 1928 HB2 MET A 128 16.677 23.918 49.694 0.00 0.00
A
ATOM 1929 CG MET A 128 16.192 25.748 48.503 0.00 0.00
A
ATOM 1930 HG1 MET A 128 15.240 25.311 48.133 0.00 0.00
A
ATOM 1931 HG2 MET A 128 15.978 26.396 49.380 0.00 0.00
A
ATOM 1932 SD MET A 128 17.015 26.600 47.173 0.00 0.00
A
ATOM 1933 CE MET A 128 16.147 28.123 46.942 0.00 0.00
A
ATOM 1934 HE1 MET A 128 15.061 28.159 46.713 0.00 0.00
A
ATOM 1935 HE2 MET A 128 16.252 28.769 47.840 0.00 0.00
A
ATOM 1936 HE3 MET A 128 16.720 28.745 46.221 0.00 0.00
A
ATOM 1937 C MET A 128 18.252 25.698 50.922 0.00 0.00
A
ATOM 1938 O MET A 128 18.155 26.911 51.074 0.00 0.00
A
ATOM 1939 N GLY A 129 18.128 24.873 51.967 0.00 0.00
A
ATOM 1940 HN GLY A 129 18.171 23.880 51.891 0.00 0.00
A
ATOM 1941 CA GLY A 129 17.661 25.355 53.304 0.00 0.00
A
ATOM 1942 HA1 GLY A 129 17.556 24.451 53.886 0.00 0.00
A
ATOM 1943 HA2 GLY A 129 16.705 25.842 53.182 0.00 0.00
A

ATOM 1944 C GLY A 129 18.605 26.265 54.035 0.00 0.00
A
ATOM 1945 O GLY A 129 18.156 27.251 54.622 0.00 0.00
A
ATOM 1946 N VAL A 130 19.911 26.056 53.790 0.00 0.00
A
ATOM 1947 HN VAL A 130 20.157 25.220 53.305 0.00 0.00
A
ATOM 1948 CA VAL A 130 20.962 26.872 54.426 0.00 0.00
A
ATOM 1949 HA VAL A 130 20.695 27.001 55.464 0.00 0.00
A
ATOM 1950 CB VAL A 130 22.352 26.240 54.246 0.00 0.00
A
ATOM 1951 HB VAL A 130 23.178 26.883 54.619 0.00 0.00
A
ATOM 1952 CG1 VAL A 130 22.369 24.974 55.131 0.00 0.00
A
ATOM 1953 HG11 VAL A 130 21.651 24.238 54.711 0.00 0.00
A
ATOM 1954 HG12 VAL A 130 23.328 24.412 55.118 0.00 0.00
A
ATOM 1955 HG13 VAL A 130 22.173 25.242 56.191 0.00 0.00
A
ATOM 1956 CG2 VAL A 130 22.688 25.850 52.798 0.00 0.00
A
ATOM 1957 HG21 VAL A 130 23.725 25.455 52.840 0.00 0.00
A
ATOM 1958 HG22 VAL A 130 22.091 25.017 52.369 0.00 0.00
A
ATOM 1959 HG23 VAL A 130 22.789 26.771 52.185 0.00 0.00
A
ATOM 1960 C VAL A 130 20.892 28.281 53.929 0.00 0.00
A
ATOM 1961 O VAL A 130 21.008 29.213 54.700 0.00 0.00
A
ATOM 1962 N LYS A 131 20.713 28.503 52.615 0.00 0.00
A
ATOM 1963 HN LYS A 131 20.573 27.638 52.138 0.00 0.00
A
ATOM 1964 CA LYS A 131 20.641 29.836 51.904 0.00 0.00
A
ATOM 1965 HA LYS A 131 21.561 30.382 52.047 0.00 0.00
A
ATOM 1966 CB LYS A 131 20.409 29.645 50.383 0.00 0.00
A
ATOM 1967 HB1 LYS A 131 19.415 29.192 50.176 0.00 0.00
A
ATOM 1968 HB2 LYS A 131 20.449 30.656 49.924 0.00 0.00
A
ATOM 1969 CG LYS A 131 21.466 28.859 49.653 0.00 0.00
A
ATOM 1970 HG1 LYS A 131 22.334 29.552 49.657 0.00 0.00
A

ATOM 1971 HG2 LYS A 131 21.623 27.941 50.259 0.00 0.00
A
ATOM 1972 CD LYS A 131 21.245 28.510 48.124 0.00 0.00
A
ATOM 1973 HD1 LYS A 131 20.359 27.853 47.993 0.00 0.00
A
ATOM 1974 HD2 LYS A 131 20.973 29.532 47.782 0.00 0.00
A
ATOM 1975 CE LYS A 131 22.488 27.942 47.471 0.00 0.00
A
ATOM 1976 HE1 LYS A 131 22.518 28.280 46.413 0.00 0.00
A
ATOM 1977 HE2 LYS A 131 23.411 28.325 47.957 0.00 0.00
A
ATOM 1978 NZ LYS A 131 22.585 26.512 47.623 0.00 0.00
A
ATOM 1979 HZ1 LYS A 131 23.482 26.063 47.349 0.00 0.00
A
ATOM 1980 HZ2 LYS A 131 22.354 26.272 48.608 0.00 0.00
A
ATOM 1981 HZ3 LYS A 131 21.808 26.100 47.067 0.00 0.00
A
ATOM 1982 C LYS A 131 19.503 30.694 52.422 0.00 0.00
A
ATOM 1983 O LYS A 131 19.640 31.897 52.731 0.00 0.00
A
ATOM 1984 N ILE A 132 18.321 30.087 52.757 0.00 0.00
A
ATOM 1985 HN ILE A 132 18.311 29.097 52.637 0.00 0.00
A
ATOM 1986 CA ILE A 132 17.131 30.673 53.286 0.00 0.00
A
ATOM 1987 HA ILE A 132 16.993 31.631 52.808 0.00 0.00
A
ATOM 1988 CB ILE A 132 15.946 29.752 53.036 0.00 0.00
A
ATOM 1989 HB ILE A 132 16.362 28.784 53.389 0.00 0.00
A
ATOM 1990 CG2 ILE A 132 14.697 30.130 53.791 0.00 0.00
A
ATOM 1991 HG21 ILE A 132 14.207 31.084 53.499 0.00 0.00
A
ATOM 1992 HG22 ILE A 132 13.912 29.358 53.643 0.00 0.00
A
ATOM 1993 HG23 ILE A 132 14.852 30.217 54.888 0.00 0.00
A
ATOM 1994 CG1 ILE A 132 15.659 29.533 51.447 0.00 0.00
A
ATOM 1995 HG11 ILE A 132 16.580 29.366 50.849 0.00 0.00
A
ATOM 1996 HG12 ILE A 132 14.975 28.682 51.241 0.00 0.00
A
ATOM 1997 CD ILE A 132 14.940 30.728 50.869 0.00 0.00
A

ATOM 1998 HD1 ILE A 132 13.960 30.900 51.363 0.00 0.00
A
ATOM 1999 HD2 ILE A 132 15.510 31.620 51.205 0.00 0.00
A
ATOM 2000 HD3 ILE A 132 14.827 30.752 49.764 0.00 0.00
A
ATOM 2001 C ILE A 132 17.349 31.032 54.717 0.00 0.00
A
ATOM 2002 O ILE A 132 17.151 32.153 55.168 0.00 0.00
A
ATOM 2003 N ALA A 133 17.907 30.123 55.586 0.00 0.00
A
ATOM 2004 HN ALA A 133 18.082 29.180 55.316 0.00 0.00
A
ATOM 2005 CA ALA A 133 18.369 30.414 56.899 0.00 0.00
A
ATOM 2006 HA ALA A 133 17.444 30.781 57.319 0.00 0.00
A
ATOM 2007 CB ALA A 133 18.886 29.099 57.621 0.00 0.00
A
ATOM 2008 HB1 ALA A 133 18.085 28.411 57.968 0.00 0.00
A
ATOM 2009 HB2 ALA A 133 19.572 28.555 56.937 0.00 0.00
A
ATOM 2010 HB3 ALA A 133 19.637 29.326 58.409 0.00 0.00
A
ATOM 2011 C ALA A 133 19.477 31.460 57.027 0.00 0.00
A
ATOM 2012 O ALA A 133 19.523 32.243 57.941 0.00 0.00
A
ATOM 2013 N GLN A 134 20.471 31.561 56.153 0.00 0.00
A
ATOM 2014 HN GLN A 134 20.534 30.813 55.497 0.00 0.00
A
ATOM 2015 CA GLN A 134 21.349 32.639 56.004 0.00 0.00
A
ATOM 2016 HA GLN A 134 21.740 32.653 57.010 0.00 0.00
A
ATOM 2017 CB GLN A 134 22.393 32.228 54.876 0.00 0.00
A
ATOM 2018 HB1 GLN A 134 22.583 31.136 54.803 0.00 0.00
A
ATOM 2019 HB2 GLN A 134 21.938 32.445 53.886 0.00 0.00
A
ATOM 2020 CG GLN A 134 23.792 32.958 54.999 0.00 0.00
A
ATOM 2021 HG1 GLN A 134 24.363 32.466 54.183 0.00 0.00
A
ATOM 2022 HG2 GLN A 134 23.726 34.050 54.810 0.00 0.00
A
ATOM 2023 CD GLN A 134 24.446 32.822 56.384 0.00 0.00
A
ATOM 2024 OE1 GLN A 134 25.287 31.907 56.544 0.00 0.00
A

ATOM 2025 NE2 GLN A 134 24.071 33.770 57.217 0.00 0.00
A
ATOM 2026 HE21 GLN A 134 24.497 33.800 58.121 0.00 0.00
A
ATOM 2027 HE22 GLN A 134 23.319 34.381 56.968 0.00 0.00
A
ATOM 2028 C GLN A 134 20.726 33.977 55.728 0.00 0.00
A
ATOM 2029 O GLN A 134 21.070 34.896 56.480 0.00 0.00
A
ATOM 2030 N SER A 135 19.769 33.995 54.782 0.00 0.00
A
ATOM 2031 HN SER A 135 19.485 33.099 54.452 0.00 0.00
A
ATOM 2032 CA SER A 135 19.047 35.162 54.451 0.00 0.00
A
ATOM 2033 HA SER A 135 19.806 35.892 54.214 0.00 0.00
A
ATOM 2034 CB SER A 135 17.976 34.906 53.329 0.00 0.00
A
ATOM 2035 HB1 SER A 135 18.391 34.382 52.441 0.00 0.00
A
ATOM 2036 HB2 SER A 135 17.244 34.157 53.701 0.00 0.00
A
ATOM 2037 OG SER A 135 17.344 36.031 52.819 0.00 0.00
A
ATOM 2038 HG1 SER A 135 16.991 35.742 51.974 0.00 0.00
A
ATOM 2039 C SER A 135 18.258 35.587 55.648 0.00 0.00
A
ATOM 2040 O SER A 135 18.306 36.814 56.022 0.00 0.00
A
ATOM 2041 N LEU A 136 17.591 34.624 56.288 0.00 0.00
A
ATOM 2042 HN LEU A 136 17.545 33.751 55.808 0.00 0.00
A
ATOM 2043 CA LEU A 136 16.757 34.900 57.437 0.00 0.00
A
ATOM 2044 HA LEU A 136 16.236 35.823 57.229 0.00 0.00
A
ATOM 2045 CB LEU A 136 15.834 33.682 57.719 0.00 0.00
A
ATOM 2046 HB1 LEU A 136 15.407 33.323 56.758 0.00 0.00
A
ATOM 2047 HB2 LEU A 136 16.476 32.806 57.956 0.00 0.00
A
ATOM 2048 CG LEU A 136 14.800 33.832 58.789 0.00 0.00
A
ATOM 2049 HG LEU A 136 15.323 34.143 59.718 0.00 0.00
A
ATOM 2050 CD1 LEU A 136 13.819 34.991 58.519 0.00 0.00
A
ATOM 2051 HD11 LEU A 136 13.535 34.992 57.445 0.00 0.00
A

```
ATOM   2052 HD12 LEU A 136      12.843  34.738  58.986  0.00  0.00
A
ATOM   2053 HD13 LEU A 136      14.230  35.975  58.829  0.00  0.00
A
ATOM   2054  CD2 LEU A 136      14.062  32.447  58.987  0.00  0.00
A
ATOM   2055 HD21 LEU A 136      14.750  31.575  59.019  0.00  0.00
A
ATOM   2056 HD22 LEU A 136      13.444  32.627  59.893  0.00  0.00
A
ATOM   2057 HD23 LEU A 136      13.388  32.346  58.110  0.00  0.00
A
ATOM   2058  C   LEU A 136      17.493  35.323  58.688  0.00  0.00
A
ATOM   2059  O   LEU A 136      17.042  36.152  59.467  0.00  0.00
A
ATOM   2060  N   SER A 137      18.717  34.716  58.976  0.00  0.00
A
ATOM   2061  HN  SER A 137      19.057  33.934  58.459  0.00  0.00
A
ATOM   2062  CA  SER A 137      19.448  34.978  60.200  0.00  0.00
A
ATOM   2063  HA  SER A 137      18.742  34.883  61.012  0.00  0.00
A
ATOM   2064  CB  SER A 137      20.689  34.012  60.414  0.00  0.00
A
ATOM   2065  HB1 SER A 137      20.289  32.981  60.310  0.00  0.00
A
ATOM   2066  HB2 SER A 137      21.366  34.123  59.540  0.00  0.00
A
ATOM   2067  OG  SER A 137      21.320  34.063  61.689  0.00  0.00
A
ATOM   2068  HG1 SER A 137      22.186  34.429  61.497  0.00  0.00
A
ATOM   2069  C   SER A 137      20.009  36.413  60.284  0.00  0.00
A
ATOM   2070  O   SER A 137      20.082  36.941  61.404  0.00  0.00
A
ATOM   2071  N   ASP A 138      20.556  36.979  59.188  0.00  0.00
A
ATOM   2072  HN  ASP A 138      20.554  36.468  58.332  0.00  0.00
A
ATOM   2073  CA  ASP A 138      21.351  38.192  59.241  0.00  0.00
A
ATOM   2074  HA  ASP A 138      20.979  38.799  60.053  0.00  0.00
A
ATOM   2075  CB  ASP A 138      22.869  37.973  59.444  0.00  0.00
A
ATOM   2076  HB1 ASP A 138      23.019  37.217  60.244  0.00  0.00
A
ATOM   2077  HB2 ASP A 138      23.371  37.570  58.538  0.00  0.00
A
ATOM   2078  CG  ASP A 138      23.538  39.249  60.023  0.00  0.00
A
```

ATOM 2079 OD1 ASP A 138 24.398 39.786 59.273 0.00 0.00
A
ATOM 2080 OD2 ASP A 138 23.301 39.766 61.146 0.00 0.00
A
ATOM 2081 C ASP A 138 21.078 39.181 58.105 0.00 0.00
A
ATOM 2082 O ASP A 138 20.830 38.796 56.967 0.00 0.00
A
ATOM 2083 N GLU A 139 21.099 40.499 58.282 0.00 0.00
A
ATOM 2084 HN GLU A 139 21.222 40.929 59.173 0.00 0.00
A
ATOM 2085 CA GLU A 139 20.837 41.467 57.200 0.00 0.00
A
ATOM 2086 HA GLU A 139 20.453 40.899 56.366 0.00 0.00
A
ATOM 2087 CB GLU A 139 19.812 42.554 57.637 0.00 0.00
A
ATOM 2088 HB1 GLU A 139 19.903 42.815 58.713 0.00 0.00
A
ATOM 2089 HB2 GLU A 139 20.042 43.495 57.093 0.00 0.00
A
ATOM 2090 CG GLU A 139 18.247 42.286 57.556 0.00 0.00
A
ATOM 2091 HG1 GLU A 139 17.940 42.444 56.500 0.00 0.00
A
ATOM 2092 HG2 GLU A 139 17.988 41.285 57.963 0.00 0.00
A
ATOM 2093 CD GLU A 139 17.420 43.331 58.350 0.00 0.00
A
ATOM 2094 OE1 GLU A 139 17.090 43.102 59.494 0.00 0.00
A
ATOM 2095 OE2 GLU A 139 17.228 44.523 57.878 0.00 0.00
A
ATOM 2096 C GLU A 139 22.169 42.240 56.740 0.00 0.00
A
ATOM 2097 O GLU A 139 22.015 43.061 55.826 0.00 0.00
A
ATOM 2098 N ASN A 140 23.312 41.928 57.332 0.00 0.00
A
ATOM 2099 HN ASN A 140 23.282 41.422 58.190 0.00 0.00
A
ATOM 2100 CA ASN A 140 24.586 42.472 56.991 0.00 0.00
A
ATOM 2101 HA ASN A 140 24.448 43.360 56.391 0.00 0.00
A
ATOM 2102 CB ASN A 140 25.293 42.982 58.301 0.00 0.00
A
ATOM 2103 HB1 ASN A 140 25.176 42.100 58.966 0.00 0.00
A
ATOM 2104 HB2 ASN A 140 26.377 43.106 58.095 0.00 0.00
A
ATOM 2105 CG ASN A 140 24.698 44.259 58.873 0.00 0.00
A

```
ATOM   2106  OD1 ASN A 140      23.599  44.375  59.414  0.00  0.00
A
ATOM   2107  ND2 ASN A 140      25.490  45.349  58.843  0.00  0.00
A
ATOM   2108 HD21 ASN A 140      25.111  46.197  59.213  0.00  0.00
A
ATOM   2109 HD22 ASN A 140      26.377  45.292  58.386  0.00  0.00
A
ATOM   2110  C   ASN A 140      25.451  41.452  56.236  0.00  0.00
A
ATOM   2111  O   ASN A 140      26.364  41.764  55.555  0.00  0.00
A
ATOM   2112  N   VAL A 141      25.138  40.148  56.441  0.00  0.00
A
ATOM   2113  HN  VAL A 141      24.631  39.821  57.234  0.00  0.00
A
ATOM   2114  CA  VAL A 141      25.800  39.080  55.622  0.00  0.00
A
ATOM   2115  HA  VAL A 141      26.808  39.306  55.309  0.00  0.00
A
ATOM   2116  CB  VAL A 141      25.757  37.685  56.201  0.00  0.00
A
ATOM   2117  HB  VAL A 141      24.711  37.494  56.523  0.00  0.00
A
ATOM   2118  CG1 VAL A 141      26.280  36.590  55.217  0.00  0.00
A
ATOM   2119 HG11 VAL A 141      26.238  35.635  55.784  0.00  0.00
A
ATOM   2120 HG12 VAL A 141      25.588  36.369  54.376  0.00  0.00
A
ATOM   2121 HG13 VAL A 141      27.250  36.802  54.719  0.00  0.00
A
ATOM   2122  CG2 VAL A 141      26.613  37.802  57.513  0.00  0.00
A
ATOM   2123 HG21 VAL A 141      27.614  38.201  57.240  0.00  0.00
A
ATOM   2124 HG22 VAL A 141      26.120  38.409  58.302  0.00  0.00
A
ATOM   2125 HG23 VAL A 141      26.792  36.763  57.864  0.00  0.00
A
ATOM   2126  C   VAL A 141      24.932  39.026  54.340  0.00  0.00
A
ATOM   2127  O   VAL A 141      23.798  38.559  54.290  0.00  0.00
A
ATOM   2128  N   LEU A 142      25.438  39.398  53.160  0.00  0.00
A
ATOM   2129  HN  LEU A 142      26.325  39.812  52.973  0.00  0.00
A
ATOM   2130  CA  LEU A 142      24.681  39.369  51.895  0.00  0.00
A
ATOM   2131  HA  LEU A 142      23.632  39.606  52.000  0.00  0.00
A
ATOM   2132  CB  LEU A 142      25.336  40.330  50.912  0.00  0.00
A
```

ATOM 2133 HB1 LEU A 142 26.440 40.263 50.806 0.00 0.00
A
ATOM 2134 HB2 LEU A 142 24.989 40.112 49.880 0.00 0.00
A
ATOM 2135 CG LEU A 142 25.045 41.828 51.136 0.00 0.00
A
ATOM 2136 HG LEU A 142 25.805 42.200 50.417 0.00 0.00
A
ATOM 2137 CD1 LEU A 142 23.674 42.321 50.693 0.00 0.00
A
ATOM 2138 HD11 LEU A 142 23.522 42.196 49.599 0.00 0.00
A
ATOM 2139 HD12 LEU A 142 22.878 41.715 51.176 0.00 0.00
A
ATOM 2140 HD13 LEU A 142 23.615 43.423 50.824 0.00 0.00
A
ATOM 2141 CD2 LEU A 142 25.450 42.442 52.479 0.00 0.00
A
ATOM 2142 HD21 LEU A 142 26.416 42.138 52.937 0.00 0.00
A
ATOM 2143 HD22 LEU A 142 25.488 43.551 52.429 0.00 0.00
A
ATOM 2144 HD23 LEU A 142 24.692 42.163 53.240 0.00 0.00
A
ATOM 2145 C LEU A 142 24.784 37.976 51.376 0.00 0.00
A
ATOM 2146 O LEU A 142 23.727 37.446 50.990 0.00 0.00
A
ATOM 2147 N ASN A 143 25.976 37.342 51.371 0.00 0.00
A
ATOM 2148 HN ASN A 143 26.766 37.779 51.794 0.00 0.00
A
ATOM 2149 CA ASN A 143 26.119 35.968 50.867 0.00 0.00
A
ATOM 2150 HA ASN A 143 25.161 35.496 51.025 0.00 0.00
A
ATOM 2151 CB ASN A 143 26.505 35.973 49.344 0.00 0.00
A
ATOM 2152 HB1 ASN A 143 25.864 36.737 48.856 0.00 0.00
A
ATOM 2153 HB2 ASN A 143 27.537 36.325 49.132 0.00 0.00
A
ATOM 2154 CG ASN A 143 26.117 34.661 48.655 0.00 0.00
A
ATOM 2155 OD1 ASN A 143 25.480 33.713 49.179 0.00 0.00
A
ATOM 2156 ND2 ASN A 143 26.482 34.586 47.338 0.00 0.00
A
ATOM 2157 HD21 ASN A 143 26.253 33.823 46.733 0.00 0.00
A
ATOM 2158 HD22 ASN A 143 26.968 35.379 46.971 0.00 0.00
A
ATOM 2159 C ASN A 143 27.162 35.146 51.700 0.00 0.00
A

ATOM 2160 O ASN A 143 28.170 35.673 52.217 0.00 0.00
A
ATOM 2161 N TYR A 144 26.956 33.805 51.867 0.00 0.00
A
ATOM 2162 HN TYR A 144 26.057 33.474 51.593 0.00 0.00
A
ATOM 2163 CA TYR A 144 27.813 33.031 52.722 0.00 0.00
A
ATOM 2164 HA TYR A 144 28.749 33.534 52.914 0.00 0.00
A
ATOM 2165 CB TYR A 144 27.201 32.681 54.098 0.00 0.00
A
ATOM 2166 HB1 TYR A 144 26.717 33.569 54.559 0.00 0.00
A
ATOM 2167 HB2 TYR A 144 26.392 31.932 53.962 0.00 0.00
A
ATOM 2168 CG TYR A 144 28.104 32.061 55.097 0.00 0.00
A
ATOM 2169 CD1 TYR A 144 28.461 30.684 55.077 0.00 0.00
A
ATOM 2170 HD1 TYR A 144 28.227 30.074 54.217 0.00 0.00
A
ATOM 2171 CE1 TYR A 144 29.268 30.139 56.067 0.00 0.00
A
ATOM 2172 HE1 TYR A 144 29.573 29.103 56.104 0.00 0.00
A
ATOM 2173 CZ TYR A 144 29.579 30.829 57.248 0.00 0.00
A
ATOM 2174 OH TYR A 144 30.241 30.141 58.317 0.00 0.00
A
ATOM 2175 HH TYR A 144 30.113 29.210 58.121 0.00 0.00
A
ATOM 2176 CD2 TYR A 144 28.596 32.805 56.254 0.00 0.00
A
ATOM 2177 HD2 TYR A 144 28.189 33.791 56.419 0.00 0.00
A
ATOM 2178 CE2 TYR A 144 29.244 32.183 57.338 0.00 0.00
A
ATOM 2179 HE2 TYR A 144 29.583 32.678 58.236 0.00 0.00
A
ATOM 2180 C TYR A 144 28.312 31.753 51.999 0.00 0.00
A
ATOM 2181 O TYR A 144 27.545 30.850 51.726 0.00 0.00
A
ATOM 2182 N ILE A 145 29.676 31.601 51.815 0.00 0.00
A
ATOM 2183 HN ILE A 145 30.241 32.324 52.206 0.00 0.00
A
ATOM 2184 CA ILE A 145 30.228 30.561 51.064 0.00 0.00
A
ATOM 2185 HA ILE A 145 29.532 29.787 50.775 0.00 0.00
A
ATOM 2186 CB ILE A 145 30.837 31.151 49.879 0.00 0.00
A

```
ATOM   2187  HB  ILE A 145      31.653  31.842  50.177  0.00  0.00
A
ATOM   2188  CG2 ILE A 145      31.576  30.064  49.091  0.00  0.00
A
ATOM   2189 HG21 ILE A 145      30.824  29.287  48.836  0.00  0.00
A
ATOM   2190 HG22 ILE A 145      31.842  30.430  48.077  0.00  0.00
A
ATOM   2191 HG23 ILE A 145      32.466  29.692  49.644  0.00  0.00
A
ATOM   2192  CG1 ILE A 145      29.922  31.882  48.900  0.00  0.00
A
ATOM   2193 HG11 ILE A 145      29.567  32.818  49.382  0.00  0.00
A
ATOM   2194 HG12 ILE A 145      30.522  32.231  48.033  0.00  0.00
A
ATOM   2195  CD  ILE A 145      28.690  31.093  48.363  0.00  0.00
A
ATOM   2196  HD1 ILE A 145      28.150  31.659  47.574  0.00  0.00
A
ATOM   2197  HD2 ILE A 145      29.037  30.139  47.911  0.00  0.00
A
ATOM   2198  HD3 ILE A 145      28.042  30.772  49.206  0.00  0.00
A
ATOM   2199  C   ILE A 145      31.064  29.823  52.105  0.00  0.00
A
ATOM   2200  O   ILE A 145      32.002  30.413  52.687  0.00  0.00
A
ATOM   2201  N   ASP A 146      30.897  28.495  52.256  0.00  0.00
A
ATOM   2202  HN  ASP A 146      30.158  27.931  51.896  0.00  0.00
A
ATOM   2203  CA  ASP A 146      31.602  27.596  53.112  0.00  0.00
A
ATOM   2204  HA  ASP A 146      32.000  28.060  54.002  0.00  0.00
A
ATOM   2205  CB  ASP A 146      30.745  26.363  53.405  0.00  0.00
A
ATOM   2206  HB1 ASP A 146      30.164  25.949  52.553  0.00  0.00
A
ATOM   2207  HB2 ASP A 146      31.244  25.494  53.885  0.00  0.00
A
ATOM   2208  CG  ASP A 146      29.682  26.795  54.415  0.00  0.00
A
ATOM   2209  OD1 ASP A 146      29.887  26.501  55.607  0.00  0.00
A
ATOM   2210  OD2 ASP A 146      28.567  27.320  54.007  0.00  0.00
A
ATOM   2211  C   ASP A 146      32.809  27.155  52.266  0.00  0.00
A
ATOM   2212  O   ASP A 146      32.606  26.507  51.235  0.00  0.00
A
ATOM   2213  N   LEU A 147      34.040  27.150  52.870  0.00  0.00
A
```

```
ATOM   2214  HN  LEU A 147      34.267  27.660  53.696  0.00  0.00
A
ATOM   2215  CA  LEU A 147      35.150  26.356  52.303  0.00  0.00
A
ATOM   2216  HA  LEU A 147      35.158  26.196  51.235  0.00  0.00
A
ATOM   2217  CB  LEU A 147      36.452  27.019  52.831  0.00  0.00
A
ATOM   2218  HB1 LEU A 147      36.228  27.372  53.860  0.00  0.00
A
ATOM   2219  HB2 LEU A 147      37.322  26.331  52.878  0.00  0.00
A
ATOM   2220  CG  LEU A 147      36.761  28.270  52.037  0.00  0.00
A
ATOM   2221  HG  LEU A 147      35.904  28.971  51.949  0.00  0.00
A
ATOM   2222  CD1 LEU A 147      37.721  29.132  52.837  0.00  0.00
A
ATOM   2223 HD11 LEU A 147      37.367  29.318  53.873  0.00  0.00
A
ATOM   2224 HD12 LEU A 147      38.672  28.564  52.929  0.00  0.00
A
ATOM   2225 HD13 LEU A 147      37.798  30.136  52.367  0.00  0.00
A
ATOM   2226  CD2 LEU A 147      37.535  28.012  50.735  0.00  0.00
A
ATOM   2227 HD21 LEU A 147      38.521  27.575  51.004  0.00  0.00
A
ATOM   2228 HD22 LEU A 147      36.925  27.359  50.076  0.00  0.00
A
ATOM   2229 HD23 LEU A 147      37.698  28.931  50.132  0.00  0.00
A
ATOM   2230  C   LEU A 147      35.088  24.991  52.956  0.00  0.00
A
ATOM   2231  O   LEU A 147      35.337  23.947  52.392  0.00  0.00
A
ATOM   2232  N   SER A 148      34.744  25.006  54.253  0.00  0.00
A
ATOM   2233  HN  SER A 148      34.446  25.859  54.675  0.00  0.00
A
ATOM   2234  CA  SER A 148      34.499  23.735  55.017  0.00  0.00
A
ATOM   2235  HA  SER A 148      34.162  22.863  54.476  0.00  0.00
A
ATOM   2236  CB  SER A 148      35.879  23.408  55.652  0.00  0.00
A
ATOM   2237  HB1 SER A 148      36.518  23.494  54.748  0.00  0.00
A
ATOM   2238  HB2 SER A 148      36.104  24.238  56.355  0.00  0.00
A
ATOM   2239  OG  SER A 148      35.963  22.103  56.250  0.00  0.00
A
ATOM   2240  HG1 SER A 148      36.647  22.223  56.913  0.00  0.00
A
```

ATOM 2241 C SER A 148 33.433 24.029 56.098 0.00 0.00
A
ATOM 2242 O SER A 148 32.860 25.105 56.150 0.00 0.00
A
ATOM 2243 N ASP A 149 33.250 22.978 56.980 0.00 0.00
A
ATOM 2244 HN ASP A 149 33.681 22.094 56.815 0.00 0.00
A
ATOM 2245 CA ASP A 149 32.303 22.992 58.078 0.00 0.00
A
ATOM 2246 HA ASP A 149 31.314 22.892 57.655 0.00 0.00
A
ATOM 2247 CB ASP A 149 32.626 21.817 58.956 0.00 0.00
A
ATOM 2248 HB1 ASP A 149 33.715 21.677 59.126 0.00 0.00
A
ATOM 2249 HB2 ASP A 149 32.164 21.910 59.962 0.00 0.00
A
ATOM 2250 CG ASP A 149 32.046 20.639 58.312 0.00 0.00
A
ATOM 2251 OD1 ASP A 149 30.871 20.692 57.926 0.00 0.00
A
ATOM 2252 OD2 ASP A 149 32.790 19.639 58.221 0.00 0.00
A
ATOM 2253 C ASP A 149 32.362 24.239 58.958 0.00 0.00
A
ATOM 2254 O ASP A 149 31.376 24.848 59.387 0.00 0.00
A
ATOM 2255 N GLU A 150 33.639 24.611 59.223 0.00 0.00
A
ATOM 2256 HN GLU A 150 34.460 24.148 58.899 0.00 0.00
A
ATOM 2257 CA GLU A 150 33.945 25.766 60.042 0.00 0.00
A
ATOM 2258 HA GLU A 150 33.119 26.399 60.329 0.00 0.00
A
ATOM 2259 CB GLU A 150 34.614 25.188 61.292 0.00 0.00
A
ATOM 2260 HB1 GLU A 150 34.945 25.989 61.987 0.00 0.00
A
ATOM 2261 HB2 GLU A 150 33.831 24.617 61.834 0.00 0.00
A
ATOM 2262 CG GLU A 150 35.877 24.375 60.924 0.00 0.00
A
ATOM 2263 HG1 GLU A 150 35.650 23.483 60.301 0.00 0.00
A
ATOM 2264 HG2 GLU A 150 36.557 25.171 60.552 0.00 0.00
A
ATOM 2265 CD GLU A 150 36.448 23.663 62.176 0.00 0.00
A
ATOM 2266 OE1 GLU A 150 36.726 22.450 62.107 0.00 0.00
A
ATOM 2267 OE2 GLU A 150 36.767 24.297 63.182 0.00 0.00
A

ATOM 2268 C GLU A 150 34.883 26.836 59.374 0.00 0.00 A
ATOM 2269 O GLU A 150 35.486 27.649 60.005 0.00 0.00 A
ATOM 2270 N TYR A 151 35.080 26.733 58.063 0.00 0.00 A
ATOM 2271 HN TYR A 151 34.687 26.041 57.462 0.00 0.00 A
ATOM 2272 CA TYR A 151 35.926 27.738 57.313 0.00 0.00 A
ATOM 2273 HA TYR A 151 36.343 28.496 57.959 0.00 0.00 A
ATOM 2274 CB TYR A 151 37.057 27.052 56.537 0.00 0.00 A
ATOM 2275 HB1 TYR A 151 36.819 26.153 55.930 0.00 0.00 A
ATOM 2276 HB2 TYR A 151 37.485 27.871 55.919 0.00 0.00 A
ATOM 2277 CG TYR A 151 38.068 26.621 57.595 0.00 0.00 A
ATOM 2278 CD1 TYR A 151 38.866 27.577 58.246 0.00 0.00 A
ATOM 2279 HD1 TYR A 151 38.738 28.628 58.032 0.00 0.00 A
ATOM 2280 CE1 TYR A 151 39.931 27.249 59.045 0.00 0.00 A
ATOM 2281 HE1 TYR A 151 40.511 27.992 59.572 0.00 0.00 A
ATOM 2282 CZ TYR A 151 40.089 25.927 59.417 0.00 0.00 A
ATOM 2283 OH TYR A 151 40.868 25.626 60.596 0.00 0.00 A
ATOM 2284 HH TYR A 151 41.721 26.061 60.532 0.00 0.00 A
ATOM 2285 CD2 TYR A 151 38.242 25.292 57.986 0.00 0.00 A
ATOM 2286 HD2 TYR A 151 37.730 24.434 57.576 0.00 0.00 A
ATOM 2287 CE2 TYR A 151 39.150 24.913 58.896 0.00 0.00 A
ATOM 2288 HE2 TYR A 151 39.320 23.862 59.078 0.00 0.00 A
ATOM 2289 C TYR A 151 35.026 28.392 56.315 0.00 0.00 A
ATOM 2290 O TYR A 151 34.278 27.776 55.625 0.00 0.00 A
ATOM 2291 N SER A 152 35.065 29.752 56.148 0.00 0.00 A
ATOM 2292 HN SER A 152 35.716 30.352 56.607 0.00 0.00 A
ATOM 2293 CA SER A 152 34.082 30.425 55.313 0.00 0.00 A
ATOM 2294 HA SER A 152 33.846 29.729 54.522 0.00 0.00 A

ATOM   2295 CB SER A 152   32.791 30.685 56.191 0.00 0.00
A
ATOM   2296 HB1 SER A 152   32.129 31.251 55.500 0.00 0.00
A
ATOM   2297 HB2 SER A 152   32.186 29.775 56.391 0.00 0.00
A
ATOM   2298 OG SER A 152   33.109 31.453 57.420 0.00 0.00
A
ATOM   2299 HG1 SER A 152   32.279 31.455 57.903 0.00 0.00
A
ATOM   2300 C   SER A 152   34.578 31.678 54.599 0.00 0.00
A
ATOM   2301 O   SER A 152   35.625 32.251 54.967 0.00 0.00
A
ATOM   2302 N   ILE A 153   33.937 32.115 53.475 0.00 0.00
A
ATOM   2303 HN ILE A 153   33.261 31.470 53.127 0.00 0.00
A
ATOM   2304 CA ILE A 153   34.190 33.316 52.763 0.00 0.00
A
ATOM   2305 HA ILE A 153   34.915 33.910 53.298 0.00 0.00
A
ATOM   2306 CB ILE A 153   34.695 33.125 51.419 0.00 0.00
A
ATOM   2307 HB ILE A 153   33.877 32.733 50.777 0.00 0.00
A
ATOM   2308 CG2 ILE A 153   35.046 34.510 50.732 0.00 0.00
A
ATOM   2309 HG21 ILE A 153   36.002 34.918 51.127 0.00 0.00
A
ATOM   2310 HG22 ILE A 153   35.336 34.369 49.669 0.00 0.00
A
ATOM   2311 HG23 ILE A 153   34.280 35.307 50.835 0.00 0.00
A
ATOM   2312 CG1 ILE A 153   35.971 32.221 51.340 0.00 0.00
A
ATOM   2313 HG11 ILE A 153   36.910 32.814 51.327 0.00 0.00
A
ATOM   2314 HG12 ILE A 153   35.921 31.572 52.241 0.00 0.00
A
ATOM   2315 CD ILE A 153   35.838 31.337 50.097 0.00 0.00
A
ATOM   2316 HD1 ILE A 153   35.757 31.825 49.103 0.00 0.00
A
ATOM   2317 HD2 ILE A 153   36.748 30.720 49.937 0.00 0.00
A
ATOM   2318 HD3 ILE A 153   34.996 30.615 50.170 0.00 0.00
A
ATOM   2319 C   ILE A 153   32.851 34.112 52.679 0.00 0.00
A
ATOM   2320 O   ILE A 153   31.837 33.792 52.032 0.00 0.00
A
ATOM   2321 N   VAL A 154   32.909 35.322 53.281 0.00 0.00
A

ATOM 2322 HN VAL A 154 33.708 35.739 53.710 0.00 0.00
A
ATOM 2323 CA VAL A 154 31.710 36.205 53.598 0.00 0.00
A
ATOM 2324 HA VAL A 154 30.780 35.805 53.220 0.00 0.00
A
ATOM 2325 CB VAL A 154 31.486 36.395 55.124 0.00 0.00
A
ATOM 2326 HB VAL A 154 32.032 37.287 55.499 0.00 0.00
A
ATOM 2327 CG1 VAL A 154 29.971 36.627 55.475 0.00 0.00
A
ATOM 2328 HG11 VAL A 154 29.691 37.598 55.013 0.00 0.00
A
ATOM 2329 HG12 VAL A 154 29.289 35.819 55.135 0.00 0.00
A
ATOM 2330 HG13 VAL A 154 29.815 36.850 56.552 0.00 0.00
A
ATOM 2331 CG2 VAL A 154 31.838 35.119 55.930 0.00 0.00
A
ATOM 2332 HG21 VAL A 154 31.300 35.209 56.898 0.00 0.00
A
ATOM 2333 HG22 VAL A 154 31.432 34.279 55.327 0.00 0.00
A
ATOM 2334 HG23 VAL A 154 32.909 34.873 56.090 0.00 0.00
A
ATOM 2335 C VAL A 154 31.605 37.601 53.050 0.00 0.00
A
ATOM 2336 O VAL A 154 32.517 38.429 53.112 0.00 0.00
A
ATOM 2337 N GLU A 155 30.411 37.920 52.427 0.00 0.00
A
ATOM 2338 HN GLU A 155 29.730 37.194 52.364 0.00 0.00
A
ATOM 2339 CA GLU A 155 30.022 39.219 51.851 0.00 0.00
A
ATOM 2340 HA GLU A 155 30.913 39.752 51.554 0.00 0.00
A
ATOM 2341 CB GLU A 155 29.239 39.261 50.521 0.00 0.00
A
ATOM 2342 HB1 GLU A 155 29.891 38.669 49.843 0.00 0.00
A
ATOM 2343 HB2 GLU A 155 28.303 38.695 50.713 0.00 0.00
A
ATOM 2344 CG GLU A 155 29.078 40.611 49.854 0.00 0.00
A
ATOM 2345 HG1 GLU A 155 28.432 41.285 50.457 0.00 0.00
A
ATOM 2346 HG2 GLU A 155 30.043 41.146 49.719 0.00 0.00
A
ATOM 2347 CD GLU A 155 28.406 40.644 48.464 0.00 0.00
A
ATOM 2348 OE1 GLU A 155 29.038 40.109 47.535 0.00 0.00
A

ATOM 2349 OE2 GLU A 155 27.273 41.216 48.348 0.00 0.00
A
ATOM 2350 C GLU A 155 29.317 40.017 52.920 0.00 0.00
A
ATOM 2351 O GLU A 155 28.209 39.629 53.328 0.00 0.00
A
ATOM 2352 N LEU A 156 30.013 41.036 53.476 0.00 0.00
A
ATOM 2353 HN LEU A 156 30.845 41.261 52.974 0.00 0.00
A
ATOM 2354 CA LEU A 156 29.702 41.624 54.778 0.00 0.00
A
ATOM 2355 HA LEU A 156 28.910 41.072 55.262 0.00 0.00
A
ATOM 2356 CB LEU A 156 30.830 41.164 55.658 0.00 0.00
A
ATOM 2357 HB1 LEU A 156 30.980 40.063 55.656 0.00 0.00
A
ATOM 2358 HB2 LEU A 156 31.802 41.518 55.251 0.00 0.00
A
ATOM 2359 CG LEU A 156 30.715 41.705 57.100 0.00 0.00
A
ATOM 2360 HG LEU A 156 30.734 42.816 57.131 0.00 0.00
A
ATOM 2361 CD1 LEU A 156 29.327 41.170 57.696 0.00 0.00
A
ATOM 2362 HD11 LEU A 156 29.252 41.359 58.788 0.00 0.00
A
ATOM 2363 HD12 LEU A 156 28.477 41.622 57.142 0.00 0.00
A
ATOM 2364 HD13 LEU A 156 29.423 40.064 57.695 0.00 0.00
A
ATOM 2365 CD2 LEU A 156 31.926 41.259 57.996 0.00 0.00
A
ATOM 2366 HD21 LEU A 156 32.857 41.595 57.491 0.00 0.00
A
ATOM 2367 HD22 LEU A 156 31.825 41.819 58.950 0.00 0.00
A
ATOM 2368 HD23 LEU A 156 31.895 40.151 58.066 0.00 0.00
A
ATOM 2369 C LEU A 156 29.651 43.074 54.708 0.00 0.00
A
ATOM 2370 O LEU A 156 30.607 43.719 54.361 0.00 0.00
A
ATOM 2371 N LEU A 157 28.534 43.658 55.136 0.00 0.00
A
ATOM 2372 HN LEU A 157 27.864 42.927 55.237 0.00 0.00
A
ATOM 2373 CA LEU A 157 28.046 45.036 55.226 0.00 0.00
A
ATOM 2374 HA LEU A 157 28.528 45.497 54.376 0.00 0.00
A
ATOM 2375 CB LEU A 157 26.465 45.034 55.109 0.00 0.00
A

ATOM 2376 HB1 LEU A 157 26.222 44.342 54.274 0.00 0.00
A
ATOM 2377 HB2 LEU A 157 26.038 44.541 56.008 0.00 0.00
A
ATOM 2378 CG LEU A 157 25.918 46.430 54.856 0.00 0.00
A
ATOM 2379 HG LEU A 157 26.259 47.017 55.736 0.00 0.00
A
ATOM 2380 CD1 LEU A 157 26.303 47.043 53.476 0.00 0.00
A
ATOM 2381 HD11 LEU A 157 25.827 46.447 52.668 0.00 0.00
A
ATOM 2382 HD12 LEU A 157 25.996 48.101 53.330 0.00 0.00
A
ATOM 2383 HD13 LEU A 157 27.401 46.914 53.369 0.00 0.00
A
ATOM 2384 CD2 LEU A 157 24.357 46.432 55.053 0.00 0.00
A
ATOM 2385 HD21 LEU A 157 24.070 45.854 55.958 0.00 0.00
A
ATOM 2386 HD22 LEU A 157 24.078 47.503 55.146 0.00 0.00
A
ATOM 2387 HD23 LEU A 157 23.861 45.997 54.159 0.00 0.00
A
ATOM 2388 C LEU A 157 28.470 45.774 56.487 0.00 0.00
A
ATOM 2389 O LEU A 157 28.007 45.528 57.598 0.00 0.00
A
ATOM 2390 N ALA A 158 29.259 46.838 56.374 0.00 0.00
A
ATOM 2391 HN ALA A 158 29.711 47.116 55.529 0.00 0.00
A
ATOM 2392 CA ALA A 158 29.849 47.583 57.448 0.00 0.00
A
ATOM 2393 HA ALA A 158 30.322 46.913 58.150 0.00 0.00
A
ATOM 2394 CB ALA A 158 31.007 48.436 56.947 0.00 0.00
A
ATOM 2395 HB1 ALA A 158 30.646 49.272 56.310 0.00 0.00
A
ATOM 2396 HB2 ALA A 158 31.674 48.766 57.772 0.00 0.00
A
ATOM 2397 HB3 ALA A 158 31.602 47.748 56.308 0.00 0.00
A
ATOM 2398 C ALA A 158 28.839 48.544 58.263 0.00 0.00
A
ATOM 2399 O ALA A 158 27.832 49.121 57.874 0.00 0.00
A
ATOM 2400 N THR A 159 29.317 48.883 59.482 0.00 0.00
A
ATOM 2401 HN THR A 159 30.174 48.486 59.802 0.00 0.00
A
ATOM 2402 CA THR A 159 28.602 49.660 60.511 0.00 0.00
A

ATOM 2403 HA THR A 159 27.601 49.922 60.202 0.00 0.00
A
ATOM 2404 CB THR A 159 28.481 48.765 61.784 0.00 0.00
A
ATOM 2405 HB THR A 159 27.621 49.168 62.359 0.00 0.00
A
ATOM 2406 OG1 THR A 159 29.684 48.660 62.599 0.00 0.00
A
ATOM 2407 HG1 THR A 159 29.425 48.141 63.364 0.00 0.00
A
ATOM 2408 CG2 THR A 159 27.982 47.338 61.449 0.00 0.00
A
ATOM 2409 HG21 THR A 159 28.838 46.818 60.969 0.00 0.00
A
ATOM 2410 HG22 THR A 159 27.858 46.849 62.439 0.00 0.00
A
ATOM 2411 HG23 THR A 159 27.012 47.294 60.909 0.00 0.00
A
ATOM 2412 C THR A 159 29.371 50.964 60.903 0.00 0.00
A
ATOM 2413 O THR A 159 30.477 51.293 60.400 0.00 0.00
A
ATOM 2414 N ARG A 160 28.764 51.761 61.767 0.00 0.00
A
ATOM 2415 HN ARG A 160 27.809 51.623 62.020 0.00 0.00
A
ATOM 2416 CA ARG A 160 29.195 53.006 62.351 0.00 0.00
A
ATOM 2417 HA ARG A 160 29.337 53.629 61.481 0.00 0.00
A
ATOM 2418 CB ARG A 160 27.944 53.592 63.165 0.00 0.00
A
ATOM 2419 HB1 ARG A 160 27.109 53.707 62.441 0.00 0.00
A
ATOM 2420 HB2 ARG A 160 27.407 52.869 63.816 0.00 0.00
A
ATOM 2421 CG ARG A 160 28.158 54.912 63.851 0.00 0.00
A
ATOM 2422 HG1 ARG A 160 27.198 55.363 64.182 0.00 0.00
A
ATOM 2423 HG2 ARG A 160 28.853 54.838 64.715 0.00 0.00
A
ATOM 2424 CD ARG A 160 28.691 56.125 62.962 0.00 0.00
A
ATOM 2425 HD1 ARG A 160 28.781 57.070 63.540 0.00 0.00
A
ATOM 2426 HD2 ARG A 160 29.687 55.827 62.569 0.00 0.00
A
ATOM 2427 NE ARG A 160 27.802 56.380 61.791 0.00 0.00
A
ATOM 2428 HE ARG A 160 27.294 55.666 61.311 0.00 0.00
A
ATOM 2429 CZ ARG A 160 27.919 57.514 61.070 0.00 0.00
A

ATOM 2430 NH1 ARG A 160 28.680 58.512 61.403 0.00 0.00
A
ATOM 2431 HH11 ARG A 160 28.596 59.360 60.879 0.00 0.00
A
ATOM 2432 HH12 ARG A 160 29.345 58.488 62.149 0.00 0.00
A
ATOM 2433 NH2 ARG A 160 27.349 57.552 59.866 0.00 0.00
A
ATOM 2434 HH21 ARG A 160 27.098 58.398 59.395 0.00 0.00
A
ATOM 2435 HH22 ARG A 160 26.913 56.795 59.380 0.00 0.00
A
ATOM 2436 C ARG A 160 30.464 52.820 63.245 0.00 0.00
A
ATOM 2437 O ARG A 160 31.331 53.641 63.248 0.00 0.00
A
ATOM 2438 N LYS A 161 30.685 51.697 63.936 0.00 0.00
A
ATOM 2439 HN LYS A 161 29.942 51.033 63.937 0.00 0.00
A
ATOM 2440 CA LYS A 161 31.874 51.334 64.642 0.00 0.00
A
ATOM 2441 HA LYS A 161 32.110 52.114 65.351 0.00 0.00
A
ATOM 2442 CB LYS A 161 31.418 50.137 65.568 0.00 0.00
A
ATOM 2443 HB1 LYS A 161 30.485 50.451 66.084 0.00 0.00
A
ATOM 2444 HB2 LYS A 161 31.097 49.300 64.911 0.00 0.00
A
ATOM 2445 CG LYS A 161 32.511 49.723 66.599 0.00 0.00
A
ATOM 2446 HG1 LYS A 161 33.355 49.172 66.131 0.00 0.00
A
ATOM 2447 HG2 LYS A 161 32.950 50.677 66.961 0.00 0.00
A
ATOM 2448 CD LYS A 161 31.920 48.953 67.796 0.00 0.00
A
ATOM 2449 HD1 LYS A 161 31.035 49.549 68.108 0.00 0.00
A
ATOM 2450 HD2 LYS A 161 31.703 47.901 67.512 0.00 0.00
A
ATOM 2451 CE LYS A 161 32.887 48.969 69.004 0.00 0.00
A
ATOM 2452 HE1 LYS A 161 33.884 48.566 68.725 0.00 0.00
A
ATOM 2453 HE2 LYS A 161 32.845 50.031 69.328 0.00 0.00
A
ATOM 2454 NZ LYS A 161 32.359 48.007 70.089 0.00 0.00
A
ATOM 2455 HZ1 LYS A 161 32.154 47.076 69.675 0.00 0.00
A
ATOM 2456 HZ2 LYS A 161 33.194 47.889 70.697 0.00 0.00
A

ATOM 2457 HZ3 LYS A 161 31.506 48.381 70.554 0.00 0.00
A
ATOM 2458 C LYS A 161 33.072 51.147 63.750 0.00 0.00
A
ATOM 2459 O LYS A 161 34.130 51.631 64.061 0.00 0.00
A
ATOM 2460 N LEU A 162 32.925 50.524 62.575 0.00 0.00
A
ATOM 2461 HN LEU A 162 32.005 50.207 62.359 0.00 0.00
A
ATOM 2462 CA LEU A 162 33.942 50.359 61.508 0.00 0.00
A
ATOM 2463 HA LEU A 162 34.914 50.223 61.960 0.00 0.00
A
ATOM 2464 CB LEU A 162 33.610 49.236 60.554 0.00 0.00
A
ATOM 2465 HB1 LEU A 162 32.674 49.567 60.056 0.00 0.00
A
ATOM 2466 HB2 LEU A 162 34.450 49.098 59.840 0.00 0.00
A
ATOM 2467 CG LEU A 162 33.534 47.844 61.207 0.00 0.00
A
ATOM 2468 HG LEU A 162 32.644 47.791 61.869 0.00 0.00
A
ATOM 2469 CD1 LEU A 162 33.253 46.788 60.155 0.00 0.00
A
ATOM 2470 HD11 LEU A 162 32.264 47.037 59.714 0.00 0.00
A
ATOM 2471 HD12 LEU A 162 34.082 46.824 59.416 0.00 0.00
A
ATOM 2472 HD13 LEU A 162 33.321 45.728 60.478 0.00 0.00
A
ATOM 2473 CD2 LEU A 162 34.750 47.613 62.050 0.00 0.00
A
ATOM 2474 HD21 LEU A 162 34.737 48.350 62.882 0.00 0.00
A
ATOM 2475 HD22 LEU A 162 34.776 46.562 62.408 0.00 0.00
A
ATOM 2476 HD23 LEU A 162 35.705 47.618 61.483 0.00 0.00
A
ATOM 2477 C LEU A 162 34.054 51.573 60.590 0.00 0.00
A
ATOM 2478 O LEU A 162 35.068 51.609 59.896 0.00 0.00
A
ATOM 2479 N ASP A 163 33.125 52.550 60.599 0.00 0.00
A
ATOM 2480 HN ASP A 163 32.256 52.446 61.075 0.00 0.00
A
ATOM 2481 CA ASP A 163 33.214 53.762 59.750 0.00 0.00
A
ATOM 2482 HA ASP A 163 33.113 53.505 58.705 0.00 0.00
A
ATOM 2483 CB ASP A 163 31.968 54.684 60.043 0.00 0.00
A

ATOM	2484	HB1	ASP	A	163	30.994	54.156	60.130	0.00	0.00	A
ATOM	2485	HB2	ASP	A	163	32.130	55.176	61.025	0.00	0.00	A
ATOM	2486	CG	ASP	A	163	31.853	55.790	59.036	0.00	0.00	A
ATOM	2487	OD1	ASP	A	163	31.333	55.660	57.929	0.00	0.00	A
ATOM	2488	OD2	ASP	A	163	32.394	56.889	59.325	0.00	0.00	A
ATOM	2489	C	ASP	A	163	34.512	54.520	59.935	0.00	0.00	A
ATOM	2490	O	ASP	A	163	34.856	54.865	61.059	0.00	0.00	A
ATOM	2491	N	SER	A	164	35.270	54.870	58.845	0.00	0.00	A
ATOM	2492	HN	SER	A	164	35.138	54.388	57.983	0.00	0.00	A
ATOM	2493	CA	SER	A	164	36.369	55.812	58.977	0.00	0.00	A
ATOM	2494	HA	SER	A	164	36.713	55.901	57.958	0.00	0.00	A
ATOM	2495	CB	SER	A	164	36.012	57.219	59.403	0.00	0.00	A
ATOM	2496	HB1	SER	A	164	35.599	57.255	60.434	0.00	0.00	A
ATOM	2497	HB2	SER	A	164	36.844	57.946	59.287	0.00	0.00	A
ATOM	2498	OG	SER	A	164	35.039	57.624	58.450	0.00	0.00	A
ATOM	2499	HG1	SER	A	164	34.295	57.031	58.582	0.00	0.00	A
ATOM	2500	C	SER	A	164	37.635	55.292	59.750	0.00	0.00	A
ATOM	2501	O	SER	A	164	38.131	55.901	60.667	0.00	0.00	A
ATOM	2502	N	LYS	A	165	37.994	54.082	59.346	0.00	0.00	A
ATOM	2503	HN	LYS	A	165	37.435	53.637	58.650	0.00	0.00	A
ATOM	2504	CA	LYS	A	165	39.370	53.587	59.696	0.00	0.00	A
ATOM	2505	HA	LYS	A	165	40.042	54.411	59.888	0.00	0.00	A
ATOM	2506	CB	LYS	A	165	39.476	52.612	60.939	0.00	0.00	A
ATOM	2507	HB1	LYS	A	165	38.956	51.639	60.811	0.00	0.00	A
ATOM	2508	HB2	LYS	A	165	40.528	52.320	61.142	0.00	0.00	A
ATOM	2509	CG	LYS	A	165	38.929	53.118	62.245	0.00	0.00	A
ATOM	2510	HG1	LYS	A	165	39.462	52.433	62.938	0.00	0.00	A

ATOM 2511 HG2 LYS A 165 39.096 54.204 62.410 0.00 0.00
A
ATOM 2512 CD LYS A 165 37.449 52.746 62.556 0.00 0.00
A
ATOM 2513 HD1 LYS A 165 36.863 52.972 61.640 0.00 0.00
A
ATOM 2514 HD2 LYS A 165 37.473 51.691 62.903 0.00 0.00
A
ATOM 2515 CE LYS A 165 36.896 53.669 63.645 0.00 0.00
A
ATOM 2516 HE1 LYS A 165 35.991 53.129 63.999 0.00 0.00
A
ATOM 2517 HE2 LYS A 165 37.677 53.739 64.432 0.00 0.00
A
ATOM 2518 NZ LYS A 165 36.647 55.025 63.249 0.00 0.00
A
ATOM 2519 HZ1 LYS A 165 36.288 55.611 64.029 0.00 0.00
A
ATOM 2520 HZ2 LYS A 165 37.544 55.432 62.914 0.00 0.00
A
ATOM 2521 HZ3 LYS A 165 35.980 54.901 62.460 0.00 0.00
A
ATOM 2522 C LYS A 165 39.929 52.858 58.515 0.00 0.00
A
ATOM 2523 O LYS A 165 39.170 52.126 57.812 0.00 0.00
A
ATOM 2524 N SER A 166 41.293 52.760 58.413 0.00 0.00
A
ATOM 2525 HN SER A 166 41.914 53.214 59.047 0.00 0.00
A
ATOM 2526 CA SER A 166 41.966 52.038 57.333 0.00 0.00
A
ATOM 2527 HA SER A 166 41.290 51.844 56.514 0.00 0.00
A
ATOM 2528 CB SER A 166 43.187 52.822 56.813 0.00 0.00
A
ATOM 2529 HB1 SER A 166 43.539 52.311 55.891 0.00 0.00
A
ATOM 2530 HB2 SER A 166 42.902 53.896 56.786 0.00 0.00
A
ATOM 2531 OG SER A 166 44.266 52.810 57.787 0.00 0.00
A
ATOM 2532 HG1 SER A 166 44.808 53.591 57.655 0.00 0.00
A
ATOM 2533 C SER A 166 42.415 50.677 57.895 0.00 0.00
A
ATOM 2534 O SER A 166 42.296 50.488 59.124 0.00 0.00
A
ATOM 2535 N ILE A 167 42.839 49.678 57.030 0.00 0.00
A
ATOM 2536 HN ILE A 167 42.814 49.863 56.051 0.00 0.00
A
ATOM 2537 CA ILE A 167 43.272 48.315 57.400 0.00 0.00
A

ATOM 2538 HA ILE A 167 42.416 47.924 57.930 0.00 0.00
A
ATOM 2539 CB ILE A 167 43.428 47.301 56.138 0.00 0.00
A
ATOM 2540 HB ILE A 167 42.574 47.534 55.467 0.00 0.00
A
ATOM 2541 CG2 ILE A 167 44.723 47.644 55.411 0.00 0.00
A
ATOM 2542 HG21 ILE A 167 44.663 47.067 54.463 0.00 0.00
A
ATOM 2543 HG22 ILE A 167 44.673 48.723 55.149 0.00 0.00
A
ATOM 2544 HG23 ILE A 167 45.658 47.410 55.963 0.00 0.00
A
ATOM 2545 CG1 ILE A 167 43.441 45.867 56.665 0.00 0.00
A
ATOM 2546 HG11 ILE A 167 43.722 45.105 55.906 0.00 0.00
A
ATOM 2547 HG12 ILE A 167 44.343 45.791 57.309 0.00 0.00
A
ATOM 2548 CD ILE A 167 42.135 45.455 57.346 0.00 0.00
A
ATOM 2549 HD1 ILE A 167 42.031 45.975 58.322 0.00 0.00
A
ATOM 2550 HD2 ILE A 167 41.198 45.782 56.847 0.00 0.00
A
ATOM 2551 HD3 ILE A 167 42.136 44.356 57.515 0.00 0.00
A
ATOM 2552 C ILE A 167 44.491 48.335 58.425 0.00 0.00
A
ATOM 2553 O ILE A 167 44.633 47.510 59.355 0.00 0.00
A
ATOM 2554 N ILE A 168 45.477 49.229 58.230 0.00 0.00
A
ATOM 2555 HN ILE A 168 45.347 49.791 57.417 0.00 0.00
A
ATOM 2556 CA ILE A 168 46.553 49.425 59.204 0.00 0.00
A
ATOM 2557 HA ILE A 168 46.926 48.435 59.422 0.00 0.00
A
ATOM 2558 CB ILE A 168 47.778 50.217 58.659 0.00 0.00
A
ATOM 2559 HB ILE A 168 48.110 49.883 57.653 0.00 0.00
A
ATOM 2560 CG2 ILE A 168 47.485 51.708 58.515 0.00 0.00
A
ATOM 2561 HG21 ILE A 168 48.417 52.162 58.116 0.00 0.00
A
ATOM 2562 HG22 ILE A 168 46.667 51.808 57.770 0.00 0.00
A
ATOM 2563 HG23 ILE A 168 47.149 52.251 59.425 0.00 0.00
A
ATOM 2564 CG1 ILE A 168 49.123 49.973 59.450 0.00 0.00
A

```
ATOM   2565 HG11 ILE A 168      49.123  50.505  60.425  0.00  0.00
A
ATOM   2566 HG12 ILE A 168      49.120  48.888  59.691  0.00  0.00
A
ATOM   2567  CD  ILE A 168      50.471  50.319  58.817  0.00  0.00
A
ATOM   2568  HD1 ILE A 168      50.452  51.281  58.262  0.00  0.00
A
ATOM   2569  HD2 ILE A 168      51.271  50.254  59.585  0.00  0.00
A
ATOM   2570  HD3 ILE A 168      50.667  49.539  58.050  0.00  0.00
A
ATOM   2571  C   ILE A 168      46.152  50.034  60.615  0.00  0.00
A
ATOM   2572  O   ILE A 168      46.702  49.634  61.681  0.00  0.00
A
ATOM   2573  N   ASP A 169      45.182  50.999  60.594  0.00  0.00
A
ATOM   2574  HN  ASP A 169      44.860  51.362  59.723  0.00  0.00
A
ATOM   2575  CA  ASP A 169      44.525  51.464  61.794  0.00  0.00
A
ATOM   2576  HA  ASP A 169      45.211  51.684  62.598  0.00  0.00
A
ATOM   2577  CB  ASP A 169      43.670  52.715  61.484  0.00  0.00
A
ATOM   2578  HB1 ASP A 169      43.018  52.486  60.614  0.00  0.00
A
ATOM   2579  HB2 ASP A 169      43.077  52.939  62.396  0.00  0.00
A
ATOM   2580  CG  ASP A 169      44.639  53.874  61.166  0.00  0.00
A
ATOM   2581  OD1 ASP A 169      44.362  54.740  60.284  0.00  0.00
A
ATOM   2582  OD2 ASP A 169      45.761  53.774  61.727  0.00  0.00
A
ATOM   2583  C   ASP A 169      43.662  50.371  62.413  0.00  0.00
A
ATOM   2584  O   ASP A 169      43.587  50.400  63.669  0.00  0.00
A
ATOM   2585  N   LEU A 170      42.952  49.400  61.737  0.00  0.00
A
ATOM   2586  HN  LEU A 170      43.060  49.313  60.750  0.00  0.00
A
ATOM   2587  CA  LEU A 170      42.143  48.308  62.351  0.00  0.00
A
ATOM   2588  HA  LEU A 170      41.628  48.679  63.225  0.00  0.00
A
ATOM   2589  CB  LEU A 170      41.197  47.599  61.365  0.00  0.00
A
ATOM   2590  HB1 LEU A 170      41.808  47.320  60.480  0.00  0.00
A
ATOM   2591  HB2 LEU A 170      40.742  46.661  61.748  0.00  0.00
A
```

```
ATOM   2592  CG  LEU A 170      40.036  48.484  60.840  0.00  0.00
A
ATOM   2593  HG  LEU A 170      40.450  49.479  60.569  0.00  0.00
A
ATOM   2594  CD1 LEU A 170      39.577  48.013  59.475  0.00  0.00
A
ATOM   2595 HD11 LEU A 170      39.249  46.957  59.584  0.00  0.00
A
ATOM   2596 HD12 LEU A 170      38.763  48.622  59.028  0.00  0.00
A
ATOM   2597 HD13 LEU A 170      40.309  47.952  58.641  0.00  0.00
A
ATOM   2598  CD2 LEU A 170      38.814  48.737  61.770  0.00  0.00
A
ATOM   2599 HD21 LEU A 170      39.061  49.525  62.513  0.00  0.00
A
ATOM   2600 HD22 LEU A 170      37.900  49.005  61.198  0.00  0.00
A
ATOM   2601 HD23 LEU A 170      38.664  47.746  62.249  0.00  0.00
A
ATOM   2602  C   LEU A 170      43.062  47.217  62.980  0.00  0.00
A
ATOM   2603  O   LEU A 170      44.291  47.198  62.859  0.00  0.00
A
ATOM   2604  N   ASN A 171      42.431  46.360  63.727  0.00  0.00
A
ATOM   2605  HN  ASN A 171      41.489  46.503  64.021  0.00  0.00
A
ATOM   2606  CA  ASN A 171      43.103  45.225  64.360  0.00  0.00
A
ATOM   2607  HA  ASN A 171      44.166  45.168  64.181  0.00  0.00
A
ATOM   2608  CB  ASN A 171      42.847  45.346  65.884  0.00  0.00
A
ATOM   2609  HB1 ASN A 171      41.774  45.463  66.148  0.00  0.00
A
ATOM   2610  HB2 ASN A 171      43.229  44.512  66.510  0.00  0.00
A
ATOM   2611  CG  ASN A 171      43.579  46.538  66.461  0.00  0.00
A
ATOM   2612  OD1 ASN A 171      44.754  46.443  66.782  0.00  0.00
A
ATOM   2613  ND2 ASN A 171      42.885  47.682  66.598  0.00  0.00
A
ATOM   2614 HD21 ASN A 171      41.941  47.653  66.270  0.00  0.00
A
ATOM   2615 HD22 ASN A 171      43.314  48.457  67.064  0.00  0.00
A
ATOM   2616  C   ASN A 171      42.709  43.861  63.870  0.00  0.00
A
ATOM   2617  O   ASN A 171      43.065  42.836  64.419  0.00  0.00
A
ATOM   2618  N   VAL A 172      42.042  43.795  62.707  0.00  0.00
A
```

ATOM 2619 HN VAL A 172 41.960 44.668 62.233 0.00 0.00
A
ATOM 2620 CA VAL A 172 41.392 42.598 62.152 0.00 0.00
A
ATOM 2621 HA VAL A 172 40.816 42.190 62.969 0.00 0.00
A
ATOM 2622 CB VAL A 172 40.383 42.914 61.006 0.00 0.00
A
ATOM 2623 HB VAL A 172 40.861 43.250 60.061 0.00 0.00
A
ATOM 2624 CG1 VAL A 172 39.577 41.697 60.507 0.00 0.00
A
ATOM 2625 HG11 VAL A 172 40.241 40.828 60.314 0.00 0.00
A
ATOM 2626 HG12 VAL A 172 38.626 41.459 61.031 0.00 0.00
A
ATOM 2627 HG13 VAL A 172 39.224 41.753 59.456 0.00 0.00
A
ATOM 2628 CG2 VAL A 172 39.288 43.898 61.564 0.00 0.00
A
ATOM 2629 HG21 VAL A 172 39.027 43.528 62.578 0.00 0.00
A
ATOM 2630 HG22 VAL A 172 39.843 44.857 61.648 0.00 0.00
A
ATOM 2631 HG23 VAL A 172 38.385 44.003 60.924 0.00 0.00
A
ATOM 2632 C VAL A 172 42.286 41.502 61.747 0.00 0.00
A
ATOM 2633 O VAL A 172 42.038 40.370 62.019 0.00 0.00
A
ATOM 2634 N ARG A 173 43.355 41.934 61.046 0.00 0.00
A
ATOM 2635 HN ARG A 173 43.418 42.894 60.786 0.00 0.00
A
ATOM 2636 CA ARG A 173 44.279 41.018 60.544 0.00 0.00
A
ATOM 2637 HA ARG A 173 43.756 40.206 60.061 0.00 0.00
A
ATOM 2638 CB ARG A 173 45.230 41.796 59.548 0.00 0.00
A
ATOM 2639 HB1 ARG A 173 45.768 42.582 60.119 0.00 0.00
A
ATOM 2640 HB2 ARG A 173 45.960 41.081 59.112 0.00 0.00
A
ATOM 2641 CG ARG A 173 44.455 42.350 58.343 0.00 0.00
A
ATOM 2642 HG1 ARG A 173 43.817 41.549 57.912 0.00 0.00
A
ATOM 2643 HG2 ARG A 173 43.767 43.207 58.503 0.00 0.00
A
ATOM 2644 CD ARG A 173 45.401 42.696 57.109 0.00 0.00
A
ATOM 2645 HD1 ARG A 173 46.076 41.850 56.861 0.00 0.00
A

ATOM 2646 HD2 ARG A 173 44.756 42.953 56.242 0.00 0.00
A
ATOM 2647 NE ARG A 173 46.344 43.867 57.426 0.00 0.00
A
ATOM 2648 HE ARG A 173 46.505 44.245 58.338 0.00 0.00
A
ATOM 2649 CZ ARG A 173 46.932 44.578 56.450 0.00 0.00
A
ATOM 2650 NH1 ARG A 173 46.712 44.316 55.202 0.00 0.00
A
ATOM 2651 HH11 ARG A 173 47.247 44.754 54.479 0.00 0.00
A
ATOM 2652 HH12 ARG A 173 46.621 43.333 55.042 0.00 0.00
A
ATOM 2653 NH2 ARG A 173 47.640 45.648 56.777 0.00 0.00
A
ATOM 2654 HH21 ARG A 173 47.960 46.280 56.072 0.00 0.00
A
ATOM 2655 HH22 ARG A 173 47.770 45.881 57.741 0.00 0.00
A
ATOM 2656 C ARG A 173 45.099 40.351 61.609 0.00 0.00
A
ATOM 2657 O ARG A 173 45.294 39.175 61.537 0.00 0.00
A
ATOM 2658 N ALA A 174 45.458 41.024 62.804 0.00 0.00
A
ATOM 2659 HN ALA A 174 45.216 41.982 62.942 0.00 0.00
A
ATOM 2660 CA ALA A 174 46.213 40.417 63.898 0.00 0.00
A
ATOM 2661 HA ALA A 174 46.879 39.706 63.432 0.00 0.00
A
ATOM 2662 CB ALA A 174 47.106 41.451 64.637 0.00 0.00
A
ATOM 2663 HB1 ALA A 174 47.900 40.922 65.207 0.00 0.00
A
ATOM 2664 HB2 ALA A 174 47.697 42.124 63.979 0.00 0.00
A
ATOM 2665 HB3 ALA A 174 46.575 42.033 65.420 0.00 0.00
A
ATOM 2666 C ALA A 174 45.394 39.667 64.969 0.00 0.00
A
ATOM 2667 O ALA A 174 45.751 38.547 65.276 0.00 0.00
A
ATOM 2668 N LYS A 175 44.337 40.265 65.510 0.00 0.00
A
ATOM 2669 HN LYS A 175 44.136 41.195 65.214 0.00 0.00
A
ATOM 2670 CA LYS A 175 43.454 39.703 66.470 0.00 0.00
A
ATOM 2671 HA LYS A 175 43.971 39.439 67.381 0.00 0.00
A
ATOM 2672 CB LYS A 175 42.449 40.828 66.883 0.00 0.00
A

ATOM 2673 HB1 LYS A 175 42.947 41.804 67.071 0.00 0.00
A
ATOM 2674 HB2 LYS A 175 41.680 40.987 66.098 0.00 0.00
A
ATOM 2675 CG LYS A 175 41.827 40.591 68.268 0.00 0.00
A
ATOM 2676 HG1 LYS A 175 41.092 39.758 68.267 0.00 0.00
A
ATOM 2677 HG2 LYS A 175 42.548 40.305 69.064 0.00 0.00
A
ATOM 2678 CD LYS A 175 41.157 41.837 68.792 0.00 0.00
A
ATOM 2679 HD1 LYS A 175 41.934 42.622 68.671 0.00 0.00
A
ATOM 2680 HD2 LYS A 175 40.258 41.981 68.155 0.00 0.00
A
ATOM 2681 CE LYS A 175 40.719 42.076 70.253 0.00 0.00
A
ATOM 2682 HE1 LYS A 175 41.589 42.370 70.878 0.00 0.00
A
ATOM 2683 HE2 LYS A 175 40.062 42.972 70.268 0.00 0.00
A
ATOM 2684 NZ LYS A 175 39.918 40.941 70.764 0.00 0.00
A
ATOM 2685 HZ1 LYS A 175 39.854 41.090 71.791 0.00 0.00
A
ATOM 2686 HZ2 LYS A 175 38.943 40.834 70.419 0.00 0.00
A
ATOM 2687 HZ3 LYS A 175 40.465 40.066 70.633 0.00 0.00
A
ATOM 2688 C LYS A 175 42.672 38.468 66.024 0.00 0.00
A
ATOM 2689 O LYS A 175 42.522 37.470 66.708 0.00 0.00
A
ATOM 2690 N TYR A 176 42.228 38.456 64.738 0.00 0.00
A
ATOM 2691 HN TYR A 176 42.321 39.273 64.175 0.00 0.00
A
ATOM 2692 CA TYR A 176 41.275 37.397 64.299 0.00 0.00
A
ATOM 2693 HA TYR A 176 41.012 36.714 65.094 0.00 0.00
A
ATOM 2694 CB TYR A 176 39.948 37.983 63.646 0.00 0.00
A
ATOM 2695 HB1 TYR A 176 40.266 38.405 62.668 0.00 0.00
A
ATOM 2696 HB2 TYR A 176 39.195 37.188 63.459 0.00 0.00
A
ATOM 2697 CG TYR A 176 39.197 38.972 64.552 0.00 0.00
A
ATOM 2698 CD1 TYR A 176 38.863 40.262 64.114 0.00 0.00
A
ATOM 2699 HD1 TYR A 176 39.089 40.539 63.095 0.00 0.00
A

ATOM 2700 CE1 TYR A 176 38.350 41.256 64.945 0.00 0.00
A
ATOM 2701 HE1 TYR A 176 38.043 42.139 64.405 0.00 0.00
A
ATOM 2702 CZ TYR A 176 38.153 40.976 66.315 0.00 0.00
A
ATOM 2703 OH TYR A 176 37.360 41.749 67.080 0.00 0.00
A
ATOM 2704 HH TYR A 176 37.463 41.503 68.002 0.00 0.00
A
ATOM 2705 CD2 TYR A 176 39.106 38.743 65.950 0.00 0.00
A
ATOM 2706 HD2 TYR A 176 39.452 37.878 66.497 0.00 0.00
A
ATOM 2707 CE2 TYR A 176 38.518 39.745 66.776 0.00 0.00
A
ATOM 2708 HE2 TYR A 176 38.447 39.497 67.825 0.00 0.00
A
ATOM 2709 C TYR A 176 41.911 36.531 63.169 0.00 0.00
A
ATOM 2710 O TYR A 176 41.375 35.387 62.966 0.00 0.00
A
ATOM 2711 N GLY A 177 42.890 37.075 62.485 0.00 0.00
A
ATOM 2712 HN GLY A 177 43.382 37.876 62.816 0.00 0.00
A
ATOM 2713 CA GLY A 177 43.609 36.290 61.448 0.00 0.00
A
ATOM 2714 HA1 GLY A 177 43.711 35.276 61.806 0.00 0.00
A
ATOM 2715 HA2 GLY A 177 44.550 36.807 61.335 0.00 0.00
A
ATOM 2716 C GLY A 177 42.902 36.251 60.134 0.00 0.00
A
ATOM 2717 O GLY A 177 43.303 35.692 59.141 0.00 0.00
A
ATOM 2718 N CYS A 178 41.777 36.979 60.084 0.00 0.00
A
ATOM 2719 HN CYS A 178 41.430 37.362 60.937 0.00 0.00
A
ATOM 2720 CA CYS A 178 41.027 37.106 58.829 0.00 0.00
A
ATOM 2721 HA CYS A 178 40.629 36.147 58.532 0.00 0.00
A
ATOM 2722 CB CYS A 178 39.761 37.961 59.100 0.00 0.00
A
ATOM 2723 HB1 CYS A 178 40.083 39.002 59.316 0.00 0.00
A
ATOM 2724 HB2 CYS A 178 39.235 38.106 58.132 0.00 0.00
A
ATOM 2725 SG CYS A 178 38.547 37.262 60.284 0.00 0.00
A
ATOM 2726 HG1 CYS A 178 38.862 35.975 60.244 0.00 0.00
A

ATOM 2727 C CYS A 178 41.834 37.725 57.687 0.00 0.00
A
ATOM 2728 O CYS A 178 42.586 38.706 57.847 0.00 0.00
A
ATOM 2729 N THR A 179 41.618 37.197 56.491 0.00 0.00
A
ATOM 2730 HN THR A 179 40.954 36.467 56.351 0.00 0.00
A
ATOM 2731 CA THR A 179 42.045 37.950 55.260 0.00 0.00
A
ATOM 2732 HA THR A 179 42.736 38.728 55.551 0.00 0.00
A
ATOM 2733 CB THR A 179 42.711 37.013 54.142 0.00 0.00
A
ATOM 2734 HB THR A 179 41.915 36.310 53.816 0.00 0.00
A
ATOM 2735 OG1 THR A 179 43.863 36.366 54.740 0.00 0.00
A
ATOM 2736 HG1 THR A 179 44.515 37.065 54.832 0.00 0.00
A
ATOM 2737 CG2 THR A 179 43.308 37.736 52.914 0.00 0.00
A
ATOM 2738 HG21 THR A 179 42.509 38.147 52.261 0.00 0.00
A
ATOM 2739 HG22 THR A 179 44.038 38.507 53.242 0.00 0.00
A
ATOM 2740 HG23 THR A 179 43.891 37.014 52.302 0.00 0.00
A
ATOM 2741 C THR A 179 40.822 38.618 54.639 0.00 0.00
A
ATOM 2742 O THR A 179 39.703 38.098 54.654 0.00 0.00
A
ATOM 2743 N ILE A 180 40.934 39.856 54.092 0.00 0.00
A
ATOM 2744 HN ILE A 180 41.794 40.355 54.153 0.00 0.00
A
ATOM 2745 CA ILE A 180 39.914 40.607 53.396 0.00 0.00
A
ATOM 2746 HA ILE A 180 38.980 40.072 53.486 0.00 0.00
A
ATOM 2747 CB ILE A 180 39.800 42.028 53.802 0.00 0.00
A
ATOM 2748 HB ILE A 180 40.826 42.453 53.770 0.00 0.00
A
ATOM 2749 CG2 ILE A 180 38.974 42.900 52.801 0.00 0.00
A
ATOM 2750 HG21 ILE A 180 38.944 43.953 53.154 0.00 0.00
A
ATOM 2751 HG22 ILE A 180 39.421 42.870 51.785 0.00 0.00
A
ATOM 2752 HG23 ILE A 180 37.966 42.443 52.697 0.00 0.00
A
ATOM 2753 CG1 ILE A 180 39.260 42.315 55.191 0.00 0.00
A

ATOM 2754 HG11 ILE A 180 39.097 43.413 55.204 0.00 0.00
A
ATOM 2755 HG12 ILE A 180 38.316 41.777 55.426 0.00 0.00
A
ATOM 2756 CD ILE A 180 40.297 42.262 56.372 0.00 0.00
A
ATOM 2757 HD1 ILE A 180 41.241 42.779 56.095 0.00 0.00
A
ATOM 2758 HD2 ILE A 180 39.840 42.786 57.239 0.00 0.00
A
ATOM 2759 HD3 ILE A 180 40.385 41.185 56.627 0.00 0.00
A
ATOM 2760 C ILE A 180 40.359 40.450 51.970 0.00 0.00
A
ATOM 2761 O ILE A 180 41.301 41.011 51.517 0.00 0.00
A
ATOM 2762 N LEU A 181 39.498 39.787 51.165 0.00 0.00
A
ATOM 2763 HN LEU A 181 38.583 39.478 51.411 0.00 0.00
A
ATOM 2764 CA LEU A 181 39.805 39.593 49.750 0.00 0.00
A
ATOM 2765 HA LEU A 181 40.866 39.410 49.665 0.00 0.00
A
ATOM 2766 CB LEU A 181 38.907 38.457 49.140 0.00 0.00
A
ATOM 2767 HB1 LEU A 181 37.876 38.868 49.195 0.00 0.00
A
ATOM 2768 HB2 LEU A 181 39.125 38.271 48.067 0.00 0.00
A
ATOM 2769 CG LEU A 181 38.954 37.085 49.868 0.00 0.00
A
ATOM 2770 HG LEU A 181 38.577 37.149 50.911 0.00 0.00
A
ATOM 2771 CD1 LEU A 181 38.165 36.045 49.117 0.00 0.00
A
ATOM 2772 HD11 LEU A 181 38.539 36.026 48.071 0.00 0.00
A
ATOM 2773 HD12 LEU A 181 38.399 35.043 49.536 0.00 0.00
A
ATOM 2774 HD13 LEU A 181 37.070 36.157 49.264 0.00 0.00
A
ATOM 2775 CD2 LEU A 181 40.427 36.683 49.943 0.00 0.00
A
ATOM 2776 HD21 LEU A 181 40.502 35.599 50.177 0.00 0.00
A
ATOM 2777 HD22 LEU A 181 40.968 36.737 48.974 0.00 0.00
A
ATOM 2778 HD23 LEU A 181 41.031 37.152 50.748 0.00 0.00
A
ATOM 2779 C LEU A 181 39.587 40.781 48.961 0.00 0.00
A
ATOM 2780 O LEU A 181 40.421 41.197 48.134 0.00 0.00
A

ATOM 2781 N ALA A 182 38.461 41.461 49.229 0.00 0.00
A
ATOM 2782 HN ALA A 182 37.834 41.194 49.957 0.00 0.00
A
ATOM 2783 CA ALA A 182 38.186 42.754 48.570 0.00 0.00
A
ATOM 2784 HA ALA A 182 39.063 43.377 48.482 0.00 0.00
A
ATOM 2785 CB ALA A 182 37.419 42.449 47.271 0.00 0.00
A
ATOM 2786 HB1 ALA A 182 36.975 43.259 46.654 0.00 0.00
A
ATOM 2787 HB2 ALA A 182 38.083 41.792 46.669 0.00 0.00
A
ATOM 2788 HB3 ALA A 182 36.501 41.880 47.531 0.00 0.00
A
ATOM 2789 C ALA A 182 37.206 43.588 49.376 0.00 0.00
A
ATOM 2790 O ALA A 182 36.654 43.161 50.387 0.00 0.00
A
ATOM 2791 N ILE A 183 37.069 44.878 48.949 0.00 0.00
A
ATOM 2792 HN ILE A 183 37.586 45.176 48.150 0.00 0.00
A
ATOM 2793 CA ILE A 183 36.021 45.773 49.332 0.00 0.00
A
ATOM 2794 HA ILE A 183 35.494 45.527 50.242 0.00 0.00
A
ATOM 2795 CB ILE A 183 36.562 47.208 49.587 0.00 0.00
A
ATOM 2796 HB ILE A 183 37.008 47.722 48.709 0.00 0.00
A
ATOM 2797 CG2 ILE A 183 35.443 48.181 50.031 0.00 0.00
A
ATOM 2798 HG21 ILE A 183 34.805 48.303 49.130 0.00 0.00
A
ATOM 2799 HG22 ILE A 183 34.801 47.636 50.755 0.00 0.00
A
ATOM 2800 HG23 ILE A 183 35.688 49.162 50.490 0.00 0.00
A
ATOM 2801 CG1 ILE A 183 37.825 47.203 50.496 0.00 0.00
A
ATOM 2802 HG11 ILE A 183 38.557 46.402 50.259 0.00 0.00
A
ATOM 2803 HG12 ILE A 183 38.400 48.097 50.171 0.00 0.00
A
ATOM 2804 CD ILE A 183 37.602 47.274 51.966 0.00 0.00
A
ATOM 2805 HD1 ILE A 183 36.917 48.107 52.235 0.00 0.00
A
ATOM 2806 HD2 ILE A 183 37.095 46.388 52.402 0.00 0.00
A
ATOM 2807 HD3 ILE A 183 38.543 47.522 52.503 0.00 0.00
A

ATOM 2808 C ILE A 183 35.092 45.768 48.056 0.00 0.00
A
ATOM 2809 O ILE A 183 35.476 45.417 46.924 0.00 0.00
A
ATOM 2810 N LYS A 184 33.773 46.030 48.128 0.00 0.00
A
ATOM 2811 HN LYS A 184 33.258 46.192 48.966 0.00 0.00
A
ATOM 2812 CA LYS A 184 32.996 46.323 46.967 0.00 0.00
A
ATOM 2813 HA LYS A 184 33.463 46.032 46.037 0.00 0.00
A
ATOM 2814 CB LYS A 184 31.663 45.471 47.062 0.00 0.00
A
ATOM 2815 HB1 LYS A 184 30.948 45.885 47.804 0.00 0.00
A
ATOM 2816 HB2 LYS A 184 31.138 45.575 46.088 0.00 0.00
A
ATOM 2817 CG LYS A 184 31.854 43.993 47.404 0.00 0.00
A
ATOM 2818 HG1 LYS A 184 32.479 43.582 46.583 0.00 0.00
A
ATOM 2819 HG2 LYS A 184 32.401 43.836 48.358 0.00 0.00
A
ATOM 2820 CD LYS A 184 30.548 43.154 47.309 0.00 0.00
A
ATOM 2821 HD1 LYS A 184 30.745 42.072 47.466 0.00 0.00
A
ATOM 2822 HD2 LYS A 184 29.798 43.514 48.045 0.00 0.00
A
ATOM 2823 CE LYS A 184 29.830 43.297 45.899 0.00 0.00
A
ATOM 2824 HE1 LYS A 184 29.569 44.342 45.629 0.00 0.00
A
ATOM 2825 HE2 LYS A 184 30.516 42.921 45.110 0.00 0.00
A
ATOM 2826 NZ LYS A 184 28.671 42.403 45.857 0.00 0.00
A
ATOM 2827 HZ1 LYS A 184 28.247 42.284 44.915 0.00 0.00
A
ATOM 2828 HZ2 LYS A 184 28.821 41.461 46.273 0.00 0.00
A
ATOM 2829 HZ3 LYS A 184 27.982 42.898 46.458 0.00 0.00
A
ATOM 2830 C LYS A 184 32.830 47.826 46.910 0.00 0.00
A
ATOM 2831 O LYS A 184 32.224 48.405 47.845 0.00 0.00
A
ATOM 2832 N HSE A 185 33.375 48.476 45.878 0.00 0.00
A
ATOM 2833 HN HSE A 185 33.822 47.984 45.135 0.00 0.00
A
ATOM 2834 CA HSE A 185 33.209 49.915 45.712 0.00 0.00
A

ATOM 2835 HA HSE A 185 32.524 50.360 46.420 0.00 0.00
A
ATOM 2836 CB HSE A 185 34.535 50.682 46.051 0.00 0.00
A
ATOM 2837 HB1 HSE A 185 35.362 49.982 45.804 0.00 0.00
A
ATOM 2838 HB2 HSE A 185 34.534 51.596 45.420 0.00 0.00
A
ATOM 2839 ND1 HSE A 185 36.042 51.399 47.928 0.00 0.00
A
ATOM 2840 CG HSE A 185 34.811 51.050 47.501 0.00 0.00
A
ATOM 2841 CE1 HSE A 185 35.881 51.679 49.237 0.00 0.00
A
ATOM 2842 HE1 HSE A 185 36.661 51.880 49.972 0.00 0.00
A
ATOM 2843 NE2 HSE A 185 34.640 51.538 49.654 0.00 0.00
A
ATOM 2844 HE2 HSE A 185 34.290 51.645 50.585 0.00 0.00
A
ATOM 2845 CD2 HSE A 185 33.925 51.102 48.563 0.00 0.00
A
ATOM 2846 HD2 HSE A 185 32.865 50.884 48.550 0.00 0.00
A
ATOM 2847 C HSE A 185 32.784 50.256 44.363 0.00 0.00
A
ATOM 2848 O HSE A 185 33.439 50.163 43.306 0.00 0.00
A
ATOM 2849 N HSE A 186 31.631 50.913 44.353 0.00 0.00
A
ATOM 2850 HN HSE A 186 31.216 51.166 45.224 0.00 0.00
A
ATOM 2851 CA HSE A 186 31.058 51.542 43.193 0.00 0.00
A
ATOM 2852 HA HSE A 186 30.112 51.968 43.495 0.00 0.00
A
ATOM 2853 CB HSE A 186 31.630 52.940 42.823 0.00 0.00
A
ATOM 2854 HB1 HSE A 186 32.552 52.753 42.232 0.00 0.00
A
ATOM 2855 HB2 HSE A 186 30.830 53.359 42.176 0.00 0.00
A
ATOM 2856 ND1 HSE A 186 30.675 53.985 44.954 0.00 0.00
A
ATOM 2857 CG HSE A 186 31.754 53.735 44.077 0.00 0.00
A
ATOM 2858 CE1 HSE A 186 31.183 54.798 45.852 0.00 0.00
A
ATOM 2859 HE1 HSE A 186 30.624 55.278 46.655 0.00 0.00
A
ATOM 2860 NE2 HSE A 186 32.519 55.050 45.711 0.00 0.00
A
ATOM 2861 HE2 HSE A 186 33.216 55.200 46.411 0.00 0.00
A

ATOM	2862	CD2	HSE	A	186	32.925	54.378	44.556	0.00	0.00	A
ATOM	2863	HD2	HSE	A	186	33.904	54.461	44.100	0.00	0.00	A
ATOM	2864	C	HSE	A	186	30.876	50.702	42.064	0.00	0.00	A
ATOM	2865	O	HSE	A	186	31.326	51.001	40.956	0.00	0.00	A
ATOM	2866	N	GLY	A	187	30.272	49.483	42.210	0.00	0.00	A
ATOM	2867	HN	GLY	A	187	29.908	49.171	43.084	0.00	0.00	A
ATOM	2868	CA	GLY	A	187	30.020	48.730	41.030	0.00	0.00	A
ATOM	2869	HA1	GLY	A	187	29.988	49.373	40.163	0.00	0.00	A
ATOM	2870	HA2	GLY	A	187	29.100	48.167	41.074	0.00	0.00	A
ATOM	2871	C	GLY	A	187	31.038	47.646	40.744	0.00	0.00	A
ATOM	2872	O	GLY	A	187	30.995	46.923	39.765	0.00	0.00	A
ATOM	2873	N	ASP	A	188	32.014	47.485	41.616	0.00	0.00	A
ATOM	2874	HN	ASP	A	188	32.165	48.238	42.253	0.00	0.00	A
ATOM	2875	CA	ASP	A	188	33.194	46.596	41.443	0.00	0.00	A
ATOM	2876	HA	ASP	A	188	32.907	45.817	40.752	0.00	0.00	A
ATOM	2877	CB	ASP	A	188	34.371	47.442	40.967	0.00	0.00	A
ATOM	2878	HB1	ASP	A	188	34.433	48.409	41.510	0.00	0.00	A
ATOM	2879	HB2	ASP	A	188	35.352	46.952	41.147	0.00	0.00	A
ATOM	2880	CG	ASP	A	188	34.360	47.591	39.465	0.00	0.00	A
ATOM	2881	OD1	ASP	A	188	34.120	48.750	38.994	0.00	0.00	A
ATOM	2882	OD2	ASP	A	188	34.416	46.593	38.743	0.00	0.00	A
ATOM	2883	C	ASP	A	188	33.574	46.062	42.725	0.00	0.00	A
ATOM	2884	O	ASP	A	188	33.357	46.609	43.797	0.00	0.00	A
ATOM	2885	N	ILE	A	189	34.001	44.791	42.668	0.00	0.00	A
ATOM	2886	HN	ILE	A	189	34.046	44.312	41.794	0.00	0.00	A
ATOM	2887	CA	ILE	A	189	34.820	44.246	43.716	0.00	0.00	A
ATOM	2888	HA	ILE	A	189	34.462	44.549	44.689	0.00	0.00	A

ATOM 2889 CB ILE A 189 34.765 42.744 43.593 0.00 0.00
A
ATOM 2890 HB ILE A 189 35.158 42.481 42.587 0.00 0.00
A
ATOM 2891 CG2 ILE A 189 35.444 41.983 44.745 0.00 0.00
A
ATOM 2892 HG21 ILE A 189 35.521 40.880 44.633 0.00 0.00
A
ATOM 2893 HG22 ILE A 189 36.512 42.268 44.628 0.00 0.00
A
ATOM 2894 HG23 ILE A 189 35.098 42.236 45.770 0.00 0.00
A
ATOM 2895 CG1 ILE A 189 33.219 42.414 43.584 0.00 0.00
A
ATOM 2896 HG11 ILE A 189 32.696 43.001 44.370 0.00 0.00
A
ATOM 2897 HG12 ILE A 189 32.798 42.695 42.595 0.00 0.00
A
ATOM 2898 CD ILE A 189 32.912 40.972 43.846 0.00 0.00
A
ATOM 2899 HD1 ILE A 189 33.337 40.462 42.955 0.00 0.00
A
ATOM 2900 HD2 ILE A 189 33.208 40.529 44.821 0.00 0.00
A
ATOM 2901 HD3 ILE A 189 31.809 40.917 43.721 0.00 0.00
A
ATOM 2902 C ILE A 189 36.164 44.802 43.618 0.00 0.00
A
ATOM 2903 O ILE A 189 36.712 44.876 42.540 0.00 0.00
A
ATOM 2904 N CYS A 190 36.714 45.274 44.700 0.00 0.00
A
ATOM 2905 HN CYS A 190 36.256 45.143 45.576 0.00 0.00
A
ATOM 2906 CA CYS A 190 37.998 46.046 44.736 0.00 0.00
A
ATOM 2907 HA CYS A 190 38.398 46.138 43.737 0.00 0.00
A
ATOM 2908 CB CYS A 190 37.676 47.513 45.332 0.00 0.00
A
ATOM 2909 HB1 CYS A 190 36.867 47.938 44.701 0.00 0.00
A
ATOM 2910 HB2 CYS A 190 37.355 47.517 46.395 0.00 0.00
A
ATOM 2911 SG CYS A 190 39.103 48.629 45.263 0.00 0.00
A
ATOM 2912 HG1 CYS A 190 40.072 47.902 45.798 0.00 0.00
A
ATOM 2913 C CYS A 190 38.954 45.361 45.665 0.00 0.00
A
ATOM 2914 O CYS A 190 38.751 45.364 46.879 0.00 0.00
A
ATOM 2915 N LEU A 191 39.983 44.867 45.059 0.00 0.00
A

ATOM   2916 HN LEU A 191   40.138 45.096 44.101 0.00 0.00
A
ATOM   2917 CA LEU A 191   40.987 44.054 45.646 0.00 0.00
A
ATOM   2918 HA LEU A 191   40.599 43.439 46.444 0.00 0.00
A
ATOM   2919 CB LEU A 191   41.785 43.216 44.632 0.00 0.00
A
ATOM   2920 HB1 LEU A 191   42.426 43.916 44.056 0.00 0.00
A
ATOM   2921 HB2 LEU A 191   42.455 42.501 45.155 0.00 0.00
A
ATOM   2922 CG LEU A 191   40.912 42.264 43.668 0.00 0.00
A
ATOM   2923 HG LEU A 191   40.529 43.016 42.945 0.00 0.00
A
ATOM   2924 CD1 LEU A 191   41.845 41.345 42.863 0.00 0.00
A
ATOM   2925 HD11 LEU A 191   42.296 40.582 43.533 0.00 0.00
A
ATOM   2926 HD12 LEU A 191   41.310 40.865 42.016 0.00 0.00
A
ATOM   2927 HD13 LEU A 191   42.632 41.963 42.381 0.00 0.00
A
ATOM   2928 CD2 LEU A 191   39.769 41.525 44.292 0.00 0.00
A
ATOM   2929 HD21 LEU A 191   39.092 42.205 44.851 0.00 0.00
A
ATOM   2930 HD22 LEU A 191   39.262 41.070 43.415 0.00 0.00
A
ATOM   2931 HD23 LEU A 191   40.238 40.724 44.902 0.00 0.00
A
ATOM   2932 C   LEU A 191   41.997 44.857 46.457 0.00 0.00
A
ATOM   2933 O   LEU A 191   42.310 46.023 46.291 0.00 0.00
A
ATOM   2934 N   SER A 192   42.593 44.100 47.476 0.00 0.00
A
ATOM   2935 HN SER A 192   42.516 43.114 47.607 0.00 0.00
A
ATOM   2936 CA SER A 192   43.853 44.455 48.172 0.00 0.00
A
ATOM   2937 HA SER A 192   44.040 43.604 48.810 0.00 0.00
A
ATOM   2938 CB SER A 192   45.062 44.558 47.145 0.00 0.00
A
ATOM   2939 HB1 SER A 192   44.872 43.750 46.408 0.00 0.00
A
ATOM   2940 HB2 SER A 192   44.995 45.506 46.569 0.00 0.00
A
ATOM   2941 OG SER A 192   46.331 44.411 47.736 0.00 0.00
A
ATOM   2942 HG1 SER A 192   46.830 45.123 47.329 0.00 0.00
A

ATOM 2943 C SER A 192 43.848 45.683 49.052 0.00 0.00
A
ATOM 2944 O SER A 192 44.588 46.578 48.716 0.00 0.00
A
ATOM 2945 N PRO A 193 43.065 45.877 50.112 0.00 0.00
A
ATOM 2946 CD PRO A 193 42.480 44.730 50.816 0.00 0.00
A
ATOM 2947 HD1 PRO A 193 41.735 44.267 50.134 0.00 0.00
A
ATOM 2948 HD2 PRO A 193 43.303 44.008 51.005 0.00 0.00
A
ATOM 2949 CA PRO A 193 43.009 47.166 50.864 0.00 0.00
A
ATOM 2950 HA PRO A 193 42.812 47.999 50.205 0.00 0.00
A
ATOM 2951 CB PRO A 193 41.846 46.888 51.808 0.00 0.00
A
ATOM 2952 HB1 PRO A 193 40.934 47.272 51.303 0.00 0.00
A
ATOM 2953 HB2 PRO A 193 42.110 47.455 52.726 0.00 0.00
A
ATOM 2954 CG PRO A 193 41.856 45.326 52.029 0.00 0.00
A
ATOM 2955 HG1 PRO A 193 40.854 44.885 52.217 0.00 0.00
A
ATOM 2956 HG2 PRO A 193 42.481 45.139 52.929 0.00 0.00
A
ATOM 2957 C PRO A 193 44.353 47.508 51.584 0.00 0.00
A
ATOM 2958 O PRO A 193 45.023 46.603 52.109 0.00 0.00
A
ATOM 2959 N ALA A 194 44.667 48.815 51.551 0.00 0.00
A
ATOM 2960 HN ALA A 194 44.118 49.484 51.055 0.00 0.00
A
ATOM 2961 CA ALA A 194 45.910 49.277 52.088 0.00 0.00
A
ATOM 2962 HA ALA A 194 46.072 48.967 53.110 0.00 0.00
A
ATOM 2963 CB ALA A 194 47.020 48.865 51.086 0.00 0.00
A
ATOM 2964 HB1 ALA A 194 47.108 47.757 51.076 0.00 0.00
A
ATOM 2965 HB2 ALA A 194 46.642 49.273 50.124 0.00 0.00
A
ATOM 2966 HB3 ALA A 194 47.999 49.306 51.369 0.00 0.00
A
ATOM 2967 C ALA A 194 45.747 50.787 52.274 0.00 0.00
A
ATOM 2968 O ALA A 194 44.920 51.431 51.652 0.00 0.00
A
ATOM 2969 N PRO A 195 46.481 51.471 53.152 0.00 0.00
A

ATOM 2970 CD PRO A 195 47.708 50.893 53.782 0.00 0.00
A
ATOM 2971 HD1 PRO A 195 47.221 50.167 54.467 0.00 0.00
A
ATOM 2972 HD2 PRO A 195 48.467 50.508 53.068 0.00 0.00
A
ATOM 2973 CA PRO A 195 46.055 52.723 53.810 0.00 0.00
A
ATOM 2974 HA PRO A 195 44.991 52.842 53.951 0.00 0.00
A
ATOM 2975 CB PRO A 195 46.957 52.797 55.083 0.00 0.00
A
ATOM 2976 HB1 PRO A 195 46.350 52.425 55.936 0.00 0.00
A
ATOM 2977 HB2 PRO A 195 47.135 53.879 55.264 0.00 0.00
A
ATOM 2978 CG PRO A 195 48.183 52.020 54.686 0.00 0.00
A
ATOM 2979 HG1 PRO A 195 48.911 51.634 55.432 0.00 0.00
A
ATOM 2980 HG2 PRO A 195 48.764 52.699 54.025 0.00 0.00
A
ATOM 2981 C PRO A 195 46.340 53.863 52.837 0.00 0.00
A
ATOM 2982 O PRO A 195 47.092 53.713 51.886 0.00 0.00
A
ATOM 2983 N GLU A 196 45.765 55.041 52.984 0.00 0.00
A
ATOM 2984 HN GLU A 196 45.957 55.630 52.202 0.00 0.00
A
ATOM 2985 CA GLU A 196 44.921 55.575 54.045 0.00 0.00
A
ATOM 2986 HA GLU A 196 45.064 55.077 54.993 0.00 0.00
A
ATOM 2987 CB GLU A 196 45.321 57.099 54.270 0.00 0.00
A
ATOM 2988 HB1 GLU A 196 45.394 57.540 53.253 0.00 0.00
A
ATOM 2989 HB2 GLU A 196 44.547 57.590 54.898 0.00 0.00
A
ATOM 2990 CG GLU A 196 46.689 57.160 54.921 0.00 0.00
A
ATOM 2991 HG1 GLU A 196 46.809 56.430 55.750 0.00 0.00
A
ATOM 2992 HG2 GLU A 196 47.433 56.872 54.148 0.00 0.00
A
ATOM 2993 CD GLU A 196 46.931 58.563 55.404 0.00 0.00
A
ATOM 2994 OE1 GLU A 196 46.811 58.872 56.626 0.00 0.00
A
ATOM 2995 OE2 GLU A 196 47.372 59.379 54.560 0.00 0.00
A
ATOM 2996 C GLU A 196 43.465 55.578 53.658 0.00 0.00
A

```
ATOM   2997  O   GLU A 196      42.637  56.040  54.395  0.00  0.00
A
ATOM   2998  N   ASP A 197      43.154  55.013  52.501  0.00  0.00
A
ATOM   2999  HN  ASP A 197      43.850  54.693  51.864  0.00  0.00
A
ATOM   3000  CA  ASP A 197      41.784  54.611  52.208  0.00  0.00
A
ATOM   3001  HA  ASP A 197      41.198  55.436  51.830  0.00  0.00
A
ATOM   3002  CB  ASP A 197      41.947  53.555  51.018  0.00  0.00
A
ATOM   3003  HB1 ASP A 197      42.636  52.758  51.370  0.00  0.00
A
ATOM   3004  HB2 ASP A 197      40.957  53.128  50.748  0.00  0.00
A
ATOM   3005  CG  ASP A 197      42.622  54.144  49.812  0.00  0.00
A
ATOM   3006  OD1 ASP A 197      41.883  54.305  48.766  0.00  0.00
A
ATOM   3007  OD2 ASP A 197      43.849  54.339  49.769  0.00  0.00
A
ATOM   3008  C   ASP A 197      41.076  53.869  53.323  0.00  0.00
A
ATOM   3009  O   ASP A 197      41.629  53.149  54.136  0.00  0.00
A
ATOM   3010  N   ILE A 198      39.755  53.967  53.285  0.00  0.00
A
ATOM   3011  HN  ILE A 198      39.316  54.398  52.500  0.00  0.00
A
ATOM   3012  CA  ILE A 198      38.975  53.564  54.466  0.00  0.00
A
ATOM   3013  HA  ILE A 198      39.477  52.778  55.011  0.00  0.00
A
ATOM   3014  CB  ILE A 198      38.472  54.741  55.323  0.00  0.00
A
ATOM   3015  HB  ILE A 198      37.848  54.311  56.135  0.00  0.00
A
ATOM   3016  CG2 ILE A 198      39.765  55.402  55.950  0.00  0.00
A
ATOM   3017 HG21 ILE A 198      39.478  56.280  56.567  0.00  0.00
A
ATOM   3018 HG22 ILE A 198      40.367  54.700  56.566  0.00  0.00
A
ATOM   3019 HG23 ILE A 198      40.401  55.815  55.139  0.00  0.00
A
ATOM   3020  CG1 ILE A 198      37.592  55.806  54.553  0.00  0.00
A
ATOM   3021 HG11 ILE A 198      38.222  56.377  53.838  0.00  0.00
A
ATOM   3022 HG12 ILE A 198      36.947  55.208  53.875  0.00  0.00
A
ATOM   3023  CD  ILE A 198      36.942  56.856  55.448  0.00  0.00
A
```

ATOM 3024 HD1 ILE A 198 36.205 56.462 56.180 0.00 0.00
A
ATOM 3025 HD2 ILE A 198 37.696 57.425 56.033 0.00 0.00
A
ATOM 3026 HD3 ILE A 198 36.321 57.504 54.794 0.00 0.00
A
ATOM 3027 C ILE A 198 37.738 52.818 54.017 0.00 0.00
A
ATOM 3028 O ILE A 198 37.476 52.667 52.841 0.00 0.00
A
ATOM 3029 N ILE A 199 37.105 52.206 55.003 0.00 0.00
A
ATOM 3030 HN ILE A 199 37.350 52.202 55.970 0.00 0.00
A
ATOM 3031 CA ILE A 199 35.776 51.587 54.920 0.00 0.00
A
ATOM 3032 HA ILE A 199 35.403 51.549 53.907 0.00 0.00
A
ATOM 3033 CB ILE A 199 35.941 50.160 55.510 0.00 0.00
A
ATOM 3034 HB ILE A 199 36.593 49.601 54.805 0.00 0.00
A
ATOM 3035 CG2 ILE A 199 36.635 50.183 56.885 0.00 0.00
A
ATOM 3036 HG21 ILE A 199 36.153 50.780 57.688 0.00 0.00
A
ATOM 3037 HG22 ILE A 199 36.728 49.136 57.244 0.00 0.00
A
ATOM 3038 HG23 ILE A 199 37.674 50.562 56.781 0.00 0.00
A
ATOM 3039 CG1 ILE A 199 34.566 49.408 55.539 0.00 0.00
A
ATOM 3040 HG11 ILE A 199 34.668 48.367 55.914 0.00 0.00
A
ATOM 3041 HG12 ILE A 199 33.950 49.871 56.340 0.00 0.00
A
ATOM 3042 CD ILE A 199 33.747 49.262 54.230 0.00 0.00
A
ATOM 3043 HD1 ILE A 199 33.233 50.202 53.936 0.00 0.00
A
ATOM 3044 HD2 ILE A 199 34.416 48.905 53.417 0.00 0.00
A
ATOM 3045 HD3 ILE A 199 33.054 48.397 54.292 0.00 0.00
A
ATOM 3046 C ILE A 199 34.869 52.444 55.792 0.00 0.00
A
ATOM 3047 O ILE A 199 35.272 53.070 56.722 0.00 0.00
A
ATOM 3048 N ARG A 200 33.618 52.634 55.288 0.00 0.00
A
ATOM 3049 HN ARG A 200 33.222 52.035 54.596 0.00 0.00
A
ATOM 3050 CA ARG A 200 32.605 53.447 55.887 0.00 0.00
A

ATOM 3051 HA ARG A 200 32.911 53.828 56.850 0.00 0.00
A
ATOM 3052 CB ARG A 200 32.351 54.809 55.051 0.00 0.00
A
ATOM 3053 HB1 ARG A 200 32.236 54.459 54.003 0.00 0.00
A
ATOM 3054 HB2 ARG A 200 31.384 55.338 55.192 0.00 0.00
A
ATOM 3055 CG ARG A 200 33.579 55.712 55.050 0.00 0.00
A
ATOM 3056 HG1 ARG A 200 34.267 55.380 55.857 0.00 0.00
A
ATOM 3057 HG2 ARG A 200 34.218 55.556 54.155 0.00 0.00
A
ATOM 3058 CD ARG A 200 33.208 57.219 54.944 0.00 0.00
A
ATOM 3059 HD1 ARG A 200 34.145 57.769 54.714 0.00 0.00
A
ATOM 3060 HD2 ARG A 200 32.441 57.333 54.147 0.00 0.00
A
ATOM 3061 NE ARG A 200 32.635 57.721 56.307 0.00 0.00
A
ATOM 3062 HE ARG A 200 32.366 57.062 57.008 0.00 0.00
A
ATOM 3063 CZ ARG A 200 32.670 58.971 56.740 0.00 0.00
A
ATOM 3064 NH1 ARG A 200 32.967 60.050 55.971 0.00 0.00
A
ATOM 3065 HH11 ARG A 200 33.098 60.956 56.373 0.00 0.00
A
ATOM 3066 HH12 ARG A 200 33.223 59.978 55.007 0.00 0.00
A
ATOM 3067 NH2 ARG A 200 32.335 59.172 58.015 0.00 0.00
A
ATOM 3068 HH21 ARG A 200 32.284 60.094 58.401 0.00 0.00
A
ATOM 3069 HH22 ARG A 200 32.150 58.346 58.546 0.00 0.00
A
ATOM 3070 C ARG A 200 31.281 52.741 56.054 0.00 0.00
A
ATOM 3071 O ARG A 200 31.122 51.707 55.450 0.00 0.00
A
ATOM 3072 N GLU A 201 30.315 53.342 56.794 0.00 0.00
A
ATOM 3073 HN GLU A 201 30.499 54.207 57.253 0.00 0.00
A
ATOM 3074 CA GLU A 201 29.050 52.690 57.083 0.00 0.00
A
ATOM 3075 HA GLU A 201 29.348 51.718 57.446 0.00 0.00
A
ATOM 3076 CB GLU A 201 28.229 53.356 58.216 0.00 0.00
A
ATOM 3077 HB1 GLU A 201 28.589 53.088 59.232 0.00 0.00
A

ATOM 3078 HB2 GLU A 201 28.350 54.457 58.131 0.00 0.00
A
ATOM 3079 CG GLU A 201 26.756 53.001 58.272 0.00 0.00
A
ATOM 3080 HG1 GLU A 201 26.204 53.516 57.458 0.00 0.00
A
ATOM 3081 HG2 GLU A 201 26.556 51.909 58.314 0.00 0.00
A
ATOM 3082 CD GLU A 201 26.030 53.522 59.565 0.00 0.00
A
ATOM 3083 OE1 GLU A 201 26.308 54.658 60.092 0.00 0.00
A
ATOM 3084 OE2 GLU A 201 25.060 52.861 59.976 0.00 0.00
A
ATOM 3085 C GLU A 201 28.174 52.508 55.838 0.00 0.00
A
ATOM 3086 O GLU A 201 27.980 53.418 55.050 0.00 0.00
A
ATOM 3087 N GLN A 202 27.647 51.275 55.607 0.00 0.00
A
ATOM 3088 HN GLN A 202 27.662 50.592 56.333 0.00 0.00
A
ATOM 3089 CA GLN A 202 26.920 50.898 54.402 0.00 0.00
A
ATOM 3090 HA GLN A 202 26.585 49.879 54.525 0.00 0.00
A
ATOM 3091 CB GLN A 202 25.667 51.655 54.035 0.00 0.00
A
ATOM 3092 HB1 GLN A 202 25.993 52.703 53.860 0.00 0.00
A
ATOM 3093 HB2 GLN A 202 25.269 51.258 53.077 0.00 0.00
A
ATOM 3094 CG GLN A 202 24.596 51.673 55.186 0.00 0.00
A
ATOM 3095 HG1 GLN A 202 24.094 50.691 55.324 0.00 0.00
A
ATOM 3096 HG2 GLN A 202 25.159 51.802 56.135 0.00 0.00
A
ATOM 3097 CD GLN A 202 23.566 52.705 55.090 0.00 0.00
A
ATOM 3098 OE1 GLN A 202 22.323 52.444 55.189 0.00 0.00
A
ATOM 3099 NE2 GLN A 202 23.966 53.984 54.891 0.00 0.00
A
ATOM 3100 HE21 GLN A 202 23.452 54.835 54.787 0.00 0.00
A
ATOM 3101 HE22 GLN A 202 24.944 54.157 54.778 0.00 0.00
A
ATOM 3102 C GLN A 202 27.821 50.697 53.172 0.00 0.00
A
ATOM 3103 O GLN A 202 27.317 50.582 52.078 0.00 0.00
A
ATOM 3104 N ASP A 203 29.022 50.184 53.415 0.00 0.00
A

ATOM 3105 HN ASP A 203 29.397 50.162 54.338 0.00 0.00
A
ATOM 3106 CA ASP A 203 29.835 49.456 52.443 0.00 0.00
A
ATOM 3107 HA ASP A 203 29.327 49.416 51.491 0.00 0.00
A
ATOM 3108 CB ASP A 203 31.108 50.173 52.194 0.00 0.00
A
ATOM 3109 HB1 ASP A 203 31.592 50.469 53.149 0.00 0.00
A
ATOM 3110 HB2 ASP A 203 31.867 49.584 51.636 0.00 0.00
A
ATOM 3111 CG ASP A 203 30.924 51.438 51.325 0.00 0.00
A
ATOM 3112 OD1 ASP A 203 31.105 52.556 51.878 0.00 0.00
A
ATOM 3113 OD2 ASP A 203 30.615 51.367 50.138 0.00 0.00
A
ATOM 3114 C ASP A 203 30.117 48.069 52.748 0.00 0.00
A
ATOM 3115 O ASP A 203 30.455 47.697 53.852 0.00 0.00
A
ATOM 3116 N CYS A 204 30.110 47.162 51.711 0.00 0.00
A
ATOM 3117 HN CYS A 204 29.966 47.442 50.765 0.00 0.00
A
ATOM 3118 CA CYS A 204 30.427 45.717 51.876 0.00 0.00
A
ATOM 3119 HA CYS A 204 30.184 45.491 52.904 0.00 0.00
A
ATOM 3120 CB CYS A 204 29.677 44.940 50.775 0.00 0.00
A
ATOM 3121 HB1 CYS A 204 29.832 45.535 49.849 0.00 0.00
A
ATOM 3122 HB2 CYS A 204 30.118 43.929 50.645 0.00 0.00
A
ATOM 3123 SG CYS A 204 27.936 44.646 51.208 0.00 0.00
A
ATOM 3124 HG1 CYS A 204 27.587 45.589 50.345 0.00 0.00
A
ATOM 3125 C CYS A 204 31.891 45.493 51.686 0.00 0.00
A
ATOM 3126 O CYS A 204 32.559 46.196 50.898 0.00 0.00
A
ATOM 3127 N LEU A 205 32.505 44.560 52.442 0.00 0.00
A
ATOM 3128 HN LEU A 205 32.003 44.211 53.230 0.00 0.00
A
ATOM 3129 CA LEU A 205 33.795 44.018 52.125 0.00 0.00
A
ATOM 3130 HA LEU A 205 34.078 44.327 51.130 0.00 0.00
A
ATOM 3131 CB LEU A 205 34.945 44.313 53.111 0.00 0.00
A

ATOM 3132 HB1 LEU A 205 35.899 43.787 52.890 0.00 0.00
A
ATOM 3133 HB2 LEU A 205 35.075 45.417 53.083 0.00 0.00
A
ATOM 3134 CG LEU A 205 34.556 44.043 54.621 0.00 0.00
A
ATOM 3135 HG LEU A 205 33.910 43.153 54.469 0.00 0.00
A
ATOM 3136 CD1 LEU A 205 35.738 43.559 55.364 0.00 0.00
A
ATOM 3137 HD11 LEU A 205 36.018 42.530 55.052 0.00 0.00
A
ATOM 3138 HD12 LEU A 205 36.583 44.263 55.207 0.00 0.00
A
ATOM 3139 HD13 LEU A 205 35.398 43.409 56.411 0.00 0.00
A
ATOM 3140 CD2 LEU A 205 33.813 45.142 55.306 0.00 0.00
A
ATOM 3141 HD21 LEU A 205 34.581 45.856 55.672 0.00 0.00
A
ATOM 3142 HD22 LEU A 205 33.126 45.668 54.609 0.00 0.00
A
ATOM 3143 HD23 LEU A 205 33.242 44.648 56.121 0.00 0.00
A
ATOM 3144 C LEU A 205 33.621 42.554 52.063 0.00 0.00
A
ATOM 3145 O LEU A 205 32.553 41.993 52.314 0.00 0.00
A
ATOM 3146 N VAL A 206 34.671 41.715 51.791 0.00 0.00
A
ATOM 3147 HN VAL A 206 35.477 42.197 51.456 0.00 0.00
A
ATOM 3148 CA VAL A 206 34.576 40.302 51.422 0.00 0.00
A
ATOM 3149 HA VAL A 206 33.660 39.850 51.773 0.00 0.00
A
ATOM 3150 CB VAL A 206 34.800 40.007 49.918 0.00 0.00
A
ATOM 3151 HB VAL A 206 35.844 40.285 49.661 0.00 0.00
A
ATOM 3152 CG1 VAL A 206 34.745 38.495 49.758 0.00 0.00
A
ATOM 3153 HG11 VAL A 206 33.843 37.989 50.164 0.00 0.00
A
ATOM 3154 HG12 VAL A 206 34.980 38.135 48.734 0.00 0.00
A
ATOM 3155 HG13 VAL A 206 35.512 38.041 50.420 0.00 0.00
A
ATOM 3156 CG2 VAL A 206 33.753 40.656 48.952 0.00 0.00
A
ATOM 3157 HG21 VAL A 206 34.171 40.611 47.924 0.00 0.00
A
ATOM 3158 HG22 VAL A 206 32.884 39.985 49.125 0.00 0.00
A

ATOM   3159 HG23 VAL A 206   33.557 41.728 49.167 0.00 0.00
A
ATOM   3160 C   VAL A 206   35.687 39.672 52.288 0.00 0.00
A
ATOM   3161 O   VAL A 206   36.858 39.949 52.034 0.00 0.00
A
ATOM   3162 N   ILE A 207   35.278 38.946 53.346 0.00 0.00
A
ATOM   3163 HN ILE A 207   34.298 38.803 53.464 0.00 0.00
A
ATOM   3164 CA ILE A 207   36.103 38.453 54.360 0.00 0.00
A
ATOM   3165 HA ILE A 207   37.133 38.725 54.183 0.00 0.00
A
ATOM   3166 CB ILE A 207   35.785 39.070 55.705 0.00 0.00
A
ATOM   3167 HB ILE A 207   35.453 40.119 55.549 0.00 0.00
A
ATOM   3168 CG2 ILE A 207   34.473 38.498 56.409 0.00 0.00
A
ATOM   3169 HG21 ILE A 207   34.220 39.052 57.339 0.00 0.00
A
ATOM   3170 HG22 ILE A 207   33.653 38.510 55.660 0.00 0.00
A
ATOM   3171 HG23 ILE A 207   34.722 37.479 56.774 0.00 0.00
A
ATOM   3172 CG1 ILE A 207   36.966 39.275 56.632 0.00 0.00
A
ATOM   3173 HG11 ILE A 207   37.421 38.314 56.954 0.00 0.00
A
ATOM   3174 HG12 ILE A 207   37.652 39.944 56.069 0.00 0.00
A
ATOM   3175 CD ILE A 207   36.574 39.975 57.953 0.00 0.00
A
ATOM   3176 HD1 ILE A 207   37.533 40.332 58.386 0.00 0.00
A
ATOM   3177 HD2 ILE A 207   35.884 40.815 57.723 0.00 0.00
A
ATOM   3178 HD3 ILE A 207   36.094 39.345 58.731 0.00 0.00
A
ATOM   3179 C   ILE A 207   36.239 36.934 54.391 0.00 0.00
A
ATOM   3180 O   ILE A 207   35.351 36.096 54.382 0.00 0.00
A
ATOM   3181 N   MET A 208   37.521 36.433 54.471 0.00 0.00
A
ATOM   3182 HN MET A 208   38.290 37.062 54.539 0.00 0.00
A
ATOM   3183 CA MET A 208   37.914 35.094 54.658 0.00 0.00
A
ATOM   3184 HA MET A 208   36.971 34.584 54.524 0.00 0.00
A
ATOM   3185 CB MET A 208   38.793 34.715 53.424 0.00 0.00
A

ATOM	3186	HB1	MET	A	208	38.272	34.864	52.454	0.00	0.00	A
ATOM	3187	HB2	MET	A	208	39.643	35.420	53.302	0.00	0.00	A
ATOM	3188	CG	MET	A	208	39.298	33.317	53.395	0.00	0.00	A
ATOM	3189	HG1	MET	A	208	39.890	33.103	54.311	0.00	0.00	A
ATOM	3190	HG2	MET	A	208	38.550	32.504	53.508	0.00	0.00	A
ATOM	3191	SD	MET	A	208	40.154	32.810	51.914	0.00	0.00	A
ATOM	3192	CE	MET	A	208	41.642	33.812	51.999	0.00	0.00	A
ATOM	3193	HE1	MET	A	208	41.503	34.828	52.427	0.00	0.00	A
ATOM	3194	HE2	MET	A	208	42.272	33.350	52.789	0.00	0.00	A
ATOM	3195	HE3	MET	A	208	42.040	33.937	50.969	0.00	0.00	A
ATOM	3196	C	MET	A	208	38.626	34.700	55.961	0.00	0.00	A
ATOM	3197	O	MET	A	208	39.507	35.448	56.451	0.00	0.00	A
ATOM	3198	N	GLY	A	209	38.321	33.571	56.559	0.00	0.00	A
ATOM	3199	HN	GLY	A	209	37.692	32.929	56.129	0.00	0.00	A
ATOM	3200	CA	GLY	A	209	38.804	33.231	57.836	0.00	0.00	A
ATOM	3201	HA1	GLY	A	209	38.692	34.084	58.488	0.00	0.00	A
ATOM	3202	HA2	GLY	A	209	39.817	32.866	57.757	0.00	0.00	A
ATOM	3203	C	GLY	A	209	38.119	31.979	58.367	0.00	0.00	A
ATOM	3204	O	GLY	A	209	37.603	31.218	57.563	0.00	0.00	A
ATOM	3205	N	HSE	A	210	38.138	31.859	59.697	0.00	0.00	A
ATOM	3206	HN	HSE	A	210	38.587	32.576	60.224	0.00	0.00	A
ATOM	3207	CA	HSE	A	210	37.494	30.771	60.512	0.00	0.00	A
ATOM	3208	HA	HSE	A	210	37.377	29.989	59.776	0.00	0.00	A
ATOM	3209	CB	HSE	A	210	38.357	30.285	61.732	0.00	0.00	A
ATOM	3210	HB1	HSE	A	210	39.342	30.037	61.282	0.00	0.00	A
ATOM	3211	HB2	HSE	A	210	38.624	31.117	62.419	0.00	0.00	A
ATOM	3212	ND1	HSE	A	210	36.799	29.402	63.407	0.00	0.00	A

ATOM 3213 CG HSE A 210 37.821 29.150 62.527 0.00 0.00
A
ATOM 3214 CE1 HSE A 210 36.433 28.249 63.869 0.00 0.00
A
ATOM 3215 HE1 HSE A 210 35.571 28.089 64.517 0.00 0.00
A
ATOM 3216 NE2 HSE A 210 37.191 27.232 63.290 0.00 0.00
A
ATOM 3217 HE2 HSE A 210 36.983 26.263 63.421 0.00 0.00
A
ATOM 3218 CD2 HSE A 210 38.052 27.811 62.412 0.00 0.00
A
ATOM 3219 HD2 HSE A 210 38.620 27.159 61.760 0.00 0.00
A
ATOM 3220 C HSE A 210 36.062 31.274 60.878 0.00 0.00
A
ATOM 3221 O HSE A 210 35.893 32.494 61.052 0.00 0.00
A
ATOM 3222 N LYS A 211 35.020 30.413 60.807 0.00 0.00
A
ATOM 3223 HN LYS A 211 35.112 29.439 60.615 0.00 0.00
A
ATOM 3224 CA LYS A 211 33.635 30.665 61.095 0.00 0.00
A
ATOM 3225 HA LYS A 211 33.366 31.484 60.443 0.00 0.00
A
ATOM 3226 CB LYS A 211 32.752 29.354 61.011 0.00 0.00
A
ATOM 3227 HB1 LYS A 211 32.932 29.027 59.965 0.00 0.00
A
ATOM 3228 HB2 LYS A 211 33.198 28.532 61.610 0.00 0.00
A
ATOM 3229 CG LYS A 211 31.263 29.581 61.367 0.00 0.00
A
ATOM 3230 HG1 LYS A 211 31.192 30.030 62.380 0.00 0.00
A
ATOM 3231 HG2 LYS A 211 30.920 30.354 60.647 0.00 0.00
A
ATOM 3232 CD LYS A 211 30.525 28.236 61.168 0.00 0.00
A
ATOM 3233 HD1 LYS A 211 30.888 27.713 60.257 0.00 0.00
A
ATOM 3234 HD2 LYS A 211 30.793 27.605 62.042 0.00 0.00
A
ATOM 3235 CE LYS A 211 29.034 28.493 61.005 0.00 0.00
A
ATOM 3236 HE1 LYS A 211 28.622 29.086 61.849 0.00 0.00
A
ATOM 3237 HE2 LYS A 211 28.895 29.133 60.107 0.00 0.00
A
ATOM 3238 NZ LYS A 211 28.241 27.251 61.004 0.00 0.00
A
ATOM 3239 HZ1 LYS A 211 28.383 26.806 60.076 0.00 0.00
A

ATOM 3240 HZ2 LYS A 211 28.521 26.575 61.743 0.00 0.00
A
ATOM 3241 HZ3 LYS A 211 27.212 27.380 61.081 0.00 0.00
A
ATOM 3242 C LYS A 211 33.497 31.385 62.490 0.00 0.00
A
ATOM 3243 O LYS A 211 32.808 32.358 62.590 0.00 0.00
A
ATOM 3244 N LYS A 212 34.156 30.907 63.581 0.00 0.00
A
ATOM 3245 HN LYS A 212 34.844 30.198 63.447 0.00 0.00
A
ATOM 3246 CA LYS A 212 34.029 31.491 64.909 0.00 0.00
A
ATOM 3247 HA LYS A 212 32.991 31.705 65.114 0.00 0.00
A
ATOM 3248 CB LYS A 212 34.599 30.605 66.013 0.00 0.00
A
ATOM 3249 HB1 LYS A 212 35.687 30.472 65.829 0.00 0.00
A
ATOM 3250 HB2 LYS A 212 34.419 31.129 66.976 0.00 0.00
A
ATOM 3251 CG LYS A 212 33.928 29.246 66.192 0.00 0.00
A
ATOM 3252 HG1 LYS A 212 32.853 29.456 66.378 0.00 0.00
A
ATOM 3253 HG2 LYS A 212 34.045 28.619 65.281 0.00 0.00
A
ATOM 3254 CD LYS A 212 34.509 28.533 67.463 0.00 0.00
A
ATOM 3255 HD1 LYS A 212 35.596 28.452 67.250 0.00 0.00
A
ATOM 3256 HD2 LYS A 212 34.392 29.238 68.314 0.00 0.00
A
ATOM 3257 CE LYS A 212 33.928 27.182 67.907 0.00 0.00
A
ATOM 3258 HE1 LYS A 212 34.302 26.690 68.831 0.00 0.00
A
ATOM 3259 HE2 LYS A 212 32.861 27.443 68.069 0.00 0.00
A
ATOM 3260 NZ LYS A 212 34.010 26.112 66.904 0.00 0.00
A
ATOM 3261 HZ1 LYS A 212 33.341 26.167 66.110 0.00 0.00
A
ATOM 3262 HZ2 LYS A 212 34.975 26.015 66.527 0.00 0.00
A
ATOM 3263 HZ3 LYS A 212 33.811 25.202 67.368 0.00 0.00
A
ATOM 3264 C LYS A 212 34.707 32.928 65.040 0.00 0.00
A
ATOM 3265 O LYS A 212 34.047 33.824 65.558 0.00 0.00
A
ATOM 3266 N ASP A 213 35.881 33.075 64.425 0.00 0.00
A

ATOM 3267 HN ASP A 213 36.224 32.294 63.909 0.00 0.00
A
ATOM 3268 CA ASP A 213 36.556 34.367 64.325 0.00 0.00
A
ATOM 3269 HA ASP A 213 36.573 34.636 65.371 0.00 0.00
A
ATOM 3270 CB ASP A 213 37.938 34.150 63.771 0.00 0.00
A
ATOM 3271 HB1 ASP A 213 37.907 33.577 62.820 0.00 0.00
A
ATOM 3272 HB2 ASP A 213 38.451 35.098 63.501 0.00 0.00
A
ATOM 3273 CG ASP A 213 38.678 33.321 64.818 0.00 0.00
A
ATOM 3274 OD1 ASP A 213 38.819 33.862 65.932 0.00 0.00
A
ATOM 3275 OD2 ASP A 213 39.104 32.172 64.606 0.00 0.00
A
ATOM 3276 C ASP A 213 35.785 35.496 63.572 0.00 0.00
A
ATOM 3277 O ASP A 213 35.735 36.643 63.970 0.00 0.00
A
ATOM 3278 N ILE A 214 35.060 35.170 62.504 0.00 0.00
A
ATOM 3279 HN ILE A 214 35.082 34.244 62.136 0.00 0.00
A
ATOM 3280 CA ILE A 214 34.285 36.119 61.684 0.00 0.00
A
ATOM 3281 HA ILE A 214 34.980 36.930 61.526 0.00 0.00
A
ATOM 3282 CB ILE A 214 33.743 35.555 60.320 0.00 0.00
A
ATOM 3283 HB ILE A 214 33.177 34.617 60.503 0.00 0.00
A
ATOM 3284 CG2 ILE A 214 32.747 36.546 59.613 0.00 0.00
A
ATOM 3285 HG21 ILE A 214 32.230 36.041 58.768 0.00 0.00
A
ATOM 3286 HG22 ILE A 214 31.873 36.736 60.272 0.00 0.00
A
ATOM 3287 HG23 ILE A 214 33.249 37.521 59.434 0.00 0.00
A
ATOM 3288 CG1 ILE A 214 34.958 35.161 59.408 0.00 0.00
A
ATOM 3289 HG11 ILE A 214 35.167 36.018 58.732 0.00 0.00
A
ATOM 3290 HG12 ILE A 214 35.743 34.904 60.151 0.00 0.00
A
ATOM 3291 CD ILE A 214 34.598 33.957 58.537 0.00 0.00
A
ATOM 3292 HD1 ILE A 214 33.613 34.202 58.085 0.00 0.00
A
ATOM 3293 HD2 ILE A 214 35.422 33.810 57.806 0.00 0.00
A

ATOM 3294 HD3 ILE A 214 34.530 33.046 59.170 0.00 0.00
A
ATOM 3295 C ILE A 214 33.084 36.591 62.530 0.00 0.00
A
ATOM 3296 O ILE A 214 32.676 37.738 62.423 0.00 0.00
A
ATOM 3297 N LYS A 215 32.430 35.734 63.357 0.00 0.00
A
ATOM 3298 HN LYS A 215 32.823 34.818 63.352 0.00 0.00
A
ATOM 3299 CA LYS A 215 31.360 36.121 64.252 0.00 0.00
A
ATOM 3300 HA LYS A 215 30.785 36.869 63.727 0.00 0.00
A
ATOM 3301 CB LYS A 215 30.391 34.916 64.592 0.00 0.00
A
ATOM 3302 HB1 LYS A 215 30.357 34.360 63.631 0.00 0.00
A
ATOM 3303 HB2 LYS A 215 30.694 34.276 65.449 0.00 0.00
A
ATOM 3304 CG LYS A 215 28.914 35.314 64.903 0.00 0.00
A
ATOM 3305 HG1 LYS A 215 28.551 34.351 65.322 0.00 0.00
A
ATOM 3306 HG2 LYS A 215 28.878 36.149 65.634 0.00 0.00
A
ATOM 3307 CD LYS A 215 28.102 35.552 63.631 0.00 0.00
A
ATOM 3308 HD1 LYS A 215 28.235 36.635 63.424 0.00 0.00
A
ATOM 3309 HD2 LYS A 215 28.534 34.895 62.846 0.00 0.00
A
ATOM 3310 CE LYS A 215 26.638 35.231 63.785 0.00 0.00
A
ATOM 3311 HE1 LYS A 215 26.219 35.163 62.758 0.00 0.00
A
ATOM 3312 HE2 LYS A 215 26.468 34.278 64.329 0.00 0.00
A
ATOM 3313 NZ LYS A 215 26.147 36.301 64.503 0.00 0.00
A
ATOM 3314 HZ1 LYS A 215 26.466 37.216 64.126 0.00 0.00
A
ATOM 3315 HZ2 LYS A 215 25.108 36.316 64.477 0.00 0.00
A
ATOM 3316 HZ3 LYS A 215 26.500 36.198 65.476 0.00 0.00
A
ATOM 3317 C LYS A 215 31.756 36.875 65.530 0.00 0.00
A
ATOM 3318 O LYS A 215 31.047 37.701 66.084 0.00 0.00
A
ATOM 3319 N ARG A 216 32.984 36.574 66.096 0.00 0.00
A
ATOM 3320 HN ARG A 216 33.492 35.764 65.812 0.00 0.00
A

ATOM   3321 CA ARG A 216   33.698 37.453 67.008 0.00 0.00
A
ATOM   3322 HA ARG A 216   33.042 37.643 67.844 0.00 0.00
A
ATOM   3323 CB ARG A 216   35.010 36.819 67.480 0.00 0.00
A
ATOM   3324 HB1 ARG A 216   34.700 35.797 67.787 0.00 0.00
A
ATOM   3325 HB2 ARG A 216   35.671 36.733 66.592 0.00 0.00
A
ATOM   3326 CG ARG A 216   35.605 37.669 68.591 0.00 0.00
A
ATOM   3327 HG1 ARG A 216   35.750 38.714 68.244 0.00 0.00
A
ATOM   3328 HG2 ARG A 216   34.882 37.751 69.431 0.00 0.00
A
ATOM   3329 CD ARG A 216   36.962 37.115 69.269 0.00 0.00
A
ATOM   3330 HD1 ARG A 216   36.830 36.039 69.512 0.00 0.00
A
ATOM   3331 HD2 ARG A 216   37.774 37.228 68.519 0.00 0.00
A
ATOM   3332 NE ARG A 216   37.142 38.072 70.472 0.00 0.00
A
ATOM   3333 HE ARG A 216   36.818 39.018 70.463 0.00 0.00
A
ATOM   3334 CZ ARG A 216   37.820 37.808 71.566 0.00 0.00
A
ATOM   3335 NH1 ARG A 216   38.554 36.766 71.835 0.00 0.00
A
ATOM   3336 HH11 ARG A 216   39.106 36.734 72.668 0.00 0.00
A
ATOM   3337 HH12 ARG A 216   38.695 35.979 71.234 0.00 0.00
A
ATOM   3338 NH2 ARG A 216   37.741 38.692 72.483 0.00 0.00
A
ATOM   3339 HH21 ARG A 216   38.121 38.565 73.399 0.00 0.00
A
ATOM   3340 HH22 ARG A 216   37.204 39.514 72.297 0.00 0.00
A
ATOM   3341 C   ARG A 216   33.990 38.891 66.356 0.00 0.00
A
ATOM   3342 O   ARG A 216   33.673 39.863 67.067 0.00 0.00
A
ATOM   3343 N   PHE A 217   34.404 38.955 65.141 0.00 0.00
A
ATOM   3344 HN PHE A 217   34.680 38.131 64.650 0.00 0.00
A
ATOM   3345 CA PHE A 217   34.490 40.332 64.482 0.00 0.00
A
ATOM   3346 HA PHE A 217   35.241 40.786 65.110 0.00 0.00
A
ATOM   3347 CB PHE A 217   34.894 40.123 62.954 0.00 0.00
A

ATOM 3348 HB1 PHE A 217 35.796 39.475 62.931 0.00 0.00
A
ATOM 3349 HB2 PHE A 217 34.117 39.490 62.475 0.00 0.00
A
ATOM 3350 CG PHE A 217 35.083 41.389 62.151 0.00 0.00
A
ATOM 3351 CD1 PHE A 217 35.690 42.513 62.732 0.00 0.00
A
ATOM 3352 HD1 PHE A 217 35.846 42.628 63.794 0.00 0.00
A
ATOM 3353 CE1 PHE A 217 35.981 43.631 61.924 0.00 0.00
A
ATOM 3354 HE1 PHE A 217 36.255 44.529 62.457 0.00 0.00
A
ATOM 3355 CZ PHE A 217 35.655 43.628 60.559 0.00 0.00
A
ATOM 3356 HZ PHE A 217 35.827 44.537 60.001 0.00 0.00
A
ATOM 3357 CD2 PHE A 217 34.849 41.354 60.751 0.00 0.00
A
ATOM 3358 HD2 PHE A 217 34.382 40.453 60.383 0.00 0.00
A
ATOM 3359 CE2 PHE A 217 35.131 42.466 59.948 0.00 0.00
A
ATOM 3360 HE2 PHE A 217 35.002 42.394 58.878 0.00 0.00
A
ATOM 3361 C PHE A 217 33.208 41.152 64.565 0.00 0.00
A
ATOM 3362 O PHE A 217 33.211 42.228 65.109 0.00 0.00
A
ATOM 3363 N GLU A 218 31.996 40.562 64.233 0.00 0.00
A
ATOM 3364 HN GLU A 218 31.936 39.682 63.767 0.00 0.00
A
ATOM 3365 CA GLU A 218 30.687 41.203 64.383 0.00 0.00
A
ATOM 3366 HA GLU A 218 30.585 42.043 63.712 0.00 0.00
A
ATOM 3367 CB GLU A 218 29.608 40.112 64.089 0.00 0.00
A
ATOM 3368 HB1 GLU A 218 29.855 39.711 63.083 0.00 0.00
A
ATOM 3369 HB2 GLU A 218 29.747 39.349 64.885 0.00 0.00
A
ATOM 3370 CG GLU A 218 28.113 40.599 64.135 0.00 0.00
A
ATOM 3371 HG1 GLU A 218 27.940 41.070 65.126 0.00 0.00
A
ATOM 3372 HG2 GLU A 218 27.978 41.393 63.370 0.00 0.00
A
ATOM 3373 CD GLU A 218 27.087 39.477 63.887 0.00 0.00
A
ATOM 3374 OE1 GLU A 218 27.104 38.842 62.827 0.00 0.00
A

ATOM 3375 OE2 GLU A 218 26.392 39.230 64.881 0.00 0.00
A
ATOM 3376 C GLU A 218 30.343 41.730 65.685 0.00 0.00
A
ATOM 3377 O GLU A 218 29.620 42.740 65.767 0.00 0.00
A
ATOM 3378 N ASN A 219 30.826 41.030 66.715 0.00 0.00
A
ATOM 3379 HN ASN A 219 31.530 40.335 66.597 0.00 0.00
A
ATOM 3380 CA ASN A 219 30.315 41.323 68.064 0.00 0.00
A
ATOM 3381 HA ASN A 219 29.509 42.041 68.039 0.00 0.00
A
ATOM 3382 CB ASN A 219 29.804 40.052 68.737 0.00 0.00
A
ATOM 3383 HB1 ASN A 219 30.402 39.155 68.470 0.00 0.00
A
ATOM 3384 HB2 ASN A 219 29.861 40.045 69.847 0.00 0.00
A
ATOM 3385 CG ASN A 219 28.288 39.827 68.428 0.00 0.00
A
ATOM 3386 OD1 ASN A 219 27.320 40.391 68.934 0.00 0.00
A
ATOM 3387 ND2 ASN A 219 28.124 39.081 67.253 0.00 0.00
A
ATOM 3388 HD21 ASN A 219 27.204 39.047 66.861 0.00 0.00
A
ATOM 3389 HD22 ASN A 219 28.929 38.724 66.779 0.00 0.00
A
ATOM 3390 C ASN A 219 31.278 42.077 68.909 0.00 0.00
A
ATOM 3391 O ASN A 219 31.018 42.295 70.114 0.00 0.00
A
ATOM 3392 N GLU A 220 32.427 42.472 68.396 0.00 0.00
A
ATOM 3393 HN GLU A 220 32.567 42.197 67.448 0.00 0.00
A
ATOM 3394 CA GLU A 220 33.416 43.092 69.271 0.00 0.00
A
ATOM 3395 HA GLU A 220 33.073 43.375 70.256 0.00 0.00
A
ATOM 3396 CB GLU A 220 34.584 42.041 69.489 0.00 0.00
A
ATOM 3397 HB1 GLU A 220 34.127 41.228 70.093 0.00 0.00
A
ATOM 3398 HB2 GLU A 220 34.892 41.645 68.498 0.00 0.00
A
ATOM 3399 CG GLU A 220 35.843 42.525 70.240 0.00 0.00
A
ATOM 3400 HG1 GLU A 220 36.403 43.380 69.805 0.00 0.00
A
ATOM 3401 HG2 GLU A 220 35.465 42.898 71.216 0.00 0.00
A

ATOM 3402 CD GLU A 220 36.796 41.417 70.543 0.00 0.00
A
ATOM 3403 OE1 GLU A 220 36.975 41.127 71.718 0.00 0.00
A
ATOM 3404 OE2 GLU A 220 37.432 40.805 69.602 0.00 0.00
A
ATOM 3405 C GLU A 220 33.969 44.298 68.585 0.00 0.00
A
ATOM 3406 O GLU A 220 33.748 45.452 68.871 0.00 0.00
A
ATOM 3407 N GLY A 221 34.829 44.026 67.531 0.00 0.00
A
ATOM 3408 HN GLY A 221 34.987 43.053 67.389 0.00 0.00
A
ATOM 3409 CA GLY A 221 35.604 45.007 66.793 0.00 0.00
A
ATOM 3410 HA1 GLY A 221 36.285 44.435 66.182 0.00 0.00
A
ATOM 3411 HA2 GLY A 221 36.159 45.674 67.437 0.00 0.00
A
ATOM 3412 C GLY A 221 34.680 45.726 65.896 0.00 0.00
A
ATOM 3413 O GLY A 221 34.827 46.962 65.853 0.00 0.00
A
ATOM 3414 N MET A 222 33.770 44.986 65.272 0.00 0.00
A
ATOM 3415 HN MET A 222 33.762 44.002 65.434 0.00 0.00
A
ATOM 3416 CA MET A 222 32.715 45.580 64.485 0.00 0.00
A
ATOM 3417 HA MET A 222 32.929 46.629 64.344 0.00 0.00
A
ATOM 3418 CB MET A 222 32.392 44.702 63.167 0.00 0.00
A
ATOM 3419 HB1 MET A 222 33.272 44.596 62.498 0.00 0.00
A
ATOM 3420 HB2 MET A 222 32.310 43.644 63.495 0.00 0.00
A
ATOM 3421 CG MET A 222 31.177 44.937 62.307 0.00 0.00
A
ATOM 3422 HG1 MET A 222 30.275 45.070 62.942 0.00 0.00
A
ATOM 3423 HG2 MET A 222 31.324 45.912 61.796 0.00 0.00
A
ATOM 3424 SD MET A 222 30.952 43.551 61.118 0.00 0.00
A
ATOM 3425 CE MET A 222 29.506 44.204 60.101 0.00 0.00
A
ATOM 3426 HE1 MET A 222 29.646 45.257 59.775 0.00 0.00
A
ATOM 3427 HE2 MET A 222 29.395 43.678 59.129 0.00 0.00
A
ATOM 3428 HE3 MET A 222 28.558 44.106 60.672 0.00 0.00
A

ATOM 3429 C MET A 222 31.458 45.434 65.304 0.00 0.00
A
ATOM 3430 O MET A 222 30.679 46.392 65.441 0.00 0.00
A
ATOM 3431 N ASN B 6 17.058 36.225 71.293 0.00 0.00
B
ATOM 3432 HT1 ASN B 6 16.318 35.505 71.409 0.00 0.00
B
ATOM 3433 HT2 ASN B 6 16.816 37.115 71.775 0.00 0.00
B
ATOM 3434 HT3 ASN B 6 17.887 35.862 71.806 0.00 0.00
B
ATOM 3435 CA ASN B 6 17.466 36.428 69.770 0.00 0.00
B
ATOM 3436 HA ASN B 6 18.025 37.350 69.694 0.00 0.00
B
ATOM 3437 CB ASN B 6 18.488 35.346 69.350 0.00 0.00
B
ATOM 3438 HB1 ASN B 6 18.029 34.336 69.412 0.00 0.00
B
ATOM 3439 HB2 ASN B 6 18.806 35.527 68.302 0.00 0.00
B
ATOM 3440 CG ASN B 6 19.759 35.467 70.174 0.00 0.00
B
ATOM 3441 OD1 ASN B 6 20.058 36.518 70.723 0.00 0.00
B
ATOM 3442 ND2 ASN B 6 20.562 34.405 70.221 0.00 0.00
B
ATOM 3443 HD21 ASN B 6 21.460 34.499 70.652 0.00 0.00
B
ATOM 3444 HD22 ASN B 6 20.244 33.577 69.759 0.00 0.00
B
ATOM 3445 C ASN B 6 16.263 36.525 68.929 0.00 0.00
B
ATOM 3446 O ASN B 6 15.332 37.117 69.391 0.00 0.00
B
ATOM 3447 N LYS B 7 16.191 36.106 67.652 0.00 0.00
B
ATOM 3448 HN LYS B 7 16.954 35.654 67.196 0.00 0.00
B
ATOM 3449 CA LYS B 7 15.049 36.266 66.795 0.00 0.00
B
ATOM 3450 HA LYS B 7 14.528 37.153 67.124 0.00 0.00
B
ATOM 3451 CB LYS B 7 15.445 36.578 65.408 0.00 0.00
B
ATOM 3452 HB1 LYS B 7 16.145 35.793 65.050 0.00 0.00
B
ATOM 3453 HB2 LYS B 7 14.594 36.590 64.695 0.00 0.00
B
ATOM 3454 CG LYS B 7 16.344 37.830 65.199 0.00 0.00
B
ATOM 3455 HG1 LYS B 7 15.817 38.797 65.345 0.00 0.00
B

ATOM	3456	HG2	LYS	B	7	17.199	37.843	65.909	0.00	0.00	B
ATOM	3457	CD	LYS	B	7	16.986	37.904	63.800	0.00	0.00	B
ATOM	3458	HD1	LYS	B	7	17.398	36.914	63.511	0.00	0.00	B
ATOM	3459	HD2	LYS	B	7	16.112	38.073	63.135	0.00	0.00	B
ATOM	3460	CE	LYS	B	7	18.058	38.931	63.705	0.00	0.00	B
ATOM	3461	HE1	LYS	B	7	17.679	39.905	64.081	0.00	0.00	B
ATOM	3462	HE2	LYS	B	7	18.927	38.592	64.309	0.00	0.00	B
ATOM	3463	NZ	LYS	B	7	18.567	39.186	62.354	0.00	0.00	B
ATOM	3464	HZ1	LYS	B	7	18.854	38.311	61.870	0.00	0.00	B
ATOM	3465	HZ2	LYS	B	7	17.789	39.624	61.821	0.00	0.00	B
ATOM	3466	HZ3	LYS	B	7	19.325	39.887	62.476	0.00	0.00	B
ATOM	3467	C	LYS	B	7	14.182	35.081	66.843	0.00	0.00	B
ATOM	3468	O	LYS	B	7	14.678	34.065	67.343	0.00	0.00	B
ATOM	3469	N	GLN	B	8	12.907	35.202	66.392	0.00	0.00	B
ATOM	3470	HN	GLN	B	8	12.744	36.123	66.046	0.00	0.00	B
ATOM	3471	CA	GLN	B	8	11.850	34.193	66.473	0.00	0.00	B
ATOM	3472	HA	GLN	B	8	12.384	33.288	66.724	0.00	0.00	B
ATOM	3473	CB	GLN	B	8	10.914	34.552	67.592	0.00	0.00	B
ATOM	3474	HB1	GLN	B	8	11.572	35.042	68.341	0.00	0.00	B
ATOM	3475	HB2	GLN	B	8	10.263	35.411	67.320	0.00	0.00	B
ATOM	3476	CG	GLN	B	8	10.152	33.362	68.164	0.00	0.00	B
ATOM	3477	HG1	GLN	B	8	9.464	32.993	67.372	0.00	0.00	B
ATOM	3478	HG2	GLN	B	8	10.718	32.438	68.407	0.00	0.00	B
ATOM	3479	CD	GLN	B	8	9.287	33.706	69.408	0.00	0.00	B
ATOM	3480	OE1	GLN	B	8	8.984	34.843	69.564	0.00	0.00	B
ATOM	3481	NE2	GLN	B	8	8.786	32.690	70.181	0.00	0.00	B
ATOM	3482	HE21	GLN	B	8	8.156	32.888	70.932	0.00	0.00	B

ATOM	3483	HE22	GLN	B	8	9.222	31.790	70.154	0.00	0.00	B
ATOM	3484	C	GLN	B	8	11.155	33.924	65.221	0.00	0.00	B
ATOM	3485	O	GLN	B	8	10.741	34.818	64.492	0.00	0.00	B
ATOM	3486	N	PHE	B	9	10.959	32.588	64.962	0.00	0.00	B
ATOM	3487	HN	PHE	B	9	11.189	31.918	65.663	0.00	0.00	B
ATOM	3488	CA	PHE	B	9	10.597	32.123	63.668	0.00	0.00	B
ATOM	3489	HA	PHE	B	9	9.975	32.840	63.153	0.00	0.00	B
ATOM	3490	CB	PHE	B	9	11.961	31.706	62.946	0.00	0.00	B
ATOM	3491	HB1	PHE	B	9	12.464	30.918	63.548	0.00	0.00	B
ATOM	3492	HB2	PHE	B	9	11.614	31.373	61.945	0.00	0.00	B
ATOM	3493	CG	PHE	B	9	12.998	32.781	62.826	0.00	0.00	B
ATOM	3494	CD1	PHE	B	9	14.333	32.597	63.215	0.00	0.00	B
ATOM	3495	HD1	PHE	B	9	14.660	31.761	63.815	0.00	0.00	B
ATOM	3496	CE1	PHE	B	9	15.195	33.663	63.232	0.00	0.00	B
ATOM	3497	HE1	PHE	B	9	16.207	33.575	63.600	0.00	0.00	B
ATOM	3498	CZ	PHE	B	9	14.846	34.894	62.641	0.00	0.00	B
ATOM	3499	HZ	PHE	B	9	15.542	35.708	62.501	0.00	0.00	B
ATOM	3500	CD2	PHE	B	9	12.677	34.049	62.226	0.00	0.00	B
ATOM	3501	HD2	PHE	B	9	11.748	34.184	61.692	0.00	0.00	B
ATOM	3502	CE2	PHE	B	9	13.559	35.095	62.160	0.00	0.00	B
ATOM	3503	HE2	PHE	B	9	13.192	36.046	61.805	0.00	0.00	B
ATOM	3504	C	PHE	B	9	9.746	30.889	63.812	0.00	0.00	B
ATOM	3505	O	PHE	B	9	9.771	30.201	64.830	0.00	0.00	B
ATOM	3506	N	ALA	B	10	8.878	30.554	62.810	0.00	0.00	B
ATOM	3507	HN	ALA	B	10	8.782	31.149	62.016	0.00	0.00	B
ATOM	3508	CA	ALA	B	10	7.987	29.484	62.690	0.00	0.00	B
ATOM	3509	HA	ALA	B	10	8.332	28.736	63.389	0.00	0.00	B

ATOM	3510	CB	ALA	B	10	6.458	29.834	62.959	0.00	0.00	B
ATOM	3511	HB1	ALA	B	10	6.391	30.425	63.897	0.00	0.00	B
ATOM	3512	HB2	ALA	B	10	5.963	30.386	62.131	0.00	0.00	B
ATOM	3513	HB3	ALA	B	10	6.034	28.815	63.088	0.00	0.00	B
ATOM	3514	C	ALA	B	10	8.200	28.965	61.371	0.00	0.00	B
ATOM	3515	O	ALA	B	10	8.390	29.719	60.450	0.00	0.00	B
ATOM	3516	N	VAL	B	11	8.440	27.631	61.247	0.00	0.00	B
ATOM	3517	HN	VAL	B	11	8.242	27.077	62.053	0.00	0.00	B
ATOM	3518	CA	VAL	B	11	8.742	26.928	60.046	0.00	0.00	B
ATOM	3519	HA	VAL	B	11	8.643	27.605	59.211	0.00	0.00	B
ATOM	3520	CB	VAL	B	11	10.157	26.325	60.012	0.00	0.00	B
ATOM	3521	HB	VAL	B	11	10.257	25.476	60.723	0.00	0.00	B
ATOM	3522	CG1	VAL	B	11	10.396	25.697	58.616	0.00	0.00	B
ATOM	3523	HG11	VAL	B	11	9.779	24.791	58.436	0.00	0.00	B
ATOM	3524	HG12	VAL	B	11	10.157	26.398	57.788	0.00	0.00	B
ATOM	3525	HG13	VAL	B	11	11.456	25.404	58.464	0.00	0.00	B
ATOM	3526	CG2	VAL	B	11	11.298	27.364	60.223	0.00	0.00	B
ATOM	3527	HG21	VAL	B	11	12.269	26.851	60.054	0.00	0.00	B
ATOM	3528	HG22	VAL	B	11	11.244	28.206	59.500	0.00	0.00	B
ATOM	3529	HG23	VAL	B	11	11.203	27.806	61.238	0.00	0.00	B
ATOM	3530	C	VAL	B	11	7.760	25.811	59.919	0.00	0.00	B
ATOM	3531	O	VAL	B	11	7.670	24.928	60.743	0.00	0.00	B
ATOM	3532	N	ILE	B	12	6.925	25.922	58.857	0.00	0.00	B
ATOM	3533	HN	ILE	B	12	7.006	26.663	58.195	0.00	0.00	B
ATOM	3534	CA	ILE	B	12	5.859	24.916	58.571	0.00	0.00	B
ATOM	3535	HA	ILE	B	12	5.600	24.378	59.471	0.00	0.00	B
ATOM	3536	CB	ILE	B	12	4.581	25.636	58.092	0.00	0.00	B

```
ATOM   3537  HB  ILE B  12       4.794  26.091  57.101  0.00  0.00
B
ATOM   3538  CG2 ILE B  12       3.380  24.590  58.005  0.00  0.00
B
ATOM   3539 HG21 ILE B  12       3.234  24.073  58.977  0.00  0.00
B
ATOM   3540 HG22 ILE B  12       2.398  25.081  57.835  0.00  0.00
B
ATOM   3541 HG23 ILE B  12       3.657  23.853  57.221  0.00  0.00
B
ATOM   3542  CG1 ILE B  12       4.328  26.831  59.104  0.00  0.00
B
ATOM   3543 HG11 ILE B  12       4.375  26.407  60.130  0.00  0.00
B
ATOM   3544 HG12 ILE B  12       5.130  27.561  58.865  0.00  0.00
B
ATOM   3545  CD  ILE B  12       2.964  27.600  58.938  0.00  0.00
B
ATOM   3546  HD1 ILE B  12       2.894  27.877  57.865  0.00  0.00
B
ATOM   3547  HD2 ILE B  12       2.105  26.933  59.166  0.00  0.00
B
ATOM   3548  HD3 ILE B  12       2.926  28.523  59.555  0.00  0.00
B
ATOM   3549  C   ILE B  12       6.275  23.876  57.530  0.00  0.00
B
ATOM   3550  O   ILE B  12       6.681  24.199  56.399  0.00  0.00
B
ATOM   3551  N   GLY B  13       6.301  22.599  57.944  0.00  0.00
B
ATOM   3552  HN  GLY B  13       6.096  22.363  58.891  0.00  0.00
B
ATOM   3553  CA  GLY B  13       6.761  21.455  57.151  0.00  0.00
B
ATOM   3554  HA1 GLY B  13       6.654  21.637  56.092  0.00  0.00
B
ATOM   3555  HA2 GLY B  13       6.101  20.654  57.451  0.00  0.00
B
ATOM   3556  C   GLY B  13       8.215  21.146  57.570  0.00  0.00
B
ATOM   3557  O   GLY B  13       9.030  22.043  57.624  0.00  0.00
B
ATOM   3558  N   LEU B  14       8.477  19.895  57.828  0.00  0.00
B
ATOM   3559  HN  LEU B  14       7.750  19.221  57.728  0.00  0.00
B
ATOM   3560  CA  LEU B  14       9.694  19.454  58.438  0.00  0.00
B
ATOM   3561  HA  LEU B  14      10.500  20.168  58.350  0.00  0.00
B
ATOM   3562  CB  LEU B  14       9.411  18.933  59.863  0.00  0.00
B
ATOM   3563  HB1 LEU B  14       8.633  18.162  59.679  0.00  0.00
B
```

ATOM	3564	HB2	LEU	B	14	10.331	18.492	60.302	0.00	0.00	B
ATOM	3565	CG	LEU	B	14	8.956	19.923	60.895	0.00	0.00	B
ATOM	3566	HG	LEU	B	14	7.962	20.218	60.496	0.00	0.00	B
ATOM	3567	CD1	LEU	B	14	8.937	19.381	62.316	0.00	0.00	B
ATOM	3568	HD11	LEU	B	14	8.935	18.281	62.472	0.00	0.00	B
ATOM	3569	HD12	LEU	B	14	9.799	19.790	62.885	0.00	0.00	B
ATOM	3570	HD13	LEU	B	14	8.064	19.794	62.864	0.00	0.00	B
ATOM	3571	CD2	LEU	B	14	9.902	21.280	60.852	0.00	0.00	B
ATOM	3572	HD21	LEU	B	14	10.948	21.101	61.179	0.00	0.00	B
ATOM	3573	HD22	LEU	B	14	9.957	21.737	59.841	0.00	0.00	B
ATOM	3574	HD23	LEU	B	14	9.352	22.011	61.482	0.00	0.00	B
ATOM	3575	C	LEU	B	14	10.242	18.280	57.661	0.00	0.00	B
ATOM	3576	O	LEU	B	14	10.956	17.374	58.096	0.00	0.00	B
ATOM	3577	N	GLY	B	15	10.009	18.310	56.332	0.00	0.00	B
ATOM	3578	HN	GLY	B	15	9.421	19.045	56.004	0.00	0.00	B
ATOM	3579	CA	GLY	B	15	10.715	17.669	55.221	0.00	0.00	B
ATOM	3580	HA1	GLY	B	15	10.225	17.985	54.312	0.00	0.00	B
ATOM	3581	HA2	GLY	B	15	10.631	16.627	55.495	0.00	0.00	B
ATOM	3582	C	GLY	B	15	12.184	18.082	55.185	0.00	0.00	B
ATOM	3583	O	GLY	B	15	12.693	18.752	56.094	0.00	0.00	B
ATOM	3584	N	ARG	B	16	12.935	17.710	54.102	0.00	0.00	B
ATOM	3585	HN	ARG	B	16	12.551	17.000	53.516	0.00	0.00	B
ATOM	3586	CA	ARG	B	16	14.369	17.944	53.879	0.00	0.00	B
ATOM	3587	HA	ARG	B	16	14.936	17.529	54.700	0.00	0.00	B
ATOM	3588	CB	ARG	B	16	14.981	17.379	52.567	0.00	0.00	B
ATOM	3589	HB1	ARG	B	16	14.487	17.603	51.597	0.00	0.00	B
ATOM	3590	HB2	ARG	B	16	16.017	17.779	52.522	0.00	0.00	B

ATOM 3591 CG ARG B 16 15.084 15.885 52.575 0.00 0.00
B
ATOM 3592 HG1 ARG B 16 15.707 15.652 53.465 0.00 0.00
B
ATOM 3593 HG2 ARG B 16 14.061 15.471 52.700 0.00 0.00
B
ATOM 3594 CD ARG B 16 15.555 15.222 51.233 0.00 0.00
B
ATOM 3595 HD1 ARG B 16 16.388 15.863 50.874 0.00 0.00
B
ATOM 3596 HD2 ARG B 16 15.942 14.228 51.545 0.00 0.00
B
ATOM 3597 NE ARG B 16 14.491 15.151 50.134 0.00 0.00
B
ATOM 3598 HE ARG B 16 13.528 15.359 50.304 0.00 0.00
B
ATOM 3599 CZ ARG B 16 14.684 14.447 49.075 0.00 0.00
B
ATOM 3600 NH1 ARG B 16 15.816 13.992 48.545 0.00 0.00
B
ATOM 3601 HH11 ARG B 16 15.887 13.486 47.686 0.00 0.00
B
ATOM 3602 HH12 ARG B 16 16.696 14.460 48.632 0.00 0.00
B
ATOM 3603 NH2 ARG B 16 13.497 13.946 48.547 0.00 0.00
B
ATOM 3604 HH21 ARG B 16 13.646 13.450 47.692 0.00 0.00
B
ATOM 3605 HH22 ARG B 16 12.653 14.458 48.709 0.00 0.00
B
ATOM 3606 C ARG B 16 14.644 19.485 53.895 0.00 0.00
B
ATOM 3607 O ARG B 16 15.650 19.975 54.512 0.00 0.00
B
ATOM 3608 N PHE B 17 13.788 20.238 53.114 0.00 0.00
B
ATOM 3609 HN PHE B 17 13.036 19.796 52.632 0.00 0.00
B
ATOM 3610 CA PHE B 17 13.870 21.692 53.002 0.00 0.00
B
ATOM 3611 HA PHE B 17 14.831 22.016 52.630 0.00 0.00
B
ATOM 3612 CB PHE B 17 12.961 22.153 51.875 0.00 0.00
B
ATOM 3613 HB1 PHE B 17 13.467 21.753 50.971 0.00 0.00
B
ATOM 3614 HB2 PHE B 17 11.907 21.840 52.038 0.00 0.00
B
ATOM 3615 CG PHE B 17 12.903 23.655 51.620 0.00 0.00
B
ATOM 3616 CD1 PHE B 17 14.091 24.422 51.463 0.00 0.00
B
ATOM 3617 HD1 PHE B 17 15.101 24.039 51.491 0.00 0.00
B

ATOM	3618	CE1	PHE	B	17	13.952	25.845	51.621	0.00	0.00	B
ATOM	3619	HE1	PHE	B	17	14.875	26.402	51.549	0.00	0.00	B
ATOM	3620	CZ	PHE	B	17	12.709	26.453	51.684	0.00	0.00	B
ATOM	3621	HZ	PHE	B	17	12.552	27.518	51.601	0.00	0.00	B
ATOM	3622	CD2	PHE	B	17	11.667	24.346	51.700	0.00	0.00	B
ATOM	3623	HD2	PHE	B	17	10.806	23.748	51.962	0.00	0.00	B
ATOM	3624	CE2	PHE	B	17	11.515	25.743	51.728	0.00	0.00	B
ATOM	3625	HE2	PHE	B	17	10.510	26.123	51.836	0.00	0.00	B
ATOM	3626	C	PHE	B	17	13.548	22.444	54.279	0.00	0.00	B
ATOM	3627	O	PHE	B	17	14.264	23.327	54.773	0.00	0.00	B
ATOM	3628	N	GLY	B	18	12.452	22.021	54.925	0.00	0.00	B
ATOM	3629	HN	GLY	B	18	11.921	21.253	54.574	0.00	0.00	B
ATOM	3630	CA	GLY	B	18	12.105	22.512	56.238	0.00	0.00	B
ATOM	3631	HA1	GLY	B	18	11.183	22.123	56.644	0.00	0.00	B
ATOM	3632	HA2	GLY	B	18	12.038	23.589	56.209	0.00	0.00	B
ATOM	3633	C	GLY	B	18	13.077	22.181	57.382	0.00	0.00	B
ATOM	3634	O	GLY	B	18	13.583	23.064	58.094	0.00	0.00	B
ATOM	3635	N	GLY	B	19	13.315	20.914	57.571	0.00	0.00	B
ATOM	3636	HN	GLY	B	19	13.006	20.240	56.905	0.00	0.00	B
ATOM	3637	CA	GLY	B	19	14.075	20.370	58.663	0.00	0.00	B
ATOM	3638	HA1	GLY	B	19	14.036	19.293	58.585	0.00	0.00	B
ATOM	3639	HA2	GLY	B	19	13.623	20.706	59.585	0.00	0.00	B
ATOM	3640	C	GLY	B	19	15.469	20.877	58.682	0.00	0.00	B
ATOM	3641	O	GLY	B	19	15.877	21.334	59.732	0.00	0.00	B
ATOM	3642	N	SER	B	20	16.094	20.926	57.476	0.00	0.00	B
ATOM	3643	HN	SER	B	20	15.554	20.663	56.680	0.00	0.00	B
ATOM	3644	CA	SER	B	20	17.377	21.634	57.424	0.00	0.00	B

ATOM 3645 HA SER B 20 18.101 21.153 58.064 0.00 0.00
B
ATOM 3646 CB SER B 20 18.088 21.746 56.027 0.00 0.00
B
ATOM 3647 HB1 SER B 20 17.438 22.404 55.412 0.00 0.00
B
ATOM 3648 HB2 SER B 20 19.080 22.241 56.112 0.00 0.00
B
ATOM 3649 OG SER B 20 18.223 20.454 55.520 0.00 0.00
B
ATOM 3650 HG1 SER B 20 17.554 20.274 54.856 0.00 0.00
B
ATOM 3651 C SER B 20 17.363 23.064 57.790 0.00 0.00
B
ATOM 3652 O SER B 20 18.324 23.514 58.420 0.00 0.00
B
ATOM 3653 N ILE B 21 16.342 23.836 57.435 0.00 0.00
B
ATOM 3654 HN ILE B 21 15.555 23.535 56.902 0.00 0.00
B
ATOM 3655 CA ILE B 21 16.143 25.252 57.789 0.00 0.00
B
ATOM 3656 HA ILE B 21 17.059 25.739 57.490 0.00 0.00
B
ATOM 3657 CB ILE B 21 14.960 25.961 57.195 0.00 0.00
B
ATOM 3658 HB ILE B 21 14.048 25.345 57.347 0.00 0.00
B
ATOM 3659 CG2 ILE B 21 14.953 27.377 57.761 0.00 0.00
B
ATOM 3660 HG21 ILE B 21 14.253 28.028 57.194 0.00 0.00
B
ATOM 3661 HG22 ILE B 21 14.559 27.528 58.788 0.00 0.00
B
ATOM 3662 HG23 ILE B 21 15.975 27.766 57.562 0.00 0.00
B
ATOM 3663 CG1 ILE B 21 15.221 26.191 55.660 0.00 0.00
B
ATOM 3664 HG11 ILE B 21 16.114 26.834 55.507 0.00 0.00
B
ATOM 3665 HG12 ILE B 21 15.611 25.233 55.255 0.00 0.00
B
ATOM 3666 CD ILE B 21 13.924 26.685 54.878 0.00 0.00
B
ATOM 3667 HD1 ILE B 21 13.495 27.635 55.259 0.00 0.00
B
ATOM 3668 HD2 ILE B 21 14.259 26.910 53.842 0.00 0.00
B
ATOM 3669 HD3 ILE B 21 13.312 25.761 54.961 0.00 0.00
B
ATOM 3670 C ILE B 21 15.992 25.355 59.326 0.00 0.00
B
ATOM 3671 O ILE B 21 16.711 26.074 60.027 0.00 0.00
B

ATOM	3672	N	VAL	B	22	15.112	24.538	59.945	0.00	0.00	B
ATOM	3673	HN	VAL	B	22	14.515	23.960	59.394	0.00	0.00	B
ATOM	3674	CA	VAL	B	22	14.881	24.485	61.419	0.00	0.00	B
ATOM	3675	HA	VAL	B	22	14.685	25.489	61.765	0.00	0.00	B
ATOM	3676	CB	VAL	B	22	13.707	23.484	61.762	0.00	0.00	B
ATOM	3677	HB	VAL	B	22	13.939	22.480	61.346	0.00	0.00	B
ATOM	3678	CG1	VAL	B	22	13.626	23.377	63.320	0.00	0.00	B
ATOM	3679	HG11	VAL	B	22	12.790	22.710	63.622	0.00	0.00	B
ATOM	3680	HG12	VAL	B	22	14.554	22.924	63.730	0.00	0.00	B
ATOM	3681	HG13	VAL	B	22	13.574	24.428	63.677	0.00	0.00	B
ATOM	3682	CG2	VAL	B	22	12.418	23.941	61.248	0.00	0.00	B
ATOM	3683	HG21	VAL	B	22	12.346	24.066	60.147	0.00	0.00	B
ATOM	3684	HG22	VAL	B	22	11.679	23.179	61.574	0.00	0.00	B
ATOM	3685	HG23	VAL	B	22	12.235	24.962	61.648	0.00	0.00	B
ATOM	3686	C	VAL	B	22	16.164	24.125	62.175	0.00	0.00	B
ATOM	3687	O	VAL	B	22	16.523	24.822	63.098	0.00	0.00	B
ATOM	3688	N	LYS	B	23	16.886	23.095	61.728	0.00	0.00	B
ATOM	3689	HN	LYS	B	23	16.398	22.500	61.094	0.00	0.00	B
ATOM	3690	CA	LYS	B	23	18.124	22.710	62.240	0.00	0.00	B
ATOM	3691	HA	LYS	B	23	17.990	22.446	63.278	0.00	0.00	B
ATOM	3692	CB	LYS	B	23	18.607	21.463	61.568	0.00	0.00	B
ATOM	3693	HB1	LYS	B	23	17.762	20.753	61.441	0.00	0.00	B
ATOM	3694	HB2	LYS	B	23	18.955	21.674	60.534	0.00	0.00	B
ATOM	3695	CG	LYS	B	23	19.773	20.696	62.163	0.00	0.00	B
ATOM	3696	HG1	LYS	B	23	20.583	21.456	62.202	0.00	0.00	B
ATOM	3697	HG2	LYS	B	23	19.509	20.373	63.192	0.00	0.00	B
ATOM	3698	CD	LYS	B	23	20.235	19.468	61.426	0.00	0.00	B

ATOM 3699 HD1 LYS B 23 19.319 18.844 61.347 0.00 0.00 B
ATOM 3700 HD2 LYS B 23 20.505 19.744 60.384 0.00 0.00 B
ATOM 3701 CE LYS B 23 21.378 18.690 62.112 0.00 0.00 B
ATOM 3702 HE1 LYS B 23 22.308 19.290 62.017 0.00 0.00 B
ATOM 3703 HE2 LYS B 23 21.218 18.533 63.200 0.00 0.00 B
ATOM 3704 NZ LYS B 23 21.598 17.371 61.550 0.00 0.00 B
ATOM 3705 HZ1 LYS B 23 22.498 16.969 61.881 0.00 0.00 B
ATOM 3706 HZ2 LYS B 23 20.788 16.739 61.709 0.00 0.00 B
ATOM 3707 HZ3 LYS B 23 21.636 17.386 60.511 0.00 0.00 B
ATOM 3708 C LYS B 23 19.169 23.864 62.116 0.00 0.00 B
ATOM 3709 O LYS B 23 19.912 24.177 63.101 0.00 0.00 B
ATOM 3710 N GLU B 24 19.323 24.557 61.036 0.00 0.00 B
ATOM 3711 HN GLU B 24 18.642 24.381 60.329 0.00 0.00 B
ATOM 3712 CA GLU B 24 20.226 25.665 60.867 0.00 0.00 B
ATOM 3713 HA GLU B 24 21.155 25.283 61.265 0.00 0.00 B
ATOM 3714 CB GLU B 24 20.381 26.116 59.375 0.00 0.00 B
ATOM 3715 HB1 GLU B 24 20.177 25.269 58.684 0.00 0.00 B
ATOM 3716 HB2 GLU B 24 19.641 26.898 59.101 0.00 0.00 B
ATOM 3717 CG GLU B 24 21.845 26.572 59.139 0.00 0.00 B
ATOM 3718 HG1 GLU B 24 21.840 26.877 58.071 0.00 0.00 B
ATOM 3719 HG2 GLU B 24 22.011 27.517 59.699 0.00 0.00 B
ATOM 3720 CD GLU B 24 22.980 25.599 59.348 0.00 0.00 B
ATOM 3721 OE1 GLU B 24 22.998 24.471 58.684 0.00 0.00 B
ATOM 3722 OE2 GLU B 24 23.795 25.823 60.241 0.00 0.00 B
ATOM 3723 C GLU B 24 19.858 26.836 61.785 0.00 0.00 B
ATOM 3724 O GLU B 24 20.775 27.474 62.290 0.00 0.00 B
ATOM 3725 N LEU B 25 18.564 27.170 61.912 0.00 0.00 B

ATOM 3726 HN LEU B 25 17.777 26.831 61.401 0.00 0.00
B
ATOM 3727 CA LEU B 25 18.270 28.287 62.763 0.00 0.00
B
ATOM 3728 HA LEU B 25 18.840 29.139 62.422 0.00 0.00
B
ATOM 3729 CB LEU B 25 16.778 28.702 62.678 0.00 0.00
B
ATOM 3730 HB1 LEU B 25 16.153 27.797 62.828 0.00 0.00
B
ATOM 3731 HB2 LEU B 25 16.501 29.273 63.590 0.00 0.00
B
ATOM 3732 CG LEU B 25 16.312 29.361 61.360 0.00 0.00
B
ATOM 3733 HG LEU B 25 16.899 28.786 60.613 0.00 0.00
B
ATOM 3734 CD1 LEU B 25 14.812 29.164 61.109 0.00 0.00
B
ATOM 3735 HD11 LEU B 25 14.615 29.620 60.115 0.00 0.00
B
ATOM 3736 HD12 LEU B 25 14.517 28.101 61.241 0.00 0.00
B
ATOM 3737 HD13 LEU B 25 14.270 29.809 61.833 0.00 0.00
B
ATOM 3738 CD2 LEU B 25 16.928 30.776 61.210 0.00 0.00
B
ATOM 3739 HD21 LEU B 25 16.547 31.621 61.822 0.00 0.00
B
ATOM 3740 HD22 LEU B 25 18.037 30.757 61.146 0.00 0.00
B
ATOM 3741 HD23 LEU B 25 16.519 31.132 60.240 0.00 0.00
B
ATOM 3742 C LEU B 25 18.493 28.034 64.265 0.00 0.00
B
ATOM 3743 O LEU B 25 18.825 28.900 64.984 0.00 0.00
B
ATOM 3744 N HSE B 26 18.410 26.778 64.795 0.00 0.00
B
ATOM 3745 HN HSE B 26 18.085 25.991 64.276 0.00 0.00
B
ATOM 3746 CA HSE B 26 18.950 26.385 66.056 0.00 0.00
B
ATOM 3747 HA HSE B 26 18.679 27.123 66.796 0.00 0.00
B
ATOM 3748 CB HSE B 26 18.188 25.082 66.316 0.00 0.00
B
ATOM 3749 HB1 HSE B 26 17.099 25.171 66.114 0.00 0.00
B
ATOM 3750 HB2 HSE B 26 18.585 24.165 65.831 0.00 0.00
B
ATOM 3751 ND1 HSE B 26 17.609 25.314 68.701 0.00 0.00
B
ATOM 3752 CG HSE B 26 18.249 24.614 67.679 0.00 0.00
B

ATOM 3753 CE1 HSE B 26 17.655 24.518 69.774 0.00 0.00
B
ATOM 3754 HE1 HSE B 26 17.084 24.703 70.684 0.00 0.00
B
ATOM 3755 NE2 HSE B 26 18.304 23.390 69.515 0.00 0.00
B
ATOM 3756 HE2 HSE B 26 18.188 22.599 70.115 0.00 0.00
B
ATOM 3757 CD2 HSE B 26 18.710 23.446 68.197 0.00 0.00
B
ATOM 3758 HD2 HSE B 26 19.162 22.598 67.698 0.00 0.00
B
ATOM 3759 C HSE B 26 20.428 26.276 66.207 0.00 0.00
B
ATOM 3760 O HSE B 26 20.925 26.526 67.287 0.00 0.00
B
ATOM 3761 N ARG B 27 21.186 26.066 65.054 0.00 0.00
B
ATOM 3762 HN ARG B 27 20.732 25.701 64.246 0.00 0.00
B
ATOM 3763 CA ARG B 27 22.658 26.172 64.981 0.00 0.00
B
ATOM 3764 HA ARG B 27 23.041 26.036 65.981 0.00 0.00
B
ATOM 3765 CB ARG B 27 23.245 25.076 64.006 0.00 0.00
B
ATOM 3766 HB1 ARG B 27 23.133 24.079 64.485 0.00 0.00
B
ATOM 3767 HB2 ARG B 27 22.652 25.201 63.075 0.00 0.00
B
ATOM 3768 CG ARG B 27 24.740 25.100 63.604 0.00 0.00
B
ATOM 3769 HG1 ARG B 27 24.961 25.980 62.962 0.00 0.00
B
ATOM 3770 HG2 ARG B 27 25.343 25.255 64.525 0.00 0.00
B
ATOM 3771 CD ARG B 27 25.213 23.799 62.986 0.00 0.00
B
ATOM 3772 HD1 ARG B 27 26.313 23.791 62.829 0.00 0.00
B
ATOM 3773 HD2 ARG B 27 25.061 22.953 63.690 0.00 0.00
B
ATOM 3774 NE ARG B 27 24.402 23.547 61.722 0.00 0.00
B
ATOM 3775 HE ARG B 27 24.181 24.370 61.198 0.00 0.00
B
ATOM 3776 CZ ARG B 27 23.958 22.360 61.309 0.00 0.00
B
ATOM 3777 NH1 ARG B 27 24.314 21.181 61.893 0.00 0.00
B
ATOM 3778 HH11 ARG B 27 24.275 20.379 61.296 0.00 0.00
B
ATOM 3779 HH12 ARG B 27 24.798 21.269 62.763 0.00 0.00
B

ATOM 3780 NH2 ARG B 27 23.325 22.206 60.131 0.00 0.00
B
ATOM 3781 HH21 ARG B 27 23.259 21.268 59.791 0.00 0.00
B
ATOM 3782 HH22 ARG B 27 23.229 23.000 59.531 0.00 0.00
B
ATOM 3783 C ARG B 27 23.181 27.572 64.790 0.00 0.00
B
ATOM 3784 O ARG B 27 24.305 27.968 65.065 0.00 0.00
B
ATOM 3785 N MET B 28 22.303 28.504 64.469 0.00 0.00
B
ATOM 3786 HN MET B 28 21.426 28.267 64.058 0.00 0.00
B
ATOM 3787 CA MET B 28 22.536 29.896 64.527 0.00 0.00
B
ATOM 3788 HA MET B 28 23.589 30.127 64.471 0.00 0.00
B
ATOM 3789 CB MET B 28 21.978 30.659 63.303 0.00 0.00
B
ATOM 3790 HB1 MET B 28 20.891 30.428 63.271 0.00 0.00
B
ATOM 3791 HB2 MET B 28 21.997 31.748 63.520 0.00 0.00
B
ATOM 3792 CG MET B 28 22.600 30.319 61.951 0.00 0.00
B
ATOM 3793 HG1 MET B 28 23.481 30.974 61.780 0.00 0.00
B
ATOM 3794 HG2 MET B 28 23.072 29.315 61.998 0.00 0.00
B
ATOM 3795 SD MET B 28 21.358 30.310 60.570 0.00 0.00
B
ATOM 3796 CE MET B 28 22.521 30.367 59.123 0.00 0.00
B
ATOM 3797 HE1 MET B 28 22.943 29.339 59.121 0.00 0.00
B
ATOM 3798 HE2 MET B 28 21.994 30.581 58.168 0.00 0.00
B
ATOM 3799 HE3 MET B 28 23.326 31.130 59.187 0.00 0.00
B
ATOM 3800 C MET B 28 21.964 30.506 65.781 0.00 0.00
B
ATOM 3801 O MET B 28 22.113 31.710 66.003 0.00 0.00
B
ATOM 3802 N GLY B 29 21.269 29.751 66.645 0.00 0.00
B
ATOM 3803 HN GLY B 29 21.118 28.784 66.453 0.00 0.00
B
ATOM 3804 CA GLY B 29 20.963 30.116 68.056 0.00 0.00
B
ATOM 3805 HA1 GLY B 29 21.857 30.652 68.338 0.00 0.00
B
ATOM 3806 HA2 GLY B 29 20.813 29.178 68.570 0.00 0.00
B

ATOM 3807 C GLY B 29 19.636 30.916 68.211 0.00 0.00
B
ATOM 3808 O GLY B 29 19.293 31.413 69.328 0.00 0.00
B
ATOM 3809 N HSE B 30 18.880 31.124 67.124 0.00 0.00
B
ATOM 3810 HN HSE B 30 19.332 30.772 66.308 0.00 0.00
B
ATOM 3811 CA HSE B 30 17.633 31.794 67.081 0.00 0.00
B
ATOM 3812 HA HSE B 30 17.736 32.660 67.717 0.00 0.00
B
ATOM 3813 CB HSE B 30 17.307 32.210 65.653 0.00 0.00
B
ATOM 3814 HB1 HSE B 30 17.230 31.372 64.928 0.00 0.00
B
ATOM 3815 HB2 HSE B 30 16.314 32.707 65.627 0.00 0.00
B
ATOM 3816 ND1 HSE B 30 18.266 34.399 65.789 0.00 0.00
B
ATOM 3817 CG HSE B 30 18.219 33.202 65.138 0.00 0.00
B
ATOM 3818 CE1 HSE B 30 19.224 35.021 65.133 0.00 0.00
B
ATOM 3819 HE1 HSE B 30 19.448 36.087 65.157 0.00 0.00
B
ATOM 3820 NE2 HSE B 30 19.813 34.334 64.171 0.00 0.00
B
ATOM 3821 HE2 HSE B 30 20.459 34.633 63.468 0.00 0.00
B
ATOM 3822 CD2 HSE B 30 19.180 33.118 64.162 0.00 0.00
B
ATOM 3823 HD2 HSE B 30 19.256 32.291 63.466 0.00 0.00
B
ATOM 3824 C HSE B 30 16.447 30.913 67.592 0.00 0.00
B
ATOM 3825 O HSE B 30 16.528 29.676 67.644 0.00 0.00
B
ATOM 3826 N GLU B 31 15.325 31.518 68.032 0.00 0.00
B
ATOM 3827 HN GLU B 31 15.083 32.483 68.102 0.00 0.00
B
ATOM 3828 CA GLU B 31 14.178 30.743 68.409 0.00 0.00
B
ATOM 3829 HA GLU B 31 14.599 29.851 68.847 0.00 0.00
B
ATOM 3830 CB GLU B 31 13.344 31.552 69.329 0.00 0.00
B
ATOM 3831 HB1 GLU B 31 12.774 32.328 68.776 0.00 0.00
B
ATOM 3832 HB2 GLU B 31 12.505 30.962 69.756 0.00 0.00
B
ATOM 3833 CG GLU B 31 14.023 32.310 70.510 0.00 0.00
B

ATOM 3834 HG1 GLU B 31 14.862 32.928 70.123 0.00 0.00
B
ATOM 3835 HG2 GLU B 31 13.336 33.024 71.013 0.00 0.00
B
ATOM 3836 CD GLU B 31 14.483 31.335 71.662 0.00 0.00
B
ATOM 3837 OE1 GLU B 31 15.668 31.149 72.000 0.00 0.00
B
ATOM 3838 OE2 GLU B 31 13.581 30.624 72.104 0.00 0.00
B
ATOM 3839 C GLU B 31 13.401 30.228 67.265 0.00 0.00
B
ATOM 3840 O GLU B 31 13.146 30.983 66.342 0.00 0.00
B
ATOM 3841 N VAL B 32 12.998 28.977 67.342 0.00 0.00
B
ATOM 3842 HN VAL B 32 13.282 28.447 68.137 0.00 0.00
B
ATOM 3843 CA VAL B 32 12.231 28.407 66.221 0.00 0.00
B
ATOM 3844 HA VAL B 32 11.734 29.236 65.740 0.00 0.00
B
ATOM 3845 CB VAL B 32 13.196 27.701 65.298 0.00 0.00
B
ATOM 3846 HB VAL B 32 14.022 28.372 64.977 0.00 0.00
B
ATOM 3847 CG1 VAL B 32 13.908 26.481 65.852 0.00 0.00
B
ATOM 3848 HG11 VAL B 32 14.252 26.709 66.884 0.00 0.00
B
ATOM 3849 HG12 VAL B 32 13.130 25.715 66.054 0.00 0.00
B
ATOM 3850 HG13 VAL B 32 14.787 26.114 65.280 0.00 0.00
B
ATOM 3851 CG2 VAL B 32 12.514 27.340 64.005 0.00 0.00
B
ATOM 3852 HG21 VAL B 32 11.614 26.697 64.110 0.00 0.00
B
ATOM 3853 HG22 VAL B 32 12.099 28.239 63.502 0.00 0.00
B
ATOM 3854 HG23 VAL B 32 13.218 26.785 63.348 0.00 0.00
B
ATOM 3855 C VAL B 32 11.116 27.558 66.685 0.00 0.00
B
ATOM 3856 O VAL B 32 11.308 26.687 67.494 0.00 0.00
B
ATOM 3857 N LEU B 33 9.924 27.795 66.182 0.00 0.00
B
ATOM 3858 HN LEU B 33 9.924 28.531 65.509 0.00 0.00
B
ATOM 3859 CA LEU B 33 8.751 26.934 66.253 0.00 0.00
B
ATOM 3860 HA LEU B 33 8.832 26.342 67.153 0.00 0.00
B

ATOM 3861 CB LEU B 33 7.513 27.881 66.239 0.00 0.00
B
ATOM 3862 HB1 LEU B 33 7.620 28.486 67.164 0.00 0.00
B
ATOM 3863 HB2 LEU B 33 7.523 28.555 65.355 0.00 0.00
B
ATOM 3864 CG LEU B 33 6.112 27.177 66.275 0.00 0.00
B
ATOM 3865 HG LEU B 33 5.976 26.505 65.400 0.00 0.00
B
ATOM 3866 CD1 LEU B 33 5.838 26.299 67.508 0.00 0.00
B
ATOM 3867 HD11 LEU B 33 5.789 26.978 68.386 0.00 0.00
B
ATOM 3868 HD12 LEU B 33 4.857 25.815 67.311 0.00 0.00
B
ATOM 3869 HD13 LEU B 33 6.596 25.524 67.752 0.00 0.00
B
ATOM 3870 CD2 LEU B 33 5.070 28.305 66.249 0.00 0.00
B
ATOM 3871 HD21 LEU B 33 5.039 28.842 67.221 0.00 0.00
B
ATOM 3872 HD22 LEU B 33 5.364 29.096 65.526 0.00 0.00
B
ATOM 3873 HD23 LEU B 33 4.040 27.985 65.984 0.00 0.00
B
ATOM 3874 C LEU B 33 8.818 25.986 65.032 0.00 0.00
B
ATOM 3875 O LEU B 33 9.049 26.451 63.916 0.00 0.00
B
ATOM 3876 N ALA B 34 8.595 24.747 65.200 0.00 0.00
B
ATOM 3877 HN ALA B 34 8.421 24.405 66.120 0.00 0.00
B
ATOM 3878 CA ALA B 34 8.536 23.749 64.190 0.00 0.00
B
ATOM 3879 HA ALA B 34 8.713 24.222 63.235 0.00 0.00
B
ATOM 3880 CB ALA B 34 9.633 22.666 64.406 0.00 0.00
B
ATOM 3881 HB1 ALA B 34 9.556 22.324 65.460 0.00 0.00
B
ATOM 3882 HB2 ALA B 34 9.600 21.849 63.655 0.00 0.00
B
ATOM 3883 HB3 ALA B 34 10.628 23.161 64.421 0.00 0.00
B
ATOM 3884 C ALA B 34 7.163 23.072 64.193 0.00 0.00
B
ATOM 3885 O ALA B 34 6.691 22.603 65.254 0.00 0.00
B
ATOM 3886 N VAL B 35 6.488 23.053 63.067 0.00 0.00
B
ATOM 3887 HN VAL B 35 6.716 23.608 62.271 0.00 0.00
B

ATOM 3888 CA VAL B 35 5.083 22.599 62.853 0.00 0.00
B
ATOM 3889 HA VAL B 35 4.668 22.014 63.660 0.00 0.00
B
ATOM 3890 CB VAL B 35 4.115 23.705 62.606 0.00 0.00
B
ATOM 3891 HB VAL B 35 4.273 24.194 61.621 0.00 0.00
B
ATOM 3892 CG1 VAL B 35 2.669 23.115 62.599 0.00 0.00
B
ATOM 3893 HG11 VAL B 35 2.439 22.126 62.149 0.00 0.00
B
ATOM 3894 HG12 VAL B 35 2.389 23.057 63.673 0.00 0.00
B
ATOM 3895 HG13 VAL B 35 2.074 23.851 62.018 0.00 0.00
B
ATOM 3896 CG2 VAL B 35 4.348 24.829 63.667 0.00 0.00
B
ATOM 3897 HG21 VAL B 35 5.199 25.513 63.458 0.00 0.00
B
ATOM 3898 HG22 VAL B 35 3.367 25.334 63.797 0.00 0.00
B
ATOM 3899 HG23 VAL B 35 4.572 24.382 64.659 0.00 0.00
B
ATOM 3900 C VAL B 35 5.148 21.602 61.629 0.00 0.00
B
ATOM 3901 O VAL B 35 5.589 21.960 60.576 0.00 0.00
B
ATOM 3902 N ASP B 36 4.573 20.405 61.766 0.00 0.00
B
ATOM 3903 HN ASP B 36 4.216 20.016 62.612 0.00 0.00
B
ATOM 3904 CA ASP B 36 4.250 19.572 60.586 0.00 0.00
B
ATOM 3905 HA ASP B 36 3.939 20.292 59.844 0.00 0.00
B
ATOM 3906 CB ASP B 36 5.463 18.643 60.284 0.00 0.00
B
ATOM 3907 HB1 ASP B 36 6.321 19.348 60.240 0.00 0.00
B
ATOM 3908 HB2 ASP B 36 5.650 18.039 61.198 0.00 0.00
B
ATOM 3909 CG ASP B 36 5.493 17.687 59.087 0.00 0.00
B
ATOM 3910 OD1 ASP B 36 6.438 17.747 58.261 0.00 0.00
B
ATOM 3911 OD2 ASP B 36 4.525 16.879 59.005 0.00 0.00
B
ATOM 3912 C ASP B 36 3.027 18.777 61.040 0.00 0.00
B
ATOM 3913 O ASP B 36 2.694 18.780 62.192 0.00 0.00
B
ATOM 3914 N ILE B 37 2.318 18.042 60.083 0.00 0.00
B

ATOM	3915	HN	ILE	B	37	2.407	17.958	59.093	0.00	0.00	B
ATOM	3916	CA	ILE	B	37	1.144	17.231	60.589	0.00	0.00	B
ATOM	3917	HA	ILE	B	37	0.690	17.608	61.494	0.00	0.00	B
ATOM	3918	CB	ILE	B	37	0.005	17.066	59.478	0.00	0.00	B
ATOM	3919	HB	ILE	B	37	-0.818	16.421	59.853	0.00	0.00	B
ATOM	3920	CG2	ILE	B	37	-0.530	18.504	59.226	0.00	0.00	B
ATOM	3921	HG21	ILE	B	37	-1.079	18.848	60.128	0.00	0.00	B
ATOM	3922	HG22	ILE	B	37	0.288	19.230	59.031	0.00	0.00	B
ATOM	3923	HG23	ILE	B	37	-1.226	18.429	58.364	0.00	0.00	B
ATOM	3924	CG1	ILE	B	37	0.569	16.527	58.157	0.00	0.00	B
ATOM	3925	HG11	ILE	B	37	-0.080	16.794	57.296	0.00	0.00	B
ATOM	3926	HG12	ILE	B	37	1.565	16.921	57.862	0.00	0.00	B
ATOM	3927	CD	ILE	B	37	0.568	14.986	58.015	0.00	0.00	B
ATOM	3928	HD1	ILE	B	37	1.003	14.614	57.062	0.00	0.00	B
ATOM	3929	HD2	ILE	B	37	1.074	14.499	58.876	0.00	0.00	B
ATOM	3930	HD3	ILE	B	37	-0.486	14.639	58.044	0.00	0.00	B
ATOM	3931	C	ILE	B	37	1.512	15.813	61.078	0.00	0.00	B
ATOM	3932	O	ILE	B	37	0.689	15.064	61.669	0.00	0.00	B
ATOM	3933	N	ASN	B	38	2.820	15.501	60.978	0.00	0.00	B
ATOM	3934	HN	ASN	B	38	3.435	16.225	60.675	0.00	0.00	B
ATOM	3935	CA	ASN	B	38	3.357	14.170	61.220	0.00	0.00	B
ATOM	3936	HA	ASN	B	38	2.567	13.446	61.351	0.00	0.00	B
ATOM	3937	CB	ASN	B	38	4.245	13.687	60.070	0.00	0.00	B
ATOM	3938	HB1	ASN	B	38	3.587	13.716	59.175	0.00	0.00	B
ATOM	3939	HB2	ASN	B	38	5.004	14.436	59.757	0.00	0.00	B
ATOM	3940	CG	ASN	B	38	4.852	12.308	60.291	0.00	0.00	B
ATOM	3941	OD1	ASN	B	38	4.684	11.672	61.326	0.00	0.00	B

ATOM 3942 ND2 ASN B 38 5.588 11.808 59.310 0.00 0.00
B
ATOM 3943 HD21 ASN B 38 6.110 10.971 59.477 0.00 0.00
B
ATOM 3944 HD22 ASN B 38 5.711 12.325 58.463 0.00 0.00
B
ATOM 3945 C ASN B 38 4.084 14.178 62.582 0.00 0.00
B
ATOM 3946 O ASN B 38 5.138 14.811 62.778 0.00 0.00
B
ATOM 3947 N GLU B 39 3.504 13.487 63.606 0.00 0.00
B
ATOM 3948 HN GLU B 39 2.583 13.133 63.468 0.00 0.00
B
ATOM 3949 CA GLU B 39 4.128 13.298 64.900 0.00 0.00
B
ATOM 3950 HA GLU B 39 4.038 14.223 65.450 0.00 0.00
B
ATOM 3951 CB GLU B 39 3.308 12.227 65.540 0.00 0.00
B
ATOM 3952 HB1 GLU B 39 2.326 12.744 65.606 0.00 0.00
B
ATOM 3953 HB2 GLU B 39 3.259 11.319 64.902 0.00 0.00
B
ATOM 3954 CG GLU B 39 3.655 11.921 67.070 0.00 0.00
B
ATOM 3955 HG1 GLU B 39 4.404 11.101 67.040 0.00 0.00
B
ATOM 3956 HG2 GLU B 39 3.973 12.844 67.601 0.00 0.00
B
ATOM 3957 CD GLU B 39 2.381 11.332 67.766 0.00 0.00
B
ATOM 3958 OE1 GLU B 39 1.616 10.632 67.082 0.00 0.00
B
ATOM 3959 OE2 GLU B 39 2.175 11.564 68.983 0.00 0.00
B
ATOM 3960 C GLU B 39 5.578 12.843 64.932 0.00 0.00
B
ATOM 3961 O GLU B 39 6.356 13.322 65.759 0.00 0.00
B
ATOM 3962 N GLU B 40 6.024 12.012 64.023 0.00 0.00
B
ATOM 3963 HN GLU B 40 5.459 11.912 63.208 0.00 0.00
B
ATOM 3964 CA GLU B 40 7.366 11.502 63.960 0.00 0.00
B
ATOM 3965 HA GLU B 40 7.544 11.002 64.901 0.00 0.00
B
ATOM 3966 CB GLU B 40 7.292 10.355 62.945 0.00 0.00
B
ATOM 3967 HB1 GLU B 40 7.082 10.758 61.931 0.00 0.00
B
ATOM 3968 HB2 GLU B 40 8.296 9.881 62.931 0.00 0.00
B

ATOM 3969 CG GLU B 40 6.271 9.225 63.129 0.00 0.00
B
ATOM 3970 HG1 GLU B 40 5.227 9.596 63.045 0.00 0.00
B
ATOM 3971 HG2 GLU B 40 6.472 8.396 62.417 0.00 0.00
B
ATOM 3972 CD GLU B 40 6.416 8.545 64.473 0.00 0.00
B
ATOM 3973 OE1 GLU B 40 7.396 7.796 64.689 0.00 0.00
B
ATOM 3974 OE2 GLU B 40 5.527 8.716 65.333 0.00 0.00
B
ATOM 3975 C GLU B 40 8.459 12.570 63.640 0.00 0.00
B
ATOM 3976 O GLU B 40 9.640 12.447 64.009 0.00 0.00
B
ATOM 3977 N LYS B 41 7.994 13.549 62.842 0.00 0.00
B
ATOM 3978 HN LYS B 41 7.061 13.553 62.489 0.00 0.00
B
ATOM 3979 CA LYS B 41 8.798 14.700 62.382 0.00 0.00
B
ATOM 3980 HA LYS B 41 9.813 14.355 62.252 0.00 0.00
B
ATOM 3981 CB LYS B 41 8.099 15.279 61.116 0.00 0.00
B
ATOM 3982 HB1 LYS B 41 7.032 15.469 61.361 0.00 0.00
B
ATOM 3983 HB2 LYS B 41 8.488 16.257 60.760 0.00 0.00
B
ATOM 3984 CG LYS B 41 8.011 14.432 59.851 0.00 0.00
B
ATOM 3985 HG1 LYS B 41 7.560 13.440 60.069 0.00 0.00
B
ATOM 3986 HG2 LYS B 41 7.392 14.816 59.012 0.00 0.00
B
ATOM 3987 CD LYS B 41 9.431 14.125 59.242 0.00 0.00
B
ATOM 3988 HD1 LYS B 41 10.051 15.042 59.345 0.00 0.00
B
ATOM 3989 HD2 LYS B 41 9.987 13.388 59.860 0.00 0.00
B
ATOM 3990 CE LYS B 41 9.408 13.745 57.759 0.00 0.00
B
ATOM 3991 HE1 LYS B 41 9.059 14.514 57.037 0.00 0.00
B
ATOM 3992 HE2 LYS B 41 10.436 13.591 57.366 0.00 0.00
B
ATOM 3993 NZ LYS B 41 8.534 12.595 57.546 0.00 0.00
B
ATOM 3994 HZ1 LYS B 41 8.814 11.997 56.743 0.00 0.00
B
ATOM 3995 HZ2 LYS B 41 8.501 12.036 58.423 0.00 0.00
B

```
ATOM   3996  HZ3 LYS B  41       7.572  12.968  57.413  0.00  0.00
B
ATOM   3997  C   LYS B  41       8.869  15.658  63.533  0.00  0.00
B
ATOM   3998  O   LYS B  41      10.007  16.212  63.796  0.00  0.00
B
ATOM   3999  N   VAL B  42       7.746  15.932  64.209  0.00  0.00
B
ATOM   4000  HN  VAL B  42       6.911  15.493  63.885  0.00  0.00
B
ATOM   4001  CA  VAL B  42       7.811  16.805  65.367  0.00  0.00
B
ATOM   4002  HA  VAL B  42       8.374  17.709  65.188  0.00  0.00
B
ATOM   4003  CB  VAL B  42       6.346  17.215  65.736  0.00  0.00
B
ATOM   4004  HB  VAL B  42       5.859  16.232  65.912  0.00  0.00
B
ATOM   4005  CG1 VAL B  42       6.259  18.016  67.088  0.00  0.00
B
ATOM   4006 HG11 VAL B  42       7.237  18.535  67.179  0.00  0.00
B
ATOM   4007 HG12 VAL B  42       5.367  18.678  67.075  0.00  0.00
B
ATOM   4008 HG13 VAL B  42       6.118  17.355  67.970  0.00  0.00
B
ATOM   4009  CG2 VAL B  42       5.720  18.035  64.602  0.00  0.00
B
ATOM   4010 HG21 VAL B  42       4.632  18.201  64.757  0.00  0.00
B
ATOM   4011 HG22 VAL B  42       6.236  19.019  64.595  0.00  0.00
B
ATOM   4012 HG23 VAL B  42       5.915  17.566  63.614  0.00  0.00
B
ATOM   4013  C   VAL B  42       8.497  16.154  66.553  0.00  0.00
B
ATOM   4014  O   VAL B  42       9.095  16.924  67.294  0.00  0.00
B
ATOM   4015  N   ASN B  43       8.602  14.796  66.697  0.00  0.00
B
ATOM   4016  HN  ASN B  43       7.932  14.168  66.309  0.00  0.00
B
ATOM   4017  CA  ASN B  43       9.560  14.149  67.646  0.00  0.00
B
ATOM   4018  HA  ASN B  43       9.482  14.375  68.699  0.00  0.00
B
ATOM   4019  CB  ASN B  43       9.299  12.645  67.624  0.00  0.00
B
ATOM   4020  HB1 ASN B  43       8.960  12.310  66.620  0.00  0.00
B
ATOM   4021  HB2 ASN B  43      10.177  12.011  67.872  0.00  0.00
B
ATOM   4022  CG  ASN B  43       8.290  12.412  68.724  0.00  0.00
B
```

ATOM 4023 OD1 ASN B 43 8.581 11.864 69.824 0.00 0.00
B
ATOM 4024 ND2 ASN B 43 7.028 12.633 68.380 0.00 0.00
B
ATOM 4025 HD21 ASN B 43 6.266 12.426 68.993 0.00 0.00
B
ATOM 4026 HD22 ASN B 43 6.898 13.182 67.554 0.00 0.00
B
ATOM 4027 C ASN B 43 11.022 14.288 67.255 0.00 0.00
B
ATOM 4028 O ASN B 43 11.954 14.446 68.040 0.00 0.00
B
ATOM 4029 N ALA B 44 11.306 14.232 65.934 0.00 0.00
B
ATOM 4030 HN ALA B 44 10.617 13.980 65.260 0.00 0.00
B
ATOM 4031 CA ALA B 44 12.660 14.188 65.388 0.00 0.00
B
ATOM 4032 HA ALA B 44 13.190 13.455 65.977 0.00 0.00
B
ATOM 4033 CB ALA B 44 12.686 13.781 63.895 0.00 0.00
B
ATOM 4034 HB1 ALA B 44 13.690 13.406 63.600 0.00 0.00
B
ATOM 4035 HB2 ALA B 44 11.853 13.082 63.666 0.00 0.00
B
ATOM 4036 HB3 ALA B 44 12.540 14.670 63.245 0.00 0.00
B
ATOM 4037 C ALA B 44 13.348 15.559 65.561 0.00 0.00
B
ATOM 4038 O ALA B 44 14.551 15.677 65.868 0.00 0.00
B
ATOM 4039 N TYR B 45 12.585 16.620 65.377 0.00 0.00
B
ATOM 4040 HN TYR B 45 11.649 16.672 65.038 0.00 0.00
B
ATOM 4041 CA TYR B 45 13.148 17.951 65.426 0.00 0.00
B
ATOM 4042 HA TYR B 45 14.224 17.935 65.343 0.00 0.00
B
ATOM 4043 CB TYR B 45 12.573 18.831 64.239 0.00 0.00
B
ATOM 4044 HB1 TYR B 45 11.492 18.579 64.201 0.00 0.00
B
ATOM 4045 HB2 TYR B 45 12.774 19.911 64.403 0.00 0.00
B
ATOM 4046 CG TYR B 45 13.184 18.475 62.941 0.00 0.00
B
ATOM 4047 CD1 TYR B 45 12.512 17.757 61.976 0.00 0.00
B
ATOM 4048 HD1 TYR B 45 11.490 17.476 62.179 0.00 0.00
B
ATOM 4049 CE1 TYR B 45 13.163 17.291 60.898 0.00 0.00
B

ATOM 4050 HE1 TYR B 45 12.594 16.713 60.185 0.00 0.00
B
ATOM 4051 CZ TYR B 45 14.471 17.642 60.628 0.00 0.00
B
ATOM 4052 OH TYR B 45 15.133 17.005 59.545 0.00 0.00
B
ATOM 4053 HH TYR B 45 14.367 16.667 59.075 0.00 0.00
B
ATOM 4054 CD2 TYR B 45 14.590 18.806 62.724 0.00 0.00
B
ATOM 4055 HD2 TYR B 45 15.184 19.380 63.419 0.00 0.00
B
ATOM 4056 CE2 TYR B 45 15.171 18.461 61.543 0.00 0.00
B
ATOM 4057 HE2 TYR B 45 16.215 18.647 61.336 0.00 0.00
B
ATOM 4058 C TYR B 45 12.750 18.631 66.668 0.00 0.00
B
ATOM 4059 O TYR B 45 12.781 19.862 66.772 0.00 0.00
B
ATOM 4060 N ALA B 46 12.318 17.896 67.738 0.00 0.00
B
ATOM 4061 HN ALA B 46 12.285 16.899 67.744 0.00 0.00
B
ATOM 4062 CA ALA B 46 12.036 18.511 69.003 0.00 0.00
B
ATOM 4063 HA ALA B 46 11.302 19.300 68.928 0.00 0.00
B
ATOM 4064 CB ALA B 46 11.554 17.371 70.000 0.00 0.00
B
ATOM 4065 HB1 ALA B 46 11.248 17.807 70.975 0.00 0.00
B
ATOM 4066 HB2 ALA B 46 10.693 16.799 69.592 0.00 0.00
B
ATOM 4067 HB3 ALA B 46 12.403 16.680 70.186 0.00 0.00
B
ATOM 4068 C ALA B 46 13.246 19.139 69.604 0.00 0.00
B
ATOM 4069 O ALA B 46 13.071 20.228 70.145 0.00 0.00
B
ATOM 4070 N SER B 47 14.445 18.551 69.441 0.00 0.00
B
ATOM 4071 HN SER B 47 14.472 17.618 69.090 0.00 0.00
B
ATOM 4072 CA SER B 47 15.632 18.971 70.178 0.00 0.00
B
ATOM 4073 HA SER B 47 15.364 19.738 70.890 0.00 0.00
B
ATOM 4074 CB SER B 47 16.542 17.861 70.756 0.00 0.00
B
ATOM 4075 HB1 SER B 47 17.218 17.349 70.038 0.00 0.00
B
ATOM 4076 HB2 SER B 47 17.232 18.291 71.513 0.00 0.00
B

ATOM	4077	OG	SER	B	47	15.701	16.885	71.353	0.00	0.00
B										
ATOM	4078	HG1	SER	B	47	15.186	16.455	70.666	0.00	0.00
B										
ATOM	4079	C	SER	B	47	16.545	19.611	69.230	0.00	0.00
B										
ATOM	4080	O	SER	B	47	17.658	20.037	69.650	0.00	0.00
B										
ATOM	4081	N	TYR	B	48	16.038	19.917	68.048	0.00	0.00
B										
ATOM	4082	HN	TYR	B	48	15.137	19.541	67.846	0.00	0.00
B										
ATOM	4083	CA	TYR	B	48	16.739	20.720	67.032	0.00	0.00
B										
ATOM	4084	HA	TYR	B	48	17.735	21.018	67.324	0.00	0.00
B										
ATOM	4085	CB	TYR	B	48	16.800	19.804	65.751	0.00	0.00
B										
ATOM	4086	HB1	TYR	B	48	15.752	19.443	65.676	0.00	0.00
B										
ATOM	4087	HB2	TYR	B	48	17.091	20.513	64.947	0.00	0.00
B										
ATOM	4088	CG	TYR	B	48	17.789	18.662	65.914	0.00	0.00
B										
ATOM	4089	CD1	TYR	B	48	17.497	17.454	66.517	0.00	0.00
B										
ATOM	4090	HD1	TYR	B	48	16.542	17.119	66.893	0.00	0.00
B										
ATOM	4091	CE1	TYR	B	48	18.539	16.470	66.650	0.00	0.00
B										
ATOM	4092	HE1	TYR	B	48	18.398	15.456	66.995	0.00	0.00
B										
ATOM	4093	CZ	TYR	B	48	19.850	16.713	66.260	0.00	0.00
B										
ATOM	4094	OH	TYR	B	48	20.949	15.802	66.244	0.00	0.00
B										
ATOM	4095	HH	TYR	B	48	20.567	14.935	66.086	0.00	0.00
B										
ATOM	4096	CD2	TYR	B	48	19.131	18.977	65.653	0.00	0.00
B										
ATOM	4097	HD2	TYR	B	48	19.409	20.001	65.451	0.00	0.00
B										
ATOM	4098	CE2	TYR	B	48	20.122	17.996	65.793	0.00	0.00
B										
ATOM	4099	HE2	TYR	B	48	21.169	18.198	65.618	0.00	0.00
B										
ATOM	4100	C	TYR	B	48	16.049	22.048	66.863	0.00	0.00
B										
ATOM	4101	O	TYR	B	48	16.289	22.796	65.879	0.00	0.00
B										
ATOM	4102	N	ALA	B	49	15.081	22.394	67.751	0.00	0.00
B										
ATOM	4103	HN	ALA	B	49	14.916	21.865	68.579	0.00	0.00
B										

ATOM 4104 CA ALA B 49 14.208 23.541 67.635 0.00 0.00
B
ATOM 4105 HA ALA B 49 14.839 24.218 67.078 0.00 0.00
B
ATOM 4106 CB ALA B 49 12.868 23.137 67.062 0.00 0.00
B
ATOM 4107 HB1 ALA B 49 12.131 23.967 67.080 0.00 0.00
B
ATOM 4108 HB2 ALA B 49 12.971 22.688 66.051 0.00 0.00
B
ATOM 4109 HB3 ALA B 49 12.342 22.368 67.666 0.00 0.00
B
ATOM 4110 C ALA B 49 14.053 24.108 69.052 0.00 0.00
B
ATOM 4111 O ALA B 49 14.622 23.520 69.952 0.00 0.00
B
ATOM 4112 N THR B 50 13.368 25.303 69.288 0.00 0.00
B
ATOM 4113 HN THR B 50 12.919 25.762 68.526 0.00 0.00
B
ATOM 4114 CA THR B 50 13.117 25.787 70.639 0.00 0.00
B
ATOM 4115 HA THR B 50 13.813 25.398 71.368 0.00 0.00
B
ATOM 4116 CB THR B 50 13.420 27.320 70.800 0.00 0.00
B
ATOM 4117 HB THR B 50 13.380 27.583 71.878 0.00 0.00
B
ATOM 4118 OG1 THR B 50 12.465 28.112 70.123 0.00 0.00
B
ATOM 4119 HG1 THR B 50 12.756 28.988 70.386 0.00 0.00
B
ATOM 4120 CG2 THR B 50 14.839 27.645 70.341 0.00 0.00
B
ATOM 4121 HG21 THR B 50 15.007 27.327 69.290 0.00 0.00
B
ATOM 4122 HG22 THR B 50 14.948 28.749 70.412 0.00 0.00
B
ATOM 4123 HG23 THR B 50 15.550 27.183 71.059 0.00 0.00
B
ATOM 4124 C THR B 50 11.700 25.387 71.013 0.00 0.00
B
ATOM 4125 O THR B 50 11.367 25.251 72.169 0.00 0.00
B
ATOM 4126 N HSE B 51 10.741 25.260 70.087 0.00 0.00
B
ATOM 4127 HN HSE B 51 10.911 25.331 69.107 0.00 0.00
B
ATOM 4128 CA HSE B 51 9.413 24.782 70.407 0.00 0.00
B
ATOM 4129 HA HSE B 51 9.459 23.955 71.101 0.00 0.00
B
ATOM 4130 CB HSE B 51 8.562 26.006 70.972 0.00 0.00
B

ATOM	4131	HB1	HSE	B	51	9.154	26.491	71.777	0.00	0.00	B
ATOM	4132	HB2	HSE	B	51	8.544	26.779	70.174	0.00	0.00	B
ATOM	4133	ND1	HSE	B	51	6.967	24.441	72.001	0.00	0.00	B
ATOM	4134	CG	HSE	B	51	7.161	25.649	71.277	0.00	0.00	B
ATOM	4135	CE1	HSE	B	51	5.625	24.342	72.032	0.00	0.00	B
ATOM	4136	HE1	HSE	B	51	5.107	23.596	72.635	0.00	0.00	B
ATOM	4137	NE2	HSE	B	51	4.978	25.376	71.400	0.00	0.00	B
ATOM	4138	HE2	HSE	B	51	3.995	25.560	71.403	0.00	0.00	B
ATOM	4139	CD2	HSE	B	51	5.956	26.192	70.897	0.00	0.00	B
ATOM	4140	HD2	HSE	B	51	5.793	27.228	70.624	0.00	0.00	B
ATOM	4141	C	HSE	B	51	8.893	24.173	69.159	0.00	0.00	B
ATOM	4142	O	HSE	B	51	9.485	24.342	68.058	0.00	0.00	B
ATOM	4143	N	ALA	B	52	7.791	23.323	69.278	0.00	0.00	B
ATOM	4144	HN	ALA	B	52	7.324	23.283	70.158	0.00	0.00	B
ATOM	4145	CA	ALA	B	52	7.239	22.563	68.185	0.00	0.00	B
ATOM	4146	HA	ALA	B	52	7.164	23.203	67.318	0.00	0.00	B
ATOM	4147	CB	ALA	B	52	8.183	21.289	67.865	0.00	0.00	B
ATOM	4148	HB1	ALA	B	52	8.289	20.660	68.775	0.00	0.00	B
ATOM	4149	HB2	ALA	B	52	7.868	20.715	66.968	0.00	0.00	B
ATOM	4150	HB3	ALA	B	52	9.231	21.634	67.730	0.00	0.00	B
ATOM	4151	C	ALA	B	52	5.939	22.178	68.578	0.00	0.00	B
ATOM	4152	O	ALA	B	52	5.496	22.136	69.762	0.00	0.00	B
ATOM	4153	N	VAL	B	53	5.120	21.836	67.638	0.00	0.00	B
ATOM	4154	HN	VAL	B	53	5.382	21.891	66.678	0.00	0.00	B
ATOM	4155	CA	VAL	B	53	3.792	21.318	67.850	0.00	0.00	B
ATOM	4156	HA	VAL	B	53	3.874	20.501	68.551	0.00	0.00	B
ATOM	4157	CB	VAL	B	53	2.729	22.329	68.259	0.00	0.00	B

ATOM 4158 HB VAL B 53 3.111 22.938 69.106 0.00 0.00
B
ATOM 4159 CG1 VAL B 53 2.227 23.209 67.026 0.00 0.00
B
ATOM 4160 HG11 VAL B 53 1.411 22.756 66.423 0.00 0.00
B
ATOM 4161 HG12 VAL B 53 1.771 24.144 67.416 0.00 0.00
B
ATOM 4162 HG13 VAL B 53 3.012 23.566 66.326 0.00 0.00
B
ATOM 4163 CG2 VAL B 53 1.515 21.656 68.903 0.00 0.00
B
ATOM 4164 HG21 VAL B 53 1.798 20.981 69.739 0.00 0.00
B
ATOM 4165 HG22 VAL B 53 0.872 22.446 69.347 0.00 0.00
B
ATOM 4166 HG23 VAL B 53 0.851 21.058 68.242 0.00 0.00
B
ATOM 4167 C VAL B 53 3.312 20.593 66.690 0.00 0.00
B
ATOM 4168 O VAL B 53 3.475 20.967 65.509 0.00 0.00
B
ATOM 4169 N ILE B 54 2.646 19.440 66.934 0.00 0.00
B
ATOM 4170 HN ILE B 54 2.395 19.222 67.874 0.00 0.00
B
ATOM 4171 CA ILE B 54 1.976 18.685 65.793 0.00 0.00
B
ATOM 4172 HA ILE B 54 2.481 18.708 64.839 0.00 0.00
B
ATOM 4173 CB ILE B 54 1.656 17.157 66.133 0.00 0.00
B
ATOM 4174 HB ILE B 54 0.928 17.068 66.968 0.00 0.00
B
ATOM 4175 CG2 ILE B 54 0.998 16.412 64.989 0.00 0.00
B
ATOM 4176 HG21 ILE B 54 0.046 16.803 64.571 0.00 0.00
B
ATOM 4177 HG22 ILE B 54 1.540 16.548 64.028 0.00 0.00
B
ATOM 4178 HG23 ILE B 54 0.949 15.310 65.114 0.00 0.00
B
ATOM 4179 CG1 ILE B 54 2.879 16.467 66.670 0.00 0.00
B
ATOM 4180 HG11 ILE B 54 3.470 16.056 65.824 0.00 0.00
B
ATOM 4181 HG12 ILE B 54 3.525 17.176 67.230 0.00 0.00
B
ATOM 4182 CD ILE B 54 2.580 15.251 67.643 0.00 0.00
B
ATOM 4183 HD1 ILE B 54 3.512 14.995 68.191 0.00 0.00
B
ATOM 4184 HD2 ILE B 54 1.754 15.472 68.352 0.00 0.00
B

```
ATOM   4185  HD3 ILE B  54       2.276  14.390  67.011  0.00  0.00
B
ATOM   4186  C   ILE B  54       0.726  19.400  65.422  0.00  0.00
B
ATOM   4187  O   ILE B  54      -0.161  19.549  66.316  0.00  0.00
B
ATOM   4188  N   ALA B  55       0.565  20.018  64.238  0.00  0.00
B
ATOM   4189  HN  ALA B  55       1.277  20.065  63.542  0.00  0.00
B
ATOM   4190  CA  ALA B  55      -0.714  20.634  63.904  0.00  0.00
B
ATOM   4191  HA  ALA B  55      -1.497  20.172  64.486  0.00  0.00
B
ATOM   4192  CB  ALA B  55      -0.650  22.089  64.325  0.00  0.00
B
ATOM   4193  HB1 ALA B  55      -0.161  22.129  65.322  0.00  0.00
B
ATOM   4194  HB2 ALA B  55       0.010  22.745  63.718  0.00  0.00
B
ATOM   4195  HB3 ALA B  55      -1.590  22.680  64.377  0.00  0.00
B
ATOM   4196  C   ALA B  55      -1.004  20.620  62.447  0.00  0.00
B
ATOM   4197  O   ALA B  55      -0.060  20.244  61.698  0.00  0.00
B
ATOM   4198  N   ASN B  56      -2.234  20.968  61.966  0.00  0.00
B
ATOM   4199  HN  ASN B  56      -3.082  20.963  62.489  0.00  0.00
B
ATOM   4200  CA  ASN B  56      -2.456  21.124  60.490  0.00  0.00
B
ATOM   4201  HA  ASN B  56      -1.560  20.762  60.009  0.00  0.00
B
ATOM   4202  CB  ASN B  56      -3.758  20.269  60.207  0.00  0.00
B
ATOM   4203  HB1 ASN B  56      -3.750  19.270  60.694  0.00  0.00
B
ATOM   4204  HB2 ASN B  56      -4.593  20.913  60.556  0.00  0.00
B
ATOM   4205  CG  ASN B  56      -3.876  20.026  58.685  0.00  0.00
B
ATOM   4206  OD1 ASN B  56      -3.706  20.795  57.772  0.00  0.00
B
ATOM   4207  ND2 ASN B  56      -4.147  18.685  58.380  0.00  0.00
B
ATOM   4208 HD21 ASN B  56      -4.436  18.371  57.476  0.00  0.00
B
ATOM   4209 HD22 ASN B  56      -3.985  17.936  59.022  0.00  0.00
B
ATOM   4210  C   ASN B  56      -2.551  22.579  60.117  0.00  0.00
B
ATOM   4211  O   ASN B  56      -3.256  23.312  60.759  0.00  0.00
B
```

ATOM	4212	N	ALA	B	57	−1.858	23.011	59.072	0.00	0.00	B
ATOM	4213	HN	ALA	B	57	−1.314	22.384	58.519	0.00	0.00	B
ATOM	4214	CA	ALA	B	57	−1.793	24.425	58.823	0.00	0.00	B
ATOM	4215	HA	ALA	B	57	−2.063	24.978	59.711	0.00	0.00	B
ATOM	4216	CB	ALA	B	57	−0.484	24.799	58.209	0.00	0.00	B
ATOM	4217	HB1	ALA	B	57	0.315	24.680	58.972	0.00	0.00	B
ATOM	4218	HB2	ALA	B	57	−0.222	24.183	57.322	0.00	0.00	B
ATOM	4219	HB3	ALA	B	57	−0.378	25.896	58.066	0.00	0.00	B
ATOM	4220	C	ALA	B	57	−2.895	24.805	57.854	0.00	0.00	B
ATOM	4221	O	ALA	B	57	−3.145	25.958	57.628	0.00	0.00	B
ATOM	4222	N	THR	B	58	−3.641	23.854	57.275	0.00	0.00	B
ATOM	4223	HN	THR	B	58	−3.383	22.903	57.423	0.00	0.00	B
ATOM	4224	CA	THR	B	58	−4.817	24.138	56.435	0.00	0.00	B
ATOM	4225	HA	THR	B	58	−4.552	25.025	55.879	0.00	0.00	B
ATOM	4226	CB	THR	B	58	−5.135	23.103	55.422	0.00	0.00	B
ATOM	4227	HB	THR	B	58	−5.898	23.604	54.789	0.00	0.00	B
ATOM	4228	OG1	THR	B	58	−5.490	21.824	55.918	0.00	0.00	B
ATOM	4229	HG1	THR	B	58	−4.724	21.381	56.289	0.00	0.00	B
ATOM	4230	CG2	THR	B	58	−3.960	22.972	54.505	0.00	0.00	B
ATOM	4231	HG21	THR	B	58	−3.763	23.910	53.943	0.00	0.00	B
ATOM	4232	HG22	THR	B	58	−2.951	22.630	54.821	0.00	0.00	B
ATOM	4233	HG23	THR	B	58	−4.274	22.400	53.605	0.00	0.00	B
ATOM	4234	C	THR	B	58	−6.089	24.420	57.247	0.00	0.00	B
ATOM	4235	O	THR	B	58	−7.149	24.943	56.828	0.00	0.00	B
ATOM	4236	N	GLU	B	59	−6.020	24.054	58.565	0.00	0.00	B
ATOM	4237	HN	GLU	B	59	−5.166	23.808	59.016	0.00	0.00	B
ATOM	4238	CA	GLU	B	59	−7.210	24.144	59.401	0.00	0.00	B

ATOM	4239	HA	GLU	B	59	-8.134	24.035	58.852	0.00	0.00	B
ATOM	4240	CB	GLU	B	59	-7.156	22.945	60.389	0.00	0.00	B
ATOM	4241	HB1	GLU	B	59	-7.243	22.027	59.768	0.00	0.00	B
ATOM	4242	HB2	GLU	B	59	-6.201	22.981	60.955	0.00	0.00	B
ATOM	4243	CG	GLU	B	59	-8.231	22.841	61.461	0.00	0.00	B
ATOM	4244	HG1	GLU	B	59	-8.286	23.752	62.093	0.00	0.00	B
ATOM	4245	HG2	GLU	B	59	-9.250	22.800	61.019	0.00	0.00	B
ATOM	4246	CD	GLU	B	59	-8.079	21.556	62.394	0.00	0.00	B
ATOM	4247	OE1	GLU	B	59	-7.638	20.535	61.811	0.00	0.00	B
ATOM	4248	OE2	GLU	B	59	-8.441	21.545	63.533	0.00	0.00	B
ATOM	4249	C	GLU	B	59	-7.199	25.425	60.271	0.00	0.00	B
ATOM	4250	O	GLU	B	59	-6.318	25.720	61.086	0.00	0.00	B
ATOM	4251	N	GLU	B	60	-8.207	26.314	59.973	0.00	0.00	B
ATOM	4252	HN	GLU	B	60	-8.940	25.975	59.389	0.00	0.00	B
ATOM	4253	CA	GLU	B	60	-8.270	27.826	60.390	0.00	0.00	B
ATOM	4254	HA	GLU	B	60	-7.528	28.382	59.836	0.00	0.00	B
ATOM	4255	CB	GLU	B	60	-9.573	28.430	59.878	0.00	0.00	B
ATOM	4256	HB1	GLU	B	60	-9.598	28.593	58.779	0.00	0.00	B
ATOM	4257	HB2	GLU	B	60	-10.367	27.664	60.011	0.00	0.00	B
ATOM	4258	CG	GLU	B	60	-9.946	29.810	60.523	0.00	0.00	B
ATOM	4259	HG1	GLU	B	60	-9.996	29.574	61.607	0.00	0.00	B
ATOM	4260	HG2	GLU	B	60	-9.104	30.533	60.480	0.00	0.00	B
ATOM	4261	CD	GLU	B	60	-11.264	30.276	59.841	0.00	0.00	B
ATOM	4262	OE1	GLU	B	60	-11.353	31.523	59.622	0.00	0.00	B
ATOM	4263	OE2	GLU	B	60	-12.155	29.432	59.540	0.00	0.00	B
ATOM	4264	C	GLU	B	60	-7.914	28.114	61.833	0.00	0.00	B
ATOM	4265	O	GLU	B	60	-6.975	28.793	62.165	0.00	0.00	B

ATOM 4266 N ASN B 61 -8.795 27.557 62.706 0.00 0.00
B
ATOM 4267 HN ASN B 61 -9.570 27.207 62.185 0.00 0.00
B
ATOM 4268 CA ASN B 61 -8.729 27.581 64.154 0.00 0.00
B
ATOM 4269 HA ASN B 61 -8.809 28.592 64.525 0.00 0.00
B
ATOM 4270 CB ASN B 61 -9.955 26.927 64.862 0.00 0.00
B
ATOM 4271 HB1 ASN B 61 -10.011 25.843 64.623 0.00 0.00
B
ATOM 4272 HB2 ASN B 61 -9.841 27.111 65.952 0.00 0.00
B
ATOM 4273 CG ASN B 61 -11.263 27.601 64.326 0.00 0.00
B
ATOM 4274 OD1 ASN B 61 -11.276 28.773 63.963 0.00 0.00
B
ATOM 4275 ND2 ASN B 61 -12.304 26.775 64.278 0.00 0.00
B
ATOM 4276 HD21 ASN B 61 -13.144 27.145 63.882 0.00 0.00
B
ATOM 4277 HD22 ASN B 61 -12.113 25.798 64.371 0.00 0.00
B
ATOM 4278 C ASN B 61 -7.495 26.989 64.833 0.00 0.00
B
ATOM 4279 O ASN B 61 -6.986 27.423 65.868 0.00 0.00
B
ATOM 4280 N GLU B 62 -7.008 25.878 64.231 0.00 0.00
B
ATOM 4281 HN GLU B 62 -7.463 25.429 63.466 0.00 0.00
B
ATOM 4282 CA GLU B 62 -5.720 25.237 64.586 0.00 0.00
B
ATOM 4283 HA GLU B 62 -5.739 25.072 65.653 0.00 0.00
B
ATOM 4284 CB GLU B 62 -5.561 23.870 63.819 0.00 0.00
B
ATOM 4285 HB1 GLU B 62 -6.490 23.277 63.961 0.00 0.00
B
ATOM 4286 HB2 GLU B 62 -5.401 24.230 62.780 0.00 0.00
B
ATOM 4287 CG GLU B 62 -4.447 22.975 64.363 0.00 0.00
B
ATOM 4288 HG1 GLU B 62 -4.288 22.077 63.729 0.00 0.00
B
ATOM 4289 HG2 GLU B 62 -3.478 23.505 64.246 0.00 0.00
B
ATOM 4290 CD GLU B 62 -4.567 22.440 65.800 0.00 0.00
B
ATOM 4291 OE1 GLU B 62 -5.598 21.845 66.166 0.00 0.00
B
ATOM 4292 OE2 GLU B 62 -3.511 22.532 66.495 0.00 0.00
B

ATOM	4293	C	GLU	B	62	−4.583	26.165	64.209	0.00	0.00	B
ATOM	4294	O	GLU	B	62	−3.721	26.370	65.047	0.00	0.00	B
ATOM	4295	N	LEU	B	63	−4.613	26.767	63.024	0.00	0.00	B
ATOM	4296	HN	LEU	B	63	−5.194	26.494	62.261	0.00	0.00	B
ATOM	4297	CA	LEU	B	63	−3.642	27.803	62.605	0.00	0.00	B
ATOM	4298	HA	LEU	B	63	−2.613	27.496	62.722	0.00	0.00	B
ATOM	4299	CB	LEU	B	63	−3.862	28.300	61.143	0.00	0.00	B
ATOM	4300	HB1	LEU	B	63	−4.036	27.466	60.431	0.00	0.00	B
ATOM	4301	HB2	LEU	B	63	−4.817	28.864	61.084	0.00	0.00	B
ATOM	4302	CG	LEU	B	63	−2.626	29.093	60.590	0.00	0.00	B
ATOM	4303	HG	LEU	B	63	−2.380	29.949	61.254	0.00	0.00	B
ATOM	4304	CD1	LEU	B	63	−1.352	28.366	60.153	0.00	0.00	B
ATOM	4305	HD11	LEU	B	63	−1.584	27.865	59.189	0.00	0.00	B
ATOM	4306	HD12	LEU	B	63	−0.535	29.066	59.877	0.00	0.00	B
ATOM	4307	HD13	LEU	B	63	−1.072	27.579	60.886	0.00	0.00	B
ATOM	4308	CD2	LEU	B	63	−3.196	29.849	59.387	0.00	0.00	B
ATOM	4309	HD21	LEU	B	63	−2.369	30.527	59.082	0.00	0.00	B
ATOM	4310	HD22	LEU	B	63	−3.470	29.123	58.592	0.00	0.00	B
ATOM	4311	HD23	LEU	B	63	−4.088	30.450	59.663	0.00	0.00	B
ATOM	4312	C	LEU	B	63	−3.677	29.039	63.397	0.00	0.00	B
ATOM	4313	O	LEU	B	63	−2.634	29.540	63.889	0.00	0.00	B
ATOM	4314	N	LEU	B	64	−4.875	29.578	63.645	0.00	0.00	B
ATOM	4315	HN	LEU	B	64	−5.680	29.185	63.207	0.00	0.00	B
ATOM	4316	CA	LEU	B	64	−5.259	30.684	64.575	0.00	0.00	B
ATOM	4317	HA	LEU	B	64	−4.755	31.564	64.206	0.00	0.00	B
ATOM	4318	CB	LEU	B	64	−6.770	30.786	64.581	0.00	0.00	B
ATOM	4319	HB1	LEU	B	64	−6.987	30.694	63.495	0.00	0.00	B

ATOM 4320 HB2 LEU B 64 -7.218 29.850 64.979 0.00 0.00
B
ATOM 4321 CG LEU B 64 -7.292 32.042 65.208 0.00 0.00
B
ATOM 4322 HG LEU B 64 -6.913 32.266 66.228 0.00 0.00
B
ATOM 4323 CD1 LEU B 64 -7.034 33.298 64.441 0.00 0.00
B
ATOM 4324 HD11 LEU B 64 -7.679 34.036 64.964 0.00 0.00
B
ATOM 4325 HD12 LEU B 64 -6.013 33.733 64.483 0.00 0.00
B
ATOM 4326 HD13 LEU B 64 -7.504 33.347 63.436 0.00 0.00
B
ATOM 4327 CD2 LEU B 64 -8.877 31.828 65.312 0.00 0.00
B
ATOM 4328 HD21 LEU B 64 -9.105 31.793 64.225 0.00 0.00
B
ATOM 4329 HD22 LEU B 64 -9.192 30.876 65.790 0.00 0.00
B
ATOM 4330 HD23 LEU B 64 -9.406 32.715 65.723 0.00 0.00
B
ATOM 4331 C LEU B 64 -4.667 30.361 66.017 0.00 0.00
B
ATOM 4332 O LEU B 64 -4.141 31.246 66.739 0.00 0.00
B
ATOM 4333 N SER B 65 -4.887 29.077 66.580 0.00 0.00
B
ATOM 4334 HN SER B 65 -5.488 28.439 66.104 0.00 0.00
B
ATOM 4335 CA SER B 65 -4.283 28.520 67.797 0.00 0.00
B
ATOM 4336 HA SER B 65 -4.618 29.246 68.523 0.00 0.00
B
ATOM 4337 CB SER B 65 -4.855 27.056 68.101 0.00 0.00
B
ATOM 4338 HB1 SER B 65 -5.132 26.630 67.113 0.00 0.00
B
ATOM 4339 HB2 SER B 65 -4.113 26.509 68.721 0.00 0.00
B
ATOM 4340 OG SER B 65 -6.078 27.051 68.872 0.00 0.00
B
ATOM 4341 HG1 SER B 65 -6.471 26.234 68.557 0.00 0.00
B
ATOM 4342 C SER B 65 -2.777 28.478 67.995 0.00 0.00
B
ATOM 4343 O SER B 65 -2.256 28.549 69.097 0.00 0.00
B
ATOM 4344 N LEU B 66 -1.950 28.396 66.945 0.00 0.00
B
ATOM 4345 HN LEU B 66 -2.430 28.334 66.073 0.00 0.00
B
ATOM 4346 CA LEU B 66 -0.484 28.482 66.973 0.00 0.00
B

ATOM 4347 HA LEU B 66 -0.207 27.608 67.543 0.00 0.00
B
ATOM 4348 CB LEU B 66 0.253 28.498 65.528 0.00 0.00
B
ATOM 4349 HB1 LEU B 66 -0.295 29.294 64.979 0.00 0.00
B
ATOM 4350 HB2 LEU B 66 1.361 28.556 65.576 0.00 0.00
B
ATOM 4351 CG LEU B 66 0.128 27.101 64.860 0.00 0.00
B
ATOM 4352 HG LEU B 66 -0.939 26.792 64.867 0.00 0.00
B
ATOM 4353 CD1 LEU B 66 0.485 27.200 63.365 0.00 0.00
B
ATOM 4354 HD11 LEU B 66 -0.087 27.996 62.842 0.00 0.00
B
ATOM 4355 HD12 LEU B 66 1.537 27.468 63.129 0.00 0.00
B
ATOM 4356 HD13 LEU B 66 0.265 26.228 62.874 0.00 0.00
B
ATOM 4357 CD2 LEU B 66 1.029 26.049 65.423 0.00 0.00
B
ATOM 4358 HD21 LEU B 66 1.038 26.040 66.534 0.00 0.00
B
ATOM 4359 HD22 LEU B 66 0.833 25.042 64.999 0.00 0.00
B
ATOM 4360 HD23 LEU B 66 2.089 26.316 65.224 0.00 0.00
B
ATOM 4361 C LEU B 66 0.107 29.684 67.687 0.00 0.00
B
ATOM 4362 O LEU B 66 0.978 29.578 68.526 0.00 0.00
B
ATOM 4363 N GLY B 67 -0.416 30.908 67.499 0.00 0.00
B
ATOM 4364 HN GLY B 67 -1.158 30.906 66.833 0.00 0.00
B
ATOM 4365 CA GLY B 67 0.102 32.071 68.177 0.00 0.00
B
ATOM 4366 HA1 GLY B 67 0.402 31.802 69.179 0.00 0.00
B
ATOM 4367 HA2 GLY B 67 -0.722 32.767 68.218 0.00 0.00
B
ATOM 4368 C GLY B 67 1.300 32.589 67.382 0.00 0.00
B
ATOM 4369 O GLY B 67 2.391 32.837 67.955 0.00 0.00
B
ATOM 4370 N ILE B 68 1.194 32.743 66.039 0.00 0.00
B
ATOM 4371 HN ILE B 68 0.272 32.580 65.695 0.00 0.00
B
ATOM 4372 CA ILE B 68 2.271 33.246 65.234 0.00 0.00
B
ATOM 4373 HA ILE B 68 3.229 33.217 65.731 0.00 0.00
B

ATOM 4374 CB ILE B 68 2.448 32.316 64.019 0.00 0.00
B
ATOM 4375 HB ILE B 68 3.181 32.852 63.379 0.00 0.00
B
ATOM 4376 CG2 ILE B 68 3.120 30.951 64.403 0.00 0.00
B
ATOM 4377 HG21 ILE B 68 3.141 30.193 63.591 0.00 0.00
B
ATOM 4378 HG22 ILE B 68 4.187 31.113 64.667 0.00 0.00
B
ATOM 4379 HG23 ILE B 68 2.625 30.552 65.314 0.00 0.00
B
ATOM 4380 CG1 ILE B 68 1.058 31.963 63.357 0.00 0.00
B
ATOM 4381 HG11 ILE B 68 0.491 31.293 64.038 0.00 0.00
B
ATOM 4382 HG12 ILE B 68 0.458 32.889 63.490 0.00 0.00
B
ATOM 4383 CD ILE B 68 1.141 31.342 61.964 0.00 0.00
B
ATOM 4384 HD1 ILE B 68 0.167 31.194 61.451 0.00 0.00
B
ATOM 4385 HD2 ILE B 68 1.752 32.020 61.330 0.00 0.00
B
ATOM 4386 HD3 ILE B 68 1.702 30.383 61.963 0.00 0.00
B
ATOM 4387 C ILE B 68 2.051 34.639 64.619 0.00 0.00
B
ATOM 4388 O ILE B 68 2.839 35.042 63.720 0.00 0.00
B
ATOM 4389 N ARG B 69 1.186 35.417 65.126 0.00 0.00
B
ATOM 4390 HN ARG B 69 0.545 35.097 65.819 0.00 0.00
B
ATOM 4391 CA ARG B 69 0.880 36.707 64.524 0.00 0.00
B
ATOM 4392 HA ARG B 69 0.836 36.470 63.471 0.00 0.00
B
ATOM 4393 CB ARG B 69 −0.553 37.170 64.895 0.00 0.00
B
ATOM 4394 HB1 ARG B 69 −0.760 38.104 64.331 0.00 0.00
B
ATOM 4395 HB2 ARG B 69 −1.285 36.343 64.767 0.00 0.00
B
ATOM 4396 CG ARG B 69 −0.902 37.436 66.462 0.00 0.00
B
ATOM 4397 HG1 ARG B 69 −0.789 36.442 66.945 0.00 0.00
B
ATOM 4398 HG2 ARG B 69 −0.101 38.108 66.836 0.00 0.00
B
ATOM 4399 CD ARG B 69 −2.338 37.953 66.646 0.00 0.00
B
ATOM 4400 HD1 ARG B 69 −2.729 38.547 65.792 0.00 0.00
B

ATOM 4401 HD2 ARG B 69 -3.078 37.125 66.687 0.00 0.00
B
ATOM 4402 NE ARG B 69 -2.349 38.718 67.939 0.00 0.00
B
ATOM 4403 HE ARG B 69 -2.734 38.304 68.763 0.00 0.00
B
ATOM 4404 CZ ARG B 69 -2.349 40.029 68.019 0.00 0.00
B
ATOM 4405 NH1 ARG B 69 -2.027 40.821 67.038 0.00 0.00
B
ATOM 4406 HH11 ARG B 69 -2.326 41.772 67.124 0.00 0.00
B
ATOM 4407 HH12 ARG B 69 -2.001 40.486 66.097 0.00 0.00
B
ATOM 4408 NH2 ARG B 69 -2.790 40.558 69.150 0.00 0.00
B
ATOM 4409 HH21 ARG B 69 -2.786 41.557 69.200 0.00 0.00
B
ATOM 4410 HH22 ARG B 69 -2.760 40.022 69.994 0.00 0.00
B
ATOM 4411 C ARG B 69 1.886 37.897 64.698 0.00 0.00
B
ATOM 4412 O ARG B 69 1.906 38.886 63.951 0.00 0.00
B
ATOM 4413 N ASN B 70 2.788 37.724 65.707 0.00 0.00
B
ATOM 4414 HN ASN B 70 2.664 36.939 66.308 0.00 0.00
B
ATOM 4415 CA ASN B 70 3.711 38.821 66.141 0.00 0.00
B
ATOM 4416 HA ASN B 70 3.576 39.727 65.568 0.00 0.00
B
ATOM 4417 CB ASN B 70 3.332 39.203 67.582 0.00 0.00
B
ATOM 4418 HB1 ASN B 70 3.394 38.369 68.314 0.00 0.00
B
ATOM 4419 HB2 ASN B 70 3.905 40.047 68.021 0.00 0.00
B
ATOM 4420 CG ASN B 70 1.989 39.784 67.614 0.00 0.00
B
ATOM 4421 OD1 ASN B 70 1.146 39.403 68.456 0.00 0.00
B
ATOM 4422 ND2 ASN B 70 1.739 40.835 66.831 0.00 0.00
B
ATOM 4423 HD21 ASN B 70 0.948 41.358 67.150 0.00 0.00
B
ATOM 4424 HD22 ASN B 70 2.441 41.193 66.215 0.00 0.00
B
ATOM 4425 C ASN B 70 5.133 38.412 65.866 0.00 0.00
B
ATOM 4426 O ASN B 70 6.115 38.968 66.299 0.00 0.00
B
ATOM 4427 N PHE B 71 5.291 37.228 65.216 0.00 0.00
B

ATOM 4428 HN PHE B 71 4.519 36.608 65.102 0.00 0.00
B
ATOM 4429 CA PHE B 71 6.467 36.837 64.529 0.00 0.00
B
ATOM 4430 HA PHE B 71 7.321 37.150 65.111 0.00 0.00
B
ATOM 4431 CB PHE B 71 6.412 35.338 64.188 0.00 0.00
B
ATOM 4432 HB1 PHE B 71 5.465 35.105 63.656 0.00 0.00
B
ATOM 4433 HB2 PHE B 71 7.083 35.113 63.332 0.00 0.00
B
ATOM 4434 CG PHE B 71 6.600 34.364 65.359 0.00 0.00
B
ATOM 4435 CD1 PHE B 71 6.960 33.096 65.016 0.00 0.00
B
ATOM 4436 HD1 PHE B 71 7.253 32.868 64.002 0.00 0.00
B
ATOM 4437 CE1 PHE B 71 7.053 32.044 66.018 0.00 0.00
B
ATOM 4438 HE1 PHE B 71 7.432 31.055 65.808 0.00 0.00
B
ATOM 4439 CZ PHE B 71 6.688 32.319 67.346 0.00 0.00
B
ATOM 4440 HZ PHE B 71 7.002 31.627 68.114 0.00 0.00
B
ATOM 4441 CD2 PHE B 71 6.147 34.661 66.708 0.00 0.00
B
ATOM 4442 HD2 PHE B 71 5.780 35.631 67.009 0.00 0.00
B
ATOM 4443 CE2 PHE B 71 6.181 33.600 67.622 0.00 0.00
B
ATOM 4444 HE2 PHE B 71 5.866 33.751 68.644 0.00 0.00
B
ATOM 4445 C PHE B 71 6.439 37.594 63.146 0.00 0.00
B
ATOM 4446 O PHE B 71 5.379 38.016 62.673 0.00 0.00
B
ATOM 4447 N GLU B 72 7.590 37.894 62.559 0.00 0.00
B
ATOM 4448 HN GLU B 72 8.508 37.694 62.895 0.00 0.00
B
ATOM 4449 CA GLU B 72 7.577 38.807 61.405 0.00 0.00
B
ATOM 4450 HA GLU B 72 6.621 39.286 61.254 0.00 0.00
B
ATOM 4451 CB GLU B 72 8.798 39.818 61.489 0.00 0.00
B
ATOM 4452 HB1 GLU B 72 9.626 39.142 61.792 0.00 0.00
B
ATOM 4453 HB2 GLU B 72 9.059 40.295 60.520 0.00 0.00
B
ATOM 4454 CG GLU B 72 8.586 40.878 62.521 0.00 0.00
B

ATOM 4455 HG1 GLU B 72 7.778 41.597 62.267 0.00 0.00
B
ATOM 4456 HG2 GLU B 72 8.192 40.481 63.481 0.00 0.00
B
ATOM 4457 CD GLU B 72 9.779 41.778 62.780 0.00 0.00
B
ATOM 4458 OE1 GLU B 72 10.864 41.210 63.170 0.00 0.00
B
ATOM 4459 OE2 GLU B 72 9.739 43.029 62.595 0.00 0.00
B
ATOM 4460 C GLU B 72 7.724 37.957 60.117 0.00 0.00
B
ATOM 4461 O GLU B 72 7.251 38.369 59.022 0.00 0.00
B
ATOM 4462 N TYR B 73 8.287 36.681 60.287 0.00 0.00
B
ATOM 4463 HN TYR B 73 8.723 36.524 61.170 0.00 0.00
B
ATOM 4464 CA TYR B 73 8.515 35.857 59.218 0.00 0.00
B
ATOM 4465 HA TYR B 73 7.937 36.237 58.389 0.00 0.00
B
ATOM 4466 CB TYR B 73 10.036 35.570 58.932 0.00 0.00
B
ATOM 4467 HB1 TYR B 73 10.464 35.219 59.895 0.00 0.00
B
ATOM 4468 HB2 TYR B 73 10.133 34.823 58.115 0.00 0.00
B
ATOM 4469 CG TYR B 73 10.756 36.826 58.526 0.00 0.00
B
ATOM 4470 CD1 TYR B 73 11.306 37.692 59.460 0.00 0.00
B
ATOM 4471 HD1 TYR B 73 11.142 37.600 60.523 0.00 0.00
B
ATOM 4472 CE1 TYR B 73 12.093 38.756 58.960 0.00 0.00
B
ATOM 4473 HE1 TYR B 73 12.516 39.464 59.658 0.00 0.00
B
ATOM 4474 CZ TYR B 73 12.191 39.024 57.605 0.00 0.00
B
ATOM 4475 OH TYR B 73 12.988 40.071 57.184 0.00 0.00
B
ATOM 4476 HH TYR B 73 12.860 40.762 57.838 0.00 0.00
B
ATOM 4477 CD2 TYR B 73 10.992 36.959 57.113 0.00 0.00
B
ATOM 4478 HD2 TYR B 73 10.804 36.170 56.400 0.00 0.00
B
ATOM 4479 CE2 TYR B 73 11.646 38.084 56.684 0.00 0.00
B
ATOM 4480 HE2 TYR B 73 11.795 38.318 55.641 0.00 0.00
B
ATOM 4481 C TYR B 73 7.981 34.361 59.395 0.00 0.00
B

ATOM 4482 O TYR B 73 8.304 33.669 60.373 0.00 0.00
B
ATOM 4483 N VAL B 74 7.177 33.843 58.406 0.00 0.00
B
ATOM 4484 HN VAL B 74 6.861 34.342 57.603 0.00 0.00
B
ATOM 4485 CA VAL B 74 6.718 32.434 58.449 0.00 0.00
B
ATOM 4486 HA VAL B 74 7.124 31.860 59.269 0.00 0.00
B
ATOM 4487 CB VAL B 74 5.246 32.266 58.749 0.00 0.00
B
ATOM 4488 HB VAL B 74 4.663 32.845 58.002 0.00 0.00
B
ATOM 4489 CG1 VAL B 74 4.748 30.793 58.678 0.00 0.00
B
ATOM 4490 HG11 VAL B 74 3.729 30.571 59.063 0.00 0.00
B
ATOM 4491 HG12 VAL B 74 4.777 30.415 57.634 0.00 0.00
B
ATOM 4492 HG13 VAL B 74 5.388 30.188 59.355 0.00 0.00
B
ATOM 4493 CG2 VAL B 74 4.977 32.674 60.233 0.00 0.00
B
ATOM 4494 HG21 VAL B 74 3.976 32.494 60.679 0.00 0.00
B
ATOM 4495 HG22 VAL B 74 5.651 31.997 60.802 0.00 0.00
B
ATOM 4496 HG23 VAL B 74 5.278 33.735 60.369 0.00 0.00
B
ATOM 4497 C VAL B 74 7.131 31.824 57.153 0.00 0.00
B
ATOM 4498 O VAL B 74 6.763 32.243 56.062 0.00 0.00
B
ATOM 4499 N ILE B 75 7.969 30.847 57.281 0.00 0.00
B
ATOM 4500 HN ILE B 75 8.110 30.429 58.175 0.00 0.00
B
ATOM 4501 CA ILE B 75 8.686 30.079 56.290 0.00 0.00
B
ATOM 4502 HA ILE B 75 8.720 30.658 55.380 0.00 0.00
B
ATOM 4503 CB ILE B 75 10.098 29.783 56.693 0.00 0.00
B
ATOM 4504 HB ILE B 75 10.044 29.081 57.553 0.00 0.00
B
ATOM 4505 CG2 ILE B 75 10.880 29.054 55.599 0.00 0.00
B
ATOM 4506 HG21 ILE B 75 10.540 29.584 54.683 0.00 0.00
B
ATOM 4507 HG22 ILE B 75 11.978 29.176 55.721 0.00 0.00
B
ATOM 4508 HG23 ILE B 75 10.542 27.996 55.574 0.00 0.00
B

ATOM 4509 CG1 ILE B 75 10.792 31.109 57.265 0.00 0.00
B
ATOM 4510 HG11 ILE B 75 10.183 31.479 58.117 0.00 0.00
B
ATOM 4511 HG12 ILE B 75 11.819 30.784 57.538 0.00 0.00
B
ATOM 4512 CD ILE B 75 10.960 32.187 56.207 0.00 0.00
B
ATOM 4513 HD1 ILE B 75 11.469 33.057 56.674 0.00 0.00
B
ATOM 4514 HD2 ILE B 75 11.538 31.784 55.348 0.00 0.00
B
ATOM 4515 HD3 ILE B 75 9.950 32.589 55.976 0.00 0.00
B
ATOM 4516 C ILE B 75 7.878 28.785 56.064 0.00 0.00
B
ATOM 4517 O ILE B 75 7.741 27.896 56.902 0.00 0.00
B
ATOM 4518 N VAL B 76 7.254 28.729 54.908 0.00 0.00
B
ATOM 4519 HN VAL B 76 7.456 29.407 54.205 0.00 0.00
B
ATOM 4520 CA VAL B 76 6.373 27.655 54.534 0.00 0.00
B
ATOM 4521 HA VAL B 76 5.942 27.088 55.346 0.00 0.00
B
ATOM 4522 CB VAL B 76 5.193 28.136 53.742 0.00 0.00
B
ATOM 4523 HB VAL B 76 5.402 28.452 52.697 0.00 0.00
B
ATOM 4524 CG1 VAL B 76 4.202 26.955 53.655 0.00 0.00
B
ATOM 4525 HG11 VAL B 76 3.333 27.393 53.119 0.00 0.00
B
ATOM 4526 HG12 VAL B 76 4.614 26.076 53.113 0.00 0.00
B
ATOM 4527 HG13 VAL B 76 3.959 26.657 54.697 0.00 0.00
B
ATOM 4528 CG2 VAL B 76 4.511 29.399 54.376 0.00 0.00
B
ATOM 4529 HG21 VAL B 76 4.033 29.138 55.344 0.00 0.00
B
ATOM 4530 HG22 VAL B 76 5.209 30.236 54.591 0.00 0.00
B
ATOM 4531 HG23 VAL B 76 3.704 29.784 53.716 0.00 0.00
B
ATOM 4532 C VAL B 76 7.133 26.703 53.630 0.00 0.00
B
ATOM 4533 O VAL B 76 7.494 26.912 52.487 0.00 0.00
B
ATOM 4534 N ALA B 77 7.570 25.516 54.191 0.00 0.00
B
ATOM 4535 HN ALA B 77 7.322 25.364 55.145 0.00 0.00
B

ATOM 4536 CA ALA B 77 8.314 24.443 53.565 0.00 0.00
B
ATOM 4537 HA ALA B 77 8.848 24.848 52.718 0.00 0.00
B
ATOM 4538 CB ALA B 77 9.471 23.900 54.477 0.00 0.00
B
ATOM 4539 HB1 ALA B 77 8.948 23.396 55.318 0.00 0.00
B
ATOM 4540 HB2 ALA B 77 10.205 23.320 53.878 0.00 0.00
B
ATOM 4541 HB3 ALA B 77 9.933 24.807 54.922 0.00 0.00
B
ATOM 4542 C ALA B 77 7.459 23.249 53.058 0.00 0.00
B
ATOM 4543 O ALA B 77 8.009 22.333 52.402 0.00 0.00
B
ATOM 4544 N ILE B 78 6.111 23.258 53.180 0.00 0.00
B
ATOM 4545 HN ILE B 78 5.597 23.975 53.644 0.00 0.00
B
ATOM 4546 CA ILE B 78 5.208 22.313 52.456 0.00 0.00
B
ATOM 4547 HA ILE B 78 5.749 21.381 52.393 0.00 0.00
B
ATOM 4548 CB ILE B 78 3.933 22.047 53.256 0.00 0.00
B
ATOM 4549 HB ILE B 78 4.201 21.379 54.102 0.00 0.00
B
ATOM 4550 CG2 ILE B 78 3.462 23.379 53.947 0.00 0.00
B
ATOM 4551 HG21 ILE B 78 2.590 23.175 54.603 0.00 0.00
B
ATOM 4552 HG22 ILE B 78 4.287 23.810 54.553 0.00 0.00
B
ATOM 4553 HG23 ILE B 78 3.188 24.130 53.176 0.00 0.00
B
ATOM 4554 CG1 ILE B 78 2.782 21.309 52.566 0.00 0.00
B
ATOM 4555 HG11 ILE B 78 1.944 21.289 53.295 0.00 0.00
B
ATOM 4556 HG12 ILE B 78 2.489 22.042 51.784 0.00 0.00
B
ATOM 4557 CD ILE B 78 3.071 19.967 51.972 0.00 0.00
B
ATOM 4558 HD1 ILE B 78 3.637 19.326 52.681 0.00 0.00
B
ATOM 4559 HD2 ILE B 78 2.137 19.447 51.669 0.00 0.00
B
ATOM 4560 HD3 ILE B 78 3.712 20.075 51.071 0.00 0.00
B
ATOM 4561 C ILE B 78 4.919 22.698 51.002 0.00 0.00
B
ATOM 4562 O ILE B 78 4.424 23.799 50.712 0.00 0.00
B

ATOM 4563 N GLY B 79 5.257 21.850 49.984 0.00 0.00
B
ATOM 4564 HN GLY B 79 5.585 20.927 50.169 0.00 0.00
B
ATOM 4565 CA GLY B 79 5.165 22.314 48.621 0.00 0.00
B
ATOM 4566 HA1 GLY B 79 6.078 22.786 48.290 0.00 0.00
B
ATOM 4567 HA2 GLY B 79 4.392 23.057 48.490 0.00 0.00
B
ATOM 4568 C GLY B 79 4.899 21.321 47.549 0.00 0.00
B
ATOM 4569 O GLY B 79 4.482 21.756 46.559 0.00 0.00
B
ATOM 4570 N ALA B 80 5.255 20.082 47.683 0.00 0.00
B
ATOM 4571 HN ALA B 80 5.614 19.825 48.577 0.00 0.00
B
ATOM 4572 CA ALA B 80 4.834 19.123 46.650 0.00 0.00
B
ATOM 4573 HA ALA B 80 5.337 19.423 45.743 0.00 0.00
B
ATOM 4574 CB ALA B 80 5.348 17.759 47.113 0.00 0.00
B
ATOM 4575 HB1 ALA B 80 6.413 17.956 47.362 0.00 0.00
B
ATOM 4576 HB2 ALA B 80 4.832 17.349 48.007 0.00 0.00
B
ATOM 4577 HB3 ALA B 80 5.193 17.006 46.311 0.00 0.00
B
ATOM 4578 C ALA B 80 3.316 18.933 46.451 0.00 0.00
B
ATOM 4579 O ALA B 80 2.768 18.902 45.350 0.00 0.00
B
ATOM 4580 N ASN B 81 2.548 18.827 47.541 0.00 0.00
B
ATOM 4581 HN ASN B 81 3.025 19.014 48.397 0.00 0.00
B
ATOM 4582 CA ASN B 81 1.095 18.748 47.506 0.00 0.00
B
ATOM 4583 HA ASN B 81 0.877 18.090 46.678 0.00 0.00
B
ATOM 4584 CB ASN B 81 0.429 17.969 48.723 0.00 0.00
B
ATOM 4585 HB1 ASN B 81 0.953 16.989 48.714 0.00 0.00
B
ATOM 4586 HB2 ASN B 81 0.687 18.568 49.623 0.00 0.00
B
ATOM 4587 CG ASN B 81 −1.043 17.824 48.581 0.00 0.00
B
ATOM 4588 OD1 ASN B 81 −1.774 18.433 49.379 0.00 0.00
B
ATOM 4589 ND2 ASN B 81 −1.428 17.169 47.516 0.00 0.00
B

ATOM	4590	HD21	ASN	B	81	-2.338	16.753	47.492	0.00	0.00	B
ATOM	4591	HD22	ASN	B	81	-0.764	16.737	46.906	0.00	0.00	B
ATOM	4592	C	ASN	B	81	0.491	20.078	47.379	0.00	0.00	B
ATOM	4593	O	ASN	B	81	0.585	20.764	48.441	0.00	0.00	B
ATOM	4594	N	ILE	B	82	-0.105	20.475	46.252	0.00	0.00	B
ATOM	4595	HN	ILE	B	82	-0.294	19.928	45.440	0.00	0.00	B
ATOM	4596	CA	ILE	B	82	-0.642	21.826	46.073	0.00	0.00	B
ATOM	4597	HA	ILE	B	82	0.100	22.500	46.476	0.00	0.00	B
ATOM	4598	CB	ILE	B	82	-0.717	22.118	44.580	0.00	0.00	B
ATOM	4599	HB	ILE	B	82	0.270	21.911	44.114	0.00	0.00	B
ATOM	4600	CG2	ILE	B	82	-1.775	21.185	43.976	0.00	0.00	B
ATOM	4601	HG21	ILE	B	82	-1.487	20.124	44.139	0.00	0.00	B
ATOM	4602	HG22	ILE	B	82	-2.804	21.339	44.364	0.00	0.00	B
ATOM	4603	HG23	ILE	B	82	-1.788	21.395	42.885	0.00	0.00	B
ATOM	4604	CG1	ILE	B	82	-1.000	23.605	44.344	0.00	0.00	B
ATOM	4605	HG11	ILE	B	82	-1.985	23.983	44.692	0.00	0.00	B
ATOM	4606	HG12	ILE	B	82	-0.168	24.123	44.867	0.00	0.00	B
ATOM	4607	CD	ILE	B	82	-1.059	23.922	42.830	0.00	0.00	B
ATOM	4608	HD1	ILE	B	82	-1.667	23.211	42.232	0.00	0.00	B
ATOM	4609	HD2	ILE	B	82	-1.457	24.959	42.809	0.00	0.00	B
ATOM	4610	HD3	ILE	B	82	-0.001	23.941	42.491	0.00	0.00	B
ATOM	4611	C	ILE	B	82	-1.816	22.048	47.040	0.00	0.00	B
ATOM	4612	O	ILE	B	82	-2.108	23.190	47.399	0.00	0.00	B
ATOM	4613	N	GLN	B	83	-2.649	21.058	47.427	0.00	0.00	B
ATOM	4614	HN	GLN	B	83	-2.511	20.103	47.177	0.00	0.00	B
ATOM	4615	CA	GLN	B	83	-3.776	21.366	48.324	0.00	0.00	B
ATOM	4616	HA	GLN	B	83	-4.316	22.191	47.885	0.00	0.00	B

ATOM 4617 CB GLN B 83 −4.759 20.117 48.397 0.00 0.00
B
ATOM 4618 HB1 GLN B 83 −5.178 20.084 47.369 0.00 0.00
B
ATOM 4619 HB2 GLN B 83 −4.234 19.172 48.653 0.00 0.00
B
ATOM 4620 CG GLN B 83 −5.864 20.397 49.428 0.00 0.00
B
ATOM 4621 HG1 GLN B 83 −5.399 20.517 50.430 0.00 0.00
B
ATOM 4622 HG2 GLN B 83 −6.337 21.364 49.153 0.00 0.00
B
ATOM 4623 CD GLN B 83 −6.978 19.376 49.546 0.00 0.00
B
ATOM 4624 OE1 GLN B 83 −7.007 18.425 48.847 0.00 0.00
B
ATOM 4625 NE2 GLN B 83 −7.878 19.602 50.519 0.00 0.00
B
ATOM 4626 HE21 GLN B 83 −8.738 19.102 50.621 0.00 0.00
B
ATOM 4627 HE22 GLN B 83 −7.613 20.233 51.249 0.00 0.00
B
ATOM 4628 C GLN B 83 −3.320 21.851 49.680 0.00 0.00
B
ATOM 4629 O GLN B 83 −3.739 22.943 50.173 0.00 0.00
B
ATOM 4630 N ALA B 84 −2.378 21.085 50.285 0.00 0.00
B
ATOM 4631 HN ALA B 84 −2.085 20.177 49.995 0.00 0.00
B
ATOM 4632 CA ALA B 84 −1.740 21.495 51.538 0.00 0.00
B
ATOM 4633 HA ALA B 84 −2.516 21.506 52.288 0.00 0.00
B
ATOM 4634 CB ALA B 84 −0.773 20.415 52.048 0.00 0.00
B
ATOM 4635 HB1 ALA B 84 −1.384 19.605 52.500 0.00 0.00
B
ATOM 4636 HB2 ALA B 84 −0.225 19.903 51.228 0.00 0.00
B
ATOM 4637 HB3 ALA B 84 0.049 20.662 52.753 0.00 0.00
B
ATOM 4638 C ALA B 84 −0.949 22.865 51.482 0.00 0.00
B
ATOM 4639 O ALA B 84 −0.994 23.755 52.339 0.00 0.00
B
ATOM 4640 N SER B 85 −0.194 23.033 50.376 0.00 0.00
B
ATOM 4641 HN SER B 85 −0.138 22.284 49.721 0.00 0.00
B
ATOM 4642 CA SER B 85 0.560 24.243 50.114 0.00 0.00
B
ATOM 4643 HA SER B 85 1.243 24.344 50.944 0.00 0.00
B

ATOM 4644 CB SER B 85 1.574 23.972 48.948 0.00 0.00
B
ATOM 4645 HB1 SER B 85 1.880 22.910 49.064 0.00 0.00
B
ATOM 4646 HB2 SER B 85 0.975 24.118 48.024 0.00 0.00
B
ATOM 4647 OG SER B 85 2.712 24.754 48.878 0.00 0.00
B
ATOM 4648 HG1 SER B 85 3.313 24.630 49.616 0.00 0.00
B
ATOM 4649 C SER B 85 -0.196 25.529 49.873 0.00 0.00
B
ATOM 4650 O SER B 85 0.093 26.544 50.503 0.00 0.00
B
ATOM 4651 N THR B 86 -1.259 25.472 49.005 0.00 0.00
B
ATOM 4652 HN THR B 86 -1.458 24.606 48.553 0.00 0.00
B
ATOM 4653 CA THR B 86 -2.135 26.669 48.738 0.00 0.00
B
ATOM 4654 HA THR B 86 -1.484 27.496 48.495 0.00 0.00
B
ATOM 4655 CB THR B 86 -2.989 26.562 47.490 0.00 0.00
B
ATOM 4656 HB THR B 86 -3.640 27.460 47.551 0.00 0.00
B
ATOM 4657 OG1 THR B 86 -3.827 25.471 47.441 0.00 0.00
B
ATOM 4658 HG1 THR B 86 -3.257 24.703 47.357 0.00 0.00
B
ATOM 4659 CG2 THR B 86 -2.128 26.545 46.189 0.00 0.00
B
ATOM 4660 HG21 THR B 86 -1.656 25.539 46.177 0.00 0.00
B
ATOM 4661 HG22 THR B 86 -2.833 26.651 45.337 0.00 0.00
B
ATOM 4662 HG23 THR B 86 -1.402 27.387 46.208 0.00 0.00
B
ATOM 4663 C THR B 86 -3.019 27.145 49.862 0.00 0.00
B
ATOM 4664 O THR B 86 -3.166 28.371 50.098 0.00 0.00
B
ATOM 4665 N LEU B 87 -3.732 26.192 50.529 0.00 0.00
B
ATOM 4666 HN LEU B 87 -3.752 25.258 50.182 0.00 0.00
B
ATOM 4667 CA LEU B 87 -4.745 26.456 51.564 0.00 0.00
B
ATOM 4668 HA LEU B 87 -5.237 27.336 51.177 0.00 0.00
B
ATOM 4669 CB LEU B 87 -5.643 25.225 51.833 0.00 0.00
B
ATOM 4670 HB1 LEU B 87 -4.950 24.361 51.921 0.00 0.00
B

ATOM	4671	HB2	LEU	B	87	-6.319	25.354	52.705	0.00	0.00	B
ATOM	4672	CG	LEU	B	87	-6.561	24.911	50.636	0.00	0.00	B
ATOM	4673	HG	LEU	B	87	-6.103	24.528	49.699	0.00	0.00	B
ATOM	4674	CD1	LEU	B	87	-7.380	23.683	51.194	0.00	0.00	B
ATOM	4675	HD11	LEU	B	87	-7.769	23.865	52.218	0.00	0.00	B
ATOM	4676	HD12	LEU	B	87	-8.259	23.397	50.578	0.00	0.00	B
ATOM	4677	HD13	LEU	B	87	-6.750	22.781	51.353	0.00	0.00	B
ATOM	4678	CD2	LEU	B	87	-7.584	26.039	50.290	0.00	0.00	B
ATOM	4679	HD21	LEU	B	87	-7.108	26.888	49.755	0.00	0.00	B
ATOM	4680	HD22	LEU	B	87	-8.195	25.610	49.468	0.00	0.00	B
ATOM	4681	HD23	LEU	B	87	-8.219	26.442	51.108	0.00	0.00	B
ATOM	4682	C	LEU	B	87	-4.062	27.007	52.828	0.00	0.00	B
ATOM	4683	O	LEU	B	87	-4.638	27.776	53.578	0.00	0.00	B
ATOM	4684	N	THR	B	88	-2.781	26.582	53.102	0.00	0.00	B
ATOM	4685	HN	THR	B	88	-2.350	25.888	52.531	0.00	0.00	B
ATOM	4686	CA	THR	B	88	-1.994	27.125	54.226	0.00	0.00	B
ATOM	4687	HA	THR	B	88	-2.617	27.043	55.104	0.00	0.00	B
ATOM	4688	CB	THR	B	88	-0.603	26.500	54.287	0.00	0.00	B
ATOM	4689	HB	THR	B	88	-0.089	26.686	53.319	0.00	0.00	B
ATOM	4690	OG1	THR	B	88	-0.580	25.130	54.548	0.00	0.00	B
ATOM	4691	HG1	THR	B	88	-0.768	24.634	53.748	0.00	0.00	B
ATOM	4692	CG2	THR	B	88	0.167	27.073	55.447	0.00	0.00	B
ATOM	4693	HG21	THR	B	88	1.120	26.504	55.474	0.00	0.00	B
ATOM	4694	HG22	THR	B	88	0.400	28.155	55.350	0.00	0.00	B
ATOM	4695	HG23	THR	B	88	-0.438	26.919	56.366	0.00	0.00	B
ATOM	4696	C	THR	B	88	-1.669	28.569	54.025	0.00	0.00	B
ATOM	4697	O	THR	B	88	-1.876	29.446	54.888	0.00	0.00	B

ATOM 4698 N THR B 89 -1.244 28.906 52.813 0.00 0.00
B
ATOM 4699 HN THR B 89 -1.283 28.220 52.090 0.00 0.00
B
ATOM 4700 CA THR B 89 -0.690 30.260 52.484 0.00 0.00
B
ATOM 4701 HA THR B 89 -0.103 30.438 53.373 0.00 0.00
B
ATOM 4702 CB THR B 89 0.187 30.420 51.221 0.00 0.00
B
ATOM 4703 HB THR B 89 0.378 31.498 51.032 0.00 0.00
B
ATOM 4704 OG1 THR B 89 -0.492 29.811 50.080 0.00 0.00
B
ATOM 4705 HG1 THR B 89 -0.179 28.916 50.230 0.00 0.00
B
ATOM 4706 CG2 THR B 89 1.503 29.687 51.534 0.00 0.00
B
ATOM 4707 HG21 THR B 89 2.145 29.987 50.678 0.00 0.00
B
ATOM 4708 HG22 THR B 89 1.870 30.179 52.460 0.00 0.00
B
ATOM 4709 HG23 THR B 89 1.330 28.605 51.714 0.00 0.00
B
ATOM 4710 C THR B 89 -1.852 31.223 52.303 0.00 0.00
B
ATOM 4711 O THR B 89 -1.697 32.385 52.746 0.00 0.00
B
ATOM 4712 N LEU B 90 -3.048 30.761 51.832 0.00 0.00
B
ATOM 4713 HN LEU B 90 -3.109 29.856 51.417 0.00 0.00
B
ATOM 4714 CA LEU B 90 -4.229 31.547 51.969 0.00 0.00
B
ATOM 4715 HA LEU B 90 -4.096 32.431 51.363 0.00 0.00
B
ATOM 4716 CB LEU B 90 -5.344 30.696 51.250 0.00 0.00
B
ATOM 4717 HB1 LEU B 90 -4.902 30.467 50.257 0.00 0.00
B
ATOM 4718 HB2 LEU B 90 -5.533 29.703 51.711 0.00 0.00
B
ATOM 4719 CG LEU B 90 -6.766 31.248 51.009 0.00 0.00
B
ATOM 4720 HG LEU B 90 -7.165 30.446 50.351 0.00 0.00
B
ATOM 4721 CD1 LEU B 90 -7.703 31.318 52.293 0.00 0.00
B
ATOM 4722 HD11 LEU B 90 -8.793 31.384 52.084 0.00 0.00
B
ATOM 4723 HD12 LEU B 90 -7.465 30.430 52.917 0.00 0.00
B
ATOM 4724 HD13 LEU B 90 -7.538 32.323 52.738 0.00 0.00
B

ATOM	4725	CD2	LEU	B	90	−6.766	32.587	50.189	0.00	0.00	B
ATOM	4726	HD21	LEU	B	90	−5.961	32.389	49.449	0.00	0.00	B
ATOM	4727	HD22	LEU	B	90	−7.737	32.776	49.683	0.00	0.00	B
ATOM	4728	HD23	LEU	B	90	−6.497	33.489	50.779	0.00	0.00	B
ATOM	4729	C	LEU	B	90	−4.744	31.851	53.431	0.00	0.00	B
ATOM	4730	O	LEU	B	90	−5.067	33.014	53.704	0.00	0.00	B
ATOM	4731	N	LEU	B	91	−4.688	30.838	54.362	0.00	0.00	B
ATOM	4732	HN	LEU	B	91	−4.369	29.949	54.041	0.00	0.00	B
ATOM	4733	CA	LEU	B	91	−4.942	31.145	55.711	0.00	0.00	B
ATOM	4734	HA	LEU	B	91	−5.756	31.854	55.709	0.00	0.00	B
ATOM	4735	CB	LEU	B	91	−5.435	30.012	56.619	0.00	0.00	B
ATOM	4736	HB1	LEU	B	91	−4.778	29.116	56.605	0.00	0.00	B
ATOM	4737	HB2	LEU	B	91	−5.488	30.399	57.659	0.00	0.00	B
ATOM	4738	CG	LEU	B	91	−6.898	29.615	56.315	0.00	0.00	B
ATOM	4739	HG	LEU	B	91	−6.983	29.468	55.217	0.00	0.00	B
ATOM	4740	CD1	LEU	B	91	−7.186	28.217	56.801	0.00	0.00	B
ATOM	4741	HD11	LEU	B	91	−6.722	28.058	57.797	0.00	0.00	B
ATOM	4742	HD12	LEU	B	91	−8.289	28.092	56.848	0.00	0.00	B
ATOM	4743	HD13	LEU	B	91	−6.754	27.448	56.125	0.00	0.00	B
ATOM	4744	CD2	LEU	B	91	−7.915	30.588	56.925	0.00	0.00	B
ATOM	4745	HD21	LEU	B	91	−7.961	30.505	58.032	0.00	0.00	B
ATOM	4746	HD22	LEU	B	91	−7.657	31.621	56.605	0.00	0.00	B
ATOM	4747	HD23	LEU	B	91	−8.945	30.413	56.547	0.00	0.00	B
ATOM	4748	C	LEU	B	91	−3.860	31.908	56.497	0.00	0.00	B
ATOM	4749	O	LEU	B	91	−4.182	32.743	57.297	0.00	0.00	B
ATOM	4750	N	LEU	B	92	−2.518	31.723	56.262	0.00	0.00	B
ATOM	4751	HN	LEU	B	92	−2.136	31.004	55.686	0.00	0.00	B

ATOM 4752 CA LEU B 92 -1.511 32.699 56.782 0.00 0.00
B
ATOM 4753 HA LEU B 92 -1.483 32.536 57.849 0.00 0.00
B
ATOM 4754 CB LEU B 92 -0.142 32.364 56.280 0.00 0.00
B
ATOM 4755 HB1 LEU B 92 -0.186 32.250 55.175 0.00 0.00
B
ATOM 4756 HB2 LEU B 92 0.611 33.156 56.482 0.00 0.00
B
ATOM 4757 CG LEU B 92 0.520 31.089 56.887 0.00 0.00
B
ATOM 4758 HG LEU B 92 -0.310 30.375 56.699 0.00 0.00
B
ATOM 4759 CD1 LEU B 92 1.745 30.578 56.212 0.00 0.00
B
ATOM 4760 HD11 LEU B 92 2.139 29.658 56.695 0.00 0.00
B
ATOM 4761 HD12 LEU B 92 1.469 30.546 55.136 0.00 0.00
B
ATOM 4762 HD13 LEU B 92 2.561 31.324 56.315 0.00 0.00
B
ATOM 4763 CD2 LEU B 92 0.809 31.066 58.436 0.00 0.00
B
ATOM 4764 HD21 LEU B 92 -0.107 31.346 59.000 0.00 0.00
B
ATOM 4765 HD22 LEU B 92 1.288 30.139 58.817 0.00 0.00
B
ATOM 4766 HD23 LEU B 92 1.605 31.829 58.571 0.00 0.00
B
ATOM 4767 C LEU B 92 -1.726 34.141 56.378 0.00 0.00
B
ATOM 4768 O LEU B 92 -1.614 35.082 57.145 0.00 0.00
B
ATOM 4769 N LYS B 93 -2.071 34.319 55.120 0.00 0.00
B
ATOM 4770 HN LYS B 93 -2.221 33.493 54.581 0.00 0.00
B
ATOM 4771 CA LYS B 93 -2.197 35.633 54.437 0.00 0.00
B
ATOM 4772 HA LYS B 93 -1.298 36.156 54.726 0.00 0.00
B
ATOM 4773 CB LYS B 93 -2.276 35.511 52.918 0.00 0.00
B
ATOM 4774 HB1 LYS B 93 -1.267 35.225 52.552 0.00 0.00
B
ATOM 4775 HB2 LYS B 93 -3.055 34.762 52.664 0.00 0.00
B
ATOM 4776 CG LYS B 93 -2.543 36.878 52.152 0.00 0.00
B
ATOM 4777 HG1 LYS B 93 -2.848 36.768 51.090 0.00 0.00
B
ATOM 4778 HG2 LYS B 93 -3.387 37.365 52.687 0.00 0.00
B

ATOM 4779 CD LYS B 93 −1.341 37.812 52.309 0.00 0.00
B
ATOM 4780 HD1 LYS B 93 −1.382 37.974 53.407 0.00 0.00
B
ATOM 4781 HD2 LYS B 93 −0.493 37.176 51.978 0.00 0.00
B
ATOM 4782 CE LYS B 93 −1.425 38.990 51.396 0.00 0.00
B
ATOM 4783 HE1 LYS B 93 −1.183 38.701 50.351 0.00 0.00
B
ATOM 4784 HE2 LYS B 93 −2.413 39.485 51.507 0.00 0.00
B
ATOM 4785 NZ LYS B 93 −0.522 40.064 51.848 0.00 0.00
B
ATOM 4786 HZ1 LYS B 93 0.485 39.809 51.800 0.00 0.00
B
ATOM 4787 HZ2 LYS B 93 −0.615 40.938 51.292 0.00 0.00
B
ATOM 4788 HZ3 LYS B 93 −0.834 40.303 52.811 0.00 0.00
B
ATOM 4789 C LYS B 93 −3.427 36.406 54.951 0.00 0.00
B
ATOM 4790 O LYS B 93 −3.337 37.642 55.210 0.00 0.00
B
ATOM 4791 N GLU B 94 −4.541 35.717 55.243 0.00 0.00
B
ATOM 4792 HN GLU B 94 −4.677 34.808 54.857 0.00 0.00
B
ATOM 4793 CA GLU B 94 −5.630 36.059 56.037 0.00 0.00
B
ATOM 4794 HA GLU B 94 −5.954 37.012 55.647 0.00 0.00
B
ATOM 4795 CB GLU B 94 −6.890 35.119 55.839 0.00 0.00
B
ATOM 4796 HB1 GLU B 94 −6.870 34.799 54.776 0.00 0.00
B
ATOM 4797 HB2 GLU B 94 −6.772 34.156 56.379 0.00 0.00
B
ATOM 4798 CG GLU B 94 −8.166 35.897 56.208 0.00 0.00
B
ATOM 4799 HG1 GLU B 94 −8.239 35.972 57.314 0.00 0.00
B
ATOM 4800 HG2 GLU B 94 −8.047 36.906 55.758 0.00 0.00
B
ATOM 4801 CD GLU B 94 −9.530 35.392 55.768 0.00 0.00
B
ATOM 4802 OE1 GLU B 94 −10.346 35.113 56.619 0.00 0.00
B
ATOM 4803 OE2 GLU B 94 −9.710 35.322 54.511 0.00 0.00
B
ATOM 4804 C GLU B 94 −5.413 36.286 57.458 0.00 0.00
B
ATOM 4805 O GLU B 94 −5.998 37.212 58.029 0.00 0.00
B

ATOM	4806	N	LEU	B	95	−4.468	35.567	58.177	0.00	0.00	B
ATOM	4807	HN	LEU	B	95	−4.025	34.829	57.674	0.00	0.00	B
ATOM	4808	CA	LEU	B	95	−4.032	36.029	59.490	0.00	0.00	B
ATOM	4809	HA	LEU	B	95	−4.914	36.243	60.076	0.00	0.00	B
ATOM	4810	CB	LEU	B	95	−3.162	34.959	60.141	0.00	0.00	B
ATOM	4811	HB1	LEU	B	95	−2.290	34.734	59.489	0.00	0.00	B
ATOM	4812	HB2	LEU	B	95	−2.726	35.340	61.089	0.00	0.00	B
ATOM	4813	CG	LEU	B	95	−3.929	33.649	60.627	0.00	0.00	B
ATOM	4814	HG	LEU	B	95	−4.243	33.162	59.680	0.00	0.00	B
ATOM	4815	CD1	LEU	B	95	−3.062	32.611	61.273	0.00	0.00	B
ATOM	4816	HD11	LEU	B	95	−3.654	31.684	61.431	0.00	0.00	B
ATOM	4817	HD12	LEU	B	95	−2.227	32.263	60.628	0.00	0.00	B
ATOM	4818	HD13	LEU	B	95	−2.561	32.969	62.197	0.00	0.00	B
ATOM	4819	CD2	LEU	B	95	−5.090	33.891	61.539	0.00	0.00	B
ATOM	4820	HD21	LEU	B	95	−5.449	32.935	61.976	0.00	0.00	B
ATOM	4821	HD22	LEU	B	95	−4.760	34.608	62.321	0.00	0.00	B
ATOM	4822	HD23	LEU	B	95	−5.979	34.405	61.116	0.00	0.00	B
ATOM	4823	C	LEU	B	95	−3.271	37.352	59.569	0.00	0.00	B
ATOM	4824	O	LEU	B	95	−3.102	37.962	60.668	0.00	0.00	B
ATOM	4825	N	ASP	B	96	−2.670	37.694	58.403	0.00	0.00	B
ATOM	4826	HN	ASP	B	96	−2.814	37.145	57.583	0.00	0.00	B
ATOM	4827	CA	ASP	B	96	−2.016	38.961	58.042	0.00	0.00	B
ATOM	4828	HA	ASP	B	96	−1.876	39.103	56.981	0.00	0.00	B
ATOM	4829	CB	ASP	B	96	−2.867	40.157	58.491	0.00	0.00	B
ATOM	4830	HB1	ASP	B	96	−3.962	40.023	58.625	0.00	0.00	B
ATOM	4831	HB2	ASP	B	96	−2.600	40.420	59.536	0.00	0.00	B
ATOM	4832	CG	ASP	B	96	−2.611	41.377	57.746	0.00	0.00	B

ATOM 4833 OD1 ASP B 96 -3.058 42.452 58.180 0.00 0.00
B
ATOM 4834 OD2 ASP B 96 -1.805 41.394 56.802 0.00 0.00
B
ATOM 4835 C ASP B 96 -0.588 38.850 58.675 0.00 0.00
B
ATOM 4836 O ASP B 96 -0.107 39.802 59.281 0.00 0.00
B
ATOM 4837 N ILE B 97 0.130 37.714 58.363 0.00 0.00
B
ATOM 4838 HN ILE B 97 -0.463 37.038 57.934 0.00 0.00
B
ATOM 4839 CA ILE B 97 1.543 37.548 58.700 0.00 0.00
B
ATOM 4840 HA ILE B 97 1.621 37.707 59.765 0.00 0.00
B
ATOM 4841 CB ILE B 97 2.137 36.181 58.410 0.00 0.00
B
ATOM 4842 HB ILE B 97 2.285 36.006 57.323 0.00 0.00
B
ATOM 4843 CG2 ILE B 97 3.483 36.024 59.183 0.00 0.00
B
ATOM 4844 HG21 ILE B 97 3.262 36.363 60.218 0.00 0.00
B
ATOM 4845 HG22 ILE B 97 3.867 34.983 59.236 0.00 0.00
B
ATOM 4846 HG23 ILE B 97 4.264 36.689 58.756 0.00 0.00
B
ATOM 4847 CG1 ILE B 97 1.216 35.045 58.852 0.00 0.00
B
ATOM 4848 HG11 ILE B 97 0.258 35.210 58.314 0.00 0.00
B
ATOM 4849 HG12 ILE B 97 1.557 34.075 58.430 0.00 0.00
B
ATOM 4850 CD ILE B 97 1.029 34.859 60.361 0.00 0.00
B
ATOM 4851 HD1 ILE B 97 0.257 34.108 60.631 0.00 0.00
B
ATOM 4852 HD2 ILE B 97 1.931 34.379 60.798 0.00 0.00
B
ATOM 4853 HD3 ILE B 97 0.732 35.816 60.842 0.00 0.00
B
ATOM 4854 C ILE B 97 2.422 38.627 57.996 0.00 0.00
B
ATOM 4855 O ILE B 97 2.199 38.700 56.754 0.00 0.00
B
ATOM 4856 N PRO B 98 3.316 39.388 58.592 0.00 0.00
B
ATOM 4857 CD PRO B 98 3.587 39.608 60.021 0.00 0.00
B
ATOM 4858 HD1 PRO B 98 2.643 39.592 60.606 0.00 0.00
B
ATOM 4859 HD2 PRO B 98 4.250 38.759 60.293 0.00 0.00
B

ATOM 4860 CA PRO B 98 4.156 40.303 57.898 0.00 0.00
B
ATOM 4861 HA PRO B 98 3.478 41.047 57.507 0.00 0.00
B
ATOM 4862 CB PRO B 98 5.031 41.055 58.908 0.00 0.00
B
ATOM 4863 HB1 PRO B 98 5.149 42.144 58.725 0.00 0.00
B
ATOM 4864 HB2 PRO B 98 5.991 40.525 59.086 0.00 0.00
B
ATOM 4865 CG PRO B 98 4.203 40.960 60.160 0.00 0.00
B
ATOM 4866 HG1 PRO B 98 3.349 41.668 60.228 0.00 0.00
B
ATOM 4867 HG2 PRO B 98 4.732 40.998 61.136 0.00 0.00
B
ATOM 4868 C PRO B 98 4.917 39.769 56.719 0.00 0.00
B
ATOM 4869 O PRO B 98 4.863 40.486 55.715 0.00 0.00
B
ATOM 4870 N ASN B 99 5.608 38.634 56.755 0.00 0.00
B
ATOM 4871 HN ASN B 99 5.889 38.309 57.654 0.00 0.00
B
ATOM 4872 CA ASN B 99 6.372 38.066 55.682 0.00 0.00
B
ATOM 4873 HA ASN B 99 6.106 38.534 54.746 0.00 0.00
B
ATOM 4874 CB ASN B 99 7.898 38.155 55.761 0.00 0.00
B
ATOM 4875 HB1 ASN B 99 8.183 37.613 56.688 0.00 0.00
B
ATOM 4876 HB2 ASN B 99 8.305 37.666 54.850 0.00 0.00
B
ATOM 4877 CG ASN B 99 8.341 39.581 55.753 0.00 0.00
B
ATOM 4878 OD1 ASN B 99 8.605 40.239 54.742 0.00 0.00
B
ATOM 4879 ND2 ASN B 99 8.438 40.193 56.942 0.00 0.00
B
ATOM 4880 HD21 ASN B 99 8.758 41.140 56.925 0.00 0.00
B
ATOM 4881 HD22 ASN B 99 8.236 39.740 57.810 0.00 0.00
B
ATOM 4882 C ASN B 99 6.030 36.612 55.507 0.00 0.00
B
ATOM 4883 O ASN B 99 6.147 35.842 56.431 0.00 0.00
B
ATOM 4884 N ILE B 100 5.599 36.150 54.291 0.00 0.00
B
ATOM 4885 HN ILE B 100 5.281 36.855 53.661 0.00 0.00
B
ATOM 4886 CA ILE B 100 5.331 34.763 53.980 0.00 0.00
B

```
ATOM   4887  HA  ILE B 100       5.530  34.152  54.849  0.00  0.00
B
ATOM   4888  CB  ILE B 100       3.868  34.513  53.566  0.00  0.00
B
ATOM   4889  HB  ILE B 100       3.791  34.997  52.568  0.00  0.00
B
ATOM   4890  CG2 ILE B 100       3.549  33.005  53.462  0.00  0.00
B
ATOM   4891 HG21 ILE B 100       4.286  32.567  52.755  0.00  0.00
B
ATOM   4892 HG22 ILE B 100       3.712  32.463  54.417  0.00  0.00
B
ATOM   4893 HG23 ILE B 100       2.564  32.796  52.992  0.00  0.00
B
ATOM   4894  CG1 ILE B 100       2.848  35.243  54.460  0.00  0.00
B
ATOM   4895 HG11 ILE B 100       2.864  34.916  55.522  0.00  0.00
B
ATOM   4896 HG12 ILE B 100       3.082  36.329  54.479  0.00  0.00
B
ATOM   4897  CD  ILE B 100       1.412  35.358  53.935  0.00  0.00
B
ATOM   4898  HD1 ILE B 100       0.722  35.819  54.673  0.00  0.00
B
ATOM   4899  HD2 ILE B 100       1.469  35.920  52.978  0.00  0.00
B
ATOM   4900  HD3 ILE B 100       0.965  34.351  53.793  0.00  0.00
B
ATOM   4901  C   ILE B 100       6.163  34.331  52.851  0.00  0.00
B
ATOM   4902  O   ILE B 100       6.202  34.884  51.744  0.00  0.00
B
ATOM   4903  N   TRP B 101       7.036  33.299  53.149  0.00  0.00
B
ATOM   4904  HN  TRP B 101       7.043  32.835  54.030  0.00  0.00
B
ATOM   4905  CA  TRP B 101       7.963  32.818  52.124  0.00  0.00
B
ATOM   4906  HA  TRP B 101       8.080  33.305  51.167  0.00  0.00
B
ATOM   4907  CB  TRP B 101       9.410  32.802  52.730  0.00  0.00
B
ATOM   4908  HB1 TRP B 101       9.421  32.255  53.697  0.00  0.00
B
ATOM   4909  HB2 TRP B 101      10.210  32.301  52.145  0.00  0.00
B
ATOM   4910  CG  TRP B 101      10.044  34.115  53.146  0.00  0.00
B
ATOM   4911  CD1 TRP B 101       9.632  35.436  53.083  0.00  0.00
B
ATOM   4912  HD1 TRP B 101       8.566  35.606  53.049  0.00  0.00
B
ATOM   4913  NE1 TRP B 101      10.554  36.291  53.665  0.00  0.00
B
```

```
ATOM   4914  HE1 TRP B 101      10.613  37.260  53.767  0.00  0.00
B
ATOM   4915  CE2 TRP B 101      11.686  35.573  53.825  0.00  0.00
B
ATOM   4916  CD2 TRP B 101      11.468  34.232  53.455  0.00  0.00
B
ATOM   4917  CE3 TRP B 101      12.461  33.256  53.508  0.00  0.00
B
ATOM   4918  HE3 TRP B 101      12.201  32.262  53.173  0.00  0.00
B
ATOM   4919  CZ3 TRP B 101      13.767  33.611  53.892  0.00  0.00
B
ATOM   4920  HZ3 TRP B 101      14.637  32.978  53.980  0.00  0.00
B
ATOM   4921  CZ2 TRP B 101      12.933  35.886  54.325  0.00  0.00
B
ATOM   4922  HZ2 TRP B 101      13.204  36.830  54.773  0.00  0.00
B
ATOM   4923  CH2 TRP B 101      13.973  34.930  54.355  0.00  0.00
B
ATOM   4924  HH2 TRP B 101      14.899  35.247  54.811  0.00  0.00
B
ATOM   4925  C   TRP B 101       7.625  31.370  51.915  0.00  0.00
B
ATOM   4926  O   TRP B 101       7.519  30.575  52.862  0.00  0.00
B
ATOM   4927  N   VAL B 102       7.417  30.934  50.598  0.00  0.00
B
ATOM   4928  HN  VAL B 102       7.491  31.541  49.810  0.00  0.00
B
ATOM   4929  CA  VAL B 102       6.891  29.610  50.348  0.00  0.00
B
ATOM   4930  HA  VAL B 102       6.882  29.064  51.280  0.00  0.00
B
ATOM   4931  CB  VAL B 102       5.395  29.805  50.034  0.00  0.00
B
ATOM   4932  HB  VAL B 102       4.930  30.431  50.825  0.00  0.00
B
ATOM   4933  CG1 VAL B 102       5.202  30.720  48.790  0.00  0.00
B
ATOM   4934 HG11 VAL B 102       5.411  31.756  49.132  0.00  0.00
B
ATOM   4935 HG12 VAL B 102       5.871  30.326  47.996  0.00  0.00
B
ATOM   4936 HG13 VAL B 102       4.133  30.602  48.510  0.00  0.00
B
ATOM   4937  CG2 VAL B 102       4.713  28.378  49.951  0.00  0.00
B
ATOM   4938 HG21 VAL B 102       5.064  27.690  50.749  0.00  0.00
B
ATOM   4939 HG22 VAL B 102       3.609  28.502  49.952  0.00  0.00
B
ATOM   4940 HG23 VAL B 102       5.041  27.815  49.051  0.00  0.00
B
```

ATOM 4941 C VAL B 102 7.544 28.745 49.252 0.00 0.00
B
ATOM 4942 O VAL B 102 7.889 29.266 48.198 0.00 0.00
B
ATOM 4943 N LYS B 103 7.834 27.466 49.532 0.00 0.00
B
ATOM 4944 HN LYS B 103 7.661 27.012 50.402 0.00 0.00
B
ATOM 4945 CA LYS B 103 8.412 26.538 48.566 0.00 0.00
B
ATOM 4946 HA LYS B 103 9.439 26.838 48.414 0.00 0.00
B
ATOM 4947 CB LYS B 103 8.517 25.205 49.385 0.00 0.00
B
ATOM 4948 HB1 LYS B 103 9.010 25.388 50.363 0.00 0.00
B
ATOM 4949 HB2 LYS B 103 7.503 24.754 49.446 0.00 0.00
B
ATOM 4950 CG LYS B 103 9.356 24.131 48.640 0.00 0.00
B
ATOM 4951 HG1 LYS B 103 8.828 23.982 47.674 0.00 0.00
B
ATOM 4952 HG2 LYS B 103 10.288 24.626 48.294 0.00 0.00
B
ATOM 4953 CD LYS B 103 9.732 22.824 49.358 0.00 0.00
B
ATOM 4954 HD1 LYS B 103 10.822 22.711 49.177 0.00 0.00
B
ATOM 4955 HD2 LYS B 103 9.619 22.938 50.457 0.00 0.00
B
ATOM 4956 CE LYS B 103 8.948 21.580 48.866 0.00 0.00
B
ATOM 4957 HE1 LYS B 103 7.949 21.469 49.340 0.00 0.00
B
ATOM 4958 HE2 LYS B 103 8.798 21.510 47.768 0.00 0.00
B
ATOM 4959 NZ LYS B 103 9.671 20.369 49.148 0.00 0.00
B
ATOM 4960 HZ1 LYS B 103 9.540 20.027 50.121 0.00 0.00
B
ATOM 4961 HZ2 LYS B 103 9.310 19.573 48.584 0.00 0.00
B
ATOM 4962 HZ3 LYS B 103 10.696 20.463 49.002 0.00 0.00
B
ATOM 4963 C LYS B 103 7.566 26.285 47.353 0.00 0.00
B
ATOM 4964 O LYS B 103 6.550 25.571 47.461 0.00 0.00
B
ATOM 4965 N ALA B 104 7.858 26.815 46.139 0.00 0.00
B
ATOM 4966 HN ALA B 104 8.639 27.424 46.028 0.00 0.00
B
ATOM 4967 CA ALA B 104 7.087 26.390 44.936 0.00 0.00
B

ATOM 4968 HA ALA B 104 6.150 26.025 45.329 0.00 0.00
B
ATOM 4969 CB ALA B 104 6.680 27.656 44.115 0.00 0.00
B
ATOM 4970 HB1 ALA B 104 6.206 27.294 43.177 0.00 0.00
B
ATOM 4971 HB2 ALA B 104 5.952 28.302 44.650 0.00 0.00
B
ATOM 4972 HB3 ALA B 104 7.570 28.262 43.840 0.00 0.00
B
ATOM 4973 C ALA B 104 7.741 25.274 44.179 0.00 0.00
B
ATOM 4974 O ALA B 104 8.943 25.373 43.810 0.00 0.00
B
ATOM 4975 N GLN B 105 7.044 24.096 44.034 0.00 0.00
B
ATOM 4976 HN GLN B 105 6.166 24.097 44.506 0.00 0.00
B
ATOM 4977 CA GLN B 105 7.446 22.905 43.337 0.00 0.00
B
ATOM 4978 HA GLN B 105 8.388 22.568 43.746 0.00 0.00
B
ATOM 4979 CB GLN B 105 6.391 21.797 43.591 0.00 0.00
B
ATOM 4980 HB1 GLN B 105 6.385 21.683 44.696 0.00 0.00
B
ATOM 4981 HB2 GLN B 105 5.340 22.055 43.339 0.00 0.00
B
ATOM 4982 CG GLN B 105 6.748 20.332 43.174 0.00 0.00
B
ATOM 4983 HG1 GLN B 105 5.870 19.763 43.549 0.00 0.00
B
ATOM 4984 HG2 GLN B 105 6.885 20.162 42.084 0.00 0.00
B
ATOM 4985 CD GLN B 105 7.977 19.703 43.948 0.00 0.00
B
ATOM 4986 OE1 GLN B 105 8.084 19.690 45.130 0.00 0.00
B
ATOM 4987 NE2 GLN B 105 8.916 19.006 43.217 0.00 0.00
B
ATOM 4988 HE21 GLN B 105 9.566 18.390 43.662 0.00 0.00
B
ATOM 4989 HE22 GLN B 105 8.919 19.038 42.218 0.00 0.00
B
ATOM 4990 C GLN B 105 7.576 23.119 41.785 0.00 0.00
B
ATOM 4991 O GLN B 105 8.429 22.500 41.157 0.00 0.00
B
ATOM 4992 N ASN B 106 6.708 23.957 41.273 0.00 0.00
B
ATOM 4993 HN ASN B 106 6.083 24.482 41.845 0.00 0.00
B
ATOM 4994 CA ASN B 106 6.601 24.265 39.836 0.00 0.00
B

ATOM 4995 HA ASN B 106 7.615 24.286 39.465 0.00 0.00 B
ATOM 4996 CB ASN B 106 5.761 23.099 39.159 0.00 0.00 B
ATOM 4997 HB1 ASN B 106 5.538 23.242 38.080 0.00 0.00 B
ATOM 4998 HB2 ASN B 106 6.367 22.167 39.145 0.00 0.00 B
ATOM 4999 CG ASN B 106 4.424 22.791 39.771 0.00 0.00 B
ATOM 5000 OD1 ASN B 106 3.532 23.635 39.726 0.00 0.00 B
ATOM 5001 ND2 ASN B 106 4.283 21.556 40.386 0.00 0.00 B
ATOM 5002 HD21 ASN B 106 3.392 21.396 40.813 0.00 0.00 B
ATOM 5003 HD22 ASN B 106 5.096 20.981 40.474 0.00 0.00 B
ATOM 5004 C ASN B 106 5.991 25.585 39.549 0.00 0.00 B
ATOM 5005 O ASN B 106 5.606 26.352 40.398 0.00 0.00 B
ATOM 5006 N TYR B 107 6.096 26.061 38.290 0.00 0.00 B
ATOM 5007 HN TYR B 107 6.839 25.619 37.792 0.00 0.00 B
ATOM 5008 CA TYR B 107 5.623 27.332 37.827 0.00 0.00 B
ATOM 5009 HA TYR B 107 6.039 27.993 38.573 0.00 0.00 B
ATOM 5010 CB TYR B 107 6.100 27.601 36.371 0.00 0.00 B
ATOM 5011 HB1 TYR B 107 5.885 26.766 35.671 0.00 0.00 B
ATOM 5012 HB2 TYR B 107 5.551 28.496 36.007 0.00 0.00 B
ATOM 5013 CG TYR B 107 7.625 27.694 36.312 0.00 0.00 B
ATOM 5014 CD1 TYR B 107 8.289 28.588 37.163 0.00 0.00 B
ATOM 5015 HD1 TYR B 107 7.864 29.302 37.852 0.00 0.00 B
ATOM 5016 CE1 TYR B 107 9.654 28.801 37.001 0.00 0.00 B
ATOM 5017 HE1 TYR B 107 10.129 29.491 37.683 0.00 0.00 B
ATOM 5018 CZ TYR B 107 10.343 28.184 35.977 0.00 0.00 B
ATOM 5019 OH TYR B 107 11.697 28.427 35.907 0.00 0.00 B
ATOM 5020 HH TYR B 107 12.054 27.799 35.275 0.00 0.00 B
ATOM 5021 CD2 TYR B 107 8.327 26.977 35.293 0.00 0.00 B

ATOM 5022 HD2 TYR B 107 7.728 26.270 34.739 0.00 0.00
B
ATOM 5023 CE2 TYR B 107 9.727 27.252 35.100 0.00 0.00
B
ATOM 5024 HE2 TYR B 107 10.367 26.687 34.439 0.00 0.00
B
ATOM 5025 C TYR B 107 4.109 27.525 37.984 0.00 0.00
B
ATOM 5026 O TYR B 107 3.665 28.559 38.620 0.00 0.00
B
ATOM 5027 N TYR B 108 3.194 26.561 37.616 0.00 0.00
B
ATOM 5028 HN TYR B 108 3.474 25.804 37.031 0.00 0.00
B
ATOM 5029 CA TYR B 108 1.784 26.694 37.777 0.00 0.00
B
ATOM 5030 HA TYR B 108 1.608 27.697 37.417 0.00 0.00
B
ATOM 5031 CB TYR B 108 0.849 25.736 37.045 0.00 0.00
B
ATOM 5032 HB1 TYR B 108 1.330 24.791 37.377 0.00 0.00
B
ATOM 5033 HB2 TYR B 108 -0.202 25.710 37.406 0.00 0.00
B
ATOM 5034 CG TYR B 108 0.914 25.822 35.557 0.00 0.00
B
ATOM 5035 CD1 TYR B 108 0.672 27.030 34.879 0.00 0.00
B
ATOM 5036 HD1 TYR B 108 0.705 27.977 35.397 0.00 0.00
B
ATOM 5037 CE1 TYR B 108 0.617 26.989 33.501 0.00 0.00
B
ATOM 5038 HE1 TYR B 108 0.257 27.868 32.987 0.00 0.00
B
ATOM 5039 CZ TYR B 108 0.445 25.780 32.773 0.00 0.00
B
ATOM 5040 OH TYR B 108 0.454 25.879 31.417 0.00 0.00
B
ATOM 5041 HH TYR B 108 0.565 25.017 31.010 0.00 0.00
B
ATOM 5042 CD2 TYR B 108 0.890 24.559 34.877 0.00 0.00
B
ATOM 5043 HD2 TYR B 108 0.796 23.625 35.411 0.00 0.00
B
ATOM 5044 CE2 TYR B 108 0.689 24.599 33.495 0.00 0.00
B
ATOM 5045 HE2 TYR B 108 0.629 23.582 33.138 0.00 0.00
B
ATOM 5046 C TYR B 108 1.408 26.838 39.270 0.00 0.00
B
ATOM 5047 O TYR B 108 0.649 27.762 39.730 0.00 0.00
B
ATOM 5048 N HSE B 109 2.068 26.039 40.178 0.00 0.00
B

ATOM	5049	HN	HSE	B	109	2.667	25.368	39.748	0.00	0.00	B
ATOM	5050	CA	HSE	B	109	1.965	26.308	41.707	0.00	0.00	B
ATOM	5051	HA	HSE	B	109	0.935	26.181	42.007	0.00	0.00	B
ATOM	5052	CB	HSE	B	109	2.878	25.222	42.335	0.00	0.00	B
ATOM	5053	HB1	HSE	B	109	2.537	24.232	41.964	0.00	0.00	B
ATOM	5054	HB2	HSE	B	109	3.921	25.480	42.054	0.00	0.00	B
ATOM	5055	ND1	HSE	B	109	4.069	24.511	44.290	0.00	0.00	B
ATOM	5056	CG	HSE	B	109	2.966	25.114	43.795	0.00	0.00	B
ATOM	5057	CE1	HSE	B	109	3.941	24.584	45.611	0.00	0.00	B
ATOM	5058	HE1	HSE	B	109	4.738	24.203	46.251	0.00	0.00	B
ATOM	5059	NE2	HSE	B	109	2.837	25.263	45.978	0.00	0.00	B
ATOM	5060	HE2	HSE	B	109	2.592	25.466	46.926	0.00	0.00	B
ATOM	5061	CD2	HSE	B	109	2.175	25.604	44.760	0.00	0.00	B
ATOM	5062	HD2	HSE	B	109	1.347	26.294	44.651	0.00	0.00	B
ATOM	5063	C	HSE	B	109	2.394	27.685	42.144	0.00	0.00	B
ATOM	5064	O	HSE	B	109	1.675	28.417	42.852	0.00	0.00	B
ATOM	5065	N	HSE	B	110	3.603	28.133	41.715	0.00	0.00	B
ATOM	5066	HN	HSE	B	110	4.215	27.532	41.207	0.00	0.00	B
ATOM	5067	CA	HSE	B	110	4.099	29.475	41.985	0.00	0.00	B
ATOM	5068	HA	HSE	B	110	4.306	29.640	43.032	0.00	0.00	B
ATOM	5069	CB	HSE	B	110	5.510	29.735	41.331	0.00	0.00	B
ATOM	5070	HB1	HSE	B	110	6.327	29.329	41.965	0.00	0.00	B
ATOM	5071	HB2	HSE	B	110	5.688	29.004	40.513	0.00	0.00	B
ATOM	5072	ND1	HSE	B	110	6.955	31.025	39.936	0.00	0.00	B
ATOM	5073	CG	HSE	B	110	5.923	31.107	40.855	0.00	0.00	B
ATOM	5074	CE1	HSE	B	110	7.306	32.304	39.764	0.00	0.00	B
ATOM	5075	HE1	HSE	B	110	7.995	32.682	39.009	0.00	0.00	B

ATOM	5076	NE2	HSE	B	110	6.567	33.119	40.449	0.00	0.00	B
ATOM	5077	HE2	HSE	B	110	6.515	34.117	40.441	0.00	0.00	B
ATOM	5078	CD2	HSE	B	110	5.634	32.411	41.186	0.00	0.00	B
ATOM	5079	HD2	HSE	B	110	4.805	32.782	41.775	0.00	0.00	B
ATOM	5080	C	HSE	B	110	3.183	30.536	41.548	0.00	0.00	B
ATOM	5081	O	HSE	B	110	2.978	31.574	42.253	0.00	0.00	B
ATOM	5082	N	LYS	B	111	2.595	30.447	40.311	0.00	0.00	B
ATOM	5083	HN	LYS	B	111	2.772	29.596	39.823	0.00	0.00	B
ATOM	5084	CA	LYS	B	111	1.511	31.394	39.852	0.00	0.00	B
ATOM	5085	HA	LYS	B	111	1.895	32.387	40.033	0.00	0.00	B
ATOM	5086	CB	LYS	B	111	1.350	31.172	38.314	0.00	0.00	B
ATOM	5087	HB1	LYS	B	111	1.265	30.078	38.140	0.00	0.00	B
ATOM	5088	HB2	LYS	B	111	0.403	31.707	38.086	0.00	0.00	B
ATOM	5089	CG	LYS	B	111	2.401	31.740	37.399	0.00	0.00	B
ATOM	5090	HG1	LYS	B	111	3.380	31.394	37.795	0.00	0.00	B
ATOM	5091	HG2	LYS	B	111	2.191	31.328	36.389	0.00	0.00	B
ATOM	5092	CD	LYS	B	111	2.270	33.262	37.346	0.00	0.00	B
ATOM	5093	HD1	LYS	B	111	1.301	33.464	36.842	0.00	0.00	B
ATOM	5094	HD2	LYS	B	111	2.171	33.605	38.398	0.00	0.00	B
ATOM	5095	CE	LYS	B	111	3.533	34.028	36.808	0.00	0.00	B
ATOM	5096	HE1	LYS	B	111	4.366	33.607	37.411	0.00	0.00	B
ATOM	5097	HE2	LYS	B	111	3.495	33.759	35.731	0.00	0.00	B
ATOM	5098	NZ	LYS	B	111	3.470	35.428	36.826	0.00	0.00	B
ATOM	5099	HZ1	LYS	B	111	3.022	35.731	37.714	0.00	0.00	B
ATOM	5100	HZ2	LYS	B	111	4.387	35.904	36.947	0.00	0.00	B
ATOM	5101	HZ3	LYS	B	111	3.019	35.939	36.041	0.00	0.00	B
ATOM	5102	C	LYS	B	111	0.205	31.249	40.587	0.00	0.00	B

ATOM 5103 O LYS B 111 -0.436 32.280 40.750 0.00 0.00
B
ATOM 5104 N VAL B 112 -0.274 30.123 41.111 0.00 0.00
B
ATOM 5105 HN VAL B 112 0.187 29.261 40.914 0.00 0.00
B
ATOM 5106 CA VAL B 112 -1.344 30.063 42.121 0.00 0.00
B
ATOM 5107 HA VAL B 112 -2.196 30.568 41.689 0.00 0.00
B
ATOM 5108 CB VAL B 112 -1.765 28.666 42.442 0.00 0.00
B
ATOM 5109 HB VAL B 112 -0.980 28.119 43.007 0.00 0.00
B
ATOM 5110 CG1 VAL B 112 -2.897 28.763 43.436 0.00 0.00
B
ATOM 5111 HG11 VAL B 112 -2.614 29.295 44.370 0.00 0.00
B
ATOM 5112 HG12 VAL B 112 -3.760 29.329 43.024 0.00 0.00
B
ATOM 5113 HG13 VAL B 112 -3.180 27.747 43.786 0.00 0.00
B
ATOM 5114 CG2 VAL B 112 -2.271 27.933 41.135 0.00 0.00
B
ATOM 5115 HG21 VAL B 112 -3.328 28.192 40.910 0.00 0.00
B
ATOM 5116 HG22 VAL B 112 -1.682 28.080 40.205 0.00 0.00
B
ATOM 5117 HG23 VAL B 112 -2.333 26.861 41.420 0.00 0.00
B
ATOM 5118 C VAL B 112 -0.890 30.842 43.426 0.00 0.00
B
ATOM 5119 O VAL B 112 -1.584 31.719 43.907 0.00 0.00
B
ATOM 5120 N LEU B 113 0.290 30.594 44.035 0.00 0.00
B
ATOM 5121 HN LEU B 113 0.822 29.783 43.806 0.00 0.00
B
ATOM 5122 CA LEU B 113 0.883 31.357 45.161 0.00 0.00
B
ATOM 5123 HA LEU B 113 0.188 31.225 45.977 0.00 0.00
B
ATOM 5124 CB LEU B 113 2.171 30.756 45.614 0.00 0.00
B
ATOM 5125 HB1 LEU B 113 2.897 30.692 44.776 0.00 0.00
B
ATOM 5126 HB2 LEU B 113 2.711 31.363 46.372 0.00 0.00
B
ATOM 5127 CG LEU B 113 2.099 29.286 46.172 0.00 0.00
B
ATOM 5128 HG LEU B 113 1.431 28.689 45.515 0.00 0.00
B
ATOM 5129 CD1 LEU B 113 3.466 28.564 46.253 0.00 0.00
B

ATOM 5130 HD11 LEU B 113 4.208 29.304 46.624 0.00 0.00
B
ATOM 5131 HD12 LEU B 113 3.394 27.662 46.898 0.00 0.00
B
ATOM 5132 HD13 LEU B 113 3.795 28.247 45.240 0.00 0.00
B
ATOM 5133 CD2 LEU B 113 1.485 29.316 47.523 0.00 0.00
B
ATOM 5134 HD21 LEU B 113 2.070 29.800 48.335 0.00 0.00
B
ATOM 5135 HD22 LEU B 113 0.484 29.793 47.589 0.00 0.00
B
ATOM 5136 HD23 LEU B 113 1.160 28.317 47.885 0.00 0.00
B
ATOM 5137 C LEU B 113 1.082 32.850 44.987 0.00 0.00
B
ATOM 5138 O LEU B 113 1.075 33.624 45.930 0.00 0.00
B
ATOM 5139 N GLU B 114 1.244 33.346 43.680 0.00 0.00
B
ATOM 5140 HN GLU B 114 1.234 32.661 42.955 0.00 0.00
B
ATOM 5141 CA GLU B 114 1.266 34.746 43.264 0.00 0.00
B
ATOM 5142 HA GLU B 114 1.922 35.280 43.936 0.00 0.00
B
ATOM 5143 CB GLU B 114 1.870 34.917 41.884 0.00 0.00
B
ATOM 5144 HB1 GLU B 114 2.670 34.163 41.723 0.00 0.00
B
ATOM 5145 HB2 GLU B 114 1.098 34.630 41.139 0.00 0.00
B
ATOM 5146 CG GLU B 114 2.322 36.310 41.565 0.00 0.00
B
ATOM 5147 HG1 GLU B 114 1.461 37.011 41.594 0.00 0.00
B
ATOM 5148 HG2 GLU B 114 3.059 36.719 42.288 0.00 0.00
B
ATOM 5149 CD GLU B 114 2.970 36.355 40.162 0.00 0.00
B
ATOM 5150 OE1 GLU B 114 2.197 36.798 39.244 0.00 0.00
B
ATOM 5151 OE2 GLU B 114 4.149 36.005 39.966 0.00 0.00
B
ATOM 5152 C GLU B 114 −0.075 35.477 43.325 0.00 0.00
B
ATOM 5153 O GLU B 114 −0.112 36.632 43.674 0.00 0.00
B
ATOM 5154 N LYS B 115 −1.178 34.815 42.857 0.00 0.00
B
ATOM 5155 HN LYS B 115 −1.029 33.987 42.322 0.00 0.00
B
ATOM 5156 CA LYS B 115 −2.495 35.357 43.008 0.00 0.00
B

ATOM 5157 HA LYS B 115 -2.615 36.370 42.653 0.00 0.00
B
ATOM 5158 CB LYS B 115 -3.543 34.494 42.216 0.00 0.00
B
ATOM 5159 HB1 LYS B 115 -3.441 33.429 42.517 0.00 0.00
B
ATOM 5160 HB2 LYS B 115 -4.621 34.691 42.400 0.00 0.00
B
ATOM 5161 CG LYS B 115 -3.302 34.446 40.708 0.00 0.00
B
ATOM 5162 HG1 LYS B 115 -2.214 34.453 40.481 0.00 0.00
B
ATOM 5163 HG2 LYS B 115 -3.776 33.483 40.423 0.00 0.00
B
ATOM 5164 CD LYS B 115 -4.123 35.685 40.096 0.00 0.00
B
ATOM 5165 HD1 LYS B 115 -5.109 35.774 40.600 0.00 0.00
B
ATOM 5166 HD2 LYS B 115 -3.632 36.646 40.361 0.00 0.00
B
ATOM 5167 CE LYS B 115 -4.509 35.474 38.639 0.00 0.00
B
ATOM 5168 HE1 LYS B 115 -4.812 34.409 38.544 0.00 0.00
B
ATOM 5169 HE2 LYS B 115 -5.455 36.044 38.517 0.00 0.00
B
ATOM 5170 NZ LYS B 115 -3.469 35.800 37.673 0.00 0.00
B
ATOM 5171 HZ1 LYS B 115 -3.888 35.799 36.721 0.00 0.00
B
ATOM 5172 HZ2 LYS B 115 -2.853 36.611 37.883 0.00 0.00
B
ATOM 5173 HZ3 LYS B 115 -2.738 35.061 37.621 0.00 0.00
B
ATOM 5174 C LYS B 115 -2.945 35.365 44.465 0.00 0.00
B
ATOM 5175 O LYS B 115 -3.561 36.312 44.991 0.00 0.00
B
ATOM 5176 N ILE B 116 -2.572 34.273 45.254 0.00 0.00
B
ATOM 5177 HN ILE B 116 -2.149 33.447 44.889 0.00 0.00
B
ATOM 5178 CA ILE B 116 -2.817 34.342 46.722 0.00 0.00
B
ATOM 5179 HA ILE B 116 -3.878 34.521 46.812 0.00 0.00
B
ATOM 5180 CB ILE B 116 -2.536 32.998 47.396 0.00 0.00
B
ATOM 5181 HB ILE B 116 -1.461 32.718 47.370 0.00 0.00
B
ATOM 5182 CG2 ILE B 116 -2.972 32.994 48.924 0.00 0.00
B
ATOM 5183 HG21 ILE B 116 -4.072 33.106 49.033 0.00 0.00
B

ATOM 5184 HG22 ILE B 116 -2.727 32.061 49.475 0.00 0.00
B
ATOM 5185 HG23 ILE B 116 -2.505 33.839 49.473 0.00 0.00
B
ATOM 5186 CG1 ILE B 116 -3.383 31.892 46.771 0.00 0.00
B
ATOM 5187 HG11 ILE B 116 -4.428 32.079 47.099 0.00 0.00
B
ATOM 5188 HG12 ILE B 116 -3.391 31.928 45.660 0.00 0.00
B
ATOM 5189 CD ILE B 116 -3.065 30.402 47.116 0.00 0.00
B
ATOM 5190 HD1 ILE B 116 -3.228 30.203 48.197 0.00 0.00
B
ATOM 5191 HD2 ILE B 116 -3.746 29.734 46.547 0.00 0.00
B
ATOM 5192 HD3 ILE B 116 -2.032 30.146 46.796 0.00 0.00
B
ATOM 5193 C ILE B 116 -2.058 35.454 47.394 0.00 0.00
B
ATOM 5194 O ILE B 116 -2.578 36.203 48.202 0.00 0.00
B
ATOM 5195 N GLY B 117 -0.742 35.542 47.058 0.00 0.00
B
ATOM 5196 HN GLY B 117 -0.279 34.791 46.595 0.00 0.00
B
ATOM 5197 CA GLY B 117 0.155 36.602 47.415 0.00 0.00
B
ATOM 5198 HA1 GLY B 117 -0.451 37.452 47.692 0.00 0.00
B
ATOM 5199 HA2 GLY B 117 0.763 36.938 46.588 0.00 0.00
B
ATOM 5200 C GLY B 117 1.083 36.206 48.423 0.00 0.00
B
ATOM 5201 O GLY B 117 1.003 36.646 49.554 0.00 0.00
B
ATOM 5202 N ALA B 118 2.053 35.466 48.025 0.00 0.00
B
ATOM 5203 HN ALA B 118 2.112 35.095 47.102 0.00 0.00
B
ATOM 5204 CA ALA B 118 3.275 35.156 48.801 0.00 0.00
B
ATOM 5205 HA ALA B 118 3.081 35.308 49.852 0.00 0.00
B
ATOM 5206 CB ALA B 118 3.611 33.664 48.819 0.00 0.00
B
ATOM 5207 HB1 ALA B 118 4.594 33.499 49.310 0.00 0.00
B
ATOM 5208 HB2 ALA B 118 2.859 33.125 49.433 0.00 0.00
B
ATOM 5209 HB3 ALA B 118 3.703 33.297 47.774 0.00 0.00
B
ATOM 5210 C ALA B 118 4.483 36.033 48.446 0.00 0.00
B

ATOM 5211 O ALA B 118 4.742 36.333 47.298 0.00 0.00
B
ATOM 5212 N ASP B 119 5.073 36.568 49.500 0.00 0.00
B
ATOM 5213 HN ASP B 119 4.996 36.150 50.402 0.00 0.00
B
ATOM 5214 CA ASP B 119 6.049 37.592 49.491 0.00 0.00
B
ATOM 5215 HA ASP B 119 5.607 38.435 48.980 0.00 0.00
B
ATOM 5216 CB ASP B 119 6.311 38.077 50.928 0.00 0.00
B
ATOM 5217 HB1 ASP B 119 6.680 37.252 51.573 0.00 0.00
B
ATOM 5218 HB2 ASP B 119 7.076 38.877 51.027 0.00 0.00
B
ATOM 5219 CG ASP B 119 4.982 38.661 51.452 0.00 0.00
B
ATOM 5220 OD1 ASP B 119 4.658 38.392 52.634 0.00 0.00
B
ATOM 5221 OD2 ASP B 119 4.283 39.436 50.745 0.00 0.00
B
ATOM 5222 C ASP B 119 7.305 37.292 48.752 0.00 0.00
B
ATOM 5223 O ASP B 119 7.865 38.093 47.994 0.00 0.00
B
ATOM 5224 N ARG B 120 7.748 36.098 48.930 0.00 0.00
B
ATOM 5225 HN ARG B 120 7.204 35.496 49.509 0.00 0.00
B
ATOM 5226 CA ARG B 120 8.964 35.614 48.288 0.00 0.00
B
ATOM 5227 HA ARG B 120 9.266 36.279 47.492 0.00 0.00
B
ATOM 5228 CB ARG B 120 10.095 35.407 49.395 0.00 0.00
B
ATOM 5229 HB1 ARG B 120 9.878 36.311 50.002 0.00 0.00
B
ATOM 5230 HB2 ARG B 120 9.816 34.496 49.967 0.00 0.00
B
ATOM 5231 CG ARG B 120 11.511 35.438 48.898 0.00 0.00
B
ATOM 5232 HG1 ARG B 120 11.794 34.518 48.342 0.00 0.00
B
ATOM 5233 HG2 ARG B 120 11.601 36.379 48.314 0.00 0.00
B
ATOM 5234 CD ARG B 120 12.522 35.519 50.016 0.00 0.00
B
ATOM 5235 HD1 ARG B 120 12.276 34.730 50.759 0.00 0.00
B
ATOM 5236 HD2 ARG B 120 13.544 35.387 49.601 0.00 0.00
B
ATOM 5237 NE ARG B 120 12.295 36.848 50.695 0.00 0.00
B

ATOM 5238 HE ARG B 120 11.440 37.341 50.538 0.00 0.00
B
ATOM 5239 CZ ARG B 120 13.290 37.636 51.211 0.00 0.00
B
ATOM 5240 NH1 ARG B 120 14.543 37.234 51.178 0.00 0.00
B
ATOM 5241 HH11 ARG B 120 15.217 37.844 51.595 0.00 0.00
B
ATOM 5242 HH12 ARG B 120 14.760 36.309 50.864 0.00 0.00
B
ATOM 5243 NH2 ARG B 120 12.928 38.820 51.710 0.00 0.00
B
ATOM 5244 HH21 ARG B 120 13.654 39.370 52.123 0.00 0.00
B
ATOM 5245 HH22 ARG B 120 11.942 38.973 51.776 0.00 0.00
B
ATOM 5246 C ARG B 120 8.699 34.122 47.732 0.00 0.00
B
ATOM 5247 O ARG B 120 8.255 33.293 48.504 0.00 0.00
B
ATOM 5248 N ILE B 121 8.851 33.869 46.386 0.00 0.00
B
ATOM 5249 HN ILE B 121 9.146 34.571 45.743 0.00 0.00
B
ATOM 5250 CA ILE B 121 8.754 32.492 45.885 0.00 0.00
B
ATOM 5251 HA ILE B 121 8.095 31.918 46.519 0.00 0.00
B
ATOM 5252 CB ILE B 121 8.165 32.414 44.519 0.00 0.00
B
ATOM 5253 HB ILE B 121 8.774 33.014 43.810 0.00 0.00
B
ATOM 5254 CG2 ILE B 121 8.180 30.898 44.101 0.00 0.00
B
ATOM 5255 HG21 ILE B 121 7.801 30.243 44.915 0.00 0.00
B
ATOM 5256 HG22 ILE B 121 7.464 30.845 43.253 0.00 0.00
B
ATOM 5257 HG23 ILE B 121 9.249 30.639 43.947 0.00 0.00
B
ATOM 5258 CG1 ILE B 121 6.646 32.973 44.516 0.00 0.00
B
ATOM 5259 HG11 ILE B 121 6.636 34.031 44.854 0.00 0.00
B
ATOM 5260 HG12 ILE B 121 6.426 32.999 43.427 0.00 0.00
B
ATOM 5261 CD ILE B 121 5.614 32.300 45.416 0.00 0.00
B
ATOM 5262 HD1 ILE B 121 5.115 31.431 44.936 0.00 0.00
B
ATOM 5263 HD2 ILE B 121 5.940 32.199 46.473 0.00 0.00
B
ATOM 5264 HD3 ILE B 121 4.836 33.088 45.497 0.00 0.00
B

ATOM 5265 C ILE B 121 10.097 31.872 45.946 0.00 0.00
B
ATOM 5266 O ILE B 121 11.098 32.477 45.564 0.00 0.00
B
ATOM 5267 N ILE B 122 10.091 30.660 46.530 0.00 0.00
B
ATOM 5268 HN ILE B 122 9.234 30.260 46.846 0.00 0.00
B
ATOM 5269 CA ILE B 122 11.251 29.786 46.850 0.00 0.00
B
ATOM 5270 HA ILE B 122 12.113 30.420 46.705 0.00 0.00
B
ATOM 5271 CB ILE B 122 11.399 29.347 48.285 0.00 0.00
B
ATOM 5272 HB ILE B 122 10.486 28.749 48.495 0.00 0.00
B
ATOM 5273 CG2 ILE B 122 12.728 28.630 48.592 0.00 0.00
B
ATOM 5274 HG21 ILE B 122 13.548 29.378 48.567 0.00 0.00
B
ATOM 5275 HG22 ILE B 122 12.864 28.144 49.582 0.00 0.00
B
ATOM 5276 HG23 ILE B 122 12.868 27.817 47.847 0.00 0.00
B
ATOM 5277 CG1 ILE B 122 11.268 30.566 49.329 0.00 0.00
B
ATOM 5278 HG11 ILE B 122 10.177 30.686 49.501 0.00 0.00
B
ATOM 5279 HG12 ILE B 122 11.823 30.308 50.255 0.00 0.00
B
ATOM 5280 CD ILE B 122 11.920 31.828 48.993 0.00 0.00
B
ATOM 5281 HD1 ILE B 122 12.867 31.615 48.451 0.00 0.00
B
ATOM 5282 HD2 ILE B 122 11.198 32.411 48.382 0.00 0.00
B
ATOM 5283 HD3 ILE B 122 12.253 32.374 49.902 0.00 0.00
B
ATOM 5284 C ILE B 122 11.359 28.589 45.985 0.00 0.00
B
ATOM 5285 O ILE B 122 10.639 27.616 46.231 0.00 0.00
B
ATOM 5286 N HSE B 123 12.241 28.644 45.010 0.00 0.00
B
ATOM 5287 HN HSE B 123 12.847 29.411 44.815 0.00 0.00
B
ATOM 5288 CA HSE B 123 12.321 27.579 44.020 0.00 0.00
B
ATOM 5289 HA HSE B 123 11.469 26.916 44.043 0.00 0.00
B
ATOM 5290 CB HSE B 123 12.403 28.221 42.591 0.00 0.00
B
ATOM 5291 HB1 HSE B 123 13.365 28.774 42.533 0.00 0.00
B

ATOM 5292 HB2 HSE B 123 12.409 27.346 41.907 0.00 0.00
B
ATOM 5293 ND1 HSE B 123 11.508 30.399 41.915 0.00 0.00
B
ATOM 5294 CG HSE B 123 11.284 29.072 42.259 0.00 0.00
B
ATOM 5295 CE1 HSE B 123 10.364 30.860 41.313 0.00 0.00
B
ATOM 5296 HE1 HSE B 123 10.202 31.864 40.921 0.00 0.00
B
ATOM 5297 NE2 HSE B 123 9.434 29.883 41.273 0.00 0.00
B
ATOM 5298 HE2 HSE B 123 8.598 29.971 40.730 0.00 0.00
B
ATOM 5299 CD2 HSE B 123 9.990 28.719 41.858 0.00 0.00
B
ATOM 5300 HD2 HSE B 123 9.607 27.711 41.758 0.00 0.00
B
ATOM 5301 C HSE B 123 13.481 26.566 44.306 0.00 0.00
B
ATOM 5302 O HSE B 123 14.626 26.893 43.886 0.00 0.00
B
ATOM 5303 N PRO B 124 13.393 25.402 44.985 0.00 0.00
B
ATOM 5304 CD PRO B 124 12.324 25.139 45.908 0.00 0.00
B
ATOM 5305 HD1 PRO B 124 12.179 26.003 46.590 0.00 0.00
B
ATOM 5306 HD2 PRO B 124 11.377 24.973 45.351 0.00 0.00
B
ATOM 5307 CA PRO B 124 14.397 24.391 45.081 0.00 0.00
B
ATOM 5308 HA PRO B 124 15.125 24.920 45.679 0.00 0.00
B
ATOM 5309 CB PRO B 124 13.626 23.214 45.751 0.00 0.00
B
ATOM 5310 HB1 PRO B 124 14.260 22.459 46.263 0.00 0.00
B
ATOM 5311 HB2 PRO B 124 13.043 22.664 44.982 0.00 0.00
B
ATOM 5312 CG PRO B 124 12.737 23.929 46.726 0.00 0.00
B
ATOM 5313 HG1 PRO B 124 13.285 24.260 47.634 0.00 0.00
B
ATOM 5314 HG2 PRO B 124 11.896 23.333 47.143 0.00 0.00
B
ATOM 5315 C PRO B 124 15.051 23.914 43.791 0.00 0.00
B
ATOM 5316 O PRO B 124 16.266 23.833 43.721 0.00 0.00
B
ATOM 5317 N GLU B 125 14.244 23.588 42.722 0.00 0.00
B
ATOM 5318 HN GLU B 125 13.253 23.692 42.767 0.00 0.00
B

ATOM 5319 CA GLU B 125 14.666 22.979 41.483 0.00 0.00
B
ATOM 5320 HA GLU B 125 14.907 21.951 41.710 0.00 0.00
B
ATOM 5321 CB GLU B 125 13.453 22.882 40.501 0.00 0.00
B
ATOM 5322 HB1 GLU B 125 12.559 22.490 41.031 0.00 0.00
B
ATOM 5323 HB2 GLU B 125 13.110 23.878 40.149 0.00 0.00
B
ATOM 5324 CG GLU B 125 13.530 21.816 39.360 0.00 0.00
B
ATOM 5325 HG1 GLU B 125 14.384 22.121 38.719 0.00 0.00
B
ATOM 5326 HG2 GLU B 125 13.805 20.823 39.778 0.00 0.00
B
ATOM 5327 CD GLU B 125 12.316 21.667 38.489 0.00 0.00
B
ATOM 5328 OE1 GLU B 125 11.706 22.690 38.162 0.00 0.00
B
ATOM 5329 OE2 GLU B 125 11.889 20.543 38.221 0.00 0.00
B
ATOM 5330 C GLU B 125 15.836 23.666 40.772 0.00 0.00
B
ATOM 5331 O GLU B 125 16.750 22.990 40.273 0.00 0.00
B
ATOM 5332 N LYS B 126 15.927 24.956 40.682 0.00 0.00
B
ATOM 5333 HN LYS B 126 15.132 25.539 40.828 0.00 0.00
B
ATOM 5334 CA LYS B 126 17.059 25.650 40.040 0.00 0.00
B
ATOM 5335 HA LYS B 126 17.148 25.214 39.056 0.00 0.00
B
ATOM 5336 CB LYS B 126 16.681 27.151 39.734 0.00 0.00
B
ATOM 5337 HB1 LYS B 126 16.025 27.149 38.838 0.00 0.00
B
ATOM 5338 HB2 LYS B 126 16.071 27.565 40.566 0.00 0.00
B
ATOM 5339 CG LYS B 126 17.836 28.189 39.473 0.00 0.00
B
ATOM 5340 HG1 LYS B 126 17.420 29.218 39.521 0.00 0.00
B
ATOM 5341 HG2 LYS B 126 18.571 28.007 40.287 0.00 0.00
B
ATOM 5342 CD LYS B 126 18.454 27.905 38.074 0.00 0.00
B
ATOM 5343 HD1 LYS B 126 18.824 26.867 37.929 0.00 0.00
B
ATOM 5344 HD2 LYS B 126 17.712 28.001 37.253 0.00 0.00
B
ATOM 5345 CE LYS B 126 19.583 28.853 37.723 0.00 0.00
B

ATOM 5346 HE1 LYS B 126 19.148 29.863 37.879 0.00 0.00
B
ATOM 5347 HE2 LYS B 126 20.489 28.614 38.320 0.00 0.00
B
ATOM 5348 NZ LYS B 126 19.966 28.906 36.268 0.00 0.00
B
ATOM 5349 HZ1 LYS B 126 19.380 29.528 35.675 0.00 0.00
B
ATOM 5350 HZ2 LYS B 126 20.974 29.115 36.123 0.00 0.00
B
ATOM 5351 HZ3 LYS B 126 19.866 27.963 35.841 0.00 0.00
B
ATOM 5352 C LYS B 126 18.408 25.494 40.801 0.00 0.00
B
ATOM 5353 O LYS B 126 19.430 25.311 40.193 0.00 0.00
B
ATOM 5354 N ASP B 127 18.354 25.545 42.172 0.00 0.00
B
ATOM 5355 HN ASP B 127 17.422 25.689 42.497 0.00 0.00
B
ATOM 5356 CA ASP B 127 19.471 25.362 43.046 0.00 0.00
B
ATOM 5357 HA ASP B 127 20.223 26.093 42.788 0.00 0.00
B
ATOM 5358 CB ASP B 127 19.018 25.573 44.523 0.00 0.00
B
ATOM 5359 HB1 ASP B 127 18.269 26.394 44.507 0.00 0.00
B
ATOM 5360 HB2 ASP B 127 18.650 24.621 44.962 0.00 0.00
B
ATOM 5361 CG ASP B 127 20.205 25.917 45.315 0.00 0.00
B
ATOM 5362 OD1 ASP B 127 20.898 26.890 45.011 0.00 0.00
B
ATOM 5363 OD2 ASP B 127 20.552 25.245 46.312 0.00 0.00
B
ATOM 5364 C ASP B 127 20.165 23.976 42.962 0.00 0.00
B
ATOM 5365 O ASP B 127 21.353 23.933 42.773 0.00 0.00
B
ATOM 5366 N MET B 128 19.307 22.960 42.881 0.00 0.00
B
ATOM 5367 HN MET B 128 18.369 23.276 42.998 0.00 0.00
B
ATOM 5368 CA MET B 128 19.532 21.511 42.665 0.00 0.00
B
ATOM 5369 HA MET B 128 20.187 21.156 43.446 0.00 0.00
B
ATOM 5370 CB MET B 128 18.290 20.603 42.605 0.00 0.00
B
ATOM 5371 HB1 MET B 128 17.509 21.219 42.110 0.00 0.00
B
ATOM 5372 HB2 MET B 128 18.413 19.630 42.082 0.00 0.00
B

ATOM 5373 CG MET B 128 17.843 20.213 44.028 0.00 0.00
B
ATOM 5374 HG1 MET B 128 18.567 19.510 44.491 0.00 0.00
B
ATOM 5375 HG2 MET B 128 17.945 21.128 44.650 0.00 0.00
B
ATOM 5376 SD MET B 128 16.166 19.685 44.208 0.00 0.00
B
ATOM 5377 CE MET B 128 16.323 19.175 45.936 0.00 0.00
B
ATOM 5378 HE1 MET B 128 17.349 19.256 46.354 0.00 0.00
B
ATOM 5379 HE2 MET B 128 15.703 19.817 46.597 0.00 0.00
B
ATOM 5380 HE3 MET B 128 15.974 18.124 46.033 0.00 0.00
B
ATOM 5381 C MET B 128 20.297 21.280 41.349 0.00 0.00
B
ATOM 5382 O MET B 128 21.104 20.343 41.173 0.00 0.00
B
ATOM 5383 N GLY B 129 19.954 22.106 40.309 0.00 0.00
B
ATOM 5384 HN GLY B 129 19.272 22.805 40.511 0.00 0.00
B
ATOM 5385 CA GLY B 129 20.527 22.057 38.902 0.00 0.00
B
ATOM 5386 HA1 GLY B 129 19.777 22.423 38.217 0.00 0.00
B
ATOM 5387 HA2 GLY B 129 20.812 21.048 38.645 0.00 0.00
B
ATOM 5388 C GLY B 129 21.705 22.956 38.667 0.00 0.00
B
ATOM 5389 O GLY B 129 22.719 22.508 38.118 0.00 0.00
B
ATOM 5390 N VAL B 130 21.730 24.210 39.044 0.00 0.00
B
ATOM 5391 HN VAL B 130 20.970 24.544 39.597 0.00 0.00
B
ATOM 5392 CA VAL B 130 22.793 25.101 38.562 0.00 0.00
B
ATOM 5393 HA VAL B 130 22.681 25.105 37.488 0.00 0.00
B
ATOM 5394 CB VAL B 130 22.340 26.518 39.005 0.00 0.00
B
ATOM 5395 HB VAL B 130 21.254 26.637 38.808 0.00 0.00
B
ATOM 5396 CG1 VAL B 130 22.488 26.891 40.560 0.00 0.00
B
ATOM 5397 HG11 VAL B 130 21.812 26.113 40.975 0.00 0.00
B
ATOM 5398 HG12 VAL B 130 23.525 26.734 40.926 0.00 0.00
B
ATOM 5399 HG13 VAL B 130 22.238 27.970 40.633 0.00 0.00
B

ATOM 5400 CG2 VAL B 130 23.053 27.571 38.129 0.00 0.00
B
ATOM 5401 HG21 VAL B 130 23.907 28.008 38.689 0.00 0.00
B
ATOM 5402 HG22 VAL B 130 23.346 27.142 37.147 0.00 0.00
B
ATOM 5403 HG23 VAL B 130 22.367 28.429 37.963 0.00 0.00
B
ATOM 5404 C VAL B 130 24.274 24.779 38.980 0.00 0.00
B
ATOM 5405 O VAL B 130 25.186 24.960 38.111 0.00 0.00
B
ATOM 5406 N LYS B 131 24.576 24.430 40.293 0.00 0.00
B
ATOM 5407 HN LYS B 131 23.788 24.115 40.816 0.00 0.00
B
ATOM 5408 CA LYS B 131 25.842 24.195 40.807 0.00 0.00
B
ATOM 5409 HA LYS B 131 26.428 25.067 40.556 0.00 0.00
B
ATOM 5410 CB LYS B 131 25.912 24.025 42.397 0.00 0.00
B
ATOM 5411 HB1 LYS B 131 25.438 24.841 42.984 0.00 0.00
B
ATOM 5412 HB2 LYS B 131 25.374 23.117 42.743 0.00 0.00
B
ATOM 5413 CG LYS B 131 27.269 23.805 43.065 0.00 0.00
B
ATOM 5414 HG1 LYS B 131 27.690 22.789 42.909 0.00 0.00
B
ATOM 5415 HG2 LYS B 131 27.942 24.502 42.521 0.00 0.00
B
ATOM 5416 CD LYS B 131 27.186 24.230 44.516 0.00 0.00
B
ATOM 5417 HD1 LYS B 131 28.198 24.126 44.962 0.00 0.00
B
ATOM 5418 HD2 LYS B 131 26.914 25.303 44.608 0.00 0.00
B
ATOM 5419 CE LYS B 131 26.327 23.406 45.436 0.00 0.00
B
ATOM 5420 HE1 LYS B 131 25.265 23.353 45.112 0.00 0.00
B
ATOM 5421 HE2 LYS B 131 26.621 22.336 45.479 0.00 0.00
B
ATOM 5422 NZ LYS B 131 26.257 23.992 46.816 0.00 0.00
B
ATOM 5423 HZ1 LYS B 131 25.476 23.621 47.394 0.00 0.00
B
ATOM 5424 HZ2 LYS B 131 27.144 23.982 47.358 0.00 0.00
B
ATOM 5425 HZ3 LYS B 131 25.989 24.987 46.672 0.00 0.00
B
ATOM 5426 C LYS B 131 26.482 23.050 40.098 0.00 0.00
B

ATOM 5427 O LYS B 131 27.634 23.216 39.713 0.00 0.00
B
ATOM 5428 N ILE B 132 25.716 21.993 39.884 0.00 0.00
B
ATOM 5429 HN ILE B 132 24.766 22.100 40.169 0.00 0.00
B
ATOM 5430 CA ILE B 132 26.221 20.762 39.264 0.00 0.00
B
ATOM 5431 HA ILE B 132 27.233 20.622 39.614 0.00 0.00
B
ATOM 5432 CB ILE B 132 25.327 19.595 39.305 0.00 0.00
B
ATOM 5433 HB ILE B 132 24.283 19.917 39.104 0.00 0.00
B
ATOM 5434 CG2 ILE B 132 25.808 18.475 38.425 0.00 0.00
B
ATOM 5435 HG21 ILE B 132 25.548 17.441 38.737 0.00 0.00
B
ATOM 5436 HG22 ILE B 132 25.534 18.602 37.356 0.00 0.00
B
ATOM 5437 HG23 ILE B 132 26.913 18.447 38.536 0.00 0.00
B
ATOM 5438 CG1 ILE B 132 25.168 19.271 40.838 0.00 0.00
B
ATOM 5439 HG11 ILE B 132 24.803 20.258 41.196 0.00 0.00
B
ATOM 5440 HG12 ILE B 132 24.360 18.514 40.747 0.00 0.00
B
ATOM 5441 CD ILE B 132 26.433 18.822 41.597 0.00 0.00
B
ATOM 5442 HD1 ILE B 132 26.851 17.876 41.192 0.00 0.00
B
ATOM 5443 HD2 ILE B 132 27.242 19.563 41.423 0.00 0.00
B
ATOM 5444 HD3 ILE B 132 26.277 18.679 42.687 0.00 0.00
B
ATOM 5445 C ILE B 132 26.535 21.071 37.783 0.00 0.00
B
ATOM 5446 O ILE B 132 27.582 20.670 37.251 0.00 0.00
B
ATOM 5447 N ALA B 133 25.685 21.910 37.011 0.00 0.00
B
ATOM 5448 HN ALA B 133 24.774 22.120 37.359 0.00 0.00
B
ATOM 5449 CA ALA B 133 25.865 22.216 35.649 0.00 0.00
B
ATOM 5450 HA ALA B 133 25.962 21.236 35.206 0.00 0.00
B
ATOM 5451 CB ALA B 133 24.643 22.949 35.041 0.00 0.00
B
ATOM 5452 HB1 ALA B 133 24.734 23.023 33.937 0.00 0.00
B
ATOM 5453 HB2 ALA B 133 23.686 22.447 35.299 0.00 0.00
B

ATOM 5454 HB3 ALA B 133 24.521 23.960 35.485 0.00 0.00
B
ATOM 5455 C ALA B 133 27.071 23.031 35.367 0.00 0.00
B
ATOM 5456 O ALA B 133 27.859 22.702 34.462 0.00 0.00
B
ATOM 5457 N GLN B 134 27.271 24.069 36.169 0.00 0.00
B
ATOM 5458 HN GLN B 134 26.531 24.295 36.798 0.00 0.00
B
ATOM 5459 CA GLN B 134 28.462 24.898 36.174 0.00 0.00
B
ATOM 5460 HA GLN B 134 28.622 25.307 35.187 0.00 0.00
B
ATOM 5461 CB GLN B 134 28.219 26.019 37.234 0.00 0.00
B
ATOM 5462 HB1 GLN B 134 27.658 25.535 38.061 0.00 0.00
B
ATOM 5463 HB2 GLN B 134 29.165 26.560 37.452 0.00 0.00
B
ATOM 5464 CG GLN B 134 27.332 27.232 36.789 0.00 0.00
B
ATOM 5465 HG1 GLN B 134 27.770 27.686 35.874 0.00 0.00
B
ATOM 5466 HG2 GLN B 134 26.278 26.936 36.599 0.00 0.00
B
ATOM 5467 CD GLN B 134 27.248 28.249 37.895 0.00 0.00
B
ATOM 5468 OE1 GLN B 134 27.078 27.930 39.118 0.00 0.00
B
ATOM 5469 NE2 GLN B 134 27.371 29.533 37.644 0.00 0.00
B
ATOM 5470 HE21 GLN B 134 27.253 30.143 38.428 0.00 0.00
B
ATOM 5471 HE22 GLN B 134 27.380 29.723 36.662 0.00 0.00
B
ATOM 5472 C GLN B 134 29.748 24.073 36.533 0.00 0.00
B
ATOM 5473 O GLN B 134 30.798 24.236 35.938 0.00 0.00
B
ATOM 5474 N SER B 135 29.704 23.140 37.579 0.00 0.00
B
ATOM 5475 HN SER B 135 28.808 23.013 37.998 0.00 0.00
B
ATOM 5476 CA SER B 135 30.833 22.353 37.896 0.00 0.00
B
ATOM 5477 HA SER B 135 31.745 22.930 37.933 0.00 0.00
B
ATOM 5478 CB SER B 135 30.645 21.521 39.088 0.00 0.00
B
ATOM 5479 HB1 SER B 135 29.796 20.814 38.971 0.00 0.00
B
ATOM 5480 HB2 SER B 135 31.604 20.972 39.208 0.00 0.00
B

```
ATOM   5481  OG  SER B 135      30.344  22.342  40.202  0.00  0.00
B
ATOM   5482  HG1 SER B 135      29.458  22.617  39.958  0.00  0.00
B
ATOM   5483  C   SER B 135      31.151  21.379  36.761  0.00  0.00
B
ATOM   5484  O   SER B 135      32.333  21.202  36.449  0.00  0.00
B
ATOM   5485  N   LEU B 136      30.077  20.747  36.194  0.00  0.00
B
ATOM   5486  HN  LEU B 136      29.160  20.984  36.507  0.00  0.00
B
ATOM   5487  CA  LEU B 136      30.294  19.742  35.127  0.00  0.00
B
ATOM   5488  HA  LEU B 136      31.007  19.068  35.580  0.00  0.00
B
ATOM   5489  CB  LEU B 136      28.913  19.135  34.795  0.00  0.00
B
ATOM   5490  HB1 LEU B 136      28.491  18.902  35.796  0.00  0.00
B
ATOM   5491  HB2 LEU B 136      28.311  19.847  34.191  0.00  0.00
B
ATOM   5492  CG  LEU B 136      28.920  17.852  33.940  0.00  0.00
B
ATOM   5493  HG  LEU B 136      29.502  17.873  32.994  0.00  0.00
B
ATOM   5494  CD1 LEU B 136      29.554  16.771  34.916  0.00  0.00
B
ATOM   5495 HD11 LEU B 136      30.655  16.915  34.962  0.00  0.00
B
ATOM   5496 HD12 LEU B 136      29.061  16.882  35.905  0.00  0.00
B
ATOM   5497 HD13 LEU B 136      29.434  15.719  34.579  0.00  0.00
B
ATOM   5498  CD2 LEU B 136      27.409  17.463  33.595  0.00  0.00
B
ATOM   5499 HD21 LEU B 136      27.156  18.281  32.887  0.00  0.00
B
ATOM   5500 HD22 LEU B 136      27.343  16.511  33.026  0.00  0.00
B
ATOM   5501 HD23 LEU B 136      26.718  17.491  34.465  0.00  0.00
B
ATOM   5502  C   LEU B 136      30.867  20.229  33.877  0.00  0.00
B
ATOM   5503  O   LEU B 136      31.449  19.504  33.055  0.00  0.00
B
ATOM   5504  N   SER B 137      30.723  21.516  33.612  0.00  0.00
B
ATOM   5505  HN  SER B 137      30.340  22.077  34.341  0.00  0.00
B
ATOM   5506  CA  SER B 137      31.133  22.112  32.331  0.00  0.00
B
ATOM   5507  HA  SER B 137      30.849  21.533  31.465  0.00  0.00
B
```

ATOM 5508 CB SER B 137 30.438 23.521 32.083 0.00 0.00
B
ATOM 5509 HB1 SER B 137 30.473 24.131 33.011 0.00 0.00
B
ATOM 5510 HB2 SER B 137 30.907 24.067 31.236 0.00 0.00
B
ATOM 5511 OG SER B 137 28.992 23.413 31.886 0.00 0.00
B
ATOM 5512 HG1 SER B 137 28.669 23.049 32.714 0.00 0.00
B
ATOM 5513 C SER B 137 32.640 22.280 32.188 0.00 0.00
B
ATOM 5514 O SER B 137 32.962 22.292 31.048 0.00 0.00
B
ATOM 5515 N ASP B 138 33.471 22.464 33.254 0.00 0.00
B
ATOM 5516 HN ASP B 138 33.105 22.473 34.181 0.00 0.00
B
ATOM 5517 CA ASP B 138 34.931 22.575 33.149 0.00 0.00
B
ATOM 5518 HA ASP B 138 35.408 22.811 32.209 0.00 0.00
B
ATOM 5519 CB ASP B 138 35.333 23.757 34.025 0.00 0.00
B
ATOM 5520 HB1 ASP B 138 34.804 23.717 35.002 0.00 0.00
B
ATOM 5521 HB2 ASP B 138 36.401 23.698 34.326 0.00 0.00
B
ATOM 5522 CG ASP B 138 35.036 25.078 33.327 0.00 0.00
B
ATOM 5523 OD1 ASP B 138 34.464 25.983 33.954 0.00 0.00
B
ATOM 5524 OD2 ASP B 138 35.485 25.330 32.187 0.00 0.00
B
ATOM 5525 C ASP B 138 35.540 21.177 33.422 0.00 0.00
B
ATOM 5526 O ASP B 138 34.836 20.256 33.808 0.00 0.00
B
ATOM 5527 N GLU B 139 36.814 20.811 33.030 0.00 0.00
B
ATOM 5528 HN GLU B 139 37.458 21.492 32.691 0.00 0.00
B
ATOM 5529 CA GLU B 139 37.475 19.528 33.221 0.00 0.00
B
ATOM 5530 HA GLU B 139 36.628 18.888 33.021 0.00 0.00
B
ATOM 5531 CB GLU B 139 38.696 19.224 32.299 0.00 0.00
B
ATOM 5532 HB1 GLU B 139 39.572 19.816 32.644 0.00 0.00
B
ATOM 5533 HB2 GLU B 139 38.804 18.151 32.566 0.00 0.00
B
ATOM 5534 CG GLU B 139 38.645 19.326 30.770 0.00 0.00
B

```
ATOM   5535  HG1 GLU B 139      37.882  18.616  30.385  0.00  0.00
B
ATOM   5536  HG2 GLU B 139      38.383  20.364  30.471  0.00  0.00
B
ATOM   5537  CD  GLU B 139      39.950  18.880  30.136  0.00  0.00
B
ATOM   5538  OE1 GLU B 139      40.169  19.213  28.948  0.00  0.00
B
ATOM   5539  OE2 GLU B 139      40.663  18.034  30.699  0.00  0.00
B
ATOM   5540  C   GLU B 139      37.760  19.252  34.683  0.00  0.00
B
ATOM   5541  O   GLU B 139      37.219  18.343  35.279  0.00  0.00
B
ATOM   5542  N   ASN B 140      38.522  20.158  35.396  0.00  0.00
B
ATOM   5543  HN  ASN B 140      38.824  20.981  34.922  0.00  0.00
B
ATOM   5544  CA  ASN B 140      38.964  19.859  36.742  0.00  0.00
B
ATOM   5545  HA  ASN B 140      39.071  18.803  36.940  0.00  0.00
B
ATOM   5546  CB  ASN B 140      40.340  20.595  36.878  0.00  0.00
B
ATOM   5547  HB1 ASN B 140      40.147  21.630  36.524  0.00  0.00
B
ATOM   5548  HB2 ASN B 140      40.589  20.597  37.961  0.00  0.00
B
ATOM   5549  CG  ASN B 140      41.481  19.906  36.200  0.00  0.00
B
ATOM   5550  OD1 ASN B 140      42.294  20.629  35.631  0.00  0.00
B
ATOM   5551  ND2 ASN B 140      41.546  18.578  36.267  0.00  0.00
B
ATOM   5552 HD21 ASN B 140      42.298  18.047  35.877  0.00  0.00
B
ATOM   5553 HD22 ASN B 140      41.023  18.115  36.982  0.00  0.00
B
ATOM   5554  C   ASN B 140      37.969  20.408  37.795  0.00  0.00
B
ATOM   5555  O   ASN B 140      38.089  20.086  38.942  0.00  0.00
B
ATOM   5556  N   VAL B 141      36.927  21.154  37.418  0.00  0.00
B
ATOM   5557  HN  VAL B 141      36.702  21.298  36.458  0.00  0.00
B
ATOM   5558  CA  VAL B 141      35.966  21.595  38.407  0.00  0.00
B
ATOM   5559  HA  VAL B 141      36.498  22.061  39.223  0.00  0.00
B
ATOM   5560  CB  VAL B 141      35.056  22.699  37.957  0.00  0.00
B
ATOM   5561  HB  VAL B 141      34.506  22.284  37.085  0.00  0.00
B
```

```
ATOM   5562  CG1 VAL B 141      34.224  23.255  39.124  0.00  0.00
B
ATOM   5563 HG11 VAL B 141      33.855  24.284  38.925  0.00  0.00
B
ATOM   5564 HG12 VAL B 141      33.382  22.553  39.302  0.00  0.00
B
ATOM   5565 HG13 VAL B 141      34.817  23.403  40.052  0.00  0.00
B
ATOM   5566  CG2 VAL B 141      35.927  23.949  37.543  0.00  0.00
B
ATOM   5567 HG21 VAL B 141      36.467  23.762  36.590  0.00  0.00
B
ATOM   5568 HG22 VAL B 141      35.234  24.793  37.340  0.00  0.00
B
ATOM   5569 HG23 VAL B 141      36.687  24.213  38.308  0.00  0.00
B
ATOM   5570  C   VAL B 141      35.179  20.488  39.188  0.00  0.00
B
ATOM   5571  O   VAL B 141      34.372  19.681  38.710  0.00  0.00
B
ATOM   5572  N   LEU B 142      35.421  20.586  40.504  0.00  0.00
B
ATOM   5573  HN  LEU B 142      36.154  21.132  40.901  0.00  0.00
B
ATOM   5574  CA  LEU B 142      34.624  19.809  41.409  0.00  0.00
B
ATOM   5575  HA  LEU B 142      34.330  18.883  40.937  0.00  0.00
B
ATOM   5576  CB  LEU B 142      35.414  19.341  42.648  0.00  0.00
B
ATOM   5577  HB1 LEU B 142      35.610  20.285  43.200  0.00  0.00
B
ATOM   5578  HB2 LEU B 142      34.774  18.733  43.323  0.00  0.00
B
ATOM   5579  CG  LEU B 142      36.669  18.562  42.333  0.00  0.00
B
ATOM   5580  HG  LEU B 142      37.430  19.101  41.729  0.00  0.00
B
ATOM   5581  CD1 LEU B 142      37.422  18.190  43.588  0.00  0.00
B
ATOM   5582 HD11 LEU B 142      37.505  19.115  44.196  0.00  0.00
B
ATOM   5583 HD12 LEU B 142      36.941  17.403  44.207  0.00  0.00
B
ATOM   5584 HD13 LEU B 142      38.424  17.739  43.427  0.00  0.00
B
ATOM   5585  CD2 LEU B 142      36.359  17.200  41.661  0.00  0.00
B
ATOM   5586 HD21 LEU B 142      37.342  16.815  41.315  0.00  0.00
B
ATOM   5587 HD22 LEU B 142      35.953  16.437  42.359  0.00  0.00
B
ATOM   5588 HD23 LEU B 142      35.569  17.172  40.882  0.00  0.00
B
```

ATOM 5589 C LEU B 142 33.415 20.612 41.874 0.00 0.00
B
ATOM 5590 O LEU B 142 32.320 20.083 42.006 0.00 0.00
B
ATOM 5591 N ASN B 143 33.607 21.868 42.286 0.00 0.00
B
ATOM 5592 HN ASN B 143 34.530 22.217 42.143 0.00 0.00
B
ATOM 5593 CA ASN B 143 32.633 22.788 42.774 0.00 0.00
B
ATOM 5594 HA ASN B 143 31.715 22.342 42.422 0.00 0.00
B
ATOM 5595 CB ASN B 143 32.629 22.845 44.304 0.00 0.00
B
ATOM 5596 HB1 ASN B 143 33.034 21.976 44.866 0.00 0.00
B
ATOM 5597 HB2 ASN B 143 33.323 23.692 44.491 0.00 0.00
B
ATOM 5598 CG ASN B 143 31.268 23.257 44.809 0.00 0.00
B
ATOM 5599 OD1 ASN B 143 30.258 23.120 44.091 0.00 0.00
B
ATOM 5600 ND2 ASN B 143 31.108 23.615 46.080 0.00 0.00
B
ATOM 5601 HD21 ASN B 143 30.139 23.720 46.302 0.00 0.00
B
ATOM 5602 HD22 ASN B 143 31.897 23.481 46.679 0.00 0.00
B
ATOM 5603 C ASN B 143 32.696 24.136 42.113 0.00 0.00
B
ATOM 5604 O ASN B 143 33.774 24.732 42.032 0.00 0.00
B
ATOM 5605 N TYR B 144 31.608 24.631 41.595 0.00 0.00
B
ATOM 5606 HN TYR B 144 30.800 24.050 41.540 0.00 0.00
B
ATOM 5607 CA TYR B 144 31.543 26.023 41.029 0.00 0.00
B
ATOM 5608 HA TYR B 144 32.530 26.454 41.106 0.00 0.00
B
ATOM 5609 CB TYR B 144 31.006 25.915 39.606 0.00 0.00
B
ATOM 5610 HB1 TYR B 144 31.500 25.047 39.119 0.00 0.00
B
ATOM 5611 HB2 TYR B 144 29.905 25.782 39.537 0.00 0.00
B
ATOM 5612 CG TYR B 144 31.375 27.135 38.810 0.00 0.00
B
ATOM 5613 CD1 TYR B 144 30.559 28.307 38.736 0.00 0.00
B
ATOM 5614 HD1 TYR B 144 29.640 28.391 39.296 0.00 0.00
B
ATOM 5615 CE1 TYR B 144 30.996 29.411 37.968 0.00 0.00
B

ATOM 5616 HE1 TYR B 144 30.352 30.278 37.946 0.00 0.00
B
ATOM 5617 CZ TYR B 144 32.087 29.236 37.150 0.00 0.00
B
ATOM 5618 OH TYR B 144 32.467 30.225 36.286 0.00 0.00
B
ATOM 5619 HH TYR B 144 33.323 30.080 35.876 0.00 0.00
B
ATOM 5620 CD2 TYR B 144 32.416 27.001 37.944 0.00 0.00
B
ATOM 5621 HD2 TYR B 144 33.052 26.132 38.022 0.00 0.00
B
ATOM 5622 CE2 TYR B 144 32.790 27.991 37.069 0.00 0.00
B
ATOM 5623 HE2 TYR B 144 33.685 27.939 36.467 0.00 0.00
B
ATOM 5624 C TYR B 144 30.622 26.967 41.754 0.00 0.00
B
ATOM 5625 O TYR B 144 29.477 26.642 41.977 0.00 0.00
B
ATOM 5626 N ILE B 145 31.117 28.144 42.272 0.00 0.00
B
ATOM 5627 HN ILE B 145 32.069 28.403 42.125 0.00 0.00
B
ATOM 5628 CA ILE B 145 30.277 29.257 42.858 0.00 0.00
B
ATOM 5629 HA ILE B 145 29.269 28.882 42.762 0.00 0.00
B
ATOM 5630 CB ILE B 145 30.765 29.510 44.337 0.00 0.00
B
ATOM 5631 HB ILE B 145 31.853 29.711 44.238 0.00 0.00
B
ATOM 5632 CG2 ILE B 145 30.142 30.764 45.001 0.00 0.00
B
ATOM 5633 HG21 ILE B 145 30.479 31.757 44.635 0.00 0.00
B
ATOM 5634 HG22 ILE B 145 29.065 30.754 44.727 0.00 0.00
B
ATOM 5635 HG23 ILE B 145 30.169 30.741 46.111 0.00 0.00
B
ATOM 5636 CG1 ILE B 145 30.585 28.266 45.259 0.00 0.00
B
ATOM 5637 HG11 ILE B 145 31.289 27.432 45.054 0.00 0.00
B
ATOM 5638 HG12 ILE B 145 30.698 28.623 46.305 0.00 0.00
B
ATOM 5639 CD ILE B 145 29.175 27.660 45.138 0.00 0.00
B
ATOM 5640 HD1 ILE B 145 28.364 28.405 45.285 0.00 0.00
B
ATOM 5641 HD2 ILE B 145 29.022 27.028 44.237 0.00 0.00
B
ATOM 5642 HD3 ILE B 145 29.030 26.952 45.982 0.00 0.00
B

ATOM 5643 C ILE B 145 30.370 30.544 42.124 0.00 0.00
B
ATOM 5644 O ILE B 145 31.463 31.029 41.860 0.00 0.00
B
ATOM 5645 N ASP B 146 29.306 31.280 41.810 0.00 0.00
B
ATOM 5646 HN ASP B 146 28.456 30.768 41.904 0.00 0.00
B
ATOM 5647 CA ASP B 146 29.244 32.646 41.484 0.00 0.00
B
ATOM 5648 HA ASP B 146 29.973 32.931 40.739 0.00 0.00
B
ATOM 5649 CB ASP B 146 27.784 32.951 40.999 0.00 0.00
B
ATOM 5650 HB1 ASP B 146 26.946 32.682 41.676 0.00 0.00
B
ATOM 5651 HB2 ASP B 146 27.568 34.036 40.895 0.00 0.00
B
ATOM 5652 CG ASP B 146 27.501 32.440 39.546 0.00 0.00
B
ATOM 5653 OD1 ASP B 146 26.613 31.603 39.282 0.00 0.00
B
ATOM 5654 OD2 ASP B 146 28.242 32.945 38.688 0.00 0.00
B
ATOM 5655 C ASP B 146 29.474 33.565 42.669 0.00 0.00
B
ATOM 5656 O ASP B 146 28.702 33.521 43.663 0.00 0.00
B
ATOM 5657 N LEU B 147 30.470 34.400 42.543 0.00 0.00
B
ATOM 5658 HN LEU B 147 31.156 34.261 41.833 0.00 0.00
B
ATOM 5659 CA LEU B 147 30.627 35.452 43.588 0.00 0.00
B
ATOM 5660 HA LEU B 147 29.993 35.191 44.423 0.00 0.00
B
ATOM 5661 CB LEU B 147 32.150 35.720 43.869 0.00 0.00
B
ATOM 5662 HB1 LEU B 147 32.516 34.844 44.445 0.00 0.00
B
ATOM 5663 HB2 LEU B 147 32.720 35.656 42.918 0.00 0.00
B
ATOM 5664 CG LEU B 147 32.569 37.005 44.553 0.00 0.00
B
ATOM 5665 HG LEU B 147 32.399 37.918 43.942 0.00 0.00
B
ATOM 5666 CD1 LEU B 147 31.894 37.409 45.913 0.00 0.00
B
ATOM 5667 HD11 LEU B 147 30.854 37.800 45.888 0.00 0.00
B
ATOM 5668 HD12 LEU B 147 32.101 36.535 46.567 0.00 0.00
B
ATOM 5669 HD13 LEU B 147 32.615 38.117 46.374 0.00 0.00
B

ATOM 5670 CD2 LEU B 147 34.112 36.874 44.812 0.00 0.00
B
ATOM 5671 HD21 LEU B 147 34.517 36.576 43.821 0.00 0.00
B
ATOM 5672 HD22 LEU B 147 34.519 37.867 45.102 0.00 0.00
B
ATOM 5673 HD23 LEU B 147 34.224 36.138 45.638 0.00 0.00
B
ATOM 5674 C LEU B 147 29.932 36.642 43.020 0.00 0.00
B
ATOM 5675 O LEU B 147 29.089 37.216 43.688 0.00 0.00
B
ATOM 5676 N SER B 148 30.186 37.006 41.778 0.00 0.00
B
ATOM 5677 HN SER B 148 30.784 36.385 41.277 0.00 0.00
B
ATOM 5678 CA SER B 148 29.601 38.122 41.065 0.00 0.00
B
ATOM 5679 HA SER B 148 28.564 38.264 41.331 0.00 0.00
B
ATOM 5680 CB SER B 148 30.381 39.536 41.095 0.00 0.00
B
ATOM 5681 HB1 SER B 148 29.622 40.328 40.920 0.00 0.00
B
ATOM 5682 HB2 SER B 148 30.889 39.669 42.074 0.00 0.00
B
ATOM 5683 OG SER B 148 31.440 39.567 40.162 0.00 0.00
B
ATOM 5684 HG1 SER B 148 31.859 40.412 40.335 0.00 0.00
B
ATOM 5685 C SER B 148 29.480 37.720 39.559 0.00 0.00
B
ATOM 5686 O SER B 148 29.898 36.644 39.060 0.00 0.00
B
ATOM 5687 N ASP B 149 28.978 38.629 38.720 0.00 0.00
B
ATOM 5688 HN ASP B 149 28.573 39.457 39.099 0.00 0.00
B
ATOM 5689 CA ASP B 149 28.831 38.654 37.282 0.00 0.00
B
ATOM 5690 HA ASP B 149 28.201 37.791 37.124 0.00 0.00
B
ATOM 5691 CB ASP B 149 28.109 39.971 36.770 0.00 0.00
B
ATOM 5692 HB1 ASP B 149 28.623 40.910 37.067 0.00 0.00
B
ATOM 5693 HB2 ASP B 149 28.030 39.959 35.662 0.00 0.00
B
ATOM 5694 CG ASP B 149 26.738 40.126 37.311 0.00 0.00
B
ATOM 5695 OD1 ASP B 149 26.547 41.106 38.101 0.00 0.00
B
ATOM 5696 OD2 ASP B 149 25.813 39.337 36.922 0.00 0.00
B

ATOM 5697 C ASP B 149 30.122 38.372 36.517 0.00 0.00
B
ATOM 5698 O ASP B 149 30.132 37.729 35.490 0.00 0.00
B
ATOM 5699 N GLU B 150 31.209 38.992 37.009 0.00 0.00
B
ATOM 5700 HN GLU B 150 31.175 39.484 37.876 0.00 0.00
B
ATOM 5701 CA GLU B 150 32.557 38.726 36.470 0.00 0.00
B
ATOM 5702 HA GLU B 150 32.316 38.061 35.655 0.00 0.00
B
ATOM 5703 CB GLU B 150 33.211 40.021 35.953 0.00 0.00
B
ATOM 5704 HB1 GLU B 150 34.205 39.828 35.494 0.00 0.00
B
ATOM 5705 HB2 GLU B 150 32.595 40.454 35.137 0.00 0.00
B
ATOM 5706 CG GLU B 150 33.255 41.164 37.023 0.00 0.00
B
ATOM 5707 HG1 GLU B 150 32.271 41.472 37.436 0.00 0.00
B
ATOM 5708 HG2 GLU B 150 33.778 40.887 37.964 0.00 0.00
B
ATOM 5709 CD GLU B 150 34.016 42.415 36.590 0.00 0.00
B
ATOM 5710 OE1 GLU B 150 35.131 42.605 37.101 0.00 0.00
B
ATOM 5711 OE2 GLU B 150 33.562 43.200 35.692 0.00 0.00
B
ATOM 5712 C GLU B 150 33.452 37.833 37.408 0.00 0.00
B
ATOM 5713 O GLU B 150 34.409 37.212 36.986 0.00 0.00
B
ATOM 5714 N TYR B 151 33.220 37.768 38.714 0.00 0.00
B
ATOM 5715 HN TYR B 151 32.395 38.211 39.057 0.00 0.00
B
ATOM 5716 CA TYR B 151 33.975 36.892 39.603 0.00 0.00
B
ATOM 5717 HA TYR B 151 34.882 36.753 39.033 0.00 0.00
B
ATOM 5718 CB TYR B 151 34.294 37.604 40.982 0.00 0.00
B
ATOM 5719 HB1 TYR B 151 33.468 38.140 41.497 0.00 0.00
B
ATOM 5720 HB2 TYR B 151 34.716 36.897 41.728 0.00 0.00
B
ATOM 5721 CG TYR B 151 35.390 38.607 40.875 0.00 0.00
B
ATOM 5722 CD1 TYR B 151 36.738 38.230 40.914 0.00 0.00
B
ATOM 5723 HD1 TYR B 151 36.864 37.158 40.955 0.00 0.00
B

ATOM 5724 CE1 TYR B 151 37.869 39.092 40.805 0.00 0.00
B
ATOM 5725 HE1 TYR B 151 38.863 38.707 40.977 0.00 0.00
B
ATOM 5726 CZ TYR B 151 37.581 40.419 40.576 0.00 0.00
B
ATOM 5727 OH TYR B 151 38.682 41.349 40.315 0.00 0.00
B
ATOM 5728 HH TYR B 151 39.465 40.801 40.232 0.00 0.00
B
ATOM 5729 CD2 TYR B 151 35.203 39.991 40.550 0.00 0.00
B
ATOM 5730 HD2 TYR B 151 34.278 40.496 40.313 0.00 0.00
B
ATOM 5731 CE2 TYR B 151 36.275 40.938 40.504 0.00 0.00
B
ATOM 5732 HE2 TYR B 151 36.163 42.001 40.344 0.00 0.00
B
ATOM 5733 C TYR B 151 33.360 35.519 39.873 0.00 0.00
B
ATOM 5734 O TYR B 151 32.181 35.317 40.075 0.00 0.00
B
ATOM 5735 N SER B 152 34.210 34.456 40.018 0.00 0.00
B
ATOM 5736 HN SER B 152 35.195 34.489 39.871 0.00 0.00
B
ATOM 5737 CA SER B 152 33.776 33.146 40.441 0.00 0.00
B
ATOM 5738 HA SER B 152 32.824 33.190 40.950 0.00 0.00
B
ATOM 5739 CB SER B 152 33.551 32.028 39.419 0.00 0.00
B
ATOM 5740 HB1 SER B 152 34.455 31.893 38.788 0.00 0.00
B
ATOM 5741 HB2 SER B 152 33.265 31.084 39.930 0.00 0.00
B
ATOM 5742 OG SER B 152 32.518 32.337 38.508 0.00 0.00
B
ATOM 5743 HG1 SER B 152 32.464 31.673 37.817 0.00 0.00
B
ATOM 5744 C SER B 152 34.731 32.663 41.506 0.00 0.00
B
ATOM 5745 O SER B 152 35.855 33.141 41.594 0.00 0.00
B
ATOM 5746 N ILE B 153 34.243 31.668 42.330 0.00 0.00
B
ATOM 5747 HN ILE B 153 33.297 31.354 42.330 0.00 0.00
B
ATOM 5748 CA ILE B 153 34.919 30.896 43.305 0.00 0.00
B
ATOM 5749 HA ILE B 153 35.984 31.073 43.308 0.00 0.00
B
ATOM 5750 CB ILE B 153 34.507 31.070 44.813 0.00 0.00
B

ATOM 5751 HB ILE B 153 33.495 30.612 44.826 0.00 0.00
B
ATOM 5752 CG2 ILE B 153 35.444 30.223 45.792 0.00 0.00
B
ATOM 5753 HG21 ILE B 153 35.452 29.141 45.542 0.00 0.00
B
ATOM 5754 HG22 ILE B 153 36.473 30.637 45.728 0.00 0.00
B
ATOM 5755 HG23 ILE B 153 35.099 30.352 46.840 0.00 0.00
B
ATOM 5756 CG1 ILE B 153 34.498 32.621 45.201 0.00 0.00
B
ATOM 5757 HG11 ILE B 153 35.531 32.848 45.542 0.00 0.00
B
ATOM 5758 HG12 ILE B 153 34.399 33.294 44.323 0.00 0.00
B
ATOM 5759 CD ILE B 153 33.334 32.977 46.166 0.00 0.00
B
ATOM 5760 HD1 ILE B 153 33.583 32.488 47.132 0.00 0.00
B
ATOM 5761 HD2 ILE B 153 33.315 34.057 46.426 0.00 0.00
B
ATOM 5762 HD3 ILE B 153 32.358 32.655 45.744 0.00 0.00
B
ATOM 5763 C ILE B 153 34.813 29.389 42.905 0.00 0.00
B
ATOM 5764 O ILE B 153 33.698 28.881 42.699 0.00 0.00
B
ATOM 5765 N VAL B 154 35.900 28.666 42.674 0.00 0.00
B
ATOM 5766 HN VAL B 154 36.831 28.988 42.826 0.00 0.00
B
ATOM 5767 CA VAL B 154 35.816 27.344 41.988 0.00 0.00
B
ATOM 5768 HA VAL B 154 34.898 26.814 42.196 0.00 0.00
B
ATOM 5769 CB VAL B 154 35.989 27.569 40.514 0.00 0.00
B
ATOM 5770 HB VAL B 154 36.994 27.890 40.166 0.00 0.00
B
ATOM 5771 CG1 VAL B 154 35.856 26.208 39.831 0.00 0.00
B
ATOM 5772 HG11 VAL B 154 34.813 25.917 39.581 0.00 0.00
B
ATOM 5773 HG12 VAL B 154 36.427 26.403 38.898 0.00 0.00
B
ATOM 5774 HG13 VAL B 154 36.391 25.432 40.417 0.00 0.00
B
ATOM 5775 CG2 VAL B 154 35.011 28.552 39.832 0.00 0.00
B
ATOM 5776 HG21 VAL B 154 35.372 28.771 38.804 0.00 0.00
B
ATOM 5777 HG22 VAL B 154 33.990 28.135 39.707 0.00 0.00
B

ATOM 5778 HG23 VAL B 154 34.924 29.505 40.396 0.00 0.00
B
ATOM 5779 C VAL B 154 36.833 26.559 42.687 0.00 0.00
B
ATOM 5780 O VAL B 154 37.860 27.053 43.085 0.00 0.00
B
ATOM 5781 N GLU B 155 36.576 25.211 42.880 0.00 0.00
B
ATOM 5782 HN GLU B 155 35.663 24.985 42.548 0.00 0.00
B
ATOM 5783 CA GLU B 155 37.450 24.263 43.500 0.00 0.00
B
ATOM 5784 HA GLU B 155 38.387 24.686 43.832 0.00 0.00
B
ATOM 5785 CB GLU B 155 36.788 23.610 44.715 0.00 0.00
B
ATOM 5786 HB1 GLU B 155 36.501 24.383 45.460 0.00 0.00
B
ATOM 5787 HB2 GLU B 155 35.811 23.145 44.464 0.00 0.00
B
ATOM 5788 CG GLU B 155 37.732 22.632 45.556 0.00 0.00
B
ATOM 5789 HG1 GLU B 155 37.960 21.857 44.793 0.00 0.00
B
ATOM 5790 HG2 GLU B 155 38.629 23.251 45.771 0.00 0.00
B
ATOM 5791 CD GLU B 155 36.993 22.204 46.782 0.00 0.00
B
ATOM 5792 OE1 GLU B 155 36.306 23.068 47.427 0.00 0.00
B
ATOM 5793 OE2 GLU B 155 37.014 21.015 47.059 0.00 0.00
B
ATOM 5794 C GLU B 155 37.842 23.251 42.419 0.00 0.00
B
ATOM 5795 O GLU B 155 36.976 22.684 41.778 0.00 0.00
B
ATOM 5796 N LEU B 156 39.136 23.030 42.265 0.00 0.00
B
ATOM 5797 HN LEU B 156 39.676 23.516 42.948 0.00 0.00
B
ATOM 5798 CA LEU B 156 39.852 22.366 41.196 0.00 0.00
B
ATOM 5799 HA LEU B 156 39.138 22.137 40.419 0.00 0.00
B
ATOM 5800 CB LEU B 156 40.975 23.329 40.617 0.00 0.00
B
ATOM 5801 HB1 LEU B 156 41.642 23.709 41.420 0.00 0.00
B
ATOM 5802 HB2 LEU B 156 41.551 22.742 39.869 0.00 0.00
B
ATOM 5803 CG LEU B 156 40.386 24.654 40.058 0.00 0.00
B
ATOM 5804 HG LEU B 156 39.859 25.214 40.861 0.00 0.00
B

ATOM 5805 CD1 LEU B 156 41.520 25.467 39.480 0.00 0.00
B
ATOM 5806 HD11 LEU B 156 42.195 24.904 38.800 0.00 0.00
B
ATOM 5807 HD12 LEU B 156 41.203 26.319 38.842 0.00 0.00
B
ATOM 5808 HD13 LEU B 156 42.144 25.977 40.244 0.00 0.00
B
ATOM 5809 CD2 LEU B 156 39.535 24.380 38.891 0.00 0.00
B
ATOM 5810 HD21 LEU B 156 39.232 25.284 38.320 0.00 0.00
B
ATOM 5811 HD22 LEU B 156 40.034 23.631 38.238 0.00 0.00
B
ATOM 5812 HD23 LEU B 156 38.599 23.898 39.246 0.00 0.00
B
ATOM 5813 C LEU B 156 40.560 21.109 41.761 0.00 0.00
B
ATOM 5814 O LEU B 156 41.412 21.249 42.660 0.00 0.00
B
ATOM 5815 N LEU B 157 40.315 19.881 41.159 0.00 0.00
B
ATOM 5816 HN LEU B 157 39.560 19.722 40.528 0.00 0.00
B
ATOM 5817 CA LEU B 157 41.092 18.725 41.560 0.00 0.00
B
ATOM 5818 HA LEU B 157 41.023 18.623 42.633 0.00 0.00
B
ATOM 5819 CB LEU B 157 40.568 17.414 40.856 0.00 0.00
B
ATOM 5820 HB1 LEU B 157 39.484 17.194 40.967 0.00 0.00
B
ATOM 5821 HB2 LEU B 157 40.759 17.452 39.762 0.00 0.00
B
ATOM 5822 CG LEU B 157 41.237 16.082 41.345 0.00 0.00
B
ATOM 5823 HG LEU B 157 42.324 15.952 41.153 0.00 0.00
B
ATOM 5824 CD1 LEU B 157 40.951 15.881 42.862 0.00 0.00
B
ATOM 5825 HD11 LEU B 157 41.428 16.589 43.574 0.00 0.00
B
ATOM 5826 HD12 LEU B 157 39.880 15.910 43.154 0.00 0.00
B
ATOM 5827 HD13 LEU B 157 41.362 14.870 43.073 0.00 0.00
B
ATOM 5828 CD2 LEU B 157 40.613 14.831 40.668 0.00 0.00
B
ATOM 5829 HD21 LEU B 157 39.514 14.934 40.794 0.00 0.00
B
ATOM 5830 HD22 LEU B 157 40.683 14.881 39.561 0.00 0.00
B
ATOM 5831 HD23 LEU B 157 41.048 13.878 41.037 0.00 0.00
B

ATOM 5832 C LEU B 157 42.522 18.936 41.089 0.00 0.00
B
ATOM 5833 O LEU B 157 42.709 19.197 39.878 0.00 0.00
B
ATOM 5834 N ALA B 158 43.533 18.674 41.924 0.00 0.00
B
ATOM 5835 HN ALA B 158 43.481 18.525 42.908 0.00 0.00
B
ATOM 5836 CA ALA B 158 44.902 18.529 41.491 0.00 0.00
B
ATOM 5837 HA ALA B 158 45.100 19.379 40.855 0.00 0.00
B
ATOM 5838 CB ALA B 158 45.794 18.536 42.760 0.00 0.00
B
ATOM 5839 HB1 ALA B 158 45.648 17.629 43.385 0.00 0.00
B
ATOM 5840 HB2 ALA B 158 46.853 18.540 42.423 0.00 0.00
B
ATOM 5841 HB3 ALA B 158 45.551 19.410 43.401 0.00 0.00
B
ATOM 5842 C ALA B 158 45.289 17.215 40.803 0.00 0.00
B
ATOM 5843 O ALA B 158 45.099 16.053 41.214 0.00 0.00
B
ATOM 5844 N THR B 159 45.733 17.478 39.569 0.00 0.00
B
ATOM 5845 HN THR B 159 45.864 18.363 39.129 0.00 0.00
B
ATOM 5846 CA THR B 159 46.153 16.356 38.696 0.00 0.00
B
ATOM 5847 HA THR B 159 45.468 15.523 38.758 0.00 0.00
B
ATOM 5848 CB THR B 159 46.108 16.829 37.271 0.00 0.00
B
ATOM 5849 HB THR B 159 46.381 15.984 36.604 0.00 0.00
B
ATOM 5850 OG1 THR B 159 46.853 18.002 37.041 0.00 0.00
B
ATOM 5851 HG1 THR B 159 46.629 18.266 36.145 0.00 0.00
B
ATOM 5852 CG2 THR B 159 44.620 17.245 37.036 0.00 0.00
B
ATOM 5853 HG21 THR B 159 44.360 17.582 36.010 0.00 0.00
B
ATOM 5854 HG22 THR B 159 43.816 16.536 37.326 0.00 0.00
B
ATOM 5855 HG23 THR B 159 44.336 18.090 37.698 0.00 0.00
B
ATOM 5856 C THR B 159 47.585 15.829 38.955 0.00 0.00
B
ATOM 5857 O THR B 159 48.388 16.497 39.647 0.00 0.00
B
ATOM 5858 N ARG B 160 48.005 14.739 38.227 0.00 0.00
B

ATOM 5859 HN ARG B 160 47.429 14.179 37.637 0.00 0.00
B
ATOM 5860 CA ARG B 160 49.413 14.361 38.266 0.00 0.00
B
ATOM 5861 HA ARG B 160 49.756 14.360 39.291 0.00 0.00
B
ATOM 5862 CB ARG B 160 49.528 12.850 37.924 0.00 0.00
B
ATOM 5863 HB1 ARG B 160 48.841 12.196 38.502 0.00 0.00
B
ATOM 5864 HB2 ARG B 160 49.104 12.776 36.900 0.00 0.00
B
ATOM 5865 CG ARG B 160 50.854 12.160 38.224 0.00 0.00
B
ATOM 5866 HG1 ARG B 160 51.691 12.714 37.746 0.00 0.00
B
ATOM 5867 HG2 ARG B 160 51.142 12.186 39.297 0.00 0.00
B
ATOM 5868 CD ARG B 160 50.878 10.663 37.728 0.00 0.00
B
ATOM 5869 HD1 ARG B 160 50.434 10.580 36.713 0.00 0.00
B
ATOM 5870 HD2 ARG B 160 51.914 10.261 37.744 0.00 0.00
B
ATOM 5871 NE ARG B 160 50.065 9.927 38.732 0.00 0.00
B
ATOM 5872 HE ARG B 160 50.008 10.187 39.696 0.00 0.00
B
ATOM 5873 CZ ARG B 160 49.357 8.801 38.500 0.00 0.00
B
ATOM 5874 NH1 ARG B 160 49.235 8.349 37.276 0.00 0.00
B
ATOM 5875 HH11 ARG B 160 48.846 7.437 37.143 0.00 0.00
B
ATOM 5876 HH12 ARG B 160 49.658 8.804 36.493 0.00 0.00
B
ATOM 5877 NH2 ARG B 160 48.793 8.212 39.548 0.00 0.00
B
ATOM 5878 HH21 ARG B 160 47.975 7.644 39.461 0.00 0.00
B
ATOM 5879 HH22 ARG B 160 48.756 8.763 40.382 0.00 0.00
B
ATOM 5880 C ARG B 160 50.527 15.237 37.642 0.00 0.00
B
ATOM 5881 O ARG B 160 51.711 15.331 37.996 0.00 0.00
B
ATOM 5882 N LYS B 161 50.054 16.020 36.683 0.00 0.00
B
ATOM 5883 HN LYS B 161 49.204 15.710 36.264 0.00 0.00
B
ATOM 5884 CA LYS B 161 50.831 17.147 36.126 0.00 0.00
B
ATOM 5885 HA LYS B 161 51.792 16.727 35.867 0.00 0.00
B

ATOM 5886 CB LYS B 161 50.174 17.691 34.846 0.00 0.00
B
ATOM 5887 HB1 LYS B 161 49.180 18.133 35.074 0.00 0.00
B
ATOM 5888 HB2 LYS B 161 50.754 18.539 34.423 0.00 0.00
B
ATOM 5889 CG LYS B 161 50.189 16.687 33.648 0.00 0.00
B
ATOM 5890 HG1 LYS B 161 51.223 16.399 33.363 0.00 0.00
B
ATOM 5891 HG2 LYS B 161 49.768 15.727 34.014 0.00 0.00
B
ATOM 5892 CD LYS B 161 49.357 17.141 32.404 0.00 0.00
B
ATOM 5893 HD1 LYS B 161 49.458 16.308 31.676 0.00 0.00
B
ATOM 5894 HD2 LYS B 161 48.295 17.238 32.715 0.00 0.00
B
ATOM 5895 CE LYS B 161 49.718 18.457 31.834 0.00 0.00
B
ATOM 5896 HE1 LYS B 161 49.124 19.149 32.468 0.00 0.00
B
ATOM 5897 HE2 LYS B 161 50.796 18.725 31.802 0.00 0.00
B
ATOM 5898 NZ LYS B 161 49.434 18.523 30.405 0.00 0.00
B
ATOM 5899 HZ1 LYS B 161 48.428 18.777 30.338 0.00 0.00
B
ATOM 5900 HZ2 LYS B 161 49.890 19.306 29.894 0.00 0.00
B
ATOM 5901 HZ3 LYS B 161 49.550 17.687 29.797 0.00 0.00
B
ATOM 5902 C LYS B 161 51.025 18.276 37.120 0.00 0.00
B
ATOM 5903 O LYS B 161 52.064 18.943 37.005 0.00 0.00
B
ATOM 5904 N LEU B 162 50.186 18.401 38.172 0.00 0.00
B
ATOM 5905 HN LEU B 162 49.457 17.749 38.364 0.00 0.00
B
ATOM 5906 CA LEU B 162 50.221 19.450 39.101 0.00 0.00
B
ATOM 5907 HA LEU B 162 50.854 20.220 38.686 0.00 0.00
B
ATOM 5908 CB LEU B 162 48.782 19.947 39.428 0.00 0.00
B
ATOM 5909 HB1 LEU B 162 48.081 19.677 38.609 0.00 0.00
B
ATOM 5910 HB2 LEU B 162 48.483 19.330 40.303 0.00 0.00
B
ATOM 5911 CG LEU B 162 48.548 21.409 39.803 0.00 0.00
B
ATOM 5912 HG LEU B 162 49.368 21.818 40.431 0.00 0.00
B

ATOM 5913 CD1 LEU B 162 48.440 22.110 38.447 0.00 0.00
B
ATOM 5914 HD11 LEU B 162 47.659 21.745 37.746 0.00 0.00
B
ATOM 5915 HD12 LEU B 162 48.388 23.202 38.647 0.00 0.00
B
ATOM 5916 HD13 LEU B 162 49.390 21.858 37.929 0.00 0.00
B
ATOM 5917 CD2 LEU B 162 47.203 21.567 40.549 0.00 0.00
B
ATOM 5918 HD21 LEU B 162 47.146 22.633 40.859 0.00 0.00
B
ATOM 5919 HD22 LEU B 162 46.346 21.305 39.892 0.00 0.00
B
ATOM 5920 HD23 LEU B 162 47.218 20.875 41.418 0.00 0.00
B
ATOM 5921 C LEU B 162 51.029 19.092 40.363 0.00 0.00
B
ATOM 5922 O LEU B 162 51.730 19.854 41.011 0.00 0.00
B
ATOM 5923 N ASP B 163 51.003 17.788 40.745 0.00 0.00
B
ATOM 5924 HN ASP B 163 50.498 17.127 40.195 0.00 0.00
B
ATOM 5925 CA ASP B 163 51.811 17.109 41.797 0.00 0.00
B
ATOM 5926 HA ASP B 163 51.396 17.444 42.736 0.00 0.00
B
ATOM 5927 CB ASP B 163 51.600 15.595 41.580 0.00 0.00
B
ATOM 5928 HB1 ASP B 163 50.498 15.474 41.513 0.00 0.00
B
ATOM 5929 HB2 ASP B 163 51.943 15.144 40.624 0.00 0.00
B
ATOM 5930 CG ASP B 163 52.228 14.755 42.694 0.00 0.00
B
ATOM 5931 OD1 ASP B 163 51.512 13.895 43.273 0.00 0.00
B
ATOM 5932 OD2 ASP B 163 53.513 14.721 42.650 0.00 0.00
B
ATOM 5933 C ASP B 163 53.306 17.499 41.784 0.00 0.00
B
ATOM 5934 O ASP B 163 53.930 17.483 40.716 0.00 0.00
B
ATOM 5935 N SER B 164 53.898 17.818 42.923 0.00 0.00
B
ATOM 5936 HN SER B 164 53.400 17.937 43.778 0.00 0.00
B
ATOM 5937 CA SER B 164 55.350 18.032 43.109 0.00 0.00
B
ATOM 5938 HA SER B 164 55.430 18.125 44.182 0.00 0.00
B
ATOM 5939 CB SER B 164 56.150 16.784 42.653 0.00 0.00
B

ATOM	5940	HB1	SER	B	164	55.911	16.612	41.582	0.00	0.00	B
ATOM	5941	HB2	SER	B	164	57.242	16.972	42.735	0.00	0.00	B
ATOM	5942	OG	SER	B	164	55.920	15.623	43.396	0.00	0.00	B
ATOM	5943	HG1	SER	B	164	55.052	15.292	43.154	0.00	0.00	B
ATOM	5944	C	SER	B	164	55.916	19.325	42.664	0.00	0.00	B
ATOM	5945	O	SER	B	164	57.090	19.574	42.652	0.00	0.00	B
ATOM	5946	N	LYS	B	165	55.052	20.314	42.365	0.00	0.00	B
ATOM	5947	HN	LYS	B	165	54.061	20.215	42.338	0.00	0.00	B
ATOM	5948	CA	LYS	B	165	55.388	21.727	42.159	0.00	0.00	B
ATOM	5949	HA	LYS	B	165	56.460	21.784	42.275	0.00	0.00	B
ATOM	5950	CB	LYS	B	165	54.863	22.240	40.841	0.00	0.00	B
ATOM	5951	HB1	LYS	B	165	53.758	22.241	40.949	0.00	0.00	B
ATOM	5952	HB2	LYS	B	165	55.151	23.301	40.681	0.00	0.00	B
ATOM	5953	CG	LYS	B	165	55.474	21.528	39.613	0.00	0.00	B
ATOM	5954	HG1	LYS	B	165	56.541	21.824	39.524	0.00	0.00	B
ATOM	5955	HG2	LYS	B	165	55.500	20.435	39.813	0.00	0.00	B
ATOM	5956	CD	LYS	B	165	54.733	21.701	38.251	0.00	0.00	B
ATOM	5957	HD1	LYS	B	165	53.697	21.338	38.426	0.00	0.00	B
ATOM	5958	HD2	LYS	B	165	54.801	22.776	37.978	0.00	0.00	B
ATOM	5959	CE	LYS	B	165	55.328	20.780	37.070	0.00	0.00	B
ATOM	5960	HE1	LYS	B	165	54.873	21.055	36.095	0.00	0.00	B
ATOM	5961	HE2	LYS	B	165	56.436	20.818	36.992	0.00	0.00	B
ATOM	5962	NZ	LYS	B	165	55.013	19.364	37.413	0.00	0.00	B
ATOM	5963	HZ1	LYS	B	165	55.427	19.037	38.309	0.00	0.00	B
ATOM	5964	HZ2	LYS	B	165	54.028	19.047	37.317	0.00	0.00	B
ATOM	5965	HZ3	LYS	B	165	55.577	18.818	36.730	0.00	0.00	B
ATOM	5966	C	LYS	B	165	54.820	22.596	43.181	0.00	0.00	B

ATOM 5967 O LYS B 165 53.796 22.360 43.760 0.00 0.00
B
ATOM 5968 N SER B 166 55.620 23.566 43.600 0.00 0.00
B
ATOM 5969 HN SER B 166 56.448 23.777 43.087 0.00 0.00
B
ATOM 5970 CA SER B 166 55.136 24.552 44.540 0.00 0.00
B
ATOM 5971 HA SER B 166 54.659 23.973 45.317 0.00 0.00
B
ATOM 5972 CB SER B 166 56.242 25.391 45.280 0.00 0.00
B
ATOM 5973 HB1 SER B 166 56.893 24.704 45.861 0.00 0.00
B
ATOM 5974 HB2 SER B 166 56.927 25.897 44.565 0.00 0.00
B
ATOM 5975 OG SER B 166 55.706 26.402 46.128 0.00 0.00
B
ATOM 5976 HG1 SER B 166 56.428 27.014 46.287 0.00 0.00
B
ATOM 5977 C SER B 166 54.070 25.387 43.897 0.00 0.00
B
ATOM 5978 O SER B 166 54.011 25.494 42.706 0.00 0.00
B
ATOM 5979 N ILE B 167 53.236 26.090 44.666 0.00 0.00
B
ATOM 5980 HN ILE B 167 53.339 25.980 45.652 0.00 0.00
B
ATOM 5981 CA ILE B 167 52.104 26.896 44.233 0.00 0.00
B
ATOM 5982 HA ILE B 167 51.500 26.340 43.532 0.00 0.00
B
ATOM 5983 CB ILE B 167 51.279 27.286 45.451 0.00 0.00
B
ATOM 5984 HB ILE B 167 50.484 28.016 45.185 0.00 0.00
B
ATOM 5985 CG2 ILE B 167 50.635 25.955 45.859 0.00 0.00
B
ATOM 5986 HG21 ILE B 167 51.364 25.391 46.479 0.00 0.00
B
ATOM 5987 HG22 ILE B 167 49.806 26.141 46.575 0.00 0.00
B
ATOM 5988 HG23 ILE B 167 50.228 25.337 45.030 0.00 0.00
B
ATOM 5989 CG1 ILE B 167 52.106 27.812 46.674 0.00 0.00
B
ATOM 5990 HG11 ILE B 167 52.716 26.997 47.119 0.00 0.00
B
ATOM 5991 HG12 ILE B 167 52.806 28.618 46.366 0.00 0.00
B
ATOM 5992 CD ILE B 167 51.209 28.376 47.757 0.00 0.00
B
ATOM 5993 HD1 ILE B 167 51.773 28.812 48.609 0.00 0.00
B

ATOM 5994 HD2 ILE B 167 50.574 29.251 47.501 0.00 0.00
B
ATOM 5995 HD3 ILE B 167 50.584 27.586 48.225 0.00 0.00
B
ATOM 5996 C ILE B 167 52.530 28.062 43.408 0.00 0.00
B
ATOM 5997 O ILE B 167 51.823 28.412 42.474 0.00 0.00
B
ATOM 5998 N ILE B 168 53.693 28.591 43.778 0.00 0.00
B
ATOM 5999 HN ILE B 168 54.174 28.170 44.542 0.00 0.00
B
ATOM 6000 CA ILE B 168 54.368 29.627 42.973 0.00 0.00
B
ATOM 6001 HA ILE B 168 53.642 30.420 42.867 0.00 0.00
B
ATOM 6002 CB ILE B 168 55.556 30.296 43.672 0.00 0.00
B
ATOM 6003 HB ILE B 168 55.297 31.021 44.472 0.00 0.00
B
ATOM 6004 CG2 ILE B 168 56.365 29.203 44.373 0.00 0.00
B
ATOM 6005 HG21 ILE B 168 55.722 28.648 45.089 0.00 0.00
B
ATOM 6006 HG22 ILE B 168 56.768 28.489 43.622 0.00 0.00
B
ATOM 6007 HG23 ILE B 168 57.273 29.533 44.921 0.00 0.00
B
ATOM 6008 CG1 ILE B 168 56.335 31.074 42.610 0.00 0.00
B
ATOM 6009 HG11 ILE B 168 56.770 30.337 41.901 0.00 0.00
B
ATOM 6010 HG12 ILE B 168 55.579 31.639 42.024 0.00 0.00
B
ATOM 6011 CD ILE B 168 57.392 31.923 43.238 0.00 0.00
B
ATOM 6012 HD1 ILE B 168 58.156 31.329 43.784 0.00 0.00
B
ATOM 6013 HD2 ILE B 168 57.934 32.621 42.564 0.00 0.00
B
ATOM 6014 HD3 ILE B 168 56.942 32.662 43.935 0.00 0.00
B
ATOM 6015 C ILE B 168 54.701 29.142 41.533 0.00 0.00
B
ATOM 6016 O ILE B 168 54.607 29.904 40.554 0.00 0.00
B
ATOM 6017 N ASP B 169 55.039 27.822 41.340 0.00 0.00
B
ATOM 6018 HN ASP B 169 55.030 27.133 42.060 0.00 0.00
B
ATOM 6019 CA ASP B 169 55.442 27.282 40.023 0.00 0.00
B
ATOM 6020 HA ASP B 169 56.057 28.050 39.578 0.00 0.00
B

ATOM 6021 CB ASP B 169 56.268 26.036 40.125 0.00 0.00
B
ATOM 6022 HB1 ASP B 169 55.663 25.170 40.472 0.00 0.00
B
ATOM 6023 HB2 ASP B 169 56.724 25.629 39.196 0.00 0.00
B
ATOM 6024 CG ASP B 169 57.450 26.230 41.027 0.00 0.00
B
ATOM 6025 OD1 ASP B 169 57.619 25.442 42.041 0.00 0.00
B
ATOM 6026 OD2 ASP B 169 58.292 27.116 40.670 0.00 0.00
B
ATOM 6027 C ASP B 169 54.246 27.037 39.109 0.00 0.00
B
ATOM 6028 O ASP B 169 54.404 26.855 37.855 0.00 0.00
B
ATOM 6029 N LEU B 170 52.992 27.084 39.622 0.00 0.00
B
ATOM 6030 HN LEU B 170 52.788 27.026 40.596 0.00 0.00
B
ATOM 6031 CA LEU B 170 51.788 26.967 38.725 0.00 0.00
B
ATOM 6032 HA LEU B 170 51.962 26.297 37.896 0.00 0.00
B
ATOM 6033 CB LEU B 170 50.604 26.541 39.658 0.00 0.00
B
ATOM 6034 HB1 LEU B 170 50.327 27.431 40.263 0.00 0.00
B
ATOM 6035 HB2 LEU B 170 49.705 26.391 39.023 0.00 0.00
B
ATOM 6036 CG LEU B 170 50.886 25.201 40.387 0.00 0.00
B
ATOM 6037 HG LEU B 170 51.693 25.504 41.088 0.00 0.00
B
ATOM 6038 CD1 LEU B 170 49.710 24.938 41.272 0.00 0.00
B
ATOM 6039 HD11 LEU B 170 48.908 24.751 40.527 0.00 0.00
B
ATOM 6040 HD12 LEU B 170 49.907 24.072 41.941 0.00 0.00
B
ATOM 6041 HD13 LEU B 170 49.491 25.780 41.963 0.00 0.00
B
ATOM 6042 CD2 LEU B 170 51.330 24.058 39.401 0.00 0.00
B
ATOM 6043 HD21 LEU B 170 50.507 23.858 38.683 0.00 0.00
B
ATOM 6044 HD22 LEU B 170 52.256 24.293 38.833 0.00 0.00
B
ATOM 6045 HD23 LEU B 170 51.606 23.216 40.071 0.00 0.00
B
ATOM 6046 C LEU B 170 51.382 28.281 38.150 0.00 0.00
B
ATOM 6047 O LEU B 170 50.630 28.358 37.224 0.00 0.00
B

ATOM 6048 N ASN B 171 51.878 29.430 38.642 0.00 0.00
B
ATOM 6049 HN ASN B 171 52.613 29.544 39.306 0.00 0.00
B
ATOM 6050 CA ASN B 171 51.463 30.755 38.100 0.00 0.00
B
ATOM 6051 HA ASN B 171 51.910 31.539 38.692 0.00 0.00
B
ATOM 6052 CB ASN B 171 51.943 31.064 36.669 0.00 0.00
B
ATOM 6053 HB1 ASN B 171 51.693 30.232 35.976 0.00 0.00
B
ATOM 6054 HB2 ASN B 171 51.600 32.042 36.270 0.00 0.00
B
ATOM 6055 CG ASN B 171 53.508 31.175 36.758 0.00 0.00
B
ATOM 6056 OD1 ASN B 171 54.179 32.194 37.041 0.00 0.00
B
ATOM 6057 ND2 ASN B 171 54.182 30.034 36.585 0.00 0.00
B
ATOM 6058 HD21 ASN B 171 55.163 30.113 36.407 0.00 0.00
B
ATOM 6059 HD22 ASN B 171 53.692 29.207 36.310 0.00 0.00
B
ATOM 6060 C ASN B 171 50.036 31.190 38.259 0.00 0.00
B
ATOM 6061 O ASN B 171 49.452 31.771 37.390 0.00 0.00
B
ATOM 6062 N VAL B 172 49.377 30.941 39.476 0.00 0.00
B
ATOM 6063 HN VAL B 172 49.962 30.523 40.167 0.00 0.00
B
ATOM 6064 CA VAL B 172 47.981 31.349 39.755 0.00 0.00
B
ATOM 6065 HA VAL B 172 47.315 30.847 39.069 0.00 0.00
B
ATOM 6066 CB VAL B 172 47.585 30.845 41.128 0.00 0.00
B
ATOM 6067 HB VAL B 172 48.064 31.356 41.990 0.00 0.00
B
ATOM 6068 CG1 VAL B 172 46.115 31.048 41.290 0.00 0.00
B
ATOM 6069 HG11 VAL B 172 45.566 30.567 40.453 0.00 0.00
B
ATOM 6070 HG12 VAL B 172 45.728 30.693 42.269 0.00 0.00
B
ATOM 6071 HG13 VAL B 172 45.824 32.117 41.207 0.00 0.00
B
ATOM 6072 CG2 VAL B 172 47.969 29.334 41.279 0.00 0.00
B
ATOM 6073 HG21 VAL B 172 47.539 28.736 40.447 0.00 0.00
B
ATOM 6074 HG22 VAL B 172 49.064 29.275 41.098 0.00 0.00
B

```
ATOM   6075 HG23 VAL B 172      47.692  29.055  42.318  0.00  0.00
B
ATOM   6076  C   VAL B 172      47.765  32.829  39.612  0.00  0.00
B
ATOM   6077  O   VAL B 172      46.822  33.246  38.951  0.00  0.00
B
ATOM   6078  N   ARG B 173      48.714  33.691  40.073  0.00  0.00
B
ATOM   6079  HN  ARG B 173      49.440  33.257  40.600  0.00  0.00
B
ATOM   6080  CA  ARG B 173      48.456  35.115  40.062  0.00  0.00
B
ATOM   6081  HA  ARG B 173      47.445  35.346  39.761  0.00  0.00
B
ATOM   6082  CB  ARG B 173      48.864  35.748  41.443  0.00  0.00
B
ATOM   6083  HB1 ARG B 173      48.400  35.102  42.218  0.00  0.00
B
ATOM   6084  HB2 ARG B 173      49.968  35.731  41.571  0.00  0.00
B
ATOM   6085  CG  ARG B 173      48.398  37.194  41.947  0.00  0.00
B
ATOM   6086  HG1 ARG B 173      47.290  37.271  41.948  0.00  0.00
B
ATOM   6087  HG2 ARG B 173      48.789  37.262  42.984  0.00  0.00
B
ATOM   6088  CD  ARG B 173      48.925  38.418  41.234  0.00  0.00
B
ATOM   6089  HD1 ARG B 173      50.021  38.319  41.080  0.00  0.00
B
ATOM   6090  HD2 ARG B 173      48.454  38.510  40.232  0.00  0.00
B
ATOM   6091  NE  ARG B 173      48.620  39.545  42.024  0.00  0.00
B
ATOM   6092  HE  ARG B 173      48.710  39.531  43.020  0.00  0.00
B
ATOM   6093  CZ  ARG B 173      48.581  40.793  41.575  0.00  0.00
B
ATOM   6094  NH1 ARG B 173      48.816  41.072  40.257  0.00  0.00
B
ATOM   6095 HH11 ARG B 173      48.790  42.019  39.935  0.00  0.00
B
ATOM   6096 HH12 ARG B 173      48.914  40.321  39.605  0.00  0.00
B
ATOM   6097  NH2 ARG B 173      48.355  41.872  42.337  0.00  0.00
B
ATOM   6098 HH21 ARG B 173      48.449  42.732  41.835  0.00  0.00
B
ATOM   6099 HH22 ARG B 173      47.994  41.695  43.252  0.00  0.00
B
ATOM   6100  C   ARG B 173      49.242  35.790  38.980  0.00  0.00
B
ATOM   6101  O   ARG B 173      48.848  36.858  38.460  0.00  0.00
B
```

ATOM 6102 N ALA B 174 50.435 35.219 38.566 0.00 0.00
B
ATOM 6103 HN ALA B 174 50.848 34.458 39.061 0.00 0.00
B
ATOM 6104 CA ALA B 174 51.318 35.809 37.626 0.00 0.00
B
ATOM 6105 HA ALA B 174 51.513 36.823 37.945 0.00 0.00
B
ATOM 6106 CB ALA B 174 52.657 35.039 37.571 0.00 0.00
B
ATOM 6107 HB1 ALA B 174 53.525 35.538 37.089 0.00 0.00
B
ATOM 6108 HB2 ALA B 174 52.928 34.732 38.604 0.00 0.00
B
ATOM 6109 HB3 ALA B 174 52.384 34.012 37.250 0.00 0.00
B
ATOM 6110 C ALA B 174 50.675 35.918 36.201 0.00 0.00
B
ATOM 6111 O ALA B 174 50.741 36.910 35.545 0.00 0.00
B
ATOM 6112 N LYS B 175 50.049 34.777 35.714 0.00 0.00
B
ATOM 6113 HN LYS B 175 49.916 33.993 36.315 0.00 0.00
B
ATOM 6114 CA LYS B 175 49.423 34.718 34.403 0.00 0.00
B
ATOM 6115 HA LYS B 175 49.815 35.477 33.743 0.00 0.00
B
ATOM 6116 CB LYS B 175 49.855 33.365 33.932 0.00 0.00
B
ATOM 6117 HB1 LYS B 175 49.510 32.518 34.563 0.00 0.00
B
ATOM 6118 HB2 LYS B 175 49.258 33.230 33.005 0.00 0.00
B
ATOM 6119 CG LYS B 175 51.295 33.167 33.426 0.00 0.00
B
ATOM 6120 HG1 LYS B 175 51.478 33.911 32.622 0.00 0.00
B
ATOM 6121 HG2 LYS B 175 51.942 33.276 34.323 0.00 0.00
B
ATOM 6122 CD LYS B 175 51.467 31.726 32.742 0.00 0.00
B
ATOM 6123 HD1 LYS B 175 51.436 30.955 33.541 0.00 0.00
B
ATOM 6124 HD2 LYS B 175 50.601 31.541 32.070 0.00 0.00
B
ATOM 6125 CE LYS B 175 52.751 31.632 31.999 0.00 0.00
B
ATOM 6126 HE1 LYS B 175 52.938 32.328 31.154 0.00 0.00
B
ATOM 6127 HE2 LYS B 175 53.654 31.761 32.633 0.00 0.00
B
ATOM 6128 NZ LYS B 175 52.848 30.241 31.457 0.00 0.00
B

ATOM 6129 HZ1 LYS B 175 52.155 30.176 30.685 0.00 0.00
B
ATOM 6130 HZ2 LYS B 175 53.795 29.991 31.106 0.00 0.00
B
ATOM 6131 HZ3 LYS B 175 52.606 29.523 32.170 0.00 0.00
B
ATOM 6132 C LYS B 175 47.940 35.009 34.362 0.00 0.00
B
ATOM 6133 O LYS B 175 47.400 35.587 33.406 0.00 0.00
B
ATOM 6134 N TYR B 176 47.157 34.613 35.379 0.00 0.00
B
ATOM 6135 HN TYR B 176 47.588 34.051 36.081 0.00 0.00
B
ATOM 6136 CA TYR B 176 45.697 34.624 35.412 0.00 0.00
B
ATOM 6137 HA TYR B 176 45.273 34.895 34.457 0.00 0.00
B
ATOM 6138 CB TYR B 176 45.118 33.198 35.892 0.00 0.00
B
ATOM 6139 HB1 TYR B 176 45.311 33.146 36.985 0.00 0.00
B
ATOM 6140 HB2 TYR B 176 44.012 33.138 35.810 0.00 0.00
B
ATOM 6141 CG TYR B 176 45.759 32.120 35.033 0.00 0.00
B
ATOM 6142 CD1 TYR B 176 46.769 31.322 35.625 0.00 0.00
B
ATOM 6143 HD1 TYR B 176 46.988 31.338 36.682 0.00 0.00
B
ATOM 6144 CE1 TYR B 176 47.662 30.631 34.810 0.00 0.00
B
ATOM 6145 HE1 TYR B 176 48.512 30.076 35.179 0.00 0.00
B
ATOM 6146 CZ TYR B 176 47.492 30.570 33.440 0.00 0.00
B
ATOM 6147 OH TYR B 176 48.455 29.996 32.660 0.00 0.00
B
ATOM 6148 HH TYR B 176 48.270 30.341 31.783 0.00 0.00
B
ATOM 6149 CD2 TYR B 176 45.513 32.019 33.665 0.00 0.00
B
ATOM 6150 HD2 TYR B 176 44.870 32.748 33.194 0.00 0.00
B
ATOM 6151 CE2 TYR B 176 46.442 31.310 32.854 0.00 0.00
B
ATOM 6152 HE2 TYR B 176 46.406 31.462 31.786 0.00 0.00
B
ATOM 6153 C TYR B 176 45.157 35.693 36.344 0.00 0.00
B
ATOM 6154 O TYR B 176 44.032 36.230 36.156 0.00 0.00
B
ATOM 6155 N GLY B 177 45.985 36.103 37.370 0.00 0.00
B

ATOM 6156 HN GLY B 177 46.796 35.578 37.616 0.00 0.00
B
ATOM 6157 CA GLY B 177 45.539 37.193 38.247 0.00 0.00
B
ATOM 6158 HA1 GLY B 177 45.048 37.969 37.677 0.00 0.00
B
ATOM 6159 HA2 GLY B 177 46.451 37.531 38.715 0.00 0.00
B
ATOM 6160 C GLY B 177 44.631 36.599 39.255 0.00 0.00
B
ATOM 6161 O GLY B 177 44.035 37.413 39.982 0.00 0.00
B
ATOM 6162 N CYS B 178 44.521 35.258 39.354 0.00 0.00
B
ATOM 6163 HN CYS B 178 45.144 34.586 38.961 0.00 0.00
B
ATOM 6164 CA CYS B 178 43.659 34.670 40.372 0.00 0.00
B
ATOM 6165 HA CYS B 178 42.734 35.225 40.413 0.00 0.00
B
ATOM 6166 CB CYS B 178 43.332 33.232 39.892 0.00 0.00
B
ATOM 6167 HB1 CYS B 178 44.244 32.645 39.653 0.00 0.00
B
ATOM 6168 HB2 CYS B 178 42.827 32.710 40.732 0.00 0.00
B
ATOM 6169 SG CYS B 178 42.345 33.299 38.400 0.00 0.00
B
ATOM 6170 HG1 CYS B 178 41.220 33.500 39.070 0.00 0.00
B
ATOM 6171 C CYS B 178 44.230 34.789 41.753 0.00 0.00
B
ATOM 6172 O CYS B 178 45.295 35.312 42.004 0.00 0.00
B
ATOM 6173 N THR B 179 43.405 34.343 42.720 0.00 0.00
B
ATOM 6174 HN THR B 179 42.461 34.043 42.608 0.00 0.00
B
ATOM 6175 CA THR B 179 43.764 34.449 44.111 0.00 0.00
B
ATOM 6176 HA THR B 179 44.815 34.683 44.191 0.00 0.00
B
ATOM 6177 CB THR B 179 42.817 35.432 44.774 0.00 0.00
B
ATOM 6178 HB THR B 179 41.779 35.036 44.779 0.00 0.00
B
ATOM 6179 OG1 THR B 179 42.971 36.692 44.186 0.00 0.00
B
ATOM 6180 HG1 THR B 179 42.231 36.955 43.633 0.00 0.00
B
ATOM 6181 CG2 THR B 179 43.084 35.774 46.208 0.00 0.00
B
ATOM 6182 HG21 THR B 179 43.006 34.821 46.774 0.00 0.00
B

ATOM 6183 HG22 THR B 179 44.130 36.118 46.357 0.00 0.00
B
ATOM 6184 HG23 THR B 179 42.306 36.362 46.739 0.00 0.00
B
ATOM 6185 C THR B 179 43.499 33.029 44.737 0.00 0.00
B
ATOM 6186 O THR B 179 42.394 32.562 44.668 0.00 0.00
B
ATOM 6187 N ILE B 180 44.428 32.390 45.388 0.00 0.00
B
ATOM 6188 HN ILE B 180 45.347 32.767 45.473 0.00 0.00
B
ATOM 6189 CA ILE B 180 44.236 31.106 46.088 0.00 0.00
B
ATOM 6190 HA ILE B 180 43.483 30.668 45.451 0.00 0.00
B
ATOM 6191 CB ILE B 180 45.544 30.330 46.243 0.00 0.00
B
ATOM 6192 HB ILE B 180 46.295 30.870 46.859 0.00 0.00
B
ATOM 6193 CG2 ILE B 180 45.248 28.994 46.793 0.00 0.00
B
ATOM 6194 HG21 ILE B 180 44.596 28.440 46.083 0.00 0.00
B
ATOM 6195 HG22 ILE B 180 46.158 28.380 46.962 0.00 0.00
B
ATOM 6196 HG23 ILE B 180 44.795 29.074 47.804 0.00 0.00
B
ATOM 6197 CG1 ILE B 180 46.256 30.051 44.884 0.00 0.00
B
ATOM 6198 HG11 ILE B 180 45.931 29.094 44.423 0.00 0.00
B
ATOM 6199 HG12 ILE B 180 46.202 30.936 44.213 0.00 0.00
B
ATOM 6200 CD ILE B 180 47.719 29.766 45.149 0.00 0.00
B
ATOM 6201 HD1 ILE B 180 48.149 30.601 45.743 0.00 0.00
B
ATOM 6202 HD2 ILE B 180 47.885 28.784 45.640 0.00 0.00
B
ATOM 6203 HD3 ILE B 180 48.313 29.771 44.210 0.00 0.00
B
ATOM 6204 C ILE B 180 43.609 31.413 47.418 0.00 0.00
B
ATOM 6205 O ILE B 180 44.039 32.209 48.188 0.00 0.00
B
ATOM 6206 N LEU B 181 42.458 30.759 47.706 0.00 0.00
B
ATOM 6207 HN LEU B 181 41.999 30.131 47.081 0.00 0.00
B
ATOM 6208 CA LEU B 181 41.719 30.883 48.932 0.00 0.00
B
ATOM 6209 HA LEU B 181 41.969 31.845 49.354 0.00 0.00
B

ATOM 6210 CB LEU B 181 40.187 30.923 48.706 0.00 0.00
B
ATOM 6211 HB1 LEU B 181 39.933 29.919 48.303 0.00 0.00
B
ATOM 6212 HB2 LEU B 181 39.618 31.119 49.640 0.00 0.00
B
ATOM 6213 CG LEU B 181 39.692 31.990 47.681 0.00 0.00
B
ATOM 6214 HG LEU B 181 39.831 31.538 46.675 0.00 0.00
B
ATOM 6215 CD1 LEU B 181 38.138 32.197 47.911 0.00 0.00
B
ATOM 6216 HD11 LEU B 181 37.611 32.607 47.022 0.00 0.00
B
ATOM 6217 HD12 LEU B 181 37.744 31.179 48.116 0.00 0.00
B
ATOM 6218 HD13 LEU B 181 37.935 32.808 48.816 0.00 0.00
B
ATOM 6219 CD2 LEU B 181 40.527 33.321 47.754 0.00 0.00
B
ATOM 6220 HD21 LEU B 181 41.533 33.159 47.312 0.00 0.00
B
ATOM 6221 HD22 LEU B 181 39.905 33.965 47.096 0.00 0.00
B
ATOM 6222 HD23 LEU B 181 40.591 33.659 48.811 0.00 0.00
B
ATOM 6223 C LEU B 181 42.129 29.760 49.872 0.00 0.00
B
ATOM 6224 O LEU B 181 42.187 29.998 51.070 0.00 0.00
B
ATOM 6225 N ALA B 182 42.394 28.531 49.367 0.00 0.00
B
ATOM 6226 HN ALA B 182 42.182 28.365 48.408 0.00 0.00
B
ATOM 6227 CA ALA B 182 42.582 27.350 50.184 0.00 0.00
B
ATOM 6228 HA ALA B 182 43.253 27.523 51.013 0.00 0.00
B
ATOM 6229 CB ALA B 182 41.303 26.898 50.862 0.00 0.00
B
ATOM 6230 HB1 ALA B 182 41.445 26.103 51.625 0.00 0.00
B
ATOM 6231 HB2 ALA B 182 40.918 27.833 51.321 0.00 0.00
B
ATOM 6232 HB3 ALA B 182 40.575 26.553 50.097 0.00 0.00
B
ATOM 6233 C ALA B 182 43.157 26.230 49.359 0.00 0.00
B
ATOM 6234 O ALA B 182 42.998 26.173 48.108 0.00 0.00
B
ATOM 6235 N ILE B 183 43.851 25.355 50.021 0.00 0.00
B
ATOM 6236 HN ILE B 183 44.023 25.567 50.980 0.00 0.00
B

ATOM 6237 CA ILE B 183 44.369 24.071 49.465 0.00 0.00
B
ATOM 6238 HA ILE B 183 44.001 23.967 48.455 0.00 0.00
B
ATOM 6239 CB ILE B 183 45.948 24.030 49.515 0.00 0.00
B
ATOM 6240 HB ILE B 183 46.411 23.800 50.499 0.00 0.00
B
ATOM 6241 CG2 ILE B 183 46.273 22.944 48.525 0.00 0.00
B
ATOM 6242 HG21 ILE B 183 47.361 22.742 48.423 0.00 0.00
B
ATOM 6243 HG22 ILE B 183 45.850 21.941 48.748 0.00 0.00
B
ATOM 6244 HG23 ILE B 183 45.987 23.188 47.480 0.00 0.00
B
ATOM 6245 CG1 ILE B 183 46.540 25.334 48.966 0.00 0.00
B
ATOM 6246 HG11 ILE B 183 46.223 25.494 47.913 0.00 0.00
B
ATOM 6247 HG12 ILE B 183 46.119 26.246 49.442 0.00 0.00
B
ATOM 6248 CD ILE B 183 48.003 25.465 49.136 0.00 0.00
B
ATOM 6249 HD1 ILE B 183 48.495 24.747 48.445 0.00 0.00
B
ATOM 6250 HD2 ILE B 183 48.388 26.470 48.863 0.00 0.00
B
ATOM 6251 HD3 ILE B 183 48.271 25.174 50.174 0.00 0.00
B
ATOM 6252 C ILE B 183 43.753 23.016 50.471 0.00 0.00
B
ATOM 6253 O ILE B 183 43.801 23.170 51.694 0.00 0.00
B
ATOM 6254 N LYS B 184 43.101 22.028 49.912 0.00 0.00
B
ATOM 6255 HN LYS B 184 42.987 21.968 48.923 0.00 0.00
B
ATOM 6256 CA LYS B 184 42.137 21.186 50.744 0.00 0.00
B
ATOM 6257 HA LYS B 184 42.221 21.220 51.820 0.00 0.00
B
ATOM 6258 CB LYS B 184 40.715 21.317 50.375 0.00 0.00
B
ATOM 6259 HB1 LYS B 184 40.670 21.385 49.267 0.00 0.00
B
ATOM 6260 HB2 LYS B 184 40.013 20.462 50.484 0.00 0.00
B
ATOM 6261 CG LYS B 184 39.914 22.519 51.088 0.00 0.00
B
ATOM 6262 HG1 LYS B 184 39.929 22.244 52.164 0.00 0.00
B
ATOM 6263 HG2 LYS B 184 40.550 23.430 51.115 0.00 0.00
B

ATOM 6264 CD LYS B 184 38.528 22.875 50.414 0.00 0.00
B
ATOM 6265 HD1 LYS B 184 38.138 23.729 51.008 0.00 0.00
B
ATOM 6266 HD2 LYS B 184 38.622 23.203 49.357 0.00 0.00
B
ATOM 6267 CE LYS B 184 37.526 21.686 50.490 0.00 0.00
B
ATOM 6268 HE1 LYS B 184 37.900 20.837 49.879 0.00 0.00
B
ATOM 6269 HE2 LYS B 184 37.326 21.350 51.530 0.00 0.00
B
ATOM 6270 NZ LYS B 184 36.163 22.098 50.101 0.00 0.00
B
ATOM 6271 HZ1 LYS B 184 35.738 22.824 50.713 0.00 0.00
B
ATOM 6272 HZ2 LYS B 184 36.227 22.316 49.086 0.00 0.00
B
ATOM 6273 HZ3 LYS B 184 35.579 21.238 50.127 0.00 0.00
B
ATOM 6274 C LYS B 184 42.440 19.771 50.567 0.00 0.00
B
ATOM 6275 O LYS B 184 42.519 19.263 49.426 0.00 0.00
B
ATOM 6276 N HSE B 185 42.680 19.010 51.710 0.00 0.00
B
ATOM 6277 HN HSE B 185 42.610 19.496 52.577 0.00 0.00
B
ATOM 6278 CA HSE B 185 43.411 17.776 51.683 0.00 0.00
B
ATOM 6279 HA HSE B 185 43.640 17.360 50.713 0.00 0.00
B
ATOM 6280 CB HSE B 185 44.650 17.928 52.510 0.00 0.00
B
ATOM 6281 HB1 HSE B 185 44.557 17.939 53.617 0.00 0.00
B
ATOM 6282 HB2 HSE B 185 45.336 17.086 52.273 0.00 0.00
B
ATOM 6283 ND1 HSE B 185 46.340 19.429 53.286 0.00 0.00
B
ATOM 6284 CG HSE B 185 45.404 19.194 52.322 0.00 0.00
B
ATOM 6285 CE1 HSE B 185 46.989 20.464 52.821 0.00 0.00
B
ATOM 6286 HE1 HSE B 185 47.888 20.870 53.285 0.00 0.00
B
ATOM 6287 NE2 HSE B 185 46.656 20.784 51.556 0.00 0.00
B
ATOM 6288 HE2 HSE B 185 47.158 21.350 50.901 0.00 0.00
B
ATOM 6289 CD2 HSE B 185 45.608 19.971 51.202 0.00 0.00
B
ATOM 6290 HD2 HSE B 185 45.072 19.946 50.261 0.00 0.00
B

ATOM 6291 C HSE B 185 42.478 16.705 52.352 0.00 0.00
B
ATOM 6292 O HSE B 185 42.945 15.671 52.902 0.00 0.00
B
ATOM 6293 N HSE B 186 41.131 16.850 52.184 0.00 0.00
B
ATOM 6294 HN HSE B 186 40.757 17.577 51.613 0.00 0.00
B
ATOM 6295 CA HSE B 186 40.053 15.965 52.670 0.00 0.00
B
ATOM 6296 HA HSE B 186 39.122 16.409 52.348 0.00 0.00
B
ATOM 6297 CB HSE B 186 40.148 14.586 52.020 0.00 0.00
B
ATOM 6298 HB1 HSE B 186 40.372 14.772 50.948 0.00 0.00
B
ATOM 6299 HB2 HSE B 186 40.961 14.031 52.534 0.00 0.00
B
ATOM 6300 ND1 HSE B 186 37.761 14.090 51.815 0.00 0.00
B
ATOM 6301 CG HSE B 186 39.042 13.631 52.100 0.00 0.00
B
ATOM 6302 CE1 HSE B 186 37.096 13.021 51.551 0.00 0.00
B
ATOM 6303 HE1 HSE B 186 36.066 12.977 51.199 0.00 0.00
B
ATOM 6304 NE2 HSE B 186 37.825 11.855 51.700 0.00 0.00
B
ATOM 6305 HE2 HSE B 186 37.580 10.955 51.339 0.00 0.00
B
ATOM 6306 CD2 HSE B 186 39.097 12.248 52.086 0.00 0.00
B
ATOM 6307 HD2 HSE B 186 39.984 11.627 52.096 0.00 0.00
B
ATOM 6308 C HSE B 186 40.114 15.857 54.197 0.00 0.00
B
ATOM 6309 O HSE B 186 40.018 14.753 54.763 0.00 0.00
B
ATOM 6310 N GLY B 187 40.122 16.999 54.861 0.00 0.00
B
ATOM 6311 HN GLY B 187 40.052 17.879 54.399 0.00 0.00
B
ATOM 6312 CA GLY B 187 40.021 16.824 56.309 0.00 0.00
B
ATOM 6313 HA1 GLY B 187 40.188 15.830 56.696 0.00 0.00
B
ATOM 6314 HA2 GLY B 187 39.077 17.185 56.689 0.00 0.00
B
ATOM 6315 C GLY B 187 41.017 17.653 57.036 0.00 0.00
B
ATOM 6316 O GLY B 187 41.124 17.513 58.226 0.00 0.00
B
ATOM 6317 N ASP B 188 41.749 18.421 56.258 0.00 0.00
B

ATOM 6318 HN ASP B 188 41.555 18.344 55.283 0.00 0.00
B
ATOM 6319 CA ASP B 188 42.816 19.253 56.697 0.00 0.00
B
ATOM 6320 HA ASP B 188 42.484 19.722 57.611 0.00 0.00
B
ATOM 6321 CB ASP B 188 44.184 18.441 56.801 0.00 0.00
B
ATOM 6322 HB1 ASP B 188 44.245 17.648 56.025 0.00 0.00
B
ATOM 6323 HB2 ASP B 188 44.968 19.140 56.439 0.00 0.00
B
ATOM 6324 CG ASP B 188 44.518 17.835 58.097 0.00 0.00
B
ATOM 6325 OD1 ASP B 188 44.935 18.636 58.981 0.00 0.00
B
ATOM 6326 OD2 ASP B 188 44.333 16.548 58.264 0.00 0.00
B
ATOM 6327 C ASP B 188 42.891 20.253 55.553 0.00 0.00
B
ATOM 6328 O ASP B 188 42.500 19.997 54.426 0.00 0.00
B
ATOM 6329 N ILE B 189 43.336 21.479 55.831 0.00 0.00
B
ATOM 6330 HN ILE B 189 43.457 21.607 56.812 0.00 0.00
B
ATOM 6331 CA ILE B 189 43.156 22.639 55.021 0.00 0.00
B
ATOM 6332 HA ILE B 189 43.094 22.360 53.979 0.00 0.00
B
ATOM 6333 CB ILE B 189 41.791 23.338 55.294 0.00 0.00
B
ATOM 6334 HB ILE B 189 41.117 22.568 54.863 0.00 0.00
B
ATOM 6335 CG2 ILE B 189 41.586 23.679 56.802 0.00 0.00
B
ATOM 6336 HG21 ILE B 189 41.592 22.768 57.437 0.00 0.00
B
ATOM 6337 HG22 ILE B 189 42.366 24.393 57.143 0.00 0.00
B
ATOM 6338 HG23 ILE B 189 40.675 24.276 57.020 0.00 0.00
B
ATOM 6339 CG1 ILE B 189 41.612 24.601 54.324 0.00 0.00
B
ATOM 6340 HG11 ILE B 189 42.135 25.433 54.842 0.00 0.00
B
ATOM 6341 HG12 ILE B 189 42.136 24.461 53.354 0.00 0.00
B
ATOM 6342 CD ILE B 189 40.182 25.143 54.051 0.00 0.00
B
ATOM 6343 HD1 ILE B 189 40.290 25.985 53.334 0.00 0.00
B
ATOM 6344 HD2 ILE B 189 39.490 24.431 53.553 0.00 0.00
B

ATOM 6345 HD3 ILE B 189 39.746 25.580 54.975 0.00 0.00 B
ATOM 6346 C ILE B 189 44.406 23.527 55.264 0.00 0.00 B
ATOM 6347 O ILE B 189 44.876 23.668 56.418 0.00 0.00 B
ATOM 6348 N CYS B 190 44.963 24.165 54.222 0.00 0.00 B
ATOM 6349 HN CYS B 190 44.692 23.893 53.301 0.00 0.00 B
ATOM 6350 CA CYS B 190 45.827 25.348 54.258 0.00 0.00 B
ATOM 6351 HA CYS B 190 46.169 25.406 55.281 0.00 0.00 B
ATOM 6352 CB CYS B 190 47.112 25.327 53.431 0.00 0.00 B
ATOM 6353 HB1 CYS B 190 47.642 24.427 53.809 0.00 0.00 B
ATOM 6354 HB2 CYS B 190 46.910 25.130 52.356 0.00 0.00 B
ATOM 6355 SG CYS B 190 48.219 26.771 53.530 0.00 0.00 B
ATOM 6356 HG1 CYS B 190 49.159 26.478 52.644 0.00 0.00 B
ATOM 6357 C CYS B 190 44.919 26.423 53.754 0.00 0.00 B
ATOM 6358 O CYS B 190 44.363 26.354 52.682 0.00 0.00 B
ATOM 6359 N LEU B 191 44.868 27.584 54.484 0.00 0.00 B
ATOM 6360 HN LEU B 191 45.345 27.722 55.349 0.00 0.00 B
ATOM 6361 CA LEU B 191 43.941 28.680 54.263 0.00 0.00 B
ATOM 6362 HA LEU B 191 43.312 28.514 53.400 0.00 0.00 B
ATOM 6363 CB LEU B 191 43.053 28.992 55.540 0.00 0.00 B
ATOM 6364 HB1 LEU B 191 42.433 28.104 55.788 0.00 0.00 B
ATOM 6365 HB2 LEU B 191 43.732 29.147 56.405 0.00 0.00 B
ATOM 6366 CG LEU B 191 42.121 30.272 55.518 0.00 0.00 B
ATOM 6367 HG LEU B 191 42.841 31.108 55.387 0.00 0.00 B
ATOM 6368 CD1 LEU B 191 41.137 30.227 54.330 0.00 0.00 B
ATOM 6369 HD11 LEU B 191 40.679 29.218 54.252 0.00 0.00 B
ATOM 6370 HD12 LEU B 191 40.368 31.022 54.423 0.00 0.00 B
ATOM 6371 HD13 LEU B 191 41.737 30.320 53.400 0.00 0.00 B

ATOM   6372 CD2 LEU B 191   41.325 30.274 56.843 0.00 0.00
B
ATOM   6373 HD21 LEU B 191   41.975 30.196 57.741 0.00 0.00
B
ATOM   6374 HD22 LEU B 191   40.674 31.175 56.848 0.00 0.00
B
ATOM   6375 HD23 LEU B 191   40.691 29.370 56.964 0.00 0.00
B
ATOM   6376 C   LEU B 191   44.696 29.947 53.998 0.00 0.00
B
ATOM   6377 O   LEU B 191   45.567 30.250 54.801 0.00 0.00
B
ATOM   6378 N   SER B 192   44.316 30.734 52.932 0.00 0.00
B
ATOM   6379 HN SER B 192   43.485 30.452 52.460 0.00 0.00
B
ATOM   6380 CA SER B 192   45.087 31.929 52.538 0.00 0.00
B
ATOM   6381 HA SER B 192   44.549 32.246 51.657 0.00 0.00
B
ATOM   6382 CB SER B 192   44.919 33.258 53.454 0.00 0.00
B
ATOM   6383 HB1 SER B 192   43.878 33.267 53.841 0.00 0.00
B
ATOM   6384 HB2 SER B 192   45.631 32.898 54.228 0.00 0.00
B
ATOM   6385 OG SER B 192   45.377 34.476 52.903 0.00 0.00
B
ATOM   6386 HG1 SER B 192   45.025 35.168 53.466 0.00 0.00
B
ATOM   6387 C   SER B 192   46.564 31.659 52.321 0.00 0.00
B
ATOM   6388 O   SER B 192   47.519 32.021 52.998 0.00 0.00
B
ATOM   6389 N   PRO B 193   46.939 30.906 51.306 0.00 0.00
B
ATOM   6390 CD PRO B 193   46.075 30.353 50.262 0.00 0.00
B
ATOM   6391 HD1 PRO B 193   45.213 29.870 50.770 0.00 0.00
B
ATOM   6392 HD2 PRO B 193   45.707 31.218 49.670 0.00 0.00
B
ATOM   6393 CA PRO B 193   48.270 30.304 51.274 0.00 0.00
B
ATOM   6394 HA PRO B 193   48.549 30.088 52.295 0.00 0.00
B
ATOM   6395 CB PRO B 193   48.047 29.056 50.365 0.00 0.00
B
ATOM   6396 HB1 PRO B 193   47.849 28.166 51.000 0.00 0.00
B
ATOM   6397 HB2 PRO B 193   48.944 28.872 49.737 0.00 0.00
B
ATOM   6398 CG PRO B 193   46.825 29.329 49.525 0.00 0.00
B

ATOM 6399 HG1 PRO B 193 46.107 28.487 49.421 0.00 0.00
B
ATOM 6400 HG2 PRO B 193 47.153 29.727 48.541 0.00 0.00
B
ATOM 6401 C PRO B 193 49.347 31.247 50.747 0.00 0.00
B
ATOM 6402 O PRO B 193 48.991 32.074 49.892 0.00 0.00
B
ATOM 6403 N ALA B 194 50.597 31.141 51.235 0.00 0.00
B
ATOM 6404 HN ALA B 194 50.796 30.371 51.837 0.00 0.00
B
ATOM 6405 CA ALA B 194 51.787 31.925 50.780 0.00 0.00
B
ATOM 6406 HA ALA B 194 51.442 32.566 49.982 0.00 0.00
B
ATOM 6407 CB ALA B 194 52.222 32.711 52.038 0.00 0.00
B
ATOM 6408 HB1 ALA B 194 51.498 33.512 52.299 0.00 0.00
B
ATOM 6409 HB2 ALA B 194 52.449 32.037 52.891 0.00 0.00
B
ATOM 6410 HB3 ALA B 194 53.230 33.115 51.803 0.00 0.00
B
ATOM 6411 C ALA B 194 52.940 30.906 50.326 0.00 0.00
B
ATOM 6412 O ALA B 194 52.986 29.809 50.839 0.00 0.00
B
ATOM 6413 N PRO B 195 53.818 31.362 49.471 0.00 0.00
B
ATOM 6414 CD PRO B 195 53.853 32.618 48.735 0.00 0.00
B
ATOM 6415 HD1 PRO B 195 52.799 32.745 48.408 0.00 0.00
B
ATOM 6416 HD2 PRO B 195 54.124 33.450 49.421 0.00 0.00
B
ATOM 6417 CA PRO B 195 55.002 30.492 49.104 0.00 0.00
B
ATOM 6418 HA PRO B 195 54.587 29.655 48.561 0.00 0.00
B
ATOM 6419 CB PRO B 195 55.843 31.348 48.123 0.00 0.00
B
ATOM 6420 HB1 PRO B 195 56.252 30.625 47.386 0.00 0.00
B
ATOM 6421 HB2 PRO B 195 56.591 31.856 48.769 0.00 0.00
B
ATOM 6422 CG PRO B 195 54.813 32.402 47.541 0.00 0.00
B
ATOM 6423 HG1 PRO B 195 54.297 31.893 46.699 0.00 0.00
B
ATOM 6424 HG2 PRO B 195 55.312 33.321 47.165 0.00 0.00
B
ATOM 6425 C PRO B 195 55.865 30.073 50.338 0.00 0.00
B

ATOM 6426 O PRO B 195 56.074 30.880 51.244 0.00 0.00
B
ATOM 6427 N GLU B 196 56.522 28.910 50.509 0.00 0.00
B
ATOM 6428 HN GLU B 196 57.151 28.831 51.279 0.00 0.00
B
ATOM 6429 CA GLU B 196 56.330 27.622 49.815 0.00 0.00
B
ATOM 6430 HA GLU B 196 56.100 27.767 48.770 0.00 0.00
B
ATOM 6431 CB GLU B 196 57.583 26.843 49.825 0.00 0.00
B
ATOM 6432 HB1 GLU B 196 58.270 27.441 49.189 0.00 0.00
B
ATOM 6433 HB2 GLU B 196 57.974 26.764 50.862 0.00 0.00
B
ATOM 6434 CG GLU B 196 57.571 25.423 49.198 0.00 0.00
B
ATOM 6435 HG1 GLU B 196 57.073 24.664 49.838 0.00 0.00
B
ATOM 6436 HG2 GLU B 196 57.069 25.507 48.210 0.00 0.00
B
ATOM 6437 CD GLU B 196 58.953 24.923 48.933 0.00 0.00
B
ATOM 6438 OE1 GLU B 196 59.534 25.116 47.859 0.00 0.00
B
ATOM 6439 OE2 GLU B 196 59.538 24.377 49.904 0.00 0.00
B
ATOM 6440 C GLU B 196 55.258 26.715 50.445 0.00 0.00
B
ATOM 6441 O GLU B 196 55.204 26.478 51.671 0.00 0.00
B
ATOM 6442 N ASP B 197 54.342 26.126 49.644 0.00 0.00
B
ATOM 6443 HN ASP B 197 54.489 26.132 48.658 0.00 0.00
B
ATOM 6444 CA ASP B 197 53.380 25.116 50.048 0.00 0.00
B
ATOM 6445 HA ASP B 197 53.744 24.537 50.884 0.00 0.00
B
ATOM 6446 CB ASP B 197 52.080 25.692 50.606 0.00 0.00
B
ATOM 6447 HB1 ASP B 197 52.317 26.633 51.147 0.00 0.00
B
ATOM 6448 HB2 ASP B 197 51.355 25.995 49.820 0.00 0.00
B
ATOM 6449 CG ASP B 197 51.524 24.843 51.698 0.00 0.00
B
ATOM 6450 OD1 ASP B 197 51.517 23.566 51.460 0.00 0.00
B
ATOM 6451 OD2 ASP B 197 51.233 25.352 52.783 0.00 0.00
B
ATOM 6452 C ASP B 197 53.241 24.287 48.775 0.00 0.00
B

```
ATOM   6453  O   ASP B 197      53.614  24.658  47.655  0.00  0.00
B
ATOM   6454  N   ILE B 198      52.709  23.044  48.892  0.00  0.00
B
ATOM   6455  HN  ILE B 198      52.257  22.765  49.736  0.00  0.00
B
ATOM   6456  CA  ILE B 198      53.049  21.970  47.960  0.00  0.00
B
ATOM   6457  HA  ILE B 198      53.670  22.420  47.200  0.00  0.00
B
ATOM   6458  CB  ILE B 198      53.919  20.862  48.547  0.00  0.00
B
ATOM   6459  HB  ILE B 198      54.869  21.329  48.884  0.00  0.00
B
ATOM   6460  CG2 ILE B 198      53.181  20.239  49.759  0.00  0.00
B
ATOM   6461 HG21 ILE B 198      52.463  19.538  49.282  0.00  0.00
B
ATOM   6462 HG22 ILE B 198      53.850  19.738  50.491  0.00  0.00
B
ATOM   6463 HG23 ILE B 198      52.568  20.989  50.304  0.00  0.00
B
ATOM   6464  CG1 ILE B 198      54.386  19.731  47.629  0.00  0.00
B
ATOM   6465 HG11 ILE B 198      55.047  19.075  48.235  0.00  0.00
B
ATOM   6466 HG12 ILE B 198      53.543  19.131  47.223  0.00  0.00
B
ATOM   6467  CD  ILE B 198      55.140  20.294  46.384  0.00  0.00
B
ATOM   6468  HD1 ILE B 198      54.464  20.683  45.593  0.00  0.00
B
ATOM   6469  HD2 ILE B 198      55.769  21.142  46.731  0.00  0.00
B
ATOM   6470  HD3 ILE B 198      55.754  19.469  45.964  0.00  0.00
B
ATOM   6471  C   ILE B 198      51.731  21.410  47.473  0.00  0.00
B
ATOM   6472  O   ILE B 198      50.749  21.126  48.191  0.00  0.00
B
ATOM   6473  N   ILE B 199      51.631  21.150  46.154  0.00  0.00
B
ATOM   6474  HN  ILE B 199      52.393  21.473  45.598  0.00  0.00
B
ATOM   6475  CA  ILE B 199      50.571  20.297  45.541  0.00  0.00
B
ATOM   6476  HA  ILE B 199      49.755  20.202  46.242  0.00  0.00
B
ATOM   6477  CB  ILE B 199      50.032  21.020  44.279  0.00  0.00
B
ATOM   6478  HB  ILE B 199      50.895  20.997  43.580  0.00  0.00
B
ATOM   6479  CG2 ILE B 199      48.863  20.271  43.601  0.00  0.00
B
```

```
ATOM   6480 HG21 ILE B 199      47.996  20.010  44.245  0.00  0.00
B
ATOM   6481 HG22 ILE B 199      48.542  20.930  42.766  0.00  0.00
B
ATOM   6482 HG23 ILE B 199      49.318  19.349  43.181  0.00  0.00
B
ATOM   6483  CG1 ILE B 199      49.709  22.513  44.513  0.00  0.00
B
ATOM   6484 HG11 ILE B 199      50.521  23.135  44.947  0.00  0.00
B
ATOM   6485 HG12 ILE B 199      49.492  22.938  43.510  0.00  0.00
B
ATOM   6486  CD  ILE B 199      48.444  22.603  45.333  0.00  0.00
B
ATOM   6487  HD1 ILE B 199      47.606  22.449  44.619  0.00  0.00
B
ATOM   6488  HD2 ILE B 199      48.424  21.836  46.136  0.00  0.00
B
ATOM   6489  HD3 ILE B 199      48.270  23.546  45.894  0.00  0.00
B
ATOM   6490  C   ILE B 199      50.952  18.896  45.244  0.00  0.00
B
ATOM   6491  O   ILE B 199      52.045  18.666  44.731  0.00  0.00
B
ATOM   6492  N   ARG B 200      50.034  18.005  45.578  0.00  0.00
B
ATOM   6493  HN  ARG B 200      49.132  18.272  45.907  0.00  0.00
B
ATOM   6494  CA  ARG B 200      50.149  16.543  45.214  0.00  0.00
B
ATOM   6495  HA  ARG B 200      51.022  16.412  44.592  0.00  0.00
B
ATOM   6496  CB  ARG B 200      50.366  15.691  46.525  0.00  0.00
B
ATOM   6497  HB1 ARG B 200      50.648  14.737  46.032  0.00  0.00
B
ATOM   6498  HB2 ARG B 200      51.260  16.013  47.101  0.00  0.00
B
ATOM   6499  CG  ARG B 200      49.227  15.724  47.547  0.00  0.00
B
ATOM   6500  HG1 ARG B 200      49.244  16.770  47.920  0.00  0.00
B
ATOM   6501  HG2 ARG B 200      48.304  15.493  46.974  0.00  0.00
B
ATOM   6502  CD  ARG B 200      49.443  14.856  48.797  0.00  0.00
B
ATOM   6503  HD1 ARG B 200      49.405  13.764  48.597  0.00  0.00
B
ATOM   6504  HD2 ARG B 200      50.362  15.157  49.344  0.00  0.00
B
ATOM   6505  NE  ARG B 200      48.127  15.127  49.560  0.00  0.00
B
ATOM   6506  HE  ARG B 200      47.583  15.894  49.220  0.00  0.00
B
```

ATOM 6507 CZ ARG B 200 47.692 14.460 50.682 0.00 0.00
B
ATOM 6508 NH1 ARG B 200 48.481 13.632 51.368 0.00 0.00
B
ATOM 6509 HH11 ARG B 200 48.038 13.035 52.037 0.00 0.00
B
ATOM 6510 HH12 ARG B 200 49.423 13.379 51.149 0.00 0.00
B
ATOM 6511 NH2 ARG B 200 46.484 14.640 51.111 0.00 0.00
B
ATOM 6512 HH21 ARG B 200 45.989 14.047 51.746 0.00 0.00
B
ATOM 6513 HH22 ARG B 200 45.976 15.156 50.422 0.00 0.00
B
ATOM 6514 C ARG B 200 48.885 16.082 44.547 0.00 0.00
B
ATOM 6515 O ARG B 200 47.808 16.628 44.670 0.00 0.00
B
ATOM 6516 N GLU B 201 49.001 15.003 43.707 0.00 0.00
B
ATOM 6517 HN GLU B 201 49.887 14.596 43.499 0.00 0.00
B
ATOM 6518 CA GLU B 201 47.777 14.473 43.063 0.00 0.00
B
ATOM 6519 HA GLU B 201 47.456 15.258 42.394 0.00 0.00
B
ATOM 6520 CB GLU B 201 47.987 13.271 42.117 0.00 0.00
B
ATOM 6521 HB1 GLU B 201 47.091 12.933 41.553 0.00 0.00
B
ATOM 6522 HB2 GLU B 201 48.717 13.617 41.354 0.00 0.00
B
ATOM 6523 CG GLU B 201 48.631 12.022 42.683 0.00 0.00
B
ATOM 6524 HG1 GLU B 201 49.556 12.239 43.257 0.00 0.00
B
ATOM 6525 HG2 GLU B 201 48.029 11.559 43.494 0.00 0.00
B
ATOM 6526 CD GLU B 201 48.742 10.821 41.805 0.00 0.00
B
ATOM 6527 OE1 GLU B 201 49.867 10.432 41.364 0.00 0.00
B
ATOM 6528 OE2 GLU B 201 47.769 10.107 41.548 0.00 0.00
B
ATOM 6529 C GLU B 201 46.614 14.158 44.053 0.00 0.00
B
ATOM 6530 O GLU B 201 46.838 13.621 45.148 0.00 0.00
B
ATOM 6531 N GLN B 202 45.445 14.750 43.757 0.00 0.00
B
ATOM 6532 HN GLN B 202 45.330 15.516 43.129 0.00 0.00
B
ATOM 6533 CA GLN B 202 44.102 14.467 44.295 0.00 0.00
B

ATOM 6534 HA GLN B 202 43.374 14.679 43.525 0.00 0.00
B
ATOM 6535 CB GLN B 202 43.896 12.983 44.696 0.00 0.00
B
ATOM 6536 HB1 GLN B 202 44.776 12.651 45.288 0.00 0.00
B
ATOM 6537 HB2 GLN B 202 43.017 12.944 45.374 0.00 0.00
B
ATOM 6538 CG GLN B 202 43.493 11.963 43.560 0.00 0.00
B
ATOM 6539 HG1 GLN B 202 43.033 11.054 44.004 0.00 0.00
B
ATOM 6540 HG2 GLN B 202 42.693 12.393 42.919 0.00 0.00
B
ATOM 6541 CD GLN B 202 44.592 11.591 42.513 0.00 0.00
B
ATOM 6542 OE1 GLN B 202 44.651 12.012 41.337 0.00 0.00
B
ATOM 6543 NE2 GLN B 202 45.528 10.812 43.088 0.00 0.00
B
ATOM 6544 HE21 GLN B 202 46.264 10.524 42.475 0.00 0.00
B
ATOM 6545 HE22 GLN B 202 45.441 10.477 44.026 0.00 0.00
B
ATOM 6546 C GLN B 202 43.681 15.318 45.505 0.00 0.00
B
ATOM 6547 O GLN B 202 42.592 15.171 46.053 0.00 0.00
B
ATOM 6548 N ASP B 203 44.554 16.301 45.856 0.00 0.00
B
ATOM 6549 HN ASP B 203 45.467 16.341 45.458 0.00 0.00
B
ATOM 6550 CA ASP B 203 44.091 17.389 46.623 0.00 0.00
B
ATOM 6551 HA ASP B 203 43.381 17.042 47.360 0.00 0.00
B
ATOM 6552 CB ASP B 203 45.210 17.990 47.493 0.00 0.00
B
ATOM 6553 HB1 ASP B 203 46.122 17.999 46.858 0.00 0.00
B
ATOM 6554 HB2 ASP B 203 45.150 19.009 47.931 0.00 0.00
B
ATOM 6555 CG ASP B 203 45.677 17.053 48.631 0.00 0.00
B
ATOM 6556 OD1 ASP B 203 46.747 17.362 49.284 0.00 0.00
B
ATOM 6557 OD2 ASP B 203 45.147 15.946 48.903 0.00 0.00
B
ATOM 6558 C ASP B 203 43.270 18.388 45.830 0.00 0.00
B
ATOM 6559 O ASP B 203 43.269 18.409 44.610 0.00 0.00
B
ATOM 6560 N CYS B 204 42.620 19.336 46.470 0.00 0.00
B

ATOM 6561 HN CYS B 204 42.778 19.359 47.454 0.00 0.00
B
ATOM 6562 CA CYS B 204 41.729 20.309 45.805 0.00 0.00
B
ATOM 6563 HA CYS B 204 41.673 20.038 44.761 0.00 0.00
B
ATOM 6564 CB CYS B 204 40.330 20.343 46.510 0.00 0.00
B
ATOM 6565 HB1 CYS B 204 40.571 20.279 47.593 0.00 0.00
B
ATOM 6566 HB2 CYS B 204 39.772 21.257 46.216 0.00 0.00
B
ATOM 6567 SG CYS B 204 39.439 18.918 46.066 0.00 0.00
B
ATOM 6568 HG1 CYS B 204 39.276 19.272 44.799 0.00 0.00
B
ATOM 6569 C CYS B 204 42.397 21.644 46.044 0.00 0.00
B
ATOM 6570 O CYS B 204 42.852 21.900 47.192 0.00 0.00
B
ATOM 6571 N LEU B 205 42.425 22.517 45.009 0.00 0.00
B
ATOM 6572 HN LEU B 205 42.068 22.215 44.129 0.00 0.00
B
ATOM 6573 CA LEU B 205 42.939 23.834 45.057 0.00 0.00
B
ATOM 6574 HA LEU B 205 43.348 24.137 46.010 0.00 0.00
B
ATOM 6575 CB LEU B 205 44.145 23.824 44.095 0.00 0.00
B
ATOM 6576 HB1 LEU B 205 44.834 23.004 44.389 0.00 0.00
B
ATOM 6577 HB2 LEU B 205 43.804 23.438 43.111 0.00 0.00
B
ATOM 6578 CG LEU B 205 44.789 25.241 43.779 0.00 0.00
B
ATOM 6579 HG LEU B 205 43.950 25.859 43.396 0.00 0.00
B
ATOM 6580 CD1 LEU B 205 45.348 25.848 45.042 0.00 0.00
B
ATOM 6581 HD11 LEU B 205 46.201 25.280 45.472 0.00 0.00
B
ATOM 6582 HD12 LEU B 205 45.777 26.861 44.893 0.00 0.00
B
ATOM 6583 HD13 LEU B 205 44.486 26.020 45.721 0.00 0.00
B
ATOM 6584 CD2 LEU B 205 45.896 25.294 42.713 0.00 0.00
B
ATOM 6585 HD21 LEU B 205 46.538 26.197 42.793 0.00 0.00
B
ATOM 6586 HD22 LEU B 205 46.595 24.436 42.615 0.00 0.00
B
ATOM 6587 HD23 LEU B 205 45.351 25.437 41.755 0.00 0.00
B

ATOM 6588 C LEU B 205 41.831 24.703 44.793 0.00 0.00
B
ATOM 6589 O LEU B 205 41.186 24.545 43.759 0.00 0.00
B
ATOM 6590 N VAL B 206 41.467 25.586 45.711 0.00 0.00
B
ATOM 6591 HN VAL B 206 41.954 25.557 46.580 0.00 0.00
B
ATOM 6592 CA VAL B 206 40.342 26.497 45.623 0.00 0.00
B
ATOM 6593 HA VAL B 206 39.670 26.211 44.827 0.00 0.00
B
ATOM 6594 CB VAL B 206 39.651 26.656 47.022 0.00 0.00
B
ATOM 6595 HB VAL B 206 40.354 27.104 47.756 0.00 0.00
B
ATOM 6596 CG1 VAL B 206 38.531 27.599 46.844 0.00 0.00
B
ATOM 6597 HG11 VAL B 206 37.821 27.201 46.088 0.00 0.00
B
ATOM 6598 HG12 VAL B 206 38.029 27.822 47.810 0.00 0.00
B
ATOM 6599 HG13 VAL B 206 38.881 28.606 46.530 0.00 0.00
B
ATOM 6600 CG2 VAL B 206 39.152 25.372 47.630 0.00 0.00
B
ATOM 6601 HG21 VAL B 206 38.171 25.052 47.219 0.00 0.00
B
ATOM 6602 HG22 VAL B 206 39.916 24.571 47.535 0.00 0.00
B
ATOM 6603 HG23 VAL B 206 38.877 25.452 48.703 0.00 0.00
B
ATOM 6604 C VAL B 206 40.946 27.895 45.295 0.00 0.00
B
ATOM 6605 O VAL B 206 41.720 28.478 46.056 0.00 0.00
B
ATOM 6606 N ILE B 207 40.503 28.451 44.130 0.00 0.00
B
ATOM 6607 HN ILE B 207 39.735 28.113 43.592 0.00 0.00
B
ATOM 6608 CA ILE B 207 40.878 29.808 43.812 0.00 0.00
B
ATOM 6609 HA ILE B 207 41.401 30.282 44.630 0.00 0.00
B
ATOM 6610 CB ILE B 207 41.739 29.836 42.529 0.00 0.00
B
ATOM 6611 HB ILE B 207 42.093 30.854 42.256 0.00 0.00
B
ATOM 6612 CG2 ILE B 207 42.992 29.025 42.694 0.00 0.00
B
ATOM 6613 HG21 ILE B 207 43.694 28.938 41.837 0.00 0.00
B
ATOM 6614 HG22 ILE B 207 43.522 29.334 43.621 0.00 0.00
B

ATOM 6615 HG23 ILE B 207 42.659 27.967 42.748 0.00 0.00
B
ATOM 6616 CG1 ILE B 207 40.818 29.401 41.272 0.00 0.00
B
ATOM 6617 HG11 ILE B 207 40.352 28.411 41.466 0.00 0.00
B
ATOM 6618 HG12 ILE B 207 39.922 30.058 41.267 0.00 0.00
B
ATOM 6619 CD ILE B 207 41.570 29.351 39.980 0.00 0.00
B
ATOM 6620 HD1 ILE B 207 40.944 29.156 39.084 0.00 0.00
B
ATOM 6621 HD2 ILE B 207 42.050 30.331 39.769 0.00 0.00
B
ATOM 6622 HD3 ILE B 207 42.398 28.612 40.045 0.00 0.00
B
ATOM 6623 C ILE B 207 39.674 30.755 43.675 0.00 0.00
B
ATOM 6624 O ILE B 207 38.524 30.427 43.407 0.00 0.00
B
ATOM 6625 N MET B 208 39.921 32.089 43.820 0.00 0.00
B
ATOM 6626 HN MET B 208 40.841 32.263 44.162 0.00 0.00
B
ATOM 6627 CA MET B 208 39.019 33.204 43.411 0.00 0.00
B
ATOM 6628 HA MET B 208 38.098 32.737 43.093 0.00 0.00
B
ATOM 6629 CB MET B 208 38.604 34.115 44.615 0.00 0.00
B
ATOM 6630 HB1 MET B 208 37.914 33.630 45.338 0.00 0.00
B
ATOM 6631 HB2 MET B 208 39.549 34.309 45.166 0.00 0.00
B
ATOM 6632 CG MET B 208 37.850 35.447 44.273 0.00 0.00
B
ATOM 6633 HG1 MET B 208 37.123 35.240 43.459 0.00 0.00
B
ATOM 6634 HG2 MET B 208 37.289 35.630 45.214 0.00 0.00
B
ATOM 6635 SD MET B 208 38.785 36.989 43.764 0.00 0.00
B
ATOM 6636 CE MET B 208 39.500 37.559 45.348 0.00 0.00
B
ATOM 6637 HE1 MET B 208 38.741 37.942 46.063 0.00 0.00
B
ATOM 6638 HE2 MET B 208 40.047 36.724 45.837 0.00 0.00
B
ATOM 6639 HE3 MET B 208 40.270 38.343 45.180 0.00 0.00
B
ATOM 6640 C MET B 208 39.540 34.035 42.269 0.00 0.00
B
ATOM 6641 O MET B 208 40.728 34.149 42.006 0.00 0.00
B

ATOM 6642 N GLY B 209 38.654 34.491 41.397 0.00 0.00
B
ATOM 6643 HN GLY B 209 37.715 34.157 41.388 0.00 0.00
B
ATOM 6644 CA GLY B 209 39.016 35.204 40.103 0.00 0.00
B
ATOM 6645 HA1 GLY B 209 39.924 34.821 39.660 0.00 0.00
B
ATOM 6646 HA2 GLY B 209 39.183 36.219 40.432 0.00 0.00
B
ATOM 6647 C GLY B 209 37.972 35.245 39.006 0.00 0.00
B
ATOM 6648 O GLY B 209 36.912 34.667 39.151 0.00 0.00
B
ATOM 6649 N HSE B 210 38.357 35.817 37.929 0.00 0.00
B
ATOM 6650 HN HSE B 210 39.233 36.290 37.867 0.00 0.00
B
ATOM 6651 CA HSE B 210 37.540 35.924 36.744 0.00 0.00
B
ATOM 6652 HA HSE B 210 36.594 36.278 37.126 0.00 0.00
B
ATOM 6653 CB HSE B 210 38.187 36.941 35.675 0.00 0.00
B
ATOM 6654 HB1 HSE B 210 39.292 36.998 35.780 0.00 0.00
B
ATOM 6655 HB2 HSE B 210 38.035 36.713 34.599 0.00 0.00
B
ATOM 6656 ND1 HSE B 210 37.827 39.323 34.910 0.00 0.00
B
ATOM 6657 CG HSE B 210 37.619 38.389 35.897 0.00 0.00
B
ATOM 6658 CE1 HSE B 210 37.286 40.411 35.330 0.00 0.00
B
ATOM 6659 HE1 HSE B 210 37.303 41.303 34.704 0.00 0.00
B
ATOM 6660 NE2 HSE B 210 36.732 40.238 36.550 0.00 0.00
B
ATOM 6661 HE2 HSE B 210 36.395 41.002 37.100 0.00 0.00
B
ATOM 6662 CD2 HSE B 210 36.976 38.951 36.959 0.00 0.00
B
ATOM 6663 HD2 HSE B 210 36.756 38.541 37.937 0.00 0.00
B
ATOM 6664 C HSE B 210 37.277 34.650 36.079 0.00 0.00
B
ATOM 6665 O HSE B 210 38.147 33.822 35.892 0.00 0.00
B
ATOM 6666 N LYS B 211 35.992 34.456 35.595 0.00 0.00
B
ATOM 6667 HN LYS B 211 35.252 35.123 35.572 0.00 0.00
B
ATOM 6668 CA LYS B 211 35.577 33.095 35.237 0.00 0.00
B

ATOM 6669 HA LYS B 211 35.816 32.488 36.097 0.00 0.00
B
ATOM 6670 CB LYS B 211 34.046 33.130 34.934 0.00 0.00
B
ATOM 6671 HB1 LYS B 211 33.947 33.394 33.860 0.00 0.00
B
ATOM 6672 HB2 LYS B 211 33.640 32.099 35.017 0.00 0.00
B
ATOM 6673 CG LYS B 211 33.190 34.025 35.857 0.00 0.00
B
ATOM 6674 HG1 LYS B 211 33.542 33.825 36.892 0.00 0.00
B
ATOM 6675 HG2 LYS B 211 33.323 35.094 35.582 0.00 0.00
B
ATOM 6676 CD LYS B 211 31.698 33.679 35.695 0.00 0.00
B
ATOM 6677 HD1 LYS B 211 31.487 33.948 34.638 0.00 0.00
B
ATOM 6678 HD2 LYS B 211 31.386 32.625 35.855 0.00 0.00
B
ATOM 6679 CE LYS B 211 30.724 34.632 36.414 0.00 0.00
B
ATOM 6680 HE1 LYS B 211 31.157 35.650 36.519 0.00 0.00
B
ATOM 6681 HE2 LYS B 211 29.707 34.596 35.970 0.00 0.00
B
ATOM 6682 NZ LYS B 211 30.607 34.218 37.848 0.00 0.00
B
ATOM 6683 HZ1 LYS B 211 29.756 33.634 37.980 0.00 0.00
B
ATOM 6684 HZ2 LYS B 211 31.389 33.585 38.111 0.00 0.00
B
ATOM 6685 HZ3 LYS B 211 30.516 35.018 38.507 0.00 0.00
B
ATOM 6686 C LYS B 211 36.292 32.536 34.003 0.00 0.00
B
ATOM 6687 O LYS B 211 36.604 31.373 33.935 0.00 0.00
B
ATOM 6688 N LYS B 212 36.523 33.361 33.011 0.00 0.00
B
ATOM 6689 HN LYS B 212 36.388 34.335 33.175 0.00 0.00
B
ATOM 6690 CA LYS B 212 37.397 33.146 31.828 0.00 0.00
B
ATOM 6691 HA LYS B 212 37.075 32.218 31.378 0.00 0.00
B
ATOM 6692 CB LYS B 212 37.274 34.286 30.785 0.00 0.00
B
ATOM 6693 HB1 LYS B 212 37.372 35.200 31.409 0.00 0.00
B
ATOM 6694 HB2 LYS B 212 38.062 34.381 30.008 0.00 0.00
B
ATOM 6695 CG LYS B 212 36.019 34.238 30.033 0.00 0.00
B

ATOM 6696 HG1 LYS B 212 36.240 33.697 29.089 0.00 0.00 B
ATOM 6697 HG2 LYS B 212 35.239 33.715 30.627 0.00 0.00 B
ATOM 6698 CD LYS B 212 35.625 35.679 29.732 0.00 0.00 B
ATOM 6699 HD1 LYS B 212 35.375 36.144 30.710 0.00 0.00 B
ATOM 6700 HD2 LYS B 212 36.597 36.146 29.465 0.00 0.00 B
ATOM 6701 CE LYS B 212 34.581 35.887 28.620 0.00 0.00 B
ATOM 6702 HE1 LYS B 212 34.390 36.975 28.503 0.00 0.00 B
ATOM 6703 HE2 LYS B 212 34.834 35.534 27.597 0.00 0.00 B
ATOM 6704 NZ LYS B 212 33.323 35.229 29.143 0.00 0.00 B
ATOM 6705 HZ1 LYS B 212 32.491 35.590 28.634 0.00 0.00 B
ATOM 6706 HZ2 LYS B 212 33.322 34.231 28.848 0.00 0.00 B
ATOM 6707 HZ3 LYS B 212 33.124 35.274 30.163 0.00 0.00 B
ATOM 6708 C LYS B 212 38.907 32.920 32.201 0.00 0.00 B
ATOM 6709 O LYS B 212 39.600 32.136 31.557 0.00 0.00 B
ATOM 6710 N ASP B 213 39.501 33.564 33.196 0.00 0.00 B
ATOM 6711 HN ASP B 213 38.926 34.135 33.777 0.00 0.00 B
ATOM 6712 CA ASP B 213 40.841 33.259 33.699 0.00 0.00 B
ATOM 6713 HA ASP B 213 41.467 33.028 32.850 0.00 0.00 B
ATOM 6714 CB ASP B 213 41.493 34.552 34.319 0.00 0.00 B
ATOM 6715 HB1 ASP B 213 40.943 34.803 35.251 0.00 0.00 B
ATOM 6716 HB2 ASP B 213 42.556 34.301 34.520 0.00 0.00 B
ATOM 6717 CG ASP B 213 41.340 35.802 33.456 0.00 0.00 B
ATOM 6718 OD1 ASP B 213 41.622 35.683 32.267 0.00 0.00 B
ATOM 6719 OD2 ASP B 213 40.968 36.916 33.923 0.00 0.00 B
ATOM 6720 C ASP B 213 40.908 32.010 34.600 0.00 0.00 B
ATOM 6721 O ASP B 213 41.908 31.348 34.541 0.00 0.00 B
ATOM 6722 N ILE B 214 39.885 31.618 35.417 0.00 0.00 B

ATOM 6723 HN ILE B 214 39.064 32.183 35.418 0.00 0.00
B
ATOM 6724 CA ILE B 214 39.824 30.283 36.013 0.00 0.00
B
ATOM 6725 HA ILE B 214 40.671 30.101 36.658 0.00 0.00
B
ATOM 6726 CB ILE B 214 38.654 30.101 37.054 0.00 0.00
B
ATOM 6727 HB ILE B 214 37.696 30.395 36.573 0.00 0.00
B
ATOM 6728 CG2 ILE B 214 38.583 28.699 37.636 0.00 0.00
B
ATOM 6729 HG21 ILE B 214 38.282 28.704 38.706 0.00 0.00
B
ATOM 6730 HG22 ILE B 214 37.741 28.172 37.140 0.00 0.00
B
ATOM 6731 HG23 ILE B 214 39.413 27.983 37.454 0.00 0.00
B
ATOM 6732 CG1 ILE B 214 38.831 31.304 38.149 0.00 0.00
B
ATOM 6733 HG11 ILE B 214 39.777 31.106 38.696 0.00 0.00
B
ATOM 6734 HG12 ILE B 214 38.918 32.264 37.597 0.00 0.00
B
ATOM 6735 CD ILE B 214 37.590 31.395 39.049 0.00 0.00
B
ATOM 6736 HD1 ILE B 214 37.978 31.663 40.056 0.00 0.00
B
ATOM 6737 HD2 ILE B 214 36.764 32.116 38.870 0.00 0.00
B
ATOM 6738 HD3 ILE B 214 37.204 30.362 39.179 0.00 0.00
B
ATOM 6739 C ILE B 214 39.806 29.085 35.034 0.00 0.00
B
ATOM 6740 O ILE B 214 40.506 28.073 35.159 0.00 0.00
B
ATOM 6741 N LYS B 215 39.030 29.221 33.856 0.00 0.00
B
ATOM 6742 HN LYS B 215 38.294 29.863 33.658 0.00 0.00
B
ATOM 6743 CA LYS B 215 39.136 28.270 32.712 0.00 0.00
B
ATOM 6744 HA LYS B 215 38.945 27.294 33.133 0.00 0.00
B
ATOM 6745 CB LYS B 215 37.979 28.613 31.647 0.00 0.00
B
ATOM 6746 HB1 LYS B 215 38.144 29.634 31.240 0.00 0.00
B
ATOM 6747 HB2 LYS B 215 38.145 27.910 30.804 0.00 0.00
B
ATOM 6748 CG LYS B 215 36.541 28.441 32.181 0.00 0.00
B
ATOM 6749 HG1 LYS B 215 36.404 27.339 32.197 0.00 0.00
B

ATOM 6750 HG2 LYS B 215 36.460 28.759 33.242 0.00 0.00
B
ATOM 6751 CD LYS B 215 35.497 29.117 31.284 0.00 0.00
B
ATOM 6752 HD1 LYS B 215 35.820 30.179 31.245 0.00 0.00
B
ATOM 6753 HD2 LYS B 215 35.623 28.621 30.298 0.00 0.00
B
ATOM 6754 CE LYS B 215 34.063 29.131 31.806 0.00 0.00
B
ATOM 6755 HE1 LYS B 215 34.140 29.720 32.745 0.00 0.00
B
ATOM 6756 HE2 LYS B 215 33.399 29.650 31.083 0.00 0.00
B
ATOM 6757 NZ LYS B 215 33.652 27.826 32.194 0.00 0.00
B
ATOM 6758 HZ1 LYS B 215 33.221 27.177 31.505 0.00 0.00
B
ATOM 6759 HZ2 LYS B 215 34.402 27.319 32.705 0.00 0.00
B
ATOM 6760 HZ3 LYS B 215 32.994 27.878 32.997 0.00 0.00
B
ATOM 6761 C LYS B 215 40.542 28.281 32.103 0.00 0.00
B
ATOM 6762 O LYS B 215 41.004 27.327 31.462 0.00 0.00
B
ATOM 6763 N ARG B 216 41.115 29.476 32.077 0.00 0.00
B
ATOM 6764 HN ARG B 216 40.628 30.300 32.356 0.00 0.00
B
ATOM 6765 CA ARG B 216 42.423 29.569 31.371 0.00 0.00
B
ATOM 6766 HA ARG B 216 42.501 28.974 30.474 0.00 0.00
B
ATOM 6767 CB ARG B 216 42.867 31.017 30.904 0.00 0.00
B
ATOM 6768 HB1 ARG B 216 42.028 31.487 30.347 0.00 0.00
B
ATOM 6769 HB2 ARG B 216 43.265 31.685 31.697 0.00 0.00
B
ATOM 6770 CG ARG B 216 44.010 31.031 29.911 0.00 0.00
B
ATOM 6771 HG1 ARG B 216 44.893 30.417 30.189 0.00 0.00
B
ATOM 6772 HG2 ARG B 216 43.677 30.526 28.979 0.00 0.00
B
ATOM 6773 CD ARG B 216 44.564 32.396 29.555 0.00 0.00
B
ATOM 6774 HD1 ARG B 216 43.783 33.131 29.264 0.00 0.00
B
ATOM 6775 HD2 ARG B 216 45.007 32.788 30.496 0.00 0.00
B
ATOM 6776 NE ARG B 216 45.642 32.237 28.526 0.00 0.00
B

ATOM	6777	HE	ARG	B	216	46.428	31.663	28.754	0.00	0.00	B
ATOM	6778	CZ	ARG	B	216	45.640	32.731	27.276	0.00	0.00	B
ATOM	6779	NH1	ARG	B	216	44.613	33.492	26.809	0.00	0.00	B
ATOM	6780	HH11	ARG	B	216	44.461	33.567	25.824	0.00	0.00	B
ATOM	6781	HH12	ARG	B	216	43.853	33.500	27.460	0.00	0.00	B
ATOM	6782	NH2	ARG	B	216	46.631	32.378	26.461	0.00	0.00	B
ATOM	6783	HH21	ARG	B	216	46.695	32.676	25.509	0.00	0.00	B
ATOM	6784	HH22	ARG	B	216	47.230	31.640	26.773	0.00	0.00	B
ATOM	6785	C	ARG	B	216	43.559	28.966	32.252	0.00	0.00	B
ATOM	6786	O	ARG	B	216	44.314	28.128	31.827	0.00	0.00	B
ATOM	6787	N	PHE	B	217	43.444	29.133	33.555	0.00	0.00	B
ATOM	6788	HN	PHE	B	217	42.742	29.792	33.812	0.00	0.00	B
ATOM	6789	CA	PHE	B	217	44.176	28.356	34.539	0.00	0.00	B
ATOM	6790	HA	PHE	B	217	45.227	28.433	34.304	0.00	0.00	B
ATOM	6791	CB	PHE	B	217	43.943	28.811	36.020	0.00	0.00	B
ATOM	6792	HB1	PHE	B	217	44.294	29.857	36.148	0.00	0.00	B
ATOM	6793	HB2	PHE	B	217	42.882	28.636	36.299	0.00	0.00	B
ATOM	6794	CG	PHE	B	217	44.840	28.121	37.012	0.00	0.00	B
ATOM	6795	CD1	PHE	B	217	46.275	28.229	36.963	0.00	0.00	B
ATOM	6796	HD1	PHE	B	217	46.617	28.742	36.076	0.00	0.00	B
ATOM	6797	CE1	PHE	B	217	47.079	27.536	37.884	0.00	0.00	B
ATOM	6798	HE1	PHE	B	217	48.148	27.415	37.787	0.00	0.00	B
ATOM	6799	CZ	PHE	B	217	46.475	26.900	38.978	0.00	0.00	B
ATOM	6800	HZ	PHE	B	217	47.103	26.461	39.739	0.00	0.00	B
ATOM	6801	CD2	PHE	B	217	44.325	27.448	38.087	0.00	0.00	B
ATOM	6802	HD2	PHE	B	217	43.261	27.265	38.097	0.00	0.00	B
ATOM	6803	CE2	PHE	B	217	45.046	26.927	39.095	0.00	0.00	B

ATOM 6804 HE2 PHE B 217 44.513 26.436 39.896 0.00 0.00
B
ATOM 6805 C PHE B 217 43.996 26.837 34.451 0.00 0.00
B
ATOM 6806 O PHE B 217 44.970 26.069 34.621 0.00 0.00
B
ATOM 6807 N GLU B 218 42.809 26.279 34.182 0.00 0.00
B
ATOM 6808 HN GLU B 218 42.077 26.920 33.965 0.00 0.00
B
ATOM 6809 CA GLU B 218 42.648 24.843 33.799 0.00 0.00
B
ATOM 6810 HA GLU B 218 43.059 24.152 34.520 0.00 0.00
B
ATOM 6811 CB GLU B 218 41.097 24.518 33.789 0.00 0.00
B
ATOM 6812 HB1 GLU B 218 40.682 24.798 34.781 0.00 0.00
B
ATOM 6813 HB2 GLU B 218 40.587 25.210 33.085 0.00 0.00
B
ATOM 6814 CG GLU B 218 40.799 23.024 33.478 0.00 0.00
B
ATOM 6815 HG1 GLU B 218 41.204 22.788 32.471 0.00 0.00
B
ATOM 6816 HG2 GLU B 218 41.226 22.324 34.227 0.00 0.00
B
ATOM 6817 CD GLU B 218 39.273 22.819 33.454 0.00 0.00
B
ATOM 6818 OE1 GLU B 218 38.704 22.892 34.570 0.00 0.00
B
ATOM 6819 OE2 GLU B 218 38.677 22.528 32.356 0.00 0.00
B
ATOM 6820 C GLU B 218 43.288 24.463 32.400 0.00 0.00
B
ATOM 6821 O GLU B 218 44.037 23.440 32.181 0.00 0.00
B
ATOM 6822 N ASN B 219 42.981 25.296 31.352 0.00 0.00
B
ATOM 6823 HN ASN B 219 42.446 26.070 31.681 0.00 0.00
B
ATOM 6824 CA ASN B 219 43.401 25.040 29.947 0.00 0.00
B
ATOM 6825 HA ASN B 219 43.155 24.006 29.758 0.00 0.00
B
ATOM 6826 CB ASN B 219 42.753 25.963 28.920 0.00 0.00
B
ATOM 6827 HB1 ASN B 219 42.659 26.962 29.398 0.00 0.00
B
ATOM 6828 HB2 ASN B 219 43.388 25.970 28.009 0.00 0.00
B
ATOM 6829 CG ASN B 219 41.398 25.393 28.412 0.00 0.00
B
ATOM 6830 OD1 ASN B 219 41.368 24.592 27.504 0.00 0.00
B

```
ATOM   6831  ND2 ASN B 219      40.287  25.836  28.959  0.00  0.00
B
ATOM   6832 HD21 ASN B 219      39.449  25.307  28.827  0.00  0.00
B
ATOM   6833 HD22 ASN B 219      40.323  26.305  29.842  0.00  0.00
B
ATOM   6834  C   ASN B 219      44.932  25.144  29.819  0.00  0.00
B
ATOM   6835  O   ASN B 219      45.583  24.255  29.354  0.00  0.00
B
ATOM   6836  N   GLU B 220      45.511  26.254  30.276  0.00  0.00
B
ATOM   6837  HN  GLU B 220      45.013  26.841  30.909  0.00  0.00
B
ATOM   6838  CA  GLU B 220      46.864  26.654  29.935  0.00  0.00
B
ATOM   6839  HA  GLU B 220      47.227  26.174  29.038  0.00  0.00
B
ATOM   6840  CB  GLU B 220      46.918  28.277  29.719  0.00  0.00
B
ATOM   6841  HB1 GLU B 220      46.315  28.513  28.816  0.00  0.00
B
ATOM   6842  HB2 GLU B 220      46.405  28.834  30.532  0.00  0.00
B
ATOM   6843  CG  GLU B 220      48.335  28.833  29.440  0.00  0.00
B
ATOM   6844  HG1 GLU B 220      49.172  28.803  30.169  0.00  0.00
B
ATOM   6845  HG2 GLU B 220      48.585  28.152  28.599  0.00  0.00
B
ATOM   6846  CD  GLU B 220      48.193  30.211  28.805  0.00  0.00
B
ATOM   6847  OE1 GLU B 220      48.059  30.262  27.577  0.00  0.00
B
ATOM   6848  OE2 GLU B 220      48.073  31.218  29.556  0.00  0.00
B
ATOM   6849  C   GLU B 220      47.942  26.238  31.010  0.00  0.00
B
ATOM   6850  O   GLU B 220      48.906  25.569  30.606  0.00  0.00
B
ATOM   6851  N   GLY B 221      47.746  26.577  32.262  0.00  0.00
B
ATOM   6852  HN  GLY B 221      46.948  27.125  32.499  0.00  0.00
B
ATOM   6853  CA  GLY B 221      48.670  26.582  33.354  0.00  0.00
B
ATOM   6854  HA1 GLY B 221      48.439  27.383  34.040  0.00  0.00
B
ATOM   6855  HA2 GLY B 221      49.663  26.645  32.933  0.00  0.00
B
ATOM   6856  C   GLY B 221      48.716  25.275  34.110  0.00  0.00
B
ATOM   6857  O   GLY B 221      49.810  24.754  34.337  0.00  0.00
B
```

ATOM 6858 N MET B 222 47.701 24.689 34.630 0.00 0.00
B
ATOM 6859 HN MET B 222 46.872 25.240 34.683 0.00 0.00
B
ATOM 6860 CA MET B 222 47.584 23.296 35.079 0.00 0.00
B
ATOM 6861 HA MET B 222 48.221 23.210 35.947 0.00 0.00
B
ATOM 6862 CB MET B 222 46.100 22.946 35.538 0.00 0.00
B
ATOM 6863 HB1 MET B 222 45.363 23.178 34.739 0.00 0.00
B
ATOM 6864 HB2 MET B 222 46.149 21.854 35.736 0.00 0.00
B
ATOM 6865 CG MET B 222 45.786 23.745 36.794 0.00 0.00
B
ATOM 6866 HG1 MET B 222 46.489 23.341 37.554 0.00 0.00
B
ATOM 6867 HG2 MET B 222 45.895 24.816 36.521 0.00 0.00
B
ATOM 6868 SD MET B 222 44.016 23.678 37.294 0.00 0.00
B
ATOM 6869 CE MET B 222 43.955 22.154 38.258 0.00 0.00
B
ATOM 6870 HE1 MET B 222 42.898 21.922 38.510 0.00 0.00
B
ATOM 6871 HE2 MET B 222 44.310 21.245 37.727 0.00 0.00
B
ATOM 6872 HE3 MET B 222 44.506 22.216 39.221 0.00 0.00
B
ATOM 6873 C MET B 222 48.103 22.181 34.136 0.00 0.00
B
ATOM 6874 O MET B 222 48.953 21.373 34.399 0.00 0.00
B
ATOM 6875 N LYS C 7 −27.795 31.439 22.878 0.00 0.00
C
ATOM 6876 HT1 LYS C 7 −28.504 32.189 22.752 0.00 0.00
C
ATOM 6877 HT2 LYS C 7 −28.203 30.894 23.665 0.00 0.00
C
ATOM 6878 HT3 LYS C 7 −27.797 30.846 22.023 0.00 0.00
C
ATOM 6879 CA LYS C 7 −26.426 31.775 23.390 0.00 0.00
C
ATOM 6880 HA LYS C 7 −25.979 32.474 22.698 0.00 0.00
C
ATOM 6881 CB LYS C 7 −26.543 32.189 24.882 0.00 0.00
C
ATOM 6882 HB1 LYS C 7 −26.821 31.318 25.513 0.00 0.00
C
ATOM 6883 HB2 LYS C 7 −25.547 32.590 25.170 0.00 0.00
C
ATOM 6884 CG LYS C 7 −27.545 33.404 25.186 0.00 0.00
C

ATOM 6885 HG1 LYS C 7 -27.414 34.210 24.432 0.00 0.00
C
ATOM 6886 HG2 LYS C 7 -28.572 33.039 24.971 0.00 0.00
C
ATOM 6887 CD LYS C 7 -27.532 33.882 26.667 0.00 0.00
C
ATOM 6888 HD1 LYS C 7 -28.185 33.135 27.167 0.00 0.00
C
ATOM 6889 HD2 LYS C 7 -26.512 33.716 27.076 0.00 0.00
C
ATOM 6890 CE LYS C 7 -27.951 35.297 26.877 0.00 0.00
C
ATOM 6891 HE1 LYS C 7 -29.052 35.297 26.726 0.00 0.00
C
ATOM 6892 HE2 LYS C 7 -27.630 35.529 27.914 0.00 0.00
C
ATOM 6893 NZ LYS C 7 -27.300 36.238 25.977 0.00 0.00
C
ATOM 6894 HZ1 LYS C 7 -27.808 36.498 25.108 0.00 0.00
C
ATOM 6895 HZ2 LYS C 7 -27.187 37.089 26.563 0.00 0.00
C
ATOM 6896 HZ3 LYS C 7 -26.387 35.855 25.658 0.00 0.00
C
ATOM 6897 C LYS C 7 -25.425 30.669 23.255 0.00 0.00
C
ATOM 6898 O LYS C 7 -25.789 29.479 23.158 0.00 0.00
C
ATOM 6899 N GLN C 8 -24.150 30.978 23.272 0.00 0.00
C
ATOM 6900 HN GLN C 8 -23.895 31.898 23.557 0.00 0.00
C
ATOM 6901 CA GLN C 8 -23.056 30.005 23.010 0.00 0.00
C
ATOM 6902 HA GLN C 8 -23.594 29.082 22.852 0.00 0.00
C
ATOM 6903 CB GLN C 8 -22.068 30.328 21.858 0.00 0.00
C
ATOM 6904 HB1 GLN C 8 -22.722 30.423 20.965 0.00 0.00
C
ATOM 6905 HB2 GLN C 8 -21.591 31.324 21.979 0.00 0.00
C
ATOM 6906 CG GLN C 8 -21.128 29.168 21.486 0.00 0.00
C
ATOM 6907 HG1 GLN C 8 -20.711 28.758 22.430 0.00 0.00
C
ATOM 6908 HG2 GLN C 8 -21.670 28.323 21.011 0.00 0.00
C
ATOM 6909 CD GLN C 8 -20.063 29.605 20.538 0.00 0.00
C
ATOM 6910 OE1 GLN C 8 -19.064 30.282 20.848 0.00 0.00
C
ATOM 6911 NE2 GLN C 8 -20.280 29.432 19.194 0.00 0.00
C

```
ATOM   6912 HE21 GLN C   8     -19.569  29.812  18.603  0.00  0.00
C
ATOM   6913 HE22 GLN C   8     -21.040  28.847  18.908  0.00  0.00
C
ATOM   6914  C   GLN C   8     -22.208  29.844  24.275  0.00  0.00
C
ATOM   6915  O   GLN C   8     -21.651  30.794  24.817  0.00  0.00
C
ATOM   6916  N   PHE C   9     -22.199  28.630  24.852  0.00  0.00
C
ATOM   6917  HN  PHE C   9     -22.761  27.847  24.600  0.00  0.00
C
ATOM   6918  CA  PHE C   9     -21.460  28.239  26.055  0.00  0.00
C
ATOM   6919  HA  PHE C   9     -20.972  29.172  26.295  0.00  0.00
C
ATOM   6920  CB  PHE C   9     -22.491  27.731  27.159  0.00  0.00
C
ATOM   6921  HB1 PHE C   9     -22.965  26.757  26.916  0.00  0.00
C
ATOM   6922  HB2 PHE C   9     -21.995  27.660  28.150  0.00  0.00
C
ATOM   6923  CG  PHE C   9     -23.545  28.835  27.393  0.00  0.00
C
ATOM   6924  CD1 PHE C   9     -23.205  29.847  28.228  0.00  0.00
C
ATOM   6925  HD1 PHE C   9     -22.178  29.985  28.532  0.00  0.00
C
ATOM   6926  CE1 PHE C   9     -24.082  30.868  28.539  0.00  0.00
C
ATOM   6927  HE1 PHE C   9     -23.778  31.700  29.158  0.00  0.00
C
ATOM   6928  CZ  PHE C   9     -25.379  30.713  28.118  0.00  0.00
C
ATOM   6929  HZ  PHE C   9     -26.069  31.443  28.515  0.00  0.00
C
ATOM   6930  CD2 PHE C   9     -24.895  28.563  27.075  0.00  0.00
C
ATOM   6931  HD2 PHE C   9     -25.036  27.648  26.519  0.00  0.00
C
ATOM   6932  CE2 PHE C   9     -25.854  29.633  27.373  0.00  0.00
C
ATOM   6933  HE2 PHE C   9     -26.903  29.483  27.168  0.00  0.00
C
ATOM   6934  C   PHE C   9     -20.389  27.187  25.907  0.00  0.00
C
ATOM   6935  O   PHE C   9     -20.545  26.366  24.995  0.00  0.00
C
ATOM   6936  N   ALA C  10     -19.339  27.191  26.784  0.00  0.00
C
ATOM   6937  HN  ALA C  10     -19.183  27.895  27.473  0.00  0.00
C
ATOM   6938  CA  ALA C  10     -18.361  26.078  26.829  0.00  0.00
C
```

ATOM 6939 HA ALA C 10 −18.501 25.241 26.160 0.00 0.00
C
ATOM 6940 CB ALA C 10 −16.965 26.632 26.574 0.00 0.00
C
ATOM 6941 HB1 ALA C 10 −16.958 27.079 25.558 0.00 0.00
C
ATOM 6942 HB2 ALA C 10 −16.802 27.425 27.335 0.00 0.00
C
ATOM 6943 HB3 ALA C 10 −16.242 25.789 26.551 0.00 0.00
C
ATOM 6944 C ALA C 10 −18.477 25.507 28.263 0.00 0.00
C
ATOM 6945 O ALA C 10 −18.744 26.262 29.172 0.00 0.00
C
ATOM 6946 N VAL C 11 −18.473 24.156 28.409 0.00 0.00
C
ATOM 6947 HN VAL C 11 −18.462 23.536 27.628 0.00 0.00
C
ATOM 6948 CA VAL C 11 −18.574 23.617 29.712 0.00 0.00
C
ATOM 6949 HA VAL C 11 −18.653 24.407 30.445 0.00 0.00
C
ATOM 6950 CB VAL C 11 −19.857 22.779 29.892 0.00 0.00
C
ATOM 6951 HB VAL C 11 −19.725 21.868 29.270 0.00 0.00
C
ATOM 6952 CG1 VAL C 11 −19.955 22.348 31.312 0.00 0.00
C
ATOM 6953 HG11 VAL C 11 −20.863 21.823 31.676 0.00 0.00
C
ATOM 6954 HG12 VAL C 11 −19.063 21.854 31.753 0.00 0.00
C
ATOM 6955 HG13 VAL C 11 −19.964 23.292 31.897 0.00 0.00
C
ATOM 6956 CG2 VAL C 11 −21.154 23.586 29.455 0.00 0.00
C
ATOM 6957 HG21 VAL C 11 −21.989 23.003 29.899 0.00 0.00
C
ATOM 6958 HG22 VAL C 11 −21.140 24.627 29.843 0.00 0.00
C
ATOM 6959 HG23 VAL C 11 −21.196 23.550 28.345 0.00 0.00
C
ATOM 6960 C VAL C 11 −17.364 22.696 29.870 0.00 0.00
C
ATOM 6961 O VAL C 11 −17.279 21.696 29.185 0.00 0.00
C
ATOM 6962 N ILE C 12 −16.474 23.116 30.830 0.00 0.00
C
ATOM 6963 HN ILE C 12 −16.722 23.940 31.335 0.00 0.00
C
ATOM 6964 CA ILE C 12 −15.302 22.380 31.243 0.00 0.00
C
ATOM 6965 HA ILE C 12 −14.998 21.762 30.412 0.00 0.00
C

```
ATOM   6966  CB  ILE C  12      -14.163  23.327  31.483  0.00  0.00
C
ATOM   6967  HB  ILE C  12      -14.385  23.983  32.352  0.00  0.00
C
ATOM   6968  CG2 ILE C  12      -12.900  22.455  31.742  0.00  0.00
C
ATOM   6969 HG21 ILE C  12      -12.719  21.848  30.829  0.00  0.00
C
ATOM   6970 HG22 ILE C  12      -12.043  23.094  32.041  0.00  0.00
C
ATOM   6971 HG23 ILE C  12      -13.049  21.667  32.511  0.00  0.00
C
ATOM   6972  CG1 ILE C  12      -13.955  24.193  30.184  0.00  0.00
C
ATOM   6973 HG11 ILE C  12      -13.859  23.565  29.273  0.00  0.00
C
ATOM   6974 HG12 ILE C  12      -14.818  24.885  30.076  0.00  0.00
C
ATOM   6975  CD  ILE C  12      -12.618  25.076  30.100  0.00  0.00
C
ATOM   6976  HD1 ILE C  12      -12.866  25.987  29.514  0.00  0.00
C
ATOM   6977  HD2 ILE C  12      -12.285  25.477  31.082  0.00  0.00
C
ATOM   6978  HD3 ILE C  12      -11.741  24.471  29.785  0.00  0.00
C
ATOM   6979  C   ILE C  12      -15.703  21.516  32.454  0.00  0.00
C
ATOM   6980  O   ILE C  12      -16.191  22.094  33.481  0.00  0.00
C
ATOM   6981  N   GLY C  13      -15.601  20.255  32.388  0.00  0.00
C
ATOM   6982  HN  GLY C  13      -15.292  19.815  31.548  0.00  0.00
C
ATOM   6983  CA  GLY C  13      -15.979  19.359  33.493  0.00  0.00
C
ATOM   6984  HA1 GLY C  13      -15.895  19.889  34.430  0.00  0.00
C
ATOM   6985  HA2 GLY C  13      -15.176  18.639  33.446  0.00  0.00
C
ATOM   6986  C   GLY C  13      -17.297  18.566  33.348  0.00  0.00
C
ATOM   6987  O   GLY C  13      -18.385  19.045  33.667  0.00  0.00
C
ATOM   6988  N   LEU C  14      -17.267  17.276  33.018  0.00  0.00
C
ATOM   6989  HN  LEU C  14      -16.450  16.751  32.791  0.00  0.00
C
ATOM   6990  CA  LEU C  14      -18.491  16.529  32.817  0.00  0.00
C
ATOM   6991  HA  LEU C  14      -19.330  17.207  32.773  0.00  0.00
C
ATOM   6992  CB  LEU C  14      -18.474  15.787  31.399  0.00  0.00
C
```

```
ATOM   6993  HB1 LEU C  14      -17.709  14.982  31.374  0.00  0.00
C
ATOM   6994  HB2 LEU C  14      -19.494  15.365  31.276  0.00  0.00
C
ATOM   6995  CG  LEU C  14      -18.357  16.696  30.142  0.00  0.00
C
ATOM   6996  HG  LEU C  14      -17.371  17.209  30.145  0.00  0.00
C
ATOM   6997  CD1 LEU C  14      -18.263  15.833  28.860  0.00  0.00
C
ATOM   6998 HD11 LEU C  14      -19.059  15.057  28.878  0.00  0.00
C
ATOM   6999 HD12 LEU C  14      -18.300  16.511  27.981  0.00  0.00
C
ATOM   7000 HD13 LEU C  14      -17.263  15.350  28.893  0.00  0.00
C
ATOM   7001  CD2 LEU C  14      -19.501  17.723  29.923  0.00  0.00
C
ATOM   7002 HD21 LEU C  14      -19.268  18.197  28.946  0.00  0.00
C
ATOM   7003 HD22 LEU C  14      -20.574  17.433  29.904  0.00  0.00
C
ATOM   7004 HD23 LEU C  14      -19.350  18.564  30.633  0.00  0.00
C
ATOM   7005  C   LEU C  14      -18.803  15.644  34.033  0.00  0.00
C
ATOM   7006  O   LEU C  14      -19.061  14.424  33.915  0.00  0.00
C
ATOM   7007  N   GLY C  15      -18.828  16.220  35.275  0.00  0.00
C
ATOM   7008  HN  GLY C  15      -18.872  17.216  35.283  0.00  0.00
C
ATOM   7009  CA  GLY C  15      -19.053  15.425  36.449  0.00  0.00
C
ATOM   7010  HA1 GLY C  15      -18.696  15.983  37.302  0.00  0.00
C
ATOM   7011  HA2 GLY C  15      -18.514  14.493  36.369  0.00  0.00
C
ATOM   7012  C   GLY C  15      -20.473  15.164  36.698  0.00  0.00
C
ATOM   7013  O   GLY C  15      -21.409  15.341  35.869  0.00  0.00
C
ATOM   7014  N   ARG C  16      -20.806  14.849  37.922  0.00  0.00
C
ATOM   7015  HN  ARG C  16      -20.093  14.812  38.619  0.00  0.00
C
ATOM   7016  CA  ARG C  16      -22.191  14.628  38.330  0.00  0.00
C
ATOM   7017  HA  ARG C  16      -22.637  13.832  37.752  0.00  0.00
C
ATOM   7018  CB  ARG C  16      -22.289  14.208  39.793  0.00  0.00
C
ATOM   7019  HB1 ARG C  16      -21.916  15.012  40.463  0.00  0.00
C
```

ATOM 7020 HB2 ARG C 16 −23.355 13.986 40.010 0.00 0.00
C
ATOM 7021 CG ARG C 16 −21.646 12.859 40.109 0.00 0.00
C
ATOM 7022 HG1 ARG C 16 −21.968 11.933 39.586 0.00 0.00
C
ATOM 7023 HG2 ARG C 16 −20.567 12.910 39.851 0.00 0.00
C
ATOM 7024 CD ARG C 16 −21.804 12.401 41.619 0.00 0.00
C
ATOM 7025 HD1 ARG C 16 −22.809 11.961 41.793 0.00 0.00
C
ATOM 7026 HD2 ARG C 16 −20.952 11.711 41.805 0.00 0.00
C
ATOM 7027 NE ARG C 16 −21.610 13.649 42.500 0.00 0.00
C
ATOM 7028 HE ARG C 16 −20.666 13.975 42.556 0.00 0.00
C
ATOM 7029 CZ ARG C 16 −22.553 14.321 43.240 0.00 0.00
C
ATOM 7030 NH1 ARG C 16 −23.873 14.061 43.180 0.00 0.00
C
ATOM 7031 HH11 ARG C 16 −24.445 14.792 43.553 0.00 0.00
C
ATOM 7032 HH12 ARG C 16 −24.192 13.563 42.373 0.00 0.00
C
ATOM 7033 NH2 ARG C 16 −22.210 15.388 43.967 0.00 0.00
C
ATOM 7034 HH21 ARG C 16 −22.893 15.928 44.458 0.00 0.00
C
ATOM 7035 HH22 ARG C 16 −21.258 15.637 43.788 0.00 0.00
C
ATOM 7036 C ARG C 16 −22.980 15.900 38.209 0.00 0.00
C
ATOM 7037 O ARG C 16 −24.083 16.031 37.646 0.00 0.00
C
ATOM 7038 N PHE C 17 −22.416 17.016 38.723 0.00 0.00
C
ATOM 7039 HN PHE C 17 −21.495 16.997 39.107 0.00 0.00
C
ATOM 7040 CA PHE C 17 −22.943 18.402 38.545 0.00 0.00
C
ATOM 7041 HA PHE C 17 −23.984 18.425 38.832 0.00 0.00
C
ATOM 7042 CB PHE C 17 −22.113 19.434 39.375 0.00 0.00
C
ATOM 7043 HB1 PHE C 17 −22.241 19.265 40.465 0.00 0.00
C
ATOM 7044 HB2 PHE C 17 −21.051 19.374 39.054 0.00 0.00
C
ATOM 7045 CG PHE C 17 −22.684 20.871 39.147 0.00 0.00
C
ATOM 7046 CD1 PHE C 17 −24.067 21.039 39.420 0.00 0.00
C

ATOM	7047	HD1	PHE	C	17	−24.663	20.289	39.920	0.00	0.00	C
ATOM	7048	CE1	PHE	C	17	−24.676	22.325	39.188	0.00	0.00	C
ATOM	7049	HE1	PHE	C	17	−25.736	22.430	39.369	0.00	0.00	C
ATOM	7050	CZ	PHE	C	17	−23.920	23.405	38.660	0.00	0.00	C
ATOM	7051	HZ	PHE	C	17	−24.294	24.379	38.382	0.00	0.00	C
ATOM	7052	CD2	PHE	C	17	−21.946	21.962	38.613	0.00	0.00	C
ATOM	7053	HD2	PHE	C	17	−20.891	21.810	38.436	0.00	0.00	C
ATOM	7054	CE2	PHE	C	17	−22.601	23.162	38.306	0.00	0.00	C
ATOM	7055	HE2	PHE	C	17	−22.033	23.988	37.902	0.00	0.00	C
ATOM	7056	C	PHE	C	17	−22.827	18.792	37.044	0.00	0.00	C
ATOM	7057	O	PHE	C	17	−23.817	19.265	36.442	0.00	0.00	C
ATOM	7058	N	GLY	C	18	−21.695	18.593	36.414	0.00	0.00	C
ATOM	7059	HN	GLY	C	18	−20.863	18.247	36.841	0.00	0.00	C
ATOM	7060	CA	GLY	C	18	−21.374	19.108	35.046	0.00	0.00	C
ATOM	7061	HA1	GLY	C	18	−20.309	18.986	34.913	0.00	0.00	C
ATOM	7062	HA2	GLY	C	18	−21.649	20.151	35.010	0.00	0.00	C
ATOM	7063	C	GLY	C	18	−22.009	18.444	33.860	0.00	0.00	C
ATOM	7064	O	GLY	C	18	−22.266	19.184	32.858	0.00	0.00	C
ATOM	7065	N	GLY	C	19	−22.333	17.152	33.919	0.00	0.00	C
ATOM	7066	HN	GLY	C	19	−22.153	16.561	34.702	0.00	0.00	C
ATOM	7067	CA	GLY	C	19	−23.094	16.411	32.880	0.00	0.00	C
ATOM	7068	HA1	GLY	C	19	−22.877	15.365	33.044	0.00	0.00	C
ATOM	7069	HA2	GLY	C	19	−22.762	16.795	31.926	0.00	0.00	C
ATOM	7070	C	GLY	C	19	−24.550	16.677	32.977	0.00	0.00	C
ATOM	7071	O	GLY	C	19	−25.431	16.615	32.035	0.00	0.00	C
ATOM	7072	N	SER	C	20	−25.041	17.222	34.107	0.00	0.00	C
ATOM	7073	HN	SER	C	20	−24.409	17.468	34.838	0.00	0.00	C

ATOM 7074 CA SER C 20 −26.422 17.684 34.227 0.00 0.00
C
ATOM 7075 HA SER C 20 −27.046 17.097 33.568 0.00 0.00
C
ATOM 7076 CB SER C 20 −26.853 17.699 35.702 0.00 0.00
C
ATOM 7077 HB1 SER C 20 −26.428 16.837 36.258 0.00 0.00
C
ATOM 7078 HB2 SER C 20 −26.363 18.582 36.165 0.00 0.00
C
ATOM 7079 OG SER C 20 −28.286 17.731 35.907 0.00 0.00
C
ATOM 7080 HG1 SER C 20 −28.595 18.619 36.100 0.00 0.00
C
ATOM 7081 C SER C 20 −26.702 19.056 33.705 0.00 0.00
C
ATOM 7082 O SER C 20 −27.747 19.340 33.154 0.00 0.00
C
ATOM 7083 N ILE C 21 −25.766 19.953 33.886 0.00 0.00
C
ATOM 7084 HN ILE C 21 −24.937 19.789 34.416 0.00 0.00
C
ATOM 7085 CA ILE C 21 −25.854 21.300 33.394 0.00 0.00
C
ATOM 7086 HA ILE C 21 −26.862 21.612 33.623 0.00 0.00
C
ATOM 7087 CB ILE C 21 −24.911 22.272 34.136 0.00 0.00
C
ATOM 7088 HB ILE C 21 −25.055 21.887 35.168 0.00 0.00
C
ATOM 7089 CG2 ILE C 21 −23.428 22.177 33.807 0.00 0.00
C
ATOM 7090 HG21 ILE C 21 −22.791 22.815 34.456 0.00 0.00
C
ATOM 7091 HG22 ILE C 21 −23.132 21.110 33.900 0.00 0.00
C
ATOM 7092 HG23 ILE C 21 −23.391 22.511 32.748 0.00 0.00
C
ATOM 7093 CG1 ILE C 21 −25.345 23.725 33.960 0.00 0.00
C
ATOM 7094 HG11 ILE C 21 −25.049 24.020 32.930 0.00 0.00
C
ATOM 7095 HG12 ILE C 21 −26.453 23.798 33.988 0.00 0.00
C
ATOM 7096 CD ILE C 21 −24.626 24.764 34.832 0.00 0.00
C
ATOM 7097 HD1 ILE C 21 −24.971 24.731 35.888 0.00 0.00
C
ATOM 7098 HD2 ILE C 21 −23.524 24.617 34.836 0.00 0.00
C
ATOM 7099 HD3 ILE C 21 −24.895 25.793 34.511 0.00 0.00
C
ATOM 7100 C ILE C 21 −25.897 21.477 31.852 0.00 0.00
C

ATOM 7101 O ILE C 21 −26.773 22.004 31.198 0.00 0.00
C
ATOM 7102 N VAL C 22 −25.008 20.784 31.134 0.00 0.00
C
ATOM 7103 HN VAL C 22 −24.277 20.273 31.578 0.00 0.00
C
ATOM 7104 CA VAL C 22 −24.903 20.579 29.703 0.00 0.00
C
ATOM 7105 HA VAL C 22 −24.567 21.489 29.229 0.00 0.00
C
ATOM 7106 CB VAL C 22 −23.869 19.531 29.407 0.00 0.00
C
ATOM 7107 HB VAL C 22 −23.898 18.817 30.258 0.00 0.00
C
ATOM 7108 CG1 VAL C 22 −24.095 18.817 28.102 0.00 0.00
C
ATOM 7109 HG11 VAL C 22 −25.151 18.502 27.959 0.00 0.00
C
ATOM 7110 HG12 VAL C 22 −23.956 19.493 27.232 0.00 0.00
C
ATOM 7111 HG13 VAL C 22 −23.372 17.994 27.917 0.00 0.00
C
ATOM 7112 CG2 VAL C 22 −22.505 20.280 29.385 0.00 0.00
C
ATOM 7113 HG21 VAL C 22 −21.694 19.531 29.260 0.00 0.00
C
ATOM 7114 HG22 VAL C 22 −22.354 21.014 28.565 0.00 0.00
C
ATOM 7115 HG23 VAL C 22 −22.256 20.753 30.359 0.00 0.00
C
ATOM 7116 C VAL C 22 −26.311 20.275 29.066 0.00 0.00
C
ATOM 7117 O VAL C 22 −26.790 20.822 28.075 0.00 0.00
C
ATOM 7118 N LYS C 23 −27.017 19.284 29.700 0.00 0.00
C
ATOM 7119 HN LYS C 23 −26.639 18.789 30.478 0.00 0.00
C
ATOM 7120 CA LYS C 23 −28.368 18.835 29.214 0.00 0.00
C
ATOM 7121 HA LYS C 23 −28.171 18.650 28.168 0.00 0.00
C
ATOM 7122 CB LYS C 23 −28.812 17.651 30.036 0.00 0.00
C
ATOM 7123 HB1 LYS C 23 −27.968 16.951 30.219 0.00 0.00
C
ATOM 7124 HB2 LYS C 23 −29.289 18.032 30.964 0.00 0.00
C
ATOM 7125 CG LYS C 23 −29.938 16.829 29.343 0.00 0.00
C
ATOM 7126 HG1 LYS C 23 −30.840 17.448 29.151 0.00 0.00
C
ATOM 7127 HG2 LYS C 23 −29.500 16.471 28.387 0.00 0.00
C

ATOM 7128 CD LYS C 23 −30.343 15.544 29.955 0.00 0.00
C
ATOM 7129 HD1 LYS C 23 −31.307 15.273 29.475 0.00 0.00
C
ATOM 7130 HD2 LYS C 23 −29.686 14.664 29.781 0.00 0.00
C
ATOM 7131 CE LYS C 23 −30.505 15.674 31.454 0.00 0.00
C
ATOM 7132 HE1 LYS C 23 −29.485 15.636 31.894 0.00 0.00
C
ATOM 7133 HE2 LYS C 23 −31.114 16.549 31.766 0.00 0.00
C
ATOM 7134 NZ LYS C 23 −31.317 14.544 31.901 0.00 0.00
C
ATOM 7135 HZ1 LYS C 23 −30.824 13.641 31.750 0.00 0.00
C
ATOM 7136 HZ2 LYS C 23 −31.383 14.655 32.933 0.00 0.00
C
ATOM 7137 HZ3 LYS C 23 −32.237 14.570 31.416 0.00 0.00
C
ATOM 7138 C LYS C 23 −29.497 19.902 29.431 0.00 0.00
C
ATOM 7139 O LYS C 23 −30.347 20.217 28.528 0.00 0.00
C
ATOM 7140 N GLU C 24 −29.441 20.635 30.547 0.00 0.00
C
ATOM 7141 HN GLU C 24 −28.656 20.498 31.146 0.00 0.00
C
ATOM 7142 CA GLU C 24 −30.354 21.744 30.853 0.00 0.00
C
ATOM 7143 HA GLU C 24 −31.358 21.411 30.632 0.00 0.00
C
ATOM 7144 CB GLU C 24 −30.229 22.370 32.254 0.00 0.00
C
ATOM 7145 HB1 GLU C 24 −29.580 21.800 32.952 0.00 0.00
C
ATOM 7146 HB2 GLU C 24 −29.684 23.325 32.091 0.00 0.00
C
ATOM 7147 CG GLU C 24 −31.503 22.656 32.999 0.00 0.00
C
ATOM 7148 HG1 GLU C 24 −31.182 23.098 33.966 0.00 0.00
C
ATOM 7149 HG2 GLU C 24 −32.139 23.330 32.387 0.00 0.00
C
ATOM 7150 CD GLU C 24 −32.249 21.371 33.235 0.00 0.00
C
ATOM 7151 OE1 GLU C 24 −31.862 20.627 34.132 0.00 0.00
C
ATOM 7152 OE2 GLU C 24 −33.329 21.178 32.576 0.00 0.00
C
ATOM 7153 C GLU C 24 −30.272 22.848 29.877 0.00 0.00
C
ATOM 7154 O GLU C 24 −31.239 23.329 29.317 0.00 0.00
C

ATOM 7155 N LEU C 25 −29.065 23.257 29.588 0.00 0.00
C
ATOM 7156 HN LEU C 25 −28.261 22.896 30.053 0.00 0.00
C
ATOM 7157 CA LEU C 25 −28.852 24.290 28.576 0.00 0.00
C
ATOM 7158 HA LEU C 25 −29.568 25.038 28.883 0.00 0.00
C
ATOM 7159 CB LEU C 25 −27.377 24.660 28.518 0.00 0.00
C
ATOM 7160 HB1 LEU C 25 −26.818 23.701 28.560 0.00 0.00
C
ATOM 7161 HB2 LEU C 25 −27.164 25.268 27.613 0.00 0.00
C
ATOM 7162 CG LEU C 25 −26.844 25.431 29.747 0.00 0.00
C
ATOM 7163 HG LEU C 25 −27.084 24.759 30.599 0.00 0.00
C
ATOM 7164 CD1 LEU C 25 −25.266 25.378 29.813 0.00 0.00
C
ATOM 7165 HD11 LEU C 25 −24.781 24.380 29.862 0.00 0.00
C
ATOM 7166 HD12 LEU C 25 −24.785 25.994 29.023 0.00 0.00
C
ATOM 7167 HD13 LEU C 25 −25.059 25.827 30.808 0.00 0.00
C
ATOM 7168 CD2 LEU C 25 −27.370 26.869 30.018 0.00 0.00
C
ATOM 7169 HD21 LEU C 25 −27.399 27.523 29.121 0.00 0.00
C
ATOM 7170 HD22 LEU C 25 −28.427 26.853 30.363 0.00 0.00
C
ATOM 7171 HD23 LEU C 25 −26.729 27.363 30.779 0.00 0.00
C
ATOM 7172 C LEU C 25 −29.256 23.794 27.189 0.00 0.00
C
ATOM 7173 O LEU C 25 −29.705 24.585 26.387 0.00 0.00
C
ATOM 7174 N HSE C 26 −29.016 22.453 26.843 0.00 0.00
C
ATOM 7175 HN HSE C 26 −28.540 21.857 27.485 0.00 0.00
C
ATOM 7176 CA HSE C 26 −29.425 21.739 25.659 0.00 0.00
C
ATOM 7177 HA HSE C 26 −29.095 22.356 24.837 0.00 0.00
C
ATOM 7178 CB HSE C 26 −28.638 20.396 25.610 0.00 0.00
C
ATOM 7179 HB1 HSE C 26 −27.580 20.725 25.696 0.00 0.00
C
ATOM 7180 HB2 HSE C 26 −28.963 19.734 26.441 0.00 0.00
C
ATOM 7181 ND1 HSE C 26 −29.677 18.677 24.261 0.00 0.00
C

ATOM 7182 CG HSE C 26 −28.633 19.574 24.436 0.00 0.00
C
ATOM 7183 CE1 HSE C 26 −29.511 18.310 22.980 0.00 0.00
C
ATOM 7184 HE1 HSE C 26 −30.301 17.753 22.475 0.00 0.00
C
ATOM 7185 NE2 HSE C 26 −28.459 18.916 22.349 0.00 0.00
C
ATOM 7186 HE2 HSE C 26 −28.375 18.974 21.354 0.00 0.00
C
ATOM 7187 CD2 HSE C 26 −27.885 19.730 23.288 0.00 0.00
C
ATOM 7188 HD2 HSE C 26 −27.094 20.429 23.046 0.00 0.00
C
ATOM 7189 C HSE C 26 −30.901 21.720 25.399 0.00 0.00
C
ATOM 7190 O HSE C 26 −31.405 22.002 24.315 0.00 0.00
C
ATOM 7191 N ARG C 27 −31.693 21.594 26.495 0.00 0.00
C
ATOM 7192 HN ARG C 27 −31.262 21.362 27.363 0.00 0.00
C
ATOM 7193 CA ARG C 27 −33.110 21.742 26.629 0.00 0.00
C
ATOM 7194 HA ARG C 27 −33.563 21.129 25.864 0.00 0.00
C
ATOM 7195 CB ARG C 27 −33.530 21.245 28.071 0.00 0.00
C
ATOM 7196 HB1 ARG C 27 −33.428 20.139 28.067 0.00 0.00
C
ATOM 7197 HB2 ARG C 27 −32.920 21.605 28.927 0.00 0.00
C
ATOM 7198 CG ARG C 27 −35.064 21.534 28.400 0.00 0.00
C
ATOM 7199 HG1 ARG C 27 −35.634 21.364 27.462 0.00 0.00
C
ATOM 7200 HG2 ARG C 27 −35.374 20.651 28.999 0.00 0.00
C
ATOM 7201 CD ARG C 27 −35.430 22.870 29.135 0.00 0.00
C
ATOM 7202 HD1 ARG C 27 −35.071 23.672 28.455 0.00 0.00
C
ATOM 7203 HD2 ARG C 27 −36.523 23.048 29.229 0.00 0.00
C
ATOM 7204 NE ARG C 27 −34.895 22.845 30.510 0.00 0.00
C
ATOM 7205 HE ARG C 27 −34.233 22.153 30.798 0.00 0.00
C
ATOM 7206 CZ ARG C 27 −35.505 23.560 31.454 0.00 0.00
C
ATOM 7207 NH1 ARG C 27 −36.418 24.432 31.302 0.00 0.00
C
ATOM 7208 HH11 ARG C 27 −36.725 25.034 32.039 0.00 0.00
C

ATOM 7209 HH12 ARG C 27 −36.780 24.645 30.394 0.00 0.00
C
ATOM 7210 NH2 ARG C 27 −35.077 23.317 32.731 0.00 0.00
C
ATOM 7211 HH21 ARG C 27 −35.561 23.593 33.561 0.00 0.00
C
ATOM 7212 HH22 ARG C 27 −34.531 22.479 32.752 0.00 0.00
C
ATOM 7213 C ARG C 27 −33.589 23.104 26.296 0.00 0.00
C
ATOM 7214 O ARG C 27 −34.686 23.307 25.745 0.00 0.00
C
ATOM 7215 N MET C 28 −32.876 24.189 26.687 0.00 0.00
C
ATOM 7216 HN MET C 28 −31.991 24.116 27.140 0.00 0.00
C
ATOM 7217 CA MET C 28 −33.209 25.615 26.505 0.00 0.00
C
ATOM 7218 HA MET C 28 −34.271 25.806 26.472 0.00 0.00
C
ATOM 7219 CB MET C 28 −32.472 26.408 27.605 0.00 0.00
C
ATOM 7220 HB1 MET C 28 −31.398 26.202 27.408 0.00 0.00
C
ATOM 7221 HB2 MET C 28 −32.519 27.501 27.411 0.00 0.00
C
ATOM 7222 CG MET C 28 −33.005 26.238 28.977 0.00 0.00
C
ATOM 7223 HG1 MET C 28 −34.031 26.636 29.129 0.00 0.00
C
ATOM 7224 HG2 MET C 28 −33.156 25.154 29.165 0.00 0.00
C
ATOM 7225 SD MET C 28 −31.738 26.953 30.129 0.00 0.00
C
ATOM 7226 CE MET C 28 −32.643 26.068 31.441 0.00 0.00
C
ATOM 7227 HE1 MET C 28 −33.469 26.624 31.933 0.00 0.00
C
ATOM 7228 HE2 MET C 28 −32.925 24.995 31.377 0.00 0.00
C
ATOM 7229 HE3 MET C 28 −31.927 26.063 32.290 0.00 0.00
C
ATOM 7230 C MET C 28 −32.653 26.021 25.174 0.00 0.00
C
ATOM 7231 O MET C 28 −32.868 27.131 24.641 0.00 0.00
C
ATOM 7232 N GLY C 29 −32.121 25.019 24.434 0.00 0.00
C
ATOM 7233 HN GLY C 29 −31.791 24.210 24.914 0.00 0.00
C
ATOM 7234 CA GLY C 29 −31.713 25.069 23.101 0.00 0.00
C
ATOM 7235 HA1 GLY C 29 −32.491 25.569 22.544 0.00 0.00
C

ATOM 7236 HA2 GLY C 29 −31.769 24.046 22.761 0.00 0.00
C
ATOM 7237 C GLY C 29 −30.383 25.693 22.834 0.00 0.00
C
ATOM 7238 O GLY C 29 −30.019 25.698 21.691 0.00 0.00
C
ATOM 7239 N HSE C 30 −29.665 26.203 23.793 0.00 0.00
C
ATOM 7240 HN HSE C 30 −29.923 26.115 24.752 0.00 0.00
C
ATOM 7241 CA HSE C 30 −28.409 26.834 23.677 0.00 0.00
C
ATOM 7242 HA HSE C 30 −28.535 27.663 22.997 0.00 0.00
C
ATOM 7243 CB HSE C 30 −27.953 27.545 24.975 0.00 0.00
C
ATOM 7244 HB1 HSE C 30 −27.801 26.793 25.779 0.00 0.00
C
ATOM 7245 HB2 HSE C 30 −26.957 28.013 24.821 0.00 0.00
C
ATOM 7246 ND1 HSE C 30 −29.201 29.697 24.839 0.00 0.00
C
ATOM 7247 CG HSE C 30 −28.896 28.566 25.592 0.00 0.00
C
ATOM 7248 CE1 HSE C 30 −30.137 30.312 25.511 0.00 0.00
C
ATOM 7249 HE1 HSE C 30 −30.695 31.188 25.181 0.00 0.00
C
ATOM 7250 NE2 HSE C 30 −30.525 29.634 26.575 0.00 0.00
C
ATOM 7251 HE2 HSE C 30 −31.251 29.842 27.230 0.00 0.00
C
ATOM 7252 CD2 HSE C 30 −29.685 28.542 26.670 0.00 0.00
C
ATOM 7253 HD2 HSE C 30 −29.762 27.724 27.377 0.00 0.00
C
ATOM 7254 C HSE C 30 −27.300 25.988 23.091 0.00 0.00
C
ATOM 7255 O HSE C 30 −27.289 24.733 23.183 0.00 0.00
C
ATOM 7256 N GLU C 31 −26.317 26.625 22.440 0.00 0.00
C
ATOM 7257 HN GLU C 31 −26.520 27.575 22.216 0.00 0.00
C
ATOM 7258 CA GLU C 31 −25.110 26.070 21.782 0.00 0.00
C
ATOM 7259 HA GLU C 31 −25.306 25.100 21.350 0.00 0.00
C
ATOM 7260 CB GLU C 31 −24.696 26.909 20.593 0.00 0.00
C
ATOM 7261 HB1 GLU C 31 −25.526 27.089 19.877 0.00 0.00
C
ATOM 7262 HB2 GLU C 31 −24.516 27.953 20.926 0.00 0.00
C

ATOM 7263 CG GLU C 31 −23.454 26.297 19.838 0.00 0.00
C
ATOM 7264 HG1 GLU C 31 −22.510 26.432 20.409 0.00 0.00
C
ATOM 7265 HG2 GLU C 31 −23.498 25.210 19.611 0.00 0.00
C
ATOM 7266 CD GLU C 31 −23.140 26.774 18.451 0.00 0.00
C
ATOM 7267 OE1 GLU C 31 −23.689 26.157 17.518 0.00 0.00
C
ATOM 7268 OE2 GLU C 31 −22.466 27.788 18.313 0.00 0.00
C
ATOM 7269 C GLU C 31 −24.009 25.765 22.855 0.00 0.00
C
ATOM 7270 O GLU C 31 −23.696 26.495 23.757 0.00 0.00
C
ATOM 7271 N VAL C 32 −23.514 24.481 22.815 0.00 0.00
C
ATOM 7272 HN VAL C 32 −23.825 23.871 22.091 0.00 0.00
C
ATOM 7273 CA VAL C 32 −22.817 23.894 24.008 0.00 0.00
C
ATOM 7274 HA VAL C 32 −22.402 24.681 24.620 0.00 0.00
C
ATOM 7275 CB VAL C 32 −23.691 22.928 24.842 0.00 0.00
C
ATOM 7276 HB VAL C 32 −24.069 22.119 24.181 0.00 0.00
C
ATOM 7277 CG1 VAL C 32 −22.929 22.259 26.024 0.00 0.00
C
ATOM 7278 HG11 VAL C 32 −23.607 21.601 26.609 0.00 0.00
C
ATOM 7279 HG12 VAL C 32 −22.097 21.617 25.662 0.00 0.00
C
ATOM 7280 HG13 VAL C 32 −22.522 23.028 26.716 0.00 0.00
C
ATOM 7281 CG2 VAL C 32 −24.908 23.707 25.427 0.00 0.00
C
ATOM 7282 HG21 VAL C 32 −25.612 23.146 26.079 0.00 0.00
C
ATOM 7283 HG22 VAL C 32 −24.493 24.625 25.896 0.00 0.00
C
ATOM 7284 HG23 VAL C 32 −25.486 23.975 24.517 0.00 0.00
C
ATOM 7285 C VAL C 32 −21.601 23.180 23.514 0.00 0.00
C
ATOM 7286 O VAL C 32 −21.600 22.327 22.676 0.00 0.00
C
ATOM 7287 N LEU C 33 −20.477 23.473 24.142 0.00 0.00
C
ATOM 7288 HN LEU C 33 −20.455 24.110 24.908 0.00 0.00
C
ATOM 7289 CA LEU C 33 −19.152 22.805 23.866 0.00 0.00
C

ATOM 7290 HA LEU C 33 -19.307 21.993 23.170 0.00 0.00
C
ATOM 7291 CB LEU C 33 -17.986 23.702 23.372 0.00 0.00
C
ATOM 7292 HB1 LEU C 33 -18.468 24.165 22.485 0.00 0.00
C
ATOM 7293 HB2 LEU C 33 -17.791 24.414 24.202 0.00 0.00
C
ATOM 7294 CG LEU C 33 -16.622 23.243 22.895 0.00 0.00
C
ATOM 7295 HG LEU C 33 -16.119 24.128 22.450 0.00 0.00
C
ATOM 7296 CD1 LEU C 33 -15.772 22.689 24.066 0.00 0.00
C
ATOM 7297 HD11 LEU C 33 -15.745 23.416 24.905 0.00 0.00
C
ATOM 7298 HD12 LEU C 33 -16.170 21.743 24.492 0.00 0.00
C
ATOM 7299 HD13 LEU C 33 -14.752 22.467 23.685 0.00 0.00
C
ATOM 7300 CD2 LEU C 33 -16.705 22.161 21.832 0.00 0.00
C
ATOM 7301 HD21 LEU C 33 -16.812 21.195 22.371 0.00 0.00
C
ATOM 7302 HD22 LEU C 33 -17.553 22.303 21.128 0.00 0.00
C
ATOM 7303 HD23 LEU C 33 -15.740 22.233 21.287 0.00 0.00
C
ATOM 7304 C LEU C 33 -18.785 22.094 25.095 0.00 0.00
C
ATOM 7305 O LEU C 33 -18.639 22.720 26.129 0.00 0.00
C
ATOM 7306 N ALA C 34 -18.527 20.795 24.951 0.00 0.00
C
ATOM 7307 HN ALA C 34 -18.595 20.378 24.048 0.00 0.00
C
ATOM 7308 CA ALA C 34 -18.201 19.977 26.133 0.00 0.00
C
ATOM 7309 HA ALA C 34 -18.331 20.501 27.068 0.00 0.00
C
ATOM 7310 CB ALA C 34 -19.320 18.843 26.179 0.00 0.00
C
ATOM 7311 HB1 ALA C 34 -19.495 18.300 25.226 0.00 0.00
C
ATOM 7312 HB2 ALA C 34 -19.201 18.092 26.989 0.00 0.00
C
ATOM 7313 HB3 ALA C 34 -20.267 19.363 26.441 0.00 0.00
C
ATOM 7314 C ALA C 34 -16.799 19.485 26.230 0.00 0.00
C
ATOM 7315 O ALA C 34 -16.350 18.784 25.316 0.00 0.00
C
ATOM 7316 N VAL C 35 -16.121 19.673 27.373 0.00 0.00
C

ATOM 7317 HN VAL C 35 -16.568 20.194 28.096 0.00 0.00
C
ATOM 7318 CA VAL C 35 -14.704 19.293 27.569 0.00 0.00
C
ATOM 7319 HA VAL C 35 -14.437 18.655 26.740 0.00 0.00
C
ATOM 7320 CB VAL C 35 -13.652 20.433 27.667 0.00 0.00
C
ATOM 7321 HB VAL C 35 -13.877 21.021 28.582 0.00 0.00
C
ATOM 7322 CG1 VAL C 35 -12.252 19.864 27.675 0.00 0.00
C
ATOM 7323 HG11 VAL C 35 -11.908 19.357 28.602 0.00 0.00
C
ATOM 7324 HG12 VAL C 35 -12.052 19.267 26.760 0.00 0.00
C
ATOM 7325 HG13 VAL C 35 -11.490 20.666 27.568 0.00 0.00
C
ATOM 7326 CG2 VAL C 35 -13.739 21.305 26.426 0.00 0.00
C
ATOM 7327 HG21 VAL C 35 -13.598 20.695 25.508 0.00 0.00
C
ATOM 7328 HG22 VAL C 35 -14.737 21.789 26.357 0.00 0.00
C
ATOM 7329 HG23 VAL C 35 -12.960 22.095 26.481 0.00 0.00
C
ATOM 7330 C VAL C 35 -14.661 18.462 28.812 0.00 0.00
C
ATOM 7331 O VAL C 35 -15.083 18.931 29.853 0.00 0.00
C
ATOM 7332 N ASP C 36 -13.973 17.317 28.684 0.00 0.00
C
ATOM 7333 HN ASP C 36 -13.637 17.040 27.787 0.00 0.00
C
ATOM 7334 CA ASP C 36 -13.574 16.622 29.870 0.00 0.00
C
ATOM 7335 HA ASP C 36 -13.464 17.313 30.693 0.00 0.00
C
ATOM 7336 CB ASP C 36 -14.569 15.577 30.230 0.00 0.00
C
ATOM 7337 HB1 ASP C 36 -15.529 16.132 30.174 0.00 0.00
C
ATOM 7338 HB2 ASP C 36 -14.501 14.655 29.614 0.00 0.00
C
ATOM 7339 CG ASP C 36 -14.568 15.027 31.714 0.00 0.00
C
ATOM 7340 OD1 ASP C 36 -15.367 15.465 32.582 0.00 0.00
C
ATOM 7341 OD2 ASP C 36 -13.619 14.305 32.053 0.00 0.00
C
ATOM 7342 C ASP C 36 -12.289 15.907 29.462 0.00 0.00
C
ATOM 7343 O ASP C 36 -12.067 15.805 28.252 0.00 0.00
C

```
ATOM   7344  N   ILE C  37     -11.459  15.405  30.447  0.00  0.00
C
ATOM   7345  HN  ILE C  37     -11.588  15.602  31.416  0.00  0.00
C
ATOM   7346  CA  ILE C  37     -10.414  14.447  30.136  0.00  0.00
C
ATOM   7347  HA  ILE C  37      -9.942  14.728  29.206  0.00  0.00
C
ATOM   7348  CB  ILE C  37      -9.236  14.652  31.118  0.00  0.00
C
ATOM   7349  HB  ILE C  37      -9.061  15.746  31.040  0.00  0.00
C
ATOM   7350  CG2 ILE C  37      -9.442  14.372  32.594  0.00  0.00
C
ATOM   7351 HG21 ILE C  37      -9.573  13.277  32.731  0.00  0.00
C
ATOM   7352 HG22 ILE C  37      -8.532  14.622  33.179  0.00  0.00
C
ATOM   7353 HG23 ILE C  37     -10.348  14.848  33.027  0.00  0.00
C
ATOM   7354  CG1 ILE C  37      -7.864  14.084  30.695  0.00  0.00
C
ATOM   7355 HG11 ILE C  37      -7.079  14.466  31.383  0.00  0.00
C
ATOM   7356 HG12 ILE C  37      -7.997  12.993  30.858  0.00  0.00
C
ATOM   7357  CD  ILE C  37      -7.359  14.349  29.319  0.00  0.00
C
ATOM   7358  HD1 ILE C  37      -6.356  13.895  29.170  0.00  0.00
C
ATOM   7359  HD2 ILE C  37      -8.113  13.988  28.587  0.00  0.00
C
ATOM   7360  HD3 ILE C  37      -7.213  15.431  29.114  0.00  0.00
C
ATOM   7361  C   ILE C  37     -10.793  12.949  29.990  0.00  0.00
C
ATOM   7362  O   ILE C  37     -10.127  12.189  29.308  0.00  0.00
C
ATOM   7363  N   ASN C  38     -11.940  12.578  30.526  0.00  0.00
C
ATOM   7364  HN  ASN C  38     -12.560  13.174  31.031  0.00  0.00
C
ATOM   7365  CA  ASN C  38     -12.432  11.217  30.496  0.00  0.00
C
ATOM   7366  HA  ASN C  38     -11.586  10.576  30.297  0.00  0.00
C
ATOM   7367  CB  ASN C  38     -13.052  10.896  31.922  0.00  0.00
C
ATOM   7368  HB1 ASN C  38     -13.633  11.774  32.278  0.00  0.00
C
ATOM   7369  HB2 ASN C  38     -13.716  10.006  31.876  0.00  0.00
C
ATOM   7370  CG  ASN C  38     -11.905  10.641  32.918  0.00  0.00
C
```

ATOM 7371 OD1 ASN C 38 −11.709 11.194 33.990 0.00 0.00
C
ATOM 7372 ND2 ASN C 38 −11.042 9.710 32.573 0.00 0.00
C
ATOM 7373 HD21 ASN C 38 −10.408 9.414 33.287 0.00 0.00
C
ATOM 7374 HD22 ASN C 38 −11.287 9.013 31.899 0.00 0.00
C
ATOM 7375 C ASN C 38 −13.353 10.952 29.383 0.00 0.00
C
ATOM 7376 O ASN C 38 −14.447 11.532 29.315 0.00 0.00
C
ATOM 7377 N GLU C 39 −12.865 10.062 28.451 0.00 0.00
C
ATOM 7378 HN GLU C 39 −11.966 9.689 28.667 0.00 0.00
C
ATOM 7379 CA GLU C 39 −13.505 9.688 27.176 0.00 0.00
C
ATOM 7380 HA GLU C 39 −13.769 10.565 26.604 0.00 0.00
C
ATOM 7381 CB GLU C 39 −12.617 8.617 26.445 0.00 0.00
C
ATOM 7382 HB1 GLU C 39 −11.615 9.077 26.307 0.00 0.00
C
ATOM 7383 HB2 GLU C 39 −12.430 7.829 27.206 0.00 0.00
C
ATOM 7384 CG GLU C 39 −13.258 7.853 25.305 0.00 0.00
C
ATOM 7385 HG1 GLU C 39 −14.093 7.143 25.491 0.00 0.00
C
ATOM 7386 HG2 GLU C 39 −13.713 8.630 24.653 0.00 0.00
C
ATOM 7387 CD GLU C 39 −12.193 6.997 24.645 0.00 0.00
C
ATOM 7388 OE1 GLU C 39 −11.810 7.267 23.440 0.00 0.00
C
ATOM 7389 OE2 GLU C 39 −11.802 6.001 25.253 0.00 0.00
C
ATOM 7390 C GLU C 39 −14.848 9.079 27.368 0.00 0.00
C
ATOM 7391 O GLU C 39 −15.771 9.463 26.693 0.00 0.00
C
ATOM 7392 N GLU C 40 −15.100 8.255 28.394 0.00 0.00
C
ATOM 7393 HN GLU C 40 −14.357 7.968 28.994 0.00 0.00
C
ATOM 7394 CA GLU C 40 −16.429 7.680 28.763 0.00 0.00
C
ATOM 7395 HA GLU C 40 −16.919 7.095 27.998 0.00 0.00
C
ATOM 7396 CB GLU C 40 −16.212 6.785 30.102 0.00 0.00
C
ATOM 7397 HB1 GLU C 40 −15.513 5.969 29.821 0.00 0.00
C

ATOM 7398 HB2 GLU C 40 −15.717 7.403 30.882 0.00 0.00
C
ATOM 7399 CG GLU C 40 −17.489 6.198 30.670 0.00 0.00
C
ATOM 7400 HG1 GLU C 40 −17.206 5.670 31.605 0.00 0.00
C
ATOM 7401 HG2 GLU C 40 −18.314 6.887 30.951 0.00 0.00
C
ATOM 7402 CD GLU C 40 −18.072 5.204 29.689 0.00 0.00
C
ATOM 7403 OE1 GLU C 40 −17.392 4.279 29.238 0.00 0.00
C
ATOM 7404 OE2 GLU C 40 −19.299 5.278 29.331 0.00 0.00
C
ATOM 7405 C GLU C 40 −17.446 8.830 29.128 0.00 0.00
C
ATOM 7406 O GLU C 40 −18.641 8.758 28.690 0.00 0.00
C
ATOM 7407 N LYS C 41 −16.986 9.905 29.799 0.00 0.00
C
ATOM 7408 HN LYS C 41 −16.026 9.943 30.065 0.00 0.00
C
ATOM 7409 CA LYS C 41 −17.893 11.121 29.977 0.00 0.00
C
ATOM 7410 HA LYS C 41 −18.913 10.824 30.173 0.00 0.00
C
ATOM 7411 CB LYS C 41 −17.445 11.981 31.149 0.00 0.00
C
ATOM 7412 HB1 LYS C 41 −16.548 12.564 30.850 0.00 0.00
C
ATOM 7413 HB2 LYS C 41 −18.278 12.651 31.452 0.00 0.00
C
ATOM 7414 CG LYS C 41 −17.067 11.043 32.340 0.00 0.00
C
ATOM 7415 HG1 LYS C 41 −17.888 10.322 32.540 0.00 0.00
C
ATOM 7416 HG2 LYS C 41 −16.117 10.487 32.185 0.00 0.00
C
ATOM 7417 CD LYS C 41 −16.771 12.049 33.533 0.00 0.00
C
ATOM 7418 HD1 LYS C 41 −15.909 12.662 33.195 0.00 0.00
C
ATOM 7419 HD2 LYS C 41 −17.542 12.808 33.786 0.00 0.00
C
ATOM 7420 CE LYS C 41 −16.443 11.241 34.855 0.00 0.00
C
ATOM 7421 HE1 LYS C 41 −17.198 10.437 34.986 0.00 0.00
C
ATOM 7422 HE2 LYS C 41 −15.439 10.771 34.780 0.00 0.00
C
ATOM 7423 NZ LYS C 41 −16.432 12.148 36.006 0.00 0.00
C
ATOM 7424 HZ1 LYS C 41 −16.137 13.081 35.656 0.00 0.00
C

ATOM 7425 HZ2 LYS C 41 −17.347 12.263 36.489 0.00 0.00
C
ATOM 7426 HZ3 LYS C 41 −15.791 11.811 36.753 0.00 0.00
C
ATOM 7427 C LYS C 41 −18.095 12.030 28.749 0.00 0.00
C
ATOM 7428 O LYS C 41 −19.190 12.436 28.494 0.00 0.00
C
ATOM 7429 N VAL C 42 −17.070 12.211 27.941 0.00 0.00
C
ATOM 7430 HN VAL C 42 −16.143 12.005 28.243 0.00 0.00
C
ATOM 7431 CA VAL C 42 −17.293 12.744 26.617 0.00 0.00
C
ATOM 7432 HA VAL C 42 −17.651 13.734 26.860 0.00 0.00
C
ATOM 7433 CB VAL C 42 −15.942 13.065 25.852 0.00 0.00
C
ATOM 7434 HB VAL C 42 −15.315 12.149 25.898 0.00 0.00
C
ATOM 7435 CG1 VAL C 42 −16.157 13.334 24.321 0.00 0.00
C
ATOM 7436 HG11 VAL C 42 −16.264 12.371 23.777 0.00 0.00
C
ATOM 7437 HG12 VAL C 42 −17.133 13.844 24.170 0.00 0.00
C
ATOM 7438 HG13 VAL C 42 −15.382 13.944 23.809 0.00 0.00
C
ATOM 7439 CG2 VAL C 42 −15.227 14.274 26.614 0.00 0.00
C
ATOM 7440 HG21 VAL C 42 −14.705 14.001 27.556 0.00 0.00
C
ATOM 7441 HG22 VAL C 42 −14.435 14.846 26.084 0.00 0.00
C
ATOM 7442 HG23 VAL C 42 −15.956 14.996 27.038 0.00 0.00
C
ATOM 7443 C VAL C 42 −18.341 11.982 25.712 0.00 0.00
C
ATOM 7444 O VAL C 42 −19.235 12.586 25.148 0.00 0.00
C
ATOM 7445 N ASN C 43 −18.268 10.663 25.696 0.00 0.00
C
ATOM 7446 HN ASN C 43 −17.434 10.233 26.033 0.00 0.00
C
ATOM 7447 CA ASN C 43 −19.324 9.818 25.176 0.00 0.00
C
ATOM 7448 HA ASN C 43 −19.654 10.142 24.200 0.00 0.00
C
ATOM 7449 CB ASN C 43 −18.935 8.321 24.971 0.00 0.00
C
ATOM 7450 HB1 ASN C 43 −18.487 7.983 25.930 0.00 0.00
C
ATOM 7451 HB2 ASN C 43 −19.825 7.691 24.758 0.00 0.00
C

ATOM 7452 CG ASN C 43 −18.012 8.436 23.827 0.00 0.00
C
ATOM 7453 OD1 ASN C 43 −18.486 8.486 22.676 0.00 0.00
C
ATOM 7454 ND2 ASN C 43 −16.673 8.493 24.039 0.00 0.00
C
ATOM 7455 HD21 ASN C 43 −15.970 8.612 23.338 0.00 0.00
C
ATOM 7456 HD22 ASN C 43 −16.471 8.680 25.000 0.00 0.00
C
ATOM 7457 C ASN C 43 −20.696 9.918 25.909 0.00 0.00
C
ATOM 7458 O ASN C 43 −21.761 9.811 25.281 0.00 0.00
C
ATOM 7459 N ALA C 44 −20.721 9.970 27.196 0.00 0.00
C
ATOM 7460 HN ALA C 44 −19.861 9.903 27.697 0.00 0.00
C
ATOM 7461 CA ALA C 44 −21.970 10.005 27.961 0.00 0.00
C
ATOM 7462 HA ALA C 44 −22.615 9.218 27.600 0.00 0.00
C
ATOM 7463 CB ALA C 44 −21.798 9.887 29.490 0.00 0.00
C
ATOM 7464 HB1 ALA C 44 −21.419 8.895 29.817 0.00 0.00
C
ATOM 7465 HB2 ALA C 44 −21.111 10.647 29.919 0.00 0.00
C
ATOM 7466 HB3 ALA C 44 −22.769 10.045 30.007 0.00 0.00
C
ATOM 7467 C ALA C 44 −22.906 11.217 27.643 0.00 0.00
C
ATOM 7468 O ALA C 44 −24.136 11.075 27.747 0.00 0.00
C
ATOM 7469 N TYR C 45 −22.320 12.333 27.256 0.00 0.00
C
ATOM 7470 HN TYR C 45 −21.324 12.348 27.218 0.00 0.00
C
ATOM 7471 CA TYR C 45 −22.996 13.603 27.086 0.00 0.00
C
ATOM 7472 HA TYR C 45 −24.066 13.455 27.079 0.00 0.00
C
ATOM 7473 CB TYR C 45 −22.640 14.620 28.183 0.00 0.00
C
ATOM 7474 HB1 TYR C 45 −21.607 15.023 28.113 0.00 0.00
C
ATOM 7475 HB2 TYR C 45 −23.331 15.489 28.160 0.00 0.00
C
ATOM 7476 CG TYR C 45 −22.712 13.961 29.562 0.00 0.00
C
ATOM 7477 CD1 TYR C 45 −23.913 13.485 30.103 0.00 0.00
C
ATOM 7478 HD1 TYR C 45 −24.864 13.631 29.612 0.00 0.00
C

ATOM 7479 CE1 TYR C 45 −24.001 12.905 31.468 0.00 0.00
C
ATOM 7480 HE1 TYR C 45 −24.866 12.439 31.919 0.00 0.00
C
ATOM 7481 CZ TYR C 45 −22.780 12.753 32.064 0.00 0.00
C
ATOM 7482 OH TYR C 45 −22.670 11.975 33.266 0.00 0.00
C
ATOM 7483 HH TYR C 45 −21.723 11.936 33.416 0.00 0.00
C
ATOM 7484 CD2 TYR C 45 −21.543 13.864 30.371 0.00 0.00
C
ATOM 7485 HD2 TYR C 45 −20.569 13.896 29.907 0.00 0.00
C
ATOM 7486 CE2 TYR C 45 −21.578 13.208 31.575 0.00 0.00
C
ATOM 7487 HE2 TYR C 45 −20.672 13.106 32.154 0.00 0.00
C
ATOM 7488 C TYR C 45 −22.759 14.201 25.678 0.00 0.00
C
ATOM 7489 O TYR C 45 −23.129 15.385 25.455 0.00 0.00
C
ATOM 7490 N ALA C 46 −22.212 13.445 24.676 0.00 0.00
C
ATOM 7491 HN ALA C 46 −21.842 12.530 24.819 0.00 0.00
C
ATOM 7492 CA ALA C 46 −22.009 13.929 23.331 0.00 0.00
C
ATOM 7493 HA ALA C 46 −21.461 14.857 23.395 0.00 0.00
C
ATOM 7494 CB ALA C 46 −21.193 12.929 22.679 0.00 0.00
C
ATOM 7495 HB1 ALA C 46 −20.955 13.305 21.661 0.00 0.00
C
ATOM 7496 HB2 ALA C 46 −20.195 12.892 23.165 0.00 0.00
C
ATOM 7497 HB3 ALA C 46 −21.687 11.940 22.569 0.00 0.00
C
ATOM 7498 C ALA C 46 −23.314 14.266 22.532 0.00 0.00
C
ATOM 7499 O ALA C 46 −23.227 14.906 21.528 0.00 0.00
C
ATOM 7500 N SER C 47 −24.545 13.811 22.992 0.00 0.00
C
ATOM 7501 HN SER C 47 −24.636 13.187 23.764 0.00 0.00
C
ATOM 7502 CA SER C 47 −25.762 14.020 22.302 0.00 0.00
C
ATOM 7503 HA SER C 47 −25.538 14.245 21.270 0.00 0.00
C
ATOM 7504 CB SER C 47 −26.673 12.769 22.277 0.00 0.00
C
ATOM 7505 HB1 SER C 47 −26.668 12.418 23.331 0.00 0.00
C

ATOM 7506 HB2 SER C 47 -27.677 12.921 21.825 0.00 0.00
C
ATOM 7507 OG SER C 47 -26.061 11.735 21.447 0.00 0.00
C
ATOM 7508 HG1 SER C 47 -26.752 11.068 21.444 0.00 0.00
C
ATOM 7509 C SER C 47 -26.502 15.154 22.955 0.00 0.00
C
ATOM 7510 O SER C 47 -27.515 15.616 22.499 0.00 0.00
C
ATOM 7511 N TYR C 48 -25.920 15.681 24.039 0.00 0.00
C
ATOM 7512 HN TYR C 48 -25.045 15.388 24.418 0.00 0.00
C
ATOM 7513 CA TYR C 48 -26.464 16.836 24.744 0.00 0.00
C
ATOM 7514 HA TYR C 48 -27.387 17.135 24.270 0.00 0.00
C
ATOM 7515 CB TYR C 48 -26.590 16.581 26.233 0.00 0.00
C
ATOM 7516 HB1 TYR C 48 -25.650 16.291 26.751 0.00 0.00
C
ATOM 7517 HB2 TYR C 48 -26.956 17.518 26.705 0.00 0.00
C
ATOM 7518 CG TYR C 48 -27.553 15.346 26.607 0.00 0.00
C
ATOM 7519 CD1 TYR C 48 -27.202 14.441 27.615 0.00 0.00
C
ATOM 7520 HD1 TYR C 48 -26.211 14.374 28.039 0.00 0.00
C
ATOM 7521 CE1 TYR C 48 -28.120 13.540 28.100 0.00 0.00
C
ATOM 7522 HE1 TYR C 48 -27.879 12.869 28.912 0.00 0.00
C
ATOM 7523 CZ TYR C 48 -29.377 13.438 27.435 0.00 0.00
C
ATOM 7524 OH TYR C 48 -30.241 12.472 27.805 0.00 0.00
C
ATOM 7525 HH TYR C 48 -31.127 12.840 27.788 0.00 0.00
C
ATOM 7526 CD2 TYR C 48 -28.817 15.217 26.029 0.00 0.00
C
ATOM 7527 HD2 TYR C 48 -28.993 15.871 25.188 0.00 0.00
C
ATOM 7528 CE2 TYR C 48 -29.736 14.240 26.396 0.00 0.00
C
ATOM 7529 HE2 TYR C 48 -30.644 14.191 25.814 0.00 0.00
C
ATOM 7530 C TYR C 48 -25.595 18.100 24.615 0.00 0.00
C
ATOM 7531 O TYR C 48 -25.877 19.131 25.145 0.00 0.00
C
ATOM 7532 N ALA C 49 -24.585 18.039 23.807 0.00 0.00
C

ATOM 7533 HN ALA C 49 −24.436 17.139 23.405 0.00 0.00
C
ATOM 7534 CA ALA C 49 −23.660 19.087 23.498 0.00 0.00
C
ATOM 7535 HA ALA C 49 −23.961 20.085 23.778 0.00 0.00
C
ATOM 7536 CB ALA C 49 −22.238 18.794 23.925 0.00 0.00
C
ATOM 7537 HB1 ALA C 49 −21.498 19.574 23.643 0.00 0.00
C
ATOM 7538 HB2 ALA C 49 −22.269 18.718 25.033 0.00 0.00
C
ATOM 7539 HB3 ALA C 49 −22.005 17.791 23.510 0.00 0.00
C
ATOM 7540 C ALA C 49 −23.649 19.166 21.929 0.00 0.00
C
ATOM 7541 O ALA C 49 −23.881 18.157 21.209 0.00 0.00
C
ATOM 7542 N THR C 50 −23.466 20.340 21.422 0.00 0.00
C
ATOM 7543 HN THR C 50 −23.221 21.156 21.941 0.00 0.00
C
ATOM 7544 CA THR C 50 −23.397 20.623 20.041 0.00 0.00
C
ATOM 7545 HA THR C 50 −24.246 20.175 19.545 0.00 0.00
C
ATOM 7546 CB THR C 50 −23.295 22.215 19.808 0.00 0.00
C
ATOM 7547 HB THR C 50 −22.401 22.622 20.328 0.00 0.00
C
ATOM 7548 OG1 THR C 50 −24.406 22.855 20.385 0.00 0.00
C
ATOM 7549 HG1 THR C 50 −25.092 22.847 19.713 0.00 0.00
C
ATOM 7550 CG2 THR C 50 −23.249 22.602 18.302 0.00 0.00
C
ATOM 7551 HG21 THR C 50 −22.319 22.196 17.850 0.00 0.00
C
ATOM 7552 HG22 THR C 50 −24.157 22.194 17.807 0.00 0.00
C
ATOM 7553 HG23 THR C 50 −23.302 23.699 18.136 0.00 0.00
C
ATOM 7554 C THR C 50 −22.146 20.026 19.306 0.00 0.00
C
ATOM 7555 O THR C 50 −22.162 19.679 18.080 0.00 0.00
C
ATOM 7556 N HSE C 51 −21.023 20.021 20.082 0.00 0.00
C
ATOM 7557 HN HSE C 51 −21.133 20.270 21.042 0.00 0.00
C
ATOM 7558 CA HSE C 51 −19.759 19.413 19.682 0.00 0.00
C
ATOM 7559 HA HSE C 51 −19.987 18.447 19.255 0.00 0.00
C

ATOM 7560 CB HSE C 51 −18.864 20.247 18.677 0.00 0.00
C
ATOM 7561 HB1 HSE C 51 −19.420 20.389 17.726 0.00 0.00
C
ATOM 7562 HB2 HSE C 51 −18.653 21.253 19.100 0.00 0.00
C
ATOM 7563 ND1 HSE C 51 −17.554 18.143 18.110 0.00 0.00
C
ATOM 7564 CG HSE C 51 −17.512 19.537 18.313 0.00 0.00
C
ATOM 7565 CE1 HSE C 51 −16.312 17.812 17.755 0.00 0.00
C
ATOM 7566 HE1 HSE C 51 −15.966 16.825 17.451 0.00 0.00
C
ATOM 7567 NE2 HSE C 51 −15.488 18.905 17.828 0.00 0.00
C
ATOM 7568 HE2 HSE C 51 −14.496 18.865 17.707 0.00 0.00
C
ATOM 7569 CD2 HSE C 51 −16.263 20.002 18.144 0.00 0.00
C
ATOM 7570 HD2 HSE C 51 −15.930 21.029 18.236 0.00 0.00
C
ATOM 7571 C HSE C 51 −19.078 19.127 21.072 0.00 0.00
C
ATOM 7572 O HSE C 51 −19.366 19.796 22.092 0.00 0.00
C
ATOM 7573 N ALA C 52 −18.118 18.228 21.160 0.00 0.00
C
ATOM 7574 HN ALA C 52 −17.839 17.909 20.257 0.00 0.00
C
ATOM 7575 CA ALA C 52 −17.518 17.718 22.383 0.00 0.00
C
ATOM 7576 HA ALA C 52 −17.496 18.494 23.135 0.00 0.00
C
ATOM 7577 CB ALA C 52 −18.200 16.442 22.832 0.00 0.00
C
ATOM 7578 HB1 ALA C 52 −17.625 15.880 23.599 0.00 0.00
C
ATOM 7579 HB2 ALA C 52 −19.264 16.547 23.132 0.00 0.00
C
ATOM 7580 HB3 ALA C 52 −18.278 15.758 21.959 0.00 0.00
C
ATOM 7581 C ALA C 52 −16.131 17.303 22.109 0.00 0.00
C
ATOM 7582 O ALA C 52 −15.837 17.025 20.994 0.00 0.00
C
ATOM 7583 N VAL C 53 −15.231 17.203 23.072 0.00 0.00
C
ATOM 7584 HN VAL C 53 −15.428 17.298 24.044 0.00 0.00
C
ATOM 7585 CA VAL C 53 −13.884 16.851 22.770 0.00 0.00
C
ATOM 7586 HA VAL C 53 −13.962 16.043 22.057 0.00 0.00
C

ATOM 7587 CB VAL C 53 −13.083 17.952 22.090 0.00 0.00
C
ATOM 7588 HB VAL C 53 −13.498 18.049 21.064 0.00 0.00
C
ATOM 7589 CG1 VAL C 53 −13.376 19.319 22.706 0.00 0.00
C
ATOM 7590 HG11 VAL C 53 −12.579 19.614 23.421 0.00 0.00
C
ATOM 7591 HG12 VAL C 53 −13.324 20.067 21.886 0.00 0.00
C
ATOM 7592 HG13 VAL C 53 −14.281 19.417 23.343 0.00 0.00
C
ATOM 7593 CG2 VAL C 53 −11.592 17.691 21.822 0.00 0.00
C
ATOM 7594 HG21 VAL C 53 −11.064 17.664 22.798 0.00 0.00
C
ATOM 7595 HG22 VAL C 53 −11.496 16.818 21.141 0.00 0.00
C
ATOM 7596 HG23 VAL C 53 −11.056 18.466 21.233 0.00 0.00
C
ATOM 7597 C VAL C 53 −13.104 16.413 24.044 0.00 0.00
C
ATOM 7598 O VAL C 53 −13.241 17.012 25.066 0.00 0.00
C
ATOM 7599 N ILE C 54 −12.385 15.288 23.989 0.00 0.00
C
ATOM 7600 HN ILE C 54 −12.531 14.738 23.170 0.00 0.00
C
ATOM 7601 CA ILE C 54 −11.406 14.781 25.066 0.00 0.00
C
ATOM 7602 HA ILE C 54 −12.003 14.971 25.946 0.00 0.00
C
ATOM 7603 CB ILE C 54 −10.974 13.276 24.789 0.00 0.00
C
ATOM 7604 HB ILE C 54 −10.512 13.389 23.785 0.00 0.00
C
ATOM 7605 CG2 ILE C 54 −9.917 12.786 25.921 0.00 0.00
C
ATOM 7606 HG21 ILE C 54 −9.683 11.712 25.757 0.00 0.00
C
ATOM 7607 HG22 ILE C 54 −9.009 13.410 25.778 0.00 0.00
C
ATOM 7608 HG23 ILE C 54 −10.257 13.128 26.922 0.00 0.00
C
ATOM 7609 CG1 ILE C 54 −12.222 12.371 24.777 0.00 0.00
C
ATOM 7610 HG11 ILE C 54 −12.414 12.121 25.842 0.00 0.00
C
ATOM 7611 HG12 ILE C 54 −13.114 12.932 24.425 0.00 0.00
C
ATOM 7612 CD ILE C 54 −12.070 11.186 23.817 0.00 0.00
C
ATOM 7613 HD1 ILE C 54 −12.974 10.542 23.841 0.00 0.00
C

ATOM 7614 HD2 ILE C 54 -12.101 11.524 22.759 0.00 0.00
C
ATOM 7615 HD3 ILE C 54 -11.176 10.553 24.007 0.00 0.00
C
ATOM 7616 C ILE C 54 -10.091 15.593 24.953 0.00 0.00
C
ATOM 7617 O ILE C 54 -9.310 15.392 24.073 0.00 0.00
C
ATOM 7618 N ALA C 55 -9.832 16.518 25.887 0.00 0.00
C
ATOM 7619 HN ALA C 55 -10.528 16.546 26.600 0.00 0.00
C
ATOM 7620 CA ALA C 55 -8.712 17.424 25.964 0.00 0.00
C
ATOM 7621 HA ALA C 55 -7.798 16.941 25.651 0.00 0.00
C
ATOM 7622 CB ALA C 55 -8.923 18.711 25.092 0.00 0.00
C
ATOM 7623 HB1 ALA C 55 -8.066 19.383 25.309 0.00 0.00
C
ATOM 7624 HB2 ALA C 55 -8.846 18.412 24.025 0.00 0.00
C
ATOM 7625 HB3 ALA C 55 -9.964 19.080 25.217 0.00 0.00
C
ATOM 7626 C ALA C 55 -8.558 17.933 27.445 0.00 0.00
C
ATOM 7627 O ALA C 55 -9.475 18.011 28.307 0.00 0.00
C
ATOM 7628 N ASN C 56 -7.329 18.332 27.786 0.00 0.00
C
ATOM 7629 HN ASN C 56 -6.560 18.267 27.156 0.00 0.00
C
ATOM 7630 CA ASN C 56 -6.964 18.688 29.123 0.00 0.00
C
ATOM 7631 HA ASN C 56 -7.648 18.214 29.811 0.00 0.00
C
ATOM 7632 CB ASN C 56 -5.517 18.405 29.463 0.00 0.00
C
ATOM 7633 HB1 ASN C 56 -5.257 17.347 29.243 0.00 0.00
C
ATOM 7634 HB2 ASN C 56 -4.963 19.020 28.722 0.00 0.00
C
ATOM 7635 CG ASN C 56 -4.996 18.755 30.843 0.00 0.00
C
ATOM 7636 OD1 ASN C 56 -5.782 19.253 31.655 0.00 0.00
C
ATOM 7637 ND2 ASN C 56 -3.689 18.572 31.050 0.00 0.00
C
ATOM 7638 HD21 ASN C 56 -3.267 18.981 31.859 0.00 0.00
C
ATOM 7639 HD22 ASN C 56 -3.274 17.895 30.442 0.00 0.00
C
ATOM 7640 C ASN C 56 -7.192 20.219 29.193 0.00 0.00
C

ATOM 7641 O ASN C 56 -6.814 20.993 28.281 0.00 0.00
C
ATOM 7642 N ALA C 57 -7.919 20.745 30.234 0.00 0.00
C
ATOM 7643 HN ALA C 57 -8.150 20.067 30.927 0.00 0.00
C
ATOM 7644 CA ALA C 57 -8.116 22.145 30.483 0.00 0.00
C
ATOM 7645 HA ALA C 57 -8.230 22.693 29.559 0.00 0.00
C
ATOM 7646 CB ALA C 57 -9.465 22.148 31.257 0.00 0.00
C
ATOM 7647 HB1 ALA C 57 -9.370 21.913 32.339 0.00 0.00
C
ATOM 7648 HB2 ALA C 57 -9.813 23.201 31.183 0.00 0.00
C
ATOM 7649 HB3 ALA C 57 -10.235 21.480 30.816 0.00 0.00
C
ATOM 7650 C ALA C 57 -7.051 22.802 31.285 0.00 0.00
C
ATOM 7651 O ALA C 57 -6.999 24.042 31.309 0.00 0.00
C
ATOM 7652 N THR C 58 -6.155 22.052 31.979 0.00 0.00
C
ATOM 7653 HN THR C 58 -6.117 21.056 31.995 0.00 0.00
C
ATOM 7654 CA THR C 58 -5.228 22.586 32.895 0.00 0.00
C
ATOM 7655 HA THR C 58 -5.691 23.413 33.413 0.00 0.00
C
ATOM 7656 CB THR C 58 -4.995 21.665 34.097 0.00 0.00
C
ATOM 7657 HB THR C 58 -4.251 22.222 34.705 0.00 0.00
C
ATOM 7658 OG1 THR C 58 -4.473 20.358 33.843 0.00 0.00
C
ATOM 7659 HG1 THR C 58 -4.884 19.936 33.085 0.00 0.00
C
ATOM 7660 CG2 THR C 58 -6.250 21.436 34.920 0.00 0.00
C
ATOM 7661 HG21 THR C 58 -7.191 21.251 34.358 0.00 0.00
C
ATOM 7662 HG22 THR C 58 -6.062 20.637 35.669 0.00 0.00
C
ATOM 7663 HG23 THR C 58 -6.467 22.301 35.584 0.00 0.00
C
ATOM 7664 C THR C 58 -3.876 22.965 32.302 0.00 0.00
C
ATOM 7665 O THR C 58 -3.005 23.505 32.989 0.00 0.00
C
ATOM 7666 N GLU C 59 -3.650 22.831 30.965 0.00 0.00
C
ATOM 7667 HN GLU C 59 -4.394 22.375 30.482 0.00 0.00
C

```
ATOM   7668  CA  GLU C  59      -2.552  23.298  30.194  0.00  0.00
C
ATOM   7669  HA  GLU C  59      -1.841  23.771  30.855  0.00  0.00
C
ATOM   7670  CB  GLU C  59      -1.866  22.083  29.363  0.00  0.00
C
ATOM   7671  HB1 GLU C  59      -1.602  21.269  30.072  0.00  0.00
C
ATOM   7672  HB2 GLU C  59      -2.627  21.677  28.663  0.00  0.00
C
ATOM   7673  CG  GLU C  59      -0.605  22.347  28.631  0.00  0.00
C
ATOM   7674  HG1 GLU C  59      -0.416  21.476  27.968  0.00  0.00
C
ATOM   7675  HG2 GLU C  59      -0.684  23.202  27.925  0.00  0.00
C
ATOM   7676  CD  GLU C  59       0.574  22.609  29.518  0.00  0.00
C
ATOM   7677  OE1 GLU C  59       1.521  21.770  29.560  0.00  0.00
C
ATOM   7678  OE2 GLU C  59       0.608  23.687  30.124  0.00  0.00
C
ATOM   7679  C   GLU C  59      -2.966  24.351  29.174  0.00  0.00
C
ATOM   7680  O   GLU C  59      -3.923  24.156  28.406  0.00  0.00
C
ATOM   7681  N   GLU C  60      -2.162  25.485  29.038  0.00  0.00
C
ATOM   7682  HN  GLU C  60      -1.460  25.679  29.720  0.00  0.00
C
ATOM   7683  CA  GLU C  60      -2.348  26.649  28.200  0.00  0.00
C
ATOM   7684  HA  GLU C  60      -3.233  27.129  28.590  0.00  0.00
C
ATOM   7685  CB  GLU C  60      -1.194  27.689  28.312  0.00  0.00
C
ATOM   7686  HB1 GLU C  60      -1.103  27.945  29.390  0.00  0.00
C
ATOM   7687  HB2 GLU C  60      -0.202  27.240  28.091  0.00  0.00
C
ATOM   7688  CG  GLU C  60      -1.317  29.023  27.643  0.00  0.00
C
ATOM   7689  HG1 GLU C  60      -1.625  28.826  26.593  0.00  0.00
C
ATOM   7690  HG2 GLU C  60      -2.155  29.591  28.101  0.00  0.00
C
ATOM   7691  CD  GLU C  60      -0.064  29.733  27.593  0.00  0.00
C
ATOM   7692  OE1 GLU C  60       0.922  29.238  27.038  0.00  0.00
C
ATOM   7693  OE2 GLU C  60      -0.045  30.901  28.028  0.00  0.00
C
ATOM   7694  C   GLU C  60      -2.469  26.321  26.725  0.00  0.00
C
```

```
ATOM   7695  O    GLU C  60      -3.342  26.817  26.045  0.00  0.00
C
ATOM   7696  N    ASN C  61      -1.532  25.492  26.217  0.00  0.00
C
ATOM   7697  HN   ASN C  61      -0.792  25.174  26.805  0.00  0.00
C
ATOM   7698  CA   ASN C  61      -1.292  25.137  24.832  0.00  0.00
C
ATOM   7699  HA   ASN C  61      -1.239  26.099  24.344  0.00  0.00
C
ATOM   7700  CB   ASN C  61      -0.030  24.155  24.667  0.00  0.00
C
ATOM   7701  HB1  ASN C  61      -0.213  23.385  25.445  0.00  0.00
C
ATOM   7702  HB2  ASN C  61       0.020  23.737  23.638  0.00  0.00
C
ATOM   7703  CG   ASN C  61       1.211  24.947  25.028  0.00  0.00
C
ATOM   7704  OD1  ASN C  61       1.351  25.376  26.166  0.00  0.00
C
ATOM   7705  ND2  ASN C  61       2.114  25.237  24.036  0.00  0.00
C
ATOM   7706 HD21  ASN C  61       2.975  25.669  24.306  0.00  0.00
C
ATOM   7707 HD22  ASN C  61       2.037  24.708  23.191  0.00  0.00
C
ATOM   7708  C    ASN C  61      -2.587  24.438  24.423  0.00  0.00
C
ATOM   7709  O    ASN C  61      -3.282  24.736  23.401  0.00  0.00
C
ATOM   7710  N    GLU C  62      -3.127  23.560  25.328  0.00  0.00
C
ATOM   7711  HN   GLU C  62      -2.672  23.244  26.157  0.00  0.00
C
ATOM   7712  CA   GLU C  62      -4.278  22.781  24.980  0.00  0.00
C
ATOM   7713  HA   GLU C  62      -4.021  22.341  24.028  0.00  0.00
C
ATOM   7714  CB   GLU C  62      -4.460  21.676  26.019  0.00  0.00
C
ATOM   7715  HB1  GLU C  62      -4.753  22.171  26.970  0.00  0.00
C
ATOM   7716  HB2  GLU C  62      -5.234  20.955  25.681  0.00  0.00
C
ATOM   7717  CG   GLU C  62      -3.225  20.829  26.294  0.00  0.00
C
ATOM   7718  HG1  GLU C  62      -2.311  21.411  26.539  0.00  0.00
C
ATOM   7719  HG2  GLU C  62      -3.495  20.091  27.079  0.00  0.00
C
ATOM   7720  CD   GLU C  62      -2.604  19.959  25.175  0.00  0.00
C
ATOM   7721  OE1  GLU C  62      -1.576  20.317  24.502  0.00  0.00
C
```

```
ATOM   7722  OE2 GLU C  62      -3.142  18.818  25.039  0.00  0.00
C
ATOM   7723  C   GLU C  62      -5.580  23.632  24.859  0.00  0.00
C
ATOM   7724  O   GLU C  62      -6.299  23.575  23.837  0.00  0.00
C
ATOM   7725  N   LEU C  63      -5.837  24.590  25.740  0.00  0.00
C
ATOM   7726  HN  LEU C  63      -5.347  24.746  26.594  0.00  0.00
C
ATOM   7727  CA  LEU C  63      -6.885  25.539  25.574  0.00  0.00
C
ATOM   7728  HA  LEU C  63      -7.762  24.924  25.439  0.00  0.00
C
ATOM   7729  CB  LEU C  63      -7.026  26.400  26.850  0.00  0.00
C
ATOM   7730  HB1 LEU C  63      -5.990  26.762  27.024  0.00  0.00
C
ATOM   7731  HB2 LEU C  63      -7.720  27.219  26.560  0.00  0.00
C
ATOM   7732  CG  LEU C  63      -7.389  25.789  28.178  0.00  0.00
C
ATOM   7733  HG  LEU C  63      -6.665  24.958  28.323  0.00  0.00
C
ATOM   7734  CD1 LEU C  63      -7.158  26.852  29.252  0.00  0.00
C
ATOM   7735 HD11 LEU C  63      -7.889  27.634  28.952  0.00  0.00
C
ATOM   7736 HD12 LEU C  63      -7.416  26.461  30.259  0.00  0.00
C
ATOM   7737 HD13 LEU C  63      -6.099  27.187  29.256  0.00  0.00
C
ATOM   7738  CD2 LEU C  63      -8.879  25.297  28.199  0.00  0.00
C
ATOM   7739 HD21 LEU C  63      -9.418  25.999  27.527  0.00  0.00
C
ATOM   7740 HD22 LEU C  63      -9.056  24.268  27.821  0.00  0.00
C
ATOM   7741 HD23 LEU C  63      -9.313  25.387  29.218  0.00  0.00
C
ATOM   7742  C   LEU C  63      -6.754  26.529  24.411  0.00  0.00
C
ATOM   7743  O   LEU C  63      -7.725  26.782  23.624  0.00  0.00
C
ATOM   7744  N   LEU C  64      -5.542  26.976  24.077  0.00  0.00
C
ATOM   7745  HN  LEU C  64      -4.762  26.661  24.612  0.00  0.00
C
ATOM   7746  CA  LEU C  64      -5.140  27.630  22.879  0.00  0.00
C
ATOM   7747  HA  LEU C  64      -5.766  28.497  22.723  0.00  0.00
C
ATOM   7748  CB  LEU C  64      -3.741  28.206  22.966  0.00  0.00
C
```

```
ATOM   7749  HB1 LEU C  64      -2.910  27.495  23.157  0.00  0.00
C
ATOM   7750  HB2 LEU C  64      -3.523  28.721  22.007  0.00  0.00
C
ATOM   7751  CG  LEU C  64      -3.598  29.407  23.939  0.00  0.00
C
ATOM   7752  HG  LEU C  64      -3.956  29.178  24.966  0.00  0.00
C
ATOM   7753  CD1 LEU C  64      -2.130  29.851  24.171  0.00  0.00
C
ATOM   7754 HD11 LEU C  64      -1.419  29.040  24.438  0.00  0.00
C
ATOM   7755 HD12 LEU C  64      -1.807  30.140  23.148  0.00  0.00
C
ATOM   7756 HD13 LEU C  64      -2.119  30.712  24.872  0.00  0.00
C
ATOM   7757  CD2 LEU C  64      -4.414  30.617  23.575  0.00  0.00
C
ATOM   7758 HD21 LEU C  64      -5.431  30.426  23.980  0.00  0.00
C
ATOM   7759 HD22 LEU C  64      -4.018  31.599  23.910  0.00  0.00
C
ATOM   7760 HD23 LEU C  64      -4.578  30.653  22.476  0.00  0.00
C
ATOM   7761  C   LEU C  64      -5.308  26.881  21.609  0.00  0.00
C
ATOM   7762  O   LEU C  64      -5.772  27.518  20.703  0.00  0.00
C
ATOM   7763  N   SER C  65      -4.955  25.562  21.568  0.00  0.00
C
ATOM   7764  HN  SER C  65      -4.576  25.078  22.353  0.00  0.00
C
ATOM   7765  CA  SER C  65      -5.245  24.645  20.466  0.00  0.00
C
ATOM   7766  HA  SER C  65      -4.829  25.040  19.551  0.00  0.00
C
ATOM   7767  CB  SER C  65      -4.534  23.306  20.715  0.00  0.00
C
ATOM   7768  HB1 SER C  65      -3.440  23.474  20.814  0.00  0.00
C
ATOM   7769  HB2 SER C  65      -4.871  22.932  21.705  0.00  0.00
C
ATOM   7770  OG  SER C  65      -4.880  22.262  19.733  0.00  0.00
C
ATOM   7771  HG1 SER C  65      -4.781  21.458  20.249  0.00  0.00
C
ATOM   7772  C   SER C  65      -6.767  24.448  20.205  0.00  0.00
C
ATOM   7773  O   SER C  65      -7.239  24.237  19.065  0.00  0.00
C
ATOM   7774  N   LEU C  66      -7.615  24.391  21.334  0.00  0.00
C
ATOM   7775  HN  LEU C  66      -7.303  24.393  22.281  0.00  0.00
C
```

ATOM 7776 CA LEU C 66 -9.124 24.282 21.290 0.00 0.00
C
ATOM 7777 HA LEU C 66 -9.261 23.455 20.609 0.00 0.00
C
ATOM 7778 CB LEU C 66 -9.465 23.947 22.742 0.00 0.00
C
ATOM 7779 HB1 LEU C 66 -8.789 24.461 23.459 0.00 0.00
C
ATOM 7780 HB2 LEU C 66 -10.482 24.330 22.971 0.00 0.00
C
ATOM 7781 CG LEU C 66 -9.343 22.439 23.172 0.00 0.00
C
ATOM 7782 HG LEU C 66 -8.302 22.146 22.917 0.00 0.00
C
ATOM 7783 CD1 LEU C 66 -9.468 22.513 24.668 0.00 0.00
C
ATOM 7784 HD11 LEU C 66 -10.488 22.796 25.006 0.00 0.00
C
ATOM 7785 HD12 LEU C 66 -9.385 21.509 25.136 0.00 0.00
C
ATOM 7786 HD13 LEU C 66 -8.667 23.138 25.115 0.00 0.00
C
ATOM 7787 CD2 LEU C 66 -10.376 21.497 22.514 0.00 0.00
C
ATOM 7788 HD21 LEU C 66 -10.259 20.494 22.976 0.00 0.00
C
ATOM 7789 HD22 LEU C 66 -11.364 21.913 22.807 0.00 0.00
C
ATOM 7790 HD23 LEU C 66 -10.300 21.339 21.417 0.00 0.00
C
ATOM 7791 C LEU C 66 -9.934 25.498 20.904 0.00 0.00
C
ATOM 7792 O LEU C 66 -11.186 25.449 20.929 0.00 0.00
C
ATOM 7793 N GLY C 67 -9.323 26.717 20.838 0.00 0.00
C
ATOM 7794 HN GLY C 67 -8.348 26.626 21.024 0.00 0.00
C
ATOM 7795 CA GLY C 67 -9.931 27.998 20.504 0.00 0.00
C
ATOM 7796 HA1 GLY C 67 -10.539 27.879 19.619 0.00 0.00
C
ATOM 7797 HA2 GLY C 67 -9.156 28.734 20.347 0.00 0.00
C
ATOM 7798 C GLY C 67 -10.741 28.626 21.641 0.00 0.00
C
ATOM 7799 O GLY C 67 -11.776 29.143 21.292 0.00 0.00
C
ATOM 7800 N ILE C 68 -10.247 28.567 22.859 0.00 0.00
C
ATOM 7801 HN ILE C 68 -9.449 27.977 22.943 0.00 0.00
C
ATOM 7802 CA ILE C 68 -10.983 28.991 24.012 0.00 0.00
C

ATOM 7803 HA ILE C 68 −11.906 28.430 24.009 0.00 0.00
C
ATOM 7804 CB ILE C 68 −10.346 28.704 25.352 0.00 0.00
C
ATOM 7805 HB ILE C 68 −9.800 27.738 25.316 0.00 0.00
C
ATOM 7806 CG2 ILE C 68 −9.182 29.652 25.564 0.00 0.00
C
ATOM 7807 HG21 ILE C 68 −8.628 29.383 26.489 0.00 0.00
C
ATOM 7808 HG22 ILE C 68 −8.461 29.595 24.721 0.00 0.00
C
ATOM 7809 HG23 ILE C 68 −9.536 30.700 25.664 0.00 0.00
C
ATOM 7810 CG1 ILE C 68 −11.327 28.653 26.651 0.00 0.00
C
ATOM 7811 HG11 ILE C 68 −10.585 28.299 27.399 0.00 0.00
C
ATOM 7812 HG12 ILE C 68 −11.770 29.615 26.985 0.00 0.00
C
ATOM 7813 CD ILE C 68 −12.323 27.508 26.528 0.00 0.00
C
ATOM 7814 HD1 ILE C 68 −11.760 26.572 26.326 0.00 0.00
C
ATOM 7815 HD2 ILE C 68 −12.826 27.414 27.514 0.00 0.00
C
ATOM 7816 HD3 ILE C 68 −12.997 27.649 25.656 0.00 0.00
C
ATOM 7817 C ILE C 68 −11.290 30.462 23.894 0.00 0.00
C
ATOM 7818 O ILE C 68 −12.267 30.988 24.407 0.00 0.00
C
ATOM 7819 N ARG C 69 −10.401 31.296 23.237 0.00 0.00
C
ATOM 7820 HN ARG C 69 −9.559 30.961 22.821 0.00 0.00
C
ATOM 7821 CA ARG C 69 −10.641 32.756 23.258 0.00 0.00
C
ATOM 7822 HA ARG C 69 −10.744 33.117 24.271 0.00 0.00
C
ATOM 7823 CB ARG C 69 −9.434 33.551 22.868 0.00 0.00
C
ATOM 7824 HB1 ARG C 69 −9.069 33.428 21.826 0.00 0.00
C
ATOM 7825 HB2 ARG C 69 −9.628 34.631 23.045 0.00 0.00
C
ATOM 7826 CG ARG C 69 −8.193 33.348 23.927 0.00 0.00
C
ATOM 7827 HG1 ARG C 69 −8.494 33.756 24.916 0.00 0.00
C
ATOM 7828 HG2 ARG C 69 −8.013 32.262 24.077 0.00 0.00
C
ATOM 7829 CD ARG C 69 −6.793 33.959 23.457 0.00 0.00
C

ATOM 7830 HD1 ARG C 69 -6.968 35.022 23.185 0.00 0.00
C
ATOM 7831 HD2 ARG C 69 -5.976 33.829 24.199 0.00 0.00
C
ATOM 7832 NE ARG C 69 -6.307 33.103 22.316 0.00 0.00
C
ATOM 7833 HE ARG C 69 -6.964 32.450 21.940 0.00 0.00
C
ATOM 7834 CZ ARG C 69 -5.150 33.317 21.680 0.00 0.00
C
ATOM 7835 NH1 ARG C 69 -4.297 34.291 21.987 0.00 0.00
C
ATOM 7836 HH11 ARG C 69 -3.577 34.465 21.315 0.00 0.00
C
ATOM 7837 HH12 ARG C 69 -4.594 34.936 22.690 0.00 0.00
C
ATOM 7838 NH2 ARG C 69 -4.836 32.481 20.690 0.00 0.00
C
ATOM 7839 HH21 ARG C 69 -3.948 32.726 20.301 0.00 0.00
C
ATOM 7840 HH22 ARG C 69 -5.517 31.867 20.291 0.00 0.00
C
ATOM 7841 C ARG C 69 -11.834 33.243 22.519 0.00 0.00
C
ATOM 7842 O ARG C 69 -12.120 34.401 22.693 0.00 0.00
C
ATOM 7843 N ASN C 70 -12.447 32.413 21.621 0.00 0.00
C
ATOM 7844 HN ASN C 70 -12.130 31.494 21.403 0.00 0.00
C
ATOM 7845 CA ASN C 70 -13.569 32.850 20.863 0.00 0.00
C
ATOM 7846 HA ASN C 70 -13.524 33.917 20.699 0.00 0.00
C
ATOM 7847 CB ASN C 70 -13.403 32.104 19.551 0.00 0.00
C
ATOM 7848 HB1 ASN C 70 -13.233 31.015 19.692 0.00 0.00
C
ATOM 7849 HB2 ASN C 70 -14.136 32.260 18.732 0.00 0.00
C
ATOM 7850 CG ASN C 70 -12.060 32.675 18.967 0.00 0.00
C
ATOM 7851 OD1 ASN C 70 -10.971 32.144 19.076 0.00 0.00
C
ATOM 7852 ND2 ASN C 70 -12.244 33.838 18.206 0.00 0.00
C
ATOM 7853 HD21 ASN C 70 -11.431 34.261 17.806 0.00 0.00
C
ATOM 7854 HD22 ASN C 70 -13.116 34.316 18.105 0.00 0.00
C
ATOM 7855 C ASN C 70 -14.909 32.440 21.650 0.00 0.00
C
ATOM 7856 O ASN C 70 -16.040 32.704 21.277 0.00 0.00
C

ATOM 7857 N PHE C 71 -14.810 31.854 22.818 0.00 0.00
C
ATOM 7858 HN PHE C 71 -13.888 31.727 23.177 0.00 0.00
C
ATOM 7859 CA PHE C 71 -15.878 31.625 23.770 0.00 0.00
C
ATOM 7860 HA PHE C 71 -16.834 31.522 23.277 0.00 0.00
C
ATOM 7861 CB PHE C 71 -15.805 30.240 24.648 0.00 0.00
C
ATOM 7862 HB1 PHE C 71 -14.867 30.048 25.211 0.00 0.00
C
ATOM 7863 HB2 PHE C 71 -16.579 30.224 25.444 0.00 0.00
C
ATOM 7864 CG PHE C 71 -16.082 29.123 23.602 0.00 0.00
C
ATOM 7865 CD1 PHE C 71 -17.404 28.726 23.244 0.00 0.00
C
ATOM 7866 HD1 PHE C 71 -18.184 29.346 23.660 0.00 0.00
C
ATOM 7867 CE1 PHE C 71 -17.673 27.549 22.499 0.00 0.00
C
ATOM 7868 HE1 PHE C 71 -18.698 27.231 22.377 0.00 0.00
C
ATOM 7869 CZ PHE C 71 -16.599 26.798 22.023 0.00 0.00
C
ATOM 7870 HZ PHE C 71 -16.712 25.960 21.350 0.00 0.00
C
ATOM 7871 CD2 PHE C 71 -15.049 28.306 23.168 0.00 0.00
C
ATOM 7872 HD2 PHE C 71 -14.021 28.587 23.342 0.00 0.00
C
ATOM 7873 CE2 PHE C 71 -15.264 27.162 22.337 0.00 0.00
C
ATOM 7874 HE2 PHE C 71 -14.411 26.583 22.016 0.00 0.00
C
ATOM 7875 C PHE C 71 -15.914 32.823 24.769 0.00 0.00
C
ATOM 7876 O PHE C 71 -14.929 33.184 25.321 0.00 0.00
C
ATOM 7877 N GLU C 72 -17.073 33.294 25.145 0.00 0.00
C
ATOM 7878 HN GLU C 72 -17.903 32.980 24.690 0.00 0.00
C
ATOM 7879 CA GLU C 72 -17.193 34.385 26.113 0.00 0.00
C
ATOM 7880 HA GLU C 72 -16.224 34.848 26.229 0.00 0.00
C
ATOM 7881 CB GLU C 72 -18.197 35.445 25.611 0.00 0.00
C
ATOM 7882 HB1 GLU C 72 -19.139 34.885 25.430 0.00 0.00
C
ATOM 7883 HB2 GLU C 72 -18.405 36.204 26.395 0.00 0.00
C

ATOM	7884	CG	GLU	C	72	−17.965	36.128	24.253	0.00	0.00	C
ATOM	7885	HG1	GLU	C	72	−17.013	36.700	24.283	0.00	0.00	C
ATOM	7886	HG2	GLU	C	72	−17.863	35.328	23.489	0.00	0.00	C
ATOM	7887	CD	GLU	C	72	−19.106	37.016	23.948	0.00	0.00	C
ATOM	7888	OE1	GLU	C	72	−18.932	38.279	24.122	0.00	0.00	C
ATOM	7889	OE2	GLU	C	72	−20.215	36.624	23.511	0.00	0.00	C
ATOM	7890	C	GLU	C	72	−17.652	33.958	27.465	0.00	0.00	C
ATOM	7891	O	GLU	C	72	−17.440	34.566	28.474	0.00	0.00	C
ATOM	7892	N	TYR	C	73	−18.415	32.867	27.530	0.00	0.00	C
ATOM	7893	HN	TYR	C	73	−18.745	32.349	26.745	0.00	0.00	C
ATOM	7894	CA	TYR	C	73	−18.848	32.295	28.759	0.00	0.00	C
ATOM	7895	HA	TYR	C	73	−18.575	32.770	29.689	0.00	0.00	C
ATOM	7896	CB	TYR	C	73	−20.378	32.098	28.657	0.00	0.00	C
ATOM	7897	HB1	TYR	C	73	−20.732	31.624	27.717	0.00	0.00	C
ATOM	7898	HB2	TYR	C	73	−20.573	31.413	29.510	0.00	0.00	C
ATOM	7899	CG	TYR	C	73	−21.207	33.395	28.692	0.00	0.00	C
ATOM	7900	CD1	TYR	C	73	−21.552	34.131	27.569	0.00	0.00	C
ATOM	7901	HD1	TYR	C	73	−21.112	33.893	26.612	0.00	0.00	C
ATOM	7902	CE1	TYR	C	73	−22.680	35.016	27.657	0.00	0.00	C
ATOM	7903	HE1	TYR	C	73	−23.156	35.475	26.803	0.00	0.00	C
ATOM	7904	CZ	TYR	C	73	−23.475	35.127	28.793	0.00	0.00	C
ATOM	7905	OH	TYR	C	73	−24.758	35.814	28.873	0.00	0.00	C
ATOM	7906	HH	TYR	C	73	−25.172	35.638	29.721	0.00	0.00	C
ATOM	7907	CD2	TYR	C	73	−21.859	33.706	29.918	0.00	0.00	C
ATOM	7908	HD2	TYR	C	73	−21.542	33.198	30.816	0.00	0.00	C
ATOM	7909	CE2	TYR	C	73	−22.955	34.587	30.000	0.00	0.00	C
ATOM	7910	HE2	TYR	C	73	−23.567	34.734	30.878	0.00	0.00	C

```
ATOM   7911  C   TYR C  73      -18.246  30.895  28.815  0.00  0.00
C
ATOM   7912  O   TYR C  73      -18.487  30.038  27.967  0.00  0.00
C
ATOM   7913  N   VAL C  74      -17.451  30.610  29.830  0.00  0.00
C
ATOM   7914  HN  VAL C  74      -17.365  31.194  30.634  0.00  0.00
C
ATOM   7915  CA  VAL C  74      -16.978  29.265  30.067  0.00  0.00
C
ATOM   7916  HA  VAL C  74      -17.408  28.498  29.440  0.00  0.00
C
ATOM   7917  CB  VAL C  74      -15.421  29.280  30.076  0.00  0.00
C
ATOM   7918  HB  VAL C  74      -15.088  29.952  30.896  0.00  0.00
C
ATOM   7919  CG1 VAL C  74      -14.911  27.862  30.430  0.00  0.00
C
ATOM   7920 HG11 VAL C  74      -13.834  27.663  30.241  0.00  0.00
C
ATOM   7921 HG12 VAL C  74      -15.006  27.603  31.506  0.00  0.00
C
ATOM   7922 HG13 VAL C  74      -15.450  27.139  29.781  0.00  0.00
C
ATOM   7923  CG2 VAL C  74      -14.943  29.732  28.705  0.00  0.00
C
ATOM   7924 HG21 VAL C  74      -13.892  30.062  28.845  0.00  0.00
C
ATOM   7925 HG22 VAL C  74      -14.871  28.874  28.003  0.00  0.00
C
ATOM   7926 HG23 VAL C  74      -15.483  30.651  28.391  0.00  0.00
C
ATOM   7927  C   VAL C  74      -17.359  28.818  31.472  0.00  0.00
C
ATOM   7928  O   VAL C  74      -17.071  29.536  32.448  0.00  0.00
C
ATOM   7929  N   ILE C  75      -17.994  27.628  31.598  0.00  0.00
C
ATOM   7930  HN  ILE C  75      -18.241  27.069  30.810  0.00  0.00
C
ATOM   7931  CA  ILE C  75      -18.352  27.063  32.863  0.00  0.00
C
ATOM   7932  HA  ILE C  75      -18.451  27.860  33.585  0.00  0.00
C
ATOM   7933  CB  ILE C  75      -19.780  26.422  32.745  0.00  0.00
C
ATOM   7934  HB  ILE C  75      -19.707  25.558  32.051  0.00  0.00
C
ATOM   7935  CG2 ILE C  75      -20.193  25.987  34.159  0.00  0.00
C
ATOM   7936 HG21 ILE C  75      -19.552  25.163  34.541  0.00  0.00
C
ATOM   7937 HG22 ILE C  75      -20.163  26.784  34.933  0.00  0.00
C
```

ATOM 7938 HG23 ILE C 75 -21.200 25.519 34.182 0.00 0.00
C
ATOM 7939 CG1 ILE C 75 -20.743 27.399 32.123 0.00 0.00
C
ATOM 7940 HG11 ILE C 75 -20.803 28.305 32.764 0.00 0.00
C
ATOM 7941 HG12 ILE C 75 -20.341 27.844 31.188 0.00 0.00
C
ATOM 7942 CD ILE C 75 -22.158 26.921 31.904 0.00 0.00
C
ATOM 7943 HD1 ILE C 75 -22.806 27.738 31.520 0.00 0.00
C
ATOM 7944 HD2 ILE C 75 -22.143 26.133 31.121 0.00 0.00
C
ATOM 7945 HD3 ILE C 75 -22.500 26.422 32.836 0.00 0.00
C
ATOM 7946 C ILE C 75 -17.341 26.073 33.376 0.00 0.00
C
ATOM 7947 O ILE C 75 -17.210 25.028 32.664 0.00 0.00
C
ATOM 7948 N VAL C 76 -16.816 26.320 34.572 0.00 0.00
C
ATOM 7949 HN VAL C 76 -17.013 27.087 35.178 0.00 0.00
C
ATOM 7950 CA VAL C 76 -15.923 25.300 35.178 0.00 0.00
C
ATOM 7951 HA VAL C 76 -15.521 24.722 34.358 0.00 0.00
C
ATOM 7952 CB VAL C 76 -14.798 26.039 35.838 0.00 0.00
C
ATOM 7953 HB VAL C 76 -15.147 26.755 36.613 0.00 0.00
C
ATOM 7954 CG1 VAL C 76 -13.789 24.998 36.491 0.00 0.00
C
ATOM 7955 HG11 VAL C 76 -13.655 24.052 35.923 0.00 0.00
C
ATOM 7956 HG12 VAL C 76 -12.802 25.501 36.574 0.00 0.00
C
ATOM 7957 HG13 VAL C 76 -14.142 24.838 37.533 0.00 0.00
C
ATOM 7958 CG2 VAL C 76 -14.102 26.811 34.772 0.00 0.00
C
ATOM 7959 HG21 VAL C 76 -14.760 27.509 34.210 0.00 0.00
C
ATOM 7960 HG22 VAL C 76 -13.302 27.429 35.233 0.00 0.00
C
ATOM 7961 HG23 VAL C 76 -13.742 26.099 33.999 0.00 0.00
C
ATOM 7962 C VAL C 76 -16.722 24.522 36.181 0.00 0.00
C
ATOM 7963 O VAL C 76 -17.071 25.056 37.226 0.00 0.00
C
ATOM 7964 N ALA C 77 -17.063 23.220 35.945 0.00 0.00
C

ATOM 7965 HN ALA C 77 −16.832 22.798 35.071 0.00 0.00
C
ATOM 7966 CA ALA C 77 −17.766 22.457 36.933 0.00 0.00
C
ATOM 7967 HA ALA C 77 −18.331 23.168 37.518 0.00 0.00
C
ATOM 7968 CB ALA C 77 −18.724 21.501 36.058 0.00 0.00
C
ATOM 7969 HB1 ALA C 77 −19.291 22.110 35.322 0.00 0.00
C
ATOM 7970 HB2 ALA C 77 −18.231 20.640 35.557 0.00 0.00
C
ATOM 7971 HB3 ALA C 77 −19.454 20.995 36.725 0.00 0.00
C
ATOM 7972 C ALA C 77 −16.829 21.572 37.704 0.00 0.00
C
ATOM 7973 O ALA C 77 −17.301 20.897 38.637 0.00 0.00
C
ATOM 7974 N ILE C 78 −15.554 21.632 37.476 0.00 0.00
C
ATOM 7975 HN ILE C 78 −15.167 22.261 36.806 0.00 0.00
C
ATOM 7976 CA ILE C 78 −14.558 20.924 38.333 0.00 0.00
C
ATOM 7977 HA ILE C 78 −14.764 19.868 38.431 0.00 0.00
C
ATOM 7978 CB ILE C 78 −13.159 21.126 37.683 0.00 0.00
C
ATOM 7979 HB ILE C 78 −12.999 22.223 37.750 0.00 0.00
C
ATOM 7980 CG2 ILE C 78 −12.097 20.259 38.527 0.00 0.00
C
ATOM 7981 HG21 ILE C 78 −11.048 20.451 38.217 0.00 0.00
C
ATOM 7982 HG22 ILE C 78 −12.150 20.490 39.613 0.00 0.00
C
ATOM 7983 HG23 ILE C 78 −12.168 19.164 38.356 0.00 0.00
C
ATOM 7984 CG1 ILE C 78 −13.127 20.793 36.169 0.00 0.00
C
ATOM 7985 HG11 ILE C 78 −13.413 19.719 36.159 0.00 0.00
C
ATOM 7986 HG12 ILE C 78 −13.937 21.354 35.656 0.00 0.00
C
ATOM 7987 CD ILE C 78 −11.841 21.055 35.456 0.00 0.00
C
ATOM 7988 HD1 ILE C 78 −11.926 20.833 34.371 0.00 0.00
C
ATOM 7989 HD2 ILE C 78 −11.520 22.116 35.522 0.00 0.00
C
ATOM 7990 HD3 ILE C 78 −11.065 20.335 35.795 0.00 0.00
C
ATOM 7991 C ILE C 78 −14.594 21.605 39.736 0.00 0.00
C

ATOM	7992	O	ILE	C	78	−14.477	22.773	39.734	0.00	0.00	C
ATOM	7993	N	GLY	C	79	−14.675	20.833	40.866	0.00	0.00	C
ATOM	7994	HN	GLY	C	79	−14.649	19.858	40.660	0.00	0.00	C
ATOM	7995	CA	GLY	C	79	−14.653	21.488	42.181	0.00	0.00	C
ATOM	7996	HA1	GLY	C	79	−15.664	21.785	42.419	0.00	0.00	C
ATOM	7997	HA2	GLY	C	79	−13.987	22.339	42.176	0.00	0.00	C
ATOM	7998	C	GLY	C	79	−14.083	20.724	43.254	0.00	0.00	C
ATOM	7999	O	GLY	C	79	−13.459	21.344	44.069	0.00	0.00	C
ATOM	8000	N	ALA	C	80	−14.208	19.391	43.203	0.00	0.00	C
ATOM	8001	HN	ALA	C	80	−14.671	18.860	42.497	0.00	0.00	C
ATOM	8002	CA	ALA	C	80	−13.740	18.555	44.327	0.00	0.00	C
ATOM	8003	HA	ALA	C	80	−14.028	19.074	45.229	0.00	0.00	C
ATOM	8004	CB	ALA	C	80	−14.447	17.213	44.393	0.00	0.00	C
ATOM	8005	HB1	ALA	C	80	−14.358	16.835	43.352	0.00	0.00	C
ATOM	8006	HB2	ALA	C	80	−13.902	16.604	45.145	0.00	0.00	C
ATOM	8007	HB3	ALA	C	80	−15.532	17.251	44.632	0.00	0.00	C
ATOM	8008	C	ALA	C	80	−12.278	18.473	44.396	0.00	0.00	C
ATOM	8009	O	ALA	C	80	−11.606	18.554	45.490	0.00	0.00	C
ATOM	8010	N	ASN	C	81	−11.631	18.225	43.220	0.00	0.00	C
ATOM	8011	HN	ASN	C	81	−12.019	18.295	42.305	0.00	0.00	C
ATOM	8012	CA	ASN	C	81	−10.132	18.236	43.141	0.00	0.00	C
ATOM	8013	HA	ASN	C	81	−9.670	17.808	44.018	0.00	0.00	C
ATOM	8014	CB	ASN	C	81	−9.782	17.415	41.910	0.00	0.00	C
ATOM	8015	HB1	ASN	C	81	−10.161	16.371	41.900	0.00	0.00	C
ATOM	8016	HB2	ASN	C	81	−10.045	18.022	41.017	0.00	0.00	C
ATOM	8017	CG	ASN	C	81	−8.215	17.244	41.808	0.00	0.00	C
ATOM	8018	OD1	ASN	C	81	−7.464	17.940	41.065	0.00	0.00	C

ATOM 8019 ND2 ASN C 81 -7.657 16.399 42.612 0.00 0.00
C
ATOM 8020 HD21 ASN C 81 -6.665 16.268 42.611 0.00 0.00
C
ATOM 8021 HD22 ASN C 81 -8.139 15.696 43.137 0.00 0.00
C
ATOM 8022 C ASN C 81 -9.598 19.647 43.063 0.00 0.00
C
ATOM 8023 O ASN C 81 -9.775 20.284 41.995 0.00 0.00
C
ATOM 8024 N ILE C 82 -8.929 20.065 44.095 0.00 0.00
C
ATOM 8025 HN ILE C 82 -8.845 19.496 44.909 0.00 0.00
C
ATOM 8026 CA ILE C 82 -8.463 21.370 44.284 0.00 0.00
C
ATOM 8027 HA ILE C 82 -9.387 21.909 44.131 0.00 0.00
C
ATOM 8028 CB ILE C 82 -7.772 21.564 45.699 0.00 0.00
C
ATOM 8029 HB ILE C 82 -6.830 20.978 45.767 0.00 0.00
C
ATOM 8030 CG2 ILE C 82 -7.397 23.027 45.871 0.00 0.00
C
ATOM 8031 HG21 ILE C 82 -8.217 23.775 45.822 0.00 0.00
C
ATOM 8032 HG22 ILE C 82 -6.853 23.225 46.819 0.00 0.00
C
ATOM 8033 HG23 ILE C 82 -6.578 23.301 45.173 0.00 0.00
C
ATOM 8034 CG1 ILE C 82 -8.601 21.027 46.905 0.00 0.00
C
ATOM 8035 HG11 ILE C 82 -8.667 19.941 46.678 0.00 0.00
C
ATOM 8036 HG12 ILE C 82 -7.982 21.272 47.795 0.00 0.00
C
ATOM 8037 CD ILE C 82 -9.957 21.637 47.017 0.00 0.00
C
ATOM 8038 HD1 ILE C 82 -10.627 21.142 46.282 0.00 0.00
C
ATOM 8039 HD2 ILE C 82 -10.353 21.388 48.025 0.00 0.00
C
ATOM 8040 HD3 ILE C 82 -9.844 22.740 46.941 0.00 0.00
C
ATOM 8041 C ILE C 82 -7.457 21.809 43.252 0.00 0.00
C
ATOM 8042 O ILE C 82 -7.535 22.959 42.698 0.00 0.00
C
ATOM 8043 N GLN C 83 -6.540 20.840 42.831 0.00 0.00
C
ATOM 8044 HN GLN C 83 -6.642 19.866 43.020 0.00 0.00
C
ATOM 8045 CA GLN C 83 -5.545 21.169 41.808 0.00 0.00
C

ATOM 8046 HA GLN C 83 -4.943 22.022 42.083 0.00 0.00
C
ATOM 8047 CB GLN C 83 -4.510 20.000 41.646 0.00 0.00
C
ATOM 8048 HB1 GLN C 83 -3.998 19.960 42.631 0.00 0.00
C
ATOM 8049 HB2 GLN C 83 -4.976 19.002 41.501 0.00 0.00
C
ATOM 8050 CG GLN C 83 -3.377 20.291 40.609 0.00 0.00
C
ATOM 8051 HG1 GLN C 83 -3.840 20.371 39.602 0.00 0.00
C
ATOM 8052 HG2 GLN C 83 -3.015 21.312 40.852 0.00 0.00
C
ATOM 8053 CD GLN C 83 -2.216 19.316 40.649 0.00 0.00
C
ATOM 8054 OE1 GLN C 83 -2.100 18.455 41.561 0.00 0.00
C
ATOM 8055 NE2 GLN C 83 -1.426 19.423 39.583 0.00 0.00
C
ATOM 8056 HE21 GLN C 83 -1.009 18.580 39.243 0.00 0.00
C
ATOM 8057 HE22 GLN C 83 -1.706 20.114 38.917 0.00 0.00
C
ATOM 8058 C GLN C 83 -6.184 21.551 40.491 0.00 0.00
C
ATOM 8059 O GLN C 83 -5.744 22.505 39.863 0.00 0.00
C
ATOM 8060 N ALA C 84 -7.148 20.793 40.073 0.00 0.00
C
ATOM 8061 HN ALA C 84 -7.394 19.965 40.571 0.00 0.00
C
ATOM 8062 CA ALA C 84 -7.679 20.901 38.768 0.00 0.00
C
ATOM 8063 HA ALA C 84 -6.819 20.950 38.116 0.00 0.00
C
ATOM 8064 CB ALA C 84 -8.377 19.627 38.351 0.00 0.00
C
ATOM 8065 HB1 ALA C 84 -7.673 18.768 38.370 0.00 0.00
C
ATOM 8066 HB2 ALA C 84 -9.070 19.324 39.164 0.00 0.00
C
ATOM 8067 HB3 ALA C 84 -8.950 19.857 37.427 0.00 0.00
C
ATOM 8068 C ALA C 84 -8.522 22.167 38.531 0.00 0.00
C
ATOM 8069 O ALA C 84 -8.374 22.911 37.523 0.00 0.00
C
ATOM 8070 N SER C 85 -9.393 22.471 39.476 0.00 0.00
C
ATOM 8071 HN SER C 85 -9.391 21.914 40.302 0.00 0.00
C
ATOM 8072 CA SER C 85 -10.286 23.619 39.493 0.00 0.00
C

```
ATOM   8073  HA  SER C  85     -10.885  23.717  38.600  0.00  0.00
C
ATOM   8074  CB  SER C  85     -11.292  23.511  40.637  0.00  0.00
C
ATOM   8075  HB1 SER C  85     -11.854  24.454  40.806  0.00  0.00
C
ATOM   8076  HB2 SER C  85     -12.085  22.829  40.264  0.00  0.00
C
ATOM   8077  OG  SER C  85     -10.779  23.135  41.892  0.00  0.00
C
ATOM   8078  HG1 SER C  85     -11.071  22.233  42.045  0.00  0.00
C
ATOM   8079  C   SER C  85      -9.597  25.020  39.607  0.00  0.00
C
ATOM   8080  O   SER C  85     -10.087  25.921  38.986  0.00  0.00
C
ATOM   8081  N   THR C  86      -8.571  25.062  40.513  0.00  0.00
C
ATOM   8082  HN  THR C  86      -8.384  24.232  41.033  0.00  0.00
C
ATOM   8083  CA  THR C  86      -7.673  26.228  40.659  0.00  0.00
C
ATOM   8084  HA  THR C  86      -8.356  27.038  40.866  0.00  0.00
C
ATOM   8085  CB  THR C  86      -6.716  26.354  41.902  0.00  0.00
C
ATOM   8086  HB  THR C  86      -5.899  27.106  41.880  0.00  0.00
C
ATOM   8087  OG1 THR C  86      -6.001  25.157  42.041  0.00  0.00
C
ATOM   8088  HG1 THR C  86      -6.620  24.508  42.384  0.00  0.00
C
ATOM   8089  CG2 THR C  86      -7.681  26.622  43.126  0.00  0.00
C
ATOM   8090 HG21 THR C  86      -8.195  27.593  42.959  0.00  0.00
C
ATOM   8091 HG22 THR C  86      -8.484  25.859  43.049  0.00  0.00
C
ATOM   8092 HG23 THR C  86      -7.120  26.537  44.081  0.00  0.00
C
ATOM   8093  C   THR C  86      -6.871  26.560  39.352  0.00  0.00
C
ATOM   8094  O   THR C  86      -7.060  27.675  38.813  0.00  0.00
C
ATOM   8095  N   LEU C  87      -6.174  25.617  38.846  0.00  0.00
C
ATOM   8096  HN  LEU C  87      -6.062  24.697  39.215  0.00  0.00
C
ATOM   8097  CA  LEU C  87      -5.274  25.916  37.716  0.00  0.00
C
ATOM   8098  HA  LEU C  87      -4.724  26.805  37.989  0.00  0.00
C
ATOM   8099  CB  LEU C  87      -4.486  24.667  37.448  0.00  0.00
C
```

ATOM   8100 HB1 LEU C 87   −5.252 23.876 37.300 0.00 0.00
C
ATOM   8101 HB2 LEU C 87   −3.941 24.736 36.483 0.00 0.00
C
ATOM   8102 CG LEU C 87   −3.370 24.335 38.501 0.00 0.00
C
ATOM   8103 HG LEU C 87   −3.942 24.278 39.452 0.00 0.00
C
ATOM   8104 CD1 LEU C 87   −2.542 23.096 38.326 0.00 0.00
C
ATOM   8105 HD11 LEU C 87   −1.910 22.951 39.229 0.00 0.00
C
ATOM   8106 HD12 LEU C 87   −3.253 22.245 38.253 0.00 0.00
C
ATOM   8107 HD13 LEU C 87   −1.775 23.157 37.525 0.00 0.00
C
ATOM   8108 CD2 LEU C 87   −2.467 25.571 38.682 0.00 0.00
C
ATOM   8109 HD21 LEU C 87   −2.202 25.888 37.650 0.00 0.00
C
ATOM   8110 HD22 LEU C 87   −3.075 26.412 39.078 0.00 0.00
C
ATOM   8111 HD23 LEU C 87   −1.566 25.414 39.313 0.00 0.00
C
ATOM   8112 C   LEU C 87   −6.027 26.290 36.361 0.00 0.00
C
ATOM   8113 O   LEU C 87   −5.518 27.149 35.585 0.00 0.00
C
ATOM   8114 N   THR C 88   −7.139 25.597 36.044 0.00 0.00
C
ATOM   8115 HN THR C 88   −7.434 24.768 36.514 0.00 0.00
C
ATOM   8116 CA THR C 88   −7.985 25.800 34.885 0.00 0.00
C
ATOM   8117 HA THR C 88   −7.477 25.767 33.933 0.00 0.00
C
ATOM   8118 CB THR C 88   −9.135 24.764 34.835 0.00 0.00
C
ATOM   8119 HB THR C 88   −9.718 24.755 35.780 0.00 0.00
C
ATOM   8120 OG1 THR C 88   −8.482 23.501 34.743 0.00 0.00
C
ATOM   8121 HG1 THR C 88   −8.498 22.946 35.526 0.00 0.00
C
ATOM   8122 CG2 THR C 88   −10.136 24.890 33.672 0.00 0.00
C
ATOM   8123 HG21 THR C 88   −10.738 25.822 33.731 0.00 0.00
C
ATOM   8124 HG22 THR C 88   −9.563 24.942 32.721 0.00 0.00
C
ATOM   8125 HG23 THR C 88   −10.725 23.956 33.557 0.00 0.00
C
ATOM   8126 C   THR C 88   −8.535 27.201 34.880 0.00 0.00
C

ATOM 8127 O THR C 88 -8.481 27.989 33.893 0.00 0.00
C
ATOM 8128 N THR C 89 -9.115 27.632 36.039 0.00 0.00
C
ATOM 8129 HN THR C 89 -9.227 26.983 36.787 0.00 0.00
C
ATOM 8130 CA THR C 89 -9.524 29.046 36.427 0.00 0.00
C
ATOM 8131 HA THR C 89 -10.288 29.314 35.713 0.00 0.00
C
ATOM 8132 CB THR C 89 -10.056 29.151 37.932 0.00 0.00
C
ATOM 8133 HB THR C 89 -9.283 28.979 38.712 0.00 0.00
C
ATOM 8134 OG1 THR C 89 -11.096 28.251 38.182 0.00 0.00
C
ATOM 8135 HG1 THR C 89 -10.744 27.398 38.445 0.00 0.00
C
ATOM 8136 CG2 THR C 89 -10.762 30.443 38.311 0.00 0.00
C
ATOM 8137 HG21 THR C 89 -11.260 30.530 39.300 0.00 0.00
C
ATOM 8138 HG22 THR C 89 -9.986 31.238 38.286 0.00 0.00
C
ATOM 8139 HG23 THR C 89 -11.468 30.734 37.503 0.00 0.00
C
ATOM 8140 C THR C 89 -8.379 30.057 36.200 0.00 0.00
C
ATOM 8141 O THR C 89 -8.572 31.049 35.524 0.00 0.00
C
ATOM 8142 N LEU C 90 -7.108 29.754 36.632 0.00 0.00
C
ATOM 8143 HN LEU C 90 -6.884 28.935 37.154 0.00 0.00
C
ATOM 8144 CA LEU C 90 -5.940 30.601 36.270 0.00 0.00
C
ATOM 8145 HA LEU C 90 -6.225 31.570 36.653 0.00 0.00
C
ATOM 8146 CB LEU C 90 -4.646 29.902 36.884 0.00 0.00
C
ATOM 8147 HB1 LEU C 90 -4.771 29.362 37.846 0.00 0.00
C
ATOM 8148 HB2 LEU C 90 -4.164 29.332 36.061 0.00 0.00
C
ATOM 8149 CG LEU C 90 -3.499 30.918 37.292 0.00 0.00
C
ATOM 8150 HG LEU C 90 -3.991 31.820 37.715 0.00 0.00
C
ATOM 8151 CD1 LEU C 90 -2.481 30.439 38.297 0.00 0.00
C
ATOM 8152 HD11 LEU C 90 -1.808 29.684 37.838 0.00 0.00
C
ATOM 8153 HD12 LEU C 90 -1.847 31.295 38.614 0.00 0.00
C

ATOM   8154 HD13 LEU C 90   -3.064 30.092 39.177 0.00 0.00
C
ATOM   8155 CD2 LEU C 90   -2.636 31.281 36.041 0.00 0.00
C
ATOM   8156 HD21 LEU C 90   -3.259 31.791 35.275 0.00 0.00
C
ATOM   8157 HD22 LEU C 90   -1.811 31.916 36.428 0.00 0.00
C
ATOM   8158 HD23 LEU C 90   -2.269 30.389 35.490 0.00 0.00
C
ATOM   8159 C   LEU C 90   -5.757 30.861 34.768 0.00 0.00
C
ATOM   8160 O   LEU C 90   -5.618 31.976 34.265 0.00 0.00
C
ATOM   8161 N   LEU C 91   -5.982 29.743 34.003 0.00 0.00
C
ATOM   8162 HN LEU C 91   -6.050 28.859 34.457 0.00 0.00
C
ATOM   8163 CA LEU C 91   -5.764 29.806 32.564 0.00 0.00
C
ATOM   8164 HA LEU C 91   -4.978 30.506 32.320 0.00 0.00
C
ATOM   8165 CB LEU C 91   -5.367 28.452 31.977 0.00 0.00
C
ATOM   8166 HB1 LEU C 91   -6.044 27.600 32.201 0.00 0.00
C
ATOM   8167 HB2 LEU C 91   -5.449 28.532 30.872 0.00 0.00
C
ATOM   8168 CG LEU C 91   -3.946 27.915 32.261 0.00 0.00
C
ATOM   8169 HG LEU C 91   -3.632 28.034 33.320 0.00 0.00
C
ATOM   8170 CD1 LEU C 91   -3.828 26.485 31.851 0.00 0.00
C
ATOM   8171 HD11 LEU C 91   -2.848 26.025 32.101 0.00 0.00
C
ATOM   8172 HD12 LEU C 91   -4.563 25.938 32.480 0.00 0.00
C
ATOM   8173 HD13 LEU C 91   -4.225 26.217 30.849 0.00 0.00
C
ATOM   8174 CD2 LEU C 91   -3.041 28.846 31.506 0.00 0.00
C
ATOM   8175 HD21 LEU C 91   -2.017 28.594 31.854 0.00 0.00
C
ATOM   8176 HD22 LEU C 91   -3.010 28.797 30.397 0.00 0.00
C
ATOM   8177 HD23 LEU C 91   -2.998 29.918 31.795 0.00 0.00
C
ATOM   8178 C   LEU C 91   -6.875 30.638 31.833 0.00 0.00
C
ATOM   8179 O   LEU C 91   -6.544 31.358 30.895 0.00 0.00
C
ATOM   8180 N   LEU C 92   -8.173 30.486 32.153 0.00 0.00
C

ATOM 8181 HN LEU C 92 -8.450 29.747 32.763 0.00 0.00
C
ATOM 8182 CA LEU C 92 -9.217 31.289 31.545 0.00 0.00
C
ATOM 8183 HA LEU C 92 -9.057 31.314 30.477 0.00 0.00
C
ATOM 8184 CB LEU C 92 -10.653 30.797 32.128 0.00 0.00
C
ATOM 8185 HB1 LEU C 92 -10.590 30.959 33.225 0.00 0.00
C
ATOM 8186 HB2 LEU C 92 -11.424 31.534 31.816 0.00 0.00
C
ATOM 8187 CG LEU C 92 -11.020 29.396 31.633 0.00 0.00
C
ATOM 8188 HG LEU C 92 -10.082 28.812 31.751 0.00 0.00
C
ATOM 8189 CD1 LEU C 92 -12.149 28.818 32.553 0.00 0.00
C
ATOM 8190 HD11 LEU C 92 -13.092 29.400 32.463 0.00 0.00
C
ATOM 8191 HD12 LEU C 92 -12.282 27.774 32.198 0.00 0.00
C
ATOM 8192 HD13 LEU C 92 -11.856 28.875 33.623 0.00 0.00
C
ATOM 8193 CD2 LEU C 92 -11.331 29.518 30.200 0.00 0.00
C
ATOM 8194 HD21 LEU C 92 -10.590 30.186 29.711 0.00 0.00
C
ATOM 8195 HD22 LEU C 92 -11.338 28.519 29.714 0.00 0.00
C
ATOM 8196 HD23 LEU C 92 -12.307 30.044 30.132 0.00 0.00
C
ATOM 8197 C LEU C 92 -9.058 32.718 31.930 0.00 0.00
C
ATOM 8198 O LEU C 92 -9.390 33.662 31.233 0.00 0.00
C
ATOM 8199 N LYS C 93 -8.560 32.944 33.147 0.00 0.00
C
ATOM 8200 HN LYS C 93 -8.342 32.191 33.764 0.00 0.00
C
ATOM 8201 CA LYS C 93 -8.298 34.310 33.692 0.00 0.00
C
ATOM 8202 HA LYS C 93 -9.147 34.971 33.610 0.00 0.00
C
ATOM 8203 CB LYS C 93 -8.111 34.281 35.227 0.00 0.00
C
ATOM 8204 HB1 LYS C 93 -9.123 34.011 35.594 0.00 0.00
C
ATOM 8205 HB2 LYS C 93 -7.369 33.548 35.609 0.00 0.00
C
ATOM 8206 CG LYS C 93 -7.852 35.580 35.911 0.00 0.00
C
ATOM 8207 HG1 LYS C 93 -7.838 35.363 37.001 0.00 0.00
C

ATOM	8208	HG2	LYS	C	93	−6.859	36.025	35.689	0.00	0.00	C
ATOM	8209	CD	LYS	C	93	−8.914	36.619	35.589	0.00	0.00	C
ATOM	8210	HD1	LYS	C	93	−8.934	36.930	34.523	0.00	0.00	C
ATOM	8211	HD2	LYS	C	93	−9.888	36.105	35.735	0.00	0.00	C
ATOM	8212	CE	LYS	C	93	−8.811	37.863	36.447	0.00	0.00	C
ATOM	8213	HE1	LYS	C	93	−8.802	37.576	37.521	0.00	0.00	C
ATOM	8214	HE2	LYS	C	93	−7.948	38.518	36.203	0.00	0.00	C
ATOM	8215	NZ	LYS	C	93	−9.954	38.630	35.984	0.00	0.00	C
ATOM	8216	HZ1	LYS	C	93	−10.704	37.910	35.955	0.00	0.00	C
ATOM	8217	HZ2	LYS	C	93	−10.177	39.424	36.617	0.00	0.00	C
ATOM	8218	HZ3	LYS	C	93	−9.772	39.066	35.057	0.00	0.00	C
ATOM	8219	C	LYS	C	93	−7.167	34.891	33.062	0.00	0.00	C
ATOM	8220	O	LYS	C	93	−7.054	36.078	32.811	0.00	0.00	C
ATOM	8221	N	GLU	C	94	−6.212	34.114	32.593	0.00	0.00	C
ATOM	8222	HN	GLU	C	94	−6.175	33.125	32.713	0.00	0.00	C
ATOM	8223	CA	GLU	C	94	−5.007	34.533	31.907	0.00	0.00	C
ATOM	8224	HA	GLU	C	94	−4.576	35.298	32.536	0.00	0.00	C
ATOM	8225	CB	GLU	C	94	−3.908	33.374	31.907	0.00	0.00	C
ATOM	8226	HB1	GLU	C	94	−3.844	32.860	32.890	0.00	0.00	C
ATOM	8227	HB2	GLU	C	94	−4.219	32.637	31.135	0.00	0.00	C
ATOM	8228	CG	GLU	C	94	−2.534	33.906	31.510	0.00	0.00	C
ATOM	8229	HG1	GLU	C	94	−1.895	32.998	31.461	0.00	0.00	C
ATOM	8230	HG2	GLU	C	94	−2.543	34.396	30.513	0.00	0.00	C
ATOM	8231	CD	GLU	C	94	−1.965	34.830	32.547	0.00	0.00	C
ATOM	8232	OE1	GLU	C	94	−1.903	34.507	33.840	0.00	0.00	C
ATOM	8233	OE2	GLU	C	94	−1.615	35.982	32.175	0.00	0.00	C
ATOM	8234	C	GLU	C	94	−5.169	35.049	30.432	0.00	0.00	C

ATOM 8235 O GLU C 94 -4.640 36.035 30.002 0.00 0.00
C
ATOM 8236 N LEU C 95 -6.027 34.288 29.713 0.00 0.00
C
ATOM 8237 HN LEU C 95 -6.282 33.393 30.072 0.00 0.00
C
ATOM 8238 CA LEU C 95 -6.458 34.603 28.416 0.00 0.00
C
ATOM 8239 HA LEU C 95 -5.574 35.102 28.048 0.00 0.00
C
ATOM 8240 CB LEU C 95 -6.727 33.266 27.663 0.00 0.00
C
ATOM 8241 HB1 LEU C 95 -7.572 32.881 28.274 0.00 0.00
C
ATOM 8242 HB2 LEU C 95 -6.968 33.537 26.613 0.00 0.00
C
ATOM 8243 CG LEU C 95 -5.507 32.262 27.681 0.00 0.00
C
ATOM 8244 HG LEU C 95 -5.164 32.169 28.734 0.00 0.00
C
ATOM 8245 CD1 LEU C 95 -5.871 30.912 27.114 0.00 0.00
C
ATOM 8246 HD11 LEU C 95 -6.633 30.496 27.808 0.00 0.00
C
ATOM 8247 HD12 LEU C 95 -6.018 30.837 26.016 0.00 0.00
C
ATOM 8248 HD13 LEU C 95 -4.974 30.256 27.138 0.00 0.00
C
ATOM 8249 CD2 LEU C 95 -4.312 32.830 26.984 0.00 0.00
C
ATOM 8250 HD21 LEU C 95 -4.476 32.742 25.889 0.00 0.00
C
ATOM 8251 HD22 LEU C 95 -4.192 33.915 27.191 0.00 0.00
C
ATOM 8252 HD23 LEU C 95 -3.421 32.271 27.340 0.00 0.00
C
ATOM 8253 C LEU C 95 -7.708 35.408 28.388 0.00 0.00
C
ATOM 8254 O LEU C 95 -8.309 35.526 27.333 0.00 0.00
C
ATOM 8255 N ASP C 96 -8.136 36.064 29.492 0.00 0.00
C
ATOM 8256 HN ASP C 96 -7.609 35.892 30.320 0.00 0.00
C
ATOM 8257 CA ASP C 96 -9.056 37.116 29.590 0.00 0.00
C
ATOM 8258 HA ASP C 96 -9.218 37.402 30.619 0.00 0.00
C
ATOM 8259 CB ASP C 96 -8.347 38.302 28.914 0.00 0.00
C
ATOM 8260 HB1 ASP C 96 -7.259 38.121 29.048 0.00 0.00
C
ATOM 8261 HB2 ASP C 96 -8.406 38.248 27.806 0.00 0.00
C

ATOM	8262	CG	ASP	C	96	−8.820	39.698	29.289	0.00	0.00	C
ATOM	8263	OD1	ASP	C	96	−9.786	40.015	29.997	0.00	0.00	C
ATOM	8264	OD2	ASP	C	96	−7.978	40.622	28.957	0.00	0.00	C
ATOM	8265	C	ASP	C	96	−10.477	36.719	29.110	0.00	0.00	C
ATOM	8266	O	ASP	C	96	−11.012	37.423	28.272	0.00	0.00	C
ATOM	8267	N	ILE	C	97	−11.101	35.609	29.449	0.00	0.00	C
ATOM	8268	HN	ILE	C	97	−10.732	34.901	30.046	0.00	0.00	C
ATOM	8269	CA	ILE	C	97	−12.433	35.235	28.985	0.00	0.00	C
ATOM	8270	HA	ILE	C	97	−12.387	35.373	27.915	0.00	0.00	C
ATOM	8271	CB	ILE	C	97	−12.831	33.801	29.107	0.00	0.00	C
ATOM	8272	HB	ILE	C	97	−12.705	33.383	30.129	0.00	0.00	C
ATOM	8273	CG2	ILE	C	97	−14.300	33.627	28.698	0.00	0.00	C
ATOM	8274	HG21	ILE	C	97	−15.012	34.188	29.341	0.00	0.00	C
ATOM	8275	HG22	ILE	C	97	−14.468	33.792	27.612	0.00	0.00	C
ATOM	8276	HG23	ILE	C	97	−14.656	32.594	28.899	0.00	0.00	C
ATOM	8277	CG1	ILE	C	97	−11.900	32.787	28.374	0.00	0.00	C
ATOM	8278	HG11	ILE	C	97	−10.963	32.921	28.957	0.00	0.00	C
ATOM	8279	HG12	ILE	C	97	−12.354	31.802	28.613	0.00	0.00	C
ATOM	8280	CD	ILE	C	97	−11.673	32.836	26.855	0.00	0.00	C
ATOM	8281	HD1	ILE	C	97	−10.960	32.074	26.473	0.00	0.00	C
ATOM	8282	HD2	ILE	C	97	−12.576	32.732	26.217	0.00	0.00	C
ATOM	8283	HD3	ILE	C	97	−11.285	33.858	26.653	0.00	0.00	C
ATOM	8284	C	ILE	C	97	−13.497	36.197	29.592	0.00	0.00	C
ATOM	8285	O	ILE	C	97	−13.330	36.433	30.739	0.00	0.00	C
ATOM	8286	N	PRO	C	98	−14.473	36.780	28.838	0.00	0.00	C
ATOM	8287	CD	PRO	C	98	−14.372	36.966	27.424	0.00	0.00	C
ATOM	8288	HD1	PRO	C	98	−13.437	37.535	27.236	0.00	0.00	C

```
ATOM   8289  HD2 PRO C  98      -14.483  35.936  27.022  0.00  0.00
C
ATOM   8290  CA  PRO C  98      -15.559  37.628  29.346  0.00  0.00
C
ATOM   8291  HA  PRO C  98      -15.158  38.612  29.538  0.00  0.00
C
ATOM   8292  CB  PRO C  98      -16.472  37.877  28.187  0.00  0.00
C
ATOM   8293  HB1 PRO C  98      -17.118  38.781  28.191  0.00  0.00
C
ATOM   8294  HB2 PRO C  98      -17.111  36.991  27.983  0.00  0.00
C
ATOM   8295  CG  PRO C  98      -15.562  37.782  26.937  0.00  0.00
C
ATOM   8296  HG1 PRO C  98      -15.300  38.762  26.483  0.00  0.00
C
ATOM   8297  HG2 PRO C  98      -16.061  37.165  26.159  0.00  0.00
C
ATOM   8298  C   PRO C  98      -16.189  37.261  30.641  0.00  0.00
C
ATOM   8299  O   PRO C  98      -16.350  38.115  31.483  0.00  0.00
C
ATOM   8300  N   ASN C  99      -16.756  35.999  30.671  0.00  0.00
C
ATOM   8301  HN  ASN C  99      -16.740  35.464  29.829  0.00  0.00
C
ATOM   8302  CA  ASN C  99      -17.620  35.528  31.836  0.00  0.00
C
ATOM   8303  HA  ASN C  99      -17.488  36.215  32.658  0.00  0.00
C
ATOM   8304  CB  ASN C  99      -19.116  35.522  31.426  0.00  0.00
C
ATOM   8305  HB1 ASN C  99      -19.375  34.651  30.787  0.00  0.00
C
ATOM   8306  HB2 ASN C  99      -19.746  35.395  32.333  0.00  0.00
C
ATOM   8307  CG  ASN C  99      -19.575  36.724  30.707  0.00  0.00
C
ATOM   8308  OD1 ASN C  99      -19.798  37.869  31.210  0.00  0.00
C
ATOM   8309  ND2 ASN C  99      -19.821  36.605  29.373  0.00  0.00
C
ATOM   8310 HD21 ASN C  99      -20.412  37.302  28.966  0.00  0.00
C
ATOM   8311 HD22 ASN C  99      -19.547  35.797  28.850  0.00  0.00
C
ATOM   8312  C   ASN C  99      -17.076  34.114  32.255  0.00  0.00
C
ATOM   8313  O   ASN C  99      -16.975  33.136  31.530  0.00  0.00
C
ATOM   8314  N   ILE C 100      -16.660  34.096  33.538  0.00  0.00
C
ATOM   8315  HN  ILE C 100      -16.627  34.907  34.116  0.00  0.00
C
```

```
ATOM   8316  CA  ILE C 100     -15.982  32.859  34.131  0.00  0.00
C
ATOM   8317  HA  ILE C 100     -16.032  32.109  33.355  0.00  0.00
C
ATOM   8318  CB  ILE C 100     -14.522  33.034  34.434  0.00  0.00
C
ATOM   8319  HB  ILE C 100     -14.457  33.782  35.253  0.00  0.00
C
ATOM   8320  CG2 ILE C 100     -13.772  31.778  34.866  0.00  0.00
C
ATOM   8321 HG21 ILE C 100     -12.724  32.060  35.103  0.00  0.00
C
ATOM   8322 HG22 ILE C 100     -14.226  31.221  35.714  0.00  0.00
C
ATOM   8323 HG23 ILE C 100     -13.636  31.064  34.026  0.00  0.00
C
ATOM   8324  CG1 ILE C 100     -13.773  33.784  33.263  0.00  0.00
C
ATOM   8325 HG11 ILE C 100     -14.006  33.153  32.380  0.00  0.00
C
ATOM   8326 HG12 ILE C 100     -14.261  34.774  33.136  0.00  0.00
C
ATOM   8327  CD  ILE C 100     -12.245  33.984  33.567  0.00  0.00
C
ATOM   8328  HD1 ILE C 100     -11.809  34.622  32.769  0.00  0.00
C
ATOM   8329  HD2 ILE C 100     -12.071  34.430  34.570  0.00  0.00
C
ATOM   8330  HD3 ILE C 100     -11.723  33.019  33.394  0.00  0.00
C
ATOM   8331  C   ILE C 100     -16.750  32.288  35.423  0.00  0.00
C
ATOM   8332  O   ILE C 100     -16.741  32.926  36.467  0.00  0.00
C
ATOM   8333  N   TRP C 101     -17.317  31.035  35.323  0.00  0.00
C
ATOM   8334  HN  TRP C 101     -17.284  30.600  34.427  0.00  0.00
C
ATOM   8335  CA  TRP C 101     -18.243  30.464  36.350  0.00  0.00
C
ATOM   8336  HA  TRP C 101     -18.423  31.206  37.114  0.00  0.00
C
ATOM   8337  CB  TRP C 101     -19.551  30.036  35.683  0.00  0.00
C
ATOM   8338  HB1 TRP C 101     -19.430  29.548  34.693  0.00  0.00
C
ATOM   8339  HB2 TRP C 101     -20.189  29.279  36.189  0.00  0.00
C
ATOM   8340  CG  TRP C 101     -20.469  31.147  35.382  0.00  0.00
C
ATOM   8341  CD1 TRP C 101     -20.244  32.232  34.520  0.00  0.00
C
ATOM   8342  HD1 TRP C 101     -19.351  32.414  33.941  0.00  0.00
C
```

ATOM 8343 NE1 TRP C 101 -21.305 33.103 34.534 0.00 0.00
C
ATOM 8344 HE1 TRP C 101 -21.385 33.980 34.112 0.00 0.00
C
ATOM 8345 CE2 TRP C 101 -22.239 32.547 35.435 0.00 0.00
C
ATOM 8346 CD2 TRP C 101 -21.704 31.360 35.962 0.00 0.00
C
ATOM 8347 CE3 TRP C 101 -22.486 30.615 36.938 0.00 0.00
C
ATOM 8348 HE3 TRP C 101 -22.040 29.737 37.382 0.00 0.00
C
ATOM 8349 CZ3 TRP C 101 -23.750 31.039 37.298 0.00 0.00
C
ATOM 8350 HZ3 TRP C 101 -24.365 30.369 37.880 0.00 0.00
C
ATOM 8351 CZ2 TRP C 101 -23.497 32.964 35.791 0.00 0.00
C
ATOM 8352 HZ2 TRP C 101 -23.993 33.820 35.358 0.00 0.00
C
ATOM 8353 CH2 TRP C 101 -24.254 32.138 36.614 0.00 0.00
C
ATOM 8354 HH2 TRP C 101 -25.306 32.352 36.723 0.00 0.00
C
ATOM 8355 C TRP C 101 -17.704 29.171 36.959 0.00 0.00
C
ATOM 8356 O TRP C 101 -17.601 28.203 36.263 0.00 0.00
C
ATOM 8357 N VAL C 102 -17.369 29.149 38.244 0.00 0.00
C
ATOM 8358 HN VAL C 102 -17.583 29.845 38.926 0.00 0.00
C
ATOM 8359 CA VAL C 102 -16.436 28.075 38.870 0.00 0.00
C
ATOM 8360 HA VAL C 102 -16.271 27.353 38.083 0.00 0.00
C
ATOM 8361 CB VAL C 102 -15.035 28.657 39.244 0.00 0.00
C
ATOM 8362 HB VAL C 102 -15.115 29.246 40.183 0.00 0.00
C
ATOM 8363 CG1 VAL C 102 -14.172 27.413 39.555 0.00 0.00
C
ATOM 8364 HG11 VAL C 102 -14.121 26.711 38.695 0.00 0.00
C
ATOM 8365 HG12 VAL C 102 -13.152 27.781 39.797 0.00 0.00
C
ATOM 8366 HG13 VAL C 102 -14.619 26.939 40.455 0.00 0.00
C
ATOM 8367 CG2 VAL C 102 -14.370 29.425 38.061 0.00 0.00
C
ATOM 8368 HG21 VAL C 102 -14.956 30.300 37.710 0.00 0.00
C
ATOM 8369 HG22 VAL C 102 -13.406 29.846 38.421 0.00 0.00
C

ATOM 8370 HG23 VAL C 102 −14.299 28.627 37.291 0.00 0.00
C
ATOM 8371 C VAL C 102 −17.209 27.297 40.013 0.00 0.00
C
ATOM 8372 O VAL C 102 −17.705 27.975 40.953 0.00 0.00
C
ATOM 8373 N LYS C 103 −17.358 25.935 39.936 0.00 0.00
C
ATOM 8374 HN LYS C 103 −17.197 25.615 39.005 0.00 0.00
C
ATOM 8375 CA LYS C 103 −17.632 25.073 41.079 0.00 0.00
C
ATOM 8376 HA LYS C 103 −18.442 25.479 41.667 0.00 0.00
C
ATOM 8377 CB LYS C 103 −18.084 23.659 40.623 0.00 0.00
C
ATOM 8378 HB1 LYS C 103 −18.858 23.782 39.836 0.00 0.00
C
ATOM 8379 HB2 LYS C 103 −17.226 23.008 40.353 0.00 0.00
C
ATOM 8380 CG LYS C 103 −18.847 22.906 41.698 0.00 0.00
C
ATOM 8381 HG1 LYS C 103 −18.290 22.918 42.659 0.00 0.00
C
ATOM 8382 HG2 LYS C 103 −19.852 23.348 41.866 0.00 0.00
C
ATOM 8383 CD LYS C 103 −19.026 21.414 41.264 0.00 0.00
C
ATOM 8384 HD1 LYS C 103 −19.588 21.300 40.312 0.00 0.00
C
ATOM 8385 HD2 LYS C 103 −18.020 20.996 41.046 0.00 0.00
C
ATOM 8386 CE LYS C 103 −19.721 20.494 42.263 0.00 0.00
C
ATOM 8387 HE1 LYS C 103 −19.204 20.615 43.239 0.00 0.00
C
ATOM 8388 HE2 LYS C 103 −20.818 20.651 42.346 0.00 0.00
C
ATOM 8389 NZ LYS C 103 −19.531 19.112 41.872 0.00 0.00
C
ATOM 8390 HZ1 LYS C 103 −19.988 18.795 40.993 0.00 0.00
C
ATOM 8391 HZ2 LYS C 103 −18.507 18.936 41.829 0.00 0.00
C
ATOM 8392 HZ3 LYS C 103 −19.783 18.484 42.661 0.00 0.00
C
ATOM 8393 C LYS C 103 −16.553 25.144 42.167 0.00 0.00
C
ATOM 8394 O LYS C 103 −15.330 25.123 41.916 0.00 0.00
C
ATOM 8395 N ALA C 104 −16.877 25.173 43.472 0.00 0.00
C
ATOM 8396 HN ALA C 104 −17.835 25.353 43.682 0.00 0.00
C

ATOM 8397 CA ALA C 104 -15.995 25.064 44.507 0.00 0.00
C
ATOM 8398 HA ALA C 104 -15.018 24.757 44.163 0.00 0.00
C
ATOM 8399 CB ALA C 104 -15.817 26.425 45.240 0.00 0.00
C
ATOM 8400 HB1 ALA C 104 -14.737 26.338 45.484 0.00 0.00
C
ATOM 8401 HB2 ALA C 104 -15.809 27.309 44.567 0.00 0.00
C
ATOM 8402 HB3 ALA C 104 -16.509 26.586 46.094 0.00 0.00
C
ATOM 8403 C ALA C 104 -16.450 24.005 45.460 0.00 0.00
C
ATOM 8404 O ALA C 104 -17.638 23.651 45.502 0.00 0.00
C
ATOM 8405 N GLN C 105 -15.523 23.502 46.280 0.00 0.00
C
ATOM 8406 HN GLN C 105 -14.555 23.739 46.264 0.00 0.00
C
ATOM 8407 CA GLN C 105 -15.646 22.479 47.288 0.00 0.00
C
ATOM 8408 HA GLN C 105 -16.501 21.871 47.032 0.00 0.00
C
ATOM 8409 CB GLN C 105 -14.315 21.645 47.279 0.00 0.00
C
ATOM 8410 HB1 GLN C 105 -14.041 21.385 46.234 0.00 0.00
C
ATOM 8411 HB2 GLN C 105 -13.627 22.424 47.671 0.00 0.00
C
ATOM 8412 CG GLN C 105 -14.225 20.499 48.323 0.00 0.00
C
ATOM 8413 HG1 GLN C 105 -13.301 19.883 48.342 0.00 0.00
C
ATOM 8414 HG2 GLN C 105 -14.418 20.959 49.315 0.00 0.00
C
ATOM 8415 CD GLN C 105 -15.345 19.482 48.071 0.00 0.00
C
ATOM 8416 OE1 GLN C 105 -15.367 18.670 47.134 0.00 0.00
C
ATOM 8417 NE2 GLN C 105 -16.300 19.586 49.021 0.00 0.00
C
ATOM 8418 HE21 GLN C 105 -16.897 18.807 49.214 0.00 0.00
C
ATOM 8419 HE22 GLN C 105 -16.523 20.424 49.517 0.00 0.00
C
ATOM 8420 C GLN C 105 -15.942 22.969 48.669 0.00 0.00
C
ATOM 8421 O GLN C 105 -16.694 22.372 49.400 0.00 0.00
C
ATOM 8422 N ASN C 106 -15.198 24.010 49.055 0.00 0.00
C
ATOM 8423 HN ASN C 106 -14.551 24.451 48.438 0.00 0.00
C

ATOM 8424 CA ASN C 106 −15.452 24.626 50.332 0.00 0.00
C
ATOM 8425 HA ASN C 106 −16.509 24.539 50.538 0.00 0.00
C
ATOM 8426 CB ASN C 106 −14.559 23.907 51.430 0.00 0.00
C
ATOM 8427 HB1 ASN C 106 −14.696 24.408 52.412 0.00 0.00
C
ATOM 8428 HB2 ASN C 106 −14.840 22.839 51.546 0.00 0.00
C
ATOM 8429 CG ASN C 106 −13.098 24.022 51.124 0.00 0.00
C
ATOM 8430 OD1 ASN C 106 −12.522 25.060 50.838 0.00 0.00
C
ATOM 8431 ND2 ASN C 106 −12.395 22.847 51.145 0.00 0.00
C
ATOM 8432 HD21 ASN C 106 −11.399 22.886 51.063 0.00 0.00
C
ATOM 8433 HD22 ASN C 106 −12.833 21.957 51.271 0.00 0.00
C
ATOM 8434 C ASN C 106 −15.222 26.184 50.304 0.00 0.00
C
ATOM 8435 O ASN C 106 −14.711 26.716 49.327 0.00 0.00
C
ATOM 8436 N TYR C 107 −15.478 26.856 51.441 0.00 0.00
C
ATOM 8437 HN TYR C 107 −15.866 26.420 52.249 0.00 0.00
C
ATOM 8438 CA TYR C 107 −15.181 28.292 51.593 0.00 0.00
C
ATOM 8439 HA TYR C 107 −15.502 28.757 50.673 0.00 0.00
C
ATOM 8440 CB TYR C 107 −15.925 28.853 52.828 0.00 0.00
C
ATOM 8441 HB1 TYR C 107 −17.002 28.620 52.695 0.00 0.00
C
ATOM 8442 HB2 TYR C 107 −15.751 28.301 53.777 0.00 0.00
C
ATOM 8443 CG TYR C 107 −15.922 30.391 53.025 0.00 0.00
C
ATOM 8444 CD1 TYR C 107 −15.707 30.891 54.321 0.00 0.00
C
ATOM 8445 HD1 TYR C 107 −15.558 30.245 55.173 0.00 0.00
C
ATOM 8446 CE1 TYR C 107 −15.710 32.279 54.546 0.00 0.00
C
ATOM 8447 HE1 TYR C 107 −15.607 32.635 55.561 0.00 0.00
C
ATOM 8448 CZ TYR C 107 −15.989 33.225 53.538 0.00 0.00
C
ATOM 8449 OH TYR C 107 −16.018 34.576 53.884 0.00 0.00
C
ATOM 8450 HH TYR C 107 −16.262 35.017 53.067 0.00 0.00
C

ATOM 8451 CD2 TYR C 107 −16.270 31.297 52.102 0.00 0.00
C
ATOM 8452 HD2 TYR C 107 −16.605 30.960 51.132 0.00 0.00
C
ATOM 8453 CE2 TYR C 107 −16.292 32.727 52.296 0.00 0.00
C
ATOM 8454 HE2 TYR C 107 −16.522 33.291 51.404 0.00 0.00
C
ATOM 8455 C TYR C 107 −13.619 28.700 51.481 0.00 0.00
C
ATOM 8456 O TYR C 107 −13.373 29.674 50.799 0.00 0.00
C
ATOM 8457 N TYR C 108 −12.639 27.939 51.972 0.00 0.00
C
ATOM 8458 HN TYR C 108 −12.914 27.089 52.415 0.00 0.00
C
ATOM 8459 CA TYR C 108 −11.245 28.217 51.850 0.00 0.00
C
ATOM 8460 HA TYR C 108 −11.028 29.226 52.168 0.00 0.00
C
ATOM 8461 CB TYR C 108 −10.327 27.347 52.770 0.00 0.00
C
ATOM 8462 HB1 TYR C 108 −10.436 26.297 52.421 0.00 0.00
C
ATOM 8463 HB2 TYR C 108 −9.317 27.799 52.680 0.00 0.00
C
ATOM 8464 CG TYR C 108 −10.881 27.447 54.154 0.00 0.00
C
ATOM 8465 CD1 TYR C 108 −10.692 28.559 54.963 0.00 0.00
C
ATOM 8466 HD1 TYR C 108 −10.037 29.343 54.613 0.00 0.00
C
ATOM 8467 CE1 TYR C 108 −11.391 28.543 56.148 0.00 0.00
C
ATOM 8468 HE1 TYR C 108 −11.252 29.362 56.838 0.00 0.00
C
ATOM 8469 CZ TYR C 108 −12.061 27.396 56.650 0.00 0.00
C
ATOM 8470 OH TYR C 108 −12.691 27.394 57.882 0.00 0.00
C
ATOM 8471 HH TYR C 108 −12.415 28.184 58.353 0.00 0.00
C
ATOM 8472 CD2 TYR C 108 −11.674 26.400 54.568 0.00 0.00
C
ATOM 8473 HD2 TYR C 108 −11.638 25.513 53.953 0.00 0.00
C
ATOM 8474 CE2 TYR C 108 −12.332 26.362 55.812 0.00 0.00
C
ATOM 8475 HE2 TYR C 108 −12.864 25.493 56.170 0.00 0.00
C
ATOM 8476 C TYR C 108 −10.751 28.180 50.368 0.00 0.00
C
ATOM 8477 O TYR C 108 −9.988 29.051 49.888 0.00 0.00
C

ATOM 8478 N HSE C 109 −11.191 27.202 49.666 0.00 0.00
C
ATOM 8479 HN HSE C 109 −11.720 26.500 50.135 0.00 0.00
C
ATOM 8480 CA HSE C 109 −10.964 26.972 48.246 0.00 0.00
C
ATOM 8481 HA HSE C 109 −9.927 26.980 47.944 0.00 0.00
C
ATOM 8482 CB HSE C 109 −11.565 25.636 47.945 0.00 0.00
C
ATOM 8483 HB1 HSE C 109 −11.056 24.843 48.533 0.00 0.00
C
ATOM 8484 HB2 HSE C 109 −12.600 25.641 48.348 0.00 0.00
C
ATOM 8485 ND1 HSE C 109 −12.663 24.375 46.183 0.00 0.00
C
ATOM 8486 CG HSE C 109 −11.637 25.166 46.545 0.00 0.00
C
ATOM 8487 CE1 HSE C 109 −12.324 23.942 44.966 0.00 0.00
C
ATOM 8488 HE1 HSE C 109 −12.885 23.229 44.363 0.00 0.00
C
ATOM 8489 NE2 HSE C 109 −11.112 24.496 44.565 0.00 0.00
C
ATOM 8490 HE2 HSE C 109 −10.534 24.306 43.771 0.00 0.00
C
ATOM 8491 CD2 HSE C 109 −10.663 25.315 45.559 0.00 0.00
C
ATOM 8492 HD2 HSE C 109 −9.779 25.933 45.654 0.00 0.00
C
ATOM 8493 C HSE C 109 −11.572 27.936 47.306 0.00 0.00
C
ATOM 8494 O HSE C 109 −11.032 28.380 46.345 0.00 0.00
C
ATOM 8495 N HSE C 110 −12.794 28.428 47.648 0.00 0.00
C
ATOM 8496 HN HSE C 110 −13.289 28.069 48.436 0.00 0.00
C
ATOM 8497 CA HSE C 110 −13.384 29.550 47.037 0.00 0.00
C
ATOM 8498 HA HSE C 110 −13.452 29.270 45.996 0.00 0.00
C
ATOM 8499 CB HSE C 110 −14.743 29.700 47.780 0.00 0.00
C
ATOM 8500 HB1 HSE C 110 −15.453 28.889 47.510 0.00 0.00
C
ATOM 8501 HB2 HSE C 110 −14.430 29.598 48.841 0.00 0.00
C
ATOM 8502 ND1 HSE C 110 −16.571 31.183 47.010 0.00 0.00
C
ATOM 8503 CG HSE C 110 −15.350 31.022 47.682 0.00 0.00
C
ATOM 8504 CE1 HSE C 110 −16.932 32.467 47.172 0.00 0.00
C

ATOM 8505 HE1 HSE C 110 -17.752 33.016 46.710 0.00 0.00
C
ATOM 8506 NE2 HSE C 110 -15.983 33.096 47.946 0.00 0.00
C
ATOM 8507 HE2 HSE C 110 -15.910 34.077 48.123 0.00 0.00
C
ATOM 8508 CD2 HSE C 110 -15.070 32.212 48.343 0.00 0.00
C
ATOM 8509 HD2 HSE C 110 -14.353 32.339 49.144 0.00 0.00
C
ATOM 8510 C HSE C 110 -12.512 30.840 47.067 0.00 0.00
C
ATOM 8511 O HSE C 110 -12.451 31.566 46.032 0.00 0.00
C
ATOM 8512 N LYS C 111 -11.918 31.183 48.216 0.00 0.00
C
ATOM 8513 HN LYS C 111 -12.060 30.726 49.091 0.00 0.00
C
ATOM 8514 CA LYS C 111 -10.944 32.320 48.270 0.00 0.00
C
ATOM 8515 HA LYS C 111 -11.521 33.148 47.887 0.00 0.00
C
ATOM 8516 CB LYS C 111 -10.447 32.487 49.822 0.00 0.00
C
ATOM 8517 HB1 LYS C 111 -9.891 31.653 50.301 0.00 0.00
C
ATOM 8518 HB2 LYS C 111 -9.677 33.287 49.791 0.00 0.00
C
ATOM 8519 CG LYS C 111 -11.587 32.781 50.837 0.00 0.00
C
ATOM 8520 HG1 LYS C 111 -12.242 33.572 50.414 0.00 0.00
C
ATOM 8521 HG2 LYS C 111 -12.193 31.884 51.088 0.00 0.00
C
ATOM 8522 CD LYS C 111 -11.177 33.314 52.223 0.00 0.00
C
ATOM 8523 HD1 LYS C 111 -10.950 32.381 52.782 0.00 0.00
C
ATOM 8524 HD2 LYS C 111 -10.176 33.796 52.246 0.00 0.00
C
ATOM 8525 CE LYS C 111 -12.306 34.042 52.865 0.00 0.00
C
ATOM 8526 HE1 LYS C 111 -12.510 35.021 52.379 0.00 0.00
C
ATOM 8527 HE2 LYS C 111 -13.309 33.601 52.686 0.00 0.00
C
ATOM 8528 NZ LYS C 111 -12.144 34.214 54.344 0.00 0.00
C
ATOM 8529 HZ1 LYS C 111 -12.231 33.250 54.722 0.00 0.00
C
ATOM 8530 HZ2 LYS C 111 -11.192 34.597 54.513 0.00 0.00
C
ATOM 8531 HZ3 LYS C 111 -12.808 34.851 54.829 0.00 0.00
C

```
ATOM   8532  C   LYS C 111      -9.756  32.143  47.406  0.00  0.00
C
ATOM   8533  O   LYS C 111      -9.413  33.030  46.633  0.00  0.00
C
ATOM   8534  N   VAL C 112      -9.053  30.940  47.404  0.00  0.00
C
ATOM   8535  HN  VAL C 112      -9.269  30.206  48.043  0.00  0.00
C
ATOM   8536  CA  VAL C 112      -8.028  30.703  46.275  0.00  0.00
C
ATOM   8537  HA  VAL C 112      -7.156  31.306  46.479  0.00  0.00
C
ATOM   8538  CB  VAL C 112      -7.497  29.267  46.469  0.00  0.00
C
ATOM   8539  HB  VAL C 112      -8.388  28.645  46.236  0.00  0.00
C
ATOM   8540  CG1 VAL C 112      -6.418  28.806  45.434  0.00  0.00
C
ATOM   8541 HG11 VAL C 112      -6.078  27.808  45.784  0.00  0.00
C
ATOM   8542 HG12 VAL C 112      -6.872  28.783  44.420  0.00  0.00
C
ATOM   8543 HG13 VAL C 112      -5.562  29.514  45.417  0.00  0.00
C
ATOM   8544  CG2 VAL C 112      -6.875  29.057  47.876  0.00  0.00
C
ATOM   8545 HG21 VAL C 112      -7.710  28.809  48.566  0.00  0.00
C
ATOM   8546 HG22 VAL C 112      -6.243  28.148  47.966  0.00  0.00
C
ATOM   8547 HG23 VAL C 112      -6.229  29.927  48.121  0.00  0.00
C
ATOM   8548  C   VAL C 112      -8.513  30.998  44.869  0.00  0.00
C
ATOM   8549  O   VAL C 112      -7.771  31.660  44.158  0.00  0.00
C
ATOM   8550  N   LEU C 113      -9.721  30.466  44.465  0.00  0.00
C
ATOM   8551  HN  LEU C 113     -10.212  29.762  44.971  0.00  0.00
C
ATOM   8552  CA  LEU C 113     -10.275  30.810  43.163  0.00  0.00
C
ATOM   8553  HA  LEU C 113      -9.607  30.521  42.365  0.00  0.00
C
ATOM   8554  CB  LEU C 113     -11.674  30.120  42.937  0.00  0.00
C
ATOM   8555  HB1 LEU C 113     -12.263  30.482  43.807  0.00  0.00
C
ATOM   8556  HB2 LEU C 113     -12.182  30.464  42.010  0.00  0.00
C
ATOM   8557  CG  LEU C 113     -11.639  28.578  42.887  0.00  0.00
C
ATOM   8558  HG  LEU C 113     -11.080  28.186  43.764  0.00  0.00
C
```

```
ATOM   8559  CD1 LEU C 113     -13.023  27.950  42.901  0.00  0.00
C
ATOM   8560 HD11 LEU C 113     -12.971  26.844  42.992  0.00  0.00
C
ATOM   8561 HD12 LEU C 113     -13.721  28.310  43.686  0.00  0.00
C
ATOM   8562 HD13 LEU C 113     -13.576  28.132  41.955  0.00  0.00
C
ATOM   8563  CD2 LEU C 113     -10.748  28.103  41.711  0.00  0.00
C
ATOM   8564 HD21 LEU C 113     -10.705  27.000  41.837  0.00  0.00
C
ATOM   8565 HD22 LEU C 113     -11.198  28.416  40.745  0.00  0.00
C
ATOM   8566 HD23 LEU C 113      -9.691  28.445  41.726  0.00  0.00
C
ATOM   8567  C   LEU C 113     -10.531  32.263  43.090  0.00  0.00
C
ATOM   8568  O   LEU C 113     -10.268  32.772  41.986  0.00  0.00
C
ATOM   8569  N   GLU C 114     -10.989  33.014  44.109  0.00  0.00
C
ATOM   8570  HN  GLU C 114     -10.938  32.557  44.994  0.00  0.00
C
ATOM   8571  CA  GLU C 114     -11.470  34.398  44.002  0.00  0.00
C
ATOM   8572  HA  GLU C 114     -12.016  34.477  43.073  0.00  0.00
C
ATOM   8573  CB  GLU C 114     -12.271  34.830  45.307  0.00  0.00
C
ATOM   8574  HB1 GLU C 114     -13.000  34.014  45.501  0.00  0.00
C
ATOM   8575  HB2 GLU C 114     -11.596  34.806  46.189  0.00  0.00
C
ATOM   8576  CG  GLU C 114     -13.069  36.121  45.171  0.00  0.00
C
ATOM   8577  HG1 GLU C 114     -12.383  36.910  44.793  0.00  0.00
C
ATOM   8578  HG2 GLU C 114     -13.952  36.005  44.507  0.00  0.00
C
ATOM   8579  CD  GLU C 114     -13.668  36.545  46.524  0.00  0.00
C
ATOM   8580  OE1 GLU C 114     -14.884  36.426  46.730  0.00  0.00
C
ATOM   8581  OE2 GLU C 114     -12.806  37.002  47.332  0.00  0.00
C
ATOM   8582  C   GLU C 114     -10.256  35.304  44.027  0.00  0.00
C
ATOM   8583  O   GLU C 114     -10.241  36.360  43.407  0.00  0.00
C
ATOM   8584  N   LYS C 115      -9.151  34.977  44.770  0.00  0.00
C
ATOM   8585  HN  LYS C 115      -9.186  34.148  45.323  0.00  0.00
C
```

ATOM 8586 CA LYS C 115 -7.838 35.517 44.649 0.00 0.00
C
ATOM 8587 HA LYS C 115 -7.872 36.583 44.818 0.00 0.00
C
ATOM 8588 CB LYS C 115 -6.732 34.887 45.580 0.00 0.00
C
ATOM 8589 HB1 LYS C 115 -6.689 33.800 45.357 0.00 0.00
C
ATOM 8590 HB2 LYS C 115 -5.843 35.313 45.069 0.00 0.00
C
ATOM 8591 CG LYS C 115 -6.812 35.197 47.058 0.00 0.00
C
ATOM 8592 HG1 LYS C 115 -7.862 35.093 47.404 0.00 0.00
C
ATOM 8593 HG2 LYS C 115 -6.149 34.600 47.721 0.00 0.00
C
ATOM 8594 CD LYS C 115 -6.542 36.675 47.316 0.00 0.00
C
ATOM 8595 HD1 LYS C 115 -5.528 36.949 46.954 0.00 0.00
C
ATOM 8596 HD2 LYS C 115 -7.335 37.255 46.798 0.00 0.00
C
ATOM 8597 CE LYS C 115 -6.515 37.080 48.801 0.00 0.00
C
ATOM 8598 HE1 LYS C 115 -7.517 36.764 49.163 0.00 0.00
C
ATOM 8599 HE2 LYS C 115 -5.719 36.631 49.432 0.00 0.00
C
ATOM 8600 NZ LYS C 115 -6.408 38.561 48.897 0.00 0.00
C
ATOM 8601 HZ1 LYS C 115 -6.612 38.927 49.849 0.00 0.00
C
ATOM 8602 HZ2 LYS C 115 -5.402 38.789 48.764 0.00 0.00
C
ATOM 8603 HZ3 LYS C 115 -7.008 38.967 48.152 0.00 0.00
C
ATOM 8604 C LYS C 115 -7.325 35.328 43.193 0.00 0.00
C
ATOM 8605 O LYS C 115 -6.706 36.305 42.708 0.00 0.00
C
ATOM 8606 N ILE C 116 -7.611 34.139 42.479 0.00 0.00
C
ATOM 8607 HN ILE C 116 -7.929 33.349 42.998 0.00 0.00
C
ATOM 8608 CA ILE C 116 -7.243 33.945 41.128 0.00 0.00
C
ATOM 8609 HA ILE C 116 -6.199 34.199 41.025 0.00 0.00
C
ATOM 8610 CB ILE C 116 -7.372 32.541 40.512 0.00 0.00
C
ATOM 8611 HB ILE C 116 -8.309 32.091 40.904 0.00 0.00
C
ATOM 8612 CG2 ILE C 116 -7.149 32.490 38.982 0.00 0.00
C

ATOM 8613 HG21 ILE C 116 -6.079 32.731 38.802 0.00 0.00
C
ATOM 8614 HG22 ILE C 116 -7.335 31.462 38.604 0.00 0.00
C
ATOM 8615 HG23 ILE C 116 -7.783 33.179 38.384 0.00 0.00
C
ATOM 8616 CG1 ILE C 116 -6.148 31.739 41.125 0.00 0.00
C
ATOM 8617 HG11 ILE C 116 -5.244 32.007 40.538 0.00 0.00
C
ATOM 8618 HG12 ILE C 116 -5.980 32.117 42.156 0.00 0.00
C
ATOM 8619 CD ILE C 116 -6.406 30.205 41.206 0.00 0.00
C
ATOM 8620 HD1 ILE C 116 -7.496 30.080 41.383 0.00 0.00
C
ATOM 8621 HD2 ILE C 116 -6.177 29.782 40.204 0.00 0.00
C
ATOM 8622 HD3 ILE C 116 -5.722 29.697 41.919 0.00 0.00
C
ATOM 8623 C ILE C 116 -8.059 34.874 40.229 0.00 0.00
C
ATOM 8624 O ILE C 116 -7.457 35.688 39.515 0.00 0.00
C
ATOM 8625 N GLY C 117 -9.402 34.894 40.470 0.00 0.00
C
ATOM 8626 HN GLY C 117 -9.812 34.235 41.095 0.00 0.00
C
ATOM 8627 CA GLY C 117 -10.385 35.782 39.895 0.00 0.00
C
ATOM 8628 HA1 GLY C 117 -9.876 36.501 39.270 0.00 0.00
C
ATOM 8629 HA2 GLY C 117 -10.895 36.292 40.699 0.00 0.00
C
ATOM 8630 C GLY C 117 -11.348 34.891 39.076 0.00 0.00
C
ATOM 8631 O GLY C 117 -11.011 34.199 38.143 0.00 0.00
C
ATOM 8632 N ALA C 118 -12.614 34.979 39.437 0.00 0.00
C
ATOM 8633 HN ALA C 118 -12.857 35.594 40.183 0.00 0.00
C
ATOM 8634 CA ALA C 118 -13.730 34.434 38.751 0.00 0.00
C
ATOM 8635 HA ALA C 118 -13.449 34.597 37.721 0.00 0.00
C
ATOM 8636 CB ALA C 118 -13.968 32.940 39.031 0.00 0.00
C
ATOM 8637 HB1 ALA C 118 -14.115 33.002 40.131 0.00 0.00
C
ATOM 8638 HB2 ALA C 118 -14.826 32.420 38.554 0.00 0.00
C
ATOM 8639 HB3 ALA C 118 -13.015 32.413 38.808 0.00 0.00
C

```
ATOM   8640  C   ALA C 118      -14.956  35.175  38.929  0.00  0.00
C
ATOM   8641  O   ALA C 118      -15.057  35.929  39.933  0.00  0.00
C
ATOM   8642  N   ASP C 119      -15.968  35.050  38.061  0.00  0.00
C
ATOM   8643  HN  ASP C 119      -16.010  34.291  37.416  0.00  0.00
C
ATOM   8644  CA  ASP C 119      -17.008  35.987  37.901  0.00  0.00
C
ATOM   8645  HA  ASP C 119      -16.739  37.026  38.026  0.00  0.00
C
ATOM   8646  CB  ASP C 119      -17.423  36.101  36.326  0.00  0.00
C
ATOM   8647  HB1 ASP C 119      -16.488  36.362  35.786  0.00  0.00
C
ATOM   8648  HB2 ASP C 119      -17.752  35.168  35.819  0.00  0.00
C
ATOM   8649  CG  ASP C 119      -18.411  37.227  36.102  0.00  0.00
C
ATOM   8650  OD1 ASP C 119      -18.059  38.395  36.149  0.00  0.00
C
ATOM   8651  OD2 ASP C 119      -19.527  36.838  35.820  0.00  0.00
C
ATOM   8652  C   ASP C 119      -18.123  35.535  38.804  0.00  0.00
C
ATOM   8653  O   ASP C 119      -18.681  36.399  39.435  0.00  0.00
C
ATOM   8654  N   ARG C 120      -18.371  34.204  38.901  0.00  0.00
C
ATOM   8655  HN  ARG C 120      -17.903  33.696  38.182  0.00  0.00
C
ATOM   8656  CA  ARG C 120      -19.340  33.577  39.763  0.00  0.00
C
ATOM   8657  HA  ARG C 120      -19.592  34.298  40.526  0.00  0.00
C
ATOM   8658  CB  ARG C 120      -20.607  32.998  39.071  0.00  0.00
C
ATOM   8659  HB1 ARG C 120      -20.306  32.464  38.144  0.00  0.00
C
ATOM   8660  HB2 ARG C 120      -21.194  32.383  39.786  0.00  0.00
C
ATOM   8661  CG  ARG C 120      -21.685  34.052  38.707  0.00  0.00
C
ATOM   8662  HG1 ARG C 120      -22.608  33.578  38.308  0.00  0.00
C
ATOM   8663  HG2 ARG C 120      -22.132  34.563  39.586  0.00  0.00
C
ATOM   8664  CD  ARG C 120      -21.281  35.198  37.750  0.00  0.00
C
ATOM   8665  HD1 ARG C 120      -20.694  35.894  38.385  0.00  0.00
C
ATOM   8666  HD2 ARG C 120      -20.614  34.891  36.916  0.00  0.00
C
```

```
ATOM   8667  NE  ARG C 120     -22.510  35.885  37.332  0.00  0.00
C
ATOM   8668  HE  ARG C 120     -23.389  35.424  37.453  0.00  0.00
C
ATOM   8669  CZ  ARG C 120     -22.582  37.189  37.360  0.00  0.00
C
ATOM   8670  NH1 ARG C 120     -21.488  37.966  37.238  0.00  0.00
C
ATOM   8671 HH11 ARG C 120     -21.509  38.880  36.834  0.00  0.00
C
ATOM   8672 HH12 ARG C 120     -20.652  37.507  36.937  0.00  0.00
C
ATOM   8673  NH2 ARG C 120     -23.814  37.714  37.192  0.00  0.00
C
ATOM   8674 HH21 ARG C 120     -23.910  38.666  36.900  0.00  0.00
C
ATOM   8675 HH22 ARG C 120     -24.516  37.015  37.325  0.00  0.00
C
ATOM   8676  C   ARG C 120     -18.720  32.323  40.376  0.00  0.00
C
ATOM   8677  O   ARG C 120     -18.286  31.462  39.604  0.00  0.00
C
ATOM   8678  N   ILE C 121     -18.780  32.206  41.704  0.00  0.00
C
ATOM   8679  HN  ILE C 121     -19.255  32.883  42.261  0.00  0.00
C
ATOM   8680  CA  ILE C 121     -18.237  31.077  42.466  0.00  0.00
C
ATOM   8681  HA  ILE C 121     -17.780  30.394  41.765  0.00  0.00
C
ATOM   8682  CB  ILE C 121     -17.056  31.412  43.416  0.00  0.00
C
ATOM   8683  HB  ILE C 121     -17.476  31.830  44.356  0.00  0.00
C
ATOM   8684  CG2 ILE C 121     -16.415  30.075  43.899  0.00  0.00
C
ATOM   8685 HG21 ILE C 121     -15.891  29.510  43.100  0.00  0.00
C
ATOM   8686 HG22 ILE C 121     -15.749  30.246  44.772  0.00  0.00
C
ATOM   8687 HG23 ILE C 121     -17.233  29.424  44.276  0.00  0.00
C
ATOM   8688  CG1 ILE C 121     -16.003  32.320  42.814  0.00  0.00
C
ATOM   8689 HG11 ILE C 121     -15.353  31.873  42.033  0.00  0.00
C
ATOM   8690 HG12 ILE C 121     -16.490  33.230  42.401  0.00  0.00
C
ATOM   8691  CD  ILE C 121     -14.840  32.751  43.737  0.00  0.00
C
ATOM   8692  HD1 ILE C 121     -14.211  31.848  43.891  0.00  0.00
C
ATOM   8693  HD2 ILE C 121     -14.299  33.567  43.211  0.00  0.00
C
```

ATOM 8694 HD3 ILE C 121 −15.307 33.112 44.678 0.00 0.00
C
ATOM 8695 C ILE C 121 −19.386 30.283 43.078 0.00 0.00
C
ATOM 8696 O ILE C 121 −20.168 30.738 43.914 0.00 0.00
C
ATOM 8697 N ILE C 122 −19.549 28.997 42.630 0.00 0.00
C
ATOM 8698 HN ILE C 122 −18.834 28.543 42.103 0.00 0.00
C
ATOM 8699 CA ILE C 122 −20.811 28.281 42.860 0.00 0.00
C
ATOM 8700 HA ILE C 122 −21.446 28.914 43.462 0.00 0.00
C
ATOM 8701 CB ILE C 122 −21.626 28.091 41.561 0.00 0.00
C
ATOM 8702 HB ILE C 122 −22.565 27.515 41.708 0.00 0.00
C
ATOM 8703 CG2 ILE C 122 −22.218 29.470 41.181 0.00 0.00
C
ATOM 8704 HG21 ILE C 122 −22.957 29.500 40.352 0.00 0.00
C
ATOM 8705 HG22 ILE C 122 −22.730 29.940 42.047 0.00 0.00
C
ATOM 8706 HG23 ILE C 122 −21.364 30.122 40.896 0.00 0.00
C
ATOM 8707 CG1 ILE C 122 −20.871 27.457 40.463 0.00 0.00
C
ATOM 8708 HG11 ILE C 122 −20.094 28.074 39.964 0.00 0.00
C
ATOM 8709 HG12 ILE C 122 −20.194 26.712 40.934 0.00 0.00
C
ATOM 8710 CD ILE C 122 −21.688 26.898 39.283 0.00 0.00
C
ATOM 8711 HD1 ILE C 122 −21.990 25.876 39.597 0.00 0.00
C
ATOM 8712 HD2 ILE C 122 −22.636 27.315 38.881 0.00 0.00
C
ATOM 8713 HD3 ILE C 122 −21.034 26.874 38.385 0.00 0.00
C
ATOM 8714 C ILE C 122 −20.643 27.055 43.720 0.00 0.00
C
ATOM 8715 O ILE C 122 −19.590 26.376 43.563 0.00 0.00
C
ATOM 8716 N HSE C 123 −21.604 26.710 44.563 0.00 0.00
C
ATOM 8717 HN HSE C 123 −22.294 27.399 44.773 0.00 0.00
C
ATOM 8718 CA HSE C 123 −21.475 25.584 45.456 0.00 0.00
C
ATOM 8719 HA HSE C 123 −20.765 24.815 45.192 0.00 0.00
C
ATOM 8720 CB HSE C 123 −21.081 25.919 46.896 0.00 0.00
C

ATOM 8721 HB1 HSE C 123 −21.881 26.470 47.435 0.00 0.00
C
ATOM 8722 HB2 HSE C 123 −20.952 24.934 47.395 0.00 0.00
C
ATOM 8723 ND1 HSE C 123 −18.991 26.436 48.029 0.00 0.00
C
ATOM 8724 CG HSE C 123 −19.901 26.870 47.177 0.00 0.00
C
ATOM 8725 CE1 HSE C 123 −18.001 27.327 47.972 0.00 0.00
C
ATOM 8726 HE1 HSE C 123 −17.078 27.302 48.552 0.00 0.00
C
ATOM 8727 NE2 HSE C 123 −18.297 28.299 47.105 0.00 0.00
C
ATOM 8728 HE2 HSE C 123 −17.692 29.057 46.862 0.00 0.00
C
ATOM 8729 CD2 HSE C 123 −19.493 27.999 46.535 0.00 0.00
C
ATOM 8730 HD2 HSE C 123 −20.090 28.622 45.879 0.00 0.00
C
ATOM 8731 C HSE C 123 −22.891 24.877 45.662 0.00 0.00
C
ATOM 8732 O HSE C 123 −23.504 24.947 46.758 0.00 0.00
C
ATOM 8733 N PRO C 124 −23.406 24.212 44.631 0.00 0.00
C
ATOM 8734 CD PRO C 124 −22.691 23.837 43.368 0.00 0.00
C
ATOM 8735 HD1 PRO C 124 −22.640 24.680 42.646 0.00 0.00
C
ATOM 8736 HD2 PRO C 124 −21.690 23.425 43.618 0.00 0.00
C
ATOM 8737 CA PRO C 124 −24.840 23.940 44.480 0.00 0.00
C
ATOM 8738 HA PRO C 124 −25.416 24.852 44.512 0.00 0.00
C
ATOM 8739 CB PRO C 124 −25.071 23.264 43.133 0.00 0.00
C
ATOM 8740 HB1 PRO C 124 −25.543 23.985 42.432 0.00 0.00
C
ATOM 8741 HB2 PRO C 124 −25.735 22.376 43.206 0.00 0.00
C
ATOM 8742 CG PRO C 124 −23.647 22.819 42.717 0.00 0.00
C
ATOM 8743 HG1 PRO C 124 −23.478 22.724 41.623 0.00 0.00
C
ATOM 8744 HG2 PRO C 124 −23.425 21.794 43.082 0.00 0.00
C
ATOM 8745 C PRO C 124 −25.408 23.112 45.589 0.00 0.00
C
ATOM 8746 O PRO C 124 −26.529 23.306 45.921 0.00 0.00
C
ATOM 8747 N GLU C 125 −24.682 22.087 45.993 0.00 0.00
C

ATOM 8748 HN GLU C 125 −23.890 21.816 45.450 0.00 0.00
C
ATOM 8749 CA GLU C 125 −25.229 21.161 47.009 0.00 0.00
C
ATOM 8750 HA GLU C 125 −26.179 20.813 46.632 0.00 0.00
C
ATOM 8751 CB GLU C 125 −24.287 19.965 47.298 0.00 0.00
C
ATOM 8752 HB1 GLU C 125 −23.244 20.335 47.396 0.00 0.00
C
ATOM 8753 HB2 GLU C 125 −24.478 19.462 48.270 0.00 0.00
C
ATOM 8754 CG GLU C 125 −24.425 18.869 46.258 0.00 0.00
C
ATOM 8755 HG1 GLU C 125 −25.417 18.391 46.108 0.00 0.00
C
ATOM 8756 HG2 GLU C 125 −24.059 19.292 45.298 0.00 0.00
C
ATOM 8757 CD GLU C 125 −23.390 17.765 46.649 0.00 0.00
C
ATOM 8758 OE1 GLU C 125 −22.457 17.560 45.817 0.00 0.00
C
ATOM 8759 OE2 GLU C 125 −23.489 17.099 47.703 0.00 0.00
C
ATOM 8760 C GLU C 125 −25.576 21.821 48.287 0.00 0.00
C
ATOM 8761 O GLU C 125 −26.655 21.762 48.935 0.00 0.00
C
ATOM 8762 N LYS C 126 −24.581 22.596 48.782 0.00 0.00
C
ATOM 8763 HN LYS C 126 −23.675 22.649 48.371 0.00 0.00
C
ATOM 8764 CA LYS C 126 −24.712 23.437 49.907 0.00 0.00
C
ATOM 8765 HA LYS C 126 −25.039 22.792 50.709 0.00 0.00
C
ATOM 8766 CB LYS C 126 −23.308 24.058 50.143 0.00 0.00
C
ATOM 8767 HB1 LYS C 126 −22.568 23.234 50.230 0.00 0.00
C
ATOM 8768 HB2 LYS C 126 −22.988 24.744 49.330 0.00 0.00
C
ATOM 8769 CG LYS C 126 −23.262 24.870 51.451 0.00 0.00
C
ATOM 8770 HG1 LYS C 126 −23.943 25.747 51.487 0.00 0.00
C
ATOM 8771 HG2 LYS C 126 −23.602 24.227 52.291 0.00 0.00
C
ATOM 8772 CD LYS C 126 −21.894 25.437 51.755 0.00 0.00
C
ATOM 8773 HD1 LYS C 126 −21.694 26.038 50.842 0.00 0.00
C
ATOM 8774 HD2 LYS C 126 −21.888 26.077 52.664 0.00 0.00
C

ATOM 8775 CE LYS C 126 −20.912 24.338 52.011 0.00 0.00
C
ATOM 8776 HE1 LYS C 126 −21.438 23.574 52.623 0.00 0.00
C
ATOM 8777 HE2 LYS C 126 −20.503 24.064 51.015 0.00 0.00
C
ATOM 8778 NZ LYS C 126 −19.818 24.749 52.851 0.00 0.00
C
ATOM 8779 HZ1 LYS C 126 −18.986 24.125 52.837 0.00 0.00
C
ATOM 8780 HZ2 LYS C 126 −19.392 25.623 52.484 0.00 0.00
C
ATOM 8781 HZ3 LYS C 126 −20.060 24.948 53.843 0.00 0.00
C
ATOM 8782 C LYS C 126 −25.709 24.635 49.763 0.00 0.00
C
ATOM 8783 O LYS C 126 −26.312 24.961 50.709 0.00 0.00
C
ATOM 8784 N ASP C 127 −25.788 25.332 48.606 0.00 0.00
C
ATOM 8785 HN ASP C 127 −25.200 25.110 47.832 0.00 0.00
C
ATOM 8786 CA ASP C 127 −26.822 26.262 48.320 0.00 0.00
C
ATOM 8787 HA ASP C 127 −26.811 27.109 48.989 0.00 0.00
C
ATOM 8788 CB ASP C 127 −26.623 26.918 46.920 0.00 0.00
C
ATOM 8789 HB1 ASP C 127 −26.239 26.146 46.220 0.00 0.00
C
ATOM 8790 HB2 ASP C 127 −27.562 27.244 46.423 0.00 0.00
C
ATOM 8791 CG ASP C 127 −25.672 28.106 46.983 0.00 0.00
C
ATOM 8792 OD1 ASP C 127 −24.932 28.269 47.957 0.00 0.00
C
ATOM 8793 OD2 ASP C 127 −25.610 28.840 45.948 0.00 0.00
C
ATOM 8794 C ASP C 127 −28.299 25.723 48.396 0.00 0.00
C
ATOM 8795 O ASP C 127 −29.099 26.301 49.132 0.00 0.00
C
ATOM 8796 N MET C 128 −28.555 24.526 47.780 0.00 0.00
C
ATOM 8797 HN MET C 128 −27.865 24.044 47.246 0.00 0.00
C
ATOM 8798 CA MET C 128 −29.808 23.881 47.839 0.00 0.00
C
ATOM 8799 HA MET C 128 −30.503 24.693 47.682 0.00 0.00
C
ATOM 8800 CB MET C 128 −29.894 22.744 46.810 0.00 0.00
C
ATOM 8801 HB1 MET C 128 −29.660 23.149 45.802 0.00 0.00
C

ATOM 8802 HB2 MET C 128 -29.044 22.072 47.053 0.00 0.00
C
ATOM 8803 CG MET C 128 -31.377 22.245 46.607 0.00 0.00
C
ATOM 8804 HG1 MET C 128 -31.336 21.262 46.091 0.00 0.00
C
ATOM 8805 HG2 MET C 128 -31.792 22.151 47.633 0.00 0.00
C
ATOM 8806 SD MET C 128 -32.350 23.472 45.743 0.00 0.00
C
ATOM 8807 CE MET C 128 -33.750 22.352 45.189 0.00 0.00
C
ATOM 8808 HE1 MET C 128 -34.713 22.906 45.208 0.00 0.00
C
ATOM 8809 HE2 MET C 128 -33.602 21.822 44.225 0.00 0.00
C
ATOM 8810 HE3 MET C 128 -33.899 21.540 45.933 0.00 0.00
C
ATOM 8811 C MET C 128 -30.104 23.323 49.247 0.00 0.00
C
ATOM 8812 O MET C 128 -31.294 23.147 49.601 0.00 0.00
C
ATOM 8813 N GLY C 129 -29.151 22.919 50.120 0.00 0.00
C
ATOM 8814 HN GLY C 129 -28.189 22.771 49.904 0.00 0.00
C
ATOM 8815 CA GLY C 129 -29.462 22.666 51.569 0.00 0.00
C
ATOM 8816 HA1 GLY C 129 -28.556 22.661 52.158 0.00 0.00
C
ATOM 8817 HA2 GLY C 129 -30.034 21.757 51.681 0.00 0.00
C
ATOM 8818 C GLY C 129 -30.373 23.711 52.199 0.00 0.00
C
ATOM 8819 O GLY C 129 -31.264 23.432 52.954 0.00 0.00
C
ATOM 8820 N VAL C 130 -30.115 25.086 51.998 0.00 0.00
C
ATOM 8821 HN VAL C 130 -29.343 25.357 51.429 0.00 0.00
C
ATOM 8822 CA VAL C 130 -30.931 26.181 52.542 0.00 0.00
C
ATOM 8823 HA VAL C 130 -30.969 26.190 53.621 0.00 0.00
C
ATOM 8824 CB VAL C 130 -30.232 27.512 52.333 0.00 0.00
C
ATOM 8825 HB VAL C 130 -30.354 27.823 51.274 0.00 0.00
C
ATOM 8826 CG1 VAL C 130 -30.748 28.664 53.229 0.00 0.00
C
ATOM 8827 HG11 VAL C 130 -30.485 28.534 54.300 0.00 0.00
C
ATOM 8828 HG12 VAL C 130 -30.201 29.596 52.969 0.00 0.00
C

ATOM 8829 HG13 VAL C 130 −31.835 28.883 53.158 0.00 0.00
C
ATOM 8830 CG2 VAL C 130 −28.658 27.280 52.492 0.00 0.00
C
ATOM 8831 HG21 VAL C 130 −28.089 28.204 52.733 0.00 0.00
C
ATOM 8832 HG22 VAL C 130 −28.345 26.614 53.324 0.00 0.00
C
ATOM 8833 HG23 VAL C 130 −28.139 26.971 51.559 0.00 0.00
C
ATOM 8834 C VAL C 130 −32.386 26.263 52.058 0.00 0.00
C
ATOM 8835 O VAL C 130 −33.318 26.604 52.830 0.00 0.00
C
ATOM 8836 N LYS C 131 −32.615 26.022 50.811 0.00 0.00
C
ATOM 8837 HN LYS C 131 −31.951 25.842 50.089 0.00 0.00
C
ATOM 8838 CA LYS C 131 −33.993 25.945 50.386 0.00 0.00
C
ATOM 8839 HA LYS C 131 −34.419 26.834 50.827 0.00 0.00
C
ATOM 8840 CB LYS C 131 −34.153 25.935 48.825 0.00 0.00
C
ATOM 8841 HB1 LYS C 131 −33.464 26.689 48.387 0.00 0.00
C
ATOM 8842 HB2 LYS C 131 −33.967 24.918 48.418 0.00 0.00
C
ATOM 8843 CG LYS C 131 −35.611 26.107 48.419 0.00 0.00
C
ATOM 8844 HG1 LYS C 131 −35.772 25.789 47.367 0.00 0.00
C
ATOM 8845 HG2 LYS C 131 −36.337 25.597 49.087 0.00 0.00
C
ATOM 8846 CD LYS C 131 −35.960 27.593 48.621 0.00 0.00
C
ATOM 8847 HD1 LYS C 131 −36.015 27.769 49.716 0.00 0.00
C
ATOM 8848 HD2 LYS C 131 −35.128 28.249 48.287 0.00 0.00
C
ATOM 8849 CE LYS C 131 −37.226 28.203 48.021 0.00 0.00
C
ATOM 8850 HE1 LYS C 131 −37.338 29.075 48.701 0.00 0.00
C
ATOM 8851 HE2 LYS C 131 −37.063 28.509 46.966 0.00 0.00
C
ATOM 8852 NZ LYS C 131 −38.358 27.284 48.140 0.00 0.00
C
ATOM 8853 HZ1 LYS C 131 −38.820 27.150 49.062 0.00 0.00
C
ATOM 8854 HZ2 LYS C 131 −39.131 27.591 47.516 0.00 0.00
C
ATOM 8855 HZ3 LYS C 131 −38.084 26.330 47.831 0.00 0.00
C

ATOM 8856 C LYS C 131 −34.732 24.697 50.933 0.00 0.00
C
ATOM 8857 O LYS C 131 −35.846 24.933 51.415 0.00 0.00
C
ATOM 8858 N ILE C 132 −34.184 23.458 50.883 0.00 0.00
C
ATOM 8859 HN ILE C 132 −33.319 23.210 50.453 0.00 0.00
C
ATOM 8860 CA ILE C 132 −34.796 22.330 51.608 0.00 0.00
C
ATOM 8861 HA ILE C 132 −35.756 22.150 51.148 0.00 0.00
C
ATOM 8862 CB ILE C 132 −33.854 21.165 51.373 0.00 0.00
C
ATOM 8863 HB ILE C 132 −32.846 21.552 51.636 0.00 0.00
C
ATOM 8864 CG2 ILE C 132 −34.213 20.040 52.248 0.00 0.00
C
ATOM 8865 HG21 ILE C 132 −35.286 19.752 52.243 0.00 0.00
C
ATOM 8866 HG22 ILE C 132 −33.639 19.118 52.014 0.00 0.00
C
ATOM 8867 HG23 ILE C 132 −33.972 20.169 53.325 0.00 0.00
C
ATOM 8868 CG1 ILE C 132 −33.849 20.807 49.865 0.00 0.00
C
ATOM 8869 HG11 ILE C 132 −33.579 21.588 49.122 0.00 0.00
C
ATOM 8870 HG12 ILE C 132 −33.142 19.953 49.802 0.00 0.00
C
ATOM 8871 CD ILE C 132 −35.199 20.274 49.243 0.00 0.00
C
ATOM 8872 HD1 ILE C 132 −36.023 20.956 49.545 0.00 0.00
C
ATOM 8873 HD2 ILE C 132 −35.096 20.335 48.139 0.00 0.00
C
ATOM 8874 HD3 ILE C 132 −35.457 19.268 49.636 0.00 0.00
C
ATOM 8875 C ILE C 132 −35.066 22.580 53.161 0.00 0.00
C
ATOM 8876 O ILE C 132 −36.113 22.171 53.615 0.00 0.00
C
ATOM 8877 N ALA C 133 −34.199 23.392 53.822 0.00 0.00
C
ATOM 8878 HN ALA C 133 −33.345 23.601 53.351 0.00 0.00
C
ATOM 8879 CA ALA C 133 −34.328 23.857 55.186 0.00 0.00
C
ATOM 8880 HA ALA C 133 −34.534 23.069 55.894 0.00 0.00
C
ATOM 8881 CB ALA C 133 −32.982 24.385 55.660 0.00 0.00
C
ATOM 8882 HB1 ALA C 133 −32.895 24.566 56.752 0.00 0.00
C

ATOM 8883 HB2 ALA C 133 −32.183 23.712 55.282 0.00 0.00
C
ATOM 8884 HB3 ALA C 133 −32.716 25.425 55.374 0.00 0.00
C
ATOM 8885 C ALA C 133 −35.457 24.758 55.415 0.00 0.00
C
ATOM 8886 O ALA C 133 −36.268 24.612 56.407 0.00 0.00
C
ATOM 8887 N GLN C 134 −35.692 25.797 54.521 0.00 0.00
C
ATOM 8888 HN GLN C 134 −35.096 25.974 53.742 0.00 0.00
C
ATOM 8889 CA GLN C 134 −36.897 26.628 54.516 0.00 0.00
C
ATOM 8890 HA GLN C 134 −37.074 27.103 55.469 0.00 0.00
C
ATOM 8891 CB GLN C 134 −36.839 27.762 53.552 0.00 0.00
C
ATOM 8892 HB1 GLN C 134 −36.557 27.316 52.574 0.00 0.00
C
ATOM 8893 HB2 GLN C 134 −37.821 28.212 53.290 0.00 0.00
C
ATOM 8894 CG GLN C 134 −35.854 28.898 53.941 0.00 0.00
C
ATOM 8895 HG1 GLN C 134 −36.352 29.567 54.675 0.00 0.00
C
ATOM 8896 HG2 GLN C 134 −34.963 28.400 54.379 0.00 0.00
C
ATOM 8897 CD GLN C 134 −35.647 29.825 52.707 0.00 0.00
C
ATOM 8898 OE1 GLN C 134 −36.249 30.889 52.536 0.00 0.00
C
ATOM 8899 NE2 GLN C 134 −34.612 29.462 51.962 0.00 0.00
C
ATOM 8900 HE21 GLN C 134 −34.311 30.037 51.201 0.00 0.00
C
ATOM 8901 HE22 GLN C 134 −33.929 28.832 52.332 0.00 0.00
C
ATOM 8902 C GLN C 134 −38.210 25.865 54.250 0.00 0.00
C
ATOM 8903 O GLN C 134 −39.252 26.053 54.867 0.00 0.00
C
ATOM 8904 N SER C 135 −38.229 24.914 53.258 0.00 0.00
C
ATOM 8905 HN SER C 135 −37.390 24.763 52.742 0.00 0.00
C
ATOM 8906 CA SER C 135 −39.235 23.925 53.011 0.00 0.00
C
ATOM 8907 HA SER C 135 −40.193 24.374 52.799 0.00 0.00
C
ATOM 8908 CB SER C 135 −38.858 22.999 51.769 0.00 0.00
C
ATOM 8909 HB1 SER C 135 −37.828 22.701 52.059 0.00 0.00
C

ATOM 8910 HB2 SER C 135 −39.535 22.173 51.460 0.00 0.00
C
ATOM 8911 OG SER C 135 −38.604 23.812 50.620 0.00 0.00
C
ATOM 8912 HG1 SER C 135 −38.640 23.287 49.816 0.00 0.00
C
ATOM 8913 C SER C 135 −39.558 22.976 54.208 0.00 0.00
C
ATOM 8914 O SER C 135 −40.727 22.540 54.387 0.00 0.00
C
ATOM 8915 N LEU C 136 −38.520 22.596 55.058 0.00 0.00
C
ATOM 8916 HN LEU C 136 −37.575 22.762 54.786 0.00 0.00
C
ATOM 8917 CA LEU C 136 −38.790 21.867 56.286 0.00 0.00
C
ATOM 8918 HA LEU C 136 −39.442 21.086 55.922 0.00 0.00
C
ATOM 8919 CB LEU C 136 −37.434 21.299 56.903 0.00 0.00
C
ATOM 8920 HB1 LEU C 136 −36.625 22.053 56.801 0.00 0.00
C
ATOM 8921 HB2 LEU C 136 −37.527 21.120 57.995 0.00 0.00
C
ATOM 8922 CG LEU C 136 −36.925 20.006 56.277 0.00 0.00
C
ATOM 8923 HG LEU C 136 −37.177 20.095 55.199 0.00 0.00
C
ATOM 8924 CD1 LEU C 136 −35.479 19.765 56.556 0.00 0.00
C
ATOM 8925 HD11 LEU C 136 −34.801 20.619 56.347 0.00 0.00
C
ATOM 8926 HD12 LEU C 136 −35.261 19.594 57.632 0.00 0.00
C
ATOM 8927 HD13 LEU C 136 −34.951 19.007 55.939 0.00 0.00
C
ATOM 8928 CD2 LEU C 136 −37.813 18.840 56.838 0.00 0.00
C
ATOM 8929 HD21 LEU C 136 −37.518 18.476 57.845 0.00 0.00
C
ATOM 8930 HD22 LEU C 136 −38.907 19.034 56.802 0.00 0.00
C
ATOM 8931 HD23 LEU C 136 −37.615 17.937 56.222 0.00 0.00
C
ATOM 8932 C LEU C 136 −39.520 22.769 57.280 0.00 0.00
C
ATOM 8933 O LEU C 136 −40.434 22.340 57.973 0.00 0.00
C
ATOM 8934 N SER C 137 −38.966 23.975 57.416 0.00 0.00
C
ATOM 8935 HN SER C 137 −38.219 24.315 56.849 0.00 0.00
C
ATOM 8936 CA SER C 137 −39.557 24.914 58.347 0.00 0.00
C

ATOM 8937 HA SER C 137 −39.439 24.503 59.339 0.00 0.00
C
ATOM 8938 CB SER C 137 −38.740 26.249 58.159 0.00 0.00
C
ATOM 8939 HB1 SER C 137 −37.699 25.869 58.238 0.00 0.00
C
ATOM 8940 HB2 SER C 137 −38.855 26.792 57.197 0.00 0.00
C
ATOM 8941 OG SER C 137 −38.865 27.215 59.247 0.00 0.00
C
ATOM 8942 HG1 SER C 137 −39.776 27.513 59.289 0.00 0.00
C
ATOM 8943 C SER C 137 −40.997 25.402 58.229 0.00 0.00
C
ATOM 8944 O SER C 137 −41.779 25.475 59.192 0.00 0.00
C
ATOM 8945 N ASP C 138 −41.445 25.619 56.952 0.00 0.00
C
ATOM 8946 HN ASP C 138 −40.786 25.560 56.206 0.00 0.00
C
ATOM 8947 CA ASP C 138 −42.758 26.145 56.548 0.00 0.00
C
ATOM 8948 HA ASP C 138 −43.439 25.925 57.358 0.00 0.00
C
ATOM 8949 CB ASP C 138 −42.672 27.582 56.119 0.00 0.00
C
ATOM 8950 HB1 ASP C 138 −41.866 27.803 55.388 0.00 0.00
C
ATOM 8951 HB2 ASP C 138 −43.650 27.907 55.704 0.00 0.00
C
ATOM 8952 CG ASP C 138 −42.375 28.477 57.336 0.00 0.00
C
ATOM 8953 OD1 ASP C 138 −43.217 28.405 58.293 0.00 0.00
C
ATOM 8954 OD2 ASP C 138 −41.430 29.287 57.364 0.00 0.00
C
ATOM 8955 C ASP C 138 −43.220 25.161 55.421 0.00 0.00
C
ATOM 8956 O ASP C 138 −42.792 25.280 54.219 0.00 0.00
C
ATOM 8957 N GLU C 139 −44.134 24.242 55.749 0.00 0.00
C
ATOM 8958 HN GLU C 139 −44.467 24.098 56.678 0.00 0.00
C
ATOM 8959 CA GLU C 139 −44.728 23.192 54.888 0.00 0.00
C
ATOM 8960 HA GLU C 139 −43.980 22.721 54.268 0.00 0.00
C
ATOM 8961 CB GLU C 139 −45.249 22.042 55.811 0.00 0.00
C
ATOM 8962 HB1 GLU C 139 −46.114 22.375 56.425 0.00 0.00
C
ATOM 8963 HB2 GLU C 139 −45.540 21.191 55.159 0.00 0.00
C

ATOM 8964 CG GLU C 139 -44.132 21.562 56.869 0.00 0.00
C
ATOM 8965 HG1 GLU C 139 -43.265 21.557 56.174 0.00 0.00
C
ATOM 8966 HG2 GLU C 139 -43.871 22.315 57.643 0.00 0.00
C
ATOM 8967 CD GLU C 139 -44.375 20.293 57.537 0.00 0.00
C
ATOM 8968 OE1 GLU C 139 -43.554 19.963 58.414 0.00 0.00
C
ATOM 8969 OE2 GLU C 139 -45.411 19.622 57.254 0.00 0.00
C
ATOM 8970 C GLU C 139 -45.831 23.639 53.919 0.00 0.00
C
ATOM 8971 O GLU C 139 -46.096 23.159 52.801 0.00 0.00
C
ATOM 8972 N ASN C 140 -46.582 24.731 54.325 0.00 0.00
C
ATOM 8973 HN ASN C 140 -46.640 24.946 55.297 0.00 0.00
C
ATOM 8974 CA ASN C 140 -47.583 25.421 53.503 0.00 0.00
C
ATOM 8975 HA ASN C 140 -47.845 24.734 52.712 0.00 0.00
C
ATOM 8976 CB ASN C 140 -48.835 25.950 54.302 0.00 0.00
C
ATOM 8977 HB1 ASN C 140 -48.524 26.697 55.063 0.00 0.00
C
ATOM 8978 HB2 ASN C 140 -49.628 26.420 53.682 0.00 0.00
C
ATOM 8979 CG ASN C 140 -49.553 24.877 54.990 0.00 0.00
C
ATOM 8980 OD1 ASN C 140 -49.786 24.895 56.196 0.00 0.00
C
ATOM 8981 ND2 ASN C 140 -49.928 23.804 54.287 0.00 0.00
C
ATOM 8982 HD21 ASN C 140 -50.718 23.297 54.633 0.00 0.00
C
ATOM 8983 HD22 ASN C 140 -49.647 23.755 53.329 0.00 0.00
C
ATOM 8984 C ASN C 140 -46.937 26.714 52.891 0.00 0.00
C
ATOM 8985 O ASN C 140 -47.707 27.561 52.393 0.00 0.00
C
ATOM 8986 N VAL C 141 -45.603 26.958 52.888 0.00 0.00
C
ATOM 8987 HN VAL C 141 -44.965 26.212 53.064 0.00 0.00
C
ATOM 8988 CA VAL C 141 -45.032 28.104 52.197 0.00 0.00
C
ATOM 8989 HA VAL C 141 -45.811 28.587 51.627 0.00 0.00
C
ATOM 8990 CB VAL C 141 -44.373 29.177 53.087 0.00 0.00
C

ATOM 8991 HB VAL C 141 −43.516 28.642 53.548 0.00 0.00
C
ATOM 8992 CG1 VAL C 141 −43.872 30.300 52.200 0.00 0.00
C
ATOM 8993 HG11 VAL C 141 −44.755 30.654 51.627 0.00 0.00
C
ATOM 8994 HG12 VAL C 141 −43.536 31.152 52.829 0.00 0.00
C
ATOM 8995 HG13 VAL C 141 −42.898 30.024 51.742 0.00 0.00
C
ATOM 8996 CG2 VAL C 141 −45.398 29.614 54.101 0.00 0.00
C
ATOM 8997 HG21 VAL C 141 −46.224 30.054 53.502 0.00 0.00
C
ATOM 8998 HG22 VAL C 141 −45.812 28.780 54.707 0.00 0.00
C
ATOM 8999 HG23 VAL C 141 −45.039 30.372 54.829 0.00 0.00
C
ATOM 9000 C VAL C 141 −44.043 27.605 51.201 0.00 0.00
C
ATOM 9001 O VAL C 141 −43.200 26.890 51.648 0.00 0.00
C
ATOM 9002 N LEU C 142 −44.127 27.860 49.873 0.00 0.00
C
ATOM 9003 HN LEU C 142 −44.880 28.440 49.572 0.00 0.00
C
ATOM 9004 CA LEU C 142 −43.296 27.109 48.931 0.00 0.00
C
ATOM 9005 HA LEU C 142 −42.906 26.187 49.337 0.00 0.00
C
ATOM 9006 CB LEU C 142 −44.189 26.617 47.815 0.00 0.00
C
ATOM 9007 HB1 LEU C 142 −44.509 27.511 47.238 0.00 0.00
C
ATOM 9008 HB2 LEU C 142 −43.538 26.034 47.129 0.00 0.00
C
ATOM 9009 CG LEU C 142 −45.398 25.662 48.178 0.00 0.00
C
ATOM 9010 HG LEU C 142 −46.036 26.043 49.004 0.00 0.00
C
ATOM 9011 CD1 LEU C 142 −46.339 25.373 46.985 0.00 0.00
C
ATOM 9012 HD11 LEU C 142 −47.050 24.596 47.339 0.00 0.00
C
ATOM 9013 HD12 LEU C 142 −46.897 26.277 46.659 0.00 0.00
C
ATOM 9014 HD13 LEU C 142 −45.646 24.920 46.244 0.00 0.00
C
ATOM 9015 CD2 LEU C 142 −44.769 24.336 48.571 0.00 0.00
C
ATOM 9016 HD21 LEU C 142 −43.977 24.464 49.340 0.00 0.00
C
ATOM 9017 HD22 LEU C 142 −45.536 23.625 48.945 0.00 0.00
C

ATOM 9018 HD23 LEU C 142 −44.305 23.883 47.669 0.00 0.00
C
ATOM 9019 C LEU C 142 −42.227 28.094 48.546 0.00 0.00
C
ATOM 9020 O LEU C 142 −41.090 27.684 48.548 0.00 0.00
C
ATOM 9021 N ASN C 143 −42.563 29.306 48.155 0.00 0.00
C
ATOM 9022 HN ASN C 143 −43.521 29.582 48.190 0.00 0.00
C
ATOM 9023 CA ASN C 143 −41.566 30.271 47.568 0.00 0.00
C
ATOM 9024 HA ASN C 143 −40.644 29.966 48.041 0.00 0.00
C
ATOM 9025 CB ASN C 143 −41.488 29.857 46.043 0.00 0.00
C
ATOM 9026 HB1 ASN C 143 −42.526 29.848 45.646 0.00 0.00
C
ATOM 9027 HB2 ASN C 143 −40.808 30.554 45.507 0.00 0.00
C
ATOM 9028 CG ASN C 143 −40.825 28.508 45.709 0.00 0.00
C
ATOM 9029 OD1 ASN C 143 −39.631 28.315 45.923 0.00 0.00
C
ATOM 9030 ND2 ASN C 143 −41.633 27.572 45.252 0.00 0.00
C
ATOM 9031 HD21 ASN C 143 −41.168 26.777 44.863 0.00 0.00
C
ATOM 9032 HD22 ASN C 143 −42.567 27.824 44.998 0.00 0.00
C
ATOM 9033 C ASN C 143 −41.868 31.760 47.841 0.00 0.00
C
ATOM 9034 O ASN C 143 −43.032 32.028 48.184 0.00 0.00
C
ATOM 9035 N TYR C 144 −40.834 32.656 47.750 0.00 0.00
C
ATOM 9036 HN TYR C 144 −39.887 32.485 47.491 0.00 0.00
C
ATOM 9037 CA TYR C 144 −41.068 34.051 48.088 0.00 0.00
C
ATOM 9038 HA TYR C 144 −41.888 34.404 47.480 0.00 0.00
C
ATOM 9039 CB TYR C 144 −41.544 34.292 49.522 0.00 0.00
C
ATOM 9040 HB1 TYR C 144 −41.610 35.367 49.792 0.00 0.00
C
ATOM 9041 HB2 TYR C 144 −42.588 33.917 49.585 0.00 0.00
C
ATOM 9042 CG TYR C 144 −40.764 33.562 50.640 0.00 0.00
C
ATOM 9043 CD1 TYR C 144 −41.263 32.463 51.290 0.00 0.00
C
ATOM 9044 HD1 TYR C 144 −42.174 32.009 50.928 0.00 0.00
C

```
ATOM   9045  CE1 TYR C 144     -40.531  31.868  52.365  0.00  0.00
C
ATOM   9046  HE1 TYR C 144     -40.922  31.005  52.883  0.00  0.00
C
ATOM   9047  CZ  TYR C 144     -39.262  32.402  52.671  0.00  0.00
C
ATOM   9048  OH  TYR C 144     -38.652  31.947  53.744  0.00  0.00
C
ATOM   9049  HH  TYR C 144     -39.080  31.120  53.977  0.00  0.00
C
ATOM   9050  CD2 TYR C 144     -39.501  34.122  51.040  0.00  0.00
C
ATOM   9051  HD2 TYR C 144     -39.124  34.963  50.477  0.00  0.00
C
ATOM   9052  CE2 TYR C 144     -38.678  33.500  51.937  0.00  0.00
C
ATOM   9053  HE2 TYR C 144     -37.782  34.037  52.212  0.00  0.00
C
ATOM   9054  C   TYR C 144     -39.990  34.946  47.763  0.00  0.00
C
ATOM   9055  O   TYR C 144     -38.777  34.681  47.831  0.00  0.00
C
ATOM   9056  N   ILE C 145     -40.451  36.092  47.199  0.00  0.00
C
ATOM   9057  HN  ILE C 145     -41.424  36.307  47.213  0.00  0.00
C
ATOM   9058  CA  ILE C 145     -39.533  37.065  46.655  0.00  0.00
C
ATOM   9059  HA  ILE C 145     -38.562  36.976  47.119  0.00  0.00
C
ATOM   9060  CB  ILE C 145     -39.570  37.213  45.127  0.00  0.00
C
ATOM   9061  HB  ILE C 145     -38.804  37.981  44.890  0.00  0.00
C
ATOM   9062  CG2 ILE C 145     -39.045  35.825  44.449  0.00  0.00
C
ATOM   9063 HG21 ILE C 145     -37.945  35.814  44.607  0.00  0.00
C
ATOM   9064 HG22 ILE C 145     -39.640  34.993  44.884  0.00  0.00
C
ATOM   9065 HG23 ILE C 145     -39.332  35.844  43.375  0.00  0.00
C
ATOM   9066  CG1 ILE C 145     -40.943  37.586  44.367  0.00  0.00
C
ATOM   9067 HG11 ILE C 145     -41.647  36.729  44.440  0.00  0.00
C
ATOM   9068 HG12 ILE C 145     -41.406  38.422  44.934  0.00  0.00
C
ATOM   9069  CD  ILE C 145     -40.889  37.791  42.821  0.00  0.00
C
ATOM   9070  HD1 ILE C 145     -40.326  38.722  42.596  0.00  0.00
C
ATOM   9071  HD2 ILE C 145     -40.299  36.934  42.431  0.00  0.00
C
```

ATOM 9072 HD3 ILE C 145 −41.886 37.884 42.341 0.00 0.00
C
ATOM 9073 C ILE C 145 −40.004 38.390 47.198 0.00 0.00
C
ATOM 9074 O ILE C 145 −41.136 38.527 47.680 0.00 0.00
C
ATOM 9075 N ASP C 146 −39.077 39.365 47.268 0.00 0.00
C
ATOM 9076 HN ASP C 146 −38.241 39.253 46.737 0.00 0.00
C
ATOM 9077 CA ASP C 146 −39.220 40.628 47.996 0.00 0.00
C
ATOM 9078 HA ASP C 146 −40.202 40.618 48.446 0.00 0.00
C
ATOM 9079 CB ASP C 146 −38.087 40.857 49.040 0.00 0.00
C
ATOM 9080 HB1 ASP C 146 −37.140 40.770 48.465 0.00 0.00
C
ATOM 9081 HB2 ASP C 146 −38.204 41.889 49.436 0.00 0.00
C
ATOM 9082 CG ASP C 146 −38.179 39.866 50.249 0.00 0.00
C
ATOM 9083 OD1 ASP C 146 −37.123 39.536 50.821 0.00 0.00
C
ATOM 9084 OD2 ASP C 146 −39.289 39.618 50.797 0.00 0.00
C
ATOM 9085 C ASP C 146 −39.361 41.769 47.012 0.00 0.00
C
ATOM 9086 O ASP C 146 −38.823 41.778 45.919 0.00 0.00
C
ATOM 9087 N LEU C 147 −40.163 42.750 47.426 0.00 0.00
C
ATOM 9088 HN LEU C 147 −40.484 42.665 48.366 0.00 0.00
C
ATOM 9089 CA LEU C 147 −40.479 43.918 46.560 0.00 0.00
C
ATOM 9090 HA LEU C 147 −40.232 43.811 45.514 0.00 0.00
C
ATOM 9091 CB LEU C 147 −42.027 44.081 46.602 0.00 0.00
C
ATOM 9092 HB1 LEU C 147 −42.327 44.130 47.671 0.00 0.00
C
ATOM 9093 HB2 LEU C 147 −42.246 45.097 46.209 0.00 0.00
C
ATOM 9094 CG LEU C 147 −42.824 42.960 45.847 0.00 0.00
C
ATOM 9095 HG LEU C 147 −42.458 42.055 46.377 0.00 0.00
C
ATOM 9096 CD1 LEU C 147 −44.265 43.186 46.220 0.00 0.00
C
ATOM 9097 HD11 LEU C 147 −44.984 42.564 45.645 0.00 0.00
C
ATOM 9098 HD12 LEU C 147 −44.447 42.912 47.281 0.00 0.00
C

ATOM 9099 HD13 LEU C 147 −44.609 44.235 46.098 0.00 0.00
C
ATOM 9100 CD2 LEU C 147 −42.544 42.905 44.370 0.00 0.00
C
ATOM 9101 HD21 LEU C 147 −42.538 43.969 44.050 0.00 0.00
C
ATOM 9102 HD22 LEU C 147 −41.643 42.284 44.179 0.00 0.00
C
ATOM 9103 HD23 LEU C 147 −43.408 42.481 43.814 0.00 0.00
C
ATOM 9104 C LEU C 147 −39.744 45.186 47.133 0.00 0.00
C
ATOM 9105 O LEU C 147 −39.312 46.055 46.404 0.00 0.00
C
ATOM 9106 N SER C 148 −39.489 45.238 48.513 0.00 0.00
C
ATOM 9107 HN SER C 148 −39.898 44.563 49.122 0.00 0.00
C
ATOM 9108 CA SER C 148 −38.600 46.182 49.165 0.00 0.00
C
ATOM 9109 HA SER C 148 −37.800 46.537 48.532 0.00 0.00
C
ATOM 9110 CB SER C 148 −39.459 47.405 49.526 0.00 0.00
C
ATOM 9111 HB1 SER C 148 −38.704 48.178 49.785 0.00 0.00
C
ATOM 9112 HB2 SER C 148 −39.982 47.711 48.595 0.00 0.00
C
ATOM 9113 OG SER C 148 −40.352 47.276 50.591 0.00 0.00
C
ATOM 9114 HG1 SER C 148 −40.655 48.181 50.692 0.00 0.00
C
ATOM 9115 C SER C 148 −38.056 45.576 50.428 0.00 0.00
C
ATOM 9116 O SER C 148 −37.701 44.416 50.454 0.00 0.00
C
ATOM 9117 N ASP C 149 −37.883 46.362 51.548 0.00 0.00
C
ATOM 9118 HN ASP C 149 −38.012 47.350 51.512 0.00 0.00
C
ATOM 9119 CA ASP C 149 −37.581 45.892 52.831 0.00 0.00
C
ATOM 9120 HA ASP C 149 −36.972 45.001 52.809 0.00 0.00
C
ATOM 9121 CB ASP C 149 −36.996 46.982 53.713 0.00 0.00
C
ATOM 9122 HB1 ASP C 149 −37.632 47.884 53.844 0.00 0.00
C
ATOM 9123 HB2 ASP C 149 −36.654 46.645 54.715 0.00 0.00
C
ATOM 9124 CG ASP C 149 −35.744 47.432 53.008 0.00 0.00
C
ATOM 9125 OD1 ASP C 149 −35.742 48.540 52.377 0.00 0.00
C

ATOM 9126 OD2 ASP C 149 −34.701 46.712 53.026 0.00 0.00
C
ATOM 9127 C ASP C 149 −38.854 45.516 53.578 0.00 0.00
C
ATOM 9128 O ASP C 149 −38.729 44.882 54.656 0.00 0.00
C
ATOM 9129 N GLU C 150 −40.043 45.773 53.107 0.00 0.00
C
ATOM 9130 HN GLU C 150 −40.143 46.181 52.202 0.00 0.00
C
ATOM 9131 CA GLU C 150 −41.217 45.732 53.918 0.00 0.00
C
ATOM 9132 HA GLU C 150 −41.030 45.188 54.832 0.00 0.00
C
ATOM 9133 CB GLU C 150 −41.658 47.101 54.473 0.00 0.00
C
ATOM 9134 HB1 GLU C 150 −41.482 47.889 53.709 0.00 0.00
C
ATOM 9135 HB2 GLU C 150 −42.719 47.052 54.798 0.00 0.00
C
ATOM 9136 CG GLU C 150 −40.714 47.477 55.662 0.00 0.00
C
ATOM 9137 HG1 GLU C 150 −40.663 46.797 56.540 0.00 0.00
C
ATOM 9138 HG2 GLU C 150 −39.698 47.570 55.224 0.00 0.00
C
ATOM 9139 CD GLU C 150 −41.038 48.840 56.235 0.00 0.00
C
ATOM 9140 OE1 GLU C 150 −40.143 49.756 56.400 0.00 0.00
C
ATOM 9141 OE2 GLU C 150 −42.224 49.029 56.615 0.00 0.00
C
ATOM 9142 C GLU C 150 −42.333 45.021 53.134 0.00 0.00
C
ATOM 9143 O GLU C 150 −43.340 44.598 53.758 0.00 0.00
C
ATOM 9144 N TYR C 151 −42.205 44.911 51.803 0.00 0.00
C
ATOM 9145 HN TYR C 151 −41.423 45.262 51.294 0.00 0.00
C
ATOM 9146 CA TYR C 151 −43.288 44.434 50.961 0.00 0.00
C
ATOM 9147 HA TYR C 151 −44.178 44.215 51.533 0.00 0.00
C
ATOM 9148 CB TYR C 151 −43.582 45.453 49.795 0.00 0.00
C
ATOM 9149 HB1 TYR C 151 −42.664 45.868 49.327 0.00 0.00
C
ATOM 9150 HB2 TYR C 151 −44.195 44.990 48.992 0.00 0.00
C
ATOM 9151 CG TYR C 151 −44.491 46.646 50.192 0.00 0.00
C
ATOM 9152 CD1 TYR C 151 −44.591 47.710 49.245 0.00 0.00
C

ATOM 9153 HD1 TYR C 151 −44.077 47.691 48.295 0.00 0.00
C
ATOM 9154 CE1 TYR C 151 −45.077 48.959 49.606 0.00 0.00
C
ATOM 9155 HE1 TYR C 151 −45.063 49.775 48.898 0.00 0.00
C
ATOM 9156 CZ TYR C 151 −45.896 48.997 50.802 0.00 0.00
C
ATOM 9157 OH TYR C 151 −46.597 50.137 50.972 0.00 0.00
C
ATOM 9158 HH TYR C 151 −46.127 50.875 50.578 0.00 0.00
C
ATOM 9159 CD2 TYR C 151 −45.186 46.844 51.462 0.00 0.00
C
ATOM 9160 HD2 TYR C 151 −45.184 46.135 52.277 0.00 0.00
C
ATOM 9161 CE2 TYR C 151 −45.918 47.941 51.707 0.00 0.00
C
ATOM 9162 HE2 TYR C 151 −46.431 48.035 52.653 0.00 0.00
C
ATOM 9163 C TYR C 151 −42.718 43.150 50.373 0.00 0.00
C
ATOM 9164 O TYR C 151 −41.525 43.153 50.032 0.00 0.00
C
ATOM 9165 N SER C 152 −43.523 42.039 50.233 0.00 0.00
C
ATOM 9166 HN SER C 152 −44.451 41.978 50.594 0.00 0.00
C
ATOM 9167 CA SER C 152 −43.102 40.836 49.681 0.00 0.00
C
ATOM 9168 HA SER C 152 −42.309 41.132 49.010 0.00 0.00
C
ATOM 9169 CB SER C 152 −42.605 39.754 50.734 0.00 0.00
C
ATOM 9170 HB1 SER C 152 −43.477 39.507 51.377 0.00 0.00
C
ATOM 9171 HB2 SER C 152 −42.223 38.872 50.177 0.00 0.00
C
ATOM 9172 OG SER C 152 −41.567 40.229 51.619 0.00 0.00
C
ATOM 9173 HG1 SER C 152 −40.715 40.035 51.221 0.00 0.00
C
ATOM 9174 C SER C 152 −44.195 40.171 48.870 0.00 0.00
C
ATOM 9175 O SER C 152 −45.375 40.491 48.982 0.00 0.00
C
ATOM 9176 N ILE C 153 −43.803 39.164 48.002 0.00 0.00
C
ATOM 9177 HN ILE C 153 −42.818 39.021 47.947 0.00 0.00
C
ATOM 9178 CA ILE C 153 −44.774 38.252 47.322 0.00 0.00
C
ATOM 9179 HA ILE C 153 −45.792 38.486 47.595 0.00 0.00
C

ATOM 9180 CB ILE C 153 −44.562 38.291 45.765 0.00 0.00
C
ATOM 9181 HB ILE C 153 −43.535 38.017 45.441 0.00 0.00
C
ATOM 9182 CG2 ILE C 153 −45.652 37.452 45.117 0.00 0.00
C
ATOM 9183 HG21 ILE C 153 −46.648 37.876 45.366 0.00 0.00
C
ATOM 9184 HG22 ILE C 153 −45.538 37.445 44.012 0.00 0.00
C
ATOM 9185 HG23 ILE C 153 −45.732 36.413 45.502 0.00 0.00
C
ATOM 9186 CG1 ILE C 153 −44.813 39.627 45.182 0.00 0.00
C
ATOM 9187 HG11 ILE C 153 −45.871 39.963 45.228 0.00 0.00
C
ATOM 9188 HG12 ILE C 153 −44.134 40.336 45.702 0.00 0.00
C
ATOM 9189 CD ILE C 153 −44.379 39.774 43.766 0.00 0.00
C
ATOM 9190 HD1 ILE C 153 −43.272 39.719 43.841 0.00 0.00
C
ATOM 9191 HD2 ILE C 153 −44.755 38.966 43.103 0.00 0.00
C
ATOM 9192 HD3 ILE C 153 −44.765 40.751 43.403 0.00 0.00
C
ATOM 9193 C ILE C 153 −44.442 36.826 47.749 0.00 0.00
C
ATOM 9194 O ILE C 153 −43.333 36.346 47.538 0.00 0.00
C
ATOM 9195 N VAL C 154 −45.500 36.147 48.273 0.00 0.00
C
ATOM 9196 HN VAL C 154 −46.432 36.494 48.345 0.00 0.00
C
ATOM 9197 CA VAL C 154 −45.318 34.800 48.830 0.00 0.00
C
ATOM 9198 HA VAL C 154 −44.297 34.455 48.755 0.00 0.00
C
ATOM 9199 CB VAL C 154 −45.739 34.853 50.272 0.00 0.00
C
ATOM 9200 HB VAL C 154 −46.755 35.291 50.370 0.00 0.00
C
ATOM 9201 CG1 VAL C 154 −45.504 33.520 50.950 0.00 0.00
C
ATOM 9202 HG11 VAL C 154 −44.427 33.251 50.979 0.00 0.00
C
ATOM 9203 HG12 VAL C 154 −45.845 33.588 52.005 0.00 0.00
C
ATOM 9204 HG13 VAL C 154 −46.162 32.695 50.602 0.00 0.00
C
ATOM 9205 CG2 VAL C 154 −44.721 35.905 50.865 0.00 0.00
C
ATOM 9206 HG21 VAL C 154 −43.699 35.476 50.790 0.00 0.00
C

ATOM 9207 HG22 VAL C 154 -44.871 36.922 50.444 0.00 0.00
C
ATOM 9208 HG23 VAL C 154 -45.088 36.019 51.907 0.00 0.00
C
ATOM 9209 C VAL C 154 -46.225 33.809 48.191 0.00 0.00
C
ATOM 9210 O VAL C 154 -47.419 33.925 48.108 0.00 0.00
C
ATOM 9211 N GLU C 155 -45.702 32.611 47.748 0.00 0.00
C
ATOM 9212 HN GLU C 155 -44.800 32.327 48.064 0.00 0.00
C
ATOM 9213 CA GLU C 155 -46.368 31.576 47.027 0.00 0.00
C
ATOM 9214 HA GLU C 155 -47.324 31.881 46.627 0.00 0.00
C
ATOM 9215 CB GLU C 155 -45.499 30.935 45.904 0.00 0.00
C
ATOM 9216 HB1 GLU C 155 -45.347 31.652 45.069 0.00 0.00
C
ATOM 9217 HB2 GLU C 155 -44.565 30.594 46.400 0.00 0.00
C
ATOM 9218 CG GLU C 155 -46.155 29.802 45.187 0.00 0.00
C
ATOM 9219 HG1 GLU C 155 -46.768 29.209 45.899 0.00 0.00
C
ATOM 9220 HG2 GLU C 155 -46.936 30.229 44.522 0.00 0.00
C
ATOM 9221 CD GLU C 155 -45.184 28.993 44.292 0.00 0.00
C
ATOM 9222 OE1 GLU C 155 -44.194 28.411 44.858 0.00 0.00
C
ATOM 9223 OE2 GLU C 155 -45.450 28.913 43.083 0.00 0.00
C
ATOM 9224 C GLU C 155 -46.724 30.505 48.046 0.00 0.00
C
ATOM 9225 O GLU C 155 -45.896 30.143 48.921 0.00 0.00
C
ATOM 9226 N LEU C 156 -48.031 30.211 48.158 0.00 0.00
C
ATOM 9227 HN LEU C 156 -48.795 30.614 47.661 0.00 0.00
C
ATOM 9228 CA LEU C 156 -48.482 29.325 49.303 0.00 0.00
C
ATOM 9229 HA LEU C 156 -47.785 29.069 50.088 0.00 0.00
C
ATOM 9230 CB LEU C 156 -49.606 29.925 50.179 0.00 0.00
C
ATOM 9231 HB1 LEU C 156 -50.509 30.032 49.542 0.00 0.00
C
ATOM 9232 HB2 LEU C 156 -49.959 29.244 50.983 0.00 0.00
C
ATOM 9233 CG LEU C 156 -49.234 31.231 50.732 0.00 0.00
C

```
ATOM   9234  HG  LEU C 156     -48.824  31.887  49.934  0.00  0.00
C
ATOM   9235  CD1 LEU C 156     -50.509  31.944  51.237  0.00  0.00
C
ATOM   9236 HD11 LEU C 156     -51.195  31.249  51.765  0.00  0.00
C
ATOM   9237 HD12 LEU C 156     -50.289  32.782  51.933  0.00  0.00
C
ATOM   9238 HD13 LEU C 156     -51.096  32.344  50.383  0.00  0.00
C
ATOM   9239  CD2 LEU C 156     -48.101  30.938  51.739  0.00  0.00
C
ATOM   9240 HD21 LEU C 156     -47.956  31.745  52.489  0.00  0.00
C
ATOM   9241 HD22 LEU C 156     -48.346  30.038  52.342  0.00  0.00
C
ATOM   9242 HD23 LEU C 156     -47.135  30.730  51.230  0.00  0.00
C
ATOM   9243  C   LEU C 156     -49.133  28.082  48.758  0.00  0.00
C
ATOM   9244  O   LEU C 156     -49.432  27.936  47.561  0.00  0.00
C
ATOM   9245  N   LEU C 157     -49.476  27.131  49.645  0.00  0.00
C
ATOM   9246  HN  LEU C 157     -49.100  27.263  50.559  0.00  0.00
C
ATOM   9247  CA  LEU C 157     -50.243  25.892  49.542  0.00  0.00
C
ATOM   9248  HA  LEU C 157     -50.642  25.781  48.544  0.00  0.00
C
ATOM   9249  CB  LEU C 157     -49.355  24.713  49.958  0.00  0.00
C
ATOM   9250  HB1 LEU C 157     -48.444  24.749  49.322  0.00  0.00
C
ATOM   9251  HB2 LEU C 157     -49.002  24.873  50.999  0.00  0.00
C
ATOM   9252  CG  LEU C 157     -49.827  23.234  49.915  0.00  0.00
C
ATOM   9253  HG  LEU C 157     -50.782  23.176  50.479  0.00  0.00
C
ATOM   9254  CD1 LEU C 157     -50.220  22.744  48.473  0.00  0.00
C
ATOM   9255 HD11 LEU C 157     -51.090  23.306  48.072  0.00  0.00
C
ATOM   9256 HD12 LEU C 157     -49.424  23.033  47.754  0.00  0.00
C
ATOM   9257 HD13 LEU C 157     -50.481  21.664  48.448  0.00  0.00
C
ATOM   9258  CD2 LEU C 157     -48.786  22.299  50.517  0.00  0.00
C
ATOM   9259 HD21 LEU C 157     -48.599  22.534  51.587  0.00  0.00
C
ATOM   9260 HD22 LEU C 157     -49.172  21.257  50.515  0.00  0.00
C
```

ATOM 9261 HD23 LEU C 157 -47.797 22.089 50.055 0.00 0.00
C
ATOM 9262 C LEU C 157 -51.428 25.940 50.456 0.00 0.00
C
ATOM 9263 O LEU C 157 -51.327 26.092 51.720 0.00 0.00
C
ATOM 9264 N ALA C 158 -52.682 25.822 49.906 0.00 0.00
C
ATOM 9265 HN ALA C 158 -52.793 25.797 48.915 0.00 0.00
C
ATOM 9266 CA ALA C 158 -53.856 25.882 50.724 0.00 0.00
C
ATOM 9267 HA ALA C 158 -53.777 26.851 51.194 0.00 0.00
C
ATOM 9268 CB ALA C 158 -55.187 25.832 49.910 0.00 0.00
C
ATOM 9269 HB1 ALA C 158 -56.036 26.244 50.497 0.00 0.00
C
ATOM 9270 HB2 ALA C 158 -55.130 26.440 48.982 0.00 0.00
C
ATOM 9271 HB3 ALA C 158 -55.406 24.778 49.635 0.00 0.00
C
ATOM 9272 C ALA C 158 -53.997 24.827 51.853 0.00 0.00
C
ATOM 9273 O ALA C 158 -53.726 23.632 51.778 0.00 0.00
C
ATOM 9274 N THR C 159 -54.468 25.368 52.982 0.00 0.00
C
ATOM 9275 HN THR C 159 -54.876 26.276 52.929 0.00 0.00
C
ATOM 9276 CA THR C 159 -54.783 24.559 54.171 0.00 0.00
C
ATOM 9277 HA THR C 159 -54.185 23.661 54.194 0.00 0.00
C
ATOM 9278 CB THR C 159 -54.396 25.437 55.393 0.00 0.00
C
ATOM 9279 HB THR C 159 -54.226 24.697 56.204 0.00 0.00
C
ATOM 9280 OG1 THR C 159 -55.336 26.512 55.620 0.00 0.00
C
ATOM 9281 HG1 THR C 159 -55.235 26.866 56.506 0.00 0.00
C
ATOM 9282 CG2 THR C 159 -53.007 26.137 55.161 0.00 0.00
C
ATOM 9283 HG21 THR C 159 -52.687 26.607 56.116 0.00 0.00
C
ATOM 9284 HG22 THR C 159 -52.279 25.440 54.694 0.00 0.00
C
ATOM 9285 HG23 THR C 159 -53.046 26.873 54.329 0.00 0.00
C
ATOM 9286 C THR C 159 -56.222 24.115 54.340 0.00 0.00
C
ATOM 9287 O THR C 159 -57.088 24.301 53.482 0.00 0.00
C

ATOM 9288 N ARG C 160 −56.482 23.250 55.349 0.00 0.00
C
ATOM 9289 HN ARG C 160 −55.807 22.928 56.010 0.00 0.00
C
ATOM 9290 CA ARG C 160 −57.898 22.831 55.615 0.00 0.00
C
ATOM 9291 HA ARG C 160 −58.251 22.452 54.667 0.00 0.00
C
ATOM 9292 CB ARG C 160 −57.912 21.781 56.706 0.00 0.00
C
ATOM 9293 HB1 ARG C 160 −57.142 21.032 56.421 0.00 0.00
C
ATOM 9294 HB2 ARG C 160 −57.556 22.165 57.686 0.00 0.00
C
ATOM 9295 CG ARG C 160 −59.256 21.020 56.968 0.00 0.00
C
ATOM 9296 HG1 ARG C 160 −59.923 21.714 57.523 0.00 0.00
C
ATOM 9297 HG2 ARG C 160 −59.686 20.892 55.952 0.00 0.00
C
ATOM 9298 CD ARG C 160 −59.191 19.626 57.636 0.00 0.00
C
ATOM 9299 HD1 ARG C 160 −58.591 19.680 58.569 0.00 0.00
C
ATOM 9300 HD2 ARG C 160 −60.214 19.265 57.878 0.00 0.00
C
ATOM 9301 NE ARG C 160 −58.433 18.808 56.574 0.00 0.00
C
ATOM 9302 HE ARG C 160 −58.339 19.124 55.630 0.00 0.00
C
ATOM 9303 CZ ARG C 160 −58.199 17.556 56.797 0.00 0.00
C
ATOM 9304 NH1 ARG C 160 −58.707 16.909 57.890 0.00 0.00
C
ATOM 9305 HH11 ARG C 160 −58.545 15.929 57.777 0.00 0.00
C
ATOM 9306 HH12 ARG C 160 −58.841 17.378 58.763 0.00 0.00
C
ATOM 9307 NH2 ARG C 160 −57.523 16.838 55.872 0.00 0.00
C
ATOM 9308 HH21 ARG C 160 −57.324 15.859 55.896 0.00 0.00
C
ATOM 9309 HH22 ARG C 160 −57.110 17.315 55.096 0.00 0.00
C
ATOM 9310 C ARG C 160 −58.749 23.963 56.011 0.00 0.00
C
ATOM 9311 O ARG C 160 −59.910 23.901 55.699 0.00 0.00
C
ATOM 9312 N LYS C 161 −58.172 24.940 56.687 0.00 0.00
C
ATOM 9313 HN LYS C 161 −57.202 24.929 56.916 0.00 0.00
C
ATOM 9314 CA LYS C 161 −58.926 26.108 56.980 0.00 0.00
C

ATOM 9315 HA LYS C 161 -59.855 25.805 57.441 0.00 0.00
C
ATOM 9316 CB LYS C 161 -58.291 27.028 58.063 0.00 0.00
C
ATOM 9317 HB1 LYS C 161 -58.213 26.334 58.928 0.00 0.00
C
ATOM 9318 HB2 LYS C 161 -57.250 27.237 57.737 0.00 0.00
C
ATOM 9319 CG LYS C 161 -58.953 28.360 58.394 0.00 0.00
C
ATOM 9320 HG1 LYS C 161 -59.222 28.842 57.430 0.00 0.00
C
ATOM 9321 HG2 LYS C 161 -59.927 28.057 58.834 0.00 0.00
C
ATOM 9322 CD LYS C 161 -58.145 29.193 59.350 0.00 0.00
C
ATOM 9323 HD1 LYS C 161 -57.920 28.445 60.140 0.00 0.00
C
ATOM 9324 HD2 LYS C 161 -57.161 29.513 58.947 0.00 0.00
C
ATOM 9325 CE LYS C 161 -58.877 30.368 60.030 0.00 0.00
C
ATOM 9326 HE1 LYS C 161 -59.193 31.184 59.346 0.00 0.00
C
ATOM 9327 HE2 LYS C 161 -59.727 29.849 60.522 0.00 0.00
C
ATOM 9328 NZ LYS C 161 -57.958 30.973 61.024 0.00 0.00
C
ATOM 9329 HZ1 LYS C 161 -57.653 31.837 60.531 0.00 0.00
C
ATOM 9330 HZ2 LYS C 161 -58.600 31.296 61.776 0.00 0.00
C
ATOM 9331 HZ3 LYS C 161 -57.081 30.440 61.192 0.00 0.00
C
ATOM 9332 C LYS C 161 -59.287 26.822 55.663 0.00 0.00
C
ATOM 9333 O LYS C 161 -60.438 27.249 55.495 0.00 0.00
C
ATOM 9334 N LEU C 162 -58.308 26.937 54.702 0.00 0.00
C
ATOM 9335 HN LEU C 162 -57.363 26.726 54.938 0.00 0.00
C
ATOM 9336 CA LEU C 162 -58.507 27.611 53.498 0.00 0.00
C
ATOM 9337 HA LEU C 162 -58.949 28.552 53.793 0.00 0.00
C
ATOM 9338 CB LEU C 162 -57.152 27.817 52.804 0.00 0.00
C
ATOM 9339 HB1 LEU C 162 -56.354 28.014 53.551 0.00 0.00
C
ATOM 9340 HB2 LEU C 162 -56.832 26.900 52.265 0.00 0.00
C
ATOM 9341 CG LEU C 162 -57.150 28.879 51.667 0.00 0.00
C

ATOM 9342 HG LEU C 162 -57.628 28.392 50.791 0.00 0.00
C
ATOM 9343 CD1 LEU C 162 -57.934 30.181 52.157 0.00 0.00
C
ATOM 9344 HD11 LEU C 162 -58.999 29.970 52.395 0.00 0.00
C
ATOM 9345 HD12 LEU C 162 -57.474 30.639 53.059 0.00 0.00
C
ATOM 9346 HD13 LEU C 162 -57.900 30.938 51.345 0.00 0.00
C
ATOM 9347 CD2 LEU C 162 -55.741 29.145 51.163 0.00 0.00
C
ATOM 9348 HD21 LEU C 162 -55.385 28.253 50.604 0.00 0.00
C
ATOM 9349 HD22 LEU C 162 -55.786 30.010 50.466 0.00 0.00
C
ATOM 9350 HD23 LEU C 162 -54.986 29.488 51.902 0.00 0.00
C
ATOM 9351 C LEU C 162 -59.480 26.919 52.633 0.00 0.00
C
ATOM 9352 O LEU C 162 -60.137 27.532 51.839 0.00 0.00
C
ATOM 9353 N ASP C 163 -59.586 25.570 52.700 0.00 0.00
C
ATOM 9354 HN ASP C 163 -58.861 25.038 53.129 0.00 0.00
C
ATOM 9355 CA ASP C 163 -60.567 24.796 51.852 0.00 0.00
C
ATOM 9356 HA ASP C 163 -60.503 24.928 50.782 0.00 0.00
C
ATOM 9357 CB ASP C 163 -60.339 23.312 52.240 0.00 0.00
C
ATOM 9358 HB1 ASP C 163 -59.283 23.028 52.046 0.00 0.00
C
ATOM 9359 HB2 ASP C 163 -60.518 23.025 53.298 0.00 0.00
C
ATOM 9360 CG ASP C 163 -61.155 22.393 51.437 0.00 0.00
C
ATOM 9361 OD1 ASP C 163 -60.888 22.065 50.272 0.00 0.00
C
ATOM 9362 OD2 ASP C 163 -62.135 21.833 52.057 0.00 0.00
C
ATOM 9363 C ASP C 163 -62.023 25.227 52.062 0.00 0.00
C
ATOM 9364 O ASP C 163 -62.536 25.305 53.194 0.00 0.00
C
ATOM 9365 N SER C 164 -62.737 25.412 50.900 0.00 0.00
C
ATOM 9366 HN SER C 164 -62.325 25.168 50.026 0.00 0.00
C
ATOM 9367 CA SER C 164 -64.168 25.674 50.882 0.00 0.00
C
ATOM 9368 HA SER C 164 -64.538 25.322 49.930 0.00 0.00
C

ATOM	9369	CB	SER	C	164	−65.113	24.865	51.915	0.00	0.00	C
ATOM	9370	HB1	SER	C	164	−64.734	23.821	51.949	0.00	0.00	C
ATOM	9371	HB2	SER	C	164	−64.903	25.304	52.914	0.00	0.00	C
ATOM	9372	OG	SER	C	164	−66.484	24.705	51.422	0.00	0.00	C
ATOM	9373	HG1	SER	C	164	−66.819	23.831	51.637	0.00	0.00	C
ATOM	9374	C	SER	C	164	−64.363	27.210	51.003	0.00	0.00	C
ATOM	9375	O	SER	C	164	−65.432	27.608	51.439	0.00	0.00	C
ATOM	9376	N	LYS	C	165	−63.350	28.045	50.679	0.00	0.00	C
ATOM	9377	HN	LYS	C	165	−62.452	27.699	50.419	0.00	0.00	C
ATOM	9378	CA	LYS	C	165	−63.479	29.502	50.803	0.00	0.00	C
ATOM	9379	HA	LYS	C	165	−64.467	29.779	51.142	0.00	0.00	C
ATOM	9380	CB	LYS	C	165	−62.529	30.169	51.766	0.00	0.00	C
ATOM	9381	HB1	LYS	C	165	−61.461	30.021	51.500	0.00	0.00	C
ATOM	9382	HB2	LYS	C	165	−62.780	31.251	51.720	0.00	0.00	C
ATOM	9383	CG	LYS	C	165	−62.807	29.633	53.159	0.00	0.00	C
ATOM	9384	HG1	LYS	C	165	−63.900	29.673	53.350	0.00	0.00	C
ATOM	9385	HG2	LYS	C	165	−62.540	28.566	53.310	0.00	0.00	C
ATOM	9386	CD	LYS	C	165	−62.112	30.578	54.179	0.00	0.00	C
ATOM	9387	HD1	LYS	C	165	−61.029	30.355	54.068	0.00	0.00	C
ATOM	9388	HD2	LYS	C	165	−62.210	31.618	53.800	0.00	0.00	C
ATOM	9389	CE	LYS	C	165	−62.552	30.440	55.597	0.00	0.00	C
ATOM	9390	HE1	LYS	C	165	−62.281	29.425	55.957	0.00	0.00	C
ATOM	9391	HE2	LYS	C	165	−61.971	30.954	56.392	0.00	0.00	C
ATOM	9392	NZ	LYS	C	165	−63.988	30.688	55.739	0.00	0.00	C
ATOM	9393	HZ1	LYS	C	165	−64.380	31.018	56.643	0.00	0.00	C
ATOM	9394	HZ2	LYS	C	165	−64.222	31.493	55.124	0.00	0.00	C
ATOM	9395	HZ3	LYS	C	165	−64.557	29.930	55.311	0.00	0.00	C

ATOM 9396 C LYS C 165 -63.444 30.140 49.348 0.00 0.00
C
ATOM 9397 O LYS C 165 -62.609 29.809 48.518 0.00 0.00
C
ATOM 9398 N SER C 166 -64.279 31.186 49.109 0.00 0.00
C
ATOM 9399 HN SER C 166 -64.918 31.497 49.809 0.00 0.00
C
ATOM 9400 CA SER C 166 -64.329 31.819 47.832 0.00 0.00
C
ATOM 9401 HA SER C 166 -63.925 31.144 47.092 0.00 0.00
C
ATOM 9402 CB SER C 166 -65.771 32.197 47.480 0.00 0.00
C
ATOM 9403 HB1 SER C 166 -66.475 31.339 47.546 0.00 0.00
C
ATOM 9404 HB2 SER C 166 -66.118 32.876 48.289 0.00 0.00
C
ATOM 9405 OG SER C 166 -65.859 32.761 46.085 0.00 0.00
C
ATOM 9406 HG1 SER C 166 -66.057 31.949 45.613 0.00 0.00
C
ATOM 9407 C SER C 166 -63.441 33.018 47.896 0.00 0.00
C
ATOM 9408 O SER C 166 -63.682 33.852 48.736 0.00 0.00
C
ATOM 9409 N ILE C 167 -62.443 33.215 47.051 0.00 0.00
C
ATOM 9410 HN ILE C 167 -62.426 32.686 46.206 0.00 0.00
C
ATOM 9411 CA ILE C 167 -61.395 34.181 47.333 0.00 0.00
C
ATOM 9412 HA ILE C 167 -60.954 34.089 48.315 0.00 0.00
C
ATOM 9413 CB ILE C 167 -60.382 34.064 46.222 0.00 0.00
C
ATOM 9414 HB ILE C 167 -61.004 34.184 45.310 0.00 0.00
C
ATOM 9415 CG2 ILE C 167 -59.269 35.152 46.414 0.00 0.00
C
ATOM 9416 HG21 ILE C 167 -58.607 35.054 47.301 0.00 0.00
C
ATOM 9417 HG22 ILE C 167 -58.601 35.115 45.527 0.00 0.00
C
ATOM 9418 HG23 ILE C 167 -59.680 36.184 46.374 0.00 0.00
C
ATOM 9419 CG1 ILE C 167 -59.784 32.643 46.122 0.00 0.00
C
ATOM 9420 HG11 ILE C 167 -60.513 31.835 45.902 0.00 0.00
C
ATOM 9421 HG12 ILE C 167 -59.033 32.887 45.340 0.00 0.00
C
ATOM 9422 CD ILE C 167 -59.095 32.261 47.382 0.00 0.00
C

ATOM 9423 HD1 ILE C 167 −59.856 31.952 48.129 0.00 0.00
C
ATOM 9424 HD2 ILE C 167 −58.504 31.330 47.246 0.00 0.00
C
ATOM 9425 HD3 ILE C 167 −58.424 33.059 47.766 0.00 0.00
C
ATOM 9426 C ILE C 167 −61.966 35.628 47.367 0.00 0.00
C
ATOM 9427 O ILE C 167 −61.468 36.494 48.088 0.00 0.00
C
ATOM 9428 N ILE C 168 −63.087 35.882 46.654 0.00 0.00
C
ATOM 9429 HN ILE C 168 −63.490 35.196 46.053 0.00 0.00
C
ATOM 9430 CA ILE C 168 −63.782 37.136 46.608 0.00 0.00
C
ATOM 9431 HA ILE C 168 −63.069 37.903 46.346 0.00 0.00
C
ATOM 9432 CB ILE C 168 −64.962 37.084 45.533 0.00 0.00
C
ATOM 9433 HB ILE C 168 −65.584 36.212 45.827 0.00 0.00
C
ATOM 9434 CG2 ILE C 168 −65.734 38.352 45.581 0.00 0.00
C
ATOM 9435 HG21 ILE C 168 −66.405 38.364 46.467 0.00 0.00
C
ATOM 9436 HG22 ILE C 168 −65.160 39.297 45.478 0.00 0.00
C
ATOM 9437 HG23 ILE C 168 −66.406 38.294 44.698 0.00 0.00
C
ATOM 9438 CG1 ILE C 168 −64.337 36.824 44.141 0.00 0.00
C
ATOM 9439 HG11 ILE C 168 −63.781 35.864 44.210 0.00 0.00
C
ATOM 9440 HG12 ILE C 168 −65.167 36.768 43.404 0.00 0.00
C
ATOM 9441 CD ILE C 168 −63.390 37.904 43.656 0.00 0.00
C
ATOM 9442 HD1 ILE C 168 −63.288 37.833 42.552 0.00 0.00
C
ATOM 9443 HD2 ILE C 168 −63.719 38.953 43.814 0.00 0.00
C
ATOM 9444 HD3 ILE C 168 −62.328 37.896 43.982 0.00 0.00
C
ATOM 9445 C ILE C 168 −64.497 37.460 48.023 0.00 0.00
C
ATOM 9446 O ILE C 168 −64.691 38.590 48.494 0.00 0.00
C
ATOM 9447 N ASP C 169 −64.981 36.388 48.751 0.00 0.00
C
ATOM 9448 HN ASP C 169 −64.865 35.475 48.367 0.00 0.00
C
ATOM 9449 CA ASP C 169 −65.572 36.476 50.050 0.00 0.00
C

ATOM 9450 HA ASP C 169 −66.110 37.402 50.192 0.00 0.00
C
ATOM 9451 CB ASP C 169 −66.399 35.228 50.209 0.00 0.00
C
ATOM 9452 HB1 ASP C 169 −65.837 34.330 49.876 0.00 0.00
C
ATOM 9453 HB2 ASP C 169 −66.855 35.035 51.203 0.00 0.00
C
ATOM 9454 CG ASP C 169 −67.658 35.352 49.347 0.00 0.00
C
ATOM 9455 OD1 ASP C 169 −67.607 35.624 48.097 0.00 0.00
C
ATOM 9456 OD2 ASP C 169 −68.694 35.188 49.962 0.00 0.00
C
ATOM 9457 C ASP C 169 −64.485 36.510 51.192 0.00 0.00
C
ATOM 9458 O ASP C 169 −64.600 37.216 52.170 0.00 0.00
C
ATOM 9459 N LEU C 170 −63.322 35.951 50.891 0.00 0.00
C
ATOM 9460 HN LEU C 170 −63.151 35.485 50.026 0.00 0.00
C
ATOM 9461 CA LEU C 170 −62.147 36.105 51.776 0.00 0.00
C
ATOM 9462 HA LEU C 170 −62.411 35.695 52.740 0.00 0.00
C
ATOM 9463 CB LEU C 170 −61.028 35.275 51.171 0.00 0.00
C
ATOM 9464 HB1 LEU C 170 −61.186 35.262 50.071 0.00 0.00
C
ATOM 9465 HB2 LEU C 170 −60.044 35.761 51.340 0.00 0.00
C
ATOM 9466 CG LEU C 170 −60.936 33.891 51.734 0.00 0.00
C
ATOM 9467 HG LEU C 170 −61.945 33.429 51.675 0.00 0.00
C
ATOM 9468 CD1 LEU C 170 −59.880 33.059 50.987 0.00 0.00
C
ATOM 9469 HD11 LEU C 170 −60.048 33.178 49.895 0.00 0.00
C
ATOM 9470 HD12 LEU C 170 −58.864 33.369 51.312 0.00 0.00
C
ATOM 9471 HD13 LEU C 170 −59.907 31.978 51.244 0.00 0.00
C
ATOM 9472 CD2 LEU C 170 −60.573 33.853 53.284 0.00 0.00
C
ATOM 9473 HD21 LEU C 170 −59.742 34.549 53.531 0.00 0.00
C
ATOM 9474 HD22 LEU C 170 −61.424 34.005 53.982 0.00 0.00
C
ATOM 9475 HD23 LEU C 170 −60.166 32.830 53.432 0.00 0.00
C
ATOM 9476 C LEU C 170 −61.763 37.543 52.030 0.00 0.00
C

ATOM 9477 O LEU C 170 -61.709 38.500 51.194 0.00 0.00
C
ATOM 9478 N ASN C 171 -61.464 37.790 53.335 0.00 0.00
C
ATOM 9479 HN ASN C 171 -61.447 37.053 54.006 0.00 0.00
C
ATOM 9480 CA ASN C 171 -61.278 39.094 53.971 0.00 0.00
C
ATOM 9481 HA ASN C 171 -62.040 39.772 53.617 0.00 0.00
C
ATOM 9482 CB ASN C 171 -61.570 39.193 55.497 0.00 0.00
C
ATOM 9483 HB1 ASN C 171 -60.699 38.670 55.947 0.00 0.00
C
ATOM 9484 HB2 ASN C 171 -61.433 40.222 55.892 0.00 0.00
C
ATOM 9485 CG ASN C 171 -62.858 38.489 55.988 0.00 0.00
C
ATOM 9486 OD1 ASN C 171 -63.030 37.287 55.939 0.00 0.00
C
ATOM 9487 ND2 ASN C 171 -63.892 39.325 56.472 0.00 0.00
C
ATOM 9488 HD21 ASN C 171 -64.764 38.920 56.746 0.00 0.00
C
ATOM 9489 HD22 ASN C 171 -63.808 40.322 56.479 0.00 0.00
C
ATOM 9490 C ASN C 171 -59.899 39.705 53.693 0.00 0.00
C
ATOM 9491 O ASN C 171 -59.559 40.692 54.333 0.00 0.00
C
ATOM 9492 N VAL C 172 -59.063 39.136 52.794 0.00 0.00
C
ATOM 9493 HN VAL C 172 -59.390 38.345 52.282 0.00 0.00
C
ATOM 9494 CA VAL C 172 -57.684 39.251 52.760 0.00 0.00
C
ATOM 9495 HA VAL C 172 -57.180 39.018 53.686 0.00 0.00
C
ATOM 9496 CB VAL C 172 -57.109 38.335 51.709 0.00 0.00
C
ATOM 9497 HB VAL C 172 -56.055 38.624 51.511 0.00 0.00
C
ATOM 9498 CG1 VAL C 172 -57.260 36.928 52.128 0.00 0.00
C
ATOM 9499 HG11 VAL C 172 -58.346 36.695 52.125 0.00 0.00
C
ATOM 9500 HG12 VAL C 172 -56.761 36.268 51.388 0.00 0.00
C
ATOM 9501 HG13 VAL C 172 -56.694 36.666 53.048 0.00 0.00
C
ATOM 9502 CG2 VAL C 172 -57.900 38.403 50.321 0.00 0.00
C
ATOM 9503 HG21 VAL C 172 -57.672 37.511 49.700 0.00 0.00
C

ATOM 9504 HG22 VAL C 172 −59.006 38.480 50.393 0.00 0.00
C
ATOM 9505 HG23 VAL C 172 −57.657 39.288 49.695 0.00 0.00
C
ATOM 9506 C VAL C 172 −57.273 40.717 52.430 0.00 0.00
C
ATOM 9507 O VAL C 172 −56.283 41.232 52.915 0.00 0.00
C
ATOM 9508 N ARG C 173 −58.004 41.367 51.502 0.00 0.00
C
ATOM 9509 HN ARG C 173 −58.865 40.972 51.193 0.00 0.00
C
ATOM 9510 CA ARG C 173 −57.842 42.768 51.168 0.00 0.00
C
ATOM 9511 HA ARG C 173 −56.787 42.892 50.973 0.00 0.00
C
ATOM 9512 CB ARG C 173 −58.439 42.978 49.720 0.00 0.00
C
ATOM 9513 HB1 ARG C 173 −57.706 42.367 49.152 0.00 0.00
C
ATOM 9514 HB2 ARG C 173 −59.446 42.532 49.574 0.00 0.00
C
ATOM 9515 CG ARG C 173 −58.622 44.446 49.272 0.00 0.00
C
ATOM 9516 HG1 ARG C 173 −59.406 44.854 49.946 0.00 0.00
C
ATOM 9517 HG2 ARG C 173 −57.666 45.010 49.322 0.00 0.00
C
ATOM 9518 CD ARG C 173 −58.997 44.551 47.810 0.00 0.00
C
ATOM 9519 HD1 ARG C 173 −58.216 44.218 47.094 0.00 0.00
C
ATOM 9520 HD2 ARG C 173 −59.947 44.016 47.595 0.00 0.00
C
ATOM 9521 NE ARG C 173 −59.333 45.968 47.530 0.00 0.00
C
ATOM 9522 HE ARG C 173 −60.278 46.287 47.604 0.00 0.00
C
ATOM 9523 CZ ARG C 173 −58.471 46.900 47.083 0.00 0.00
C
ATOM 9524 NH1 ARG C 173 −57.271 46.613 46.676 0.00 0.00
C
ATOM 9525 HH11 ARG C 173 −56.684 47.385 46.432 0.00 0.00
C
ATOM 9526 HH12 ARG C 173 −56.800 45.895 47.189 0.00 0.00
C
ATOM 9527 NH2 ARG C 173 −58.951 48.124 46.841 0.00 0.00
C
ATOM 9528 HH21 ARG C 173 −58.363 48.794 46.388 0.00 0.00
C
ATOM 9529 HH22 ARG C 173 −59.779 48.514 47.244 0.00 0.00
C
ATOM 9530 C ARG C 173 −58.378 43.688 52.215 0.00 0.00
C

```
ATOM   9531  O   ARG C 173     -57.877  44.765  52.491  0.00  0.00
C
ATOM   9532  N   ALA C 174     -59.470  43.242  52.862  0.00  0.00
C
ATOM   9533  HN  ALA C 174     -59.748  42.309  52.648  0.00  0.00
C
ATOM   9534  CA  ALA C 174     -60.105  44.086  53.877  0.00  0.00
C
ATOM   9535  HA  ALA C 174     -60.211  45.099  53.518  0.00  0.00
C
ATOM   9536  CB  ALA C 174     -61.568  43.400  54.164  0.00  0.00
C
ATOM   9537  HB1 ALA C 174     -62.135  43.315  53.212  0.00  0.00
C
ATOM   9538  HB2 ALA C 174     -61.375  42.376  54.549  0.00  0.00
C
ATOM   9539  HB3 ALA C 174     -62.083  44.023  54.927  0.00  0.00
C
ATOM   9540  C   ALA C 174     -59.339  44.254  55.211  0.00  0.00
C
ATOM   9541  O   ALA C 174     -59.127  45.334  55.805  0.00  0.00
C
ATOM   9542  N   LYS C 175     -58.917  43.069  55.745  0.00  0.00
C
ATOM   9543  HN  LYS C 175     -59.143  42.298  55.155  0.00  0.00
C
ATOM   9544  CA  LYS C 175     -58.127  42.895  56.908  0.00  0.00
C
ATOM   9545  HA  LYS C 175     -58.486  43.574  57.666  0.00  0.00
C
ATOM   9546  CB  LYS C 175     -58.197  41.393  57.389  0.00  0.00
C
ATOM   9547  HB1 LYS C 175     -59.272  41.147  57.255  0.00  0.00
C
ATOM   9548  HB2 LYS C 175     -57.647  40.663  56.758  0.00  0.00
C
ATOM   9549  CG  LYS C 175     -57.933  41.139  58.888  0.00  0.00
C
ATOM   9550  HG1 LYS C 175     -56.829  41.122  59.017  0.00  0.00
C
ATOM   9551  HG2 LYS C 175     -58.320  42.022  59.441  0.00  0.00
C
ATOM   9552  CD  LYS C 175     -58.555  39.806  59.432  0.00  0.00
C
ATOM   9553  HD1 LYS C 175     -59.620  39.940  59.143  0.00  0.00
C
ATOM   9554  HD2 LYS C 175     -58.273  39.036  58.682  0.00  0.00
C
ATOM   9555  CE  LYS C 175     -58.414  39.498  60.971  0.00  0.00
C
ATOM   9556  HE1 LYS C 175     -57.367  39.239  61.238  0.00  0.00
C
ATOM   9557  HE2 LYS C 175     -58.712  40.418  61.519  0.00  0.00
C
```

ATOM 9558 NZ LYS C 175 -59.297 38.419 61.226 0.00 0.00
C
ATOM 9559 HZ1 LYS C 175 -59.146 38.018 62.174 0.00 0.00
C
ATOM 9560 HZ2 LYS C 175 -60.270 38.779 61.153 0.00 0.00
C
ATOM 9561 HZ3 LYS C 175 -59.269 37.663 60.513 0.00 0.00
C
ATOM 9562 C LYS C 175 -56.697 43.180 56.719 0.00 0.00
C
ATOM 9563 O LYS C 175 -56.159 43.904 57.498 0.00 0.00
C
ATOM 9564 N TYR C 176 -56.061 42.539 55.759 0.00 0.00
C
ATOM 9565 HN TYR C 176 -56.577 41.970 55.123 0.00 0.00
C
ATOM 9566 CA TYR C 176 -54.603 42.495 55.608 0.00 0.00
C
ATOM 9567 HA TYR C 176 -54.144 42.926 56.486 0.00 0.00
C
ATOM 9568 CB TYR C 176 -54.188 40.995 55.567 0.00 0.00
C
ATOM 9569 HB1 TYR C 176 -54.691 40.476 54.723 0.00 0.00
C
ATOM 9570 HB2 TYR C 176 -53.081 40.996 55.473 0.00 0.00
C
ATOM 9571 CG TYR C 176 -54.537 40.233 56.832 0.00 0.00
C
ATOM 9572 CD1 TYR C 176 -55.502 39.271 56.751 0.00 0.00
C
ATOM 9573 HD1 TYR C 176 -56.025 39.085 55.825 0.00 0.00
C
ATOM 9574 CE1 TYR C 176 -55.652 38.328 57.785 0.00 0.00
C
ATOM 9575 HE1 TYR C 176 -56.315 37.482 57.684 0.00 0.00
C
ATOM 9576 CZ TYR C 176 -54.952 38.347 58.986 0.00 0.00
C
ATOM 9577 OH TYR C 176 -55.200 37.416 59.975 0.00 0.00
C
ATOM 9578 HH TYR C 176 -55.788 36.700 59.723 0.00 0.00
C
ATOM 9579 CD2 TYR C 176 -53.853 40.411 58.107 0.00 0.00
C
ATOM 9580 HD2 TYR C 176 -53.173 41.243 58.219 0.00 0.00
C
ATOM 9581 CE2 TYR C 176 -54.092 39.465 59.166 0.00 0.00
C
ATOM 9582 HE2 TYR C 176 -53.622 39.530 60.136 0.00 0.00
C
ATOM 9583 C TYR C 176 -54.059 43.316 54.463 0.00 0.00
C
ATOM 9584 O TYR C 176 -52.849 43.451 54.280 0.00 0.00
C

ATOM 9585 N GLY C 177 −54.872 44.014 53.670 0.00 0.00
C
ATOM 9586 HN GLY C 177 −55.864 43.914 53.691 0.00 0.00
C
ATOM 9587 CA GLY C 177 −54.339 44.943 52.650 0.00 0.00
C
ATOM 9588 HA1 GLY C 177 −53.519 45.503 53.076 0.00 0.00
C
ATOM 9589 HA2 GLY C 177 −55.233 45.510 52.439 0.00 0.00
C
ATOM 9590 C GLY C 177 −53.713 44.280 51.452 0.00 0.00
C
ATOM 9591 O GLY C 177 −52.876 44.929 50.767 0.00 0.00
C
ATOM 9592 N CYS C 178 −53.968 42.967 51.243 0.00 0.00
C
ATOM 9593 HN CYS C 178 −54.385 42.446 51.983 0.00 0.00
C
ATOM 9594 CA CYS C 178 −53.285 42.161 50.246 0.00 0.00
C
ATOM 9595 HA CYS C 178 −52.330 42.626 50.055 0.00 0.00
C
ATOM 9596 CB CYS C 178 −52.860 40.838 50.949 0.00 0.00
C
ATOM 9597 HB1 CYS C 178 −53.741 40.246 51.276 0.00 0.00
C
ATOM 9598 HB2 CYS C 178 −52.272 40.209 50.247 0.00 0.00
C
ATOM 9599 SG CYS C 178 −51.618 41.072 52.358 0.00 0.00
C
ATOM 9600 HG1 CYS C 178 −50.586 40.846 51.557 0.00 0.00
C
ATOM 9601 C CYS C 178 −53.927 41.814 48.905 0.00 0.00
C
ATOM 9602 O CYS C 178 −55.086 41.721 48.776 0.00 0.00
C
ATOM 9603 N THR C 179 −53.102 41.689 47.823 0.00 0.00
C
ATOM 9604 HN THR C 179 −52.132 41.828 48.009 0.00 0.00
C
ATOM 9605 CA THR C 179 −53.598 41.271 46.535 0.00 0.00
C
ATOM 9606 HA THR C 179 −54.675 41.350 46.526 0.00 0.00
C
ATOM 9607 CB THR C 179 −53.041 41.903 45.315 0.00 0.00
C
ATOM 9608 HB THR C 179 −51.973 41.603 45.265 0.00 0.00
C
ATOM 9609 OG1 THR C 179 −53.117 43.308 45.648 0.00 0.00
C
ATOM 9610 HG1 THR C 179 −52.398 43.395 46.278 0.00 0.00
C
ATOM 9611 CG2 THR C 179 −53.855 41.657 44.013 0.00 0.00
C

```
ATOM   9612 HG21 THR C 179     -54.828  42.153  44.216  0.00  0.00
C
ATOM   9613 HG22 THR C 179     -53.243  42.069  43.182  0.00  0.00
C
ATOM   9614 HG23 THR C 179     -54.007  40.559  43.938  0.00  0.00
C
ATOM   9615  C   THR C 179     -53.366  39.784  46.347  0.00  0.00
C
ATOM   9616  O   THR C 179     -52.248  39.302  46.531  0.00  0.00
C
ATOM   9617  N   ILE C 180     -54.441  39.001  46.022  0.00  0.00
C
ATOM   9618  HN  ILE C 180     -55.364  39.377  46.043  0.00  0.00
C
ATOM   9619  CA  ILE C 180     -54.296  37.610  45.570  0.00  0.00
C
ATOM   9620  HA  ILE C 180     -53.391  37.181  45.977  0.00  0.00
C
ATOM   9621  CB  ILE C 180     -55.513  36.772  45.969  0.00  0.00
C
ATOM   9622  HB  ILE C 180     -56.455  37.088  45.474  0.00  0.00
C
ATOM   9623  CG2 ILE C 180     -55.254  35.346  45.424  0.00  0.00
C
ATOM   9624 HG21 ILE C 180     -55.988  34.645  45.875  0.00  0.00
C
ATOM   9625 HG22 ILE C 180     -55.289  35.330  44.313  0.00  0.00
C
ATOM   9626 HG23 ILE C 180     -54.256  34.989  45.756  0.00  0.00
C
ATOM   9627  CG1 ILE C 180     -55.766  36.713  47.455  0.00  0.00
C
ATOM   9628 HG11 ILE C 180     -55.835  37.737  47.881  0.00  0.00
C
ATOM   9629 HG12 ILE C 180     -56.775  36.391  47.791  0.00  0.00
C
ATOM   9630  CD  ILE C 180     -54.722  35.927  48.255  0.00  0.00
C
ATOM   9631  HD1 ILE C 180     -54.645  34.854  47.978  0.00  0.00
C
ATOM   9632  HD2 ILE C 180     -53.756  36.456  48.107  0.00  0.00
C
ATOM   9633  HD3 ILE C 180     -54.953  35.963  49.341  0.00  0.00
C
ATOM   9634  C   ILE C 180     -54.103  37.641  44.076  0.00  0.00
C
ATOM   9635  O   ILE C 180     -55.003  38.043  43.325  0.00  0.00
C
ATOM   9636  N   LEU C 181     -52.935  37.228  43.546  0.00  0.00
C
ATOM   9637  HN  LEU C 181     -52.229  36.801  44.106  0.00  0.00
C
ATOM   9638  CA  LEU C 181     -52.600  37.345  42.204  0.00  0.00
C
```

ATOM 9639 HA LEU C 181 -53.156 38.164 41.773 0.00 0.00
C
ATOM 9640 CB LEU C 181 -51.089 37.565 41.936 0.00 0.00
C
ATOM 9641 HB1 LEU C 181 -50.645 36.559 42.096 0.00 0.00
C
ATOM 9642 HB2 LEU C 181 -50.845 37.814 40.881 0.00 0.00
C
ATOM 9643 CG LEU C 181 -50.423 38.592 42.798 0.00 0.00
C
ATOM 9644 HG LEU C 181 -50.634 38.472 43.883 0.00 0.00
C
ATOM 9645 CD1 LEU C 181 -48.949 38.567 42.597 0.00 0.00
C
ATOM 9646 HD11 LEU C 181 -48.450 37.634 42.935 0.00 0.00
C
ATOM 9647 HD12 LEU C 181 -48.819 38.553 41.494 0.00 0.00
C
ATOM 9648 HD13 LEU C 181 -48.440 39.457 43.025 0.00 0.00
C
ATOM 9649 CD2 LEU C 181 -50.909 40.020 42.570 0.00 0.00
C
ATOM 9650 HD21 LEU C 181 -50.347 40.708 43.237 0.00 0.00
C
ATOM 9651 HD22 LEU C 181 -50.898 40.347 41.508 0.00 0.00
C
ATOM 9652 HD23 LEU C 181 -51.985 40.122 42.828 0.00 0.00
C
ATOM 9653 C LEU C 181 -52.952 36.072 41.385 0.00 0.00
C
ATOM 9654 O LEU C 181 -52.946 36.049 40.119 0.00 0.00
C
ATOM 9655 N ALA C 182 -53.324 34.968 42.037 0.00 0.00
C
ATOM 9656 HN ALA C 182 -53.534 34.870 43.007 0.00 0.00
C
ATOM 9657 CA ALA C 182 -53.506 33.620 41.398 0.00 0.00
C
ATOM 9658 HA ALA C 182 -54.191 33.736 40.571 0.00 0.00
C
ATOM 9659 CB ALA C 182 -52.109 33.127 40.879 0.00 0.00
C
ATOM 9660 HB1 ALA C 182 -51.273 33.145 41.611 0.00 0.00
C
ATOM 9661 HB2 ALA C 182 -52.195 32.116 40.427 0.00 0.00
C
ATOM 9662 HB3 ALA C 182 -51.739 33.792 40.069 0.00 0.00
C
ATOM 9663 C ALA C 182 -54.009 32.574 42.446 0.00 0.00
C
ATOM 9664 O ALA C 182 -53.612 32.682 43.590 0.00 0.00
C
ATOM 9665 N ILE C 183 -54.757 31.527 41.974 0.00 0.00
C

ATOM 9666 HN ILE C 183 −55.060 31.632 41.030 0.00 0.00
C
ATOM 9667 CA ILE C 183 −55.085 30.262 42.723 0.00 0.00
C
ATOM 9668 HA ILE C 183 −54.284 30.084 43.425 0.00 0.00
C
ATOM 9669 CB ILE C 183 −56.484 30.129 43.405 0.00 0.00
C
ATOM 9670 HB ILE C 183 −56.490 29.125 43.882 0.00 0.00
C
ATOM 9671 CG2 ILE C 183 −56.632 31.230 44.452 0.00 0.00
C
ATOM 9672 HG21 ILE C 183 −56.418 32.254 44.076 0.00 0.00
C
ATOM 9673 HG22 ILE C 183 −57.610 31.168 44.976 0.00 0.00
C
ATOM 9674 HG23 ILE C 183 −55.795 31.115 45.173 0.00 0.00
C
ATOM 9675 CG1 ILE C 183 −57.660 30.124 42.444 0.00 0.00
C
ATOM 9676 HG11 ILE C 183 −57.686 31.004 41.767 0.00 0.00
C
ATOM 9677 HG12 ILE C 183 −57.673 29.218 41.800 0.00 0.00
C
ATOM 9678 CD ILE C 183 −58.932 29.987 43.233 0.00 0.00
C
ATOM 9679 HD1 ILE C 183 −59.759 29.642 42.577 0.00 0.00
C
ATOM 9680 HD2 ILE C 183 −58.873 29.334 44.130 0.00 0.00
C
ATOM 9681 HD3 ILE C 183 −59.154 30.942 43.757 0.00 0.00
C
ATOM 9682 C ILE C 183 −55.152 29.073 41.661 0.00 0.00
C
ATOM 9683 O ILE C 183 −55.363 29.324 40.418 0.00 0.00
C
ATOM 9684 N LYS C 184 −54.984 27.845 42.084 0.00 0.00
C
ATOM 9685 HN LYS C 184 −54.726 27.732 43.041 0.00 0.00
C
ATOM 9686 CA LYS C 184 −55.009 26.598 41.344 0.00 0.00
C
ATOM 9687 HA LYS C 184 −54.697 26.792 40.329 0.00 0.00
C
ATOM 9688 CB LYS C 184 −53.958 25.649 41.908 0.00 0.00
C
ATOM 9689 HB1 LYS C 184 −53.016 26.229 41.799 0.00 0.00
C
ATOM 9690 HB2 LYS C 184 −54.188 25.541 42.989 0.00 0.00
C
ATOM 9691 CG LYS C 184 −53.793 24.348 41.205 0.00 0.00
C
ATOM 9692 HG1 LYS C 184 −54.711 23.728 41.290 0.00 0.00
C

ATOM 9693 HG2 LYS C 184 −53.546 24.491 40.131 0.00 0.00
C
ATOM 9694 CD LYS C 184 −52.576 23.505 41.749 0.00 0.00
C
ATOM 9695 HD1 LYS C 184 −52.609 23.612 42.854 0.00 0.00
C
ATOM 9696 HD2 LYS C 184 −52.796 22.460 41.443 0.00 0.00
C
ATOM 9697 CE LYS C 184 −51.162 23.857 41.216 0.00 0.00
C
ATOM 9698 HE1 LYS C 184 −51.097 24.006 40.117 0.00 0.00
C
ATOM 9699 HE2 LYS C 184 −50.863 24.800 41.722 0.00 0.00
C
ATOM 9700 NZ LYS C 184 −50.109 22.865 41.606 0.00 0.00
C
ATOM 9701 HZ1 LYS C 184 −50.195 21.975 41.075 0.00 0.00
C
ATOM 9702 HZ2 LYS C 184 −49.174 23.316 41.539 0.00 0.00
C
ATOM 9703 HZ3 LYS C 184 −50.339 22.641 42.595 0.00 0.00
C
ATOM 9704 C LYS C 184 −56.441 26.006 41.468 0.00 0.00
C
ATOM 9705 O LYS C 184 −57.042 25.678 42.475 0.00 0.00
C
ATOM 9706 N HSE C 185 −57.023 25.832 40.239 0.00 0.00
C
ATOM 9707 HN HSE C 185 −56.667 26.260 39.411 0.00 0.00
C
ATOM 9708 CA HSE C 185 −58.230 25.136 40.008 0.00 0.00
C
ATOM 9709 HA HSE C 185 −58.429 24.541 40.887 0.00 0.00
C
ATOM 9710 CB HSE C 185 −59.504 25.974 39.729 0.00 0.00
C
ATOM 9711 HB1 HSE C 185 −59.438 26.545 38.778 0.00 0.00
C
ATOM 9712 HB2 HSE C 185 −60.301 25.256 39.441 0.00 0.00
C
ATOM 9713 ND1 HSE C 185 −61.056 27.673 40.464 0.00 0.00
C
ATOM 9714 CG HSE C 185 −60.079 26.740 40.787 0.00 0.00
C
ATOM 9715 CE1 HSE C 185 −61.494 28.158 41.671 0.00 0.00
C
ATOM 9716 HE1 HSE C 185 −62.396 28.754 41.816 0.00 0.00
C
ATOM 9717 NE2 HSE C 185 −60.941 27.513 42.681 0.00 0.00
C
ATOM 9718 HE2 HSE C 185 −61.232 27.365 43.626 0.00 0.00
C
ATOM 9719 CD2 HSE C 185 −59.996 26.624 42.151 0.00 0.00
C

```
ATOM   9720  HD2 HSE C 185     -59.396  25.918  42.711  0.00  0.00
C
ATOM   9721  C   HSE C 185     -57.988  24.000  39.005  0.00  0.00
C
ATOM   9722  O   HSE C 185     -57.300  24.198  38.000  0.00  0.00
C
ATOM   9723  N   HSE C 186     -58.386  22.762  39.292  0.00  0.00
C
ATOM   9724  HN  HSE C 186     -58.888  22.593  40.136  0.00  0.00
C
ATOM   9725  CA  HSE C 186     -58.296  21.636  38.359  0.00  0.00
C
ATOM   9726  HA  HSE C 186     -58.764  20.797  38.853  0.00  0.00
C
ATOM   9727  CB  HSE C 186     -59.029  21.847  37.012  0.00  0.00
C
ATOM   9728  HB1 HSE C 186     -59.012  22.940  36.811  0.00  0.00
C
ATOM   9729  HB2 HSE C 186     -58.582  21.371  36.113  0.00  0.00
C
ATOM   9730  ND1 HSE C 186     -60.980  20.382  37.285  0.00  0.00
C
ATOM   9731  CG  HSE C 186     -60.585  21.713  37.134  0.00  0.00
C
ATOM   9732  CE1 HSE C 186     -62.321  20.519  37.253  0.00  0.00
C
ATOM   9733  HE1 HSE C 186     -62.930  19.616  37.234  0.00  0.00
C
ATOM   9734  NE2 HSE C 186     -62.710  21.807  37.031  0.00  0.00
C
ATOM   9735  HE2 HSE C 186     -63.658  22.115  36.961  0.00  0.00
C
ATOM   9736  CD2 HSE C 186     -61.585  22.564  36.877  0.00  0.00
C
ATOM   9737  HD2 HSE C 186     -61.581  23.598  36.557  0.00  0.00
C
ATOM   9738  C   HSE C 186     -56.841  21.199  38.105  0.00  0.00
C
ATOM   9739  O   HSE C 186     -56.525  20.462  37.201  0.00  0.00
C
ATOM   9740  N   GLY C 187     -55.914  21.670  38.966  0.00  0.00
C
ATOM   9741  HN  GLY C 187     -56.126  22.274  39.731  0.00  0.00
C
ATOM   9742  CA  GLY C 187     -54.486  21.481  38.716  0.00  0.00
C
ATOM   9743  HA1 GLY C 187     -54.213  20.558  38.227  0.00  0.00
C
ATOM   9744  HA2 GLY C 187     -54.045  21.545  39.700  0.00  0.00
C
ATOM   9745  C   GLY C 187     -53.852  22.475  37.789  0.00  0.00
C
ATOM   9746  O   GLY C 187     -52.677  22.336  37.457  0.00  0.00
C
```

ATOM 9747 N ASP C 188 −54.629 23.484 37.297 0.00 0.00
C
ATOM 9748 HN ASP C 188 −55.603 23.641 37.439 0.00 0.00
C
ATOM 9749 CA ASP C 188 −54.062 24.511 36.416 0.00 0.00
C
ATOM 9750 HA ASP C 188 −53.122 24.267 35.945 0.00 0.00
C
ATOM 9751 CB ASP C 188 −55.079 24.802 35.200 0.00 0.00
C
ATOM 9752 HB1 ASP C 188 −56.089 25.049 35.591 0.00 0.00
C
ATOM 9753 HB2 ASP C 188 −54.740 25.615 34.523 0.00 0.00
C
ATOM 9754 CG ASP C 188 −55.395 23.654 34.257 0.00 0.00
C
ATOM 9755 OD1 ASP C 188 −54.760 22.558 34.357 0.00 0.00
C
ATOM 9756 OD2 ASP C 188 −56.175 23.935 33.327 0.00 0.00
C
ATOM 9757 C ASP C 188 −53.970 25.782 37.161 0.00 0.00
C
ATOM 9758 O ASP C 188 −54.794 26.089 38.026 0.00 0.00
C
ATOM 9759 N ILE C 189 −53.163 26.790 36.763 0.00 0.00
C
ATOM 9760 HN ILE C 189 −52.438 26.524 36.132 0.00 0.00
C
ATOM 9761 CA ILE C 189 −53.252 28.137 37.231 0.00 0.00
C
ATOM 9762 HA ILE C 189 −53.542 27.982 38.260 0.00 0.00
C
ATOM 9763 CB ILE C 189 −51.961 28.923 36.989 0.00 0.00
C
ATOM 9764 HB ILE C 189 −51.804 29.173 35.918 0.00 0.00
C
ATOM 9765 CG2 ILE C 189 −52.002 30.264 37.795 0.00 0.00
C
ATOM 9766 HG21 ILE C 189 −52.700 30.989 37.324 0.00 0.00
C
ATOM 9767 HG22 ILE C 189 −52.330 30.076 38.839 0.00 0.00
C
ATOM 9768 HG23 ILE C 189 −50.952 30.616 37.706 0.00 0.00
C
ATOM 9769 CG1 ILE C 189 −50.720 27.972 37.323 0.00 0.00
C
ATOM 9770 HG11 ILE C 189 −50.740 26.979 36.825 0.00 0.00
C
ATOM 9771 HG12 ILE C 189 −49.816 28.408 36.846 0.00 0.00
C
ATOM 9772 CD ILE C 189 −50.502 27.661 38.793 0.00 0.00
C
ATOM 9773 HD1 ILE C 189 −49.576 27.126 39.092 0.00 0.00
C

```
ATOM   9774  HD2 ILE C 189     -50.569  28.624  39.344  0.00  0.00
C
ATOM   9775  HD3 ILE C 189     -51.300  26.987  39.173  0.00  0.00
C
ATOM   9776  C   ILE C 189     -54.343  28.942  36.592  0.00  0.00
C
ATOM   9777  O   ILE C 189     -54.509  29.119  35.395  0.00  0.00
C
ATOM   9778  N   CYS C 190     -55.184  29.500  37.493  0.00  0.00
C
ATOM   9779  HN  CYS C 190     -55.045  29.191  38.430  0.00  0.00
C
ATOM   9780  CA  CYS C 190     -56.104  30.536  37.214  0.00  0.00
C
ATOM   9781  HA  CYS C 190     -56.095  30.738  36.153  0.00  0.00
C
ATOM   9782  CB  CYS C 190     -57.542  30.098  37.424  0.00  0.00
C
ATOM   9783  HB1 CYS C 190     -58.199  30.993  37.414  0.00  0.00
C
ATOM   9784  HB2 CYS C 190     -57.739  29.533  36.487  0.00  0.00
C
ATOM   9785  SG  CYS C 190     -57.882  28.955  38.728  0.00  0.00
C
ATOM   9786  HG1 CYS C 190     -58.147  29.844  39.674  0.00  0.00
C
ATOM   9787  C   CYS C 190     -55.731  31.911  37.901  0.00  0.00
C
ATOM   9788  O   CYS C 190     -55.667  32.125  39.090  0.00  0.00
C
ATOM   9789  N   LEU C 191     -55.421  32.950  37.093  0.00  0.00
C
ATOM   9790  HN  LEU C 191     -55.529  32.869  36.105  0.00  0.00
C
ATOM   9791  CA  LEU C 191     -54.857  34.219  37.484  0.00  0.00
C
ATOM   9792  HA  LEU C 191     -54.261  33.986  38.354  0.00  0.00
C
ATOM   9793  CB  LEU C 191     -53.927  34.887  36.454  0.00  0.00
C
ATOM   9794  HB1 LEU C 191     -54.459  34.978  35.483  0.00  0.00
C
ATOM   9795  HB2 LEU C 191     -53.623  35.879  36.852  0.00  0.00
C
ATOM   9796  CG  LEU C 191     -52.606  34.067  36.053  0.00  0.00
C
ATOM   9797  HG  LEU C 191     -53.004  33.095  35.694  0.00  0.00
C
ATOM   9798  CD1 LEU C 191     -51.957  34.701  34.783  0.00  0.00
C
ATOM   9799 HD11 LEU C 191     -51.640  35.741  35.014  0.00  0.00
C
ATOM   9800 HD12 LEU C 191     -51.015  34.165  34.539  0.00  0.00
C
```

ATOM 9801 HD13 LEU C 191 -52.749 34.592 34.012 0.00 0.00
C
ATOM 9802 CD2 LEU C 191 -51.663 33.854 37.196 0.00 0.00
C
ATOM 9803 HD21 LEU C 191 -52.104 33.221 37.995 0.00 0.00
C
ATOM 9804 HD22 LEU C 191 -50.652 33.421 37.040 0.00 0.00
C
ATOM 9805 HD23 LEU C 191 -51.568 34.813 37.749 0.00 0.00
C
ATOM 9806 C LEU C 191 -55.965 35.238 37.839 0.00 0.00
C
ATOM 9807 O LEU C 191 -57.029 35.218 37.208 0.00 0.00
C
ATOM 9808 N SER C 192 -55.631 36.084 38.813 0.00 0.00
C
ATOM 9809 HN SER C 192 -54.765 35.843 39.244 0.00 0.00
C
ATOM 9810 CA SER C 192 -56.326 37.220 39.416 0.00 0.00
C
ATOM 9811 HA SER C 192 -55.892 37.525 40.357 0.00 0.00
C
ATOM 9812 CB SER C 192 -56.281 38.540 38.586 0.00 0.00
C
ATOM 9813 HB1 SER C 192 -56.671 38.357 37.562 0.00 0.00
C
ATOM 9814 HB2 SER C 192 -56.961 39.265 39.083 0.00 0.00
C
ATOM 9815 OG SER C 192 -54.963 39.154 38.588 0.00 0.00
C
ATOM 9816 HG1 SER C 192 -54.537 38.829 37.791 0.00 0.00
C
ATOM 9817 C SER C 192 -57.797 36.823 39.659 0.00 0.00
C
ATOM 9818 O SER C 192 -58.717 37.193 38.902 0.00 0.00
C
ATOM 9819 N PRO C 193 -58.093 36.037 40.691 0.00 0.00
C
ATOM 9820 CD PRO C 193 -57.116 35.746 41.781 0.00 0.00
C
ATOM 9821 HD1 PRO C 193 -56.041 35.582 41.550 0.00 0.00
C
ATOM 9822 HD2 PRO C 193 -57.173 36.647 42.428 0.00 0.00
C
ATOM 9823 CA PRO C 193 -59.410 35.422 40.919 0.00 0.00
C
ATOM 9824 HA PRO C 193 -59.411 34.638 40.177 0.00 0.00
C
ATOM 9825 CB PRO C 193 -59.295 34.778 42.343 0.00 0.00
C
ATOM 9826 HB1 PRO C 193 -59.826 33.805 42.415 0.00 0.00
C
ATOM 9827 HB2 PRO C 193 -59.668 35.607 42.982 0.00 0.00
C

ATOM 9828 CG PRO C 193 -57.789 34.613 42.519 0.00 0.00
C
ATOM 9829 HG1 PRO C 193 -57.541 33.605 42.124 0.00 0.00
C
ATOM 9830 HG2 PRO C 193 -57.544 34.521 43.599 0.00 0.00
C
ATOM 9831 C PRO C 193 -60.642 36.186 40.636 0.00 0.00
C
ATOM 9832 O PRO C 193 -60.712 37.346 40.998 0.00 0.00
C
ATOM 9833 N ALA C 194 -61.600 35.563 39.975 0.00 0.00
C
ATOM 9834 HN ALA C 194 -61.357 34.637 39.699 0.00 0.00
C
ATOM 9835 CA ALA C 194 -63.016 35.947 39.674 0.00 0.00
C
ATOM 9836 HA ALA C 194 -63.223 36.948 40.021 0.00 0.00
C
ATOM 9837 CB ALA C 194 -63.329 36.000 38.176 0.00 0.00
C
ATOM 9838 HB1 ALA C 194 -63.083 35.084 37.597 0.00 0.00
C
ATOM 9839 HB2 ALA C 194 -64.337 36.350 37.868 0.00 0.00
C
ATOM 9840 HB3 ALA C 194 -62.533 36.666 37.779 0.00 0.00
C
ATOM 9841 C ALA C 194 -64.185 35.144 40.238 0.00 0.00
C
ATOM 9842 O ALA C 194 -63.957 33.941 40.442 0.00 0.00
C
ATOM 9843 N PRO C 195 -65.321 35.705 40.619 0.00 0.00
C
ATOM 9844 CD PRO C 195 -65.813 37.062 40.081 0.00 0.00
C
ATOM 9845 HD1 PRO C 195 -65.095 37.896 40.237 0.00 0.00
C
ATOM 9846 HD2 PRO C 195 -66.120 36.910 39.025 0.00 0.00
C
ATOM 9847 CA PRO C 195 -66.384 34.967 41.265 0.00 0.00
C
ATOM 9848 HA PRO C 195 -65.960 34.657 42.209 0.00 0.00
C
ATOM 9849 CB PRO C 195 -67.565 35.973 41.350 0.00 0.00
C
ATOM 9850 HB1 PRO C 195 -67.956 36.001 42.390 0.00 0.00
C
ATOM 9851 HB2 PRO C 195 -68.366 35.846 40.590 0.00 0.00
C
ATOM 9852 CG PRO C 195 -66.901 37.335 41.057 0.00 0.00
C
ATOM 9853 HG1 PRO C 195 -66.458 37.600 42.040 0.00 0.00
C
ATOM 9854 HG2 PRO C 195 -67.682 37.987 40.609 0.00 0.00
C

ATOM 9855 C PRO C 195 −66.951 33.669 40.518 0.00 0.00
C
ATOM 9856 O PRO C 195 −67.173 33.752 39.305 0.00 0.00
C
ATOM 9857 N GLU C 196 −67.133 32.488 41.129 0.00 0.00
C
ATOM 9858 HN GLU C 196 −67.553 31.787 40.557 0.00 0.00
C
ATOM 9859 CA GLU C 196 −67.306 32.352 42.543 0.00 0.00
C
ATOM 9860 HA GLU C 196 −66.826 33.163 43.072 0.00 0.00
C
ATOM 9861 CB GLU C 196 −68.795 32.293 43.014 0.00 0.00
C
ATOM 9862 HB1 GLU C 196 −69.189 31.362 42.553 0.00 0.00
C
ATOM 9863 HB2 GLU C 196 −68.860 32.218 44.121 0.00 0.00
C
ATOM 9864 CG GLU C 196 −69.799 33.451 42.592 0.00 0.00
C
ATOM 9865 HG1 GLU C 196 −69.437 34.434 42.964 0.00 0.00
C
ATOM 9866 HG2 GLU C 196 −70.008 33.515 41.503 0.00 0.00
C
ATOM 9867 CD GLU C 196 −71.143 33.072 43.237 0.00 0.00
C
ATOM 9868 OE1 GLU C 196 −71.778 32.030 42.960 0.00 0.00
C
ATOM 9869 OE2 GLU C 196 −71.589 33.848 44.085 0.00 0.00
C
ATOM 9870 C GLU C 196 −66.715 31.073 43.105 0.00 0.00
C
ATOM 9871 O GLU C 196 −66.828 30.887 44.305 0.00 0.00
C
ATOM 9872 N ASP C 197 −66.143 30.104 42.322 0.00 0.00
C
ATOM 9873 HN ASP C 197 −66.072 30.228 41.335 0.00 0.00
C
ATOM 9874 CA ASP C 197 −65.577 28.839 42.720 0.00 0.00
C
ATOM 9875 HA ASP C 197 −66.447 28.272 43.018 0.00 0.00
C
ATOM 9876 CB ASP C 197 −64.834 28.197 41.546 0.00 0.00
C
ATOM 9877 HB1 ASP C 197 −64.060 28.873 41.123 0.00 0.00
C
ATOM 9878 HB2 ASP C 197 −64.423 27.204 41.829 0.00 0.00
C
ATOM 9879 CG ASP C 197 −65.904 28.058 40.455 0.00 0.00
C
ATOM 9880 OD1 ASP C 197 −66.793 27.209 40.630 0.00 0.00
C
ATOM 9881 OD2 ASP C 197 −65.911 28.636 39.399 0.00 0.00
C

ATOM 9882 C ASP C 197 −64.641 28.803 43.934 0.00 0.00
C
ATOM 9883 O ASP C 197 −63.722 29.628 43.933 0.00 0.00
C
ATOM 9884 N ILE C 198 −64.843 27.776 44.868 0.00 0.00
C
ATOM 9885 HN ILE C 198 −65.562 27.086 44.839 0.00 0.00
C
ATOM 9886 CA ILE C 198 −64.014 27.708 46.094 0.00 0.00
C
ATOM 9887 HA ILE C 198 −64.060 28.596 46.707 0.00 0.00
C
ATOM 9888 CB ILE C 198 −64.639 26.688 47.039 0.00 0.00
C
ATOM 9889 HB ILE C 198 −63.973 26.520 47.912 0.00 0.00
C
ATOM 9890 CG2 ILE C 198 −65.893 27.462 47.636 0.00 0.00
C
ATOM 9891 HG21 ILE C 198 −65.697 28.524 47.894 0.00 0.00
C
ATOM 9892 HG22 ILE C 198 −66.776 27.384 46.966 0.00 0.00
C
ATOM 9893 HG23 ILE C 198 −66.172 27.032 48.621 0.00 0.00
C
ATOM 9894 CG1 ILE C 198 −65.056 25.372 46.313 0.00 0.00
C
ATOM 9895 HG11 ILE C 198 −65.860 25.678 45.611 0.00 0.00
C
ATOM 9896 HG12 ILE C 198 −64.233 24.953 45.695 0.00 0.00
C
ATOM 9897 CD ILE C 198 −65.681 24.256 47.217 0.00 0.00
C
ATOM 9898 HD1 ILE C 198 −64.921 24.063 48.004 0.00 0.00
C
ATOM 9899 HD2 ILE C 198 −66.625 24.659 47.643 0.00 0.00
C
ATOM 9900 HD3 ILE C 198 −66.095 23.470 46.550 0.00 0.00
C
ATOM 9901 C ILE C 198 −62.524 27.366 45.834 0.00 0.00
C
ATOM 9902 O ILE C 198 −62.154 26.606 44.934 0.00 0.00
C
ATOM 9903 N ILE C 199 −61.488 27.888 46.611 0.00 0.00
C
ATOM 9904 HN ILE C 199 −61.757 28.473 47.372 0.00 0.00
C
ATOM 9905 CA ILE C 199 −60.191 27.277 46.724 0.00 0.00
C
ATOM 9906 HA ILE C 199 −59.910 26.967 45.728 0.00 0.00
C
ATOM 9907 CB ILE C 199 −59.072 28.270 47.221 0.00 0.00
C
ATOM 9908 HB ILE C 199 −59.080 29.164 46.561 0.00 0.00
C

ATOM 9909 CG2 ILE C 199 -59.261 28.705 48.687 0.00 0.00
C
ATOM 9910 HG21 ILE C 199 -58.555 29.484 49.046 0.00 0.00
C
ATOM 9911 HG22 ILE C 199 -60.241 29.142 48.977 0.00 0.00
C
ATOM 9912 HG23 ILE C 199 -59.086 27.826 49.344 0.00 0.00
C
ATOM 9913 CG1 ILE C 199 -57.625 27.698 46.818 0.00 0.00
C
ATOM 9914 HG11 ILE C 199 -57.507 26.680 47.247 0.00 0.00
C
ATOM 9915 HG12 ILE C 199 -57.465 27.533 45.731 0.00 0.00
C
ATOM 9916 CD ILE C 199 -56.429 28.625 47.078 0.00 0.00
C
ATOM 9917 HD1 ILE C 199 -56.352 29.010 48.117 0.00 0.00
C
ATOM 9918 HD2 ILE C 199 -55.503 28.119 46.730 0.00 0.00
C
ATOM 9919 HD3 ILE C 199 -56.500 29.571 46.500 0.00 0.00
C
ATOM 9920 C ILE C 199 -60.439 25.987 47.557 0.00 0.00
C
ATOM 9921 O ILE C 199 -61.206 25.960 48.550 0.00 0.00
C
ATOM 9922 N ARG C 200 -59.619 24.931 47.335 0.00 0.00
C
ATOM 9923 HN ARG C 200 -59.002 24.955 46.551 0.00 0.00
C
ATOM 9924 CA ARG C 200 -59.560 23.700 48.090 0.00 0.00
C
ATOM 9925 HA ARG C 200 -60.362 23.730 48.813 0.00 0.00
C
ATOM 9926 CB ARG C 200 -60.225 22.548 47.270 0.00 0.00
C
ATOM 9927 HB1 ARG C 200 -59.731 22.325 46.301 0.00 0.00
C
ATOM 9928 HB2 ARG C 200 -60.178 21.669 47.948 0.00 0.00
C
ATOM 9929 CG ARG C 200 -61.776 22.800 46.986 0.00 0.00
C
ATOM 9930 HG1 ARG C 200 -62.094 23.694 47.563 0.00 0.00
C
ATOM 9931 HG2 ARG C 200 -61.908 23.051 45.912 0.00 0.00
C
ATOM 9932 CD ARG C 200 -62.853 21.729 47.189 0.00 0.00
C
ATOM 9933 HD1 ARG C 200 -63.801 22.071 46.723 0.00 0.00
C
ATOM 9934 HD2 ARG C 200 -62.448 20.795 46.742 0.00 0.00
C
ATOM 9935 NE ARG C 200 -62.941 21.372 48.680 0.00 0.00
C

ATOM 9936 HE ARG C 200 −62.272 21.731 49.331 0.00 0.00
C
ATOM 9937 CZ ARG C 200 −64.066 20.975 49.299 0.00 0.00
C
ATOM 9938 NH1 ARG C 200 −65.104 20.532 48.621 0.00 0.00
C
ATOM 9939 HH11 ARG C 200 −65.928 20.170 49.057 0.00 0.00
C
ATOM 9940 HH12 ARG C 200 −65.119 20.531 47.621 0.00 0.00
C
ATOM 9941 NH2 ARG C 200 −64.057 20.920 50.615 0.00 0.00
C
ATOM 9942 HH21 ARG C 200 −64.564 20.126 50.953 0.00 0.00
C
ATOM 9943 HH22 ARG C 200 −63.244 21.139 51.155 0.00 0.00
C
ATOM 9944 C ARG C 200 −58.214 23.299 48.694 0.00 0.00
C
ATOM 9945 O ARG C 200 −57.147 23.694 48.239 0.00 0.00
C
ATOM 9946 N GLU C 201 −58.229 22.435 49.715 0.00 0.00
C
ATOM 9947 HN GLU C 201 −59.132 22.251 50.096 0.00 0.00
C
ATOM 9948 CA GLU C 201 −57.089 21.962 50.408 0.00 0.00
C
ATOM 9949 HA GLU C 201 −56.614 22.790 50.914 0.00 0.00
C
ATOM 9950 CB GLU C 201 −57.682 20.870 51.446 0.00 0.00
C
ATOM 9951 HB1 GLU C 201 −58.282 21.350 52.249 0.00 0.00
C
ATOM 9952 HB2 GLU C 201 −58.408 20.188 50.953 0.00 0.00
C
ATOM 9953 CG GLU C 201 −56.622 20.055 52.197 0.00 0.00
C
ATOM 9954 HG1 GLU C 201 −56.114 19.426 51.436 0.00 0.00
C
ATOM 9955 HG2 GLU C 201 −55.874 20.757 52.623 0.00 0.00
C
ATOM 9956 CD GLU C 201 −57.127 19.220 53.346 0.00 0.00
C
ATOM 9957 OE1 GLU C 201 −58.347 19.209 53.708 0.00 0.00
C
ATOM 9958 OE2 GLU C 201 −56.297 18.463 53.912 0.00 0.00
C
ATOM 9959 C GLU C 201 −56.003 21.400 49.555 0.00 0.00
C
ATOM 9960 O GLU C 201 −56.196 20.541 48.730 0.00 0.00
C
ATOM 9961 N GLN C 202 −54.746 21.825 49.787 0.00 0.00
C
ATOM 9962 HN GLN C 202 −54.419 22.418 50.519 0.00 0.00
C

ATOM 9963 CA GLN C 202 -53.521 21.408 49.075 0.00 0.00
C
ATOM 9964 HA GLN C 202 -52.697 21.881 49.589 0.00 0.00
C
ATOM 9965 CB GLN C 202 -53.114 19.879 49.220 0.00 0.00
C
ATOM 9966 HB1 GLN C 202 -53.889 19.346 48.628 0.00 0.00
C
ATOM 9967 HB2 GLN C 202 -52.093 19.734 48.807 0.00 0.00
C
ATOM 9968 CG GLN C 202 -53.156 19.274 50.658 0.00 0.00
C
ATOM 9969 HG1 GLN C 202 -54.174 19.521 51.027 0.00 0.00
C
ATOM 9970 HG2 GLN C 202 -53.066 18.170 50.573 0.00 0.00
C
ATOM 9971 CD GLN C 202 -52.080 19.585 51.743 0.00 0.00
C
ATOM 9972 OE1 GLN C 202 -50.906 19.398 51.600 0.00 0.00
C
ATOM 9973 NE2 GLN C 202 -52.576 20.111 52.879 0.00 0.00
C
ATOM 9974 HE21 GLN C 202 -51.973 20.347 53.642 0.00 0.00
C
ATOM 9975 HE22 GLN C 202 -53.572 20.196 52.902 0.00 0.00
C
ATOM 9976 C GLN C 202 -53.471 21.903 47.651 0.00 0.00
C
ATOM 9977 O GLN C 202 -52.862 21.230 46.756 0.00 0.00
C
ATOM 9978 N ASP C 203 -53.987 23.072 47.382 0.00 0.00
C
ATOM 9979 HN ASP C 203 -54.527 23.522 48.089 0.00 0.00
C
ATOM 9980 CA ASP C 203 -53.771 23.724 46.117 0.00 0.00
C
ATOM 9981 HA ASP C 203 -53.148 23.095 45.499 0.00 0.00
C
ATOM 9982 CB ASP C 203 -55.111 23.934 45.308 0.00 0.00
C
ATOM 9983 HB1 ASP C 203 -55.929 24.334 45.943 0.00 0.00
C
ATOM 9984 HB2 ASP C 203 -54.967 24.561 44.402 0.00 0.00
C
ATOM 9985 CG ASP C 203 -55.623 22.587 44.752 0.00 0.00
C
ATOM 9986 OD1 ASP C 203 -56.803 22.185 44.946 0.00 0.00
C
ATOM 9987 OD2 ASP C 203 -54.861 22.055 43.907 0.00 0.00
C
ATOM 9988 C ASP C 203 -53.036 25.012 46.266 0.00 0.00
C
ATOM 9989 O ASP C 203 -53.021 25.756 47.242 0.00 0.00
C

```
ATOM   9990  N   CYS C 204     -52.177  25.310  45.283  0.00  0.00
C
ATOM   9991  HN  CYS C 204     -51.942  24.667  44.559  0.00  0.00
C
ATOM   9992  CA  CYS C 204     -51.363  26.506  45.323  0.00  0.00
C
ATOM   9993  HA  CYS C 204     -50.806  26.449  46.247  0.00  0.00
C
ATOM   9994  CB  CYS C 204     -50.166  26.541  44.302  0.00  0.00
C
ATOM   9995  HB1 CYS C 204     -50.618  26.594  43.288  0.00  0.00
C
ATOM   9996  HB2 CYS C 204     -49.608  27.490  44.452  0.00  0.00
C
ATOM   9997  SG  CYS C 204     -49.078  25.067  44.581  0.00  0.00
C
ATOM   9998  HG1 CYS C 204     -48.479  25.639  45.615  0.00  0.00
C
ATOM   9999  C   CYS C 204     -52.114  27.812  45.155  0.00  0.00
C
ATOM  10000  O   CYS C 204     -53.182  27.889  44.560  0.00  0.00
C
ATOM  10001  N   LEU C 205     -51.569  28.910  45.720  0.00  0.00
C
ATOM  10002  HN  LEU C 205     -50.722  28.916  46.247  0.00  0.00
C
ATOM  10003  CA  LEU C 205     -52.027  30.229  45.515  0.00  0.00
C
ATOM  10004  HA  LEU C 205     -52.406  30.251  44.504  0.00  0.00
C
ATOM  10005  CB  LEU C 205     -53.161  30.654  46.477  0.00  0.00
C
ATOM  10006  HB1 LEU C 205     -53.514  31.670  46.200  0.00  0.00
C
ATOM  10007  HB2 LEU C 205     -53.989  29.933  46.304  0.00  0.00
C
ATOM  10008  CG  LEU C 205     -52.844  30.520  48.044  0.00  0.00
C
ATOM  10009  HG  LEU C 205     -51.757  30.677  48.211  0.00  0.00
C
ATOM  10010  CD1 LEU C 205     -53.496  31.623  48.880  0.00  0.00
C
ATOM  10011 HD11 LEU C 205     -53.380  32.653  48.478  0.00  0.00
C
ATOM  10012 HD12 LEU C 205     -54.600  31.510  48.909  0.00  0.00
C
ATOM  10013 HD13 LEU C 205     -53.160  31.588  49.938  0.00  0.00
C
ATOM  10014  CD2 LEU C 205     -53.005  29.129  48.659  0.00  0.00
C
ATOM  10015 HD21 LEU C 205     -54.030  28.715  48.550  0.00  0.00
C
ATOM  10016 HD22 LEU C 205     -52.241  28.429  48.258  0.00  0.00
C
```

ATOM 10017 HD23 LEU C 205   -52.644 29.305 49.695 0.00 0.00
C
ATOM 10018 C   LEU C 205   -50.848 31.163 45.610 0.00 0.00
C
ATOM 10019 O   LEU C 205   -49.754 30.690 45.820 0.00 0.00
C
ATOM 10020 N   VAL C 206   -51.070 32.450 45.198 0.00 0.00
C
ATOM 10021 HN VAL C 206   -51.975 32.725 44.883 0.00 0.00
C
ATOM 10022 CA VAL C 206   -50.046 33.467 45.429 0.00 0.00
C
ATOM 10023 HA VAL C 206   -49.299 33.014 46.065 0.00 0.00
C
ATOM 10024 CB VAL C 206   -49.435 34.040 44.186 0.00 0.00
C
ATOM 10025 HB VAL C 206   -50.206 34.452 43.499 0.00 0.00
C
ATOM 10026 CG1 VAL C 206   -48.484 35.295 44.318 0.00 0.00
C
ATOM 10027 HG11 VAL C 206   -48.102 35.496 43.294 0.00 0.00
C
ATOM 10028 HG12 VAL C 206   -49.108 36.179 44.568 0.00 0.00
C
ATOM 10029 HG13 VAL C 206   -47.567 35.088 44.910 0.00 0.00
C
ATOM 10030 CG2 VAL C 206   -48.678 32.821 43.618 0.00 0.00
C
ATOM 10031 HG21 VAL C 206   -48.151 33.174 42.706 0.00 0.00
C
ATOM 10032 HG22 VAL C 206   -47.915 32.446 44.333 0.00 0.00
C
ATOM 10033 HG23 VAL C 206   -49.318 31.947 43.373 0.00 0.00
C
ATOM 10034 C   VAL C 206   -50.682 34.660 46.190 0.00 0.00
C
ATOM 10035 O   VAL C 206   -51.640 35.236 45.678 0.00 0.00
C
ATOM 10036 N   ILE C 207   -50.088 35.093 47.317 0.00 0.00
C
ATOM 10037 HN ILE C 207   -49.360 34.629 47.815 0.00 0.00
C
ATOM 10038 CA ILE C 207   -50.487 36.373 48.012 0.00 0.00
C
ATOM 10039 HA ILE C 207   -51.339 36.842 47.542 0.00 0.00
C
ATOM 10040 CB ILE C 207   -50.823 36.165 49.477 0.00 0.00
C
ATOM 10041 HB ILE C 207   -51.604 35.377 49.520 0.00 0.00
C
ATOM 10042 CG2 ILE C 207   -49.698 35.686 50.342 0.00 0.00
C
ATOM 10043 HG21 ILE C 207   -49.143 34.812 49.941 0.00 0.00
C

ATOM 10044 HG22 ILE C 207 −48.996 36.544 50.270 0.00 0.00
C
ATOM 10045 HG23 ILE C 207 −49.914 35.630 51.430 0.00 0.00
C
ATOM 10046 CG1 ILE C 207 −51.542 37.333 50.118 0.00 0.00
C
ATOM 10047 HG11 ILE C 207 −50.805 38.157 50.231 0.00 0.00
C
ATOM 10048 HG12 ILE C 207 −52.147 37.826 49.326 0.00 0.00
C
ATOM 10049 CD ILE C 207 −52.362 37.022 51.368 0.00 0.00
C
ATOM 10050 HD1 ILE C 207 −53.278 36.478 51.054 0.00 0.00
C
ATOM 10051 HD2 ILE C 207 −51.879 36.314 52.075 0.00 0.00
C
ATOM 10052 HD3 ILE C 207 −52.688 37.977 51.833 0.00 0.00
C
ATOM 10053 C ILE C 207 −49.369 37.447 47.854 0.00 0.00
C
ATOM 10054 O ILE C 207 −48.181 37.154 47.807 0.00 0.00
C
ATOM 10055 N MET C 208 −49.725 38.748 47.595 0.00 0.00
C
ATOM 10056 HN MET C 208 −50.667 39.029 47.761 0.00 0.00
C
ATOM 10057 CA MET C 208 −48.771 39.858 47.489 0.00 0.00
C
ATOM 10058 HA MET C 208 −47.729 39.611 47.628 0.00 0.00
C
ATOM 10059 CB MET C 208 −48.849 40.374 46.057 0.00 0.00
C
ATOM 10060 HB1 MET C 208 −48.406 39.563 45.440 0.00 0.00
C
ATOM 10061 HB2 MET C 208 −49.940 40.458 45.866 0.00 0.00
C
ATOM 10062 CG MET C 208 −48.211 41.754 45.758 0.00 0.00
C
ATOM 10063 HG1 MET C 208 −48.965 42.489 46.113 0.00 0.00
C
ATOM 10064 HG2 MET C 208 −47.279 41.883 46.349 0.00 0.00
C
ATOM 10065 SD MET C 208 −47.860 42.047 43.929 0.00 0.00
C
ATOM 10066 CE MET C 208 −47.303 43.783 44.157 0.00 0.00
C
ATOM 10067 HE1 MET C 208 −48.205 44.272 44.585 0.00 0.00
C
ATOM 10068 HE2 MET C 208 −46.478 43.833 44.899 0.00 0.00
C
ATOM 10069 HE3 MET C 208 −47.058 44.196 43.155 0.00 0.00
C
ATOM 10070 C MET C 208 −49.171 40.920 48.505 0.00 0.00
C

ATOM 10071 O MET C 208 -50.317 41.229 48.679 0.00 0.00
C
ATOM 10072 N GLY C 209 -48.251 41.474 49.270 0.00 0.00
C
ATOM 10073 HN GLY C 209 -47.279 41.360 49.080 0.00 0.00
C
ATOM 10074 CA GLY C 209 -48.582 42.448 50.349 0.00 0.00
C
ATOM 10075 HA1 GLY C 209 -49.355 41.967 50.931 0.00 0.00
C
ATOM 10076 HA2 GLY C 209 -48.815 43.431 49.966 0.00 0.00
C
ATOM 10077 C GLY C 209 -47.392 42.622 51.310 0.00 0.00
C
ATOM 10078 O GLY C 209 -46.223 42.391 50.967 0.00 0.00
C
ATOM 10079 N HSE C 210 -47.610 43.054 52.515 0.00 0.00
C
ATOM 10080 HN HSE C 210 -48.546 43.154 52.842 0.00 0.00
C
ATOM 10081 CA HSE C 210 -46.607 43.445 53.461 0.00 0.00
C
ATOM 10082 HA HSE C 210 -45.750 43.889 52.975 0.00 0.00
C
ATOM 10083 CB HSE C 210 -47.113 44.394 54.491 0.00 0.00
C
ATOM 10084 HB1 HSE C 210 -47.501 45.313 54.003 0.00 0.00
C
ATOM 10085 HB2 HSE C 210 -47.966 44.067 55.122 0.00 0.00
C
ATOM 10086 ND1 HSE C 210 -45.935 44.300 56.685 0.00 0.00
C
ATOM 10087 CG HSE C 210 -46.145 44.972 55.492 0.00 0.00
C
ATOM 10088 CE1 HSE C 210 -45.247 45.144 57.383 0.00 0.00
C
ATOM 10089 HE1 HSE C 210 -44.813 44.902 58.353 0.00 0.00
C
ATOM 10090 NE2 HSE C 210 -45.063 46.328 56.741 0.00 0.00
C
ATOM 10091 HE2 HSE C 210 -44.400 47.043 56.964 0.00 0.00
C
ATOM 10092 CD2 HSE C 210 -45.709 46.236 55.568 0.00 0.00
C
ATOM 10093 HD2 HSE C 210 -45.692 46.973 54.775 0.00 0.00
C
ATOM 10094 C HSE C 210 -46.072 42.173 54.130 0.00 0.00
C
ATOM 10095 O HSE C 210 -46.836 41.305 54.495 0.00 0.00
C
ATOM 10096 N LYS C 211 -44.795 41.995 54.407 0.00 0.00
C
ATOM 10097 HN LYS C 211 -44.158 42.722 54.162 0.00 0.00
C

```
ATOM  10098  CA  LYS C 211     −44.189  40.816  55.085  0.00  0.00
C
ATOM  10099  HA  LYS C 211     −44.321  39.910  54.512  0.00  0.00
C
ATOM  10100  CB  LYS C 211     −42.604  40.971  55.080  0.00  0.00
C
ATOM  10101  HB1 LYS C 211     −42.215  39.985  55.412  0.00  0.00
C
ATOM  10102  HB2 LYS C 211     −42.261  41.098  54.031  0.00  0.00
C
ATOM  10103  CG  LYS C 211     −41.969  42.079  55.993  0.00  0.00
C
ATOM  10104  HG1 LYS C 211     −42.241  43.117  55.703  0.00  0.00
C
ATOM  10105  HG2 LYS C 211     −42.158  41.871  57.067  0.00  0.00
C
ATOM  10106  CD  LYS C 211     −40.438  42.060  55.759  0.00  0.00
C
ATOM  10107  HD1 LYS C 211     −40.176  40.985  55.658  0.00  0.00
C
ATOM  10108  HD2 LYS C 211     −40.312  42.485  54.740  0.00  0.00
C
ATOM  10109  CE  LYS C 211     −39.557  42.722  56.874  0.00  0.00
C
ATOM  10110  HE1 LYS C 211     −40.003  43.730  57.018  0.00  0.00
C
ATOM  10111  HE2 LYS C 211     −39.610  42.059  57.764  0.00  0.00
C
ATOM  10112  NZ  LYS C 211     −38.215  42.886  56.486  0.00  0.00
C
ATOM  10113  HZ1 LYS C 211     −38.084  43.585  55.728  0.00  0.00
C
ATOM  10114  HZ2 LYS C 211     −37.496  43.125  57.198  0.00  0.00
C
ATOM  10115  HZ3 LYS C 211     −37.885  41.944  56.193  0.00  0.00
C
ATOM  10116  C   LYS C 211     −44.688  40.435  56.459  0.00  0.00
C
ATOM  10117  O   LYS C 211     −44.957  39.227  56.669  0.00  0.00
C
ATOM  10118  N   LYS C 212     −44.917  41.458  57.342  0.00  0.00
C
ATOM  10119  HN  LYS C 212     −44.881  42.388  56.986  0.00  0.00
C
ATOM  10120  CA  LYS C 212     −45.601  41.241  58.567  0.00  0.00
C
ATOM  10121  HA  LYS C 212     −45.090  40.455  59.102  0.00  0.00
C
ATOM  10122  CB  LYS C 212     −45.550  42.519  59.509  0.00  0.00
C
ATOM  10123  HB1 LYS C 212     −44.506  42.898  59.501  0.00  0.00
C
ATOM  10124  HB2 LYS C 212     −46.156  43.304  59.008  0.00  0.00
C
```

ATOM 10125 CG LYS C 212 −45.988 42.344 60.934 0.00 0.00
C
ATOM 10126 HG1 LYS C 212 −46.464 43.296 61.250 0.00 0.00
C
ATOM 10127 HG2 LYS C 212 −46.832 41.621 60.939 0.00 0.00
C
ATOM 10128 CD LYS C 212 −44.986 41.935 62.031 0.00 0.00
C
ATOM 10129 HD1 LYS C 212 −45.557 41.553 62.904 0.00 0.00
C
ATOM 10130 HD2 LYS C 212 −44.390 41.033 61.775 0.00 0.00
C
ATOM 10131 CE LYS C 212 −44.136 43.043 62.470 0.00 0.00
C
ATOM 10132 HE1 LYS C 212 −43.656 43.425 61.544 0.00 0.00
C
ATOM 10133 HE2 LYS C 212 −44.788 43.795 62.964 0.00 0.00
C
ATOM 10134 NZ LYS C 212 −43.113 42.513 63.536 0.00 0.00
C
ATOM 10135 HZ1 LYS C 212 −43.565 42.478 64.472 0.00 0.00
C
ATOM 10136 HZ2 LYS C 212 −42.772 41.552 63.334 0.00 0.00
C
ATOM 10137 HZ3 LYS C 212 −42.253 43.084 63.660 0.00 0.00
C
ATOM 10138 C LYS C 212 −47.057 40.821 58.454 0.00 0.00
C
ATOM 10139 O LYS C 212 −47.450 39.720 58.963 0.00 0.00
C
ATOM 10140 N ASP C 213 −47.829 41.514 57.657 0.00 0.00
C
ATOM 10141 HN ASP C 213 −47.481 42.295 57.144 0.00 0.00
C
ATOM 10142 CA ASP C 213 −49.248 41.189 57.457 0.00 0.00
C
ATOM 10143 HA ASP C 213 −49.728 41.062 58.416 0.00 0.00
C
ATOM 10144 CB ASP C 213 −50.052 42.226 56.610 0.00 0.00
C
ATOM 10145 HB1 ASP C 213 −49.572 42.441 55.632 0.00 0.00
C
ATOM 10146 HB2 ASP C 213 −51.044 41.771 56.399 0.00 0.00
C
ATOM 10147 CG ASP C 213 −50.090 43.541 57.297 0.00 0.00
C
ATOM 10148 OD1 ASP C 213 −50.332 43.587 58.550 0.00 0.00
C
ATOM 10149 OD2 ASP C 213 −49.909 44.572 56.586 0.00 0.00
C
ATOM 10150 C ASP C 213 −49.417 39.780 56.779 0.00 0.00
C
ATOM 10151 O ASP C 213 −50.260 39.034 57.218 0.00 0.00
C

```
ATOM  10152  N   ILE C 214     -48.562  39.360  55.835  0.00  0.00
C
ATOM  10153  HN  ILE C 214     -47.773  39.922  55.598  0.00  0.00
C
ATOM  10154  CA  ILE C 214     -48.614  37.977  55.307  0.00  0.00
C
ATOM  10155  HA  ILE C 214     -49.646  37.755  55.081  0.00  0.00
C
ATOM  10156  CB  ILE C 214     -47.747  37.738  54.080  0.00  0.00
C
ATOM  10157  HB  ILE C 214     -46.691  37.898  54.389  0.00  0.00
C
ATOM  10158  CG2 ILE C 214     -47.797  36.260  53.657  0.00  0.00
C
ATOM  10159 HG21 ILE C 214     -47.289  35.564  54.357  0.00  0.00
C
ATOM  10160 HG22 ILE C 214     -48.891  36.079  53.732  0.00  0.00
C
ATOM  10161 HG23 ILE C 214     -47.395  36.173  52.625  0.00  0.00
C
ATOM  10162  CG1 ILE C 214     -48.049  38.659  52.824  0.00  0.00
C
ATOM  10163 HG11 ILE C 214     -48.964  38.208  52.385  0.00  0.00
C
ATOM  10164 HG12 ILE C 214     -48.218  39.733  53.054  0.00  0.00
C
ATOM  10165  CD  ILE C 214     -47.002  38.685  51.726  0.00  0.00
C
ATOM  10166  HD1 ILE C 214     -47.179  39.419  50.911  0.00  0.00
C
ATOM  10167  HD2 ILE C 214     -46.057  38.935  52.254  0.00  0.00
C
ATOM  10168  HD3 ILE C 214     -46.826  37.672  51.305  0.00  0.00
C
ATOM  10169  C   ILE C 214     -48.231  36.962  56.380  0.00  0.00
C
ATOM  10170  O   ILE C 214     -48.892  35.971  56.497  0.00  0.00
C
ATOM  10171  N   LYS C 215     -47.266  37.262  57.255  0.00  0.00
C
ATOM  10172  HN  LYS C 215     -46.733  38.104  57.293  0.00  0.00
C
ATOM  10173  CA  LYS C 215     -46.897  36.398  58.317  0.00  0.00
C
ATOM  10174  HA  LYS C 215     -46.666  35.419  57.924  0.00  0.00
C
ATOM  10175  CB  LYS C 215     -45.674  36.897  59.081  0.00  0.00
C
ATOM  10176  HB1 LYS C 215     -44.826  36.989  58.369  0.00  0.00
C
ATOM  10177  HB2 LYS C 215     -45.775  37.928  59.485  0.00  0.00
C
ATOM  10178  CG  LYS C 215     -45.152  35.992  60.231  0.00  0.00
C
```

ATOM 10179 HG1 LYS C 215 −44.353 36.513 60.800 0.00 0.00
C
ATOM 10180 HG2 LYS C 215 −45.906 35.816 61.027 0.00 0.00
C
ATOM 10181 CD LYS C 215 −44.690 34.547 59.745 0.00 0.00
C
ATOM 10182 HD1 LYS C 215 −45.478 33.880 59.336 0.00 0.00
C
ATOM 10183 HD2 LYS C 215 −43.860 34.635 59.012 0.00 0.00
C
ATOM 10184 CE LYS C 215 −44.221 33.784 60.969 0.00 0.00
C
ATOM 10185 HE1 LYS C 215 −43.217 34.259 60.958 0.00 0.00
C
ATOM 10186 HE2 LYS C 215 −44.831 33.918 61.888 0.00 0.00
C
ATOM 10187 NZ LYS C 215 −44.102 32.371 60.621 0.00 0.00
C
ATOM 10188 HZ1 LYS C 215 −44.917 31.938 60.141 0.00 0.00
C
ATOM 10189 HZ2 LYS C 215 −43.388 32.086 59.921 0.00 0.00
C
ATOM 10190 HZ3 LYS C 215 −43.857 31.799 61.454 0.00 0.00
C
ATOM 10191 C LYS C 215 −47.989 36.188 59.387 0.00 0.00
C
ATOM 10192 O LYS C 215 −48.058 35.188 59.989 0.00 0.00
C
ATOM 10193 N ARG C 216 −48.833 37.193 59.614 0.00 0.00
C
ATOM 10194 HN ARG C 216 −48.572 38.069 59.217 0.00 0.00
C
ATOM 10195 CA ARG C 216 −49.908 37.133 60.549 0.00 0.00
C
ATOM 10196 HA ARG C 216 −49.652 36.738 61.521 0.00 0.00
C
ATOM 10197 CB ARG C 216 −50.553 38.530 60.705 0.00 0.00
C
ATOM 10198 HB1 ARG C 216 −50.868 38.923 59.715 0.00 0.00
C
ATOM 10199 HB2 ARG C 216 −51.499 38.405 61.274 0.00 0.00
C
ATOM 10200 CG ARG C 216 −49.718 39.595 61.470 0.00 0.00
C
ATOM 10201 HG1 ARG C 216 −49.655 39.432 62.568 0.00 0.00
C
ATOM 10202 HG2 ARG C 216 −48.683 39.682 61.076 0.00 0.00
C
ATOM 10203 CD ARG C 216 −50.347 40.997 61.265 0.00 0.00
C
ATOM 10204 HD1 ARG C 216 −49.826 41.669 61.980 0.00 0.00
C
ATOM 10205 HD2 ARG C 216 −50.204 41.429 60.251 0.00 0.00
C

```
ATOM  10206  NE  ARG C 216     −51.791  40.880  61.637  0.00  0.00
C
ATOM  10207  HE  ARG C 216     −52.189  40.114  62.142  0.00  0.00
C
ATOM  10208  CZ  ARG C 216     −52.673  41.863  61.454  0.00  0.00
C
ATOM  10209  NH1 ARG C 216     −52.400  42.874  60.639  0.00  0.00
C
ATOM  10210 HH11 ARG C 216     −53.104  43.570  60.497  0.00  0.00
C
ATOM  10211 HH12 ARG C 216     −51.557  42.928  60.104  0.00  0.00
C
ATOM  10212  NH2 ARG C 216     −53.856  41.729  61.970  0.00  0.00
C
ATOM  10213 HH21 ARG C 216     −54.497  42.469  61.770  0.00  0.00
C
ATOM  10214 HH22 ARG C 216     −53.866  40.972  62.624  0.00  0.00
C
ATOM  10215  C   ARG C 216     −50.966  36.236  59.868  0.00  0.00
C
ATOM  10216  O   ARG C 216     −51.793  35.557  60.483  0.00  0.00
C
ATOM  10217  N   PHE C 217     −51.038  36.277  58.514  0.00  0.00
C
ATOM  10218  HN  PHE C 217     −50.333  36.789  58.030  0.00  0.00
C
ATOM  10219  CA  PHE C 217     −52.029  35.512  57.734  0.00  0.00
C
ATOM  10220  HA  PHE C 217     −52.967  35.557  58.266  0.00  0.00
C
ATOM  10221  CB  PHE C 217     −52.324  36.233  56.348  0.00  0.00
C
ATOM  10222  HB1 PHE C 217     −53.050  37.028  56.622  0.00  0.00
C
ATOM  10223  HB2 PHE C 217     −51.497  36.731  55.797  0.00  0.00
C
ATOM  10224  CG  PHE C 217     −52.994  35.372  55.352  0.00  0.00
C
ATOM  10225  CD1 PHE C 217     −54.383  35.486  55.159  0.00  0.00
C
ATOM  10226  HD1 PHE C 217     −55.027  36.179  55.680  0.00  0.00
C
ATOM  10227  CE1 PHE C 217     −55.005  34.769  54.112  0.00  0.00
C
ATOM  10228  HE1 PHE C 217     −56.069  34.749  53.925  0.00  0.00
C
ATOM  10229  CZ  PHE C 217     −54.167  33.798  53.383  0.00  0.00
C
ATOM  10230  HZ  PHE C 217     −54.581  33.262  52.541  0.00  0.00
C
ATOM  10231  CD2 PHE C 217     −52.264  34.471  54.592  0.00  0.00
C
ATOM  10232  HD2 PHE C 217     −51.223  34.286  54.809  0.00  0.00
C
```

ATOM 10233 CE2 PHE C 217 -52.813 33.726 53.582 0.00 0.00
C
ATOM 10234 HE2 PHE C 217 -52.213 33.037 53.005 0.00 0.00
C
ATOM 10235 C PHE C 217 -51.610 34.084 57.765 0.00 0.00
C
ATOM 10236 O PHE C 217 -52.341 33.104 57.862 0.00 0.00
C
ATOM 10237 N GLU C 218 -50.247 33.853 57.792 0.00 0.00
C
ATOM 10238 HN GLU C 218 -49.589 34.567 57.566 0.00 0.00
C
ATOM 10239 CA GLU C 218 -49.544 32.545 57.939 0.00 0.00
C
ATOM 10240 HA GLU C 218 -50.074 31.890 57.264 0.00 0.00
C
ATOM 10241 CB GLU C 218 -48.041 32.739 57.636 0.00 0.00
C
ATOM 10242 HB1 GLU C 218 -48.118 33.338 56.704 0.00 0.00
C
ATOM 10243 HB2 GLU C 218 -47.562 33.388 58.399 0.00 0.00
C
ATOM 10244 CG GLU C 218 -47.232 31.435 57.370 0.00 0.00
C
ATOM 10245 HG1 GLU C 218 -47.790 30.782 56.666 0.00 0.00
C
ATOM 10246 HG2 GLU C 218 -46.232 31.654 56.940 0.00 0.00
C
ATOM 10247 CD GLU C 218 -46.824 30.669 58.664 0.00 0.00
C
ATOM 10248 OE1 GLU C 218 -46.945 29.395 58.675 0.00 0.00
C
ATOM 10249 OE2 GLU C 218 -46.382 31.265 59.615 0.00 0.00
C
ATOM 10250 C GLU C 218 -49.868 31.902 59.267 0.00 0.00
C
ATOM 10251 O GLU C 218 -50.480 30.864 59.403 0.00 0.00
C
ATOM 10252 N ASN C 219 -49.642 32.637 60.349 0.00 0.00
C
ATOM 10253 HN ASN C 219 -49.093 33.466 60.271 0.00 0.00
C
ATOM 10254 CA ASN C 219 -49.880 32.317 61.783 0.00 0.00
C
ATOM 10255 HA ASN C 219 -49.258 31.487 62.083 0.00 0.00
C
ATOM 10256 CB ASN C 219 -49.303 33.399 62.708 0.00 0.00
C
ATOM 10257 HB1 ASN C 219 -48.315 33.611 62.245 0.00 0.00
C
ATOM 10258 HB2 ASN C 219 -49.849 34.366 62.732 0.00 0.00
C
ATOM 10259 CG ASN C 219 -48.914 32.863 64.128 0.00 0.00
C

ATOM 10260 OD1 ASN C 219   -49.756 32.641 64.993 0.00 0.00
C
ATOM 10261 ND2 ASN C 219   -47.605 32.726 64.403 0.00 0.00
C
ATOM 10262 HD21 ASN C 219   -47.290 32.559 65.337 0.00 0.00
C
ATOM 10263 HD22 ASN C 219   -46.896 32.877 63.714 0.00 0.00
C
ATOM 10264 C   ASN C 219   -51.290 31.945 62.178 0.00 0.00
C
ATOM 10265 O   ASN C 219   -51.532 30.999 62.982 0.00 0.00
C
ATOM 10266 N   GLU C 220   -52.323 32.546 61.511 0.00 0.00
C
ATOM 10267 HN GLU C 220   -52.160 33.234 60.809 0.00 0.00
C
ATOM 10268 CA GLU C 220   -53.723 32.185 61.743 0.00 0.00
C
ATOM 10269 HA GLU C 220   -53.802 31.881 62.776 0.00 0.00
C
ATOM 10270 CB GLU C 220   -54.712 33.347 61.405 0.00 0.00
C
ATOM 10271 HB1 GLU C 220   -55.687 33.070 61.860 0.00 0.00
C
ATOM 10272 HB2 GLU C 220   -54.389 34.295 61.887 0.00 0.00
C
ATOM 10273 CG GLU C 220   -54.924 33.646 59.914 0.00 0.00
C
ATOM 10274 HG1 GLU C 220   -54.145 34.420 59.743 0.00 0.00
C
ATOM 10275 HG2 GLU C 220   -54.705 32.809 59.217 0.00 0.00
C
ATOM 10276 CD GLU C 220   -56.292 34.177 59.680 0.00 0.00
C
ATOM 10277 OE1 GLU C 220   -57.228 33.297 59.921 0.00 0.00
C
ATOM 10278 OE2 GLU C 220   -56.603 35.329 59.378 0.00 0.00
C
ATOM 10279 C   GLU C 220   -54.157 30.960 61.036 0.00 0.00
C
ATOM 10280 O   GLU C 220   -55.253 30.460 61.240 0.00 0.00
C
ATOM 10281 N   GLY C 221   -53.331 30.416 60.067 0.00 0.00
C
ATOM 10282 HN GLY C 221   -52.437 30.826 59.903 0.00 0.00
C
ATOM 10283 CA GLY C 221   -53.580 29.146 59.343 0.00 0.00
C
ATOM 10284 HA1 GLY C 221   -54.301 28.575 59.910 0.00 0.00
C
ATOM 10285 HA2 GLY C 221   -52.593 28.718 59.247 0.00 0.00
C
ATOM 10286 C   GLY C 221   -54.171 29.248 57.988 0.00 0.00
C

ATOM 10287 O GLY C 221 −54.894 28.335 57.583 0.00 0.00
C
ATOM 10288 N MET C 222 −53.872 30.381 57.322 0.00 0.00
C
ATOM 10289 HN MET C 222 −53.191 30.993 57.716 0.00 0.00
C
ATOM 10290 CA MET C 222 −54.501 30.859 56.047 0.00 0.00
C
ATOM 10291 HA MET C 222 −54.417 31.932 55.960 0.00 0.00
C
ATOM 10292 CB MET C 222 −53.794 30.227 54.842 0.00 0.00
C
ATOM 10293 HB1 MET C 222 −53.895 29.137 54.648 0.00 0.00
C
ATOM 10294 HB2 MET C 222 −54.173 30.782 53.957 0.00 0.00
C
ATOM 10295 CG MET C 222 −52.273 30.404 55.027 0.00 0.00
C
ATOM 10296 HG1 MET C 222 −51.990 31.477 55.060 0.00 0.00
C
ATOM 10297 HG2 MET C 222 −52.000 29.909 55.983 0.00 0.00
C
ATOM 10298 SD MET C 222 −51.310 29.584 53.720 0.00 0.00
C
ATOM 10299 CE MET C 222 −49.833 29.715 54.752 0.00 0.00
C
ATOM 10300 HE1 MET C 222 −49.965 29.163 55.707 0.00 0.00
C
ATOM 10301 HE2 MET C 222 −48.943 29.233 54.294 0.00 0.00
C
ATOM 10302 HE3 MET C 222 −49.510 30.750 54.995 0.00 0.00
C
ATOM 10303 C MET C 222 −56.001 30.645 55.837 0.00 0.00
C
ATOM 10304 O MET C 222 −56.839 31.491 56.222 0.00 0.00
C
ATOM 10305 N ASN D 6 −40.414 25.380 69.178 0.00 0.00
D
ATOM 10306 HT1 ASN D 6 −40.714 24.502 69.647 0.00 0.00
D
ATOM 10307 HT2 ASN D 6 −41.032 26.135 69.540 0.00 0.00
D
ATOM 10308 HT3 ASN D 6 −39.445 25.651 69.442 0.00 0.00
D
ATOM 10309 CA ASN D 6 −40.594 25.262 67.685 0.00 0.00
D
ATOM 10310 HA ASN D 6 −41.570 25.614 67.383 0.00 0.00
D
ATOM 10311 CB ASN D 6 −39.517 25.985 66.898 0.00 0.00
D
ATOM 10312 HB1 ASN D 6 −38.607 25.349 66.856 0.00 0.00
D
ATOM 10313 HB2 ASN D 6 −39.940 26.094 65.876 0.00 0.00
D

ATOM 10314 CG ASN D 6 −39.452 27.404 67.336 0.00 0.00
D
ATOM 10315 OD1 ASN D 6 −40.409 27.976 67.866 0.00 0.00
D
ATOM 10316 ND2 ASN D 6 −38.255 28.021 67.242 0.00 0.00
D
ATOM 10317 HD21 ASN D 6 −38.220 28.986 67.504 0.00 0.00
D
ATOM 10318 HD22 ASN D 6 −37.461 27.592 66.813 0.00 0.00
D
ATOM 10319 C ASN D 6 −40.586 23.893 67.301 0.00 0.00
D
ATOM 10320 O ASN D 6 −41.176 23.084 68.062 0.00 0.00
D
ATOM 10321 N LYS D 7 −40.167 23.490 66.060 0.00 0.00
D
ATOM 10322 HN LYS D 7 −39.815 24.091 65.346 0.00 0.00
D
ATOM 10323 CA LYS D 7 −40.190 22.155 65.557 0.00 0.00
D
ATOM 10324 HA LYS D 7 −40.983 21.535 65.948 0.00 0.00
D
ATOM 10325 CB LYS D 7 −40.254 22.276 64.012 0.00 0.00
D
ATOM 10326 HB1 LYS D 7 −39.341 22.873 63.801 0.00 0.00
D
ATOM 10327 HB2 LYS D 7 −40.102 21.257 63.596 0.00 0.00
D
ATOM 10328 CG LYS D 7 −41.557 22.921 63.503 0.00 0.00
D
ATOM 10329 HG1 LYS D 7 −42.387 22.357 63.980 0.00 0.00
D
ATOM 10330 HG2 LYS D 7 −41.615 23.923 63.979 0.00 0.00
D
ATOM 10331 CD LYS D 7 −41.614 23.004 61.957 0.00 0.00
D
ATOM 10332 HD1 LYS D 7 −42.192 23.934 61.769 0.00 0.00
D
ATOM 10333 HD2 LYS D 7 −40.620 23.291 61.553 0.00 0.00
D
ATOM 10334 CE LYS D 7 −42.168 21.707 61.371 0.00 0.00
D
ATOM 10335 HE1 LYS D 7 −41.511 20.822 61.515 0.00 0.00
D
ATOM 10336 HE2 LYS D 7 −43.174 21.379 61.709 0.00 0.00
D
ATOM 10337 NZ LYS D 7 −42.224 21.833 59.865 0.00 0.00
D
ATOM 10338 HZ1 LYS D 7 −42.684 21.025 59.399 0.00 0.00
D
ATOM 10339 HZ2 LYS D 7 −42.782 22.691 59.679 0.00 0.00
D
ATOM 10340 HZ3 LYS D 7 −41.242 21.911 59.529 0.00 0.00
D

ATOM 10341 C LYS D 7 −38.862 21.443 65.983 0.00 0.00
D
ATOM 10342 O LYS D 7 −37.927 22.028 66.572 0.00 0.00
D
ATOM 10343 N GLN D 8 −38.810 20.103 65.662 0.00 0.00
D
ATOM 10344 HN GLN D 8 −39.501 19.693 65.071 0.00 0.00
D
ATOM 10345 CA GLN D 8 −37.581 19.354 65.978 0.00 0.00
D
ATOM 10346 HA GLN D 8 −36.710 19.991 65.952 0.00 0.00
D
ATOM 10347 CB GLN D 8 −37.700 18.629 67.263 0.00 0.00
D
ATOM 10348 HB1 GLN D 8 −36.790 18.019 67.446 0.00 0.00
D
ATOM 10349 HB2 GLN D 8 −37.795 19.349 68.103 0.00 0.00
D
ATOM 10350 CG GLN D 8 −38.930 17.619 67.445 0.00 0.00
D
ATOM 10351 HG1 GLN D 8 −39.775 18.335 67.365 0.00 0.00
D
ATOM 10352 HG2 GLN D 8 −39.124 16.841 66.676 0.00 0.00
D
ATOM 10353 CD GLN D 8 −39.040 16.938 68.835 0.00 0.00
D
ATOM 10354 OE1 GLN D 8 −39.914 17.383 69.634 0.00 0.00
D
ATOM 10355 NE2 GLN D 8 −38.255 15.877 69.130 0.00 0.00
D
ATOM 10356 HE21 GLN D 8 −38.350 15.449 70.029 0.00 0.00
D
ATOM 10357 HE22 GLN D 8 −37.568 15.541 68.485 0.00 0.00
D
ATOM 10358 C GLN D 8 −37.378 18.398 64.806 0.00 0.00
D
ATOM 10359 O GLN D 8 −38.294 17.810 64.235 0.00 0.00
D
ATOM 10360 N PHE D 9 −36.114 18.235 64.407 0.00 0.00
D
ATOM 10361 HN PHE D 9 −35.415 18.792 64.850 0.00 0.00
D
ATOM 10362 CA PHE D 9 −35.642 17.589 63.251 0.00 0.00
D
ATOM 10363 HA PHE D 9 −36.517 17.008 63.001 0.00 0.00
D
ATOM 10364 CB PHE D 9 −35.199 18.537 62.052 0.00 0.00
D
ATOM 10365 HB1 PHE D 9 −34.262 19.099 62.255 0.00 0.00
D
ATOM 10366 HB2 PHE D 9 −34.904 17.861 61.222 0.00 0.00
D
ATOM 10367 CG PHE D 9 −36.308 19.468 61.554 0.00 0.00
D

ATOM 10368 CD1 PHE D 9 -37.249 19.115 60.633 0.00 0.00
D
ATOM 10369 HD1 PHE D 9 -37.235 18.119 60.216 0.00 0.00
D
ATOM 10370 CE1 PHE D 9 -38.377 19.930 60.325 0.00 0.00
D
ATOM 10371 HE1 PHE D 9 -39.024 19.601 59.525 0.00 0.00
D
ATOM 10372 CZ PHE D 9 -38.364 21.257 60.852 0.00 0.00
D
ATOM 10373 HZ PHE D 9 -39.084 21.900 60.368 0.00 0.00
D
ATOM 10374 CD2 PHE D 9 -36.377 20.789 62.142 0.00 0.00
D
ATOM 10375 HD2 PHE D 9 -35.692 21.167 62.887 0.00 0.00
D
ATOM 10376 CE2 PHE D 9 -37.318 21.721 61.641 0.00 0.00
D
ATOM 10377 HE2 PHE D 9 -37.280 22.747 61.975 0.00 0.00
D
ATOM 10378 C PHE D 9 -34.520 16.644 63.519 0.00 0.00
D
ATOM 10379 O PHE D 9 -34.009 16.597 64.641 0.00 0.00
D
ATOM 10380 N ALA D 10 -34.140 15.765 62.577 0.00 0.00
D
ATOM 10381 HN ALA D 10 -34.766 15.490 61.852 0.00 0.00
D
ATOM 10382 CA ALA D 10 -32.897 15.011 62.597 0.00 0.00
D
ATOM 10383 HA ALA D 10 -32.220 15.624 63.173 0.00 0.00
D
ATOM 10384 CB ALA D 10 -33.276 13.570 63.147 0.00 0.00
D
ATOM 10385 HB1 ALA D 10 -34.008 13.111 62.449 0.00 0.00
D
ATOM 10386 HB2 ALA D 10 -32.474 12.823 63.329 0.00 0.00
D
ATOM 10387 HB3 ALA D 10 -33.769 13.751 64.126 0.00 0.00
D
ATOM 10388 C ALA D 10 -32.275 14.950 61.215 0.00 0.00
D
ATOM 10389 O ALA D 10 -32.951 14.931 60.238 0.00 0.00
D
ATOM 10390 N VAL D 11 -30.985 14.982 61.178 0.00 0.00
D
ATOM 10391 HN VAL D 11 -30.484 14.934 62.038 0.00 0.00
D
ATOM 10392 CA VAL D 11 -30.251 14.903 59.952 0.00 0.00
D
ATOM 10393 HA VAL D 11 -30.916 14.762 59.112 0.00 0.00
D
ATOM 10394 CB VAL D 11 -29.551 16.237 59.595 0.00 0.00
D

```
ATOM  10395  HB  VAL D  11     -28.969  16.474  60.511  0.00  0.00
D
ATOM  10396  CG1 VAL D  11     -28.550  16.259  58.395  0.00  0.00
D
ATOM  10397 HG11 VAL D  11     -28.005  17.217  58.258  0.00  0.00
D
ATOM  10398 HG12 VAL D  11     -27.808  15.433  58.440  0.00  0.00
D
ATOM  10399 HG13 VAL D  11     -29.122  16.204  57.444  0.00  0.00
D
ATOM  10400  CG2 VAL D  11     -30.561  17.310  59.425  0.00  0.00
D
ATOM  10401 HG21 VAL D  11     -31.215  17.491  60.304  0.00  0.00
D
ATOM  10402 HG22 VAL D  11     -30.140  18.242  58.991  0.00  0.00
D
ATOM  10403 HG23 VAL D  11     -31.255  16.912  58.655  0.00  0.00
D
ATOM  10404  C   VAL D  11     -29.201  13.784  60.021  0.00  0.00
D
ATOM  10405  O   VAL D  11     -28.290  13.916  60.861  0.00  0.00
D
ATOM  10406  N   ILE D  12     -29.300  12.691  59.247  0.00  0.00
D
ATOM  10407  HN  ILE D  12     -29.962  12.541  58.517  0.00  0.00
D
ATOM  10408  CA  ILE D  12     -28.208  11.789  59.133  0.00  0.00
D
ATOM  10409  HA  ILE D  12     -27.532  11.867  59.972  0.00  0.00
D
ATOM  10410  CB  ILE D  12     -28.720  10.401  58.874  0.00  0.00
D
ATOM  10411  HB  ILE D  12     -29.462  10.361  58.048  0.00  0.00
D
ATOM  10412  CG2 ILE D  12     -27.582   9.445  58.474  0.00  0.00
D
ATOM  10413 HG21 ILE D  12     -27.272   9.547  57.412  0.00  0.00
D
ATOM  10414 HG22 ILE D  12     -26.744   9.520  59.200  0.00  0.00
D
ATOM  10415 HG23 ILE D  12     -28.047   8.436  58.442  0.00  0.00
D
ATOM  10416  CG1 ILE D  12     -29.455   9.753  60.122  0.00  0.00
D
ATOM  10417 HG11 ILE D  12     -28.599   9.424  60.749  0.00  0.00
D
ATOM  10418 HG12 ILE D  12     -30.004  10.555  60.660  0.00  0.00
D
ATOM  10419  CD  ILE D  12     -30.431   8.635  59.819  0.00  0.00
D
ATOM  10420  HD1 ILE D  12     -30.000   7.656  59.518  0.00  0.00
D
ATOM  10421  HD2 ILE D  12     -31.000   8.314  60.717  0.00  0.00
D
```

ATOM 10422 HD3 ILE D 12 -31.095 8.970 58.993 0.00 0.00
D
ATOM 10423 C ILE D 12 -27.389 12.209 57.950 0.00 0.00
D
ATOM 10424 O ILE D 12 -27.855 12.262 56.818 0.00 0.00
D
ATOM 10425 N GLY D 13 -26.072 12.425 58.123 0.00 0.00
D
ATOM 10426 HN GLY D 13 -25.694 12.349 59.043 0.00 0.00
D
ATOM 10427 CA GLY D 13 -25.062 12.636 57.075 0.00 0.00
D
ATOM 10428 HA1 GLY D 13 -25.375 12.247 56.117 0.00 0.00
D
ATOM 10429 HA2 GLY D 13 -24.215 12.048 57.395 0.00 0.00
D
ATOM 10430 C GLY D 13 -24.703 14.147 57.026 0.00 0.00
D
ATOM 10431 O GLY D 13 -25.548 14.936 56.662 0.00 0.00
D
ATOM 10432 N LEU D 14 -23.461 14.592 57.350 0.00 0.00
D
ATOM 10433 HN LEU D 14 -22.748 13.931 57.568 0.00 0.00
D
ATOM 10434 CA LEU D 14 -23.207 15.994 57.692 0.00 0.00
D
ATOM 10435 HA LEU D 14 -24.128 16.557 57.736 0.00 0.00
D
ATOM 10436 CB LEU D 14 -22.608 16.161 59.123 0.00 0.00
D
ATOM 10437 HB1 LEU D 14 -21.818 15.406 59.324 0.00 0.00
D
ATOM 10438 HB2 LEU D 14 -22.215 17.169 59.379 0.00 0.00
D
ATOM 10439 CG LEU D 14 -23.675 15.927 60.224 0.00 0.00
D
ATOM 10440 HG LEU D 14 -24.228 14.975 60.078 0.00 0.00
D
ATOM 10441 CD1 LEU D 14 -23.156 15.727 61.682 0.00 0.00
D
ATOM 10442 HD11 LEU D 14 -22.703 16.665 62.069 0.00 0.00
D
ATOM 10443 HD12 LEU D 14 -24.053 15.542 62.311 0.00 0.00
D
ATOM 10444 HD13 LEU D 14 -22.416 14.899 61.632 0.00 0.00
D
ATOM 10445 CD2 LEU D 14 -24.827 17.041 60.180 0.00 0.00
D
ATOM 10446 HD21 LEU D 14 -25.544 16.691 60.953 0.00 0.00
D
ATOM 10447 HD22 LEU D 14 -24.436 18.010 60.557 0.00 0.00
D
ATOM 10448 HD23 LEU D 14 -25.368 17.160 59.217 0.00 0.00
D

ATOM 10449 C LEU D 14 −22.388 16.702 56.671 0.00 0.00
D
ATOM 10450 O LEU D 14 −21.932 17.870 56.934 0.00 0.00
D
ATOM 10451 N GLY D 15 −22.116 16.158 55.463 0.00 0.00
D
ATOM 10452 HN GLY D 15 −22.503 15.264 55.249 0.00 0.00
D
ATOM 10453 CA GLY D 15 −21.361 16.738 54.422 0.00 0.00
D
ATOM 10454 HA1 GLY D 15 −21.296 15.962 53.673 0.00 0.00
D
ATOM 10455 HA2 GLY D 15 −20.470 17.043 54.949 0.00 0.00
D
ATOM 10456 C GLY D 15 −21.928 18.014 53.751 0.00 0.00
D
ATOM 10457 O GLY D 15 −22.446 18.853 54.518 0.00 0.00
D
ATOM 10458 N ARG D 16 −21.704 18.236 52.389 0.00 0.00
D
ATOM 10459 HN ARG D 16 −21.166 17.639 51.800 0.00 0.00
D
ATOM 10460 CA ARG D 16 −22.170 19.440 51.714 0.00 0.00
D
ATOM 10461 HA ARG D 16 −21.670 20.265 52.200 0.00 0.00
D
ATOM 10462 CB ARG D 16 −21.878 19.547 50.186 0.00 0.00
D
ATOM 10463 HB1 ARG D 16 −22.601 18.849 49.712 0.00 0.00
D
ATOM 10464 HB2 ARG D 16 −22.041 20.582 49.815 0.00 0.00
D
ATOM 10465 CG ARG D 16 −20.442 19.188 49.747 0.00 0.00
D
ATOM 10466 HG1 ARG D 16 −19.643 19.793 50.227 0.00 0.00
D
ATOM 10467 HG2 ARG D 16 −20.309 18.107 49.968 0.00 0.00
D
ATOM 10468 CD ARG D 16 −20.399 19.090 48.202 0.00 0.00
D
ATOM 10469 HD1 ARG D 16 −21.372 18.584 48.023 0.00 0.00
D
ATOM 10470 HD2 ARG D 16 −20.204 20.027 47.638 0.00 0.00
D
ATOM 10471 NE ARG D 16 −19.249 18.141 47.835 0.00 0.00
D
ATOM 10472 HE ARG D 16 −18.684 17.680 48.519 0.00 0.00
D
ATOM 10473 CZ ARG D 16 −18.988 17.828 46.574 0.00 0.00
D
ATOM 10474 NH1 ARG D 16 −19.874 18.050 45.530 0.00 0.00
D
ATOM 10475 HH11 ARG D 16 −19.639 17.478 44.744 0.00 0.00
D

ATOM 10476 HH12 ARG D 16 -20.850 18.109 45.738 0.00 0.00
D
ATOM 10477 NH2 ARG D 16 -17.919 17.098 46.277 0.00 0.00
D
ATOM 10478 HH21 ARG D 16 -17.881 16.950 45.289 0.00 0.00
D
ATOM 10479 HH22 ARG D 16 -17.437 16.663 47.038 0.00 0.00
D
ATOM 10480 C ARG D 16 -23.623 19.911 51.932 0.00 0.00
D
ATOM 10481 O ARG D 16 -23.823 20.931 52.523 0.00 0.00
D
ATOM 10482 N PHE D 17 -24.508 18.993 51.495 0.00 0.00
D
ATOM 10483 HN PHE D 17 -24.154 18.190 51.022 0.00 0.00
D
ATOM 10484 CA PHE D 17 -25.928 19.209 51.506 0.00 0.00
D
ATOM 10485 HA PHE D 17 -26.061 20.152 50.996 0.00 0.00
D
ATOM 10486 CB PHE D 17 -26.567 18.129 50.656 0.00 0.00
D
ATOM 10487 HB1 PHE D 17 -26.238 18.196 49.597 0.00 0.00
D
ATOM 10488 HB2 PHE D 17 -26.267 17.167 51.125 0.00 0.00
D
ATOM 10489 CG PHE D 17 -28.065 18.155 50.659 0.00 0.00
D
ATOM 10490 CD1 PHE D 17 -28.740 19.251 50.118 0.00 0.00
D
ATOM 10491 HD1 PHE D 17 -28.180 20.068 49.687 0.00 0.00
D
ATOM 10492 CE1 PHE D 17 -30.143 19.257 50.096 0.00 0.00
D
ATOM 10493 HE1 PHE D 17 -30.634 20.070 49.582 0.00 0.00
D
ATOM 10494 CZ PHE D 17 -30.879 18.231 50.655 0.00 0.00
D
ATOM 10495 HZ PHE D 17 -31.937 18.271 50.869 0.00 0.00
D
ATOM 10496 CD2 PHE D 17 -28.781 17.066 51.181 0.00 0.00
D
ATOM 10497 HD2 PHE D 17 -28.272 16.176 51.521 0.00 0.00
D
ATOM 10498 CE2 PHE D 17 -30.166 17.119 51.206 0.00 0.00
D
ATOM 10499 HE2 PHE D 17 -30.580 16.234 51.666 0.00 0.00
D
ATOM 10500 C PHE D 17 -26.446 19.309 52.951 0.00 0.00
D
ATOM 10501 O PHE D 17 -27.169 20.232 53.335 0.00 0.00
D
ATOM 10502 N GLY D 18 -26.034 18.323 53.750 0.00 0.00
D

ATOM 10503 HN GLY D 18 -25.661 17.473 53.386 0.00 0.00
D
ATOM 10504 CA GLY D 18 -26.534 18.023 55.019 0.00 0.00
D
ATOM 10505 HA1 GLY D 18 -26.214 16.994 55.088 0.00 0.00
D
ATOM 10506 HA2 GLY D 18 -27.614 18.045 55.041 0.00 0.00
D
ATOM 10507 C GLY D 18 -26.052 18.820 56.104 0.00 0.00
D
ATOM 10508 O GLY D 18 -26.811 19.112 56.985 0.00 0.00
D
ATOM 10509 N GLY D 19 -24.756 19.225 56.149 0.00 0.00
D
ATOM 10510 HN GLY D 19 -24.061 18.953 55.488 0.00 0.00
D
ATOM 10511 CA GLY D 19 -24.246 20.196 57.137 0.00 0.00
D
ATOM 10512 HA1 GLY D 19 -23.167 20.162 57.172 0.00 0.00
D
ATOM 10513 HA2 GLY D 19 -24.498 19.726 58.076 0.00 0.00
D
ATOM 10514 C GLY D 19 -24.932 21.533 57.025 0.00 0.00
D
ATOM 10515 O GLY D 19 -25.274 22.085 58.080 0.00 0.00
D
ATOM 10516 N SER D 20 -25.217 22.103 55.809 0.00 0.00
D
ATOM 10517 HN SER D 20 -24.784 21.671 55.022 0.00 0.00
D
ATOM 10518 CA SER D 20 -25.946 23.314 55.552 0.00 0.00
D
ATOM 10519 HA SER D 20 -25.378 24.147 55.937 0.00 0.00
D
ATOM 10520 CB SER D 20 -25.860 23.550 54.022 0.00 0.00
D
ATOM 10521 HB1 SER D 20 -24.765 23.701 53.909 0.00 0.00
D
ATOM 10522 HB2 SER D 20 -26.308 22.682 53.493 0.00 0.00
D
ATOM 10523 OG SER D 20 -26.636 24.695 53.622 0.00 0.00
D
ATOM 10524 HG1 SER D 20 -26.615 24.830 52.672 0.00 0.00
D
ATOM 10525 C SER D 20 -27.394 23.372 56.150 0.00 0.00
D
ATOM 10526 O SER D 20 -27.872 24.338 56.705 0.00 0.00
D
ATOM 10527 N ILE D 21 -28.162 22.242 56.102 0.00 0.00
D
ATOM 10528 HN ILE D 21 -27.742 21.485 55.607 0.00 0.00
D
ATOM 10529 CA ILE D 21 -29.482 22.079 56.607 0.00 0.00
D

ATOM 10530 HA ILE D 21 −30.044 22.931 56.251 0.00 0.00
D
ATOM 10531 CB ILE D 21 −30.078 20.713 56.222 0.00 0.00
D
ATOM 10532 HB ILE D 21 −29.294 19.926 56.245 0.00 0.00
D
ATOM 10533 CG2 ILE D 21 −31.310 20.352 57.024 0.00 0.00
D
ATOM 10534 HG21 ILE D 21 −32.121 21.111 57.043 0.00 0.00
D
ATOM 10535 HG22 ILE D 21 −31.696 19.419 56.559 0.00 0.00
D
ATOM 10536 HG23 ILE D 21 −31.137 20.172 58.107 0.00 0.00
D
ATOM 10537 CG1 ILE D 21 −30.606 20.876 54.716 0.00 0.00
D
ATOM 10538 HG11 ILE D 21 −31.609 21.348 54.785 0.00 0.00
D
ATOM 10539 HG12 ILE D 21 −29.880 21.502 54.154 0.00 0.00
D
ATOM 10540 CD ILE D 21 −30.764 19.484 54.082 0.00 0.00
D
ATOM 10541 HD1 ILE D 21 −29.788 19.076 53.742 0.00 0.00
D
ATOM 10542 HD2 ILE D 21 −31.215 18.686 54.711 0.00 0.00
D
ATOM 10543 HD3 ILE D 21 −31.453 19.610 53.220 0.00 0.00
D
ATOM 10544 C ILE D 21 −29.498 22.295 58.081 0.00 0.00
D
ATOM 10545 O ILE D 21 −30.182 23.190 58.554 0.00 0.00
D
ATOM 10546 N VAL D 22 −28.621 21.651 58.904 0.00 0.00
D
ATOM 10547 HN VAL D 22 −28.010 20.972 58.504 0.00 0.00
D
ATOM 10548 CA VAL D 22 −28.372 21.798 60.391 0.00 0.00
D
ATOM 10549 HA VAL D 22 −29.337 21.526 60.792 0.00 0.00
D
ATOM 10550 CB VAL D 22 −27.199 20.940 60.875 0.00 0.00
D
ATOM 10551 HB VAL D 22 −26.306 21.374 60.376 0.00 0.00
D
ATOM 10552 CG1 VAL D 22 −27.052 20.947 62.414 0.00 0.00
D
ATOM 10553 HG11 VAL D 22 −28.001 20.536 62.819 0.00 0.00
D
ATOM 10554 HG12 VAL D 22 −26.174 20.352 62.744 0.00 0.00
D
ATOM 10555 HG13 VAL D 22 −27.011 21.954 62.883 0.00 0.00
D
ATOM 10556 CG2 VAL D 22 −27.271 19.537 60.355 0.00 0.00
D

ATOM 10557 HG21 VAL D 22   −26.802 19.439 59.352 0.00 0.00
D
ATOM 10558 HG22 VAL D 22   −26.733 18.977 61.150 0.00 0.00
D
ATOM 10559 HG23 VAL D 22   −28.274 19.063 60.412 0.00 0.00
D
ATOM 10560 C   VAL D 22   −28.062 23.304 60.758 0.00 0.00
D
ATOM 10561 O   VAL D 22   −28.616 23.881 61.692 0.00 0.00
D
ATOM 10562 N   LYS D 23   −27.155 23.887 59.908 0.00 0.00
D
ATOM 10563 HN LYS D 23   −26.758 23.379 59.148 0.00 0.00
D
ATOM 10564 CA LYS D 23   −26.761 25.210 60.079 0.00 0.00
D
ATOM 10565 HA LYS D 23   −26.339 25.266 61.071 0.00 0.00
D
ATOM 10566 CB LYS D 23   −25.604 25.468 59.104 0.00 0.00
D
ATOM 10567 HB1 LYS D 23   −24.893 24.631 59.272 0.00 0.00
D
ATOM 10568 HB2 LYS D 23   −25.952 25.471 58.048 0.00 0.00
D
ATOM 10569 CG LYS D 23   −24.972 26.819 59.366 0.00 0.00
D
ATOM 10570 HG1 LYS D 23   −25.559 27.749 59.204 0.00 0.00
D
ATOM 10571 HG2 LYS D 23   −24.589 26.814 60.409 0.00 0.00
D
ATOM 10572 CD LYS D 23   −23.722 26.992 58.483 0.00 0.00
D
ATOM 10573 HD1 LYS D 23   −23.085 26.111 58.712 0.00 0.00
D
ATOM 10574 HD2 LYS D 23   −24.042 26.936 57.420 0.00 0.00
D
ATOM 10575 CE LYS D 23   −22.919 28.356 58.782 0.00 0.00
D
ATOM 10576 HE1 LYS D 23   −23.708 29.138 58.759 0.00 0.00
D
ATOM 10577 HE2 LYS D 23   −22.367 28.452 59.742 0.00 0.00
D
ATOM 10578 NZ LYS D 23   −21.972 28.704 57.737 0.00 0.00
D
ATOM 10579 HZ1 LYS D 23   −21.668 29.689 57.872 0.00 0.00
D
ATOM 10580 HZ2 LYS D 23   −21.093 28.149 57.754 0.00 0.00
D
ATOM 10581 HZ3 LYS D 23   −22.414 28.672 56.796 0.00 0.00
D
ATOM 10582 C   LYS D 23   −27.842 26.283 59.939 0.00 0.00
D
ATOM 10583 O   LYS D 23   −27.985 27.169 60.760 0.00 0.00
D

ATOM 10584 N GLU D 24 −28.686 26.151 58.961 0.00 0.00
D
ATOM 10585 HN GLU D 24 −28.414 25.595 58.180 0.00 0.00
D
ATOM 10586 CA GLU D 24 −29.869 26.996 58.807 0.00 0.00
D
ATOM 10587 HA GLU D 24 −29.451 27.976 58.985 0.00 0.00
D
ATOM 10588 CB GLU D 24 −30.405 26.857 57.283 0.00 0.00
D
ATOM 10589 HB1 GLU D 24 −29.494 27.065 56.683 0.00 0.00
D
ATOM 10590 HB2 GLU D 24 −30.689 25.804 57.072 0.00 0.00
D
ATOM 10591 CG GLU D 24 −31.494 27.811 56.983 0.00 0.00
D
ATOM 10592 HG1 GLU D 24 −31.835 27.593 55.948 0.00 0.00
D
ATOM 10593 HG2 GLU D 24 −32.352 27.697 57.679 0.00 0.00
D
ATOM 10594 CD GLU D 24 −31.115 29.288 57.018 0.00 0.00
D
ATOM 10595 OE1 GLU D 24 −31.979 30.081 56.727 0.00 0.00
D
ATOM 10596 OE2 GLU D 24 −29.948 29.635 57.279 0.00 0.00
D
ATOM 10597 C GLU D 24 −30.984 26.758 59.757 0.00 0.00
D
ATOM 10598 O GLU D 24 −31.592 27.766 60.229 0.00 0.00
D
ATOM 10599 N LEU D 25 −31.374 25.479 60.048 0.00 0.00
D
ATOM 10600 HN LEU D 25 −31.009 24.770 59.449 0.00 0.00
D
ATOM 10601 CA LEU D 25 −32.420 25.075 61.060 0.00 0.00
D
ATOM 10602 HA LEU D 25 −33.286 25.602 60.688 0.00 0.00
D
ATOM 10603 CB LEU D 25 −32.762 23.535 61.026 0.00 0.00
D
ATOM 10604 HB1 LEU D 25 −31.775 23.045 61.165 0.00 0.00
D
ATOM 10605 HB2 LEU D 25 −33.410 23.305 61.898 0.00 0.00
D
ATOM 10606 CG LEU D 25 −33.456 23.083 59.663 0.00 0.00
D
ATOM 10607 HG LEU D 25 −32.713 23.353 58.881 0.00 0.00
D
ATOM 10608 CD1 LEU D 25 −33.587 21.574 59.580 0.00 0.00
D
ATOM 10609 HD11 LEU D 25 −33.829 21.299 58.531 0.00 0.00
D
ATOM 10610 HD12 LEU D 25 −32.702 21.064 60.017 0.00 0.00
D

ATOM 10611 HD13 LEU D 25 −34.417 21.195 60.216 0.00 0.00
D
ATOM 10612 CD2 LEU D 25 −34.798 23.808 59.370 0.00 0.00
D
ATOM 10613 HD21 LEU D 25 −35.029 23.476 58.335 0.00 0.00
D
ATOM 10614 HD22 LEU D 25 −35.614 23.406 60.007 0.00 0.00
D
ATOM 10615 HD23 LEU D 25 −34.721 24.914 59.298 0.00 0.00
D
ATOM 10616 C LEU D 25 −32.052 25.599 62.442 0.00 0.00
D
ATOM 10617 O LEU D 25 −32.927 26.130 63.121 0.00 0.00
D
ATOM 10618 N HSE D 26 −30.734 25.508 62.895 0.00 0.00
D
ATOM 10619 HN HSE D 26 −30.039 25.009 62.383 0.00 0.00
D
ATOM 10620 CA HSE D 26 −30.255 26.177 64.098 0.00 0.00
D
ATOM 10621 HA HSE D 26 −30.802 25.707 64.902 0.00 0.00
D
ATOM 10622 CB HSE D 26 −28.710 25.906 64.254 0.00 0.00
D
ATOM 10623 HB1 HSE D 26 −28.612 24.810 64.405 0.00 0.00
D
ATOM 10624 HB2 HSE D 26 −28.211 26.089 63.278 0.00 0.00
D
ATOM 10625 ND1 HSE D 26 −27.392 27.610 65.362 0.00 0.00
D
ATOM 10626 CG HSE D 26 −28.103 26.530 65.495 0.00 0.00
D
ATOM 10627 CE1 HSE D 26 −26.838 27.857 66.513 0.00 0.00
D
ATOM 10628 HE1 HSE D 26 −26.381 28.803 66.801 0.00 0.00
D
ATOM 10629 NE2 HSE D 26 −27.107 26.866 67.393 0.00 0.00
D
ATOM 10630 HE2 HSE D 26 −26.679 26.683 68.278 0.00 0.00
D
ATOM 10631 CD2 HSE D 26 −27.969 26.044 66.751 0.00 0.00
D
ATOM 10632 HD2 HSE D 26 −28.474 25.214 67.229 0.00 0.00
D
ATOM 10633 C HSE D 26 −30.504 27.675 64.106 0.00 0.00
D
ATOM 10634 O HSE D 26 −30.831 28.276 65.103 0.00 0.00
D
ATOM 10635 N ARG D 27 −30.403 28.311 62.907 0.00 0.00
D
ATOM 10636 HN ARG D 27 −30.306 27.821 62.044 0.00 0.00
D
ATOM 10637 CA ARG D 27 −30.459 29.761 62.760 0.00 0.00
D

ATOM 10638 HA ARG D 27 −29.926 30.360 63.485 0.00 0.00
D
ATOM 10639 CB ARG D 27 −30.154 30.121 61.272 0.00 0.00
D
ATOM 10640 HB1 ARG D 27 −29.307 29.490 60.926 0.00 0.00
D
ATOM 10641 HB2 ARG D 27 −31.057 29.996 60.638 0.00 0.00
D
ATOM 10642 CG ARG D 27 −29.665 31.573 61.135 0.00 0.00
D
ATOM 10643 HG1 ARG D 27 −30.589 32.122 61.417 0.00 0.00
D
ATOM 10644 HG2 ARG D 27 −28.893 31.869 61.876 0.00 0.00
D
ATOM 10645 CD ARG D 27 −29.085 32.163 59.856 0.00 0.00
D
ATOM 10646 HD1 ARG D 27 −28.845 33.242 59.965 0.00 0.00
D
ATOM 10647 HD2 ARG D 27 −28.231 31.576 59.455 0.00 0.00
D
ATOM 10648 NE ARG D 27 −30.098 32.036 58.738 0.00 0.00
D
ATOM 10649 HE ARG D 27 −30.272 31.122 58.371 0.00 0.00
D
ATOM 10650 CZ ARG D 27 −30.876 32.974 58.218 0.00 0.00
D
ATOM 10651 NH1 ARG D 27 −30.804 34.294 58.430 0.00 0.00
D
ATOM 10652 HH11 ARG D 27 −31.490 34.825 57.933 0.00 0.00
D
ATOM 10653 HH12 ARG D 27 −30.256 34.617 59.201 0.00 0.00
D
ATOM 10654 NH2 ARG D 27 −31.630 32.587 57.197 0.00 0.00
D
ATOM 10655 HH21 ARG D 27 −32.389 33.207 56.997 0.00 0.00
D
ATOM 10656 HH22 ARG D 27 −31.597 31.607 57.001 0.00 0.00
D
ATOM 10657 C ARG D 27 −31.906 30.210 62.930 0.00 0.00
D
ATOM 10658 O ARG D 27 −32.189 31.334 63.467 0.00 0.00
D
ATOM 10659 N MET D 28 −32.854 29.242 62.684 0.00 0.00
D
ATOM 10660 HN MET D 28 −32.482 28.406 62.288 0.00 0.00
D
ATOM 10661 CA MET D 28 −34.278 29.428 62.926 0.00 0.00
D
ATOM 10662 HA MET D 28 −34.471 30.486 62.832 0.00 0.00
D
ATOM 10663 CB MET D 28 −35.002 28.499 61.923 0.00 0.00
D
ATOM 10664 HB1 MET D 28 −34.623 27.461 62.029 0.00 0.00
D

ATOM 10665 HB2 MET D 28 −36.095 28.493 62.124 0.00 0.00
D
ATOM 10666 CG MET D 28 −34.781 28.821 60.451 0.00 0.00
D
ATOM 10667 HG1 MET D 28 −35.457 29.668 60.206 0.00 0.00
D
ATOM 10668 HG2 MET D 28 −33.708 28.935 60.187 0.00 0.00
D
ATOM 10669 SD MET D 28 −35.298 27.415 59.389 0.00 0.00
D
ATOM 10670 CE MET D 28 −35.395 28.261 57.797 0.00 0.00
D
ATOM 10671 HE1 MET D 28 −34.342 28.464 57.508 0.00 0.00
D
ATOM 10672 HE2 MET D 28 −35.786 27.470 57.121 0.00 0.00
D
ATOM 10673 HE3 MET D 28 −35.986 29.194 57.912 0.00 0.00
D
ATOM 10674 C MET D 28 −34.792 29.024 64.303 0.00 0.00
D
ATOM 10675 O MET D 28 −36.000 29.115 64.562 0.00 0.00
D
ATOM 10676 N GLY D 29 −33.890 28.396 65.152 0.00 0.00
D
ATOM 10677 HN GLY D 29 −32.935 28.269 64.898 0.00 0.00
D
ATOM 10678 CA GLY D 29 −34.278 27.940 66.519 0.00 0.00
D
ATOM 10679 HA1 GLY D 29 −35.001 28.647 66.900 0.00 0.00
D
ATOM 10680 HA2 GLY D 29 −33.402 27.960 67.150 0.00 0.00
D
ATOM 10681 C GLY D 29 −34.938 26.605 66.490 0.00 0.00
D
ATOM 10682 O GLY D 29 −35.594 26.297 67.466 0.00 0.00
D
ATOM 10683 N HSE D 30 −34.913 25.822 65.360 0.00 0.00
D
ATOM 10684 HN HSE D 30 −34.384 26.133 64.574 0.00 0.00
D
ATOM 10685 CA HSE D 30 −35.516 24.593 65.354 0.00 0.00
D
ATOM 10686 HA HSE D 30 −36.363 24.619 66.024 0.00 0.00
D
ATOM 10687 CB HSE D 30 −36.058 24.101 64.003 0.00 0.00
D
ATOM 10688 HB1 HSE D 30 −35.226 23.927 63.287 0.00 0.00
D
ATOM 10689 HB2 HSE D 30 −36.551 23.116 64.146 0.00 0.00
D
ATOM 10690 ND1 HSE D 30 −38.202 25.412 63.980 0.00 0.00
D
ATOM 10691 CG HSE D 30 −37.008 25.050 63.354 0.00 0.00
D

ATOM 10692 CE1 HSE D 30 −38.845 26.117 63.040 0.00 0.00
D
ATOM 10693 HE1 HSE D 30 −39.871 26.474 63.119 0.00 0.00
D
ATOM 10694 NE2 HSE D 30 −38.105 26.220 61.896 0.00 0.00
D
ATOM 10695 HE2 HSE D 30 −38.393 26.692 61.062 0.00 0.00
D
ATOM 10696 CD2 HSE D 30 −36.943 25.566 62.066 0.00 0.00
D
ATOM 10697 HD2 HSE D 30 −36.205 25.459 61.280 0.00 0.00
D
ATOM 10698 C HSE D 30 −34.511 23.500 65.787 0.00 0.00
D
ATOM 10699 O HSE D 30 −33.303 23.635 65.482 0.00 0.00
D
ATOM 10700 N GLU D 31 −34.882 22.464 66.552 0.00 0.00
D
ATOM 10701 HN GLU D 31 −35.864 22.381 66.702 0.00 0.00
D
ATOM 10702 CA GLU D 31 −33.920 21.469 67.016 0.00 0.00
D
ATOM 10703 HA GLU D 31 −33.065 22.020 67.379 0.00 0.00
D
ATOM 10704 CB GLU D 31 −34.536 20.637 68.214 0.00 0.00
D
ATOM 10705 HB1 GLU D 31 −35.467 20.111 67.913 0.00 0.00
D
ATOM 10706 HB2 GLU D 31 −33.780 19.934 68.627 0.00 0.00
D
ATOM 10707 CG GLU D 31 −34.940 21.594 69.347 0.00 0.00
D
ATOM 10708 HG1 GLU D 31 −33.988 22.045 69.699 0.00 0.00
D
ATOM 10709 HG2 GLU D 31 −35.627 22.449 69.170 0.00 0.00
D
ATOM 10710 CD GLU D 31 −35.615 20.793 70.434 0.00 0.00
D
ATOM 10711 OE1 GLU D 31 −34.813 20.403 71.371 0.00 0.00
D
ATOM 10712 OE2 GLU D 31 −36.884 20.733 70.541 0.00 0.00
D
ATOM 10713 C GLU D 31 −33.475 20.534 65.941 0.00 0.00
D
ATOM 10714 O GLU D 31 −34.181 20.022 65.071 0.00 0.00
D
ATOM 10715 N VAL D 32 −32.255 20.159 66.115 0.00 0.00
D
ATOM 10716 HN VAL D 32 −31.622 20.677 66.685 0.00 0.00
D
ATOM 10717 CA VAL D 32 −31.641 19.221 65.233 0.00 0.00
D
ATOM 10718 HA VAL D 32 −32.289 18.632 64.600 0.00 0.00
D

ATOM 10719 CB VAL D 32 −30.676 19.948 64.238 0.00 0.00
D
ATOM 10720 HB VAL D 32 −29.797 20.396 64.748 0.00 0.00
D
ATOM 10721 CG1 VAL D 32 −30.375 19.032 63.091 0.00 0.00
D
ATOM 10722 HG11 VAL D 32 −29.866 19.695 62.359 0.00 0.00
D
ATOM 10723 HG12 VAL D 32 −29.836 18.102 63.373 0.00 0.00
D
ATOM 10724 HG13 VAL D 32 −31.318 18.611 62.683 0.00 0.00
D
ATOM 10725 CG2 VAL D 32 −31.295 21.234 63.594 0.00 0.00
D
ATOM 10726 HG21 VAL D 32 −31.597 22.015 64.323 0.00 0.00
D
ATOM 10727 HG22 VAL D 32 −30.571 21.744 62.922 0.00 0.00
D
ATOM 10728 HG23 VAL D 32 −32.104 20.885 62.918 0.00 0.00
D
ATOM 10729 C VAL D 32 −30.830 18.172 65.888 0.00 0.00
D
ATOM 10730 O VAL D 32 −29.725 18.467 66.496 0.00 0.00
D
ATOM 10731 N LEU D 33 −31.154 16.909 65.745 0.00 0.00
D
ATOM 10732 HN LEU D 33 −32.085 16.594 65.573 0.00 0.00
D
ATOM 10733 CA LEU D 33 −30.243 15.787 65.923 0.00 0.00
D
ATOM 10734 HA LEU D 33 −29.705 15.953 66.845 0.00 0.00
D
ATOM 10735 CB LEU D 33 −30.984 14.465 66.009 0.00 0.00
D
ATOM 10736 HB1 LEU D 33 −31.685 14.581 66.864 0.00 0.00
D
ATOM 10737 HB2 LEU D 33 −31.521 14.236 65.064 0.00 0.00
D
ATOM 10738 CG LEU D 33 −30.105 13.209 66.132 0.00 0.00
D
ATOM 10739 HG LEU D 33 −29.528 13.253 65.184 0.00 0.00
D
ATOM 10740 CD1 LEU D 33 −29.106 13.001 67.305 0.00 0.00
D
ATOM 10741 HD11 LEU D 33 −28.735 11.974 67.510 0.00 0.00
D
ATOM 10742 HD12 LEU D 33 −28.186 13.593 67.113 0.00 0.00
D
ATOM 10743 HD13 LEU D 33 −29.587 13.442 68.205 0.00 0.00
D
ATOM 10744 CD2 LEU D 33 −30.988 11.920 66.156 0.00 0.00
D
ATOM 10745 HD21 LEU D 33 −30.253 11.123 66.399 0.00 0.00
D

ATOM 10746 HD22 LEU D 33 −31.816 11.936 66.897 0.00 0.00
D
ATOM 10747 HD23 LEU D 33 −31.368 11.724 65.131 0.00 0.00
D
ATOM 10748 C LEU D 33 −29.292 15.640 64.758 0.00 0.00
D
ATOM 10749 O LEU D 33 −29.741 15.367 63.662 0.00 0.00
D
ATOM 10750 N ALA D 34 −28.004 15.891 64.932 0.00 0.00
D
ATOM 10751 HN ALA D 34 −27.554 15.949 65.820 0.00 0.00
D
ATOM 10752 CA ALA D 34 −26.999 15.754 63.858 0.00 0.00
D
ATOM 10753 HA ALA D 34 −27.396 15.756 62.854 0.00 0.00
D
ATOM 10754 CB ALA D 34 −26.165 17.023 64.009 0.00 0.00
D
ATOM 10755 HB1 ALA D 34 −26.727 17.764 64.618 0.00 0.00
D
ATOM 10756 HB2 ALA D 34 −25.184 16.835 64.494 0.00 0.00
D
ATOM 10757 HB3 ALA D 34 −25.809 17.478 63.060 0.00 0.00
D
ATOM 10758 C ALA D 34 −26.120 14.520 64.014 0.00 0.00
D
ATOM 10759 O ALA D 34 −25.511 14.365 65.039 0.00 0.00
D
ATOM 10760 N VAL D 35 −26.212 13.581 63.024 0.00 0.00
D
ATOM 10761 HN VAL D 35 −26.800 13.691 62.226 0.00 0.00
D
ATOM 10762 CA VAL D 35 −25.575 12.295 62.984 0.00 0.00
D
ATOM 10763 HA VAL D 35 −25.000 12.084 63.873 0.00 0.00
D
ATOM 10764 CB VAL D 35 −26.723 11.229 62.912 0.00 0.00
D
ATOM 10765 HB VAL D 35 −27.218 11.551 61.971 0.00 0.00
D
ATOM 10766 CG1 VAL D 35 −26.244 9.733 62.885 0.00 0.00
D
ATOM 10767 HG11 VAL D 35 −25.649 9.457 63.782 0.00 0.00
D
ATOM 10768 HG12 VAL D 35 −27.081 9.003 62.837 0.00 0.00
D
ATOM 10769 HG13 VAL D 35 −25.636 9.533 61.977 0.00 0.00
D
ATOM 10770 CG2 VAL D 35 −27.737 11.559 64.000 0.00 0.00
D
ATOM 10771 HG21 VAL D 35 −28.533 10.801 63.839 0.00 0.00
D
ATOM 10772 HG22 VAL D 35 −27.212 11.476 64.976 0.00 0.00
D

```
ATOM  10773 HG23 VAL D  35      -28.193  12.572  64.020  0.00  0.00
D
ATOM  10774  C   VAL D  35      -24.697  12.123  61.728  0.00  0.00
D
ATOM  10775  O   VAL D  35      -25.178  12.185  60.594  0.00  0.00
D
ATOM  10776  N   ASP D  36      -23.406  11.808  61.853  0.00  0.00
D
ATOM  10777  HN  ASP D  36      -23.074  11.605  62.771  0.00  0.00
D
ATOM  10778  CA  ASP D  36      -22.530  11.333  60.807  0.00  0.00
D
ATOM  10779  HA  ASP D  36      -23.151  10.783  60.115  0.00  0.00
D
ATOM  10780  CB  ASP D  36      -21.791  12.562  60.224  0.00  0.00
D
ATOM  10781  HB1 ASP D  36      -22.544  13.309  59.892  0.00  0.00
D
ATOM  10782  HB2 ASP D  36      -21.066  13.020  60.930  0.00  0.00
D
ATOM  10783  CG  ASP D  36      -21.077  12.294  58.863  0.00  0.00
D
ATOM  10784  OD1 ASP D  36      -21.638  12.694  57.815  0.00  0.00
D
ATOM  10785  OD2 ASP D  36      -19.937  11.700  58.895  0.00  0.00
D
ATOM  10786  C   ASP D  36      -21.586  10.228  61.517  0.00  0.00
D
ATOM  10787  O   ASP D  36      -21.710   9.859  62.663  0.00  0.00
D
ATOM  10788  N   ILE D  37      -20.613   9.794  60.719  0.00  0.00
D
ATOM  10789  HN  ILE D  37      -20.568  10.179  59.801  0.00  0.00
D
ATOM  10790  CA  ILE D  37      -19.618   8.842  61.198  0.00  0.00
D
ATOM  10791  HA  ILE D  37      -19.949   8.550  62.184  0.00  0.00
D
ATOM  10792  CB  ILE D  37      -19.477   7.682  60.296  0.00  0.00
D
ATOM  10793  HB  ILE D  37      -18.572   7.075  60.513  0.00  0.00
D
ATOM  10794  CG2 ILE D  37      -20.605   6.710  60.631  0.00  0.00
D
ATOM  10795 HG21 ILE D  37      -20.322   6.332  61.636  0.00  0.00
D
ATOM  10796 HG22 ILE D  37      -21.571   7.259  60.627  0.00  0.00
D
ATOM  10797 HG23 ILE D  37      -20.618   5.892  59.879  0.00  0.00
D
ATOM  10798  CG1 ILE D  37      -19.386   8.113  58.791  0.00  0.00
D
ATOM  10799 HG11 ILE D  37      -20.314   8.669  58.540  0.00  0.00
D
```

ATOM	10800	HG12	ILE	D	37	−18.500	8.782	58.737	0.00	0.00	D
ATOM	10801	CD	ILE	D	37	−19.214	7.003	57.719	0.00	0.00	D
ATOM	10802	HD1	ILE	D	37	−19.331	7.408	56.691	0.00	0.00	D
ATOM	10803	HD2	ILE	D	37	−18.320	6.344	57.760	0.00	0.00	D
ATOM	10804	HD3	ILE	D	37	−20.094	6.353	57.913	0.00	0.00	D
ATOM	10805	C	ILE	D	37	−18.356	9.514	61.390	0.00	0.00	D
ATOM	10806	O	ILE	D	37	−17.485	8.910	62.146	0.00	0.00	D
ATOM	10807	N	ASN	D	38	−18.066	10.686	60.732	0.00	0.00	D
ATOM	10808	HN	ASN	D	38	−18.783	11.240	60.316	0.00	0.00	D
ATOM	10809	CA	ASN	D	38	−16.770	11.311	60.767	0.00	0.00	D
ATOM	10810	HA	ASN	D	38	−15.985	10.576	60.873	0.00	0.00	D
ATOM	10811	CB	ASN	D	38	−16.567	12.075	59.405	0.00	0.00	D
ATOM	10812	HB1	ASN	D	38	−16.655	11.459	58.485	0.00	0.00	D
ATOM	10813	HB2	ASN	D	38	−17.380	12.825	59.298	0.00	0.00	D
ATOM	10814	CG	ASN	D	38	−15.233	12.898	59.358	0.00	0.00	D
ATOM	10815	OD1	ASN	D	38	−15.228	14.097	59.609	0.00	0.00	D
ATOM	10816	ND2	ASN	D	38	−14.094	12.246	59.128	0.00	0.00	D
ATOM	10817	HD21	ASN	D	38	−13.223	12.720	59.259	0.00	0.00	D
ATOM	10818	HD22	ASN	D	38	−14.170	11.289	58.845	0.00	0.00	D
ATOM	10819	C	ASN	D	38	−16.715	12.280	61.951	0.00	0.00	D
ATOM	10820	O	ASN	D	38	−17.607	13.096	62.131	0.00	0.00	D
ATOM	10821	N	GLU	D	39	−15.655	12.191	62.783	0.00	0.00	D
ATOM	10822	HN	GLU	D	39	−14.987	11.476	62.589	0.00	0.00	D
ATOM	10823	CA	GLU	D	39	−15.585	12.984	64.055	0.00	0.00	D
ATOM	10824	HA	GLU	D	39	−16.502	12.851	64.610	0.00	0.00	D
ATOM	10825	CB	GLU	D	39	−14.522	12.383	65.003	0.00	0.00	D
ATOM	10826	HB1	GLU	D	39	−14.830	11.322	65.123	0.00	0.00	D

ATOM 10827 HB2 GLU D 39 −13.548 12.468 64.475 0.00 0.00
D
ATOM 10828 CG GLU D 39 −14.517 13.079 66.327 0.00 0.00
D
ATOM 10829 HG1 GLU D 39 −14.278 14.163 66.286 0.00 0.00
D
ATOM 10830 HG2 GLU D 39 −15.552 13.083 66.731 0.00 0.00
D
ATOM 10831 CD GLU D 39 −13.665 12.362 67.347 0.00 0.00
D
ATOM 10832 OE1 GLU D 39 −14.127 11.345 67.898 0.00 0.00
D
ATOM 10833 OE2 GLU D 39 −12.468 12.840 67.563 0.00 0.00
D
ATOM 10834 C GLU D 39 −15.428 14.487 63.800 0.00 0.00
D
ATOM 10835 O GLU D 39 −16.070 15.281 64.535 0.00 0.00
D
ATOM 10836 N GLU D 40 −14.684 14.948 62.806 0.00 0.00
D
ATOM 10837 HN GLU D 40 −14.112 14.271 62.349 0.00 0.00
D
ATOM 10838 CA GLU D 40 −14.560 16.329 62.453 0.00 0.00
D
ATOM 10839 HA GLU D 40 −14.145 16.864 63.294 0.00 0.00
D
ATOM 10840 CB GLU D 40 −13.433 16.476 61.377 0.00 0.00
D
ATOM 10841 HB1 GLU D 40 −12.556 15.902 61.745 0.00 0.00
D
ATOM 10842 HB2 GLU D 40 −13.892 16.139 60.423 0.00 0.00
D
ATOM 10843 CG GLU D 40 −13.152 17.953 61.069 0.00 0.00
D
ATOM 10844 HG1 GLU D 40 −14.054 18.411 60.610 0.00 0.00
D
ATOM 10845 HG2 GLU D 40 −12.962 18.479 62.029 0.00 0.00
D
ATOM 10846 CD GLU D 40 −11.976 18.180 60.133 0.00 0.00
D
ATOM 10847 OE1 GLU D 40 −11.792 17.324 59.192 0.00 0.00
D
ATOM 10848 OE2 GLU D 40 −11.262 19.175 60.398 0.00 0.00
D
ATOM 10849 C GLU D 40 −15.892 16.985 62.117 0.00 0.00
D
ATOM 10850 O GLU D 40 −16.219 18.060 62.579 0.00 0.00
D
ATOM 10851 N LYS D 41 −16.566 16.321 61.155 0.00 0.00
D
ATOM 10852 HN LYS D 41 −16.087 15.662 60.581 0.00 0.00
D
ATOM 10853 CA LYS D 41 −17.872 16.706 60.744 0.00 0.00
D

ATOM 10854 HA LYS D 41 −17.968 17.727 60.407 0.00 0.00
D
ATOM 10855 CB LYS D 41 −18.429 15.815 59.608 0.00 0.00
D
ATOM 10856 HB1 LYS D 41 −18.301 14.734 59.833 0.00 0.00
D
ATOM 10857 HB2 LYS D 41 −19.538 15.848 59.543 0.00 0.00
D
ATOM 10858 CG LYS D 41 −17.781 16.193 58.245 0.00 0.00
D
ATOM 10859 HG1 LYS D 41 −18.073 17.259 58.127 0.00 0.00
D
ATOM 10860 HG2 LYS D 41 −16.700 16.094 58.481 0.00 0.00
D
ATOM 10861 CD LYS D 41 −18.257 15.370 57.026 0.00 0.00
D
ATOM 10862 HD1 LYS D 41 −18.136 14.318 57.362 0.00 0.00
D
ATOM 10863 HD2 LYS D 41 −19.354 15.512 56.925 0.00 0.00
D
ATOM 10864 CE LYS D 41 −17.631 15.617 55.614 0.00 0.00
D
ATOM 10865 HE1 LYS D 41 −17.955 16.604 55.222 0.00 0.00
D
ATOM 10866 HE2 LYS D 41 −16.523 15.640 55.695 0.00 0.00
D
ATOM 10867 NZ LYS D 41 −18.118 14.641 54.591 0.00 0.00
D
ATOM 10868 HZ1 LYS D 41 −17.436 13.878 54.409 0.00 0.00
D
ATOM 10869 HZ2 LYS D 41 −18.968 14.155 54.942 0.00 0.00
D
ATOM 10870 HZ3 LYS D 41 −18.415 15.138 53.727 0.00 0.00
D
ATOM 10871 C LYS D 41 −19.000 16.799 61.757 0.00 0.00
D
ATOM 10872 O LYS D 41 −19.817 17.662 61.693 0.00 0.00
D
ATOM 10873 N VAL D 42 −19.070 15.914 62.752 0.00 0.00
D
ATOM 10874 HN VAL D 42 −18.325 15.251 62.754 0.00 0.00
D
ATOM 10875 CA VAL D 42 −19.959 16.012 63.950 0.00 0.00
D
ATOM 10876 HA VAL D 42 −20.926 16.383 63.645 0.00 0.00
D
ATOM 10877 CB VAL D 42 −19.980 14.668 64.713 0.00 0.00
D
ATOM 10878 HB VAL D 42 −18.944 14.356 64.966 0.00 0.00
D
ATOM 10879 CG1 VAL D 42 −20.749 14.765 66.093 0.00 0.00
D
ATOM 10880 HG11 VAL D 42 −20.181 15.412 66.795 0.00 0.00
D

ATOM 10881 HG12 VAL D 42 -21.713 15.241 65.814 0.00 0.00
D
ATOM 10882 HG13 VAL D 42 -20.933 13.775 66.563 0.00 0.00
D
ATOM 10883 CG2 VAL D 42 -20.562 13.570 63.759 0.00 0.00
D
ATOM 10884 HG21 VAL D 42 -21.650 13.764 63.648 0.00 0.00
D
ATOM 10885 HG22 VAL D 42 -20.130 13.358 62.757 0.00 0.00
D
ATOM 10886 HG23 VAL D 42 -20.547 12.578 64.258 0.00 0.00
D
ATOM 10887 C VAL D 42 -19.490 17.151 64.868 0.00 0.00
D
ATOM 10888 O VAL D 42 -20.273 17.893 65.473 0.00 0.00
D
ATOM 10889 N ASN D 43 -18.132 17.226 65.015 0.00 0.00
D
ATOM 10890 HN ASN D 43 -17.553 16.509 64.635 0.00 0.00
D
ATOM 10891 CA ASN D 43 -17.604 18.238 65.969 0.00 0.00
D
ATOM 10892 HA ASN D 43 -18.189 18.135 66.872 0.00 0.00
D
ATOM 10893 CB ASN D 43 -16.085 17.936 66.163 0.00 0.00
D
ATOM 10894 HB1 ASN D 43 -15.957 16.868 66.439 0.00 0.00
D
ATOM 10895 HB2 ASN D 43 -15.442 18.010 65.260 0.00 0.00
D
ATOM 10896 CG ASN D 43 -15.560 18.701 67.329 0.00 0.00
D
ATOM 10897 OD1 ASN D 43 -16.226 18.858 68.347 0.00 0.00
D
ATOM 10898 ND2 ASN D 43 -14.438 19.466 67.184 0.00 0.00
D
ATOM 10899 HD21 ASN D 43 -14.018 19.992 67.924 0.00 0.00
D
ATOM 10900 HD22 ASN D 43 -13.994 19.467 66.288 0.00 0.00
D
ATOM 10901 C ASN D 43 -17.799 19.647 65.486 0.00 0.00
D
ATOM 10902 O ASN D 43 -17.881 20.528 66.359 0.00 0.00
D
ATOM 10903 N ALA D 44 -17.929 19.894 64.144 0.00 0.00
D
ATOM 10904 HN ALA D 44 -17.731 19.159 63.501 0.00 0.00
D
ATOM 10905 CA ALA D 44 -18.293 21.099 63.463 0.00 0.00
D
ATOM 10906 HA ALA D 44 -17.594 21.890 63.693 0.00 0.00
D
ATOM 10907 CB ALA D 44 -18.088 20.956 61.938 0.00 0.00
D

ATOM 10908 HB1 ALA D 44 −17.019 21.072 61.657 0.00 0.00
D
ATOM 10909 HB2 ALA D 44 −18.529 19.986 61.625 0.00 0.00
D
ATOM 10910 HB3 ALA D 44 −18.653 21.784 61.461 0.00 0.00
D
ATOM 10911 C ALA D 44 −19.675 21.530 63.804 0.00 0.00
D
ATOM 10912 O ALA D 44 −19.852 22.706 63.814 0.00 0.00
D
ATOM 10913 N TYR D 45 −20.664 20.578 64.131 0.00 0.00
D
ATOM 10914 HN TYR D 45 −20.384 19.621 64.115 0.00 0.00
D
ATOM 10915 CA TYR D 45 −22.062 20.912 64.504 0.00 0.00
D
ATOM 10916 HA TYR D 45 −22.171 21.975 64.352 0.00 0.00
D
ATOM 10917 CB TYR D 45 −23.124 20.166 63.592 0.00 0.00
D
ATOM 10918 HB1 TYR D 45 −22.907 19.107 63.849 0.00 0.00
D
ATOM 10919 HB2 TYR D 45 −24.177 20.316 63.912 0.00 0.00
D
ATOM 10920 CG TYR D 45 −22.938 20.622 62.105 0.00 0.00
D
ATOM 10921 CD1 TYR D 45 −22.202 19.836 61.285 0.00 0.00
D
ATOM 10922 HD1 TYR D 45 −21.814 18.933 61.731 0.00 0.00
D
ATOM 10923 CE1 TYR D 45 −21.665 20.140 60.047 0.00 0.00
D
ATOM 10924 HE1 TYR D 45 −20.962 19.505 59.529 0.00 0.00
D
ATOM 10925 CZ TYR D 45 −21.959 21.466 59.552 0.00 0.00
D
ATOM 10926 OH TYR D 45 −21.566 21.829 58.302 0.00 0.00
D
ATOM 10927 HH TYR D 45 −21.026 21.153 57.886 0.00 0.00
D
ATOM 10928 CD2 TYR D 45 −23.305 21.856 61.505 0.00 0.00
D
ATOM 10929 HD2 TYR D 45 −24.021 22.433 62.071 0.00 0.00
D
ATOM 10930 CE2 TYR D 45 −22.889 22.267 60.200 0.00 0.00
D
ATOM 10931 HE2 TYR D 45 −23.243 23.215 59.821 0.00 0.00
D
ATOM 10932 C TYR D 45 −22.453 20.669 65.935 0.00 0.00
D
ATOM 10933 O TYR D 45 −23.609 20.779 66.218 0.00 0.00
D
ATOM 10934 N ALA D 46 −21.469 20.372 66.881 0.00 0.00
D

ATOM 10935 HN ALA D 46 −20.540 20.149 66.596 0.00 0.00
D
ATOM 10936 CA ALA D 46 −21.575 20.355 68.258 0.00 0.00
D
ATOM 10937 HA ALA D 46 −22.232 19.545 68.540 0.00 0.00
D
ATOM 10938 CB ALA D 46 −20.179 20.220 68.832 0.00 0.00
D
ATOM 10939 HB1 ALA D 46 −19.909 19.168 68.601 0.00 0.00
D
ATOM 10940 HB2 ALA D 46 −19.538 21.006 68.376 0.00 0.00
D
ATOM 10941 HB3 ALA D 46 −20.049 20.457 69.909 0.00 0.00
D
ATOM 10942 C ALA D 46 −22.236 21.559 68.917 0.00 0.00
D
ATOM 10943 O ALA D 46 −23.131 21.451 69.788 0.00 0.00
D
ATOM 10944 N SER D 47 −21.979 22.790 68.476 0.00 0.00
D
ATOM 10945 HN SER D 47 −21.285 22.818 67.761 0.00 0.00
D
ATOM 10946 CA SER D 47 −22.628 24.067 68.919 0.00 0.00
D
ATOM 10947 HA SER D 47 −22.860 24.021 69.973 0.00 0.00
D
ATOM 10948 CB SER D 47 −21.775 25.285 68.585 0.00 0.00
D
ATOM 10949 HB1 SER D 47 −22.345 26.179 68.919 0.00 0.00
D
ATOM 10950 HB2 SER D 47 −20.795 25.247 69.107 0.00 0.00
D
ATOM 10951 OG SER D 47 −21.503 25.281 67.177 0.00 0.00
D
ATOM 10952 HG1 SER D 47 −20.775 24.678 67.007 0.00 0.00
D
ATOM 10953 C SER D 47 −24.025 24.263 68.254 0.00 0.00
D
ATOM 10954 O SER D 47 −24.908 24.891 68.846 0.00 0.00
D
ATOM 10955 N TYR D 48 −24.183 23.871 66.955 0.00 0.00
D
ATOM 10956 HN TYR D 48 −23.430 23.433 66.470 0.00 0.00
D
ATOM 10957 CA TYR D 48 −25.363 24.052 66.149 0.00 0.00
D
ATOM 10958 HA TYR D 48 −25.742 25.059 66.241 0.00 0.00
D
ATOM 10959 CB TYR D 48 −25.009 23.881 64.638 0.00 0.00
D
ATOM 10960 HB1 TYR D 48 −24.493 22.899 64.699 0.00 0.00
D
ATOM 10961 HB2 TYR D 48 −25.946 23.853 64.043 0.00 0.00
D

ATOM 10962 CG TYR D 48 -24.168 25.065 64.195 0.00 0.00
D
ATOM 10963 CD1 TYR D 48 -22.784 24.925 63.904 0.00 0.00
D
ATOM 10964 HD1 TYR D 48 -22.227 24.015 64.073 0.00 0.00
D
ATOM 10965 CE1 TYR D 48 -21.993 25.965 63.295 0.00 0.00
D
ATOM 10966 HE1 TYR D 48 -20.920 25.892 63.199 0.00 0.00
D
ATOM 10967 CZ TYR D 48 -22.611 27.102 62.894 0.00 0.00
D
ATOM 10968 OH TYR D 48 -21.839 28.096 62.203 0.00 0.00
D
ATOM 10969 HH TYR D 48 -20.922 27.934 62.436 0.00 0.00
D
ATOM 10970 CD2 TYR D 48 -24.686 26.310 63.773 0.00 0.00
D
ATOM 10971 HD2 TYR D 48 -25.764 26.327 63.842 0.00 0.00
D
ATOM 10972 CE2 TYR D 48 -23.981 27.269 63.128 0.00 0.00
D
ATOM 10973 HE2 TYR D 48 -24.586 28.099 62.793 0.00 0.00
D
ATOM 10974 C TYR D 48 -26.546 23.013 66.482 0.00 0.00
D
ATOM 10975 O TYR D 48 -27.723 23.221 66.182 0.00 0.00
D
ATOM 10976 N ALA D 49 -26.194 21.812 66.980 0.00 0.00
D
ATOM 10977 HN ALA D 49 -25.249 21.539 67.142 0.00 0.00
D
ATOM 10978 CA ALA D 49 -27.037 20.671 67.239 0.00 0.00
D
ATOM 10979 HA ALA D 49 -27.895 20.839 66.604 0.00 0.00
D
ATOM 10980 CB ALA D 49 -26.345 19.306 66.981 0.00 0.00
D
ATOM 10981 HB1 ALA D 49 -25.851 19.331 65.986 0.00 0.00
D
ATOM 10982 HB2 ALA D 49 -25.757 19.027 67.882 0.00 0.00
D
ATOM 10983 HB3 ALA D 49 -27.158 18.550 66.995 0.00 0.00
D
ATOM 10984 C ALA D 49 -27.387 20.688 68.698 0.00 0.00
D
ATOM 10985 O ALA D 49 -26.572 20.757 69.631 0.00 0.00
D
ATOM 10986 N THR D 50 -28.702 20.368 69.048 0.00 0.00
D
ATOM 10987 HN THR D 50 -29.386 19.963 68.446 0.00 0.00
D
ATOM 10988 CA THR D 50 -29.130 19.931 70.437 0.00 0.00
D

ATOM	10989	HA	THR	D	50	−28.915	20.687	71.178	0.00	0.00	D
ATOM	10990	CB	THR	D	50	−30.572	19.815	70.672	0.00	0.00	D
ATOM	10991	HB	THR	D	50	−30.805	19.448	71.694	0.00	0.00	D
ATOM	10992	OG1	THR	D	50	−31.208	19.016	69.660	0.00	0.00	D
ATOM	10993	HG1	THR	D	50	−32.143	18.905	69.845	0.00	0.00	D
ATOM	10994	CG2	THR	D	50	−31.107	21.214	70.540	0.00	0.00	D
ATOM	10995	HG21	THR	D	50	−31.150	21.458	69.457	0.00	0.00	D
ATOM	10996	HG22	THR	D	50	−32.158	21.265	70.896	0.00	0.00	D
ATOM	10997	HG23	THR	D	50	−30.476	21.921	71.121	0.00	0.00	D
ATOM	10998	C	THR	D	50	−28.477	18.680	70.865	0.00	0.00	D
ATOM	10999	O	THR	D	50	−27.868	18.537	71.979	0.00	0.00	D
ATOM	11000	N	HSE	D	51	−28.458	17.723	69.977	0.00	0.00	D
ATOM	11001	HN	HSE	D	51	−28.979	17.835	69.135	0.00	0.00	D
ATOM	11002	CA	HSE	D	51	−27.684	16.468	70.118	0.00	0.00	D
ATOM	11003	HA	HSE	D	51	−27.082	16.541	71.012	0.00	0.00	D
ATOM	11004	CB	HSE	D	51	−28.513	15.188	70.478	0.00	0.00	D
ATOM	11005	HB1	HSE	D	51	−29.364	15.480	71.129	0.00	0.00	D
ATOM	11006	HB2	HSE	D	51	−29.047	14.777	69.594	0.00	0.00	D
ATOM	11007	ND1	HSE	D	51	−27.491	13.936	72.275	0.00	0.00	D
ATOM	11008	CG	HSE	D	51	−27.715	14.073	70.949	0.00	0.00	D
ATOM	11009	CE1	HSE	D	51	−26.401	13.171	72.387	0.00	0.00	D
ATOM	11010	HE1	HSE	D	51	−25.890	12.776	73.266	0.00	0.00	D
ATOM	11011	NE2	HSE	D	51	−25.911	12.912	71.140	0.00	0.00	D
ATOM	11012	HE2	HSE	D	51	−25.107	12.319	71.091	0.00	0.00	D
ATOM	11013	CD2	HSE	D	51	−26.790	13.446	70.231	0.00	0.00	D
ATOM	11014	HD2	HSE	D	51	−26.698	13.525	69.155	0.00	0.00	D
ATOM	11015	C	HSE	D	51	−26.876	16.256	68.912	0.00	0.00	D

```
ATOM  11016  O   HSE D  51      -27.347  16.041  67.794  0.00  0.00
D
ATOM  11017  N   ALA D  52      -25.522  16.242  69.070  0.00  0.00
D
ATOM  11018  HN  ALA D  52      -25.179  16.711  69.881  0.00  0.00
D
ATOM  11019  CA  ALA D  52      -24.633  15.857  67.973  0.00  0.00
D
ATOM  11020  HA  ALA D  52      -25.285  15.718  67.123  0.00  0.00
D
ATOM  11021  CB  ALA D  52      -23.567  16.976  67.650  0.00  0.00
D
ATOM  11022  HB1 ALA D  52      -24.198  17.865  67.438  0.00  0.00
D
ATOM  11023  HB2 ALA D  52      -22.804  17.160  68.437  0.00  0.00
D
ATOM  11024  HB3 ALA D  52      -23.297  16.683  66.613  0.00  0.00
D
ATOM  11025  C   ALA D  52      -23.939  14.532  68.248  0.00  0.00
D
ATOM  11026  O   ALA D  52      -23.287  14.465  69.314  0.00  0.00
D
ATOM  11027  N   VAL D  53      -24.004  13.528  67.379  0.00  0.00
D
ATOM  11028  HN  VAL D  53      -24.440  13.631  66.488  0.00  0.00
D
ATOM  11029  CA  VAL D  53      -23.514  12.196  67.611  0.00  0.00
D
ATOM  11030  HA  VAL D  53      -22.773  12.201  68.397  0.00  0.00
D
ATOM  11031  CB  VAL D  53      -24.669  11.274  68.058  0.00  0.00
D
ATOM  11032  HB  VAL D  53      -25.066  11.859  68.915  0.00  0.00
D
ATOM  11033  CG1 VAL D  53      -25.829  11.055  67.019  0.00  0.00
D
ATOM  11034 HG11 VAL D  53      -25.604  10.349  66.191  0.00  0.00
D
ATOM  11035 HG12 VAL D  53      -26.679  10.706  67.644  0.00  0.00
D
ATOM  11036 HG13 VAL D  53      -26.064  12.094  66.703  0.00  0.00
D
ATOM  11037  CG2 VAL D  53      -24.195   9.932  68.674  0.00  0.00
D
ATOM  11038 HG21 VAL D  53      -25.008   9.443  69.250  0.00  0.00
D
ATOM  11039 HG22 VAL D  53      -23.874   9.135  67.970  0.00  0.00
D
ATOM  11040 HG23 VAL D  53      -23.384  10.144  69.403  0.00  0.00
D
ATOM  11041  C   VAL D  53      -22.763  11.512  66.451  0.00  0.00
D
ATOM  11042  O   VAL D  53      -22.990  11.849  65.269  0.00  0.00
D
```

ATOM 11043 N ILE D 54 −21.737 10.658 66.730 0.00 0.00
D
ATOM 11044 HN ILE D 54 −21.536 10.503 67.695 0.00 0.00
D
ATOM 11045 CA ILE D 54 −21.001 9.821 65.818 0.00 0.00
D
ATOM 11046 HA ILE D 54 −21.149 10.309 64.866 0.00 0.00
D
ATOM 11047 CB ILE D 54 −19.555 9.666 66.295 0.00 0.00
D
ATOM 11048 HB ILE D 54 −19.596 9.132 67.268 0.00 0.00
D
ATOM 11049 CG2 ILE D 54 −18.912 8.809 65.247 0.00 0.00
D
ATOM 11050 HG21 ILE D 54 −17.821 8.608 65.316 0.00 0.00
D
ATOM 11051 HG22 ILE D 54 −19.265 7.757 65.301 0.00 0.00
D
ATOM 11052 HG23 ILE D 54 −19.153 9.151 64.218 0.00 0.00
D
ATOM 11053 CG1 ILE D 54 −18.908 11.044 66.460 0.00 0.00
D
ATOM 11054 HG11 ILE D 54 −18.564 11.324 65.442 0.00 0.00
D
ATOM 11055 HG12 ILE D 54 −19.503 11.790 67.029 0.00 0.00
D
ATOM 11056 CD ILE D 54 −17.592 10.929 67.210 0.00 0.00
D
ATOM 11057 HD1 ILE D 54 −17.578 10.214 68.060 0.00 0.00
D
ATOM 11058 HD2 ILE D 54 −16.709 10.734 66.565 0.00 0.00
D
ATOM 11059 HD3 ILE D 54 −17.244 11.897 67.629 0.00 0.00
D
ATOM 11060 C ILE D 54 −21.736 8.481 65.871 0.00 0.00
D
ATOM 11061 O ILE D 54 −21.859 7.908 66.956 0.00 0.00
D
ATOM 11062 N ALA D 55 −22.199 7.953 64.753 0.00 0.00
D
ATOM 11063 HN ALA D 55 −22.212 8.476 63.905 0.00 0.00
D
ATOM 11064 CA ALA D 55 −22.942 6.699 64.725 0.00 0.00
D
ATOM 11065 HA ALA D 55 −22.320 5.961 65.209 0.00 0.00
D
ATOM 11066 CB ALA D 55 −24.304 6.749 65.419 0.00 0.00
D
ATOM 11067 HB1 ALA D 55 −25.001 7.311 64.761 0.00 0.00
D
ATOM 11068 HB2 ALA D 55 −24.815 5.762 65.429 0.00 0.00
D
ATOM 11069 HB3 ALA D 55 −24.389 7.259 66.403 0.00 0.00
D

ATOM 11070 C ALA D 55 −23.256 6.288 63.314 0.00 0.00
D
ATOM 11071 O ALA D 55 −23.667 7.066 62.470 0.00 0.00
D
ATOM 11072 N ASN D 56 −22.990 5.007 62.940 0.00 0.00
D
ATOM 11073 HN ASN D 56 −22.709 4.463 63.726 0.00 0.00
D
ATOM 11074 CA ASN D 56 −23.287 4.320 61.686 0.00 0.00
D
ATOM 11075 HA ASN D 56 −23.029 4.888 60.805 0.00 0.00
D
ATOM 11076 CB ASN D 56 −22.568 2.920 61.549 0.00 0.00
D
ATOM 11077 HB1 ASN D 56 −22.859 2.130 62.275 0.00 0.00
D
ATOM 11078 HB2 ASN D 56 −22.848 2.607 60.520 0.00 0.00
D
ATOM 11079 CG ASN D 56 −21.078 3.066 61.437 0.00 0.00
D
ATOM 11080 OD1 ASN D 56 −20.485 3.289 60.378 0.00 0.00
D
ATOM 11081 ND2 ASN D 56 −20.449 3.214 62.635 0.00 0.00
D
ATOM 11082 HD21 ASN D 56 −19.548 3.614 62.802 0.00 0.00
D
ATOM 11083 HD22 ASN D 56 −20.950 2.959 63.461 0.00 0.00
D
ATOM 11084 C ASN D 56 −24.843 4.253 61.523 0.00 0.00
D
ATOM 11085 O ASN D 56 −25.580 3.794 62.427 0.00 0.00
D
ATOM 11086 N ALA D 57 −25.395 4.728 60.365 0.00 0.00
D
ATOM 11087 HN ALA D 57 −24.881 5.391 59.825 0.00 0.00
D
ATOM 11088 CA ALA D 57 −26.859 4.759 60.205 0.00 0.00
D
ATOM 11089 HA ALA D 57 −27.267 4.588 61.191 0.00 0.00
D
ATOM 11090 CB ALA D 57 −27.372 6.158 59.625 0.00 0.00
D
ATOM 11091 HB1 ALA D 57 −26.890 6.913 60.283 0.00 0.00
D
ATOM 11092 HB2 ALA D 57 −26.935 6.259 58.608 0.00 0.00
D
ATOM 11093 HB3 ALA D 57 −28.477 6.261 59.596 0.00 0.00
D
ATOM 11094 C ALA D 57 −27.173 3.569 59.330 0.00 0.00
D
ATOM 11095 O ALA D 57 −28.373 3.283 59.067 0.00 0.00
D
ATOM 11096 N THR D 58 −26.167 2.767 58.837 0.00 0.00
D

ATOM 11097 HN THR D 58 −25.205 2.957 59.021 0.00 0.00
D
ATOM 11098 CA THR D 58 −26.343 1.602 57.968 0.00 0.00
D
ATOM 11099 HA THR D 58 −27.351 1.595 57.581 0.00 0.00
D
ATOM 11100 CB THR D 58 −25.397 1.396 56.727 0.00 0.00
D
ATOM 11101 HB THR D 58 −25.564 0.397 56.272 0.00 0.00
D
ATOM 11102 OG1 THR D 58 −24.035 1.563 57.154 0.00 0.00
D
ATOM 11103 HG1 THR D 58 −23.465 1.593 56.382 0.00 0.00
D
ATOM 11104 CG2 THR D 58 −25.654 2.578 55.768 0.00 0.00
D
ATOM 11105 HG21 THR D 58 −24.762 2.584 55.104 0.00 0.00
D
ATOM 11106 HG22 THR D 58 −26.552 2.285 55.183 0.00 0.00
D
ATOM 11107 HG23 THR D 58 −25.881 3.549 56.256 0.00 0.00
D
ATOM 11108 C THR D 58 −26.321 0.327 58.928 0.00 0.00
D
ATOM 11109 O THR D 58 −26.231 −0.785 58.464 0.00 0.00
D
ATOM 11110 N GLU D 59 −26.532 0.604 60.195 0.00 0.00
D
ATOM 11111 HN GLU D 59 −26.620 1.580 60.377 0.00 0.00
D
ATOM 11112 CA GLU D 59 −26.644 −0.475 61.189 0.00 0.00
D
ATOM 11113 HA GLU D 59 −26.666 −1.448 60.720 0.00 0.00
D
ATOM 11114 CB GLU D 59 −25.347 −0.707 61.968 0.00 0.00
D
ATOM 11115 HB1 GLU D 59 −25.082 0.122 62.659 0.00 0.00
D
ATOM 11116 HB2 GLU D 59 −25.514 −1.699 62.440 0.00 0.00
D
ATOM 11117 CG GLU D 59 −24.013 −0.907 61.177 0.00 0.00
D
ATOM 11118 HG1 GLU D 59 −24.267 −1.482 60.261 0.00 0.00
D
ATOM 11119 HG2 GLU D 59 −23.463 0.033 60.956 0.00 0.00
D
ATOM 11120 CD GLU D 59 −22.969 −1.725 61.937 0.00 0.00
D
ATOM 11121 OE1 GLU D 59 −23.176 −2.926 62.208 0.00 0.00
D
ATOM 11122 OE2 GLU D 59 −21.862 −1.126 62.148 0.00 0.00
D
ATOM 11123 C GLU D 59 −27.862 −0.373 62.106 0.00 0.00
D

```
ATOM  11124  O    GLU D  59      -28.133   0.624  62.762  0.00  0.00
D
ATOM  11125  N    GLU D  60      -28.636  -1.478  62.145  0.00  0.00
D
ATOM  11126  HN   GLU D  60      -28.447  -2.273  61.573  0.00  0.00
D
ATOM  11127  CA   GLU D  60      -29.951  -1.460  62.756  0.00  0.00
D
ATOM  11128  HA   GLU D  60      -30.599  -0.813  62.183  0.00  0.00
D
ATOM  11129  CB   GLU D  60      -30.572  -2.874  62.723  0.00  0.00
D
ATOM  11130  HB1  GLU D  60      -30.663  -3.368  61.731  0.00  0.00
D
ATOM  11131  HB2  GLU D  60      -29.917  -3.522  63.345  0.00  0.00
D
ATOM  11132  CG   GLU D  60      -32.015  -3.010  63.305  0.00  0.00
D
ATOM  11133  HG1  GLU D  60      -32.097  -2.623  64.343  0.00  0.00
D
ATOM  11134  HG2  GLU D  60      -32.690  -2.389  62.678  0.00  0.00
D
ATOM  11135  CD   GLU D  60      -32.527  -4.400  63.335  0.00  0.00
D
ATOM  11136  OE1  GLU D  60      -33.569  -4.634  62.635  0.00  0.00
D
ATOM  11137  OE2  GLU D  60      -32.006  -5.332  64.024  0.00  0.00
D
ATOM  11138  C    GLU D  60      -29.997  -1.018  64.212  0.00  0.00
D
ATOM  11139  O    GLU D  60      -30.713  -0.046  64.546  0.00  0.00
D
ATOM  11140  N    ASN D  61      -29.219  -1.539  65.113  0.00  0.00
D
ATOM  11141  HN   ASN D  61      -28.596  -2.294  64.926  0.00  0.00
D
ATOM  11142  CA   ASN D  61      -29.145  -1.177  66.521  0.00  0.00
D
ATOM  11143  HA   ASN D  61      -30.187  -1.112  66.798  0.00  0.00
D
ATOM  11144  CB   ASN D  61      -28.497  -2.244  67.420  0.00  0.00
D
ATOM  11145  HB1  ASN D  61      -27.410  -2.210  67.193  0.00  0.00
D
ATOM  11146  HB2  ASN D  61      -28.651  -2.097  68.511  0.00  0.00
D
ATOM  11147  CG   ASN D  61      -29.173  -3.702  67.162  0.00  0.00
D
ATOM  11148  OD1  ASN D  61      -28.870  -4.446  66.203  0.00  0.00
D
ATOM  11149  ND2  ASN D  61      -30.293  -3.983  67.908  0.00  0.00
D
ATOM  11150 HD21  ASN D  61      -30.877  -4.733  67.598  0.00  0.00
D
```

ATOM	11151	HD22	ASN	D	61	−30.581	−3.467	68.715	0.00	0.00	D
ATOM	11152	C	ASN	D	61	−28.507	0.147	66.747	0.00	0.00	D
ATOM	11153	O	ASN	D	61	−28.986	0.987	67.510	0.00	0.00	D
ATOM	11154	N	GLU	D	62	−27.369	0.487	66.050	0.00	0.00	D
ATOM	11155	HN	GLU	D	62	−26.864	−0.218	65.558	0.00	0.00	D
ATOM	11156	CA	GLU	D	62	−26.714	1.676	65.962	0.00	0.00	D
ATOM	11157	HA	GLU	D	62	−26.362	1.910	66.956	0.00	0.00	D
ATOM	11158	CB	GLU	D	62	−25.552	1.663	64.958	0.00	0.00	D
ATOM	11159	HB1	GLU	D	62	−25.942	1.492	63.932	0.00	0.00	D
ATOM	11160	HB2	GLU	D	62	−25.035	2.645	64.983	0.00	0.00	D
ATOM	11161	CG	GLU	D	62	−24.436	0.542	65.189	0.00	0.00	D
ATOM	11162	HG1	GLU	D	62	−24.810	−0.504	65.195	0.00	0.00	D
ATOM	11163	HG2	GLU	D	62	−23.671	0.530	64.383	0.00	0.00	D
ATOM	11164	CD	GLU	D	62	−23.668	0.481	66.523	0.00	0.00	D
ATOM	11165	OE1	GLU	D	62	−23.240	1.541	66.977	0.00	0.00	D
ATOM	11166	OE2	GLU	D	62	−23.595	−0.604	67.111	0.00	0.00	D
ATOM	11167	C	GLU	D	62	−27.732	2.824	65.599	0.00	0.00	D
ATOM	11168	O	GLU	D	62	−27.818	3.851	66.263	0.00	0.00	D
ATOM	11169	N	LEU	D	63	−28.579	2.663	64.474	0.00	0.00	D
ATOM	11170	HN	LEU	D	63	−28.503	1.819	63.948	0.00	0.00	D
ATOM	11171	CA	LEU	D	63	−29.627	3.636	64.046	0.00	0.00	D
ATOM	11172	HA	LEU	D	63	−29.122	4.573	63.863	0.00	0.00	D
ATOM	11173	CB	LEU	D	63	−30.259	3.134	62.725	0.00	0.00	D
ATOM	11174	HB1	LEU	D	63	−29.434	2.966	62.001	0.00	0.00	D
ATOM	11175	HB2	LEU	D	63	−30.702	2.129	62.892	0.00	0.00	D
ATOM	11176	CG	LEU	D	63	−31.365	4.069	62.153	0.00	0.00	D
ATOM	11177	HG	LEU	D	63	−31.713	4.742	62.965	0.00	0.00	D

ATOM 11178 CD1 LEU D 63   -30.791   5.031 61.085 0.00 0.00
D
ATOM 11179 HD11 LEU D 63   -31.374   5.935 60.809 0.00 0.00
D
ATOM 11180 HD12 LEU D 63   -29.786   5.379 61.406 0.00 0.00
D
ATOM 11181 HD13 LEU D 63   -30.763   4.354 60.204 0.00 0.00
D
ATOM 11182 CD2 LEU D 63   -32.486   3.298 61.574 0.00 0.00
D
ATOM 11183 HD21 LEU D 63   -33.326   3.997 61.373 0.00 0.00
D
ATOM 11184 HD22 LEU D 63   -32.218   2.652 60.711 0.00 0.00
D
ATOM 11185 HD23 LEU D 63   -32.796   2.487 62.267 0.00 0.00
D
ATOM 11186 C   LEU D 63   -30.726   3.844 65.082 0.00 0.00
D
ATOM 11187 O   LEU D 63   -31.177   4.964 65.250 0.00 0.00
D
ATOM 11188 N   LEU D 64   -31.203   2.810 65.748 0.00 0.00
D
ATOM 11189 HN LEU D 64   -30.771   1.929 65.570 0.00 0.00
D
ATOM 11190 CA LEU D 64   -32.263   2.868 66.776 0.00 0.00
D
ATOM 11191 HA LEU D 64   -33.137   3.336 66.348 0.00 0.00
D
ATOM 11192 CB LEU D 64   -32.653   1.432 67.271 0.00 0.00
D
ATOM 11193 HB1 LEU D 64   -32.927   0.957 66.304 0.00 0.00
D
ATOM 11194 HB2 LEU D 64   -31.744   0.955 67.695 0.00 0.00
D
ATOM 11195 CG LEU D 64   -33.788   1.311 68.196 0.00 0.00
D
ATOM 11196 HG LEU D 64   -33.452   2.040 68.964 0.00 0.00
D
ATOM 11197 CD1 LEU D 64   -35.230   1.797 67.606 0.00 0.00
D
ATOM 11198 HD11 LEU D 64   -35.971   1.705 68.429 0.00 0.00
D
ATOM 11199 HD12 LEU D 64   -35.178   2.897 67.459 0.00 0.00
D
ATOM 11200 HD13 LEU D 64   -35.475   1.312 66.636 0.00 0.00
D
ATOM 11201 CD2 LEU D 64   -33.916 -0.066 68.794 0.00 0.00
D
ATOM 11202 HD21 LEU D 64   -33.557 -0.849 68.092 0.00 0.00
D
ATOM 11203 HD22 LEU D 64   -33.292 -0.316 69.679 0.00 0.00
D
ATOM 11204 HD23 LEU D 64   -34.924 -0.367 69.151 0.00 0.00
D

ATOM 11205 C LEU D 64 −31.932 3.753 67.975 0.00 0.00
D
ATOM 11206 O LEU D 64 −32.826 4.411 68.468 0.00 0.00
D
ATOM 11207 N SER D 65 −30.733 3.729 68.492 0.00 0.00
D
ATOM 11208 HN SER D 65 −30.061 3.208 67.972 0.00 0.00
D
ATOM 11209 CA SER D 65 −30.182 4.330 69.697 0.00 0.00
D
ATOM 11210 HA SER D 65 −30.779 4.148 70.578 0.00 0.00
D
ATOM 11211 CB SER D 65 −28.750 3.789 69.870 0.00 0.00
D
ATOM 11212 HB1 SER D 65 −28.193 4.199 69.001 0.00 0.00
D
ATOM 11213 HB2 SER D 65 −28.334 4.369 70.721 0.00 0.00
D
ATOM 11214 OG SER D 65 −28.765 2.387 70.049 0.00 0.00
D
ATOM 11215 HG1 SER D 65 −29.223 1.985 69.306 0.00 0.00
D
ATOM 11216 C SER D 65 −29.987 5.753 69.494 0.00 0.00
D
ATOM 11217 O SER D 65 −29.839 6.491 70.445 0.00 0.00
D
ATOM 11218 N LEU D 66 −30.057 6.317 68.262 0.00 0.00
D
ATOM 11219 HN LEU D 66 −30.267 5.680 67.524 0.00 0.00
D
ATOM 11220 CA LEU D 66 −30.272 7.700 67.941 0.00 0.00
D
ATOM 11221 HA LEU D 66 −29.498 8.171 68.529 0.00 0.00
D
ATOM 11222 CB LEU D 66 −30.380 7.980 66.413 0.00 0.00
D
ATOM 11223 HB1 LEU D 66 −31.172 7.525 65.781 0.00 0.00
D
ATOM 11224 HB2 LEU D 66 −30.363 9.086 66.309 0.00 0.00
D
ATOM 11225 CG LEU D 66 −29.072 7.443 65.759 0.00 0.00
D
ATOM 11226 HG LEU D 66 −29.032 6.336 65.843 0.00 0.00
D
ATOM 11227 CD1 LEU D 66 −29.138 7.896 64.297 0.00 0.00
D
ATOM 11228 HD11 LEU D 66 −28.135 7.650 63.887 0.00 0.00
D
ATOM 11229 HD12 LEU D 66 −29.868 7.189 63.848 0.00 0.00
D
ATOM 11230 HD13 LEU D 66 −29.421 8.968 64.230 0.00 0.00
D
ATOM 11231 CD2 LEU D 66 −27.720 7.905 66.418 0.00 0.00
D

ATOM 11232 HD21 LEU D 66   -26.855   7.411 65.925 0.00 0.00
D
ATOM 11233 HD22 LEU D 66   -27.705   9.015 66.386 0.00 0.00
D
ATOM 11234 HD23 LEU D 66   -27.738   7.617 67.491 0.00 0.00
D
ATOM 11235 C   LEU D 66   -31.551   8.307 68.625 0.00 0.00
D
ATOM 11236 O   LEU D 66   -31.475   9.459 68.902 0.00 0.00
D
ATOM 11237 N   GLY D 67   -32.572   7.445 68.830 0.00 0.00
D
ATOM 11238 HN GLY D 67   -32.371   6.470 68.777 0.00 0.00
D
ATOM 11239 CA GLY D 67   -33.847   7.985 69.141 0.00 0.00
D
ATOM 11240 HA1 GLY D 67   -33.805   8.678 69.969 0.00 0.00
D
ATOM 11241 HA2 GLY D 67   -34.400   7.074 69.313 0.00 0.00
D
ATOM 11242 C   GLY D 67   -34.575   8.649 68.039 0.00 0.00
D
ATOM 11243 O   GLY D 67   -35.310   9.673 68.243 0.00 0.00
D
ATOM 11244 N   ILE D 68   -34.457   8.096 66.837 0.00 0.00
D
ATOM 11245 HN ILE D 68   -33.925   7.254 66.805 0.00 0.00
D
ATOM 11246 CA ILE D 68   -35.104   8.628 65.600 0.00 0.00
D
ATOM 11247 HA ILE D 68   -35.062   9.706 65.655 0.00 0.00
D
ATOM 11248 CB ILE D 68   -34.326   8.206 64.323 0.00 0.00
D
ATOM 11249 HB ILE D 68   -34.976   8.482 63.465 0.00 0.00
D
ATOM 11250 CG2 ILE D 68   -33.076   9.099 64.065 0.00 0.00
D
ATOM 11251 HG21 ILE D 68   -33.384 10.167 64.061 0.00 0.00
D
ATOM 11252 HG22 ILE D 68   -32.265   9.054 64.823 0.00 0.00
D
ATOM 11253 HG23 ILE D 68   -32.568   8.826 63.116 0.00 0.00
D
ATOM 11254 CG1 ILE D 68   -33.916   6.678 64.130 0.00 0.00
D
ATOM 11255 HG11 ILE D 68   -33.410   6.581 63.146 0.00 0.00
D
ATOM 11256 HG12 ILE D 68   -33.169   6.478 64.928 0.00 0.00
D
ATOM 11257 CD ILE D 68   -35.015   5.600 64.256 0.00 0.00
D
ATOM 11258 HD1 ILE D 68   -35.842   5.752 63.529 0.00 0.00
D

ATOM 11259 HD2 ILE D 68 −34.498 4.623 64.138 0.00 0.00
D
ATOM 11260 HD3 ILE D 68 −35.371 5.466 65.300 0.00 0.00
D
ATOM 11261 C ILE D 68 −36.570 8.347 65.460 0.00 0.00
D
ATOM 11262 O ILE D 68 −37.296 8.912 64.606 0.00 0.00
D
ATOM 11263 N ARG D 69 −37.099 7.432 66.246 0.00 0.00
D
ATOM 11264 HN ARG D 69 −36.535 7.015 66.954 0.00 0.00
D
ATOM 11265 CA ARG D 69 −38.525 7.168 66.215 0.00 0.00
D
ATOM 11266 HA ARG D 69 −38.943 7.367 65.239 0.00 0.00
D
ATOM 11267 CB ARG D 69 −38.771 5.683 66.660 0.00 0.00
D
ATOM 11268 HB1 ARG D 69 −37.923 5.127 66.206 0.00 0.00
D
ATOM 11269 HB2 ARG D 69 −38.619 5.569 67.754 0.00 0.00
D
ATOM 11270 CG ARG D 69 −40.137 5.159 66.376 0.00 0.00
D
ATOM 11271 HG1 ARG D 69 −40.877 5.833 66.857 0.00 0.00
D
ATOM 11272 HG2 ARG D 69 −40.225 5.210 65.270 0.00 0.00
D
ATOM 11273 CD ARG D 69 −40.221 3.717 66.762 0.00 0.00
D
ATOM 11274 HD1 ARG D 69 −41.250 3.323 66.613 0.00 0.00
D
ATOM 11275 HD2 ARG D 69 −39.424 3.070 66.336 0.00 0.00
D
ATOM 11276 NE ARG D 69 −40.026 3.700 68.264 0.00 0.00
D
ATOM 11277 HE ARG D 69 −40.264 4.534 68.762 0.00 0.00
D
ATOM 11278 CZ ARG D 69 −39.328 2.767 68.961 0.00 0.00
D
ATOM 11279 NH1 ARG D 69 −38.663 1.740 68.424 0.00 0.00
D
ATOM 11280 HH11 ARG D 69 −38.582 1.009 69.102 0.00 0.00
D
ATOM 11281 HH12 ARG D 69 −39.028 1.414 67.552 0.00 0.00
D
ATOM 11282 NH2 ARG D 69 −39.152 3.041 70.240 0.00 0.00
D
ATOM 11283 HH21 ARG D 69 −38.518 2.500 70.792 0.00 0.00
D
ATOM 11284 HH22 ARG D 69 −39.555 3.929 70.460 0.00 0.00
D
ATOM 11285 C ARG D 69 −39.292 8.087 67.238 0.00 0.00
D

ATOM 11286 O ARG D 69 −40.500 8.187 67.220 0.00 0.00
D
ATOM 11287 N ASN D 70 −38.567 8.955 67.980 0.00 0.00
D
ATOM 11288 HN ASN D 70 −37.585 8.784 67.965 0.00 0.00
D
ATOM 11289 CA ASN D 70 −39.241 9.957 68.763 0.00 0.00
D
ATOM 11290 HA ASN D 70 −40.263 9.719 69.018 0.00 0.00
D
ATOM 11291 CB ASN D 70 −38.566 10.291 70.119 0.00 0.00
D
ATOM 11292 HB1 ASN D 70 −37.548 10.644 69.847 0.00 0.00
D
ATOM 11293 HB2 ASN D 70 −39.028 11.119 70.698 0.00 0.00
D
ATOM 11294 CG ASN D 70 −38.476 9.053 70.991 0.00 0.00
D
ATOM 11295 OD1 ASN D 70 −39.034 8.017 70.725 0.00 0.00
D
ATOM 11296 ND2 ASN D 70 −37.637 9.131 72.051 0.00 0.00
D
ATOM 11297 HD21 ASN D 70 −37.511 8.496 72.812 0.00 0.00
D
ATOM 11298 HD22 ASN D 70 −37.148 10.003 72.025 0.00 0.00
D
ATOM 11299 C ASN D 70 −39.162 11.287 67.925 0.00 0.00
D
ATOM 11300 O ASN D 70 −39.259 12.418 68.386 0.00 0.00
D
ATOM 11301 N PHE D 71 −38.919 11.155 66.614 0.00 0.00
D
ATOM 11302 HN PHE D 71 −38.722 10.344 66.069 0.00 0.00
D
ATOM 11303 CA PHE D 71 −38.985 12.290 65.696 0.00 0.00
D
ATOM 11304 HA PHE D 71 −39.476 13.187 66.045 0.00 0.00
D
ATOM 11305 CB PHE D 71 −37.582 12.622 65.044 0.00 0.00
D
ATOM 11306 HB1 PHE D 71 −37.077 11.754 64.570 0.00 0.00
D
ATOM 11307 HB2 PHE D 71 −37.728 13.432 64.298 0.00 0.00
D
ATOM 11308 CG PHE D 71 −36.729 13.208 66.114 0.00 0.00
D
ATOM 11309 CD1 PHE D 71 −36.095 12.355 66.982 0.00 0.00
D
ATOM 11310 HD1 PHE D 71 −36.308 11.297 66.929 0.00 0.00
D
ATOM 11311 CE1 PHE D 71 −35.206 12.883 67.914 0.00 0.00
D
ATOM 11312 HE1 PHE D 71 −34.748 12.190 68.604 0.00 0.00
D

ATOM 11313 CZ PHE D 71 −34.885 14.223 67.916 0.00 0.00
D
ATOM 11314 HZ PHE D 71 −34.164 14.661 68.591 0.00 0.00
D
ATOM 11315 CD2 PHE D 71 −36.349 14.540 66.004 0.00 0.00
D
ATOM 11316 HD2 PHE D 71 −36.686 15.187 65.207 0.00 0.00
D
ATOM 11317 CE2 PHE D 71 −35.433 15.073 66.934 0.00 0.00
D
ATOM 11318 HE2 PHE D 71 −35.043 16.077 66.861 0.00 0.00
D
ATOM 11319 C PHE D 71 −39.859 11.792 64.580 0.00 0.00
D
ATOM 11320 O PHE D 71 −40.372 10.666 64.388 0.00 0.00
D
ATOM 11321 N GLU D 72 −40.274 12.747 63.766 0.00 0.00
D
ATOM 11322 HN GLU D 72 −40.086 13.696 64.010 0.00 0.00
D
ATOM 11323 CA GLU D 72 −41.085 12.468 62.595 0.00 0.00
D
ATOM 11324 HA GLU D 72 −41.498 11.480 62.453 0.00 0.00
D
ATOM 11325 CB GLU D 72 −42.307 13.389 62.557 0.00 0.00
D
ATOM 11326 HB1 GLU D 72 −41.877 14.408 62.651 0.00 0.00
D
ATOM 11327 HB2 GLU D 72 −42.846 13.246 61.596 0.00 0.00
D
ATOM 11328 CG GLU D 72 −43.339 13.190 63.680 0.00 0.00
D
ATOM 11329 HG1 GLU D 72 −43.838 12.203 63.570 0.00 0.00
D
ATOM 11330 HG2 GLU D 72 −42.831 13.124 64.666 0.00 0.00
D
ATOM 11331 CD GLU D 72 −44.343 14.301 63.714 0.00 0.00
D
ATOM 11332 OE1 GLU D 72 −45.515 14.147 63.388 0.00 0.00
D
ATOM 11333 OE2 GLU D 72 −44.035 15.338 64.329 0.00 0.00
D
ATOM 11334 C GLU D 72 −40.323 12.794 61.401 0.00 0.00
D
ATOM 11335 O GLU D 72 −40.596 12.293 60.336 0.00 0.00
D
ATOM 11336 N TYR D 73 −39.291 13.620 61.447 0.00 0.00
D
ATOM 11337 HN TYR D 73 −38.897 14.022 62.270 0.00 0.00
D
ATOM 11338 CA TYR D 73 −38.599 14.080 60.244 0.00 0.00
D
ATOM 11339 HA TYR D 73 −38.993 13.675 59.324 0.00 0.00
D

ATOM 11340 CB TYR D 73 −38.542 15.626 60.144 0.00 0.00
D
ATOM 11341 HB1 TYR D 73 −38.603 16.136 61.129 0.00 0.00
D
ATOM 11342 HB2 TYR D 73 −37.613 15.916 59.609 0.00 0.00
D
ATOM 11343 CG TYR D 73 −39.707 16.132 59.377 0.00 0.00
D
ATOM 11344 CD1 TYR D 73 −40.801 16.723 60.020 0.00 0.00
D
ATOM 11345 HD1 TYR D 73 −40.847 16.821 61.095 0.00 0.00
D
ATOM 11346 CE1 TYR D 73 −41.784 17.345 59.264 0.00 0.00
D
ATOM 11347 HE1 TYR D 73 −42.588 17.857 59.771 0.00 0.00
D
ATOM 11348 CZ TYR D 73 −41.794 17.264 57.864 0.00 0.00
D
ATOM 11349 OH TYR D 73 −42.761 17.874 57.057 0.00 0.00
D
ATOM 11350 HH TYR D 73 −43.090 18.711 57.394 0.00 0.00
D
ATOM 11351 CD2 TYR D 73 −39.653 15.997 58.005 0.00 0.00
D
ATOM 11352 HD2 TYR D 73 −38.867 15.464 57.491 0.00 0.00
D
ATOM 11353 CE2 TYR D 73 −40.697 16.686 57.225 0.00 0.00
D
ATOM 11354 HE2 TYR D 73 −40.635 16.631 56.148 0.00 0.00
D
ATOM 11355 C TYR D 73 −37.097 13.663 60.336 0.00 0.00
D
ATOM 11356 O TYR D 73 −36.246 14.265 60.926 0.00 0.00
D
ATOM 11357 N VAL D 74 −36.812 12.575 59.562 0.00 0.00
D
ATOM 11358 HN VAL D 74 −37.490 12.017 59.090 0.00 0.00
D
ATOM 11359 CA VAL D 74 −35.403 12.095 59.366 0.00 0.00
D
ATOM 11360 HA VAL D 74 −34.825 12.727 60.024 0.00 0.00
D
ATOM 11361 CB VAL D 74 −35.079 10.700 59.856 0.00 0.00
D
ATOM 11362 HB VAL D 74 −35.684 10.006 59.234 0.00 0.00
D
ATOM 11363 CG1 VAL D 74 −33.533 10.501 59.714 0.00 0.00
D
ATOM 11364 HG11 VAL D 74 −33.061 10.327 58.724 0.00 0.00
D
ATOM 11365 HG12 VAL D 74 −32.891 11.268 60.197 0.00 0.00
D
ATOM 11366 HG13 VAL D 74 −33.291 9.529 60.197 0.00 0.00
D

ATOM 11367 CG2 VAL D 74 −35.673 10.458 61.310 0.00 0.00
D
ATOM 11368 HG21 VAL D 74 −36.761 10.237 61.266 0.00 0.00
D
ATOM 11369 HG22 VAL D 74 −35.239 9.514 61.703 0.00 0.00
D
ATOM 11370 HG23 VAL D 74 −35.460 11.267 62.041 0.00 0.00
D
ATOM 11371 C VAL D 74 −34.984 12.479 57.995 0.00 0.00
D
ATOM 11372 O VAL D 74 −35.637 12.064 56.964 0.00 0.00
D
ATOM 11373 N ILE D 75 −34.001 13.351 57.942 0.00 0.00
D
ATOM 11374 HN ILE D 75 −33.660 13.739 58.795 0.00 0.00
D
ATOM 11375 CA ILE D 75 −33.423 13.752 56.718 0.00 0.00
D
ATOM 11376 HA ILE D 75 −34.034 13.424 55.890 0.00 0.00
D
ATOM 11377 CB ILE D 75 −33.196 15.252 56.576 0.00 0.00
D
ATOM 11378 HB ILE D 75 −32.503 15.541 57.395 0.00 0.00
D
ATOM 11379 CG2 ILE D 75 −32.683 15.522 55.174 0.00 0.00
D
ATOM 11380 HG21 ILE D 75 −31.928 14.755 54.896 0.00 0.00
D
ATOM 11381 HG22 ILE D 75 −33.365 15.479 54.299 0.00 0.00
D
ATOM 11382 HG23 ILE D 75 −32.187 16.512 55.257 0.00 0.00
D
ATOM 11383 CG1 ILE D 75 −34.481 16.018 56.756 0.00 0.00
D
ATOM 11384 HG11 ILE D 75 −34.285 17.056 56.411 0.00 0.00
D
ATOM 11385 HG12 ILE D 75 −35.384 15.712 56.186 0.00 0.00
D
ATOM 11386 CD ILE D 75 −35.024 16.318 58.199 0.00 0.00
D
ATOM 11387 HD1 ILE D 75 −35.814 17.099 58.232 0.00 0.00
D
ATOM 11388 HD2 ILE D 75 −35.461 15.406 58.660 0.00 0.00
D
ATOM 11389 HD3 ILE D 75 −34.071 16.613 58.687 0.00 0.00
D
ATOM 11390 C ILE D 75 −32.115 12.956 56.511 0.00 0.00
D
ATOM 11391 O ILE D 75 −31.138 13.112 57.235 0.00 0.00
D
ATOM 11392 N VAL D 76 −32.101 12.082 55.498 0.00 0.00
D
ATOM 11393 HN VAL D 76 −32.983 12.021 55.036 0.00 0.00
D

ATOM 11394 CA VAL D 76 −30.862 11.397 55.133 0.00 0.00
D
ATOM 11395 HA VAL D 76 −30.223 11.381 56.003 0.00 0.00
D
ATOM 11396 CB VAL D 76 −31.251 9.910 54.642 0.00 0.00
D
ATOM 11397 HB VAL D 76 −31.895 10.008 53.741 0.00 0.00
D
ATOM 11398 CG1 VAL D 76 −30.079 8.982 54.237 0.00 0.00
D
ATOM 11399 HG11 VAL D 76 −30.479 8.164 53.601 0.00 0.00
D
ATOM 11400 HG12 VAL D 76 −29.269 9.477 53.659 0.00 0.00
D
ATOM 11401 HG13 VAL D 76 −29.712 8.497 55.166 0.00 0.00
D
ATOM 11402 CG2 VAL D 76 −32.024 9.199 55.802 0.00 0.00
D
ATOM 11403 HG21 VAL D 76 −31.980 8.089 55.789 0.00 0.00
D
ATOM 11404 HG22 VAL D 76 −31.741 9.487 56.838 0.00 0.00
D
ATOM 11405 HG23 VAL D 76 −33.042 9.627 55.683 0.00 0.00
D
ATOM 11406 C VAL D 76 −30.158 12.152 54.056 0.00 0.00
D
ATOM 11407 O VAL D 76 −30.679 12.282 52.925 0.00 0.00
D
ATOM 11408 N ALA D 77 −28.990 12.672 54.360 0.00 0.00
D
ATOM 11409 HN ALA D 77 −28.529 12.479 55.222 0.00 0.00
D
ATOM 11410 CA ALA D 77 −28.214 13.534 53.488 0.00 0.00
D
ATOM 11411 HA ALA D 77 −28.788 13.793 52.611 0.00 0.00
D
ATOM 11412 CB ALA D 77 −27.924 14.694 54.371 0.00 0.00
D
ATOM 11413 HB1 ALA D 77 −27.648 14.315 55.379 0.00 0.00
D
ATOM 11414 HB2 ALA D 77 −27.156 15.388 53.967 0.00 0.00
D
ATOM 11415 HB3 ALA D 77 −28.877 15.257 54.286 0.00 0.00
D
ATOM 11416 C ALA D 77 −27.072 12.759 52.987 0.00 0.00
D
ATOM 11417 O ALA D 77 −26.164 13.309 52.355 0.00 0.00
D
ATOM 11418 N ILE D 78 −27.104 11.425 53.085 0.00 0.00
D
ATOM 11419 HN ILE D 78 −27.897 10.968 53.480 0.00 0.00
D
ATOM 11420 CA ILE D 78 −26.168 10.498 52.519 0.00 0.00
D

ATOM 11421 HA ILE D 78   -25.163 10.872 52.645 0.00 0.00
D
ATOM 11422 CB ILE D 78   -26.249   9.092 53.174 0.00 0.00
D
ATOM 11423 HB ILE D 78   -27.309   8.780 53.054 0.00 0.00
D
ATOM 11424 CG2 ILE D 78   -25.302   8.067 52.490 0.00 0.00
D
ATOM 11425 HG21 ILE D 78   -25.550   7.939 51.415 0.00 0.00
D
ATOM 11426 HG22 ILE D 78   -24.258   8.421 52.626 0.00 0.00
D
ATOM 11427 HG23 ILE D 78   -25.448   7.121 53.054 0.00 0.00
D
ATOM 11428 CG1 ILE D 78   -25.931   9.336 54.683 0.00 0.00
D
ATOM 11429 HG11 ILE D 78   -24.888   9.681 54.845 0.00 0.00
D
ATOM 11430 HG12 ILE D 78   -26.601 10.095 55.140 0.00 0.00
D
ATOM 11431 CD ILE D 78   -26.041   8.036 55.538 0.00 0.00
D
ATOM 11432 HD1 ILE D 78   -27.077   7.635 55.503 0.00 0.00
D
ATOM 11433 HD2 ILE D 78   -25.332   7.248 55.206 0.00 0.00
D
ATOM 11434 HD3 ILE D 78   -25.801   8.356 56.575 0.00 0.00
D
ATOM 11435 C   ILE D 78   -26.619 10.363 51.017 0.00 0.00
D
ATOM 11436 O   ILE D 78   -27.801 10.184 50.709 0.00 0.00
D
ATOM 11437 N   GLY D 79   -25.668 10.369 50.038 0.00 0.00
D
ATOM 11438 HN GLY D 79   -24.734 10.658 50.234 0.00 0.00
D
ATOM 11439 CA GLY D 79   -26.211 10.435 48.628 0.00 0.00
D
ATOM 11440 HA1 GLY D 79   -26.657 11.411 48.503 0.00 0.00
D
ATOM 11441 HA2 GLY D 79   -26.960   9.658 48.591 0.00 0.00
D
ATOM 11442 C   GLY D 79   -25.210 10.179 47.567 0.00 0.00
D
ATOM 11443 O   GLY D 79   -25.597   9.850 46.437 0.00 0.00
D
ATOM 11444 N   ALA D 80   -23.918 10.387 47.914 0.00 0.00
D
ATOM 11445 HN ALA D 80   -23.800 10.853 48.787 0.00 0.00
D
ATOM 11446 CA ALA D 80   -22.805 10.066 47.044 0.00 0.00
D
ATOM 11447 HA ALA D 80   -23.039 10.491 46.079 0.00 0.00
D

```
ATOM  11448  CB  ALA D  80     -21.495  10.668  47.606  0.00  0.00
D
ATOM  11449  HB1 ALA D  80     -20.675  10.359  46.924  0.00  0.00
D
ATOM  11450  HB2 ALA D  80     -21.666  11.754  47.444  0.00  0.00
D
ATOM  11451  HB3 ALA D  80     -21.300  10.455  48.679  0.00  0.00
D
ATOM  11452  C   ALA D  80     -22.616   8.592  46.838  0.00  0.00
D
ATOM  11453  O   ALA D  80     -22.191   8.196  45.775  0.00  0.00
D
ATOM  11454  N   ASN D  81     -22.927   7.709  47.749  0.00  0.00
D
ATOM  11455  HN  ASN D  81     -23.169   8.098  48.634  0.00  0.00
D
ATOM  11456  CA  ASN D  81     -22.851   6.263  47.644  0.00  0.00
D
ATOM  11457  HA  ASN D  81     -22.644   6.133  46.592  0.00  0.00
D
ATOM  11458  CB  ASN D  81     -21.765   5.755  48.651  0.00  0.00
D
ATOM  11459  HB1 ASN D  81     -20.792   6.245  48.431  0.00  0.00
D
ATOM  11460  HB2 ASN D  81     -22.211   5.977  49.644  0.00  0.00
D
ATOM  11461  CG  ASN D  81     -21.706   4.199  48.498  0.00  0.00
D
ATOM  11462  OD1 ASN D  81     -22.424   3.465  49.182  0.00  0.00
D
ATOM  11463  ND2 ASN D  81     -20.818   3.770  47.535  0.00  0.00
D
ATOM  11464 HD21 ASN D  81     -20.824   2.802  47.286  0.00  0.00
D
ATOM  11465 HD22 ASN D  81     -20.311   4.460  47.019  0.00  0.00
D
ATOM  11466  C   ASN D  81     -24.173   5.633  47.864  0.00  0.00
D
ATOM  11467  O   ASN D  81     -24.710   5.669  48.967  0.00  0.00
D
ATOM  11468  N   ILE D  82     -24.820   5.070  46.752  0.00  0.00
D
ATOM  11469  HN  ILE D  82     -24.364   5.113  45.866  0.00  0.00
D
ATOM  11470  CA  ILE D  82     -26.164   4.514  46.648  0.00  0.00
D
ATOM  11471  HA  ILE D  82     -26.876   5.249  46.993  0.00  0.00
D
ATOM  11472  CB  ILE D  82     -26.525   4.115  45.264  0.00  0.00
D
ATOM  11473  HB  ILE D  82     -25.927   3.277  44.845  0.00  0.00
D
ATOM  11474  CG2 ILE D  82     -27.993   3.449  45.301  0.00  0.00
D
```

```
ATOM  11475 HG21 ILE D  82      -28.742   4.115  45.780  0.00  0.00
D
ATOM  11476 HG22 ILE D  82      -28.249   3.187  44.252  0.00  0.00
D
ATOM  11477 HG23 ILE D  82      -28.002   2.524  45.916  0.00  0.00
D
ATOM  11478  CG1 ILE D  82      -26.640   5.298  44.364  0.00  0.00
D
ATOM  11479 HG11 ILE D  82      -27.399   6.000  44.772  0.00  0.00
D
ATOM  11480 HG12 ILE D  82      -25.702   5.873  44.516  0.00  0.00
D
ATOM  11481  CD  ILE D  82      -26.779   5.041  42.826  0.00  0.00
D
ATOM  11482  HD1 ILE D  82      -27.622   4.336  42.660  0.00  0.00
D
ATOM  11483  HD2 ILE D  82      -27.160   5.969  42.349  0.00  0.00
D
ATOM  11484  HD3 ILE D  82      -25.822   4.792  42.319  0.00  0.00
D
ATOM  11485  C   ILE D  82      -26.308   3.353  47.632  0.00  0.00
D
ATOM  11486  O   ILE D  82      -27.378   3.315  48.293  0.00  0.00
D
ATOM  11487  N   GLN D  83      -25.311   2.400  47.815  0.00  0.00
D
ATOM  11488  HN  GLN D  83      -24.559   2.425  47.160  0.00  0.00
D
ATOM  11489  CA  GLN D  83      -25.417   1.408  48.883  0.00  0.00
D
ATOM  11490  HA  GLN D  83      -26.170   0.694  48.583  0.00  0.00
D
ATOM  11491  CB  GLN D  83      -24.059   0.579  48.919  0.00  0.00
D
ATOM  11492  HB1 GLN D  83      -23.933   0.330  47.844  0.00  0.00
D
ATOM  11493  HB2 GLN D  83      -23.175   1.191  49.200  0.00  0.00
D
ATOM  11494  CG  GLN D  83      -24.211  -0.733  49.640  0.00  0.00
D
ATOM  11495  HG1 GLN D  83      -24.021  -0.605  50.727  0.00  0.00
D
ATOM  11496  HG2 GLN D  83      -25.198  -1.220  49.490  0.00  0.00
D
ATOM  11497  CD  GLN D  83      -23.201  -1.805  49.161  0.00  0.00
D
ATOM  11498  OE1 GLN D  83      -23.043  -2.158  47.981  0.00  0.00
D
ATOM  11499  NE2 GLN D  83      -22.554  -2.403  50.201  0.00  0.00
D
ATOM  11500 HE21 GLN D  83      -21.807  -3.024  49.965  0.00  0.00
D
ATOM  11501 HE22 GLN D  83      -22.916  -2.213  51.114  0.00  0.00
D
```

ATOM	11502	C	GLN	D	83	−25.645	1.955	50.243	0.00	0.00	D
ATOM	11503	O	GLN	D	83	−26.593	1.591	50.905	0.00	0.00	D
ATOM	11504	N	ALA	D	84	−24.837	2.940	50.767	0.00	0.00	D
ATOM	11505	HN	ALA	D	84	−24.093	3.292	50.204	0.00	0.00	D
ATOM	11506	CA	ALA	D	84	−25.030	3.514	52.074	0.00	0.00	D
ATOM	11507	HA	ALA	D	84	−25.082	2.598	52.645	0.00	0.00	D
ATOM	11508	CB	ALA	D	84	−23.832	4.442	52.484	0.00	0.00	D
ATOM	11509	HB1	ALA	D	84	−23.964	5.448	52.033	0.00	0.00	D
ATOM	11510	HB2	ALA	D	84	−23.620	4.509	53.573	0.00	0.00	D
ATOM	11511	HB3	ALA	D	84	−22.901	3.977	52.094	0.00	0.00	D
ATOM	11512	C	ALA	D	84	−26.331	4.280	52.216	0.00	0.00	D
ATOM	11513	O	ALA	D	84	−27.119	4.129	53.197	0.00	0.00	D
ATOM	11514	N	SER	D	85	−26.642	5.183	51.201	0.00	0.00	D
ATOM	11515	HN	SER	D	85	−25.938	5.310	50.506	0.00	0.00	D
ATOM	11516	CA	SER	D	85	−27.896	5.927	51.206	0.00	0.00	D
ATOM	11517	HA	SER	D	85	−27.891	6.557	52.083	0.00	0.00	D
ATOM	11518	CB	SER	D	85	−27.908	6.835	49.954	0.00	0.00	D
ATOM	11519	HB1	SER	D	85	−26.950	7.381	49.812	0.00	0.00	D
ATOM	11520	HB2	SER	D	85	−28.102	6.235	49.040	0.00	0.00	D
ATOM	11521	OG	SER	D	85	−29.030	7.754	49.907	0.00	0.00	D
ATOM	11522	HG1	SER	D	85	−28.680	8.648	49.906	0.00	0.00	D
ATOM	11523	C	SER	D	85	−29.151	4.981	51.220	0.00	0.00	D
ATOM	11524	O	SER	D	85	−30.088	5.242	51.980	0.00	0.00	D
ATOM	11525	N	THR	D	86	−29.233	4.001	50.395	0.00	0.00	D
ATOM	11526	HN	THR	D	86	−28.373	3.757	49.953	0.00	0.00	D
ATOM	11527	CA	THR	D	86	−30.428	3.115	50.239	0.00	0.00	D
ATOM	11528	HA	THR	D	86	−31.342	3.682	50.337	0.00	0.00	D

ATOM 11529 CB THR D 86 −30.585 2.386 48.881 0.00 0.00
D
ATOM 11530 HB THR D 86 −31.374 1.609 48.962 0.00 0.00
D
ATOM 11531 OG1 THR D 86 −29.400 1.586 48.537 0.00 0.00
D
ATOM 11532 HG1 THR D 86 −28.630 2.147 48.655 0.00 0.00
D
ATOM 11533 CG2 THR D 86 −30.918 3.319 47.705 0.00 0.00
D
ATOM 11534 HG21 THR D 86 −30.029 3.984 47.669 0.00 0.00
D
ATOM 11535 HG22 THR D 86 −30.992 2.696 46.788 0.00 0.00
D
ATOM 11536 HG23 THR D 86 −31.885 3.807 47.951 0.00 0.00
D
ATOM 11537 C THR D 86 −30.635 2.142 51.377 0.00 0.00
D
ATOM 11538 O THR D 86 −31.745 1.830 51.716 0.00 0.00
D
ATOM 11539 N LEU D 87 −29.547 1.783 52.130 0.00 0.00
D
ATOM 11540 HN LEU D 87 −28.632 2.092 51.882 0.00 0.00
D
ATOM 11541 CA LEU D 87 −29.638 1.069 53.437 0.00 0.00
D
ATOM 11542 HA LEU D 87 −30.383 0.291 53.360 0.00 0.00
D
ATOM 11543 CB LEU D 87 −28.303 0.396 53.929 0.00 0.00
D
ATOM 11544 HB1 LEU D 87 −27.534 1.183 54.081 0.00 0.00
D
ATOM 11545 HB2 LEU D 87 −28.572 0.057 54.952 0.00 0.00
D
ATOM 11546 CG LEU D 87 −27.823 −0.823 53.121 0.00 0.00
D
ATOM 11547 HG LEU D 87 −27.584 −0.501 52.085 0.00 0.00
D
ATOM 11548 CD1 LEU D 87 −26.523 −1.324 53.846 0.00 0.00
D
ATOM 11549 HD11 LEU D 87 −25.640 −0.679 53.647 0.00 0.00
D
ATOM 11550 HD12 LEU D 87 −26.680 −1.404 54.943 0.00 0.00
D
ATOM 11551 HD13 LEU D 87 −26.223 −2.335 53.496 0.00 0.00
D
ATOM 11552 CD2 LEU D 87 −28.792 −1.956 52.813 0.00 0.00
D
ATOM 11553 HD21 LEU D 87 −29.604 −1.678 52.107 0.00 0.00
D
ATOM 11554 HD22 LEU D 87 −28.199 −2.820 52.445 0.00 0.00
D
ATOM 11555 HD23 LEU D 87 −29.257 −2.138 53.805 0.00 0.00
D

ATOM	11556	C	LEU	D	87	−30.118	2.012	54.572	0.00	0.00	D
ATOM	11557	O	LEU	D	87	−31.013	1.571	55.295	0.00	0.00	D
ATOM	11558	N	THR	D	88	−29.679	3.255	54.712	0.00	0.00	D
ATOM	11559	HN	THR	D	88	−28.894	3.478	54.139	0.00	0.00	D
ATOM	11560	CA	THR	D	88	−30.183	4.119	55.775	0.00	0.00	D
ATOM	11561	HA	THR	D	88	−29.903	3.642	56.703	0.00	0.00	D
ATOM	11562	CB	THR	D	88	−29.491	5.536	55.731	0.00	0.00	D
ATOM	11563	HB	THR	D	88	−29.793	6.128	54.840	0.00	0.00	D
ATOM	11564	OG1	THR	D	88	−28.030	5.329	55.539	0.00	0.00	D
ATOM	11565	HG1	THR	D	88	−27.838	4.862	54.723	0.00	0.00	D
ATOM	11566	CG2	THR	D	88	−29.765	6.393	56.941	0.00	0.00	D
ATOM	11567	HG21	THR	D	88	−29.313	7.401	56.828	0.00	0.00	D
ATOM	11568	HG22	THR	D	88	−30.848	6.358	57.186	0.00	0.00	D
ATOM	11569	HG23	THR	D	88	−29.210	5.830	57.722	0.00	0.00	D
ATOM	11570	C	THR	D	88	−31.716	4.331	55.707	0.00	0.00	D
ATOM	11571	O	THR	D	88	−32.461	4.133	56.665	0.00	0.00	D
ATOM	11572	N	THR	D	89	−32.257	4.636	54.459	0.00	0.00	D
ATOM	11573	HN	THR	D	89	−31.604	4.756	53.715	0.00	0.00	D
ATOM	11574	CA	THR	D	89	−33.639	4.685	53.974	0.00	0.00	D
ATOM	11575	HA	THR	D	89	−34.061	5.543	54.475	0.00	0.00	D
ATOM	11576	CB	THR	D	89	−33.726	4.932	52.465	0.00	0.00	D
ATOM	11577	HB	THR	D	89	−33.554	4.021	51.854	0.00	0.00	D
ATOM	11578	OG1	THR	D	89	−32.917	6.045	52.012	0.00	0.00	D
ATOM	11579	HG1	THR	D	89	−31.994	5.783	51.968	0.00	0.00	D
ATOM	11580	CG2	THR	D	89	−35.132	5.216	52.161	0.00	0.00	D
ATOM	11581	HG21	THR	D	89	−35.832	4.354	52.131	0.00	0.00	D
ATOM	11582	HG22	THR	D	89	−35.521	5.990	52.857	0.00	0.00	D

```
ATOM  11583 HG23 THR D  89     -35.134   5.741  51.182  0.00  0.00
D
ATOM  11584  C   THR D  89     -34.308   3.446  54.350  0.00  0.00
D
ATOM  11585  O   THR D  89     -35.366   3.429  54.971  0.00  0.00
D
ATOM  11586  N   LEU D  90     -33.693   2.275  53.978  0.00  0.00
D
ATOM  11587  HN  LEU D  90     -32.919   2.336  53.353  0.00  0.00
D
ATOM  11588  CA  LEU D  90     -34.356   1.038  54.154  0.00  0.00
D
ATOM  11589  HA  LEU D  90     -35.314   1.119  53.662  0.00  0.00
D
ATOM  11590  CB  LEU D  90     -33.366  -0.044  53.704  0.00  0.00
D
ATOM  11591  HB1 LEU D  90     -33.054   0.267  52.685  0.00  0.00
D
ATOM  11592  HB2 LEU D  90     -32.520  -0.003  54.424  0.00  0.00
D
ATOM  11593  CG  LEU D  90     -34.057  -1.446  53.699  0.00  0.00
D
ATOM  11594  HG  LEU D  90     -34.806  -1.620  54.501  0.00  0.00
D
ATOM  11595  CD1 LEU D  90     -34.890  -1.671  52.427  0.00  0.00
D
ATOM  11596 HD11 LEU D  90     -34.247  -1.337  51.585  0.00  0.00
D
ATOM  11597 HD12 LEU D  90     -35.226  -2.722  52.293  0.00  0.00
D
ATOM  11598 HD13 LEU D  90     -35.751  -0.973  52.510  0.00  0.00
D
ATOM  11599  CD2 LEU D  90     -33.013  -2.527  53.874  0.00  0.00
D
ATOM  11600 HD21 LEU D  90     -33.430  -3.557  53.840  0.00  0.00
D
ATOM  11601 HD22 LEU D  90     -32.273  -2.450  53.049  0.00  0.00
D
ATOM  11602 HD23 LEU D  90     -32.426  -2.400  54.809  0.00  0.00
D
ATOM  11603  C   LEU D  90     -34.640   0.766  55.660  0.00  0.00
D
ATOM  11604  O   LEU D  90     -35.728   0.375  56.132  0.00  0.00
D
ATOM  11605  N   LEU D  91     -33.658   0.940  56.563  0.00  0.00
D
ATOM  11606  HN  LEU D  91     -32.791   1.269  56.196  0.00  0.00
D
ATOM  11607  CA  LEU D  91     -33.869   0.689  57.980  0.00  0.00
D
ATOM  11608  HA  LEU D  91     -34.318  -0.284  58.113  0.00  0.00
D
ATOM  11609  CB  LEU D  91     -32.499   0.738  58.680  0.00  0.00
D
```

ATOM 11610 HB1 LEU D 91 −32.111 1.763 58.496 0.00 0.00
D
ATOM 11611 HB2 LEU D 91 −32.651 0.600 59.772 0.00 0.00
D
ATOM 11612 CG LEU D 91 −31.503 −0.315 58.174 0.00 0.00
D
ATOM 11613 HG LEU D 91 −31.333 −0.166 57.087 0.00 0.00
D
ATOM 11614 CD1 LEU D 91 −30.258 0.099 58.939 0.00 0.00
D
ATOM 11615 HD11 LEU D 91 −30.322 0.008 60.044 0.00 0.00
D
ATOM 11616 HD12 LEU D 91 −29.353 −0.518 58.748 0.00 0.00
D
ATOM 11617 HD13 LEU D 91 −30.005 1.159 58.726 0.00 0.00
D
ATOM 11618 CD2 LEU D 91 −31.868 −1.764 58.640 0.00 0.00
D
ATOM 11619 HD21 LEU D 91 −30.921 −2.343 58.612 0.00 0.00
D
ATOM 11620 HD22 LEU D 91 −32.291 −1.791 59.667 0.00 0.00
D
ATOM 11621 HD23 LEU D 91 −32.521 −2.309 57.925 0.00 0.00
D
ATOM 11622 C LEU D 91 −34.793 1.668 58.721 0.00 0.00
D
ATOM 11623 O LEU D 91 −35.657 1.137 59.442 0.00 0.00
D
ATOM 11624 N LEU D 92 −34.815 2.974 58.401 0.00 0.00
D
ATOM 11625 HN LEU D 92 −34.245 3.384 57.694 0.00 0.00
D
ATOM 11626 CA LEU D 92 −35.870 3.948 58.844 0.00 0.00
D
ATOM 11627 HA LEU D 92 −35.660 3.953 59.904 0.00 0.00
D
ATOM 11628 CB LEU D 92 −35.418 5.331 58.228 0.00 0.00
D
ATOM 11629 HB1 LEU D 92 −35.120 5.127 57.177 0.00 0.00
D
ATOM 11630 HB2 LEU D 92 −36.266 6.046 58.163 0.00 0.00
D
ATOM 11631 CG LEU D 92 −34.264 6.134 58.900 0.00 0.00
D
ATOM 11632 HG LEU D 92 −33.347 5.508 58.845 0.00 0.00
D
ATOM 11633 CD1 LEU D 92 −33.915 7.441 58.078 0.00 0.00
D
ATOM 11634 HD11 LEU D 92 −33.115 8.002 58.606 0.00 0.00
D
ATOM 11635 HD12 LEU D 92 −33.797 7.222 56.996 0.00 0.00
D
ATOM 11636 HD13 LEU D 92 −34.774 8.143 58.123 0.00 0.00
D

ATOM 11637 CD2 LEU D 92 −34.418 6.629 60.362 0.00 0.00
D
ATOM 11638 HD21 LEU D 92 −33.366 6.883 60.610 0.00 0.00
D
ATOM 11639 HD22 LEU D 92 −35.131 7.481 60.341 0.00 0.00
D
ATOM 11640 HD23 LEU D 92 −34.716 5.878 61.124 0.00 0.00
D
ATOM 11641 C LEU D 92 −37.222 3.506 58.322 0.00 0.00
D
ATOM 11642 O LEU D 92 −38.197 3.475 59.064 0.00 0.00
D
ATOM 11643 N LYS D 93 −37.374 3.020 57.061 0.00 0.00
D
ATOM 11644 HN LYS D 93 −36.649 2.839 56.401 0.00 0.00
D
ATOM 11645 CA LYS D 93 −38.712 2.547 56.544 0.00 0.00
D
ATOM 11646 HA LYS D 93 −39.451 3.286 56.815 0.00 0.00
D
ATOM 11647 CB LYS D 93 −38.725 2.436 54.979 0.00 0.00
D
ATOM 11648 HB1 LYS D 93 −38.522 3.470 54.628 0.00 0.00
D
ATOM 11649 HB2 LYS D 93 −37.872 1.771 54.725 0.00 0.00
D
ATOM 11650 CG LYS D 93 −40.059 1.873 54.381 0.00 0.00
D
ATOM 11651 HG1 LYS D 93 −39.910 1.722 53.291 0.00 0.00
D
ATOM 11652 HG2 LYS D 93 −40.173 0.862 54.827 0.00 0.00
D
ATOM 11653 CD LYS D 93 −41.276 2.740 54.524 0.00 0.00
D
ATOM 11654 HD1 LYS D 93 −41.504 2.689 55.610 0.00 0.00
D
ATOM 11655 HD2 LYS D 93 −41.024 3.770 54.193 0.00 0.00
D
ATOM 11656 CE LYS D 93 −42.493 2.148 53.800 0.00 0.00
D
ATOM 11657 HE1 LYS D 93 −42.406 2.417 52.726 0.00 0.00
D
ATOM 11658 HE2 LYS D 93 −42.495 1.064 54.042 0.00 0.00
D
ATOM 11659 NZ LYS D 93 −43.690 2.761 54.401 0.00 0.00
D
ATOM 11660 HZ1 LYS D 93 −44.577 2.385 54.009 0.00 0.00
D
ATOM 11661 HZ2 LYS D 93 −43.692 2.570 55.423 0.00 0.00
D
ATOM 11662 HZ3 LYS D 93 −43.764 3.784 54.228 0.00 0.00
D
ATOM 11663 C LYS D 93 −39.188 1.197 57.165 0.00 0.00
D

ATOM 11664 O LYS D 93 −40.444 1.029 57.281 0.00 0.00
D
ATOM 11665 N GLU D 94 −38.229 0.310 57.560 0.00 0.00
D
ATOM 11666 HN GLU D 94 −37.262 0.520 57.439 0.00 0.00
D
ATOM 11667 CA GLU D 94 −38.653 −0.844 58.381 0.00 0.00
D
ATOM 11668 HA GLU D 94 −39.429 −1.344 57.820 0.00 0.00
D
ATOM 11669 CB GLU D 94 −37.542 −1.843 58.467 0.00 0.00
D
ATOM 11670 HB1 GLU D 94 −36.614 −1.346 58.823 0.00 0.00
D
ATOM 11671 HB2 GLU D 94 −37.799 −2.535 59.296 0.00 0.00
D
ATOM 11672 CG GLU D 94 −37.204 −2.551 57.161 0.00 0.00
D
ATOM 11673 HG1 GLU D 94 −36.952 −1.831 56.354 0.00 0.00
D
ATOM 11674 HG2 GLU D 94 −36.255 −3.114 57.292 0.00 0.00
D
ATOM 11675 CD GLU D 94 −38.203 −3.541 56.691 0.00 0.00
D
ATOM 11676 OE1 GLU D 94 −38.558 −4.454 57.496 0.00 0.00
D
ATOM 11677 OE2 GLU D 94 −38.671 −3.334 55.570 0.00 0.00
D
ATOM 11678 C GLU D 94 −39.227 −0.504 59.750 0.00 0.00
D
ATOM 11679 O GLU D 94 −40.081 −1.195 60.256 0.00 0.00
D
ATOM 11680 N LEU D 95 −38.799 0.684 60.371 0.00 0.00
D
ATOM 11681 HN LEU D 95 −37.973 1.153 60.068 0.00 0.00
D
ATOM 11682 CA LEU D 95 −39.330 0.966 61.708 0.00 0.00
D
ATOM 11683 HA LEU D 95 −39.529 0.075 62.284 0.00 0.00
D
ATOM 11684 CB LEU D 95 −38.290 1.766 62.575 0.00 0.00
D
ATOM 11685 HB1 LEU D 95 −38.092 2.751 62.101 0.00 0.00
D
ATOM 11686 HB2 LEU D 95 −38.814 2.009 63.524 0.00 0.00
D
ATOM 11687 CG LEU D 95 −37.007 0.998 62.827 0.00 0.00
D
ATOM 11688 HG LEU D 95 −36.769 0.549 61.839 0.00 0.00
D
ATOM 11689 CD1 LEU D 95 −35.881 1.984 63.261 0.00 0.00
D
ATOM 11690 HD11 LEU D 95 −34.860 1.551 63.318 0.00 0.00
D

ATOM 11691 HD12 LEU D 95   -35.934   2.873 62.597 0.00 0.00
D
ATOM 11692 HD13 LEU D 95   -36.106   2.381 64.274 0.00 0.00
D
ATOM 11693 CD2 LEU D 95   -37.224 -0.244 63.749 0.00 0.00
D
ATOM 11694 HD21 LEU D 95   -36.296 -0.850 63.831 0.00 0.00
D
ATOM 11695 HD22 LEU D 95   -37.571 -0.023 64.781 0.00 0.00
D
ATOM 11696 HD23 LEU D 95   -37.854 -1.025 63.274 0.00 0.00
D
ATOM 11697 C   LEU D 95   -40.677   1.753 61.513 0.00 0.00
D
ATOM 11698 O   LEU D 95   -41.315   2.151 62.507 0.00 0.00
D
ATOM 11699 N   ASP D 96   -40.930   2.039 60.175 0.00 0.00
D
ATOM 11700 HN ASP D 96   -40.418   1.508 59.504 0.00 0.00
D
ATOM 11701 CA ASP D 96   -41.891   2.886 59.603 0.00 0.00
D
ATOM 11702 HA ASP D 96   -41.537   3.014 58.591 0.00 0.00
D
ATOM 11703 CB ASP D 96   -43.184   2.087 59.430 0.00 0.00
D
ATOM 11704 HB1 ASP D 96   -42.882   1.033 59.254 0.00 0.00
D
ATOM 11705 HB2 ASP D 96   -43.661   2.174 60.429 0.00 0.00
D
ATOM 11706 CG ASP D 96   -44.124   2.645 58.366 0.00 0.00
D
ATOM 11707 OD1 ASP D 96   -45.212   3.112 58.764 0.00 0.00
D
ATOM 11708 OD2 ASP D 96   -43.800   2.640 57.175 0.00 0.00
D
ATOM 11709 C   ASP D 96   -42.107   4.229 60.169 0.00 0.00
D
ATOM 11710 O   ASP D 96   -43.175   4.577 60.623 0.00 0.00
D
ATOM 11711 N   ILE D 97   -40.962   4.941 60.273 0.00 0.00
D
ATOM 11712 HN ILE D 97   -40.163   4.572 59.805 0.00 0.00
D
ATOM 11713 CA ILE D 97   -40.912   6.367 60.621 0.00 0.00
D
ATOM 11714 HA ILE D 97   -41.230   6.402 61.653 0.00 0.00
D
ATOM 11715 CB ILE D 97   -39.401   6.890 60.441 0.00 0.00
D
ATOM 11716 HB ILE D 97   -39.138   6.793 59.366 0.00 0.00
D
ATOM 11717 CG2 ILE D 97   -39.100   8.312 60.911 0.00 0.00
D

ATOM	11718	HG21	ILE	D	97	−39.452	8.580	61.930	0.00	0.00	D
ATOM	11719	HG22	ILE	D	97	−37.998	8.449	60.923	0.00	0.00	D
ATOM	11720	HG23	ILE	D	97	−39.463	9.072	60.186	0.00	0.00	D
ATOM	11721	CG1	ILE	D	97	−38.413	5.966	61.230	0.00	0.00	D
ATOM	11722	HG11	ILE	D	97	−38.266	4.917	60.895	0.00	0.00	D
ATOM	11723	HG12	ILE	D	97	−37.498	6.563	61.028	0.00	0.00	D
ATOM	11724	CD	ILE	D	97	−38.582	5.965	62.788	0.00	0.00	D
ATOM	11725	HD1	ILE	D	97	−38.415	6.984	63.197	0.00	0.00	D
ATOM	11726	HD2	ILE	D	97	−39.581	5.570	63.072	0.00	0.00	D
ATOM	11727	HD3	ILE	D	97	−37.842	5.270	63.241	0.00	0.00	D
ATOM	11728	C	ILE	D	97	−41.727	7.211	59.577	0.00	0.00	D
ATOM	11729	O	ILE	D	97	−41.500	7.094	58.392	0.00	0.00	D
ATOM	11730	N	PRO	D	98	−42.580	8.171	60.043	0.00	0.00	D
ATOM	11731	CD	PRO	D	98	−42.632	8.490	61.507	0.00	0.00	D
ATOM	11732	HD1	PRO	D	98	−42.731	7.577	62.133	0.00	0.00	D
ATOM	11733	HD2	PRO	D	98	−41.676	8.983	61.783	0.00	0.00	D
ATOM	11734	CA	PRO	D	98	−43.365	9.128	59.230	0.00	0.00	D
ATOM	11735	HA	PRO	D	98	−44.242	8.602	58.882	0.00	0.00	D
ATOM	11736	CB	PRO	D	98	−43.763	10.207	60.272	0.00	0.00	D
ATOM	11737	HB1	PRO	D	98	−44.713	10.749	60.080	0.00	0.00	D
ATOM	11738	HB2	PRO	D	98	−43.094	11.054	60.535	0.00	0.00	D
ATOM	11739	CG	PRO	D	98	−43.808	9.436	61.553	0.00	0.00	D
ATOM	11740	HG1	PRO	D	98	−44.811	8.960	61.556	0.00	0.00	D
ATOM	11741	HG2	PRO	D	98	−43.649	10.107	62.424	0.00	0.00	D
ATOM	11742	C	PRO	D	98	−42.854	9.742	57.998	0.00	0.00	D
ATOM	11743	O	PRO	D	98	−43.247	9.380	56.952	0.00	0.00	D
ATOM	11744	N	ASN	D	99	−41.803	10.648	58.000	0.00	0.00	D

ATOM 11745 HN ASN D 99 −41.324 10.917 58.832 0.00 0.00
D
ATOM 11746 CA ASN D 99 −41.478 11.360 56.804 0.00 0.00
D
ATOM 11747 HA ASN D 99 −41.990 11.061 55.902 0.00 0.00
D
ATOM 11748 CB ASN D 99 −41.490 12.897 56.875 0.00 0.00
D
ATOM 11749 HB1 ASN D 99 −40.865 13.041 57.781 0.00 0.00
D
ATOM 11750 HB2 ASN D 99 −41.063 13.363 55.962 0.00 0.00
D
ATOM 11751 CG ASN D 99 −42.885 13.348 57.133 0.00 0.00
D
ATOM 11752 OD1 ASN D 99 −43.812 12.956 56.327 0.00 0.00
D
ATOM 11753 ND2 ASN D 99 −43.136 14.051 58.202 0.00 0.00
D
ATOM 11754 HD21 ASN D 99 −44.078 14.292 58.433 0.00 0.00
D
ATOM 11755 HD22 ASN D 99 −42.415 14.210 58.876 0.00 0.00
D
ATOM 11756 C ASN D 99 −39.998 11.054 56.620 0.00 0.00
D
ATOM 11757 O ASN D 99 −39.106 11.403 57.422 0.00 0.00
D
ATOM 11758 N ILE D 100 −39.639 10.356 55.525 0.00 0.00
D
ATOM 11759 HN ILE D 100 −40.257 9.791 54.983 0.00 0.00
D
ATOM 11760 CA ILE D 100 −38.275 10.034 55.140 0.00 0.00
D
ATOM 11761 HA ILE D 100 −37.657 10.174 56.014 0.00 0.00
D
ATOM 11762 CB ILE D 100 −38.022 8.530 54.959 0.00 0.00
D
ATOM 11763 HB ILE D 100 −38.764 8.242 54.184 0.00 0.00
D
ATOM 11764 CG2 ILE D 100 −36.545 8.162 54.665 0.00 0.00
D
ATOM 11765 HG21 ILE D 100 −36.330 7.097 54.430 0.00 0.00
D
ATOM 11766 HG22 ILE D 100 −36.206 8.763 53.794 0.00 0.00
D
ATOM 11767 HG23 ILE D 100 −35.932 8.639 55.459 0.00 0.00
D
ATOM 11768 CG1 ILE D 100 −38.533 7.728 56.232 0.00 0.00
D
ATOM 11769 HG11 ILE D 100 −38.042 8.169 57.126 0.00 0.00
D
ATOM 11770 HG12 ILE D 100 −39.580 8.066 56.390 0.00 0.00
D
ATOM 11771 CD ILE D 100 −38.386 6.233 56.151 0.00 0.00
D

ATOM 11772 HD1 ILE D 100 −38.909 5.683 56.962 0.00 0.00
D
ATOM 11773 HD2 ILE D 100 −38.736 5.943 55.138 0.00 0.00
D
ATOM 11774 HD3 ILE D 100 −37.351 5.828 56.166 0.00 0.00
D
ATOM 11775 C ILE D 100 −37.739 10.756 54.019 0.00 0.00
D
ATOM 11776 O ILE D 100 −38.276 10.524 52.943 0.00 0.00
D
ATOM 11777 N TRP D 101 −36.804 11.694 54.188 0.00 0.00
D
ATOM 11778 HN TRP D 101 −36.554 12.048 55.086 0.00 0.00
D
ATOM 11779 CA TRP D 101 −36.353 12.553 53.157 0.00 0.00
D
ATOM 11780 HA TRP D 101 −36.990 12.371 52.304 0.00 0.00
D
ATOM 11781 CB TRP D 101 −36.432 14.061 53.497 0.00 0.00
D
ATOM 11782 HB1 TRP D 101 −35.925 14.349 54.443 0.00 0.00
D
ATOM 11783 HB2 TRP D 101 −35.900 14.710 52.770 0.00 0.00
D
ATOM 11784 CG TRP D 101 −37.824 14.686 53.583 0.00 0.00
D
ATOM 11785 CD1 TRP D 101 −39.018 14.198 54.042 0.00 0.00
D
ATOM 11786 HD1 TRP D 101 −39.194 13.134 53.986 0.00 0.00
D
ATOM 11787 NE1 TRP D 101 −40.031 15.197 53.930 0.00 0.00
D
ATOM 11788 HE1 TRP D 101 −40.985 15.136 54.126 0.00 0.00
D
ATOM 11789 CE2 TRP D 101 −39.482 16.324 53.638 0.00 0.00
D
ATOM 11790 CD2 TRP D 101 −38.102 16.096 53.411 0.00 0.00
D
ATOM 11791 CE3 TRP D 101 −37.225 17.119 53.089 0.00 0.00
D
ATOM 11792 HE3 TRP D 101 −36.155 17.014 52.983 0.00 0.00
D
ATOM 11793 CZ3 TRP D 101 −37.817 18.358 52.922 0.00 0.00
D
ATOM 11794 HZ3 TRP D 101 −37.118 19.093 52.551 0.00 0.00
D
ATOM 11795 CZ2 TRP D 101 −40.065 17.670 53.515 0.00 0.00
D
ATOM 11796 HZ2 TRP D 101 −41.127 17.702 53.708 0.00 0.00
D
ATOM 11797 CH2 TRP D 101 −39.174 18.695 53.191 0.00 0.00
D
ATOM 11798 HH2 TRP D 101 −39.489 19.722 53.083 0.00 0.00
D

ATOM 11799 C TRP D 101 −34.936 12.288 52.727 0.00 0.00
D
ATOM 11800 O TRP D 101 −33.997 12.475 53.502 0.00 0.00
D
ATOM 11801 N VAL D 102 −34.628 11.938 51.442 0.00 0.00
D
ATOM 11802 HN VAL D 102 −35.310 12.174 50.755 0.00 0.00
D
ATOM 11803 CA VAL D 102 −33.410 11.314 51.054 0.00 0.00
D
ATOM 11804 HA VAL D 102 −32.655 11.461 51.813 0.00 0.00
D
ATOM 11805 CB VAL D 102 −33.687 9.872 50.623 0.00 0.00
D
ATOM 11806 HB VAL D 102 −34.020 9.896 49.563 0.00 0.00
D
ATOM 11807 CG1 VAL D 102 −32.385 9.055 50.582 0.00 0.00
D
ATOM 11808 HG11 VAL D 102 −31.743 9.321 51.449 0.00 0.00
D
ATOM 11809 HG12 VAL D 102 −32.553 7.957 50.579 0.00 0.00
D
ATOM 11810 HG13 VAL D 102 −31.836 9.250 49.636 0.00 0.00
D
ATOM 11811 CG2 VAL D 102 −34.779 9.177 51.549 0.00 0.00
D
ATOM 11812 HG21 VAL D 102 −34.568 8.088 51.602 0.00 0.00
D
ATOM 11813 HG22 VAL D 102 −34.591 9.552 52.577 0.00 0.00
D
ATOM 11814 HG23 VAL D 102 −35.828 9.395 51.253 0.00 0.00
D
ATOM 11815 C VAL D 102 −32.738 11.921 49.844 0.00 0.00
D
ATOM 11816 O VAL D 102 −33.344 12.066 48.773 0.00 0.00
D
ATOM 11817 N LYS D 103 −31.446 12.337 49.887 0.00 0.00
D
ATOM 11818 HN LYS D 103 −30.929 12.157 50.720 0.00 0.00
D
ATOM 11819 CA LYS D 103 −30.572 12.596 48.781 0.00 0.00
D
ATOM 11820 HA LYS D 103 −30.987 13.438 48.247 0.00 0.00
D
ATOM 11821 CB LYS D 103 −29.124 12.984 49.221 0.00 0.00
D
ATOM 11822 HB1 LYS D 103 −29.229 13.575 50.156 0.00 0.00
D
ATOM 11823 HB2 LYS D 103 −28.530 12.109 49.560 0.00 0.00
D
ATOM 11824 CG LYS D 103 −28.306 13.823 48.257 0.00 0.00
D
ATOM 11825 HG1 LYS D 103 −28.214 13.202 47.340 0.00 0.00
D

ATOM 11826 HG2 LYS D 103   -28.872 14.727 47.945 0.00 0.00
D
ATOM 11827 CD LYS D 103   -26.976 14.419 48.882 0.00 0.00
D
ATOM 11828 HD1 LYS D 103   -27.362 14.876 49.818 0.00 0.00
D
ATOM 11829 HD2 LYS D 103   -26.356 13.527 49.115 0.00 0.00
D
ATOM 11830 CE LYS D 103   -26.192 15.374 48.076 0.00 0.00
D
ATOM 11831 HE1 LYS D 103   -26.750 16.313 47.874 0.00 0.00
D
ATOM 11832 HE2 LYS D 103   -25.233 15.591 48.593 0.00 0.00
D
ATOM 11833 NZ LYS D 103   -25.826 14.739 46.791 0.00 0.00
D
ATOM 11834 HZ1 LYS D 103   -26.535 14.117 46.352 0.00 0.00
D
ATOM 11835 HZ2 LYS D 103   -25.503 15.349 46.013 0.00 0.00
D
ATOM 11836 HZ3 LYS D 103   -25.011 14.170 47.096 0.00 0.00
D
ATOM 11837 C   LYS D 103   -30.354 11.581 47.764 0.00 0.00
D
ATOM 11838 O   LYS D 103   -29.944 10.446 48.041 0.00 0.00
D
ATOM 11839 N   ALA D 104   -30.666 11.912 46.534 0.00 0.00
D
ATOM 11840 HN ALA D 104   -31.164 12.737 46.278 0.00 0.00
D
ATOM 11841 CA ALA D 104   -30.489 10.951 45.433 0.00 0.00
D
ATOM 11842 HA ALA D 104   -29.852 10.140 45.753 0.00 0.00
D
ATOM 11843 CB ALA D 104   -31.741 10.335 44.825 0.00 0.00
D
ATOM 11844 HB1 ALA D 104   -32.505 10.175 45.616 0.00 0.00
D
ATOM 11845 HB2 ALA D 104   -32.131 11.107 44.128 0.00 0.00
D
ATOM 11846 HB3 ALA D 104   -31.458   9.439 44.232 0.00 0.00
D
ATOM 11847 C   ALA D 104   -29.774 11.588 44.272 0.00 0.00
D
ATOM 11848 O   ALA D 104   -29.750 12.787 44.175 0.00 0.00
D
ATOM 11849 N   GLN D 105   -28.991 10.845 43.544 0.00 0.00
D
ATOM 11850 HN GLN D 105   -28.880   9.892 43.818 0.00 0.00
D
ATOM 11851 CA GLN D 105   -28.097 11.534 42.601 0.00 0.00
D
ATOM 11852 HA GLN D 105   -28.174 12.611 42.599 0.00 0.00
D

ATOM 11853 CB GLN D 105 −26.576 11.198 42.866 0.00 0.00
D
ATOM 11854 HB1 GLN D 105 −25.976 11.755 42.115 0.00 0.00
D
ATOM 11855 HB2 GLN D 105 −26.442 11.569 43.904 0.00 0.00
D
ATOM 11856 CG GLN D 105 −26.242 9.746 42.806 0.00 0.00
D
ATOM 11857 HG1 GLN D 105 −26.729 9.195 43.639 0.00 0.00
D
ATOM 11858 HG2 GLN D 105 −26.638 9.285 41.876 0.00 0.00
D
ATOM 11859 CD GLN D 105 −24.777 9.503 42.696 0.00 0.00
D
ATOM 11860 OE1 GLN D 105 −24.124 9.613 41.654 0.00 0.00
D
ATOM 11861 NE2 GLN D 105 −24.173 9.154 43.865 0.00 0.00
D
ATOM 11862 HE21 GLN D 105 −23.208 8.903 43.788 0.00 0.00
D
ATOM 11863 HE22 GLN D 105 −24.766 9.030 44.660 0.00 0.00
D
ATOM 11864 C GLN D 105 −28.443 11.197 41.166 0.00 0.00
D
ATOM 11865 O GLN D 105 −27.843 11.700 40.278 0.00 0.00
D
ATOM 11866 N ASN D 106 −29.371 10.179 40.915 0.00 0.00
D
ATOM 11867 HN ASN D 106 −29.782 9.652 41.655 0.00 0.00
D
ATOM 11868 CA ASN D 106 −29.790 9.883 39.618 0.00 0.00
D
ATOM 11869 HA ASN D 106 −30.016 10.732 38.990 0.00 0.00
D
ATOM 11870 CB ASN D 106 −28.634 9.072 38.847 0.00 0.00
D
ATOM 11871 HB1 ASN D 106 −29.019 8.991 37.808 0.00 0.00
D
ATOM 11872 HB2 ASN D 106 −27.709 9.685 38.789 0.00 0.00
D
ATOM 11873 CG ASN D 106 −28.378 7.727 39.468 0.00 0.00
D
ATOM 11874 OD1 ASN D 106 −29.135 7.081 40.237 0.00 0.00
D
ATOM 11875 ND2 ASN D 106 −27.085 7.313 39.211 0.00 0.00
D
ATOM 11876 HD21 ASN D 106 −26.782 6.376 39.385 0.00 0.00
D
ATOM 11877 HD22 ASN D 106 −26.427 7.959 38.824 0.00 0.00
D
ATOM 11878 C ASN D 106 −31.183 9.227 39.572 0.00 0.00
D
ATOM 11879 O ASN D 106 −31.855 9.063 40.601 0.00 0.00
D

ATOM 11880 N TYR D 107 -31.606 8.891 38.342 0.00 0.00
D
ATOM 11881 HN TYR D 107 -31.025 8.950 37.533 0.00 0.00
D
ATOM 11882 CA TYR D 107 -32.978 8.312 38.102 0.00 0.00
D
ATOM 11883 HA TYR D 107 -33.622 9.023 38.598 0.00 0.00
D
ATOM 11884 CB TYR D 107 -33.370 8.307 36.541 0.00 0.00
D
ATOM 11885 HB1 TYR D 107 -33.340 9.381 36.259 0.00 0.00
D
ATOM 11886 HB2 TYR D 107 -32.636 7.754 35.916 0.00 0.00
D
ATOM 11887 CG TYR D 107 -34.663 7.685 36.339 0.00 0.00
D
ATOM 11888 CD1 TYR D 107 -35.853 8.477 36.513 0.00 0.00
D
ATOM 11889 HD1 TYR D 107 -35.778 9.541 36.684 0.00 0.00
D
ATOM 11890 CE1 TYR D 107 -37.070 7.833 36.172 0.00 0.00
D
ATOM 11891 HE1 TYR D 107 -38.053 8.267 36.283 0.00 0.00
D
ATOM 11892 CZ TYR D 107 -37.063 6.487 35.708 0.00 0.00
D
ATOM 11893 OH TYR D 107 -38.337 5.845 35.453 0.00 0.00
D
ATOM 11894 HH TYR D 107 -38.142 5.125 34.849 0.00 0.00
D
ATOM 11895 CD2 TYR D 107 -34.737 6.417 35.726 0.00 0.00
D
ATOM 11896 HD2 TYR D 107 -33.790 5.917 35.582 0.00 0.00
D
ATOM 11897 CE2 TYR D 107 -35.909 5.793 35.464 0.00 0.00
D
ATOM 11898 HE2 TYR D 107 -35.975 4.825 34.988 0.00 0.00
D
ATOM 11899 C TYR D 107 -33.152 6.959 38.749 0.00 0.00
D
ATOM 11900 O TYR D 107 -34.181 6.768 39.440 0.00 0.00
D
ATOM 11901 N TYR D 108 -32.223 6.047 38.592 0.00 0.00
D
ATOM 11902 HN TYR D 108 -31.339 6.236 38.172 0.00 0.00
D
ATOM 11903 CA TYR D 108 -32.424 4.771 39.266 0.00 0.00
D
ATOM 11904 HA TYR D 108 -33.472 4.543 39.137 0.00 0.00
D
ATOM 11905 CB TYR D 108 -31.526 3.700 38.538 0.00 0.00
D
ATOM 11906 HB1 TYR D 108 -30.447 3.960 38.596 0.00 0.00
D

ATOM 11907 HB2 TYR D 108 −31.600 2.690 38.994 0.00 0.00
D
ATOM 11908 CG TYR D 108 −31.868 3.531 37.126 0.00 0.00
D
ATOM 11909 CD1 TYR D 108 −33.235 3.215 36.810 0.00 0.00
D
ATOM 11910 HD1 TYR D 108 −33.973 2.963 37.556 0.00 0.00
D
ATOM 11911 CE1 TYR D 108 −33.618 3.008 35.511 0.00 0.00
D
ATOM 11912 HE1 TYR D 108 −34.625 2.765 35.206 0.00 0.00
D
ATOM 11913 CZ TYR D 108 −32.693 2.984 34.459 0.00 0.00
D
ATOM 11914 OH TYR D 108 −33.217 2.750 33.164 0.00 0.00
D
ATOM 11915 HH TYR D 108 −32.533 2.797 32.492 0.00 0.00
D
ATOM 11916 CD2 TYR D 108 −30.994 3.522 36.043 0.00 0.00
D
ATOM 11917 HD2 TYR D 108 −29.921 3.508 36.167 0.00 0.00
D
ATOM 11918 CE2 TYR D 108 −31.353 3.156 34.715 0.00 0.00
D
ATOM 11919 HE2 TYR D 108 −30.680 3.244 33.875 0.00 0.00
D
ATOM 11920 C TYR D 108 −32.217 4.838 40.762 0.00 0.00
D
ATOM 11921 O TYR D 108 −32.872 4.150 41.556 0.00 0.00
D
ATOM 11922 N HSE D 109 −31.428 5.737 41.367 0.00 0.00
D
ATOM 11923 HN HSE D 109 −30.976 6.354 40.728 0.00 0.00
D
ATOM 11924 CA HSE D 109 −31.402 6.005 42.790 0.00 0.00
D
ATOM 11925 HA HSE D 109 −31.160 5.099 43.326 0.00 0.00
D
ATOM 11926 CB HSE D 109 −30.268 6.980 43.038 0.00 0.00
D
ATOM 11927 HB1 HSE D 109 −29.338 6.537 42.621 0.00 0.00
D
ATOM 11928 HB2 HSE D 109 −30.457 7.953 42.535 0.00 0.00
D
ATOM 11929 ND1 HSE D 109 −28.952 8.116 44.929 0.00 0.00
D
ATOM 11930 CG HSE D 109 −29.938 7.242 44.508 0.00 0.00
D
ATOM 11931 CE1 HSE D 109 −28.870 7.905 46.233 0.00 0.00
D
ATOM 11932 HE1 HSE D 109 −28.164 8.513 46.799 0.00 0.00
D
ATOM 11933 NE2 HSE D 109 −29.736 6.972 46.619 0.00 0.00
D

ATOM 11934 HE2 HSE D 109 -29.973 6.663 47.540 0.00 0.00
D
ATOM 11935 CD2 HSE D 109 -30.405 6.503 45.519 0.00 0.00
D
ATOM 11936 HD2 HSE D 109 -31.215 5.784 45.500 0.00 0.00
D
ATOM 11937 C HSE D 109 -32.838 6.367 43.275 0.00 0.00
D
ATOM 11938 O HSE D 109 -33.237 5.858 44.341 0.00 0.00
D
ATOM 11939 N HSE D 110 -33.533 7.237 42.547 0.00 0.00
D
ATOM 11940 HN HSE D 110 -33.131 7.751 41.794 0.00 0.00
D
ATOM 11941 CA HSE D 110 -34.924 7.561 42.867 0.00 0.00
D
ATOM 11942 HA HSE D 110 -34.948 7.874 43.900 0.00 0.00
D
ATOM 11943 CB HSE D 110 -35.516 8.628 41.984 0.00 0.00
D
ATOM 11944 HB1 HSE D 110 -34.869 9.506 42.198 0.00 0.00
D
ATOM 11945 HB2 HSE D 110 -35.334 8.338 40.927 0.00 0.00
D
ATOM 11946 ND1 HSE D 110 -37.111 10.525 42.038 0.00 0.00
D
ATOM 11947 CG HSE D 110 -36.880 9.150 42.326 0.00 0.00
D
ATOM 11948 CE1 HSE D 110 -38.451 10.667 42.236 0.00 0.00
D
ATOM 11949 HE1 HSE D 110 -39.033 11.587 42.185 0.00 0.00
D
ATOM 11950 NE2 HSE D 110 -39.026 9.471 42.584 0.00 0.00
D
ATOM 11951 HE2 HSE D 110 -39.980 9.454 42.883 0.00 0.00
D
ATOM 11952 CD2 HSE D 110 -38.033 8.504 42.575 0.00 0.00
D
ATOM 11953 HD2 HSE D 110 -38.390 7.507 42.803 0.00 0.00
D
ATOM 11954 C HSE D 110 -35.830 6.363 42.814 0.00 0.00
D
ATOM 11955 O HSE D 110 -36.647 6.051 43.658 0.00 0.00
D
ATOM 11956 N LYS D 111 -35.676 5.411 41.807 0.00 0.00
D
ATOM 11957 HN LYS D 111 -35.000 5.629 41.108 0.00 0.00
D
ATOM 11958 CA LYS D 111 -36.510 4.239 41.755 0.00 0.00
D
ATOM 11959 HA LYS D 111 -37.545 4.506 41.910 0.00 0.00
D
ATOM 11960 CB LYS D 111 -36.361 3.579 40.420 0.00 0.00
D

ATOM 11961 HB1 LYS D 111 −35.269 3.418 40.299 0.00 0.00
D
ATOM 11962 HB2 LYS D 111 −36.908 2.612 40.400 0.00 0.00
D
ATOM 11963 CG LYS D 111 −36.817 4.411 39.193 0.00 0.00
D
ATOM 11964 HG1 LYS D 111 −35.977 5.084 38.919 0.00 0.00
D
ATOM 11965 HG2 LYS D 111 −36.894 3.645 38.392 0.00 0.00
D
ATOM 11966 CD LYS D 111 −38.121 5.157 39.434 0.00 0.00
D
ATOM 11967 HD1 LYS D 111 −38.281 5.665 40.409 0.00 0.00
D
ATOM 11968 HD2 LYS D 111 −38.269 5.830 38.562 0.00 0.00
D
ATOM 11969 CE LYS D 111 −39.308 4.238 39.144 0.00 0.00
D
ATOM 11970 HE1 LYS D 111 −39.197 3.588 38.251 0.00 0.00
D
ATOM 11971 HE2 LYS D 111 −39.271 3.454 39.930 0.00 0.00
D
ATOM 11972 NZ LYS D 111 −40.573 4.853 39.193 0.00 0.00
D
ATOM 11973 HZ1 LYS D 111 −41.190 4.061 38.921 0.00 0.00
D
ATOM 11974 HZ2 LYS D 111 −40.684 5.158 40.181 0.00 0.00
D
ATOM 11975 HZ3 LYS D 111 −40.755 5.694 38.609 0.00 0.00
D
ATOM 11976 C LYS D 111 −36.269 3.248 42.847 0.00 0.00
D
ATOM 11977 O LYS D 111 −37.150 2.493 43.185 0.00 0.00
D
ATOM 11978 N VAL D 112 −34.981 3.051 43.370 0.00 0.00
D
ATOM 11979 HN VAL D 112 −34.221 3.630 43.084 0.00 0.00
D
ATOM 11980 CA VAL D 112 −34.739 2.185 44.503 0.00 0.00
D
ATOM 11981 HA VAL D 112 −35.135 1.203 44.289 0.00 0.00
D
ATOM 11982 CB VAL D 112 −33.259 2.077 44.763 0.00 0.00
D
ATOM 11983 HB VAL D 112 −32.814 3.092 44.689 0.00 0.00
D
ATOM 11984 CG1 VAL D 112 −32.923 1.314 46.003 0.00 0.00
D
ATOM 11985 HG11 VAL D 112 −33.057 2.115 46.762 0.00 0.00
D
ATOM 11986 HG12 VAL D 112 −33.616 0.462 46.166 0.00 0.00
D
ATOM 11987 HG13 VAL D 112 −31.865 0.976 45.985 0.00 0.00
D

ATOM 11988 CG2 VAL D 112   −32.750   1.320 43.514 0.00 0.00
D
ATOM 11989 HG21 VAL D 112   −32.508   1.973 42.648 0.00 0.00
D
ATOM 11990 HG22 VAL D 112   −31.899   0.631 43.702 0.00 0.00
D
ATOM 11991 HG23 VAL D 112   −33.548   0.586 43.273 0.00 0.00
D
ATOM 11992 C   VAL D 112   −35.467   2.767 45.748 0.00 0.00
D
ATOM 11993 O   VAL D 112   −36.217   2.094 46.492 0.00 0.00
D
ATOM 11994 N   LEU D 113   −35.362   4.066 45.891 0.00 0.00
D
ATOM 11995 HN LEU D 113   −34.845   4.701 45.322 0.00 0.00
D
ATOM 11996 CA LEU D 113   −35.912   4.725 47.094 0.00 0.00
D
ATOM 11997 HA LEU D 113   −35.735   4.202 48.022 0.00 0.00
D
ATOM 11998 CB LEU D 113   −35.220   6.112 47.264 0.00 0.00
D
ATOM 11999 HB1 LEU D 113   −35.223   6.662 46.299 0.00 0.00
D
ATOM 12000 HB2 LEU D 113   −35.745   6.616 48.103 0.00 0.00
D
ATOM 12001 CG LEU D 113   −33.664   6.121 47.569 0.00 0.00
D
ATOM 12002 HG LEU D 113   −33.193   5.621 46.695 0.00 0.00
D
ATOM 12003 CD1 LEU D 113   −33.182   7.574 47.439 0.00 0.00
D
ATOM 12004 HD11 LEU D 113   −32.965   7.701 46.357 0.00 0.00
D
ATOM 12005 HD12 LEU D 113   −33.980   8.245 47.823 0.00 0.00
D
ATOM 12006 HD13 LEU D 113   −32.193   7.797 47.895 0.00 0.00
D
ATOM 12007 CD2 LEU D 113   −33.281   5.570 48.973 0.00 0.00
D
ATOM 12008 HD21 LEU D 113   −33.878   6.149 49.710 0.00 0.00
D
ATOM 12009 HD22 LEU D 113   −33.552   4.523 49.229 0.00 0.00
D
ATOM 12010 HD23 LEU D 113   −32.215   5.722 49.245 0.00 0.00
D
ATOM 12011 C   LEU D 113   −37.399   4.762 47.007 0.00 0.00
D
ATOM 12012 O   LEU D 113   −38.020   4.277 47.924 0.00 0.00
D
ATOM 12013 N   GLU D 114   −38.009   4.964 45.802 0.00 0.00
D
ATOM 12014 HN GLU D 114   −37.592   5.382 44.999 0.00 0.00
D

ATOM 12015 CA GLU D 114 −39.420 4.585 45.563 0.00 0.00
D
ATOM 12016 HA GLU D 114 −40.038 5.197 46.205 0.00 0.00
D
ATOM 12017 CB GLU D 114 −39.765 4.922 44.135 0.00 0.00
D
ATOM 12018 HB1 GLU D 114 −39.474 5.977 43.944 0.00 0.00
D
ATOM 12019 HB2 GLU D 114 −39.169 4.268 43.462 0.00 0.00
D
ATOM 12020 CG GLU D 114 −41.266 4.799 43.673 0.00 0.00
D
ATOM 12021 HG1 GLU D 114 −41.587 3.745 43.813 0.00 0.00
D
ATOM 12022 HG2 GLU D 114 −41.891 5.524 44.238 0.00 0.00
D
ATOM 12023 CD GLU D 114 −41.555 5.097 42.203 0.00 0.00
D
ATOM 12024 OE1 GLU D 114 −42.337 4.386 41.581 0.00 0.00
D
ATOM 12025 OE2 GLU D 114 −40.881 6.077 41.657 0.00 0.00
D
ATOM 12026 C GLU D 114 −39.896 3.100 45.842 0.00 0.00
D
ATOM 12027 O GLU D 114 −40.866 2.943 46.566 0.00 0.00
D
ATOM 12028 N LYS D 115 −39.192 2.089 45.431 0.00 0.00
D
ATOM 12029 HN LYS D 115 −38.405 2.098 44.820 0.00 0.00
D
ATOM 12030 CA LYS D 115 −39.598 0.741 45.809 0.00 0.00
D
ATOM 12031 HA LYS D 115 −40.646 0.589 45.598 0.00 0.00
D
ATOM 12032 CB LYS D 115 −38.846 −0.314 44.980 0.00 0.00
D
ATOM 12033 HB1 LYS D 115 −37.749 −0.149 45.032 0.00 0.00
D
ATOM 12034 HB2 LYS D 115 −39.111 −1.325 45.357 0.00 0.00
D
ATOM 12035 CG LYS D 115 −39.096 −0.354 43.427 0.00 0.00
D
ATOM 12036 HG1 LYS D 115 −38.295 0.294 43.012 0.00 0.00
D
ATOM 12037 HG2 LYS D 115 −38.846 −1.364 43.037 0.00 0.00
D
ATOM 12038 CD LYS D 115 −40.463 −0.004 42.819 0.00 0.00
D
ATOM 12039 HD1 LYS D 115 −40.518 −0.653 41.920 0.00 0.00
D
ATOM 12040 HD2 LYS D 115 −41.258 −0.263 43.551 0.00 0.00
D
ATOM 12041 CE LYS D 115 −40.550 1.454 42.293 0.00 0.00
D

ATOM 12042 HE1 LYS D 115   −40.286   2.149 43.119 0.00 0.00
D
ATOM 12043 HE2 LYS D 115   −39.836   1.697 41.477 0.00 0.00
D
ATOM 12044 NZ LYS D 115   −41.968   1.589 41.875 0.00 0.00
D
ATOM 12045 HZ1 LYS D 115   −42.057   1.114 40.955 0.00 0.00
D
ATOM 12046 HZ2 LYS D 115   −42.533   1.178 42.645 0.00 0.00
D
ATOM 12047 HZ3 LYS D 115   −42.260   2.576 41.727 0.00 0.00
D
ATOM 12048 C   LYS D 115   −39.480   0.433 47.338 0.00 0.00
D
ATOM 12049 O   LYS D 115   −40.378 −0.216 47.957 0.00 0.00
D
ATOM 12050 N   ILE D 116   −38.404   0.930 48.003 0.00 0.00
D
ATOM 12051 HN ILE D 116   −37.739   1.501 47.529 0.00 0.00
D
ATOM 12052 CA ILE D 116   −38.164   0.803 49.401 0.00 0.00
D
ATOM 12053 HA ILE D 116   −38.093 −0.240 49.673 0.00 0.00
D
ATOM 12054 CB ILE D 116   −36.870   1.511 49.862 0.00 0.00
D
ATOM 12055 HB ILE D 116   −36.846   2.449 49.266 0.00 0.00
D
ATOM 12056 CG2 ILE D 116   −36.775   1.746 51.358 0.00 0.00
D
ATOM 12057 HG21 ILE D 116   −37.550   2.490 51.642 0.00 0.00
D
ATOM 12058 HG22 ILE D 116   −36.857   0.834 51.986 0.00 0.00
D
ATOM 12059 HG23 ILE D 116   −35.867   2.374 51.485 0.00 0.00
D
ATOM 12060 CG1 ILE D 116   −35.665   0.570 49.451 0.00 0.00
D
ATOM 12061 HG11 ILE D 116   −35.731 −0.383 50.018 0.00 0.00
D
ATOM 12062 HG12 ILE D 116   −35.807   0.312 48.380 0.00 0.00
D
ATOM 12063 CD ILE D 116   −34.318   1.221 49.560 0.00 0.00
D
ATOM 12064 HD1 ILE D 116   −34.088   1.781 50.492 0.00 0.00
D
ATOM 12065 HD2 ILE D 116   −33.489   0.500 49.394 0.00 0.00
D
ATOM 12066 HD3 ILE D 116   −34.184   1.875 48.672 0.00 0.00
D
ATOM 12067 C   ILE D 116   −39.359   1.559 50.162 0.00 0.00
D
ATOM 12068 O   ILE D 116   −39.923   1.022 51.090 0.00 0.00
D

ATOM 12069 N GLY D 117 −39.840 2.707 49.576 0.00 0.00
D
ATOM 12070 HN GLY D 117 −39.292 3.153 48.873 0.00 0.00
D
ATOM 12071 CA GLY D 117 −40.865 3.423 50.214 0.00 0.00
D
ATOM 12072 HA1 GLY D 117 −41.357 2.867 50.999 0.00 0.00
D
ATOM 12073 HA2 GLY D 117 −41.481 3.621 49.349 0.00 0.00
D
ATOM 12074 C GLY D 117 −40.598 4.815 50.843 0.00 0.00
D
ATOM 12075 O GLY D 117 −41.233 5.100 51.847 0.00 0.00
D
ATOM 12076 N ALA D 118 −39.607 5.553 50.270 0.00 0.00
D
ATOM 12077 HN ALA D 118 −39.141 5.228 49.451 0.00 0.00
D
ATOM 12078 CA ALA D 118 −39.196 6.877 50.619 0.00 0.00
D
ATOM 12079 HA ALA D 118 −39.083 6.976 51.688 0.00 0.00
D
ATOM 12080 CB ALA D 118 −37.831 7.200 49.864 0.00 0.00
D
ATOM 12081 HB1 ALA D 118 −37.198 6.298 50.001 0.00 0.00
D
ATOM 12082 HB2 ALA D 118 −38.115 7.394 48.807 0.00 0.00
D
ATOM 12083 HB3 ALA D 118 −37.349 8.124 50.249 0.00 0.00
D
ATOM 12084 C ALA D 118 −40.287 7.896 50.228 0.00 0.00
D
ATOM 12085 O ALA D 118 −40.761 7.866 49.112 0.00 0.00
D
ATOM 12086 N ASP D 119 −40.672 8.772 51.256 0.00 0.00
D
ATOM 12087 HN ASP D 119 −40.343 8.651 52.189 0.00 0.00
D
ATOM 12088 CA ASP D 119 −41.727 9.756 51.132 0.00 0.00
D
ATOM 12089 HA ASP D 119 −42.620 9.345 50.684 0.00 0.00
D
ATOM 12090 CB ASP D 119 −42.181 10.402 52.521 0.00 0.00
D
ATOM 12091 HB1 ASP D 119 −41.261 10.888 52.911 0.00 0.00
D
ATOM 12092 HB2 ASP D 119 −42.991 11.163 52.512 0.00 0.00
D
ATOM 12093 CG ASP D 119 −42.476 9.394 53.624 0.00 0.00
D
ATOM 12094 OD1 ASP D 119 −41.500 8.849 54.233 0.00 0.00
D
ATOM 12095 OD2 ASP D 119 −43.733 9.327 53.982 0.00 0.00
D

ATOM 12096 C ASP D 119 −41.272 10.885 50.262 0.00 0.00
D
ATOM 12097 O ASP D 119 −42.055 11.339 49.440 0.00 0.00
D
ATOM 12098 N ARG D 120 −40.039 11.351 50.376 0.00 0.00
D
ATOM 12099 HN ARG D 120 −39.397 11.126 51.105 0.00 0.00
D
ATOM 12100 CA ARG D 120 −39.543 12.339 49.446 0.00 0.00
D
ATOM 12101 HA ARG D 120 −40.035 12.435 48.489 0.00 0.00
D
ATOM 12102 CB ARG D 120 −39.626 13.746 50.188 0.00 0.00
D
ATOM 12103 HB1 ARG D 120 −40.655 13.842 50.594 0.00 0.00
D
ATOM 12104 HB2 ARG D 120 −38.879 13.747 51.011 0.00 0.00
D
ATOM 12105 CG ARG D 120 −39.368 14.843 49.145 0.00 0.00
D
ATOM 12106 HG1 ARG D 120 −38.406 14.575 48.658 0.00 0.00
D
ATOM 12107 HG2 ARG D 120 −40.243 14.878 48.462 0.00 0.00
D
ATOM 12108 CD ARG D 120 −39.263 16.265 49.786 0.00 0.00
D
ATOM 12109 HD1 ARG D 120 −40.272 16.504 50.184 0.00 0.00
D
ATOM 12110 HD2 ARG D 120 −38.503 16.322 50.594 0.00 0.00
D
ATOM 12111 NE ARG D 120 −38.895 17.085 48.617 0.00 0.00
D
ATOM 12112 HE ARG D 120 −38.508 16.611 47.826 0.00 0.00
D
ATOM 12113 CZ ARG D 120 −38.562 18.363 48.688 0.00 0.00
D
ATOM 12114 NH1 ARG D 120 −39.007 19.231 49.600 0.00 0.00
D
ATOM 12115 HH11 ARG D 120 −38.842 20.169 49.294 0.00 0.00
D
ATOM 12116 HH12 ARG D 120 −39.897 18.954 49.962 0.00 0.00
D
ATOM 12117 NH2 ARG D 120 −37.598 18.711 47.871 0.00 0.00
D
ATOM 12118 HH21 ARG D 120 −37.395 19.677 47.715 0.00 0.00
D
ATOM 12119 HH22 ARG D 120 −37.319 18.084 47.144 0.00 0.00
D
ATOM 12120 C ARG D 120 −38.112 12.125 49.088 0.00 0.00
D
ATOM 12121 O ARG D 120 −37.187 12.012 49.833 0.00 0.00
D
ATOM 12122 N ILE D 121 −37.834 12.132 47.755 0.00 0.00
D

ATOM 12123 HN ILE D 121 −38.570 12.390 47.133 0.00 0.00
D
ATOM 12124 CA ILE D 121 −36.520 12.005 47.151 0.00 0.00
D
ATOM 12125 HA ILE D 121 −35.857 11.741 47.962 0.00 0.00
D
ATOM 12126 CB ILE D 121 −36.603 10.885 46.084 0.00 0.00
D
ATOM 12127 HB ILE D 121 −37.336 11.204 45.313 0.00 0.00
D
ATOM 12128 CG2 ILE D 121 −35.214 10.801 45.508 0.00 0.00
D
ATOM 12129 HG21 ILE D 121 −34.428 10.692 46.285 0.00 0.00
D
ATOM 12130 HG22 ILE D 121 −35.048 9.857 44.947 0.00 0.00
D
ATOM 12131 HG23 ILE D 121 −34.926 11.707 44.933 0.00 0.00
D
ATOM 12132 CG1 ILE D 121 −37.057 9.564 46.752 0.00 0.00
D
ATOM 12133 HG11 ILE D 121 −36.170 9.079 47.213 0.00 0.00
D
ATOM 12134 HG12 ILE D 121 −37.803 9.718 47.561 0.00 0.00
D
ATOM 12135 CD ILE D 121 −37.634 8.597 45.751 0.00 0.00
D
ATOM 12136 HD1 ILE D 121 −38.174 7.845 46.364 0.00 0.00
D
ATOM 12137 HD2 ILE D 121 −38.322 9.082 45.025 0.00 0.00
D
ATOM 12138 HD3 ILE D 121 −36.830 8.178 45.109 0.00 0.00
D
ATOM 12139 C ILE D 121 −36.079 13.325 46.506 0.00 0.00
D
ATOM 12140 O ILE D 121 −36.769 13.926 45.728 0.00 0.00
D
ATOM 12141 N ILE D 122 −34.844 13.773 46.888 0.00 0.00
D
ATOM 12142 HN ILE D 122 −34.184 13.146 47.295 0.00 0.00
D
ATOM 12143 CA ILE D 122 −34.326 15.044 46.608 0.00 0.00
D
ATOM 12144 HA ILE D 122 −35.136 15.628 46.197 0.00 0.00
D
ATOM 12145 CB ILE D 122 −33.939 15.709 47.925 0.00 0.00
D
ATOM 12146 HB ILE D 122 −33.011 15.241 48.317 0.00 0.00
D
ATOM 12147 CG2 ILE D 122 −33.529 17.217 47.653 0.00 0.00
D
ATOM 12148 HG21 ILE D 122 −33.031 17.616 48.562 0.00 0.00
D
ATOM 12149 HG22 ILE D 122 −32.808 17.461 46.845 0.00 0.00
D

ATOM 12150 HG23 ILE D 122   -34.463 17.778 47.434 0.00 0.00
D
ATOM 12151 CG1 ILE D 122   -34.851 15.591 49.141 0.00 0.00
D
ATOM 12152 HG11 ILE D 122   -35.676 16.258 48.810 0.00 0.00
D
ATOM 12153 HG12 ILE D 122   -35.283 14.568 49.157 0.00 0.00
D
ATOM 12154 CD ILE D 122   -34.248 16.090 50.424 0.00 0.00
D
ATOM 12155 HD1 ILE D 122   -33.360 15.513 50.761 0.00 0.00
D
ATOM 12156 HD2 ILE D 122   -33.772 17.085 50.298 0.00 0.00
D
ATOM 12157 HD3 ILE D 122   -34.928 16.088 51.303 0.00 0.00
D
ATOM 12158 C   ILE D 122   -33.095 14.976 45.706 0.00 0.00
D
ATOM 12159 O   ILE D 122   -32.321 14.074 45.866 0.00 0.00
D
ATOM 12160 N   HSE D 123   -32.878 15.892 44.734 0.00 0.00
D
ATOM 12161 HN HSE D 123   -33.474 16.690 44.694 0.00 0.00
D
ATOM 12162 CA HSE D 123   -31.878 15.811 43.731 0.00 0.00
D
ATOM 12163 HA HSE D 123   -31.195 14.985 43.863 0.00 0.00
D
ATOM 12164 CB HSE D 123   -32.535 15.430 42.412 0.00 0.00
D
ATOM 12165 HB1 HSE D 123   -33.087 16.294 41.985 0.00 0.00
D
ATOM 12166 HB2 HSE D 123   -31.793 15.171 41.626 0.00 0.00
D
ATOM 12167 ND1 HSE D 123   -32.872 13.045 41.996 0.00 0.00
D
ATOM 12168 CG HSE D 123   -33.373 14.203 42.458 0.00 0.00
D
ATOM 12169 CE1 HSE D 123   -33.799 12.155 42.057 0.00 0.00
D
ATOM 12170 HE1 HSE D 123   -33.680 11.087 41.876 0.00 0.00
D
ATOM 12171 NE2 HSE D 123   -34.960 12.726 42.468 0.00 0.00
D
ATOM 12172 HE2 HSE D 123   -35.850 12.271 42.500 0.00 0.00
D
ATOM 12173 CD2 HSE D 123   -34.666 14.044 42.790 0.00 0.00
D
ATOM 12174 HD2 HSE D 123   -35.463 14.744 43.007 0.00 0.00
D
ATOM 12175 C   HSE D 123   -31.018 17.065 43.549 0.00 0.00
D
ATOM 12176 O   HSE D 123   -31.058 17.663 42.474 0.00 0.00
D

ATOM 12177 N PRO D 124 −30.314 17.482 44.647 0.00 0.00
D
ATOM 12178 CD PRO D 124 −29.925 16.598 45.734 0.00 0.00
D
ATOM 12179 HD1 PRO D 124 −30.774 16.551 46.448 0.00 0.00
D
ATOM 12180 HD2 PRO D 124 −29.807 15.564 45.346 0.00 0.00
D
ATOM 12181 CA PRO D 124 −29.820 18.858 44.876 0.00 0.00
D
ATOM 12182 HA PRO D 124 −30.736 19.422 44.967 0.00 0.00
D
ATOM 12183 CB PRO D 124 −29.001 18.798 46.204 0.00 0.00
D
ATOM 12184 HB1 PRO D 124 −29.597 19.147 47.074 0.00 0.00
D
ATOM 12185 HB2 PRO D 124 −28.097 19.444 46.189 0.00 0.00
D
ATOM 12186 CG PRO D 124 −28.782 17.321 46.437 0.00 0.00
D
ATOM 12187 HG1 PRO D 124 −28.689 16.972 47.488 0.00 0.00
D
ATOM 12188 HG2 PRO D 124 −27.892 16.961 45.877 0.00 0.00
D
ATOM 12189 C PRO D 124 −29.136 19.415 43.710 0.00 0.00
D
ATOM 12190 O PRO D 124 −29.484 20.502 43.261 0.00 0.00
D
ATOM 12191 N GLU D 125 −28.122 18.702 43.173 0.00 0.00
D
ATOM 12192 HN GLU D 125 −27.784 17.794 43.409 0.00 0.00
D
ATOM 12193 CA GLU D 125 −27.333 19.201 42.091 0.00 0.00
D
ATOM 12194 HA GLU D 125 −26.907 20.126 42.451 0.00 0.00
D
ATOM 12195 CB GLU D 125 −26.197 18.156 41.694 0.00 0.00
D
ATOM 12196 HB1 GLU D 125 −26.618 17.293 41.134 0.00 0.00
D
ATOM 12197 HB2 GLU D 125 −25.541 18.741 41.014 0.00 0.00
D
ATOM 12198 CG GLU D 125 −25.302 17.647 42.864 0.00 0.00
D
ATOM 12199 HG1 GLU D 125 −24.431 17.207 42.334 0.00 0.00
D
ATOM 12200 HG2 GLU D 125 −25.059 18.457 43.585 0.00 0.00
D
ATOM 12201 CD GLU D 125 −25.850 16.431 43.631 0.00 0.00
D
ATOM 12202 OE1 GLU D 125 −27.059 16.111 43.414 0.00 0.00
D
ATOM 12203 OE2 GLU D 125 −25.196 15.821 44.511 0.00 0.00
D

ATOM 12204 C GLU D 125 −28.138 19.437 40.828 0.00 0.00
D
ATOM 12205 O GLU D 125 −28.074 20.527 40.256 0.00 0.00
D
ATOM 12206 N LYS D 126 −29.041 18.527 40.390 0.00 0.00
D
ATOM 12207 HN LYS D 126 −29.016 17.653 40.870 0.00 0.00
D
ATOM 12208 CA LYS D 126 −29.951 18.623 39.343 0.00 0.00
D
ATOM 12209 HA LYS D 126 −29.288 18.794 38.508 0.00 0.00
D
ATOM 12210 CB LYS D 126 −30.652 17.239 39.234 0.00 0.00
D
ATOM 12211 HB1 LYS D 126 −29.878 16.454 39.096 0.00 0.00
D
ATOM 12212 HB2 LYS D 126 −31.193 16.844 40.120 0.00 0.00
D
ATOM 12213 CG LYS D 126 −31.592 17.118 38.073 0.00 0.00
D
ATOM 12214 HG1 LYS D 126 −32.360 17.919 38.011 0.00 0.00
D
ATOM 12215 HG2 LYS D 126 −30.900 17.182 37.207 0.00 0.00
D
ATOM 12216 CD LYS D 126 −32.345 15.792 38.150 0.00 0.00
D
ATOM 12217 HD1 LYS D 126 −31.546 15.084 38.458 0.00 0.00
D
ATOM 12218 HD2 LYS D 126 −33.150 15.858 38.912 0.00 0.00
D
ATOM 12219 CE LYS D 126 −32.895 15.410 36.781 0.00 0.00
D
ATOM 12220 HE1 LYS D 126 −33.730 16.068 36.459 0.00 0.00
D
ATOM 12221 HE2 LYS D 126 −32.179 15.475 35.934 0.00 0.00
D
ATOM 12222 NZ LYS D 126 −33.493 14.088 36.716 0.00 0.00
D
ATOM 12223 HZ1 LYS D 126 −34.236 14.002 35.993 0.00 0.00
D
ATOM 12224 HZ2 LYS D 126 −32.709 13.439 36.503 0.00 0.00
D
ATOM 12225 HZ3 LYS D 126 −33.855 13.774 37.639 0.00 0.00
D
ATOM 12226 C LYS D 126 −30.894 19.778 39.455 0.00 0.00
D
ATOM 12227 O LYS D 126 −30.896 20.669 38.563 0.00 0.00
D
ATOM 12228 N ASP D 127 −31.604 19.895 40.566 0.00 0.00
D
ATOM 12229 HN ASP D 127 −31.314 19.397 41.380 0.00 0.00
D
ATOM 12230 CA ASP D 127 −32.599 20.881 40.796 0.00 0.00
D

```
ATOM  12231  HA  ASP D 127     −33.050  21.067  39.833  0.00  0.00
D
ATOM  12232  CB  ASP D 127     −33.633  20.456  41.873  0.00  0.00
D
ATOM  12233  HB1 ASP D 127     −33.179  20.297  42.874  0.00  0.00
D
ATOM  12234  HB2 ASP D 127     −34.335  21.310  41.989  0.00  0.00
D
ATOM  12235  CG  ASP D 127     −34.515  19.310  41.369  0.00  0.00
D
ATOM  12236  OD1 ASP D 127     −35.020  19.302  40.219  0.00  0.00
D
ATOM  12237  OD2 ASP D 127     −34.714  18.452  42.227  0.00  0.00
D
ATOM  12238  C   ASP D 127     −32.129  22.253  41.001  0.00  0.00
D
ATOM  12239  O   ASP D 127     −32.639  23.235  40.486  0.00  0.00
D
ATOM  12240  N   MET D 128     −31.018  22.394  41.784  0.00  0.00
D
ATOM  12241  HN  MET D 128     −30.722  21.597  42.304  0.00  0.00
D
ATOM  12242  CA  MET D 128     −30.118  23.595  41.805  0.00  0.00
D
ATOM  12243  HA  MET D 128     −30.752  24.440  42.033  0.00  0.00
D
ATOM  12244  CB  MET D 128     −29.028  23.334  42.813  0.00  0.00
D
ATOM  12245  HB1 MET D 128     −29.462  22.907  43.742  0.00  0.00
D
ATOM  12246  HB2 MET D 128     −28.302  22.544  42.525  0.00  0.00
D
ATOM  12247  CG  MET D 128     −28.368  24.614  43.316  0.00  0.00
D
ATOM  12248  HG1 MET D 128     −27.430  24.472  43.895  0.00  0.00
D
ATOM  12249  HG2 MET D 128     −28.008  25.024  42.348  0.00  0.00
D
ATOM  12250  SD  MET D 128     −29.449  25.755  44.281  0.00  0.00
D
ATOM  12251  CE  MET D 128     −29.049  27.376  43.463  0.00  0.00
D
ATOM  12252  HE1 MET D 128     −29.654  28.190  43.916  0.00  0.00
D
ATOM  12253  HE2 MET D 128     −27.995  27.631  43.705  0.00  0.00
D
ATOM  12254  HE3 MET D 128     −29.200  27.333  42.363  0.00  0.00
D
ATOM  12255  C   MET D 128     −29.573  24.050  40.457  0.00  0.00
D
ATOM  12256  O   MET D 128     −29.545  25.202  40.088  0.00  0.00
D
ATOM  12257  N   GLY D 129     −29.255  23.055  39.607  0.00  0.00
D
```

ATOM 12258 HN GLY D 129 −29.204 22.120 39.949 0.00 0.00
D
ATOM 12259 CA GLY D 129 −28.566 23.338 38.330 0.00 0.00
D
ATOM 12260 HA1 GLY D 129 −28.187 22.427 37.891 0.00 0.00
D
ATOM 12261 HA2 GLY D 129 −27.773 24.065 38.426 0.00 0.00
D
ATOM 12262 C GLY D 129 −29.542 23.804 37.260 0.00 0.00
D
ATOM 12263 O GLY D 129 −29.112 24.258 36.179 0.00 0.00
D
ATOM 12264 N VAL D 130 −30.806 23.590 37.508 0.00 0.00
D
ATOM 12265 HN VAL D 130 −31.100 22.921 38.187 0.00 0.00
D
ATOM 12266 CA VAL D 130 −31.850 24.229 36.727 0.00 0.00
D
ATOM 12267 HA VAL D 130 −31.574 24.151 35.685 0.00 0.00
D
ATOM 12268 CB VAL D 130 −33.234 23.807 37.053 0.00 0.00
D
ATOM 12269 HB VAL D 130 −33.608 24.231 38.009 0.00 0.00
D
ATOM 12270 CG1 VAL D 130 −34.191 24.193 35.944 0.00 0.00
D
ATOM 12271 HG11 VAL D 130 −35.195 23.752 36.119 0.00 0.00
D
ATOM 12272 HG12 VAL D 130 −34.363 25.284 35.823 0.00 0.00
D
ATOM 12273 HG13 VAL D 130 −33.770 23.844 34.977 0.00 0.00
D
ATOM 12274 CG2 VAL D 130 −33.172 22.265 37.111 0.00 0.00
D
ATOM 12275 HG21 VAL D 130 −32.733 21.853 36.177 0.00 0.00
D
ATOM 12276 HG22 VAL D 130 −32.889 21.748 38.052 0.00 0.00
D
ATOM 12277 HG23 VAL D 130 −34.247 21.989 37.052 0.00 0.00
D
ATOM 12278 C VAL D 130 −31.781 25.760 36.892 0.00 0.00
D
ATOM 12279 O VAL D 130 −31.707 26.474 35.923 0.00 0.00
D
ATOM 12280 N LYS D 131 −31.807 26.183 38.179 0.00 0.00
D
ATOM 12281 HN LYS D 131 −31.867 25.544 38.942 0.00 0.00
D
ATOM 12282 CA LYS D 131 −31.650 27.577 38.507 0.00 0.00
D
ATOM 12283 HA LYS D 131 −32.438 28.139 38.027 0.00 0.00
D
ATOM 12284 CB LYS D 131 −31.799 27.630 40.061 0.00 0.00
D

ATOM 12285 HB1 LYS D 131 −31.087 26.900 40.501 0.00 0.00
D
ATOM 12286 HB2 LYS D 131 −31.597 28.659 40.426 0.00 0.00
D
ATOM 12287 CG LYS D 131 −33.142 27.212 40.653 0.00 0.00
D
ATOM 12288 HG1 LYS D 131 −33.979 27.722 40.129 0.00 0.00
D
ATOM 12289 HG2 LYS D 131 −33.294 26.148 40.371 0.00 0.00
D
ATOM 12290 CD LYS D 131 −33.157 27.462 42.173 0.00 0.00
D
ATOM 12291 HD1 LYS D 131 −32.169 27.163 42.584 0.00 0.00
D
ATOM 12292 HD2 LYS D 131 −33.400 28.545 42.214 0.00 0.00
D
ATOM 12293 CE LYS D 131 −34.346 26.756 42.884 0.00 0.00
D
ATOM 12294 HE1 LYS D 131 −35.250 27.126 42.355 0.00 0.00
D
ATOM 12295 HE2 LYS D 131 −34.303 25.665 42.678 0.00 0.00
D
ATOM 12296 NZ LYS D 131 −34.465 27.032 44.358 0.00 0.00
D
ATOM 12297 HZ1 LYS D 131 −33.499 26.960 44.738 0.00 0.00
D
ATOM 12298 HZ2 LYS D 131 −34.711 28.009 44.614 0.00 0.00
D
ATOM 12299 HZ3 LYS D 131 −35.117 26.307 44.721 0.00 0.00
D
ATOM 12300 C LYS D 131 −30.322 28.244 38.104 0.00 0.00
D
ATOM 12301 O LYS D 131 −30.342 29.341 37.529 0.00 0.00
D
ATOM 12302 N ILE D 132 −29.216 27.467 38.129 0.00 0.00
D
ATOM 12303 HN ILE D 132 −29.305 26.544 38.496 0.00 0.00
D
ATOM 12304 CA ILE D 132 −27.959 27.927 37.574 0.00 0.00
D
ATOM 12305 HA ILE D 132 −27.812 28.913 37.989 0.00 0.00
D
ATOM 12306 CB ILE D 132 −26.808 27.026 38.060 0.00 0.00
D
ATOM 12307 HB ILE D 132 −27.179 26.019 37.773 0.00 0.00
D
ATOM 12308 CG2 ILE D 132 −25.511 27.353 37.260 0.00 0.00
D
ATOM 12309 HG21 ILE D 132 −25.716 27.023 36.218 0.00 0.00
D
ATOM 12310 HG22 ILE D 132 −25.221 28.418 37.384 0.00 0.00
D
ATOM 12311 HG23 ILE D 132 −24.690 26.744 37.694 0.00 0.00
D

ATOM 12312 CG1 ILE D 132   -26.577 27.235 39.548 0.00 0.00
D
ATOM 12313 HG11 ILE D 132   -26.288 28.244 39.910 0.00 0.00
D
ATOM 12314 HG12 ILE D 132   -27.530 26.936 40.034 0.00 0.00
D
ATOM 12315 CD ILE D 132   -25.585 26.190 40.099 0.00 0.00
D
ATOM 12316 HD1 ILE D 132   -25.538 26.257 41.207 0.00 0.00
D
ATOM 12317 HD2 ILE D 132   -25.986 25.158 40.007 0.00 0.00
D
ATOM 12318 HD3 ILE D 132   -24.587 26.443 39.682 0.00 0.00
D
ATOM 12319 C   ILE D 132   -27.935 28.103 36.004 0.00 0.00
D
ATOM 12320 O   ILE D 132   -27.691 29.186 35.434 0.00 0.00
D
ATOM 12321 N   ALA D 133   -28.484 27.090 35.265 0.00 0.00
D
ATOM 12322 HN ALA D 133   -28.897 26.304 35.718 0.00 0.00
D
ATOM 12323 CA ALA D 133   -28.747 27.162 33.895 0.00 0.00
D
ATOM 12324 HA ALA D 133   -27.799 27.357 33.414 0.00 0.00
D
ATOM 12325 CB ALA D 133   -29.082 25.741 33.432 0.00 0.00
D
ATOM 12326 HB1 ALA D 133   -28.454 24.909 33.815 0.00 0.00
D
ATOM 12327 HB2 ALA D 133   -30.145 25.529 33.680 0.00 0.00
D
ATOM 12328 HB3 ALA D 133   -29.154 25.689 32.325 0.00 0.00
D
ATOM 12329 C   ALA D 133   -29.822 28.212 33.359 0.00 0.00
D
ATOM 12330 O   ALA D 133   -29.670 28.732 32.288 0.00 0.00
D
ATOM 12331 N   GLN D 134   -30.832 28.641 34.200 0.00 0.00
D
ATOM 12332 HN GLN D 134   -30.817 28.259 35.120 0.00 0.00
D
ATOM 12333 CA GLN D 134   -31.732 29.786 33.943 0.00 0.00
D
ATOM 12334 HA GLN D 134   -32.039 29.716 32.910 0.00 0.00
D
ATOM 12335 CB GLN D 134   -32.890 29.824 34.901 0.00 0.00
D
ATOM 12336 HB1 GLN D 134   -32.402 29.728 35.895 0.00 0.00
D
ATOM 12337 HB2 GLN D 134   -33.513 30.737 34.793 0.00 0.00
D
ATOM 12338 CG GLN D 134   -33.877 28.627 34.857 0.00 0.00
D

ATOM 12339 HG1 GLN D 134 −33.312 27.674 34.927 0.00 0.00
D
ATOM 12340 HG2 GLN D 134 −34.479 28.648 35.790 0.00 0.00
D
ATOM 12341 CD GLN D 134 −34.808 28.632 33.641 0.00 0.00
D
ATOM 12342 OE1 GLN D 134 −34.351 29.118 32.602 0.00 0.00
D
ATOM 12343 NE2 GLN D 134 −36.074 28.094 33.643 0.00 0.00
D
ATOM 12344 HE21 GLN D 134 −36.491 28.196 32.740 0.00 0.00
D
ATOM 12345 HE22 GLN D 134 −36.520 27.733 34.462 0.00 0.00
D
ATOM 12346 C GLN D 134 −30.888 31.148 34.028 0.00 0.00
D
ATOM 12347 O GLN D 134 −31.082 32.077 33.216 0.00 0.00
D
ATOM 12348 N SER D 135 −29.922 31.247 34.992 0.00 0.00
D
ATOM 12349 HN SER D 135 −29.717 30.570 35.694 0.00 0.00
D
ATOM 12350 CA SER D 135 −29.073 32.335 35.201 0.00 0.00
D
ATOM 12351 HA SER D 135 −29.747 33.172 35.310 0.00 0.00
D
ATOM 12352 CB SER D 135 −28.180 32.308 36.529 0.00 0.00
D
ATOM 12353 HB1 SER D 135 −28.830 31.796 37.271 0.00 0.00
D
ATOM 12354 HB2 SER D 135 −27.281 31.670 36.392 0.00 0.00
D
ATOM 12355 OG SER D 135 −27.782 33.623 37.013 0.00 0.00
D
ATOM 12356 HG1 SER D 135 −27.404 33.501 37.887 0.00 0.00
D
ATOM 12357 C SER D 135 −28.160 32.584 34.057 0.00 0.00
D
ATOM 12358 O SER D 135 −27.852 33.736 33.708 0.00 0.00
D
ATOM 12359 N LEU D 136 −27.678 31.457 33.458 0.00 0.00
D
ATOM 12360 HN LEU D 136 −27.870 30.569 33.868 0.00 0.00
D
ATOM 12361 CA LEU D 136 −26.955 31.517 32.196 0.00 0.00
D
ATOM 12362 HA LEU D 136 −26.136 32.216 32.267 0.00 0.00
D
ATOM 12363 CB LEU D 136 −26.361 30.109 31.945 0.00 0.00
D
ATOM 12364 HB1 LEU D 136 −27.109 29.302 32.099 0.00 0.00
D
ATOM 12365 HB2 LEU D 136 −26.018 29.891 30.911 0.00 0.00
D

ATOM 12366 CG LEU D 136   -25.146 29.730 32.930 0.00 0.00
D
ATOM 12367 HG LEU D 136   -25.508 30.228 33.855 0.00 0.00
D
ATOM 12368 CD1 LEU D 136   -24.937 28.180 33.115 0.00 0.00
D
ATOM 12369 HD11 LEU D 136   -25.024 27.684 32.125 0.00 0.00
D
ATOM 12370 HD12 LEU D 136   -24.025 27.858 33.662 0.00 0.00
D
ATOM 12371 HD13 LEU D 136   -25.790 27.687 33.629 0.00 0.00
D
ATOM 12372 CD2 LEU D 136   -23.851 30.360 32.468 0.00 0.00
D
ATOM 12373 HD21 LEU D 136   -23.708 30.156 31.385 0.00 0.00
D
ATOM 12374 HD22 LEU D 136   -23.973 31.457 32.595 0.00 0.00
D
ATOM 12375 HD23 LEU D 136   -22.933 30.108 33.040 0.00 0.00
D
ATOM 12376 C   LEU D 136   -27.877 31.899 31.022 0.00 0.00
D
ATOM 12377 O   LEU D 136   -27.444 32.744 30.249 0.00 0.00
D
ATOM 12378 N   SER D 137   -29.080 31.268 30.860 0.00 0.00
D
ATOM 12379 HN SER D 137   -29.296 30.559 31.526 0.00 0.00
D
ATOM 12380 CA SER D 137   -29.915 31.498 29.691 0.00 0.00
D
ATOM 12381 HA SER D 137   -29.413 31.070 28.835 0.00 0.00
D
ATOM 12382 CB SER D 137   -31.114 30.602 29.768 0.00 0.00
D
ATOM 12383 HB1 SER D 137   -30.594 29.650 29.529 0.00 0.00
D
ATOM 12384 HB2 SER D 137   -31.598 30.625 30.768 0.00 0.00
D
ATOM 12385 OG SER D 137   -32.045 30.846 28.719 0.00 0.00
D
ATOM 12386 HG1 SER D 137   -32.895 30.449 28.920 0.00 0.00
D
ATOM 12387 C   SER D 137   -30.329 32.943 29.439 0.00 0.00
D
ATOM 12388 O   SER D 137   -30.265 33.486 28.316 0.00 0.00
D
ATOM 12389 N   ASP D 138   -30.787 33.560 30.522 0.00 0.00
D
ATOM 12390 HN ASP D 138   -31.026 33.184 31.414 0.00 0.00
D
ATOM 12391 CA ASP D 138   -31.304 34.932 30.601 0.00 0.00
D
ATOM 12392 HA ASP D 138   -31.610 35.295 29.631 0.00 0.00
D

ATOM 12393 CB ASP D 138 −32.465 34.835 31.632 0.00 0.00
D
ATOM 12394 HB1 ASP D 138 −32.218 34.124 32.449 0.00 0.00
D
ATOM 12395 HB2 ASP D 138 −32.670 35.767 32.200 0.00 0.00
D
ATOM 12396 CG ASP D 138 −33.789 34.489 31.058 0.00 0.00
D
ATOM 12397 OD1 ASP D 138 −34.501 33.511 31.424 0.00 0.00
D
ATOM 12398 OD2 ASP D 138 −34.212 35.152 30.091 0.00 0.00
D
ATOM 12399 C ASP D 138 −30.285 35.913 31.203 0.00 0.00
D
ATOM 12400 O ASP D 138 −29.229 35.551 31.762 0.00 0.00
D
ATOM 12401 N GLU D 139 −30.564 37.247 31.019 0.00 0.00
D
ATOM 12402 HN GLU D 139 −31.422 37.411 30.537 0.00 0.00
D
ATOM 12403 CA GLU D 139 −29.673 38.266 31.518 0.00 0.00
D
ATOM 12404 HA GLU D 139 −28.998 37.875 32.265 0.00 0.00
D
ATOM 12405 CB GLU D 139 −28.933 38.862 30.307 0.00 0.00
D
ATOM 12406 HB1 GLU D 139 −29.590 39.095 29.442 0.00 0.00
D
ATOM 12407 HB2 GLU D 139 −28.472 39.830 30.598 0.00 0.00
D
ATOM 12408 CG GLU D 139 −27.694 38.036 29.898 0.00 0.00
D
ATOM 12409 HG1 GLU D 139 −26.906 37.963 30.678 0.00 0.00
D
ATOM 12410 HG2 GLU D 139 −27.994 36.993 29.660 0.00 0.00
D
ATOM 12411 CD GLU D 139 −27.031 38.707 28.723 0.00 0.00
D
ATOM 12412 OE1 GLU D 139 −26.067 39.483 28.904 0.00 0.00
D
ATOM 12413 OE2 GLU D 139 −27.379 38.454 27.571 0.00 0.00
D
ATOM 12414 C GLU D 139 −30.539 39.404 32.134 0.00 0.00
D
ATOM 12415 O GLU D 139 −30.079 40.326 32.819 0.00 0.00
D
ATOM 12416 N ASN D 140 −31.875 39.422 31.954 0.00 0.00
D
ATOM 12417 HN ASN D 140 −32.360 38.759 31.389 0.00 0.00
D
ATOM 12418 CA ASN D 140 −32.655 40.533 32.450 0.00 0.00
D
ATOM 12419 HA ASN D 140 −32.120 41.448 32.660 0.00 0.00
D

ATOM 12420 CB ASN D 140 −33.774 40.825 31.424 0.00 0.00
D
ATOM 12421 HB1 ASN D 140 −34.304 39.859 31.278 0.00 0.00
D
ATOM 12422 HB2 ASN D 140 −34.485 41.514 31.928 0.00 0.00
D
ATOM 12423 CG ASN D 140 −33.172 41.358 30.072 0.00 0.00
D
ATOM 12424 OD1 ASN D 140 −33.117 42.586 29.808 0.00 0.00
D
ATOM 12425 ND2 ASN D 140 −32.682 40.470 29.199 0.00 0.00
D
ATOM 12426 HD21 ASN D 140 −32.055 40.876 28.535 0.00 0.00
D
ATOM 12427 HD22 ASN D 140 −32.984 39.520 29.124 0.00 0.00
D
ATOM 12428 C ASN D 140 −33.260 40.123 33.828 0.00 0.00
D
ATOM 12429 O ASN D 140 −33.296 40.835 34.872 0.00 0.00
D
ATOM 12430 N VAL D 141 −33.823 38.906 33.788 0.00 0.00
D
ATOM 12431 HN VAL D 141 −33.742 38.401 32.932 0.00 0.00
D
ATOM 12432 CA VAL D 141 −34.588 38.352 34.904 0.00 0.00
D
ATOM 12433 HA VAL D 141 −35.373 39.028 35.209 0.00 0.00
D
ATOM 12434 CB VAL D 141 −35.277 37.032 34.547 0.00 0.00
D
ATOM 12435 HB VAL D 141 −34.465 36.325 34.273 0.00 0.00
D
ATOM 12436 CG1 VAL D 141 −35.972 36.434 35.816 0.00 0.00
D
ATOM 12437 HG11 VAL D 141 −35.265 35.940 36.516 0.00 0.00
D
ATOM 12438 HG12 VAL D 141 −36.597 37.149 36.391 0.00 0.00
D
ATOM 12439 HG13 VAL D 141 −36.537 35.522 35.525 0.00 0.00
D
ATOM 12440 CG2 VAL D 141 −36.264 37.136 33.402 0.00 0.00
D
ATOM 12441 HG21 VAL D 141 −37.180 37.751 33.531 0.00 0.00
D
ATOM 12442 HG22 VAL D 141 −35.780 37.577 32.505 0.00 0.00
D
ATOM 12443 HG23 VAL D 141 −36.568 36.131 33.039 0.00 0.00
D
ATOM 12444 C VAL D 141 −33.601 38.123 36.127 0.00 0.00
D
ATOM 12445 O VAL D 141 −32.483 37.588 36.005 0.00 0.00
D
ATOM 12446 N LEU D 142 −33.971 38.544 37.307 0.00 0.00
D

ATOM 12447 HN LEU D 142 -34.858 38.973 37.464 0.00 0.00
D
ATOM 12448 CA LEU D 142 -33.116 38.520 38.469 0.00 0.00
D
ATOM 12449 HA LEU D 142 -32.126 38.218 38.162 0.00 0.00
D
ATOM 12450 CB LEU D 142 -33.164 39.916 39.125 0.00 0.00
D
ATOM 12451 HB1 LEU D 142 -34.192 40.188 39.447 0.00 0.00
D
ATOM 12452 HB2 LEU D 142 -32.603 39.725 40.065 0.00 0.00
D
ATOM 12453 CG LEU D 142 -32.652 41.115 38.320 0.00 0.00
D
ATOM 12454 HG LEU D 142 -33.318 41.274 37.446 0.00 0.00
D
ATOM 12455 CD1 LEU D 142 -32.850 42.427 39.104 0.00 0.00
D
ATOM 12456 HD11 LEU D 142 -32.655 43.341 38.503 0.00 0.00
D
ATOM 12457 HD12 LEU D 142 -33.906 42.546 39.427 0.00 0.00
D
ATOM 12458 HD13 LEU D 142 -32.218 42.436 40.018 0.00 0.00
D
ATOM 12459 CD2 LEU D 142 -31.250 40.942 37.941 0.00 0.00
D
ATOM 12460 HD21 LEU D 142 -31.197 39.995 37.363 0.00 0.00
D
ATOM 12461 HD22 LEU D 142 -30.820 41.796 37.374 0.00 0.00
D
ATOM 12462 HD23 LEU D 142 -30.615 40.826 38.846 0.00 0.00
D
ATOM 12463 C LEU D 142 -33.525 37.541 39.584 0.00 0.00
D
ATOM 12464 O LEU D 142 -32.649 36.947 40.201 0.00 0.00
D
ATOM 12465 N ASN D 143 -34.795 37.324 39.923 0.00 0.00
D
ATOM 12466 HN ASN D 143 -35.489 37.913 39.517 0.00 0.00
D
ATOM 12467 CA ASN D 143 -35.523 36.474 40.860 0.00 0.00
D
ATOM 12468 HA ASN D 143 -35.075 35.492 40.847 0.00 0.00
D
ATOM 12469 CB ASN D 143 -35.784 37.088 42.272 0.00 0.00
D
ATOM 12470 HB1 ASN D 143 -36.419 37.983 42.098 0.00 0.00
D
ATOM 12471 HB2 ASN D 143 -36.193 36.352 42.998 0.00 0.00
D
ATOM 12472 CG ASN D 143 -34.444 37.421 42.874 0.00 0.00
D
ATOM 12473 OD1 ASN D 143 -33.708 36.565 43.334 0.00 0.00
D

ATOM 12474 ND2 ASN D 143 −33.984 38.712 42.807 0.00 0.00
D
ATOM 12475 HD21 ASN D 143 −33.081 39.019 43.107 0.00 0.00
D
ATOM 12476 HD22 ASN D 143 −34.616 39.418 42.489 0.00 0.00
D
ATOM 12477 C ASN D 143 −36.905 36.232 40.245 0.00 0.00
D
ATOM 12478 O ASN D 143 −37.274 37.071 39.475 0.00 0.00
D
ATOM 12479 N TYR D 144 −37.615 35.100 40.466 0.00 0.00
D
ATOM 12480 HN TYR D 144 −37.156 34.440 41.055 0.00 0.00
D
ATOM 12481 CA TYR D 144 −38.862 34.782 39.803 0.00 0.00
D
ATOM 12482 HA TYR D 144 −39.459 35.638 40.081 0.00 0.00
D
ATOM 12483 CB TYR D 144 −38.747 34.533 38.241 0.00 0.00
D
ATOM 12484 HB1 TYR D 144 −39.722 34.248 37.792 0.00 0.00
D
ATOM 12485 HB2 TYR D 144 −38.385 35.447 37.723 0.00 0.00
D
ATOM 12486 CG TYR D 144 −37.654 33.474 37.973 0.00 0.00
D
ATOM 12487 CD1 TYR D 144 −36.270 33.610 38.018 0.00 0.00
D
ATOM 12488 HD1 TYR D 144 −35.784 34.573 38.047 0.00 0.00
D
ATOM 12489 CE1 TYR D 144 −35.401 32.549 37.905 0.00 0.00
D
ATOM 12490 HE1 TYR D 144 −34.332 32.697 37.853 0.00 0.00
D
ATOM 12491 CZ TYR D 144 −35.943 31.253 37.690 0.00 0.00
D
ATOM 12492 OH TYR D 144 −35.022 30.225 37.703 0.00 0.00
D
ATOM 12493 HH TYR D 144 −35.548 29.456 37.935 0.00 0.00
D
ATOM 12494 CD2 TYR D 144 −38.142 32.165 37.811 0.00 0.00
D
ATOM 12495 HD2 TYR D 144 −39.202 32.103 37.612 0.00 0.00
D
ATOM 12496 CE2 TYR D 144 −37.290 31.013 37.633 0.00 0.00
D
ATOM 12497 HE2 TYR D 144 −37.672 30.074 37.262 0.00 0.00
D
ATOM 12498 C TYR D 144 −39.408 33.647 40.653 0.00 0.00
D
ATOM 12499 O TYR D 144 −38.696 32.845 41.314 0.00 0.00
D
ATOM 12500 N ILE D 145 −40.749 33.551 40.650 0.00 0.00
D

ATOM 12501 HN ILE D 145 -41.199 34.303 40.174 0.00 0.00
D
ATOM 12502 CA ILE D 145 -41.600 32.481 41.118 0.00 0.00
D
ATOM 12503 HA ILE D 145 -40.950 31.829 41.682 0.00 0.00
D
ATOM 12504 CB ILE D 145 -42.743 33.054 41.987 0.00 0.00
D
ATOM 12505 HB ILE D 145 -43.192 33.955 41.517 0.00 0.00
D
ATOM 12506 CG2 ILE D 145 -43.904 32.007 42.132 0.00 0.00
D
ATOM 12507 HG21 ILE D 145 -44.321 31.770 41.129 0.00 0.00
D
ATOM 12508 HG22 ILE D 145 -43.542 31.023 42.499 0.00 0.00
D
ATOM 12509 HG23 ILE D 145 -44.735 32.403 42.754 0.00 0.00
D
ATOM 12510 CG1 ILE D 145 -42.192 33.437 43.384 0.00 0.00
D
ATOM 12511 HG11 ILE D 145 -42.168 32.623 44.140 0.00 0.00
D
ATOM 12512 HG12 ILE D 145 -41.161 33.847 43.444 0.00 0.00
D
ATOM 12513 CD ILE D 145 -43.175 34.367 44.129 0.00 0.00
D
ATOM 12514 HD1 ILE D 145 -43.147 35.300 43.527 0.00 0.00
D
ATOM 12515 HD2 ILE D 145 -44.223 34.003 44.068 0.00 0.00
D
ATOM 12516 HD3 ILE D 145 -42.833 34.628 45.153 0.00 0.00
D
ATOM 12517 C ILE D 145 -42.043 31.704 39.910 0.00 0.00
D
ATOM 12518 O ILE D 145 -42.899 32.173 39.139 0.00 0.00
D
ATOM 12519 N ASP D 146 -41.569 30.424 39.733 0.00 0.00
D
ATOM 12520 HN ASP D 146 -40.978 29.911 40.350 0.00 0.00
D
ATOM 12521 CA ASP D 146 -41.878 29.614 38.648 0.00 0.00
D
ATOM 12522 HA ASP D 146 -41.905 30.223 37.756 0.00 0.00
D
ATOM 12523 CB ASP D 146 -40.782 28.491 38.644 0.00 0.00
D
ATOM 12524 HB1 ASP D 146 -39.749 28.843 38.853 0.00 0.00
D
ATOM 12525 HB2 ASP D 146 -41.078 27.825 39.482 0.00 0.00
D
ATOM 12526 CG ASP D 146 -40.809 27.767 37.304 0.00 0.00
D
ATOM 12527 OD1 ASP D 146 -39.763 27.229 36.863 0.00 0.00
D

ATOM 12528 OD2 ASP D 146   −41.893 27.560 36.773 0.00 0.00
D
ATOM 12529 C   ASP D 146   −43.266 29.076 39.087 0.00 0.00
D
ATOM 12530 O   ASP D 146   −43.469 28.176 39.903 0.00 0.00
D
ATOM 12531 N   LEU D 147   −44.366 29.622 38.432 0.00 0.00
D
ATOM 12532 HN LEU D 147   −44.172 30.133 37.598 0.00 0.00
D
ATOM 12533 CA LEU D 147   −45.708 29.488 38.982 0.00 0.00
D
ATOM 12534 HA LEU D 147   −45.641 29.450 40.060 0.00 0.00
D
ATOM 12535 CB LEU D 147   −46.363 30.813 38.707 0.00 0.00
D
ATOM 12536 HB1 LEU D 147   −45.751 31.620 39.166 0.00 0.00
D
ATOM 12537 HB2 LEU D 147   −46.238 31.133 37.650 0.00 0.00
D
ATOM 12538 CG LEU D 147   −47.772 31.003 39.201 0.00 0.00
D
ATOM 12539 HG LEU D 147   −48.361 30.070 39.077 0.00 0.00
D
ATOM 12540 CD1 LEU D 147   −47.777 31.331 40.632 0.00 0.00
D
ATOM 12541 HD11 LEU D 147   −47.639 30.477 41.329 0.00 0.00
D
ATOM 12542 HD12 LEU D 147   −47.064 32.128 40.933 0.00 0.00
D
ATOM 12543 HD13 LEU D 147   −48.812 31.545 40.974 0.00 0.00
D
ATOM 12544 CD2 LEU D 147   −48.524 32.137 38.433 0.00 0.00
D
ATOM 12545 HD21 LEU D 147   −49.626 32.014 38.501 0.00 0.00
D
ATOM 12546 HD22 LEU D 147   −48.288 33.189 38.701 0.00 0.00
D
ATOM 12547 HD23 LEU D 147   −48.312 32.069 37.344 0.00 0.00
D
ATOM 12548 C   LEU D 147   −46.458 28.263 38.468 0.00 0.00
D
ATOM 12549 O   LEU D 147   −47.180 27.583 39.163 0.00 0.00
D
ATOM 12550 N   SER D 148   −46.172 27.946 37.197 0.00 0.00
D
ATOM 12551 HN SER D 148   −45.585 28.367 36.509 0.00 0.00
D
ATOM 12552 CA SER D 148   −46.374 26.591 36.675 0.00 0.00
D
ATOM 12553 HA SER D 148   −45.997 25.906 37.421 0.00 0.00
D
ATOM 12554 CB SER D 148   −47.753 26.289 36.228 0.00 0.00
D

ATOM 12555 HB1 SER D 148   −47.886 25.199 36.060 0.00 0.00
D
ATOM 12556 HB2 SER D 148   −48.429 26.574 37.063 0.00 0.00
D
ATOM 12557 OG SER D 148   −48.182 26.965 35.012 0.00 0.00
D
ATOM 12558 HG1 SER D 148   −49.055 26.628 34.798 0.00 0.00
D
ATOM 12559 C   SER D 148   −45.422 26.382 35.532 0.00 0.00
D
ATOM 12560 O   SER D 148   −44.737 27.328 35.149 0.00 0.00
D
ATOM 12561 N   ASP D 149   −45.450 25.173 34.935 0.00 0.00
D
ATOM 12562 HN ASP D 149   −46.015 24.483 35.381 0.00 0.00
D
ATOM 12563 CA ASP D 149   −44.756 24.804 33.703 0.00 0.00
D
ATOM 12564 HA ASP D 149   −43.724 25.086 33.846 0.00 0.00
D
ATOM 12565 CB ASP D 149   −44.897 23.236 33.344 0.00 0.00
D
ATOM 12566 HB1 ASP D 149   −45.842 23.087 32.779 0.00 0.00
D
ATOM 12567 HB2 ASP D 149   −44.056 22.984 32.663 0.00 0.00
D
ATOM 12568 CG ASP D 149   −44.888 22.429 34.534 0.00 0.00
D
ATOM 12569 OD1 ASP D 149   −45.821 21.588 34.704 0.00 0.00
D
ATOM 12570 OD2 ASP D 149   −43.943 22.523 35.336 0.00 0.00
D
ATOM 12571 C   ASP D 149   −45.249 25.700 32.468 0.00 0.00
D
ATOM 12572 O   ASP D 149   −44.558 25.789 31.477 0.00 0.00
D
ATOM 12573 N   GLU D 150   −46.310 26.461 32.683 0.00 0.00
D
ATOM 12574 HN GLU D 150   −46.834 26.337 33.522 0.00 0.00
D
ATOM 12575 CA GLU D 150   −46.758 27.476 31.689 0.00 0.00
D
ATOM 12576 HA GLU D 150   −46.143 27.313 30.816 0.00 0.00
D
ATOM 12577 CB GLU D 150   −48.260 27.192 31.406 0.00 0.00
D
ATOM 12578 HB1 GLU D 150   −48.994 27.385 32.218 0.00 0.00
D
ATOM 12579 HB2 GLU D 150   −48.589 27.925 30.638 0.00 0.00
D
ATOM 12580 CG GLU D 150   −48.510 25.811 30.769 0.00 0.00
D
ATOM 12581 HG1 GLU D 150   −48.269 24.900 31.358 0.00 0.00
D

ATOM 12582 HG2 GLU D 150 −49.610 25.724 30.647 0.00 0.00
D
ATOM 12583 CD GLU D 150 −47.979 25.532 29.342 0.00 0.00
D
ATOM 12584 OE1 GLU D 150 −47.922 26.566 28.613 0.00 0.00
D
ATOM 12585 OE2 GLU D 150 −47.875 24.347 28.993 0.00 0.00
D
ATOM 12586 C GLU D 150 −46.482 28.911 32.217 0.00 0.00
D
ATOM 12587 O GLU D 150 −46.468 29.824 31.393 0.00 0.00
D
ATOM 12588 N TYR D 151 −46.394 29.197 33.517 0.00 0.00
D
ATOM 12589 HN TYR D 151 −46.484 28.483 34.206 0.00 0.00
D
ATOM 12590 CA TYR D 151 −46.552 30.623 33.987 0.00 0.00
D
ATOM 12591 HA TYR D 151 −46.459 31.246 33.110 0.00 0.00
D
ATOM 12592 CB TYR D 151 −47.967 30.846 34.631 0.00 0.00
D
ATOM 12593 HB1 TYR D 151 −48.151 30.268 35.562 0.00 0.00
D
ATOM 12594 HB2 TYR D 151 −47.992 31.920 34.916 0.00 0.00
D
ATOM 12595 CG TYR D 151 −49.140 30.699 33.784 0.00 0.00
D
ATOM 12596 CD1 TYR D 151 −49.528 31.751 32.926 0.00 0.00
D
ATOM 12597 HD1 TYR D 151 −48.951 32.641 33.133 0.00 0.00
D
ATOM 12598 CE1 TYR D 151 −50.459 31.595 31.913 0.00 0.00
D
ATOM 12599 HE1 TYR D 151 −50.623 32.475 31.309 0.00 0.00
D
ATOM 12600 CZ TYR D 151 −51.170 30.391 31.774 0.00 0.00
D
ATOM 12601 OH TYR D 151 −52.038 30.123 30.734 0.00 0.00
D
ATOM 12602 HH TYR D 151 −51.832 30.757 30.043 0.00 0.00
D
ATOM 12603 CD2 TYR D 151 −49.800 29.461 33.632 0.00 0.00
D
ATOM 12604 HD2 TYR D 151 −49.544 28.553 34.158 0.00 0.00
D
ATOM 12605 CE2 TYR D 151 −50.891 29.345 32.748 0.00 0.00
D
ATOM 12606 HE2 TYR D 151 −51.550 28.500 32.611 0.00 0.00
D
ATOM 12607 C TYR D 151 −45.450 31.068 34.900 0.00 0.00
D
ATOM 12608 O TYR D 151 −44.844 30.164 35.479 0.00 0.00
D

ATOM 12609 N SER D 152 -45.179 32.356 35.062 0.00 0.00
D
ATOM 12610 HN SER D 152 -45.590 33.080 34.514 0.00 0.00
D
ATOM 12611 CA SER D 152 -44.224 32.824 36.053 0.00 0.00
D
ATOM 12612 HA SER D 152 -44.225 32.144 36.892 0.00 0.00
D
ATOM 12613 CB SER D 152 -42.719 32.867 35.580 0.00 0.00
D
ATOM 12614 HB1 SER D 152 -42.668 33.391 34.602 0.00 0.00
D
ATOM 12615 HB2 SER D 152 -42.066 33.351 36.338 0.00 0.00
D
ATOM 12616 OG SER D 152 -42.105 31.616 35.259 0.00 0.00
D
ATOM 12617 HG1 SER D 152 -41.306 31.953 34.848 0.00 0.00
D
ATOM 12618 C SER D 152 -44.648 34.149 36.545 0.00 0.00
D
ATOM 12619 O SER D 152 -45.267 34.913 35.766 0.00 0.00
D
ATOM 12620 N ILE D 153 -44.140 34.490 37.780 0.00 0.00
D
ATOM 12621 HN ILE D 153 -43.791 33.818 38.429 0.00 0.00
D
ATOM 12622 CA ILE D 153 -44.021 35.839 38.340 0.00 0.00
D
ATOM 12623 HA ILE D 153 -44.497 36.572 37.706 0.00 0.00
D
ATOM 12624 CB ILE D 153 -44.774 35.977 39.684 0.00 0.00
D
ATOM 12625 HB ILE D 153 -44.423 35.241 40.439 0.00 0.00
D
ATOM 12626 CG2 ILE D 153 -44.381 37.327 40.383 0.00 0.00
D
ATOM 12627 HG21 ILE D 153 -43.283 37.279 40.548 0.00 0.00
D
ATOM 12628 HG22 ILE D 153 -44.648 38.161 39.699 0.00 0.00
D
ATOM 12629 HG23 ILE D 153 -44.887 37.372 41.371 0.00 0.00
D
ATOM 12630 CG1 ILE D 153 -46.305 35.684 39.581 0.00 0.00
D
ATOM 12631 HG11 ILE D 153 -46.808 36.552 39.105 0.00 0.00
D
ATOM 12632 HG12 ILE D 153 -46.407 34.880 38.821 0.00 0.00
D
ATOM 12633 CD ILE D 153 -47.027 35.544 40.941 0.00 0.00
D
ATOM 12634 HD1 ILE D 153 -46.513 34.683 41.420 0.00 0.00
D
ATOM 12635 HD2 ILE D 153 -46.921 36.431 41.601 0.00 0.00
D

ATOM 12636 HD3 ILE D 153 −48.100 35.279 40.826 0.00 0.00
D
ATOM 12637 C ILE D 153 −42.562 36.271 38.346 0.00 0.00
D
ATOM 12638 O ILE D 153 −41.752 35.611 38.934 0.00 0.00
D
ATOM 12639 N VAL D 154 −42.228 37.291 37.514 0.00 0.00
D
ATOM 12640 HN VAL D 154 −42.892 37.674 36.876 0.00 0.00
D
ATOM 12641 CA VAL D 154 −40.884 37.624 37.085 0.00 0.00
D
ATOM 12642 HA VAL D 154 −40.296 36.842 37.542 0.00 0.00
D
ATOM 12643 CB VAL D 154 −40.626 37.489 35.595 0.00 0.00
D
ATOM 12644 HB VAL D 154 −41.221 38.228 35.017 0.00 0.00
D
ATOM 12645 CG1 VAL D 154 −39.188 37.757 35.168 0.00 0.00
D
ATOM 12646 HG11 VAL D 154 −38.551 36.996 35.667 0.00 0.00
D
ATOM 12647 HG12 VAL D 154 −39.173 37.507 34.085 0.00 0.00
D
ATOM 12648 HG13 VAL D 154 −38.820 38.741 35.530 0.00 0.00
D
ATOM 12649 CG2 VAL D 154 −40.957 36.037 35.279 0.00 0.00
D
ATOM 12650 HG21 VAL D 154 −40.604 35.340 36.068 0.00 0.00
D
ATOM 12651 HG22 VAL D 154 −42.052 35.872 35.193 0.00 0.00
D
ATOM 12652 HG23 VAL D 154 −40.407 35.794 34.344 0.00 0.00
D
ATOM 12653 C VAL D 154 −40.484 38.959 37.653 0.00 0.00
D
ATOM 12654 O VAL D 154 −41.143 39.940 37.483 0.00 0.00
D
ATOM 12655 N GLU D 155 −39.282 38.968 38.287 0.00 0.00
D
ATOM 12656 HN GLU D 155 −38.644 38.204 38.343 0.00 0.00
D
ATOM 12657 CA GLU D 155 −38.589 40.265 38.564 0.00 0.00
D
ATOM 12658 HA GLU D 155 −39.307 41.064 38.452 0.00 0.00
D
ATOM 12659 CB GLU D 155 −37.932 40.233 39.928 0.00 0.00
D
ATOM 12660 HB1 GLU D 155 −38.768 39.996 40.621 0.00 0.00
D
ATOM 12661 HB2 GLU D 155 −37.173 39.430 40.038 0.00 0.00
D
ATOM 12662 CG GLU D 155 −37.329 41.637 40.282 0.00 0.00
D

ATOM 12663 HG1 GLU D 155   -36.395 41.704 39.686 0.00 0.00
D
ATOM 12664 HG2 GLU D 155   -38.092 42.412 40.055 0.00 0.00
D
ATOM 12665 CD GLU D 155   -36.897 41.816 41.727 0.00 0.00
D
ATOM 12666 OE1 GLU D 155   -36.037 41.022 42.225 0.00 0.00
D
ATOM 12667 OE2 GLU D 155   -37.532 42.659 42.412 0.00 0.00
D
ATOM 12668 C   GLU D 155   -37.499 40.527 37.527 0.00 0.00
D
ATOM 12669 O   GLU D 155   -36.709 39.582 37.310 0.00 0.00
D
ATOM 12670 N   LEU D 156   -37.332 41.732 36.986 0.00 0.00
D
ATOM 12671 HN LEU D 156   -38.104 42.359 37.051 0.00 0.00
D
ATOM 12672 CA LEU D 156   -36.326 41.999 35.974 0.00 0.00
D
ATOM 12673 HA LEU D 156   -35.383 41.481 36.076 0.00 0.00
D
ATOM 12674 CB LEU D 156   -36.746 41.473 34.554 0.00 0.00
D
ATOM 12675 HB1 LEU D 156   -36.042 41.969 33.852 0.00 0.00
D
ATOM 12676 HB2 LEU D 156   -36.594 40.374 34.492 0.00 0.00
D
ATOM 12677 CG LEU D 156   -38.211 41.577 34.192 0.00 0.00
D
ATOM 12678 HG LEU D 156   -38.873 41.308 35.042 0.00 0.00
D
ATOM 12679 CD1 LEU D 156   -38.681 43.025 33.854 0.00 0.00
D
ATOM 12680 HD11 LEU D 156   -39.643 43.012 33.297 0.00 0.00
D
ATOM 12681 HD12 LEU D 156   -38.933 43.472 34.840 0.00 0.00
D
ATOM 12682 HD13 LEU D 156   -37.972 43.689 33.315 0.00 0.00
D
ATOM 12683 CD2 LEU D 156   -38.473 40.619 33.012 0.00 0.00
D
ATOM 12684 HD21 LEU D 156   -37.838 40.935 32.157 0.00 0.00
D
ATOM 12685 HD22 LEU D 156   -38.139 39.570 33.162 0.00 0.00
D
ATOM 12686 HD23 LEU D 156   -39.478 40.720 32.548 0.00 0.00
D
ATOM 12687 C   LEU D 156   -35.893 43.418 35.911 0.00 0.00
D
ATOM 12688 O   LEU D 156   -36.519 44.313 36.526 0.00 0.00
D
ATOM 12689 N   LEU D 157   -34.720 43.761 35.318 0.00 0.00
D

ATOM 12690 HN LEU D 157   -34.143 42.997 35.040 0.00 0.00
D
ATOM 12691 CA LEU D 157   -34.317 45.179 35.019 0.00 0.00
D
ATOM 12692 HA LEU D 157   -34.438 45.767 35.916 0.00 0.00
D
ATOM 12693 CB LEU D 157   -32.805 45.130 34.540 0.00 0.00
D
ATOM 12694 HB1 LEU D 157   -32.817 44.410 33.694 0.00 0.00
D
ATOM 12695 HB2 LEU D 157   -32.619 46.137 34.108 0.00 0.00
D
ATOM 12696 CG LEU D 157   -31.729 44.699 35.392 0.00 0.00
D
ATOM 12697 HG LEU D 157   -31.893 43.606 35.500 0.00 0.00
D
ATOM 12698 CD1 LEU D 157   -30.414 44.843 34.638 0.00 0.00
D
ATOM 12699 HD11 LEU D 157   -30.444 44.298 33.670 0.00 0.00
D
ATOM 12700 HD12 LEU D 157   -30.268 45.888 34.291 0.00 0.00
D
ATOM 12701 HD13 LEU D 157   -29.560 44.542 35.281 0.00 0.00
D
ATOM 12702 CD2 LEU D 157   -31.639 45.282 36.831 0.00 0.00
D
ATOM 12703 HD21 LEU D 157   -31.414 46.370 36.817 0.00 0.00
D
ATOM 12704 HD22 LEU D 157   -32.577 45.073 37.388 0.00 0.00
D
ATOM 12705 HD23 LEU D 157   -30.886 44.694 37.399 0.00 0.00
D
ATOM 12706 C   LEU D 157   -35.092 45.776 33.810 0.00 0.00
D
ATOM 12707 O   LEU D 157   -35.033 45.280 32.698 0.00 0.00
D
ATOM 12708 N   ALA D 158   -35.694 46.969 33.995 0.00 0.00
D
ATOM 12709 HN ALA D 158   -35.781 47.429 34.875 0.00 0.00
D
ATOM 12710 CA ALA D 158   -36.113 47.837 32.892 0.00 0.00
D
ATOM 12711 HA ALA D 158   -36.924 47.364 32.359 0.00 0.00
D
ATOM 12712 CB ALA D 158   -36.936 49.108 33.239 0.00 0.00
D
ATOM 12713 HB1 ALA D 158   -37.621 49.445 32.432 0.00 0.00
D
ATOM 12714 HB2 ALA D 158   -37.574 48.759 34.078 0.00 0.00
D
ATOM 12715 HB3 ALA D 158   -36.378 50.032 33.504 0.00 0.00
D
ATOM 12716 C   ALA D 158   -34.972 48.260 31.910 0.00 0.00
D

ATOM	12717	O	ALA	D	158	−33.837	48.414	32.240	0.00	0.00	D
ATOM	12718	N	THR	D	159	−35.413	48.395	30.616	0.00	0.00	D
ATOM	12719	HN	THR	D	159	−36.357	48.145	30.415	0.00	0.00	D
ATOM	12720	CA	THR	D	159	−34.557	48.520	29.467	0.00	0.00	D
ATOM	12721	HA	THR	D	159	−33.552	48.251	29.757	0.00	0.00	D
ATOM	12722	CB	THR	D	159	−34.996	47.719	28.257	0.00	0.00	D
ATOM	12723	HB	THR	D	159	−34.321	47.905	27.394	0.00	0.00	D
ATOM	12724	OG1	THR	D	159	−36.337	47.999	27.922	0.00	0.00	D
ATOM	12725	HG1	THR	D	159	−36.514	47.382	27.208	0.00	0.00	D
ATOM	12726	CG2	THR	D	159	−34.867	46.252	28.628	0.00	0.00	D
ATOM	12727	HG21	THR	D	159	−34.885	45.424	27.888	0.00	0.00	D
ATOM	12728	HG22	THR	D	159	−33.880	46.058	29.100	0.00	0.00	D
ATOM	12729	HG23	THR	D	159	−35.558	45.992	29.458	0.00	0.00	D
ATOM	12730	C	THR	D	159	−34.499	49.982	29.062	0.00	0.00	D
ATOM	12731	O	THR	D	159	−35.324	50.863	29.324	0.00	0.00	D
ATOM	12732	N	ARG	D	160	−33.509	50.403	28.171	0.00	0.00	D
ATOM	12733	HN	ARG	D	160	−32.802	49.748	27.916	0.00	0.00	D
ATOM	12734	CA	ARG	D	160	−33.385	51.763	27.596	0.00	0.00	D
ATOM	12735	HA	ARG	D	160	−33.390	52.458	28.423	0.00	0.00	D
ATOM	12736	CB	ARG	D	160	−31.971	51.931	26.923	0.00	0.00	D
ATOM	12737	HB1	ARG	D	160	−31.193	51.639	27.661	0.00	0.00	D
ATOM	12738	HB2	ARG	D	160	−31.982	51.186	26.099	0.00	0.00	D
ATOM	12739	CG	ARG	D	160	−31.624	53.281	26.296	0.00	0.00	D
ATOM	12740	HG1	ARG	D	160	−30.657	53.246	25.750	0.00	0.00	D
ATOM	12741	HG2	ARG	D	160	−32.505	53.450	25.640	0.00	0.00	D
ATOM	12742	CD	ARG	D	160	−31.498	54.405	27.291	0.00	0.00	D
ATOM	12743	HD1	ARG	D	160	−32.424	54.521	27.894	0.00	0.00	D

ATOM 12744 HD2 ARG D 160 −30.609 54.184 27.920 0.00 0.00
D
ATOM 12745 NE ARG D 160 −31.317 55.749 26.575 0.00 0.00
D
ATOM 12746 HE ARG D 160 −31.184 55.774 25.584 0.00 0.00
D
ATOM 12747 CZ ARG D 160 −31.331 56.894 27.265 0.00 0.00
D
ATOM 12748 NH1 ARG D 160 −31.348 56.928 28.608 0.00 0.00
D
ATOM 12749 HH11 ARG D 160 −31.715 57.776 28.991 0.00 0.00
D
ATOM 12750 HH12 ARG D 160 −31.476 56.040 29.050 0.00 0.00
D
ATOM 12751 NH2 ARG D 160 −31.061 58.053 26.677 0.00 0.00
D
ATOM 12752 HH21 ARG D 160 −30.927 58.895 27.198 0.00 0.00
D
ATOM 12753 HH22 ARG D 160 −30.634 58.000 25.774 0.00 0.00
D
ATOM 12754 C ARG D 160 −34.534 52.007 26.635 0.00 0.00
D
ATOM 12755 O ARG D 160 −35.046 53.154 26.596 0.00 0.00
D
ATOM 12756 N LYS D 161 −34.990 50.931 25.902 0.00 0.00
D
ATOM 12757 HN LYS D 161 −34.551 50.037 25.950 0.00 0.00
D
ATOM 12758 CA LYS D 161 −36.193 50.980 25.165 0.00 0.00
D
ATOM 12759 HA LYS D 161 −36.024 51.777 24.456 0.00 0.00
D
ATOM 12760 CB LYS D 161 −36.406 49.639 24.446 0.00 0.00
D
ATOM 12761 HB1 LYS D 161 −35.500 49.469 23.825 0.00 0.00
D
ATOM 12762 HB2 LYS D 161 −36.486 48.849 25.223 0.00 0.00
D
ATOM 12763 CG LYS D 161 −37.577 49.690 23.516 0.00 0.00
D
ATOM 12764 HG1 LYS D 161 −38.442 49.317 24.106 0.00 0.00
D
ATOM 12765 HG2 LYS D 161 −37.786 50.658 23.013 0.00 0.00
D
ATOM 12766 CD LYS D 161 −37.235 48.775 22.342 0.00 0.00
D
ATOM 12767 HD1 LYS D 161 −38.072 49.163 21.724 0.00 0.00
D
ATOM 12768 HD2 LYS D 161 −36.263 49.116 21.925 0.00 0.00
D
ATOM 12769 CE LYS D 161 −37.235 47.274 22.509 0.00 0.00
D
ATOM 12770 HE1 LYS D 161 −36.282 46.925 22.960 0.00 0.00
D

ATOM 12771 HE2 LYS D 161 −37.998 46.853 23.199 0.00 0.00
D
ATOM 12772 NZ LYS D 161 −37.427 46.609 21.187 0.00 0.00
D
ATOM 12773 HZ1 LYS D 161 −36.815 47.109 20.511 0.00 0.00
D
ATOM 12774 HZ2 LYS D 161 −37.214 45.592 21.209 0.00 0.00
D
ATOM 12775 HZ3 LYS D 161 −38.367 46.831 20.801 0.00 0.00
D
ATOM 12776 C LYS D 161 −37.467 51.332 25.891 0.00 0.00
D
ATOM 12777 O LYS D 161 −38.316 52.035 25.325 0.00 0.00
D
ATOM 12778 N LEU D 162 −37.639 51.036 27.180 0.00 0.00
D
ATOM 12779 HN LEU D 162 −36.937 50.507 27.650 0.00 0.00
D
ATOM 12780 CA LEU D 162 −38.864 51.344 27.916 0.00 0.00
D
ATOM 12781 HA LEU D 162 −39.662 51.524 27.210 0.00 0.00
D
ATOM 12782 CB LEU D 162 −39.408 50.156 28.806 0.00 0.00
D
ATOM 12783 HB1 LEU D 162 −39.248 49.187 28.288 0.00 0.00
D
ATOM 12784 HB2 LEU D 162 −38.855 50.217 29.768 0.00 0.00
D
ATOM 12785 CG LEU D 162 −41.003 50.097 29.112 0.00 0.00
D
ATOM 12786 HG LEU D 162 −41.419 51.113 29.283 0.00 0.00
D
ATOM 12787 CD1 LEU D 162 −41.830 49.515 27.974 0.00 0.00
D
ATOM 12788 HD11 LEU D 162 −41.638 49.940 26.965 0.00 0.00
D
ATOM 12789 HD12 LEU D 162 −41.511 48.469 27.782 0.00 0.00
D
ATOM 12790 HD13 LEU D 162 −42.915 49.569 28.207 0.00 0.00
D
ATOM 12791 CD2 LEU D 162 −41.059 49.194 30.350 0.00 0.00
D
ATOM 12792 HD21 LEU D 162 −40.627 48.177 30.234 0.00 0.00
D
ATOM 12793 HD22 LEU D 162 −40.359 49.640 31.089 0.00 0.00
D
ATOM 12794 HD23 LEU D 162 −42.123 49.191 30.668 0.00 0.00
D
ATOM 12795 C LEU D 162 −38.778 52.627 28.764 0.00 0.00
D
ATOM 12796 O LEU D 162 −39.686 53.039 29.503 0.00 0.00
D
ATOM 12797 N ASP D 163 −37.573 53.245 28.699 0.00 0.00
D

ATOM 12798 HN ASP D 163 -36.809 52.842 28.202 0.00 0.00
D
ATOM 12799 CA ASP D 163 -37.111 54.371 29.465 0.00 0.00
D
ATOM 12800 HA ASP D 163 -37.316 54.148 30.501 0.00 0.00
D
ATOM 12801 CB ASP D 163 -35.626 54.687 29.254 0.00 0.00
D
ATOM 12802 HB1 ASP D 163 -34.973 53.791 29.327 0.00 0.00
D
ATOM 12803 HB2 ASP D 163 -35.355 54.960 28.212 0.00 0.00
D
ATOM 12804 CG ASP D 163 -35.110 55.804 30.160 0.00 0.00
D
ATOM 12805 OD1 ASP D 163 -34.417 56.744 29.697 0.00 0.00
D
ATOM 12806 OD2 ASP D 163 -35.461 55.764 31.372 0.00 0.00
D
ATOM 12807 C ASP D 163 -37.927 55.701 29.139 0.00 0.00
D
ATOM 12808 O ASP D 163 -37.949 56.202 28.030 0.00 0.00
D
ATOM 12809 N SER D 164 -38.600 56.339 30.229 0.00 0.00
D
ATOM 12810 HN SER D 164 -38.406 55.999 31.146 0.00 0.00
D
ATOM 12811 CA SER D 164 -39.318 57.605 30.159 0.00 0.00
D
ATOM 12812 HA SER D 164 -39.480 57.894 31.186 0.00 0.00
D
ATOM 12813 CB SER D 164 -38.571 58.785 29.524 0.00 0.00
D
ATOM 12814 HB1 SER D 164 -37.462 58.814 29.584 0.00 0.00
D
ATOM 12815 HB2 SER D 164 -38.814 58.785 28.440 0.00 0.00
D
ATOM 12816 OG SER D 164 -38.928 59.996 30.176 0.00 0.00
D
ATOM 12817 HG1 SER D 164 -38.675 60.760 29.653 0.00 0.00
D
ATOM 12818 C SER D 164 -40.648 57.374 29.564 0.00 0.00
D
ATOM 12819 O SER D 164 -41.136 58.093 28.706 0.00 0.00
D
ATOM 12820 N LYS D 165 -41.367 56.329 30.050 0.00 0.00
D
ATOM 12821 HN LYS D 165 -41.095 55.867 30.890 0.00 0.00
D
ATOM 12822 CA LYS D 165 -42.685 56.033 29.428 0.00 0.00
D
ATOM 12823 HA LYS D 165 -42.925 56.713 28.624 0.00 0.00
D
ATOM 12824 CB LYS D 165 -42.496 54.715 28.703 0.00 0.00
D

ATOM 12825 HB1 LYS D 165 −41.762 54.878 27.885 0.00 0.00
D
ATOM 12826 HB2 LYS D 165 −42.083 53.861 29.282 0.00 0.00
D
ATOM 12827 CG LYS D 165 −43.762 54.178 28.048 0.00 0.00
D
ATOM 12828 HG1 LYS D 165 −43.553 53.218 27.531 0.00 0.00
D
ATOM 12829 HG2 LYS D 165 −44.320 54.015 28.995 0.00 0.00
D
ATOM 12830 CD LYS D 165 −44.409 55.042 26.971 0.00 0.00
D
ATOM 12831 HD1 LYS D 165 −45.480 54.776 26.845 0.00 0.00
D
ATOM 12832 HD2 LYS D 165 −44.545 56.084 27.331 0.00 0.00
D
ATOM 12833 CE LYS D 165 −43.749 55.216 25.590 0.00 0.00
D
ATOM 12834 HE1 LYS D 165 −42.736 55.637 25.760 0.00 0.00
D
ATOM 12835 HE2 LYS D 165 −43.597 54.205 25.155 0.00 0.00
D
ATOM 12836 NZ LYS D 165 −44.527 55.958 24.614 0.00 0.00
D
ATOM 12837 HZ1 LYS D 165 −44.069 55.932 23.681 0.00 0.00
D
ATOM 12838 HZ2 LYS D 165 −45.450 55.492 24.497 0.00 0.00
D
ATOM 12839 HZ3 LYS D 165 −44.628 56.950 24.908 0.00 0.00
D
ATOM 12840 C LYS D 165 −43.686 55.867 30.567 0.00 0.00
D
ATOM 12841 O LYS D 165 −43.527 55.090 31.464 0.00 0.00
D
ATOM 12842 N SER D 166 −44.863 56.508 30.429 0.00 0.00
D
ATOM 12843 HN SER D 166 −44.895 57.123 29.645 0.00 0.00
D
ATOM 12844 CA SER D 166 −46.106 56.219 31.156 0.00 0.00
D
ATOM 12845 HA SER D 166 −46.009 56.269 32.231 0.00 0.00
D
ATOM 12846 CB SER D 166 −47.134 57.245 30.897 0.00 0.00
D
ATOM 12847 HB1 SER D 166 −46.594 58.209 30.782 0.00 0.00
D
ATOM 12848 HB2 SER D 166 −47.577 57.111 29.887 0.00 0.00
D
ATOM 12849 OG SER D 166 −48.060 57.384 31.948 0.00 0.00
D
ATOM 12850 HG1 SER D 166 −48.436 58.260 31.832 0.00 0.00
D
ATOM 12851 C SER D 166 −46.777 54.894 30.935 0.00 0.00
D

ATOM 12852 O SER D 166 −46.706 54.212 29.925 0.00 0.00 D
ATOM 12853 N ILE D 167 −47.478 54.453 31.991 0.00 0.00 D
ATOM 12854 HN ILE D 167 −47.587 54.907 32.872 0.00 0.00 D
ATOM 12855 CA ILE D 167 −48.093 53.135 31.944 0.00 0.00 D
ATOM 12856 HA ILE D 167 −47.397 52.481 31.439 0.00 0.00 D
ATOM 12857 CB ILE D 167 −48.252 52.548 33.317 0.00 0.00 D
ATOM 12858 HB ILE D 167 −47.272 52.801 33.777 0.00 0.00 D
ATOM 12859 CG2 ILE D 167 −49.433 53.204 34.032 0.00 0.00 D
ATOM 12860 HG21 ILE D 167 −49.367 52.930 35.107 0.00 0.00 D
ATOM 12861 HG22 ILE D 167 −49.232 54.296 34.001 0.00 0.00 D
ATOM 12862 HG23 ILE D 167 −50.389 52.837 33.600 0.00 0.00 D
ATOM 12863 CG1 ILE D 167 −48.497 51.055 33.437 0.00 0.00 D
ATOM 12864 HG11 ILE D 167 −49.066 50.786 34.353 0.00 0.00 D
ATOM 12865 HG12 ILE D 167 −49.183 50.800 32.601 0.00 0.00 D
ATOM 12866 CD ILE D 167 −47.322 50.192 33.206 0.00 0.00 D
ATOM 12867 HD1 ILE D 167 −46.576 50.613 33.913 0.00 0.00 D
ATOM 12868 HD2 ILE D 167 −47.506 49.155 33.559 0.00 0.00 D
ATOM 12869 HD3 ILE D 167 −46.913 50.042 32.184 0.00 0.00 D
ATOM 12870 C ILE D 167 −49.458 53.087 31.164 0.00 0.00 D
ATOM 12871 O ILE D 167 −49.975 52.028 30.785 0.00 0.00 D
ATOM 12872 N ILE D 168 −50.102 54.207 30.889 0.00 0.00 D
ATOM 12873 HN ILE D 168 −49.766 55.038 31.325 0.00 0.00 D
ATOM 12874 CA ILE D 168 −51.358 54.286 30.194 0.00 0.00 D
ATOM 12875 HA ILE D 168 −51.921 53.492 30.662 0.00 0.00 D
ATOM 12876 CB ILE D 168 −51.929 55.737 30.329 0.00 0.00 D
ATOM 12877 HB ILE D 168 −52.053 56.005 31.401 0.00 0.00 D
ATOM 12878 CG2 ILE D 168 −50.933 56.726 29.718 0.00 0.00 D

```
ATOM  12879 HG21 ILE D 168     -51.138  57.800  29.917  0.00  0.00
D
ATOM  12880 HG22 ILE D 168     -49.901  56.493  30.059  0.00  0.00
D
ATOM  12881 HG23 ILE D 168     -50.985  56.670  28.610  0.00  0.00
D
ATOM  12882  CG1 ILE D 168     -53.369  55.702  29.823  0.00  0.00
D
ATOM  12883 HG11 ILE D 168     -53.787  56.711  30.022  0.00  0.00
D
ATOM  12884 HG12 ILE D 168     -53.421  55.395  28.756  0.00  0.00
D
ATOM  12885  CD  ILE D 168     -54.378  54.778  30.538  0.00  0.00
D
ATOM  12886  HD1 ILE D 168     -54.208  53.681  30.500  0.00  0.00
D
ATOM  12887  HD2 ILE D 168     -54.306  55.061  31.610  0.00  0.00
D
ATOM  12888  HD3 ILE D 168     -55.419  55.057  30.266  0.00  0.00
D
ATOM  12889  C   ILE D 168     -51.219  53.835  28.760  0.00  0.00
D
ATOM  12890  O   ILE D 168     -52.088  53.125  28.253  0.00  0.00
D
ATOM  12891  N   ASP D 169     -50.078  54.197  28.115  0.00  0.00
D
ATOM  12892  HN  ASP D 169     -49.478  54.793  28.644  0.00  0.00
D
ATOM  12893  CA  ASP D 169     -49.605  53.961  26.776  0.00  0.00
D
ATOM  12894  HA  ASP D 169     -50.445  54.162  26.127  0.00  0.00
D
ATOM  12895  CB  ASP D 169     -48.402  54.779  26.538  0.00  0.00
D
ATOM  12896  HB1 ASP D 169     -48.547  55.812  26.923  0.00  0.00
D
ATOM  12897  HB2 ASP D 169     -47.568  54.350  27.133  0.00  0.00
D
ATOM  12898  CG  ASP D 169     -48.021  55.037  25.048  0.00  0.00
D
ATOM  12899  OD1 ASP D 169     -46.949  54.558  24.631  0.00  0.00
D
ATOM  12900  OD2 ASP D 169     -48.779  55.795  24.370  0.00  0.00
D
ATOM  12901  C   ASP D 169     -49.333  52.486  26.518  0.00  0.00
D
ATOM  12902  O   ASP D 169     -49.667  51.968  25.448  0.00  0.00
D
ATOM  12903  N   LEU D 170     -48.682  51.689  27.425  0.00  0.00
D
ATOM  12904  HN  LEU D 170     -48.488  52.214  28.250  0.00  0.00
D
ATOM  12905  CA  LEU D 170     -48.201  50.389  27.266  0.00  0.00
D
```

ATOM 12906 HA LEU D 170 -47.616 50.285 26.365 0.00 0.00
D
ATOM 12907 CB LEU D 170 -47.304 49.904 28.485 0.00 0.00
D
ATOM 12908 HB1 LEU D 170 -47.881 49.900 29.435 0.00 0.00
D
ATOM 12909 HB2 LEU D 170 -47.004 48.852 28.293 0.00 0.00
D
ATOM 12910 CG LEU D 170 -46.040 50.823 28.542 0.00 0.00
D
ATOM 12911 HG LEU D 170 -46.360 51.887 28.540 0.00 0.00
D
ATOM 12912 CD1 LEU D 170 -45.303 50.451 29.803 0.00 0.00
D
ATOM 12913 HD11 LEU D 170 -45.934 50.462 30.718 0.00 0.00
D
ATOM 12914 HD12 LEU D 170 -44.718 49.506 29.810 0.00 0.00
D
ATOM 12915 HD13 LEU D 170 -44.650 51.343 29.917 0.00 0.00
D
ATOM 12916 CD2 LEU D 170 -45.209 50.597 27.282 0.00 0.00
D
ATOM 12917 HD21 LEU D 170 -44.725 49.615 27.097 0.00 0.00
D
ATOM 12918 HD22 LEU D 170 -45.712 50.837 26.320 0.00 0.00
D
ATOM 12919 HD23 LEU D 170 -44.378 51.333 27.252 0.00 0.00
D
ATOM 12920 C LEU D 170 -49.304 49.392 26.910 0.00 0.00
D
ATOM 12921 O LEU D 170 -50.453 49.642 27.277 0.00 0.00
D
ATOM 12922 N ASN D 171 -48.959 48.361 26.078 0.00 0.00
D
ATOM 12923 HN ASN D 171 -47.979 48.239 25.942 0.00 0.00
D
ATOM 12924 CA ASN D 171 -49.842 47.454 25.367 0.00 0.00
D
ATOM 12925 HA ASN D 171 -50.895 47.648 25.508 0.00 0.00
D
ATOM 12926 CB ASN D 171 -49.494 47.388 23.824 0.00 0.00
D
ATOM 12927 HB1 ASN D 171 -48.404 47.290 23.630 0.00 0.00
D
ATOM 12928 HB2 ASN D 171 -50.242 46.712 23.358 0.00 0.00
D
ATOM 12929 CG ASN D 171 -49.846 48.786 23.215 0.00 0.00
D
ATOM 12930 OD1 ASN D 171 -48.945 49.477 22.696 0.00 0.00
D
ATOM 12931 ND2 ASN D 171 -51.100 49.232 23.288 0.00 0.00
D
ATOM 12932 HD21 ASN D 171 -51.219 50.163 22.942 0.00 0.00
D

ATOM 12933 HD22 ASN D 171 −51.852 48.889 23.851 0.00 0.00
D
ATOM 12934 C ASN D 171 −49.695 46.022 25.845 0.00 0.00
D
ATOM 12935 O ASN D 171 −49.791 44.985 25.182 0.00 0.00
D
ATOM 12936 N VAL D 172 −49.405 45.980 27.156 0.00 0.00
D
ATOM 12937 HN VAL D 172 −49.268 46.867 27.590 0.00 0.00
D
ATOM 12938 CA VAL D 172 −49.193 44.774 28.028 0.00 0.00
D
ATOM 12939 HA VAL D 172 −48.393 44.171 27.627 0.00 0.00
D
ATOM 12940 CB VAL D 172 −48.830 45.175 29.498 0.00 0.00
D
ATOM 12941 HB VAL D 172 −48.957 44.335 30.214 0.00 0.00
D
ATOM 12942 CG1 VAL D 172 −47.349 45.525 29.281 0.00 0.00
D
ATOM 12943 HG11 VAL D 172 −47.111 46.288 28.509 0.00 0.00
D
ATOM 12944 HG12 VAL D 172 −46.893 45.852 30.239 0.00 0.00
D
ATOM 12945 HG13 VAL D 172 −46.782 44.626 28.957 0.00 0.00
D
ATOM 12946 CG2 VAL D 172 −49.589 46.375 30.103 0.00 0.00
D
ATOM 12947 HG21 VAL D 172 −49.305 46.445 31.175 0.00 0.00
D
ATOM 12948 HG22 VAL D 172 −49.256 47.301 29.585 0.00 0.00
D
ATOM 12949 HG23 VAL D 172 −50.695 46.331 30.014 0.00 0.00
D
ATOM 12950 C VAL D 172 −50.372 43.871 27.873 0.00 0.00
D
ATOM 12951 O VAL D 172 −50.188 42.682 27.772 0.00 0.00
D
ATOM 12952 N ARG D 173 −51.653 44.376 27.961 0.00 0.00
D
ATOM 12953 HN ARG D 173 −51.698 45.355 28.143 0.00 0.00
D
ATOM 12954 CA ARG D 173 −52.842 43.494 27.791 0.00 0.00
D
ATOM 12955 HA ARG D 173 −52.752 42.579 28.358 0.00 0.00
D
ATOM 12956 CB ARG D 173 −53.954 44.298 28.468 0.00 0.00
D
ATOM 12957 HB1 ARG D 173 −53.443 44.741 29.349 0.00 0.00
D
ATOM 12958 HB2 ARG D 173 −54.157 45.078 27.704 0.00 0.00
D
ATOM 12959 CG ARG D 173 −55.149 43.441 28.859 0.00 0.00
D

ATOM 12960 HG1 ARG D 173 -55.526 42.979 27.921 0.00 0.00
D
ATOM 12961 HG2 ARG D 173 -54.769 42.642 29.531 0.00 0.00
D
ATOM 12962 CD ARG D 173 -56.204 44.254 29.638 0.00 0.00
D
ATOM 12963 HD1 ARG D 173 -56.595 45.224 29.264 0.00 0.00
D
ATOM 12964 HD2 ARG D 173 -57.119 43.624 29.646 0.00 0.00
D
ATOM 12965 NE ARG D 173 -55.715 44.352 31.109 0.00 0.00
D
ATOM 12966 HE ARG D 173 -54.900 43.855 31.406 0.00 0.00
D
ATOM 12967 CZ ARG D 173 -56.532 44.771 32.109 0.00 0.00
D
ATOM 12968 NH1 ARG D 173 -57.630 45.433 31.877 0.00 0.00
D
ATOM 12969 HH11 ARG D 173 -58.082 45.899 32.638 0.00 0.00
D
ATOM 12970 HH12 ARG D 173 -57.779 45.863 30.987 0.00 0.00
D
ATOM 12971 NH2 ARG D 173 -56.137 44.532 33.345 0.00 0.00
D
ATOM 12972 HH21 ARG D 173 -56.749 44.841 34.074 0.00 0.00
D
ATOM 12973 HH22 ARG D 173 -55.483 43.784 33.455 0.00 0.00
D
ATOM 12974 C ARG D 173 -53.142 43.100 26.344 0.00 0.00
D
ATOM 12975 O ARG D 173 -53.308 41.959 26.033 0.00 0.00
D
ATOM 12976 N ALA D 174 -53.228 44.066 25.420 0.00 0.00
D
ATOM 12977 HN ALA D 174 -53.332 45.017 25.700 0.00 0.00
D
ATOM 12978 CA ALA D 174 -53.714 43.805 24.067 0.00 0.00
D
ATOM 12979 HA ALA D 174 -54.740 43.476 24.141 0.00 0.00
D
ATOM 12980 CB ALA D 174 -53.880 45.116 23.255 0.00 0.00
D
ATOM 12981 HB1 ALA D 174 -54.459 45.853 23.851 0.00 0.00
D
ATOM 12982 HB2 ALA D 174 -52.889 45.496 22.927 0.00 0.00
D
ATOM 12983 HB3 ALA D 174 -54.473 44.971 22.327 0.00 0.00
D
ATOM 12984 C ALA D 174 -52.844 42.786 23.322 0.00 0.00
D
ATOM 12985 O ALA D 174 -53.338 41.795 22.764 0.00 0.00
D
ATOM 12986 N LYS D 175 -51.493 42.909 23.397 0.00 0.00
D

ATOM 12987 HN LYS D 175 −50.992 43.633 23.864 0.00 0.00
D
ATOM 12988 CA LYS D 175 −50.534 42.150 22.633 0.00 0.00
D
ATOM 12989 HA LYS D 175 −50.923 41.862 21.668 0.00 0.00
D
ATOM 12990 CB LYS D 175 −49.291 43.050 22.310 0.00 0.00
D
ATOM 12991 HB1 LYS D 175 −48.701 43.355 23.201 0.00 0.00
D
ATOM 12992 HB2 LYS D 175 −48.567 42.589 21.605 0.00 0.00
D
ATOM 12993 CG LYS D 175 −49.729 44.169 21.326 0.00 0.00
D
ATOM 12994 HG1 LYS D 175 −50.239 43.717 20.449 0.00 0.00
D
ATOM 12995 HG2 LYS D 175 −50.498 44.785 21.840 0.00 0.00
D
ATOM 12996 CD LYS D 175 −48.516 45.013 20.820 0.00 0.00
D
ATOM 12997 HD1 LYS D 175 −47.934 45.289 21.725 0.00 0.00
D
ATOM 12998 HD2 LYS D 175 −47.901 44.244 20.306 0.00 0.00
D
ATOM 12999 CE LYS D 175 −48.780 46.258 19.951 0.00 0.00
D
ATOM 13000 HE1 LYS D 175 −49.312 47.063 20.502 0.00 0.00
D
ATOM 13001 HE2 LYS D 175 −47.857 46.684 19.503 0.00 0.00
D
ATOM 13002 NZ LYS D 175 −49.711 45.905 18.841 0.00 0.00
D
ATOM 13003 HZ1 LYS D 175 −49.494 44.941 18.514 0.00 0.00
D
ATOM 13004 HZ2 LYS D 175 −50.680 45.951 19.216 0.00 0.00
D
ATOM 13005 HZ3 LYS D 175 −49.634 46.553 18.031 0.00 0.00
D
ATOM 13006 C LYS D 175 −49.916 41.017 23.323 0.00 0.00
D
ATOM 13007 O LYS D 175 −49.616 39.950 22.830 0.00 0.00
D
ATOM 13008 N TYR D 176 −49.630 41.168 24.616 0.00 0.00
D
ATOM 13009 HN TYR D 176 −49.770 42.070 25.017 0.00 0.00
D
ATOM 13010 CA TYR D 176 −48.923 40.083 25.307 0.00 0.00
D
ATOM 13011 HA TYR D 176 −48.580 39.275 24.677 0.00 0.00
D
ATOM 13012 CB TYR D 176 −47.715 40.715 26.008 0.00 0.00
D
ATOM 13013 HB1 TYR D 176 −48.122 41.156 26.943 0.00 0.00
D

ATOM 13014 HB2 TYR D 176 −47.034 39.876 26.265 0.00 0.00
D
ATOM 13015 CG TYR D 176 −46.872 41.682 25.205 0.00 0.00
D
ATOM 13016 CD1 TYR D 176 −47.042 43.074 25.408 0.00 0.00
D
ATOM 13017 HD1 TYR D 176 −47.781 43.424 26.114 0.00 0.00
D
ATOM 13018 CE1 TYR D 176 −46.334 43.928 24.592 0.00 0.00
D
ATOM 13019 HE1 TYR D 176 −46.476 44.985 24.758 0.00 0.00
D
ATOM 13020 CZ TYR D 176 −45.563 43.419 23.487 0.00 0.00
D
ATOM 13021 OH TYR D 176 −45.047 44.166 22.509 0.00 0.00
D
ATOM 13022 HH TYR D 176 −44.991 43.683 21.681 0.00 0.00
D
ATOM 13023 CD2 TYR D 176 −46.066 41.191 24.176 0.00 0.00
D
ATOM 13024 HD2 TYR D 176 −46.060 40.140 23.928 0.00 0.00
D
ATOM 13025 CE2 TYR D 176 −45.436 42.039 23.323 0.00 0.00
D
ATOM 13026 HE2 TYR D 176 −44.751 41.622 22.599 0.00 0.00
D
ATOM 13027 C TYR D 176 −49.708 39.318 26.337 0.00 0.00
D
ATOM 13028 O TYR D 176 −49.245 38.257 26.732 0.00 0.00
D
ATOM 13029 N GLY D 177 −50.896 39.825 26.842 0.00 0.00
D
ATOM 13030 HN GLY D 177 −51.334 40.574 26.351 0.00 0.00
D
ATOM 13031 CA GLY D 177 −51.715 39.189 27.885 0.00 0.00
D
ATOM 13032 HA1 GLY D 177 −51.937 38.171 27.599 0.00 0.00
D
ATOM 13033 HA2 GLY D 177 −52.640 39.746 27.916 0.00 0.00
D
ATOM 13034 C GLY D 177 −51.191 39.252 29.258 0.00 0.00
D
ATOM 13035 O GLY D 177 −51.356 38.405 30.144 0.00 0.00
D
ATOM 13036 N CYS D 178 −50.355 40.306 29.430 0.00 0.00
D
ATOM 13037 HN CYS D 178 −50.289 41.022 28.740 0.00 0.00
D
ATOM 13038 CA CYS D 178 −49.450 40.477 30.578 0.00 0.00
D
ATOM 13039 HA CYS D 178 −49.462 39.504 31.048 0.00 0.00
D
ATOM 13040 CB CYS D 178 −47.981 40.789 30.089 0.00 0.00
D

ATOM 13041 HB1 CYS D 178 -48.129 41.446 29.206 0.00 0.00
D
ATOM 13042 HB2 CYS D 178 -47.424 41.270 30.921 0.00 0.00
D
ATOM 13043 SG CYS D 178 -47.030 39.372 29.527 0.00 0.00
D
ATOM 13044 HG1 CYS D 178 -47.938 38.877 28.700 0.00 0.00
D
ATOM 13045 C CYS D 178 -49.851 41.487 31.591 0.00 0.00
D
ATOM 13046 O CYS D 178 -50.638 42.380 31.203 0.00 0.00
D
ATOM 13047 N THR D 179 -49.435 41.329 32.795 0.00 0.00
D
ATOM 13048 HN THR D 179 -48.862 40.574 33.103 0.00 0.00
D
ATOM 13049 CA THR D 179 -49.849 42.266 33.814 0.00 0.00
D
ATOM 13050 HA THR D 179 -50.280 43.125 33.321 0.00 0.00
D
ATOM 13051 CB THR D 179 -50.903 41.698 34.830 0.00 0.00
D
ATOM 13052 HB THR D 179 -50.594 40.839 35.464 0.00 0.00
D
ATOM 13053 OG1 THR D 179 -52.011 41.210 34.186 0.00 0.00
D
ATOM 13054 HG1 THR D 179 -51.709 40.372 33.828 0.00 0.00
D
ATOM 13055 CG2 THR D 179 -51.406 42.784 35.740 0.00 0.00
D
ATOM 13056 HG21 THR D 179 -51.726 43.655 35.130 0.00 0.00
D
ATOM 13057 HG22 THR D 179 -52.202 42.336 36.373 0.00 0.00
D
ATOM 13058 HG23 THR D 179 -50.607 43.146 36.422 0.00 0.00
D
ATOM 13059 C THR D 179 -48.656 42.802 34.611 0.00 0.00
D
ATOM 13060 O THR D 179 -47.877 41.970 35.083 0.00 0.00
D
ATOM 13061 N ILE D 180 -48.401 44.139 34.811 0.00 0.00
D
ATOM 13062 HN ILE D 180 -48.965 44.854 34.405 0.00 0.00
D
ATOM 13063 CA ILE D 180 -47.231 44.725 35.547 0.00 0.00
D
ATOM 13064 HA ILE D 180 -46.430 44.002 35.515 0.00 0.00
D
ATOM 13065 CB ILE D 180 -46.745 46.041 34.853 0.00 0.00
D
ATOM 13066 HB ILE D 180 -47.511 46.841 34.938 0.00 0.00
D
ATOM 13067 CG2 ILE D 180 -45.488 46.614 35.573 0.00 0.00
D

ATOM 13068 HG21 ILE D 180   −44.669 45.928 35.269 0.00 0.00
D
ATOM 13069 HG22 ILE D 180   −45.288 47.590 35.082 0.00 0.00
D
ATOM 13070 HG23 ILE D 180   −45.768 46.696 36.645 0.00 0.00
D
ATOM 13071 CG1 ILE D 180   −46.693 45.797 33.308 0.00 0.00
D
ATOM 13072 HG11 ILE D 180   −47.691 45.690 32.830 0.00 0.00
D
ATOM 13073 HG12 ILE D 180   −46.166 46.703 32.939 0.00 0.00
D
ATOM 13074 CD ILE D 180   −45.829 44.578 32.967 0.00 0.00
D
ATOM 13075 HD1 ILE D 180   −46.412 43.634 32.923 0.00 0.00
D
ATOM 13076 HD2 ILE D 180   −45.287 44.567 31.997 0.00 0.00
D
ATOM 13077 HD3 ILE D 180   −45.038 44.289 33.692 0.00 0.00
D
ATOM 13078 C   ILE D 180   −47.692 44.862 36.971 0.00 0.00
D
ATOM 13079 O   ILE D 180   −48.551 45.666 37.218 0.00 0.00
D
ATOM 13080 N   LEU D 181   −47.260 44.006 37.939 0.00 0.00
D
ATOM 13081 HN LEU D 181   −46.549 43.337 37.739 0.00 0.00
D
ATOM 13082 CA LEU D 181   −47.581 43.948 39.351 0.00 0.00
D
ATOM 13083 HA LEU D 181   −48.656 43.920 39.458 0.00 0.00
D
ATOM 13084 CB LEU D 181   −47.016 42.669 39.898 0.00 0.00
D
ATOM 13085 HB1 LEU D 181   −45.910 42.734 39.977 0.00 0.00
D
ATOM 13086 HB2 LEU D 181   −47.395 42.535 40.934 0.00 0.00
D
ATOM 13087 CG LEU D 181   −47.392 41.320 39.276 0.00 0.00
D
ATOM 13088 HG LEU D 181   −46.935 41.379 38.265 0.00 0.00
D
ATOM 13089 CD1 LEU D 181   −46.763 40.173 40.063 0.00 0.00
D
ATOM 13090 HD11 LEU D 181   −46.854 40.474 41.129 0.00 0.00
D
ATOM 13091 HD12 LEU D 181   −47.461 39.323 39.904 0.00 0.00
D
ATOM 13092 HD13 LEU D 181   −45.706 39.950 39.804 0.00 0.00
D
ATOM 13093 CD2 LEU D 181   −48.886 41.003 39.243 0.00 0.00
D
ATOM 13094 HD21 LEU D 181   −49.211 40.054 38.765 0.00 0.00
D

ATOM 13095 HD22 LEU D 181 −49.307 40.841 40.259 0.00 0.00
D
ATOM 13096 HD23 LEU D 181 −49.491 41.824 38.802 0.00 0.00
D
ATOM 13097 C LEU D 181 −47.018 45.148 40.066 0.00 0.00
D
ATOM 13098 O LEU D 181 −47.816 45.760 40.832 0.00 0.00
D
ATOM 13099 N ALA D 182 −45.680 45.491 39.815 0.00 0.00
D
ATOM 13100 HN ALA D 182 −45.186 45.069 39.058 0.00 0.00
D
ATOM 13101 CA ALA D 182 −45.033 46.485 40.598 0.00 0.00
D
ATOM 13102 HA ALA D 182 −45.773 47.188 40.950 0.00 0.00
D
ATOM 13103 CB ALA D 182 −44.332 45.898 41.886 0.00 0.00
D
ATOM 13104 HB1 ALA D 182 −43.725 46.657 42.425 0.00 0.00
D
ATOM 13105 HB2 ALA D 182 −45.117 45.404 42.498 0.00 0.00
D
ATOM 13106 HB3 ALA D 182 −43.782 44.955 41.683 0.00 0.00
D
ATOM 13107 C ALA D 182 −44.055 47.229 39.744 0.00 0.00
D
ATOM 13108 O ALA D 182 −43.724 46.779 38.660 0.00 0.00
D
ATOM 13109 N ILE D 183 −43.626 48.423 40.120 0.00 0.00
D
ATOM 13110 HN ILE D 183 −43.966 48.667 41.025 0.00 0.00
D
ATOM 13111 CA ILE D 183 −42.659 49.248 39.523 0.00 0.00
D
ATOM 13112 HA ILE D 183 −41.900 48.681 39.005 0.00 0.00
D
ATOM 13113 CB ILE D 183 −43.273 50.355 38.505 0.00 0.00
D
ATOM 13114 HB ILE D 183 −43.869 51.048 39.136 0.00 0.00
D
ATOM 13115 CG2 ILE D 183 −42.251 51.237 37.797 0.00 0.00
D
ATOM 13116 HG21 ILE D 183 −41.419 51.570 38.454 0.00 0.00
D
ATOM 13117 HG22 ILE D 183 −41.721 50.788 36.930 0.00 0.00
D
ATOM 13118 HG23 ILE D 183 −42.754 52.162 37.444 0.00 0.00
D
ATOM 13119 CG1 ILE D 183 −44.202 49.668 37.429 0.00 0.00
D
ATOM 13120 HG11 ILE D 183 −43.619 48.819 37.012 0.00 0.00
D
ATOM 13121 HG12 ILE D 183 −45.035 49.303 38.066 0.00 0.00
D

ATOM 13122 CD ILE D 183 −44.922 50.479 36.327 0.00 0.00
D
ATOM 13123 HD1 ILE D 183 −44.255 50.529 35.440 0.00 0.00
D
ATOM 13124 HD2 ILE D 183 −45.924 50.109 36.024 0.00 0.00
D
ATOM 13125 HD3 ILE D 183 −45.088 51.521 36.673 0.00 0.00
D
ATOM 13126 C ILE D 183 −41.962 49.845 40.747 0.00 0.00
D
ATOM 13127 O ILE D 183 −42.562 49.827 41.861 0.00 0.00
D
ATOM 13128 N LYS D 184 −40.692 50.414 40.541 0.00 0.00
D
ATOM 13129 HN LYS D 184 −40.122 49.971 39.854 0.00 0.00
D
ATOM 13130 CA LYS D 184 −39.971 51.182 41.494 0.00 0.00
D
ATOM 13131 HA LYS D 184 −40.622 51.642 42.222 0.00 0.00
D
ATOM 13132 CB LYS D 184 −38.649 50.557 41.955 0.00 0.00
D
ATOM 13133 HB1 LYS D 184 −38.873 49.504 42.230 0.00 0.00
D
ATOM 13134 HB2 LYS D 184 −37.992 50.483 41.062 0.00 0.00
D
ATOM 13135 CG LYS D 184 −37.843 51.105 43.170 0.00 0.00
D
ATOM 13136 HG1 LYS D 184 −37.471 52.048 42.715 0.00 0.00
D
ATOM 13137 HG2 LYS D 184 −38.511 51.327 44.030 0.00 0.00
D
ATOM 13138 CD LYS D 184 −36.665 50.173 43.618 0.00 0.00
D
ATOM 13139 HD1 LYS D 184 −36.089 50.798 44.335 0.00 0.00
D
ATOM 13140 HD2 LYS D 184 −37.110 49.339 44.202 0.00 0.00
D
ATOM 13141 CE LYS D 184 −35.777 49.614 42.506 0.00 0.00
D
ATOM 13142 HE1 LYS D 184 −36.217 48.877 41.801 0.00 0.00
D
ATOM 13143 HE2 LYS D 184 −35.458 50.518 41.944 0.00 0.00
D
ATOM 13144 NZ LYS D 184 −34.555 49.008 43.026 0.00 0.00
D
ATOM 13145 HZ1 LYS D 184 −34.043 49.530 43.765 0.00 0.00
D
ATOM 13146 HZ2 LYS D 184 −34.733 48.096 43.494 0.00 0.00
D
ATOM 13147 HZ3 LYS D 184 −33.837 48.882 42.283 0.00 0.00
D
ATOM 13148 C LYS D 184 −39.569 52.431 40.722 0.00 0.00
D

ATOM 13149 O LYS D 184 −38.856 52.539 39.760 0.00 0.00
D
ATOM 13150 N HSE D 185 −40.019 53.493 41.259 0.00 0.00
D
ATOM 13151 HN HSE D 185 −40.650 53.451 42.029 0.00 0.00
D
ATOM 13152 CA HSE D 185 −39.891 54.883 40.817 0.00 0.00
D
ATOM 13153 HA HSE D 185 −39.396 54.936 39.859 0.00 0.00
D
ATOM 13154 CB HSE D 185 −41.252 55.507 40.791 0.00 0.00
D
ATOM 13155 HB1 HSE D 185 −41.781 55.273 41.740 0.00 0.00
D
ATOM 13156 HB2 HSE D 185 −41.317 56.595 40.580 0.00 0.00
D
ATOM 13157 ND1 HSE D 185 −42.171 55.080 38.437 0.00 0.00
D
ATOM 13158 CG HSE D 185 −42.128 54.761 39.806 0.00 0.00
D
ATOM 13159 CE1 HSE D 185 −43.356 54.630 38.070 0.00 0.00
D
ATOM 13160 HE1 HSE D 185 −43.729 54.604 37.046 0.00 0.00
D
ATOM 13161 NE2 HSE D 185 −43.929 53.911 39.040 0.00 0.00
D
ATOM 13162 HE2 HSE D 185 −44.762 53.381 38.885 0.00 0.00
D
ATOM 13163 CD2 HSE D 185 −43.232 54.035 40.146 0.00 0.00
D
ATOM 13164 HD2 HSE D 185 −43.568 53.677 41.111 0.00 0.00
D
ATOM 13165 C HSE D 185 −39.088 55.709 41.792 0.00 0.00
D
ATOM 13166 O HSE D 185 −39.569 56.151 42.854 0.00 0.00
D
ATOM 13167 N HSE D 186 −37.735 55.942 41.510 0.00 0.00
D
ATOM 13168 HN HSE D 186 −37.369 55.730 40.607 0.00 0.00
D
ATOM 13169 CA HSE D 186 −36.763 56.806 42.251 0.00 0.00
D
ATOM 13170 HA HSE D 186 −35.859 56.736 41.664 0.00 0.00
D
ATOM 13171 CB HSE D 186 −37.228 58.312 42.324 0.00 0.00
D
ATOM 13172 HB1 HSE D 186 −37.851 58.413 43.238 0.00 0.00
D
ATOM 13173 HB2 HSE D 186 −36.322 58.949 42.403 0.00 0.00
D
ATOM 13174 ND1 HSE D 186 −37.483 58.909 39.974 0.00 0.00
D
ATOM 13175 CG HSE D 186 −38.099 58.645 41.169 0.00 0.00
D

ATOM 13176 CE1 HSE D 186 −38.471 59.221 39.075 0.00 0.00
D
ATOM 13177 HE1 HSE D 186 −38.325 59.467 38.024 0.00 0.00
D
ATOM 13178 NE2 HSE D 186 −39.704 59.086 39.688 0.00 0.00
D
ATOM 13179 HE2 HSE D 186 −40.583 59.338 39.284 0.00 0.00
D
ATOM 13180 CD2 HSE D 186 −39.424 58.827 41.060 0.00 0.00
D
ATOM 13181 HD2 HSE D 186 −40.245 58.696 41.753 0.00 0.00
D
ATOM 13182 C HSE D 186 −36.521 56.229 43.603 0.00 0.00
D
ATOM 13183 O HSE D 186 −36.550 56.894 44.678 0.00 0.00
D
ATOM 13184 N GLY D 187 −36.319 54.929 43.706 0.00 0.00
D
ATOM 13185 HN GLY D 187 −36.344 54.351 42.893 0.00 0.00
D
ATOM 13186 CA GLY D 187 −36.021 54.186 44.893 0.00 0.00
D
ATOM 13187 HA1 GLY D 187 −35.309 54.687 45.532 0.00 0.00
D
ATOM 13188 HA2 GLY D 187 −35.761 53.184 44.587 0.00 0.00
D
ATOM 13189 C GLY D 187 −37.269 53.923 45.640 0.00 0.00
D
ATOM 13190 O GLY D 187 −37.253 53.323 46.692 0.00 0.00
D
ATOM 13191 N ASP D 188 −38.488 54.380 45.178 0.00 0.00
D
ATOM 13192 HN ASP D 188 −38.514 54.861 44.304 0.00 0.00
D
ATOM 13193 CA ASP D 188 −39.721 54.316 45.894 0.00 0.00
D
ATOM 13194 HA ASP D 188 −39.503 53.825 46.831 0.00 0.00
D
ATOM 13195 CB ASP D 188 −40.394 55.664 45.965 0.00 0.00
D
ATOM 13196 HB1 ASP D 188 −40.487 56.111 44.952 0.00 0.00
D
ATOM 13197 HB2 ASP D 188 −41.431 55.621 46.361 0.00 0.00
D
ATOM 13198 CG ASP D 188 −39.571 56.583 46.784 0.00 0.00
D
ATOM 13199 OD1 ASP D 188 −39.290 57.725 46.422 0.00 0.00
D
ATOM 13200 OD2 ASP D 188 −39.267 56.147 47.930 0.00 0.00
D
ATOM 13201 C ASP D 188 −40.588 53.265 45.149 0.00 0.00
D
ATOM 13202 O ASP D 188 −41.006 53.518 43.996 0.00 0.00
D

ATOM 13203 N ILE D 189 -40.795 52.091 45.764 0.00 0.00
D
ATOM 13204 HN ILE D 189 -40.294 51.769 46.564 0.00 0.00
D
ATOM 13205 CA ILE D 189 -41.682 51.040 45.298 0.00 0.00
D
ATOM 13206 HA ILE D 189 -41.318 50.726 44.331 0.00 0.00
D
ATOM 13207 CB ILE D 189 -41.563 49.851 46.303 0.00 0.00
D
ATOM 13208 HB ILE D 189 -40.484 49.590 46.340 0.00 0.00
D
ATOM 13209 CG2 ILE D 189 -42.145 50.214 47.721 0.00 0.00
D
ATOM 13210 HG21 ILE D 189 -43.251 50.314 47.745 0.00 0.00
D
ATOM 13211 HG22 ILE D 189 -41.817 49.352 48.339 0.00 0.00
D
ATOM 13212 HG23 ILE D 189 -41.771 51.176 48.132 0.00 0.00
D
ATOM 13213 CG1 ILE D 189 -42.368 48.654 45.706 0.00 0.00
D
ATOM 13214 HG11 ILE D 189 -42.485 47.761 46.357 0.00 0.00
D
ATOM 13215 HG12 ILE D 189 -43.448 48.908 45.658 0.00 0.00
D
ATOM 13216 CD ILE D 189 -41.729 48.169 44.385 0.00 0.00
D
ATOM 13217 HD1 ILE D 189 -40.709 47.757 44.538 0.00 0.00
D
ATOM 13218 HD2 ILE D 189 -42.440 47.397 44.021 0.00 0.00
D
ATOM 13219 HD3 ILE D 189 -41.678 48.950 43.597 0.00 0.00
D
ATOM 13220 C ILE D 189 -43.069 51.548 45.133 0.00 0.00
D
ATOM 13221 O ILE D 189 -43.552 52.385 45.840 0.00 0.00
D
ATOM 13222 N CYS D 190 -43.812 50.966 44.152 0.00 0.00
D
ATOM 13223 HN CYS D 190 -43.410 50.401 43.435 0.00 0.00
D
ATOM 13224 CA CYS D 190 -45.228 51.166 43.881 0.00 0.00
D
ATOM 13225 HA CYS D 190 -45.718 51.593 44.744 0.00 0.00
D
ATOM 13226 CB CYS D 190 -45.401 52.136 42.634 0.00 0.00
D
ATOM 13227 HB1 CYS D 190 -45.002 53.100 43.013 0.00 0.00
D
ATOM 13228 HB2 CYS D 190 -44.683 51.717 41.896 0.00 0.00
D
ATOM 13229 SG CYS D 190 -47.092 52.504 42.115 0.00 0.00
D

ATOM 13230 HG1 CYS D 190 −46.843 53.386 41.158 0.00 0.00
D
ATOM 13231 C CYS D 190 −45.943 49.854 43.573 0.00 0.00
D
ATOM 13232 O CYS D 190 −45.419 49.000 42.838 0.00 0.00
D
ATOM 13233 N LEU D 191 −47.075 49.621 44.198 0.00 0.00
D
ATOM 13234 HN LEU D 191 −47.396 50.254 44.897 0.00 0.00
D
ATOM 13235 CA LEU D 191 −47.876 48.385 44.072 0.00 0.00
D
ATOM 13236 HA LEU D 191 −47.431 47.543 43.563 0.00 0.00
D
ATOM 13237 CB LEU D 191 −48.350 47.846 45.462 0.00 0.00
D
ATOM 13238 HB1 LEU D 191 −49.106 48.478 45.975 0.00 0.00
D
ATOM 13239 HB2 LEU D 191 −48.742 46.827 45.258 0.00 0.00
D
ATOM 13240 CG LEU D 191 −47.252 47.615 46.533 0.00 0.00
D
ATOM 13241 HG LEU D 191 −46.826 48.600 46.821 0.00 0.00
D
ATOM 13242 CD1 LEU D 191 −47.850 46.989 47.768 0.00 0.00
D
ATOM 13243 HD11 LEU D 191 −48.750 47.561 48.079 0.00 0.00
D
ATOM 13244 HD12 LEU D 191 −48.034 45.937 47.461 0.00 0.00
D
ATOM 13245 HD13 LEU D 191 −47.065 46.967 48.555 0.00 0.00
D
ATOM 13246 CD2 LEU D 191 −46.135 46.647 46.000 0.00 0.00
D
ATOM 13247 HD21 LEU D 191 −45.635 47.148 45.144 0.00 0.00
D
ATOM 13248 HD22 LEU D 191 −45.468 46.249 46.794 0.00 0.00
D
ATOM 13249 HD23 LEU D 191 −46.681 45.756 45.623 0.00 0.00
D
ATOM 13250 C LEU D 191 −49.119 48.491 43.209 0.00 0.00
D
ATOM 13251 O LEU D 191 −49.872 47.523 42.961 0.00 0.00
D
ATOM 13252 N SER D 192 −49.301 49.699 42.707 0.00 0.00
D
ATOM 13253 HN SER D 192 −48.632 50.409 42.912 0.00 0.00
D
ATOM 13254 CA SER D 192 −50.470 50.057 41.845 0.00 0.00
D
ATOM 13255 HA SER D 192 −50.803 49.122 41.419 0.00 0.00
D
ATOM 13256 CB SER D 192 −51.621 50.651 42.695 0.00 0.00
D

ATOM 13257 HB1 SER D 192 −51.682 50.069 43.639 0.00 0.00
D
ATOM 13258 HB2 SER D 192 −51.472 51.664 43.127 0.00 0.00
D
ATOM 13259 OG SER D 192 −52.952 50.538 42.060 0.00 0.00
D
ATOM 13260 HG1 SER D 192 −53.145 51.193 41.385 0.00 0.00
D
ATOM 13261 C SER D 192 −50.184 50.998 40.724 0.00 0.00
D
ATOM 13262 O SER D 192 −50.357 52.213 40.882 0.00 0.00
D
ATOM 13263 N PRO D 193 −49.626 50.589 39.571 0.00 0.00
D
ATOM 13264 CD PRO D 193 −48.926 49.309 39.478 0.00 0.00
D
ATOM 13265 HD1 PRO D 193 −48.646 48.877 40.463 0.00 0.00
D
ATOM 13266 HD2 PRO D 193 −49.631 48.592 39.006 0.00 0.00
D
ATOM 13267 CA PRO D 193 −49.032 51.477 38.542 0.00 0.00
D
ATOM 13268 HA PRO D 193 −48.169 51.971 38.963 0.00 0.00
D
ATOM 13269 CB PRO D 193 −48.456 50.480 37.563 0.00 0.00
D
ATOM 13270 HB1 PRO D 193 −47.881 51.015 36.777 0.00 0.00
D
ATOM 13271 HB2 PRO D 193 −49.239 49.959 36.973 0.00 0.00
D
ATOM 13272 CG PRO D 193 −47.827 49.399 38.483 0.00 0.00
D
ATOM 13273 HG1 PRO D 193 −46.949 49.894 38.951 0.00 0.00
D
ATOM 13274 HG2 PRO D 193 −47.663 48.398 38.030 0.00 0.00
D
ATOM 13275 C PRO D 193 −50.020 52.458 38.036 0.00 0.00
D
ATOM 13276 O PRO D 193 −51.142 52.146 37.732 0.00 0.00
D
ATOM 13277 N ALA D 194 −49.607 53.726 37.903 0.00 0.00
D
ATOM 13278 HN ALA D 194 −48.725 53.946 38.313 0.00 0.00
D
ATOM 13279 CA ALA D 194 −50.620 54.755 37.599 0.00 0.00
D
ATOM 13280 HA ALA D 194 −51.378 54.336 36.953 0.00 0.00
D
ATOM 13281 CB ALA D 194 −51.248 55.530 38.775 0.00 0.00
D
ATOM 13282 HB1 ALA D 194 −50.464 56.064 39.353 0.00 0.00
D
ATOM 13283 HB2 ALA D 194 −51.975 56.342 38.564 0.00 0.00
D

ATOM 13284 HB3 ALA D 194 -51.664 54.786 39.488 0.00 0.00
D
ATOM 13285 C ALA D 194 -49.883 55.906 36.859 0.00 0.00
D
ATOM 13286 O ALA D 194 -48.654 56.109 36.835 0.00 0.00
D
ATOM 13287 N PRO D 195 -50.701 56.552 35.942 0.00 0.00
D
ATOM 13288 CD PRO D 195 -51.979 56.114 35.283 0.00 0.00
D
ATOM 13289 HD1 PRO D 195 -52.057 55.054 34.958 0.00 0.00
D
ATOM 13290 HD2 PRO D 195 -52.729 56.257 36.090 0.00 0.00
D
ATOM 13291 CA PRO D 195 -50.365 57.858 35.376 0.00 0.00
D
ATOM 13292 HA PRO D 195 -49.541 57.611 34.722 0.00 0.00
D
ATOM 13293 CB PRO D 195 -51.654 58.410 34.733 0.00 0.00
D
ATOM 13294 HB1 PRO D 195 -51.630 59.066 33.837 0.00 0.00
D
ATOM 13295 HB2 PRO D 195 -52.262 58.829 35.563 0.00 0.00
D
ATOM 13296 CG PRO D 195 -52.384 57.133 34.219 0.00 0.00
D
ATOM 13297 HG1 PRO D 195 -51.915 56.879 33.244 0.00 0.00
D
ATOM 13298 HG2 PRO D 195 -53.483 57.116 34.060 0.00 0.00
D
ATOM 13299 C PRO D 195 -49.845 58.944 36.394 0.00 0.00
D
ATOM 13300 O PRO D 195 -50.177 58.816 37.550 0.00 0.00
D
ATOM 13301 N GLU D 196 -48.978 59.920 36.016 0.00 0.00
D
ATOM 13302 HN GLU D 196 -48.708 60.503 36.780 0.00 0.00
D
ATOM 13303 CA GLU D 196 -48.380 60.224 34.716 0.00 0.00
D
ATOM 13304 HA GLU D 196 -48.673 59.478 33.992 0.00 0.00
D
ATOM 13305 CB GLU D 196 -48.643 61.640 34.288 0.00 0.00
D
ATOM 13306 HB1 GLU D 196 -47.967 62.404 34.728 0.00 0.00
D
ATOM 13307 HB2 GLU D 196 -48.313 61.827 33.244 0.00 0.00
D
ATOM 13308 CG GLU D 196 -50.133 62.080 34.196 0.00 0.00
D
ATOM 13309 HG1 GLU D 196 -50.603 61.387 33.465 0.00 0.00
D
ATOM 13310 HG2 GLU D 196 -50.592 61.922 35.195 0.00 0.00
D

ATOM 13311 CD GLU D 196 -50.444 63.488 33.625 0.00 0.00
D
ATOM 13312 OE1 GLU D 196 -49.571 64.008 32.886 0.00 0.00
D
ATOM 13313 OE2 GLU D 196 -51.551 63.985 33.919 0.00 0.00
D
ATOM 13314 C GLU D 196 -46.896 59.927 34.799 0.00 0.00
D
ATOM 13315 O GLU D 196 -46.200 60.285 33.868 0.00 0.00
D
ATOM 13316 N ASP D 197 -46.391 59.399 35.942 0.00 0.00
D
ATOM 13317 HN ASP D 197 -47.061 59.282 36.672 0.00 0.00
D
ATOM 13318 CA ASP D 197 -44.995 59.113 36.220 0.00 0.00
D
ATOM 13319 HA ASP D 197 -44.446 60.034 36.090 0.00 0.00
D
ATOM 13320 CB ASP D 197 -44.846 58.475 37.665 0.00 0.00
D
ATOM 13321 HB1 ASP D 197 -45.383 57.511 37.795 0.00 0.00
D
ATOM 13322 HB2 ASP D 197 -43.774 58.247 37.851 0.00 0.00
D
ATOM 13323 CG ASP D 197 -45.226 59.591 38.566 0.00 0.00
D
ATOM 13324 OD1 ASP D 197 -46.175 59.545 39.343 0.00 0.00
D
ATOM 13325 OD2 ASP D 197 -44.508 60.639 38.395 0.00 0.00
D
ATOM 13326 C ASP D 197 -44.497 57.990 35.276 0.00 0.00
D
ATOM 13327 O ASP D 197 -45.209 57.225 34.640 0.00 0.00
D
ATOM 13328 N ILE D 198 -43.183 58.017 35.123 0.00 0.00
D
ATOM 13329 HN ILE D 198 -42.730 58.651 35.745 0.00 0.00
D
ATOM 13330 CA ILE D 198 -42.458 57.379 34.058 0.00 0.00
D
ATOM 13331 HA ILE D 198 -43.206 56.950 33.408 0.00 0.00
D
ATOM 13332 CB ILE D 198 -41.626 58.298 33.250 0.00 0.00
D
ATOM 13333 HB ILE D 198 -40.970 57.674 32.605 0.00 0.00
D
ATOM 13334 CG2 ILE D 198 -42.432 59.199 32.306 0.00 0.00
D
ATOM 13335 HG21 ILE D 198 -42.880 60.026 32.898 0.00 0.00
D
ATOM 13336 HG22 ILE D 198 -41.851 59.736 31.527 0.00 0.00
D
ATOM 13337 HG23 ILE D 198 -43.240 58.528 31.944 0.00 0.00
D

ATOM 13338 CG1 ILE D 198   -40.753 59.215 34.248 0.00 0.00
D
ATOM 13339 HG11 ILE D 198   -41.379 59.976 34.759 0.00 0.00
D
ATOM 13340 HG12 ILE D 198   -40.160 58.661 35.006 0.00 0.00
D
ATOM 13341 CD ILE D 198   -39.795 60.015 33.399 0.00 0.00
D
ATOM 13342 HD1 ILE D 198   -40.286 60.671 32.648 0.00 0.00
D
ATOM 13343 HD2 ILE D 198   -38.956 60.508 33.936 0.00 0.00
D
ATOM 13344 HD3 ILE D 198   -39.292 59.302 32.711 0.00 0.00
D
ATOM 13345 C   ILE D 198   -41.579 56.200 34.525 0.00 0.00
D
ATOM 13346 O   ILE D 198   -40.812 56.339 35.489 0.00 0.00
D
ATOM 13347 N   ILE D 199   -41.651 55.039 33.885 0.00 0.00
D
ATOM 13348 HN ILE D 199   -42.498 55.016 33.359 0.00 0.00
D
ATOM 13349 CA ILE D 199   -40.781 53.939 33.987 0.00 0.00
D
ATOM 13350 HA ILE D 199   -40.782 53.544 34.992 0.00 0.00
D
ATOM 13351 CB ILE D 199   -41.290 52.684 33.216 0.00 0.00
D
ATOM 13352 HB ILE D 199   -41.179 52.848 32.122 0.00 0.00
D
ATOM 13353 CG2 ILE D 199   -40.529 51.427 33.684 0.00 0.00
D
ATOM 13354 HG21 ILE D 199   -41.151 50.808 34.366 0.00 0.00
D
ATOM 13355 HG22 ILE D 199   -40.231 50.771 32.839 0.00 0.00
D
ATOM 13356 HG23 ILE D 199   -39.639 51.699 34.290 0.00 0.00
D
ATOM 13357 CG1 ILE D 199   -42.792 52.410 33.490 0.00 0.00
D
ATOM 13358 HG11 ILE D 199   -42.991 52.149 34.551 0.00 0.00
D
ATOM 13359 HG12 ILE D 199   -43.398 53.306 33.233 0.00 0.00
D
ATOM 13360 CD ILE D 199   -43.322 51.181 32.709 0.00 0.00
D
ATOM 13361 HD1 ILE D 199   -44.388 51.008 32.973 0.00 0.00
D
ATOM 13362 HD2 ILE D 199   -43.191 51.469 31.644 0.00 0.00
D
ATOM 13363 HD3 ILE D 199   -42.711 50.253 32.696 0.00 0.00
D
ATOM 13364 C   ILE D 199   -39.354 54.390 33.635 0.00 0.00
D

ATOM 13365 O ILE D 199 −39.135 55.100 32.627 0.00 0.00
D
ATOM 13366 N ARG D 200 −38.260 53.970 34.322 0.00 0.00
D
ATOM 13367 HN ARG D 200 −38.420 53.441 35.152 0.00 0.00
D
ATOM 13368 CA ARG D 200 −36.901 54.334 33.955 0.00 0.00
D
ATOM 13369 HA ARG D 200 −36.947 54.751 32.960 0.00 0.00
D
ATOM 13370 CB ARG D 200 −36.202 55.388 34.877 0.00 0.00
D
ATOM 13371 HB1 ARG D 200 −35.923 54.822 35.792 0.00 0.00
D
ATOM 13372 HB2 ARG D 200 −35.249 55.614 34.352 0.00 0.00
D
ATOM 13373 CG ARG D 200 −36.946 56.705 35.255 0.00 0.00
D
ATOM 13374 HG1 ARG D 200 −37.902 56.457 35.765 0.00 0.00
D
ATOM 13375 HG2 ARG D 200 −36.337 57.348 35.925 0.00 0.00
D
ATOM 13376 CD ARG D 200 −37.408 57.559 34.087 0.00 0.00
D
ATOM 13377 HD1 ARG D 200 −38.159 56.989 33.499 0.00 0.00
D
ATOM 13378 HD2 ARG D 200 −37.941 58.471 34.432 0.00 0.00
D
ATOM 13379 NE ARG D 200 −36.304 57.663 33.077 0.00 0.00
D
ATOM 13380 HE ARG D 200 −35.930 56.886 32.570 0.00 0.00
D
ATOM 13381 CZ ARG D 200 −35.693 58.744 32.711 0.00 0.00
D
ATOM 13382 NH1 ARG D 200 −35.796 59.879 33.426 0.00 0.00
D
ATOM 13383 HH11 ARG D 200 −35.456 60.742 33.052 0.00 0.00
D
ATOM 13384 HH12 ARG D 200 −36.149 59.799 34.358 0.00 0.00
D
ATOM 13385 NH2 ARG D 200 −34.908 58.799 31.629 0.00 0.00
D
ATOM 13386 HH21 ARG D 200 −34.402 59.658 31.553 0.00 0.00
D
ATOM 13387 HH22 ARG D 200 −34.678 58.017 31.049 0.00 0.00
D
ATOM 13388 C ARG D 200 −35.961 53.180 33.913 0.00 0.00
D
ATOM 13389 O ARG D 200 −36.108 52.100 34.499 0.00 0.00
D
ATOM 13390 N GLU D 201 −34.987 53.389 33.072 0.00 0.00
D
ATOM 13391 HN GLU D 201 −35.018 54.159 32.439 0.00 0.00
D

```
ATOM  13392  CA  GLU D 201     -33.989  52.419  32.669  0.00  0.00
D
ATOM  13393  HA  GLU D 201     -34.385  51.519  32.223  0.00  0.00
D
ATOM  13394  CB  GLU D 201     -33.072  53.309  31.759  0.00  0.00
D
ATOM  13395  HB1 GLU D 201     -33.595  53.865  30.952  0.00  0.00
D
ATOM  13396  HB2 GLU D 201     -32.662  54.195  32.288  0.00  0.00
D
ATOM  13397  CG  GLU D 201     -32.017  52.495  31.012  0.00  0.00
D
ATOM  13398  HG1 GLU D 201     -31.442  51.822  31.684  0.00  0.00
D
ATOM  13399  HG2 GLU D 201     -32.474  51.899  30.194  0.00  0.00
D
ATOM  13400  CD  GLU D 201     -30.987  53.426  30.432  0.00  0.00
D
ATOM  13401  OE1 GLU D 201     -29.824  53.040  30.164  0.00  0.00
D
ATOM  13402  OE2 GLU D 201     -31.302  54.597  30.229  0.00  0.00
D
ATOM  13403  C   GLU D 201     -33.146  51.960  33.832  0.00  0.00
D
ATOM  13404  O   GLU D 201     -32.784  52.698  34.792  0.00  0.00
D
ATOM  13405  N   GLN D 202     -32.815  50.672  33.730  0.00  0.00
D
ATOM  13406  HN  GLN D 202     -33.202  50.142  32.979  0.00  0.00
D
ATOM  13407  CA  GLN D 202     -31.867  50.009  34.600  0.00  0.00
D
ATOM  13408  HA  GLN D 202     -31.823  49.059  34.087  0.00  0.00
D
ATOM  13409  CB  GLN D 202     -30.403  50.482  34.506  0.00  0.00
D
ATOM  13410  HB1 GLN D 202     -30.263  50.839  33.463  0.00  0.00
D
ATOM  13411  HB2 GLN D 202     -30.136  51.415  35.048  0.00  0.00
D
ATOM  13412  CG  GLN D 202     -29.361  49.418  34.812  0.00  0.00
D
ATOM  13413  HG1 GLN D 202     -29.384  49.062  35.864  0.00  0.00
D
ATOM  13414  HG2 GLN D 202     -29.426  48.496  34.195  0.00  0.00
D
ATOM  13415  CD  GLN D 202     -27.979  49.893  34.559  0.00  0.00
D
ATOM  13416  OE1 GLN D 202     -27.173  50.270  35.415  0.00  0.00
D
ATOM  13417  NE2 GLN D 202     -27.532  49.836  33.266  0.00  0.00
D
ATOM  13418 HE21 GLN D 202     -26.719  50.365  33.023  0.00  0.00
D
```

ATOM 13419 HE22 GLN D 202 -28.100 49.382 32.580 0.00 0.00
D
ATOM 13420 C GLN D 202 -32.303 49.922 36.049 0.00 0.00
D
ATOM 13421 O GLN D 202 -31.516 50.044 36.955 0.00 0.00
D
ATOM 13422 N ASP D 203 -33.512 49.493 36.399 0.00 0.00
D
ATOM 13423 HN ASP D 203 -34.261 49.386 35.749 0.00 0.00
D
ATOM 13424 CA ASP D 203 -33.780 49.016 37.708 0.00 0.00
D
ATOM 13425 HA ASP D 203 -32.970 48.352 37.974 0.00 0.00
D
ATOM 13426 CB ASP D 203 -34.129 50.130 38.807 0.00 0.00
D
ATOM 13427 HB1 ASP D 203 -33.710 51.103 38.470 0.00 0.00
D
ATOM 13428 HB2 ASP D 203 -35.199 50.183 39.100 0.00 0.00
D
ATOM 13429 CG ASP D 203 -33.423 49.807 40.066 0.00 0.00
D
ATOM 13430 OD1 ASP D 203 -33.590 48.702 40.662 0.00 0.00
D
ATOM 13431 OD2 ASP D 203 -32.555 50.639 40.421 0.00 0.00
D
ATOM 13432 C ASP D 203 -35.064 48.147 37.678 0.00 0.00
D
ATOM 13433 O ASP D 203 -35.722 48.118 36.631 0.00 0.00
D
ATOM 13434 N CYS D 204 -35.364 47.449 38.788 0.00 0.00
D
ATOM 13435 HN CYS D 204 -34.719 47.538 39.543 0.00 0.00
D
ATOM 13436 CA CYS D 204 -36.495 46.488 39.047 0.00 0.00
D
ATOM 13437 HA CYS D 204 -36.207 45.563 38.571 0.00 0.00
D
ATOM 13438 CB CYS D 204 -36.568 46.115 40.587 0.00 0.00
D
ATOM 13439 HB1 CYS D 204 -37.028 46.933 41.183 0.00 0.00
D
ATOM 13440 HB2 CYS D 204 -37.224 45.242 40.790 0.00 0.00
D
ATOM 13441 SG CYS D 204 -35.047 45.533 41.210 0.00 0.00
D
ATOM 13442 HG1 CYS D 204 -35.556 45.015 42.318 0.00 0.00
D
ATOM 13443 C CYS D 204 -37.844 46.741 38.423 0.00 0.00
D
ATOM 13444 O CYS D 204 -38.350 47.849 38.492 0.00 0.00
D
ATOM 13445 N LEU D 205 -38.421 45.746 37.777 0.00 0.00
D

ATOM 13446 HN LEU D 205   -37.963 44.869 37.649 0.00 0.00
D
ATOM 13447 CA LEU D 205   -39.763 45.876 37.233 0.00 0.00
D
ATOM 13448 HA LEU D 205   -40.324 46.515 37.898 0.00 0.00
D
ATOM 13449 CB LEU D 205   -39.643 46.437 35.761 0.00 0.00
D
ATOM 13450 HB1 LEU D 205   -38.967 47.317 35.726 0.00 0.00
D
ATOM 13451 HB2 LEU D 205   -39.161 45.629 35.171 0.00 0.00
D
ATOM 13452 CG LEU D 205   -40.983 46.687 35.122 0.00 0.00
D
ATOM 13453 HG LEU D 205   -41.438 45.687 34.959 0.00 0.00
D
ATOM 13454 CD1 LEU D 205   -41.860 47.670 35.902 0.00 0.00
D
ATOM 13455 HD11 LEU D 205   -41.304 48.625 36.020 0.00 0.00
D
ATOM 13456 HD12 LEU D 205   -42.708 47.838 35.204 0.00 0.00
D
ATOM 13457 HD13 LEU D 205   -42.230 47.234 36.854 0.00 0.00
D
ATOM 13458 CD2 LEU D 205   -40.639 47.168 33.692 0.00 0.00
D
ATOM 13459 HD21 LEU D 205   -40.234 48.199 33.787 0.00 0.00
D
ATOM 13460 HD22 LEU D 205   -39.948 46.449 33.202 0.00 0.00
D
ATOM 13461 HD23 LEU D 205   -41.647 47.342 33.258 0.00 0.00
D
ATOM 13462 C   LEU D 205   -40.386 44.473 37.337 0.00 0.00
D
ATOM 13463 O   LEU D 205   -39.793 43.455 36.995 0.00 0.00
D
ATOM 13464 N   VAL D 206   -41.600 44.332 37.867 0.00 0.00
D
ATOM 13465 HN VAL D 206   -42.135 45.145 38.082 0.00 0.00
D
ATOM 13466 CA VAL D 206   -42.159 43.025 38.144 0.00 0.00
D
ATOM 13467 HA VAL D 206   -41.464 42.206 38.030 0.00 0.00
D
ATOM 13468 CB VAL D 206   -42.514 42.895 39.658 0.00 0.00
D
ATOM 13469 HB VAL D 206   -43.257 43.667 39.952 0.00 0.00
D
ATOM 13470 CG1 VAL D 206   -42.891 41.439 40.005 0.00 0.00
D
ATOM 13471 HG11 VAL D 206   -43.601 41.062 39.239 0.00 0.00
D
ATOM 13472 HG12 VAL D 206   -41.990 40.792 40.066 0.00 0.00
D

ATOM 13473 HG13 VAL D 206   −43.433 41.477 40.974 0.00 0.00
D
ATOM 13474 CG2 VAL D 206   −41.260 43.237 40.450 0.00 0.00
D
ATOM 13475 HG21 VAL D 206   −40.458 42.498 40.239 0.00 0.00
D
ATOM 13476 HG22 VAL D 206   −40.829 44.231 40.205 0.00 0.00
D
ATOM 13477 HG23 VAL D 206   −41.639 43.319 41.491 0.00 0.00
D
ATOM 13478 C   VAL D 206   −43.376 42.761 37.264 0.00 0.00
D
ATOM 13479 O   VAL D 206   −44.325 43.589 37.249 0.00 0.00
D
ATOM 13480 N   ILE D 207   −43.317 41.726 36.404 0.00 0.00
D
ATOM 13481 HN ILE D 207   −42.639 41.013 36.564 0.00 0.00
D
ATOM 13482 CA ILE D 207   −44.274 41.441 35.396 0.00 0.00
D
ATOM 13483 HA ILE D 207   −45.141 42.083 35.347 0.00 0.00
D
ATOM 13484 CB ILE D 207   −43.568 41.520 34.087 0.00 0.00
D
ATOM 13485 HB ILE D 207   −43.371 42.580 33.819 0.00 0.00
D
ATOM 13486 CG2 ILE D 207   −42.244 40.668 34.054 0.00 0.00
D
ATOM 13487 HG21 ILE D 207   −41.560 40.804 33.190 0.00 0.00
D
ATOM 13488 HG22 ILE D 207   −41.623 40.723 34.974 0.00 0.00
D
ATOM 13489 HG23 ILE D 207   −42.568 39.606 34.034 0.00 0.00
D
ATOM 13490 CG1 ILE D 207   −44.538 41.142 32.889 0.00 0.00
D
ATOM 13491 HG11 ILE D 207   −44.676 40.044 32.794 0.00 0.00
D
ATOM 13492 HG12 ILE D 207   −45.555 41.575 33.001 0.00 0.00
D
ATOM 13493 CD ILE D 207   −44.019 41.515 31.505 0.00 0.00
D
ATOM 13494 HD1 ILE D 207   −43.744 42.591 31.531 0.00 0.00
D
ATOM 13495 HD2 ILE D 207   −43.287 40.779 31.109 0.00 0.00
D
ATOM 13496 HD3 ILE D 207   −44.890 41.436 30.820 0.00 0.00
D
ATOM 13497 C   ILE D 207   −44.697 40.021 35.640 0.00 0.00
D
ATOM 13498 O   ILE D 207   −43.990 39.176 36.143 0.00 0.00
D
ATOM 13499 N   MET D 208   −46.026 39.722 35.495 0.00 0.00
D

ATOM 13500 HN MET D 208 −46.567 40.385 34.984 0.00 0.00
D
ATOM 13501 CA MET D 208 −46.593 38.372 35.638 0.00 0.00
D
ATOM 13502 HA MET D 208 −45.830 37.620 35.769 0.00 0.00
D
ATOM 13503 CB MET D 208 −47.624 38.409 36.722 0.00 0.00
D
ATOM 13504 HB1 MET D 208 −47.313 38.623 37.767 0.00 0.00
D
ATOM 13505 HB2 MET D 208 −48.291 39.252 36.441 0.00 0.00
D
ATOM 13506 CG MET D 208 −48.633 37.209 36.823 0.00 0.00
D
ATOM 13507 HG1 MET D 208 −49.262 37.163 35.908 0.00 0.00
D
ATOM 13508 HG2 MET D 208 −48.082 36.247 36.744 0.00 0.00
D
ATOM 13509 SD MET D 208 −49.687 37.044 38.262 0.00 0.00
D
ATOM 13510 CE MET D 208 −51.215 37.849 37.585 0.00 0.00
D
ATOM 13511 HE1 MET D 208 −51.027 38.932 37.424 0.00 0.00
D
ATOM 13512 HE2 MET D 208 −51.570 37.391 36.637 0.00 0.00
D
ATOM 13513 HE3 MET D 208 −52.090 37.812 38.269 0.00 0.00
D
ATOM 13514 C MET D 208 −47.261 38.062 34.305 0.00 0.00
D
ATOM 13515 O MET D 208 −47.962 38.807 33.617 0.00 0.00
D
ATOM 13516 N GLY D 209 −47.060 36.813 33.863 0.00 0.00
D
ATOM 13517 HN GLY D 209 −46.617 36.143 34.454 0.00 0.00
D
ATOM 13518 CA GLY D 209 −47.536 36.325 32.593 0.00 0.00
D
ATOM 13519 HA1 GLY D 209 −47.187 36.950 31.784 0.00 0.00
D
ATOM 13520 HA2 GLY D 209 −48.616 36.320 32.619 0.00 0.00
D
ATOM 13521 C GLY D 209 −47.118 34.901 32.225 0.00 0.00
D
ATOM 13522 O GLY D 209 −46.357 34.270 32.962 0.00 0.00
D
ATOM 13523 N HSE D 210 −47.609 34.356 31.064 0.00 0.00
D
ATOM 13524 HN HSE D 210 −48.420 34.790 30.678 0.00 0.00
D
ATOM 13525 CA HSE D 210 −47.086 33.138 30.419 0.00 0.00
D
ATOM 13526 HA HSE D 210 −47.212 32.340 31.137 0.00 0.00
D

ATOM 13527 CB HSE D 210   -47.786 32.719 29.106 0.00 0.00
D
ATOM 13528 HB1 HSE D 210   -48.859 32.692 29.393 0.00 0.00
D
ATOM 13529 HB2 HSE D 210   -47.543 33.415 28.275 0.00 0.00
D
ATOM 13530 ND1 HSE D 210   -46.362 31.118 27.878 0.00 0.00
D
ATOM 13531 CG HSE D 210   -47.511 31.387 28.611 0.00 0.00
D
ATOM 13532 CE1 HSE D 210   -46.290 29.865 27.656 0.00 0.00
D
ATOM 13533 HE1 HSE D 210   -45.478 29.361 27.133 0.00 0.00
D
ATOM 13534 NE2 HSE D 210   -47.326 29.310 28.274 0.00 0.00
D
ATOM 13535 HE2 HSE D 210   -47.553 28.336 28.312 0.00 0.00
D
ATOM 13536 CD2 HSE D 210   -48.108 30.253 28.868 0.00 0.00
D
ATOM 13537 HD2 HSE D 210   -48.961 29.924 29.448 0.00 0.00
D
ATOM 13538 C   HSE D 210   -45.621 33.309 30.110 0.00 0.00
D
ATOM 13539 O   HSE D 210   -45.147 34.359 29.694 0.00 0.00
D
ATOM 13540 N   LYS D 211   -44.851 32.245 30.302 0.00 0.00
D
ATOM 13541 HN LYS D 211   -45.354 31.420 30.550 0.00 0.00
D
ATOM 13542 CA LYS D 211   -43.323 32.097 30.117 0.00 0.00
D
ATOM 13543 HA LYS D 211   -42.787 32.687 30.846 0.00 0.00
D
ATOM 13544 CB LYS D 211   -42.802 30.608 30.178 0.00 0.00
D
ATOM 13545 HB1 LYS D 211   -43.423 29.899 29.590 0.00 0.00
D
ATOM 13546 HB2 LYS D 211   -41.702 30.589 30.024 0.00 0.00
D
ATOM 13547 CG LYS D 211   -42.843 30.077 31.643 0.00 0.00
D
ATOM 13548 HG1 LYS D 211   -42.521 30.779 32.442 0.00 0.00
D
ATOM 13549 HG2 LYS D 211   -43.890 30.004 32.007 0.00 0.00
D
ATOM 13550 CD LYS D 211   -42.143 28.769 31.754 0.00 0.00
D
ATOM 13551 HD1 LYS D 211   -42.486 28.133 30.910 0.00 0.00
D
ATOM 13552 HD2 LYS D 211   -41.080 29.015 31.548 0.00 0.00
D
ATOM 13553 CE LYS D 211   -42.282 27.871 33.031 0.00 0.00
D

ATOM 13554 HE1 LYS D 211 −43.209 27.259 33.039 0.00 0.00
D
ATOM 13555 HE2 LYS D 211 −41.379 27.226 32.999 0.00 0.00
D
ATOM 13556 NZ LYS D 211 −42.030 28.702 34.214 0.00 0.00
D
ATOM 13557 HZ1 LYS D 211 −42.080 28.189 35.117 0.00 0.00
D
ATOM 13558 HZ2 LYS D 211 −41.183 29.272 34.014 0.00 0.00
D
ATOM 13559 HZ3 LYS D 211 −42.805 29.391 34.290 0.00 0.00
D
ATOM 13560 C LYS D 211 −42.847 32.683 28.791 0.00 0.00
D
ATOM 13561 O LYS D 211 −41.929 33.496 28.769 0.00 0.00
D
ATOM 13562 N LYS D 212 −43.379 32.188 27.660 0.00 0.00
D
ATOM 13563 HN LYS D 212 −44.123 31.543 27.819 0.00 0.00
D
ATOM 13564 CA LYS D 212 −43.153 32.634 26.317 0.00 0.00
D
ATOM 13565 HA LYS D 212 −42.101 32.447 26.157 0.00 0.00
D
ATOM 13566 CB LYS D 212 −43.829 31.650 25.357 0.00 0.00
D
ATOM 13567 HB1 LYS D 212 −43.262 30.747 25.665 0.00 0.00
D
ATOM 13568 HB2 LYS D 212 −44.914 31.690 25.595 0.00 0.00
D
ATOM 13569 CG LYS D 212 −43.723 31.957 23.876 0.00 0.00
D
ATOM 13570 HG1 LYS D 212 −44.224 32.910 23.602 0.00 0.00
D
ATOM 13571 HG2 LYS D 212 −42.660 32.104 23.587 0.00 0.00
D
ATOM 13572 CD LYS D 212 −44.138 30.758 22.994 0.00 0.00
D
ATOM 13573 HD1 LYS D 212 −43.395 29.968 23.234 0.00 0.00
D
ATOM 13574 HD2 LYS D 212 −45.182 30.481 23.256 0.00 0.00
D
ATOM 13575 CE LYS D 212 −44.124 31.167 21.536 0.00 0.00
D
ATOM 13576 HE1 LYS D 212 −44.812 32.021 21.359 0.00 0.00
D
ATOM 13577 HE2 LYS D 212 −43.069 31.464 21.358 0.00 0.00
D
ATOM 13578 NZ LYS D 212 −44.478 30.078 20.658 0.00 0.00
D
ATOM 13579 HZ1 LYS D 212 −44.616 30.401 19.679 0.00 0.00
D
ATOM 13580 HZ2 LYS D 212 −43.783 29.305 20.617 0.00 0.00
D

ATOM 13581 HZ3 LYS D 212   -45.395 29.671 20.934 0.00 0.00
D
ATOM 13582 C   LYS D 212   -43.534 34.132 26.062 0.00 0.00
D
ATOM 13583 O   LYS D 212   -42.805 34.862 25.388 0.00 0.00
D
ATOM 13584 N   ASP D 213   -44.622 34.531 26.753 0.00 0.00
D
ATOM 13585 HN ASP D 213   -45.035 33.954 27.454 0.00 0.00
D
ATOM 13586 CA ASP D 213   -45.113 35.915 26.603 0.00 0.00
D
ATOM 13587 HA ASP D 213   -44.850 36.289 25.625 0.00 0.00
D
ATOM 13588 CB ASP D 213   -46.615 36.069 26.843 0.00 0.00
D
ATOM 13589 HB1 ASP D 213   -46.910 35.669 27.837 0.00 0.00
D
ATOM 13590 HB2 ASP D 213   -46.933 37.134 26.838 0.00 0.00
D
ATOM 13591 CG ASP D 213   -47.428 35.326 25.788 0.00 0.00
D
ATOM 13592 OD1 ASP D 213   -48.387 34.603 26.225 0.00 0.00
D
ATOM 13593 OD2 ASP D 213   -47.275 35.530 24.581 0.00 0.00
D
ATOM 13594 C   ASP D 213   -44.345 36.923 27.384 0.00 0.00
D
ATOM 13595 O   ASP D 213   -44.176 38.076 26.942 0.00 0.00
D
ATOM 13596 N   ILE D 214   -43.762 36.530 28.498 0.00 0.00
D
ATOM 13597 HN ILE D 214   -43.995 35.641 28.886 0.00 0.00
D
ATOM 13598 CA ILE D 214   -42.729 37.417 29.138 0.00 0.00
D
ATOM 13599 HA ILE D 214   -43.227 38.368 29.254 0.00 0.00
D
ATOM 13600 CB ILE D 214   -42.401 36.858 30.545 0.00 0.00
D
ATOM 13601 HB ILE D 214   -41.993 35.850 30.317 0.00 0.00
D
ATOM 13602 CG2 ILE D 214   -41.278 37.718 31.413 0.00 0.00
D
ATOM 13603 HG21 ILE D 214   -41.048 37.206 32.372 0.00 0.00
D
ATOM 13604 HG22 ILE D 214   -40.308 37.809 30.879 0.00 0.00
D
ATOM 13605 HG23 ILE D 214   -41.676 38.735 31.618 0.00 0.00
D
ATOM 13606 CG1 ILE D 214   -43.722 36.893 31.409 0.00 0.00
D
ATOM 13607 HG11 ILE D 214   -43.827 37.927 31.801 0.00 0.00
D

ATOM 13608 HG12 ILE D 214 −44.628 36.572 30.853 0.00 0.00
D
ATOM 13609 CD ILE D 214 −43.564 36.049 32.644 0.00 0.00
D
ATOM 13610 HD1 ILE D 214 −42.618 36.290 33.176 0.00 0.00
D
ATOM 13611 HD2 ILE D 214 −44.466 36.165 33.282 0.00 0.00
D
ATOM 13612 HD3 ILE D 214 −43.530 34.960 32.429 0.00 0.00
D
ATOM 13613 C ILE D 214 −41.446 37.566 28.274 0.00 0.00
D
ATOM 13614 O ILE D 214 −40.976 38.690 28.089 0.00 0.00
D
ATOM 13615 N LYS D 215 −40.922 36.483 27.673 0.00 0.00
D
ATOM 13616 HN LYS D 215 −41.338 35.581 27.765 0.00 0.00
D
ATOM 13617 CA LYS D 215 −39.803 36.551 26.735 0.00 0.00
D
ATOM 13618 HA LYS D 215 −38.948 36.979 27.237 0.00 0.00
D
ATOM 13619 CB LYS D 215 −39.413 35.108 26.284 0.00 0.00
D
ATOM 13620 HB1 LYS D 215 −39.250 34.629 27.273 0.00 0.00
D
ATOM 13621 HB2 LYS D 215 −40.228 34.609 25.719 0.00 0.00
D
ATOM 13622 CG LYS D 215 −38.214 34.825 25.468 0.00 0.00
D
ATOM 13623 HG1 LYS D 215 −37.987 33.783 25.157 0.00 0.00
D
ATOM 13624 HG2 LYS D 215 −38.386 35.335 24.496 0.00 0.00
D
ATOM 13625 CD LYS D 215 −36.915 35.404 25.900 0.00 0.00
D
ATOM 13626 HD1 LYS D 215 −36.118 35.316 25.130 0.00 0.00
D
ATOM 13627 HD2 LYS D 215 −37.067 36.502 25.829 0.00 0.00
D
ATOM 13628 CE LYS D 215 −36.517 35.116 27.331 0.00 0.00
D
ATOM 13629 HE1 LYS D 215 −37.205 35.649 28.022 0.00 0.00
D
ATOM 13630 HE2 LYS D 215 −36.589 34.013 27.439 0.00 0.00
D
ATOM 13631 NZ LYS D 215 −35.148 35.564 27.638 0.00 0.00
D
ATOM 13632 HZ1 LYS D 215 −34.859 35.044 28.492 0.00 0.00
D
ATOM 13633 HZ2 LYS D 215 −34.507 35.365 26.844 0.00 0.00
D
ATOM 13634 HZ3 LYS D 215 −35.246 36.587 27.794 0.00 0.00
D

ATOM 13635 C LYS D 215 −40.033 37.435 25.574 0.00 0.00
D
ATOM 13636 O LYS D 215 −39.124 38.106 25.100 0.00 0.00
D
ATOM 13637 N ARG D 216 −41.227 37.344 24.935 0.00 0.00
D
ATOM 13638 HN ARG D 216 −41.910 36.686 25.242 0.00 0.00
D
ATOM 13639 CA ARG D 216 −41.678 38.097 23.849 0.00 0.00
D
ATOM 13640 HA ARG D 216 −40.983 38.058 23.024 0.00 0.00
D
ATOM 13641 CB ARG D 216 −43.047 37.671 23.265 0.00 0.00
D
ATOM 13642 HB1 ARG D 216 −42.944 36.581 23.076 0.00 0.00
D
ATOM 13643 HB2 ARG D 216 −43.864 37.747 24.013 0.00 0.00
D
ATOM 13644 CG ARG D 216 −43.612 38.593 22.139 0.00 0.00
D
ATOM 13645 HG1 ARG D 216 −43.642 39.631 22.533 0.00 0.00
D
ATOM 13646 HG2 ARG D 216 −42.764 38.609 21.421 0.00 0.00
D
ATOM 13647 CD ARG D 216 −44.893 38.107 21.605 0.00 0.00
D
ATOM 13648 HD1 ARG D 216 −44.813 37.214 20.950 0.00 0.00
D
ATOM 13649 HD2 ARG D 216 −45.571 37.832 22.442 0.00 0.00
D
ATOM 13650 NE ARG D 216 −45.376 39.342 20.815 0.00 0.00
D
ATOM 13651 HE ARG D 216 −44.694 39.978 20.455 0.00 0.00
D
ATOM 13652 CZ ARG D 216 −46.646 39.625 20.493 0.00 0.00
D
ATOM 13653 NH1 ARG D 216 −47.681 38.811 20.620 0.00 0.00
D
ATOM 13654 HH11 ARG D 216 −48.588 39.194 20.441 0.00 0.00
D
ATOM 13655 HH12 ARG D 216 −47.514 37.984 21.156 0.00 0.00
D
ATOM 13656 NH2 ARG D 216 −46.881 40.743 19.882 0.00 0.00
D
ATOM 13657 HH21 ARG D 216 −47.752 40.960 19.440 0.00 0.00
D
ATOM 13658 HH22 ARG D 216 −46.215 41.481 19.985 0.00 0.00
D
ATOM 13659 C ARG D 216 −41.815 39.575 24.275 0.00 0.00
D
ATOM 13660 O ARG D 216 −41.388 40.402 23.502 0.00 0.00
D
ATOM 13661 N PHE D 217 −42.336 39.912 25.505 0.00 0.00
D

ATOM	13662	HN	PHE	D	217	−42.882	39.292	26.063	0.00	0.00	D
ATOM	13663	CA	PHE	D	217	−42.283	41.186	26.044	0.00	0.00	D
ATOM	13664	HA	PHE	D	217	−42.802	41.785	25.311	0.00	0.00	D
ATOM	13665	CB	PHE	D	217	−43.055	41.357	27.380	0.00	0.00	D
ATOM	13666	HB1	PHE	D	217	−44.044	40.881	27.202	0.00	0.00	D
ATOM	13667	HB2	PHE	D	217	−42.535	40.766	28.164	0.00	0.00	D
ATOM	13668	CG	PHE	D	217	−43.338	42.731	27.903	0.00	0.00	D
ATOM	13669	CD1	PHE	D	217	−42.689	43.178	29.036	0.00	0.00	D
ATOM	13670	HD1	PHE	D	217	−42.062	42.640	29.731	0.00	0.00	D
ATOM	13671	CE1	PHE	D	217	−42.768	44.506	29.460	0.00	0.00	D
ATOM	13672	HE1	PHE	D	217	−42.206	44.860	30.311	0.00	0.00	D
ATOM	13673	CZ	PHE	D	217	−43.303	45.456	28.576	0.00	0.00	D
ATOM	13674	HZ	PHE	D	217	−43.412	46.470	28.931	0.00	0.00	D
ATOM	13675	CD2	PHE	D	217	−43.922	43.666	27.107	0.00	0.00	D
ATOM	13676	HD2	PHE	D	217	−44.346	43.247	26.206	0.00	0.00	D
ATOM	13677	CE2	PHE	D	217	−43.825	45.100	27.325	0.00	0.00	D
ATOM	13678	HE2	PHE	D	217	−44.236	45.801	26.614	0.00	0.00	D
ATOM	13679	C	PHE	D	217	−40.831	41.815	26.140	0.00	0.00	D
ATOM	13680	O	PHE	D	217	−40.494	42.871	25.541	0.00	0.00	D
ATOM	13681	N	GLU	D	218	−39.923	41.109	26.872	0.00	0.00	D
ATOM	13682	HN	GLU	D	218	−40.065	40.268	27.389	0.00	0.00	D
ATOM	13683	CA	GLU	D	218	−38.547	41.458	26.900	0.00	0.00	D
ATOM	13684	HA	GLU	D	218	−38.425	42.364	27.475	0.00	0.00	D
ATOM	13685	CB	GLU	D	218	−37.890	40.388	27.685	0.00	0.00	D
ATOM	13686	HB1	GLU	D	218	−38.357	40.251	28.684	0.00	0.00	D
ATOM	13687	HB2	GLU	D	218	−38.156	39.410	27.229	0.00	0.00	D
ATOM	13688	CG	GLU	D	218	−36.370	40.363	27.784	0.00	0.00	D

ATOM 13689 HG1 GLU D 218 −36.022 40.338 26.729 0.00 0.00
D
ATOM 13690 HG2 GLU D 218 −35.979 41.211 28.387 0.00 0.00
D
ATOM 13691 CD GLU D 218 −35.887 39.056 28.452 0.00 0.00
D
ATOM 13692 OE1 GLU D 218 −35.994 38.852 29.715 0.00 0.00
D
ATOM 13693 OE2 GLU D 218 −35.322 38.183 27.694 0.00 0.00
D
ATOM 13694 C GLU D 218 −37.757 41.799 25.601 0.00 0.00
D
ATOM 13695 O GLU D 218 −36.960 42.763 25.499 0.00 0.00
D
ATOM 13696 N ASN D 219 −37.930 40.958 24.534 0.00 0.00
D
ATOM 13697 HN ASN D 219 −38.529 40.161 24.529 0.00 0.00
D
ATOM 13698 CA ASN D 219 −37.331 41.243 23.266 0.00 0.00
D
ATOM 13699 HA ASN D 219 −36.350 41.681 23.374 0.00 0.00
D
ATOM 13700 CB ASN D 219 −37.445 39.956 22.339 0.00 0.00
D
ATOM 13701 HB1 ASN D 219 −38.473 39.534 22.310 0.00 0.00
D
ATOM 13702 HB2 ASN D 219 −37.085 40.080 21.296 0.00 0.00
D
ATOM 13703 CG ASN D 219 −36.576 38.924 22.978 0.00 0.00
D
ATOM 13704 OD1 ASN D 219 −35.469 39.188 23.481 0.00 0.00
D
ATOM 13705 ND2 ASN D 219 −36.928 37.595 22.836 0.00 0.00
D
ATOM 13706 HD21 ASN D 219 −36.338 36.911 23.265 0.00 0.00
D
ATOM 13707 HD22 ASN D 219 −37.809 37.375 22.417 0.00 0.00
D
ATOM 13708 C ASN D 219 −38.070 42.357 22.506 0.00 0.00
D
ATOM 13709 O ASN D 219 −37.463 43.131 21.742 0.00 0.00
D
ATOM 13710 N GLU D 220 −39.423 42.398 22.626 0.00 0.00
D
ATOM 13711 HN GLU D 220 −39.914 41.787 23.242 0.00 0.00
D
ATOM 13712 CA GLU D 220 −40.170 43.135 21.561 0.00 0.00
D
ATOM 13713 HA GLU D 220 −39.644 43.321 20.635 0.00 0.00
D
ATOM 13714 CB GLU D 220 −41.516 42.459 21.223 0.00 0.00
D
ATOM 13715 HB1 GLU D 220 −41.370 41.401 20.916 0.00 0.00
D

ATOM 13716 HB2 GLU D 220 −42.015 42.491 22.215 0.00 0.00
D
ATOM 13717 CG GLU D 220 −42.451 43.088 20.210 0.00 0.00
D
ATOM 13718 HG1 GLU D 220 −42.762 44.124 20.462 0.00 0.00
D
ATOM 13719 HG2 GLU D 220 −41.960 43.300 19.236 0.00 0.00
D
ATOM 13720 CD GLU D 220 −43.700 42.222 19.869 0.00 0.00
D
ATOM 13721 OE1 GLU D 220 −44.809 42.647 20.332 0.00 0.00
D
ATOM 13722 OE2 GLU D 220 −43.701 41.215 19.136 0.00 0.00
D
ATOM 13723 C GLU D 220 −40.402 44.534 22.047 0.00 0.00
D
ATOM 13724 O GLU D 220 −39.985 45.541 21.411 0.00 0.00
D
ATOM 13725 N GLY D 221 −41.149 44.675 23.157 0.00 0.00
D
ATOM 13726 HN GLY D 221 −41.486 43.885 23.662 0.00 0.00
D
ATOM 13727 CA GLY D 221 −41.674 46.003 23.607 0.00 0.00
D
ATOM 13728 HA1 GLY D 221 −42.701 45.789 23.862 0.00 0.00
D
ATOM 13729 HA2 GLY D 221 −41.546 46.726 22.815 0.00 0.00
D
ATOM 13730 C GLY D 221 −40.967 46.487 24.717 0.00 0.00
D
ATOM 13731 O GLY D 221 −40.876 47.681 24.925 0.00 0.00
D
ATOM 13732 N MET D 222 −40.312 45.654 25.511 0.00 0.00
D
ATOM 13733 HN MET D 222 −40.538 44.685 25.434 0.00 0.00
D
ATOM 13734 CA MET D 222 −39.284 46.020 26.435 0.00 0.00
D
ATOM 13735 HA MET D 222 −39.238 47.086 26.601 0.00 0.00
D
ATOM 13736 CB MET D 222 −39.523 45.337 27.873 0.00 0.00
D
ATOM 13737 HB1 MET D 222 −40.518 45.663 28.245 0.00 0.00
D
ATOM 13738 HB2 MET D 222 −39.444 44.244 27.691 0.00 0.00
D
ATOM 13739 CG MET D 222 −38.629 45.912 28.992 0.00 0.00
D
ATOM 13740 HG1 MET D 222 −37.551 45.994 28.736 0.00 0.00
D
ATOM 13741 HG2 MET D 222 −38.998 46.956 29.084 0.00 0.00
D
ATOM 13742 SD MET D 222 −38.677 44.965 30.527 0.00 0.00
D

ATOM 13743 CE MET D 222   -37.412 43.857 30.268 0.00 0.00
D
ATOM 13744 HE1 MET D 222   -37.541 43.010 30.975 0.00 0.00
D
ATOM 13745 HE2 MET D 222   -37.432 43.456 29.232 0.00 0.00
D
ATOM 13746 HE3 MET D 222   -36.402 44.274 30.469 0.00 0.00
D
ATOM 13747 C   MET D 222   -38.002 45.608 25.910 0.00 0.00
D
ATOM 13748 O   MET D 222   -37.044 46.401 25.847 0.00 0.00
D
ATOM 13749 N   LYS E   7   -33.676 -25.054 26.140 0.00 0.00
E
ATOM 13750 HT1 LYS E   7   -33.068 -25.649 26.738 0.00 0.00
E
ATOM 13751 HT2 LYS E   7   -33.091 -24.776 25.326 0.00 0.00
E
ATOM 13752 HT3 LYS E   7   -34.440 -25.698 25.851 0.00 0.00
E
ATOM 13753 CA LYS E   7   -34.247 -23.847 26.790 0.00 0.00
E
ATOM 13754 HA LYS E   7   -35.070 -23.334 26.314 0.00 0.00
E
ATOM 13755 CB LYS E   7   -34.778 -24.034 28.228 0.00 0.00
E
ATOM 13756 HB1 LYS E   7   -34.028 -24.565 28.853 0.00 0.00
E
ATOM 13757 HB2 LYS E   7   -34.908 -22.998 28.609 0.00 0.00
E
ATOM 13758 CG LYS E   7   -35.961 -24.904 28.124 0.00 0.00
E
ATOM 13759 HG1 LYS E   7   -36.756 -24.516 27.451 0.00 0.00
E
ATOM 13760 HG2 LYS E   7   -35.628 -25.898 27.757 0.00 0.00
E
ATOM 13761 CD LYS E   7   -36.520 -25.121 29.575 0.00 0.00
E
ATOM 13762 HD1 LYS E   7   -35.821 -25.741 30.177 0.00 0.00
E
ATOM 13763 HD2 LYS E   7   -36.483 -24.106 30.025 0.00 0.00
E
ATOM 13764 CE LYS E   7   -37.920 -25.714 29.749 0.00 0.00
E
ATOM 13765 HE1 LYS E   7   -38.596 -25.194 29.037 0.00 0.00
E
ATOM 13766 HE2 LYS E   7   -37.895 -26.818 29.625 0.00 0.00
E
ATOM 13767 NZ LYS E   7   -38.377 -25.606 31.132 0.00 0.00
E
ATOM 13768 HZ1 LYS E   7   -38.258 -24.619 31.434 0.00 0.00
E
ATOM 13769 HZ2 LYS E   7   -39.379 -25.875 31.196 0.00 0.00
E

ATOM 13770 HZ3 LYS E 7 −37.913 −26.272 31.783 0.00 0.00
E
ATOM 13771 C LYS E 7 −33.184 −22.778 26.694 0.00 0.00
E
ATOM 13772 O LYS E 7 −32.092 −22.970 26.200 0.00 0.00
E
ATOM 13773 N GLN E 8 −33.379 −21.479 27.187 0.00 0.00
E
ATOM 13774 HN GLN E 8 −34.152 −21.236 27.767 0.00 0.00
E
ATOM 13775 CA GLN E 8 −32.355 −20.409 27.073 0.00 0.00
E
ATOM 13776 HA GLN E 8 −31.396 −20.877 26.906 0.00 0.00
E
ATOM 13777 CB GLN E 8 −32.694 −19.471 25.873 0.00 0.00
E
ATOM 13778 HB1 GLN E 8 −33.582 −18.864 26.152 0.00 0.00
E
ATOM 13779 HB2 GLN E 8 −31.877 −18.722 25.785 0.00 0.00
E
ATOM 13780 CG GLN E 8 −33.120 −20.103 24.563 0.00 0.00
E
ATOM 13781 HG1 GLN E 8 −32.588 −21.019 24.230 0.00 0.00
E
ATOM 13782 HG2 GLN E 8 −34.201 −20.358 24.609 0.00 0.00
E
ATOM 13783 CD GLN E 8 −32.939 −19.122 23.374 0.00 0.00
E
ATOM 13784 OE1 GLN E 8 −32.298 −19.470 22.416 0.00 0.00
E
ATOM 13785 NE2 GLN E 8 −33.648 −17.978 23.346 0.00 0.00
E
ATOM 13786 HE21 GLN E 8 −33.493 −17.328 22.602 0.00 0.00
E
ATOM 13787 HE22 GLN E 8 −34.294 −17.838 24.096 0.00 0.00
E
ATOM 13788 C GLN E 8 −32.231 −19.584 28.333 0.00 0.00
E
ATOM 13789 O GLN E 8 −33.223 −19.168 28.910 0.00 0.00
E
ATOM 13790 N PHE E 9 −31.081 −19.229 28.780 0.00 0.00
E
ATOM 13791 HN PHE E 9 −30.266 −19.432 28.242 0.00 0.00
E
ATOM 13792 CA PHE E 9 −30.812 −18.575 30.073 0.00 0.00
E
ATOM 13793 HA PHE E 9 −31.712 −18.069 30.389 0.00 0.00
E
ATOM 13794 CB PHE E 9 −30.211 −19.627 31.062 0.00 0.00
E
ATOM 13795 HB1 PHE E 9 −29.208 −20.016 30.785 0.00 0.00
E
ATOM 13796 HB2 PHE E 9 −30.087 −19.210 32.084 0.00 0.00
E

ATOM 13797 CG PHE E   9   −31.096 −20.775 31.316 0.00 0.00
E
ATOM 13798 CD1 PHE E   9   −32.124 −20.626 32.222 0.00 0.00
E
ATOM 13799 HD1 PHE E   9   −32.186 −19.655 32.692 0.00 0.00
E
ATOM 13800 CE1 PHE E   9   −33.050 −21.671 32.416 0.00 0.00
E
ATOM 13801 HE1 PHE E   9   −33.799 −21.494 33.174 0.00 0.00
E
ATOM 13802 CZ PHE E   9   −32.925 −22.858 31.670 0.00 0.00
E
ATOM 13803 HZ PHE E   9   −33.617 −23.669 31.842 0.00 0.00
E
ATOM 13804 CD2 PHE E   9   −30.949 −21.971 30.600 0.00 0.00
E
ATOM 13805 HD2 PHE E   9   −30.136 −22.129 29.907 0.00 0.00
E
ATOM 13806 CE2 PHE E   9   −31.796 −23.032 30.838 0.00 0.00
E
ATOM 13807 HE2 PHE E   9   −31.630 −23.957 30.307 0.00 0.00
E
ATOM 13808 C   PHE E   9   −29.604 −17.559 30.044 0.00 0.00
E
ATOM 13809 O   PHE E   9   −28.636 −17.709 29.265 0.00 0.00
E
ATOM 13810 N   ALA E 10   −29.640 −16.622 30.923 0.00 0.00
E
ATOM 13811 HN ALA E 10   −30.468 −16.407 31.434 0.00 0.00
E
ATOM 13812 CA ALA E 10   −28.523 −15.811 31.256 0.00 0.00
E
ATOM 13813 HA ALA E 10   −27.738 −16.016 30.544 0.00 0.00
E
ATOM 13814 CB ALA E 10   −28.806 −14.383 30.777 0.00 0.00
E
ATOM 13815 HB1 ALA E 10   −29.576 −13.791 31.316 0.00 0.00
E
ATOM 13816 HB2 ALA E 10   −27.900 −13.741 30.783 0.00 0.00
E
ATOM 13817 HB3 ALA E 10   −29.093 −14.361 29.704 0.00 0.00
E
ATOM 13818 C   ALA E 10   −27.998 −15.968 32.657 0.00 0.00
E
ATOM 13819 O   ALA E 10   −28.698 −16.090 33.671 0.00 0.00
E
ATOM 13820 N   VAL E 11   −26.711 −15.869 32.754 0.00 0.00
E
ATOM 13821 HN VAL E 11   −26.187 −15.615 31.945 0.00 0.00
E
ATOM 13822 CA VAL E 11   −25.969 −15.802 33.932 0.00 0.00
E
ATOM 13823 HA VAL E 11   −26.638 −15.866 34.777 0.00 0.00
E

ATOM 13824 CB VAL E 11 -24.930 -16.921 33.964 0.00 0.00
E
ATOM 13825 HB VAL E 11 -24.136 -16.863 33.189 0.00 0.00
E
ATOM 13826 CG1 VAL E 11 -24.249 -16.995 35.292 0.00 0.00
E
ATOM 13827 HG11 VAL E 11 -24.953 -16.748 36.115 0.00 0.00
E
ATOM 13828 HG12 VAL E 11 -23.818 -18.011 35.416 0.00 0.00
E
ATOM 13829 HG13 VAL E 11 -23.444 -16.233 35.219 0.00 0.00
E
ATOM 13830 CG2 VAL E 11 -25.789 -18.224 33.876 0.00 0.00
E
ATOM 13831 HG21 VAL E 11 -26.543 -18.108 34.684 0.00 0.00
E
ATOM 13832 HG22 VAL E 11 -26.290 -18.372 32.895 0.00 0.00
E
ATOM 13833 HG23 VAL E 11 -25.230 -19.178 33.983 0.00 0.00
E
ATOM 13834 C VAL E 11 -25.187 -14.559 33.979 0.00 0.00
E
ATOM 13835 O VAL E 11 -24.361 -14.396 33.124 0.00 0.00
E
ATOM 13836 N ILE E 12 -25.344 -13.740 35.010 0.00 0.00
E
ATOM 13837 HN ILE E 12 -25.985 -14.015 35.722 0.00 0.00
E
ATOM 13838 CA ILE E 12 -24.707 -12.510 35.030 0.00 0.00
E
ATOM 13839 HA ILE E 12 -24.010 -12.344 34.222 0.00 0.00
E
ATOM 13840 CB ILE E 12 -25.743 -11.396 35.173 0.00 0.00
E
ATOM 13841 HB ILE E 12 -26.339 -11.576 36.093 0.00 0.00
E
ATOM 13842 CG2 ILE E 12 -24.974 -10.094 35.353 0.00 0.00
E
ATOM 13843 HG21 ILE E 12 -25.694 -9.250 35.405 0.00 0.00
E
ATOM 13844 HG22 ILE E 12 -24.276 -10.062 36.217 0.00 0.00
E
ATOM 13845 HG23 ILE E 12 -24.443 -9.967 34.386 0.00 0.00
E
ATOM 13846 CG1 ILE E 12 -26.955 -11.561 34.175 0.00 0.00
E
ATOM 13847 HG11 ILE E 12 -27.402 -12.554 34.397 0.00 0.00
E
ATOM 13848 HG12 ILE E 12 -27.762 -10.882 34.526 0.00 0.00
E
ATOM 13849 CD ILE E 12 -26.703 -11.335 32.700 0.00 0.00
E
ATOM 13850 HD1 ILE E 12 -27.615 -11.442 32.074 0.00 0.00
E

```
ATOM  13851  HD2 ILE E  12      -26.270 -10.325  32.540  0.00  0.00
E
ATOM  13852  HD3 ILE E  12      -25.943 -12.023  32.272  0.00  0.00
E
ATOM  13853  C   ILE E  12      -23.827 -12.626  36.320  0.00  0.00
E
ATOM  13854  O   ILE E  12      -24.460 -13.055  37.332  0.00  0.00
E
ATOM  13855  N   GLY E  13      -22.503 -12.463  36.283  0.00  0.00
E
ATOM  13856  HN  GLY E  13      -22.021 -12.314  35.423  0.00  0.00
E
ATOM  13857  CA  GLY E  13      -21.702 -12.644  37.463  0.00  0.00
E
ATOM  13858  HA1 GLY E  13      -22.346 -12.506  38.319  0.00  0.00
E
ATOM  13859  HA2 GLY E  13      -20.903 -11.917  37.446  0.00  0.00
E
ATOM  13860  C   GLY E  13      -21.114 -14.073  37.408  0.00  0.00
E
ATOM  13861  O   GLY E  13      -21.836 -14.949  37.904  0.00  0.00
E
ATOM  13862  N   LEU E  14      -19.897 -14.261  36.928  0.00  0.00
E
ATOM  13863  HN  LEU E  14      -19.308 -13.524  36.607  0.00  0.00
E
ATOM  13864  CA  LEU E  14      -19.315 -15.621  36.669  0.00  0.00
E
ATOM  13865  HA  LEU E  14      -20.091 -16.371  36.621  0.00  0.00
E
ATOM  13866  CB  LEU E  14      -18.701 -15.740  35.313  0.00  0.00
E
ATOM  13867  HB1 LEU E  14      -17.886 -14.994  35.193  0.00  0.00
E
ATOM  13868  HB2 LEU E  14      -18.349 -16.787  35.197  0.00  0.00
E
ATOM  13869  CG  LEU E  14      -19.670 -15.436  34.134  0.00  0.00
E
ATOM  13870  HG  LEU E  14      -20.179 -14.496  34.440  0.00  0.00
E
ATOM  13871  CD1 LEU E  14      -18.900 -15.260  32.873  0.00  0.00
E
ATOM  13872 HD11 LEU E  14      -18.005 -14.642  33.100  0.00  0.00
E
ATOM  13873 HD12 LEU E  14      -18.546 -16.219  32.438  0.00  0.00
E
ATOM  13874 HD13 LEU E  14      -19.526 -14.753  32.107  0.00  0.00
E
ATOM  13875  CD2 LEU E  14      -20.696 -16.548  33.999  0.00  0.00
E
ATOM  13876 HD21 LEU E  14      -21.668 -16.220  33.573  0.00  0.00
E
ATOM  13877 HD22 LEU E  14      -20.277 -17.283  33.279  0.00  0.00
E
```

ATOM 13878 HD23 LEU E 14 −20.866 −16.994 35.002 0.00 0.00
E
ATOM 13879 C LEU E 14 −18.283 −15.982 37.727 0.00 0.00
E
ATOM 13880 O LEU E 14 −17.142 −16.287 37.455 0.00 0.00
E
ATOM 13881 N GLY E 15 −18.744 −15.878 38.977 0.00 0.00
E
ATOM 13882 HN GLY E 15 −19.562 −15.331 39.139 0.00 0.00
E
ATOM 13883 CA GLY E 15 −18.020 −16.194 40.177 0.00 0.00
E
ATOM 13884 HA1 GLY E 15 −18.733 −16.031 40.971 0.00 0.00
E
ATOM 13885 HA2 GLY E 15 −17.154 −15.553 40.258 0.00 0.00
E
ATOM 13886 C GLY E 15 −17.528 −17.583 40.355 0.00 0.00
E
ATOM 13887 O GLY E 15 −17.460 −18.434 39.441 0.00 0.00
E
ATOM 13888 N ARG E 16 −17.177 −17.936 41.597 0.00 0.00
E
ATOM 13889 HN ARG E 16 −17.168 −17.207 42.277 0.00 0.00
E
ATOM 13890 CA ARG E 16 −17.007 −19.332 41.923 0.00 0.00
E
ATOM 13891 HA ARG E 16 −16.230 −19.732 41.289 0.00 0.00
E
ATOM 13892 CB ARG E 16 −16.485 −19.351 43.411 0.00 0.00
E
ATOM 13893 HB1 ARG E 16 −17.205 −18.920 44.139 0.00 0.00
E
ATOM 13894 HB2 ARG E 16 −16.275 −20.413 43.662 0.00 0.00
E
ATOM 13895 CG ARG E 16 −15.152 −18.520 43.583 0.00 0.00
E
ATOM 13896 HG1 ARG E 16 −14.499 −18.974 42.807 0.00 0.00
E
ATOM 13897 HG2 ARG E 16 −15.421 −17.458 43.401 0.00 0.00
E
ATOM 13898 CD ARG E 16 −14.278 −18.703 44.792 0.00 0.00
E
ATOM 13899 HD1 ARG E 16 −14.071 −19.751 45.098 0.00 0.00
E
ATOM 13900 HD2 ARG E 16 −13.271 −18.238 44.740 0.00 0.00
E
ATOM 13901 NE ARG E 16 −15.081 −17.999 45.785 0.00 0.00
E
ATOM 13902 HE ARG E 16 −16.008 −17.789 45.475 0.00 0.00
E
ATOM 13903 CZ ARG E 16 −14.742 −17.879 47.108 0.00 0.00
E
ATOM 13904 NH1 ARG E 16 −13.650 −18.351 47.623 0.00 0.00
E

```
ATOM  13905 HH11 ARG E  16      -13.371 -18.102  48.551  0.00  0.00
E
ATOM  13906 HH12 ARG E  16      -13.153 -18.866  46.925  0.00  0.00
E
ATOM  13907  NH2 ARG E  16      -15.639 -17.284  47.906  0.00  0.00
E
ATOM  13908 HH21 ARG E  16      -15.609 -17.364  48.902  0.00  0.00
E
ATOM  13909 HH22 ARG E  16      -16.540 -17.185  47.484  0.00  0.00
E
ATOM  13910  C   ARG E  16      -18.353 -20.130  41.731  0.00  0.00
E
ATOM  13911  O   ARG E  16      -18.240 -21.282  41.382  0.00  0.00
E
ATOM  13912  N   PHE E  17      -19.473 -19.679  42.191  0.00  0.00
E
ATOM  13913  HN  PHE E  17      -19.534 -18.771  42.597  0.00  0.00
E
ATOM  13914  CA  PHE E  17      -20.722 -20.223  41.784  0.00  0.00
E
ATOM  13915  HA  PHE E  17      -20.721 -21.302  41.746  0.00  0.00
E
ATOM  13916  CB  PHE E  17      -21.712 -19.651  42.800  0.00  0.00
E
ATOM  13917  HB1 PHE E  17      -21.335 -19.931  43.807  0.00  0.00
E
ATOM  13918  HB2 PHE E  17      -21.647 -18.542  42.802  0.00  0.00
E
ATOM  13919  CG  PHE E  17      -23.113 -20.097  42.606  0.00  0.00
E
ATOM  13920  CD1 PHE E  17      -23.508 -21.298  43.201  0.00  0.00
E
ATOM  13921  HD1 PHE E  17      -22.821 -21.840  43.834  0.00  0.00
E
ATOM  13922  CE1 PHE E  17      -24.793 -21.749  43.165  0.00  0.00
E
ATOM  13923  HE1 PHE E  17      -25.058 -22.671  43.660  0.00  0.00
E
ATOM  13924  CZ  PHE E  17      -25.776 -21.014  42.517  0.00  0.00
E
ATOM  13925  HZ  PHE E  17      -26.810 -21.323  42.463  0.00  0.00
E
ATOM  13926  CD2 PHE E  17      -24.060 -19.358  41.926  0.00  0.00
E
ATOM  13927  HD2 PHE E  17      -23.783 -18.503  41.329  0.00  0.00
E
ATOM  13928  CE2 PHE E  17      -25.363 -19.793  41.857  0.00  0.00
E
ATOM  13929  HE2 PHE E  17      -26.131 -19.149  41.456  0.00  0.00
E
ATOM  13930  C   PHE E  17      -21.064 -20.013  40.286  0.00  0.00
E
ATOM  13931  O   PHE E  17      -21.479 -20.957  39.551  0.00  0.00
E
```

ATOM 13932 N GLY E 18 -20.869 -18.746 39.759 0.00 0.00
E
ATOM 13933 HN GLY E 18 -20.368 -18.015 40.217 0.00 0.00
E
ATOM 13934 CA GLY E 18 -21.506 -18.328 38.512 0.00 0.00
E
ATOM 13935 HA1 GLY E 18 -21.451 -17.261 38.355 0.00 0.00
E
ATOM 13936 HA2 GLY E 18 -22.574 -18.485 38.540 0.00 0.00
E
ATOM 13937 C GLY E 18 -20.915 -18.969 37.328 0.00 0.00
E
ATOM 13938 O GLY E 18 -21.551 -19.278 36.384 0.00 0.00
E
ATOM 13939 N GLY E 19 -19.581 -19.187 37.371 0.00 0.00
E
ATOM 13940 HN GLY E 19 -19.046 -18.825 38.130 0.00 0.00
E
ATOM 13941 CA GLY E 19 -19.021 -20.055 36.302 0.00 0.00
E
ATOM 13942 HA1 GLY E 19 -17.943 -19.995 36.338 0.00 0.00
E
ATOM 13943 HA2 GLY E 19 -19.255 -19.736 35.298 0.00 0.00
E
ATOM 13944 C GLY E 19 -19.357 -21.504 36.336 0.00 0.00
E
ATOM 13945 O GLY E 19 -19.262 -22.150 35.288 0.00 0.00
E
ATOM 13946 N SER E 20 -19.691 -22.073 37.490 0.00 0.00
E
ATOM 13947 HN SER E 20 -19.576 -21.531 38.319 0.00 0.00
E
ATOM 13948 CA SER E 20 -20.238 -23.409 37.742 0.00 0.00
E
ATOM 13949 HA SER E 20 -19.596 -24.000 37.106 0.00 0.00
E
ATOM 13950 CB SER E 20 -20.129 -23.972 39.183 0.00 0.00
E
ATOM 13951 HB1 SER E 20 -19.164 -23.579 39.568 0.00 0.00
E
ATOM 13952 HB2 SER E 20 -20.922 -23.731 39.922 0.00 0.00
E
ATOM 13953 OG SER E 20 -20.236 -25.418 39.173 0.00 0.00
E
ATOM 13954 HG1 SER E 20 -20.405 -25.641 40.091 0.00 0.00
E
ATOM 13955 C SER E 20 -21.660 -23.622 37.212 0.00 0.00
E
ATOM 13956 O SER E 20 -21.851 -24.521 36.416 0.00 0.00
E
ATOM 13957 N ILE E 21 -22.613 -22.687 37.451 0.00 0.00
E
ATOM 13958 HN ILE E 21 -22.421 -22.046 38.190 0.00 0.00
E

```
ATOM  13959  CA  ILE E  21      -24.007 -22.790  36.921  0.00  0.00
E
ATOM  13960  HA  ILE E  21      -24.361 -23.798  37.081  0.00  0.00
E
ATOM  13961  CB  ILE E  21      -25.106 -21.917  37.537  0.00  0.00
E
ATOM  13962  HB  ILE E  21      -26.003 -21.747  36.904  0.00  0.00
E
ATOM  13963  CG2 ILE E  21      -25.506 -22.470  38.894  0.00  0.00
E
ATOM  13964 HG21 ILE E  21      -24.567 -22.553  39.482  0.00  0.00
E
ATOM  13965 HG22 ILE E  21      -26.296 -21.879  39.405  0.00  0.00
E
ATOM  13966 HG23 ILE E  21      -25.901 -23.490  38.700  0.00  0.00
E
ATOM  13967  CG1 ILE E  21      -24.560 -20.523  37.608  0.00  0.00
E
ATOM  13968 HG11 ILE E  21      -23.880 -20.470  38.485  0.00  0.00
E
ATOM  13969 HG12 ILE E  21      -24.009 -20.366  36.656  0.00  0.00
E
ATOM  13970  CD  ILE E  21      -25.737 -19.488  37.776  0.00  0.00
E
ATOM  13971  HD1 ILE E  21      -26.534 -19.627  37.014  0.00  0.00
E
ATOM  13972  HD2 ILE E  21      -26.104 -19.421  38.822  0.00  0.00
E
ATOM  13973  HD3 ILE E  21      -25.325 -18.475  37.581  0.00  0.00
E
ATOM  13974  C   ILE E  21      -24.106 -22.718  35.399  0.00  0.00
E
ATOM  13975  O   ILE E  21      -24.883 -23.482  34.813  0.00  0.00
E
ATOM  13976  N   VAL E  22      -23.172 -22.033  34.744  0.00  0.00
E
ATOM  13977  HN  VAL E  22      -22.620 -21.402  35.285  0.00  0.00
E
ATOM  13978  CA  VAL E  22      -22.984 -22.049  33.354  0.00  0.00
E
ATOM  13979  HA  VAL E  22      -23.956 -21.805  32.950  0.00  0.00
E
ATOM  13980  CB  VAL E  22      -22.022 -20.931  32.981  0.00  0.00
E
ATOM  13981  HB  VAL E  22      -21.150 -21.066  33.657  0.00  0.00
E
ATOM  13982  CG1 VAL E  22      -21.575 -20.850  31.523  0.00  0.00
E
ATOM  13983 HG11 VAL E  22      -20.467 -20.789  31.576  0.00  0.00
E
ATOM  13984 HG12 VAL E  22      -21.863 -21.774  30.977  0.00  0.00
E
ATOM  13985 HG13 VAL E  22      -21.994 -19.952  31.022  0.00  0.00
E
```

ATOM 13986 CG2 VAL E 22 −22.587 −19.551 33.373 0.00 0.00
E
ATOM 13987 HG21 VAL E 22 −23.226 −19.090 32.590 0.00 0.00
E
ATOM 13988 HG22 VAL E 22 −23.213 −19.528 34.291 0.00 0.00
E
ATOM 13989 HG23 VAL E 22 −21.774 −18.802 33.488 0.00 0.00
E
ATOM 13990 C VAL E 22 −22.600 −23.368 32.682 0.00 0.00
E
ATOM 13991 O VAL E 22 −23.287 −23.859 31.794 0.00 0.00
E
ATOM 13992 N LYS E 23 −21.600 −23.971 33.316 0.00 0.00
E
ATOM 13993 HN LYS E 23 −21.202 −23.558 34.132 0.00 0.00
E
ATOM 13994 CA LYS E 23 −21.105 −25.362 32.962 0.00 0.00
E
ATOM 13995 HA LYS E 23 −20.770 −25.233 31.944 0.00 0.00
E
ATOM 13996 CB LYS E 23 −20.030 −25.916 33.971 0.00 0.00
E
ATOM 13997 HB1 LYS E 23 −20.487 −25.825 34.979 0.00 0.00
E
ATOM 13998 HB2 LYS E 23 −19.894 −26.989 33.715 0.00 0.00
E
ATOM 13999 CG LYS E 23 −18.768 −25.118 33.983 0.00 0.00
E
ATOM 14000 HG1 LYS E 23 −18.215 −25.217 33.024 0.00 0.00
E
ATOM 14001 HG2 LYS E 23 −19.140 −24.103 34.241 0.00 0.00
E
ATOM 14002 CD LYS E 23 −17.873 −25.628 35.136 0.00 0.00
E
ATOM 14003 HD1 LYS E 23 −18.508 −25.796 36.032 0.00 0.00
E
ATOM 14004 HD2 LYS E 23 −17.460 −26.597 34.781 0.00 0.00
E
ATOM 14005 CE LYS E 23 −16.809 −24.555 35.317 0.00 0.00
E
ATOM 14006 HE1 LYS E 23 −16.397 −24.237 34.335 0.00 0.00
E
ATOM 14007 HE2 LYS E 23 −17.216 −23.727 35.937 0.00 0.00
E
ATOM 14008 NZ LYS E 23 −15.656 −25.043 36.085 0.00 0.00
E
ATOM 14009 HZ1 LYS E 23 −15.997 −25.456 36.976 0.00 0.00
E
ATOM 14010 HZ2 LYS E 23 −15.295 −25.868 35.564 0.00 0.00
E
ATOM 14011 HZ3 LYS E 23 −14.854 −24.392 36.203 0.00 0.00
E
ATOM 14012 C LYS E 23 −22.199 −26.342 32.934 0.00 0.00
E

ATOM 14013 O LYS E 23 −22.428 −27.050 31.963 0.00 0.00
E
ATOM 14014 N GLU E 24 −22.914 −26.487 34.039 0.00 0.00
E
ATOM 14015 HN GLU E 24 −22.621 −26.013 34.865 0.00 0.00
E
ATOM 14016 CA GLU E 24 −24.003 −27.379 34.234 0.00 0.00
E
ATOM 14017 HA GLU E 24 −23.732 −28.392 33.974 0.00 0.00
E
ATOM 14018 CB GLU E 24 −24.573 −27.230 35.624 0.00 0.00
E
ATOM 14019 HB1 GLU E 24 −25.028 −26.253 35.893 0.00 0.00
E
ATOM 14020 HB2 GLU E 24 −25.392 −27.976 35.721 0.00 0.00
E
ATOM 14021 CG GLU E 24 −23.563 −27.603 36.711 0.00 0.00
E
ATOM 14022 HG1 GLU E 24 −22.630 −27.003 36.641 0.00 0.00
E
ATOM 14023 HG2 GLU E 24 −23.973 −27.358 37.714 0.00 0.00
E
ATOM 14024 CD GLU E 24 −23.045 −29.019 36.593 0.00 0.00
E
ATOM 14025 OE1 GLU E 24 −21.851 −29.301 36.863 0.00 0.00
E
ATOM 14026 OE2 GLU E 24 −23.797 −29.866 36.064 0.00 0.00
E
ATOM 14027 C GLU E 24 −25.185 −27.056 33.272 0.00 0.00
E
ATOM 14028 O GLU E 24 −25.626 −27.960 32.563 0.00 0.00
E
ATOM 14029 N LEU E 25 −25.599 −25.812 33.009 0.00 0.00
E
ATOM 14030 HN LEU E 25 −25.359 −25.014 33.556 0.00 0.00
E
ATOM 14031 CA LEU E 25 −26.626 −25.520 31.963 0.00 0.00
E
ATOM 14032 HA LEU E 25 −27.503 −26.143 32.054 0.00 0.00
E
ATOM 14033 CB LEU E 25 −27.000 −24.029 31.989 0.00 0.00
E
ATOM 14034 HB1 LEU E 25 −26.036 −23.481 31.921 0.00 0.00
E
ATOM 14035 HB2 LEU E 25 −27.460 −23.769 31.011 0.00 0.00
E
ATOM 14036 CG LEU E 25 −27.827 −23.450 33.170 0.00 0.00
E
ATOM 14037 HG LEU E 25 −27.235 −23.609 34.097 0.00 0.00
E
ATOM 14038 CD1 LEU E 25 −28.035 −22.006 32.975 0.00 0.00
E
ATOM 14039 HD11 LEU E 25 −28.662 −21.558 33.775 0.00 0.00
E

```
ATOM  14040 HD12 LEU E  25      -27.097 -21.417  33.071  0.00  0.00
E
ATOM  14041 HD13 LEU E  25      -28.549 -21.856  32.002  0.00  0.00
E
ATOM  14042  CD2 LEU E  25      -29.162 -24.247  33.245  0.00  0.00
E
ATOM  14043 HD21 LEU E  25      -28.952 -25.315  33.470  0.00  0.00
E
ATOM  14044 HD22 LEU E  25      -29.796 -23.737  34.001  0.00  0.00
E
ATOM  14045 HD23 LEU E  25      -29.731 -24.260  32.290  0.00  0.00
E
ATOM  14046  C   LEU E  25      -26.077 -25.960  30.590  0.00  0.00
E
ATOM  14047  O   LEU E  25      -26.933 -26.528  29.935  0.00  0.00
E
ATOM  14048  N   HSE E  26      -24.770 -25.773  30.213  0.00  0.00
E
ATOM  14049  HN  HSE E  26      -24.166 -25.415  30.920  0.00  0.00
E
ATOM  14050  CA  HSE E  26      -24.180 -26.146  28.947  0.00  0.00
E
ATOM  14051  HA  HSE E  26      -24.876 -25.756  28.219  0.00  0.00
E
ATOM  14052  CB  HSE E  26      -22.804 -25.526  28.787  0.00  0.00
E
ATOM  14053  HB1 HSE E  26      -22.922 -24.467  29.100  0.00  0.00
E
ATOM  14054  HB2 HSE E  26      -22.004 -25.877  29.474  0.00  0.00
E
ATOM  14055  ND1 HSE E  26      -23.061 -24.590  26.515  0.00  0.00
E
ATOM  14056  CG  HSE E  26      -22.370 -25.456  27.366  0.00  0.00
E
ATOM  14057  CE1 HSE E  26      -22.470 -24.759  25.337  0.00  0.00
E
ATOM  14058  HE1 HSE E  26      -22.450 -24.164  24.424  0.00  0.00
E
ATOM  14059  NE2 HSE E  26      -21.464 -25.651  25.476  0.00  0.00
E
ATOM  14060  HE2 HSE E  26      -20.845 -25.910  24.735  0.00  0.00
E
ATOM  14061  CD2 HSE E  26      -21.373 -26.089  26.714  0.00  0.00
E
ATOM  14062  HD2 HSE E  26      -20.575 -26.737  27.056  0.00  0.00
E
ATOM  14063  C   HSE E  26      -23.978 -27.643  28.785  0.00  0.00
E
ATOM  14064  O   HSE E  26      -24.019 -28.132  27.670  0.00  0.00
E
ATOM  14065  N   ARG E  27      -23.839 -28.418  29.931  0.00  0.00
E
ATOM  14066  HN  ARG E  27      -23.510 -27.965  30.756  0.00  0.00
E
```

ATOM 14067 CA ARG E 27 −23.774 −29.836 29.920 0.00 0.00
E
ATOM 14068 HA ARG E 27 −23.024 −30.131 29.201 0.00 0.00
E
ATOM 14069 CB ARG E 27 −23.429 −30.375 31.377 0.00 0.00
E
ATOM 14070 HB1 ARG E 27 −22.616 −29.731 31.774 0.00 0.00
E
ATOM 14071 HB2 ARG E 27 −24.275 −30.092 32.039 0.00 0.00
E
ATOM 14072 CG ARG E 27 −23.141 −31.869 31.618 0.00 0.00
E
ATOM 14073 HG1 ARG E 27 −24.141 −32.291 31.380 0.00 0.00
E
ATOM 14074 HG2 ARG E 27 −22.299 −32.207 30.977 0.00 0.00
E
ATOM 14075 CD ARG E 27 −22.688 −32.194 33.027 0.00 0.00
E
ATOM 14076 HD1 ARG E 27 −22.497 −33.275 33.196 0.00 0.00
E
ATOM 14077 HD2 ARG E 27 −21.745 −31.671 33.294 0.00 0.00
E
ATOM 14078 NE ARG E 27 −23.657 −31.709 34.084 0.00 0.00
E
ATOM 14079 HE ARG E 27 −23.484 −30.881 34.617 0.00 0.00
E
ATOM 14080 CZ ARG E 27 −24.906 −32.161 34.147 0.00 0.00
E
ATOM 14081 NH1 ARG E 27 −25.432 −33.148 33.436 0.00 0.00
E
ATOM 14082 HH11 ARG E 27 −26.409 −33.361 33.431 0.00 0.00
E
ATOM 14083 HH12 ARG E 27 −24.866 −33.800 32.932 0.00 0.00
E
ATOM 14084 NH2 ARG E 27 −25.773 −31.431 34.881 0.00 0.00
E
ATOM 14085 HH21 ARG E 27 −26.576 −31.886 35.266 0.00 0.00
E
ATOM 14086 HH22 ARG E 27 −25.303 −30.779 35.477 0.00 0.00
E
ATOM 14087 C ARG E 27 −25.128 −30.373 29.607 0.00 0.00
E
ATOM 14088 O ARG E 27 −25.253 −31.361 28.913 0.00 0.00
E
ATOM 14089 N MET E 28 −26.195 −29.699 30.060 0.00 0.00
E
ATOM 14090 HN MET E 28 −26.074 −28.904 30.649 0.00 0.00
E
ATOM 14091 CA MET E 28 −27.623 −30.247 29.777 0.00 0.00
E
ATOM 14092 HA MET E 28 −27.752 −31.304 29.956 0.00 0.00
E
ATOM 14093 CB MET E 28 −28.532 −29.604 30.791 0.00 0.00
E

ATOM 14094 HB1 MET E 28   -28.456 -28.501 30.897 0.00 0.00
E
ATOM 14095 HB2 MET E 28   -29.596 -29.849 30.585 0.00 0.00
E
ATOM 14096 CG MET E 28   -28.408 -30.189 32.224 0.00 0.00
E
ATOM 14097 HG1 MET E 28   -28.389 -31.292 32.095 0.00 0.00
E
ATOM 14098 HG2 MET E 28   -27.444 -29.803 32.620 0.00 0.00
E
ATOM 14099 SD MET E 28   -29.796 -29.936 33.303 0.00 0.00
E
ATOM 14100 CE MET E 28   -29.364 -28.216 33.757 0.00 0.00
E
ATOM 14101 HE1 MET E 28   -30.078 -27.947 34.565 0.00 0.00
E
ATOM 14102 HE2 MET E 28   -28.310 -28.090 34.085 0.00 0.00
E
ATOM 14103 HE3 MET E 28   -29.427 -27.505 32.905 0.00 0.00
E
ATOM 14104 C   MET E 28   -28.136 -29.971 28.360 0.00 0.00
E
ATOM 14105 O   MET E 28   -29.107 -30.640 28.033 0.00 0.00
E
ATOM 14106 N   GLY E 29   -27.536 -29.028 27.556 0.00 0.00
E
ATOM 14107 HN GLY E 29   -26.718 -28.602 27.935 0.00 0.00
E
ATOM 14108 CA GLY E 29   -27.989 -28.712 26.189 0.00 0.00
E
ATOM 14109 HA1 GLY E 29   -28.420 -29.597 25.743 0.00 0.00
E
ATOM 14110 HA2 GLY E 29   -27.062 -28.428 25.714 0.00 0.00
E
ATOM 14111 C   GLY E 29   -29.014 -27.686 26.084 0.00 0.00
E
ATOM 14112 O   GLY E 29   -30.013 -27.729 25.413 0.00 0.00
E
ATOM 14113 N   HSE E 30   -28.707 -26.523 26.756 0.00 0.00
E
ATOM 14114 HN HSE E 30   -27.885 -26.541 27.320 0.00 0.00
E
ATOM 14115 CA HSE E 30   -29.542 -25.294 26.709 0.00 0.00
E
ATOM 14116 HA HSE E 30   -30.259 -25.384 25.907 0.00 0.00
E
ATOM 14117 CB HSE E 30   -30.170 -25.030 28.087 0.00 0.00
E
ATOM 14118 HB1 HSE E 30   -29.483 -24.805 28.930 0.00 0.00
E
ATOM 14119 HB2 HSE E 30   -30.921 -24.235 27.891 0.00 0.00
E
ATOM 14120 ND1 HSE E 30   -32.009 -26.587 27.886 0.00 0.00
E

ATOM	14121	CG	HSE	E	30	−30.933	−26.186	28.591	0.00	0.00	E
ATOM	14122	CE1	HSE	E	30	−32.471	−27.650	28.425	0.00	0.00	E
ATOM	14123	HE1	HSE	E	30	−33.345	−28.254	28.179	0.00	0.00	E
ATOM	14124	NE2	HSE	E	30	−31.722	−27.963	29.516	0.00	0.00	E
ATOM	14125	HE2	HSE	E	30	−31.865	−28.757	30.107	0.00	0.00	E
ATOM	14126	CD2	HSE	E	30	−30.777	−26.985	29.592	0.00	0.00	E
ATOM	14127	HD2	HSE	E	30	−30.021	−26.960	30.367	0.00	0.00	E
ATOM	14128	C	HSE	E	30	−28.749	−24.155	26.368	0.00	0.00	E
ATOM	14129	O	HSE	E	30	−27.574	−24.140	26.741	0.00	0.00	E
ATOM	14130	N	GLU	E	31	−29.310	−23.149	25.757	0.00	0.00	E
ATOM	14131	HN	GLU	E	31	−30.295	−23.075	25.618	0.00	0.00	E
ATOM	14132	CA	GLU	E	31	−28.564	−21.998	25.253	0.00	0.00	E
ATOM	14133	HA	GLU	E	31	−27.625	−22.340	24.843	0.00	0.00	E
ATOM	14134	CB	GLU	E	31	−29.463	−21.072	24.423	0.00	0.00	E
ATOM	14135	HB1	GLU	E	31	−30.348	−20.795	25.035	0.00	0.00	E
ATOM	14136	HB2	GLU	E	31	−28.930	−20.115	24.239	0.00	0.00	E
ATOM	14137	CG	GLU	E	31	−29.783	−21.753	23.042	0.00	0.00	E
ATOM	14138	HG1	GLU	E	31	−30.345	−22.699	23.195	0.00	0.00	E
ATOM	14139	HG2	GLU	E	31	−30.417	−21.158	22.351	0.00	0.00	E
ATOM	14140	CD	GLU	E	31	−28.500	−22.135	22.393	0.00	0.00	E
ATOM	14141	OE1	GLU	E	31	−28.309	−23.314	22.002	0.00	0.00	E
ATOM	14142	OE2	GLU	E	31	−27.627	−21.183	22.208	0.00	0.00	E
ATOM	14143	C	GLU	E	31	−28.161	−21.184	26.512	0.00	0.00	E
ATOM	14144	O	GLU	E	31	−29.005	−20.905	27.375	0.00	0.00	E
ATOM	14145	N	VAL	E	32	−26.891	−20.827	26.673	0.00	0.00	E
ATOM	14146	HN	VAL	E	32	−26.292	−20.923	25.882	0.00	0.00	E
ATOM	14147	CA	VAL	E	32	−26.370	−20.191	27.923	0.00	0.00	E

ATOM 14148 HA VAL E 32 -27.185 -19.884 28.561 0.00 0.00
E
ATOM 14149 CB VAL E 32 -25.301 -21.084 28.598 0.00 0.00
E
ATOM 14150 HB VAL E 32 -24.298 -21.168 28.129 0.00 0.00
E
ATOM 14151 CG1 VAL E 32 -25.116 -20.470 30.041 0.00 0.00
E
ATOM 14152 HG11 VAL E 32 -24.395 -21.078 30.629 0.00 0.00
E
ATOM 14153 HG12 VAL E 32 -24.697 -19.441 30.054 0.00 0.00
E
ATOM 14154 HG13 VAL E 32 -26.103 -20.575 30.541 0.00 0.00
E
ATOM 14155 CG2 VAL E 32 -25.742 -22.513 28.833 0.00 0.00
E
ATOM 14156 HG21 VAL E 32 -25.642 -23.130 27.915 0.00 0.00
E
ATOM 14157 HG22 VAL E 32 -25.146 -22.955 29.660 0.00 0.00
E
ATOM 14158 HG23 VAL E 32 -26.830 -22.600 29.038 0.00 0.00
E
ATOM 14159 C VAL E 32 -25.649 -18.963 27.411 0.00 0.00
E
ATOM 14160 O VAL E 32 -24.707 -19.039 26.667 0.00 0.00
E
ATOM 14161 N LEU E 33 -26.087 -17.760 27.806 0.00 0.00
E
ATOM 14162 HN LEU E 33 -26.825 -17.727 28.476 0.00 0.00
E
ATOM 14163 CA LEU E 33 -25.384 -16.589 27.527 0.00 0.00
E
ATOM 14164 HA LEU E 33 -24.651 -16.721 26.745 0.00 0.00
E
ATOM 14165 CB LEU E 33 -26.350 -15.521 27.009 0.00 0.00
E
ATOM 14166 HB1 LEU E 33 -26.920 -15.923 26.144 0.00 0.00
E
ATOM 14167 HB2 LEU E 33 -27.076 -15.318 27.825 0.00 0.00
E
ATOM 14168 CG LEU E 33 -25.741 -14.145 26.521 0.00 0.00
E
ATOM 14169 HG LEU E 33 -24.770 -14.514 26.128 0.00 0.00
E
ATOM 14170 CD1 LEU E 33 -26.678 -13.521 25.528 0.00 0.00
E
ATOM 14171 HD11 LEU E 33 -27.726 -13.436 25.888 0.00 0.00
E
ATOM 14172 HD12 LEU E 33 -26.396 -12.534 25.101 0.00 0.00
E
ATOM 14173 HD13 LEU E 33 -26.837 -14.226 24.684 0.00 0.00
E
ATOM 14174 CD2 LEU E 33 -25.554 -13.106 27.619 0.00 0.00
E

ATOM 14175 HD21 LEU E 33 −26.485 −12.745 28.108 0.00 0.00
E
ATOM 14176 HD22 LEU E 33 −24.741 −13.384 28.323 0.00 0.00
E
ATOM 14177 HD23 LEU E 33 −25.288 −12.144 27.132 0.00 0.00
E
ATOM 14178 C LEU E 33 −24.732 −16.067 28.835 0.00 0.00
E
ATOM 14179 O LEU E 33 −25.401 −15.555 29.768 0.00 0.00
E
ATOM 14180 N ALA E 34 −23.398 −16.110 28.910 0.00 0.00
E
ATOM 14181 HN ALA E 34 −22.868 −16.413 28.121 0.00 0.00
E
ATOM 14182 CA ALA E 34 −22.682 −15.780 30.171 0.00 0.00
E
ATOM 14183 HA ALA E 34 −23.301 −15.847 31.053 0.00 0.00
E
ATOM 14184 CB ALA E 34 −21.441 −16.630 30.395 0.00 0.00
E
ATOM 14185 HB1 ALA E 34 −20.692 −16.008 30.931 0.00 0.00
E
ATOM 14186 HB2 ALA E 34 −21.776 −17.438 31.079 0.00 0.00
E
ATOM 14187 HB3 ALA E 34 −20.979 −17.018 29.462 0.00 0.00
E
ATOM 14188 C ALA E 34 −22.252 −14.289 30.156 0.00 0.00
E
ATOM 14189 O ALA E 34 −21.655 −13.831 29.170 0.00 0.00
E
ATOM 14190 N VAL E 35 −22.515 −13.454 31.173 0.00 0.00
E
ATOM 14191 HN VAL E 35 −23.343 −13.646 31.695 0.00 0.00
E
ATOM 14192 CA VAL E 35 −21.891 −12.108 31.328 0.00 0.00
E
ATOM 14193 HA VAL E 35 −21.247 −11.848 30.501 0.00 0.00
E
ATOM 14194 CB VAL E 35 −22.907 −10.939 31.450 0.00 0.00
E
ATOM 14195 HB VAL E 35 −23.615 −11.181 32.272 0.00 0.00
E
ATOM 14196 CG1 VAL E 35 −22.387 −9.506 31.608 0.00 0.00
E
ATOM 14197 HG11 VAL E 35 −23.265 −8.831 31.695 0.00 0.00
E
ATOM 14198 HG12 VAL E 35 −21.876 −9.362 32.584 0.00 0.00
E
ATOM 14199 HG13 VAL E 35 −21.653 −9.190 30.835 0.00 0.00
E
ATOM 14200 CG2 VAL E 35 −23.724 −11.025 30.208 0.00 0.00
E
ATOM 14201 HG21 VAL E 35 −24.636 −10.400 30.324 0.00 0.00
E

```
ATOM  14202 HG22 VAL E  35      -23.106 -10.716  29.339  0.00  0.00
E
ATOM  14203 HG23 VAL E  35      -24.035 -12.048  29.906  0.00  0.00
E
ATOM  14204  C   VAL E  35      -20.994 -11.977  32.571  0.00  0.00
E
ATOM  14205  O   VAL E  35      -21.383 -12.220  33.677  0.00  0.00
E
ATOM  14206  N   ASP E  36      -19.877 -11.380  32.322  0.00  0.00
E
ATOM  14207  HN  ASP E  36      -19.626 -11.171  31.380  0.00  0.00
E
ATOM  14208  CA  ASP E  36      -19.125 -10.805  33.426  0.00  0.00
E
ATOM  14209  HA  ASP E  36      -19.797 -10.505  34.216  0.00  0.00
E
ATOM  14210  CB  ASP E  36      -18.071 -11.838  34.057  0.00  0.00
E
ATOM  14211  HB1 ASP E  36      -18.522 -12.845  34.190  0.00  0.00
E
ATOM  14212  HB2 ASP E  36      -17.086 -11.979  33.564  0.00  0.00
E
ATOM  14213  CG  ASP E  36      -17.712 -11.516  35.496  0.00  0.00
E
ATOM  14214  OD1 ASP E  36      -18.201 -12.212  36.429  0.00  0.00
E
ATOM  14215  OD2 ASP E  36      -16.841 -10.615  35.612  0.00  0.00
E
ATOM  14216  C   ASP E  36      -18.478  -9.542  32.876  0.00  0.00
E
ATOM  14217  O   ASP E  36      -18.610  -9.224  31.667  0.00  0.00
E
ATOM  14218  N   ILE E  37      -17.791  -8.714  33.681  0.00  0.00
E
ATOM  14219  HN  ILE E  37      -17.769  -9.039  34.624  0.00  0.00
E
ATOM  14220  CA  ILE E  37      -16.987  -7.606  33.344  0.00  0.00
E
ATOM  14221  HA  ILE E  37      -17.222  -7.297  32.336  0.00  0.00
E
ATOM  14222  CB  ILE E  37      -17.314  -6.431  34.365  0.00  0.00
E
ATOM  14223  HB  ILE E  37      -18.415  -6.297  34.291  0.00  0.00
E
ATOM  14224  CG2 ILE E  37      -16.904  -6.870  35.758  0.00  0.00
E
ATOM  14225 HG21 ILE E  37      -17.002  -6.020  36.468  0.00  0.00
E
ATOM  14226 HG22 ILE E  37      -17.465  -7.760  36.116  0.00  0.00
E
ATOM  14227 HG23 ILE E  37      -15.834  -7.157  35.681  0.00  0.00
E
ATOM  14228  CG1 ILE E  37      -16.678  -5.072  33.979  0.00  0.00
E
```

ATOM 14229 HG11 ILE E 37   -17.138 -4.311 34.645 0.00 0.00
E
ATOM 14230 HG12 ILE E 37   -15.597 -5.182 34.211 0.00 0.00
E
ATOM 14231 CD ILE E 37   -16.756 -4.580 32.546 0.00 0.00
E
ATOM 14232 HD1 ILE E 37   -16.420 -3.523 32.489 0.00 0.00
E
ATOM 14233 HD2 ILE E 37   -16.133 -5.191 31.859 0.00 0.00
E
ATOM 14234 HD3 ILE E 37   -17.825 -4.563 32.242 0.00 0.00
E
ATOM 14235 C   ILE E 37   -15.503 -8.033 33.411 0.00 0.00
E
ATOM 14236 O   ILE E 37   -14.654 -7.443 32.713 0.00 0.00
E
ATOM 14237 N   ASN E 38   -15.212 -9.164 34.077 0.00 0.00
E
ATOM 14238 HN ASN E 38   -15.889 -9.585 34.676 0.00 0.00
E
ATOM 14239 CA ASN E 38   -13.873 -9.722 34.028 0.00 0.00
E
ATOM 14240 HA ASN E 38   -13.151 -8.919 34.009 0.00 0.00
E
ATOM 14241 CB ASN E 38   -13.539 -10.575 35.288 0.00 0.00
E
ATOM 14242 HB1 ASN E 38   -13.709 -9.946 36.188 0.00 0.00
E
ATOM 14243 HB2 ASN E 38   -14.175 -11.483 35.362 0.00 0.00
E
ATOM 14244 CG ASN E 38   -12.071 -11.095 35.423 0.00 0.00
E
ATOM 14245 OD1 ASN E 38   -11.328 -11.395 34.481 0.00 0.00
E
ATOM 14246 ND2 ASN E 38   -11.726 -11.300 36.737 0.00 0.00
E
ATOM 14247 HD21 ASN E 38   -10.865 -11.755 36.965 0.00 0.00
E
ATOM 14248 HD22 ASN E 38   -12.320 -11.089 37.513 0.00 0.00
E
ATOM 14249 C   ASN E 38   -13.624 -10.439 32.747 0.00 0.00
E
ATOM 14250 O   ASN E 38   -14.052 -11.544 32.534 0.00 0.00
E
ATOM 14251 N   GLU E 39   -12.696 -9.798 31.944 0.00 0.00
E
ATOM 14252 HN GLU E 39   -12.319 -8.935 32.271 0.00 0.00
E
ATOM 14253 CA GLU E 39   -12.371 -10.252 30.587 0.00 0.00
E
ATOM 14254 HA GLU E 39   -13.234 -10.156 29.945 0.00 0.00
E
ATOM 14255 CB GLU E 39   -11.185 -9.383 30.139 0.00 0.00
E

ATOM 14256 HB1 GLU E 39   −11.441 −8.307 30.240 0.00 0.00
E
ATOM 14257 HB2 GLU E 39   −10.389 −9.567 30.892 0.00 0.00
E
ATOM 14258 CG GLU E 39   −10.689 −9.700 28.694 0.00 0.00
E
ATOM 14259 HG1 GLU E 39   −9.902 −10.483 28.742 0.00 0.00
E
ATOM 14260 HG2 GLU E 39   −11.524 −10.141 28.109 0.00 0.00
E
ATOM 14261 CD GLU E 39   −10.078 −8.548 27.903 0.00 0.00
E
ATOM 14262 OE1 GLU E 39   −9.630 −7.465 28.361 0.00 0.00
E
ATOM 14263 OE2 GLU E 39   −10.116 −8.799 26.664 0.00 0.00
E
ATOM 14264 C   GLU E 39   −11.813 −11.618 30.592 0.00 0.00
E
ATOM 14265 O   GLU E 39   −12.280 −12.441 29.791 0.00 0.00
E
ATOM 14266 N   GLU E 40   −10.898 −11.968 31.544 0.00 0.00
E
ATOM 14267 HN GLU E 40   −10.637 −11.286 32.223 0.00 0.00
E
ATOM 14268 CA GLU E 40   −10.251 −13.283 31.564 0.00 0.00
E
ATOM 14269 HA GLU E 40   −9.921 −13.401 30.543 0.00 0.00
E
ATOM 14270 CB GLU E 40   −9.029 −13.350 32.483 0.00 0.00
E
ATOM 14271 HB1 GLU E 40   −9.506 −13.467 33.480 0.00 0.00
E
ATOM 14272 HB2 GLU E 40   −8.424 −14.270 32.334 0.00 0.00
E
ATOM 14273 CG GLU E 40   −8.172 −12.110 32.521 0.00 0.00
E
ATOM 14274 HG1 GLU E 40   −7.596 −12.092 31.571 0.00 0.00
E
ATOM 14275 HG2 GLU E 40   −8.771 −11.196 32.723 0.00 0.00
E
ATOM 14276 CD GLU E 40   −7.077 −12.239 33.540 0.00 0.00
E
ATOM 14277 OE1 GLU E 40   −6.176 −13.151 33.472 0.00 0.00
E
ATOM 14278 OE2 GLU E 40   −7.148 −11.424 34.604 0.00 0.00
E
ATOM 14279 C   GLU E 40   −11.232 −14.427 31.892 0.00 0.00
E
ATOM 14280 O   GLU E 40   −11.226 −15.604 31.419 0.00 0.00
E
ATOM 14281 N   LYS E 41   −12.257 −14.056 32.696 0.00 0.00
E
ATOM 14282 HN LYS E 41   −12.271 −13.127 33.059 0.00 0.00
E

ATOM 14283 CA LYS E 41 -13.287 -14.948 33.133 0.00 0.00
E
ATOM 14284 HA LYS E 41 -12.719 -15.814 33.443 0.00 0.00
E
ATOM 14285 CB LYS E 41 -14.079 -14.393 34.320 0.00 0.00
E
ATOM 14286 HB1 LYS E 41 -14.310 -13.307 34.280 0.00 0.00
E
ATOM 14287 HB2 LYS E 41 -15.004 -14.954 34.573 0.00 0.00
E
ATOM 14288 CG LYS E 41 -13.361 -14.498 35.649 0.00 0.00
E
ATOM 14289 HG1 LYS E 41 -12.883 -15.500 35.681 0.00 0.00
E
ATOM 14290 HG2 LYS E 41 -12.472 -13.849 35.495 0.00 0.00
E
ATOM 14291 CD LYS E 41 -14.211 -14.323 36.883 0.00 0.00
E
ATOM 14292 HD1 LYS E 41 -14.757 -13.356 36.915 0.00 0.00
E
ATOM 14293 HD2 LYS E 41 -14.974 -15.128 36.948 0.00 0.00
E
ATOM 14294 CE LYS E 41 -13.343 -14.260 38.164 0.00 0.00
E
ATOM 14295 HE1 LYS E 41 -12.871 -15.241 38.383 0.00 0.00
E
ATOM 14296 HE2 LYS E 41 -12.559 -13.476 38.091 0.00 0.00
E
ATOM 14297 NZ LYS E 41 -14.176 -13.896 39.424 0.00 0.00
E
ATOM 14298 HZ1 LYS E 41 -14.812 -14.639 39.780 0.00 0.00
E
ATOM 14299 HZ2 LYS E 41 -13.658 -13.458 40.212 0.00 0.00
E
ATOM 14300 HZ3 LYS E 41 -14.752 -13.093 39.100 0.00 0.00
E
ATOM 14301 C LYS E 41 -14.205 -15.218 31.997 0.00 0.00
E
ATOM 14302 O LYS E 41 -14.480 -16.374 31.711 0.00 0.00
E
ATOM 14303 N VAL E 42 -14.694 -14.218 31.276 0.00 0.00
E
ATOM 14304 HN VAL E 42 -14.301 -13.324 31.475 0.00 0.00
E
ATOM 14305 CA VAL E 42 -15.603 -14.311 30.155 0.00 0.00
E
ATOM 14306 HA VAL E 42 -16.546 -14.789 30.374 0.00 0.00
E
ATOM 14307 CB VAL E 42 -15.971 -13.039 29.502 0.00 0.00
E
ATOM 14308 HB VAL E 42 -15.038 -12.509 29.213 0.00 0.00
E
ATOM 14309 CG1 VAL E 42 -16.805 -13.290 28.203 0.00 0.00
E

ATOM 14310 HG11 VAL E 42   -17.076 -12.341 27.693 0.00 0.00
E
ATOM 14311 HG12 VAL E 42   -16.257 -13.792 27.377 0.00 0.00
E
ATOM 14312 HG13 VAL E 42   -17.706 -13.915 28.383 0.00 0.00
E
ATOM 14313 CG2 VAL E 42   -16.808 -12.174 30.383 0.00 0.00
E
ATOM 14314 HG21 VAL E 42   -16.618 -11.120 30.089 0.00 0.00
E
ATOM 14315 HG22 VAL E 42   -17.905 -12.342 30.434 0.00 0.00
E
ATOM 14316 HG23 VAL E 42   -16.436 -12.226 31.429 0.00 0.00
E
ATOM 14317 C   VAL E 42   -15.081 -15.245 29.014 0.00 0.00
E
ATOM 14318 O   VAL E 42   -15.728 -16.022 28.342 0.00 0.00
E
ATOM 14319 N   ASN E 43   -13.718 -15.140 28.885 0.00 0.00
E
ATOM 14320 HN ASN E 43   -13.207 -14.493 29.444 0.00 0.00
E
ATOM 14321 CA ASN E 43   -12.835 -15.853 27.930 0.00 0.00
E
ATOM 14322 HA ASN E 43   -13.325 -15.918 26.970 0.00 0.00
E
ATOM 14323 CB ASN E 43   -11.543 -15.113 27.798 0.00 0.00
E
ATOM 14324 HB1 ASN E 43   -11.341 -14.542 28.729 0.00 0.00
E
ATOM 14325 HB2 ASN E 43   -10.739 -15.842 27.559 0.00 0.00
E
ATOM 14326 CG ASN E 43   -11.640 -14.122 26.576 0.00 0.00
E
ATOM 14327 OD1 ASN E 43   -11.499 -14.715 25.480 0.00 0.00
E
ATOM 14328 ND2 ASN E 43   -11.789 -12.826 26.844 0.00 0.00
E
ATOM 14329 HD21 ASN E 43   -11.716 -12.172 26.091 0.00 0.00
E
ATOM 14330 HD22 ASN E 43   -12.110 -12.507 27.735 0.00 0.00
E
ATOM 14331 C   ASN E 43   -12.731 -17.280 28.341 0.00 0.00
E
ATOM 14332 O   ASN E 43   -12.878 -18.186 27.535 0.00 0.00
E
ATOM 14333 N   ALA E 44   -12.575 -17.566 29.609 0.00 0.00
E
ATOM 14334 HN ALA E 44   -12.551 -16.779 30.221 0.00 0.00
E
ATOM 14335 CA ALA E 44   -12.562 -18.898 30.172 0.00 0.00
E
ATOM 14336 HA ALA E 44   -11.840 -19.513 29.656 0.00 0.00
E

ATOM 14337 CB ALA E 44 -12.350 -18.885 31.673 0.00 0.00
E
ATOM 14338 HB1 ALA E 44 -13.160 -18.481 32.318 0.00 0.00
E
ATOM 14339 HB2 ALA E 44 -12.101 -19.885 32.087 0.00 0.00
E
ATOM 14340 HB3 ALA E 44 -11.474 -18.299 32.025 0.00 0.00
E
ATOM 14341 C ALA E 44 -13.829 -19.657 29.988 0.00 0.00
E
ATOM 14342 O ALA E 44 -13.841 -20.770 29.470 0.00 0.00
E
ATOM 14343 N TYR E 45 -15.005 -18.973 30.213 0.00 0.00
E
ATOM 14344 HN TYR E 45 -14.842 -18.104 30.673 0.00 0.00
E
ATOM 14345 CA TYR E 45 -16.267 -19.584 30.268 0.00 0.00
E
ATOM 14346 HA TYR E 45 -16.130 -20.610 30.576 0.00 0.00
E
ATOM 14347 CB TYR E 45 -17.187 -19.016 31.410 0.00 0.00
E
ATOM 14348 HB1 TYR E 45 -17.335 -17.935 31.196 0.00 0.00
E
ATOM 14349 HB2 TYR E 45 -18.191 -19.486 31.338 0.00 0.00
E
ATOM 14350 CG TYR E 45 -16.634 -19.142 32.762 0.00 0.00
E
ATOM 14351 CD1 TYR E 45 -16.470 -20.475 33.189 0.00 0.00
E
ATOM 14352 HD1 TYR E 45 -16.659 -21.234 32.445 0.00 0.00
E
ATOM 14353 CE1 TYR E 45 -15.860 -20.687 34.440 0.00 0.00
E
ATOM 14354 HE1 TYR E 45 -15.481 -21.670 34.677 0.00 0.00
E
ATOM 14355 CZ TYR E 45 -15.416 -19.644 35.226 0.00 0.00
E
ATOM 14356 OH TYR E 45 -14.675 -19.896 36.457 0.00 0.00
E
ATOM 14357 HH TYR E 45 -14.696 -19.073 36.950 0.00 0.00
E
ATOM 14358 CD2 TYR E 45 -16.360 -18.053 33.567 0.00 0.00
E
ATOM 14359 HD2 TYR E 45 -16.405 -17.037 33.203 0.00 0.00
E
ATOM 14360 CE2 TYR E 45 -15.771 -18.314 34.861 0.00 0.00
E
ATOM 14361 HE2 TYR E 45 -15.524 -17.515 35.545 0.00 0.00
E
ATOM 14362 C TYR E 45 -17.019 -19.509 28.995 0.00 0.00
E
ATOM 14363 O TYR E 45 -18.206 -19.952 28.910 0.00 0.00
E

ATOM 14364 N ALA E 46 −16.347 −18.954 27.966 0.00 0.00
E
ATOM 14365 HN ALA E 46 −15.436 −18.555 28.031 0.00 0.00
E
ATOM 14366 CA ALA E 46 −16.836 −18.934 26.621 0.00 0.00
E
ATOM 14367 HA ALA E 46 −17.906 −18.792 26.663 0.00 0.00
E
ATOM 14368 CB ALA E 46 −16.014 −17.960 25.785 0.00 0.00
E
ATOM 14369 HB1 ALA E 46 −14.927 −18.094 25.975 0.00 0.00
E
ATOM 14370 HB2 ALA E 46 −16.303 −17.934 24.713 0.00 0.00
E
ATOM 14371 HB3 ALA E 46 −16.185 −16.905 26.087 0.00 0.00
E
ATOM 14372 C ALA E 46 −16.828 −20.275 25.974 0.00 0.00
E
ATOM 14373 O ALA E 46 −17.518 −20.606 24.988 0.00 0.00
E
ATOM 14374 N SER E 47 −15.994 −21.113 26.617 0.00 0.00
E
ATOM 14375 HN SER E 47 −15.193 −20.730 27.072 0.00 0.00
E
ATOM 14376 CA SER E 47 −15.886 −22.513 26.151 0.00 0.00
E
ATOM 14377 HA SER E 47 −15.848 −22.646 25.080 0.00 0.00
E
ATOM 14378 CB SER E 47 −14.542 −22.942 26.675 0.00 0.00
E
ATOM 14379 HB1 SER E 47 −14.453 −22.702 27.756 0.00 0.00
E
ATOM 14380 HB2 SER E 47 −14.477 −24.048 26.601 0.00 0.00
E
ATOM 14381 OG SER E 47 −13.508 −22.285 25.937 0.00 0.00
E
ATOM 14382 HG1 SER E 47 −12.763 −22.867 26.104 0.00 0.00
E
ATOM 14383 C SER E 47 −16.948 −23.349 26.837 0.00 0.00
E
ATOM 14384 O SER E 47 −17.226 −24.521 26.544 0.00 0.00
E
ATOM 14385 N TYR E 48 −17.664 −22.746 27.793 0.00 0.00
E
ATOM 14386 HN TYR E 48 −17.395 −21.789 27.872 0.00 0.00
E
ATOM 14387 CA TYR E 48 −18.728 −23.376 28.539 0.00 0.00
E
ATOM 14388 HA TYR E 48 −18.874 −24.337 28.069 0.00 0.00
E
ATOM 14389 CB TYR E 48 −18.524 −23.438 30.043 0.00 0.00
E
ATOM 14390 HB1 TYR E 48 −18.352 −22.446 30.513 0.00 0.00
E

ATOM 14391 HB2 TYR E 48 −19.353 −23.953 30.575 0.00 0.00
E
ATOM 14392 CG TYR E 48 −17.246 −24.235 30.319 0.00 0.00
E
ATOM 14393 CD1 TYR E 48 −16.223 −23.794 31.136 0.00 0.00
E
ATOM 14394 HD1 TYR E 48 −16.235 −22.840 31.642 0.00 0.00
E
ATOM 14395 CE1 TYR E 48 −15.073 −24.626 31.332 0.00 0.00
E
ATOM 14396 HE1 TYR E 48 −14.240 −24.288 31.932 0.00 0.00
E
ATOM 14397 CZ TYR E 48 −14.872 −25.787 30.637 0.00 0.00
E
ATOM 14398 OH TYR E 48 −13.636 −26.467 30.808 0.00 0.00
E
ATOM 14399 HH TYR E 48 −13.699 −27.422 30.736 0.00 0.00
E
ATOM 14400 CD2 TYR E 48 −17.155 −25.494 29.746 0.00 0.00
E
ATOM 14401 HD2 TYR E 48 −17.984 −25.906 29.189 0.00 0.00
E
ATOM 14402 CE2 TYR E 48 −15.933 −26.239 29.760 0.00 0.00
E
ATOM 14403 HE2 TYR E 48 −15.837 −27.165 29.214 0.00 0.00
E
ATOM 14404 C TYR E 48 −20.051 −22.666 28.328 0.00 0.00
E
ATOM 14405 O TYR E 48 −20.992 −22.731 29.129 0.00 0.00
E
ATOM 14406 N ALA E 49 −20.260 −21.891 27.262 0.00 0.00
E
ATOM 14407 HN ALA E 49 −19.657 −21.760 26.479 0.00 0.00
E
ATOM 14408 CA ALA E 49 −21.543 −21.333 27.044 0.00 0.00
E
ATOM 14409 HA ALA E 49 −22.350 −21.901 27.483 0.00 0.00
E
ATOM 14410 CB ALA E 49 −21.541 −19.941 27.752 0.00 0.00
E
ATOM 14411 HB1 ALA E 49 −20.794 −19.243 27.318 0.00 0.00
E
ATOM 14412 HB2 ALA E 49 −22.536 −19.479 27.580 0.00 0.00
E
ATOM 14413 HB3 ALA E 49 −21.453 −20.001 28.858 0.00 0.00
E
ATOM 14414 C ALA E 49 −21.687 −21.286 25.573 0.00 0.00
E
ATOM 14415 O ALA E 49 −20.699 −21.522 24.952 0.00 0.00
E
ATOM 14416 N THR E 50 −22.861 −21.041 25.129 0.00 0.00
E
ATOM 14417 HN THR E 50 −23.598 −20.922 25.790 0.00 0.00
E

ATOM 14418 CA THR E 50 -23.244 -21.001 23.677 0.00 0.00
E
ATOM 14419 HA THR E 50 -22.598 -21.662 23.118 0.00 0.00
E
ATOM 14420 CB THR E 50 -24.709 -21.550 23.383 0.00 0.00
E
ATOM 14421 HB THR E 50 -24.888 -21.613 22.288 0.00 0.00
E
ATOM 14422 OG1 THR E 50 -25.748 -20.717 23.912 0.00 0.00
E
ATOM 14423 HG1 THR E 50 -26.492 -20.781 23.309 0.00 0.00
E
ATOM 14424 CG2 THR E 50 -24.790 -23.001 23.955 0.00 0.00
E
ATOM 14425 HG21 THR E 50 -24.068 -23.638 23.402 0.00 0.00
E
ATOM 14426 HG22 THR E 50 -24.519 -23.044 25.032 0.00 0.00
E
ATOM 14427 HG23 THR E 50 -25.824 -23.370 23.781 0.00 0.00
E
ATOM 14428 C THR E 50 -23.122 -19.544 23.218 0.00 0.00
E
ATOM 14429 O THR E 50 -23.097 -19.157 22.063 0.00 0.00
E
ATOM 14430 N HSE E 51 -22.882 -18.573 24.205 0.00 0.00
E
ATOM 14431 HN HSE E 51 -23.035 -18.779 25.168 0.00 0.00
E
ATOM 14432 CA HSE E 51 -22.541 -17.169 23.981 0.00 0.00
E
ATOM 14433 HA HSE E 51 -21.742 -17.162 23.254 0.00 0.00
E
ATOM 14434 CB HSE E 51 -23.707 -16.243 23.421 0.00 0.00
E
ATOM 14435 HB1 HSE E 51 -23.978 -16.786 22.491 0.00 0.00
E
ATOM 14436 HB2 HSE E 51 -24.518 -16.419 24.160 0.00 0.00
E
ATOM 14437 ND1 HSE E 51 -22.151 -14.829 22.211 0.00 0.00
E
ATOM 14438 CG HSE E 51 -23.291 -14.922 23.032 0.00 0.00
E
ATOM 14439 CE1 HSE E 51 -22.024 -13.481 22.016 0.00 0.00
E
ATOM 14440 HE1 HSE E 51 -21.113 -13.084 21.569 0.00 0.00
E
ATOM 14441 NE2 HSE E 51 -22.930 -12.751 22.613 0.00 0.00
E
ATOM 14442 HE2 HSE E 51 -23.018 -11.755 22.596 0.00 0.00
E
ATOM 14443 CD2 HSE E 51 -23.703 -13.635 23.321 0.00 0.00
E
ATOM 14444 HD2 HSE E 51 -24.514 -13.228 23.912 0.00 0.00
E

```
ATOM  14445  C   HSE E  51      -22.004 -16.625  25.244  0.00  0.00
E
ATOM  14446  O   HSE E  51      -22.273 -17.161  26.358  0.00  0.00
E
ATOM  14447  N   ALA E  52      -21.199 -15.605  25.066  0.00  0.00
E
ATOM  14448  HN  ALA E  52      -21.157 -15.196  24.157  0.00  0.00
E
ATOM  14449  CA  ALA E  52      -20.533 -15.046  26.234  0.00  0.00
E
ATOM  14450  HA  ALA E  52      -21.246 -14.956  27.040  0.00  0.00
E
ATOM  14451  CB  ALA E  52      -19.234 -15.860  26.707  0.00  0.00
E
ATOM  14452  HB1 ALA E  52      -19.609 -16.905  26.745  0.00  0.00
E
ATOM  14453  HB2 ALA E  52      -18.324 -15.738  26.081  0.00  0.00
E
ATOM  14454  HB3 ALA E  52      -18.963 -15.444  27.701  0.00  0.00
E
ATOM  14455  C   ALA E  52      -20.155 -13.599  25.840  0.00  0.00
E
ATOM  14456  O   ALA E  52      -19.919 -13.284  24.672  0.00  0.00
E
ATOM  14457  N   VAL E  53      -20.306 -12.616  26.760  0.00  0.00
E
ATOM  14458  HN  VAL E  53      -20.464 -12.821  27.723  0.00  0.00
E
ATOM  14459  CA  VAL E  53      -20.231 -11.133  26.593  0.00  0.00
E
ATOM  14460  HA  VAL E  53      -19.650 -10.995  25.693  0.00  0.00
E
ATOM  14461  CB  VAL E  53      -21.519 -10.372  26.591  0.00  0.00
E
ATOM  14462  HB  VAL E  53      -22.007 -10.438  27.587  0.00  0.00
E
ATOM  14463  CG1 VAL E  53      -21.246  -8.872  26.363  0.00  0.00
E
ATOM  14464 HG11 VAL E  53      -20.892  -8.310  27.254  0.00  0.00
E
ATOM  14465 HG12 VAL E  53      -20.376  -8.694  25.695  0.00  0.00
E
ATOM  14466 HG13 VAL E  53      -22.162  -8.369  25.989  0.00  0.00
E
ATOM  14467  CG2 VAL E  53      -22.520 -10.922  25.661  0.00  0.00
E
ATOM  14468 HG21 VAL E  53      -22.013 -10.986  24.674  0.00  0.00
E
ATOM  14469 HG22 VAL E  53      -22.878 -11.957  25.845  0.00  0.00
E
ATOM  14470 HG23 VAL E  53      -23.384 -10.224  25.655  0.00  0.00
E
ATOM  14471  C   VAL E  53      -19.360 -10.488  27.673  0.00  0.00
E
```

ATOM 14472 O   VAL E 53   -19.443 -10.961 28.837 0.00 0.00
E
ATOM 14473 N   ILE E 54   -18.509 -9.526 27.435 0.00 0.00
E
ATOM 14474 HN ILE E 54   -18.392 -9.204 26.499 0.00 0.00
E
ATOM 14475 CA ILE E 54   -17.786 -8.730 28.374 0.00 0.00
E
ATOM 14476 HA ILE E 54   -17.783 -9.242 29.325 0.00 0.00
E
ATOM 14477 CB ILE E 54   -16.406 -8.395 27.943 0.00 0.00
E
ATOM 14478 HB ILE E 54   -16.458 -7.866 26.968 0.00 0.00
E
ATOM 14479 CG2 ILE E 54   -15.714 -7.456 28.951 0.00 0.00
E
ATOM 14480 HG21 ILE E 54   -14.623 -7.374 28.758 0.00 0.00
E
ATOM 14481 HG22 ILE E 54   -16.191 -6.453 28.961 0.00 0.00
E
ATOM 14482 HG23 ILE E 54   -15.930 -7.841 29.970 0.00 0.00
E
ATOM 14483 CG1 ILE E 54   -15.416 -9.585 27.633 0.00 0.00
E
ATOM 14484 HG11 ILE E 54   -14.933 -9.914 28.578 0.00 0.00
E
ATOM 14485 HG12 ILE E 54   -16.033 -10.445 27.296 0.00 0.00
E
ATOM 14486 CD ILE E 54   -14.163 -9.219 26.752 0.00 0.00
E
ATOM 14487 HD1 ILE E 54   -13.402 -8.664 27.342 0.00 0.00
E
ATOM 14488 HD2 ILE E 54   -13.577 -10.139 26.540 0.00 0.00
E
ATOM 14489 HD3 ILE E 54   -14.319 -8.678 25.795 0.00 0.00
E
ATOM 14490 C   ILE E 54   -18.593 -7.396 28.569 0.00 0.00
E
ATOM 14491 O   ILE E 54   -18.640 -6.579 27.666 0.00 0.00
E
ATOM 14492 N   ALA E 55   -19.271 -7.198 29.722 0.00 0.00
E
ATOM 14493 HN ALA E 55   -19.110 -7.854 30.456 0.00 0.00
E
ATOM 14494 CA ALA E 55   -20.034 -6.010 29.865 0.00 0.00
E
ATOM 14495 HA ALA E 55   -19.380 -5.201 29.575 0.00 0.00
E
ATOM 14496 CB ALA E 55   -21.259 -6.128 28.901 0.00 0.00
E
ATOM 14497 HB1 ALA E 55   -20.948 -5.936 27.852 0.00 0.00
E
ATOM 14498 HB2 ALA E 55   -21.607 -7.178 29.000 0.00 0.00
E

ATOM 14499 HB3 ALA E 55 −22.094 −5.436 29.141 0.00 0.00
E
ATOM 14500 C ALA E 55 −20.531 −5.718 31.241 0.00 0.00
E
ATOM 14501 O ALA E 55 −20.441 −6.635 32.084 0.00 0.00
E
ATOM 14502 N ASN E 56 −20.733 −4.372 31.600 0.00 0.00
E
ATOM 14503 HN ASN E 56 −20.580 −3.740 30.845 0.00 0.00
E
ATOM 14504 CA ASN E 56 −21.263 −3.975 32.832 0.00 0.00
E
ATOM 14505 HA ASN E 56 −20.802 −4.676 33.511 0.00 0.00
E
ATOM 14506 CB ASN E 56 −21.012 −2.425 32.990 0.00 0.00
E
ATOM 14507 HB1 ASN E 56 −21.358 −2.041 32.007 0.00 0.00
E
ATOM 14508 HB2 ASN E 56 −21.503 −1.983 33.883 0.00 0.00
E
ATOM 14509 CG ASN E 56 −19.536 −2.146 33.258 0.00 0.00
E
ATOM 14510 OD1 ASN E 56 −19.144 −2.172 34.442 0.00 0.00
E
ATOM 14511 ND2 ASN E 56 −18.792 −1.851 32.166 0.00 0.00
E
ATOM 14512 HD21 ASN E 56 −17.796 −1.767 32.195 0.00 0.00
E
ATOM 14513 HD22 ASN E 56 −19.281 −1.872 31.294 0.00 0.00
E
ATOM 14514 C ASN E 56 −22.773 −4.062 32.737 0.00 0.00
E
ATOM 14515 O ASN E 56 −23.303 −3.349 31.906 0.00 0.00
E
ATOM 14516 N ALA E 57 −23.447 −5.032 33.401 0.00 0.00
E
ATOM 14517 HN ALA E 57 −22.890 −5.733 33.839 0.00 0.00
E
ATOM 14518 CA ALA E 57 −24.921 −5.155 33.310 0.00 0.00
E
ATOM 14519 HA ALA E 57 −25.224 −4.981 32.288 0.00 0.00
E
ATOM 14520 CB ALA E 57 −25.231 −6.588 33.778 0.00 0.00
E
ATOM 14521 HB1 ALA E 57 −25.235 −6.744 34.878 0.00 0.00
E
ATOM 14522 HB2 ALA E 57 −26.211 −6.814 33.306 0.00 0.00
E
ATOM 14523 HB3 ALA E 57 −24.405 −7.158 33.301 0.00 0.00
E
ATOM 14524 C ALA E 57 −25.628 −4.174 34.204 0.00 0.00
E
ATOM 14525 O ALA E 57 −26.862 −4.209 34.329 0.00 0.00
E

ATOM 14526 N THR E 58 −24.885 −3.262 34.845 0.00 0.00
E
ATOM 14527 HN THR E 58 −23.891 −3.302 34.782 0.00 0.00
E
ATOM 14528 CA THR E 58 −25.294 −2.195 35.724 0.00 0.00
E
ATOM 14529 HA THR E 58 −26.225 −2.525 36.160 0.00 0.00
E
ATOM 14530 CB THR E 58 −24.241 −1.977 36.769 0.00 0.00
E
ATOM 14531 HB THR E 58 −24.538 −1.128 37.422 0.00 0.00
E
ATOM 14532 OG1 THR E 58 −23.002 −1.671 36.145 0.00 0.00
E
ATOM 14533 HG1 THR E 58 −22.509 −1.171 36.799 0.00 0.00
E
ATOM 14534 CG2 THR E 58 −24.054 −3.289 37.462 0.00 0.00
E
ATOM 14535 HG21 THR E 58 −25.017 −3.759 37.755 0.00 0.00
E
ATOM 14536 HG22 THR E 58 −23.520 −4.101 36.922 0.00 0.00
E
ATOM 14537 HG23 THR E 58 −23.426 −3.154 38.369 0.00 0.00
E
ATOM 14538 C THR E 58 −25.553 −0.925 34.959 0.00 0.00
E
ATOM 14539 O THR E 58 −25.821 0.150 35.421 0.00 0.00
E
ATOM 14540 N GLU E 59 −25.455 −1.081 33.601 0.00 0.00
E
ATOM 14541 HN GLU E 59 −25.273 −1.991 33.238 0.00 0.00
E
ATOM 14542 CA GLU E 59 −25.650 0.060 32.795 0.00 0.00
E
ATOM 14543 HA GLU E 59 −26.107 0.905 33.288 0.00 0.00
E
ATOM 14544 CB GLU E 59 −24.252 0.704 32.338 0.00 0.00
E
ATOM 14545 HB1 GLU E 59 −23.696 −0.058 31.750 0.00 0.00
E
ATOM 14546 HB2 GLU E 59 −24.625 1.529 31.694 0.00 0.00
E
ATOM 14547 CG GLU E 59 −23.465 1.252 33.512 0.00 0.00
E
ATOM 14548 HG1 GLU E 59 −24.011 1.879 34.250 0.00 0.00
E
ATOM 14549 HG2 GLU E 59 −22.932 0.384 33.955 0.00 0.00
E
ATOM 14550 CD GLU E 59 −22.419 2.113 32.921 0.00 0.00
E
ATOM 14551 OE1 GLU E 59 −21.204 1.821 33.198 0.00 0.00
E
ATOM 14552 OE2 GLU E 59 −22.694 3.050 32.078 0.00 0.00
E

ATOM	14553	C	GLU	E	59	−26.533	−0.143	31.584	0.00	0.00	E
ATOM	14554	O	GLU	E	59	−26.448	−1.124	30.839	0.00	0.00	E
ATOM	14555	N	GLU	E	60	−27.355	0.884	31.308	0.00	0.00	E
ATOM	14556	HN	GLU	E	60	−27.155	1.812	31.615	0.00	0.00	E
ATOM	14557	CA	GLU	E	60	−28.470	0.782	30.448	0.00	0.00	E
ATOM	14558	HA	GLU	E	60	−29.083	0.045	30.945	0.00	0.00	E
ATOM	14559	CB	GLU	E	60	−29.234	2.104	30.599	0.00	0.00	E
ATOM	14560	HB1	GLU	E	60	−29.295	2.336	31.683	0.00	0.00	E
ATOM	14561	HB2	GLU	E	60	−28.716	3.018	30.237	0.00	0.00	E
ATOM	14562	CG	GLU	E	60	−30.671	2.007	30.001	0.00	0.00	E
ATOM	14563	HG1	GLU	E	60	−30.700	1.513	29.006	0.00	0.00	E
ATOM	14564	HG2	GLU	E	60	−31.365	1.476	30.686	0.00	0.00	E
ATOM	14565	CD	GLU	E	60	−31.249	3.382	29.931	0.00	0.00	E
ATOM	14566	OE1	GLU	E	60	−31.543	3.861	28.798	0.00	0.00	E
ATOM	14567	OE2	GLU	E	60	−31.427	4.050	30.991	0.00	0.00	E
ATOM	14568	C	GLU	E	60	−28.260	0.311	28.994	0.00	0.00	E
ATOM	14569	O	GLU	E	60	−28.861	−0.740	28.643	0.00	0.00	E
ATOM	14570	N	ASN	E	61	−27.283	0.887	28.311	0.00	0.00	E
ATOM	14571	HN	ASN	E	61	−26.801	1.707	28.611	0.00	0.00	E
ATOM	14572	CA	ASN	E	61	−27.111	0.397	26.974	0.00	0.00	E
ATOM	14573	HA	ASN	E	61	−28.078	0.088	26.606	0.00	0.00	E
ATOM	14574	CB	ASN	E	61	−26.392	1.491	26.160	0.00	0.00	E
ATOM	14575	HB1	ASN	E	61	−25.567	1.929	26.762	0.00	0.00	E
ATOM	14576	HB2	ASN	E	61	−25.858	1.082	25.275	0.00	0.00	E
ATOM	14577	CG	ASN	E	61	−27.303	2.579	25.729	0.00	0.00	E
ATOM	14578	OD1	ASN	E	61	−27.270	3.690	26.294	0.00	0.00	E
ATOM	14579	ND2	ASN	E	61	−28.266	2.310	24.839	0.00	0.00	E

ATOM 14580 HD21 ASN E 61 -28.952 3.020 24.678 0.00 0.00
E
ATOM 14581 HD22 ASN E 61 -28.299 1.379 24.477 0.00 0.00
E
ATOM 14582 C ASN E 61 -26.194 -0.794 26.983 0.00 0.00
E
ATOM 14583 O ASN E 61 -26.245 -1.572 26.049 0.00 0.00
E
ATOM 14584 N GLU E 62 -25.314 -1.067 28.015 0.00 0.00
E
ATOM 14585 HN GLU E 62 -25.352 -0.450 28.797 0.00 0.00
E
ATOM 14586 CA GLU E 62 -24.581 -2.328 27.972 0.00 0.00
E
ATOM 14587 HA GLU E 62 -24.156 -2.269 26.981 0.00 0.00
E
ATOM 14588 CB GLU E 62 -23.381 -2.231 28.939 0.00 0.00
E
ATOM 14589 HB1 GLU E 62 -23.915 -1.743 29.782 0.00 0.00
E
ATOM 14590 HB2 GLU E 62 -23.001 -3.170 29.395 0.00 0.00
E
ATOM 14591 CG GLU E 62 -22.171 -1.509 28.401 0.00 0.00
E
ATOM 14592 HG1 GLU E 62 -21.880 -1.818 27.374 0.00 0.00
E
ATOM 14593 HG2 GLU E 62 -22.526 -0.468 28.242 0.00 0.00
E
ATOM 14594 CD GLU E 62 -20.902 -1.453 29.315 0.00 0.00
E
ATOM 14595 OE1 GLU E 62 -20.225 -2.497 29.462 0.00 0.00
E
ATOM 14596 OE2 GLU E 62 -20.596 -0.299 29.821 0.00 0.00
E
ATOM 14597 C GLU E 62 -25.607 -3.476 28.171 0.00 0.00
E
ATOM 14598 O GLU E 62 -25.586 -4.473 27.390 0.00 0.00
E
ATOM 14599 N LEU E 63 -26.439 -3.358 29.140 0.00 0.00
E
ATOM 14600 HN LEU E 63 -26.375 -2.605 29.791 0.00 0.00
E
ATOM 14601 CA LEU E 63 -27.438 -4.350 29.392 0.00 0.00
E
ATOM 14602 HA LEU E 63 -26.985 -5.328 29.473 0.00 0.00
E
ATOM 14603 CB LEU E 63 -28.129 -4.039 30.777 0.00 0.00
E
ATOM 14604 HB1 LEU E 63 -27.267 -4.141 31.471 0.00 0.00
E
ATOM 14605 HB2 LEU E 63 -28.481 -2.986 30.806 0.00 0.00
E
ATOM 14606 CG LEU E 63 -29.321 -5.012 31.110 0.00 0.00
E

ATOM 14607 HG LEU E 63   -29.124 -5.880 30.446 0.00 0.00
E
ATOM 14608 CD1 LEU E 63   -29.303 -5.499 32.570 0.00 0.00
E
ATOM 14609 HD11 LEU E 63   -28.472 -6.216 32.741 0.00 0.00
E
ATOM 14610 HD12 LEU E 63   -29.071 -4.675 33.279 0.00 0.00
E
ATOM 14611 HD13 LEU E 63   -30.300 -5.873 32.885 0.00 0.00
E
ATOM 14612 CD2 LEU E 63   -30.679 -4.383 30.813 0.00 0.00
E
ATOM 14613 HD21 LEU E 63   -30.799 -4.215 29.721 0.00 0.00
E
ATOM 14614 HD22 LEU E 63   -31.614 -4.930 31.057 0.00 0.00
E
ATOM 14615 HD23 LEU E 63   -30.794 -3.371 31.256 0.00 0.00
E
ATOM 14616 C   LEU E 63   -28.544 -4.432 28.252 0.00 0.00
E
ATOM 14617 O   LEU E 63   -28.874 -5.588 27.948 0.00 0.00
E
ATOM 14618 N   LEU E 64   -28.991 -3.382 27.607 0.00 0.00
E
ATOM 14619 HN LEU E 64   -28.900 -2.446 27.937 0.00 0.00
E
ATOM 14620 CA LEU E 64   -29.922 -3.523 26.451 0.00 0.00
E
ATOM 14621 HA LEU E 64   -30.814 -4.049 26.759 0.00 0.00
E
ATOM 14622 CB LEU E 64   -30.388 -2.155 25.807 0.00 0.00
E
ATOM 14623 HB1 LEU E 64   -29.512 -1.493 25.637 0.00 0.00
E
ATOM 14624 HB2 LEU E 64   -30.964 -2.432 24.898 0.00 0.00
E
ATOM 14625 CG LEU E 64   -31.373 -1.351 26.663 0.00 0.00
E
ATOM 14626 HG LEU E 64   -31.025 -1.391 27.717 0.00 0.00
E
ATOM 14627 CD1 LEU E 64   -31.415   0.095 26.235 0.00 0.00
E
ATOM 14628 HD11 LEU E 64   -30.408   0.468 25.950 0.00 0.00
E
ATOM 14629 HD12 LEU E 64   -32.146   0.194 25.405 0.00 0.00
E
ATOM 14630 HD13 LEU E 64   -31.787   0.755 27.048 0.00 0.00
E
ATOM 14631 CD2 LEU E 64   -32.850 -1.918 26.713 0.00 0.00
E
ATOM 14632 HD21 LEU E 64   -33.359 -1.666 25.758 0.00 0.00
E
ATOM 14633 HD22 LEU E 64   -32.969 -2.989 26.984 0.00 0.00
E

ATOM	14634	HD23	LEU	E	64	−33.440	−1.436	27.520	0.00	0.00	E
ATOM	14635	C	LEU	E	64	−29.195	−4.282	25.254	0.00	0.00	E
ATOM	14636	O	LEU	E	64	−29.784	−5.044	24.479	0.00	0.00	E
ATOM	14637	N	SER	E	65	−27.850	−4.132	25.084	0.00	0.00	E
ATOM	14638	HN	SER	E	65	−27.387	−3.638	25.816	0.00	0.00	E
ATOM	14639	CA	SER	E	65	−27.049	−4.786	24.072	0.00	0.00	E
ATOM	14640	HA	SER	E	65	−27.579	−4.839	23.133	0.00	0.00	E
ATOM	14641	CB	SER	E	65	−25.690	−4.060	23.794	0.00	0.00	E
ATOM	14642	HB1	SER	E	65	−25.189	−4.161	24.781	0.00	0.00	E
ATOM	14643	HB2	SER	E	65	−25.089	−4.540	22.992	0.00	0.00	E
ATOM	14644	OG	SER	E	65	−25.852	−2.677	23.491	0.00	0.00	E
ATOM	14645	HG1	SER	E	65	−26.258	−2.120	24.159	0.00	0.00	E
ATOM	14646	C	SER	E	65	−26.785	−6.202	24.363	0.00	0.00	E
ATOM	14647	O	SER	E	65	−26.300	−6.978	23.524	0.00	0.00	E
ATOM	14648	N	LEU	E	66	−27.113	−6.731	25.583	0.00	0.00	E
ATOM	14649	HN	LEU	E	66	−27.508	−6.121	26.265	0.00	0.00	E
ATOM	14650	CA	LEU	E	66	−26.855	−8.146	25.931	0.00	0.00	E
ATOM	14651	HA	LEU	E	66	−25.827	−8.414	25.735	0.00	0.00	E
ATOM	14652	CB	LEU	E	66	−27.133	−8.514	27.419	0.00	0.00	E
ATOM	14653	HB1	LEU	E	66	−28.193	−8.205	27.542	0.00	0.00	E
ATOM	14654	HB2	LEU	E	66	−27.198	−9.609	27.596	0.00	0.00	E
ATOM	14655	CG	LEU	E	66	−26.170	−7.738	28.403	0.00	0.00	E
ATOM	14656	HG	LEU	E	66	−26.276	−6.671	28.114	0.00	0.00	E
ATOM	14657	CD1	LEU	E	66	−26.693	−8.029	29.851	0.00	0.00	E
ATOM	14658	HD11	LEU	E	66	−27.796	−7.897	29.844	0.00	0.00	E
ATOM	14659	HD12	LEU	E	66	−26.425	−9.033	30.244	0.00	0.00	E
ATOM	14660	HD13	LEU	E	66	−26.165	−7.365	30.569	0.00	0.00	E

```
ATOM  14661  CD2 LEU E  66      -24.711  -8.140  28.308  0.00  0.00
E
ATOM  14662 HD21 LEU E  66      -24.246  -7.577  27.472  0.00  0.00
E
ATOM  14663 HD22 LEU E  66      -24.045  -7.723  29.093  0.00  0.00
E
ATOM  14664 HD23 LEU E  66      -24.599  -9.245  28.266  0.00  0.00
E
ATOM  14665  C   LEU E  66      -27.621  -9.151  25.039  0.00  0.00
E
ATOM  14666  O   LEU E  66      -27.162 -10.213  24.660  0.00  0.00
E
ATOM  14667  N   GLY E  67      -28.916  -8.785  24.701  0.00  0.00
E
ATOM  14668  HN  GLY E  67      -29.197  -7.876  24.998  0.00  0.00
E
ATOM  14669  CA  GLY E  67      -29.851  -9.724  24.020  0.00  0.00
E
ATOM  14670  HA1 GLY E  67      -29.303 -10.133  23.184  0.00  0.00
E
ATOM  14671  HA2 GLY E  67      -30.714  -9.186  23.658  0.00  0.00
E
ATOM  14672  C   GLY E  67      -30.459 -10.838  24.850  0.00  0.00
E
ATOM  14673  O   GLY E  67      -30.631 -11.905  24.377  0.00  0.00
E
ATOM  14674  N   ILE E  68      -30.924 -10.464  26.017  0.00  0.00
E
ATOM  14675  HN  ILE E  68      -30.724  -9.587  26.445  0.00  0.00
E
ATOM  14676  CA  ILE E  68      -31.507 -11.375  26.947  0.00  0.00
E
ATOM  14677  HA  ILE E  68      -31.455 -12.377  26.548  0.00  0.00
E
ATOM  14678  CB  ILE E  68      -30.603 -11.321  28.207  0.00  0.00
E
ATOM  14679  HB  ILE E  68      -31.009 -12.195  28.761  0.00  0.00
E
ATOM  14680  CG2 ILE E  68      -29.163 -11.854  27.937  0.00  0.00
E
ATOM  14681 HG21 ILE E  68      -28.504 -12.190  28.766  0.00  0.00
E
ATOM  14682 HG22 ILE E  68      -29.466 -12.720  27.310  0.00  0.00
E
ATOM  14683 HG23 ILE E  68      -28.613 -11.099  27.334  0.00  0.00
E
ATOM  14684  CG1 ILE E  68      -30.552 -10.098  29.007  0.00  0.00
E
ATOM  14685 HG11 ILE E  68      -30.073  -9.263  28.452  0.00  0.00
E
ATOM  14686 HG12 ILE E  68      -31.540  -9.657  29.259  0.00  0.00
E
ATOM  14687  CD  ILE E  68      -29.866 -10.291  30.398  0.00  0.00
E
```

ATOM	14688	HD1	ILE	E	68	−30.121	−9.488	31.122	0.00	0.00	E
ATOM	14689	HD2	ILE	E	68	−30.127	−11.279	30.834	0.00	0.00	E
ATOM	14690	HD3	ILE	E	68	−28.766	−10.305	30.248	0.00	0.00	E
ATOM	14691	C	ILE	E	68	−33.014	−11.077	27.183	0.00	0.00	E
ATOM	14692	O	ILE	E	68	−33.638	−11.486	28.167	0.00	0.00	E
ATOM	14693	N	ARG	E	69	−33.628	−10.249	26.315	0.00	0.00	E
ATOM	14694	HN	ARG	E	69	−33.169	−10.361	25.437	0.00	0.00	E
ATOM	14695	CA	ARG	E	69	−34.995	−9.819	26.387	0.00	0.00	E
ATOM	14696	HA	ARG	E	69	−35.164	−9.480	27.399	0.00	0.00	E
ATOM	14697	CB	ARG	E	69	−35.225	−8.613	25.418	0.00	0.00	E
ATOM	14698	HB1	ARG	E	69	−34.400	−7.875	25.517	0.00	0.00	E
ATOM	14699	HB2	ARG	E	69	−35.310	−8.913	24.352	0.00	0.00	E
ATOM	14700	CG	ARG	E	69	−36.489	−7.781	25.693	0.00	0.00	E
ATOM	14701	HG1	ARG	E	69	−37.368	−8.254	25.204	0.00	0.00	E
ATOM	14702	HG2	ARG	E	69	−36.925	−7.750	26.715	0.00	0.00	E
ATOM	14703	CD	ARG	E	69	−36.462	−6.300	25.146	0.00	0.00	E
ATOM	14704	HD1	ARG	E	69	−35.594	−5.706	25.505	0.00	0.00	E
ATOM	14705	HD2	ARG	E	69	−36.451	−6.591	24.074	0.00	0.00	E
ATOM	14706	NE	ARG	E	69	−37.774	−5.655	25.566	0.00	0.00	E
ATOM	14707	HE	ARG	E	69	−38.416	−6.154	26.149	0.00	0.00	E
ATOM	14708	CZ	ARG	E	69	−38.289	−4.539	25.070	0.00	0.00	E
ATOM	14709	NH1	ARG	E	69	−37.493	−3.629	24.523	0.00	0.00	E
ATOM	14710	HH11	ARG	E	69	−37.859	−2.748	24.223	0.00	0.00	E
ATOM	14711	HH12	ARG	E	69	−36.518	−3.851	24.499	0.00	0.00	E
ATOM	14712	NH2	ARG	E	69	−39.567	−4.236	25.367	0.00	0.00	E
ATOM	14713	HH21	ARG	E	69	−39.929	−3.391	24.974	0.00	0.00	E
ATOM	14714	HH22	ARG	E	69	−40.031	−4.876	25.979	0.00	0.00	E

ATOM	14715	C	ARG	E	69	−36.071	−10.822	26.118	0.00	0.00	E
ATOM	14716	O	ARG	E	69	−37.051	−10.844	26.815	0.00	0.00	E
ATOM	14717	N	ASN	E	70	−35.948	−11.752	25.134	0.00	0.00	E
ATOM	14718	HN	ASN	E	70	−35.106	−11.688	24.604	0.00	0.00	E
ATOM	14719	CA	ASN	E	70	−36.850	−12.858	24.850	0.00	0.00	E
ATOM	14720	HA	ASN	E	70	−37.805	−12.471	25.174	0.00	0.00	E
ATOM	14721	CB	ASN	E	70	−36.772	−13.252	23.381	0.00	0.00	E
ATOM	14722	HB1	ASN	E	70	−35.728	−13.430	23.045	0.00	0.00	E
ATOM	14723	HB2	ASN	E	70	−37.259	−14.239	23.232	0.00	0.00	E
ATOM	14724	CG	ASN	E	70	−37.359	−12.239	22.428	0.00	0.00	E
ATOM	14725	OD1	ASN	E	70	−36.611	−11.559	21.699	0.00	0.00	E
ATOM	14726	ND2	ASN	E	70	−38.663	−11.983	22.395	0.00	0.00	E
ATOM	14727	HD21	ASN	E	70	−39.022	−11.266	21.797	0.00	0.00	E
ATOM	14728	HD22	ASN	E	70	−39.309	−12.571	22.881	0.00	0.00	E
ATOM	14729	C	ASN	E	70	−36.564	−14.123	25.729	0.00	0.00	E
ATOM	14730	O	ASN	E	70	−36.884	−15.265	25.444	0.00	0.00	E
ATOM	14731	N	PHE	E	71	−36.052	−13.926	27.014	0.00	0.00	E
ATOM	14732	HN	PHE	E	71	−35.765	−13.010	27.283	0.00	0.00	E
ATOM	14733	CA	PHE	E	71	−35.683	−14.998	27.907	0.00	0.00	E
ATOM	14734	HA	PHE	E	71	−35.863	−15.998	27.543	0.00	0.00	E
ATOM	14735	CB	PHE	E	71	−34.270	−14.810	28.545	0.00	0.00	E
ATOM	14736	HB1	PHE	E	71	−34.032	−13.803	28.948	0.00	0.00	E
ATOM	14737	HB2	PHE	E	71	−34.112	−15.385	29.482	0.00	0.00	E
ATOM	14738	CG	PHE	E	71	−33.176	−15.240	27.595	0.00	0.00	E
ATOM	14739	CD1	PHE	E	71	−33.362	−15.581	26.248	0.00	0.00	E
ATOM	14740	HD1	PHE	E	71	−34.259	−15.563	25.647	0.00	0.00	E
ATOM	14741	CE1	PHE	E	71	−32.216	−15.706	25.417	0.00	0.00	E

ATOM	14742	HE1	PHE	E	71	−32.401	−15.768	24.355	0.00	0.00	E
ATOM	14743	CZ	PHE	E	71	−30.975	−15.703	26.002	0.00	0.00	E
ATOM	14744	HZ	PHE	E	71	−30.081	−15.884	25.424	0.00	0.00	E
ATOM	14745	CD2	PHE	E	71	−31.922	−15.409	28.247	0.00	0.00	E
ATOM	14746	HD2	PHE	E	71	−31.653	−15.233	29.278	0.00	0.00	E
ATOM	14747	CE2	PHE	E	71	−30.830	−15.650	27.389	0.00	0.00	E
ATOM	14748	HE2	PHE	E	71	−29.827	−15.609	27.787	0.00	0.00	E
ATOM	14749	C	PHE	E	71	−36.710	−14.926	29.031	0.00	0.00	E
ATOM	14750	O	PHE	E	71	−37.178	−13.823	29.500	0.00	0.00	E
ATOM	14751	N	GLU	E	72	−37.058	−16.169	29.476	0.00	0.00	E
ATOM	14752	HN	GLU	E	72	−36.722	−16.959	28.969	0.00	0.00	E
ATOM	14753	CA	GLU	E	72	−37.692	−16.356	30.698	0.00	0.00	E
ATOM	14754	HA	GLU	E	72	−38.494	−15.633	30.691	0.00	0.00	E
ATOM	14755	CB	GLU	E	72	−38.528	−17.680	30.670	0.00	0.00	E
ATOM	14756	HB1	GLU	E	72	−37.809	−18.527	30.695	0.00	0.00	E
ATOM	14757	HB2	GLU	E	72	−39.086	−17.739	31.629	0.00	0.00	E
ATOM	14758	CG	GLU	E	72	−39.482	−17.798	29.443	0.00	0.00	E
ATOM	14759	HG1	GLU	E	72	−40.007	−16.846	29.218	0.00	0.00	E
ATOM	14760	HG2	GLU	E	72	−38.743	−17.986	28.635	0.00	0.00	E
ATOM	14761	CD	GLU	E	72	−40.492	−18.938	29.391	0.00	0.00	E
ATOM	14762	OE1	GLU	E	72	−41.401	−18.980	30.270	0.00	0.00	E
ATOM	14763	OE2	GLU	E	72	−40.387	−19.760	28.506	0.00	0.00	E
ATOM	14764	C	GLU	E	72	−36.827	−16.143	31.940	0.00	0.00	E
ATOM	14765	O	GLU	E	72	−37.228	−15.448	32.834	0.00	0.00	E
ATOM	14766	N	TYR	E	73	−35.631	−16.663	32.001	0.00	0.00	E
ATOM	14767	HN	TYR	E	73	−35.124	−17.026	31.223	0.00	0.00	E
ATOM	14768	CA	TYR	E	73	−34.980	−16.828	33.302	0.00	0.00	E

ATOM 14769 HA TYR E 73 -35.601 -16.369 34.057 0.00 0.00
E
ATOM 14770 CB TYR E 73 -34.626 -18.367 33.718 0.00 0.00
E
ATOM 14771 HB1 TYR E 73 -33.948 -18.804 32.954 0.00 0.00
E
ATOM 14772 HB2 TYR E 73 -33.946 -18.397 34.597 0.00 0.00
E
ATOM 14773 CG TYR E 73 -35.815 -19.246 33.975 0.00 0.00
E
ATOM 14774 CD1 TYR E 73 -36.413 -19.809 32.837 0.00 0.00
E
ATOM 14775 HD1 TYR E 73 -36.014 -19.503 31.881 0.00 0.00
E
ATOM 14776 CE1 TYR E 73 -37.606 -20.529 33.025 0.00 0.00
E
ATOM 14777 HE1 TYR E 73 -38.060 -20.985 32.158 0.00 0.00
E
ATOM 14778 CZ TYR E 73 -38.127 -20.739 34.307 0.00 0.00
E
ATOM 14779 OH TYR E 73 -39.229 -21.556 34.444 0.00 0.00
E
ATOM 14780 HH TYR E 73 -39.582 -21.581 33.552 0.00 0.00
E
ATOM 14781 CD2 TYR E 73 -36.239 -19.642 35.265 0.00 0.00
E
ATOM 14782 HD2 TYR E 73 -35.639 -19.287 36.090 0.00 0.00
E
ATOM 14783 CE2 TYR E 73 -37.424 -20.310 35.421 0.00 0.00
E
ATOM 14784 HE2 TYR E 73 -37.759 -20.480 36.434 0.00 0.00
E
ATOM 14785 C TYR E 73 -33.684 -16.149 33.272 0.00 0.00
E
ATOM 14786 O TYR E 73 -32.846 -16.251 32.372 0.00 0.00
E
ATOM 14787 N VAL E 74 -33.455 -15.264 34.212 0.00 0.00
E
ATOM 14788 HN VAL E 74 -34.067 -15.039 34.966 0.00 0.00
E
ATOM 14789 CA VAL E 74 -32.103 -14.656 34.344 0.00 0.00
E
ATOM 14790 HA VAL E 74 -31.375 -15.173 33.736 0.00 0.00
E
ATOM 14791 CB VAL E 74 -32.068 -13.178 33.840 0.00 0.00
E
ATOM 14792 HB VAL E 74 -32.705 -12.531 34.480 0.00 0.00
E
ATOM 14793 CG1 VAL E 74 -30.618 -12.708 33.992 0.00 0.00
E
ATOM 14794 HG11 VAL E 74 -30.490 -11.717 33.507 0.00 0.00
E
ATOM 14795 HG12 VAL E 74 -30.210 -12.649 35.024 0.00 0.00
E

ATOM 14796 HG13 VAL E 74   -29.903 -13.354 33.439 0.00 0.00
E
ATOM 14797 CG2 VAL E 74   -32.385 -13.193 32.324 0.00 0.00
E
ATOM 14798 HG21 VAL E 74   -31.697 -13.792 31.690 0.00 0.00
E
ATOM 14799 HG22 VAL E 74   -33.440 -13.486 32.134 0.00 0.00
E
ATOM 14800 HG23 VAL E 74   -32.350 -12.138 31.979 0.00 0.00
E
ATOM 14801 C   VAL E 74   -31.738 -14.715 35.793 0.00 0.00
E
ATOM 14802 O   VAL E 74   -32.468 -14.307 36.745 0.00 0.00
E
ATOM 14803 N   ILE E 75   -30.623 -15.403 35.977 0.00 0.00
E
ATOM 14804 HN ILE E 75   -30.134 -15.843 35.228 0.00 0.00
E
ATOM 14805 CA ILE E 75   -29.926 -15.549 37.264 0.00 0.00
E
ATOM 14806 HA ILE E 75   -30.591 -15.424 38.106 0.00 0.00
E
ATOM 14807 CB ILE E 75   -29.568 -16.969 37.445 0.00 0.00
E
ATOM 14808 HB ILE E 75   -30.545 -17.499 37.415 0.00 0.00
E
ATOM 14809 CG2 ILE E 75   -28.748 -17.584 36.342 0.00 0.00
E
ATOM 14810 HG21 ILE E 75   -28.233 -18.528 36.622 0.00 0.00
E
ATOM 14811 HG22 ILE E 75   -29.321 -17.777 35.410 0.00 0.00
E
ATOM 14812 HG23 ILE E 75   -27.904 -16.893 36.130 0.00 0.00
E
ATOM 14813 CG1 ILE E 75   -28.862 -17.169 38.819 0.00 0.00
E
ATOM 14814 HG11 ILE E 75   -27.766 -17.100 38.653 0.00 0.00
E
ATOM 14815 HG12 ILE E 75   -29.263 -16.462 39.576 0.00 0.00
E
ATOM 14816 CD ILE E 75   -29.202 -18.583 39.383 0.00 0.00
E
ATOM 14817 HD1 ILE E 75   -28.844 -19.374 38.690 0.00 0.00
E
ATOM 14818 HD2 ILE E 75   -28.685 -18.785 40.345 0.00 0.00
E
ATOM 14819 HD3 ILE E 75   -30.291 -18.653 39.593 0.00 0.00
E
ATOM 14820 C   ILE E 75   -28.811 -14.479 37.508 0.00 0.00
E
ATOM 14821 O   ILE E 75   -27.842 -14.398 36.850 0.00 0.00
E
ATOM 14822 N   VAL E 76   -28.969 -13.674 38.543 0.00 0.00
E

ATOM	14823	HN	VAL	E	76	−29.812	−13.668	39.075	0.00	0.00	E
ATOM	14824	CA	VAL	E	76	−28.081	−12.620	38.870	0.00	0.00	E
ATOM	14825	HA	VAL	E	76	−27.490	−12.242	38.048	0.00	0.00	E
ATOM	14826	CB	VAL	E	76	−28.830	−11.442	39.263	0.00	0.00	E
ATOM	14827	HB	VAL	E	76	−29.436	−11.646	40.171	0.00	0.00	E
ATOM	14828	CG1	VAL	E	76	−27.969	−10.194	39.605	0.00	0.00	E
ATOM	14829	HG11	VAL	E	76	−27.591	−9.739	38.665	0.00	0.00	E
ATOM	14830	HG12	VAL	E	76	−28.709	−9.483	40.030	0.00	0.00	E
ATOM	14831	HG13	VAL	E	76	−27.128	−10.339	40.317	0.00	0.00	E
ATOM	14832	CG2	VAL	E	76	−29.743	−11.073	38.134	0.00	0.00	E
ATOM	14833	HG21	VAL	E	76	−30.211	−10.082	38.312	0.00	0.00	E
ATOM	14834	HG22	VAL	E	76	−29.293	−11.105	37.118	0.00	0.00	E
ATOM	14835	HG23	VAL	E	76	−30.700	−11.615	37.972	0.00	0.00	E
ATOM	14836	C	VAL	E	76	−27.111	−12.956	39.987	0.00	0.00	E
ATOM	14837	O	VAL	E	76	−27.556	−13.088	41.069	0.00	0.00	E
ATOM	14838	N	ALA	E	77	−25.922	−13.374	39.678	0.00	0.00	E
ATOM	14839	HN	ALA	E	77	−25.673	−13.363	38.712	0.00	0.00	E
ATOM	14840	CA	ALA	E	77	−25.084	−14.115	40.584	0.00	0.00	E
ATOM	14841	HA	ALA	E	77	−25.606	−14.285	41.514	0.00	0.00	E
ATOM	14842	CB	ALA	E	77	−24.614	−15.359	39.858	0.00	0.00	E
ATOM	14843	HB1	ALA	E	77	−25.497	−15.937	39.510	0.00	0.00	E
ATOM	14844	HB2	ALA	E	77	−24.021	−15.103	38.955	0.00	0.00	E
ATOM	14845	HB3	ALA	E	77	−24.027	−15.975	40.573	0.00	0.00	E
ATOM	14846	C	ALA	E	77	−23.813	−13.333	41.018	0.00	0.00	E
ATOM	14847	O	ALA	E	77	−22.872	−13.866	41.549	0.00	0.00	E
ATOM	14848	N	ILE	E	78	−23.912	−12.007	40.766	0.00	0.00	E
ATOM	14849	HN	ILE	E	78	−24.714	−11.603	40.334	0.00	0.00	E

ATOM 14850 CA ILE E 78   -22.970 -11.058 41.364 0.00 0.00
E
ATOM 14851 HA ILE E 78   -22.060 -11.616 41.529 0.00 0.00
E
ATOM 14852 CB ILE E 78   -22.603 -9.781 40.575 0.00 0.00
E
ATOM 14853 HB ILE E 78   -21.803 -9.949 39.822 0.00 0.00
E
ATOM 14854 CG2 ILE E 78   -23.844 -9.251 39.829 0.00 0.00
E
ATOM 14855 HG21 ILE E 78   -23.624 -8.392 39.160 0.00 0.00
E
ATOM 14856 HG22 ILE E 78   -24.007 -9.973 39.000 0.00 0.00
E
ATOM 14857 HG23 ILE E 78   -24.714 -9.006 40.476 0.00 0.00
E
ATOM 14858 CG1 ILE E 78   -22.087 -8.584 41.388 0.00 0.00
E
ATOM 14859 HG11 ILE E 78   -21.872 -7.738 40.701 0.00 0.00
E
ATOM 14860 HG12 ILE E 78   -22.933 -8.356 42.072 0.00 0.00
E
ATOM 14861 CD ILE E 78   -20.797 -8.870 42.155 0.00 0.00
E
ATOM 14862 HD1 ILE E 78   -21.078 -9.607 42.938 0.00 0.00
E
ATOM 14863 HD2 ILE E 78   -20.024 -9.245 41.451 0.00 0.00
E
ATOM 14864 HD3 ILE E 78   -20.438 -7.980 42.715 0.00 0.00
E
ATOM 14865 C   ILE E 78   -23.525 -10.836 42.756 0.00 0.00
E
ATOM 14866 O   ILE E 78   -24.487 -10.040 42.966 0.00 0.00
E
ATOM 14867 N   GLY E 79   -22.892 -11.447 43.778 0.00 0.00
E
ATOM 14868 HN GLY E 79   -22.023 -11.937 43.779 0.00 0.00
E
ATOM 14869 CA GLY E 79   -23.449 -11.382 45.122 0.00 0.00
E
ATOM 14870 HA1 GLY E 79   -23.571 -12.368 45.544 0.00 0.00
E
ATOM 14871 HA2 GLY E 79   -24.316 -10.738 45.147 0.00 0.00
E
ATOM 14872 C   GLY E 79   -22.577 -10.625 46.130 0.00 0.00
E
ATOM 14873 O   GLY E 79   -23.073 -10.182 47.188 0.00 0.00
E
ATOM 14874 N   ALA E 80   -21.276 -10.482 45.846 0.00 0.00
E
ATOM 14875 HN ALA E 80   -20.798 -11.061 45.191 0.00 0.00
E
ATOM 14876 CA ALA E 80   -20.418 -9.673 46.634 0.00 0.00
E

ATOM 14877 HA ALA E 80   -20.300 -10.129 47.606 0.00 0.00
E
ATOM 14878 CB ALA E 80   -18.996 -9.667 46.007 0.00 0.00
E
ATOM 14879 HB1 ALA E 80   -18.357 -8.962 46.581 0.00 0.00
E
ATOM 14880 HB2 ALA E 80   -18.622 -10.713 46.035 0.00 0.00
E
ATOM 14881 HB3 ALA E 80   -19.107 -9.305 44.962 0.00 0.00
E
ATOM 14882 C   ALA E 80   -20.710 -8.202 46.926 0.00 0.00
E
ATOM 14883 O   ALA E 80   -20.494 -7.656 48.033 0.00 0.00
E
ATOM 14884 N   ASN E 81   -21.258 -7.435 46.011 0.00 0.00
E
ATOM 14885 HN ASN E 81   -21.291 -7.776 45.075 0.00 0.00
E
ATOM 14886 CA ASN E 81   -21.560 -6.016 46.254 0.00 0.00
E
ATOM 14887 HA ASN E 81   -21.361 -5.712 47.271 0.00 0.00
E
ATOM 14888 CB ASN E 81   -20.635 -5.148 45.355 0.00 0.00
E
ATOM 14889 HB1 ASN E 81   -19.611 -5.309 45.755 0.00 0.00
E
ATOM 14890 HB2 ASN E 81   -20.793 -5.524 44.322 0.00 0.00
E
ATOM 14891 CG ASN E 81   -20.939 -3.616 45.350 0.00 0.00
E
ATOM 14892 OD1 ASN E 81   -21.660 -3.103 44.437 0.00 0.00
E
ATOM 14893 ND2 ASN E 81   -20.418 -2.969 46.332 0.00 0.00
E
ATOM 14894 HD21 ASN E 81   -20.594 -1.984 46.317 0.00 0.00
E
ATOM 14895 HD22 ASN E 81   -19.915 -3.454 47.048 0.00 0.00
E
ATOM 14896 C   ASN E 81   -23.051 -5.823 46.053 0.00 0.00
E
ATOM 14897 O   ASN E 81   -23.556 -6.101 44.937 0.00 0.00
E
ATOM 14898 N   ILE E 82   -23.741 -5.247 47.083 0.00 0.00
E
ATOM 14899 HN ILE E 82   -23.271 -5.063 47.943 0.00 0.00
E
ATOM 14900 CA ILE E 82   -25.223 -5.003 47.143 0.00 0.00
E
ATOM 14901 HA ILE E 82   -25.702 -5.965 47.039 0.00 0.00
E
ATOM 14902 CB ILE E 82   -25.716 -4.278 48.462 0.00 0.00
E
ATOM 14903 HB ILE E 82   -25.246 -3.276 48.555 0.00 0.00
E

ATOM 14904 CG2 ILE E 82   -27.257 -4.105 48.309 0.00 0.00
E
ATOM 14905 HG21 ILE E 82   -27.775 -5.086 48.256 0.00 0.00
E
ATOM 14906 HG22 ILE E 82   -27.662 -3.434 49.097 0.00 0.00
E
ATOM 14907 HG23 ILE E 82   -27.462 -3.505 47.397 0.00 0.00
E
ATOM 14908 CG1 ILE E 82   -25.190 -5.005 49.743 0.00 0.00
E
ATOM 14909 HG11 ILE E 82   -25.595 -6.036 49.818 0.00 0.00
E
ATOM 14910 HG12 ILE E 82   -24.085 -5.087 49.658 0.00 0.00
E
ATOM 14911 CD ILE E 82   -25.531 -4.391 51.124 0.00 0.00
E
ATOM 14912 HD1 ILE E 82   -26.576 -4.687 51.360 0.00 0.00
E
ATOM 14913 HD2 ILE E 82   -24.752 -4.661 51.868 0.00 0.00
E
ATOM 14914 HD3 ILE E 82   -25.561 -3.289 50.986 0.00 0.00
E
ATOM 14915 C   ILE E 82   -25.672 -4.105 46.013 0.00 0.00
E
ATOM 14916 O   ILE E 82   -26.538 -4.504 45.246 0.00 0.00
E
ATOM 14917 N   GLN E 83   -24.841 -3.031 45.852 0.00 0.00
E
ATOM 14918 HN GLN E 83   -24.163 -2.806 46.547 0.00 0.00
E
ATOM 14919 CA GLN E 83   -25.061 -2.044 44.770 0.00 0.00
E
ATOM 14920 HA GLN E 83   -25.966 -1.579 45.130 0.00 0.00
E
ATOM 14921 CB GLN E 83   -24.124 -0.836 44.902 0.00 0.00
E
ATOM 14922 HB1 GLN E 83   -24.068 -0.573 45.980 0.00 0.00
E
ATOM 14923 HB2 GLN E 83   -23.139 -1.065 44.441 0.00 0.00
E
ATOM 14924 CG GLN E 83   -24.646   0.411 44.143 0.00 0.00
E
ATOM 14925 HG1 GLN E 83   -24.626   0.296 43.038 0.00 0.00
E
ATOM 14926 HG2 GLN E 83   -25.692   0.656 44.429 0.00 0.00
E
ATOM 14927 CD GLN E 83   -23.727   1.624 44.499 0.00 0.00
E
ATOM 14928 OE1 GLN E 83   -23.506   1.835 45.672 0.00 0.00
E
ATOM 14929 NE2 GLN E 83   -23.169   2.387 43.533 0.00 0.00
E
ATOM 14930 HE21 GLN E 83   -22.574   3.126 43.850 0.00 0.00
E

ATOM 14931 HE22 GLN E 83 −23.340 2.027 42.616 0.00 0.00
E
ATOM 14932 C GLN E 83 −25.098 −2.525 43.287 0.00 0.00
E
ATOM 14933 O GLN E 83 −26.025 −2.276 42.525 0.00 0.00
E
ATOM 14934 N ALA E 84 −24.091 −3.304 42.867 0.00 0.00
E
ATOM 14935 HN ALA E 84 −23.304 −3.350 43.478 0.00 0.00
E
ATOM 14936 CA ALA E 84 −24.075 −4.054 41.619 0.00 0.00
E
ATOM 14937 HA ALA E 84 −24.238 −3.344 40.822 0.00 0.00
E
ATOM 14938 CB ALA E 84 −22.739 −4.775 41.584 0.00 0.00
E
ATOM 14939 HB1 ALA E 84 −22.806 −5.425 42.482 0.00 0.00
E
ATOM 14940 HB2 ALA E 84 −22.608 −5.379 40.661 0.00 0.00
E
ATOM 14941 HB3 ALA E 84 −21.933 −4.026 41.737 0.00 0.00
E
ATOM 14942 C ALA E 84 −25.187 −5.027 41.409 0.00 0.00
E
ATOM 14943 O ALA E 84 −25.894 −4.986 40.394 0.00 0.00
E
ATOM 14944 N SER E 85 −25.506 −5.906 42.374 0.00 0.00
E
ATOM 14945 HN SER E 85 −24.834 −5.845 43.108 0.00 0.00
E
ATOM 14946 CA SER E 85 −26.562 −6.979 42.337 0.00 0.00
E
ATOM 14947 HA SER E 85 −26.377 −7.683 41.540 0.00 0.00
E
ATOM 14948 CB SER E 85 −26.578 −7.544 43.807 0.00 0.00
E
ATOM 14949 HB1 SER E 85 −25.555 −7.358 44.198 0.00 0.00
E
ATOM 14950 HB2 SER E 85 −27.177 −6.898 44.484 0.00 0.00
E
ATOM 14951 OG SER E 85 −26.934 −8.927 44.006 0.00 0.00
E
ATOM 14952 HG1 SER E 85 −26.198 −9.392 43.599 0.00 0.00
E
ATOM 14953 C SER E 85 −27.946 −6.376 42.165 0.00 0.00
E
ATOM 14954 O SER E 85 −28.888 −6.916 41.539 0.00 0.00
E
ATOM 14955 N THR E 86 −28.227 −5.213 42.838 0.00 0.00
E
ATOM 14956 HN THR E 86 −27.549 −4.775 43.424 0.00 0.00
E
ATOM 14957 CA THR E 86 −29.554 −4.618 42.882 0.00 0.00
E

ATOM 14958 HA THR E 86 -30.233 -5.451 42.984 0.00 0.00
E
ATOM 14959 CB THR E 86 -29.769 -3.682 44.120 0.00 0.00
E
ATOM 14960 HB THR E 86 -30.661 -3.019 44.108 0.00 0.00
E
ATOM 14961 OG1 THR E 86 -28.622 -2.838 44.183 0.00 0.00
E
ATOM 14962 HG1 THR E 86 -28.112 -3.254 44.882 0.00 0.00
E
ATOM 14963 CG2 THR E 86 -29.921 -4.516 45.310 0.00 0.00
E
ATOM 14964 HG21 THR E 86 -30.879 -5.074 45.235 0.00 0.00
E
ATOM 14965 HG22 THR E 86 -29.049 -5.203 45.343 0.00 0.00
E
ATOM 14966 HG23 THR E 86 -29.847 -3.998 46.290 0.00 0.00
E
ATOM 14967 C THR E 86 -29.877 -3.784 41.649 0.00 0.00
E
ATOM 14968 O THR E 86 -31.006 -3.810 41.156 0.00 0.00
E
ATOM 14969 N LEU E 87 -28.857 -3.070 41.202 0.00 0.00
E
ATOM 14970 HN LEU E 87 -27.963 -3.147 41.637 0.00 0.00
E
ATOM 14971 CA LEU E 87 -29.056 -2.229 40.028 0.00 0.00
E
ATOM 14972 HA LEU E 87 -29.888 -1.543 40.096 0.00 0.00
E
ATOM 14973 CB LEU E 87 -27.874 -1.211 39.895 0.00 0.00
E
ATOM 14974 HB1 LEU E 87 -27.801 -0.606 40.824 0.00 0.00
E
ATOM 14975 HB2 LEU E 87 -26.873 -1.685 39.810 0.00 0.00
E
ATOM 14976 CG LEU E 87 -27.872 -0.098 38.742 0.00 0.00
E
ATOM 14977 HG LEU E 87 -27.737 -0.629 37.776 0.00 0.00
E
ATOM 14978 CD1 LEU E 87 -29.162 0.693 38.764 0.00 0.00
E
ATOM 14979 HD11 LEU E 87 -30.092 0.117 38.568 0.00 0.00
E
ATOM 14980 HD12 LEU E 87 -29.228 1.183 39.759 0.00 0.00
E
ATOM 14981 HD13 LEU E 87 -29.210 1.520 38.024 0.00 0.00
E
ATOM 14982 CD2 LEU E 87 -26.749 0.879 38.914 0.00 0.00
E
ATOM 14983 HD21 LEU E 87 -26.804 1.332 39.927 0.00 0.00
E
ATOM 14984 HD22 LEU E 87 -25.755 0.385 38.939 0.00 0.00
E

ATOM 14985 HD23 LEU E 87 −26.748 1.626 38.091 0.00 0.00
E
ATOM 14986 C LEU E 87 −29.191 −3.084 38.732 0.00 0.00
E
ATOM 14987 O LEU E 87 −30.195 −2.890 38.053 0.00 0.00
E
ATOM 14988 N THR E 88 −28.348 −4.123 38.454 0.00 0.00
E
ATOM 14989 HN THR E 88 −27.457 −4.146 38.900 0.00 0.00
E
ATOM 14990 CA THR E 88 −28.579 −5.231 37.514 0.00 0.00
E
ATOM 14991 HA THR E 88 −28.477 −4.913 36.487 0.00 0.00
E
ATOM 14992 CB THR E 88 −27.651 −6.353 37.738 0.00 0.00
E
ATOM 14993 HB THR E 88 −27.883 −6.768 38.742 0.00 0.00
E
ATOM 14994 OG1 THR E 88 −26.289 −5.859 37.790 0.00 0.00
E
ATOM 14995 HG1 THR E 88 −26.028 −5.664 38.693 0.00 0.00
E
ATOM 14996 CG2 THR E 88 −27.604 −7.281 36.493 0.00 0.00
E
ATOM 14997 HG21 THR E 88 −26.670 −7.872 36.377 0.00 0.00
E
ATOM 14998 HG22 THR E 88 −28.438 −8.000 36.351 0.00 0.00
E
ATOM 14999 HG23 THR E 88 −27.591 −6.592 35.622 0.00 0.00
E
ATOM 15000 C THR E 88 −29.993 −5.888 37.624 0.00 0.00
E
ATOM 15001 O THR E 88 −30.713 −5.994 36.610 0.00 0.00
E
ATOM 15002 N THR E 89 −30.414 −6.136 38.849 0.00 0.00
E
ATOM 15003 HN THR E 89 −29.922 −5.839 39.664 0.00 0.00
E
ATOM 15004 CA THR E 89 −31.810 −6.696 39.000 0.00 0.00
E
ATOM 15005 HA THR E 89 −31.949 −7.539 38.339 0.00 0.00
E
ATOM 15006 CB THR E 89 −32.102 −7.175 40.494 0.00 0.00
E
ATOM 15007 HB THR E 89 −32.106 −6.381 41.271 0.00 0.00
E
ATOM 15008 OG1 THR E 89 −31.193 −8.166 40.882 0.00 0.00
E
ATOM 15009 HG1 THR E 89 −30.407 −7.681 41.146 0.00 0.00
E
ATOM 15010 CG2 THR E 89 −33.488 −7.789 40.700 0.00 0.00
E
ATOM 15011 HG21 THR E 89 −33.643 −7.989 41.782 0.00 0.00
E

ATOM	15012	HG22	THR	E	89	−34.356	−7.229	40.290	0.00	0.00	E
ATOM	15013	HG23	THR	E	89	−33.467	−8.751	40.146	0.00	0.00	E
ATOM	15014	C	THR	E	89	−33.006	−5.767	38.552	0.00	0.00	E
ATOM	15015	O	THR	E	89	−33.926	−6.205	37.898	0.00	0.00	E
ATOM	15016	N	LEU	E	90	−32.861	−4.433	38.875	0.00	0.00	E
ATOM	15017	HN	LEU	E	90	−32.243	−4.130	39.597	0.00	0.00	E
ATOM	15018	CA	LEU	E	90	−33.749	−3.310	38.405	0.00	0.00	E
ATOM	15019	HA	LEU	E	90	−34.763	−3.627	38.598	0.00	0.00	E
ATOM	15020	CB	LEU	E	90	−33.493	−1.988	39.169	0.00	0.00	E
ATOM	15021	HB1	LEU	E	90	−33.379	−2.213	40.251	0.00	0.00	E
ATOM	15022	HB2	LEU	E	90	−32.471	−1.719	38.827	0.00	0.00	E
ATOM	15023	CG	LEU	E	90	−34.391	−0.761	39.002	0.00	0.00	E
ATOM	15024	HG	LEU	E	90	−34.404	−0.396	37.953	0.00	0.00	E
ATOM	15025	CD1	LEU	E	90	−35.859	−1.147	39.323	0.00	0.00	E
ATOM	15026	HD11	LEU	E	90	−36.448	−0.226	39.522	0.00	0.00	E
ATOM	15027	HD12	LEU	E	90	−36.300	−1.661	38.442	0.00	0.00	E
ATOM	15028	HD13	LEU	E	90	−35.866	−1.823	40.204	0.00	0.00	E
ATOM	15029	CD2	LEU	E	90	−33.859	0.341	39.954	0.00	0.00	E
ATOM	15030	HD21	LEU	E	90	−33.762	−0.010	41.003	0.00	0.00	E
ATOM	15031	HD22	LEU	E	90	−32.848	0.684	39.649	0.00	0.00	E
ATOM	15032	HD23	LEU	E	90	−34.504	1.245	39.920	0.00	0.00	E
ATOM	15033	C	LEU	E	90	−33.640	−3.046	36.951	0.00	0.00	E
ATOM	15034	O	LEU	E	90	−34.654	−2.578	36.367	0.00	0.00	E
ATOM	15035	N	LEU	E	91	−32.495	−3.244	36.327	0.00	0.00	E
ATOM	15036	HN	LEU	E	91	−31.692	−3.539	36.838	0.00	0.00	E
ATOM	15037	CA	LEU	E	91	−32.398	−3.036	34.881	0.00	0.00	E
ATOM	15038	HA	LEU	E	91	−32.987	−2.194	34.548	0.00	0.00	E

ATOM 15039 CB LEU E 91 −30.950 −2.809 34.348 0.00 0.00
E
ATOM 15040 HB1 LEU E 91 −30.280 −3.622 34.699 0.00 0.00
E
ATOM 15041 HB2 LEU E 91 −30.990 −2.787 33.238 0.00 0.00
E
ATOM 15042 CG LEU E 91 −30.351 −1.419 34.700 0.00 0.00
E
ATOM 15043 HG LEU E 91 −30.429 −1.174 35.781 0.00 0.00
E
ATOM 15044 CD1 LEU E 91 −28.887 −1.266 34.383 0.00 0.00
E
ATOM 15045 HD11 LEU E 91 −28.482 −0.290 34.728 0.00 0.00
E
ATOM 15046 HD12 LEU E 91 −28.273 −2.026 34.912 0.00 0.00
E
ATOM 15047 HD13 LEU E 91 −28.780 −1.340 33.280 0.00 0.00
E
ATOM 15048 CD2 LEU E 91 −31.051 −0.244 33.983 0.00 0.00
E
ATOM 15049 HD21 LEU E 91 −30.959 −0.414 32.889 0.00 0.00
E
ATOM 15050 HD22 LEU E 91 −32.070 −0.144 34.415 0.00 0.00
E
ATOM 15051 HD23 LEU E 91 −30.443 0.665 34.182 0.00 0.00
E
ATOM 15052 C LEU E 91 −32.928 −4.193 34.063 0.00 0.00
E
ATOM 15053 O LEU E 91 −33.470 −3.950 32.984 0.00 0.00
E
ATOM 15054 N LEU E 92 −32.838 −5.459 34.470 0.00 0.00
E
ATOM 15055 HN LEU E 92 −32.222 −5.628 35.236 0.00 0.00
E
ATOM 15056 CA LEU E 92 −33.579 −6.577 33.886 0.00 0.00
E
ATOM 15057 HA LEU E 92 −33.276 −6.759 32.865 0.00 0.00
E
ATOM 15058 CB LEU E 92 −33.285 −7.912 34.716 0.00 0.00
E
ATOM 15059 HB1 LEU E 92 −33.559 −7.730 35.778 0.00 0.00
E
ATOM 15060 HB2 LEU E 92 −34.005 −8.671 34.343 0.00 0.00
E
ATOM 15061 CG LEU E 92 −31.856 −8.448 34.600 0.00 0.00
E
ATOM 15062 HG LEU E 92 −31.124 −7.617 34.691 0.00 0.00
E
ATOM 15063 CD1 LEU E 92 −31.687 −9.517 35.727 0.00 0.00
E
ATOM 15064 HD11 LEU E 92 −31.564 −9.137 36.763 0.00 0.00
E
ATOM 15065 HD12 LEU E 92 −32.463 −10.311 35.696 0.00 0.00
E

ATOM 15066 HD13 LEU E 92 −30.683 −9.942 35.512 0.00 0.00
E
ATOM 15067 CD2 LEU E 92 −31.598 −9.130 33.298 0.00 0.00
E
ATOM 15068 HD21 LEU E 92 −31.877 −8.489 32.435 0.00 0.00
E
ATOM 15069 HD22 LEU E 92 −30.520 −9.370 33.178 0.00 0.00
E
ATOM 15070 HD23 LEU E 92 −32.221 −10.018 33.059 0.00 0.00
E
ATOM 15071 C LEU E 92 −35.112 −6.485 33.921 0.00 0.00
E
ATOM 15072 O LEU E 92 −35.785 −6.880 33.007 0.00 0.00
E
ATOM 15073 N LYS E 93 −35.678 −5.932 35.034 0.00 0.00
E
ATOM 15074 HN LYS E 93 −35.187 −5.761 35.885 0.00 0.00
E
ATOM 15075 CA LYS E 93 −37.071 −5.510 35.079 0.00 0.00
E
ATOM 15076 HA LYS E 93 −37.743 −6.349 34.970 0.00 0.00
E
ATOM 15077 CB LYS E 93 −37.289 −4.870 36.442 0.00 0.00
E
ATOM 15078 HB1 LYS E 93 −36.372 −4.268 36.620 0.00 0.00
E
ATOM 15079 HB2 LYS E 93 −38.192 −4.227 36.511 0.00 0.00
E
ATOM 15080 CG LYS E 93 −37.236 −5.888 37.578 0.00 0.00
E
ATOM 15081 HG1 LYS E 93 −36.619 −6.767 37.293 0.00 0.00
E
ATOM 15082 HG2 LYS E 93 −36.926 −5.386 38.520 0.00 0.00
E
ATOM 15083 CD LYS E 93 −38.584 −6.475 37.791 0.00 0.00
E
ATOM 15084 HD1 LYS E 93 −39.438 −5.831 37.493 0.00 0.00
E
ATOM 15085 HD2 LYS E 93 −38.656 −7.275 37.024 0.00 0.00
E
ATOM 15086 CE LYS E 93 −38.828 −6.950 39.178 0.00 0.00
E
ATOM 15087 HE1 LYS E 93 −37.961 −7.497 39.605 0.00 0.00
E
ATOM 15088 HE2 LYS E 93 −39.028 −6.113 39.881 0.00 0.00
E
ATOM 15089 NZ LYS E 93 −40.059 −7.799 39.319 0.00 0.00
E
ATOM 15090 HZ1 LYS E 93 −40.948 −7.346 39.027 0.00 0.00
E
ATOM 15091 HZ2 LYS E 93 −39.898 −8.723 38.870 0.00 0.00
E
ATOM 15092 HZ3 LYS E 93 −40.281 −7.993 40.317 0.00 0.00
E

ATOM	15093	C	LYS	E	93	−37.560	−4.578	34.062	0.00	0.00	E
ATOM	15094	O	LYS	E	93	−38.760	−4.674	33.700	0.00	0.00	E
ATOM	15095	N	GLU	E	94	−36.728	−3.708	33.550	0.00	0.00	E
ATOM	15096	HN	GLU	E	94	−35.777	−3.603	33.831	0.00	0.00	E
ATOM	15097	CA	GLU	E	94	−37.068	−2.870	32.446	0.00	0.00	E
ATOM	15098	HA	GLU	E	94	−38.071	−2.552	32.690	0.00	0.00	E
ATOM	15099	CB	GLU	E	94	−36.148	−1.713	32.672	0.00	0.00	E
ATOM	15100	HB1	GLU	E	94	−36.279	−1.353	33.715	0.00	0.00	E
ATOM	15101	HB2	GLU	E	94	−35.097	−2.043	32.521	0.00	0.00	E
ATOM	15102	CG	GLU	E	94	−36.389	−0.492	31.770	0.00	0.00	E
ATOM	15103	HG1	GLU	E	94	−35.554	0.235	31.862	0.00	0.00	E
ATOM	15104	HG2	GLU	E	94	−36.350	−0.659	30.672	0.00	0.00	E
ATOM	15105	CD	GLU	E	94	−37.682	0.244	32.064	0.00	0.00	E
ATOM	15106	OE1	GLU	E	94	−37.991	0.430	33.274	0.00	0.00	E
ATOM	15107	OE2	GLU	E	94	−38.393	0.612	31.145	0.00	0.00	E
ATOM	15108	C	GLU	E	94	−37.059	−3.566	31.135	0.00	0.00	E
ATOM	15109	O	GLU	E	94	−37.520	−3.057	30.152	0.00	0.00	E
ATOM	15110	N	LEU	E	95	−36.657	−4.849	31.116	0.00	0.00	E
ATOM	15111	HN	LEU	E	95	−36.546	−5.368	31.959	0.00	0.00	E
ATOM	15112	CA	LEU	E	95	−36.749	−5.550	29.850	0.00	0.00	E
ATOM	15113	HA	LEU	E	95	−36.820	−4.926	28.971	0.00	0.00	E
ATOM	15114	CB	LEU	E	95	−35.546	−6.507	29.558	0.00	0.00	E
ATOM	15115	HB1	LEU	E	95	−35.422	−7.202	30.417	0.00	0.00	E
ATOM	15116	HB2	LEU	E	95	−35.794	−7.124	28.668	0.00	0.00	E
ATOM	15117	CG	LEU	E	95	−34.144	−5.876	29.337	0.00	0.00	E
ATOM	15118	HG	LEU	E	95	−33.985	−5.007	30.009	0.00	0.00	E
ATOM	15119	CD1	LEU	E	95	−33.045	−6.837	29.753	0.00	0.00	E

ATOM 15120 HD11 LEU E 95 -31.997 -6.545 29.526 0.00 0.00
E
ATOM 15121 HD12 LEU E 95 -33.034 -7.027 30.848 0.00 0.00
E
ATOM 15122 HD13 LEU E 95 -33.181 -7.835 29.284 0.00 0.00
E
ATOM 15123 CD2 LEU E 95 -33.844 -5.333 27.873 0.00 0.00
E
ATOM 15124 HD21 LEU E 95 -33.993 -6.092 27.075 0.00 0.00
E
ATOM 15125 HD22 LEU E 95 -34.556 -4.486 27.774 0.00 0.00
E
ATOM 15126 HD23 LEU E 95 -32.806 -4.942 27.810 0.00 0.00
E
ATOM 15127 C LEU E 95 -38.047 -6.357 29.963 0.00 0.00
E
ATOM 15128 O LEU E 95 -38.364 -7.090 28.976 0.00 0.00
E
ATOM 15129 N ASP E 96 -38.671 -6.477 31.205 0.00 0.00
E
ATOM 15130 HN ASP E 96 -38.558 -5.803 31.932 0.00 0.00
E
ATOM 15131 CA ASP E 96 -39.773 -7.466 31.386 0.00 0.00
E
ATOM 15132 HA ASP E 96 -40.206 -7.260 32.354 0.00 0.00
E
ATOM 15133 CB ASP E 96 -40.981 -7.308 30.414 0.00 0.00
E
ATOM 15134 HB1 ASP E 96 -40.663 -7.452 29.359 0.00 0.00
E
ATOM 15135 HB2 ASP E 96 -41.740 -8.067 30.700 0.00 0.00
E
ATOM 15136 CG ASP E 96 -41.482 -5.878 30.591 0.00 0.00
E
ATOM 15137 OD1 ASP E 96 -42.330 -5.769 31.431 0.00 0.00
E
ATOM 15138 OD2 ASP E 96 -41.105 -4.980 29.791 0.00 0.00
E
ATOM 15139 C ASP E 96 -39.333 -8.896 31.408 0.00 0.00
E
ATOM 15140 O ASP E 96 -39.981 -9.741 30.752 0.00 0.00
E
ATOM 15141 N ILE E 97 -38.257 -9.209 32.153 0.00 0.00
E
ATOM 15142 HN ILE E 97 -37.616 -8.510 32.461 0.00 0.00
E
ATOM 15143 CA ILE E 97 -37.864 -10.628 32.374 0.00 0.00
E
ATOM 15144 HA ILE E 97 -37.897 -11.029 31.372 0.00 0.00
E
ATOM 15145 CB ILE E 97 -36.459 -10.742 32.918 0.00 0.00
E
ATOM 15146 HB ILE E 97 -36.430 -10.247 33.912 0.00 0.00
E

```
ATOM  15147  CG2 ILE E  97      -36.092 -12.179  33.116  0.00  0.00
E
ATOM  15148 HG21 ILE E  97      -36.207 -12.726  32.156  0.00  0.00
E
ATOM  15149 HG22 ILE E  97      -35.071 -12.278  33.542  0.00  0.00
E
ATOM  15150 HG23 ILE E  97      -36.707 -12.671  33.900  0.00  0.00
E
ATOM  15151  CG1 ILE E  97      -35.341 -10.062  32.053  0.00  0.00
E
ATOM  15152 HG11 ILE E  97      -35.479  -8.961  32.004  0.00  0.00
E
ATOM  15153 HG12 ILE E  97      -34.381 -10.389  32.509  0.00  0.00
E
ATOM  15154  CD  ILE E  97      -35.195 -10.709  30.644  0.00  0.00
E
ATOM  15155  HD1 ILE E  97      -35.114 -11.817  30.648  0.00  0.00
E
ATOM  15156  HD2 ILE E  97      -36.114 -10.527  30.046  0.00  0.00
E
ATOM  15157  HD3 ILE E  97      -34.377 -10.195  30.094  0.00  0.00
E
ATOM  15158  C   ILE E  97      -38.750 -11.399  33.392  0.00  0.00
E
ATOM  15159  O   ILE E  97      -38.693 -10.957  34.538  0.00  0.00
E
ATOM  15160  N   PRO E  98      -39.422 -12.495  33.135  0.00  0.00
E
ATOM  15161  CD  PRO E  98      -39.565 -13.090  31.821  0.00  0.00
E
ATOM  15162  HD1 PRO E  98      -39.918 -12.308  31.114  0.00  0.00
E
ATOM  15163  HD2 PRO E  98      -38.654 -13.503  31.337  0.00  0.00
E
ATOM  15164  CA  PRO E  98      -40.377 -13.077  34.089  0.00  0.00
E
ATOM  15165  HA  PRO E  98      -41.015 -12.310  34.504  0.00  0.00
E
ATOM  15166  CB  PRO E  98      -41.337 -13.967  33.268  0.00  0.00
E
ATOM  15167  HB1 PRO E  98      -42.236 -13.403  32.938  0.00  0.00
E
ATOM  15168  HB2 PRO E  98      -41.808 -14.882  33.689  0.00  0.00
E
ATOM  15169  CG  PRO E  98      -40.602 -14.220  31.945  0.00  0.00
E
ATOM  15170  HG1 PRO E  98      -41.330 -14.247  31.106  0.00  0.00
E
ATOM  15171  HG2 PRO E  98      -40.001 -15.154  31.971  0.00  0.00
E
ATOM  15172  C   PRO E  98      -39.838 -13.662  35.315  0.00  0.00
E
ATOM  15173  O   PRO E  98      -40.520 -13.517  36.350  0.00  0.00
E
```

ATOM 15174 N ASN E 99 −38.752 −14.401 35.321 0.00 0.00
E
ATOM 15175 HN ASN E 99 −38.131 −14.431 34.542 0.00 0.00
E
ATOM 15176 CA ASN E 99 −38.326 −15.030 36.557 0.00 0.00
E
ATOM 15177 HA ASN E 99 −38.859 −14.762 37.457 0.00 0.00
E
ATOM 15178 CB ASN E 99 −38.276 −16.605 36.355 0.00 0.00
E
ATOM 15179 HB1 ASN E 99 −37.696 −16.956 35.476 0.00 0.00
E
ATOM 15180 HB2 ASN E 99 −37.627 −17.079 37.122 0.00 0.00
E
ATOM 15181 CG ASN E 99 −39.677 −17.191 36.240 0.00 0.00
E
ATOM 15182 OD1 ASN E 99 −40.659 −16.912 36.952 0.00 0.00
E
ATOM 15183 ND2 ASN E 99 −39.823 −18.144 35.334 0.00 0.00
E
ATOM 15184 HD21 ASN E 99 −40.735 −18.472 35.089 0.00 0.00
E
ATOM 15185 HD22 ASN E 99 −39.048 −18.431 34.770 0.00 0.00
E
ATOM 15186 C ASN E 99 −36.926 −14.576 36.828 0.00 0.00
E
ATOM 15187 O ASN E 99 −35.961 −15.023 36.192 0.00 0.00
E
ATOM 15188 N ILE E 100 −36.739 −13.666 37.902 0.00 0.00
E
ATOM 15189 HN ILE E 100 −37.473 −13.199 38.390 0.00 0.00
E
ATOM 15190 CA ILE E 100 −35.383 −13.186 38.286 0.00 0.00
E
ATOM 15191 HA ILE E 100 −34.676 −13.430 37.507 0.00 0.00
E
ATOM 15192 CB ILE E 100 −35.293 −11.594 38.460 0.00 0.00
E
ATOM 15193 HB ILE E 100 −35.897 −11.289 39.341 0.00 0.00
E
ATOM 15194 CG2 ILE E 100 −33.850 −11.173 38.585 0.00 0.00
E
ATOM 15195 HG21 ILE E 100 −33.340 −11.500 37.653 0.00 0.00
E
ATOM 15196 HG22 ILE E 100 −33.797 −10.064 38.638 0.00 0.00
E
ATOM 15197 HG23 ILE E 100 −33.397 −11.588 39.511 0.00 0.00
E
ATOM 15198 CG1 ILE E 100 −35.955 −10.905 37.208 0.00 0.00
E
ATOM 15199 HG11 ILE E 100 −35.387 −11.325 36.350 0.00 0.00
E
ATOM 15200 HG12 ILE E 100 −36.995 −11.263 37.054 0.00 0.00
E

ATOM 15201 CD ILE E 100 −35.914 −9.380 37.252 0.00 0.00
E
ATOM 15202 HD1 ILE E 100 −36.408 −9.015 36.326 0.00 0.00
E
ATOM 15203 HD2 ILE E 100 −36.359 −8.985 38.190 0.00 0.00
E
ATOM 15204 HD3 ILE E 100 −34.895 −8.946 37.167 0.00 0.00
E
ATOM 15205 C ILE E 100 −34.820 −13.792 39.617 0.00 0.00
E
ATOM 15206 O ILE E 100 −35.185 −13.469 40.725 0.00 0.00
E
ATOM 15207 N TRP E 101 −33.787 −14.701 39.521 0.00 0.00
E
ATOM 15208 HN TRP E 101 −33.501 −14.897 38.586 0.00 0.00
E
ATOM 15209 CA TRP E 101 −33.183 −15.364 40.616 0.00 0.00
E
ATOM 15210 HA TRP E 101 −33.856 −15.288 41.456 0.00 0.00
E
ATOM 15211 CB TRP E 101 −32.846 −16.866 40.387 0.00 0.00
E
ATOM 15212 HB1 TRP E 101 −32.160 −16.995 39.523 0.00 0.00
E
ATOM 15213 HB2 TRP E 101 −32.390 −17.294 41.305 0.00 0.00
E
ATOM 15214 CG TRP E 101 −34.033 −17.762 39.966 0.00 0.00
E
ATOM 15215 CD1 TRP E 101 −35.284 −17.489 39.551 0.00 0.00
E
ATOM 15216 HD1 TRP E 101 −35.598 −16.492 39.279 0.00 0.00
E
ATOM 15217 NE1 TRP E 101 −36.089 −18.605 39.469 0.00 0.00
E
ATOM 15218 HE1 TRP E 101 −37.045 −18.591 39.273 0.00 0.00
E
ATOM 15219 CE2 TRP E 101 −35.350 −19.683 39.907 0.00 0.00
E
ATOM 15220 CD2 TRP E 101 −34.032 −19.204 40.237 0.00 0.00
E
ATOM 15221 CE3 TRP E 101 −32.987 −20.047 40.625 0.00 0.00
E
ATOM 15222 HE3 TRP E 101 −31.994 −19.689 40.855 0.00 0.00
E
ATOM 15223 CZ3 TRP E 101 −33.311 −21.389 40.798 0.00 0.00
E
ATOM 15224 HZ3 TRP E 101 −32.522 −22.000 41.213 0.00 0.00
E
ATOM 15225 CZ2 TRP E 101 −35.596 −21.015 40.041 0.00 0.00
E
ATOM 15226 HZ2 TRP E 101 −36.507 −21.523 39.762 0.00 0.00
E
ATOM 15227 CH2 TRP E 101 −34.607 −21.866 40.563 0.00 0.00
E

ATOM 15228 HH2 TRP E 101 −34.843 −22.919 40.611 0.00 0.00
E
ATOM 15229 C TRP E 101 −31.899 −14.561 40.919 0.00 0.00
E
ATOM 15230 O TRP E 101 −31.196 −14.060 40.078 0.00 0.00
E
ATOM 15231 N VAL E 102 −31.555 −14.365 42.207 0.00 0.00
E
ATOM 15232 HN VAL E 102 −32.010 −14.754 43.005 0.00 0.00
E
ATOM 15233 CA VAL E 102 −30.402 −13.511 42.572 0.00 0.00
E
ATOM 15234 HA VAL E 102 −29.734 −13.486 41.723 0.00 0.00
E
ATOM 15235 CB VAL E 102 −30.844 −12.051 42.894 0.00 0.00
E
ATOM 15236 HB VAL E 102 −31.365 −12.053 43.875 0.00 0.00
E
ATOM 15237 CG1 VAL E 102 −29.631 −11.108 43.099 0.00 0.00
E
ATOM 15238 HG11 VAL E 102 −29.127 −11.369 44.054 0.00 0.00
E
ATOM 15239 HG12 VAL E 102 −28.925 −11.153 42.243 0.00 0.00
E
ATOM 15240 HG13 VAL E 102 −30.030 −10.081 43.246 0.00 0.00
E
ATOM 15241 CG2 VAL E 102 −31.777 −11.466 41.805 0.00 0.00
E
ATOM 15242 HG21 VAL E 102 −31.870 −10.379 42.015 0.00 0.00
E
ATOM 15243 HG22 VAL E 102 −31.410 −11.426 40.757 0.00 0.00
E
ATOM 15244 HG23 VAL E 102 −32.760 −11.983 41.816 0.00 0.00
E
ATOM 15245 C VAL E 102 −29.712 −14.083 43.727 0.00 0.00
E
ATOM 15246 O VAL E 102 −30.353 −14.494 44.727 0.00 0.00
E
ATOM 15247 N LYS E 103 −28.401 −14.103 43.715 0.00 0.00
E
ATOM 15248 HN LYS E 103 −28.058 −13.611 42.919 0.00 0.00
E
ATOM 15249 CA LYS E 103 −27.476 −14.592 44.764 0.00 0.00
E
ATOM 15250 HA LYS E 103 −27.906 −15.463 45.237 0.00 0.00
E
ATOM 15251 CB LYS E 103 −26.108 −15.067 44.097 0.00 0.00
E
ATOM 15252 HB1 LYS E 103 −26.323 −15.731 43.233 0.00 0.00
E
ATOM 15253 HB2 LYS E 103 −25.555 −14.199 43.677 0.00 0.00
E
ATOM 15254 CG LYS E 103 −25.259 −15.957 44.996 0.00 0.00
E

ATOM 15255 HG1 LYS E 103 −25.022 −15.645 46.036 0.00 0.00
E
ATOM 15256 HG2 LYS E 103 −25.849 −16.872 45.219 0.00 0.00
E
ATOM 15257 CD LYS E 103 −23.899 −16.256 44.286 0.00 0.00
E
ATOM 15258 HD1 LYS E 103 −24.084 −17.003 43.485 0.00 0.00
E
ATOM 15259 HD2 LYS E 103 −23.564 −15.369 43.707 0.00 0.00
E
ATOM 15260 CE LYS E 103 −22.847 −16.869 45.205 0.00 0.00
E
ATOM 15261 HE1 LYS E 103 −23.204 −17.861 45.557 0.00 0.00
E
ATOM 15262 HE2 LYS E 103 −21.935 −17.117 44.622 0.00 0.00
E
ATOM 15263 NZ LYS E 103 −22.404 −15.992 46.266 0.00 0.00
E
ATOM 15264 HZ1 LYS E 103 −22.403 −14.989 45.991 0.00 0.00
E
ATOM 15265 HZ2 LYS E 103 −22.971 −16.145 47.125 0.00 0.00
E
ATOM 15266 HZ3 LYS E 103 −21.440 −16.299 46.505 0.00 0.00
E
ATOM 15267 C LYS E 103 −27.280 −13.530 45.869 0.00 0.00
E
ATOM 15268 O LYS E 103 −27.028 −12.333 45.496 0.00 0.00
E
ATOM 15269 N ALA E 104 −27.525 −13.898 47.150 0.00 0.00
E
ATOM 15270 HN ALA E 104 −27.981 −14.773 47.293 0.00 0.00
E
ATOM 15271 CA ALA E 104 −27.515 −12.961 48.218 0.00 0.00
E
ATOM 15272 HA ALA E 104 −26.947 −12.097 47.907 0.00 0.00
E
ATOM 15273 CB ALA E 104 −28.832 −12.630 48.766 0.00 0.00
E
ATOM 15274 HB1 ALA E 104 −28.728 −11.929 49.622 0.00 0.00
E
ATOM 15275 HB2 ALA E 104 −29.515 −12.218 47.992 0.00 0.00
E
ATOM 15276 HB3 ALA E 104 −29.151 −13.596 49.212 0.00 0.00
E
ATOM 15277 C ALA E 104 −26.599 −13.512 49.357 0.00 0.00
E
ATOM 15278 O ALA E 104 −26.520 −14.696 49.567 0.00 0.00
E
ATOM 15279 N GLN E 105 −25.952 −12.589 50.097 0.00 0.00
E
ATOM 15280 HN GLN E 105 −25.840 −11.625 49.869 0.00 0.00
E
ATOM 15281 CA GLN E 105 −25.096 −13.068 51.188 0.00 0.00
E

ATOM 15282 HA GLN E 105 -24.800 -14.097 51.044 0.00 0.00
E
ATOM 15283 CB GLN E 105 -23.697 -12.352 51.044 0.00 0.00
E
ATOM 15284 HB1 GLN E 105 -23.725 -11.248 51.161 0.00 0.00
E
ATOM 15285 HB2 GLN E 105 -23.015 -12.729 51.835 0.00 0.00
E
ATOM 15286 CG GLN E 105 -23.047 -12.517 49.655 0.00 0.00
E
ATOM 15287 HG1 GLN E 105 -23.296 -13.533 49.281 0.00 0.00
E
ATOM 15288 HG2 GLN E 105 -23.409 -11.693 49.004 0.00 0.00
E
ATOM 15289 CD GLN E 105 -21.562 -12.149 49.601 0.00 0.00
E
ATOM 15290 OE1 GLN E 105 -21.117 -11.331 50.400 0.00 0.00
E
ATOM 15291 NE2 GLN E 105 -20.792 -12.703 48.689 0.00 0.00
E
ATOM 15292 HE21 GLN E 105 -19.810 -12.527 48.749 0.00 0.00
E
ATOM 15293 HE22 GLN E 105 -20.988 -13.039 47.768 0.00 0.00
E
ATOM 15294 C GLN E 105 -25.602 -12.815 52.603 0.00 0.00
E
ATOM 15295 O GLN E 105 -25.185 -13.514 53.476 0.00 0.00
E
ATOM 15296 N ASN E 106 -26.561 -11.878 52.823 0.00 0.00
E
ATOM 15297 HN ASN E 106 -27.064 -11.488 52.056 0.00 0.00
E
ATOM 15298 CA ASN E 106 -26.919 -11.559 54.192 0.00 0.00
E
ATOM 15299 HA ASN E 106 -26.845 -12.383 54.887 0.00 0.00
E
ATOM 15300 CB ASN E 106 -25.769 -10.579 54.717 0.00 0.00
E
ATOM 15301 HB1 ASN E 106 -26.141 -10.113 55.655 0.00 0.00
E
ATOM 15302 HB2 ASN E 106 -24.847 -11.138 54.986 0.00 0.00
E
ATOM 15303 CG ASN E 106 -25.402 -9.343 53.852 0.00 0.00
E
ATOM 15304 OD1 ASN E 106 -26.272 -8.581 53.416 0.00 0.00
E
ATOM 15305 ND2 ASN E 106 -24.101 -9.090 53.678 0.00 0.00
E
ATOM 15306 HD21 ASN E 106 -23.829 -8.235 53.237 0.00 0.00
E
ATOM 15307 HD22 ASN E 106 -23.341 -9.548 54.140 0.00 0.00
E
ATOM 15308 C ASN E 106 -28.228 -10.792 54.202 0.00 0.00
E

```
ATOM   15309  O   ASN E 106     -28.840 -10.545  53.140  0.00  0.00
E
ATOM   15310  N   TYR E 107     -28.621 -10.393  55.399  0.00  0.00
E
ATOM   15311  HN  TYR E 107     -28.081 -10.728  56.168  0.00  0.00
E
ATOM   15312  CA  TYR E 107     -29.939  -9.761  55.683  0.00  0.00
E
ATOM   15313  HA  TYR E 107     -30.643 -10.487  55.303  0.00  0.00
E
ATOM   15314  CB  TYR E 107     -30.088  -9.429  57.187  0.00  0.00
E
ATOM   15315  HB1 TYR E 107     -29.863 -10.350  57.766  0.00  0.00
E
ATOM   15316  HB2 TYR E 107     -29.341  -8.613  57.297  0.00  0.00
E
ATOM   15317  CG  TYR E 107     -31.479  -8.958  57.509  0.00  0.00
E
ATOM   15318  CD1 TYR E 107     -32.465  -9.994  57.312  0.00  0.00
E
ATOM   15319  HD1 TYR E 107     -32.197 -11.009  57.056  0.00  0.00
E
ATOM   15320  CE1 TYR E 107     -33.836  -9.574  57.515  0.00  0.00
E
ATOM   15321  HE1 TYR E 107     -34.668 -10.249  57.379  0.00  0.00
E
ATOM   15322  CZ  TYR E 107     -34.124  -8.247  57.862  0.00  0.00
E
ATOM   15323  OH  TYR E 107     -35.490  -7.833  58.221  0.00  0.00
E
ATOM   15324  HH  TYR E 107     -35.463  -6.900  58.448  0.00  0.00
E
ATOM   15325  CD2 TYR E 107     -31.788  -7.631  57.962  0.00  0.00
E
ATOM   15326  HD2 TYR E 107     -30.904  -7.070  58.227  0.00  0.00
E
ATOM   15327  CE2 TYR E 107     -33.134  -7.236  58.097  0.00  0.00
E
ATOM   15328  HE2 TYR E 107     -33.318  -6.200  58.340  0.00  0.00
E
ATOM   15329  C   TYR E 107     -30.126  -8.441  54.987  0.00  0.00
E
ATOM   15330  O   TYR E 107     -31.134  -8.130  54.439  0.00  0.00
E
ATOM   15331  N   TYR E 108     -29.043  -7.555  54.935  0.00  0.00
E
ATOM   15332  HN  TYR E 108     -28.240  -7.747  55.493  0.00  0.00
E
ATOM   15333  CA  TYR E 108     -29.073  -6.283  54.265  0.00  0.00
E
ATOM   15334  HA  TYR E 108     -29.792  -5.631  54.738  0.00  0.00
E
ATOM   15335  CB  TYR E 108     -27.787  -5.438  54.572  0.00  0.00
E
```

ATOM	15336	HB1	TYR	E	108	−26.822	−5.886	54.252	0.00	0.00	E
ATOM	15337	HB2	TYR	E	108	−28.030	−4.512	54.008	0.00	0.00	E
ATOM	15338	CG	TYR	E	108	−27.758	−5.042	55.974	0.00	0.00	E
ATOM	15339	CD1	TYR	E	108	−28.299	−3.834	56.397	0.00	0.00	E
ATOM	15340	HD1	TYR	E	108	−28.794	−3.145	55.729	0.00	0.00	E
ATOM	15341	CE1	TYR	E	108	−28.234	−3.482	57.778	0.00	0.00	E
ATOM	15342	HE1	TYR	E	108	−28.565	−2.481	58.015	0.00	0.00	E
ATOM	15343	CZ	TYR	E	108	−27.551	−4.332	58.703	0.00	0.00	E
ATOM	15344	OH	TYR	E	108	−27.489	−3.886	60.083	0.00	0.00	E
ATOM	15345	HH	TYR	E	108	−26.757	−4.297	60.549	0.00	0.00	E
ATOM	15346	CD2	TYR	E	108	−27.024	−5.848	56.938	0.00	0.00	E
ATOM	15347	HD2	TYR	E	108	−26.465	−6.717	56.622	0.00	0.00	E
ATOM	15348	CE2	TYR	E	108	−27.007	−5.508	58.291	0.00	0.00	E
ATOM	15349	HE2	TYR	E	108	−26.416	−6.086	58.985	0.00	0.00	E
ATOM	15350	C	TYR	E	108	−29.341	−6.394	52.753	0.00	0.00	E
ATOM	15351	O	TYR	E	108	−30.252	−5.787	52.293	0.00	0.00	E
ATOM	15352	N	HSE	E	109	−28.519	−7.288	52.185	0.00	0.00	E
ATOM	15353	HN	HSE	E	109	−27.895	−7.812	52.762	0.00	0.00	E
ATOM	15354	CA	HSE	E	109	−28.522	−7.660	50.803	0.00	0.00	E
ATOM	15355	HA	HSE	E	109	−28.346	−6.797	50.178	0.00	0.00	E
ATOM	15356	CB	HSE	E	109	−27.358	−8.708	50.627	0.00	0.00	E
ATOM	15357	HB1	HSE	E	109	−26.404	−8.338	51.062	0.00	0.00	E
ATOM	15358	HB2	HSE	E	109	−27.481	−9.648	51.206	0.00	0.00	E
ATOM	15359	ND1	HSE	E	109	−26.032	−9.849	48.920	0.00	0.00	E
ATOM	15360	CG	HSE	E	109	−27.083	−8.977	49.170	0.00	0.00	E
ATOM	15361	CE1	HSE	E	109	−25.949	−9.940	47.577	0.00	0.00	E
ATOM	15362	HE1	HSE	E	109	−25.146	−10.456	47.052	0.00	0.00	E

ATOM 15363 NE2 HSE E 109 −26.917 −9.162 46.969 0.00 0.00
E
ATOM 15364 HE2 HSE E 109 −26.848 −8.870 46.016 0.00 0.00
E
ATOM 15365 CD2 HSE E 109 −27.577 −8.532 48.045 0.00 0.00
E
ATOM 15366 HD2 HSE E 109 −28.355 −7.849 47.725 0.00 0.00
E
ATOM 15367 C HSE E 109 −29.829 −8.363 50.430 0.00 0.00
E
ATOM 15368 O HSE E 109 −30.356 −8.147 49.374 0.00 0.00
E
ATOM 15369 N HSE E 110 −30.451 −9.123 51.298 0.00 0.00
E
ATOM 15370 HN HSE E 110 −30.090 −9.289 52.212 0.00 0.00
E
ATOM 15371 CA HSE E 110 −31.667 −9.777 51.057 0.00 0.00
E
ATOM 15372 HA HSE E 110 −31.549 −10.171 50.058 0.00 0.00
E
ATOM 15373 CB HSE E 110 −31.724 −11.012 51.996 0.00 0.00
E
ATOM 15374 HB1 HSE E 110 −30.751 −11.548 51.973 0.00 0.00
E
ATOM 15375 HB2 HSE E 110 −31.760 −10.652 53.046 0.00 0.00
E
ATOM 15376 ND1 HSE E 110 −32.261 −13.516 52.022 0.00 0.00
E
ATOM 15377 CG HSE E 110 −32.641 −12.215 51.719 0.00 0.00
E
ATOM 15378 CE1 HSE E 110 −33.338 −14.234 51.954 0.00 0.00
E
ATOM 15379 HE1 HSE E 110 −33.411 −15.306 52.138 0.00 0.00
E
ATOM 15380 NE2 HSE E 110 −34.392 −13.496 51.627 0.00 0.00
E
ATOM 15381 HE2 HSE E 110 −35.318 −13.853 51.504 0.00 0.00
E
ATOM 15382 CD2 HSE E 110 −33.962 −12.202 51.516 0.00 0.00
E
ATOM 15383 HD2 HSE E 110 −34.735 −11.463 51.344 0.00 0.00
E
ATOM 15384 C HSE E 110 −32.875 −8.820 51.093 0.00 0.00
E
ATOM 15385 O HSE E 110 −33.740 −8.862 50.267 0.00 0.00
E
ATOM 15386 N LYS E 111 −32.960 −7.975 52.101 0.00 0.00
E
ATOM 15387 HN LYS E 111 −32.196 −8.052 52.736 0.00 0.00
E
ATOM 15388 CA LYS E 111 −34.088 −7.122 52.293 0.00 0.00
E
ATOM 15389 HA LYS E 111 −34.991 −7.704 52.185 0.00 0.00
E

```
ATOM  15390  CB  LYS E 111     -34.162  -6.427  53.740  0.00  0.00
E
ATOM  15391  HB1 LYS E 111     -33.908  -7.049  54.625  0.00  0.00
E
ATOM  15392  HB2 LYS E 111     -33.386  -5.632  53.758  0.00  0.00
E
ATOM  15393  CG  LYS E 111     -35.573  -5.880  54.145  0.00  0.00
E
ATOM  15394  HG1 LYS E 111     -35.450  -5.559  55.201  0.00  0.00
E
ATOM  15395  HG2 LYS E 111     -35.831  -5.111  53.386  0.00  0.00
E
ATOM  15396  CD  LYS E 111     -36.793  -6.911  54.120  0.00  0.00
E
ATOM  15397  HD1 LYS E 111     -37.530  -6.292  54.676  0.00  0.00
E
ATOM  15398  HD2 LYS E 111     -37.146  -7.057  53.076  0.00  0.00
E
ATOM  15399  CE  LYS E 111     -36.518  -8.226  54.869  0.00  0.00
E
ATOM  15400  HE1 LYS E 111     -35.788  -8.748  54.214  0.00  0.00
E
ATOM  15401  HE2 LYS E 111     -36.139  -8.097  55.905  0.00  0.00
E
ATOM  15402  NZ  LYS E 111     -37.812  -8.967  54.955  0.00  0.00
E
ATOM  15403  HZ1 LYS E 111     -38.190  -8.976  53.987  0.00  0.00
E
ATOM  15404  HZ2 LYS E 111     -37.576  -9.939  55.240  0.00  0.00
E
ATOM  15405  HZ3 LYS E 111     -38.529  -8.666  55.646  0.00  0.00
E
ATOM  15406  C   LYS E 111     -34.273  -6.051  51.209  0.00  0.00
E
ATOM  15407  O   LYS E 111     -35.319  -5.883  50.635  0.00  0.00
E
ATOM  15408  N   VAL E 112     -33.183  -5.274  50.797  0.00  0.00
E
ATOM  15409  HN  VAL E 112     -32.286  -5.509  51.165  0.00  0.00
E
ATOM  15410  CA  VAL E 112     -33.300  -4.469  49.634  0.00  0.00
E
ATOM  15411  HA  VAL E 112     -34.172  -3.837  49.712  0.00  0.00
E
ATOM  15412  CB  VAL E 112     -32.018  -3.732  49.413  0.00  0.00
E
ATOM  15413  HB  VAL E 112     -31.843  -3.054  50.276  0.00  0.00
E
ATOM  15414  CG1 VAL E 112     -30.687  -4.533  49.287  0.00  0.00
E
ATOM  15415 HG11 VAL E 112     -30.656  -5.361  50.027  0.00  0.00
E
ATOM  15416 HG12 VAL E 112     -30.524  -5.048  48.316  0.00  0.00
E
```

```
ATOM  15417 HG13 VAL E 112     -29.845  -3.815  49.382  0.00  0.00
E
ATOM  15418  CG2 VAL E 112     -32.188  -2.647  48.233  0.00  0.00
E
ATOM  15419 HG21 VAL E 112     -31.274  -2.032  48.091  0.00  0.00
E
ATOM  15420 HG22 VAL E 112     -32.248  -3.336  47.364  0.00  0.00
E
ATOM  15421 HG23 VAL E 112     -33.094  -2.026  48.400  0.00  0.00
E
ATOM  15422  C   VAL E 112     -33.678  -5.302  48.389  0.00  0.00
E
ATOM  15423  O   VAL E 112     -34.564  -4.898  47.644  0.00  0.00
E
ATOM  15424  N   LEU E 113     -33.087  -6.500  48.061  0.00  0.00
E
ATOM  15425  HN  LEU E 113     -32.396  -6.945  48.626  0.00  0.00
E
ATOM  15426  CA  LEU E 113     -33.437  -7.340  46.879  0.00  0.00
E
ATOM  15427  HA  LEU E 113     -33.368  -6.717  46.000  0.00  0.00
E
ATOM  15428  CB  LEU E 113     -32.672  -8.563  46.858  0.00  0.00
E
ATOM  15429  HB1 LEU E 113     -32.630  -8.866  47.925  0.00  0.00
E
ATOM  15430  HB2 LEU E 113     -33.218  -9.344  46.288  0.00  0.00
E
ATOM  15431  CG  LEU E 113     -31.260  -8.362  46.234  0.00  0.00
E
ATOM  15432  HG  LEU E 113     -30.816  -7.473  46.730  0.00  0.00
E
ATOM  15433  CD1 LEU E 113     -30.542  -9.704  46.298  0.00  0.00
E
ATOM  15434 HD11 LEU E 113     -29.802  -9.759  45.471  0.00  0.00
E
ATOM  15435 HD12 LEU E 113     -30.015  -9.905  47.255  0.00  0.00
E
ATOM  15436 HD13 LEU E 113     -31.246 -10.508  45.994  0.00  0.00
E
ATOM  15437  CD2 LEU E 113     -31.492  -8.047  44.708  0.00  0.00
E
ATOM  15438 HD21 LEU E 113     -32.010  -7.078  44.540  0.00  0.00
E
ATOM  15439 HD22 LEU E 113     -30.454  -7.882  44.348  0.00  0.00
E
ATOM  15440 HD23 LEU E 113     -31.981  -8.781  44.033  0.00  0.00
E
ATOM  15441  C   LEU E 113     -34.949  -7.765  46.918  0.00  0.00
E
ATOM  15442  O   LEU E 113     -35.633  -7.748  45.870  0.00  0.00
E
ATOM  15443  N   GLU E 114     -35.405  -7.983  48.123  0.00  0.00
E
```

ATOM 15444 HN GLU E 114 −34.792 −8.087 48.902 0.00 0.00
E
ATOM 15445 CA GLU E 114 −36.834 −8.256 48.394 0.00 0.00
E
ATOM 15446 HA GLU E 114 −37.111 −9.089 47.765 0.00 0.00
E
ATOM 15447 CB GLU E 114 −36.982 −8.587 49.876 0.00 0.00
E
ATOM 15448 HB1 GLU E 114 −36.343 −9.449 50.164 0.00 0.00
E
ATOM 15449 HB2 GLU E 114 −36.639 −7.739 50.506 0.00 0.00
E
ATOM 15450 CG GLU E 114 −38.431 −9.053 50.205 0.00 0.00
E
ATOM 15451 HG1 GLU E 114 −39.081 −8.157 50.111 0.00 0.00
E
ATOM 15452 HG2 GLU E 114 −38.740 −9.865 49.512 0.00 0.00
E
ATOM 15453 CD GLU E 114 −38.528 −9.601 51.623 0.00 0.00
E
ATOM 15454 OE1 GLU E 114 −38.087 −10.690 51.968 0.00 0.00
E
ATOM 15455 OE2 GLU E 114 −39.134 −8.906 52.501 0.00 0.00
E
ATOM 15456 C GLU E 114 −37.755 −7.059 47.989 0.00 0.00
E
ATOM 15457 O GLU E 114 −38.863 −7.205 47.483 0.00 0.00
E
ATOM 15458 N LYS E 115 −37.387 −5.834 48.371 0.00 0.00
E
ATOM 15459 HN LYS E 115 −36.571 −5.717 48.931 0.00 0.00
E
ATOM 15460 CA LYS E 115 −38.248 −4.679 48.006 0.00 0.00
E
ATOM 15461 HA LYS E 115 −39.282 −4.990 48.023 0.00 0.00
E
ATOM 15462 CB LYS E 115 −38.141 −3.494 48.968 0.00 0.00
E
ATOM 15463 HB1 LYS E 115 −37.154 −2.984 48.945 0.00 0.00
E
ATOM 15464 HB2 LYS E 115 −38.866 −2.742 48.591 0.00 0.00
E
ATOM 15465 CG LYS E 115 −38.342 −3.794 50.406 0.00 0.00
E
ATOM 15466 HG1 LYS E 115 −39.322 −4.308 50.496 0.00 0.00
E
ATOM 15467 HG2 LYS E 115 −37.476 −4.406 50.738 0.00 0.00
E
ATOM 15468 CD LYS E 115 −38.475 −2.474 51.209 0.00 0.00
E
ATOM 15469 HD1 LYS E 115 −37.575 −1.847 51.037 0.00 0.00
E
ATOM 15470 HD2 LYS E 115 −39.301 −1.891 50.749 0.00 0.00
E

ATOM 15471 CE LYS E 115 −38.639 −2.591 52.691 0.00 0.00
E
ATOM 15472 HE1 LYS E 115 −37.729 −3.043 53.140 0.00 0.00
E
ATOM 15473 HE2 LYS E 115 −38.998 −1.669 53.197 0.00 0.00
E
ATOM 15474 NZ LYS E 115 −39.789 −3.559 53.007 0.00 0.00
E
ATOM 15475 HZ1 LYS E 115 −40.615 −3.003 52.706 0.00 0.00
E
ATOM 15476 HZ2 LYS E 115 −39.719 −4.401 52.401 0.00 0.00
E
ATOM 15477 HZ3 LYS E 115 −39.762 −3.865 54.000 0.00 0.00
E
ATOM 15478 C LYS E 115 −38.217 −4.296 46.557 0.00 0.00
E
ATOM 15479 O LYS E 115 −39.274 −4.019 45.950 0.00 0.00
E
ATOM 15480 N ILE E 116 −37.014 −4.372 45.964 0.00 0.00
E
ATOM 15481 HN ILE E 116 −36.214 −4.635 46.497 0.00 0.00
E
ATOM 15482 CA ILE E 116 −36.785 −4.113 44.469 0.00 0.00
E
ATOM 15483 HA ILE E 116 −37.208 −3.147 44.234 0.00 0.00
E
ATOM 15484 CB ILE E 116 −35.251 −4.271 44.153 0.00 0.00
E
ATOM 15485 HB ILE E 116 −34.871 −5.264 44.477 0.00 0.00
E
ATOM 15486 CG2 ILE E 116 −35.060 −4.277 42.627 0.00 0.00
E
ATOM 15487 HG21 ILE E 116 −33.971 −4.288 42.411 0.00 0.00
E
ATOM 15488 HG22 ILE E 116 −35.529 −5.160 42.144 0.00 0.00
E
ATOM 15489 HG23 ILE E 116 −35.502 −3.410 42.090 0.00 0.00
E
ATOM 15490 CG1 ILE E 116 −34.321 −3.213 44.774 0.00 0.00
E
ATOM 15491 HG11 ILE E 116 −34.360 −3.237 45.884 0.00 0.00
E
ATOM 15492 HG12 ILE E 116 −33.274 −3.443 44.483 0.00 0.00
E
ATOM 15493 CD ILE E 116 −34.633 −1.854 44.267 0.00 0.00
E
ATOM 15494 HD1 ILE E 116 −34.092 −1.026 44.773 0.00 0.00
E
ATOM 15495 HD2 ILE E 116 −34.266 −1.729 43.226 0.00 0.00
E
ATOM 15496 HD3 ILE E 116 −35.681 −1.552 44.480 0.00 0.00
E
ATOM 15497 C ILE E 116 −37.596 −5.148 43.549 0.00 0.00
E

ATOM 15498 O ILE E 116 -38.305 -4.845 42.586 0.00 0.00
E
ATOM 15499 N GLY E 117 -37.511 -6.364 43.976 0.00 0.00
E
ATOM 15500 HN GLY E 117 -36.827 -6.532 44.681 0.00 0.00
E
ATOM 15501 CA GLY E 117 -38.207 -7.496 43.352 0.00 0.00
E
ATOM 15502 HA1 GLY E 117 -38.708 -7.162 42.455 0.00 0.00
E
ATOM 15503 HA2 GLY E 117 -38.790 -7.893 44.170 0.00 0.00
E
ATOM 15504 C GLY E 117 -37.403 -8.580 42.770 0.00 0.00
E
ATOM 15505 O GLY E 117 -36.716 -8.362 41.774 0.00 0.00
E
ATOM 15506 N ALA E 118 -37.537 -9.823 43.327 0.00 0.00
E
ATOM 15507 HN ALA E 118 -37.953 -9.921 44.228 0.00 0.00
E
ATOM 15508 CA ALA E 118 -36.895 -10.987 42.788 0.00 0.00
E
ATOM 15509 HA ALA E 118 -36.848 -10.864 41.716 0.00 0.00
E
ATOM 15510 CB ALA E 118 -35.403 -11.104 43.358 0.00 0.00
E
ATOM 15511 HB1 ALA E 118 -34.943 -10.094 43.334 0.00 0.00
E
ATOM 15512 HB2 ALA E 118 -35.397 -11.451 44.413 0.00 0.00
E
ATOM 15513 HB3 ALA E 118 -34.746 -11.728 42.715 0.00 0.00
E
ATOM 15514 C ALA E 118 -37.664 -12.167 43.238 0.00 0.00
E
ATOM 15515 O ALA E 118 -38.160 -12.296 44.325 0.00 0.00
E
ATOM 15516 N ASP E 119 -37.710 -13.138 42.256 0.00 0.00
E
ATOM 15517 HN ASP E 119 -37.178 -13.032 41.420 0.00 0.00
E
ATOM 15518 CA ASP E 119 -38.535 -14.375 42.293 0.00 0.00
E
ATOM 15519 HA ASP E 119 -39.395 -14.067 42.869 0.00 0.00
E
ATOM 15520 CB ASP E 119 -38.937 -14.700 40.834 0.00 0.00
E
ATOM 15521 HB1 ASP E 119 -38.071 -14.973 40.195 0.00 0.00
E
ATOM 15522 HB2 ASP E 119 -39.624 -15.567 40.725 0.00 0.00
E
ATOM 15523 CG ASP E 119 -39.528 -13.479 40.214 0.00 0.00
E
ATOM 15524 OD1 ASP E 119 -40.754 -13.186 40.401 0.00 0.00
E

ATOM 15525 OD2 ASP E 119 −38.809 −12.715 39.435 0.00 0.00
E
ATOM 15526 C ASP E 119 −37.792 −15.568 42.938 0.00 0.00
E
ATOM 15527 O ASP E 119 −38.265 −16.663 42.963 0.00 0.00
E
ATOM 15528 N ARG E 120 −36.544 −15.346 43.357 0.00 0.00
E
ATOM 15529 HN ARG E 120 −36.158 −14.483 43.042 0.00 0.00
E
ATOM 15530 CA ARG E 120 −35.741 −16.260 44.112 0.00 0.00
E
ATOM 15531 HA ARG E 120 −36.428 −16.599 44.874 0.00 0.00
E
ATOM 15532 CB ARG E 120 −35.182 −17.509 43.338 0.00 0.00
E
ATOM 15533 HB1 ARG E 120 −35.959 −17.824 42.610 0.00 0.00
E
ATOM 15534 HB2 ARG E 120 −34.329 −17.203 42.694 0.00 0.00
E
ATOM 15535 CG ARG E 120 −34.858 −18.639 44.295 0.00 0.00
E
ATOM 15536 HG1 ARG E 120 −34.381 −19.360 43.597 0.00 0.00
E
ATOM 15537 HG2 ARG E 120 −34.164 −18.402 45.129 0.00 0.00
E
ATOM 15538 CD ARG E 120 −36.137 −19.254 44.878 0.00 0.00
E
ATOM 15539 HD1 ARG E 120 −35.865 −19.697 45.860 0.00 0.00
E
ATOM 15540 HD2 ARG E 120 −36.942 −18.489 44.874 0.00 0.00
E
ATOM 15541 NE ARG E 120 −36.691 −20.291 43.947 0.00 0.00
E
ATOM 15542 HE ARG E 120 −37.499 −20.073 43.400 0.00 0.00
E
ATOM 15543 CZ ARG E 120 −36.198 −21.495 43.846 0.00 0.00
E
ATOM 15544 NH1 ARG E 120 −35.038 −21.784 44.471 0.00 0.00
E
ATOM 15545 HH11 ARG E 120 −34.823 −22.760 44.486 0.00 0.00
E
ATOM 15546 HH12 ARG E 120 −34.588 −21.258 45.192 0.00 0.00
E
ATOM 15547 NH2 ARG E 120 −36.785 −22.422 43.054 0.00 0.00
E
ATOM 15548 HH21 ARG E 120 −36.336 −23.308 42.935 0.00 0.00
E
ATOM 15549 HH22 ARG E 120 −37.634 −22.184 42.582 0.00 0.00
E
ATOM 15550 C ARG E 120 −34.589 −15.438 44.674 0.00 0.00
E
ATOM 15551 O ARG E 120 −33.806 −14.859 43.971 0.00 0.00
E

ATOM 15552 N   ILE E 121   -34.344 -15.595 45.987 0.00 0.00
E
ATOM 15553 HN ILE E 121   -34.964 -16.093 46.588 0.00 0.00
E
ATOM 15554 CA ILE E 121   -33.138 -15.114 46.637 0.00 0.00
E
ATOM 15555 HA ILE E 121   -32.466 -14.577 45.983 0.00 0.00
E
ATOM 15556 CB ILE E 121   -33.494 -14.233 47.809 0.00 0.00
E
ATOM 15557 HB ILE E 121   -34.229 -14.746 48.465 0.00 0.00
E
ATOM 15558 CG2 ILE E 121   -32.182 -13.893 48.633 0.00 0.00
E
ATOM 15559 HG21 ILE E 121   -32.379 -12.975 49.228 0.00 0.00
E
ATOM 15560 HG22 ILE E 121   -31.771 -14.724 49.244 0.00 0.00
E
ATOM 15561 HG23 ILE E 121   -31.478 -13.599 47.825 0.00 0.00
E
ATOM 15562 CG1 ILE E 121   -34.210 -12.982 47.295 0.00 0.00
E
ATOM 15563 HG11 ILE E 121   -33.671 -12.407 46.511 0.00 0.00
E
ATOM 15564 HG12 ILE E 121   -35.091 -13.318 46.707 0.00 0.00
E
ATOM 15565 CD ILE E 121   -34.862 -12.053 48.300 0.00 0.00
E
ATOM 15566 HD1 ILE E 121   -35.829 -12.464 48.662 0.00 0.00
E
ATOM 15567 HD2 ILE E 121   -34.117 -11.781 49.077 0.00 0.00
E
ATOM 15568 HD3 ILE E 121   -35.080 -11.139 47.708 0.00 0.00
E
ATOM 15569 C   ILE E 121   -32.399 -16.358 47.070 0.00 0.00
E
ATOM 15570 O   ILE E 121   -32.891 -17.309 47.670 0.00 0.00
E
ATOM 15571 N   ILE E 122   -31.139 -16.530 46.630 0.00 0.00
E
ATOM 15572 HN ILE E 122   -30.719 -15.758 46.161 0.00 0.00
E
ATOM 15573 CA ILE E 122   -30.367 -17.724 46.798 0.00 0.00
E
ATOM 15574 HA ILE E 122   -30.960 -18.454 47.328 0.00 0.00
E
ATOM 15575 CB ILE E 122   -29.819 -18.375 45.561 0.00 0.00
E
ATOM 15576 HB ILE E 122   -29.159 -17.654 45.033 0.00 0.00
E
ATOM 15577 CG2 ILE E 122   -29.179 -19.701 45.862 0.00 0.00
E
ATOM 15578 HG21 ILE E 122   -29.963 -20.435 46.148 0.00 0.00
E

ATOM 15579 HG22 ILE E 122   -28.641 -20.028 44.947 0.00 0.00
E
ATOM 15580 HG23 ILE E 122   -28.444 -19.654 46.694 0.00 0.00
E
ATOM 15581 CG1 ILE E 122   -30.944 -18.525 44.453 0.00 0.00
E
ATOM 15582 HG11 ILE E 122   -31.771 -19.176 44.807 0.00 0.00
E
ATOM 15583 HG12 ILE E 122   -31.472 -17.552 44.360 0.00 0.00
E
ATOM 15584 CD ILE E 122   -30.383 -18.945 43.141 0.00 0.00
E
ATOM 15585 HD1 ILE E 122   -29.449 -19.545 43.102 0.00 0.00
E
ATOM 15586 HD2 ILE E 122   -31.179 -19.482 42.583 0.00 0.00
E
ATOM 15587 HD3 ILE E 122   -30.066 -18.078 42.523 0.00 0.00
E
ATOM 15588 C   ILE E 122   -29.224 -17.441 47.752 0.00 0.00
E
ATOM 15589 O   ILE E 122   -28.582 -16.449 47.459 0.00 0.00
E
ATOM 15590 N   HSE E 123   -28.898 -18.226 48.791 0.00 0.00
E
ATOM 15591 HN HSE E 123   -29.475 -19.010 49.007 0.00 0.00
E
ATOM 15592 CA HSE E 123   -27.538 -18.207 49.407 0.00 0.00
E
ATOM 15593 HA HSE E 123   -26.835 -17.521 48.958 0.00 0.00
E
ATOM 15594 CB HSE E 123   -27.721 -17.939 50.944 0.00 0.00
E
ATOM 15595 HB1 HSE E 123   -28.493 -18.650 51.309 0.00 0.00
E
ATOM 15596 HB2 HSE E 123   -26.792 -18.217 51.487 0.00 0.00
E
ATOM 15597 ND1 HSE E 123   -27.405 -15.975 52.398 0.00 0.00
E
ATOM 15598 CG HSE E 123   -28.112 -16.590 51.427 0.00 0.00
E
ATOM 15599 CE1 HSE E 123   -28.031 -14.753 52.539 0.00 0.00
E
ATOM 15600 HE1 HSE E 123   -27.895 -14.012 53.327 0.00 0.00
E
ATOM 15601 NE2 HSE E 123   -29.070 -14.616 51.615 0.00 0.00
E
ATOM 15602 HE2 HSE E 123   -29.788 -13.921 51.620 0.00 0.00
E
ATOM 15603 CD2 HSE E 123   -29.155 -15.832 50.960 0.00 0.00
E
ATOM 15604 HD2 HSE E 123   -29.906 -15.979 50.193 0.00 0.00
E
ATOM 15605 C   HSE E 123   -26.999 -19.646 49.359 0.00 0.00
E

ATOM 15606 O   HSE E 123   -27.384 -20.447 50.202 0.00 0.00
E
ATOM 15607 N   PRO E 124   -26.065 -20.096 48.461 0.00 0.00
E
ATOM 15608 CD PRO E 124   -25.390 -19.159 47.561 0.00 0.00
E
ATOM 15609 HD1 PRO E 124   -26.108 -18.454 47.089 0.00 0.00
E
ATOM 15610 HD2 PRO E 124   -24.565 -18.652 48.105 0.00 0.00
E
ATOM 15611 CA PRO E 124   -26.086 -21.445 47.983 0.00 0.00
E
ATOM 15612 HA PRO E 124   -27.094 -21.811 48.113 0.00 0.00
E
ATOM 15613 CB PRO E 124   -25.556 -21.362 46.582 0.00 0.00
E
ATOM 15614 HB1 PRO E 124   -26.436 -21.211 45.921 0.00 0.00
E
ATOM 15615 HB2 PRO E 124   -24.941 -22.250 46.323 0.00 0.00
E
ATOM 15616 CG PRO E 124   -24.792 -20.023 46.452 0.00 0.00
E
ATOM 15617 HG1 PRO E 124   -24.885 -19.652 45.409 0.00 0.00
E
ATOM 15618 HG2 PRO E 124   -23.692 -20.154 46.537 0.00 0.00
E
ATOM 15619 C   PRO E 124   -25.275 -22.348 48.852 0.00 0.00
E
ATOM 15620 O   PRO E 124   -25.719 -23.359 49.254 0.00 0.00
E
ATOM 15621 N   GLU E 125   -24.143 -21.895 49.319 0.00 0.00
E
ATOM 15622 HN GLU E 125   -23.822 -21.015 48.977 0.00 0.00
E
ATOM 15623 CA GLU E 125   -23.229 -22.428 50.295 0.00 0.00
E
ATOM 15624 HA GLU E 125   -22.995 -23.399 49.883 0.00 0.00
E
ATOM 15625 CB GLU E 125   -21.898 -21.616 50.347 0.00 0.00
E
ATOM 15626 HB1 GLU E 125   -21.213 -22.302 50.890 0.00 0.00
E
ATOM 15627 HB2 GLU E 125   -21.471 -21.524 49.326 0.00 0.00
E
ATOM 15628 CG GLU E 125   -21.862 -20.209 51.068 0.00 0.00
E
ATOM 15629 HG1 GLU E 125   -22.152 -20.322 52.135 0.00 0.00
E
ATOM 15630 HG2 GLU E 125   -20.803 -19.887 50.980 0.00 0.00
E
ATOM 15631 CD GLU E 125   -22.774 -19.109 50.533 0.00 0.00
E
ATOM 15632 OE1 GLU E 125   -24.050 -19.316 50.677 0.00 0.00
E

ATOM 15633 OE2 GLU E 125 −22.299 −18.011 50.104 0.00 0.00
E
ATOM 15634 C GLU E 125 −23.905 −22.591 51.635 0.00 0.00
E
ATOM 15635 O GLU E 125 −23.632 −23.652 52.127 0.00 0.00
E
ATOM 15636 N LYS E 126 −24.690 −21.677 52.197 0.00 0.00
E
ATOM 15637 HN LYS E 126 −24.936 −20.854 51.692 0.00 0.00
E
ATOM 15638 CA LYS E 126 −25.380 −21.982 53.429 0.00 0.00
E
ATOM 15639 HA LYS E 126 −24.676 −22.167 54.227 0.00 0.00
E
ATOM 15640 CB LYS E 126 −26.164 −20.697 53.869 0.00 0.00
E
ATOM 15641 HB1 LYS E 126 −26.976 −20.370 53.185 0.00 0.00
E
ATOM 15642 HB2 LYS E 126 −26.653 −21.160 54.752 0.00 0.00
E
ATOM 15643 CG LYS E 126 −25.130 −19.520 54.175 0.00 0.00
E
ATOM 15644 HG1 LYS E 126 −24.352 −19.942 54.847 0.00 0.00
E
ATOM 15645 HG2 LYS E 126 −24.640 −19.036 53.303 0.00 0.00
E
ATOM 15646 CD LYS E 126 −25.823 −18.269 54.711 0.00 0.00
E
ATOM 15647 HD1 LYS E 126 −26.436 −17.875 53.872 0.00 0.00
E
ATOM 15648 HD2 LYS E 126 −26.547 −18.349 55.550 0.00 0.00
E
ATOM 15649 CE LYS E 126 −24.771 −17.208 54.987 0.00 0.00
E
ATOM 15650 HE1 LYS E 126 −24.128 −17.588 55.809 0.00 0.00
E
ATOM 15651 HE2 LYS E 126 −23.996 −17.162 54.192 0.00 0.00
E
ATOM 15652 NZ LYS E 126 −25.315 −15.881 55.243 0.00 0.00
E
ATOM 15653 HZ1 LYS E 126 −24.647 −15.163 55.589 0.00 0.00
E
ATOM 15654 HZ2 LYS E 126 −25.642 −15.377 54.395 0.00 0.00
E
ATOM 15655 HZ3 LYS E 126 −26.185 −15.951 55.807 0.00 0.00
E
ATOM 15656 C LYS E 126 −26.377 −23.165 53.293 0.00 0.00
E
ATOM 15657 O LYS E 126 −26.568 −23.945 54.257 0.00 0.00
E
ATOM 15658 N ASP E 127 −27.122 −23.357 52.196 0.00 0.00
E
ATOM 15659 HN ASP E 127 −27.030 −22.709 51.443 0.00 0.00
E

ATOM 15660 CA ASP E 127 -27.974 -24.561 52.088 0.00 0.00
E
ATOM 15661 HA ASP E 127 -28.708 -24.642 52.875 0.00 0.00
E
ATOM 15662 CB ASP E 127 -28.692 -24.576 50.740 0.00 0.00
E
ATOM 15663 HB1 ASP E 127 -28.073 -24.436 49.828 0.00 0.00
E
ATOM 15664 HB2 ASP E 127 -29.232 -25.514 50.488 0.00 0.00
E
ATOM 15665 CG ASP E 127 -29.894 -23.643 50.594 0.00 0.00
E
ATOM 15666 OD1 ASP E 127 -30.180 -23.134 49.489 0.00 0.00
E
ATOM 15667 OD2 ASP E 127 -30.580 -23.378 51.603 0.00 0.00
E
ATOM 15668 C ASP E 127 -27.193 -25.883 52.019 0.00 0.00
E
ATOM 15669 O ASP E 127 -27.576 -26.951 52.465 0.00 0.00
E
ATOM 15670 N MET E 128 -25.984 -25.832 51.453 0.00 0.00
E
ATOM 15671 HN MET E 128 -25.782 -24.932 51.076 0.00 0.00
E
ATOM 15672 CA MET E 128 -24.972 -26.908 51.446 0.00 0.00
E
ATOM 15673 HA MET E 128 -25.435 -27.851 51.194 0.00 0.00
E
ATOM 15674 CB MET E 128 -23.871 -26.590 50.424 0.00 0.00
E
ATOM 15675 HB1 MET E 128 -24.358 -26.418 49.441 0.00 0.00
E
ATOM 15676 HB2 MET E 128 -23.327 -25.643 50.633 0.00 0.00
E
ATOM 15677 CG MET E 128 -22.979 -27.834 50.064 0.00 0.00
E
ATOM 15678 HG1 MET E 128 -22.069 -27.569 49.484 0.00 0.00
E
ATOM 15679 HG2 MET E 128 -22.671 -28.368 50.988 0.00 0.00
E
ATOM 15680 SD MET E 128 -23.867 -29.045 49.174 0.00 0.00
E
ATOM 15681 CE MET E 128 -22.608 -30.408 49.090 0.00 0.00
E
ATOM 15682 HE1 MET E 128 -23.122 -31.338 48.767 0.00 0.00
E
ATOM 15683 HE2 MET E 128 -21.855 -30.339 48.276 0.00 0.00
E
ATOM 15684 HE3 MET E 128 -22.039 -30.574 50.030 0.00 0.00
E
ATOM 15685 C MET E 128 -24.455 -27.150 52.848 0.00 0.00
E
ATOM 15686 O MET E 128 -24.175 -28.251 53.223 0.00 0.00
E

ATOM 15687 N GLY E 129 −24.214 −26.060 53.546 0.00 0.00
E
ATOM 15688 HN GLY E 129 −24.196 −25.214 53.019 0.00 0.00
E
ATOM 15689 CA GLY E 129 −23.794 −25.979 54.953 0.00 0.00
E
ATOM 15690 HA1 GLY E 129 −23.883 −24.956 55.289 0.00 0.00
E
ATOM 15691 HA2 GLY E 129 −22.791 −26.374 55.016 0.00 0.00
E
ATOM 15692 C GLY E 129 −24.681 −26.780 55.876 0.00 0.00
E
ATOM 15693 O GLY E 129 −24.252 −27.505 56.812 0.00 0.00
E
ATOM 15694 N VAL E 130 −25.976 −26.686 55.531 0.00 0.00
E
ATOM 15695 HN VAL E 130 −26.249 −26.034 54.828 0.00 0.00
E
ATOM 15696 CA VAL E 130 −27.117 −27.273 56.219 0.00 0.00
E
ATOM 15697 HA VAL E 130 −26.943 −27.231 57.285 0.00 0.00
E
ATOM 15698 CB VAL E 130 −28.378 −26.607 55.872 0.00 0.00
E
ATOM 15699 HB VAL E 130 −28.539 −26.520 54.776 0.00 0.00
E
ATOM 15700 CG1 VAL E 130 −29.621 −27.332 56.469 0.00 0.00
E
ATOM 15701 HG11 VAL E 130 −29.746 −27.309 57.573 0.00 0.00
E
ATOM 15702 HG12 VAL E 130 −30.541 −26.820 56.115 0.00 0.00
E
ATOM 15703 HG13 VAL E 130 −29.770 −28.390 56.163 0.00 0.00
E
ATOM 15704 CG2 VAL E 130 −28.352 −25.240 56.641 0.00 0.00
E
ATOM 15705 HG21 VAL E 130 −29.362 −24.788 56.543 0.00 0.00
E
ATOM 15706 HG22 VAL E 130 −28.175 −25.538 57.697 0.00 0.00
E
ATOM 15707 HG23 VAL E 130 −27.621 −24.488 56.275 0.00 0.00
E
ATOM 15708 C VAL E 130 −27.153 −28.734 55.965 0.00 0.00
E
ATOM 15709 O VAL E 130 −27.327 −29.587 56.879 0.00 0.00
E
ATOM 15710 N LYS E 131 −26.945 −29.043 54.714 0.00 0.00
E
ATOM 15711 HN LYS E 131 −26.835 −28.275 54.088 0.00 0.00
E
ATOM 15712 CA LYS E 131 −26.919 −30.422 54.179 0.00 0.00
E
ATOM 15713 HA LYS E 131 −27.853 −30.850 54.512 0.00 0.00
E

ATOM 15714 CB LYS E 131 -27.038 -30.382 52.640 0.00 0.00
E
ATOM 15715 HB1 LYS E 131 -27.857 -29.646 52.485 0.00 0.00
E
ATOM 15716 HB2 LYS E 131 -26.263 -29.701 52.227 0.00 0.00
E
ATOM 15717 CG LYS E 131 -27.431 -31.678 51.927 0.00 0.00
E
ATOM 15718 HG1 LYS E 131 -26.701 -32.491 52.125 0.00 0.00
E
ATOM 15719 HG2 LYS E 131 -28.410 -32.041 52.307 0.00 0.00
E
ATOM 15720 CD LYS E 131 -27.504 -31.424 50.458 0.00 0.00
E
ATOM 15721 HD1 LYS E 131 -28.176 -30.569 50.229 0.00 0.00
E
ATOM 15722 HD2 LYS E 131 -26.526 -31.099 50.044 0.00 0.00
E
ATOM 15723 CE LYS E 131 -27.901 -32.641 49.644 0.00 0.00
E
ATOM 15724 HE1 LYS E 131 -28.957 -32.892 49.883 0.00 0.00
E
ATOM 15725 HE2 LYS E 131 -27.854 -32.338 48.576 0.00 0.00
E
ATOM 15726 NZ LYS E 131 -26.962 -33.789 49.827 0.00 0.00
E
ATOM 15727 HZ1 LYS E 131 -26.028 -33.674 49.385 0.00 0.00
E
ATOM 15728 HZ2 LYS E 131 -26.722 -33.939 50.828 0.00 0.00
E
ATOM 15729 HZ3 LYS E 131 -27.245 -34.708 49.432 0.00 0.00
E
ATOM 15730 C LYS E 131 -25.768 -31.293 54.728 0.00 0.00
E
ATOM 15731 O LYS E 131 -26.009 -32.432 55.217 0.00 0.00
E
ATOM 15732 N ILE E 132 -24.499 -30.733 54.887 0.00 0.00
E
ATOM 15733 HN ILE E 132 -24.113 -29.969 54.375 0.00 0.00
E
ATOM 15734 CA ILE E 132 -23.442 -31.508 55.559 0.00 0.00
E
ATOM 15735 HA ILE E 132 -23.397 -32.472 55.074 0.00 0.00
E
ATOM 15736 CB ILE E 132 -22.136 -30.772 55.344 0.00 0.00
E
ATOM 15737 HB ILE E 132 -22.156 -29.725 55.717 0.00 0.00
E
ATOM 15738 CG2 ILE E 132 -20.940 -31.482 56.043 0.00 0.00
E
ATOM 15739 HG21 ILE E 132 -20.837 -31.320 57.138 0.00 0.00
E
ATOM 15740 HG22 ILE E 132 -20.951 -32.570 55.818 0.00 0.00
E

```
ATOM  15741 HG23 ILE E 132     -19.999 -31.077  55.614  0.00  0.00
E
ATOM  15742  CG1 ILE E 132     -21.889 -30.748  53.754  0.00  0.00
E
ATOM  15743 HG11 ILE E 132     -22.721 -30.157  53.314  0.00  0.00
E
ATOM  15744 HG12 ILE E 132     -21.032 -30.042  53.701  0.00  0.00
E
ATOM  15745  CD  ILE E 132     -21.614 -32.054  53.013  0.00  0.00
E
ATOM  15746  HD1 ILE E 132     -21.370 -31.880  51.943  0.00  0.00
E
ATOM  15747  HD2 ILE E 132     -20.733 -32.522  53.502  0.00  0.00
E
ATOM  15748  HD3 ILE E 132     -22.432 -32.800  53.102  0.00  0.00
E
ATOM  15749  C   ILE E 132     -23.678 -31.782  57.017  0.00  0.00
E
ATOM  15750  O   ILE E 132     -23.553 -32.908  57.527  0.00  0.00
E
ATOM  15751  N   ALA E 133     -24.146 -30.835  57.741  0.00  0.00
E
ATOM  15752  HN  ALA E 133     -24.065 -29.902  57.399  0.00  0.00
E
ATOM  15753  CA  ALA E 133     -24.623 -30.926  59.090  0.00  0.00
E
ATOM  15754  HA  ALA E 133     -23.819 -31.285  59.716  0.00  0.00
E
ATOM  15755  CB  ALA E 133     -25.145 -29.622  59.700  0.00  0.00
E
ATOM  15756  HB1 ALA E 133     -25.973 -29.246  59.061  0.00  0.00
E
ATOM  15757  HB2 ALA E 133     -25.575 -29.770  60.714  0.00  0.00
E
ATOM  15758  HB3 ALA E 133     -24.304 -28.910  59.839  0.00  0.00
E
ATOM  15759  C   ALA E 133     -25.727 -31.909  59.281  0.00  0.00
E
ATOM  15760  O   ALA E 133     -25.581 -32.681  60.236  0.00  0.00
E
ATOM  15761  N   GLN E 134     -26.741 -31.941  58.355  0.00  0.00
E
ATOM  15762  HN  GLN E 134     -26.868 -31.212  57.688  0.00  0.00
E
ATOM  15763  CA  GLN E 134     -27.765 -32.940  58.460  0.00  0.00
E
ATOM  15764  HA  GLN E 134     -28.019 -33.011  59.508  0.00  0.00
E
ATOM  15765  CB  GLN E 134     -28.968 -32.545  57.461  0.00  0.00
E
ATOM  15766  HB1 GLN E 134     -29.440 -31.582  57.752  0.00  0.00
E
ATOM  15767  HB2 GLN E 134     -28.653 -32.564  56.396  0.00  0.00
E
```

ATOM 15768 CG GLN E 134 -30.164 -33.458 57.603 0.00 0.00
E
ATOM 15769 HG1 GLN E 134 -30.990 -32.932 57.079 0.00 0.00
E
ATOM 15770 HG2 GLN E 134 -30.186 -34.452 57.109 0.00 0.00
E
ATOM 15771 CD GLN E 134 -30.707 -33.608 59.046 0.00 0.00
E
ATOM 15772 OE1 GLN E 134 -30.989 -32.652 59.784 0.00 0.00
E
ATOM 15773 NE2 GLN E 134 -30.657 -34.818 59.547 0.00 0.00
E
ATOM 15774 HE21 GLN E 134 -30.846 -34.904 60.525 0.00 0.00
E
ATOM 15775 HE22 GLN E 134 -30.230 -35.568 59.042 0.00 0.00
E
ATOM 15776 C GLN E 134 -27.187 -34.311 58.083 0.00 0.00
E
ATOM 15777 O GLN E 134 -27.529 -35.348 58.680 0.00 0.00
E
ATOM 15778 N SER E 135 -26.230 -34.428 57.185 0.00 0.00
E
ATOM 15779 HN SER E 135 -26.111 -33.699 56.515 0.00 0.00
E
ATOM 15780 CA SER E 135 -25.499 -35.654 56.977 0.00 0.00
E
ATOM 15781 HA SER E 135 -26.114 -36.491 56.684 0.00 0.00
E
ATOM 15782 CB SER E 135 -24.462 -35.521 55.835 0.00 0.00
E
ATOM 15783 HB1 SER E 135 -24.916 -35.151 54.891 0.00 0.00
E
ATOM 15784 HB2 SER E 135 -23.737 -34.716 56.081 0.00 0.00
E
ATOM 15785 OG SER E 135 -23.870 -36.759 55.555 0.00 0.00
E
ATOM 15786 HG1 SER E 135 -23.812 -36.842 54.600 0.00 0.00
E
ATOM 15787 C SER E 135 -24.798 -36.169 58.216 0.00 0.00
E
ATOM 15788 O SER E 135 -24.820 -37.393 58.493 0.00 0.00
E
ATOM 15789 N LEU E 136 -24.182 -35.301 58.997 0.00 0.00
E
ATOM 15790 HN LEU E 136 -24.145 -34.371 58.640 0.00 0.00
E
ATOM 15791 CA LEU E 136 -23.491 -35.653 60.245 0.00 0.00
E
ATOM 15792 HA LEU E 136 -22.903 -36.535 60.038 0.00 0.00
E
ATOM 15793 CB LEU E 136 -22.552 -34.507 60.658 0.00 0.00
E
ATOM 15794 HB1 LEU E 136 -23.258 -33.650 60.701 0.00 0.00
E

ATOM 15795 HB2 LEU E 136 -22.257 -34.597 61.726 0.00 0.00
E
ATOM 15796 CG LEU E 136 -21.381 -34.203 59.677 0.00 0.00
E
ATOM 15797 HG LEU E 136 -21.748 -34.215 58.628 0.00 0.00
E
ATOM 15798 CD1 LEU E 136 -20.877 -32.740 59.742 0.00 0.00
E
ATOM 15799 HD11 LEU E 136 -21.816 -32.157 59.626 0.00 0.00
E
ATOM 15800 HD12 LEU E 136 -20.485 -32.673 60.780 0.00 0.00
E
ATOM 15801 HD13 LEU E 136 -20.094 -32.502 58.991 0.00 0.00
E
ATOM 15802 CD2 LEU E 136 -20.248 -35.204 59.606 0.00 0.00
E
ATOM 15803 HD21 LEU E 136 -19.395 -34.819 59.006 0.00 0.00
E
ATOM 15804 HD22 LEU E 136 -19.907 -35.249 60.662 0.00 0.00
E
ATOM 15805 HD23 LEU E 136 -20.633 -36.208 59.329 0.00 0.00
E
ATOM 15806 C LEU E 136 -24.346 -36.082 61.347 0.00 0.00
E
ATOM 15807 O LEU E 136 -23.918 -36.912 62.108 0.00 0.00
E
ATOM 15808 N SER E 137 -25.539 -35.547 61.455 0.00 0.00
E
ATOM 15809 HN SER E 137 -25.738 -34.885 60.737 0.00 0.00
E
ATOM 15810 CA SER E 137 -26.477 -35.800 62.538 0.00 0.00
E
ATOM 15811 HA SER E 137 -26.103 -35.366 63.453 0.00 0.00
E
ATOM 15812 CB SER E 137 -27.687 -34.949 62.170 0.00 0.00
E
ATOM 15813 HB1 SER E 137 -27.278 -33.928 62.011 0.00 0.00
E
ATOM 15814 HB2 SER E 137 -28.176 -35.287 61.232 0.00 0.00
E
ATOM 15815 OG SER E 137 -28.595 -34.726 63.204 0.00 0.00
E
ATOM 15816 HG1 SER E 137 -29.423 -34.977 62.789 0.00 0.00
E
ATOM 15817 C SER E 137 -27.026 -37.113 62.637 0.00 0.00
E
ATOM 15818 O SER E 137 -27.292 -37.584 63.751 0.00 0.00
E
ATOM 15819 N ASP E 138 -27.281 -37.842 61.527 0.00 0.00
E
ATOM 15820 HN ASP E 138 -27.186 -37.378 60.650 0.00 0.00
E
ATOM 15821 CA ASP E 138 -27.799 -39.214 61.445 0.00 0.00
E

ATOM	15822	HA	ASP	E	138	-28.019	-39.556	62.445	0.00	0.00	E
ATOM	15823	CB	ASP	E	138	-29.075	-39.267	60.703	0.00	0.00	E
ATOM	15824	HB1	ASP	E	138	-28.993	-38.633	59.794	0.00	0.00	E
ATOM	15825	HB2	ASP	E	138	-29.151	-40.314	60.341	0.00	0.00	E
ATOM	15826	CG	ASP	E	138	-30.315	-38.919	61.503	0.00	0.00	E
ATOM	15827	OD1	ASP	E	138	-30.554	-39.745	62.425	0.00	0.00	E
ATOM	15828	OD2	ASP	E	138	-30.940	-37.862	61.405	0.00	0.00	E
ATOM	15829	C	ASP	E	138	-26.731	-40.248	60.874	0.00	0.00	E
ATOM	15830	O	ASP	E	138	-26.253	-40.165	59.736	0.00	0.00	E
ATOM	15831	N	GLU	E	139	-26.407	-41.266	61.692	0.00	0.00	E
ATOM	15832	HN	GLU	E	139	-26.899	-41.419	62.545	0.00	0.00	E
ATOM	15833	CA	GLU	E	139	-25.295	-42.189	61.383	0.00	0.00	E
ATOM	15834	HA	GLU	E	139	-24.489	-41.621	60.943	0.00	0.00	E
ATOM	15835	CB	GLU	E	139	-24.786	-42.722	62.684	0.00	0.00	E
ATOM	15836	HB1	GLU	E	139	-24.526	-41.865	63.341	0.00	0.00	E
ATOM	15837	HB2	GLU	E	139	-25.643	-43.158	63.242	0.00	0.00	E
ATOM	15838	CG	GLU	E	139	-23.569	-43.635	62.600	0.00	0.00	E
ATOM	15839	HG1	GLU	E	139	-23.768	-44.557	62.012	0.00	0.00	E
ATOM	15840	HG2	GLU	E	139	-22.840	-43.177	61.898	0.00	0.00	E
ATOM	15841	CD	GLU	E	139	-22.992	-43.862	63.973	0.00	0.00	E
ATOM	15842	OE1	GLU	E	139	-23.637	-44.677	64.693	0.00	0.00	E
ATOM	15843	OE2	GLU	E	139	-21.943	-43.359	64.329	0.00	0.00	E
ATOM	15844	C	GLU	E	139	-25.596	-43.141	60.259	0.00	0.00	E
ATOM	15845	O	GLU	E	139	-24.725	-43.519	59.466	0.00	0.00	E
ATOM	15846	N	ASN	E	140	-26.840	-43.604	60.106	0.00	0.00	E
ATOM	15847	HN	ASN	E	140	-27.447	-43.301	60.837	0.00	0.00	E
ATOM	15848	CA	ASN	E	140	-27.220	-44.754	59.349	0.00	0.00	E

ATOM 15849 HA ASN E 140 -26.293 -45.070 58.894 0.00 0.00
E
ATOM 15850 CB ASN E 140 -27.869 -45.897 60.139 0.00 0.00
E
ATOM 15851 HB1 ASN E 140 -28.728 -45.500 60.721 0.00 0.00
E
ATOM 15852 HB2 ASN E 140 -28.250 -46.709 59.483 0.00 0.00
E
ATOM 15853 CG ASN E 140 -26.883 -46.497 61.140 0.00 0.00
E
ATOM 15854 OD1 ASN E 140 -26.059 -47.335 60.737 0.00 0.00
E
ATOM 15855 ND2 ASN E 140 -26.822 -46.096 62.443 0.00 0.00
E
ATOM 15856 HD21 ASN E 140 -26.108 -46.501 63.015 0.00 0.00
E
ATOM 15857 HD22 ASN E 140 -27.637 -45.723 62.886 0.00 0.00
E
ATOM 15858 C ASN E 140 -28.077 -44.275 58.135 0.00 0.00
E
ATOM 15859 O ASN E 140 -28.506 -45.156 57.384 0.00 0.00
E
ATOM 15860 N VAL E 141 -28.216 -42.962 57.912 0.00 0.00
E
ATOM 15861 HN VAL E 141 -27.642 -42.316 58.411 0.00 0.00
E
ATOM 15862 CA VAL E 141 -29.127 -42.484 56.843 0.00 0.00
E
ATOM 15863 HA VAL E 141 -29.713 -43.301 56.447 0.00 0.00
E
ATOM 15864 CB VAL E 141 -30.025 -41.335 57.318 0.00 0.00
E
ATOM 15865 HB VAL E 141 -29.404 -40.573 57.835 0.00 0.00
E
ATOM 15866 CG1 VAL E 141 -30.781 -40.611 56.201 0.00 0.00
E
ATOM 15867 HG11 VAL E 141 -31.418 -41.379 55.713 0.00 0.00
E
ATOM 15868 HG12 VAL E 141 -31.458 -39.843 56.631 0.00 0.00
E
ATOM 15869 HG13 VAL E 141 -30.190 -40.041 55.452 0.00 0.00
E
ATOM 15870 CG2 VAL E 141 -31.036 -41.681 58.418 0.00 0.00
E
ATOM 15871 HG21 VAL E 141 -30.615 -42.261 59.267 0.00 0.00
E
ATOM 15872 HG22 VAL E 141 -31.519 -40.759 58.806 0.00 0.00
E
ATOM 15873 HG23 VAL E 141 -31.881 -42.303 58.055 0.00 0.00
E
ATOM 15874 C VAL E 141 -28.358 -41.889 55.680 0.00 0.00
E
ATOM 15875 O VAL E 141 -27.790 -40.837 55.760 0.00 0.00
E

ATOM 15876 N LEU E 142 -28.276 -42.608 54.636 0.00 0.00
E
ATOM 15877 HN LEU E 142 -28.695 -43.512 54.605 0.00 0.00
E
ATOM 15878 CA LEU E 142 -27.436 -42.278 53.481 0.00 0.00
E
ATOM 15879 HA LEU E 142 -26.541 -41.796 53.847 0.00 0.00
E
ATOM 15880 CB LEU E 142 -27.029 -43.533 52.690 0.00 0.00
E
ATOM 15881 HB1 LEU E 142 -27.906 -44.106 52.319 0.00 0.00
E
ATOM 15882 HB2 LEU E 142 -26.582 -43.212 51.725 0.00 0.00
E
ATOM 15883 CG LEU E 142 -26.013 -44.448 53.392 0.00 0.00
E
ATOM 15884 HG LEU E 142 -26.462 -44.803 54.344 0.00 0.00
E
ATOM 15885 CD1 LEU E 142 -25.830 -45.704 52.600 0.00 0.00
E
ATOM 15886 HD11 LEU E 142 -25.837 -45.374 51.540 0.00 0.00
E
ATOM 15887 HD12 LEU E 142 -24.803 -46.070 52.814 0.00 0.00
E
ATOM 15888 HD13 LEU E 142 -26.600 -46.482 52.789 0.00 0.00
E
ATOM 15889 CD2 LEU E 142 -24.667 -43.842 53.658 0.00 0.00
E
ATOM 15890 HD21 LEU E 142 -24.100 -43.873 52.702 0.00 0.00
E
ATOM 15891 HD22 LEU E 142 -24.623 -42.862 54.179 0.00 0.00
E
ATOM 15892 HD23 LEU E 142 -24.189 -44.561 54.357 0.00 0.00
E
ATOM 15893 C LEU E 142 -28.035 -41.231 52.594 0.00 0.00
E
ATOM 15894 O LEU E 142 -27.386 -40.308 52.053 0.00 0.00
E
ATOM 15895 N ASN E 143 -29.370 -41.129 52.448 0.00 0.00
E
ATOM 15896 HN ASN E 143 -29.923 -41.870 52.821 0.00 0.00
E
ATOM 15897 CA ASN E 143 -29.941 -39.978 51.701 0.00 0.00
E
ATOM 15898 HA ASN E 143 -29.573 -39.005 51.991 0.00 0.00
E
ATOM 15899 CB ASN E 143 -29.899 -40.287 50.096 0.00 0.00
E
ATOM 15900 HB1 ASN E 143 -28.950 -40.765 49.771 0.00 0.00
E
ATOM 15901 HB2 ASN E 143 -30.705 -40.996 49.809 0.00 0.00
E
ATOM 15902 CG ASN E 143 -30.089 -39.010 49.327 0.00 0.00
E

ATOM 15903 OD1 ASN E 143 −30.288 −37.919 49.911 0.00 0.00
E
ATOM 15904 ND2 ASN E 143 −30.113 −39.030 47.976 0.00 0.00
E
ATOM 15905 HD21 ASN E 143 −30.198 −38.161 47.488 0.00 0.00
E
ATOM 15906 HD22 ASN E 143 −30.241 −39.927 47.553 0.00 0.00
E
ATOM 15907 C ASN E 143 −31.364 −39.947 52.079 0.00 0.00
E
ATOM 15908 O ASN E 143 −31.911 −40.933 52.610 0.00 0.00
E
ATOM 15909 N TYR E 144 −32.044 −38.816 51.901 0.00 0.00
E
ATOM 15910 HN TYR E 144 −31.571 −38.093 51.404 0.00 0.00
E
ATOM 15911 CA TYR E 144 −33.392 −38.647 52.417 0.00 0.00
E
ATOM 15912 HA TYR E 144 −33.952 −39.571 52.408 0.00 0.00
E
ATOM 15913 CB TYR E 144 −33.240 −37.919 53.748 0.00 0.00
E
ATOM 15914 HB1 TYR E 144 −34.235 −37.620 54.142 0.00 0.00
E
ATOM 15915 HB2 TYR E 144 −32.832 −38.529 54.582 0.00 0.00
E
ATOM 15916 CG TYR E 144 −32.525 −36.547 53.772 0.00 0.00
E
ATOM 15917 CD1 TYR E 144 −33.237 −35.356 53.566 0.00 0.00
E
ATOM 15918 HD1 TYR E 144 −34.310 −35.472 53.531 0.00 0.00
E
ATOM 15919 CE1 TYR E 144 −32.620 −34.078 53.567 0.00 0.00
E
ATOM 15920 HE1 TYR E 144 −33.149 −33.167 53.330 0.00 0.00
E
ATOM 15921 CZ TYR E 144 −31.260 −33.990 53.691 0.00 0.00
E
ATOM 15922 OH TYR E 144 −30.539 −32.814 53.625 0.00 0.00
E
ATOM 15923 HH TYR E 144 −31.054 −32.021 53.792 0.00 0.00
E
ATOM 15924 CD2 TYR E 144 −31.130 −36.428 53.909 0.00 0.00
E
ATOM 15925 HD2 TYR E 144 −30.537 −37.284 54.194 0.00 0.00
E
ATOM 15926 CE2 TYR E 144 −30.541 −35.189 53.888 0.00 0.00
E
ATOM 15927 HE2 TYR E 144 −29.480 −35.254 54.074 0.00 0.00
E
ATOM 15928 C TYR E 144 −34.321 −37.718 51.537 0.00 0.00
E
ATOM 15929 O TYR E 144 −33.851 −36.892 50.718 0.00 0.00
E

ATOM 15930 N   ILE E 145   -35.629 -37.705 51.807 0.00 0.00
E
ATOM 15931 HN ILE E 145   -35.964 -38.433 52.401 0.00 0.00
E
ATOM 15932 CA ILE E 145   -36.487 -36.560 51.428 0.00 0.00
E
ATOM 15933 HA ILE E 145   -35.942 -35.627 51.423 0.00 0.00
E
ATOM 15934 CB ILE E 145   -36.982 -36.734 50.005 0.00 0.00
E
ATOM 15935 HB ILE E 145   -36.028 -36.570 49.459 0.00 0.00
E
ATOM 15936 CG2 ILE E 145   -37.597 -38.157 49.762 0.00 0.00
E
ATOM 15937 HG21 ILE E 145   -37.497 -38.454 48.696 0.00 0.00
E
ATOM 15938 HG22 ILE E 145   -37.064 -38.941 50.342 0.00 0.00
E
ATOM 15939 HG23 ILE E 145   -38.672 -38.122 50.039 0.00 0.00
E
ATOM 15940 CG1 ILE E 145   -37.897 -35.604 49.555 0.00 0.00
E
ATOM 15941 HG11 ILE E 145   -38.880 -35.713 50.061 0.00 0.00
E
ATOM 15942 HG12 ILE E 145   -37.397 -34.652 49.834 0.00 0.00
E
ATOM 15943 CD ILE E 145   -38.032 -35.605 48.028 0.00 0.00
E
ATOM 15944 HD1 ILE E 145   -37.055 -35.650 47.501 0.00 0.00
E
ATOM 15945 HD2 ILE E 145   -38.613 -36.459 47.619 0.00 0.00
E
ATOM 15946 HD3 ILE E 145   -38.485 -34.617 47.793 0.00 0.00
E
ATOM 15947 C   ILE E 145   -37.636 -36.381 52.413 0.00 0.00
E
ATOM 15948 O   ILE E 145   -38.379 -37.289 52.829 0.00 0.00
E
ATOM 15949 N   ASP E 146   -37.848 -35.102 52.800 0.00 0.00
E
ATOM 15950 HN ASP E 146   -37.244 -34.338 52.587 0.00 0.00
E
ATOM 15951 CA ASP E 146   -39.021 -34.755 53.596 0.00 0.00
E
ATOM 15952 HA ASP E 146   -39.206 -35.567 54.283 0.00 0.00
E
ATOM 15953 CB ASP E 146   -38.714 -33.375 54.374 0.00 0.00
E
ATOM 15954 HB1 ASP E 146   -38.391 -32.607 53.639 0.00 0.00
E
ATOM 15955 HB2 ASP E 146   -39.707 -33.083 54.778 0.00 0.00
E
ATOM 15956 CG ASP E 146   -37.746 -33.565 55.418 0.00 0.00
E

```
ATOM  15957  OD1 ASP E 146     -36.592 -32.911  55.407  0.00  0.00
E
ATOM  15958  OD2 ASP E 146     -38.016 -34.289  56.448  0.00  0.00
E
ATOM  15959  C   ASP E 146     -40.194 -34.675  52.700  0.00  0.00
E
ATOM  15960  O   ASP E 146     -40.237 -34.066  51.622  0.00  0.00
E
ATOM  15961  N   LEU E 147     -41.224 -35.222  53.327  0.00  0.00
E
ATOM  15962  HN  LEU E 147     -41.062 -35.733  54.168  0.00  0.00
E
ATOM  15963  CA  LEU E 147     -42.537 -35.150  52.839  0.00  0.00
E
ATOM  15964  HA  LEU E 147     -42.448 -34.902  51.792  0.00  0.00
E
ATOM  15965  CB  LEU E 147     -43.299 -36.479  52.966  0.00  0.00
E
ATOM  15966  HB1 LEU E 147     -43.603 -36.622  54.025  0.00  0.00
E
ATOM  15967  HB2 LEU E 147     -44.070 -36.489  52.166  0.00  0.00
E
ATOM  15968  CG  LEU E 147     -42.447 -37.704  52.505  0.00  0.00
E
ATOM  15969  HG  LEU E 147     -41.565 -37.800  53.174  0.00  0.00
E
ATOM  15970  CD1 LEU E 147     -43.325 -38.932  52.726  0.00  0.00
E
ATOM  15971 HD11 LEU E 147     -43.899 -39.112  51.793  0.00  0.00
E
ATOM  15972 HD12 LEU E 147     -42.588 -39.758  52.631  0.00  0.00
E
ATOM  15973 HD13 LEU E 147     -43.784 -39.035  53.733  0.00  0.00
E
ATOM  15974  CD2 LEU E 147     -41.897 -37.581  51.111  0.00  0.00
E
ATOM  15975 HD21 LEU E 147     -42.678 -37.225  50.405  0.00  0.00
E
ATOM  15976 HD22 LEU E 147     -41.012 -36.912  51.046  0.00  0.00
E
ATOM  15977 HD23 LEU E 147     -41.506 -38.538  50.706  0.00  0.00
E
ATOM  15978  C   LEU E 147     -43.325 -34.085  53.586  0.00  0.00
E
ATOM  15979  O   LEU E 147     -44.196 -33.356  53.087  0.00  0.00
E
ATOM  15980  N   SER E 148     -43.049 -33.995  54.891  0.00  0.00
E
ATOM  15981  HN  SER E 148     -42.341 -34.510  55.367  0.00  0.00
E
ATOM  15982  CA  SER E 148     -43.686 -33.054  55.800  0.00  0.00
E
ATOM  15983  HA  SER E 148     -43.777 -32.144  55.225  0.00  0.00
E
```

ATOM	15984	CB	SER	E	148	−45.105	−33.442	56.230	0.00	0.00	E
ATOM	15985	HB1	SER	E	148	−45.458	−32.479	56.656	0.00	0.00	E
ATOM	15986	HB2	SER	E	148	−45.862	−33.653	55.444	0.00	0.00	E
ATOM	15987	OG	SER	E	148	−45.149	−34.464	57.156	0.00	0.00	E
ATOM	15988	HG1	SER	E	148	−46.084	−34.571	57.347	0.00	0.00	E
ATOM	15989	C	SER	E	148	−42.780	−32.768	57.004	0.00	0.00	E
ATOM	15990	O	SER	E	148	−41.690	−33.301	57.095	0.00	0.00	E
ATOM	15991	N	ASP	E	149	−43.157	−31.905	57.961	0.00	0.00	E
ATOM	15992	HN	ASP	E	149	−43.924	−31.275	57.861	0.00	0.00	E
ATOM	15993	CA	ASP	E	149	−42.584	−31.846	59.286	0.00	0.00	E
ATOM	15994	HA	ASP	E	149	−41.538	−31.680	59.074	0.00	0.00	E
ATOM	15995	CB	ASP	E	149	−43.151	−30.671	60.145	0.00	0.00	E
ATOM	15996	HB1	ASP	E	149	−44.260	−30.733	60.132	0.00	0.00	E
ATOM	15997	HB2	ASP	E	149	−42.916	−30.776	61.226	0.00	0.00	E
ATOM	15998	CG	ASP	E	149	−42.722	−29.342	59.710	0.00	0.00	E
ATOM	15999	OD1	ASP	E	149	−43.625	−28.467	59.472	0.00	0.00	E
ATOM	16000	OD2	ASP	E	149	−41.461	−29.198	59.498	0.00	0.00	E
ATOM	16001	C	ASP	E	149	−42.682	−33.146	60.066	0.00	0.00	E
ATOM	16002	O	ASP	E	149	−41.733	−33.516	60.624	0.00	0.00	E
ATOM	16003	N	GLU	E	150	−43.814	−33.870	60.060	0.00	0.00	E
ATOM	16004	HN	GLU	E	150	−44.598	−33.433	59.625	0.00	0.00	E
ATOM	16005	CA	GLU	E	150	−43.913	−35.160	60.642	0.00	0.00	E
ATOM	16006	HA	GLU	E	150	−43.362	−35.137	61.570	0.00	0.00	E
ATOM	16007	CB	GLU	E	150	−45.432	−35.620	60.892	0.00	0.00	E
ATOM	16008	HB1	GLU	E	150	−45.892	−35.713	59.885	0.00	0.00	E
ATOM	16009	HB2	GLU	E	150	−45.308	−36.643	61.307	0.00	0.00	E
ATOM	16010	CG	GLU	E	150	−46.233	−34.541	61.660	0.00	0.00	E

ATOM 16011 HG1 GLU E 150 -45.982 -33.493 61.389 0.00 0.00
E
ATOM 16012 HG2 GLU E 150 -47.296 -34.768 61.433 0.00 0.00
E
ATOM 16013 CD GLU E 150 -46.046 -34.710 63.202 0.00 0.00
E
ATOM 16014 OE1 GLU E 150 -45.113 -34.080 63.784 0.00 0.00
E
ATOM 16015 OE2 GLU E 150 -46.816 -35.430 63.806 0.00 0.00
E
ATOM 16016 C GLU E 150 -43.276 -36.266 59.849 0.00 0.00
E
ATOM 16017 O GLU E 150 -42.526 -37.068 60.422 0.00 0.00
E
ATOM 16018 N TYR E 151 -43.532 -36.357 58.496 0.00 0.00
E
ATOM 16019 HN TYR E 151 -44.076 -35.607 58.126 0.00 0.00
E
ATOM 16020 CA TYR E 151 -43.277 -37.546 57.659 0.00 0.00
E
ATOM 16021 HA TYR E 151 -42.869 -38.328 58.282 0.00 0.00
E
ATOM 16022 CB TYR E 151 -44.494 -37.931 56.807 0.00 0.00
E
ATOM 16023 HB1 TYR E 151 -44.674 -37.147 56.041 0.00 0.00
E
ATOM 16024 HB2 TYR E 151 -44.349 -38.912 56.306 0.00 0.00
E
ATOM 16025 CG TYR E 151 -45.693 -38.040 57.800 0.00 0.00
E
ATOM 16026 CD1 TYR E 151 -46.834 -37.128 57.802 0.00 0.00
E
ATOM 16027 HD1 TYR E 151 -46.939 -36.342 57.068 0.00 0.00
E
ATOM 16028 CE1 TYR E 151 -47.739 -37.154 58.877 0.00 0.00
E
ATOM 16029 HE1 TYR E 151 -48.655 -36.581 58.873 0.00 0.00
E
ATOM 16030 CZ TYR E 151 -47.600 -38.192 59.841 0.00 0.00
E
ATOM 16031 OH TYR E 151 -48.442 -38.215 60.961 0.00 0.00
E
ATOM 16032 HH TYR E 151 -49.166 -37.663 60.659 0.00 0.00
E
ATOM 16033 CD2 TYR E 151 -45.588 -39.112 58.749 0.00 0.00
E
ATOM 16034 HD2 TYR E 151 -44.840 -39.889 58.693 0.00 0.00
E
ATOM 16035 CE2 TYR E 151 -46.586 -39.164 59.754 0.00 0.00
E
ATOM 16036 HE2 TYR E 151 -46.409 -39.889 60.535 0.00 0.00
E
ATOM 16037 C TYR E 151 -42.095 -37.329 56.626 0.00 0.00
E

```
ATOM  16038  O   TYR E 151     -41.981 -36.294  55.983  0.00  0.00
E
ATOM  16039  N   SER E 152     -41.243 -38.360  56.537  0.00  0.00
E
ATOM  16040  HN  SER E 152     -41.415 -39.216  57.019  0.00  0.00
E
ATOM  16041  CA  SER E 152     -40.059 -38.369  55.655  0.00  0.00
E
ATOM  16042  HA  SER E 152     -40.267 -38.017  54.655  0.00  0.00
E
ATOM  16043  CB  SER E 152     -38.770 -37.720  56.231  0.00  0.00
E
ATOM  16044  HB1 SER E 152     -37.937 -37.675  55.497  0.00  0.00
E
ATOM  16045  HB2 SER E 152     -38.926 -36.639  56.438  0.00  0.00
E
ATOM  16046  OG  SER E 152     -38.330 -38.345  57.423  0.00  0.00
E
ATOM  16047  HG1 SER E 152     -37.493 -38.019  57.762  0.00  0.00
E
ATOM  16048  C   SER E 152     -39.692 -39.811  55.246  0.00  0.00
E
ATOM  16049  O   SER E 152     -39.962 -40.863  55.852  0.00  0.00
E
ATOM  16050  N   ILE E 153     -38.946 -39.867  54.105  0.00  0.00
E
ATOM  16051  HN  ILE E 153     -38.715 -39.021  53.631  0.00  0.00
E
ATOM  16052  CA  ILE E 153     -38.298 -40.992  53.558  0.00  0.00
E
ATOM  16053  HA  ILE E 153     -38.700 -41.860  54.060  0.00  0.00
E
ATOM  16054  CB  ILE E 153     -38.526 -40.986  52.022  0.00  0.00
E
ATOM  16055  HB  ILE E 153     -38.060 -40.074  51.593  0.00  0.00
E
ATOM  16056  CG2 ILE E 153     -37.897 -42.241  51.475  0.00  0.00
E
ATOM  16057 HG21 ILE E 153     -36.806 -42.163  51.668  0.00  0.00
E
ATOM  16058 HG22 ILE E 153     -38.276 -43.155  51.981  0.00  0.00
E
ATOM  16059 HG23 ILE E 153     -38.016 -42.392  50.381  0.00  0.00
E
ATOM  16060  CG1 ILE E 153     -40.013 -41.032  51.561  0.00  0.00
E
ATOM  16061 HG11 ILE E 153     -40.461 -42.015  51.822  0.00  0.00
E
ATOM  16062 HG12 ILE E 153     -40.625 -40.270  52.088  0.00  0.00
E
ATOM  16063  CD  ILE E 153     -40.166 -40.736  50.099  0.00  0.00
E
ATOM  16064  HD1 ILE E 153     -39.718 -39.741  49.888  0.00  0.00
E
```

ATOM 16065 HD2 ILE E 153   −39.924 −41.524 49.354 0.00 0.00
E
ATOM 16066 HD3 ILE E 153   −41.249 −40.661 49.861 0.00 0.00
E
ATOM 16067 C   ILE E 153   −36.822 −41.064 53.763 0.00 0.00
E
ATOM 16068 O   ILE E 153   −36.134 −40.157 53.344 0.00 0.00
E
ATOM 16069 N   VAL E 154   −36.291 −42.102 54.356 0.00 0.00
E
ATOM 16070 HN VAL E 154   −36.835 −42.850 54.728 0.00 0.00
E
ATOM 16071 CA VAL E 154   −34.790 −42.253 54.536 0.00 0.00
E
ATOM 16072 HA VAL E 154   −34.258 −41.472 54.013 0.00 0.00
E
ATOM 16073 CB VAL E 154   −34.390 −42.234 55.996 0.00 0.00
E
ATOM 16074 HB VAL E 154   −33.314 −42.498 56.074 0.00 0.00
E
ATOM 16075 CG1 VAL E 154   −34.450 −40.874 56.642 0.00 0.00
E
ATOM 16076 HG11 VAL E 154   −33.924 −40.924 57.619 0.00 0.00
E
ATOM 16077 HG12 VAL E 154   −33.788 −40.237 56.016 0.00 0.00
E
ATOM 16078 HG13 VAL E 154   −35.455 −40.401 56.665 0.00 0.00
E
ATOM 16079 CG2 VAL E 154   −35.200 −43.164 56.868 0.00 0.00
E
ATOM 16080 HG21 VAL E 154   −35.147 −44.225 56.543 0.00 0.00
E
ATOM 16081 HG22 VAL E 154   −34.940 −43.063 57.943 0.00 0.00
E
ATOM 16082 HG23 VAL E 154   −36.247 −42.867 56.643 0.00 0.00
E
ATOM 16083 C   VAL E 154   −34.251 −43.564 53.927 0.00 0.00
E
ATOM 16084 O   VAL E 154   −34.882 −44.625 53.831 0.00 0.00
E
ATOM 16085 N   GLU E 155   −33.088 −43.467 53.264 0.00 0.00
E
ATOM 16086 HN GLU E 155   −32.726 −42.557 53.078 0.00 0.00
E
ATOM 16087 CA GLU E 155   −32.366 −44.592 52.813 0.00 0.00
E
ATOM 16088 HA GLU E 155   −33.012 −45.432 52.606 0.00 0.00
E
ATOM 16089 CB GLU E 155   −31.631 −44.228 51.517 0.00 0.00
E
ATOM 16090 HB1 GLU E 155   −32.420 −43.912 50.801 0.00 0.00
E
ATOM 16091 HB2 GLU E 155   −31.039 −43.326 51.784 0.00 0.00
E

ATOM 16092 CG GLU E 155 -30.775 -45.369 50.921 0.00 0.00
E
ATOM 16093 HG1 GLU E 155 -29.934 -45.547 51.625 0.00 0.00
E
ATOM 16094 HG2 GLU E 155 -31.261 -46.367 50.963 0.00 0.00
E
ATOM 16095 CD GLU E 155 -30.275 -45.138 49.503 0.00 0.00
E
ATOM 16096 OE1 GLU E 155 -31.071 -44.689 48.648 0.00 0.00
E
ATOM 16097 OE2 GLU E 155 -29.070 -45.421 49.308 0.00 0.00
E
ATOM 16098 C GLU E 155 -31.378 -45.014 53.904 0.00 0.00
E
ATOM 16099 O GLU E 155 -30.260 -44.503 54.184 0.00 0.00
E
ATOM 16100 N LEU E 156 -31.763 -46.146 54.528 0.00 0.00
E
ATOM 16101 HN LEU E 156 -32.560 -46.683 54.262 0.00 0.00
E
ATOM 16102 CA LEU E 156 -31.080 -46.567 55.715 0.00 0.00
E
ATOM 16103 HA LEU E 156 -30.522 -45.695 56.023 0.00 0.00
E
ATOM 16104 CB LEU E 156 -32.100 -47.109 56.756 0.00 0.00
E
ATOM 16105 HB1 LEU E 156 -32.779 -47.783 56.191 0.00 0.00
E
ATOM 16106 HB2 LEU E 156 -31.618 -47.658 57.593 0.00 0.00
E
ATOM 16107 CG LEU E 156 -32.929 -46.069 57.415 0.00 0.00
E
ATOM 16108 HG LEU E 156 -33.428 -45.351 56.729 0.00 0.00
E
ATOM 16109 CD1 LEU E 156 -34.014 -46.743 58.253 0.00 0.00
E
ATOM 16110 HD11 LEU E 156 -34.802 -47.153 57.586 0.00 0.00
E
ATOM 16111 HD12 LEU E 156 -33.547 -47.505 58.913 0.00 0.00
E
ATOM 16112 HD13 LEU E 156 -34.456 -45.953 58.899 0.00 0.00
E
ATOM 16113 CD2 LEU E 156 -32.091 -45.231 58.467 0.00 0.00
E
ATOM 16114 HD21 LEU E 156 -31.104 -44.876 58.100 0.00 0.00
E
ATOM 16115 HD22 LEU E 156 -32.535 -44.297 58.871 0.00 0.00
E
ATOM 16116 HD23 LEU E 156 -31.924 -45.918 59.325 0.00 0.00
E
ATOM 16117 C LEU E 156 -30.134 -47.667 55.387 0.00 0.00
E
ATOM 16118 O LEU E 156 -30.459 -48.535 54.679 0.00 0.00
E

ATOM 16119 N LEU E 157 -28.931 -47.663 55.937 0.00 0.00
E
ATOM 16120 HN LEU E 157 -28.797 -46.783 56.386 0.00 0.00
E
ATOM 16121 CA LEU E 157 -27.930 -48.714 55.783 0.00 0.00
E
ATOM 16122 HA LEU E 157 -28.057 -49.179 54.817 0.00 0.00
E
ATOM 16123 CB LEU E 157 -26.532 -47.963 55.689 0.00 0.00
E
ATOM 16124 HB1 LEU E 157 -26.466 -47.180 54.904 0.00 0.00
E
ATOM 16125 HB2 LEU E 157 -26.257 -47.490 56.656 0.00 0.00
E
ATOM 16126 CG LEU E 157 -25.377 -48.761 55.287 0.00 0.00
E
ATOM 16127 HG LEU E 157 -25.220 -49.411 56.174 0.00 0.00
E
ATOM 16128 CD1 LEU E 157 -25.583 -49.502 53.963 0.00 0.00
E
ATOM 16129 HD11 LEU E 157 -26.424 -50.223 54.051 0.00 0.00
E
ATOM 16130 HD12 LEU E 157 -25.738 -48.771 53.141 0.00 0.00
E
ATOM 16131 HD13 LEU E 157 -24.582 -49.947 53.779 0.00 0.00
E
ATOM 16132 CD2 LEU E 157 -24.122 -47.887 55.164 0.00 0.00
E
ATOM 16133 HD21 LEU E 157 -23.247 -48.524 54.915 0.00 0.00
E
ATOM 16134 HD22 LEU E 157 -24.168 -47.081 54.401 0.00 0.00
E
ATOM 16135 HD23 LEU E 157 -24.034 -47.328 56.120 0.00 0.00
E
ATOM 16136 C LEU E 157 -27.948 -49.817 56.934 0.00 0.00
E
ATOM 16137 O LEU E 157 -27.675 -49.624 58.137 0.00 0.00
E
ATOM 16138 N ALA E 158 -28.226 -51.050 56.571 0.00 0.00
E
ATOM 16139 HN ALA E 158 -28.472 -51.179 55.613 0.00 0.00
E
ATOM 16140 CA ALA E 158 -28.367 -52.202 57.320 0.00 0.00
E
ATOM 16141 HA ALA E 158 -29.072 -52.002 58.114 0.00 0.00
E
ATOM 16142 CB ALA E 158 -28.953 -53.272 56.469 0.00 0.00
E
ATOM 16143 HB1 ALA E 158 -28.275 -53.389 55.597 0.00 0.00
E
ATOM 16144 HB2 ALA E 158 -28.947 -54.219 57.051 0.00 0.00
E
ATOM 16145 HB3 ALA E 158 -30.011 -53.091 56.185 0.00 0.00
E

ATOM 16146 C ALA E 158 -27.022 -52.605 57.976 0.00 0.00
E
ATOM 16147 O ALA E 158 -25.993 -52.797 57.388 0.00 0.00
E
ATOM 16148 N THR E 159 -27.110 -52.788 59.328 0.00 0.00
E
ATOM 16149 HN THR E 159 -27.965 -52.613 59.810 0.00 0.00
E
ATOM 16150 CA THR E 159 -26.063 -53.304 60.156 0.00 0.00
E
ATOM 16151 HA THR E 159 -25.077 -53.029 59.812 0.00 0.00
E
ATOM 16152 CB THR E 159 -26.314 -52.909 61.654 0.00 0.00
E
ATOM 16153 HB THR E 159 -25.484 -53.381 62.222 0.00 0.00
E
ATOM 16154 OG1 THR E 159 -27.634 -53.259 62.183 0.00 0.00
E
ATOM 16155 HG1 THR E 159 -27.671 -52.864 63.057 0.00 0.00
E
ATOM 16156 CG2 THR E 159 -26.146 -51.426 61.848 0.00 0.00
E
ATOM 16157 HG21 THR E 159 -25.281 -51.139 61.212 0.00 0.00
E
ATOM 16158 HG22 THR E 159 -26.951 -50.755 61.480 0.00 0.00
E
ATOM 16159 HG23 THR E 159 -25.984 -51.082 62.891 0.00 0.00
E
ATOM 16160 C THR E 159 -26.131 -54.857 60.102 0.00 0.00
E
ATOM 16161 O THR E 159 -27.091 -55.473 59.501 0.00 0.00
E
ATOM 16162 N ARG E 160 -25.101 -55.555 60.589 0.00 0.00
E
ATOM 16163 HN ARG E 160 -24.396 -55.110 61.135 0.00 0.00
E
ATOM 16164 CA ARG E 160 -25.032 -57.010 60.595 0.00 0.00
E
ATOM 16165 HA ARG E 160 -25.134 -57.314 59.564 0.00 0.00
E
ATOM 16166 CB ARG E 160 -23.685 -57.420 61.199 0.00 0.00
E
ATOM 16167 HB1 ARG E 160 -22.873 -56.792 60.773 0.00 0.00
E
ATOM 16168 HB2 ARG E 160 -23.505 -57.262 62.283 0.00 0.00
E
ATOM 16169 CG ARG E 160 -23.278 -58.899 60.834 0.00 0.00
E
ATOM 16170 HG1 ARG E 160 -23.982 -59.632 61.282 0.00 0.00
E
ATOM 16171 HG2 ARG E 160 -23.484 -59.022 59.749 0.00 0.00
E
ATOM 16172 CD ARG E 160 -21.860 -59.318 61.189 0.00 0.00
E

ATOM 16173 HD1 ARG E 160 −21.677 −58.973 62.230 0.00 0.00
E
ATOM 16174 HD2 ARG E 160 −21.820 −60.425 61.105 0.00 0.00
E
ATOM 16175 NE ARG E 160 −20.942 −58.679 60.249 0.00 0.00
E
ATOM 16176 HE ARG E 160 −21.368 −58.195 59.484 0.00 0.00
E
ATOM 16177 CZ ARG E 160 −19.695 −58.257 60.493 0.00 0.00
E
ATOM 16178 NH1 ARG E 160 −18.996 −58.817 61.518 0.00 0.00
E
ATOM 16179 HH11 ARG E 160 −18.289 −58.280 61.979 0.00 0.00
E
ATOM 16180 HH12 ARG E 160 −19.591 −59.371 62.099 0.00 0.00
E
ATOM 16181 NH2 ARG E 160 −19.085 −57.458 59.677 0.00 0.00
E
ATOM 16182 HH21 ARG E 160 −18.252 −57.053 60.055 0.00 0.00
E
ATOM 16183 HH22 ARG E 160 −19.650 −57.067 58.950 0.00 0.00
E
ATOM 16184 C ARG E 160 −26.084 −57.651 61.507 0.00 0.00
E
ATOM 16185 O ARG E 160 −26.154 −58.868 61.584 0.00 0.00
E
ATOM 16186 N LYS E 161 −26.876 −56.914 62.331 0.00 0.00
E
ATOM 16187 HN LYS E 161 −26.620 −55.951 62.344 0.00 0.00
E
ATOM 16188 CA LYS E 161 −27.920 −57.352 63.197 0.00 0.00
E
ATOM 16189 HA LYS E 161 −27.588 −58.175 63.813 0.00 0.00
E
ATOM 16190 CB LYS E 161 −28.323 −56.205 64.123 0.00 0.00
E
ATOM 16191 HB1 LYS E 161 −28.596 −55.252 63.621 0.00 0.00
E
ATOM 16192 HB2 LYS E 161 −29.276 −56.445 64.642 0.00 0.00
E
ATOM 16193 CG LYS E 161 −27.226 −55.861 65.138 0.00 0.00
E
ATOM 16194 HG1 LYS E 161 −26.412 −55.570 64.441 0.00 0.00
E
ATOM 16195 HG2 LYS E 161 −27.465 −54.940 65.711 0.00 0.00
E
ATOM 16196 CD LYS E 161 −26.751 −57.015 66.095 0.00 0.00
E
ATOM 16197 HD1 LYS E 161 −27.575 −57.373 66.748 0.00 0.00
E
ATOM 16198 HD2 LYS E 161 −26.686 −57.953 65.503 0.00 0.00
E
ATOM 16199 CE LYS E 161 −25.443 −56.885 66.871 0.00 0.00
E

ATOM 16200 HE1 LYS E 161 −24.560 −56.676 66.230 0.00 0.00
E
ATOM 16201 HE2 LYS E 161 −25.562 −56.180 67.721 0.00 0.00
E
ATOM 16202 NZ LYS E 161 −25.189 −58.285 67.556 0.00 0.00
E
ATOM 16203 HZ1 LYS E 161 −24.171 −58.327 67.762 0.00 0.00
E
ATOM 16204 HZ2 LYS E 161 −25.800 −58.352 68.395 0.00 0.00
E
ATOM 16205 HZ3 LYS E 161 −25.446 −58.991 66.837 0.00 0.00
E
ATOM 16206 C LYS E 161 −29.140 −57.834 62.356 0.00 0.00
E
ATOM 16207 O LYS E 161 −29.859 −58.819 62.621 0.00 0.00
E
ATOM 16208 N LEU E 162 −29.419 −57.061 61.272 0.00 0.00
E
ATOM 16209 HN LEU E 162 −28.913 −56.248 60.994 0.00 0.00
E
ATOM 16210 CA LEU E 162 −30.471 −57.385 60.326 0.00 0.00
E
ATOM 16211 HA LEU E 162 −31.335 −57.747 60.863 0.00 0.00
E
ATOM 16212 CB LEU E 162 −30.862 −56.079 59.560 0.00 0.00
E
ATOM 16213 HB1 LEU E 162 −30.044 −55.638 58.950 0.00 0.00
E
ATOM 16214 HB2 LEU E 162 −31.679 −56.371 58.866 0.00 0.00
E
ATOM 16215 CG LEU E 162 −31.382 −54.998 60.489 0.00 0.00
E
ATOM 16216 HG LEU E 162 −30.512 −54.437 60.892 0.00 0.00
E
ATOM 16217 CD1 LEU E 162 −32.117 −53.961 59.548 0.00 0.00
E
ATOM 16218 HD11 LEU E 162 −32.441 −53.026 60.054 0.00 0.00
E
ATOM 16219 HD12 LEU E 162 −31.463 −53.708 58.686 0.00 0.00
E
ATOM 16220 HD13 LEU E 162 −32.989 −54.465 59.081 0.00 0.00
E
ATOM 16221 CD2 LEU E 162 −32.364 −55.402 61.548 0.00 0.00
E
ATOM 16222 HD21 LEU E 162 −33.241 −55.962 61.159 0.00 0.00
E
ATOM 16223 HD22 LEU E 162 −31.859 −55.959 62.367 0.00 0.00
E
ATOM 16224 HD23 LEU E 162 −32.894 −54.498 61.916 0.00 0.00
E
ATOM 16225 C LEU E 162 −30.215 −58.461 59.299 0.00 0.00
E
ATOM 16226 O LEU E 162 −31.148 −58.957 58.606 0.00 0.00
E

ATOM	16227	N	ASP	E	163	−28.979	−58.901	59.134	0.00	0.00	E
ATOM	16228	HN	ASP	E	163	−28.211	−58.560	59.671	0.00	0.00	E
ATOM	16229	CA	ASP	E	163	−28.546	−60.080	58.320	0.00	0.00	E
ATOM	16230	HA	ASP	E	163	−28.759	−59.830	57.291	0.00	0.00	E
ATOM	16231	CB	ASP	E	163	−27.078	−60.388	58.464	0.00	0.00	E
ATOM	16232	HB1	ASP	E	163	−26.547	−59.439	58.237	0.00	0.00	E
ATOM	16233	HB2	ASP	E	163	−26.839	−60.823	59.458	0.00	0.00	E
ATOM	16234	CG	ASP	E	163	−26.635	−61.402	57.299	0.00	0.00	E
ATOM	16235	OD1	ASP	E	163	−26.549	−60.938	56.131	0.00	0.00	E
ATOM	16236	OD2	ASP	E	163	−26.330	−62.595	57.622	0.00	0.00	E
ATOM	16237	C	ASP	E	163	−29.303	−61.287	58.632	0.00	0.00	E
ATOM	16238	O	ASP	E	163	−29.198	−61.943	59.727	0.00	0.00	E
ATOM	16239	N	SER	E	164	−30.094	−61.757	57.631	0.00	0.00	E
ATOM	16240	HN	SER	E	164	−30.259	−61.190	56.827	0.00	0.00	E
ATOM	16241	CA	SER	E	164	−30.694	−63.084	57.473	0.00	0.00	E
ATOM	16242	HA	SER	E	164	−30.982	−63.158	56.434	0.00	0.00	E
ATOM	16243	CB	SER	E	164	−29.695	−64.230	57.820	0.00	0.00	E
ATOM	16244	HB1	SER	E	164	−29.450	−64.072	58.892	0.00	0.00	E
ATOM	16245	HB2	SER	E	164	−30.224	−65.196	57.677	0.00	0.00	E
ATOM	16246	OG	SER	E	164	−28.518	−64.180	57.043	0.00	0.00	E
ATOM	16247	HG1	SER	E	164	−27.875	−63.535	57.344	0.00	0.00	E
ATOM	16248	C	SER	E	164	−32.037	−63.160	58.203	0.00	0.00	E
ATOM	16249	O	SER	E	164	−32.463	−64.299	58.497	0.00	0.00	E
ATOM	16250	N	LYS	E	165	−32.758	−62.055	58.417	0.00	0.00	E
ATOM	16251	HN	LYS	E	165	−32.514	−61.192	57.982	0.00	0.00	E
ATOM	16252	CA	LYS	E	165	−34.097	−62.040	58.921	0.00	0.00	E
ATOM	16253	HA	LYS	E	165	−34.533	−63.027	58.965	0.00	0.00	E

ATOM	16254	CB	LYS	E	165	−34.261	−61.399	60.366	0.00	0.00	E
ATOM	16255	HB1	LYS	E	165	−34.046	−60.327	60.171	0.00	0.00	E
ATOM	16256	HB2	LYS	E	165	−35.290	−61.530	60.766	0.00	0.00	E
ATOM	16257	CG	LYS	E	165	−33.248	−61.911	61.341	0.00	0.00	E
ATOM	16258	HG1	LYS	E	165	−33.235	−63.020	61.414	0.00	0.00	E
ATOM	16259	HG2	LYS	E	165	−32.241	−61.659	60.945	0.00	0.00	E
ATOM	16260	CD	LYS	E	165	−33.544	−61.431	62.722	0.00	0.00	E
ATOM	16261	HD1	LYS	E	165	−34.615	−61.663	62.906	0.00	0.00	E
ATOM	16262	HD2	LYS	E	165	−32.990	−62.126	63.389	0.00	0.00	E
ATOM	16263	CE	LYS	E	165	−33.081	−60.015	63.052	0.00	0.00	E
ATOM	16264	HE1	LYS	E	165	−31.972	−59.979	63.001	0.00	0.00	E
ATOM	16265	HE2	LYS	E	165	−33.514	−59.217	62.413	0.00	0.00	E
ATOM	16266	NZ	LYS	E	165	−33.442	−59.621	64.497	0.00	0.00	E
ATOM	16267	HZ1	LYS	E	165	−34.466	−59.518	64.649	0.00	0.00	E
ATOM	16268	HZ2	LYS	E	165	−33.037	−60.265	65.206	0.00	0.00	E
ATOM	16269	HZ3	LYS	E	165	−33.103	−58.674	64.762	0.00	0.00	E
ATOM	16270	C	LYS	E	165	−34.894	−61.260	57.880	0.00	0.00	E
ATOM	16271	O	LYS	E	165	−34.248	−60.437	57.208	0.00	0.00	E
ATOM	16272	N	SER	E	166	−36.212	−61.628	57.763	0.00	0.00	E
ATOM	16273	HN	SER	E	166	−36.629	−62.287	58.385	0.00	0.00	E
ATOM	16274	CA	SER	E	166	−37.190	−61.047	56.785	0.00	0.00	E
ATOM	16275	HA	SER	E	166	−36.494	−60.617	56.080	0.00	0.00	E
ATOM	16276	CB	SER	E	166	−38.095	−61.962	55.894	0.00	0.00	E
ATOM	16277	HB1	SER	E	166	−37.549	−62.874	55.571	0.00	0.00	E
ATOM	16278	HB2	SER	E	166	−38.880	−62.428	56.527	0.00	0.00	E
ATOM	16279	OG	SER	E	166	−38.714	−61.319	54.786	0.00	0.00	E
ATOM	16280	HG1	SER	E	166	−38.101	−61.163	54.064	0.00	0.00	E

ATOM 16281 C SER E 166 -37.902 -59.891 57.467 0.00 0.00
E
ATOM 16282 O SER E 166 -37.769 -59.604 58.654 0.00 0.00
E
ATOM 16283 N ILE E 167 -38.808 -59.113 56.767 0.00 0.00
E
ATOM 16284 HN ILE E 167 -39.006 -59.281 55.804 0.00 0.00
E
ATOM 16285 CA ILE E 167 -39.539 -57.951 57.223 0.00 0.00
E
ATOM 16286 HA ILE E 167 -38.814 -57.197 57.491 0.00 0.00
E
ATOM 16287 CB ILE E 167 -40.267 -57.215 56.108 0.00 0.00
E
ATOM 16288 HB ILE E 167 -41.286 -57.616 55.924 0.00 0.00
E
ATOM 16289 CG2 ILE E 167 -40.392 -55.787 56.575 0.00 0.00
E
ATOM 16290 HG21 ILE E 167 -40.615 -55.134 55.704 0.00 0.00
E
ATOM 16291 HG22 ILE E 167 -41.135 -55.714 57.398 0.00 0.00
E
ATOM 16292 HG23 ILE E 167 -39.418 -55.398 56.943 0.00 0.00
E
ATOM 16293 CG1 ILE E 167 -39.462 -57.187 54.727 0.00 0.00
E
ATOM 16294 HG11 ILE E 167 -39.609 -56.248 54.152 0.00 0.00
E
ATOM 16295 HG12 ILE E 167 -38.412 -57.123 55.084 0.00 0.00
E
ATOM 16296 CD ILE E 167 -39.578 -58.330 53.740 0.00 0.00
E
ATOM 16297 HD1 ILE E 167 -40.595 -58.778 53.749 0.00 0.00
E
ATOM 16298 HD2 ILE E 167 -39.319 -58.066 52.693 0.00 0.00
E
ATOM 16299 HD3 ILE E 167 -38.951 -59.209 54.002 0.00 0.00
E
ATOM 16300 C ILE E 167 -40.524 -58.337 58.344 0.00 0.00
E
ATOM 16301 O ILE E 167 -40.714 -57.690 59.407 0.00 0.00
E
ATOM 16302 N ILE E 168 -41.230 -59.452 58.189 0.00 0.00
E
ATOM 16303 HN ILE E 168 -41.075 -60.008 57.376 0.00 0.00
E
ATOM 16304 CA ILE E 168 -42.236 -59.834 59.121 0.00 0.00
E
ATOM 16305 HA ILE E 168 -42.734 -58.925 59.423 0.00 0.00
E
ATOM 16306 CB ILE E 168 -43.241 -60.806 58.518 0.00 0.00
E
ATOM 16307 HB ILE E 168 -43.920 -61.132 59.336 0.00 0.00
E

ATOM 16308 CG2 ILE E 168 −43.952 −59.945 57.458 0.00 0.00
E
ATOM 16309 HG21 ILE E 168 −44.522 −59.077 57.855 0.00 0.00
E
ATOM 16310 HG22 ILE E 168 −43.286 −59.558 56.658 0.00 0.00
E
ATOM 16311 HG23 ILE E 168 −44.797 −60.570 57.098 0.00 0.00
E
ATOM 16312 CG1 ILE E 168 −42.638 −62.145 57.944 0.00 0.00
E
ATOM 16313 HG11 ILE E 168 −42.210 −61.991 56.930 0.00 0.00
E
ATOM 16314 HG12 ILE E 168 −41.809 −62.496 58.596 0.00 0.00
E
ATOM 16315 CD ILE E 168 −43.653 −63.269 57.855 0.00 0.00
E
ATOM 16316 HD1 ILE E 168 −43.288 −64.128 57.252 0.00 0.00
E
ATOM 16317 HD2 ILE E 168 −43.921 −63.518 58.904 0.00 0.00
E
ATOM 16318 HD3 ILE E 168 −44.594 −62.900 57.395 0.00 0.00
E
ATOM 16319 C ILE E 168 −41.597 −60.573 60.313 0.00 0.00
E
ATOM 16320 O ILE E 168 −42.229 −60.978 61.220 0.00 0.00
E
ATOM 16321 N ASP E 169 −40.286 −60.730 60.375 0.00 0.00
E
ATOM 16322 HN ASP E 169 −39.686 −60.321 59.691 0.00 0.00
E
ATOM 16323 CA ASP E 169 −39.594 −61.351 61.504 0.00 0.00
E
ATOM 16324 HA ASP E 169 −40.243 −62.129 61.878 0.00 0.00
E
ATOM 16325 CB ASP E 169 −38.193 −61.906 61.027 0.00 0.00
E
ATOM 16326 HB1 ASP E 169 −37.721 −61.054 60.492 0.00 0.00
E
ATOM 16327 HB2 ASP E 169 −37.603 −62.133 61.940 0.00 0.00
E
ATOM 16328 CG ASP E 169 −38.245 −63.230 60.259 0.00 0.00
E
ATOM 16329 OD1 ASP E 169 −37.958 −63.298 59.085 0.00 0.00
E
ATOM 16330 OD2 ASP E 169 −38.612 −64.219 61.016 0.00 0.00
E
ATOM 16331 C ASP E 169 −39.436 −60.279 62.629 0.00 0.00
E
ATOM 16332 O ASP E 169 −39.653 −60.512 63.813 0.00 0.00
E
ATOM 16333 N LEU E 170 −39.253 −59.030 62.242 0.00 0.00
E
ATOM 16334 HN LEU E 170 −38.955 −58.792 61.321 0.00 0.00
E

ATOM 16335 CA LEU E 170 -39.265 -57.932 63.197 0.00 0.00
E
ATOM 16336 HA LEU E 170 -39.010 -58.324 64.170 0.00 0.00
E
ATOM 16337 CB LEU E 170 -38.254 -56.920 62.628 0.00 0.00
E
ATOM 16338 HB1 LEU E 170 -37.423 -57.458 62.124 0.00 0.00
E
ATOM 16339 HB2 LEU E 170 -38.794 -56.389 61.815 0.00 0.00
E
ATOM 16340 CG LEU E 170 -37.659 -55.900 63.612 0.00 0.00
E
ATOM 16341 HG LEU E 170 -38.168 -56.089 64.582 0.00 0.00
E
ATOM 16342 CD1 LEU E 170 -36.126 -56.248 63.688 0.00 0.00
E
ATOM 16343 HD11 LEU E 170 -35.740 -56.385 62.656 0.00 0.00
E
ATOM 16344 HD12 LEU E 170 -35.421 -55.555 64.194 0.00 0.00
E
ATOM 16345 HD13 LEU E 170 -36.107 -57.283 64.091 0.00 0.00
E
ATOM 16346 CD2 LEU E 170 -37.901 -54.471 63.146 0.00 0.00
E
ATOM 16347 HD21 LEU E 170 -37.659 -54.439 62.063 0.00 0.00
E
ATOM 16348 HD22 LEU E 170 -38.983 -54.277 63.306 0.00 0.00
E
ATOM 16349 HD23 LEU E 170 -37.263 -53.823 63.785 0.00 0.00
E
ATOM 16350 C LEU E 170 -40.652 -57.285 63.265 0.00 0.00
E
ATOM 16351 O LEU E 170 -40.969 -56.533 64.212 0.00 0.00
E
ATOM 16352 N ASN E 171 -41.481 -57.657 62.316 0.00 0.00
E
ATOM 16353 HN ASN E 171 -41.188 -58.307 61.619 0.00 0.00
E
ATOM 16354 CA ASN E 171 -42.880 -57.156 62.158 0.00 0.00
E
ATOM 16355 HA ASN E 171 -43.167 -57.531 61.187 0.00 0.00
E
ATOM 16356 CB ASN E 171 -43.782 -57.522 63.269 0.00 0.00
E
ATOM 16357 HB1 ASN E 171 -43.422 -56.991 64.176 0.00 0.00
E
ATOM 16358 HB2 ASN E 171 -44.847 -57.371 62.990 0.00 0.00
E
ATOM 16359 CG ASN E 171 -43.650 -59.039 63.593 0.00 0.00
E
ATOM 16360 OD1 ASN E 171 -42.864 -59.443 64.445 0.00 0.00
E
ATOM 16361 ND2 ASN E 171 -44.491 -59.775 63.005 0.00 0.00
E

```
ATOM  16362 HD21 ASN E 171     -44.581 -60.751  63.199  0.00  0.00
E
ATOM  16363 HD22 ASN E 171     -45.113 -59.358  62.341  0.00  0.00
E
ATOM  16364  C   ASN E 171     -42.862 -55.588  62.052  0.00  0.00
E
ATOM  16365  O   ASN E 171     -43.482 -54.869  62.793  0.00  0.00
E
ATOM  16366  N   VAL E 172     -42.098 -55.053  61.064  0.00  0.00
E
ATOM  16367  HN  VAL E 172     -41.617 -55.661  60.437  0.00  0.00
E
ATOM  16368  CA  VAL E 172     -41.666 -53.671  60.903  0.00  0.00
E
ATOM  16369  HA  VAL E 172     -41.066 -53.444  61.771  0.00  0.00
E
ATOM  16370  CB  VAL E 172     -40.718 -53.482  59.727  0.00  0.00
E
ATOM  16371  HB  VAL E 172     -41.120 -54.147  58.932  0.00  0.00
E
ATOM  16372  CG1 VAL E 172     -40.732 -52.014  59.298  0.00  0.00
E
ATOM  16373 HG11 VAL E 172     -40.695 -51.353  60.191  0.00  0.00
E
ATOM  16374 HG12 VAL E 172     -39.905 -51.768  58.599  0.00  0.00
E
ATOM  16375 HG13 VAL E 172     -41.638 -51.789  58.695  0.00  0.00
E
ATOM  16376  CG2 VAL E 172     -39.277 -53.901  60.001  0.00  0.00
E
ATOM  16377 HG21 VAL E 172     -39.295 -54.942  60.389  0.00  0.00
E
ATOM  16378 HG22 VAL E 172     -38.700 -53.837  59.054  0.00  0.00
E
ATOM  16379 HG23 VAL E 172     -38.792 -53.195  60.709  0.00  0.00
E
ATOM  16380  C   VAL E 172     -42.915 -52.793  60.861  0.00  0.00
E
ATOM  16381  O   VAL E 172     -42.978 -51.807  61.600  0.00  0.00
E
ATOM  16382  N   ARG E 173     -43.909 -53.107  60.076  0.00  0.00
E
ATOM  16383  HN  ARG E 173     -43.967 -53.969  59.578  0.00  0.00
E
ATOM  16384  CA  ARG E 173     -45.103 -52.316  59.881  0.00  0.00
E
ATOM  16385  HA  ARG E 173     -44.900 -51.255  59.887  0.00  0.00
E
ATOM  16386  CB  ARG E 173     -45.860 -52.724  58.585  0.00  0.00
E
ATOM  16387  HB1 ARG E 173     -45.068 -52.428  57.864  0.00  0.00
E
ATOM  16388  HB2 ARG E 173     -46.112 -53.799  58.709  0.00  0.00
E
```

ATOM 16389 CG ARG E 173 −47.016 −51.779 58.335 0.00 0.00
E
ATOM 16390 HG1 ARG E 173 −47.759 −51.559 59.130 0.00 0.00
E
ATOM 16391 HG2 ARG E 173 −46.492 −50.799 58.328 0.00 0.00
E
ATOM 16392 CD ARG E 173 −47.764 −51.929 56.952 0.00 0.00
E
ATOM 16393 HD1 ARG E 173 −46.990 −51.765 56.172 0.00 0.00
E
ATOM 16394 HD2 ARG E 173 −48.264 −52.911 56.812 0.00 0.00
E
ATOM 16395 NE ARG E 173 −48.785 −50.865 56.915 0.00 0.00
E
ATOM 16396 HE ARG E 173 −49.067 −50.565 57.826 0.00 0.00
E
ATOM 16397 CZ ARG E 173 −49.394 −50.193 55.900 0.00 0.00
E
ATOM 16398 NH1 ARG E 173 −49.122 −50.632 54.646 0.00 0.00
E
ATOM 16399 HH11 ARG E 173 −49.634 −50.140 53.942 0.00 0.00
E
ATOM 16400 HH12 ARG E 173 −48.938 −51.611 54.555 0.00 0.00
E
ATOM 16401 NH2 ARG E 173 −50.271 −49.226 55.973 0.00 0.00
E
ATOM 16402 HH21 ARG E 173 −50.479 −48.826 55.081 0.00 0.00
E
ATOM 16403 HH22 ARG E 173 −50.149 −48.577 56.724 0.00 0.00
E
ATOM 16404 C ARG E 173 −46.078 −52.385 61.067 0.00 0.00
E
ATOM 16405 O ARG E 173 −46.729 −51.357 61.432 0.00 0.00
E
ATOM 16406 N ALA E 174 −46.181 −53.534 61.698 0.00 0.00
E
ATOM 16407 HN ALA E 174 −45.751 −54.393 61.433 0.00 0.00
E
ATOM 16408 CA ALA E 174 −47.024 −53.699 62.845 0.00 0.00
E
ATOM 16409 HA ALA E 174 −48.053 −53.396 62.722 0.00 0.00
E
ATOM 16410 CB ALA E 174 −47.031 −55.218 63.187 0.00 0.00
E
ATOM 16411 HB1 ALA E 174 −46.087 −55.530 63.683 0.00 0.00
E
ATOM 16412 HB2 ALA E 174 −47.957 −55.439 63.760 0.00 0.00
E
ATOM 16413 HB3 ALA E 174 −47.067 −55.788 62.234 0.00 0.00
E
ATOM 16414 C ALA E 174 −46.541 −52.984 64.164 0.00 0.00
E
ATOM 16415 O ALA E 174 −47.306 −52.438 64.909 0.00 0.00
E

ATOM 16416 N LYS E 175 −45.215 −52.954 64.317 0.00 0.00
E
ATOM 16417 HN LYS E 175 −44.678 −53.466 63.652 0.00 0.00
E
ATOM 16418 CA LYS E 175 −44.571 −52.484 65.470 0.00 0.00
E
ATOM 16419 HA LYS E 175 −45.298 −52.426 66.266 0.00 0.00
E
ATOM 16420 CB LYS E 175 −43.443 −53.477 65.865 0.00 0.00
E
ATOM 16421 HB1 LYS E 175 −42.915 −53.917 64.992 0.00 0.00
E
ATOM 16422 HB2 LYS E 175 −42.659 −53.122 66.568 0.00 0.00
E
ATOM 16423 CG LYS E 175 −44.065 −54.738 66.575 0.00 0.00
E
ATOM 16424 HG1 LYS E 175 −44.702 −54.426 67.430 0.00 0.00
E
ATOM 16425 HG2 LYS E 175 −44.768 −55.246 65.880 0.00 0.00
E
ATOM 16426 CD LYS E 175 −43.071 −55.764 67.044 0.00 0.00
E
ATOM 16427 HD1 LYS E 175 −42.598 −56.240 66.158 0.00 0.00
E
ATOM 16428 HD2 LYS E 175 −42.254 −55.290 67.629 0.00 0.00
E
ATOM 16429 CE LYS E 175 −43.644 −56.934 67.766 0.00 0.00
E
ATOM 16430 HE1 LYS E 175 −44.138 −56.592 68.700 0.00 0.00
E
ATOM 16431 HE2 LYS E 175 −44.463 −57.357 67.147 0.00 0.00
E
ATOM 16432 NZ LYS E 175 −42.612 −57.950 68.103 0.00 0.00
E
ATOM 16433 HZ1 LYS E 175 −42.064 −58.104 67.233 0.00 0.00
E
ATOM 16434 HZ2 LYS E 175 −41.803 −57.534 68.609 0.00 0.00
E
ATOM 16435 HZ3 LYS E 175 −42.892 −58.871 68.497 0.00 0.00
E
ATOM 16436 C LYS E 175 −43.949 −51.110 65.312 0.00 0.00
E
ATOM 16437 O LYS E 175 −43.843 −50.415 66.324 0.00 0.00
E
ATOM 16438 N TYR E 176 −43.675 −50.619 64.126 0.00 0.00
E
ATOM 16439 HN TYR E 176 −43.674 −51.278 63.378 0.00 0.00
E
ATOM 16440 CA TYR E 176 −42.979 −49.328 63.887 0.00 0.00
E
ATOM 16441 HA TYR E 176 −43.166 −48.834 64.829 0.00 0.00
E
ATOM 16442 CB TYR E 176 −41.530 −49.481 63.489 0.00 0.00
E

ATOM 16443 HB1 TYR E 176 −41.470 −49.951 62.485 0.00 0.00
E
ATOM 16444 HB2 TYR E 176 −41.029 −48.499 63.349 0.00 0.00
E
ATOM 16445 CG TYR E 176 −40.747 −50.221 64.496 0.00 0.00
E
ATOM 16446 CD1 TYR E 176 −40.537 −51.595 64.317 0.00 0.00
E
ATOM 16447 HD1 TYR E 176 −40.940 −52.029 63.413 0.00 0.00
E
ATOM 16448 CE1 TYR E 176 −39.937 −52.372 65.375 0.00 0.00
E
ATOM 16449 HE1 TYR E 176 −39.922 −53.441 65.222 0.00 0.00
E
ATOM 16450 CZ TYR E 176 −39.532 −51.745 66.550 0.00 0.00
E
ATOM 16451 OH TYR E 176 −38.983 −52.624 67.551 0.00 0.00
E
ATOM 16452 HH TYR E 176 −39.070 −52.205 68.411 0.00 0.00
E
ATOM 16453 CD2 TYR E 176 −40.331 −49.650 65.712 0.00 0.00
E
ATOM 16454 HD2 TYR E 176 −40.600 −48.615 65.862 0.00 0.00
E
ATOM 16455 CE2 TYR E 176 −39.764 −50.374 66.745 0.00 0.00
E
ATOM 16456 HE2 TYR E 176 −39.589 −49.837 67.665 0.00 0.00
E
ATOM 16457 C TYR E 176 −43.686 −48.404 62.902 0.00 0.00
E
ATOM 16458 O TYR E 176 −43.153 −47.329 62.542 0.00 0.00
E
ATOM 16459 N GLY E 177 −44.838 −48.878 62.363 0.00 0.00
E
ATOM 16460 HN GLY E 177 −45.241 −49.754 62.619 0.00 0.00
E
ATOM 16461 CA GLY E 177 −45.751 −48.058 61.509 0.00 0.00
E
ATOM 16462 HA1 GLY E 177 −46.135 −47.241 62.103 0.00 0.00
E
ATOM 16463 HA2 GLY E 177 −46.620 −48.669 61.316 0.00 0.00
E
ATOM 16464 C GLY E 177 −45.171 −47.591 60.271 0.00 0.00
E
ATOM 16465 O GLY E 177 −45.607 −46.503 59.759 0.00 0.00
E
ATOM 16466 N CYS E 178 −44.139 −48.351 59.764 0.00 0.00
E
ATOM 16467 HN CYS E 178 −43.841 −49.163 60.260 0.00 0.00
E
ATOM 16468 CA CYS E 178 −43.403 −47.880 58.612 0.00 0.00
E
ATOM 16469 HA CYS E 178 −43.707 −46.905 58.260 0.00 0.00
E

ATOM 16470 CB CYS E 178 −41.875 −47.681 58.864 0.00 0.00
E
ATOM 16471 HB1 CYS E 178 −41.561 −48.705 59.159 0.00 0.00
E
ATOM 16472 HB2 CYS E 178 −41.395 −47.388 57.906 0.00 0.00
E
ATOM 16473 SG CYS E 178 −41.317 −46.517 60.184 0.00 0.00
E
ATOM 16474 HG1 CYS E 178 −41.723 −47.252 61.209 0.00 0.00
E
ATOM 16475 C CYS E 178 −43.483 −48.855 57.460 0.00 0.00
E
ATOM 16476 O CYS E 178 −43.544 −50.063 57.716 0.00 0.00
E
ATOM 16477 N THR E 179 −43.454 −48.393 56.223 0.00 0.00
E
ATOM 16478 HN THR E 179 −43.436 −47.418 56.018 0.00 0.00
E
ATOM 16479 CA THR E 179 −43.429 −49.233 55.081 0.00 0.00
E
ATOM 16480 HA THR E 179 −43.491 −50.283 55.324 0.00 0.00
E
ATOM 16481 CB THR E 179 −44.580 −49.029 54.077 0.00 0.00
E
ATOM 16482 HB THR E 179 −44.484 −49.784 53.268 0.00 0.00
E
ATOM 16483 OG1 THR E 179 −44.692 −47.725 53.589 0.00 0.00
E
ATOM 16484 HG1 THR E 179 −45.357 −47.759 52.897 0.00 0.00
E
ATOM 16485 CG2 THR E 179 −45.962 −49.240 54.790 0.00 0.00
E
ATOM 16486 HG21 THR E 179 −46.824 −49.243 54.089 0.00 0.00
E
ATOM 16487 HG22 THR E 179 −45.979 −50.294 55.143 0.00 0.00
E
ATOM 16488 HG23 THR E 179 −46.210 −48.573 55.644 0.00 0.00
E
ATOM 16489 C THR E 179 −42.137 −49.039 54.342 0.00 0.00
E
ATOM 16490 O THR E 179 −41.717 −47.850 54.138 0.00 0.00
E
ATOM 16491 N ILE E 180 −41.558 −50.151 53.809 0.00 0.00
E
ATOM 16492 HN ILE E 180 −41.964 −51.044 53.988 0.00 0.00
E
ATOM 16493 CA ILE E 180 −40.283 −50.212 53.082 0.00 0.00
E
ATOM 16494 HA ILE E 180 −39.836 −49.257 53.315 0.00 0.00
E
ATOM 16495 CB ILE E 180 −39.363 −51.364 53.545 0.00 0.00
E
ATOM 16496 HB ILE E 180 −39.871 −52.260 53.129 0.00 0.00
E

ATOM 16497 CG2 ILE E 180   -37.940 -51.238 52.871 0.00 0.00
E
ATOM 16498 HG21 ILE E 180   -37.232 -52.001 53.258 0.00 0.00
E
ATOM 16499 HG22 ILE E 180   -38.114 -51.314 51.776 0.00 0.00
E
ATOM 16500 HG23 ILE E 180   -37.474 -50.232 52.948 0.00 0.00
E
ATOM 16501 CG1 ILE E 180   -39.329 -51.532 55.086 0.00 0.00
E
ATOM 16502 HG11 ILE E 180   -39.130 -50.531 55.523 0.00 0.00
E
ATOM 16503 HG12 ILE E 180   -40.331 -51.857 55.441 0.00 0.00
E
ATOM 16504 CD ILE E 180   -38.337 -52.515 55.645 0.00 0.00
E
ATOM 16505 HD1 ILE E 180   -38.338 -53.447 55.040 0.00 0.00
E
ATOM 16506 HD2 ILE E 180   -37.325 -52.070 55.533 0.00 0.00
E
ATOM 16507 HD3 ILE E 180   -38.438 -52.813 56.710 0.00 0.00
E
ATOM 16508 C   ILE E 180   -40.632 -50.138 51.595 0.00 0.00
E
ATOM 16509 O   ILE E 180   -41.257 -51.085 51.029 0.00 0.00
E
ATOM 16510 N   LEU E 181   -40.217 -48.973 50.911 0.00 0.00
E
ATOM 16511 HN LEU E 181   -39.811 -48.217 51.419 0.00 0.00
E
ATOM 16512 CA LEU E 181   -40.479 -48.676 49.502 0.00 0.00
E
ATOM 16513 HA LEU E 181   -41.469 -49.068 49.321 0.00 0.00
E
ATOM 16514 CB LEU E 181   -40.246 -47.174 49.137 0.00 0.00
E
ATOM 16515 HB1 LEU E 181   -39.200 -46.996 49.466 0.00 0.00
E
ATOM 16516 HB2 LEU E 181   -40.165 -46.946 48.052 0.00 0.00
E
ATOM 16517 CG LEU E 181   -41.233 -46.207 49.857 0.00 0.00
E
ATOM 16518 HG LEU E 181   -41.436 -46.581 50.883 0.00 0.00
E
ATOM 16519 CD1 LEU E 181   -40.823 -44.805 50.024 0.00 0.00
E
ATOM 16520 HD11 LEU E 181   -41.670 -44.210 50.428 0.00 0.00
E
ATOM 16521 HD12 LEU E 181   -39.932 -44.643 50.668 0.00 0.00
E
ATOM 16522 HD13 LEU E 181   -40.546 -44.493 48.994 0.00 0.00
E
ATOM 16523 CD2 LEU E 181   -42.632 -46.182 49.240 0.00 0.00
E

ATOM 16524 HD21 LEU E 181 −43.380 −46.022 50.045 0.00 0.00
E
ATOM 16525 HD22 LEU E 181 −42.740 −45.450 48.411 0.00 0.00
E
ATOM 16526 HD23 LEU E 181 −42.775 −47.134 48.685 0.00 0.00
E
ATOM 16527 C LEU E 181 −39.560 −49.441 48.589 0.00 0.00
E
ATOM 16528 O LEU E 181 −39.904 −49.747 47.429 0.00 0.00
E
ATOM 16529 N ALA E 182 −38.341 −49.720 48.973 0.00 0.00
E
ATOM 16530 HN ALA E 182 −38.037 −49.661 49.920 0.00 0.00
E
ATOM 16531 CA ALA E 182 −37.404 −50.460 48.198 0.00 0.00
E
ATOM 16532 HA ALA E 182 −37.949 −51.278 47.753 0.00 0.00
E
ATOM 16533 CB ALA E 182 −36.732 −49.591 47.179 0.00 0.00
E
ATOM 16534 HB1 ALA E 182 −36.150 −50.142 46.409 0.00 0.00
E
ATOM 16535 HB2 ALA E 182 −37.561 −48.998 46.738 0.00 0.00
E
ATOM 16536 HB3 ALA E 182 −36.230 −48.748 47.700 0.00 0.00
E
ATOM 16537 C ALA E 182 −36.309 −50.919 49.064 0.00 0.00
E
ATOM 16538 O ALA E 182 −36.189 −50.592 50.317 0.00 0.00
E
ATOM 16539 N ILE E 183 −35.460 −51.780 48.404 0.00 0.00
E
ATOM 16540 HN ILE E 183 −35.569 −52.053 47.452 0.00 0.00
E
ATOM 16541 CA ILE E 183 −34.295 −52.350 49.063 0.00 0.00
E
ATOM 16542 HA ILE E 183 −34.025 −51.748 49.918 0.00 0.00
E
ATOM 16543 CB ILE E 183 −34.575 −53.613 49.859 0.00 0.00
E
ATOM 16544 HB ILE E 183 −35.315 −53.412 50.663 0.00 0.00
E
ATOM 16545 CG2 ILE E 183 −35.278 −54.664 48.941 0.00 0.00
E
ATOM 16546 HG21 ILE E 183 −35.471 −55.640 49.436 0.00 0.00
E
ATOM 16547 HG22 ILE E 183 −36.256 −54.415 48.476 0.00 0.00
E
ATOM 16548 HG23 ILE E 183 −34.604 −54.880 48.085 0.00 0.00
E
ATOM 16549 CG1 ILE E 183 −33.391 −54.297 50.549 0.00 0.00
E
ATOM 16550 HG11 ILE E 183 −32.788 −54.878 49.819 0.00 0.00
E

```
ATOM  16551 HG12 ILE E 183      -32.566 -53.635  50.888  0.00  0.00
E
ATOM  16552  CD  ILE E 183      -33.803 -55.237  51.711  0.00  0.00
E
ATOM  16553  HD1 ILE E 183      -34.586 -54.739  52.323  0.00  0.00
E
ATOM  16554  HD2 ILE E 183      -34.305 -56.077  51.184  0.00  0.00
E
ATOM  16555  HD3 ILE E 183      -32.925 -55.549  52.316  0.00  0.00
E
ATOM  16556  C   ILE E 183      -33.133 -52.580  48.064  0.00  0.00
E
ATOM  16557  O   ILE E 183      -33.298 -53.184  46.982  0.00  0.00
E
ATOM  16558  N   LYS E 184      -31.977 -52.062  48.300  0.00  0.00
E
ATOM  16559  HN  LYS E 184      -31.730 -51.695  49.193  0.00  0.00
E
ATOM  16560  CA  LYS E 184      -31.076 -51.757  47.206  0.00  0.00
E
ATOM  16561  HA  LYS E 184      -31.495 -52.108  46.274  0.00  0.00
E
ATOM  16562  CB  LYS E 184      -30.772 -50.216  47.164  0.00  0.00
E
ATOM  16563  HB1 LYS E 184      -30.356 -49.823  48.117  0.00  0.00
E
ATOM  16564  HB2 LYS E 184      -30.087 -49.982  46.322  0.00  0.00
E
ATOM  16565  CG  LYS E 184      -32.049 -49.452  46.854  0.00  0.00
E
ATOM  16566  HG1 LYS E 184      -32.291 -49.536  45.773  0.00  0.00
E
ATOM  16567  HG2 LYS E 184      -32.812 -49.867  47.546  0.00  0.00
E
ATOM  16568  CD  LYS E 184      -31.828 -47.970  47.209  0.00  0.00
E
ATOM  16569  HD1 LYS E 184      -32.768 -47.378  47.208  0.00  0.00
E
ATOM  16570  HD2 LYS E 184      -31.485 -47.954  48.266  0.00  0.00
E
ATOM  16571  CE  LYS E 184      -30.687 -47.347  46.334  0.00  0.00
E
ATOM  16572  HE1 LYS E 184      -29.753 -47.738  46.791  0.00  0.00
E
ATOM  16573  HE2 LYS E 184      -30.717 -47.582  45.248  0.00  0.00
E
ATOM  16574  NZ  LYS E 184      -30.802 -45.921  46.306  0.00  0.00
E
ATOM  16575  HZ1 LYS E 184      -31.741 -45.519  46.111  0.00  0.00
E
ATOM  16576  HZ2 LYS E 184      -30.482 -45.525  47.213  0.00  0.00
E
ATOM  16577  HZ3 LYS E 184      -30.250 -45.574  45.496  0.00  0.00
E
```

ATOM 16578 C LYS E 184 -29.765 -52.396 47.451 0.00 0.00
E
ATOM 16579 O LYS E 184 -29.383 -52.455 48.606 0.00 0.00
E
ATOM 16580 N HSE E 185 -28.993 -52.867 46.403 0.00 0.00
E
ATOM 16581 HN HSE E 185 -29.420 -52.760 45.508 0.00 0.00
E
ATOM 16582 CA HSE E 185 -27.574 -53.175 46.556 0.00 0.00
E
ATOM 16583 HA HSE E 185 -27.178 -52.477 47.278 0.00 0.00
E
ATOM 16584 CB HSE E 185 -27.217 -54.548 47.185 0.00 0.00
E
ATOM 16585 HB1 HSE E 185 -27.857 -54.865 48.036 0.00 0.00
E
ATOM 16586 HB2 HSE E 185 -27.272 -55.440 46.525 0.00 0.00
E
ATOM 16587 ND1 HSE E 185 -24.761 -54.466 47.177 0.00 0.00
E
ATOM 16588 CG HSE E 185 -25.958 -54.559 47.857 0.00 0.00
E
ATOM 16589 CE1 HSE E 185 -23.846 -54.319 48.092 0.00 0.00
E
ATOM 16590 HE1 HSE E 185 -22.766 -54.321 47.951 0.00 0.00
E
ATOM 16591 NE2 HSE E 185 -24.293 -54.299 49.351 0.00 0.00
E
ATOM 16592 HE2 HSE E 185 -23.759 -54.427 50.187 0.00 0.00
E
ATOM 16593 CD2 HSE E 185 -25.662 -54.412 49.231 0.00 0.00
E
ATOM 16594 HD2 HSE E 185 -26.330 -54.374 50.082 0.00 0.00
E
ATOM 16595 C HSE E 185 -26.922 -52.960 45.270 0.00 0.00
E
ATOM 16596 O HSE E 185 -27.108 -53.708 44.315 0.00 0.00
E
ATOM 16597 N HSE E 186 -25.982 -51.912 45.179 0.00 0.00
E
ATOM 16598 HN HSE E 186 -25.895 -51.366 46.009 0.00 0.00
E
ATOM 16599 CA HSE E 186 -25.091 -51.657 44.092 0.00 0.00
E
ATOM 16600 HA HSE E 186 -24.794 -50.619 44.102 0.00 0.00
E
ATOM 16601 CB HSE E 186 -23.836 -52.533 44.191 0.00 0.00
E
ATOM 16602 HB1 HSE E 186 -24.236 -53.569 44.211 0.00 0.00
E
ATOM 16603 HB2 HSE E 186 -23.350 -52.476 43.193 0.00 0.00
E
ATOM 16604 ND1 HSE E 186 -23.009 -50.961 46.058 0.00 0.00
E

ATOM 16605 CG HSE E 186 −22.956 −52.198 45.362 0.00 0.00
E
ATOM 16606 CE1 HSE E 186 −22.071 −51.068 46.998 0.00 0.00
E
ATOM 16607 HE1 HSE E 186 −21.943 −50.353 47.812 0.00 0.00
E
ATOM 16608 NE2 HSE E 186 −21.431 −52.254 46.957 0.00 0.00
E
ATOM 16609 HE2 HSE E 186 −20.848 −52.707 47.631 0.00 0.00
E
ATOM 16610 CD2 HSE E 186 −21.962 −52.955 45.922 0.00 0.00
E
ATOM 16611 HD2 HSE E 186 −21.826 −53.990 45.635 0.00 0.00
E
ATOM 16612 C HSE E 186 −25.574 −51.564 42.683 0.00 0.00
E
ATOM 16613 O HSE E 186 −25.061 −52.134 41.753 0.00 0.00
E
ATOM 16614 N GLY E 187 −26.694 −50.850 42.513 0.00 0.00
E
ATOM 16615 HN GLY E 187 −27.207 −50.494 43.290 0.00 0.00
E
ATOM 16616 CA GLY E 187 −27.279 −50.695 41.196 0.00 0.00
E
ATOM 16617 HA1 GLY E 187 −26.490 −50.855 40.477 0.00 0.00
E
ATOM 16618 HA2 GLY E 187 −27.796 −49.751 41.096 0.00 0.00
E
ATOM 16619 C GLY E 187 −28.391 −51.636 40.868 0.00 0.00
E
ATOM 16620 O GLY E 187 −29.133 −51.394 39.947 0.00 0.00
E
ATOM 16621 N ASP E 188 −28.477 −52.762 41.688 0.00 0.00
E
ATOM 16622 HN ASP E 188 −27.854 −52.945 42.444 0.00 0.00
E
ATOM 16623 CA ASP E 188 −29.650 −53.545 41.715 0.00 0.00
E
ATOM 16624 HA ASP E 188 −30.126 −53.430 40.752 0.00 0.00
E
ATOM 16625 CB ASP E 188 −29.350 −54.994 42.104 0.00 0.00
E
ATOM 16626 HB1 ASP E 188 −28.830 −55.105 43.079 0.00 0.00
E
ATOM 16627 HB2 ASP E 188 −30.204 −55.689 42.254 0.00 0.00
E
ATOM 16628 CG ASP E 188 −28.550 −55.643 40.963 0.00 0.00
E
ATOM 16629 OD1 ASP E 188 −29.037 −55.737 39.801 0.00 0.00
E
ATOM 16630 OD2 ASP E 188 −27.353 −55.987 41.180 0.00 0.00
E
ATOM 16631 C ASP E 188 −30.598 −53.022 42.725 0.00 0.00
E

```
ATOM  16632  O   ASP E 188     -30.238 -52.405  43.730  0.00  0.00
E
ATOM  16633  N   ILE E 189     -31.921 -53.181  42.449  0.00  0.00
E
ATOM  16634  HN  ILE E 189     -32.309 -53.874  41.845  0.00  0.00
E
ATOM  16635  CA  ILE E 189     -32.993 -52.646  43.162  0.00  0.00
E
ATOM  16636  HA  ILE E 189     -32.621 -52.549  44.171  0.00  0.00
E
ATOM  16637  CB  ILE E 189     -33.378 -51.290  42.687  0.00  0.00
E
ATOM  16638  HB  ILE E 189     -32.518 -50.597  42.802  0.00  0.00
E
ATOM  16639  CG2 ILE E 189     -33.841 -51.258  41.259  0.00  0.00
E
ATOM  16640 HG21 ILE E 189     -33.207 -51.935  40.648  0.00  0.00
E
ATOM  16641 HG22 ILE E 189     -34.935 -51.440  41.310  0.00  0.00
E
ATOM  16642 HG23 ILE E 189     -33.710 -50.217  40.892  0.00  0.00
E
ATOM  16643  CG1 ILE E 189     -34.469 -50.696  43.579  0.00  0.00
E
ATOM  16644 HG11 ILE E 189     -35.463 -51.127  43.335  0.00  0.00
E
ATOM  16645 HG12 ILE E 189     -34.276 -51.011  44.627  0.00  0.00
E
ATOM  16646  CD  ILE E 189     -34.564 -49.178  43.633  0.00  0.00
E
ATOM  16647  HD1 ILE E 189     -33.666 -48.737  44.115  0.00  0.00
E
ATOM  16648  HD2 ILE E 189     -34.637 -48.593  42.691  0.00  0.00
E
ATOM  16649  HD3 ILE E 189     -35.309 -48.916  44.415  0.00  0.00
E
ATOM  16650  C   ILE E 189     -34.124 -53.619  43.089  0.00  0.00
E
ATOM  16651  O   ILE E 189     -34.436 -54.175  42.057  0.00  0.00
E
ATOM  16652  N   CYS E 190     -34.735 -53.958  44.273  0.00  0.00
E
ATOM  16653  HN  CYS E 190     -34.428 -53.654  45.172  0.00  0.00
E
ATOM  16654  CA  CYS E 190     -36.059 -54.620  44.387  0.00  0.00
E
ATOM  16655  HA  CYS E 190     -36.368 -54.900  43.391  0.00  0.00
E
ATOM  16656  CB  CYS E 190     -35.905 -55.811  45.405  0.00  0.00
E
ATOM  16657  HB1 CYS E 190     -35.213 -56.525  44.909  0.00  0.00
E
ATOM  16658  HB2 CYS E 190     -35.425 -55.491  46.354  0.00  0.00
E
```

ATOM 16659 SG CYS E 190 −37.475 −56.610 45.768 0.00 0.00
E
ATOM 16660 HG1 CYS E 190 −37.050 −57.370 46.768 0.00 0.00
E
ATOM 16661 C CYS E 190 −36.953 −53.519 44.919 0.00 0.00
E
ATOM 16662 O CYS E 190 −36.694 −52.971 46.012 0.00 0.00
E
ATOM 16663 N LEU E 191 −38.042 −52.998 44.211 0.00 0.00
E
ATOM 16664 HN LEU E 191 −38.213 −53.513 43.374 0.00 0.00
E
ATOM 16665 CA LEU E 191 −39.018 −52.095 44.736 0.00 0.00
E
ATOM 16666 HA LEU E 191 −38.656 −51.337 45.415 0.00 0.00
E
ATOM 16667 CB LEU E 191 −39.731 −51.379 43.603 0.00 0.00
E
ATOM 16668 HB1 LEU E 191 −40.220 −52.035 42.851 0.00 0.00
E
ATOM 16669 HB2 LEU E 191 −40.530 −50.763 44.068 0.00 0.00
E
ATOM 16670 CG LEU E 191 −38.879 −50.444 42.728 0.00 0.00
E
ATOM 16671 HG LEU E 191 −38.150 −51.152 42.278 0.00 0.00
E
ATOM 16672 CD1 LEU E 191 −39.760 −49.804 41.665 0.00 0.00
E
ATOM 16673 HD11 LEU E 191 −40.444 −50.546 41.201 0.00 0.00
E
ATOM 16674 HD12 LEU E 191 −40.425 −49.000 42.047 0.00 0.00
E
ATOM 16675 HD13 LEU E 191 −39.083 −49.359 40.905 0.00 0.00
E
ATOM 16676 CD2 LEU E 191 −38.225 −49.402 43.637 0.00 0.00
E
ATOM 16677 HD21 LEU E 191 −38.938 −48.744 44.178 0.00 0.00
E
ATOM 16678 HD22 LEU E 191 −37.587 −49.938 44.372 0.00 0.00
E
ATOM 16679 HD23 LEU E 191 −37.541 −48.743 43.060 0.00 0.00
E
ATOM 16680 C LEU E 191 −40.012 −52.984 45.456 0.00 0.00
E
ATOM 16681 O LEU E 191 −40.443 −54.001 44.880 0.00 0.00
E
ATOM 16682 N SER E 192 −40.486 −52.550 46.675 0.00 0.00
E
ATOM 16683 HN SER E 192 −40.275 −51.603 46.906 0.00 0.00
E
ATOM 16684 CA SER E 192 −41.469 −53.210 47.501 0.00 0.00
E
ATOM 16685 HA SER E 192 −41.579 −52.679 48.435 0.00 0.00
E

ATOM 16686 CB SER E 192 −42.912 −53.053 47.026 0.00 0.00
E
ATOM 16687 HB1 SER E 192 −43.027 −53.480 46.007 0.00 0.00
E
ATOM 16688 HB2 SER E 192 −43.584 −53.638 47.689 0.00 0.00
E
ATOM 16689 OG SER E 192 −43.312 −51.686 46.884 0.00 0.00
E
ATOM 16690 HG1 SER E 192 −44.173 −51.842 46.489 0.00 0.00
E
ATOM 16691 C SER E 192 −41.166 −54.674 47.951 0.00 0.00
E
ATOM 16692 O SER E 192 −41.950 −55.556 47.479 0.00 0.00
E
ATOM 16693 N PRO E 193 −40.088 −54.990 48.733 0.00 0.00
E
ATOM 16694 CD PRO E 193 −39.499 −53.939 49.510 0.00 0.00
E
ATOM 16695 HD1 PRO E 193 −38.816 −53.307 48.903 0.00 0.00
E
ATOM 16696 HD2 PRO E 193 −40.236 −53.296 50.035 0.00 0.00
E
ATOM 16697 CA PRO E 193 −39.675 −56.296 49.182 0.00 0.00
E
ATOM 16698 HA PRO E 193 −39.370 −56.834 48.297 0.00 0.00
E
ATOM 16699 CB PRO E 193 −38.461 −56.022 50.006 0.00 0.00
E
ATOM 16700 HB1 PRO E 193 −37.654 −55.837 49.264 0.00 0.00
E
ATOM 16701 HB2 PRO E 193 −38.174 −56.848 50.692 0.00 0.00
E
ATOM 16702 CG PRO E 193 −38.784 −54.678 50.625 0.00 0.00
E
ATOM 16703 HG1 PRO E 193 −37.876 −54.146 50.980 0.00 0.00
E
ATOM 16704 HG2 PRO E 193 −39.590 −54.735 51.388 0.00 0.00
E
ATOM 16705 C PRO E 193 −40.829 −57.000 49.897 0.00 0.00
E
ATOM 16706 O PRO E 193 −41.741 −56.398 50.382 0.00 0.00
E
ATOM 16707 N ALA E 194 −40.874 −58.329 49.972 0.00 0.00
E
ATOM 16708 HN ALA E 194 −40.213 −58.835 49.424 0.00 0.00
E
ATOM 16709 CA ALA E 194 −41.928 −59.010 50.700 0.00 0.00
E
ATOM 16710 HA ALA E 194 −42.290 −58.369 51.491 0.00 0.00
E
ATOM 16711 CB ALA E 194 −43.171 −59.242 49.789 0.00 0.00
E
ATOM 16712 HB1 ALA E 194 −43.483 −58.296 49.298 0.00 0.00
E

ATOM 16713 HB2 ALA E 194 -42.979 -60.008 49.008 0.00 0.00
E
ATOM 16714 HB3 ALA E 194 -43.990 -59.654 50.418 0.00 0.00
E
ATOM 16715 C ALA E 194 -41.440 -60.323 51.315 0.00 0.00
E
ATOM 16716 O ALA E 194 -40.324 -60.752 51.027 0.00 0.00
E
ATOM 16717 N PRO E 195 -42.141 -60.994 52.207 0.00 0.00
E
ATOM 16718 CD PRO E 195 -43.025 -60.325 53.185 0.00 0.00
E
ATOM 16719 HD1 PRO E 195 -42.586 -59.325 53.390 0.00 0.00
E
ATOM 16720 HD2 PRO E 195 -44.077 -60.327 52.826 0.00 0.00
E
ATOM 16721 CA PRO E 195 -41.733 -62.237 52.669 0.00 0.00
E
ATOM 16722 HA PRO E 195 -40.739 -62.066 53.056 0.00 0.00
E
ATOM 16723 CB PRO E 195 -42.675 -62.541 53.817 0.00 0.00
E
ATOM 16724 HB1 PRO E 195 -42.238 -63.179 54.614 0.00 0.00
E
ATOM 16725 HB2 PRO E 195 -43.697 -62.874 53.533 0.00 0.00
E
ATOM 16726 CG PRO E 195 -42.983 -61.204 54.395 0.00 0.00
E
ATOM 16727 HG1 PRO E 195 -42.174 -60.963 55.117 0.00 0.00
E
ATOM 16728 HG2 PRO E 195 -43.963 -61.059 54.899 0.00 0.00
E
ATOM 16729 C PRO E 195 -41.697 -63.231 51.524 0.00 0.00
E
ATOM 16730 O PRO E 195 -42.542 -63.080 50.605 0.00 0.00
E
ATOM 16731 N GLU E 196 -40.780 -64.263 51.504 0.00 0.00
E
ATOM 16732 HN GLU E 196 -40.875 -64.854 50.707 0.00 0.00
E
ATOM 16733 CA GLU E 196 -39.892 -64.637 52.576 0.00 0.00
E
ATOM 16734 HA GLU E 196 -40.227 -64.283 53.539 0.00 0.00
E
ATOM 16735 CB GLU E 196 -39.705 -66.201 52.705 0.00 0.00
E
ATOM 16736 HB1 GLU E 196 -39.039 -66.384 53.574 0.00 0.00
E
ATOM 16737 HB2 GLU E 196 -40.681 -66.670 52.956 0.00 0.00
E
ATOM 16738 CG GLU E 196 -39.159 -66.949 51.396 0.00 0.00
E
ATOM 16739 HG1 GLU E 196 -39.735 -66.790 50.460 0.00 0.00
E

ATOM 16740 HG2 GLU E 196   −38.162 −66.605 51.047 0.00 0.00
E
ATOM 16741 CD GLU E 196   −39.173 −68.392 51.740 0.00 0.00
E
ATOM 16742 OE1 GLU E 196   −39.762 −68.769 52.749 0.00 0.00
E
ATOM 16743 OE2 GLU E 196   −38.985 −69.130 50.787 0.00 0.00
E
ATOM 16744 C   GLU E 196   −38.465 −64.016 52.515 0.00 0.00
E
ATOM 16745 O   GLU E 196   −37.560 −64.454 53.146 0.00 0.00
E
ATOM 16746 N   ASP E 197   −38.294 −62.955 51.743 0.00 0.00
E
ATOM 16747 HN ASP E 197   −39.087 −62.616 51.242 0.00 0.00
E
ATOM 16748 CA ASP E 197   −36.931 −62.584 51.366 0.00 0.00
E
ATOM 16749 HA ASP E 197   −36.364 −63.458 51.080 0.00 0.00
E
ATOM 16750 CB ASP E 197   −37.068 −61.644 50.106 0.00 0.00
E
ATOM 16751 HB1 ASP E 197   −37.652 −60.721 50.310 0.00 0.00
E
ATOM 16752 HB2 ASP E 197   −36.052 −61.385 49.741 0.00 0.00
E
ATOM 16753 CG ASP E 197   −37.737 −62.366 48.928 0.00 0.00
E
ATOM 16754 OD1 ASP E 197   −38.688 −61.871 48.365 0.00 0.00
E
ATOM 16755 OD2 ASP E 197   −37.053 −63.318 48.495 0.00 0.00
E
ATOM 16756 C   ASP E 197   −36.154 −61.858 52.458 0.00 0.00
E
ATOM 16757 O   ASP E 197   −36.629 −60.840 52.999 0.00 0.00
E
ATOM 16758 N   ILE E 198   −34.962 −62.383 52.847 0.00 0.00
E
ATOM 16759 HN ILE E 198   −34.681 −63.224 52.392 0.00 0.00
E
ATOM 16760 CA ILE E 198   −34.323 −61.820 54.017 0.00 0.00
E
ATOM 16761 HA ILE E 198   −35.153 −61.559 54.656 0.00 0.00
E
ATOM 16762 CB ILE E 198   −33.496 −62.845 54.677 0.00 0.00
E
ATOM 16763 HB ILE E 198   −32.957 −62.378 55.529 0.00 0.00
E
ATOM 16764 CG2 ILE E 198   −34.438 −63.928 55.237 0.00 0.00
E
ATOM 16765 HG21 ILE E 198   −33.974 −64.608 55.983 0.00 0.00
E
ATOM 16766 HG22 ILE E 198   −35.261 −63.377 55.742 0.00 0.00
E

ATOM 16767 HG23 ILE E 198   -34.868 -64.517 54.400 0.00 0.00
E
ATOM 16768 CG1 ILE E 198   -32.424 -63.511 53.837 0.00 0.00
E
ATOM 16769 HG11 ILE E 198   -31.966 -64.244 54.535 0.00 0.00
E
ATOM 16770 HG12 ILE E 198   -32.786 -64.119 52.981 0.00 0.00
E
ATOM 16771 CD ILE E 198   -31.286 -62.576 53.542 0.00 0.00
E
ATOM 16772 HD1 ILE E 198   -31.613 -61.886 52.735 0.00 0.00
E
ATOM 16773 HD2 ILE E 198   -31.026 -62.033 54.475 0.00 0.00
E
ATOM 16774 HD3 ILE E 198   -30.429 -63.169 53.156 0.00 0.00
E
ATOM 16775 C   ILE E 198   -33.568 -60.489 53.687 0.00 0.00
E
ATOM 16776 O   ILE E 198   -33.370 -60.116 52.465 0.00 0.00
E
ATOM 16777 N   ILE E 199   -33.228 -59.722 54.714 0.00 0.00
E
ATOM 16778 HN ILE E 199   -33.504 -59.955 55.644 0.00 0.00
E
ATOM 16779 CA ILE E 199   -32.447 -58.441 54.726 0.00 0.00
E
ATOM 16780 HA ILE E 199   -32.441 -57.987 53.746 0.00 0.00
E
ATOM 16781 CB ILE E 199   -32.977 -57.440 55.816 0.00 0.00
E
ATOM 16782 HB ILE E 199   -32.774 -58.001 56.753 0.00 0.00
E
ATOM 16783 CG2 ILE E 199   -32.296 -56.084 55.637 0.00 0.00
E
ATOM 16784 HG21 ILE E 199   -32.696 -55.315 56.332 0.00 0.00
E
ATOM 16785 HG22 ILE E 199   -31.203 -56.086 55.836 0.00 0.00
E
ATOM 16786 HG23 ILE E 199   -32.343 -55.802 54.563 0.00 0.00
E
ATOM 16787 CG1 ILE E 199   -34.498 -57.321 55.664 0.00 0.00
E
ATOM 16788 HG11 ILE E 199   -34.775 -56.756 54.748 0.00 0.00
E
ATOM 16789 HG12 ILE E 199   -34.996 -58.302 55.515 0.00 0.00
E
ATOM 16790 CD ILE E 199   -35.210 -56.513 56.850 0.00 0.00
E
ATOM 16791 HD1 ILE E 199   -34.552 -56.669 57.731 0.00 0.00
E
ATOM 16792 HD2 ILE E 199   -35.301 -55.442 56.570 0.00 0.00
E
ATOM 16793 HD3 ILE E 199   -36.235 -56.899 57.039 0.00 0.00
E

ATOM 16794 C ILE E 199 -30.952 -58.832 55.079 0.00 0.00
E
ATOM 16795 O ILE E 199 -30.700 -59.756 55.866 0.00 0.00
E
ATOM 16796 N ARG E 200 -29.958 -58.145 54.498 0.00 0.00
E
ATOM 16797 HN ARG E 200 -30.188 -57.327 53.977 0.00 0.00
E
ATOM 16798 CA ARG E 200 -28.573 -58.491 54.668 0.00 0.00
E
ATOM 16799 HA ARG E 200 -28.337 -59.350 55.280 0.00 0.00
E
ATOM 16800 CB ARG E 200 -28.074 -58.945 53.289 0.00 0.00
E
ATOM 16801 HB1 ARG E 200 -27.946 -58.095 52.586 0.00 0.00
E
ATOM 16802 HB2 ARG E 200 -26.999 -59.226 53.288 0.00 0.00
E
ATOM 16803 CG ARG E 200 -28.893 -60.071 52.582 0.00 0.00
E
ATOM 16804 HG1 ARG E 200 -28.817 -60.941 53.269 0.00 0.00
E
ATOM 16805 HG2 ARG E 200 -29.947 -59.719 52.580 0.00 0.00
E
ATOM 16806 CD ARG E 200 -28.533 -60.418 51.145 0.00 0.00
E
ATOM 16807 HD1 ARG E 200 -27.503 -60.826 51.065 0.00 0.00
E
ATOM 16808 HD2 ARG E 200 -29.209 -61.203 50.743 0.00 0.00
E
ATOM 16809 NE ARG E 200 -28.605 -59.139 50.431 0.00 0.00
E
ATOM 16810 HE ARG E 200 -28.935 -58.382 50.996 0.00 0.00
E
ATOM 16811 CZ ARG E 200 -27.920 -58.898 49.369 0.00 0.00
E
ATOM 16812 NH1 ARG E 200 -27.574 -59.830 48.483 0.00 0.00
E
ATOM 16813 HH11 ARG E 200 -27.180 -59.413 47.663 0.00 0.00
E
ATOM 16814 HH12 ARG E 200 -27.924 -60.765 48.525 0.00 0.00
E
ATOM 16815 NH2 ARG E 200 -27.674 -57.671 48.977 0.00 0.00
E
ATOM 16816 HH21 ARG E 200 -26.932 -57.595 48.310 0.00 0.00
E
ATOM 16817 HH22 ARG E 200 -27.902 -57.060 49.735 0.00 0.00
E
ATOM 16818 C ARG E 200 -27.709 -57.370 55.149 0.00 0.00
E
ATOM 16819 O ARG E 200 -28.074 -56.192 55.151 0.00 0.00
E
ATOM 16820 N GLU E 201 -26.467 -57.635 55.607 0.00 0.00
E

ATOM 16821 HN GLU E 201 -26.013 -58.513 55.479 0.00 0.00
E
ATOM 16822 CA GLU E 201 -25.534 -56.556 56.022 0.00 0.00
E
ATOM 16823 HA GLU E 201 -26.064 -55.824 56.612 0.00 0.00
E
ATOM 16824 CB GLU E 201 -24.283 -57.156 56.634 0.00 0.00
E
ATOM 16825 HB1 GLU E 201 -24.609 -57.775 57.496 0.00 0.00
E
ATOM 16826 HB2 GLU E 201 -23.795 -57.800 55.872 0.00 0.00
E
ATOM 16827 CG GLU E 201 -23.223 -56.141 57.127 0.00 0.00
E
ATOM 16828 HG1 GLU E 201 -22.791 -55.395 56.427 0.00 0.00
E
ATOM 16829 HG2 GLU E 201 -23.640 -55.544 57.966 0.00 0.00
E
ATOM 16830 CD GLU E 201 -21.918 -56.771 57.636 0.00 0.00
E
ATOM 16831 OE1 GLU E 201 -21.770 -58.039 57.679 0.00 0.00
E
ATOM 16832 OE2 GLU E 201 -21.097 -56.015 58.140 0.00 0.00
E
ATOM 16833 C GLU E 201 -25.148 -55.671 54.818 0.00 0.00
E
ATOM 16834 O GLU E 201 -24.969 -56.171 53.714 0.00 0.00
E
ATOM 16835 N GLN E 202 -25.248 -54.345 55.027 0.00 0.00
E
ATOM 16836 HN GLN E 202 -25.433 -54.177 55.992 0.00 0.00
E
ATOM 16837 CA GLN E 202 -24.959 -53.309 54.079 0.00 0.00
E
ATOM 16838 HA GLN E 202 -24.925 -52.408 54.674 0.00 0.00
E
ATOM 16839 CB GLN E 202 -23.474 -53.343 53.500 0.00 0.00
E
ATOM 16840 HB1 GLN E 202 -22.885 -54.083 54.083 0.00 0.00
E
ATOM 16841 HB2 GLN E 202 -23.607 -53.897 52.547 0.00 0.00
E
ATOM 16842 CG GLN E 202 -22.813 -52.005 53.417 0.00 0.00
E
ATOM 16843 HG1 GLN E 202 -23.230 -51.453 52.548 0.00 0.00
E
ATOM 16844 HG2 GLN E 202 -22.950 -51.323 54.284 0.00 0.00
E
ATOM 16845 CD GLN E 202 -21.318 -52.189 53.353 0.00 0.00
E
ATOM 16846 OE1 GLN E 202 -20.715 -52.497 52.283 0.00 0.00
E
ATOM 16847 NE2 GLN E 202 -20.624 -52.005 54.467 0.00 0.00
E

```
ATOM  16848 HE21 GLN E 202     -19.641 -52.186  54.465  0.00  0.00
E
ATOM  16849 HE22 GLN E 202     -21.038 -51.443  55.183  0.00  0.00
E
ATOM  16850  C   GLN E 202     -25.977 -53.098  52.936  0.00  0.00
E
ATOM  16851  O   GLN E 202     -25.812 -52.255  52.063  0.00  0.00
E
ATOM  16852  N   ASP E 203     -27.150 -53.869  52.988  0.00  0.00
E
ATOM  16853  HN  ASP E 203     -27.258 -54.556  53.703  0.00  0.00
E
ATOM  16854  CA  ASP E 203     -28.388 -53.568  52.286  0.00  0.00
E
ATOM  16855  HA  ASP E 203     -28.174 -53.739  51.241  0.00  0.00
E
ATOM  16856  CB  ASP E 203     -29.520 -54.540  52.791  0.00  0.00
E
ATOM  16857  HB1 ASP E 203     -29.424 -54.872  53.847  0.00  0.00
E
ATOM  16858  HB2 ASP E 203     -30.462 -53.954  52.719  0.00  0.00
E
ATOM  16859  CG  ASP E 203     -29.564 -55.814  52.002  0.00  0.00
E
ATOM  16860  OD1 ASP E 203     -28.624 -56.076  51.125  0.00  0.00
E
ATOM  16861  OD2 ASP E 203     -30.530 -56.595  52.164  0.00  0.00
E
ATOM  16862  C   ASP E 203     -28.834 -52.161  52.580  0.00  0.00
E
ATOM  16863  O   ASP E 203     -28.857 -51.714  53.743  0.00  0.00
E
ATOM  16864  N   CYS E 204     -29.176 -51.408  51.572  0.00  0.00
E
ATOM  16865  HN  CYS E 204     -29.184 -51.738  50.631  0.00  0.00
E
ATOM  16866  CA  CYS E 204     -29.818 -50.104  51.869  0.00  0.00
E
ATOM  16867  HA  CYS E 204     -29.528 -49.688  52.823  0.00  0.00
E
ATOM  16868  CB  CYS E 204     -29.454 -49.160  50.760  0.00  0.00
E
ATOM  16869  HB1 CYS E 204     -29.536 -49.783  49.844  0.00  0.00
E
ATOM  16870  HB2 CYS E 204     -30.159 -48.332  50.533  0.00  0.00
E
ATOM  16871  SG  CYS E 204     -27.770 -48.581  50.955  0.00  0.00
E
ATOM  16872  HG1 CYS E 204     -27.423 -49.470  51.875  0.00  0.00
E
ATOM  16873  C   CYS E 204     -31.376 -50.314  51.849  0.00  0.00
E
ATOM  16874  O   CYS E 204     -31.946 -50.886  50.890  0.00  0.00
E
```

ATOM 16875 N LEU E 205 -32.131 -49.868 52.858 0.00 0.00
E
ATOM 16876 HN LEU E 205 -31.688 -49.356 53.590 0.00 0.00
E
ATOM 16877 CA LEU E 205 -33.518 -50.054 52.900 0.00 0.00
E
ATOM 16878 HA LEU E 205 -33.870 -50.794 52.196 0.00 0.00
E
ATOM 16879 CB LEU E 205 -33.898 -50.315 54.430 0.00 0.00
E
ATOM 16880 HB1 LEU E 205 -33.352 -49.490 54.936 0.00 0.00
E
ATOM 16881 HB2 LEU E 205 -35.003 -50.239 54.523 0.00 0.00
E
ATOM 16882 CG LEU E 205 -33.529 -51.685 55.037 0.00 0.00
E
ATOM 16883 HG LEU E 205 -32.461 -51.858 54.785 0.00 0.00
E
ATOM 16884 CD1 LEU E 205 -33.502 -51.642 56.655 0.00 0.00
E
ATOM 16885 HD11 LEU E 205 -34.482 -51.504 57.159 0.00 0.00
E
ATOM 16886 HD12 LEU E 205 -32.992 -52.571 56.991 0.00 0.00
E
ATOM 16887 HD13 LEU E 205 -32.871 -50.763 56.906 0.00 0.00
E
ATOM 16888 CD2 LEU E 205 -34.447 -52.754 54.522 0.00 0.00
E
ATOM 16889 HD21 LEU E 205 -35.455 -52.550 54.944 0.00 0.00
E
ATOM 16890 HD22 LEU E 205 -34.440 -52.885 53.418 0.00 0.00
E
ATOM 16891 HD23 LEU E 205 -34.078 -53.718 54.932 0.00 0.00
E
ATOM 16892 C LEU E 205 -34.129 -48.721 52.642 0.00 0.00
E
ATOM 16893 O LEU E 205 -33.736 -47.742 53.366 0.00 0.00
E
ATOM 16894 N VAL E 206 -35.092 -48.498 51.664 0.00 0.00
E
ATOM 16895 HN VAL E 206 -35.432 -49.167 51.008 0.00 0.00
E
ATOM 16896 CA VAL E 206 -35.619 -47.138 51.552 0.00 0.00
E
ATOM 16897 HA VAL E 206 -35.020 -46.396 52.058 0.00 0.00
E
ATOM 16898 CB VAL E 206 -35.904 -46.752 50.157 0.00 0.00
E
ATOM 16899 HB VAL E 206 -36.662 -47.385 49.649 0.00 0.00
E
ATOM 16900 CG1 VAL E 206 -36.376 -45.263 50.123 0.00 0.00
E
ATOM 16901 HG11 VAL E 206 -35.724 -44.508 50.613 0.00 0.00
E

```
ATOM  16902 HG12 VAL E 206      -36.448 -44.874  49.085  0.00  0.00
E
ATOM  16903 HG13 VAL E 206      -37.338 -45.138  50.665  0.00  0.00
E
ATOM  16904  CG2 VAL E 206      -34.571 -46.947  49.423  0.00  0.00
E
ATOM  16905 HG21 VAL E 206      -33.759 -46.440  49.987  0.00  0.00
E
ATOM  16906 HG22 VAL E 206      -34.385 -48.042  49.410  0.00  0.00
E
ATOM  16907 HG23 VAL E 206      -34.513 -46.474  48.420  0.00  0.00
E
ATOM  16908  C   VAL E 206      -36.945 -47.086  52.299  0.00  0.00
E
ATOM  16909  O   VAL E 206      -37.980 -47.721  51.984  0.00  0.00
E
ATOM  16910  N   ILE E 207      -37.005 -46.371  53.495  0.00  0.00
E
ATOM  16911  HN  ILE E 207      -36.270 -45.756  53.770  0.00  0.00
E
ATOM  16912  CA  ILE E 207      -38.047 -46.452  54.426  0.00  0.00
E
ATOM  16913  HA  ILE E 207      -38.745 -47.157  53.998  0.00  0.00
E
ATOM  16914  CB  ILE E 207      -37.670 -47.111  55.711  0.00  0.00
E
ATOM  16915  HB  ILE E 207      -36.900 -46.478  56.203  0.00  0.00
E
ATOM  16916  CG2 ILE E 207      -38.982 -47.385  56.520  0.00  0.00
E
ATOM  16917 HG21 ILE E 207      -39.715 -48.060  56.029  0.00  0.00
E
ATOM  16918 HG22 ILE E 207      -38.637 -47.799  57.491  0.00  0.00
E
ATOM  16919 HG23 ILE E 207      -39.514 -46.437  56.750  0.00  0.00
E
ATOM  16920  CG1 ILE E 207      -36.971 -48.494  55.403  0.00  0.00
E
ATOM  16921 HG11 ILE E 207      -37.711 -49.060  54.798  0.00  0.00
E
ATOM  16922 HG12 ILE E 207      -36.029 -48.351  54.832  0.00  0.00
E
ATOM  16923  CD  ILE E 207      -36.685 -49.298  56.653  0.00  0.00
E
ATOM  16924  HD1 ILE E 207      -36.365 -48.633  57.482  0.00  0.00
E
ATOM  16925  HD2 ILE E 207      -37.589 -49.850  56.989  0.00  0.00
E
ATOM  16926  HD3 ILE E 207      -35.957 -50.120  56.482  0.00  0.00
E
ATOM  16927  C   ILE E 207      -38.760 -45.136  54.640  0.00  0.00
E
ATOM  16928  O   ILE E 207      -38.085 -44.081  55.016  0.00  0.00
E
```

ATOM 16929 N MET E 208 -40.101 -45.090 54.366 0.00 0.00
E
ATOM 16930 HN MET E 208 -40.553 -45.951 54.150 0.00 0.00
E
ATOM 16931 CA MET E 208 -40.943 -43.954 54.708 0.00 0.00
E
ATOM 16932 HA MET E 208 -40.304 -43.084 54.723 0.00 0.00
E
ATOM 16933 CB MET E 208 -42.043 -43.731 53.661 0.00 0.00
E
ATOM 16934 HB1 MET E 208 -41.526 -43.501 52.705 0.00 0.00
E
ATOM 16935 HB2 MET E 208 -42.634 -44.613 53.333 0.00 0.00
E
ATOM 16936 CG MET E 208 -42.875 -42.427 53.848 0.00 0.00
E
ATOM 16937 HG1 MET E 208 -43.631 -42.541 54.654 0.00 0.00
E
ATOM 16938 HG2 MET E 208 -42.061 -41.689 54.010 0.00 0.00
E
ATOM 16939 SD MET E 208 -43.854 -42.172 52.289 0.00 0.00
E
ATOM 16940 CE MET E 208 -45.097 -43.445 52.197 0.00 0.00
E
ATOM 16941 HE1 MET E 208 -45.278 -43.623 53.278 0.00 0.00
E
ATOM 16942 HE2 MET E 208 -46.006 -43.321 51.570 0.00 0.00
E
ATOM 16943 HE3 MET E 208 -44.560 -44.367 51.887 0.00 0.00
E
ATOM 16944 C MET E 208 -41.622 -44.066 56.042 0.00 0.00
E
ATOM 16945 O MET E 208 -42.268 -45.059 56.309 0.00 0.00
E
ATOM 16946 N GLY E 209 -41.532 -43.022 56.908 0.00 0.00
E
ATOM 16947 HN GLY E 209 -40.965 -42.220 56.736 0.00 0.00
E
ATOM 16948 CA GLY E 209 -42.315 -43.024 58.104 0.00 0.00
E
ATOM 16949 HA1 GLY E 209 -41.730 -43.670 58.741 0.00 0.00
E
ATOM 16950 HA2 GLY E 209 -43.344 -43.250 57.867 0.00 0.00
E
ATOM 16951 C GLY E 209 -42.433 -41.710 58.765 0.00 0.00
E
ATOM 16952 O GLY E 209 -42.423 -40.682 58.111 0.00 0.00
E
ATOM 16953 N HSE E 210 -42.616 -41.741 60.085 0.00 0.00
E
ATOM 16954 HN HSE E 210 -42.627 -42.637 60.523 0.00 0.00
E
ATOM 16955 CA HSE E 210 -42.537 -40.560 60.873 0.00 0.00
E

```
ATOM  16956  HA  HSE E 210     -42.721 -39.691  60.258  0.00  0.00
E
ATOM  16957  CB  HSE E 210     -43.596 -40.645  61.984  0.00  0.00
E
ATOM  16958  HB1 HSE E 210     -44.593 -40.847  61.537  0.00  0.00
E
ATOM  16959  HB2 HSE E 210     -43.318 -41.525  62.603  0.00  0.00
E
ATOM  16960  ND1 HSE E 210     -42.903 -39.363  64.121  0.00  0.00
E
ATOM  16961  CG  HSE E 210     -43.767 -39.540  63.040  0.00  0.00
E
ATOM  16962  CE1 HSE E 210     -43.465 -38.377  64.821  0.00  0.00
E
ATOM  16963  HE1 HSE E 210     -43.081 -37.959  65.752  0.00  0.00
E
ATOM  16964  NE2 HSE E 210     -44.621 -37.951  64.244  0.00  0.00
E
ATOM  16965  HE2 HSE E 210     -45.139 -37.147  64.535  0.00  0.00
E
ATOM  16966  CD2 HSE E 210     -44.801 -38.694  63.071  0.00  0.00
E
ATOM  16967  HD2 HSE E 210     -45.652 -38.599  62.408  0.00  0.00
E
ATOM  16968  C   HSE E 210     -41.193 -40.416  61.562  0.00  0.00
E
ATOM  16969  O   HSE E 210     -40.452 -41.386  61.734  0.00  0.00
E
ATOM  16970  N   LYS E 211     -40.687 -39.201  61.866  0.00  0.00
E
ATOM  16971  HN  LYS E 211     -41.194 -38.370  61.651  0.00  0.00
E
ATOM  16972  CA  LYS E 211     -39.381 -39.035  62.364  0.00  0.00
E
ATOM  16973  HA  LYS E 211     -38.717 -39.483  61.639  0.00  0.00
E
ATOM  16974  CB  LYS E 211     -39.104 -37.509  62.319  0.00  0.00
E
ATOM  16975  HB1 LYS E 211     -39.889 -36.974  62.894  0.00  0.00
E
ATOM  16976  HB2 LYS E 211     -38.125 -37.343  62.818  0.00  0.00
E
ATOM  16977  CG  LYS E 211     -38.865 -37.118  60.868  0.00  0.00
E
ATOM  16978  HG1 LYS E 211     -37.974 -37.587  60.398  0.00  0.00
E
ATOM  16979  HG2 LYS E 211     -39.794 -37.369  60.313  0.00  0.00
E
ATOM  16980  CD  LYS E 211     -38.682 -35.639  60.747  0.00  0.00
E
ATOM  16981  HD1 LYS E 211     -39.560 -35.233  61.293  0.00  0.00
E
ATOM  16982  HD2 LYS E 211     -37.807 -35.268  61.324  0.00  0.00
E
```

ATOM 16983 CE LYS E 211 −38.603 −35.026 59.342 0.00 0.00
E
ATOM 16984 HE1 LYS E 211 −39.456 −35.470 58.785 0.00 0.00
E
ATOM 16985 HE2 LYS E 211 −38.604 −33.922 59.464 0.00 0.00
E
ATOM 16986 NZ LYS E 211 −37.403 −35.447 58.730 0.00 0.00
E
ATOM 16987 HZ1 LYS E 211 −36.558 −34.966 59.099 0.00 0.00
E
ATOM 16988 HZ2 LYS E 211 −37.190 −36.464 58.770 0.00 0.00
E
ATOM 16989 HZ3 LYS E 211 −37.443 −35.115 57.745 0.00 0.00
E
ATOM 16990 C LYS E 211 −39.061 −39.702 63.716 0.00 0.00
E
ATOM 16991 O LYS E 211 −37.991 −40.325 63.820 0.00 0.00
E
ATOM 16992 N LYS E 212 −40.081 −39.772 64.700 0.00 0.00
E
ATOM 16993 HN LYS E 212 −40.913 −39.263 64.491 0.00 0.00
E
ATOM 16994 CA LYS E 212 −39.761 −40.345 65.997 0.00 0.00
E
ATOM 16995 HA LYS E 212 −38.805 −39.991 66.353 0.00 0.00
E
ATOM 16996 CB LYS E 212 −40.703 −39.850 67.019 0.00 0.00
E
ATOM 16997 HB1 LYS E 212 −41.744 −40.095 66.718 0.00 0.00
E
ATOM 16998 HB2 LYS E 212 −40.602 −40.408 67.974 0.00 0.00
E
ATOM 16999 CG LYS E 212 −40.718 −38.374 67.330 0.00 0.00
E
ATOM 17000 HG1 LYS E 212 −39.770 −38.090 67.834 0.00 0.00
E
ATOM 17001 HG2 LYS E 212 −40.891 −37.743 66.432 0.00 0.00
E
ATOM 17002 CD LYS E 212 −41.980 −38.089 68.303 0.00 0.00
E
ATOM 17003 HD1 LYS E 212 −42.767 −38.430 67.597 0.00 0.00
E
ATOM 17004 HD2 LYS E 212 −41.999 −38.833 69.129 0.00 0.00
E
ATOM 17005 CE LYS E 212 −42.277 −36.690 68.742 0.00 0.00
E
ATOM 17006 HE1 LYS E 212 −43.343 −36.378 68.779 0.00 0.00
E
ATOM 17007 HE2 LYS E 212 −41.806 −36.388 69.702 0.00 0.00
E
ATOM 17008 NZ LYS E 212 −41.776 −35.755 67.654 0.00 0.00
E
ATOM 17009 HZ1 LYS E 212 −42.243 −34.848 67.853 0.00 0.00
E

ATOM 17010 HZ2 LYS E 212   −40.745 −35.666 67.764 0.00 0.00
E
ATOM 17011 HZ3 LYS E 212   −42.130 −36.060 66.726 0.00 0.00
E
ATOM 17012 C   LYS E 212   −39.760 −41.918 65.889 0.00 0.00
E
ATOM 17013 O   LYS E 212   −38.870 −42.608 66.396 0.00 0.00
E
ATOM 17014 N   ASP E 213   −40.588 −42.466 65.041 0.00 0.00
E
ATOM 17015 HN ASP E 213   −41.202 −41.910 64.486 0.00 0.00
E
ATOM 17016 CA ASP E 213   −40.574 −43.930 64.689 0.00 0.00
E
ATOM 17017 HA ASP E 213   −40.614 −44.462 65.628 0.00 0.00
E
ATOM 17018 CB ASP E 213   −41.909 −44.317 64.031 0.00 0.00
E
ATOM 17019 HB1 ASP E 213   −42.006 −43.754 63.078 0.00 0.00
E
ATOM 17020 HB2 ASP E 213   −41.929 −45.402 63.795 0.00 0.00
E
ATOM 17021 CG ASP E 213   −43.000 −44.040 64.945 0.00 0.00
E
ATOM 17022 OD1 ASP E 213   −42.782 −43.994 66.136 0.00 0.00
E
ATOM 17023 OD2 ASP E 213   −44.071 −43.689 64.416 0.00 0.00
E
ATOM 17024 C   ASP E 213   −39.311 −44.463 63.991 0.00 0.00
E
ATOM 17025 O   ASP E 213   −38.731 −45.495 64.436 0.00 0.00
E
ATOM 17026 N   ILE E 214   −38.818 −43.670 62.959 0.00 0.00
E
ATOM 17027 HN ILE E 214   −39.238 −42.895 62.493 0.00 0.00
E
ATOM 17028 CA ILE E 214   −37.589 −43.949 62.276 0.00 0.00
E
ATOM 17029 HA ILE E 214   −37.714 −44.955 61.904 0.00 0.00
E
ATOM 17030 CB ILE E 214   −37.439 −43.077 61.074 0.00 0.00
E
ATOM 17031 HB ILE E 214   −37.977 −42.158 61.390 0.00 0.00
E
ATOM 17032 CG2 ILE E 214   −35.962 −42.679 60.790 0.00 0.00
E
ATOM 17033 HG21 ILE E 214   −35.599 −42.075 61.648 0.00 0.00
E
ATOM 17034 HG22 ILE E 214   −35.323 −43.575 60.637 0.00 0.00
E
ATOM 17035 HG23 ILE E 214   −35.837 −41.940 59.970 0.00 0.00
E
ATOM 17036 CG1 ILE E 214   −38.093 −43.646 59.809 0.00 0.00
E

ATOM 17037 HG11 ILE E 214 -37.377 -44.436 59.496 0.00 0.00
E
ATOM 17038 HG12 ILE E 214 -38.993 -44.221 60.115 0.00 0.00
E
ATOM 17039 CD ILE E 214 -38.312 -42.664 58.786 0.00 0.00
E
ATOM 17040 HD1 ILE E 214 -37.455 -42.030 58.473 0.00 0.00
E
ATOM 17041 HD2 ILE E 214 -38.608 -43.265 57.900 0.00 0.00
E
ATOM 17042 HD3 ILE E 214 -39.070 -41.913 59.099 0.00 0.00
E
ATOM 17043 C ILE E 214 -36.429 -43.950 63.234 0.00 0.00
E
ATOM 17044 O ILE E 214 -35.440 -44.687 63.133 0.00 0.00
E
ATOM 17045 N LYS E 215 -36.454 -43.043 64.247 0.00 0.00
E
ATOM 17046 HN LYS E 215 -37.030 -42.231 64.187 0.00 0.00
E
ATOM 17047 CA LYS E 215 -35.348 -43.084 65.178 0.00 0.00
E
ATOM 17048 HA LYS E 215 -34.399 -43.258 64.692 0.00 0.00
E
ATOM 17049 CB LYS E 215 -35.163 -41.700 65.890 0.00 0.00
E
ATOM 17050 HB1 LYS E 215 -35.119 -40.958 65.064 0.00 0.00
E
ATOM 17051 HB2 LYS E 215 -36.053 -41.427 66.497 0.00 0.00
E
ATOM 17052 CG LYS E 215 -33.915 -41.635 66.813 0.00 0.00
E
ATOM 17053 HG1 LYS E 215 -33.654 -40.621 67.184 0.00 0.00
E
ATOM 17054 HG2 LYS E 215 -34.106 -42.286 67.693 0.00 0.00
E
ATOM 17055 CD LYS E 215 -32.530 -42.001 66.139 0.00 0.00
E
ATOM 17056 HD1 LYS E 215 -31.793 -42.249 66.932 0.00 0.00
E
ATOM 17057 HD2 LYS E 215 -32.522 -42.944 65.551 0.00 0.00
E
ATOM 17058 CE LYS E 215 -31.885 -40.853 65.350 0.00 0.00
E
ATOM 17059 HE1 LYS E 215 -32.617 -40.552 64.571 0.00 0.00
E
ATOM 17060 HE2 LYS E 215 -31.748 -39.988 66.033 0.00 0.00
E
ATOM 17061 NZ LYS E 215 -30.680 -41.287 64.511 0.00 0.00
E
ATOM 17062 HZ1 LYS E 215 -31.029 -42.181 64.111 0.00 0.00
E
ATOM 17063 HZ2 LYS E 215 -30.457 -40.529 63.834 0.00 0.00
E

ATOM 17064 HZ3 LYS E 215 −29.870 −41.450 65.142 0.00 0.00
E
ATOM 17065 C LYS E 215 −35.438 −44.170 66.146 0.00 0.00
E
ATOM 17066 O LYS E 215 −34.416 −44.822 66.482 0.00 0.00
E
ATOM 17067 N ARG E 216 −36.705 −44.430 66.600 0.00 0.00
E
ATOM 17068 HN ARG E 216 −37.478 −43.951 66.191 0.00 0.00
E
ATOM 17069 CA ARG E 216 −37.051 −45.604 67.336 0.00 0.00
E
ATOM 17070 HA ARG E 216 −36.554 −45.592 68.294 0.00 0.00
E
ATOM 17071 CB ARG E 216 −38.572 −45.717 67.653 0.00 0.00
E
ATOM 17072 HB1 ARG E 216 −38.950 −44.780 68.115 0.00 0.00
E
ATOM 17073 HB2 ARG E 216 −39.252 −45.908 66.795 0.00 0.00
E
ATOM 17074 CG ARG E 216 −38.867 −46.878 68.565 0.00 0.00
E
ATOM 17075 HG1 ARG E 216 −38.672 −47.852 68.068 0.00 0.00
E
ATOM 17076 HG2 ARG E 216 −38.085 −46.968 69.350 0.00 0.00
E
ATOM 17077 CD ARG E 216 −40.318 −46.964 69.162 0.00 0.00
E
ATOM 17078 HD1 ARG E 216 −40.564 −46.410 70.093 0.00 0.00
E
ATOM 17079 HD2 ARG E 216 −40.991 −46.673 68.327 0.00 0.00
E
ATOM 17080 NE ARG E 216 −40.682 −48.369 69.553 0.00 0.00
E
ATOM 17081 HE ARG E 216 −40.063 −48.911 70.122 0.00 0.00
E
ATOM 17082 CZ ARG E 216 −41.846 −48.950 69.500 0.00 0.00
E
ATOM 17083 NH1 ARG E 216 −42.912 −48.279 68.987 0.00 0.00
E
ATOM 17084 HH11 ARG E 216 −43.742 −48.833 68.924 0.00 0.00
E
ATOM 17085 HH12 ARG E 216 −42.780 −47.357 68.623 0.00 0.00
E
ATOM 17086 NH2 ARG E 216 −41.951 −50.240 69.818 0.00 0.00
E
ATOM 17087 HH21 ARG E 216 −42.774 −50.670 69.446 0.00 0.00
E
ATOM 17088 HH22 ARG E 216 −41.140 −50.823 69.770 0.00 0.00
E
ATOM 17089 C ARG E 216 −36.577 −46.937 66.729 0.00 0.00
E
ATOM 17090 O ARG E 216 −36.036 −47.781 67.383 0.00 0.00
E

ATOM 17091 N PHE E 217 -36.799 -47.130 65.410 0.00 0.00
E
ATOM 17092 HN PHE E 217 -37.191 -46.344 64.938 0.00 0.00
E
ATOM 17093 CA PHE E 217 -36.399 -48.255 64.551 0.00 0.00
E
ATOM 17094 HA PHE E 217 -36.905 -49.173 64.809 0.00 0.00
E
ATOM 17095 CB PHE E 217 -36.770 -48.014 63.071 0.00 0.00
E
ATOM 17096 HB1 PHE E 217 -37.862 -47.995 62.866 0.00 0.00
E
ATOM 17097 HB2 PHE E 217 -36.419 -47.014 62.737 0.00 0.00
E
ATOM 17098 CG PHE E 217 -36.326 -49.084 62.061 0.00 0.00
E
ATOM 17099 CD1 PHE E 217 -35.387 -48.790 61.067 0.00 0.00
E
ATOM 17100 HD1 PHE E 217 -35.044 -47.769 60.993 0.00 0.00
E
ATOM 17101 CE1 PHE E 217 -34.956 -49.777 60.220 0.00 0.00
E
ATOM 17102 HE1 PHE E 217 -34.346 -49.464 59.385 0.00 0.00
E
ATOM 17103 CZ PHE E 217 -35.357 -51.134 60.498 0.00 0.00
E
ATOM 17104 HZ PHE E 217 -35.224 -51.890 59.738 0.00 0.00
E
ATOM 17105 CD2 PHE E 217 -36.777 -50.385 62.244 0.00 0.00
E
ATOM 17106 HD2 PHE E 217 -37.443 -50.631 63.057 0.00 0.00
E
ATOM 17107 CE2 PHE E 217 -36.312 -51.406 61.472 0.00 0.00
E
ATOM 17108 HE2 PHE E 217 -36.674 -52.424 61.491 0.00 0.00
E
ATOM 17109 C PHE E 217 -34.856 -48.502 64.665 0.00 0.00
E
ATOM 17110 O PHE E 217 -34.400 -49.623 64.885 0.00 0.00
E
ATOM 17111 N GLU E 218 -34.117 -47.420 64.456 0.00 0.00
E
ATOM 17112 HN GLU E 218 -34.653 -46.579 64.443 0.00 0.00
E
ATOM 17113 CA GLU E 218 -32.600 -47.416 64.521 0.00 0.00
E
ATOM 17114 HA GLU E 218 -32.292 -48.228 63.879 0.00 0.00
E
ATOM 17115 CB GLU E 218 -32.133 -45.989 64.143 0.00 0.00
E
ATOM 17116 HB1 GLU E 218 -32.243 -45.874 63.043 0.00 0.00
E
ATOM 17117 HB2 GLU E 218 -32.756 -45.250 64.690 0.00 0.00
E

ATOM 17118 CG GLU E 218 −30.585 −45.833 64.435 0.00 0.00
E
ATOM 17119 HG1 GLU E 218 −30.496 −45.767 65.541 0.00 0.00
E
ATOM 17120 HG2 GLU E 218 −30.071 −46.734 64.036 0.00 0.00
E
ATOM 17121 CD GLU E 218 −30.154 −44.553 63.822 0.00 0.00
E
ATOM 17122 OE1 GLU E 218 −30.903 −43.714 63.319 0.00 0.00
E
ATOM 17123 OE2 GLU E 218 −28.912 −44.466 63.764 0.00 0.00
E
ATOM 17124 C GLU E 218 −32.007 −47.794 65.875 0.00 0.00
E
ATOM 17125 O GLU E 218 −31.090 −48.613 65.931 0.00 0.00
E
ATOM 17126 N ASN E 219 −32.588 −47.262 67.013 0.00 0.00
E
ATOM 17127 HN ASN E 219 −33.297 −46.564 66.945 0.00 0.00
E
ATOM 17128 CA ASN E 219 −32.268 −47.802 68.343 0.00 0.00
E
ATOM 17129 HA ASN E 219 −31.209 −47.978 68.458 0.00 0.00
E
ATOM 17130 CB ASN E 219 −32.810 −46.861 69.385 0.00 0.00
E
ATOM 17131 HB1 ASN E 219 −33.847 −46.496 69.223 0.00 0.00
E
ATOM 17132 HB2 ASN E 219 −32.595 −47.208 70.418 0.00 0.00
E
ATOM 17133 CG ASN E 219 −31.953 −45.637 69.231 0.00 0.00
E
ATOM 17134 OD1 ASN E 219 −30.775 −45.773 69.334 0.00 0.00
E
ATOM 17135 ND2 ASN E 219 −32.569 −44.449 68.893 0.00 0.00
E
ATOM 17136 HD21 ASN E 219 −31.976 −43.644 68.937 0.00 0.00
E
ATOM 17137 HD22 ASN E 219 −33.561 −44.369 68.802 0.00 0.00
E
ATOM 17138 C ASN E 219 −32.845 −49.109 68.679 0.00 0.00
E
ATOM 17139 O ASN E 219 −32.134 −50.005 69.156 0.00 0.00
E
ATOM 17140 N GLU E 220 −34.169 −49.277 68.566 0.00 0.00
E
ATOM 17141 HN GLU E 220 −34.701 −48.509 68.219 0.00 0.00
E
ATOM 17142 CA GLU E 220 −34.876 −50.505 69.069 0.00 0.00
E
ATOM 17143 HA GLU E 220 −34.443 −50.716 70.036 0.00 0.00
E
ATOM 17144 CB GLU E 220 −36.406 −50.291 69.165 0.00 0.00
E

ATOM	17145	HB1	GLU	E	220	−36.518	−49.217	69.428	0.00	0.00	E
ATOM	17146	HB2	GLU	E	220	−36.939	−50.540	68.223	0.00	0.00	E
ATOM	17147	CG	GLU	E	220	−37.187	−51.236	70.174	0.00	0.00	E
ATOM	17148	HG1	GLU	E	220	−37.057	−52.301	69.884	0.00	0.00	E
ATOM	17149	HG2	GLU	E	220	−36.680	−51.164	71.160	0.00	0.00	E
ATOM	17150	CD	GLU	E	220	−38.704	−50.967	70.363	0.00	0.00	E
ATOM	17151	OE1	GLU	E	220	−39.063	−49.899	70.989	0.00	0.00	E
ATOM	17152	OE2	GLU	E	220	−39.578	−51.741	69.829	0.00	0.00	E
ATOM	17153	C	GLU	E	220	−34.603	−51.765	68.214	0.00	0.00	E
ATOM	17154	O	GLU	E	220	−34.322	−52.831	68.738	0.00	0.00	E
ATOM	17155	N	GLY	E	221	−34.658	−51.686	66.871	0.00	0.00	E
ATOM	17156	HN	GLY	E	221	−34.980	−50.825	66.485	0.00	0.00	E
ATOM	17157	CA	GLY	E	221	−34.688	−52.891	66.018	0.00	0.00	E
ATOM	17158	HA1	GLY	E	221	−35.377	−52.678	65.214	0.00	0.00	E
ATOM	17159	HA2	GLY	E	221	−34.964	−53.815	66.504	0.00	0.00	E
ATOM	17160	C	GLY	E	221	−33.345	−53.122	65.277	0.00	0.00	E
ATOM	17161	O	GLY	E	221	−32.961	−54.298	65.003	0.00	0.00	E
ATOM	17162	N	MET	E	222	−32.684	−52.068	64.788	0.00	0.00	E
ATOM	17163	HN	MET	E	222	−32.972	−51.137	64.999	0.00	0.00	E
ATOM	17164	CA	MET	E	222	−31.394	−52.000	64.151	0.00	0.00	E
ATOM	17165	HA	MET	E	222	−31.183	−52.947	63.677	0.00	0.00	E
ATOM	17166	CB	MET	E	222	−31.302	−50.893	63.073	0.00	0.00	E
ATOM	17167	HB1	MET	E	222	−32.101	−50.923	62.302	0.00	0.00	E
ATOM	17168	HB2	MET	E	222	−31.362	−49.953	63.663	0.00	0.00	E
ATOM	17169	CG	MET	E	222	−29.951	−50.637	62.393	0.00	0.00	E
ATOM	17170	HG1	MET	E	222	−29.049	−50.413	63.003	0.00	0.00	E
ATOM	17171	HG2	MET	E	222	−29.712	−51.630	61.956	0.00	0.00	E

ATOM 17172 SD MET E 222   -29.882 -49.302 61.145 0.00 0.00
E
ATOM 17173 CE MET E 222   -30.925 -50.149 59.848 0.00 0.00
E
ATOM 17174 HE1 MET E 222   -32.027 -50.170 59.983 0.00 0.00
E
ATOM 17175 HE2 MET E 222   -30.585 -49.769 58.861 0.00 0.00
E
ATOM 17176 HE3 MET E 222   -30.531 -51.179 59.987 0.00 0.00
E
ATOM 17177 C   MET E 222   -30.165 -51.915 65.155 0.00 0.00
E
ATOM 17178 O   MET E 222   -29.988 -52.875 65.880 0.00 0.00
E
ATOM 17179 N   ASN F   6   -23.027 -35.722 73.368 0.00 0.00
F
ATOM 17180 HT1 ASN F   6   -23.451 -35.023 72.725 0.00 0.00
F
ATOM 17181 HT2 ASN F   6   -22.014 -35.503 73.455 0.00 0.00
F
ATOM 17182 HT3 ASN F   6   -23.509 -35.652 74.287 0.00 0.00
F
ATOM 17183 CA ASN F   6   -23.175 -37.180 72.857 0.00 0.00
F
ATOM 17184 HA ASN F   6   -22.700 -37.796 73.607 0.00 0.00
F
ATOM 17185 CB ASN F   6   -24.637 -37.540 72.729 0.00 0.00
F
ATOM 17186 HB1 ASN F   6   -25.319 -37.213 73.544 0.00 0.00
F
ATOM 17187 HB2 ASN F   6   -25.172 -36.968 71.940 0.00 0.00
F
ATOM 17188 CG ASN F   6   -24.874 -39.048 72.361 0.00 0.00
F
ATOM 17189 OD1 ASN F   6   -24.141 -39.989 72.556 0.00 0.00
F
ATOM 17190 ND2 ASN F   6   -26.051 -39.243 71.778 0.00 0.00
F
ATOM 17191 HD21 ASN F   6   -26.341 -40.122 71.401 0.00 0.00
F
ATOM 17192 HD22 ASN F   6   -26.759 -38.552 71.928 0.00 0.00
F
ATOM 17193 C   ASN F   6   -22.381 -37.386 71.512 0.00 0.00
F
ATOM 17194 O   ASN F   6   -21.232 -37.795 71.563 0.00 0.00
F
ATOM 17195 N   LYS F   7   -22.937 -36.984 70.313 0.00 0.00
F
ATOM 17196 HN LYS F   7   -23.846 -36.597 70.176 0.00 0.00
F
ATOM 17197 CA LYS F   7   -22.123 -37.015 69.013 0.00 0.00
F
ATOM 17198 HA LYS F   7   -21.343 -37.723 69.251 0.00 0.00
F

ATOM	17199	CB	LYS	F	7	−23.041	−37.471	67.805	0.00	0.00	F
ATOM	17200	HB1	LYS	F	7	−23.784	−38.222	68.150	0.00	0.00	F
ATOM	17201	HB2	LYS	F	7	−23.514	−36.584	67.331	0.00	0.00	F
ATOM	17202	CG	LYS	F	7	−22.334	−38.101	66.617	0.00	0.00	F
ATOM	17203	HG1	LYS	F	7	−22.986	−38.185	65.722	0.00	0.00	F
ATOM	17204	HG2	LYS	F	7	−21.470	−37.466	66.326	0.00	0.00	F
ATOM	17205	CD	LYS	F	7	−21.834	−39.519	66.873	0.00	0.00	F
ATOM	17206	HD1	LYS	F	7	−21.403	−39.591	67.894	0.00	0.00	F
ATOM	17207	HD2	LYS	F	7	−22.599	−40.324	66.830	0.00	0.00	F
ATOM	17208	CE	LYS	F	7	−20.833	−40.050	65.806	0.00	0.00	F
ATOM	17209	HE1	LYS	F	7	−21.468	−40.147	64.900	0.00	0.00	F
ATOM	17210	HE2	LYS	F	7	−19.862	−39.513	65.762	0.00	0.00	F
ATOM	17211	NZ	LYS	F	7	−20.423	−41.427	66.180	0.00	0.00	F
ATOM	17212	HZ1	LYS	F	7	−20.100	−41.507	67.165	0.00	0.00	F
ATOM	17213	HZ2	LYS	F	7	−21.174	−42.140	66.078	0.00	0.00	F
ATOM	17214	HZ3	LYS	F	7	−19.571	−41.692	65.645	0.00	0.00	F
ATOM	17215	C	LYS	F	7	−21.421	−35.669	68.851	0.00	0.00	F
ATOM	17216	O	LYS	F	7	−21.773	−34.590	69.276	0.00	0.00	F
ATOM	17217	N	GLN	F	8	−20.179	−35.730	68.269	0.00	0.00	F
ATOM	17218	HN	GLN	F	8	−19.819	−36.614	67.981	0.00	0.00	F
ATOM	17219	CA	GLN	F	8	−19.167	−34.692	68.431	0.00	0.00	F
ATOM	17220	HA	GLN	F	8	−19.751	−33.837	68.738	0.00	0.00	F
ATOM	17221	CB	GLN	F	8	−18.014	−35.166	69.398	0.00	0.00	F
ATOM	17222	HB1	GLN	F	8	−18.417	−35.608	70.335	0.00	0.00	F
ATOM	17223	HB2	GLN	F	8	−17.666	−36.111	68.929	0.00	0.00	F
ATOM	17224	CG	GLN	F	8	−16.912	−34.131	69.756	0.00	0.00	F
ATOM	17225	HG1	GLN	F	8	−16.453	−33.672	68.854	0.00	0.00	F

ATOM 17226 HG2 GLN F 8 −17.412 −33.317 70.324 0.00 0.00
F
ATOM 17227 CD GLN F 8 −15.796 −34.883 70.491 0.00 0.00
F
ATOM 17228 OE1 GLN F 8 −15.007 −35.710 69.951 0.00 0.00
F
ATOM 17229 NE2 GLN F 8 −15.820 −34.601 71.814 0.00 0.00
F
ATOM 17230 HE21 GLN F 8 −15.100 −35.031 72.359 0.00 0.00
F
ATOM 17231 HE22 GLN F 8 −16.341 −33.835 72.190 0.00 0.00
F
ATOM 17232 C GLN F 8 −18.485 −34.364 67.100 0.00 0.00
F
ATOM 17233 O GLN F 8 −18.006 −35.151 66.329 0.00 0.00
F
ATOM 17234 N PHE F 9 −18.407 −33.061 66.764 0.00 0.00
F
ATOM 17235 HN PHE F 9 −18.707 −32.270 67.293 0.00 0.00
F
ATOM 17236 CA PHE F 9 −18.063 −32.519 65.489 0.00 0.00
F
ATOM 17237 HA PHE F 9 −17.555 −33.228 64.852 0.00 0.00
F
ATOM 17238 CB PHE F 9 −19.322 −32.060 64.679 0.00 0.00
F
ATOM 17239 HB1 PHE F 9 −19.745 −31.267 65.332 0.00 0.00
F
ATOM 17240 HB2 PHE F 9 −19.056 −31.762 63.642 0.00 0.00
F
ATOM 17241 CG PHE F 9 −20.258 −33.261 64.472 0.00 0.00
F
ATOM 17242 CD1 PHE F 9 −19.757 −34.248 63.569 0.00 0.00
F
ATOM 17243 HD1 PHE F 9 −18.819 −34.056 63.069 0.00 0.00
F
ATOM 17244 CE1 PHE F 9 −20.345 −35.515 63.513 0.00 0.00
F
ATOM 17245 HE1 PHE F 9 −19.949 −36.268 62.847 0.00 0.00
F
ATOM 17246 CZ PHE F 9 −21.537 −35.766 64.204 0.00 0.00
F
ATOM 17247 HZ PHE F 9 −22.043 −36.717 64.125 0.00 0.00
F
ATOM 17248 CD2 PHE F 9 −21.442 −33.511 65.172 0.00 0.00
F
ATOM 17249 HD2 PHE F 9 −21.804 −32.989 66.045 0.00 0.00
F
ATOM 17250 CE2 PHE F 9 −22.043 −34.829 65.071 0.00 0.00
F
ATOM 17251 HE2 PHE F 9 −22.868 −35.068 65.726 0.00 0.00
F
ATOM 17252 C PHE F 9 −17.071 −31.351 65.704 0.00 0.00
F

```
ATOM  17253  O   PHE F   9      -17.175 -30.540  66.649  0.00  0.00
F
ATOM  17254  N   ALA F  10      -16.127 -31.300  64.754  0.00  0.00
F
ATOM  17255  HN  ALA F  10      -15.857 -31.971  64.068  0.00  0.00
F
ATOM  17256  CA  ALA F  10      -15.269 -30.098  64.663  0.00  0.00
F
ATOM  17257  HA  ALA F  10      -15.797 -29.339  65.221  0.00  0.00
F
ATOM  17258  CB  ALA F  10      -13.798 -30.360  65.102  0.00  0.00
F
ATOM  17259  HB1 ALA F  10      -13.406 -31.296  64.650  0.00  0.00
F
ATOM  17260  HB2 ALA F  10      -13.084 -29.541  64.871  0.00  0.00
F
ATOM  17261  HB3 ALA F  10      -14.035 -30.502  66.178  0.00  0.00
F
ATOM  17262  C   ALA F  10      -15.305 -29.519  63.273  0.00  0.00
F
ATOM  17263  O   ALA F  10      -15.391 -30.325  62.366  0.00  0.00
F
ATOM  17264  N   VAL F  11      -15.335 -28.213  63.151  0.00  0.00
F
ATOM  17265  HN  VAL F  11      -15.394 -27.612  63.944  0.00  0.00
F
ATOM  17266  CA  VAL F  11      -15.322 -27.560  61.849  0.00  0.00
F
ATOM  17267  HA  VAL F  11      -15.060 -28.256  61.065  0.00  0.00
F
ATOM  17268  CB  VAL F  11      -16.670 -26.836  61.510  0.00  0.00
F
ATOM  17269  HB  VAL F  11      -16.870 -26.125  62.339  0.00  0.00
F
ATOM  17270  CG1 VAL F  11      -16.681 -26.155  60.124  0.00  0.00
F
ATOM  17271 HG11 VAL F  11      -17.503 -25.425  59.965  0.00  0.00
F
ATOM  17272 HG12 VAL F  11      -15.706 -25.676  59.890  0.00  0.00
F
ATOM  17273 HG13 VAL F  11      -16.589 -26.902  59.307  0.00  0.00
F
ATOM  17274  CG2 VAL F  11      -17.788 -27.993  61.567  0.00  0.00
F
ATOM  17275 HG21 VAL F  11      -17.521 -28.899  60.982  0.00  0.00
F
ATOM  17276 HG22 VAL F  11      -17.897 -28.341  62.617  0.00  0.00
F
ATOM  17277 HG23 VAL F  11      -18.748 -27.592  61.178  0.00  0.00
F
ATOM  17278  C   VAL F  11      -14.186 -26.530  61.874  0.00  0.00
F
ATOM  17279  O   VAL F  11      -14.163 -25.676  62.770  0.00  0.00
F
```

ATOM 17280 N   ILE F 12   -13.172 -26.691 60.984 0.00 0.00
F
ATOM 17281 HN ILE F 12   -13.312 -27.392 60.289 0.00 0.00
F
ATOM 17282 CA ILE F 12   -12.010 -25.820 60.873 0.00 0.00
F
ATOM 17283 HA ILE F 12   -11.986 -25.145 61.716 0.00 0.00
F
ATOM 17284 CB ILE F 12   -10.648 -26.655 60.638 0.00 0.00
F
ATOM 17285 HB ILE F 12   -10.818 -27.225 59.700 0.00 0.00
F
ATOM 17286 CG2 ILE F 12   -9.486 -25.688 60.349 0.00 0.00
F
ATOM 17287 HG21 ILE F 12   -9.337 -24.979 61.191 0.00 0.00
F
ATOM 17288 HG22 ILE F 12   -8.550 -26.200 60.038 0.00 0.00
F
ATOM 17289 HG23 ILE F 12   -9.785 -25.109 59.449 0.00 0.00
F
ATOM 17290 CG1 ILE F 12   -10.265 -27.514 61.877 0.00 0.00
F
ATOM 17291 HG11 ILE F 12   -10.189 -26.933 62.821 0.00 0.00
F
ATOM 17292 HG12 ILE F 12   -11.043 -28.243 62.191 0.00 0.00
F
ATOM 17293 CD ILE F 12   -9.048 -28.399 61.534 0.00 0.00
F
ATOM 17294 HD1 ILE F 12   -8.102 -27.868 61.771 0.00 0.00
F
ATOM 17295 HD2 ILE F 12   -9.197 -29.286 62.186 0.00 0.00
F
ATOM 17296 HD3 ILE F 12   -9.081 -28.889 60.538 0.00 0.00
F
ATOM 17297 C   ILE F 12   -12.268 -24.880 59.715 0.00 0.00
F
ATOM 17298 O   ILE F 12   -12.227 -25.267 58.528 0.00 0.00
F
ATOM 17299 N   GLY F 13   -12.421 -23.567 59.978 0.00 0.00
F
ATOM 17300 HN GLY F 13   -12.536 -23.290 60.929 0.00 0.00
F
ATOM 17301 CA GLY F 13   -12.775 -22.577 58.960 0.00 0.00
F
ATOM 17302 HA1 GLY F 13   -12.557 -22.920 57.959 0.00 0.00
F
ATOM 17303 HA2 GLY F 13   -12.221 -21.701 59.260 0.00 0.00
F
ATOM 17304 C   GLY F 13   -14.241 -22.184 58.907 0.00 0.00
F
ATOM 17305 O   GLY F 13   -15.050 -22.734 58.204 0.00 0.00
F
ATOM 17306 N   LEU F 14   -14.586 -21.089 59.602 0.00 0.00
F

ATOM 17307 HN LEU F 14 -13.968 -20.605 60.217 0.00 0.00
F
ATOM 17308 CA LEU F 14 -15.970 -20.717 59.707 0.00 0.00
F
ATOM 17309 HA LEU F 14 -16.536 -21.599 59.447 0.00 0.00
F
ATOM 17310 CB LEU F 14 -16.467 -20.303 61.123 0.00 0.00
F
ATOM 17311 HB1 LEU F 14 -16.167 -19.240 61.245 0.00 0.00
F
ATOM 17312 HB2 LEU F 14 -17.575 -20.385 61.092 0.00 0.00
F
ATOM 17313 CG LEU F 14 -15.725 -21.079 62.212 0.00 0.00
F
ATOM 17314 HG LEU F 14 -14.630 -20.950 62.076 0.00 0.00
F
ATOM 17315 CD1 LEU F 14 -16.138 -20.555 63.648 0.00 0.00
F
ATOM 17316 HD11 LEU F 14 -17.169 -20.898 63.879 0.00 0.00
F
ATOM 17317 HD12 LEU F 14 -15.472 -21.180 64.281 0.00 0.00
F
ATOM 17318 HD13 LEU F 14 -15.915 -19.484 63.837 0.00 0.00
F
ATOM 17319 CD2 LEU F 14 -16.073 -22.564 62.158 0.00 0.00
F
ATOM 17320 HD21 LEU F 14 -15.745 -23.058 61.218 0.00 0.00
F
ATOM 17321 HD22 LEU F 14 -15.558 -23.186 62.921 0.00 0.00
F
ATOM 17322 HD23 LEU F 14 -17.160 -22.716 62.329 0.00 0.00
F
ATOM 17323 C LEU F 14 -16.387 -19.717 58.635 0.00 0.00
F
ATOM 17324 O LEU F 14 -16.808 -18.646 58.928 0.00 0.00
F
ATOM 17325 N GLY F 15 -16.364 -20.102 57.329 0.00 0.00
F
ATOM 17326 HN GLY F 15 -15.887 -20.950 57.112 0.00 0.00
F
ATOM 17327 CA GLY F 15 -17.000 -19.402 56.248 0.00 0.00
F
ATOM 17328 HA1 GLY F 15 -16.756 -19.893 55.318 0.00 0.00
F
ATOM 17329 HA2 GLY F 15 -16.573 -18.418 56.117 0.00 0.00
F
ATOM 17330 C GLY F 15 -18.453 -19.317 56.339 0.00 0.00
F
ATOM 17331 O GLY F 15 -19.172 -19.716 57.295 0.00 0.00
F
ATOM 17332 N ARG F 16 -19.072 -18.825 55.231 0.00 0.00
F
ATOM 17333 HN ARG F 16 -18.521 -18.599 54.431 0.00 0.00
F

```
ATOM  17334  CA  ARG F  16      -20.499 -18.776  54.992  0.00  0.00
F
ATOM  17335  HA  ARG F  16      -20.987 -18.309  55.835  0.00  0.00
F
ATOM  17336  CB  ARG F  16      -20.955 -18.034  53.783  0.00  0.00
F
ATOM  17337  HB1 ARG F  16      -20.283 -18.305  52.941  0.00  0.00
F
ATOM  17338  HB2 ARG F  16      -22.010 -18.283  53.538  0.00  0.00
F
ATOM  17339  CG  ARG F  16      -20.734 -16.537  53.989  0.00  0.00
F
ATOM  17340  HG1 ARG F  16      -21.519 -16.285  54.734  0.00  0.00
F
ATOM  17341  HG2 ARG F  16      -19.725 -16.290  54.382  0.00  0.00
F
ATOM  17342  CD  ARG F  16      -20.942 -15.741  52.700  0.00  0.00
F
ATOM  17343  HD1 ARG F  16      -21.755 -16.170  52.075  0.00  0.00
F
ATOM  17344  HD2 ARG F  16      -20.983 -14.664  52.970  0.00  0.00
F
ATOM  17345  NE  ARG F  16      -19.758 -15.938  52.006  0.00  0.00
F
ATOM  17346  HE  ARG F  16      -18.926 -15.795  52.542  0.00  0.00
F
ATOM  17347  CZ  ARG F  16      -19.652 -15.702  50.726  0.00  0.00
F
ATOM  17348  NH1 ARG F  16      -20.691 -15.970  49.870  0.00  0.00
F
ATOM  17349 HH11 ARG F  16      -20.515 -15.897  48.889  0.00  0.00
F
ATOM  17350 HH12 ARG F  16      -21.338 -16.621  50.268  0.00  0.00
F
ATOM  17351  NH2 ARG F  16      -18.630 -15.087  50.265  0.00  0.00
F
ATOM  17352 HH21 ARG F  16      -18.497 -15.189  49.279  0.00  0.00
F
ATOM  17353 HH22 ARG F  16      -17.855 -14.833  50.844  0.00  0.00
F
ATOM  17354  C   ARG F  16      -21.030 -20.262  55.027  0.00  0.00
F
ATOM  17355  O   ARG F  16      -21.977 -20.589  55.714  0.00  0.00
F
ATOM  17356  N   PHE F  17      -20.309 -21.232  54.356  0.00  0.00
F
ATOM  17357  HN  PHE F  17      -19.651 -21.041  53.631  0.00  0.00
F
ATOM  17358  CA  PHE F  17      -20.503 -22.665  54.332  0.00  0.00
F
ATOM  17359  HA  PHE F  17      -21.536 -22.758  54.031  0.00  0.00
F
ATOM  17360  CB  PHE F  17      -19.503 -23.200  53.343  0.00  0.00
F
```

ATOM 17361 HB1 PHE F 17 −19.666 −22.808 52.316 0.00 0.00
F
ATOM 17362 HB2 PHE F 17 −18.479 −22.812 53.529 0.00 0.00
F
ATOM 17363 CG PHE F 17 −19.547 −24.633 53.236 0.00 0.00
F
ATOM 17364 CD1 PHE F 17 −20.766 −25.357 53.263 0.00 0.00
F
ATOM 17365 HD1 PHE F 17 −21.724 −24.862 53.213 0.00 0.00
F
ATOM 17366 CE1 PHE F 17 −20.759 −26.735 53.456 0.00 0.00
F
ATOM 17367 HE1 PHE F 17 −21.693 −27.272 53.532 0.00 0.00
F
ATOM 17368 CZ PHE F 17 −19.562 −27.426 53.614 0.00 0.00
F
ATOM 17369 HZ PHE F 17 −19.630 −28.467 53.893 0.00 0.00
F
ATOM 17370 CD2 PHE F 17 −18.384 −25.355 53.456 0.00 0.00
F
ATOM 17371 HD2 PHE F 17 −17.456 −24.808 53.533 0.00 0.00
F
ATOM 17372 CE2 PHE F 17 −18.326 −26.744 53.623 0.00 0.00
F
ATOM 17373 HE2 PHE F 17 −17.415 −27.288 53.823 0.00 0.00
F
ATOM 17374 C PHE F 17 −20.305 −23.134 55.749 0.00 0.00
F
ATOM 17375 O PHE F 17 −21.094 −23.981 56.213 0.00 0.00
F
ATOM 17376 N GLY F 18 −19.243 −22.658 56.488 0.00 0.00
F
ATOM 17377 HN GLY F 18 −18.651 −21.951 56.110 0.00 0.00
F
ATOM 17378 CA GLY F 18 −18.624 −23.423 57.606 0.00 0.00
F
ATOM 17379 HA1 GLY F 18 −17.620 −23.030 57.667 0.00 0.00
F
ATOM 17380 HA2 GLY F 18 −18.661 −24.489 57.432 0.00 0.00
F
ATOM 17381 C GLY F 18 −19.353 −23.089 58.896 0.00 0.00
F
ATOM 17382 O GLY F 18 −19.593 −23.967 59.712 0.00 0.00
F
ATOM 17383 N GLY F 19 −19.741 −21.814 59.157 0.00 0.00
F
ATOM 17384 HN GLY F 19 −19.343 −21.172 58.506 0.00 0.00
F
ATOM 17385 CA GLY F 19 −20.631 −21.306 60.251 0.00 0.00
F
ATOM 17386 HA1 GLY F 19 −20.763 −20.235 60.221 0.00 0.00
F
ATOM 17387 HA2 GLY F 19 −20.249 −21.686 61.187 0.00 0.00
F

ATOM 17388 C GLY F 19 −22.009 −21.935 60.137 0.00 0.00
F
ATOM 17389 O GLY F 19 −22.649 −22.067 61.158 0.00 0.00
F
ATOM 17390 N SER F 20 −22.489 −22.268 58.917 0.00 0.00
F
ATOM 17391 HN SER F 20 −22.089 −21.999 58.045 0.00 0.00
F
ATOM 17392 CA SER F 20 −23.712 −22.945 58.835 0.00 0.00
F
ATOM 17393 HA SER F 20 −24.412 −22.328 59.380 0.00 0.00
F
ATOM 17394 CB SER F 20 −24.183 −23.130 57.322 0.00 0.00
F
ATOM 17395 HB1 SER F 20 −24.165 −22.099 56.907 0.00 0.00
F
ATOM 17396 HB2 SER F 20 −23.486 −23.831 56.814 0.00 0.00
F
ATOM 17397 OG SER F 20 −25.526 −23.498 57.114 0.00 0.00
F
ATOM 17398 HG1 SER F 20 −25.660 −23.811 56.217 0.00 0.00
F
ATOM 17399 C SER F 20 −23.772 −24.350 59.483 0.00 0.00
F
ATOM 17400 O SER F 20 −24.834 −24.719 59.997 0.00 0.00
F
ATOM 17401 N ILE F 21 −22.723 −25.146 59.260 0.00 0.00
F
ATOM 17402 HN ILE F 21 −21.987 −24.841 58.661 0.00 0.00
F
ATOM 17403 CA ILE F 21 −22.568 −26.461 59.810 0.00 0.00
F
ATOM 17404 HA ILE F 21 −23.415 −27.046 59.483 0.00 0.00
F
ATOM 17405 CB ILE F 21 −21.173 −27.087 59.408 0.00 0.00
F
ATOM 17406 HB ILE F 21 −20.372 −26.437 59.821 0.00 0.00
F
ATOM 17407 CG2 ILE F 21 −21.124 −28.499 60.026 0.00 0.00
F
ATOM 17408 HG21 ILE F 21 −21.903 −29.121 59.535 0.00 0.00
F
ATOM 17409 HG22 ILE F 21 −20.179 −29.080 59.967 0.00 0.00
F
ATOM 17410 HG23 ILE F 21 −21.519 −28.528 61.064 0.00 0.00
F
ATOM 17411 CG1 ILE F 21 −21.135 −27.144 57.866 0.00 0.00
F
ATOM 17412 HG11 ILE F 21 −21.950 −27.799 57.491 0.00 0.00
F
ATOM 17413 HG12 ILE F 21 −21.092 −26.107 57.469 0.00 0.00
F
ATOM 17414 CD ILE F 21 −19.863 −27.749 57.353 0.00 0.00
F

ATOM 17415 HD1 ILE F 21 −18.998 −27.579 58.029 0.00 0.00
F
ATOM 17416 HD2 ILE F 21 −19.933 −28.845 57.186 0.00 0.00
F
ATOM 17417 HD3 ILE F 21 −19.661 −27.368 56.329 0.00 0.00
F
ATOM 17418 C ILE F 21 −22.627 −26.439 61.322 0.00 0.00
F
ATOM 17419 O ILE F 21 −23.292 −27.204 61.952 0.00 0.00
F
ATOM 17420 N VAL F 22 −21.944 −25.460 61.992 0.00 0.00
F
ATOM 17421 HN VAL F 22 −21.429 −24.773 61.486 0.00 0.00
F
ATOM 17422 CA VAL F 22 −21.871 −25.463 63.472 0.00 0.00
F
ATOM 17423 HA VAL F 22 −21.901 −26.506 63.750 0.00 0.00
F
ATOM 17424 CB VAL F 22 −20.620 −24.835 64.142 0.00 0.00
F
ATOM 17425 HB VAL F 22 −20.678 −24.959 65.245 0.00 0.00
F
ATOM 17426 CG1 VAL F 22 −19.422 −25.531 63.495 0.00 0.00
F
ATOM 17427 HG11 VAL F 22 −19.523 −26.637 63.469 0.00 0.00
F
ATOM 17428 HG12 VAL F 22 −19.311 −25.129 62.466 0.00 0.00
F
ATOM 17429 HG13 VAL F 22 −18.544 −25.168 64.071 0.00 0.00
F
ATOM 17430 CG2 VAL F 22 −20.547 −23.307 63.881 0.00 0.00
F
ATOM 17431 HG21 VAL F 22 −21.264 −22.759 64.528 0.00 0.00
F
ATOM 17432 HG22 VAL F 22 −19.493 −23.023 64.086 0.00 0.00
F
ATOM 17433 HG23 VAL F 22 −20.823 −23.108 62.823 0.00 0.00
F
ATOM 17434 C VAL F 22 −23.250 −24.985 64.015 0.00 0.00
F
ATOM 17435 O VAL F 22 −23.738 −25.504 65.074 0.00 0.00
F
ATOM 17436 N LYS F 23 −23.889 −23.936 63.374 0.00 0.00
F
ATOM 17437 HN LYS F 23 −23.317 −23.396 62.762 0.00 0.00
F
ATOM 17438 CA LYS F 23 −25.223 −23.508 63.650 0.00 0.00
F
ATOM 17439 HA LYS F 23 −25.150 −23.154 64.668 0.00 0.00
F
ATOM 17440 CB LYS F 23 −25.654 −22.336 62.671 0.00 0.00
F
ATOM 17441 HB1 LYS F 23 −24.861 −21.559 62.630 0.00 0.00
F

ATOM	17442	HB2	LYS	F	23	−25.827	−22.699	61.635	0.00	0.00	F
ATOM	17443	CG	LYS	F	23	−26.839	−21.544	63.231	0.00	0.00	F
ATOM	17444	HG1	LYS	F	23	−27.794	−22.113	63.234	0.00	0.00	F
ATOM	17445	HG2	LYS	F	23	−26.702	−21.440	64.328	0.00	0.00	F
ATOM	17446	CD	LYS	F	23	−27.137	−20.173	62.557	0.00	0.00	F
ATOM	17447	HD1	LYS	F	23	−27.749	−19.608	63.291	0.00	0.00	F
ATOM	17448	HD2	LYS	F	23	−26.177	−19.623	62.661	0.00	0.00	F
ATOM	17449	CE	LYS	F	23	−27.602	−20.128	61.079	0.00	0.00	F
ATOM	17450	HE1	LYS	F	23	−26.734	−20.363	60.426	0.00	0.00	F
ATOM	17451	HE2	LYS	F	23	−28.547	−20.709	61.022	0.00	0.00	F
ATOM	17452	NZ	LYS	F	23	−28.138	−18.806	60.779	0.00	0.00	F
ATOM	17453	HZ1	LYS	F	23	−28.414	−18.716	59.780	0.00	0.00	F
ATOM	17454	HZ2	LYS	F	23	−28.944	−18.540	61.380	0.00	0.00	F
ATOM	17455	HZ3	LYS	F	23	−27.430	−18.058	60.920	0.00	0.00	F
ATOM	17456	C	LYS	F	23	−26.232	−24.613	63.708	0.00	0.00	F
ATOM	17457	O	LYS	F	23	−26.991	−24.686	64.689	0.00	0.00	F
ATOM	17458	N	GLU	F	24	−26.272	−25.383	62.657	0.00	0.00	F
ATOM	17459	HN	GLU	F	24	−25.557	−25.216	61.983	0.00	0.00	F
ATOM	17460	CA	GLU	F	24	−27.304	−26.311	62.545	0.00	0.00	F
ATOM	17461	HA	GLU	F	24	−28.240	−25.838	62.804	0.00	0.00	F
ATOM	17462	CB	GLU	F	24	−27.360	−26.668	60.986	0.00	0.00	F
ATOM	17463	HB1	GLU	F	24	−27.530	−25.699	60.468	0.00	0.00	F
ATOM	17464	HB2	GLU	F	24	−26.356	−27.102	60.795	0.00	0.00	F
ATOM	17465	CG	GLU	F	24	−28.410	−27.808	60.650	0.00	0.00	F
ATOM	17466	HG1	GLU	F	24	−28.284	−28.020	59.567	0.00	0.00	F
ATOM	17467	HG2	GLU	F	24	−28.056	−28.762	61.096	0.00	0.00	F
ATOM	17468	CD	GLU	F	24	−29.824	−27.459	60.853	0.00	0.00	F

ATOM 17469 OE1 GLU F 24 −30.142 −26.242 61.101 0.00 0.00
F
ATOM 17470 OE2 GLU F 24 −30.690 −28.326 60.832 0.00 0.00
F
ATOM 17471 C GLU F 24 −27.045 −27.521 63.454 0.00 0.00
F
ATOM 17472 O GLU F 24 −28.010 −27.974 64.074 0.00 0.00
F
ATOM 17473 N LEU F 25 −25.806 −28.007 63.728 0.00 0.00
F
ATOM 17474 HN LEU F 25 −24.976 −27.722 63.255 0.00 0.00
F
ATOM 17475 CA LEU F 25 −25.499 −29.044 64.752 0.00 0.00
F
ATOM 17476 HA LEU F 25 −26.108 −29.910 64.536 0.00 0.00
F
ATOM 17477 CB LEU F 25 −24.019 −29.525 64.683 0.00 0.00
F
ATOM 17478 HB1 LEU F 25 −23.374 −28.665 64.962 0.00 0.00
F
ATOM 17479 HB2 LEU F 25 −23.911 −30.263 65.506 0.00 0.00
F
ATOM 17480 CG LEU F 25 −23.713 −30.205 63.399 0.00 0.00
F
ATOM 17481 HG LEU F 25 −24.071 −29.641 62.511 0.00 0.00
F
ATOM 17482 CD1 LEU F 25 −22.166 −30.164 63.367 0.00 0.00
F
ATOM 17483 HD11 LEU F 25 −21.834 −30.809 62.526 0.00 0.00
F
ATOM 17484 HD12 LEU F 25 −21.776 −29.124 63.348 0.00 0.00
F
ATOM 17485 HD13 LEU F 25 −21.775 −30.647 64.289 0.00 0.00
F
ATOM 17486 CD2 LEU F 25 −24.219 −31.636 63.332 0.00 0.00
F
ATOM 17487 HD21 LEU F 25 −23.620 −32.199 62.585 0.00 0.00
F
ATOM 17488 HD22 LEU F 25 −24.140 −32.125 64.327 0.00 0.00
F
ATOM 17489 HD23 LEU F 25 −25.295 −31.703 63.064 0.00 0.00
F
ATOM 17490 C LEU F 25 −25.901 −28.616 66.102 0.00 0.00
F
ATOM 17491 O LEU F 25 −26.288 −29.432 66.889 0.00 0.00
F
ATOM 17492 N HSE F 26 −25.732 −27.363 66.435 0.00 0.00
F
ATOM 17493 HN HSE F 26 −25.332 −26.783 65.730 0.00 0.00
F
ATOM 17494 CA HSE F 26 −26.119 −26.774 67.647 0.00 0.00
F
ATOM 17495 HA HSE F 26 −25.590 −27.288 68.437 0.00 0.00
F

ATOM 17496 CB HSE F 26 −25.761 −25.268 67.690 0.00 0.00
F
ATOM 17497 HB1 HSE F 26 −24.714 −25.282 67.317 0.00 0.00
F
ATOM 17498 HB2 HSE F 26 −26.470 −24.713 67.040 0.00 0.00
F
ATOM 17499 ND1 HSE F 26 −25.020 −25.345 70.071 0.00 0.00
F
ATOM 17500 CG HSE F 26 −25.598 −24.604 69.034 0.00 0.00
F
ATOM 17501 CE1 HSE F 26 −24.989 −24.502 71.098 0.00 0.00
F
ATOM 17502 HE1 HSE F 26 −24.467 −24.799 72.008 0.00 0.00
F
ATOM 17503 NE2 HSE F 26 −25.581 −23.267 70.821 0.00 0.00
F
ATOM 17504 HE2 HSE F 26 −25.683 −22.429 71.356 0.00 0.00
F
ATOM 17505 CD2 HSE F 26 −26.042 −23.384 69.512 0.00 0.00
F
ATOM 17506 HD2 HSE F 26 −26.442 −22.527 68.984 0.00 0.00
F
ATOM 17507 C HSE F 26 −27.679 −26.827 67.812 0.00 0.00
F
ATOM 17508 O HSE F 26 −28.227 −27.105 68.919 0.00 0.00
F
ATOM 17509 N ARG F 27 −28.458 −26.507 66.719 0.00 0.00
F
ATOM 17510 HN ARG F 27 −27.819 −26.375 65.965 0.00 0.00
F
ATOM 17511 CA ARG F 27 −29.839 −26.489 66.569 0.00 0.00
F
ATOM 17512 HA ARG F 27 −30.157 −25.849 67.379 0.00 0.00
F
ATOM 17513 CB ARG F 27 −30.292 −26.041 65.103 0.00 0.00
F
ATOM 17514 HB1 ARG F 27 −29.684 −25.190 64.729 0.00 0.00
F
ATOM 17515 HB2 ARG F 27 −30.102 −26.890 64.413 0.00 0.00
F
ATOM 17516 CG ARG F 27 −31.766 −25.622 64.907 0.00 0.00
F
ATOM 17517 HG1 ARG F 27 −32.368 −26.512 65.192 0.00 0.00
F
ATOM 17518 HG2 ARG F 27 −31.983 −24.813 65.636 0.00 0.00
F
ATOM 17519 CD ARG F 27 −32.181 −25.224 63.489 0.00 0.00
F
ATOM 17520 HD1 ARG F 27 −33.127 −24.660 63.635 0.00 0.00
F
ATOM 17521 HD2 ARG F 27 −31.476 −24.524 62.992 0.00 0.00
F
ATOM 17522 NE ARG F 27 −32.358 −26.462 62.695 0.00 0.00
F

ATOM 17523 HE ARG F 27 −31.589 −26.747 62.123 0.00 0.00
F
ATOM 17524 CZ ARG F 27 −33.202 −27.475 62.856 0.00 0.00
F
ATOM 17525 NH1 ARG F 27 −34.194 −27.503 63.721 0.00 0.00
F
ATOM 17526 HH11 ARG F 27 −34.850 −28.229 63.517 0.00 0.00
F
ATOM 17527 HH12 ARG F 27 −34.679 −26.662 63.960 0.00 0.00
F
ATOM 17528 NH2 ARG F 27 −32.953 −28.581 62.170 0.00 0.00
F
ATOM 17529 HH21 ARG F 27 −33.473 −29.426 62.289 0.00 0.00
F
ATOM 17530 HH22 ARG F 27 −32.130 −28.564 61.601 0.00 0.00
F
ATOM 17531 C ARG F 27 −30.402 −27.809 66.909 0.00 0.00
F
ATOM 17532 O ARG F 27 −31.480 −27.918 67.519 0.00 0.00
F
ATOM 17533 N MET F 28 −29.731 −28.889 66.506 0.00 0.00
F
ATOM 17534 HN MET F 28 −28.980 −28.593 65.922 0.00 0.00
F
ATOM 17535 CA MET F 28 −30.151 −30.320 66.735 0.00 0.00
F
ATOM 17536 HA MET F 28 −31.221 −30.302 66.882 0.00 0.00
F
ATOM 17537 CB MET F 28 −29.877 −31.305 65.494 0.00 0.00
F
ATOM 17538 HB1 MET F 28 −28.776 −31.286 65.348 0.00 0.00
F
ATOM 17539 HB2 MET F 28 −30.314 −32.282 65.792 0.00 0.00
F
ATOM 17540 CG MET F 28 −30.598 −30.822 64.213 0.00 0.00
F
ATOM 17541 HG1 MET F 28 −31.676 −30.654 64.425 0.00 0.00
F
ATOM 17542 HG2 MET F 28 −30.312 −29.758 64.069 0.00 0.00
F
ATOM 17543 SD MET F 28 −30.260 −31.960 62.851 0.00 0.00
F
ATOM 17544 CE MET F 28 −28.684 −31.288 62.201 0.00 0.00
F
ATOM 17545 HE1 MET F 28 −28.754 −30.180 62.153 0.00 0.00
F
ATOM 17546 HE2 MET F 28 −27.777 −31.690 62.701 0.00 0.00
F
ATOM 17547 HE3 MET F 28 −28.592 −31.681 61.166 0.00 0.00
F
ATOM 17548 C MET F 28 −29.479 −30.875 67.976 0.00 0.00
F
ATOM 17549 O MET F 28 −29.782 −32.024 68.377 0.00 0.00
F

ATOM	17550	N	GLY	F	29	−28.616	−30.116	68.738	0.00	0.00	F
ATOM	17551	HN	GLY	F	29	−28.462	−29.151	68.542	0.00	0.00	F
ATOM	17552	CA	GLY	F	29	−28.028	−30.534	70.055	0.00	0.00	F
ATOM	17553	HA1	GLY	F	29	−28.721	−31.060	70.696	0.00	0.00	F
ATOM	17554	HA2	GLY	F	29	−27.552	−29.682	70.516	0.00	0.00	F
ATOM	17555	C	GLY	F	29	−26.919	−31.554	69.980	0.00	0.00	F
ATOM	17556	O	GLY	F	29	−26.777	−32.588	70.696	0.00	0.00	F
ATOM	17557	N	HSE	F	30	−25.998	−31.376	69.011	0.00	0.00	F
ATOM	17558	HN	HSE	F	30	−26.170	−30.734	68.268	0.00	0.00	F
ATOM	17559	CA	HSE	F	30	−24.723	−32.181	68.922	0.00	0.00	F
ATOM	17560	HA	HSE	F	30	−24.825	−32.941	69.682	0.00	0.00	F
ATOM	17561	CB	HSE	F	30	−24.455	−32.837	67.557	0.00	0.00	F
ATOM	17562	HB1	HSE	F	30	−24.140	−32.119	66.769	0.00	0.00	F
ATOM	17563	HB2	HSE	F	30	−23.669	−33.611	67.688	0.00	0.00	F
ATOM	17564	ND1	HSE	F	30	−26.002	−34.651	67.565	0.00	0.00	F
ATOM	17565	CG	HSE	F	30	−25.624	−33.475	66.989	0.00	0.00	F
ATOM	17566	CE1	HSE	F	30	−26.943	−35.138	66.772	0.00	0.00	F
ATOM	17567	HE1	HSE	F	30	−27.506	−36.051	66.964	0.00	0.00	F
ATOM	17568	NE2	HSE	F	30	−27.204	−34.310	65.673	0.00	0.00	F
ATOM	17569	HE2	HSE	F	30	−27.839	−34.364	64.902	0.00	0.00	F
ATOM	17570	CD2	HSE	F	30	−26.322	−33.270	65.819	0.00	0.00	F
ATOM	17571	HD2	HSE	F	30	−26.263	−32.446	65.120	0.00	0.00	F
ATOM	17572	C	HSE	F	30	−23.527	−31.319	69.327	0.00	0.00	F
ATOM	17573	O	HSE	F	30	−23.583	−30.078	69.261	0.00	0.00	F
ATOM	17574	N	GLU	F	31	−22.430	−31.948	69.853	0.00	0.00	F
ATOM	17575	HN	GLU	F	31	−22.291	−32.932	69.942	0.00	0.00	F
ATOM	17576	CA	GLU	F	31	−21.319	−31.238	70.476	0.00	0.00	F

ATOM 17577 HA GLU F 31   -21.595 -30.361 71.043 0.00 0.00
F
ATOM 17578 CB GLU F 31   -20.409 -32.224 71.263 0.00 0.00
F
ATOM 17579 HB1 GLU F 31   -20.994 -32.613 72.123 0.00 0.00
F
ATOM 17580 HB2 GLU F 31   -20.104 -33.037 70.571 0.00 0.00
F
ATOM 17581 CG GLU F 31   -19.095 -31.568 71.804 0.00 0.00
F
ATOM 17582 HG1 GLU F 31   -18.432 -31.335 70.943 0.00 0.00
F
ATOM 17583 HG2 GLU F 31   -19.383 -30.645 72.351 0.00 0.00
F
ATOM 17584 CD GLU F 31   -18.362 -32.468 72.801 0.00 0.00
F
ATOM 17585 OE1 GLU F 31   -17.133 -32.479 72.921 0.00 0.00
F
ATOM 17586 OE2 GLU F 31   -19.084 -33.227 73.516 0.00 0.00
F
ATOM 17587 C   GLU F 31   -20.461 -30.676 69.367 0.00 0.00
F
ATOM 17588 O   GLU F 31   -20.097 -31.470 68.519 0.00 0.00
F
ATOM 17589 N   VAL F 32   -20.340 -29.327 69.166 0.00 0.00
F
ATOM 17590 HN VAL F 32   -20.513 -28.809 70.000 0.00 0.00
F
ATOM 17591 CA VAL F 32   -19.754 -28.688 67.937 0.00 0.00
F
ATOM 17592 HA VAL F 32   -19.312 -29.465 67.331 0.00 0.00
F
ATOM 17593 CB VAL F 32   -20.864 -28.270 66.961 0.00 0.00
F
ATOM 17594 HB VAL F 32   -21.500 -29.145 66.709 0.00 0.00
F
ATOM 17595 CG1 VAL F 32   -21.674 -27.095 67.464 0.00 0.00
F
ATOM 17596 HG11 VAL F 32   -22.367 -26.710 66.686 0.00 0.00
F
ATOM 17597 HG12 VAL F 32   -22.281 -27.312 68.369 0.00 0.00
F
ATOM 17598 HG13 VAL F 32   -21.026 -26.223 67.697 0.00 0.00
F
ATOM 17599 CG2 VAL F 32   -20.187 -28.130 65.561 0.00 0.00
F
ATOM 17600 HG21 VAL F 32   -19.703 -29.078 65.240 0.00 0.00
F
ATOM 17601 HG22 VAL F 32   -20.972 -27.903 64.809 0.00 0.00
F
ATOM 17602 HG23 VAL F 32   -19.403 -27.343 65.571 0.00 0.00
F
ATOM 17603 C   VAL F 32   -18.613 -27.654 68.283 0.00 0.00
F

```
ATOM  17604  O   VAL F  32      -18.871 -26.663  68.927  0.00  0.00
F
ATOM  17605  N   LEU F  33      -17.417 -28.002  67.924  0.00  0.00
F
ATOM  17606  HN  LEU F  33      -17.271 -28.845  67.413  0.00  0.00
F
ATOM  17607  CA  LEU F  33      -16.247 -27.231  68.230  0.00  0.00
F
ATOM  17608  HA  LEU F  33      -16.441 -26.679  69.138  0.00  0.00
F
ATOM  17609  CB  LEU F  33      -15.080 -28.167  68.515  0.00  0.00
F
ATOM  17610  HB1 LEU F  33      -15.428 -28.847  69.321  0.00  0.00
F
ATOM  17611  HB2 LEU F  33      -14.952 -28.807  67.616  0.00  0.00
F
ATOM  17612  CG  LEU F  33      -13.852 -27.450  68.991  0.00  0.00
F
ATOM  17613  HG  LEU F  33      -13.644 -26.663  68.234  0.00  0.00
F
ATOM  17614  CD1 LEU F  33      -13.922 -26.753  70.336  0.00  0.00
F
ATOM  17615 HD11 LEU F  33      -14.866 -26.183  70.471  0.00  0.00
F
ATOM  17616 HD12 LEU F  33      -13.831 -27.461  71.187  0.00  0.00
F
ATOM  17617 HD13 LEU F  33      -12.975 -26.189  70.466  0.00  0.00
F
ATOM  17618  CD2 LEU F  33      -12.664 -28.381  69.077  0.00  0.00
F
ATOM  17619 HD21 LEU F  33      -12.911 -29.107  69.881  0.00  0.00
F
ATOM  17620 HD22 LEU F  33      -12.626 -28.919  68.106  0.00  0.00
F
ATOM  17621 HD23 LEU F  33      -11.707 -27.925  69.411  0.00  0.00
F
ATOM  17622  C   LEU F  33      -15.905 -26.376  67.036  0.00  0.00
F
ATOM  17623  O   LEU F  33      -15.462 -26.916  66.044  0.00  0.00
F
ATOM  17624  N   ALA F  34      -16.178 -25.111  67.080  0.00  0.00
F
ATOM  17625  HN  ALA F  34      -16.355 -24.704  67.973  0.00  0.00
F
ATOM  17626  CA  ALA F  34      -15.994 -24.175  66.018  0.00  0.00
F
ATOM  17627  HA  ALA F  34      -15.990 -24.750  65.103  0.00  0.00
F
ATOM  17628  CB  ALA F  34      -17.188 -23.174  66.079  0.00  0.00
F
ATOM  17629  HB1 ALA F  34      -17.084 -22.504  66.959  0.00  0.00
F
ATOM  17630  HB2 ALA F  34      -17.120 -22.581  65.142  0.00  0.00
F
```

ATOM 17631 HB3 ALA F 34   −18.173 −23.684 66.025 0.00 0.00
F
ATOM 17632 C   ALA F 34   −14.630 −23.540 66.082 0.00 0.00
F
ATOM 17633 O   ALA F 34   −14.397 −22.747 66.996 0.00 0.00
F
ATOM 17634 N   VAL F 35   −13.695 −23.887 65.051 0.00 0.00
F
ATOM 17635 HN VAL F 35   −13.941 −24.484 64.292 0.00 0.00
F
ATOM 17636 CA VAL F 35   −12.303 −23.546 65.155 0.00 0.00
F
ATOM 17637 HA VAL F 35   −12.061 −23.052 66.084 0.00 0.00
F
ATOM 17638 CB VAL F 35   −11.444 −24.837 65.090 0.00 0.00
F
ATOM 17639 HB VAL F 35   −11.602 −25.221 64.060 0.00 0.00
F
ATOM 17640 CG1 VAL F 35   −9.966 −24.563 65.175 0.00 0.00
F
ATOM 17641 HG11 VAL F 35   −9.500 −25.568 65.264 0.00 0.00
F
ATOM 17642 HG12 VAL F 35   −9.721 −24.198 64.154 0.00 0.00
F
ATOM 17643 HG13 VAL F 35   −9.609 −23.884 65.979 0.00 0.00
F
ATOM 17644 CG2 VAL F 35   −11.869 −25.859 66.109 0.00 0.00
F
ATOM 17645 HG21 VAL F 35   −11.430 −26.871 65.974 0.00 0.00
F
ATOM 17646 HG22 VAL F 35   −11.566 −25.622 67.152 0.00 0.00
F
ATOM 17647 HG23 VAL F 35   −12.967 −26.027 66.120 0.00 0.00
F
ATOM 17648 C   VAL F 35   −12.031 −22.552 63.963 0.00 0.00
F
ATOM 17649 O   VAL F 35   −12.070 −22.856 62.762 0.00 0.00
F
ATOM 17650 N   ASP F 36   −11.494 −21.333 64.291 0.00 0.00
F
ATOM 17651 HN ASP F 36   −11.265 −21.084 65.229 0.00 0.00
F
ATOM 17652 CA ASP F 36   −11.040 −20.419 63.203 0.00 0.00
F
ATOM 17653 HA ASP F 36   −10.788 −20.975 62.312 0.00 0.00
F
ATOM 17654 CB ASP F 36   −12.160 −19.404 62.888 0.00 0.00
F
ATOM 17655 HB1 ASP F 36   −13.083 −19.997 63.062 0.00 0.00
F
ATOM 17656 HB2 ASP F 36   −12.152 −18.496 63.527 0.00 0.00
F
ATOM 17657 CG ASP F 36   −12.099 −18.925 61.405 0.00 0.00
F

ATOM 17658 OD1 ASP F 36 -13.007 -19.295 60.658 0.00 0.00
F
ATOM 17659 OD2 ASP F 36 -11.167 -18.215 61.105 0.00 0.00
F
ATOM 17660 C ASP F 36 -9.809 -19.602 63.721 0.00 0.00
F
ATOM 17661 O ASP F 36 -9.514 -19.474 64.935 0.00 0.00
F
ATOM 17662 N ILE F 37 -9.125 -19.029 62.735 0.00 0.00
F
ATOM 17663 HN ILE F 37 -9.520 -18.950 61.823 0.00 0.00
F
ATOM 17664 CA ILE F 37 -7.917 -18.298 62.987 0.00 0.00
F
ATOM 17665 HA ILE F 37 -7.422 -18.563 63.909 0.00 0.00
F
ATOM 17666 CB ILE F 37 -6.876 -18.660 61.980 0.00 0.00
F
ATOM 17667 HB ILE F 37 -7.021 -19.761 61.943 0.00 0.00
F
ATOM 17668 CG2 ILE F 37 -7.207 -18.028 60.576 0.00 0.00
F
ATOM 17669 HG21 ILE F 37 -6.544 -18.437 59.784 0.00 0.00
F
ATOM 17670 HG22 ILE F 37 -8.283 -18.231 60.391 0.00 0.00
F
ATOM 17671 HG23 ILE F 37 -7.117 -16.921 60.553 0.00 0.00
F
ATOM 17672 CG1 ILE F 37 -5.449 -18.440 62.414 0.00 0.00
F
ATOM 17673 HG11 ILE F 37 -5.357 -17.335 62.477 0.00 0.00
F
ATOM 17674 HG12 ILE F 37 -5.367 -18.896 63.423 0.00 0.00
F
ATOM 17675 CD ILE F 37 -4.418 -19.000 61.450 0.00 0.00
F
ATOM 17676 HD1 ILE F 37 -4.406 -18.469 60.474 0.00 0.00
F
ATOM 17677 HD2 ILE F 37 -3.375 -18.978 61.831 0.00 0.00
F
ATOM 17678 HD3 ILE F 37 -4.574 -20.065 61.174 0.00 0.00
F
ATOM 17679 C ILE F 37 -8.207 -16.747 63.142 0.00 0.00
F
ATOM 17680 O ILE F 37 -7.316 -16.026 63.656 0.00 0.00
F
ATOM 17681 N ASN F 38 -9.455 -16.409 62.837 0.00 0.00
F
ATOM 17682 HN ASN F 38 -10.087 -17.068 62.436 0.00 0.00
F
ATOM 17683 CA ASN F 38 -10.087 -15.128 62.884 0.00 0.00
F
ATOM 17684 HA ASN F 38 -9.309 -14.401 63.066 0.00 0.00
F

ATOM 17685 CB ASN F 38 -10.800 -14.788 61.552 0.00 0.00
F
ATOM 17686 HB1 ASN F 38 -10.128 -14.965 60.686 0.00 0.00
F
ATOM 17687 HB2 ASN F 38 -11.649 -15.502 61.487 0.00 0.00
F
ATOM 17688 CG ASN F 38 -11.470 -13.468 61.582 0.00 0.00
F
ATOM 17689 OD1 ASN F 38 -11.415 -12.640 62.474 0.00 0.00
F
ATOM 17690 ND2 ASN F 38 -12.386 -13.264 60.551 0.00 0.00
F
ATOM 17691 HD21 ASN F 38 -12.962 -12.449 60.501 0.00 0.00
F
ATOM 17692 HD22 ASN F 38 -12.456 -14.020 59.900 0.00 0.00
F
ATOM 17693 C ASN F 38 -10.966 -15.101 64.052 0.00 0.00
F
ATOM 17694 O ASN F 38 -11.968 -15.798 64.203 0.00 0.00
F
ATOM 17695 N GLU F 39 -10.592 -14.219 64.998 0.00 0.00
F
ATOM 17696 HN GLU F 39 -9.909 -13.573 64.670 0.00 0.00
F
ATOM 17697 CA GLU F 39 -11.273 -13.940 66.242 0.00 0.00
F
ATOM 17698 HA GLU F 39 -11.276 -14.794 66.903 0.00 0.00
F
ATOM 17699 CB GLU F 39 -10.557 -12.737 66.927 0.00 0.00
F
ATOM 17700 HB1 GLU F 39 -9.508 -13.103 66.971 0.00 0.00
F
ATOM 17701 HB2 GLU F 39 -10.697 -11.863 66.257 0.00 0.00
F
ATOM 17702 CG GLU F 39 -11.056 -12.368 68.350 0.00 0.00
F
ATOM 17703 HG1 GLU F 39 -12.010 -11.813 68.222 0.00 0.00
F
ATOM 17704 HG2 GLU F 39 -11.303 -13.279 68.936 0.00 0.00
F
ATOM 17705 CD GLU F 39 -10.254 -11.326 69.094 0.00 0.00
F
ATOM 17706 OE1 GLU F 39 -9.875 -10.264 68.486 0.00 0.00
F
ATOM 17707 OE2 GLU F 39 -9.814 -11.549 70.246 0.00 0.00
F
ATOM 17708 C GLU F 39 -12.723 -13.657 66.081 0.00 0.00
F
ATOM 17709 O GLU F 39 -13.550 -13.990 66.926 0.00 0.00
F
ATOM 17710 N GLU F 40 -13.087 -12.885 65.076 0.00 0.00
F
ATOM 17711 HN GLU F 40 -12.451 -12.415 64.469 0.00 0.00
F

ATOM 17712 CA GLU F 40 −14.477 −12.366 64.849 0.00 0.00
F
ATOM 17713 HA GLU F 40 −14.795 −11.843 65.739 0.00 0.00
F
ATOM 17714 CB GLU F 40 −14.673 −11.404 63.670 0.00 0.00
F
ATOM 17715 HB1 GLU F 40 −14.494 −11.960 62.725 0.00 0.00
F
ATOM 17716 HB2 GLU F 40 −15.700 −10.984 63.725 0.00 0.00
F
ATOM 17717 CG GLU F 40 −13.711 −10.243 63.594 0.00 0.00
F
ATOM 17718 HG1 GLU F 40 −13.786 −9.727 64.575 0.00 0.00
F
ATOM 17719 HG2 GLU F 40 −12.681 −10.569 63.332 0.00 0.00
F
ATOM 17720 CD GLU F 40 −14.030 −9.178 62.578 0.00 0.00
F
ATOM 17721 OE1 GLU F 40 −13.183 −8.211 62.452 0.00 0.00
F
ATOM 17722 OE2 GLU F 40 −15.048 −9.304 61.884 0.00 0.00
F
ATOM 17723 C GLU F 40 −15.423 −13.550 64.684 0.00 0.00
F
ATOM 17724 O GLU F 40 −16.530 −13.512 65.124 0.00 0.00
F
ATOM 17725 N LYS F 41 −15.004 −14.604 63.948 0.00 0.00
F
ATOM 17726 HN LYS F 41 −14.018 −14.656 63.811 0.00 0.00
F
ATOM 17727 CA LYS F 41 −15.794 −15.756 63.638 0.00 0.00
F
ATOM 17728 HA LYS F 41 −16.759 −15.375 63.336 0.00 0.00
F
ATOM 17729 CB LYS F 41 −15.051 −16.687 62.631 0.00 0.00
F
ATOM 17730 HB1 LYS F 41 −14.066 −16.936 63.082 0.00 0.00
F
ATOM 17731 HB2 LYS F 41 −15.678 −17.604 62.620 0.00 0.00
F
ATOM 17732 CG LYS F 41 −14.831 −16.127 61.252 0.00 0.00
F
ATOM 17733 HG1 LYS F 41 −14.032 −15.363 61.139 0.00 0.00
F
ATOM 17734 HG2 LYS F 41 −14.571 −16.971 60.578 0.00 0.00
F
ATOM 17735 CD LYS F 41 −16.066 −15.559 60.677 0.00 0.00
F
ATOM 17736 HD1 LYS F 41 −16.747 −16.435 60.722 0.00 0.00
F
ATOM 17737 HD2 LYS F 41 −16.541 −14.782 61.313 0.00 0.00
F
ATOM 17738 CE LYS F 41 −16.069 −15.076 59.229 0.00 0.00
F

ATOM 17739 HE1 LYS F 41 −17.142 −14.845 59.058 0.00 0.00
F
ATOM 17740 HE2 LYS F 41 −15.446 −14.160 59.141 0.00 0.00
F
ATOM 17741 NZ LYS F 41 −15.725 −16.095 58.311 0.00 0.00
F
ATOM 17742 HZ1 LYS F 41 −15.848 −15.790 57.324 0.00 0.00
F
ATOM 17743 HZ2 LYS F 41 −14.736 −16.411 58.357 0.00 0.00
F
ATOM 17744 HZ3 LYS F 41 −16.330 −16.939 58.371 0.00 0.00
F
ATOM 17745 C LYS F 41 −15.983 −16.588 64.887 0.00 0.00
F
ATOM 17746 O LYS F 41 −17.102 −17.046 65.197 0.00 0.00
F
ATOM 17747 N VAL F 42 −14.947 −16.919 65.702 0.00 0.00
F
ATOM 17748 HN VAL F 42 −14.020 −16.660 65.444 0.00 0.00
F
ATOM 17749 CA VAL F 42 −14.991 −17.559 66.955 0.00 0.00
F
ATOM 17750 HA VAL F 42 −15.433 −18.544 66.958 0.00 0.00
F
ATOM 17751 CB VAL F 42 −13.602 −17.645 67.507 0.00 0.00
F
ATOM 17752 HB VAL F 42 −13.194 −16.619 67.621 0.00 0.00
F
ATOM 17753 CG1 VAL F 42 −13.513 −18.334 68.849 0.00 0.00
F
ATOM 17754 HG11 VAL F 42 −14.000 −19.331 68.915 0.00 0.00
F
ATOM 17755 HG12 VAL F 42 −12.458 −18.366 69.196 0.00 0.00
F
ATOM 17756 HG13 VAL F 42 −13.972 −17.719 69.651 0.00 0.00
F
ATOM 17757 CG2 VAL F 42 −12.563 −18.362 66.556 0.00 0.00
F
ATOM 17758 HG21 VAL F 42 −11.630 −18.423 67.156 0.00 0.00
F
ATOM 17759 HG22 VAL F 42 −12.842 −19.434 66.467 0.00 0.00
F
ATOM 17760 HG23 VAL F 42 −12.341 −17.919 65.561 0.00 0.00
F
ATOM 17761 C VAL F 42 −15.852 −16.698 67.961 0.00 0.00
F
ATOM 17762 O VAL F 42 −16.552 −17.305 68.777 0.00 0.00
F
ATOM 17763 N ASN F 43 −15.801 −15.335 67.901 0.00 0.00
F
ATOM 17764 HN ASN F 43 −15.118 −14.784 67.428 0.00 0.00
F
ATOM 17765 CA ASN F 43 −16.733 −14.588 68.735 0.00 0.00
F

ATOM 17766 HA ASN F 43 −16.708 −15.023 69.723 0.00 0.00
F
ATOM 17767 CB ASN F 43 −16.315 −13.107 68.674 0.00 0.00
F
ATOM 17768 HB1 ASN F 43 −15.907 −12.707 67.720 0.00 0.00
F
ATOM 17769 HB2 ASN F 43 −17.173 −12.550 69.107 0.00 0.00
F
ATOM 17770 CG ASN F 43 −15.046 −12.935 69.559 0.00 0.00
F
ATOM 17771 OD1 ASN F 43 −14.352 −13.858 69.881 0.00 0.00
F
ATOM 17772 ND2 ASN F 43 −14.797 −11.672 69.990 0.00 0.00
F
ATOM 17773 HD21 ASN F 43 −13.935 −11.451 70.448 0.00 0.00
F
ATOM 17774 HD22 ASN F 43 −15.471 −10.958 69.804 0.00 0.00
F
ATOM 17775 C ASN F 43 −18.234 −14.740 68.363 0.00 0.00
F
ATOM 17776 O ASN F 43 −19.122 −15.031 69.133 0.00 0.00
F
ATOM 17777 N ALA F 44 −18.425 −14.529 67.049 0.00 0.00
F
ATOM 17778 HN ALA F 44 −17.662 −14.120 66.556 0.00 0.00
F
ATOM 17779 CA ALA F 44 −19.726 −14.584 66.399 0.00 0.00
F
ATOM 17780 HA ALA F 44 −20.359 −13.782 66.749 0.00 0.00
F
ATOM 17781 CB ALA F 44 −19.525 −14.343 64.895 0.00 0.00
F
ATOM 17782 HB1 ALA F 44 −20.505 −14.355 64.372 0.00 0.00
F
ATOM 17783 HB2 ALA F 44 −18.941 −13.411 64.734 0.00 0.00
F
ATOM 17784 HB3 ALA F 44 −18.829 −15.116 64.505 0.00 0.00
F
ATOM 17785 C ALA F 44 −20.632 −15.835 66.544 0.00 0.00
F
ATOM 17786 O ALA F 44 −21.837 −15.690 66.624 0.00 0.00
F
ATOM 17787 N TYR F 45 −20.051 −17.008 66.607 0.00 0.00
F
ATOM 17788 HN TYR F 45 −19.071 −17.067 66.434 0.00 0.00
F
ATOM 17789 CA TYR F 45 −20.728 −18.248 66.735 0.00 0.00
F
ATOM 17790 HA TYR F 45 −21.760 −18.031 66.501 0.00 0.00
F
ATOM 17791 CB TYR F 45 −20.115 −19.223 65.693 0.00 0.00
F
ATOM 17792 HB1 TYR F 45 −19.017 −19.333 65.825 0.00 0.00
F

ATOM 17793 HB2 TYR F 45 -20.470 -20.272 65.780 0.00 0.00
F
ATOM 17794 CG TYR F 45 -20.351 -18.809 64.274 0.00 0.00
F
ATOM 17795 CD1 TYR F 45 -19.403 -18.473 63.298 0.00 0.00
F
ATOM 17796 HD1 TYR F 45 -18.375 -18.539 63.623 0.00 0.00
F
ATOM 17797 CE1 TYR F 45 -19.734 -17.831 62.124 0.00 0.00
F
ATOM 17798 HE1 TYR F 45 -19.047 -17.482 61.367 0.00 0.00
F
ATOM 17799 CZ TYR F 45 -21.128 -17.538 61.877 0.00 0.00
F
ATOM 17800 OH TYR F 45 -21.538 -16.732 60.805 0.00 0.00
F
ATOM 17801 HH TYR F 45 -20.758 -16.693 60.246 0.00 0.00
F
ATOM 17802 CD2 TYR F 45 -21.710 -18.669 63.904 0.00 0.00
F
ATOM 17803 HD2 TYR F 45 -22.498 -18.836 64.623 0.00 0.00
F
ATOM 17804 CE2 TYR F 45 -22.130 -18.065 62.726 0.00 0.00
F
ATOM 17805 HE2 TYR F 45 -23.168 -17.823 62.553 0.00 0.00
F
ATOM 17806 C TYR F 45 -20.730 -18.891 68.075 0.00 0.00
F
ATOM 17807 O TYR F 45 -21.161 -20.034 68.241 0.00 0.00
F
ATOM 17808 N ALA F 46 -20.398 -18.027 69.120 0.00 0.00
F
ATOM 17809 HN ALA F 46 -20.089 -17.106 68.894 0.00 0.00
F
ATOM 17810 CA ALA F 46 -20.278 -18.447 70.456 0.00 0.00
F
ATOM 17811 HA ALA F 46 -19.748 -19.380 70.328 0.00 0.00
F
ATOM 17812 CB ALA F 46 -19.616 -17.380 71.386 0.00 0.00
F
ATOM 17813 HB1 ALA F 46 -19.970 -16.352 71.155 0.00 0.00
F
ATOM 17814 HB2 ALA F 46 -19.881 -17.660 72.427 0.00 0.00
F
ATOM 17815 HB3 ALA F 46 -18.508 -17.385 71.304 0.00 0.00
F
ATOM 17816 C ALA F 46 -21.609 -18.926 71.113 0.00 0.00
F
ATOM 17817 O ALA F 46 -21.589 -19.920 71.898 0.00 0.00
F
ATOM 17818 N SER F 47 -22.822 -18.313 70.809 0.00 0.00
F
ATOM 17819 HN SER F 47 -22.766 -17.526 70.200 0.00 0.00
F

ATOM 17820 CA SER F 47 -24.103 -18.760 71.360 0.00 0.00
F
ATOM 17821 HA SER F 47 -24.073 -19.500 72.146 0.00 0.00
F
ATOM 17822 CB SER F 47 -25.041 -17.607 71.905 0.00 0.00
F
ATOM 17823 HB1 SER F 47 -25.065 -16.843 71.099 0.00 0.00
F
ATOM 17824 HB2 SER F 47 -26.059 -17.880 72.254 0.00 0.00
F
ATOM 17825 OG SER F 47 -24.521 -17.236 73.136 0.00 0.00
F
ATOM 17826 HG1 SER F 47 -24.464 -16.278 73.130 0.00 0.00
F
ATOM 17827 C SER F 47 -24.873 -19.536 70.263 0.00 0.00
F
ATOM 17828 O SER F 47 -26.020 -19.872 70.474 0.00 0.00
F
ATOM 17829 N TYR F 48 -24.188 -19.738 69.060 0.00 0.00
F
ATOM 17830 HN TYR F 48 -23.258 -19.462 68.827 0.00 0.00
F
ATOM 17831 CA TYR F 48 -24.701 -20.549 67.940 0.00 0.00
F
ATOM 17832 HA TYR F 48 -25.705 -20.877 68.164 0.00 0.00
F
ATOM 17833 CB TYR F 48 -24.555 -19.677 66.645 0.00 0.00
F
ATOM 17834 HB1 TYR F 48 -23.564 -19.177 66.673 0.00 0.00
F
ATOM 17835 HB2 TYR F 48 -24.590 -20.259 65.700 0.00 0.00
F
ATOM 17836 CG TYR F 48 -25.529 -18.580 66.472 0.00 0.00
F
ATOM 17837 CD1 TYR F 48 -25.048 -17.344 66.079 0.00 0.00
F
ATOM 17838 HD1 TYR F 48 -23.997 -17.101 66.017 0.00 0.00
F
ATOM 17839 CE1 TYR F 48 -25.865 -16.233 65.917 0.00 0.00
F
ATOM 17840 HE1 TYR F 48 -25.401 -15.322 65.569 0.00 0.00
F
ATOM 17841 CZ TYR F 48 -27.255 -16.413 65.931 0.00 0.00
F
ATOM 17842 OH TYR F 48 -28.086 -15.365 65.611 0.00 0.00
F
ATOM 17843 HH TYR F 48 -27.559 -14.720 65.134 0.00 0.00
F
ATOM 17844 CD2 TYR F 48 -26.934 -18.740 66.488 0.00 0.00
F
ATOM 17845 HD2 TYR F 48 -27.311 -19.708 66.785 0.00 0.00
F
ATOM 17846 CE2 TYR F 48 -27.803 -17.672 66.180 0.00 0.00
F

ATOM 17847 HE2 TYR F 48 −28.880 −17.753 66.138 0.00 0.00
F
ATOM 17848 C TYR F 48 −23.818 −21.889 67.781 0.00 0.00
F
ATOM 17849 O TYR F 48 −23.826 −22.460 66.716 0.00 0.00
F
ATOM 17850 N ALA F 49 −22.992 −22.245 68.810 0.00 0.00
F
ATOM 17851 HN ALA F 49 −22.927 −21.612 69.577 0.00 0.00
F
ATOM 17852 CA ALA F 49 −22.016 −23.357 68.976 0.00 0.00
F
ATOM 17853 HA ALA F 49 −22.470 −24.287 68.668 0.00 0.00
F
ATOM 17854 CB ALA F 49 −20.733 −22.991 68.237 0.00 0.00
F
ATOM 17855 HB1 ALA F 49 −20.042 −23.860 68.187 0.00 0.00
F
ATOM 17856 HB2 ALA F 49 −20.903 −22.654 67.192 0.00 0.00
F
ATOM 17857 HB3 ALA F 49 −20.105 −22.292 68.830 0.00 0.00
F
ATOM 17858 C ALA F 49 −21.694 −23.560 70.458 0.00 0.00
F
ATOM 17859 O ALA F 49 −21.757 −22.715 71.317 0.00 0.00
F
ATOM 17860 N THR F 50 −21.420 −24.840 70.847 0.00 0.00
F
ATOM 17861 HN THR F 50 −21.275 −25.538 70.150 0.00 0.00
F
ATOM 17862 CA THR F 50 −21.134 −25.391 72.163 0.00 0.00
F
ATOM 17863 HA THR F 50 −21.782 −24.827 72.817 0.00 0.00
F
ATOM 17864 CB THR F 50 −21.391 −26.931 72.438 0.00 0.00
F
ATOM 17865 HB THR F 50 −21.172 −27.261 73.476 0.00 0.00
F
ATOM 17866 OG1 THR F 50 −20.698 −27.774 71.464 0.00 0.00
F
ATOM 17867 HG1 THR F 50 −19.754 −27.666 71.603 0.00 0.00
F
ATOM 17868 CG2 THR F 50 −22.900 −27.198 72.281 0.00 0.00
F
ATOM 17869 HG21 THR F 50 −23.086 −28.272 72.498 0.00 0.00
F
ATOM 17870 HG22 THR F 50 −23.393 −26.452 72.941 0.00 0.00
F
ATOM 17871 HG23 THR F 50 −23.204 −26.933 71.246 0.00 0.00
F
ATOM 17872 C THR F 50 −19.860 −24.958 72.790 0.00 0.00
F
ATOM 17873 O THR F 50 −19.815 −24.494 73.898 0.00 0.00
F

ATOM 17874 N HSE F 51 −18.816 −24.933 72.003 0.00 0.00
F
ATOM 17875 HN HSE F 51 −18.900 −25.196 71.045 0.00 0.00
F
ATOM 17876 CA HSE F 51 −17.632 −24.231 72.344 0.00 0.00
F
ATOM 17877 HA HSE F 51 −17.868 −23.366 72.946 0.00 0.00
F
ATOM 17878 CB HSE F 51 −16.655 −25.137 73.097 0.00 0.00
F
ATOM 17879 HB1 HSE F 51 −17.228 −25.470 73.989 0.00 0.00
F
ATOM 17880 HB2 HSE F 51 −16.293 −25.998 72.495 0.00 0.00
F
ATOM 17881 ND1 HSE F 51 −15.648 −23.251 74.365 0.00 0.00
F
ATOM 17882 CG HSE F 51 −15.443 −24.448 73.646 0.00 0.00
F
ATOM 17883 CE1 HSE F 51 −14.439 −22.964 74.778 0.00 0.00
F
ATOM 17884 HE1 HSE F 51 −14.116 −22.061 75.296 0.00 0.00
F
ATOM 17885 NE2 HSE F 51 −13.534 −23.881 74.372 0.00 0.00
F
ATOM 17886 HE2 HSE F 51 −12.637 −23.936 74.812 0.00 0.00
F
ATOM 17887 CD2 HSE F 51 −14.176 −24.867 73.684 0.00 0.00
F
ATOM 17888 HD2 HSE F 51 −13.791 −25.762 73.210 0.00 0.00
F
ATOM 17889 C HSE F 51 −16.988 −23.729 71.089 0.00 0.00
F
ATOM 17890 O HSE F 51 −16.903 −24.363 70.048 0.00 0.00
F
ATOM 17891 N ALA F 52 −16.460 −22.483 71.077 0.00 0.00
F
ATOM 17892 HN ALA F 52 −16.568 −21.967 71.923 0.00 0.00
F
ATOM 17893 CA ALA F 52 −15.704 −21.894 70.010 0.00 0.00
F
ATOM 17894 HA ALA F 52 −15.575 −22.572 69.179 0.00 0.00
F
ATOM 17895 CB ALA F 52 −16.549 −20.702 69.377 0.00 0.00
F
ATOM 17896 HB1 ALA F 52 −16.516 −19.798 70.022 0.00 0.00
F
ATOM 17897 HB2 ALA F 52 −16.115 −20.430 68.391 0.00 0.00
F
ATOM 17898 HB3 ALA F 52 −17.581 −21.062 69.183 0.00 0.00
F
ATOM 17899 C ALA F 52 −14.320 −21.508 70.463 0.00 0.00
F
ATOM 17900 O ALA F 52 −14.105 −21.088 71.597 0.00 0.00
F

```
ATOM  17901  N   VAL F  53     -13.295 -21.796  69.648  0.00  0.00
F
ATOM  17902  HN  VAL F  53     -13.416 -22.072  68.697  0.00  0.00
F
ATOM  17903  CA  VAL F  53     -11.860 -21.595  69.978  0.00  0.00
F
ATOM  17904  HA  VAL F  53     -11.947 -20.946  70.837  0.00  0.00
F
ATOM  17905  CB  VAL F  53     -11.182 -22.858  70.337  0.00  0.00
F
ATOM  17906  HB  VAL F  53     -11.194 -23.530  69.453  0.00  0.00
F
ATOM  17907  CG1 VAL F  53      -9.745 -22.587  70.798  0.00  0.00
F
ATOM  17908 HG11 VAL F  53      -9.211 -23.472  71.204  0.00  0.00
F
ATOM  17909 HG12 VAL F  53      -9.101 -22.362  69.921  0.00  0.00
F
ATOM  17910 HG13 VAL F  53      -9.641 -21.762  71.535  0.00  0.00
F
ATOM  17911  CG2 VAL F  53     -11.851 -23.646  71.494  0.00  0.00
F
ATOM  17912 HG21 VAL F  53     -11.328 -24.614  71.643  0.00  0.00
F
ATOM  17913 HG22 VAL F  53     -11.666 -23.074  72.428  0.00  0.00
F
ATOM  17914 HG23 VAL F  53     -12.951 -23.758  71.378  0.00  0.00
F
ATOM  17915  C   VAL F  53     -11.073 -20.878  68.821  0.00  0.00
F
ATOM  17916  O   VAL F  53     -11.195 -21.213  67.671  0.00  0.00
F
ATOM  17917  N   ILE F  54     -10.169 -19.958  69.172  0.00  0.00
F
ATOM  17918  HN  ILE F  54     -10.035 -19.674  70.119  0.00  0.00
F
ATOM  17919  CA  ILE F  54      -9.257 -19.299  68.269  0.00  0.00
F
ATOM  17920  HA  ILE F  54      -9.700 -19.291  67.284  0.00  0.00
F
ATOM  17921  CB  ILE F  54      -8.863 -17.880  68.695  0.00  0.00
F
ATOM  17922  HB  ILE F  54      -8.003 -17.858  69.399  0.00  0.00
F
ATOM  17923  CG2 ILE F  54      -8.283 -17.264  67.354  0.00  0.00
F
ATOM  17924 HG21 ILE F  54      -7.792 -16.314  67.655  0.00  0.00
F
ATOM  17925 HG22 ILE F  54      -7.509 -17.927  66.911  0.00  0.00
F
ATOM  17926 HG23 ILE F  54      -9.055 -17.265  66.554  0.00  0.00
F
ATOM  17927  CG1 ILE F  54     -10.002 -17.011  69.234  0.00  0.00
F
```

ATOM 17928 HG11 ILE F 54   −10.769 −17.067 68.433 0.00 0.00
F
ATOM 17929 HG12 ILE F 54   −10.613 −17.367 70.091 0.00 0.00
F
ATOM 17930 CD ILE F 54   −9.591 −15.555 69.537 0.00 0.00
F
ATOM 17931 HD1 ILE F 54   −8.803 −15.583 70.320 0.00 0.00
F
ATOM 17932 HD2 ILE F 54   −9.284 −14.956 68.654 0.00 0.00
F
ATOM 17933 HD3 ILE F 54   −10.466 −15.063 70.012 0.00 0.00
F
ATOM 17934 C   ILE F 54   −8.054 −20.215 68.174 0.00 0.00
F
ATOM 17935 O   ILE F 54   −7.343 −20.417 69.126 0.00 0.00
F
ATOM 17936 N   ALA F 55   −7.843 −20.730 66.973 0.00 0.00
F
ATOM 17937 HN ALA F 55   −8.436 −20.496 66.206 0.00 0.00
F
ATOM 17938 CA ALA F 55   −6.559 −21.512 66.819 0.00 0.00
F
ATOM 17939 HA ALA F 55   −5.781 −20.972 67.338 0.00 0.00
F
ATOM 17940 CB ALA F 55   −6.651 −22.882 67.540 0.00 0.00
F
ATOM 17941 HB1 ALA F 55   −6.930 −22.678 68.597 0.00 0.00
F
ATOM 17942 HB2 ALA F 55   −7.585 −23.367 67.184 0.00 0.00
F
ATOM 17943 HB3 ALA F 55   −5.788 −23.559 67.368 0.00 0.00
F
ATOM 17944 C   ALA F 55   −6.038 −21.631 65.345 0.00 0.00
F
ATOM 17945 O   ALA F 55   −6.831 −21.763 64.419 0.00 0.00
F
ATOM 17946 N   ASN F 56   −4.720 −21.724 65.253 0.00 0.00
F
ATOM 17947 HN ASN F 56   −4.187 −21.662 66.094 0.00 0.00
F
ATOM 17948 CA ASN F 56   −3.961 −21.928 64.038 0.00 0.00
F
ATOM 17949 HA ASN F 56   −4.430 −21.419 63.208 0.00 0.00
F
ATOM 17950 CB ASN F 56   −2.611 −21.182 64.137 0.00 0.00
F
ATOM 17951 HB1 ASN F 56   −2.961 −20.154 64.370 0.00 0.00
F
ATOM 17952 HB2 ASN F 56   −2.043 −21.540 65.022 0.00 0.00
F
ATOM 17953 CG ASN F 56   −1.910 −21.158 62.771 0.00 0.00
F
ATOM 17954 OD1 ASN F 56   −2.302 −21.663 61.707 0.00 0.00
F

ATOM 17955 ND2 ASN F 56   -0.667 -20.671 62.807 0.00 0.00
F
ATOM 17956 HD21 ASN F 56   -0.156 -20.486 61.968 0.00 0.00
F
ATOM 17957 HD22 ASN F 56   -0.335 -20.256 63.654 0.00 0.00
F
ATOM 17958 C   ASN F 56   -3.821 -23.396 63.736 0.00 0.00
F
ATOM 17959 O   ASN F 56   -3.134 -24.088 64.468 0.00 0.00
F
ATOM 17960 N   ALA F 57   -4.475 -23.994 62.715 0.00 0.00
F
ATOM 17961 HN ALA F 57   -5.017 -23.378 62.148 0.00 0.00
F
ATOM 17962 CA ALA F 57   -4.633 -25.412 62.406 0.00 0.00
F
ATOM 17963 HA ALA F 57   -4.566 -26.002 63.308 0.00 0.00
F
ATOM 17964 CB ALA F 57   -6.004 -25.480 61.686 0.00 0.00
F
ATOM 17965 HB1 ALA F 57   -6.945 -25.183 62.198 0.00 0.00
F
ATOM 17966 HB2 ALA F 57   -5.740 -24.838 60.819 0.00 0.00
F
ATOM 17967 HB3 ALA F 57   -6.110 -26.533 61.348 0.00 0.00
F
ATOM 17968 C   ALA F 57   -3.435 -25.898 61.559 0.00 0.00
F
ATOM 17969 O   ALA F 57   -3.185 -27.097 61.386 0.00 0.00
F
ATOM 17970 N   THR F 58   -2.602 -25.034 60.950 0.00 0.00
F
ATOM 17971 HN THR F 58   -2.832 -24.065 60.902 0.00 0.00
F
ATOM 17972 CA THR F 58   -1.394 -25.474 60.255 0.00 0.00
F
ATOM 17973 HA THR F 58   -1.418 -26.529 60.022 0.00 0.00
F
ATOM 17974 CB THR F 58   -1.108 -24.708 58.927 0.00 0.00
F
ATOM 17975 HB THR F 58   -0.162 -25.039 58.449 0.00 0.00
F
ATOM 17976 OG1 THR F 58   -1.026 -23.330 59.189 0.00 0.00
F
ATOM 17977 HG1 THR F 58   -0.980 -22.756 58.420 0.00 0.00
F
ATOM 17978 CG2 THR F 58   -2.362 -24.969 58.060 0.00 0.00
F
ATOM 17979 HG21 THR F 58   -2.115 -24.425 57.123 0.00 0.00
F
ATOM 17980 HG22 THR F 58   -2.521 -26.047 57.844 0.00 0.00
F
ATOM 17981 HG23 THR F 58   -3.297 -24.544 58.484 0.00 0.00
F

ATOM 17982 C THR F 58 −0.192 −25.534 61.072 0.00 0.00
F
ATOM 17983 O THR F 58 0.865 −25.979 60.620 0.00 0.00
F
ATOM 17984 N GLU F 59 −0.290 −25.171 62.357 0.00 0.00
F
ATOM 17985 HN GLU F 59 −1.224 −25.009 62.665 0.00 0.00
F
ATOM 17986 CA GLU F 59 0.808 −25.370 63.329 0.00 0.00
F
ATOM 17987 HA GLU F 59 1.719 −25.680 62.838 0.00 0.00
F
ATOM 17988 CB GLU F 59 0.903 −24.134 64.167 0.00 0.00
F
ATOM 17989 HB1 GLU F 59 1.104 −23.198 63.603 0.00 0.00
F
ATOM 17990 HB2 GLU F 59 −0.154 −23.984 64.473 0.00 0.00
F
ATOM 17991 CG GLU F 59 1.733 −24.221 65.529 0.00 0.00
F
ATOM 17992 HG1 GLU F 59 1.866 −23.227 66.008 0.00 0.00
F
ATOM 17993 HG2 GLU F 59 1.270 −24.939 66.239 0.00 0.00
F
ATOM 17994 CD GLU F 59 3.114 −24.772 65.310 0.00 0.00
F
ATOM 17995 OE1 GLU F 59 3.504 −25.733 66.064 0.00 0.00
F
ATOM 17996 OE2 GLU F 59 3.837 −24.249 64.420 0.00 0.00
F
ATOM 17997 C GLU F 59 0.569 −26.576 64.262 0.00 0.00
F
ATOM 17998 O GLU F 59 −0.482 −26.744 64.873 0.00 0.00
F
ATOM 17999 N GLU F 60 1.597 −27.534 64.314 0.00 0.00
F
ATOM 18000 HN GLU F 60 2.436 −27.212 63.884 0.00 0.00
F
ATOM 18001 CA GLU F 60 1.497 −28.877 64.908 0.00 0.00
F
ATOM 18002 HA GLU F 60 0.648 −29.399 64.491 0.00 0.00
F
ATOM 18003 CB GLU F 60 2.648 −29.762 64.473 0.00 0.00
F
ATOM 18004 HB1 GLU F 60 2.644 −29.798 63.363 0.00 0.00
F
ATOM 18005 HB2 GLU F 60 3.634 −29.437 64.866 0.00 0.00
F
ATOM 18006 CG GLU F 60 2.426 −31.257 64.967 0.00 0.00
F
ATOM 18007 HG1 GLU F 60 2.590 −31.431 66.052 0.00 0.00
F
ATOM 18008 HG2 GLU F 60 1.364 −31.520 64.778 0.00 0.00
F

ATOM 18009 CD GLU F 60   3.225 -32.281 64.254 0.00 0.00
F
ATOM 18010 OE1 GLU F 60   4.469 -32.253 64.149 0.00 0.00
F
ATOM 18011 OE2 GLU F 60   2.604 -33.249 63.715 0.00 0.00
F
ATOM 18012 C   GLU F 60   1.261 -28.836 66.405 0.00 0.00
F
ATOM 18013 O   GLU F 60   0.463 -29.645 66.907 0.00 0.00
F
ATOM 18014 N   ASN F 61   1.862 -27.865 67.148 0.00 0.00
F
ATOM 18015 HN ASN F 61   2.459 -27.170 66.755 0.00 0.00
F
ATOM 18016 CA ASN F 61   1.832 -27.859 68.589 0.00 0.00
F
ATOM 18017 HA ASN F 61   1.997 -28.892 68.855 0.00 0.00
F
ATOM 18018 CB ASN F 61   2.856 -26.918 69.228 0.00 0.00
F
ATOM 18019 HB1 ASN F 61   2.952 -25.942 68.705 0.00 0.00
F
ATOM 18020 HB2 ASN F 61   2.673 -26.684 70.299 0.00 0.00
F
ATOM 18021 CG ASN F 61   4.305 -27.446 69.121 0.00 0.00
F
ATOM 18022 OD1 ASN F 61   4.413 -28.583 68.600 0.00 0.00
F
ATOM 18023 ND2 ASN F 61   5.331 -26.730 69.626 0.00 0.00
F
ATOM 18024 HD21 ASN F 61   6.227 -27.166 69.712 0.00 0.00
F
ATOM 18025 HD22 ASN F 61   5.350 -25.828 70.058 0.00 0.00
F
ATOM 18026 C   ASN F 61   0.469 -27.298 69.065 0.00 0.00
F
ATOM 18027 O   ASN F 61   -0.123 -27.745 70.047 0.00 0.00
F
ATOM 18028 N   GLU F 62   -0.034 -26.293 68.348 0.00 0.00
F
ATOM 18029 HN GLU F 62   0.336 -26.015 67.464 0.00 0.00
F
ATOM 18030 CA GLU F 62   -1.234 -25.533 68.639 0.00 0.00
F
ATOM 18031 HA GLU F 62   -1.241 -25.188 69.662 0.00 0.00
F
ATOM 18032 CB GLU F 62   -1.446 -24.450 67.586 0.00 0.00
F
ATOM 18033 HB1 GLU F 62   -0.548 -23.829 67.795 0.00 0.00
F
ATOM 18034 HB2 GLU F 62   -1.429 -24.882 66.562 0.00 0.00
F
ATOM 18035 CG GLU F 62   -2.708 -23.571 67.777 0.00 0.00
F

ATOM 18036 HG1 GLU F 62   −2.818 −22.888 66.908 0.00 0.00
F
ATOM 18037 HG2 GLU F 62   −3.602 −24.229 67.835 0.00 0.00
F
ATOM 18038 CD GLU F 62   −2.690 −22.704 69.017 0.00 0.00
F
ATOM 18039 OE1 GLU F 62   −3.420 −23.042 70.047 0.00 0.00
F
ATOM 18040 OE2 GLU F 62   −2.026 −21.665 69.013 0.00 0.00
F
ATOM 18041 C   GLU F 62   −2.422 −26.522 68.514 0.00 0.00
F
ATOM 18042 O   GLU F 62   −3.262 −26.518 69.362 0.00 0.00
F
ATOM 18043 N   LEU F 63   −2.382 −27.340 67.424 0.00 0.00
F
ATOM 18044 HN LEU F 63   −1.689 −27.222 66.717 0.00 0.00
F
ATOM 18045 CA LEU F 63   −3.323 −28.392 67.106 0.00 0.00
F
ATOM 18046 HA LEU F 63   −4.307 −27.947 67.127 0.00 0.00
F
ATOM 18047 CB LEU F 63   −2.865 −28.808 65.697 0.00 0.00
F
ATOM 18048 HB1 LEU F 63   −2.665 −27.873 65.132 0.00 0.00
F
ATOM 18049 HB2 LEU F 63   −1.883 −29.292 65.510 0.00 0.00
F
ATOM 18050 CG LEU F 63   −3.848 −29.706 65.025 0.00 0.00
F
ATOM 18051 HG LEU F 63   −3.792 −30.654 65.601 0.00 0.00
F
ATOM 18052 CD1 LEU F 63   −5.290 −29.201 65.062 0.00 0.00
F
ATOM 18053 HD11 LEU F 63   −5.244 −28.200 64.583 0.00 0.00
F
ATOM 18054 HD12 LEU F 63   −6.026 −29.884 64.587 0.00 0.00
F
ATOM 18055 HD13 LEU F 63   −5.643 −28.974 66.091 0.00 0.00
F
ATOM 18056 CD2 LEU F 63   −3.520 −30.074 63.507 0.00 0.00
F
ATOM 18057 HD21 LEU F 63   −2.411 −30.008 63.479 0.00 0.00
F
ATOM 18058 HD22 LEU F 63   −3.944 −31.057 63.209 0.00 0.00
F
ATOM 18059 HD23 LEU F 63   −3.870 −29.279 62.815 0.00 0.00
F
ATOM 18060 C   LEU F 63   −3.341 −29.498 68.186 0.00 0.00
F
ATOM 18061 O   LEU F 63   −4.392 −29.979 68.647 0.00 0.00
F
ATOM 18062 N   LEU F 64   −2.138 −30.004 68.557 0.00 0.00
F

```
ATOM  18063  HN  LEU F  64      -1.308 -29.731  68.076  0.00  0.00
F
ATOM  18064  CA  LEU F  64      -2.066 -30.991  69.636  0.00  0.00
F
ATOM  18065  HA  LEU F  64      -2.714 -31.836  69.452  0.00  0.00
F
ATOM  18066  CB  LEU F  64      -0.567 -31.279  69.830  0.00  0.00
F
ATOM  18067  HB1 LEU F  64      -0.023 -30.311  69.834  0.00  0.00
F
ATOM  18068  HB2 LEU F  64      -0.454 -31.763  70.824  0.00  0.00
F
ATOM  18069  CG  LEU F  64       0.065 -32.230  68.755  0.00  0.00
F
ATOM  18070  HG  LEU F  64      -0.257 -31.987  67.720  0.00  0.00
F
ATOM  18071  CD1 LEU F  64       1.600 -32.152  68.809  0.00  0.00
F
ATOM  18072 HD11 LEU F  64       1.926 -32.358  69.851  0.00  0.00
F
ATOM  18073 HD12 LEU F  64       1.968 -32.905  68.080  0.00  0.00
F
ATOM  18074 HD13 LEU F  64       1.887 -31.156  68.407  0.00  0.00
F
ATOM  18075  CD2 LEU F  64      -0.271 -33.700  69.044  0.00  0.00
F
ATOM  18076 HD21 LEU F  64      -1.347 -33.977  69.022  0.00  0.00
F
ATOM  18077 HD22 LEU F  64       0.283 -34.348  68.331  0.00  0.00
F
ATOM  18078 HD23 LEU F  64       0.138 -33.926  70.053  0.00  0.00
F
ATOM  18079  C   LEU F  64      -2.635 -30.409  71.024  0.00  0.00
F
ATOM  18080  O   LEU F  64      -3.392 -31.062  71.712  0.00  0.00
F
ATOM  18081  N   SER F  65      -2.285 -29.153  71.306  0.00  0.00
F
ATOM  18082  HN  SER F  65      -1.526 -28.816  70.755  0.00  0.00
F
ATOM  18083  CA  SER F  65      -2.806 -28.288  72.435  0.00  0.00
F
ATOM  18084  HA  SER F  65      -2.624 -28.818  73.358  0.00  0.00
F
ATOM  18085  CB  SER F  65      -2.094 -26.880  72.579  0.00  0.00
F
ATOM  18086  HB1 SER F  65      -2.318 -26.253  71.689  0.00  0.00
F
ATOM  18087  HB2 SER F  65      -2.449 -26.328  73.475  0.00  0.00
F
ATOM  18088  OG  SER F  65      -0.734 -27.068  72.687  0.00  0.00
F
ATOM  18089  HG1 SER F  65      -0.444 -27.309  71.804  0.00  0.00
F
```

```
ATOM  18090  C   SER F  65      -4.352 -28.105  72.396  0.00  0.00
F
ATOM  18091  O   SER F  65      -5.028 -27.975  73.468  0.00  0.00
F
ATOM  18092  N   LEU F  66      -4.955 -28.132  71.184  0.00  0.00
F
ATOM  18093  HN  LEU F  66      -4.371 -27.927  70.402  0.00  0.00
F
ATOM  18094  CA  LEU F  66      -6.386 -27.872  70.978  0.00  0.00
F
ATOM  18095  HA  LEU F  66      -6.608 -27.018  71.601  0.00  0.00
F
ATOM  18096  CB  LEU F  66      -6.640 -27.595  69.439  0.00  0.00
F
ATOM  18097  HB1 LEU F  66      -6.033 -26.698  69.191  0.00  0.00
F
ATOM  18098  HB2 LEU F  66      -6.242 -28.472  68.886  0.00  0.00
F
ATOM  18099  CG  LEU F  66      -8.098 -27.402  68.993  0.00  0.00
F
ATOM  18100  HG  LEU F  66      -8.692 -28.338  69.074  0.00  0.00
F
ATOM  18101  CD1 LEU F  66      -8.834 -26.224  69.676  0.00  0.00
F
ATOM  18102 HD11 LEU F  66      -8.787 -26.384  70.775  0.00  0.00
F
ATOM  18103 HD12 LEU F  66      -8.319 -25.261  69.470  0.00  0.00
F
ATOM  18104 HD13 LEU F  66      -9.834 -26.101  69.208  0.00  0.00
F
ATOM  18105  CD2 LEU F  66      -8.033 -27.046  67.483  0.00  0.00
F
ATOM  18106 HD21 LEU F  66      -7.122 -27.610  67.187  0.00  0.00
F
ATOM  18107 HD22 LEU F  66      -8.957 -27.483  67.048  0.00  0.00
F
ATOM  18108 HD23 LEU F  66      -7.847 -25.977  67.244  0.00  0.00
F
ATOM  18109  C   LEU F  66      -7.161 -29.022  71.534  0.00  0.00
F
ATOM  18110  O   LEU F  66      -8.156 -28.776  72.176  0.00  0.00
F
ATOM  18111  N   GLY F  67      -6.733 -30.296  71.302  0.00  0.00
F
ATOM  18112  HN  GLY F  67      -5.834 -30.435  70.894  0.00  0.00
F
ATOM  18113  CA  GLY F  67      -7.429 -31.466  71.837  0.00  0.00
F
ATOM  18114  HA1 GLY F  67      -8.039 -31.188  72.684  0.00  0.00
F
ATOM  18115  HA2 GLY F  67      -6.751 -32.232  72.184  0.00  0.00
F
ATOM  18116  C   GLY F  67      -8.366 -32.182  70.867  0.00  0.00
F
```

ATOM 18117 O   GLY F 67   -9.432 -32.758 71.208 0.00 0.00
F
ATOM 18118 N   ILE F 68   -8.029 -32.051 69.551 0.00 0.00
F
ATOM 18119 HN ILE F 68   -7.240 -31.489 69.314 0.00 0.00
F
ATOM 18120 CA ILE F 68   -8.663 -32.620 68.431 0.00 0.00
F
ATOM 18121 HA ILE F 68   -9.706 -32.670 68.707 0.00 0.00
F
ATOM 18122 CB ILE F 68   -8.641 -31.574 67.331 0.00 0.00
F
ATOM 18123 HB ILE F 68   -8.672 -30.573 67.813 0.00 0.00
F
ATOM 18124 CG2 ILE F 68   -7.329 -31.711 66.552 0.00 0.00
F
ATOM 18125 HG21 ILE F 68   -6.522 -31.860 67.300 0.00 0.00
F
ATOM 18126 HG22 ILE F 68   -7.316 -32.568 65.845 0.00 0.00
F
ATOM 18127 HG23 ILE F 68   -7.116 -30.871 65.856 0.00 0.00
F
ATOM 18128 CG1 ILE F 68   -9.894 -31.667 66.327 0.00 0.00
F
ATOM 18129 HG11 ILE F 68   -9.432 -32.298 65.538 0.00 0.00
F
ATOM 18130 HG12 ILE F 68   -10.829 -32.102 66.741 0.00 0.00
F
ATOM 18131 CD ILE F 68   -10.259 -30.334 65.745 0.00 0.00
F
ATOM 18132 HD1 ILE F 68   -10.996 -30.450 64.921 0.00 0.00
F
ATOM 18133 HD2 ILE F 68   -10.503 -29.584 66.528 0.00 0.00
F
ATOM 18134 HD3 ILE F 68   -9.365 -29.854 65.293 0.00 0.00
F
ATOM 18135 C   ILE F 68   -8.252 -34.033 67.920 0.00 0.00
F
ATOM 18136 O   ILE F 68   -8.899 -34.695 67.119 0.00 0.00
F
ATOM 18137 N   ARG F 69   -7.109 -34.558 68.484 0.00 0.00
F
ATOM 18138 HN ARG F 69   -6.678 -33.955 69.150 0.00 0.00
F
ATOM 18139 CA ARG F 69   -6.629 -35.921 68.257 0.00 0.00
F
ATOM 18140 HA ARG F 69   -6.292 -35.971 67.232 0.00 0.00
F
ATOM 18141 CB ARG F 69   -5.404 -36.079 69.203 0.00 0.00
F
ATOM 18142 HB1 ARG F 69   -4.732 -35.244 68.910 0.00 0.00
F
ATOM 18143 HB2 ARG F 69   -5.614 -35.853 70.271 0.00 0.00
F

ATOM	18144	CG	ARG	F	69	−4.609	−37.435	69.138	0.00	0.00	F
ATOM	18145	HG1	ARG	F	69	−5.332	−38.247	69.368	0.00	0.00	F
ATOM	18146	HG2	ARG	F	69	−4.129	−37.554	68.143	0.00	0.00	F
ATOM	18147	CD	ARG	F	69	−3.571	−37.460	70.146	0.00	0.00	F
ATOM	18148	HD1	ARG	F	69	−2.760	−36.737	69.916	0.00	0.00	F
ATOM	18149	HD2	ARG	F	69	−3.915	−37.156	71.158	0.00	0.00	F
ATOM	18150	NE	ARG	F	69	−3.089	−38.886	70.127	0.00	0.00	F
ATOM	18151	HE	ARG	F	69	−3.238	−39.482	69.338	0.00	0.00	F
ATOM	18152	CZ	ARG	F	69	−2.229	−39.548	70.905	0.00	0.00	F
ATOM	18153	NH1	ARG	F	69	−1.746	−39.080	72.004	0.00	0.00	F
ATOM	18154	HH11	ARG	F	69	−1.396	−39.687	72.718	0.00	0.00	F
ATOM	18155	HH12	ARG	F	69	−2.172	−38.223	72.295	0.00	0.00	F
ATOM	18156	NH2	ARG	F	69	−1.858	−40.773	70.521	0.00	0.00	F
ATOM	18157	HH21	ARG	F	69	−1.152	−41.138	71.128	0.00	0.00	F
ATOM	18158	HH22	ARG	F	69	−2.239	−41.268	69.740	0.00	0.00	F
ATOM	18159	C	ARG	F	69	−7.582	−37.001	68.593	0.00	0.00	F
ATOM	18160	O	ARG	F	69	−7.523	−38.066	68.122	0.00	0.00	F
ATOM	18161	N	ASN	F	70	−8.581	−36.689	69.480	0.00	0.00	F
ATOM	18162	HN	ASN	F	70	−8.457	−35.838	69.983	0.00	0.00	F
ATOM	18163	CA	ASN	F	70	−9.479	−37.723	69.915	0.00	0.00	F
ATOM	18164	HA	ASN	F	70	−9.006	−38.647	69.616	0.00	0.00	F
ATOM	18165	CB	ASN	F	70	−9.702	−37.823	71.403	0.00	0.00	F
ATOM	18166	HB1	ASN	F	70	−10.288	−36.944	71.746	0.00	0.00	F
ATOM	18167	HB2	ASN	F	70	−10.355	−38.688	71.647	0.00	0.00	F
ATOM	18168	CG	ASN	F	70	−8.463	−37.831	72.280	0.00	0.00	F
ATOM	18169	OD1	ASN	F	70	−7.544	−38.598	72.024	0.00	0.00	F
ATOM	18170	ND2	ASN	F	70	−8.389	−37.118	73.373	0.00	0.00	F

```
ATOM  18171 HD21 ASN F  70      -9.083 -36.459  73.662  0.00  0.00
F
ATOM  18172 HD22 ASN F  70      -7.514 -37.138  73.857  0.00  0.00
F
ATOM  18173  C   ASN F  70     -10.763 -37.785  69.120  0.00  0.00
F
ATOM  18174  O   ASN F  70     -11.573 -38.662  69.252  0.00  0.00
F
ATOM  18175  N   PHE F  71     -10.868 -36.768  68.270  0.00  0.00
F
ATOM  18176  HN  PHE F  71     -10.150 -36.115  68.039  0.00  0.00
F
ATOM  18177  CA  PHE F  71     -12.121 -36.648  67.491  0.00  0.00
F
ATOM  18178  HA  PHE F  71     -13.017 -36.658  68.093  0.00  0.00
F
ATOM  18179  CB  PHE F  71     -12.192 -35.316  66.669  0.00  0.00
F
ATOM  18180  HB1 PHE F  71     -11.257 -35.088  66.115  0.00  0.00
F
ATOM  18181  HB2 PHE F  71     -13.048 -35.399  65.965  0.00  0.00
F
ATOM  18182  CG  PHE F  71     -12.642 -34.122  67.496  0.00  0.00
F
ATOM  18183  CD1 PHE F  71     -13.805 -33.379  67.201  0.00  0.00
F
ATOM  18184  HD1 PHE F  71     -14.279 -33.415  66.231  0.00  0.00
F
ATOM  18185  CE1 PHE F  71     -14.227 -32.370  68.033  0.00  0.00
F
ATOM  18186  HE1 PHE F  71     -15.043 -31.762  67.674  0.00  0.00
F
ATOM  18187  CZ  PHE F  71     -13.515 -32.066  69.185  0.00  0.00
F
ATOM  18188  HZ  PHE F  71     -13.871 -31.228  69.766  0.00  0.00
F
ATOM  18189  CD2 PHE F  71     -11.967 -33.868  68.791  0.00  0.00
F
ATOM  18190  HD2 PHE F  71     -11.111 -34.452  69.094  0.00  0.00
F
ATOM  18191  CE2 PHE F  71     -12.403 -32.777  69.549  0.00  0.00
F
ATOM  18192  HE2 PHE F  71     -11.900 -32.532  70.473  0.00  0.00
F
ATOM  18193  C   PHE F  71     -12.359 -37.787  66.542  0.00  0.00
F
ATOM  18194  O   PHE F  71     -11.357 -38.366  66.097  0.00  0.00
F
ATOM  18195  N   GLU F  72     -13.663 -38.160  66.276  0.00  0.00
F
ATOM  18196  HN  GLU F  72     -14.346 -37.724  66.857  0.00  0.00
F
ATOM  18197  CA  GLU F  72     -14.095 -39.097  65.319  0.00  0.00
F
```

ATOM 18198 HA GLU F 72 −13.330 −39.811 65.052 0.00 0.00
F
ATOM 18199 CB GLU F 72 −15.393 −39.750 65.862 0.00 0.00
F
ATOM 18200 HB1 GLU F 72 −15.093 −40.260 66.802 0.00 0.00
F
ATOM 18201 HB2 GLU F 72 −16.152 −38.946 65.970 0.00 0.00
F
ATOM 18202 CG GLU F 72 −16.093 −40.913 65.011 0.00 0.00
F
ATOM 18203 HG1 GLU F 72 −16.434 −40.434 64.068 0.00 0.00
F
ATOM 18204 HG2 GLU F 72 −15.452 −41.798 64.810 0.00 0.00
F
ATOM 18205 CD GLU F 72 −17.361 −41.467 65.651 0.00 0.00
F
ATOM 18206 OE1 GLU F 72 −17.457 −41.476 66.926 0.00 0.00
F
ATOM 18207 OE2 GLU F 72 −18.134 −42.147 64.926 0.00 0.00
F
ATOM 18208 C GLU F 72 −14.340 −38.315 64.049 0.00 0.00
F
ATOM 18209 O GLU F 72 −14.101 −38.825 62.972 0.00 0.00
F
ATOM 18210 N TYR F 73 −14.832 −37.046 64.060 0.00 0.00
F
ATOM 18211 HN TYR F 73 −15.213 −36.659 64.896 0.00 0.00
F
ATOM 18212 CA TYR F 73 −15.159 −36.205 62.867 0.00 0.00
F
ATOM 18213 HA TYR F 73 −14.660 −36.805 62.120 0.00 0.00
F
ATOM 18214 CB TYR F 73 −16.669 −35.931 62.685 0.00 0.00
F
ATOM 18215 HB1 TYR F 73 −17.181 −35.706 63.645 0.00 0.00
F
ATOM 18216 HB2 TYR F 73 −16.967 −35.156 61.947 0.00 0.00
F
ATOM 18217 CG TYR F 73 −17.346 −37.197 62.258 0.00 0.00
F
ATOM 18218 CD1 TYR F 73 −17.964 −38.023 63.186 0.00 0.00
F
ATOM 18219 HD1 TYR F 73 −17.867 −37.747 64.226 0.00 0.00
F
ATOM 18220 CE1 TYR F 73 −18.538 −39.180 62.697 0.00 0.00
F
ATOM 18221 HE1 TYR F 73 −18.983 −39.904 63.363 0.00 0.00
F
ATOM 18222 CZ TYR F 73 −18.644 −39.472 61.342 0.00 0.00
F
ATOM 18223 OH TYR F 73 −19.173 −40.579 60.804 0.00 0.00
F
ATOM 18224 HH TYR F 73 −19.608 −41.058 61.514 0.00 0.00
F

ATOM 18225 CD2 TYR F 73 −17.567 −37.327 60.904 0.00 0.00
F
ATOM 18226 HD2 TYR F 73 −17.110 −36.663 60.185 0.00 0.00
F
ATOM 18227 CE2 TYR F 73 −18.198 −38.491 60.418 0.00 0.00
F
ATOM 18228 HE2 TYR F 73 −18.315 −38.666 59.359 0.00 0.00
F
ATOM 18229 C TYR F 73 −14.548 −34.743 62.811 0.00 0.00
F
ATOM 18230 O TYR F 73 −14.930 −33.878 63.598 0.00 0.00
F
ATOM 18231 N VAL F 74 −13.684 −34.513 61.908 0.00 0.00
F
ATOM 18232 HN VAL F 74 −13.376 −35.256 61.319 0.00 0.00
F
ATOM 18233 CA VAL F 74 −13.223 −33.184 61.659 0.00 0.00
F
ATOM 18234 HA VAL F 74 −13.715 −32.409 62.229 0.00 0.00
F
ATOM 18235 CB VAL F 74 −11.615 −33.078 61.813 0.00 0.00
F
ATOM 18236 HB VAL F 74 −11.208 −33.897 61.182 0.00 0.00
F
ATOM 18237 CG1 VAL F 74 −11.061 −31.700 61.428 0.00 0.00
F
ATOM 18238 HG11 VAL F 74 −11.705 −30.895 61.842 0.00 0.00
F
ATOM 18239 HG12 VAL F 74 −10.038 −31.619 61.853 0.00 0.00
F
ATOM 18240 HG13 VAL F 74 −11.032 −31.633 60.319 0.00 0.00
F
ATOM 18241 CG2 VAL F 74 −11.227 −33.481 63.296 0.00 0.00
F
ATOM 18242 HG21 VAL F 74 −10.169 −33.309 63.591 0.00 0.00
F
ATOM 18243 HG22 VAL F 74 −11.813 −32.854 64.001 0.00 0.00
F
ATOM 18244 HG23 VAL F 74 −11.457 −34.525 63.598 0.00 0.00
F
ATOM 18245 C VAL F 74 −13.561 −32.833 60.273 0.00 0.00
F
ATOM 18246 O VAL F 74 −13.280 −33.472 59.233 0.00 0.00
F
ATOM 18247 N ILE F 75 −14.158 −31.681 60.146 0.00 0.00
F
ATOM 18248 HN ILE F 75 −14.363 −31.115 60.941 0.00 0.00
F
ATOM 18249 CA ILE F 75 −14.481 −31.121 58.831 0.00 0.00
F
ATOM 18250 HA ILE F 75 −14.332 −31.787 57.993 0.00 0.00
F
ATOM 18251 CB ILE F 75 −15.934 −30.674 58.812 0.00 0.00
F

```
ATOM  18252  HB  ILE F  75      -16.211 -29.994  59.646  0.00  0.00
F
ATOM  18253  CG2 ILE F  75      -16.286 -30.094  57.397  0.00  0.00
F
ATOM  18254 HG21 ILE F  75      -15.839 -30.717  56.593  0.00  0.00
F
ATOM  18255 HG22 ILE F  75      -17.386 -30.170  57.260  0.00  0.00
F
ATOM  18256 HG23 ILE F  75      -15.868 -29.075  57.252  0.00  0.00
F
ATOM  18257  CG1 ILE F  75      -16.706 -32.014  58.980  0.00  0.00
F
ATOM  18258 HG11 ILE F  75      -17.637 -32.054  58.375  0.00  0.00
F
ATOM  18259 HG12 ILE F  75      -16.076 -32.863  58.637  0.00  0.00
F
ATOM  18260  CD  ILE F  75      -17.136 -32.370  60.470  0.00  0.00
F
ATOM  18261  HD1 ILE F  75      -17.882 -31.690  60.935  0.00  0.00
F
ATOM  18262  HD2 ILE F  75      -17.553 -33.399  60.447  0.00  0.00
F
ATOM  18263  HD3 ILE F  75      -16.264 -32.399  61.159  0.00  0.00
F
ATOM  18264  C   ILE F  75      -13.614 -29.913  58.661  0.00  0.00
F
ATOM  18265  O   ILE F  75      -13.582 -28.994  59.461  0.00  0.00
F
ATOM  18266  N   VAL F  76      -12.822 -29.916  57.542  0.00  0.00
F
ATOM  18267  HN  VAL F  76      -12.904 -30.716  56.953  0.00  0.00
F
ATOM  18268  CA  VAL F  76      -12.176 -28.686  57.156  0.00  0.00
F
ATOM  18269  HA  VAL F  76      -12.039 -28.088  58.045  0.00  0.00
F
ATOM  18270  CB  VAL F  76      -10.809 -29.064  56.526  0.00  0.00
F
ATOM  18271  HB  VAL F  76      -10.936 -29.627  55.576  0.00  0.00
F
ATOM  18272  CG1 VAL F  76       -9.874 -27.900  56.264  0.00  0.00
F
ATOM  18273 HG11 VAL F  76      -10.417 -27.077  55.752  0.00  0.00
F
ATOM  18274 HG12 VAL F  76       -9.647 -27.431  57.246  0.00  0.00
F
ATOM  18275 HG13 VAL F  76       -8.923 -28.211  55.783  0.00  0.00
F
ATOM  18276  CG2 VAL F  76      -10.023 -29.931  57.538  0.00  0.00
F
ATOM  18277 HG21 VAL F  76       -9.096 -30.271  57.028  0.00  0.00
F
ATOM  18278 HG22 VAL F  76       -9.802 -29.277  58.409  0.00  0.00
F
```

ATOM 18279 HG23 VAL F 76   -10.576 -30.762 58.025 0.00 0.00
F
ATOM 18280 C   VAL F 76   -13.068 -27.935 56.198 0.00 0.00
F
ATOM 18281 O   VAL F 76   -13.495 -28.487 55.154 0.00 0.00
F
ATOM 18282 N   ALA F 77   -13.382 -26.650 56.541 0.00 0.00
F
ATOM 18283 HN ALA F 77   -13.009 -26.224 57.361 0.00 0.00
F
ATOM 18284 CA ALA F 77   -14.233 -25.791 55.722 0.00 0.00
F
ATOM 18285 HA ALA F 77   -14.705 -26.276 54.880 0.00 0.00
F
ATOM 18286 CB ALA F 77   -15.367 -25.340 56.664 0.00 0.00
F
ATOM 18287 HB1 ALA F 77   -14.948 -24.857 57.573 0.00 0.00
F
ATOM 18288 HB2 ALA F 77   -16.051 -24.734 56.032 0.00 0.00
F
ATOM 18289 HB3 ALA F 77   -15.946 -26.239 56.964 0.00 0.00
F
ATOM 18290 C   ALA F 77   -13.500 -24.677 55.025 0.00 0.00
F
ATOM 18291 O   ALA F 77   -14.217 -23.772 54.555 0.00 0.00
F
ATOM 18292 N   ILE F 78   -12.156 -24.604 55.017 0.00 0.00
F
ATOM 18293 HN ILE F 78   -11.623 -25.290 55.507 0.00 0.00
F
ATOM 18294 CA ILE F 78   -11.308 -23.775 54.238 0.00 0.00
F
ATOM 18295 HA ILE F 78   -12.099 -23.165 53.826 0.00 0.00
F
ATOM 18296 CB ILE F 78   -10.243 -23.027 55.167 0.00 0.00
F
ATOM 18297 HB ILE F 78   -10.837 -22.322 55.788 0.00 0.00
F
ATOM 18298 CG2 ILE F 78   -9.352 -23.975 56.025 0.00 0.00
F
ATOM 18299 HG21 ILE F 78   -8.584 -23.491 56.666 0.00 0.00
F
ATOM 18300 HG22 ILE F 78   -9.946 -24.732 56.581 0.00 0.00
F
ATOM 18301 HG23 ILE F 78   -8.767 -24.740 55.471 0.00 0.00
F
ATOM 18302 CG1 ILE F 78   -9.219 -22.117 54.453 0.00 0.00
F
ATOM 18303 HG11 ILE F 78   -8.594 -21.616 55.223 0.00 0.00
F
ATOM 18304 HG12 ILE F 78   -8.471 -22.764 53.947 0.00 0.00
F
ATOM 18305 CD ILE F 78   -9.653 -21.007 53.444 0.00 0.00
F

ATOM 18306 HD1 ILE F 78 −10.442 −20.345 53.861 0.00 0.00
F
ATOM 18307 HD2 ILE F 78 −8.751 −20.465 53.089 0.00 0.00
F
ATOM 18308 HD3 ILE F 78 −10.114 −21.426 52.524 0.00 0.00
F
ATOM 18309 C ILE F 78 −10.799 −24.620 53.086 0.00 0.00
F
ATOM 18310 O ILE F 78 −9.819 −25.377 53.140 0.00 0.00
F
ATOM 18311 N GLY F 79 −11.352 −24.355 51.855 0.00 0.00
F
ATOM 18312 HN GLY F 79 −11.974 −23.583 51.749 0.00 0.00
F
ATOM 18313 CA GLY F 79 −10.936 −24.924 50.610 0.00 0.00
F
ATOM 18314 HA1 GLY F 79 −11.862 −25.133 50.096 0.00 0.00
F
ATOM 18315 HA2 GLY F 79 −10.166 −25.660 50.788 0.00 0.00
F
ATOM 18316 C GLY F 79 −10.259 −23.968 49.765 0.00 0.00
F
ATOM 18317 O GLY F 79 −9.559 −24.275 48.781 0.00 0.00
F
ATOM 18318 N ALA F 80 −10.537 −22.624 49.933 0.00 0.00
F
ATOM 18319 HN ALA F 80 −11.285 −22.471 50.575 0.00 0.00
F
ATOM 18320 CA ALA F 80 −10.053 −21.550 49.153 0.00 0.00
F
ATOM 18321 HA ALA F 80 −10.232 −21.739 48.104 0.00 0.00
F
ATOM 18322 CB ALA F 80 −10.676 −20.277 49.647 0.00 0.00
F
ATOM 18323 HB1 ALA F 80 −10.636 −20.291 50.757 0.00 0.00
F
ATOM 18324 HB2 ALA F 80 −10.139 −19.405 49.217 0.00 0.00
F
ATOM 18325 HB3 ALA F 80 −11.746 −20.199 49.360 0.00 0.00
F
ATOM 18326 C ALA F 80 −8.570 −21.507 49.153 0.00 0.00
F
ATOM 18327 O ALA F 80 −7.840 −21.213 48.207 0.00 0.00
F
ATOM 18328 N ASN F 81 −7.948 −21.803 50.372 0.00 0.00
F
ATOM 18329 HN ASN F 81 −8.411 −22.122 51.195 0.00 0.00
F
ATOM 18330 CA ASN F 81 −6.440 −21.800 50.579 0.00 0.00
F
ATOM 18331 HA ASN F 81 −6.035 −21.047 49.920 0.00 0.00
F
ATOM 18332 CB ASN F 81 −6.147 −21.173 51.952 0.00 0.00
F

ATOM 18333 HB1 ASN F 81   -6.736 -20.243 52.104 0.00 0.00
F
ATOM 18334 HB2 ASN F 81   -6.255 -21.957 52.732 0.00 0.00
F
ATOM 18335 CG ASN F 81   -4.681 -20.759 51.987 0.00 0.00
F
ATOM 18336 OD1 ASN F 81   -3.916 -21.400 52.684 0.00 0.00
F
ATOM 18337 ND2 ASN F 81   -4.213 -19.727 51.263 0.00 0.00
F
ATOM 18338 HD21 ASN F 81   -3.231 -19.605 51.112 0.00 0.00
F
ATOM 18339 HD22 ASN F 81   -4.778 -19.287 50.565 0.00 0.00
F
ATOM 18340 C   ASN F 81   -5.846 -23.214 50.388 0.00 0.00
F
ATOM 18341 O   ASN F 81   -6.191 -24.144 51.169 0.00 0.00
F
ATOM 18342 N   ILE F 82   -5.010 -23.453 49.293 0.00 0.00
F
ATOM 18343 HN ILE F 82   -4.833 -22.804 48.556 0.00 0.00
F
ATOM 18344 CA ILE F 82   -4.355 -24.709 49.110 0.00 0.00
F
ATOM 18345 HA ILE F 82   -5.131 -25.454 49.200 0.00 0.00
F
ATOM 18346 CB ILE F 82   -3.607 -24.843 47.762 0.00 0.00
F
ATOM 18347 HB ILE F 82   -2.842 -24.038 47.724 0.00 0.00
F
ATOM 18348 CG2 ILE F 82   -3.078 -26.303 47.566 0.00 0.00
F
ATOM 18349 HG21 ILE F 82   -2.446 -26.671 48.402 0.00 0.00
F
ATOM 18350 HG22 ILE F 82   -3.978 -26.954 47.580 0.00 0.00
F
ATOM 18351 HG23 ILE F 82   -2.620 -26.480 46.569 0.00 0.00
F
ATOM 18352 CG1 ILE F 82   -4.606 -24.402 46.549 0.00 0.00
F
ATOM 18353 HG11 ILE F 82   -5.449 -25.113 46.682 0.00 0.00
F
ATOM 18354 HG12 ILE F 82   -4.922 -23.339 46.620 0.00 0.00
F
ATOM 18355 CD ILE F 82   -4.027 -24.617 45.107 0.00 0.00
F
ATOM 18356 HD1 ILE F 82   -4.210 -23.661 44.571 0.00 0.00
F
ATOM 18357 HD2 ILE F 82   -2.939 -24.751 45.289 0.00 0.00
F
ATOM 18358 HD3 ILE F 82   -4.524 -25.497 44.646 0.00 0.00
F
ATOM 18359 C   ILE F 82   -3.341 -24.967 50.241 0.00 0.00
F

ATOM 18360 O ILE F 82 -3.260 -26.104 50.695 0.00 0.00
F
ATOM 18361 N GLN F 83 -2.657 -23.952 50.739 0.00 0.00
F
ATOM 18362 HN GLN F 83 -2.891 -23.045 50.396 0.00 0.00
F
ATOM 18363 CA GLN F 83 -1.593 -24.098 51.744 0.00 0.00
F
ATOM 18364 HA GLN F 83 -0.847 -24.787 51.379 0.00 0.00
F
ATOM 18365 CB GLN F 83 -0.910 -22.762 52.042 0.00 0.00
F
ATOM 18366 HB1 GLN F 83 -0.344 -22.449 51.138 0.00 0.00
F
ATOM 18367 HB2 GLN F 83 -1.679 -22.023 52.351 0.00 0.00
F
ATOM 18368 CG GLN F 83 0.254 -22.960 53.118 0.00 0.00
F
ATOM 18369 HG1 GLN F 83 -0.083 -23.125 54.164 0.00 0.00
F
ATOM 18370 HG2 GLN F 83 1.004 -23.696 52.759 0.00 0.00
F
ATOM 18371 CD GLN F 83 1.083 -21.678 53.268 0.00 0.00
F
ATOM 18372 OE1 GLN F 83 2.103 -21.590 52.639 0.00 0.00
F
ATOM 18373 NE2 GLN F 83 0.577 -20.666 53.947 0.00 0.00
F
ATOM 18374 HE21 GLN F 83 1.066 -19.794 53.937 0.00 0.00
F
ATOM 18375 HE22 GLN F 83 -0.350 -20.750 54.311 0.00 0.00
F
ATOM 18376 C GLN F 83 -2.179 -24.610 53.042 0.00 0.00
F
ATOM 18377 O GLN F 83 -1.858 -25.645 53.617 0.00 0.00
F
ATOM 18378 N ALA F 84 -3.190 -23.891 53.634 0.00 0.00
F
ATOM 18379 HN ALA F 84 -3.479 -23.061 53.162 0.00 0.00
F
ATOM 18380 CA ALA F 84 -3.865 -24.435 54.771 0.00 0.00
F
ATOM 18381 HA ALA F 84 -3.047 -24.739 55.407 0.00 0.00
F
ATOM 18382 CB ALA F 84 -4.822 -23.469 55.500 0.00 0.00
F
ATOM 18383 HB1 ALA F 84 -4.453 -22.487 55.865 0.00 0.00
F
ATOM 18384 HB2 ALA F 84 -5.742 -23.265 54.912 0.00 0.00
F
ATOM 18385 HB3 ALA F 84 -5.033 -24.008 56.448 0.00 0.00
F
ATOM 18386 C ALA F 84 -4.654 -25.754 54.620 0.00 0.00
F

ATOM 18387 O   ALA F 84   -4.681 -26.484 55.628 0.00 0.00
F
ATOM 18388 N   SER F 85   -5.292 -26.062 53.533 0.00 0.00
F
ATOM 18389 HN SER F 85   -5.295 -25.379 52.806 0.00 0.00
F
ATOM 18390 CA SER F 85   -6.004 -27.326 53.340 0.00 0.00
F
ATOM 18391 HA SER F 85   -6.590 -27.387 54.245 0.00 0.00
F
ATOM 18392 CB SER F 85   -6.943 -27.305 52.107 0.00 0.00
F
ATOM 18393 HB1 SER F 85   -7.311 -28.344 51.969 0.00 0.00
F
ATOM 18394 HB2 SER F 85   -7.784 -26.629 52.372 0.00 0.00
F
ATOM 18395 OG SER F 85   -6.321 -26.791 50.839 0.00 0.00
F
ATOM 18396 HG1 SER F 85   -6.359 -25.832 50.826 0.00 0.00
F
ATOM 18397 C   SER F 85   -5.067 -28.538 53.304 0.00 0.00
F
ATOM 18398 O   SER F 85   -5.207 -29.469 54.091 0.00 0.00
F
ATOM 18399 N   THR F 86   -4.024 -28.469 52.459 0.00 0.00
F
ATOM 18400 HN THR F 86   -3.857 -27.618 51.967 0.00 0.00
F
ATOM 18401 CA THR F 86   -3.006 -29.521 52.347 0.00 0.00
F
ATOM 18402 HA THR F 86   -3.425 -30.505 52.199 0.00 0.00
F
ATOM 18403 CB THR F 86   -1.999 -29.285 51.252 0.00 0.00
F
ATOM 18404 HB THR F 86   -1.154 -30.005 51.293 0.00 0.00
F
ATOM 18405 OG1 THR F 86   -1.464 -27.944 51.351 0.00 0.00
F
ATOM 18406 HG1 THR F 86   -2.174 -27.322 51.174 0.00 0.00
F
ATOM 18407 CG2 THR F 86   -2.690 -29.379 49.942 0.00 0.00
F
ATOM 18408 HG21 THR F 86   -3.435 -28.558 49.862 0.00 0.00
F
ATOM 18409 HG22 THR F 86   -2.000 -29.254 49.081 0.00 0.00
F
ATOM 18410 HG23 THR F 86   -3.246 -30.326 49.771 0.00 0.00
F
ATOM 18411 C   THR F 86   -2.275 -29.744 53.601 0.00 0.00
F
ATOM 18412 O   THR F 86   -2.182 -30.854 54.131 0.00 0.00
F
ATOM 18413 N   LEU F 87   -1.795 -28.694 54.327 0.00 0.00
F

ATOM 18414 HN LEU F 87   -1.956 -27.757 54.028 0.00 0.00
F
ATOM 18415 CA LEU F 87   -1.257 -28.861 55.680 0.00 0.00
F
ATOM 18416 HA LEU F 87   -0.434 -29.558 55.613 0.00 0.00
F
ATOM 18417 CB LEU F 87   -0.617 -27.526 56.109 0.00 0.00
F
ATOM 18418 HB1 LEU F 87   -1.388 -26.729 56.042 0.00 0.00
F
ATOM 18419 HB2 LEU F 87   -0.373 -27.557 57.193 0.00 0.00
F
ATOM 18420 CG LEU F 87   0.662 -27.079 55.325 0.00 0.00
F
ATOM 18421 HG LEU F 87   0.431 -26.875 54.258 0.00 0.00
F
ATOM 18422 CD1 LEU F 87   1.213 -25.744 55.925 0.00 0.00
F
ATOM 18423 HD11 LEU F 87   1.566 -25.920 56.964 0.00 0.00
F
ATOM 18424 HD12 LEU F 87   1.945 -25.219 55.274 0.00 0.00
F
ATOM 18425 HD13 LEU F 87   0.347 -25.049 55.940 0.00 0.00
F
ATOM 18426 CD2 LEU F 87   1.809 -28.169 55.388 0.00 0.00
F
ATOM 18427 HD21 LEU F 87   2.773 -27.939 54.887 0.00 0.00
F
ATOM 18428 HD22 LEU F 87   2.075 -28.275 56.462 0.00 0.00
F
ATOM 18429 HD23 LEU F 87   1.376 -29.058 54.882 0.00 0.00
F
ATOM 18430 C   LEU F 87   -2.170 -29.374 56.736 0.00 0.00
F
ATOM 18431 O   LEU F 87   -1.768 -30.318 57.444 0.00 0.00
F
ATOM 18432 N   THR F 88   -3.462 -28.812 56.813 0.00 0.00
F
ATOM 18433 HN THR F 88   -3.710 -28.158 56.103 0.00 0.00
F
ATOM 18434 CA THR F 88   -4.435 -29.226 57.812 0.00 0.00
F
ATOM 18435 HA THR F 88   -3.992 -29.156 58.794 0.00 0.00
F
ATOM 18436 CB THR F 88   -5.745 -28.416 57.798 0.00 0.00
F
ATOM 18437 HB THR F 88   -6.247 -28.450 56.807 0.00 0.00
F
ATOM 18438 OG1 THR F 88   -5.470 -27.088 58.074 0.00 0.00
F
ATOM 18439 HG1 THR F 88   -4.932 -26.720 57.369 0.00 0.00
F
ATOM 18440 CG2 THR F 88   -6.719 -28.887 58.862 0.00 0.00
F

```
ATOM  18441 HG21 THR F  88      -6.355 -28.668  59.889  0.00  0.00
F
ATOM  18442 HG22 THR F  88      -7.679 -28.337  58.756  0.00  0.00
F
ATOM  18443 HG23 THR F  88      -6.908 -29.980  58.802  0.00  0.00
F
ATOM  18444  C   THR F  88      -4.716 -30.741  57.583  0.00  0.00
F
ATOM  18445  O   THR F  88      -4.765 -31.490  58.599  0.00  0.00
F
ATOM  18446  N   THR F  89      -4.938 -31.162  56.275  0.00  0.00
F
ATOM  18447  HN  THR F  89      -5.093 -30.539  55.512  0.00  0.00
F
ATOM  18448  CA  THR F  89      -5.152 -32.588  55.897  0.00  0.00
F
ATOM  18449  HA  THR F  89      -6.008 -32.904  56.474  0.00  0.00
F
ATOM  18450  CB  THR F  89      -5.288 -32.701  54.360  0.00  0.00
F
ATOM  18451  HB  THR F  89      -4.353 -32.384  53.850  0.00  0.00
F
ATOM  18452  OG1 THR F  89      -6.322 -31.827  53.890  0.00  0.00
F
ATOM  18453  HG1 THR F  89      -6.036 -30.911  53.916  0.00  0.00
F
ATOM  18454  CG2 THR F  89      -5.697 -34.059  53.894  0.00  0.00
F
ATOM  18455 HG21 THR F  89      -4.927 -34.850  54.023  0.00  0.00
F
ATOM  18456 HG22 THR F  89      -6.697 -34.419  54.219  0.00  0.00
F
ATOM  18457 HG23 THR F  89      -5.901 -33.898  52.814  0.00  0.00
F
ATOM  18458  C   THR F  89      -3.947 -33.494  56.149  0.00  0.00
F
ATOM  18459  O   THR F  89      -4.109 -34.628  56.648  0.00  0.00
F
ATOM  18460  N   LEU F  90      -2.684 -33.036  55.845  0.00  0.00
F
ATOM  18461  HN  LEU F  90      -2.590 -32.187  55.331  0.00  0.00
F
ATOM  18462  CA  LEU F  90      -1.512 -33.699  56.406  0.00  0.00
F
ATOM  18463  HA  LEU F  90      -1.447 -34.717  56.051  0.00  0.00
F
ATOM  18464  CB  LEU F  90      -0.179 -32.902  56.127  0.00  0.00
F
ATOM  18465  HB1 LEU F  90      -0.219 -32.734  55.029  0.00  0.00
F
ATOM  18466  HB2 LEU F  90      -0.243 -31.950  56.696  0.00  0.00
F
ATOM  18467  CG  LEU F  90       1.156 -33.627  56.527  0.00  0.00
F
```

ATOM 18468 HG LEU F 90   1.174 -33.592 57.637 0.00 0.00
F
ATOM 18469 CD1 LEU F 90   1.335 -35.104 56.091 0.00 0.00
F
ATOM 18470 HD11 LEU F 90   0.607 -35.875 56.423 0.00 0.00
F
ATOM 18471 HD12 LEU F 90   1.385 -35.202 54.985 0.00 0.00
F
ATOM 18472 HD13 LEU F 90   2.238 -35.532 56.576 0.00 0.00
F
ATOM 18473 CD2 LEU F 90   2.300 -32.728 56.022 0.00 0.00
F
ATOM 18474 HD21 LEU F 90   3.307 -32.955 56.431 0.00 0.00
F
ATOM 18475 HD22 LEU F 90   2.474 -32.839 54.930 0.00 0.00
F
ATOM 18476 HD23 LEU F 90   2.115 -31.698 56.397 0.00 0.00
F
ATOM 18477 C   LEU F 90   -1.598 -34.007 57.901 0.00 0.00
F
ATOM 18478 O   LEU F 90   -1.559 -35.135 58.378 0.00 0.00
F
ATOM 18479 N   LEU F 91   -1.864 -32.927 58.680 0.00 0.00
F
ATOM 18480 HN LEU F 91   -2.062 -32.078 58.198 0.00 0.00
F
ATOM 18481 CA LEU F 91   -1.870 -33.024 60.104 0.00 0.00
F
ATOM 18482 HA LEU F 91   -0.981 -33.570 60.381 0.00 0.00
F
ATOM 18483 CB LEU F 91   -1.874 -31.715 60.820 0.00 0.00
F
ATOM 18484 HB1 LEU F 91   -2.811 -31.131 60.696 0.00 0.00
F
ATOM 18485 HB2 LEU F 91   -1.721 -31.787 61.918 0.00 0.00
F
ATOM 18486 CG LEU F 91   -0.652 -30.802 60.381 0.00 0.00
F
ATOM 18487 HG LEU F 91   -0.721 -30.755 59.273 0.00 0.00
F
ATOM 18488 CD1 LEU F 91   -0.821 -29.340 60.846 0.00 0.00
F
ATOM 18489 HD11 LEU F 91   -1.706 -28.955 60.296 0.00 0.00
F
ATOM 18490 HD12 LEU F 91   -0.966 -29.215 61.941 0.00 0.00
F
ATOM 18491 HD13 LEU F 91   0.017 -28.667 60.565 0.00 0.00
F
ATOM 18492 CD2 LEU F 91   0.669 -31.335 60.924 0.00 0.00
F
ATOM 18493 HD21 LEU F 91   0.794 -32.397 60.622 0.00 0.00
F
ATOM 18494 HD22 LEU F 91   1.564 -30.806 60.531 0.00 0.00
F

ATOM 18495 HD23 LEU F 91 0.693 -31.410 62.032 0.00 0.00
F
ATOM 18496 C LEU F 91 -2.975 -33.882 60.707 0.00 0.00
F
ATOM 18497 O LEU F 91 -2.649 -34.726 61.567 0.00 0.00
F
ATOM 18498 N LEU F 92 -4.286 -33.827 60.354 0.00 0.00
F
ATOM 18499 HN LEU F 92 -4.609 -33.085 59.771 0.00 0.00
F
ATOM 18500 CA LEU F 92 -5.267 -34.734 60.901 0.00 0.00
F
ATOM 18501 HA LEU F 92 -5.368 -34.564 61.962 0.00 0.00
F
ATOM 18502 CB LEU F 92 -6.705 -34.369 60.266 0.00 0.00
F
ATOM 18503 HB1 LEU F 92 -6.514 -34.364 59.172 0.00 0.00
F
ATOM 18504 HB2 LEU F 92 -7.417 -35.150 60.609 0.00 0.00
F
ATOM 18505 CG LEU F 92 -7.164 -32.917 60.652 0.00 0.00
F
ATOM 18506 HG LEU F 92 -6.386 -32.144 60.478 0.00 0.00
F
ATOM 18507 CD1 LEU F 92 -8.270 -32.598 59.684 0.00 0.00
F
ATOM 18508 HD11 LEU F 92 -9.119 -33.301 59.822 0.00 0.00
F
ATOM 18509 HD12 LEU F 92 -8.740 -31.595 59.766 0.00 0.00
F
ATOM 18510 HD13 LEU F 92 -7.887 -32.648 58.642 0.00 0.00
F
ATOM 18511 CD2 LEU F 92 -7.536 -32.865 62.204 0.00 0.00
F
ATOM 18512 HD21 LEU F 92 -6.587 -33.003 62.764 0.00 0.00
F
ATOM 18513 HD22 LEU F 92 -7.728 -31.799 62.449 0.00 0.00
F
ATOM 18514 HD23 LEU F 92 -8.304 -33.603 62.521 0.00 0.00
F
ATOM 18515 C LEU F 92 -4.915 -36.248 60.544 0.00 0.00
F
ATOM 18516 O LEU F 92 -5.310 -37.190 61.270 0.00 0.00
F
ATOM 18517 N LYS F 93 -4.280 -36.559 59.453 0.00 0.00
F
ATOM 18518 HN LYS F 93 -4.010 -35.778 58.896 0.00 0.00
F
ATOM 18519 CA LYS F 93 -3.919 -37.933 59.028 0.00 0.00
F
ATOM 18520 HA LYS F 93 -4.716 -38.631 59.239 0.00 0.00
F
ATOM 18521 CB LYS F 93 -3.622 -37.925 57.479 0.00 0.00
F

```
ATOM   18522  HB1 LYS F  93      -4.340 -37.233  56.989  0.00  0.00
F
ATOM   18523  HB2 LYS F  93      -2.616 -37.494  57.290  0.00  0.00
F
ATOM   18524  CG  LYS F  93      -3.779 -39.268  56.794  0.00  0.00
F
ATOM   18525  HG1 LYS F  93      -3.480 -39.322  55.726  0.00  0.00
F
ATOM   18526  HG2 LYS F  93      -3.240 -40.065  57.348  0.00  0.00
F
ATOM   18527  CD  LYS F  93      -5.252 -39.816  56.783  0.00  0.00
F
ATOM   18528  HD1 LYS F  93      -5.707 -39.563  57.764  0.00  0.00
F
ATOM   18529  HD2 LYS F  93      -5.946 -39.233  56.141  0.00  0.00
F
ATOM   18530  CE  LYS F  93      -5.354 -41.348  56.481  0.00  0.00
F
ATOM   18531  HE1 LYS F  93      -5.136 -41.544  55.410  0.00  0.00
F
ATOM   18532  HE2 LYS F  93      -4.585 -42.018  56.922  0.00  0.00
F
ATOM   18533  NZ  LYS F  93      -6.706 -41.795  56.777  0.00  0.00
F
ATOM   18534  HZ1 LYS F  93      -6.592 -42.817  56.932  0.00  0.00
F
ATOM   18535  HZ2 LYS F  93      -7.109 -41.383  57.643  0.00  0.00
F
ATOM   18536  HZ3 LYS F  93      -7.411 -41.681  56.021  0.00  0.00
F
ATOM   18537  C   LYS F  93      -2.703 -38.465  59.772  0.00  0.00
F
ATOM   18538  O   LYS F  93      -2.574 -39.688  59.966  0.00  0.00
F
ATOM   18539  N   GLU F  94      -1.818 -37.558  60.322  0.00  0.00
F
ATOM   18540  HN  GLU F  94      -2.005 -36.608  60.081  0.00  0.00
F
ATOM   18541  CA  GLU F  94      -0.777 -37.843  61.311  0.00  0.00
F
ATOM   18542  HA  GLU F  94      -0.228 -38.750  61.105  0.00  0.00
F
ATOM   18543  CB  GLU F  94       0.345 -36.685  61.372  0.00  0.00
F
ATOM   18544  HB1 GLU F  94      -0.144 -35.688  61.353  0.00  0.00
F
ATOM   18545  HB2 GLU F  94       0.925 -36.782  62.314  0.00  0.00
F
ATOM   18546  CG  GLU F  94       1.365 -36.678  60.266  0.00  0.00
F
ATOM   18547  HG1 GLU F  94       0.692 -36.679  59.382  0.00  0.00
F
ATOM   18548  HG2 GLU F  94       1.950 -35.746  60.112  0.00  0.00
F
```

ATOM 18549 CD GLU F 94   2.258 -37.910 60.139 0.00 0.00
F
ATOM 18550 OE1 GLU F 94   1.893 -38.763 59.295 0.00 0.00
F
ATOM 18551 OE2 GLU F 94   3.278 -37.993 60.832 0.00 0.00
F
ATOM 18552 C   GLU F 94   -1.287 -37.886 62.795 0.00 0.00
F
ATOM 18553 O   GLU F 94   -0.633 -38.533 63.640 0.00 0.00
F
ATOM 18554 N   LEU F 95   -2.518 -37.319 63.113 0.00 0.00
F
ATOM 18555 HN LEU F 95   -2.966 -36.598 62.589 0.00 0.00
F
ATOM 18556 CA LEU F 95   -3.053 -37.552 64.459 0.00 0.00
F
ATOM 18557 HA LEU F 95   -2.311 -37.813 65.200 0.00 0.00
F
ATOM 18558 CB LEU F 95   -3.912 -36.339 64.907 0.00 0.00
F
ATOM 18559 HB1 LEU F 95   -4.712 -36.221 64.144 0.00 0.00
F
ATOM 18560 HB2 LEU F 95   -4.436 -36.512 65.871 0.00 0.00
F
ATOM 18561 CG LEU F 95   -3.291 -34.990 65.172 0.00 0.00
F
ATOM 18562 HG LEU F 95   -2.927 -34.640 64.182 0.00 0.00
F
ATOM 18563 CD1 LEU F 95   -4.500 -34.019 65.576 0.00 0.00
F
ATOM 18564 HD11 LEU F 95   -4.889 -34.354 66.561 0.00 0.00
F
ATOM 18565 HD12 LEU F 95   -4.176 -32.963 65.460 0.00 0.00
F
ATOM 18566 HD13 LEU F 95   -5.308 -34.248 64.847 0.00 0.00
F
ATOM 18567 CD2 LEU F 95   -2.297 -34.941 66.346 0.00 0.00
F
ATOM 18568 HD21 LEU F 95   -2.839 -35.446 67.175 0.00 0.00
F
ATOM 18569 HD22 LEU F 95   -1.428 -35.563 66.042 0.00 0.00
F
ATOM 18570 HD23 LEU F 95   -1.930 -33.899 66.462 0.00 0.00
F
ATOM 18571 C   LEU F 95   -4.053 -38.753 64.413 0.00 0.00
F
ATOM 18572 O   LEU F 95   -4.683 -39.050 65.398 0.00 0.00
F
ATOM 18573 N   ASP F 96   -4.234 -39.325 63.265 0.00 0.00
F
ATOM 18574 HN ASP F 96   -3.845 -38.917 62.443 0.00 0.00
F
ATOM 18575 CA ASP F 96   -4.903 -40.549 62.971 0.00 0.00
F

ATOM 18576 HA ASP F 96   -4.743 -40.794 61.931 0.00 0.00
F
ATOM 18577 CB ASP F 96   -4.325 -41.742 63.754 0.00 0.00
F
ATOM 18578 HB1 ASP F 96   -4.068 -41.626 64.829 0.00 0.00
F
ATOM 18579 HB2 ASP F 96   -5.038 -42.586 63.639 0.00 0.00
F
ATOM 18580 CG ASP F 96   -3.107 -42.315 63.124 0.00 0.00
F
ATOM 18581 OD1 ASP F 96   -3.196 -43.092 62.184 0.00 0.00
F
ATOM 18582 OD2 ASP F 96   -1.995 -41.932 63.531 0.00 0.00
F
ATOM 18583 C   ASP F 96   -6.431 -40.439 63.229 0.00 0.00
F
ATOM 18584 O   ASP F 96   -6.963 -41.271 63.974 0.00 0.00
F
ATOM 18585 N   ILE F 97   -7.155 -39.458 62.593 0.00 0.00
F
ATOM 18586 HN ILE F 97   -6.613 -38.669 62.313 0.00 0.00
F
ATOM 18587 CA ILE F 97   -8.571 -39.279 62.788 0.00 0.00
F
ATOM 18588 HA ILE F 97   -8.876 -39.434 63.813 0.00 0.00
F
ATOM 18589 CB ILE F 97   -9.006 -37.865 62.513 0.00 0.00
F
ATOM 18590 HB ILE F 97   -8.706 -37.691 61.457 0.00 0.00
F
ATOM 18591 CG2 ILE F 97   -10.535 -37.758 62.578 0.00 0.00
F
ATOM 18592 HG21 ILE F 97   -10.862 -37.816 63.638 0.00 0.00
F
ATOM 18593 HG22 ILE F 97   -10.765 -36.689 62.380 0.00 0.00
F
ATOM 18594 HG23 ILE F 97   -11.211 -38.447 62.028 0.00 0.00
F
ATOM 18595 CG1 ILE F 97   -8.187 -36.766 63.257 0.00 0.00
F
ATOM 18596 HG11 ILE F 97   -7.169 -36.676 62.820 0.00 0.00
F
ATOM 18597 HG12 ILE F 97   -8.657 -35.790 63.012 0.00 0.00
F
ATOM 18598 CD ILE F 97   -8.144 -36.950 64.762 0.00 0.00
F
ATOM 18599 HD1 ILE F 97   -7.618 -36.120 65.281 0.00 0.00
F
ATOM 18600 HD2 ILE F 97   -9.189 -36.973 65.138 0.00 0.00
F
ATOM 18601 HD3 ILE F 97   -7.718 -37.942 65.026 0.00 0.00
F
ATOM 18602 C   ILE F 97   -9.364 -40.222 61.989 0.00 0.00
F

```
ATOM  18603  O   ILE F  97      -8.997 -40.393  60.821  0.00  0.00
F
ATOM  18604  N   PRO F  98     -10.403 -40.941  62.490  0.00  0.00
F
ATOM  18605  CD  PRO F  98     -10.592 -41.302  63.899  0.00  0.00
F
ATOM  18606  HD1 PRO F  98      -9.640 -41.645  64.359  0.00  0.00
F
ATOM  18607  HD2 PRO F  98     -10.919 -40.388  64.440  0.00  0.00
F
ATOM  18608  CA  PRO F  98     -11.302 -41.795  61.637  0.00  0.00
F
ATOM  18609  HA  PRO F  98     -10.603 -42.501  61.214  0.00  0.00
F
ATOM  18610  CB  PRO F  98     -12.328 -42.275  62.625  0.00  0.00
F
ATOM  18611  HB1 PRO F  98     -12.656 -43.302  62.357  0.00  0.00
F
ATOM  18612  HB2 PRO F  98     -13.265 -41.680  62.657  0.00  0.00
F
ATOM  18613  CG  PRO F  98     -11.567 -42.466  63.924  0.00  0.00
F
ATOM  18614  HG1 PRO F  98     -10.943 -43.380  63.833  0.00  0.00
F
ATOM  18615  HG2 PRO F  98     -12.127 -42.448  64.884  0.00  0.00
F
ATOM  18616  C   PRO F  98     -12.054 -41.143  60.465  0.00  0.00
F
ATOM  18617  O   PRO F  98     -12.212 -41.741  59.402  0.00  0.00
F
ATOM  18618  N   ASN F  99     -12.585 -39.905  60.612  0.00  0.00
F
ATOM  18619  HN  ASN F  99     -12.403 -39.353  61.422  0.00  0.00
F
ATOM  18620  CA  ASN F  99     -13.289 -39.199  59.545  0.00  0.00
F
ATOM  18621  HA  ASN F  99     -13.005 -39.689  58.626  0.00  0.00
F
ATOM  18622  CB  ASN F  99     -14.758 -39.095  59.773  0.00  0.00
F
ATOM  18623  HB1 ASN F  99     -15.080 -38.547  60.684  0.00  0.00
F
ATOM  18624  HB2 ASN F  99     -15.246 -38.731  58.844  0.00  0.00
F
ATOM  18625  CG  ASN F  99     -15.422 -40.478  59.900  0.00  0.00
F
ATOM  18626  OD1 ASN F  99     -15.868 -41.143  58.963  0.00  0.00
F
ATOM  18627  ND2 ASN F  99     -15.626 -40.949  61.160  0.00  0.00
F
ATOM  18628 HD21 ASN F  99     -16.147 -41.791  61.303  0.00  0.00
F
ATOM  18629 HD22 ASN F  99     -15.294 -40.446  61.958  0.00  0.00
F
```

ATOM 18630 C   ASN F 99   -12.738 -37.778 59.366 0.00 0.00
F
ATOM 18631 O   ASN F 99   -13.023 -36.865 60.115 0.00 0.00
F
ATOM 18632 N   ILE F 100   -12.064 -37.620 58.238 0.00 0.00
F
ATOM 18633 HN ILE F 100   -11.817 -38.425 57.703 0.00 0.00
F
ATOM 18634 CA ILE F 100   -11.409 -36.381 57.728 0.00 0.00
F
ATOM 18635 HA ILE F 100   -11.492 -35.638 58.507 0.00 0.00
F
ATOM 18636 CB ILE F 100   -9.966 -36.547 57.395 0.00 0.00
F
ATOM 18637 HB ILE F 100   -9.882 -37.411 56.702 0.00 0.00
F
ATOM 18638 CG2 ILE F 100   -9.322 -35.256 56.838 0.00 0.00
F
ATOM 18639 HG21 ILE F 100   -9.720 -35.043 55.822 0.00 0.00
F
ATOM 18640 HG22 ILE F 100   -9.531 -34.453 57.577 0.00 0.00
F
ATOM 18641 HG23 ILE F 100   -8.218 -35.276 56.716 0.00 0.00
F
ATOM 18642 CG1 ILE F 100   -9.124 -37.258 58.587 0.00 0.00
F
ATOM 18643 HG11 ILE F 100   -9.379 -36.792 59.563 0.00 0.00
F
ATOM 18644 HG12 ILE F 100   -9.595 -38.260 58.671 0.00 0.00
F
ATOM 18645 CD ILE F 100   -7.598 -37.375 58.289 0.00 0.00
F
ATOM 18646 HD1 ILE F 100   -7.322 -37.796 57.298 0.00 0.00
F
ATOM 18647 HD2 ILE F 100   -7.058 -36.407 58.353 0.00 0.00
F
ATOM 18648 HD3 ILE F 100   -7.068 -37.947 59.081 0.00 0.00
F
ATOM 18649 C   ILE F 100   -12.126 -35.869 56.524 0.00 0.00
F
ATOM 18650 O   ILE F 100   -12.151 -36.553 55.483 0.00 0.00
F
ATOM 18651 N   TRP F 101   -12.872 -34.774 56.614 0.00 0.00
F
ATOM 18652 HN TRP F 101   -12.782 -34.274 57.472 0.00 0.00
F
ATOM 18653 CA TRP F 101   -13.798 -34.364 55.586 0.00 0.00
F
ATOM 18654 HA TRP F 101   -13.669 -34.912 54.664 0.00 0.00
F
ATOM 18655 CB TRP F 101   -15.240 -34.377 56.117 0.00 0.00
F
ATOM 18656 HB1 TRP F 101   -15.298 -33.934 57.135 0.00 0.00
F

```
ATOM  18657  HB2 TRP F 101     -15.954 -33.853  55.446  0.00  0.00
F
ATOM  18658  CG  TRP F 101     -16.013 -35.680  56.242  0.00  0.00
F
ATOM  18659  CD1 TRP F 101     -15.525 -36.952  56.471  0.00  0.00
F
ATOM  18660  HD1 TRP F 101     -14.478 -37.214  56.458  0.00  0.00
F
ATOM  18661  NE1 TRP F 101     -16.535 -37.934  56.404  0.00  0.00
F
ATOM  18662  HE1 TRP F 101     -16.416 -38.900  56.325  0.00  0.00
F
ATOM  18663  CE2 TRP F 101     -17.711 -37.263  56.118  0.00  0.00
F
ATOM  18664  CD2 TRP F 101     -17.444 -35.850  56.059  0.00  0.00
F
ATOM  18665  CE3 TRP F 101     -18.470 -34.904  55.757  0.00  0.00
F
ATOM  18666  HE3 TRP F 101     -18.300 -33.838  55.767  0.00  0.00
F
ATOM  18667  CZ3 TRP F 101     -19.775 -35.425  55.686  0.00  0.00
F
ATOM  18668  HZ3 TRP F 101     -20.625 -34.803  55.451  0.00  0.00
F
ATOM  18669  CZ2 TRP F 101     -19.060 -37.753  56.153  0.00  0.00
F
ATOM  18670  HZ2 TRP F 101     -19.193 -38.825  56.143  0.00  0.00
F
ATOM  18671  CH2 TRP F 101     -20.049 -36.781  55.969  0.00  0.00
F
ATOM  18672  HH2 TRP F 101     -21.086 -37.079  55.913  0.00  0.00
F
ATOM  18673  C   TRP F 101     -13.277 -32.926  55.154  0.00  0.00
F
ATOM  18674  O   TRP F 101     -13.251 -32.109  56.047  0.00  0.00
F
ATOM  18675  N   VAL F 102     -12.942 -32.756  53.853  0.00  0.00
F
ATOM  18676  HN  VAL F 102     -12.979 -33.557  53.260  0.00  0.00
F
ATOM  18677  CA  VAL F 102     -12.215 -31.643  53.458  0.00  0.00
F
ATOM  18678  HA  VAL F 102     -12.095 -30.856  54.189  0.00  0.00
F
ATOM  18679  CB  VAL F 102     -10.743 -32.020  53.099  0.00  0.00
F
ATOM  18680  HB  VAL F 102     -10.792 -32.659  52.192  0.00  0.00
F
ATOM  18681  CG1 VAL F 102      -9.820 -30.828  52.889  0.00  0.00
F
ATOM  18682 HG11 VAL F 102      -8.818 -31.220  52.615  0.00  0.00
F
ATOM  18683 HG12 VAL F 102     -10.204 -30.131  52.113  0.00  0.00
F
```

```
ATOM  18684 HG13 VAL F 102      -9.693 -30.412  53.911  0.00  0.00
F
ATOM  18685  CG2 VAL F 102     -10.125 -32.897  54.243  0.00  0.00
F
ATOM  18686 HG21 VAL F 102      -9.075 -33.077  53.928  0.00  0.00
F
ATOM  18687 HG22 VAL F 102     -10.260 -32.353  55.202  0.00  0.00
F
ATOM  18688 HG23 VAL F 102     -10.761 -33.802  54.343  0.00  0.00
F
ATOM  18689  C   VAL F 102     -12.919 -30.818  52.311  0.00  0.00
F
ATOM  18690  O   VAL F 102     -13.285 -31.340  51.262  0.00  0.00
F
ATOM  18691  N   LYS F 103     -13.257 -29.550  52.409  0.00  0.00
F
ATOM  18692  HN  LYS F 103     -13.340 -29.196  53.337  0.00  0.00
F
ATOM  18693  CA  LYS F 103     -13.628 -28.599  51.350  0.00  0.00
F
ATOM  18694  HA  LYS F 103     -14.491 -29.087  50.921  0.00  0.00
F
ATOM  18695  CB  LYS F 103     -14.133 -27.314  52.065  0.00  0.00
F
ATOM  18696  HB1 LYS F 103     -14.739 -27.677  52.922  0.00  0.00
F
ATOM  18697  HB2 LYS F 103     -13.283 -26.826  52.588  0.00  0.00
F
ATOM  18698  CG  LYS F 103     -14.793 -26.254  51.097  0.00  0.00
F
ATOM  18699  HG1 LYS F 103     -14.133 -26.099  50.217  0.00  0.00
F
ATOM  18700  HG2 LYS F 103     -15.742 -26.665  50.691  0.00  0.00
F
ATOM  18701  CD  LYS F 103     -15.024 -24.948  51.806  0.00  0.00
F
ATOM  18702  HD1 LYS F 103     -15.282 -25.167  52.864  0.00  0.00
F
ATOM  18703  HD2 LYS F 103     -14.046 -24.442  51.960  0.00  0.00
F
ATOM  18704  CE  LYS F 103     -16.018 -23.927  51.130  0.00  0.00
F
ATOM  18705  HE1 LYS F 103     -17.070 -24.282  51.150  0.00  0.00
F
ATOM  18706  HE2 LYS F 103     -15.983 -23.051  51.813  0.00  0.00
F
ATOM  18707  NZ  LYS F 103     -15.561 -23.512  49.818  0.00  0.00
F
ATOM  18708  HZ1 LYS F 103     -14.794 -22.811  49.865  0.00  0.00
F
ATOM  18709  HZ2 LYS F 103     -15.330 -24.320  49.206  0.00  0.00
F
ATOM  18710  HZ3 LYS F 103     -16.320 -23.019  49.306  0.00  0.00
F
```

```
ATOM  18711  C   LYS F 103     -12.577 -28.350  50.290  0.00  0.00
F
ATOM  18712  O   LYS F 103     -11.481 -27.928  50.598  0.00  0.00
F
ATOM  18713  N   ALA F 104     -12.849 -28.592  49.003  0.00  0.00
F
ATOM  18714  HN  ALA F 104     -13.603 -29.203  48.772  0.00  0.00
F
ATOM  18715  CA  ALA F 104     -11.879 -28.393  47.997  0.00  0.00
F
ATOM  18716  HA  ALA F 104     -10.924 -28.072  48.387  0.00  0.00
F
ATOM  18717  CB  ALA F 104     -11.639 -29.761  47.292  0.00  0.00
F
ATOM  18718  HB1 ALA F 104     -11.209 -30.486  48.016  0.00  0.00
F
ATOM  18719  HB2 ALA F 104     -12.594 -30.161  46.887  0.00  0.00
F
ATOM  18720  HB3 ALA F 104     -10.922 -29.645  46.451  0.00  0.00
F
ATOM  18721  C   ALA F 104     -12.512 -27.439  47.024  0.00  0.00
F
ATOM  18722  O   ALA F 104     -13.767 -27.497  46.837  0.00  0.00
F
ATOM  18723  N   GLN F 105     -11.790 -26.537  46.309  0.00  0.00
F
ATOM  18724  HN  GLN F 105     -10.833 -26.450  46.576  0.00  0.00
F
ATOM  18725  CA  GLN F 105     -12.449 -25.615  45.421  0.00  0.00
F
ATOM  18726  HA  GLN F 105     -13.444 -25.371  45.764  0.00  0.00
F
ATOM  18727  CB  GLN F 105     -11.659 -24.272  45.295  0.00  0.00
F
ATOM  18728  HB1 GLN F 105     -10.967 -24.111  46.149  0.00  0.00
F
ATOM  18729  HB2 GLN F 105     -10.927 -24.437  44.476  0.00  0.00
F
ATOM  18730  CG  GLN F 105     -12.480 -22.982  45.063  0.00  0.00
F
ATOM  18731  HG1 GLN F 105     -13.063 -22.836  44.129  0.00  0.00
F
ATOM  18732  HG2 GLN F 105     -13.214 -22.935  45.896  0.00  0.00
F
ATOM  18733  CD  GLN F 105     -11.552 -21.767  45.374  0.00  0.00
F
ATOM  18734  OE1 GLN F 105     -11.642 -21.092  46.436  0.00  0.00
F
ATOM  18735  NE2 GLN F 105     -10.611 -21.488  44.459  0.00  0.00
F
ATOM  18736 HE21 GLN F 105      -9.995 -20.721  44.638  0.00  0.00
F
ATOM  18737 HE22 GLN F 105     -10.632 -21.876  43.538  0.00  0.00
F
```

```
ATOM  18738  C   GLN F 105     -12.663 -26.199  44.005  0.00  0.00
F
ATOM  18739  O   GLN F 105     -13.562 -25.755  43.261  0.00  0.00
F
ATOM  18740  N   ASN F 106     -11.830 -27.174  43.557  0.00  0.00
F
ATOM  18741  HN  ASN F 106     -10.984 -27.397  44.035  0.00  0.00
F
ATOM  18742  CA  ASN F 106     -11.855 -27.750  42.230  0.00  0.00
F
ATOM  18743  HA  ASN F 106     -12.887 -27.795  41.915  0.00  0.00
F
ATOM  18744  CB  ASN F 106     -11.010 -26.962  41.173  0.00  0.00
F
ATOM  18745  HB1 ASN F 106     -10.931 -27.480  40.193  0.00  0.00
F
ATOM  18746  HB2 ASN F 106     -11.393 -25.930  41.019  0.00  0.00
F
ATOM  18747  CG  ASN F 106      -9.548 -26.865  41.545  0.00  0.00
F
ATOM  18748  OD1 ASN F 106      -8.808 -27.823  41.789  0.00  0.00
F
ATOM  18749  ND2 ASN F 106      -9.158 -25.545  41.609  0.00  0.00
F
ATOM  18750 HD21 ASN F 106      -8.232 -25.346  41.929  0.00  0.00
F
ATOM  18751 HD22 ASN F 106      -9.791 -24.842  41.283  0.00  0.00
F
ATOM  18752  C   ASN F 106     -11.383 -29.252  42.307  0.00  0.00
F
ATOM  18753  O   ASN F 106     -10.839 -29.759  43.316  0.00  0.00
F
ATOM  18754  N   TYR F 107     -11.603 -29.919  41.195  0.00  0.00
F
ATOM  18755  HN  TYR F 107     -12.015 -29.532  40.373  0.00  0.00
F
ATOM  18756  CA  TYR F 107     -11.208 -31.350  41.138  0.00  0.00
F
ATOM  18757  HA  TYR F 107     -11.644 -32.013  41.870  0.00  0.00
F
ATOM  18758  CB  TYR F 107     -11.475 -31.926  39.719  0.00  0.00
F
ATOM  18759  HB1 TYR F 107     -11.061 -31.231  38.957  0.00  0.00
F
ATOM  18760  HB2 TYR F 107     -11.091 -32.958  39.576  0.00  0.00
F
ATOM  18761  CG  TYR F 107     -12.915 -32.107  39.395  0.00  0.00
F
ATOM  18762  CD1 TYR F 107     -13.511 -31.740  38.294  0.00  0.00
F
ATOM  18763  HD1 TYR F 107     -12.958 -31.198  37.542  0.00  0.00
F
ATOM  18764  CE1 TYR F 107     -14.858 -32.006  38.087  0.00  0.00
F
```

ATOM	18765	HE1	TYR	F	107	−15.291	−31.503	37.235	0.00	0.00	F
ATOM	18766	CZ	TYR	F	107	−15.587	−32.745	38.963	0.00	0.00	F
ATOM	18767	OH	TYR	F	107	−16.918	−33.216	38.808	0.00	0.00	F
ATOM	18768	HH	TYR	F	107	−17.002	−33.928	39.446	0.00	0.00	F
ATOM	18769	CD2	TYR	F	107	−13.588	−32.823	40.379	0.00	0.00	F
ATOM	18770	HD2	TYR	F	107	−13.116	−33.385	41.171	0.00	0.00	F
ATOM	18771	CE2	TYR	F	107	−14.949	−33.155	40.142	0.00	0.00	F
ATOM	18772	HE2	TYR	F	107	−15.512	−33.683	40.897	0.00	0.00	F
ATOM	18773	C	TYR	F	107	−9.719	−31.722	41.218	0.00	0.00	F
ATOM	18774	O	TYR	F	107	−9.293	−32.676	41.811	0.00	0.00	F
ATOM	18775	N	TYR	F	108	−8.918	−30.885	40.556	0.00	0.00	F
ATOM	18776	HN	TYR	F	108	−9.213	−30.070	40.063	0.00	0.00	F
ATOM	18777	CA	TYR	F	108	−7.475	−30.919	40.749	0.00	0.00	F
ATOM	18778	HA	TYR	F	108	−7.172	−31.917	40.470	0.00	0.00	F
ATOM	18779	CB	TYR	F	108	−6.673	−29.878	39.931	0.00	0.00	F
ATOM	18780	HB1	TYR	F	108	−6.735	−28.804	40.207	0.00	0.00	F
ATOM	18781	HB2	TYR	F	108	−5.599	−30.146	40.026	0.00	0.00	F
ATOM	18782	CG	TYR	F	108	−7.091	−30.042	38.453	0.00	0.00	F
ATOM	18783	CD1	TYR	F	108	−7.988	−29.121	37.853	0.00	0.00	F
ATOM	18784	HD1	TYR	F	108	−8.347	−28.252	38.383	0.00	0.00	F
ATOM	18785	CE1	TYR	F	108	−8.244	−29.232	36.449	0.00	0.00	F
ATOM	18786	HE1	TYR	F	108	−8.718	−28.366	36.011	0.00	0.00	F
ATOM	18787	CZ	TYR	F	108	−7.757	−30.378	35.724	0.00	0.00	F
ATOM	18788	OH	TYR	F	108	−7.924	−30.432	34.401	0.00	0.00	F
ATOM	18789	HH	TYR	F	108	−7.777	−31.355	34.180	0.00	0.00	F
ATOM	18790	CD2	TYR	F	108	−6.734	−31.223	37.749	0.00	0.00	F
ATOM	18791	HD2	TYR	F	108	−6.284	−32.091	38.207	0.00	0.00	F

ATOM	18792	CE2	TYR	F	108	−6.999	−31.369	36.355	0.00	0.00	F
ATOM	18793	HE2	TYR	F	108	−6.582	−32.122	35.703	0.00	0.00	F
ATOM	18794	C	TYR	F	108	−6.973	−30.835	42.232	0.00	0.00	F
ATOM	18795	O	TYR	F	108	−6.215	−31.704	42.725	0.00	0.00	F
ATOM	18796	N	HSE	F	109	−7.410	−29.846	42.962	0.00	0.00	F
ATOM	18797	HN	HSE	F	109	−7.913	−29.092	42.547	0.00	0.00	F
ATOM	18798	CA	HSE	F	109	−7.212	−29.663	44.383	0.00	0.00	F
ATOM	18799	HA	HSE	F	109	−6.165	−29.720	44.642	0.00	0.00	F
ATOM	18800	CB	HSE	F	109	−7.907	−28.419	44.796	0.00	0.00	F
ATOM	18801	HB1	HSE	F	109	−7.403	−27.629	44.199	0.00	0.00	F
ATOM	18802	HB2	HSE	F	109	−8.990	−28.487	44.556	0.00	0.00	F
ATOM	18803	ND1	HSE	F	109	−8.829	−27.261	46.808	0.00	0.00	F
ATOM	18804	CG	HSE	F	109	−7.734	−27.995	46.262	0.00	0.00	F
ATOM	18805	CE1	HSE	F	109	−8.413	−27.006	48.058	0.00	0.00	F
ATOM	18806	HE1	HSE	F	109	−8.921	−26.390	48.800	0.00	0.00	F
ATOM	18807	NE2	HSE	F	109	−7.167	−27.481	48.267	0.00	0.00	F
ATOM	18808	HE2	HSE	F	109	−6.700	−27.314	49.136	0.00	0.00	F
ATOM	18809	CD2	HSE	F	109	−6.742	−28.170	47.182	0.00	0.00	F
ATOM	18810	HD2	HSE	F	109	−5.759	−28.613	47.076	0.00	0.00	F
ATOM	18811	C	HSE	F	109	−7.675	−30.803	45.317	0.00	0.00	F
ATOM	18812	O	HSE	F	109	−7.069	−31.158	46.306	0.00	0.00	F
ATOM	18813	N	HSE	F	110	−8.762	−31.526	44.939	0.00	0.00	F
ATOM	18814	HN	HSE	F	110	−9.135	−31.172	44.085	0.00	0.00	F
ATOM	18815	CA	HSE	F	110	−9.396	−32.667	45.509	0.00	0.00	F
ATOM	18816	HA	HSE	F	110	−9.547	−32.375	46.538	0.00	0.00	F
ATOM	18817	CB	HSE	F	110	−10.775	−32.761	44.826	0.00	0.00	F
ATOM	18818	HB1	HSE	F	110	−11.269	−31.809	45.117	0.00	0.00	F

ATOM 18819 HB2 HSE F 110   -10.677 -32.740 43.719 0.00 0.00
F
ATOM 18820 ND1 HSE F 110   -12.818 -34.092 44.183 0.00 0.00
F
ATOM 18821 CG HSE F 110   -11.619 -34.069 44.899 0.00 0.00
F
ATOM 18822 CE1 HSE F 110   -13.342 -35.264 44.462 0.00 0.00
F
ATOM 18823 HE1 HSE F 110   -14.351 -35.600 44.220 0.00 0.00
F
ATOM 18824 NE2 HSE F 110   -12.552 -36.062 45.171 0.00 0.00
F
ATOM 18825 HE2 HSE F 110   -12.664 -37.018 45.441 0.00 0.00
F
ATOM 18826 CD2 HSE F 110   -11.484 -35.292 45.487 0.00 0.00
F
ATOM 18827 HD2 HSE F 110   -10.714 -35.653 46.158 0.00 0.00
F
ATOM 18828 C   HSE F 110   -8.601 -33.966 45.407 0.00 0.00
F
ATOM 18829 O   HSE F 110   -8.651 -34.808 46.308 0.00 0.00
F
ATOM 18830 N   LYS F 111   -7.871 -34.117 44.240 0.00 0.00
F
ATOM 18831 HN LYS F 111   -7.890 -33.493 43.463 0.00 0.00
F
ATOM 18832 CA LYS F 111   -7.098 -35.334 44.030 0.00 0.00
F
ATOM 18833 HA LYS F 111   -7.748 -36.088 44.449 0.00 0.00
F
ATOM 18834 CB LYS F 111   -6.775 -35.380 42.557 0.00 0.00
F
ATOM 18835 HB1 LYS F 111   -7.718 -35.294 41.976 0.00 0.00
F
ATOM 18836 HB2 LYS F 111   -6.259 -34.431 42.296 0.00 0.00
F
ATOM 18837 CG LYS F 111   -5.944 -36.639 42.119 0.00 0.00
F
ATOM 18838 HG1 LYS F 111   -4.854 -36.610 42.334 0.00 0.00
F
ATOM 18839 HG2 LYS F 111   -6.316 -37.365 42.873 0.00 0.00
F
ATOM 18840 CD LYS F 111   -6.139 -37.276 40.777 0.00 0.00
F
ATOM 18841 HD1 LYS F 111   -5.587 -38.227 40.622 0.00 0.00
F
ATOM 18842 HD2 LYS F 111   -7.212 -37.532 40.645 0.00 0.00
F
ATOM 18843 CE LYS F 111   -5.503 -36.336 39.717 0.00 0.00
F
ATOM 18844 HE1 LYS F 111   -5.958 -35.328 39.828 0.00 0.00
F
ATOM 18845 HE2 LYS F 111   -4.398 -36.319 39.833 0.00 0.00
F

ATOM 18846 NZ LYS F 111   -5.785 -36.753 38.333 0.00 0.00
F
ATOM 18847 HZ1 LYS F 111   -5.123 -37.533 38.147 0.00 0.00
F
ATOM 18848 HZ2 LYS F 111   -6.788 -37.025 38.282 0.00 0.00
F
ATOM 18849 HZ3 LYS F 111   -5.572 -35.945 37.714 0.00 0.00
F
ATOM 18850 C   LYS F 111   -5.809 -35.308 44.833 0.00 0.00
F
ATOM 18851 O   LYS F 111   -5.419 -36.271 45.487 0.00 0.00
F
ATOM 18852 N   VAL F 112   -5.220 -34.129 45.045 0.00 0.00
F
ATOM 18853 HN VAL F 112   -5.648 -33.298 44.698 0.00 0.00
F
ATOM 18854 CA VAL F 112   -4.250 -33.939 46.076 0.00 0.00
F
ATOM 18855 HA VAL F 112   -3.383 -34.549 45.870 0.00 0.00
F
ATOM 18856 CB VAL F 112   -3.712 -32.465 46.057 0.00 0.00
F
ATOM 18857 HB VAL F 112   -4.623 -31.849 46.210 0.00 0.00
F
ATOM 18858 CG1 VAL F 112   -2.714 -32.231 47.173 0.00 0.00
F
ATOM 18859 HG11 VAL F 112   -3.256 -32.202 48.142 0.00 0.00
F
ATOM 18860 HG12 VAL F 112   -1.964 -33.049 47.233 0.00 0.00
F
ATOM 18861 HG13 VAL F 112   -2.227 -31.251 46.981 0.00 0.00
F
ATOM 18862 CG2 VAL F 112   -3.318 -32.049 44.638 0.00 0.00
F
ATOM 18863 HG21 VAL F 112   -4.204 -32.135 43.974 0.00 0.00
F
ATOM 18864 HG22 VAL F 112   -2.917 -31.015 44.584 0.00 0.00
F
ATOM 18865 HG23 VAL F 112   -2.410 -32.590 44.294 0.00 0.00
F
ATOM 18866 C   VAL F 112   -4.749 -34.252 47.472 0.00 0.00
F
ATOM 18867 O   VAL F 112   -4.044 -34.981 48.266 0.00 0.00
F
ATOM 18868 N   LEU F 113   -5.926 -33.860 47.966 0.00 0.00
F
ATOM 18869 HN LEU F 113   -6.432 -33.234 47.379 0.00 0.00
F
ATOM 18870 CA LEU F 113   -6.349 -34.011 49.268 0.00 0.00
F
ATOM 18871 HA LEU F 113   -5.549 -33.827 49.970 0.00 0.00
F
ATOM 18872 CB LEU F 113   -7.534 -33.044 49.455 0.00 0.00
F

ATOM 18873 HB1 LEU F 113   -8.230 -33.171 48.598 0.00 0.00
F
ATOM 18874 HB2 LEU F 113   -8.016 -33.206 50.443 0.00 0.00
F
ATOM 18875 CG LEU F 113   -7.211 -31.480 49.535 0.00 0.00
F
ATOM 18876 HG LEU F 113   -6.507 -31.336 48.688 0.00 0.00
F
ATOM 18877 CD1 LEU F 113   -8.550 -30.613 49.508 0.00 0.00
F
ATOM 18878 HD11 LEU F 113   -8.282 -29.541 49.624 0.00 0.00
F
ATOM 18879 HD12 LEU F 113   -9.038 -30.839 48.536 0.00 0.00
F
ATOM 18880 HD13 LEU F 113   -9.222 -30.931 50.334 0.00 0.00
F
ATOM 18881 CD2 LEU F 113   -6.372 -31.197 50.779 0.00 0.00
F
ATOM 18882 HD21 LEU F 113   -5.407 -31.731 50.647 0.00 0.00
F
ATOM 18883 HD22 LEU F 113   -6.229 -30.104 50.918 0.00 0.00
F
ATOM 18884 HD23 LEU F 113   -6.978 -31.448 51.676 0.00 0.00
F
ATOM 18885 C   LEU F 113   -6.708 -35.499 49.536 0.00 0.00
F
ATOM 18886 O   LEU F 113   -6.355 -36.094 50.573 0.00 0.00
F
ATOM 18887 N   GLU F 114   -7.350 -36.196 48.590 0.00 0.00
F
ATOM 18888 HN GLU F 114   -7.865 -35.727 47.877 0.00 0.00
F
ATOM 18889 CA GLU F 114   -7.571 -37.601 48.617 0.00 0.00
F
ATOM 18890 HA GLU F 114   -8.189 -37.730 49.494 0.00 0.00
F
ATOM 18891 CB GLU F 114   -8.292 -38.028 47.310 0.00 0.00
F
ATOM 18892 HB1 GLU F 114   -9.303 -37.572 47.246 0.00 0.00
F
ATOM 18893 HB2 GLU F 114   -7.714 -37.656 46.438 0.00 0.00
F
ATOM 18894 CG GLU F 114   -8.463 -39.553 47.177 0.00 0.00
F
ATOM 18895 HG1 GLU F 114   -7.616 -40.082 46.689 0.00 0.00
F
ATOM 18896 HG2 GLU F 114   -8.442 -40.037 48.177 0.00 0.00
F
ATOM 18897 CD GLU F 114   -9.777 -40.027 46.565 0.00 0.00
F
ATOM 18898 OE1 GLU F 114   -10.875 -39.896 47.144 0.00 0.00
F
ATOM 18899 OE2 GLU F 114   -9.778 -40.664 45.436 0.00 0.00
F

ATOM 18900 C GLU F 114 -6.286 -38.453 48.919 0.00 0.00
F
ATOM 18901 O GLU F 114 -6.339 -39.405 49.686 0.00 0.00
F
ATOM 18902 N LYS F 115 -5.133 -38.131 48.284 0.00 0.00
F
ATOM 18903 HN LYS F 115 -5.200 -37.370 47.642 0.00 0.00
F
ATOM 18904 CA LYS F 115 -3.932 -38.980 48.372 0.00 0.00
F
ATOM 18905 HA LYS F 115 -4.327 -39.985 48.408 0.00 0.00
F
ATOM 18906 CB LYS F 115 -3.183 -38.729 47.043 0.00 0.00
F
ATOM 18907 HB1 LYS F 115 -3.235 -37.619 47.061 0.00 0.00
F
ATOM 18908 HB2 LYS F 115 -2.108 -39.000 47.113 0.00 0.00
F
ATOM 18909 CG LYS F 115 -3.976 -39.234 45.849 0.00 0.00
F
ATOM 18910 HG1 LYS F 115 -4.070 -40.337 45.937 0.00 0.00
F
ATOM 18911 HG2 LYS F 115 -4.979 -38.769 45.748 0.00 0.00
F
ATOM 18912 CD LYS F 115 -3.123 -38.972 44.631 0.00 0.00
F
ATOM 18913 HD1 LYS F 115 -3.090 -37.880 44.427 0.00 0.00
F
ATOM 18914 HD2 LYS F 115 -2.139 -39.447 44.833 0.00 0.00
F
ATOM 18915 CE LYS F 115 -3.806 -39.613 43.357 0.00 0.00
F
ATOM 18916 HE1 LYS F 115 -4.700 -39.084 42.965 0.00 0.00
F
ATOM 18917 HE2 LYS F 115 -3.055 -39.502 42.546 0.00 0.00
F
ATOM 18918 NZ LYS F 115 -3.978 -41.128 43.519 0.00 0.00
F
ATOM 18919 HZ1 LYS F 115 -3.075 -41.544 43.824 0.00 0.00
F
ATOM 18920 HZ2 LYS F 115 -4.699 -41.379 44.225 0.00 0.00
F
ATOM 18921 HZ3 LYS F 115 -4.285 -41.510 42.601 0.00 0.00
F
ATOM 18922 C LYS F 115 -2.962 -38.611 49.561 0.00 0.00
F
ATOM 18923 O LYS F 115 -2.134 -39.342 50.047 0.00 0.00
F
ATOM 18924 N ILE F 116 -3.232 -37.377 50.098 0.00 0.00
F
ATOM 18925 HN ILE F 116 -3.866 -36.817 49.572 0.00 0.00
F
ATOM 18926 CA ILE F 116 -2.740 -36.976 51.398 0.00 0.00
F

ATOM 18927 HA ILE F 116   -1.704 -37.267 51.486 0.00 0.00
F
ATOM 18928 CB ILE F 116   -2.800 -35.405 51.626 0.00 0.00
F
ATOM 18929 HB ILE F 116   -3.863 -35.086 51.693 0.00 0.00
F
ATOM 18930 CG2 ILE F 116   -2.188 -35.054 52.929 0.00 0.00
F
ATOM 18931 HG21 ILE F 116   -2.607 -35.670 53.753 0.00 0.00
F
ATOM 18932 HG22 ILE F 116   -1.153 -35.453 52.889 0.00 0.00
F
ATOM 18933 HG23 ILE F 116   -2.295 -33.964 53.117 0.00 0.00
F
ATOM 18934 CG1 ILE F 116   -1.985 -34.535 50.516 0.00 0.00
F
ATOM 18935 HG11 ILE F 116   -0.950 -34.482 50.914 0.00 0.00
F
ATOM 18936 HG12 ILE F 116   -1.967 -35.059 49.536 0.00 0.00
F
ATOM 18937 CD ILE F 116   -2.381 -33.089 50.414 0.00 0.00
F
ATOM 18938 HD1 ILE F 116   -3.405 -32.976 49.997 0.00 0.00
F
ATOM 18939 HD2 ILE F 116   -2.371 -32.473 51.338 0.00 0.00
F
ATOM 18940 HD3 ILE F 116   -1.649 -32.728 49.660 0.00 0.00
F
ATOM 18941 C   ILE F 116   -3.464 -37.655 52.527 0.00 0.00
F
ATOM 18942 O   ILE F 116   -2.884 -38.352 53.331 0.00 0.00
F
ATOM 18943 N   GLY F 117   -4.800 -37.669 52.497 0.00 0.00
F
ATOM 18944 HN GLY F 117   -5.358 -37.005 52.004 0.00 0.00
F
ATOM 18945 CA GLY F 117   -5.590 -38.517 53.415 0.00 0.00
F
ATOM 18946 HA1 GLY F 117   -5.215 -38.341 54.412 0.00 0.00
F
ATOM 18947 HA2 GLY F 117   -5.419 -39.533 53.089 0.00 0.00
F
ATOM 18948 C   GLY F 117   -7.100 -38.257 53.579 0.00 0.00
F
ATOM 18949 O   GLY F 117   -7.604 -38.877 54.506 0.00 0.00
F
ATOM 18950 N   ALA F 118   -7.830 -37.490 52.725 0.00 0.00
F
ATOM 18951 HN ALA F 118   -7.324 -36.914 52.088 0.00 0.00
F
ATOM 18952 CA ALA F 118   -9.229 -37.244 52.852 0.00 0.00
F
ATOM 18953 HA ALA F 118   -9.398 -36.713 53.777 0.00 0.00
F

ATOM 18954 CB ALA F 118   -9.901 -36.354 51.810 0.00 0.00
F
ATOM 18955 HB1 ALA F 118   -10.115 -36.872 50.851 0.00 0.00
F
ATOM 18956 HB2 ALA F 118   -10.837 -35.837 52.111 0.00 0.00
F
ATOM 18957 HB3 ALA F 118   -9.221 -35.506 51.579 0.00 0.00
F
ATOM 18958 C   ALA F 118   -10.131 -38.451 52.712 0.00 0.00
F
ATOM 18959 O   ALA F 118   -9.917 -39.247 51.863 0.00 0.00
F
ATOM 18960 N   ASP F 119   -11.183 -38.538 53.611 0.00 0.00
F
ATOM 18961 HN ASP F 119   -11.302 -37.953 54.409 0.00 0.00
F
ATOM 18962 CA ASP F 119   -12.214 -39.573 53.459 0.00 0.00
F
ATOM 18963 HA ASP F 119   -11.814 -40.470 53.010 0.00 0.00
F
ATOM 18964 CB ASP F 119   -12.676 -39.996 54.836 0.00 0.00
F
ATOM 18965 HB1 ASP F 119   -13.029 -39.078 55.353 0.00 0.00
F
ATOM 18966 HB2 ASP F 119   -13.511 -40.721 54.731 0.00 0.00
F
ATOM 18967 CG ASP F 119   -11.549 -40.615 55.677 0.00 0.00
F
ATOM 18968 OD1 ASP F 119   -11.194 -40.215 56.836 0.00 0.00
F
ATOM 18969 OD2 ASP F 119   -11.071 -41.681 55.193 0.00 0.00
F
ATOM 18970 C   ASP F 119   -13.354 -39.178 52.642 0.00 0.00
F
ATOM 18971 O   ASP F 119   -14.043 -39.904 51.962 0.00 0.00
F
ATOM 18972 N   ARG F 120   -13.642 -37.822 52.748 0.00 0.00
F
ATOM 18973 HN ARG F 120   -13.137 -37.193 53.335 0.00 0.00
F
ATOM 18974 CA ARG F 120   -14.683 -37.232 52.001 0.00 0.00
F
ATOM 18975 HA ARG F 120   -14.841 -37.776 51.082 0.00 0.00
F
ATOM 18976 CB ARG F 120   -16.048 -37.186 52.790 0.00 0.00
F
ATOM 18977 HB1 ARG F 120   -16.305 -38.242 53.018 0.00 0.00
F
ATOM 18978 HB2 ARG F 120   -16.017 -36.641 53.758 0.00 0.00
F
ATOM 18979 CG ARG F 120   -17.192 -36.459 51.993 0.00 0.00
F
ATOM 18980 HG1 ARG F 120   -16.894 -35.411 51.776 0.00 0.00
F

ATOM 18981 HG2 ARG F 120   −17.249 −36.875 50.964 0.00 0.00
F
ATOM 18982 CD ARG F 120   −18.543 −36.486 52.568 0.00 0.00
F
ATOM 18983 HD1 ARG F 120   −18.874 −37.428 53.057 0.00 0.00
F
ATOM 18984 HD2 ARG F 120   −18.711 −35.608 53.227 0.00 0.00
F
ATOM 18985 NE ARG F 120   −19.503 −36.236 51.473 0.00 0.00
F
ATOM 18986 HE ARG F 120   −19.521 −35.259 51.264 0.00 0.00
F
ATOM 18987 CZ ARG F 120   −20.146 −37.128 50.706 0.00 0.00
F
ATOM 18988 NH1 ARG F 120   −19.949 −38.468 50.872 0.00 0.00
F
ATOM 18989 HH11 ARG F 120   −20.234 −39.107 50.158 0.00 0.00
F
ATOM 18990 HH12 ARG F 120   −19.178 −38.807 51.412 0.00 0.00
F
ATOM 18991 NH2 ARG F 120   −20.850 −36.668 49.638 0.00 0.00
F
ATOM 18992 HH21 ARG F 120   −21.137 −37.234 48.864 0.00 0.00
F
ATOM 18993 HH22 ARG F 120   −20.924 −35.688 49.453 0.00 0.00
F
ATOM 18994 C   ARG F 120   −14.224 −35.811 51.591 0.00 0.00
F
ATOM 18995 O   ARG F 120   −13.599 −35.125 52.450 0.00 0.00
F
ATOM 18996 N   ILE F 121   −14.524 −35.347 50.397 0.00 0.00
F
ATOM 18997 HN ILE F 121   −14.822 −36.064 49.772 0.00 0.00
F
ATOM 18998 CA ILE F 121   −14.373 −34.042 49.880 0.00 0.00
F
ATOM 18999 HA ILE F 121   −13.854 −33.430 50.603 0.00 0.00
F
ATOM 19000 CB ILE F 121   −13.564 −34.003 48.505 0.00 0.00
F
ATOM 19001 HB ILE F 121   −14.220 −34.370 47.686 0.00 0.00
F
ATOM 19002 CG2 ILE F 121   −13.314 −32.600 48.005 0.00 0.00
F
ATOM 19003 HG21 ILE F 121   −14.254 −32.036 47.826 0.00 0.00
F
ATOM 19004 HG22 ILE F 121   −12.881 −32.102 48.899 0.00 0.00
F
ATOM 19005 HG23 ILE F 121   −12.538 −32.562 47.211 0.00 0.00
F
ATOM 19006 CG1 ILE F 121   −12.180 −34.800 48.735 0.00 0.00
F
ATOM 19007 HG11 ILE F 121   −12.503 −35.856 48.859 0.00 0.00
F

```
ATOM  19008 HG12 ILE F 121     -11.610 -34.829  47.782  0.00  0.00
F
ATOM  19009  CD  ILE F 121     -11.355 -34.149  49.830  0.00  0.00
F
ATOM  19010  HD1 ILE F 121     -10.344 -34.602  49.908  0.00  0.00
F
ATOM  19011  HD2 ILE F 121     -11.179 -33.085  49.563  0.00  0.00
F
ATOM  19012  HD3 ILE F 121     -11.738 -34.257  50.868  0.00  0.00
F
ATOM  19013  C   ILE F 121     -15.735 -33.454  49.622  0.00  0.00
F
ATOM  19014  O   ILE F 121     -16.644 -34.166  49.116  0.00  0.00
F
ATOM  19015  N   ILE F 122     -15.892 -32.179  49.962  0.00  0.00
F
ATOM  19016  HN  ILE F 122     -15.185 -31.765  50.531  0.00  0.00
F
ATOM  19017  CA  ILE F 122     -17.062 -31.422  49.723  0.00  0.00
F
ATOM  19018  HA  ILE F 122     -17.804 -32.016  49.210  0.00  0.00
F
ATOM  19019  CB  ILE F 122     -17.644 -30.718  50.993  0.00  0.00
F
ATOM  19020  HB  ILE F 122     -16.799 -30.103  51.369  0.00  0.00
F
ATOM  19021  CG2 ILE F 122     -18.974 -29.971  50.761  0.00  0.00
F
ATOM  19022 HG21 ILE F 122     -19.794 -30.715  50.663  0.00  0.00
F
ATOM  19023 HG22 ILE F 122     -19.191 -29.274  51.599  0.00  0.00
F
ATOM  19024 HG23 ILE F 122     -19.005 -29.496  49.758  0.00  0.00
F
ATOM  19025  CG1 ILE F 122     -17.794 -31.772  52.206  0.00  0.00
F
ATOM  19026 HG11 ILE F 122     -18.532 -31.244  52.847  0.00  0.00
F
ATOM  19027 HG12 ILE F 122     -18.397 -32.618  51.811  0.00  0.00
F
ATOM  19028  CD  ILE F 122     -16.582 -32.168  53.036  0.00  0.00
F
ATOM  19029  HD1 ILE F 122     -16.051 -31.273  53.425  0.00  0.00
F
ATOM  19030  HD2 ILE F 122     -16.974 -32.812  53.852  0.00  0.00
F
ATOM  19031  HD3 ILE F 122     -15.826 -32.701  52.420  0.00  0.00
F
ATOM  19032  C   ILE F 122     -16.704 -30.240  48.781  0.00  0.00
F
ATOM  19033  O   ILE F 122     -15.821 -29.425  49.044  0.00  0.00
F
ATOM  19034  N   HSE F 123     -17.465 -30.220  47.655  0.00  0.00
F
```

ATOM 19035 HN HSE F 123 -17.787 -31.065 47.234 0.00 0.00
F
ATOM 19036 CA HSE F 123 -17.470 -28.998 46.822 0.00 0.00
F
ATOM 19037 HA HSE F 123 -16.897 -28.150 47.167 0.00 0.00
F
ATOM 19038 CB HSE F 123 -16.990 -29.320 45.429 0.00 0.00
F
ATOM 19039 HB1 HSE F 123 -17.698 -30.119 45.122 0.00 0.00
F
ATOM 19040 HB2 HSE F 123 -17.087 -28.502 44.684 0.00 0.00
F
ATOM 19041 ND1 HSE F 123 -15.546 -31.353 45.459 0.00 0.00
F
ATOM 19042 CG HSE F 123 -15.669 -29.974 45.155 0.00 0.00
F
ATOM 19043 CE1 HSE F 123 -14.401 -31.757 44.950 0.00 0.00
F
ATOM 19044 HE1 HSE F 123 -13.971 -32.752 45.065 0.00 0.00
F
ATOM 19045 NE2 HSE F 123 -13.690 -30.716 44.502 0.00 0.00
F
ATOM 19046 HE2 HSE F 123 -12.771 -30.790 44.113 0.00 0.00
F
ATOM 19047 CD2 HSE F 123 -14.545 -29.571 44.607 0.00 0.00
F
ATOM 19048 HD2 HSE F 123 -14.242 -28.640 44.144 0.00 0.00
F
ATOM 19049 C HSE F 123 -18.861 -28.453 46.772 0.00 0.00
F
ATOM 19050 O HSE F 123 -19.674 -28.941 46.013 0.00 0.00
F
ATOM 19051 N PRO F 124 -19.157 -27.345 47.442 0.00 0.00
F
ATOM 19052 CD PRO F 124 -18.468 -26.902 48.626 0.00 0.00
F
ATOM 19053 HD1 PRO F 124 -17.991 -27.688 49.249 0.00 0.00
F
ATOM 19054 HD2 PRO F 124 -17.794 -26.078 48.308 0.00 0.00
F
ATOM 19055 CA PRO F 124 -20.453 -26.679 47.344 0.00 0.00
F
ATOM 19056 HA PRO F 124 -21.132 -27.509 47.474 0.00 0.00
F
ATOM 19057 CB PRO F 124 -20.453 -25.670 48.435 0.00 0.00
F
ATOM 19058 HB1 PRO F 124 -21.439 -25.312 48.801 0.00 0.00
F
ATOM 19059 HB2 PRO F 124 -19.959 -24.743 48.074 0.00 0.00
F
ATOM 19060 CG PRO F 124 -19.561 -26.290 49.527 0.00 0.00
F
ATOM 19061 HG1 PRO F 124 -20.126 -27.038 50.123 0.00 0.00
F

ATOM 19062 HG2 PRO F 124 −19.161 −25.492 50.189 0.00 0.00
F
ATOM 19063 C PRO F 124 −20.769 −26.071 46.013 0.00 0.00
F
ATOM 19064 O PRO F 124 −21.900 −26.198 45.569 0.00 0.00
F
ATOM 19065 N GLU F 125 −19.869 −25.475 45.274 0.00 0.00
F
ATOM 19066 HN GLU F 125 −18.935 −25.428 45.620 0.00 0.00
F
ATOM 19067 CA GLU F 125 −20.160 −24.814 44.065 0.00 0.00
F
ATOM 19068 HA GLU F 125 −20.990 −24.125 44.114 0.00 0.00
F
ATOM 19069 CB GLU F 125 −18.975 −23.887 43.705 0.00 0.00
F
ATOM 19070 HB1 GLU F 125 −18.247 −24.493 43.124 0.00 0.00
F
ATOM 19071 HB2 GLU F 125 −19.328 −23.038 43.081 0.00 0.00
F
ATOM 19072 CG GLU F 125 −18.292 −23.085 44.871 0.00 0.00
F
ATOM 19073 HG1 GLU F 125 −17.843 −22.175 44.419 0.00 0.00
F
ATOM 19074 HG2 GLU F 125 −19.005 −22.676 45.618 0.00 0.00
F
ATOM 19075 CD GLU F 125 −17.141 −23.864 45.539 0.00 0.00
F
ATOM 19076 OE1 GLU F 125 −16.025 −23.410 45.337 0.00 0.00
F
ATOM 19077 OE2 GLU F 125 −17.314 −24.899 46.289 0.00 0.00
F
ATOM 19078 C GLU F 125 −20.546 −25.820 42.968 0.00 0.00
F
ATOM 19079 O GLU F 125 −21.168 −25.556 41.963 0.00 0.00
F
ATOM 19080 N LYS F 126 −20.033 −27.047 43.154 0.00 0.00
F
ATOM 19081 HN LYS F 126 −19.641 −27.210 44.056 0.00 0.00
F
ATOM 19082 CA LYS F 126 −20.328 −28.163 42.280 0.00 0.00
F
ATOM 19083 HA LYS F 126 −20.378 −27.876 41.240 0.00 0.00
F
ATOM 19084 CB LYS F 126 −19.264 −29.255 42.389 0.00 0.00
F
ATOM 19085 HB1 LYS F 126 −18.371 −28.756 41.956 0.00 0.00
F
ATOM 19086 HB2 LYS F 126 −19.032 −29.441 43.459 0.00 0.00
F
ATOM 19087 CG LYS F 126 −19.664 −30.571 41.792 0.00 0.00
F
ATOM 19088 HG1 LYS F 126 −18.890 −31.321 42.061 0.00 0.00
F

ATOM 19089 HG2 LYS F 126 -20.612 -30.825 42.313 0.00 0.00
F
ATOM 19090 CD LYS F 126 -19.755 -30.589 40.255 0.00 0.00
F
ATOM 19091 HD1 LYS F 126 -20.035 -29.695 39.657 0.00 0.00
F
ATOM 19092 HD2 LYS F 126 -18.748 -30.917 39.918 0.00 0.00
F
ATOM 19093 CE LYS F 126 -20.648 -31.722 39.729 0.00 0.00
F
ATOM 19094 HE1 LYS F 126 -20.204 -32.711 39.972 0.00 0.00
F
ATOM 19095 HE2 LYS F 126 -21.730 -31.646 39.971 0.00 0.00
F
ATOM 19096 NZ LYS F 126 -20.665 -31.765 38.253 0.00 0.00
F
ATOM 19097 HZ1 LYS F 126 -19.924 -32.412 37.916 0.00 0.00
F
ATOM 19098 HZ2 LYS F 126 -21.626 -32.052 37.979 0.00 0.00
F
ATOM 19099 HZ3 LYS F 126 -20.606 -30.757 38.005 0.00 0.00
F
ATOM 19100 C LYS F 126 -21.727 -28.703 42.548 0.00 0.00
F
ATOM 19101 O LYS F 126 -22.575 -28.586 41.696 0.00 0.00
F
ATOM 19102 N ASP F 127 -22.059 -29.116 43.797 0.00 0.00
F
ATOM 19103 HN ASP F 127 -21.355 -29.155 44.502 0.00 0.00
F
ATOM 19104 CA ASP F 127 -23.274 -29.546 44.222 0.00 0.00
F
ATOM 19105 HA ASP F 127 -23.609 -30.357 43.591 0.00 0.00
F
ATOM 19106 CB ASP F 127 -23.156 -30.019 45.657 0.00 0.00
F
ATOM 19107 HB1 ASP F 127 -22.410 -29.347 46.131 0.00 0.00
F
ATOM 19108 HB2 ASP F 127 -24.097 -29.930 46.241 0.00 0.00
F
ATOM 19109 CG ASP F 127 -22.807 -31.428 45.563 0.00 0.00
F
ATOM 19110 OD1 ASP F 127 -23.736 -32.245 45.466 0.00 0.00
F
ATOM 19111 OD2 ASP F 127 -21.568 -31.757 45.524 0.00 0.00
F
ATOM 19112 C ASP F 127 -24.404 -28.510 44.120 0.00 0.00
F
ATOM 19113 O ASP F 127 -25.504 -28.839 43.637 0.00 0.00
F
ATOM 19114 N MET F 128 -24.066 -27.239 44.410 0.00 0.00
F
ATOM 19115 HN MET F 128 -23.273 -26.893 44.906 0.00 0.00
F

ATOM 19116 CA MET F 128 −25.038 −26.143 44.397 0.00 0.00
F
ATOM 19117 HA MET F 128 −26.059 −26.443 44.580 0.00 0.00
F
ATOM 19118 CB MET F 128 −24.731 −25.074 45.530 0.00 0.00
F
ATOM 19119 HB1 MET F 128 −23.659 −24.782 45.508 0.00 0.00
F
ATOM 19120 HB2 MET F 128 −25.410 −24.196 45.489 0.00 0.00
F
ATOM 19121 CG MET F 128 −25.008 −25.742 46.916 0.00 0.00
F
ATOM 19122 HG1 MET F 128 −24.269 −26.552 47.097 0.00 0.00
F
ATOM 19123 HG2 MET F 128 −24.812 −24.971 47.692 0.00 0.00
F
ATOM 19124 SD MET F 128 −26.641 −26.468 47.350 0.00 0.00
F
ATOM 19125 CE MET F 128 −27.572 −24.972 46.922 0.00 0.00
F
ATOM 19126 HE1 MET F 128 −27.634 −24.208 47.727 0.00 0.00
F
ATOM 19127 HE2 MET F 128 −27.283 −24.532 45.944 0.00 0.00
F
ATOM 19128 HE3 MET F 128 −28.642 −25.268 46.872 0.00 0.00
F
ATOM 19129 C MET F 128 −25.046 −25.542 42.998 0.00 0.00
F
ATOM 19130 O MET F 128 −26.042 −24.993 42.553 0.00 0.00
F
ATOM 19131 N GLY F 129 −24.025 −25.931 42.124 0.00 0.00
F
ATOM 19132 HN GLY F 129 −23.303 −26.567 42.384 0.00 0.00
F
ATOM 19133 CA GLY F 129 −24.220 −25.474 40.680 0.00 0.00
F
ATOM 19134 HA1 GLY F 129 −23.195 −25.637 40.382 0.00 0.00
F
ATOM 19135 HA2 GLY F 129 −24.596 −24.462 40.646 0.00 0.00
F
ATOM 19136 C GLY F 129 −25.299 −26.429 40.166 0.00 0.00
F
ATOM 19137 O GLY F 129 −26.173 −25.900 39.499 0.00 0.00
F
ATOM 19138 N VAL F 130 −25.100 −27.744 40.408 0.00 0.00
F
ATOM 19139 HN VAL F 130 −24.218 −27.996 40.799 0.00 0.00
F
ATOM 19140 CA VAL F 130 −26.031 −28.812 39.976 0.00 0.00
F
ATOM 19141 HA VAL F 130 −26.046 −28.821 38.896 0.00 0.00
F
ATOM 19142 CB VAL F 130 −25.636 −30.228 40.275 0.00 0.00
F

ATOM 19143 HB VAL F 130   −25.611 −30.280 41.385 0.00 0.00
F
ATOM 19144 CG1 VAL F 130   −26.768 −31.124 39.921 0.00 0.00
F
ATOM 19145 HG11 VAL F 130   −27.724 −30.810 40.390 0.00 0.00
F
ATOM 19146 HG12 VAL F 130   −26.969 −31.212 38.832 0.00 0.00
F
ATOM 19147 HG13 VAL F 130   −26.639 −32.171 40.269 0.00 0.00
F
ATOM 19148 CG2 VAL F 130   −24.325 −30.642 39.628 0.00 0.00
F
ATOM 19149 HG21 VAL F 130   −24.480 −30.486 38.539 0.00 0.00
F
ATOM 19150 HG22 VAL F 130   −23.388 −30.108 39.896 0.00 0.00
F
ATOM 19151 HG23 VAL F 130   −24.170 −31.703 39.918 0.00 0.00
F
ATOM 19152 C   VAL F 130   −27.419 −28.474 40.533 0.00 0.00
F
ATOM 19153 O   VAL F 130   −28.386 −28.478 39.785 0.00 0.00
F
ATOM 19154 N   LYS F 131   −27.657 −28.130 41.821 0.00 0.00
F
ATOM 19155 HN LYS F 131   −26.910 −28.126 42.482 0.00 0.00
F
ATOM 19156 CA LYS F 131   −29.008 −27.870 42.317 0.00 0.00
F
ATOM 19157 HA LYS F 131   −29.666 −28.650 41.965 0.00 0.00
F
ATOM 19158 CB LYS F 131   −29.200 −27.839 43.837 0.00 0.00
F
ATOM 19159 HB1 LYS F 131   −28.700 −28.787 44.129 0.00 0.00
F
ATOM 19160 HB2 LYS F 131   −28.660 −26.981 44.291 0.00 0.00
F
ATOM 19161 CG LYS F 131   −30.605 −27.683 44.414 0.00 0.00
F
ATOM 19162 HG1 LYS F 131   −30.448 −27.433 45.485 0.00 0.00
F
ATOM 19163 HG2 LYS F 131   −31.081 −26.867 43.830 0.00 0.00
F
ATOM 19164 CD LYS F 131   −31.474 −28.853 44.328 0.00 0.00
F
ATOM 19165 HD1 LYS F 131   −31.630 −29.090 43.254 0.00 0.00
F
ATOM 19166 HD2 LYS F 131   −30.844 −29.689 44.701 0.00 0.00
F
ATOM 19167 CE LYS F 131   −32.885 −28.850 45.058 0.00 0.00
F
ATOM 19168 HE1 LYS F 131   −32.735 −28.655 46.142 0.00 0.00
F
ATOM 19169 HE2 LYS F 131   −33.393 −27.951 44.648 0.00 0.00
F

ATOM 19170 NZ LYS F 131 -33.685 -29.982 44.824 0.00 0.00
F
ATOM 19171 HZ1 LYS F 131 -34.610 -29.923 45.297 0.00 0.00
F
ATOM 19172 HZ2 LYS F 131 -33.857 -29.931 43.800 0.00 0.00
F
ATOM 19173 HZ3 LYS F 131 -33.257 -30.919 44.968 0.00 0.00
F
ATOM 19174 C LYS F 131 -29.630 -26.663 41.707 0.00 0.00
F
ATOM 19175 O LYS F 131 -30.787 -26.655 41.332 0.00 0.00
F
ATOM 19176 N ILE F 132 -28.866 -25.574 41.421 0.00 0.00
F
ATOM 19177 HN ILE F 132 -27.895 -25.580 41.647 0.00 0.00
F
ATOM 19178 CA ILE F 132 -29.500 -24.440 40.845 0.00 0.00
F
ATOM 19179 HA ILE F 132 -30.442 -24.263 41.344 0.00 0.00
F
ATOM 19180 CB ILE F 132 -28.736 -23.155 41.163 0.00 0.00
F
ATOM 19181 HB ILE F 132 -27.670 -23.369 41.391 0.00 0.00
F
ATOM 19182 CG2 ILE F 132 -28.815 -22.100 40.127 0.00 0.00
F
ATOM 19183 HG21 ILE F 132 -28.217 -21.217 40.440 0.00 0.00
F
ATOM 19184 HG22 ILE F 132 -28.379 -22.468 39.174 0.00 0.00
F
ATOM 19185 HG23 ILE F 132 -29.870 -21.911 39.832 0.00 0.00
F
ATOM 19186 CG1 ILE F 132 -29.380 -22.498 42.402 0.00 0.00
F
ATOM 19187 HG11 ILE F 132 -28.903 -21.509 42.568 0.00 0.00
F
ATOM 19188 HG12 ILE F 132 -30.428 -22.256 42.122 0.00 0.00
F
ATOM 19189 CD ILE F 132 -29.269 -23.143 43.770 0.00 0.00
F
ATOM 19190 HD1 ILE F 132 -28.291 -23.085 44.295 0.00 0.00
F
ATOM 19191 HD2 ILE F 132 -29.873 -22.538 44.481 0.00 0.00
F
ATOM 19192 HD3 ILE F 132 -29.517 -24.226 43.775 0.00 0.00
F
ATOM 19193 C ILE F 132 -29.802 -24.591 39.346 0.00 0.00
F
ATOM 19194 O ILE F 132 -30.877 -24.295 38.858 0.00 0.00
F
ATOM 19195 N ALA F 133 -28.906 -25.225 38.601 0.00 0.00
F
ATOM 19196 HN ALA F 133 -28.000 -25.479 38.930 0.00 0.00
F

ATOM 19197 CA   ALA F 133   -29.058 -25.454 37.211 0.00 0.00
F
ATOM 19198 HA   ALA F 133   -29.313 -24.513 36.745 0.00 0.00
F
ATOM 19199 CB   ALA F 133   -27.730 -25.891 36.550 0.00 0.00
F
ATOM 19200 HB1 ALA F 133   -27.442 -26.854 37.023 0.00 0.00
F
ATOM 19201 HB2 ALA F 133   -27.850 -25.997 35.450 0.00 0.00
F
ATOM 19202 HB3 ALA F 133   -26.920 -25.169 36.787 0.00 0.00
F
ATOM 19203 C   ALA F 133   -30.166 -26.456 36.973 0.00 0.00
F
ATOM 19204 O   ALA F 133   -30.997 -26.266 36.023 0.00 0.00
F
ATOM 19205 N   GLN F 134   -30.231 -27.541 37.742 0.00 0.00
F
ATOM 19206 HN   GLN F 134   -29.511 -27.622 38.427 0.00 0.00
F
ATOM 19207 CA   GLN F 134   -31.323 -28.591 37.727 0.00 0.00
F
ATOM 19208 HA   GLN F 134   -31.389 -29.031 36.743 0.00 0.00
F
ATOM 19209 CB   GLN F 134   -31.107 -29.699 38.875 0.00 0.00
F
ATOM 19210 HB1 GLN F 134   -30.060 -30.065 38.805 0.00 0.00
F
ATOM 19211 HB2 GLN F 134   -31.130 -29.159 39.845 0.00 0.00
F
ATOM 19212 CG   GLN F 134   -32.082 -30.864 38.868 0.00 0.00
F
ATOM 19213 HG1 GLN F 134   -33.094 -30.409 38.912 0.00 0.00
F
ATOM 19214 HG2 GLN F 134   -32.042 -31.275 37.836 0.00 0.00
F
ATOM 19215 CD   GLN F 134   -31.943 -31.799 40.015 0.00 0.00
F
ATOM 19216 OE1 GLN F 134   -31.797 -31.304 41.144 0.00 0.00
F
ATOM 19217 NE2 GLN F 134   -32.019 -33.126 39.774 0.00 0.00
F
ATOM 19218 HE21 GLN F 134   -32.099 -33.705 40.586 0.00 0.00
F
ATOM 19219 HE22 GLN F 134   -31.957 -33.556 38.874 0.00 0.00
F
ATOM 19220 C   GLN F 134   -32.733 -27.938 37.991 0.00 0.00
F
ATOM 19221 O   GLN F 134   -33.757 -28.202 37.314 0.00 0.00
F
ATOM 19222 N   SER F 135   -32.881 -27.036 38.972 0.00 0.00
F
ATOM 19223 HN   SER F 135   -32.129 -26.984 39.626 0.00 0.00
F

ATOM 19224 CA SER F 135   -34.142 -26.368 39.321 0.00 0.00
F
ATOM 19225 HA SER F 135   -34.937 -27.099 39.306 0.00 0.00
F
ATOM 19226 CB SER F 135   -34.067 -25.718 40.700 0.00 0.00
F
ATOM 19227 HB1 SER F 135   -33.598 -26.420 41.422 0.00 0.00
F
ATOM 19228 HB2 SER F 135   -33.529 -24.746 40.676 0.00 0.00
F
ATOM 19229 OG SER F 135   -35.434 -25.461 41.152 0.00 0.00
F
ATOM 19230 HG1 SER F 135   -35.943 -26.150 40.717 0.00 0.00
F
ATOM 19231 C   SER F 135   -34.503 -25.290 38.360 0.00 0.00
F
ATOM 19232 O   SER F 135   -35.676 -25.028 38.041 0.00 0.00
F
ATOM 19233 N   LEU F 136   -33.474 -24.670 37.761 0.00 0.00
F
ATOM 19234 HN LEU F 136   -32.546 -24.938 38.006 0.00 0.00
F
ATOM 19235 CA LEU F 136   -33.687 -23.779 36.635 0.00 0.00
F
ATOM 19236 HA LEU F 136   -34.346 -23.041 37.069 0.00 0.00
F
ATOM 19237 CB LEU F 136   -32.358 -23.102 36.217 0.00 0.00
F
ATOM 19238 HB1 LEU F 136   -31.468 -23.762 36.298 0.00 0.00
F
ATOM 19239 HB2 LEU F 136   -32.315 -22.845 35.137 0.00 0.00
F
ATOM 19240 CG LEU F 136   -32.077 -21.848 37.034 0.00 0.00
F
ATOM 19241 HG LEU F 136   -32.204 -22.140 38.098 0.00 0.00
F
ATOM 19242 CD1 LEU F 136   -30.590 -21.497 36.931 0.00 0.00
F
ATOM 19243 HD11 LEU F 136   -30.341 -20.541 37.440 0.00 0.00
F
ATOM 19244 HD12 LEU F 136   -30.047 -22.237 37.556 0.00 0.00
F
ATOM 19245 HD13 LEU F 136   -30.113 -21.455 35.929 0.00 0.00
F
ATOM 19246 CD2 LEU F 136   -32.905 -20.593 36.800 0.00 0.00
F
ATOM 19247 HD21 LEU F 136   -33.872 -21.001 37.164 0.00 0.00
F
ATOM 19248 HD22 LEU F 136   -32.608 -19.715 37.412 0.00 0.00
F
ATOM 19249 HD23 LEU F 136   -32.763 -20.348 35.725 0.00 0.00
F
ATOM 19250 C   LEU F 136   -34.381 -24.376 35.372 0.00 0.00
F

ATOM 19251 O LEU F 136 -35.296 -23.831 34.739 0.00 0.00
F
ATOM 19252 N SER F 137 -33.895 -25.607 34.990 0.00 0.00
F
ATOM 19253 HN SER F 137 -33.240 -26.027 35.614 0.00 0.00
F
ATOM 19254 CA SER F 137 -34.425 -26.327 33.859 0.00 0.00
F
ATOM 19255 HA SER F 137 -34.610 -25.685 33.010 0.00 0.00
F
ATOM 19256 CB SER F 137 -33.485 -27.491 33.451 0.00 0.00
F
ATOM 19257 HB1 SER F 137 -32.489 -27.011 33.345 0.00 0.00
F
ATOM 19258 HB2 SER F 137 -33.425 -28.242 34.267 0.00 0.00
F
ATOM 19259 OG SER F 137 -33.771 -28.139 32.218 0.00 0.00
F
ATOM 19260 HG1 SER F 137 -33.517 -29.064 32.230 0.00 0.00
F
ATOM 19261 C SER F 137 -35.764 -26.890 33.991 0.00 0.00
F
ATOM 19262 O SER F 137 -36.624 -26.839 33.131 0.00 0.00
F
ATOM 19263 N ASP F 138 -36.035 -27.521 35.157 0.00 0.00
F
ATOM 19264 HN ASP F 138 -35.426 -27.592 35.943 0.00 0.00
F
ATOM 19265 CA ASP F 138 -37.231 -28.154 35.572 0.00 0.00
F
ATOM 19266 HA ASP F 138 -37.701 -28.461 34.650 0.00 0.00
F
ATOM 19267 CB ASP F 138 -36.973 -29.397 36.456 0.00 0.00
F
ATOM 19268 HB1 ASP F 138 -36.053 -29.235 37.058 0.00 0.00
F
ATOM 19269 HB2 ASP F 138 -37.815 -29.620 37.146 0.00 0.00
F
ATOM 19270 CG ASP F 138 -36.757 -30.562 35.579 0.00 0.00
F
ATOM 19271 OD1 ASP F 138 -37.609 -30.821 34.719 0.00 0.00
F
ATOM 19272 OD2 ASP F 138 -35.703 -31.197 35.689 0.00 0.00
F
ATOM 19273 C ASP F 138 -38.285 -27.212 36.183 0.00 0.00
F
ATOM 19274 O ASP F 138 -38.143 -26.624 37.223 0.00 0.00
F
ATOM 19275 N GLU F 139 -39.336 -26.968 35.368 0.00 0.00
F
ATOM 19276 HN GLU F 139 -39.237 -27.309 34.436 0.00 0.00
F
ATOM 19277 CA GLU F 139 -40.408 -26.029 35.567 0.00 0.00
F

ATOM 19278 HA GLU F 139 -40.053 -25.145 36.076 0.00 0.00
F
ATOM 19279 CB GLU F 139 -40.832 -25.427 34.164 0.00 0.00
F
ATOM 19280 HB1 GLU F 139 -41.621 -24.664 34.338 0.00 0.00
F
ATOM 19281 HB2 GLU F 139 -40.006 -24.865 33.678 0.00 0.00
F
ATOM 19282 CG GLU F 139 -41.543 -26.355 33.192 0.00 0.00
F
ATOM 19283 HG1 GLU F 139 -40.812 -27.084 32.781 0.00 0.00
F
ATOM 19284 HG2 GLU F 139 -42.422 -26.845 33.662 0.00 0.00
F
ATOM 19285 CD GLU F 139 -41.907 -25.603 31.926 0.00 0.00
F
ATOM 19286 OE1 GLU F 139 -41.049 -25.395 31.075 0.00 0.00
F
ATOM 19287 OE2 GLU F 139 -42.985 -25.046 31.884 0.00 0.00
F
ATOM 19288 C GLU F 139 -41.601 -26.557 36.216 0.00 0.00
F
ATOM 19289 O GLU F 139 -42.404 -25.799 36.701 0.00 0.00
F
ATOM 19290 N ASN F 140 -41.864 -27.828 36.188 0.00 0.00
F
ATOM 19291 HN ASN F 140 -41.235 -28.445 35.722 0.00 0.00
F
ATOM 19292 CA ASN F 140 -42.989 -28.544 36.873 0.00 0.00
F
ATOM 19293 HA ASN F 140 -43.739 -27.829 37.174 0.00 0.00
F
ATOM 19294 CB ASN F 140 -43.671 -29.626 36.019 0.00 0.00
F
ATOM 19295 HB1 ASN F 140 -43.035 -30.453 35.639 0.00 0.00
F
ATOM 19296 HB2 ASN F 140 -44.482 -30.072 36.633 0.00 0.00
F
ATOM 19297 CG ASN F 140 -44.213 -28.924 34.864 0.00 0.00
F
ATOM 19298 OD1 ASN F 140 -43.866 -29.113 33.711 0.00 0.00
F
ATOM 19299 ND2 ASN F 140 -45.079 -27.913 35.105 0.00 0.00
F
ATOM 19300 HD21 ASN F 140 -45.580 -27.432 34.385 0.00 0.00
F
ATOM 19301 HD22 ASN F 140 -45.468 -27.752 36.012 0.00 0.00
F
ATOM 19302 C ASN F 140 -42.510 -29.268 38.052 0.00 0.00
F
ATOM 19303 O ASN F 140 -43.120 -29.236 39.125 0.00 0.00
F
ATOM 19304 N VAL F 141 -41.281 -29.869 37.963 0.00 0.00
F

ATOM 19305 HN VAL F 141   -40.772 -29.953 37.110 0.00 0.00
F
ATOM 19306 CA VAL F 141   -40.734 -30.647 39.058 0.00 0.00
F
ATOM 19307 HA VAL F 141   -41.569 -31.116 39.557 0.00 0.00
F
ATOM 19308 CB VAL F 141   -39.835 -31.720 38.517 0.00 0.00
F
ATOM 19309 HB VAL F 141   -39.087 -31.199 37.882 0.00 0.00
F
ATOM 19310 CG1 VAL F 141   -39.151 -32.486 39.707 0.00 0.00
F
ATOM 19311 HG11 VAL F 141   -38.425 -31.809 40.206 0.00 0.00
F
ATOM 19312 HG12 VAL F 141   -39.857 -32.852 40.483 0.00 0.00
F
ATOM 19313 HG13 VAL F 141   -38.593 -33.326 39.241 0.00 0.00
F
ATOM 19314 CG2 VAL F 141   -40.624 -32.733 37.679 0.00 0.00
F
ATOM 19315 HG21 VAL F 141   -41.338 -33.335 38.282 0.00 0.00
F
ATOM 19316 HG22 VAL F 141   -41.081 -32.162 36.843 0.00 0.00
F
ATOM 19317 HG23 VAL F 141   -39.906 -33.399 37.154 0.00 0.00
F
ATOM 19318 C   VAL F 141   -40.008 -29.829 40.048 0.00 0.00
F
ATOM 19319 O   VAL F 141   -39.026 -29.212 39.784 0.00 0.00
F
ATOM 19320 N   LEU F 142   -40.547 -29.796 41.281 0.00 0.00
F
ATOM 19321 HN LEU F 142   -41.308 -30.419 41.446 0.00 0.00
F
ATOM 19322 CA LEU F 142   -40.004 -28.991 42.320 0.00 0.00
F
ATOM 19323 HA LEU F 142   -39.871 -27.968 42.001 0.00 0.00
F
ATOM 19324 CB LEU F 142   -40.928 -28.804 43.513 0.00 0.00
F
ATOM 19325 HB1 LEU F 142   -41.009 -29.801 43.998 0.00 0.00
F
ATOM 19326 HB2 LEU F 142   -40.457 -28.089 44.221 0.00 0.00
F
ATOM 19327 CG LEU F 142   -42.338 -28.379 43.263 0.00 0.00
F
ATOM 19328 HG LEU F 142   -42.911 -29.111 42.655 0.00 0.00
F
ATOM 19329 CD1 LEU F 142   -43.054 -28.204 44.567 0.00 0.00
F
ATOM 19330 HD11 LEU F 142   -42.695 -28.931 45.327 0.00 0.00
F
ATOM 19331 HD12 LEU F 142   -42.850 -27.189 44.969 0.00 0.00
F

```
ATOM  19332 HD13 LEU F 142      -44.132 -28.304  44.318  0.00  0.00
F
ATOM  19333  CD2 LEU F 142      -42.378 -27.103  42.374  0.00  0.00
F
ATOM  19334 HD21 LEU F 142      -42.022 -27.175  41.324  0.00  0.00
F
ATOM  19335 HD22 LEU F 142      -43.381 -26.625  42.391  0.00  0.00
F
ATOM  19336 HD23 LEU F 142      -41.682 -26.364  42.826  0.00  0.00
F
ATOM  19337  C   LEU F 142      -38.639 -29.602  42.803  0.00  0.00
F
ATOM  19338  O   LEU F 142      -37.692 -28.874  43.052  0.00  0.00
F
ATOM  19339  N   ASN F 143      -38.651 -30.949  43.002  0.00  0.00
F
ATOM  19340  HN  ASN F 143      -39.457 -31.519  42.863  0.00  0.00
F
ATOM  19341  CA  ASN F 143      -37.545 -31.578  43.678  0.00  0.00
F
ATOM  19342  HA  ASN F 143      -36.677 -30.984  43.430  0.00  0.00
F
ATOM  19343  CB  ASN F 143      -37.860 -31.601  45.183  0.00  0.00
F
ATOM  19344  HB1 ASN F 143      -38.083 -30.557  45.490  0.00  0.00
F
ATOM  19345  HB2 ASN F 143      -38.780 -32.184  45.402  0.00  0.00
F
ATOM  19346  CG  ASN F 143      -36.656 -32.022  45.955  0.00  0.00
F
ATOM  19347  OD1 ASN F 143      -35.538 -32.252  45.494  0.00  0.00
F
ATOM  19348  ND2 ASN F 143      -36.818 -32.220  47.275  0.00  0.00
F
ATOM  19349 HD21 ASN F 143      -35.971 -32.275  47.804  0.00  0.00
F
ATOM  19350 HD22 ASN F 143      -37.713 -32.270  47.718  0.00  0.00
F
ATOM  19351  C   ASN F 143      -37.460 -32.995  43.026  0.00  0.00
F
ATOM  19352  O   ASN F 143      -38.425 -33.651  42.582  0.00  0.00
F
ATOM  19353  N   TYR F 144      -36.196 -33.538  43.020  0.00  0.00
F
ATOM  19354  HN  TYR F 144      -35.454 -33.054  43.478  0.00  0.00
F
ATOM  19355  CA  TYR F 144      -35.890 -34.775  42.286  0.00  0.00
F
ATOM  19356  HA  TYR F 144      -36.795 -35.353  42.165  0.00  0.00
F
ATOM  19357  CB  TYR F 144      -35.244 -34.304  40.903  0.00  0.00
F
ATOM  19358  HB1 TYR F 144      -35.824 -33.443  40.509  0.00  0.00
F
```

ATOM 19359 HB2 TYR F 144   -34.307 -33.778 41.185 0.00 0.00
F
ATOM 19360 CG TYR F 144   -35.056 -35.437 39.932 0.00 0.00
F
ATOM 19361 CD1 TYR F 144   -33.945 -36.335 40.052 0.00 0.00
F
ATOM 19362 HD1 TYR F 144   -33.292 -36.124 40.885 0.00 0.00
F
ATOM 19363 CE1 TYR F 144   -33.741 -37.295 39.134 0.00 0.00
F
ATOM 19364 HE1 TYR F 144   -32.999 -38.064 39.289 0.00 0.00
F
ATOM 19365 CZ TYR F 144   -34.482 -37.250 37.916 0.00 0.00
F
ATOM 19366 OH TYR F 144   -34.384 -38.408 37.065 0.00 0.00
F
ATOM 19367 HH TYR F 144   -35.096 -38.391 36.420 0.00 0.00
F
ATOM 19368 CD2 TYR F 144   -35.827 -35.429 38.772 0.00 0.00
F
ATOM 19369 HD2 TYR F 144   -36.682 -34.778 38.659 0.00 0.00
F
ATOM 19370 CE2 TYR F 144   -35.528 -36.341 37.672 0.00 0.00
F
ATOM 19371 HE2 TYR F 144   -36.217 -36.450 36.848 0.00 0.00
F
ATOM 19372 C   TYR F 144   -34.887 -35.521 43.154 0.00 0.00
F
ATOM 19373 O   TYR F 144   -33.829 -35.054 43.590 0.00 0.00
F
ATOM 19374 N   ILE F 145   -35.245 -36.790 43.382 0.00 0.00
F
ATOM 19375 HN ILE F 145   -35.970 -37.198 42.832 0.00 0.00
F
ATOM 19376 CA ILE F 145   -34.306 -37.720 44.145 0.00 0.00
F
ATOM 19377 HA ILE F 145   -33.323 -37.280 44.219 0.00 0.00
F
ATOM 19378 CB ILE F 145   -34.683 -37.790 45.677 0.00 0.00
F
ATOM 19379 HB ILE F 145   -34.458 -36.745 45.980 0.00 0.00
F
ATOM 19380 CG2 ILE F 145   -36.136 -38.092 45.932 0.00 0.00
F
ATOM 19381 HG21 ILE F 145   -36.285 -39.157 45.652 0.00 0.00
F
ATOM 19382 HG22 ILE F 145   -36.349 -38.040 47.021 0.00 0.00
F
ATOM 19383 HG23 ILE F 145   -36.786 -37.447 45.303 0.00 0.00
F
ATOM 19384 CG1 ILE F 145   -33.694 -38.700 46.406 0.00 0.00
F
ATOM 19385 HG11 ILE F 145   -33.887 -39.761 46.140 0.00 0.00
F

```
ATOM  19386 HG12 ILE F 145     -32.683 -38.532  45.978  0.00  0.00
F
ATOM  19387  CD  ILE F 145     -33.705 -38.432  47.922  0.00  0.00
F
ATOM  19388  HD1 ILE F 145     -33.983 -37.379  48.141  0.00  0.00
F
ATOM  19389  HD2 ILE F 145     -34.586 -38.995  48.297  0.00  0.00
F
ATOM  19390  HD3 ILE F 145     -32.802 -38.702  48.510  0.00  0.00
F
ATOM  19391  C   ILE F 145     -34.220 -39.026  43.411  0.00  0.00
F
ATOM  19392  O   ILE F 145     -35.223 -39.641  43.000  0.00  0.00
F
ATOM  19393  N   ASP F 146     -32.957 -39.519  43.157  0.00  0.00
F
ATOM  19394  HN  ASP F 146     -32.111 -39.000  43.244  0.00  0.00
F
ATOM  19395  CA  ASP F 146     -32.739 -40.745  42.407  0.00  0.00
F
ATOM  19396  HA  ASP F 146     -33.436 -40.640  41.589  0.00  0.00
F
ATOM  19397  CB  ASP F 146     -31.371 -41.116  41.894  0.00  0.00
F
ATOM  19398  HB1 ASP F 146     -30.598 -40.943  42.673  0.00  0.00
F
ATOM  19399  HB2 ASP F 146     -31.278 -42.191  41.630  0.00  0.00
F
ATOM  19400  CG  ASP F 146     -30.973 -40.272  40.731  0.00  0.00
F
ATOM  19401  OD1 ASP F 146     -30.326 -39.250  41.020  0.00  0.00
F
ATOM  19402  OD2 ASP F 146     -30.988 -40.776  39.610  0.00  0.00
F
ATOM  19403  C   ASP F 146     -33.095 -41.837  43.443  0.00  0.00
F
ATOM  19404  O   ASP F 146     -32.907 -41.706  44.618  0.00  0.00
F
ATOM  19405  N   LEU F 147     -33.740 -42.953  42.947  0.00  0.00
F
ATOM  19406  HN  LEU F 147     -33.776 -42.913  41.952  0.00  0.00
F
ATOM  19407  CA  LEU F 147     -34.136 -44.105  43.717  0.00  0.00
F
ATOM  19408  HA  LEU F 147     -34.101 -43.878  44.773  0.00  0.00
F
ATOM  19409  CB  LEU F 147     -35.615 -44.438  43.398  0.00  0.00
F
ATOM  19410  HB1 LEU F 147     -36.058 -43.486  43.759  0.00  0.00
F
ATOM  19411  HB2 LEU F 147     -35.556 -44.560  42.295  0.00  0.00
F
ATOM  19412  CG  LEU F 147     -36.284 -45.663  44.085  0.00  0.00
F
```

ATOM 19413 HG LEU F 147   -35.822 -46.634 43.803 0.00 0.00
F
ATOM 19414 CD1 LEU F 147   -36.076 -45.526 45.630 0.00 0.00
F
ATOM 19415 HD11 LEU F 147   -36.627 -46.402 46.033 0.00 0.00
F
ATOM 19416 HD12 LEU F 147   -34.989 -45.680 45.803 0.00 0.00
F
ATOM 19417 HD13 LEU F 147   -36.343 -44.521 46.021 0.00 0.00
F
ATOM 19418 CD2 LEU F 147   -37.744 -45.600 43.776 0.00 0.00
F
ATOM 19419 HD21 LEU F 147   -38.179 -44.657 44.169 0.00 0.00
F
ATOM 19420 HD22 LEU F 147   -38.011 -45.764 42.711 0.00 0.00
F
ATOM 19421 HD23 LEU F 147   -38.293 -46.360 44.373 0.00 0.00
F
ATOM 19422 C   LEU F 147   -33.108 -45.248 43.344 0.00 0.00
F
ATOM 19423 O   LEU F 147   -32.896 -46.150 44.153 0.00 0.00
F
ATOM 19424 N   SER F 148   -32.471 -45.070 42.240 0.00 0.00
F
ATOM 19425 HN SER F 148   -32.774 -44.323 41.653 0.00 0.00
F
ATOM 19426 CA SER F 148   -31.462 -45.977 41.716 0.00 0.00
F
ATOM 19427 HA SER F 148   -30.568 -45.688 42.249 0.00 0.00
F
ATOM 19428 CB SER F 148   -31.787 -47.515 41.805 0.00 0.00
F
ATOM 19429 HB1 SER F 148   -31.868 -47.863 42.857 0.00 0.00
F
ATOM 19430 HB2 SER F 148   -32.748 -47.793 41.322 0.00 0.00
F
ATOM 19431 OG SER F 148   -30.722 -48.325 41.261 0.00 0.00
F
ATOM 19432 HG1 SER F 148   -30.452 -48.776 42.064 0.00 0.00
F
ATOM 19433 C   SER F 148   -31.304 -45.447 40.301 0.00 0.00
F
ATOM 19434 O   SER F 148   -31.791 -44.401 39.983 0.00 0.00
F
ATOM 19435 N   ASP F 149   -30.431 -46.152 39.500 0.00 0.00
F
ATOM 19436 HN ASP F 149   -30.079 -47.019 39.843 0.00 0.00
F
ATOM 19437 CA ASP F 149   -30.200 -45.879 38.171 0.00 0.00
F
ATOM 19438 HA ASP F 149   -29.534 -45.030 38.120 0.00 0.00
F
ATOM 19439 CB ASP F 149   -29.347 -46.989 37.528 0.00 0.00
F

ATOM 19440 HB1 ASP F 149   −28.516 −47.313 38.189 0.00 0.00
F
ATOM 19441 HB2 ASP F 149   −30.017 −47.826 37.235 0.00 0.00
F
ATOM 19442 CG ASP F 149   −28.620 −46.651 36.255 0.00 0.00
F
ATOM 19443 OD1 ASP F 149   −27.711 −47.405 35.839 0.00 0.00
F
ATOM 19444 OD2 ASP F 149   −28.911 −45.534 35.718 0.00 0.00
F
ATOM 19445 C   ASP F 149   −31.465 −45.668 37.353 0.00 0.00
F
ATOM 19446 O   ASP F 149   −31.682 −44.625 36.700 0.00 0.00
F
ATOM 19447 N   GLU F 150   −32.423 −46.605 37.368 0.00 0.00
F
ATOM 19448 HN GLU F 150   −32.292 −47.403 37.952 0.00 0.00
F
ATOM 19449 CA GLU F 150   −33.499 −46.669 36.431 0.00 0.00
F
ATOM 19450 HA GLU F 150   −33.188 −46.289 35.469 0.00 0.00
F
ATOM 19451 CB GLU F 150   −33.931 −48.071 36.047 0.00 0.00
F
ATOM 19452 HB1 GLU F 150   −34.375 −48.653 36.883 0.00 0.00
F
ATOM 19453 HB2 GLU F 150   −34.794 −48.076 35.348 0.00 0.00
F
ATOM 19454 CG GLU F 150   −32.787 −48.929 35.460 0.00 0.00
F
ATOM 19455 HG1 GLU F 150   −31.994 −48.951 36.238 0.00 0.00
F
ATOM 19456 HG2 GLU F 150   −33.136 −49.970 35.293 0.00 0.00
F
ATOM 19457 CD GLU F 150   −32.332 −48.496 34.081 0.00 0.00
F
ATOM 19458 OE1 GLU F 150   −33.272 −48.309 33.237 0.00 0.00
F
ATOM 19459 OE2 GLU F 150   −31.118 −48.394 33.783 0.00 0.00
F
ATOM 19460 C   GLU F 150   −34.802 −45.917 36.869 0.00 0.00
F
ATOM 19461 O   GLU F 150   −35.701 −45.580 36.071 0.00 0.00
F
ATOM 19462 N   TYR F 151   −34.894 −45.548 38.137 0.00 0.00
F
ATOM 19463 HN TYR F 151   −34.059 −45.742 38.646 0.00 0.00
F
ATOM 19464 CA TYR F 151   −36.081 −45.062 38.756 0.00 0.00
F
ATOM 19465 HA TYR F 151   −36.864 −44.855 38.040 0.00 0.00
F
ATOM 19466 CB TYR F 151   −36.548 −46.108 39.850 0.00 0.00
F

ATOM 19467 HB1 TYR F 151 -35.782 -46.218 40.647 0.00 0.00
F
ATOM 19468 HB2 TYR F 151 -37.537 -45.735 40.194 0.00 0.00
F
ATOM 19469 CG TYR F 151 -36.762 -47.394 39.194 0.00 0.00
F
ATOM 19470 CD1 TYR F 151 -37.955 -47.656 38.471 0.00 0.00
F
ATOM 19471 HD1 TYR F 151 -38.771 -46.959 38.351 0.00 0.00
F
ATOM 19472 CE1 TYR F 151 -38.064 -48.856 37.853 0.00 0.00
F
ATOM 19473 HE1 TYR F 151 -39.007 -49.108 37.391 0.00 0.00
F
ATOM 19474 CZ TYR F 151 -37.189 -49.859 38.121 0.00 0.00
F
ATOM 19475 OH TYR F 151 -37.249 -51.115 37.513 0.00 0.00
F
ATOM 19476 HH TYR F 151 -37.858 -50.916 36.798 0.00 0.00
F
ATOM 19477 CD2 TYR F 151 -35.838 -48.460 39.402 0.00 0.00
F
ATOM 19478 HD2 TYR F 151 -34.921 -48.237 39.928 0.00 0.00
F
ATOM 19479 CE2 TYR F 151 -36.098 -49.723 38.945 0.00 0.00
F
ATOM 19480 HE2 TYR F 151 -35.332 -50.476 39.053 0.00 0.00
F
ATOM 19481 C TYR F 151 -35.843 -43.750 39.456 0.00 0.00
F
ATOM 19482 O TYR F 151 -34.671 -43.497 39.828 0.00 0.00
F
ATOM 19483 N SER F 152 -36.820 -42.834 39.539 0.00 0.00
F
ATOM 19484 HN SER F 152 -37.662 -43.216 39.166 0.00 0.00
F
ATOM 19485 CA SER F 152 -36.631 -41.624 40.235 0.00 0.00
F
ATOM 19486 HA SER F 152 -35.867 -41.862 40.960 0.00 0.00
F
ATOM 19487 CB SER F 152 -36.248 -40.420 39.325 0.00 0.00
F
ATOM 19488 HB1 SER F 152 -36.969 -40.241 38.498 0.00 0.00
F
ATOM 19489 HB2 SER F 152 -36.100 -39.542 39.989 0.00 0.00
F
ATOM 19490 OG SER F 152 -34.964 -40.660 38.690 0.00 0.00
F
ATOM 19491 HG1 SER F 152 -34.701 -39.839 38.269 0.00 0.00
F
ATOM 19492 C SER F 152 -37.833 -41.182 40.962 0.00 0.00
F
ATOM 19493 O SER F 152 -38.947 -41.545 40.564 0.00 0.00
F

```
ATOM  19494  N   ILE F 153     -37.721 -40.593  42.143  0.00  0.00
F
ATOM  19495  HN  ILE F 153     -36.847 -40.361  42.563  0.00  0.00
F
ATOM  19496  CA  ILE F 153     -38.849 -40.045  42.939  0.00  0.00
F
ATOM  19497  HA  ILE F 153     -39.798 -40.393  42.559  0.00  0.00
F
ATOM  19498  CB  ILE F 153     -38.782 -40.358  44.357  0.00  0.00
F
ATOM  19499  HB  ILE F 153     -37.896 -39.803  44.735  0.00  0.00
F
ATOM  19500  CG2 ILE F 153     -40.055 -39.880  45.194  0.00  0.00
F
ATOM  19501 HG21 ILE F 153     -39.860 -40.058  46.273  0.00  0.00
F
ATOM  19502 HG22 ILE F 153     -40.414 -38.855  44.960  0.00  0.00
F
ATOM  19503 HG23 ILE F 153     -40.814 -40.602  44.822  0.00  0.00
F
ATOM  19504  CG1 ILE F 153     -38.687 -41.888  44.662  0.00  0.00
F
ATOM  19505 HG11 ILE F 153     -39.647 -42.436  44.546  0.00  0.00
F
ATOM  19506 HG12 ILE F 153     -38.121 -42.444  43.884  0.00  0.00
F
ATOM  19507  CD  ILE F 153     -37.980 -42.172  45.962  0.00  0.00
F
ATOM  19508  HD1 ILE F 153     -36.911 -41.886  46.061  0.00  0.00
F
ATOM  19509  HD2 ILE F 153     -38.494 -41.561  46.735  0.00  0.00
F
ATOM  19510  HD3 ILE F 153     -38.077 -43.245  46.235  0.00  0.00
F
ATOM  19511  C   ILE F 153     -38.810 -38.530  42.800  0.00  0.00
F
ATOM  19512  O   ILE F 153     -37.750 -37.897  42.971  0.00  0.00
F
ATOM  19513  N   VAL F 154     -40.006 -37.956  42.486  0.00  0.00
F
ATOM  19514  HN  VAL F 154     -40.907 -38.325  42.273  0.00  0.00
F
ATOM  19515  CA  VAL F 154     -39.971 -36.526  42.243  0.00  0.00
F
ATOM  19516  HA  VAL F 154     -39.076 -36.051  42.618  0.00  0.00
F
ATOM  19517  CB  VAL F 154     -40.055 -36.087  40.748  0.00  0.00
F
ATOM  19518  HB  VAL F 154     -40.140 -34.981  40.690  0.00  0.00
F
ATOM  19519  CG1 VAL F 154     -38.793 -36.455  39.975  0.00  0.00
F
ATOM  19520 HG11 VAL F 154     -38.846 -35.945  38.989  0.00  0.00
F
```

```
ATOM  19521 HG12 VAL F 154      -37.891 -36.286  40.602  0.00  0.00
F
ATOM  19522 HG13 VAL F 154      -38.738 -37.533  39.712  0.00  0.00
F
ATOM  19523  CG2 VAL F 154      -41.278 -36.531  39.950  0.00  0.00
F
ATOM  19524 HG21 VAL F 154      -41.249 -36.429  38.844  0.00  0.00
F
ATOM  19525 HG22 VAL F 154      -41.496 -37.594  40.186  0.00  0.00
F
ATOM  19526 HG23 VAL F 154      -42.206 -36.033  40.305  0.00  0.00
F
ATOM  19527  C   VAL F 154      -41.146 -35.882  42.914  0.00  0.00
F
ATOM  19528  O   VAL F 154      -42.211 -36.483  43.115  0.00  0.00
F
ATOM  19529  N   GLU F 155      -40.949 -34.589  43.373  0.00  0.00
F
ATOM  19530  HN  GLU F 155      -40.061 -34.137  43.324  0.00  0.00
F
ATOM  19531  CA  GLU F 155      -42.094 -33.713  43.855  0.00  0.00
F
ATOM  19532  HA  GLU F 155      -42.930 -34.331  44.148  0.00  0.00
F
ATOM  19533  CB  GLU F 155      -41.738 -32.842  45.112  0.00  0.00
F
ATOM  19534  HB1 GLU F 155      -41.248 -33.514  45.848  0.00  0.00
F
ATOM  19535  HB2 GLU F 155      -41.014 -32.083  44.746  0.00  0.00
F
ATOM  19536  CG  GLU F 155      -42.997 -32.222  45.638  0.00  0.00
F
ATOM  19537  HG1 GLU F 155      -43.411 -31.600  44.815  0.00  0.00
F
ATOM  19538  HG2 GLU F 155      -43.667 -33.044  45.968  0.00  0.00
F
ATOM  19539  CD  GLU F 155      -42.774 -31.357  46.852  0.00  0.00
F
ATOM  19540  OE1 GLU F 155      -41.705 -31.340  47.538  0.00  0.00
F
ATOM  19541  OE2 GLU F 155      -43.664 -30.619  47.278  0.00  0.00
F
ATOM  19542  C   GLU F 155      -42.645 -32.884  42.705  0.00  0.00
F
ATOM  19543  O   GLU F 155      -41.994 -32.092  42.042  0.00  0.00
F
ATOM  19544  N   LEU F 156      -43.924 -33.016  42.302  0.00  0.00
F
ATOM  19545  HN  LEU F 156      -44.574 -33.624  42.752  0.00  0.00
F
ATOM  19546  CA  LEU F 156      -44.397 -32.432  41.070  0.00  0.00
F
ATOM  19547  HA  LEU F 156      -43.693 -31.792  40.560  0.00  0.00
F
```

ATOM	19548	CB	LEU	F	156	−44.921	−33.668	40.229	0.00	0.00	F
ATOM	19549	HB1	LEU	F	156	−43.962	−34.188	40.016	0.00	0.00	F
ATOM	19550	HB2	LEU	F	156	−45.631	−34.266	40.839	0.00	0.00	F
ATOM	19551	CG	LEU	F	156	−45.683	−33.300	38.964	0.00	0.00	F
ATOM	19552	HG	LEU	F	156	−46.652	−32.807	39.190	0.00	0.00	F
ATOM	19553	CD1	LEU	F	156	−44.956	−32.418	37.971	0.00	0.00	F
ATOM	19554	HD11	LEU	F	156	−44.708	−31.427	38.409	0.00	0.00	F
ATOM	19555	HD12	LEU	F	156	−43.935	−32.774	37.715	0.00	0.00	F
ATOM	19556	HD13	LEU	F	156	−45.554	−32.301	37.041	0.00	0.00	F
ATOM	19557	CD2	LEU	F	156	−46.066	−34.667	38.267	0.00	0.00	F
ATOM	19558	HD21	LEU	F	156	−45.091	−34.968	37.826	0.00	0.00	F
ATOM	19559	HD22	LEU	F	156	−46.332	−35.398	39.060	0.00	0.00	F
ATOM	19560	HD23	LEU	F	156	−46.793	−34.469	37.450	0.00	0.00	F
ATOM	19561	C	LEU	F	156	−45.561	−31.547	41.334	0.00	0.00	F
ATOM	19562	O	LEU	F	156	−46.340	−31.794	42.254	0.00	0.00	F
ATOM	19563	N	LEU	F	157	−45.711	−30.350	40.740	0.00	0.00	F
ATOM	19564	HN	LEU	F	157	−45.212	−30.120	39.908	0.00	0.00	F
ATOM	19565	CA	LEU	F	157	−46.779	−29.420	41.054	0.00	0.00	F
ATOM	19566	HA	LEU	F	157	−47.311	−29.798	41.915	0.00	0.00	F
ATOM	19567	CB	LEU	F	157	−46.234	−27.976	41.233	0.00	0.00	F
ATOM	19568	HB1	LEU	F	157	−45.477	−27.990	42.045	0.00	0.00	F
ATOM	19569	HB2	LEU	F	157	−45.711	−27.705	40.291	0.00	0.00	F
ATOM	19570	CG	LEU	F	157	−47.234	−26.853	41.554	0.00	0.00	F
ATOM	19571	HG	LEU	F	157	−48.100	−26.827	40.860	0.00	0.00	F
ATOM	19572	CD1	LEU	F	157	−47.775	−27.028	42.984	0.00	0.00	F
ATOM	19573	HD11	LEU	F	157	−46.908	−27.105	43.674	0.00	0.00	F
ATOM	19574	HD12	LEU	F	157	−48.450	−26.170	43.192	0.00	0.00	F

ATOM 19575 HD13 LEU F 157   -48.430 -27.925 42.995 0.00 0.00
F
ATOM 19576 CD2 LEU F 157   -46.582 -25.425 41.651 0.00 0.00
F
ATOM 19577 HD21 LEU F 157   -45.858 -25.431 42.494 0.00 0.00
F
ATOM 19578 HD22 LEU F 157   -46.132 -25.216 40.657 0.00 0.00
F
ATOM 19579 HD23 LEU F 157   -47.376 -24.674 41.851 0.00 0.00
F
ATOM 19580 C   LEU F 157   -47.695 -29.416 39.834 0.00 0.00
F
ATOM 19581 O   LEU F 157   -47.313 -29.168 38.700 0.00 0.00
F
ATOM 19582 N   ALA F 158   -49.009 -29.663 40.100 0.00 0.00
F
ATOM 19583 HN ALA F 158   -49.269 -29.578 41.059 0.00 0.00
F
ATOM 19584 CA ALA F 158   -50.076 -29.916 39.082 0.00 0.00
F
ATOM 19585 HA ALA F 158   -49.689 -30.672 38.415 0.00 0.00
F
ATOM 19586 CB ALA F 158   -51.330 -30.471 39.800 0.00 0.00
F
ATOM 19587 HB1 ALA F 158   -51.636 -29.873 40.685 0.00 0.00
F
ATOM 19588 HB2 ALA F 158   -52.065 -30.711 39.001 0.00 0.00
F
ATOM 19589 HB3 ALA F 158   -51.070 -31.489 40.161 0.00 0.00
F
ATOM 19590 C   ALA F 158   -50.383 -28.766 38.242 0.00 0.00
F
ATOM 19591 O   ALA F 158   -50.377 -27.639 38.646 0.00 0.00
F
ATOM 19592 N   THR F 159   -50.598 -28.958 36.954 0.00 0.00
F
ATOM 19593 HN THR F 159   -50.503 -29.892 36.616 0.00 0.00
F
ATOM 19594 CA THR F 159   -51.069 -27.963 35.964 0.00 0.00
F
ATOM 19595 HA THR F 159   -50.672 -26.992 36.221 0.00 0.00
F
ATOM 19596 CB THR F 159   -50.680 -28.335 34.560 0.00 0.00
F
ATOM 19597 HB THR F 159   -51.245 -27.691 33.853 0.00 0.00
F
ATOM 19598 OG1 THR F 159   -51.005 -29.704 34.252 0.00 0.00
F
ATOM 19599 HG1 THR F 159   -50.583 -29.890 33.410 0.00 0.00
F
ATOM 19600 CG2 THR F 159   -49.203 -28.223 34.387 0.00 0.00
F
ATOM 19601 HG21 THR F 159   -48.722 -28.898 35.126 0.00 0.00
F

ATOM	19602	HG22	THR	F	159	−48.832	−28.347	33.347	0.00	0.00	F
ATOM	19603	HG23	THR	F	159	−48.968	−27.172	34.658	0.00	0.00	F
ATOM	19604	C	THR	F	159	−52.669	−27.798	35.905	0.00	0.00	F
ATOM	19605	O	THR	F	159	−53.421	−28.592	36.446	0.00	0.00	F
ATOM	19606	N	ARG	F	160	−53.153	−26.779	35.194	0.00	0.00	F
ATOM	19607	HN	ARG	F	160	−52.549	−26.137	34.729	0.00	0.00	F
ATOM	19608	CA	ARG	F	160	−54.583	−26.519	34.986	0.00	0.00	F
ATOM	19609	HA	ARG	F	160	−55.128	−26.417	35.913	0.00	0.00	F
ATOM	19610	CB	ARG	F	160	−54.683	−25.118	34.231	0.00	0.00	F
ATOM	19611	HB1	ARG	F	160	−53.815	−24.462	34.457	0.00	0.00	F
ATOM	19612	HB2	ARG	F	160	−54.672	−25.295	33.134	0.00	0.00	F
ATOM	19613	CG	ARG	F	160	−55.911	−24.270	34.620	0.00	0.00	F
ATOM	19614	HG1	ARG	F	160	−56.791	−24.534	33.995	0.00	0.00	F
ATOM	19615	HG2	ARG	F	160	−56.199	−24.353	35.690	0.00	0.00	F
ATOM	19616	CD	ARG	F	160	−55.833	−22.729	34.263	0.00	0.00	F
ATOM	19617	HD1	ARG	F	160	−55.729	−22.492	33.183	0.00	0.00	F
ATOM	19618	HD2	ARG	F	160	−56.672	−22.130	34.680	0.00	0.00	F
ATOM	19619	NE	ARG	F	160	−54.693	−22.297	35.060	0.00	0.00	F
ATOM	19620	HE	ARG	F	160	−54.361	−22.887	35.796	0.00	0.00	F
ATOM	19621	CZ	ARG	F	160	−54.101	−21.085	34.984	0.00	0.00	F
ATOM	19622	NH1	ARG	F	160	−54.450	−20.182	34.059	0.00	0.00	F
ATOM	19623	HH11	ARG	F	160	−53.839	−19.394	33.988	0.00	0.00	F
ATOM	19624	HH12	ARG	F	160	−55.291	−20.293	33.530	0.00	0.00	F
ATOM	19625	NH2	ARG	F	160	−53.161	−20.762	35.869	0.00	0.00	F
ATOM	19626	HH21	ARG	F	160	−52.570	−19.958	35.805	0.00	0.00	F
ATOM	19627	HH22	ARG	F	160	−52.961	−21.456	36.560	0.00	0.00	F
ATOM	19628	C	ARG	F	160	−55.419	−27.519	34.191	0.00	0.00	F

ATOM 19629 O ARG F 160 -56.659 -27.567 34.123 0.00 0.00
F
ATOM 19630 N LYS F 161 -54.766 -28.490 33.482 0.00 0.00
F
ATOM 19631 HN LYS F 161 -53.782 -28.331 33.489 0.00 0.00
F
ATOM 19632 CA LYS F 161 -55.295 -29.679 32.807 0.00 0.00
F
ATOM 19633 HA LYS F 161 -56.270 -29.558 32.360 0.00 0.00
F
ATOM 19634 CB LYS F 161 -54.272 -30.058 31.646 0.00 0.00
F
ATOM 19635 HB1 LYS F 161 -53.218 -29.996 31.991 0.00 0.00
F
ATOM 19636 HB2 LYS F 161 -54.383 -31.094 31.261 0.00 0.00
F
ATOM 19637 CG LYS F 161 -54.429 -29.158 30.445 0.00 0.00
F
ATOM 19638 HG1 LYS F 161 -55.386 -29.351 29.914 0.00 0.00
F
ATOM 19639 HG2 LYS F 161 -54.331 -28.124 30.840 0.00 0.00
F
ATOM 19640 CD LYS F 161 -53.221 -29.358 29.455 0.00 0.00
F
ATOM 19641 HD1 LYS F 161 -52.294 -29.213 30.051 0.00 0.00
F
ATOM 19642 HD2 LYS F 161 -53.259 -30.444 29.223 0.00 0.00
F
ATOM 19643 CE LYS F 161 -53.261 -28.284 28.297 0.00 0.00
F
ATOM 19644 HE1 LYS F 161 -53.412 -27.241 28.649 0.00 0.00
F
ATOM 19645 HE2 LYS F 161 -52.277 -28.217 27.787 0.00 0.00
F
ATOM 19646 NZ LYS F 161 -54.262 -28.579 27.272 0.00 0.00
F
ATOM 19647 HZ1 LYS F 161 -54.324 -29.603 27.102 0.00 0.00
F
ATOM 19648 HZ2 LYS F 161 -55.132 -28.138 27.632 0.00 0.00
F
ATOM 19649 HZ3 LYS F 161 -53.995 -28.128 26.374 0.00 0.00
F
ATOM 19650 C LYS F 161 -55.461 -30.801 33.806 0.00 0.00
F
ATOM 19651 O LYS F 161 -56.309 -31.674 33.609 0.00 0.00
F
ATOM 19652 N LEU F 162 -54.642 -30.815 34.916 0.00 0.00
F
ATOM 19653 HN LEU F 162 -53.970 -30.110 35.127 0.00 0.00
F
ATOM 19654 CA LEU F 162 -54.739 -31.801 35.929 0.00 0.00
F
ATOM 19655 HA LEU F 162 -55.227 -32.722 35.646 0.00 0.00
F

ATOM 19656 CB LEU F 162   -53.280 -32.158 36.413 0.00 0.00
F
ATOM 19657 HB1 LEU F 162   -52.705 -32.390 35.491 0.00 0.00
F
ATOM 19658 HB2 LEU F 162   -52.883 -31.165 36.713 0.00 0.00
F
ATOM 19659 CG LEU F 162   -53.048 -33.310 37.456 0.00 0.00
F
ATOM 19660 HG LEU F 162   -53.559 -32.987 38.388 0.00 0.00
F
ATOM 19661 CD1 LEU F 162   -53.655 -34.685 37.142 0.00 0.00
F
ATOM 19662 HD11 LEU F 162   -53.428 -35.373 37.985 0.00 0.00
F
ATOM 19663 HD12 LEU F 162   -54.732 -34.469 36.979 0.00 0.00
F
ATOM 19664 HD13 LEU F 162   -53.258 -35.083 36.183 0.00 0.00
F
ATOM 19665 CD2 LEU F 162   -51.534 -33.435 37.625 0.00 0.00
F
ATOM 19666 HD21 LEU F 162   -51.046 -33.665 36.654 0.00 0.00
F
ATOM 19667 HD22 LEU F 162   -51.079 -32.620 38.228 0.00 0.00
F
ATOM 19668 HD23 LEU F 162   -51.292 -34.322 38.248 0.00 0.00
F
ATOM 19669 C   LEU F 162   -55.585 -31.276 37.140 0.00 0.00
F
ATOM 19670 O   LEU F 162   -55.583 -31.873 38.199 0.00 0.00
F
ATOM 19671 N   ASP F 163   -56.387 -30.212 36.883 0.00 0.00
F
ATOM 19672 HN ASP F 163   -56.225 -29.764 36.007 0.00 0.00
F
ATOM 19673 CA ASP F 163   -57.163 -29.391 37.779 0.00 0.00
F
ATOM 19674 HA ASP F 163   -56.681 -29.273 38.739 0.00 0.00
F
ATOM 19675 CB ASP F 163   -57.373 -28.020 37.193 0.00 0.00
F
ATOM 19676 HB1 ASP F 163   -56.362 -27.573 37.089 0.00 0.00
F
ATOM 19677 HB2 ASP F 163   -57.860 -27.995 36.194 0.00 0.00
F
ATOM 19678 CG ASP F 163   -58.162 -27.137 38.059 0.00 0.00
F
ATOM 19679 OD1 ASP F 163   -57.838 -26.953 39.268 0.00 0.00
F
ATOM 19680 OD2 ASP F 163   -59.126 -26.502 37.497 0.00 0.00
F
ATOM 19681 C   ASP F 163   -58.516 -30.125 37.990 0.00 0.00
F
ATOM 19682 O   ASP F 163   -59.146 -30.267 36.971 0.00 0.00
F

```
ATOM  19683  N   SER F 164      -58.871 -30.560  39.201  0.00  0.00
F
ATOM  19684  HN  SER F 164      -58.209 -30.686  39.936  0.00  0.00
F
ATOM  19685  CA  SER F 164      -60.059 -31.387  39.532  0.00  0.00
F
ATOM  19686  HA  SER F 164      -60.071 -31.566  40.597  0.00  0.00
F
ATOM  19687  CB  SER F 164      -61.451 -30.836  39.104  0.00  0.00
F
ATOM  19688  HB1 SER F 164      -61.497 -30.782  37.995  0.00  0.00
F
ATOM  19689  HB2 SER F 164      -62.265 -31.455  39.538  0.00  0.00
F
ATOM  19690  OG  SER F 164      -61.603 -29.569  39.730  0.00  0.00
F
ATOM  19691  HG1 SER F 164      -61.631 -28.946  39.000  0.00  0.00
F
ATOM  19692  C   SER F 164      -60.091 -32.775  39.069  0.00  0.00
F
ATOM  19693  O   SER F 164      -61.191 -33.277  38.717  0.00  0.00
F
ATOM  19694  N   LYS F 165      -58.955 -33.495  39.073  0.00  0.00
F
ATOM  19695  HN  LYS F 165      -58.170 -33.084  39.530  0.00  0.00
F
ATOM  19696  CA  LYS F 165      -58.878 -34.863  38.544  0.00  0.00
F
ATOM  19697  HA  LYS F 165      -59.729 -35.051  37.906  0.00  0.00
F
ATOM  19698  CB  LYS F 165      -57.590 -35.122  37.782  0.00  0.00
F
ATOM  19699  HB1 LYS F 165      -56.736 -34.831  38.430  0.00  0.00
F
ATOM  19700  HB2 LYS F 165      -57.438 -36.222  37.742  0.00  0.00
F
ATOM  19701  CG  LYS F 165      -57.422 -34.445  36.360  0.00  0.00
F
ATOM  19702  HG1 LYS F 165      -57.734 -33.393  36.536  0.00  0.00
F
ATOM  19703  HG2 LYS F 165      -56.365 -34.583  36.047  0.00  0.00
F
ATOM  19704  CD  LYS F 165      -58.428 -35.054  35.400  0.00  0.00
F
ATOM  19705  HD1 LYS F 165      -58.416 -36.152  35.570  0.00  0.00
F
ATOM  19706  HD2 LYS F 165      -59.459 -34.653  35.498  0.00  0.00
F
ATOM  19707  CE  LYS F 165      -58.176 -34.885  33.900  0.00  0.00
F
ATOM  19708  HE1 LYS F 165      -57.124 -35.054  33.588  0.00  0.00
F
ATOM  19709  HE2 LYS F 165      -58.854 -35.504  33.275  0.00  0.00
F
```

ATOM 19710 NZ LYS F 165   -58.409 -33.497 33.451 0.00 0.00
F
ATOM 19711 HZ1 LYS F 165   -59.319 -33.228 33.876 0.00 0.00
F
ATOM 19712 HZ2 LYS F 165   -57.623 -32.850 33.663 0.00 0.00
F
ATOM 19713 HZ3 LYS F 165   -58.452 -33.399 32.416 0.00 0.00
F
ATOM 19714 C   LYS F 165   -58.875 -35.822 39.770 0.00 0.00
F
ATOM 19715 O   LYS F 165   -58.050 -35.791 40.638 0.00 0.00
F
ATOM 19716 N   SER F 166   -59.683 -36.822 39.600 0.00 0.00
F
ATOM 19717 HN SER F 166   -60.288 -36.841 38.807 0.00 0.00
F
ATOM 19718 CA SER F 166   -59.749 -38.064 40.388 0.00 0.00
F
ATOM 19719 HA SER F 166   -59.380 -37.789 41.365 0.00 0.00
F
ATOM 19720 CB SER F 166   -61.150 -38.750 40.676 0.00 0.00
F
ATOM 19721 HB1 SER F 166   -61.831 -38.090 41.254 0.00 0.00
F
ATOM 19722 HB2 SER F 166   -61.671 -38.919 39.709 0.00 0.00
F
ATOM 19723 OG SER F 166   -60.999 -40.051 41.401 0.00 0.00
F
ATOM 19724 HG1 SER F 166   -61.851 -40.484 41.492 0.00 0.00
F
ATOM 19725 C   SER F 166   -58.749 -39.030 39.825 0.00 0.00
F
ATOM 19726 O   SER F 166   -58.537 -39.166 38.662 0.00 0.00
F
ATOM 19727 N   ILE F 167   -57.985 -39.643 40.771 0.00 0.00
F
ATOM 19728 HN ILE F 167   -58.149 -39.581 41.752 0.00 0.00
F
ATOM 19729 CA ILE F 167   -56.821 -40.324 40.363 0.00 0.00
F
ATOM 19730 HA ILE F 167   -56.196 -39.719 39.722 0.00 0.00
F
ATOM 19731 CB ILE F 167   -55.886 -40.626 41.533 0.00 0.00
F
ATOM 19732 HB ILE F 167   -55.065 -41.229 41.091 0.00 0.00
F
ATOM 19733 CG2 ILE F 167   -55.111 -39.385 41.861 0.00 0.00
F
ATOM 19734 HG21 ILE F 167   -55.813 -38.607 42.229 0.00 0.00
F
ATOM 19735 HG22 ILE F 167   -54.240 -39.607 42.513 0.00 0.00
F
ATOM 19736 HG23 ILE F 167   -54.678 -38.949 40.935 0.00 0.00
F

ATOM 19737 CG1 ILE F 167 -56.513 -41.324 42.767 0.00 0.00
F
ATOM 19738 HG11 ILE F 167 -57.179 -40.617 43.306 0.00 0.00
F
ATOM 19739 HG12 ILE F 167 -57.038 -42.232 42.398 0.00 0.00
F
ATOM 19740 CD ILE F 167 -55.376 -41.879 43.599 0.00 0.00
F
ATOM 19741 HD1 ILE F 167 -55.701 -42.653 44.327 0.00 0.00
F
ATOM 19742 HD2 ILE F 167 -54.461 -42.230 43.075 0.00 0.00
F
ATOM 19743 HD3 ILE F 167 -55.123 -41.120 44.369 0.00 0.00
F
ATOM 19744 C ILE F 167 -56.994 -41.602 39.537 0.00 0.00
F
ATOM 19745 O ILE F 167 -56.032 -42.090 38.952 0.00 0.00
F
ATOM 19746 N ILE F 168 -58.218 -42.097 39.425 0.00 0.00
F
ATOM 19747 HN ILE F 168 -59.015 -41.558 39.685 0.00 0.00
F
ATOM 19748 CA ILE F 168 -58.489 -43.435 38.848 0.00 0.00
F
ATOM 19749 HA ILE F 168 -57.496 -43.839 38.713 0.00 0.00
F
ATOM 19750 CB ILE F 168 -59.256 -44.548 39.560 0.00 0.00
F
ATOM 19751 HB ILE F 168 -59.633 -45.314 38.849 0.00 0.00
F
ATOM 19752 CG2 ILE F 168 -58.055 -45.157 40.495 0.00 0.00
F
ATOM 19753 HG21 ILE F 168 -57.290 -45.669 39.873 0.00 0.00
F
ATOM 19754 HG22 ILE F 168 -57.613 -44.326 41.084 0.00 0.00
F
ATOM 19755 HG23 ILE F 168 -58.472 -45.972 41.125 0.00 0.00
F
ATOM 19756 CG1 ILE F 168 -60.463 -44.066 40.389 0.00 0.00
F
ATOM 19757 HG11 ILE F 168 -60.842 -44.889 41.030 0.00 0.00
F
ATOM 19758 HG12 ILE F 168 -60.128 -43.291 41.111 0.00 0.00
F
ATOM 19759 CD ILE F 168 -61.593 -43.560 39.497 0.00 0.00
F
ATOM 19760 HD1 ILE F 168 -62.643 -43.595 39.860 0.00 0.00
F
ATOM 19761 HD2 ILE F 168 -61.390 -42.528 39.138 0.00 0.00
F
ATOM 19762 HD3 ILE F 168 -61.614 -44.232 38.613 0.00 0.00
F
ATOM 19763 C ILE F 168 -58.989 -43.327 37.411 0.00 0.00
F

ATOM 19764 O   ILE F 168   -58.841 -44.256 36.652 0.00 0.00
F
ATOM 19765 N   ASP F 169   -59.321 -42.063 37.101 0.00 0.00
F
ATOM 19766 HN ASP F 169   -59.322 -41.402 37.848 0.00 0.00
F
ATOM 19767 CA ASP F 169   -59.575 -41.506 35.769 0.00 0.00
F
ATOM 19768 HA ASP F 169   -60.077 -42.296 35.229 0.00 0.00
F
ATOM 19769 CB ASP F 169   -60.325 -40.162 35.914 0.00 0.00
F
ATOM 19770 HB1 ASP F 169   -59.823 -39.580 36.716 0.00 0.00
F
ATOM 19771 HB2 ASP F 169   -60.353 -39.595 34.959 0.00 0.00
F
ATOM 19772 CG ASP F 169   -61.748 -40.407 36.275 0.00 0.00
F
ATOM 19773 OD1 ASP F 169   -62.511 -40.895 35.409 0.00 0.00
F
ATOM 19774 OD2 ASP F 169   -62.113 -40.066 37.478 0.00 0.00
F
ATOM 19775 C   ASP F 169   -58.254 -41.288 35.044 0.00 0.00
F
ATOM 19776 O   ASP F 169   -58.168 -41.446 33.828 0.00 0.00
F
ATOM 19777 N   LEU F 170   -57.170 -40.967 35.754 0.00 0.00
F
ATOM 19778 HN LEU F 170   -57.212 -40.864 36.745 0.00 0.00
F
ATOM 19779 CA LEU F 170   -55.834 -41.061 35.171 0.00 0.00
F
ATOM 19780 HA LEU F 170   -55.807 -40.419 34.304 0.00 0.00
F
ATOM 19781 CB LEU F 170   -54.816 -40.462 36.204 0.00 0.00
F
ATOM 19782 HB1 LEU F 170   -54.631 -41.139 37.066 0.00 0.00
F
ATOM 19783 HB2 LEU F 170   -53.810 -40.412 35.737 0.00 0.00
F
ATOM 19784 CG LEU F 170   -55.083 -39.002 36.743 0.00 0.00
F
ATOM 19785 HG LEU F 170   -56.006 -39.073 37.357 0.00 0.00
F
ATOM 19786 CD1 LEU F 170   -54.025 -38.603 37.863 0.00 0.00
F
ATOM 19787 HD11 LEU F 170   -53.091 -38.393 37.299 0.00 0.00
F
ATOM 19788 HD12 LEU F 170   -54.203 -37.671 38.440 0.00 0.00
F
ATOM 19789 HD13 LEU F 170   -53.683 -39.394 38.564 0.00 0.00
F
ATOM 19790 CD2 LEU F 170   -55.086 -38.001 35.583 0.00 0.00
F

ATOM 19791 HD21 LEU F 170   -55.973 -38.246 34.960 0.00 0.00
F
ATOM 19792 HD22 LEU F 170   -55.191 -36.979 36.006 0.00 0.00
F
ATOM 19793 HD23 LEU F 170   -54.140 -38.005 35.001 0.00 0.00
F
ATOM 19794 C   LEU F 170   -55.142 -42.359 34.690 0.00 0.00
F
ATOM 19795 O   LEU F 170   -55.552 -43.459 34.997 0.00 0.00
F
ATOM 19796 N   ASN F 171   -54.157 -42.242 33.810 0.00 0.00
F
ATOM 19797 HN ASN F 171   -53.878 -41.292 33.693 0.00 0.00
F
ATOM 19798 CA ASN F 171   -53.495 -43.309 33.141 0.00 0.00
F
ATOM 19799 HA ASN F 171   -54.112 -44.188 33.251 0.00 0.00
F
ATOM 19800 CB ASN F 171   -53.249 -42.898 31.708 0.00 0.00
F
ATOM 19801 HB1 ASN F 171   -52.811 -41.880 31.789 0.00 0.00
F
ATOM 19802 HB2 ASN F 171   -52.541 -43.530 31.130 0.00 0.00
F
ATOM 19803 CG ASN F 171   -54.602 -43.016 31.052 0.00 0.00
F
ATOM 19804 OD1 ASN F 171   -55.154 -44.126 30.958 0.00 0.00
F
ATOM 19805 ND2 ASN F 171   -55.142 -41.846 30.632 0.00 0.00
F
ATOM 19806 HD21 ASN F 171   -54.598 -41.006 30.632 0.00 0.00
F
ATOM 19807 HD22 ASN F 171   -56.086 -41.846 30.302 0.00 0.00
F
ATOM 19808 C   ASN F 171   -52.239 -43.695 33.812 0.00 0.00
F
ATOM 19809 O   ASN F 171   -51.429 -44.448 33.308 0.00 0.00
F
ATOM 19810 N   VAL F 172   -52.042 -43.145 35.038 0.00 0.00
F
ATOM 19811 HN VAL F 172   -52.736 -42.592 35.492 0.00 0.00
F
ATOM 19812 CA VAL F 172   -50.864 -43.236 35.848 0.00 0.00
F
ATOM 19813 HA VAL F 172   -50.030 -42.827 35.298 0.00 0.00
F
ATOM 19814 CB VAL F 172   -51.054 -42.325 37.019 0.00 0.00
F
ATOM 19815 HB VAL F 172   -51.336 -41.355 36.557 0.00 0.00
F
ATOM 19816 CG1 VAL F 172   -52.065 -42.860 37.986 0.00 0.00
F
ATOM 19817 HG11 VAL F 172   -51.841 -43.889 38.342 0.00 0.00
F

ATOM 19818 HG12 VAL F 172   -52.178 -42.126 38.812 0.00 0.00
F
ATOM 19819 HG13 VAL F 172   -53.077 -42.868 37.529 0.00 0.00
F
ATOM 19820 CG2 VAL F 172   -49.763 -41.960 37.828 0.00 0.00
F
ATOM 19821 HG21 VAL F 172   -49.969 -41.013 38.370 0.00 0.00
F
ATOM 19822 HG22 VAL F 172   -49.541 -42.823 38.492 0.00 0.00
F
ATOM 19823 HG23 VAL F 172   -48.911 -41.851 37.124 0.00 0.00
F
ATOM 19824 C   VAL F 172   -50.390 -44.604 36.156 0.00 0.00
F
ATOM 19825 O   VAL F 172   -49.174 -44.894 36.085 0.00 0.00
F
ATOM 19826 N   ARG F 173   -51.269 -45.573 36.511 0.00 0.00
F
ATOM 19827 HN ARG F 173   -52.196 -45.368 36.814 0.00 0.00
F
ATOM 19828 CA ARG F 173   -51.048 -46.989 36.757 0.00 0.00
F
ATOM 19829 HA ARG F 173   -50.017 -47.262 36.925 0.00 0.00
F
ATOM 19830 CB ARG F 173   -51.862 -47.467 37.974 0.00 0.00
F
ATOM 19831 HB1 ARG F 173   -52.951 -47.246 37.981 0.00 0.00
F
ATOM 19832 HB2 ARG F 173   -51.669 -48.544 38.171 0.00 0.00
F
ATOM 19833 CG ARG F 173   -51.324 -46.787 39.248 0.00 0.00
F
ATOM 19834 HG1 ARG F 173   -50.238 -47.018 39.232 0.00 0.00
F
ATOM 19835 HG2 ARG F 173   -51.396 -45.681 39.324 0.00 0.00
F
ATOM 19836 CD ARG F 173   -52.011 -47.500 40.456 0.00 0.00
F
ATOM 19837 HD1 ARG F 173   -52.034 -46.742 41.268 0.00 0.00
F
ATOM 19838 HD2 ARG F 173   -53.044 -47.783 40.159 0.00 0.00
F
ATOM 19839 NE ARG F 173   -51.253 -48.753 40.855 0.00 0.00
F
ATOM 19840 HE ARG F 173   -50.257 -48.765 40.770 0.00 0.00
F
ATOM 19841 CZ ARG F 173   -51.833 -49.904 41.054 0.00 0.00
F
ATOM 19842 NH1 ARG F 173   -53.147 -49.998 41.180 0.00 0.00
F
ATOM 19843 HH11 ARG F 173   -53.461 -50.858 41.581 0.00 0.00
F
ATOM 19844 HH12 ARG F 173   -53.726 -49.226 40.917 0.00 0.00
F

ATOM 19845 NH2 ARG F 173   -51.144 -51.066 41.236 0.00 0.00
F
ATOM 19846 HH21 ARG F 173   -51.717 -51.870 41.400 0.00 0.00
F
ATOM 19847 HH22 ARG F 173   -50.180 -51.180 40.996 0.00 0.00
F
ATOM 19848 C   ARG F 173   -51.527 -47.785 35.550 0.00 0.00
F
ATOM 19849 O   ARG F 173   -50.933 -48.760 35.111 0.00 0.00
F
ATOM 19850 N   ALA F 174   -52.682 -47.425 34.981 0.00 0.00
F
ATOM 19851 HN ALA F 174   -53.172 -46.627 35.324 0.00 0.00
F
ATOM 19852 CA ALA F 174   -53.255 -48.012 33.809 0.00 0.00
F
ATOM 19853 HA ALA F 174   -53.512 -49.017 34.111 0.00 0.00
F
ATOM 19854 CB ALA F 174   -54.675 -47.556 33.684 0.00 0.00
F
ATOM 19855 HB1 ALA F 174   -54.695 -46.506 33.323 0.00 0.00
F
ATOM 19856 HB2 ALA F 174   -55.215 -48.077 32.866 0.00 0.00
F
ATOM 19857 HB3 ALA F 174   -55.251 -47.514 34.633 0.00 0.00
F
ATOM 19858 C   ALA F 174   -52.416 -48.047 32.584 0.00 0.00
F
ATOM 19859 O   ALA F 174   -52.296 -49.003 31.815 0.00 0.00
F
ATOM 19860 N   LYS F 175   -51.747 -46.936 32.225 0.00 0.00
F
ATOM 19861 HN LYS F 175   -51.929 -46.029 32.596 0.00 0.00
F
ATOM 19862 CA LYS F 175   -50.785 -46.931 31.129 0.00 0.00
F
ATOM 19863 HA LYS F 175   -50.934 -47.770 30.466 0.00 0.00
F
ATOM 19864 CB LYS F 175   -50.964 -45.555 30.359 0.00 0.00
F
ATOM 19865 HB1 LYS F 175   -52.037 -45.510 30.075 0.00 0.00
F
ATOM 19866 HB2 LYS F 175   -50.749 -44.667 30.991 0.00 0.00
F
ATOM 19867 CG LYS F 175   -50.058 -45.519 29.090 0.00 0.00
F
ATOM 19868 HG1 LYS F 175   -49.010 -45.414 29.443 0.00 0.00
F
ATOM 19869 HG2 LYS F 175   -50.229 -46.467 28.535 0.00 0.00
F
ATOM 19870 CD LYS F 175   -50.254 -44.341 28.180 0.00 0.00
F
ATOM 19871 HD1 LYS F 175   -51.178 -44.445 27.573 0.00 0.00
F

ATOM 19872 HD2 LYS F 175 −50.398 −43.426 28.794 0.00 0.00
F
ATOM 19873 CE LYS F 175 −49.088 −44.044 27.184 0.00 0.00
F
ATOM 19874 HE1 LYS F 175 −49.150 −43.119 26.571 0.00 0.00
F
ATOM 19875 HE2 LYS F 175 −48.090 −43.940 27.659 0.00 0.00
F
ATOM 19876 NZ LYS F 175 −49.036 −45.214 26.222 0.00 0.00
F
ATOM 19877 HZ1 LYS F 175 −48.304 −44.982 25.521 0.00 0.00
F
ATOM 19878 HZ2 LYS F 175 −48.695 −46.030 26.769 0.00 0.00
F
ATOM 19879 HZ3 LYS F 175 −50.005 −45.296 25.854 0.00 0.00
F
ATOM 19880 C LYS F 175 −49.337 −47.035 31.647 0.00 0.00
F
ATOM 19881 O LYS F 175 −48.690 −48.069 31.515 0.00 0.00
F
ATOM 19882 N TYR F 176 −48.861 −45.925 32.367 0.00 0.00
F
ATOM 19883 HN TYR F 176 −49.442 −45.124 32.488 0.00 0.00
F
ATOM 19884 CA TYR F 176 −47.482 −45.720 32.691 0.00 0.00
F
ATOM 19885 HA TYR F 176 −46.950 −45.655 31.753 0.00 0.00
F
ATOM 19886 CB TYR F 176 −47.222 −44.329 33.285 0.00 0.00
F
ATOM 19887 HB1 TYR F 176 −47.851 −44.234 34.196 0.00 0.00
F
ATOM 19888 HB2 TYR F 176 −46.137 −44.132 33.422 0.00 0.00
F
ATOM 19889 CG TYR F 176 −47.655 −43.388 32.244 0.00 0.00
F
ATOM 19890 CD1 TYR F 176 −48.818 −42.612 32.460 0.00 0.00
F
ATOM 19891 HD1 TYR F 176 −49.329 −42.630 33.412 0.00 0.00
F
ATOM 19892 CE1 TYR F 176 −49.446 −41.927 31.436 0.00 0.00
F
ATOM 19893 HE1 TYR F 176 −50.408 −41.463 31.593 0.00 0.00
F
ATOM 19894 CZ TYR F 176 −48.703 −41.716 30.276 0.00 0.00
F
ATOM 19895 OH TYR F 176 −49.257 −40.912 29.297 0.00 0.00
F
ATOM 19896 HH TYR F 176 −48.615 −40.948 28.585 0.00 0.00
F
ATOM 19897 CD2 TYR F 176 −46.949 −43.207 31.044 0.00 0.00
F
ATOM 19898 HD2 TYR F 176 −46.134 −43.876 30.807 0.00 0.00
F

ATOM 19899 CE2 TYR F 176 −47.496 −42.344 30.089 0.00 0.00
F
ATOM 19900 HE2 TYR F 176 −46.985 −42.161 29.155 0.00 0.00
F
ATOM 19901 C TYR F 176 −46.859 −46.829 33.585 0.00 0.00
F
ATOM 19902 O TYR F 176 −45.673 −47.210 33.498 0.00 0.00
F
ATOM 19903 N GLY F 177 −47.704 −47.368 34.504 0.00 0.00
F
ATOM 19904 HN GLY F 177 −48.662 −47.094 34.472 0.00 0.00
F
ATOM 19905 CA GLY F 177 −47.372 −48.288 35.490 0.00 0.00
F
ATOM 19906 HA1 GLY F 177 −46.730 −49.044 35.062 0.00 0.00
F
ATOM 19907 HA2 GLY F 177 −48.313 −48.585 35.930 0.00 0.00
F
ATOM 19908 C GLY F 177 −46.504 −47.725 36.634 0.00 0.00
F
ATOM 19909 O GLY F 177 −45.655 −48.359 37.174 0.00 0.00
F
ATOM 19910 N CYS F 178 −46.729 −46.474 37.000 0.00 0.00
F
ATOM 19911 HN CYS F 178 −47.546 −46.162 36.522 0.00 0.00
F
ATOM 19912 CA CYS F 178 −46.157 −45.629 38.096 0.00 0.00
F
ATOM 19913 HA CYS F 178 −45.166 −45.920 38.412 0.00 0.00
F
ATOM 19914 CB CYS F 178 −46.090 −44.038 37.675 0.00 0.00
F
ATOM 19915 HB1 CYS F 178 −47.093 −43.574 37.565 0.00 0.00
F
ATOM 19916 HB2 CYS F 178 −45.584 −43.485 38.496 0.00 0.00
F
ATOM 19917 SG CYS F 178 −45.130 −43.690 36.203 0.00 0.00
F
ATOM 19918 HG1 CYS F 178 −45.706 −42.505 36.058 0.00 0.00
F
ATOM 19919 C CYS F 178 −46.952 −45.786 39.405 0.00 0.00
F
ATOM 19920 O CYS F 178 −47.964 −46.480 39.379 0.00 0.00
F
ATOM 19921 N THR F 179 −46.634 −45.060 40.493 0.00 0.00
F
ATOM 19922 HN THR F 179 −45.825 −44.491 40.618 0.00 0.00
F
ATOM 19923 CA THR F 179 −47.524 −44.931 41.649 0.00 0.00
F
ATOM 19924 HA THR F 179 −48.511 −45.178 41.284 0.00 0.00
F
ATOM 19925 CB THR F 179 −47.252 −45.940 42.731 0.00 0.00
F

```
ATOM  19926  HB  THR F 179     -47.289 -46.938  42.243  0.00  0.00
F
ATOM  19927  OG1 THR F 179     -48.232 -45.926  43.840  0.00  0.00
F
ATOM  19928  HG1 THR F 179     -47.902 -46.634  44.398  0.00  0.00
F
ATOM  19929  CG2 THR F 179     -45.902 -45.734  43.399  0.00  0.00
F
ATOM  19930 HG21 THR F 179     -45.611 -46.674  43.916  0.00  0.00
F
ATOM  19931 HG22 THR F 179     -45.027 -45.419  42.791  0.00  0.00
F
ATOM  19932 HG23 THR F 179     -45.929 -44.962  44.197  0.00  0.00
F
ATOM  19933  C   THR F 179     -47.510 -43.505  42.233  0.00  0.00
F
ATOM  19934  O   THR F 179     -46.454 -42.853  42.369  0.00  0.00
F
ATOM  19935  N   ILE F 180     -48.595 -42.904  42.628  0.00  0.00
F
ATOM  19936  HN  ILE F 180     -49.494 -43.298  42.449  0.00  0.00
F
ATOM  19937  CA  ILE F 180     -48.580 -41.581  43.360  0.00  0.00
F
ATOM  19938  HA  ILE F 180     -47.855 -40.913  42.917  0.00  0.00
F
ATOM  19939  CB  ILE F 180     -49.902 -40.765  43.348  0.00  0.00
F
ATOM  19940  HB  ILE F 180     -50.776 -41.341  43.720  0.00  0.00
F
ATOM  19941  CG2 ILE F 180     -49.678 -39.548  44.235  0.00  0.00
F
ATOM  19942 HG21 ILE F 180     -49.485 -39.741  45.312  0.00  0.00
F
ATOM  19943 HG22 ILE F 180     -48.770 -38.984  43.930  0.00  0.00
F
ATOM  19944 HG23 ILE F 180     -50.557 -38.872  44.175  0.00  0.00
F
ATOM  19945  CG1 ILE F 180     -50.135 -40.458  41.905  0.00  0.00
F
ATOM  19946 HG11 ILE F 180     -49.236 -40.043  41.402  0.00  0.00
F
ATOM  19947 HG12 ILE F 180     -50.350 -41.425  41.402  0.00  0.00
F
ATOM  19948  CD  ILE F 180     -51.343 -39.611  41.613  0.00  0.00
F
ATOM  19949  HD1 ILE F 180     -51.242 -38.574  41.998  0.00  0.00
F
ATOM  19950  HD2 ILE F 180     -51.464 -39.477  40.516  0.00  0.00
F
ATOM  19951  HD3 ILE F 180     -52.272 -39.995  42.085  0.00  0.00
F
ATOM  19952  C   ILE F 180     -48.195 -41.891  44.809  0.00  0.00
F
```

```
ATOM  19953  O   ILE F 180      -49.072 -42.352  45.528  0.00  0.00
F
ATOM  19954  N   LEU F 181      -46.949 -41.536  45.249  0.00  0.00
F
ATOM  19955  HN  LEU F 181      -46.301 -41.198  44.572  0.00  0.00
F
ATOM  19956  CA  LEU F 181      -46.508 -41.815  46.635  0.00  0.00
F
ATOM  19957  HA  LEU F 181      -46.791 -42.845  46.797  0.00  0.00
F
ATOM  19958  CB  LEU F 181      -44.932 -41.592  46.770  0.00  0.00
F
ATOM  19959  HB1 LEU F 181      -44.409 -42.136  45.955  0.00  0.00
F
ATOM  19960  HB2 LEU F 181      -44.830 -40.492  46.652  0.00  0.00
F
ATOM  19961  CG  LEU F 181      -44.258 -42.166  48.080  0.00  0.00
F
ATOM  19962  HG  LEU F 181      -44.554 -41.442  48.870  0.00  0.00
F
ATOM  19963  CD1 LEU F 181      -44.708 -43.573  48.420  0.00  0.00
F
ATOM  19964 HD11 LEU F 181      -45.817 -43.497  48.435  0.00  0.00
F
ATOM  19965 HD12 LEU F 181      -44.579 -44.429  47.723  0.00  0.00
F
ATOM  19966 HD13 LEU F 181      -44.212 -43.830  49.380  0.00  0.00
F
ATOM  19967  CD2 LEU F 181      -42.669 -42.153  48.028  0.00  0.00
F
ATOM  19968 HD21 LEU F 181      -42.410 -42.951  47.299  0.00  0.00
F
ATOM  19969 HD22 LEU F 181      -42.380 -41.092  47.874  0.00  0.00
F
ATOM  19970 HD23 LEU F 181      -42.275 -42.433  49.028  0.00  0.00
F
ATOM  19971  C   LEU F 181      -47.236 -40.961  47.783  0.00  0.00
F
ATOM  19972  O   LEU F 181      -47.548 -41.496  48.878  0.00  0.00
F
ATOM  19973  N   ALA F 182      -47.544 -39.650  47.437  0.00  0.00
F
ATOM  19974  HN  ALA F 182      -47.307 -39.239  46.560  0.00  0.00
F
ATOM  19975  CA  ALA F 182      -48.127 -38.849  48.456  0.00  0.00
F
ATOM  19976  HA  ALA F 182      -48.937 -39.389  48.924  0.00  0.00
F
ATOM  19977  CB  ALA F 182      -47.213 -38.183  49.553  0.00  0.00
F
ATOM  19978  HB1 ALA F 182      -47.812 -37.487  50.178  0.00  0.00
F
ATOM  19979  HB2 ALA F 182      -46.831 -39.041  50.146  0.00  0.00
F
```

ATOM 19980 HB3 ALA F 182 -46.345 -37.636 49.127 0.00 0.00
F
ATOM 19981 C ALA F 182 -48.748 -37.571 47.694 0.00 0.00
F
ATOM 19982 O ALA F 182 -48.258 -37.171 46.636 0.00 0.00
F
ATOM 19983 N ILE F 183 -49.837 -36.925 48.310 0.00 0.00
F
ATOM 19984 HN ILE F 183 -50.356 -37.291 49.079 0.00 0.00
F
ATOM 19985 CA ILE F 183 -50.323 -35.630 47.909 0.00 0.00
F
ATOM 19986 HA ILE F 183 -49.588 -35.155 47.276 0.00 0.00
F
ATOM 19987 CB ILE F 183 -51.471 -35.776 46.947 0.00 0.00
F
ATOM 19988 HB ILE F 183 -51.222 -36.646 46.303 0.00 0.00
F
ATOM 19989 CG2 ILE F 183 -52.802 -36.112 47.606 0.00 0.00
F
ATOM 19990 HG21 ILE F 183 -53.549 -36.331 46.814 0.00 0.00
F
ATOM 19991 HG22 ILE F 183 -52.695 -36.992 48.275 0.00 0.00
F
ATOM 19992 HG23 ILE F 183 -53.047 -35.141 48.088 0.00 0.00
F
ATOM 19993 CG1 ILE F 183 -51.492 -34.595 46.057 0.00 0.00
F
ATOM 19994 HG11 ILE F 183 -51.883 -33.666 46.522 0.00 0.00
F
ATOM 19995 HG12 ILE F 183 -50.460 -34.444 45.672 0.00 0.00
F
ATOM 19996 CD ILE F 183 -52.340 -34.834 44.833 0.00 0.00
F
ATOM 19997 HD1 ILE F 183 -52.087 -34.136 44.007 0.00 0.00
F
ATOM 19998 HD2 ILE F 183 -52.360 -35.915 44.578 0.00 0.00
F
ATOM 19999 HD3 ILE F 183 -53.396 -34.618 45.103 0.00 0.00
F
ATOM 20000 C ILE F 183 -50.627 -34.578 48.966 0.00 0.00
F
ATOM 20001 O ILE F 183 -51.205 -34.740 50.010 0.00 0.00
F
ATOM 20002 N LYS F 184 -50.123 -33.332 48.726 0.00 0.00
F
ATOM 20003 HN LYS F 184 -49.724 -33.195 47.823 0.00 0.00
F
ATOM 20004 CA LYS F 184 -50.009 -32.169 49.624 0.00 0.00
F
ATOM 20005 HA LYS F 184 -50.156 -32.591 50.607 0.00 0.00
F
ATOM 20006 CB LYS F 184 -48.668 -31.392 49.405 0.00 0.00
F

ATOM 20007 HB1 LYS F 184   -48.740 -30.893 48.415 0.00 0.00
F
ATOM 20008 HB2 LYS F 184   -48.656 -30.661 50.242 0.00 0.00
F
ATOM 20009 CG LYS F 184   -47.434 -32.315 49.509 0.00 0.00
F
ATOM 20010 HG1 LYS F 184   -47.486 -32.585 50.585 0.00 0.00
F
ATOM 20011 HG2 LYS F 184   -47.517 -33.204 48.847 0.00 0.00
F
ATOM 20012 CD LYS F 184   -46.084 -31.626 49.495 0.00 0.00
F
ATOM 20013 HD1 LYS F 184   -45.944 -31.145 48.503 0.00 0.00
F
ATOM 20014 HD2 LYS F 184   -46.180 -30.907 50.337 0.00 0.00
F
ATOM 20015 CE LYS F 184   -44.913 -32.509 49.734 0.00 0.00
F
ATOM 20016 HE1 LYS F 184   -45.079 -32.774 50.800 0.00 0.00
F
ATOM 20017 HE2 LYS F 184   -44.962 -33.379 49.044 0.00 0.00
F
ATOM 20018 NZ LYS F 184   -43.607 -31.842 49.637 0.00 0.00
F
ATOM 20019 HZ1 LYS F 184   -43.438 -31.371 48.725 0.00 0.00
F
ATOM 20020 HZ2 LYS F 184   -43.403 -31.145 50.382 0.00 0.00
F
ATOM 20021 HZ3 LYS F 184   -42.875 -32.573 49.741 0.00 0.00
F
ATOM 20022 C   LYS F 184   -51.159 -31.215 49.342 0.00 0.00
F
ATOM 20023 O   LYS F 184   -51.242 -30.659 48.246 0.00 0.00
F
ATOM 20024 N   HSE F 185   -52.169 -31.203 50.247 0.00 0.00
F
ATOM 20025 HN HSE F 185   -52.191 -31.844 51.010 0.00 0.00
F
ATOM 20026 CA HSE F 185   -53.253 -30.151 50.162 0.00 0.00
F
ATOM 20027 HA HSE F 185   -53.130 -29.493 49.314 0.00 0.00
F
ATOM 20028 CB HSE F 185   -54.517 -30.822 49.661 0.00 0.00
F
ATOM 20029 HB1 HSE F 185   -55.134 -29.920 49.461 0.00 0.00
F
ATOM 20030 HB2 HSE F 185   -54.316 -31.323 48.690 0.00 0.00
F
ATOM 20031 ND1 HSE F 185   -56.538 -31.928 50.266 0.00 0.00
F
ATOM 20032 CG HSE F 185   -55.264 -31.721 50.588 0.00 0.00
F
ATOM 20033 CE1 HSE F 185   -56.937 -32.906 51.112 0.00 0.00
F

ATOM 20034 HE1 HSE F 185 -57.949 -33.304 51.034 0.00 0.00
F
ATOM 20035 NE2 HSE F 185 -55.957 -33.201 52.004 0.00 0.00
F
ATOM 20036 HE2 HSE F 185 -56.122 -33.690 52.860 0.00 0.00
F
ATOM 20037 CD2 HSE F 185 -54.911 -32.415 51.682 0.00 0.00
F
ATOM 20038 HD2 HSE F 185 -53.864 -32.519 51.940 0.00 0.00
F
ATOM 20039 C HSE F 185 -53.514 -29.249 51.337 0.00 0.00
F
ATOM 20040 O HSE F 185 -53.882 -29.735 52.390 0.00 0.00
F
ATOM 20041 N HSE F 186 -53.377 -27.947 51.220 0.00 0.00
F
ATOM 20042 HN HSE F 186 -53.058 -27.656 50.321 0.00 0.00
F
ATOM 20043 CA HSE F 186 -53.678 -27.052 52.332 0.00 0.00
F
ATOM 20044 HA HSE F 186 -53.191 -26.128 52.055 0.00 0.00
F
ATOM 20045 CB HSE F 186 -55.217 -26.634 52.366 0.00 0.00
F
ATOM 20046 HB1 HSE F 186 -55.891 -27.517 52.352 0.00 0.00
F
ATOM 20047 HB2 HSE F 186 -55.549 -26.098 53.281 0.00 0.00
F
ATOM 20048 ND1 HSE F 186 -55.464 -26.619 49.854 0.00 0.00
F
ATOM 20049 CG HSE F 186 -55.569 -26.024 51.079 0.00 0.00
F
ATOM 20050 CE1 HSE F 186 -55.652 -25.703 48.961 0.00 0.00
F
ATOM 20051 HE1 HSE F 186 -55.731 -25.846 47.884 0.00 0.00
F
ATOM 20052 NE2 HSE F 186 -55.882 -24.499 49.531 0.00 0.00
F
ATOM 20053 HE2 HSE F 186 -56.222 -23.669 49.089 0.00 0.00
F
ATOM 20054 CD2 HSE F 186 -55.826 -24.655 50.865 0.00 0.00
F
ATOM 20055 HD2 HSE F 186 -55.948 -23.917 51.648 0.00 0.00
F
ATOM 20056 C HSE F 186 -53.176 -27.368 53.703 0.00 0.00
F
ATOM 20057 O HSE F 186 -53.827 -27.176 54.691 0.00 0.00
F
ATOM 20058 N GLY F 187 -51.890 -27.795 53.841 0.00 0.00
F
ATOM 20059 HN GLY F 187 -51.343 -28.056 53.050 0.00 0.00
F
ATOM 20060 CA GLY F 187 -51.112 -27.937 55.018 0.00 0.00
F

ATOM 20061 HA1 GLY F 187 −51.541 −27.248 55.731 0.00 0.00
F
ATOM 20062 HA2 GLY F 187 −50.125 −27.558 54.800 0.00 0.00
F
ATOM 20063 C GLY F 187 −51.136 −29.342 55.563 0.00 0.00
F
ATOM 20064 O GLY F 187 −50.521 −29.641 56.588 0.00 0.00
F
ATOM 20065 N ASP F 188 −51.847 −30.244 54.872 0.00 0.00
F
ATOM 20066 HN ASP F 188 −52.208 −29.947 53.992 0.00 0.00
F
ATOM 20067 CA ASP F 188 −52.131 −31.571 55.277 0.00 0.00
F
ATOM 20068 HA ASP F 188 −51.676 −31.776 56.235 0.00 0.00
F
ATOM 20069 CB ASP F 188 −53.645 −31.931 55.364 0.00 0.00
F
ATOM 20070 HB1 ASP F 188 −54.147 −31.592 54.433 0.00 0.00
F
ATOM 20071 HB2 ASP F 188 −53.673 −33.016 55.604 0.00 0.00
F
ATOM 20072 CG ASP F 188 −54.370 −31.162 56.472 0.00 0.00
F
ATOM 20073 OD1 ASP F 188 −53.873 −31.355 57.598 0.00 0.00
F
ATOM 20074 OD2 ASP F 188 −55.363 −30.469 56.219 0.00 0.00
F
ATOM 20075 C ASP F 188 −51.479 −32.447 54.203 0.00 0.00
F
ATOM 20076 O ASP F 188 −51.143 −31.960 53.125 0.00 0.00
F
ATOM 20077 N ILE F 189 −51.268 −33.754 54.462 0.00 0.00
F
ATOM 20078 HN ILE F 189 −51.628 −34.139 55.309 0.00 0.00
F
ATOM 20079 CA ILE F 189 −50.667 −34.681 53.470 0.00 0.00
F
ATOM 20080 HA ILE F 189 −50.907 −34.260 52.506 0.00 0.00
F
ATOM 20081 CB ILE F 189 −49.132 −34.839 53.465 0.00 0.00
F
ATOM 20082 HB ILE F 189 −48.674 −33.832 53.567 0.00 0.00
F
ATOM 20083 CG2 ILE F 189 −48.720 −35.443 54.793 0.00 0.00
F
ATOM 20084 HG21 ILE F 189 −47.638 −35.266 54.977 0.00 0.00
F
ATOM 20085 HG22 ILE F 189 −49.373 −35.037 55.594 0.00 0.00
F
ATOM 20086 HG23 ILE F 189 −48.811 −36.550 54.824 0.00 0.00
F
ATOM 20087 CG1 ILE F 189 −48.502 −35.572 52.291 0.00 0.00
F

ATOM 20088 HG11 ILE F 189   −48.665 −36.671 52.285 0.00 0.00
F
ATOM 20089 HG12 ILE F 189   −49.013 −35.151 51.398 0.00 0.00
F
ATOM 20090 CD ILE F 189   −46.953 −35.430 52.118 0.00 0.00
F
ATOM 20091 HD1 ILE F 189   −46.416 −35.858 52.992 0.00 0.00
F
ATOM 20092 HD2 ILE F 189   −46.713 −36.121 51.281 0.00 0.00
F
ATOM 20093 HD3 ILE F 189   −46.697 −34.424 51.723 0.00 0.00
F
ATOM 20094 C   ILE F 189   −51.327 −36.031 53.407 0.00 0.00
F
ATOM 20095 O   ILE F 189   −51.520 −36.627 54.470 0.00 0.00
F
ATOM 20096 N   CYS F 190   −51.657 −36.483 52.165 0.00 0.00
F
ATOM 20097 HN CYS F 190   −51.669 −35.830 51.411 0.00 0.00
F
ATOM 20098 CA CYS F 190   −52.228 −37.831 51.902 0.00 0.00
F
ATOM 20099 HA CYS F 190   −52.611 −38.221 52.834 0.00 0.00
F
ATOM 20100 CB CYS F 190   −53.433 −37.811 50.946 0.00 0.00
F
ATOM 20101 HB1 CYS F 190   −53.009 −37.293 50.059 0.00 0.00
F
ATOM 20102 HB2 CYS F 190   −53.845 −38.795 50.636 0.00 0.00
F
ATOM 20103 SG CYS F 190   −54.825 −36.857 51.777 0.00 0.00
F
ATOM 20104 HG1 CYS F 190   −55.874 −36.851 50.967 0.00 0.00
F
ATOM 20105 C   CYS F 190   −51.184 −38.783 51.443 0.00 0.00
F
ATOM 20106 O   CYS F 190   −50.805 −38.744 50.287 0.00 0.00
F
ATOM 20107 N   LEU F 191   −50.773 −39.711 52.352 0.00 0.00
F
ATOM 20108 HN LEU F 191   −51.303 −39.657 53.195 0.00 0.00
F
ATOM 20109 CA LEU F 191   −49.759 −40.745 51.958 0.00 0.00
F
ATOM 20110 HA LEU F 191   −49.136 −40.335 51.177 0.00 0.00
F
ATOM 20111 CB LEU F 191   −48.851 −41.125 53.192 0.00 0.00
F
ATOM 20112 HB1 LEU F 191   −49.552 −41.585 53.921 0.00 0.00
F
ATOM 20113 HB2 LEU F 191   −48.101 −41.877 52.866 0.00 0.00
F
ATOM 20114 CG LEU F 191   −48.193 −39.877 53.879 0.00 0.00
F

```
ATOM  20115  HG  LEU F 191     -49.000 -39.186  54.204  0.00  0.00
F
ATOM  20116  CD1 LEU F 191     -47.436 -40.360  55.073  0.00  0.00
F
ATOM  20117 HD11 LEU F 191     -47.269 -39.584  55.851  0.00  0.00
F
ATOM  20118 HD12 LEU F 191     -48.025 -41.189  55.519  0.00  0.00
F
ATOM  20119 HD13 LEU F 191     -46.445 -40.765  54.778  0.00  0.00
F
ATOM  20120  CD2 LEU F 191     -47.301 -39.100  52.952  0.00  0.00
F
ATOM  20121 HD21 LEU F 191     -46.484 -39.661  52.451  0.00  0.00
F
ATOM  20122 HD22 LEU F 191     -47.965 -38.537  52.262  0.00  0.00
F
ATOM  20123 HD23 LEU F 191     -46.768 -38.327  53.547  0.00  0.00
F
ATOM  20124  C   LEU F 191     -50.454 -41.979  51.406  0.00  0.00
F
ATOM  20125  O   LEU F 191     -51.385 -42.553  51.988  0.00  0.00
F
ATOM  20126  N   SER F 192     -49.970 -42.354  50.239  0.00  0.00
F
ATOM  20127  HN  SER F 192     -49.131 -41.977  49.854  0.00  0.00
F
ATOM  20128  CA  SER F 192     -50.593 -43.356  49.367  0.00  0.00
F
ATOM  20129  HA  SER F 192     -50.062 -43.439  48.430  0.00  0.00
F
ATOM  20130  CB  SER F 192     -50.286 -44.745  50.022  0.00  0.00
F
ATOM  20131  HB1 SER F 192     -49.339 -44.788  50.601  0.00  0.00
F
ATOM  20132  HB2 SER F 192     -50.989 -44.851  50.876  0.00  0.00
F
ATOM  20133  OG  SER F 192     -50.455 -45.896  49.252  0.00  0.00
F
ATOM  20134  HG1 SER F 192     -49.734 -45.769  48.631  0.00  0.00
F
ATOM  20135  C   SER F 192     -52.070 -43.108  49.032  0.00  0.00
F
ATOM  20136  O   SER F 192     -52.967 -43.834  49.359  0.00  0.00
F
ATOM  20137  N   PRO F 193     -52.338 -41.971  48.228  0.00  0.00
F
ATOM  20138  CD  PRO F 193     -51.336 -41.063  47.682  0.00  0.00
F
ATOM  20139  HD1 PRO F 193     -50.925 -40.508  48.553  0.00  0.00
F
ATOM  20140  HD2 PRO F 193     -50.574 -41.730  47.225  0.00  0.00
F
ATOM  20141  CA  PRO F 193     -53.743 -41.592  47.996  0.00  0.00
F
```

ATOM 20142 HA PRO F 193 −54.221 −41.464 48.955 0.00 0.00
F
ATOM 20143 CB PRO F 193 −53.695 −40.276 47.283 0.00 0.00
F
ATOM 20144 HB1 PRO F 193 −53.795 −39.418 47.981 0.00 0.00
F
ATOM 20145 HB2 PRO F 193 −54.467 −40.191 46.489 0.00 0.00
F
ATOM 20146 CG PRO F 193 −52.246 −40.291 46.753 0.00 0.00
F
ATOM 20147 HG1 PRO F 193 −51.916 −39.272 46.457 0.00 0.00
F
ATOM 20148 HG2 PRO F 193 −52.110 −40.706 45.731 0.00 0.00
F
ATOM 20149 C PRO F 193 −54.572 −42.613 47.312 0.00 0.00
F
ATOM 20150 O PRO F 193 −54.043 −43.391 46.471 0.00 0.00
F
ATOM 20151 N ALA F 194 −55.860 −42.667 47.640 0.00 0.00
F
ATOM 20152 HN ALA F 194 −56.356 −41.998 48.189 0.00 0.00
F
ATOM 20153 CA ALA F 194 −56.874 −43.528 47.080 0.00 0.00
F
ATOM 20154 HA ALA F 194 −56.421 −44.163 46.333 0.00 0.00
F
ATOM 20155 CB ALA F 194 −57.479 −44.385 48.206 0.00 0.00
F
ATOM 20156 HB1 ALA F 194 −58.417 −43.943 48.605 0.00 0.00
F
ATOM 20157 HB2 ALA F 194 −57.765 −45.390 47.831 0.00 0.00
F
ATOM 20158 HB3 ALA F 194 −56.722 −44.558 49.001 0.00 0.00
F
ATOM 20159 C ALA F 194 −57.976 −42.720 46.469 0.00 0.00
F
ATOM 20160 O ALA F 194 −58.012 −41.548 46.673 0.00 0.00
F
ATOM 20161 N PRO F 195 −58.910 −43.265 45.689 0.00 0.00
F
ATOM 20162 CD PRO F 195 −58.892 −44.595 45.173 0.00 0.00
F
ATOM 20163 HD1 PRO F 195 −57.853 −44.892 44.915 0.00 0.00
F
ATOM 20164 HD2 PRO F 195 −59.273 −45.290 45.951 0.00 0.00
F
ATOM 20165 CA PRO F 195 −60.167 −42.551 45.215 0.00 0.00
F
ATOM 20166 HA PRO F 195 −59.897 −41.627 44.725 0.00 0.00
F
ATOM 20167 CB PRO F 195 −60.919 −43.544 44.215 0.00 0.00
F
ATOM 20168 HB1 PRO F 195 −61.291 −43.045 43.295 0.00 0.00
F

ATOM 20169 HB2 PRO F 195 −61.777 −44.002 44.751 0.00 0.00
F
ATOM 20170 CG PRO F 195 −59.812 −44.593 43.971 0.00 0.00
F
ATOM 20171 HG1 PRO F 195 −59.223 −44.388 43.051 0.00 0.00
F
ATOM 20172 HG2 PRO F 195 −60.221 −45.612 43.806 0.00 0.00
F
ATOM 20173 C PRO F 195 −60.988 −42.113 46.455 0.00 0.00
F
ATOM 20174 O PRO F 195 −61.127 −42.967 47.387 0.00 0.00
F
ATOM 20175 N GLU F 196 −61.638 −40.968 46.507 0.00 0.00
F
ATOM 20176 HN GLU F 196 −61.908 −40.793 47.451 0.00 0.00
F
ATOM 20177 CA GLU F 196 −62.175 −40.284 45.362 0.00 0.00
F
ATOM 20178 HA GLU F 196 −62.070 −40.810 44.425 0.00 0.00
F
ATOM 20179 CB GLU F 196 −63.665 −39.960 45.698 0.00 0.00
F
ATOM 20180 HB1 GLU F 196 −63.763 −39.203 46.505 0.00 0.00
F
ATOM 20181 HB2 GLU F 196 −64.054 −39.459 44.786 0.00 0.00
F
ATOM 20182 CG GLU F 196 −64.527 −41.186 46.082 0.00 0.00
F
ATOM 20183 HG1 GLU F 196 −64.448 −42.088 45.438 0.00 0.00
F
ATOM 20184 HG2 GLU F 196 −64.308 −41.515 47.120 0.00 0.00
F
ATOM 20185 CD GLU F 196 −66.041 −40.741 46.039 0.00 0.00
F
ATOM 20186 OE1 GLU F 196 −66.420 −39.607 45.745 0.00 0.00
F
ATOM 20187 OE2 GLU F 196 −66.823 −41.667 46.278 0.00 0.00
F
ATOM 20188 C GLU F 196 −61.574 −38.929 45.216 0.00 0.00
F
ATOM 20189 O GLU F 196 −61.831 −38.304 44.214 0.00 0.00
F
ATOM 20190 N ASP F 197 −60.766 −38.490 46.224 0.00 0.00
F
ATOM 20191 HN ASP F 197 −60.729 −38.946 47.110 0.00 0.00
F
ATOM 20192 CA ASP F 197 −60.232 −37.094 46.444 0.00 0.00
F
ATOM 20193 HA ASP F 197 −61.034 −36.507 46.866 0.00 0.00
F
ATOM 20194 CB ASP F 197 −59.231 −37.122 47.585 0.00 0.00
F
ATOM 20195 HB1 ASP F 197 −58.481 −37.931 47.451 0.00 0.00
F

ATOM 20196 HB2 ASP F 197   -58.718 -36.138 47.646 0.00 0.00
F
ATOM 20197 CG ASP F 197   -59.935 -37.437 48.896 0.00 0.00
F
ATOM 20198 OD1 ASP F 197   -59.741 -38.536 49.456 0.00 0.00
F
ATOM 20199 OD2 ASP F 197   -60.690 -36.591 49.441 0.00 0.00
F
ATOM 20200 C   ASP F 197   -59.602 -36.527 45.195 0.00 0.00
F
ATOM 20201 O   ASP F 197   -58.776 -37.154 44.642 0.00 0.00
F
ATOM 20202 N   ILE F 198   -60.037 -35.356 44.678 0.00 0.00
F
ATOM 20203 HN ILE F 198   -60.803 -34.924 45.148 0.00 0.00
F
ATOM 20204 CA ILE F 198   -59.483 -34.720 43.520 0.00 0.00
F
ATOM 20205 HA ILE F 198   -59.363 -35.492 42.775 0.00 0.00
F
ATOM 20206 CB ILE F 198   -60.490 -33.719 42.826 0.00 0.00
F
ATOM 20207 HB ILE F 198   -60.045 -33.235 41.930 0.00 0.00
F
ATOM 20208 CG2 ILE F 198   -61.706 -34.562 42.356 0.00 0.00
F
ATOM 20209 HG21 ILE F 198   -62.411 -34.036 41.677 0.00 0.00
F
ATOM 20210 HG22 ILE F 198   -61.325 -35.456 41.817 0.00 0.00
F
ATOM 20211 HG23 ILE F 198   -62.198 -34.839 43.313 0.00 0.00
F
ATOM 20212 CG1 ILE F 198   -60.918 -32.467 43.740 0.00 0.00
F
ATOM 20213 HG11 ILE F 198   -61.177 -32.849 44.750 0.00 0.00
F
ATOM 20214 HG12 ILE F 198   -60.043 -31.791 43.843 0.00 0.00
F
ATOM 20215 CD ILE F 198   -62.067 -31.639 43.126 0.00 0.00
F
ATOM 20216 HD1 ILE F 198   -61.791 -31.348 42.090 0.00 0.00
F
ATOM 20217 HD2 ILE F 198   -63.032 -32.181 43.224 0.00 0.00
F
ATOM 20218 HD3 ILE F 198   -62.131 -30.715 43.741 0.00 0.00
F
ATOM 20219 C   ILE F 198   -58.121 -34.132 43.716 0.00 0.00
F
ATOM 20220 O   ILE F 198   -57.614 -33.875 44.806 0.00 0.00
F
ATOM 20221 N   ILE F 199   -57.377 -34.002 42.555 0.00 0.00
F
ATOM 20222 HN ILE F 199   -57.710 -34.409 41.708 0.00 0.00
F

ATOM	20223	CA	ILE	F	199	-56.089	-33.214	42.518	0.00	0.00	F
ATOM	20224	HA	ILE	F	199	-55.765	-33.031	43.532	0.00	0.00	F
ATOM	20225	CB	ILE	F	199	-54.998	-33.853	41.720	0.00	0.00	F
ATOM	20226	HB	ILE	F	199	-55.337	-33.968	40.668	0.00	0.00	F
ATOM	20227	CG2	ILE	F	199	-53.745	-32.996	41.641	0.00	0.00	F
ATOM	20228	HG21	ILE	F	199	-53.949	-31.990	41.217	0.00	0.00	F
ATOM	20229	HG22	ILE	F	199	-53.436	-32.843	42.698	0.00	0.00	F
ATOM	20230	HG23	ILE	F	199	-52.941	-33.455	41.027	0.00	0.00	F
ATOM	20231	CG1	ILE	F	199	-54.798	-35.341	42.181	0.00	0.00	F
ATOM	20232	HG11	ILE	F	199	-55.047	-35.320	43.263	0.00	0.00	F
ATOM	20233	HG12	ILE	F	199	-55.531	-35.940	41.598	0.00	0.00	F
ATOM	20234	CD	ILE	F	199	-53.438	-35.987	41.774	0.00	0.00	F
ATOM	20235	HD1	ILE	F	199	-53.426	-37.035	42.144	0.00	0.00	F
ATOM	20236	HD2	ILE	F	199	-53.281	-35.960	40.674	0.00	0.00	F
ATOM	20237	HD3	ILE	F	199	-52.485	-35.616	42.208	0.00	0.00	F
ATOM	20238	C	ILE	F	199	-56.578	-31.929	41.808	0.00	0.00	F
ATOM	20239	O	ILE	F	199	-57.488	-31.875	41.026	0.00	0.00	F
ATOM	20240	N	ARG	F	200	-55.997	-30.771	42.098	0.00	0.00	F
ATOM	20241	HN	ARG	F	200	-55.351	-30.574	42.831	0.00	0.00	F
ATOM	20242	CA	ARG	F	200	-56.346	-29.377	41.557	0.00	0.00	F
ATOM	20243	HA	ARG	F	200	-57.035	-29.535	40.740	0.00	0.00	F
ATOM	20244	CB	ARG	F	200	-56.962	-28.497	42.681	0.00	0.00	F
ATOM	20245	HB1	ARG	F	200	-56.170	-28.299	43.434	0.00	0.00	F
ATOM	20246	HB2	ARG	F	200	-57.205	-27.518	42.215	0.00	0.00	F
ATOM	20247	CG	ARG	F	200	-58.256	-29.045	43.226	0.00	0.00	F
ATOM	20248	HG1	ARG	F	200	-58.992	-29.055	42.394	0.00	0.00	F
ATOM	20249	HG2	ARG	F	200	-58.011	-30.099	43.479	0.00	0.00	F

ATOM 20250 CD ARG F 200 −58.714 −28.259 44.498 0.00 0.00
F
ATOM 20251 HD1 ARG F 200 −58.934 −27.192 44.280 0.00 0.00
F
ATOM 20252 HD2 ARG F 200 −59.733 −28.643 44.716 0.00 0.00
F
ATOM 20253 NE ARG F 200 −57.503 −28.452 45.541 0.00 0.00
F
ATOM 20254 HE ARG F 200 −56.658 −28.805 45.141 0.00 0.00
F
ATOM 20255 CZ ARG F 200 −57.399 −28.156 46.863 0.00 0.00
F
ATOM 20256 NH1 ARG F 200 −58.392 −27.538 47.490 0.00 0.00
F
ATOM 20257 HH11 ARG F 200 −58.587 −27.546 48.470 0.00 0.00
F
ATOM 20258 HH12 ARG F 200 −59.256 −27.525 46.987 0.00 0.00
F
ATOM 20259 NH2 ARG F 200 −56.198 −28.376 47.462 0.00 0.00
F
ATOM 20260 HH21 ARG F 200 −56.027 −28.032 48.385 0.00 0.00
F
ATOM 20261 HH22 ARG F 200 −55.434 −28.635 46.870 0.00 0.00
F
ATOM 20262 C ARG F 200 −55.047 −28.737 41.124 0.00 0.00
F
ATOM 20263 O ARG F 200 −53.946 −29.232 41.363 0.00 0.00
F
ATOM 20264 N GLU F 201 −55.150 −27.563 40.382 0.00 0.00
F
ATOM 20265 HN GLU F 201 −56.047 −27.187 40.159 0.00 0.00
F
ATOM 20266 CA GLU F 201 −54.026 −26.708 40.168 0.00 0.00
F
ATOM 20267 HA GLU F 201 −53.224 −27.221 39.657 0.00 0.00
F
ATOM 20268 CB GLU F 201 −54.454 −25.561 39.366 0.00 0.00
F
ATOM 20269 HB1 GLU F 201 −55.036 −26.000 38.528 0.00 0.00
F
ATOM 20270 HB2 GLU F 201 −55.237 −24.887 39.774 0.00 0.00
F
ATOM 20271 CG GLU F 201 −53.278 −24.824 38.702 0.00 0.00
F
ATOM 20272 HG1 GLU F 201 −52.538 −24.447 39.440 0.00 0.00
F
ATOM 20273 HG2 GLU F 201 −52.788 −25.462 37.936 0.00 0.00
F
ATOM 20274 CD GLU F 201 −53.655 −23.499 38.036 0.00 0.00
F
ATOM 20275 OE1 GLU F 201 −52.805 −22.841 37.413 0.00 0.00
F
ATOM 20276 OE2 GLU F 201 −54.834 −23.121 38.091 0.00 0.00
F

ATOM 20277 C GLU F 201 -53.405 -26.170 41.431 0.00 0.00
F
ATOM 20278 O GLU F 201 -54.073 -25.776 42.408 0.00 0.00
F
ATOM 20279 N GLN F 202 -52.106 -26.192 41.426 0.00 0.00
F
ATOM 20280 HN GLN F 202 -51.706 -26.464 40.554 0.00 0.00
F
ATOM 20281 CA GLN F 202 -51.174 -25.826 42.471 0.00 0.00
F
ATOM 20282 HA GLN F 202 -50.173 -25.833 42.065 0.00 0.00
F
ATOM 20283 CB GLN F 202 -51.391 -24.360 42.988 0.00 0.00
F
ATOM 20284 HB1 GLN F 202 -52.103 -23.779 42.363 0.00 0.00
F
ATOM 20285 HB2 GLN F 202 -51.740 -24.353 44.042 0.00 0.00
F
ATOM 20286 CG GLN F 202 -50.048 -23.496 43.133 0.00 0.00
F
ATOM 20287 HG1 GLN F 202 -50.118 -22.648 43.847 0.00 0.00
F
ATOM 20288 HG2 GLN F 202 -49.341 -24.086 43.753 0.00 0.00
F
ATOM 20289 CD GLN F 202 -49.535 -22.985 41.836 0.00 0.00
F
ATOM 20290 OE1 GLN F 202 -49.270 -23.742 40.847 0.00 0.00
F
ATOM 20291 NE2 GLN F 202 -49.393 -21.630 41.758 0.00 0.00
F
ATOM 20292 HE21 GLN F 202 -48.914 -21.390 40.914 0.00 0.00
F
ATOM 20293 HE22 GLN F 202 -49.753 -20.990 42.437 0.00 0.00
F
ATOM 20294 C GLN F 202 -51.173 -26.800 43.667 0.00 0.00
F
ATOM 20295 O GLN F 202 -50.638 -26.529 44.739 0.00 0.00
F
ATOM 20296 N ASP F 203 -51.641 -28.004 43.431 0.00 0.00
F
ATOM 20297 HN ASP F 203 -52.090 -28.333 42.604 0.00 0.00
F
ATOM 20298 CA ASP F 203 -51.495 -29.064 44.468 0.00 0.00
F
ATOM 20299 HA ASP F 203 -51.312 -28.676 45.459 0.00 0.00
F
ATOM 20300 CB ASP F 203 -52.676 -30.080 44.536 0.00 0.00
F
ATOM 20301 HB1 ASP F 203 -52.983 -30.357 43.505 0.00 0.00
F
ATOM 20302 HB2 ASP F 203 -52.373 -31.040 45.004 0.00 0.00
F
ATOM 20303 CG ASP F 203 -53.978 -29.628 45.239 0.00 0.00
F

```
ATOM  20304  OD1 ASP F 203     -55.062 -30.299  44.998  0.00  0.00
F
ATOM  20305  OD2 ASP F 203     -53.927 -28.601  45.940  0.00  0.00
F
ATOM  20306  C   ASP F 203     -50.216 -29.792  44.068  0.00  0.00
F
ATOM  20307  O   ASP F 203     -49.993 -30.179  42.865  0.00  0.00
F
ATOM  20308  N   CYS F 204     -49.247 -30.041  45.067  0.00  0.00
F
ATOM  20309  HN  CYS F 204     -49.632 -29.864  45.969  0.00  0.00
F
ATOM  20310  CA  CYS F 204     -48.071 -30.780  44.958  0.00  0.00
F
ATOM  20311  HA  CYS F 204     -47.645 -30.624  43.978  0.00  0.00
F
ATOM  20312  CB  CYS F 204     -46.995 -30.449  46.068  0.00  0.00
F
ATOM  20313  HB1 CYS F 204     -47.372 -30.749  47.069  0.00  0.00
F
ATOM  20314  HB2 CYS F 204     -46.019 -30.962  45.931  0.00  0.00
F
ATOM  20315  SG  CYS F 204     -46.429 -28.697  46.099  0.00  0.00
F
ATOM  20316  HG1 CYS F 204     -45.907 -28.613  47.314  0.00  0.00
F
ATOM  20317  C   CYS F 204     -48.318 -32.224  45.175  0.00  0.00
F
ATOM  20318  O   CYS F 204     -48.866 -32.649  46.175  0.00  0.00
F
ATOM  20319  N   LEU F 205     -47.915 -33.110  44.166  0.00  0.00
F
ATOM  20320  HN  LEU F 205     -47.407 -32.616  43.465  0.00  0.00
F
ATOM  20321  CA  LEU F 205     -47.921 -34.570  44.267  0.00  0.00
F
ATOM  20322  HA  LEU F 205     -48.137 -34.931  45.261  0.00  0.00
F
ATOM  20323  CB  LEU F 205     -48.956 -35.138  43.295  0.00  0.00
F
ATOM  20324  HB1 LEU F 205     -49.067 -36.226  43.492  0.00  0.00
F
ATOM  20325  HB2 LEU F 205     -49.976 -34.743  43.487  0.00  0.00
F
ATOM  20326  CG  LEU F 205     -48.664 -35.073  41.726  0.00  0.00
F
ATOM  20327  HG  LEU F 205     -47.564 -34.989  41.589  0.00  0.00
F
ATOM  20328  CD1 LEU F 205     -49.238 -36.247  40.993  0.00  0.00
F
ATOM  20329 HD11 LEU F 205     -48.853 -37.182  41.454  0.00  0.00
F
ATOM  20330 HD12 LEU F 205     -50.330 -36.077  40.888  0.00  0.00
F
```

ATOM 20331 HD13 LEU F 205   −48.816 −36.232 39.965 0.00 0.00
F
ATOM 20332 CD2 LEU F 205   −49.178 −33.656 41.196 0.00 0.00
F
ATOM 20333 HD21 LEU F 205   −49.062 −33.586 40.094 0.00 0.00
F
ATOM 20334 HD22 LEU F 205   −50.271 −33.657 41.397 0.00 0.00
F
ATOM 20335 HD23 LEU F 205   −48.584 −32.833 41.647 0.00 0.00
F
ATOM 20336 C   LEU F 205   −46.574 −35.172 44.102 0.00 0.00
F
ATOM 20337 O   LEU F 205   −45.750 −34.791 43.309 0.00 0.00
F
ATOM 20338 N   VAL F 206   −46.264 −36.262 44.880 0.00 0.00
F
ATOM 20339 HN VAL F 206   −46.885 −36.726 45.506 0.00 0.00
F
ATOM 20340 CA VAL F 206   −44.953 −36.983 44.886 0.00 0.00
F
ATOM 20341 HA VAL F 206   −44.150 −36.424 44.427 0.00 0.00
F
ATOM 20342 CB VAL F 206   −44.377 −37.368 46.264 0.00 0.00
F
ATOM 20343 HB VAL F 206   −45.110 −38.073 46.711 0.00 0.00
F
ATOM 20344 CG1 VAL F 206   −42.946 −38.041 46.158 0.00 0.00
F
ATOM 20345 HG11 VAL F 206   −42.517 −38.335 47.140 0.00 0.00
F
ATOM 20346 HG12 VAL F 206   −43.022 −39.059 45.719 0.00 0.00
F
ATOM 20347 HG13 VAL F 206   −42.202 −37.495 45.541 0.00 0.00
F
ATOM 20348 CG2 VAL F 206   −44.401 −36.021 47.076 0.00 0.00
F
ATOM 20349 HG21 VAL F 206   −44.138 −36.331 48.110 0.00 0.00
F
ATOM 20350 HG22 VAL F 206   −43.584 −35.386 46.672 0.00 0.00
F
ATOM 20351 HG23 VAL F 206   −45.307 −35.381 47.019 0.00 0.00
F
ATOM 20352 C   VAL F 206   −45.133 −38.140 44.002 0.00 0.00
F
ATOM 20353 O   VAL F 206   −45.968 −38.983 44.341 0.00 0.00
F
ATOM 20354 N   ILE F 207   −44.471 −38.100 42.877 0.00 0.00
F
ATOM 20355 HN ILE F 207   −43.705 −37.472 42.759 0.00 0.00
F
ATOM 20356 CA ILE F 207   −44.754 −39.170 41.903 0.00 0.00
F
ATOM 20357 HA ILE F 207   −45.461 −39.821 42.395 0.00 0.00
F

ATOM 20358 CB ILE F 207 −45.456 −38.692 40.598 0.00 0.00
F
ATOM 20359 HB ILE F 207 −46.321 −38.028 40.808 0.00 0.00
F
ATOM 20360 CG2 ILE F 207 −44.457 −37.927 39.729 0.00 0.00
F
ATOM 20361 HG21 ILE F 207 −45.082 −37.520 38.905 0.00 0.00
F
ATOM 20362 HG22 ILE F 207 −44.035 −36.995 40.162 0.00 0.00
F
ATOM 20363 HG23 ILE F 207 −43.653 −38.504 39.225 0.00 0.00
F
ATOM 20364 CG1 ILE F 207 −46.095 −39.927 39.881 0.00 0.00
F
ATOM 20365 HG11 ILE F 207 −45.322 −40.353 39.206 0.00 0.00
F
ATOM 20366 HG12 ILE F 207 −46.494 −40.649 40.625 0.00 0.00
F
ATOM 20367 CD ILE F 207 −47.241 −39.466 39.018 0.00 0.00
F
ATOM 20368 HD1 ILE F 207 −47.878 −38.698 39.508 0.00 0.00
F
ATOM 20369 HD2 ILE F 207 −47.065 −38.950 38.050 0.00 0.00
F
ATOM 20370 HD3 ILE F 207 −47.923 −40.329 38.867 0.00 0.00
F
ATOM 20371 C ILE F 207 −43.528 −40.064 41.607 0.00 0.00
F
ATOM 20372 O ILE F 207 −42.375 −39.669 41.561 0.00 0.00
F
ATOM 20373 N MET F 208 −43.764 −41.388 41.500 0.00 0.00
F
ATOM 20374 HN MET F 208 −44.665 −41.813 41.526 0.00 0.00
F
ATOM 20375 CA MET F 208 −42.688 −42.334 41.678 0.00 0.00
F
ATOM 20376 HA MET F 208 −41.720 −41.864 41.586 0.00 0.00
F
ATOM 20377 CB MET F 208 −42.639 −43.137 43.078 0.00 0.00
F
ATOM 20378 HB1 MET F 208 −42.476 −42.370 43.866 0.00 0.00
F
ATOM 20379 HB2 MET F 208 −43.468 −43.823 43.351 0.00 0.00
F
ATOM 20380 CG MET F 208 −41.485 −44.153 43.150 0.00 0.00
F
ATOM 20381 HG1 MET F 208 −41.752 −45.072 42.584 0.00 0.00
F
ATOM 20382 HG2 MET F 208 −40.587 −43.804 42.597 0.00 0.00
F
ATOM 20383 SD MET F 208 −41.025 −44.656 44.882 0.00 0.00
F
ATOM 20384 CE MET F 208 −42.557 −45.508 45.288 0.00 0.00
F

ATOM 20385 HE1 MET F 208 −43.448 −44.861 45.142 0.00 0.00
F
ATOM 20386 HE2 MET F 208 −42.693 −46.299 44.519 0.00 0.00
F
ATOM 20387 HE3 MET F 208 −42.726 −46.000 46.269 0.00 0.00
F
ATOM 20388 C MET F 208 −42.782 −43.236 40.471 0.00 0.00
F
ATOM 20389 O MET F 208 −43.859 −43.827 40.221 0.00 0.00
F
ATOM 20390 N GLY F 209 −41.701 −43.496 39.752 0.00 0.00
F
ATOM 20391 HN GLY F 209 −40.849 −43.020 39.956 0.00 0.00
F
ATOM 20392 CA GLY F 209 −41.656 −44.510 38.688 0.00 0.00
F
ATOM 20393 HA1 GLY F 209 −42.297 −44.179 37.883 0.00 0.00
F
ATOM 20394 HA2 GLY F 209 −41.892 −45.446 39.172 0.00 0.00
F
ATOM 20395 C GLY F 209 −40.284 −44.603 38.097 0.00 0.00
F
ATOM 20396 O GLY F 209 −39.342 −44.082 38.585 0.00 0.00
F
ATOM 20397 N HSE F 210 −40.218 −45.386 37.020 0.00 0.00
F
ATOM 20398 HN HSE F 210 −40.896 −46.116 36.975 0.00 0.00
F
ATOM 20399 CA HSE F 210 −39.065 −45.349 36.122 0.00 0.00
F
ATOM 20400 HA HSE F 210 −38.128 −45.530 36.627 0.00 0.00
F
ATOM 20401 CB HSE F 210 −39.248 −46.486 35.092 0.00 0.00
F
ATOM 20402 HB1 HSE F 210 −39.334 −47.403 35.714 0.00 0.00
F
ATOM 20403 HB2 HSE F 210 −40.173 −46.354 34.492 0.00 0.00
F
ATOM 20404 ND1 HSE F 210 −38.311 −45.915 32.958 0.00 0.00
F
ATOM 20405 CG HSE F 210 −38.219 −46.683 34.093 0.00 0.00
F
ATOM 20406 CE1 HSE F 210 −37.210 −46.150 32.290 0.00 0.00
F
ATOM 20407 HE1 HSE F 210 −36.895 −45.697 31.349 0.00 0.00
F
ATOM 20408 NE2 HSE F 210 −36.488 −47.147 32.799 0.00 0.00
F
ATOM 20409 HE2 HSE F 210 −35.597 −47.513 32.529 0.00 0.00
F
ATOM 20410 CD2 HSE F 210 −37.112 −47.481 33.980 0.00 0.00
F
ATOM 20411 HD2 HSE F 210 −36.740 −48.329 34.542 0.00 0.00
F

ATOM 20412 C HSE F 210 −38.893 −44.041 35.469 0.00 0.00
F
ATOM 20413 O HSE F 210 −39.807 −43.275 35.266 0.00 0.00
F
ATOM 20414 N LYS F 211 −37.587 −43.652 35.173 0.00 0.00
F
ATOM 20415 HN LYS F 211 −36.873 −44.348 35.174 0.00 0.00
F
ATOM 20416 CA LYS F 211 −37.182 −42.408 34.602 0.00 0.00
F
ATOM 20417 HA LYS F 211 −37.217 −41.624 35.344 0.00 0.00
F
ATOM 20418 CB LYS F 211 −35.692 −42.477 34.287 0.00 0.00
F
ATOM 20419 HB1 LYS F 211 −35.438 −43.357 33.658 0.00 0.00
F
ATOM 20420 HB2 LYS F 211 −35.395 −41.500 33.850 0.00 0.00
F
ATOM 20421 CG LYS F 211 −34.836 −42.438 35.547 0.00 0.00
F
ATOM 20422 HG1 LYS F 211 −35.082 −41.530 36.138 0.00 0.00
F
ATOM 20423 HG2 LYS F 211 −35.055 −43.300 36.213 0.00 0.00
F
ATOM 20424 CD LYS F 211 −33.336 −42.290 35.318 0.00 0.00
F
ATOM 20425 HD1 LYS F 211 −32.867 −43.273 35.101 0.00 0.00
F
ATOM 20426 HD2 LYS F 211 −33.136 −41.683 34.409 0.00 0.00
F
ATOM 20427 CE LYS F 211 −32.592 −41.532 36.481 0.00 0.00
F
ATOM 20428 HE1 LYS F 211 −31.566 −41.332 36.104 0.00 0.00
F
ATOM 20429 HE2 LYS F 211 −33.161 −40.586 36.603 0.00 0.00
F
ATOM 20430 NZ LYS F 211 −32.505 −42.246 37.708 0.00 0.00
F
ATOM 20431 HZ1 LYS F 211 −33.422 −42.167 38.192 0.00 0.00
F
ATOM 20432 HZ2 LYS F 211 −32.279 −43.249 37.553 0.00 0.00
F
ATOM 20433 HZ3 LYS F 211 −31.769 −41.815 38.303 0.00 0.00
F
ATOM 20434 C LYS F 211 −37.891 −41.973 33.326 0.00 0.00
F
ATOM 20435 O LYS F 211 −38.127 −40.768 33.175 0.00 0.00
F
ATOM 20436 N LYS F 212 −38.148 −42.813 32.364 0.00 0.00
F
ATOM 20437 HN LYS F 212 −37.908 −43.766 32.530 0.00 0.00
F
ATOM 20438 CA LYS F 212 −38.960 −42.573 31.141 0.00 0.00
F

ATOM 20439 HA LYS F 212   −38.650 −41.596 30.799 0.00 0.00
F
ATOM 20440 CB LYS F 212   −38.679 −43.520 29.929 0.00 0.00
F
ATOM 20441 HB1 LYS F 212   −38.690 −44.557 30.327 0.00 0.00
F
ATOM 20442 HB2 LYS F 212   −39.455 −43.309 29.163 0.00 0.00
F
ATOM 20443 CG LYS F 212   −37.321 −43.112 29.299 0.00 0.00
F
ATOM 20444 HG1 LYS F 212   −36.537 −42.949 30.069 0.00 0.00
F
ATOM 20445 HG2 LYS F 212   −36.964 −43.985 28.713 0.00 0.00
F
ATOM 20446 CD LYS F 212   −37.408 −41.772 28.474 0.00 0.00
F
ATOM 20447 HD1 LYS F 212   −38.373 −41.900 27.937 0.00 0.00
F
ATOM 20448 HD2 LYS F 212   −37.643 −40.910 29.134 0.00 0.00
F
ATOM 20449 CE LYS F 212   −36.218 −41.560 27.625 0.00 0.00
F
ATOM 20450 HE1 LYS F 212   −35.845 −40.519 27.734 0.00 0.00
F
ATOM 20451 HE2 LYS F 212   −35.517 −42.359 27.949 0.00 0.00
F
ATOM 20452 NZ LYS F 212   −36.486 −41.842 26.189 0.00 0.00
F
ATOM 20453 HZ1 LYS F 212   −36.528 −42.861 25.982 0.00 0.00
F
ATOM 20454 HZ2 LYS F 212   −37.391 −41.390 25.949 0.00 0.00
F
ATOM 20455 HZ3 LYS F 212   −35.893 −41.312 25.519 0.00 0.00
F
ATOM 20456 C   LYS F 212   −40.461 −42.517 31.476 0.00 0.00
F
ATOM 20457 O   LYS F 212   −41.060 −41.465 31.074 0.00 0.00
F
ATOM 20458 N   ASP F 213   −40.967 −43.458 32.274 0.00 0.00
F
ATOM 20459 HN ASP F 213   −40.518 −44.283 32.608 0.00 0.00
F
ATOM 20460 CA ASP F 213   −42.373 −43.349 32.693 0.00 0.00
F
ATOM 20461 HA ASP F 213   −42.902 −43.226 31.759 0.00 0.00
F
ATOM 20462 CB ASP F 213   −43.011 −44.635 33.238 0.00 0.00
F
ATOM 20463 HB1 ASP F 213   −42.470 −44.779 34.198 0.00 0.00
F
ATOM 20464 HB2 ASP F 213   −44.117 −44.627 33.347 0.00 0.00
F
ATOM 20465 CG ASP F 213   −42.703 −45.721 32.267 0.00 0.00
F

ATOM 20466 OD1 ASP F 213 −42.980 −45.679 31.051 0.00 0.00
F
ATOM 20467 OD2 ASP F 213 −42.180 −46.774 32.683 0.00 0.00
F
ATOM 20468 C ASP F 213 −42.826 −42.106 33.495 0.00 0.00
F
ATOM 20469 O ASP F 213 −43.880 −41.543 33.197 0.00 0.00
F
ATOM 20470 N ILE F 214 −42.183 −41.613 34.532 0.00 0.00
F
ATOM 20471 HN ILE F 214 −41.429 −42.066 35.003 0.00 0.00
F
ATOM 20472 CA ILE F 214 −42.504 −40.265 34.996 0.00 0.00
F
ATOM 20473 HA ILE F 214 −43.568 −40.187 35.161 0.00 0.00
F
ATOM 20474 CB ILE F 214 −41.935 −39.895 36.377 0.00 0.00
F
ATOM 20475 HB ILE F 214 −42.344 −38.886 36.599 0.00 0.00
F
ATOM 20476 CG2 ILE F 214 −42.638 −40.762 37.437 0.00 0.00
F
ATOM 20477 HG21 ILE F 214 −43.736 −40.862 37.302 0.00 0.00
F
ATOM 20478 HG22 ILE F 214 −42.374 −41.841 37.437 0.00 0.00
F
ATOM 20479 HG23 ILE F 214 −42.356 −40.391 38.445 0.00 0.00
F
ATOM 20480 CG1 ILE F 214 −40.396 −39.662 36.443 0.00 0.00
F
ATOM 20481 HG11 ILE F 214 −39.876 −40.588 36.116 0.00 0.00
F
ATOM 20482 HG12 ILE F 214 −40.263 −38.869 35.676 0.00 0.00
F
ATOM 20483 CD ILE F 214 −40.024 −39.189 37.836 0.00 0.00
F
ATOM 20484 HD1 ILE F 214 −38.922 −39.075 37.927 0.00 0.00
F
ATOM 20485 HD2 ILE F 214 −40.521 −38.202 37.947 0.00 0.00
F
ATOM 20486 HD3 ILE F 214 −40.389 −39.849 38.652 0.00 0.00
F
ATOM 20487 C ILE F 214 −42.260 −39.196 33.987 0.00 0.00
F
ATOM 20488 O ILE F 214 −43.052 −38.259 33.918 0.00 0.00
F
ATOM 20489 N LYS F 215 −41.207 −39.274 33.098 0.00 0.00
F
ATOM 20490 HN LYS F 215 −40.559 −40.031 33.127 0.00 0.00
F
ATOM 20491 CA LYS F 215 −41.096 −38.203 32.049 0.00 0.00
F
ATOM 20492 HA LYS F 215 −41.167 −37.271 32.591 0.00 0.00
F

ATOM 20493 CB LYS F 215 −39.749 −38.085 31.357 0.00 0.00
F
ATOM 20494 HB1 LYS F 215 −38.958 −38.109 32.136 0.00 0.00
F
ATOM 20495 HB2 LYS F 215 −39.794 −39.038 30.788 0.00 0.00
F
ATOM 20496 CG LYS F 215 −39.488 −36.843 30.453 0.00 0.00
F
ATOM 20497 HG1 LYS F 215 −38.754 −37.082 29.654 0.00 0.00
F
ATOM 20498 HG2 LYS F 215 −40.442 −36.572 29.951 0.00 0.00
F
ATOM 20499 CD LYS F 215 −39.022 −35.543 31.155 0.00 0.00
F
ATOM 20500 HD1 LYS F 215 −39.860 −35.306 31.845 0.00 0.00
F
ATOM 20501 HD2 LYS F 215 −38.129 −35.613 31.812 0.00 0.00
F
ATOM 20502 CE LYS F 215 −38.748 −34.373 30.237 0.00 0.00
F
ATOM 20503 HE1 LYS F 215 −37.861 −34.576 29.600 0.00 0.00
F
ATOM 20504 HE2 LYS F 215 −39.632 −34.216 29.583 0.00 0.00
F
ATOM 20505 NZ LYS F 215 −38.388 −33.199 31.083 0.00 0.00
F
ATOM 20506 HZ1 LYS F 215 −39.228 −33.081 31.685 0.00 0.00
F
ATOM 20507 HZ2 LYS F 215 −37.513 −33.339 31.628 0.00 0.00
F
ATOM 20508 HZ3 LYS F 215 −38.190 −32.314 30.574 0.00 0.00
F
ATOM 20509 C LYS F 215 −42.264 −38.348 31.046 0.00 0.00
F
ATOM 20510 O LYS F 215 −42.842 −37.391 30.633 0.00 0.00
F
ATOM 20511 N ARG F 216 −42.783 −39.550 30.647 0.00 0.00
F
ATOM 20512 HN ARG F 216 −42.340 −40.415 30.868 0.00 0.00
F
ATOM 20513 CA ARG F 216 −43.933 −39.604 29.762 0.00 0.00
F
ATOM 20514 HA ARG F 216 −43.748 −39.083 28.834 0.00 0.00
F
ATOM 20515 CB ARG F 216 −44.368 −41.049 29.431 0.00 0.00
F
ATOM 20516 HB1 ARG F 216 −44.601 −41.614 30.360 0.00 0.00
F
ATOM 20517 HB2 ARG F 216 −45.310 −40.907 28.860 0.00 0.00
F
ATOM 20518 CG ARG F 216 −43.380 −41.852 28.580 0.00 0.00
F
ATOM 20519 HG1 ARG F 216 −43.358 −41.345 27.591 0.00 0.00
F

ATOM 20520 HG2 ARG F 216 −42.364 −41.802 29.027 0.00 0.00
F
ATOM 20521 CD ARG F 216 −43.828 −43.261 28.486 0.00 0.00
F
ATOM 20522 HD1 ARG F 216 −43.704 −43.756 29.473 0.00 0.00
F
ATOM 20523 HD2 ARG F 216 −44.865 −43.382 28.106 0.00 0.00
F
ATOM 20524 NE ARG F 216 −42.974 −43.840 27.430 0.00 0.00
F
ATOM 20525 HE ARG F 216 −43.138 −43.730 26.450 0.00 0.00
F
ATOM 20526 CZ ARG F 216 −41.895 −44.575 27.638 0.00 0.00
F
ATOM 20527 NH1 ARG F 216 −41.451 −45.028 28.815 0.00 0.00
F
ATOM 20528 HH11 ARG F 216 −40.763 −45.753 28.834 0.00 0.00
F
ATOM 20529 HH12 ARG F 216 −42.072 −45.068 29.599 0.00 0.00
F
ATOM 20530 NH2 ARG F 216 −41.273 −45.088 26.582 0.00 0.00
F
ATOM 20531 HH21 ARG F 216 −40.292 −45.265 26.661 0.00 0.00
F
ATOM 20532 HH22 ARG F 216 −41.623 −44.780 25.698 0.00 0.00
F
ATOM 20533 C ARG F 216 −45.245 −38.925 30.346 0.00 0.00
F
ATOM 20534 O ARG F 216 −45.989 −38.185 29.690 0.00 0.00
F
ATOM 20535 N PHE F 217 −45.372 −39.104 31.689 0.00 0.00
F
ATOM 20536 HN PHE F 217 −44.662 −39.622 32.160 0.00 0.00
F
ATOM 20537 CA PHE F 217 −46.370 −38.506 32.601 0.00 0.00
F
ATOM 20538 HA PHE F 217 −47.336 −38.772 32.199 0.00 0.00
F
ATOM 20539 CB PHE F 217 −46.245 −39.091 34.093 0.00 0.00
F
ATOM 20540 HB1 PHE F 217 −46.367 −40.194 34.050 0.00 0.00
F
ATOM 20541 HB2 PHE F 217 −45.254 −38.717 34.432 0.00 0.00
F
ATOM 20542 CG PHE F 217 −47.356 −38.542 35.040 0.00 0.00
F
ATOM 20543 CD1 PHE F 217 −47.176 −37.496 35.978 0.00 0.00
F
ATOM 20544 HD1 PHE F 217 −46.185 −37.085 36.103 0.00 0.00
F
ATOM 20545 CE1 PHE F 217 −48.293 −36.950 36.605 0.00 0.00
F
ATOM 20546 HE1 PHE F 217 −48.101 −36.195 37.352 0.00 0.00
F

ATOM 20547 CZ PHE F 217 -49.563 -37.503 36.446 0.00 0.00
F
ATOM 20548 HZ PHE F 217 -50.430 -37.116 36.960 0.00 0.00
F
ATOM 20549 CD2 PHE F 217 -48.661 -39.114 34.861 0.00 0.00
F
ATOM 20550 HD2 PHE F 217 -48.795 -39.952 34.192 0.00 0.00
F
ATOM 20551 CE2 PHE F 217 -49.788 -38.555 35.574 0.00 0.00
F
ATOM 20552 HE2 PHE F 217 -50.750 -38.995 35.356 0.00 0.00
F
ATOM 20553 C PHE F 217 -46.246 -37.025 32.616 0.00 0.00
F
ATOM 20554 O PHE F 217 -47.247 -36.276 32.641 0.00 0.00
F
ATOM 20555 N GLU F 218 -45.009 -36.426 32.630 0.00 0.00
F
ATOM 20556 HN GLU F 218 -44.165 -36.939 32.494 0.00 0.00
F
ATOM 20557 CA GLU F 218 -44.744 -35.000 32.573 0.00 0.00
F
ATOM 20558 HA GLU F 218 -45.288 -34.554 33.393 0.00 0.00
F
ATOM 20559 CB GLU F 218 -43.226 -34.727 32.918 0.00 0.00
F
ATOM 20560 HB1 GLU F 218 -43.135 -35.195 33.921 0.00 0.00
F
ATOM 20561 HB2 GLU F 218 -42.608 -35.279 32.178 0.00 0.00
F
ATOM 20562 CG GLU F 218 -42.865 -33.255 32.899 0.00 0.00
F
ATOM 20563 HG1 GLU F 218 -43.055 -32.784 31.911 0.00 0.00
F
ATOM 20564 HG2 GLU F 218 -43.425 -32.681 33.668 0.00 0.00
F
ATOM 20565 CD GLU F 218 -41.426 -33.090 33.187 0.00 0.00
F
ATOM 20566 OE1 GLU F 218 -40.939 -33.340 34.377 0.00 0.00
F
ATOM 20567 OE2 GLU F 218 -40.620 -32.543 32.299 0.00 0.00
F
ATOM 20568 C GLU F 218 -45.108 -34.481 31.172 0.00 0.00
F
ATOM 20569 O GLU F 218 -45.646 -33.416 30.897 0.00 0.00
F
ATOM 20570 N ASN F 219 -44.841 -35.298 30.105 0.00 0.00
F
ATOM 20571 HN ASN F 219 -44.326 -36.150 30.158 0.00 0.00
F
ATOM 20572 CA ASN F 219 -45.200 -34.909 28.730 0.00 0.00
F
ATOM 20573 HA ASN F 219 -44.870 -33.886 28.630 0.00 0.00
F

ATOM 20574 CB ASN F 219 -44.428 -35.877 27.809 0.00 0.00
F
ATOM 20575 HB1 ASN F 219 -44.600 -36.908 28.186 0.00 0.00
F
ATOM 20576 HB2 ASN F 219 -44.749 -35.706 26.759 0.00 0.00
F
ATOM 20577 CG ASN F 219 -42.912 -35.580 27.880 0.00 0.00
F
ATOM 20578 OD1 ASN F 219 -42.443 -34.582 28.397 0.00 0.00
F
ATOM 20579 ND2 ASN F 219 -42.102 -36.460 27.302 0.00 0.00
F
ATOM 20580 HD21 ASN F 219 -41.152 -36.232 27.088 0.00 0.00
F
ATOM 20581 HD22 ASN F 219 -42.431 -37.283 26.838 0.00 0.00
F
ATOM 20582 C ASN F 219 -46.657 -34.865 28.380 0.00 0.00
F
ATOM 20583 O ASN F 219 -46.995 -33.917 27.687 0.00 0.00
F
ATOM 20584 N GLU F 220 -47.458 -35.873 28.746 0.00 0.00
F
ATOM 20585 HN GLU F 220 -47.056 -36.580 29.322 0.00 0.00
F
ATOM 20586 CA GLU F 220 -48.795 -36.089 28.222 0.00 0.00
F
ATOM 20587 HA GLU F 220 -49.176 -35.154 27.841 0.00 0.00
F
ATOM 20588 CB GLU F 220 -48.909 -37.231 27.100 0.00 0.00
F
ATOM 20589 HB1 GLU F 220 -48.650 -38.231 27.509 0.00 0.00
F
ATOM 20590 HB2 GLU F 220 -49.995 -37.418 26.955 0.00 0.00
F
ATOM 20591 CG GLU F 220 -48.047 -36.949 25.838 0.00 0.00
F
ATOM 20592 HG1 GLU F 220 -48.093 -35.899 25.477 0.00 0.00
F
ATOM 20593 HG2 GLU F 220 -46.988 -37.181 26.079 0.00 0.00
F
ATOM 20594 CD GLU F 220 -48.319 -37.748 24.584 0.00 0.00
F
ATOM 20595 OE1 GLU F 220 -48.389 -37.176 23.464 0.00 0.00
F
ATOM 20596 OE2 GLU F 220 -48.461 -39.007 24.592 0.00 0.00
F
ATOM 20597 C GLU F 220 -49.783 -36.626 29.352 0.00 0.00
F
ATOM 20598 O GLU F 220 -50.960 -36.855 29.114 0.00 0.00
F
ATOM 20599 N GLY F 221 -49.305 -36.736 30.582 0.00 0.00
F
ATOM 20600 HN GLY F 221 -48.357 -36.498 30.780 0.00 0.00
F

ATOM 20601 CA GLY F 221   -50.067 -37.462 31.556 0.00 0.00
F
ATOM 20602 HA1 GLY F 221   -49.330 -37.850 32.243 0.00 0.00
F
ATOM 20603 HA2 GLY F 221   -50.709 -38.248 31.186 0.00 0.00
F
ATOM 20604 C   GLY F 221   -50.898 -36.542 32.390 0.00 0.00
F
ATOM 20605 O   GLY F 221   -52.020 -36.945 32.770 0.00 0.00
F
ATOM 20606 N   MET F 222   -50.475 -35.294 32.645 0.00 0.00
F
ATOM 20607 HN MET F 222   -49.569 -35.028 32.325 0.00 0.00
F
ATOM 20608 CA MET F 222   -51.218 -34.332 33.420 0.00 0.00
F
ATOM 20609 HA MET F 222   -51.607 -34.786 34.319 0.00 0.00
F
ATOM 20610 CB MET F 222   -50.171 -33.227 33.738 0.00 0.00
F
ATOM 20611 HB1 MET F 222   -49.816 -32.769 32.790 0.00 0.00
F
ATOM 20612 HB2 MET F 222   -50.651 -32.463 34.386 0.00 0.00
F
ATOM 20613 CG MET F 222   -48.920 -33.793 34.573 0.00 0.00
F
ATOM 20614 HG1 MET F 222   -49.303 -34.507 35.332 0.00 0.00
F
ATOM 20615 HG2 MET F 222   -48.177 -34.297 33.919 0.00 0.00
F
ATOM 20616 SD MET F 222   -48.201 -32.427 35.487 0.00 0.00
F
ATOM 20617 CE MET F 222   -47.348 -31.594 34.096 0.00 0.00
F
ATOM 20618 HE1 MET F 222   -46.547 -30.874 34.369 0.00 0.00
F
ATOM 20619 HE2 MET F 222   -47.056 -32.220 33.225 0.00 0.00
F
ATOM 20620 HE3 MET F 222   -48.106 -31.007 33.534 0.00 0.00
F
ATOM 20621 C   MET F 222   -52.358 -33.774 32.697 0.00 0.00
F
ATOM 20622 O   MET F 222   -53.404 -34.445 32.729 0.00 0.00
F
ATOM 20623 N   LYS G   7   22.060 -36.505 27.754 0.00 0.00
G
ATOM 20624 HT1 LYS G   7   22.274 -35.829 28.515 0.00 0.00
G
ATOM 20625 HT2 LYS G   7   22.483 -36.150 26.873 0.00 0.00
G
ATOM 20626 HT3 LYS G   7   22.536 -37.384 28.042 0.00 0.00
G
ATOM 20627 CA LYS G   7   20.569 -36.655 27.655 0.00 0.00
G

ATOM 20628 HA LYS G 7 20.237 -37.327 26.878 0.00 0.00
G
ATOM 20629 CB LYS G 7 20.040 -37.372 28.919 0.00 0.00
G
ATOM 20630 HB1 LYS G 7 20.123 -36.741 29.830 0.00 0.00
G
ATOM 20631 HB2 LYS G 7 18.954 -37.566 28.786 0.00 0.00
G
ATOM 20632 CG LYS G 7 20.657 -38.798 29.196 0.00 0.00
G
ATOM 20633 HG1 LYS G 7 20.561 -39.490 28.332 0.00 0.00
G
ATOM 20634 HG2 LYS G 7 21.689 -38.636 29.573 0.00 0.00
G
ATOM 20635 CD LYS G 7 19.938 -39.567 30.418 0.00 0.00
G
ATOM 20636 HD1 LYS G 7 19.950 -38.843 31.260 0.00 0.00
G
ATOM 20637 HD2 LYS G 7 18.910 -39.731 30.031 0.00 0.00
G
ATOM 20638 CE LYS G 7 20.659 -40.760 30.980 0.00 0.00
G
ATOM 20639 HE1 LYS G 7 20.865 -41.625 30.314 0.00 0.00
G
ATOM 20640 HE2 LYS G 7 21.654 -40.364 31.276 0.00 0.00
G
ATOM 20641 NZ LYS G 7 19.937 -41.383 32.097 0.00 0.00
G
ATOM 20642 HZ1 LYS G 7 20.276 -42.338 32.333 0.00 0.00
G
ATOM 20643 HZ2 LYS G 7 19.863 -40.734 32.906 0.00 0.00
G
ATOM 20644 HZ3 LYS G 7 18.987 -41.569 31.718 0.00 0.00
G
ATOM 20645 C LYS G 7 19.706 -35.408 27.405 0.00 0.00
G
ATOM 20646 O LYS G 7 20.172 -34.275 27.317 0.00 0.00
G
ATOM 20647 N GLN G 8 18.371 -35.660 27.313 0.00 0.00
G
ATOM 20648 HN GLN G 8 18.181 -36.621 27.128 0.00 0.00
G
ATOM 20649 CA GLN G 8 17.321 -34.622 27.283 0.00 0.00
G
ATOM 20650 HA GLN G 8 17.740 -33.671 26.988 0.00 0.00
G
ATOM 20651 CB GLN G 8 16.218 -34.975 26.217 0.00 0.00
G
ATOM 20652 HB1 GLN G 8 16.304 -36.077 26.102 0.00 0.00
G
ATOM 20653 HB2 GLN G 8 15.234 -34.562 26.524 0.00 0.00
G
ATOM 20654 CG GLN G 8 16.532 -34.389 24.799 0.00 0.00
G

ATOM 20655 HG1 GLN G 8 16.489 -33.291 24.965 0.00 0.00
G
ATOM 20656 HG2 GLN G 8 17.564 -34.673 24.502 0.00 0.00
G
ATOM 20657 CD GLN G 8 15.511 -34.778 23.696 0.00 0.00
G
ATOM 20658 OE1 GLN G 8 14.337 -34.962 24.041 0.00 0.00
G
ATOM 20659 NE2 GLN G 8 15.957 -34.911 22.431 0.00 0.00
G
ATOM 20660 HE21 GLN G 8 15.452 -35.443 21.752 0.00 0.00
G
ATOM 20661 HE22 GLN G 8 16.901 -34.660 22.219 0.00 0.00
G
ATOM 20662 C GLN G 8 16.650 -34.480 28.653 0.00 0.00
G
ATOM 20663 O GLN G 8 15.992 -35.392 29.105 0.00 0.00
G
ATOM 20664 N PHE G 9 16.789 -33.313 29.282 0.00 0.00
G
ATOM 20665 HN PHE G 9 17.267 -32.551 28.852 0.00 0.00
G
ATOM 20666 CA PHE G 9 16.226 -33.070 30.644 0.00 0.00
G
ATOM 20667 HA PHE G 9 15.631 -33.900 30.996 0.00 0.00
G
ATOM 20668 CB PHE G 9 17.350 -32.813 31.717 0.00 0.00
G
ATOM 20669 HB1 PHE G 9 17.999 -31.997 31.332 0.00 0.00
G
ATOM 20670 HB2 PHE G 9 16.923 -32.466 32.683 0.00 0.00
G
ATOM 20671 CG PHE G 9 18.098 -34.054 31.953 0.00 0.00
G
ATOM 20672 CD1 PHE G 9 17.649 -34.866 33.095 0.00 0.00
G
ATOM 20673 HD1 PHE G 9 16.758 -34.482 33.570 0.00 0.00
G
ATOM 20674 CE1 PHE G 9 18.542 -35.826 33.655 0.00 0.00
G
ATOM 20675 HE1 PHE G 9 18.203 -36.442 34.475 0.00 0.00
G
ATOM 20676 CZ PHE G 9 19.782 -36.029 33.116 0.00 0.00
G
ATOM 20677 HZ PHE G 9 20.386 -36.790 33.587 0.00 0.00
G
ATOM 20678 CD2 PHE G 9 19.418 -34.366 31.418 0.00 0.00
G
ATOM 20679 HD2 PHE G 9 19.742 -33.729 30.609 0.00 0.00
G
ATOM 20680 CE2 PHE G 9 20.216 -35.305 31.994 0.00 0.00
G
ATOM 20681 HE2 PHE G 9 21.190 -35.513 31.577 0.00 0.00
G

```
ATOM  20682  C   PHE G   9      15.279 -31.926  30.628  0.00  0.00
G
ATOM  20683  O   PHE G   9      15.297 -31.198  29.668  0.00  0.00
G
ATOM  20684  N   ALA G  10      14.521 -31.626  31.695  0.00  0.00
G
ATOM  20685  HN  ALA G  10      14.329 -32.278  32.425  0.00  0.00
G
ATOM  20686  CA  ALA G  10      13.666 -30.510  31.705  0.00  0.00
G
ATOM  20687  HA  ALA G  10      13.923 -29.823  30.913  0.00  0.00
G
ATOM  20688  CB  ALA G  10      12.244 -31.138  31.393  0.00  0.00
G
ATOM  20689  HB1 ALA G  10      11.453 -30.380  31.576  0.00  0.00
G
ATOM  20690  HB2 ALA G  10      12.159 -31.564  30.370  0.00  0.00
G
ATOM  20691  HB3 ALA G  10      11.987 -31.961  32.094  0.00  0.00
G
ATOM  20692  C   ALA G  10      13.551 -29.910  33.057  0.00  0.00
G
ATOM  20693  O   ALA G  10      13.700 -30.547  34.114  0.00  0.00
G
ATOM  20694  N   VAL G  11      13.356 -28.585  33.052  0.00  0.00
G
ATOM  20695  HN  VAL G  11      13.323 -28.045  32.215  0.00  0.00
G
ATOM  20696  CA  VAL G  11      13.130 -27.742  34.266  0.00  0.00
G
ATOM  20697  HA  VAL G  11      12.902 -28.300  35.162  0.00  0.00
G
ATOM  20698  CB  VAL G  11      14.299 -26.882  34.603  0.00  0.00
G
ATOM  20699  HB  VAL G  11      14.496 -26.088  33.851  0.00  0.00
G
ATOM  20700  CG1 VAL G  11      14.116 -26.032  35.943  0.00  0.00
G
ATOM  20701 HG11 VAL G  11      13.136 -25.521  36.062  0.00  0.00
G
ATOM  20702 HG12 VAL G  11      14.127 -26.744  36.796  0.00  0.00
G
ATOM  20703 HG13 VAL G  11      14.942 -25.295  36.034  0.00  0.00
G
ATOM  20704  CG2 VAL G  11      15.554 -27.769  34.659  0.00  0.00
G
ATOM  20705 HG21 VAL G  11      16.008 -27.971  33.665  0.00  0.00
G
ATOM  20706 HG22 VAL G  11      16.222 -27.058  35.190  0.00  0.00
G
ATOM  20707 HG23 VAL G  11      15.422 -28.692  35.263  0.00  0.00
G
ATOM  20708  C   VAL G  11      11.840 -26.900  34.155  0.00  0.00
G
```

```
ATOM  20709  O   VAL G  11      11.693 -26.151  33.187  0.00  0.00
G
ATOM  20710  N   ILE G  12      10.910 -27.048  35.137  0.00  0.00
G
ATOM  20711  HN  ILE G  12      11.036 -27.732  35.851  0.00  0.00
G
ATOM  20712  CA  ILE G  12       9.659 -26.236  35.256  0.00  0.00
G
ATOM  20713  HA  ILE G  12       9.617 -25.750  34.293  0.00  0.00
G
ATOM  20714  CB  ILE G  12       8.456 -27.150  35.552  0.00  0.00
G
ATOM  20715  HB  ILE G  12       8.644 -27.645  36.529  0.00  0.00
G
ATOM  20716  CG2 ILE G  12       7.203 -26.200  35.624  0.00  0.00
G
ATOM  20717 HG21 ILE G  12       6.315 -26.866  35.589  0.00  0.00
G
ATOM  20718 HG22 ILE G  12       7.290 -25.645  36.582  0.00  0.00
G
ATOM  20719 HG23 ILE G  12       7.134 -25.451  34.806  0.00  0.00
G
ATOM  20720  CG1 ILE G  12       8.346 -28.327  34.672  0.00  0.00
G
ATOM  20721 HG11 ILE G  12       9.233 -28.996  34.708  0.00  0.00
G
ATOM  20722 HG12 ILE G  12       7.395 -28.833  34.946  0.00  0.00
G
ATOM  20723  CD  ILE G  12       8.116 -27.926  33.215  0.00  0.00
G
ATOM  20724  HD1 ILE G  12       7.426 -28.686  32.790  0.00  0.00
G
ATOM  20725  HD2 ILE G  12       7.591 -26.967  33.015  0.00  0.00
G
ATOM  20726  HD3 ILE G  12       9.042 -27.766  32.622  0.00  0.00
G
ATOM  20727  C   ILE G  12       9.954 -25.308  36.347  0.00  0.00
G
ATOM  20728  O   ILE G  12      10.190 -25.755  37.468  0.00  0.00
G
ATOM  20729  N   GLY G  13      10.006 -24.001  35.974  0.00  0.00
G
ATOM  20730  HN  GLY G  13       9.872 -23.783  35.011  0.00  0.00
G
ATOM  20731  CA  GLY G  13      10.253 -22.820  36.866  0.00  0.00
G
ATOM  20732  HA1 GLY G  13      10.151 -23.240  37.856  0.00  0.00
G
ATOM  20733  HA2 GLY G  13       9.674 -21.978  36.515  0.00  0.00
G
ATOM  20734  C   GLY G  13      11.732 -22.356  36.828  0.00  0.00
G
ATOM  20735  O   GLY G  13      12.627 -23.094  37.172  0.00  0.00
G
```

ATOM 20736 N LEU G 14 12.083 -21.122 36.479 0.00 0.00
G
ATOM 20737 HN LEU G 14 11.372 -20.477 36.208 0.00 0.00
G
ATOM 20738 CA LEU G 14 13.368 -20.600 36.297 0.00 0.00
G
ATOM 20739 HA LEU G 14 14.089 -21.400 36.379 0.00 0.00
G
ATOM 20740 CB LEU G 14 13.648 -19.971 34.912 0.00 0.00
G
ATOM 20741 HB1 LEU G 14 12.764 -19.354 34.642 0.00 0.00
G
ATOM 20742 HB2 LEU G 14 14.395 -19.163 35.062 0.00 0.00
G
ATOM 20743 CG LEU G 14 13.992 -20.859 33.767 0.00 0.00
G
ATOM 20744 HG LEU G 14 15.081 -21.070 33.697 0.00 0.00
G
ATOM 20745 CD1 LEU G 14 13.329 -22.195 33.866 0.00 0.00
G
ATOM 20746 HD11 LEU G 14 12.288 -21.892 34.109 0.00 0.00
G
ATOM 20747 HD12 LEU G 14 13.203 -22.784 32.932 0.00 0.00
G
ATOM 20748 HD13 LEU G 14 13.789 -22.874 34.616 0.00 0.00
G
ATOM 20749 CD2 LEU G 14 13.649 -20.025 32.530 0.00 0.00
G
ATOM 20750 HD21 LEU G 14 14.278 -19.110 32.498 0.00 0.00
G
ATOM 20751 HD22 LEU G 14 13.899 -20.601 31.613 0.00 0.00
G
ATOM 20752 HD23 LEU G 14 12.558 -19.818 32.560 0.00 0.00
G
ATOM 20753 C LEU G 14 13.618 -19.737 37.540 0.00 0.00
G
ATOM 20754 O LEU G 14 14.064 -18.578 37.394 0.00 0.00
G
ATOM 20755 N GLY G 15 13.423 -20.145 38.794 0.00 0.00
G
ATOM 20756 HN GLY G 15 13.281 -21.123 38.922 0.00 0.00
G
ATOM 20757 CA GLY G 15 13.872 -19.388 39.987 0.00 0.00
G
ATOM 20758 HA1 GLY G 15 13.308 -19.730 40.842 0.00 0.00
G
ATOM 20759 HA2 GLY G 15 13.717 -18.358 39.701 0.00 0.00
G
ATOM 20760 C GLY G 15 15.360 -19.496 40.122 0.00 0.00
G
ATOM 20761 O GLY G 15 16.035 -20.003 39.245 0.00 0.00
G
ATOM 20762 N ARG G 16 15.951 -19.034 41.208 0.00 0.00
G

ATOM 20763 HN ARG G 16   15.424 -18.676 41.975 0.00 0.00
G
ATOM 20764 CA ARG G 16   17.357 -19.205 41.474 0.00 0.00
G
ATOM 20765 HA ARG G 16   17.913 -18.690 40.705 0.00 0.00
G
ATOM 20766 CB ARG G 16   17.790 -18.600 42.842 0.00 0.00
G
ATOM 20767 HB1 ARG G 16   17.221 -19.113 43.646 0.00 0.00
G
ATOM 20768 HB2 ARG G 16   18.861 -18.810 43.049 0.00 0.00
G
ATOM 20769 CG ARG G 16   17.544 -17.121 42.963 0.00 0.00
G
ATOM 20770 HG1 ARG G 16   18.310 -16.543 42.403 0.00 0.00
G
ATOM 20771 HG2 ARG G 16   16.523 -16.806 42.658 0.00 0.00
G
ATOM 20772 CD ARG G 16   17.711 -16.628 44.423 0.00 0.00
G
ATOM 20773 HD1 ARG G 16   18.655 -16.993 44.880 0.00 0.00
G
ATOM 20774 HD2 ARG G 16   17.748 -15.517 44.406 0.00 0.00
G
ATOM 20775 NE ARG G 16   16.594 -17.098 45.262 0.00 0.00
G
ATOM 20776 HE ARG G 16   15.943 -17.709 44.812 0.00 0.00
G
ATOM 20777 CZ ARG G 16   16.144 -16.644 46.401 0.00 0.00
G
ATOM 20778 NH1 ARG G 16   16.802 -15.840 47.185 0.00 0.00
G
ATOM 20779 HH11 ARG G 16   16.402 -15.647 48.081 0.00 0.00
G
ATOM 20780 HH12 ARG G 16   17.775 -15.703 47.002 0.00 0.00
G
ATOM 20781 NH2 ARG G 16   14.906 -16.964 46.755 0.00 0.00
G
ATOM 20782 HH21 ARG G 16   14.588 -16.623 47.640 0.00 0.00
G
ATOM 20783 HH22 ARG G 16   14.708 -17.906 46.484 0.00 0.00
G
ATOM 20784 C   ARG G 16   17.914 -20.684 41.428 0.00 0.00
G
ATOM 20785 O   ARG G 16   18.888 -20.972 40.796 0.00 0.00
G
ATOM 20786 N   PHE G 17   17.168 -21.613 42.077 0.00 0.00
G
ATOM 20787 HN PHE G 17   16.534 -21.257 42.760 0.00 0.00
G
ATOM 20788 CA PHE G 17   17.366 -23.101 42.007 0.00 0.00
G
ATOM 20789 HA PHE G 17   18.376 -23.256 42.357 0.00 0.00
G

ATOM 20790 CB PHE G 17 16.478 -23.809 43.064 0.00 0.00
G
ATOM 20791 HB1 PHE G 17 16.691 -23.190 43.961 0.00 0.00
G
ATOM 20792 HB2 PHE G 17 15.383 -23.910 42.906 0.00 0.00
G
ATOM 20793 CG PHE G 17 17.075 -25.206 43.284 0.00 0.00
G
ATOM 20794 CD1 PHE G 17 18.434 -25.582 43.468 0.00 0.00
G
ATOM 20795 HD1 PHE G 17 19.240 -24.873 43.581 0.00 0.00
G
ATOM 20796 CE1 PHE G 17 18.792 -26.949 43.646 0.00 0.00
G
ATOM 20797 HE1 PHE G 17 19.822 -27.274 43.664 0.00 0.00
G
ATOM 20798 CZ PHE G 17 17.798 -27.887 43.806 0.00 0.00
G
ATOM 20799 HZ PHE G 17 17.954 -28.928 44.049 0.00 0.00
G
ATOM 20800 CD2 PHE G 17 16.142 -26.246 43.280 0.00 0.00
G
ATOM 20801 HD2 PHE G 17 15.098 -25.972 43.254 0.00 0.00
G
ATOM 20802 CE2 PHE G 17 16.477 -27.563 43.674 0.00 0.00
G
ATOM 20803 HE2 PHE G 17 15.657 -28.238 43.868 0.00 0.00
G
ATOM 20804 C PHE G 17 17.276 -23.746 40.606 0.00 0.00
G
ATOM 20805 O PHE G 17 17.974 -24.628 40.208 0.00 0.00
G
ATOM 20806 N GLY G 18 16.266 -23.234 39.845 0.00 0.00
G
ATOM 20807 HN GLY G 18 15.732 -22.523 40.295 0.00 0.00
G
ATOM 20808 CA GLY G 18 15.811 -23.791 38.600 0.00 0.00
G
ATOM 20809 HA1 GLY G 18 14.976 -23.181 38.289 0.00 0.00
G
ATOM 20810 HA2 GLY G 18 15.740 -24.866 38.533 0.00 0.00
G
ATOM 20811 C GLY G 18 16.860 -23.353 37.512 0.00 0.00
G
ATOM 20812 O GLY G 18 17.269 -24.126 36.630 0.00 0.00
G
ATOM 20813 N GLY G 19 17.308 -22.098 37.695 0.00 0.00
G
ATOM 20814 HN GLY G 19 16.892 -21.557 38.422 0.00 0.00
G
ATOM 20815 CA GLY G 19 18.291 -21.435 36.855 0.00 0.00
G
ATOM 20816 HA1 GLY G 19 18.374 -20.413 37.194 0.00 0.00
G

```
ATOM  20817  HA2 GLY G  19      17.924 -21.598  35.852  0.00  0.00
G
ATOM  20818  C   GLY G  19      19.657 -22.039  37.040  0.00  0.00
G
ATOM  20819  O   GLY G  19      20.394 -22.095  36.077  0.00  0.00
G
ATOM  20820  N   SER G  20      19.931 -22.517  38.281  0.00  0.00
G
ATOM  20821  HN  SER G  20      19.222 -22.422  38.975  0.00  0.00
G
ATOM  20822  CA  SER G  20      21.089 -23.296  38.620  0.00  0.00
G
ATOM  20823  HA  SER G  20      22.006 -22.730  38.545  0.00  0.00
G
ATOM  20824  CB  SER G  20      21.259 -23.667  40.156  0.00  0.00
G
ATOM  20825  HB1 SER G  20      21.017 -22.783  40.783  0.00  0.00
G
ATOM  20826  HB2 SER G  20      20.612 -24.516  40.465  0.00  0.00
G
ATOM  20827  OG  SER G  20      22.556 -24.079  40.525  0.00  0.00
G
ATOM  20828  HG1 SER G  20      22.700 -24.194  41.467  0.00  0.00
G
ATOM  20829  C   SER G  20      21.094 -24.525  37.780  0.00  0.00
G
ATOM  20830  O   SER G  20      22.138 -24.860  37.167  0.00  0.00
G
ATOM  20831  N   ILE G  21      19.996 -25.264  37.741  0.00  0.00
G
ATOM  20832  HN  ILE G  21      19.198 -25.180  38.332  0.00  0.00
G
ATOM  20833  CA  ILE G  21      19.829 -26.479  36.914  0.00  0.00
G
ATOM  20834  HA  ILE G  21      20.711 -27.099  36.978  0.00  0.00
G
ATOM  20835  CB  ILE G  21      18.631 -27.232  37.413  0.00  0.00
G
ATOM  20836  HB  ILE G  21      17.751 -26.569  37.559  0.00  0.00
G
ATOM  20837  CG2 ILE G  21      18.231 -28.322  36.438  0.00  0.00
G
ATOM  20838 HG21 ILE G  21      17.210 -28.655  36.722  0.00  0.00
G
ATOM  20839 HG22 ILE G  21      18.222 -28.064  35.357  0.00  0.00
G
ATOM  20840 HG23 ILE G  21      18.781 -29.281  36.544  0.00  0.00
G
ATOM  20841  CG1 ILE G  21      18.942 -27.766  38.798  0.00  0.00
G
ATOM  20842 HG11 ILE G  21      19.915 -28.301  38.757  0.00  0.00
G
ATOM  20843 HG12 ILE G  21      19.135 -26.875  39.432  0.00  0.00
G
```

ATOM 20844 CD ILE G 21   17.818 -28.598 39.454 0.00 0.00
G
ATOM 20845 HD1 ILE G 21   16.925 -27.988 39.707 0.00 0.00
G
ATOM 20846 HD2 ILE G 21   17.507 -29.513 38.905 0.00 0.00
G
ATOM 20847 HD3 ILE G 21   18.190 -28.918 40.451 0.00 0.00
G
ATOM 20848 C   ILE G 21   19.977 -26.295 35.475 0.00 0.00
G
ATOM 20849 O   ILE G 21   20.835 -26.960 34.889 0.00 0.00
G
ATOM 20850 N   VAL G 22   19.261 -25.296 34.901 0.00 0.00
G
ATOM 20851 HN VAL G 22   18.608 -24.810 35.476 0.00 0.00
G
ATOM 20852 CA VAL G 22   19.452 -24.980 33.519 0.00 0.00
G
ATOM 20853 HA VAL G 22   19.162 -25.825 32.913 0.00 0.00
G
ATOM 20854 CB VAL G 22   18.611 -23.736 33.148 0.00 0.00
G
ATOM 20855 HB VAL G 22   18.848 -22.953 33.899 0.00 0.00
G
ATOM 20856 CG1 VAL G 22   19.061 -23.215 31.765 0.00 0.00
G
ATOM 20857 HG11 VAL G 22   18.983 -24.005 30.988 0.00 0.00
G
ATOM 20858 HG12 VAL G 22   18.394 -22.408 31.392 0.00 0.00
G
ATOM 20859 HG13 VAL G 22   20.039 -22.691 31.803 0.00 0.00
G
ATOM 20860 CG2 VAL G 22   17.101 -24.015 33.176 0.00 0.00
G
ATOM 20861 HG21 VAL G 22   16.688 -24.178 34.194 0.00 0.00
G
ATOM 20862 HG22 VAL G 22   16.489 -23.248 32.654 0.00 0.00
G
ATOM 20863 HG23 VAL G 22   16.854 -24.867 32.507 0.00 0.00
G
ATOM 20864 C   VAL G 22   20.840 -24.731 33.105 0.00 0.00
G
ATOM 20865 O   VAL G 22   21.356 -25.336 32.153 0.00 0.00
G
ATOM 20866 N   LYS G 23   21.511 -23.888 33.968 0.00 0.00
G
ATOM 20867 HN LYS G 23   21.076 -23.618 34.823 0.00 0.00
G
ATOM 20868 CA LYS G 23   22.777 -23.434 33.821 0.00 0.00
G
ATOM 20869 HA LYS G 23   22.897 -23.123 32.794 0.00 0.00
G
ATOM 20870 CB LYS G 23   23.130 -22.159 34.537 0.00 0.00
G

ATOM 20871 HB1 LYS G 23 22.255 -21.487 34.404 0.00 0.00
G
ATOM 20872 HB2 LYS G 23 23.107 -22.134 35.648 0.00 0.00
G
ATOM 20873 CG LYS G 23 24.416 -21.401 34.122 0.00 0.00
G
ATOM 20874 HG1 LYS G 23 24.415 -20.549 34.835 0.00 0.00
G
ATOM 20875 HG2 LYS G 23 25.321 -22.018 34.308 0.00 0.00
G
ATOM 20876 CD LYS G 23 24.621 -20.871 32.680 0.00 0.00
G
ATOM 20877 HD1 LYS G 23 24.663 -21.747 31.998 0.00 0.00
G
ATOM 20878 HD2 LYS G 23 23.750 -20.330 32.253 0.00 0.00
G
ATOM 20879 CE LYS G 23 25.880 -19.987 32.489 0.00 0.00
G
ATOM 20880 HE1 LYS G 23 25.898 -19.470 31.506 0.00 0.00
G
ATOM 20881 HE2 LYS G 23 26.034 -19.173 33.229 0.00 0.00
G
ATOM 20882 NZ LYS G 23 27.136 -20.822 32.512 0.00 0.00
G
ATOM 20883 HZ1 LYS G 23 27.484 -20.897 33.490 0.00 0.00
G
ATOM 20884 HZ2 LYS G 23 27.068 -21.840 32.311 0.00 0.00
G
ATOM 20885 HZ3 LYS G 23 27.908 -20.493 31.898 0.00 0.00
G
ATOM 20886 C LYS G 23 23.835 -24.573 34.032 0.00 0.00
G
ATOM 20887 O LYS G 23 24.655 -24.815 33.145 0.00 0.00
G
ATOM 20888 N GLU G 24 23.810 -25.372 35.106 0.00 0.00
G
ATOM 20889 HN GLU G 24 23.110 -25.147 35.779 0.00 0.00
G
ATOM 20890 CA GLU G 24 24.512 -26.625 35.402 0.00 0.00
G
ATOM 20891 HA GLU G 24 25.557 -26.373 35.300 0.00 0.00
G
ATOM 20892 CB GLU G 24 24.278 -27.105 36.867 0.00 0.00
G
ATOM 20893 HB1 GLU G 24 24.254 -26.131 37.400 0.00 0.00
G
ATOM 20894 HB2 GLU G 24 23.379 -27.709 37.115 0.00 0.00
G
ATOM 20895 CG GLU G 24 25.501 -27.825 37.537 0.00 0.00
G
ATOM 20896 HG1 GLU G 24 25.180 -28.195 38.535 0.00 0.00
G
ATOM 20897 HG2 GLU G 24 25.900 -28.672 36.940 0.00 0.00
G

ATOM 20898 CD GLU G 24 26.639 -26.862 37.846 0.00 0.00
G
ATOM 20899 OE1 GLU G 24 27.761 -27.150 37.343 0.00 0.00
G
ATOM 20900 OE2 GLU G 24 26.419 -25.887 38.576 0.00 0.00
G
ATOM 20901 C GLU G 24 24.410 -27.787 34.356 0.00 0.00
G
ATOM 20902 O GLU G 24 25.416 -28.339 33.910 0.00 0.00
G
ATOM 20903 N LEU G 25 23.156 -28.033 33.790 0.00 0.00
G
ATOM 20904 HN LEU G 25 22.286 -27.723 34.165 0.00 0.00
G
ATOM 20905 CA LEU G 25 23.095 -29.139 32.894 0.00 0.00
G
ATOM 20906 HA LEU G 25 23.830 -29.888 33.145 0.00 0.00
G
ATOM 20907 CB LEU G 25 21.660 -29.738 32.736 0.00 0.00
G
ATOM 20908 HB1 LEU G 25 21.059 -28.832 32.503 0.00 0.00
G
ATOM 20909 HB2 LEU G 25 21.637 -30.496 31.924 0.00 0.00
G
ATOM 20910 CG LEU G 25 21.095 -30.558 33.908 0.00 0.00
G
ATOM 20911 HG LEU G 25 21.345 -30.013 34.843 0.00 0.00
G
ATOM 20912 CD1 LEU G 25 19.567 -30.548 33.751 0.00 0.00
G
ATOM 20913 HD11 LEU G 25 19.075 -31.254 34.454 0.00 0.00
G
ATOM 20914 HD12 LEU G 25 19.166 -29.520 33.880 0.00 0.00
G
ATOM 20915 HD13 LEU G 25 19.262 -30.689 32.693 0.00 0.00
G
ATOM 20916 CD2 LEU G 25 21.738 -31.971 33.990 0.00 0.00
G
ATOM 20917 HD21 LEU G 25 21.438 -32.532 33.079 0.00 0.00
G
ATOM 20918 HD22 LEU G 25 22.825 -31.895 34.204 0.00 0.00
G
ATOM 20919 HD23 LEU G 25 21.266 -32.471 34.863 0.00 0.00
G
ATOM 20920 C LEU G 25 23.538 -28.770 31.476 0.00 0.00
G
ATOM 20921 O LEU G 25 23.974 -29.666 30.752 0.00 0.00
G
ATOM 20922 N HSE G 26 23.441 -27.469 31.081 0.00 0.00
G
ATOM 20923 HN HSE G 26 22.997 -26.738 31.593 0.00 0.00
G
ATOM 20924 CA HSE G 26 24.085 -26.942 29.817 0.00 0.00
G

ATOM 20925 HA HSE G 26 23.711 -27.563 29.017 0.00 0.00
G
ATOM 20926 CB HSE G 26 23.643 -25.515 29.577 0.00 0.00
G
ATOM 20927 HB1 HSE G 26 22.614 -25.453 29.165 0.00 0.00
G
ATOM 20928 HB2 HSE G 26 23.639 -24.971 30.547 0.00 0.00
G
ATOM 20929 ND1 HSE G 26 25.577 -23.896 28.978 0.00 0.00
G
ATOM 20930 CG HSE G 26 24.449 -24.681 28.598 0.00 0.00
G
ATOM 20931 CE1 HSE G 26 25.742 -23.011 28.026 0.00 0.00
G
ATOM 20932 HE1 HSE G 26 26.543 -22.273 28.003 0.00 0.00
G
ATOM 20933 NE2 HSE G 26 24.801 -23.085 27.128 0.00 0.00
G
ATOM 20934 HE2 HSE G 26 24.605 -22.418 26.409 0.00 0.00
G
ATOM 20935 CD2 HSE G 26 23.931 -24.101 27.463 0.00 0.00
G
ATOM 20936 HD2 HSE G 26 23.074 -24.429 26.888 0.00 0.00
G
ATOM 20937 C HSE G 26 25.594 -27.086 29.907 0.00 0.00
G
ATOM 20938 O HSE G 26 26.244 -27.370 28.898 0.00 0.00
G
ATOM 20939 N ARG G 27 26.121 -26.868 31.073 0.00 0.00
G
ATOM 20940 HN ARG G 27 25.589 -26.603 31.873 0.00 0.00
G
ATOM 20941 CA ARG G 27 27.573 -26.946 31.317 0.00 0.00
G
ATOM 20942 HA ARG G 27 28.064 -26.335 30.574 0.00 0.00
G
ATOM 20943 CB ARG G 27 27.954 -26.398 32.672 0.00 0.00
G
ATOM 20944 HB1 ARG G 27 27.334 -25.503 32.892 0.00 0.00
G
ATOM 20945 HB2 ARG G 27 27.564 -27.139 33.401 0.00 0.00
G
ATOM 20946 CG ARG G 27 29.407 -25.933 32.879 0.00 0.00
G
ATOM 20947 HG1 ARG G 27 30.189 -26.710 32.739 0.00 0.00
G
ATOM 20948 HG2 ARG G 27 29.716 -25.193 32.111 0.00 0.00
G
ATOM 20949 CD ARG G 27 29.735 -25.395 34.325 0.00 0.00
G
ATOM 20950 HD1 ARG G 27 30.755 -24.953 34.312 0.00 0.00
G
ATOM 20951 HD2 ARG G 27 29.051 -24.573 34.625 0.00 0.00
G

ATOM 20952 NE ARG G 27 29.504 −26.468 35.320 0.00 0.00
G
ATOM 20953 HE ARG G 27 28.658 −26.578 35.842 0.00 0.00
G
ATOM 20954 CZ ARG G 27 30.293 −27.527 35.521 0.00 0.00
G
ATOM 20955 NH1 ARG G 27 31.329 −27.911 34.813 0.00 0.00
G
ATOM 20956 HH11 ARG G 27 31.519 −28.892 34.787 0.00 0.00
G
ATOM 20957 HH12 ARG G 27 31.503 −27.377 33.985 0.00 0.00
G
ATOM 20958 NH2 ARG G 27 29.888 −28.306 36.538 0.00 0.00
G
ATOM 20959 HH21 ARG G 27 30.446 −29.005 36.984 0.00 0.00
G
ATOM 20960 HH22 ARG G 27 29.126 −27.922 37.060 0.00 0.00
G
ATOM 20961 C ARG G 27 28.069 −28.396 31.162 0.00 0.00
G
ATOM 20962 O ARG G 27 29.125 −28.673 30.580 0.00 0.00
G
ATOM 20963 N MET G 28 27.291 −29.349 31.708 0.00 0.00
G
ATOM 20964 HN MET G 28 26.501 −29.111 32.267 0.00 0.00
G
ATOM 20965 CA MET G 28 27.621 −30.779 31.700 0.00 0.00
G
ATOM 20966 HA MET G 28 28.669 −30.799 31.963 0.00 0.00
G
ATOM 20967 CB MET G 28 26.834 −31.468 32.785 0.00 0.00
G
ATOM 20968 HB1 MET G 28 25.780 −31.151 32.635 0.00 0.00
G
ATOM 20969 HB2 MET G 28 26.789 −32.552 32.542 0.00 0.00
G
ATOM 20970 CG MET G 28 27.311 −31.307 34.248 0.00 0.00
G
ATOM 20971 HG1 MET G 28 28.366 −31.618 34.406 0.00 0.00
G
ATOM 20972 HG2 MET G 28 27.297 −30.225 34.500 0.00 0.00
G
ATOM 20973 SD MET G 28 26.388 −32.111 35.590 0.00 0.00
G
ATOM 20974 CE MET G 28 27.655 −31.459 36.733 0.00 0.00
G
ATOM 20975 HE1 MET G 28 27.456 −31.568 37.821 0.00 0.00
G
ATOM 20976 HE2 MET G 28 28.667 −31.887 36.570 0.00 0.00
G
ATOM 20977 HE3 MET G 28 27.615 −30.350 36.686 0.00 0.00
G
ATOM 20978 C MET G 28 27.357 −31.502 30.375 0.00 0.00
G

ATOM 20979 O MET G 28 27.748 −32.591 30.178 0.00 0.00
G
ATOM 20980 N GLY G 29 26.769 −30.819 29.337 0.00 0.00
G
ATOM 20981 HN GLY G 29 26.325 −29.972 29.618 0.00 0.00
G
ATOM 20982 CA GLY G 29 26.742 −31.375 27.937 0.00 0.00
G
ATOM 20983 HA1 GLY G 29 27.578 −32.015 27.696 0.00 0.00
G
ATOM 20984 HA2 GLY G 29 26.765 −30.496 27.310 0.00 0.00
G
ATOM 20985 C GLY G 29 25.477 −32.215 27.645 0.00 0.00
G
ATOM 20986 O GLY G 29 25.488 −33.066 26.786 0.00 0.00
G
ATOM 20987 N HSE G 30 24.414 −31.899 28.317 0.00 0.00
G
ATOM 20988 HN HSE G 30 24.483 −31.302 29.112 0.00 0.00
G
ATOM 20989 CA HSE G 30 23.051 −32.320 28.118 0.00 0.00
G
ATOM 20990 HA HSE G 30 23.048 −33.254 27.576 0.00 0.00
G
ATOM 20991 CB HSE G 30 22.464 −32.579 29.506 0.00 0.00
G
ATOM 20992 HB1 HSE G 30 22.339 −31.750 30.235 0.00 0.00
G
ATOM 20993 HB2 HSE G 30 21.419 −32.896 29.303 0.00 0.00
G
ATOM 20994 ND1 HSE G 30 22.852 −35.021 29.820 0.00 0.00
G
ATOM 20995 CG HSE G 30 23.122 −33.738 30.266 0.00 0.00
G
ATOM 20996 CE1 HSE G 30 23.418 −35.818 30.693 0.00 0.00
G
ATOM 20997 HE1 HSE G 30 23.185 −36.881 30.744 0.00 0.00
G
ATOM 20998 NE2 HSE G 30 23.874 −35.129 31.766 0.00 0.00
G
ATOM 20999 HE2 HSE G 30 24.422 −35.463 32.532 0.00 0.00
G
ATOM 21000 CD2 HSE G 30 23.712 −33.805 31.505 0.00 0.00
G
ATOM 21001 HD2 HSE G 30 23.896 −32.960 32.156 0.00 0.00
G
ATOM 21002 C HSE G 30 22.281 −31.222 27.346 0.00 0.00
G
ATOM 21003 O HSE G 30 22.828 −30.217 26.920 0.00 0.00
G
ATOM 21004 N GLU G 31 20.987 −31.464 27.112 0.00 0.00
G
ATOM 21005 HN GLU G 31 20.738 −32.428 27.165 0.00 0.00
G

ATOM 21006 CA GLU G 31 19.999 -30.593 26.559 0.00 0.00
G
ATOM 21007 HA GLU G 31 20.438 -29.612 26.456 0.00 0.00
G
ATOM 21008 CB GLU G 31 19.505 -30.938 25.111 0.00 0.00
G
ATOM 21009 HB1 GLU G 31 18.720 -31.721 25.030 0.00 0.00
G
ATOM 21010 HB2 GLU G 31 18.991 -30.025 24.740 0.00 0.00
G
ATOM 21011 CG GLU G 31 20.585 -31.109 24.025 0.00 0.00
G
ATOM 21012 HG1 GLU G 31 21.325 -30.281 23.988 0.00 0.00
G
ATOM 21013 HG2 GLU G 31 21.062 -32.112 24.057 0.00 0.00
G
ATOM 21014 CD GLU G 31 19.984 -31.142 22.629 0.00 0.00
G
ATOM 21015 OE1 GLU G 31 20.351 -32.049 21.799 0.00 0.00
G
ATOM 21016 OE2 GLU G 31 19.345 -30.088 22.269 0.00 0.00
G
ATOM 21017 C GLU G 31 18.747 -30.368 27.443 0.00 0.00
G
ATOM 21018 O GLU G 31 18.180 -31.349 27.886 0.00 0.00
G
ATOM 21019 N VAL G 32 18.351 -29.155 27.630 0.00 0.00
G
ATOM 21020 HN VAL G 32 18.863 -28.416 27.200 0.00 0.00
G
ATOM 21021 CA VAL G 32 17.356 -28.769 28.692 0.00 0.00
G
ATOM 21022 HA VAL G 32 16.978 -29.649 29.191 0.00 0.00
G
ATOM 21023 CB VAL G 32 18.085 -27.870 29.678 0.00 0.00
G
ATOM 21024 HB VAL G 32 18.470 -26.927 29.235 0.00 0.00
G
ATOM 21025 CG1 VAL G 32 17.087 -27.550 30.870 0.00 0.00
G
ATOM 21026 HG11 VAL G 32 17.656 -27.387 31.810 0.00 0.00
G
ATOM 21027 HG12 VAL G 32 16.286 -26.802 30.691 0.00 0.00
G
ATOM 21028 HG13 VAL G 32 16.485 -28.471 31.026 0.00 0.00
G
ATOM 21029 CG2 VAL G 32 19.425 -28.499 30.117 0.00 0.00
G
ATOM 21030 HG21 VAL G 32 19.936 -27.870 30.877 0.00 0.00
G
ATOM 21031 HG22 VAL G 32 19.366 -29.589 30.324 0.00 0.00
G
ATOM 21032 HG23 VAL G 32 20.244 -28.528 29.367 0.00 0.00
G

```
ATOM  21033  C   VAL G  32      16.176 -28.038  28.138  0.00  0.00
G
ATOM  21034  O   VAL G  32      16.292 -26.972  27.508  0.00  0.00
G
ATOM  21035  N   LEU G  33      15.002 -28.612  28.309  0.00  0.00
G
ATOM  21036  HN  LEU G  33      15.034 -29.561  28.612  0.00  0.00
G
ATOM  21037  CA  LEU G  33      13.658 -27.972  27.936  0.00  0.00
G
ATOM  21038  HA  LEU G  33      13.792 -27.361  27.055  0.00  0.00
G
ATOM  21039  CB  LEU G  33      12.499 -28.981  27.637  0.00  0.00
G
ATOM  21040  HB1 LEU G  33      12.795 -29.516  26.709  0.00  0.00
G
ATOM  21041  HB2 LEU G  33      12.416 -29.564  28.579  0.00  0.00
G
ATOM  21042  CG  LEU G  33      11.044 -28.275  27.476  0.00  0.00
G
ATOM  21043  HG  LEU G  33      10.731 -27.775  28.418  0.00  0.00
G
ATOM  21044  CD1 LEU G  33      10.893 -27.298  26.288  0.00  0.00
G
ATOM  21045 HD11 LEU G  33       9.824 -26.996  26.310  0.00  0.00
G
ATOM  21046 HD12 LEU G  33      11.589 -26.441  26.418  0.00  0.00
G
ATOM  21047 HD13 LEU G  33      11.108 -27.861  25.355  0.00  0.00
G
ATOM  21048  CD2 LEU G  33      10.163 -29.481  27.197  0.00  0.00
G
ATOM  21049 HD21 LEU G  33       9.880 -29.998  28.139  0.00  0.00
G
ATOM  21050 HD22 LEU G  33       9.197 -29.120  26.785  0.00  0.00
G
ATOM  21051 HD23 LEU G  33      10.513 -30.122  26.360  0.00  0.00
G
ATOM  21052  C   LEU G  33      13.332 -27.037  29.131  0.00  0.00
G
ATOM  21053  O   LEU G  33      13.200 -27.498  30.290  0.00  0.00
G
ATOM  21054  N   ALA G  34      13.160 -25.709  28.861  0.00  0.00
G
ATOM  21055  HN  ALA G  34      13.451 -25.286  28.006  0.00  0.00
G
ATOM  21056  CA  ALA G  34      12.855 -24.759  29.929  0.00  0.00
G
ATOM  21057  HA  ALA G  34      12.776 -25.186  30.918  0.00  0.00
G
ATOM  21058  CB  ALA G  34      13.978 -23.697  29.799  0.00  0.00
G
ATOM  21059  HB1 ALA G  34      14.021 -22.997  30.660  0.00  0.00
G
```

ATOM 21060 HB2 ALA G 34 14.947 -24.234 29.707 0.00 0.00
G
ATOM 21061 HB3 ALA G 34 13.836 -23.046 28.910 0.00 0.00
G
ATOM 21062 C ALA G 34 11.481 -24.197 29.742 0.00 0.00
G
ATOM 21063 O ALA G 34 11.029 -23.699 28.654 0.00 0.00
G
ATOM 21064 N VAL G 35 10.740 -24.128 30.858 0.00 0.00
G
ATOM 21065 HN VAL G 35 10.973 -24.534 31.738 0.00 0.00
G
ATOM 21066 CA VAL G 35 9.385 -23.629 30.922 0.00 0.00
G
ATOM 21067 HA VAL G 35 9.233 -22.876 30.163 0.00 0.00
G
ATOM 21068 CB VAL G 35 8.283 -24.706 30.951 0.00 0.00
G
ATOM 21069 HB VAL G 35 8.338 -25.137 31.974 0.00 0.00
G
ATOM 21070 CG1 VAL G 35 6.808 -24.143 30.694 0.00 0.00
G
ATOM 21071 HG11 VAL G 35 5.997 -24.901 30.712 0.00 0.00
G
ATOM 21072 HG12 VAL G 35 6.509 -23.332 31.393 0.00 0.00
G
ATOM 21073 HG13 VAL G 35 6.776 -23.712 29.671 0.00 0.00
G
ATOM 21074 CG2 VAL G 35 8.562 -25.941 29.961 0.00 0.00
G
ATOM 21075 HG21 VAL G 35 7.827 -26.750 30.160 0.00 0.00
G
ATOM 21076 HG22 VAL G 35 8.424 -25.631 28.903 0.00 0.00
G
ATOM 21077 HG23 VAL G 35 9.576 -26.391 30.027 0.00 0.00
G
ATOM 21078 C VAL G 35 9.300 -22.796 32.200 0.00 0.00
G
ATOM 21079 O VAL G 35 9.605 -23.282 33.280 0.00 0.00
G
ATOM 21080 N ASP G 36 8.828 -21.591 32.094 0.00 0.00
G
ATOM 21081 HN ASP G 36 8.559 -21.222 31.207 0.00 0.00
G
ATOM 21082 CA ASP G 36 8.414 -20.798 33.267 0.00 0.00
G
ATOM 21083 HA ASP G 36 7.970 -21.393 34.051 0.00 0.00
G
ATOM 21084 CB ASP G 36 9.525 -19.864 33.898 0.00 0.00
G
ATOM 21085 HB1 ASP G 36 10.471 -20.434 34.021 0.00 0.00
G
ATOM 21086 HB2 ASP G 36 9.839 -19.092 33.163 0.00 0.00
G

ATOM 21087 CG ASP G 36   9.130 -19.250 35.232 0.00 0.00
G
ATOM 21088 OD1 ASP G 36   9.798 -19.561 36.230 0.00 0.00
G
ATOM 21089 OD2 ASP G 36   8.136 -18.491 35.335 0.00 0.00
G
ATOM 21090 C   ASP G 36   7.254 -20.071 32.651 0.00 0.00
G
ATOM 21091 O   ASP G 36   7.075 -20.101 31.437 0.00 0.00
G
ATOM 21092 N   ILE G 37   6.385 -19.418 33.437 0.00 0.00
G
ATOM 21093 HN ILE G 37   6.651 -19.398 34.398 0.00 0.00
G
ATOM 21094 CA ILE G 37   5.316 -18.537 32.919 0.00 0.00
G
ATOM 21095 HA ILE G 37   4.992 -18.891 31.952 0.00 0.00
G
ATOM 21096 CB ILE G 37   4.095 -18.626 33.825 0.00 0.00
G
ATOM 21097 HB ILE G 37   3.893 -19.699 34.029 0.00 0.00
G
ATOM 21098 CG2 ILE G 37   4.406 -18.046 35.201 0.00 0.00
G
ATOM 21099 HG21 ILE G 37   5.313 -18.534 35.617 0.00 0.00
G
ATOM 21100 HG22 ILE G 37   4.573 -16.953 35.092 0.00 0.00
G
ATOM 21101 HG23 ILE G 37   3.541 -18.315 35.845 0.00 0.00
G
ATOM 21102 CG1 ILE G 37   2.752 -18.108 33.178 0.00 0.00
G
ATOM 21103 HG11 ILE G 37   2.864 -17.066 32.808 0.00 0.00
G
ATOM 21104 HG12 ILE G 37   2.526 -18.732 32.287 0.00 0.00
G
ATOM 21105 CD ILE G 37   1.460 -18.217 33.989 0.00 0.00
G
ATOM 21106 HD1 ILE G 37   0.497 -17.906 33.531 0.00 0.00
G
ATOM 21107 HD2 ILE G 37   1.419 -19.296 34.251 0.00 0.00
G
ATOM 21108 HD3 ILE G 37   1.612 -17.519 34.840 0.00 0.00
G
ATOM 21109 C   ILE G 37   5.808 -17.120 32.662 0.00 0.00
G
ATOM 21110 O   ILE G 37   5.287 -16.324 31.792 0.00 0.00
G
ATOM 21111 N   ASN G 38   6.931 -16.699 33.258 0.00 0.00
G
ATOM 21112 HN ASN G 38   7.425 -17.286 33.894 0.00 0.00
G
ATOM 21113 CA ASN G 38   7.388 -15.364 33.302 0.00 0.00
G

```
ATOM  21114  HA  ASN G  38       6.568 -14.691  33.097  0.00  0.00
G
ATOM  21115  CB  ASN G  38       8.053 -14.953  34.629  0.00  0.00
G
ATOM  21116  HB1 ASN G  38       8.717 -15.761  35.004  0.00  0.00
G
ATOM  21117  HB2 ASN G  38       8.537 -13.965  34.477  0.00  0.00
G
ATOM  21118  CG  ASN G  38       6.929 -14.690  35.597  0.00  0.00
G
ATOM  21119  OD1 ASN G  38       6.224 -13.698  35.427  0.00  0.00
G
ATOM  21120  ND2 ASN G  38       6.793 -15.607  36.654  0.00  0.00
G
ATOM  21121 HD21 ASN G  38       6.180 -15.361  37.405  0.00  0.00
G
ATOM  21122 HD22 ASN G  38       7.109 -16.552  36.574  0.00  0.00
G
ATOM  21123  C   ASN G  38       8.360 -15.123  32.161  0.00  0.00
G
ATOM  21124  O   ASN G  38       9.512 -15.585  32.236  0.00  0.00
G
ATOM  21125  N   GLU G  39       7.978 -14.260  31.131  0.00  0.00
G
ATOM  21126  HN  GLU G  39       7.041 -13.933  31.036  0.00  0.00
G
ATOM  21127  CA  GLU G  39       8.827 -13.879  30.027  0.00  0.00
G
ATOM  21128  HA  GLU G  39       8.953 -14.751  29.403  0.00  0.00
G
ATOM  21129  CB  GLU G  39       8.022 -12.775  29.222  0.00  0.00
G
ATOM  21130  HB1 GLU G  39       7.232 -13.320  28.663  0.00  0.00
G
ATOM  21131  HB2 GLU G  39       7.552 -12.239  30.075  0.00  0.00
G
ATOM  21132  CG  GLU G  39       8.724 -11.826  28.149  0.00  0.00
G
ATOM  21133  HG1 GLU G  39       9.557 -11.285  28.646  0.00  0.00
G
ATOM  21134  HG2 GLU G  39       9.078 -12.485  27.328  0.00  0.00
G
ATOM  21135  CD  GLU G  39       7.822 -10.745  27.623  0.00  0.00
G
ATOM  21136  OE1 GLU G  39       8.173 -10.089  26.584  0.00  0.00
G
ATOM  21137  OE2 GLU G  39       6.703 -10.407  28.152  0.00  0.00
G
ATOM  21138  C   GLU G  39      10.107 -13.425  30.434  0.00  0.00
G
ATOM  21139  O   GLU G  39      11.118 -13.852  29.909  0.00  0.00
G
ATOM  21140  N   GLU G  40      10.109 -12.490  31.408  0.00  0.00
G
```

```
ATOM  21141  HN  GLU G  40       9.211 -12.205  31.735  0.00  0.00
G
ATOM  21142  CA  GLU G  40      11.365 -11.870  31.821  0.00  0.00
G
ATOM  21143  HA  GLU G  40      11.828 -11.415  30.958  0.00  0.00
G
ATOM  21144  CB  GLU G  40      11.188 -10.732  32.806  0.00  0.00
G
ATOM  21145  HB1 GLU G  40      10.467 -11.126  33.554  0.00  0.00
G
ATOM  21146  HB2 GLU G  40      12.208 -10.544  33.206  0.00  0.00
G
ATOM  21147  CG  GLU G  40      10.628  -9.456  32.158  0.00  0.00
G
ATOM  21148  HG1 GLU G  40      11.220  -9.156  31.267  0.00  0.00
G
ATOM  21149  HG2 GLU G  40       9.583  -9.670  31.847  0.00  0.00
G
ATOM  21150  CD  GLU G  40      10.660  -8.202  32.974  0.00  0.00
G
ATOM  21151  OE1 GLU G  40      11.735  -7.565  33.048  0.00  0.00
G
ATOM  21152  OE2 GLU G  40       9.635  -7.881  33.565  0.00  0.00
G
ATOM  21153  C   GLU G  40      12.336 -12.936  32.388  0.00  0.00
G
ATOM  21154  O   GLU G  40      13.582 -12.848  32.187  0.00  0.00
G
ATOM  21155  N   LYS G  41      11.852 -13.926  33.163  0.00  0.00
G
ATOM  21156  HN  LYS G  41      10.872 -13.980  33.335  0.00  0.00
G
ATOM  21157  CA  LYS G  41      12.644 -15.040  33.667  0.00  0.00
G
ATOM  21158  HA  LYS G  41      13.571 -14.655  34.065  0.00  0.00
G
ATOM  21159  CB  LYS G  41      12.018 -15.882  34.837  0.00  0.00
G
ATOM  21160  HB1 LYS G  41      11.032 -16.316  34.565  0.00  0.00
G
ATOM  21161  HB2 LYS G  41      12.576 -16.812  35.077  0.00  0.00
G
ATOM  21162  CG  LYS G  41      11.779 -15.126  36.185  0.00  0.00
G
ATOM  21163  HG1 LYS G  41      12.787 -14.732  36.432  0.00  0.00
G
ATOM  21164  HG2 LYS G  41      11.154 -14.246  35.924  0.00  0.00
G
ATOM  21165  CD  LYS G  41      11.373 -16.031  37.371  0.00  0.00
G
ATOM  21166  HD1 LYS G  41      10.309 -16.310  37.220  0.00  0.00
G
ATOM  21167  HD2 LYS G  41      11.998 -16.943  37.256  0.00  0.00
G
```

```
ATOM  21168  CE  LYS G  41      11.489 -15.343  38.720  0.00  0.00
G
ATOM  21169  HE1 LYS G  41      12.511 -14.957  38.923  0.00  0.00
G
ATOM  21170  HE2 LYS G  41      10.739 -14.523  38.752  0.00  0.00
G
ATOM  21171  NZ  LYS G  41      11.207 -16.311  39.800  0.00  0.00
G
ATOM  21172  HZ1 LYS G  41      10.820 -17.210  39.448  0.00  0.00
G
ATOM  21173  HZ2 LYS G  41      12.076 -16.593  40.296  0.00  0.00
G
ATOM  21174  HZ3 LYS G  41      10.473 -15.928  40.430  0.00  0.00
G
ATOM  21175  C   LYS G  41      13.045 -15.976  32.581  0.00  0.00
G
ATOM  21176  O   LYS G  41      14.232 -16.255  32.557  0.00  0.00
G
ATOM  21177  N   VAL G  42      12.169 -16.398  31.581  0.00  0.00
G
ATOM  21178  HN  VAL G  42      11.212 -16.123  31.636  0.00  0.00
G
ATOM  21179  CA  VAL G  42      12.573 -17.165  30.405  0.00  0.00
G
ATOM  21180  HA  VAL G  42      12.970 -18.104  30.762  0.00  0.00
G
ATOM  21181  CB  VAL G  42      11.306 -17.438  29.539  0.00  0.00
G
ATOM  21182  HB  VAL G  42      10.784 -16.467  29.403  0.00  0.00
G
ATOM  21183  CG1 VAL G  42      11.635 -18.033  28.155  0.00  0.00
G
ATOM  21184 HG11 VAL G  42      12.539 -18.666  28.287  0.00  0.00
G
ATOM  21185 HG12 VAL G  42      10.849 -18.734  27.801  0.00  0.00
G
ATOM  21186 HG13 VAL G  42      11.736 -17.312  27.316  0.00  0.00
G
ATOM  21187  CG2 VAL G  42      10.419 -18.452  30.335  0.00  0.00
G
ATOM  21188 HG21 VAL G  42       9.435 -18.545  29.828  0.00  0.00
G
ATOM  21189 HG22 VAL G  42      10.964 -19.418  30.271  0.00  0.00
G
ATOM  21190 HG23 VAL G  42      10.198 -18.134  31.376  0.00  0.00
G
ATOM  21191  C   VAL G  42      13.687 -16.433  29.629  0.00  0.00
G
ATOM  21192  O   VAL G  42      14.655 -17.104  29.291  0.00  0.00
G
ATOM  21193  N   ASN G  43      13.608 -15.087  29.368  0.00  0.00
G
ATOM  21194  HN  ASN G  43      12.782 -14.543  29.497  0.00  0.00
G
```

ATOM 21195 CA ASN G 43 14.663 −14.365 28.655 0.00 0.00
G
ATOM 21196 HA ASN G 43 14.857 −14.830 27.700 0.00 0.00
G
ATOM 21197 CB ASN G 43 14.203 −12.933 28.396 0.00 0.00
G
ATOM 21198 HB1 ASN G 43 13.878 −12.442 29.338 0.00 0.00
G
ATOM 21199 HB2 ASN G 43 15.060 −12.327 28.034 0.00 0.00
G
ATOM 21200 CG ASN G 43 13.113 −12.886 27.295 0.00 0.00
G
ATOM 21201 OD1 ASN G 43 13.163 −13.605 26.314 0.00 0.00
G
ATOM 21202 ND2 ASN G 43 12.391 −11.736 27.306 0.00 0.00
G
ATOM 21203 HD21 ASN G 43 11.520 −11.683 26.819 0.00 0.00
G
ATOM 21204 HD22 ASN G 43 12.686 −10.922 27.807 0.00 0.00
G
ATOM 21205 C ASN G 43 16.094 −14.361 29.259 0.00 0.00
G
ATOM 21206 O ASN G 43 17.085 −14.369 28.550 0.00 0.00
G
ATOM 21207 N ALA G 44 16.124 −14.514 30.547 0.00 0.00
G
ATOM 21208 HN ALA G 44 15.235 −14.498 31.000 0.00 0.00
G
ATOM 21209 CA ALA G 44 17.332 −14.602 31.235 0.00 0.00
G
ATOM 21210 HA ALA G 44 17.940 −13.733 31.035 0.00 0.00
G
ATOM 21211 CB ALA G 44 17.142 −14.562 32.772 0.00 0.00
G
ATOM 21212 HB1 ALA G 44 18.163 −14.575 33.209 0.00 0.00
G
ATOM 21213 HB2 ALA G 44 16.525 −13.719 33.150 0.00 0.00
G
ATOM 21214 HB3 ALA G 44 16.710 −15.490 33.204 0.00 0.00
G
ATOM 21215 C ALA G 44 18.123 −15.933 30.924 0.00 0.00
G
ATOM 21216 O ALA G 44 19.307 −15.898 30.734 0.00 0.00
G
ATOM 21217 N TYR G 45 17.437 −17.071 30.847 0.00 0.00
G
ATOM 21218 HN TYR G 45 16.440 −17.075 30.825 0.00 0.00
G
ATOM 21219 CA TYR G 45 17.894 −18.434 30.607 0.00 0.00
G
ATOM 21220 HA TYR G 45 18.965 −18.293 30.624 0.00 0.00
G
ATOM 21221 CB TYR G 45 17.406 −19.386 31.756 0.00 0.00
G

ATOM 21222 HB1 TYR G 45 16.313 -19.263 31.913 0.00 0.00
G
ATOM 21223 HB2 TYR G 45 17.603 -20.468 31.598 0.00 0.00
G
ATOM 21224 CG TYR G 45 18.079 -18.901 33.085 0.00 0.00
G
ATOM 21225 CD1 TYR G 45 17.325 -18.449 34.189 0.00 0.00
G
ATOM 21226 HD1 TYR G 45 16.264 -18.306 34.043 0.00 0.00
G
ATOM 21227 CE1 TYR G 45 17.925 -17.834 35.323 0.00 0.00
G
ATOM 21228 HE1 TYR G 45 17.328 -17.355 36.084 0.00 0.00
G
ATOM 21229 CZ TYR G 45 19.271 -17.934 35.463 0.00 0.00
G
ATOM 21230 OH TYR G 45 19.864 -17.444 36.643 0.00 0.00
G
ATOM 21231 HH TYR G 45 20.617 -18.029 36.754 0.00 0.00
G
ATOM 21232 CD2 TYR G 45 19.495 -18.958 33.324 0.00 0.00
G
ATOM 21233 HD2 TYR G 45 20.114 -19.545 32.662 0.00 0.00
G
ATOM 21234 CE2 TYR G 45 20.040 -18.409 34.457 0.00 0.00
G
ATOM 21235 HE2 TYR G 45 21.112 -18.346 34.569 0.00 0.00
G
ATOM 21236 C TYR G 45 17.586 -18.992 29.201 0.00 0.00
G
ATOM 21237 O TYR G 45 17.777 -20.178 28.875 0.00 0.00
G
ATOM 21238 N ALA G 46 17.208 -18.147 28.227 0.00 0.00
G
ATOM 21239 HN ALA G 46 17.160 -17.169 28.412 0.00 0.00
G
ATOM 21240 CA ALA G 46 16.831 -18.626 26.959 0.00 0.00
G
ATOM 21241 HA ALA G 46 16.139 -19.447 27.074 0.00 0.00
G
ATOM 21242 CB ALA G 46 15.933 -17.569 26.252 0.00 0.00
G
ATOM 21243 HB1 ALA G 46 14.991 -17.370 26.806 0.00 0.00
G
ATOM 21244 HB2 ALA G 46 16.566 -16.679 26.050 0.00 0.00
G
ATOM 21245 HB3 ALA G 46 15.572 -17.829 25.234 0.00 0.00
G
ATOM 21246 C ALA G 46 17.964 -19.071 26.059 0.00 0.00
G
ATOM 21247 O ALA G 46 17.750 -20.028 25.287 0.00 0.00
G
ATOM 21248 N SER G 47 19.221 -18.543 26.180 0.00 0.00
G

ATOM 21249 HN SER G 47 19.470 −17.729 26.698 0.00 0.00
G
ATOM 21250 CA SER G 47 20.358 −18.928 25.386 0.00 0.00
G
ATOM 21251 HA SER G 47 20.050 −19.278 24.412 0.00 0.00
G
ATOM 21252 CB SER G 47 21.494 −17.812 25.218 0.00 0.00
G
ATOM 21253 HB1 SER G 47 21.750 −17.337 26.189 0.00 0.00
G
ATOM 21254 HB2 SER G 47 22.403 −18.372 24.910 0.00 0.00
G
ATOM 21255 OG SER G 47 21.111 −16.965 24.163 0.00 0.00
G
ATOM 21256 HG1 SER G 47 21.720 −16.228 24.075 0.00 0.00
G
ATOM 21257 C SER G 47 21.067 −20.146 26.067 0.00 0.00
G
ATOM 21258 O SER G 47 21.886 −20.810 25.426 0.00 0.00
G
ATOM 21259 N TYR G 48 20.737 −20.545 27.312 0.00 0.00
G
ATOM 21260 HN TYR G 48 20.114 −19.939 27.799 0.00 0.00
G
ATOM 21261 CA TYR G 48 21.173 −21.693 28.151 0.00 0.00
G
ATOM 21262 HA TYR G 48 22.158 −21.967 27.803 0.00 0.00
G
ATOM 21263 CB TYR G 48 21.282 −21.414 29.600 0.00 0.00
G
ATOM 21264 HB1 TYR G 48 20.304 −21.059 29.989 0.00 0.00
G
ATOM 21265 HB2 TYR G 48 21.540 −22.285 30.240 0.00 0.00
G
ATOM 21266 CG TYR G 48 22.289 −20.334 29.836 0.00 0.00
G
ATOM 21267 CD1 TYR G 48 23.622 −20.457 29.387 0.00 0.00
G
ATOM 21268 HD1 TYR G 48 23.988 −21.415 29.047 0.00 0.00
G
ATOM 21269 CE1 TYR G 48 24.534 −19.401 29.459 0.00 0.00
G
ATOM 21270 HE1 TYR G 48 25.533 −19.598 29.098 0.00 0.00
G
ATOM 21271 CZ TYR G 48 24.154 −18.161 29.989 0.00 0.00
G
ATOM 21272 OH TYR G 48 25.081 −17.121 30.033 0.00 0.00
G
ATOM 21273 HH TYR G 48 24.709 −16.277 30.298 0.00 0.00
G
ATOM 21274 CD2 TYR G 48 21.925 −19.058 30.299 0.00 0.00
G
ATOM 21275 HD2 TYR G 48 20.915 −18.781 30.561 0.00 0.00
G

ATOM 21276 CE2 TYR G 48 22.831 −18.018 30.415 0.00 0.00
G
ATOM 21277 HE2 TYR G 48 22.475 −17.019 30.618 0.00 0.00
G
ATOM 21278 C TYR G 48 20.262 −22.968 27.850 0.00 0.00
G
ATOM 21279 O TYR G 48 20.518 −24.092 28.245 0.00 0.00
G
ATOM 21280 N ALA G 49 19.164 −22.771 27.078 0.00 0.00
G
ATOM 21281 HN ALA G 49 18.912 −21.943 26.583 0.00 0.00
G
ATOM 21282 CA ALA G 49 18.148 −23.780 26.931 0.00 0.00
G
ATOM 21283 HA ALA G 49 18.353 −24.665 27.515 0.00 0.00
G
ATOM 21284 CB ALA G 49 16.810 −23.101 27.345 0.00 0.00
G
ATOM 21285 HB1 ALA G 49 16.550 −22.345 26.574 0.00 0.00
G
ATOM 21286 HB2 ALA G 49 16.021 −23.882 27.317 0.00 0.00
G
ATOM 21287 HB3 ALA G 49 16.971 −22.638 28.342 0.00 0.00
G
ATOM 21288 C ALA G 49 18.018 −24.319 25.500 0.00 0.00
G
ATOM 21289 O ALA G 49 18.123 −23.644 24.485 0.00 0.00
G
ATOM 21290 N THR G 50 17.791 −25.652 25.251 0.00 0.00
G
ATOM 21291 HN THR G 50 17.651 −26.312 25.985 0.00 0.00
G
ATOM 21292 CA THR G 50 17.567 −26.172 23.902 0.00 0.00
G
ATOM 21293 HA THR G 50 18.350 −25.824 23.244 0.00 0.00
G
ATOM 21294 CB THR G 50 17.708 −27.655 23.720 0.00 0.00
G
ATOM 21295 HB THR G 50 18.603 −27.984 24.291 0.00 0.00
G
ATOM 21296 OG1 THR G 50 17.750 −28.151 22.396 0.00 0.00
G
ATOM 21297 HG1 THR G 50 18.148 −29.016 22.511 0.00 0.00
G
ATOM 21298 CG2 THR G 50 16.491 −28.385 24.337 0.00 0.00
G
ATOM 21299 HG21 THR G 50 16.608 −29.480 24.186 0.00 0.00
G
ATOM 21300 HG22 THR G 50 16.411 −28.195 25.428 0.00 0.00
G
ATOM 21301 HG23 THR G 50 15.551 −28.016 23.874 0.00 0.00
G
ATOM 21302 C THR G 50 16.332 −25.730 23.222 0.00 0.00
G

ATOM 21303 O THR G 50 16.294 -25.611 22.007 0.00 0.00
G
ATOM 21304 N HSE G 51 15.290 -25.448 24.009 0.00 0.00
G
ATOM 21305 HN HSE G 51 15.294 -25.482 25.005 0.00 0.00
G
ATOM 21306 CA HSE G 51 14.146 -24.631 23.563 0.00 0.00
G
ATOM 21307 HA HSE G 51 14.501 -23.816 22.952 0.00 0.00
G
ATOM 21308 CB HSE G 51 13.108 -25.433 22.790 0.00 0.00
G
ATOM 21309 HB1 HSE G 51 13.754 -25.824 21.975 0.00 0.00
G
ATOM 21310 HB2 HSE G 51 12.818 -26.252 23.481 0.00 0.00
G
ATOM 21311 ND1 HSE G 51 12.244 -23.416 21.688 0.00 0.00
G
ATOM 21312 CG HSE G 51 12.030 -24.708 22.177 0.00 0.00
G
ATOM 21313 CE1 HSE G 51 11.083 -23.014 21.144 0.00 0.00
G
ATOM 21314 HE1 HSE G 51 10.840 -22.011 20.796 0.00 0.00
G
ATOM 21315 NE2 HSE G 51 10.166 -23.982 21.348 0.00 0.00
G
ATOM 21316 HE2 HSE G 51 9.251 -24.092 20.960 0.00 0.00
G
ATOM 21317 CD2 HSE G 51 10.755 -25.050 22.023 0.00 0.00
G
ATOM 21318 HD2 HSE G 51 10.386 -26.051 22.208 0.00 0.00
G
ATOM 21319 C HSE G 51 13.536 -24.152 24.810 0.00 0.00
G
ATOM 21320 O HSE G 51 13.731 -24.754 25.883 0.00 0.00
G
ATOM 21321 N ALA G 52 12.906 -22.964 24.788 0.00 0.00
G
ATOM 21322 HN ALA G 52 12.897 -22.450 23.933 0.00 0.00
G
ATOM 21323 CA ALA G 52 12.341 -22.342 25.925 0.00 0.00
G
ATOM 21324 HA ALA G 52 12.038 -23.187 26.525 0.00 0.00
G
ATOM 21325 CB ALA G 52 13.420 -21.331 26.492 0.00 0.00
G
ATOM 21326 HB1 ALA G 52 13.035 -20.843 27.412 0.00 0.00
G
ATOM 21327 HB2 ALA G 52 14.419 -21.765 26.708 0.00 0.00
G
ATOM 21328 HB3 ALA G 52 13.648 -20.590 25.696 0.00 0.00
G
ATOM 21329 C ALA G 52 10.998 -21.708 25.485 0.00 0.00
G

ATOM 21330 O ALA G 52 10.949 -20.951 24.515 0.00 0.00
G
ATOM 21331 N VAL G 53 9.950 -22.138 26.184 0.00 0.00
G
ATOM 21332 HN VAL G 53 10.085 -22.710 26.989 0.00 0.00
G
ATOM 21333 CA VAL G 53 8.531 -21.671 25.980 0.00 0.00
G
ATOM 21334 HA VAL G 53 8.616 -20.884 25.245 0.00 0.00
G
ATOM 21335 CB VAL G 53 7.600 -22.720 25.446 0.00 0.00
G
ATOM 21336 HB VAL G 53 6.564 -22.328 25.535 0.00 0.00
G
ATOM 21337 CG1 VAL G 53 7.901 -22.967 23.936 0.00 0.00
G
ATOM 21338 HG11 VAL G 53 6.998 -23.465 23.523 0.00 0.00
G
ATOM 21339 HG12 VAL G 53 7.979 -22.008 23.382 0.00 0.00
G
ATOM 21340 HG13 VAL G 53 8.844 -23.534 23.785 0.00 0.00
G
ATOM 21341 CG2 VAL G 53 7.762 -23.970 26.286 0.00 0.00
G
ATOM 21342 HG21 VAL G 53 8.778 -24.390 26.120 0.00 0.00
G
ATOM 21343 HG22 VAL G 53 7.613 -23.768 27.368 0.00 0.00
G
ATOM 21344 HG23 VAL G 53 7.077 -24.770 25.932 0.00 0.00
G
ATOM 21345 C VAL G 53 7.953 -21.044 27.271 0.00 0.00
G
ATOM 21346 O VAL G 53 8.160 -21.523 28.345 0.00 0.00
G
ATOM 21347 N ILE G 54 7.294 -19.888 27.149 0.00 0.00
G
ATOM 21348 HN ILE G 54 7.258 -19.370 26.298 0.00 0.00
G
ATOM 21349 CA ILE G 54 6.487 -19.163 28.154 0.00 0.00
G
ATOM 21350 HA ILE G 54 6.926 -19.171 29.141 0.00 0.00
G
ATOM 21351 CB ILE G 54 6.417 -17.661 27.713 0.00 0.00
G
ATOM 21352 HB ILE G 54 6.056 -17.492 26.676 0.00 0.00
G
ATOM 21353 CG2 ILE G 54 5.522 -17.023 28.755 0.00 0.00
G
ATOM 21354 HG21 ILE G 54 4.455 -17.154 28.472 0.00 0.00
G
ATOM 21355 HG22 ILE G 54 5.805 -17.400 29.761 0.00 0.00
G
ATOM 21356 HG23 ILE G 54 5.707 -15.929 28.794 0.00 0.00
G

ATOM 21357 CG1 ILE G 54   7.837 -17.026 27.875 0.00 0.00
G
ATOM 21358 HG11 ILE G 54   7.987 -16.643 28.908 0.00 0.00
G
ATOM 21359 HG12 ILE G 54   8.577 -17.790 27.556 0.00 0.00
G
ATOM 21360 CD ILE G 54   8.072 -15.794 26.913 0.00 0.00
G
ATOM 21361 HD1 ILE G 54   7.975 -16.130 25.859 0.00 0.00
G
ATOM 21362 HD2 ILE G 54   7.232 -15.126 27.202 0.00 0.00
G
ATOM 21363 HD3 ILE G 54   9.005 -15.278 27.224 0.00 0.00
G
ATOM 21364 C   ILE G 54   5.187 -19.800 28.267 0.00 0.00
G
ATOM 21365 O   ILE G 54   4.274 -19.555 27.495 0.00 0.00
G
ATOM 21366 N   ALA G 55   4.933 -20.707 29.283 0.00 0.00
G
ATOM 21367 HN ALA G 55   5.520 -20.816 30.082 0.00 0.00
G
ATOM 21368 CA ALA G 55   3.766 -21.453 29.274 0.00 0.00
G
ATOM 21369 HA ALA G 55   3.008 -21.077 28.603 0.00 0.00
G
ATOM 21370 CB ALA G 55   3.934 -22.878 28.665 0.00 0.00
G
ATOM 21371 HB1 ALA G 55   4.276 -22.825 27.609 0.00 0.00
G
ATOM 21372 HB2 ALA G 55   4.746 -23.429 29.186 0.00 0.00
G
ATOM 21373 HB3 ALA G 55   2.984 -23.451 28.711 0.00 0.00
G
ATOM 21374 C   ALA G 55   3.269 -21.638 30.693 0.00 0.00
G
ATOM 21375 O   ALA G 55   4.020 -21.423 31.634 0.00 0.00
G
ATOM 21376 N   ASN G 56   1.996 -22.021 30.885 0.00 0.00
G
ATOM 21377 HN ASN G 56   1.428 -22.140 30.074 0.00 0.00
G
ATOM 21378 CA ASN G 56   1.480 -22.549 32.087 0.00 0.00
G
ATOM 21379 HA ASN G 56   2.029 -22.140 32.922 0.00 0.00
G
ATOM 21380 CB ASN G 56   0.030 -22.073 32.392 0.00 0.00
G
ATOM 21381 HB1 ASN G 56   0.110 -20.977 32.229 0.00 0.00
G
ATOM 21382 HB2 ASN G 56   -0.787 -22.612 31.868 0.00 0.00
G
ATOM 21383 CG ASN G 56   -0.262 -22.348 33.883 0.00 0.00
G

ATOM 21384 OD1 ASN G 56   0.212 -23.282 34.481 0.00 0.00
G
ATOM 21385 ND2 ASN G 56   -1.003 -21.469 34.494 0.00 0.00
G
ATOM 21386 HD21 ASN G 56   -1.245 -21.576 35.458 0.00 0.00
G
ATOM 21387 HD22 ASN G 56   -1.139 -20.599 34.019 0.00 0.00
G
ATOM 21388 C   ASN G 56   1.735 -24.087 32.190 0.00 0.00
G
ATOM 21389 O   ASN G 56   1.338 -24.880 31.306 0.00 0.00
G
ATOM 21390 N   ALA G 57   2.467 -24.542 33.193 0.00 0.00
G
ATOM 21391 HN ALA G 57   2.942 -23.862 33.747 0.00 0.00
G
ATOM 21392 CA ALA G 57   2.707 -25.909 33.506 0.00 0.00
G
ATOM 21393 HA ALA G 57   2.541 -26.460 32.592 0.00 0.00
G
ATOM 21394 CB ALA G 57   4.200 -26.119 33.793 0.00 0.00
G
ATOM 21395 HB1 ALA G 57   4.460 -25.696 34.787 0.00 0.00
G
ATOM 21396 HB2 ALA G 57   4.462 -27.198 33.839 0.00 0.00
G
ATOM 21397 HB3 ALA G 57   4.852 -25.574 33.077 0.00 0.00
G
ATOM 21398 C   ALA G 57   1.886 -26.528 34.630 0.00 0.00
G
ATOM 21399 O   ALA G 57   2.261 -27.625 35.016 0.00 0.00
G
ATOM 21400 N   THR G 58   0.922 -25.792 35.230 0.00 0.00
G
ATOM 21401 HN THR G 58   0.725 -24.847 34.981 0.00 0.00
G
ATOM 21402 CA THR G 58   0.075 -26.232 36.334 0.00 0.00
G
ATOM 21403 HA THR G 58   0.292 -27.266 36.557 0.00 0.00
G
ATOM 21404 CB THR G 58   0.049 -25.368 37.625 0.00 0.00
G
ATOM 21405 HB THR G 58   -0.689 -25.804 38.332 0.00 0.00
G
ATOM 21406 OG1 THR G 58   -0.311 -23.954 37.319 0.00 0.00
G
ATOM 21407 HG1 THR G 58   0.171 -23.814 36.500 0.00 0.00
G
ATOM 21408 CG2 THR G 58   1.426 -25.201 38.351 0.00 0.00
G
ATOM 21409 HG21 THR G 58   1.739 -26.248 38.551 0.00 0.00
G
ATOM 21410 HG22 THR G 58   2.221 -24.756 37.716 0.00 0.00
G

ATOM 21411 HG23 THR G 58 1.284 -24.605 39.278 0.00 0.00
G
ATOM 21412 C THR G 58 -1.402 -26.172 35.872 0.00 0.00
G
ATOM 21413 O THR G 58 -2.272 -26.555 36.553 0.00 0.00
G
ATOM 21414 N GLU G 59 -1.629 -25.785 34.555 0.00 0.00
G
ATOM 21415 HN GLU G 59 -0.864 -25.455 34.007 0.00 0.00
G
ATOM 21416 CA GLU G 59 -2.857 -26.106 33.779 0.00 0.00
G
ATOM 21417 HA GLU G 59 -3.649 -26.241 34.502 0.00 0.00
G
ATOM 21418 CB GLU G 59 -3.194 -24.881 32.966 0.00 0.00
G
ATOM 21419 HB1 GLU G 59 -3.433 -24.130 33.749 0.00 0.00
G
ATOM 21420 HB2 GLU G 59 -2.374 -24.581 32.279 0.00 0.00
G
ATOM 21421 CG GLU G 59 -4.465 -24.961 32.007 0.00 0.00
G
ATOM 21422 HG1 GLU G 59 -4.695 -23.975 31.548 0.00 0.00
G
ATOM 21423 HG2 GLU G 59 -4.249 -25.584 31.113 0.00 0.00
G
ATOM 21424 CD GLU G 59 -5.730 -25.607 32.642 0.00 0.00
G
ATOM 21425 OE1 GLU G 59 -6.478 -24.831 33.233 0.00 0.00
G
ATOM 21426 OE2 GLU G 59 -6.009 -26.805 32.470 0.00 0.00
G
ATOM 21427 C GLU G 59 -2.799 -27.387 32.980 0.00 0.00
G
ATOM 21428 O GLU G 59 -1.828 -27.595 32.195 0.00 0.00
G
ATOM 21429 N GLU G 60 -3.625 -28.350 33.188 0.00 0.00
G
ATOM 21430 HN GLU G 60 -4.418 -28.254 33.785 0.00 0.00
G
ATOM 21431 CA GLU G 60 -3.581 -29.684 32.582 0.00 0.00
G
ATOM 21432 HA GLU G 60 -2.711 -30.205 32.952 0.00 0.00
G
ATOM 21433 CB GLU G 60 -4.755 -30.472 33.119 0.00 0.00
G
ATOM 21434 HB1 GLU G 60 -4.800 -30.458 34.229 0.00 0.00
G
ATOM 21435 HB2 GLU G 60 -5.639 -29.911 32.746 0.00 0.00
G
ATOM 21436 CG GLU G 60 -4.911 -31.909 32.451 0.00 0.00
G
ATOM 21437 HG1 GLU G 60 -5.165 -31.645 31.402 0.00 0.00
G

ATOM 21438 HG2 GLU G 60   -3.882 -32.327 32.436 0.00 0.00
G
ATOM 21439 CD GLU G 60   -6.015 -32.804 33.060 0.00 0.00
G
ATOM 21440 OE1 GLU G 60   -7.206 -32.518 33.011 0.00 0.00
G
ATOM 21441 OE2 GLU G 60   -5.582 -33.790 33.743 0.00 0.00
G
ATOM 21442 C   GLU G 60   -3.519 -29.708 31.065 0.00 0.00
G
ATOM 21443 O   GLU G 60   -2.631 -30.250 30.367 0.00 0.00
G
ATOM 21444 N   ASN G 61   -4.473 -28.948 30.542 0.00 0.00
G
ATOM 21445 HN ASN G 61   -5.046 -28.378 31.126 0.00 0.00
G
ATOM 21446 CA ASN G 61   -4.632 -28.807 29.131 0.00 0.00
G
ATOM 21447 HA ASN G 61   -4.503 -29.764 28.647 0.00 0.00
G
ATOM 21448 CB ASN G 61   -6.035 -28.340 28.788 0.00 0.00
G
ATOM 21449 HB1 ASN G 61   -6.314 -27.454 29.398 0.00 0.00
G
ATOM 21450 HB2 ASN G 61   -6.186 -28.040 27.729 0.00 0.00
G
ATOM 21451 CG ASN G 61   -7.001 -29.506 29.032 0.00 0.00
G
ATOM 21452 OD1 ASN G 61   -6.643 -30.650 28.861 0.00 0.00
G
ATOM 21453 ND2 ASN G 61   -8.207 -29.160 29.458 0.00 0.00
G
ATOM 21454 HD21 ASN G 61   -8.956 -29.815 29.358 0.00 0.00
G
ATOM 21455 HD22 ASN G 61   -8.399 -28.269 29.871 0.00 0.00
G
ATOM 21456 C   ASN G 61   -3.642 -27.844 28.502 0.00 0.00
G
ATOM 21457 O   ASN G 61   -3.404 -27.878 27.244 0.00 0.00
G
ATOM 21458 N   GLU G 62   -2.977 -27.000 29.259 0.00 0.00
G
ATOM 21459 HN GLU G 62   -3.110 -26.919 30.244 0.00 0.00
G
ATOM 21460 CA GLU G 62   -1.850 -26.265 28.671 0.00 0.00
G
ATOM 21461 HA GLU G 62   -1.948 -26.041 27.619 0.00 0.00
G
ATOM 21462 CB GLU G 62   -1.313 -25.012 29.415 0.00 0.00
G
ATOM 21463 HB1 GLU G 62   -1.141 -25.237 30.489 0.00 0.00
G
ATOM 21464 HB2 GLU G 62   -0.338 -24.717 28.972 0.00 0.00
G

ATOM 21465 CG GLU G 62   -2.216 -23.719 29.478 0.00 0.00
G
ATOM 21466 HG1 GLU G 62   -3.175 -23.895 30.011 0.00 0.00
G
ATOM 21467 HG2 GLU G 62   -1.727 -22.891 30.034 0.00 0.00
G
ATOM 21468 CD GLU G 62   -2.616 -23.251 28.071 0.00 0.00
G
ATOM 21469 OE1 GLU G 62   -1.708 -23.028 27.200 0.00 0.00
G
ATOM 21470 OE2 GLU G 62   -3.848 -22.987 27.830 0.00 0.00
G
ATOM 21471 C   GLU G 62   -0.638 -27.257 28.637 0.00 0.00
G
ATOM 21472 O   GLU G 62   0.108 -27.292 27.675 0.00 0.00
G
ATOM 21473 N   LEU G 63   -0.481 -28.092 29.632 0.00 0.00
G
ATOM 21474 HN LEU G 63   -1.121 -28.075 30.396 0.00 0.00
G
ATOM 21475 CA LEU G 63   0.665 -29.052 29.781 0.00 0.00
G
ATOM 21476 HA LEU G 63   1.534 -28.487 29.478 0.00 0.00
G
ATOM 21477 CB LEU G 63   0.868 -29.443 31.278 0.00 0.00
G
ATOM 21478 HB1 LEU G 63   1.118 -28.562 31.907 0.00 0.00
G
ATOM 21479 HB2 LEU G 63   -0.108 -29.876 31.586 0.00 0.00
G
ATOM 21480 CG LEU G 63   1.976 -30.527 31.533 0.00 0.00
G
ATOM 21481 HG LEU G 63   1.807 -31.303 30.756 0.00 0.00
G
ATOM 21482 CD1 LEU G 63   3.340 -29.904 31.319 0.00 0.00
G
ATOM 21483 HD11 LEU G 63   3.557 -29.210 32.159 0.00 0.00
G
ATOM 21484 HD12 LEU G 63   4.101 -30.713 31.293 0.00 0.00
G
ATOM 21485 HD13 LEU G 63   3.408 -29.348 30.359 0.00 0.00
G
ATOM 21486 CD2 LEU G 63   1.725 -31.086 32.909 0.00 0.00
G
ATOM 21487 HD21 LEU G 63   2.083 -32.107 33.160 0.00 0.00
G
ATOM 21488 HD22 LEU G 63   2.069 -30.407 33.717 0.00 0.00
G
ATOM 21489 HD23 LEU G 63   0.621 -31.135 33.026 0.00 0.00
G
ATOM 21490 C   LEU G 63   0.631 -30.241 28.811 0.00 0.00
G
ATOM 21491 O   LEU G 63   1.623 -30.596 28.203 0.00 0.00
G

ATOM 21492 N LEU G 64 -0.555 -30.800 28.559 0.00 0.00
G
ATOM 21493 HN LEU G 64 -1.366 -30.730 29.134 0.00 0.00
G
ATOM 21494 CA LEU G 64 -0.901 -31.547 27.304 0.00 0.00
G
ATOM 21495 HA LEU G 64 -0.351 -32.475 27.248 0.00 0.00
G
ATOM 21496 CB LEU G 64 -2.401 -31.923 27.276 0.00 0.00
G
ATOM 21497 HB1 LEU G 64 -3.105 -31.071 27.167 0.00 0.00
G
ATOM 21498 HB2 LEU G 64 -2.571 -32.490 26.335 0.00 0.00
G
ATOM 21499 CG LEU G 64 -2.743 -32.889 28.458 0.00 0.00
G
ATOM 21500 HG LEU G 64 -2.510 -32.311 29.378 0.00 0.00
G
ATOM 21501 CD1 LEU G 64 -4.260 -33.379 28.629 0.00 0.00
G
ATOM 21502 HD11 LEU G 64 -4.885 -32.463 28.549 0.00 0.00
G
ATOM 21503 HD12 LEU G 64 -4.621 -34.129 27.893 0.00 0.00
G
ATOM 21504 HD13 LEU G 64 -4.469 -33.819 29.627 0.00 0.00
G
ATOM 21505 CD2 LEU G 64 -1.832 -34.180 28.515 0.00 0.00
G
ATOM 21506 HD21 LEU G 64 -2.029 -34.724 27.567 0.00 0.00
G
ATOM 21507 HD22 LEU G 64 -0.743 -33.960 28.505 0.00 0.00
G
ATOM 21508 HD23 LEU G 64 -1.983 -34.790 29.431 0.00 0.00
G
ATOM 21509 C LEU G 64 -0.538 -30.817 26.009 0.00 0.00
G
ATOM 21510 O LEU G 64 -0.059 -31.406 25.076 0.00 0.00
G
ATOM 21511 N SER G 65 -0.779 -29.534 25.955 0.00 0.00
G
ATOM 21512 HN SER G 65 -1.117 -28.991 26.720 0.00 0.00
G
ATOM 21513 CA SER G 65 -0.363 -28.755 24.712 0.00 0.00
G
ATOM 21514 HA SER G 65 -0.757 -29.240 23.831 0.00 0.00
G
ATOM 21515 CB SER G 65 -0.805 -27.299 24.706 0.00 0.00
G
ATOM 21516 HB1 SER G 65 -0.436 -26.747 25.597 0.00 0.00
G
ATOM 21517 HB2 SER G 65 -0.331 -26.786 23.843 0.00 0.00
G
ATOM 21518 OG SER G 65 -2.227 -27.201 24.485 0.00 0.00
G

```
ATOM  21519  HG1 SER G  65      -2.330 -26.253  24.376  0.00  0.00
G
ATOM  21520  C   SER G  65       1.089 -28.760  24.513  0.00  0.00
G
ATOM  21521  O   SER G  65       1.573 -28.774  23.336  0.00  0.00
G
ATOM  21522  N   LEU G  66       1.942 -28.793  25.532  0.00  0.00
G
ATOM  21523  HN  LEU G  66       1.652 -28.712  26.483  0.00  0.00
G
ATOM  21524  CA  LEU G  66       3.325 -28.989  25.346  0.00  0.00
G
ATOM  21525  HA  LEU G  66       3.691 -28.241  24.658  0.00  0.00
G
ATOM  21526  CB  LEU G  66       4.146 -28.589  26.594  0.00  0.00
G
ATOM  21527  HB1 LEU G  66       4.009 -29.320  27.420  0.00  0.00
G
ATOM  21528  HB2 LEU G  66       5.239 -28.544  26.402  0.00  0.00
G
ATOM  21529  CG  LEU G  66       3.799 -27.184  27.203  0.00  0.00
G
ATOM  21530  HG  LEU G  66       2.689 -27.212  27.222  0.00  0.00
G
ATOM  21531  CD1 LEU G  66       4.487 -26.996  28.596  0.00  0.00
G
ATOM  21532 HD11 LEU G  66       4.014 -26.059  28.960  0.00  0.00
G
ATOM  21533 HD12 LEU G  66       4.158 -27.835  29.245  0.00  0.00
G
ATOM  21534 HD13 LEU G  66       5.587 -26.894  28.473  0.00  0.00
G
ATOM  21535  CD2 LEU G  66       4.297 -26.056  26.325  0.00  0.00
G
ATOM  21536 HD21 LEU G  66       5.408 -26.028  26.356  0.00  0.00
G
ATOM  21537 HD22 LEU G  66       4.073 -26.222  25.249  0.00  0.00
G
ATOM  21538 HD23 LEU G  66       3.767 -25.126  26.622  0.00  0.00
G
ATOM  21539  C   LEU G  66       3.756 -30.366  24.970  0.00  0.00
G
ATOM  21540  O   LEU G  66       4.616 -30.485  24.066  0.00  0.00
G
ATOM  21541  N   GLY G  67       3.188 -31.426  25.587  0.00  0.00
G
ATOM  21542  HN  GLY G  67       2.533 -31.213  26.308  0.00  0.00
G
ATOM  21543  CA  GLY G  67       3.534 -32.781  25.251  0.00  0.00
G
ATOM  21544  HA1 GLY G  67       3.584 -32.920  24.181  0.00  0.00
G
ATOM  21545  HA2 GLY G  67       2.793 -33.375  25.766  0.00  0.00
G
```

ATOM 21546 C   GLY G 67   4.831 -33.266 25.760 0.00 0.00
G
ATOM 21547 O   GLY G 67   5.669 -33.648 24.979 0.00 0.00
G
ATOM 21548 N   ILE G 68   5.107 -33.235 27.098 0.00 0.00
G
ATOM 21549 HN ILE G 68   4.432 -33.096 27.818 0.00 0.00
G
ATOM 21550 CA ILE G 68   6.345 -33.526 27.632 0.00 0.00
G
ATOM 21551 HA ILE G 68   7.076 -33.477 26.839 0.00 0.00
G
ATOM 21552 CB ILE G 68   6.773 -32.403 28.609 0.00 0.00
G
ATOM 21553 HB ILE G 68   7.698 -32.725 29.133 0.00 0.00
G
ATOM 21554 CG2 ILE G 68   7.077 -31.119 27.921 0.00 0.00
G
ATOM 21555 HG21 ILE G 68   6.159 -30.658 27.497 0.00 0.00
G
ATOM 21556 HG22 ILE G 68   7.503 -30.300 28.540 0.00 0.00
G
ATOM 21557 HG23 ILE G 68   7.845 -31.320 27.144 0.00 0.00
G
ATOM 21558 CG1 ILE G 68   5.828 -32.038 29.805 0.00 0.00
G
ATOM 21559 HG11 ILE G 68   6.304 -31.171 30.310 0.00 0.00
G
ATOM 21560 HG12 ILE G 68   4.811 -31.770 29.449 0.00 0.00
G
ATOM 21561 CD ILE G 68   5.593 -33.137 30.872 0.00 0.00
G
ATOM 21562 HD1 ILE G 68   6.485 -33.384 31.487 0.00 0.00
G
ATOM 21563 HD2 ILE G 68   4.771 -32.763 31.518 0.00 0.00
G
ATOM 21564 HD3 ILE G 68   5.162 -33.985 30.298 0.00 0.00
G
ATOM 21565 C   ILE G 68   6.463 -34.944 28.300 0.00 0.00
G
ATOM 21566 O   ILE G 68   7.340 -35.113 29.133 0.00 0.00
G
ATOM 21567 N   ARG G 69   5.596 -35.920 27.865 0.00 0.00
G
ATOM 21568 HN ARG G 69   4.985 -35.548 27.170 0.00 0.00
G
ATOM 21569 CA ARG G 69   5.561 -37.335 28.177 0.00 0.00
G
ATOM 21570 HA ARG G 69   5.460 -37.407 29.250 0.00 0.00
G
ATOM 21571 CB ARG G 69   4.264 -37.926 27.502 0.00 0.00
G
ATOM 21572 HB1 ARG G 69   4.045 -38.967 27.822 0.00 0.00
G

ATOM 21573 HB2 ARG G 69 3.409 -37.341 27.904 0.00 0.00
G
ATOM 21574 CG ARG G 69 4.270 -37.932 26.000 0.00 0.00
G
ATOM 21575 HG1 ARG G 69 3.916 -36.960 25.594 0.00 0.00
G
ATOM 21576 HG2 ARG G 69 5.297 -37.993 25.580 0.00 0.00
G
ATOM 21577 CD ARG G 69 3.456 -39.031 25.335 0.00 0.00
G
ATOM 21578 HD1 ARG G 69 3.867 -40.045 25.528 0.00 0.00
G
ATOM 21579 HD2 ARG G 69 2.416 -38.966 25.721 0.00 0.00
G
ATOM 21580 NE ARG G 69 3.427 -38.783 23.882 0.00 0.00
G
ATOM 21581 HE ARG G 69 3.317 -37.854 23.528 0.00 0.00
G
ATOM 21582 CZ ARG G 69 3.079 -39.688 22.971 0.00 0.00
G
ATOM 21583 NH1 ARG G 69 3.339 -41.008 22.995 0.00 0.00
G
ATOM 21584 HH11 ARG G 69 2.958 -41.635 22.316 0.00 0.00
G
ATOM 21585 HH12 ARG G 69 3.645 -41.487 23.818 0.00 0.00
G
ATOM 21586 NH2 ARG G 69 2.655 -39.151 21.829 0.00 0.00
G
ATOM 21587 HH21 ARG G 69 2.627 -39.612 20.942 0.00 0.00
G
ATOM 21588 HH22 ARG G 69 2.598 -38.160 21.712 0.00 0.00
G
ATOM 21589 C ARG G 69 6.809 -38.117 27.735 0.00 0.00
G
ATOM 21590 O ARG G 69 7.047 -39.243 28.136 0.00 0.00
G
ATOM 21591 N ASN G 70 7.615 -37.482 26.873 0.00 0.00
G
ATOM 21592 HN ASN G 70 7.392 -36.534 26.659 0.00 0.00
G
ATOM 21593 CA ASN G 70 8.765 -38.189 26.349 0.00 0.00
G
ATOM 21594 HA ASN G 70 8.517 -39.216 26.125 0.00 0.00
G
ATOM 21595 CB ASN G 70 9.268 -37.481 25.056 0.00 0.00
G
ATOM 21596 HB1 ASN G 70 10.163 -38.005 24.656 0.00 0.00
G
ATOM 21597 HB2 ASN G 70 8.398 -37.780 24.434 0.00 0.00
G
ATOM 21598 CG ASN G 70 9.333 -36.005 25.106 0.00 0.00
G
ATOM 21599 OD1 ASN G 70 8.325 -35.246 25.115 0.00 0.00
G

ATOM 21600 ND2 ASN G 70   10.563 -35.551 25.275 0.00 0.00
G
ATOM 21601 HD21 ASN G 70   10.782 -34.575 25.300 0.00 0.00
G
ATOM 21602 HD22 ASN G 70   11.267 -36.253 25.174 0.00 0.00
G
ATOM 21603 C   ASN G 70   9.936 -38.263 27.355 0.00 0.00
G
ATOM 21604 O   ASN G 70   10.846 -39.127 27.258 0.00 0.00
G
ATOM 21605 N   PHE G 71   9.975 -37.329 28.244 0.00 0.00
G
ATOM 21606 HN PHE G 71   9.239 -36.668 28.362 0.00 0.00
G
ATOM 21607 CA PHE G 71   10.974 -37.164 29.270 0.00 0.00
G
ATOM 21608 HA PHE G 71   11.972 -37.224 28.862 0.00 0.00
G
ATOM 21609 CB PHE G 71   11.000 -35.762 29.980 0.00 0.00
G
ATOM 21610 HB1 PHE G 71   9.998 -35.493 30.376 0.00 0.00
G
ATOM 21611 HB2 PHE G 71   11.798 -35.842 30.749 0.00 0.00
G
ATOM 21612 CG PHE G 71   11.465 -34.603 29.096 0.00 0.00
G
ATOM 21613 CD1 PHE G 71   12.802 -34.296 28.839 0.00 0.00
G
ATOM 21614 HD1 PHE G 71   13.656 -34.771 29.298 0.00 0.00
G
ATOM 21615 CE1 PHE G 71   13.148 -33.411 27.829 0.00 0.00
G
ATOM 21616 HE1 PHE G 71   14.174 -33.076 27.802 0.00 0.00
G
ATOM 21617 CZ PHE G 71   12.244 -32.732 27.098 0.00 0.00
G
ATOM 21618 HZ PHE G 71   12.598 -31.947 26.445 0.00 0.00
G
ATOM 21619 CD2 PHE G 71   10.469 -33.806 28.435 0.00 0.00
G
ATOM 21620 HD2 PHE G 71   9.452 -34.137 28.588 0.00 0.00
G
ATOM 21621 CE2 PHE G 71   10.838 -32.878 27.447 0.00 0.00
G
ATOM 21622 HE2 PHE G 71   10.051 -32.332 26.947 0.00 0.00
G
ATOM 21623 C   PHE G 71   10.892 -38.293 30.283 0.00 0.00
G
ATOM 21624 O   PHE G 71   9.890 -38.983 30.470 0.00 0.00
G
ATOM 21625 N   GLU G 72   12.066 -38.471 30.922 0.00 0.00
G
ATOM 21626 HN GLU G 72   12.857 -37.968 30.582 0.00 0.00
G

ATOM 21627 CA GLU G 72 12.267 -39.415 32.000 0.00 0.00
G
ATOM 21628 HA GLU G 72 11.394 -39.980 32.291 0.00 0.00
G
ATOM 21629 CB GLU G 72 13.473 -40.380 31.599 0.00 0.00
G
ATOM 21630 HB1 GLU G 72 13.976 -39.755 30.831 0.00 0.00
G
ATOM 21631 HB2 GLU G 72 14.131 -40.622 32.461 0.00 0.00
G
ATOM 21632 CG GLU G 72 12.905 -41.690 30.985 0.00 0.00
G
ATOM 21633 HG1 GLU G 72 12.102 -42.144 31.604 0.00 0.00
G
ATOM 21634 HG2 GLU G 72 12.529 -41.651 29.940 0.00 0.00
G
ATOM 21635 CD GLU G 72 14.013 -42.729 30.801 0.00 0.00
G
ATOM 21636 OE1 GLU G 72 15.185 -42.242 30.721 0.00 0.00
G
ATOM 21637 OE2 GLU G 72 13.762 -43.909 30.759 0.00 0.00
G
ATOM 21638 C GLU G 72 12.639 -38.803 33.329 0.00 0.00
G
ATOM 21639 O GLU G 72 12.112 -39.295 34.334 0.00 0.00
G
ATOM 21640 N TYR G 73 13.435 -37.684 33.350 0.00 0.00
G
ATOM 21641 HN TYR G 73 13.813 -37.426 32.464 0.00 0.00
G
ATOM 21642 CA TYR G 73 13.769 -36.946 34.479 0.00 0.00
G
ATOM 21643 HA TYR G 73 13.236 -37.365 35.319 0.00 0.00
G
ATOM 21644 CB TYR G 73 15.290 -37.028 34.791 0.00 0.00
G
ATOM 21645 HB1 TYR G 73 15.953 -36.979 33.900 0.00 0.00
G
ATOM 21646 HB2 TYR G 73 15.542 -36.127 35.390 0.00 0.00
G
ATOM 21647 CG TYR G 73 15.704 -38.265 35.602 0.00 0.00
G
ATOM 21648 CD1 TYR G 73 15.930 -39.520 35.050 0.00 0.00
G
ATOM 21649 HD1 TYR G 73 15.674 -39.754 34.027 0.00 0.00
G
ATOM 21650 CE1 TYR G 73 16.269 -40.633 35.910 0.00 0.00
G
ATOM 21651 HE1 TYR G 73 16.242 -41.619 35.469 0.00 0.00
G
ATOM 21652 CZ TYR G 73 16.445 -40.415 37.306 0.00 0.00
G
ATOM 21653 OH TYR G 73 16.720 -41.508 38.083 0.00 0.00
G

ATOM 21654 HH   TYR G 73   17.125 −41.137 38.870 0.00 0.00
G
ATOM 21655 CD2 TYR G 73   16.016 −38.072 36.923 0.00 0.00
G
ATOM 21656 HD2 TYR G 73   15.864 −37.091 37.347 0.00 0.00
G
ATOM 21657 CE2 TYR G 73   16.312 −39.128 37.847 0.00 0.00
G
ATOM 21658 HE2 TYR G 73   16.551 −38.883 38.871 0.00 0.00
G
ATOM 21659 C   TYR G 73   13.334 −35.471 34.422 0.00 0.00
G
ATOM 21660 O   TYR G 73   13.619 −34.833 33.410 0.00 0.00
G
ATOM 21661 N   VAL G 74   12.554 −34.945 35.384 0.00 0.00
G
ATOM 21662 HN VAL G 74   12.313 −35.522 36.161 0.00 0.00
G
ATOM 21663 CA VAL G 74   11.888 −33.613 35.171 0.00 0.00
G
ATOM 21664 HA VAL G 74   12.302 −33.068 34.336 0.00 0.00
G
ATOM 21665 CB VAL G 74   10.383 −33.601 34.681 0.00 0.00
G
ATOM 21666 HB VAL G 74   9.674 −34.152 35.336 0.00 0.00
G
ATOM 21667 CG1 VAL G 74   9.851 −32.169 34.499 0.00 0.00
G
ATOM 21668 HG11 VAL G 74   8.792 −32.224 34.166 0.00 0.00
G
ATOM 21669 HG12 VAL G 74   10.021 −31.584 35.428 0.00 0.00
G
ATOM 21670 HG13 VAL G 74   10.506 −31.678 33.747 0.00 0.00
G
ATOM 21671 CG2 VAL G 74   10.314 −34.256 33.303 0.00 0.00
G
ATOM 21672 HG21 VAL G 74   10.552 −35.337 33.393 0.00 0.00
G
ATOM 21673 HG22 VAL G 74   9.281 −34.114 32.920 0.00 0.00
G
ATOM 21674 HG23 VAL G 74   10.915 −33.765 32.508 0.00 0.00
G
ATOM 21675 C   VAL G 74   11.967 −32.767 36.383 0.00 0.00
G
ATOM 21676 O   VAL G 74   11.334 −33.055 37.426 0.00 0.00
G
ATOM 21677 N   ILE G 75   12.706 −31.651 36.361 0.00 0.00
G
ATOM 21678 HN ILE G 75   13.053 −31.191 35.547 0.00 0.00
G
ATOM 21679 CA ILE G 75   12.994 −30.849 37.563 0.00 0.00
G
ATOM 21680 HA ILE G 75   13.152 −31.506 38.406 0.00 0.00
G

```
ATOM  21681  CB  ILE G  75      14.392 -30.150  37.387  0.00  0.00
G
ATOM  21682  HB  ILE G  75      14.186 -29.670  36.407  0.00  0.00
G
ATOM  21683  CG2 ILE G  75      14.564 -29.147  38.570  0.00  0.00
G
ATOM  21684 HG21 ILE G  75      14.792 -29.665  39.526  0.00  0.00
G
ATOM  21685 HG22 ILE G  75      15.353 -28.381  38.411  0.00  0.00
G
ATOM  21686 HG23 ILE G  75      13.638 -28.561  38.746  0.00  0.00
G
ATOM  21687  CG1 ILE G  75      15.510 -31.222  37.212  0.00  0.00
G
ATOM  21688 HG11 ILE G  75      15.268 -31.800  36.295  0.00  0.00
G
ATOM  21689 HG12 ILE G  75      16.548 -30.832  37.147  0.00  0.00
G
ATOM  21690  CD  ILE G  75      15.582 -32.113  38.413  0.00  0.00
G
ATOM  21691  HD1 ILE G  75      14.711 -32.801  38.462  0.00  0.00
G
ATOM  21692  HD2 ILE G  75      16.471 -32.778  38.382  0.00  0.00
G
ATOM  21693  HD3 ILE G  75      15.625 -31.522  39.353  0.00  0.00
G
ATOM  21694  C   ILE G  75      11.889 -29.872  37.780  0.00  0.00
G
ATOM  21695  O   ILE G  75      11.446 -29.089  36.858  0.00  0.00
G
ATOM  21696  N   VAL G  76      11.309 -29.796  38.996  0.00  0.00
G
ATOM  21697  HN  VAL G  76      11.665 -30.361  39.736  0.00  0.00
G
ATOM  21698  CA  VAL G  76      10.196 -28.889  39.362  0.00  0.00
G
ATOM  21699  HA  VAL G  76      10.099 -28.168  38.564  0.00  0.00
G
ATOM  21700  CB  VAL G  76       8.924 -29.505  39.813  0.00  0.00
G
ATOM  21701  HB  VAL G  76       9.118 -30.213  40.647  0.00  0.00
G
ATOM  21702  CG1 VAL G  76       7.797 -28.534  40.101  0.00  0.00
G
ATOM  21703 HG11 VAL G  76       7.404 -28.035  39.190  0.00  0.00
G
ATOM  21704 HG12 VAL G  76       6.957 -29.163  40.466  0.00  0.00
G
ATOM  21705 HG13 VAL G  76       7.913 -27.743  40.873  0.00  0.00
G
ATOM  21706  CG2 VAL G  76       8.499 -30.372  38.638  0.00  0.00
G
ATOM  21707 HG21 VAL G  76       9.326 -30.974  38.204  0.00  0.00
G
```

```
ATOM  21708 HG22 VAL G  76       7.609 -31.002  38.854  0.00  0.00
G
ATOM  21709 HG23 VAL G  76       8.177 -29.695  37.818  0.00  0.00
G
ATOM  21710  C   VAL G  76      10.852 -28.090  40.512  0.00  0.00
G
ATOM  21711  O   VAL G  76      10.974 -28.481  41.676  0.00  0.00
G
ATOM  21712  N   ALA G  77      11.297 -26.868  40.190  0.00  0.00
G
ATOM  21713  HN  ALA G  77      11.079 -26.464  39.305  0.00  0.00
G
ATOM  21714  CA  ALA G  77      12.060 -26.021  41.084  0.00  0.00
G
ATOM  21715  HA  ALA G  77      12.558 -26.611  41.839  0.00  0.00
G
ATOM  21716  CB  ALA G  77      13.159 -25.354  40.224  0.00  0.00
G
ATOM  21717  HB1 ALA G  77      13.678 -26.199  39.721  0.00  0.00
G
ATOM  21718  HB2 ALA G  77      12.733 -24.642  39.485  0.00  0.00
G
ATOM  21719  HB3 ALA G  77      13.674 -24.682  40.943  0.00  0.00
G
ATOM  21720  C   ALA G  77      11.225 -25.024  41.803  0.00  0.00
G
ATOM  21721  O   ALA G  77      11.660 -24.008  42.316  0.00  0.00
G
ATOM  21722  N   ILE G  78       9.944 -25.329  41.946  0.00  0.00
G
ATOM  21723  HN  ILE G  78       9.611 -26.203  41.600  0.00  0.00
G
ATOM  21724  CA  ILE G  78       9.087 -24.445  42.726  0.00  0.00
G
ATOM  21725  HA  ILE G  78       9.393 -23.418  42.588  0.00  0.00
G
ATOM  21726  CB  ILE G  78       7.750 -24.685  42.125  0.00  0.00
G
ATOM  21727  HB  ILE G  78       7.332 -25.690  42.348  0.00  0.00
G
ATOM  21728  CG2 ILE G  78       6.712 -23.651  42.729  0.00  0.00
G
ATOM  21729 HG21 ILE G  78       7.273 -22.694  42.788  0.00  0.00
G
ATOM  21730 HG22 ILE G  78       5.848 -23.302  42.124  0.00  0.00
G
ATOM  21731 HG23 ILE G  78       6.371 -23.978  43.735  0.00  0.00
G
ATOM  21732  CG1 ILE G  78       7.599 -24.535  40.600  0.00  0.00
G
ATOM  21733 HG11 ILE G  78       7.511 -23.477  40.271  0.00  0.00
G
ATOM  21734 HG12 ILE G  78       8.479 -25.020  40.127  0.00  0.00
G
```

ATOM 21735 CD ILE G 78 6.369 -25.237 40.112 0.00 0.00
G
ATOM 21736 HD1 ILE G 78 6.498 -26.318 40.332 0.00 0.00
G
ATOM 21737 HD2 ILE G 78 5.428 -24.876 40.580 0.00 0.00
G
ATOM 21738 HD3 ILE G 78 6.249 -25.086 39.018 0.00 0.00
G
ATOM 21739 C ILE G 78 9.077 -24.808 44.243 0.00 0.00
G
ATOM 21740 O ILE G 78 8.597 -25.875 44.712 0.00 0.00
G
ATOM 21741 N GLY G 79 9.582 -23.876 45.080 0.00 0.00
G
ATOM 21742 HN GLY G 79 10.071 -23.078 44.738 0.00 0.00
G
ATOM 21743 CA GLY G 79 9.892 -24.124 46.463 0.00 0.00
G
ATOM 21744 HA1 GLY G 79 10.893 -23.763 46.649 0.00 0.00
G
ATOM 21745 HA2 GLY G 79 9.872 -25.181 46.683 0.00 0.00
G
ATOM 21746 C GLY G 79 9.150 -23.404 47.485 0.00 0.00
G
ATOM 21747 O GLY G 79 9.102 -23.841 48.613 0.00 0.00
G
ATOM 21748 N ALA G 80 8.548 -22.211 47.231 0.00 0.00
G
ATOM 21749 HN ALA G 80 8.612 -21.790 46.329 0.00 0.00
G
ATOM 21750 CA ALA G 80 7.921 -21.399 48.163 0.00 0.00
G
ATOM 21751 HA ALA G 80 8.007 -21.889 49.122 0.00 0.00
G
ATOM 21752 CB ALA G 80 8.690 -20.052 48.103 0.00 0.00
G
ATOM 21753 HB1 ALA G 80 8.059 -19.350 48.689 0.00 0.00
G
ATOM 21754 HB2 ALA G 80 9.660 -19.953 48.636 0.00 0.00
G
ATOM 21755 HB3 ALA G 80 8.787 -19.632 47.079 0.00 0.00
G
ATOM 21756 C ALA G 80 6.484 -21.307 47.878 0.00 0.00
G
ATOM 21757 O ALA G 80 5.657 -20.984 48.699 0.00 0.00
G
ATOM 21758 N ASN G 81 6.048 -21.557 46.633 0.00 0.00
G
ATOM 21759 HN ASN G 81 6.737 -21.817 45.962 0.00 0.00
G
ATOM 21760 CA ASN G 81 4.673 -21.522 46.246 0.00 0.00
G
ATOM 21761 HA ASN G 81 4.137 -20.995 47.021 0.00 0.00
G

ATOM 21762 CB ASN G 81   4.436 −20.697 44.952 0.00 0.00
G
ATOM 21763 HB1 ASN G 81   5.058 −21.043 44.100 0.00 0.00
G
ATOM 21764 HB2 ASN G 81   3.372 −20.800 44.647 0.00 0.00
G
ATOM 21765 CG ASN G 81   4.785 −19.223 45.139 0.00 0.00
G
ATOM 21766 OD1 ASN G 81   3.997 −18.312 45.270 0.00 0.00
G
ATOM 21767 ND2 ASN G 81   6.070 −18.996 45.246 0.00 0.00
G
ATOM 21768 HD21 ASN G 81   6.379 −18.074 45.478 0.00 0.00
G
ATOM 21769 HD22 ASN G 81   6.673 −19.793 45.262 0.00 0.00
G
ATOM 21770 C   ASN G 81   4.238 −23.036 46.257 0.00 0.00
G
ATOM 21771 O   ASN G 81   4.180 −23.740 45.242 0.00 0.00
G
ATOM 21772 N   ILE G 82   3.654 −23.513 47.414 0.00 0.00
G
ATOM 21773 HN ILE G 82   3.655 −22.922 48.216 0.00 0.00
G
ATOM 21774 CA ILE G 82   2.907 −24.746 47.521 0.00 0.00
G
ATOM 21775 HA ILE G 82   3.586 −25.547 47.267 0.00 0.00
G
ATOM 21776 CB ILE G 82   2.449 −24.949 48.980 0.00 0.00
G
ATOM 21777 HB ILE G 82   3.336 −24.693 49.599 0.00 0.00
G
ATOM 21778 CG2 ILE G 82   1.239 −24.056 49.289 0.00 0.00
G
ATOM 21779 HG21 ILE G 82   0.306 −24.535 48.921 0.00 0.00
G
ATOM 21780 HG22 ILE G 82   1.338 −23.905 50.385 0.00 0.00
G
ATOM 21781 HG23 ILE G 82   1.245 −23.058 48.802 0.00 0.00
G
ATOM 21782 CG1 ILE G 82   2.141 −26.452 49.257 0.00 0.00
G
ATOM 21783 HG11 ILE G 82   1.437 −26.837 48.488 0.00 0.00
G
ATOM 21784 HG12 ILE G 82   3.060 −27.058 49.107 0.00 0.00
G
ATOM 21785 CD ILE G 82   1.569 −26.768 50.677 0.00 0.00
G
ATOM 21786 HD1 ILE G 82   1.376 −27.848 50.850 0.00 0.00
G
ATOM 21787 HD2 ILE G 82   2.180 −26.386 51.522 0.00 0.00
G
ATOM 21788 HD3 ILE G 82   0.625 −26.190 50.772 0.00 0.00
G

```
ATOM  21789  C   ILE G  82       1.785 -24.962  46.491  0.00  0.00
G
ATOM  21790  O   ILE G  82       1.608 -26.104  46.026  0.00  0.00
G
ATOM  21791  N   GLN G  83       0.965 -23.894  46.113  0.00  0.00
G
ATOM  21792  HN  GLN G  83       1.024 -23.044  46.631  0.00  0.00
G
ATOM  21793  CA  GLN G  83       0.006 -23.938  45.092  0.00  0.00
G
ATOM  21794  HA  GLN G  83      -0.654 -24.699  45.483  0.00  0.00
G
ATOM  21795  CB  GLN G  83      -0.756 -22.518  44.872  0.00  0.00
G
ATOM  21796  HB1 GLN G  83      -1.374 -22.206  45.741  0.00  0.00
G
ATOM  21797  HB2 GLN G  83       0.110 -21.822  44.851  0.00  0.00
G
ATOM  21798  CG  GLN G  83      -1.346 -22.448  43.399  0.00  0.00
G
ATOM  21799  HG1 GLN G  83      -0.529 -22.226  42.680  0.00  0.00
G
ATOM  21800  HG2 GLN G  83      -1.691 -23.470  43.135  0.00  0.00
G
ATOM  21801  CD  GLN G  83      -2.459 -21.451  43.276  0.00  0.00
G
ATOM  21802  OE1 GLN G  83      -3.383 -21.478  44.084  0.00  0.00
G
ATOM  21803  NE2 GLN G  83      -2.468 -20.497  42.410  0.00  0.00
G
ATOM  21804 HE21 GLN G  83      -3.121 -19.739  42.425  0.00  0.00
G
ATOM  21805 HE22 GLN G  83      -1.706 -20.385  41.773  0.00  0.00
G
ATOM  21806  C   GLN G  83       0.583 -24.506  43.832  0.00  0.00
G
ATOM  21807  O   GLN G  83      -0.016 -25.380  43.198  0.00  0.00
G
ATOM  21808  N   ALA G  84       1.698 -23.992  43.297  0.00  0.00
G
ATOM  21809  HN  ALA G  84       2.223 -23.382  43.884  0.00  0.00
G
ATOM  21810  CA  ALA G  84       2.289 -24.234  42.047  0.00  0.00
G
ATOM  21811  HA  ALA G  84       1.514 -24.361  41.306  0.00  0.00
G
ATOM  21812  CB  ALA G  84       3.178 -23.103  41.653  0.00  0.00
G
ATOM  21813  HB1 ALA G  84       2.514 -22.216  41.564  0.00  0.00
G
ATOM  21814  HB2 ALA G  84       3.947 -22.690  42.340  0.00  0.00
G
ATOM  21815  HB3 ALA G  84       3.588 -23.301  40.639  0.00  0.00
G
```

```
ATOM  21816  C   ALA G  84       2.989 -25.571  42.075  0.00  0.00
G
ATOM  21817  O   ALA G  84       2.754 -26.400  41.198  0.00  0.00
G
ATOM  21818  N   SER G  85       3.775 -25.811  43.140  0.00  0.00
G
ATOM  21819  HN  SER G  85       4.051 -25.074  43.753  0.00  0.00
G
ATOM  21820  CA  SER G  85       4.424 -27.086  43.338  0.00  0.00
G
ATOM  21821  HA  SER G  85       5.009 -27.248  42.445  0.00  0.00
G
ATOM  21822  CB  SER G  85       5.548 -27.049  44.377  0.00  0.00
G
ATOM  21823  HB1 SER G  85       6.191 -27.956  44.352  0.00  0.00
G
ATOM  21824  HB2 SER G  85       6.079 -26.091  44.192  0.00  0.00
G
ATOM  21825  OG  SER G  85       4.985 -27.028  45.683  0.00  0.00
G
ATOM  21826  HG1 SER G  85       5.735 -27.338  46.196  0.00  0.00
G
ATOM  21827  C   SER G  85       3.589 -28.275  43.486  0.00  0.00
G
ATOM  21828  O   SER G  85       3.873 -29.248  42.804  0.00  0.00
G
ATOM  21829  N   THR G  86       2.482 -28.304  44.210  0.00  0.00
G
ATOM  21830  HN  THR G  86       2.169 -27.535  44.763  0.00  0.00
G
ATOM  21831  CA  THR G  86       1.597 -29.426  44.344  0.00  0.00
G
ATOM  21832  HA  THR G  86       2.178 -30.304  44.586  0.00  0.00
G
ATOM  21833  CB  THR G  86       0.393 -29.257  45.337  0.00  0.00
G
ATOM  21834  HB  THR G  86      -0.285 -30.135  45.276  0.00  0.00
G
ATOM  21835  OG1 THR G  86      -0.299 -27.995  45.098  0.00  0.00
G
ATOM  21836  HG1 THR G  86       0.391 -27.341  45.229  0.00  0.00
G
ATOM  21837  CG2 THR G  86       0.992 -29.175  46.800  0.00  0.00
G
ATOM  21838 HG21 THR G  86       1.864 -28.493  46.890  0.00  0.00
G
ATOM  21839 HG22 THR G  86       0.148 -28.966  47.491  0.00  0.00
G
ATOM  21840 HG23 THR G  86       1.289 -30.229  46.988  0.00  0.00
G
ATOM  21841  C   THR G  86       0.898 -29.820  43.071  0.00  0.00
G
ATOM  21842  O   THR G  86       0.834 -31.046  42.838  0.00  0.00
G
```

ATOM 21843 N   LEU G 87   0.403 -28.822 42.270 0.00 0.00
G
ATOM 21844 HN LEU G 87   0.607 -27.874 42.499 0.00 0.00
G
ATOM 21845 CA LEU G 87   -0.267 -28.988 41.021 0.00 0.00
G
ATOM 21846 HA LEU G 87   -0.958 -29.803 41.180 0.00 0.00
G
ATOM 21847 CB LEU G 87   -1.053 -27.777 40.561 0.00 0.00
G
ATOM 21848 HB1 LEU G 87   -0.464 -26.835 40.531 0.00 0.00
G
ATOM 21849 HB2 LEU G 87   -1.287 -27.872 39.479 0.00 0.00
G
ATOM 21850 CG LEU G 87   -2.334 -27.592 41.388 0.00 0.00
G
ATOM 21851 HG LEU G 87   -2.131 -27.751 42.468 0.00 0.00
G
ATOM 21852 CD1 LEU G 87   -2.780 -26.120 41.276 0.00 0.00
G
ATOM 21853 HD11 LEU G 87   -3.706 -26.149 41.889 0.00 0.00
G
ATOM 21854 HD12 LEU G 87   -2.056 -25.376 41.671 0.00 0.00
G
ATOM 21855 HD13 LEU G 87   -2.904 -25.878 40.199 0.00 0.00
G
ATOM 21856 CD2 LEU G 87   -3.536 -28.442 40.910 0.00 0.00
G
ATOM 21857 HD21 LEU G 87   -3.346 -29.529 40.781 0.00 0.00
G
ATOM 21858 HD22 LEU G 87   -4.434 -28.160 41.501 0.00 0.00
G
ATOM 21859 HD23 LEU G 87   -3.817 -28.050 39.909 0.00 0.00
G
ATOM 21860 C   LEU G 87   0.637 -29.447 39.885 0.00 0.00
G
ATOM 21861 O   LEU G 87   0.311 -30.392 39.147 0.00 0.00
G
ATOM 21862 N   THR G 88   1.783 -28.789 39.771 0.00 0.00
G
ATOM 21863 HN THR G 88   2.065 -27.994 40.303 0.00 0.00
G
ATOM 21864 CA THR G 88   2.791 -29.256 38.840 0.00 0.00
G
ATOM 21865 HA THR G 88   2.451 -29.118 37.824 0.00 0.00
G
ATOM 21866 CB THR G 88   4.100 -28.439 38.775 0.00 0.00
G
ATOM 21867 HB THR G 88   4.657 -28.620 39.719 0.00 0.00
G
ATOM 21868 OG1 THR G 88   3.945 -27.066 38.582 0.00 0.00
G
ATOM 21869 HG1 THR G 88   3.593 -26.716 39.403 0.00 0.00
G

ATOM 21870 CG2 THR G 88   4.963 -28.843 37.581 0.00 0.00
G
ATOM 21871 HG21 THR G 88   5.909 -28.261 37.575 0.00 0.00
G
ATOM 21872 HG22 THR G 88   5.186 -29.911 37.791 0.00 0.00
G
ATOM 21873 HG23 THR G 88   4.435 -28.790 36.605 0.00 0.00
G
ATOM 21874 C   THR G 88   3.188 -30.677 39.069 0.00 0.00
G
ATOM 21875 O   THR G 88   3.066 -31.522 38.166 0.00 0.00
G
ATOM 21876 N   THR G 89   3.445 -31.080 40.330 0.00 0.00
G
ATOM 21877 HN THR G 89   3.377 -30.462 41.110 0.00 0.00
G
ATOM 21878 CA THR G 89   3.766 -32.419 40.652 0.00 0.00
G
ATOM 21879 HA THR G 89   4.648 -32.748 40.122 0.00 0.00
G
ATOM 21880 CB THR G 89   4.037 -32.534 42.192 0.00 0.00
G
ATOM 21881 HB THR G 89   3.101 -32.323 42.752 0.00 0.00
G
ATOM 21882 OG1 THR G 89   5.043 -31.621 42.596 0.00 0.00
G
ATOM 21883 HG1 THR G 89   4.573 -30.794 42.729 0.00 0.00
G
ATOM 21884 CG2 THR G 89   4.606 -33.926 42.599 0.00 0.00
G
ATOM 21885 HG21 THR G 89   5.476 -34.091 41.927 0.00 0.00
G
ATOM 21886 HG22 THR G 89   4.874 -33.990 43.675 0.00 0.00
G
ATOM 21887 HG23 THR G 89   4.012 -34.827 42.334 0.00 0.00
G
ATOM 21888 C   THR G 89   2.708 -33.467 40.321 0.00 0.00
G
ATOM 21889 O   THR G 89   3.054 -34.564 39.868 0.00 0.00
G
ATOM 21890 N   LEU G 90   1.465 -33.045 40.489 0.00 0.00
G
ATOM 21891 HN LEU G 90   1.199 -32.192 40.932 0.00 0.00
G
ATOM 21892 CA LEU G 90   0.293 -33.925 40.228 0.00 0.00
G
ATOM 21893 HA LEU G 90   0.301 -34.903 40.686 0.00 0.00
G
ATOM 21894 CB LEU G 90   -0.923 -33.202 40.830 0.00 0.00
G
ATOM 21895 HB1 LEU G 90   -0.656 -32.939 41.876 0.00 0.00
G
ATOM 21896 HB2 LEU G 90   -1.252 -32.269 40.323 0.00 0.00
G

```
ATOM  21897  CG  LEU G  90      -2.281 -33.958  40.910  0.00  0.00
G
ATOM  21898  HG  LEU G  90      -2.393 -34.551  39.977  0.00  0.00
G
ATOM  21899  CD1 LEU G  90      -2.356 -35.016  42.099  0.00  0.00
G
ATOM  21900 HD11 LEU G  90      -2.375 -34.452  43.055  0.00  0.00
G
ATOM  21901 HD12 LEU G  90      -3.373 -35.452  41.997  0.00  0.00
G
ATOM  21902 HD13 LEU G  90      -1.549 -35.778  42.148  0.00  0.00
G
ATOM  21903  CD2 LEU G  90      -3.510 -32.975  40.969  0.00  0.00
G
ATOM  21904 HD21 LEU G  90      -3.269 -32.067  41.563  0.00  0.00
G
ATOM  21905 HD22 LEU G  90      -3.821 -32.667  39.947  0.00  0.00
G
ATOM  21906 HD23 LEU G  90      -4.446 -33.449  41.335  0.00  0.00
G
ATOM  21907  C   LEU G  90       0.027 -34.221  38.790  0.00  0.00
G
ATOM  21908  O   LEU G  90      -0.213 -35.382  38.382  0.00  0.00
G
ATOM  21909  N   LEU G  91       0.148 -33.239  37.847  0.00  0.00
G
ATOM  21910  HN  LEU G  91       0.290 -32.315  38.191  0.00  0.00
G
ATOM  21911  CA  LEU G  91      -0.144 -33.447  36.448  0.00  0.00
G
ATOM  21912  HA  LEU G  91      -1.117 -33.916  36.437  0.00  0.00
G
ATOM  21913  CB  LEU G  91      -0.195 -32.149  35.688  0.00  0.00
G
ATOM  21914  HB1 LEU G  91       0.804 -31.681  35.817  0.00  0.00
G
ATOM  21915  HB2 LEU G  91      -0.327 -32.286  34.593  0.00  0.00
G
ATOM  21916  CG  LEU G  91      -1.294 -31.142  36.146  0.00  0.00
G
ATOM  21917  HG  LEU G  91      -1.183 -31.190  37.250  0.00  0.00
G
ATOM  21918  CD1 LEU G  91      -0.913 -29.761  35.566  0.00  0.00
G
ATOM  21919 HD11 LEU G  91      -1.037 -29.685  34.465  0.00  0.00
G
ATOM  21920 HD12 LEU G  91      -1.527 -29.025  36.128  0.00  0.00
G
ATOM  21921 HD13 LEU G  91       0.115 -29.546  35.931  0.00  0.00
G
ATOM  21922  CD2 LEU G  91      -2.723 -31.548  35.819  0.00  0.00
G
ATOM  21923 HD21 LEU G  91      -3.431 -30.693  35.861  0.00  0.00
G
```

ATOM 21924 HD22 LEU G 91 -2.916 -32.011 34.827 0.00 0.00
G
ATOM 21925 HD23 LEU G 91 -3.090 -32.333 36.514 0.00 0.00
G
ATOM 21926 C LEU G 91 0.937 -34.394 35.792 0.00 0.00
G
ATOM 21927 O LEU G 91 0.605 -35.350 35.043 0.00 0.00
G
ATOM 21928 N LEU G 92 2.139 -34.221 36.125 0.00 0.00
G
ATOM 21929 HN LEU G 92 2.418 -33.414 36.640 0.00 0.00
G
ATOM 21930 CA LEU G 92 3.238 -35.015 35.729 0.00 0.00
G
ATOM 21931 HA LEU G 92 3.263 -35.005 34.650 0.00 0.00
G
ATOM 21932 CB LEU G 92 4.603 -34.470 36.282 0.00 0.00
G
ATOM 21933 HB1 LEU G 92 4.359 -34.317 37.355 0.00 0.00
G
ATOM 21934 HB2 LEU G 92 5.474 -35.147 36.154 0.00 0.00
G
ATOM 21935 CG LEU G 92 5.102 -33.110 35.809 0.00 0.00
G
ATOM 21936 HG LEU G 92 4.342 -32.323 36.001 0.00 0.00
G
ATOM 21937 CD1 LEU G 92 6.403 -32.659 36.622 0.00 0.00
G
ATOM 21938 HD11 LEU G 92 7.127 -33.497 36.537 0.00 0.00
G
ATOM 21939 HD12 LEU G 92 6.771 -31.704 36.190 0.00 0.00
G
ATOM 21940 HD13 LEU G 92 6.098 -32.510 37.680 0.00 0.00
G
ATOM 21941 CD2 LEU G 92 5.591 -33.100 34.364 0.00 0.00
G
ATOM 21942 HD21 LEU G 92 5.865 -32.095 33.980 0.00 0.00
G
ATOM 21943 HD22 LEU G 92 6.468 -33.772 34.243 0.00 0.00
G
ATOM 21944 HD23 LEU G 92 4.785 -33.535 33.735 0.00 0.00
G
ATOM 21945 C LEU G 92 3.272 -36.513 36.204 0.00 0.00
G
ATOM 21946 O LEU G 92 3.743 -37.294 35.414 0.00 0.00
G
ATOM 21947 N LYS G 93 2.804 -36.841 37.395 0.00 0.00
G
ATOM 21948 HN LYS G 93 2.552 -36.264 38.167 0.00 0.00
G
ATOM 21949 CA LYS G 93 2.563 -38.179 37.833 0.00 0.00
G
ATOM 21950 HA LYS G 93 3.451 -38.735 37.570 0.00 0.00
G

```
ATOM  21951  CB  LYS G  93       2.123 -38.209  39.364  0.00  0.00
G
ATOM  21952  HB1 LYS G  93       2.710 -37.457  39.934  0.00  0.00
G
ATOM  21953  HB2 LYS G  93       1.048 -37.930  39.406  0.00  0.00
G
ATOM  21954  CG  LYS G  93       2.365 -39.514  40.049  0.00  0.00
G
ATOM  21955  HG1 LYS G  93       2.010 -39.464  41.100  0.00  0.00
G
ATOM  21956  HG2 LYS G  93       1.829 -40.319  39.503  0.00  0.00
G
ATOM  21957  CD  LYS G  93       3.801 -39.945  40.184  0.00  0.00
G
ATOM  21958  HD1 LYS G  93       4.177 -40.078  39.148  0.00  0.00
G
ATOM  21959  HD2 LYS G  93       4.334 -39.122  40.707  0.00  0.00
G
ATOM  21960  CE  LYS G  93       3.837 -41.331  40.848  0.00  0.00
G
ATOM  21961  HE1 LYS G  93       3.442 -41.456  41.879  0.00  0.00
G
ATOM  21962  HE2 LYS G  93       3.127 -41.914  40.223  0.00  0.00
G
ATOM  21963  NZ  LYS G  93       5.151 -42.023  40.809  0.00  0.00
G
ATOM  21964  HZ1 LYS G  93       5.454 -42.101  39.817  0.00  0.00
G
ATOM  21965  HZ2 LYS G  93       5.899 -41.575  41.377  0.00  0.00
G
ATOM  21966  HZ3 LYS G  93       4.994 -42.954  41.243  0.00  0.00
G
ATOM  21967  C   LYS G  93       1.473 -38.862  36.983  0.00  0.00
G
ATOM  21968  O   LYS G  93       1.635 -39.985  36.580  0.00  0.00
G
ATOM  21969  N   GLU G  94       0.412 -38.164  36.587  0.00  0.00
G
ATOM  21970  HN  GLU G  94       0.401 -37.190  36.803  0.00  0.00
G
ATOM  21971  CA  GLU G  94      -0.739 -38.617  35.810  0.00  0.00
G
ATOM  21972  HA  GLU G  94      -1.260 -39.354  36.404  0.00  0.00
G
ATOM  21973  CB  GLU G  94      -1.825 -37.509  35.649  0.00  0.00
G
ATOM  21974  HB1 GLU G  94      -2.032 -36.998  36.613  0.00  0.00
G
ATOM  21975  HB2 GLU G  94      -1.511 -36.749  34.902  0.00  0.00
G
ATOM  21976  CG  GLU G  94      -3.254 -38.064  35.172  0.00  0.00
G
ATOM  21977  HG1 GLU G  94      -3.993 -37.240  35.091  0.00  0.00
G
```

```
ATOM  21978  HG2 GLU G  94      -3.216 -38.538  34.168  0.00  0.00
G
ATOM  21979  CD  GLU G  94      -3.908 -38.992  36.167  0.00  0.00
G
ATOM  21980  OE1 GLU G  94      -4.090 -40.219  35.861  0.00  0.00
G
ATOM  21981  OE2 GLU G  94      -4.177 -38.599  37.349  0.00  0.00
G
ATOM  21982  C   GLU G  94      -0.331 -39.012  34.326  0.00  0.00
G
ATOM  21983  O   GLU G  94      -0.680 -40.020  33.819  0.00  0.00
G
ATOM  21984  N   LEU G  95       0.651 -38.254  33.745  0.00  0.00
G
ATOM  21985  HN  LEU G  95       0.867 -37.421  34.248  0.00  0.00
G
ATOM  21986  CA  LEU G  95       1.381 -38.474  32.503  0.00  0.00
G
ATOM  21987  HA  LEU G  95       0.740 -38.692  31.662  0.00  0.00
G
ATOM  21988  CB  LEU G  95       2.230 -37.215  32.240  0.00  0.00
G
ATOM  21989  HB1 LEU G  95       2.877 -36.897  33.085  0.00  0.00
G
ATOM  21990  HB2 LEU G  95       2.889 -37.495  31.390  0.00  0.00
G
ATOM  21991  CG  LEU G  95       1.390 -35.967  31.954  0.00  0.00
G
ATOM  21992  HG  LEU G  95       0.672 -35.740  32.770  0.00  0.00
G
ATOM  21993  CD1 LEU G  95       2.344 -34.776  31.797  0.00  0.00
G
ATOM  21994 HD11 LEU G  95       2.977 -34.875  30.889  0.00  0.00
G
ATOM  21995 HD12 LEU G  95       1.898 -33.759  31.839  0.00  0.00
G
ATOM  21996 HD13 LEU G  95       3.035 -34.843  32.664  0.00  0.00
G
ATOM  21997  CD2 LEU G  95       0.592 -36.250  30.654  0.00  0.00
G
ATOM  21998 HD21 LEU G  95       0.202 -35.267  30.315  0.00  0.00
G
ATOM  21999 HD22 LEU G  95       1.252 -36.588  29.826  0.00  0.00
G
ATOM  22000 HD23 LEU G  95      -0.234 -36.956  30.886  0.00  0.00
G
ATOM  22001  C   LEU G  95       2.300 -39.674  32.524  0.00  0.00
G
ATOM  22002  O   LEU G  95       2.866 -40.035  31.489  0.00  0.00
G
ATOM  22003  N   ASP G  96       2.530 -40.339  33.670  0.00  0.00
G
ATOM  22004  HN  ASP G  96       2.129 -40.042  34.533  0.00  0.00
G
```

```
ATOM  22005  CA  ASP G  96       3.460 -41.348  33.978  0.00  0.00
G
ATOM  22006  HA  ASP G  96       3.456 -41.495  35.048  0.00  0.00
G
ATOM  22007  CB  ASP G  96       3.127 -42.680  33.377  0.00  0.00
G
ATOM  22008  HB1 ASP G  96       2.981 -42.650  32.276  0.00  0.00
G
ATOM  22009  HB2 ASP G  96       3.945 -43.422  33.506  0.00  0.00
G
ATOM  22010  CG  ASP G  96       1.878 -43.221  34.003  0.00  0.00
G
ATOM  22011  OD1 ASP G  96       0.833 -43.398  33.326  0.00  0.00
G
ATOM  22012  OD2 ASP G  96       1.977 -43.566  35.240  0.00  0.00
G
ATOM  22013  C   ASP G  96       4.897 -40.976  33.594  0.00  0.00
G
ATOM  22014  O   ASP G  96       5.683 -41.748  33.037  0.00  0.00
G
ATOM  22015  N   ILE G  97       5.334 -39.764  34.133  0.00  0.00
G
ATOM  22016  HN  ILE G  97       4.747 -39.235  34.741  0.00  0.00
G
ATOM  22017  CA  ILE G  97       6.750 -39.347  34.117  0.00  0.00
G
ATOM  22018  HA  ILE G  97       7.089 -39.706  33.156  0.00  0.00
G
ATOM  22019  CB  ILE G  97       6.936 -37.889  34.352  0.00  0.00
G
ATOM  22020  HB  ILE G  97       6.463 -37.641  35.326  0.00  0.00
G
ATOM  22021  CG2 ILE G  97       8.399 -37.434  34.596  0.00  0.00
G
ATOM  22022 HG21 ILE G  97       9.068 -37.974  33.894  0.00  0.00
G
ATOM  22023 HG22 ILE G  97       8.493 -36.328  34.551  0.00  0.00
G
ATOM  22024 HG23 ILE G  97       8.709 -37.676  35.635  0.00  0.00
G
ATOM  22025  CG1 ILE G  97       6.254 -37.121  33.267  0.00  0.00
G
ATOM  22026 HG11 ILE G  97       5.196 -37.453  33.327  0.00  0.00
G
ATOM  22027 HG12 ILE G  97       6.385 -36.052  33.540  0.00  0.00
G
ATOM  22028  CD  ILE G  97       6.832 -37.216  31.863  0.00  0.00
G
ATOM  22029  HD1 ILE G  97       6.891 -38.292  31.592  0.00  0.00
G
ATOM  22030  HD2 ILE G  97       6.232 -36.626  31.138  0.00  0.00
G
ATOM  22031  HD3 ILE G  97       7.856 -36.784  31.872  0.00  0.00
G
```

ATOM 22032 C ILE G 97 7.431 -40.212 35.247 0.00 0.00
G
ATOM 22033 O ILE G 97 6.875 -40.370 36.331 0.00 0.00
G
ATOM 22034 N PRO G 98 8.594 -40.845 35.081 0.00 0.00
G
ATOM 22035 CD PRO G 98 9.302 -40.946 33.820 0.00 0.00
G
ATOM 22036 HD1 PRO G 98 8.584 -41.266 33.035 0.00 0.00
G
ATOM 22037 HD2 PRO G 98 9.726 -39.966 33.515 0.00 0.00
G
ATOM 22038 CA PRO G 98 9.023 -41.817 36.026 0.00 0.00
G
ATOM 22039 HA PRO G 98 8.210 -42.312 36.537 0.00 0.00
G
ATOM 22040 CB PRO G 98 9.738 -42.848 35.242 0.00 0.00
G
ATOM 22041 HB1 PRO G 98 8.972 -43.578 34.904 0.00 0.00
G
ATOM 22042 HB2 PRO G 98 10.546 -43.365 35.802 0.00 0.00
G
ATOM 22043 CG PRO G 98 10.316 -42.035 34.104 0.00 0.00
G
ATOM 22044 HG1 PRO G 98 10.703 -42.502 33.173 0.00 0.00
G
ATOM 22045 HG2 PRO G 98 11.246 -41.587 34.512 0.00 0.00
G
ATOM 22046 C PRO G 98 9.839 -41.190 37.134 0.00 0.00
G
ATOM 22047 O PRO G 98 9.948 -41.880 38.141 0.00 0.00
G
ATOM 22048 N ASN G 99 10.553 -40.043 36.956 0.00 0.00
G
ATOM 22049 HN ASN G 99 10.468 -39.631 36.052 0.00 0.00
G
ATOM 22050 CA ASN G 99 11.336 -39.387 37.999 0.00 0.00
G
ATOM 22051 HA ASN G 99 11.060 -39.778 38.966 0.00 0.00
G
ATOM 22052 CB ASN G 99 12.858 -39.618 37.734 0.00 0.00
G
ATOM 22053 HB1 ASN G 99 13.136 -39.200 36.743 0.00 0.00
G
ATOM 22054 HB2 ASN G 99 13.428 -39.060 38.507 0.00 0.00
G
ATOM 22055 CG ASN G 99 13.105 -41.126 37.700 0.00 0.00
G
ATOM 22056 OD1 ASN G 99 13.094 -41.748 38.762 0.00 0.00
G
ATOM 22057 ND2 ASN G 99 13.335 -41.693 36.506 0.00 0.00
G
ATOM 22058 HD21 ASN G 99 13.339 -42.686 36.386 0.00 0.00
G

```
ATOM  22059 HD22 ASN G  99      13.361 -41.075  35.720  0.00  0.00
G
ATOM  22060  C   ASN G  99      11.056 -37.874  37.997  0.00  0.00
G
ATOM  22061  O   ASN G  99      11.659 -37.111  37.208  0.00  0.00
G
ATOM  22062  N   ILE G 100      10.321 -37.403  39.027  0.00  0.00
G
ATOM  22063  HN  ILE G 100      10.009 -37.984  39.774  0.00  0.00
G
ATOM  22064  CA  ILE G 100       9.877 -35.995  39.244  0.00  0.00
G
ATOM  22065  HA  ILE G 100      10.108 -35.365  38.398  0.00  0.00
G
ATOM  22066  CB  ILE G 100       8.374 -35.994  39.556  0.00  0.00
G
ATOM  22067  HB  ILE G 100       8.248 -36.669  40.430  0.00  0.00
G
ATOM  22068  CG2 ILE G 100       7.856 -34.570  39.898  0.00  0.00
G
ATOM  22069 HG21 ILE G 100       8.247 -34.156  40.852  0.00  0.00
G
ATOM  22070 HG22 ILE G 100       8.114 -33.864  39.079  0.00  0.00
G
ATOM  22071 HG23 ILE G 100       6.750 -34.623  39.991  0.00  0.00
G
ATOM  22072  CG1 ILE G 100       7.513 -36.620  38.391  0.00  0.00
G
ATOM  22073 HG11 ILE G 100       7.584 -35.956  37.503  0.00  0.00
G
ATOM  22074 HG12 ILE G 100       8.051 -37.511  38.005  0.00  0.00
G
ATOM  22075  CD  ILE G 100       6.117 -37.104  38.726  0.00  0.00
G
ATOM  22076  HD1 ILE G 100       5.726 -37.749  37.911  0.00  0.00
G
ATOM  22077  HD2 ILE G 100       6.338 -37.717  39.626  0.00  0.00
G
ATOM  22078  HD3 ILE G 100       5.391 -36.279  38.886  0.00  0.00
G
ATOM  22079  C   ILE G 100      10.530 -35.544  40.464  0.00  0.00
G
ATOM  22080  O   ILE G 100      10.466 -36.154  41.549  0.00  0.00
G
ATOM  22081  N   TRP G 101      11.368 -34.460  40.395  0.00  0.00
G
ATOM  22082  HN  TRP G 101      11.607 -33.930  39.585  0.00  0.00
G
ATOM  22083  CA  TRP G 101      12.107 -33.998  41.579  0.00  0.00
G
ATOM  22084  HA  TRP G 101      11.884 -34.635  42.421  0.00  0.00
G
ATOM  22085  CB  TRP G 101      13.644 -34.048  41.379  0.00  0.00
G
```

ATOM 22086 HB1 TRP G 101   13.990 -33.212 40.734 0.00 0.00
G
ATOM 22087 HB2 TRP G 101   14.057 -33.896 42.399 0.00 0.00
G
ATOM 22088 CG TRP G 101   14.377 -35.290 41.119 0.00 0.00
G
ATOM 22089 CD1 TRP G 101   13.905 -36.576 41.100 0.00 0.00
G
ATOM 22090 HD1 TRP G 101   12.862 -36.792 41.279 0.00 0.00
G
ATOM 22091 NE1 TRP G 101   15.055 -37.477 40.897 0.00 0.00
G
ATOM 22092 HE1 TRP G 101   15.024 -38.421 41.143 0.00 0.00
G
ATOM 22093 CE2 TRP G 101   16.184 -36.674 40.851 0.00 0.00
G
ATOM 22094 CD2 TRP G 101   15.821 -35.374 41.041 0.00 0.00
G
ATOM 22095 CE3 TRP G 101   16.783 -34.339 41.034 0.00 0.00
G
ATOM 22096 HE3 TRP G 101   16.447 -33.337 41.258 0.00 0.00
G
ATOM 22097 CZ3 TRP G 101   18.123 -34.649 40.991 0.00 0.00
G
ATOM 22098 HZ3 TRP G 101   18.835 -33.839 41.040 0.00 0.00
G
ATOM 22099 CZ2 TRP G 101   17.491 -37.000 40.738 0.00 0.00
G
ATOM 22100 HZ2 TRP G 101   17.705 -38.058 40.782 0.00 0.00
G
ATOM 22101 CH2 TRP G 101   18.470 -36.001 40.908 0.00 0.00
G
ATOM 22102 HH2 TRP G 101   19.487 -36.345 41.024 0.00 0.00
G
ATOM 22103 C   TRP G 101   11.689 -32.602 41.939 0.00 0.00
G
ATOM 22104 O   TRP G 101   11.826 -31.638 41.218 0.00 0.00
G
ATOM 22105 N   VAL G 102   11.251 -32.488 43.187 0.00 0.00
G
ATOM 22106 HN VAL G 102   11.297 -33.220 43.862 0.00 0.00
G
ATOM 22107 CA VAL G 102   10.519 -31.205 43.554 0.00 0.00
G
ATOM 22108 HA VAL G 102   10.619 -30.488 42.753 0.00 0.00
G
ATOM 22109 CB VAL G 102   9.072 -31.449 43.885 0.00 0.00
G
ATOM 22110 HB VAL G 102   9.016 -31.876 44.909 0.00 0.00
G
ATOM 22111 CG1 VAL G 102   8.228 -30.157 43.756 0.00 0.00
G
ATOM 22112 HG11 VAL G 102   7.205 -30.521 43.523 0.00 0.00
G

```
ATOM  22113 HG12 VAL G 102       8.320 -29.599  44.713  0.00  0.00
G
ATOM  22114 HG13 VAL G 102       8.650 -29.530  42.942  0.00  0.00
G
ATOM  22115  CG2 VAL G 102       8.511 -32.543  42.930  0.00  0.00
G
ATOM  22116 HG21 VAL G 102       8.751 -33.558  43.311  0.00  0.00
G
ATOM  22117 HG22 VAL G 102       7.408 -32.459  43.034  0.00  0.00
G
ATOM  22118 HG23 VAL G 102       8.721 -32.429  41.845  0.00  0.00
G
ATOM  22119  C   VAL G 102      11.224 -30.429  44.653  0.00  0.00
G
ATOM  22120  O   VAL G 102      11.669 -31.065  45.574  0.00  0.00
G
ATOM  22121  N   LYS G 103      11.400 -29.110  44.556  0.00  0.00
G
ATOM  22122  HN  LYS G 103      11.046 -28.678  43.730  0.00  0.00
G
ATOM  22123  CA  LYS G 103      11.963 -28.293  45.613  0.00  0.00
G
ATOM  22124  HA  LYS G 103      12.841 -28.769  46.023  0.00  0.00
G
ATOM  22125  CB  LYS G 103      12.335 -26.823  45.208  0.00  0.00
G
ATOM  22126  HB1 LYS G 103      12.662 -26.954  44.154  0.00  0.00
G
ATOM  22127  HB2 LYS G 103      11.433 -26.201  45.026  0.00  0.00
G
ATOM  22128  CG  LYS G 103      13.415 -26.117  45.964  0.00  0.00
G
ATOM  22129  HG1 LYS G 103      13.067 -26.097  47.019  0.00  0.00
G
ATOM  22130  HG2 LYS G 103      14.424 -26.563  45.834  0.00  0.00
G
ATOM  22131  CD  LYS G 103      13.519 -24.633  45.427  0.00  0.00
G
ATOM  22132  HD1 LYS G 103      13.914 -24.628  44.388  0.00  0.00
G
ATOM  22133  HD2 LYS G 103      12.583 -24.034  45.416  0.00  0.00
G
ATOM  22134  CE  LYS G 103      14.561 -23.874  46.291  0.00  0.00
G
ATOM  22135  HE1 LYS G 103      14.340 -24.128  47.350  0.00  0.00
G
ATOM  22136  HE2 LYS G 103      15.536 -24.395  46.179  0.00  0.00
G
ATOM  22137  NZ  LYS G 103      14.596 -22.415  46.148  0.00  0.00
G
ATOM  22138  HZ1 LYS G 103      13.597 -22.128  46.143  0.00  0.00
G
ATOM  22139  HZ2 LYS G 103      15.135 -22.020  46.946  0.00  0.00
G
```

ATOM 22140 HZ3 LYS G 103 14.944 -22.069 45.232 0.00 0.00
G
ATOM 22141 C LYS G 103 10.963 -28.243 46.888 0.00 0.00
G
ATOM 22142 O LYS G 103 9.733 -28.427 46.727 0.00 0.00
G
ATOM 22143 N ALA G 104 11.501 -28.064 48.139 0.00 0.00
G
ATOM 22144 HN ALA G 104 12.470 -28.084 48.374 0.00 0.00
G
ATOM 22145 CA ALA G 104 10.684 -27.824 49.252 0.00 0.00
G
ATOM 22146 HA ALA G 104 9.858 -27.176 48.996 0.00 0.00
G
ATOM 22147 CB ALA G 104 10.304 -29.118 49.892 0.00 0.00
G
ATOM 22148 HB1 ALA G 104 9.682 -29.024 50.808 0.00 0.00
G
ATOM 22149 HB2 ALA G 104 9.754 -29.743 49.157 0.00 0.00
G
ATOM 22150 HB3 ALA G 104 11.162 -29.713 50.272 0.00 0.00
G
ATOM 22151 C ALA G 104 11.550 -27.031 50.231 0.00 0.00
G
ATOM 22152 O ALA G 104 12.690 -26.744 50.004 0.00 0.00
G
ATOM 22153 N GLN G 105 10.929 -26.680 51.361 0.00 0.00
G
ATOM 22154 HN GLN G 105 9.961 -26.850 51.532 0.00 0.00
G
ATOM 22155 CA GLN G 105 11.683 -25.976 52.441 0.00 0.00
G
ATOM 22156 HA GLN G 105 12.752 -26.065 52.313 0.00 0.00
G
ATOM 22157 CB GLN G 105 11.456 -24.483 52.422 0.00 0.00
G
ATOM 22158 HB1 GLN G 105 12.314 -23.998 52.934 0.00 0.00
G
ATOM 22159 HB2 GLN G 105 11.502 -24.081 51.387 0.00 0.00
G
ATOM 22160 CG GLN G 105 10.067 -24.061 52.899 0.00 0.00
G
ATOM 22161 HG1 GLN G 105 9.388 -24.422 52.097 0.00 0.00
G
ATOM 22162 HG2 GLN G 105 9.811 -24.532 53.872 0.00 0.00
G
ATOM 22163 CD GLN G 105 9.953 -22.529 52.954 0.00 0.00
G
ATOM 22164 OE1 GLN G 105 10.038 -21.851 53.979 0.00 0.00
G
ATOM 22165 NE2 GLN G 105 9.950 -21.959 51.725 0.00 0.00
G
ATOM 22166 HE21 GLN G 105 10.096 -20.972 51.655 0.00 0.00
G

ATOM 22167 HE22 GLN G 105 9.824 -22.450 50.863 0.00 0.00
G
ATOM 22168 C GLN G 105 11.459 -26.418 53.861 0.00 0.00
G
ATOM 22169 O GLN G 105 12.063 -25.831 54.767 0.00 0.00
G
ATOM 22170 N ASN G 106 10.584 -27.421 54.079 0.00 0.00
G
ATOM 22171 HN ASN G 106 10.045 -27.910 53.397 0.00 0.00
G
ATOM 22172 CA ASN G 106 10.400 -27.936 55.366 0.00 0.00
G
ATOM 22173 HA ASN G 106 11.386 -28.176 55.737 0.00 0.00
G
ATOM 22174 CB ASN G 106 9.663 -26.912 56.280 0.00 0.00
G
ATOM 22175 HB1 ASN G 106 9.741 -27.260 57.333 0.00 0.00
G
ATOM 22176 HB2 ASN G 106 10.115 -25.899 56.222 0.00 0.00
G
ATOM 22177 CG ASN G 106 8.152 -26.841 56.032 0.00 0.00
G
ATOM 22178 OD1 ASN G 106 7.410 -27.824 56.198 0.00 0.00
G
ATOM 22179 ND2 ASN G 106 7.652 -25.637 55.658 0.00 0.00
G
ATOM 22180 HD21 ASN G 106 6.697 -25.435 55.874 0.00 0.00
G
ATOM 22181 HD22 ASN G 106 8.273 -24.866 55.797 0.00 0.00
G
ATOM 22182 C ASN G 106 9.737 -29.333 55.278 0.00 0.00
G
ATOM 22183 O ASN G 106 9.565 -29.856 54.148 0.00 0.00
G
ATOM 22184 N TYR G 107 9.543 -29.985 56.507 0.00 0.00
G
ATOM 22185 HN TYR G 107 9.902 -29.513 57.307 0.00 0.00
G
ATOM 22186 CA TYR G 107 9.118 -31.360 56.642 0.00 0.00
G
ATOM 22187 HA TYR G 107 9.715 -31.962 55.973 0.00 0.00
G
ATOM 22188 CB TYR G 107 9.448 -31.719 58.073 0.00 0.00
G
ATOM 22189 HB1 TYR G 107 10.544 -31.569 58.174 0.00 0.00
G
ATOM 22190 HB2 TYR G 107 8.971 -31.061 58.830 0.00 0.00
G
ATOM 22191 CG TYR G 107 9.063 -33.191 58.317 0.00 0.00
G
ATOM 22192 CD1 TYR G 107 7.976 -33.459 59.173 0.00 0.00
G
ATOM 22193 HD1 TYR G 107 7.428 -32.624 59.584 0.00 0.00
G

ATOM 22194 CE1 TYR G 107   7.566 -34.785 59.340 0.00 0.00
G
ATOM 22195 HE1 TYR G 107   6.733 -35.000 59.993 0.00 0.00
G
ATOM 22196 CZ TYR G 107   8.376 -35.827 58.974 0.00 0.00
G
ATOM 22197 OH TYR G 107   7.995 -37.168 59.310 0.00 0.00
G
ATOM 22198 HH TYR G 107   7.192 -37.133 59.836 0.00 0.00
G
ATOM 22199 CD2 TYR G 107   9.891 -34.227 57.847 0.00 0.00
G
ATOM 22200 HD2 TYR G 107   10.731 -34.030 57.198 0.00 0.00
G
ATOM 22201 CE2 TYR G 107   9.480 -35.553 58.165 0.00 0.00
G
ATOM 22202 HE2 TYR G 107   10.143 -36.320 57.793 0.00 0.00
G
ATOM 22203 C   TYR G 107   7.708 -31.547 56.312 0.00 0.00
G
ATOM 22204 O   TYR G 107   7.338 -32.551 55.713 0.00 0.00
G
ATOM 22205 N   TYR G 108   6.792 -30.603 56.667 0.00 0.00
G
ATOM 22206 HN TYR G 108   7.158 -29.854 57.215 0.00 0.00
G
ATOM 22207 CA TYR G 108   5.334 -30.617 56.360 0.00 0.00
G
ATOM 22208 HA TYR G 108   4.938 -31.592 56.599 0.00 0.00
G
ATOM 22209 CB TYR G 108   4.619 -29.495 57.275 0.00 0.00
G
ATOM 22210 HB1 TYR G 108   4.960 -28.507 56.899 0.00 0.00
G
ATOM 22211 HB2 TYR G 108   3.511 -29.516 57.195 0.00 0.00
G
ATOM 22212 CG TYR G 108   4.922 -29.662 58.837 0.00 0.00
G
ATOM 22213 CD1 TYR G 108   4.177 -30.553 59.563 0.00 0.00
G
ATOM 22214 HD1 TYR G 108   3.364 -31.083 59.090 0.00 0.00
G
ATOM 22215 CE1 TYR G 108   4.475 -30.691 60.916 0.00 0.00
G
ATOM 22216 HE1 TYR G 108   3.903 -31.443 61.440 0.00 0.00
G
ATOM 22217 CZ TYR G 108   5.458 -30.007 61.555 0.00 0.00
G
ATOM 22218 OH TYR G 108   5.649 -30.253 62.973 0.00 0.00
G
ATOM 22219 HH TYR G 108   4.995 -30.876 63.299 0.00 0.00
G
ATOM 22220 CD2 TYR G 108   6.010 -28.935 59.445 0.00 0.00
G

ATOM 22221 HD2 TYR G 108 6.734 −28.338 58.910 0.00 0.00
G
ATOM 22222 CE2 TYR G 108 6.313 −29.148 60.816 0.00 0.00
G
ATOM 22223 HE2 TYR G 108 7.155 −28.667 61.291 0.00 0.00
G
ATOM 22224 C TYR G 108 5.038 −30.418 54.944 0.00 0.00
G
ATOM 22225 O TYR G 108 4.210 −31.126 54.368 0.00 0.00
G
ATOM 22226 N HSE G 109 5.693 −29.433 54.317 0.00 0.00
G
ATOM 22227 HN HSE G 109 6.418 −28.970 54.821 0.00 0.00
G
ATOM 22228 CA HSE G 109 5.755 −29.125 52.951 0.00 0.00
G
ATOM 22229 HA HSE G 109 4.741 −28.879 52.672 0.00 0.00
G
ATOM 22230 CB HSE G 109 6.740 −27.899 52.864 0.00 0.00
G
ATOM 22231 HB1 HSE G 109 6.352 −27.147 53.585 0.00 0.00
G
ATOM 22232 HB2 HSE G 109 7.759 −28.259 53.122 0.00 0.00
G
ATOM 22233 ND1 HSE G 109 8.043 −26.523 51.356 0.00 0.00
G
ATOM 22234 CG HSE G 109 6.909 −27.334 51.471 0.00 0.00
G
ATOM 22235 CE1 HSE G 109 8.066 −26.148 50.103 0.00 0.00
G
ATOM 22236 HE1 HSE G 109 8.755 −25.503 49.559 0.00 0.00
G
ATOM 22237 NE2 HSE G 109 7.027 −26.754 49.423 0.00 0.00
G
ATOM 22238 HE2 HSE G 109 7.097 −26.779 48.425 0.00 0.00
G
ATOM 22239 CD2 HSE G 109 6.339 −27.541 50.278 0.00 0.00
G
ATOM 22240 HD2 HSE G 109 5.422 −28.011 49.944 0.00 0.00
G
ATOM 22241 C HSE G 109 6.247 −30.303 52.176 0.00 0.00
G
ATOM 22242 O HSE G 109 5.810 −30.667 51.110 0.00 0.00
G
ATOM 22243 N HSE G 110 7.356 −30.958 52.657 0.00 0.00
G
ATOM 22244 HN HSE G 110 7.895 −30.757 53.471 0.00 0.00
G
ATOM 22245 CA HSE G 110 7.946 −32.148 51.998 0.00 0.00
G
ATOM 22246 HA HSE G 110 8.335 −31.958 51.009 0.00 0.00
G
ATOM 22247 CB HSE G 110 9.122 −32.627 52.888 0.00 0.00
G

ATOM 22248 HB1 HSE G 110 9.878 -31.826 53.032 0.00 0.00
G
ATOM 22249 HB2 HSE G 110 8.806 -32.938 53.906 0.00 0.00
G
ATOM 22250 ND1 HSE G 110 11.308 -33.613 51.980 0.00 0.00
G
ATOM 22251 CG HSE G 110 9.899 -33.773 52.335 0.00 0.00
G
ATOM 22252 CE1 HSE G 110 11.718 -34.860 51.802 0.00 0.00
G
ATOM 22253 HE1 HSE G 110 12.754 -35.063 51.529 0.00 0.00
G
ATOM 22254 NE2 HSE G 110 10.676 -35.737 51.845 0.00 0.00
G
ATOM 22255 HE2 HSE G 110 10.667 -36.719 51.654 0.00 0.00
G
ATOM 22256 CD2 HSE G 110 9.508 -35.048 52.249 0.00 0.00
G
ATOM 22257 HD2 HSE G 110 8.570 -35.544 52.469 0.00 0.00
G
ATOM 22258 C HSE G 110 6.996 -33.365 51.987 0.00 0.00
G
ATOM 22259 O HSE G 110 6.888 -34.088 50.928 0.00 0.00
G
ATOM 22260 N LYS G 111 6.323 -33.714 53.147 0.00 0.00
G
ATOM 22261 HN LYS G 111 6.215 -33.098 53.924 0.00 0.00
G
ATOM 22262 CA LYS G 111 5.356 -34.791 53.362 0.00 0.00
G
ATOM 22263 HA LYS G 111 5.878 -35.648 52.962 0.00 0.00
G
ATOM 22264 CB LYS G 111 5.011 -34.869 54.843 0.00 0.00
G
ATOM 22265 HB1 LYS G 111 5.084 -33.844 55.264 0.00 0.00
G
ATOM 22266 HB2 LYS G 111 4.012 -35.294 55.079 0.00 0.00
G
ATOM 22267 CG LYS G 111 6.081 -35.643 55.518 0.00 0.00
G
ATOM 22268 HG1 LYS G 111 7.081 -35.240 55.249 0.00 0.00
G
ATOM 22269 HG2 LYS G 111 6.034 -35.441 56.609 0.00 0.00
G
ATOM 22270 CD LYS G 111 6.168 -37.107 55.259 0.00 0.00
G
ATOM 22271 HD1 LYS G 111 5.360 -37.744 55.678 0.00 0.00
G
ATOM 22272 HD2 LYS G 111 5.972 -37.312 54.185 0.00 0.00
G
ATOM 22273 CE LYS G 111 7.632 -37.634 55.610 0.00 0.00
G
ATOM 22274 HE1 LYS G 111 8.412 -37.035 55.093 0.00 0.00
G

ATOM 22275 HE2 LYS G 111   7.873 -37.493 56.685 0.00 0.00
G
ATOM 22276 NZ LYS G 111   7.777 -39.063 55.220 0.00 0.00
G
ATOM 22277 HZ1 LYS G 111   7.197 -39.687 55.817 0.00 0.00
G
ATOM 22278 HZ2 LYS G 111   7.505 -39.141 54.219 0.00 0.00
G
ATOM 22279 HZ3 LYS G 111   8.747 -39.402 55.380 0.00 0.00
G
ATOM 22280 C   LYS G 111   4.169 -34.635 52.392 0.00 0.00
G
ATOM 22281 O   LYS G 111   3.741 -35.542 51.718 0.00 0.00
G
ATOM 22282 N   VAL G 112   3.666 -33.368 52.124 0.00 0.00
G
ATOM 22283 HN VAL G 112   4.032 -32.613 52.663 0.00 0.00
G
ATOM 22284 CA VAL G 112   2.645 -33.177 51.025 0.00 0.00
G
ATOM 22285 HA VAL G 112   1.792 -33.792 51.270 0.00 0.00
G
ATOM 22286 CB VAL G 112   2.290 -31.688 50.941 0.00 0.00
G
ATOM 22287 HB VAL G 112   3.208 -31.105 50.712 0.00 0.00
G
ATOM 22288 CG1 VAL G 112   1.284 -31.323 49.814 0.00 0.00
G
ATOM 22289 HG11 VAL G 112   1.654 -31.591 48.802 0.00 0.00
G
ATOM 22290 HG12 VAL G 112   0.393 -31.982 49.900 0.00 0.00
G
ATOM 22291 HG13 VAL G 112   1.030 -30.256 49.987 0.00 0.00
G
ATOM 22292 CG2 VAL G 112   1.693 -31.277 52.350 0.00 0.00
G
ATOM 22293 HG21 VAL G 112   1.347 -32.188 52.883 0.00 0.00
G
ATOM 22294 HG22 VAL G 112   2.498 -30.975 53.053 0.00 0.00
G
ATOM 22295 HG23 VAL G 112   0.874 -30.536 52.228 0.00 0.00
G
ATOM 22296 C   VAL G 112   3.098 -33.693 49.654 0.00 0.00
G
ATOM 22297 O   VAL G 112   2.375 -34.443 49.014 0.00 0.00
G
ATOM 22298 N   LEU G 113   4.281 -33.345 49.149 0.00 0.00
G
ATOM 22299 HN LEU G 113   4.762 -32.830 49.854 0.00 0.00
G
ATOM 22300 CA LEU G 113   4.803 -33.741 47.869 0.00 0.00
G
ATOM 22301 HA LEU G 113   4.031 -33.605 47.126 0.00 0.00
G

```
ATOM  22302  CB  LEU G 113       6.065 -32.890  47.538  0.00  0.00
G
ATOM  22303  HB1 LEU G 113       6.907 -33.119  48.226  0.00  0.00
G
ATOM  22304  HB2 LEU G 113       6.319 -33.040  46.467  0.00  0.00
G
ATOM  22305  CG  LEU G 113       5.899 -31.324  47.641  0.00  0.00
G
ATOM  22306  HG  LEU G 113       5.070 -31.050  48.329  0.00  0.00
G
ATOM  22307  CD1 LEU G 113       7.182 -30.543  47.994  0.00  0.00
G
ATOM  22308 HD11 LEU G 113       7.655 -30.804  48.965  0.00  0.00
G
ATOM  22309 HD12 LEU G 113       7.867 -30.720  47.137  0.00  0.00
G
ATOM  22310 HD13 LEU G 113       6.995 -29.450  48.073  0.00  0.00
G
ATOM  22311  CD2 LEU G 113       5.410 -30.856  46.281  0.00  0.00
G
ATOM  22312 HD21 LEU G 113       4.664 -31.536  45.817  0.00  0.00
G
ATOM  22313 HD22 LEU G 113       5.085 -29.794  46.324  0.00  0.00
G
ATOM  22314 HD23 LEU G 113       6.297 -30.835  45.613  0.00  0.00
G
ATOM  22315  C   LEU G 113       5.087 -35.258  47.793  0.00  0.00
G
ATOM  22316  O   LEU G 113       4.744 -35.904  46.819  0.00  0.00
G
ATOM  22317  N   GLU G 114       5.679 -35.797  48.869  0.00  0.00
G
ATOM  22318  HN  GLU G 114       6.134 -35.253  49.570  0.00  0.00
G
ATOM  22319  CA  GLU G 114       5.841 -37.227  49.055  0.00  0.00
G
ATOM  22320  HA  GLU G 114       6.481 -37.610  48.273  0.00  0.00
G
ATOM  22321  CB  GLU G 114       6.554 -37.598  50.343  0.00  0.00
G
ATOM  22322  HB1 GLU G 114       7.580 -37.180  50.436  0.00  0.00
G
ATOM  22323  HB2 GLU G 114       6.043 -37.096  51.192  0.00  0.00
G
ATOM  22324  CG  GLU G 114       6.765 -39.139  50.611  0.00  0.00
G
ATOM  22325  HG1 GLU G 114       5.761 -39.600  50.725  0.00  0.00
G
ATOM  22326  HG2 GLU G 114       7.159 -39.607  49.683  0.00  0.00
G
ATOM  22327  CD  GLU G 114       7.642 -39.410  51.789  0.00  0.00
G
ATOM  22328  OE1 GLU G 114       8.913 -39.378  51.731  0.00  0.00
G
```

ATOM 22329 OE2 GLU G 114 6.975 −39.809 52.812 0.00 0.00
G
ATOM 22330 C GLU G 114 4.532 −38.064 48.842 0.00 0.00
G
ATOM 22331 O GLU G 114 4.637 −39.155 48.291 0.00 0.00
G
ATOM 22332 N LYS G 115 3.418 −37.553 49.284 0.00 0.00
G
ATOM 22333 HN LYS G 115 3.381 −36.753 49.877 0.00 0.00
G
ATOM 22334 CA LYS G 115 2.182 −38.363 49.125 0.00 0.00
G
ATOM 22335 HA LYS G 115 2.516 −39.387 49.204 0.00 0.00
G
ATOM 22336 CB LYS G 115 1.224 −38.236 50.308 0.00 0.00
G
ATOM 22337 HB1 LYS G 115 0.766 −37.238 50.141 0.00 0.00
G
ATOM 22338 HB2 LYS G 115 0.512 −39.063 50.099 0.00 0.00
G
ATOM 22339 CG LYS G 115 1.918 −38.508 51.644 0.00 0.00
G
ATOM 22340 HG1 LYS G 115 2.482 −39.465 51.621 0.00 0.00
G
ATOM 22341 HG2 LYS G 115 2.721 −37.772 51.864 0.00 0.00
G
ATOM 22342 CD LYS G 115 0.830 −38.542 52.675 0.00 0.00
G
ATOM 22343 HD1 LYS G 115 0.169 −37.663 52.513 0.00 0.00
G
ATOM 22344 HD2 LYS G 115 0.212 −39.442 52.471 0.00 0.00
G
ATOM 22345 CE LYS G 115 1.119 −38.478 54.151 0.00 0.00
G
ATOM 22346 HE1 LYS G 115 1.665 −37.559 54.456 0.00 0.00
G
ATOM 22347 HE2 LYS G 115 0.197 −38.654 54.747 0.00 0.00
G
ATOM 22348 NZ LYS G 115 2.073 −39.576 54.539 0.00 0.00
G
ATOM 22349 HZ1 LYS G 115 2.983 −39.515 54.040 0.00 0.00
G
ATOM 22350 HZ2 LYS G 115 2.318 −39.448 55.541 0.00 0.00
G
ATOM 22351 HZ3 LYS G 115 1.662 −40.531 54.504 0.00 0.00
G
ATOM 22352 C LYS G 115 1.409 −38.246 47.790 0.00 0.00
G
ATOM 22353 O LYS G 115 0.566 −39.063 47.391 0.00 0.00
G
ATOM 22354 N ILE G 116 1.707 −37.213 47.074 0.00 0.00
G
ATOM 22355 HN ILE G 116 2.139 −36.436 47.525 0.00 0.00
G

ATOM 22356 CA ILE G 116   1.165 -37.004 45.826 0.00 0.00
G
ATOM 22357 HA ILE G 116   0.206 -37.480 45.686 0.00 0.00
G
ATOM 22358 CB ILE G 116   0.761 -35.544 45.585 0.00 0.00
G
ATOM 22359 HB ILE G 116   0.143 -35.399 44.673 0.00 0.00
G
ATOM 22360 CG2 ILE G 116   -0.149 -35.082 46.714 0.00 0.00
G
ATOM 22361 HG21 ILE G 116   -1.033 -35.744 46.839 0.00 0.00
G
ATOM 22362 HG22 ILE G 116   0.377 -35.075 47.692 0.00 0.00
G
ATOM 22363 HG23 ILE G 116   -0.648 -34.108 46.522 0.00 0.00
G
ATOM 22364 CG1 ILE G 116   1.933 -34.548 45.451 0.00 0.00
G
ATOM 22365 HG11 ILE G 116   2.676 -34.843 46.223 0.00 0.00
G
ATOM 22366 HG12 ILE G 116   2.390 -34.584 44.439 0.00 0.00
G
ATOM 22367 CD ILE G 116   1.692 -33.033 45.710 0.00 0.00
G
ATOM 22368 HD1 ILE G 116   2.561 -32.352 45.589 0.00 0.00
G
ATOM 22369 HD2 ILE G 116   0.946 -32.758 44.934 0.00 0.00
G
ATOM 22370 HD3 ILE G 116   1.295 -32.949 46.745 0.00 0.00
G
ATOM 22371 C   ILE G 116   2.017 -37.378 44.671 0.00 0.00
G
ATOM 22372 O   ILE G 116   1.609 -37.424 43.524 0.00 0.00
G
ATOM 22373 N   GLY G 117   3.143 -37.962 45.005 0.00 0.00
G
ATOM 22374 HN GLY G 117   3.337 -38.008 45.982 0.00 0.00
G
ATOM 22375 CA GLY G 117   3.989 -38.791 44.119 0.00 0.00
G
ATOM 22376 HA1 GLY G 117   3.436 -39.198 43.285 0.00 0.00
G
ATOM 22377 HA2 GLY G 117   4.281 -39.602 44.768 0.00 0.00
G
ATOM 22378 C   GLY G 117   5.226 -38.019 43.561 0.00 0.00
G
ATOM 22379 O   GLY G 117   5.823 -38.291 42.472 0.00 0.00
G
ATOM 22380 N   ALA G 118   5.802 -37.129 44.391 0.00 0.00
G
ATOM 22381 HN ALA G 118   5.398 -36.849 45.258 0.00 0.00
G
ATOM 22382 CA ALA G 118   7.228 -36.657 44.219 0.00 0.00
G

ATOM 22383 HA ALA G 118   7.365 -36.331 43.198 0.00 0.00
G
ATOM 22384 CB ALA G 118   7.444 -35.388 45.043 0.00 0.00
G
ATOM 22385 HB1 ALA G 118   7.001 -35.424 46.061 0.00 0.00
G
ATOM 22386 HB2 ALA G 118   8.532 -35.225 45.199 0.00 0.00
G
ATOM 22387 HB3 ALA G 118   7.032 -34.531 44.469 0.00 0.00
G
ATOM 22388 C   ALA G 118   8.272 -37.756 44.600 0.00 0.00
G
ATOM 22389 O   ALA G 118   8.406 -38.217 45.774 0.00 0.00
G
ATOM 22390 N   ASP G 119   9.151 -38.156 43.630 0.00 0.00
G
ATOM 22391 HN ASP G 119   9.212 -37.725 42.732 0.00 0.00
G
ATOM 22392 CA ASP G 119   10.247 -39.071 43.852 0.00 0.00
G
ATOM 22393 HA ASP G 119   9.854 -39.871 44.461 0.00 0.00
G
ATOM 22394 CB ASP G 119   10.857 -39.584 42.551 0.00 0.00
G
ATOM 22395 HB1 ASP G 119   11.368 -38.754 42.018 0.00 0.00
G
ATOM 22396 HB2 ASP G 119   11.580 -40.403 42.755 0.00 0.00
G
ATOM 22397 CG ASP G 119   9.740 -40.098 41.654 0.00 0.00
G
ATOM 22398 OD1 ASP G 119   9.238 -39.266 40.851 0.00 0.00
G
ATOM 22399 OD2 ASP G 119   9.315 -41.246 41.849 0.00 0.00
G
ATOM 22400 C   ASP G 119   11.476 -38.588 44.701 0.00 0.00
G
ATOM 22401 O   ASP G 119   11.980 -39.391 45.491 0.00 0.00
G
ATOM 22402 N   ARG G 120   11.963 -37.330 44.594 0.00 0.00
G
ATOM 22403 HN ARG G 120   11.576 -36.799 43.844 0.00 0.00
G
ATOM 22404 CA ARG G 120   13.017 -36.821 45.437 0.00 0.00
G
ATOM 22405 HA ARG G 120   13.007 -37.319 46.395 0.00 0.00
G
ATOM 22406 CB ARG G 120   14.485 -37.013 44.783 0.00 0.00
G
ATOM 22407 HB1 ARG G 120   14.716 -38.099 44.815 0.00 0.00
G
ATOM 22408 HB2 ARG G 120   14.665 -36.709 43.730 0.00 0.00
G
ATOM 22409 CG ARG G 120   15.516 -36.320 45.596 0.00 0.00
G

```
ATOM  22410  HG1 ARG G 120      15.270 -35.237  45.615  0.00  0.00
G
ATOM  22411  HG2 ARG G 120      15.450 -36.703  46.637  0.00  0.00
G
ATOM  22412  CD  ARG G 120      16.984 -36.477  45.044  0.00  0.00
G
ATOM  22413  HD1 ARG G 120      17.308 -37.486  45.378  0.00  0.00
G
ATOM  22414  HD2 ARG G 120      16.910 -36.497  43.936  0.00  0.00
G
ATOM  22415  NE  ARG G 120      17.850 -35.490  45.778  0.00  0.00
G
ATOM  22416  HE  ARG G 120      17.373 -34.923  46.450  0.00  0.00
G
ATOM  22417  CZ  ARG G 120      19.210 -35.662  45.660  0.00  0.00
G
ATOM  22418  NH1 ARG G 120      19.744 -36.554  44.757  0.00  0.00
G
ATOM  22419 HH11 ARG G 120      20.727 -36.713  44.672  0.00  0.00
G
ATOM  22420 HH12 ARG G 120      19.090 -37.206  44.372  0.00  0.00
G
ATOM  22421  NH2 ARG G 120      20.018 -34.841  46.341  0.00  0.00
G
ATOM  22422 HH21 ARG G 120      20.999 -34.949  46.178  0.00  0.00
G
ATOM  22423 HH22 ARG G 120      19.678 -34.458  47.200  0.00  0.00
G
ATOM  22424  C   ARG G 120      12.815 -35.354  45.707  0.00  0.00
G
ATOM  22425  O   ARG G 120      12.459 -34.611  44.795  0.00  0.00
G
ATOM  22426  N   ILE G 121      12.867 -34.880  46.977  0.00  0.00
G
ATOM  22427  HN  ILE G 121      13.033 -35.488  47.749  0.00  0.00
G
ATOM  22428  CA  ILE G 121      12.383 -33.607  47.389  0.00  0.00
G
ATOM  22429  HA  ILE G 121      12.127 -33.128  46.455  0.00  0.00
G
ATOM  22430  CB  ILE G 121      11.149 -33.822  48.309  0.00  0.00
G
ATOM  22431  HB  ILE G 121      11.430 -34.143  49.335  0.00  0.00
G
ATOM  22432  CG2 ILE G 121      10.544 -32.412  48.465  0.00  0.00
G
ATOM  22433 HG21 ILE G 121       9.815 -32.448  49.302  0.00  0.00
G
ATOM  22434 HG22 ILE G 121      11.291 -31.687  48.854  0.00  0.00
G
ATOM  22435 HG23 ILE G 121      10.152 -32.059  47.487  0.00  0.00
G
ATOM  22436  CG1 ILE G 121      10.056 -34.781  47.696  0.00  0.00
G
```

```
ATOM  22437 HG11 ILE G 121       9.516 -34.114  46.991  0.00  0.00
G
ATOM  22438 HG12 ILE G 121      10.550 -35.669  47.246  0.00  0.00
G
ATOM  22439  CD  ILE G 121       9.064 -35.216  48.744  0.00  0.00
G
ATOM  22440  HD1 ILE G 121       8.447 -35.956  48.190  0.00  0.00
G
ATOM  22441  HD2 ILE G 121       9.491 -35.909  49.500  0.00  0.00
G
ATOM  22442  HD3 ILE G 121       8.529 -34.378  49.241  0.00  0.00
G
ATOM  22443  C   ILE G 121      13.537 -32.923  47.989  0.00  0.00
G
ATOM  22444  O   ILE G 121      14.248 -33.518  48.816  0.00  0.00
G
ATOM  22445  N   ILE G 122      13.914 -31.726  47.498  0.00  0.00
G
ATOM  22446  HN  ILE G 122      13.282 -31.327  46.839  0.00  0.00
G
ATOM  22447  CA  ILE G 122      15.257 -31.119  47.639  0.00  0.00
G
ATOM  22448  HA  ILE G 122      15.855 -31.797  48.230  0.00  0.00
G
ATOM  22449  CB  ILE G 122      15.961 -30.986  46.242  0.00  0.00
G
ATOM  22450  HB  ILE G 122      15.615 -30.020  45.815  0.00  0.00
G
ATOM  22451  CG2 ILE G 122      17.474 -30.818  46.667  0.00  0.00
G
ATOM  22452 HG21 ILE G 122      18.093 -30.730  45.749  0.00  0.00
G
ATOM  22453 HG22 ILE G 122      17.656 -29.985  47.379  0.00  0.00
G
ATOM  22454 HG23 ILE G 122      17.606 -31.858  47.034  0.00  0.00
G
ATOM  22455  CG1 ILE G 122      15.734 -32.092  45.235  0.00  0.00
G
ATOM  22456 HG11 ILE G 122      16.714 -32.488  44.893  0.00  0.00
G
ATOM  22457 HG12 ILE G 122      15.152 -32.964  45.604  0.00  0.00
G
ATOM  22458  CD  ILE G 122      15.060 -31.803  43.880  0.00  0.00
G
ATOM  22459  HD1 ILE G 122      15.442 -30.859  43.436  0.00  0.00
G
ATOM  22460  HD2 ILE G 122      15.407 -32.570  43.155  0.00  0.00
G
ATOM  22461  HD3 ILE G 122      13.969 -31.636  44.003  0.00  0.00
G
ATOM  22462  C   ILE G 122      15.059 -29.780  48.395  0.00  0.00
G
ATOM  22463  O   ILE G 122      14.340 -28.936  47.951  0.00  0.00
G
```

ATOM 22464 N HSE G 123 15.894 -29.608 49.483 0.00 0.00
G
ATOM 22465 HN HSE G 123 16.530 -30.320 49.772 0.00 0.00
G
ATOM 22466 CA HSE G 123 15.852 -28.376 50.259 0.00 0.00
G
ATOM 22467 HA HSE G 123 15.090 -27.695 49.909 0.00 0.00
G
ATOM 22468 CB HSE G 123 15.546 -28.657 51.782 0.00 0.00
G
ATOM 22469 HB1 HSE G 123 16.442 -29.043 52.313 0.00 0.00
G
ATOM 22470 HB2 HSE G 123 15.301 -27.686 52.262 0.00 0.00
G
ATOM 22471 ND1 HSE G 123 13.147 -28.998 52.245 0.00 0.00
G
ATOM 22472 CG HSE G 123 14.322 -29.567 51.869 0.00 0.00
G
ATOM 22473 CE1 HSE G 123 12.273 -30.000 52.319 0.00 0.00
G
ATOM 22474 HE1 HSE G 123 11.209 -29.986 52.555 0.00 0.00
G
ATOM 22475 NE2 HSE G 123 12.937 -31.186 52.061 0.00 0.00
G
ATOM 22476 HE2 HSE G 123 12.483 -32.077 52.073 0.00 0.00
G
ATOM 22477 CD2 HSE G 123 14.240 -30.864 51.754 0.00 0.00
G
ATOM 22478 HD2 HSE G 123 14.947 -31.647 51.508 0.00 0.00
G
ATOM 22479 C HSE G 123 17.229 -27.793 50.149 0.00 0.00
G
ATOM 22480 O HSE G 123 18.058 -28.042 51.064 0.00 0.00
G
ATOM 22481 N PRO G 124 17.599 -27.137 49.122 0.00 0.00
G
ATOM 22482 CD PRO G 124 16.767 -26.830 48.042 0.00 0.00
G
ATOM 22483 HD1 PRO G 124 16.721 -27.796 47.495 0.00 0.00
G
ATOM 22484 HD2 PRO G 124 15.772 -26.438 48.341 0.00 0.00
G
ATOM 22485 CA PRO G 124 19.048 -26.810 48.816 0.00 0.00
G
ATOM 22486 HA PRO G 124 19.626 -27.714 48.942 0.00 0.00
G
ATOM 22487 CB PRO G 124 19.004 -26.375 47.314 0.00 0.00
G
ATOM 22488 HB1 PRO G 124 19.338 -27.223 46.678 0.00 0.00
G
ATOM 22489 HB2 PRO G 124 19.632 -25.463 47.218 0.00 0.00
G
ATOM 22490 CG PRO G 124 17.593 -25.848 47.176 0.00 0.00
G

ATOM 22491 HG1 PRO G 124 17.199 -25.812 46.138 0.00 0.00
G
ATOM 22492 HG2 PRO G 124 17.444 -24.805 47.528 0.00 0.00
G
ATOM 22493 C PRO G 124 19.605 -25.799 49.793 0.00 0.00
G
ATOM 22494 O PRO G 124 20.795 -25.847 50.099 0.00 0.00
G
ATOM 22495 N GLU G 125 18.838 -24.786 50.245 0.00 0.00
G
ATOM 22496 HN GLU G 125 17.913 -24.619 49.912 0.00 0.00
G
ATOM 22497 CA GLU G 125 19.419 -23.859 51.160 0.00 0.00
G
ATOM 22498 HA GLU G 125 20.264 -23.298 50.788 0.00 0.00
G
ATOM 22499 CB GLU G 125 18.327 -22.761 51.323 0.00 0.00
G
ATOM 22500 HB1 GLU G 125 17.390 -23.051 51.846 0.00 0.00
G
ATOM 22501 HB2 GLU G 125 18.862 -21.964 51.883 0.00 0.00
G
ATOM 22502 CG GLU G 125 17.829 -21.919 50.126 0.00 0.00
G
ATOM 22503 HG1 GLU G 125 17.396 -21.013 50.602 0.00 0.00
G
ATOM 22504 HG2 GLU G 125 18.671 -21.814 49.409 0.00 0.00
G
ATOM 22505 CD GLU G 125 16.643 -22.622 49.484 0.00 0.00
G
ATOM 22506 OE1 GLU G 125 16.361 -23.770 49.786 0.00 0.00
G
ATOM 22507 OE2 GLU G 125 16.048 -22.017 48.551 0.00 0.00
G
ATOM 22508 C GLU G 125 19.677 -24.439 52.517 0.00 0.00
G
ATOM 22509 O GLU G 125 20.666 -24.165 53.194 0.00 0.00
G
ATOM 22510 N LYS G 126 18.719 -25.342 52.977 0.00 0.00
G
ATOM 22511 HN LYS G 126 18.035 -25.670 52.330 0.00 0.00
G
ATOM 22512 CA LYS G 126 18.868 -26.168 54.132 0.00 0.00
G
ATOM 22513 HA LYS G 126 19.172 -25.517 54.939 0.00 0.00
G
ATOM 22514 CB LYS G 126 17.547 -26.808 54.485 0.00 0.00
G
ATOM 22515 HB1 LYS G 126 16.798 -25.988 54.529 0.00 0.00
G
ATOM 22516 HB2 LYS G 126 17.340 -27.528 53.665 0.00 0.00
G
ATOM 22517 CG LYS G 126 17.506 -27.599 55.817 0.00 0.00
G

ATOM 22518 HG1 LYS G 126 18.201 -28.459 55.927 0.00 0.00
G
ATOM 22519 HG2 LYS G 126 17.796 -26.817 56.550 0.00 0.00
G
ATOM 22520 CD LYS G 126 16.077 -28.163 56.119 0.00 0.00
G
ATOM 22521 HD1 LYS G 126 15.747 -28.755 55.239 0.00 0.00
G
ATOM 22522 HD2 LYS G 126 15.974 -28.862 56.976 0.00 0.00
G
ATOM 22523 CE LYS G 126 14.951 -27.117 56.318 0.00 0.00
G
ATOM 22524 HE1 LYS G 126 14.911 -26.620 55.325 0.00 0.00
G
ATOM 22525 HE2 LYS G 126 13.999 -27.680 56.422 0.00 0.00
G
ATOM 22526 NZ LYS G 126 15.248 -26.265 57.449 0.00 0.00
G
ATOM 22527 HZ1 LYS G 126 14.288 -26.009 57.754 0.00 0.00
G
ATOM 22528 HZ2 LYS G 126 15.582 -26.824 58.260 0.00 0.00
G
ATOM 22529 HZ3 LYS G 126 16.034 -25.583 57.464 0.00 0.00
G
ATOM 22530 C LYS G 126 20.051 -27.202 54.058 0.00 0.00
G
ATOM 22531 O LYS G 126 20.808 -27.443 54.990 0.00 0.00
G
ATOM 22532 N ASP G 127 20.133 -27.908 52.938 0.00 0.00
G
ATOM 22533 HN ASP G 127 19.563 -27.843 52.123 0.00 0.00
G
ATOM 22534 CA ASP G 127 21.148 -28.950 52.631 0.00 0.00
G
ATOM 22535 HA ASP G 127 21.098 -29.596 53.495 0.00 0.00
G
ATOM 22536 CB ASP G 127 20.772 -29.690 51.364 0.00 0.00
G
ATOM 22537 HB1 ASP G 127 19.687 -29.925 51.413 0.00 0.00
G
ATOM 22538 HB2 ASP G 127 21.013 -29.077 50.470 0.00 0.00
G
ATOM 22539 CG ASP G 127 21.461 -31.074 51.177 0.00 0.00
G
ATOM 22540 OD1 ASP G 127 21.076 -31.733 50.183 0.00 0.00
G
ATOM 22541 OD2 ASP G 127 22.373 -31.413 52.014 0.00 0.00
G
ATOM 22542 C ASP G 127 22.610 -28.431 52.577 0.00 0.00
G
ATOM 22543 O ASP G 127 23.462 -29.024 53.261 0.00 0.00
G
ATOM 22544 N MET G 128 22.798 -27.256 51.919 0.00 0.00
G

ATOM 22545 HN MET G 128   22.171 -26.820 51.277 0.00 0.00
G
ATOM 22546 CA MET G 128   23.977 -26.425 52.044 0.00 0.00
G
ATOM 22547 HA MET G 128   24.860 -27.004 51.819 0.00 0.00
G
ATOM 22548 CB MET G 128   23.972 -25.217 51.139 0.00 0.00
G
ATOM 22549 HB1 MET G 128   23.930 -25.506 50.067 0.00 0.00
G
ATOM 22550 HB2 MET G 128   23.062 -24.582 51.186 0.00 0.00
G
ATOM 22551 CG MET G 128   25.210 -24.263 51.146 0.00 0.00
G
ATOM 22552 HG1 MET G 128   24.966 -23.285 50.679 0.00 0.00
G
ATOM 22553 HG2 MET G 128   25.459 -24.006 52.197 0.00 0.00
G
ATOM 22554 SD MET G 128   26.589 -24.983 50.465 0.00 0.00
G
ATOM 22555 CE MET G 128   27.749 -23.664 51.038 0.00 0.00
G
ATOM 22556 HE1 MET G 128   27.190 -22.776 51.403 0.00 0.00
G
ATOM 22557 HE2 MET G 128   28.365 -24.000 51.900 0.00 0.00
G
ATOM 22558 HE3 MET G 128   28.311 -23.345 50.134 0.00 0.00
G
ATOM 22559 C   MET G 128   24.221 -25.851 53.459 0.00 0.00
G
ATOM 22560 O   MET G 128   25.257 -25.821 54.135 0.00 0.00
G
ATOM 22561 N   GLY G 129   23.134 -25.347 54.139 0.00 0.00
G
ATOM 22562 HN GLY G 129   22.213 -25.420 53.766 0.00 0.00
G
ATOM 22563 CA GLY G 129   23.209 -24.794 55.457 0.00 0.00
G
ATOM 22564 HA1 GLY G 129   22.202 -24.421 55.573 0.00 0.00
G
ATOM 22565 HA2 GLY G 129   23.958 -24.017 55.518 0.00 0.00
G
ATOM 22566 C   GLY G 129   23.639 -25.778 56.531 0.00 0.00
G
ATOM 22567 O   GLY G 129   24.419 -25.412 57.470 0.00 0.00
G
ATOM 22568 N   VAL G 130   23.208 -27.052 56.476 0.00 0.00
G
ATOM 22569 HN VAL G 130   22.416 -27.181 55.884 0.00 0.00
G
ATOM 22570 CA VAL G 130   23.703 -28.154 57.363 0.00 0.00
G
ATOM 22571 HA VAL G 130   23.620 -27.684 58.332 0.00 0.00
G

```
ATOM  22572  CB  VAL G 130      22.873 -29.356  57.643  0.00  0.00
G
ATOM  22573  HB  VAL G 130      23.096 -29.816  58.629  0.00  0.00
G
ATOM  22574  CG1 VAL G 130      21.373 -29.037  57.791  0.00  0.00
G
ATOM  22575 HG11 VAL G 130      20.841 -29.987  58.012  0.00  0.00
G
ATOM  22576 HG12 VAL G 130      21.217 -28.282  58.592  0.00  0.00
G
ATOM  22577 HG13 VAL G 130      20.873 -28.686  56.863  0.00  0.00
G
ATOM  22578  CG2 VAL G 130      23.129 -30.434  56.542  0.00  0.00
G
ATOM  22579 HG21 VAL G 130      24.214 -30.669  56.517  0.00  0.00
G
ATOM  22580 HG22 VAL G 130      22.616 -31.391  56.779  0.00  0.00
G
ATOM  22581 HG23 VAL G 130      22.773 -30.007  55.580  0.00  0.00
G
ATOM  22582  C   VAL G 130      25.170 -28.463  57.333  0.00  0.00
G
ATOM  22583  O   VAL G 130      25.837 -28.776  58.350  0.00  0.00
G
ATOM  22584  N   LYS G 131      25.668 -28.458  56.083  0.00  0.00
G
ATOM  22585  HN  LYS G 131      25.027 -28.354  55.326  0.00  0.00
G
ATOM  22586  CA  LYS G 131      26.994 -28.854  55.747  0.00  0.00
G
ATOM  22587  HA  LYS G 131      27.210 -29.804  56.215  0.00  0.00
G
ATOM  22588  CB  LYS G 131      27.227 -29.044  54.246  0.00  0.00
G
ATOM  22589  HB1 LYS G 131      26.750 -28.233  53.656  0.00  0.00
G
ATOM  22590  HB2 LYS G 131      28.331 -29.038  54.123  0.00  0.00
G
ATOM  22591  CG  LYS G 131      26.637 -30.400  53.715  0.00  0.00
G
ATOM  22592  HG1 LYS G 131      27.426 -31.158  53.905  0.00  0.00
G
ATOM  22593  HG2 LYS G 131      25.700 -30.728  54.212  0.00  0.00
G
ATOM  22594  CD  LYS G 131      26.392 -30.268  52.157  0.00  0.00
G
ATOM  22595  HD1 LYS G 131      25.584 -29.524  51.987  0.00  0.00
G
ATOM  22596  HD2 LYS G 131      27.303 -29.807  51.719  0.00  0.00
G
ATOM  22597  CE  LYS G 131      26.030 -31.512  51.390  0.00  0.00
G
ATOM  22598  HE1 LYS G 131      25.773 -31.450  50.310  0.00  0.00
G
```

ATOM 22599 HE2 LYS G 131   26.886 -32.218 51.435 0.00 0.00
G
ATOM 22600 NZ LYS G 131   24.890 -32.185 52.046 0.00 0.00
G
ATOM 22601 HZ1 LYS G 131   25.113 -32.568 52.987 0.00 0.00
G
ATOM 22602 HZ2 LYS G 131   23.994 -31.663 52.118 0.00 0.00
G
ATOM 22603 HZ3 LYS G 131   24.670 -32.983 51.416 0.00 0.00
G
ATOM 22604 C   LYS G 131   27.934 -27.783 56.347 0.00 0.00
G
ATOM 22605 O   LYS G 131   28.978 -27.965 56.947 0.00 0.00
G
ATOM 22606 N   ILE G 132   27.662 -26.463 56.143 0.00 0.00
G
ATOM 22607 HN ILE G 132   26.971 -26.186 55.480 0.00 0.00
G
ATOM 22608 CA ILE G 132   28.465 -25.437 56.857 0.00 0.00
G
ATOM 22609 HA ILE G 132   29.500 -25.739 56.912 0.00 0.00
G
ATOM 22610 CB ILE G 132   28.500 -24.107 56.211 0.00 0.00
G
ATOM 22611 HB ILE G 132   29.123 -23.392 56.790 0.00 0.00
G
ATOM 22612 CG2 ILE G 132   29.238 -24.226 54.864 0.00 0.00
G
ATOM 22613 HG21 ILE G 132   29.437 -23.237 54.397 0.00 0.00
G
ATOM 22614 HG22 ILE G 132   30.123 -24.893 54.933 0.00 0.00
G
ATOM 22615 HG23 ILE G 132   28.626 -24.787 54.127 0.00 0.00
G
ATOM 22616 CG1 ILE G 132   27.084 -23.411 56.167 0.00 0.00
G
ATOM 22617 HG11 ILE G 132   26.719 -23.675 55.152 0.00 0.00
G
ATOM 22618 HG12 ILE G 132   26.538 -23.882 57.012 0.00 0.00
G
ATOM 22619 CD ILE G 132   27.205 -21.952 56.354 0.00 0.00
G
ATOM 22620 HD1 ILE G 132   27.903 -21.494 55.620 0.00 0.00
G
ATOM 22621 HD2 ILE G 132   26.192 -21.524 56.197 0.00 0.00
G
ATOM 22622 HD3 ILE G 132   27.496 -21.663 57.386 0.00 0.00
G
ATOM 22623 C   ILE G 132   28.181 -25.269 58.349 0.00 0.00
G
ATOM 22624 O   ILE G 132   29.125 -24.959 59.065 0.00 0.00
G
ATOM 22625 N   ALA G 133   26.963 -25.540 58.909 0.00 0.00
G

ATOM 22626 HN ALA G 133 26.171 -25.837 58.382 0.00 0.00
G
ATOM 22627 CA ALA G 133 26.761 -25.599 60.372 0.00 0.00
G
ATOM 22628 HA ALA G 133 26.977 -24.634 60.805 0.00 0.00
G
ATOM 22629 CB ALA G 133 25.270 -25.690 60.769 0.00 0.00
G
ATOM 22630 HB1 ALA G 133 24.777 -24.702 60.644 0.00 0.00
G
ATOM 22631 HB2 ALA G 133 24.722 -26.421 60.137 0.00 0.00
G
ATOM 22632 HB3 ALA G 133 25.225 -25.990 61.837 0.00 0.00
G
ATOM 22633 C ALA G 133 27.493 -26.651 61.122 0.00 0.00
G
ATOM 22634 O ALA G 133 28.138 -26.446 62.177 0.00 0.00
G
ATOM 22635 N GLN G 134 27.500 -27.904 60.617 0.00 0.00
G
ATOM 22636 HN GLN G 134 26.883 -28.017 59.842 0.00 0.00
G
ATOM 22637 CA GLN G 134 28.377 -29.001 61.141 0.00 0.00
G
ATOM 22638 HA GLN G 134 28.206 -29.041 62.206 0.00 0.00
G
ATOM 22639 CB GLN G 134 27.901 -30.368 60.538 0.00 0.00
G
ATOM 22640 HB1 GLN G 134 28.026 -30.542 59.447 0.00 0.00
G
ATOM 22641 HB2 GLN G 134 28.587 -31.137 60.952 0.00 0.00
G
ATOM 22642 CG GLN G 134 26.508 -30.787 61.163 0.00 0.00
G
ATOM 22643 HG1 GLN G 134 26.780 -31.134 62.182 0.00 0.00
G
ATOM 22644 HG2 GLN G 134 25.864 -29.883 61.217 0.00 0.00
G
ATOM 22645 CD GLN G 134 25.791 -31.741 60.222 0.00 0.00
G
ATOM 22646 OE1 GLN G 134 25.471 -32.799 60.751 0.00 0.00
G
ATOM 22647 NE2 GLN G 134 25.485 -31.411 58.981 0.00 0.00
G
ATOM 22648 HE21 GLN G 134 24.912 -32.026 58.440 0.00 0.00
G
ATOM 22649 HE22 GLN G 134 25.622 -30.462 58.697 0.00 0.00
G
ATOM 22650 C GLN G 134 29.858 -28.781 60.910 0.00 0.00
G
ATOM 22651 O GLN G 134 30.716 -29.162 61.723 0.00 0.00
G
ATOM 22652 N SER G 135 30.281 -28.073 59.842 0.00 0.00
G

ATOM 22653 HN SER G 135 29.587 -27.856 59.160 0.00 0.00
G
ATOM 22654 CA SER G 135 31.656 -27.516 59.596 0.00 0.00
G
ATOM 22655 HA SER G 135 32.291 -28.379 59.731 0.00 0.00
G
ATOM 22656 CB SER G 135 31.931 -26.946 58.151 0.00 0.00
G
ATOM 22657 HB1 SER G 135 31.350 -26.002 58.088 0.00 0.00
G
ATOM 22658 HB2 SER G 135 33.028 -26.813 58.038 0.00 0.00
G
ATOM 22659 OG SER G 135 31.575 -27.827 57.104 0.00 0.00
G
ATOM 22660 HG1 SER G 135 30.616 -27.866 57.114 0.00 0.00
G
ATOM 22661 C SER G 135 32.030 -26.474 60.668 0.00 0.00
G
ATOM 22662 O SER G 135 33.176 -26.402 61.075 0.00 0.00
G
ATOM 22663 N LEU G 136 31.008 -25.645 61.097 0.00 0.00
G
ATOM 22664 HN LEU G 136 30.155 -25.573 60.587 0.00 0.00
G
ATOM 22665 CA LEU G 136 31.180 -24.702 62.230 0.00 0.00
G
ATOM 22666 HA LEU G 136 32.168 -24.266 62.197 0.00 0.00
G
ATOM 22667 CB LEU G 136 30.200 -23.490 61.989 0.00 0.00
G
ATOM 22668 HB1 LEU G 136 29.160 -23.880 61.956 0.00 0.00
G
ATOM 22669 HB2 LEU G 136 30.212 -22.912 62.938 0.00 0.00
G
ATOM 22670 CG LEU G 136 30.406 -22.472 60.923 0.00 0.00
G
ATOM 22671 HG LEU G 136 30.558 -22.997 59.956 0.00 0.00
G
ATOM 22672 CD1 LEU G 136 29.151 -21.465 60.831 0.00 0.00
G
ATOM 22673 HD11 LEU G 136 28.239 -22.095 60.759 0.00 0.00
G
ATOM 22674 HD12 LEU G 136 29.111 -20.756 61.685 0.00 0.00
G
ATOM 22675 HD13 LEU G 136 29.315 -20.840 59.927 0.00 0.00
G
ATOM 22676 CD2 LEU G 136 31.712 -21.627 61.165 0.00 0.00
G
ATOM 22677 HD21 LEU G 136 32.561 -22.311 61.377 0.00 0.00
G
ATOM 22678 HD22 LEU G 136 32.101 -21.078 60.281 0.00 0.00
G
ATOM 22679 HD23 LEU G 136 31.698 -20.947 62.044 0.00 0.00
G

ATOM 22680 C LEU G 136 30.900 -25.197 63.671 0.00 0.00
G
ATOM 22681 O LEU G 136 31.057 -24.494 64.644 0.00 0.00
G
ATOM 22682 N SER G 137 30.591 -26.530 63.811 0.00 0.00
G
ATOM 22683 HN SER G 137 30.396 -27.059 62.989 0.00 0.00
G
ATOM 22684 CA SER G 137 30.419 -27.193 65.073 0.00 0.00
G
ATOM 22685 HA SER G 137 29.695 -26.618 65.632 0.00 0.00
G
ATOM 22686 CB SER G 137 29.955 -28.673 64.983 0.00 0.00
G
ATOM 22687 HB1 SER G 137 29.058 -28.730 64.328 0.00 0.00
G
ATOM 22688 HB2 SER G 137 30.889 -29.193 64.682 0.00 0.00
G
ATOM 22689 OG SER G 137 29.655 -29.107 66.280 0.00 0.00
G
ATOM 22690 HG1 SER G 137 29.847 -30.047 66.241 0.00 0.00
G
ATOM 22691 C SER G 137 31.749 -27.221 66.005 0.00 0.00
G
ATOM 22692 O SER G 137 31.621 -27.232 67.241 0.00 0.00
G
ATOM 22693 N ASP G 138 32.987 -27.315 65.388 0.00 0.00
G
ATOM 22694 HN ASP G 138 33.064 -27.186 64.403 0.00 0.00
G
ATOM 22695 CA ASP G 138 34.219 -27.118 66.106 0.00 0.00
G
ATOM 22696 HA ASP G 138 34.044 -26.568 67.019 0.00 0.00
G
ATOM 22697 CB ASP G 138 34.796 -28.427 66.611 0.00 0.00
G
ATOM 22698 HB1 ASP G 138 34.031 -28.830 67.308 0.00 0.00
G
ATOM 22699 HB2 ASP G 138 34.787 -29.211 65.825 0.00 0.00
G
ATOM 22700 CG ASP G 138 36.124 -28.514 67.341 0.00 0.00
G
ATOM 22701 OD1 ASP G 138 37.141 -28.616 66.708 0.00 0.00
G
ATOM 22702 OD2 ASP G 138 36.127 -28.507 68.613 0.00 0.00
G
ATOM 22703 C ASP G 138 35.332 -26.441 65.236 0.00 0.00
G
ATOM 22704 O ASP G 138 35.251 -26.421 64.004 0.00 0.00
G
ATOM 22705 N GLU G 139 36.394 -25.887 65.898 0.00 0.00
G
ATOM 22706 HN GLU G 139 36.401 -25.842 66.894 0.00 0.00
G

ATOM 22707 CA GLU G 139   37.266 -24.912 65.261 0.00 0.00
G
ATOM 22708 HA GLU G 139   36.608 -24.217 64.761 0.00 0.00
G
ATOM 22709 CB GLU G 139   38.070 -24.129 66.356 0.00 0.00
G
ATOM 22710 HB1 GLU G 139   37.324 -23.709 67.064 0.00 0.00
G
ATOM 22711 HB2 GLU G 139   38.679 -24.902 66.871 0.00 0.00
G
ATOM 22712 CG GLU G 139   39.104 -23.070 65.858 0.00 0.00
G
ATOM 22713 HG1 GLU G 139   39.819 -23.603 65.195 0.00 0.00
G
ATOM 22714 HG2 GLU G 139   38.673 -22.348 65.132 0.00 0.00
G
ATOM 22715 CD GLU G 139   39.705 -22.471 67.038 0.00 0.00
G
ATOM 22716 OE1 GLU G 139   39.009 -21.905 67.936 0.00 0.00
G
ATOM 22717 OE2 GLU G 139   40.947 -22.467 67.101 0.00 0.00
G
ATOM 22718 C   GLU G 139   38.205 -25.575 64.299 0.00 0.00
G
ATOM 22719 O   GLU G 139   38.837 -25.005 63.447 0.00 0.00
G
ATOM 22720 N   ASN G 140   38.433 -26.891 64.425 0.00 0.00
G
ATOM 22721 HN ASN G 140   38.095 -27.427 65.195 0.00 0.00
G
ATOM 22722 CA ASN G 140   39.434 -27.705 63.696 0.00 0.00
G
ATOM 22723 HA ASN G 140   40.073 -27.045 63.128 0.00 0.00
G
ATOM 22724 CB ASN G 140   40.144 -28.605 64.686 0.00 0.00
G
ATOM 22725 HB1 ASN G 140   39.439 -29.318 65.164 0.00 0.00
G
ATOM 22726 HB2 ASN G 140   41.068 -29.090 64.303 0.00 0.00
G
ATOM 22727 CG ASN G 140   40.683 -27.926 65.930 0.00 0.00
G
ATOM 22728 OD1 ASN G 140   41.729 -27.217 65.779 0.00 0.00
G
ATOM 22729 ND2 ASN G 140   39.953 -27.972 67.055 0.00 0.00
G
ATOM 22730 HD21 ASN G 140   40.306 -27.281 67.685 0.00 0.00
G
ATOM 22731 HD22 ASN G 140   38.976 -28.172 66.991 0.00 0.00
G
ATOM 22732 C   ASN G 140   38.822 -28.547 62.670 0.00 0.00
G
ATOM 22733 O   ASN G 140   39.464 -29.321 61.975 0.00 0.00
G

ATOM 22734 N   VAL G 141   37.489 -28.488 62.505 0.00 0.00
G
ATOM 22735 HN VAL G 141   37.018 -27.821 63.078 0.00 0.00
G
ATOM 22736 CA VAL G 141   36.690 -29.014 61.468 0.00 0.00
G
ATOM 22737 HA VAL G 141   37.116 -29.954 61.148 0.00 0.00
G
ATOM 22738 CB VAL G 141   35.220 -29.223 61.817 0.00 0.00
G
ATOM 22739 HB VAL G 141   34.623 -28.288 61.867 0.00 0.00
G
ATOM 22740 CG1 VAL G 141   34.617 -30.038 60.655 0.00 0.00
G
ATOM 22741 HG11 VAL G 141   34.541 -29.373 59.769 0.00 0.00
G
ATOM 22742 HG12 VAL G 141   35.201 -30.947 60.398 0.00 0.00
G
ATOM 22743 HG13 VAL G 141   33.570 -30.277 60.941 0.00 0.00
G
ATOM 22744 CG2 VAL G 141   35.078 -29.965 63.112 0.00 0.00
G
ATOM 22745 HG21 VAL G 141   35.491 -30.990 62.993 0.00 0.00
G
ATOM 22746 HG22 VAL G 141   35.685 -29.491 63.913 0.00 0.00
G
ATOM 22747 HG23 VAL G 141   34.015 -30.046 63.428 0.00 0.00
G
ATOM 22748 C   VAL G 141   36.746 -28.102 60.317 0.00 0.00
G
ATOM 22749 O   VAL G 141   36.023 -27.105 60.274 0.00 0.00
G
ATOM 22750 N   LEU G 142   37.602 -28.422 59.344 0.00 0.00
G
ATOM 22751 HN LEU G 142   38.255 -29.175 59.323 0.00 0.00
G
ATOM 22752 CA LEU G 142   37.864 -27.561 58.231 0.00 0.00
G
ATOM 22753 HA LEU G 142   37.927 -26.545 58.589 0.00 0.00
G
ATOM 22754 CB LEU G 142   39.210 -27.848 57.612 0.00 0.00
G
ATOM 22755 HB1 LEU G 142   39.277 -28.908 57.283 0.00 0.00
G
ATOM 22756 HB2 LEU G 142   39.364 -27.141 56.770 0.00 0.00
G
ATOM 22757 CG LEU G 142   40.399 -27.709 58.577 0.00 0.00
G
ATOM 22758 HG LEU G 142   40.329 -28.564 59.283 0.00 0.00
G
ATOM 22759 CD1 LEU G 142   41.624 -27.886 57.754 0.00 0.00
G
ATOM 22760 HD11 LEU G 142   41.458 -28.778 57.114 0.00 0.00
G

ATOM 22761 HD12 LEU G 142   41.757 -26.998 57.100 0.00 0.00
G
ATOM 22762 HD13 LEU G 142   42.584 -28.009 58.301 0.00 0.00
G
ATOM 22763 CD2 LEU G 142   40.356 -26.470 59.494 0.00 0.00
G
ATOM 22764 HD21 LEU G 142   39.387 -26.349 60.023 0.00 0.00
G
ATOM 22765 HD22 LEU G 142   41.123 -26.428 60.297 0.00 0.00
G
ATOM 22766 HD23 LEU G 142   40.487 -25.536 58.907 0.00 0.00
G
ATOM 22767 C   LEU G 142   36.717 -27.761 57.258 0.00 0.00
G
ATOM 22768 O   LEU G 142   36.199 -26.825 56.655 0.00 0.00
G
ATOM 22769 N   ASN G 143   36.289 -29.042 57.019 0.00 0.00
G
ATOM 22770 HN ASN G 143   36.519 -29.909 57.455 0.00 0.00
G
ATOM 22771 CA ASN G 143   35.131 -29.307 56.248 0.00 0.00
G
ATOM 22772 HA ASN G 143   34.395 -28.517 56.246 0.00 0.00
G
ATOM 22773 CB ASN G 143   35.532 -29.601 54.793 0.00 0.00
G
ATOM 22774 HB1 ASN G 143   36.204 -28.804 54.409 0.00 0.00
G
ATOM 22775 HB2 ASN G 143   36.135 -30.529 54.703 0.00 0.00
G
ATOM 22776 CG ASN G 143   34.363 -29.567 53.886 0.00 0.00
G
ATOM 22777 OD1 ASN G 143   33.502 -28.712 54.001 0.00 0.00
G
ATOM 22778 ND2 ASN G 143   34.228 -30.562 53.003 0.00 0.00
G
ATOM 22779 HD21 ASN G 143   33.547 -30.339 52.305 0.00 0.00
G
ATOM 22780 HD22 ASN G 143   34.777 -31.388 52.875 0.00 0.00
G
ATOM 22781 C   ASN G 143   34.390 -30.546 56.850 0.00 0.00
G
ATOM 22782 O   ASN G 143   34.992 -31.512 57.169 0.00 0.00
G
ATOM 22783 N   TYR G 144   33.032 -30.485 56.967 0.00 0.00
G
ATOM 22784 HN TYR G 144   32.492 -29.663 56.802 0.00 0.00
G
ATOM 22785 CA TYR G 144   32.259 -31.662 57.356 0.00 0.00
G
ATOM 22786 HA TYR G 144   32.946 -32.485 57.490 0.00 0.00
G
ATOM 22787 CB TYR G 144   31.431 -31.193 58.545 0.00 0.00
G

ATOM 22788 HB1 TYR G 144   32.030 -30.646 59.305 0.00 0.00
G
ATOM 22789 HB2 TYR G 144   30.627 -30.502 58.214 0.00 0.00
G
ATOM 22790 CG TYR G 144   30.751 -32.392 59.252 0.00 0.00
G
ATOM 22791 CD1 TYR G 144   31.246 -32.835 60.458 0.00 0.00
G
ATOM 22792 HD1 TYR G 144   32.063 -32.331 60.953 0.00 0.00
G
ATOM 22793 CE1 TYR G 144   30.823 -34.020 61.066 0.00 0.00
G
ATOM 22794 HE1 TYR G 144   31.288 -34.554 61.882 0.00 0.00
G
ATOM 22795 CZ TYR G 144   29.647 -34.674 60.570 0.00 0.00
G
ATOM 22796 OH TYR G 144   29.237 -35.710 61.323 0.00 0.00
G
ATOM 22797 HH TYR G 144   29.631 -35.711 62.199 0.00 0.00
G
ATOM 22798 CD2 TYR G 144   29.584 -33.008 58.773 0.00 0.00
G
ATOM 22799 HD2 TYR G 144   29.217 -32.668 57.816 0.00 0.00
G
ATOM 22800 CE2 TYR G 144   28.964 -34.134 59.483 0.00 0.00
G
ATOM 22801 HE2 TYR G 144   28.067 -34.640 59.160 0.00 0.00
G
ATOM 22802 C   TYR G 144   31.345 -31.909 56.194 0.00 0.00
G
ATOM 22803 O   TYR G 144   30.605 -31.035 55.804 0.00 0.00
G
ATOM 22804 N   ILE G 145   31.454 -33.102 55.538 0.00 0.00
G
ATOM 22805 HN ILE G 145   32.025 -33.862 55.836 0.00 0.00
G
ATOM 22806 CA ILE G 145   30.431 -33.422 54.459 0.00 0.00
G
ATOM 22807 HA ILE G 145   29.675 -32.653 54.393 0.00 0.00
G
ATOM 22808 CB ILE G 145   31.063 -33.321 53.060 0.00 0.00
G
ATOM 22809 HB ILE G 145   31.355 -32.253 52.961 0.00 0.00
G
ATOM 22810 CG2 ILE G 145   32.222 -34.389 52.867 0.00 0.00
G
ATOM 22811 HG21 ILE G 145   32.715 -34.077 51.921 0.00 0.00
G
ATOM 22812 HG22 ILE G 145   33.036 -34.296 53.618 0.00 0.00
G
ATOM 22813 HG23 ILE G 145   31.922 -35.453 52.758 0.00 0.00
G
ATOM 22814 CG1 ILE G 145   30.029 -33.629 51.934 0.00 0.00
G

ATOM 22815 HG11 ILE G 145 29.787 -34.708 51.823 0.00 0.00
G
ATOM 22816 HG12 ILE G 145 29.062 -33.177 52.243 0.00 0.00
G
ATOM 22817 CD ILE G 145 30.395 -33.270 50.539 0.00 0.00
G
ATOM 22818 HD1 ILE G 145 29.575 -33.503 49.826 0.00 0.00
G
ATOM 22819 HD2 ILE G 145 30.478 -32.173 50.384 0.00 0.00
G
ATOM 22820 HD3 ILE G 145 31.390 -33.571 50.148 0.00 0.00
G
ATOM 22821 C ILE G 145 29.632 -34.756 54.692 0.00 0.00
G
ATOM 22822 O ILE G 145 30.224 -35.843 54.892 0.00 0.00
G
ATOM 22823 N ASP G 146 28.231 -34.758 54.776 0.00 0.00
G
ATOM 22824 HN ASP G 146 27.711 -33.911 54.855 0.00 0.00
G
ATOM 22825 CA ASP G 146 27.454 -35.938 54.685 0.00 0.00
G
ATOM 22826 HA ASP G 146 27.884 -36.587 55.434 0.00 0.00
G
ATOM 22827 CB ASP G 146 25.979 -35.712 55.010 0.00 0.00
G
ATOM 22828 HB1 ASP G 146 25.365 -36.627 54.868 0.00 0.00
G
ATOM 22829 HB2 ASP G 146 25.943 -35.430 56.085 0.00 0.00
G
ATOM 22830 CG ASP G 146 25.219 -34.654 54.198 0.00 0.00
G
ATOM 22831 OD1 ASP G 146 25.474 -33.409 54.374 0.00 0.00
G
ATOM 22832 OD2 ASP G 146 24.466 -35.049 53.272 0.00 0.00
G
ATOM 22833 C ASP G 146 27.665 -36.769 53.423 0.00 0.00
G
ATOM 22834 O ASP G 146 27.267 -36.548 52.304 0.00 0.00
G
ATOM 22835 N LEU G 147 28.280 -37.947 53.638 0.00 0.00
G
ATOM 22836 HN LEU G 147 28.603 -38.219 54.541 0.00 0.00
G
ATOM 22837 CA LEU G 147 28.267 -39.069 52.707 0.00 0.00
G
ATOM 22838 HA LEU G 147 28.400 -38.780 51.675 0.00 0.00
G
ATOM 22839 CB LEU G 147 29.520 -39.961 52.963 0.00 0.00
G
ATOM 22840 HB1 LEU G 147 30.459 -39.374 52.865 0.00 0.00
G
ATOM 22841 HB2 LEU G 147 29.516 -40.249 54.036 0.00 0.00
G

ATOM 22842 CG LEU G 147   29.733 -41.123 51.952 0.00 0.00
G
ATOM 22843 HG LEU G 147   28.816 -41.749 51.896 0.00 0.00
G
ATOM 22844 CD1 LEU G 147   29.839 -40.580 50.520 0.00 0.00
G
ATOM 22845 HD11 LEU G 147   30.118 -41.362 49.781 0.00 0.00
G
ATOM 22846 HD12 LEU G 147   29.054 -39.917 50.097 0.00 0.00
G
ATOM 22847 HD13 LEU G 147   30.717 -39.904 50.435 0.00 0.00
G
ATOM 22848 CD2 LEU G 147   30.956 -42.029 52.363 0.00 0.00
G
ATOM 22849 HD21 LEU G 147   31.147 -42.669 51.476 0.00 0.00
G
ATOM 22850 HD22 LEU G 147   31.906 -41.530 52.650 0.00 0.00
G
ATOM 22851 HD23 LEU G 147   30.600 -42.652 53.211 0.00 0.00
G
ATOM 22852 C   LEU G 147   26.992 -39.939 52.748 0.00 0.00
G
ATOM 22853 O   LEU G 147   26.543 -40.594 51.791 0.00 0.00
G
ATOM 22854 N   SER G 148   26.405 -40.037 53.969 0.00 0.00
G
ATOM 22855 HN SER G 148   26.780 -39.606 54.786 0.00 0.00
G
ATOM 22856 CA SER G 148   25.301 -40.885 54.229 0.00 0.00
G
ATOM 22857 HA SER G 148   24.665 -40.873 53.356 0.00 0.00
G
ATOM 22858 CB SER G 148   25.674 -42.395 54.481 0.00 0.00
G
ATOM 22859 HB1 SER G 148   26.424 -42.731 53.733 0.00 0.00
G
ATOM 22860 HB2 SER G 148   26.211 -42.388 55.453 0.00 0.00
G
ATOM 22861 OG SER G 148   24.553 -43.270 54.562 0.00 0.00
G
ATOM 22862 HG1 SER G 148   24.320 -43.565 53.679 0.00 0.00
G
ATOM 22863 C   SER G 148   24.564 -40.259 55.442 0.00 0.00
G
ATOM 22864 O   SER G 148   24.978 -39.360 56.180 0.00 0.00
G
ATOM 22865 N   ASP G 149   23.444 -40.894 55.892 0.00 0.00
G
ATOM 22866 HN ASP G 149   23.097 -41.638 55.327 0.00 0.00
G
ATOM 22867 CA ASP G 149   22.776 -40.461 57.097 0.00 0.00
G
ATOM 22868 HA ASP G 149   22.570 -39.404 57.015 0.00 0.00
G

ATOM 22869 CB ASP G 149 21.413 -41.231 57.156 0.00 0.00
G
ATOM 22870 HB1 ASP G 149 21.504 -42.336 57.086 0.00 0.00
G
ATOM 22871 HB2 ASP G 149 20.859 -40.942 58.075 0.00 0.00
G
ATOM 22872 CG ASP G 149 20.656 -40.710 56.005 0.00 0.00
G
ATOM 22873 OD1 ASP G 149 20.338 -39.383 55.860 0.00 0.00
G
ATOM 22874 OD2 ASP G 149 20.395 -41.515 55.082 0.00 0.00
G
ATOM 22875 C ASP G 149 23.609 -40.747 58.355 0.00 0.00
G
ATOM 22876 O ASP G 149 23.921 -39.791 59.068 0.00 0.00
G
ATOM 22877 N GLU G 150 24.025 -41.983 58.567 0.00 0.00
G
ATOM 22878 HN GLU G 150 23.694 -42.781 58.070 0.00 0.00
G
ATOM 22879 CA GLU G 150 24.938 -42.294 59.704 0.00 0.00
G
ATOM 22880 HA GLU G 150 24.826 -41.540 60.469 0.00 0.00
G
ATOM 22881 CB GLU G 150 24.633 -43.692 60.303 0.00 0.00
G
ATOM 22882 HB1 GLU G 150 24.693 -44.367 59.423 0.00 0.00
G
ATOM 22883 HB2 GLU G 150 25.352 -44.010 61.088 0.00 0.00
G
ATOM 22884 CG GLU G 150 23.198 -43.668 60.992 0.00 0.00
G
ATOM 22885 HG1 GLU G 150 23.250 -42.903 61.796 0.00 0.00
G
ATOM 22886 HG2 GLU G 150 22.320 -43.495 60.334 0.00 0.00
G
ATOM 22887 CD GLU G 150 22.825 -44.999 61.534 0.00 0.00
G
ATOM 22888 OE1 GLU G 150 23.536 -45.324 62.555 0.00 0.00
G
ATOM 22889 OE2 GLU G 150 21.993 -45.718 61.052 0.00 0.00
G
ATOM 22890 C GLU G 150 26.399 -42.171 59.403 0.00 0.00
G
ATOM 22891 O GLU G 150 27.156 -42.543 60.306 0.00 0.00
G
ATOM 22892 N TYR G 151 26.883 -41.788 58.170 0.00 0.00
G
ATOM 22893 HN TYR G 151 26.278 -41.366 57.499 0.00 0.00
G
ATOM 22894 CA TYR G 151 28.333 -41.797 57.910 0.00 0.00
G
ATOM 22895 HA TYR G 151 28.896 -42.008 58.808 0.00 0.00
G

ATOM 22896 CB TYR G 151 28.766 -42.964 56.993 0.00 0.00
G
ATOM 22897 HB1 TYR G 151 28.167 -42.910 56.058 0.00 0.00
G
ATOM 22898 HB2 TYR G 151 29.759 -42.869 56.506 0.00 0.00
G
ATOM 22899 CG TYR G 151 28.579 -44.390 57.596 0.00 0.00
G
ATOM 22900 CD1 TYR G 151 29.676 -44.887 58.380 0.00 0.00
G
ATOM 22901 HD1 TYR G 151 30.612 -44.349 58.355 0.00 0.00
G
ATOM 22902 CE1 TYR G 151 29.393 -46.053 59.143 0.00 0.00
G
ATOM 22903 HE1 TYR G 151 30.230 -46.462 59.689 0.00 0.00
G
ATOM 22904 CZ TYR G 151 28.110 -46.580 59.310 0.00 0.00
G
ATOM 22905 OH TYR G 151 27.928 -47.674 60.182 0.00 0.00
G
ATOM 22906 HH TYR G 151 28.737 -47.743 60.693 0.00 0.00
G
ATOM 22907 CD2 TYR G 151 27.260 -44.947 57.659 0.00 0.00
G
ATOM 22908 HD2 TYR G 151 26.429 -44.527 57.111 0.00 0.00
G
ATOM 22909 CE2 TYR G 151 27.039 -45.971 58.641 0.00 0.00
G
ATOM 22910 HE2 TYR G 151 26.044 -46.378 58.750 0.00 0.00
G
ATOM 22911 C TYR G 151 28.698 -40.470 57.274 0.00 0.00
G
ATOM 22912 O TYR G 151 28.047 -40.048 56.336 0.00 0.00
G
ATOM 22913 N SER G 152 29.793 -39.894 57.655 0.00 0.00
G
ATOM 22914 HN SER G 152 30.394 -40.228 58.377 0.00 0.00
G
ATOM 22915 CA SER G 152 30.131 -38.572 57.259 0.00 0.00
G
ATOM 22916 HA SER G 152 29.641 -38.218 56.364 0.00 0.00
G
ATOM 22917 CB SER G 152 29.853 -37.522 58.369 0.00 0.00
G
ATOM 22918 HB1 SER G 152 30.591 -37.643 59.190 0.00 0.00
G
ATOM 22919 HB2 SER G 152 29.891 -36.487 57.966 0.00 0.00
G
ATOM 22920 OG SER G 152 28.482 -37.699 58.747 0.00 0.00
G
ATOM 22921 HG1 SER G 152 28.419 -37.109 59.502 0.00 0.00
G
ATOM 22922 C SER G 152 31.604 -38.523 57.013 0.00 0.00
G

ATOM 22923 O SER G 152 32.319 -39.446 57.443 0.00 0.00
G
ATOM 22924 N ILE G 153 32.008 -37.484 56.276 0.00 0.00
G
ATOM 22925 HN ILE G 153 31.317 -36.839 55.960 0.00 0.00
G
ATOM 22926 CA ILE G 153 33.438 -37.363 56.014 0.00 0.00
G
ATOM 22927 HA ILE G 153 34.115 -38.077 56.459 0.00 0.00
G
ATOM 22928 CB ILE G 153 33.802 -37.498 54.483 0.00 0.00
G
ATOM 22929 HB ILE G 153 33.394 -36.561 54.047 0.00 0.00
G
ATOM 22930 CG2 ILE G 153 35.290 -37.407 54.122 0.00 0.00
G
ATOM 22931 HG21 ILE G 153 35.792 -38.333 54.473 0.00 0.00
G
ATOM 22932 HG22 ILE G 153 35.499 -37.382 53.031 0.00 0.00
G
ATOM 22933 HG23 ILE G 153 35.879 -36.584 54.580 0.00 0.00
G
ATOM 22934 CG1 ILE G 153 33.142 -38.808 53.927 0.00 0.00
G
ATOM 22935 HG11 ILE G 153 33.746 -39.603 54.416 0.00 0.00
G
ATOM 22936 HG12 ILE G 153 32.064 -38.941 54.156 0.00 0.00
G
ATOM 22937 CD ILE G 153 33.100 -38.792 52.370 0.00 0.00
G
ATOM 22938 HD1 ILE G 153 34.113 -38.628 51.945 0.00 0.00
G
ATOM 22939 HD2 ILE G 153 32.754 -39.813 52.101 0.00 0.00
G
ATOM 22940 HD3 ILE G 153 32.373 -38.046 51.983 0.00 0.00
G
ATOM 22941 C ILE G 153 33.867 -36.048 56.630 0.00 0.00
G
ATOM 22942 O ILE G 153 33.272 -34.993 56.348 0.00 0.00
G
ATOM 22943 N VAL G 154 34.856 -36.102 57.550 0.00 0.00
G
ATOM 22944 HN VAL G 154 35.429 -36.912 57.643 0.00 0.00
G
ATOM 22945 CA VAL G 154 35.339 -34.882 58.208 0.00 0.00
G
ATOM 22946 HA VAL G 154 34.739 -34.050 57.871 0.00 0.00
G
ATOM 22947 CB VAL G 154 35.113 -35.074 59.767 0.00 0.00
G
ATOM 22948 HB VAL G 154 34.059 -35.314 60.023 0.00 0.00
G
ATOM 22949 CG1 VAL G 154 36.157 -35.979 60.364 0.00 0.00
G

ATOM 22950 HG11 VAL G 154   36.295 -36.876 59.722 0.00 0.00
G
ATOM 22951 HG12 VAL G 154   37.137 -35.513 60.604 0.00 0.00
G
ATOM 22952 HG13 VAL G 154   35.869 -36.237 61.405 0.00 0.00
G
ATOM 22953 CG2 VAL G 154   35.199 -33.688 60.395 0.00 0.00
G
ATOM 22954 HG21 VAL G 154   36.184 -33.186 60.284 0.00 0.00
G
ATOM 22955 HG22 VAL G 154   34.455 -32.941 60.047 0.00 0.00
G
ATOM 22956 HG23 VAL G 154   34.998 -33.819 61.479 0.00 0.00
G
ATOM 22957 C   VAL G 154   36.686 -34.557 57.695 0.00 0.00
G
ATOM 22958 O   VAL G 154   37.431 -35.467 57.385 0.00 0.00
G
ATOM 22959 N   GLU G 155   36.977 -33.287 57.499 0.00 0.00
G
ATOM 22960 HN GLU G 155   36.283 -32.594 57.675 0.00 0.00
G
ATOM 22961 CA GLU G 155   38.316 -32.905 57.192 0.00 0.00
G
ATOM 22962 HA GLU G 155   38.957 -33.774 57.176 0.00 0.00
G
ATOM 22963 CB GLU G 155   38.215 -32.313 55.757 0.00 0.00
G
ATOM 22964 HB1 GLU G 155   37.908 -33.056 54.991 0.00 0.00
G
ATOM 22965 HB2 GLU G 155   37.496 -31.471 55.851 0.00 0.00
G
ATOM 22966 CG GLU G 155   39.569 -31.698 55.179 0.00 0.00
G
ATOM 22967 HG1 GLU G 155   39.905 -30.834 55.791 0.00 0.00
G
ATOM 22968 HG2 GLU G 155   40.303 -32.530 55.225 0.00 0.00
G
ATOM 22969 CD GLU G 155   39.511 -31.236 53.721 0.00 0.00
G
ATOM 22970 OE1 GLU G 155   38.819 -31.915 52.868 0.00 0.00
G
ATOM 22971 OE2 GLU G 155   40.170 -30.272 53.352 0.00 0.00
G
ATOM 22972 C   GLU G 155   38.894 -31.927 58.145 0.00 0.00
G
ATOM 22973 O   GLU G 155   38.299 -30.938 58.593 0.00 0.00
G
ATOM 22974 N   LEU G 156   40.077 -32.228 58.763 0.00 0.00
G
ATOM 22975 HN LEU G 156   40.651 -32.906 58.312 0.00 0.00
G
ATOM 22976 CA LEU G 156   40.567 -32.079 60.109 0.00 0.00
G

ATOM 22977 HA LEU G 156 39.905 −31.410 60.639 0.00 0.00
G
ATOM 22978 CB LEU G 156 40.533 −33.481 60.772 0.00 0.00
G
ATOM 22979 HB1 LEU G 156 39.553 −33.988 60.638 0.00 0.00
G
ATOM 22980 HB2 LEU G 156 41.307 −34.144 60.331 0.00 0.00
G
ATOM 22981 CG LEU G 156 40.755 −33.417 62.267 0.00 0.00
G
ATOM 22982 HG LEU G 156 41.682 −32.854 62.508 0.00 0.00
G
ATOM 22983 CD1 LEU G 156 39.697 −32.672 63.096 0.00 0.00
G
ATOM 22984 HD11 LEU G 156 39.276 −31.802 62.548 0.00 0.00
G
ATOM 22985 HD12 LEU G 156 38.788 −33.283 63.285 0.00 0.00
G
ATOM 22986 HD13 LEU G 156 40.107 −32.427 64.098 0.00 0.00
G
ATOM 22987 CD2 LEU G 156 40.905 −34.834 62.821 0.00 0.00
G
ATOM 22988 HD21 LEU G 156 40.806 −34.875 63.927 0.00 0.00
G
ATOM 22989 HD22 LEU G 156 39.993 −35.338 62.435 0.00 0.00
G
ATOM 22990 HD23 LEU G 156 41.758 −35.449 62.464 0.00 0.00
G
ATOM 22991 C LEU G 156 41.959 −31.501 60.092 0.00 0.00
G
ATOM 22992 O LEU G 156 42.889 −31.929 59.342 0.00 0.00
G
ATOM 22993 N LEU G 157 42.094 −30.488 60.896 0.00 0.00
G
ATOM 22994 HN LEU G 157 41.300 −30.369 61.486 0.00 0.00
G
ATOM 22995 CA LEU G 157 43.360 −29.814 61.092 0.00 0.00
G
ATOM 22996 HA LEU G 157 43.969 −29.902 60.204 0.00 0.00
G
ATOM 22997 CB LEU G 157 43.025 −28.325 61.413 0.00 0.00
G
ATOM 22998 HB1 LEU G 157 42.404 −27.917 60.587 0.00 0.00
G
ATOM 22999 HB2 LEU G 157 42.485 −28.115 62.361 0.00 0.00
G
ATOM 23000 CG LEU G 157 44.300 −27.362 61.352 0.00 0.00
G
ATOM 23001 HG LEU G 157 45.190 −27.811 61.843 0.00 0.00
G
ATOM 23002 CD1 LEU G 157 44.624 −27.167 59.857 0.00 0.00
G
ATOM 23003 HD11 LEU G 157 43.850 −26.653 59.249 0.00 0.00
G

ATOM 23004 HD12 LEU G 157   45.552 -26.565 59.756 0.00 0.00
G
ATOM 23005 HD13 LEU G 157   44.808 -28.137 59.348 0.00 0.00
G
ATOM 23006 CD2 LEU G 157   43.887 -25.979 62.078 0.00 0.00
G
ATOM 23007 HD21 LEU G 157   44.829 -25.395 62.158 0.00 0.00
G
ATOM 23008 HD22 LEU G 157   43.119 -25.447 61.476 0.00 0.00
G
ATOM 23009 HD23 LEU G 157   43.459 -26.140 63.090 0.00 0.00
G
ATOM 23010 C   LEU G 157   44.207 -30.448 62.194 0.00 0.00
G
ATOM 23011 O   LEU G 157   43.705 -30.908 63.177 0.00 0.00
G
ATOM 23012 N   ALA G 158   45.548 -30.571 61.950 0.00 0.00
G
ATOM 23013 HN ALA G 158   46.014 -30.377 61.090 0.00 0.00
G
ATOM 23014 CA ALA G 158   46.619 -30.953 62.855 0.00 0.00
G
ATOM 23015 HA ALA G 158   46.167 -31.577 63.612 0.00 0.00
G
ATOM 23016 CB ALA G 158   47.695 -31.783 62.083 0.00 0.00
G
ATOM 23017 HB1 ALA G 158   48.327 -31.218 61.366 0.00 0.00
G
ATOM 23018 HB2 ALA G 158   48.428 -32.298 62.741 0.00 0.00
G
ATOM 23019 HB3 ALA G 158   47.244 -32.683 61.613 0.00 0.00
G
ATOM 23020 C   ALA G 158   47.145 -29.789 63.531 0.00 0.00
G
ATOM 23021 O   ALA G 158   47.615 -28.810 62.909 0.00 0.00
G
ATOM 23022 N   THR G 159   46.996 -29.820 64.838 0.00 0.00
G
ATOM 23023 HN THR G 159   46.588 -30.637 65.237 0.00 0.00
G
ATOM 23024 CA THR G 159   47.395 -28.796 65.766 0.00 0.00
G
ATOM 23025 HA THR G 159   47.563 -27.845 65.282 0.00 0.00
G
ATOM 23026 CB THR G 159   46.426 -28.792 66.949 0.00 0.00
G
ATOM 23027 HB THR G 159   46.729 -27.980 67.644 0.00 0.00
G
ATOM 23028 OG1 THR G 159   46.182 -30.066 67.437 0.00 0.00
G
ATOM 23029 HG1 THR G 159   45.444 -29.945 68.038 0.00 0.00
G
ATOM 23030 CG2 THR G 159   45.037 -28.396 66.448 0.00 0.00
G

ATOM 23031 HG21 THR G 159   44.325 -28.312 67.297 0.00 0.00
G
ATOM 23032 HG22 THR G 159   44.955 -27.449 65.874 0.00 0.00
G
ATOM 23033 HG23 THR G 159   44.632 -29.210 65.808 0.00 0.00
G
ATOM 23034 C   THR G 159   48.778 -29.052 66.315 0.00 0.00
G
ATOM 23035 O   THR G 159   49.455 -30.047 66.086 0.00 0.00
G
ATOM 23036 N   ARG G 160   49.249 -28.090 67.144 0.00 0.00
G
ATOM 23037 HN ARG G 160   48.779 -27.214 67.222 0.00 0.00
G
ATOM 23038 CA ARG G 160   50.548 -28.215 67.745 0.00 0.00
G
ATOM 23039 HA ARG G 160   51.272 -28.407 66.967 0.00 0.00
G
ATOM 23040 CB ARG G 160   50.978 -26.869 68.335 0.00 0.00
G
ATOM 23041 HB1 ARG G 160   50.247 -26.468 69.069 0.00 0.00
G
ATOM 23042 HB2 ARG G 160   51.959 -26.996 68.840 0.00 0.00
G
ATOM 23043 CG ARG G 160   51.268 -25.849 67.230 0.00 0.00
G
ATOM 23044 HG1 ARG G 160   52.121 -26.171 66.594 0.00 0.00
G
ATOM 23045 HG2 ARG G 160   50.375 -25.674 66.592 0.00 0.00
G
ATOM 23046 CD ARG G 160   51.455 -24.487 67.830 0.00 0.00
G
ATOM 23047 HD1 ARG G 160   50.476 -24.149 68.233 0.00 0.00
G
ATOM 23048 HD2 ARG G 160   52.230 -24.497 68.625 0.00 0.00
G
ATOM 23049 NE ARG G 160   51.950 -23.613 66.673 0.00 0.00
G
ATOM 23050 HE ARG G 160   52.276 -23.997 65.809 0.00 0.00
G
ATOM 23051 CZ ARG G 160   51.907 -22.291 66.561 0.00 0.00
G
ATOM 23052 NH1 ARG G 160   51.260 -21.539 67.448 0.00 0.00
G
ATOM 23053 HH11 ARG G 160   51.515 -20.573 67.416 0.00 0.00
G
ATOM 23054 HH12 ARG G 160   50.935 -21.971 68.289 0.00 0.00
G
ATOM 23055 NH2 ARG G 160   52.503 -21.737 65.438 0.00 0.00
G
ATOM 23056 HH21 ARG G 160   52.108 -20.879 65.112 0.00 0.00
G
ATOM 23057 HH22 ARG G 160   52.985 -22.424 64.892 0.00 0.00
G

ATOM 23058 C ARG G 160 50.545 −29.334 68.802 0.00 0.00
G
ATOM 23059 O ARG G 160 51.504 −30.021 69.058 0.00 0.00
G
ATOM 23060 N LYS G 161 49.377 −29.562 69.435 0.00 0.00
G
ATOM 23061 HN LYS G 161 48.734 −28.801 69.398 0.00 0.00
G
ATOM 23062 CA LYS G 161 49.131 −30.641 70.343 0.00 0.00
G
ATOM 23063 HA LYS G 161 49.853 −30.498 71.133 0.00 0.00
G
ATOM 23064 CB LYS G 161 47.710 −30.489 70.902 0.00 0.00
G
ATOM 23065 HB1 LYS G 161 47.603 −29.461 71.310 0.00 0.00
G
ATOM 23066 HB2 LYS G 161 46.885 −30.405 70.163 0.00 0.00
G
ATOM 23067 CG LYS G 161 47.191 −31.517 71.954 0.00 0.00
G
ATOM 23068 HG1 LYS G 161 46.210 −31.252 72.404 0.00 0.00
G
ATOM 23069 HG2 LYS G 161 46.901 −32.439 71.406 0.00 0.00
G
ATOM 23070 CD LYS G 161 48.080 −31.736 73.077 0.00 0.00
G
ATOM 23071 HD1 LYS G 161 49.081 −32.140 72.817 0.00 0.00
G
ATOM 23072 HD2 LYS G 161 48.240 −30.774 73.609 0.00 0.00
G
ATOM 23073 CE LYS G 161 47.487 −32.714 74.127 0.00 0.00
G
ATOM 23074 HE1 LYS G 161 46.470 −32.425 74.467 0.00 0.00
G
ATOM 23075 HE2 LYS G 161 47.371 −33.643 73.528 0.00 0.00
G
ATOM 23076 NZ LYS G 161 48.449 −32.915 75.154 0.00 0.00
G
ATOM 23077 HZ1 LYS G 161 48.158 −33.560 75.916 0.00 0.00
G
ATOM 23078 HZ2 LYS G 161 49.163 −33.469 74.640 0.00 0.00
G
ATOM 23079 HZ3 LYS G 161 48.949 −32.047 75.435 0.00 0.00
G
ATOM 23080 C LYS G 161 49.295 −31.950 69.636 0.00 0.00
G
ATOM 23081 O LYS G 161 50.098 −32.758 70.158 0.00 0.00
G
ATOM 23082 N LEU G 162 48.602 −32.152 68.463 0.00 0.00
G
ATOM 23083 HN LEU G 162 47.982 −31.412 68.215 0.00 0.00
G
ATOM 23084 CA LEU G 162 48.849 −33.270 67.633 0.00 0.00
G

ATOM 23085 HA LEU G 162 48.699 -34.156 68.233 0.00 0.00
G
ATOM 23086 CB LEU G 162 47.876 -33.301 66.445 0.00 0.00
G
ATOM 23087 HB1 LEU G 162 46.823 -33.090 66.728 0.00 0.00
G
ATOM 23088 HB2 LEU G 162 48.331 -32.569 65.744 0.00 0.00
G
ATOM 23089 CG LEU G 162 47.748 -34.714 65.731 0.00 0.00
G
ATOM 23090 HG LEU G 162 48.733 -34.967 65.285 0.00 0.00
G
ATOM 23091 CD1 LEU G 162 47.355 -35.911 66.717 0.00 0.00
G
ATOM 23092 HD11 LEU G 162 47.333 -36.801 66.053 0.00 0.00
G
ATOM 23093 HD12 LEU G 162 48.112 -36.177 67.485 0.00 0.00
G
ATOM 23094 HD13 LEU G 162 46.398 -35.704 67.242 0.00 0.00
G
ATOM 23095 CD2 LEU G 162 46.743 -34.671 64.610 0.00 0.00
G
ATOM 23096 HD21 LEU G 162 45.742 -34.446 65.037 0.00 0.00
G
ATOM 23097 HD22 LEU G 162 47.132 -34.012 63.805 0.00 0.00
G
ATOM 23098 HD23 LEU G 162 46.600 -35.664 64.132 0.00 0.00
G
ATOM 23099 C LEU G 162 50.312 -33.573 67.096 0.00 0.00
G
ATOM 23100 O LEU G 162 50.806 -34.723 67.020 0.00 0.00
G
ATOM 23101 N ASP G 163 51.001 -32.508 66.707 0.00 0.00
G
ATOM 23102 HN ASP G 163 50.609 -31.655 67.044 0.00 0.00
G
ATOM 23103 CA ASP G 163 52.379 -32.489 66.143 0.00 0.00
G
ATOM 23104 HA ASP G 163 52.169 -32.728 65.111 0.00 0.00
G
ATOM 23105 CB ASP G 163 52.895 -31.033 66.293 0.00 0.00
G
ATOM 23106 HB1 ASP G 163 52.071 -30.410 65.884 0.00 0.00
G
ATOM 23107 HB2 ASP G 163 53.002 -30.884 67.388 0.00 0.00
G
ATOM 23108 CG ASP G 163 54.211 -30.834 65.601 0.00 0.00
G
ATOM 23109 OD1 ASP G 163 55.195 -31.381 66.070 0.00 0.00
G
ATOM 23110 OD2 ASP G 163 54.326 -30.108 64.568 0.00 0.00
G
ATOM 23111 C ASP G 163 53.408 -33.507 66.834 0.00 0.00
G

ATOM 23112 O ASP G 163 53.497 -33.712 68.026 0.00 0.00
G
ATOM 23113 N SER G 164 54.270 -34.171 66.048 0.00 0.00
G
ATOM 23114 HN SER G 164 54.302 -33.919 65.084 0.00 0.00
G
ATOM 23115 CA SER G 164 55.373 -35.084 66.512 0.00 0.00
G
ATOM 23116 HA SER G 164 55.979 -35.362 65.662 0.00 0.00
G
ATOM 23117 CB SER G 164 56.296 -34.500 67.633 0.00 0.00
G
ATOM 23118 HB1 SER G 164 56.361 -33.398 67.512 0.00 0.00
G
ATOM 23119 HB2 SER G 164 55.800 -34.629 68.619 0.00 0.00
G
ATOM 23120 OG SER G 164 57.593 -34.981 67.457 0.00 0.00
G
ATOM 23121 HG1 SER G 164 57.656 -35.780 67.985 0.00 0.00
G
ATOM 23122 C SER G 164 54.818 -36.445 66.965 0.00 0.00
G
ATOM 23123 O SER G 164 55.622 -37.302 67.426 0.00 0.00
G
ATOM 23124 N LYS G 165 53.597 -36.878 66.596 0.00 0.00
G
ATOM 23125 HN LYS G 165 52.883 -36.340 66.154 0.00 0.00
G
ATOM 23126 CA LYS G 165 53.295 -38.290 66.531 0.00 0.00
G
ATOM 23127 HA LYS G 165 53.817 -38.827 67.309 0.00 0.00
G
ATOM 23128 CB LYS G 165 51.751 -38.486 66.649 0.00 0.00
G
ATOM 23129 HB1 LYS G 165 51.282 -37.723 65.991 0.00 0.00
G
ATOM 23130 HB2 LYS G 165 51.313 -39.469 66.373 0.00 0.00
G
ATOM 23131 CG LYS G 165 51.164 -38.369 68.055 0.00 0.00
G
ATOM 23132 HG1 LYS G 165 51.593 -37.479 68.564 0.00 0.00
G
ATOM 23133 HG2 LYS G 165 50.113 -38.044 67.898 0.00 0.00
G
ATOM 23134 CD LYS G 165 51.278 -39.646 68.930 0.00 0.00
G
ATOM 23135 HD1 LYS G 165 50.974 -40.506 68.294 0.00 0.00
G
ATOM 23136 HD2 LYS G 165 52.344 -39.679 69.241 0.00 0.00
G
ATOM 23137 CE LYS G 165 50.405 -39.737 70.219 0.00 0.00
G
ATOM 23138 HE1 LYS G 165 49.366 -40.065 70.004 0.00 0.00
G

ATOM 23139 HE2 LYS G 165 50.869 -40.430 70.954 0.00 0.00
G
ATOM 23140 NZ LYS G 165 50.290 -38.426 70.907 0.00 0.00
G
ATOM 23141 HZ1 LYS G 165 49.751 -38.596 71.780 0.00 0.00
G
ATOM 23142 HZ2 LYS G 165 51.279 -38.128 71.028 0.00 0.00
G
ATOM 23143 HZ3 LYS G 165 49.809 -37.718 70.317 0.00 0.00
G
ATOM 23144 C LYS G 165 53.479 -38.886 65.135 0.00 0.00
G
ATOM 23145 O LYS G 165 53.328 -38.279 64.064 0.00 0.00
G
ATOM 23146 N SER G 166 53.821 -40.168 65.054 0.00 0.00
G
ATOM 23147 HN SER G 166 53.861 -40.688 65.904 0.00 0.00
G
ATOM 23148 CA SER G 166 53.919 -40.803 63.743 0.00 0.00
G
ATOM 23149 HA SER G 166 54.354 -40.070 63.080 0.00 0.00
G
ATOM 23150 CB SER G 166 54.818 -42.047 63.836 0.00 0.00
G
ATOM 23151 HB1 SER G 166 55.816 -41.717 64.196 0.00 0.00
G
ATOM 23152 HB2 SER G 166 54.351 -42.782 64.526 0.00 0.00
G
ATOM 23153 OG SER G 166 55.055 -42.645 62.554 0.00 0.00
G
ATOM 23154 HG1 SER G 166 55.752 -43.242 62.833 0.00 0.00
G
ATOM 23155 C SER G 166 52.587 -41.179 63.024 0.00 0.00
G
ATOM 23156 O SER G 166 51.610 -41.497 63.689 0.00 0.00
G
ATOM 23157 N ILE G 167 52.514 -41.192 61.722 0.00 0.00
G
ATOM 23158 HN ILE G 167 53.175 -40.683 61.176 0.00 0.00
G
ATOM 23159 CA ILE G 167 51.188 -41.409 61.097 0.00 0.00
G
ATOM 23160 HA ILE G 167 50.564 -40.747 61.680 0.00 0.00
G
ATOM 23161 CB ILE G 167 51.014 -40.935 59.663 0.00 0.00
G
ATOM 23162 HB ILE G 167 51.512 -39.949 59.542 0.00 0.00
G
ATOM 23163 CG2 ILE G 167 51.631 -41.840 58.605 0.00 0.00
G
ATOM 23164 HG21 ILE G 167 52.557 -42.303 59.009 0.00 0.00
G
ATOM 23165 HG22 ILE G 167 50.953 -42.678 58.338 0.00 0.00
G

```
ATOM  23166 HG23 ILE G 167      51.807 -41.195  57.718  0.00  0.00
G
ATOM  23167  CG1 ILE G 167      49.519 -40.748  59.276  0.00  0.00
G
ATOM  23168 HG11 ILE G 167      49.103 -41.770  59.147  0.00  0.00
G
ATOM  23169 HG12 ILE G 167      48.937 -40.377  60.146  0.00  0.00
G
ATOM  23170  CD  ILE G 167      49.332 -39.828  58.183  0.00  0.00
G
ATOM  23171  HD1 ILE G 167      49.644 -40.434  57.306  0.00  0.00
G
ATOM  23172  HD2 ILE G 167      48.282 -39.518  57.994  0.00  0.00
G
ATOM  23173  HD3 ILE G 167      49.893 -38.869  58.218  0.00  0.00
G
ATOM  23174  C   ILE G 167      50.610 -42.853  61.267  0.00  0.00
G
ATOM  23175  O   ILE G 167      49.406 -43.084  61.259  0.00  0.00
G
ATOM  23176  N   ILE G 168      51.495 -43.750  61.573  0.00  0.00
G
ATOM  23177  HN  ILE G 168      52.385 -43.351  61.781  0.00  0.00
G
ATOM  23178  CA  ILE G 168      51.109 -45.173  61.820  0.00  0.00
G
ATOM  23179  HA  ILE G 168      50.215 -45.401  61.258  0.00  0.00
G
ATOM  23180  CB  ILE G 168      52.135 -46.046  61.246  0.00  0.00
G
ATOM  23181  HB  ILE G 168      51.847 -47.110  61.390  0.00  0.00
G
ATOM  23182  CG2 ILE G 168      52.082 -45.969  59.749  0.00  0.00
G
ATOM  23183 HG21 ILE G 168      52.335 -44.924  59.467  0.00  0.00
G
ATOM  23184 HG22 ILE G 168      52.745 -46.653  59.178  0.00  0.00
G
ATOM  23185 HG23 ILE G 168      51.018 -46.138  59.481  0.00  0.00
G
ATOM  23186  CG1 ILE G 168      53.566 -45.874  61.764  0.00  0.00
G
ATOM  23187 HG11 ILE G 168      53.794 -44.786  61.778  0.00  0.00
G
ATOM  23188 HG12 ILE G 168      53.660 -46.218  62.816  0.00  0.00
G
ATOM  23189  CD  ILE G 168      54.635 -46.555  60.971  0.00  0.00
G
ATOM  23190  HD1 ILE G 168      55.073 -45.912  60.178  0.00  0.00
G
ATOM  23191  HD2 ILE G 168      55.502 -46.732  61.643  0.00  0.00
G
ATOM  23192  HD3 ILE G 168      54.272 -47.524  60.566  0.00  0.00
G
```

ATOM 23193 C   ILE G 168   50.877 -45.522 63.297 0.00 0.00
G
ATOM 23194 O   ILE G 168   50.704 -46.700 63.598 0.00 0.00
G
ATOM 23195 N   ASP G 169   51.012 -44.577 64.239 0.00 0.00
G
ATOM 23196 HN ASP G 169   51.406 -43.676 64.072 0.00 0.00
G
ATOM 23197 CA ASP G 169   50.948 -44.834 65.600 0.00 0.00
G
ATOM 23198 HA ASP G 169   50.978 -45.882 65.861 0.00 0.00
G
ATOM 23199 CB ASP G 169   52.055 -43.997 66.235 0.00 0.00
G
ATOM 23200 HB1 ASP G 169   52.956 -44.216 65.623 0.00 0.00
G
ATOM 23201 HB2 ASP G 169   51.902 -42.898 66.292 0.00 0.00
G
ATOM 23202 CG ASP G 169   52.616 -44.449 67.528 0.00 0.00
G
ATOM 23203 OD1 ASP G 169   52.428 -43.599 68.487 0.00 0.00
G
ATOM 23204 OD2 ASP G 169   53.222 -45.542 67.700 0.00 0.00
G
ATOM 23205 C   ASP G 169   49.608 -44.314 66.130 0.00 0.00
G
ATOM 23206 O   ASP G 169   49.113 -44.441 67.258 0.00 0.00
G
ATOM 23207 N   LEU G 170   48.842 -43.615 65.172 0.00 0.00
G
ATOM 23208 HN LEU G 170   49.271 -43.541 64.275 0.00 0.00
G
ATOM 23209 CA LEU G 170   47.485 -43.229 65.458 0.00 0.00
G
ATOM 23210 HA LEU G 170   47.500 -42.695 66.396 0.00 0.00
G
ATOM 23211 CB LEU G 170   46.919 -42.348 64.249 0.00 0.00
G
ATOM 23212 HB1 LEU G 170   46.992 -42.907 63.291 0.00 0.00
G
ATOM 23213 HB2 LEU G 170   45.826 -42.207 64.393 0.00 0.00
G
ATOM 23214 CG LEU G 170   47.577 -40.957 64.036 0.00 0.00
G
ATOM 23215 HG LEU G 170   48.677 -41.108 64.064 0.00 0.00
G
ATOM 23216 CD1 LEU G 170   47.079 -40.438 62.771 0.00 0.00
G
ATOM 23217 HD11 LEU G 170   47.220 -39.337 62.735 0.00 0.00
G
ATOM 23218 HD12 LEU G 170   47.611 -40.989 61.966 0.00 0.00
G
ATOM 23219 HD13 LEU G 170   46.014 -40.661 62.551 0.00 0.00
G

ATOM 23220 CD2 LEU G 170 47.332 -39.779 65.108 0.00 0.00
G
ATOM 23221 HD21 LEU G 170 47.683 -40.120 66.105 0.00 0.00
G
ATOM 23222 HD22 LEU G 170 48.008 -38.945 64.822 0.00 0.00
G
ATOM 23223 HD23 LEU G 170 46.293 -39.384 65.126 0.00 0.00
G
ATOM 23224 C LEU G 170 46.487 -44.346 65.500 0.00 0.00
G
ATOM 23225 O LEU G 170 46.572 -45.386 64.728 0.00 0.00
G
ATOM 23226 N ASN G 171 45.544 -44.239 66.435 0.00 0.00
G
ATOM 23227 HN ASN G 171 45.473 -43.521 67.123 0.00 0.00
G
ATOM 23228 CA ASN G 171 44.607 -45.254 66.693 0.00 0.00
G
ATOM 23229 HA ASN G 171 44.938 -46.207 66.307 0.00 0.00
G
ATOM 23230 CB ASN G 171 44.590 -45.458 68.210 0.00 0.00
G
ATOM 23231 HB1 ASN G 171 44.507 -44.483 68.735 0.00 0.00
G
ATOM 23232 HB2 ASN G 171 43.600 -45.882 68.482 0.00 0.00
G
ATOM 23233 CG ASN G 171 45.736 -46.351 68.727 0.00 0.00
G
ATOM 23234 OD1 ASN G 171 45.701 -47.554 68.580 0.00 0.00
G
ATOM 23235 ND2 ASN G 171 46.764 -45.705 69.328 0.00 0.00
G
ATOM 23236 HD21 ASN G 171 47.551 -46.188 69.711 0.00 0.00
G
ATOM 23237 HD22 ASN G 171 46.695 -44.732 69.546 0.00 0.00
G
ATOM 23238 C ASN G 171 43.148 -44.934 66.362 0.00 0.00
G
ATOM 23239 O ASN G 171 42.198 -45.440 66.910 0.00 0.00
G
ATOM 23240 N VAL G 172 42.964 -44.040 65.314 0.00 0.00
G
ATOM 23241 HN VAL G 172 43.662 -43.638 64.726 0.00 0.00
G
ATOM 23242 CA VAL G 172 41.692 -43.482 64.940 0.00 0.00
G
ATOM 23243 HA VAL G 172 41.183 -43.352 65.884 0.00 0.00
G
ATOM 23244 CB VAL G 172 41.901 -42.330 64.068 0.00 0.00
G
ATOM 23245 HB VAL G 172 42.376 -42.701 63.135 0.00 0.00
G
ATOM 23246 CG1 VAL G 172 40.517 -41.691 63.658 0.00 0.00
G

ATOM 23247 HG11 VAL G 172 39.966 -42.338 62.943 0.00 0.00
G
ATOM 23248 HG12 VAL G 172 39.795 -41.536 64.489 0.00 0.00
G
ATOM 23249 HG13 VAL G 172 40.775 -40.764 63.104 0.00 0.00
G
ATOM 23250 CG2 VAL G 172 42.815 -41.315 64.799 0.00 0.00
G
ATOM 23251 HG21 VAL G 172 42.695 -41.308 65.904 0.00 0.00
G
ATOM 23252 HG22 VAL G 172 43.913 -41.437 64.681 0.00 0.00
G
ATOM 23253 HG23 VAL G 172 42.500 -40.328 64.399 0.00 0.00
G
ATOM 23254 C VAL G 172 40.817 -44.523 64.322 0.00 0.00
G
ATOM 23255 O VAL G 172 39.641 -44.748 64.695 0.00 0.00
G
ATOM 23256 N ARG G 173 41.430 -45.279 63.430 0.00 0.00
G
ATOM 23257 HN ARG G 173 42.335 -45.039 63.088 0.00 0.00
G
ATOM 23258 CA ARG G 173 40.792 -46.545 62.986 0.00 0.00
G
ATOM 23259 HA ARG G 173 39.799 -46.153 62.826 0.00 0.00
G
ATOM 23260 CB ARG G 173 41.450 -46.969 61.685 0.00 0.00
G
ATOM 23261 HB1 ARG G 173 41.283 -46.143 60.961 0.00 0.00
G
ATOM 23262 HB2 ARG G 173 42.525 -47.102 61.934 0.00 0.00
G
ATOM 23263 CG ARG G 173 40.922 -48.189 61.110 0.00 0.00
G
ATOM 23264 HG1 ARG G 173 41.084 -49.139 61.663 0.00 0.00
G
ATOM 23265 HG2 ARG G 173 39.835 -48.161 60.882 0.00 0.00
G
ATOM 23266 CD ARG G 173 41.710 -48.643 59.811 0.00 0.00
G
ATOM 23267 HD1 ARG G 173 41.559 -47.883 59.015 0.00 0.00
G
ATOM 23268 HD2 ARG G 173 42.781 -48.814 60.055 0.00 0.00
G
ATOM 23269 NE ARG G 173 41.076 -49.913 59.283 0.00 0.00
G
ATOM 23270 HE ARG G 173 40.237 -50.234 59.722 0.00 0.00
G
ATOM 23271 CZ ARG G 173 41.526 -50.583 58.250 0.00 0.00
G
ATOM 23272 NH1 ARG G 173 42.494 -50.136 57.474 0.00 0.00
G
ATOM 23273 HH11 ARG G 173 42.550 -50.513 56.549 0.00 0.00
G

ATOM 23274 HH12 ARG G 173   42.820 -49.206 57.645 0.00 0.00
G
ATOM 23275 NH2 ARG G 173   40.988 -51.773 57.908 0.00 0.00
G
ATOM 23276 HH21 ARG G 173   41.515 -52.390 57.324 0.00 0.00
G
ATOM 23277 HH22 ARG G 173   40.386 -52.123 58.626 0.00 0.00
G
ATOM 23278 C   ARG G 173   40.715 -47.609 63.991 0.00 0.00
G
ATOM 23279 O   ARG G 173   39.833 -48.454 63.909 0.00 0.00
G
ATOM 23280 N   ALA G 174   41.616 -47.713 64.995 0.00 0.00
G
ATOM 23281 HN ALA G 174   42.349 -47.038 65.037 0.00 0.00
G
ATOM 23282 CA ALA G 174   41.427 -48.706 66.026 0.00 0.00
G
ATOM 23283 HA ALA G 174   41.281 -49.687 65.599 0.00 0.00
G
ATOM 23284 CB ALA G 174   42.724 -48.750 66.927 0.00 0.00
G
ATOM 23285 HB1 ALA G 174   43.558 -48.446 66.258 0.00 0.00
G
ATOM 23286 HB2 ALA G 174   42.592 -47.918 67.651 0.00 0.00
G
ATOM 23287 HB3 ALA G 174   42.806 -49.768 67.363 0.00 0.00
G
ATOM 23288 C   ALA G 174   40.217 -48.544 66.954 0.00 0.00
G
ATOM 23289 O   ALA G 174   39.461 -49.497 67.208 0.00 0.00
G
ATOM 23290 N   LYS G 175   39.968 -47.290 67.424 0.00 0.00
G
ATOM 23291 HN LYS G 175   40.464 -46.532 67.008 0.00 0.00
G
ATOM 23292 CA LYS G 175   38.935 -47.037 68.431 0.00 0.00
G
ATOM 23293 HA LYS G 175   38.701 -47.886 69.056 0.00 0.00
G
ATOM 23294 CB LYS G 175   39.559 -45.899 69.349 0.00 0.00
G
ATOM 23295 HB1 LYS G 175   40.665 -45.972 69.418 0.00 0.00
G
ATOM 23296 HB2 LYS G 175   39.393 -44.865 68.976 0.00 0.00
G
ATOM 23297 CG LYS G 175   38.974 -45.822 70.776 0.00 0.00
G
ATOM 23298 HG1 LYS G 175   37.966 -45.360 70.713 0.00 0.00
G
ATOM 23299 HG2 LYS G 175   38.893 -46.828 71.240 0.00 0.00
G
ATOM 23300 CD LYS G 175   39.830 -44.952 71.682 0.00 0.00
G

ATOM 23301 HD1 LYS G 175 40.848 -45.360 71.504 0.00 0.00
G
ATOM 23302 HD2 LYS G 175 39.899 -43.925 71.264 0.00 0.00
G
ATOM 23303 CE LYS G 175 39.473 -44.886 73.148 0.00 0.00
G
ATOM 23304 HE1 LYS G 175 39.657 -45.903 73.554 0.00 0.00
G
ATOM 23305 HE2 LYS G 175 40.255 -44.253 73.618 0.00 0.00
G
ATOM 23306 NZ LYS G 175 38.122 -44.354 73.518 0.00 0.00
G
ATOM 23307 HZ1 LYS G 175 37.429 -44.552 72.768 0.00 0.00
G
ATOM 23308 HZ2 LYS G 175 37.800 -44.743 74.427 0.00 0.00
G
ATOM 23309 HZ3 LYS G 175 38.103 -43.318 73.609 0.00 0.00
G
ATOM 23310 C LYS G 175 37.582 -46.537 67.931 0.00 0.00
G
ATOM 23311 O LYS G 175 36.486 -46.950 68.489 0.00 0.00
G
ATOM 23312 N TYR G 176 37.598 -45.654 66.985 0.00 0.00
G
ATOM 23313 HN TYR G 176 38.487 -45.439 66.589 0.00 0.00
G
ATOM 23314 CA TYR G 176 36.445 -44.976 66.438 0.00 0.00
G
ATOM 23315 HA TYR G 176 35.544 -45.098 67.021 0.00 0.00
G
ATOM 23316 CB TYR G 176 36.716 -43.448 66.061 0.00 0.00
G
ATOM 23317 HB1 TYR G 176 37.300 -43.236 65.140 0.00 0.00
G
ATOM 23318 HB2 TYR G 176 35.792 -42.868 65.851 0.00 0.00
G
ATOM 23319 CG TYR G 176 37.384 -42.858 67.213 0.00 0.00
G
ATOM 23320 CD1 TYR G 176 38.716 -42.469 67.155 0.00 0.00
G
ATOM 23321 HD1 TYR G 176 39.251 -42.736 66.255 0.00 0.00
G
ATOM 23322 CE1 TYR G 176 39.401 -41.928 68.253 0.00 0.00
G
ATOM 23323 HE1 TYR G 176 40.444 -41.670 68.142 0.00 0.00
G
ATOM 23324 CZ TYR G 176 38.723 -41.737 69.437 0.00 0.00
G
ATOM 23325 OH TYR G 176 39.344 -41.175 70.561 0.00 0.00
G
ATOM 23326 HH TYR G 176 38.885 -41.474 71.350 0.00 0.00
G
ATOM 23327 CD2 TYR G 176 36.670 -42.637 68.441 0.00 0.00
G

ATOM 23328 HD2 TYR G 176   35.635 -42.944 68.471 0.00 0.00
G
ATOM 23329 CE2 TYR G 176   37.367 -42.072 69.529 0.00 0.00
G
ATOM 23330 HE2 TYR G 176   36.743 -41.881 70.390 0.00 0.00
G
ATOM 23331 C   TYR G 176   36.036 -45.633 65.140 0.00 0.00
G
ATOM 23332 O   TYR G 176   34.921 -45.491 64.763 0.00 0.00
G
ATOM 23333 N   GLY G 177   36.834 -46.389 64.380 0.00 0.00
G
ATOM 23334 HN GLY G 177   37.746 -46.484 64.771 0.00 0.00
G
ATOM 23335 CA GLY G 177   36.591 -47.199 63.171 0.00 0.00
G
ATOM 23336 HA1 GLY G 177   35.660 -47.736 63.277 0.00 0.00
G
ATOM 23337 HA2 GLY G 177   37.376 -47.926 63.023 0.00 0.00
G
ATOM 23338 C   GLY G 177   36.575 -46.293 61.960 0.00 0.00
G
ATOM 23339 O   GLY G 177   36.254 -46.583 60.851 0.00 0.00
G
ATOM 23340 N   CYS G 178   37.028 -45.055 62.220 0.00 0.00
G
ATOM 23341 HN CYS G 178   37.402 -44.796 63.108 0.00 0.00
G
ATOM 23342 CA CYS G 178   37.109 -44.020 61.227 0.00 0.00
G
ATOM 23343 HA CYS G 178   36.259 -44.175 60.580 0.00 0.00
G
ATOM 23344 CB CYS G 178   36.979 -42.684 61.904 0.00 0.00
G
ATOM 23345 HB1 CYS G 178   37.882 -42.616 62.547 0.00 0.00
G
ATOM 23346 HB2 CYS G 178   36.908 -41.874 61.147 0.00 0.00
G
ATOM 23347 SG CYS G 178   35.503 -42.499 63.030 0.00 0.00
G
ATOM 23348 HG1 CYS G 178   35.711 -41.266 63.468 0.00 0.00
G
ATOM 23349 C   CYS G 178   38.401 -44.134 60.399 0.00 0.00
G
ATOM 23350 O   CYS G 178   39.514 -44.110 60.904 0.00 0.00
G
ATOM 23351 N   THR G 179   38.180 -44.433 59.122 0.00 0.00
G
ATOM 23352 HN THR G 179   37.249 -44.435 58.765 0.00 0.00
G
ATOM 23353 CA THR G 179   39.234 -44.611 58.168 0.00 0.00
G
ATOM 23354 HA THR G 179   40.051 -45.052 58.719 0.00 0.00
G

```
ATOM  23355  CB  THR G 179      38.763 -45.401  56.960  0.00  0.00
G
ATOM  23356  HB  THR G 179      38.452 -46.431  57.238  0.00  0.00
G
ATOM  23357  OG1 THR G 179      39.922 -45.796  56.175  0.00  0.00
G
ATOM  23358  HG1 THR G 179      39.787 -46.603  55.672  0.00  0.00
G
ATOM  23359  CG2 THR G 179      37.702 -44.672  56.099  0.00  0.00
G
ATOM  23360 HG21 THR G 179      38.199 -43.891  55.484  0.00  0.00
G
ATOM  23361 HG22 THR G 179      37.240 -45.381  55.380  0.00  0.00
G
ATOM  23362 HG23 THR G 179      36.929 -44.229  56.762  0.00  0.00
G
ATOM  23363  C   THR G 179      39.807 -43.301  57.685  0.00  0.00
G
ATOM  23364  O   THR G 179      39.086 -42.336  57.527  0.00  0.00
G
ATOM  23365  N   ILE G 180      41.161 -43.273  57.457  0.00  0.00
G
ATOM  23366  HN  ILE G 180      41.783 -44.002  57.734  0.00  0.00
G
ATOM  23367  CA  ILE G 180      41.788 -41.985  57.034  0.00  0.00
G
ATOM  23368  HA  ILE G 180      41.201 -41.089  57.168  0.00  0.00
G
ATOM  23369  CB  ILE G 180      43.283 -41.779  57.595  0.00  0.00
G
ATOM  23370  HB  ILE G 180      43.903 -42.666  57.341  0.00  0.00
G
ATOM  23371  CG2 ILE G 180      43.969 -40.498  56.967  0.00  0.00
G
ATOM  23372 HG21 ILE G 180      43.320 -39.608  57.112  0.00  0.00
G
ATOM  23373 HG22 ILE G 180      44.928 -40.333  57.503  0.00  0.00
G
ATOM  23374 HG23 ILE G 180      44.023 -40.660  55.869  0.00  0.00
G
ATOM  23375  CG1 ILE G 180      43.213 -41.768  59.133  0.00  0.00
G
ATOM  23376 HG11 ILE G 180      42.359 -41.153  59.490  0.00  0.00
G
ATOM  23377 HG12 ILE G 180      42.929 -42.807  59.406  0.00  0.00
G
ATOM  23378  CD  ILE G 180      44.581 -41.454  59.801  0.00  0.00
G
ATOM  23379  HD1 ILE G 180      44.757 -40.360  59.725  0.00  0.00
G
ATOM  23380  HD2 ILE G 180      44.625 -41.816  60.851  0.00  0.00
G
ATOM  23381  HD3 ILE G 180      45.384 -41.965  59.228  0.00  0.00
G
```

ATOM 23382 C   ILE G 180   41.966 -42.037 55.544 0.00 0.00
G
ATOM 23383 O   ILE G 180   42.702 -42.861 54.964 0.00 0.00
G
ATOM 23384 N   LEU G 181   41.137 -41.308 54.796 0.00 0.00
G
ATOM 23385 HN LEU G 181   40.411 -40.711 55.129 0.00 0.00
G
ATOM 23386 CA LEU G 181   41.226 -41.190 53.337 0.00 0.00
G
ATOM 23387 HA LEU G 181   41.157 -42.153 52.853 0.00 0.00
G
ATOM 23388 CB LEU G 181   40.119 -40.121 52.979 0.00 0.00
G
ATOM 23389 HB1 LEU G 181   40.374 -39.101 53.338 0.00 0.00
G
ATOM 23390 HB2 LEU G 181   40.066 -40.036 51.872 0.00 0.00
G
ATOM 23391 CG LEU G 181   38.725 -40.460 53.499 0.00 0.00
G
ATOM 23392 HG LEU G 181   38.699 -40.358 54.605 0.00 0.00
G
ATOM 23393 CD1 LEU G 181   37.867 -39.446 52.772 0.00 0.00
G
ATOM 23394 HD11 LEU G 181   36.782 -39.632 52.921 0.00 0.00
G
ATOM 23395 HD12 LEU G 181   37.949 -38.362 53.001 0.00 0.00
G
ATOM 23396 HD13 LEU G 181   38.100 -39.672 51.710 0.00 0.00
G
ATOM 23397 CD2 LEU G 181   38.240 -41.879 53.172 0.00 0.00
G
ATOM 23398 HD21 LEU G 181   38.743 -42.581 53.871 0.00 0.00
G
ATOM 23399 HD22 LEU G 181   37.146 -42.024 53.301 0.00 0.00
G
ATOM 23400 HD23 LEU G 181   38.500 -42.251 52.158 0.00 0.00
G
ATOM 23401 C   LEU G 181   42.517 -40.648 52.921 0.00 0.00
G
ATOM 23402 O   LEU G 181   43.240 -41.164 52.035 0.00 0.00
G
ATOM 23403 N   ALA G 182   43.024 -39.571 53.604 0.00 0.00
G
ATOM 23404 HN ALA G 182   42.672 -39.349 54.510 0.00 0.00
G
ATOM 23405 CA ALA G 182   44.237 -38.932 53.268 0.00 0.00
G
ATOM 23406 HA ALA G 182   44.987 -39.694 53.117 0.00 0.00
G
ATOM 23407 CB ALA G 182   44.015 -37.974 52.108 0.00 0.00
G
ATOM 23408 HB1 ALA G 182   43.178 -37.311 52.416 0.00 0.00
G

ATOM 23409 HB2 ALA G 182   44.833 -37.336 51.710 0.00 0.00
G
ATOM 23410 HB3 ALA G 182   43.743 -38.626 51.251 0.00 0.00
G
ATOM 23411 C   ALA G 182   44.989 -38.096 54.174 0.00 0.00
G
ATOM 23412 O   ALA G 182   44.405 -37.790 55.274 0.00 0.00
G
ATOM 23413 N   ILE G 183   46.261 -37.703 53.774 0.00 0.00
G
ATOM 23414 HN ILE G 183   46.692 -37.969 52.915 0.00 0.00
G
ATOM 23415 CA ILE G 183   46.980 -36.599 54.468 0.00 0.00
G
ATOM 23416 HA ILE G 183   46.414 -36.168 55.281 0.00 0.00
G
ATOM 23417 CB ILE G 183   48.319 -37.125 54.912 0.00 0.00
G
ATOM 23418 HB ILE G 183   48.159 -37.913 55.680 0.00 0.00
G
ATOM 23419 CG2 ILE G 183   49.099 -37.841 53.785 0.00 0.00
G
ATOM 23420 HG21 ILE G 183   50.084 -38.269 54.071 0.00 0.00
G
ATOM 23421 HG22 ILE G 183   48.603 -38.632 53.183 0.00 0.00
G
ATOM 23422 HG23 ILE G 183   49.358 -37.051 53.047 0.00 0.00
G
ATOM 23423 CG1 ILE G 183   49.309 -36.101 55.491 0.00 0.00
G
ATOM 23424 HG11 ILE G 183   50.352 -36.473 55.581 0.00 0.00
G
ATOM 23425 HG12 ILE G 183   49.358 -35.230 54.804 0.00 0.00
G
ATOM 23426 CD ILE G 183   48.975 -35.599 56.897 0.00 0.00
G
ATOM 23427 HD1 ILE G 183   47.888 -35.367 56.914 0.00 0.00
G
ATOM 23428 HD2 ILE G 183   49.200 -36.392 57.641 0.00 0.00
G
ATOM 23429 HD3 ILE G 183   49.575 -34.708 57.182 0.00 0.00
G
ATOM 23430 C   ILE G 183   47.092 -35.596 53.400 0.00 0.00
G
ATOM 23431 O   ILE G 183   47.227 -35.893 52.233 0.00 0.00
G
ATOM 23432 N   LYS G 184   47.034 -34.239 53.721 0.00 0.00
G
ATOM 23433 HN LYS G 184   46.719 -33.937 54.618 0.00 0.00
G
ATOM 23434 CA LYS G 184   47.159 -33.224 52.707 0.00 0.00
G
ATOM 23435 HA LYS G 184   47.595 -33.577 51.784 0.00 0.00
G

ATOM 23436 CB LYS G 184 45.769 -32.680 52.220 0.00 0.00
G
ATOM 23437 HB1 LYS G 184 46.020 -31.845 51.532 0.00 0.00
G
ATOM 23438 HB2 LYS G 184 45.351 -33.590 51.739 0.00 0.00
G
ATOM 23439 CG LYS G 184 44.813 -32.135 53.347 0.00 0.00
G
ATOM 23440 HG1 LYS G 184 44.725 -32.935 54.113 0.00 0.00
G
ATOM 23441 HG2 LYS G 184 45.241 -31.179 53.718 0.00 0.00
G
ATOM 23442 CD LYS G 184 43.479 -31.633 52.805 0.00 0.00
G
ATOM 23443 HD1 LYS G 184 42.916 -32.361 52.182 0.00 0.00
G
ATOM 23444 HD2 LYS G 184 42.727 -31.290 53.547 0.00 0.00
G
ATOM 23445 CE LYS G 184 43.603 -30.339 51.942 0.00 0.00
G
ATOM 23446 HE1 LYS G 184 44.285 -29.616 52.438 0.00 0.00
G
ATOM 23447 HE2 LYS G 184 43.968 -30.766 50.983 0.00 0.00
G
ATOM 23448 NZ LYS G 184 42.309 -29.699 51.780 0.00 0.00
G
ATOM 23449 HZ1 LYS G 184 41.526 -29.970 52.407 0.00 0.00
G
ATOM 23450 HZ2 LYS G 184 42.475 -28.684 51.937 0.00 0.00
G
ATOM 23451 HZ3 LYS G 184 41.960 -29.816 50.807 0.00 0.00
G
ATOM 23452 C LYS G 184 48.135 -32.145 53.310 0.00 0.00
G
ATOM 23453 O LYS G 184 47.873 -31.533 54.375 0.00 0.00
G
ATOM 23454 N HSE G 185 49.264 -32.032 52.569 0.00 0.00
G
ATOM 23455 HN HSE G 185 49.394 -32.479 51.686 0.00 0.00
G
ATOM 23456 CA HSE G 185 50.440 -31.336 52.820 0.00 0.00
G
ATOM 23457 HA HSE G 185 50.376 -30.800 53.756 0.00 0.00
G
ATOM 23458 CB HSE G 185 51.559 -32.296 52.961 0.00 0.00
G
ATOM 23459 HB1 HSE G 185 51.321 -33.031 53.760 0.00 0.00
G
ATOM 23460 HB2 HSE G 185 51.681 -32.879 52.024 0.00 0.00
G
ATOM 23461 ND1 HSE G 185 53.455 -31.042 54.197 0.00 0.00
G
ATOM 23462 CG HSE G 185 52.975 -31.718 53.118 0.00 0.00
G

ATOM 23463 CE1 HSE G 185 54.678 -30.743 53.940 0.00 0.00
G
ATOM 23464 HE1 HSE G 185 55.414 -30.132 54.461 0.00 0.00
G
ATOM 23465 NE2 HSE G 185 55.035 -31.121 52.740 0.00 0.00
G
ATOM 23466 HE2 HSE G 185 55.912 -30.803 52.378 0.00 0.00
G
ATOM 23467 CD2 HSE G 185 54.006 -31.797 52.192 0.00 0.00
G
ATOM 23468 HD2 HSE G 185 53.900 -32.271 51.224 0.00 0.00
G
ATOM 23469 C HSE G 185 50.829 -30.297 51.726 0.00 0.00
G
ATOM 23470 O HSE G 185 51.165 -30.656 50.605 0.00 0.00
G
ATOM 23471 N HSE G 186 50.926 -28.976 52.088 0.00 0.00
G
ATOM 23472 HN HSE G 186 50.353 -28.794 52.884 0.00 0.00
G
ATOM 23473 CA HSE G 186 51.358 -27.927 51.141 0.00 0.00
G
ATOM 23474 HA HSE G 186 51.238 -26.959 51.604 0.00 0.00
G
ATOM 23475 CB HSE G 186 52.897 -27.804 50.885 0.00 0.00
G
ATOM 23476 HB1 HSE G 186 53.554 -27.860 51.779 0.00 0.00
G
ATOM 23477 HB2 HSE G 186 53.217 -28.630 50.215 0.00 0.00
G
ATOM 23478 ND1 HSE G 186 53.049 -25.381 50.555 0.00 0.00
G
ATOM 23479 CG HSE G 186 53.215 -26.618 50.013 0.00 0.00
G
ATOM 23480 CE1 HSE G 186 53.441 -24.527 49.619 0.00 0.00
G
ATOM 23481 HE1 HSE G 186 53.684 -23.494 49.867 0.00 0.00
G
ATOM 23482 NE2 HSE G 186 53.808 -25.180 48.479 0.00 0.00
G
ATOM 23483 HE2 HSE G 186 54.471 -24.916 47.778 0.00 0.00
G
ATOM 23484 CD2 HSE G 186 53.645 -26.557 48.751 0.00 0.00
G
ATOM 23485 HD2 HSE G 186 53.773 -27.364 48.040 0.00 0.00
G
ATOM 23486 C HSE G 186 50.478 -27.900 49.907 0.00 0.00
G
ATOM 23487 O HSE G 186 50.827 -27.820 48.745 0.00 0.00
G
ATOM 23488 N GLY G 187 49.117 -28.009 50.091 0.00 0.00
G
ATOM 23489 HN GLY G 187 48.646 -27.941 50.967 0.00 0.00
G

ATOM 23490 CA GLY G 187 48.176 -28.127 48.973 0.00 0.00
G
ATOM 23491 HA1 GLY G 187 48.417 -27.410 48.203 0.00 0.00
G
ATOM 23492 HA2 GLY G 187 47.165 -27.793 49.153 0.00 0.00
G
ATOM 23493 C GLY G 187 48.126 -29.447 48.211 0.00 0.00
G
ATOM 23494 O GLY G 187 47.193 -29.663 47.414 0.00 0.00
G
ATOM 23495 N ASP G 188 49.075 -30.364 48.493 0.00 0.00
G
ATOM 23496 HN ASP G 188 49.801 -30.171 49.149 0.00 0.00
G
ATOM 23497 CA ASP G 188 49.212 -31.684 47.947 0.00 0.00
G
ATOM 23498 HA ASP G 188 48.682 -31.680 47.006 0.00 0.00
G
ATOM 23499 CB ASP G 188 50.662 -31.840 47.477 0.00 0.00
G
ATOM 23500 HB1 ASP G 188 50.785 -31.025 46.733 0.00 0.00
G
ATOM 23501 HB2 ASP G 188 51.374 -31.697 48.318 0.00 0.00
G
ATOM 23502 CG ASP G 188 50.885 -33.114 46.721 0.00 0.00
G
ATOM 23503 OD1 ASP G 188 51.469 -34.040 47.349 0.00 0.00
G
ATOM 23504 OD2 ASP G 188 50.345 -33.287 45.622 0.00 0.00
G
ATOM 23505 C ASP G 188 48.665 -32.860 48.859 0.00 0.00
G
ATOM 23506 O ASP G 188 48.925 -32.993 50.027 0.00 0.00
G
ATOM 23507 N ILE G 189 47.761 -33.627 48.284 0.00 0.00
G
ATOM 23508 HN ILE G 189 47.707 -33.599 47.289 0.00 0.00
G
ATOM 23509 CA ILE G 189 47.081 -34.686 48.980 0.00 0.00
G
ATOM 23510 HA ILE G 189 47.346 -34.631 50.026 0.00 0.00
G
ATOM 23511 CB ILE G 189 45.538 -34.672 48.794 0.00 0.00
G
ATOM 23512 HB ILE G 189 45.290 -33.610 49.006 0.00 0.00
G
ATOM 23513 CG2 ILE G 189 45.175 -34.919 47.286 0.00 0.00
G
ATOM 23514 HG21 ILE G 189 45.649 -34.120 46.678 0.00 0.00
G
ATOM 23515 HG22 ILE G 189 45.470 -35.905 46.869 0.00 0.00
G
ATOM 23516 HG23 ILE G 189 44.079 -34.773 47.175 0.00 0.00
G

ATOM 23517 CG1 ILE G 189   44.730 -35.665 49.677 0.00 0.00
G
ATOM 23518 HG11 ILE G 189   44.729 -36.702 49.279 0.00 0.00
G
ATOM 23519 HG12 ILE G 189   45.169 -35.576 50.694 0.00 0.00
G
ATOM 23520 CD ILE G 189   43.197 -35.336 49.763 0.00 0.00
G
ATOM 23521 HD1 ILE G 189   42.971 -34.306 50.114 0.00 0.00
G
ATOM 23522 HD2 ILE G 189   42.753 -35.466 48.753 0.00 0.00
G
ATOM 23523 HD3 ILE G 189   42.721 -36.097 50.417 0.00 0.00
G
ATOM 23524 C   ILE G 189   47.583 -36.096 48.536 0.00 0.00
G
ATOM 23525 O   ILE G 189   47.928 -36.330 47.358 0.00 0.00
G
ATOM 23526 N   CYS G 190   47.790 -36.893 49.554 0.00 0.00
G
ATOM 23527 HN CYS G 190   47.427 -36.658 50.452 0.00 0.00
G
ATOM 23528 CA CYS G 190   48.263 -38.207 49.409 0.00 0.00
G
ATOM 23529 HA CYS G 190   48.359 -38.421 48.355 0.00 0.00
G
ATOM 23530 CB CYS G 190   49.683 -38.300 49.914 0.00 0.00
G
ATOM 23531 HB1 CYS G 190   50.194 -37.508 49.327 0.00 0.00
G
ATOM 23532 HB2 CYS G 190   49.670 -38.158 51.016 0.00 0.00
G
ATOM 23533 SG CYS G 190   50.527 -39.974 49.680 0.00 0.00
G
ATOM 23534 HG1 CYS G 190   50.197 -40.138 48.408 0.00 0.00
G
ATOM 23535 C   CYS G 190   47.237 -39.190 49.978 0.00 0.00
G
ATOM 23536 O   CYS G 190   46.755 -39.124 51.088 0.00 0.00
G
ATOM 23537 N   LEU G 191   46.789 -40.063 49.073 0.00 0.00
G
ATOM 23538 HN LEU G 191   47.005 -40.006 48.101 0.00 0.00
G
ATOM 23539 CA LEU G 191   45.711 -41.000 49.301 0.00 0.00
G
ATOM 23540 HA LEU G 191   44.996 -40.539 49.968 0.00 0.00
G
ATOM 23541 CB LEU G 191   44.986 -41.275 48.000 0.00 0.00
G
ATOM 23542 HB1 LEU G 191   45.680 -41.424 47.146 0.00 0.00
G
ATOM 23543 HB2 LEU G 191   44.487 -42.257 48.144 0.00 0.00
G

ATOM 23544 CG LEU G 191   43.989 -40.148 47.563 0.00 0.00
G
ATOM 23545 HG LEU G 191   44.545 -39.186 47.563 0.00 0.00
G
ATOM 23546 CD1 LEU G 191   43.398 -40.347 46.151 0.00 0.00
G
ATOM 23547 HD11 LEU G 191   44.148 -40.270 45.335 0.00 0.00
G
ATOM 23548 HD12 LEU G 191   42.793 -41.263 45.976 0.00 0.00
G
ATOM 23549 HD13 LEU G 191   42.651 -39.556 45.927 0.00 0.00
G
ATOM 23550 CD2 LEU G 191   42.790 -39.937 48.428 0.00 0.00
G
ATOM 23551 HD21 LEU G 191   41.895 -40.563 48.227 0.00 0.00
G
ATOM 23552 HD22 LEU G 191   43.059 -40.180 49.479 0.00 0.00
G
ATOM 23553 HD23 LEU G 191   42.541 -38.856 48.370 0.00 0.00
G
ATOM 23554 C   LEU G 191   46.153 -42.273 49.938 0.00 0.00
G
ATOM 23555 O   LEU G 191   47.315 -42.715 49.670 0.00 0.00
G
ATOM 23556 N   SER G 192   45.302 -42.810 50.806 0.00 0.00
G
ATOM 23557 HN SER G 192   44.507 -42.304 51.129 0.00 0.00
G
ATOM 23558 CA SER G 192   45.536 -43.867 51.809 0.00 0.00
G
ATOM 23559 HA SER G 192   44.874 -43.552 52.602 0.00 0.00
G
ATOM 23560 CB SER G 192   45.121 -45.349 51.400 0.00 0.00
G
ATOM 23561 HB1 SER G 192   45.756 -45.615 50.528 0.00 0.00
G
ATOM 23562 HB2 SER G 192   45.548 -45.983 52.206 0.00 0.00
G
ATOM 23563 OG SER G 192   43.659 -45.558 51.141 0.00 0.00
G
ATOM 23564 HG1 SER G 192   43.601 -46.425 50.733 0.00 0.00
G
ATOM 23565 C   SER G 192   46.948 -43.941 52.390 0.00 0.00
G
ATOM 23566 O   SER G 192   47.789 -44.696 51.804 0.00 0.00
G
ATOM 23567 N   PRO G 193   47.184 -43.261 53.514 0.00 0.00
G
ATOM 23568 CD PRO G 193   46.309 -42.289 54.009 0.00 0.00
G
ATOM 23569 HD1 PRO G 193   45.804 -41.729 53.195 0.00 0.00
G
ATOM 23570 HD2 PRO G 193   45.447 -42.758 54.530 0.00 0.00
G

ATOM 23571 CA PRO G 193 48.422 -43.226 54.274 0.00 0.00
G
ATOM 23572 HA PRO G 193 49.041 -42.800 53.499 0.00 0.00
G
ATOM 23573 CB PRO G 193 48.205 -42.296 55.455 0.00 0.00
G
ATOM 23574 HB1 PRO G 193 49.070 -41.663 55.748 0.00 0.00
G
ATOM 23575 HB2 PRO G 193 47.896 -42.854 56.366 0.00 0.00
G
ATOM 23576 CG PRO G 193 47.062 -41.439 55.054 0.00 0.00
G
ATOM 23577 HG1 PRO G 193 47.527 -40.530 54.616 0.00 0.00
G
ATOM 23578 HG2 PRO G 193 46.489 -41.125 55.952 0.00 0.00
G
ATOM 23579 C PRO G 193 49.201 -44.544 54.617 0.00 0.00
G
ATOM 23580 O PRO G 193 48.623 -45.628 54.500 0.00 0.00
G
ATOM 23581 N ALA G 194 50.498 -44.460 55.023 0.00 0.00
G
ATOM 23582 HN ALA G 194 50.925 -43.562 55.103 0.00 0.00
G
ATOM 23583 CA ALA G 194 51.538 -45.609 55.164 0.00 0.00
G
ATOM 23584 HA ALA G 194 50.970 -46.412 55.609 0.00 0.00
G
ATOM 23585 CB ALA G 194 52.115 -46.033 53.746 0.00 0.00
G
ATOM 23586 HB1 ALA G 194 51.304 -46.300 53.035 0.00 0.00
G
ATOM 23587 HB2 ALA G 194 52.625 -45.147 53.310 0.00 0.00
G
ATOM 23588 HB3 ALA G 194 52.888 -46.822 53.868 0.00 0.00
G
ATOM 23589 C ALA G 194 52.703 -45.209 56.048 0.00 0.00
G
ATOM 23590 O ALA G 194 52.719 -43.994 56.416 0.00 0.00
G
ATOM 23591 N PRO G 195 53.638 -45.996 56.519 0.00 0.00
G
ATOM 23592 CD PRO G 195 53.586 -47.454 56.470 0.00 0.00
G
ATOM 23593 HD1 PRO G 195 52.691 -47.837 57.005 0.00 0.00
G
ATOM 23594 HD2 PRO G 195 53.745 -47.944 55.486 0.00 0.00
G
ATOM 23595 CA PRO G 195 54.785 -45.527 57.250 0.00 0.00
G
ATOM 23596 HA PRO G 195 54.377 -45.259 58.213 0.00 0.00
G
ATOM 23597 CB PRO G 195 55.641 -46.766 57.265 0.00 0.00
G

ATOM 23598 HB1 PRO G 195 56.280 -46.658 58.167 0.00 0.00
G
ATOM 23599 HB2 PRO G 195 56.246 -46.911 56.345 0.00 0.00
G
ATOM 23600 CG PRO G 195 54.710 -47.974 57.401 0.00 0.00
G
ATOM 23601 HG1 PRO G 195 54.327 -48.193 58.421 0.00 0.00
G
ATOM 23602 HG2 PRO G 195 55.150 -48.935 57.059 0.00 0.00
G
ATOM 23603 C PRO G 195 55.616 -44.406 56.559 0.00 0.00
G
ATOM 23604 O PRO G 195 55.697 -44.438 55.350 0.00 0.00
G
ATOM 23605 N GLU G 196 56.161 -43.430 57.251 0.00 0.00
G
ATOM 23606 HN GLU G 196 56.608 -42.759 56.664 0.00 0.00
G
ATOM 23607 CA GLU G 196 56.476 -43.289 58.684 0.00 0.00
G
ATOM 23608 HA GLU G 196 55.595 -43.580 59.238 0.00 0.00
G
ATOM 23609 CB GLU G 196 57.629 -44.196 59.158 0.00 0.00
G
ATOM 23610 HB1 GLU G 196 57.837 -43.969 60.226 0.00 0.00
G
ATOM 23611 HB2 GLU G 196 57.253 -45.236 59.059 0.00 0.00
G
ATOM 23612 CG GLU G 196 58.928 -44.119 58.281 0.00 0.00
G
ATOM 23613 HG1 GLU G 196 58.710 -44.438 57.239 0.00 0.00
G
ATOM 23614 HG2 GLU G 196 59.380 -43.110 58.167 0.00 0.00
G
ATOM 23615 CD GLU G 196 60.091 -44.922 58.936 0.00 0.00
G
ATOM 23616 OE1 GLU G 196 60.417 -44.662 60.146 0.00 0.00
G
ATOM 23617 OE2 GLU G 196 60.756 -45.751 58.248 0.00 0.00
G
ATOM 23618 C GLU G 196 56.678 -41.822 59.086 0.00 0.00
G
ATOM 23619 O GLU G 196 57.370 -41.504 60.004 0.00 0.00
G
ATOM 23620 N ASP G 197 56.064 -40.924 58.317 0.00 0.00
G
ATOM 23621 HN ASP G 197 55.456 -41.195 57.575 0.00 0.00
G
ATOM 23622 CA ASP G 197 56.295 -39.478 58.440 0.00 0.00
G
ATOM 23623 HA ASP G 197 57.291 -39.269 58.800 0.00 0.00
G
ATOM 23624 CB ASP G 197 56.028 -38.753 57.053 0.00 0.00
G

```
ATOM  23625  HB1 ASP G 197      55.232 -39.164  56.397  0.00  0.00
G
ATOM  23626  HB2 ASP G 197      55.854 -37.712  57.400  0.00  0.00
G
ATOM  23627  CG  ASP G 197      57.289 -38.681  56.280  0.00  0.00
G
ATOM  23628  OD1 ASP G 197      58.360 -38.160  56.720  0.00  0.00
G
ATOM  23629  OD2 ASP G 197      57.338 -39.092  55.080  0.00  0.00
G
ATOM  23630  C   ASP G 197      55.352 -38.821  59.445  0.00  0.00
G
ATOM  23631  O   ASP G 197      54.136 -39.093  59.614  0.00  0.00
G
ATOM  23632  N   ILE G 198      56.035 -37.952  60.314  0.00  0.00
G
ATOM  23633  HN  ILE G 198      56.956 -37.681  60.043  0.00  0.00
G
ATOM  23634  CA  ILE G 198      55.385 -37.420  61.496  0.00  0.00
G
ATOM  23635  HA  ILE G 198      54.847 -38.165  62.064  0.00  0.00
G
ATOM  23636  CB  ILE G 198      56.435 -36.793  62.484  0.00  0.00
G
ATOM  23637  HB  ILE G 198      55.876 -36.613  63.427  0.00  0.00
G
ATOM  23638  CG2 ILE G 198      57.506 -37.824  62.611  0.00  0.00
G
ATOM  23639 HG21 ILE G 198      57.061 -38.776  62.972  0.00  0.00
G
ATOM  23640 HG22 ILE G 198      58.209 -37.848  61.751  0.00  0.00
G
ATOM  23641 HG23 ILE G 198      58.132 -37.552  63.488  0.00  0.00
G
ATOM  23642  CG1 ILE G 198      57.063 -35.378  62.043  0.00  0.00
G
ATOM  23643 HG11 ILE G 198      57.572 -35.467  61.059  0.00  0.00
G
ATOM  23644 HG12 ILE G 198      56.291 -34.629  61.765  0.00  0.00
G
ATOM  23645  CD  ILE G 198      58.118 -34.771  62.971  0.00  0.00
G
ATOM  23646  HD1 ILE G 198      57.621 -34.768  63.965  0.00  0.00
G
ATOM  23647  HD2 ILE G 198      59.052 -35.352  62.816  0.00  0.00
G
ATOM  23648  HD3 ILE G 198      58.338 -33.734  62.639  0.00  0.00
G
ATOM  23649  C   ILE G 198      54.282 -36.414  61.196  0.00  0.00
G
ATOM  23650  O   ILE G 198      54.339 -35.611  60.269  0.00  0.00
G
ATOM  23651  N   ILE G 199      53.168 -36.372  61.989  0.00  0.00
G
```

ATOM 23652 HN ILE G 199 52.919 -37.055 62.671 0.00 0.00
G
ATOM 23653 CA ILE G 199 52.260 -35.236 61.864 0.00 0.00
G
ATOM 23654 HA ILE G 199 51.990 -35.172 60.820 0.00 0.00
G
ATOM 23655 CB ILE G 199 51.015 -35.641 62.591 0.00 0.00
G
ATOM 23656 HB ILE G 199 51.271 -35.834 63.655 0.00 0.00
G
ATOM 23657 CG2 ILE G 199 49.965 -34.517 62.598 0.00 0.00
G
ATOM 23658 HG21 ILE G 199 49.852 -34.272 61.521 0.00 0.00
G
ATOM 23659 HG22 ILE G 199 48.990 -34.818 63.039 0.00 0.00
G
ATOM 23660 HG23 ILE G 199 50.285 -33.605 63.147 0.00 0.00
G
ATOM 23661 CG1 ILE G 199 50.284 -36.882 61.964 0.00 0.00
G
ATOM 23662 HG11 ILE G 199 50.889 -37.784 62.197 0.00 0.00
G
ATOM 23663 HG12 ILE G 199 49.322 -36.958 62.515 0.00 0.00
G
ATOM 23664 CD ILE G 199 50.014 -36.789 60.448 0.00 0.00
G
ATOM 23665 HD1 ILE G 199 51.013 -36.830 59.963 0.00 0.00
G
ATOM 23666 HD2 ILE G 199 49.376 -37.578 59.995 0.00 0.00
G
ATOM 23667 HD3 ILE G 199 49.440 -35.848 60.309 0.00 0.00
G
ATOM 23668 C ILE G 199 52.891 -33.935 62.270 0.00 0.00
G
ATOM 23669 O ILE G 199 53.543 -33.829 63.311 0.00 0.00
G
ATOM 23670 N ARG G 200 52.643 -32.859 61.535 0.00 0.00
G
ATOM 23671 HN ARG G 200 52.089 -32.961 60.712 0.00 0.00
G
ATOM 23672 CA ARG G 200 53.153 -31.565 61.792 0.00 0.00
G
ATOM 23673 HA ARG G 200 53.886 -31.674 62.578 0.00 0.00
G
ATOM 23674 CB ARG G 200 53.924 -30.999 60.608 0.00 0.00
G
ATOM 23675 HB1 ARG G 200 54.648 -31.756 60.238 0.00 0.00
G
ATOM 23676 HB2 ARG G 200 53.259 -30.755 59.752 0.00 0.00
G
ATOM 23677 CG ARG G 200 54.846 -29.747 60.916 0.00 0.00
G
ATOM 23678 HG1 ARG G 200 54.931 -29.179 59.965 0.00 0.00
G

ATOM 23679 HG2 ARG G 200 54.384 -29.063 61.659 0.00 0.00
G
ATOM 23680 CD ARG G 200 56.285 -30.278 61.345 0.00 0.00
G
ATOM 23681 HD1 ARG G 200 56.751 -31.083 60.738 0.00 0.00
G
ATOM 23682 HD2 ARG G 200 57.011 -29.460 61.149 0.00 0.00
G
ATOM 23683 NE ARG G 200 56.416 -30.525 62.801 0.00 0.00
G
ATOM 23684 HE ARG G 200 55.666 -30.416 63.453 0.00 0.00
G
ATOM 23685 CZ ARG G 200 57.422 -31.254 63.315 0.00 0.00
G
ATOM 23686 NH1 ARG G 200 58.580 -31.252 62.735 0.00 0.00
G
ATOM 23687 HH11 ARG G 200 59.399 -31.569 63.212 0.00 0.00
G
ATOM 23688 HH12 ARG G 200 58.783 -30.497 62.111 0.00 0.00
G
ATOM 23689 NH2 ARG G 200 57.292 -31.849 64.469 0.00 0.00
G
ATOM 23690 HH21 ARG G 200 58.049 -32.267 64.972 0.00 0.00
G
ATOM 23691 HH22 ARG G 200 56.377 -31.789 64.866 0.00 0.00
G
ATOM 23692 C ARG G 200 51.991 -30.610 62.078 0.00 0.00
G
ATOM 23693 O ARG G 200 50.873 -30.812 61.649 0.00 0.00
G
ATOM 23694 N GLU G 201 52.240 -29.405 62.757 0.00 0.00
G
ATOM 23695 HN GLU G 201 53.023 -29.377 63.373 0.00 0.00
G
ATOM 23696 CA GLU G 201 51.219 -28.363 62.817 0.00 0.00
G
ATOM 23697 HA GLU G 201 50.322 -28.751 63.279 0.00 0.00
G
ATOM 23698 CB GLU G 201 51.664 -27.151 63.648 0.00 0.00
G
ATOM 23699 HB1 GLU G 201 50.775 -26.485 63.619 0.00 0.00
G
ATOM 23700 HB2 GLU G 201 51.946 -27.467 64.675 0.00 0.00
G
ATOM 23701 CG GLU G 201 52.969 -26.453 63.076 0.00 0.00
G
ATOM 23702 HG1 GLU G 201 53.709 -27.282 63.064 0.00 0.00
G
ATOM 23703 HG2 GLU G 201 52.899 -26.128 62.016 0.00 0.00
G
ATOM 23704 CD GLU G 201 53.510 -25.300 64.020 0.00 0.00
G
ATOM 23705 OE1 GLU G 201 54.637 -25.533 64.527 0.00 0.00
G

ATOM 23706 OE2 GLU G 201   52.940 -24.218 64.234 0.00 0.00
G
ATOM 23707 C   GLU G 201   50.730 -27.909 61.447 0.00 0.00
G
ATOM 23708 O   GLU G 201   51.464 -27.764 60.470 0.00 0.00
G
ATOM 23709 N   GLN G 202   49.437 -27.703 61.255 0.00 0.00
G
ATOM 23710 HN GLN G 202   48.837 -27.838 62.040 0.00 0.00
G
ATOM 23711 CA GLN G 202   48.707 -27.377 60.037 0.00 0.00
G
ATOM 23712 HA GLN G 202   47.704 -27.580 60.384 0.00 0.00
G
ATOM 23713 CB GLN G 202   48.616 -25.788 59.717 0.00 0.00
G
ATOM 23714 HB1 GLN G 202   49.634 -25.420 59.470 0.00 0.00
G
ATOM 23715 HB2 GLN G 202   48.008 -25.676 58.794 0.00 0.00
G
ATOM 23716 CG GLN G 202   47.997 -25.018 60.908 0.00 0.00
G
ATOM 23717 HG1 GLN G 202   47.155 -25.533 61.418 0.00 0.00
G
ATOM 23718 HG2 GLN G 202   48.807 -24.991 61.667 0.00 0.00
G
ATOM 23719 CD GLN G 202   47.652 -23.547 60.556 0.00 0.00
G
ATOM 23720 OE1 GLN G 202   48.451 -22.661 60.765 0.00 0.00
G
ATOM 23721 NE2 GLN G 202   46.608 -23.433 59.707 0.00 0.00
G
ATOM 23722 HE21 GLN G 202   46.308 -22.550 59.347 0.00 0.00
G
ATOM 23723 HE22 GLN G 202   46.020 -24.231 59.844 0.00 0.00
G
ATOM 23724 C   GLN G 202   48.960 -28.235 58.879 0.00 0.00
G
ATOM 23725 O   GLN G 202   48.819 -27.903 57.711 0.00 0.00
G
ATOM 23726 N   ASP G 203   49.176 -29.569 59.087 0.00 0.00
G
ATOM 23727 HN ASP G 203   49.576 -29.914 59.933 0.00 0.00
G
ATOM 23728 CA ASP G 203   48.861 -30.604 58.099 0.00 0.00
G
ATOM 23729 HA ASP G 203   49.083 -30.273 57.095 0.00 0.00
G
ATOM 23730 CB ASP G 203   49.608 -31.940 58.426 0.00 0.00
G
ATOM 23731 HB1 ASP G 203   49.599 -32.172 59.513 0.00 0.00
G
ATOM 23732 HB2 ASP G 203   49.268 -32.818 57.836 0.00 0.00
G

ATOM 23733 CG ASP G 203 51.060 -31.735 58.056 0.00 0.00
G
ATOM 23734 OD1 ASP G 203 51.503 -31.011 57.122 0.00 0.00
G
ATOM 23735 OD2 ASP G 203 51.843 -32.457 58.677 0.00 0.00
G
ATOM 23736 C ASP G 203 47.358 -30.806 58.177 0.00 0.00
G
ATOM 23737 O ASP G 203 46.739 -30.536 59.168 0.00 0.00
G
ATOM 23738 N CYS G 204 46.774 -31.495 57.224 0.00 0.00
G
ATOM 23739 HN CYS G 204 47.316 -31.843 56.463 0.00 0.00
G
ATOM 23740 CA CYS G 204 45.358 -31.790 57.356 0.00 0.00
G
ATOM 23741 HA CYS G 204 44.994 -31.634 58.361 0.00 0.00
G
ATOM 23742 CB CYS G 204 44.334 -30.883 56.597 0.00 0.00
G
ATOM 23743 HB1 CYS G 204 44.044 -31.373 55.643 0.00 0.00
G
ATOM 23744 HB2 CYS G 204 43.404 -31.013 57.192 0.00 0.00
G
ATOM 23745 SG CYS G 204 44.729 -29.069 56.357 0.00 0.00
G
ATOM 23746 HG1 CYS G 204 45.468 -29.040 57.457 0.00 0.00
G
ATOM 23747 C CYS G 204 45.204 -33.333 57.032 0.00 0.00
G
ATOM 23748 O CYS G 204 45.876 -33.847 56.122 0.00 0.00
G
ATOM 23749 N LEU G 205 44.423 -34.076 57.824 0.00 0.00
G
ATOM 23750 HN LEU G 205 44.107 -33.615 58.649 0.00 0.00
G
ATOM 23751 CA LEU G 205 43.899 -35.444 57.629 0.00 0.00
G
ATOM 23752 HA LEU G 205 44.484 -35.771 56.782 0.00 0.00
G
ATOM 23753 CB LEU G 205 43.848 -36.345 58.910 0.00 0.00
G
ATOM 23754 HB1 LEU G 205 43.133 -35.858 59.607 0.00 0.00
G
ATOM 23755 HB2 LEU G 205 43.508 -37.382 58.702 0.00 0.00
G
ATOM 23756 CG LEU G 205 45.203 -36.629 59.734 0.00 0.00
G
ATOM 23757 HG LEU G 205 44.860 -37.247 60.590 0.00 0.00
G
ATOM 23758 CD1 LEU G 205 46.248 -37.412 58.999 0.00 0.00
G
ATOM 23759 HD11 LEU G 205 46.978 -37.830 59.726 0.00 0.00
G

ATOM 23760 HD12 LEU G 205   45.857 -38.244 58.376 0.00 0.00
G
ATOM 23761 HD13 LEU G 205   46.724 -36.690 58.301 0.00 0.00
G
ATOM 23762 CD2 LEU G 205   45.633 -35.331 60.473 0.00 0.00
G
ATOM 23763 HD21 LEU G 205   45.895 -34.490 59.795 0.00 0.00
G
ATOM 23764 HD22 LEU G 205   44.857 -34.921 61.154 0.00 0.00
G
ATOM 23765 HD23 LEU G 205   46.535 -35.546 61.084 0.00 0.00
G
ATOM 23766 C   LEU G 205   42.550 -35.318 57.138 0.00 0.00
G
ATOM 23767 O   LEU G 205   41.874 -34.329 57.454 0.00 0.00
G
ATOM 23768 N   VAL G 206   42.066 -36.326 56.426 0.00 0.00
G
ATOM 23769 HN VAL G 206   42.739 -36.938 56.019 0.00 0.00
G
ATOM 23770 CA VAL G 206   40.706 -36.418 55.971 0.00 0.00
G
ATOM 23771 HA VAL G 206   40.135 -35.693 56.532 0.00 0.00
G
ATOM 23772 CB VAL G 206   40.754 -36.224 54.467 0.00 0.00
G
ATOM 23773 HB VAL G 206   41.243 -37.043 53.898 0.00 0.00
G
ATOM 23774 CG1 VAL G 206   39.289 -36.102 54.148 0.00 0.00
G
ATOM 23775 HG11 VAL G 206   38.911 -35.186 54.650 0.00 0.00
G
ATOM 23776 HG12 VAL G 206   39.161 -36.062 53.045 0.00 0.00
G
ATOM 23777 HG13 VAL G 206   38.714 -36.987 54.494 0.00 0.00
G
ATOM 23778 CG2 VAL G 206   41.656 -35.006 53.994 0.00 0.00
G
ATOM 23779 HG21 VAL G 206   41.218 -34.121 54.504 0.00 0.00
G
ATOM 23780 HG22 VAL G 206   42.692 -35.271 54.295 0.00 0.00
G
ATOM 23781 HG23 VAL G 206   41.590 -34.809 52.902 0.00 0.00
G
ATOM 23782 C   VAL G 206   40.235 -37.824 56.321 0.00 0.00
G
ATOM 23783 O   VAL G 206   40.886 -38.800 55.959 0.00 0.00
G
ATOM 23784 N   ILE G 207   39.125 -37.874 57.052 0.00 0.00
G
ATOM 23785 HN ILE G 207   38.703 -37.012 57.321 0.00 0.00
G
ATOM 23786 CA ILE G 207   38.710 -39.035 57.819 0.00 0.00
G

ATOM 23787 HA ILE G 207 39.165 -39.969 57.522 0.00 0.00
G
ATOM 23788 CB ILE G 207 38.982 -38.654 59.298 0.00 0.00
G
ATOM 23789 HB ILE G 207 38.417 -37.715 59.481 0.00 0.00
G
ATOM 23790 CG2 ILE G 207 38.335 -39.630 60.316 0.00 0.00
G
ATOM 23791 HG21 ILE G 207 37.293 -39.877 60.020 0.00 0.00
G
ATOM 23792 HG22 ILE G 207 38.842 -40.608 60.171 0.00 0.00
G
ATOM 23793 HG23 ILE G 207 38.334 -39.245 61.359 0.00 0.00
G
ATOM 23794 CG1 ILE G 207 40.511 -38.602 59.652 0.00 0.00
G
ATOM 23795 HG11 ILE G 207 40.943 -39.557 59.284 0.00 0.00
G
ATOM 23796 HG12 ILE G 207 40.855 -37.794 58.972 0.00 0.00
G
ATOM 23797 CD ILE G 207 40.786 -38.300 61.116 0.00 0.00
G
ATOM 23798 HD1 ILE G 207 41.863 -38.047 61.215 0.00 0.00
G
ATOM 23799 HD2 ILE G 207 40.175 -37.446 61.480 0.00 0.00
G
ATOM 23800 HD3 ILE G 207 40.733 -39.191 61.777 0.00 0.00
G
ATOM 23801 C ILE G 207 37.266 -39.363 57.531 0.00 0.00
G
ATOM 23802 O ILE G 207 36.454 -38.467 57.437 0.00 0.00
G
ATOM 23803 N MET G 208 36.944 -40.626 57.276 0.00 0.00
G
ATOM 23804 HN MET G 208 37.638 -41.338 57.200 0.00 0.00
G
ATOM 23805 CA MET G 208 35.570 -41.045 57.049 0.00 0.00
G
ATOM 23806 HA MET G 208 34.970 -40.148 57.041 0.00 0.00
G
ATOM 23807 CB MET G 208 35.593 -41.809 55.672 0.00 0.00
G
ATOM 23808 HB1 MET G 208 35.929 -41.083 54.901 0.00 0.00
G
ATOM 23809 HB2 MET G 208 36.426 -42.544 55.692 0.00 0.00
G
ATOM 23810 CG MET G 208 34.314 -42.428 55.186 0.00 0.00
G
ATOM 23811 HG1 MET G 208 34.035 -43.143 55.990 0.00 0.00
G
ATOM 23812 HG2 MET G 208 33.602 -41.584 55.062 0.00 0.00
G
ATOM 23813 SD MET G 208 34.479 -43.424 53.617 0.00 0.00
G

ATOM 23814 CE MET G 208   32.834 -44.169 53.994 0.00 0.00
G
ATOM 23815 HE1 MET G 208   32.811 -44.942 54.792 0.00 0.00
G
ATOM 23816 HE2 MET G 208   32.140 -43.377 54.347 0.00 0.00
G
ATOM 23817 HE3 MET G 208   32.386 -44.605 53.075 0.00 0.00
G
ATOM 23818 C   MET G 208   35.047 -41.923 58.167 0.00 0.00
G
ATOM 23819 O   MET G 208   35.738 -42.823 58.543 0.00 0.00
G
ATOM 23820 N   GLY G 209   33.857 -41.756 58.817 0.00 0.00
G
ATOM 23821 HN GLY G 209   33.295 -40.946 58.667 0.00 0.00
G
ATOM 23822 CA GLY G 209   33.416 -42.735 59.772 0.00 0.00
G
ATOM 23823 HA1 GLY G 209   34.001 -42.542 60.660 0.00 0.00
G
ATOM 23824 HA2 GLY G 209   33.499 -43.705 59.305 0.00 0.00
G
ATOM 23825 C   GLY G 209   31.974 -42.595 60.088 0.00 0.00
G
ATOM 23826 O   GLY G 209   31.299 -41.688 59.563 0.00 0.00
G
ATOM 23827 N   HSE G 210   31.454 -43.480 60.967 0.00 0.00
G
ATOM 23828 HN HSE G 210   32.017 -44.223 61.319 0.00 0.00
G
ATOM 23829 CA HSE G 210   30.117 -43.252 61.507 0.00 0.00
G
ATOM 23830 HA HSE G 210   29.377 -43.303 60.722 0.00 0.00
G
ATOM 23831 CB HSE G 210   29.680 -44.429 62.472 0.00 0.00
G
ATOM 23832 HB1 HSE G 210   29.850 -45.291 61.792 0.00 0.00
G
ATOM 23833 HB2 HSE G 210   30.404 -44.503 63.311 0.00 0.00
G
ATOM 23834 ND1 HSE G 210   27.924 -43.557 64.054 0.00 0.00
G
ATOM 23835 CG HSE G 210   28.315 -44.479 63.140 0.00 0.00
G
ATOM 23836 CE1 HSE G 210   26.590 -43.696 64.115 0.00 0.00
G
ATOM 23837 HE1 HSE G 210   26.044 -42.955 64.699 0.00 0.00
G
ATOM 23838 NE2 HSE G 210   26.086 -44.715 63.358 0.00 0.00
G
ATOM 23839 HE2 HSE G 210   25.127 -44.954 63.204 0.00 0.00
G
ATOM 23840 CD2 HSE G 210   27.185 -45.170 62.718 0.00 0.00
G

ATOM 23841 HD2 HSE G 210 27.189 -45.894 61.913 0.00 0.00
G
ATOM 23842 C HSE G 210 29.969 -41.953 62.302 0.00 0.00
G
ATOM 23843 O HSE G 210 30.909 -41.608 62.993 0.00 0.00
G
ATOM 23844 N LYS G 211 28.795 -41.184 62.241 0.00 0.00
G
ATOM 23845 HN LYS G 211 28.081 -41.574 61.666 0.00 0.00
G
ATOM 23846 CA LYS G 211 28.595 -39.845 62.834 0.00 0.00
G
ATOM 23847 HA LYS G 211 29.213 -39.135 62.304 0.00 0.00
G
ATOM 23848 CB LYS G 211 27.187 -39.466 62.476 0.00 0.00
G
ATOM 23849 HB1 LYS G 211 27.057 -39.493 61.373 0.00 0.00
G
ATOM 23850 HB2 LYS G 211 26.478 -40.272 62.762 0.00 0.00
G
ATOM 23851 CG LYS G 211 26.697 -37.994 62.829 0.00 0.00
G
ATOM 23852 HG1 LYS G 211 26.934 -37.804 63.898 0.00 0.00
G
ATOM 23853 HG2 LYS G 211 27.269 -37.141 62.406 0.00 0.00
G
ATOM 23854 CD LYS G 211 25.211 -37.858 62.718 0.00 0.00
G
ATOM 23855 HD1 LYS G 211 24.845 -38.865 63.013 0.00 0.00
G
ATOM 23856 HD2 LYS G 211 24.846 -37.095 63.438 0.00 0.00
G
ATOM 23857 CE LYS G 211 24.837 -37.398 61.324 0.00 0.00
G
ATOM 23858 HE1 LYS G 211 25.461 -36.538 61.000 0.00 0.00
G
ATOM 23859 HE2 LYS G 211 24.928 -38.230 60.592 0.00 0.00
G
ATOM 23860 NZ LYS G 211 23.465 -36.969 61.177 0.00 0.00
G
ATOM 23861 HZ1 LYS G 211 22.787 -37.752 61.090 0.00 0.00
G
ATOM 23862 HZ2 LYS G 211 23.240 -36.353 61.985 0.00 0.00
G
ATOM 23863 HZ3 LYS G 211 23.453 -36.350 60.341 0.00 0.00
G
ATOM 23864 C LYS G 211 28.833 -39.688 64.327 0.00 0.00
G
ATOM 23865 O LYS G 211 29.468 -38.750 64.874 0.00 0.00
G
ATOM 23866 N LYS G 212 28.354 -40.664 65.135 0.00 0.00
G
ATOM 23867 HN LYS G 212 28.201 -41.514 64.636 0.00 0.00
G

ATOM 23868 CA LYS G 212 28.285 -40.700 66.585 0.00 0.00
G
ATOM 23869 HA LYS G 212 28.047 -39.744 67.027 0.00 0.00
G
ATOM 23870 CB LYS G 212 27.323 -41.791 67.194 0.00 0.00
G
ATOM 23871 HB1 LYS G 212 26.333 -41.743 66.692 0.00 0.00
G
ATOM 23872 HB2 LYS G 212 27.738 -42.705 66.717 0.00 0.00
G
ATOM 23873 CG LYS G 212 27.292 -42.107 68.731 0.00 0.00
G
ATOM 23874 HG1 LYS G 212 28.031 -42.929 68.847 0.00 0.00
G
ATOM 23875 HG2 LYS G 212 27.680 -41.257 69.331 0.00 0.00
G
ATOM 23876 CD LYS G 212 25.911 -42.504 69.084 0.00 0.00
G
ATOM 23877 HD1 LYS G 212 25.232 -41.628 69.011 0.00 0.00
G
ATOM 23878 HD2 LYS G 212 25.660 -43.351 68.410 0.00 0.00
G
ATOM 23879 CE LYS G 212 25.836 -43.004 70.576 0.00 0.00
G
ATOM 23880 HE1 LYS G 212 26.648 -43.754 70.685 0.00 0.00
G
ATOM 23881 HE2 LYS G 212 26.044 -42.215 71.329 0.00 0.00
G
ATOM 23882 NZ LYS G 212 24.548 -43.683 70.911 0.00 0.00
G
ATOM 23883 HZ1 LYS G 212 24.269 -44.404 70.216 0.00 0.00
G
ATOM 23884 HZ2 LYS G 212 24.721 -44.216 71.788 0.00 0.00
G
ATOM 23885 HZ3 LYS G 212 23.733 -43.064 71.093 0.00 0.00
G
ATOM 23886 C LYS G 212 29.675 -40.943 67.061 0.00 0.00
G
ATOM 23887 O LYS G 212 30.210 -40.373 68.038 0.00 0.00
G
ATOM 23888 N ASP G 213 30.390 -41.753 66.239 0.00 0.00
G
ATOM 23889 HN ASP G 213 29.920 -42.206 65.485 0.00 0.00
G
ATOM 23890 CA ASP G 213 31.781 -42.088 66.419 0.00 0.00
G
ATOM 23891 HA ASP G 213 32.053 -42.128 67.463 0.00 0.00
G
ATOM 23892 CB ASP G 213 32.241 -43.371 65.722 0.00 0.00
G
ATOM 23893 HB1 ASP G 213 32.082 -43.381 64.622 0.00 0.00
G
ATOM 23894 HB2 ASP G 213 33.325 -43.548 65.890 0.00 0.00
G

ATOM 23895 CG ASP G 213   31.576 -44.480 66.408 0.00 0.00
G
ATOM 23896 OD1 ASP G 213   31.589 -44.576 67.656 0.00 0.00
G
ATOM 23897 OD2 ASP G 213   31.032 -45.383 65.738 0.00 0.00
G
ATOM 23898 C   ASP G 213   32.702 -40.919 66.066 0.00 0.00
G
ATOM 23899 O   ASP G 213   33.673 -40.706 66.739 0.00 0.00
G
ATOM 23900 N   ILE G 214   32.352 -40.046 65.104 0.00 0.00
G
ATOM 23901 HN ILE G 214   31.544 -40.273 64.566 0.00 0.00
G
ATOM 23902 CA ILE G 214   33.085 -38.854 64.730 0.00 0.00
G
ATOM 23903 HA ILE G 214   34.155 -38.997 64.752 0.00 0.00
G
ATOM 23904 CB ILE G 214   32.577 -38.331 63.400 0.00 0.00
G
ATOM 23905 HB ILE G 214   31.467 -38.367 63.449 0.00 0.00
G
ATOM 23906 CG2 ILE G 214   33.116 -36.870 63.242 0.00 0.00
G
ATOM 23907 HG21 ILE G 214   32.599 -36.210 63.971 0.00 0.00
G
ATOM 23908 HG22 ILE G 214   34.210 -36.864 63.436 0.00 0.00
G
ATOM 23909 HG23 ILE G 214   32.955 -36.414 62.242 0.00 0.00
G
ATOM 23910 CG1 ILE G 214   33.199 -39.208 62.260 0.00 0.00
G
ATOM 23911 HG11 ILE G 214   34.287 -38.995 62.188 0.00 0.00
G
ATOM 23912 HG12 ILE G 214   33.160 -40.303 62.445 0.00 0.00
G
ATOM 23913 CD ILE G 214   32.507 -38.823 60.906 0.00 0.00
G
ATOM 23914 HD1 ILE G 214   32.771 -37.796 60.575 0.00 0.00
G
ATOM 23915 HD2 ILE G 214   32.677 -39.513 60.051 0.00 0.00
G
ATOM 23916 HD3 ILE G 214   31.423 -38.669 61.095 0.00 0.00
G
ATOM 23917 C   ILE G 214   32.917 -37.761 65.821 0.00 0.00
G
ATOM 23918 O   ILE G 214   33.792 -36.918 66.137 0.00 0.00
G
ATOM 23919 N   LYS G 215   31.711 -37.733 66.459 0.00 0.00
G
ATOM 23920 HN LYS G 215   30.953 -38.199 66.009 0.00 0.00
G
ATOM 23921 CA LYS G 215   31.289 -36.821 67.545 0.00 0.00
G

ATOM 23922 HA LYS G 215 31.674 -35.837 67.325 0.00 0.00
G
ATOM 23923 CB LYS G 215 29.766 -36.645 67.713 0.00 0.00
G
ATOM 23924 HB1 LYS G 215 29.149 -36.441 66.813 0.00 0.00
G
ATOM 23925 HB2 LYS G 215 29.394 -37.640 68.039 0.00 0.00
G
ATOM 23926 CG LYS G 215 29.339 -35.663 68.850 0.00 0.00
G
ATOM 23927 HG1 LYS G 215 29.873 -35.971 69.774 0.00 0.00
G
ATOM 23928 HG2 LYS G 215 29.597 -34.602 68.645 0.00 0.00
G
ATOM 23929 CD LYS G 215 27.783 -35.560 69.023 0.00 0.00
G
ATOM 23930 HD1 LYS G 215 27.327 -35.195 68.078 0.00 0.00
G
ATOM 23931 HD2 LYS G 215 27.594 -36.626 69.270 0.00 0.00
G
ATOM 23932 CE LYS G 215 27.397 -34.791 70.236 0.00 0.00
G
ATOM 23933 HE1 LYS G 215 26.302 -34.819 70.422 0.00 0.00
G
ATOM 23934 HE2 LYS G 215 27.985 -35.202 71.084 0.00 0.00
G
ATOM 23935 NZ LYS G 215 27.790 -33.417 70.088 0.00 0.00
G
ATOM 23936 HZ1 LYS G 215 27.331 -32.839 69.355 0.00 0.00
G
ATOM 23937 HZ2 LYS G 215 27.656 -32.967 71.015 0.00 0.00
G
ATOM 23938 HZ3 LYS G 215 28.825 -33.479 70.004 0.00 0.00
G
ATOM 23939 C LYS G 215 31.913 -37.142 68.937 0.00 0.00
G
ATOM 23940 O LYS G 215 32.364 -36.376 69.737 0.00 0.00
G
ATOM 23941 N ARG G 216 32.178 -38.486 69.190 0.00 0.00
G
ATOM 23942 HN ARG G 216 31.877 -39.238 68.608 0.00 0.00
G
ATOM 23943 CA ARG G 216 32.885 -38.828 70.411 0.00 0.00
G
ATOM 23944 HA ARG G 216 32.475 -38.185 71.176 0.00 0.00
G
ATOM 23945 CB ARG G 216 32.564 -40.234 70.817 0.00 0.00
G
ATOM 23946 HB1 ARG G 216 31.464 -40.292 70.959 0.00 0.00
G
ATOM 23947 HB2 ARG G 216 32.789 -40.933 69.984 0.00 0.00
G
ATOM 23948 CG ARG G 216 33.415 -40.689 72.093 0.00 0.00
G

ATOM 23949 HG1 ARG G 216 33.347 -41.778 72.302 0.00 0.00
G
ATOM 23950 HG2 ARG G 216 34.460 -40.379 71.877 0.00 0.00
G
ATOM 23951 CD ARG G 216 33.053 -39.923 73.369 0.00 0.00
G
ATOM 23952 HD1 ARG G 216 33.150 -38.821 73.258 0.00 0.00
G
ATOM 23953 HD2 ARG G 216 32.029 -40.081 73.771 0.00 0.00
G
ATOM 23954 NE ARG G 216 34.110 -40.384 74.375 0.00 0.00
G
ATOM 23955 HE ARG G 216 34.512 -41.290 74.245 0.00 0.00
G
ATOM 23956 CZ ARG G 216 34.574 -39.610 75.432 0.00 0.00
G
ATOM 23957 NH1 ARG G 216 33.966 -38.582 75.889 0.00 0.00
G
ATOM 23958 HH11 ARG G 216 34.176 -38.256 76.811 0.00 0.00
G
ATOM 23959 HH12 ARG G 216 33.058 -38.329 75.553 0.00 0.00
G
ATOM 23960 NH2 ARG G 216 35.577 -39.998 76.214 0.00 0.00
G
ATOM 23961 HH21 ARG G 216 35.774 -39.299 76.902 0.00 0.00
G
ATOM 23962 HH22 ARG G 216 36.347 -40.414 75.730 0.00 0.00
G
ATOM 23963 C ARG G 216 34.420 -38.513 70.204 0.00 0.00
G
ATOM 23964 O ARG G 216 35.063 -37.889 71.075 0.00 0.00
G
ATOM 23965 N PHE G 217 34.921 -38.831 68.965 0.00 0.00
G
ATOM 23966 HN PHE G 217 34.268 -39.198 68.307 0.00 0.00
G
ATOM 23967 CA PHE G 217 36.288 -38.439 68.609 0.00 0.00
G
ATOM 23968 HA PHE G 217 37.043 -38.721 69.327 0.00 0.00
G
ATOM 23969 CB PHE G 217 36.466 -38.931 67.103 0.00 0.00
G
ATOM 23970 HB1 PHE G 217 36.256 -40.019 67.028 0.00 0.00
G
ATOM 23971 HB2 PHE G 217 35.731 -38.416 66.447 0.00 0.00
G
ATOM 23972 CG PHE G 217 37.774 -38.674 66.328 0.00 0.00
G
ATOM 23973 CD1 PHE G 217 38.983 -38.858 66.967 0.00 0.00
G
ATOM 23974 HD1 PHE G 217 39.061 -39.144 68.005 0.00 0.00
G
ATOM 23975 CE1 PHE G 217 40.118 -38.727 66.233 0.00 0.00
G

ATOM 23976 HE1 PHE G 217 41.054 −39.087 66.632 0.00 0.00
G
ATOM 23977 CZ PHE G 217 40.147 −38.422 64.854 0.00 0.00
G
ATOM 23978 HZ PHE G 217 41.115 −38.458 64.375 0.00 0.00
G
ATOM 23979 CD2 PHE G 217 37.797 −38.241 64.985 0.00 0.00
G
ATOM 23980 HD2 PHE G 217 36.851 −38.048 64.501 0.00 0.00
G
ATOM 23981 CE2 PHE G 217 38.949 −38.144 64.242 0.00 0.00
G
ATOM 23982 HE2 PHE G 217 38.914 −37.850 63.204 0.00 0.00
G
ATOM 23983 C PHE G 217 36.629 −36.917 68.645 0.00 0.00
G
ATOM 23984 O PHE G 217 37.666 −36.476 69.192 0.00 0.00
G
ATOM 23985 N GLU G 218 35.734 −36.110 68.152 0.00 0.00
G
ATOM 23986 HN GLU G 218 34.974 −36.569 67.698 0.00 0.00
G
ATOM 23987 CA GLU G 218 35.657 −34.650 68.280 0.00 0.00
G
ATOM 23988 HA GLU G 218 36.470 −34.215 67.716 0.00 0.00
G
ATOM 23989 CB GLU G 218 34.278 −34.062 67.789 0.00 0.00
G
ATOM 23990 HB1 GLU G 218 34.211 −34.623 66.832 0.00 0.00
G
ATOM 23991 HB2 GLU G 218 33.503 −34.537 68.426 0.00 0.00
G
ATOM 23992 CG GLU G 218 34.126 −32.541 67.749 0.00 0.00
G
ATOM 23993 HG1 GLU G 218 34.215 −32.176 68.795 0.00 0.00
G
ATOM 23994 HG2 GLU G 218 34.809 −32.086 67.000 0.00 0.00
G
ATOM 23995 CD GLU G 218 32.781 −32.128 67.192 0.00 0.00
G
ATOM 23996 OE1 GLU G 218 31.836 −31.631 67.867 0.00 0.00
G
ATOM 23997 OE2 GLU G 218 32.618 −32.263 65.977 0.00 0.00
G
ATOM 23998 C GLU G 218 35.835 −34.163 69.746 0.00 0.00
G
ATOM 23999 O GLU G 218 36.525 −33.205 70.017 0.00 0.00
G
ATOM 24000 N ASN G 219 35.137 −34.783 70.651 0.00 0.00
G
ATOM 24001 HN ASN G 219 34.507 −35.494 70.348 0.00 0.00
G
ATOM 24002 CA ASN G 219 35.152 −34.534 72.121 0.00 0.00
G

ATOM 24003 HA ASN G 219 35.284 -33.469 72.238 0.00 0.00
G
ATOM 24004 CB ASN G 219 33.916 -35.148 72.862 0.00 0.00
G
ATOM 24005 HB1 ASN G 219 33.893 -36.240 72.655 0.00 0.00
G
ATOM 24006 HB2 ASN G 219 34.033 -34.958 73.950 0.00 0.00
G
ATOM 24007 CG ASN G 219 32.635 -34.389 72.478 0.00 0.00
G
ATOM 24008 OD1 ASN G 219 32.641 -33.217 71.996 0.00 0.00
G
ATOM 24009 ND2 ASN G 219 31.429 -35.062 72.705 0.00 0.00
G
ATOM 24010 HD21 ASN G 219 30.579 -34.718 72.309 0.00 0.00
G
ATOM 24011 HD22 ASN G 219 31.549 -35.911 73.220 0.00 0.00
G
ATOM 24012 C ASN G 219 36.382 -35.051 72.790 0.00 0.00
G
ATOM 24013 O ASN G 219 36.976 -34.336 73.648 0.00 0.00
G
ATOM 24014 N GLU G 220 36.812 -36.293 72.447 0.00 0.00
G
ATOM 24015 HN GLU G 220 36.274 -36.823 71.796 0.00 0.00
G
ATOM 24016 CA GLU G 220 37.821 -37.045 73.105 0.00 0.00
G
ATOM 24017 HA GLU G 220 37.628 -36.889 74.156 0.00 0.00
G
ATOM 24018 CB GLU G 220 37.625 -38.669 72.932 0.00 0.00
G
ATOM 24019 HB1 GLU G 220 36.573 -38.894 73.211 0.00 0.00
G
ATOM 24020 HB2 GLU G 220 37.810 -39.166 71.956 0.00 0.00
G
ATOM 24021 CG GLU G 220 38.436 -39.574 73.923 0.00 0.00
G
ATOM 24022 HG1 GLU G 220 39.514 -39.491 73.666 0.00 0.00
G
ATOM 24023 HG2 GLU G 220 38.284 -39.215 74.963 0.00 0.00
G
ATOM 24024 CD GLU G 220 37.920 -41.024 73.911 0.00 0.00
G
ATOM 24025 OE1 GLU G 220 37.251 -41.496 74.843 0.00 0.00
G
ATOM 24026 OE2 GLU G 220 38.338 -41.739 72.986 0.00 0.00
G
ATOM 24027 C GLU G 220 39.180 -36.593 72.628 0.00 0.00
G
ATOM 24028 O GLU G 220 40.095 -36.755 73.338 0.00 0.00
G
ATOM 24029 N GLY G 221 39.375 -35.991 71.480 0.00 0.00
G

ATOM 24030 HN GLY G 221   38.551 -35.884 70.929 0.00 0.00
G
ATOM 24031 CA GLY G 221   40.600 -35.443 70.961 0.00 0.00
G
ATOM 24032 HA1 GLY G 221   41.194 -35.025 71.760 0.00 0.00
G
ATOM 24033 HA2 GLY G 221   40.319 -34.734 70.197 0.00 0.00
G
ATOM 24034 C   GLY G 221   41.391 -36.495 70.345 0.00 0.00
G
ATOM 24035 O   GLY G 221   41.391 -37.655 70.715 0.00 0.00
G
ATOM 24036 N   MET G 222   42.186 -36.061 69.297 0.00 0.00
G
ATOM 24037 HN MET G 222   42.144 -35.117 68.978 0.00 0.00
G
ATOM 24038 CA MET G 222   42.873 -36.987 68.403 0.00 0.00
G
ATOM 24039 HA MET G 222   42.475 -37.987 68.486 0.00 0.00
G
ATOM 24040 CB MET G 222   42.868 -36.402 66.960 0.00 0.00
G
ATOM 24041 HB1 MET G 222   41.814 -36.195 66.675 0.00 0.00
G
ATOM 24042 HB2 MET G 222   43.394 -35.428 67.057 0.00 0.00
G
ATOM 24043 CG MET G 222   43.694 -37.290 66.053 0.00 0.00
G
ATOM 24044 HG1 MET G 222   44.778 -37.271 66.294 0.00 0.00
G
ATOM 24045 HG2 MET G 222   43.323 -38.332 66.152 0.00 0.00
G
ATOM 24046 SD MET G 222   43.488 -36.765 64.289 0.00 0.00
G
ATOM 24047 CE MET G 222   44.252 -38.275 63.575 0.00 0.00
G
ATOM 24048 HE1 MET G 222   44.030 -39.096 64.290 0.00 0.00
G
ATOM 24049 HE2 MET G 222   43.830 -38.553 62.586 0.00 0.00
G
ATOM 24050 HE3 MET G 222   45.337 -38.113 63.400 0.00 0.00
G
ATOM 24051 C   MET G 222   44.280 -37.222 68.879 0.00 0.00
G
ATOM 24052 O   MET G 222   44.597 -37.700 70.005 0.00 0.00
G
ATOM 24053 N   ASN H   6   32.811 -23.501 73.258 0.00 0.00
H
ATOM 24054 HT1 ASN H   6   31.885 -23.779 73.642 0.00 0.00
H
ATOM 24055 HT2 ASN H   6   33.171 -22.657 73.748 0.00 0.00
H
ATOM 24056 HT3 ASN H   6   33.487 -24.283 73.373 0.00 0.00
H

ATOM 24057 CA ASN H   6   32.724 -23.336 71.843 0.00 0.00
H
ATOM 24058 HA ASN H   6   33.731 -23.462 71.476 0.00 0.00
H
ATOM 24059 CB ASN H   6   31.759 -24.391 71.251 0.00 0.00
H
ATOM 24060 HB1 ASN H   6   30.781 -24.319 71.772 0.00 0.00
H
ATOM 24061 HB2 ASN H   6   31.602 -24.145 70.179 0.00 0.00
H
ATOM 24062 CG ASN H   6   32.476 -25.752 71.272 0.00 0.00
H
ATOM 24063 OD1 ASN H   6   32.914 -26.177 72.363 0.00 0.00
H
ATOM 24064 ND2 ASN H   6   32.677 -26.429 70.114 0.00 0.00
H
ATOM 24065 HD21 ASN H   6   33.321 -27.190 70.184 0.00 0.00
H
ATOM 24066 HD22 ASN H   6   32.304 -26.145 69.230 0.00 0.00
H
ATOM 24067 C   ASN H   6   32.357 -21.924 71.380 0.00 0.00
H
ATOM 24068 O   ASN H   6   31.868 -21.070 72.101 0.00 0.00
H
ATOM 24069 N   LYS H   7   32.735 -21.555 70.147 0.00 0.00
H
ATOM 24070 HN LYS H   7   32.928 -22.332 69.552 0.00 0.00
H
ATOM 24071 CA LYS H   7   32.524 -20.189 69.698 0.00 0.00
H
ATOM 24072 HA LYS H   7   32.895 -19.467 70.410 0.00 0.00
H
ATOM 24073 CB LYS H   7   33.178 -19.917 68.235 0.00 0.00
H
ATOM 24074 HB1 LYS H   7   32.834 -20.676 67.501 0.00 0.00
H
ATOM 24075 HB2 LYS H   7   32.954 -18.870 67.936 0.00 0.00
H
ATOM 24076 CG LYS H   7   34.676 -20.146 68.260 0.00 0.00
H
ATOM 24077 HG1 LYS H   7   35.149 -19.503 69.032 0.00 0.00
H
ATOM 24078 HG2 LYS H   7   34.924 -21.191 68.544 0.00 0.00
H
ATOM 24079 CD LYS H   7   35.220 -19.607 66.929 0.00 0.00
H
ATOM 24080 HD1 LYS H   7   34.639 -20.003 66.069 0.00 0.00
H
ATOM 24081 HD2 LYS H   7   35.284 -18.502 66.830 0.00 0.00
H
ATOM 24082 CE LYS H   7   36.555 -20.232 66.569 0.00 0.00
H
ATOM 24083 HE1 LYS H   7   36.453 -21.286 66.907 0.00 0.00
H

ATOM 24084 HE2 LYS H   7   36.886 -20.239 65.508 0.00 0.00
H
ATOM 24085 NZ LYS H   7   37.619 -19.624 67.394 0.00 0.00
H
ATOM 24086 HZ1 LYS H   7   38.347 -20.362 67.474 0.00 0.00
H
ATOM 24087 HZ2 LYS H   7   38.046 -18.782 66.956 0.00 0.00
H
ATOM 24088 HZ3 LYS H   7   37.298 -19.242 68.307 0.00 0.00
H
ATOM 24089 C   LYS H   7   31.094 -19.772 69.599 0.00 0.00
H
ATOM 24090 O   LYS H   7   30.239 -20.692 69.536 0.00 0.00
H
ATOM 24091 N   GLN H   8   30.776 -18.495 69.816 0.00 0.00
H
ATOM 24092 HN GLN H   8   31.546 -17.884 69.988 0.00 0.00
H
ATOM 24093 CA GLN H   8   29.466 -17.954 69.815 0.00 0.00
H
ATOM 24094 HA GLN H   8   28.806 -18.796 69.963 0.00 0.00
H
ATOM 24095 CB GLN H   8   29.169 -17.107 71.023 0.00 0.00
H
ATOM 24096 HB1 GLN H   8   28.136 -16.698 71.027 0.00 0.00
H
ATOM 24097 HB2 GLN H   8   29.142 -17.770 71.914 0.00 0.00
H
ATOM 24098 CG GLN H   8   30.105 -15.834 71.267 0.00 0.00
H
ATOM 24099 HG1 GLN H   8   31.105 -16.317 71.227 0.00 0.00
H
ATOM 24100 HG2 GLN H   8   29.999 -15.133 70.412 0.00 0.00
H
ATOM 24101 CD GLN H   8   29.809 -15.271 72.647 0.00 0.00
H
ATOM 24102 OE1 GLN H   8   30.265 -15.998 73.596 0.00 0.00
H
ATOM 24103 NE2 GLN H   8   28.969 -14.214 72.789 0.00 0.00
H
ATOM 24104 HE21 GLN H   8   28.746 -13.836 73.688 0.00 0.00
H
ATOM 24105 HE22 GLN H   8   28.527 -13.791 71.998 0.00 0.00
H
ATOM 24106 C   GLN H   8   29.077 -17.338 68.448 0.00 0.00
H
ATOM 24107 O   GLN H   8   29.911 -16.697 67.808 0.00 0.00
H
ATOM 24108 N   PHE H   9   27.885 -17.592 68.003 0.00 0.00
H
ATOM 24109 HN PHE H   9   27.244 -18.092 68.580 0.00 0.00
H
ATOM 24110 CA PHE H   9   27.513 -17.221 66.666 0.00 0.00
H

ATOM 24111 HA PHE H 9 28.368 −16.818 66.144 0.00 0.00
H
ATOM 24112 CB PHE H 9 26.925 −18.468 65.991 0.00 0.00
H
ATOM 24113 HB1 PHE H 9 26.197 −18.987 66.651 0.00 0.00
H
ATOM 24114 HB2 PHE H 9 26.419 −18.265 65.023 0.00 0.00
H
ATOM 24115 CG PHE H 9 28.138 −19.409 65.624 0.00 0.00
H
ATOM 24116 CD1 PHE H 9 28.851 −19.107 64.445 0.00 0.00
H
ATOM 24117 HD1 PHE H 9 28.539 −18.254 63.860 0.00 0.00
H
ATOM 24118 CE1 PHE H 9 29.993 −19.838 64.053 0.00 0.00
H
ATOM 24119 HE1 PHE H 9 30.553 −19.647 63.149 0.00 0.00
H
ATOM 24120 CZ PHE H 9 30.283 −20.978 64.817 0.00 0.00
H
ATOM 24121 HZ PHE H 9 31.137 −21.534 64.458 0.00 0.00
H
ATOM 24122 CD2 PHE H 9 28.465 −20.587 66.314 0.00 0.00
H
ATOM 24123 HD2 PHE H 9 27.784 −20.797 67.126 0.00 0.00
H
ATOM 24124 CE2 PHE H 9 29.546 −21.409 65.946 0.00 0.00
H
ATOM 24125 HE2 PHE H 9 29.893 −22.175 66.623 0.00 0.00
H
ATOM 24126 C PHE H 9 26.440 −16.154 66.756 0.00 0.00
H
ATOM 24127 O PHE H 9 25.500 −16.152 67.528 0.00 0.00
H
ATOM 24128 N ALA H 10 26.535 −15.132 65.835 0.00 0.00
H
ATOM 24129 HN ALA H 10 27.247 −15.024 65.146 0.00 0.00
H
ATOM 24130 CA ALA H 10 25.439 −14.131 65.599 0.00 0.00
H
ATOM 24131 HA ALA H 10 24.553 −14.353 66.175 0.00 0.00
H
ATOM 24132 CB ALA H 10 25.876 −12.725 66.052 0.00 0.00
H
ATOM 24133 HB1 ALA H 10 26.100 −12.713 67.140 0.00 0.00
H
ATOM 24134 HB2 ALA H 10 26.724 −12.377 65.424 0.00 0.00
H
ATOM 24135 HB3 ALA H 10 24.967 −12.099 65.922 0.00 0.00
H
ATOM 24136 C ALA H 10 24.935 −14.177 64.079 0.00 0.00
H
ATOM 24137 O ALA H 10 25.809 −14.079 63.190 0.00 0.00
H

```
ATOM  24138  N   VAL H  11      23.650 -14.382  63.779  0.00  0.00
H
ATOM  24139  HN  VAL H  11      23.094 -14.596  64.578  0.00  0.00
H
ATOM  24140  CA  VAL H  11      23.147 -14.758  62.490  0.00  0.00
H
ATOM  24141  HA  VAL H  11      23.909 -14.654  61.732  0.00  0.00
H
ATOM  24142  CB  VAL H  11      22.535 -16.138  62.453  0.00  0.00
H
ATOM  24143  HB  VAL H  11      21.565 -16.201  62.992  0.00  0.00
H
ATOM  24144  CG1 VAL H  11      22.059 -16.404  60.974  0.00  0.00
H
ATOM  24145 HG11 VAL H  11      21.177 -15.774  60.731  0.00  0.00
H
ATOM  24146 HG12 VAL H  11      22.913 -16.353  60.266  0.00  0.00
H
ATOM  24147 HG13 VAL H  11      21.580 -17.404  60.905  0.00  0.00
H
ATOM  24148  CG2 VAL H  11      23.396 -17.275  63.002  0.00  0.00
H
ATOM  24149 HG21 VAL H  11      23.653 -17.031  64.055  0.00  0.00
H
ATOM  24150 HG22 VAL H  11      22.876 -18.256  63.053  0.00  0.00
H
ATOM  24151 HG23 VAL H  11      24.300 -17.374  62.364  0.00  0.00
H
ATOM  24152  C   VAL H  11      22.108 -13.768  62.191  0.00  0.00
H
ATOM  24153  O   VAL H  11      21.105 -13.528  62.983  0.00  0.00
H
ATOM  24154  N   ILE H  12      22.243 -13.031  61.097  0.00  0.00
H
ATOM  24155  HN  ILE H  12      23.005 -13.185  60.472  0.00  0.00
H
ATOM  24156  CA  ILE H  12      21.309 -11.953  60.757  0.00  0.00
H
ATOM  24157  HA  ILE H  12      20.525 -11.781  61.479  0.00  0.00
H
ATOM  24158  CB  ILE H  12      22.119 -10.746  60.383  0.00  0.00
H
ATOM  24159  HB  ILE H  12      22.644 -11.085  59.464  0.00  0.00
H
ATOM  24160  CG2 ILE H  12      21.298  -9.571  59.947  0.00  0.00
H
ATOM  24161 HG21 ILE H  12      20.555  -9.902  59.190  0.00  0.00
H
ATOM  24162 HG22 ILE H  12      20.641  -9.179  60.752  0.00  0.00
H
ATOM  24163 HG23 ILE H  12      21.962  -8.773  59.551  0.00  0.00
H
ATOM  24164  CG1 ILE H  12      23.218 -10.362  61.465  0.00  0.00
H
```

ATOM 24165 HG11 ILE H 12   22.748 -10.168 62.453 0.00 0.00
H
ATOM 24166 HG12 ILE H 12   23.816 -11.274 61.676 0.00 0.00
H
ATOM 24167 CD ILE H 12   24.067 -9.152 60.979 0.00 0.00
H
ATOM 24168 HD1 ILE H 12   24.670 -9.319 60.060 0.00 0.00
H
ATOM 24169 HD2 ILE H 12   23.401 -8.291 60.757 0.00 0.00
H
ATOM 24170 HD3 ILE H 12   24.799 -8.946 61.789 0.00 0.00
H
ATOM 24171 C   ILE H 12   20.516 -12.387 59.565 0.00 0.00
H
ATOM 24172 O   ILE H 12   21.091 -12.621 58.488 0.00 0.00
H
ATOM 24173 N   GLY H 13   19.146 -12.511 59.726 0.00 0.00
H
ATOM 24174 HN GLY H 13   18.766 -12.320 60.627 0.00 0.00
H
ATOM 24175 CA GLY H 13   18.049 -12.771 58.744 0.00 0.00
H
ATOM 24176 HA1 GLY H 13   18.492 -12.497 57.798 0.00 0.00
H
ATOM 24177 HA2 GLY H 13   17.222 -12.178 59.106 0.00 0.00
H
ATOM 24178 C   GLY H 13   17.673 -14.256 58.824 0.00 0.00
H
ATOM 24179 O   GLY H 13   18.495 -15.169 58.784 0.00 0.00
H
ATOM 24180 N   LEU H 14   16.412 -14.599 59.001 0.00 0.00
H
ATOM 24181 HN LEU H 14   15.727 -13.876 59.049 0.00 0.00
H
ATOM 24182 CA LEU H 14   15.917 -15.834 59.463 0.00 0.00
H
ATOM 24183 HA LEU H 14   16.717 -16.552 59.569 0.00 0.00
H
ATOM 24184 CB LEU H 14   15.235 -15.561 60.833 0.00 0.00
H
ATOM 24185 HB1 LEU H 14   14.407 -14.824 60.759 0.00 0.00
H
ATOM 24186 HB2 LEU H 14   14.854 -16.536 61.203 0.00 0.00
H
ATOM 24187 CG LEU H 14   16.269 -15.051 61.879 0.00 0.00
H
ATOM 24188 HG LEU H 14   16.659 -14.048 61.603 0.00 0.00
H
ATOM 24189 CD1 LEU H 14   15.454 -14.832 63.175 0.00 0.00
H
ATOM 24190 HD11 LEU H 14   14.648 -14.068 63.143 0.00 0.00
H
ATOM 24191 HD12 LEU H 14   15.059 -15.755 63.652 0.00 0.00
H

```
ATOM  24192 HD13 LEU H  14      16.143 -14.388  63.925  0.00  0.00
H
ATOM  24193  CD2 LEU H  14      17.455 -15.952  62.081  0.00  0.00
H
ATOM  24194 HD21 LEU H  14      18.039 -15.454  62.884  0.00  0.00
H
ATOM  24195 HD22 LEU H  14      17.166 -16.992  62.345  0.00  0.00
H
ATOM  24196 HD23 LEU H  14      18.141 -15.945  61.207  0.00  0.00
H
ATOM  24197  C   LEU H  14      15.054 -16.488  58.421  0.00  0.00
H
ATOM  24198  O   LEU H  14      14.101 -17.194  58.753  0.00  0.00
H
ATOM  24199  N   GLY H  15      15.470 -16.316  57.143  0.00  0.00
H
ATOM  24200  HN  GLY H  15      16.181 -15.647  56.944  0.00  0.00
H
ATOM  24201  CA  GLY H  15      14.882 -16.980  56.034  0.00  0.00
H
ATOM  24202  HA1 GLY H  15      15.075 -16.429  55.126  0.00  0.00
H
ATOM  24203  HA2 GLY H  15      13.810 -16.998  56.164  0.00  0.00
H
ATOM  24204  C   GLY H  15      15.275 -18.470  55.910  0.00  0.00
H
ATOM  24205  O   GLY H  15      15.561 -19.121  56.861  0.00  0.00
H
ATOM  24206  N   ARG H  16      15.220 -19.022  54.717  0.00  0.00
H
ATOM  24207  HN  ARG H  16      15.001 -18.420  53.954  0.00  0.00
H
ATOM  24208  CA  ARG H  16      15.442 -20.437  54.440  0.00  0.00
H
ATOM  24209  HA  ARG H  16      14.736 -20.942  55.083  0.00  0.00
H
ATOM  24210  CB  ARG H  16      15.128 -20.724  53.007  0.00  0.00
H
ATOM  24211  HB1 ARG H  16      15.623 -20.009  52.315  0.00  0.00
H
ATOM  24212  HB2 ARG H  16      15.391 -21.759  52.702  0.00  0.00
H
ATOM  24213  CG  ARG H  16      13.672 -20.611  52.654  0.00  0.00
H
ATOM  24214  HG1 ARG H  16      12.977 -21.080  53.383  0.00  0.00
H
ATOM  24215  HG2 ARG H  16      13.452 -19.522  52.692  0.00  0.00
H
ATOM  24216  CD  ARG H  16      13.384 -21.163  51.233  0.00  0.00
H
ATOM  24217  HD1 ARG H  16      14.331 -21.394  50.699  0.00  0.00
H
ATOM  24218  HD2 ARG H  16      12.695 -22.032  51.287  0.00  0.00
H
```

```
ATOM  24219  NE  ARG H  16      12.752 -19.979  50.395  0.00  0.00
H
ATOM  24220  HE  ARG H  16      12.847 -19.022  50.670  0.00  0.00
H
ATOM  24221  CZ  ARG H  16      12.482 -20.169  49.098  0.00  0.00
H
ATOM  24222  NH1 ARG H  16      12.285 -21.392  48.558  0.00  0.00
H
ATOM  24223 HH11 ARG H  16      12.221 -21.457  47.562  0.00  0.00
H
ATOM  24224 HH12 ARG H  16      12.662 -22.199  49.013  0.00  0.00
H
ATOM  24225  NH2 ARG H  16      12.497 -19.129  48.273  0.00  0.00
H
ATOM  24226 HH21 ARG H  16      12.063 -19.134  47.372  0.00  0.00
H
ATOM  24227 HH22 ARG H  16      12.711 -18.270  48.739  0.00  0.00
H
ATOM  24228  C   ARG H  16      16.837 -20.954  54.729  0.00  0.00
H
ATOM  24229  O   ARG H  16      17.000 -21.937  55.413  0.00  0.00
H
ATOM  24230  N   PHE H  17      17.850 -20.248  54.236  0.00  0.00
H
ATOM  24231  HN  PHE H  17      17.694 -19.436  53.679  0.00  0.00
H
ATOM  24232  CA  PHE H  17      19.203 -20.561  54.630  0.00  0.00
H
ATOM  24233  HA  PHE H  17      19.327 -21.628  54.739  0.00  0.00
H
ATOM  24234  CB  PHE H  17      20.080 -19.868  53.480  0.00  0.00
H
ATOM  24235  HB1 PHE H  17      19.919 -20.206  52.434  0.00  0.00
H
ATOM  24236  HB2 PHE H  17      19.984 -18.762  53.537  0.00  0.00
H
ATOM  24237  CG  PHE H  17      21.531 -20.186  53.785  0.00  0.00
H
ATOM  24238  CD1 PHE H  17      22.198 -21.322  53.369  0.00  0.00
H
ATOM  24239  HD1 PHE H  17      21.741 -22.170  52.880  0.00  0.00
H
ATOM  24240  CE1 PHE H  17      23.566 -21.408  53.601  0.00  0.00
H
ATOM  24241  HE1 PHE H  17      24.050 -22.364  53.463  0.00  0.00
H
ATOM  24242  CZ  PHE H  17      24.362 -20.371  54.029  0.00  0.00
H
ATOM  24243  HZ  PHE H  17      25.438 -20.375  54.113  0.00  0.00
H
ATOM  24244  CD2 PHE H  17      22.338 -19.164  54.374  0.00  0.00
H
ATOM  24245  HD2 PHE H  17      21.761 -18.332  54.749  0.00  0.00
H
```

ATOM 24246 CE2 PHE H 17 23.723 −19.229 54.562 0.00 0.00
H
ATOM 24247 HE2 PHE H 17 24.261 −18.422 55.038 0.00 0.00
H
ATOM 24248 C PHE H 17 19.522 −20.128 56.111 0.00 0.00
H
ATOM 24249 O PHE H 17 20.206 −20.879 56.767 0.00 0.00
H
ATOM 24250 N GLY H 18 18.982 −18.944 56.505 0.00 0.00
H
ATOM 24251 HN GLY H 18 18.429 −18.321 55.958 0.00 0.00
H
ATOM 24252 CA GLY H 18 19.287 −18.456 57.809 0.00 0.00
H
ATOM 24253 HA1 GLY H 18 18.945 −17.432 57.794 0.00 0.00
H
ATOM 24254 HA2 GLY H 18 20.358 −18.365 57.920 0.00 0.00
H
ATOM 24255 C GLY H 18 18.767 −19.128 59.044 0.00 0.00
H
ATOM 24256 O GLY H 18 19.536 −19.231 59.990 0.00 0.00
H
ATOM 24257 N GLY H 19 17.456 −19.561 59.048 0.00 0.00
H
ATOM 24258 HN GLY H 19 16.936 −19.358 58.222 0.00 0.00
H
ATOM 24259 CA GLY H 19 16.826 −20.346 60.109 0.00 0.00
H
ATOM 24260 HA1 GLY H 19 15.770 −20.328 59.883 0.00 0.00
H
ATOM 24261 HA2 GLY H 19 17.053 −19.797 61.011 0.00 0.00
H
ATOM 24262 C GLY H 19 17.431 −21.726 60.210 0.00 0.00
H
ATOM 24263 O GLY H 19 17.575 −22.385 61.265 0.00 0.00
H
ATOM 24264 N SER H 20 17.867 −22.217 59.038 0.00 0.00
H
ATOM 24265 HN SER H 20 17.770 −21.684 58.202 0.00 0.00
H
ATOM 24266 CA SER H 20 18.531 −23.527 59.025 0.00 0.00
H
ATOM 24267 HA SER H 20 17.910 −24.199 59.598 0.00 0.00
H
ATOM 24268 CB SER H 20 18.702 −24.021 57.628 0.00 0.00
H
ATOM 24269 HB1 SER H 20 19.381 −23.290 57.139 0.00 0.00
H
ATOM 24270 HB2 SER H 20 19.239 −24.994 57.601 0.00 0.00
H
ATOM 24271 OG SER H 20 17.480 −24.198 56.900 0.00 0.00
H
ATOM 24272 HG1 SER H 20 17.269 −23.402 56.407 0.00 0.00
H

ATOM 24273 C   SER H 20   19.844 -23.562 59.769 0.00 0.00
H
ATOM 24274 O   SER H 20   20.198 -24.515 60.435 0.00 0.00
H
ATOM 24275 N   ILE H 21   20.652 -22.472 59.708 0.00 0.00
H
ATOM 24276 HN ILE H 21   20.416 -21.725 59.091 0.00 0.00
H
ATOM 24277 CA ILE H 21   21.817 -22.195 60.558 0.00 0.00
H
ATOM 24278 HA ILE H 21   22.546 -22.981 60.428 0.00 0.00
H
ATOM 24279 CB ILE H 21   22.546 -20.913 60.155 0.00 0.00
H
ATOM 24280 HB ILE H 21   21.801 -20.093 60.080 0.00 0.00
H
ATOM 24281 CG2 ILE H 21   23.707 -20.587 61.118 0.00 0.00
H
ATOM 24282 HG21 ILE H 21   24.346 -19.762 60.738 0.00 0.00
H
ATOM 24283 HG22 ILE H 21   23.425 -20.320 62.158 0.00 0.00
H
ATOM 24284 HG23 ILE H 21   24.508 -21.356 61.144 0.00 0.00
H
ATOM 24285 CG1 ILE H 21   23.012 -21.028 58.686 0.00 0.00
H
ATOM 24286 HG11 ILE H 21   22.200 -21.022 57.929 0.00 0.00
H
ATOM 24287 HG12 ILE H 21   23.515 -20.047 58.544 0.00 0.00
H
ATOM 24288 CD ILE H 21   23.927 -22.197 58.229 0.00 0.00
H
ATOM 24289 HD1 ILE H 21   24.822 -22.220 58.888 0.00 0.00
H
ATOM 24290 HD2 ILE H 21   23.365 -23.126 58.463 0.00 0.00
H
ATOM 24291 HD3 ILE H 21   24.295 -22.050 57.191 0.00 0.00
H
ATOM 24292 C   ILE H 21   21.457 -22.161 62.035 0.00 0.00
H
ATOM 24293 O   ILE H 21   21.970 -22.855 62.908 0.00 0.00
H
ATOM 24294 N   VAL H 22   20.459 -21.266 62.364 0.00 0.00
H
ATOM 24295 HN VAL H 22   20.047 -20.737 61.626 0.00 0.00
H
ATOM 24296 CA VAL H 22   20.053 -20.903 63.702 0.00 0.00
H
ATOM 24297 HA VAL H 22   20.913 -20.486 64.203 0.00 0.00
H
ATOM 24298 CB VAL H 22   18.971 -19.893 63.632 0.00 0.00
H
ATOM 24299 HB VAL H 22   18.201 -20.253 62.916 0.00 0.00
H

```
ATOM  24300  CG1 VAL H  22      18.275 -19.571  64.991  0.00  0.00
H
ATOM  24301 HG11 VAL H  22      19.052 -19.323  65.745  0.00  0.00
H
ATOM  24302 HG12 VAL H  22      17.515 -18.774  64.847  0.00  0.00
H
ATOM  24303 HG13 VAL H  22      17.841 -20.497  65.426  0.00  0.00
H
ATOM  24304  CG2 VAL H  22      19.626 -18.573  63.124  0.00  0.00
H
ATOM  24305 HG21 VAL H  22      18.849 -17.780  63.101  0.00  0.00
H
ATOM  24306 HG22 VAL H  22      20.427 -18.254  63.825  0.00  0.00
H
ATOM  24307 HG23 VAL H  22      19.878 -18.657  62.045  0.00  0.00
H
ATOM  24308  C   VAL H  22      19.669 -22.200  64.499  0.00  0.00
H
ATOM  24309  O   VAL H  22      20.169 -22.494  65.570  0.00  0.00
H
ATOM  24310  N   LYS H  23      18.723 -22.930  63.975  0.00  0.00
H
ATOM  24311  HN  LYS H  23      18.296 -22.713  63.101  0.00  0.00
H
ATOM  24312  CA  LYS H  23      18.317 -24.169  64.582  0.00  0.00
H
ATOM  24313  HA  LYS H  23      18.206 -23.957  65.635  0.00  0.00
H
ATOM  24314  CB  LYS H  23      16.994 -24.689  63.826  0.00  0.00
H
ATOM  24315  HB1 LYS H  23      16.272 -23.846  63.762  0.00  0.00
H
ATOM  24316  HB2 LYS H  23      17.304 -24.965  62.796  0.00  0.00
H
ATOM  24317  CG  LYS H  23      16.208 -25.910  64.560  0.00  0.00
H
ATOM  24318  HG1 LYS H  23      16.769 -26.855  64.395  0.00  0.00
H
ATOM  24319  HG2 LYS H  23      16.062 -25.828  65.659  0.00  0.00
H
ATOM  24320  CD  LYS H  23      14.854 -26.217  63.917  0.00  0.00
H
ATOM  24321  HD1 LYS H  23      14.321 -27.007  64.488  0.00  0.00
H
ATOM  24322  HD2 LYS H  23      14.192 -25.330  64.023  0.00  0.00
H
ATOM  24323  CE  LYS H  23      15.005 -26.460  62.432  0.00  0.00
H
ATOM  24324  HE1 LYS H  23      15.050 -25.471  61.928  0.00  0.00
H
ATOM  24325  HE2 LYS H  23      15.868 -27.126  62.221  0.00  0.00
H
ATOM  24326  NZ  LYS H  23      13.692 -27.043  61.961  0.00  0.00
H
```

ATOM 24327 HZ1 LYS H 23   12.869 -26.470 62.236 0.00 0.00
H
ATOM 24328 HZ2 LYS H 23   13.605 -27.031 60.924 0.00 0.00
H
ATOM 24329 HZ3 LYS H 23   13.548 -28.010 62.315 0.00 0.00
H
ATOM 24330 C   LYS H 23   19.351 -25.307 64.560 0.00 0.00
H
ATOM 24331 O   LYS H 23   19.508 -26.079 65.500 0.00 0.00
H
ATOM 24332 N   GLU H 24   20.063 -25.543 63.428 0.00 0.00
H
ATOM 24333 HN GLU H 24   19.990 -24.990 62.601 0.00 0.00
H
ATOM 24334 CA GLU H 24   21.086 -26.607 63.415 0.00 0.00
H
ATOM 24335 HA GLU H 24   20.641 -27.538 63.732 0.00 0.00
H
ATOM 24336 CB GLU H 24   21.762 -26.759 62.064 0.00 0.00
H
ATOM 24337 HB1 GLU H 24   21.017 -26.900 61.253 0.00 0.00
H
ATOM 24338 HB2 GLU H 24   22.398 -25.911 61.729 0.00 0.00
H
ATOM 24339 CG GLU H 24   22.638 -28.083 61.896 0.00 0.00
H
ATOM 24340 HG1 GLU H 24   23.061 -28.076 60.868 0.00 0.00
H
ATOM 24341 HG2 GLU H 24   23.479 -28.086 62.622 0.00 0.00
H
ATOM 24342 CD GLU H 24   21.923 -29.427 61.968 0.00 0.00
H
ATOM 24343 OE1 GLU H 24   22.526 -30.354 61.375 0.00 0.00
H
ATOM 24344 OE2 GLU H 24   20.748 -29.495 62.472 0.00 0.00
H
ATOM 24345 C   GLU H 24   22.177 -26.424 64.493 0.00 0.00
H
ATOM 24346 O   GLU H 24   22.579 -27.330 65.283 0.00 0.00
H
ATOM 24347 N   LEU H 25   22.717 -25.234 64.633 0.00 0.00
H
ATOM 24348 HN LEU H 25   22.374 -24.463 64.101 0.00 0.00
H
ATOM 24349 CA LEU H 25   23.774 -24.875 65.609 0.00 0.00
H
ATOM 24350 HA LEU H 25   24.595 -25.574 65.547 0.00 0.00
H
ATOM 24351 CB LEU H 25   24.278 -23.440 65.421 0.00 0.00
H
ATOM 24352 HB1 LEU H 25   23.371 -22.811 65.297 0.00 0.00
H
ATOM 24353 HB2 LEU H 25   24.951 -23.100 66.238 0.00 0.00
H

ATOM 24354 CG LEU H 25   25.195 -23.243 64.181 0.00 0.00
H
ATOM 24355 HG LEU H 25   24.708 -23.596 63.247 0.00 0.00
H
ATOM 24356 CD1 LEU H 25   25.450 -21.711 63.967 0.00 0.00
H
ATOM 24357 HD11 LEU H 25   25.991 -21.265 64.829 0.00 0.00
H
ATOM 24358 HD12 LEU H 25   26.153 -21.507 63.131 0.00 0.00
H
ATOM 24359 HD13 LEU H 25   24.486 -21.160 63.955 0.00 0.00
H
ATOM 24360 CD2 LEU H 25   26.501 -24.061 64.389 0.00 0.00
H
ATOM 24361 HD21 LEU H 25   26.361 -25.136 64.145 0.00 0.00
H
ATOM 24362 HD22 LEU H 25   27.366 -23.615 63.852 0.00 0.00
H
ATOM 24363 HD23 LEU H 25   26.812 -24.081 65.455 0.00 0.00
H
ATOM 24364 C   LEU H 25   23.403 -25.053 67.010 0.00 0.00
H
ATOM 24365 O   LEU H 25   24.090 -25.742 67.710 0.00 0.00
H
ATOM 24366 N   HSE H 26   22.158 -24.515 67.337 0.00 0.00
H
ATOM 24367 HN HSE H 26   21.534 -23.971 66.782 0.00 0.00
H
ATOM 24368 CA HSE H 26   21.395 -24.741 68.627 0.00 0.00
H
ATOM 24369 HA HSE H 26   21.981 -24.393 69.465 0.00 0.00
H
ATOM 24370 CB HSE H 26   20.081 -23.929 68.709 0.00 0.00
H
ATOM 24371 HB1 HSE H 26   20.285 -22.838 68.672 0.00 0.00
H
ATOM 24372 HB2 HSE H 26   19.469 -24.225 67.830 0.00 0.00
H
ATOM 24373 ND1 HSE H 26   19.671 -23.459 71.155 0.00 0.00
H
ATOM 24374 CG HSE H 26   19.250 -24.092 69.996 0.00 0.00
H
ATOM 24375 CE1 HSE H 26   18.865 -24.012 72.107 0.00 0.00
H
ATOM 24376 HE1 HSE H 26   19.179 -23.839 73.136 0.00 0.00
H
ATOM 24377 NE2 HSE H 26   17.974 -24.866 71.606 0.00 0.00
H
ATOM 24378 HE2 HSE H 26   17.347 -25.391 72.182 0.00 0.00
H
ATOM 24379 CD2 HSE H 26   18.203 -24.934 70.233 0.00 0.00
H
ATOM 24380 HD2 HSE H 26   17.661 -25.638 69.614 0.00 0.00
H

ATOM 24381 C   HSE H 26   20.985 -26.182 68.872 0.00 0.00
H
ATOM 24382 O   HSE H 26   21.091 -26.646 69.979 0.00 0.00
H
ATOM 24383 N   ARG H 27   20.765 -27.071 67.863 0.00 0.00
H
ATOM 24384 HN ARG H 27   20.756 -26.727 66.927 0.00 0.00
H
ATOM 24385 CA ARG H 27   20.644 -28.525 68.018 0.00 0.00
H
ATOM 24386 HA ARG H 27   19.780 -28.763 68.621 0.00 0.00
H
ATOM 24387 CB ARG H 27   20.376 -29.217 66.667 0.00 0.00
H
ATOM 24388 HB1 ARG H 27   19.608 -28.646 66.103 0.00 0.00
H
ATOM 24389 HB2 ARG H 27   21.266 -29.150 66.006 0.00 0.00
H
ATOM 24390 CG ARG H 27   19.932 -30.754 66.827 0.00 0.00
H
ATOM 24391 HG1 ARG H 27   20.695 -31.363 67.356 0.00 0.00
H
ATOM 24392 HG2 ARG H 27   18.943 -30.738 67.332 0.00 0.00
H
ATOM 24393 CD ARG H 27   19.757 -31.476 65.513 0.00 0.00
H
ATOM 24394 HD1 ARG H 27   19.557 -32.545 65.739 0.00 0.00
H
ATOM 24395 HD2 ARG H 27   18.842 -31.136 64.982 0.00 0.00
H
ATOM 24396 NE ARG H 27   20.931 -31.238 64.653 0.00 0.00
H
ATOM 24397 HE ARG H 27   20.780 -30.582 63.913 0.00 0.00
H
ATOM 24398 CZ ARG H 27   22.042 -31.970 64.692 0.00 0.00
H
ATOM 24399 NH1 ARG H 27   22.474 -32.803 65.673 0.00 0.00
H
ATOM 24400 HH11 ARG H 27   23.451 -32.994 65.761 0.00 0.00
H
ATOM 24401 HH12 ARG H 27   21.935 -32.929 66.506 0.00 0.00
H
ATOM 24402 NH2 ARG H 27   22.888 -31.900 63.704 0.00 0.00
H
ATOM 24403 HH21 ARG H 27   23.621 -32.552 63.510 0.00 0.00
H
ATOM 24404 HH22 ARG H 27   22.653 -31.284 62.952 0.00 0.00
H
ATOM 24405 C   ARG H 27   21.943 -29.210 68.536 0.00 0.00
H
ATOM 24406 O   ARG H 27   21.875 -30.117 69.369 0.00 0.00
H
ATOM 24407 N   MET H 28   23.142 -28.873 68.069 0.00 0.00
H

ATOM 24408 HN MET H 28   23.126 -28.144 67.389 0.00 0.00
H
ATOM 24409 CA MET H 28   24.427 -29.368 68.572 0.00 0.00
H
ATOM 24410 HA MET H 28   24.387 -30.423 68.799 0.00 0.00
H
ATOM 24411 CB MET H 28   25.552 -29.212 67.522 0.00 0.00
H
ATOM 24412 HB1 MET H 28   25.636 -28.133 67.269 0.00 0.00
H
ATOM 24413 HB2 MET H 28   26.490 -29.611 67.963 0.00 0.00
H
ATOM 24414 CG MET H 28   25.076 -29.958 66.207 0.00 0.00
H
ATOM 24415 HG1 MET H 28   24.798 -30.944 66.635 0.00 0.00
H
ATOM 24416 HG2 MET H 28   24.262 -29.473 65.625 0.00 0.00
H
ATOM 24417 SD MET H 28   26.474 -30.014 64.925 0.00 0.00
H
ATOM 24418 CE MET H 28   25.806 -28.610 63.987 0.00 0.00
H
ATOM 24419 HE1 MET H 28   25.198 -27.899 64.585 0.00 0.00
H
ATOM 24420 HE2 MET H 28   26.633 -28.040 63.512 0.00 0.00
H
ATOM 24421 HE3 MET H 28   25.150 -29.049 63.205 0.00 0.00
H
ATOM 24422 C   MET H 28   24.831 -28.635 69.827 0.00 0.00
H
ATOM 24423 O   MET H 28   25.670 -29.109 70.601 0.00 0.00
H
ATOM 24424 N   GLY H 29   24.202 -27.444 70.066 0.00 0.00
H
ATOM 24425 HN GLY H 29   23.674 -27.032 69.328 0.00 0.00
H
ATOM 24426 CA GLY H 29   24.232 -26.724 71.398 0.00 0.00
H
ATOM 24427 HA1 GLY H 29   24.309 -27.480 72.165 0.00 0.00
H
ATOM 24428 HA2 GLY H 29   23.276 -26.247 71.561 0.00 0.00
H
ATOM 24429 C   GLY H 29   25.328 -25.745 71.578 0.00 0.00
H
ATOM 24430 O   GLY H 29   25.866 -25.595 72.653 0.00 0.00
H
ATOM 24431 N   HSE H 30   25.539 -25.027 70.429 0.00 0.00
H
ATOM 24432 HN HSE H 30   25.000 -25.221 69.613 0.00 0.00
H
ATOM 24433 CA HSE H 30   26.378 -23.870 70.289 0.00 0.00
H
ATOM 24434 HA HSE H 30   27.111 -23.925 71.081 0.00 0.00
H

ATOM 24435 CB HSE H 30 27.007 -23.758 68.843 0.00 0.00
H
ATOM 24436 HB1 HSE H 30 26.139 -23.695 68.152 0.00 0.00
H
ATOM 24437 HB2 HSE H 30 27.793 -22.993 68.665 0.00 0.00
H
ATOM 24438 ND1 HSE H 30 28.886 -25.259 69.393 0.00 0.00
H
ATOM 24439 CG HSE H 30 27.806 -25.063 68.640 0.00 0.00
H
ATOM 24440 CE1 HSE H 30 29.338 -26.460 69.088 0.00 0.00
H
ATOM 24441 HE1 HSE H 30 30.281 -26.863 69.457 0.00 0.00
H
ATOM 24442 NE2 HSE H 30 28.547 -27.047 68.177 0.00 0.00
H
ATOM 24443 HE2 HSE H 30 28.881 -27.746 67.546 0.00 0.00
H
ATOM 24444 CD2 HSE H 30 27.471 -26.169 67.901 0.00 0.00
H
ATOM 24445 HD2 HSE H 30 26.698 -26.498 67.218 0.00 0.00
H
ATOM 24446 C HSE H 30 25.575 -22.668 70.492 0.00 0.00
H
ATOM 24447 O HSE H 30 24.405 -22.628 70.109 0.00 0.00
H
ATOM 24448 N GLU H 31 26.119 -21.590 71.153 0.00 0.00
H
ATOM 24449 HN GLU H 31 27.090 -21.643 71.370 0.00 0.00
H
ATOM 24450 CA GLU H 31 25.354 -20.432 71.419 0.00 0.00
H
ATOM 24451 HA GLU H 31 24.399 -20.694 71.851 0.00 0.00
H
ATOM 24452 CB GLU H 31 26.118 -19.558 72.398 0.00 0.00
H
ATOM 24453 HB1 GLU H 31 26.136 -20.191 73.312 0.00 0.00
H
ATOM 24454 HB2 GLU H 31 27.175 -19.388 72.102 0.00 0.00
H
ATOM 24455 CG GLU H 31 25.400 -18.256 72.821 0.00 0.00
H
ATOM 24456 HG1 GLU H 31 26.122 -17.592 73.344 0.00 0.00
H
ATOM 24457 HG2 GLU H 31 24.948 -17.680 71.986 0.00 0.00
H
ATOM 24458 CD GLU H 31 24.292 -18.499 73.837 0.00 0.00
H
ATOM 24459 OE1 GLU H 31 23.975 -19.670 74.189 0.00 0.00
H
ATOM 24460 OE2 GLU H 31 23.718 -17.453 74.289 0.00 0.00
H
ATOM 24461 C GLU H 31 25.118 -19.584 70.155 0.00 0.00
H

```
ATOM  24462  O   GLU H  31      25.974 -19.329  69.340  0.00  0.00
H
ATOM  24463  N   VAL H  32      23.832 -19.116  70.040  0.00  0.00
H
ATOM  24464  HN  VAL H  32      23.067 -19.463  70.578  0.00  0.00
H
ATOM  24465  CA  VAL H  32      23.430 -18.360  68.861  0.00  0.00
H
ATOM  24466  HA  VAL H  32      24.240 -17.931  68.291  0.00  0.00
H
ATOM  24467  CB  VAL H  32      22.654 -19.394  67.922  0.00  0.00
H
ATOM  24468  HB  VAL H  32      21.840 -19.816  68.549  0.00  0.00
H
ATOM  24469  CG1 VAL H  32      22.016 -18.724  66.706  0.00  0.00
H
ATOM  24470 HG11 VAL H  32      21.194 -18.045  67.019  0.00  0.00
H
ATOM  24471 HG12 VAL H  32      22.813 -18.207  66.130  0.00  0.00
H
ATOM  24472 HG13 VAL H  32      21.686 -19.483  65.965  0.00  0.00
H
ATOM  24473  CG2 VAL H  32      23.610 -20.600  67.448  0.00  0.00
H
ATOM  24474 HG21 VAL H  32      23.067 -21.131  66.637  0.00  0.00
H
ATOM  24475 HG22 VAL H  32      24.621 -20.231  67.172  0.00  0.00
H
ATOM  24476 HG23 VAL H  32      23.736 -21.182  68.386  0.00  0.00
H
ATOM  24477  C   VAL H  32      22.520 -17.210  69.212  0.00  0.00
H
ATOM  24478  O   VAL H  32      21.619 -17.418  70.008  0.00  0.00
H
ATOM  24479  N   LEU H  33      22.726 -16.075  68.571  0.00  0.00
H
ATOM  24480  HN  LEU H  33      23.582 -15.932  68.079  0.00  0.00
H
ATOM  24481  CA  LEU H  33      21.727 -15.005  68.638  0.00  0.00
H
ATOM  24482  HA  LEU H  33      21.053 -15.203  69.458  0.00  0.00
H
ATOM  24483  CB  LEU H  33      22.552 -13.641  69.007  0.00  0.00
H
ATOM  24484  HB1 LEU H  33      22.925 -13.862  70.030  0.00  0.00
H
ATOM  24485  HB2 LEU H  33      23.492 -13.463  68.442  0.00  0.00
H
ATOM  24486  CG  LEU H  33      21.818 -12.307  69.170  0.00  0.00
H
ATOM  24487  HG  LEU H  33      22.434 -11.586  69.750  0.00  0.00
H
ATOM  24488  CD1 LEU H  33      21.365 -11.618  67.815  0.00  0.00
H
```

ATOM 24489 HD11 LEU H 33   21.866 −12.080 66.938 0.00 0.00
H
ATOM 24490 HD12 LEU H 33   20.286 −11.871 67.735 0.00 0.00
H
ATOM 24491 HD13 LEU H 33   21.423 −10.513 67.705 0.00 0.00
H
ATOM 24492 CD2 LEU H 33   20.488 −12.329 70.006 0.00 0.00
H
ATOM 24493 HD21 LEU H 33   20.760 −12.865 70.941 0.00 0.00
H
ATOM 24494 HD22 LEU H 33   20.083 −11.382 70.422 0.00 0.00
H
ATOM 24495 HD23 LEU H 33   19.732 −12.961 69.493 0.00 0.00
H
ATOM 24496 C   LEU H 33   21.118 −14.930 67.222 0.00 0.00
H
ATOM 24497 O   LEU H 33   21.789 −15.168 66.210 0.00 0.00
H
ATOM 24498 N   ALA H 34   19.809 −14.734 67.169 0.00 0.00
H
ATOM 24499 HN ALA H 34   19.298 −14.853 68.017 0.00 0.00
H
ATOM 24500 CA ALA H 34   19.073 −14.642 65.971 0.00 0.00
H
ATOM 24501 HA ALA H 34   19.737 −14.688 65.120 0.00 0.00
H
ATOM 24502 CB ALA H 34   18.255 −15.923 65.960 0.00 0.00
H
ATOM 24503 HB1 ALA H 34   18.982 −16.763 65.969 0.00 0.00
H
ATOM 24504 HB2 ALA H 34   17.568 −16.021 66.827 0.00 0.00
H
ATOM 24505 HB3 ALA H 34   17.646 −16.013 65.035 0.00 0.00
H
ATOM 24506 C   ALA H 34   18.322 −13.372 65.872 0.00 0.00
H
ATOM 24507 O   ALA H 34   17.389 −12.968 66.568 0.00 0.00
H
ATOM 24508 N   VAL H 35   18.744 −12.612 64.780 0.00 0.00
H
ATOM 24509 HN VAL H 35   19.447 −12.929 64.148 0.00 0.00
H
ATOM 24510 CA VAL H 35   18.111 −11.278 64.584 0.00 0.00
H
ATOM 24511 HA VAL H 35   17.292 −11.098 65.265 0.00 0.00
H
ATOM 24512 CB VAL H 35   19.059 −10.114 64.930 0.00 0.00
H
ATOM 24513 HB VAL H 35   19.492 −10.290 65.938 0.00 0.00
H
ATOM 24514 CG1 VAL H 35   20.234 −10.170 63.889 0.00 0.00
H
ATOM 24515 HG11 VAL H 35   19.864 −9.747 62.931 0.00 0.00
H

ATOM 24516 HG12 VAL H 35   21.047 -9.482 64.205 0.00 0.00
H
ATOM 24517 HG13 VAL H 35   20.582 -11.184 63.599 0.00 0.00
H
ATOM 24518 CG2 VAL H 35   18.430 -8.706 65.061 0.00 0.00
H
ATOM 24519 HG21 VAL H 35   17.484 -8.833 65.630 0.00 0.00
H
ATOM 24520 HG22 VAL H 35   19.149 -8.041 65.585 0.00 0.00
H
ATOM 24521 HG23 VAL H 35   18.198 -8.355 64.033 0.00 0.00
H
ATOM 24522 C   VAL H 35   17.495 -11.192 63.118 0.00 0.00
H
ATOM 24523 O   VAL H 35   18.089 -11.590 62.126 0.00 0.00
H
ATOM 24524 N   ASP H 36   16.250 -10.770 63.002 0.00 0.00
H
ATOM 24525 HN ASP H 36   15.745 -10.614 63.848 0.00 0.00
H
ATOM 24526 CA ASP H 36   15.541 -10.390 61.814 0.00 0.00
H
ATOM 24527 HA ASP H 36   16.235 -10.339 60.988 0.00 0.00
H
ATOM 24528 CB ASP H 36   14.389 -11.402 61.484 0.00 0.00
H
ATOM 24529 HB1 ASP H 36   14.705 -12.406 61.841 0.00 0.00
H
ATOM 24530 HB2 ASP H 36   13.453 -11.264 62.068 0.00 0.00
H
ATOM 24531 CG ASP H 36   14.149 -11.385 60.028 0.00 0.00
H
ATOM 24532 OD1 ASP H 36   14.414 -12.479 59.335 0.00 0.00
H
ATOM 24533 OD2 ASP H 36   13.608 -10.404 59.482 0.00 0.00
H
ATOM 24534 C   ASP H 36   14.977 -9.043 62.257 0.00 0.00
H
ATOM 24535 O   ASP H 36   14.890 -8.650 63.439 0.00 0.00
H
ATOM 24536 N   ILE H 37   14.476 -8.287 61.248 0.00 0.00
H
ATOM 24537 HN ILE H 37   14.535 -8.613 60.307 0.00 0.00
H
ATOM 24538 CA ILE H 37   13.559 -7.218 61.422 0.00 0.00
H
ATOM 24539 HA ILE H 37   13.708 -6.735 62.376 0.00 0.00
H
ATOM 24540 CB ILE H 37   13.708 -6.149 60.305 0.00 0.00
H
ATOM 24541 HB ILE H 37   14.800 -5.952 60.367 0.00 0.00
H
ATOM 24542 CG2 ILE H 37   13.386 -6.636 58.880 0.00 0.00
H

```
ATOM  24543 HG21 ILE H  37      13.990  -7.548  58.681  0.00  0.00
H
ATOM  24544 HG22 ILE H  37      12.339  -7.006  58.863  0.00  0.00
H
ATOM  24545 HG23 ILE H  37      13.660  -5.920  58.076  0.00  0.00
H
ATOM  24546  CG1 ILE H  37      12.977  -4.827  60.674  0.00  0.00
H
ATOM  24547 HG11 ILE H  37      13.282  -4.161  59.839  0.00  0.00
H
ATOM  24548 HG12 ILE H  37      11.871  -4.938  60.655  0.00  0.00
H
ATOM  24549  CD  ILE H  37      13.383  -4.111  61.984  0.00  0.00
H
ATOM  24550  HD1 ILE H  37      14.434  -3.769  61.865  0.00  0.00
H
ATOM  24551  HD2 ILE H  37      12.784  -3.191  62.149  0.00  0.00
H
ATOM  24552  HD3 ILE H  37      13.175  -4.655  62.930  0.00  0.00
H
ATOM  24553  C   ILE H  37      12.101  -7.816  61.531  0.00  0.00
H
ATOM  24554  O   ILE H  37      11.224  -7.206  62.122  0.00  0.00
H
ATOM  24555  N   ASN H  38      11.874  -9.107  61.201  0.00  0.00
H
ATOM  24556  HN  ASN H  38      12.578  -9.680  60.791  0.00  0.00
H
ATOM  24557  CA  ASN H  38      10.627  -9.730  61.250  0.00  0.00
H
ATOM  24558  HA  ASN H  38       9.840  -9.050  60.957  0.00  0.00
H
ATOM  24559  CB  ASN H  38      10.690 -10.821  60.128  0.00  0.00
H
ATOM  24560  HB1 ASN H  38      11.188 -10.424  59.217  0.00  0.00
H
ATOM  24561  HB2 ASN H  38      11.267 -11.684  60.524  0.00  0.00
H
ATOM  24562  CG  ASN H  38       9.308 -11.334  59.675  0.00  0.00
H
ATOM  24563  OD1 ASN H  38       8.346 -11.027  60.359  0.00  0.00
H
ATOM  24564  ND2 ASN H  38       9.223 -12.026  58.500  0.00  0.00
H
ATOM  24565 HD21 ASN H  38      10.014 -12.221  57.920  0.00  0.00
H
ATOM  24566 HD22 ASN H  38       8.301 -12.322  58.251  0.00  0.00
H
ATOM  24567  C   ASN H  38      10.260 -10.319  62.587  0.00  0.00
H
ATOM  24568  O   ASN H  38      10.902 -11.274  63.099  0.00  0.00
H
ATOM  24569  N   GLU H  39       9.219  -9.772  63.219  0.00  0.00
H
```

ATOM 24570 HN GLU H 39   8.810 -8.933 62.867 0.00 0.00
H
ATOM 24571 CA GLU H 39   8.571 -10.309 64.360 0.00 0.00
H
ATOM 24572 HA GLU H 39   9.243 -10.095 65.178 0.00 0.00
H
ATOM 24573 CB GLU H 39   7.212 -9.482 64.802 0.00 0.00
H
ATOM 24574 HB1 GLU H 39   7.390 -8.388 64.876 0.00 0.00
H
ATOM 24575 HB2 GLU H 39   6.515 -9.610 63.946 0.00 0.00
H
ATOM 24576 CG GLU H 39   6.506 -9.866 66.079 0.00 0.00
H
ATOM 24577 HG1 GLU H 39   6.115 -10.897 65.944 0.00 0.00
H
ATOM 24578 HG2 GLU H 39   7.165 -9.877 66.973 0.00 0.00
H
ATOM 24579 CD GLU H 39   5.324 -8.932 66.366 0.00 0.00
H
ATOM 24580 OE1 GLU H 39   4.352 -8.648 65.612 0.00 0.00
H
ATOM 24581 OE2 GLU H 39   5.466 -8.489 67.549 0.00 0.00
H
ATOM 24582 C   GLU H 39   8.238 -11.817 64.266 0.00 0.00
H
ATOM 24583 O   GLU H 39   8.712 -12.541 65.098 0.00 0.00
H
ATOM 24584 N   GLU H 40   7.490 -12.175 63.234 0.00 0.00
H
ATOM 24585 HN GLU H 40   7.233 -11.485 62.562 0.00 0.00
H
ATOM 24586 CA GLU H 40   7.000 -13.490 62.958 0.00 0.00
H
ATOM 24587 HA GLU H 40   6.205 -13.773 63.631 0.00 0.00
H
ATOM 24588 CB GLU H 40   6.275 -13.447 61.553 0.00 0.00
H
ATOM 24589 HB1 GLU H 40   6.988 -13.162 60.750 0.00 0.00
H
ATOM 24590 HB2 GLU H 40   5.886 -14.456 61.301 0.00 0.00
H
ATOM 24591 CG GLU H 40   5.092 -12.437 61.615 0.00 0.00
H
ATOM 24592 HG1 GLU H 40   4.709 -12.383 62.656 0.00 0.00
H
ATOM 24593 HG2 GLU H 40   5.367 -11.397 61.338 0.00 0.00
H
ATOM 24594 CD GLU H 40   4.009 -12.894 60.733 0.00 0.00
H
ATOM 24595 OE1 GLU H 40   4.242 -12.802 59.477 0.00 0.00
H
ATOM 24596 OE2 GLU H 40   2.953 -13.424 61.122 0.00 0.00
H

```
ATOM  24597  C   GLU H  40       8.034 -14.503  63.008  0.00  0.00
H
ATOM  24598  O   GLU H  40       7.830 -15.641  63.450  0.00  0.00
H
ATOM  24599  N   LYS H  41       9.221 -14.197  62.500  0.00  0.00
H
ATOM  24600  HN  LYS H  41       9.446 -13.246  62.304  0.00  0.00
H
ATOM  24601  CA  LYS H  41      10.365 -15.131  62.679  0.00  0.00
H
ATOM  24602  HA  LYS H  41      10.139 -16.146  62.386  0.00  0.00
H
ATOM  24603  CB  LYS H  41      11.473 -14.625  61.627  0.00  0.00
H
ATOM  24604  HB1 LYS H  41      11.730 -13.587  61.927  0.00  0.00
H
ATOM  24605  HB2 LYS H  41      12.355 -15.300  61.643  0.00  0.00
H
ATOM  24606  CG  LYS H  41      10.969 -14.578  60.183  0.00  0.00
H
ATOM  24607  HG1 LYS H  41      10.059 -13.942  60.140  0.00  0.00
H
ATOM  24608  HG2 LYS H  41      11.744 -14.050  59.587  0.00  0.00
H
ATOM  24609  CD  LYS H  41      10.697 -16.022  59.714  0.00  0.00
H
ATOM  24610  HD1 LYS H  41      11.374 -16.699  60.279  0.00  0.00
H
ATOM  24611  HD2 LYS H  41       9.658 -16.254  60.032  0.00  0.00
H
ATOM  24612  CE  LYS H  41      10.851 -16.282  58.178  0.00  0.00
H
ATOM  24613  HE1 LYS H  41      10.183 -15.613  57.593  0.00  0.00
H
ATOM  24614  HE2 LYS H  41      11.893 -16.106  57.836  0.00  0.00
H
ATOM  24615  NZ  LYS H  41      10.478 -17.663  57.959  0.00  0.00
H
ATOM  24616  HZ1 LYS H  41      11.137 -18.362  58.358  0.00  0.00
H
ATOM  24617  HZ2 LYS H  41       9.586 -17.933  58.420  0.00  0.00
H
ATOM  24618  HZ3 LYS H  41      10.378 -17.909  56.953  0.00  0.00
H
ATOM  24619  C   LYS H  41      11.006 -15.123  64.106  0.00  0.00
H
ATOM  24620  O   LYS H  41      11.453 -16.089  64.659  0.00  0.00
H
ATOM  24621  N   VAL H  42      11.144 -13.970  64.723  0.00  0.00
H
ATOM  24622  HN  VAL H  42      10.880 -13.126  64.263  0.00  0.00
H
ATOM  24623  CA  VAL H  42      11.732 -13.807  66.028  0.00  0.00
H
```

ATOM 24624 HA VAL H 42 12.609 −14.437 66.010 0.00 0.00
H
ATOM 24625 CB VAL H 42 12.060 −12.296 66.321 0.00 0.00
H
ATOM 24626 HB VAL H 42 11.151 −11.701 66.086 0.00 0.00
H
ATOM 24627 CG1 VAL H 42 12.466 −12.193 67.734 0.00 0.00
H
ATOM 24628 HG11 VAL H 42 12.692 −11.130 67.964 0.00 0.00
H
ATOM 24629 HG12 VAL H 42 11.564 −12.444 68.333 0.00 0.00
H
ATOM 24630 HG13 VAL H 42 13.282 −12.882 68.037 0.00 0.00
H
ATOM 24631 CG2 VAL H 42 13.223 −11.868 65.351 0.00 0.00
H
ATOM 24632 HG21 VAL H 42 14.155 −12.441 65.542 0.00 0.00
H
ATOM 24633 HG22 VAL H 42 12.996 −11.929 64.265 0.00 0.00
H
ATOM 24634 HG23 VAL H 42 13.478 −10.815 65.596 0.00 0.00
H
ATOM 24635 C VAL H 42 10.877 −14.473 67.019 0.00 0.00
H
ATOM 24636 O VAL H 42 11.345 −15.102 67.963 0.00 0.00
H
ATOM 24637 N ASN H 43 9.570 −14.507 66.860 0.00 0.00
H
ATOM 24638 HN ASN H 43 9.184 −13.821 66.248 0.00 0.00
H
ATOM 24639 CA ASN H 43 8.617 −15.384 67.629 0.00 0.00
H
ATOM 24640 HA ASN H 43 8.705 −15.240 68.696 0.00 0.00
H
ATOM 24641 CB ASN H 43 7.111 −15.151 67.230 0.00 0.00
H
ATOM 24642 HB1 ASN H 43 7.032 −15.267 66.128 0.00 0.00
H
ATOM 24643 HB2 ASN H 43 6.492 −15.979 67.637 0.00 0.00
H
ATOM 24644 CG ASN H 43 6.710 −13.736 67.688 0.00 0.00
H
ATOM 24645 OD1 ASN H 43 7.455 −13.027 68.352 0.00 0.00
H
ATOM 24646 ND2 ASN H 43 5.495 −13.384 67.238 0.00 0.00
H
ATOM 24647 HD21 ASN H 43 5.149 −12.491 67.523 0.00 0.00
H
ATOM 24648 HD22 ASN H 43 5.006 −13.846 66.498 0.00 0.00
H
ATOM 24649 C ASN H 43 8.908 −16.862 67.388 0.00 0.00
H
ATOM 24650 O ASN H 43 8.874 −17.703 68.321 0.00 0.00
H

```
ATOM  24651  N   ALA H  44       9.185 -17.222  66.141  0.00  0.00
H
ATOM  24652  HN  ALA H  44       9.024 -16.671  65.326  0.00  0.00
H
ATOM  24653  CA  ALA H  44       9.399 -18.610  65.691  0.00  0.00
H
ATOM  24654  HA  ALA H  44       8.545 -19.181  66.024  0.00  0.00
H
ATOM  24655  CB  ALA H  44       9.434 -18.627  64.153  0.00  0.00
H
ATOM  24656  HB1 ALA H  44       8.517 -18.147  63.748  0.00  0.00
H
ATOM  24657  HB2 ALA H  44      10.291 -18.114  63.666  0.00  0.00
H
ATOM  24658  HB3 ALA H  44       9.266 -19.601  63.647  0.00  0.00
H
ATOM  24659  C   ALA H  44      10.641 -19.211  66.294  0.00  0.00
H
ATOM  24660  O   ALA H  44      10.618 -20.330  66.868  0.00  0.00
H
ATOM  24661  N   TYR H  45      11.714 -18.436  66.233  0.00  0.00
H
ATOM  24662  HN  TYR H  45      11.669 -17.484  65.942  0.00  0.00
H
ATOM  24663  CA  TYR H  45      13.043 -18.906  66.462  0.00  0.00
H
ATOM  24664  HA  TYR H  45      13.110 -19.984  66.443  0.00  0.00
H
ATOM  24665  CB  TYR H  45      14.073 -18.226  65.473  0.00  0.00
H
ATOM  24666  HB1 TYR H  45      13.741 -17.201  65.206  0.00  0.00
H
ATOM  24667  HB2 TYR H  45      15.100 -18.278  65.895  0.00  0.00
H
ATOM  24668  CG  TYR H  45      14.039 -18.969  64.156  0.00  0.00
H
ATOM  24669  CD1 TYR H  45      14.791 -20.117  64.105  0.00  0.00
H
ATOM  24670  HD1 TYR H  45      15.323 -20.333  65.020  0.00  0.00
H
ATOM  24671  CE1 TYR H  45      14.639 -21.054  63.065  0.00  0.00
H
ATOM  24672  HE1 TYR H  45      15.062 -22.038  63.205  0.00  0.00
H
ATOM  24673  CZ  TYR H  45      13.930 -20.600  61.930  0.00  0.00
H
ATOM  24674  OH  TYR H  45      13.737 -21.569  60.944  0.00  0.00
H
ATOM  24675  HH  TYR H  45      12.954 -21.233  60.502  0.00  0.00
H
ATOM  24676  CD2 TYR H  45      13.425 -18.532  62.980  0.00  0.00
H
ATOM  24677  HD2 TYR H  45      12.864 -17.609  63.005  0.00  0.00
H
```

ATOM	24678	CE2	TYR	H	45	13.370	−19.314	61.836	0.00	0.00	H
ATOM	24679	HE2	TYR	H	45	12.824	−18.976	60.968	0.00	0.00	H
ATOM	24680	C	TYR	H	45	13.443	−18.573	67.949	0.00	0.00	H
ATOM	24681	O	TYR	H	45	14.581	−18.809	68.356	0.00	0.00	H
ATOM	24682	N	ALA	H	46	12.554	−18.118	68.823	0.00	0.00	H
ATOM	24683	HN	ALA	H	46	11.684	−17.717	68.545	0.00	0.00	H
ATOM	24684	CA	ALA	H	46	12.763	−17.905	70.218	0.00	0.00	H
ATOM	24685	HA	ALA	H	46	13.741	−17.497	70.425	0.00	0.00	H
ATOM	24686	CB	ALA	H	46	11.691	−17.039	70.828	0.00	0.00	H
ATOM	24687	HB1	ALA	H	46	10.728	−17.588	70.898	0.00	0.00	H
ATOM	24688	HB2	ALA	H	46	11.986	−16.747	71.859	0.00	0.00	H
ATOM	24689	HB3	ALA	H	46	11.605	−16.087	70.261	0.00	0.00	H
ATOM	24690	C	ALA	H	46	12.738	−19.255	70.933	0.00	0.00	H
ATOM	24691	O	ALA	H	46	13.226	−19.386	71.993	0.00	0.00	H
ATOM	24692	N	SER	H	47	12.170	−20.268	70.238	0.00	0.00	H
ATOM	24693	HN	SER	H	47	11.568	−20.020	69.483	0.00	0.00	H
ATOM	24694	CA	SER	H	47	12.090	−21.584	70.829	0.00	0.00	H
ATOM	24695	HA	SER	H	47	12.202	−21.465	71.896	0.00	0.00	H
ATOM	24696	CB	SER	H	47	10.671	−22.216	70.641	0.00	0.00	H
ATOM	24697	HB1	SER	H	47	10.413	−22.355	69.570	0.00	0.00	H
ATOM	24698	HB2	SER	H	47	10.665	−23.140	71.258	0.00	0.00	H
ATOM	24699	OG	SER	H	47	9.708	−21.433	71.268	0.00	0.00	H
ATOM	24700	HG1	SER	H	47	8.903	−21.819	70.915	0.00	0.00	H
ATOM	24701	C	SER	H	47	13.082	−22.557	70.216	0.00	0.00	H
ATOM	24702	O	SER	H	47	13.170	−23.686	70.545	0.00	0.00	H
ATOM	24703	N	TYR	H	48	13.963	−22.094	69.267	0.00	0.00	H
ATOM	24704	HN	TYR	H	48	14.023	−21.148	68.957	0.00	0.00	H

ATOM 24705 CA TYR H 48   14.749 -22.854 68.330 0.00 0.00
H
ATOM 24706 HA TYR H 48   14.913 -23.838 68.744 0.00 0.00
H
ATOM 24707 CB TYR H 48   14.367 -22.682 66.830 0.00 0.00
H
ATOM 24708 HB1 TYR H 48   14.331 -21.605 66.558 0.00 0.00
H
ATOM 24709 HB2 TYR H 48   15.104 -23.085 66.103 0.00 0.00
H
ATOM 24710 CG TYR H 48   13.059 -23.354 66.386 0.00 0.00
H
ATOM 24711 CD1 TYR H 48   12.673 -24.609 66.961 0.00 0.00
H
ATOM 24712 HD1 TYR H 48   13.407 -25.178 67.512 0.00 0.00
H
ATOM 24713 CE1 TYR H 48   11.455 -25.237 66.548 0.00 0.00
H
ATOM 24714 HE1 TYR H 48   11.274 -26.288 66.719 0.00 0.00
H
ATOM 24715 CZ TYR H 48   10.577 -24.528 65.660 0.00 0.00
H
ATOM 24716 OH TYR H 48   9.358 -25.136 65.430 0.00 0.00
H
ATOM 24717 HH TYR H 48   9.018 -24.906 64.562 0.00 0.00
H
ATOM 24718 CD2 TYR H 48   12.192 -22.722 65.551 0.00 0.00
H
ATOM 24719 HD2 TYR H 48   12.468 -21.818 65.028 0.00 0.00
H
ATOM 24720 CE2 TYR H 48   10.953 -23.261 65.171 0.00 0.00
H
ATOM 24721 HE2 TYR H 48   10.282 -22.708 64.530 0.00 0.00
H
ATOM 24722 C   TYR H 48   16.115 -22.284 68.421 0.00 0.00
H
ATOM 24723 O   TYR H 48   16.968 -22.712 67.654 0.00 0.00
H
ATOM 24724 N   ALA H 49   16.361 -21.383 69.350 0.00 0.00
H
ATOM 24725 HN ALA H 49   15.739 -21.151 70.094 0.00 0.00
H
ATOM 24726 CA ALA H 49   17.680 -20.920 69.643 0.00 0.00
H
ATOM 24727 HA ALA H 49   18.316 -21.786 69.534 0.00 0.00
H
ATOM 24728 CB ALA H 49   18.214 -19.785 68.781 0.00 0.00
H
ATOM 24729 HB1 ALA H 49   19.254 -19.503 69.052 0.00 0.00
H
ATOM 24730 HB2 ALA H 49   18.093 -20.087 67.718 0.00 0.00
H
ATOM 24731 HB3 ALA H 49   17.579 -18.882 68.902 0.00 0.00
H

ATOM 24732 C   ALA H 49   17.727 -20.409 71.034 0.00 0.00
H
ATOM 24733 O   ALA H 49   16.696 -20.037 71.629 0.00 0.00
H
ATOM 24734 N   THR H 50   18.916 -20.311 71.668 0.00 0.00
H
ATOM 24735 HN THR H 50   19.690 -20.746 71.215 0.00 0.00
H
ATOM 24736 CA THR H 50   19.187 -19.655 72.982 0.00 0.00
H
ATOM 24737 HA THR H 50   18.604 -20.190 73.717 0.00 0.00
H
ATOM 24738 CB THR H 50   20.641 -19.741 73.547 0.00 0.00
H
ATOM 24739 HB THR H 50   20.685 -19.019 74.391 0.00 0.00
H
ATOM 24740 OG1 THR H 50   21.600 -19.367 72.575 0.00 0.00
H
ATOM 24741 HG1 THR H 50   22.447 -19.316 73.023 0.00 0.00
H
ATOM 24742 CG2 THR H 50   20.923 -21.246 74.019 0.00 0.00
H
ATOM 24743 HG21 THR H 50   22.014 -21.347 74.200 0.00 0.00
H
ATOM 24744 HG22 THR H 50   20.424 -21.362 75.005 0.00 0.00
H
ATOM 24745 HG23 THR H 50   20.647 -21.949 73.203 0.00 0.00
H
ATOM 24746 C   THR H 50   18.783 -18.157 73.002 0.00 0.00
H
ATOM 24747 O   THR H 50   18.353 -17.668 74.123 0.00 0.00
H
ATOM 24748 N   HSE H 51   18.867 -17.325 71.897 0.00 0.00
H
ATOM 24749 HN HSE H 51   19.202 -17.766 71.068 0.00 0.00
H
ATOM 24750 CA HSE H 51   18.159 -16.057 71.935 0.00 0.00
H
ATOM 24751 HA HSE H 51   17.202 -16.221 72.407 0.00 0.00
H
ATOM 24752 CB HSE H 51   18.976 -14.954 72.754 0.00 0.00
H
ATOM 24753 HB1 HSE H 51   19.347 -15.429 73.688 0.00 0.00
H
ATOM 24754 HB2 HSE H 51   19.795 -14.487 72.166 0.00 0.00
H
ATOM 24755 ND1 HSE H 51   16.752 -14.016 73.434 0.00 0.00
H
ATOM 24756 CG HSE H 51   18.039 -13.797 73.092 0.00 0.00
H
ATOM 24757 CE1 HSE H 51   16.262 -12.795 73.686 0.00 0.00
H
ATOM 24758 HE1 HSE H 51   15.247 -12.481 73.928 0.00 0.00
H

ATOM 24759 NE2 HSE H 51   17.220 −11.860 73.522 0.00 0.00
H
ATOM 24760 HE2 HSE H 51   17.017 −10.882 73.562 0.00 0.00
H
ATOM 24761 CD2 HSE H 51   18.301 −12.449 73.136 0.00 0.00
H
ATOM 24762 HD2 HSE H 51   19.172 −11.838 72.933 0.00 0.00
H
ATOM 24763 C   HSE H 51   17.838 −15.601 70.470 0.00 0.00
H
ATOM 24764 O   HSE H 51   18.434 −16.029 69.496 0.00 0.00
H
ATOM 24765 N   ALA H 52   16.828 −14.709 70.401 0.00 0.00
H
ATOM 24766 HN ALA H 52   16.447 −14.424 71.277 0.00 0.00
H
ATOM 24767 CA ALA H 52   16.238 −14.121 69.167 0.00 0.00
H
ATOM 24768 HA ALA H 52   16.930 −14.091 68.338 0.00 0.00
H
ATOM 24769 CB ALA H 52   15.153 −15.107 68.705 0.00 0.00
H
ATOM 24770 HB1 ALA H 52   14.410 −15.333 69.499 0.00 0.00
H
ATOM 24771 HB2 ALA H 52   14.586 −14.568 67.917 0.00 0.00
H
ATOM 24772 HB3 ALA H 52   15.663 −15.957 68.203 0.00 0.00
H
ATOM 24773 C   ALA H 52   15.641 −12.743 69.590 0.00 0.00
H
ATOM 24774 O   ALA H 52   14.840 −12.593 70.554 0.00 0.00
H
ATOM 24775 N   VAL H 53   15.949 −11.615 68.792 0.00 0.00
H
ATOM 24776 HN VAL H 53   16.604 −11.762 68.055 0.00 0.00
H
ATOM 24777 CA VAL H 53   15.507 −10.276 69.124 0.00 0.00
H
ATOM 24778 HA VAL H 53   14.688 −10.259 69.828 0.00 0.00
H
ATOM 24779 CB VAL H 53   16.724 −9.464 69.774 0.00 0.00
H
ATOM 24780 HB VAL H 53   16.994 −10.079 70.659 0.00 0.00
H
ATOM 24781 CG1 VAL H 53   17.944 −9.393 68.883 0.00 0.00
H
ATOM 24782 HG11 VAL H 53   18.791 −8.943 69.443 0.00 0.00
H
ATOM 24783 HG12 VAL H 53   18.187 −10.400 68.482 0.00 0.00
H
ATOM 24784 HG13 VAL H 53   17.704 −8.679 68.066 0.00 0.00
H
ATOM 24785 CG2 VAL H 53   16.291 −8.089 70.463 0.00 0.00
H

```
ATOM  24786 HG21 VAL H  53      15.687  -7.522  69.723  0.00  0.00
H
ATOM  24787 HG22 VAL H  53      15.657  -8.308  71.348  0.00  0.00
H
ATOM  24788 HG23 VAL H  53      17.090  -7.455  70.904  0.00  0.00
H
ATOM  24789  C   VAL H  53      15.169  -9.746  67.800  0.00  0.00
H
ATOM  24790  O   VAL H  53      15.687 -10.128  66.762  0.00  0.00
H
ATOM  24791  N   ILE H  54      14.299  -8.766  67.799  0.00  0.00
H
ATOM  24792  HN  ILE H  54      13.766  -8.651  68.633  0.00  0.00
H
ATOM  24793  CA  ILE H  54      13.906  -7.948  66.614  0.00  0.00
H
ATOM  24794  HA  ILE H  54      14.052  -8.607  65.771  0.00  0.00
H
ATOM  24795  CB  ILE H  54      12.459  -7.578  66.541  0.00  0.00
H
ATOM  24796  HB  ILE H  54      12.273  -6.975  67.456  0.00  0.00
H
ATOM  24797  CG2 ILE H  54      12.221  -6.805  65.243  0.00  0.00
H
ATOM  24798 HG21 ILE H  54      12.323  -7.435  64.334  0.00  0.00
H
ATOM  24799 HG22 ILE H  54      11.159  -6.486  65.183  0.00  0.00
H
ATOM  24800 HG23 ILE H  54      12.879  -5.912  65.180  0.00  0.00
H
ATOM  24801  CG1 ILE H  54      11.436  -8.698  66.686  0.00  0.00
H
ATOM  24802 HG11 ILE H  54      11.249  -9.222  65.725  0.00  0.00
H
ATOM  24803 HG12 ILE H  54      11.793  -9.419  67.453  0.00  0.00
H
ATOM  24804  CD  ILE H  54      10.088  -8.226  67.236  0.00  0.00
H
ATOM  24805  HD1 ILE H  54       9.602  -7.562  66.490  0.00  0.00
H
ATOM  24806  HD2 ILE H  54       9.362  -9.003  67.558  0.00  0.00
H
ATOM  24807  HD3 ILE H  54      10.373  -7.594  68.104  0.00  0.00
H
ATOM  24808  C   ILE H  54      14.922  -6.763  66.484  0.00  0.00
H
ATOM  24809  O   ILE H  54      14.861  -5.920  67.395  0.00  0.00
H
ATOM  24810  N   ALA H  55      15.691  -6.636  65.420  0.00  0.00
H
ATOM  24811  HN  ALA H  55      15.567  -7.231  64.630  0.00  0.00
H
ATOM  24812  CA  ALA H  55      16.300  -5.348  65.192  0.00  0.00
H
```

ATOM 24813 HA ALA H 55 15.572 -4.573 65.386 0.00 0.00
H
ATOM 24814 CB ALA H 55 17.593 -5.086 66.108 0.00 0.00
H
ATOM 24815 HB1 ALA H 55 17.342 -5.247 67.178 0.00 0.00
H
ATOM 24816 HB2 ALA H 55 18.382 -5.825 65.854 0.00 0.00
H
ATOM 24817 HB3 ALA H 55 17.898 -4.039 65.898 0.00 0.00
H
ATOM 24818 C ALA H 55 16.715 -5.218 63.771 0.00 0.00
H
ATOM 24819 O ALA H 55 16.816 -6.223 62.995 0.00 0.00
H
ATOM 24820 N ASN H 56 16.931 -3.936 63.299 0.00 0.00
H
ATOM 24821 HN ASN H 56 16.704 -3.248 63.984 0.00 0.00
H
ATOM 24822 CA ASN H 56 17.682 -3.547 62.118 0.00 0.00
H
ATOM 24823 HA ASN H 56 17.289 -4.221 61.372 0.00 0.00
H
ATOM 24824 CB ASN H 56 17.554 -2.038 61.714 0.00 0.00
H
ATOM 24825 HB1 ASN H 56 17.893 -1.355 62.522 0.00 0.00
H
ATOM 24826 HB2 ASN H 56 18.219 -1.735 60.877 0.00 0.00
H
ATOM 24827 CG ASN H 56 16.111 -1.629 61.330 0.00 0.00
H
ATOM 24828 OD1 ASN H 56 15.682 -2.008 60.209 0.00 0.00
H
ATOM 24829 ND2 ASN H 56 15.441 -0.841 62.197 0.00 0.00
H
ATOM 24830 HD21 ASN H 56 14.524 -0.523 61.953 0.00 0.00
H
ATOM 24831 HD22 ASN H 56 15.728 -0.870 63.154 0.00 0.00
H
ATOM 24832 C ASN H 56 19.180 -3.797 62.265 0.00 0.00
H
ATOM 24833 O ASN H 56 19.720 -3.496 63.335 0.00 0.00
H
ATOM 24834 N ALA H 57 19.875 -4.276 61.259 0.00 0.00
H
ATOM 24835 HN ALA H 57 19.397 -4.676 60.481 0.00 0.00
H
ATOM 24836 CA ALA H 57 21.326 -4.465 61.271 0.00 0.00
H
ATOM 24837 HA ALA H 57 21.705 -4.027 62.182 0.00 0.00
H
ATOM 24838 CB ALA H 57 21.572 -5.970 61.290 0.00 0.00
H
ATOM 24839 HB1 ALA H 57 20.905 -6.488 60.568 0.00 0.00
H

ATOM 24840 HB2 ALA H 57 22.655 -6.162 61.128 0.00 0.00
H
ATOM 24841 HB3 ALA H 57 21.358 -6.419 62.283 0.00 0.00
H
ATOM 24842 C ALA H 57 21.954 -3.691 60.109 0.00 0.00
H
ATOM 24843 O ALA H 57 23.128 -3.946 59.803 0.00 0.00
H
ATOM 24844 N THR H 58 21.245 -2.673 59.584 0.00 0.00
H
ATOM 24845 HN THR H 58 20.268 -2.572 59.755 0.00 0.00
H
ATOM 24846 CA THR H 58 21.614 -1.875 58.423 0.00 0.00
H
ATOM 24847 HA THR H 58 22.504 -2.269 57.954 0.00 0.00
H
ATOM 24848 CB THR H 58 20.533 -1.945 57.279 0.00 0.00
H
ATOM 24849 HB THR H 58 20.848 -1.223 56.495 0.00 0.00
H
ATOM 24850 OG1 THR H 58 19.229 -1.677 57.783 0.00 0.00
H
ATOM 24851 HG1 THR H 58 18.712 -1.536 56.987 0.00 0.00
H
ATOM 24852 CG2 THR H 58 20.416 -3.318 56.652 0.00 0.00
H
ATOM 24853 HG21 THR H 58 19.498 -3.290 56.026 0.00 0.00
H
ATOM 24854 HG22 THR H 58 21.272 -3.446 55.955 0.00 0.00
H
ATOM 24855 HG23 THR H 58 20.351 -4.115 57.423 0.00 0.00
H
ATOM 24856 C THR H 58 21.703 -0.423 58.863 0.00 0.00
H
ATOM 24857 O THR H 58 21.747 0.452 58.010 0.00 0.00
H
ATOM 24858 N GLU H 59 21.672 -0.197 60.223 0.00 0.00
H
ATOM 24859 HN GLU H 59 21.609 -1.011 60.795 0.00 0.00
H
ATOM 24860 CA GLU H 59 21.718 1.049 60.962 0.00 0.00
H
ATOM 24861 HA GLU H 59 21.854 1.901 60.313 0.00 0.00
H
ATOM 24862 CB GLU H 59 20.406 1.177 61.758 0.00 0.00
H
ATOM 24863 HB1 GLU H 59 20.113 0.178 62.148 0.00 0.00
H
ATOM 24864 HB2 GLU H 59 20.592 1.824 62.642 0.00 0.00
H
ATOM 24865 CG GLU H 59 19.253 1.769 60.954 0.00 0.00
H
ATOM 24866 HG1 GLU H 59 19.016 1.289 59.981 0.00 0.00
H

ATOM 24867 HG2 GLU H 59 18.397 1.616 61.645 0.00 0.00
H
ATOM 24868 CD GLU H 59 19.242 3.249 60.839 0.00 0.00
H
ATOM 24869 OE1 GLU H 59 20.093 4.016 61.394 0.00 0.00
H
ATOM 24870 OE2 GLU H 59 18.274 3.776 60.176 0.00 0.00
H
ATOM 24871 C GLU H 59 22.913 1.054 61.798 0.00 0.00
H
ATOM 24872 O GLU H 59 23.199 0.123 62.459 0.00 0.00
H
ATOM 24873 N GLU H 60 23.690 2.168 61.921 0.00 0.00
H
ATOM 24874 HN GLU H 60 23.338 2.958 61.425 0.00 0.00
H
ATOM 24875 CA GLU H 60 24.986 2.284 62.540 0.00 0.00
H
ATOM 24876 HA GLU H 60 25.504 1.396 62.209 0.00 0.00
H
ATOM 24877 CB GLU H 60 25.761 3.626 62.270 0.00 0.00
H
ATOM 24878 HB1 GLU H 60 25.702 3.761 61.169 0.00 0.00
H
ATOM 24879 HB2 GLU H 60 25.113 4.300 62.871 0.00 0.00
H
ATOM 24880 CG GLU H 60 27.233 3.678 62.864 0.00 0.00
H
ATOM 24881 HG1 GLU H 60 27.114 3.655 63.968 0.00 0.00
H
ATOM 24882 HG2 GLU H 60 27.647 2.703 62.530 0.00 0.00
H
ATOM 24883 CD GLU H 60 27.961 4.900 62.269 0.00 0.00
H
ATOM 24884 OE1 GLU H 60 27.585 6.073 62.565 0.00 0.00
H
ATOM 24885 OE2 GLU H 60 28.817 4.751 61.360 0.00 0.00
H
ATOM 24886 C GLU H 60 24.802 1.994 64.010 0.00 0.00
H
ATOM 24887 O GLU H 60 25.377 1.126 64.630 0.00 0.00
H
ATOM 24888 N ASN H 61 23.873 2.610 64.718 0.00 0.00
H
ATOM 24889 HN ASN H 61 23.263 3.213 64.209 0.00 0.00
H
ATOM 24890 CA ASN H 61 23.654 2.591 66.077 0.00 0.00
H
ATOM 24891 HA ASN H 61 24.574 2.445 66.623 0.00 0.00
H
ATOM 24892 CB ASN H 61 23.085 3.918 66.674 0.00 0.00
H
ATOM 24893 HB1 ASN H 61 22.340 4.386 65.995 0.00 0.00
H

ATOM 24894 HB2 ASN H 61 22.697 3.745 67.701 0.00 0.00
H
ATOM 24895 CG ASN H 61 24.229 4.894 66.701 0.00 0.00
H
ATOM 24896 OD1 ASN H 61 24.706 5.250 65.589 0.00 0.00
H
ATOM 24897 ND2 ASN H 61 24.777 5.312 67.873 0.00 0.00
H
ATOM 24898 HD21 ASN H 61 25.653 5.794 67.874 0.00 0.00
H
ATOM 24899 HD22 ASN H 61 24.355 5.149 68.764 0.00 0.00
H
ATOM 24900 C ASN H 61 22.747 1.479 66.387 0.00 0.00
H
ATOM 24901 O ASN H 61 22.894 0.817 67.441 0.00 0.00
H
ATOM 24902 N GLU H 62 21.803 1.061 65.461 0.00 0.00
H
ATOM 24903 HN GLU H 62 21.749 1.516 64.576 0.00 0.00
H
ATOM 24904 CA GLU H 62 20.914 -0.050 65.789 0.00 0.00
H
ATOM 24905 HA GLU H 62 20.691 0.100 66.835 0.00 0.00
H
ATOM 24906 CB GLU H 62 19.529 -0.086 65.162 0.00 0.00
H
ATOM 24907 HB1 GLU H 62 19.115 0.931 64.994 0.00 0.00
H
ATOM 24908 HB2 GLU H 62 19.696 -0.550 64.166 0.00 0.00
H
ATOM 24909 CG GLU H 62 18.446 -0.854 65.909 0.00 0.00
H
ATOM 24910 HG1 GLU H 62 18.673 -1.933 66.045 0.00 0.00
H
ATOM 24911 HG2 GLU H 62 18.358 -0.346 66.893 0.00 0.00
H
ATOM 24912 CD GLU H 62 17.043 -0.741 65.319 0.00 0.00
H
ATOM 24913 OE1 GLU H 62 16.320 -1.695 64.991 0.00 0.00
H
ATOM 24914 OE2 GLU H 62 16.581 0.411 65.141 0.00 0.00
H
ATOM 24915 C GLU H 62 21.591 -1.395 65.726 0.00 0.00
H
ATOM 24916 O GLU H 62 21.324 -2.310 66.514 0.00 0.00
H
ATOM 24917 N LEU H 63 22.684 -1.393 64.871 0.00 0.00
H
ATOM 24918 HN LEU H 63 22.854 -0.560 64.351 0.00 0.00
H
ATOM 24919 CA LEU H 63 23.731 -2.450 64.941 0.00 0.00
H
ATOM 24920 HA LEU H 63 23.227 -3.361 65.229 0.00 0.00
H

```
ATOM  24921  CB  LEU H  63      24.400  -2.617  63.611  0.00  0.00
H
ATOM  24922  HB1 LEU H  63      23.639  -3.015  62.906  0.00  0.00
H
ATOM  24923  HB2 LEU H  63      24.716  -1.626  63.222  0.00  0.00
H
ATOM  24924  CG  LEU H  63      25.588  -3.637  63.553  0.00  0.00
H
ATOM  24925  HG  LEU H  63      26.304  -3.546  64.398  0.00  0.00
H
ATOM  24926  CD1 LEU H  63      25.142  -5.140  63.644  0.00  0.00
H
ATOM  24927 HD11 LEU H  63      24.401  -5.202  62.819  0.00  0.00
H
ATOM  24928 HD12 LEU H  63      25.958  -5.882  63.503  0.00  0.00
H
ATOM  24929 HD13 LEU H  63      24.618  -5.225  64.620  0.00  0.00
H
ATOM  24930  CD2 LEU H  63      26.198  -3.362  62.215  0.00  0.00
H
ATOM  24931 HD21 LEU H  63      26.708  -2.381  62.321  0.00  0.00
H
ATOM  24932 HD22 LEU H  63      27.006  -4.089  61.988  0.00  0.00
H
ATOM  24933 HD23 LEU H  63      25.385  -3.371  61.458  0.00  0.00
H
ATOM  24934  C   LEU H  63      24.812  -2.262  66.011  0.00  0.00
H
ATOM  24935  O   LEU H  63      25.274  -3.224  66.656  0.00  0.00
H
ATOM  24936  N   LEU H  64      25.207  -1.080  66.345  0.00  0.00
H
ATOM  24937  HN  LEU H  64      24.916  -0.310  65.783  0.00  0.00
H
ATOM  24938  CA  LEU H  64      26.104  -0.804  67.442  0.00  0.00
H
ATOM  24939  HA  LEU H  64      26.996  -1.371  67.219  0.00  0.00
H
ATOM  24940  CB  LEU H  64      26.383   0.702  67.576  0.00  0.00
H
ATOM  24941  HB1 LEU H  64      26.645   1.115  66.578  0.00  0.00
H
ATOM  24942  HB2 LEU H  64      25.348   1.083  67.706  0.00  0.00
H
ATOM  24943  CG  LEU H  64      27.467   1.162  68.562  0.00  0.00
H
ATOM  24944  HG  LEU H  64      27.255   0.710  69.555  0.00  0.00
H
ATOM  24945  CD1 LEU H  64      28.857   0.735  68.197  0.00  0.00
H
ATOM  24946 HD11 LEU H  64      28.956  -0.332  68.491  0.00  0.00
H
ATOM  24947 HD12 LEU H  64      29.046   0.993  67.133  0.00  0.00
H
```

ATOM 24948 HD13 LEU H 64   29.679   1.233 68.756 0.00 0.00
H
ATOM 24949 CD2 LEU H 64   27.472   2.675 68.638 0.00 0.00
H
ATOM 24950 HD21 LEU H 64   26.501   3.047 69.029 0.00 0.00
H
ATOM 24951 HD22 LEU H 64   28.175   2.819 69.486 0.00 0.00
H
ATOM 24952 HD23 LEU H 64   27.769   3.099 67.655 0.00 0.00
H
ATOM 24953 C   LEU H 64   25.612 -1.247 68.870 0.00 0.00
H
ATOM 24954 O   LEU H 64   26.328 -1.978 69.554 0.00 0.00
H
ATOM 24955 N   SER H 65   24.341 -0.959 69.235 0.00 0.00
H
ATOM 24956 HN SER H 65   23.867 -0.424 68.540 0.00 0.00
H
ATOM 24957 CA SER H 65   23.709 -1.301 70.512 0.00 0.00
H
ATOM 24958 HA SER H 65   24.490 -1.298 71.258 0.00 0.00
H
ATOM 24959 CB SER H 65   22.559 -0.309 70.994 0.00 0.00
H
ATOM 24960 HB1 SER H 65   21.785 -0.281 70.197 0.00 0.00
H
ATOM 24961 HB2 SER H 65   22.041 -0.799 71.845 0.00 0.00
H
ATOM 24962 OG SER H 65   23.007   0.999 71.379 0.00 0.00
H
ATOM 24963 HG1 SER H 65   22.471   1.633 70.898 0.00 0.00
H
ATOM 24964 C   SER H 65   22.997 -2.640 70.395 0.00 0.00
H
ATOM 24965 O   SER H 65   22.315 -3.082 71.289 0.00 0.00
H
ATOM 24966 N   LEU H 66   23.194 -3.439 69.327 0.00 0.00
H
ATOM 24967 HN LEU H 66   23.769 -3.161 68.561 0.00 0.00
H
ATOM 24968 CA LEU H 66   22.597 -4.771 69.193 0.00 0.00
H
ATOM 24969 HA LEU H 66   21.539 -4.658 69.376 0.00 0.00
H
ATOM 24970 CB LEU H 66   22.904 -5.398 67.732 0.00 0.00
H
ATOM 24971 HB1 LEU H 66   22.466 -4.764 66.932 0.00 0.00
H
ATOM 24972 HB2 LEU H 66   24.002 -5.385 67.562 0.00 0.00
H
ATOM 24973 CG LEU H 66   22.276 -6.781 67.440 0.00 0.00
H
ATOM 24974 HG LEU H 66   22.739 -7.557 68.086 0.00 0.00
H

```
ATOM  24975  CD1 LEU H  66      20.809  -6.837  67.744  0.00  0.00
H
ATOM  24976 HD11 LEU H  66      20.541  -7.914  67.787  0.00  0.00
H
ATOM  24977 HD12 LEU H  66      20.596  -6.403  68.744  0.00  0.00
H
ATOM  24978 HD13 LEU H  66      20.067  -6.340  67.082  0.00  0.00
H
ATOM  24979  CD2 LEU H  66      22.414  -7.194  65.976  0.00  0.00
H
ATOM  24980 HD21 LEU H  66      21.727  -6.599  65.338  0.00  0.00
H
ATOM  24981 HD22 LEU H  66      23.406  -6.999  65.514  0.00  0.00
H
ATOM  24982 HD23 LEU H  66      22.102  -8.228  65.714  0.00  0.00
H
ATOM  24983  C   LEU H  66      23.125  -5.669  70.249  0.00  0.00
H
ATOM  24984  O   LEU H  66      22.464  -6.539  70.824  0.00  0.00
H
ATOM  24985  N   GLY H  67      24.482  -5.548  70.578  0.00  0.00
H
ATOM  24986  HN  GLY H  67      24.883  -4.783  70.081  0.00  0.00
H
ATOM  24987  CA  GLY H  67      25.174  -6.449  71.426  0.00  0.00
H
ATOM  24988  HA1 GLY H  67      24.525  -6.992  72.097  0.00  0.00
H
ATOM  24989  HA2 GLY H  67      25.833  -5.845  72.033  0.00  0.00
H
ATOM  24990  C   GLY H  67      25.990  -7.527  70.766  0.00  0.00
H
ATOM  24991  O   GLY H  67      26.465  -8.469  71.462  0.00  0.00
H
ATOM  24992  N   ILE H  68      26.180  -7.567  69.437  0.00  0.00
H
ATOM  24993  HN  ILE H  68      25.953  -6.801  68.840  0.00  0.00
H
ATOM  24994  CA  ILE H  68      27.004  -8.579  68.770  0.00  0.00
H
ATOM  24995  HA  ILE H  68      27.074  -9.410  69.456  0.00  0.00
H
ATOM  24996  CB  ILE H  68      26.376  -9.236  67.536  0.00  0.00
H
ATOM  24997  HB  ILE H  68      26.869 -10.196  67.271  0.00  0.00
H
ATOM  24998  CG2 ILE H  68      24.950  -9.660  67.921  0.00  0.00
H
ATOM  24999 HG21 ILE H  68      24.953 -10.391  68.758  0.00  0.00
H
ATOM  25000 HG22 ILE H  68      24.302  -8.785  68.138  0.00  0.00
H
ATOM  25001 HG23 ILE H  68      24.426 -10.210  67.110  0.00  0.00
H
```

ATOM 25002 CG1 ILE H 68 26.285 -8.314 66.284 0.00 0.00
H
ATOM 25003 HG11 ILE H 68 25.707 -7.413 66.584 0.00 0.00
H
ATOM 25004 HG12 ILE H 68 27.309 -7.938 66.075 0.00 0.00
H
ATOM 25005 CD ILE H 68 25.829 -8.998 64.974 0.00 0.00
H
ATOM 25006 HD1 ILE H 68 26.530 -8.810 64.132 0.00 0.00
H
ATOM 25007 HD2 ILE H 68 25.939 -10.090 65.145 0.00 0.00
H
ATOM 25008 HD3 ILE H 68 24.808 -8.684 64.667 0.00 0.00
H
ATOM 25009 C ILE H 68 28.349 -8.005 68.376 0.00 0.00
H
ATOM 25010 O ILE H 68 29.019 -8.592 67.586 0.00 0.00
H
ATOM 25011 N ARG H 69 28.789 -6.850 68.877 0.00 0.00
H
ATOM 25012 HN ARG H 69 28.221 -6.301 69.485 0.00 0.00
H
ATOM 25013 CA ARG H 69 30.059 -6.211 68.525 0.00 0.00
H
ATOM 25014 HA ARG H 69 30.172 -5.997 67.472 0.00 0.00
H
ATOM 25015 CB ARG H 69 30.254 -4.900 69.134 0.00 0.00
H
ATOM 25016 HB1 ARG H 69 31.208 -4.439 68.800 0.00 0.00
H
ATOM 25017 HB2 ARG H 69 29.438 -4.220 68.807 0.00 0.00
H
ATOM 25018 CG ARG H 69 30.070 -4.786 70.693 0.00 0.00
H
ATOM 25019 HG1 ARG H 69 29.413 -5.609 71.046 0.00 0.00
H
ATOM 25020 HG2 ARG H 69 31.024 -5.053 71.196 0.00 0.00
H
ATOM 25021 CD ARG H 69 29.478 -3.559 71.379 0.00 0.00
H
ATOM 25022 HD1 ARG H 69 28.617 -3.288 70.732 0.00 0.00
H
ATOM 25023 HD2 ARG H 69 29.166 -3.742 72.430 0.00 0.00
H
ATOM 25024 NE ARG H 69 30.563 -2.499 71.204 0.00 0.00
H
ATOM 25025 HE ARG H 69 31.514 -2.773 71.067 0.00 0.00
H
ATOM 25026 CZ ARG H 69 30.229 -1.228 71.461 0.00 0.00
H
ATOM 25027 NH1 ARG H 69 29.004 -0.790 71.798 0.00 0.00
H
ATOM 25028 HH11 ARG H 69 28.835 0.191 71.898 0.00 0.00
H

```
ATOM  25029 HH12 ARG H  69      28.229  -1.421  71.843  0.00  0.00
H
ATOM  25030  NH2 ARG H  69      31.096  -0.239  71.465  0.00  0.00
H
ATOM  25031 HH21 ARG H  69      30.840   0.634  71.880  0.00  0.00
H
ATOM  25032 HH22 ARG H  69      32.037  -0.525  71.284  0.00  0.00
H
ATOM  25033  C   ARG H  69      31.294  -6.959  68.946  0.00  0.00
H
ATOM  25034  O   ARG H  69      32.415  -6.834  68.468  0.00  0.00
H
ATOM  25035  N   ASN H  70      31.104  -7.893  69.953  0.00  0.00
H
ATOM  25036  HN  ASN H  70      30.281  -8.082  70.483  0.00  0.00
H
ATOM  25037  CA  ASN H  70      32.311  -8.631  70.414  0.00  0.00
H
ATOM  25038  HA  ASN H  70      33.232  -8.162  70.098  0.00  0.00
H
ATOM  25039  CB  ASN H  70      32.390  -8.578  71.988  0.00  0.00
H
ATOM  25040  HB1 ASN H  70      31.475  -9.074  72.378  0.00  0.00
H
ATOM  25041  HB2 ASN H  70      33.383  -8.984  72.277  0.00  0.00
H
ATOM  25042  CG  ASN H  70      32.236  -7.141  72.562  0.00  0.00
H
ATOM  25043  OD1 ASN H  70      33.005  -6.182  72.328  0.00  0.00
H
ATOM  25044  ND2 ASN H  70      31.190  -7.076  73.465  0.00  0.00
H
ATOM  25045 HD21 ASN H  70      31.022  -6.258  74.016  0.00  0.00
H
ATOM  25046 HD22 ASN H  70      30.680  -7.926  73.594  0.00  0.00
H
ATOM  25047  C   ASN H  70      32.405 -10.032  69.843  0.00  0.00
H
ATOM  25048  O   ASN H  70      33.280 -10.882  70.177  0.00  0.00
H
ATOM  25049  N   PHE H  71      31.546 -10.412  68.952  0.00  0.00
H
ATOM  25050  HN  PHE H  71      30.836  -9.753  68.714  0.00  0.00
H
ATOM  25051  CA  PHE H  71      31.520 -11.710  68.236  0.00  0.00
H
ATOM  25052  HA  PHE H  71      31.760 -12.423  69.011  0.00  0.00
H
ATOM  25053  CB  PHE H  71      30.132 -11.938  67.499  0.00  0.00
H
ATOM  25054  HB1 PHE H  71      29.827 -11.050  66.905  0.00  0.00
H
ATOM  25055  HB2 PHE H  71      30.268 -12.739  66.741  0.00  0.00
H
```

ATOM 25056 CG PHE H 71 29.076 -12.405 68.530 0.00 0.00
H
ATOM 25057 CD1 PHE H 71 28.603 -11.585 69.555 0.00 0.00
H
ATOM 25058 HD1 PHE H 71 28.997 -10.604 69.779 0.00 0.00
H
ATOM 25059 CE1 PHE H 71 27.493 -12.003 70.351 0.00 0.00
H
ATOM 25060 HE1 PHE H 71 27.063 -11.264 71.012 0.00 0.00
H
ATOM 25061 CZ PHE H 71 26.921 -13.247 70.190 0.00 0.00
H
ATOM 25062 HZ PHE H 71 26.219 -13.640 70.911 0.00 0.00
H
ATOM 25063 CD2 PHE H 71 28.468 -13.679 68.409 0.00 0.00
H
ATOM 25064 HD2 PHE H 71 28.869 -14.294 67.616 0.00 0.00
H
ATOM 25065 CE2 PHE H 71 27.518 -14.136 69.289 0.00 0.00
H
ATOM 25066 HE2 PHE H 71 27.115 -15.137 69.256 0.00 0.00
H
ATOM 25067 C PHE H 71 32.723 -11.814 67.276 0.00 0.00
H
ATOM 25068 O PHE H 71 33.166 -10.888 66.537 0.00 0.00
H
ATOM 25069 N GLU H 72 33.283 -13.136 67.236 0.00 0.00
H
ATOM 25070 HN GLU H 72 32.845 -13.842 67.788 0.00 0.00
H
ATOM 25071 CA GLU H 72 34.358 -13.566 66.344 0.00 0.00
H
ATOM 25072 HA GLU H 72 35.038 -12.727 66.355 0.00 0.00
H
ATOM 25073 CB GLU H 72 35.003 -14.861 66.897 0.00 0.00
H
ATOM 25074 HB1 GLU H 72 34.994 -14.732 68.001 0.00 0.00
H
ATOM 25075 HB2 GLU H 72 34.258 -15.683 66.842 0.00 0.00
H
ATOM 25076 CG GLU H 72 36.408 -15.039 66.279 0.00 0.00
H
ATOM 25077 HG1 GLU H 72 36.176 -15.380 65.247 0.00 0.00
H
ATOM 25078 HG2 GLU H 72 36.968 -14.083 66.370 0.00 0.00
H
ATOM 25079 CD GLU H 72 37.148 -16.186 66.954 0.00 0.00
H
ATOM 25080 OE1 GLU H 72 37.495 -16.161 68.173 0.00 0.00
H
ATOM 25081 OE2 GLU H 72 37.577 -17.093 66.194 0.00 0.00
H
ATOM 25082 C GLU H 72 33.725 -13.773 64.938 0.00 0.00
H

ATOM 25083 O GLU H 72 34.154 -13.305 63.887 0.00 0.00
H
ATOM 25084 N TYR H 73 32.544 -14.508 64.968 0.00 0.00
H
ATOM 25085 HN TYR H 73 32.076 -14.767 65.809 0.00 0.00
H
ATOM 25086 CA TYR H 73 31.818 -14.846 63.798 0.00 0.00
H
ATOM 25087 HA TYR H 73 32.413 -14.662 62.915 0.00 0.00
H
ATOM 25088 CB TYR H 73 31.558 -16.347 63.730 0.00 0.00
H
ATOM 25089 HB1 TYR H 73 31.401 -16.735 64.760 0.00 0.00
H
ATOM 25090 HB2 TYR H 73 30.687 -16.545 63.069 0.00 0.00
H
ATOM 25091 CG TYR H 73 32.724 -17.118 63.115 0.00 0.00
H
ATOM 25092 CD1 TYR H 73 33.909 -17.474 63.896 0.00 0.00
H
ATOM 25093 HD1 TYR H 73 34.088 -17.174 64.918 0.00 0.00
H
ATOM 25094 CE1 TYR H 73 35.021 -18.020 63.367 0.00 0.00
H
ATOM 25095 HE1 TYR H 73 35.850 -18.314 63.994 0.00 0.00
H
ATOM 25096 CZ TYR H 73 34.939 -18.453 62.051 0.00 0.00
H
ATOM 25097 OH TYR H 73 36.010 -19.186 61.642 0.00 0.00
H
ATOM 25098 HH TYR H 73 35.918 -19.272 60.691 0.00 0.00
H
ATOM 25099 CD2 TYR H 73 32.723 -17.483 61.744 0.00 0.00
H
ATOM 25100 HD2 TYR H 73 31.865 -17.321 61.108 0.00 0.00
H
ATOM 25101 CE2 TYR H 73 33.875 -18.079 61.229 0.00 0.00
H
ATOM 25102 HE2 TYR H 73 33.808 -18.493 60.234 0.00 0.00
H
ATOM 25103 C TYR H 73 30.490 -14.118 63.740 0.00 0.00
H
ATOM 25104 O TYR H 73 29.745 -14.164 64.688 0.00 0.00
H
ATOM 25105 N VAL H 74 30.215 -13.572 62.533 0.00 0.00
H
ATOM 25106 HN VAL H 74 30.793 -13.657 61.725 0.00 0.00
H
ATOM 25107 CA VAL H 74 28.925 -13.078 62.099 0.00 0.00
H
ATOM 25108 HA VAL H 74 28.161 -13.515 62.724 0.00 0.00
H
ATOM 25109 CB VAL H 74 28.733 -11.538 62.113 0.00 0.00
H

ATOM 25110 HB VAL H 74   29.458 -11.052 61.426 0.00 0.00
H
ATOM 25111 CG1 VAL H 74   27.265 -11.285 61.718 0.00 0.00
H
ATOM 25112 HG11 VAL H 74   26.524 -11.915 62.255 0.00 0.00
H
ATOM 25113 HG12 VAL H 74   27.044 -10.207 61.873 0.00 0.00
H
ATOM 25114 HG13 VAL H 74   27.093 -11.455 60.633 0.00 0.00
H
ATOM 25115 CG2 VAL H 74   28.916 -10.767 63.491 0.00 0.00
H
ATOM 25116 HG21 VAL H 74   28.050 -10.789 64.187 0.00 0.00
H
ATOM 25117 HG22 VAL H 74   29.763 -11.236 64.036 0.00 0.00
H
ATOM 25118 HG23 VAL H 74   29.144 -9.686 63.374 0.00 0.00
H
ATOM 25119 C   VAL H 74   28.527 -13.539 60.725 0.00 0.00
H
ATOM 25120 O   VAL H 74   29.138 -13.497 59.602 0.00 0.00
H
ATOM 25121 N   ILE H 75   27.353 -14.192 60.698 0.00 0.00
H
ATOM 25122 HN ILE H 75   26.778 -14.330 61.500 0.00 0.00
H
ATOM 25123 CA ILE H 75   26.835 -14.946 59.549 0.00 0.00
H
ATOM 25124 HA ILE H 75   27.567 -14.871 58.759 0.00 0.00
H
ATOM 25125 CB ILE H 75   26.418 -16.332 59.987 0.00 0.00
H
ATOM 25126 HB ILE H 75   25.364 -16.226 60.320 0.00 0.00
H
ATOM 25127 CG2 ILE H 75   26.508 -17.289 58.772 0.00 0.00
H
ATOM 25128 HG21 ILE H 75   25.958 -16.859 57.907 0.00 0.00
H
ATOM 25129 HG22 ILE H 75   27.586 -17.490 58.599 0.00 0.00
H
ATOM 25130 HG23 ILE H 75   26.138 -18.305 59.029 0.00 0.00
H
ATOM 25131 CG1 ILE H 75   27.045 -16.923 61.346 0.00 0.00
H
ATOM 25132 HG11 ILE H 75   27.004 -16.124 62.116 0.00 0.00
H
ATOM 25133 HG12 ILE H 75   26.612 -17.920 61.578 0.00 0.00
H
ATOM 25134 CD ILE H 75   28.583 -17.271 61.121 0.00 0.00
H
ATOM 25135 HD1 ILE H 75   29.143 -17.143 62.072 0.00 0.00
H
ATOM 25136 HD2 ILE H 75   28.520 -18.305 60.720 0.00 0.00
H

```
ATOM  25137  HD3 ILE H  75      28.960 -16.604  60.316  0.00  0.00
H
ATOM  25138  C   ILE H  75      25.647 -14.148  59.020  0.00  0.00
H
ATOM  25139  O   ILE H  75      24.548 -14.162  59.574  0.00  0.00
H
ATOM  25140  N   VAL H  76      25.724 -13.587  57.750  0.00  0.00
H
ATOM  25141  HN  VAL H  76      26.545 -13.665  57.190  0.00  0.00
H
ATOM  25142  CA  VAL H  76      24.662 -12.778  57.181  0.00  0.00
H
ATOM  25143  HA  VAL H  76      23.992 -12.397  57.938  0.00  0.00
H
ATOM  25144  CB  VAL H  76      25.218 -11.509  56.418  0.00  0.00
H
ATOM  25145  HB  VAL H  76      26.050 -11.946  55.825  0.00  0.00
H
ATOM  25146  CG1 VAL H  76      24.297 -10.676  55.565  0.00  0.00
H
ATOM  25147 HG11 VAL H  76      23.880 -11.277  54.729  0.00  0.00
H
ATOM  25148 HG12 VAL H  76      23.508 -10.362  56.282  0.00  0.00
H
ATOM  25149 HG13 VAL H  76      24.820  -9.789  55.146  0.00  0.00
H
ATOM  25150  CG2 VAL H  76      25.918 -10.574  57.417  0.00  0.00
H
ATOM  25151 HG21 VAL H  76      26.330  -9.658  56.943  0.00  0.00
H
ATOM  25152 HG22 VAL H  76      25.093 -10.239  58.082  0.00  0.00
H
ATOM  25153 HG23 VAL H  76      26.768 -11.043  57.956  0.00  0.00
H
ATOM  25154  C   VAL H  76      23.870 -13.618  56.199  0.00  0.00
H
ATOM  25155  O   VAL H  76      24.368 -13.959  55.144  0.00  0.00
H
ATOM  25156  N   ALA H  77      22.608 -13.956  56.537  0.00  0.00
H
ATOM  25157  HN  ALA H  77      22.189 -13.579  57.359  0.00  0.00
H
ATOM  25158  CA  ALA H  77      21.860 -14.822  55.596  0.00  0.00
H
ATOM  25159  HA  ALA H  77      22.392 -15.373  54.835  0.00  0.00
H
ATOM  25160  CB  ALA H  77      21.065 -15.756  56.546  0.00  0.00
H
ATOM  25161  HB1 ALA H  77      20.303 -16.241  55.900  0.00  0.00
H
ATOM  25162  HB2 ALA H  77      21.635 -16.568  57.047  0.00  0.00
H
ATOM  25163  HB3 ALA H  77      20.603 -15.127  57.337  0.00  0.00
H
```

ATOM 25164 C ALA H 77 20.938 -13.961 54.718 0.00 0.00
H
ATOM 25165 O ALA H 77 20.253 -14.404 53.847 0.00 0.00
H
ATOM 25166 N ILE H 78 20.820 -12.650 54.967 0.00 0.00
H
ATOM 25167 HN ILE H 78 21.281 -12.267 55.764 0.00 0.00
H
ATOM 25168 CA ILE H 78 20.111 -11.699 54.047 0.00 0.00
H
ATOM 25169 HA ILE H 78 19.215 -12.151 53.648 0.00 0.00
H
ATOM 25170 CB ILE H 78 19.751 -10.368 54.598 0.00 0.00
H
ATOM 25171 HB ILE H 78 19.005 -10.448 55.417 0.00 0.00
H
ATOM 25172 CG2 ILE H 78 20.993 -9.725 55.406 0.00 0.00
H
ATOM 25173 HG21 ILE H 78 21.810 -9.483 54.692 0.00 0.00
H
ATOM 25174 HG22 ILE H 78 20.742 -8.699 55.751 0.00 0.00
H
ATOM 25175 HG23 ILE H 78 21.252 -10.335 56.297 0.00 0.00
H
ATOM 25176 CG1 ILE H 78 19.095 -9.445 53.575 0.00 0.00
H
ATOM 25177 HG11 ILE H 78 19.910 -8.935 53.019 0.00 0.00
H
ATOM 25178 HG12 ILE H 78 18.490 -10.075 52.888 0.00 0.00
H
ATOM 25179 CD ILE H 78 18.260 -8.435 54.267 0.00 0.00
H
ATOM 25180 HD1 ILE H 78 18.756 -7.733 54.971 0.00 0.00
H
ATOM 25181 HD2 ILE H 78 17.653 -7.825 53.565 0.00 0.00
H
ATOM 25182 HD3 ILE H 78 17.428 -8.884 54.851 0.00 0.00
H
ATOM 25183 C ILE H 78 20.953 -11.574 52.748 0.00 0.00
H
ATOM 25184 O ILE H 78 22.167 -11.327 52.693 0.00 0.00
H
ATOM 25185 N GLY H 79 20.378 -11.809 51.561 0.00 0.00
H
ATOM 25186 HN GLY H 79 19.403 -12.019 51.568 0.00 0.00
H
ATOM 25187 CA GLY H 79 21.224 -12.021 50.375 0.00 0.00
H
ATOM 25188 HA1 GLY H 79 21.715 -12.982 50.429 0.00 0.00
H
ATOM 25189 HA2 GLY H 79 21.894 -11.175 50.326 0.00 0.00
H
ATOM 25190 C GLY H 79 20.573 -12.050 49.051 0.00 0.00
H

ATOM	25191	O	GLY	H	79	21.273	−11.956	48.055	0.00	0.00	H
ATOM	25192	N	ALA	H	80	19.237	−12.186	48.856	0.00	0.00	H
ATOM	25193	HN	ALA	H	80	18.559	−12.370	49.563	0.00	0.00	H
ATOM	25194	CA	ALA	H	80	18.742	−11.743	47.592	0.00	0.00	H
ATOM	25195	HA	ALA	H	80	19.081	−12.315	46.740	0.00	0.00	H
ATOM	25196	CB	ALA	H	80	17.291	−12.193	47.596	0.00	0.00	H
ATOM	25197	HB1	ALA	H	80	17.239	−13.303	47.626	0.00	0.00	H
ATOM	25198	HB2	ALA	H	80	16.846	−11.834	48.548	0.00	0.00	H
ATOM	25199	HB3	ALA	H	80	16.714	−11.888	46.697	0.00	0.00	H
ATOM	25200	C	ALA	H	80	18.870	−10.241	47.311	0.00	0.00	H
ATOM	25201	O	ALA	H	80	19.267	−9.743	46.292	0.00	0.00	H
ATOM	25202	N	ASN	H	81	18.572	−9.333	48.292	0.00	0.00	H
ATOM	25203	HN	ASN	H	81	18.124	−9.778	49.063	0.00	0.00	H
ATOM	25204	CA	ASN	H	81	18.801	−7.967	48.206	0.00	0.00	H
ATOM	25205	HA	ASN	H	81	18.383	−7.557	47.298	0.00	0.00	H
ATOM	25206	CB	ASN	H	81	17.990	−7.271	49.357	0.00	0.00	H
ATOM	25207	HB1	ASN	H	81	16.978	−7.727	49.296	0.00	0.00	H
ATOM	25208	HB2	ASN	H	81	18.423	−7.471	50.360	0.00	0.00	H
ATOM	25209	CG	ASN	H	81	17.913	−5.772	49.256	0.00	0.00	H
ATOM	25210	OD1	ASN	H	81	18.843	−4.964	49.124	0.00	0.00	H
ATOM	25211	ND2	ASN	H	81	16.688	−5.206	49.428	0.00	0.00	H
ATOM	25212	HD21	ASN	H	81	16.475	−4.230	49.389	0.00	0.00	H
ATOM	25213	HD22	ASN	H	81	15.837	−5.715	49.301	0.00	0.00	H
ATOM	25214	C	ASN	H	81	20.316	−7.619	48.216	0.00	0.00	H
ATOM	25215	O	ASN	H	81	21.096	−7.822	49.151	0.00	0.00	H
ATOM	25216	N	ILE	H	82	20.775	−6.955	47.215	0.00	0.00	H
ATOM	25217	HN	ILE	H	82	20.217	−6.588	46.474	0.00	0.00	H

ATOM 25218 CA ILE H 82 22.235 -6.643 47.038 0.00 0.00
H
ATOM 25219 HA ILE H 82 22.869 -7.493 47.241 0.00 0.00
H
ATOM 25220 CB ILE H 82 22.569 -6.237 45.528 0.00 0.00
H
ATOM 25221 HB ILE H 82 21.991 -5.293 45.433 0.00 0.00
H
ATOM 25222 CG2 ILE H 82 24.105 -5.896 45.454 0.00 0.00
H
ATOM 25223 HG21 ILE H 82 24.732 -6.796 45.632 0.00 0.00
H
ATOM 25224 HG22 ILE H 82 24.324 -5.580 44.412 0.00 0.00
H
ATOM 25225 HG23 ILE H 82 24.298 -5.045 46.141 0.00 0.00
H
ATOM 25226 CG1 ILE H 82 22.140 -7.419 44.582 0.00 0.00
H
ATOM 25227 HG11 ILE H 82 22.550 -8.390 44.935 0.00 0.00
H
ATOM 25228 HG12 ILE H 82 21.054 -7.640 44.666 0.00 0.00
H
ATOM 25229 CD ILE H 82 22.432 -7.350 43.083 0.00 0.00
H
ATOM 25230 HD1 ILE H 82 23.495 -7.171 42.815 0.00 0.00
H
ATOM 25231 HD2 ILE H 82 22.027 -8.249 42.570 0.00 0.00
H
ATOM 25232 HD3 ILE H 82 21.725 -6.589 42.688 0.00 0.00
H
ATOM 25233 C ILE H 82 22.639 -5.490 48.011 0.00 0.00
H
ATOM 25234 O ILE H 82 23.647 -5.548 48.714 0.00 0.00
H
ATOM 25235 N GLN H 83 21.877 -4.351 48.103 0.00 0.00
H
ATOM 25236 HN GLN H 83 21.059 -4.230 47.545 0.00 0.00
H
ATOM 25237 CA GLN H 83 22.123 -3.235 48.970 0.00 0.00
H
ATOM 25238 HA GLN H 83 23.142 -2.953 48.749 0.00 0.00
H
ATOM 25239 CB GLN H 83 21.149 -2.068 48.735 0.00 0.00
H
ATOM 25240 HB1 GLN H 83 21.269 -1.822 47.658 0.00 0.00
H
ATOM 25241 HB2 GLN H 83 20.106 -2.391 48.939 0.00 0.00
H
ATOM 25242 CG GLN H 83 21.363 -0.769 49.621 0.00 0.00
H
ATOM 25243 HG1 GLN H 83 20.730 -0.019 49.100 0.00 0.00
H
ATOM 25244 HG2 GLN H 83 20.955 -0.791 50.654 0.00 0.00
H

ATOM 25245 CD GLN H 83 22.722 -0.021 49.510 0.00 0.00
H
ATOM 25246 OE1 GLN H 83 23.426 -0.248 48.501 0.00 0.00
H
ATOM 25247 NE2 GLN H 83 23.084 0.833 50.486 0.00 0.00
H
ATOM 25248 HE21 GLN H 83 22.493 0.790 51.292 0.00 0.00
H
ATOM 25249 HE22 GLN H 83 24.048 0.968 50.717 0.00 0.00
H
ATOM 25250 C GLN H 83 22.046 -3.633 50.424 0.00 0.00
H
ATOM 25251 O GLN H 83 23.002 -3.236 51.163 0.00 0.00
H
ATOM 25252 N ALA H 84 20.994 -4.383 50.830 0.00 0.00
H
ATOM 25253 HN ALA H 84 20.334 -4.789 50.202 0.00 0.00
H
ATOM 25254 CA ALA H 84 20.895 -4.735 52.232 0.00 0.00
H
ATOM 25255 HA ALA H 84 20.910 -3.771 52.719 0.00 0.00
H
ATOM 25256 CB ALA H 84 19.575 -5.392 52.631 0.00 0.00
H
ATOM 25257 HB1 ALA H 84 19.547 -5.712 53.695 0.00 0.00
H
ATOM 25258 HB2 ALA H 84 18.829 -4.577 52.512 0.00 0.00
H
ATOM 25259 HB3 ALA H 84 19.337 -6.278 52.004 0.00 0.00
H
ATOM 25260 C ALA H 84 21.978 -5.616 52.764 0.00 0.00
H
ATOM 25261 O ALA H 84 22.538 -5.434 53.816 0.00 0.00
H
ATOM 25262 N SER H 85 22.337 -6.657 51.978 0.00 0.00
H
ATOM 25263 HN SER H 85 21.704 -6.759 51.214 0.00 0.00
H
ATOM 25264 CA SER H 85 23.483 -7.560 52.195 0.00 0.00
H
ATOM 25265 HA SER H 85 23.334 -8.012 53.164 0.00 0.00
H
ATOM 25266 CB SER H 85 23.501 -8.647 51.066 0.00 0.00
H
ATOM 25267 HB1 SER H 85 22.559 -9.236 51.031 0.00 0.00
H
ATOM 25268 HB2 SER H 85 23.603 -8.113 50.097 0.00 0.00
H
ATOM 25269 OG SER H 85 24.560 -9.600 51.217 0.00 0.00
H
ATOM 25270 HG1 SER H 85 24.167 -10.389 51.599 0.00 0.00
H
ATOM 25271 C SER H 85 24.868 -6.861 52.198 0.00 0.00
H

ATOM	25272	O	SER	H	85	25.661	-6.882	53.176	0.00	0.00	H
ATOM	25273	N	THR	H	86	25.207	-6.026	51.153	0.00	0.00	H
ATOM	25274	HN	THR	H	86	24.492	-6.056	50.459	0.00	0.00	H
ATOM	25275	CA	THR	H	86	26.441	-5.278	51.032	0.00	0.00	H
ATOM	25276	HA	THR	H	86	27.292	-5.920	51.209	0.00	0.00	H
ATOM	25277	CB	THR	H	86	26.607	-4.426	49.771	0.00	0.00	H
ATOM	25278	HB	THR	H	86	27.378	-3.626	49.803	0.00	0.00	H
ATOM	25279	OG1	THR	H	86	25.413	-3.731	49.525	0.00	0.00	H
ATOM	25280	HG1	THR	H	86	24.778	-4.432	49.361	0.00	0.00	H
ATOM	25281	CG2	THR	H	86	26.821	-5.228	48.486	0.00	0.00	H
ATOM	25282	HG21	THR	H	86	27.788	-5.776	48.487	0.00	0.00	H
ATOM	25283	HG22	THR	H	86	26.041	-6.016	48.417	0.00	0.00	H
ATOM	25284	HG23	THR	H	86	26.697	-4.718	47.507	0.00	0.00	H
ATOM	25285	C	THR	H	86	26.553	-4.232	52.130	0.00	0.00	H
ATOM	25286	O	THR	H	86	27.683	-4.183	52.666	0.00	0.00	H
ATOM	25287	N	LEU	H	87	25.479	-3.505	52.454	0.00	0.00	H
ATOM	25288	HN	LEU	H	87	24.670	-3.571	51.876	0.00	0.00	H
ATOM	25289	CA	LEU	H	87	25.469	-2.526	53.496	0.00	0.00	H
ATOM	25290	HA	LEU	H	87	26.262	-1.836	53.249	0.00	0.00	H
ATOM	25291	CB	LEU	H	87	24.084	-1.762	53.681	0.00	0.00	H
ATOM	25292	HB1	LEU	H	87	23.906	-1.312	52.681	0.00	0.00	H
ATOM	25293	HB2	LEU	H	87	23.311	-2.555	53.774	0.00	0.00	H
ATOM	25294	CG	LEU	H	87	24.142	-0.616	54.745	0.00	0.00	H
ATOM	25295	HG	LEU	H	87	24.252	-1.051	55.762	0.00	0.00	H
ATOM	25296	CD1	LEU	H	87	25.237	0.368	54.465	0.00	0.00	H
ATOM	25297	HD11	LEU	H	87	26.183	-0.207	54.369	0.00	0.00	H
ATOM	25298	HD12	LEU	H	87	25.062	0.826	53.468	0.00	0.00	H

ATOM 25299 HD13 LEU H 87   25.264   1.218 55.180 0.00 0.00
H
ATOM 25300 CD2 LEU H 87   22.744   0.011 54.732 0.00 0.00
H
ATOM 25301 HD21 LEU H 87   22.665   0.629 53.812 0.00 0.00
H
ATOM 25302 HD22 LEU H 87   21.943 −0.750 54.845 0.00 0.00
H
ATOM 25303 HD23 LEU H 87   22.584   0.745 55.551 0.00 0.00
H
ATOM 25304 C   LEU H 87   25.827 −3.192 54.897 0.00 0.00
H
ATOM 25305 O   LEU H 87   26.732 −2.729 55.608 0.00 0.00
H
ATOM 25306 N   THR H 88   25.097 −4.253 55.316 0.00 0.00
H
ATOM 25307 HN THR H 88   24.307 −4.674 54.877 0.00 0.00
H
ATOM 25308 CA THR H 88   25.322 −4.814 56.686 0.00 0.00
H
ATOM 25309 HA THR H 88   25.404 −3.989 57.378 0.00 0.00
H
ATOM 25310 CB THR H 88   24.144 −5.749 57.309 0.00 0.00
H
ATOM 25311 HB THR H 88   23.176 −5.204 57.271 0.00 0.00
H
ATOM 25312 OG1 THR H 88   24.518 −6.148 58.613 0.00 0.00
H
ATOM 25313 HG1 THR H 88   24.378 −5.348 59.126 0.00 0.00
H
ATOM 25314 CG2 THR H 88   24.026 −6.983 56.405 0.00 0.00
H
ATOM 25315 HG21 THR H 88   23.412 −7.811 56.818 0.00 0.00
H
ATOM 25316 HG22 THR H 88   23.547 −6.842 55.413 0.00 0.00
H
ATOM 25317 HG23 THR H 88   25.008 −7.501 56.378 0.00 0.00
H
ATOM 25318 C   THR H 88   26.703 −5.399 56.847 0.00 0.00
H
ATOM 25319 O   THR H 88   27.368 −5.213 57.848 0.00 0.00
H
ATOM 25320 N   THR H 89   27.202 −6.057 55.839 0.00 0.00
H
ATOM 25321 HN THR H 89   26.781 −6.183 54.944 0.00 0.00
H
ATOM 25322 CA THR H 89   28.591 −6.540 55.800 0.00 0.00
H
ATOM 25323 HA THR H 89   28.716 −7.100 56.715 0.00 0.00
H
ATOM 25324 CB THR H 89   28.784 −7.510 54.636 0.00 0.00
H
ATOM 25325 HB THR H 89   29.836 −7.868 54.650 0.00 0.00
H

ATOM 25326 OG1 THR H 89 28.422 -6.996 53.375 0.00 0.00
H
ATOM 25327 HG1 THR H 89 27.476 -7.149 53.315 0.00 0.00
H
ATOM 25328 CG2 THR H 89 27.995 -8.817 54.809 0.00 0.00
H
ATOM 25329 HG21 THR H 89 28.303 -9.676 54.176 0.00 0.00
H
ATOM 25330 HG22 THR H 89 28.095 -9.189 55.851 0.00 0.00
H
ATOM 25331 HG23 THR H 89 26.909 -8.705 54.603 0.00 0.00
H
ATOM 25332 C THR H 89 29.656 -5.416 55.863 0.00 0.00
H
ATOM 25333 O THR H 89 30.696 -5.512 56.512 0.00 0.00
H
ATOM 25334 N LEU H 90 29.439 -4.310 55.185 0.00 0.00
H
ATOM 25335 HN LEU H 90 28.665 -4.178 54.570 0.00 0.00
H
ATOM 25336 CA LEU H 90 30.380 -3.102 55.281 0.00 0.00
H
ATOM 25337 HA LEU H 90 31.358 -3.556 55.350 0.00 0.00
H
ATOM 25338 CB LEU H 90 30.122 -2.095 54.141 0.00 0.00
H
ATOM 25339 HB1 LEU H 90 30.348 -2.544 53.150 0.00 0.00
H
ATOM 25340 HB2 LEU H 90 29.069 -1.743 54.177 0.00 0.00
H
ATOM 25341 CG LEU H 90 31.074 -0.861 54.188 0.00 0.00
H
ATOM 25342 HG LEU H 90 31.061 -0.306 55.150 0.00 0.00
H
ATOM 25343 CD1 LEU H 90 32.542 -1.284 53.990 0.00 0.00
H
ATOM 25344 HD11 LEU H 90 32.722 -1.819 53.032 0.00 0.00
H
ATOM 25345 HD12 LEU H 90 33.180 -0.376 53.933 0.00 0.00
H
ATOM 25346 HD13 LEU H 90 32.869 -1.906 54.851 0.00 0.00
H
ATOM 25347 CD2 LEU H 90 30.562 0.011 53.004 0.00 0.00
H
ATOM 25348 HD21 LEU H 90 30.591 -0.738 52.184 0.00 0.00
H
ATOM 25349 HD22 LEU H 90 29.527 0.353 53.221 0.00 0.00
H
ATOM 25350 HD23 LEU H 90 31.225 0.887 52.835 0.00 0.00
H
ATOM 25351 C LEU H 90 30.297 -2.463 56.630 0.00 0.00
H
ATOM 25352 O LEU H 90 31.329 -1.959 57.090 0.00 0.00
H

ATOM	25353	N	LEU	H	91	29.116	-2.364	57.256	0.00	0.00	H
ATOM	25354	HN	LEU	H	91	28.310	-2.723	56.791	0.00	0.00	H
ATOM	25355	CA	LEU	H	91	28.966	-1.816	58.603	0.00	0.00	H
ATOM	25356	HA	LEU	H	91	29.244	-0.772	58.613	0.00	0.00	H
ATOM	25357	CB	LEU	H	91	27.416	-1.805	58.987	0.00	0.00	H
ATOM	25358	HB1	LEU	H	91	27.067	-2.824	58.716	0.00	0.00	H
ATOM	25359	HB2	LEU	H	91	27.277	-1.781	60.089	0.00	0.00	H
ATOM	25360	CG	LEU	H	91	26.514	-0.761	58.310	0.00	0.00	H
ATOM	25361	HG	LEU	H	91	26.490	-0.902	57.208	0.00	0.00	H
ATOM	25362	CD1	LEU	H	91	25.090	-0.898	58.693	0.00	0.00	H
ATOM	25363	HD11	LEU	H	91	24.674	-1.780	58.161	0.00	0.00	H
ATOM	25364	HD12	LEU	H	91	24.928	-0.960	59.790	0.00	0.00	H
ATOM	25365	HD13	LEU	H	91	24.490	-0.028	58.351	0.00	0.00	H
ATOM	25366	CD2	LEU	H	91	27.032	0.650	58.653	0.00	0.00	H
ATOM	25367	HD21	LEU	H	91	28.036	0.946	58.281	0.00	0.00	H
ATOM	25368	HD22	LEU	H	91	26.406	1.513	58.343	0.00	0.00	H
ATOM	25369	HD23	LEU	H	91	27.126	0.631	59.760	0.00	0.00	H
ATOM	25370	C	LEU	H	91	29.787	-2.714	59.617	0.00	0.00	H
ATOM	25371	O	LEU	H	91	30.398	-2.142	60.522	0.00	0.00	H
ATOM	25372	N	LEU	H	92	29.667	-4.085	59.502	0.00	0.00	H
ATOM	25373	HN	LEU	H	92	29.210	-4.594	58.777	0.00	0.00	H
ATOM	25374	CA	LEU	H	92	30.368	-5.014	60.463	0.00	0.00	H
ATOM	25375	HA	LEU	H	92	30.069	-4.746	61.466	0.00	0.00	H
ATOM	25376	CB	LEU	H	92	29.908	-6.426	60.138	0.00	0.00	H
ATOM	25377	HB1	LEU	H	92	30.055	-6.654	59.060	0.00	0.00	H
ATOM	25378	HB2	LEU	H	92	30.479	-7.157	60.749	0.00	0.00	H
ATOM	25379	CG	LEU	H	92	28.384	-6.709	60.607	0.00	0.00	H

ATOM 25380 HG LEU H 92   27.651 -5.920 60.331 0.00 0.00
H
ATOM 25381 CD1 LEU H 92   27.953 -8.002 59.919 0.00 0.00
H
ATOM 25382 HD11 LEU H 92   28.659 -8.785 60.270 0.00 0.00
H
ATOM 25383 HD12 LEU H 92   26.882 -8.297 59.952 0.00 0.00
H
ATOM 25384 HD13 LEU H 92   28.081 -7.876 58.823 0.00 0.00
H
ATOM 25385 CD2 LEU H 92   28.338 -6.875 62.104 0.00 0.00
H
ATOM 25386 HD21 LEU H 92   27.365 -7.123 62.579 0.00 0.00
H
ATOM 25387 HD22 LEU H 92   28.862 -7.829 62.329 0.00 0.00
H
ATOM 25388 HD23 LEU H 92   28.879 -6.095 62.681 0.00 0.00
H
ATOM 25389 C   LEU H 92   31.873 -5.046 60.308 0.00 0.00
H
ATOM 25390 O   LEU H 92   32.672 -5.131 61.228 0.00 0.00
H
ATOM 25391 N   LYS H 93   32.354 -4.766 59.057 0.00 0.00
H
ATOM 25392 HN LYS H 93   31.797 -4.572 58.253 0.00 0.00
H
ATOM 25393 CA LYS H 93   33.773 -4.648 58.729 0.00 0.00
H
ATOM 25394 HA LYS H 93   34.223 -5.567 59.077 0.00 0.00
H
ATOM 25395 CB LYS H 93   34.112 -4.736 57.182 0.00 0.00
H
ATOM 25396 HB1 LYS H 93   33.511 -5.538 56.702 0.00 0.00
H
ATOM 25397 HB2 LYS H 93   33.711 -3.838 56.664 0.00 0.00
H
ATOM 25398 CG LYS H 93   35.580 -4.952 56.763 0.00 0.00
H
ATOM 25399 HG1 LYS H 93   35.692 -4.929 55.658 0.00 0.00
H
ATOM 25400 HG2 LYS H 93   36.189 -4.171 57.266 0.00 0.00
H
ATOM 25401 CD LYS H 93   36.110 -6.383 57.052 0.00 0.00
H
ATOM 25402 HD1 LYS H 93   35.935 -6.556 58.136 0.00 0.00
H
ATOM 25403 HD2 LYS H 93   35.505 -7.177 56.563 0.00 0.00
H
ATOM 25404 CE LYS H 93   37.550 -6.612 56.734 0.00 0.00
H
ATOM 25405 HE1 LYS H 93   37.632 -6.881 55.660 0.00 0.00
H
ATOM 25406 HE2 LYS H 93   38.190 -5.721 56.906 0.00 0.00
H

ATOM 25407 NZ LYS H 93 38.079 -7.829 57.439 0.00 0.00
H
ATOM 25408 HZ1 LYS H 93 37.359 -8.578 57.480 0.00 0.00
H
ATOM 25409 HZ2 LYS H 93 38.934 -8.190 56.970 0.00 0.00
H
ATOM 25410 HZ3 LYS H 93 38.329 -7.483 58.387 0.00 0.00
H
ATOM 25411 C LYS H 93 34.372 -3.435 59.402 0.00 0.00
H
ATOM 25412 O LYS H 93 35.433 -3.419 60.015 0.00 0.00
H
ATOM 25413 N GLU H 94 33.568 -2.338 59.490 0.00 0.00
H
ATOM 25414 HN GLU H 94 32.660 -2.163 59.117 0.00 0.00
H
ATOM 25415 CA GLU H 94 33.898 -1.127 60.323 0.00 0.00
H
ATOM 25416 HA GLU H 94 34.889 -0.799 60.049 0.00 0.00
H
ATOM 25417 CB GLU H 94 33.000 -0.078 59.802 0.00 0.00
H
ATOM 25418 HB1 GLU H 94 33.170 0.019 58.709 0.00 0.00
H
ATOM 25419 HB2 GLU H 94 31.914 -0.268 59.945 0.00 0.00
H
ATOM 25420 CG GLU H 94 33.309 1.225 60.552 0.00 0.00
H
ATOM 25421 HG1 GLU H 94 32.708 1.291 61.485 0.00 0.00
H
ATOM 25422 HG2 GLU H 94 34.401 1.336 60.724 0.00 0.00
H
ATOM 25423 CD GLU H 94 32.828 2.399 59.757 0.00 0.00
H
ATOM 25424 OE1 GLU H 94 31.947 3.232 60.144 0.00 0.00
H
ATOM 25425 OE2 GLU H 94 33.305 2.508 58.661 0.00 0.00
H
ATOM 25426 C GLU H 94 33.854 -1.429 61.883 0.00 0.00
H
ATOM 25427 O GLU H 94 34.653 -0.958 62.705 0.00 0.00
H
ATOM 25428 N LEU H 95 32.911 -2.277 62.347 0.00 0.00
H
ATOM 25429 HN LEU H 95 32.338 -2.681 61.639 0.00 0.00
H
ATOM 25430 CA LEU H 95 32.712 -2.643 63.747 0.00 0.00
H
ATOM 25431 HA LEU H 95 32.794 -1.737 64.329 0.00 0.00
H
ATOM 25432 CB LEU H 95 31.320 -3.275 63.791 0.00 0.00
H
ATOM 25433 HB1 LEU H 95 30.500 -2.736 63.270 0.00 0.00
H

ATOM 25434 HB2 LEU H 95 31.361 -4.190 63.163 0.00 0.00
H
ATOM 25435 CG LEU H 95 30.833 -3.455 65.238 0.00 0.00
H
ATOM 25436 HG LEU H 95 31.621 -3.777 65.952 0.00 0.00
H
ATOM 25437 CD1 LEU H 95 30.293 -2.185 65.844 0.00 0.00
H
ATOM 25438 HD11 LEU H 95 29.538 -1.681 65.203 0.00 0.00
H
ATOM 25439 HD12 LEU H 95 29.662 -2.393 66.734 0.00 0.00
H
ATOM 25440 HD13 LEU H 95 31.074 -1.419 66.039 0.00 0.00
H
ATOM 25441 CD2 LEU H 95 29.762 -4.633 65.175 0.00 0.00
H
ATOM 25442 HD21 LEU H 95 30.172 -5.629 64.902 0.00 0.00
H
ATOM 25443 HD22 LEU H 95 29.237 -4.689 66.153 0.00 0.00
H
ATOM 25444 HD23 LEU H 95 28.943 -4.517 64.433 0.00 0.00
H
ATOM 25445 C LEU H 95 33.849 -3.639 64.194 0.00 0.00
H
ATOM 25446 O LEU H 95 33.984 -4.057 65.346 0.00 0.00
H
ATOM 25447 N ASP H 96 34.724 -3.943 63.194 0.00 0.00
H
ATOM 25448 HN ASP H 96 34.499 -3.707 62.252 0.00 0.00
H
ATOM 25449 CA ASP H 96 35.904 -4.840 63.374 0.00 0.00
H
ATOM 25450 HA ASP H 96 36.178 -4.954 62.335 0.00 0.00
H
ATOM 25451 CB ASP H 96 37.027 -4.147 64.069 0.00 0.00
H
ATOM 25452 HB1 ASP H 96 36.561 -3.578 64.901 0.00 0.00
H
ATOM 25453 HB2 ASP H 96 37.741 -4.902 64.462 0.00 0.00
H
ATOM 25454 CG ASP H 96 37.948 -3.269 63.213 0.00 0.00
H
ATOM 25455 OD1 ASP H 96 38.467 -2.162 63.663 0.00 0.00
H
ATOM 25456 OD2 ASP H 96 38.340 -3.666 62.097 0.00 0.00
H
ATOM 25457 C ASP H 96 35.686 -6.295 63.706 0.00 0.00
H
ATOM 25458 O ASP H 96 36.343 -6.856 64.561 0.00 0.00
H
ATOM 25459 N ILE H 97 34.704 -6.893 63.071 0.00 0.00
H
ATOM 25460 HN ILE H 97 34.148 -6.356 62.442 0.00 0.00
H

ATOM 25461 CA ILE H 97 34.315 -8.323 63.249 0.00 0.00
H
ATOM 25462 HA ILE H 97 34.387 -8.481 64.315 0.00 0.00
H
ATOM 25463 CB ILE H 97 32.827 -8.578 62.930 0.00 0.00
H
ATOM 25464 HB ILE H 97 32.730 -8.318 61.854 0.00 0.00
H
ATOM 25465 CG2 ILE H 97 32.515 -10.066 63.206 0.00 0.00
H
ATOM 25466 HG21 ILE H 97 33.110 -10.619 62.449 0.00 0.00
H
ATOM 25467 HG22 ILE H 97 32.766 -10.410 64.233 0.00 0.00
H
ATOM 25468 HG23 ILE H 97 31.483 -10.445 63.044 0.00 0.00
H
ATOM 25469 CG1 ILE H 97 31.862 -7.742 63.782 0.00 0.00
H
ATOM 25470 HG11 ILE H 97 32.000 -6.665 63.548 0.00 0.00
H
ATOM 25471 HG12 ILE H 97 30.811 -7.844 63.437 0.00 0.00
H
ATOM 25472 CD ILE H 97 31.806 -7.965 65.312 0.00 0.00
H
ATOM 25473 HD1 ILE H 97 31.672 -9.060 65.444 0.00 0.00
H
ATOM 25474 HD2 ILE H 97 32.627 -7.447 65.854 0.00 0.00
H
ATOM 25475 HD3 ILE H 97 30.892 -7.485 65.721 0.00 0.00
H
ATOM 25476 C ILE H 97 35.259 -9.193 62.428 0.00 0.00
H
ATOM 25477 O ILE H 97 35.328 -9.049 61.212 0.00 0.00
H
ATOM 25478 N PRO H 98 35.999 -10.157 63.012 0.00 0.00
H
ATOM 25479 CD PRO H 98 36.214 -10.185 64.460 0.00 0.00
H
ATOM 25480 HD1 PRO H 98 36.048 -9.216 64.978 0.00 0.00
H
ATOM 25481 HD2 PRO H 98 35.517 -10.908 64.936 0.00 0.00
H
ATOM 25482 CA PRO H 98 36.978 -11.064 62.294 0.00 0.00
H
ATOM 25483 HA PRO H 98 37.759 -10.474 61.838 0.00 0.00
H
ATOM 25484 CB PRO H 98 37.571 -11.928 63.427 0.00 0.00
H
ATOM 25485 HB1 PRO H 98 38.571 -12.316 63.137 0.00 0.00
H
ATOM 25486 HB2 PRO H 98 36.955 -12.833 63.615 0.00 0.00
H
ATOM 25487 CG PRO H 98 37.560 -10.973 64.688 0.00 0.00
H

ATOM 25488 HG1 PRO H 98   38.421 -10.281 64.569 0.00 0.00
H
ATOM 25489 HG2 PRO H 98   37.643 -11.516 65.653 0.00 0.00
H
ATOM 25490 C   PRO H 98   36.545 -11.885 61.169 0.00 0.00
H
ATOM 25491 O   PRO H 98   37.345 -12.123 60.255 0.00 0.00
H
ATOM 25492 N   ASN H 99   35.263 -12.312 61.165 0.00 0.00
H
ATOM 25493 HN ASN H 99   34.673 -12.059 61.928 0.00 0.00
H
ATOM 25494 CA ASN H 99   34.835 -13.237 60.237 0.00 0.00
H
ATOM 25495 HA ASN H 99   35.435 -13.148 59.343 0.00 0.00
H
ATOM 25496 CB ASN H 99   34.731 -14.674 60.939 0.00 0.00
H
ATOM 25497 HB1 ASN H 99   33.955 -14.749 61.731 0.00 0.00
H
ATOM 25498 HB2 ASN H 99   34.380 -15.427 60.201 0.00 0.00
H
ATOM 25499 CG ASN H 99   36.094 -15.131 61.381 0.00 0.00
H
ATOM 25500 OD1 ASN H 99   36.895 -15.616 60.584 0.00 0.00
H
ATOM 25501 ND2 ASN H 99   36.324 -15.103 62.725 0.00 0.00
H
ATOM 25502 HD21 ASN H 99   37.013 -15.636 63.217 0.00 0.00
H
ATOM 25503 HD22 ASN H 99   35.559 -14.748 63.262 0.00 0.00
H
ATOM 25504 C   ASN H 99   33.382 -12.856 59.944 0.00 0.00
H
ATOM 25505 O   ASN H 99   32.485 -13.013 60.759 0.00 0.00
H
ATOM 25506 N   ILE H 100   33.115 -12.397 58.707 0.00 0.00
H
ATOM 25507 HN ILE H 100   33.931 -12.159 58.186 0.00 0.00
H
ATOM 25508 CA ILE H 100   31.821 -12.054 58.156 0.00 0.00
H
ATOM 25509 HA ILE H 100   31.047 -12.249 58.883 0.00 0.00
H
ATOM 25510 CB ILE H 100   31.919 -10.545 57.759 0.00 0.00
H
ATOM 25511 HB ILE H 100   32.732 -10.387 57.018 0.00 0.00
H
ATOM 25512 CG2 ILE H 100   30.614 -10.031 57.150 0.00 0.00
H
ATOM 25513 HG21 ILE H 100   30.576 -8.986 56.773 0.00 0.00
H
ATOM 25514 HG22 ILE H 100   30.322 -10.622 56.255 0.00 0.00
H

ATOM 25515 HG23 ILE H 100 29.736 -10.042 57.831 0.00 0.00
H
ATOM 25516 CG1 ILE H 100 32.205 -9.517 58.904 0.00 0.00
H
ATOM 25517 HG11 ILE H 100 31.253 -9.304 59.437 0.00 0.00
H
ATOM 25518 HG12 ILE H 100 32.842 -9.834 59.757 0.00 0.00
H
ATOM 25519 CD ILE H 100 32.825 -8.178 58.375 0.00 0.00
H
ATOM 25520 HD1 ILE H 100 32.200 -7.615 57.650 0.00 0.00
H
ATOM 25521 HD2 ILE H 100 33.058 -7.462 59.193 0.00 0.00
H
ATOM 25522 HD3 ILE H 100 33.800 -8.399 57.890 0.00 0.00
H
ATOM 25523 C ILE H 100 31.598 -12.969 56.909 0.00 0.00
H
ATOM 25524 O ILE H 100 32.353 -12.953 55.949 0.00 0.00
H
ATOM 25525 N TRP H 101 30.506 -13.747 56.969 0.00 0.00
H
ATOM 25526 HN TRP H 101 29.949 -13.768 57.796 0.00 0.00
H
ATOM 25527 CA TRP H 101 29.973 -14.738 56.107 0.00 0.00
H
ATOM 25528 HA TRP H 101 30.712 -14.800 55.322 0.00 0.00
H
ATOM 25529 CB TRP H 101 29.795 -16.032 56.856 0.00 0.00
H
ATOM 25530 HB1 TRP H 101 29.382 -15.821 57.866 0.00 0.00
H
ATOM 25531 HB2 TRP H 101 29.115 -16.725 56.316 0.00 0.00
H
ATOM 25532 CG TRP H 101 31.090 -16.859 57.164 0.00 0.00
H
ATOM 25533 CD1 TRP H 101 32.459 -16.473 57.142 0.00 0.00
H
ATOM 25534 HD1 TRP H 101 32.691 -15.439 56.932 0.00 0.00
H
ATOM 25535 NE1 TRP H 101 33.237 -17.529 57.507 0.00 0.00
H
ATOM 25536 HE1 TRP H 101 34.207 -17.541 57.399 0.00 0.00
H
ATOM 25537 CE2 TRP H 101 32.467 -18.677 57.593 0.00 0.00
H
ATOM 25538 CD2 TRP H 101 31.133 -18.287 57.340 0.00 0.00
H
ATOM 25539 CE3 TRP H 101 30.072 -19.216 57.283 0.00 0.00
H
ATOM 25540 HE3 TRP H 101 29.065 -18.907 57.044 0.00 0.00
H
ATOM 25541 CZ3 TRP H 101 30.521 -20.539 57.448 0.00 0.00
H

```
ATOM  25542  HZ3 TRP H 101      29.767 -21.296  57.288  0.00  0.00
H
ATOM  25543  CZ2 TRP H 101      32.818 -20.025  57.744  0.00  0.00
H
ATOM  25544  HZ2 TRP H 101      33.851 -20.312  57.872  0.00  0.00
H
ATOM  25545  CH2 TRP H 101      31.832 -21.031  57.703  0.00  0.00
H
ATOM  25546  HH2 TRP H 101      32.206 -22.044  57.719  0.00  0.00
H
ATOM  25547  C   TRP H 101      28.671 -14.272  55.603  0.00  0.00
H
ATOM  25548  O   TRP H 101      27.867 -13.753  56.366  0.00  0.00
H
ATOM  25549  N   VAL H 102      28.416 -14.359  54.296  0.00  0.00
H
ATOM  25550  HN  VAL H 102      29.176 -14.508  53.668  0.00  0.00
H
ATOM  25551  CA  VAL H 102      27.200 -13.745  53.730  0.00  0.00
H
ATOM  25552  HA  VAL H 102      26.488 -13.784  54.541  0.00  0.00
H
ATOM  25553  CB  VAL H 102      27.629 -12.345  53.396  0.00  0.00
H
ATOM  25554  HB  VAL H 102      28.233 -11.955  54.244  0.00  0.00
H
ATOM  25555  CG1 VAL H 102      28.655 -12.209  52.241  0.00  0.00
H
ATOM  25556 HG11 VAL H 102      29.031 -11.167  52.152  0.00  0.00
H
ATOM  25557 HG12 VAL H 102      29.572 -12.824  52.367  0.00  0.00
H
ATOM  25558 HG13 VAL H 102      28.197 -12.424  51.252  0.00  0.00
H
ATOM  25559  CG2 VAL H 102      26.387 -11.480  52.944  0.00  0.00
H
ATOM  25560 HG21 VAL H 102      26.082 -11.760  51.913  0.00  0.00
H
ATOM  25561 HG22 VAL H 102      25.482 -11.689  53.554  0.00  0.00
H
ATOM  25562 HG23 VAL H 102      26.611 -10.404  52.785  0.00  0.00
H
ATOM  25563  C   VAL H 102      26.583 -14.430  52.469  0.00  0.00
H
ATOM  25564  O   VAL H 102      27.266 -14.822  51.524  0.00  0.00
H
ATOM  25565  N   LYS H 103      25.259 -14.561  52.468  0.00  0.00
H
ATOM  25566  HN  LYS H 103      24.679 -14.331  53.246  0.00  0.00
H
ATOM  25567  CA  LYS H 103      24.567 -15.024  51.276  0.00  0.00
H
ATOM  25568  HA  LYS H 103      25.084 -15.920  50.967  0.00  0.00
H
```

ATOM 25569 CB LYS H 103   23.203 -15.586 51.552 0.00 0.00
H
ATOM 25570 HB1 LYS H 103   23.297 -16.383 52.320 0.00 0.00
H
ATOM 25571 HB2 LYS H 103   22.543 -14.820 52.012 0.00 0.00
H
ATOM 25572 CG LYS H 103   22.522 -16.328 50.339 0.00 0.00
H
ATOM 25573 HG1 LYS H 103   22.487 -15.678 49.439 0.00 0.00
H
ATOM 25574 HG2 LYS H 103   23.098 -17.237 50.061 0.00 0.00
H
ATOM 25575 CD LYS H 103   21.048 -16.776 50.532 0.00 0.00
H
ATOM 25576 HD1 LYS H 103   20.970 -17.644 49.843 0.00 0.00
H
ATOM 25577 HD2 LYS H 103   20.948 -17.229 51.542 0.00 0.00
H
ATOM 25578 CE LYS H 103   19.959 -15.716 50.346 0.00 0.00
H
ATOM 25579 HE1 LYS H 103   19.976 -14.985 51.183 0.00 0.00
H
ATOM 25580 HE2 LYS H 103   20.229 -15.336 49.338 0.00 0.00
H
ATOM 25581 NZ LYS H 103   18.611 -16.244 50.421 0.00 0.00
H
ATOM 25582 HZ1 LYS H 103   18.440 -16.799 51.284 0.00 0.00
H
ATOM 25583 HZ2 LYS H 103   17.807 -15.598 50.289 0.00 0.00
H
ATOM 25584 HZ3 LYS H 103   18.642 -16.969 49.676 0.00 0.00
H
ATOM 25585 C   LYS H 103   24.551 -14.011 50.093 0.00 0.00
H
ATOM 25586 O   LYS H 103   24.211 -12.846 50.251 0.00 0.00
H
ATOM 25587 N   ALA H 104   25.017 -14.420 48.884 0.00 0.00
H
ATOM 25588 HN ALA H 104   25.359 -15.348 48.755 0.00 0.00
H
ATOM 25589 CA ALA H 104   25.153 -13.533 47.716 0.00 0.00
H
ATOM 25590 HA ALA H 104   24.798 -12.534 47.926 0.00 0.00
H
ATOM 25591 CB ALA H 104   26.569 -13.430 47.128 0.00 0.00
H
ATOM 25592 HB1 ALA H 104   26.867 -14.245 46.433 0.00 0.00
H
ATOM 25593 HB2 ALA H 104   26.594 -12.541 46.462 0.00 0.00
H
ATOM 25594 HB3 ALA H 104   27.325 -13.273 47.926 0.00 0.00
H
ATOM 25595 C   ALA H 104   24.235 -14.026 46.584 0.00 0.00
H

ATOM 25596 O ALA H 104 24.081 -15.190 46.349 0.00 0.00
H
ATOM 25597 N GLN H 105 23.620 -13.100 45.846 0.00 0.00
H
ATOM 25598 HN GLN H 105 23.703 -12.129 46.056 0.00 0.00
H
ATOM 25599 CA GLN H 105 22.737 -13.378 44.759 0.00 0.00
H
ATOM 25600 HA GLN H 105 22.110 -14.144 45.189 0.00 0.00
H
ATOM 25601 CB GLN H 105 21.906 -12.113 44.279 0.00 0.00
H
ATOM 25602 HB1 GLN H 105 21.413 -11.648 45.159 0.00 0.00
H
ATOM 25603 HB2 GLN H 105 22.666 -11.392 43.907 0.00 0.00
H
ATOM 25604 CG GLN H 105 20.975 -12.420 42.998 0.00 0.00
H
ATOM 25605 HG1 GLN H 105 20.410 -11.561 42.578 0.00 0.00
H
ATOM 25606 HG2 GLN H 105 21.394 -12.986 42.138 0.00 0.00
H
ATOM 25607 CD GLN H 105 19.748 -13.304 43.412 0.00 0.00
H
ATOM 25608 OE1 GLN H 105 19.570 -14.420 42.948 0.00 0.00
H
ATOM 25609 NE2 GLN H 105 18.789 -12.662 44.178 0.00 0.00
H
ATOM 25610 HE21 GLN H 105 18.962 -11.731 44.502 0.00 0.00
H
ATOM 25611 HE22 GLN H 105 17.966 -13.213 44.319 0.00 0.00
H
ATOM 25612 C GLN H 105 23.493 -13.883 43.517 0.00 0.00
H
ATOM 25613 O GLN H 105 23.121 -14.860 42.825 0.00 0.00
H
ATOM 25614 N ASN H 106 24.544 -13.198 43.180 0.00 0.00
H
ATOM 25615 HN ASN H 106 24.864 -12.510 43.827 0.00 0.00
H
ATOM 25616 CA ASN H 106 25.080 -13.064 41.811 0.00 0.00
H
ATOM 25617 HA ASN H 106 25.029 -14.079 41.445 0.00 0.00
H
ATOM 25618 CB ASN H 106 24.262 -12.266 40.768 0.00 0.00
H
ATOM 25619 HB1 ASN H 106 24.556 -12.519 39.727 0.00 0.00
H
ATOM 25620 HB2 ASN H 106 23.241 -12.703 40.782 0.00 0.00
H
ATOM 25621 CG ASN H 106 24.180 -10.748 40.883 0.00 0.00
H
ATOM 25622 OD1 ASN H 106 24.598 -10.199 41.948 0.00 0.00
H

ATOM 25623 ND2 ASN H 106   23.590 −10.075 39.904 0.00 0.00
H
ATOM 25624 HD21 ASN H 106   23.448 −9.097 40.055 0.00 0.00
H
ATOM 25625 HD22 ASN H 106   23.248 −10.452 39.043 0.00 0.00
H
ATOM 25626 C   ASN H 106   26.501 −12.569 41.833 0.00 0.00
H
ATOM 25627 O   ASN H 106   27.063 −12.205 42.861 0.00 0.00
H
ATOM 25628 N   TYR H 107   27.147 −12.607 40.641 0.00 0.00
H
ATOM 25629 HN TYR H 107   26.741 −12.825 39.757 0.00 0.00
H
ATOM 25630 CA TYR H 107   28.561 −12.276 40.412 0.00 0.00
H
ATOM 25631 HA TYR H 107   29.049 −12.847 41.188 0.00 0.00
H
ATOM 25632 CB TYR H 107   28.977 −12.767 39.009 0.00 0.00
H
ATOM 25633 HB1 TYR H 107   28.665 −13.825 38.875 0.00 0.00
H
ATOM 25634 HB2 TYR H 107   28.488 −12.169 38.211 0.00 0.00
H
ATOM 25635 CG TYR H 107   30.522 −12.841 38.797 0.00 0.00
H
ATOM 25636 CD1 TYR H 107   31.395 −13.327 39.837 0.00 0.00
H
ATOM 25637 HD1 TYR H 107   30.880 −13.763 40.679 0.00 0.00
H
ATOM 25638 CE1 TYR H 107   32.785 −13.389 39.692 0.00 0.00
H
ATOM 25639 HE1 TYR H 107   33.395 −13.793 40.486 0.00 0.00
H
ATOM 25640 CZ TYR H 107   33.300 −12.931 38.482 0.00 0.00
H
ATOM 25641 OH TYR H 107   34.703 −12.954 38.368 0.00 0.00
H
ATOM 25642 HH TYR H 107   35.054 −12.957 39.262 0.00 0.00
H
ATOM 25643 CD2 TYR H 107   31.111 −12.474 37.585 0.00 0.00
H
ATOM 25644 HD2 TYR H 107   30.441 −12.138 36.807 0.00 0.00
H
ATOM 25645 CE2 TYR H 107   32.515 −12.500 37.426 0.00 0.00
H
ATOM 25646 HE2 TYR H 107   32.989 −12.264 36.485 0.00 0.00
H
ATOM 25647 C   TYR H 107   28.733 −10.807 40.621 0.00 0.00
H
ATOM 25648 O   TYR H 107   29.671 −10.482 41.291 0.00 0.00
H
ATOM 25649 N   TYR H 108   27.923 −9.843 40.150 0.00 0.00
H

ATOM 25650 HN TYR H 108 27.140 -9.992 39.552 0.00 0.00
H
ATOM 25651 CA TYR H 108 28.069 -8.427 40.552 0.00 0.00
H
ATOM 25652 HA TYR H 108 29.048 -8.056 40.288 0.00 0.00
H
ATOM 25653 CB TYR H 108 27.034 -7.463 39.930 0.00 0.00
H
ATOM 25654 HB1 TYR H 108 26.072 -7.759 40.401 0.00 0.00
H
ATOM 25655 HB2 TYR H 108 27.199 -6.377 40.097 0.00 0.00
H
ATOM 25656 CG TYR H 108 27.039 -7.621 38.430 0.00 0.00
H
ATOM 25657 CD1 TYR H 108 28.132 -8.055 37.594 0.00 0.00
H
ATOM 25658 HD1 TYR H 108 29.102 -8.244 38.031 0.00 0.00
H
ATOM 25659 CE1 TYR H 108 27.956 -8.098 36.172 0.00 0.00
H
ATOM 25660 HE1 TYR H 108 28.662 -8.612 35.535 0.00 0.00
H
ATOM 25661 CZ TYR H 108 26.735 -7.749 35.541 0.00 0.00
H
ATOM 25662 OH TYR H 108 26.551 -7.643 34.124 0.00 0.00
H
ATOM 25663 HH TYR H 108 25.600 -7.532 34.056 0.00 0.00
H
ATOM 25664 CD2 TYR H 108 25.864 -7.198 37.771 0.00 0.00
H
ATOM 25665 HD2 TYR H 108 25.044 -6.858 38.387 0.00 0.00
H
ATOM 25666 CE2 TYR H 108 25.755 -7.192 36.361 0.00 0.00
H
ATOM 25667 HE2 TYR H 108 24.784 -6.951 35.953 0.00 0.00
H
ATOM 25668 C TYR H 108 28.020 -8.126 41.983 0.00 0.00
H
ATOM 25669 O TYR H 108 28.876 -7.387 42.472 0.00 0.00
H
ATOM 25670 N HSE H 109 27.149 -8.724 42.755 0.00 0.00
H
ATOM 25671 HN HSE H 109 26.398 -9.245 42.357 0.00 0.00
H
ATOM 25672 CA HSE H 109 27.176 -8.759 44.171 0.00 0.00
H
ATOM 25673 HA HSE H 109 26.902 -7.759 44.472 0.00 0.00
H
ATOM 25674 CB HSE H 109 25.971 -9.579 44.755 0.00 0.00
H
ATOM 25675 HB1 HSE H 109 25.140 -9.138 44.164 0.00 0.00
H
ATOM 25676 HB2 HSE H 109 26.109 -10.641 44.462 0.00 0.00
H

```
ATOM  25677  ND1 HSE H 109      24.691 -10.296  46.678  0.00  0.00
H
ATOM  25678  CG  HSE H 109      25.730  -9.540  46.201  0.00  0.00
H
ATOM  25679  CE1 HSE H 109      24.516  -9.902  47.921  0.00  0.00
H
ATOM  25680  HE1 HSE H 109      23.887 -10.324  48.704  0.00  0.00
H
ATOM  25681  NE2 HSE H 109      25.449  -8.852  48.249  0.00  0.00
H
ATOM  25682  HE2 HSE H 109      25.479  -8.384  49.132  0.00  0.00
H
ATOM  25683  CD2 HSE H 109      26.202  -8.614  47.130  0.00  0.00
H
ATOM  25684  HD2 HSE H 109      26.955  -7.836  47.155  0.00  0.00
H
ATOM  25685  C   HSE H 109      28.399  -9.273  44.858  0.00  0.00
H
ATOM  25686  O   HSE H 109      28.930  -8.690  45.782  0.00  0.00
H
ATOM  25687  N   HSE H 110      28.909 -10.394  44.330  0.00  0.00
H
ATOM  25688  HN  HSE H 110      28.459 -10.952  43.637  0.00  0.00
H
ATOM  25689  CA  HSE H 110      30.084 -11.091  44.730  0.00  0.00
H
ATOM  25690  HA  HSE H 110      29.998 -11.296  45.786  0.00  0.00
H
ATOM  25691  CB  HSE H 110      30.219 -12.433  43.821  0.00  0.00
H
ATOM  25692  HB1 HSE H 110      29.184 -12.830  43.747  0.00  0.00
H
ATOM  25693  HB2 HSE H 110      30.546 -12.182  42.789  0.00  0.00
H
ATOM  25694  ND1 HSE H 110      31.009 -14.740  43.875  0.00  0.00
H
ATOM  25695  CG  HSE H 110      31.161 -13.474  44.386  0.00  0.00
H
ATOM  25696  CE1 HSE H 110      31.738 -15.488  44.665  0.00  0.00
H
ATOM  25697  HE1 HSE H 110      31.746 -16.578  44.678  0.00  0.00
H
ATOM  25698  NE2 HSE H 110      32.478 -14.808  45.518  0.00  0.00
H
ATOM  25699  HE2 HSE H 110      33.167 -15.157  46.154  0.00  0.00
H
ATOM  25700  CD2 HSE H 110      32.098 -13.522  45.450  0.00  0.00
H
ATOM  25701  HD2 HSE H 110      32.337 -12.766  46.188  0.00  0.00
H
ATOM  25702  C   HSE H 110      31.316 -10.230  44.559  0.00  0.00
H
ATOM  25703  O   HSE H 110      32.070 -10.114  45.479  0.00  0.00
H
```

ATOM 25704 N LYS H 111 31.463 -9.455 43.474 0.00 0.00
H
ATOM 25705 HN LYS H 111 30.823 -9.586 42.721 0.00 0.00
H
ATOM 25706 CA LYS H 111 32.439 -8.486 43.245 0.00 0.00
H
ATOM 25707 HA LYS H 111 33.351 -9.035 43.423 0.00 0.00
H
ATOM 25708 CB LYS H 111 32.529 -8.068 41.734 0.00 0.00
H
ATOM 25709 HB1 LYS H 111 31.624 -7.485 41.461 0.00 0.00
H
ATOM 25710 HB2 LYS H 111 33.421 -7.412 41.641 0.00 0.00
H
ATOM 25711 CG LYS H 111 32.766 -9.267 40.816 0.00 0.00
H
ATOM 25712 HG1 LYS H 111 33.429 -10.041 41.258 0.00 0.00
H
ATOM 25713 HG2 LYS H 111 31.800 -9.700 40.476 0.00 0.00
H
ATOM 25714 CD LYS H 111 33.526 -9.014 39.460 0.00 0.00
H
ATOM 25715 HD1 LYS H 111 34.577 -8.753 39.711 0.00 0.00
H
ATOM 25716 HD2 LYS H 111 33.515 -10.019 38.987 0.00 0.00
H
ATOM 25717 CE LYS H 111 32.978 -8.130 38.374 0.00 0.00
H
ATOM 25718 HE1 LYS H 111 32.054 -8.496 37.879 0.00 0.00
H
ATOM 25719 HE2 LYS H 111 32.896 -7.163 38.914 0.00 0.00
H
ATOM 25720 NZ LYS H 111 33.845 -7.987 37.255 0.00 0.00
H
ATOM 25721 HZ1 LYS H 111 34.716 -7.442 37.416 0.00 0.00
H
ATOM 25722 HZ2 LYS H 111 34.196 -8.916 36.948 0.00 0.00
H
ATOM 25723 HZ3 LYS H 111 33.412 -7.497 36.446 0.00 0.00
H
ATOM 25724 C LYS H 111 32.439 -7.257 44.222 0.00 0.00
H
ATOM 25725 O LYS H 111 33.488 -6.921 44.752 0.00 0.00
H
ATOM 25726 N VAL H 112 31.245 -6.708 44.705 0.00 0.00
H
ATOM 25727 HN VAL H 112 30.424 -6.950 44.193 0.00 0.00
H
ATOM 25728 CA VAL H 112 31.228 -5.620 45.630 0.00 0.00
H
ATOM 25729 HA VAL H 112 32.063 -5.020 45.300 0.00 0.00
H
ATOM 25730 CB VAL H 112 30.011 -4.755 45.479 0.00 0.00
H

ATOM 25731 HB VAL H 112   30.256 -4.194 44.552 0.00 0.00
H
ATOM 25732 CG1 VAL H 112   28.695 -5.548 45.384 0.00 0.00
H
ATOM 25733 HG11 VAL H 112   28.369 -5.936 46.373 0.00 0.00
H
ATOM 25734 HG12 VAL H 112   27.957 -4.860 44.920 0.00 0.00
H
ATOM 25735 HG13 VAL H 112   28.798 -6.413 44.694 0.00 0.00
H
ATOM 25736 CG2 VAL H 112   29.994 -3.763 46.657 0.00 0.00
H
ATOM 25737 HG21 VAL H 112   29.692 -4.296 47.584 0.00 0.00
H
ATOM 25738 HG22 VAL H 112   31.032 -3.400 46.810 0.00 0.00
H
ATOM 25739 HG23 VAL H 112   29.247 -2.968 46.446 0.00 0.00
H
ATOM 25740 C   VAL H 112   31.395 -6.173 47.078 0.00 0.00
H
ATOM 25741 O   VAL H 112   31.910 -5.543 47.934 0.00 0.00
H
ATOM 25742 N   LEU H 113   30.958 -7.420 47.435 0.00 0.00
H
ATOM 25743 HN LEU H 113   30.286 -7.975 46.951 0.00 0.00
H
ATOM 25744 CA LEU H 113   31.365 -8.022 48.662 0.00 0.00
H
ATOM 25745 HA LEU H 113   31.055 -7.389 49.481 0.00 0.00
H
ATOM 25746 CB LEU H 113   30.747 -9.380 48.901 0.00 0.00
H
ATOM 25747 HB1 LEU H 113   30.900 -10.114 48.081 0.00 0.00
H
ATOM 25748 HB2 LEU H 113   31.043 -9.744 49.908 0.00 0.00
H
ATOM 25749 CG LEU H 113   29.121 -9.320 48.882 0.00 0.00
H
ATOM 25750 HG LEU H 113   28.789 -8.778 47.972 0.00 0.00
H
ATOM 25751 CD1 LEU H 113   28.485 -10.700 48.790 0.00 0.00
H
ATOM 25752 HD11 LEU H 113   28.862 -11.152 47.847 0.00 0.00
H
ATOM 25753 HD12 LEU H 113   28.692 -11.280 49.714 0.00 0.00
H
ATOM 25754 HD13 LEU H 113   27.386 -10.552 48.721 0.00 0.00
H
ATOM 25755 CD2 LEU H 113   28.585 -8.547 50.135 0.00 0.00
H
ATOM 25756 HD21 LEU H 113   27.503 -8.573 50.386 0.00 0.00
H
ATOM 25757 HD22 LEU H 113   29.017 -9.102 50.995 0.00 0.00
H

ATOM 25758 HD23 LEU H 113   28.894 -7.480 50.143 0.00 0.00
H
ATOM 25759 C   LEU H 113   32.815 -8.291 48.867 0.00 0.00
H
ATOM 25760 O   LEU H 113   33.388 -8.109 49.952 0.00 0.00
H
ATOM 25761 N   GLU H 114   33.485 -8.767 47.791 0.00 0.00
H
ATOM 25762 HN GLU H 114   32.910 -9.061 47.031 0.00 0.00
H
ATOM 25763 CA GLU H 114   34.938 -8.968 47.730 0.00 0.00
H
ATOM 25764 HA GLU H 114   35.243 -9.588 48.561 0.00 0.00
H
ATOM 25765 CB GLU H 114   35.347 -9.578 46.401 0.00 0.00
H
ATOM 25766 HB1 GLU H 114   34.716 -10.482 46.260 0.00 0.00
H
ATOM 25767 HB2 GLU H 114   35.030 -8.967 45.528 0.00 0.00
H
ATOM 25768 CG GLU H 114   36.744 -10.145 46.261 0.00 0.00
H
ATOM 25769 HG1 GLU H 114   37.447 -9.301 46.426 0.00 0.00
H
ATOM 25770 HG2 GLU H 114   36.918 -10.954 47.002 0.00 0.00
H
ATOM 25771 CD GLU H 114   36.939 -10.729 44.867 0.00 0.00
H
ATOM 25772 OE1 GLU H 114   37.463 -10.005 44.004 0.00 0.00
H
ATOM 25773 OE2 GLU H 114   36.650 -11.944 44.712 0.00 0.00
H
ATOM 25774 C   GLU H 114   35.721 -7.588 47.979 0.00 0.00
H
ATOM 25775 O   GLU H 114   36.733 -7.516 48.659 0.00 0.00
H
ATOM 25776 N   LYS H 115   35.252 -6.558 47.319 0.00 0.00
H
ATOM 25777 HN LYS H 115   34.653 -6.720 46.539 0.00 0.00
H
ATOM 25778 CA LYS H 115   35.599 -5.160 47.482 0.00 0.00
H
ATOM 25779 HA LYS H 115   36.614 -5.217 47.117 0.00 0.00
H
ATOM 25780 CB LYS H 115   34.755 -4.244 46.621 0.00 0.00
H
ATOM 25781 HB1 LYS H 115   34.718 -4.605 45.571 0.00 0.00
H
ATOM 25782 HB2 LYS H 115   33.706 -4.243 46.987 0.00 0.00
H
ATOM 25783 CG LYS H 115   35.249 -2.759 46.590 0.00 0.00
H
ATOM 25784 HG1 LYS H 115   34.572 -2.110 45.994 0.00 0.00
H

```
ATOM  25785  HG2 LYS H 115      35.259  -2.414  47.646  0.00  0.00
H
ATOM  25786  CD  LYS H 115      36.629  -2.580  46.020  0.00  0.00
H
ATOM  25787  HD1 LYS H 115      37.342  -3.289  46.494  0.00  0.00
H
ATOM  25788  HD2 LYS H 115      36.678  -2.851  44.944  0.00  0.00
H
ATOM  25789  CE  LYS H 115      37.276  -1.221  46.136  0.00  0.00
H
ATOM  25790  HE1 LYS H 115      37.091  -1.057  47.219  0.00  0.00
H
ATOM  25791  HE2 LYS H 115      38.379  -1.265  46.009  0.00  0.00
H
ATOM  25792  NZ  LYS H 115      36.593  -0.206  45.350  0.00  0.00
H
ATOM  25793  HZ1 LYS H 115      37.355   0.350  44.914  0.00  0.00
H
ATOM  25794  HZ2 LYS H 115      35.973  -0.628  44.630  0.00  0.00
H
ATOM  25795  HZ3 LYS H 115      36.083   0.410  46.015  0.00  0.00
H
ATOM  25796  C   LYS H 115      35.378  -4.628  48.969  0.00  0.00
H
ATOM  25797  O   LYS H 115      36.245  -3.908  49.496  0.00  0.00
H
ATOM  25798  N   ILE H 116      34.268  -4.965  49.710  0.00  0.00
H
ATOM  25799  HN  ILE H 116      33.530  -5.453  49.249  0.00  0.00
H
ATOM  25800  CA  ILE H 116      34.109  -4.817  51.149  0.00  0.00
H
ATOM  25801  HA  ILE H 116      34.260  -3.809  51.506  0.00  0.00
H
ATOM  25802  CB  ILE H 116      32.671  -5.122  51.671  0.00  0.00
H
ATOM  25803  HB  ILE H 116      32.453  -6.160  51.341  0.00  0.00
H
ATOM  25804  CG2 ILE H 116      32.638  -5.065  53.142  0.00  0.00
H
ATOM  25805 HG21 ILE H 116      32.994  -4.157  53.673  0.00  0.00
H
ATOM  25806 HG22 ILE H 116      31.580  -5.085  53.483  0.00  0.00
H
ATOM  25807 HG23 ILE H 116      33.074  -6.032  53.474  0.00  0.00
H
ATOM  25808  CG1 ILE H 116      31.685  -4.183  50.920  0.00  0.00
H
ATOM  25809 HG11 ILE H 116      31.799  -3.185  51.396  0.00  0.00
H
ATOM  25810 HG12 ILE H 116      31.977  -4.030  49.859  0.00  0.00
H
ATOM  25811  CD  ILE H 116      30.194  -4.640  50.957  0.00  0.00
H
```

ATOM 25812 HD1 ILE H 116 30.106 -5.402 50.153 0.00 0.00
H
ATOM 25813 HD2 ILE H 116 29.959 -5.231 51.868 0.00 0.00
H
ATOM 25814 HD3 ILE H 116 29.583 -3.734 50.758 0.00 0.00
H
ATOM 25815 C ILE H 116 35.069 -5.677 51.973 0.00 0.00
H
ATOM 25816 O ILE H 116 35.596 -5.288 53.049 0.00 0.00
H
ATOM 25817 N GLY H 117 35.282 -6.882 51.536 0.00 0.00
H
ATOM 25818 HN GLY H 117 34.788 -7.202 50.731 0.00 0.00
H
ATOM 25819 CA GLY H 117 36.204 -7.868 52.042 0.00 0.00
H
ATOM 25820 HA1 GLY H 117 36.779 -7.264 52.728 0.00 0.00
H
ATOM 25821 HA2 GLY H 117 36.779 -8.339 51.258 0.00 0.00
H
ATOM 25822 C GLY H 117 35.717 -9.001 52.782 0.00 0.00
H
ATOM 25823 O GLY H 117 36.436 -9.511 53.643 0.00 0.00
H
ATOM 25824 N ALA H 118 34.465 -9.343 52.529 0.00 0.00
H
ATOM 25825 HN ALA H 118 34.036 -8.947 51.722 0.00 0.00
H
ATOM 25826 CA ALA H 118 33.768 -10.456 53.306 0.00 0.00
H
ATOM 25827 HA ALA H 118 33.755 -10.119 54.332 0.00 0.00
H
ATOM 25828 CB ALA H 118 32.273 -10.572 52.997 0.00 0.00
H
ATOM 25829 HB1 ALA H 118 32.201 -10.822 51.917 0.00 0.00
H
ATOM 25830 HB2 ALA H 118 31.730 -11.343 53.585 0.00 0.00
H
ATOM 25831 HB3 ALA H 118 31.863 -9.542 53.064 0.00 0.00
H
ATOM 25832 C ALA H 118 34.491 -11.834 53.239 0.00 0.00
H
ATOM 25833 O ALA H 118 34.936 -12.286 52.154 0.00 0.00
H
ATOM 25834 N ASP H 119 34.698 -12.426 54.394 0.00 0.00
H
ATOM 25835 HN ASP H 119 34.261 -12.037 55.202 0.00 0.00
H
ATOM 25836 CA ASP H 119 35.566 -13.583 54.633 0.00 0.00
H
ATOM 25837 HA ASP H 119 36.550 -13.279 54.309 0.00 0.00
H
ATOM 25838 CB ASP H 119 35.705 -13.826 56.120 0.00 0.00
H

ATOM 25839 HB1 ASP H 119 34.754 −14.190 56.564 0.00 0.00
H
ATOM 25840 HB2 ASP H 119 36.479 −14.620 56.198 0.00 0.00
H
ATOM 25841 CG ASP H 119 36.244 −12.679 56.924 0.00 0.00
H
ATOM 25842 OD1 ASP H 119 35.447 −11.732 57.214 0.00 0.00
H
ATOM 25843 OD2 ASP H 119 37.500 −12.676 57.144 0.00 0.00
H
ATOM 25844 C ASP H 119 35.238 −14.907 53.909 0.00 0.00
H
ATOM 25845 O ASP H 119 36.137 −15.623 53.494 0.00 0.00
H
ATOM 25846 N ARG H 120 33.872 −15.182 53.789 0.00 0.00
H
ATOM 25847 HN ARG H 120 33.231 −14.534 54.194 0.00 0.00
H
ATOM 25848 CA ARG H 120 33.375 −16.230 52.912 0.00 0.00
H
ATOM 25849 HA ARG H 120 34.076 −16.468 52.127 0.00 0.00
H
ATOM 25850 CB ARG H 120 33.250 −17.608 53.642 0.00 0.00
H
ATOM 25851 HB1 ARG H 120 34.225 −18.003 54.002 0.00 0.00
H
ATOM 25852 HB2 ARG H 120 32.573 −17.514 54.517 0.00 0.00
H
ATOM 25853 CG ARG H 120 32.683 −18.800 52.855 0.00 0.00
H
ATOM 25854 HG1 ARG H 120 31.730 −18.502 52.368 0.00 0.00
H
ATOM 25855 HG2 ARG H 120 33.384 −19.036 52.026 0.00 0.00
H
ATOM 25856 CD ARG H 120 32.377 −20.047 53.740 0.00 0.00
H
ATOM 25857 HD1 ARG H 120 33.321 −20.271 54.281 0.00 0.00
H
ATOM 25858 HD2 ARG H 120 31.516 −19.930 54.431 0.00 0.00
H
ATOM 25859 NE ARG H 120 32.176 −21.178 52.771 0.00 0.00
H
ATOM 25860 HE ARG H 120 31.580 −20.955 52.000 0.00 0.00
H
ATOM 25861 CZ ARG H 120 32.423 −22.432 53.000 0.00 0.00
H
ATOM 25862 NH1 ARG H 120 32.908 −22.936 54.122 0.00 0.00
H
ATOM 25863 HH11 ARG H 120 33.079 −23.917 54.217 0.00 0.00
H
ATOM 25864 HH12 ARG H 120 33.316 −22.388 54.852 0.00 0.00
H
ATOM 25865 NH2 ARG H 120 32.105 −23.296 52.026 0.00 0.00
H

ATOM 25866 HH21 ARG H 120 32.496 -24.215 52.086 0.00 0.00
H
ATOM 25867 HH22 ARG H 120 32.047 -22.957 51.087 0.00 0.00
H
ATOM 25868 C ARG H 120 32.127 -15.750 52.277 0.00 0.00
H
ATOM 25869 O ARG H 120 31.365 -15.082 52.994 0.00 0.00
H
ATOM 25870 N ILE H 121 31.881 -15.958 51.035 0.00 0.00
H
ATOM 25871 HN ILE H 121 32.323 -16.675 50.503 0.00 0.00
H
ATOM 25872 CA ILE H 121 30.792 -15.400 50.323 0.00 0.00
H
ATOM 25873 HA ILE H 121 30.061 -14.904 50.945 0.00 0.00
H
ATOM 25874 CB ILE H 121 31.173 -14.558 49.140 0.00 0.00
H
ATOM 25875 HB ILE H 121 31.884 -15.013 48.418 0.00 0.00
H
ATOM 25876 CG2 ILE H 121 29.995 -14.455 48.137 0.00 0.00
H
ATOM 25877 HG21 ILE H 121 29.017 -14.159 48.573 0.00 0.00
H
ATOM 25878 HG22 ILE H 121 30.220 -13.722 47.333 0.00 0.00
H
ATOM 25879 HG23 ILE H 121 29.762 -15.348 47.517 0.00 0.00
H
ATOM 25880 CG1 ILE H 121 31.600 -13.151 49.468 0.00 0.00
H
ATOM 25881 HG11 ILE H 121 31.052 -12.439 48.815 0.00 0.00
H
ATOM 25882 HG12 ILE H 121 31.134 -12.935 50.453 0.00 0.00
H
ATOM 25883 CD ILE H 121 33.130 -12.930 49.499 0.00 0.00
H
ATOM 25884 HD1 ILE H 121 33.526 -13.624 50.271 0.00 0.00
H
ATOM 25885 HD2 ILE H 121 33.584 -13.209 48.524 0.00 0.00
H
ATOM 25886 HD3 ILE H 121 33.445 -11.953 49.924 0.00 0.00
H
ATOM 25887 C ILE H 121 30.152 -16.604 49.810 0.00 0.00
H
ATOM 25888 O ILE H 121 30.845 -17.613 49.453 0.00 0.00
H
ATOM 25889 N ILE H 122 28.778 -16.756 49.909 0.00 0.00
H
ATOM 25890 HN ILE H 122 28.202 -15.991 50.187 0.00 0.00
H
ATOM 25891 CA ILE H 122 28.121 -18.028 49.810 0.00 0.00
H
ATOM 25892 HA ILE H 122 28.829 -18.784 49.505 0.00 0.00
H

ATOM 25893 CB ILE H 122   27.612 -18.420 51.194 0.00 0.00
H
ATOM 25894 HB ILE H 122   26.826 -17.672 51.433 0.00 0.00
H
ATOM 25895 CG2 ILE H 122   27.102 -19.784 51.022 0.00 0.00
H
ATOM 25896 HG21 ILE H 122   26.652 -20.060 51.999 0.00 0.00
H
ATOM 25897 HG22 ILE H 122   26.317 -19.832 50.237 0.00 0.00
H
ATOM 25898 HG23 ILE H 122   27.889 -20.505 50.712 0.00 0.00
H
ATOM 25899 CG1 ILE H 122   28.697 -18.414 52.257 0.00 0.00
H
ATOM 25900 HG11 ILE H 122   29.558 -19.031 51.923 0.00 0.00
H
ATOM 25901 HG12 ILE H 122   28.953 -17.335 52.326 0.00 0.00
H
ATOM 25902 CD ILE H 122   28.209 -18.804 53.654 0.00 0.00
H
ATOM 25903 HD1 ILE H 122   27.399 -18.129 54.006 0.00 0.00
H
ATOM 25904 HD2 ILE H 122   27.780 -19.827 53.717 0.00 0.00
H
ATOM 25905 HD3 ILE H 122   29.032 -18.790 54.399 0.00 0.00
H
ATOM 25906 C   ILE H 122   26.949 -17.985 48.885 0.00 0.00
H
ATOM 25907 O   ILE H 122   26.091 -17.098 49.010 0.00 0.00
H
ATOM 25908 N   HSE H 123   26.932 -18.868 47.903 0.00 0.00
H
ATOM 25909 HN HSE H 123   27.732 -19.449 47.777 0.00 0.00
H
ATOM 25910 CA HSE H 123   25.862 -18.981 46.998 0.00 0.00
H
ATOM 25911 HA HSE H 123   25.102 -18.277 47.304 0.00 0.00
H
ATOM 25912 CB HSE H 123   26.284 -18.706 45.517 0.00 0.00
H
ATOM 25913 HB1 HSE H 123   27.063 -19.421 45.175 0.00 0.00
H
ATOM 25914 HB2 HSE H 123   25.336 -18.830 44.951 0.00 0.00
H
ATOM 25915 ND1 HSE H 123   26.370 -16.388 44.655 0.00 0.00
H
ATOM 25916 CG HSE H 123   27.019 -17.405 45.245 0.00 0.00
H
ATOM 25917 CE1 HSE H 123   27.269 -15.499 44.376 0.00 0.00
H
ATOM 25918 HE1 HSE H 123   27.030 -14.550 43.896 0.00 0.00
H
ATOM 25919 NE2 HSE H 123   28.497 -15.892 44.701 0.00 0.00
H

ATOM 25920 HE2 HSE H 123 29.361 -15.461 44.441 0.00 0.00
H
ATOM 25921 CD2 HSE H 123 28.378 -17.157 45.278 0.00 0.00
H
ATOM 25922 HD2 HSE H 123 29.168 -17.699 45.785 0.00 0.00
H
ATOM 25923 C HSE H 123 25.404 -20.438 47.047 0.00 0.00
H
ATOM 25924 O HSE H 123 26.004 -21.267 46.374 0.00 0.00
H
ATOM 25925 N PRO H 124 24.315 -20.797 47.839 0.00 0.00
H
ATOM 25926 CD PRO H 124 23.787 -19.951 48.931 0.00 0.00
H
ATOM 25927 HD1 PRO H 124 24.482 -19.180 49.326 0.00 0.00
H
ATOM 25928 HD2 PRO H 124 23.026 -19.315 48.429 0.00 0.00
H
ATOM 25929 CA PRO H 124 23.945 -22.187 48.101 0.00 0.00
H
ATOM 25930 HA PRO H 124 24.832 -22.754 48.344 0.00 0.00
H
ATOM 25931 CB PRO H 124 22.933 -22.129 49.261 0.00 0.00
H
ATOM 25932 HB1 PRO H 124 22.758 -23.097 49.776 0.00 0.00
H
ATOM 25933 HB2 PRO H 124 21.962 -21.780 48.849 0.00 0.00
H
ATOM 25934 CG PRO H 124 23.304 -20.857 50.032 0.00 0.00
H
ATOM 25935 HG1 PRO H 124 24.152 -21.056 50.722 0.00 0.00
H
ATOM 25936 HG2 PRO H 124 22.463 -20.472 50.647 0.00 0.00
H
ATOM 25937 C PRO H 124 23.342 -22.942 46.970 0.00 0.00
H
ATOM 25938 O PRO H 124 23.595 -24.124 46.853 0.00 0.00
H
ATOM 25939 N GLU H 125 22.600 -22.234 46.154 0.00 0.00
H
ATOM 25940 HN GLU H 125 22.400 -21.259 46.211 0.00 0.00
H
ATOM 25941 CA GLU H 125 21.932 -22.817 45.043 0.00 0.00
H
ATOM 25942 HA GLU H 125 21.466 -23.766 45.259 0.00 0.00
H
ATOM 25943 CB GLU H 125 20.866 -21.942 44.385 0.00 0.00
H
ATOM 25944 HB1 GLU H 125 20.305 -22.535 43.631 0.00 0.00
H
ATOM 25945 HB2 GLU H 125 20.113 -21.643 45.144 0.00 0.00
H
ATOM 25946 CG GLU H 125 21.259 -20.624 43.769 0.00 0.00
H

```
ATOM  25947  HG1 GLU H 125      21.856 -20.763  42.842  0.00  0.00
H
ATOM  25948  HG2 GLU H 125      20.364 -20.138  43.325  0.00  0.00
H
ATOM  25949  CD  GLU H 125      21.850 -19.547  44.634  0.00  0.00
H
ATOM  25950  OE1 GLU H 125      22.399 -18.523  44.090  0.00  0.00
H
ATOM  25951  OE2 GLU H 125      21.851 -19.743  45.865  0.00  0.00
H
ATOM  25952  C   GLU H 125      22.925 -23.266  43.994  0.00  0.00
H
ATOM  25953  O   GLU H 125      22.672 -24.230  43.241  0.00  0.00
H
ATOM  25954  N   LYS H 126      24.100 -22.586  43.815  0.00  0.00
H
ATOM  25955  HN  LYS H 126      24.049 -21.679  44.226  0.00  0.00
H
ATOM  25956  CA  LYS H 126      25.322 -22.890  42.929  0.00  0.00
H
ATOM  25957  HA  LYS H 126      24.933 -23.379  42.048  0.00  0.00
H
ATOM  25958  CB  LYS H 126      26.093 -21.578  42.633  0.00  0.00
H
ATOM  25959  HB1 LYS H 126      26.729 -21.399  43.526  0.00  0.00
H
ATOM  25960  HB2 LYS H 126      26.760 -21.694  41.752  0.00  0.00
H
ATOM  25961  CG  LYS H 126      25.148 -20.386  42.336  0.00  0.00
H
ATOM  25962  HG1 LYS H 126      24.682 -20.714  41.382  0.00  0.00
H
ATOM  25963  HG2 LYS H 126      24.357 -20.234  43.102  0.00  0.00
H
ATOM  25964  CD  LYS H 126      25.904 -19.058  42.081  0.00  0.00
H
ATOM  25965  HD1 LYS H 126      26.469 -18.811  43.005  0.00  0.00
H
ATOM  25966  HD2 LYS H 126      26.549 -19.070  41.177  0.00  0.00
H
ATOM  25967  CE  LYS H 126      25.110 -17.867  41.551  0.00  0.00
H
ATOM  25968  HE1 LYS H 126      25.866 -17.123  41.222  0.00  0.00
H
ATOM  25969  HE2 LYS H 126      24.541 -18.199  40.656  0.00  0.00
H
ATOM  25970  NZ  LYS H 126      24.231 -17.313  42.547  0.00  0.00
H
ATOM  25971  HZ1 LYS H 126      23.531 -16.684  42.103  0.00  0.00
H
ATOM  25972  HZ2 LYS H 126      23.560 -17.939  43.037  0.00  0.00
H
ATOM  25973  HZ3 LYS H 126      24.752 -16.857  43.323  0.00  0.00
H
```

ATOM 25974 C LYS H 126 26.214 −24.018 43.603 0.00 0.00
H
ATOM 25975 O LYS H 126 26.546 −24.950 42.938 0.00 0.00
H
ATOM 25976 N ASP H 127 26.479 −23.949 44.916 0.00 0.00
H
ATOM 25977 HN ASP H 127 26.251 −23.140 45.453 0.00 0.00
H
ATOM 25978 CA ASP H 127 27.267 −24.972 45.560 0.00 0.00
H
ATOM 25979 HA ASP H 127 28.203 −24.988 45.022 0.00 0.00
H
ATOM 25980 CB ASP H 127 27.684 −24.637 47.070 0.00 0.00
H
ATOM 25981 HB1 ASP H 127 27.952 −23.560 47.127 0.00 0.00
H
ATOM 25982 HB2 ASP H 127 26.775 −24.627 47.709 0.00 0.00
H
ATOM 25983 CG ASP H 127 28.749 −25.445 47.784 0.00 0.00
H
ATOM 25984 OD1 ASP H 127 29.889 −24.907 47.954 0.00 0.00
H
ATOM 25985 OD2 ASP H 127 28.552 −26.624 48.197 0.00 0.00
H
ATOM 25986 C ASP H 127 26.616 −26.303 45.549 0.00 0.00
H
ATOM 25987 O ASP H 127 27.227 −27.326 45.278 0.00 0.00
H
ATOM 25988 N MET H 128 25.286 −26.312 45.779 0.00 0.00
H
ATOM 25989 HN MET H 128 24.883 −25.483 46.157 0.00 0.00
H
ATOM 25990 CA MET H 128 24.556 −27.541 45.634 0.00 0.00
H
ATOM 25991 HA MET H 128 25.107 −28.375 46.042 0.00 0.00
H
ATOM 25992 CB MET H 128 23.144 −27.463 46.373 0.00 0.00
H
ATOM 25993 HB1 MET H 128 22.616 −26.513 46.147 0.00 0.00
H
ATOM 25994 HB2 MET H 128 22.577 −28.280 45.878 0.00 0.00
H
ATOM 25995 CG MET H 128 23.282 −27.700 47.908 0.00 0.00
H
ATOM 25996 HG1 MET H 128 23.733 −26.826 48.425 0.00 0.00
H
ATOM 25997 HG2 MET H 128 22.272 −27.826 48.353 0.00 0.00
H
ATOM 25998 SD MET H 128 24.175 −29.140 48.440 0.00 0.00
H
ATOM 25999 CE MET H 128 23.225 −30.427 47.640 0.00 0.00
H
ATOM 26000 HE1 MET H 128 22.128 −30.494 47.806 0.00 0.00
H

ATOM 26001 HE2 MET H 128   23.469 -30.315 46.562 0.00 0.00
H
ATOM 26002 HE3 MET H 128   23.503 -31.475 47.883 0.00 0.00
H
ATOM 26003 C   MET H 128   24.212 -27.903 44.228 0.00 0.00
H
ATOM 26004 O   MET H 128   24.040 -29.085 43.889 0.00 0.00
H
ATOM 26005 N   GLY H 129   24.226 -26.962 43.331 0.00 0.00
H
ATOM 26006 HN GLY H 129   24.382 -26.033 43.659 0.00 0.00
H
ATOM 26007 CA GLY H 129   23.849 -27.125 41.913 0.00 0.00
H
ATOM 26008 HA1 GLY H 129   24.237 -26.305 41.327 0.00 0.00
H
ATOM 26009 HA2 GLY H 129   22.771 -27.186 41.892 0.00 0.00
H
ATOM 26010 C   GLY H 129   24.577 -28.277 41.204 0.00 0.00
H
ATOM 26011 O   GLY H 129   23.885 -29.137 40.668 0.00 0.00
H
ATOM 26012 N   VAL H 130   25.847 -28.288 41.317 0.00 0.00
H
ATOM 26013 HN VAL H 130   26.402 -27.628 41.817 0.00 0.00
H
ATOM 26014 CA VAL H 130   26.724 -29.225 40.628 0.00 0.00
H
ATOM 26015 HA VAL H 130   26.411 -29.407 39.611 0.00 0.00
H
ATOM 26016 CB VAL H 130   28.156 -28.567 40.567 0.00 0.00
H
ATOM 26017 HB VAL H 130   28.039 -27.565 40.103 0.00 0.00
H
ATOM 26018 CG1 VAL H 130   28.793 -28.407 41.977 0.00 0.00
H
ATOM 26019 HG11 VAL H 130   29.132 -29.351 42.455 0.00 0.00
H
ATOM 26020 HG12 VAL H 130   29.739 -27.836 41.862 0.00 0.00
H
ATOM 26021 HG13 VAL H 130   28.089 -27.905 42.675 0.00 0.00
H
ATOM 26022 CG2 VAL H 130   29.043 -29.465 39.629 0.00 0.00
H
ATOM 26023 HG21 VAL H 130   28.508 -29.560 38.660 0.00 0.00
H
ATOM 26024 HG22 VAL H 130   30.014 -28.951 39.462 0.00 0.00
H
ATOM 26025 HG23 VAL H 130   29.277 -30.479 40.017 0.00 0.00
H
ATOM 26026 C   VAL H 130   26.589 -30.650 41.285 0.00 0.00
H
ATOM 26027 O   VAL H 130   26.654 -31.744 40.657 0.00 0.00
H

ATOM 26028 N LYS H 131 26.286 -30.634 42.650 0.00 0.00
H
ATOM 26029 HN LYS H 131 26.193 -29.819 43.216 0.00 0.00
H
ATOM 26030 CA LYS H 131 26.002 -31.892 43.392 0.00 0.00
H
ATOM 26031 HA LYS H 131 26.854 -32.506 43.142 0.00 0.00
H
ATOM 26032 CB LYS H 131 26.052 -31.573 44.877 0.00 0.00
H
ATOM 26033 HB1 LYS H 131 25.223 -30.896 45.175 0.00 0.00
H
ATOM 26034 HB2 LYS H 131 26.024 -32.502 45.485 0.00 0.00
H
ATOM 26035 CG LYS H 131 27.515 -31.100 45.306 0.00 0.00
H
ATOM 26036 HG1 LYS H 131 28.304 -31.871 45.172 0.00 0.00
H
ATOM 26037 HG2 LYS H 131 27.875 -30.233 44.711 0.00 0.00
H
ATOM 26038 CD LYS H 131 27.386 -30.802 46.769 0.00 0.00
H
ATOM 26039 HD1 LYS H 131 26.624 -30.014 46.953 0.00 0.00
H
ATOM 26040 HD2 LYS H 131 27.102 -31.669 47.402 0.00 0.00
H
ATOM 26041 CE LYS H 131 28.705 -30.227 47.455 0.00 0.00
H
ATOM 26042 HE1 LYS H 131 28.492 -30.015 48.525 0.00 0.00
H
ATOM 26043 HE2 LYS H 131 29.553 -30.930 47.310 0.00 0.00
H
ATOM 26044 NZ LYS H 131 29.163 -28.933 46.885 0.00 0.00
H
ATOM 26045 HZ1 LYS H 131 28.497 -28.137 46.812 0.00 0.00
H
ATOM 26046 HZ2 LYS H 131 29.947 -28.617 47.490 0.00 0.00
H
ATOM 26047 HZ3 LYS H 131 29.451 -29.166 45.913 0.00 0.00
H
ATOM 26048 C LYS H 131 24.736 -32.610 42.946 0.00 0.00
H
ATOM 26049 O LYS H 131 24.671 -33.789 42.645 0.00 0.00
H
ATOM 26050 N ILE H 132 23.715 -31.737 42.873 0.00 0.00
H
ATOM 26051 HN ILE H 132 23.919 -30.761 42.884 0.00 0.00
H
ATOM 26052 CA ILE H 132 22.380 -32.123 42.402 0.00 0.00
H
ATOM 26053 HA ILE H 132 22.002 -32.935 43.004 0.00 0.00
H
ATOM 26054 CB ILE H 132 21.357 -31.008 42.525 0.00 0.00
H

ATOM 26055 HB ILE H 132 21.673 -30.115 41.944 0.00 0.00
H
ATOM 26056 CG2 ILE H 132 20.003 -31.401 41.912 0.00 0.00
H
ATOM 26057 HG21 ILE H 132 19.514 -32.273 42.396 0.00 0.00
H
ATOM 26058 HG22 ILE H 132 19.275 -30.582 42.096 0.00 0.00
H
ATOM 26059 HG23 ILE H 132 19.939 -31.550 40.813 0.00 0.00
H
ATOM 26060 CG1 ILE H 132 21.203 -30.649 44.031 0.00 0.00
H
ATOM 26061 HG11 ILE H 132 22.074 -30.042 44.358 0.00 0.00
H
ATOM 26062 HG12 ILE H 132 20.343 -29.959 44.169 0.00 0.00
H
ATOM 26063 CD ILE H 132 20.845 -31.759 45.088 0.00 0.00
H
ATOM 26064 HD1 ILE H 132 21.722 -32.401 45.317 0.00 0.00
H
ATOM 26065 HD2 ILE H 132 20.450 -31.428 46.072 0.00 0.00
H
ATOM 26066 HD3 ILE H 132 20.106 -32.448 44.626 0.00 0.00
H
ATOM 26067 C ILE H 132 22.482 -32.484 40.912 0.00 0.00
H
ATOM 26068 O ILE H 132 22.009 -33.584 40.601 0.00 0.00
H
ATOM 26069 N ALA H 133 23.109 -31.694 40.007 0.00 0.00
H
ATOM 26070 HN ALA H 133 23.386 -30.805 40.362 0.00 0.00
H
ATOM 26071 CA ALA H 133 23.247 -31.931 38.607 0.00 0.00
H
ATOM 26072 HA ALA H 133 22.246 -32.172 38.284 0.00 0.00
H
ATOM 26073 CB ALA H 133 23.756 -30.752 37.780 0.00 0.00
H
ATOM 26074 HB1 ALA H 133 23.892 -31.041 36.716 0.00 0.00
H
ATOM 26075 HB2 ALA H 133 23.081 -29.875 37.688 0.00 0.00
H
ATOM 26076 HB3 ALA H 133 24.702 -30.321 38.171 0.00 0.00
H
ATOM 26077 C ALA H 133 23.931 -33.190 38.252 0.00 0.00
H
ATOM 26078 O ALA H 133 23.635 -33.868 37.287 0.00 0.00
H
ATOM 26079 N GLN H 134 25.099 -33.474 38.956 0.00 0.00
H
ATOM 26080 HN GLN H 134 25.379 -32.948 39.755 0.00 0.00
H
ATOM 26081 CA GLN H 134 25.939 -34.630 38.784 0.00 0.00
H

```
ATOM  26082  HA  GLN H 134      26.356 -34.502  37.796  0.00  0.00
H
ATOM  26083  CB  GLN H 134      27.114 -34.675  39.784  0.00  0.00
H
ATOM  26084  HB1 GLN H 134      27.495 -33.638  39.900  0.00  0.00
H
ATOM  26085  HB2 GLN H 134      26.691 -34.914  40.783  0.00  0.00
H
ATOM  26086  CG  GLN H 134      28.125 -35.762  39.390  0.00  0.00
H
ATOM  26087  HG1 GLN H 134      27.988 -36.552  40.159  0.00  0.00
H
ATOM  26088  HG2 GLN H 134      27.757 -36.142  38.413  0.00  0.00
H
ATOM  26089  CD  GLN H 134      29.563 -35.371  39.157  0.00  0.00
H
ATOM  26090  OE1 GLN H 134      30.171 -35.690  38.140  0.00  0.00
H
ATOM  26091  NE2 GLN H 134      30.097 -34.513  40.026  0.00  0.00
H
ATOM  26092 HE21 GLN H 134      31.023 -34.191  39.829  0.00  0.00
H
ATOM  26093 HE22 GLN H 134      29.620 -34.286  40.875  0.00  0.00
H
ATOM  26094  C   GLN H 134      25.081 -35.972  39.025  0.00  0.00
H
ATOM  26095  O   GLN H 134      25.171 -36.868  38.229  0.00  0.00
H
ATOM  26096  N   SER H 135      24.263 -36.034  40.079  0.00  0.00
H
ATOM  26097  HN  SER H 135      24.239 -35.315  40.769  0.00  0.00
H
ATOM  26098  CA  SER H 135      23.340 -37.164  40.460  0.00  0.00
H
ATOM  26099  HA  SER H 135      24.023 -38.000  40.424  0.00  0.00
H
ATOM  26100  CB  SER H 135      22.668 -37.039  41.875  0.00  0.00
H
ATOM  26101  HB1 SER H 135      22.602 -35.939  42.020  0.00  0.00
H
ATOM  26102  HB2 SER H 135      21.694 -37.556  42.010  0.00  0.00
H
ATOM  26103  OG  SER H 135      23.560 -37.529  42.826  0.00  0.00
H
ATOM  26104  HG1 SER H 135      23.285 -37.603  43.743  0.00  0.00
H
ATOM  26105  C   SER H 135      22.164 -37.335  39.542  0.00  0.00
H
ATOM  26106  O   SER H 135      21.716 -38.426  39.206  0.00  0.00
H
ATOM  26107  N   LEU H 136      21.572 -36.278  39.144  0.00  0.00
H
ATOM  26108  HN  LEU H 136      21.889 -35.383  39.447  0.00  0.00
H
```

ATOM 26109 CA LEU H 136   20.620 -36.160 38.015 0.00 0.00
H
ATOM 26110 HA LEU H 136   19.658 -36.565 38.292 0.00 0.00
H
ATOM 26111 CB LEU H 136   20.312 -34.647 37.845 0.00 0.00
H
ATOM 26112 HB1 LEU H 136   20.131 -34.261 38.871 0.00 0.00
H
ATOM 26113 HB2 LEU H 136   21.252 -34.211 37.445 0.00 0.00
H
ATOM 26114 CG LEU H 136   19.237 -34.163 36.878 0.00 0.00
H
ATOM 26115 HG LEU H 136   19.516 -34.329 35.815 0.00 0.00
H
ATOM 26116 CD1 LEU H 136   17.922 -34.801 37.107 0.00 0.00
H
ATOM 26117 HD11 LEU H 136   17.087 -34.182 36.714 0.00 0.00
H
ATOM 26118 HD12 LEU H 136   17.877 -35.839 36.713 0.00 0.00
H
ATOM 26119 HD13 LEU H 136   17.617 -34.823 38.175 0.00 0.00
H
ATOM 26120 CD2 LEU H 136   19.182 -32.613 36.934 0.00 0.00
H
ATOM 26121 HD21 LEU H 136   20.081 -32.124 36.502 0.00 0.00
H
ATOM 26122 HD22 LEU H 136   18.306 -32.301 36.326 0.00 0.00
H
ATOM 26123 HD23 LEU H 136   19.087 -32.151 37.940 0.00 0.00
H
ATOM 26124 C   LEU H 136   21.214 -36.796 36.685 0.00 0.00
H
ATOM 26125 O   LEU H 136   20.557 -37.611 36.011 0.00 0.00
H
ATOM 26126 N   SER H 137   22.432 -36.399 36.346 0.00 0.00
H
ATOM 26127 HN SER H 137   22.803 -35.736 36.992 0.00 0.00
H
ATOM 26128 CA SER H 137   23.327 -36.807 35.267 0.00 0.00
H
ATOM 26129 HA SER H 137   22.766 -36.627 34.362 0.00 0.00
H
ATOM 26130 CB SER H 137   24.654 -36.000 35.210 0.00 0.00
H
ATOM 26131 HB1 SER H 137   24.418 -34.918 35.122 0.00 0.00
H
ATOM 26132 HB2 SER H 137   25.236 -36.100 36.152 0.00 0.00
H
ATOM 26133 OG SER H 137   25.515 -36.390 34.175 0.00 0.00
H
ATOM 26134 HG1 SER H 137   26.018 -37.114 34.556 0.00 0.00
H
ATOM 26135 C   SER H 137   23.617 -38.227 35.180 0.00 0.00
H

ATOM 26136 O SER H 137 23.598 −38.806 34.114 0.00 0.00
H
ATOM 26137 N ASP H 138 23.954 −38.880 36.286 0.00 0.00
H
ATOM 26138 HN ASP H 138 23.980 −38.374 37.145 0.00 0.00
H
ATOM 26139 CA ASP H 138 24.481 −40.264 36.204 0.00 0.00
H
ATOM 26140 HA ASP H 138 24.554 −40.592 35.178 0.00 0.00
H
ATOM 26141 CB ASP H 138 25.997 −40.403 36.737 0.00 0.00
H
ATOM 26142 HB1 ASP H 138 25.900 −39.922 37.733 0.00 0.00
H
ATOM 26143 HB2 ASP H 138 26.316 −41.467 36.779 0.00 0.00
H
ATOM 26144 CG ASP H 138 26.899 −39.676 35.809 0.00 0.00
H
ATOM 26145 OD1 ASP H 138 27.042 −38.403 35.887 0.00 0.00
H
ATOM 26146 OD2 ASP H 138 27.507 −40.338 34.925 0.00 0.00
H
ATOM 26147 C ASP H 138 23.502 −41.200 36.899 0.00 0.00
H
ATOM 26148 O ASP H 138 23.279 −41.100 38.100 0.00 0.00
H
ATOM 26149 N GLU H 139 22.885 −42.138 36.130 0.00 0.00
H
ATOM 26150 HN GLU H 139 22.959 −42.264 35.143 0.00 0.00
H
ATOM 26151 CA GLU H 139 21.836 −43.001 36.692 0.00 0.00
H
ATOM 26152 HA GLU H 139 21.199 −42.396 37.320 0.00 0.00
H
ATOM 26153 CB GLU H 139 20.867 −43.662 35.647 0.00 0.00
H
ATOM 26154 HB1 GLU H 139 20.257 −44.461 36.120 0.00 0.00
H
ATOM 26155 HB2 GLU H 139 20.151 −42.907 35.256 0.00 0.00
H
ATOM 26156 CG GLU H 139 21.460 −44.447 34.507 0.00 0.00
H
ATOM 26157 HG1 GLU H 139 22.353 −44.995 34.876 0.00 0.00
H
ATOM 26158 HG2 GLU H 139 20.737 −45.190 34.108 0.00 0.00
H
ATOM 26159 CD GLU H 139 21.883 −43.612 33.329 0.00 0.00
H
ATOM 26160 OE1 GLU H 139 22.761 −42.719 33.466 0.00 0.00
H
ATOM 26161 OE2 GLU H 139 21.278 −43.755 32.218 0.00 0.00
H
ATOM 26162 C GLU H 139 22.347 −43.914 37.646 0.00 0.00
H

ATOM 26163 O GLU H 139 21.719 -44.244 38.631 0.00 0.00
H
ATOM 26164 N ASN H 140 23.569 -44.478 37.527 0.00 0.00
H
ATOM 26165 HN ASN H 140 24.145 -44.249 36.746 0.00 0.00
H
ATOM 26166 CA ASN H 140 24.061 -45.577 38.317 0.00 0.00
H
ATOM 26167 HA ASN H 140 23.235 -46.174 38.676 0.00 0.00
H
ATOM 26168 CB ASN H 140 24.924 -46.593 37.421 0.00 0.00
H
ATOM 26169 HB1 ASN H 140 25.641 -45.902 36.927 0.00 0.00
H
ATOM 26170 HB2 ASN H 140 25.399 -47.378 38.048 0.00 0.00
H
ATOM 26171 CG ASN H 140 23.998 -47.205 36.364 0.00 0.00
H
ATOM 26172 OD1 ASN H 140 22.957 -47.917 36.585 0.00 0.00
H
ATOM 26173 ND2 ASN H 140 24.361 -46.810 35.093 0.00 0.00
H
ATOM 26174 HD21 ASN H 140 23.819 -47.162 34.331 0.00 0.00
H
ATOM 26175 HD22 ASN H 140 25.084 -46.152 34.883 0.00 0.00
H
ATOM 26176 C ASN H 140 24.696 -45.058 39.550 0.00 0.00
H
ATOM 26177 O ASN H 140 25.370 -45.851 40.261 0.00 0.00
H
ATOM 26178 N VAL H 141 24.637 -43.768 39.831 0.00 0.00
H
ATOM 26179 HN VAL H 141 24.041 -43.317 39.172 0.00 0.00
H
ATOM 26180 CA VAL H 141 25.424 -43.010 40.819 0.00 0.00
H
ATOM 26181 HA VAL H 141 26.180 -43.593 41.324 0.00 0.00
H
ATOM 26182 CB VAL H 141 26.147 -41.767 40.235 0.00 0.00
H
ATOM 26183 HB VAL H 141 25.415 -41.101 39.731 0.00 0.00
H
ATOM 26184 CG1 VAL H 141 26.841 -40.958 41.316 0.00 0.00
H
ATOM 26185 HG11 VAL H 141 27.601 -41.561 41.859 0.00 0.00
H
ATOM 26186 HG12 VAL H 141 27.372 -40.090 40.871 0.00 0.00
H
ATOM 26187 HG13 VAL H 141 26.098 -40.525 42.019 0.00 0.00
H
ATOM 26188 CG2 VAL H 141 27.207 -42.276 39.258 0.00 0.00
H
ATOM 26189 HG21 VAL H 141 27.868 -43.008 39.771 0.00 0.00
H

ATOM 26190 HG22 VAL H 141 26.690 −42.834 38.449 0.00 0.00
H
ATOM 26191 HG23 VAL H 141 27.941 −41.534 38.877 0.00 0.00
H
ATOM 26192 C VAL H 141 24.380 −42.488 41.810 0.00 0.00
H
ATOM 26193 O VAL H 141 23.358 −41.829 41.529 0.00 0.00
H
ATOM 26194 N LEU H 142 24.510 −42.878 43.137 0.00 0.00
H
ATOM 26195 HN LEU H 142 25.270 −43.439 43.457 0.00 0.00
H
ATOM 26196 CA LEU H 142 23.586 −42.339 44.127 0.00 0.00
H
ATOM 26197 HA LEU H 142 22.636 −42.049 43.703 0.00 0.00
H
ATOM 26198 CB LEU H 142 23.337 −43.377 45.197 0.00 0.00
H
ATOM 26199 HB1 LEU H 142 24.280 −43.742 45.657 0.00 0.00
H
ATOM 26200 HB2 LEU H 142 22.720 −43.037 46.056 0.00 0.00
H
ATOM 26201 CG LEU H 142 22.713 −44.708 44.694 0.00 0.00
H
ATOM 26202 HG LEU H 142 23.251 −45.207 43.859 0.00 0.00
H
ATOM 26203 CD1 LEU H 142 22.413 −45.610 45.940 0.00 0.00
H
ATOM 26204 HD11 LEU H 142 21.970 −46.563 45.579 0.00 0.00
H
ATOM 26205 HD12 LEU H 142 23.334 −45.841 46.516 0.00 0.00
H
ATOM 26206 HD13 LEU H 142 21.589 −45.085 46.469 0.00 0.00
H
ATOM 26207 CD2 LEU H 142 21.345 −44.487 44.008 0.00 0.00
H
ATOM 26208 HD21 LEU H 142 21.421 −43.924 43.053 0.00 0.00
H
ATOM 26209 HD22 LEU H 142 20.757 −45.417 43.854 0.00 0.00
H
ATOM 26210 HD23 LEU H 142 20.626 −43.891 44.610 0.00 0.00
H
ATOM 26211 C LEU H 142 24.111 −41.101 44.891 0.00 0.00
H
ATOM 26212 O LEU H 142 23.293 −40.257 45.246 0.00 0.00
H
ATOM 26213 N ASN H 143 25.397 −40.955 45.091 0.00 0.00
H
ATOM 26214 HN ASN H 143 25.969 −41.732 44.838 0.00 0.00
H
ATOM 26215 CA ASN H 143 26.048 −39.806 45.680 0.00 0.00
H
ATOM 26216 HA ASN H 143 25.550 −38.889 45.403 0.00 0.00
H

ATOM 26217 CB ASN H 143 26.119 -39.843 47.257 0.00 0.00
H
ATOM 26218 HB1 ASN H 143 25.166 -40.217 47.689 0.00 0.00
H
ATOM 26219 HB2 ASN H 143 26.996 -40.418 47.623 0.00 0.00
H
ATOM 26220 CG ASN H 143 26.243 -38.487 47.979 0.00 0.00
H
ATOM 26221 OD1 ASN H 143 26.116 -37.409 47.446 0.00 0.00
H
ATOM 26222 ND2 ASN H 143 26.500 -38.543 49.302 0.00 0.00
H
ATOM 26223 HD21 ASN H 143 26.308 -37.725 49.846 0.00 0.00
H
ATOM 26224 HD22 ASN H 143 26.474 -39.410 49.800 0.00 0.00
H
ATOM 26225 C ASN H 143 27.387 -39.645 45.015 0.00 0.00
H
ATOM 26226 O ASN H 143 28.119 -40.613 44.714 0.00 0.00
H
ATOM 26227 N TYR H 144 27.821 -38.372 44.865 0.00 0.00
H
ATOM 26228 HN TYR H 144 27.127 -37.755 45.229 0.00 0.00
H
ATOM 26229 CA TYR H 144 29.089 -37.975 44.229 0.00 0.00
H
ATOM 26230 HA TYR H 144 29.686 -38.875 44.237 0.00 0.00
H
ATOM 26231 CB TYR H 144 28.977 -37.684 42.762 0.00 0.00
H
ATOM 26232 HB1 TYR H 144 28.332 -38.450 42.279 0.00 0.00
H
ATOM 26233 HB2 TYR H 144 28.481 -36.709 42.564 0.00 0.00
H
ATOM 26234 CG TYR H 144 30.332 -37.687 42.012 0.00 0.00
H
ATOM 26235 CD1 TYR H 144 30.597 -38.600 41.006 0.00 0.00
H
ATOM 26236 HD1 TYR H 144 29.926 -39.427 40.827 0.00 0.00
H
ATOM 26237 CE1 TYR H 144 31.794 -38.557 40.195 0.00 0.00
H
ATOM 26238 HE1 TYR H 144 31.941 -39.311 39.435 0.00 0.00
H
ATOM 26239 CZ TYR H 144 32.638 -37.428 40.378 0.00 0.00
H
ATOM 26240 OH TYR H 144 33.874 -37.282 39.684 0.00 0.00
H
ATOM 26241 HH TYR H 144 33.982 -37.932 38.986 0.00 0.00
H
ATOM 26242 CD2 TYR H 144 31.148 -36.542 42.092 0.00 0.00
H
ATOM 26243 HD2 TYR H 144 30.924 -35.604 42.578 0.00 0.00
H

ATOM 26244 CE2 TYR H 144 32.303 -36.475 41.319 0.00 0.00
H
ATOM 26245 HE2 TYR H 144 32.956 -35.617 41.379 0.00 0.00
H
ATOM 26246 C TYR H 144 29.726 -36.840 44.938 0.00 0.00
H
ATOM 26247 O TYR H 144 29.175 -35.712 44.994 0.00 0.00
H
ATOM 26248 N ILE H 145 30.978 -37.085 45.419 0.00 0.00
H
ATOM 26249 HN ILE H 145 31.525 -37.917 45.364 0.00 0.00
H
ATOM 26250 CA ILE H 145 31.704 -36.107 46.199 0.00 0.00
H
ATOM 26251 HA ILE H 145 31.182 -35.181 46.005 0.00 0.00
H
ATOM 26252 CB ILE H 145 31.812 -36.538 47.659 0.00 0.00
H
ATOM 26253 HB ILE H 145 32.377 -37.480 47.830 0.00 0.00
H
ATOM 26254 CG2 ILE H 145 32.754 -35.474 48.439 0.00 0.00
H
ATOM 26255 HG21 ILE H 145 33.794 -35.444 48.050 0.00 0.00
H
ATOM 26256 HG22 ILE H 145 32.218 -34.506 48.335 0.00 0.00
H
ATOM 26257 HG23 ILE H 145 32.757 -35.774 49.509 0.00 0.00
H
ATOM 26258 CG1 ILE H 145 30.395 -36.593 48.334 0.00 0.00
H
ATOM 26259 HG11 ILE H 145 30.007 -35.553 48.287 0.00 0.00
H
ATOM 26260 HG12 ILE H 145 29.682 -37.218 47.754 0.00 0.00
H
ATOM 26261 CD ILE H 145 30.342 -37.116 49.767 0.00 0.00
H
ATOM 26262 HD1 ILE H 145 29.445 -37.770 49.819 0.00 0.00
H
ATOM 26263 HD2 ILE H 145 31.231 -37.709 50.068 0.00 0.00
H
ATOM 26264 HD3 ILE H 145 30.133 -36.196 50.354 0.00 0.00
H
ATOM 26265 C ILE H 145 33.064 -35.876 45.618 0.00 0.00
H
ATOM 26266 O ILE H 145 33.794 -36.890 45.563 0.00 0.00
H
ATOM 26267 N ASP H 146 33.329 -34.600 45.179 0.00 0.00
H
ATOM 26268 HN ASP H 146 32.669 -33.858 45.085 0.00 0.00
H
ATOM 26269 CA ASP H 146 34.551 -34.064 44.637 0.00 0.00
H
ATOM 26270 HA ASP H 146 34.956 -34.769 43.926 0.00 0.00
H

ATOM 26271 CB ASP H 146 34.208 −32.696 43.967 0.00 0.00
H
ATOM 26272 HB1 ASP H 146 33.453 −32.134 44.556 0.00 0.00
H
ATOM 26273 HB2 ASP H 146 35.101 −32.037 44.022 0.00 0.00
H
ATOM 26274 CG ASP H 146 34.047 −32.902 42.455 0.00 0.00
H
ATOM 26275 OD1 ASP H 146 32.856 −32.737 42.089 0.00 0.00
H
ATOM 26276 OD2 ASP H 146 34.965 −33.294 41.707 0.00 0.00
H
ATOM 26277 C ASP H 146 35.537 −33.900 45.699 0.00 0.00
H
ATOM 26278 O ASP H 146 35.329 −33.189 46.666 0.00 0.00
H
ATOM 26279 N LEU H 147 36.751 −34.638 45.687 0.00 0.00
H
ATOM 26280 HN LEU H 147 36.888 −35.212 44.884 0.00 0.00
H
ATOM 26281 CA LEU H 147 37.704 −34.635 46.795 0.00 0.00
H
ATOM 26282 HA LEU H 147 37.398 −34.123 47.695 0.00 0.00
H
ATOM 26283 CB LEU H 147 38.342 −36.052 47.094 0.00 0.00
H
ATOM 26284 HB1 LEU H 147 37.585 −36.862 47.014 0.00 0.00
H
ATOM 26285 HB2 LEU H 147 39.114 −36.247 46.320 0.00 0.00
H
ATOM 26286 CG LEU H 147 39.066 −36.141 48.451 0.00 0.00
H
ATOM 26287 HG LEU H 147 39.867 −35.378 48.353 0.00 0.00
H
ATOM 26288 CD1 LEU H 147 38.090 −35.818 49.550 0.00 0.00
H
ATOM 26289 HD11 LEU H 147 37.866 −34.730 49.567 0.00 0.00
H
ATOM 26290 HD12 LEU H 147 37.119 −36.357 49.571 0.00 0.00
H
ATOM 26291 HD13 LEU H 147 38.592 −36.001 50.524 0.00 0.00
H
ATOM 26292 CD2 LEU H 147 39.847 −37.485 48.563 0.00 0.00
H
ATOM 26293 HD21 LEU H 147 40.567 −37.612 47.726 0.00 0.00
H
ATOM 26294 HD22 LEU H 147 40.465 −37.490 49.486 0.00 0.00
H
ATOM 26295 HD23 LEU H 147 39.155 −38.354 48.598 0.00 0.00
H
ATOM 26296 C LEU H 147 38.817 −33.686 46.370 0.00 0.00
H
ATOM 26297 O LEU H 147 39.287 −32.848 47.169 0.00 0.00
H

ATOM 26298 N SER H 148 39.232 -33.716 45.099 0.00 0.00
H
ATOM 26299 HN SER H 148 38.694 -34.251 44.453 0.00 0.00
H
ATOM 26300 CA SER H 148 40.228 -32.820 44.591 0.00 0.00
H
ATOM 26301 HA SER H 148 40.016 -31.816 44.930 0.00 0.00
H
ATOM 26302 CB SER H 148 41.662 -33.288 44.935 0.00 0.00
H
ATOM 26303 HB1 SER H 148 42.414 -32.500 44.714 0.00 0.00
H
ATOM 26304 HB2 SER H 148 41.690 -33.614 45.996 0.00 0.00
H
ATOM 26305 OG SER H 148 42.037 -34.459 44.211 0.00 0.00
H
ATOM 26306 HG1 SER H 148 42.993 -34.527 44.158 0.00 0.00
H
ATOM 26307 C SER H 148 39.990 -32.712 43.089 0.00 0.00
H
ATOM 26308 O SER H 148 39.008 -33.188 42.546 0.00 0.00
H
ATOM 26309 N ASP H 149 40.814 -31.981 42.314 0.00 0.00
H
ATOM 26310 HN ASP H 149 41.506 -31.402 42.737 0.00 0.00
H
ATOM 26311 CA ASP H 149 40.745 -31.777 40.871 0.00 0.00
H
ATOM 26312 HA ASP H 149 39.731 -31.514 40.609 0.00 0.00
H
ATOM 26313 CB ASP H 149 41.813 -30.686 40.344 0.00 0.00
H
ATOM 26314 HB1 ASP H 149 42.803 -31.103 40.627 0.00 0.00
H
ATOM 26315 HB2 ASP H 149 41.781 -30.437 39.261 0.00 0.00
H
ATOM 26316 CG ASP H 149 41.719 -29.449 41.143 0.00 0.00
H
ATOM 26317 OD1 ASP H 149 42.771 -28.886 41.552 0.00 0.00
H
ATOM 26318 OD2 ASP H 149 40.590 -28.866 41.362 0.00 0.00
H
ATOM 26319 C ASP H 149 41.012 -33.015 40.196 0.00 0.00
H
ATOM 26320 O ASP H 149 40.563 -33.238 39.095 0.00 0.00
H
ATOM 26321 N GLU H 150 41.771 -33.932 40.851 0.00 0.00
H
ATOM 26322 HN GLU H 150 42.289 -33.718 41.676 0.00 0.00
H
ATOM 26323 CA GLU H 150 41.978 -35.332 40.389 0.00 0.00
H
ATOM 26324 HA GLU H 150 41.962 -35.304 39.309 0.00 0.00
H

ATOM 26325 CB GLU H 150 43.450 -35.766 40.792 0.00 0.00
H
ATOM 26326 HB1 GLU H 150 43.736 -35.679 41.862 0.00 0.00
H
ATOM 26327 HB2 GLU H 150 43.583 -36.845 40.567 0.00 0.00
H
ATOM 26328 CG GLU H 150 44.536 -34.978 39.949 0.00 0.00
H
ATOM 26329 HG1 GLU H 150 44.359 -33.888 40.073 0.00 0.00
H
ATOM 26330 HG2 GLU H 150 45.555 -35.207 40.329 0.00 0.00
H
ATOM 26331 CD GLU H 150 44.687 -35.288 38.499 0.00 0.00
H
ATOM 26332 OE1 GLU H 150 45.314 -36.374 38.247 0.00 0.00
H
ATOM 26333 OE2 GLU H 150 44.245 -34.526 37.597 0.00 0.00
H
ATOM 26334 C GLU H 150 41.065 -36.461 40.875 0.00 0.00
H
ATOM 26335 O GLU H 150 40.758 -37.409 40.100 0.00 0.00
H
ATOM 26336 N TYR H 151 40.620 -36.456 42.111 0.00 0.00
H
ATOM 26337 HN TYR H 151 40.697 -35.649 42.692 0.00 0.00
H
ATOM 26338 CA TYR H 151 40.046 -37.554 42.852 0.00 0.00
H
ATOM 26339 HA TYR H 151 39.953 -38.391 42.177 0.00 0.00
H
ATOM 26340 CB TYR H 151 40.986 -37.778 44.122 0.00 0.00
H
ATOM 26341 HB1 TYR H 151 40.874 -36.857 44.733 0.00 0.00
H
ATOM 26342 HB2 TYR H 151 40.657 -38.700 44.647 0.00 0.00
H
ATOM 26343 CG TYR H 151 42.429 -37.959 43.737 0.00 0.00
H
ATOM 26344 CD1 TYR H 151 43.364 -37.303 44.551 0.00 0.00
H
ATOM 26345 HD1 TYR H 151 43.087 -36.535 45.257 0.00 0.00
H
ATOM 26346 CE1 TYR H 151 44.721 -37.414 44.190 0.00 0.00
H
ATOM 26347 HE1 TYR H 151 45.461 -36.819 44.705 0.00 0.00
H
ATOM 26348 CZ TYR H 151 45.123 -38.297 43.191 0.00 0.00
H
ATOM 26349 OH TYR H 151 46.526 -38.389 42.918 0.00 0.00
H
ATOM 26350 HH TYR H 151 46.755 -39.169 42.408 0.00 0.00
H
ATOM 26351 CD2 TYR H 151 42.879 -38.783 42.692 0.00 0.00
H

ATOM 26352 HD2 TYR H 151   42.153 -39.265 42.055 0.00 0.00
H
ATOM 26353 CE2 TYR H 151   44.222 -38.948 42.385 0.00 0.00
H
ATOM 26354 HE2 TYR H 151   44.512 -39.689 41.654 0.00 0.00
H
ATOM 26355 C   TYR H 151   38.639 -37.294 43.293 0.00 0.00
H
ATOM 26356 O   TYR H 151   38.147 -36.181 43.502 0.00 0.00
H
ATOM 26357 N   SER H 152   37.948 -38.413 43.432 0.00 0.00
H
ATOM 26358 HN SER H 152   38.442 -39.237 43.163 0.00 0.00
H
ATOM 26359 CA SER H 152   36.587 -38.418 43.886 0.00 0.00
H
ATOM 26360 HA SER H 152   36.455 -37.594 44.572 0.00 0.00
H
ATOM 26361 CB SER H 152   35.563 -38.391 42.715 0.00 0.00
H
ATOM 26362 HB1 SER H 152   35.605 -39.286 42.058 0.00 0.00
H
ATOM 26363 HB2 SER H 152   34.518 -38.311 43.082 0.00 0.00
H
ATOM 26364 OG SER H 152   35.628 -37.211 41.947 0.00 0.00
H
ATOM 26365 HG1 SER H 152   34.773 -37.109 41.522 0.00 0.00
H
ATOM 26366 C   SER H 152   36.230 -39.569 44.790 0.00 0.00
H
ATOM 26367 O   SER H 152   36.946 -40.549 44.853 0.00 0.00
H
ATOM 26368 N   ILE H 153   35.149 -39.444 45.540 0.00 0.00
H
ATOM 26369 HN ILE H 153   34.734 -38.537 45.526 0.00 0.00
H
ATOM 26370 CA ILE H 153   34.502 -40.464 46.316 0.00 0.00
H
ATOM 26371 HA ILE H 153   35.139 -41.335 46.273 0.00 0.00
H
ATOM 26372 CB ILE H 153   34.468 -40.019 47.866 0.00 0.00
H
ATOM 26373 HB ILE H 153   33.862 -39.088 47.831 0.00 0.00
H
ATOM 26374 CG2 ILE H 153   33.740 -41.111 48.793 0.00 0.00
H
ATOM 26375 HG21 ILE H 153   32.661 -41.097 48.530 0.00 0.00
H
ATOM 26376 HG22 ILE H 153   34.191 -42.114 48.636 0.00 0.00
H
ATOM 26377 HG23 ILE H 153   33.727 -40.815 49.864 0.00 0.00
H
ATOM 26378 CG1 ILE H 153   35.966 -39.784 48.364 0.00 0.00
H

ATOM 26379 HG11 ILE H 153   36.483 -40.760 48.245 0.00 0.00
H
ATOM 26380 HG12 ILE H 153   36.453 -39.189 47.562 0.00 0.00
H
ATOM 26381 CD ILE H 153   36.204 -39.027 49.687 0.00 0.00
H
ATOM 26382 HD1 ILE H 153   35.810 -37.989 49.733 0.00 0.00
H
ATOM 26383 HD2 ILE H 153   35.929 -39.694 50.532 0.00 0.00
H
ATOM 26384 HD3 ILE H 153   37.288 -38.828 49.830 0.00 0.00
H
ATOM 26385 C   ILE H 153   33.068 -40.614 45.750 0.00 0.00
H
ATOM 26386 O   ILE H 153   32.452 -39.561 45.588 0.00 0.00
H
ATOM 26387 N   VAL H 154   32.655 -41.860 45.438 0.00 0.00
H
ATOM 26388 HN VAL H 154   33.319 -42.601 45.373 0.00 0.00
H
ATOM 26389 CA VAL H 154   31.446 -42.010 44.705 0.00 0.00
H
ATOM 26390 HA VAL H 154   30.795 -41.154 44.795 0.00 0.00
H
ATOM 26391 CB VAL H 154   31.616 -42.246 43.240 0.00 0.00
H
ATOM 26392 HB VAL H 154   32.097 -43.232 43.066 0.00 0.00
H
ATOM 26393 CG1 VAL H 154   30.328 -42.224 42.400 0.00 0.00
H
ATOM 26394 HG11 VAL H 154   29.775 -43.172 42.572 0.00 0.00
H
ATOM 26395 HG12 VAL H 154   29.618 -41.372 42.470 0.00 0.00
H
ATOM 26396 HG13 VAL H 154   30.653 -42.178 41.338 0.00 0.00
H
ATOM 26397 CG2 VAL H 154   32.574 -41.217 42.689 0.00 0.00
H
ATOM 26398 HG21 VAL H 154   33.625 -41.383 43.007 0.00 0.00
H
ATOM 26399 HG22 VAL H 154   32.568 -41.492 41.612 0.00 0.00
H
ATOM 26400 HG23 VAL H 154   32.230 -40.169 42.823 0.00 0.00
H
ATOM 26401 C   VAL H 154   30.654 -43.201 45.202 0.00 0.00
H
ATOM 26402 O   VAL H 154   31.372 -44.183 45.462 0.00 0.00
H
ATOM 26403 N   GLU H 155   29.364 -43.139 45.434 0.00 0.00
H
ATOM 26404 HN GLU H 155   28.855 -42.283 45.385 0.00 0.00
H
ATOM 26405 CA GLU H 155   28.514 -44.339 45.772 0.00 0.00
H

ATOM 26406 HA GLU H 155 29.086 -45.219 46.024 0.00 0.00
H
ATOM 26407 CB GLU H 155 27.514 -44.008 47.022 0.00 0.00
H
ATOM 26408 HB1 GLU H 155 27.996 -43.847 48.010 0.00 0.00
H
ATOM 26409 HB2 GLU H 155 26.843 -43.144 46.833 0.00 0.00
H
ATOM 26410 CG GLU H 155 26.508 -45.125 47.539 0.00 0.00
H
ATOM 26411 HG1 GLU H 155 25.795 -45.330 46.712 0.00 0.00
H
ATOM 26412 HG2 GLU H 155 27.064 -46.050 47.805 0.00 0.00
H
ATOM 26413 CD GLU H 155 25.541 -44.722 48.695 0.00 0.00
H
ATOM 26414 OE1 GLU H 155 25.978 -44.467 49.870 0.00 0.00
H
ATOM 26415 OE2 GLU H 155 24.296 -44.699 48.443 0.00 0.00
H
ATOM 26416 C GLU H 155 27.657 -44.826 44.614 0.00 0.00
H
ATOM 26417 O GLU H 155 26.854 -44.086 44.039 0.00 0.00
H
ATOM 26418 N LEU H 156 27.797 -46.118 44.310 0.00 0.00
H
ATOM 26419 HN LEU H 156 28.271 -46.705 44.961 0.00 0.00
H
ATOM 26420 CA LEU H 156 27.380 -46.637 43.010 0.00 0.00
H
ATOM 26421 HA LEU H 156 26.951 -45.847 42.412 0.00 0.00
H
ATOM 26422 CB LEU H 156 28.490 -47.378 42.307 0.00 0.00
H
ATOM 26423 HB1 LEU H 156 28.804 -48.299 42.842 0.00 0.00
H
ATOM 26424 HB2 LEU H 156 28.067 -47.706 41.333 0.00 0.00
H
ATOM 26425 CG LEU H 156 29.707 -46.481 41.915 0.00 0.00
H
ATOM 26426 HG LEU H 156 29.879 -45.746 42.730 0.00 0.00
H
ATOM 26427 CD1 LEU H 156 31.079 -47.224 41.826 0.00 0.00
H
ATOM 26428 HD11 LEU H 156 31.332 -47.693 42.800 0.00 0.00
H
ATOM 26429 HD12 LEU H 156 30.999 -48.016 41.051 0.00 0.00
H
ATOM 26430 HD13 LEU H 156 31.891 -46.489 41.641 0.00 0.00
H
ATOM 26431 CD2 LEU H 156 29.412 -45.780 40.672 0.00 0.00
H
ATOM 26432 HD21 LEU H 156 30.218 -45.091 40.341 0.00 0.00
H

ATOM 26433 HD22 LEU H 156 29.226 −46.551 39.893 0.00 0.00
H
ATOM 26434 HD23 LEU H 156 28.483 −45.179 40.765 0.00 0.00
H
ATOM 26435 C LEU H 156 26.312 −47.694 43.300 0.00 0.00
H
ATOM 26436 O LEU H 156 26.461 −48.351 44.329 0.00 0.00
H
ATOM 26437 N LEU H 157 25.297 −47.843 42.380 0.00 0.00
H
ATOM 26438 HN LEU H 157 25.150 −47.174 41.655 0.00 0.00
H
ATOM 26439 CA LEU H 157 24.286 −48.829 42.505 0.00 0.00
H
ATOM 26440 HA LEU H 157 24.247 −49.294 43.480 0.00 0.00
H
ATOM 26441 CB LEU H 157 22.946 −48.087 42.268 0.00 0.00
H
ATOM 26442 HB1 LEU H 157 22.728 −47.336 43.057 0.00 0.00
H
ATOM 26443 HB2 LEU H 157 22.972 −47.586 41.277 0.00 0.00
H
ATOM 26444 CG LEU H 157 21.659 −48.996 42.135 0.00 0.00
H
ATOM 26445 HG LEU H 157 21.976 −49.789 41.424 0.00 0.00
H
ATOM 26446 CD1 LEU H 157 21.160 −49.466 43.488 0.00 0.00
H
ATOM 26447 HD11 LEU H 157 20.311 −50.142 43.254 0.00 0.00
H
ATOM 26448 HD12 LEU H 157 21.994 −49.947 44.042 0.00 0.00
H
ATOM 26449 HD13 LEU H 157 20.769 −48.573 44.022 0.00 0.00
H
ATOM 26450 CD2 LEU H 157 20.442 −48.218 41.519 0.00 0.00
H
ATOM 26451 HD21 LEU H 157 19.558 −48.888 41.467 0.00 0.00
H
ATOM 26452 HD22 LEU H 157 20.155 −47.429 42.247 0.00 0.00
H
ATOM 26453 HD23 LEU H 157 20.655 −47.816 40.505 0.00 0.00
H
ATOM 26454 C LEU H 157 24.584 −49.951 41.560 0.00 0.00
H
ATOM 26455 O LEU H 157 24.511 −49.861 40.303 0.00 0.00
H
ATOM 26456 N ALA H 158 24.962 −51.122 42.198 0.00 0.00
H
ATOM 26457 HN ALA H 158 24.879 −51.214 43.187 0.00 0.00
H
ATOM 26458 CA ALA H 158 25.321 −52.310 41.447 0.00 0.00
H
ATOM 26459 HA ALA H 158 26.142 −52.006 40.814 0.00 0.00
H

ATOM 26460 CB ALA H 158 25.957 -53.340 42.401 0.00 0.00
H
ATOM 26461 HB1 ALA H 158 25.233 -53.829 43.088 0.00 0.00
H
ATOM 26462 HB2 ALA H 158 26.416 -54.113 41.748 0.00 0.00
H
ATOM 26463 HB3 ALA H 158 26.771 -52.839 42.967 0.00 0.00
H
ATOM 26464 C ALA H 158 24.258 -52.932 40.552 0.00 0.00
H
ATOM 26465 O ALA H 158 23.112 -52.983 40.888 0.00 0.00
H
ATOM 26466 N THR H 159 24.709 -53.513 39.388 0.00 0.00
H
ATOM 26467 HN THR H 159 25.605 -53.371 38.974 0.00 0.00
H
ATOM 26468 CA THR H 159 23.889 -54.118 38.443 0.00 0.00
H
ATOM 26469 HA THR H 159 23.349 -54.862 39.010 0.00 0.00
H
ATOM 26470 CB THR H 159 22.801 -53.167 37.775 0.00 0.00
H
ATOM 26471 HB THR H 159 22.393 -52.402 38.468 0.00 0.00
H
ATOM 26472 OG1 THR H 159 21.775 -53.866 37.166 0.00 0.00
H
ATOM 26473 HG1 THR H 159 21.045 -53.281 36.952 0.00 0.00
H
ATOM 26474 CG2 THR H 159 23.434 -52.225 36.719 0.00 0.00
H
ATOM 26475 HG21 THR H 159 23.445 -52.572 35.664 0.00 0.00
H
ATOM 26476 HG22 THR H 159 22.841 -51.285 36.740 0.00 0.00
H
ATOM 26477 HG23 THR H 159 24.440 -51.883 37.044 0.00 0.00
H
ATOM 26478 C THR H 159 24.760 -54.936 37.527 0.00 0.00
H
ATOM 26479 O THR H 159 25.987 -54.719 37.482 0.00 0.00
H
ATOM 26480 N ARG H 160 24.243 -55.966 36.833 0.00 0.00
H
ATOM 26481 HN ARG H 160 23.352 -56.337 37.084 0.00 0.00
H
ATOM 26482 CA ARG H 160 24.809 -56.462 35.578 0.00 0.00
H
ATOM 26483 HA ARG H 160 24.078 -57.174 35.224 0.00 0.00
H
ATOM 26484 CB ARG H 160 24.806 -55.300 34.499 0.00 0.00
H
ATOM 26485 HB1 ARG H 160 23.791 -54.853 34.437 0.00 0.00
H
ATOM 26486 HB2 ARG H 160 25.537 -54.500 34.745 0.00 0.00
H

ATOM 26487 CG ARG H 160 25.173 -55.721 33.047 0.00 0.00
H
ATOM 26488 HG1 ARG H 160 25.174 -54.747 32.512 0.00 0.00
H
ATOM 26489 HG2 ARG H 160 26.250 -55.993 33.062 0.00 0.00
H
ATOM 26490 CD ARG H 160 24.440 -56.974 32.386 0.00 0.00
H
ATOM 26491 HD1 ARG H 160 24.590 -57.805 33.109 0.00 0.00
H
ATOM 26492 HD2 ARG H 160 23.374 -56.694 32.249 0.00 0.00
H
ATOM 26493 NE ARG H 160 25.141 -57.267 31.062 0.00 0.00
H
ATOM 26494 HE ARG H 160 25.477 -56.552 30.448 0.00 0.00
H
ATOM 26495 CZ ARG H 160 25.537 -58.471 30.693 0.00 0.00
H
ATOM 26496 NH1 ARG H 160 25.175 -59.617 31.175 0.00 0.00
H
ATOM 26497 HH11 ARG H 160 25.863 -60.328 31.027 0.00 0.00
H
ATOM 26498 HH12 ARG H 160 24.676 -59.600 32.041 0.00 0.00
H
ATOM 26499 NH2 ARG H 160 26.250 -58.465 29.563 0.00 0.00
H
ATOM 26500 HH21 ARG H 160 26.296 -59.321 29.048 0.00 0.00
H
ATOM 26501 HH22 ARG H 160 26.052 -57.673 28.986 0.00 0.00
H
ATOM 26502 C ARG H 160 26.111 -57.294 35.596 0.00 0.00
H
ATOM 26503 O ARG H 160 26.173 -58.519 35.714 0.00 0.00
H
ATOM 26504 N LYS H 161 27.238 -56.572 35.538 0.00 0.00
H
ATOM 26505 HN LYS H 161 27.183 -55.582 35.435 0.00 0.00
H
ATOM 26506 CA LYS H 161 28.508 -57.117 35.642 0.00 0.00
H
ATOM 26507 HA LYS H 161 28.488 -58.196 35.604 0.00 0.00
H
ATOM 26508 CB LYS H 161 29.388 -56.844 34.387 0.00 0.00
H
ATOM 26509 HB1 LYS H 161 29.110 -55.858 33.958 0.00 0.00
H
ATOM 26510 HB2 LYS H 161 30.468 -56.785 34.640 0.00 0.00
H
ATOM 26511 CG LYS H 161 29.106 -57.720 33.161 0.00 0.00
H
ATOM 26512 HG1 LYS H 161 29.619 -58.697 33.293 0.00 0.00
H
ATOM 26513 HG2 LYS H 161 28.014 -57.771 32.964 0.00 0.00
H

ATOM 26514 CD LYS H 161   29.610 -57.057 31.843 0.00 0.00
H
ATOM 26515 HD1 LYS H 161   29.459 -57.763 30.998 0.00 0.00
H
ATOM 26516 HD2 LYS H 161   29.028 -56.121 31.703 0.00 0.00
H
ATOM 26517 CE LYS H 161   31.121 -56.808 31.944 0.00 0.00
H
ATOM 26518 HE1 LYS H 161   31.305 -56.285 32.907 0.00 0.00
H
ATOM 26519 HE2 LYS H 161   31.588 -57.811 31.852 0.00 0.00
H
ATOM 26520 NZ LYS H 161   31.569 -56.008 30.841 0.00 0.00
H
ATOM 26521 HZ1 LYS H 161   32.567 -55.749 30.704 0.00 0.00
H
ATOM 26522 HZ2 LYS H 161   31.358 -56.329 29.875 0.00 0.00
H
ATOM 26523 HZ3 LYS H 161   31.286 -55.016 30.976 0.00 0.00
H
ATOM 26524 C   LYS H 161   29.135 -56.788 36.993 0.00 0.00
H
ATOM 26525 O   LYS H 161   30.305 -57.114 37.159 0.00 0.00
H
ATOM 26526 N   LEU H 162   28.394 -56.080 37.939 0.00 0.00
H
ATOM 26527 HN LEU H 162   27.580 -55.572 37.670 0.00 0.00
H
ATOM 26528 CA LEU H 162   28.791 -56.216 39.306 0.00 0.00
H
ATOM 26529 HA LEU H 162   29.776 -56.651 39.383 0.00 0.00
H
ATOM 26530 CB LEU H 162   28.723 -54.791 39.907 0.00 0.00
H
ATOM 26531 HB1 LEU H 162   27.624 -54.633 39.953 0.00 0.00
H
ATOM 26532 HB2 LEU H 162   29.029 -54.660 40.967 0.00 0.00
H
ATOM 26533 CG LEU H 162   29.351 -53.665 39.049 0.00 0.00
H
ATOM 26534 HG LEU H 162   28.705 -53.517 38.157 0.00 0.00
H
ATOM 26535 CD1 LEU H 162   29.266 -52.420 39.901 0.00 0.00
H
ATOM 26536 HD11 LEU H 162   30.068 -52.411 40.670 0.00 0.00
H
ATOM 26537 HD12 LEU H 162   29.577 -51.529 39.315 0.00 0.00
H
ATOM 26538 HD13 LEU H 162   28.265 -52.232 40.344 0.00 0.00
H
ATOM 26539 CD2 LEU H 162   30.795 -53.935 38.631 0.00 0.00
H
ATOM 26540 HD21 LEU H 162   30.890 -54.905 38.097 0.00 0.00
H

ATOM 26541 HD22 LEU H 162 31.435 −53.143 38.187 0.00 0.00
H
ATOM 26542 HD23 LEU H 162 31.347 −54.118 39.578 0.00 0.00
H
ATOM 26543 C LEU H 162 27.905 −57.230 40.107 0.00 0.00
H
ATOM 26544 O LEU H 162 28.119 −57.435 41.306 0.00 0.00
H
ATOM 26545 N ASP H 163 26.891 −57.846 39.467 0.00 0.00
H
ATOM 26546 HN ASP H 163 26.694 −57.699 38.501 0.00 0.00
H
ATOM 26547 CA ASP H 163 26.032 −58.799 40.083 0.00 0.00
H
ATOM 26548 HA ASP H 163 25.783 −58.455 41.075 0.00 0.00
H
ATOM 26549 CB ASP H 163 24.829 −58.765 39.095 0.00 0.00
H
ATOM 26550 HB1 ASP H 163 24.380 −57.751 39.019 0.00 0.00
H
ATOM 26551 HB2 ASP H 163 25.129 −59.064 38.068 0.00 0.00
H
ATOM 26552 CG ASP H 163 23.866 −59.715 39.656 0.00 0.00
H
ATOM 26553 OD1 ASP H 163 23.579 −59.770 40.881 0.00 0.00
H
ATOM 26554 OD2 ASP H 163 23.322 −60.549 38.855 0.00 0.00
H
ATOM 26555 C ASP H 163 26.773 −60.128 40.155 0.00 0.00
H
ATOM 26556 O ASP H 163 27.522 −60.451 39.246 0.00 0.00
H
ATOM 26557 N SER H 164 26.512 −60.973 41.185 0.00 0.00
H
ATOM 26558 HN SER H 164 25.895 −60.675 41.909 0.00 0.00
H
ATOM 26559 CA SER H 164 26.936 −62.357 41.235 0.00 0.00
H
ATOM 26560 HA SER H 164 26.690 −62.640 42.247 0.00 0.00
H
ATOM 26561 CB SER H 164 26.172 −63.255 40.312 0.00 0.00
H
ATOM 26562 HB1 SER H 164 26.229 −62.852 39.278 0.00 0.00
H
ATOM 26563 HB2 SER H 164 26.637 −64.264 40.324 0.00 0.00
H
ATOM 26564 OG SER H 164 24.800 −63.277 40.848 0.00 0.00
H
ATOM 26565 HG1 SER H 164 24.193 −63.418 40.117 0.00 0.00
H
ATOM 26566 C SER H 164 28.379 −62.526 41.227 0.00 0.00
H
ATOM 26567 O SER H 164 28.922 −63.417 40.624 0.00 0.00
H

ATOM 26568 N LYS H 165 29.180 −61.633 41.879 0.00 0.00
H
ATOM 26569 HN LYS H 165 28.712 −61.058 42.545 0.00 0.00
H
ATOM 26570 CA LYS H 165 30.643 −61.491 41.817 0.00 0.00
H
ATOM 26571 HA LYS H 165 31.162 −62.404 41.566 0.00 0.00
H
ATOM 26572 CB LYS H 165 31.029 −60.405 40.905 0.00 0.00
H
ATOM 26573 HB1 LYS H 165 30.574 −60.652 39.921 0.00 0.00
H
ATOM 26574 HB2 LYS H 165 30.603 −59.412 41.163 0.00 0.00
H
ATOM 26575 CG LYS H 165 32.552 −60.319 40.662 0.00 0.00
H
ATOM 26576 HG1 LYS H 165 33.035 −59.761 41.492 0.00 0.00
H
ATOM 26577 HG2 LYS H 165 32.877 −61.373 40.534 0.00 0.00
H
ATOM 26578 CD LYS H 165 32.845 −59.405 39.376 0.00 0.00
H
ATOM 26579 HD1 LYS H 165 32.135 −59.623 38.550 0.00 0.00
H
ATOM 26580 HD2 LYS H 165 32.727 −58.312 39.539 0.00 0.00
H
ATOM 26581 CE LYS H 165 34.240 −59.478 38.754 0.00 0.00
H
ATOM 26582 HE1 LYS H 165 34.289 −58.587 38.092 0.00 0.00
H
ATOM 26583 HE2 LYS H 165 34.951 −59.278 39.584 0.00 0.00
H
ATOM 26584 NZ LYS H 165 34.518 −60.788 38.093 0.00 0.00
H
ATOM 26585 HZ1 LYS H 165 34.819 −61.527 38.759 0.00 0.00
H
ATOM 26586 HZ2 LYS H 165 33.660 −61.186 37.660 0.00 0.00
H
ATOM 26587 HZ3 LYS H 165 35.102 −60.735 37.234 0.00 0.00
H
ATOM 26588 C LYS H 165 31.007 −61.190 43.163 0.00 0.00
H
ATOM 26589 O LYS H 165 30.323 −60.511 43.918 0.00 0.00
H
ATOM 26590 N SER H 166 32.119 −61.739 43.746 0.00 0.00
H
ATOM 26591 HN SER H 166 32.587 −62.440 43.213 0.00 0.00
H
ATOM 26592 CA SER H 166 32.549 −61.280 45.083 0.00 0.00
H
ATOM 26593 HA SER H 166 31.659 −61.360 45.689 0.00 0.00
H
ATOM 26594 CB SER H 166 33.618 −62.286 45.692 0.00 0.00
H

ATOM 26595 HB1 SER H 166   33.201 -63.311 45.593 0.00 0.00
H
ATOM 26596 HB2 SER H 166   34.578 -62.190 45.140 0.00 0.00
H
ATOM 26597 OG SER H 166   33.749 -62.046 47.072 0.00 0.00
H
ATOM 26598 HG1 SER H 166   34.122 -62.872 47.387 0.00 0.00
H
ATOM 26599 C   SER H 166   33.058 -59.879 45.143 0.00 0.00
H
ATOM 26600 O   SER H 166   33.730 -59.439 44.205 0.00 0.00
H
ATOM 26601 N   ILE H 167   32.835 -59.153 46.266 0.00 0.00
H
ATOM 26602 HN ILE H 167   32.270 -59.469 47.024 0.00 0.00
H
ATOM 26603 CA ILE H 167   33.444 -57.892 46.616 0.00 0.00
H
ATOM 26604 HA ILE H 167   33.054 -57.132 45.954 0.00 0.00
H
ATOM 26605 CB ILE H 167   33.044 -57.557 47.994 0.00 0.00
H
ATOM 26606 HB ILE H 167   31.933 -57.523 47.989 0.00 0.00
H
ATOM 26607 CG2 ILE H 167   33.481 -58.631 48.998 0.00 0.00
H
ATOM 26608 HG21 ILE H 167   33.362 -59.700 48.718 0.00 0.00
H
ATOM 26609 HG22 ILE H 167   34.515 -58.508 49.386 0.00 0.00
H
ATOM 26610 HG23 ILE H 167   32.824 -58.435 49.872 0.00 0.00
H
ATOM 26611 CG1 ILE H 167   33.377 -56.117 48.484 0.00 0.00
H
ATOM 26612 HG11 ILE H 167   34.425 -55.798 48.298 0.00 0.00
H
ATOM 26613 HG12 ILE H 167   32.722 -55.490 47.841 0.00 0.00
H
ATOM 26614 CD ILE H 167   33.093 -55.853 49.991 0.00 0.00
H
ATOM 26615 HD1 ILE H 167   32.184 -56.348 50.395 0.00 0.00
H
ATOM 26616 HD2 ILE H 167   33.935 -56.409 50.458 0.00 0.00
H
ATOM 26617 HD3 ILE H 167   33.065 -54.792 50.320 0.00 0.00
H
ATOM 26618 C   ILE H 167   35.024 -57.943 46.661 0.00 0.00
H
ATOM 26619 O   ILE H 167   35.692 -56.927 46.629 0.00 0.00
H
ATOM 26620 N   ILE H 168   35.749 -59.077 46.708 0.00 0.00
H
ATOM 26621 HN ILE H 168   35.266 -59.944 46.808 0.00 0.00
H

ATOM 26622 CA ILE H 168 37.235 -59.165 46.462 0.00 0.00
H
ATOM 26623 HA ILE H 168 37.781 -58.233 46.469 0.00 0.00
H
ATOM 26624 CB ILE H 168 37.957 -60.034 47.489 0.00 0.00
H
ATOM 26625 HB ILE H 168 39.030 -59.942 47.218 0.00 0.00
H
ATOM 26626 CG2 ILE H 168 37.813 -59.538 48.920 0.00 0.00
H
ATOM 26627 HG21 ILE H 168 37.765 -58.431 48.845 0.00 0.00
H
ATOM 26628 HG22 ILE H 168 36.836 -59.912 49.294 0.00 0.00
H
ATOM 26629 HG23 ILE H 168 38.670 -59.933 49.507 0.00 0.00
H
ATOM 26630 CG1 ILE H 168 37.545 -61.569 47.482 0.00 0.00
H
ATOM 26631 HG11 ILE H 168 36.463 -61.622 47.729 0.00 0.00
H
ATOM 26632 HG12 ILE H 168 37.626 -61.952 46.442 0.00 0.00
H
ATOM 26633 CD ILE H 168 38.396 -62.434 48.449 0.00 0.00
H
ATOM 26634 HD1 ILE H 168 38.032 -63.449 48.182 0.00 0.00
H
ATOM 26635 HD2 ILE H 168 39.482 -62.305 48.252 0.00 0.00
H
ATOM 26636 HD3 ILE H 168 38.191 -62.200 49.516 0.00 0.00
H
ATOM 26637 C ILE H 168 37.578 -59.741 45.130 0.00 0.00
H
ATOM 26638 O ILE H 168 38.749 -59.821 44.805 0.00 0.00
H
ATOM 26639 N ASP H 169 36.617 -60.131 44.278 0.00 0.00
H
ATOM 26640 HN ASP H 169 35.637 -60.204 44.450 0.00 0.00
H
ATOM 26641 CA ASP H 169 36.924 -60.556 42.943 0.00 0.00
H
ATOM 26642 HA ASP H 169 37.834 -61.137 42.908 0.00 0.00
H
ATOM 26643 CB ASP H 169 35.741 -61.405 42.432 0.00 0.00
H
ATOM 26644 HB1 ASP H 169 35.439 -61.985 43.331 0.00 0.00
H
ATOM 26645 HB2 ASP H 169 34.837 -60.815 42.171 0.00 0.00
H
ATOM 26646 CG ASP H 169 36.117 -62.295 41.308 0.00 0.00
H
ATOM 26647 OD1 ASP H 169 35.484 -62.170 40.183 0.00 0.00
H
ATOM 26648 OD2 ASP H 169 37.024 -63.146 41.449 0.00 0.00
H

```
ATOM  26649  C   ASP H 169      37.037 -59.373  42.022  0.00  0.00
H
ATOM  26650  O   ASP H 169      37.473 -59.521  40.893  0.00  0.00
H
ATOM  26651  N   LEU H 170      36.692 -58.109  42.440  0.00  0.00
H
ATOM  26652  HN  LEU H 170      36.283 -58.042  43.347  0.00  0.00
H
ATOM  26653  CA  LEU H 170      36.856 -56.899  41.632  0.00  0.00
H
ATOM  26654  HA  LEU H 170      36.250 -57.044  40.750  0.00  0.00
H
ATOM  26655  CB  LEU H 170      36.347 -55.681  42.389  0.00  0.00
H
ATOM  26656  HB1 LEU H 170      36.951 -55.732  43.320  0.00  0.00
H
ATOM  26657  HB2 LEU H 170      36.500 -54.828  41.694  0.00  0.00
H
ATOM  26658  CG  LEU H 170      34.827 -55.669  42.765  0.00  0.00
H
ATOM  26659  HG  LEU H 170      34.583 -56.611  43.303  0.00  0.00
H
ATOM  26660  CD1 LEU H 170      34.423 -54.556  43.762  0.00  0.00
H
ATOM  26661 HD11 LEU H 170      33.341 -54.569  44.014  0.00  0.00
H
ATOM  26662 HD12 LEU H 170      34.995 -54.701  44.703  0.00  0.00
H
ATOM  26663 HD13 LEU H 170      34.712 -53.543  43.410  0.00  0.00
H
ATOM  26664  CD2 LEU H 170      34.023 -55.475  41.437  0.00  0.00
H
ATOM  26665 HD21 LEU H 170      32.983 -55.762  41.703  0.00  0.00
H
ATOM  26666 HD22 LEU H 170      33.955 -54.510  40.890  0.00  0.00
H
ATOM  26667 HD23 LEU H 170      34.209 -56.303  40.720  0.00  0.00
H
ATOM  26668  C   LEU H 170      38.271 -56.659  41.189  0.00  0.00
H
ATOM  26669  O   LEU H 170      39.238 -56.865  41.940  0.00  0.00
H
ATOM  26670  N   ASN H 171      38.416 -56.224  39.955  0.00  0.00
H
ATOM  26671  HN  ASN H 171      37.624 -56.225  39.349  0.00  0.00
H
ATOM  26672  CA  ASN H 171      39.646 -55.948  39.338  0.00  0.00
H
ATOM  26673  HA  ASN H 171      40.407 -56.483  39.888  0.00  0.00
H
ATOM  26674  CB  ASN H 171      39.442 -56.197  37.797  0.00  0.00
H
ATOM  26675  HB1 ASN H 171      38.590 -55.558  37.482  0.00  0.00
H
```

ATOM 26676 HB2 ASN H 171 40.340 -55.996 37.174 0.00 0.00
H
ATOM 26677 CG ASN H 171 39.304 -57.658 37.571 0.00 0.00
H
ATOM 26678 OD1 ASN H 171 40.170 -58.396 37.985 0.00 0.00
H
ATOM 26679 ND2 ASN H 171 38.253 -58.111 36.891 0.00 0.00
H
ATOM 26680 HD21 ASN H 171 38.197 -59.078 36.643 0.00 0.00
H
ATOM 26681 HD22 ASN H 171 37.498 -57.519 36.607 0.00 0.00
H
ATOM 26682 C ASN H 171 39.981 -54.437 39.447 0.00 0.00
H
ATOM 26683 O ASN H 171 40.964 -54.042 38.864 0.00 0.00
H
ATOM 26684 N VAL H 172 39.351 -53.714 40.375 0.00 0.00
H
ATOM 26685 HN VAL H 172 38.726 -54.190 40.989 0.00 0.00
H
ATOM 26686 CA VAL H 172 39.597 -52.265 40.716 0.00 0.00
H
ATOM 26687 HA VAL H 172 39.444 -51.873 39.721 0.00 0.00
H
ATOM 26688 CB VAL H 172 38.621 -51.696 41.714 0.00 0.00
H
ATOM 26689 HB VAL H 172 38.909 -50.649 41.947 0.00 0.00
H
ATOM 26690 CG1 VAL H 172 37.192 -51.763 41.070 0.00 0.00
H
ATOM 26691 HG11 VAL H 172 36.504 -51.478 41.894 0.00 0.00
H
ATOM 26692 HG12 VAL H 172 37.085 -51.067 40.211 0.00 0.00
H
ATOM 26693 HG13 VAL H 172 36.891 -52.771 40.714 0.00 0.00
H
ATOM 26694 CG2 VAL H 172 38.615 -52.531 43.051 0.00 0.00
H
ATOM 26695 HG21 VAL H 172 38.346 -53.571 42.766 0.00 0.00
H
ATOM 26696 HG22 VAL H 172 39.602 -52.587 43.559 0.00 0.00
H
ATOM 26697 HG23 VAL H 172 37.931 -52.131 43.829 0.00 0.00
H
ATOM 26698 C VAL H 172 41.056 -51.840 41.081 0.00 0.00
H
ATOM 26699 O VAL H 172 41.533 -50.783 40.572 0.00 0.00
H
ATOM 26700 N ARG H 173 41.689 -52.596 41.960 0.00 0.00
H
ATOM 26701 HN ARG H 173 41.302 -53.453 42.291 0.00 0.00
H
ATOM 26702 CA ARG H 173 43.005 -52.186 42.460 0.00 0.00
H

ATOM 26703 HA ARG H 173 42.882 -51.156 42.758 0.00 0.00
H
ATOM 26704 CB ARG H 173 43.574 -52.970 43.667 0.00 0.00
H
ATOM 26705 HB1 ARG H 173 42.786 -52.759 44.422 0.00 0.00
H
ATOM 26706 HB2 ARG H 173 43.526 -54.058 43.449 0.00 0.00
H
ATOM 26707 CG ARG H 173 44.961 -52.630 44.204 0.00 0.00
H
ATOM 26708 HG1 ARG H 173 45.289 -53.316 45.014 0.00 0.00
H
ATOM 26709 HG2 ARG H 173 45.734 -52.979 43.487 0.00 0.00
H
ATOM 26710 CD ARG H 173 45.400 -51.218 44.632 0.00 0.00
H
ATOM 26711 HD1 ARG H 173 46.481 -51.254 44.887 0.00 0.00
H
ATOM 26712 HD2 ARG H 173 45.239 -50.461 43.834 0.00 0.00
H
ATOM 26713 NE ARG H 173 44.476 -50.719 45.754 0.00 0.00
H
ATOM 26714 HE ARG H 173 43.682 -51.256 46.039 0.00 0.00
H
ATOM 26715 CZ ARG H 173 44.793 -49.718 46.620 0.00 0.00
H
ATOM 26716 NH1 ARG H 173 45.879 -48.966 46.408 0.00 0.00
H
ATOM 26717 HH11 ARG H 173 46.011 -48.799 45.431 0.00 0.00
H
ATOM 26718 HH12 ARG H 173 46.081 -48.221 47.043 0.00 0.00
H
ATOM 26719 NH2 ARG H 173 43.993 -49.650 47.684 0.00 0.00
H
ATOM 26720 HH21 ARG H 173 43.852 -48.741 48.077 0.00 0.00
H
ATOM 26721 HH22 ARG H 173 43.311 -50.378 47.744 0.00 0.00
H
ATOM 26722 C ARG H 173 44.115 -52.080 41.399 0.00 0.00
H
ATOM 26723 O ARG H 173 44.796 -51.085 41.175 0.00 0.00
H
ATOM 26724 N ALA H 174 44.250 -53.103 40.534 0.00 0.00
H
ATOM 26725 HN ALA H 174 43.580 -53.829 40.672 0.00 0.00
H
ATOM 26726 CA ALA H 174 45.223 -53.164 39.467 0.00 0.00
H
ATOM 26727 HA ALA H 174 46.090 -52.704 39.918 0.00 0.00
H
ATOM 26728 CB ALA H 174 45.680 -54.609 39.139 0.00 0.00
H
ATOM 26729 HB1 ALA H 174 46.322 -54.912 39.994 0.00 0.00
H

ATOM 26730 HB2 ALA H 174 44.870 -55.317 38.861 0.00 0.00
H
ATOM 26731 HB3 ALA H 174 46.354 -54.564 38.256 0.00 0.00
H
ATOM 26732 C ALA H 174 44.823 -52.427 38.180 0.00 0.00
H
ATOM 26733 O ALA H 174 45.621 -51.796 37.518 0.00 0.00
H
ATOM 26734 N LYS H 175 43.534 -52.607 37.743 0.00 0.00
H
ATOM 26735 HN LYS H 175 43.037 -53.268 38.300 0.00 0.00
H
ATOM 26736 CA LYS H 175 43.005 -52.118 36.471 0.00 0.00
H
ATOM 26737 HA LYS H 175 43.757 -52.272 35.712 0.00 0.00
H
ATOM 26738 CB LYS H 175 41.781 -52.899 35.961 0.00 0.00
H
ATOM 26739 HB1 LYS H 175 42.126 -53.954 36.007 0.00 0.00
H
ATOM 26740 HB2 LYS H 175 40.950 -52.737 36.681 0.00 0.00
H
ATOM 26741 CG LYS H 175 41.455 -52.535 34.442 0.00 0.00
H
ATOM 26742 HG1 LYS H 175 41.036 -51.508 34.378 0.00 0.00
H
ATOM 26743 HG2 LYS H 175 42.423 -52.633 33.907 0.00 0.00
H
ATOM 26744 CD LYS H 175 40.471 -53.465 33.822 0.00 0.00
H
ATOM 26745 HD1 LYS H 175 40.891 -54.492 33.881 0.00 0.00
H
ATOM 26746 HD2 LYS H 175 39.473 -53.473 34.310 0.00 0.00
H
ATOM 26747 CE LYS H 175 40.056 -53.140 32.341 0.00 0.00
H
ATOM 26748 HE1 LYS H 175 39.676 -52.102 32.236 0.00 0.00
H
ATOM 26749 HE2 LYS H 175 40.841 -53.313 31.575 0.00 0.00
H
ATOM 26750 NZ LYS H 175 39.031 -54.133 32.048 0.00 0.00
H
ATOM 26751 HZ1 LYS H 175 38.963 -54.113 31.010 0.00 0.00
H
ATOM 26752 HZ2 LYS H 175 39.383 -55.050 32.388 0.00 0.00
H
ATOM 26753 HZ3 LYS H 175 38.091 -53.893 32.420 0.00 0.00
H
ATOM 26754 C LYS H 175 42.698 -50.624 36.493 0.00 0.00
H
ATOM 26755 O LYS H 175 43.000 -49.792 35.594 0.00 0.00
H
ATOM 26756 N TYR H 176 42.150 -50.222 37.630 0.00 0.00
H

ATOM 26757 HN TYR H 176 41.829 -50.787 38.386 0.00 0.00
H
ATOM 26758 CA TYR H 176 41.743 -48.855 37.707 0.00 0.00
H
ATOM 26759 HA TYR H 176 42.005 -48.287 36.826 0.00 0.00
H
ATOM 26760 CB TYR H 176 40.180 -48.890 37.945 0.00 0.00
H
ATOM 26761 HB1 TYR H 176 39.878 -49.515 38.812 0.00 0.00
H
ATOM 26762 HB2 TYR H 176 39.815 -47.850 38.086 0.00 0.00
H
ATOM 26763 CG TYR H 176 39.325 -49.423 36.794 0.00 0.00
H
ATOM 26764 CD1 TYR H 176 38.712 -50.661 36.887 0.00 0.00
H
ATOM 26765 HD1 TYR H 176 38.850 -51.177 37.825 0.00 0.00
H
ATOM 26766 CE1 TYR H 176 37.991 -51.161 35.876 0.00 0.00
H
ATOM 26767 HE1 TYR H 176 37.457 -52.099 35.911 0.00 0.00
H
ATOM 26768 CZ TYR H 176 38.015 -50.493 34.639 0.00 0.00
H
ATOM 26769 OH TYR H 176 37.355 -50.879 33.467 0.00 0.00
H
ATOM 26770 HH TYR H 176 36.880 -51.713 33.505 0.00 0.00
H
ATOM 26771 CD2 TYR H 176 39.400 -48.760 35.513 0.00 0.00
H
ATOM 26772 HD2 TYR H 176 39.980 -47.866 35.338 0.00 0.00
H
ATOM 26773 CE2 TYR H 176 38.736 -49.385 34.450 0.00 0.00
H
ATOM 26774 HE2 TYR H 176 38.694 -49.042 33.426 0.00 0.00
H
ATOM 26775 C TYR H 176 42.366 -48.024 38.775 0.00 0.00
H
ATOM 26776 O TYR H 176 42.059 -46.802 38.916 0.00 0.00
H
ATOM 26777 N GLY H 177 43.232 -48.536 39.660 0.00 0.00
H
ATOM 26778 HN GLY H 177 43.404 -49.483 39.398 0.00 0.00
H
ATOM 26779 CA GLY H 177 43.858 -47.785 40.836 0.00 0.00
H
ATOM 26780 HA1 GLY H 177 44.396 -46.935 40.440 0.00 0.00
H
ATOM 26781 HA2 GLY H 177 44.574 -48.399 41.361 0.00 0.00
H
ATOM 26782 C GLY H 177 42.836 -47.253 41.817 0.00 0.00
H
ATOM 26783 O GLY H 177 43.009 -46.220 42.441 0.00 0.00
H

ATOM 26784 N CYS H 178 41.793 -48.042 42.086 0.00 0.00
H
ATOM 26785 HN CYS H 178 41.773 -48.873 41.535 0.00 0.00
H
ATOM 26786 CA CYS H 178 40.725 -47.632 42.967 0.00 0.00
H
ATOM 26787 HA CYS H 178 40.814 -46.585 43.217 0.00 0.00
H
ATOM 26788 CB CYS H 178 39.396 -47.615 42.174 0.00 0.00
H
ATOM 26789 HB1 CYS H 178 39.147 -48.622 41.777 0.00 0.00
H
ATOM 26790 HB2 CYS H 178 38.552 -47.442 42.876 0.00 0.00
H
ATOM 26791 SG CYS H 178 39.353 -46.327 40.853 0.00 0.00
H
ATOM 26792 HG1 CYS H 178 40.554 -46.417 40.300 0.00 0.00
H
ATOM 26793 C CYS H 178 40.694 -48.424 44.291 0.00 0.00
H
ATOM 26794 O CYS H 178 41.339 -49.450 44.463 0.00 0.00
H
ATOM 26795 N THR H 179 39.828 -48.034 45.234 0.00 0.00
H
ATOM 26796 HN THR H 179 39.298 -47.204 45.077 0.00 0.00
H
ATOM 26797 CA THR H 179 39.628 -48.666 46.540 0.00 0.00
H
ATOM 26798 HA THR H 179 39.800 -49.713 46.338 0.00 0.00
H
ATOM 26799 CB THR H 179 40.589 -48.256 47.646 0.00 0.00
H
ATOM 26800 HB THR H 179 41.630 -48.175 47.268 0.00 0.00
H
ATOM 26801 OG1 THR H 179 40.741 -49.247 48.633 0.00 0.00
H
ATOM 26802 HG1 THR H 179 41.453 -48.936 49.196 0.00 0.00
H
ATOM 26803 CG2 THR H 179 40.039 -47.021 48.335 0.00 0.00
H
ATOM 26804 HG21 THR H 179 39.126 -47.328 48.889 0.00 0.00
H
ATOM 26805 HG22 THR H 179 40.787 -46.491 48.963 0.00 0.00
H
ATOM 26806 HG23 THR H 179 39.731 -46.310 47.539 0.00 0.00
H
ATOM 26807 C THR H 179 38.174 -48.619 46.894 0.00 0.00
H
ATOM 26808 O THR H 179 37.501 -47.695 46.466 0.00 0.00
H
ATOM 26809 N ILE H 180 37.745 -49.649 47.637 0.00 0.00
H
ATOM 26810 HN ILE H 180 38.391 -50.360 47.904 0.00 0.00
H

ATOM 26811 CA ILE H 180 36.334 -49.745 48.041 0.00 0.00
H
ATOM 26812 HA ILE H 180 35.697 -49.028 47.545 0.00 0.00
H
ATOM 26813 CB ILE H 180 35.803 -51.164 47.734 0.00 0.00
H
ATOM 26814 HB ILE H 180 36.208 -51.869 48.491 0.00 0.00
H
ATOM 26815 CG2 ILE H 180 34.233 -51.286 47.994 0.00 0.00
H
ATOM 26816 HG21 ILE H 180 33.819 -52.255 47.642 0.00 0.00
H
ATOM 26817 HG22 ILE H 180 34.060 -51.150 49.083 0.00 0.00
H
ATOM 26818 HG23 ILE H 180 33.749 -50.483 47.398 0.00 0.00
H
ATOM 26819 CG1 ILE H 180 36.127 -51.665 46.271 0.00 0.00
H
ATOM 26820 HG11 ILE H 180 37.224 -51.725 46.106 0.00 0.00
H
ATOM 26821 HG12 ILE H 180 35.728 -52.701 46.299 0.00 0.00
H
ATOM 26822 CD ILE H 180 35.342 -50.888 45.270 0.00 0.00
H
ATOM 26823 HD1 ILE H 180 35.318 -49.785 45.402 0.00 0.00
H
ATOM 26824 HD2 ILE H 180 35.818 -51.096 44.288 0.00 0.00
H
ATOM 26825 HD3 ILE H 180 34.269 -51.155 45.161 0.00 0.00
H
ATOM 26826 C ILE H 180 36.220 -49.562 49.512 0.00 0.00
H
ATOM 26827 O ILE H 180 36.770 -50.313 50.335 0.00 0.00
H
ATOM 26828 N LEU H 181 35.513 -48.471 49.906 0.00 0.00
H
ATOM 26829 HN LEU H 181 35.179 -47.939 49.132 0.00 0.00
H
ATOM 26830 CA LEU H 181 35.535 -47.929 51.308 0.00 0.00
H
ATOM 26831 HA LEU H 181 36.451 -48.094 51.856 0.00 0.00
H
ATOM 26832 CB LEU H 181 35.274 -46.437 51.389 0.00 0.00
H
ATOM 26833 HB1 LEU H 181 34.332 -46.261 50.826 0.00 0.00
H
ATOM 26834 HB2 LEU H 181 35.000 -46.126 52.419 0.00 0.00
H
ATOM 26835 CG LEU H 181 36.443 -45.581 50.848 0.00 0.00
H
ATOM 26836 HG LEU H 181 37.035 -46.072 50.046 0.00 0.00
H
ATOM 26837 CD1 LEU H 181 35.954 -44.276 50.330 0.00 0.00
H

ATOM 26838 HD11 LEU H 181   35.273 −44.316 49.453 0.00 0.00
H
ATOM 26839 HD12 LEU H 181   35.296 −43.784 51.078 0.00 0.00
H
ATOM 26840 HD13 LEU H 181   36.820 −43.647 50.031 0.00 0.00
H
ATOM 26841 CD2 LEU H 181   37.422 −45.314 51.982 0.00 0.00
H
ATOM 26842 HD21 LEU H 181   36.870 −45.099 52.921 0.00 0.00
H
ATOM 26843 HD22 LEU H 181   38.045 −46.219 52.151 0.00 0.00
H
ATOM 26844 HD23 LEU H 181   38.216 −44.555 51.819 0.00 0.00
H
ATOM 26845 C   LEU H 181   34.481 −48.507 52.220 0.00 0.00
H
ATOM 26846 O   LEU H 181   34.610 −48.556 53.477 0.00 0.00
H
ATOM 26847 N   ALA H 182   33.420 −48.987 51.577 0.00 0.00
H
ATOM 26848 HN ALA H 182   33.214 −48.905 50.605 0.00 0.00
H
ATOM 26849 CA ALA H 182   32.302 −49.512 52.285 0.00 0.00
H
ATOM 26850 HA ALA H 182   32.585 −50.198 53.070 0.00 0.00
H
ATOM 26851 CB ALA H 182   31.408 −48.369 52.876 0.00 0.00
H
ATOM 26852 HB1 ALA H 182   31.143 −47.655 52.067 0.00 0.00
H
ATOM 26853 HB2 ALA H 182   30.490 −48.879 53.236 0.00 0.00
H
ATOM 26854 HB3 ALA H 182   31.836 −47.861 53.766 0.00 0.00
H
ATOM 26855 C   ALA H 182   31.381 −50.196 51.234 0.00 0.00
H
ATOM 26856 O   ALA H 182   31.614 −50.100 50.023 0.00 0.00
H
ATOM 26857 N   ILE H 183   30.436 −51.008 51.728 0.00 0.00
H
ATOM 26858 HN ILE H 183   30.494 −51.237 52.696 0.00 0.00
H
ATOM 26859 CA ILE H 183   29.296 −51.616 51.038 0.00 0.00
H
ATOM 26860 HA ILE H 183   28.983 −50.953 50.245 0.00 0.00
H
ATOM 26861 CB ILE H 183   29.749 −52.905 50.438 0.00 0.00
H
ATOM 26862 HB ILE H 183   30.539 −52.660 49.696 0.00 0.00
H
ATOM 26863 CG2 ILE H 183   30.327 −53.951 51.372 0.00 0.00
H
ATOM 26864 HG21 ILE H 183   30.589 −54.926 50.907 0.00 0.00
H

ATOM 26865 HG22 ILE H 183   31.312 -53.621 51.768 0.00 0.00
H
ATOM 26866 HG23 ILE H 183   29.796 -54.065 52.341 0.00 0.00
H
ATOM 26867 CG1 ILE H 183   28.700 -53.546 49.497 0.00 0.00
H
ATOM 26868 HG11 ILE H 183   27.957 -54.065 50.140 0.00 0.00
H
ATOM 26869 HG12 ILE H 183   28.159 -52.775 48.907 0.00 0.00
H
ATOM 26870 CD ILE H 183   29.393 -54.478 48.443 0.00 0.00
H
ATOM 26871 HD1 ILE H 183   28.647 -54.937 47.760 0.00 0.00
H
ATOM 26872 HD2 ILE H 183   30.056 -53.776 47.893 0.00 0.00
H
ATOM 26873 HD3 ILE H 183   29.947 -55.290 48.961 0.00 0.00
H
ATOM 26874 C   ILE H 183   28.089 -51.837 51.914 0.00 0.00
H
ATOM 26875 O   ILE H 183   28.345 -52.071 53.097 0.00 0.00
H
ATOM 26876 N   LYS H 184   26.835 -51.761 51.444 0.00 0.00
H
ATOM 26877 HN LYS H 184   26.710 -51.848 50.458 0.00 0.00
H
ATOM 26878 CA LYS H 184   25.676 -51.971 52.277 0.00 0.00
H
ATOM 26879 HA LYS H 184   25.928 -51.578 53.250 0.00 0.00
H
ATOM 26880 CB LYS H 184   24.565 -50.978 51.800 0.00 0.00
H
ATOM 26881 HB1 LYS H 184   25.068 -49.988 51.754 0.00 0.00
H
ATOM 26882 HB2 LYS H 184   24.262 -51.206 50.756 0.00 0.00
H
ATOM 26883 CG LYS H 184   23.320 -50.788 52.673 0.00 0.00
H
ATOM 26884 HG1 LYS H 184   22.699 -51.701 52.548 0.00 0.00
H
ATOM 26885 HG2 LYS H 184   23.666 -50.819 53.729 0.00 0.00
H
ATOM 26886 CD LYS H 184   22.608 -49.430 52.482 0.00 0.00
H
ATOM 26887 HD1 LYS H 184   21.929 -49.210 53.333 0.00 0.00
H
ATOM 26888 HD2 LYS H 184   23.356 -48.611 52.410 0.00 0.00
H
ATOM 26889 CE LYS H 184   21.674 -49.162 51.277 0.00 0.00
H
ATOM 26890 HE1 LYS H 184   22.286 -49.316 50.363 0.00 0.00
H
ATOM 26891 HE2 LYS H 184   20.948 -50.002 51.312 0.00 0.00
H

ATOM 26892 NZ LYS H 184 21.015 -47.865 51.280 0.00 0.00
H
ATOM 26893 HZ1 LYS H 184 20.549 -47.824 50.352 0.00 0.00
H
ATOM 26894 HZ2 LYS H 184 20.363 -47.765 52.084 0.00 0.00
H
ATOM 26895 HZ3 LYS H 184 21.776 -47.161 51.206 0.00 0.00
H
ATOM 26896 C LYS H 184 25.159 -53.344 52.205 0.00 0.00
H
ATOM 26897 O LYS H 184 25.024 -53.916 51.108 0.00 0.00
H
ATOM 26898 N HSE H 185 24.841 -53.932 53.346 0.00 0.00
H
ATOM 26899 HN HSE H 185 24.865 -53.435 54.210 0.00 0.00
H
ATOM 26900 CA HSE H 185 24.307 -55.263 53.323 0.00 0.00
H
ATOM 26901 HA HSE H 185 24.098 -55.502 52.291 0.00 0.00
H
ATOM 26902 CB HSE H 185 25.347 -56.335 53.785 0.00 0.00
H
ATOM 26903 HB1 HSE H 185 25.818 -56.047 54.749 0.00 0.00
H
ATOM 26904 HB2 HSE H 185 24.831 -57.314 53.883 0.00 0.00
H
ATOM 26905 ND1 HSE H 185 26.455 -57.814 52.277 0.00 0.00
H
ATOM 26906 CG HSE H 185 26.501 -56.538 52.827 0.00 0.00
H
ATOM 26907 CE1 HSE H 185 27.338 -57.789 51.297 0.00 0.00
H
ATOM 26908 HE1 HSE H 185 27.411 -58.572 50.542 0.00 0.00
H
ATOM 26909 NE2 HSE H 185 27.928 -56.583 51.239 0.00 0.00
H
ATOM 26910 HE2 HSE H 185 28.579 -56.349 50.517 0.00 0.00
H
ATOM 26911 CD2 HSE H 185 27.442 -55.805 52.263 0.00 0.00
H
ATOM 26912 HD2 HSE H 185 27.750 -54.777 52.404 0.00 0.00
H
ATOM 26913 C HSE H 185 23.081 -55.339 54.101 0.00 0.00
H
ATOM 26914 O HSE H 185 23.107 -55.275 55.321 0.00 0.00
H
ATOM 26915 N HSE H 186 21.934 -55.637 53.461 0.00 0.00
H
ATOM 26916 HN HSE H 186 21.910 -55.778 52.475 0.00 0.00
H
ATOM 26917 CA HSE H 186 20.585 -55.778 54.000 0.00 0.00
H
ATOM 26918 HA HSE H 186 19.937 -55.501 53.181 0.00 0.00
H

ATOM 26919 CB HSE H 186 20.406 -57.234 54.535 0.00 0.00
H
ATOM 26920 HB1 HSE H 186 20.907 -58.014 53.924 0.00 0.00
H
ATOM 26921 HB2 HSE H 186 20.948 -57.290 55.503 0.00 0.00
H
ATOM 26922 ND1 HSE H 186 18.066 -57.330 53.591 0.00 0.00
H
ATOM 26923 CG HSE H 186 18.941 -57.612 54.668 0.00 0.00
H
ATOM 26924 CE1 HSE H 186 16.911 -57.931 53.929 0.00 0.00
H
ATOM 26925 HE1 HSE H 186 16.021 -57.813 53.311 0.00 0.00
H
ATOM 26926 NE2 HSE H 186 17.008 -58.450 55.191 0.00 0.00
H
ATOM 26927 HE2 HSE H 186 16.208 -58.593 55.774 0.00 0.00
H
ATOM 26928 CD2 HSE H 186 18.286 -58.214 55.655 0.00 0.00
H
ATOM 26929 HD2 HSE H 186 18.604 -58.390 56.675 0.00 0.00
H
ATOM 26930 C HSE H 186 20.247 -54.738 55.065 0.00 0.00
H
ATOM 26931 O HSE H 186 19.766 -55.082 56.170 0.00 0.00
H
ATOM 26932 N GLY H 187 20.408 -53.471 54.678 0.00 0.00
H
ATOM 26933 HN GLY H 187 20.859 -53.434 53.789 0.00 0.00
H
ATOM 26934 CA GLY H 187 20.057 -52.296 55.411 0.00 0.00
H
ATOM 26935 HA1 GLY H 187 19.273 -52.438 56.139 0.00 0.00
H
ATOM 26936 HA2 GLY H 187 19.761 -51.490 54.756 0.00 0.00
H
ATOM 26937 C GLY H 187 21.184 -51.735 56.246 0.00 0.00
H
ATOM 26938 O GLY H 187 21.042 -50.680 56.839 0.00 0.00
H
ATOM 26939 N ASP H 188 22.368 -52.413 56.361 0.00 0.00
H
ATOM 26940 HN ASP H 188 22.468 -53.309 55.934 0.00 0.00
H
ATOM 26941 CA ASP H 188 23.411 -52.063 57.264 0.00 0.00
H
ATOM 26942 HA ASP H 188 23.164 -51.137 57.762 0.00 0.00
H
ATOM 26943 CB ASP H 188 23.524 -53.194 58.330 0.00 0.00
H
ATOM 26944 HB1 ASP H 188 22.491 -53.399 58.682 0.00 0.00
H
ATOM 26945 HB2 ASP H 188 23.829 -54.144 57.843 0.00 0.00
H

ATOM 26946 CG ASP H 188 24.429 -52.902 59.534 0.00 0.00
H
ATOM 26947 OD1 ASP H 188 24.638 -51.751 59.995 0.00 0.00
H
ATOM 26948 OD2 ASP H 188 24.934 -53.853 60.104 0.00 0.00
H
ATOM 26949 C ASP H 188 24.767 -51.847 56.475 0.00 0.00
H
ATOM 26950 O ASP H 188 25.215 -52.728 55.703 0.00 0.00
H
ATOM 26951 N ILE H 189 25.355 -50.591 56.571 0.00 0.00
H
ATOM 26952 HN ILE H 189 24.867 -49.861 57.042 0.00 0.00
H
ATOM 26953 CA ILE H 189 26.641 -50.220 55.992 0.00 0.00
H
ATOM 26954 HA ILE H 189 26.574 -50.633 54.996 0.00 0.00
H
ATOM 26955 CB ILE H 189 26.824 -48.683 55.921 0.00 0.00
H
ATOM 26956 HB ILE H 189 26.228 -48.156 56.697 0.00 0.00
H
ATOM 26957 CG2 ILE H 189 28.277 -48.309 55.944 0.00 0.00
H
ATOM 26958 HG21 ILE H 189 28.913 -48.720 55.131 0.00 0.00
H
ATOM 26959 HG22 ILE H 189 28.415 -47.207 55.913 0.00 0.00
H
ATOM 26960 HG23 ILE H 189 28.878 -48.603 56.831 0.00 0.00
H
ATOM 26961 CG1 ILE H 189 26.215 -48.321 54.596 0.00 0.00
H
ATOM 26962 HG11 ILE H 189 26.774 -48.891 53.824 0.00 0.00
H
ATOM 26963 HG12 ILE H 189 25.168 -48.692 54.571 0.00 0.00
H
ATOM 26964 CD ILE H 189 26.197 -46.784 54.360 0.00 0.00
H
ATOM 26965 HD1 ILE H 189 26.014 -46.609 53.278 0.00 0.00
H
ATOM 26966 HD2 ILE H 189 25.448 -46.374 55.070 0.00 0.00
H
ATOM 26967 HD3 ILE H 189 27.115 -46.249 54.685 0.00 0.00
H
ATOM 26968 C ILE H 189 27.765 -51.035 56.642 0.00 0.00
H
ATOM 26969 O ILE H 189 27.966 -51.014 57.840 0.00 0.00
H
ATOM 26970 N CYS H 190 28.560 -51.778 55.863 0.00 0.00
H
ATOM 26971 HN CYS H 190 28.462 -51.762 54.871 0.00 0.00
H
ATOM 26972 CA CYS H 190 29.838 -52.360 56.440 0.00 0.00
H

ATOM 26973 HA CYS H 190   29.868 −52.336 57.520 0.00 0.00
H
ATOM 26974 CB CYS H 190   30.169 −53.776 55.954 0.00 0.00
H
ATOM 26975 HB1 CYS H 190   29.352 −54.417 56.348 0.00 0.00
H
ATOM 26976 HB2 CYS H 190   30.292 −53.938 54.862 0.00 0.00
H
ATOM 26977 SG CYS H 190   31.627 −54.685 56.654 0.00 0.00
H
ATOM 26978 HG1 CYS H 190   31.702 −54.245 57.901 0.00 0.00
H
ATOM 26979 C   CYS H 190   30.922 −51.450 55.958 0.00 0.00
H
ATOM 26980 O   CYS H 190   31.376 −51.440 54.791 0.00 0.00
H
ATOM 26981 N   LEU H 191   31.436 −50.650 56.960 0.00 0.00
H
ATOM 26982 HN LEU H 191   30.914 −50.634 57.810 0.00 0.00
H
ATOM 26983 CA LEU H 191   32.420 −49.644 56.723 0.00 0.00
H
ATOM 26984 HA LEU H 191   32.238 −49.211 55.751 0.00 0.00
H
ATOM 26985 CB LEU H 191   32.259 −48.593 57.797 0.00 0.00
H
ATOM 26986 HB1 LEU H 191   31.203 −48.250 57.772 0.00 0.00
H
ATOM 26987 HB2 LEU H 191   32.391 −49.255 58.679 0.00 0.00
H
ATOM 26988 CG LEU H 191   33.354 −47.521 57.860 0.00 0.00
H
ATOM 26989 HG LEU H 191   34.408 −47.872 57.857 0.00 0.00
H
ATOM 26990 CD1 LEU H 191   33.350 −46.611 56.632 0.00 0.00
H
ATOM 26991 HD11 LEU H 191   32.408 −46.044 56.477 0.00 0.00
H
ATOM 26992 HD12 LEU H 191   34.236 −45.941 56.650 0.00 0.00
H
ATOM 26993 HD13 LEU H 191   33.524 −47.187 55.698 0.00 0.00
H
ATOM 26994 CD2 LEU H 191   33.235 −46.614 59.043 0.00 0.00
H
ATOM 26995 HD21 LEU H 191   33.282 −47.277 59.934 0.00 0.00
H
ATOM 26996 HD22 LEU H 191   34.041 −45.866 59.203 0.00 0.00
H
ATOM 26997 HD23 LEU H 191   32.310 −46.020 59.203 0.00 0.00
H
ATOM 26998 C   LEU H 191   33.805 −50.382 56.748 0.00 0.00
H
ATOM 26999 O   LEU H 191   34.229 −50.931 57.774 0.00 0.00
H

```
ATOM  27000  N   SER H 192      34.587 -50.225  55.637  0.00  0.00
H
ATOM  27001  HN  SER H 192      34.187 -49.738  54.865  0.00  0.00
H
ATOM  27002  CA  SER H 192      35.876 -50.900  55.502  0.00  0.00
H
ATOM  27003  HA  SER H 192      36.302 -50.547  54.574  0.00  0.00
H
ATOM  27004  CB  SER H 192      36.964 -50.322  56.471  0.00  0.00
H
ATOM  27005  HB1 SER H 192      36.586 -50.519  57.497  0.00  0.00
H
ATOM  27006  HB2 SER H 192      37.876 -50.949  56.374  0.00  0.00
H
ATOM  27007  OG  SER H 192      37.265 -48.939  56.194  0.00  0.00
H
ATOM  27008  HG1 SER H 192      37.258 -48.555  57.074  0.00  0.00
H
ATOM  27009  C   SER H 192      35.926 -52.429  55.598  0.00  0.00
H
ATOM  27010  O   SER H 192      36.410 -53.024  56.578  0.00  0.00
H
ATOM  27011  N   PRO H 193      35.546 -53.178  54.587  0.00  0.00
H
ATOM  27012  CD  PRO H 193      35.441 -52.614  53.254  0.00  0.00
H
ATOM  27013  HD1 PRO H 193      34.618 -51.869  53.210  0.00  0.00
H
ATOM  27014  HD2 PRO H 193      36.383 -52.090  52.984  0.00  0.00
H
ATOM  27015  CA  PRO H 193      35.171 -54.646  54.639  0.00  0.00
H
ATOM  27016  HA  PRO H 193      34.527 -54.884  55.473  0.00  0.00
H
ATOM  27017  CB  PRO H 193      34.501 -54.792  53.244  0.00  0.00
H
ATOM  27018  HB1 PRO H 193      33.436 -54.494  53.349  0.00  0.00
H
ATOM  27019  HB2 PRO H 193      34.465 -55.860  52.942  0.00  0.00
H
ATOM  27020  CG  PRO H 193      35.135 -53.791  52.314  0.00  0.00
H
ATOM  27021  HG1 PRO H 193      34.495 -53.679  51.412  0.00  0.00
H
ATOM  27022  HG2 PRO H 193      36.092 -54.226  51.953  0.00  0.00
H
ATOM  27023  C   PRO H 193      36.334 -55.644  54.774  0.00  0.00
H
ATOM  27024  O   PRO H 193      37.522 -55.248  54.817  0.00  0.00
H
ATOM  27025  N   ALA H 194      35.919 -56.943  54.864  0.00  0.00
H
ATOM  27026  HN  ALA H 194      34.930 -57.044  54.788  0.00  0.00
H
```

ATOM 27027 CA ALA H 194 36.915 -57.946 54.908 0.00 0.00
H
ATOM 27028 HA ALA H 194 37.914 -57.547 55.005 0.00 0.00
H
ATOM 27029 CB ALA H 194 36.881 -58.732 56.257 0.00 0.00
H
ATOM 27030 HB1 ALA H 194 37.720 -59.355 56.634 0.00 0.00
H
ATOM 27031 HB2 ALA H 194 36.728 -57.857 56.925 0.00 0.00
H
ATOM 27032 HB3 ALA H 194 35.985 -59.343 56.496 0.00 0.00
H
ATOM 27033 C ALA H 194 36.660 -58.945 53.786 0.00 0.00
H
ATOM 27034 O ALA H 194 35.527 -58.880 53.296 0.00 0.00
H
ATOM 27035 N PRO H 195 37.560 -59.844 53.447 0.00 0.00
H
ATOM 27036 CD PRO H 195 39.027 -59.816 53.693 0.00 0.00
H
ATOM 27037 HD1 PRO H 195 39.464 -58.827 53.436 0.00 0.00
H
ATOM 27038 HD2 PRO H 195 39.206 -60.180 54.727 0.00 0.00
H
ATOM 27039 CA PRO H 195 37.202 -61.016 52.671 0.00 0.00
H
ATOM 27040 HA PRO H 195 36.827 -60.715 51.704 0.00 0.00
H
ATOM 27041 CB PRO H 195 38.472 -61.830 52.494 0.00 0.00
H
ATOM 27042 HB1 PRO H 195 38.488 -62.259 51.469 0.00 0.00
H
ATOM 27043 HB2 PRO H 195 38.543 -62.556 53.332 0.00 0.00
H
ATOM 27044 CG PRO H 195 39.562 -60.695 52.555 0.00 0.00
H
ATOM 27045 HG1 PRO H 195 39.435 -60.098 51.626 0.00 0.00
H
ATOM 27046 HG2 PRO H 195 40.616 -61.042 52.602 0.00 0.00
H
ATOM 27047 C PRO H 195 36.125 -61.834 53.329 0.00 0.00
H
ATOM 27048 O PRO H 195 36.294 -62.326 54.406 0.00 0.00
H
ATOM 27049 N GLU H 196 34.934 -62.099 52.755 0.00 0.00
H
ATOM 27050 HN GLU H 196 34.418 -62.857 53.146 0.00 0.00
H
ATOM 27051 CA GLU H 196 34.446 -61.597 51.517 0.00 0.00
H
ATOM 27052 HA GLU H 196 34.556 -60.522 51.496 0.00 0.00
H
ATOM 27053 CB GLU H 196 35.129 -62.323 50.318 0.00 0.00
H

ATOM 27054 HB1 GLU H 196   34.837 −61.834 49.365 0.00 0.00
H
ATOM 27055 HB2 GLU H 196   36.219 −62.156 50.460 0.00 0.00
H
ATOM 27056 CG GLU H 196   34.870 −63.860 50.204 0.00 0.00
H
ATOM 27057 HG1 GLU H 196   35.343 −64.313 51.101 0.00 0.00
H
ATOM 27058 HG2 GLU H 196   33.788 −64.109 50.238 0.00 0.00
H
ATOM 27059 CD GLU H 196   35.459 −64.513 48.978 0.00 0.00
H
ATOM 27060 OE1 GLU H 196   36.541 −65.127 49.096 0.00 0.00
H
ATOM 27061 OE2 GLU H 196   34.881 −64.365 47.881 0.00 0.00
H
ATOM 27062 C   GLU H 196   32.959 −61.954 51.460 0.00 0.00
H
ATOM 27063 O   GLU H 196   32.545 −62.855 52.244 0.00 0.00
H
ATOM 27064 N   ASP H 197   32.112 −61.378 50.556 0.00 0.00
H
ATOM 27065 HN ASP H 197   32.470 −60.630 50.002 0.00 0.00
H
ATOM 27066 CA ASP H 197   30.737 −61.637 50.348 0.00 0.00
H
ATOM 27067 HA ASP H 197   30.643 −62.705 50.223 0.00 0.00
H
ATOM 27068 CB ASP H 197   29.814 −61.019 51.524 0.00 0.00
H
ATOM 27069 HB1 ASP H 197   30.383 −61.158 52.467 0.00 0.00
H
ATOM 27070 HB2 ASP H 197   29.703 −59.935 51.310 0.00 0.00
H
ATOM 27071 CG ASP H 197   28.376 −61.623 51.463 0.00 0.00
H
ATOM 27072 OD1 ASP H 197   28.112 −62.591 50.729 0.00 0.00
H
ATOM 27073 OD2 ASP H 197   27.443 −61.148 52.178 0.00 0.00
H
ATOM 27074 C   ASP H 197   30.311 −61.141 48.944 0.00 0.00
H
ATOM 27075 O   ASP H 197   30.979 −60.365 48.180 0.00 0.00
H
ATOM 27076 N   ILE H 198   29.212 −61.721 48.387 0.00 0.00
H
ATOM 27077 HN ILE H 198   28.709 −62.409 48.905 0.00 0.00
H
ATOM 27078 CA ILE H 198   28.875 −61.599 46.953 0.00 0.00
H
ATOM 27079 HA ILE H 198   29.762 −61.438 46.359 0.00 0.00
H
ATOM 27080 CB ILE H 198   28.219 −62.790 46.407 0.00 0.00
H

```
ATOM  27081  HB  ILE H 198      27.232 -62.918  46.901  0.00  0.00
H
ATOM  27082  CG2 ILE H 198      27.916 -62.725  44.917  0.00  0.00
H
ATOM  27083 HG21 ILE H 198      27.258 -63.548  44.565  0.00  0.00
H
ATOM  27084 HG22 ILE H 198      27.331 -61.809  44.689  0.00  0.00
H
ATOM  27085 HG23 ILE H 198      28.799 -62.661  44.246  0.00  0.00
H
ATOM  27086  CG1 ILE H 198      29.022 -64.063  46.807  0.00  0.00
H
ATOM  27087 HG11 ILE H 198      28.981 -64.207  47.907  0.00  0.00
H
ATOM  27088 HG12 ILE H 198      28.499 -64.955  46.401  0.00  0.00
H
ATOM  27089  CD  ILE H 198      30.399 -64.171  46.278  0.00  0.00
H
ATOM  27090  HD1 ILE H 198      30.871 -65.143  46.537  0.00  0.00
H
ATOM  27091  HD2 ILE H 198      30.390 -63.976  45.185  0.00  0.00
H
ATOM  27092  HD3 ILE H 198      31.086 -63.436  46.749  0.00  0.00
H
ATOM  27093  C   ILE H 198      27.920 -60.332  46.701  0.00  0.00
H
ATOM  27094  O   ILE H 198      26.877 -60.136  47.321  0.00  0.00
H
ATOM  27095  N   ILE H 199      28.471 -59.441  45.895  0.00  0.00
H
ATOM  27096  HN  ILE H 199      29.115 -59.726  45.189  0.00  0.00
H
ATOM  27097  CA  ILE H 199      27.764 -58.320  45.459  0.00  0.00
H
ATOM  27098  HA  ILE H 199      27.363 -57.782  46.305  0.00  0.00
H
ATOM  27099  CB  ILE H 199      28.582 -57.257  44.812  0.00  0.00
H
ATOM  27100  HB  ILE H 199      28.749 -57.497  43.740  0.00  0.00
H
ATOM  27101  CG2 ILE H 199      27.748 -56.012  44.626  0.00  0.00
H
ATOM  27102 HG21 ILE H 199      27.178 -56.117  43.678  0.00  0.00
H
ATOM  27103 HG22 ILE H 199      26.981 -55.985  45.429  0.00  0.00
H
ATOM  27104 HG23 ILE H 199      28.347 -55.102  44.409  0.00  0.00
H
ATOM  27105  CG1 ILE H 199      29.947 -57.041  45.585  0.00  0.00
H
ATOM  27106 HG11 ILE H 199      29.619 -56.987  46.645  0.00  0.00
H
ATOM  27107 HG12 ILE H 199      30.547 -57.976  45.597  0.00  0.00
H
```

```
ATOM  27108  CD  ILE H 199      30.759 -55.834  45.236  0.00  0.00
H
ATOM  27109  HD1 ILE H 199      31.172 -55.914  44.208  0.00  0.00
H
ATOM  27110  HD2 ILE H 199      30.111 -54.932  45.231  0.00  0.00
H
ATOM  27111  HD3 ILE H 199      31.566 -55.714  45.990  0.00  0.00
H
ATOM  27112  C   ILE H 199      26.695 -58.716  44.392  0.00  0.00
H
ATOM  27113  O   ILE H 199      26.948 -59.488  43.440  0.00  0.00
H
ATOM  27114  N   ARG H 200      25.396 -58.214  44.610  0.00  0.00
H
ATOM  27115  HN  ARG H 200      25.117 -57.550  45.300  0.00  0.00
H
ATOM  27116  CA  ARG H 200      24.277 -58.611  43.785  0.00  0.00
H
ATOM  27117  HA  ARG H 200      24.659 -59.101  42.902  0.00  0.00
H
ATOM  27118  CB  ARG H 200      23.291 -59.553  44.512  0.00  0.00
H
ATOM  27119  HB1 ARG H 200      23.078 -59.151  45.525  0.00  0.00
H
ATOM  27120  HB2 ARG H 200      22.342 -59.518  43.935  0.00  0.00
H
ATOM  27121  CG  ARG H 200      23.769 -61.061  44.490  0.00  0.00
H
ATOM  27122  HG1 ARG H 200      24.679 -61.122  43.855  0.00  0.00
H
ATOM  27123  HG2 ARG H 200      24.050 -61.322  45.533  0.00  0.00
H
ATOM  27124  CD  ARG H 200      22.718 -62.012  43.918  0.00  0.00
H
ATOM  27125  HD1 ARG H 200      23.163 -63.029  43.964  0.00  0.00
H
ATOM  27126  HD2 ARG H 200      21.764 -61.944  44.482  0.00  0.00
H
ATOM  27127  NE  ARG H 200      22.512 -61.725  42.446  0.00  0.00
H
ATOM  27128  HE  ARG H 200      23.060 -61.071  41.925  0.00  0.00
H
ATOM  27129  CZ  ARG H 200      21.742 -62.409  41.609  0.00  0.00
H
ATOM  27130  NH1 ARG H 200      20.970 -63.385  41.993  0.00  0.00
H
ATOM  27131 HH11 ARG H 200      20.248 -63.748  41.404  0.00  0.00
H
ATOM  27132 HH12 ARG H 200      21.003 -63.717  42.936  0.00  0.00
H
ATOM  27133  NH2 ARG H 200      21.803 -62.053  40.343  0.00  0.00
H
ATOM  27134 HH21 ARG H 200      21.149 -62.525  39.752  0.00  0.00
H
```

ATOM 27135 HH22 ARG H 200   22.514 -61.490 39.922 0.00 0.00
H
ATOM 27136 C   ARG H 200   23.608 -57.384 43.238 0.00 0.00
H
ATOM 27137 O   ARG H 200   23.851 -56.257 43.717 0.00 0.00
H
ATOM 27138 N   GLU H 201   22.750 -57.459 42.244 0.00 0.00
H
ATOM 27139 HN GLU H 201   22.845 -58.193 41.576 0.00 0.00
H
ATOM 27140 CA GLU H 201   21.955 -56.335 41.799 0.00 0.00
H
ATOM 27141 HA GLU H 201   22.544 -55.629 41.232 0.00 0.00
H
ATOM 27142 CB GLU H 201   20.846 -56.948 40.871 0.00 0.00
H
ATOM 27143 HB1 GLU H 201   21.451 -57.359 40.035 0.00 0.00
H
ATOM 27144 HB2 GLU H 201   20.216 -57.786 41.238 0.00 0.00
H
ATOM 27145 CG GLU H 201   19.986 -55.927 40.087 0.00 0.00
H
ATOM 27146 HG1 GLU H 201   19.517 -55.178 40.759 0.00 0.00
H
ATOM 27147 HG2 GLU H 201   20.645 -55.394 39.369 0.00 0.00
H
ATOM 27148 CD GLU H 201   18.945 -56.624 39.262 0.00 0.00
H
ATOM 27149 OE1 GLU H 201   19.331 -57.669 38.625 0.00 0.00
H
ATOM 27150 OE2 GLU H 201   17.801 -56.133 39.133 0.00 0.00
H
ATOM 27151 C   GLU H 201   21.219 -55.597 42.915 0.00 0.00
H
ATOM 27152 O   GLU H 201   20.608 -56.172 43.746 0.00 0.00
H
ATOM 27153 N   GLN H 202   21.323 -54.231 42.843 0.00 0.00
H
ATOM 27154 HN GLN H 202   21.849 -53.871 42.076 0.00 0.00
H
ATOM 27155 CA GLN H 202   20.652 -53.247 43.711 0.00 0.00
H
ATOM 27156 HA GLN H 202   20.836 -52.344 43.147 0.00 0.00
H
ATOM 27157 CB GLN H 202   19.170 -53.454 44.088 0.00 0.00
H
ATOM 27158 HB1 GLN H 202   19.012 -54.405 44.639 0.00 0.00
H
ATOM 27159 HB2 GLN H 202   18.845 -52.586 44.701 0.00 0.00
H
ATOM 27160 CG GLN H 202   18.302 -53.639 42.856 0.00 0.00
H
ATOM 27161 HG1 GLN H 202   18.478 -52.812 42.135 0.00 0.00
H

ATOM 27162 HG2 GLN H 202   18.484 −54.647 42.426 0.00 0.00
H
ATOM 27163 CD GLN H 202   16.801 −53.591 43.209 0.00 0.00
H
ATOM 27164 OE1 GLN H 202   15.999 −52.941 42.542 0.00 0.00
H
ATOM 27165 NE2 GLN H 202   16.503 −54.332 44.317 0.00 0.00
H
ATOM 27166 HE21 GLN H 202   15.583 −54.304 44.709 0.00 0.00
H
ATOM 27167 HE22 GLN H 202   17.176 −54.874 44.820 0.00 0.00
H
ATOM 27168 C   GLN H 202   21.460 −52.969 44.965 0.00 0.00
H
ATOM 27169 O   GLN H 202   20.993 −52.261 45.885 0.00 0.00
H
ATOM 27170 N   ASP H 203   22.734 −53.486 45.108 0.00 0.00
H
ATOM 27171 HN ASP H 203   22.982 −54.162 44.417 0.00 0.00
H
ATOM 27172 CA ASP H 203   23.601 −53.146 46.286 0.00 0.00
H
ATOM 27173 HA ASP H 203   23.088 −52.964 47.218 0.00 0.00
H
ATOM 27174 CB ASP H 203   24.761 −54.179 46.417 0.00 0.00
H
ATOM 27175 HB1 ASP H 203   25.310 −54.435 45.486 0.00 0.00
H
ATOM 27176 HB2 ASP H 203   25.517 −53.848 47.160 0.00 0.00
H
ATOM 27177 CG ASP H 203   24.198 −55.543 46.964 0.00 0.00
H
ATOM 27178 OD1 ASP H 203   24.869 −56.567 46.924 0.00 0.00
H
ATOM 27179 OD2 ASP H 203   23.161 −55.484 47.667 0.00 0.00
H
ATOM 27180 C   ASP H 203   24.267 −51.800 46.048 0.00 0.00
H
ATOM 27181 O   ASP H 203   24.537 −51.408 44.982 0.00 0.00
H
ATOM 27182 N   CYS H 204   24.365 −51.045 47.141 0.00 0.00
H
ATOM 27183 HN CYS H 204   24.141 −51.440 48.029 0.00 0.00
H
ATOM 27184 CA CYS H 204   25.030 −49.723 47.169 0.00 0.00
H
ATOM 27185 HA CYS H 204   25.235 −49.312 46.191 0.00 0.00
H
ATOM 27186 CB CYS H 204   24.287 −48.677 48.112 0.00 0.00
H
ATOM 27187 HB1 CYS H 204   24.360 −48.964 49.183 0.00 0.00
H
ATOM 27188 HB2 CYS H 204   24.891 −47.774 47.880 0.00 0.00
H

ATOM 27189 SG CYS H 204 22.515 -48.451 47.633 0.00 0.00
H
ATOM 27190 HG1 CYS H 204 22.573 -48.776 46.349 0.00 0.00
H
ATOM 27191 C CYS H 204 26.327 -49.902 47.852 0.00 0.00
H
ATOM 27192 O CYS H 204 26.427 -50.627 48.858 0.00 0.00
H
ATOM 27193 N LEU H 205 27.296 -49.248 47.381 0.00 0.00
H
ATOM 27194 HN LEU H 205 27.125 -48.639 46.611 0.00 0.00
H
ATOM 27195 CA LEU H 205 28.692 -49.370 47.753 0.00 0.00
H
ATOM 27196 HA LEU H 205 28.699 -49.424 48.832 0.00 0.00
H
ATOM 27197 CB LEU H 205 29.548 -50.523 47.100 0.00 0.00
H
ATOM 27198 HB1 LEU H 205 30.560 -50.653 47.540 0.00 0.00
H
ATOM 27199 HB2 LEU H 205 29.121 -51.526 47.314 0.00 0.00
H
ATOM 27200 CG LEU H 205 29.748 -50.564 45.568 0.00 0.00
H
ATOM 27201 HG LEU H 205 29.947 -49.528 45.220 0.00 0.00
H
ATOM 27202 CD1 LEU H 205 31.045 -51.340 45.242 0.00 0.00
H
ATOM 27203 HD11 LEU H 205 31.918 -50.896 45.765 0.00 0.00
H
ATOM 27204 HD12 LEU H 205 30.959 -52.381 45.621 0.00 0.00
H
ATOM 27205 HD13 LEU H 205 31.211 -51.344 44.144 0.00 0.00
H
ATOM 27206 CD2 LEU H 205 28.542 -51.189 44.874 0.00 0.00
H
ATOM 27207 HD21 LEU H 205 27.568 -50.872 45.304 0.00 0.00
H
ATOM 27208 HD22 LEU H 205 28.578 -50.974 43.785 0.00 0.00
H
ATOM 27209 HD23 LEU H 205 28.623 -52.284 45.043 0.00 0.00
H
ATOM 27210 C LEU H 205 29.441 -48.131 47.352 0.00 0.00
H
ATOM 27211 O LEU H 205 28.920 -47.396 46.553 0.00 0.00
H
ATOM 27212 N VAL H 206 30.601 -47.882 48.013 0.00 0.00
H
ATOM 27213 HN VAL H 206 30.966 -48.554 48.653 0.00 0.00
H
ATOM 27214 CA VAL H 206 31.288 -46.612 47.993 0.00 0.00
H
ATOM 27215 HA VAL H 206 30.896 -46.065 47.148 0.00 0.00
H

ATOM 27216 CB VAL H 206 31.349 -45.893 49.293 0.00 0.00
H
ATOM 27217 HB VAL H 206 32.120 -46.437 49.879 0.00 0.00
H
ATOM 27218 CG1 VAL H 206 31.881 -44.402 49.226 0.00 0.00
H
ATOM 27219 HG11 VAL H 206 32.247 -43.988 50.190 0.00 0.00
H
ATOM 27220 HG12 VAL H 206 32.554 -44.232 48.359 0.00 0.00
H
ATOM 27221 HG13 VAL H 206 31.004 -43.841 48.837 0.00 0.00
H
ATOM 27222 CG2 VAL H 206 29.938 -45.891 50.003 0.00 0.00
H
ATOM 27223 HG21 VAL H 206 29.520 -46.899 50.212 0.00 0.00
H
ATOM 27224 HG22 VAL H 206 30.113 -45.430 50.999 0.00 0.00
H
ATOM 27225 HG23 VAL H 206 29.152 -45.293 49.493 0.00 0.00
H
ATOM 27226 C VAL H 206 32.707 -46.746 47.491 0.00 0.00
H
ATOM 27227 O VAL H 206 33.464 -47.505 48.083 0.00 0.00
H
ATOM 27228 N ILE H 207 33.144 -46.043 46.406 0.00 0.00
H
ATOM 27229 HN ILE H 207 32.516 -45.409 45.961 0.00 0.00
H
ATOM 27230 CA ILE H 207 34.479 -46.157 45.734 0.00 0.00
H
ATOM 27231 HA ILE H 207 34.942 -47.004 46.217 0.00 0.00
H
ATOM 27232 CB ILE H 207 34.404 -46.649 44.254 0.00 0.00
H
ATOM 27233 HB ILE H 207 33.687 -47.497 44.215 0.00 0.00
H
ATOM 27234 CG2 ILE H 207 33.825 -45.585 43.331 0.00 0.00
H
ATOM 27235 HG21 ILE H 207 33.596 -46.107 42.377 0.00 0.00
H
ATOM 27236 HG22 ILE H 207 32.824 -45.259 43.687 0.00 0.00
H
ATOM 27237 HG23 ILE H 207 34.527 -44.730 43.227 0.00 0.00
H
ATOM 27238 CG1 ILE H 207 35.728 -47.180 43.698 0.00 0.00
H
ATOM 27239 HG11 ILE H 207 36.515 -46.424 43.488 0.00 0.00
H
ATOM 27240 HG12 ILE H 207 36.079 -47.771 44.571 0.00 0.00
H
ATOM 27241 CD ILE H 207 35.548 -48.227 42.480 0.00 0.00
H
ATOM 27242 HD1 ILE H 207 36.439 -48.888 42.416 0.00 0.00
H

ATOM 27243 HD2 ILE H 207 34.686 -48.915 42.612 0.00 0.00
H
ATOM 27244 HD3 ILE H 207 35.431 -47.621 41.557 0.00 0.00
H
ATOM 27245 C ILE H 207 35.243 -44.889 45.764 0.00 0.00
H
ATOM 27246 O ILE H 207 34.621 -43.836 45.668 0.00 0.00
H
ATOM 27247 N MET H 208 36.548 -44.859 45.989 0.00 0.00
H
ATOM 27248 HN MET H 208 36.959 -45.723 46.271 0.00 0.00
H
ATOM 27249 CA MET H 208 37.353 -43.685 45.921 0.00 0.00
H
ATOM 27250 HA MET H 208 36.867 -42.867 45.409 0.00 0.00
H
ATOM 27251 CB MET H 208 37.931 -43.364 47.273 0.00 0.00
H
ATOM 27252 HB1 MET H 208 37.127 -43.094 47.991 0.00 0.00
H
ATOM 27253 HB2 MET H 208 38.421 -44.284 47.657 0.00 0.00
H
ATOM 27254 CG MET H 208 39.015 -42.286 47.319 0.00 0.00
H
ATOM 27255 HG1 MET H 208 39.877 -42.837 46.886 0.00 0.00
H
ATOM 27256 HG2 MET H 208 38.826 -41.405 46.669 0.00 0.00
H
ATOM 27257 SD MET H 208 39.464 -41.652 48.926 0.00 0.00
H
ATOM 27258 CE MET H 208 40.122 -43.183 49.722 0.00 0.00
H
ATOM 27259 HE1 MET H 208 40.630 -43.802 48.952 0.00 0.00
H
ATOM 27260 HE2 MET H 208 40.828 -43.002 50.561 0.00 0.00
H
ATOM 27261 HE3 MET H 208 39.233 -43.759 50.056 0.00 0.00
H
ATOM 27262 C MET H 208 38.527 -43.952 44.950 0.00 0.00
H
ATOM 27263 O MET H 208 39.021 -45.080 44.823 0.00 0.00
H
ATOM 27264 N GLY H 209 38.986 -42.961 44.211 0.00 0.00
H
ATOM 27265 HN GLY H 209 38.599 -42.058 44.384 0.00 0.00
H
ATOM 27266 CA GLY H 209 40.130 -43.067 43.247 0.00 0.00
H
ATOM 27267 HA1 GLY H 209 39.966 -43.975 42.686 0.00 0.00
H
ATOM 27268 HA2 GLY H 209 41.082 -43.070 43.757 0.00 0.00
H
ATOM 27269 C GLY H 209 40.125 -41.909 42.318 0.00 0.00
H

ATOM 27270 O   GLY H 209   39.611 -40.862 42.620 0.00 0.00
H
ATOM 27271 N   HSE H 210   40.613 -42.082 41.079 0.00 0.00
H
ATOM 27272 HN HSE H 210   40.996 -42.978 40.870 0.00 0.00
H
ATOM 27273 CA HSE H 210   40.592 -41.052 39.978 0.00 0.00
H
ATOM 27274 HA HSE H 210   40.715 -40.064 40.398 0.00 0.00
H
ATOM 27275 CB HSE H 210   41.508 -41.357 38.774 0.00 0.00
H
ATOM 27276 HB1 HSE H 210   42.419 -41.893 39.115 0.00 0.00
H
ATOM 27277 HB2 HSE H 210   40.983 -41.947 37.993 0.00 0.00
H
ATOM 27278 ND1 HSE H 210   41.456 -39.566 37.017 0.00 0.00
H
ATOM 27279 CG HSE H 210   41.958 -40.058 38.176 0.00 0.00
H
ATOM 27280 CE1 HSE H 210   42.181 -38.561 36.729 0.00 0.00
H
ATOM 27281 HE1 HSE H 210   42.044 -37.949 35.837 0.00 0.00
H
ATOM 27282 NE2 HSE H 210   43.154 -38.304 37.672 0.00 0.00
H
ATOM 27283 HE2 HSE H 210   43.866 -37.605 37.727 0.00 0.00
H
ATOM 27284 CD2 HSE H 210   42.983 -39.286 38.654 0.00 0.00
H
ATOM 27285 HD2 HSE H 210   43.625 -39.453 39.510 0.00 0.00
H
ATOM 27286 C   HSE H 210   39.152 -41.027 39.273 0.00 0.00
H
ATOM 27287 O   HSE H 210   38.470 -42.002 39.048 0.00 0.00
H
ATOM 27288 N   LYS H 211   38.697 -39.785 38.909 0.00 0.00
H
ATOM 27289 HN LYS H 211   39.197 -38.957 39.151 0.00 0.00
H
ATOM 27290 CA LYS H 211   37.425 -39.634 38.275 0.00 0.00
H
ATOM 27291 HA LYS H 211   36.686 -39.881 39.022 0.00 0.00
H
ATOM 27292 CB LYS H 211   37.273 -38.110 37.941 0.00 0.00
H
ATOM 27293 HB1 LYS H 211   37.976 -37.878 37.112 0.00 0.00
H
ATOM 27294 HB2 LYS H 211   36.189 -38.028 37.714 0.00 0.00
H
ATOM 27295 CG LYS H 211   37.615 -37.062 39.083 0.00 0.00
H
ATOM 27296 HG1 LYS H 211   37.240 -37.476 40.044 0.00 0.00
H

ATOM 27297 HG2 LYS H 211 38.714 -36.913 39.132 0.00 0.00
H
ATOM 27298 CD LYS H 211 37.103 -35.651 38.872 0.00 0.00
H
ATOM 27299 HD1 LYS H 211 37.461 -35.309 37.878 0.00 0.00
H
ATOM 27300 HD2 LYS H 211 36.009 -35.566 39.047 0.00 0.00
H
ATOM 27301 CE LYS H 211 37.783 -34.750 39.878 0.00 0.00
H
ATOM 27302 HE1 LYS H 211 38.797 -35.155 40.084 0.00 0.00
H
ATOM 27303 HE2 LYS H 211 37.883 -33.688 39.567 0.00 0.00
H
ATOM 27304 NZ LYS H 211 37.091 -34.694 41.189 0.00 0.00
H
ATOM 27305 HZ1 LYS H 211 37.698 -34.133 41.820 0.00 0.00
H
ATOM 27306 HZ2 LYS H 211 36.165 -34.240 41.057 0.00 0.00
H
ATOM 27307 HZ3 LYS H 211 36.888 -35.632 41.591 0.00 0.00
H
ATOM 27308 C LYS H 211 37.127 -40.365 37.002 0.00 0.00
H
ATOM 27309 O LYS H 211 36.131 -40.982 36.898 0.00 0.00
H
ATOM 27310 N LYS H 212 38.134 -40.393 36.083 0.00 0.00
H
ATOM 27311 HN LYS H 212 38.997 -39.912 36.219 0.00 0.00
H
ATOM 27312 CA LYS H 212 38.088 -41.073 34.821 0.00 0.00
H
ATOM 27313 HA LYS H 212 37.155 -40.785 34.360 0.00 0.00
H
ATOM 27314 CB LYS H 212 39.287 -40.781 33.919 0.00 0.00
H
ATOM 27315 HB1 LYS H 212 39.233 -39.675 33.828 0.00 0.00
H
ATOM 27316 HB2 LYS H 212 40.218 -40.989 34.488 0.00 0.00
H
ATOM 27317 CG LYS H 212 39.277 -41.171 32.457 0.00 0.00
H
ATOM 27318 HG1 LYS H 212 39.276 -42.282 32.445 0.00 0.00
H
ATOM 27319 HG2 LYS H 212 38.301 -40.891 32.006 0.00 0.00
H
ATOM 27320 CD LYS H 212 40.310 -40.442 31.654 0.00 0.00
H
ATOM 27321 HD1 LYS H 212 40.527 -39.415 32.018 0.00 0.00
H
ATOM 27322 HD2 LYS H 212 41.235 -41.026 31.845 0.00 0.00
H
ATOM 27323 CE LYS H 212 39.983 -40.430 30.174 0.00 0.00
H

ATOM 27324 HE1 LYS H 212   39.815 -41.377 29.617 0.00 0.00
H
ATOM 27325 HE2 LYS H 212   39.105 -39.786 29.954 0.00 0.00
H
ATOM 27326 NZ LYS H 212   41.077 -39.741 29.459 0.00 0.00
H
ATOM 27327 HZ1 LYS H 212   40.798 -39.400 28.517 0.00 0.00
H
ATOM 27328 HZ2 LYS H 212   41.473 -38.986 30.054 0.00 0.00
H
ATOM 27329 HZ3 LYS H 212   41.833 -40.430 29.275 0.00 0.00
H
ATOM 27330 C   LYS H 212   38.036 -42.596 34.979 0.00 0.00
H
ATOM 27331 O   LYS H 212   37.289 -43.317 34.387 0.00 0.00
H
ATOM 27332 N   ASP H 213   38.802 -43.100 35.872 0.00 0.00
H
ATOM 27333 HN ASP H 213   39.309 -42.451 36.434 0.00 0.00
H
ATOM 27334 CA ASP H 213   38.945 -44.469 36.145 0.00 0.00
H
ATOM 27335 HA ASP H 213   39.075 -44.870 35.151 0.00 0.00
H
ATOM 27336 CB ASP H 213   40.284 -44.810 36.983 0.00 0.00
H
ATOM 27337 HB1 ASP H 213   40.332 -44.249 37.941 0.00 0.00
H
ATOM 27338 HB2 ASP H 213   40.213 -45.879 37.276 0.00 0.00
H
ATOM 27339 CG ASP H 213   41.546 -44.716 36.085 0.00 0.00
H
ATOM 27340 OD1 ASP H 213   41.898 -43.559 35.760 0.00 0.00
H
ATOM 27341 OD2 ASP H 213   42.193 -45.761 35.838 0.00 0.00
H
ATOM 27342 C   ASP H 213   37.716 -45.136 36.788 0.00 0.00
H
ATOM 27343 O   ASP H 213   37.387 -46.256 36.441 0.00 0.00
H
ATOM 27344 N   ILE H 214   36.931 -44.387 37.652 0.00 0.00
H
ATOM 27345 HN ILE H 214   37.166 -43.429 37.796 0.00 0.00
H
ATOM 27346 CA ILE H 214   35.560 -44.691 38.166 0.00 0.00
H
ATOM 27347 HA ILE H 214   35.656 -45.699 38.541 0.00 0.00
H
ATOM 27348 CB ILE H 214   35.231 -43.837 39.337 0.00 0.00
H
ATOM 27349 HB ILE H 214   35.328 -42.784 38.997 0.00 0.00
H
ATOM 27350 CG2 ILE H 214   33.756 -44.125 39.788 0.00 0.00
H

```
ATOM  27351 HG21 ILE H 214      33.468 -43.417  40.595  0.00  0.00
H
ATOM  27352 HG22 ILE H 214      33.083 -43.908  38.931  0.00  0.00
H
ATOM  27353 HG23 ILE H 214      33.588 -45.198  40.022  0.00  0.00
H
ATOM  27354  CG1 ILE H 214      36.287 -43.880  40.461  0.00  0.00
H
ATOM  27355 HG11 ILE H 214      36.098 -44.826  41.012  0.00  0.00
H
ATOM  27356 HG12 ILE H 214      37.347 -43.986  40.146  0.00  0.00
H
ATOM  27357  CD  ILE H 214      36.248 -42.620  41.408  0.00  0.00
H
ATOM  27358  HD1 ILE H 214      36.551 -42.925  42.433  0.00  0.00
H
ATOM  27359  HD2 ILE H 214      36.945 -41.861  40.993  0.00  0.00
H
ATOM  27360  HD3 ILE H 214      35.200 -42.253  41.424  0.00  0.00
H
ATOM  27361  C   ILE H 214      34.524 -44.834  37.092  0.00  0.00
H
ATOM  27362  O   ILE H 214      33.702 -45.753  37.057  0.00  0.00
H
ATOM  27363  N   LYS H 215      34.638 -43.931  36.145  0.00  0.00
H
ATOM  27364  HN  LYS H 215      35.356 -43.239  36.150  0.00  0.00
H
ATOM  27365  CA  LYS H 215      33.776 -43.958  35.048  0.00  0.00
H
ATOM  27366  HA  LYS H 215      32.795 -44.208  35.425  0.00  0.00
H
ATOM  27367  CB  LYS H 215      33.796 -42.610  34.287  0.00  0.00
H
ATOM  27368  HB1 LYS H 215      34.100 -41.757  34.931  0.00  0.00
H
ATOM  27369  HB2 LYS H 215      34.617 -42.648  33.540  0.00  0.00
H
ATOM  27370  CG  LYS H 215      32.582 -42.284  33.387  0.00  0.00
H
ATOM  27371  HG1 LYS H 215      32.734 -41.251  33.008  0.00  0.00
H
ATOM  27372  HG2 LYS H 215      32.529 -42.892  32.458  0.00  0.00
H
ATOM  27373  CD  LYS H 215      31.179 -42.372  34.144  0.00  0.00
H
ATOM  27374  HD1 LYS H 215      30.520 -42.171  33.272  0.00  0.00
H
ATOM  27375  HD2 LYS H 215      31.048 -43.389  34.571  0.00  0.00
H
ATOM  27376  CE  LYS H 215      31.009 -41.256  35.201  0.00  0.00
H
ATOM  27377  HE1 LYS H 215      31.771 -41.495  35.973  0.00  0.00
H
```

ATOM 27378 HE2 LYS H 215   31.129 -40.214 34.835 0.00 0.00
H
ATOM 27379 NZ LYS H 215   29.642 -41.479 35.859 0.00 0.00
H
ATOM 27380 HZ1 LYS H 215   28.876 -40.943 35.402 0.00 0.00
H
ATOM 27381 HZ2 LYS H 215   29.378 -42.484 35.812 0.00 0.00
H
ATOM 27382 HZ3 LYS H 215   29.856 -41.118 36.811 0.00 0.00
H
ATOM 27383 C   LYS H 215   34.005 -45.113 34.093 0.00 0.00
H
ATOM 27384 O   LYS H 215   33.061 -45.871 33.713 0.00 0.00
H
ATOM 27385 N   ARG H 216   35.246 -45.432 33.810 0.00 0.00
H
ATOM 27386 HN ARG H 216   35.954 -44.742 33.939 0.00 0.00
H
ATOM 27387 CA ARG H 216   35.628 -46.660 33.116 0.00 0.00
H
ATOM 27388 HA ARG H 216   35.058 -46.653 32.199 0.00 0.00
H
ATOM 27389 CB ARG H 216   37.114 -46.475 32.702 0.00 0.00
H
ATOM 27390 HB1 ARG H 216   37.871 -46.392 33.511 0.00 0.00
H
ATOM 27391 HB2 ARG H 216   37.248 -47.382 32.074 0.00 0.00
H
ATOM 27392 CG ARG H 216   37.294 -45.370 31.658 0.00 0.00
H
ATOM 27393 HG1 ARG H 216   36.616 -45.559 30.798 0.00 0.00
H
ATOM 27394 HG2 ARG H 216   37.092 -44.320 31.958 0.00 0.00
H
ATOM 27395 CD ARG H 216   38.735 -45.309 31.142 0.00 0.00
H
ATOM 27396 HD1 ARG H 216   39.446 -44.813 31.837 0.00 0.00
H
ATOM 27397 HD2 ARG H 216   39.131 -46.288 30.797 0.00 0.00
H
ATOM 27398 NE ARG H 216   38.629 -44.603 29.902 0.00 0.00
H
ATOM 27399 HE ARG H 216   37.727 -44.449 29.499 0.00 0.00
H
ATOM 27400 CZ ARG H 216   39.706 -44.249 29.181 0.00 0.00
H
ATOM 27401 NH1 ARG H 216   40.896 -44.220 29.739 0.00 0.00
H
ATOM 27402 HH11 ARG H 216   41.564 -43.875 29.079 0.00 0.00
H
ATOM 27403 HH12 ARG H 216   40.970 -44.468 30.705 0.00 0.00
H
ATOM 27404 NH2 ARG H 216   39.626 -44.004 27.863 0.00 0.00
H

ATOM 27405 HH21 ARG H 216   40.488 −43.840 27.384 0.00 0.00
H
ATOM 27406 HH22 ARG H 216   38.852 −44.354 27.335 0.00 0.00
H
ATOM 27407 C   ARG H 216   35.222 −47.982 33.714 0.00 0.00
H
ATOM 27408 O   ARG H 216   34.975 −49.025 33.117 0.00 0.00
H
ATOM 27409 N   PHE H 217   35.148 −48.019 35.071 0.00 0.00
H
ATOM 27410 HN PHE H 217   35.722 −47.365 35.557 0.00 0.00
H
ATOM 27411 CA PHE H 217   34.683 −49.161 35.820 0.00 0.00
H
ATOM 27412 HA PHE H 217   35.036 −50.097 35.414 0.00 0.00
H
ATOM 27413 CB PHE H 217   35.284 −49.127 37.264 0.00 0.00
H
ATOM 27414 HB1 PHE H 217   36.372 −49.337 37.178 0.00 0.00
H
ATOM 27415 HB2 PHE H 217   35.195 −48.074 37.606 0.00 0.00
H
ATOM 27416 CG PHE H 217   34.721 −50.097 38.222 0.00 0.00
H
ATOM 27417 CD1 PHE H 217   35.008 −51.429 38.024 0.00 0.00
H
ATOM 27418 HD1 PHE H 217   35.600 −51.749 37.179 0.00 0.00
H
ATOM 27419 CE1 PHE H 217   34.274 −52.400 38.738 0.00 0.00
H
ATOM 27420 HE1 PHE H 217   34.259 −53.462 38.541 0.00 0.00
H
ATOM 27421 CZ PHE H 217   33.426 −51.968 39.816 0.00 0.00
H
ATOM 27422 HZ PHE H 217   32.818 −52.704 40.320 0.00 0.00
H
ATOM 27423 CD2 PHE H 217   33.968 −49.647 39.301 0.00 0.00
H
ATOM 27424 HD2 PHE H 217   33.693 −48.603 39.275 0.00 0.00
H
ATOM 27425 CE2 PHE H 217   33.262 −50.622 40.112 0.00 0.00
H
ATOM 27426 HE2 PHE H 217   32.608 −50.252 40.888 0.00 0.00
H
ATOM 27427 C   PHE H 217   33.153 −49.256 35.794 0.00 0.00
H
ATOM 27428 O   PHE H 217   32.589 −50.318 35.431 0.00 0.00
H
ATOM 27429 N   GLU H 218   32.416 −48.167 35.865 0.00 0.00
H
ATOM 27430 HN GLU H 218   32.907 −47.382 36.235 0.00 0.00
H
ATOM 27431 CA GLU H 218   30.944 −47.967 35.568 0.00 0.00
H

ATOM 27432 HA GLU H 218 30.295 -48.579 36.177 0.00 0.00
H
ATOM 27433 CB GLU H 218 30.462 -46.518 35.903 0.00 0.00
H
ATOM 27434 HB1 GLU H 218 31.053 -46.255 36.806 0.00 0.00
H
ATOM 27435 HB2 GLU H 218 30.885 -45.869 35.107 0.00 0.00
H
ATOM 27436 CG GLU H 218 28.926 -46.256 35.944 0.00 0.00
H
ATOM 27437 HG1 GLU H 218 28.425 -46.895 35.186 0.00 0.00
H
ATOM 27438 HG2 GLU H 218 28.529 -46.496 36.954 0.00 0.00
H
ATOM 27439 CD GLU H 218 28.691 -44.778 35.735 0.00 0.00
H
ATOM 27440 OE1 GLU H 218 29.050 -43.986 36.657 0.00 0.00
H
ATOM 27441 OE2 GLU H 218 28.062 -44.443 34.671 0.00 0.00
H
ATOM 27442 C GLU H 218 30.595 -48.312 34.132 0.00 0.00
H
ATOM 27443 O GLU H 218 29.658 -49.026 33.880 0.00 0.00
H
ATOM 27444 N ASN H 219 31.331 -47.908 33.130 0.00 0.00
H
ATOM 27445 HN ASN H 219 32.121 -47.322 33.293 0.00 0.00
H
ATOM 27446 CA ASN H 219 31.243 -48.268 31.690 0.00 0.00
H
ATOM 27447 HA ASN H 219 30.223 -47.974 31.493 0.00 0.00
H
ATOM 27448 CB ASN H 219 32.309 -47.569 30.872 0.00 0.00
H
ATOM 27449 HB1 ASN H 219 33.283 -47.730 31.382 0.00 0.00
H
ATOM 27450 HB2 ASN H 219 32.227 -47.909 29.817 0.00 0.00
H
ATOM 27451 CG ASN H 219 32.208 -45.997 30.820 0.00 0.00
H
ATOM 27452 OD1 ASN H 219 31.204 -45.419 31.167 0.00 0.00
H
ATOM 27453 ND2 ASN H 219 33.296 -45.456 30.244 0.00 0.00
H
ATOM 27454 HD21 ASN H 219 33.296 -44.468 30.088 0.00 0.00
H
ATOM 27455 HD22 ASN H 219 34.071 -45.950 29.849 0.00 0.00
H
ATOM 27456 C ASN H 219 31.395 -49.729 31.433 0.00 0.00
H
ATOM 27457 O ASN H 219 30.606 -50.350 30.787 0.00 0.00
H
ATOM 27458 N GLU H 220 32.419 -50.411 32.002 0.00 0.00
H

ATOM 27459 HN GLU H 220 33.002 -49.884 32.615 0.00 0.00
H
ATOM 27460 CA GLU H 220 32.528 -51.854 31.859 0.00 0.00
H
ATOM 27461 HA GLU H 220 32.531 -52.085 30.804 0.00 0.00
H
ATOM 27462 CB GLU H 220 34.030 -52.211 32.340 0.00 0.00
H
ATOM 27463 HB1 GLU H 220 34.674 -51.647 31.631 0.00 0.00
H
ATOM 27464 HB2 GLU H 220 34.132 -51.838 33.381 0.00 0.00
H
ATOM 27465 CG GLU H 220 34.329 -53.745 32.236 0.00 0.00
H
ATOM 27466 HG1 GLU H 220 33.783 -54.341 32.998 0.00 0.00
H
ATOM 27467 HG2 GLU H 220 33.866 -53.935 31.244 0.00 0.00
H
ATOM 27468 CD GLU H 220 35.751 -54.101 32.232 0.00 0.00
H
ATOM 27469 OE1 GLU H 220 35.998 -55.329 32.016 0.00 0.00
H
ATOM 27470 OE2 GLU H 220 36.592 -53.254 32.579 0.00 0.00
H
ATOM 27471 C GLU H 220 31.497 -52.623 32.634 0.00 0.00
H
ATOM 27472 O GLU H 220 30.980 -53.544 32.051 0.00 0.00
H
ATOM 27473 N GLY H 221 31.187 -52.230 33.880 0.00 0.00
H
ATOM 27474 HN GLY H 221 31.628 -51.422 34.263 0.00 0.00
H
ATOM 27475 CA GLY H 221 30.326 -53.040 34.739 0.00 0.00
H
ATOM 27476 HA1 GLY H 221 30.680 -52.846 35.741 0.00 0.00
H
ATOM 27477 HA2 GLY H 221 30.417 -54.104 34.580 0.00 0.00
H
ATOM 27478 C GLY H 221 28.876 -52.833 34.874 0.00 0.00
H
ATOM 27479 O GLY H 221 28.203 -53.765 35.256 0.00 0.00
H
ATOM 27480 N MET H 222 28.286 -51.580 34.617 0.00 0.00
H
ATOM 27481 HN MET H 222 28.720 -50.758 34.256 0.00 0.00
H
ATOM 27482 CA MET H 222 26.897 -51.502 34.675 0.00 0.00
H
ATOM 27483 HA MET H 222 26.568 -52.150 35.474 0.00 0.00
H
ATOM 27484 CB MET H 222 26.513 -50.046 34.992 0.00 0.00
H
ATOM 27485 HB1 MET H 222 26.787 -49.387 34.140 0.00 0.00
H

ATOM 27486 HB2 MET H 222   25.404 -50.010 34.945 0.00 0.00
H
ATOM 27487 CG MET H 222   27.177 -49.436 36.276 0.00 0.00
H
ATOM 27488 HG1 MET H 222   28.227 -49.164 36.033 0.00 0.00
H
ATOM 27489 HG2 MET H 222   26.678 -48.464 36.476 0.00 0.00
H
ATOM 27490 SD MET H 222   26.881 -50.391 37.844 0.00 0.00
H
ATOM 27491 CE MET H 222   27.602 -49.206 39.035 0.00 0.00
H
ATOM 27492 HE1 MET H 222   26.860 -48.428 39.315 0.00 0.00
H
ATOM 27493 HE2 MET H 222   27.733 -49.795 39.969 0.00 0.00
H
ATOM 27494 HE3 MET H 222   28.603 -48.842 38.720 0.00 0.00
H
ATOM 27495 C   MET H 222   26.366 -51.950 33.306 0.00 0.00
H
ATOM 27496 O   MET H 222   25.130 -51.915 33.043 0.00 0.00
H
ATOM 27497 N   THR I 35   -21.739 16.946   9.483 0.00 0.00
I
ATOM 27498 HT1 THR I 35   -22.394 16.219   9.130 0.00 0.00
I
ATOM 27499 HT2 THR I 35   -22.022 17.880   9.123 0.00 0.00
I
ATOM 27500 HT3 THR I 35   -21.991 16.934 10.492 0.00 0.00
I
ATOM 27501 CA THR I 35   -20.238 16.616   9.308 0.00 0.00
I
ATOM 27502 HA THR I 35   -19.805 16.759 10.287 0.00 0.00
I
ATOM 27503 CB THR I 35   -20.043 15.125   9.006 0.00 0.00
I
ATOM 27504 HB THR I 35   -18.969 14.920   8.809 0.00 0.00
I
ATOM 27505 OG1 THR I 35   -20.793 14.682   7.859 0.00 0.00
I
ATOM 27506 HG1 THR I 35   -20.628 13.737   7.878 0.00 0.00
I
ATOM 27507 CG2 THR I 35   -20.515 14.257 10.164 0.00 0.00
I
ATOM 27508 HG21 THR I 35   -21.626 14.288 10.178 0.00 0.00
I
ATOM 27509 HG22 THR I 35   -20.275 13.199   9.923 0.00 0.00
I
ATOM 27510 HG23 THR I 35   -19.926 14.494 11.075 0.00 0.00
I
ATOM 27511 C   THR I 35   -19.367 17.464   8.346 0.00 0.00
I
ATOM 27512 O   THR I 35   -19.656 17.473   7.188 0.00 0.00
I

ATOM	27513	N	PRO	I	36	−18.376	18.199	8.810	0.00	0.00	I
ATOM	27514	CD	PRO	I	36	−18.303	18.695	10.168	0.00	0.00	I
ATOM	27515	HD1	PRO	I	36	−19.186	19.336	10.378	0.00	0.00	I
ATOM	27516	HD2	PRO	I	36	−18.323	17.840	10.877	0.00	0.00	I
ATOM	27517	CA	PRO	I	36	−17.327	18.806	8.015	0.00	0.00	I
ATOM	27518	HA	PRO	I	36	−17.916	19.631	7.643	0.00	0.00	I
ATOM	27519	CB	PRO	I	36	−16.202	19.258	9.027	0.00	0.00	I
ATOM	27520	HB1	PRO	I	36	−15.581	20.072	8.595	0.00	0.00	I
ATOM	27521	HB2	PRO	I	36	−15.636	18.408	9.464	0.00	0.00	I
ATOM	27522	CG	PRO	I	36	−17.143	19.713	10.141	0.00	0.00	I
ATOM	27523	HG1	PRO	I	36	−17.591	20.699	9.891	0.00	0.00	I
ATOM	27524	HG2	PRO	I	36	−16.555	19.891	11.066	0.00	0.00	I
ATOM	27525	C	PRO	I	36	−16.772	18.093	6.842	0.00	0.00	I
ATOM	27526	O	PRO	I	36	−16.717	18.755	5.731	0.00	0.00	I
ATOM	27527	N	PRO	I	37	−16.417	16.798	6.867	0.00	0.00	I
ATOM	27528	CD	PRO	I	37	−16.115	16.006	8.079	0.00	0.00	I
ATOM	27529	HD1	PRO	I	37	−15.125	16.319	8.475	0.00	0.00	I
ATOM	27530	HD2	PRO	I	37	−16.850	16.201	8.889	0.00	0.00	I
ATOM	27531	CA	PRO	I	37	−16.041	16.049	5.688	0.00	0.00	I
ATOM	27532	HA	PRO	I	37	−15.329	16.581	5.073	0.00	0.00	I
ATOM	27533	CB	PRO	I	37	−15.509	14.690	6.208	0.00	0.00	I
ATOM	27534	HB1	PRO	I	37	−14.417	14.775	6.394	0.00	0.00	I
ATOM	27535	HB2	PRO	I	37	−15.732	13.786	5.603	0.00	0.00	I
ATOM	27536	CG	PRO	I	37	−16.088	14.599	7.620	0.00	0.00	I
ATOM	27537	HG1	PRO	I	37	−15.555	13.951	8.349	0.00	0.00	I
ATOM	27538	HG2	PRO	I	37	−17.043	14.107	7.340	0.00	0.00	I
ATOM	27539	C	PRO	I	37	−17.157	15.956	4.728	0.00	0.00	I

ATOM 27540 O   PRO I 37   -16.914 16.203   3.552 0.00 0.00
I
ATOM 27541 N   GLN I 38   -18.444 15.633   4.986 0.00 0.00
I
ATOM 27542 HN GLN I 38   -18.715 15.174   5.829 0.00 0.00
I
ATOM 27543 CA GLN I 38   -19.506 15.693   3.999 0.00 0.00
I
ATOM 27544 HA GLN I 38   -19.334 15.054   3.145 0.00 0.00
I
ATOM 27545 CB GLN I 38   -20.790 15.290   4.774 0.00 0.00
I
ATOM 27546 HB1 GLN I 38   -20.596 14.400   5.410 0.00 0.00
I
ATOM 27547 HB2 GLN I 38   -21.257 16.064   5.420 0.00 0.00
I
ATOM 27548 CG GLN I 38   -21.932 14.879   3.816 0.00 0.00
I
ATOM 27549 HG1 GLN I 38   -22.155 15.642   3.040 0.00 0.00
I
ATOM 27550 HG2 GLN I 38   -21.478 14.025   3.270 0.00 0.00
I
ATOM 27551 CD GLN I 38   -23.227 14.453   4.531 0.00 0.00
I
ATOM 27552 OE1 GLN I 38   -23.987 15.200   5.068 0.00 0.00
I
ATOM 27553 NE2 GLN I 38   -23.364 13.137   4.609 0.00 0.00
I
ATOM 27554 HE21 GLN I 38   -23.911 12.805   5.377 0.00 0.00
I
ATOM 27555 HE22 GLN I 38   -22.545 12.626   4.348 0.00 0.00
I
ATOM 27556 C   GLN I 38   -19.639 17.055   3.467 0.00 0.00
I
ATOM 27557 O   GLN I 38   -19.697 17.185   2.221 0.00 0.00
I
ATOM 27558 N   VAL I 39   -19.680 18.199   4.233 0.00 0.00
I
ATOM 27559 HN VAL I 39   -19.582 18.065   5.216 0.00 0.00
I
ATOM 27560 CA VAL I 39   -19.693 19.526   3.622 0.00 0.00
I
ATOM 27561 HA VAL I 39   -20.556 19.560   2.973 0.00 0.00
I
ATOM 27562 CB VAL I 39   -20.004 20.568   4.671 0.00 0.00
I
ATOM 27563 HB VAL I 39   -19.179 20.726   5.398 0.00 0.00
I
ATOM 27564 CG1 VAL I 39   -20.392 21.929   3.976 0.00 0.00
I
ATOM 27565 HG11 VAL I 39   -20.569 22.543   4.885 0.00 0.00
I
ATOM 27566 HG12 VAL I 39   -19.529 22.261   3.360 0.00 0.00
I

ATOM 27567 HG13 VAL I 39 -21.344 21.758 3.430 0.00 0.00
I
ATOM 27568 CG2 VAL I 39 -21.169 20.174 5.546 0.00 0.00
I
ATOM 27569 HG21 VAL I 39 -21.551 20.947 6.247 0.00 0.00
I
ATOM 27570 HG22 VAL I 39 -22.004 19.747 4.950 0.00 0.00
I
ATOM 27571 HG23 VAL I 39 -20.789 19.403 6.249 0.00 0.00
I
ATOM 27572 C VAL I 39 -18.467 19.833 2.751 0.00 0.00
I
ATOM 27573 O VAL I 39 -18.608 20.465 1.746 0.00 0.00
I
ATOM 27574 N LEU I 40 -17.235 19.399 3.118 0.00 0.00
I
ATOM 27575 HN LEU I 40 -16.968 19.023 4.002 0.00 0.00
I
ATOM 27576 CA LEU I 40 -16.148 19.521 2.195 0.00 0.00
I
ATOM 27577 HA LEU I 40 -16.246 20.559 1.915 0.00 0.00
I
ATOM 27578 CB LEU I 40 -14.798 19.224 2.875 0.00 0.00
I
ATOM 27579 HB1 LEU I 40 -14.867 19.743 3.856 0.00 0.00
I
ATOM 27580 HB2 LEU I 40 -14.850 18.142 3.121 0.00 0.00
I
ATOM 27581 CG LEU I 40 -13.452 19.743 2.251 0.00 0.00
I
ATOM 27582 HG LEU I 40 -13.468 19.504 1.166 0.00 0.00
I
ATOM 27583 CD1 LEU I 40 -13.203 21.209 2.464 0.00 0.00
I
ATOM 27584 HD11 LEU I 40 -13.784 21.907 1.824 0.00 0.00
I
ATOM 27585 HD12 LEU I 40 -13.410 21.398 3.539 0.00 0.00
I
ATOM 27586 HD13 LEU I 40 -12.117 21.379 2.301 0.00 0.00
I
ATOM 27587 CD2 LEU I 40 -12.308 19.012 2.979 0.00 0.00
I
ATOM 27588 HD21 LEU I 40 -12.445 17.911 2.923 0.00 0.00
I
ATOM 27589 HD22 LEU I 40 -11.349 19.143 2.433 0.00 0.00
I
ATOM 27590 HD23 LEU I 40 -12.023 19.420 3.972 0.00 0.00
I
ATOM 27591 C LEU I 40 -16.294 18.722 0.879 0.00 0.00
I
ATOM 27592 O LEU I 40 -16.096 19.318 -0.156 0.00 0.00
I
ATOM 27593 N ALA I 41 -16.814 17.421 0.911 0.00 0.00
I

ATOM 27594 HN ALA I 41 -16.981 17.163 1.860 0.00 0.00
I
ATOM 27595 CA ALA I 41 -17.024 16.523 -0.254 0.00 0.00
I
ATOM 27596 HA ALA I 41 -16.105 16.513 -0.822 0.00 0.00
I
ATOM 27597 CB ALA I 41 -17.283 15.070 0.282 0.00 0.00
I
ATOM 27598 HB1 ALA I 41 -16.480 14.963 1.041 0.00 0.00
I
ATOM 27599 HB2 ALA I 41 -18.262 14.797 0.732 0.00 0.00
I
ATOM 27600 HB3 ALA I 41 -17.001 14.524 -0.644 0.00 0.00
I
ATOM 27601 C ALA I 41 -18.081 17.035 -1.168 0.00 0.00
I
ATOM 27602 O ALA I 41 -17.964 17.096 -2.408 0.00 0.00
I
ATOM 27603 N ILE I 42 -19.189 17.542 -0.606 0.00 0.00
I
ATOM 27604 HN ILE I 42 -19.202 17.492 0.390 0.00 0.00
I
ATOM 27605 CA ILE I 42 -20.253 18.346 -1.337 0.00 0.00
I
ATOM 27606 HA ILE I 42 -20.554 17.833 -2.238 0.00 0.00
I
ATOM 27607 CB ILE I 42 -21.420 18.664 -0.463 0.00 0.00
I
ATOM 27608 HB ILE I 42 -21.097 19.253 0.422 0.00 0.00
I
ATOM 27609 CG2 ILE I 42 -22.381 19.692 -1.175 0.00 0.00
I
ATOM 27610 HG21 ILE I 42 -21.857 20.649 -1.386 0.00 0.00
I
ATOM 27611 HG22 ILE I 42 -22.758 19.204 -2.099 0.00 0.00
I
ATOM 27612 HG23 ILE I 42 -23.294 19.826 -0.558 0.00 0.00
I
ATOM 27613 CG1 ILE I 42 -22.137 17.429 0.087 0.00 0.00
I
ATOM 27614 HG11 ILE I 42 -22.613 16.819 -0.711 0.00 0.00
I
ATOM 27615 HG12 ILE I 42 -21.356 16.847 0.619 0.00 0.00
I
ATOM 27616 CD ILE I 42 -23.229 17.764 1.156 0.00 0.00
I
ATOM 27617 HD1 ILE I 42 -22.754 18.264 2.027 0.00 0.00
I
ATOM 27618 HD2 ILE I 42 -24.125 18.304 0.781 0.00 0.00
I
ATOM 27619 HD3 ILE I 42 -23.648 16.805 1.527 0.00 0.00
I
ATOM 27620 C ILE I 42 -19.644 19.700 -1.842 0.00 0.00
I

ATOM	27621	O	ILE	I	42	−19.926	19.983	−3.034	0.00	0.00	I
ATOM	27622	N	GLY	I	43	−18.753	20.346	−1.076	0.00	0.00	I
ATOM	27623	HN	GLY	I	43	−18.778	20.132	−0.103	0.00	0.00	I
ATOM	27624	CA	GLY	I	43	−17.999	21.521	−1.453	0.00	0.00	I
ATOM	27625	HA1	GLY	I	43	−17.380	21.928	−0.668	0.00	0.00	I
ATOM	27626	HA2	GLY	I	43	−18.836	22.197	−1.554	0.00	0.00	I
ATOM	27627	C	GLY	I	43	−17.174	21.378	−2.720	0.00	0.00	I
ATOM	27628	O	GLY	I	43	−17.240	22.103	−3.653	0.00	0.00	I
ATOM	27629	N	PHE	I	44	−16.405	20.266	−2.796	0.00	0.00	I
ATOM	27630	HN	PHE	I	44	−16.317	19.570	−2.088	0.00	0.00	I
ATOM	27631	CA	PHE	I	44	−15.652	19.898	−3.993	0.00	0.00	I
ATOM	27632	HA	PHE	I	44	−14.936	20.666	−4.248	0.00	0.00	I
ATOM	27633	CB	PHE	I	44	−14.814	18.591	−3.829	0.00	0.00	I
ATOM	27634	HB1	PHE	I	44	−15.497	17.813	−3.425	0.00	0.00	I
ATOM	27635	HB2	PHE	I	44	−14.356	18.259	−4.785	0.00	0.00	I
ATOM	27636	CG	PHE	I	44	−13.636	18.834	−2.947	0.00	0.00	I
ATOM	27637	CD1	PHE	I	44	−12.782	19.915	−3.177	0.00	0.00	I
ATOM	27638	HD1	PHE	I	44	−12.956	20.635	−3.963	0.00	0.00	I
ATOM	27639	CE1	PHE	I	44	−11.812	20.333	−2.329	0.00	0.00	I
ATOM	27640	HE1	PHE	I	44	−11.096	21.124	−2.498	0.00	0.00	I
ATOM	27641	CZ	PHE	I	44	−11.647	19.545	−1.106	0.00	0.00	I
ATOM	27642	HZ	PHE	I	44	−10.857	19.860	−0.440	0.00	0.00	I
ATOM	27643	CD2	PHE	I	44	−13.441	18.078	−1.806	0.00	0.00	I
ATOM	27644	HD2	PHE	I	44	−14.137	17.320	−1.478	0.00	0.00	I
ATOM	27645	CE2	PHE	I	44	−12.460	18.420	−0.877	0.00	0.00	I
ATOM	27646	HE2	PHE	I	44	−12.162	17.797	−0.047	0.00	0.00	I
ATOM	27647	C	PHE	I	44	−16.561	19.736	−5.229	0.00	0.00	I

ATOM 27648 O PHE I 44 −16.223 20.336 −6.263 0.00 0.00
I
ATOM 27649 N PHE I 45 −17.706 19.041 −5.069 0.00 0.00
I
ATOM 27650 HN PHE I 45 −17.865 18.646 −4.168 0.00 0.00
I
ATOM 27651 CA PHE I 45 −18.744 18.815 −6.155 0.00 0.00
I
ATOM 27652 HA PHE I 45 −18.214 18.242 −6.902 0.00 0.00
I
ATOM 27653 CB PHE I 45 −19.909 17.907 −5.629 0.00 0.00
I
ATOM 27654 HB1 PHE I 45 −19.524 17.045 −5.042 0.00 0.00
I
ATOM 27655 HB2 PHE I 45 −20.494 18.542 −4.930 0.00 0.00
I
ATOM 27656 CG PHE I 45 −20.928 17.307 −6.564 0.00 0.00
I
ATOM 27657 CD1 PHE I 45 −20.488 16.052 −7.118 0.00 0.00
I
ATOM 27658 HD1 PHE I 45 −19.531 15.623 −6.860 0.00 0.00
I
ATOM 27659 CE1 PHE I 45 −21.218 15.432 −8.104 0.00 0.00
I
ATOM 27660 HE1 PHE I 45 −20.831 14.469 −8.403 0.00 0.00
I
ATOM 27661 CZ PHE I 45 −22.281 16.135 −8.672 0.00 0.00
I
ATOM 27662 HZ PHE I 45 −22.845 15.614 −9.431 0.00 0.00
I
ATOM 27663 CD2 PHE I 45 −22.003 17.951 −7.101 0.00 0.00
I
ATOM 27664 HD2 PHE I 45 −22.384 18.850 −6.640 0.00 0.00
I
ATOM 27665 CE2 PHE I 45 −22.715 17.387 −8.175 0.00 0.00
I
ATOM 27666 HE2 PHE I 45 −23.577 17.883 −8.595 0.00 0.00
I
ATOM 27667 C PHE I 45 −19.428 20.085 −6.691 0.00 0.00
I
ATOM 27668 O PHE I 45 −19.433 20.268 −7.864 0.00 0.00
I
ATOM 27669 N LEU I 46 −19.775 20.974 −5.767 0.00 0.00
I
ATOM 27670 HN LEU I 46 −19.632 20.746 −4.807 0.00 0.00
I
ATOM 27671 CA LEU I 46 −20.226 22.303 −6.054 0.00 0.00
I
ATOM 27672 HA LEU I 46 −21.135 22.136 −6.614 0.00 0.00
I
ATOM 27673 CB LEU I 46 −20.590 23.122 −4.751 0.00 0.00
I
ATOM 27674 HB1 LEU I 46 −19.703 22.965 −4.100 0.00 0.00
I

ATOM 27675 HB2 LEU I 46   −20.707 24.223 −4.833 0.00 0.00
I
ATOM 27676 CG LEU I 46   −21.861 22.514 −4.082 0.00 0.00
I
ATOM 27677 HG LEU I 46   −21.860 21.405 −4.014 0.00 0.00
I
ATOM 27678 CD1 LEU I 46   −21.998 23.095 −2.694 0.00 0.00
I
ATOM 27679 HD11 LEU I 46   −21.177 22.785 −2.013 0.00 0.00
I
ATOM 27680 HD12 LEU I 46   −22.186 24.190 −2.711 0.00 0.00
I
ATOM 27681 HD13 LEU I 46   −22.839 22.611 −2.153 0.00 0.00
I
ATOM 27682 CD2 LEU I 46   −23.096 22.809 −4.850 0.00 0.00
I
ATOM 27683 HD21 LEU I 46   −23.195 23.888 −5.096 0.00 0.00
I
ATOM 27684 HD22 LEU I 46   −23.076 22.290 −5.832 0.00 0.00
I
ATOM 27685 HD23 LEU I 46   −24.038 22.309 −4.539 0.00 0.00
I
ATOM 27686 C   LEU I 46   −19.246 23.138 −6.804 0.00 0.00
I
ATOM 27687 O   LEU I 46   −19.569 23.935 −7.680 0.00 0.00
I
ATOM 27688 N   THR I 47   −17.938 23.019 −6.462 0.00 0.00
I
ATOM 27689 HN THR I 47   −17.752 22.319 −5.776 0.00 0.00
I
ATOM 27690 CA THR I 47   −16.854 23.736 −7.154 0.00 0.00
I
ATOM 27691 HA THR I 47   −17.217 24.753 −7.155 0.00 0.00
I
ATOM 27692 CB THR I 47   −15.559 23.543 −6.533 0.00 0.00
I
ATOM 27693 HB THR I 47   −15.113 22.526 −6.491 0.00 0.00
I
ATOM 27694 OG1 THR I 47   −15.662 23.868 −5.128 0.00 0.00
I
ATOM 27695 HG1 THR I 47   −16.355 23.279 −4.821 0.00 0.00
I
ATOM 27696 CG2 THR I 47   −14.457 24.562 −7.013 0.00 0.00
I
ATOM 27697 HG21 THR I 47   −14.080 24.411 −8.048 0.00 0.00
I
ATOM 27698 HG22 THR I 47   −14.916 25.571 −6.940 0.00 0.00
I
ATOM 27699 HG23 THR I 47   −13.531 24.575 −6.400 0.00 0.00
I
ATOM 27700 C   THR I 47   −16.628 23.288 −8.529 0.00 0.00
I
ATOM 27701 O   THR I 47   −16.464 24.097 −9.457 0.00 0.00
I

ATOM 27702 N   ILE I 48   -16.716 21.981 -8.687 0.00 0.00
I
ATOM 27703 HN ILE I 48   -16.609 21.390 -7.892 0.00 0.00
I
ATOM 27704 CA ILE I 48   -16.697 21.407 -10.113 0.00 0.00
I
ATOM 27705 HA ILE I 48   -15.875 21.833 -10.668 0.00 0.00
I
ATOM 27706 CB ILE I 48   -16.480 19.842 -10.040 0.00 0.00
I
ATOM 27707 HB ILE I 48   -17.192 19.505 -9.256 0.00 0.00
I
ATOM 27708 CG2 ILE I 48   -16.868 19.139 -11.333 0.00 0.00
I
ATOM 27709 HG21 ILE I 48   -16.690 18.043 -11.277 0.00 0.00
I
ATOM 27710 HG22 ILE I 48   -17.973 19.036 -11.378 0.00 0.00
I
ATOM 27711 HG23 ILE I 48   -16.545 19.587 -12.296 0.00 0.00
I
ATOM 27712 CG1 ILE I 48   -15.105 19.511 -9.584 0.00 0.00
I
ATOM 27713 HG11 ILE I 48   -14.475 19.747 -10.469 0.00 0.00
I
ATOM 27714 HG12 ILE I 48   -14.802 20.173 -8.745 0.00 0.00
I
ATOM 27715 CD ILE I 48   -14.934 18.058 -9.172 0.00 0.00
I
ATOM 27716 HD1 ILE I 48   -15.670 17.878 -8.359 0.00 0.00
I
ATOM 27717 HD2 ILE I 48   -15.247 17.309 -9.930 0.00 0.00
I
ATOM 27718 HD3 ILE I 48   -13.933 17.722 -8.826 0.00 0.00
I
ATOM 27719 C   ILE I 48   -17.867 21.797 -10.975 0.00 0.00
I
ATOM 27720 O   ILE I 48   -17.606 22.183 -12.120 0.00 0.00
I
ATOM 27721 N   ILE I 49   -19.138 21.809 -10.441 0.00 0.00
I
ATOM 27722 HN ILE I 49   -19.405 21.554 -9.515 0.00 0.00
I
ATOM 27723 CA ILE I 49   -20.245 21.985 -11.355 0.00 0.00
I
ATOM 27724 HA ILE I 49   -20.049 21.501 -12.301 0.00 0.00
I
ATOM 27725 CB ILE I 49   -21.650 21.539 -10.735 0.00 0.00
I
ATOM 27726 HB ILE I 49   -22.433 21.738 -11.497 0.00 0.00
I
ATOM 27727 CG2 ILE I 49   -21.460 20.010 -10.652 0.00 0.00
I
ATOM 27728 HG21 ILE I 49   -22.396 19.532 -10.293 0.00 0.00
I

```
ATOM  27729 HG22 ILE I  49      -21.269  19.482 -11.610  0.00  0.00
I
ATOM  27730 HG23 ILE I  49      -20.638  19.801  -9.934  0.00  0.00
I
ATOM  27731  CG1 ILE I  49      -22.019  22.293  -9.472  0.00  0.00
I
ATOM  27732 HG11 ILE I  49      -21.559  21.731  -8.631  0.00  0.00
I
ATOM  27733 HG12 ILE I  49      -21.780  23.378  -9.455  0.00  0.00
I
ATOM  27734  CD  ILE I  49      -23.566  22.131  -9.261  0.00  0.00
I
ATOM  27735  HD1 ILE I  49      -24.148  22.494 -10.134  0.00  0.00
I
ATOM  27736  HD2 ILE I  49      -23.847  21.060  -9.175  0.00  0.00
I
ATOM  27737  HD3 ILE I  49      -23.770  22.596  -8.272  0.00  0.00
I
ATOM  27738  C   ILE I  49      -20.330  23.366 -12.036  0.00  0.00
I
ATOM  27739  O   ILE I  49      -20.560  23.448 -13.215  0.00  0.00
I
ATOM  27740  N   ILE I  50      -20.029  24.497 -11.317  0.00  0.00
I
ATOM  27741  HN  ILE I  50      -19.819  24.403 -10.347  0.00  0.00
I
ATOM  27742  CA  ILE I  50      -19.808  25.772 -11.836  0.00  0.00
I
ATOM  27743  HA  ILE I  50      -20.631  25.997 -12.499  0.00  0.00
I
ATOM  27744  CB  ILE I  50      -20.160  26.822 -10.774  0.00  0.00
I
ATOM  27745  HB  ILE I  50      -21.218  26.728 -10.449  0.00  0.00
I
ATOM  27746  CG2 ILE I  50      -19.177  26.850  -9.608  0.00  0.00
I
ATOM  27747 HG21 ILE I  50      -19.545  27.682  -8.971  0.00  0.00
I
ATOM  27748 HG22 ILE I  50      -19.124  25.942  -8.969  0.00  0.00
I
ATOM  27749 HG23 ILE I  50      -18.143  27.030  -9.973  0.00  0.00
I
ATOM  27750  CG1 ILE I  50      -20.232  28.237 -11.476  0.00  0.00
I
ATOM  27751 HG11 ILE I  50      -20.316  29.041 -10.714  0.00  0.00
I
ATOM  27752 HG12 ILE I  50      -19.196  28.360 -11.857  0.00  0.00
I
ATOM  27753  CD  ILE I  50      -21.330  28.311 -12.517  0.00  0.00
I
ATOM  27754  HD1 ILE I  50      -21.234  27.588 -13.355  0.00  0.00
I
ATOM  27755  HD2 ILE I  50      -22.249  27.887 -12.058  0.00  0.00
I
```

ATOM 27756 HD3 ILE I 50   -21.565 29.334 -12.883 0.00 0.00
I
ATOM 27757 C   ILE I 50   -18.513 26.090 -12.595 0.00 0.00
I
ATOM 27758 O   ILE I 50   -18.635 26.765 -13.612 0.00 0.00
I
ATOM 27759 N   GLY I 51   -17.388 25.511 -12.277 0.00 0.00
I
ATOM 27760 HN GLY I 51   -17.339 25.011 -11.416 0.00 0.00
I
ATOM 27761 CA GLY I 51   -16.193 25.759 -13.079 0.00 0.00
I
ATOM 27762 HA1 GLY I 51   -15.505 25.138 -12.524 0.00 0.00
I
ATOM 27763 HA2 GLY I 51   -16.025 26.826 -13.090 0.00 0.00
I
ATOM 27764 C   GLY I 51   -16.230 25.164 -14.534 0.00 0.00
I
ATOM 27765 O   GLY I 51   -15.587 25.661 -15.522 0.00 0.00
I
ATOM 27766 N   ALA I 52   -16.976 24.061 -14.670 0.00 0.00
I
ATOM 27767 HN ALA I 52   -17.417 23.711 -13.848 0.00 0.00
I
ATOM 27768 CA ALA I 52   -17.259 23.403 -15.932 0.00 0.00
I
ATOM 27769 HA ALA I 52   -16.317 23.169 -16.406 0.00 0.00
I
ATOM 27770 CB ALA I 52   -17.888 22.071 -15.741 0.00 0.00
I
ATOM 27771 HB1 ALA I 52   -17.136 21.415 -15.252 0.00 0.00
I
ATOM 27772 HB2 ALA I 52   -18.741 22.194 -15.040 0.00 0.00
I
ATOM 27773 HB3 ALA I 52   -18.136 21.599 -16.716 0.00 0.00
I
ATOM 27774 C   ALA I 52   -18.143 24.164 -16.872 0.00 0.00
I
ATOM 27775 O   ALA I 52   -18.063 24.133 -18.076 0.00 0.00
I
ATOM 27776 N   VAL I 53   -19.066 24.991 -16.315 0.00 0.00
I
ATOM 27777 HN VAL I 53   -19.329 25.086 -15.358 0.00 0.00
I
ATOM 27778 CA VAL I 53   -19.791 25.995 -17.056 0.00 0.00
I
ATOM 27779 HA VAL I 53   -20.400 25.555 -17.832 0.00 0.00
I
ATOM 27780 CB VAL I 53   -20.829 26.474 -16.137 0.00 0.00
I
ATOM 27781 HB VAL I 53   -20.425 26.978 -15.233 0.00 0.00
I
ATOM 27782 CG1 VAL I 53   -21.481 27.705 -16.959 0.00 0.00
I

```
ATOM  27783 HG11 VAL I  53     -21.873  27.452 -17.967  0.00  0.00
I
ATOM  27784 HG12 VAL I  53     -22.356  28.008 -16.345  0.00  0.00
I
ATOM  27785 HG13 VAL I  53     -20.792  28.565 -17.100  0.00  0.00
I
ATOM  27786  CG2 VAL I  53     -21.855  25.417 -15.831  0.00  0.00
I
ATOM  27787 HG21 VAL I  53     -21.415  24.766 -15.046  0.00  0.00
I
ATOM  27788 HG22 VAL I  53     -22.840  25.871 -15.588  0.00  0.00
I
ATOM  27789 HG23 VAL I  53     -22.013  24.700 -16.665  0.00  0.00
I
ATOM  27790  C   VAL I  53     -18.842  27.149 -17.578  0.00  0.00
I
ATOM  27791  O   VAL I  53     -19.033  27.658 -18.626  0.00  0.00
I
ATOM  27792  N   LEU I  54     -17.764  27.506 -16.825  0.00  0.00
I
ATOM  27793  HN  LEU I  54     -17.700  27.124 -15.906  0.00  0.00
I
ATOM  27794  CA  LEU I  54     -16.899  28.591 -17.133  0.00  0.00
I
ATOM  27795  HA  LEU I  54     -17.449  29.485 -17.389  0.00  0.00
I
ATOM  27796  CB  LEU I  54     -16.046  28.909 -15.877  0.00  0.00
I
ATOM  27797  HB1 LEU I  54     -15.559  27.993 -15.478  0.00  0.00
I
ATOM  27798  HB2 LEU I  54     -15.300  29.672 -16.188  0.00  0.00
I
ATOM  27799  CG  LEU I  54     -16.848  29.521 -14.651  0.00  0.00
I
ATOM  27800  HG  LEU I  54     -17.611  28.803 -14.280  0.00  0.00
I
ATOM  27801  CD1 LEU I  54     -15.934  30.063 -13.544  0.00  0.00
I
ATOM  27802 HD11 LEU I  54     -16.364  30.723 -12.761  0.00  0.00
I
ATOM  27803 HD12 LEU I  54     -15.503  29.168 -13.047  0.00  0.00
I
ATOM  27804 HD13 LEU I  54     -15.069  30.592 -13.998  0.00  0.00
I
ATOM  27805  CD2 LEU I  54     -17.862  30.606 -15.042  0.00  0.00
I
ATOM  27806 HD21 LEU I  54     -17.352  31.424 -15.593  0.00  0.00
I
ATOM  27807 HD22 LEU I  54     -18.818  30.365 -15.555  0.00  0.00
I
ATOM  27808 HD23 LEU I  54     -18.175  31.143 -14.121  0.00  0.00
I
ATOM  27809  C   LEU I  54     -15.947  28.257 -18.247  0.00  0.00
I
```

ATOM 27810 O LEU I 54 -15.490 29.103 -18.945 0.00 0.00
I
ATOM 27811 N LEU I 55 -15.667 26.972 -18.472 0.00 0.00
I
ATOM 27812 HN LEU I 55 -16.215 26.337 -17.934 0.00 0.00
I
ATOM 27813 CA LEU I 55 -14.918 26.498 -19.620 0.00 0.00
I
ATOM 27814 HA LEU I 55 -14.130 27.221 -19.775 0.00 0.00
I
ATOM 27815 CB LEU I 55 -14.347 25.205 -19.286 0.00 0.00
I
ATOM 27816 HB1 LEU I 55 -15.176 24.570 -18.907 0.00 0.00
I
ATOM 27817 HB2 LEU I 55 -13.990 24.689 -20.203 0.00 0.00
I
ATOM 27818 CG LEU I 55 -13.200 25.140 -18.218 0.00 0.00
I
ATOM 27819 HG LEU I 55 -13.556 25.312 -17.180 0.00 0.00
I
ATOM 27820 CD1 LEU I 55 -12.611 23.709 -18.281 0.00 0.00
I
ATOM 27821 HD11 LEU I 55 -12.222 23.517 -19.304 0.00 0.00
I
ATOM 27822 HD12 LEU I 55 -11.749 23.422 -17.641 0.00 0.00
I
ATOM 27823 HD13 LEU I 55 -13.448 23.004 -18.092 0.00 0.00
I
ATOM 27824 CD2 LEU I 55 -12.086 26.162 -18.423 0.00 0.00
I
ATOM 27825 HD21 LEU I 55 -11.274 25.853 -17.731 0.00 0.00
I
ATOM 27826 HD22 LEU I 55 -11.790 26.190 -19.493 0.00 0.00
I
ATOM 27827 HD23 LEU I 55 -12.500 27.163 -18.175 0.00 0.00
I
ATOM 27828 C LEU I 55 -15.610 26.445 -20.952 0.00 0.00
I
ATOM 27829 O LEU I 55 -14.907 26.568 -21.949 0.00 0.00
I
ATOM 27830 N MET I 56 -17.024 26.317 -20.938 0.00 0.00
I
ATOM 27831 HN MET I 56 -17.467 26.042 -20.088 0.00 0.00
I
ATOM 27832 CA MET I 56 -17.737 26.100 -22.229 0.00 0.00
I
ATOM 27833 HA MET I 56 -17.059 25.975 -23.060 0.00 0.00
I
ATOM 27834 CB MET I 56 -18.814 25.006 -22.185 0.00 0.00
I
ATOM 27835 HB1 MET I 56 -19.335 24.884 -23.158 0.00 0.00
I
ATOM 27836 HB2 MET I 56 -18.280 24.049 -22.005 0.00 0.00
I

ATOM 27837 CG MET I 56   -19.851 25.171 -21.010 0.00 0.00
I
ATOM 27838 HG1 MET I 56   -19.237 25.143 -20.085 0.00 0.00
I
ATOM 27839 HG2 MET I 56   -20.351 26.158 -21.106 0.00 0.00
I
ATOM 27840 SD MET I 56   -21.240 24.029 -21.091 0.00 0.00
I
ATOM 27841 CE MET I 56   -21.726 24.114 -22.777 0.00 0.00
I
ATOM 27842 HE1 MET I 56   -22.714 23.625 -22.910 0.00 0.00
I
ATOM 27843 HE2 MET I 56   -21.628 25.146 -23.177 0.00 0.00
I
ATOM 27844 HE3 MET I 56   -21.030 23.553 -23.436 0.00 0.00
I
ATOM 27845 C   MET I 56   -18.436 27.371 -22.758 0.00 0.00
I
ATOM 27846 O   MET I 56   -19.010 27.352 -23.852 0.00 0.00
I
ATOM 27847 N   LEU I 57   -18.359 28.529 -22.095 0.00 0.00
I
ATOM 27848 HN LEU I 57   -17.908 28.419 -21.213 0.00 0.00
I
ATOM 27849 CA LEU I 57   -18.856 29.815 -22.576 0.00 0.00
I
ATOM 27850 HA LEU I 57   -19.881 29.601 -22.839 0.00 0.00
I
ATOM 27851 CB LEU I 57   -18.767 30.777 -21.446 0.00 0.00
I
ATOM 27852 HB1 LEU I 57   -17.812 30.488 -20.956 0.00 0.00
I
ATOM 27853 HB2 LEU I 57   -18.586 31.836 -21.726 0.00 0.00
I
ATOM 27854 CG LEU I 57   -19.959 30.788 -20.397 0.00 0.00
I
ATOM 27855 HG LEU I 57   -19.999 29.798 -19.893 0.00 0.00
I
ATOM 27856 CD1 LEU I 57   -19.666 31.728 -19.240 0.00 0.00
I
ATOM 27857 HD11 LEU I 57   -19.887 32.763 -19.578 0.00 0.00
I
ATOM 27858 HD12 LEU I 57   -20.302 31.384 -18.396 0.00 0.00
I
ATOM 27859 HD13 LEU I 57   -18.587 31.737 -18.975 0.00 0.00
I
ATOM 27860 CD2 LEU I 57   -21.360 31.046 -21.069 0.00 0.00
I
ATOM 27861 HD21 LEU I 57   -22.187 30.804 -20.367 0.00 0.00
I
ATOM 27862 HD22 LEU I 57   -21.472 32.134 -21.267 0.00 0.00
I
ATOM 27863 HD23 LEU I 57   -21.476 30.426 -21.983 0.00 0.00
I

ATOM 27864 C   LEU I 57   -18.080 30.334 -23.758 0.00 0.00
I
ATOM 27865 O   LEU I 57   -16.874 30.053 -23.832 0.00 0.00
I
ATOM 27866 N   PRO I 58   -18.738 31.093 -24.687 0.00 0.00
I
ATOM 27867 CD PRO I 58   -20.111 31.552 -24.552 0.00 0.00
I
ATOM 27868 HD1 PRO I 58   -20.804 30.688 -24.469 0.00 0.00
I
ATOM 27869 HD2 PRO I 58   -20.104 32.101 -23.585 0.00 0.00
I
ATOM 27870 CA PRO I 58   -18.190 31.395 -25.969 0.00 0.00
I
ATOM 27871 HA PRO I 58   -17.735 30.556 -26.473 0.00 0.00
I
ATOM 27872 CB PRO I 58   -19.327 31.911 -26.829 0.00 0.00
I
ATOM 27873 HB1 PRO I 58   -19.712 31.005 -27.345 0.00 0.00
I
ATOM 27874 HB2 PRO I 58   -19.019 32.619 -27.628 0.00 0.00
I
ATOM 27875 CG PRO I 58   -20.377 32.390 -25.769 0.00 0.00
I
ATOM 27876 HG1 PRO I 58   -20.123 33.407 -25.399 0.00 0.00
I
ATOM 27877 HG2 PRO I 58   -21.409 32.278 -26.165 0.00 0.00
I
ATOM 27878 C   PRO I 58   -17.012 32.362 -25.994 0.00 0.00
I
ATOM 27879 O   PRO I 58   -16.564 32.781 -27.033 0.00 0.00
I
ATOM 27880 N   ILE I 59   -16.642 32.917 -24.840 0.00 0.00
I
ATOM 27881 HN ILE I 59   -17.330 32.814 -24.125 0.00 0.00
I
ATOM 27882 CA ILE I 59   -15.374 33.667 -24.557 0.00 0.00
I
ATOM 27883 HA ILE I 59   -15.120 34.227 -25.444 0.00 0.00
I
ATOM 27884 CB ILE I 59   -15.585 34.733 -23.600 0.00 0.00
I
ATOM 27885 HB ILE I 59   -14.596 35.192 -23.385 0.00 0.00
I
ATOM 27886 CG2 ILE I 59   -16.375 35.927 -24.367 0.00 0.00
I
ATOM 27887 HG21 ILE I 59   -16.689 36.790 -23.741 0.00 0.00
I
ATOM 27888 HG22 ILE I 59   -15.710 36.233 -25.203 0.00 0.00
I
ATOM 27889 HG23 ILE I 59   -17.309 35.452 -24.736 0.00 0.00
I
ATOM 27890 CG1 ILE I 59   -16.291 34.247 -22.364 0.00 0.00
I

```
ATOM  27891 HG11 ILE I  59      -17.356  34.077 -22.630  0.00  0.00
I
ATOM  27892 HG12 ILE I  59      -15.764  33.302 -22.112  0.00  0.00
I
ATOM  27893  CD  ILE I  59      -16.309  35.254 -21.130  0.00  0.00
I
ATOM  27894  HD1 ILE I  59      -16.766  34.671 -20.302  0.00  0.00
I
ATOM  27895  HD2 ILE I  59      -15.261  35.509 -20.863  0.00  0.00
I
ATOM  27896  HD3 ILE I  59      -16.955  36.125 -21.370  0.00  0.00
I
ATOM  27897  C   ILE I  59      -14.218  32.736 -24.255  0.00  0.00
I
ATOM  27898  O   ILE I  59      -13.048  33.174 -24.344  0.00  0.00
I
ATOM  27899  N   SER I  60      -14.511  31.541 -23.783  0.00  0.00
I
ATOM  27900  HN  SER I  60      -15.476  31.304 -23.716  0.00  0.00
I
ATOM  27901  CA  SER I  60      -13.555  30.735 -23.052  0.00  0.00
I
ATOM  27902  HA  SER I  60      -12.731  31.296 -22.636  0.00  0.00
I
ATOM  27903  CB  SER I  60      -14.296  30.042 -21.874  0.00  0.00
I
ATOM  27904  HB1 SER I  60      -15.113  29.335 -22.132  0.00  0.00
I
ATOM  27905  HB2 SER I  60      -13.539  29.411 -21.362  0.00  0.00
I
ATOM  27906  OG  SER I  60      -14.901  30.856 -20.868  0.00  0.00
I
ATOM  27907  HG1 SER I  60      -15.174  30.319 -20.121  0.00  0.00
I
ATOM  27908  C   SER I  60      -12.985  29.611 -23.911  0.00  0.00
I
ATOM  27909  O   SER I  60      -11.933  29.086 -23.742  0.00  0.00
I
ATOM  27910  N   THR I  61      -13.725  29.055 -24.846  0.00  0.00
I
ATOM  27911  HN  THR I  61      -14.693  29.293 -24.874  0.00  0.00
I
ATOM  27912  CA  THR I  61      -13.368  28.019 -25.677  0.00  0.00
I
ATOM  27913  HA  THR I  61      -12.348  27.730 -25.473  0.00  0.00
I
ATOM  27914  CB  THR I  61      -14.217  26.738 -25.539  0.00  0.00
I
ATOM  27915  HB  THR I  61      -14.260  26.512 -24.453  0.00  0.00
I
ATOM  27916  OG1 THR I  61      -13.657  25.639 -26.206  0.00  0.00
I
ATOM  27917  HG1 THR I  61      -13.946  25.683 -27.121  0.00  0.00
I
```

ATOM 27918 CG2 THR I 61   -15.629 26.888 -25.972 0.00 0.00
I
ATOM 27919 HG21 THR I 61   -16.127 27.722 -25.432 0.00 0.00
I
ATOM 27920 HG22 THR I 61   -15.792 26.914 -27.071 0.00 0.00
I
ATOM 27921 HG23 THR I 61   -16.197 25.962 -25.738 0.00 0.00
I
ATOM 27922 C   THR I 61   -13.315 28.510 -27.106 0.00 0.00
I
ATOM 27923 O   THR I 61   -14.152 29.322 -27.593 0.00 0.00
I
ATOM 27924 N   THR I 62   -12.228 28.143 -27.876 0.00 0.00
I
ATOM 27925 HN THR I 62   -11.567 27.489 -27.518 0.00 0.00
I
ATOM 27926 CA THR I 62   -11.936 28.729 -29.151 0.00 0.00
I
ATOM 27927 HA THR I 62   -12.695 29.432 -29.461 0.00 0.00
I
ATOM 27928 CB THR I 62   -10.576 29.407 -29.246 0.00 0.00
I
ATOM 27929 HB THR I 62   -10.396 30.033 -28.346 0.00 0.00
I
ATOM 27930 OG1 THR I 62   -10.520 30.314 -30.359 0.00 0.00
I
ATOM 27931 HG1 THR I 62   -9.689 30.773 -30.218 0.00 0.00
I
ATOM 27932 CG2 THR I 62   -9.434 28.545 -29.564 0.00 0.00
I
ATOM 27933 HG21 THR I 62   -9.606 28.305 -30.636 0.00 0.00
I
ATOM 27934 HG22 THR I 62   -8.438 29.036 -29.528 0.00 0.00
I
ATOM 27935 HG23 THR I 62   -9.428 27.546 -29.078 0.00 0.00
I
ATOM 27936 C   THR I 62   -12.054 27.728 -30.281 0.00 0.00
I
ATOM 27937 O   THR I 62   -12.340 28.088 -31.374 0.00 0.00
I
ATOM 27938 N   LYS I 63   -12.068 26.399 -29.935 0.00 0.00
I
ATOM 27939 HN LYS I 63   -11.626 26.037 -29.118 0.00 0.00
I
ATOM 27940 CA LYS I 63   -12.589 25.387 -30.821 0.00 0.00
I
ATOM 27941 HA LYS I 63   -12.764 25.792 -31.806 0.00 0.00
I
ATOM 27942 CB LYS I 63   -11.653 24.118 -30.843 0.00 0.00
I
ATOM 27943 HB1 LYS I 63   -11.533 23.844 -29.773 0.00 0.00
I
ATOM 27944 HB2 LYS I 63   -12.096 23.394 -31.560 0.00 0.00
I

ATOM 27945 CG LYS I 63 -10.185 24.382 -31.323 0.00 0.00
I
ATOM 27946 HG1 LYS I 63 -9.663 25.010 -30.570 0.00 0.00
I
ATOM 27947 HG2 LYS I 63 -9.570 23.457 -31.311 0.00 0.00
I
ATOM 27948 CD LYS I 63 -10.083 25.092 -32.729 0.00 0.00
I
ATOM 27949 HD1 LYS I 63 -10.769 25.965 -32.777 0.00 0.00
I
ATOM 27950 HD2 LYS I 63 -9.063 25.510 -32.864 0.00 0.00
I
ATOM 27951 CE LYS I 63 -10.272 24.050 -33.861 0.00 0.00
I
ATOM 27952 HE1 LYS I 63 -9.429 23.326 -33.853 0.00 0.00
I
ATOM 27953 HE2 LYS I 63 -11.164 23.389 -33.820 0.00 0.00
I
ATOM 27954 NZ LYS I 63 -10.219 24.675 -35.221 0.00 0.00
I
ATOM 27955 HZ1 LYS I 63 -10.211 23.837 -35.837 0.00 0.00
I
ATOM 27956 HZ2 LYS I 63 -10.990 25.342 -35.425 0.00 0.00
I
ATOM 27957 HZ3 LYS I 63 -9.261 25.033 -35.412 0.00 0.00
I
ATOM 27958 C LYS I 63 -13.972 24.888 -30.263 0.00 0.00
I
ATOM 27959 O LYS I 63 -14.300 25.425 -29.193 0.00 0.00
I
ATOM 27960 N PRO I 64 -14.773 24.054 -30.904 0.00 0.00
I
ATOM 27961 CD PRO I 64 -14.755 23.839 -32.389 0.00 0.00
I
ATOM 27962 HD1 PRO I 64 -14.654 24.781 -32.969 0.00 0.00
I
ATOM 27963 HD2 PRO I 64 -13.999 23.063 -32.635 0.00 0.00
I
ATOM 27964 CA PRO I 64 -15.981 23.443 -30.319 0.00 0.00
I
ATOM 27965 HA PRO I 64 -16.462 24.308 -29.886 0.00 0.00
I
ATOM 27966 CB PRO I 64 -16.726 22.775 -31.417 0.00 0.00
I
ATOM 27967 HB1 PRO I 64 -17.820 22.960 -31.475 0.00 0.00
I
ATOM 27968 HB2 PRO I 64 -16.507 21.686 -31.394 0.00 0.00
I
ATOM 27969 CG PRO I 64 -16.173 23.491 -32.723 0.00 0.00
I
ATOM 27970 HG1 PRO I 64 -16.150 22.701 -33.505 0.00 0.00
I
ATOM 27971 HG2 PRO I 64 -16.855 24.357 -32.862 0.00 0.00
I

```
ATOM  27972  C   PRO I  64      -15.688  22.499 -29.154  0.00  0.00
I
ATOM  27973  O   PRO I  64      -14.773  21.666 -29.066  0.00  0.00
I
ATOM  27974  N   LEU I  65      -16.517  22.562 -28.107  0.00  0.00
I
ATOM  27975  HN  LEU I  65      -17.254  23.230 -28.175  0.00  0.00
I
ATOM  27976  CA  LEU I  65      -16.309  21.916 -26.842  0.00  0.00
I
ATOM  27977  HA  LEU I  65      -15.639  21.079 -26.971  0.00  0.00
I
ATOM  27978  CB  LEU I  65      -15.848  22.979 -25.818  0.00  0.00
I
ATOM  27979  HB1 LEU I  65      -14.873  23.369 -26.181  0.00  0.00
I
ATOM  27980  HB2 LEU I  65      -16.526  23.859 -25.803  0.00  0.00
I
ATOM  27981  CG  LEU I  65      -15.576  22.532 -24.385  0.00  0.00
I
ATOM  27982  HG  LEU I  65      -16.569  22.142 -24.076  0.00  0.00
I
ATOM  27983  CD1 LEU I  65      -14.497  21.451 -24.210  0.00  0.00
I
ATOM  27984 HD11 LEU I  65      -14.514  20.579 -24.898  0.00  0.00
I
ATOM  27985 HD12 LEU I  65      -13.501  21.914 -24.374  0.00  0.00
I
ATOM  27986 HD13 LEU I  65      -14.448  21.208 -23.127  0.00  0.00
I
ATOM  27987  CD2 LEU I  65      -15.146  23.793 -23.631  0.00  0.00
I
ATOM  27988 HD21 LEU I  65      -14.144  24.204 -23.880  0.00  0.00
I
ATOM  27989 HD22 LEU I  65      -15.948  24.551 -23.764  0.00  0.00
I
ATOM  27990 HD23 LEU I  65      -15.002  23.652 -22.538  0.00  0.00
I
ATOM  27991  C   LEU I  65      -17.636  21.377 -26.383  0.00  0.00
I
ATOM  27992  O   LEU I  65      -18.770  21.933 -26.461  0.00  0.00
I
ATOM  27993  N   SER I  66      -17.522  20.125 -25.923  0.00  0.00
I
ATOM  27994  HN  SER I  66      -16.680  19.647 -26.159  0.00  0.00
I
ATOM  27995  CA  SER I  66      -18.429  19.366 -25.054  0.00  0.00
I
ATOM  27996  HA  SER I  66      -19.446  19.558 -25.362  0.00  0.00
I
ATOM  27997  CB  SER I  66      -18.209  17.843 -25.401  0.00  0.00
I
ATOM  27998  HB1 SER I  66      -17.961  17.748 -26.480  0.00  0.00
I
```

ATOM 27999 HB2 SER I 66 −17.407 17.350 −24.811 0.00 0.00
I
ATOM 28000 OG SER I 66 −19.457 17.089 −25.295 0.00 0.00
I
ATOM 28001 HG1 SER I 66 −19.347 16.350 −25.898 0.00 0.00
I
ATOM 28002 C SER I 66 −18.352 19.569 −23.600 0.00 0.00
I
ATOM 28003 O SER I 66 −17.323 19.809 −23.027 0.00 0.00
I
ATOM 28004 N TRP I 67 −19.405 19.345 −22.837 0.00 0.00
I
ATOM 28005 HN TRP I 67 −20.225 19.072 −23.335 0.00 0.00
I
ATOM 28006 CA TRP I 67 −19.568 19.306 −21.429 0.00 0.00
I
ATOM 28007 HA TRP I 67 −19.143 20.224 −21.051 0.00 0.00
I
ATOM 28008 CB TRP I 67 −21.124 19.199 −20.977 0.00 0.00
I
ATOM 28009 HB1 TRP I 67 −21.709 20.014 −21.454 0.00 0.00
I
ATOM 28010 HB2 TRP I 67 −21.596 18.283 −21.393 0.00 0.00
I
ATOM 28011 CG TRP I 67 −21.427 19.045 −19.550 0.00 0.00
I
ATOM 28012 CD1 TRP I 67 −22.060 18.061 −18.894 0.00 0.00
I
ATOM 28013 HD1 TRP I 67 −22.311 17.144 −19.406 0.00 0.00
I
ATOM 28014 NE1 TRP I 67 −22.236 18.386 −17.561 0.00 0.00
I
ATOM 28015 HE1 TRP I 67 −22.373 17.701 −16.880 0.00 0.00
I
ATOM 28016 CE2 TRP I 67 −21.874 19.684 −17.383 0.00 0.00
I
ATOM 28017 CD2 TRP I 67 −21.412 20.177 −18.648 0.00 0.00
I
ATOM 28018 CE3 TRP I 67 −20.887 21.426 −18.734 0.00 0.00
I
ATOM 28019 HE3 TRP I 67 −20.537 21.831 −19.672 0.00 0.00
I
ATOM 28020 CZ3 TRP I 67 −20.923 22.284 −17.600 0.00 0.00
I
ATOM 28021 HZ3 TRP I 67 −20.579 23.302 −17.710 0.00 0.00
I
ATOM 28022 CZ2 TRP I 67 −21.935 20.511 −16.279 0.00 0.00
I
ATOM 28023 HZ2 TRP I 67 −22.325 20.199 −15.321 0.00 0.00
I
ATOM 28024 CH2 TRP I 67 −21.453 21.845 −16.419 0.00 0.00
I
ATOM 28025 HH2 TRP I 67 −21.643 22.619 −15.690 0.00 0.00
I

ATOM 28026 C   TRP I 67   -18.707 18.279 -20.723 0.00 0.00
I
ATOM 28027 O   TRP I 67   -18.094 18.657 -19.710 0.00 0.00
I
ATOM 28028 N   ILE I 68   -18.620 17.016 -21.221 0.00 0.00
I
ATOM 28029 HN ILE I 68   -19.337 16.649 -21.809 0.00 0.00
I
ATOM 28030 CA ILE I 68   -17.744 15.999 -20.721 0.00 0.00
I
ATOM 28031 HA ILE I 68   -17.929 15.871 -19.665 0.00 0.00
I
ATOM 28032 CB ILE I 68   -18.084 14.653 -21.369 0.00 0.00
I
ATOM 28033 HB ILE I 68   -19.160 14.376 -21.350 0.00 0.00
I
ATOM 28034 CG2 ILE I 68   -17.851 14.676 -22.886 0.00 0.00
I
ATOM 28035 HG21 ILE I 68   -18.417 13.823 -23.319 0.00 0.00
I
ATOM 28036 HG22 ILE I 68   -18.144 15.610 -23.412 0.00 0.00
I
ATOM 28037 HG23 ILE I 68   -16.768 14.512 -23.073 0.00 0.00
I
ATOM 28038 CG1 ILE I 68   -17.461 13.532 -20.556 0.00 0.00
I
ATOM 28039 HG11 ILE I 68   -17.508 12.581 -21.129 0.00 0.00
I
ATOM 28040 HG12 ILE I 68   -16.385 13.637 -20.302 0.00 0.00
I
ATOM 28041 CD ILE I 68   -18.111 13.196 -19.198 0.00 0.00
I
ATOM 28042 HD1 ILE I 68   -18.038 14.044 -18.483 0.00 0.00
I
ATOM 28043 HD2 ILE I 68   -19.182 12.931 -19.329 0.00 0.00
I
ATOM 28044 HD3 ILE I 68   -17.590 12.322 -18.751 0.00 0.00
I
ATOM 28045 C   ILE I 68   -16.276 16.357 -20.717 0.00 0.00
I
ATOM 28046 O   ILE I 68   -15.555 16.220 -19.788 0.00 0.00
I
ATOM 28047 N   ASP I 69   -15.688 16.847 -21.827 0.00 0.00
I
ATOM 28048 HN ASP I 69   -16.237 16.956 -22.653 0.00 0.00
I
ATOM 28049 CA ASP I 69   -14.341 17.346 -21.981 0.00 0.00
I
ATOM 28050 HA ASP I 69   -13.672 16.547 -21.699 0.00 0.00
I
ATOM 28051 CB ASP I 69   -13.972 17.721 -23.440 0.00 0.00
I
ATOM 28052 HB1 ASP I 69   -14.783 18.433 -23.702 0.00 0.00
I

ATOM 28053 HB2 ASP I 69 -12.983 18.206 -23.584 0.00 0.00
I
ATOM 28054 CG ASP I 69 -14.091 16.479 -24.284 0.00 0.00
I
ATOM 28055 OD1 ASP I 69 -14.953 16.433 -25.246 0.00 0.00
I
ATOM 28056 OD2 ASP I 69 -13.379 15.486 -24.026 0.00 0.00
I
ATOM 28057 C ASP I 69 -14.088 18.503 -20.977 0.00 0.00
I
ATOM 28058 O ASP I 69 -13.051 18.535 -20.334 0.00 0.00
I
ATOM 28059 N ALA I 70 -15.063 19.481 -20.909 0.00 0.00
I
ATOM 28060 HN ALA I 70 -15.869 19.485 -21.496 0.00 0.00
I
ATOM 28061 CA ALA I 70 -15.001 20.611 -19.999 0.00 0.00
I
ATOM 28062 HA ALA I 70 -14.052 21.108 -20.130 0.00 0.00
I
ATOM 28063 CB ALA I 70 -16.229 21.557 -20.266 0.00 0.00
I
ATOM 28064 HB1 ALA I 70 -16.281 21.825 -21.343 0.00 0.00
I
ATOM 28065 HB2 ALA I 70 -17.142 20.993 -19.979 0.00 0.00
I
ATOM 28066 HB3 ALA I 70 -16.107 22.512 -19.711 0.00 0.00
I
ATOM 28067 C ALA I 70 -15.054 20.210 -18.508 0.00 0.00
I
ATOM 28068 O ALA I 70 -14.244 20.690 -17.736 0.00 0.00
I
ATOM 28069 N LEU I 71 -15.968 19.235 -18.154 0.00 0.00
I
ATOM 28070 HN LEU I 71 -16.651 18.922 -18.810 0.00 0.00
I
ATOM 28071 CA LEU I 71 -16.160 18.671 -16.851 0.00 0.00
I
ATOM 28072 HA LEU I 71 -16.326 19.454 -16.127 0.00 0.00
I
ATOM 28073 CB LEU I 71 -17.370 17.707 -16.818 0.00 0.00
I
ATOM 28074 HB1 LEU I 71 -18.277 18.144 -17.288 0.00 0.00
I
ATOM 28075 HB2 LEU I 71 -17.110 16.966 -17.604 0.00 0.00
I
ATOM 28076 CG LEU I 71 -17.613 17.034 -15.432 0.00 0.00
I
ATOM 28077 HG LEU I 71 -16.698 16.439 -15.227 0.00 0.00
I
ATOM 28078 CD1 LEU I 71 -17.832 18.067 -14.414 0.00 0.00
I
ATOM 28079 HD11 LEU I 71 -18.425 18.839 -14.950 0.00 0.00
I

ATOM 28080 HD12 LEU I 71   -18.406 17.757 -13.514 0.00 0.00
I
ATOM 28081 HD13 LEU I 71   -16.869 18.407 -13.977 0.00 0.00
I
ATOM 28082 CD2 LEU I 71   -18.745 15.994 -15.537 0.00 0.00
I
ATOM 28083 HD21 LEU I 71   -18.916 15.338 -14.657 0.00 0.00
I
ATOM 28084 HD22 LEU I 71   -19.731 16.470 -15.726 0.00 0.00
I
ATOM 28085 HD23 LEU I 71   -18.706 15.332 -16.427 0.00 0.00
I
ATOM 28086 C   LEU I 71   -14.901 17.933 -16.511 0.00 0.00
I
ATOM 28087 O   LEU I 71   -14.467 18.025 -15.404 0.00 0.00
I
ATOM 28088 N   PHE I 72   -14.315 17.107 -17.412 0.00 0.00
I
ATOM 28089 HN PHE I 72   -14.625 16.992 -18.352 0.00 0.00
I
ATOM 28090 CA PHE I 72   -13.060 16.349 -17.195 0.00 0.00
I
ATOM 28091 HA PHE I 72   -13.154 15.651 -16.376 0.00 0.00
I
ATOM 28092 CB PHE I 72   -12.656 15.519 -18.417 0.00 0.00
I
ATOM 28093 HB1 PHE I 72   -13.488 15.004 -18.943 0.00 0.00
I
ATOM 28094 HB2 PHE I 72   -12.122 16.079 -19.214 0.00 0.00
I
ATOM 28095 CG PHE I 72   -11.552 14.530 -18.017 0.00 0.00
I
ATOM 28096 CD1 PHE I 72   -10.182 15.003 -17.995 0.00 0.00
I
ATOM 28097 HD1 PHE I 72   -10.020 15.981 -18.425 0.00 0.00
I
ATOM 28098 CE1 PHE I 72   -9.165 14.036 -17.835 0.00 0.00
I
ATOM 28099 HE1 PHE I 72   -8.132 14.348 -17.884 0.00 0.00
I
ATOM 28100 CZ PHE I 72   -9.455 12.704 -17.770 0.00 0.00
I
ATOM 28101 HZ PHE I 72   -8.607 12.036 -17.797 0.00 0.00
I
ATOM 28102 CD2 PHE I 72   -11.853 13.159 -17.706 0.00 0.00
I
ATOM 28103 HD2 PHE I 72   -12.864 12.854 -17.484 0.00 0.00
I
ATOM 28104 CE2 PHE I 72   -10.811 12.228 -17.679 0.00 0.00
I
ATOM 28105 HE2 PHE I 72   -11.045 11.211 -17.402 0.00 0.00
I
ATOM 28106 C   PHE I 72   -12.049 17.335 -16.921 0.00 0.00
I

ATOM	28107	O	PHE	I	72	-11.369	17.297	-15.904	0.00	0.00	I
ATOM	28108	N	THR	I	73	-11.920	18.393	-17.734	0.00	0.00	I
ATOM	28109	HN	THR	I	73	-12.420	18.386	-18.596	0.00	0.00	I
ATOM	28110	CA	THR	I	73	-10.876	19.379	-17.590	0.00	0.00	I
ATOM	28111	HA	THR	I	73	-9.947	18.834	-17.505	0.00	0.00	I
ATOM	28112	CB	THR	I	73	-10.861	20.463	-18.645	0.00	0.00	I
ATOM	28113	HB	THR	I	73	-11.851	20.966	-18.618	0.00	0.00	I
ATOM	28114	OG1	THR	I	73	-10.704	19.888	-19.920	0.00	0.00	I
ATOM	28115	HG1	THR	I	73	-11.482	19.364	-20.126	0.00	0.00	I
ATOM	28116	CG2	THR	I	73	-9.651	21.457	-18.451	0.00	0.00	I
ATOM	28117	HG21	THR	I	73	-9.869	22.012	-17.514	0.00	0.00	I
ATOM	28118	HG22	THR	I	73	-8.666	20.957	-18.328	0.00	0.00	I
ATOM	28119	HG23	THR	I	73	-9.602	22.169	-19.302	0.00	0.00	I
ATOM	28120	C	THR	I	73	-11.004	20.131	-16.213	0.00	0.00	I
ATOM	28121	O	THR	I	73	-10.020	20.250	-15.510	0.00	0.00	I
ATOM	28122	N	ALA	I	74	-12.230	20.654	-15.825	0.00	0.00	I
ATOM	28123	HN	ALA	I	74	-12.939	20.392	-16.476	0.00	0.00	I
ATOM	28124	CA	ALA	I	74	-12.465	21.290	-14.588	0.00	0.00	I
ATOM	28125	HA	ALA	I	74	-11.878	22.190	-14.479	0.00	0.00	I
ATOM	28126	CB	ALA	I	74	-13.929	21.803	-14.617	0.00	0.00	I
ATOM	28127	HB1	ALA	I	74	-14.072	22.487	-15.481	0.00	0.00	I
ATOM	28128	HB2	ALA	I	74	-14.578	20.921	-14.802	0.00	0.00	I
ATOM	28129	HB3	ALA	I	74	-14.119	22.283	-13.633	0.00	0.00	I
ATOM	28130	C	ALA	I	74	-12.220	20.455	-13.287	0.00	0.00	I
ATOM	28131	O	ALA	I	74	-11.663	20.991	-12.368	0.00	0.00	I
ATOM	28132	N	ALA	I	75	-12.789	19.219	-13.274	0.00	0.00	I
ATOM	28133	HN	ALA	I	75	-13.347	18.836	-14.006	0.00	0.00	I

ATOM 28134 CA ALA I 75 −12.534 18.253 −12.266 0.00 0.00
I
ATOM 28135 HA ALA I 75 −12.753 18.699 −11.308 0.00 0.00
I
ATOM 28136 CB ALA I 75 −13.406 16.992 −12.453 0.00 0.00
I
ATOM 28137 HB1 ALA I 75 −13.238 16.425 −13.393 0.00 0.00
I
ATOM 28138 HB2 ALA I 75 −13.271 16.282 −11.609 0.00 0.00
I
ATOM 28139 HB3 ALA I 75 −14.429 17.412 −12.348 0.00 0.00
I
ATOM 28140 C ALA I 75 −11.045 17.959 −12.219 0.00 0.00
I
ATOM 28141 O ALA I 75 −10.526 17.899 −11.083 0.00 0.00
I
ATOM 28142 N SER I 76 −10.330 17.722 −13.322 0.00 0.00
I
ATOM 28143 HN SER I 76 −10.790 17.769 −14.205 0.00 0.00
I
ATOM 28144 CA SER I 76 −8.898 17.414 −13.248 0.00 0.00
I
ATOM 28145 HA SER I 76 −8.769 16.531 −12.640 0.00 0.00
I
ATOM 28146 CB SER I 76 −8.304 17.107 −14.629 0.00 0.00
I
ATOM 28147 HB1 SER I 76 −8.776 16.179 −15.016 0.00 0.00
I
ATOM 28148 HB2 SER I 76 −8.579 17.903 −15.354 0.00 0.00
I
ATOM 28149 OG SER I 76 −6.975 16.803 −14.626 0.00 0.00
I
ATOM 28150 HG1 SER I 76 −6.729 16.781 −15.554 0.00 0.00
I
ATOM 28151 C SER I 76 −8.075 18.495 −12.593 0.00 0.00
I
ATOM 28152 O SER I 76 −7.095 18.328 −11.952 0.00 0.00
I
ATOM 28153 N ALA I 77 −8.431 19.771 −12.974 0.00 0.00
I
ATOM 28154 HN ALA I 77 −9.073 19.964 −13.712 0.00 0.00
I
ATOM 28155 CA ALA I 77 −7.784 20.945 −12.407 0.00 0.00
I
ATOM 28156 HA ALA I 77 −6.727 20.727 −12.433 0.00 0.00
I
ATOM 28157 CB ALA I 77 −8.259 22.181 −13.238 0.00 0.00
I
ATOM 28158 HB1 ALA I 77 −7.712 23.111 −12.978 0.00 0.00
I
ATOM 28159 HB2 ALA I 77 −8.071 21.911 −14.299 0.00 0.00
I
ATOM 28160 HB3 ALA I 77 −9.360 22.183 −13.089 0.00 0.00
I

```
ATOM  28161  C   ALA I  77      -8.124  21.073 -10.926  0.00  0.00
I
ATOM  28162  O   ALA I  77      -7.230  21.264 -10.118  0.00  0.00
I
ATOM  28163  N   THR I  78      -9.437  20.942 -10.566  0.00  0.00
I
ATOM  28164  HN  THR I  78     -10.041  20.682 -11.316  0.00  0.00
I
ATOM  28165  CA  THR I  78     -10.012  21.189  -9.152  0.00  0.00
I
ATOM  28166  HA  THR I  78      -9.525  22.116  -8.888  0.00  0.00
I
ATOM  28167  CB  THR I  78     -11.558  21.241  -9.120  0.00  0.00
I
ATOM  28168  HB  THR I  78     -12.035  20.375  -9.626  0.00  0.00
I
ATOM  28169  OG1 THR I  78     -11.933  22.281  -9.999  0.00  0.00
I
ATOM  28170  HG1 THR I  78     -11.710  22.031 -10.898  0.00  0.00
I
ATOM  28171  CG2 THR I  78     -12.235  21.417  -7.729  0.00  0.00
I
ATOM  28172 HG21 THR I  78     -12.166  20.568  -7.016  0.00  0.00
I
ATOM  28173 HG22 THR I  78     -11.726  22.266  -7.225  0.00  0.00
I
ATOM  28174 HG23 THR I  78     -13.261  21.795  -7.923  0.00  0.00
I
ATOM  28175  C   THR I  78      -9.525  20.161  -8.130  0.00  0.00
I
ATOM  28176  O   THR I  78      -9.424  20.471  -6.941  0.00  0.00
I
ATOM  28177  N   THR I  79      -9.175  18.927  -8.632  0.00  0.00
I
ATOM  28178  HN  THR I  79      -9.270  18.673  -9.591  0.00  0.00
I
ATOM  28179  CA  THR I  79      -8.552  17.843  -7.836  0.00  0.00
I
ATOM  28180  HA  THR I  79      -8.954  17.750  -6.837  0.00  0.00
I
ATOM  28181  CB  THR I  79      -8.904  16.474  -8.521  0.00  0.00
I
ATOM  28182  HB  THR I  79      -8.291  15.688  -8.031  0.00  0.00
I
ATOM  28183  OG1 THR I  79      -8.567  16.465  -9.905  0.00  0.00
I
ATOM  28184  HG1 THR I  79      -9.358  16.778 -10.350  0.00  0.00
I
ATOM  28185  CG2 THR I  79     -10.345  16.063  -8.281  0.00  0.00
I
ATOM  28186 HG21 THR I  79     -10.667  15.062  -8.641  0.00  0.00
I
ATOM  28187 HG22 THR I  79     -10.652  16.249  -7.229  0.00  0.00
I
```

ATOM 28188 HG23 THR I 79   -11.047 16.687 -8.875 0.00 0.00
I
ATOM 28189 C   THR I 79   -7.045 17.879 -7.614 0.00 0.00
I
ATOM 28190 O   THR I 79   -6.525 17.131 -6.837 0.00 0.00
I
ATOM 28191 N   VAL I 80   -6.434 18.776 -8.399 0.00 0.00
I
ATOM 28192 HN VAL I 80   -6.954 19.415 -8.962 0.00 0.00
I
ATOM 28193 CA VAL I 80   -4.971 19.019 -8.600 0.00 0.00
I
ATOM 28194 HA VAL I 80   -4.908 19.736 -9.405 0.00 0.00
I
ATOM 28195 CB VAL I 80   -4.171 19.639 -7.449 0.00 0.00
I
ATOM 28196 HB VAL I 80   -3.193 19.980 -7.850 0.00 0.00
I
ATOM 28197 CG1 VAL I 80   -4.822 21.047 -7.250 0.00 0.00
I
ATOM 28198 HG11 VAL I 80   -4.887 21.483 -8.270 0.00 0.00
I
ATOM 28199 HG12 VAL I 80   -5.824 20.912 -6.790 0.00 0.00
I
ATOM 28200 HG13 VAL I 80   -4.323 21.718 -6.519 0.00 0.00
I
ATOM 28201 CG2 VAL I 80   -4.008 18.824 -6.103 0.00 0.00
I
ATOM 28202 HG21 VAL I 80   -3.506 17.868 -6.364 0.00 0.00
I
ATOM 28203 HG22 VAL I 80   -3.390 19.183 -5.252 0.00 0.00
I
ATOM 28204 HG23 VAL I 80   -5.011 18.574 -5.696 0.00 0.00
I
ATOM 28205 C   VAL I 80   -4.243 17.824 -9.265 0.00 0.00
I
ATOM 28206 O   VAL I 80   -3.163 17.484 -8.777 0.00 0.00
I
ATOM 28207 N   THR I 81   -4.791 17.218 -10.318 0.00 0.00
I
ATOM 28208 HN THR I 81   -5.693 17.445 -10.678 0.00 0.00
I
ATOM 28209 CA THR I 81   -4.088 16.138 -10.961 0.00 0.00
I
ATOM 28210 HA THR I 81   -3.549 15.625 -10.179 0.00 0.00
I
ATOM 28211 CB THR I 81   -4.961 15.027 -11.491 0.00 0.00
I
ATOM 28212 HB THR I 81   -5.645 15.449 -12.258 0.00 0.00
I
ATOM 28213 OG1 THR I 81   -5.767 14.550 -10.475 0.00 0.00
I
ATOM 28214 HG1 THR I 81   -6.389 15.247 -10.252 0.00 0.00
I

```
ATOM  28215  CG2 THR I  81      -4.279  13.717 -11.792  0.00  0.00
I
ATOM  28216 HG21 THR I  81      -5.075  13.000 -12.086  0.00  0.00
I
ATOM  28217 HG22 THR I  81      -3.589  13.773 -12.662  0.00  0.00
I
ATOM  28218 HG23 THR I  81      -3.763  13.339 -10.884  0.00  0.00
I
ATOM  28219  C   THR I  81      -3.108  16.536 -12.019  0.00  0.00
I
ATOM  28220  O   THR I  81      -1.931  16.116 -11.969  0.00  0.00
I
ATOM  28221  N   GLY I  82      -3.450  17.325 -13.079  0.00  0.00
I
ATOM  28222  HN  GLY I  82      -4.278  17.875 -13.002  0.00  0.00
I
ATOM  28223  CA  GLY I  82      -2.601  17.437 -14.304  0.00  0.00
I
ATOM  28224  HA1 GLY I  82      -1.567  17.156 -14.162  0.00  0.00
I
ATOM  28225  HA2 GLY I  82      -2.688  18.473 -14.596  0.00  0.00
I
ATOM  28226  C   GLY I  82      -3.007  16.680 -15.524  0.00  0.00
I
ATOM  28227  O   GLY I  82      -2.490  16.914 -16.577  0.00  0.00
I
ATOM  28228  N   LEU I  83      -4.072  15.885 -15.550  0.00  0.00
I
ATOM  28229  HN  LEU I  83      -4.614  15.719 -14.730  0.00  0.00
I
ATOM  28230  CA  LEU I  83      -4.548  15.183 -16.700  0.00  0.00
I
ATOM  28231  HA  LEU I  83      -3.729  14.817 -17.301  0.00  0.00
I
ATOM  28232  CB  LEU I  83      -5.326  13.897 -16.248  0.00  0.00
I
ATOM  28233  HB1 LEU I  83      -6.264  14.107 -15.691  0.00  0.00
I
ATOM  28234  HB2 LEU I  83      -5.714  13.381 -17.152  0.00  0.00
I
ATOM  28235  CG  LEU I  83      -4.608  12.810 -15.482  0.00  0.00
I
ATOM  28236  HG  LEU I  83      -4.209  13.305 -14.572  0.00  0.00
I
ATOM  28237  CD1 LEU I  83      -5.552  11.665 -14.933  0.00  0.00
I
ATOM  28238 HD11 LEU I  83      -6.372  12.182 -14.391  0.00  0.00
I
ATOM  28239 HD12 LEU I  83      -5.910  11.041 -15.779  0.00  0.00
I
ATOM  28240 HD13 LEU I  83      -5.152  10.918 -14.215  0.00  0.00
I
ATOM  28241  CD2 LEU I  83      -3.419  12.323 -16.358  0.00  0.00
I
```

```
ATOM  28242 HD21 LEU I  83      -2.656  13.102 -16.570  0.00  0.00
I
ATOM  28243 HD22 LEU I  83      -2.846  11.534 -15.827  0.00  0.00
I
ATOM  28244 HD23 LEU I  83      -3.806  11.987 -17.344  0.00  0.00
I
ATOM  28245  C   LEU I  83      -5.401  16.080 -17.691  0.00  0.00
I
ATOM  28246  O   LEU I  83      -6.380  16.749 -17.332  0.00  0.00
I
ATOM  28247  N   ALA I  84      -5.010  16.001 -19.004  0.00  0.00
I
ATOM  28248  HN  ALA I  84      -4.229  15.416 -19.208  0.00  0.00
I
ATOM  28249  CA  ALA I  84      -5.587  16.929 -20.016  0.00  0.00
I
ATOM  28250  HA  ALA I  84      -6.387  17.533 -19.615  0.00  0.00
I
ATOM  28251  CB  ALA I  84      -4.429  17.861 -20.538  0.00  0.00
I
ATOM  28252  HB1 ALA I  84      -3.960  18.392 -19.682  0.00  0.00
I
ATOM  28253  HB2 ALA I  84      -3.626  17.204 -20.933  0.00  0.00
I
ATOM  28254  HB3 ALA I  84      -4.828  18.513 -21.344  0.00  0.00
I
ATOM  28255  C   ALA I  84      -6.061  16.105 -21.215  0.00  0.00
I
ATOM  28256  O   ALA I  84      -5.340  15.319 -21.821  0.00  0.00
I
ATOM  28257  N   VAL I  85      -7.339  16.140 -21.491  0.00  0.00
I
ATOM  28258  HN  VAL I  85      -7.827  16.781 -20.904  0.00  0.00
I
ATOM  28259  CA  VAL I  85      -7.951  15.491 -22.555  0.00  0.00
I
ATOM  28260  HA  VAL I  85      -7.448  14.577 -22.835  0.00  0.00
I
ATOM  28261  CB  VAL I  85      -9.369  15.060 -22.367  0.00  0.00
I
ATOM  28262  HB  VAL I  85      -9.725  14.878 -23.403  0.00  0.00
I
ATOM  28263  CG1 VAL I  85      -9.459  13.830 -21.387  0.00  0.00
I
ATOM  28264 HG11 VAL I  85      -8.933  14.078 -20.441  0.00  0.00
I
ATOM  28265 HG12 VAL I  85     -10.476  13.562 -21.028  0.00  0.00
I
ATOM  28266 HG13 VAL I  85      -8.981  12.969 -21.902  0.00  0.00
I
ATOM  28267  CG2 VAL I  85     -10.262  16.334 -22.094  0.00  0.00
I
ATOM  28268 HG21 VAL I  85     -10.036  17.151 -22.813  0.00  0.00
I
```

```
ATOM  28269 HG22 VAL I  85     -11.338  16.069 -22.166  0.00  0.00
I
ATOM  28270 HG23 VAL I  85     -10.147  16.703 -21.052  0.00  0.00
I
ATOM  28271  C   VAL I  85      -7.826  16.420 -23.816  0.00  0.00
I
ATOM  28272  O   VAL I  85      -7.741  15.952 -24.934  0.00  0.00
I
ATOM  28273  N   VAL I  86      -7.858  17.764 -23.620  0.00  0.00
I
ATOM  28274  HN  VAL I  86      -7.985  18.130 -22.701  0.00  0.00
I
ATOM  28275  CA  VAL I  86      -7.818  18.749 -24.671  0.00  0.00
I
ATOM  28276  HA  VAL I  86      -7.648  18.265 -25.622  0.00  0.00
I
ATOM  28277  CB  VAL I  86      -8.980  19.706 -24.844  0.00  0.00
I
ATOM  28278  HB  VAL I  86      -8.750  20.582 -25.488  0.00  0.00
I
ATOM  28279  CG1 VAL I  86     -10.160  18.794 -25.326  0.00  0.00
I
ATOM  28280 HG11 VAL I  86     -10.003  18.408 -26.356  0.00  0.00
I
ATOM  28281 HG12 VAL I  86     -10.487  18.048 -24.571  0.00  0.00
I
ATOM  28282 HG13 VAL I  86     -11.007  19.514 -25.323  0.00  0.00
I
ATOM  28283  CG2 VAL I  86      -9.452  20.208 -23.428  0.00  0.00
I
ATOM  28284 HG21 VAL I  86      -9.825  19.467 -22.689  0.00  0.00
I
ATOM  28285 HG22 VAL I  86      -8.618  20.674 -22.862  0.00  0.00
I
ATOM  28286 HG23 VAL I  86     -10.309  20.914 -23.471  0.00  0.00
I
ATOM  28287  C   VAL I  86      -6.633  19.581 -24.465  0.00  0.00
I
ATOM  28288  O   VAL I  86      -6.209  19.845 -23.277  0.00  0.00
I
ATOM  28289  N   ASP I  87      -6.015  20.103 -25.559  0.00  0.00
I
ATOM  28290  HN  ASP I  87      -6.488  20.076 -26.436  0.00  0.00
I
ATOM  28291  CA  ASP I  87      -4.671  20.749 -25.445  0.00  0.00
I
ATOM  28292  HA  ASP I  87      -4.058  20.304 -24.675  0.00  0.00
I
ATOM  28293  CB  ASP I  87      -3.967  20.488 -26.806  0.00  0.00
I
ATOM  28294  HB1 ASP I  87      -4.677  20.792 -27.605  0.00  0.00
I
ATOM  28295  HB2 ASP I  87      -3.005  21.035 -26.911  0.00  0.00
I
```

ATOM	28296	CG	ASP	I	87	-3.888	18.997	-27.009	0.00	0.00	I
ATOM	28297	OD1	ASP	I	87	-4.349	18.439	-28.076	0.00	0.00	I
ATOM	28298	OD2	ASP	I	87	-3.380	18.338	-26.116	0.00	0.00	I
ATOM	28299	C	ASP	I	87	-4.765	22.210	-25.127	0.00	0.00	I
ATOM	28300	O	ASP	I	87	-5.464	23.054	-25.732	0.00	0.00	I
ATOM	28301	N	THR	I	88	-4.200	22.634	-23.910	0.00	0.00	I
ATOM	28302	HN	THR	I	88	-3.699	21.977	-23.352	0.00	0.00	I
ATOM	28303	CA	THR	I	88	-4.508	23.914	-23.137	0.00	0.00	I
ATOM	28304	HA	THR	I	88	-5.556	24.007	-22.896	0.00	0.00	I
ATOM	28305	CB	THR	I	88	-3.723	23.941	-21.768	0.00	0.00	I
ATOM	28306	HB	THR	I	88	-2.675	24.216	-22.015	0.00	0.00	I
ATOM	28307	OG1	THR	I	88	-3.567	22.610	-21.205	0.00	0.00	I
ATOM	28308	HG1	THR	I	88	-2.923	22.657	-20.494	0.00	0.00	I
ATOM	28309	CG2	THR	I	88	-4.314	24.836	-20.754	0.00	0.00	I
ATOM	28310	HG21	THR	I	88	-5.406	24.647	-20.680	0.00	0.00	I
ATOM	28311	HG22	THR	I	88	-3.830	24.726	-19.760	0.00	0.00	I
ATOM	28312	HG23	THR	I	88	-4.226	25.901	-21.058	0.00	0.00	I
ATOM	28313	C	THR	I	88	-4.182	25.165	-23.902	0.00	0.00	I
ATOM	28314	O	THR	I	88	-4.893	26.124	-23.843	0.00	0.00	I
ATOM	28315	N	GLY	I	89	-3.117	25.243	-24.646	0.00	0.00	I
ATOM	28316	HN	GLY	I	89	-2.519	24.446	-24.671	0.00	0.00	I
ATOM	28317	CA	GLY	I	89	-2.884	26.390	-25.485	0.00	0.00	I
ATOM	28318	HA1	GLY	I	89	-1.831	26.448	-25.717	0.00	0.00	I
ATOM	28319	HA2	GLY	I	89	-3.049	27.308	-24.941	0.00	0.00	I
ATOM	28320	C	GLY	I	89	-3.664	26.415	-26.829	0.00	0.00	I
ATOM	28321	O	GLY	I	89	-3.573	27.332	-27.610	0.00	0.00	I
ATOM	28322	N	THR	I	90	-4.423	25.342	-27.120	0.00	0.00	I

ATOM 28323 HN THR I 90 −4.493 24.587 −26.473 0.00 0.00
I
ATOM 28324 CA THR I 90 −5.063 25.254 −28.381 0.00 0.00
I
ATOM 28325 HA THR I 90 −4.732 25.968 −29.121 0.00 0.00
I
ATOM 28326 CB THR I 90 −4.752 23.948 −29.050 0.00 0.00
I
ATOM 28327 HB THR I 90 −5.220 23.173 −28.406 0.00 0.00
I
ATOM 28328 OG1 THR I 90 −3.339 23.716 −29.262 0.00 0.00
I
ATOM 28329 HG1 THR I 90 −3.329 22.937 −29.823 0.00 0.00
I
ATOM 28330 CG2 THR I 90 −5.407 23.867 −30.473 0.00 0.00
I
ATOM 28331 HG21 THR I 90 −5.001 24.678 −31.114 0.00 0.00
I
ATOM 28332 HG22 THR I 90 −5.166 22.838 −30.813 0.00 0.00
I
ATOM 28333 HG23 THR I 90 −6.518 23.859 −30.456 0.00 0.00
I
ATOM 28334 C THR I 90 −6.534 25.343 −28.286 0.00 0.00
I
ATOM 28335 O THR I 90 −7.173 26.129 −29.014 0.00 0.00
I
ATOM 28336 N GLN I 91 −7.178 24.600 −27.317 0.00 0.00
I
ATOM 28337 HN GLN I 91 −6.709 23.857 −26.847 0.00 0.00
I
ATOM 28338 CA GLN I 91 −8.621 24.523 −27.314 0.00 0.00
I
ATOM 28339 HA GLN I 91 −8.946 24.437 −28.340 0.00 0.00
I
ATOM 28340 CB GLN I 91 −9.038 23.332 −26.385 0.00 0.00
I
ATOM 28341 HB1 GLN I 91 −8.575 22.378 −26.717 0.00 0.00
I
ATOM 28342 HB2 GLN I 91 −8.585 23.673 −25.430 0.00 0.00
I
ATOM 28343 CG GLN I 91 −10.572 23.097 −26.084 0.00 0.00
I
ATOM 28344 HG1 GLN I 91 −10.574 22.373 −25.241 0.00 0.00
I
ATOM 28345 HG2 GLN I 91 −11.082 24.012 −25.714 0.00 0.00
I
ATOM 28346 CD GLN I 91 −11.340 22.468 −27.257 0.00 0.00
I
ATOM 28347 OE1 GLN I 91 −10.840 21.868 −28.193 0.00 0.00
I
ATOM 28348 NE2 GLN I 91 −12.679 22.646 −27.231 0.00 0.00
I
ATOM 28349 HE21 GLN I 91 −13.259 22.182 −27.901 0.00 0.00
I

ATOM 28350 HE22 GLN I 91   -13.090 23.186 -26.497 0.00 0.00
I
ATOM 28351 C   GLN I 91   -9.227 25.736 -26.662 0.00 0.00
I
ATOM 28352 O   GLN I 91   -10.366 26.078 -27.001 0.00 0.00
I
ATOM 28353 N   PHE I 92   -8.538 26.433 -25.770 0.00 0.00
I
ATOM 28354 HN PHE I 92   -7.577 26.202 -25.637 0.00 0.00
I
ATOM 28355 CA PHE I 92   -9.113 27.387 -24.834 0.00 0.00
I
ATOM 28356 HA PHE I 92   -10.191 27.402 -24.903 0.00 0.00
I
ATOM 28357 CB PHE I 92   -8.643 27.117 -23.406 0.00 0.00
I
ATOM 28358 HB1 PHE I 92   -7.534 27.077 -23.437 0.00 0.00
I
ATOM 28359 HB2 PHE I 92   -8.832 27.991 -22.745 0.00 0.00
I
ATOM 28360 CG PHE I 92   -9.186 25.824 -22.811 0.00 0.00
I
ATOM 28361 CD1 PHE I 92   -10.566 25.593 -22.750 0.00 0.00
I
ATOM 28362 HD1 PHE I 92   -11.258 26.272 -23.227 0.00 0.00
I
ATOM 28363 CE1 PHE I 92   -10.957 24.371 -22.247 0.00 0.00
I
ATOM 28364 HE1 PHE I 92   -12.027 24.221 -22.244 0.00 0.00
I
ATOM 28365 CZ PHE I 92   -10.069 23.455 -21.653 0.00 0.00
I
ATOM 28366 HZ PHE I 92   -10.406 22.567 -21.140 0.00 0.00
I
ATOM 28367 CD2 PHE I 92   -8.284 24.944 -22.285 0.00 0.00
I
ATOM 28368 HD2 PHE I 92   -7.258 25.277 -22.342 0.00 0.00
I
ATOM 28369 CE2 PHE I 92   -8.736 23.762 -21.654 0.00 0.00
I
ATOM 28370 HE2 PHE I 92   -8.013 23.093 -21.213 0.00 0.00
I
ATOM 28371 C   PHE I 92   -8.561 28.712 -25.289 0.00 0.00
I
ATOM 28372 O   PHE I 92   -7.655 28.841 -26.098 0.00 0.00
I
ATOM 28373 N   THR I 93   -9.175 29.766 -24.808 0.00 0.00
I
ATOM 28374 HN THR I 93   -10.041 29.519 -24.381 0.00 0.00
I
ATOM 28375 CA THR I 93   -8.685 31.113 -24.922 0.00 0.00
I
ATOM 28376 HA THR I 93   -8.013 31.279 -25.751 0.00 0.00
I

ATOM 28377 CB THR I 93   -9.803 32.145 -25.093 0.00 0.00
I
ATOM 28378 HB THR I 93   -9.497 33.211 -25.162 0.00 0.00
I
ATOM 28379 OG1 THR I 93   -10.747 31.929 -24.107 0.00 0.00
I
ATOM 28380 HG1 THR I 93   -11.418 32.614 -24.153 0.00 0.00
I
ATOM 28381 CG2 THR I 93   -10.505 31.909 -26.398 0.00 0.00
I
ATOM 28382 HG21 THR I 93   -11.207 32.671 -26.798 0.00 0.00
I
ATOM 28383 HG22 THR I 93   -9.745 31.720 -27.186 0.00 0.00
I
ATOM 28384 HG23 THR I 93   -11.150 31.017 -26.247 0.00 0.00
I
ATOM 28385 C   THR I 93   -7.930 31.399 -23.581 0.00 0.00
I
ATOM 28386 O   THR I 93   -7.982 30.667 -22.606 0.00 0.00
I
ATOM 28387 N   VAL I 94   -7.353 32.580 -23.405 0.00 0.00
I
ATOM 28388 HN VAL I 94   -7.344 33.275 -24.120 0.00 0.00
I
ATOM 28389 CA VAL I 94   -6.673 33.002 -22.175 0.00 0.00
I
ATOM 28390 HA VAL I 94   -6.054 32.156 -21.913 0.00 0.00
I
ATOM 28391 CB VAL I 94   -5.880 34.206 -22.461 0.00 0.00
I
ATOM 28392 HB VAL I 94   -6.443 35.106 -22.788 0.00 0.00
I
ATOM 28393 CG1 VAL I 94   -5.219 34.709 -21.143 0.00 0.00
I
ATOM 28394 HG11 VAL I 94   -4.396 35.444 -21.276 0.00 0.00
I
ATOM 28395 HG12 VAL I 94   -5.840 35.255 -20.401 0.00 0.00
I
ATOM 28396 HG13 VAL I 94   -4.714 33.890 -20.588 0.00 0.00
I
ATOM 28397 CG2 VAL I 94   -4.791 33.949 -23.465 0.00 0.00
I
ATOM 28398 HG21 VAL I 94   -4.087 33.152 -23.142 0.00 0.00
I
ATOM 28399 HG22 VAL I 94   -5.146 33.693 -24.486 0.00 0.00
I
ATOM 28400 HG23 VAL I 94   -4.205 34.881 -23.615 0.00 0.00
I
ATOM 28401 C   VAL I 94   -7.706 33.204 -21.082 0.00 0.00
I
ATOM 28402 O   VAL I 94   -7.414 32.933 -19.891 0.00 0.00
I
ATOM 28403 N   PHE I 95   -8.940 33.644 -21.416 0.00 0.00
I

ATOM 28404 HN PHE I 95 -9.094 33.955 -22.351 0.00 0.00
I
ATOM 28405 CA PHE I 95 -10.044 33.593 -20.445 0.00 0.00
I
ATOM 28406 HA PHE I 95 -9.963 34.212 -19.563 0.00 0.00
I
ATOM 28407 CB PHE I 95 -11.452 33.926 -21.070 0.00 0.00
I
ATOM 28408 HB1 PHE I 95 -11.594 33.449 -22.064 0.00 0.00
I
ATOM 28409 HB2 PHE I 95 -12.299 33.719 -20.382 0.00 0.00
I
ATOM 28410 CG PHE I 95 -11.547 35.429 -21.322 0.00 0.00
I
ATOM 28411 CD1 PHE I 95 -12.199 35.857 -22.454 0.00 0.00
I
ATOM 28412 HD1 PHE I 95 -12.569 35.105 -23.135 0.00 0.00
I
ATOM 28413 CE1 PHE I 95 -12.461 37.223 -22.662 0.00 0.00
I
ATOM 28414 HE1 PHE I 95 -12.936 37.321 -23.627 0.00 0.00
I
ATOM 28415 CZ PHE I 95 -12.086 38.178 -21.715 0.00 0.00
I
ATOM 28416 HZ PHE I 95 -12.358 39.218 -21.821 0.00 0.00
I
ATOM 28417 CD2 PHE I 95 -11.257 36.394 -20.326 0.00 0.00
I
ATOM 28418 HD2 PHE I 95 -10.858 36.010 -19.399 0.00 0.00
I
ATOM 28419 CE2 PHE I 95 -11.468 37.807 -20.520 0.00 0.00
I
ATOM 28420 HE2 PHE I 95 -11.141 38.589 -19.852 0.00 0.00
I
ATOM 28421 C PHE I 95 -10.422 32.241 -19.812 0.00 0.00
I
ATOM 28422 O PHE I 95 -10.524 32.079 -18.606 0.00 0.00
I
ATOM 28423 N GLY I 96 -10.360 31.157 -20.585 0.00 0.00
I
ATOM 28424 HN GLY I 96 -10.238 31.272 -21.567 0.00 0.00
I
ATOM 28425 CA GLY I 96 -10.496 29.753 -20.170 0.00 0.00
I
ATOM 28426 HA1 GLY I 96 -10.554 29.199 -21.095 0.00 0.00
I
ATOM 28427 HA2 GLY I 96 -11.244 29.734 -19.391 0.00 0.00
I
ATOM 28428 C GLY I 96 -9.244 29.203 -19.452 0.00 0.00
I
ATOM 28429 O GLY I 96 -9.358 28.614 -18.363 0.00 0.00
I
ATOM 28430 N GLN I 97 -8.041 29.597 -19.969 0.00 0.00
I

```
ATOM  28431  HN  GLN I  97      -7.957  30.114 -20.817  0.00  0.00
I
ATOM  28432  CA  GLN I  97      -6.849  29.205 -19.250  0.00  0.00
I
ATOM  28433  HA  GLN I  97      -6.899  28.137 -19.102  0.00  0.00
I
ATOM  28434  CB  GLN I  97      -5.448  29.577 -19.907  0.00  0.00
I
ATOM  28435  HB1 GLN I  97      -5.381  30.683 -19.990  0.00  0.00
I
ATOM  28436  HB2 GLN I  97      -4.579  29.228 -19.310  0.00  0.00
I
ATOM  28437  CG  GLN I  97      -5.246  28.938 -21.249  0.00  0.00
I
ATOM  28438  HG1 GLN I  97      -4.858  27.908 -21.102  0.00  0.00
I
ATOM  28439  HG2 GLN I  97      -6.191  28.951 -21.834  0.00  0.00
I
ATOM  28440  CD  GLN I  97      -4.288  29.577 -22.204  0.00  0.00
I
ATOM  28441  OE1 GLN I  97      -3.226  30.104 -21.864  0.00  0.00
I
ATOM  28442  NE2 GLN I  97      -4.663  29.702 -23.521  0.00  0.00
I
ATOM  28443 HE21 GLN I  97      -3.938  29.978 -24.152  0.00  0.00
I
ATOM  28444 HE22 GLN I  97      -5.482  29.209 -23.817  0.00  0.00
I
ATOM  28445  C   GLN I  97      -6.667  29.866 -17.916  0.00  0.00
I
ATOM  28446  O   GLN I  97      -6.211  29.229 -16.950  0.00  0.00
I
ATOM  28447  N   THR I  98      -7.129  31.100 -17.692  0.00  0.00
I
ATOM  28448  HN  THR I  98      -7.410  31.634 -18.485  0.00  0.00
I
ATOM  28449  CA  THR I  98      -7.431  31.774 -16.409  0.00  0.00
I
ATOM  28450  HA  THR I  98      -6.519  31.826 -15.833  0.00  0.00
I
ATOM  28451  CB  THR I  98      -8.016  33.156 -16.690  0.00  0.00
I
ATOM  28452  HB  THR I  98      -9.046  33.088 -17.100  0.00  0.00
I
ATOM  28453  OG1 THR I  98      -7.183  34.025 -17.489  0.00  0.00
I
ATOM  28454  HG1 THR I  98      -7.185  33.690 -18.388  0.00  0.00
I
ATOM  28455  CG2 THR I  98      -8.133  34.027 -15.379  0.00  0.00
I
ATOM  28456 HG21 THR I  98      -9.016  33.777 -14.753  0.00  0.00
I
ATOM  28457 HG22 THR I  98      -7.196  33.892 -14.797  0.00  0.00
I
```

```
ATOM  28458 HG23 THR I  98      -8.267  35.084 -15.693  0.00  0.00
I
ATOM  28459  C   THR I  98      -8.442  31.061 -15.507  0.00  0.00
I
ATOM  28460  O   THR I  98      -8.141  30.843 -14.295  0.00  0.00
I
ATOM  28461  N   VAL I  99      -9.575  30.515 -16.116  0.00  0.00
I
ATOM  28462  HN  VAL I  99      -9.815  30.752 -17.054  0.00  0.00
I
ATOM  28463  CA  VAL I  99     -10.548  29.620 -15.447  0.00  0.00
I
ATOM  28464  HA  VAL I  99     -10.717  30.181 -14.540  0.00  0.00
I
ATOM  28465  CB  VAL I  99     -11.747  29.186 -16.364  0.00  0.00
I
ATOM  28466  HB  VAL I  99     -11.337  28.813 -17.327  0.00  0.00
I
ATOM  28467  CG1 VAL I  99     -12.521  28.074 -15.620  0.00  0.00
I
ATOM  28468 HG11 VAL I  99     -13.492  27.807 -16.089  0.00  0.00
I
ATOM  28469 HG12 VAL I  99     -11.940  27.136 -15.487  0.00  0.00
I
ATOM  28470 HG13 VAL I  99     -12.804  28.405 -14.599  0.00  0.00
I
ATOM  28471  CG2 VAL I  99     -12.668  30.423 -16.707  0.00  0.00
I
ATOM  28472 HG21 VAL I  99     -13.485  30.136 -17.404  0.00  0.00
I
ATOM  28473 HG22 VAL I  99     -13.231  30.615 -15.769  0.00  0.00
I
ATOM  28474 HG23 VAL I  99     -12.122  31.325 -17.057  0.00  0.00
I
ATOM  28475  C   VAL I  99      -9.870  28.389 -14.785  0.00  0.00
I
ATOM  28476  O   VAL I  99      -9.943  28.167 -13.566  0.00  0.00
I
ATOM  28477  N   ILE I 100      -9.025  27.673 -15.594  0.00  0.00
I
ATOM  28478  HN  ILE I 100      -8.809  27.838 -16.554  0.00  0.00
I
ATOM  28479  CA  ILE I 100      -8.284  26.577 -14.970  0.00  0.00
I
ATOM  28480  HA  ILE I 100      -9.051  25.912 -14.601  0.00  0.00
I
ATOM  28481  CB  ILE I 100      -7.459  25.998 -16.084  0.00  0.00
I
ATOM  28482  HB  ILE I 100      -6.869  26.828 -16.528  0.00  0.00
I
ATOM  28483  CG2 ILE I 100      -6.566  24.863 -15.452  0.00  0.00
I
ATOM  28484 HG21 ILE I 100      -5.809  25.301 -14.768  0.00  0.00
I
```

```
ATOM  28485 HG22 ILE I 100      -7.150  24.159 -14.822  0.00  0.00
I
ATOM  28486 HG23 ILE I 100      -6.074  24.256 -16.242  0.00  0.00
I
ATOM  28487  CG1 ILE I 100      -8.203  25.508 -17.300  0.00  0.00
I
ATOM  28488 HG11 ILE I 100      -9.173  25.048 -17.012  0.00  0.00
I
ATOM  28489 HG12 ILE I 100      -8.591  26.382 -17.866  0.00  0.00
I
ATOM  28490  CD  ILE I 100      -7.458  24.568 -18.347  0.00  0.00
I
ATOM  28491  HD1 ILE I 100      -7.446  23.595 -17.811  0.00  0.00
I
ATOM  28492  HD2 ILE I 100      -8.156  24.563 -19.211  0.00  0.00
I
ATOM  28493  HD3 ILE I 100      -6.583  25.062 -18.820  0.00  0.00
I
ATOM  28494  C   ILE I 100      -7.390  26.972 -13.802  0.00  0.00
I
ATOM  28495  O   ILE I 100      -7.428  26.390 -12.763  0.00  0.00
I
ATOM  28496  N   MET I 101      -6.590  28.052 -14.041  0.00  0.00
I
ATOM  28497  HN  MET I 101      -6.671  28.510 -14.923  0.00  0.00
I
ATOM  28498  CA  MET I 101      -5.613  28.591 -13.116  0.00  0.00
I
ATOM  28499  HA  MET I 101      -4.973  27.762 -12.853  0.00  0.00
I
ATOM  28500  CB  MET I 101      -4.747  29.550 -13.910  0.00  0.00
I
ATOM  28501  HB1 MET I 101      -4.515  29.019 -14.858  0.00  0.00
I
ATOM  28502  HB2 MET I 101      -5.331  30.411 -14.299  0.00  0.00
I
ATOM  28503  CG  MET I 101      -3.444  30.065 -13.277  0.00  0.00
I
ATOM  28504  HG1 MET I 101      -3.611  30.481 -12.260  0.00  0.00
I
ATOM  28505  HG2 MET I 101      -2.789  29.177 -13.150  0.00  0.00
I
ATOM  28506  SD  MET I 101      -2.623  31.281 -14.384  0.00  0.00
I
ATOM  28507  CE  MET I 101      -3.585  32.743 -13.900  0.00  0.00
I
ATOM  28508  HE1 MET I 101      -4.005  32.558 -12.888  0.00  0.00
I
ATOM  28509  HE2 MET I 101      -3.047  33.713 -13.834  0.00  0.00
I
ATOM  28510  HE3 MET I 101      -4.384  32.851 -14.664  0.00  0.00
I
ATOM  28511  C   MET I 101      -6.185  29.183 -11.808  0.00  0.00
I
```

ATOM 28512 O   MET I 101   -5.499 29.188 -10.805 0.00 0.00
I
ATOM 28513 N   GLY I 102   -7.506 29.627 -11.785 0.00 0.00
I
ATOM 28514 HN GLY I 102   -7.814 29.820 -12.713 0.00 0.00
I
ATOM 28515 CA GLY I 102   -8.304 30.098 -10.668 0.00 0.00
I
ATOM 28516 HA1 GLY I 102   -9.178 30.620 -11.027 0.00 0.00
I
ATOM 28517 HA2 GLY I 102   -7.692 30.693 -10.006 0.00 0.00
I
ATOM 28518 C   GLY I 102   -8.943 28.917 -9.933 0.00 0.00
I
ATOM 28519 O   GLY I 102   -8.992 29.053 -8.734 0.00 0.00
I
ATOM 28520 N   LEU I 103   -9.357 27.851 -10.602 0.00 0.00
I
ATOM 28521 HN LEU I 103   -9.314 27.880 -11.597 0.00 0.00
I
ATOM 28522 CA LEU I 103   -10.032 26.705 -9.949 0.00 0.00
I
ATOM 28523 HA LEU I 103   -10.928 27.012 -9.430 0.00 0.00
I
ATOM 28524 CB LEU I 103   -10.525 25.658 -11.044 0.00 0.00
I
ATOM 28525 HB1 LEU I 103   -9.672 25.574 -11.752 0.00 0.00
I
ATOM 28526 HB2 LEU I 103   -10.613 24.670 -10.544 0.00 0.00
I
ATOM 28527 CG LEU I 103   -11.916 25.884 -11.759 0.00 0.00
I
ATOM 28528 HG LEU I 103   -11.843 26.891 -12.223 0.00 0.00
I
ATOM 28529 CD1 LEU I 103   -12.215 25.082 -13.054 0.00 0.00
I
ATOM 28530 HD11 LEU I 103   -11.382 25.374 -13.729 0.00 0.00
I
ATOM 28531 HD12 LEU I 103   -12.369 24.007 -12.821 0.00 0.00
I
ATOM 28532 HD13 LEU I 103   -13.081 25.499 -13.611 0.00 0.00
I
ATOM 28533 CD2 LEU I 103   -13.052 25.990 -10.714 0.00 0.00
I
ATOM 28534 HD21 LEU I 103   -12.935 25.303 -9.849 0.00 0.00
I
ATOM 28535 HD22 LEU I 103   -13.143 27.027 -10.324 0.00 0.00
I
ATOM 28536 HD23 LEU I 103   -14.067 25.912 -11.158 0.00 0.00
I
ATOM 28537 C   LEU I 103   -9.025 25.996 -9.007 0.00 0.00
I
ATOM 28538 O   LEU I 103   -9.346 25.444 -7.925 0.00 0.00
I

ATOM 28539 N   ILE I 104   -7.754 25.905 -9.442 0.00 0.00
I
ATOM 28540 HN ILE I 104   -7.438 26.483 -10.190 0.00 0.00
I
ATOM 28541 CA ILE I 104   -6.710 25.281 -8.548 0.00 0.00
I
ATOM 28542 HA ILE I 104   -7.099 24.310 -8.281 0.00 0.00
I
ATOM 28543 CB ILE I 104   -5.411 25.194 -9.421 0.00 0.00
I
ATOM 28544 HB ILE I 104   -5.199 26.201 -9.841 0.00 0.00
I
ATOM 28545 CG2 ILE I 104   -4.236 24.809 -8.508 0.00 0.00
I
ATOM 28546 HG21 ILE I 104   -4.192 23.720 -8.296 0.00 0.00
I
ATOM 28547 HG22 ILE I 104   -3.249 24.970 -8.992 0.00 0.00
I
ATOM 28548 HG23 ILE I 104   -4.156 25.434 -7.592 0.00 0.00
I
ATOM 28549 CG1 ILE I 104   -5.617 24.199 -10.541 0.00 0.00
I
ATOM 28550 HG11 ILE I 104   -5.552 23.247 -9.973 0.00 0.00
I
ATOM 28551 HG12 ILE I 104   -6.593 24.269 -11.066 0.00 0.00
I
ATOM 28552 CD ILE I 104   -4.558 24.352 -11.605 0.00 0.00
I
ATOM 28553 HD1 ILE I 104   -4.708 25.309 -12.148 0.00 0.00
I
ATOM 28554 HD2 ILE I 104   -3.526 24.295 -11.197 0.00 0.00
I
ATOM 28555 HD3 ILE I 104   -4.624 23.505 -12.322 0.00 0.00
I
ATOM 28556 C   ILE I 104   -6.448 25.993 -7.179 0.00 0.00
I
ATOM 28557 O   ILE I 104   -6.262 25.324 -6.148 0.00 0.00
I
ATOM 28558 N   GLN I 105   -6.603 27.328 -7.096 0.00 0.00
I
ATOM 28559 HN GLN I 105   -6.900 27.791 -7.928 0.00 0.00
I
ATOM 28560 CA GLN I 105   -6.698 28.063 -5.814 0.00 0.00
I
ATOM 28561 HA GLN I 105   -5.888 27.755 -5.169 0.00 0.00
I
ATOM 28562 CB GLN I 105   -6.570 29.586 -6.013 0.00 0.00
I
ATOM 28563 HB1 GLN I 105   -7.557 29.961 -6.358 0.00 0.00
I
ATOM 28564 HB2 GLN I 105   -6.383 30.192 -5.101 0.00 0.00
I
ATOM 28565 CG GLN I 105   -5.549 30.025 -7.064 0.00 0.00
I

```
ATOM  28566  HG1 GLN I 105      -5.714  29.533  -8.046  0.00  0.00
I
ATOM  28567  HG2 GLN I 105      -5.749  31.097  -7.276  0.00  0.00
I
ATOM  28568  CD  GLN I 105      -4.058  29.881  -6.790  0.00  0.00
I
ATOM  28569  OE1 GLN I 105      -3.535  29.162  -5.952  0.00  0.00
I
ATOM  28570  NE2 GLN I 105      -3.311  30.732  -7.541  0.00  0.00
I
ATOM  28571 HE21 GLN I 105      -2.350  30.937  -7.355  0.00  0.00
I
ATOM  28572 HE22 GLN I 105      -3.762  31.455  -8.065  0.00  0.00
I
ATOM  28573  C   GLN I 105      -7.908  27.670  -4.999  0.00  0.00
I
ATOM  28574  O   GLN I 105      -7.830  27.253  -3.857  0.00  0.00
I
ATOM  28575  N   ILE I 106      -9.107  27.660  -5.631  0.00  0.00
I
ATOM  28576  HN  ILE I 106      -9.192  28.115  -6.514  0.00  0.00
I
ATOM  28577  CA  ILE I 106     -10.422  27.351  -4.990  0.00  0.00
I
ATOM  28578  HA  ILE I 106     -10.532  28.012  -4.143  0.00  0.00
I
ATOM  28579  CB  ILE I 106     -11.583  27.594  -5.955  0.00  0.00
I
ATOM  28580  HB  ILE I 106     -11.505  26.957  -6.862  0.00  0.00
I
ATOM  28581  CG2 ILE I 106     -12.927  27.307  -5.154  0.00  0.00
I
ATOM  28582 HG21 ILE I 106     -12.961  26.268  -4.764  0.00  0.00
I
ATOM  28583 HG22 ILE I 106     -13.002  28.048  -4.330  0.00  0.00
I
ATOM  28584 HG23 ILE I 106     -13.704  27.463  -5.934  0.00  0.00
I
ATOM  28585  CG1 ILE I 106     -11.696  29.045  -6.463  0.00  0.00
I
ATOM  28586 HG11 ILE I 106     -12.165  29.695  -5.694  0.00  0.00
I
ATOM  28587 HG12 ILE I 106     -10.686  29.467  -6.659  0.00  0.00
I
ATOM  28588  CD  ILE I 106     -12.605  29.240  -7.655  0.00  0.00
I
ATOM  28589  HD1 ILE I 106     -12.603  30.250  -8.117  0.00  0.00
I
ATOM  28590  HD2 ILE I 106     -12.296  28.517  -8.440  0.00  0.00
I
ATOM  28591  HD3 ILE I 106     -13.672  29.061  -7.406  0.00  0.00
I
ATOM  28592  C   ILE I 106     -10.471  25.934  -4.457  0.00  0.00
I
```

ATOM 28593 O   ILE I 106   -11.012 25.554 -3.463 0.00 0.00
I
ATOM 28594 N   GLY I 107   -9.797 25.031 -5.206 0.00 0.00
I
ATOM 28595 HN GLY I 107   -9.475 25.268 -6.119 0.00 0.00
I
ATOM 28596 CA GLY I 107   -9.735 23.614 -4.766 0.00 0.00
I
ATOM 28597 HA1 GLY I 107   -9.242 23.080 -5.565 0.00 0.00
I
ATOM 28598 HA2 GLY I 107   -10.727 23.222 -4.597 0.00 0.00
I
ATOM 28599 C   GLY I 107   -8.853 23.330 -3.550 0.00 0.00
I
ATOM 28600 O   GLY I 107   -9.044 22.440 -2.780 0.00 0.00
I
ATOM 28601 N   GLY I 108   -7.795 24.190 -3.442 0.00 0.00
I
ATOM 28602 HN GLY I 108   -7.670 24.785 -4.232 0.00 0.00
I
ATOM 28603 CA GLY I 108   -6.724 24.085 -2.394 0.00 0.00
I
ATOM 28604 HA1 GLY I 108   -5.796 24.558 -2.679 0.00 0.00
I
ATOM 28605 HA2 GLY I 108   -6.684 23.006 -2.409 0.00 0.00
I
ATOM 28606 C   GLY I 108   -7.129 24.496 -1.007 0.00 0.00
I
ATOM 28607 O   GLY I 108   -6.698 23.912 -0.027 0.00 0.00
I
ATOM 28608 N   LEU I 109   -7.973 25.528 -0.961 0.00 0.00
I
ATOM 28609 HN LEU I 109   -8.209 25.991 -1.812 0.00 0.00
I
ATOM 28610 CA LEU I 109   -8.694 25.933   0.206 0.00 0.00
I
ATOM 28611 HA LEU I 109   -8.069 25.727   1.063 0.00 0.00
I
ATOM 28612 CB LEU I 109   -8.994 27.453   0.166 0.00 0.00
I
ATOM 28613 HB1 LEU I 109   -9.416 27.721 -0.826 0.00 0.00
I
ATOM 28614 HB2 LEU I 109   -9.555 27.786   1.065 0.00 0.00
I
ATOM 28615 CG LEU I 109   -7.652 28.345   0.305 0.00 0.00
I
ATOM 28616 HG LEU I 109   -7.081 28.324 -0.647 0.00 0.00
I
ATOM 28617 CD1 LEU I 109   -8.013 29.889   0.386 0.00 0.00
I
ATOM 28618 HD11 LEU I 109   -8.391 30.075   1.414 0.00 0.00
I
ATOM 28619 HD12 LEU I 109   -7.059 30.457   0.338 0.00 0.00
I

```
ATOM  28620 HD13 LEU I 109      -8.728  30.246  -0.386  0.00  0.00
I
ATOM  28621  CD2 LEU I 109      -6.751  27.907   1.444  0.00  0.00
I
ATOM  28622 HD21 LEU I 109      -5.877  28.547   1.690  0.00  0.00
I
ATOM  28623 HD22 LEU I 109      -7.390  27.943   2.352  0.00  0.00
I
ATOM  28624 HD23 LEU I 109      -6.393  26.866   1.293  0.00  0.00
I
ATOM  28625  C   LEU I 109     -10.012  25.222   0.502  0.00  0.00
I
ATOM  28626  O   LEU I 109     -10.450  25.022   1.643  0.00  0.00
I
ATOM  28627  N   GLY I 110     -10.765  24.813  -0.591  0.00  0.00
I
ATOM  28628  HN  GLY I 110     -10.559  24.952  -1.556  0.00  0.00
I
ATOM  28629  CA  GLY I 110     -12.182  24.241  -0.442  0.00  0.00
I
ATOM  28630  HA1 GLY I 110     -12.162  23.896   0.581  0.00  0.00
I
ATOM  28631  HA2 GLY I 110     -12.342  23.481  -1.193  0.00  0.00
I
ATOM  28632  C   GLY I 110     -13.231  25.282  -0.474  0.00  0.00
I
ATOM  28633  O   GLY I 110     -13.076  26.392   0.011  0.00  0.00
I
ATOM  28634  N   PHE I 111     -14.380  25.003  -1.147  0.00  0.00
I
ATOM  28635  HN  PHE I 111     -14.534  24.048  -1.390  0.00  0.00
I
ATOM  28636  CA  PHE I 111     -15.419  25.934  -1.530  0.00  0.00
I
ATOM  28637  HA  PHE I 111     -15.088  26.579  -2.332  0.00  0.00
I
ATOM  28638  CB  PHE I 111     -16.501  25.060  -2.186  0.00  0.00
I
ATOM  28639  HB1 PHE I 111     -16.064  24.246  -2.803  0.00  0.00
I
ATOM  28640  HB2 PHE I 111     -17.073  24.453  -1.451  0.00  0.00
I
ATOM  28641  CG  PHE I 111     -17.511  25.853  -3.032  0.00  0.00
I
ATOM  28642  CD1 PHE I 111     -17.105  26.589  -4.085  0.00  0.00
I
ATOM  28643  HD1 PHE I 111     -16.046  26.682  -4.274  0.00  0.00
I
ATOM  28644  CE1 PHE I 111     -18.022  27.371  -4.767  0.00  0.00
I
ATOM  28645  HE1 PHE I 111     -17.738  27.966  -5.622  0.00  0.00
I
ATOM  28646  CZ  PHE I 111     -19.394  27.322  -4.516  0.00  0.00
I
```

ATOM 28647 HZ PHE I 111 -20.196 27.795 -5.062 0.00 0.00
I
ATOM 28648 CD2 PHE I 111 -18.855 25.788 -2.701 0.00 0.00
I
ATOM 28649 HD2 PHE I 111 -19.146 25.157 -1.875 0.00 0.00
I
ATOM 28650 CE2 PHE I 111 -19.804 26.488 -3.410 0.00 0.00
I
ATOM 28651 HE2 PHE I 111 -20.828 26.376 -3.085 0.00 0.00
I
ATOM 28652 C PHE I 111 -15.998 26.850 -0.425 0.00 0.00
I
ATOM 28653 O PHE I 111 -16.022 28.042 -0.523 0.00 0.00
I
ATOM 28654 N MET I 112 -16.387 26.340 0.716 0.00 0.00
I
ATOM 28655 HN MET I 112 -16.284 25.382 0.973 0.00 0.00
I
ATOM 28656 CA MET I 112 -17.054 27.044 1.714 0.00 0.00
I
ATOM 28657 HA MET I 112 -17.886 27.514 1.210 0.00 0.00
I
ATOM 28658 CB MET I 112 -17.600 25.958 2.643 0.00 0.00
I
ATOM 28659 HB1 MET I 112 -16.690 25.446 3.022 0.00 0.00
I
ATOM 28660 HB2 MET I 112 -18.021 26.483 3.527 0.00 0.00
I
ATOM 28661 CG MET I 112 -18.544 24.886 2.006 0.00 0.00
I
ATOM 28662 HG1 MET I 112 -17.956 24.219 1.340 0.00 0.00
I
ATOM 28663 HG2 MET I 112 -18.936 24.306 2.868 0.00 0.00
I
ATOM 28664 SD MET I 112 -20.035 25.521 1.067 0.00 0.00
I
ATOM 28665 CE MET I 112 -20.675 23.911 0.509 0.00 0.00
I
ATOM 28666 HE1 MET I 112 -21.727 23.982 0.159 0.00 0.00
I
ATOM 28667 HE2 MET I 112 -20.100 23.521 -0.358 0.00 0.00
I
ATOM 28668 HE3 MET I 112 -20.428 23.075 1.197 0.00 0.00
I
ATOM 28669 C MET I 112 -16.226 27.940 2.586 0.00 0.00
I
ATOM 28670 O MET I 112 -16.712 28.896 3.182 0.00 0.00
I
ATOM 28671 N THR I 113 -14.898 27.662 2.685 0.00 0.00
I
ATOM 28672 HN THR I 113 -14.556 26.851 2.217 0.00 0.00
I
ATOM 28673 CA THR I 113 -13.722 28.271 3.416 0.00 0.00
I

```
ATOM  28674  HA  THR I 113     -13.798  28.156   4.487  0.00  0.00
I
ATOM  28675  CB  THR I 113     -12.401  27.651   2.985  0.00  0.00
I
ATOM  28676  HB  THR I 113     -12.139  28.149   2.028  0.00  0.00
I
ATOM  28677  OG1 THR I 113     -12.564  26.266   2.782  0.00  0.00
I
ATOM  28678  HG1 THR I 113     -11.884  25.937   2.190  0.00  0.00
I
ATOM  28679  CG2 THR I 113     -11.335  27.891   4.152  0.00  0.00
I
ATOM  28680 HG21 THR I 113     -10.941  28.930   4.159  0.00  0.00
I
ATOM  28681 HG22 THR I 113     -11.858  27.641   5.100  0.00  0.00
I
ATOM  28682 HG23 THR I 113     -10.455  27.214   4.117  0.00  0.00
I
ATOM  28683  C   THR I 113     -13.717  29.747   3.157  0.00  0.00
I
ATOM  28684  O   THR I 113     -13.840  30.559   4.073  0.00  0.00
I
ATOM  28685  N   PHE I 114     -13.823  30.176   1.897  0.00  0.00
I
ATOM  28686  HN  PHE I 114     -13.702  29.491   1.184  0.00  0.00
I
ATOM  28687  CA  PHE I 114     -13.841  31.509   1.434  0.00  0.00
I
ATOM  28688  HA  PHE I 114     -12.952  32.004   1.796  0.00  0.00
I
ATOM  28689  CB  PHE I 114     -14.020  31.645  -0.060  0.00  0.00
I
ATOM  28690  HB1 PHE I 114     -15.020  31.283  -0.381  0.00  0.00
I
ATOM  28691  HB2 PHE I 114     -14.056  32.724  -0.325  0.00  0.00
I
ATOM  28692  CG  PHE I 114     -12.856  30.993  -0.777  0.00  0.00
I
ATOM  28693  CD1 PHE I 114     -12.874  29.625  -1.082  0.00  0.00
I
ATOM  28694  HD1 PHE I 114     -13.649  28.950  -0.751  0.00  0.00
I
ATOM  28695  CE1 PHE I 114     -11.815  29.071  -1.827  0.00  0.00
I
ATOM  28696  HE1 PHE I 114     -11.796  28.002  -1.976  0.00  0.00
I
ATOM  28697  CZ  PHE I 114     -10.734  29.871  -2.270  0.00  0.00
I
ATOM  28698  HZ  PHE I 114      -9.881  29.446  -2.778  0.00  0.00
I
ATOM  28699  CD2 PHE I 114     -11.811  31.767  -1.206  0.00  0.00
I
ATOM  28700  HD2 PHE I 114     -11.857  32.831  -1.030  0.00  0.00
I
```

ATOM 28701 CE2 PHE I 114   -10.797 31.249 -2.046 0.00 0.00
I
ATOM 28702 HE2 PHE I 114   -9.967 31.880 -2.329 0.00 0.00
I
ATOM 28703 C   PHE I 114   -14.979 32.464   2.053 0.00 0.00
I
ATOM 28704 O   PHE I 114   -14.835 33.700   2.264 0.00 0.00
I
ATOM 28705 N   ALA I 115   -16.127 31.877   2.429 0.00 0.00
I
ATOM 28706 HN ALA I 115   -16.107 30.882   2.376 0.00 0.00
I
ATOM 28707 CA ALA I 115   -17.222 32.570   3.039 0.00 0.00
I
ATOM 28708 HA ALA I 115   -17.334 33.554   2.608 0.00 0.00
I
ATOM 28709 CB ALA I 115   -18.484 31.674   2.918 0.00 0.00
I
ATOM 28710 HB1 ALA I 115   -19.342 32.151   3.439 0.00 0.00
I
ATOM 28711 HB2 ALA I 115   -18.888 31.538   1.892 0.00 0.00
I
ATOM 28712 HB3 ALA I 115   -18.434 30.732   3.504 0.00 0.00
I
ATOM 28713 C   ALA I 115   -16.961 32.750   4.558 0.00 0.00
I
ATOM 28714 O   ALA I 115   -17.213 33.807   5.070 0.00 0.00
I
ATOM 28715 N   VAL I 116   -16.401 31.714   5.169 0.00 0.00
I
ATOM 28716 HN VAL I 116   -16.163 30.889   4.663 0.00 0.00
I
ATOM 28717 CA VAL I 116   -16.068 31.724   6.585 0.00 0.00
I
ATOM 28718 HA VAL I 116   -17.010 31.854   7.096 0.00 0.00
I
ATOM 28719 CB VAL I 116   -15.387 30.485   7.066 0.00 0.00
I
ATOM 28720 HB VAL I 116   -14.296 30.423   6.864 0.00 0.00
I
ATOM 28721 CG1 VAL I 116   -15.583 30.472   8.622 0.00 0.00
I
ATOM 28722 HG11 VAL I 116   -15.001 29.623   9.041 0.00 0.00
I
ATOM 28723 HG12 VAL I 116   -15.197 31.400   9.097 0.00 0.00
I
ATOM 28724 HG13 VAL I 116   -16.635 30.327   8.947 0.00 0.00
I
ATOM 28725 CG2 VAL I 116   -16.068 29.229   6.426 0.00 0.00
I
ATOM 28726 HG21 VAL I 116   -17.146 29.497   6.409 0.00 0.00
I
ATOM 28727 HG22 VAL I 116   -15.581 29.013   5.451 0.00 0.00
I

ATOM 28728 HG23 VAL I 116   -15.868 28.313   7.022 0.00 0.00
I
ATOM 28729 C   VAL I 116   -15.141 32.912   6.940 0.00 0.00
I
ATOM 28730 O   VAL I 116   -15.118 33.478   7.975 0.00 0.00
I
ATOM 28731 N   LEU I 117   -14.215 33.225   5.998 0.00 0.00
I
ATOM 28732 HN LEU I 117   -14.125 32.515   5.304 0.00 0.00
I
ATOM 28733 CA LEU I 117   -13.149 34.231   6.212 0.00 0.00
I
ATOM 28734 HA LEU I 117   -12.483 33.930   7.007 0.00 0.00
I
ATOM 28735 CB LEU I 117   -12.133 34.272   4.983 0.00 0.00
I
ATOM 28736 HB1 LEU I 117   -12.070 33.219   4.634 0.00 0.00
I
ATOM 28737 HB2 LEU I 117   -12.639 34.739   4.111 0.00 0.00
I
ATOM 28738 CG LEU I 117   -10.709 34.849   5.079 0.00 0.00
I
ATOM 28739 HG LEU I 117   -10.195 34.670   6.048 0.00 0.00
I
ATOM 28740 CD1 LEU I 117   -10.018 34.225   3.896 0.00 0.00
I
ATOM 28741 HD11 LEU I 117   -10.590 34.339   2.950 0.00 0.00
I
ATOM 28742 HD12 LEU I 117   -9.005 34.594   3.625 0.00 0.00
I
ATOM 28743 HD13 LEU I 117   -9.864 33.133   4.029 0.00 0.00
I
ATOM 28744 CD2 LEU I 117   -10.769 36.391   4.897 0.00 0.00
I
ATOM 28745 HD21 LEU I 117   -11.056 36.956   5.809 0.00 0.00
I
ATOM 28746 HD22 LEU I 117   -9.701 36.585   4.657 0.00 0.00
I
ATOM 28747 HD23 LEU I 117   -11.447 36.736   4.087 0.00 0.00
I
ATOM 28748 C   LEU I 117   -13.587 35.608   6.463 0.00 0.00
I
ATOM 28749 O   LEU I 117   -13.203 36.243   7.446 0.00 0.00
I
ATOM 28750 N   ILE I 118   -14.585 36.129   5.669 0.00 0.00
I
ATOM 28751 HN ILE I 118   -15.007 35.623   4.921 0.00 0.00
I
ATOM 28752 CA ILE I 118   -14.914 37.589   5.732 0.00 0.00
I
ATOM 28753 HA ILE I 118   -14.083 38.118   6.175 0.00 0.00
I
ATOM 28754 CB ILE I 118   -14.994 38.065   4.329 0.00 0.00
I

ATOM 28755 HB ILE I 118   -14.005 37.794   3.901 0.00 0.00
I
ATOM 28756 CG2 ILE I 118   -16.071 37.339   3.490 0.00 0.00
I
ATOM 28757 HG21 ILE I 118   -17.078 37.435   3.949 0.00 0.00
I
ATOM 28758 HG22 ILE I 118   -16.074 37.772   2.467 0.00 0.00
I
ATOM 28759 HG23 ILE I 118   -15.696 36.301   3.365 0.00 0.00
I
ATOM 28760 CG1 ILE I 118   -15.127 39.647   4.169 0.00 0.00
I
ATOM 28761 HG11 ILE I 118   -15.306 39.929   3.109 0.00 0.00
I
ATOM 28762 HG12 ILE I 118   -16.048 40.017   4.670 0.00 0.00
I
ATOM 28763 CD ILE I 118   -13.878 40.373   4.559 0.00 0.00
I
ATOM 28764 HD1 ILE I 118   -13.718 40.131   5.631 0.00 0.00
I
ATOM 28765 HD2 ILE I 118   -12.920 40.070   4.086 0.00 0.00
I
ATOM 28766 HD3 ILE I 118   -13.858 41.481   4.478 0.00 0.00
I
ATOM 28767 C   ILE I 118   -16.208 37.754   6.675 0.00 0.00
I
ATOM 28768 O   ILE I 118   -16.886 38.763   6.522 0.00 0.00
I
ATOM 28769 N   VAL I 119   -16.569 36.772   7.590 0.00 0.00
I
ATOM 28770 HN VAL I 119   -16.127 35.885   7.480 0.00 0.00
I
ATOM 28771 CA VAL I 119   -17.581 36.869   8.567 0.00 0.00
I
ATOM 28772 HA VAL I 119   -18.180 37.766   8.515 0.00 0.00
I
ATOM 28773 CB VAL I 119   -18.582 35.767   8.413 0.00 0.00
I
ATOM 28774 HB VAL I 119   -18.039 34.799   8.359 0.00 0.00
I
ATOM 28775 CG1 VAL I 119   -19.587 35.704   9.574 0.00 0.00
I
ATOM 28776 HG11 VAL I 119   -19.068 35.281 10.461 0.00 0.00
I
ATOM 28777 HG12 VAL I 119   -20.006 36.716   9.759 0.00 0.00
I
ATOM 28778 HG13 VAL I 119   -20.383 34.970   9.326 0.00 0.00
I
ATOM 28779 CG2 VAL I 119   -19.362 35.955   7.078 0.00 0.00
I
ATOM 28780 HG21 VAL I 119   -18.622 36.000   6.250 0.00 0.00
I
ATOM 28781 HG22 VAL I 119   -20.097 35.162   6.825 0.00 0.00
I

ATOM	28782	HG23	VAL	I	119	−19.951	36.896	7.039	0.00	0.00	I
ATOM	28783	C	VAL	I	119	−16.842	36.817	9.915	0.00	0.00	I
ATOM	28784	O	VAL	I	119	−16.065	35.892	10.275	0.00	0.00	I
ATOM	28785	N	MET	I	120	−17.104	37.848	10.800	0.00	0.00	I
ATOM	28786	HN	MET	I	120	−17.491	38.688	10.428	0.00	0.00	I
ATOM	28787	CA	MET	I	120	−16.727	37.934	12.228	0.00	0.00	I
ATOM	28788	HA	MET	I	120	−15.671	37.791	12.405	0.00	0.00	I
ATOM	28789	CB	MET	I	120	−17.348	39.259	12.746	0.00	0.00	I
ATOM	28790	HB1	MET	I	120	−16.963	40.042	12.057	0.00	0.00	I
ATOM	28791	HB2	MET	I	120	−18.423	39.291	12.470	0.00	0.00	I
ATOM	28792	CG	MET	I	120	−16.868	39.715	14.139	0.00	0.00	I
ATOM	28793	HG1	MET	I	120	−17.260	38.914	14.801	0.00	0.00	I
ATOM	28794	HG2	MET	I	120	−15.762	39.726	14.242	0.00	0.00	I
ATOM	28795	SD	MET	I	120	−17.456	41.369	14.608	0.00	0.00	I
ATOM	28796	CE	MET	I	120	−16.302	42.280	13.562	0.00	0.00	I
ATOM	28797	HE1	MET	I	120	−15.306	41.791	13.509	0.00	0.00	I
ATOM	28798	HE2	MET	I	120	−16.662	42.278	12.511	0.00	0.00	I
ATOM	28799	HE3	MET	I	120	−16.217	43.352	13.843	0.00	0.00	I
ATOM	28800	C	MET	I	120	−17.167	36.735	13.096	0.00	0.00	I
ATOM	28801	O	MET	I	120	−18.256	36.221	12.969	0.00	0.00	I
ATOM	28802	N	ILE	I	121	−16.268	36.250	14.028	0.00	0.00	I
ATOM	28803	HN	ILE	I	121	−15.425	36.721	14.276	0.00	0.00	I
ATOM	28804	CA	ILE	I	121	−16.460	35.176	14.947	0.00	0.00	I
ATOM	28805	HA	ILE	I	121	−17.039	34.420	14.436	0.00	0.00	I
ATOM	28806	CB	ILE	I	121	−15.075	34.596	15.453	0.00	0.00	I
ATOM	28807	HB	ILE	I	121	−14.553	35.491	15.855	0.00	0.00	I
ATOM	28808	CG2	ILE	I	121	−15.334	33.525	16.551	0.00	0.00	I

```
ATOM  28809 HG21 ILE I 121      -15.879  32.716  16.020  0.00  0.00
I
ATOM  28810 HG22 ILE I 121      -14.374  33.102  16.916  0.00  0.00
I
ATOM  28811 HG23 ILE I 121      -15.972  33.827  17.410  0.00  0.00
I
ATOM  28812  CG1 ILE I 121      -14.262  34.005  14.256  0.00  0.00
I
ATOM  28813 HG11 ILE I 121      -14.699  33.132  13.725  0.00  0.00
I
ATOM  28814 HG12 ILE I 121      -14.029  34.799  13.515  0.00  0.00
I
ATOM  28815  CD  ILE I 121      -12.975  33.260  14.595  0.00  0.00
I
ATOM  28816  HD1 ILE I 121      -12.192  33.985  14.904  0.00  0.00
I
ATOM  28817  HD2 ILE I 121      -13.079  32.445  15.342  0.00  0.00
I
ATOM  28818  HD3 ILE I 121      -12.478  32.750  13.742  0.00  0.00
I
ATOM  28819  C   ILE I 121      -17.378  35.607  16.029  0.00  0.00
I
ATOM  28820  O   ILE I 121      -18.194  34.836  16.578  0.00  0.00
I
ATOM  28821  N   LEU I 122      -17.274  36.941  16.451  0.00  0.00
I
ATOM  28822  HN  LEU I 122      -16.518  37.540  16.198  0.00  0.00
I
ATOM  28823  CA  LEU I 122      -18.188  37.445  17.428  0.00  0.00
I
ATOM  28824  HA  LEU I 122      -18.119  36.780  18.276  0.00  0.00
I
ATOM  28825  CB  LEU I 122      -17.861  38.964  17.826  0.00  0.00
I
ATOM  28826  HB1 LEU I 122      -18.287  39.587  17.011  0.00  0.00
I
ATOM  28827  HB2 LEU I 122      -18.292  39.278  18.800  0.00  0.00
I
ATOM  28828  CG  LEU I 122      -16.342  39.357  17.958  0.00  0.00
I
ATOM  28829  HG  LEU I 122      -15.862  39.043  17.007  0.00  0.00
I
ATOM  28830  CD1 LEU I 122      -16.221  40.847  18.191  0.00  0.00
I
ATOM  28831 HD11 LEU I 122      -16.480  41.217  19.206  0.00  0.00
I
ATOM  28832 HD12 LEU I 122      -15.198  41.204  17.945  0.00  0.00
I
ATOM  28833 HD13 LEU I 122      -16.806  41.373  17.407  0.00  0.00
I
ATOM  28834  CD2 LEU I 122      -15.564  38.802  19.078  0.00  0.00
I
ATOM  28835 HD21 LEU I 122      -14.455  38.869  19.076  0.00  0.00
I
```

ATOM 28836 HD22 LEU I 122   -16.051 39.110 20.027 0.00 0.00
I
ATOM 28837 HD23 LEU I 122   -15.679 37.700 19.158 0.00 0.00
I
ATOM 28838 C   LEU I 122   -19.679 37.355 17.070 0.00 0.00
I
ATOM 28839 O   LEU I 122   -19.954 37.773 15.965 0.00 0.00
I
ATOM 28840 N   GLY I 123   -20.529 37.010 18.027 0.00 0.00
I
ATOM 28841 HN GLY I 123   -20.230 36.780 18.950 0.00 0.00
I
ATOM 28842 CA GLY I 123   -21.942 36.812 17.872 0.00 0.00
I
ATOM 28843 HA1 GLY I 123   -22.445 37.602 17.333 0.00 0.00
I
ATOM 28844 HA2 GLY I 123   -22.339 36.645 18.862 0.00 0.00
I
ATOM 28845 C   GLY I 123   -22.273 35.575 17.168 0.00 0.00
I
ATOM 28846 O   GLY I 123   -21.577 34.582 17.240 0.00 0.00
I
ATOM 28847 N   LYS I 124   -23.448 35.511 16.568 0.00 0.00
I
ATOM 28848 HN LYS I 124   -24.118 36.246 16.633 0.00 0.00
I
ATOM 28849 CA LYS I 124   -24.025 34.256 16.049 0.00 0.00
I
ATOM 28850 HA LYS I 124   -23.505 33.436 16.522 0.00 0.00
I
ATOM 28851 CB LYS I 124   -25.477 34.298 16.591 0.00 0.00
I
ATOM 28852 HB1 LYS I 124   -25.918 33.298 16.394 0.00 0.00
I
ATOM 28853 HB2 LYS I 124   -25.466 34.329 17.701 0.00 0.00
I
ATOM 28854 CG LYS I 124   -26.432 35.380 16.115 0.00 0.00
I
ATOM 28855 HG1 LYS I 124   -26.059 36.327 16.560 0.00 0.00
I
ATOM 28856 HG2 LYS I 124   -26.485 35.388 15.006 0.00 0.00
I
ATOM 28857 CD LYS I 124   -27.821 35.204 16.803 0.00 0.00
I
ATOM 28858 HD1 LYS I 124   -28.120 34.145 16.651 0.00 0.00
I
ATOM 28859 HD2 LYS I 124   -27.678 35.417 17.884 0.00 0.00
I
ATOM 28860 CE LYS I 124   -28.890 36.159 16.265 0.00 0.00
I
ATOM 28861 HE1 LYS I 124   -28.858 36.084 15.157 0.00 0.00
I
ATOM 28862 HE2 LYS I 124   -29.882 35.965 16.727 0.00 0.00
I

ATOM	28863	NZ	LYS	I	124	-28.542	37.513	16.540	0.00	0.00	I
ATOM	28864	HZ1	LYS	I	124	-28.313	37.646	17.546	0.00	0.00	I
ATOM	28865	HZ2	LYS	I	124	-27.878	37.939	15.862	0.00	0.00	I
ATOM	28866	HZ3	LYS	I	124	-29.448	38.020	16.486	0.00	0.00	I
ATOM	28867	C	LYS	I	124	-24.059	34.001	14.568	0.00	0.00	I
ATOM	28868	O	LYS	I	124	-24.764	33.136	14.015	0.00	0.00	I
ATOM	28869	N	LYS	I	125	-23.224	34.743	13.825	0.00	0.00	I
ATOM	28870	HN	LYS	I	125	-22.633	35.252	14.445	0.00	0.00	I
ATOM	28871	CA	LYS	I	125	-22.997	34.875	12.389	0.00	0.00	I
ATOM	28872	HA	LYS	I	125	-23.987	35.030	11.985	0.00	0.00	I
ATOM	28873	CB	LYS	I	125	-22.046	36.053	12.091	0.00	0.00	I
ATOM	28874	HB1	LYS	I	125	-20.984	35.798	12.296	0.00	0.00	I
ATOM	28875	HB2	LYS	I	125	-22.163	36.363	11.030	0.00	0.00	I
ATOM	28876	CG	LYS	I	125	-22.231	37.243	12.978	0.00	0.00	I
ATOM	28877	HG1	LYS	I	125	-23.314	37.474	12.890	0.00	0.00	I
ATOM	28878	HG2	LYS	I	125	-22.203	36.965	14.053	0.00	0.00	I
ATOM	28879	CD	LYS	I	125	-21.414	38.534	12.706	0.00	0.00	I
ATOM	28880	HD1	LYS	I	125	-20.330	38.297	12.644	0.00	0.00	I
ATOM	28881	HD2	LYS	I	125	-21.773	38.784	11.685	0.00	0.00	I
ATOM	28882	CE	LYS	I	125	-21.680	39.697	13.658	0.00	0.00	I
ATOM	28883	HE1	LYS	I	125	-20.802	40.372	13.573	0.00	0.00	I
ATOM	28884	HE2	LYS	I	125	-22.662	40.087	13.313	0.00	0.00	I
ATOM	28885	NZ	LYS	I	125	-21.986	39.345	15.055	0.00	0.00	I
ATOM	28886	HZ1	LYS	I	125	-21.208	38.777	15.446	0.00	0.00	I
ATOM	28887	HZ2	LYS	I	125	-22.237	40.155	15.658	0.00	0.00	I
ATOM	28888	HZ3	LYS	I	125	-22.792	38.690	15.107	0.00	0.00	I
ATOM	28889	C	LYS	I	125	-22.392	33.571	11.828	0.00	0.00	I

```
ATOM  28890  O   LYS I 125     -22.735  32.997  10.792  0.00  0.00
I
ATOM  28891  N   ILE I 126     -21.445  33.122  12.575  0.00  0.00
I
ATOM  28892  HN  ILE I 126     -21.088  33.680  13.320  0.00  0.00
I
ATOM  28893  CA  ILE I 126     -20.862  31.766  12.364  0.00  0.00
I
ATOM  28894  HA  ILE I 126     -21.448  31.127  11.720  0.00  0.00
I
ATOM  28895  CB  ILE I 126     -19.452  31.896  11.822  0.00  0.00
I
ATOM  28896  HB  ILE I 126     -18.908  30.928  11.797  0.00  0.00
I
ATOM  28897  CG2 ILE I 126     -19.710  32.276  10.359  0.00  0.00
I
ATOM  28898 HG21 ILE I 126     -20.366  33.173  10.363  0.00  0.00
I
ATOM  28899 HG22 ILE I 126     -18.774  32.235   9.763  0.00  0.00
I
ATOM  28900 HG23 ILE I 126     -20.363  31.491   9.922  0.00  0.00
I
ATOM  28901  CG1 ILE I 126     -18.506  32.935  12.390  0.00  0.00
I
ATOM  28902 HG11 ILE I 126     -18.930  33.953  12.259  0.00  0.00
I
ATOM  28903 HG12 ILE I 126     -18.459  32.855  13.497  0.00  0.00
I
ATOM  28904  CD  ILE I 126     -17.074  32.849  11.753  0.00  0.00
I
ATOM  28905  HD1 ILE I 126     -17.087  33.059  10.662  0.00  0.00
I
ATOM  28906  HD2 ILE I 126     -16.507  33.686  12.213  0.00  0.00
I
ATOM  28907  HD3 ILE I 126     -16.573  31.871  11.917  0.00  0.00
I
ATOM  28908  C   ILE I 126     -20.752  31.064  13.700  0.00  0.00
I
ATOM  28909  O   ILE I 126     -20.353  31.597  14.756  0.00  0.00
I
ATOM  28910  N   GLY I 127     -21.042  29.744  13.559  0.00  0.00
I
ATOM  28911  HN  GLY I 127     -21.207  29.350  12.658  0.00  0.00
I
ATOM  28912  CA  GLY I 127     -20.922  28.742  14.606  0.00  0.00
I
ATOM  28913  HA1 GLY I 127     -21.778  28.093  14.493  0.00  0.00
I
ATOM  28914  HA2 GLY I 127     -20.970  29.233  15.567  0.00  0.00
I
ATOM  28915  C   GLY I 127     -19.642  27.858  14.533  0.00  0.00
I
ATOM  28916  O   GLY I 127     -18.747  28.107  13.774  0.00  0.00
I
```

ATOM 28917 N   LEU I 128   -19.590 26.919 15.504 0.00 0.00
I
ATOM 28918 HN LEU I 128   -20.339 26.862 16.160 0.00 0.00
I
ATOM 28919 CA LEU I 128   -18.481 26.040 15.673 0.00 0.00
I
ATOM 28920 HA LEU I 128   -17.599 26.638 15.853 0.00 0.00
I
ATOM 28921 CB LEU I 128   -18.784 25.136 16.866 0.00 0.00
I
ATOM 28922 HB1 LEU I 128   -19.556 24.415 16.522 0.00 0.00
I
ATOM 28923 HB2 LEU I 128   -17.903 24.495 17.083 0.00 0.00
I
ATOM 28924 CG LEU I 128   -19.297 25.868 18.097 0.00 0.00
I
ATOM 28925 HG LEU I 128   -20.109 26.527 17.724 0.00 0.00
I
ATOM 28926 CD1 LEU I 128   -19.800 24.774 19.080 0.00 0.00
I
ATOM 28927 HD11 LEU I 128   -20.508 24.180 18.463 0.00 0.00
I
ATOM 28928 HD12 LEU I 128   -18.901 24.203 19.395 0.00 0.00
I
ATOM 28929 HD13 LEU I 128   -20.234 25.145 20.032 0.00 0.00
I
ATOM 28930 CD2 LEU I 128   -18.157 26.608 18.712 0.00 0.00
I
ATOM 28931 HD21 LEU I 128   -18.342 26.930 19.760 0.00 0.00
I
ATOM 28932 HD22 LEU I 128   -17.280 25.930 18.789 0.00 0.00
I
ATOM 28933 HD23 LEU I 128   -17.685 27.424 18.124 0.00 0.00
I
ATOM 28934 C   LEU I 128   -18.208 25.157 14.522 0.00 0.00
I
ATOM 28935 O   LEU I 128   -17.050 24.986 14.149 0.00 0.00
I
ATOM 28936 N   LYS I 129   -19.310 24.527 13.947 0.00 0.00
I
ATOM 28937 HN LYS I 129   -20.232 24.790 14.221 0.00 0.00
I
ATOM 28938 CA LYS I 129   -19.322 23.486 12.852 0.00 0.00
I
ATOM 28939 HA LYS I 129   -18.788 22.641 13.260 0.00 0.00
I
ATOM 28940 CB LYS I 129   -20.842 23.053 12.598 0.00 0.00
I
ATOM 28941 HB1 LYS I 129   -21.357 24.038 12.605 0.00 0.00
I
ATOM 28942 HB2 LYS I 129   -20.832 22.547 11.609 0.00 0.00
I
ATOM 28943 CG LYS I 129   -21.422 22.210 13.719 0.00 0.00
I

```
ATOM  28944  HG1 LYS I 129     -20.878  21.273  13.965  0.00  0.00
I
ATOM  28945  HG2 LYS I 129     -21.373  22.665  14.732  0.00  0.00
I
ATOM  28946  CD  LYS I 129     -22.750  21.603  13.419  0.00  0.00
I
ATOM  28947  HD1 LYS I 129     -22.769  21.216  12.378  0.00  0.00
I
ATOM  28948  HD2 LYS I 129     -22.913  20.703  14.049  0.00  0.00
I
ATOM  28949  CE  LYS I 129     -23.974  22.492  13.393  0.00  0.00
I
ATOM  28950  HE1 LYS I 129     -23.869  23.290  12.627  0.00  0.00
I
ATOM  28951  HE2 LYS I 129     -24.933  21.970  13.187  0.00  0.00
I
ATOM  28952  NZ  LYS I 129     -24.043  23.128  14.685  0.00  0.00
I
ATOM  28953  HZ1 LYS I 129     -24.222  22.420  15.425  0.00  0.00
I
ATOM  28954  HZ2 LYS I 129     -23.214  23.716  14.903  0.00  0.00
I
ATOM  28955  HZ3 LYS I 129     -24.900  23.711  14.598  0.00  0.00
I
ATOM  28956  C   LYS I 129     -18.626  23.939  11.572  0.00  0.00
I
ATOM  28957  O   LYS I 129     -17.770  23.232  11.065  0.00  0.00
I
ATOM  28958  N   GLU I 130     -18.905  25.232  11.167  0.00  0.00
I
ATOM  28959  HN  GLU I 130     -19.587  25.811  11.607  0.00  0.00
I
ATOM  28960  CA  GLU I 130     -18.197  25.953  10.089  0.00  0.00
I
ATOM  28961  HA  GLU I 130     -18.126  25.176   9.342  0.00  0.00
I
ATOM  28962  CB  GLU I 130     -19.051  26.980   9.414  0.00  0.00
I
ATOM  28963  HB1 GLU I 130     -18.544  27.631   8.671  0.00  0.00
I
ATOM  28964  HB2 GLU I 130     -19.876  26.396   8.953  0.00  0.00
I
ATOM  28965  CG  GLU I 130     -19.655  28.080  10.270  0.00  0.00
I
ATOM  28966  HG1 GLU I 130     -19.023  28.312  11.154  0.00  0.00
I
ATOM  28967  HG2 GLU I 130     -19.638  29.000   9.647  0.00  0.00
I
ATOM  28968  CD  GLU I 130     -21.118  27.958  10.852  0.00  0.00
I
ATOM  28969  OE1 GLU I 130     -21.392  26.840  11.395  0.00  0.00
I
ATOM  28970  OE2 GLU I 130     -22.005  28.863  10.745  0.00  0.00
I
```

```
ATOM  28971  C   GLU I 130     -16.902  26.415  10.323  0.00  0.00
I
ATOM  28972  O   GLU I 130     -16.043  26.424   9.404  0.00  0.00
I
ATOM  28973  N   ARG I 131     -16.479  26.821  11.583  0.00  0.00
I
ATOM  28974  HN  ARG I 131     -17.153  27.018  12.292  0.00  0.00
I
ATOM  28975  CA  ARG I 131     -15.113  26.909  11.997  0.00  0.00
I
ATOM  28976  HA  ARG I 131     -14.592  27.423  11.203  0.00  0.00
I
ATOM  28977  CB  ARG I 131     -14.927  27.690  13.296  0.00  0.00
I
ATOM  28978  HB1 ARG I 131     -15.585  27.127  13.992  0.00  0.00
I
ATOM  28979  HB2 ARG I 131     -13.858  27.748  13.592  0.00  0.00
I
ATOM  28980  CG  ARG I 131     -15.261  29.182  13.056  0.00  0.00
I
ATOM  28981  HG1 ARG I 131     -14.377  29.742  12.681  0.00  0.00
I
ATOM  28982  HG2 ARG I 131     -16.036  29.375  12.284  0.00  0.00
I
ATOM  28983  CD  ARG I 131     -15.813  29.895  14.286  0.00  0.00
I
ATOM  28984  HD1 ARG I 131     -16.087  30.945  14.051  0.00  0.00
I
ATOM  28985  HD2 ARG I 131     -16.650  29.283  14.685  0.00  0.00
I
ATOM  28986  NE  ARG I 131     -14.617  30.019  15.225  0.00  0.00
I
ATOM  28987  HE  ARG I 131     -13.703  29.837  14.863  0.00  0.00
I
ATOM  28988  CZ  ARG I 131     -14.744  29.627  16.515  0.00  0.00
I
ATOM  28989  NH1 ARG I 131     -13.656  29.191  17.189  0.00  0.00
I
ATOM  28990 HH11 ARG I 131     -13.859  28.802  18.088  0.00  0.00
I
ATOM  28991 HH12 ARG I 131     -12.956  28.764  16.616  0.00  0.00
I
ATOM  28992  NH2 ARG I 131     -15.850  29.692  17.183  0.00  0.00
I
ATOM  28993 HH21 ARG I 131     -15.719  29.807  18.167  0.00  0.00
I
ATOM  28994 HH22 ARG I 131     -16.628  30.178  16.785  0.00  0.00
I
ATOM  28995  C   ARG I 131     -14.379  25.590  11.986  0.00  0.00
I
ATOM  28996  O   ARG I 131     -13.193  25.468  11.648  0.00  0.00
I
ATOM  28997  N   MET I 132     -14.968  24.464  12.347  0.00  0.00
I
```

```
ATOM  28998  HN  MET I 132     -15.850  24.396  12.807  0.00  0.00
I
ATOM  28999  CA  MET I 132     -14.440  23.046  12.249  0.00  0.00
I
ATOM  29000  HA  MET I 132     -13.549  22.997  12.856  0.00  0.00
I
ATOM  29001  CB  MET I 132     -15.384  22.066  13.024  0.00  0.00
I
ATOM  29002  HB1 MET I 132     -15.601  22.532  14.009  0.00  0.00
I
ATOM  29003  HB2 MET I 132     -16.416  22.074  12.611  0.00  0.00
I
ATOM  29004  CG  MET I 132     -14.868  20.674  13.205  0.00  0.00
I
ATOM  29005  HG1 MET I 132     -14.689  20.246  12.195  0.00  0.00
I
ATOM  29006  HG2 MET I 132     -13.830  20.634  13.601  0.00  0.00
I
ATOM  29007  SD  MET I 132     -15.918  19.512  14.109  0.00  0.00
I
ATOM  29008  CE  MET I 132     -17.396  20.525  14.555  0.00  0.00
I
ATOM  29009  HE1 MET I 132     -18.050  19.911  15.210  0.00  0.00
I
ATOM  29010  HE2 MET I 132     -17.159  21.382  15.221  0.00  0.00
I
ATOM  29011  HE3 MET I 132     -18.085  20.923  13.779  0.00  0.00
I
ATOM  29012  C   MET I 132     -14.188  22.627  10.798  0.00  0.00
I
ATOM  29013  O   MET I 132     -13.253  21.931  10.459  0.00  0.00
I
ATOM  29014  N   LEU I 133     -15.042  23.170   9.932  0.00  0.00
I
ATOM  29015  HN  LEU I 133     -15.833  23.593  10.367  0.00  0.00
I
ATOM  29016  CA  LEU I 133     -14.896  22.999   8.489  0.00  0.00
I
ATOM  29017  HA  LEU I 133     -14.945  21.943   8.271  0.00  0.00
I
ATOM  29018  CB  LEU I 133     -16.181  23.540   7.812  0.00  0.00
I
ATOM  29019  HB1 LEU I 133     -17.136  23.348   8.345  0.00  0.00
I
ATOM  29020  HB2 LEU I 133     -16.136  24.633   8.006  0.00  0.00
I
ATOM  29021  CG  LEU I 133     -16.368  23.311   6.246  0.00  0.00
I
ATOM  29022  HG  LEU I 133     -15.579  23.907   5.740  0.00  0.00
I
ATOM  29023  CD1 LEU I 133     -16.132  21.856   5.869  0.00  0.00
I
ATOM  29024 HD11 LEU I 133     -16.086  21.726   4.767  0.00  0.00
I
```

ATOM 29025 HD12 LEU I 133   −15.104 21.538   6.147 0.00 0.00
I
ATOM 29026 HD13 LEU I 133   −16.967 21.271   6.310 0.00 0.00
I
ATOM 29027 CD2 LEU I 133   −17.676 23.694   5.716 0.00 0.00
I
ATOM 29028 HD21 LEU I 133   −17.749 24.797   5.607 0.00 0.00
I
ATOM 29029 HD22 LEU I 133   −17.860 23.274   4.704 0.00 0.00
I
ATOM 29030 HD23 LEU I 133   −18.631 23.462   6.236 0.00 0.00
I
ATOM 29031 C   LEU I 133   −13.644 23.627   7.914 0.00 0.00
I
ATOM 29032 O   LEU I 133   −12.926 22.996   7.078 0.00 0.00
I
ATOM 29033 N   VAL I 134   −13.208 24.844   8.430 0.00 0.00
I
ATOM 29034 HN VAL I 134   −13.869 25.341   8.987 0.00 0.00
I
ATOM 29035 CA VAL I 134   −11.898 25.517   8.209 0.00 0.00
I
ATOM 29036 HA VAL I 134   −11.746 25.525   7.140 0.00 0.00
I
ATOM 29037 CB VAL I 134   −11.817 26.954   8.718 0.00 0.00
I
ATOM 29038 HB VAL I 134   −11.859 26.950   9.828 0.00 0.00
I
ATOM 29039 CG1 VAL I 134   −10.422 27.480   8.300 0.00 0.00
I
ATOM 29040 HG11 VAL I 134   −10.449 28.591   8.311 0.00 0.00
I
ATOM 29041 HG12 VAL I 134   −9.642 27.062   8.971 0.00 0.00
I
ATOM 29042 HG13 VAL I 134   −10.191 27.190   7.252 0.00 0.00
I
ATOM 29043 CG2 VAL I 134   −12.937 27.751   8.109 0.00 0.00
I
ATOM 29044 HG21 VAL I 134   −12.702 28.836   8.157 0.00 0.00
I
ATOM 29045 HG22 VAL I 134   −13.150 27.433   7.066 0.00 0.00
I
ATOM 29046 HG23 VAL I 134   −13.912 27.631   8.628 0.00 0.00
I
ATOM 29047 C   VAL I 134   −10.704 24.699   8.741 0.00 0.00
I
ATOM 29048 O   VAL I 134   −9.746 24.455   7.968 0.00 0.00
I
ATOM 29049 N   GLN I 135   −10.734 24.152   9.929 0.00 0.00
I
ATOM 29050 HN GLN I 135   −11.420 24.399 10.610 0.00 0.00
I
ATOM 29051 CA GLN I 135   −9.654 23.347 10.462 0.00 0.00
I

```
ATOM  29052  HA  GLN I 135      -8.671  23.792  10.405  0.00  0.00
I
ATOM  29053  CB  GLN I 135      -9.978  23.005  11.914  0.00  0.00
I
ATOM  29054  HB1 GLN I 135     -10.124  23.850  12.621  0.00  0.00
I
ATOM  29055  HB2 GLN I 135     -11.004  22.593  11.808  0.00  0.00
I
ATOM  29056  CG  GLN I 135      -9.122  21.973  12.641  0.00  0.00
I
ATOM  29057  HG1 GLN I 135      -9.012  21.014  12.091  0.00  0.00
I
ATOM  29058  HG2 GLN I 135      -8.132  22.455  12.793  0.00  0.00
I
ATOM  29059  CD  GLN I 135      -9.678  21.388  13.952  0.00  0.00
I
ATOM  29060  OE1 GLN I 135     -10.883  21.546  14.212  0.00  0.00
I
ATOM  29061  NE2 GLN I 135      -8.817  20.650  14.702  0.00  0.00
I
ATOM  29062 HE21 GLN I 135      -9.187  20.203  15.517  0.00  0.00
I
ATOM  29063 HE22 GLN I 135      -7.856  20.580  14.436  0.00  0.00
I
ATOM  29064  C   GLN I 135      -9.501  22.030   9.615  0.00  0.00
I
ATOM  29065  O   GLN I 135      -8.405  21.642   9.216  0.00  0.00
I
ATOM  29066  N   GLU I 136     -10.674  21.429   9.179  0.00  0.00
I
ATOM  29067  HN  GLU I 136     -11.559  21.636   9.589  0.00  0.00
I
ATOM  29068  CA  GLU I 136     -10.571  20.252   8.328  0.00  0.00
I
ATOM  29069  HA  GLU I 136      -9.775  19.573   8.596  0.00  0.00
I
ATOM  29070  CB  GLU I 136     -11.951  19.590   8.397  0.00  0.00
I
ATOM  29071  HB1 GLU I 136     -11.967  19.273   9.461  0.00  0.00
I
ATOM  29072  HB2 GLU I 136     -12.619  20.441   8.143  0.00  0.00
I
ATOM  29073  CG  GLU I 136     -12.309  18.307   7.530  0.00  0.00
I
ATOM  29074  HG1 GLU I 136     -13.352  17.933   7.606  0.00  0.00
I
ATOM  29075  HG2 GLU I 136     -12.114  18.548   6.464  0.00  0.00
I
ATOM  29076  CD  GLU I 136     -11.421  17.150   7.841  0.00  0.00
I
ATOM  29077  OE1 GLU I 136     -10.476  16.930   7.058  0.00  0.00
I
ATOM  29078  OE2 GLU I 136     -11.641  16.463   8.870  0.00  0.00
I
```

ATOM 29079 C   GLU I 136   -10.171 20.599   6.898 0.00 0.00
I
ATOM 29080 O   GLU I 136   -9.339 19.968   6.277 0.00 0.00
I
ATOM 29081 N   ALA I 137   -10.591 21.751   6.318 0.00 0.00
I
ATOM 29082 HN ALA I 137   -11.371 22.131   6.811 0.00 0.00
I
ATOM 29083 CA ALA I 137   -10.134 22.217   5.050 0.00 0.00
I
ATOM 29084 HA ALA I 137   -10.268 21.476   4.276 0.00 0.00
I
ATOM 29085 CB ALA I 137   -10.985 23.461   4.774 0.00 0.00
I
ATOM 29086 HB1 ALA I 137   -12.057 23.276   5.003 0.00 0.00
I
ATOM 29087 HB2 ALA I 137   -10.739 24.279   5.485 0.00 0.00
I
ATOM 29088 HB3 ALA I 137   -10.880 23.750   3.707 0.00 0.00
I
ATOM 29089 C   ALA I 137   -8.592 22.594   4.957 0.00 0.00
I
ATOM 29090 O   ALA I 137   -7.849 22.297   4.059 0.00 0.00
I
ATOM 29091 N   LEU I 138   -8.007 23.198   6.008 0.00 0.00
I
ATOM 29092 HN LEU I 138   -8.570 23.442   6.794 0.00 0.00
I
ATOM 29093 CA LEU I 138   -6.619 23.616   6.168 0.00 0.00
I
ATOM 29094 HA LEU I 138   -6.283 23.780   5.155 0.00 0.00
I
ATOM 29095 CB LEU I 138   -6.555 24.951   6.972 0.00 0.00
I
ATOM 29096 HB1 LEU I 138   -6.983 24.871   7.994 0.00 0.00
I
ATOM 29097 HB2 LEU I 138   -5.509 25.089   7.322 0.00 0.00
I
ATOM 29098 CG LEU I 138   -7.003 26.229   6.302 0.00 0.00
I
ATOM 29099 HG LEU I 138   -8.107 26.326   6.216 0.00 0.00
I
ATOM 29100 CD1 LEU I 138   -6.553 27.358   7.203 0.00 0.00
I
ATOM 29101 HD11 LEU I 138   -6.492 28.322   6.653 0.00 0.00
I
ATOM 29102 HD12 LEU I 138   -7.329 27.444   7.993 0.00 0.00
I
ATOM 29103 HD13 LEU I 138   -5.543 27.164   7.624 0.00 0.00
I
ATOM 29104 CD2 LEU I 138   -6.317 26.415   4.952 0.00 0.00
I
ATOM 29105 HD21 LEU I 138   -6.715 27.381   4.573 0.00 0.00
I

ATOM 29106 HD22 LEU I 138   -5.220 26.332   5.104 0.00 0.00
I
ATOM 29107 HD23 LEU I 138   -6.596 25.578   4.277 0.00 0.00
I
ATOM 29108 C   LEU I 138   -5.775 22.535   6.697 0.00 0.00
I
ATOM 29109 O   LEU I 138   -4.560 22.740   6.859 0.00 0.00
I
ATOM 29110 N   ASN I 139   -6.355 21.331   7.066 0.00 0.00
I
ATOM 29111 HN ASN I 139   -7.331 21.232   6.884 0.00 0.00
I
ATOM 29112 CA ASN I 139   -5.688 20.187   7.633 0.00 0.00
I
ATOM 29113 HA ASN I 139   -6.504 19.523   7.877 0.00 0.00
I
ATOM 29114 CB ASN I 139   -4.947 19.286   6.633 0.00 0.00
I
ATOM 29115 HB1 ASN I 139   -4.035 19.781   6.235 0.00 0.00
I
ATOM 29116 HB2 ASN I 139   -4.498 18.401   7.132 0.00 0.00
I
ATOM 29117 CG ASN I 139   -5.892 18.883   5.475 0.00 0.00
I
ATOM 29118 OD1 ASN I 139   -6.515 17.845   5.520 0.00 0.00
I
ATOM 29119 ND2 ASN I 139   -5.939 19.706   4.433 0.00 0.00
I
ATOM 29120 HD21 ASN I 139   -5.486 20.596   4.384 0.00 0.00
I
ATOM 29121 HD22 ASN I 139   -6.384 19.323   3.624 0.00 0.00
I
ATOM 29122 C   ASN I 139   -5.019 20.328   8.963 0.00 0.00
I
ATOM 29123 O   ASN I 139   -3.932 19.792   9.176 0.00 0.00
I
ATOM 29124 N   GLN I 140   -5.625 21.002   9.922 0.00 0.00
I
ATOM 29125 HN GLN I 140   -6.586 21.247   9.813 0.00 0.00
I
ATOM 29126 CA GLN I 140   -4.975 21.422 11.126 0.00 0.00
I
ATOM 29127 HA GLN I 140   -3.936 21.304 10.856 0.00 0.00
I
ATOM 29128 CB GLN I 140   -5.185 22.899 11.497 0.00 0.00
I
ATOM 29129 HB1 GLN I 140   -6.286 22.955 11.635 0.00 0.00
I
ATOM 29130 HB2 GLN I 140   -4.628 23.167 12.420 0.00 0.00
I
ATOM 29131 CG GLN I 140   -4.831 24.006 10.366 0.00 0.00
I
ATOM 29132 HG1 GLN I 140   -5.300 23.717   9.402 0.00 0.00
I

ATOM 29133 HG2 GLN I 140   -5.317 24.927 10.754 0.00 0.00
I
ATOM 29134 CD GLN I 140   -3.347 24.180 10.173 0.00 0.00
I
ATOM 29135 OE1 GLN I 140   -2.688 24.576 11.131 0.00 0.00
I
ATOM 29136 NE2 GLN I 140   -2.847 23.832   9.021 0.00 0.00
I
ATOM 29137 HE21 GLN I 140   -1.860 23.985   8.976 0.00 0.00
I
ATOM 29138 HE22 GLN I 140   -3.436 23.404   8.335 0.00 0.00
I
ATOM 29139 C   GLN I 140   -5.234 20.613 12.339 0.00 0.00
I
ATOM 29140 O   GLN I 140   -6.352 20.027 12.495 0.00 0.00
I
ATOM 29141 N   PRO I 141   -4.196 20.344 13.229 0.00 0.00
I
ATOM 29142 CD PRO I 141   -2.778 20.439 12.949 0.00 0.00
I
ATOM 29143 HD1 PRO I 141   -2.647 19.696 12.133 0.00 0.00
I
ATOM 29144 HD2 PRO I 141   -2.475 21.406 12.493 0.00 0.00
I
ATOM 29145 CA PRO I 141   -4.505 20.004 14.581 0.00 0.00
I
ATOM 29146 HA PRO I 141   -5.313 19.287 14.578 0.00 0.00
I
ATOM 29147 CB PRO I 141   -3.188 19.507 15.138 0.00 0.00
I
ATOM 29148 HB1 PRO I 141   -2.996 18.414 15.078 0.00 0.00
I
ATOM 29149 HB2 PRO I 141   -3.052 19.839 16.189 0.00 0.00
I
ATOM 29150 CG PRO I 141   -2.069 20.179 14.287 0.00 0.00
I
ATOM 29151 HG1 PRO I 141   -1.176 19.520 14.247 0.00 0.00
I
ATOM 29152 HG2 PRO I 141   -1.809 21.183 14.687 0.00 0.00
I
ATOM 29153 C   PRO I 141   -5.091 21.074 15.498 0.00 0.00
I
ATOM 29154 O   PRO I 141   -5.723 20.806 16.517 0.00 0.00
I
ATOM 29155 N   THR I 142   -5.022 22.435 15.190 0.00 0.00
I
ATOM 29156 HN THR I 142   -4.719 22.756 14.296 0.00 0.00
I
ATOM 29157 CA THR I 142   -5.575 23.489 15.995 0.00 0.00
I
ATOM 29158 HA THR I 142   -5.781 23.288 17.036 0.00 0.00
I
ATOM 29159 CB THR I 142   -4.568 24.708 16.183 0.00 0.00
I

ATOM 29160 HB THR I 142   -3.724 24.165 16.660 0.00 0.00
I
ATOM 29161 OG1 THR I 142   -4.974 25.714 17.128 0.00 0.00
I
ATOM 29162 HG1 THR I 142   -4.183 26.255 17.174 0.00 0.00
I
ATOM 29163 CG2 THR I 142   -4.046 25.371 14.930 0.00 0.00
I
ATOM 29164 HG21 THR I 142   -4.790 26.044 14.453 0.00 0.00
I
ATOM 29165 HG22 THR I 142   -3.286 26.156 15.130 0.00 0.00
I
ATOM 29166 HG23 THR I 142   -3.778 24.591 14.185 0.00 0.00
I
ATOM 29167 C   THR I 142   -6.762 24.056 15.274 0.00 0.00
I
ATOM 29168 O   THR I 142   -6.830 24.091 14.045 0.00 0.00
I
ATOM 29169 N   ILE I 143   -7.799 24.509 16.023 0.00 0.00
I
ATOM 29170 HN ILE I 143   -7.777 24.208 16.974 0.00 0.00
I
ATOM 29171 CA ILE I 143   -8.946 25.287 15.546 0.00 0.00
I
ATOM 29172 HA ILE I 143   -8.990 25.352 14.469 0.00 0.00
I
ATOM 29173 CB ILE I 143   -10.326 24.789 16.017 0.00 0.00
I
ATOM 29174 HB ILE I 143   -10.291 23.715 15.736 0.00 0.00
I
ATOM 29175 CG2 ILE I 143   -10.409 24.989 17.556 0.00 0.00
I
ATOM 29176 HG21 ILE I 143   -9.431 25.031 18.081 0.00 0.00
I
ATOM 29177 HG22 ILE I 143   -10.889 25.984 17.675 0.00 0.00
I
ATOM 29178 HG23 ILE I 143   -11.074 24.278 18.091 0.00 0.00
I
ATOM 29179 CG1 ILE I 143   -11.574 25.404 15.345 0.00 0.00
I
ATOM 29180 HG11 ILE I 143   -11.767 26.462 15.626 0.00 0.00
I
ATOM 29181 HG12 ILE I 143   -11.431 25.315 14.247 0.00 0.00
I
ATOM 29182 CD ILE I 143   -12.927 24.732 15.615 0.00 0.00
I
ATOM 29183 HD1 ILE I 143   -13.379 24.727 16.630 0.00 0.00
I
ATOM 29184 HD2 ILE I 143   -13.588 25.023 14.771 0.00 0.00
I
ATOM 29185 HD3 ILE I 143   -12.769 23.639 15.490 0.00 0.00
I
ATOM 29186 C   ILE I 143   -8.751 26.714 16.055 0.00 0.00
I

ATOM 29187 O   ILE I 143   -9.566 27.599 15.871 0.00 0.00
I
ATOM 29188 N   GLY I 144   -7.620 26.934 16.845 0.00 0.00
I
ATOM 29189 HN GLY I 144   -6.966 26.226 17.098 0.00 0.00
I
ATOM 29190 CA GLY I 144   -7.194 28.230 17.380 0.00 0.00
I
ATOM 29191 HA1 GLY I 144   -6.563 28.122 18.251 0.00 0.00
I
ATOM 29192 HA2 GLY I 144   -8.100 28.786 17.573 0.00 0.00
I
ATOM 29193 C   GLY I 144   -6.360 28.916 16.362 0.00 0.00
I
ATOM 29194 O   GLY I 144   -5.355 28.367 15.946 0.00 0.00
I
ATOM 29195 N   GLY I 145   -6.807 30.128 15.912 0.00 0.00
I
ATOM 29196 HN GLY I 145   -7.651 30.569 16.208 0.00 0.00
I
ATOM 29197 CA GLY I 145   -6.081 30.904 14.974 0.00 0.00
I
ATOM 29198 HA1 GLY I 145   -5.014 30.963 15.124 0.00 0.00
I
ATOM 29199 HA2 GLY I 145   -6.426 31.926 15.032 0.00 0.00
I
ATOM 29200 C   GLY I 145   -6.387 30.525 13.554 0.00 0.00
I
ATOM 29201 O   GLY I 145   -5.728 31.105 12.710 0.00 0.00
I
ATOM 29202 N   VAL I 146   -7.335 29.590 13.311 0.00 0.00
I
ATOM 29203 HN VAL I 146   -7.961 29.246 14.006 0.00 0.00
I
ATOM 29204 CA VAL I 146   -7.557 29.124 11.934 0.00 0.00
I
ATOM 29205 HA VAL I 146   -6.616 28.824 11.498 0.00 0.00
I
ATOM 29206 CB VAL I 146   -8.256 27.778 11.718 0.00 0.00
I
ATOM 29207 HB VAL I 146   -8.372 27.552 10.636 0.00 0.00
I
ATOM 29208 CG1 VAL I 146   -7.440 26.668 12.299 0.00 0.00
I
ATOM 29209 HG11 VAL I 146   -7.346 26.878 13.386 0.00 0.00
I
ATOM 29210 HG12 VAL I 146   -8.060 25.752 12.197 0.00 0.00
I
ATOM 29211 HG13 VAL I 146   -6.453 26.699 11.789 0.00 0.00
I
ATOM 29212 CG2 VAL I 146   -9.624 27.832 12.441 0.00 0.00
I
ATOM 29213 HG21 VAL I 146   -9.477 27.921 13.539 0.00 0.00
I

ATOM 29214 HG22 VAL I 146   -10.358 28.613 12.148 0.00 0.00
I
ATOM 29215 HG23 VAL I 146   -10.152 26.858 12.358 0.00 0.00
I
ATOM 29216 C   VAL I 146   -8.149 30.139 10.874 0.00 0.00
I
ATOM 29217 O   VAL I 146   -7.741 30.137   9.686 0.00 0.00
I
ATOM 29218 N   ILE I 147   -9.140 30.994 11.306 0.00 0.00
I
ATOM 29219 HN ILE I 147   -9.481 30.979 12.242 0.00 0.00
I
ATOM 29220 CA ILE I 147   -9.733 31.943 10.435 0.00 0.00
I
ATOM 29221 HA ILE I 147   -10.031 31.421   9.537 0.00 0.00
I
ATOM 29222 CB ILE I 147   -11.100 32.452 10.839 0.00 0.00
I
ATOM 29223 HB ILE I 147   -10.933 33.100 11.725 0.00 0.00
I
ATOM 29224 CG2 ILE I 147   -11.759 33.322   9.755 0.00 0.00
I
ATOM 29225 HG21 ILE I 147   -12.851 33.499   9.857 0.00 0.00
I
ATOM 29226 HG22 ILE I 147   -11.275 34.308   9.591 0.00 0.00
I
ATOM 29227 HG23 ILE I 147   -11.689 32.780   8.787 0.00 0.00
I
ATOM 29228 CG1 ILE I 147   -12.026 31.333 11.316 0.00 0.00
I
ATOM 29229 HG11 ILE I 147   -11.663 30.867 12.257 0.00 0.00
I
ATOM 29230 HG12 ILE I 147   -12.988 31.866 11.475 0.00 0.00
I
ATOM 29231 CD ILE I 147   -12.261 30.185 10.365 0.00 0.00
I
ATOM 29232 HD1 ILE I 147   -11.371 29.521 10.324 0.00 0.00
I
ATOM 29233 HD2 ILE I 147   -13.158 29.548 10.523 0.00 0.00
I
ATOM 29234 HD3 ILE I 147   -12.548 30.578   9.366 0.00 0.00
I
ATOM 29235 C   ILE I 147   -8.771 33.046   9.911 0.00 0.00
I
ATOM 29236 O   ILE I 147   -8.808 33.449   8.711 0.00 0.00
I
ATOM 29237 N   GLY I 148   -7.833 33.439 10.716 0.00 0.00
I
ATOM 29238 HN GLY I 148   -7.763 33.115 11.657 0.00 0.00
I
ATOM 29239 CA GLY I 148   -6.692 34.298 10.356 0.00 0.00
I
ATOM 29240 HA1 GLY I 148   -6.195 34.586 11.271 0.00 0.00
I

ATOM 29241 HA2 GLY I 148 -7.142 35.172 9.909 0.00 0.00
I
ATOM 29242 C GLY I 148 -5.589 33.633 9.600 0.00 0.00
I
ATOM 29243 O GLY I 148 -4.909 34.254 8.829 0.00 0.00
I
ATOM 29244 N LEU I 149 -5.467 32.310 9.624 0.00 0.00
I
ATOM 29245 HN LEU I 149 -5.988 31.679 10.194 0.00 0.00
I
ATOM 29246 CA LEU I 149 -4.521 31.573 8.780 0.00 0.00
I
ATOM 29247 HA LEU I 149 -3.641 32.185 8.907 0.00 0.00
I
ATOM 29248 CB LEU I 149 -4.313 30.172 9.262 0.00 0.00
I
ATOM 29249 HB1 LEU I 149 -5.274 29.661 9.486 0.00 0.00
I
ATOM 29250 HB2 LEU I 149 -3.979 29.530 8.419 0.00 0.00
I
ATOM 29251 CG LEU I 149 -3.345 29.958 10.460 0.00 0.00
I
ATOM 29252 HG LEU I 149 -3.827 30.510 11.295 0.00 0.00
I
ATOM 29253 CD1 LEU I 149 -3.300 28.428 10.687 0.00 0.00
I
ATOM 29254 HD11 LEU I 149 -2.930 27.909 9.778 0.00 0.00
I
ATOM 29255 HD12 LEU I 149 -2.613 28.124 11.506 0.00 0.00
I
ATOM 29256 HD13 LEU I 149 -4.282 28.087 11.077 0.00 0.00
I
ATOM 29257 CD2 LEU I 149 -2.017 30.390 10.232 0.00 0.00
I
ATOM 29258 HD21 LEU I 149 -1.566 29.913 9.335 0.00 0.00
I
ATOM 29259 HD22 LEU I 149 -2.006 31.442 9.876 0.00 0.00
I
ATOM 29260 HD23 LEU I 149 -1.381 30.233 11.129 0.00 0.00
I
ATOM 29261 C LEU I 149 -4.765 31.529 7.290 0.00 0.00
I
ATOM 29262 O LEU I 149 -3.812 31.568 6.485 0.00 0.00
I
ATOM 29263 N VAL I 150 -6.058 31.467 6.917 0.00 0.00
I
ATOM 29264 HN VAL I 150 -6.744 31.295 7.621 0.00 0.00
I
ATOM 29265 CA VAL I 150 -6.413 31.642 5.544 0.00 0.00
I
ATOM 29266 HA VAL I 150 -6.007 30.799 5.005 0.00 0.00
I
ATOM 29267 CB VAL I 150 -7.945 31.581 5.292 0.00 0.00
I

ATOM 29268 HB VAL I 150   -8.376 32.593   5.449 0.00 0.00
I
ATOM 29269 CG1 VAL I 150   -8.266 30.973   3.909 0.00 0.00
I
ATOM 29270 HG11 VAL I 150   -9.328 30.690   3.743 0.00 0.00
I
ATOM 29271 HG12 VAL I 150   -8.102 31.731   3.114 0.00 0.00
I
ATOM 29272 HG13 VAL I 150   -7.663 30.054   3.748 0.00 0.00
I
ATOM 29273 CG2 VAL I 150   -8.658 30.656   6.352 0.00 0.00
I
ATOM 29274 HG21 VAL I 150   -9.752 30.648   6.156 0.00 0.00
I
ATOM 29275 HG22 VAL I 150   -8.260 29.622   6.276 0.00 0.00
I
ATOM 29276 HG23 VAL I 150   -8.534 31.181   7.323 0.00 0.00
I
ATOM 29277 C   VAL I 150   -5.859 32.925   4.788 0.00 0.00
I
ATOM 29278 O   VAL I 150   -5.332 32.917   3.666 0.00 0.00
I
ATOM 29279 N   LYS I 151   -5.953 34.069   5.536 0.00 0.00
I
ATOM 29280 HN LYS I 151   -6.295 33.892   6.456 0.00 0.00
I
ATOM 29281 CA LYS I 151   -5.385 35.356   5.194 0.00 0.00
I
ATOM 29282 HA LYS I 151   -5.858 35.685   4.281 0.00 0.00
I
ATOM 29283 CB LYS I 151   -5.667 36.248   6.402 0.00 0.00
I
ATOM 29284 HB1 LYS I 151   -5.115 36.019   7.339 0.00 0.00
I
ATOM 29285 HB2 LYS I 151   -5.310 37.273   6.161 0.00 0.00
I
ATOM 29286 CG LYS I 151   -7.125 36.404   6.831 0.00 0.00
I
ATOM 29287 HG1 LYS I 151   -7.638 36.969   6.023 0.00 0.00
I
ATOM 29288 HG2 LYS I 151   -7.569 35.424   7.107 0.00 0.00
I
ATOM 29289 CD LYS I 151   -7.381 37.398   8.078 0.00 0.00
I
ATOM 29290 HD1 LYS I 151   -6.750 37.131   8.953 0.00 0.00
I
ATOM 29291 HD2 LYS I 151   -7.307 38.470   7.796 0.00 0.00
I
ATOM 29292 CE LYS I 151   -8.779 37.208   8.660 0.00 0.00
I
ATOM 29293 HE1 LYS I 151   -9.479 37.480   7.841 0.00 0.00
I
ATOM 29294 HE2 LYS I 151   -8.907 36.128   8.884 0.00 0.00
I

ATOM 29295 NZ LYS I 151   -9.146 38.036   9.835 0.00 0.00
I
ATOM 29296 HZ1 LYS I 151   -9.139 39.060   9.652 0.00 0.00
I
ATOM 29297 HZ2 LYS I 151   -9.976 37.669 10.342 0.00 0.00
I
ATOM 29298 HZ3 LYS I 151   -8.403 37.945 10.558 0.00 0.00
I
ATOM 29299 C   LYS I 151   -3.885 35.348   5.052 0.00 0.00
I
ATOM 29300 O   LYS I 151   -3.341 35.892   4.055 0.00 0.00
I
ATOM 29301 N   VAL I 152   -3.121 34.776   5.986 0.00 0.00
I
ATOM 29302 HN VAL I 152   -3.570 34.351   6.768 0.00 0.00
I
ATOM 29303 CA VAL I 152   -1.703 34.510   6.003 0.00 0.00
I
ATOM 29304 HA VAL I 152   -1.191 35.460   6.049 0.00 0.00
I
ATOM 29305 CB VAL I 152   -1.260 33.692   7.271 0.00 0.00
I
ATOM 29306 HB VAL I 152   -1.982 32.855   7.383 0.00 0.00
I
ATOM 29307 CG1 VAL I 152   0.206 33.137   7.117 0.00 0.00
I
ATOM 29308 HG11 VAL I 152   0.496 32.668   8.082 0.00 0.00
I
ATOM 29309 HG12 VAL I 152   0.235 32.456   6.239 0.00 0.00
I
ATOM 29310 HG13 VAL I 152   0.900 33.983   6.923 0.00 0.00
I
ATOM 29311 CG2 VAL I 152   -1.408 34.511   8.581 0.00 0.00
I
ATOM 29312 HG21 VAL I 152   -0.694 35.355   8.475 0.00 0.00
I
ATOM 29313 HG22 VAL I 152   -2.456 34.859   8.707 0.00 0.00
I
ATOM 29314 HG23 VAL I 152   -1.182 33.869   9.460 0.00 0.00
I
ATOM 29315 C   VAL I 152   -1.281 33.750   4.739 0.00 0.00
I
ATOM 29316 O   VAL I 152   -0.288 34.137   4.104 0.00 0.00
I
ATOM 29317 N   LEU I 153   -2.060 32.652   4.446 0.00 0.00
I
ATOM 29318 HN LEU I 153   -2.720 32.373   5.139 0.00 0.00
I
ATOM 29319 CA LEU I 153   -1.811 31.858   3.265 0.00 0.00
I
ATOM 29320 HA LEU I 153   -0.759 31.616   3.283 0.00 0.00
I
ATOM 29321 CB LEU I 153   -2.633 30.536   3.333 0.00 0.00
I

ATOM 29322 HB1 LEU I 153   -2.348 29.993   4.259 0.00 0.00
I
ATOM 29323 HB2 LEU I 153   -3.697 30.855   3.296 0.00 0.00
I
ATOM 29324 CG LEU I 153   -2.285 29.521   2.306 0.00 0.00
I
ATOM 29325 HG LEU I 153   -2.615 29.877   1.307 0.00 0.00
I
ATOM 29326 CD1 LEU I 153   -0.741 29.295   2.229 0.00 0.00
I
ATOM 29327 HD11 LEU I 153   -0.569 28.450   1.528 0.00 0.00
I
ATOM 29328 HD12 LEU I 153   -0.299 30.179   1.721 0.00 0.00
I
ATOM 29329 HD13 LEU I 153   -0.403 29.133   3.274 0.00 0.00
I
ATOM 29330 CD2 LEU I 153   -2.907 28.160   2.616 0.00 0.00
I
ATOM 29331 HD21 LEU I 153   -2.357 27.615   3.413 0.00 0.00
I
ATOM 29332 HD22 LEU I 153   -3.989 28.252   2.852 0.00 0.00
I
ATOM 29333 HD23 LEU I 153   -2.789 27.604   1.662 0.00 0.00
I
ATOM 29334 C   LEU I 153   -2.015 32.496   1.935 0.00 0.00
I
ATOM 29335 O   LEU I 153   -1.191 32.278   1.049 0.00 0.00
I
ATOM 29336 N   PHE I 154   -3.093 33.234   1.730 0.00 0.00
I
ATOM 29337 HN PHE I 154   -3.757 33.279   2.473 0.00 0.00
I
ATOM 29338 CA PHE I 154   -3.364 33.934   0.447 0.00 0.00
I
ATOM 29339 HA PHE I 154   -3.345 33.231 -0.372 0.00 0.00
I
ATOM 29340 CB PHE I 154   -4.856 34.505   0.655 0.00 0.00
I
ATOM 29341 HB1 PHE I 154   -5.454 33.651   1.039 0.00 0.00
I
ATOM 29342 HB2 PHE I 154   -4.894 35.367   1.355 0.00 0.00
I
ATOM 29343 CG PHE I 154   -5.554 35.044 -0.620 0.00 0.00
I
ATOM 29344 CD1 PHE I 154   -5.636 36.407 -0.973 0.00 0.00
I
ATOM 29345 HD1 PHE I 154   -5.169 37.177 -0.376 0.00 0.00
I
ATOM 29346 CE1 PHE I 154   -6.461 36.836 -2.008 0.00 0.00
I
ATOM 29347 HE1 PHE I 154   -6.654 37.894 -2.109 0.00 0.00
I
ATOM 29348 CZ PHE I 154   -7.080 35.859 -2.795 0.00 0.00
I

```
ATOM  29349  HZ  PHE I 154      -7.741  36.083  -3.620  0.00  0.00
I
ATOM  29350  CD2 PHE I 154      -6.253  34.096  -1.408  0.00  0.00
I
ATOM  29351  HD2 PHE I 154      -6.211  33.071  -1.071  0.00  0.00
I
ATOM  29352  CE2 PHE I 154      -6.966  34.449  -2.518  0.00  0.00
I
ATOM  29353  HE2 PHE I 154      -7.429  33.706  -3.151  0.00  0.00
I
ATOM  29354  C   PHE I 154      -2.445  35.076   0.131  0.00  0.00
I
ATOM  29355  O   PHE I 154      -1.994  35.262  -0.969  0.00  0.00
I
ATOM  29356  N   LEU I 155      -1.951  35.765   1.171  0.00  0.00
I
ATOM  29357  HN  LEU I 155      -2.407  35.689   2.054  0.00  0.00
I
ATOM  29358  CA  LEU I 155      -0.753  36.579   1.074  0.00  0.00
I
ATOM  29359  HA  LEU I 155      -0.788  37.329   0.298  0.00  0.00
I
ATOM  29360  CB  LEU I 155      -0.416  37.171   2.411  0.00  0.00
I
ATOM  29361  HB1 LEU I 155      -1.353  37.655   2.759  0.00  0.00
I
ATOM  29362  HB2 LEU I 155      -0.119  36.349   3.097  0.00  0.00
I
ATOM  29363  CG  LEU I 155       0.702  38.253   2.382  0.00  0.00
I
ATOM  29364  HG  LEU I 155       1.747  37.897   2.259  0.00  0.00
I
ATOM  29365  CD1 LEU I 155       0.181  39.431   1.576  0.00  0.00
I
ATOM  29366 HD11 LEU I 155       0.797  40.352   1.661  0.00  0.00
I
ATOM  29367 HD12 LEU I 155       0.324  39.157   0.509  0.00  0.00
I
ATOM  29368 HD13 LEU I 155      -0.926  39.449   1.661  0.00  0.00
I
ATOM  29369  CD2 LEU I 155       0.856  38.861   3.824  0.00  0.00
I
ATOM  29370 HD21 LEU I 155       1.715  39.561   3.741  0.00  0.00
I
ATOM  29371 HD22 LEU I 155       0.059  39.585   4.097  0.00  0.00
I
ATOM  29372 HD23 LEU I 155       1.095  38.035   4.528  0.00  0.00
I
ATOM  29373  C   LEU I 155       0.527  35.867   0.633  0.00  0.00
I
ATOM  29374  O   LEU I 155       1.200  36.362  -0.271  0.00  0.00
I
ATOM  29375  N   PHE I 156       0.874  34.679   1.105  0.00  0.00
I
```

ATOM	29376	HN	PHE	I	156	0.256	34.156	1.688	0.00	0.00	I
ATOM	29377	CA	PHE	I	156	2.000	33.897	0.603	0.00	0.00	I
ATOM	29378	HA	PHE	I	156	2.888	34.510	0.552	0.00	0.00	I
ATOM	29379	CB	PHE	I	156	2.234	32.710	1.579	0.00	0.00	I
ATOM	29380	HB1	PHE	I	156	2.442	33.124	2.589	0.00	0.00	I
ATOM	29381	HB2	PHE	I	156	1.432	31.950	1.698	0.00	0.00	I
ATOM	29382	CG	PHE	I	156	3.460	31.956	1.189	0.00	0.00	I
ATOM	29383	CD1	PHE	I	156	4.682	32.540	1.257	0.00	0.00	I
ATOM	29384	HD1	PHE	I	156	4.763	33.595	1.476	0.00	0.00	I
ATOM	29385	CE1	PHE	I	156	5.888	31.816	1.070	0.00	0.00	I
ATOM	29386	HE1	PHE	I	156	6.841	32.310	1.185	0.00	0.00	I
ATOM	29387	CZ	PHE	I	156	5.783	30.462	0.852	0.00	0.00	I
ATOM	29388	HZ	PHE	I	156	6.681	29.900	0.647	0.00	0.00	I
ATOM	29389	CD2	PHE	I	156	3.330	30.606	0.843	0.00	0.00	I
ATOM	29390	HD2	PHE	I	156	2.346	30.195	0.678	0.00	0.00	I
ATOM	29391	CE2	PHE	I	156	4.556	29.874	0.577	0.00	0.00	I
ATOM	29392	HE2	PHE	I	156	4.469	28.836	0.291	0.00	0.00	I
ATOM	29393	C	PHE	I	156	1.855	33.498	−0.835	0.00	0.00	I
ATOM	29394	O	PHE	I	156	2.759	33.756	−1.669	0.00	0.00	I
ATOM	29395	N	SER	I	157	0.685	32.917	−1.223	0.00	0.00	I
ATOM	29396	HN	SER	I	157	−0.005	32.799	−0.513	0.00	0.00	I
ATOM	29397	CA	SER	I	157	0.309	32.344	−2.492	0.00	0.00	I
ATOM	29398	HA	SER	I	157	1.016	31.545	−2.660	0.00	0.00	I
ATOM	29399	CB	SER	I	157	−1.185	31.769	−2.466	0.00	0.00	I
ATOM	29400	HB1	SER	I	157	−1.994	32.525	−2.376	0.00	0.00	I
ATOM	29401	HB2	SER	I	157	−1.414	31.278	−3.436	0.00	0.00	I
ATOM	29402	OG	SER	I	157	−1.308	30.748	−1.406	0.00	0.00	I

ATOM 29403 HG1 SER I 157   -1.331 31.164 -0.541 0.00 0.00
I
ATOM 29404 C   SER I 157   0.437 33.357 -3.599 0.00 0.00
I
ATOM 29405 O   SER I 157   1.112 33.098 -4.610 0.00 0.00
I
ATOM 29406 N   ILE I 158   -0.039 34.600 -3.443 0.00 0.00
I
ATOM 29407 HN ILE I 158   -0.531 34.816 -2.603 0.00 0.00
I
ATOM 29408 CA ILE I 158   0.094 35.684 -4.387 0.00 0.00
I
ATOM 29409 HA ILE I 158   -0.283 35.265 -5.309 0.00 0.00
I
ATOM 29410 CB ILE I 158   -0.769 36.911 -3.967 0.00 0.00
I
ATOM 29411 HB ILE I 158   -0.623 37.122 -2.886 0.00 0.00
I
ATOM 29412 CG2 ILE I 158   -0.486 38.177 -4.831 0.00 0.00
I
ATOM 29413 HG21 ILE I 158   -0.435 38.060 -5.935 0.00 0.00
I
ATOM 29414 HG22 ILE I 158   -1.215 39.012 -4.760 0.00 0.00
I
ATOM 29415 HG23 ILE I 158   0.534 38.489 -4.521 0.00 0.00
I
ATOM 29416 CG1 ILE I 158   -2.260 36.534 -4.005 0.00 0.00
I
ATOM 29417 HG11 ILE I 158   -2.579 36.389 -5.060 0.00 0.00
I
ATOM 29418 HG12 ILE I 158   -2.401 35.529 -3.553 0.00 0.00
I
ATOM 29419 CD ILE I 158   -3.088 37.599 -3.296 0.00 0.00
I
ATOM 29420 HD1 ILE I 158   -4.103 37.241 -3.021 0.00 0.00
I
ATOM 29421 HD2 ILE I 158   -2.625 37.785 -2.304 0.00 0.00
I
ATOM 29422 HD3 ILE I 158   -3.252 38.518 -3.898 0.00 0.00
I
ATOM 29423 C   ILE I 158   1.526 36.159 -4.660 0.00 0.00
I
ATOM 29424 O   ILE I 158   1.949 36.328 -5.773 0.00 0.00
I
ATOM 29425 N   SER I 159   2.311 36.276 -3.520 0.00 0.00
I
ATOM 29426 HN SER I 159   1.889 36.023 -2.653 0.00 0.00
I
ATOM 29427 CA SER I 159   3.737 36.609 -3.413 0.00 0.00
I
ATOM 29428 HA SER I 159   3.792 37.520 -3.991 0.00 0.00
I
ATOM 29429 CB SER I 159   4.313 36.943 -2.028 0.00 0.00
I

ATOM 29430 HB1 SER I 159   4.383 36.025 -1.407 0.00 0.00
I
ATOM 29431 HB2 SER I 159   5.355 37.310 -2.148 0.00 0.00
I
ATOM 29432 OG SER I 159   3.537 37.903 -1.330 0.00 0.00
I
ATOM 29433 HG1 SER I 159   2.806 37.490 -0.865 0.00 0.00
I
ATOM 29434 C   SER I 159   4.634 35.644 -4.052 0.00 0.00
I
ATOM 29435 O   SER I 159   5.568 36.001 -4.823 0.00 0.00
I
ATOM 29436 N   ILE I 160   4.433 34.290 -3.740 0.00 0.00
I
ATOM 29437 HN ILE I 160   3.822 34.123 -2.970 0.00 0.00
I
ATOM 29438 CA ILE I 160   5.327 33.226 -4.154 0.00 0.00
I
ATOM 29439 HA ILE I 160   6.254 33.766 -4.278 0.00 0.00
I
ATOM 29440 CB ILE I 160   5.308 32.029 -3.166 0.00 0.00
I
ATOM 29441 HB ILE I 160   5.171 32.332 -2.106 0.00 0.00
I
ATOM 29442 CG2 ILE I 160   4.097 31.140 -3.396 0.00 0.00
I
ATOM 29443 HG21 ILE I 160   3.150 31.711 -3.291 0.00 0.00
I
ATOM 29444 HG22 ILE I 160   4.244 30.615 -4.364 0.00 0.00
I
ATOM 29445 HG23 ILE I 160   4.116 30.377 -2.589 0.00 0.00
I
ATOM 29446 CG1 ILE I 160   6.659 31.243 -3.176 0.00 0.00
I
ATOM 29447 HG11 ILE I 160   6.637 30.325 -2.551 0.00 0.00
I
ATOM 29448 HG12 ILE I 160   6.905 30.865 -4.191 0.00 0.00
I
ATOM 29449 CD ILE I 160   7.768 32.096 -2.570 0.00 0.00
I
ATOM 29450 HD1 ILE I 160   7.669 32.305 -1.484 0.00 0.00
I
ATOM 29451 HD2 ILE I 160   8.689 31.477 -2.628 0.00 0.00
I
ATOM 29452 HD3 ILE I 160   7.990 33.070 -3.056 0.00 0.00
I
ATOM 29453 C   ILE I 160   5.122 32.939 -5.602 0.00 0.00
I
ATOM 29454 O   ILE I 160   6.125 32.825 -6.344 0.00 0.00
I
ATOM 29455 N   GLU I 161   3.839 32.868 -6.065 0.00 0.00
I
ATOM 29456 HN GLU I 161   3.066 32.905 -5.438 0.00 0.00
I

ATOM 29457 CA GLU I 161 3.468 32.845 -7.505 0.00 0.00
I
ATOM 29458 HA GLU I 161 3.987 32.030 -7.989 0.00 0.00
I
ATOM 29459 CB GLU I 161 1.944 32.642 -7.770 0.00 0.00
I
ATOM 29460 HB1 GLU I 161 1.823 32.064 -8.711 0.00 0.00
I
ATOM 29461 HB2 GLU I 161 1.526 31.925 -7.031 0.00 0.00
I
ATOM 29462 CG GLU I 161 0.999 33.905 -7.827 0.00 0.00
I
ATOM 29463 HG1 GLU I 161 1.235 34.584 -6.980 0.00 0.00
I
ATOM 29464 HG2 GLU I 161 1.213 34.513 -8.732 0.00 0.00
I
ATOM 29465 CD GLU I 161 -0.412 33.698 -7.729 0.00 0.00
I
ATOM 29466 OE1 GLU I 161 -1.132 34.710 -7.939 0.00 0.00
I
ATOM 29467 OE2 GLU I 161 -0.898 32.595 -7.416 0.00 0.00
I
ATOM 29468 C GLU I 161 3.973 34.097 -8.289 0.00 0.00
I
ATOM 29469 O GLU I 161 4.366 33.935 -9.434 0.00 0.00
I
ATOM 29470 N LEU I 162 4.020 35.328 -7.673 0.00 0.00
I
ATOM 29471 HN LEU I 162 3.762 35.356 -6.710 0.00 0.00
I
ATOM 29472 CA LEU I 162 4.612 36.518 -8.235 0.00 0.00
I
ATOM 29473 HA LEU I 162 4.220 36.410 -9.236 0.00 0.00
I
ATOM 29474 CB LEU I 162 4.285 37.870 -7.513 0.00 0.00
I
ATOM 29475 HB1 LEU I 162 3.212 37.911 -7.229 0.00 0.00
I
ATOM 29476 HB2 LEU I 162 4.894 37.974 -6.590 0.00 0.00
I
ATOM 29477 CG LEU I 162 4.495 39.184 -8.305 0.00 0.00
I
ATOM 29478 HG LEU I 162 5.596 39.325 -8.352 0.00 0.00
I
ATOM 29479 CD1 LEU I 162 4.033 39.146 -9.740 0.00 0.00
I
ATOM 29480 HD11 LEU I 162 4.404 40.017 -10.322 0.00 0.00
I
ATOM 29481 HD12 LEU I 162 4.268 38.232 -10.327 0.00 0.00
I
ATOM 29482 HD13 LEU I 162 2.932 39.234 -9.862 0.00 0.00
I
ATOM 29483 CD2 LEU I 162 3.943 40.395 -7.565 0.00 0.00
I

ATOM 29484 HD21 LEU I 162   4.406 40.394 -6.555 0.00 0.00
I
ATOM 29485 HD22 LEU I 162   4.152 41.348 -8.095 0.00 0.00
I
ATOM 29486 HD23 LEU I 162   2.850 40.280 -7.400 0.00 0.00
I
ATOM 29487 C   LEU I 162   6.076 36.427 -8.405 0.00 0.00
I
ATOM 29488 O   LEU I 162   6.654 36.777 -9.485 0.00 0.00
I
ATOM 29489 N   ILE I 163   6.730 35.826 -7.417 0.00 0.00
I
ATOM 29490 HN ILE I 163   6.369 35.770 -6.489 0.00 0.00
I
ATOM 29491 CA ILE I 163   8.192 35.612 -7.510 0.00 0.00
I
ATOM 29492 HA ILE I 163   8.662 36.563 -7.712 0.00 0.00
I
ATOM 29493 CB ILE I 163   8.815 35.409 -6.093 0.00 0.00
I
ATOM 29494 HB ILE I 163   8.108 34.683 -5.637 0.00 0.00
I
ATOM 29495 CG2 ILE I 163   10.173 34.684 -6.056 0.00 0.00
I
ATOM 29496 HG21 ILE I 163   10.596 34.758 -5.031 0.00 0.00
I
ATOM 29497 HG22 ILE I 163   10.082 33.610 -6.326 0.00 0.00
I
ATOM 29498 HG23 ILE I 163   10.934 35.042 -6.782 0.00 0.00
I
ATOM 29499 CG1 ILE I 163   8.814 36.707 -5.230 0.00 0.00
I
ATOM 29500 HG11 ILE I 163   9.578 37.466 -5.502 0.00 0.00
I
ATOM 29501 HG12 ILE I 163   7.942 37.364 -5.438 0.00 0.00
I
ATOM 29502 CD ILE I 163   8.630 36.478 -3.772 0.00 0.00
I
ATOM 29503 HD1 ILE I 163   8.594 37.428 -3.197 0.00 0.00
I
ATOM 29504 HD2 ILE I 163   7.675 35.919 -3.665 0.00 0.00
I
ATOM 29505 HD3 ILE I 163   9.481 35.854 -3.425 0.00 0.00
I
ATOM 29506 C   ILE I 163   8.508 34.586 -8.545 0.00 0.00
I
ATOM 29507 O   ILE I 163   9.386 34.883 -9.349 0.00 0.00
I
ATOM 29508 N   ALA I 164   7.836 33.438 -8.491 0.00 0.00
I
ATOM 29509 HN ALA I 164   7.201 33.110 -7.796 0.00 0.00
I
ATOM 29510 CA ALA I 164   7.937 32.452 -9.620 0.00 0.00
I

ATOM 29511 HA ALA I 164 8.977 32.221 -9.793 0.00 0.00
I
ATOM 29512 CB ALA I 164 7.244 31.107 -9.176 0.00 0.00
I
ATOM 29513 HB1 ALA I 164 7.494 30.259 -9.850 0.00 0.00
I
ATOM 29514 HB2 ALA I 164 7.728 30.831 -8.215 0.00 0.00
I
ATOM 29515 HB3 ALA I 164 6.165 31.262 -8.960 0.00 0.00
I
ATOM 29516 C ALA I 164 7.558 32.943 -11.036 0.00 0.00
I
ATOM 29517 O ALA I 164 8.187 32.551 -12.044 0.00 0.00
I
ATOM 29518 N ALA I 165 6.665 33.875 -11.157 0.00 0.00
I
ATOM 29519 HN ALA I 165 6.065 34.009 -10.372 0.00 0.00
I
ATOM 29520 CA ALA I 165 6.242 34.490 -12.361 0.00 0.00
I
ATOM 29521 HA ALA I 165 5.998 33.769 -13.127 0.00 0.00
I
ATOM 29522 CB ALA I 165 4.998 35.330 -12.207 0.00 0.00
I
ATOM 29523 HB1 ALA I 165 4.143 34.823 -11.711 0.00 0.00
I
ATOM 29524 HB2 ALA I 165 5.235 36.176 -11.526 0.00 0.00
I
ATOM 29525 HB3 ALA I 165 4.507 35.802 -13.084 0.00 0.00
I
ATOM 29526 C ALA I 165 7.235 35.368 -13.045 0.00 0.00
I
ATOM 29527 O ALA I 165 7.379 35.359 -14.279 0.00 0.00
I
ATOM 29528 N LEU I 166 8.058 36.126 -12.253 0.00 0.00
I
ATOM 29529 HN LEU I 166 7.923 36.040 -11.268 0.00 0.00
I
ATOM 29530 CA LEU I 166 8.851 37.259 -12.696 0.00 0.00
I
ATOM 29531 HA LEU I 166 8.745 37.463 -13.751 0.00 0.00
I
ATOM 29532 CB LEU I 166 8.452 38.490 -11.900 0.00 0.00
I
ATOM 29533 HB1 LEU I 166 7.341 38.508 -11.920 0.00 0.00
I
ATOM 29534 HB2 LEU I 166 8.643 38.362 -10.813 0.00 0.00
I
ATOM 29535 CG LEU I 166 8.965 39.804 -12.508 0.00 0.00
I
ATOM 29536 HG LEU I 166 10.071 39.822 -12.613 0.00 0.00
I
ATOM 29537 CD1 LEU I 166 8.350 40.167 -13.894 0.00 0.00
I

```
ATOM  29538 HD11 LEU I 166       8.969  39.554 -14.582  0.00  0.00
I
ATOM  29539 HD12 LEU I 166       7.303  39.794 -13.883  0.00  0.00
I
ATOM  29540 HD13 LEU I 166       8.534  41.238 -14.123  0.00  0.00
I
ATOM  29541  CD2 LEU I 166       8.660  40.960 -11.513  0.00  0.00
I
ATOM  29542 HD21 LEU I 166       7.553  41.041 -11.560  0.00  0.00
I
ATOM  29543 HD22 LEU I 166       8.919  40.602 -10.494  0.00  0.00
I
ATOM  29544 HD23 LEU I 166       9.127  41.920 -11.820  0.00  0.00
I
ATOM  29545  C   LEU I 166      10.315  36.977 -12.482  0.00  0.00
I
ATOM  29546  O   LEU I 166      11.112  36.820 -13.408  0.00  0.00
I
ATOM  29547  N   ILE I 167      10.769  36.969 -11.204  0.00  0.00
I
ATOM  29548  HN  ILE I 167      10.203  36.801 -10.401  0.00  0.00
I
ATOM  29549  CA  ILE I 167      12.165  37.105 -10.932  0.00  0.00
I
ATOM  29550  HA  ILE I 167      12.624  37.784 -11.636  0.00  0.00
I
ATOM  29551  CB  ILE I 167      12.380  37.805  -9.563  0.00  0.00
I
ATOM  29552  HB  ILE I 167      11.605  38.587  -9.412  0.00  0.00
I
ATOM  29553  CG2 ILE I 167      12.172  36.772  -8.433  0.00  0.00
I
ATOM  29554 HG21 ILE I 167      13.080  36.145  -8.299  0.00  0.00
I
ATOM  29555 HG22 ILE I 167      11.956  37.226  -7.443  0.00  0.00
I
ATOM  29556 HG23 ILE I 167      11.327  36.069  -8.597  0.00  0.00
I
ATOM  29557  CG1 ILE I 167      13.750  38.564  -9.495  0.00  0.00
I
ATOM  29558 HG11 ILE I 167      13.985  38.967  -8.487  0.00  0.00
I
ATOM  29559 HG12 ILE I 167      14.613  37.945  -9.820  0.00  0.00
I
ATOM  29560  CD  ILE I 167      13.718  39.789 -10.440  0.00  0.00
I
ATOM  29561  HD1 ILE I 167      13.759  39.449 -11.497  0.00  0.00
I
ATOM  29562  HD2 ILE I 167      12.754  40.313 -10.265  0.00  0.00
I
ATOM  29563  HD3 ILE I 167      14.551  40.506 -10.278  0.00  0.00
I
ATOM  29564  C   ILE I 167      12.886  35.822 -11.100  0.00  0.00
I
```

ATOM 29565 O   ILE I 167   14.076 35.757 −11.419 0.00 0.00
I
ATOM 29566 N   LEU I 168   12.185 34.700 −10.901 0.00 0.00
I
ATOM 29567 HN LEU I 168   11.264 34.829 −10.540 0.00 0.00
I
ATOM 29568 CA LEU I 168   12.837 33.398 −10.999 0.00 0.00
I
ATOM 29569 HA LEU I 168   13.878 33.566 −10.762 0.00 0.00
I
ATOM 29570 CB LEU I 168   12.151 32.229 −10.260 0.00 0.00
I
ATOM 29571 HB1 LEU I 168   11.110 32.104 −10.627 0.00 0.00
I
ATOM 29572 HB2 LEU I 168   12.775 31.332 −10.459 0.00 0.00
I
ATOM 29573 CG LEU I 168   12.244 32.483 −8.665 0.00 0.00
I
ATOM 29574 HG LEU I 168   11.741 33.439 −8.405 0.00 0.00
I
ATOM 29575 CD1 LEU I 168   11.582 31.328 −7.843 0.00 0.00
I
ATOM 29576 HD11 LEU I 168   12.235 30.431 −7.893 0.00 0.00
I
ATOM 29577 HD12 LEU I 168   11.495 31.513 −6.751 0.00 0.00
I
ATOM 29578 HD13 LEU I 168   10.525 31.119 −8.115 0.00 0.00
I
ATOM 29579 CD2 LEU I 168   13.727 32.598 −8.186 0.00 0.00
I
ATOM 29580 HD21 LEU I 168   14.264 33.444 −8.665 0.00 0.00
I
ATOM 29581 HD22 LEU I 168   13.781 32.843 −7.104 0.00 0.00
I
ATOM 29582 HD23 LEU I 168   14.250 31.645 −8.418 0.00 0.00
I
ATOM 29583 C   LEU I 168   12.892 32.997 −12.525 0.00 0.00
I
ATOM 29584 O   LEU I 168   13.710 32.147 −12.883 0.00 0.00
I
ATOM 29585 N   SER I 169   12.082 33.580 −13.384 0.00 0.00
I
ATOM 29586 HN SER I 169   11.573 34.373 −13.058 0.00 0.00
I
ATOM 29587 CA SER I 169   11.775 33.179 −14.718 0.00 0.00
I
ATOM 29588 HA SER I 169   12.204 32.194 −14.835 0.00 0.00
I
ATOM 29589 CB SER I 169   10.293 33.182 −14.937 0.00 0.00
I
ATOM 29590 HB1 SER I 169   9.787 33.920 −14.278 0.00 0.00
I
ATOM 29591 HB2 SER I 169   9.967 33.531 −15.940 0.00 0.00
I

ATOM 29592 OG SER I 169   9.760 31.900 -14.636 0.00 0.00
I
ATOM 29593 HG1 SER I 169   8.822 32.104 -14.660 0.00 0.00
I
ATOM 29594 C   SER I 169   12.377 34.038 -15.767 0.00 0.00
I
ATOM 29595 O   SER I 169   12.031 34.024 -16.968 0.00 0.00
I
ATOM 29596 N   ILE I 170   13.289 34.952 -15.258 0.00 0.00
I
ATOM 29597 HN ILE I 170   13.575 35.076 -14.311 0.00 0.00
I
ATOM 29598 CA ILE I 170   13.978 35.988 -16.075 0.00 0.00
I
ATOM 29599 HA ILE I 170   13.200 36.561 -16.559 0.00 0.00
I
ATOM 29600 CB ILE I 170   14.705 37.021 -15.187 0.00 0.00
I
ATOM 29601 HB ILE I 170   14.047 37.268 -14.326 0.00 0.00
I
ATOM 29602 CG2 ILE I 170   16.030 36.440 -14.612 0.00 0.00
I
ATOM 29603 HG21 ILE I 170   16.713 37.189 -14.157 0.00 0.00
I
ATOM 29604 HG22 ILE I 170   15.855 35.649 -13.852 0.00 0.00
I
ATOM 29605 HG23 ILE I 170   16.526 35.936 -15.469 0.00 0.00
I
ATOM 29606 CG1 ILE I 170   14.790 38.439 -15.842 0.00 0.00
I
ATOM 29607 HG11 ILE I 170   15.621 38.407 -16.579 0.00 0.00
I
ATOM 29608 HG12 ILE I 170   13.869 38.691 -16.410 0.00 0.00
I
ATOM 29609 CD ILE I 170   15.127 39.496 -14.789 0.00 0.00
I
ATOM 29610 HD1 ILE I 170   14.284 39.532 -14.066 0.00 0.00
I
ATOM 29611 HD2 ILE I 170   16.116 39.339 -14.306 0.00 0.00
I
ATOM 29612 HD3 ILE I 170   15.124 40.475 -15.312 0.00 0.00
I
ATOM 29613 C   ILE I 170   14.813 35.489 -17.276 0.00 0.00
I
ATOM 29614 O   ILE I 170   14.922 36.185 -18.289 0.00 0.00
I
ATOM 29615 N   ARG I 171   15.406 34.254 -17.196 0.00 0.00
I
ATOM 29616 HN ARG I 171   15.452 33.779 -16.321 0.00 0.00
I
ATOM 29617 CA ARG I 171   16.133 33.673 -18.367 0.00 0.00
I
ATOM 29618 HA ARG I 171   16.718 34.412 -18.895 0.00 0.00
I

ATOM 29619 CB ARG I 171 16.910 32.490 -17.891 0.00 0.00
I
ATOM 29620 HB1 ARG I 171 16.358 31.943 -17.098 0.00 0.00
I
ATOM 29621 HB2 ARG I 171 16.969 31.695 -18.666 0.00 0.00
I
ATOM 29622 CG ARG I 171 18.242 32.938 -17.309 0.00 0.00
I
ATOM 29623 HG1 ARG I 171 18.919 33.448 -18.027 0.00 0.00
I
ATOM 29624 HG2 ARG I 171 18.023 33.735 -16.566 0.00 0.00
I
ATOM 29625 CD ARG I 171 18.942 31.678 -16.679 0.00 0.00
I
ATOM 29626 HD1 ARG I 171 19.835 32.001 -16.102 0.00 0.00
I
ATOM 29627 HD2 ARG I 171 18.321 31.152 -15.922 0.00 0.00
I
ATOM 29628 NE ARG I 171 19.455 30.780 -17.721 0.00 0.00
I
ATOM 29629 HE ARG I 171 19.908 31.247 -18.480 0.00 0.00
I
ATOM 29630 CZ ARG I 171 19.410 29.418 -17.647 0.00 0.00
I
ATOM 29631 NH1 ARG I 171 18.724 28.705 -16.797 0.00 0.00
I
ATOM 29632 HH11 ARG I 171 18.750 27.710 -16.888 0.00 0.00
I
ATOM 29633 HH12 ARG I 171 17.915 29.098 -16.359 0.00 0.00
I
ATOM 29634 NH2 ARG I 171 20.113 28.704 -18.530 0.00 0.00
I
ATOM 29635 HH21 ARG I 171 20.260 27.739 -18.311 0.00 0.00
I
ATOM 29636 HH22 ARG I 171 20.689 29.175 -19.198 0.00 0.00
I
ATOM 29637 C ARG I 171 15.074 33.253 -19.357 0.00 0.00
I
ATOM 29638 O ARG I 171 15.204 33.400 -20.561 0.00 0.00
I
ATOM 29639 N LEU I 172 13.966 32.778 -18.733 0.00 0.00
I
ATOM 29640 HN LEU I 172 13.781 32.871 -17.757 0.00 0.00
I
ATOM 29641 CA LEU I 172 12.944 32.013 -19.480 0.00 0.00
I
ATOM 29642 HA LEU I 172 13.566 31.444 -20.156 0.00 0.00
I
ATOM 29643 CB LEU I 172 12.129 30.951 -18.631 0.00 0.00
I
ATOM 29644 HB1 LEU I 172 11.753 31.525 -17.758 0.00 0.00
I
ATOM 29645 HB2 LEU I 172 11.272 30.572 -19.228 0.00 0.00
I

```
ATOM  29646  CG  LEU I 172      12.863  29.663 -18.243  0.00  0.00
I
ATOM  29647  HG  LEU I 172      13.369  29.188 -19.111  0.00  0.00
I
ATOM  29648  CD1 LEU I 172      14.004  29.790 -17.230  0.00  0.00
I
ATOM  29649 HD11 LEU I 172      14.394  28.818 -16.860  0.00  0.00
I
ATOM  29650 HD12 LEU I 172      14.847  30.440 -17.548  0.00  0.00
I
ATOM  29651 HD13 LEU I 172      13.556  30.323 -16.364  0.00  0.00
I
ATOM  29652  CD2 LEU I 172      11.954  28.652 -17.601  0.00  0.00
I
ATOM  29653 HD21 LEU I 172      11.619  28.915 -16.574  0.00  0.00
I
ATOM  29654 HD22 LEU I 172      11.063  28.480 -18.242  0.00  0.00
I
ATOM  29655 HD23 LEU I 172      12.556  27.733 -17.435  0.00  0.00
I
ATOM  29656  C   LEU I 172      11.968  32.802 -20.375  0.00  0.00
I
ATOM  29657  O   LEU I 172      11.750  32.438 -21.494  0.00  0.00
I
ATOM  29658  N   VAL I 173      11.337  33.854 -19.865  0.00  0.00
I
ATOM  29659  HN  VAL I 173      11.803  34.260 -19.082  0.00  0.00
I
ATOM  29660  CA  VAL I 173      10.341  34.692 -20.544  0.00  0.00
I
ATOM  29661  HA  VAL I 173       9.561  34.006 -20.838  0.00  0.00
I
ATOM  29662  CB  VAL I 173       9.660  35.580 -19.512  0.00  0.00
I
ATOM  29663  HB  VAL I 173      10.394  36.314 -19.116  0.00  0.00
I
ATOM  29664  CG1 VAL I 173       8.598  36.526 -20.086  0.00  0.00
I
ATOM  29665 HG11 VAL I 173       7.778  35.940 -20.554  0.00  0.00
I
ATOM  29666 HG12 VAL I 173       8.071  37.020 -19.241  0.00  0.00
I
ATOM  29667 HG13 VAL I 173       9.081  37.236 -20.792  0.00  0.00
I
ATOM  29668  CG2 VAL I 173       8.928  34.661 -18.519  0.00  0.00
I
ATOM  29669 HG21 VAL I 173       9.659  33.893 -18.186  0.00  0.00
I
ATOM  29670 HG22 VAL I 173       8.526  35.151 -17.607  0.00  0.00
I
ATOM  29671 HG23 VAL I 173       8.073  34.174 -19.035  0.00  0.00
I
ATOM  29672  C   VAL I 173      10.921  35.409 -21.720  0.00  0.00
I
```

ATOM 29673 O VAL I 173 10.255 35.354 −22.746 0.00 0.00
I
ATOM 29674 N PRO I 174 12.162 35.972 −21.840 0.00 0.00
I
ATOM 29675 CD PRO I 174 12.721 36.684 −20.697 0.00 0.00
I
ATOM 29676 HD1 PRO I 174 11.904 37.190 −20.139 0.00 0.00
I
ATOM 29677 HD2 PRO I 174 13.239 35.951 −20.042 0.00 0.00
I
ATOM 29678 CA PRO I 174 12.758 36.499 −23.002 0.00 0.00
I
ATOM 29679 HA PRO I 174 12.065 37.241 −23.369 0.00 0.00
I
ATOM 29680 CB PRO I 174 14.128 37.126 −22.473 0.00 0.00
I
ATOM 29681 HB1 PRO I 174 14.604 37.856 −23.162 0.00 0.00
I
ATOM 29682 HB2 PRO I 174 14.820 36.316 −22.157 0.00 0.00
I
ATOM 29683 CG PRO I 174 13.721 37.675 −21.149 0.00 0.00
I
ATOM 29684 HG1 PRO I 174 13.284 38.670 −21.378 0.00 0.00
I
ATOM 29685 HG2 PRO I 174 14.588 37.846 −20.475 0.00 0.00
I
ATOM 29686 C PRO I 174 13.012 35.563 −24.058 0.00 0.00
I
ATOM 29687 O PRO I 174 12.623 35.875 −25.194 0.00 0.00
I
ATOM 29688 N GLN I 175 13.453 34.286 −23.731 0.00 0.00
I
ATOM 29689 HN GLN I 175 13.682 34.022 −22.797 0.00 0.00
I
ATOM 29690 CA GLN I 175 13.550 33.223 −24.722 0.00 0.00
I
ATOM 29691 HA GLN I 175 14.405 33.426 −25.350 0.00 0.00
I
ATOM 29692 CB GLN I 175 13.820 31.843 −24.017 0.00 0.00
I
ATOM 29693 HB1 GLN I 175 14.694 31.890 −23.332 0.00 0.00
I
ATOM 29694 HB2 GLN I 175 13.013 31.781 −23.256 0.00 0.00
I
ATOM 29695 CG GLN I 175 13.915 30.515 −24.804 0.00 0.00
I
ATOM 29696 HG1 GLN I 175 14.062 29.717 −24.046 0.00 0.00
I
ATOM 29697 HG2 GLN I 175 12.930 30.397 −25.304 0.00 0.00
I
ATOM 29698 CD GLN I 175 14.964 30.272 −25.933 0.00 0.00
I
ATOM 29699 OE1 GLN I 175 14.595 30.281 −27.100 0.00 0.00
I

ATOM 29700 NE2 GLN I 175   16.224 30.245 -25.558 0.00 0.00
I
ATOM 29701 HE21 GLN I 175   16.920 29.850 -26.158 0.00 0.00
I
ATOM 29702 HE22 GLN I 175   16.524 30.604 -24.674 0.00 0.00
I
ATOM 29703 C   GLN I 175   12.286 32.943 -25.531 0.00 0.00
I
ATOM 29704 O   GLN I 175   12.302 32.817 -26.748 0.00 0.00
I
ATOM 29705 N   TYR I 176   11.143 32.908 -24.870 0.00 0.00
I
ATOM 29706 HN TYR I 176   11.045 32.811 -23.882 0.00 0.00
I
ATOM 29707 CA TYR I 176   9.880 32.627 -25.498 0.00 0.00
I
ATOM 29708 HA TYR I 176   9.989 32.115 -26.443 0.00 0.00
I
ATOM 29709 CB TYR I 176   9.061 31.679 -24.632 0.00 0.00
I
ATOM 29710 HB1 TYR I 176   8.980 32.263 -23.690 0.00 0.00
I
ATOM 29711 HB2 TYR I 176   8.003 31.504 -24.925 0.00 0.00
I
ATOM 29712 CG TYR I 176   9.807 30.443 -24.463 0.00 0.00
I
ATOM 29713 CD1 TYR I 176   10.120 29.690 -25.588 0.00 0.00
I
ATOM 29714 HD1 TYR I 176   9.955 29.944 -26.625 0.00 0.00
I
ATOM 29715 CE1 TYR I 176   10.767 28.442 -25.367 0.00 0.00
I
ATOM 29716 HE1 TYR I 176   10.895 27.722 -26.162 0.00 0.00
I
ATOM 29717 CZ TYR I 176   11.206 28.125 -24.089 0.00 0.00
I
ATOM 29718 OH TYR I 176   11.864 26.860 -23.854 0.00 0.00
I
ATOM 29719 HH TYR I 176   12.157 26.497 -24.693 0.00 0.00
I
ATOM 29720 CD2 TYR I 176   10.323 30.089 -23.143 0.00 0.00
I
ATOM 29721 HD2 TYR I 176   10.151 30.754 -22.309 0.00 0.00
I
ATOM 29722 CE2 TYR I 176   11.124 28.929 -22.949 0.00 0.00
I
ATOM 29723 HE2 TYR I 176   11.560 28.665 -21.997 0.00 0.00
I
ATOM 29724 C   TYR I 176   9.103 33.817 -25.874 0.00 0.00
I
ATOM 29725 O   TYR I 176   8.207 33.751 -26.674 0.00 0.00
I
ATOM 29726 N   GLY I 177   9.401 35.013 -25.360 0.00 0.00
I

```
ATOM  29727  HN  GLY I 177      10.118  34.976 -24.669  0.00  0.00
I
ATOM  29728  CA  GLY I 177       8.605  36.258 -25.474  0.00  0.00
I
ATOM  29729  HA1 GLY I 177       7.980  36.214 -26.354  0.00  0.00
I
ATOM  29730  HA2 GLY I 177       9.303  37.073 -25.598  0.00  0.00
I
ATOM  29731  C   GLY I 177       7.646  36.677 -24.416  0.00  0.00
I
ATOM  29732  O   GLY I 177       6.837  35.933 -23.835  0.00  0.00
I
ATOM  29733  N   TRP I 178       7.725  37.942 -24.127  0.00  0.00
I
ATOM  29734  HN  TRP I 178       8.417  38.589 -24.439  0.00  0.00
I
ATOM  29735  CA  TRP I 178       6.903  38.471 -23.033  0.00  0.00
I
ATOM  29736  HA  TRP I 178       7.040  37.754 -22.237  0.00  0.00
I
ATOM  29737  CB  TRP I 178       7.389  39.975 -22.663  0.00  0.00
I
ATOM  29738  HB1 TRP I 178       7.207  40.736 -23.451  0.00  0.00
I
ATOM  29739  HB2 TRP I 178       6.735  40.302 -21.826  0.00  0.00
I
ATOM  29740  CG  TRP I 178       8.808  40.010 -22.190  0.00  0.00
I
ATOM  29741  CD1 TRP I 178       9.899  40.104 -22.936  0.00  0.00
I
ATOM  29742  HD1 TRP I 178       9.897  40.152 -24.015  0.00  0.00
I
ATOM  29743  NE1 TRP I 178      11.015  40.151 -22.105  0.00  0.00
I
ATOM  29744  HE1 TRP I 178      11.979  40.196 -22.251  0.00  0.00
I
ATOM  29745  CE2 TRP I 178      10.687  40.037 -20.826  0.00  0.00
I
ATOM  29746  CD2 TRP I 178       9.263  40.018 -20.870  0.00  0.00
I
ATOM  29747  CE3 TRP I 178       8.600  40.053 -19.645  0.00  0.00
I
ATOM  29748  HE3 TRP I 178       7.528  40.040 -19.519  0.00  0.00
I
ATOM  29749  CZ3 TRP I 178       9.367  40.054 -18.424  0.00  0.00
I
ATOM  29750  HZ3 TRP I 178       8.799  40.148 -17.510  0.00  0.00
I
ATOM  29751  CZ2 TRP I 178      11.427  40.128 -19.660  0.00  0.00
I
ATOM  29752  HZ2 TRP I 178      12.502  40.142 -19.766  0.00  0.00
I
ATOM  29753  CH2 TRP I 178      10.791  40.102 -18.464  0.00  0.00
I
```

ATOM 29754 HH2 TRP I 178 11.406 40.217 −17.584 0.00 0.00
I
ATOM 29755 C TRP I 178 5.405 38.504 −23.332 0.00 0.00
I
ATOM 29756 O TRP I 178 4.551 38.408 −22.428 0.00 0.00
I
ATOM 29757 N SER I 179 5.090 38.468 −24.579 0.00 0.00
I
ATOM 29758 HN SER I 179 5.810 38.425 −25.267 0.00 0.00
I
ATOM 29759 CA SER I 179 3.814 38.551 −25.218 0.00 0.00
I
ATOM 29760 HA SER I 179 3.413 39.522 −24.967 0.00 0.00
I
ATOM 29761 CB SER I 179 3.833 38.540 −26.729 0.00 0.00
I
ATOM 29762 HB1 SER I 179 4.494 37.747 −27.139 0.00 0.00
I
ATOM 29763 HB2 SER I 179 2.851 38.355 −27.216 0.00 0.00
I
ATOM 29764 OG SER I 179 4.222 39.790 −27.242 0.00 0.00
I
ATOM 29765 HG1 SER I 179 4.173 39.767 −28.200 0.00 0.00
I
ATOM 29766 C SER I 179 2.878 37.358 −24.785 0.00 0.00
I
ATOM 29767 O SER I 179 1.775 37.683 −24.293 0.00 0.00
I
ATOM 29768 N SER I 180 3.327 36.017 −24.847 0.00 0.00
I
ATOM 29769 HN SER I 180 4.110 35.780 −25.417 0.00 0.00
I
ATOM 29770 CA SER I 180 2.576 34.962 −24.203 0.00 0.00
I
ATOM 29771 HA SER I 180 1.568 35.286 −23.991 0.00 0.00
I
ATOM 29772 CB SER I 180 2.492 33.611 −25.086 0.00 0.00
I
ATOM 29773 HB1 SER I 180 2.206 34.049 −26.066 0.00 0.00
I
ATOM 29774 HB2 SER I 180 3.485 33.124 −25.195 0.00 0.00
I
ATOM 29775 OG SER I 180 1.451 32.768 −24.576 0.00 0.00
I
ATOM 29776 HG1 SER I 180 1.837 32.324 −23.817 0.00 0.00
I
ATOM 29777 C SER I 180 3.199 34.491 −22.818 0.00 0.00
I
ATOM 29778 O SER I 180 2.664 33.622 −22.139 0.00 0.00
I
ATOM 29779 N GLY I 181 4.440 34.869 −22.599 0.00 0.00
I
ATOM 29780 HN GLY I 181 4.853 35.591 −23.148 0.00 0.00
I

```
ATOM  29781  CA  GLY I 181       5.277  34.130 -21.652  0.00  0.00
I
ATOM  29782  HA1 GLY I 181       6.326  34.104 -21.906  0.00  0.00
I
ATOM  29783  HA2 GLY I 181       4.874  33.148 -21.450  0.00  0.00
I
ATOM  29784  C   GLY I 181       5.272  34.684 -20.333  0.00  0.00
I
ATOM  29785  O   GLY I 181       5.561  33.976 -19.348  0.00  0.00
I
ATOM  29786  N   LEU I 182       4.769  35.866 -20.180  0.00  0.00
I
ATOM  29787  HN  LEU I 182       4.453  36.334 -21.002  0.00  0.00
I
ATOM  29788  CA  LEU I 182       4.533  36.357 -18.800  0.00  0.00
I
ATOM  29789  HA  LEU I 182       5.377  36.080 -18.185  0.00  0.00
I
ATOM  29790  CB  LEU I 182       4.223  37.961 -18.856  0.00  0.00
I
ATOM  29791  HB1 LEU I 182       5.025  38.532 -19.372  0.00  0.00
I
ATOM  29792  HB2 LEU I 182       3.272  38.190 -19.382  0.00  0.00
I
ATOM  29793  CG  LEU I 182       4.055  38.465 -17.347  0.00  0.00
I
ATOM  29794  HG  LEU I 182       3.248  37.887 -16.848  0.00  0.00
I
ATOM  29795  CD1 LEU I 182       5.399  38.443 -16.513  0.00  0.00
I
ATOM  29796 HD11 LEU I 182       5.107  38.770 -15.492  0.00  0.00
I
ATOM  29797 HD12 LEU I 182       5.771  37.402 -16.397  0.00  0.00
I
ATOM  29798 HD13 LEU I 182       6.240  39.091 -16.840  0.00  0.00
I
ATOM  29799  CD2 LEU I 182       3.492  39.884 -17.377  0.00  0.00
I
ATOM  29800 HD21 LEU I 182       2.634  39.993 -18.074  0.00  0.00
I
ATOM  29801 HD22 LEU I 182       3.273  40.046 -16.300  0.00  0.00
I
ATOM  29802 HD23 LEU I 182       4.270  40.602 -17.712  0.00  0.00
I
ATOM  29803  C   LEU I 182       3.301  35.644 -18.186  0.00  0.00
I
ATOM  29804  O   LEU I 182       3.234  35.074 -17.063  0.00  0.00
I
ATOM  29805  N   PHE I 183       2.326  35.514 -19.055  0.00  0.00
I
ATOM  29806  HN  PHE I 183       2.316  35.964 -19.945  0.00  0.00
I
ATOM  29807  CA  PHE I 183       1.157  34.686 -18.718  0.00  0.00
I
```

ATOM 29808 HA PHE I 183 0.736 35.016 -17.779 0.00 0.00
I
ATOM 29809 CB PHE I 183 -0.024 34.974 -19.763 0.00 0.00
I
ATOM 29810 HB1 PHE I 183 -0.136 36.079 -19.746 0.00 0.00
I
ATOM 29811 HB2 PHE I 183 0.256 34.582 -20.765 0.00 0.00
I
ATOM 29812 CG PHE I 183 -1.293 34.298 -19.293 0.00 0.00
I
ATOM 29813 CD1 PHE I 183 -1.862 33.285 -20.089 0.00 0.00
I
ATOM 29814 HD1 PHE I 183 -1.523 33.069 -21.092 0.00 0.00
I
ATOM 29815 CE1 PHE I 183 -2.899 32.499 -19.526 0.00 0.00
I
ATOM 29816 HE1 PHE I 183 -3.250 31.726 -20.194 0.00 0.00
I
ATOM 29817 CZ PHE I 183 -3.378 32.703 -18.228 0.00 0.00
I
ATOM 29818 HZ PHE I 183 -4.163 32.079 -17.829 0.00 0.00
I
ATOM 29819 CD2 PHE I 183 -1.936 34.627 -18.024 0.00 0.00
I
ATOM 29820 HD2 PHE I 183 -1.574 35.400 -17.363 0.00 0.00
I
ATOM 29821 CE2 PHE I 183 -2.992 33.852 -17.529 0.00 0.00
I
ATOM 29822 HE2 PHE I 183 -3.464 34.126 -16.597 0.00 0.00
I
ATOM 29823 C PHE I 183 1.378 33.120 -18.546 0.00 0.00
I
ATOM 29824 O PHE I 183 1.014 32.528 -17.532 0.00 0.00
I
ATOM 29825 N ALA I 184 2.121 32.520 -19.520 0.00 0.00
I
ATOM 29826 HN ALA I 184 2.357 33.063 -20.322 0.00 0.00
I
ATOM 29827 CA ALA I 184 2.530 31.092 -19.343 0.00 0.00
I
ATOM 29828 HA ALA I 184 1.648 30.495 -19.161 0.00 0.00
I
ATOM 29829 CB ALA I 184 3.251 30.644 -20.597 0.00 0.00
I
ATOM 29830 HB1 ALA I 184 3.396 29.560 -20.791 0.00 0.00
I
ATOM 29831 HB2 ALA I 184 2.819 31.160 -21.481 0.00 0.00
I
ATOM 29832 HB3 ALA I 184 4.277 31.067 -20.539 0.00 0.00
I
ATOM 29833 C ALA I 184 3.445 30.882 -18.125 0.00 0.00
I
ATOM 29834 O ALA I 184 3.446 29.923 -17.322 0.00 0.00
I

```
ATOM  29835  N   SER I 185       4.371  31.829 -17.727  0.00  0.00
I
ATOM  29836  HN  SER I 185       4.628  32.677 -18.185  0.00  0.00
I
ATOM  29837  CA  SER I 185       5.144  31.753 -16.600  0.00  0.00
I
ATOM  29838  HA  SER I 185       5.797  30.893 -16.616  0.00  0.00
I
ATOM  29839  CB  SER I 185       6.007  33.072 -16.522  0.00  0.00
I
ATOM  29840  HB1 SER I 185       6.594  33.098 -17.465  0.00  0.00
I
ATOM  29841  HB2 SER I 185       5.353  33.960 -16.651  0.00  0.00
I
ATOM  29842  OG  SER I 185       6.973  32.997 -15.457  0.00  0.00
I
ATOM  29843  HG1 SER I 185       7.258  33.882 -15.217  0.00  0.00
I
ATOM  29844  C   SER I 185       4.329  31.803 -15.295  0.00  0.00
I
ATOM  29845  O   SER I 185       4.609  31.147 -14.289  0.00  0.00
I
ATOM  29846  N   LEU I 186       3.232  32.664 -15.282  0.00  0.00
I
ATOM  29847  HN  LEU I 186       2.913  32.975 -16.174  0.00  0.00
I
ATOM  29848  CA  LEU I 186       2.371  32.967 -14.165  0.00  0.00
I
ATOM  29849  HA  LEU I 186       2.931  33.295 -13.302  0.00  0.00
I
ATOM  29850  CB  LEU I 186       1.305  34.021 -14.634  0.00  0.00
I
ATOM  29851  HB1 LEU I 186       1.978  34.902 -14.714  0.00  0.00
I
ATOM  29852  HB2 LEU I 186       0.751  33.589 -15.494  0.00  0.00
I
ATOM  29853  CG  LEU I 186       0.178  34.426 -13.590  0.00  0.00
I
ATOM  29854  HG  LEU I 186      -0.438  33.531 -13.360  0.00  0.00
I
ATOM  29855  CD1 LEU I 186       0.934  34.897 -12.365  0.00  0.00
I
ATOM  29856 HD11 LEU I 186       1.743  35.600 -12.660  0.00  0.00
I
ATOM  29857 HD12 LEU I 186       0.252  35.376 -11.631  0.00  0.00
I
ATOM  29858 HD13 LEU I 186       1.285  33.952 -11.899  0.00  0.00
I
ATOM  29859  CD2 LEU I 186      -0.598  35.569 -14.299  0.00  0.00
I
ATOM  29860 HD21 LEU I 186      -1.033  36.269 -13.553  0.00  0.00
I
ATOM  29861 HD22 LEU I 186       0.047  36.239 -14.908  0.00  0.00
I
```

ATOM 29862 HD23 LEU I 186   -1.373 35.065 -14.915 0.00 0.00
I
ATOM 29863 C   LEU I 186   1.616 31.695 -13.827 0.00 0.00
I
ATOM 29864 O   LEU I 186   1.489 31.332 -12.643 0.00 0.00
I
ATOM 29865 N   PHE I 187   1.034 31.027 -14.880 0.00 0.00
I
ATOM 29866 HN PHE I 187   1.108 31.358 -15.818 0.00 0.00
I
ATOM 29867 CA PHE I 187   0.345 29.722 -14.867 0.00 0.00
I
ATOM 29868 HA PHE I 187   -0.446 29.728 -14.130 0.00 0.00
I
ATOM 29869 CB PHE I 187   -0.264 29.393 -16.279 0.00 0.00
I
ATOM 29870 HB1 PHE I 187   -0.881 30.248 -16.630 0.00 0.00
I
ATOM 29871 HB2 PHE I 187   0.592 29.208 -16.963 0.00 0.00
I
ATOM 29872 CG PHE I 187   -1.157 28.259 -16.323 0.00 0.00
I
ATOM 29873 CD1 PHE I 187   -0.540 27.024 -16.449 0.00 0.00
I
ATOM 29874 HD1 PHE I 187   0.540 27.027 -16.406 0.00 0.00
I
ATOM 29875 CE1 PHE I 187   -1.246 25.892 -16.899 0.00 0.00
I
ATOM 29876 HE1 PHE I 187   -0.746 24.935 -16.875 0.00 0.00
I
ATOM 29877 CZ PHE I 187   -2.605 25.972 -17.153 0.00 0.00
I
ATOM 29878 HZ PHE I 187   -3.259 25.154 -17.415 0.00 0.00
I
ATOM 29879 CD2 PHE I 187   -2.532 28.294 -16.585 0.00 0.00
I
ATOM 29880 HD2 PHE I 187   -3.067 29.232 -16.562 0.00 0.00
I
ATOM 29881 CE2 PHE I 187   -3.263 27.186 -16.977 0.00 0.00
I
ATOM 29882 HE2 PHE I 187   -4.306 27.294 -17.235 0.00 0.00
I
ATOM 29883 C   PHE I 187   1.290 28.570 -14.317 0.00 0.00
I
ATOM 29884 O   PHE I 187   0.843 27.761 -13.534 0.00 0.00
I
ATOM 29885 N   HSE I 188   2.564 28.437 -14.685 0.00 0.00
I
ATOM 29886 HN HSE I 188   2.934 29.099 -15.333 0.00 0.00
I
ATOM 29887 CA HSE I 188   3.589 27.525 -14.131 0.00 0.00
I
ATOM 29888 HA HSE I 188   3.114 26.558 -14.206 0.00 0.00
I

```
ATOM  29889  CB  HSE I 188       4.905  27.525 -14.932  0.00  0.00
I
ATOM  29890  HB1 HSE I 188       4.647  27.723 -15.994  0.00  0.00
I
ATOM  29891  HB2 HSE I 188       5.577  28.347 -14.605  0.00  0.00
I
ATOM  29892  ND1 HSE I 188       6.852  26.022 -14.448  0.00  0.00
I
ATOM  29893  CG  HSE I 188       5.645  26.233 -15.043  0.00  0.00
I
ATOM  29894  CE1 HSE I 188       7.171  24.733 -14.736  0.00  0.00
I
ATOM  29895  HE1 HSE I 188       8.128  24.274 -14.484  0.00  0.00
I
ATOM  29896  NE2 HSE I 188       6.168  24.097 -15.465  0.00  0.00
I
ATOM  29897  HE2 HSE I 188       6.217  23.190 -15.884  0.00  0.00
I
ATOM  29898  CD2 HSE I 188       5.235  25.059 -15.662  0.00  0.00
I
ATOM  29899  HD2 HSE I 188       4.342  24.849 -16.236  0.00  0.00
I
ATOM  29900  C   HSE I 188       3.904  27.767 -12.717  0.00  0.00
I
ATOM  29901  O   HSE I 188       3.990  26.811 -11.942  0.00  0.00
I
ATOM  29902  N   ALA I 189       3.943  29.044 -12.294  0.00  0.00
I
ATOM  29903  HN  ALA I 189       3.932  29.815 -12.926  0.00  0.00
I
ATOM  29904  CA  ALA I 189       4.206  29.402 -10.898  0.00  0.00
I
ATOM  29905  HA  ALA I 189       5.209  29.009 -10.829  0.00  0.00
I
ATOM  29906  CB  ALA I 189       4.187  30.852 -10.692  0.00  0.00
I
ATOM  29907  HB1 ALA I 189       5.044  31.322 -11.221  0.00  0.00
I
ATOM  29908  HB2 ALA I 189       3.253  31.426 -10.871  0.00  0.00
I
ATOM  29909  HB3 ALA I 189       4.506  31.100  -9.657  0.00  0.00
I
ATOM  29910  C   ALA I 189       3.143  28.824  -9.938  0.00  0.00
I
ATOM  29911  O   ALA I 189       3.492  28.038  -9.048  0.00  0.00
I
ATOM  29912  N   ILE I 190       1.841  29.049 -10.185  0.00  0.00
I
ATOM  29913  HN  ILE I 190       1.561  29.772 -10.812  0.00  0.00
I
ATOM  29914  CA  ILE I 190       0.732  28.366  -9.423  0.00  0.00
I
ATOM  29915  HA  ILE I 190       0.865  28.670  -8.396  0.00  0.00
I
```

ATOM 29916 CB ILE I 190   -0.508 28.933 -10.113 0.00 0.00
I
ATOM 29917 HB ILE I 190   -0.601 28.744 -11.204 0.00 0.00
I
ATOM 29918 CG2 ILE I 190   -1.764 28.273 -9.562 0.00 0.00
I
ATOM 29919 HG21 ILE I 190   -1.686 27.164 -9.580 0.00 0.00
I
ATOM 29920 HG22 ILE I 190   -1.910 28.623 -8.518 0.00 0.00
I
ATOM 29921 HG23 ILE I 190   -2.683 28.606 -10.089 0.00 0.00
I
ATOM 29922 CG1 ILE I 190   -0.705 30.485 -9.796 0.00 0.00
I
ATOM 29923 HG11 ILE I 190   -1.154 30.523 -8.780 0.00 0.00
I
ATOM 29924 HG12 ILE I 190   0.283 30.983 -9.892 0.00 0.00
I
ATOM 29925 CD ILE I 190   -1.531 31.209 -10.743 0.00 0.00
I
ATOM 29926 HD1 ILE I 190   -1.184 31.191 -11.798 0.00 0.00
I
ATOM 29927 HD2 ILE I 190   -2.560 30.790 -10.769 0.00 0.00
I
ATOM 29928 HD3 ILE I 190   -1.612 32.304 -10.577 0.00 0.00
I
ATOM 29929 C   ILE I 190   0.758 26.900 -9.658 0.00 0.00
I
ATOM 29930 O   ILE I 190   0.532 26.092 -8.810 0.00 0.00
I
ATOM 29931 N   SER I 191   1.076 26.423 -10.880 0.00 0.00
I
ATOM 29932 HN SER I 191   1.147 27.060 -11.645 0.00 0.00
I
ATOM 29933 CA SER I 191   1.045 24.983 -11.210 0.00 0.00
I
ATOM 29934 HA SER I 191   0.056 24.623 -10.965 0.00 0.00
I
ATOM 29935 CB SER I 191   1.219 24.683 -12.733 0.00 0.00
I
ATOM 29936 HB1 SER I 191   0.433 25.266 -13.260 0.00 0.00
I
ATOM 29937 HB2 SER I 191   2.245 24.874 -13.116 0.00 0.00
I
ATOM 29938 OG SER I 191   0.986 23.325 -13.059 0.00 0.00
I
ATOM 29939 HG1 SER I 191   1.272 23.238 -13.971 0.00 0.00
I
ATOM 29940 C   SER I 191   2.123 24.193 -10.530 0.00 0.00
I
ATOM 29941 O   SER I 191   1.789 23.098 -10.053 0.00 0.00
I
ATOM 29942 N   ALA I 192   3.336 24.701 -10.513 0.00 0.00
I

ATOM 29943 HN ALA I 192 3.412 25.541 −11.044 0.00 0.00
I
ATOM 29944 CA ALA I 192 4.469 24.222 −9.812 0.00 0.00
I
ATOM 29945 HA ALA I 192 4.793 23.268 −10.202 0.00 0.00
I
ATOM 29946 CB ALA I 192 5.734 25.182 −9.925 0.00 0.00
I
ATOM 29947 HB1 ALA I 192 5.594 26.228 −9.578 0.00 0.00
I
ATOM 29948 HB2 ALA I 192 6.588 24.798 −9.327 0.00 0.00
I
ATOM 29949 HB3 ALA I 192 5.936 25.368 −11.002 0.00 0.00
I
ATOM 29950 C ALA I 192 4.185 24.041 −8.375 0.00 0.00
I
ATOM 29951 O ALA I 192 4.181 22.973 −7.762 0.00 0.00
I
ATOM 29952 N PHE I 193 3.775 25.137 −7.658 0.00 0.00
I
ATOM 29953 HN PHE I 193 3.694 25.962 −8.213 0.00 0.00
I
ATOM 29954 CA PHE I 193 3.671 25.118 −6.234 0.00 0.00
I
ATOM 29955 HA PHE I 193 4.581 24.710 −5.817 0.00 0.00
I
ATOM 29956 CB PHE I 193 3.465 26.622 −5.786 0.00 0.00
I
ATOM 29957 HB1 PHE I 193 4.034 27.193 −6.551 0.00 0.00
I
ATOM 29958 HB2 PHE I 193 2.424 26.981 −5.928 0.00 0.00
I
ATOM 29959 CG PHE I 193 3.963 26.916 −4.400 0.00 0.00
I
ATOM 29960 CD1 PHE I 193 5.354 26.668 −4.174 0.00 0.00
I
ATOM 29961 HD1 PHE I 193 5.989 26.415 −5.010 0.00 0.00
I
ATOM 29962 CE1 PHE I 193 5.864 26.740 −2.863 0.00 0.00
I
ATOM 29963 HE1 PHE I 193 6.905 26.554 −2.644 0.00 0.00
I
ATOM 29964 CZ PHE I 193 5.035 27.116 −1.844 0.00 0.00
I
ATOM 29965 HZ PHE I 193 5.505 27.284 −0.886 0.00 0.00
I
ATOM 29966 CD2 PHE I 193 3.106 27.243 −3.296 0.00 0.00
I
ATOM 29967 HD2 PHE I 193 2.044 27.310 −3.479 0.00 0.00
I
ATOM 29968 CE2 PHE I 193 3.715 27.391 −2.019 0.00 0.00
I
ATOM 29969 HE2 PHE I 193 3.150 27.762 −1.177 0.00 0.00
I

```
ATOM  29970  C   PHE I 193       2.481  24.341  -5.595  0.00  0.00
I
ATOM  29971  O   PHE I 193       2.544  23.991  -4.451  0.00  0.00
I
ATOM  29972  N   ASN I 194       1.381  24.058  -6.294  0.00  0.00
I
ATOM  29973  HN  ASN I 194       1.224  24.509  -7.169  0.00  0.00
I
ATOM  29974  CA  ASN I 194       0.328  23.203  -5.694  0.00  0.00
I
ATOM  29975  HA  ASN I 194       0.383  23.195  -4.616  0.00  0.00
I
ATOM  29976  CB  ASN I 194      -1.080  23.667  -6.184  0.00  0.00
I
ATOM  29977  HB1 ASN I 194      -0.938  23.786  -7.279  0.00  0.00
I
ATOM  29978  HB2 ASN I 194      -1.899  22.952  -5.955  0.00  0.00
I
ATOM  29979  CG  ASN I 194      -1.432  25.005  -5.584  0.00  0.00
I
ATOM  29980  OD1 ASN I 194      -2.168  25.033  -4.551  0.00  0.00
I
ATOM  29981  ND2 ASN I 194      -0.993  26.111  -6.193  0.00  0.00
I
ATOM  29982 HD21 ASN I 194      -1.370  27.011  -5.973  0.00  0.00
I
ATOM  29983 HD22 ASN I 194      -0.363  25.992  -6.960  0.00  0.00
I
ATOM  29984  C   ASN I 194       0.514  21.817  -6.235  0.00  0.00
I
ATOM  29985  O   ASN I 194      -0.351  21.043  -5.943  0.00  0.00
I
ATOM  29986  N   ASN I 195       1.596  21.532  -6.977  0.00  0.00
I
ATOM  29987  HN  ASN I 195       2.310  22.209  -7.137  0.00  0.00
I
ATOM  29988  CA  ASN I 195       1.721  20.266  -7.744  0.00  0.00
I
ATOM  29989  HA  ASN I 195       2.605  20.326  -8.361  0.00  0.00
I
ATOM  29990  CB  ASN I 195       2.062  19.044  -6.816  0.00  0.00
I
ATOM  29991  HB1 ASN I 195       1.293  19.040  -6.014  0.00  0.00
I
ATOM  29992  HB2 ASN I 195       2.148  18.136  -7.451  0.00  0.00
I
ATOM  29993  CG  ASN I 195       3.409  19.333  -6.165  0.00  0.00
I
ATOM  29994  OD1 ASN I 195       4.480  18.862  -6.545  0.00  0.00
I
ATOM  29995  ND2 ASN I 195       3.450  20.089  -5.061  0.00  0.00
I
ATOM  29996 HD21 ASN I 195       4.339  20.419  -4.745  0.00  0.00
I
```

```
ATOM  29997 HD22 ASN I 195       2.568  20.409  -4.715  0.00  0.00
I
ATOM  29998  C   ASN I 195       0.546  19.991  -8.744  0.00  0.00
I
ATOM  29999  O   ASN I 195      -0.125  18.974  -8.699  0.00  0.00
I
ATOM  30000  N   ALA I 196       0.236  20.933  -9.630  0.00  0.00
I
ATOM  30001  HN  ALA I 196       0.858  21.701  -9.767  0.00  0.00
I
ATOM  30002  CA  ALA I 196      -0.945  20.738 -10.505  0.00  0.00
I
ATOM  30003  HA  ALA I 196      -1.651  20.078 -10.023  0.00  0.00
I
ATOM  30004  CB  ALA I 196      -1.730  22.112 -10.624  0.00  0.00
I
ATOM  30005  HB1 ALA I 196      -2.753  21.939 -11.021  0.00  0.00
I
ATOM  30006  HB2 ALA I 196      -1.887  22.516  -9.601  0.00  0.00
I
ATOM  30007  HB3 ALA I 196      -1.120  22.864 -11.169  0.00  0.00
I
ATOM  30008  C   ALA I 196      -0.604  20.223 -11.859  0.00  0.00
I
ATOM  30009  O   ALA I 196      -1.562  19.831 -12.542  0.00  0.00
I
ATOM  30010  N   GLY I 197       0.679  20.127 -12.258  0.00  0.00
I
ATOM  30011  HN  GLY I 197       1.393  20.543 -11.700  0.00  0.00
I
ATOM  30012  CA  GLY I 197       1.105  19.283 -13.367  0.00  0.00
I
ATOM  30013  HA1 GLY I 197       0.418  18.457 -13.259  0.00  0.00
I
ATOM  30014  HA2 GLY I 197       2.130  18.947 -13.304  0.00  0.00
I
ATOM  30015  C   GLY I 197       1.012  19.860 -14.755  0.00  0.00
I
ATOM  30016  O   GLY I 197       1.553  19.241 -15.674  0.00  0.00
I
ATOM  30017  N   PHE I 198       0.390  21.051 -14.882  0.00  0.00
I
ATOM  30018  HN  PHE I 198       0.051  21.432 -14.025  0.00  0.00
I
ATOM  30019  CA  PHE I 198      -0.045  21.618 -16.171  0.00  0.00
I
ATOM  30020  HA  PHE I 198      -0.207  20.867 -16.931  0.00  0.00
I
ATOM  30021  CB  PHE I 198      -1.389  22.407 -16.040  0.00  0.00
I
ATOM  30022  HB1 PHE I 198      -1.282  23.224 -15.295  0.00  0.00
I
ATOM  30023  HB2 PHE I 198      -1.588  22.853 -17.038  0.00  0.00
I
```

ATOM 30024 CG PHE I 198 −2.642 21.604 −15.699 0.00 0.00
I
ATOM 30025 CD1 PHE I 198 −3.300 20.907 −16.702 0.00 0.00
I
ATOM 30026 HD1 PHE I 198 −2.823 20.861 −17.670 0.00 0.00
I
ATOM 30027 CE1 PHE I 198 −4.531 20.232 −16.464 0.00 0.00
I
ATOM 30028 HE1 PHE I 198 −5.052 19.549 −17.118 0.00 0.00
I
ATOM 30029 CZ PHE I 198 −5.037 20.246 −15.140 0.00 0.00
I
ATOM 30030 HZ PHE I 198 −5.953 19.690 −15.009 0.00 0.00
I
ATOM 30031 CD2 PHE I 198 −3.207 21.579 −14.377 0.00 0.00
I
ATOM 30032 HD2 PHE I 198 −2.850 22.143 −13.528 0.00 0.00
I
ATOM 30033 CE2 PHE I 198 −4.395 20.941 −14.072 0.00 0.00
I
ATOM 30034 HE2 PHE I 198 −4.785 20.921 −13.065 0.00 0.00
I
ATOM 30035 C PHE I 198 1.035 22.592 −16.656 0.00 0.00
I
ATOM 30036 O PHE I 198 1.420 23.575 −15.998 0.00 0.00
I
ATOM 30037 N SER I 199 1.593 22.442 −17.866 0.00 0.00
I
ATOM 30038 HN SER I 199 1.515 21.587 −18.373 0.00 0.00
I
ATOM 30039 CA SER I 199 2.482 23.407 −18.454 0.00 0.00
I
ATOM 30040 HA SER I 199 2.694 24.178 −17.729 0.00 0.00
I
ATOM 30041 CB SER I 199 3.791 22.732 −18.723 0.00 0.00
I
ATOM 30042 HB1 SER I 199 4.144 22.280 −17.771 0.00 0.00
I
ATOM 30043 HB2 SER I 199 3.813 21.999 −19.557 0.00 0.00
I
ATOM 30044 OG SER I 199 4.875 23.691 −18.915 0.00 0.00
I
ATOM 30045 HG1 SER I 199 5.695 23.300 −18.605 0.00 0.00
I
ATOM 30046 C SER I 199 2.047 24.039 −19.710 0.00 0.00
I
ATOM 30047 O SER I 199 1.423 23.294 −20.501 0.00 0.00
I
ATOM 30048 N LEU I 200 2.229 25.322 −19.839 0.00 0.00
I
ATOM 30049 HN LEU I 200 2.511 25.889 −19.069 0.00 0.00
I
ATOM 30050 CA LEU I 200 1.992 26.112 −21.114 0.00 0.00
I

ATOM 30051 HA LEU I 200 1.545 25.442 -21.833 0.00 0.00
I
ATOM 30052 CB LEU I 200 1.044 27.234 -20.855 0.00 0.00
I
ATOM 30053 HB1 LEU I 200 1.284 27.722 -19.886 0.00 0.00
I
ATOM 30054 HB2 LEU I 200 1.294 28.048 -21.568 0.00 0.00
I
ATOM 30055 CG LEU I 200 -0.420 26.914 -20.892 0.00 0.00
I
ATOM 30056 HG LEU I 200 -0.635 26.109 -20.157 0.00 0.00
I
ATOM 30057 CD1 LEU I 200 -1.173 28.109 -20.397 0.00 0.00
I
ATOM 30058 HD11 LEU I 200 -0.993 28.407 -19.342 0.00 0.00
I
ATOM 30059 HD12 LEU I 200 -0.985 28.988 -21.050 0.00 0.00
I
ATOM 30060 HD13 LEU I 200 -2.262 27.889 -20.439 0.00 0.00
I
ATOM 30061 CD2 LEU I 200 -0.948 26.468 -22.318 0.00 0.00
I
ATOM 30062 HD21 LEU I 200 -0.815 25.375 -22.461 0.00 0.00
I
ATOM 30063 HD22 LEU I 200 -2.054 26.571 -22.309 0.00 0.00
I
ATOM 30064 HD23 LEU I 200 -0.387 27.115 -23.027 0.00 0.00
I
ATOM 30065 C LEU I 200 3.332 26.548 -21.732 0.00 0.00
I
ATOM 30066 O LEU I 200 3.275 27.323 -22.668 0.00 0.00
I
ATOM 30067 N TRP I 201 4.463 26.108 -21.193 0.00 0.00
I
ATOM 30068 HN TRP I 201 4.345 25.629 -20.327 0.00 0.00
I
ATOM 30069 CA TRP I 201 5.723 26.310 -21.884 0.00 0.00
I
ATOM 30070 HA TRP I 201 5.797 27.386 -21.928 0.00 0.00
I
ATOM 30071 CB TRP I 201 7.001 25.938 -21.000 0.00 0.00
I
ATOM 30072 HB1 TRP I 201 6.940 24.841 -20.836 0.00 0.00
I
ATOM 30073 HB2 TRP I 201 7.936 26.046 -21.591 0.00 0.00
I
ATOM 30074 CG TRP I 201 7.140 26.696 -19.807 0.00 0.00
I
ATOM 30075 CD1 TRP I 201 7.054 26.191 -18.567 0.00 0.00
I
ATOM 30076 HD1 TRP I 201 6.995 25.145 -18.307 0.00 0.00
I
ATOM 30077 NE1 TRP I 201 7.355 27.106 -17.593 0.00 0.00
I

ATOM 30078 HE1 TRP I 201 7.822 26.931 -16.754 0.00 0.00
I
ATOM 30079 CE2 TRP I 201 7.650 28.372 -18.287 0.00 0.00
I
ATOM 30080 CD2 TRP I 201 7.518 28.116 -19.681 0.00 0.00
I
ATOM 30081 CE3 TRP I 201 7.690 29.233 -20.610 0.00 0.00
I
ATOM 30082 HE3 TRP I 201 7.547 29.159 -21.678 0.00 0.00
I
ATOM 30083 CZ3 TRP I 201 7.975 30.509 -20.028 0.00 0.00
I
ATOM 30084 HZ3 TRP I 201 8.039 31.355 -20.696 0.00 0.00
I
ATOM 30085 CZ2 TRP I 201 7.999 29.543 -17.797 0.00 0.00
I
ATOM 30086 HZ2 TRP I 201 8.178 29.648 -16.737 0.00 0.00
I
ATOM 30087 CH2 TRP I 201 8.257 30.649 -18.708 0.00 0.00
I
ATOM 30088 HH2 TRP I 201 8.534 31.552 -18.185 0.00 0.00
I
ATOM 30089 C TRP I 201 5.777 25.586 -23.220 0.00 0.00
I
ATOM 30090 O TRP I 201 5.444 24.414 -23.267 0.00 0.00
I
ATOM 30091 N PRO I 202 6.013 26.300 -24.401 0.00 0.00
I
ATOM 30092 CD PRO I 202 6.505 27.684 -24.485 0.00 0.00
I
ATOM 30093 HD1 PRO I 202 6.036 28.304 -23.690 0.00 0.00
I
ATOM 30094 HD2 PRO I 202 7.616 27.699 -24.494 0.00 0.00
I
ATOM 30095 CA PRO I 202 5.815 25.769 -25.706 0.00 0.00
I
ATOM 30096 HA PRO I 202 4.766 25.514 -25.751 0.00 0.00
I
ATOM 30097 CB PRO I 202 6.347 26.843 -26.688 0.00 0.00
I
ATOM 30098 HB1 PRO I 202 5.878 26.703 -27.685 0.00 0.00
I
ATOM 30099 HB2 PRO I 202 7.429 26.685 -26.884 0.00 0.00
I
ATOM 30100 CG PRO I 202 6.136 28.144 -25.932 0.00 0.00
I
ATOM 30101 HG1 PRO I 202 5.101 28.544 -25.991 0.00 0.00
I
ATOM 30102 HG2 PRO I 202 6.781 29.025 -26.138 0.00 0.00
I
ATOM 30103 C PRO I 202 6.364 24.396 -26.043 0.00 0.00
I
ATOM 30104 O PRO I 202 5.605 23.578 -26.648 0.00 0.00
I

```
ATOM  30105  N   ASP I 203       7.607  24.053 -25.734  0.00  0.00
I
ATOM  30106  HN  ASP I 203       8.179  24.692 -25.226  0.00  0.00
I
ATOM  30107  CA  ASP I 203       8.366  22.858 -26.077  0.00  0.00
I
ATOM  30108  HA  ASP I 203       7.856  22.118 -26.676  0.00  0.00
I
ATOM  30109  CB  ASP I 203       9.553  23.173 -27.091  0.00  0.00
I
ATOM  30110  HB1 ASP I 203      10.222  22.298 -27.239  0.00  0.00
I
ATOM  30111  HB2 ASP I 203       9.175  23.370 -28.117  0.00  0.00
I
ATOM  30112  CG  ASP I 203      10.289  24.390 -26.759  0.00  0.00
I
ATOM  30113  OD1 ASP I 203      10.610  25.182 -27.687  0.00  0.00
I
ATOM  30114  OD2 ASP I 203      10.678  24.666 -25.605  0.00  0.00
I
ATOM  30115  C   ASP I 203       8.706  22.171 -24.756  0.00  0.00
I
ATOM  30116  O   ASP I 203       9.541  21.243 -24.672  0.00  0.00
I
ATOM  30117  N   ASN I 204       7.938  22.533 -23.711  0.00  0.00
I
ATOM  30118  HN  ASN I 204       7.161  23.114 -23.941  0.00  0.00
I
ATOM  30119  CA  ASN I 204       8.024  22.189 -22.334  0.00  0.00
I
ATOM  30120  HA  ASN I 204       7.319  22.782 -21.770  0.00  0.00
I
ATOM  30121  CB  ASN I 204       7.490  20.755 -22.181  0.00  0.00
I
ATOM  30122  HB1 ASN I 204       7.995  20.065 -22.891  0.00  0.00
I
ATOM  30123  HB2 ASN I 204       7.553  20.408 -21.127  0.00  0.00
I
ATOM  30124  CG  ASN I 204       5.936  20.781 -22.335  0.00  0.00
I
ATOM  30125  OD1 ASN I 204       5.248  20.672 -21.285  0.00  0.00
I
ATOM  30126  ND2 ASN I 204       5.337  20.820 -23.548  0.00  0.00
I
ATOM  30127 HD21 ASN I 204       4.343  20.922 -23.595  0.00  0.00
I
ATOM  30128 HD22 ASN I 204       5.869  20.577 -24.359  0.00  0.00
I
ATOM  30129  C   ASN I 204       9.360  22.295 -21.740  0.00  0.00
I
ATOM  30130  O   ASN I 204       9.888  21.487 -20.989  0.00  0.00
I
ATOM  30131  N   LEU I 205      10.006  23.402 -22.120  0.00  0.00
I
```

ATOM 30132 HN LEU I 205   9.441 23.943 -22.738 0.00 0.00
I
ATOM 30133 CA LEU I 205   11.374 23.922 -22.010 0.00 0.00
I
ATOM 30134 HA LEU I 205   11.366 24.890 -22.489 0.00 0.00
I
ATOM 30135 CB LEU I 205   11.741 24.116 -20.512 0.00 0.00
I
ATOM 30136 HB1 LEU I 205   11.835 23.047 -20.224 0.00 0.00
I
ATOM 30137 HB2 LEU I 205   12.732 24.597 -20.370 0.00 0.00
I
ATOM 30138 CG LEU I 205   10.764 24.944 -19.611 0.00 0.00
I
ATOM 30139 HG LEU I 205   9.776 24.490 -19.838 0.00 0.00
I
ATOM 30140 CD1 LEU I 205   11.156 24.777 -18.118 0.00 0.00
I
ATOM 30141 HD11 LEU I 205   11.063 23.690 -17.910 0.00 0.00
I
ATOM 30142 HD12 LEU I 205   12.222 25.056 -17.982 0.00 0.00
I
ATOM 30143 HD13 LEU I 205   10.507 25.405 -17.470 0.00 0.00
I
ATOM 30144 CD2 LEU I 205   10.687 26.381 -19.993 0.00 0.00
I
ATOM 30145 HD21 LEU I 205   9.999 26.829 -19.245 0.00 0.00
I
ATOM 30146 HD22 LEU I 205   11.700 26.815 -19.850 0.00 0.00
I
ATOM 30147 HD23 LEU I 205   10.230 26.537 -20.994 0.00 0.00
I
ATOM 30148 C   LEU I 205   12.475 23.019 -22.619 0.00 0.00
I
ATOM 30149 O   LEU I 205   13.373 22.581 -21.920 0.00 0.00
I
ATOM 30150 N   MET I 206   12.406 22.910 -23.952 0.00 0.00
I
ATOM 30151 HN MET I 206   11.776 23.430 -24.523 0.00 0.00
I
ATOM 30152 CA MET I 206   13.385 22.010 -24.606 0.00 0.00
I
ATOM 30153 HA MET I 206   13.339 20.957 -24.369 0.00 0.00
I
ATOM 30154 CB MET I 206   12.919 21.927 -26.090 0.00 0.00
I
ATOM 30155 HB1 MET I 206   12.029 21.269 -26.185 0.00 0.00
I
ATOM 30156 HB2 MET I 206   12.647 22.860 -26.630 0.00 0.00
I
ATOM 30157 CG MET I 206   13.884 21.358 -27.148 0.00 0.00
I
ATOM 30158 HG1 MET I 206   13.488 21.818 -28.079 0.00 0.00
I

ATOM 30159 HG2 MET I 206   14.908 21.754 -26.975 0.00 0.00
I
ATOM 30160 SD MET I 206   14.169 19.447 -27.111 0.00 0.00
I
ATOM 30161 CE MET I 206   15.208 19.411 -28.551 0.00 0.00
I
ATOM 30162 HE1 MET I 206   14.596 19.503 -29.474 0.00 0.00
I
ATOM 30163 HE2 MET I 206   15.807 20.332 -28.386 0.00 0.00
I
ATOM 30164 HE3 MET I 206   15.900 18.549 -28.662 0.00 0.00
I
ATOM 30165 C   MET I 206   14.834 22.386 -24.522 0.00 0.00
I
ATOM 30166 O   MET I 206   15.738 21.553 -24.295 0.00 0.00
I
ATOM 30167 N   SER I 207   15.058 23.676 -24.571 0.00 0.00
I
ATOM 30168 HN SER I 207   14.431 24.306 -25.024 0.00 0.00
I
ATOM 30169 CA SER I 207   16.278 24.363 -24.287 0.00 0.00
I
ATOM 30170 HA SER I 207   16.957 24.158 -25.102 0.00 0.00
I
ATOM 30171 CB SER I 207   16.089 25.989 -24.337 0.00 0.00
I
ATOM 30172 HB1 SER I 207   15.380 26.199 -23.508 0.00 0.00
I
ATOM 30173 HB2 SER I 207   17.041 26.456 -24.005 0.00 0.00
I
ATOM 30174 OG SER I 207   15.602 26.423 -25.635 0.00 0.00
I
ATOM 30175 HG1 SER I 207   14.647 26.512 -25.593 0.00 0.00
I
ATOM 30176 C   SER I 207   16.988 24.050 -22.990 0.00 0.00
I
ATOM 30177 O   SER I 207   18.202 23.911 -22.871 0.00 0.00
I
ATOM 30178 N   TYR I 208   16.078 23.883 -21.984 0.00 0.00
I
ATOM 30179 HN TYR I 208   15.115 23.820 -22.234 0.00 0.00
I
ATOM 30180 CA TYR I 208   16.504 23.753 -20.601 0.00 0.00
I
ATOM 30181 HA TYR I 208   17.552 24.005 -20.543 0.00 0.00
I
ATOM 30182 CB TYR I 208   15.759 24.717 -19.718 0.00 0.00
I
ATOM 30183 HB1 TYR I 208   14.677 24.465 -19.721 0.00 0.00
I
ATOM 30184 HB2 TYR I 208   15.942 24.586 -18.630 0.00 0.00
I
ATOM 30185 CG TYR I 208   15.776 26.197 -20.068 0.00 0.00
I

ATOM 30186 CD1 TYR I 208 16.871 26.951 -19.731 0.00 0.00
I
ATOM 30187 HD1 TYR I 208 17.858 26.673 -19.393 0.00 0.00
I
ATOM 30188 CE1 TYR I 208 16.926 28.291 -19.994 0.00 0.00
I
ATOM 30189 HE1 TYR I 208 17.834 28.801 -19.708 0.00 0.00
I
ATOM 30190 CZ TYR I 208 15.874 28.960 -20.538 0.00 0.00
I
ATOM 30191 OH TYR I 208 15.883 30.359 -20.404 0.00 0.00
I
ATOM 30192 HH TYR I 208 16.776 30.664 -20.578 0.00 0.00
I
ATOM 30193 CD2 TYR I 208 14.663 26.880 -20.653 0.00 0.00
I
ATOM 30194 HD2 TYR I 208 13.722 26.370 -20.792 0.00 0.00
I
ATOM 30195 CE2 TYR I 208 14.696 28.260 -20.838 0.00 0.00
I
ATOM 30196 HE2 TYR I 208 13.876 28.826 -21.256 0.00 0.00
I
ATOM 30197 C TYR I 208 16.379 22.329 -19.948 0.00 0.00
I
ATOM 30198 O TYR I 208 16.546 22.262 -18.669 0.00 0.00
I
ATOM 30199 N VAL I 209 16.332 21.279 -20.714 0.00 0.00
I
ATOM 30200 HN VAL I 209 16.102 21.206 -21.681 0.00 0.00
I
ATOM 30201 CA VAL I 209 16.182 19.946 -20.066 0.00 0.00
I
ATOM 30202 HA VAL I 209 15.403 19.969 -19.319 0.00 0.00
I
ATOM 30203 CB VAL I 209 15.771 18.948 -21.120 0.00 0.00
I
ATOM 30204 HB VAL I 209 14.797 19.327 -21.496 0.00 0.00
I
ATOM 30205 CG1 VAL I 209 16.749 18.831 -22.217 0.00 0.00
I
ATOM 30206 HG11 VAL I 209 17.041 19.857 -22.530 0.00 0.00
I
ATOM 30207 HG12 VAL I 209 17.632 18.224 -21.924 0.00 0.00
I
ATOM 30208 HG13 VAL I 209 16.442 18.262 -23.120 0.00 0.00
I
ATOM 30209 CG2 VAL I 209 15.435 17.592 -20.502 0.00 0.00
I
ATOM 30210 HG21 VAL I 209 15.051 16.967 -21.337 0.00 0.00
I
ATOM 30211 HG22 VAL I 209 16.357 17.105 -20.120 0.00 0.00
I
ATOM 30212 HG23 VAL I 209 14.649 17.743 -19.731 0.00 0.00
I

ATOM 30213 C VAL I 209 17.399 19.432 -19.249 0.00 0.00
I
ATOM 30214 O VAL I 209 17.267 18.818 -18.217 0.00 0.00
I
ATOM 30215 N GLY I 210 18.607 19.677 -19.634 0.00 0.00
I
ATOM 30216 HN GLY I 210 18.626 20.190 -20.489 0.00 0.00
I
ATOM 30217 CA GLY I 210 19.863 19.366 -18.959 0.00 0.00
I
ATOM 30218 HA1 GLY I 210 20.568 19.309 -19.775 0.00 0.00
I
ATOM 30219 HA2 GLY I 210 19.727 18.510 -18.315 0.00 0.00
I
ATOM 30220 C GLY I 210 20.384 20.546 -18.135 0.00 0.00
I
ATOM 30221 O GLY I 210 21.321 20.378 -17.347 0.00 0.00
I
ATOM 30222 N ASP I 211 19.672 21.705 -18.233 0.00 0.00
I
ATOM 30223 HN ASP I 211 18.936 21.829 -18.894 0.00 0.00
I
ATOM 30224 CA ASP I 211 20.123 22.925 -17.525 0.00 0.00
I
ATOM 30225 HA ASP I 211 21.166 23.028 -17.785 0.00 0.00
I
ATOM 30226 CB ASP I 211 19.357 24.088 -18.199 0.00 0.00
I
ATOM 30227 HB1 ASP I 211 19.339 24.140 -19.308 0.00 0.00
I
ATOM 30228 HB2 ASP I 211 18.300 24.095 -17.855 0.00 0.00
I
ATOM 30229 CG ASP I 211 20.003 25.413 -17.715 0.00 0.00
I
ATOM 30230 OD1 ASP I 211 19.380 26.032 -16.811 0.00 0.00
I
ATOM 30231 OD2 ASP I 211 21.052 25.836 -18.204 0.00 0.00
I
ATOM 30232 C ASP I 211 20.168 22.877 -15.959 0.00 0.00
I
ATOM 30233 O ASP I 211 19.183 22.405 -15.447 0.00 0.00
I
ATOM 30234 N PRO I 212 21.262 23.153 -15.213 0.00 0.00
I
ATOM 30235 CD PRO I 212 22.509 23.742 -15.713 0.00 0.00
I
ATOM 30236 HD1 PRO I 212 22.944 23.002 -16.418 0.00 0.00
I
ATOM 30237 HD2 PRO I 212 22.301 24.688 -16.257 0.00 0.00
I
ATOM 30238 CA PRO I 212 21.379 22.654 -13.855 0.00 0.00
I
ATOM 30239 HA PRO I 212 20.979 21.653 -13.917 0.00 0.00
I

ATOM 30240 CB PRO I 212   22.887 22.793 -13.624 0.00 0.00
I
ATOM 30241 HB1 PRO I 212   23.345 21.817 -13.894 0.00 0.00
I
ATOM 30242 HB2 PRO I 212   23.188 23.019 -12.579 0.00 0.00
I
ATOM 30243 CG PRO I 212   23.349 23.987 -14.538 0.00 0.00
I
ATOM 30244 HG1 PRO I 212   24.439 23.839 -14.691 0.00 0.00
I
ATOM 30245 HG2 PRO I 212   23.065 24.956 -14.074 0.00 0.00
I
ATOM 30246 C   PRO I 212   20.471 23.494 -12.957 0.00 0.00
I
ATOM 30247 O   PRO I 212   19.801 22.993 -12.066 0.00 0.00
I
ATOM 30248 N   THR I 213   20.343 24.812 -13.135 0.00 0.00
I
ATOM 30249 HN THR I 213   20.717 25.281 -13.931 0.00 0.00
I
ATOM 30250 CA THR I 213   19.532 25.763 -12.393 0.00 0.00
I
ATOM 30251 HA THR I 213   19.758 25.650 -11.343 0.00 0.00
I
ATOM 30252 CB THR I 213   19.644 27.293 -12.693 0.00 0.00
I
ATOM 30253 HB THR I 213   19.367 27.581 -13.730 0.00 0.00
I
ATOM 30254 OG1 THR I 213   20.985 27.800 -12.536 0.00 0.00
I
ATOM 30255 HG1 THR I 213   21.358 27.539 -13.381 0.00 0.00
I
ATOM 30256 CG2 THR I 213   18.887 28.212 -11.669 0.00 0.00
I
ATOM 30257 HG21 THR I 213   18.968 27.838 -10.627 0.00 0.00
I
ATOM 30258 HG22 THR I 213   19.272 29.226 -11.914 0.00 0.00
I
ATOM 30259 HG23 THR I 213   17.806 28.347 -11.889 0.00 0.00
I
ATOM 30260 C   THR I 213   18.043 25.565 -12.437 0.00 0.00
I
ATOM 30261 O   THR I 213   17.246 25.640 -11.545 0.00 0.00
I
ATOM 30262 N   VAL I 214   17.592 25.321 -13.664 0.00 0.00
I
ATOM 30263 HN VAL I 214   18.234 25.263 -14.425 0.00 0.00
I
ATOM 30264 CA VAL I 214   16.233 25.006 -14.029 0.00 0.00
I
ATOM 30265 HA VAL I 214   15.596 25.717 -13.522 0.00 0.00
I
ATOM 30266 CB VAL I 214   15.956 25.056 -15.568 0.00 0.00
I

ATOM 30267 HB VAL I 214   16.661 24.424 -16.149 0.00 0.00
I
ATOM 30268 CG1 VAL I 214   14.449 24.519 -15.808 0.00 0.00
I
ATOM 30269 HG11 VAL I 214   14.375 23.479 -15.424 0.00 0.00
I
ATOM 30270 HG12 VAL I 214   13.659 25.219 -15.461 0.00 0.00
I
ATOM 30271 HG13 VAL I 214   14.286 24.501 -16.907 0.00 0.00
I
ATOM 30272 CG2 VAL I 214   16.141 26.494 -15.955 0.00 0.00
I
ATOM 30273 HG21 VAL I 214   15.420 26.857 -16.718 0.00 0.00
I
ATOM 30274 HG22 VAL I 214   15.993 27.102 -15.038 0.00 0.00
I
ATOM 30275 HG23 VAL I 214   17.154 26.744 -16.337 0.00 0.00
I
ATOM 30276 C   VAL I 214   15.787 23.733 -13.378 0.00 0.00
I
ATOM 30277 O   VAL I 214   14.776 23.698 -12.696 0.00 0.00
I
ATOM 30278 N   ASN I 215   16.597 22.644 -13.335 0.00 0.00
I
ATOM 30279 HN ASN I 215   17.373 22.657 -13.961 0.00 0.00
I
ATOM 30280 CA ASN I 215   16.299 21.429 -12.653 0.00 0.00
I
ATOM 30281 HA ASN I 215   15.262 21.216 -12.866 0.00 0.00
I
ATOM 30282 CB ASN I 215   17.137 20.247 -13.167 0.00 0.00
I
ATOM 30283 HB1 ASN I 215   18.244 20.326 -13.121 0.00 0.00
I
ATOM 30284 HB2 ASN I 215   16.980 19.311 -12.589 0.00 0.00
I
ATOM 30285 CG ASN I 215   16.861 19.891 -14.640 0.00 0.00
I
ATOM 30286 OD1 ASN I 215   15.793 19.371 -14.930 0.00 0.00
I
ATOM 30287 ND2 ASN I 215   17.809 20.033 -15.512 0.00 0.00
I
ATOM 30288 HD21 ASN I 215   17.625 19.663 -16.423 0.00 0.00
I
ATOM 30289 HD22 ASN I 215   18.364 20.840 -15.310 0.00 0.00
I
ATOM 30290 C   ASN I 215   16.371 21.482 -11.142 0.00 0.00
I
ATOM 30291 O   ASN I 215   15.617 20.776 -10.459 0.00 0.00
I
ATOM 30292 N   LEU I 216   17.246 22.308 -10.561 0.00 0.00
I
ATOM 30293 HN LEU I 216   17.894 22.774 -11.158 0.00 0.00
I

ATOM 30294 CA LEU I 216   17.386 22.468 -9.147 0.00 0.00
I
ATOM 30295 HA LEU I 216   17.177 21.531 -8.652 0.00 0.00
I
ATOM 30296 CB LEU I 216   18.731 23.112 -8.897 0.00 0.00
I
ATOM 30297 HB1 LEU I 216   19.502 22.701 -9.583 0.00 0.00
I
ATOM 30298 HB2 LEU I 216   18.688 24.206 -9.087 0.00 0.00
I
ATOM 30299 CG LEU I 216   19.313 22.922 -7.438 0.00 0.00
I
ATOM 30300 HG LEU I 216   18.512 23.089 -6.686 0.00 0.00
I
ATOM 30301 CD1 LEU I 216   19.862 21.528 -7.239 0.00 0.00
I
ATOM 30302 HD11 LEU I 216   19.000 20.907 -6.913 0.00 0.00
I
ATOM 30303 HD12 LEU I 216   20.473 21.139 -8.082 0.00 0.00
I
ATOM 30304 HD13 LEU I 216   20.423 21.563 -6.281 0.00 0.00
I
ATOM 30305 CD2 LEU I 216   20.379 23.907 -7.289 0.00 0.00
I
ATOM 30306 HD21 LEU I 216   20.589 24.082 -6.212 0.00 0.00
I
ATOM 30307 HD22 LEU I 216   21.315 23.529 -7.752 0.00 0.00
I
ATOM 30308 HD23 LEU I 216   20.131 24.912 -7.694 0.00 0.00
I
ATOM 30309 C   LEU I 216   16.256 23.357 -8.626 0.00 0.00
I
ATOM 30310 O   LEU I 216   15.540 22.960 -7.713 0.00 0.00
I
ATOM 30311 N   VAL I 217   15.901 24.472 -9.315 0.00 0.00
I
ATOM 30312 HN VAL I 217   16.408 24.857 -10.083 0.00 0.00
I
ATOM 30313 CA VAL I 217   14.851 25.308 -8.699 0.00 0.00
I
ATOM 30314 HA VAL I 217   14.981 25.434 -7.634 0.00 0.00
I
ATOM 30315 CB VAL I 217   14.740 26.538 -9.539 0.00 0.00
I
ATOM 30316 HB VAL I 217   14.723 26.238 -10.608 0.00 0.00
I
ATOM 30317 CG1 VAL I 217   13.474 27.369 -9.245 0.00 0.00
I
ATOM 30318 HG11 VAL I 217   13.400 27.482 -8.142 0.00 0.00
I
ATOM 30319 HG12 VAL I 217   13.549 28.290 -9.862 0.00 0.00
I
ATOM 30320 HG13 VAL I 217   12.533 26.901 -9.605 0.00 0.00
I

ATOM 30321 CG2 VAL I 217   15.942 27.437 -9.186 0.00 0.00
I
ATOM 30322 HG21 VAL I 217   16.904 26.922 -9.395 0.00 0.00
I
ATOM 30323 HG22 VAL I 217   15.972 28.455 -9.632 0.00 0.00
I
ATOM 30324 HG23 VAL I 217   15.913 27.528 -8.079 0.00 0.00
I
ATOM 30325 C   VAL I 217   13.426 24.704 -8.712 0.00 0.00
I
ATOM 30326 O   VAL I 217   12.694 24.745 -7.729 0.00 0.00
I
ATOM 30327 N   ILE I 218   12.975 24.080 -9.802 0.00 0.00
I
ATOM 30328 HN ILE I 218   13.537 24.104 -10.625 0.00 0.00
I
ATOM 30329 CA ILE I 218   11.657 23.571 -10.041 0.00 0.00
I
ATOM 30330 HA ILE I 218   10.904 24.246 -9.661 0.00 0.00
I
ATOM 30331 CB ILE I 218   11.459 23.420 -11.533 0.00 0.00
I
ATOM 30332 HB ILE I 218   12.251 22.722 -11.879 0.00 0.00
I
ATOM 30333 CG2 ILE I 218   10.181 22.717 -11.999 0.00 0.00
I
ATOM 30334 HG21 ILE I 218   10.043 22.659 -13.100 0.00 0.00
I
ATOM 30335 HG22 ILE I 218   10.189 21.655 -11.671 0.00 0.00
I
ATOM 30336 HG23 ILE I 218   9.263 23.195 -11.597 0.00 0.00
I
ATOM 30337 CG1 ILE I 218   11.590 24.850 -12.217 0.00 0.00
I
ATOM 30338 HG11 ILE I 218   10.808 25.500 -11.769 0.00 0.00
I
ATOM 30339 HG12 ILE I 218   12.614 25.252 -12.060 0.00 0.00
I
ATOM 30340 CD ILE I 218   11.325 24.691 -13.648 0.00 0.00
I
ATOM 30341 HD1 ILE I 218   11.764 25.537 -14.219 0.00 0.00
I
ATOM 30342 HD2 ILE I 218   11.884 23.787 -13.974 0.00 0.00
I
ATOM 30343 HD3 ILE I 218   10.314 24.540 -14.083 0.00 0.00
I
ATOM 30344 C   ILE I 218   11.409 22.304 -9.258 0.00 0.00
I
ATOM 30345 O   ILE I 218   10.319 22.196 -8.718 0.00 0.00
I
ATOM 30346 N   THR I 219   12.461 21.453 -9.066 0.00 0.00
I
ATOM 30347 HN THR I 219   13.338 21.668 -9.489 0.00 0.00
I

ATOM 30348 CA THR I 219 12.301 20.358 -8.113 0.00 0.00
I
ATOM 30349 HA THR I 219 11.332 19.929 -8.318 0.00 0.00
I
ATOM 30350 CB THR I 219 13.263 19.243 -8.254 0.00 0.00
I
ATOM 30351 HB THR I 219 13.048 18.505 -7.452 0.00 0.00
I
ATOM 30352 OG1 THR I 219 14.585 19.727 -8.177 0.00 0.00
I
ATOM 30353 HG1 THR I 219 14.743 20.243 -8.971 0.00 0.00
I
ATOM 30354 CG2 THR I 219 13.035 18.519 -9.597 0.00 0.00
I
ATOM 30355 HG21 THR I 219 11.947 18.293 -9.556 0.00 0.00
I
ATOM 30356 HG22 THR I 219 13.226 19.241 -10.419 0.00 0.00
I
ATOM 30357 HG23 THR I 219 13.676 17.614 -9.670 0.00 0.00
I
ATOM 30358 C THR I 219 12.210 20.804 -6.644 0.00 0.00
I
ATOM 30359 O THR I 219 11.534 20.168 -5.918 0.00 0.00
I
ATOM 30360 N PHE I 220 12.953 21.925 -6.232 0.00 0.00
I
ATOM 30361 HN PHE I 220 13.610 22.270 -6.897 0.00 0.00
I
ATOM 30362 CA PHE I 220 12.755 22.556 -5.034 0.00 0.00
I
ATOM 30363 HA PHE I 220 12.684 21.778 -4.289 0.00 0.00
I
ATOM 30364 CB PHE I 220 13.824 23.675 -4.865 0.00 0.00
I
ATOM 30365 HB1 PHE I 220 14.747 23.319 -5.370 0.00 0.00
I
ATOM 30366 HB2 PHE I 220 13.595 24.629 -5.387 0.00 0.00
I
ATOM 30367 CG PHE I 220 14.186 23.966 -3.424 0.00 0.00
I
ATOM 30368 CD1 PHE I 220 14.610 22.892 -2.664 0.00 0.00
I
ATOM 30369 HD1 PHE I 220 14.664 21.887 -3.057 0.00 0.00
I
ATOM 30370 CE1 PHE I 220 14.968 23.036 -1.297 0.00 0.00
I
ATOM 30371 HE1 PHE I 220 15.280 22.312 -0.559 0.00 0.00
I
ATOM 30372 CZ PHE I 220 14.949 24.365 -0.747 0.00 0.00
I
ATOM 30373 HZ PHE I 220 15.366 24.478 0.243 0.00 0.00
I
ATOM 30374 CD2 PHE I 220 14.015 25.256 -2.858 0.00 0.00
I

ATOM 30375 HD2 PHE I 220   13.503 26.033 -3.407 0.00 0.00
I
ATOM 30376 CE2 PHE I 220   14.532 25.453 -1.540 0.00 0.00
I
ATOM 30377 HE2 PHE I 220   14.573 26.435 -1.091 0.00 0.00
I
ATOM 30378 C   PHE I 220   11.380 23.142 -4.957 0.00 0.00
I
ATOM 30379 O   PHE I 220   10.785 23.137 -3.928 0.00 0.00
I
ATOM 30380 N   LEU I 221   10.752 23.611 -6.037 0.00 0.00
I
ATOM 30381 HN LEU I 221   11.323 23.845 -6.820 0.00 0.00
I
ATOM 30382 CA LEU I 221   9.381 24.156 -5.904 0.00 0.00
I
ATOM 30383 HA LEU I 221   9.211 24.753 -5.020 0.00 0.00
I
ATOM 30384 CB LEU I 221   8.860 25.083 -7.015 0.00 0.00
I
ATOM 30385 HB1 LEU I 221   8.929 24.529 -7.975 0.00 0.00
I
ATOM 30386 HB2 LEU I 221   7.781 25.311 -6.878 0.00 0.00
I
ATOM 30387 CG LEU I 221   9.594 26.484 -7.106 0.00 0.00
I
ATOM 30388 HG LEU I 221   10.245 26.575 -8.001 0.00 0.00
I
ATOM 30389 CD1 LEU I 221   8.558 27.620 -7.422 0.00 0.00
I
ATOM 30390 HD11 LEU I 221   8.040 27.430 -8.386 0.00 0.00
I
ATOM 30391 HD12 LEU I 221   7.901 27.796 -6.544 0.00 0.00
I
ATOM 30392 HD13 LEU I 221   9.060 28.611 -7.448 0.00 0.00
I
ATOM 30393 CD2 LEU I 221   10.449 26.887 -5.892 0.00 0.00
I
ATOM 30394 HD21 LEU I 221   10.974 27.855 -6.040 0.00 0.00
I
ATOM 30395 HD22 LEU I 221   9.918 26.902 -4.916 0.00 0.00
I
ATOM 30396 HD23 LEU I 221   11.309 26.194 -5.773 0.00 0.00
I
ATOM 30397 C   LEU I 221   8.376 23.012 -5.774 0.00 0.00
I
ATOM 30398 O   LEU I 221   7.326 23.129 -5.199 0.00 0.00
I
ATOM 30399 N   PHE I 222   8.612 21.800 -6.288 0.00 0.00
I
ATOM 30400 HN PHE I 222   9.365 21.672 -6.928 0.00 0.00
I
ATOM 30401 CA PHE I 222   7.812 20.617 -5.998 0.00 0.00
I

```
ATOM  30402  HA  PHE I 222       6.787  20.941  -6.107  0.00  0.00
I
ATOM  30403  CB  PHE I 222       8.001  19.438  -6.942  0.00  0.00
I
ATOM  30404  HB1 PHE I 222       8.993  18.939  -6.892  0.00  0.00
I
ATOM  30405  HB2 PHE I 222       7.238  18.660  -6.727  0.00  0.00
I
ATOM  30406  CG  PHE I 222       7.966  19.822  -8.360  0.00  0.00
I
ATOM  30407  CD1 PHE I 222       6.994  20.774  -8.803  0.00  0.00
I
ATOM  30408  HD1 PHE I 222       6.315  21.254  -8.114  0.00  0.00
I
ATOM  30409  CE1 PHE I 222       6.954  21.135 -10.156  0.00  0.00
I
ATOM  30410  HE1 PHE I 222       6.181  21.835 -10.437  0.00  0.00
I
ATOM  30411  CZ  PHE I 222       7.730  20.484 -11.088  0.00  0.00
I
ATOM  30412  HZ  PHE I 222       7.694  20.717 -12.142  0.00  0.00
I
ATOM  30413  CD2 PHE I 222       8.681  19.116  -9.345  0.00  0.00
I
ATOM  30414  HD2 PHE I 222       9.304  18.270  -9.093  0.00  0.00
I
ATOM  30415  CE2 PHE I 222       8.610  19.518 -10.702  0.00  0.00
I
ATOM  30416  HE2 PHE I 222       9.237  19.116 -11.485  0.00  0.00
I
ATOM  30417  C   PHE I 222       7.901  20.144  -4.518  0.00  0.00
I
ATOM  30418  O   PHE I 222       6.911  19.791  -3.873  0.00  0.00
I
ATOM  30419  N   ILE I 223       9.150  20.218  -3.990  0.00  0.00
I
ATOM  30420  HN  ILE I 223       9.901  20.423  -4.613  0.00  0.00
I
ATOM  30421  CA  ILE I 223       9.348  19.744  -2.644  0.00  0.00
I
ATOM  30422  HA  ILE I 223       8.876  18.787  -2.479  0.00  0.00
I
ATOM  30423  CB  ILE I 223      10.778  19.481  -2.300  0.00  0.00
I
ATOM  30424  HB  ILE I 223      11.424  20.261  -2.756  0.00  0.00
I
ATOM  30425  CG2 ILE I 223      11.069  19.424  -0.798  0.00  0.00
I
ATOM  30426 HG21 ILE I 223      11.957  18.774  -0.639  0.00  0.00
I
ATOM  30427 HG22 ILE I 223      11.191  20.447  -0.382  0.00  0.00
I
ATOM  30428 HG23 ILE I 223      10.164  18.993  -0.320  0.00  0.00
I
```

ATOM 30429 CG1 ILE I 223 11.265 18.155 -2.931 0.00 0.00
I
ATOM 30430 HG11 ILE I 223 11.054 17.339 -2.207 0.00 0.00
I
ATOM 30431 HG12 ILE I 223 10.672 17.995 -3.857 0.00 0.00
I
ATOM 30432 CD ILE I 223 12.759 18.246 -3.391 0.00 0.00
I
ATOM 30433 HD1 ILE I 223 12.778 19.068 -4.138 0.00 0.00
I
ATOM 30434 HD2 ILE I 223 13.468 18.440 -2.558 0.00 0.00
I
ATOM 30435 HD3 ILE I 223 13.116 17.404 -4.021 0.00 0.00
I
ATOM 30436 C ILE I 223 8.782 20.835 -1.678 0.00 0.00
I
ATOM 30437 O ILE I 223 8.077 20.599 -0.690 0.00 0.00
I
ATOM 30438 N THR I 224 9.002 22.133 -2.025 0.00 0.00
I
ATOM 30439 HN THR I 224 9.465 22.603 -2.772 0.00 0.00
I
ATOM 30440 CA THR I 224 8.565 23.184 -1.111 0.00 0.00
I
ATOM 30441 HA THR I 224 8.755 22.899 -0.087 0.00 0.00
I
ATOM 30442 CB THR I 224 9.301 24.558 -1.137 0.00 0.00
I
ATOM 30443 HB THR I 224 8.663 25.310 -0.624 0.00 0.00
I
ATOM 30444 OG1 THR I 224 9.472 24.998 -2.453 0.00 0.00
I
ATOM 30445 HG1 THR I 224 10.228 24.573 -2.865 0.00 0.00
I
ATOM 30446 CG2 THR I 224 10.725 24.450 -0.577 0.00 0.00
I
ATOM 30447 HG21 THR I 224 11.270 25.410 -0.704 0.00 0.00
I
ATOM 30448 HG22 THR I 224 10.636 24.059 0.459 0.00 0.00
I
ATOM 30449 HG23 THR I 224 11.337 23.639 -1.026 0.00 0.00
I
ATOM 30450 C THR I 224 7.076 23.500 -1.159 0.00 0.00
I
ATOM 30451 O THR I 224 6.517 23.798 -0.158 0.00 0.00
I
ATOM 30452 N GLY I 225 6.427 23.308 -2.329 0.00 0.00
I
ATOM 30453 HN GLY I 225 6.917 23.340 -3.196 0.00 0.00
I
ATOM 30454 CA GLY I 225 5.017 23.416 -2.432 0.00 0.00
I
ATOM 30455 HA1 GLY I 225 4.881 23.607 -3.486 0.00 0.00
I

ATOM	30456	HA2	GLY	I	225	4.686	24.182	-1.747	0.00	0.00	I
ATOM	30457	C	GLY	I	225	4.306	22.069	-2.110	0.00	0.00	I
ATOM	30458	O	GLY	I	225	3.101	22.031	-1.972	0.00	0.00	I
ATOM	30459	N	GLY	I	226	5.094	20.927	-1.869	0.00	0.00	I
ATOM	30460	HN	GLY	I	226	6.088	20.996	-1.868	0.00	0.00	I
ATOM	30461	CA	GLY	I	226	4.461	19.614	-1.486	0.00	0.00	I
ATOM	30462	HA1	GLY	I	226	5.165	18.930	-1.938	0.00	0.00	I
ATOM	30463	HA2	GLY	I	226	3.443	19.552	-1.842	0.00	0.00	I
ATOM	30464	C	GLY	I	226	4.433	19.465	0.056	0.00	0.00	I
ATOM	30465	O	GLY	I	226	3.601	18.698	0.580	0.00	0.00	I
ATOM	30466	N	ILE	I	227	5.330	20.009	0.833	0.00	0.00	I
ATOM	30467	HN	ILE	I	227	5.936	20.685	0.421	0.00	0.00	I
ATOM	30468	CA	ILE	I	227	5.369	20.034	2.310	0.00	0.00	I
ATOM	30469	HA	ILE	I	227	5.133	19.052	2.694	0.00	0.00	I
ATOM	30470	CB	ILE	I	227	6.828	20.303	2.822	0.00	0.00	I
ATOM	30471	HB	ILE	I	227	7.464	19.533	2.335	0.00	0.00	I
ATOM	30472	CG2	ILE	I	227	7.318	21.651	2.374	0.00	0.00	I
ATOM	30473	HG21	ILE	I	227	8.344	21.935	2.691	0.00	0.00	I
ATOM	30474	HG22	ILE	I	227	7.501	21.767	1.285	0.00	0.00	I
ATOM	30475	HG23	ILE	I	227	6.663	22.484	2.708	0.00	0.00	I
ATOM	30476	CG1	ILE	I	227	6.828	20.121	4.364	0.00	0.00	I
ATOM	30477	HG11	ILE	I	227	6.181	20.948	4.727	0.00	0.00	I
ATOM	30478	HG12	ILE	I	227	6.347	19.176	4.695	0.00	0.00	I
ATOM	30479	CD	ILE	I	227	8.178	20.262	5.003	0.00	0.00	I
ATOM	30480	HD1	ILE	I	227	7.959	20.310	6.091	0.00	0.00	I
ATOM	30481	HD2	ILE	I	227	8.892	19.470	4.690	0.00	0.00	I
ATOM	30482	HD3	ILE	I	227	8.745	21.178	4.731	0.00	0.00	I

ATOM 30483 C ILE I 227 4.260 21.035 2.763 0.00 0.00
I
ATOM 30484 O ILE I 227 4.116 22.057 2.212 0.00 0.00
I
ATOM 30485 N GLY I 228 3.446 20.642 3.774 0.00 0.00
I
ATOM 30486 HN GLY I 228 3.613 19.733 4.149 0.00 0.00
I
ATOM 30487 CA GLY I 228 2.345 21.480 4.360 0.00 0.00
I
ATOM 30488 HA1 GLY I 228 2.147 21.005 5.309 0.00 0.00
I
ATOM 30489 HA2 GLY I 228 1.526 21.489 3.655 0.00 0.00
I
ATOM 30490 C GLY I 228 2.685 22.847 4.803 0.00 0.00
I
ATOM 30491 O GLY I 228 3.777 23.100 5.350 0.00 0.00
I
ATOM 30492 N PHE I 229 1.823 23.908 4.644 0.00 0.00
I
ATOM 30493 HN PHE I 229 0.890 23.829 4.301 0.00 0.00
I
ATOM 30494 CA PHE I 229 2.268 25.293 4.882 0.00 0.00
I
ATOM 30495 HA PHE I 229 3.069 25.363 4.161 0.00 0.00
I
ATOM 30496 CB PHE I 229 1.233 26.368 4.312 0.00 0.00
I
ATOM 30497 HB1 PHE I 229 1.645 27.374 4.542 0.00 0.00
I
ATOM 30498 HB2 PHE I 229 1.068 26.090 3.249 0.00 0.00
I
ATOM 30499 CG PHE I 229 −0.100 26.316 5.050 0.00 0.00
I
ATOM 30500 CD1 PHE I 229 −0.292 27.236 6.090 0.00 0.00
I
ATOM 30501 HD1 PHE I 229 0.517 27.945 6.191 0.00 0.00
I
ATOM 30502 CE1 PHE I 229 −1.533 27.287 6.781 0.00 0.00
I
ATOM 30503 HE1 PHE I 229 −1.779 27.990 7.563 0.00 0.00
I
ATOM 30504 CZ PHE I 229 −2.486 26.328 6.504 0.00 0.00
I
ATOM 30505 HZ PHE I 229 −3.348 26.380 7.153 0.00 0.00
I
ATOM 30506 CD2 PHE I 229 −1.166 25.460 4.760 0.00 0.00
I
ATOM 30507 HD2 PHE I 229 −1.078 24.861 3.865 0.00 0.00
I
ATOM 30508 CE2 PHE I 229 −2.321 25.344 5.560 0.00 0.00
I
ATOM 30509 HE2 PHE I 229 −3.116 24.669 5.281 0.00 0.00
I

ATOM 30510 C   PHE I 229   2.807 25.732   6.197 0.00 0.00
I
ATOM 30511 O   PHE I 229   3.760 26.517   6.295 0.00 0.00
I
ATOM 30512 N   THR I 230   2.263 25.229   7.411 0.00 0.00
I
ATOM 30513 HN THR I 230   1.575 24.521   7.270 0.00 0.00
I
ATOM 30514 CA THR I 230   2.679 25.650   8.743 0.00 0.00
I
ATOM 30515 HA THR I 230   2.837 26.704   8.916 0.00 0.00
I
ATOM 30516 CB THR I 230   1.633 25.106   9.833 0.00 0.00
I
ATOM 30517 HB THR I 230   2.157 25.414 10.763 0.00 0.00
I
ATOM 30518 OG1 THR I 230   1.477 23.706   9.790 0.00 0.00
I
ATOM 30519 HG1 THR I 230   1.269 23.449 10.692 0.00 0.00
I
ATOM 30520 CG2 THR I 230   0.247 25.765   9.535 0.00 0.00
I
ATOM 30521 HG21 THR I 230   0.029 25.792   8.446 0.00 0.00
I
ATOM 30522 HG22 THR I 230   -0.550 25.347 10.187 0.00 0.00
I
ATOM 30523 HG23 THR I 230   0.419 26.805   9.887 0.00 0.00
I
ATOM 30524 C   THR I 230   4.058 25.055   9.056 0.00 0.00
I
ATOM 30525 O   THR I 230   4.818 25.627   9.761 0.00 0.00
I
ATOM 30526 N   VAL I 231   4.309 23.811   8.506 0.00 0.00
I
ATOM 30527 HN VAL I 231   3.573 23.283   8.090 0.00 0.00
I
ATOM 30528 CA VAL I 231   5.583 23.032   8.714 0.00 0.00
I
ATOM 30529 HA VAL I 231   5.914 23.160   9.734 0.00 0.00
I
ATOM 30530 CB VAL I 231   5.353 21.552   8.200 0.00 0.00
I
ATOM 30531 HB VAL I 231   5.086 21.573   7.122 0.00 0.00
I
ATOM 30532 CG1 VAL I 231   6.550 20.791   8.442 0.00 0.00
I
ATOM 30533 HG11 VAL I 231   7.365 21.169   7.789 0.00 0.00
I
ATOM 30534 HG12 VAL I 231   6.822 21.033   9.491 0.00 0.00
I
ATOM 30535 HG13 VAL I 231   6.439 19.704   8.237 0.00 0.00
I
ATOM 30536 CG2 VAL I 231   4.068 20.867   8.811 0.00 0.00
I

```
ATOM  30537 HG21 VAL I 231       3.120  21.310   8.439  0.00  0.00
I
ATOM  30538 HG22 VAL I 231       4.021  19.776   8.606  0.00  0.00
I
ATOM  30539 HG23 VAL I 231       4.108  20.758   9.916  0.00  0.00
I
ATOM  30540  C   VAL I 231       6.673  23.619   7.830  0.00  0.00
I
ATOM  30541  O   VAL I 231       7.831  23.613   8.112  0.00  0.00
I
ATOM  30542  N   LEU I 232       6.279  24.157   6.629  0.00  0.00
I
ATOM  30543  HN  LEU I 232       5.370  24.049   6.234  0.00  0.00
I
ATOM  30544  CA  LEU I 232       7.151  24.953   5.773  0.00  0.00
I
ATOM  30545  HA  LEU I 232       8.094  24.448   5.621  0.00  0.00
I
ATOM  30546  CB  LEU I 232       6.520  25.515   4.463  0.00  0.00
I
ATOM  30547  HB1 LEU I 232       6.132  24.711   3.802  0.00  0.00
I
ATOM  30548  HB2 LEU I 232       5.655  26.197   4.609  0.00  0.00
I
ATOM  30549  CG  LEU I 232       7.545  26.235   3.505  0.00  0.00
I
ATOM  30550  HG  LEU I 232       7.918  27.158   3.997  0.00  0.00
I
ATOM  30551  CD1 LEU I 232       8.677  25.405   3.024  0.00  0.00
I
ATOM  30552 HD11 LEU I 232       9.232  25.070   3.926  0.00  0.00
I
ATOM  30553 HD12 LEU I 232       8.310  24.469   2.551  0.00  0.00
I
ATOM  30554 HD13 LEU I 232       9.451  25.861   2.371  0.00  0.00
I
ATOM  30555  CD2 LEU I 232       6.750  26.815   2.342  0.00  0.00
I
ATOM  30556 HD21 LEU I 232       5.867  27.384   2.703  0.00  0.00
I
ATOM  30557 HD22 LEU I 232       7.412  27.530   1.809  0.00  0.00
I
ATOM  30558 HD23 LEU I 232       6.363  26.036   1.650  0.00  0.00
I
ATOM  30559  C   LEU I 232       7.648  26.201   6.537  0.00  0.00
I
ATOM  30560  O   LEU I 232       8.862  26.391   6.712  0.00  0.00
I
ATOM  30561  N   PHE I 233       6.717  26.938   7.136  0.00  0.00
I
ATOM  30562  HN  PHE I 233       5.775  26.631   7.020  0.00  0.00
I
ATOM  30563  CA  PHE I 233       7.011  28.112   7.924  0.00  0.00
I
```

ATOM 30564 HA PHE I 233 7.673 28.733 7.339 0.00 0.00
I
ATOM 30565 CB PHE I 233 5.739 28.834 8.468 0.00 0.00
I
ATOM 30566 HB1 PHE I 233 5.200 28.171 9.178 0.00 0.00
I
ATOM 30567 HB2 PHE I 233 6.069 29.769 8.969 0.00 0.00
I
ATOM 30568 CG PHE I 233 4.675 29.286 7.447 0.00 0.00
I
ATOM 30569 CD1 PHE I 233 4.947 29.620 6.141 0.00 0.00
I
ATOM 30570 HD1 PHE I 233 5.896 29.445 5.657 0.00 0.00
I
ATOM 30571 CE1 PHE I 233 3.893 30.003 5.248 0.00 0.00
I
ATOM 30572 HE1 PHE I 233 4.068 30.206 4.202 0.00 0.00
I
ATOM 30573 CZ PHE I 233 2.575 30.042 5.705 0.00 0.00
I
ATOM 30574 HZ PHE I 233 1.790 30.406 5.059 0.00 0.00
I
ATOM 30575 CD2 PHE I 233 3.370 29.398 7.921 0.00 0.00
I
ATOM 30576 HD2 PHE I 233 3.165 29.290 8.976 0.00 0.00
I
ATOM 30577 CE2 PHE I 233 2.383 29.826 7.085 0.00 0.00
I
ATOM 30578 HE2 PHE I 233 1.410 29.978 7.530 0.00 0.00
I
ATOM 30579 C PHE I 233 7.881 27.812 9.166 0.00 0.00
I
ATOM 30580 O PHE I 233 8.805 28.560 9.547 0.00 0.00
I
ATOM 30581 N ASP I 234 7.650 26.636 9.775 0.00 0.00
I
ATOM 30582 HN ASP I 234 6.890 26.076 9.454 0.00 0.00
I
ATOM 30583 CA ASP I 234 8.499 26.104 10.826 0.00 0.00
I
ATOM 30584 HA ASP I 234 8.618 26.800 11.643 0.00 0.00
I
ATOM 30585 CB ASP I 234 7.839 24.847 11.491 0.00 0.00
I
ATOM 30586 HB1 ASP I 234 7.379 24.095 10.814 0.00 0.00
I
ATOM 30587 HB2 ASP I 234 8.548 24.287 12.136 0.00 0.00
I
ATOM 30588 CG ASP I 234 6.794 25.226 12.564 0.00 0.00
I
ATOM 30589 OD1 ASP I 234 5.703 24.579 12.472 0.00 0.00
I
ATOM 30590 OD2 ASP I 234 7.112 25.995 13.556 0.00 0.00
I

```
ATOM  30591  C   ASP I 234       9.900  25.817  10.427  0.00  0.00
I
ATOM  30592  O   ASP I 234      10.910  26.251  11.011  0.00  0.00
I
ATOM  30593  N   VAL I 235      10.003  25.068   9.334  0.00  0.00
I
ATOM  30594  HN  VAL I 235       9.147  24.753   8.930  0.00  0.00
I
ATOM  30595  CA  VAL I 235      11.244  24.719   8.654  0.00  0.00
I
ATOM  30596  HA  VAL I 235      11.766  24.249   9.475  0.00  0.00
I
ATOM  30597  CB  VAL I 235      11.062  23.656   7.514  0.00  0.00
I
ATOM  30598  HB  VAL I 235      10.157  23.908   6.920  0.00  0.00
I
ATOM  30599  CG1 VAL I 235      12.311  23.487   6.660  0.00  0.00
I
ATOM  30600 HG11 VAL I 235      12.381  24.390   6.016  0.00  0.00
I
ATOM  30601 HG12 VAL I 235      13.246  23.238   7.206  0.00  0.00
I
ATOM  30602 HG13 VAL I 235      12.165  22.618   5.984  0.00  0.00
I
ATOM  30603  CG2 VAL I 235      10.871  22.343   8.267  0.00  0.00
I
ATOM  30604 HG21 VAL I 235      10.067  22.436   9.027  0.00  0.00
I
ATOM  30605 HG22 VAL I 235      10.527  21.573   7.543  0.00  0.00
I
ATOM  30606 HG23 VAL I 235      11.794  21.945   8.739  0.00  0.00
I
ATOM  30607  C   VAL I 235      12.118  25.916   8.329  0.00  0.00
I
ATOM  30608  O   VAL I 235      13.318  25.885   8.615  0.00  0.00
I
ATOM  30609  N   MET I 236      11.502  27.064   7.938  0.00  0.00
I
ATOM  30610  HN  MET I 236      10.560  27.015   7.614  0.00  0.00
I
ATOM  30611  CA  MET I 236      12.192  28.309   7.685  0.00  0.00
I
ATOM  30612  HA  MET I 236      13.218  28.051   7.468  0.00  0.00
I
ATOM  30613  CB  MET I 236      11.736  29.059   6.412  0.00  0.00
I
ATOM  30614  HB1 MET I 236      10.762  29.581   6.529  0.00  0.00
I
ATOM  30615  HB2 MET I 236      12.531  29.805   6.200  0.00  0.00
I
ATOM  30616  CG  MET I 236      11.442  28.219   5.130  0.00  0.00
I
ATOM  30617  HG1 MET I 236      10.482  27.744   5.424  0.00  0.00
I
```

ATOM 30618 HG2 MET I 236   11.275 28.939   4.300 0.00 0.00
I
ATOM 30619 SD MET I 236   12.851 26.945   4.787 0.00 0.00
I
ATOM 30620 CE MET I 236   12.098 25.921   3.557 0.00 0.00
I
ATOM 30621 HE1 MET I 236   11.260 25.380   4.046 0.00 0.00
I
ATOM 30622 HE2 MET I 236   11.637 26.539   2.757 0.00 0.00
I
ATOM 30623 HE3 MET I 236   12.774 25.227   3.013 0.00 0.00
I
ATOM 30624 C   MET I 236   12.368 29.266   8.833 0.00 0.00
I
ATOM 30625 O   MET I 236   13.083 30.303   8.694 0.00 0.00
I
ATOM 30626 N   LYS I 237   11.744 28.995 10.025 0.00 0.00
I
ATOM 30627 HN LYS I 237   11.171 28.195 10.190 0.00 0.00
I
ATOM 30628 CA LYS I 237   11.747 29.978 11.058 0.00 0.00
I
ATOM 30629 HA LYS I 237   12.369 30.822 10.798 0.00 0.00
I
ATOM 30630 CB LYS I 237   10.298 30.493 11.279 0.00 0.00
I
ATOM 30631 HB1 LYS I 237   9.785 30.619 10.302 0.00 0.00
I
ATOM 30632 HB2 LYS I 237   9.732 29.747 11.877 0.00 0.00
I
ATOM 30633 CG LYS I 237   10.265 31.737 12.166 0.00 0.00
I
ATOM 30634 HG1 LYS I 237   10.691 31.549 13.175 0.00 0.00
I
ATOM 30635 HG2 LYS I 237   10.927 32.486 11.682 0.00 0.00
I
ATOM 30636 CD LYS I 237   8.899 32.358 12.357 0.00 0.00
I
ATOM 30637 HD1 LYS I 237   9.131 33.411 12.627 0.00 0.00
I
ATOM 30638 HD2 LYS I 237   8.313 32.298 11.416 0.00 0.00
I
ATOM 30639 CE LYS I 237   8.067 31.705 13.423 0.00 0.00
I
ATOM 30640 HE1 LYS I 237   7.855 30.659 13.116 0.00 0.00
I
ATOM 30641 HE2 LYS I 237   8.613 31.743 14.390 0.00 0.00
I
ATOM 30642 NZ LYS I 237   6.705 32.354 13.524 0.00 0.00
I
ATOM 30643 HZ1 LYS I 237   6.134 31.885 14.255 0.00 0.00
I
ATOM 30644 HZ2 LYS I 237   6.845 33.344 13.810 0.00 0.00
I

ATOM	30645	HZ3	LYS	I	237	6.216	32.323	12.606	0.00	0.00	I
ATOM	30646	C	LYS	I	237	12.374	29.440	12.288	0.00	0.00	I
ATOM	30647	O	LYS	I	237	13.163	30.179	12.882	0.00	0.00	I
ATOM	30648	N	ASN	I	238	12.273	28.136	12.620	0.00	0.00	I
ATOM	30649	HN	ASN	I	238	11.504	27.629	12.237	0.00	0.00	I
ATOM	30650	CA	ASN	I	238	13.207	27.478	13.531	0.00	0.00	I
ATOM	30651	HA	ASN	I	238	13.361	28.205	14.314	0.00	0.00	I
ATOM	30652	CB	ASN	I	238	12.733	26.160	14.090	0.00	0.00	I
ATOM	30653	HB1	ASN	I	238	12.609	25.398	13.291	0.00	0.00	I
ATOM	30654	HB2	ASN	I	238	13.368	25.771	14.914	0.00	0.00	I
ATOM	30655	CG	ASN	I	238	11.297	26.183	14.600	0.00	0.00	I
ATOM	30656	OD1	ASN	I	238	10.309	26.044	13.845	0.00	0.00	I
ATOM	30657	ND2	ASN	I	238	11.147	26.448	15.937	0.00	0.00	I
ATOM	30658	HD21	ASN	I	238	11.856	26.997	16.379	0.00	0.00	I
ATOM	30659	HD22	ASN	I	238	10.215	26.419	16.299	0.00	0.00	I
ATOM	30660	C	ASN	I	238	14.627	27.200	12.907	0.00	0.00	I
ATOM	30661	O	ASN	I	238	15.640	27.330	13.571	0.00	0.00	I
ATOM	30662	N	ARG	I	239	14.671	26.721	11.619	0.00	0.00	I
ATOM	30663	HN	ARG	I	239	13.849	26.754	11.056	0.00	0.00	I
ATOM	30664	CA	ARG	I	239	15.964	26.430	10.978	0.00	0.00	I
ATOM	30665	HA	ARG	I	239	15.784	26.060	9.980	0.00	0.00	I
ATOM	30666	CB	ARG	I	239	16.830	27.763	10.585	0.00	0.00	I
ATOM	30667	HB1	ARG	I	239	17.255	28.286	11.468	0.00	0.00	I
ATOM	30668	HB2	ARG	I	239	17.541	27.480	9.780	0.00	0.00	I
ATOM	30669	CG	ARG	I	239	16.007	28.933	10.067	0.00	0.00	I
ATOM	30670	HG1	ARG	I	239	15.324	28.659	9.235	0.00	0.00	I
ATOM	30671	HG2	ARG	I	239	15.273	29.182	10.863	0.00	0.00	I

ATOM 30672 CD ARG I 239   16.734 30.256   9.897 0.00 0.00
I
ATOM 30673 HD1 ARG I 239   17.397 30.413 10.774 0.00 0.00
I
ATOM 30674 HD2 ARG I 239   17.295 30.126   8.947 0.00 0.00
I
ATOM 30675 NE ARG I 239   15.716 31.406   9.678 0.00 0.00
I
ATOM 30676 HE ARG I 239   14.731 31.272   9.780 0.00 0.00
I
ATOM 30677 CZ ARG I 239   15.934 32.690 10.009 0.00 0.00
I
ATOM 30678 NH1 ARG I 239   17.112 33.240 10.322 0.00 0.00
I
ATOM 30679 HH11 ARG I 239   17.148 34.239 10.336 0.00 0.00
I
ATOM 30680 HH12 ARG I 239   17.951 32.719 10.162 0.00 0.00
I
ATOM 30681 NH2 ARG I 239   14.932 33.544 10.097 0.00 0.00
I
ATOM 30682 HH21 ARG I 239   15.093 34.529 10.163 0.00 0.00
I
ATOM 30683 HH22 ARG I 239   14.068 33.429   9.606 0.00 0.00
I
ATOM 30684 C   ARG I 239   16.844 25.491 11.735 0.00 0.00
I
ATOM 30685 O   ARG I 239   18.038 25.326 11.361 0.00 0.00
I
ATOM 30686 N   ARG I 240   16.348 24.688 12.692 0.00 0.00
I
ATOM 30687 HN ARG I 240   15.367 24.718 12.871 0.00 0.00
I
ATOM 30688 CA ARG I 240   17.112 23.642 13.446 0.00 0.00
I
ATOM 30689 HA ARG I 240   18.160 23.545 13.201 0.00 0.00
I
ATOM 30690 CB ARG I 240   17.160 24.100 14.931 0.00 0.00
I
ATOM 30691 HB1 ARG I 240   17.586 25.125 14.989 0.00 0.00
I
ATOM 30692 HB2 ARG I 240   16.121 24.184 15.315 0.00 0.00
I
ATOM 30693 CG ARG I 240   17.997 23.244 15.971 0.00 0.00
I
ATOM 30694 HG1 ARG I 240   17.461 22.296 16.191 0.00 0.00
I
ATOM 30695 HG2 ARG I 240   18.998 22.885 15.649 0.00 0.00
I
ATOM 30696 CD ARG I 240   18.170 23.893 17.387 0.00 0.00
I
ATOM 30697 HD1 ARG I 240   18.923 24.698 17.253 0.00 0.00
I
ATOM 30698 HD2 ARG I 240   17.227 24.388 17.703 0.00 0.00
I

ATOM 30699 NE ARG I 240   18.595 22.750 18.351 0.00 0.00
I
ATOM 30700 HE ARG I 240   19.568 22.520 18.368 0.00 0.00
I
ATOM 30701 CZ ARG I 240   17.778 22.195 19.285 0.00 0.00
I
ATOM 30702 NH1 ARG I 240   16.476 22.343 19.308 0.00 0.00
I
ATOM 30703 HH11 ARG I 240   15.924 21.815 19.953 0.00 0.00
I
ATOM 30704 HH12 ARG I 240   15.976 22.881 18.629 0.00 0.00
I
ATOM 30705 NH2 ARG I 240   18.407 21.526 20.274 0.00 0.00
I
ATOM 30706 HH21 ARG I 240   17.907 21.009 20.969 0.00 0.00
I
ATOM 30707 HH22 ARG I 240   19.382 21.688 20.426 0.00 0.00
I
ATOM 30708 C   ARG I 240   16.514 22.209 13.373 0.00 0.00
I
ATOM 30709 O   ARG I 240   15.409 21.942 13.711 0.00 0.00
I
ATOM 30710 N   PHE I 241   17.366 21.326 12.759 0.00 0.00
I
ATOM 30711 HN PHE I 241   18.274 21.541 12.407 0.00 0.00
I
ATOM 30712 CA PHE I 241   16.980 19.937 12.361 0.00 0.00
I
ATOM 30713 HA PHE I 241   16.062 19.931 11.792 0.00 0.00
I
ATOM 30714 CB PHE I 241   18.252 19.193 11.686 0.00 0.00
I
ATOM 30715 HB1 PHE I 241   18.646 19.953 10.977 0.00 0.00
I
ATOM 30716 HB2 PHE I 241   19.057 18.889 12.389 0.00 0.00
I
ATOM 30717 CG PHE I 241   17.800 18.042 10.812 0.00 0.00
I
ATOM 30718 CD1 PHE I 241   16.948 18.182   9.676 0.00 0.00
I
ATOM 30719 HD1 PHE I 241   16.596 19.160   9.384 0.00 0.00
I
ATOM 30720 CE1 PHE I 241   16.517 17.065   8.986 0.00 0.00
I
ATOM 30721 HE1 PHE I 241   15.822 17.196   8.170 0.00 0.00
I
ATOM 30722 CZ PHE I 241   17.080 15.779   9.304 0.00 0.00
I
ATOM 30723 HZ PHE I 241   16.744 14.940   8.712 0.00 0.00
I
ATOM 30724 CD2 PHE I 241   18.307 16.752 11.091 0.00 0.00
I
ATOM 30725 HD2 PHE I 241   18.980 16.612 11.924 0.00 0.00
I

ATOM 30726 CE2 PHE I 241   17.914 15.678 10.410 0.00 0.00
I
ATOM 30727 HE2 PHE I 241   18.366 14.715 10.597 0.00 0.00
I
ATOM 30728 C   PHE I 241   16.489 19.206 13.642 0.00 0.00
I
ATOM 30729 O   PHE I 241   15.419 18.617 13.641 0.00 0.00
I
ATOM 30730 N   LYS I 242   17.148 19.342 14.831 0.00 0.00
I
ATOM 30731 HN LYS I 242   17.965 19.914 14.830 0.00 0.00
I
ATOM 30732 CA LYS I 242   16.753 18.764 16.080 0.00 0.00
I
ATOM 30733 HA LYS I 242   16.766 17.695 15.933 0.00 0.00
I
ATOM 30734 CB LYS I 242   17.731 19.033 17.294 0.00 0.00
I
ATOM 30735 HB1 LYS I 242   17.734 20.109 17.570 0.00 0.00
I
ATOM 30736 HB2 LYS I 242   17.346 18.459 18.163 0.00 0.00
I
ATOM 30737 CG LYS I 242   19.092 18.452 17.123 0.00 0.00
I
ATOM 30738 HG1 LYS I 242   18.958 17.349 17.125 0.00 0.00
I
ATOM 30739 HG2 LYS I 242   19.617 18.676 16.170 0.00 0.00
I
ATOM 30740 CD LYS I 242   20.044 18.822 18.206 0.00 0.00
I
ATOM 30741 HD1 LYS I 242   20.238 19.915 18.240 0.00 0.00
I
ATOM 30742 HD2 LYS I 242   19.735 18.544 19.236 0.00 0.00
I
ATOM 30743 CE LYS I 242   21.444 18.190 17.898 0.00 0.00
I
ATOM 30744 HE1 LYS I 242   21.276 17.095 17.982 0.00 0.00
I
ATOM 30745 HE2 LYS I 242   21.795 18.383 16.862 0.00 0.00
I
ATOM 30746 NZ LYS I 242   22.436 18.506 18.945 0.00 0.00
I
ATOM 30747 HZ1 LYS I 242   23.236 17.842 18.905 0.00 0.00
I
ATOM 30748 HZ2 LYS I 242   22.867 19.452 18.968 0.00 0.00
I
ATOM 30749 HZ3 LYS I 242   22.028 18.331 19.886 0.00 0.00
I
ATOM 30750 C   LYS I 242   15.372 19.231 16.601 0.00 0.00
I
ATOM 30751 O   LYS I 242   14.743 18.471 17.352 0.00 0.00
I
ATOM 30752 N   THR I 243   15.053 20.509 16.385 0.00 0.00
I

ATOM 30753 HN THR I 243   15.626 21.105 15.828 0.00 0.00
I
ATOM 30754 CA THR I 243   13.823 21.138 16.714 0.00 0.00
I
ATOM 30755 HA THR I 243   13.662 21.049 17.778 0.00 0.00
I
ATOM 30756 CB THR I 243   13.791 22.644 16.490 0.00 0.00
I
ATOM 30757 HB THR I 243   13.955 22.788 15.400 0.00 0.00
I
ATOM 30758 OG1 THR I 243   14.941 23.159 17.146 0.00 0.00
I
ATOM 30759 HG1 THR I 243   14.896 24.111 17.259 0.00 0.00
I
ATOM 30760 CG2 THR I 243   12.476 23.353 16.945 0.00 0.00
I
ATOM 30761 HG21 THR I 243   12.417 23.226 18.047 0.00 0.00
I
ATOM 30762 HG22 THR I 243   12.621 24.436 16.744 0.00 0.00
I
ATOM 30763 HG23 THR I 243   11.568 22.964 16.436 0.00 0.00
I
ATOM 30764 C   THR I 243   12.732 20.477 15.889 0.00 0.00
I
ATOM 30765 O   THR I 243   11.573 20.304 16.331 0.00 0.00
I
ATOM 30766 N   PHE I 244   12.964 20.209 14.572 0.00 0.00
I
ATOM 30767 HN PHE I 244   13.852 20.318 14.132 0.00 0.00
I
ATOM 30768 CA PHE I 244   11.937 19.648 13.670 0.00 0.00
I
ATOM 30769 HA PHE I 244   11.096 20.324 13.641 0.00 0.00
I
ATOM 30770 CB PHE I 244   12.577 19.448 12.255 0.00 0.00
I
ATOM 30771 HB1 PHE I 244   13.438 18.764 12.413 0.00 0.00
I
ATOM 30772 HB2 PHE I 244   11.844 18.990 11.556 0.00 0.00
I
ATOM 30773 CG PHE I 244   13.112 20.687 11.678 0.00 0.00
I
ATOM 30774 CD1 PHE I 244   12.838 22.029 12.022 0.00 0.00
I
ATOM 30775 HD1 PHE I 244   12.138 22.203 12.826 0.00 0.00
I
ATOM 30776 CE1 PHE I 244   13.361 23.117 11.314 0.00 0.00
I
ATOM 30777 HE1 PHE I 244   13.136 24.136 11.590 0.00 0.00
I
ATOM 30778 CZ PHE I 244   14.205 22.827 10.187 0.00 0.00
I
ATOM 30779 HZ PHE I 244   14.514 23.612   9.513 0.00 0.00
I

```
ATOM  30780  CD2 PHE I 244      14.094  20.511  10.685  0.00  0.00
I
ATOM  30781  HD2 PHE I 244      14.429  19.504  10.483  0.00  0.00
I
ATOM  30782  CE2 PHE I 244      14.642  21.535   9.911  0.00  0.00
I
ATOM  30783  HE2 PHE I 244      15.416  21.390   9.172  0.00  0.00
I
ATOM  30784  C   PHE I 244      11.235  18.349  14.219  0.00  0.00
I
ATOM  30785  O   PHE I 244      11.896  17.415  14.730  0.00  0.00
I
ATOM  30786  N   SER I 245       9.902  18.358  14.234  0.00  0.00
I
ATOM  30787  HN  SER I 245       9.471  19.219  13.977  0.00  0.00
I
ATOM  30788  CA  SER I 245       8.983  17.292  14.520  0.00  0.00
I
ATOM  30789  HA  SER I 245       8.912  17.122  15.584  0.00  0.00
I
ATOM  30790  CB  SER I 245       7.500  17.642  14.200  0.00  0.00
I
ATOM  30791  HB1 SER I 245       6.885  17.054  14.915  0.00  0.00
I
ATOM  30792  HB2 SER I 245       7.261  18.673  14.541  0.00  0.00
I
ATOM  30793  OG  SER I 245       7.061  17.532  12.825  0.00  0.00
I
ATOM  30794  HG1 SER I 245       6.192  17.933  12.751  0.00  0.00
I
ATOM  30795  C   SER I 245       9.348  15.939  13.866  0.00  0.00
I
ATOM  30796  O   SER I 245      10.033  15.921  12.829  0.00  0.00
I
ATOM  30797  N   LEU I 246       8.926  14.799  14.355  0.00  0.00
I
ATOM  30798  HN  LEU I 246       8.449  14.791  15.230  0.00  0.00
I
ATOM  30799  CA  LEU I 246       8.895  13.517  13.616  0.00  0.00
I
ATOM  30800  HA  LEU I 246       9.956  13.337  13.526  0.00  0.00
I
ATOM  30801  CB  LEU I 246       8.245  12.385  14.538  0.00  0.00
I
ATOM  30802  HB1 LEU I 246       8.819  12.434  15.487  0.00  0.00
I
ATOM  30803  HB2 LEU I 246       7.171  12.646  14.654  0.00  0.00
I
ATOM  30804  CG  LEU I 246       8.337  10.969  13.891  0.00  0.00
I
ATOM  30805  HG  LEU I 246       7.832  10.992  12.902  0.00  0.00
I
ATOM  30806  CD1 LEU I 246       9.778  10.615  13.660  0.00  0.00
I
```

ATOM 30807 HD11 LEU I 246   9.886   9.559 13.331 0.00 0.00
I
ATOM 30808 HD12 LEU I 246   10.164 11.312 12.886 0.00 0.00
I
ATOM 30809 HD13 LEU I 246   10.279 10.778 14.638 0.00 0.00
I
ATOM 30810 CD2 LEU I 246   7.709   9.940 14.759 0.00 0.00
I
ATOM 30811 HD21 LEU I 246   6.717 10.244 15.155 0.00 0.00
I
ATOM 30812 HD22 LEU I 246   7.499   9.090 14.075 0.00 0.00
I
ATOM 30813 HD23 LEU I 246   8.356   9.657 15.617 0.00 0.00
I
ATOM 30814 C   LEU I 246   8.140 13.564 12.292 0.00 0.00
I
ATOM 30815 O   LEU I 246   8.681 13.096 11.248 0.00 0.00
I
ATOM 30816 N   HSE I 247   6.972 14.229 12.276 0.00 0.00
I
ATOM 30817 HN HSE I 247   6.385 14.341 13.073 0.00 0.00
I
ATOM 30818 CA HSE I 247   6.238 14.531 11.015 0.00 0.00
I
ATOM 30819 HA HSE I 247   5.870 13.629 10.548 0.00 0.00
I
ATOM 30820 CB HSE I 247   4.838 15.354 11.329 0.00 0.00
I
ATOM 30821 HB1 HSE I 247   4.230 14.683 11.972 0.00 0.00
I
ATOM 30822 HB2 HSE I 247   5.150 16.255 11.898 0.00 0.00
I
ATOM 30823 ND1 HSE I 247   3.640 14.760   9.205 0.00 0.00
I
ATOM 30824 CG HSE I 247   4.010 15.704 10.147 0.00 0.00
I
ATOM 30825 CE1 HSE I 247   2.939 15.432   8.317 0.00 0.00
I
ATOM 30826 HE1 HSE I 247   2.389 14.939   7.515 0.00 0.00
I
ATOM 30827 NE2 HSE I 247   2.800 16.725   8.622 0.00 0.00
I
ATOM 30828 HE2 HSE I 247   2.295 17.496   8.234 0.00 0.00
I
ATOM 30829 CD2 HSE I 247   3.495 16.929   9.792 0.00 0.00
I
ATOM 30830 HD2 HSE I 247   3.764 17.894 10.203 0.00 0.00
I
ATOM 30831 C   HSE I 247   7.037 15.329   9.939 0.00 0.00
I
ATOM 30832 O   HSE I 247   6.921 15.085   8.732 0.00 0.00
I
ATOM 30833 N   THR I 248   7.818 16.407 10.363 0.00 0.00
I

ATOM 30834 HN THR I 248 7.607 16.571 11.323 0.00 0.00
I
ATOM 30835 CA THR I 248 8.695 17.193 9.532 0.00 0.00
I
ATOM 30836 HA THR I 248 8.167 17.478 8.634 0.00 0.00
I
ATOM 30837 CB THR I 248 9.287 18.372 10.322 0.00 0.00
I
ATOM 30838 HB THR I 248 9.895 17.919 11.135 0.00 0.00
I
ATOM 30839 OG1 THR I 248 8.318 19.229 10.900 0.00 0.00
I
ATOM 30840 HG1 THR I 248 7.870 18.643 11.513 0.00 0.00
I
ATOM 30841 CG2 THR I 248 10.060 19.310 9.402 0.00 0.00
I
ATOM 30842 HG21 THR I 248 11.048 18.906 9.092 0.00 0.00
I
ATOM 30843 HG22 THR I 248 9.524 19.616 8.478 0.00 0.00
I
ATOM 30844 HG23 THR I 248 10.425 20.203 9.954 0.00 0.00
I
ATOM 30845 C THR I 248 9.882 16.414 8.965 0.00 0.00
I
ATOM 30846 O THR I 248 9.978 16.223 7.807 0.00 0.00
I
ATOM 30847 N LYS I 249 10.552 15.741 9.883 0.00 0.00
I
ATOM 30848 HN LYS I 249 10.266 15.903 10.824 0.00 0.00
I
ATOM 30849 CA LYS I 249 11.639 14.800 9.589 0.00 0.00
I
ATOM 30850 HA LYS I 249 12.412 15.292 9.018 0.00 0.00
I
ATOM 30851 CB LYS I 249 12.395 14.325 10.937 0.00 0.00
I
ATOM 30852 HB1 LYS I 249 12.698 15.244 11.483 0.00 0.00
I
ATOM 30853 HB2 LYS I 249 11.788 13.647 11.574 0.00 0.00
I
ATOM 30854 CG LYS I 249 13.868 13.783 10.569 0.00 0.00
I
ATOM 30855 HG1 LYS I 249 13.703 12.722 10.285 0.00 0.00
I
ATOM 30856 HG2 LYS I 249 14.122 14.340 9.642 0.00 0.00
I
ATOM 30857 CD LYS I 249 14.887 13.798 11.668 0.00 0.00
I
ATOM 30858 HD1 LYS I 249 15.832 13.300 11.361 0.00 0.00
I
ATOM 30859 HD2 LYS I 249 15.176 14.871 11.666 0.00 0.00
I
ATOM 30860 CE LYS I 249 14.433 13.333 12.973 0.00 0.00
I

```
ATOM  30861  HE1 LYS I 249      13.424  13.628  13.331  0.00  0.00
I
ATOM  30862  HE2 LYS I 249      14.400  12.228  12.864  0.00  0.00
I
ATOM  30863  NZ  LYS I 249      15.379  13.768  14.084  0.00  0.00
I
ATOM  30864  HZ1 LYS I 249      15.253  14.798  14.156  0.00  0.00
I
ATOM  30865  HZ2 LYS I 249      15.188  13.401  15.038  0.00  0.00
I
ATOM  30866  HZ3 LYS I 249      16.346  13.522  13.789  0.00  0.00
I
ATOM  30867  C   LYS I 249      11.224  13.622   8.691  0.00  0.00
I
ATOM  30868  O   LYS I 249      12.002  13.371   7.755  0.00  0.00
I
ATOM  30869  N   LEU I 250      10.114  12.885   8.927  0.00  0.00
I
ATOM  30870  HN  LEU I 250       9.521  13.084   9.703  0.00  0.00
I
ATOM  30871  CA  LEU I 250       9.580  11.786   8.084  0.00  0.00
I
ATOM  30872  HA  LEU I 250      10.355  11.037   8.019  0.00  0.00
I
ATOM  30873  CB  LEU I 250       8.282  11.202   8.746  0.00  0.00
I
ATOM  30874  HB1 LEU I 250       7.735  11.990   9.308  0.00  0.00
I
ATOM  30875  HB2 LEU I 250       7.703  10.715   7.932  0.00  0.00
I
ATOM  30876  CG  LEU I 250       8.591  10.179   9.876  0.00  0.00
I
ATOM  30877  HG  LEU I 250       8.917  10.796  10.741  0.00  0.00
I
ATOM  30878  CD1 LEU I 250       7.311   9.527  10.121  0.00  0.00
I
ATOM  30879 HD11 LEU I 250       6.842   8.984   9.273  0.00  0.00
I
ATOM  30880 HD12 LEU I 250       7.470   8.794  10.941  0.00  0.00
I
ATOM  30881 HD13 LEU I 250       6.607  10.249  10.590  0.00  0.00
I
ATOM  30882  CD2 LEU I 250       9.675   9.077   9.567  0.00  0.00
I
ATOM  30883 HD21 LEU I 250       9.496   8.616   8.572  0.00  0.00
I
ATOM  30884 HD22 LEU I 250      10.687   9.533   9.531  0.00  0.00
I
ATOM  30885 HD23 LEU I 250       9.716   8.331  10.389  0.00  0.00
I
ATOM  30886  C   LEU I 250       9.310  12.321   6.649  0.00  0.00
I
ATOM  30887  O   LEU I 250       9.360  11.572   5.671  0.00  0.00
I
```

```
ATOM  30888  N   MET I 251       8.991  13.615   6.422  0.00  0.00
I
ATOM  30889  HN  MET I 251       8.882  14.097   7.288  0.00  0.00
I
ATOM  30890  CA  MET I 251       8.908  14.168   5.054  0.00  0.00
I
ATOM  30891  HA  MET I 251       8.368  13.455   4.448  0.00  0.00
I
ATOM  30892  CB  MET I 251       8.058  15.504   5.162  0.00  0.00
I
ATOM  30893  HB1 MET I 251       7.046  15.259   5.549  0.00  0.00
I
ATOM  30894  HB2 MET I 251       8.427  16.119   6.010  0.00  0.00
I
ATOM  30895  CG  MET I 251       7.850  16.352   3.841  0.00  0.00
I
ATOM  30896  HG1 MET I 251       7.053  17.124   3.897  0.00  0.00
I
ATOM  30897  HG2 MET I 251       8.762  16.941   3.604  0.00  0.00
I
ATOM  30898  SD  MET I 251       7.258  15.346   2.390  0.00  0.00
I
ATOM  30899  CE  MET I 251       8.034  16.405   1.072  0.00  0.00
I
ATOM  30900  HE1 MET I 251       9.057  16.766   1.311  0.00  0.00
I
ATOM  30901  HE2 MET I 251       8.006  15.907   0.080  0.00  0.00
I
ATOM  30902  HE3 MET I 251       7.523  17.368   0.856  0.00  0.00
I
ATOM  30903  C   MET I 251      10.159  14.484   4.497  0.00  0.00
I
ATOM  30904  O   MET I 251      10.552  14.031   3.405  0.00  0.00
I
ATOM  30905  N   LEU I 252      10.983  15.311   5.258  0.00  0.00
I
ATOM  30906  HN  LEU I 252      10.643  15.615   6.145  0.00  0.00
I
ATOM  30907  CA  LEU I 252      12.384  15.617   4.899  0.00  0.00
I
ATOM  30908  HA  LEU I 252      12.291  16.142   3.960  0.00  0.00
I
ATOM  30909  CB  LEU I 252      12.949  16.579   5.950  0.00  0.00
I
ATOM  30910  HB1 LEU I 252      13.022  16.020   6.907  0.00  0.00
I
ATOM  30911  HB2 LEU I 252      13.987  16.795   5.617  0.00  0.00
I
ATOM  30912  CG  LEU I 252      12.343  17.977   6.126  0.00  0.00
I
ATOM  30913  HG  LEU I 252      11.433  17.831   6.747  0.00  0.00
I
ATOM  30914  CD1 LEU I 252      13.283  18.757   7.019  0.00  0.00
I
```

ATOM 30915 HD11 LEU I 252   14.210 19.032   6.472 0.00 0.00
I
ATOM 30916 HD12 LEU I 252   12.878 19.725   7.383 0.00 0.00
I
ATOM 30917 HD13 LEU I 252   13.560 18.089   7.863 0.00 0.00
I
ATOM 30918 CD2 LEU I 252   12.031 18.708   4.837 0.00 0.00
I
ATOM 30919 HD21 LEU I 252   12.930 18.688   4.184 0.00 0.00
I
ATOM 30920 HD22 LEU I 252   11.232 18.274   4.198 0.00 0.00
I
ATOM 30921 HD23 LEU I 252   11.674 19.713   5.147 0.00 0.00
I
ATOM 30922 C   LEU I 252   13.304 14.478   4.657 0.00 0.00
I
ATOM 30923 O   LEU I 252   13.945 14.438   3.589 0.00 0.00
I
ATOM 30924 N   THR I 253   13.328 13.457   5.547 0.00 0.00
I
ATOM 30925 HN THR I 253   12.743 13.393   6.352 0.00 0.00
I
ATOM 30926 CA THR I 253   14.305 12.400   5.366 0.00 0.00
I
ATOM 30927 HA THR I 253   15.200 12.910   5.044 0.00 0.00
I
ATOM 30928 CB THR I 253   14.856 11.658   6.607 0.00 0.00
I
ATOM 30929 HB THR I 253   15.487 10.771   6.384 0.00 0.00
I
ATOM 30930 OG1 THR I 253   13.751 11.172   7.284 0.00 0.00
I
ATOM 30931 HG1 THR I 253   13.420 11.940   7.754 0.00 0.00
I
ATOM 30932 CG2 THR I 253   15.466 12.688   7.494 0.00 0.00
I
ATOM 30933 HG21 THR I 253   16.277 13.337   7.099 0.00 0.00
I
ATOM 30934 HG22 THR I 253   14.708 13.422   7.843 0.00 0.00
I
ATOM 30935 HG23 THR I 253   15.917 12.201   8.385 0.00 0.00
I
ATOM 30936 C   THR I 253   13.789 11.371   4.326 0.00 0.00
I
ATOM 30937 O   THR I 253   14.581 10.889   3.592 0.00 0.00
I
ATOM 30938 N   GLY I 254   12.449 11.084   4.286 0.00 0.00
I
ATOM 30939 HN GLY I 254   11.865 11.551   4.945 0.00 0.00
I
ATOM 30940 CA GLY I 254   11.927 10.152   3.300 0.00 0.00
I
ATOM 30941 HA1 GLY I 254   10.868 10.025   3.471 0.00 0.00
I

ATOM 30942 HA2 GLY I 254   12.442   9.207   3.384 0.00 0.00
I
ATOM 30943 C   GLY I 254   11.921 10.597   1.870 0.00 0.00
I
ATOM 30944 O   GLY I 254   12.076   9.704   0.993 0.00 0.00
I
ATOM 30945 N   THR I 255   11.859 11.911   1.584 0.00 0.00
I
ATOM 30946 HN THR I 255   11.585 12.569   2.281 0.00 0.00
I
ATOM 30947 CA THR I 255   12.144 12.646   0.394 0.00 0.00
I
ATOM 30948 HA THR I 255   11.548 12.191 -0.383 0.00 0.00
I
ATOM 30949 CB THR I 255   11.785 14.099   0.400 0.00 0.00
I
ATOM 30950 HB THR I 255   12.363 14.626   1.189 0.00 0.00
I
ATOM 30951 OG1 THR I 255   10.411 14.237   0.765 0.00 0.00
I
ATOM 30952 HG1 THR I 255   10.372 14.247   1.724 0.00 0.00
I
ATOM 30953 CG2 THR I 255   12.066 14.661 -0.998 0.00 0.00
I
ATOM 30954 HG21 THR I 255   11.672 15.698 -0.954 0.00 0.00
I
ATOM 30955 HG22 THR I 255   13.150 14.730 -1.230 0.00 0.00
I
ATOM 30956 HG23 THR I 255   11.518 14.162 -1.825 0.00 0.00
I
ATOM 30957 C   THR I 255   13.561 12.537   0.004 0.00 0.00
I
ATOM 30958 O   THR I 255   13.891 12.081 -1.109 0.00 0.00
I
ATOM 30959 N   LEU I 256   14.530 12.845   0.880 0.00 0.00
I
ATOM 30960 HN LEU I 256   14.316 13.088   1.823 0.00 0.00
I
ATOM 30961 CA LEU I 256   15.998 12.788   0.646 0.00 0.00
I
ATOM 30962 HA LEU I 256   16.372 13.517 -0.057 0.00 0.00
I
ATOM 30963 CB LEU I 256   16.650 13.110   2.041 0.00 0.00
I
ATOM 30964 HB1 LEU I 256   16.225 14.082   2.370 0.00 0.00
I
ATOM 30965 HB2 LEU I 256   16.290 12.427   2.840 0.00 0.00
I
ATOM 30966 CG LEU I 256   18.222 13.178   2.056 0.00 0.00
I
ATOM 30967 HG LEU I 256   18.662 12.192   1.795 0.00 0.00
I
ATOM 30968 CD1 LEU I 256   18.745 14.264   1.030 0.00 0.00
I

ATOM 30969 HD11 LEU I 256   18.499 13.869   0.021 0.00 0.00
I
ATOM 30970 HD12 LEU I 256   18.194 15.206   1.239 0.00 0.00
I
ATOM 30971 HD13 LEU I 256   19.850 14.359   1.102 0.00 0.00
I
ATOM 30972 CD2 LEU I 256   18.608 13.469   3.478 0.00 0.00
I
ATOM 30973 HD21 LEU I 256   18.100 12.814   4.218 0.00 0.00
I
ATOM 30974 HD22 LEU I 256   19.715 13.471   3.580 0.00 0.00
I
ATOM 30975 HD23 LEU I 256   18.289 14.516   3.669 0.00 0.00
I
ATOM 30976 C   LEU I 256   16.493 11.396   0.245 0.00 0.00
I
ATOM 30977 O   LEU I 256   17.244 11.291 -0.718 0.00 0.00
I
ATOM 30978 N   MET I 257   16.038 10.382   0.945 0.00 0.00
I
ATOM 30979 HN MET I 257   15.381 10.526   1.682 0.00 0.00
I
ATOM 30980 CA MET I 257   16.240   8.979   0.665 0.00 0.00
I
ATOM 30981 HA MET I 257   17.293   8.763   0.561 0.00 0.00
I
ATOM 30982 CB MET I 257   15.524   8.258   1.873 0.00 0.00
I
ATOM 30983 HB1 MET I 257   15.945   8.620   2.835 0.00 0.00
I
ATOM 30984 HB2 MET I 257   14.449   8.533   1.926 0.00 0.00
I
ATOM 30985 CG MET I 257   15.794   6.722   1.859 0.00 0.00
I
ATOM 30986 HG1 MET I 257   15.649   6.296   0.843 0.00 0.00
I
ATOM 30987 HG2 MET I 257   16.867   6.558   2.096 0.00 0.00
I
ATOM 30988 SD MET I 257   14.770   5.703   3.044 0.00 0.00
I
ATOM 30989 CE MET I 257   15.186   6.172   4.698 0.00 0.00
I
ATOM 30990 HE1 MET I 257   14.907   7.240   4.830 0.00 0.00
I
ATOM 30991 HE2 MET I 257   14.641   5.514   5.408 0.00 0.00
I
ATOM 30992 HE3 MET I 257   16.292   6.067   4.729 0.00 0.00
I
ATOM 30993 C   MET I 257   15.614   8.455 -0.658 0.00 0.00
I
ATOM 30994 O   MET I 257   16.290   7.858 -1.506 0.00 0.00
I
ATOM 30995 N   LEU I 258   14.305   8.780 -1.022 0.00 0.00
I

ATOM 30996 HN LEU I 258   13.592   9.070 -0.388 0.00 0.00
I
ATOM 30997 CA LEU I 258   13.693   8.252 -2.198 0.00 0.00
I
ATOM 30998 HA LEU I 258   13.994   7.225 -2.341 0.00 0.00
I
ATOM 30999 CB LEU I 258   12.149   8.260 -2.006 0.00 0.00
I
ATOM 31000 HB1 LEU I 258   11.955   7.849 -0.993 0.00 0.00
I
ATOM 31001 HB2 LEU I 258   11.700   9.276 -2.024 0.00 0.00
I
ATOM 31002 CG LEU I 258   11.329   7.416 -2.996 0.00 0.00
I
ATOM 31003 HG LEU I 258   11.260   7.898 -3.995 0.00 0.00
I
ATOM 31004 CD1 LEU I 258   11.784   5.949 -3.107 0.00 0.00
I
ATOM 31005 HD11 LEU I 258   12.849   5.860 -3.412 0.00 0.00
I
ATOM 31006 HD12 LEU I 258   11.733   5.407 -2.139 0.00 0.00
I
ATOM 31007 HD13 LEU I 258   11.095   5.480 -3.841 0.00 0.00
I
ATOM 31008 CD2 LEU I 258   9.854   7.529 -2.479 0.00 0.00
I
ATOM 31009 HD21 LEU I 258   9.580   8.605 -2.458 0.00 0.00
I
ATOM 31010 HD22 LEU I 258   9.098   7.018 -3.113 0.00 0.00
I
ATOM 31011 HD23 LEU I 258   9.961   7.003 -1.506 0.00 0.00
I
ATOM 31012 C   LEU I 258   14.113   9.021 -3.493 0.00 0.00
I
ATOM 31013 O   LEU I 258   14.113   8.468 -4.606 0.00 0.00
I
ATOM 31014 N   ASN I 259   14.508 10.270 -3.340 0.00 0.00
I
ATOM 31015 HN ASN I 259   14.397 10.637 -2.420 0.00 0.00
I
ATOM 31016 CA ASN I 259   15.326 11.111 -4.295 0.00 0.00
I
ATOM 31017 HA ASN I 259   15.050 11.002 -5.333 0.00 0.00
I
ATOM 31018 CB ASN I 259   15.274 12.572 -3.902 0.00 0.00
I
ATOM 31019 HB1 ASN I 259   15.331 12.770 -2.810 0.00 0.00
I
ATOM 31020 HB2 ASN I 259   16.107 13.098 -4.414 0.00 0.00
I
ATOM 31021 CG ASN I 259   13.891 13.035 -4.442 0.00 0.00
I
ATOM 31022 OD1 ASN I 259   12.999 12.321 -4.969 0.00 0.00
I

ATOM 31023 ND2 ASN I 259   13.712 14.368 -4.299 0.00 0.00
I
ATOM 31024 HD21 ASN I 259   12.995 14.769 -4.869 0.00 0.00
I
ATOM 31025 HD22 ASN I 259   14.404 14.889 -3.801 0.00 0.00
I
ATOM 31026 C   ASN I 259   16.706 10.565 -4.536 0.00 0.00
I
ATOM 31027 O   ASN I 259   17.044 10.356 -5.641 0.00 0.00
I
ATOM 31028 N   ALA I 260   17.497 10.254 -3.530 0.00 0.00
I
ATOM 31029 HN ALA I 260   17.334 10.461 -2.569 0.00 0.00
I
ATOM 31030 CA ALA I 260   18.727   9.544 -3.693 0.00 0.00
I
ATOM 31031 HA ALA I 260   19.410 10.140 -4.279 0.00 0.00
I
ATOM 31032 CB ALA I 260   19.476   9.540 -2.370 0.00 0.00
I
ATOM 31033 HB1 ALA I 260   19.033   8.791 -1.679 0.00 0.00
I
ATOM 31034 HB2 ALA I 260   20.538   9.274 -2.558 0.00 0.00
I
ATOM 31035 HB3 ALA I 260   19.555 10.479 -1.781 0.00 0.00
I
ATOM 31036 C   ALA I 260   18.672   8.096 -4.313 0.00 0.00
I
ATOM 31037 O   ALA I 260   19.427   7.839 -5.236 0.00 0.00
I
ATOM 31038 N   ILE I 261   17.759   7.281 -3.861 0.00 0.00
I
ATOM 31039 HN ILE I 261   17.211   7.492 -3.055 0.00 0.00
I
ATOM 31040 CA ILE I 261   17.629   5.913 -4.373 0.00 0.00
I
ATOM 31041 HA ILE I 261   18.615   5.473 -4.398 0.00 0.00
I
ATOM 31042 CB ILE I 261   16.667   4.996 -3.584 0.00 0.00
I
ATOM 31043 HB ILE I 261   15.683   5.508 -3.527 0.00 0.00
I
ATOM 31044 CG2 ILE I 261   16.376   3.632 -4.373 0.00 0.00
I
ATOM 31045 HG21 ILE I 261   15.761   2.923 -3.777 0.00 0.00
I
ATOM 31046 HG22 ILE I 261   15.961   3.734 -5.398 0.00 0.00
I
ATOM 31047 HG23 ILE I 261   17.383   3.168 -4.445 0.00 0.00
I
ATOM 31048 CG1 ILE I 261   17.169   4.784 -2.101 0.00 0.00
I
ATOM 31049 HG11 ILE I 261   18.192   4.365 -1.991 0.00 0.00
I

ATOM	31050	HG12	ILE	I	261	17.135	5.783	-1.617	0.00	0.00	I
ATOM	31051	CD	ILE	I	261	16.200	3.881	-1.305	0.00	0.00	I
ATOM	31052	HD1	ILE	I	261	16.274	2.778	-1.410	0.00	0.00	I
ATOM	31053	HD2	ILE	I	261	16.392	4.133	-0.241	0.00	0.00	I
ATOM	31054	HD3	ILE	I	261	15.166	4.128	-1.628	0.00	0.00	I
ATOM	31055	C	ILE	I	261	17.125	6.035	-5.842	0.00	0.00	I
ATOM	31056	O	ILE	I	261	17.702	5.371	-6.723	0.00	0.00	I
ATOM	31057	N	ALA	I	262	16.069	6.845	-6.142	0.00	0.00	I
ATOM	31058	HN	ALA	I	262	15.693	7.437	-5.434	0.00	0.00	I
ATOM	31059	CA	ALA	I	262	15.618	6.899	-7.393	0.00	0.00	I
ATOM	31060	HA	ALA	I	262	15.402	5.869	-7.635	0.00	0.00	I
ATOM	31061	CB	ALA	I	262	14.346	7.739	-7.508	0.00	0.00	I
ATOM	31062	HB1	ALA	I	262	13.563	7.405	-6.793	0.00	0.00	I
ATOM	31063	HB2	ALA	I	262	14.663	8.757	-7.198	0.00	0.00	I
ATOM	31064	HB3	ALA	I	262	13.979	7.773	-8.556	0.00	0.00	I
ATOM	31065	C	ALA	I	262	16.641	7.453	-8.354	0.00	0.00	I
ATOM	31066	O	ALA	I	262	16.757	6.856	-9.436	0.00	0.00	I
ATOM	31067	N	MET	I	263	17.337	8.617	-8.043	0.00	0.00	I
ATOM	31068	HN	MET	I	263	17.427	8.920	-7.097	0.00	0.00	I
ATOM	31069	CA	MET	I	263	18.254	9.196	-9.057	0.00	0.00	I
ATOM	31070	HA	MET	I	263	17.629	9.580	-9.849	0.00	0.00	I
ATOM	31071	CB	MET	I	263	18.922	10.490	-8.394	0.00	0.00	I
ATOM	31072	HB1	MET	I	263	18.040	11.157	-8.298	0.00	0.00	I
ATOM	31073	HB2	MET	I	263	19.295	10.245	-7.377	0.00	0.00	I
ATOM	31074	CG	MET	I	263	20.128	11.185	-9.094	0.00	0.00	I
ATOM	31075	HG1	MET	I	263	20.973	10.504	-8.857	0.00	0.00	I
ATOM	31076	HG2	MET	I	263	19.927	11.019	-10.174	0.00	0.00	I

ATOM 31077 SD MET I 263   20.478 12.869 -8.626 0.00 0.00
I
ATOM 31078 CE MET I 263   21.314 13.301 -10.151 0.00 0.00
I
ATOM 31079 HE1 MET I 263   20.756 13.078 -11.086 0.00 0.00
I
ATOM 31080 HE2 MET I 263   21.394 14.406 -10.064 0.00 0.00
I
ATOM 31081 HE3 MET I 263   22.363 12.954 -10.262 0.00 0.00
I
ATOM 31082 C   MET I 263   19.308   8.293 -9.677 0.00 0.00
I
ATOM 31083 O   MET I 263   19.568   8.329 -10.889 0.00 0.00
I
ATOM 31084 N   LEU I 264   19.898   7.450 -8.763 0.00 0.00
I
ATOM 31085 HN LEU I 264   19.573   7.464 -7.820 0.00 0.00
I
ATOM 31086 CA LEU I 264   20.923   6.483 -9.107 0.00 0.00
I
ATOM 31087 HA LEU I 264   21.622   7.041 -9.712 0.00 0.00
I
ATOM 31088 CB LEU I 264   21.569   6.002 -7.844 0.00 0.00
I
ATOM 31089 HB1 LEU I 264   21.955   6.878 -7.281 0.00 0.00
I
ATOM 31090 HB2 LEU I 264   20.750   5.571 -7.230 0.00 0.00
I
ATOM 31091 CG LEU I 264   22.589   4.834 -7.985 0.00 0.00
I
ATOM 31092 HG LEU I 264   22.227   3.884 -8.433 0.00 0.00
I
ATOM 31093 CD1 LEU I 264   23.779   5.278 -8.901 0.00 0.00
I
ATOM 31094 HD11 LEU I 264   23.284   5.617 -9.836 0.00 0.00
I
ATOM 31095 HD12 LEU I 264   24.224   6.185 -8.439 0.00 0.00
I
ATOM 31096 HD13 LEU I 264   24.564   4.506 -9.049 0.00 0.00
I
ATOM 31097 CD2 LEU I 264   23.086   4.554 -6.554 0.00 0.00
I
ATOM 31098 HD21 LEU I 264   22.229   4.178 -5.955 0.00 0.00
I
ATOM 31099 HD22 LEU I 264   23.831   3.733 -6.629 0.00 0.00
I
ATOM 31100 HD23 LEU I 264   23.453   5.523 -6.154 0.00 0.00
I
ATOM 31101 C   LEU I 264   20.371   5.398 -10.015 0.00 0.00
I
ATOM 31102 O   LEU I 264   20.874   5.076 -11.059 0.00 0.00
I
ATOM 31103 N   THR I 265   19.262   4.884 -9.584 0.00 0.00
I

ATOM 31104 HN THR I 265 18.923 5.137 −8.681 0.00 0.00
I
ATOM 31105 CA THR I 265 18.586 3.801 −10.316 0.00 0.00
I
ATOM 31106 HA THR I 265 19.264 2.960 −10.320 0.00 0.00
I
ATOM 31107 CB THR I 265 17.356 3.397 −9.541 0.00 0.00
I
ATOM 31108 HB THR I 265 16.622 4.229 −9.491 0.00 0.00
I
ATOM 31109 OG1 THR I 265 17.500 3.030 −8.130 0.00 0.00
I
ATOM 31110 HG1 THR I 265 17.443 3.831 −7.605 0.00 0.00
I
ATOM 31111 CG2 THR I 265 16.833 2.117 −10.165 0.00 0.00
I
ATOM 31112 HG21 THR I 265 17.632 1.356 −10.289 0.00 0.00
I
ATOM 31113 HG22 THR I 265 16.050 1.699 −9.497 0.00 0.00
I
ATOM 31114 HG23 THR I 265 16.472 2.342 −11.192 0.00 0.00
I
ATOM 31115 C THR I 265 18.109 4.123 −11.718 0.00 0.00
I
ATOM 31116 O THR I 265 18.396 3.410 −12.639 0.00 0.00
I
ATOM 31117 N VAL I 266 17.347 5.204 −11.841 0.00 0.00
I
ATOM 31118 HN VAL I 266 17.294 5.763 −11.018 0.00 0.00
I
ATOM 31119 CA VAL I 266 16.672 5.669 −13.003 0.00 0.00
I
ATOM 31120 HA VAL I 266 16.002 4.918 −13.396 0.00 0.00
I
ATOM 31121 CB VAL I 266 15.906 7.030 −12.671 0.00 0.00
I
ATOM 31122 HB VAL I 266 16.506 7.774 −12.105 0.00 0.00
I
ATOM 31123 CG1 VAL I 266 15.129 7.671 −13.862 0.00 0.00
I
ATOM 31124 HG11 VAL I 266 14.445 6.888 −14.255 0.00 0.00
I
ATOM 31125 HG12 VAL I 266 14.508 8.564 −13.636 0.00 0.00
I
ATOM 31126 HG13 VAL I 266 15.835 7.953 −14.671 0.00 0.00
I
ATOM 31127 CG2 VAL I 266 14.878 6.512 −11.674 0.00 0.00
I
ATOM 31128 HG21 VAL I 266 15.274 5.932 −10.813 0.00 0.00
I
ATOM 31129 HG22 VAL I 266 14.276 7.365 −11.296 0.00 0.00
I
ATOM 31130 HG23 VAL I 266 14.144 5.930 −12.271 0.00 0.00
I

ATOM 31131 C   VAL I 266   17.614   5.994 -14.139 0.00 0.00
I
ATOM 31132 O   VAL I 266   17.366   5.622 -15.300 0.00 0.00
I
ATOM 31133 N   PHE I 267   18.780   6.562 -13.846 0.00 0.00
I
ATOM 31134 HN PHE I 267   19.007   7.003 -12.981 0.00 0.00
I
ATOM 31135 CA PHE I 267   19.826   6.786 -14.698 0.00 0.00
I
ATOM 31136 HA PHE I 267   19.543   7.163 -15.670 0.00 0.00
I
ATOM 31137 CB PHE I 267   20.798   7.638 -13.865 0.00 0.00
I
ATOM 31138 HB1 PHE I 267   20.223   8.504 -13.473 0.00 0.00
I
ATOM 31139 HB2 PHE I 267   21.029   7.061 -12.944 0.00 0.00
I
ATOM 31140 CG PHE I 267   22.009   8.054 -14.686 0.00 0.00
I
ATOM 31141 CD1 PHE I 267   21.823   8.770 -15.877 0.00 0.00
I
ATOM 31142 HD1 PHE I 267   20.896   9.222 -16.200 0.00 0.00
I
ATOM 31143 CE1 PHE I 267   22.937   9.062 -16.636 0.00 0.00
I
ATOM 31144 HE1 PHE I 267   22.773   9.572 -17.574 0.00 0.00
I
ATOM 31145 CZ PHE I 267   24.254   8.614 -16.183 0.00 0.00
I
ATOM 31146 HZ PHE I 267   25.129   8.747 -16.803 0.00 0.00
I
ATOM 31147 CD2 PHE I 267   23.204   7.617 -14.264 0.00 0.00
I
ATOM 31148 HD2 PHE I 267   23.334   6.990 -13.394 0.00 0.00
I
ATOM 31149 CE2 PHE I 267   24.395   7.843 -15.018 0.00 0.00
I
ATOM 31150 HE2 PHE I 267   25.361   7.543 -14.639 0.00 0.00
I
ATOM 31151 C   PHE I 267   20.380   5.465 -15.102 0.00 0.00
I
ATOM 31152 O   PHE I 267   20.562   5.234 -16.303 0.00 0.00
I
ATOM 31153 N   ILE I 268   20.663   4.505 -14.218 0.00 0.00
I
ATOM 31154 HN ILE I 268   20.634   4.742 -13.250 0.00 0.00
I
ATOM 31155 CA ILE I 268   21.307   3.257 -14.513 0.00 0.00
I
ATOM 31156 HA ILE I 268   22.159   3.450 -15.149 0.00 0.00
I
ATOM 31157 CB ILE I 268   21.883   2.510 -13.281 0.00 0.00
I

```
ATOM  31158  HB  ILE I 268      21.003   2.522 -12.603  0.00  0.00
I
ATOM  31159  CG2 ILE I 268      22.242   1.012 -13.540  0.00  0.00
I
ATOM  31160 HG21 ILE I 268      23.037   0.972 -14.315  0.00  0.00
I
ATOM  31161 HG22 ILE I 268      22.743   0.588 -12.643  0.00  0.00
I
ATOM  31162 HG23 ILE I 268      21.346   0.413 -13.806  0.00  0.00
I
ATOM  31163  CG1 ILE I 268      22.967   3.241 -12.584  0.00  0.00
I
ATOM  31164 HG11 ILE I 268      22.637   4.231 -12.205  0.00  0.00
I
ATOM  31165 HG12 ILE I 268      23.223   2.617 -11.701  0.00  0.00
I
ATOM  31166  CD  ILE I 268      24.249   3.352 -13.446  0.00  0.00
I
ATOM  31167  HD1 ILE I 268      23.944   4.154 -14.152  0.00  0.00
I
ATOM  31168  HD2 ILE I 268      25.122   3.667 -12.835  0.00  0.00
I
ATOM  31169  HD3 ILE I 268      24.481   2.433 -14.027  0.00  0.00
I
ATOM  31170  C   ILE I 268      20.404   2.295 -15.364  0.00  0.00
I
ATOM  31171  O   ILE I 268      20.841   1.534 -16.171  0.00  0.00
I
ATOM  31172  N   LEU I 269      19.067   2.395 -15.018  0.00  0.00
I
ATOM  31173  HN  LEU I 269      18.931   3.059 -14.287  0.00  0.00
I
ATOM  31174  CA  LEU I 269      17.900   1.777 -15.702  0.00  0.00
I
ATOM  31175  HA  LEU I 269      18.043   0.711 -15.611  0.00  0.00
I
ATOM  31176  CB  LEU I 269      16.690   2.183 -14.877  0.00  0.00
I
ATOM  31177  HB1 LEU I 269      16.912   1.959 -13.812  0.00  0.00
I
ATOM  31178  HB2 LEU I 269      16.428   3.261 -14.943  0.00  0.00
I
ATOM  31179  CG  LEU I 269      15.294   1.526 -15.233  0.00  0.00
I
ATOM  31180  HG  LEU I 269      14.914   2.017 -16.153  0.00  0.00
I
ATOM  31181  CD1 LEU I 269      15.144  -0.027 -15.445  0.00  0.00
I
ATOM  31182 HD11 LEU I 269      14.061  -0.178 -15.640  0.00  0.00
I
ATOM  31183 HD12 LEU I 269      15.748  -0.361 -16.315  0.00  0.00
I
ATOM  31184 HD13 LEU I 269      15.446  -0.699 -14.613  0.00  0.00
I
```

ATOM 31185 CD2 LEU I 269   14.373   1.984 −14.084 0.00 0.00
I
ATOM 31186 HD21 LEU I 269   14.237   3.078 −14.218 0.00 0.00
I
ATOM 31187 HD22 LEU I 269   13.365   1.518 −14.120 0.00 0.00
I
ATOM 31188 HD23 LEU I 269   14.769   1.699 −13.086 0.00 0.00
I
ATOM 31189 C   LEU I 269   17.756   2.181 −17.205 0.00 0.00
I
ATOM 31190 O   LEU I 269   17.416   1.349 −18.061 0.00 0.00
I
ATOM 31191 N   GLU I 270   18.007   3.470 −17.534 0.00 0.00
I
ATOM 31192 HN GLU I 270   18.195   4.083 −16.770 0.00 0.00
I
ATOM 31193 CA GLU I 270   17.885   4.090 −18.842 0.00 0.00
I
ATOM 31194 HA GLU I 270   17.143   3.573 −19.433 0.00 0.00
I
ATOM 31195 CB GLU I 270   17.285   5.477 −18.711 0.00 0.00
I
ATOM 31196 HB1 GLU I 270   17.712   6.141 −17.928 0.00 0.00
I
ATOM 31197 HB2 GLU I 270   17.392   6.003 −19.684 0.00 0.00
I
ATOM 31198 CG GLU I 270   15.742   5.328 −18.321 0.00 0.00
I
ATOM 31199 HG1 GLU I 270   15.660   4.734 −17.386 0.00 0.00
I
ATOM 31200 HG2 GLU I 270   15.294   6.329 −18.144 0.00 0.00
I
ATOM 31201 CD GLU I 270   14.819   4.730 −19.452 0.00 0.00
I
ATOM 31202 OE1 GLU I 270   14.005   3.789 −19.187 0.00 0.00
I
ATOM 31203 OE2 GLU I 270   14.978   5.207 −20.594 0.00 0.00
I
ATOM 31204 C   GLU I 270   19.185   4.082 −19.646 0.00 0.00
I
ATOM 31205 O   GLU I 270   19.261   4.627 −20.797 0.00 0.00
I
ATOM 31206 N   TYR I 271   20.291   3.504 −19.152 0.00 0.00
I
ATOM 31207 HN TYR I 271   20.141   3.019 −18.293 0.00 0.00
I
ATOM 31208 CA TYR I 271   21.656   3.678 −19.636 0.00 0.00
I
ATOM 31209 HA TYR I 271   21.893   4.730 −19.698 0.00 0.00
I
ATOM 31210 CB TYR I 271   22.508   2.889 −18.675 0.00 0.00
I
ATOM 31211 HB1 TYR I 271   22.065   2.932 −17.657 0.00 0.00
I

ATOM 31212 HB2 TYR I 271 22.591 1.838 −19.026 0.00 0.00
I
ATOM 31213 CG TYR I 271 23.959 3.419 −18.573 0.00 0.00
I
ATOM 31214 CD1 TYR I 271 24.985 3.013 −19.445 0.00 0.00
I
ATOM 31215 HD1 TYR I 271 24.708 2.247 −20.154 0.00 0.00
I
ATOM 31216 CE1 TYR I 271 26.228 3.638 −19.455 0.00 0.00
I
ATOM 31217 HE1 TYR I 271 26.981 3.326 −20.163 0.00 0.00
I
ATOM 31218 CZ TYR I 271 26.487 4.595 −18.415 0.00 0.00
I
ATOM 31219 OH TYR I 271 27.763 5.172 −18.435 0.00 0.00
I
ATOM 31220 HH TYR I 271 28.297 4.970 −19.206 0.00 0.00
I
ATOM 31221 CD2 TYR I 271 24.177 4.442 −17.645 0.00 0.00
I
ATOM 31222 HD2 TYR I 271 23.394 4.842 −17.017 0.00 0.00
I
ATOM 31223 CE2 TYR I 271 25.479 4.953 −17.525 0.00 0.00
I
ATOM 31224 HE2 TYR I 271 25.722 5.759 −16.848 0.00 0.00
I
ATOM 31225 C TYR I 271 21.973 3.178 −21.047 0.00 0.00
I
ATOM 31226 O TYR I 271 22.915 3.566 −21.717 0.00 0.00
I
ATOM 31227 N SER I 272 21.100 2.309 −21.557 0.00 0.00
I
ATOM 31228 HN SER I 272 20.356 1.962 −20.991 0.00 0.00
I
ATOM 31229 CA SER I 272 21.305 1.566 −22.785 0.00 0.00
I
ATOM 31230 HA SER I 272 22.138 2.081 −23.240 0.00 0.00
I
ATOM 31231 CB SER I 272 21.524 0.052 −22.583 0.00 0.00
I
ATOM 31232 HB1 SER I 272 20.550 −0.262 −22.151 0.00 0.00
I
ATOM 31233 HB2 SER I 272 21.812 −0.366 −23.571 0.00 0.00
I
ATOM 31234 OG SER I 272 22.548 −0.282 −21.687 0.00 0.00
I
ATOM 31235 HG1 SER I 272 22.587 −1.234 −21.573 0.00 0.00
I
ATOM 31236 C SER I 272 20.124 1.858 −23.766 0.00 0.00
I
ATOM 31237 O SER I 272 20.056 1.222 −24.820 0.00 0.00
I
ATOM 31238 N ASN I 273 19.227 2.823 −23.419 0.00 0.00
I

ATOM 31239 HN ASN I 273   19.377   3.308 -22.561 0.00 0.00
I
[truncated: 443,247 more chars]
