# Supplementary material for: Structural basis and synergism of ATP and Na+ activation in bacterial K+ uptake system KtrAB
Source: Nat Commun. 2024 May 8;15:3850. doi: 10.1038/s41467-024-48057-y (PMC11078986; doi:10.1038/s41467-024-48057-y)
Supplement: Supplementary file 6 — Supplementary Data 3 [file 41467_2024_48057_MOESM6_ESM.pdf]

```
CRYST1  200.000  200.000  194.000  90.00  90.00  90.00 P 1
1
ATOM      1  N   THR I  35      20.192 -17.706  15.253  1.00  0.00
I    N
ATOM      2  HT1 THR I  35      21.050 -17.574  15.749  1.00  0.00
I    H
ATOM      3  HT2 THR I  35      20.332 -18.334  14.488  1.00  0.00
I    H
ATOM      4  HT3 THR I  35      19.494 -18.070  15.870  1.00  0.00
I    H
ATOM      5  CA  THR I  35      19.723 -16.365  14.712  1.00  0.00
I    C
ATOM      6  HA  THR I  35      19.508 -15.756  15.580  1.00  0.00
I    H
ATOM      7  CB  THR I  35      20.807 -15.769  13.798  1.00  0.00
I    C
ATOM      8  HB  THR I  35      20.277 -15.072  13.103  1.00  0.00
I    H
ATOM      9  OG1 THR I  35      21.441 -16.757  12.987  1.00  0.00
I    O
ATOM     10  HG1 THR I  35      22.100 -16.281  12.471  1.00  0.00
I    H
ATOM     11  CG2 THR I  35      21.908 -15.130  14.652  1.00  0.00
I    C
ATOM     12 HG21 THR I  35      22.642 -14.595  14.013  1.00  0.00
I    H
ATOM     13 HG22 THR I  35      21.471 -14.394  15.360  1.00  0.00
I    H
ATOM     14 HG23 THR I  35      22.455 -15.897  15.241  1.00  0.00
I    H
ATOM     15  C   THR I  35      18.415 -16.502  13.945  1.00  0.00
I    C
ATOM     16  O   THR I  35      18.463 -16.636  12.728  1.00  0.00
I    O
ATOM     17  N   PRO I  36      17.235 -16.544  14.576  1.00  0.00
I    N
ATOM     18  CD  PRO I  36      17.000 -16.121  15.961  1.00  0.00
I    C
ATOM     19  HD1 PRO I  36      17.744 -16.924  15.755  1.00  0.00
I    H
ATOM     20  HD2 PRO I  36      17.357 -15.460  16.783  1.00  0.00
I    H
ATOM     21  CA  PRO I  36      15.982 -16.969  13.932  1.00  0.00
I    C
ATOM     22  HA  PRO I  36      16.042 -18.046  13.838  1.00  0.00
I    H
ATOM     23  CB  PRO I  36      14.888 -16.809  15.005  1.00  0.00
I    C
ATOM     24  HB1 PRO I  36      14.863 -17.724  15.638  1.00  0.00
I    H
ATOM     25  HB2 PRO I  36      13.881 -16.645  14.570  1.00  0.00
I    H
ATOM     26  CG  PRO I  36      15.651 -16.741  16.336  1.00  0.00
I    C
```

```
ATOM     27  HG1 PRO I  36      15.030 -17.033  17.205  1.00  0.00
I    H
ATOM     28  HG2 PRO I  36      16.035 -15.705  16.485  1.00  0.00
I    H
ATOM     29  C   PRO I  36      15.583 -16.368  12.581  1.00  0.00
I    C
ATOM     30  O   PRO I  36      15.300 -17.197  11.714  1.00  0.00
I    O
ATOM     31  N   PRO I  37      15.534 -15.066  12.283  1.00  0.00
I    N
ATOM     32  CD  PRO I  37      15.735 -13.960  13.225  1.00  0.00
I    C
ATOM     33  HD1 PRO I  37      14.975 -14.016  14.037  1.00  0.00
I    H
ATOM     34  HD2 PRO I  37      16.764 -13.980  13.652  1.00  0.00
I    H
ATOM     35  CA  PRO I  37      15.067 -14.600  10.984  1.00  0.00
I    C
ATOM     36  HA  PRO I  37      14.212 -15.172  10.641  1.00  0.00
I    H
ATOM     37  CB  PRO I  37      14.637 -13.148  11.253  1.00  0.00
I    C
ATOM     38  HB1 PRO I  37      13.591 -13.143  11.636  1.00  0.00
I    H
ATOM     39  HB2 PRO I  37      14.691 -12.510  10.347  1.00  0.00
I    H
ATOM     40  CG  PRO I  37      15.533 -12.683  12.406  1.00  0.00
I    C
ATOM     41  HG1 PRO I  37      15.097 -11.843  12.981  1.00  0.00
I    H
ATOM     42  HG2 PRO I  37      16.525 -12.379  12.000  1.00  0.00
I    H
ATOM     43  C   PRO I  37      16.186 -14.718   9.964  1.00  0.00
I    C
ATOM     44  O   PRO I  37      15.900 -14.915   8.783  1.00  0.00
I    O
ATOM     45  N   GLN I  38      17.472 -14.632  10.379  1.00  0.00
I    N
ATOM     46  HN  GLN I  38      17.708 -14.392  11.320  1.00  0.00
I    H
ATOM     47  CA  GLN I  38      18.614 -14.873   9.517  1.00  0.00
I    C
ATOM     48  HA  GLN I  38      18.498 -14.223   8.659  1.00  0.00
I    H
ATOM     49  CB  GLN I  38      19.981 -14.593  10.201  1.00  0.00
I    C
ATOM     50  HB1 GLN I  38      19.979 -15.150  11.166  1.00  0.00
I    H
ATOM     51  HB2 GLN I  38      20.799 -15.036   9.588  1.00  0.00
I    H
ATOM     52  CG  GLN I  38      20.304 -13.102  10.499  1.00  0.00
I    C
ATOM     53  HG1 GLN I  38      21.402 -12.953  10.588  1.00  0.00
I    H
```

```
ATOM     54  HG2 GLN I  38      19.939 -12.450   9.679  1.00  0.00
I    H
ATOM     55  CD  GLN I  38      19.724 -12.553  11.808  1.00  0.00
I    C
ATOM     56  OE1 GLN I  38      18.982 -13.219  12.535  1.00  0.00
I    O
ATOM     57  NE2 GLN I  38      20.110 -11.298  12.149  1.00  0.00
I    N
ATOM     58 HE21 GLN I  38      19.744 -10.930  12.999  1.00  0.00
I    H
ATOM     59 HE22 GLN I  38      20.717 -10.788  11.550  1.00  0.00
I    H
ATOM     60  C   GLN I  38      18.647 -16.305   9.006  1.00  0.00
I    C
ATOM     61  O   GLN I  38      18.893 -16.532   7.827  1.00  0.00
I    O
ATOM     62  N   VAL I  39      18.361 -17.306   9.871  1.00  0.00
I    N
ATOM     63  HN  VAL I  39      18.166 -17.123  10.830  1.00  0.00
I    H
ATOM     64  CA  VAL I  39      18.310 -18.724   9.502  1.00  0.00
I    C
ATOM     65  HA  VAL I  39      19.289 -18.991   9.125  1.00  0.00
I    H
ATOM     66  CB  VAL I  39      17.929 -19.603  10.705  1.00  0.00
I    C
ATOM     67  HB  VAL I  39      17.064 -19.116  11.219  1.00  0.00
I    H
ATOM     68  CG1 VAL I  39      17.499 -21.037  10.301  1.00  0.00
I    C
ATOM     69 HG11 VAL I  39      17.272 -21.641  11.205  1.00  0.00
I    H
ATOM     70 HG12 VAL I  39      16.585 -21.028   9.672  1.00  0.00
I    H
ATOM     71 HG13 VAL I  39      18.312 -21.548   9.741  1.00  0.00
I    H
ATOM     72  CG2 VAL I  39      19.140 -19.697  11.653  1.00  0.00
I    C
ATOM     73 HG21 VAL I  39      18.913 -20.359  12.515  1.00  0.00
I    H
ATOM     74 HG22 VAL I  39      20.023 -20.109  11.118  1.00  0.00
I    H
ATOM     75 HG23 VAL I  39      19.412 -18.700  12.057  1.00  0.00
I    H
ATOM     76  C   VAL I  39      17.311 -18.974   8.392  1.00  0.00
I    C
ATOM     77  O   VAL I  39      17.632 -19.584   7.371  1.00  0.00
I    O
ATOM     78  N   LEU I  40      16.081 -18.443   8.543  1.00  0.00
I    N
ATOM     79  HN  LEU I  40      15.788 -17.945   9.357  1.00  0.00
I    H
ATOM     80  CA  LEU I  40      15.069 -18.555   7.520  1.00  0.00
I    C
```

```
ATOM     81  HA  LEU I  40      15.007 -19.607   7.273  1.00  0.00
I    H
ATOM     82  CB  LEU I  40      13.699 -18.115   8.089  1.00  0.00
I    C
ATOM     83  HB1 LEU I  40      13.460 -18.799   8.937  1.00  0.00
I    H
ATOM     84  HB2 LEU I  40      13.800 -17.100   8.532  1.00  0.00
I    H
ATOM     85  CG  LEU I  40      12.533 -18.152   7.080  1.00  0.00
I    C
ATOM     86  HG  LEU I  40      12.848 -18.816   6.239  1.00  0.00
I    H
ATOM     87  CD1 LEU I  40      11.259 -18.677   7.738  1.00  0.00
I    C
ATOM     88 HD11 LEU I  40      10.412 -18.655   7.019  1.00  0.00
I    H
ATOM     89 HD12 LEU I  40      11.399 -19.724   8.081  1.00  0.00
I    H
ATOM     90 HD13 LEU I  40      10.984 -18.053   8.616  1.00  0.00
I    H
ATOM     91  CD2 LEU I  40      12.237 -16.769   6.505  1.00  0.00
I    C
ATOM     92 HD21 LEU I  40      11.406 -16.826   5.767  1.00  0.00
I    H
ATOM     93 HD22 LEU I  40      11.936 -16.051   7.295  1.00  0.00
I    H
ATOM     94 HD23 LEU I  40      13.133 -16.362   5.987  1.00  0.00
I    H
ATOM     95  C   LEU I  40      15.439 -17.806   6.248  1.00  0.00
I    C
ATOM     96  O   LEU I  40      15.424 -18.394   5.173  1.00  0.00
I    O
ATOM     97  N   ALA I  41      15.855 -16.524   6.318  1.00  0.00
I    N
ATOM     98  HN  ALA I  41      15.972 -16.053   7.192  1.00  0.00
I    H
ATOM     99  CA  ALA I  41      16.163 -15.734   5.138  1.00  0.00
I    C
ATOM    100  HA  ALA I  41      15.307 -15.748   4.472  1.00  0.00
I    H
ATOM    101  CB  ALA I  41      16.398 -14.266   5.550  1.00  0.00
I    C
ATOM    102  HB1 ALA I  41      15.510 -13.886   6.099  1.00  0.00
I    H
ATOM    103  HB2 ALA I  41      17.279 -14.176   6.222  1.00  0.00
I    H
ATOM    104  HB3 ALA I  41      16.560 -13.612   4.666  1.00  0.00
I    H
ATOM    105  C   ALA I  41      17.355 -16.244   4.321  1.00  0.00
I    C
ATOM    106  O   ALA I  41      17.285 -16.343   3.096  1.00  0.00
I    O
ATOM    107  N   ILE I  42      18.468 -16.618   4.996  1.00  0.00
I    N
```

ATOM 108 HN ILE I 42 18.540 −16.515 5.986 1.00 0.00
I H
ATOM 109 CA ILE I 42 19.651 −17.197 4.368 1.00 0.00
I C
ATOM 110 HA ILE I 42 19.915 −16.545 3.545 1.00 0.00
I H
ATOM 111 CB ILE I 42 20.822 −17.312 5.357 1.00 0.00
I C
ATOM 112 HB ILE I 42 20.398 −17.873 6.228 1.00 0.00
I H
ATOM 113 CG2 ILE I 42 22.022 −18.070 4.736 1.00 0.00
I C
ATOM 114 HG21 ILE I 42 22.793 −18.281 5.507 1.00 0.00
I H
ATOM 115 HG22 ILE I 42 21.698 −19.043 4.310 1.00 0.00
I H
ATOM 116 HG23 ILE I 42 22.495 −17.473 3.927 1.00 0.00
I H
ATOM 117 CG1 ILE I 42 21.257 −15.921 5.905 1.00 0.00
I C
ATOM 118 HG11 ILE I 42 20.359 −15.337 6.206 1.00 0.00
I H
ATOM 119 HG12 ILE I 42 21.874 −16.072 6.821 1.00 0.00
I H
ATOM 120 CD ILE I 42 22.082 −15.063 4.937 1.00 0.00
I C
ATOM 121 HD1 ILE I 42 22.395 −14.118 5.430 1.00 0.00
I H
ATOM 122 HD2 ILE I 42 22.995 −15.607 4.613 1.00 0.00
I H
ATOM 123 HD3 ILE I 42 21.490 −14.800 4.035 1.00 0.00
I H
ATOM 124 C ILE I 42 19.326 −18.556 3.768 1.00 0.00
I C
ATOM 125 O ILE I 42 19.627 −18.820 2.604 1.00 0.00
I O
ATOM 126 N GLY I 43 18.622 −19.443 4.513 1.00 0.00
I N
ATOM 127 HN GLY I 43 18.310 −19.238 5.439 1.00 0.00
I H
ATOM 128 CA GLY I 43 18.271 −20.767 4.008 1.00 0.00
I C
ATOM 129 HA1 GLY I 43 17.785 −21.285 4.823 1.00 0.00
I H
ATOM 130 HA2 GLY I 43 19.193 −21.225 3.676 1.00 0.00
I H
ATOM 131 C GLY I 43 17.319 −20.741 2.840 1.00 0.00
I C
ATOM 132 O GLY I 43 17.423 −21.541 1.916 1.00 0.00
I O
ATOM 133 N PHE I 44 16.382 −19.776 2.829 1.00 0.00
I N
ATOM 134 HN PHE I 44 16.277 −19.130 3.582 1.00 0.00
I H

```
ATOM    135  CA  PHE I  44      15.449 -19.583   1.740  1.00  0.00
I    C
ATOM    136  HA  PHE I  44      15.006 -20.556   1.564  1.00  0.00
I    H
ATOM    137  CB  PHE I  44      14.368 -18.553   2.155  1.00  0.00
I    C
ATOM    138  HB1 PHE I  44      14.325 -18.497   3.265  1.00  0.00
I    H
ATOM    139  HB2 PHE I  44      14.623 -17.536   1.786  1.00  0.00
I    H
ATOM    140  CG  PHE I  44      13.008 -18.967   1.673  1.00  0.00
I    C
ATOM    141  CD1 PHE I  44      12.059 -19.433   2.601  1.00  0.00
I    C
ATOM    142  HD1 PHE I  44      12.322 -19.498   3.648  1.00  0.00
I    H
ATOM    143  CE1 PHE I  44      10.780 -19.818   2.183  1.00  0.00
I    C
ATOM    144  HE1 PHE I  44      10.044 -20.127   2.912  1.00  0.00
I    H
ATOM    145  CZ  PHE I  44      10.459 -19.795   0.822  1.00  0.00
I    C
ATOM    146  HZ  PHE I  44       9.472 -20.088   0.493  1.00  0.00
I    H
ATOM    147  CD2 PHE I  44      12.669 -18.929   0.313  1.00  0.00
I    C
ATOM    148  HD2 PHE I  44      13.386 -18.570  -0.413  1.00  0.00
I    H
ATOM    149  CE2 PHE I  44      11.415 -19.383  -0.115  1.00  0.00
I    C
ATOM    150  HE2 PHE I  44      11.185 -19.415  -1.170  1.00  0.00
I    H
ATOM    151  C   PHE I  44      16.130 -19.162   0.445  1.00  0.00
I    C
ATOM    152  O   PHE I  44      15.832 -19.703  -0.621  1.00  0.00
I    O
ATOM    153  N   PHE I  45      17.102 -18.216   0.499  1.00  0.00
I    N
ATOM    154  HN  PHE I  45      17.388 -17.779   1.350  1.00  0.00
I    H
ATOM    155  CA  PHE I  45      17.816 -17.755  -0.685  1.00  0.00
I    C
ATOM    156  HA  PHE I  45      17.055 -17.426  -1.382  1.00  0.00
I    H
ATOM    157  CB  PHE I  45      18.774 -16.566  -0.360  1.00  0.00
I    C
ATOM    158  HB1 PHE I  45      18.207 -15.781   0.187  1.00  0.00
I    H
ATOM    159  HB2 PHE I  45      19.604 -16.892   0.304  1.00  0.00
I    H
ATOM    160  CG  PHE I  45      19.375 -15.860  -1.573  1.00  0.00
I    C
ATOM    161  CD1 PHE I  45      18.868 -15.955  -2.890  1.00  0.00
I    C
```

```
ATOM    162  HD1 PHE I  45      17.994 -16.562  -3.084  1.00  0.00
I    H
ATOM    163  CE1 PHE I  45      19.485 -15.280  -3.953  1.00  0.00
I    C
ATOM    164  HE1 PHE I  45      19.093 -15.378  -4.955  1.00  0.00
I    H
ATOM    165  CZ  PHE I  45      20.602 -14.473  -3.716  1.00  0.00
I    C
ATOM    166  HZ  PHE I  45      21.071 -13.939  -4.530  1.00  0.00
I    H
ATOM    167  CD2 PHE I  45      20.500 -15.043  -1.361  1.00  0.00
I    C
ATOM    168  HD2 PHE I  45      20.926 -14.957  -0.371  1.00  0.00
I    H
ATOM    169  CE2 PHE I  45      21.106 -14.350  -2.417  1.00  0.00
I    C
ATOM    170  HE2 PHE I  45      21.965 -13.722  -2.226  1.00  0.00
I    H
ATOM    171  C   PHE I  45      18.584 -18.891  -1.363  1.00  0.00
I    C
ATOM    172  O   PHE I  45      18.506 -19.075  -2.577  1.00  0.00
I    O
ATOM    173  N   LEU I  46      19.276 -19.733  -0.569  1.00  0.00
I    N
ATOM    174  HN  LEU I  46      19.323 -19.592   0.418  1.00  0.00
I    H
ATOM    175  CA  LEU I  46      20.006 -20.900  -1.042  1.00  0.00
I    C
ATOM    176  HA  LEU I  46      20.721 -20.537  -1.769  1.00  0.00
I    H
ATOM    177  CB  LEU I  46      20.758 -21.573   0.138  1.00  0.00
I    C
ATOM    178  HB1 LEU I  46      20.115 -21.465   1.042  1.00  0.00
I    H
ATOM    179  HB2 LEU I  46      20.833 -22.666  -0.057  1.00  0.00
I    H
ATOM    180  CG  LEU I  46      22.163 -20.988   0.461  1.00  0.00
I    C
ATOM    181  HG  LEU I  46      22.338 -21.373   1.494  1.00  0.00
I    H
ATOM    182  CD1 LEU I  46      23.239 -21.525  -0.498  1.00  0.00
I    C
ATOM    183 HD11 LEU I  46      24.223 -21.059  -0.280  1.00  0.00
I    H
ATOM    184 HD12 LEU I  46      23.342 -22.626  -0.392  1.00  0.00
I    H
ATOM    185 HD13 LEU I  46      22.974 -21.297  -1.552  1.00  0.00
I    H
ATOM    186  CD2 LEU I  46      22.245 -19.450   0.506  1.00  0.00
I    C
ATOM    187 HD21 LEU I  46      23.277 -19.126   0.761  1.00  0.00
I    H
ATOM    188 HD22 LEU I  46      21.982 -18.997  -0.471  1.00  0.00
I    H
```

```
ATOM    189 HD23 LEU I  46      21.553 -19.046   1.277  1.00  0.00
I    H
ATOM    190  C   LEU I  46      19.095 -21.900  -1.750  1.00  0.00
I    C
ATOM    191  O   LEU I  46      19.413 -22.382  -2.835  1.00  0.00
I    O
ATOM    192  N   THR I  47      17.892 -22.172  -1.206  1.00  0.00
I    N
ATOM    193  HN  THR I  47      17.630 -21.821  -0.307  1.00  0.00
I    H
ATOM    194  CA  THR I  47      16.863 -22.993  -1.856  1.00  0.00
I    C
ATOM    195  HA  THR I  47      17.346 -23.941  -2.051  1.00  0.00
I    H
ATOM    196  CB  THR I  47      15.628 -23.179  -0.979  1.00  0.00
I    C
ATOM    197  HB  THR I  47      15.227 -22.154  -0.788  1.00  0.00
I    H
ATOM    198  OG1 THR I  47      16.019 -23.740   0.262  1.00  0.00
I    O
ATOM    199  HG1 THR I  47      15.203 -23.845   0.763  1.00  0.00
I    H
ATOM    200  CG2 THR I  47      14.636 -24.191  -1.574  1.00  0.00
I    C
ATOM    201 HG21 THR I  47      13.699 -24.223  -0.978  1.00  0.00
I    H
ATOM    202 HG22 THR I  47      14.368 -23.905  -2.615  1.00  0.00
I    H
ATOM    203 HG23 THR I  47      15.070 -25.213  -1.595  1.00  0.00
I    H
ATOM    204  C   THR I  47      16.412 -22.448  -3.209  1.00  0.00
I    C
ATOM    205  O   THR I  47      16.248 -23.203  -4.169  1.00  0.00
I    O
ATOM    206  N   ILE I  48      16.233 -21.111  -3.348  1.00  0.00
I    N
ATOM    207  HN  ILE I  48      16.320 -20.483  -2.577  1.00  0.00
I    H
ATOM    208  CA  ILE I  48      15.899 -20.460  -4.618  1.00  0.00
I    C
ATOM    209  HA  ILE I  48      15.001 -20.950  -4.970  1.00  0.00
I    H
ATOM    210  CB  ILE I  48      15.653 -18.949  -4.440  1.00  0.00
I    C
ATOM    211  HB  ILE I  48      16.549 -18.583  -3.877  1.00  0.00
I    H
ATOM    212  CG2 ILE I  48      15.404 -18.283  -5.814  1.00  0.00
I    C
ATOM    213 HG21 ILE I  48      15.381 -17.177  -5.714  1.00  0.00
I    H
ATOM    214 HG22 ILE I  48      16.212 -18.539  -6.530  1.00  0.00
I    H
ATOM    215 HG23 ILE I  48      14.435 -18.612  -6.244  1.00  0.00
I    H
```

```
ATOM    216  CG1 ILE I  48      14.425 -18.696  -3.536  1.00  0.00
I    C
ATOM    217 HG11 ILE I  48      13.503 -19.054  -4.044  1.00  0.00
I    H
ATOM    218 HG12 ILE I  48      14.537 -19.291  -2.600  1.00  0.00
I    H
ATOM    219  CD  ILE I  48      14.196 -17.238  -3.116  1.00  0.00
I    C
ATOM    220  HD1 ILE I  48      13.329 -17.168  -2.424  1.00  0.00
I    H
ATOM    221  HD2 ILE I  48      15.091 -16.835  -2.596  1.00  0.00
I    H
ATOM    222  HD3 ILE I  48      13.984 -16.599  -3.998  1.00  0.00
I    H
ATOM    223  C   ILE I  48      16.968 -20.673  -5.690  1.00  0.00
I    C
ATOM    224  O   ILE I  48      16.670 -21.118  -6.801  1.00  0.00
I    O
ATOM    225  N   ILE I  49      18.257 -20.410  -5.383  1.00  0.00
I    N
ATOM    226  HN  ILE I  49      18.573 -20.107  -4.486  1.00  0.00
I    H
ATOM    227  CA  ILE I  49      19.320 -20.556  -6.373  1.00  0.00
I    C
ATOM    228  HA  ILE I  49      18.911 -20.162  -7.295  1.00  0.00
I    H
ATOM    229  CB  ILE I  49      20.537 -19.645  -6.164  1.00  0.00
I    C
ATOM    230  HB  ILE I  49      21.329 -20.079  -6.826  1.00  0.00
I    H
ATOM    231  CG2 ILE I  49      20.094 -18.208  -6.537  1.00  0.00
I    C
ATOM    232 HG21 ILE I  49      20.968 -17.523  -6.558  1.00  0.00
I    H
ATOM    233 HG22 ILE I  49      19.625 -18.187  -7.543  1.00  0.00
I    H
ATOM    234 HG23 ILE I  49      19.362 -17.814  -5.801  1.00  0.00
I    H
ATOM    235  CG1 ILE I  49      21.152 -19.666  -4.745  1.00  0.00
I    C
ATOM    236 HG11 ILE I  49      21.734 -18.733  -4.573  1.00  0.00
I    H
ATOM    237 HG12 ILE I  49      20.329 -19.683  -3.995  1.00  0.00
I    H
ATOM    238  CD  ILE I  49      22.067 -20.861  -4.446  1.00  0.00
I    C
ATOM    239  HD1 ILE I  49      22.415 -20.825  -3.391  1.00  0.00
I    H
ATOM    240  HD2 ILE I  49      21.524 -21.819  -4.600  1.00  0.00
I    H
ATOM    241  HD3 ILE I  49      22.961 -20.853  -5.104  1.00  0.00
I    H
ATOM    242  C   ILE I  49      19.697 -22.005  -6.643  1.00  0.00
I    C
```

```
ATOM    243  O   ILE I  49      20.074 -22.343  -7.763  1.00  0.00
I    O
ATOM    244  N   ILE I  50      19.526 -22.931  -5.671  1.00  0.00
I    N
ATOM    245  HN  ILE I  50      19.299 -22.689  -4.730  1.00  0.00
I    H
ATOM    246  CA  ILE I  50      19.657 -24.370  -5.904  1.00  0.00
I    C
ATOM    247  HA  ILE I  50      20.625 -24.516  -6.366  1.00  0.00
I    H
ATOM    248  CB  ILE I  50      19.605 -25.163  -4.590  1.00  0.00
I    C
ATOM    249  HB  ILE I  50      18.823 -24.643  -3.981  1.00  0.00
I    H
ATOM    250  CG2 ILE I  50      19.280 -26.663  -4.810  1.00  0.00
I    C
ATOM    251 HG21 ILE I  50      19.097 -27.170  -3.839  1.00  0.00
I    H
ATOM    252 HG22 ILE I  50      18.368 -26.784  -5.430  1.00  0.00
I    H
ATOM    253 HG23 ILE I  50      20.122 -27.181  -5.317  1.00  0.00
I    H
ATOM    254  CG1 ILE I  50      20.958 -24.996  -3.853  1.00  0.00
I    C
ATOM    255 HG11 ILE I  50      21.766 -25.499  -4.431  1.00  0.00
I    H
ATOM    256 HG12 ILE I  50      21.213 -23.912  -3.808  1.00  0.00
I    H
ATOM    257  CD  ILE I  50      20.959 -25.544  -2.420  1.00  0.00
I    C
ATOM    258  HD1 ILE I  50      21.935 -25.341  -1.930  1.00  0.00
I    H
ATOM    259  HD2 ILE I  50      20.160 -25.062  -1.816  1.00  0.00
I    H
ATOM    260  HD3 ILE I  50      20.792 -26.641  -2.413  1.00  0.00
I    H
ATOM    261  C   ILE I  50      18.617 -24.865  -6.895  1.00  0.00
I    C
ATOM    262  O   ILE I  50      18.937 -25.575  -7.849  1.00  0.00
I    O
ATOM    263  N   GLY I  51      17.341 -24.440  -6.752  1.00  0.00
I    N
ATOM    264  HN  GLY I  51      17.048 -23.873  -5.984  1.00  0.00
I    H
ATOM    265  CA  GLY I  51      16.301 -24.783  -7.716  1.00  0.00
I    C
ATOM    266  HA1 GLY I  51      15.385 -24.352  -7.337  1.00  0.00
I    H
ATOM    267  HA2 GLY I  51      16.290 -25.863  -7.783  1.00  0.00
I    H
ATOM    268  C   GLY I  51      16.537 -24.235  -9.100  1.00  0.00
I    C
ATOM    269  O   GLY I  51      16.215 -24.881 -10.092  1.00  0.00
I    O
```

```
ATOM    270  N   ALA I  52      17.155 -23.044  -9.205  1.00  0.00
I    N
ATOM    271  HN  ALA I  52      17.364 -22.513  -8.383  1.00  0.00
I    H
ATOM    272  CA  ALA I  52      17.561 -22.436 -10.458  1.00  0.00
I    C
ATOM    273  HA  ALA I  52      16.706 -22.358 -11.121  1.00  0.00
I    H
ATOM    274  CB  ALA I  52      18.053 -20.995 -10.195  1.00  0.00
I    C
ATOM    275  HB1 ALA I  52      17.250 -20.412  -9.697  1.00  0.00
I    H
ATOM    276  HB2 ALA I  52      18.942 -20.993  -9.528  1.00  0.00
I    H
ATOM    277  HB3 ALA I  52      18.319 -20.475 -11.141  1.00  0.00
I    H
ATOM    278  C   ALA I  52      18.612 -23.256 -11.206  1.00  0.00
I    C
ATOM    279  O   ALA I  52      18.507 -23.462 -12.413  1.00  0.00
I    O
ATOM    280  N   VAL I  53      19.622 -23.799 -10.486  1.00  0.00
I    N
ATOM    281  HN  VAL I  53      19.747 -23.585  -9.520  1.00  0.00
I    H
ATOM    282  CA  VAL I  53      20.609 -24.730 -11.031  1.00  0.00
I    C
ATOM    283  HA  VAL I  53      21.062 -24.245 -11.886  1.00  0.00
I    H
ATOM    284  CB  VAL I  53      21.715 -25.049 -10.018  1.00  0.00
I    C
ATOM    285  HB  VAL I  53      21.226 -25.467  -9.105  1.00  0.00
I    H
ATOM    286  CG1 VAL I  53      22.706 -26.097 -10.577  1.00  0.00
I    C
ATOM    287 HG11 VAL I  53      23.521 -26.285  -9.846  1.00  0.00
I    H
ATOM    288 HG12 VAL I  53      22.208 -27.068 -10.775  1.00  0.00
I    H
ATOM    289 HG13 VAL I  53      23.169 -25.735 -11.521  1.00  0.00
I    H
ATOM    290  CG2 VAL I  53      22.486 -23.752  -9.695  1.00  0.00
I    C
ATOM    291 HG21 VAL I  53      23.322 -23.957  -8.992  1.00  0.00
I    H
ATOM    292 HG22 VAL I  53      22.910 -23.307 -10.621  1.00  0.00
I    H
ATOM    293 HG23 VAL I  53      21.825 -22.999  -9.218  1.00  0.00
I    H
ATOM    294  C   VAL I  53      19.966 -26.027 -11.527  1.00  0.00
I    C
ATOM    295  O   VAL I  53      20.226 -26.476 -12.641  1.00  0.00
I    O
ATOM    296  N   LEU I  54      19.045 -26.629 -10.738  1.00  0.00
I    N
```

ATOM 297 HN LEU I 54 18.782 -26.250 -9.854 1.00 0.00
I H
ATOM 298 CA LEU I 54 18.355 -27.870 -11.091 1.00 0.00
I C
ATOM 299 HA LEU I 54 19.138 -28.576 -11.336 1.00 0.00
I H
ATOM 300 CB LEU I 54 17.554 -28.457 -9.889 1.00 0.00
I C
ATOM 301 HB1 LEU I 54 17.189 -27.595 -9.285 1.00 0.00
I H
ATOM 302 HB2 LEU I 54 16.645 -28.969 -10.276 1.00 0.00
I H
ATOM 303 CG LEU I 54 18.357 -29.407 -8.964 1.00 0.00
I C
ATOM 304 HG LEU I 54 18.899 -30.102 -9.650 1.00 0.00
I H
ATOM 305 CD1 LEU I 54 19.350 -28.682 -8.047 1.00 0.00
I C
ATOM 306 HD11 LEU I 54 19.861 -29.405 -7.375 1.00 0.00
I H
ATOM 307 HD12 LEU I 54 20.125 -28.156 -8.646 1.00 0.00
I H
ATOM 308 HD13 LEU I 54 18.827 -27.932 -7.416 1.00 0.00
I H
ATOM 309 CD2 LEU I 54 17.391 -30.243 -8.108 1.00 0.00
I C
ATOM 310 HD21 LEU I 54 17.959 -30.940 -7.454 1.00 0.00
I H
ATOM 311 HD22 LEU I 54 16.766 -29.603 -7.453 1.00 0.00
I H
ATOM 312 HD23 LEU I 54 16.717 -30.844 -8.756 1.00 0.00
I H
ATOM 313 C LEU I 54 17.440 -27.745 -12.319 1.00 0.00
I C
ATOM 314 O LEU I 54 17.175 -28.716 -13.031 1.00 0.00
I O
ATOM 315 N LEU I 55 16.939 -26.533 -12.619 1.00 0.00
I N
ATOM 316 HN LEU I 55 17.150 -25.715 -12.087 1.00 0.00
I H
ATOM 317 CA LEU I 55 16.046 -26.303 -13.740 1.00 0.00
I C
ATOM 318 HA LEU I 55 15.556 -27.253 -13.914 1.00 0.00
I H
ATOM 319 CB LEU I 55 15.065 -25.162 -13.398 1.00 0.00
I C
ATOM 320 HB1 LEU I 55 15.671 -24.311 -13.012 1.00 0.00
I H
ATOM 321 HB2 LEU I 55 14.589 -24.800 -14.336 1.00 0.00
I H
ATOM 322 CG LEU I 55 14.011 -25.560 -12.351 1.00 0.00
I C
ATOM 323 HG LEU I 55 14.548 -26.108 -11.539 1.00 0.00
I H

```
ATOM    324  CD1 LEU I  55      13.367 -24.292 -11.777  1.00  0.00
I    C
ATOM    325 HD11 LEU I  55      12.568 -24.555 -11.051  1.00  0.00
I    H
ATOM    326 HD12 LEU I  55      14.125 -23.673 -11.251  1.00  0.00
I    H
ATOM    327 HD13 LEU I  55      12.914 -23.681 -12.586  1.00  0.00
I    H
ATOM    328  CD2 LEU I  55      12.938 -26.497 -12.928  1.00  0.00
I    C
ATOM    329 HD21 LEU I  55      12.206 -26.778 -12.140  1.00  0.00
I    H
ATOM    330 HD22 LEU I  55      12.376 -26.015 -13.754  1.00  0.00
I    H
ATOM    331 HD23 LEU I  55      13.403 -27.428 -13.318  1.00  0.00
I    H
ATOM    332  C   LEU I  55      16.747 -25.907 -15.027  1.00  0.00
I    C
ATOM    333  O   LEU I  55      16.117 -25.909 -16.079  1.00  0.00
I    O
ATOM    334  N   MET I  56      18.055 -25.583 -15.007  1.00  0.00
I    N
ATOM    335  HN  MET I  56      18.641 -25.647 -14.202  1.00  0.00
I    H
ATOM    336  CA  MET I  56      18.728 -25.098 -16.205  1.00  0.00
I    C
ATOM    337  HA  MET I  56      17.955 -24.762 -16.885  1.00  0.00
I    H
ATOM    338  CB  MET I  56      19.683 -23.949 -15.810  1.00  0.00
I    C
ATOM    339  HB1 MET I  56      19.287 -23.509 -14.865  1.00  0.00
I    H
ATOM    340  HB2 MET I  56      20.683 -24.369 -15.563  1.00  0.00
I    H
ATOM    341  CG  MET I  56      19.797 -22.839 -16.873  1.00  0.00
I    C
ATOM    342  HG1 MET I  56      20.054 -23.276 -17.861  1.00  0.00
I    H
ATOM    343  HG2 MET I  56      18.820 -22.320 -16.979  1.00  0.00
I    H
ATOM    344  SD  MET I  56      21.061 -21.571 -16.514  1.00  0.00
I    S
ATOM    345  CE  MET I  56      20.689 -21.275 -14.759  1.00  0.00
I    C
ATOM    346  HE1 MET I  56      21.364 -20.501 -14.336  1.00  0.00
I    H
ATOM    347  HE2 MET I  56      19.643 -20.926 -14.626  1.00  0.00
I    H
ATOM    348  HE3 MET I  56      20.818 -22.204 -14.161  1.00  0.00
I    H
ATOM    349  C   MET I  56      19.505 -26.204 -16.904  1.00  0.00
I    C
ATOM    350  O   MET I  56      20.087 -26.025 -17.972  1.00  0.00
I    O
```

ATOM 351 N LEU I 57 19.521 -27.407 -16.301 1.00 0.00
I N
ATOM 352 HN LEU I 57 19.120 -27.538 -15.397 1.00 0.00
I H
ATOM 353 CA LEU I 57 20.099 -28.603 -16.875 1.00 0.00
I C
ATOM 354 HA LEU I 57 21.112 -28.338 -17.150 1.00 0.00
I H
ATOM 355 CB LEU I 57 20.084 -29.756 -15.842 1.00 0.00
I C
ATOM 356 HB1 LEU I 57 19.020 -29.936 -15.566 1.00 0.00
I H
ATOM 357 HB2 LEU I 57 20.437 -30.689 -16.336 1.00 0.00
I H
ATOM 358 CG LEU I 57 20.895 -29.476 -14.559 1.00 0.00
I C
ATOM 359 HG LEU I 57 20.555 -28.481 -14.185 1.00 0.00
I H
ATOM 360 CD1 LEU I 57 20.603 -30.556 -13.505 1.00 0.00
I C
ATOM 361 HD11 LEU I 57 21.219 -30.389 -12.596 1.00 0.00
I H
ATOM 362 HD12 LEU I 57 19.532 -30.535 -13.210 1.00 0.00
I H
ATOM 363 HD13 LEU I 57 20.837 -31.566 -13.902 1.00 0.00
I H
ATOM 364 CD2 LEU I 57 22.407 -29.391 -14.834 1.00 0.00
I C
ATOM 365 HD21 LEU I 57 22.958 -29.170 -13.895 1.00 0.00
I H
ATOM 366 HD22 LEU I 57 22.803 -30.342 -15.242 1.00 0.00
I H
ATOM 367 HD23 LEU I 57 22.625 -28.579 -15.563 1.00 0.00
I H
ATOM 368 C LEU I 57 19.379 -29.072 -18.152 1.00 0.00
I C
ATOM 369 O LEU I 57 18.149 -29.020 -18.173 1.00 0.00
I O
ATOM 370 N PRO I 58 20.033 -29.582 -19.212 1.00 0.00
I N
ATOM 371 CD PRO I 58 21.494 -29.622 -19.337 1.00 0.00
I C
ATOM 372 HD1 PRO I 58 21.003 -28.673 -19.020 1.00 0.00
I H
ATOM 373 HD2 PRO I 58 22.448 -29.771 -18.781 1.00 0.00
I H
ATOM 374 CA PRO I 58 19.378 -29.863 -20.498 1.00 0.00
I C
ATOM 375 HA PRO I 58 18.921 -28.950 -20.863 1.00 0.00
I H
ATOM 376 CB PRO I 58 20.530 -30.279 -21.433 1.00 0.00
I C
ATOM 377 HB1 PRO I 58 20.993 -29.365 -21.868 1.00 0.00
I H

```
ATOM    378  HB2 PRO I  58      20.196 -30.940 -22.257  1.00  0.00
I    H
ATOM    379  CG  PRO I  58      21.764 -29.595 -20.843  1.00  0.00
I    C
ATOM    380  HG1 PRO I  58      22.572 -29.446 -21.585  1.00  0.00
I    H
ATOM    381  HG2 PRO I  58      22.149 -30.203 -19.992  1.00  0.00
I    H
ATOM    382  C   PRO I  58      18.329 -30.961 -20.455  1.00  0.00
I    C
ATOM    383  O   PRO I  58      17.541 -31.084 -21.382  1.00  0.00
I    O
ATOM    384  N   ILE I  59      18.335 -31.804 -19.407  1.00  0.00
I    N
ATOM    385  HN  ILE I  59      19.090 -31.789 -18.755  1.00  0.00
I    H
ATOM    386  CA  ILE I  59      17.307 -32.788 -19.106  1.00  0.00
I    C
ATOM    387  HA  ILE I  59      17.171 -33.352 -20.019  1.00  0.00
I    H
ATOM    388  CB  ILE I  59      17.778 -33.724 -17.979  1.00  0.00
I    C
ATOM    389  HB  ILE I  59      18.650 -34.271 -18.417  1.00  0.00
I    H
ATOM    390  CG2 ILE I  59      18.252 -32.921 -16.738  1.00  0.00
I    C
ATOM    391 HG21 ILE I  59      18.732 -33.594 -15.997  1.00  0.00
I    H
ATOM    392 HG22 ILE I  59      18.997 -32.150 -17.025  1.00  0.00
I    H
ATOM    393 HG23 ILE I  59      17.397 -32.414 -16.242  1.00  0.00
I    H
ATOM    394  CG1 ILE I  59      16.715 -34.803 -17.639  1.00  0.00
I    C
ATOM    395 HG11 ILE I  59      15.774 -34.312 -17.304  1.00  0.00
I    H
ATOM    396 HG12 ILE I  59      16.475 -35.377 -18.563  1.00  0.00
I    H
ATOM    397  CD  ILE I  59      17.158 -35.804 -16.565  1.00  0.00
I    C
ATOM    398  HD1 ILE I  59      16.375 -36.579 -16.413  1.00  0.00
I    H
ATOM    399  HD2 ILE I  59      18.097 -36.315 -16.867  1.00  0.00
I    H
ATOM    400  HD3 ILE I  59      17.330 -35.296 -15.592  1.00  0.00
I    H
ATOM    401  C   ILE I  59      15.953 -32.158 -18.767  1.00  0.00
I    C
ATOM    402  O   ILE I  59      14.893 -32.668 -19.128  1.00  0.00
I    O
ATOM    403  N   SER I  60      15.958 -31.026 -18.035  1.00  0.00
I    N
ATOM    404  HN  SER I  60      16.810 -30.557 -17.806  1.00  0.00
I    H
```

```
ATOM     405  CA  SER I  60      14.765 -30.382 -17.512  1.00  0.00
I    C
ATOM     406  HA  SER I  60      14.147 -31.190 -17.145  1.00  0.00
I    H
ATOM     407  CB  SER I  60      15.092 -29.381 -16.370  1.00  0.00
I    C
ATOM     408  HB1 SER I  60      15.846 -28.654 -16.752  1.00  0.00
I    H
ATOM     409  HB2 SER I  60      14.185 -28.796 -16.095  1.00  0.00
I    H
ATOM     410  OG  SER I  60      15.633 -30.096 -15.257  1.00  0.00
I    O
ATOM     411  HG1 SER I  60      16.028 -29.443 -14.666  1.00  0.00
I    H
ATOM     412  C   SER I  60      13.971 -29.652 -18.566  1.00  0.00
I    C
ATOM     413  O   SER I  60      12.747 -29.570 -18.465  1.00  0.00
I    O
ATOM     414  N   THR I  61      14.646 -29.081 -19.585  1.00  0.00
I    N
ATOM     415  HN  THR I  61      15.603 -29.302 -19.768  1.00  0.00
I    H
ATOM     416  CA  THR I  61      14.071 -28.106 -20.505  1.00  0.00
I    C
ATOM     417  HA  THR I  61      13.039 -27.996 -20.199  1.00  0.00
I    H
ATOM     418  CB  THR I  61      14.756 -26.731 -20.447  1.00  0.00
I    C
ATOM     419  HB  THR I  61      14.144 -26.052 -21.090  1.00  0.00
I    H
ATOM     420  OG1 THR I  61      16.067 -26.695 -20.989  1.00  0.00
I    O
ATOM     421  HG1 THR I  61      16.367 -25.786 -20.874  1.00  0.00
I    H
ATOM     422  CG2 THR I  61      14.933 -26.324 -18.981  1.00  0.00
I    C
ATOM     423 HG21 THR I  61      15.321 -25.286 -18.903  1.00  0.00
I    H
ATOM     424 HG22 THR I  61      13.959 -26.364 -18.448  1.00  0.00
I    H
ATOM     425 HG23 THR I  61      15.643 -27.001 -18.461  1.00  0.00
I    H
ATOM     426  C   THR I  61      14.046 -28.613 -21.942  1.00  0.00
I    C
ATOM     427  O   THR I  61      15.003 -29.196 -22.435  1.00  0.00
I    O
ATOM     428  N   THR I  62      12.917 -28.443 -22.671  1.00  0.00
I    N
ATOM     429  HN  THR I  62      12.073 -28.045 -22.313  1.00  0.00
I    H
ATOM     430  CA  THR I  62      12.843 -28.843 -24.089  1.00  0.00
I    C
ATOM     431  HA  THR I  62      13.445 -29.741 -24.142  1.00  0.00
I    H
```

```
ATOM    432  CB  THR I  62      11.429 -29.107 -24.613  1.00  0.00
I    C
ATOM    433  HB  THR I  62      11.489 -29.015 -25.726  1.00  0.00
I    H
ATOM    434  OG1 THR I  62      10.464 -28.148 -24.189  1.00  0.00
I    O
ATOM    435  HG1 THR I  62       9.630 -28.436 -24.576  1.00  0.00
I    H
ATOM    436  CG2 THR I  62      10.968 -30.428 -24.022  1.00  0.00
I    C
ATOM    437 HG21 THR I  62       9.992 -30.735 -24.453  1.00  0.00
I    H
ATOM    438 HG22 THR I  62      11.705 -31.229 -24.241  1.00  0.00
I    H
ATOM    439 HG23 THR I  62      10.853 -30.354 -22.919  1.00  0.00
I    H
ATOM    440  C   THR I  62      13.444 -27.865 -25.064  1.00  0.00
I    C
ATOM    441  O   THR I  62      14.213 -28.223 -25.947  1.00  0.00
I    O
ATOM    442  N   LYS I  63      13.081 -26.581 -24.930  1.00  0.00
I    N
ATOM    443  HN  LYS I  63      12.380 -26.306 -24.274  1.00  0.00
I    H
ATOM    444  CA  LYS I  63      13.654 -25.502 -25.704  1.00  0.00
I    C
ATOM    445  HA  LYS I  63      14.138 -25.907 -26.584  1.00  0.00
I    H
ATOM    446  CB  LYS I  63      12.618 -24.404 -26.101  1.00  0.00
I    C
ATOM    447  HB1 LYS I  63      12.119 -24.069 -25.163  1.00  0.00
I    H
ATOM    448  HB2 LYS I  63      13.163 -23.519 -26.499  1.00  0.00
I    H
ATOM    449  CG  LYS I  63      11.523 -24.841 -27.101  1.00  0.00
I    C
ATOM    450  HG1 LYS I  63      11.092 -23.936 -27.586  1.00  0.00
I    H
ATOM    451  HG2 LYS I  63      11.989 -25.458 -27.904  1.00  0.00
I    H
ATOM    452  CD  LYS I  63      10.334 -25.638 -26.528  1.00  0.00
I    C
ATOM    453  HD1 LYS I  63       9.602 -25.818 -27.348  1.00  0.00
I    H
ATOM    454  HD2 LYS I  63      10.712 -26.632 -26.194  1.00  0.00
I    H
ATOM    455  CE  LYS I  63       9.645 -24.930 -25.359  1.00  0.00
I    C
ATOM    456  HE1 LYS I  63      10.366 -24.756 -24.528  1.00  0.00
I    H
ATOM    457  HE2 LYS I  63       9.238 -23.947 -25.683  1.00  0.00
I    H
ATOM    458  NZ  LYS I  63       8.530 -25.731 -24.829  1.00  0.00
I    N
```

```
ATOM    459  HZ1 LYS I  63       8.078 -25.232 -24.036  1.00  0.00
I    H
ATOM    460  HZ2 LYS I  63       7.830 -25.891 -25.582  1.00  0.00
I    H
ATOM    461  HZ3 LYS I  63       8.891 -26.648 -24.498  1.00  0.00
I    H
ATOM    462  C   LYS I  63      14.738 -24.896 -24.828  1.00  0.00
I    C
ATOM    463  O   LYS I  63      14.669 -25.075 -23.606  1.00  0.00
I    O
ATOM    464  N   PRO I  64      15.748 -24.191 -25.345  1.00  0.00
I    N
ATOM    465  CD  PRO I  64      15.958 -23.960 -26.776  1.00  0.00
I    C
ATOM    466  HD1 PRO I  64      16.011 -24.986 -26.347  1.00  0.00
I    H
ATOM    467  HD2 PRO I  64      15.487 -23.983 -27.785  1.00  0.00
I    H
ATOM    468  CA  PRO I  64      16.697 -23.435 -24.530  1.00  0.00
I    C
ATOM    469  HA  PRO I  64      17.405 -24.122 -24.081  1.00  0.00
I    H
ATOM    470  CB  PRO I  64      17.477 -22.582 -25.550  1.00  0.00
I    C
ATOM    471  HB1 PRO I  64      18.314 -23.187 -25.964  1.00  0.00
I    H
ATOM    472  HB2 PRO I  64      17.890 -21.653 -25.106  1.00  0.00
I    H
ATOM    473  CG  PRO I  64      17.358 -23.358 -26.867  1.00  0.00
I    C
ATOM    474  HG1 PRO I  64      18.170 -23.123 -27.582  1.00  0.00
I    H
ATOM    475  HG2 PRO I  64      17.600 -24.102 -27.489  1.00  0.00
I    H
ATOM    476  C   PRO I  64      16.052 -22.595 -23.435  1.00  0.00
I    C
ATOM    477  O   PRO I  64      15.057 -21.921 -23.697  1.00  0.00
I    O
ATOM    478  N   LEU I  65      16.583 -22.627 -22.200  1.00  0.00
I    N
ATOM    479  HN  LEU I  65      17.385 -23.166 -21.953  1.00  0.00
I    H
ATOM    480  CA  LEU I  65      16.022 -21.864 -21.110  1.00  0.00
I    C
ATOM    481  HA  LEU I  65      15.152 -21.334 -21.476  1.00  0.00
I    H
ATOM    482  CB  LEU I  65      15.641 -22.761 -19.908  1.00  0.00
I    C
ATOM    483  HB1 LEU I  65      15.029 -23.603 -20.307  1.00  0.00
I    H
ATOM    484  HB2 LEU I  65      16.565 -23.222 -19.493  1.00  0.00
I    H
ATOM    485  CG  LEU I  65      14.841 -22.050 -18.790  1.00  0.00
I    C
```

```
ATOM    486  HG  LEU I  65      15.464 -21.178 -18.476  1.00  0.00
I    H
ATOM    487  CD1 LEU I  65      13.451 -21.580 -19.252  1.00  0.00
I    C
ATOM    488 HD11 LEU I  65      12.894 -21.119 -18.407  1.00  0.00
I    H
ATOM    489 HD12 LEU I  65      13.543 -20.826 -20.062  1.00  0.00
I    H
ATOM    490 HD13 LEU I  65      12.855 -22.435 -19.635  1.00  0.00
I    H
ATOM    491  CD2 LEU I  65      14.672 -22.975 -17.576  1.00  0.00
I    C
ATOM    492 HD21 LEU I  65      14.113 -22.454 -16.769  1.00  0.00
I    H
ATOM    493 HD22 LEU I  65      14.108 -23.894 -17.837  1.00  0.00
I    H
ATOM    494 HD23 LEU I  65      15.664 -23.275 -17.175  1.00  0.00
I    H
ATOM    495  C   LEU I  65      17.049 -20.833 -20.710  1.00  0.00
I    C
ATOM    496  O   LEU I  65      18.211 -21.140 -20.458  1.00  0.00
I    O
ATOM    497  N   SER I  66      16.641 -19.555 -20.694  1.00  0.00
I    N
ATOM    498  HN  SER I  66      15.725 -19.295 -20.998  1.00  0.00
I    H
ATOM    499  CA  SER I  66      17.454 -18.443 -20.248  1.00  0.00
I    C
ATOM    500  HA  SER I  66      18.402 -18.546 -20.760  1.00  0.00
I    H
ATOM    501  CB  SER I  66      16.775 -17.087 -20.598  1.00  0.00
I    C
ATOM    502  HB1 SER I  66      17.503 -16.271 -20.388  1.00  0.00
I    H
ATOM    503  HB2 SER I  66      16.536 -17.042 -21.685  1.00  0.00
I    H
ATOM    504  OG  SER I  66      15.618 -16.926 -19.779  1.00  0.00
I    O
ATOM    505  HG1 SER I  66      15.348 -16.002 -19.849  1.00  0.00
I    H
ATOM    506  C   SER I  66      17.738 -18.515 -18.748  1.00  0.00
I    C
ATOM    507  O   SER I  66      16.976 -19.081 -17.968  1.00  0.00
I    O
ATOM    508  N   TRP I  67      18.853 -17.909 -18.294  1.00  0.00
I    N
ATOM    509  HN  TRP I  67      19.507 -17.486 -18.917  1.00  0.00
I    H
ATOM    510  CA  TRP I  67      19.198 -17.820 -16.884  1.00  0.00
I    C
ATOM    511  HA  TRP I  67      19.278 -18.837 -16.519  1.00  0.00
I    H
ATOM    512  CB  TRP I  67      20.563 -17.069 -16.785  1.00  0.00
I    C
```

ATOM 513 HB1 TRP I 67 21.362 -17.769 -17.114 1.00 0.00
I H
ATOM 514 HB2 TRP I 67 20.575 -16.211 -17.493 1.00 0.00
I H
ATOM 515 CG TRP I 67 20.968 -16.580 -15.397 1.00 0.00
I C
ATOM 516 CD1 TRP I 67 21.343 -17.304 -14.300 1.00 0.00
I C
ATOM 517 HD1 TRP I 67 21.523 -18.371 -14.309 1.00 0.00
I H
ATOM 518 NE1 TRP I 67 21.498 -16.475 -13.207 1.00 0.00
I N
ATOM 519 HE1 TRP I 67 21.794 -16.734 -12.312 1.00 0.00
I H
ATOM 520 CE2 TRP I 67 21.210 -15.184 -13.594 1.00 0.00
I C
ATOM 521 CD2 TRP I 67 20.876 -15.208 -14.964 1.00 0.00
I C
ATOM 522 CE3 TRP I 67 20.524 -14.040 -15.640 1.00 0.00
I C
ATOM 523 HE3 TRP I 67 20.245 -14.047 -16.685 1.00 0.00
I H
ATOM 524 CZ3 TRP I 67 20.531 -12.836 -14.920 1.00 0.00
I C
ATOM 525 HZ3 TRP I 67 20.268 -11.915 -15.421 1.00 0.00
I H
ATOM 526 CZ2 TRP I 67 21.207 -13.991 -12.879 1.00 0.00
I C
ATOM 527 HZ2 TRP I 67 21.456 -13.957 -11.829 1.00 0.00
I H
ATOM 528 CH2 TRP I 67 20.869 -12.810 -13.560 1.00 0.00
I C
ATOM 529 HH2 TRP I 67 20.869 -11.868 -13.028 1.00 0.00
I H
ATOM 530 C TRP I 67 18.115 -17.127 -16.045 1.00 0.00
I C
ATOM 531 O TRP I 67 17.702 -17.629 -14.996 1.00 0.00
I O
ATOM 532 N ILE I 68 17.586 -15.980 -16.520 1.00 0.00
I N
ATOM 533 HN ILE I 68 17.879 -15.575 -17.385 1.00 0.00
I H
ATOM 534 CA ILE I 68 16.553 -15.228 -15.831 1.00 0.00
I C
ATOM 535 HA ILE I 68 16.918 -15.089 -14.821 1.00 0.00
I H
ATOM 536 CB ILE I 68 16.361 -13.819 -16.406 1.00 0.00
I C
ATOM 537 HB ILE I 68 17.355 -13.323 -16.270 1.00 0.00
I H
ATOM 538 CG2 ILE I 68 15.972 -13.849 -17.906 1.00 0.00
I C
ATOM 539 HG21 ILE I 68 15.992 -12.826 -18.335 1.00 0.00
I H

```
ATOM    540 HG22 ILE I  68       16.683 -14.473 -18.487  1.00  0.00
I    H
ATOM    541 HG23 ILE I  68       14.950 -14.263 -18.042  1.00  0.00
I    H
ATOM    542  CG1 ILE I  68       15.347 -12.981 -15.582  1.00  0.00
I    C
ATOM    543 HG11 ILE I  68       15.209 -11.983 -16.057  1.00  0.00
I    H
ATOM    544 HG12 ILE I  68       14.358 -13.493 -15.600  1.00  0.00
I    H
ATOM    545  CD  ILE I  68       15.741 -12.774 -14.111  1.00  0.00
I    C
ATOM    546  HD1 ILE I  68       14.944 -12.217 -13.573  1.00  0.00
I    H
ATOM    547  HD2 ILE I  68       15.888 -13.751 -13.603  1.00  0.00
I    H
ATOM    548  HD3 ILE I  68       16.682 -12.190 -14.029  1.00  0.00
I    H
ATOM    549  C   ILE I  68       15.240 -15.989 -15.722  1.00  0.00
I    C
ATOM    550  O   ILE I  68       14.637 -16.043 -14.650  1.00  0.00
I    O
ATOM    551  N   ASP I  69       14.785 -16.670 -16.796  1.00  0.00
I    N
ATOM    552  HN  ASP I  69       15.269 -16.774 -17.661  1.00  0.00
I    H
ATOM    553  CA  ASP I  69       13.504 -17.337 -16.784  1.00  0.00
I    C
ATOM    554  HA  ASP I  69       12.830 -16.729 -16.192  1.00  0.00
I    H
ATOM    555  CB  ASP I  69       12.877 -17.381 -18.217  1.00  0.00
I    C
ATOM    556  HB1 ASP I  69       13.514 -18.005 -18.876  1.00  0.00
I    H
ATOM    557  HB2 ASP I  69       11.863 -17.828 -18.186  1.00  0.00
I    H
ATOM    558  CG  ASP I  69       12.766 -16.002 -18.883  1.00  0.00
I    C
ATOM    559  OD1 ASP I  69       12.187 -15.049 -18.299  1.00  0.00
I    O
ATOM    560  OD2 ASP I  69       13.264 -15.851 -20.030  1.00  0.00
I    O
ATOM    561  C   ASP I  69       13.611 -18.708 -16.071  1.00  0.00
I    C
ATOM    562  O   ASP I  69       12.608 -19.314 -15.692  1.00  0.00
I    O
ATOM    563  N   ALA I  70       14.845 -19.204 -15.791  1.00  0.00
I    N
ATOM    564  HN  ALA I  70       15.656 -18.830 -16.241  1.00  0.00
I    H
ATOM    565  CA  ALA I  70       15.095 -20.281 -14.848  1.00  0.00
I    C
ATOM    566  HA  ALA I  70       14.331 -21.043 -14.953  1.00  0.00
I    H
```

```
ATOM    567  CB  ALA I  70      16.468 -20.928 -15.138  1.00  0.00
I    C
ATOM    568  HB1 ALA I  70      16.488 -21.308 -16.183  1.00  0.00
I    H
ATOM    569  HB2 ALA I  70      17.291 -20.188 -15.028  1.00  0.00
I    H
ATOM    570  HB3 ALA I  70      16.669 -21.784 -14.459  1.00  0.00
I    H
ATOM    571  C   ALA I  70      15.050 -19.823 -13.389  1.00  0.00
I    C
ATOM    572  O   ALA I  70      14.355 -20.418 -12.567  1.00  0.00
I    O
ATOM    573  N   LEU I  71      15.751 -18.721 -13.027  1.00  0.00
I    N
ATOM    574  HN  LEU I  71      16.323 -18.219 -13.673  1.00  0.00
I    H
ATOM    575  CA  LEU I  71      15.739 -18.164 -11.678  1.00  0.00
I    C
ATOM    576  HA  LEU I  71      15.998 -18.979 -11.016  1.00  0.00
I    H
ATOM    577  CB  LEU I  71      16.839 -17.081 -11.514  1.00  0.00
I    C
ATOM    578  HB1 LEU I  71      17.805 -17.542 -11.825  1.00  0.00
I    H
ATOM    579  HB2 LEU I  71      16.645 -16.257 -12.235  1.00  0.00
I    H
ATOM    580  CG  LEU I  71      17.026 -16.493 -10.090  1.00  0.00
I    C
ATOM    581  HG  LEU I  71      16.126 -15.855  -9.919  1.00  0.00
I    H
ATOM    582  CD1 LEU I  71      17.153 -17.558  -8.986  1.00  0.00
I    C
ATOM    583 HD11 LEU I  71      17.333 -17.077  -8.001  1.00  0.00
I    H
ATOM    584 HD12 LEU I  71      16.224 -18.163  -8.916  1.00  0.00
I    H
ATOM    585 HD13 LEU I  71      18.003 -18.243  -9.197  1.00  0.00
I    H
ATOM    586  CD2 LEU I  71      18.268 -15.588 -10.042  1.00  0.00
I    C
ATOM    587 HD21 LEU I  71      18.389 -15.153  -9.026  1.00  0.00
I    H
ATOM    588 HD22 LEU I  71      19.193 -16.148 -10.281  1.00  0.00
I    H
ATOM    589 HD23 LEU I  71      18.168 -14.752 -10.768  1.00  0.00
I    H
ATOM    590  C   LEU I  71      14.361 -17.655 -11.264  1.00  0.00
I    C
ATOM    591  O   LEU I  71      13.923 -17.854 -10.136  1.00  0.00
I    O
ATOM    592  N   PHE I  72      13.609 -17.036 -12.202  1.00  0.00
I    N
ATOM    593  HN  PHE I  72      13.966 -16.810 -13.106  1.00  0.00
I    H
```

```
ATOM    594  CA  PHE I  72      12.226 -16.647 -11.984  1.00  0.00
I    C
ATOM    595  HA  PHE I  72      12.239 -16.022 -11.100  1.00  0.00
I    H
ATOM    596  CB  PHE I  72      11.706 -15.915 -13.253  1.00  0.00
I    C
ATOM    597  HB1 PHE I  72      12.449 -15.147 -13.559  1.00  0.00
I    H
ATOM    598  HB2 PHE I  72      11.593 -16.620 -14.104  1.00  0.00
I    H
ATOM    599  CG  PHE I  72      10.409 -15.195 -12.997  1.00  0.00
I    C
ATOM    600  CD1 PHE I  72       9.178 -15.879 -12.977  1.00  0.00
I    C
ATOM    601  HD1 PHE I  72       9.156 -16.946 -13.151  1.00  0.00
I    H
ATOM    602  CE1 PHE I  72       7.985 -15.189 -12.740  1.00  0.00
I    C
ATOM    603  HE1 PHE I  72       7.048 -15.725 -12.714  1.00  0.00
I    H
ATOM    604  CZ  PHE I  72       8.006 -13.806 -12.529  1.00  0.00
I    C
ATOM    605  HZ  PHE I  72       7.087 -13.273 -12.333  1.00  0.00
I    H
ATOM    606  CD2 PHE I  72      10.416 -13.809 -12.783  1.00  0.00
I    C
ATOM    607  HD2 PHE I  72      11.348 -13.263 -12.808  1.00  0.00
I    H
ATOM    608  CE2 PHE I  72       9.222 -13.116 -12.562  1.00  0.00
I    C
ATOM    609  HE2 PHE I  72       9.240 -12.044 -12.419  1.00  0.00
I    H
ATOM    610  C   PHE I  72      11.307 -17.829 -11.684  1.00  0.00
I    C
ATOM    611  O   PHE I  72      10.553 -17.818 -10.714  1.00  0.00
I    O
ATOM    612  N   THR I  73      11.370 -18.904 -12.498  1.00  0.00
I    N
ATOM    613  HN  THR I  73      11.972 -18.933 -13.296  1.00  0.00
I    H
ATOM    614  CA  THR I  73      10.578 -20.119 -12.298  1.00  0.00
I    C
ATOM    615  HA  THR I  73       9.555 -19.783 -12.194  1.00  0.00
I    H
ATOM    616  CB  THR I  73      10.680 -21.084 -13.469  1.00  0.00
I    C
ATOM    617  HB  THR I  73      11.771 -21.227 -13.660  1.00  0.00
I    H
ATOM    618  OG1 THR I  73      10.102 -20.471 -14.610  1.00  0.00
I    O
ATOM    619  HG1 THR I  73      10.178 -21.120 -15.317  1.00  0.00
I    H
ATOM    620  CG2 THR I  73       9.878 -22.374 -13.247  1.00  0.00
I    C
```

```
ATOM    621 HG21 THR I  73      10.063 -23.101 -14.065  1.00  0.00
I    H
ATOM    622 HG22 THR I  73      10.173 -22.853 -12.289  1.00  0.00
I    H
ATOM    623 HG23 THR I  73       8.787 -22.165 -13.209  1.00  0.00
I    H
ATOM    624  C   THR I  73      10.934 -20.823 -11.005  1.00  0.00
I    C
ATOM    625  O   THR I  73      10.057 -21.251 -10.259  1.00  0.00
I    O
ATOM    626  N   ALA I  74      12.242 -20.905 -10.673  1.00  0.00
I    N
ATOM    627  HN  ALA I  74      12.956 -20.596 -11.299  1.00  0.00
I    H
ATOM    628  CA  ALA I  74      12.712 -21.434  -9.407  1.00  0.00
I    C
ATOM    629  HA  ALA I  74      12.357 -22.452  -9.286  1.00  0.00
I    H
ATOM    630  CB  ALA I  74      14.252 -21.406  -9.374  1.00  0.00
I    C
ATOM    631  HB1 ALA I  74      14.654 -22.009 -10.216  1.00  0.00
I    H
ATOM    632  HB2 ALA I  74      14.635 -20.368  -9.483  1.00  0.00
I    H
ATOM    633  HB3 ALA I  74      14.649 -21.832  -8.428  1.00  0.00
I    H
ATOM    634  C   ALA I  74      12.205 -20.657  -8.201  1.00  0.00
I    C
ATOM    635  O   ALA I  74      11.660 -21.248  -7.264  1.00  0.00
I    O
ATOM    636  N   ALA I  75      12.313 -19.307  -8.228  1.00  0.00
I    N
ATOM    637  HN  ALA I  75      12.748 -18.852  -9.005  1.00  0.00
I    H
ATOM    638  CA  ALA I  75      11.832 -18.427  -7.183  1.00  0.00
I    C
ATOM    639  HA  ALA I  75      12.276 -18.690  -6.230  1.00  0.00
I    H
ATOM    640  CB  ALA I  75      12.234 -16.961  -7.474  1.00  0.00
I    C
ATOM    641  HB1 ALA I  75      13.340 -16.886  -7.551  1.00  0.00
I    H
ATOM    642  HB2 ALA I  75      11.800 -16.610  -8.436  1.00  0.00
I    H
ATOM    643  HB3 ALA I  75      11.900 -16.277  -6.664  1.00  0.00
I    H
ATOM    644  C   ALA I  75      10.331 -18.551  -7.005  1.00  0.00
I    C
ATOM    645  O   ALA I  75       9.868 -18.823  -5.908  1.00  0.00
I    O
ATOM    646  N   SER I  76       9.562 -18.507  -8.113  1.00  0.00
I    N
ATOM    647  HN  SER I  76       9.981 -18.386  -9.012  1.00  0.00
I    H
```

```
ATOM    648  CA  SER I  76       8.106 -18.622  -8.142  1.00  0.00
I    C
ATOM    649  HA  SER I  76       7.751 -17.814  -7.515  1.00  0.00
I    H
ATOM    650  CB  SER I  76       7.591 -18.522  -9.608  1.00  0.00
I    C
ATOM    651  HB1 SER I  76       8.070 -17.635 -10.082  1.00  0.00
I    H
ATOM    652  HB2 SER I  76       7.907 -19.417 -10.191  1.00  0.00
I    H
ATOM    653  OG  SER I  76       6.175 -18.354  -9.694  1.00  0.00
I    O
ATOM    654  HG1 SER I  76       5.976 -18.081 -10.598  1.00  0.00
I    H
ATOM    655  C   SER I  76       7.580 -19.916  -7.551  1.00  0.00
I    C
ATOM    656  O   SER I  76       6.503 -19.964  -6.950  1.00  0.00
I    O
ATOM    657  N   ALA I  77       8.326 -21.021  -7.742  1.00  0.00
I    N
ATOM    658  HN  ALA I  77       9.144 -20.997  -8.317  1.00  0.00
I    H
ATOM    659  CA  ALA I  77       8.020 -22.301  -7.154  1.00  0.00
I    C
ATOM    660  HA  ALA I  77       6.967 -22.507  -7.310  1.00  0.00
I    H
ATOM    661  CB  ALA I  77       8.815 -23.373  -7.920  1.00  0.00
I    C
ATOM    662  HB1 ALA I  77       8.584 -23.304  -9.005  1.00  0.00
I    H
ATOM    663  HB2 ALA I  77       9.910 -23.224  -7.794  1.00  0.00
I    H
ATOM    664  HB3 ALA I  77       8.555 -24.398  -7.578  1.00  0.00
I    H
ATOM    665  C   ALA I  77       8.264 -22.411  -5.644  1.00  0.00
I    C
ATOM    666  O   ALA I  77       7.393 -22.861  -4.913  1.00  0.00
I    O
ATOM    667  N   THR I  78       9.448 -21.991  -5.132  1.00  0.00
I    N
ATOM    668  HN  THR I  78      10.178 -21.602  -5.692  1.00  0.00
I    H
ATOM    669  CA  THR I  78       9.763 -22.072  -3.694  1.00  0.00
I    C
ATOM    670  HA  THR I  78       9.270 -22.971  -3.348  1.00  0.00
I    H
ATOM    671  CB  THR I  78      11.257 -22.286  -3.412  1.00  0.00
I    C
ATOM    672  HB  THR I  78      11.526 -23.259  -3.891  1.00  0.00
I    H
ATOM    673  OG1 THR I  78      11.561 -22.424  -2.032  1.00  0.00
I    O
ATOM    674  HG1 THR I  78      12.517 -22.539  -1.991  1.00  0.00
I    H
```

```
ATOM    675  CG2 THR I  78      12.101 -21.125  -3.913  1.00  0.00
I    C
ATOM    676 HG21 THR I  78      13.184 -21.348  -3.804  1.00  0.00
I    H
ATOM    677 HG22 THR I  78      11.899 -20.935  -4.989  1.00  0.00
I    H
ATOM    678 HG23 THR I  78      11.879 -20.194  -3.348  1.00  0.00
I    H
ATOM    679  C   THR I  78       9.177 -20.914  -2.883  1.00  0.00
I    C
ATOM    680  O   THR I  78       8.617 -21.116  -1.803  1.00  0.00
I    O
ATOM    681  N   THR I  79       9.211 -19.654  -3.391  1.00  0.00
I    N
ATOM    682  HN  THR I  79       9.793 -19.403  -4.164  1.00  0.00
I    H
ATOM    683  CA  THR I  79       8.406 -18.550  -2.860  1.00  0.00
I    C
ATOM    684  HA  THR I  79       8.287 -18.714  -1.797  1.00  0.00
I    H
ATOM    685  CB  THR I  79       8.930 -17.096  -2.986  1.00  0.00
I    C
ATOM    686  HB  THR I  79       8.138 -16.442  -2.546  1.00  0.00
I    H
ATOM    687  OG1 THR I  79       9.126 -16.595  -4.295  1.00  0.00
I    O
ATOM    688  HG1 THR I  79       9.458 -15.698  -4.180  1.00  0.00
I    H
ATOM    689  CG2 THR I  79      10.302 -16.980  -2.327  1.00  0.00
I    C
ATOM    690 HG21 THR I  79      10.643 -15.924  -2.306  1.00  0.00
I    H
ATOM    691 HG22 THR I  79      10.260 -17.346  -1.279  1.00  0.00
I    H
ATOM    692 HG23 THR I  79      11.061 -17.580  -2.875  1.00  0.00
I    H
ATOM    693  C   THR I  79       7.045 -18.672  -3.465  1.00  0.00
I    C
ATOM    694  O   THR I  79       6.700 -17.995  -4.430  1.00  0.00
I    O
ATOM    695  N   VAL I  80       6.238 -19.612  -2.932  1.00  0.00
I    N
ATOM    696  HN  VAL I  80       6.367 -19.977  -2.014  1.00  0.00
I    H
ATOM    697  CA  VAL I  80       5.116 -20.172  -3.645  1.00  0.00
I    C
ATOM    698  HA  VAL I  80       5.534 -20.670  -4.511  1.00  0.00
I    H
ATOM    699  CB  VAL I  80       4.410 -21.281  -2.879  1.00  0.00
I    C
ATOM    700  HB  VAL I  80       3.565 -20.816  -2.316  1.00  0.00
I    H
ATOM    701  CG1 VAL I  80       3.913 -22.248  -3.951  1.00  0.00
I    C
```

```
ATOM    702 HG11 VAL I  80       3.412 -23.121  -3.479  1.00  0.00
I    H
ATOM    703 HG12 VAL I  80       3.174 -21.768  -4.625  1.00  0.00
I    H
ATOM    704 HG13 VAL I  80       4.760 -22.627  -4.562  1.00  0.00
I    H
ATOM    705  CG2 VAL I  80       5.348 -22.082  -1.957  1.00  0.00
I    C
ATOM    706 HG21 VAL I  80       4.795 -22.903  -1.452  1.00  0.00
I    H
ATOM    707 HG22 VAL I  80       6.182 -22.530  -2.540  1.00  0.00
I    H
ATOM    708 HG23 VAL I  80       5.783 -21.436  -1.167  1.00  0.00
I    H
ATOM    709  C   VAL I  80       4.112 -19.141  -4.151  1.00  0.00
I    C
ATOM    710  O   VAL I  80       3.386 -18.525  -3.375  1.00  0.00
I    O
ATOM    711  N   THR I  81       4.133 -18.901  -5.478  1.00  0.00
I    N
ATOM    712  HN  THR I  81       4.615 -19.499  -6.117  1.00  0.00
I    H
ATOM    713  CA  THR I  81       3.469 -17.768  -6.106  1.00  0.00
I    C
ATOM    714  HA  THR I  81       2.730 -17.410  -5.401  1.00  0.00
I    H
ATOM    715  CB  THR I  81       4.424 -16.680  -6.619  1.00  0.00
I    C
ATOM    716  HB  THR I  81       5.133 -17.193  -7.315  1.00  0.00
I    H
ATOM    717  OG1 THR I  81       5.216 -16.106  -5.579  1.00  0.00
I    O
ATOM    718  HG1 THR I  81       5.761 -15.439  -6.010  1.00  0.00
I    H
ATOM    719  CG2 THR I  81       3.636 -15.539  -7.293  1.00  0.00
I    C
ATOM    720 HG21 THR I  81       4.325 -14.807  -7.765  1.00  0.00
I    H
ATOM    721 HG22 THR I  81       2.971 -15.943  -8.085  1.00  0.00
I    H
ATOM    722 HG23 THR I  81       3.006 -14.997  -6.554  1.00  0.00
I    H
ATOM    723  C   THR I  81       2.708 -18.248  -7.317  1.00  0.00
I    C
ATOM    724  O   THR I  81       1.506 -18.056  -7.403  1.00  0.00
I    O
ATOM    725  N   GLY I  82       3.376 -18.863  -8.321  1.00  0.00
I    N
ATOM    726  HN  GLY I  82       4.363 -19.018  -8.293  1.00  0.00
I    H
ATOM    727  CA  GLY I  82       2.692 -19.346  -9.525  1.00  0.00
I    C
ATOM    728  HA1 GLY I  82       1.650 -19.427  -9.250  1.00  0.00
I    H
```

```
ATOM    729  HA2 GLY I  82       3.162 -20.289  -9.769  1.00  0.00
I    H
ATOM    730  C   GLY I  82       2.787 -18.456 -10.730  1.00  0.00
I    C
ATOM    731  O   GLY I  82       2.331 -18.824 -11.807  1.00  0.00
I    O
ATOM    732  N   LEU I  83       3.439 -17.275 -10.621  1.00  0.00
I    N
ATOM    733  HN  LEU I  83       3.736 -16.893  -9.748  1.00  0.00
I    H
ATOM    734  CA  LEU I  83       3.765 -16.450 -11.779  1.00  0.00
I    C
ATOM    735  HA  LEU I  83       2.834 -16.254 -12.294  1.00  0.00
I    H
ATOM    736  CB  LEU I  83       4.467 -15.111 -11.402  1.00  0.00
I    C
ATOM    737  HB1 LEU I  83       5.300 -15.361 -10.706  1.00  0.00
I    H
ATOM    738  HB2 LEU I  83       4.943 -14.683 -12.312  1.00  0.00
I    H
ATOM    739  CG  LEU I  83       3.555 -14.066 -10.728  1.00  0.00
I    C
ATOM    740  HG  LEU I  83       3.070 -14.617  -9.887  1.00  0.00
I    H
ATOM    741  CD1 LEU I  83       4.322 -12.841 -10.205  1.00  0.00
I    C
ATOM    742 HD11 LEU I  83       3.619 -12.097  -9.772  1.00  0.00
I    H
ATOM    743 HD12 LEU I  83       5.045 -13.140  -9.415  1.00  0.00
I    H
ATOM    744 HD13 LEU I  83       4.884 -12.350 -11.027  1.00  0.00
I    H
ATOM    745  CD2 LEU I  83       2.449 -13.576 -11.675  1.00  0.00
I    C
ATOM    746 HD21 LEU I  83       1.796 -12.840 -11.159  1.00  0.00
I    H
ATOM    747 HD22 LEU I  83       2.868 -13.082 -12.574  1.00  0.00
I    H
ATOM    748 HD23 LEU I  83       1.816 -14.427 -12.008  1.00  0.00
I    H
ATOM    749  C   LEU I  83       4.679 -17.196 -12.747  1.00  0.00
I    C
ATOM    750  O   LEU I  83       5.648 -17.838 -12.336  1.00  0.00
I    O
ATOM    751  N   ALA I  84       4.389 -17.126 -14.058  1.00  0.00
I    N
ATOM    752  HN  ALA I  84       3.677 -16.519 -14.411  1.00  0.00
I    H
ATOM    753  CA  ALA I  84       5.076 -17.916 -15.050  1.00  0.00
I    C
ATOM    754  HA  ALA I  84       5.958 -18.384 -14.627  1.00  0.00
I    H
ATOM    755  CB  ALA I  84       4.121 -19.000 -15.587  1.00  0.00
I    C
```

ATOM   756 HB1 ALA I 84   3.810 -19.668 -14.756 1.00 0.00
I   H
ATOM   757 HB2 ALA I 84   3.205 -18.545 -16.021 1.00 0.00
I   H
ATOM   758 HB3 ALA I 84   4.609 -19.625 -16.366 1.00 0.00
I   H
ATOM   759 C   ALA I 84   5.563 -17.037 -16.190 1.00 0.00
I   C
ATOM   760 O   ALA I 84   4.782 -16.453 -16.937 1.00 0.00
I   O
ATOM   761 N   VAL I 85   6.897 -16.929 -16.337 1.00 0.00
I   N
ATOM   762 HN VAL I 85   7.506 -17.261 -15.622 1.00 0.00
I   H
ATOM   763 CA VAL I 85   7.575 -16.354 -17.485 1.00 0.00
I   C
ATOM   764 HA VAL I 85   7.056 -15.435 -17.727 1.00 0.00
I   H
ATOM   765 CB VAL I 85   9.028 -16.035 -17.149 1.00 0.00
I   C
ATOM   766 HB VAL I 85   9.569 -15.873 -18.113 1.00 0.00
I   H
ATOM   767 CG1 VAL I 85   9.043 -14.752 -16.300 1.00 0.00
I   C
ATOM   768 HG11 VAL I 85   10.082 -14.502 -15.999 1.00 0.00
I   H
ATOM   769 HG12 VAL I 85   8.645 -13.884 -16.864 1.00 0.00
I   H
ATOM   770 HG13 VAL I 85   8.440 -14.885 -15.377 1.00 0.00
I   H
ATOM   771 CG2 VAL I 85   9.732 -17.194 -16.408 1.00 0.00
I   C
ATOM   772 HG21 VAL I 85   10.774 -16.914 -16.143 1.00 0.00
I   H
ATOM   773 HG22 VAL I 85   9.189 -17.445 -15.472 1.00 0.00
I   H
ATOM   774 HG23 VAL I 85   9.779 -18.105 -17.039 1.00 0.00
I   H
ATOM   775 C   VAL I 85   7.508 -17.251 -18.708 1.00 0.00
I   C
ATOM   776 O   VAL I 85   7.377 -16.786 -19.836 1.00 0.00
I   O
ATOM   777 N   VAL I 86   7.579 -18.579 -18.497 1.00 0.00
I   N
ATOM   778 HN VAL I 86   7.685 -18.941 -17.574 1.00 0.00
I   H
ATOM   779 CA VAL I 86   7.514 -19.587 -19.530 1.00 0.00
I   C
ATOM   780 HA VAL I 86   7.164 -19.138 -20.452 1.00 0.00
I   H
ATOM   781 CB VAL I 86   8.844 -20.323 -19.752 1.00 0.00
I   C
ATOM   782 HB VAL I 86   8.674 -21.099 -20.539 1.00 0.00
I   H

ATOM 783 CG1 VAL I 86 9.910 −19.317 −20.234 1.00 0.00
I C
ATOM 784 HG11 VAL I 86 10.890 −19.826 −20.362 1.00 0.00
I H
ATOM 785 HG12 VAL I 86 9.639 −18.870 −21.212 1.00 0.00
I H
ATOM 786 HG13 VAL I 86 10.041 −18.500 −19.493 1.00 0.00
I H
ATOM 787 CG2 VAL I 86 9.330 −21.054 −18.476 1.00 0.00
I C
ATOM 788 HG21 VAL I 86 10.311 −21.543 −18.652 1.00 0.00
I H
ATOM 789 HG22 VAL I 86 9.443 −20.335 −17.634 1.00 0.00
I H
ATOM 790 HG23 VAL I 86 8.612 −21.842 −18.166 1.00 0.00
I H
ATOM 791 C VAL I 86 6.468 −20.584 −19.086 1.00 0.00
I C
ATOM 792 O VAL I 86 6.167 −20.687 −17.899 1.00 0.00
I O
ATOM 793 N ASP I 87 5.873 −21.335 −20.032 1.00 0.00
I N
ATOM 794 HN ASP I 87 6.041 −21.210 −21.006 1.00 0.00
I H
ATOM 795 CA ASP I 87 4.931 −22.398 −19.754 1.00 0.00
I C
ATOM 796 HA ASP I 87 4.153 −21.964 −19.137 1.00 0.00
I H
ATOM 797 CB ASP I 87 4.397 −22.904 −21.117 1.00 0.00
I C
ATOM 798 HB1 ASP I 87 4.173 −22.034 −21.767 1.00 0.00
I H
ATOM 799 HB2 ASP I 87 5.155 −23.533 −21.625 1.00 0.00
I H
ATOM 800 CG ASP I 87 3.117 −23.711 −21.002 1.00 0.00
I C
ATOM 801 OD1 ASP I 87 3.083 −24.652 −20.172 1.00 0.00
I O
ATOM 802 OD2 ASP I 87 2.214 −23.467 −21.834 1.00 0.00
I O
ATOM 803 C ASP I 87 5.552 −23.545 −18.943 1.00 0.00
I C
ATOM 804 O ASP I 87 6.682 −23.965 −19.186 1.00 0.00
I O
ATOM 805 N THR I 88 4.810 −24.095 −17.969 1.00 0.00
I N
ATOM 806 HN THR I 88 3.900 −23.755 −17.734 1.00 0.00
I H
ATOM 807 CA THR I 88 5.252 −25.232 −17.166 1.00 0.00
I C
ATOM 808 HA THR I 88 6.309 −25.074 −17.003 1.00 0.00
I H
ATOM 809 CB THR I 88 4.544 −25.323 −15.821 1.00 0.00
I C

```
ATOM    810  HB  THR I  88       3.462 -25.131 -16.026  1.00  0.00
I    H
ATOM    811  OG1 THR I  88       5.038 -24.286 -14.990  1.00  0.00
I    O
ATOM    812  HG1 THR I  88       4.575 -24.387 -14.151  1.00  0.00
I    H
ATOM    813  CG2 THR I  88       4.830 -26.628 -15.056  1.00  0.00
I    C
ATOM    814 HG21 THR I  88       4.205 -26.695 -14.141  1.00  0.00
I    H
ATOM    815 HG22 THR I  88       4.599 -27.510 -15.692  1.00  0.00
I    H
ATOM    816 HG23 THR I  88       5.898 -26.689 -14.753  1.00  0.00
I    H
ATOM    817  C   THR I  88       5.121 -26.541 -17.903  1.00  0.00
I    C
ATOM    818  O   THR I  88       6.005 -27.393 -17.836  1.00  0.00
I    O
ATOM    819  N   GLY I  89       4.022 -26.749 -18.655  1.00  0.00
I    N
ATOM    820  HN  GLY I  89       3.346 -26.031 -18.812  1.00  0.00
I    H
ATOM    821  CA  GLY I  89       3.742 -28.028 -19.288  1.00  0.00
I    C
ATOM    822  HA1 GLY I  89       2.697 -28.013 -19.566  1.00  0.00
I    H
ATOM    823  HA2 GLY I  89       4.000 -28.796 -18.573  1.00  0.00
I    H
ATOM    824  C   GLY I  89       4.546 -28.263 -20.528  1.00  0.00
I    C
ATOM    825  O   GLY I  89       4.866 -29.394 -20.880  1.00  0.00
I    O
ATOM    826  N   THR I  90       4.864 -27.173 -21.252  1.00  0.00
I    N
ATOM    827  HN  THR I  90       4.642 -26.237 -20.979  1.00  0.00
I    H
ATOM    828  CA  THR I  90       5.576 -27.275 -22.527  1.00  0.00
I    C
ATOM    829  HA  THR I  90       5.380 -28.281 -22.873  1.00  0.00
I    H
ATOM    830  CB  THR I  90       5.073 -26.352 -23.653  1.00  0.00
I    C
ATOM    831  HB  THR I  90       5.378 -26.838 -24.611  1.00  0.00
I    H
ATOM    832  OG1 THR I  90       5.652 -25.051 -23.690  1.00  0.00
I    O
ATOM    833  HG1 THR I  90       5.229 -24.604 -24.431  1.00  0.00
I    H
ATOM    834  CG2 THR I  90       3.562 -26.157 -23.527  1.00  0.00
I    C
ATOM    835 HG21 THR I  90       3.163 -25.588 -24.393  1.00  0.00
I    H
ATOM    836 HG22 THR I  90       3.045 -27.140 -23.497  1.00  0.00
I    H
```

```
ATOM    837 HG23 THR I  90       3.306 -25.601 -22.599  1.00  0.00
I    H
ATOM    838  C   THR I  90       7.080 -27.148 -22.402  1.00  0.00
I    C
ATOM    839  O   THR I  90       7.833 -27.831 -23.101  1.00  0.00
I    O
ATOM    840  N   GLN I  91       7.582 -26.205 -21.572  1.00  0.00
I    N
ATOM    841  HN  GLN I  91       6.980 -25.624 -21.028  1.00  0.00
I    H
ATOM    842  CA  GLN I  91       9.005 -25.942 -21.385  1.00  0.00
I    C
ATOM    843  HA  GLN I  91       9.440 -25.874 -22.374  1.00  0.00
I    H
ATOM    844  CB  GLN I  91       9.260 -24.611 -20.631  1.00  0.00
I    C
ATOM    845  HB1 GLN I  91       8.618 -23.839 -21.115  1.00  0.00
I    H
ATOM    846  HB2 GLN I  91       8.896 -24.705 -19.583  1.00  0.00
I    H
ATOM    847  CG  GLN I  91      10.732 -24.123 -20.647  1.00  0.00
I    C
ATOM    848  HG1 GLN I  91      10.886 -23.333 -19.881  1.00  0.00
I    H
ATOM    849  HG2 GLN I  91      11.425 -24.958 -20.419  1.00  0.00
I    H
ATOM    850  CD  GLN I  91      11.124 -23.507 -21.990  1.00  0.00
I    C
ATOM    851  OE1 GLN I  91      10.279 -23.200 -22.834  1.00  0.00
I    O
ATOM    852  NE2 GLN I  91      12.446 -23.338 -22.218  1.00  0.00
I    N
ATOM    853 HE21 GLN I  91      12.704 -22.934 -23.090  1.00  0.00
I    H
ATOM    854 HE22 GLN I  91      13.106 -23.629 -21.534  1.00  0.00
I    H
ATOM    855  C   GLN I  91       9.697 -27.066 -20.661  1.00  0.00
I    C
ATOM    856  O   GLN I  91      10.784 -27.495 -21.046  1.00  0.00
I    O
ATOM    857  N   PHE I  92       9.043 -27.588 -19.620  1.00  0.00
I    N
ATOM    858  HN  PHE I  92       8.112 -27.306 -19.395  1.00  0.00
I    H
ATOM    859  CA  PHE I  92       9.592 -28.576 -18.742  1.00  0.00
I    C
ATOM    860  HA  PHE I  92      10.636 -28.710 -18.997  1.00  0.00
I    H
ATOM    861  CB  PHE I  92       9.324 -28.176 -17.270  1.00  0.00
I    C
ATOM    862  HB1 PHE I  92       8.233 -28.005 -17.132  1.00  0.00
I    H
ATOM    863  HB2 PHE I  92       9.619 -28.991 -16.574  1.00  0.00
I    H
```

```
ATOM    864  CG  PHE I  92      10.043 -26.891 -16.952  1.00  0.00
I    C
ATOM    865  CD1 PHE I  92      11.416 -26.896 -16.659  1.00  0.00
I    C
ATOM    866  HD1 PHE I  92      11.952 -27.834 -16.623  1.00  0.00
I    H
ATOM    867  CE1 PHE I  92      12.098 -25.699 -16.406  1.00  0.00
I    C
ATOM    868  HE1 PHE I  92      13.157 -25.716 -16.190  1.00  0.00
I    H
ATOM    869  CZ  PHE I  92      11.409 -24.480 -16.439  1.00  0.00
I    C
ATOM    870  HZ  PHE I  92      11.933 -23.556 -16.247  1.00  0.00
I    H
ATOM    871  CD2 PHE I  92       9.366 -25.661 -16.988  1.00  0.00
I    C
ATOM    872  HD2 PHE I  92       8.305 -25.634 -17.195  1.00  0.00
I    H
ATOM    873  CE2 PHE I  92      10.040 -24.461 -16.731  1.00  0.00
I    C
ATOM    874  HE2 PHE I  92       9.502 -23.523 -16.759  1.00  0.00
I    H
ATOM    875  C   PHE I  92       8.892 -29.873 -19.055  1.00  0.00
I    C
ATOM    876  O   PHE I  92       7.671 -29.916 -19.179  1.00  0.00
I    O
ATOM    877  N   THR I  93       9.641 -30.978 -19.200  1.00  0.00
I    N
ATOM    878  HN  THR I  93      10.640 -30.960 -19.237  1.00  0.00
I    H
ATOM    879  CA  THR I  93       9.059 -32.314 -19.317  1.00  0.00
I    C
ATOM    880  HA  THR I  93       8.051 -32.195 -19.688  1.00  0.00
I    H
ATOM    881  CB  THR I  93       9.823 -33.276 -20.231  1.00  0.00
I    C
ATOM    882  HB  THR I  93       9.290 -34.256 -20.164  1.00  0.00
I    H
ATOM    883  OG1 THR I  93      11.151 -33.494 -19.781  1.00  0.00
I    O
ATOM    884  HG1 THR I  93      11.543 -34.096 -20.423  1.00  0.00
I    H
ATOM    885  CG2 THR I  93       9.913 -32.633 -21.614  1.00  0.00
I    C
ATOM    886 HG21 THR I  93      10.358 -33.336 -22.349  1.00  0.00
I    H
ATOM    887 HG22 THR I  93       8.901 -32.354 -21.978  1.00  0.00
I    H
ATOM    888 HG23 THR I  93      10.539 -31.714 -21.588  1.00  0.00
I    H
ATOM    889  C   THR I  93       8.939 -32.890 -17.924  1.00  0.00
I    C
ATOM    890  O   THR I  93       9.087 -32.172 -16.933  1.00  0.00
I    O
```

```
ATOM    891  N   VAL I  94       8.650 -34.190 -17.770  1.00  0.00
I    N
ATOM    892  HN  VAL I  94       8.643 -34.815 -18.547  1.00  0.00
I    H
ATOM    893  CA  VAL I  94       8.326 -34.811 -16.493  1.00  0.00
I    C
ATOM    894  HA  VAL I  94       7.426 -34.320 -16.144  1.00  0.00
I    H
ATOM    895  CB  VAL I  94       7.965 -36.288 -16.659  1.00  0.00
I    C
ATOM    896  HB  VAL I  94       8.921 -36.865 -16.712  1.00  0.00
I    H
ATOM    897  CG1 VAL I  94       7.144 -36.729 -15.429  1.00  0.00
I    C
ATOM    898 HG11 VAL I  94       6.830 -37.790 -15.534  1.00  0.00
I    H
ATOM    899 HG12 VAL I  94       7.734 -36.651 -14.492  1.00  0.00
I    H
ATOM    900 HG13 VAL I  94       6.227 -36.110 -15.325  1.00  0.00
I    H
ATOM    901  CG2 VAL I  94       7.133 -36.505 -17.945  1.00  0.00
I    C
ATOM    902 HG21 VAL I  94       6.836 -37.570 -18.047  1.00  0.00
I    H
ATOM    903 HG22 VAL I  94       6.210 -35.885 -17.922  1.00  0.00
I    H
ATOM    904 HG23 VAL I  94       7.713 -36.231 -18.850  1.00  0.00
I    H
ATOM    905  C   VAL I  94       9.402 -34.632 -15.405  1.00  0.00
I    C
ATOM    906  O   VAL I  94       9.092 -34.351 -14.250  1.00  0.00
I    O
ATOM    907  N   PHE I  95      10.711 -34.715 -15.769  1.00  0.00
I    N
ATOM    908  HN  PHE I  95      10.992 -35.019 -16.678  1.00  0.00
I    H
ATOM    909  CA  PHE I  95      11.816 -34.373 -14.877  1.00  0.00
I    C
ATOM    910  HA  PHE I  95      11.665 -34.962 -13.982  1.00  0.00
I    H
ATOM    911  CB  PHE I  95      13.199 -34.745 -15.497  1.00  0.00
I    C
ATOM    912  HB1 PHE I  95      13.205 -35.829 -15.748  1.00  0.00
I    H
ATOM    913  HB2 PHE I  95      13.376 -34.189 -16.443  1.00  0.00
I    H
ATOM    914  CG  PHE I  95      14.303 -34.492 -14.493  1.00  0.00
I    C
ATOM    915  CD1 PHE I  95      14.407 -35.263 -13.320  1.00  0.00
I    C
ATOM    916  HD1 PHE I  95      13.732 -36.093 -13.163  1.00  0.00
I    H
ATOM    917  CE1 PHE I  95      15.374 -34.962 -12.350  1.00  0.00
I    C
```

```
ATOM    918  HE1 PHE I  95      15.450 -35.565 -11.457  1.00  0.00
I    H
ATOM    919  CZ  PHE I  95      16.246 -33.883 -12.543  1.00  0.00
I    C
ATOM    920  HZ  PHE I  95      17.008 -33.664 -11.809  1.00  0.00
I    H
ATOM    921  CD2 PHE I  95      15.167 -33.398 -14.658  1.00  0.00
I    C
ATOM    922  HD2 PHE I  95      15.076 -32.764 -15.529  1.00  0.00
I    H
ATOM    923  CE2 PHE I  95      16.135 -33.094 -13.693  1.00  0.00
I    C
ATOM    924  HE2 PHE I  95      16.794 -32.249 -13.839  1.00  0.00
I    H
ATOM    925  C   PHE I  95      11.783 -32.896 -14.455  1.00  0.00
I    C
ATOM    926  O   PHE I  95      11.854 -32.571 -13.272  1.00  0.00
I    O
ATOM    927  N   GLY I  96      11.588 -31.949 -15.395  1.00  0.00
I    N
ATOM    928  HN  GLY I  96      11.464 -32.161 -16.364  1.00  0.00
I    H
ATOM    929  CA  GLY I  96      11.546 -30.533 -15.048  1.00  0.00
I    C
ATOM    930  HA1 GLY I  96      11.468 -29.986 -15.978  1.00  0.00
I    H
ATOM    931  HA2 GLY I  96      12.443 -30.320 -14.481  1.00  0.00
I    H
ATOM    932  C   GLY I  96      10.355 -30.170 -14.190  1.00  0.00
I    C
ATOM    933  O   GLY I  96      10.445 -29.353 -13.285  1.00  0.00
I    O
ATOM    934  N   GLN I  97       9.201 -30.819 -14.436  1.00  0.00
I    N
ATOM    935  HN  GLN I  97       9.138 -31.484 -15.177  1.00  0.00
I    H
ATOM    936  CA  GLN I  97       7.972 -30.637 -13.686  1.00  0.00
I    C
ATOM    937  HA  GLN I  97       7.827 -29.566 -13.621  1.00  0.00
I    H
ATOM    938  CB  GLN I  97       6.778 -31.246 -14.457  1.00  0.00
I    C
ATOM    939  HB1 GLN I  97       7.003 -32.328 -14.598  1.00  0.00
I    H
ATOM    940  HB2 GLN I  97       5.865 -31.199 -13.820  1.00  0.00
I    H
ATOM    941  CG  GLN I  97       6.524 -30.586 -15.837  1.00  0.00
I    C
ATOM    942  HG1 GLN I  97       6.077 -29.577 -15.708  1.00  0.00
I    H
ATOM    943  HG2 GLN I  97       7.475 -30.466 -16.396  1.00  0.00
I    H
ATOM    944  CD  GLN I  97       5.543 -31.398 -16.686  1.00  0.00
I    C
```

```
ATOM    945  OE1 GLN I  97       4.972 -32.404 -16.270  1.00  0.00
I    O
ATOM    946  NE2 GLN I  97       5.347 -30.964 -17.949  1.00  0.00
I    N
ATOM    947 HE21 GLN I  97       4.707 -31.484 -18.509  1.00  0.00
I    H
ATOM    948 HE22 GLN I  97       5.845 -30.171 -18.281  1.00  0.00
I    H
ATOM    949  C   GLN I  97       8.022 -31.185 -12.254  1.00  0.00
I    C
ATOM    950  O   GLN I  97       7.424 -30.624 -11.337  1.00  0.00
I    O
ATOM    951  N   THR I  98       8.746 -32.301 -12.001  1.00  0.00
I    N
ATOM    952  HN  THR I  98       9.195 -32.827 -12.722  1.00  0.00
I    H
ATOM    953  CA  THR I  98       8.939 -32.842 -10.648  1.00  0.00
I    C
ATOM    954  HA  THR I  98       7.963 -32.803 -10.181  1.00  0.00
I    H
ATOM    955  CB  THR I  98       9.318 -34.330 -10.593  1.00  0.00
I    C
ATOM    956  HB  THR I  98       8.511 -34.876 -11.139  1.00  0.00
I    H
ATOM    957  OG1 THR I  98       9.334 -34.842  -9.270  1.00  0.00
I    O
ATOM    958  HG1 THR I  98       9.593 -35.765  -9.355  1.00  0.00
I    H
ATOM    959  CG2 THR I  98      10.690 -34.605 -11.206  1.00  0.00
I    C
ATOM    960 HG21 THR I  98      10.890 -35.696 -11.256  1.00  0.00
I    H
ATOM    961 HG22 THR I  98      10.742 -34.201 -12.240  1.00  0.00
I    H
ATOM    962 HG23 THR I  98      11.498 -34.132 -10.607  1.00  0.00
I    H
ATOM    963  C   THR I  98       9.862 -31.971  -9.797  1.00  0.00
I    C
ATOM    964  O   THR I  98       9.655 -31.821  -8.596  1.00  0.00
I    O
ATOM    965  N   VAL I  99      10.872 -31.287 -10.392  1.00  0.00
I    N
ATOM    966  HN  VAL I  99      11.130 -31.421 -11.345  1.00  0.00
I    H
ATOM    967  CA  VAL I  99      11.674 -30.285  -9.681  1.00  0.00
I    C
ATOM    968  HA  VAL I  99      12.100 -30.773  -8.814  1.00  0.00
I    H
ATOM    969  CB  VAL I  99      12.821 -29.748 -10.540  1.00  0.00
I    C
ATOM    970  HB  VAL I  99      12.370 -29.263 -11.441  1.00  0.00
I    H
ATOM    971  CG1 VAL I  99      13.659 -28.696  -9.776  1.00  0.00
I    C
```

```
ATOM    972 HG11 VAL I  99      14.508 -28.349 -10.405  1.00  0.00
I    H
ATOM    973 HG12 VAL I  99      13.055 -27.802  -9.516  1.00  0.00
I    H
ATOM    974 HG13 VAL I  99      14.077 -29.128  -8.842  1.00  0.00
I    H
ATOM    975  CG2 VAL I  99      13.741 -30.917 -10.943  1.00  0.00
I    C
ATOM    976 HG21 VAL I  99      14.607 -30.550 -11.535  1.00  0.00
I    H
ATOM    977 HG22 VAL I  99      14.130 -31.436 -10.041  1.00  0.00
I    H
ATOM    978 HG23 VAL I  99      13.200 -31.658 -11.567  1.00  0.00
I    H
ATOM    979  C   VAL I  99      10.813 -29.126  -9.178  1.00  0.00
I    C
ATOM    980  O   VAL I  99      10.964 -28.651  -8.054  1.00  0.00
I    O
ATOM    981  N   ILE I 100       9.834 -28.688  -9.997  1.00  0.00
I    N
ATOM    982  HN  ILE I 100       9.765 -29.008 -10.939  1.00  0.00
I    H
ATOM    983  CA  ILE I 100       8.799 -27.731  -9.613  1.00  0.00
I    C
ATOM    984  HA  ILE I 100       9.330 -26.859  -9.252  1.00  0.00
I    H
ATOM    985  CB  ILE I 100       7.943 -27.323 -10.815  1.00  0.00
I    C
ATOM    986  HB  ILE I 100       7.554 -28.290 -11.223  1.00  0.00
I    H
ATOM    987  CG2 ILE I 100       6.788 -26.374 -10.416  1.00  0.00
I    C
ATOM    988 HG21 ILE I 100       6.096 -26.219 -11.270  1.00  0.00
I    H
ATOM    989 HG22 ILE I 100       6.198 -26.799  -9.577  1.00  0.00
I    H
ATOM    990 HG23 ILE I 100       7.179 -25.382 -10.103  1.00  0.00
I    H
ATOM    991  CG1 ILE I 100       8.810 -26.690 -11.931  1.00  0.00
I    C
ATOM    992 HG11 ILE I 100       9.514 -27.450 -12.338  1.00  0.00
I    H
ATOM    993 HG12 ILE I 100       8.146 -26.375 -12.769  1.00  0.00
I    H
ATOM    994  CD  ILE I 100       9.624 -25.462 -11.506  1.00  0.00
I    C
ATOM    995  HD1 ILE I 100      10.169 -25.039 -12.378  1.00  0.00
I    H
ATOM    996  HD2 ILE I 100       8.957 -24.672 -11.096  1.00  0.00
I    H
ATOM    997  HD3 ILE I 100      10.372 -25.729 -10.730  1.00  0.00
I    H
ATOM    998  C   ILE I 100       7.926 -28.224  -8.463  1.00  0.00
I    C
```

```
ATOM    999  O   ILE I 100       7.726 -27.493  -7.496  1.00  0.00
I    O
ATOM   1000  N   MET I 101       7.446 -29.491  -8.468  1.00  0.00
I    N
ATOM   1001  HN  MET I 101       7.527 -30.105  -9.251  1.00  0.00
I    H
ATOM   1002  CA  MET I 101       6.761 -30.073  -7.313  1.00  0.00
I    C
ATOM   1003  HA  MET I 101       5.913 -29.430  -7.109  1.00  0.00
I    H
ATOM   1004  CB  MET I 101       6.346 -31.539  -7.563  1.00  0.00
I    C
ATOM   1005  HB1 MET I 101       7.135 -31.993  -8.209  1.00  0.00
I    H
ATOM   1006  HB2 MET I 101       6.371 -32.099  -6.602  1.00  0.00
I    H
ATOM   1007  CG  MET I 101       4.986 -31.679  -8.251  1.00  0.00
I    C
ATOM   1008  HG1 MET I 101       4.166 -31.482  -7.529  1.00  0.00
I    H
ATOM   1009  HG2 MET I 101       4.901 -30.929  -9.068  1.00  0.00
I    H
ATOM   1010  SD  MET I 101       4.665 -33.325  -8.976  1.00  0.00
I    S
ATOM   1011  CE  MET I 101       5.706 -34.406  -7.946  1.00  0.00
I    C
ATOM   1012  HE1 MET I 101       5.626 -35.464  -8.276  1.00  0.00
I    H
ATOM   1013  HE2 MET I 101       6.775 -34.108  -8.010  1.00  0.00
I    H
ATOM   1014  HE3 MET I 101       5.400 -34.357  -6.879  1.00  0.00
I    H
ATOM   1015  C   MET I 101       7.619 -30.103  -6.069  1.00  0.00
I    C
ATOM   1016  O   MET I 101       7.159 -29.742  -4.989  1.00  0.00
I    O
ATOM   1017  N   GLY I 102       8.901 -30.507  -6.207  1.00  0.00
I    N
ATOM   1018  HN  GLY I 102       9.208 -30.911  -7.067  1.00  0.00
I    H
ATOM   1019  CA  GLY I 102       9.913 -30.398  -5.165  1.00  0.00
I    C
ATOM   1020  HA1 GLY I 102      10.864 -30.614  -5.632  1.00  0.00
I    H
ATOM   1021  HA2 GLY I 102       9.636 -31.090  -4.382  1.00  0.00
I    H
ATOM   1022  C   GLY I 102       9.993 -29.026  -4.563  1.00  0.00
I    C
ATOM   1023  O   GLY I 102       9.789 -28.867  -3.366  1.00  0.00
I    O
ATOM   1024  N   LEU I 103      10.225 -27.980  -5.370  1.00  0.00
I    N
ATOM   1025  HN  LEU I 103      10.371 -28.088  -6.352  1.00  0.00
I    H
```

```
ATOM   1026  CA  LEU I 103      10.287 -26.605  -4.905  1.00  0.00
I    C
ATOM   1027  HA  LEU I 103      11.065 -26.598  -4.152  1.00  0.00
I    H
ATOM   1028  CB  LEU I 103      10.667 -25.680  -6.081  1.00  0.00
I    C
ATOM   1029  HB1 LEU I 103       9.982 -25.923  -6.925  1.00  0.00
I    H
ATOM   1030  HB2 LEU I 103      10.444 -24.627  -5.798  1.00  0.00
I    H
ATOM   1031  CG  LEU I 103      12.127 -25.834  -6.548  1.00  0.00
I    C
ATOM   1032  HG  LEU I 103      12.364 -26.925  -6.521  1.00  0.00
I    H
ATOM   1033  CD1 LEU I 103      12.277 -25.309  -7.983  1.00  0.00
I    C
ATOM   1034 HD11 LEU I 103      13.337 -25.371  -8.312  1.00  0.00
I    H
ATOM   1035 HD12 LEU I 103      11.657 -25.907  -8.686  1.00  0.00
I    H
ATOM   1036 HD13 LEU I 103      11.956 -24.247  -8.049  1.00  0.00
I    H
ATOM   1037  CD2 LEU I 103      13.101 -25.113  -5.600  1.00  0.00
I    C
ATOM   1038 HD21 LEU I 103      14.149 -25.253  -5.944  1.00  0.00
I    H
ATOM   1039 HD22 LEU I 103      12.902 -24.023  -5.560  1.00  0.00
I    H
ATOM   1040 HD23 LEU I 103      13.017 -25.522  -4.570  1.00  0.00
I    H
ATOM   1041  C   LEU I 103       9.004 -26.094  -4.249  1.00  0.00
I    C
ATOM   1042  O   LEU I 103       9.063 -25.486  -3.184  1.00  0.00
I    O
ATOM   1043  N   ILE I 104       7.816 -26.381  -4.838  1.00  0.00
I    N
ATOM   1044  HN  ILE I 104       7.770 -26.854  -5.716  1.00  0.00
I    H
ATOM   1045  CA  ILE I 104       6.508 -26.040  -4.271  1.00  0.00
I    C
ATOM   1046  HA  ILE I 104       6.523 -24.972  -4.095  1.00  0.00
I    H
ATOM   1047  CB  ILE I 104       5.360 -26.460  -5.206  1.00  0.00
I    C
ATOM   1048  HB  ILE I 104       5.624 -27.501  -5.523  1.00  0.00
I    H
ATOM   1049  CG2 ILE I 104       3.987 -26.373  -4.491  1.00  0.00
I    C
ATOM   1050 HG21 ILE I 104       3.190 -26.824  -5.120  1.00  0.00
I    H
ATOM   1051 HG22 ILE I 104       4.006 -26.924  -3.527  1.00  0.00
I    H
ATOM   1052 HG23 ILE I 104       3.711 -25.318  -4.286  1.00  0.00
I    H
```

```
ATOM   1053  CG1 ILE I 104       5.351 -25.593  -6.489  1.00  0.00
I    C
ATOM   1054 HG11 ILE I 104       5.043 -24.553  -6.238  1.00  0.00
I    H
ATOM   1055 HG12 ILE I 104       6.386 -25.540  -6.897  1.00  0.00
I    H
ATOM   1056  CD  ILE I 104       4.424 -26.150  -7.580  1.00  0.00
I    C
ATOM   1057  HD1 ILE I 104       4.494 -25.529  -8.499  1.00  0.00
I    H
ATOM   1058  HD2 ILE I 104       4.709 -27.192  -7.841  1.00  0.00
I    H
ATOM   1059  HD3 ILE I 104       3.366 -26.147  -7.243  1.00  0.00
I    H
ATOM   1060  C   ILE I 104       6.298 -26.703  -2.923  1.00  0.00
I    C
ATOM   1061  O   ILE I 104       5.918 -26.057  -1.943  1.00  0.00
I    O
ATOM   1062  N   GLN I 105       6.587 -28.020  -2.833  1.00  0.00
I    N
ATOM   1063  HN  GLN I 105       6.885 -28.562  -3.616  1.00  0.00
I    H
ATOM   1064  CA  GLN I 105       6.490 -28.761  -1.595  1.00  0.00
I    C
ATOM   1065  HA  GLN I 105       5.499 -28.580  -1.197  1.00  0.00
I    H
ATOM   1066  CB  GLN I 105       6.697 -30.282  -1.856  1.00  0.00
I    C
ATOM   1067  HB1 GLN I 105       5.967 -30.575  -2.646  1.00  0.00
I    H
ATOM   1068  HB2 GLN I 105       7.709 -30.447  -2.291  1.00  0.00
I    H
ATOM   1069  CG  GLN I 105       6.495 -31.218  -0.629  1.00  0.00
I    C
ATOM   1070  HG1 GLN I 105       6.820 -32.252  -0.873  1.00  0.00
I    H
ATOM   1071  HG2 GLN I 105       7.096 -30.868   0.235  1.00  0.00
I    H
ATOM   1072  CD  GLN I 105       5.054 -31.391  -0.112  1.00  0.00
I    C
ATOM   1073  OE1 GLN I 105       4.873 -31.869   1.003  1.00  0.00
I    O
ATOM   1074  NE2 GLN I 105       4.050 -31.048  -0.937  1.00  0.00
I    N
ATOM   1075 HE21 GLN I 105       3.123 -31.161  -0.589  1.00  0.00
I    H
ATOM   1076 HE22 GLN I 105       4.253 -30.716  -1.852  1.00  0.00
I    H
ATOM   1077  C   GLN I 105       7.494 -28.261  -0.566  1.00  0.00
I    C
ATOM   1078  O   GLN I 105       7.104 -27.845   0.516  1.00  0.00
I    O
ATOM   1079  N   ILE I 106       8.804 -28.187  -0.897  1.00  0.00
I    N
```

```
ATOM   1080  HN  ILE I 106       9.131 -28.407  -1.813  1.00  0.00
I    H
ATOM   1081  CA  ILE I 106       9.857 -27.785   0.035  1.00  0.00
I    C
ATOM   1082  HA  ILE I 106       9.764 -28.446   0.887  1.00  0.00
I    H
ATOM   1083  CB  ILE I 106      11.249 -27.910  -0.605  1.00  0.00
I    C
ATOM   1084  HB  ILE I 106      11.147 -27.404  -1.599  1.00  0.00
I    H
ATOM   1085  CG2 ILE I 106      12.354 -27.275   0.280  1.00  0.00
I    C
ATOM   1086 HG21 ILE I 106      13.319 -27.238  -0.267  1.00  0.00
I    H
ATOM   1087 HG22 ILE I 106      12.086 -26.235   0.562  1.00  0.00
I    H
ATOM   1088 HG23 ILE I 106      12.505 -27.863   1.210  1.00  0.00
I    H
ATOM   1089  CG1 ILE I 106      11.601 -29.391  -0.897  1.00  0.00
I    C
ATOM   1090 HG11 ILE I 106      11.827 -29.920   0.055  1.00  0.00
I    H
ATOM   1091 HG12 ILE I 106      10.713 -29.891  -1.348  1.00  0.00
I    H
ATOM   1092  CD  ILE I 106      12.790 -29.528  -1.860  1.00  0.00
I    C
ATOM   1093  HD1 ILE I 106      12.977 -30.599  -2.092  1.00  0.00
I    H
ATOM   1094  HD2 ILE I 106      12.584 -28.996  -2.814  1.00  0.00
I    H
ATOM   1095  HD3 ILE I 106      13.714 -29.107  -1.413  1.00  0.00
I    H
ATOM   1096  C   ILE I 106       9.667 -26.366   0.556  1.00  0.00
I    C
ATOM   1097  O   ILE I 106       9.726 -26.123   1.761  1.00  0.00
I    O
ATOM   1098  N   GLY I 107       9.384 -25.389  -0.332  1.00  0.00
I    N
ATOM   1099  HN  GLY I 107       9.246 -25.584  -1.302  1.00  0.00
I    H
ATOM   1100  CA  GLY I 107       9.258 -23.987   0.053  1.00  0.00
I    C
ATOM   1101  HA1 GLY I 107       9.300 -23.416  -0.865  1.00  0.00
I    H
ATOM   1102  HA2 GLY I 107      10.068 -23.783   0.740  1.00  0.00
I    H
ATOM   1103  C   GLY I 107       7.964 -23.658   0.748  1.00  0.00
I    C
ATOM   1104  O   GLY I 107       7.927 -22.888   1.709  1.00  0.00
I    O
ATOM   1105  N   GLY I 108       6.843 -24.262   0.300  1.00  0.00
I    N
ATOM   1106  HN  GLY I 108       6.858 -24.896  -0.472  1.00  0.00
I    H
```

```
ATOM   1107  CA  GLY I 108       5.537 -24.036   0.906  1.00  0.00
I    C
ATOM   1108  HA1 GLY I 108       4.806 -24.436   0.217  1.00  0.00
I    H
ATOM   1109  HA2 GLY I 108       5.452 -22.971   1.075  1.00  0.00
I    H
ATOM   1110  C   GLY I 108       5.359 -24.730   2.226  1.00  0.00
I    C
ATOM   1111  O   GLY I 108       4.875 -24.131   3.182  1.00  0.00
I    O
ATOM   1112  N   LEU I 109       5.785 -26.008   2.328  1.00  0.00
I    N
ATOM   1113  HN  LEU I 109       6.109 -26.521   1.537  1.00  0.00
I    H
ATOM   1114  CA  LEU I 109       5.814 -26.756   3.578  1.00  0.00
I    C
ATOM   1115  HA  LEU I 109       4.827 -26.688   4.016  1.00  0.00
I    H
ATOM   1116  CB  LEU I 109       6.240 -28.223   3.285  1.00  0.00
I    C
ATOM   1117  HB1 LEU I 109       5.598 -28.592   2.453  1.00  0.00
I    H
ATOM   1118  HB2 LEU I 109       7.282 -28.226   2.894  1.00  0.00
I    H
ATOM   1119  CG  LEU I 109       6.159 -29.296   4.392  1.00  0.00
I    C
ATOM   1120  HG  LEU I 109       5.166 -29.200   4.895  1.00  0.00
I    H
ATOM   1121  CD1 LEU I 109       6.307 -30.665   3.722  1.00  0.00
I    C
ATOM   1122 HD11 LEU I 109       6.304 -31.474   4.483  1.00  0.00
I    H
ATOM   1123 HD12 LEU I 109       5.469 -30.847   3.016  1.00  0.00
I    H
ATOM   1124 HD13 LEU I 109       7.261 -30.725   3.157  1.00  0.00
I    H
ATOM   1125  CD2 LEU I 109       7.247 -29.186   5.468  1.00  0.00
I    C
ATOM   1126 HD21 LEU I 109       7.112 -29.978   6.237  1.00  0.00
I    H
ATOM   1127 HD22 LEU I 109       8.262 -29.305   5.038  1.00  0.00
I    H
ATOM   1128 HD23 LEU I 109       7.195 -28.198   5.974  1.00  0.00
I    H
ATOM   1129  C   LEU I 109       6.789 -26.112   4.544  1.00  0.00
I    C
ATOM   1130  O   LEU I 109       6.466 -25.855   5.702  1.00  0.00
I    O
ATOM   1131  N   GLY I 110       8.005 -25.765   4.063  1.00  0.00
I    N
ATOM   1132  HN  GLY I 110       8.226 -25.867   3.094  1.00  0.00
I    H
ATOM   1133  CA  GLY I 110       9.069 -25.227   4.897  1.00  0.00
I    C
```

ATOM 1134 HA1 GLY I 110 9.928 -25.103 4.252 1.00 0.00
I H
ATOM 1135 HA2 GLY I 110 9.217 -25.934 5.702 1.00 0.00
I H
ATOM 1136 C GLY I 110 8.750 -23.898 5.503 1.00 0.00
I C
ATOM 1137 O GLY I 110 9.056 -23.646 6.658 1.00 0.00
I O
ATOM 1138 N PHE I 111 8.047 -23.005 4.785 1.00 0.00
I N
ATOM 1139 HN PHE I 111 7.807 -23.103 3.821 1.00 0.00
I H
ATOM 1140 CA PHE I 111 7.569 -21.781 5.403 1.00 0.00
I C
ATOM 1141 HA PHE I 111 8.442 -21.327 5.855 1.00 0.00
I H
ATOM 1142 CB PHE I 111 6.972 -20.825 4.343 1.00 0.00
I C
ATOM 1143 HB1 PHE I 111 7.691 -20.721 3.500 1.00 0.00
I H
ATOM 1144 HB2 PHE I 111 6.029 -21.235 3.920 1.00 0.00
I H
ATOM 1145 CG PHE I 111 6.753 -19.447 4.939 1.00 0.00
I C
ATOM 1146 CD1 PHE I 111 7.837 -18.558 5.061 1.00 0.00
I C
ATOM 1147 HD1 PHE I 111 8.806 -18.840 4.673 1.00 0.00
I H
ATOM 1148 CE1 PHE I 111 7.677 -17.314 5.686 1.00 0.00
I C
ATOM 1149 HE1 PHE I 111 8.519 -16.643 5.778 1.00 0.00
I H
ATOM 1150 CZ PHE I 111 6.426 -16.936 6.184 1.00 0.00
I C
ATOM 1151 HZ PHE I 111 6.295 -15.964 6.638 1.00 0.00
I H
ATOM 1152 CD2 PHE I 111 5.507 -19.067 5.478 1.00 0.00
I C
ATOM 1153 HD2 PHE I 111 4.672 -19.752 5.445 1.00 0.00
I H
ATOM 1154 CE2 PHE I 111 5.342 -17.814 6.085 1.00 0.00
I C
ATOM 1155 HE2 PHE I 111 4.376 -17.529 6.478 1.00 0.00
I H
ATOM 1156 C PHE I 111 6.558 -22.031 6.527 1.00 0.00
I C
ATOM 1157 O PHE I 111 6.620 -21.384 7.570 1.00 0.00
I O
ATOM 1158 N MET I 112 5.629 -23.005 6.362 1.00 0.00
I N
ATOM 1159 HN MET I 112 5.516 -23.510 5.510 1.00 0.00
I H
ATOM 1160 CA MET I 112 4.713 -23.399 7.421 1.00 0.00
I C

```
ATOM   1161  HA  MET I 112       4.154 -22.509   7.684  1.00  0.00
I    H
ATOM   1162  CB  MET I 112       3.737 -24.529   7.011  1.00  0.00
I    C
ATOM   1163  HB1 MET I 112       4.251 -25.132   6.226  1.00  0.00
I    H
ATOM   1164  HB2 MET I 112       3.580 -25.213   7.876  1.00  0.00
I    H
ATOM   1165  CG  MET I 112       2.407 -24.007   6.464  1.00  0.00
I    C
ATOM   1166  HG1 MET I 112       2.095 -23.094   7.013  1.00  0.00
I    H
ATOM   1167  HG2 MET I 112       2.526 -23.731   5.393  1.00  0.00
I    H
ATOM   1168  SD  MET I 112       1.033 -25.198   6.587  1.00  0.00
I    S
ATOM   1169  CE  MET I 112       1.816 -26.665   5.848  1.00  0.00
I    C
ATOM   1170  HE1 MET I 112       1.109 -27.523   5.823  1.00  0.00
I    H
ATOM   1171  HE2 MET I 112       2.141 -26.461   4.804  1.00  0.00
I    H
ATOM   1172  HE3 MET I 112       2.710 -26.977   6.430  1.00  0.00
I    H
ATOM   1173  C   MET I 112       5.417 -23.879   8.665  1.00  0.00
I    C
ATOM   1174  O   MET I 112       5.021 -23.501   9.759  1.00  0.00
I    O
ATOM   1175  N   THR I 113       6.488 -24.699   8.546  1.00  0.00
I    N
ATOM   1176  HN  THR I 113       6.865 -25.021   7.678  1.00  0.00
I    H
ATOM   1177  CA  THR I 113       7.195 -25.191   9.729  1.00  0.00
I    C
ATOM   1178  HA  THR I 113       6.442 -25.670  10.340  1.00  0.00
I    H
ATOM   1179  CB  THR I 113       8.289 -26.240   9.464  1.00  0.00
I    C
ATOM   1180  HB  THR I 113       8.709 -26.509  10.464  1.00  0.00
I    H
ATOM   1181  OG1 THR I 113       9.381 -25.760   8.697  1.00  0.00
I    O
ATOM   1182  HG1 THR I 113       9.975 -26.512   8.599  1.00  0.00
I    H
ATOM   1183  CG2 THR I 113       7.690 -27.391   8.654  1.00  0.00
I    C
ATOM   1184 HG21 THR I 113       8.423 -28.218   8.542  1.00  0.00
I    H
ATOM   1185 HG22 THR I 113       6.791 -27.797   9.165  1.00  0.00
I    H
ATOM   1186 HG23 THR I 113       7.390 -27.053   7.639  1.00  0.00
I    H
ATOM   1187  C   THR I 113       7.763 -24.056  10.555  1.00  0.00
I    C
```

```
ATOM   1188  O   THR I 113       7.449 -23.929  11.739  1.00  0.00
I    O
ATOM   1189  N   PHE I 114       8.509 -23.128   9.940  1.00  0.00
I    N
ATOM   1190  HN  PHE I 114       8.744 -23.192   8.971  1.00  0.00
I    H
ATOM   1191  CA  PHE I 114       9.039 -21.956  10.611  1.00  0.00
I    C
ATOM   1192  HA  PHE I 114       9.559 -22.349  11.475  1.00  0.00
I    H
ATOM   1193  CB  PHE I 114       9.990 -21.175   9.687  1.00  0.00
I    C
ATOM   1194  HB1 PHE I 114       9.538 -21.104   8.674  1.00  0.00
I    H
ATOM   1195  HB2 PHE I 114      10.139 -20.136  10.052  1.00  0.00
I    H
ATOM   1196  CG  PHE I 114      11.314 -21.874   9.534  1.00  0.00
I    C
ATOM   1197  CD1 PHE I 114      12.319 -21.726  10.506  1.00  0.00
I    C
ATOM   1198  HD1 PHE I 114      12.123 -21.138  11.392  1.00  0.00
I    H
ATOM   1199  CE1 PHE I 114      13.574 -22.327  10.331  1.00  0.00
I    C
ATOM   1200  HE1 PHE I 114      14.341 -22.205  11.083  1.00  0.00
I    H
ATOM   1201  CZ  PHE I 114      13.833 -23.092   9.186  1.00  0.00
I    C
ATOM   1202  HZ  PHE I 114      14.792 -23.575   9.061  1.00  0.00
I    H
ATOM   1203  CD2 PHE I 114      11.604 -22.616   8.381  1.00  0.00
I    C
ATOM   1204  HD2 PHE I 114      10.868 -22.699   7.593  1.00  0.00
I    H
ATOM   1205  CE2 PHE I 114      12.845 -23.238   8.208  1.00  0.00
I    C
ATOM   1206  HE2 PHE I 114      13.033 -23.826   7.320  1.00  0.00
I    H
ATOM   1207  C   PHE I 114       7.985 -20.979  11.128  1.00  0.00
I    C
ATOM   1208  O   PHE I 114       8.107 -20.459  12.236  1.00  0.00
I    O
ATOM   1209  N   ALA I 115       6.916 -20.703  10.348  1.00  0.00
I    N
ATOM   1210  HN  ALA I 115       6.843 -21.084   9.427  1.00  0.00
I    H
ATOM   1211  CA  ALA I 115       5.811 -19.855  10.762  1.00  0.00
I    C
ATOM   1212  HA  ALA I 115       6.187 -18.885  11.069  1.00  0.00
I    H
ATOM   1213  CB  ALA I 115       4.842 -19.610   9.585  1.00  0.00
I    C
ATOM   1214  HB1 ALA I 115       5.395 -19.154   8.736  1.00  0.00
I    H
```

```
ATOM   1215  HB2 ALA I 115       4.397 -20.565   9.229  1.00  0.00
I    H
ATOM   1216  HB3 ALA I 115       4.018 -18.920   9.869  1.00  0.00
I    H
ATOM   1217  C   ALA I 115       5.047 -20.419  11.959  1.00  0.00
I    C
ATOM   1218  O   ALA I 115       4.713 -19.696  12.895  1.00  0.00
I    O
ATOM   1219  N   VAL I 116       4.813 -21.752  11.983  1.00  0.00
I    N
ATOM   1220  HN  VAL I 116       4.988 -22.311  11.176  1.00  0.00
I    H
ATOM   1221  CA  VAL I 116       4.304 -22.492  13.133  1.00  0.00
I    C
ATOM   1222  HA  VAL I 116       3.353 -22.050  13.403  1.00  0.00
I    H
ATOM   1223  CB  VAL I 116       4.035 -23.959  12.769  1.00  0.00
I    C
ATOM   1224  HB  VAL I 116       4.900 -24.319  12.161  1.00  0.00
I    H
ATOM   1225  CG1 VAL I 116       3.902 -24.895  13.993  1.00  0.00
I    C
ATOM   1226 HG11 VAL I 116       3.670 -25.930  13.663  1.00  0.00
I    H
ATOM   1227 HG12 VAL I 116       4.843 -24.938  14.580  1.00  0.00
I    H
ATOM   1228 HG13 VAL I 116       3.081 -24.555  14.660  1.00  0.00
I    H
ATOM   1229  CG2 VAL I 116       2.735 -23.987  11.939  1.00  0.00
I    C
ATOM   1230 HG21 VAL I 116       2.460 -25.030  11.675  1.00  0.00
I    H
ATOM   1231 HG22 VAL I 116       1.895 -23.539  12.514  1.00  0.00
I    H
ATOM   1232 HG23 VAL I 116       2.850 -23.420  10.991  1.00  0.00
I    H
ATOM   1233  C   VAL I 116       5.228 -22.360  14.336  1.00  0.00
I    C
ATOM   1234  O   VAL I 116       4.766 -22.080  15.440  1.00  0.00
I    O
ATOM   1235  N   LEU I 117       6.565 -22.471  14.169  1.00  0.00
I    N
ATOM   1236  HN  LEU I 117       6.990 -22.708  13.298  1.00  0.00
I    H
ATOM   1237  CA  LEU I 117       7.512 -22.251  15.258  1.00  0.00
I    C
ATOM   1238  HA  LEU I 117       7.220 -22.943  16.038  1.00  0.00
I    H
ATOM   1239  CB  LEU I 117       8.975 -22.603  14.878  1.00  0.00
I    C
ATOM   1240  HB1 LEU I 117       9.247 -21.974  13.998  1.00  0.00
I    H
ATOM   1241  HB2 LEU I 117       9.650 -22.286  15.704  1.00  0.00
I    H
```

```
ATOM   1242  CG  LEU I 117       9.207 -24.090  14.522  1.00  0.00
I    C
ATOM   1243  HG  LEU I 117       8.470 -24.254  13.699  1.00  0.00
I    H
ATOM   1244  CD1 LEU I 117      10.629 -24.317  13.982  1.00  0.00
I    C
ATOM   1245 HD11 LEU I 117      10.800 -25.395  13.774  1.00  0.00
I    H
ATOM   1246 HD12 LEU I 117      10.783 -23.752  13.037  1.00  0.00
I    H
ATOM   1247 HD13 LEU I 117      11.387 -23.981  14.720  1.00  0.00
I    H
ATOM   1248  CD2 LEU I 117       8.876 -25.071  15.660  1.00  0.00
I    C
ATOM   1249 HD21 LEU I 117       9.047 -26.118  15.329  1.00  0.00
I    H
ATOM   1250 HD22 LEU I 117       9.509 -24.892  16.553  1.00  0.00
I    H
ATOM   1251 HD23 LEU I 117       7.811 -24.971  15.962  1.00  0.00
I    H
ATOM   1252  C   LEU I 117       7.460 -20.848  15.845  1.00  0.00
I    C
ATOM   1253  O   LEU I 117       7.493 -20.692  17.064  1.00  0.00
I    O
ATOM   1254  N   ILE I 118       7.312 -19.791  15.019  1.00  0.00
I    N
ATOM   1255  HN  ILE I 118       7.353 -19.863  14.024  1.00  0.00
I    H
ATOM   1256  CA  ILE I 118       7.074 -18.432  15.515  1.00  0.00
I    C
ATOM   1257  HA  ILE I 118       7.890 -18.219  16.195  1.00  0.00
I    H
ATOM   1258  CB  ILE I 118       7.066 -17.387  14.390  1.00  0.00
I    C
ATOM   1259  HB  ILE I 118       6.362 -17.804  13.626  1.00  0.00
I    H
ATOM   1260  CG2 ILE I 118       6.644 -15.989  14.920  1.00  0.00
I    C
ATOM   1261 HG21 ILE I 118       6.490 -15.282  14.078  1.00  0.00
I    H
ATOM   1262 HG22 ILE I 118       5.690 -16.050  15.486  1.00  0.00
I    H
ATOM   1263 HG23 ILE I 118       7.422 -15.566  15.590  1.00  0.00
I    H
ATOM   1264  CG1 ILE I 118       8.461 -17.307  13.719  1.00  0.00
I    C
ATOM   1265 HG11 ILE I 118       9.200 -16.886  14.437  1.00  0.00
I    H
ATOM   1266 HG12 ILE I 118       8.799 -18.337  13.465  1.00  0.00
I    H
ATOM   1267  CD  ILE I 118       8.488 -16.469  12.432  1.00  0.00
I    C
ATOM   1268  HD1 ILE I 118       9.500 -16.500  11.972  1.00  0.00
I    H
```

ATOM 1269 HD2 ILE I 118 7.758 -16.862 11.693 1.00 0.00
I H
ATOM 1270 HD3 ILE I 118 8.239 -15.407 12.642 1.00 0.00
I H
ATOM 1271 C ILE I 118 5.784 -18.329 16.326 1.00 0.00
I C
ATOM 1272 O ILE I 118 5.771 -17.767 17.420 1.00 0.00
I O
ATOM 1273 N VAL I 119 4.673 -18.924 15.847 1.00 0.00
I N
ATOM 1274 HN VAL I 119 4.661 -19.363 14.952 1.00 0.00
I H
ATOM 1275 CA VAL I 119 3.404 -18.988 16.566 1.00 0.00
I C
ATOM 1276 HA VAL I 119 3.115 -17.967 16.781 1.00 0.00
I H
ATOM 1277 CB VAL I 119 2.336 -19.622 15.679 1.00 0.00
I C
ATOM 1278 HB VAL I 119 2.744 -20.591 15.302 1.00 0.00
I H
ATOM 1279 CG1 VAL I 119 1.037 -19.906 16.453 1.00 0.00
I C
ATOM 1280 HG11 VAL I 119 0.268 -20.331 15.772 1.00 0.00
I H
ATOM 1281 HG12 VAL I 119 1.197 -20.641 17.269 1.00 0.00
I H
ATOM 1282 HG13 VAL I 119 0.628 -18.971 16.891 1.00 0.00
I H
ATOM 1283 CG2 VAL I 119 2.025 -18.653 14.521 1.00 0.00
I C
ATOM 1284 HG21 VAL I 119 1.221 -19.061 13.872 1.00 0.00
I H
ATOM 1285 HG22 VAL I 119 1.691 -17.668 14.915 1.00 0.00
I H
ATOM 1286 HG23 VAL I 119 2.919 -18.488 13.885 1.00 0.00
I H
ATOM 1287 C VAL I 119 3.521 -19.720 17.901 1.00 0.00
I C
ATOM 1288 O VAL I 119 3.017 -19.273 18.933 1.00 0.00
I O
ATOM 1289 N MET I 120 4.256 -20.847 17.936 1.00 0.00
I N
ATOM 1290 HN MET I 120 4.626 -21.264 17.109 1.00 0.00
I H
ATOM 1291 CA MET I 120 4.576 -21.555 19.158 1.00 0.00
I C
ATOM 1292 HA MET I 120 3.626 -21.780 19.628 1.00 0.00
I H
ATOM 1293 CB MET I 120 5.352 -22.850 18.857 1.00 0.00
I C
ATOM 1294 HB1 MET I 120 6.209 -22.569 18.201 1.00 0.00
I H
ATOM 1295 HB2 MET I 120 5.796 -23.242 19.799 1.00 0.00
I H

```
ATOM   1296  CG  MET I 120       4.522 -23.937  18.153  1.00  0.00
I    C
ATOM   1297  HG1 MET I 120       3.738 -24.328  18.836  1.00  0.00
I    H
ATOM   1298  HG2 MET I 120       4.011 -23.501  17.267  1.00  0.00
I    H
ATOM   1299  SD  MET I 120       5.536 -25.342  17.609  1.00  0.00
I    S
ATOM   1300  CE  MET I 120       5.946 -25.946  19.272  1.00  0.00
I    C
ATOM   1301  HE1 MET I 120       6.601 -26.842  19.218  1.00  0.00
I    H
ATOM   1302  HE2 MET I 120       6.480 -25.168  19.859  1.00  0.00
I    H
ATOM   1303  HE3 MET I 120       5.029 -26.228  19.833  1.00  0.00
I    H
ATOM   1304  C   MET I 120       5.396 -20.736  20.147  1.00  0.00
I    C
ATOM   1305  O   MET I 120       5.126 -20.761  21.345  1.00  0.00
I    O
ATOM   1306  N   ILE I 121       6.407 -19.971  19.686  1.00  0.00
I    N
ATOM   1307  HN  ILE I 121       6.689 -19.994  18.729  1.00  0.00
I    H
ATOM   1308  CA  ILE I 121       7.182 -19.047  20.516  1.00  0.00
I    C
ATOM   1309  HA  ILE I 121       7.573 -19.636  21.336  1.00  0.00
I    H
ATOM   1310  CB  ILE I 121       8.355 -18.428  19.749  1.00  0.00
I    C
ATOM   1311  HB  ILE I 121       7.904 -18.067  18.790  1.00  0.00
I    H
ATOM   1312  CG2 ILE I 121       9.046 -17.309  20.571  1.00  0.00
I    C
ATOM   1313 HG21 ILE I 121       9.787 -16.768  19.948  1.00  0.00
I    H
ATOM   1314 HG22 ILE I 121       8.303 -16.567  20.931  1.00  0.00
I    H
ATOM   1315 HG23 ILE I 121       9.575 -17.731  21.451  1.00  0.00
I    H
ATOM   1316  CG1 ILE I 121       9.393 -19.515  19.374  1.00  0.00
I    C
ATOM   1317 HG11 ILE I 121       9.953 -19.830  20.283  1.00  0.00
I    H
ATOM   1318 HG12 ILE I 121       8.854 -20.413  18.992  1.00  0.00
I    H
ATOM   1319  CD  ILE I 121      10.389 -19.055  18.299  1.00  0.00
I    C
ATOM   1320  HD1 ILE I 121      11.073 -19.887  18.024  1.00  0.00
I    H
ATOM   1321  HD2 ILE I 121       9.852 -18.728  17.381  1.00  0.00
I    H
ATOM   1322  HD3 ILE I 121      11.007 -18.209  18.665  1.00  0.00
I    H
```

```
ATOM   1323  C   ILE I 121       6.301 -17.966  21.130  1.00  0.00
I    C
ATOM   1324  O   ILE I 121       6.413 -17.660  22.317  1.00  0.00
I    O
ATOM   1325  N   LEU I 122       5.351 -17.396  20.356  1.00  0.00
I    N
ATOM   1326  HN  LEU I 122       5.262 -17.589  19.382  1.00  0.00
I    H
ATOM   1327  CA  LEU I 122       4.372 -16.451  20.870  1.00  0.00
I    C
ATOM   1328  HA  LEU I 122       4.943 -15.646  21.315  1.00  0.00
I    H
ATOM   1329  CB  LEU I 122       3.495 -15.879  19.724  1.00  0.00
I    C
ATOM   1330  HB1 LEU I 122       3.020 -16.746  19.210  1.00  0.00
I    H
ATOM   1331  HB2 LEU I 122       2.662 -15.289  20.166  1.00  0.00
I    H
ATOM   1332  CG  LEU I 122       4.277 -15.041  18.686  1.00  0.00
I    C
ATOM   1333  HG  LEU I 122       5.142 -15.672  18.368  1.00  0.00
I    H
ATOM   1334  CD1 LEU I 122       3.384 -14.733  17.474  1.00  0.00
I    C
ATOM   1335 HD11 LEU I 122       3.924 -14.090  16.745  1.00  0.00
I    H
ATOM   1336 HD12 LEU I 122       3.088 -15.672  16.957  1.00  0.00
I    H
ATOM   1337 HD13 LEU I 122       2.462 -14.200  17.790  1.00  0.00
I    H
ATOM   1338  CD2 LEU I 122       4.834 -13.738  19.286  1.00  0.00
I    C
ATOM   1339 HD21 LEU I 122       5.403 -13.171  18.517  1.00  0.00
I    H
ATOM   1340 HD22 LEU I 122       4.024 -13.078  19.657  1.00  0.00
I    H
ATOM   1341 HD23 LEU I 122       5.519 -13.960  20.133  1.00  0.00
I    H
ATOM   1342  C   LEU I 122       3.487 -17.045  21.961  1.00  0.00
I    C
ATOM   1343  O   LEU I 122       3.324 -16.446  23.025  1.00  0.00
I    O
ATOM   1344  N   GLY I 123       2.969 -18.280  21.774  1.00  0.00
I    N
ATOM   1345  HN  GLY I 123       3.097 -18.778  20.916  1.00  0.00
I    H
ATOM   1346  CA  GLY I 123       2.191 -18.977  22.800  1.00  0.00
I    C
ATOM   1347  HA1 GLY I 123       1.872 -19.907  22.349  1.00  0.00
I    H
ATOM   1348  HA2 GLY I 123       1.381 -18.312  23.065  1.00  0.00
I    H
ATOM   1349  C   GLY I 123       2.954 -19.306  24.061  1.00  0.00
I    C
```

```
ATOM   1350  O   GLY I 123       2.431 -19.224  25.170  1.00  0.00
I    O
ATOM   1351  N   LYS I 124       4.250 -19.643  23.932  1.00  0.00
I    N
ATOM   1352  HN  LYS I 124       4.660 -19.794  23.034  1.00  0.00
I    H
ATOM   1353  CA  LYS I 124       5.152 -19.814  25.057  1.00  0.00
I    C
ATOM   1354  HA  LYS I 124       4.672 -20.518  25.727  1.00  0.00
I    H
ATOM   1355  CB  LYS I 124       6.522 -20.364  24.579  1.00  0.00
I    C
ATOM   1356  HB1 LYS I 124       6.884 -19.686  23.772  1.00  0.00
I    H
ATOM   1357  HB2 LYS I 124       7.257 -20.280  25.412  1.00  0.00
I    H
ATOM   1358  CG  LYS I 124       6.456 -21.805  24.044  1.00  0.00
I    C
ATOM   1359  HG1 LYS I 124       6.268 -22.502  24.892  1.00  0.00
I    H
ATOM   1360  HG2 LYS I 124       5.592 -21.894  23.345  1.00  0.00
I    H
ATOM   1361  CD  LYS I 124       7.755 -22.237  23.330  1.00  0.00
I    C
ATOM   1362  HD1 LYS I 124       7.924 -21.557  22.465  1.00  0.00
I    H
ATOM   1363  HD2 LYS I 124       8.604 -22.093  24.039  1.00  0.00
I    H
ATOM   1364  CE  LYS I 124       7.718 -23.699  22.856  1.00  0.00
I    C
ATOM   1365  HE1 LYS I 124       7.320 -24.360  23.658  1.00  0.00
I    H
ATOM   1366  HE2 LYS I 124       7.067 -23.803  21.960  1.00  0.00
I    H
ATOM   1367  NZ  LYS I 124       9.072 -24.194  22.492  1.00  0.00
I    N
ATOM   1368  HZ1 LYS I 124       9.016 -25.184  22.176  1.00  0.00
I    H
ATOM   1369  HZ2 LYS I 124       9.461 -23.609  21.725  1.00  0.00
I    H
ATOM   1370  HZ3 LYS I 124       9.697 -24.132  23.321  1.00  0.00
I    H
ATOM   1371  C   LYS I 124       5.380 -18.527  25.839  1.00  0.00
I    C
ATOM   1372  O   LYS I 124       5.398 -18.526  27.069  1.00  0.00
I    O
ATOM   1373  N   LYS I 125       5.543 -17.391  25.134  1.00  0.00
I    N
ATOM   1374  HN  LYS I 125       5.575 -17.422  24.136  1.00  0.00
I    H
ATOM   1375  CA  LYS I 125       5.683 -16.071  25.714  1.00  0.00
I    C
ATOM   1376  HA  LYS I 125       6.506 -16.140  26.415  1.00  0.00
I    H
```

```
ATOM   1377  CB  LYS I 125       6.021 -15.053  24.596  1.00  0.00
I    C
ATOM   1378  HB1 LYS I 125       6.903 -15.454  24.044  1.00  0.00
I    H
ATOM   1379  HB2 LYS I 125       5.181 -15.026  23.865  1.00  0.00
I    H
ATOM   1380  CG  LYS I 125       6.347 -13.639  25.106  1.00  0.00
I    C
ATOM   1381  HG1 LYS I 125       5.452 -13.223  25.623  1.00  0.00
I    H
ATOM   1382  HG2 LYS I 125       7.165 -13.705  25.859  1.00  0.00
I    H
ATOM   1383  CD  LYS I 125       6.750 -12.689  23.964  1.00  0.00
I    C
ATOM   1384  HD1 LYS I 125       7.601 -13.147  23.410  1.00  0.00
I    H
ATOM   1385  HD2 LYS I 125       5.892 -12.614  23.257  1.00  0.00
I    H
ATOM   1386  CE  LYS I 125       7.139 -11.284  24.437  1.00  0.00
I    C
ATOM   1387  HE1 LYS I 125       6.272 -10.781  24.920  1.00  0.00
I    H
ATOM   1388  HE2 LYS I 125       7.971 -11.336  25.174  1.00  0.00
I    H
ATOM   1389  NZ  LYS I 125       7.582 -10.453  23.291  1.00  0.00
I    N
ATOM   1390  HZ1 LYS I 125       7.843  -9.502  23.621  1.00  0.00
I    H
ATOM   1391  HZ2 LYS I 125       8.404 -10.899  22.836  1.00  0.00
I    H
ATOM   1392  HZ3 LYS I 125       6.808 -10.379  22.600  1.00  0.00
I    H
ATOM   1393  C   LYS I 125       4.458 -15.611  26.494  1.00  0.00
I    C
ATOM   1394  O   LYS I 125       4.572 -14.979  27.542  1.00  0.00
I    O
ATOM   1395  N   ILE I 126       3.240 -15.908  26.010  1.00  0.00
I    N
ATOM   1396  HN  ILE I 126       3.109 -16.335  25.118  1.00  0.00
I    H
ATOM   1397  CA  ILE I 126       2.000 -15.638  26.733  1.00  0.00
I    C
ATOM   1398  HA  ILE I 126       2.046 -14.593  27.012  1.00  0.00
I    H
ATOM   1399  CB  ILE I 126       0.817 -15.937  25.852  1.00  0.00
I    C
ATOM   1400  HB  ILE I 126       1.017 -16.964  25.455  1.00  0.00
I    H
ATOM   1401  CG2 ILE I 126      -0.545 -15.830  26.585  1.00  0.00
I    C
ATOM   1402 HG21 ILE I 126      -1.366 -16.209  25.941  1.00  0.00
I    H
ATOM   1403 HG22 ILE I 126      -0.542 -16.433  27.516  1.00  0.00
I    H
```

```
ATOM   1404 HG23 ILE I 126      -0.772 -14.775  26.850  1.00  0.00
I    H
ATOM   1405  CG1 ILE I 126       0.881 -14.948  24.684  1.00  0.00
I    C
ATOM   1406 HG11 ILE I 126       0.463 -13.965  24.999  1.00  0.00
I    H
ATOM   1407 HG12 ILE I 126       1.948 -14.780  24.411  1.00  0.00
I    H
ATOM   1408  CD  ILE I 126       0.122 -15.506  23.498  1.00  0.00
I    C
ATOM   1409  HD1 ILE I 126       0.212 -14.822  22.627  1.00  0.00
I    H
ATOM   1410  HD2 ILE I 126       0.529 -16.498  23.205  1.00  0.00
I    H
ATOM   1411  HD3 ILE I 126      -0.957 -15.622  23.735  1.00  0.00
I    H
ATOM   1412  C   ILE I 126       1.892 -16.422  28.008  1.00  0.00
I    C
ATOM   1413  O   ILE I 126       1.641 -15.850  29.068  1.00  0.00
I    O
ATOM   1414  N   GLY I 127       2.186 -17.738  27.958  1.00  0.00
I    N
ATOM   1415  HN  GLY I 127       2.421 -18.203  27.105  1.00  0.00
I    H
ATOM   1416  CA  GLY I 127       2.182 -18.571  29.150  1.00  0.00
I    C
ATOM   1417  HA1 GLY I 127       2.431 -19.574  28.829  1.00  0.00
I    H
ATOM   1418  HA2 GLY I 127       1.200 -18.479  29.595  1.00  0.00
I    H
ATOM   1419  C   GLY I 127       3.199 -18.146  30.177  1.00  0.00
I    C
ATOM   1420  O   GLY I 127       2.984 -18.270  31.373  1.00  0.00
I    O
ATOM   1421  N   LEU I 128       4.342 -17.572  29.754  1.00  0.00
I    N
ATOM   1422  HN  LEU I 128       4.644 -17.580  28.802  1.00  0.00
I    H
ATOM   1423  CA  LEU I 128       5.246 -16.884  30.662  1.00  0.00
I    C
ATOM   1424  HA  LEU I 128       5.457 -17.596  31.450  1.00  0.00
I    H
ATOM   1425  CB  LEU I 128       6.554 -16.457  29.960  1.00  0.00
I    C
ATOM   1426  HB1 LEU I 128       6.266 -15.931  29.021  1.00  0.00
I    H
ATOM   1427  HB2 LEU I 128       7.072 -15.701  30.591  1.00  0.00
I    H
ATOM   1428  CG  LEU I 128       7.499 -17.618  29.613  1.00  0.00
I    C
ATOM   1429  HG  LEU I 128       6.933 -18.377  29.021  1.00  0.00
I    H
ATOM   1430  CD1 LEU I 128       8.658 -17.073  28.764  1.00  0.00
I    C
```

```
ATOM   1431 HD11 LEU I 128       9.387 -17.880  28.536  1.00  0.00
I    H
ATOM   1432 HD12 LEU I 128       8.280 -16.662  27.803  1.00  0.00
I    H
ATOM   1433 HD13 LEU I 128       9.194 -16.265  29.305  1.00  0.00
I    H
ATOM   1434  CD2 LEU I 128       8.015 -18.301  30.891  1.00  0.00
I    C
ATOM   1435 HD21 LEU I 128       8.683 -19.150  30.632  1.00  0.00
I    H
ATOM   1436 HD22 LEU I 128       8.594 -17.599  31.526  1.00  0.00
I    H
ATOM   1437 HD23 LEU I 128       7.167 -18.696  31.491  1.00  0.00
I    H
ATOM   1438  C   LEU I 128       4.651 -15.645  31.311  1.00  0.00
I    C
ATOM   1439  O   LEU I 128       4.775 -15.461  32.515  1.00  0.00
I    O
ATOM   1440  N   LYS I 129       3.958 -14.780  30.544  1.00  0.00
I    N
ATOM   1441  HN  LYS I 129       3.858 -14.914  29.559  1.00  0.00
I    H
ATOM   1442  CA  LYS I 129       3.306 -13.594  31.075  1.00  0.00
I    C
ATOM   1443  HA  LYS I 129       4.075 -13.033  31.592  1.00  0.00
I    H
ATOM   1444  CB  LYS I 129       2.667 -12.745  29.956  1.00  0.00
I    C
ATOM   1445  HB1 LYS I 129       2.023 -13.427  29.355  1.00  0.00
I    H
ATOM   1446  HB2 LYS I 129       1.986 -11.995  30.416  1.00  0.00
I    H
ATOM   1447  CG  LYS I 129       3.681 -12.066  29.030  1.00  0.00
I    C
ATOM   1448  HG1 LYS I 129       4.272 -11.327  29.617  1.00  0.00
I    H
ATOM   1449  HG2 LYS I 129       4.394 -12.833  28.649  1.00  0.00
I    H
ATOM   1450  CD  LYS I 129       2.935 -11.367  27.888  1.00  0.00
I    C
ATOM   1451  HD1 LYS I 129       2.304 -12.122  27.365  1.00  0.00
I    H
ATOM   1452  HD2 LYS I 129       2.248 -10.610  28.334  1.00  0.00
I    H
ATOM   1453  CE  LYS I 129       3.836 -10.677  26.874  1.00  0.00
I    C
ATOM   1454  HE1 LYS I 129       4.423  -9.867  27.362  1.00  0.00
I    H
ATOM   1455  HE2 LYS I 129       4.547 -11.405  26.425  1.00  0.00
I    H
ATOM   1456  NZ  LYS I 129       2.983 -10.099  25.813  1.00  0.00
I    N
ATOM   1457  HZ1 LYS I 129       3.573  -9.618  25.104  1.00  0.00
I    H
```

ATOM 1458 HZ2 LYS I 129 2.439 −10.857 25.352 1.00 0.00
I H
ATOM 1459 HZ3 LYS I 129 2.324 −9.413 26.234 1.00 0.00
I H
ATOM 1460 C LYS I 129 2.220 −13.908 32.084 1.00 0.00
I C
ATOM 1461 O LYS I 129 2.153 −13.275 33.134 1.00 0.00
I O
ATOM 1462 N GLU I 130 1.381 −14.931 31.826 1.00 0.00
I N
ATOM 1463 HN GLU I 130 1.402 −15.432 30.964 1.00 0.00
I H
ATOM 1464 CA GLU I 130 0.374 −15.405 32.759 1.00 0.00
I C
ATOM 1465 HA GLU I 130 −0.294 −14.579 32.973 1.00 0.00
I H
ATOM 1466 CB GLU I 130 −0.393 −16.587 32.129 1.00 0.00
I C
ATOM 1467 HB1 GLU I 130 0.353 −17.344 31.790 1.00 0.00
I H
ATOM 1468 HB2 GLU I 130 −1.010 −17.093 32.905 1.00 0.00
I H
ATOM 1469 CG GLU I 130 −1.279 −16.170 30.929 1.00 0.00
I C
ATOM 1470 HG1 GLU I 130 −2.119 −15.549 31.289 1.00 0.00
I H
ATOM 1471 HG2 GLU I 130 −0.693 −15.596 30.184 1.00 0.00
I H
ATOM 1472 CD GLU I 130 −1.921 −17.367 30.225 1.00 0.00
I C
ATOM 1473 OE1 GLU I 130 −1.503 −18.521 30.497 1.00 0.00
I O
ATOM 1474 OE2 GLU I 130 −2.828 −17.115 29.392 1.00 0.00
I O
ATOM 1475 C GLU I 130 0.988 −15.843 34.082 1.00 0.00
I C
ATOM 1476 O GLU I 130 0.553 −15.461 35.162 1.00 0.00
I O
ATOM 1477 N ARG I 131 2.102 −16.588 34.018 1.00 0.00
I N
ATOM 1478 HN ARG I 131 2.425 −16.941 33.144 1.00 0.00
I H
ATOM 1479 CA ARG I 131 2.892 −16.922 35.187 1.00 0.00
I C
ATOM 1480 HA ARG I 131 2.212 −17.370 35.902 1.00 0.00
I H
ATOM 1481 CB ARG I 131 4.014 −17.887 34.819 1.00 0.00
I C
ATOM 1482 HB1 ARG I 131 4.626 −17.376 34.038 1.00 0.00
I H
ATOM 1483 HB2 ARG I 131 4.688 −18.025 35.694 1.00 0.00
I H
ATOM 1484 CG ARG I 131 3.493 −19.209 34.274 1.00 0.00
I C

ATOM 1485 HG1 ARG I 131 2.884 -19.702 35.063 1.00 0.00
I H
ATOM 1486 HG2 ARG I 131 2.788 -19.003 33.436 1.00 0.00
I H
ATOM 1487 CD ARG I 131 4.668 -20.016 33.789 1.00 0.00
I C
ATOM 1488 HD1 ARG I 131 5.193 -19.456 32.982 1.00 0.00
I H
ATOM 1489 HD2 ARG I 131 5.391 -20.220 34.612 1.00 0.00
I H
ATOM 1490 NE ARG I 131 4.103 -21.293 33.297 1.00 0.00
I N
ATOM 1491 HE ARG I 131 3.102 -21.266 33.185 1.00 0.00
I H
ATOM 1492 CZ ARG I 131 4.896 -22.335 33.049 1.00 0.00
I C
ATOM 1493 NH1 ARG I 131 4.390 -23.537 32.819 1.00 0.00
I N
ATOM 1494 HH11 ARG I 131 4.984 -24.313 32.661 1.00 0.00
I H
ATOM 1495 HH12 ARG I 131 3.401 -23.635 32.952 1.00 0.00
I H
ATOM 1496 NH2 ARG I 131 6.213 -22.146 33.058 1.00 0.00
I N
ATOM 1497 HH21 ARG I 131 6.820 -22.881 32.792 1.00 0.00
I H
ATOM 1498 HH22 ARG I 131 6.530 -21.209 33.130 1.00 0.00
I H
ATOM 1499 C ARG I 131 3.535 -15.753 35.882 1.00 0.00
I C
ATOM 1500 O ARG I 131 3.627 -15.752 37.115 1.00 0.00
I O
ATOM 1501 N MET I 132 4.014 -14.722 35.190 1.00 0.00
I N
ATOM 1502 HN MET I 132 4.085 -14.696 34.196 1.00 0.00
I H
ATOM 1503 CA MET I 132 4.481 -13.523 35.855 1.00 0.00
I C
ATOM 1504 HA MET I 132 5.223 -13.848 36.574 1.00 0.00
I H
ATOM 1505 CB MET I 132 5.153 -12.570 34.855 1.00 0.00
I C
ATOM 1506 HB1 MET I 132 4.423 -12.398 34.029 1.00 0.00
I H
ATOM 1507 HB2 MET I 132 5.315 -11.581 35.338 1.00 0.00
I H
ATOM 1508 CG MET I 132 6.460 -13.144 34.282 1.00 0.00
I C
ATOM 1509 HG1 MET I 132 7.245 -13.178 35.067 1.00 0.00
I H
ATOM 1510 HG2 MET I 132 6.290 -14.186 33.932 1.00 0.00
I H
ATOM 1511 SD MET I 132 7.122 -12.177 32.892 1.00 0.00
I S

```
ATOM   1512  CE  MET I 132       7.646 -10.745  33.879  1.00  0.00
I    C
ATOM   1513  HE1 MET I 132       8.099  -9.963  33.233  1.00  0.00
I    H
ATOM   1514  HE2 MET I 132       6.783 -10.291  34.412  1.00  0.00
I    H
ATOM   1515  HE3 MET I 132       8.402 -11.038  34.639  1.00  0.00
I    H
ATOM   1516  C   MET I 132       3.382 -12.786  36.625  1.00  0.00
I    C
ATOM   1517  O   MET I 132       3.628 -12.225  37.683  1.00  0.00
I    O
ATOM   1518  N   LEU I 133       2.137 -12.788  36.093  1.00  0.00
I    N
ATOM   1519  HN  LEU I 133       1.956 -13.151  35.181  1.00  0.00
I    H
ATOM   1520  CA  LEU I 133       0.961 -12.272  36.778  1.00  0.00
I    C
ATOM   1521  HA  LEU I 133       1.235 -11.268  37.074  1.00  0.00
I    H
ATOM   1522  CB  LEU I 133      -0.259 -12.241  35.815  1.00  0.00
I    C
ATOM   1523  HB1 LEU I 133      -0.411 -13.280  35.443  1.00  0.00
I    H
ATOM   1524  HB2 LEU I 133      -1.173 -11.986  36.397  1.00  0.00
I    H
ATOM   1525  CG  LEU I 133      -0.109 -11.298  34.604  1.00  0.00
I    C
ATOM   1526  HG  LEU I 133       0.885 -11.526  34.151  1.00  0.00
I    H
ATOM   1527  CD1 LEU I 133      -1.221 -11.569  33.578  1.00  0.00
I    C
ATOM   1528 HD11 LEU I 133      -1.147 -10.861  32.726  1.00  0.00
I    H
ATOM   1529 HD12 LEU I 133      -1.142 -12.604  33.180  1.00  0.00
I    H
ATOM   1530 HD13 LEU I 133      -2.223 -11.449  34.043  1.00  0.00
I    H
ATOM   1531  CD2 LEU I 133      -0.113  -9.819  35.020  1.00  0.00
I    C
ATOM   1532 HD21 LEU I 133       0.015  -9.167  34.128  1.00  0.00
I    H
ATOM   1533 HD22 LEU I 133      -1.065  -9.534  35.511  1.00  0.00
I    H
ATOM   1534 HD23 LEU I 133       0.720  -9.610  35.725  1.00  0.00
I    H
ATOM   1535  C   LEU I 133       0.545 -13.030  38.044  1.00  0.00
I    C
ATOM   1536  O   LEU I 133       0.238 -12.392  39.053  1.00  0.00
I    O
ATOM   1537  N   VAL I 134       0.493 -14.387  38.043  1.00  0.00
I    N
ATOM   1538  HN  VAL I 134       0.812 -14.955  37.289  1.00  0.00
I    H
```

```
ATOM   1539  CA  VAL I 134      -0.052 -15.140  39.184  1.00  0.00
I    C
ATOM   1540  HA  VAL I 134      -0.360 -14.382  39.894  1.00  0.00
I    H
ATOM   1541  CB  VAL I 134      -1.294 -15.969  38.821  1.00  0.00
I    C
ATOM   1542  HB  VAL I 134      -1.607 -16.526  39.738  1.00  0.00
I    H
ATOM   1543  CG1 VAL I 134      -2.414 -15.013  38.376  1.00  0.00
I    C
ATOM   1544 HG11 VAL I 134      -3.314 -15.590  38.070  1.00  0.00
I    H
ATOM   1545 HG12 VAL I 134      -2.722 -14.332  39.195  1.00  0.00
I    H
ATOM   1546 HG13 VAL I 134      -2.086 -14.401  37.509  1.00  0.00
I    H
ATOM   1547  CG2 VAL I 134      -1.012 -17.018  37.725  1.00  0.00
I    C
ATOM   1548 HG21 VAL I 134      -1.939 -17.569  37.461  1.00  0.00
I    H
ATOM   1549 HG22 VAL I 134      -0.623 -16.527  36.806  1.00  0.00
I    H
ATOM   1550 HG23 VAL I 134      -0.263 -17.763  38.065  1.00  0.00
I    H
ATOM   1551  C   VAL I 134       0.912 -16.082  39.908  1.00  0.00
I    C
ATOM   1552  O   VAL I 134       0.590 -16.582  40.974  1.00  0.00
I    O
ATOM   1553  N   GLN I 135       2.122 -16.321  39.348  1.00  0.00
I    N
ATOM   1554  HN  GLN I 135       2.437 -15.746  38.594  1.00  0.00
I    H
ATOM   1555  CA  GLN I 135       3.060 -17.370  39.739  1.00  0.00
I    C
ATOM   1556  HA  GLN I 135       3.970 -17.177  39.184  1.00  0.00
I    H
ATOM   1557  CB  GLN I 135       3.630 -17.221  41.182  1.00  0.00
I    C
ATOM   1558  HB1 GLN I 135       3.930 -16.154  41.302  1.00  0.00
I    H
ATOM   1559  HB2 GLN I 135       2.814 -17.398  41.921  1.00  0.00
I    H
ATOM   1560  CG  GLN I 135       4.854 -18.109  41.548  1.00  0.00
I    C
ATOM   1561  HG1 GLN I 135       5.168 -17.923  42.597  1.00  0.00
I    H
ATOM   1562  HG2 GLN I 135       4.601 -19.185  41.455  1.00  0.00
I    H
ATOM   1563  CD  GLN I 135       6.081 -17.823  40.678  1.00  0.00
I    C
ATOM   1564  OE1 GLN I 135       6.556 -16.694  40.570  1.00  0.00
I    O
ATOM   1565  NE2 GLN I 135       6.652 -18.875  40.044  1.00  0.00
I    N
```

ATOM   1566 HE21 GLN I 135   7.452 -18.683 39.484 1.00 0.00
I   H
ATOM   1567 HE22 GLN I 135   6.257 -19.784 40.139 1.00 0.00
I   H
ATOM   1568 C   GLN I 135   2.588 -18.780 39.376 1.00 0.00
I   C
ATOM   1569 O   GLN I 135   1.400 -19.077 39.286 1.00 0.00
I   O
ATOM   1570 N   GLU I 136   3.520 -19.705 39.092 1.00 0.00
I   N
ATOM   1571 HN GLU I 136   4.501 -19.533 39.103 1.00 0.00
I   H
ATOM   1572 CA GLU I 136   3.166 -21.056 38.739 1.00 0.00
I   C
ATOM   1573 HA GLU I 136   2.197 -21.297 39.157 1.00 0.00
I   H
ATOM   1574 CB GLU I 136   3.145 -21.143 37.198 1.00 0.00
I   C
ATOM   1575 HB1 GLU I 136   2.436 -20.374 36.816 1.00 0.00
I   H
ATOM   1576 HB2 GLU I 136   4.143 -20.867 36.791 1.00 0.00
I   H
ATOM   1577 CG GLU I 136   2.739 -22.479 36.538 1.00 0.00
I   C
ATOM   1578 HG1 GLU I 136   2.559 -22.317 35.459 1.00 0.00
I   H
ATOM   1579 HG2 GLU I 136   3.531 -23.243 36.665 1.00 0.00
I   H
ATOM   1580 CD GLU I 136   1.439 -23.067 37.071 1.00 0.00
I   C
ATOM   1581 OE1 GLU I 136   1.477 -23.607 38.207 1.00 0.00
I   O
ATOM   1582 OE2 GLU I 136   0.444 -23.048 36.309 1.00 0.00
I   O
ATOM   1583 C   GLU I 136   4.173 -21.989 39.374 1.00 0.00
I   C
ATOM   1584 O   GLU I 136   5.294 -21.580 39.694 1.00 0.00
I   O
ATOM   1585 N   ALA I 137   3.767 -23.249 39.608 1.00 0.00
I   N
ATOM   1586 HN ALA I 137   2.842 -23.513 39.334 1.00 0.00
I   H
ATOM   1587 CA ALA I 137   4.553 -24.292 40.233 1.00 0.00
I   C
ATOM   1588 HA ALA I 137   5.428 -23.873 40.717 1.00 0.00
I   H
ATOM   1589 CB ALA I 137   3.647 -25.045 41.233 1.00 0.00
I   C
ATOM   1590 HB1 ALA I 137   3.303 -24.345 42.024 1.00 0.00
I   H
ATOM   1591 HB2 ALA I 137   2.747 -25.456 40.726 1.00 0.00
I   H
ATOM   1592 HB3 ALA I 137   4.188 -25.882 41.726 1.00 0.00
I   H

```
ATOM   1593  C   ALA I 137       5.070 -25.287  39.192  1.00  0.00
I    C
ATOM   1594  O   ALA I 137       5.836 -26.199  39.491  1.00  0.00
I    O
ATOM   1595  N   LEU I 138       4.656 -25.110  37.924  1.00  0.00
I    N
ATOM   1596  HN  LEU I 138       4.070 -24.334  37.697  1.00  0.00
I    H
ATOM   1597  CA  LEU I 138       4.983 -25.960  36.795  1.00  0.00
I    C
ATOM   1598  HA  LEU I 138       5.231 -26.923  37.221  1.00  0.00
I    H
ATOM   1599  CB  LEU I 138       3.823 -26.017  35.768  1.00  0.00
I    C
ATOM   1600  HB1 LEU I 138       3.713 -24.994  35.339  1.00  0.00
I    H
ATOM   1601  HB2 LEU I 138       4.120 -26.675  34.922  1.00  0.00
I    H
ATOM   1602  CG  LEU I 138       2.469 -26.459  36.346  1.00  0.00
I    C
ATOM   1603  HG  LEU I 138       2.274 -25.800  37.226  1.00  0.00
I    H
ATOM   1604  CD1 LEU I 138       1.370 -26.244  35.295  1.00  0.00
I    C
ATOM   1605 HD11 LEU I 138       0.390 -26.603  35.676  1.00  0.00
I    H
ATOM   1606 HD12 LEU I 138       1.274 -25.166  35.045  1.00  0.00
I    H
ATOM   1607 HD13 LEU I 138       1.605 -26.801  34.362  1.00  0.00
I    H
ATOM   1608  CD2 LEU I 138       2.489 -27.914  36.836  1.00  0.00
I    C
ATOM   1609 HD21 LEU I 138       1.501 -28.191  37.263  1.00  0.00
I    H
ATOM   1610 HD22 LEU I 138       2.710 -28.621  36.012  1.00  0.00
I    H
ATOM   1611 HD23 LEU I 138       3.258 -28.047  37.627  1.00  0.00
I    H
ATOM   1612  C   LEU I 138       6.188 -25.488  35.995  1.00  0.00
I    C
ATOM   1613  O   LEU I 138       6.441 -24.297  35.807  1.00  0.00
I    O
ATOM   1614  N   ASN I 139       6.913 -26.456  35.396  1.00  0.00
I    N
ATOM   1615  HN  ASN I 139       6.796 -27.414  35.653  1.00  0.00
I    H
ATOM   1616  CA  ASN I 139       7.904 -26.226  34.356  1.00  0.00
I    C
ATOM   1617  HA  ASN I 139       8.635 -25.560  34.804  1.00  0.00
I    H
ATOM   1618  CB  ASN I 139       8.524 -27.553  33.845  1.00  0.00
I    C
ATOM   1619  HB1 ASN I 139       7.692 -28.212  33.515  1.00  0.00
I    H
```

```
ATOM   1620  HB2 ASN I 139       9.169 -27.363  32.964  1.00  0.00
I    H
ATOM   1621  CG  ASN I 139       9.288 -28.227  34.973  1.00  0.00
I    C
ATOM   1622  OD1 ASN I 139       9.891 -27.572  35.819  1.00  0.00
I    O
ATOM   1623  ND2 ASN I 139       9.287 -29.578  34.987  1.00  0.00
I    N
ATOM   1624 HD21 ASN I 139       9.776 -30.029  35.730  1.00  0.00
I    H
ATOM   1625 HD22 ASN I 139       8.786 -30.083  34.291  1.00  0.00
I    H
ATOM   1626  C   ASN I 139       7.329 -25.524  33.131  1.00  0.00
I    C
ATOM   1627  O   ASN I 139       6.165 -25.675  32.764  1.00  0.00
I    O
ATOM   1628  N   GLN I 140       8.138 -24.694  32.445  1.00  0.00
I    N
ATOM   1629  HN  GLN I 140       9.024 -24.379  32.780  1.00  0.00
I    H
ATOM   1630  CA  GLN I 140       7.772 -24.181  31.134  1.00  0.00
I    C
ATOM   1631  HA  GLN I 140       6.791 -23.733  31.232  1.00  0.00
I    H
ATOM   1632  CB  GLN I 140       8.747 -23.060  30.705  1.00  0.00
I    C
ATOM   1633  HB1 GLN I 140       9.106 -22.571  31.640  1.00  0.00
I    H
ATOM   1634  HB2 GLN I 140       9.644 -23.515  30.228  1.00  0.00
I    H
ATOM   1635  CG  GLN I 140       8.112 -21.999  29.781  1.00  0.00
I    C
ATOM   1636  HG1 GLN I 140       7.538 -22.488  28.966  1.00  0.00
I    H
ATOM   1637  HG2 GLN I 140       7.411 -21.352  30.349  1.00  0.00
I    H
ATOM   1638  CD  GLN I 140       9.232 -21.175  29.160  1.00  0.00
I    C
ATOM   1639  OE1 GLN I 140      10.383 -21.224  29.612  1.00  0.00
I    O
ATOM   1640  NE2 GLN I 140       8.942 -20.403  28.100  1.00  0.00
I    N
ATOM   1641 HE21 GLN I 140       9.691 -19.877  27.708  1.00  0.00
I    H
ATOM   1642 HE22 GLN I 140       8.009 -20.366  27.757  1.00  0.00
I    H
ATOM   1643  C   GLN I 140       7.664 -25.315  30.104  1.00  0.00
I    C
ATOM   1644  O   GLN I 140       8.601 -26.112  30.037  1.00  0.00
I    O
ATOM   1645  N   PRO I 141       6.622 -25.490  29.277  1.00  0.00
I    N
ATOM   1646  CD  PRO I 141       5.337 -24.782  29.380  1.00  0.00
I    C
```

ATOM 1647 HD1 PRO I 141 5.020 -24.751 30.448 1.00 0.00
I H
ATOM 1648 HD2 PRO I 141 5.417 -23.750 28.968 1.00 0.00
I H
ATOM 1649 CA PRO I 141 6.473 -26.752 28.556 1.00 0.00
I C
ATOM 1650 HA PRO I 141 7.016 -27.577 29.001 1.00 0.00
I H
ATOM 1651 CB PRO I 141 4.951 -26.997 28.574 1.00 0.00
I C
ATOM 1652 HB1 PRO I 141 4.677 -27.502 29.527 1.00 0.00
I H
ATOM 1653 HB2 PRO I 141 4.608 -27.620 27.724 1.00 0.00
I H
ATOM 1654 CG PRO I 141 4.332 -25.598 28.559 1.00 0.00
I C
ATOM 1655 HG1 PRO I 141 3.304 -25.575 28.971 1.00 0.00
I H
ATOM 1656 HG2 PRO I 141 4.320 -25.214 27.513 1.00 0.00
I H
ATOM 1657 C PRO I 141 7.028 -26.481 27.175 1.00 0.00
I C
ATOM 1658 O PRO I 141 6.335 -26.525 26.160 1.00 0.00
I O
ATOM 1659 N THR I 142 8.328 -26.153 27.122 1.00 0.00
I N
ATOM 1660 HN THR I 142 8.914 -26.227 27.928 1.00 0.00
I H
ATOM 1661 CA THR I 142 9.005 -25.673 25.937 1.00 0.00
I C
ATOM 1662 HA THR I 142 8.220 -25.472 25.221 1.00 0.00
I H
ATOM 1663 CB THR I 142 9.871 -24.469 26.240 1.00 0.00
I C
ATOM 1664 HB THR I 142 10.587 -24.380 25.387 1.00 0.00
I H
ATOM 1665 OG1 THR I 142 10.646 -24.653 27.414 1.00 0.00
I O
ATOM 1666 HG1 THR I 142 11.145 -23.837 27.522 1.00 0.00
I H
ATOM 1667 CG2 THR I 142 8.891 -23.353 26.557 1.00 0.00
I C
ATOM 1668 HG21 THR I 142 9.421 -22.386 26.685 1.00 0.00
I H
ATOM 1669 HG22 THR I 142 8.159 -23.235 25.730 1.00 0.00
I H
ATOM 1670 HG23 THR I 142 8.328 -23.568 27.490 1.00 0.00
I H
ATOM 1671 C THR I 142 9.882 -26.711 25.323 1.00 0.00
I C
ATOM 1672 O THR I 142 10.429 -26.481 24.247 1.00 0.00
I O
ATOM 1673 N ILE I 143 9.940 -27.882 25.979 1.00 0.00
I N

```
ATOM   1674  HN  ILE I 143       9.278 -28.048  26.708  1.00  0.00
I    H
ATOM   1675  CA  ILE I 143      10.868 -28.973  25.761  1.00  0.00
I    C
ATOM   1676  HA  ILE I 143      11.753 -28.533  25.319  1.00  0.00
I    H
ATOM   1677  CB  ILE I 143      11.141 -29.678  27.095  1.00  0.00
I    C
ATOM   1678  HB  ILE I 143      11.732 -30.587  26.815  1.00  0.00
I    H
ATOM   1679  CG2 ILE I 143      11.893 -28.687  28.014  1.00  0.00
I    C
ATOM   1680 HG21 ILE I 143      12.251 -29.199  28.931  1.00  0.00
I    H
ATOM   1681 HG22 ILE I 143      12.780 -28.263  27.498  1.00  0.00
I    H
ATOM   1682 HG23 ILE I 143      11.231 -27.849  28.320  1.00  0.00
I    H
ATOM   1683  CG1 ILE I 143       9.845 -30.203  27.770  1.00  0.00
I    C
ATOM   1684 HG11 ILE I 143       9.229 -29.345  28.121  1.00  0.00
I    H
ATOM   1685 HG12 ILE I 143       9.241 -30.753  27.012  1.00  0.00
I    H
ATOM   1686  CD  ILE I 143      10.126 -31.146  28.947  1.00  0.00
I    C
ATOM   1687  HD1 ILE I 143       9.173 -31.541  29.360  1.00  0.00
I    H
ATOM   1688  HD2 ILE I 143      10.747 -32.008  28.619  1.00  0.00
I    H
ATOM   1689  HD3 ILE I 143      10.661 -30.616  29.763  1.00  0.00
I    H
ATOM   1690  C   ILE I 143      10.324 -29.973  24.752  1.00  0.00
I    C
ATOM   1691  O   ILE I 143      10.875 -31.050  24.545  1.00  0.00
I    O
ATOM   1692  N   GLY I 144       9.201 -29.627  24.081  1.00  0.00
I    N
ATOM   1693  HN  GLY I 144       8.703 -28.799  24.335  1.00  0.00
I    H
ATOM   1694  CA  GLY I 144       8.633 -30.390  22.974  1.00  0.00
I    C
ATOM   1695  HA1 GLY I 144       7.787 -29.814  22.625  1.00  0.00
I    H
ATOM   1696  HA2 GLY I 144       8.373 -31.357  23.383  1.00  0.00
I    H
ATOM   1697  C   GLY I 144       9.562 -30.615  21.801  1.00  0.00
I    C
ATOM   1698  O   GLY I 144      10.357 -29.755  21.424  1.00  0.00
I    O
ATOM   1699  N   GLY I 145       9.447 -31.788  21.148  1.00  0.00
I    N
ATOM   1700  HN  GLY I 145       8.746 -32.457  21.392  1.00  0.00
I    H
```

```
ATOM   1701  CA  GLY I 145      10.325 -32.169  20.051  1.00  0.00
I    C
ATOM   1702  HA1 GLY I 145      10.185 -33.230  19.902  1.00  0.00
I    H
ATOM   1703  HA2 GLY I 145      11.328 -31.890  20.345  1.00  0.00
I    H
ATOM   1704  C   GLY I 145      10.012 -31.472  18.757  1.00  0.00
I    C
ATOM   1705  O   GLY I 145       9.135 -31.904  18.018  1.00  0.00
I    O
ATOM   1706  N   VAL I 146      10.773 -30.409  18.417  1.00  0.00
I    N
ATOM   1707  HN  VAL I 146      11.526 -30.121  19.003  1.00  0.00
I    H
ATOM   1708  CA  VAL I 146      10.596 -29.594  17.214  1.00  0.00
I    C
ATOM   1709  HA  VAL I 146       9.617 -29.139  17.288  1.00  0.00
I    H
ATOM   1710  CB  VAL I 146      11.674 -28.504  17.143  1.00  0.00
I    C
ATOM   1711  HB  VAL I 146      12.670 -29.012  17.164  1.00  0.00
I    H
ATOM   1712  CG1 VAL I 146      11.560 -27.683  15.840  1.00  0.00
I    C
ATOM   1713 HG11 VAL I 146      12.323 -26.875  15.825  1.00  0.00
I    H
ATOM   1714 HG12 VAL I 146      11.730 -28.313  14.943  1.00  0.00
I    H
ATOM   1715 HG13 VAL I 146      10.558 -27.210  15.760  1.00  0.00
I    H
ATOM   1716  CG2 VAL I 146      11.523 -27.565  18.360  1.00  0.00
I    C
ATOM   1717 HG21 VAL I 146      12.267 -26.741  18.314  1.00  0.00
I    H
ATOM   1718 HG22 VAL I 146      10.506 -27.118  18.384  1.00  0.00
I    H
ATOM   1719 HG23 VAL I 146      11.686 -28.111  19.312  1.00  0.00
I    H
ATOM   1720  C   VAL I 146      10.621 -30.421  15.936  1.00  0.00
I    C
ATOM   1721  O   VAL I 146       9.745 -30.309  15.082  1.00  0.00
I    O
ATOM   1722  N   ILE I 147      11.591 -31.350  15.813  1.00  0.00
I    N
ATOM   1723  HN  ILE I 147      12.303 -31.445  16.505  1.00  0.00
I    H
ATOM   1724  CA  ILE I 147      11.711 -32.287  14.700  1.00  0.00
I    C
ATOM   1725  HA  ILE I 147      11.729 -31.679  13.805  1.00  0.00
I    H
ATOM   1726  CB  ILE I 147      13.003 -33.094  14.797  1.00  0.00
I    C
ATOM   1727  HB  ILE I 147      12.997 -33.510  15.836  1.00  0.00
I    H
```

```
ATOM   1728  CG2 ILE I 147      13.053 -34.174  13.685  1.00  0.00
I    C
ATOM   1729 HG21 ILE I 147      13.911 -34.860  13.846  1.00  0.00
I    H
ATOM   1730 HG22 ILE I 147      12.127 -34.787  13.686  1.00  0.00
I    H
ATOM   1731 HG23 ILE I 147      13.164 -33.708  12.683  1.00  0.00
I    H
ATOM   1732  CG1 ILE I 147      14.218 -32.136  14.693  1.00  0.00
I    C
ATOM   1733 HG11 ILE I 147      14.263 -31.693  13.673  1.00  0.00
I    H
ATOM   1734 HG12 ILE I 147      14.078 -31.296  15.410  1.00  0.00
I    H
ATOM   1735  CD  ILE I 147      15.558 -32.814  15.004  1.00  0.00
I    C
ATOM   1736  HD1 ILE I 147      16.384 -32.070  14.970  1.00  0.00
I    H
ATOM   1737  HD2 ILE I 147      15.541 -33.269  16.018  1.00  0.00
I    H
ATOM   1738  HD3 ILE I 147      15.784 -33.610  14.263  1.00  0.00
I    H
ATOM   1739  C   ILE I 147      10.502 -33.212  14.580  1.00  0.00
I    C
ATOM   1740  O   ILE I 147      10.033 -33.531  13.487  1.00  0.00
I    O
ATOM   1741  N   GLY I 148       9.927 -33.644  15.721  1.00  0.00
I    N
ATOM   1742  HN  GLY I 148      10.300 -33.421  16.620  1.00  0.00
I    H
ATOM   1743  CA  GLY I 148       8.723 -34.465  15.726  1.00  0.00
I    C
ATOM   1744  HA1 GLY I 148       8.539 -34.752  16.752  1.00  0.00
I    H
ATOM   1745  HA2 GLY I 148       8.897 -35.296  15.055  1.00  0.00
I    H
ATOM   1746  C   GLY I 148       7.517 -33.712  15.229  1.00  0.00
I    C
ATOM   1747  O   GLY I 148       6.725 -34.242  14.460  1.00  0.00
I    O
ATOM   1748  N   LEU I 149       7.377 -32.430  15.605  1.00  0.00
I    N
ATOM   1749  HN  LEU I 149       7.991 -31.994  16.261  1.00  0.00
I    H
ATOM   1750  CA  LEU I 149       6.331 -31.561  15.099  1.00  0.00
I    C
ATOM   1751  HA  LEU I 149       5.408 -32.099  15.271  1.00  0.00
I    H
ATOM   1752  CB  LEU I 149       6.304 -30.232  15.872  1.00  0.00
I    C
ATOM   1753  HB1 LEU I 149       7.291 -29.738  15.714  1.00  0.00
I    H
ATOM   1754  HB2 LEU I 149       5.547 -29.560  15.411  1.00  0.00
I    H
```

```
ATOM   1755  CG  LEU I 149       6.053 -30.420  17.378  1.00  0.00
I    C
ATOM   1756  HG  LEU I 149       6.801 -31.160  17.752  1.00  0.00
I    H
ATOM   1757  CD1 LEU I 149       6.249 -29.077  18.069  1.00  0.00
I    C
ATOM   1758 HD11 LEU I 149       6.029 -29.161  19.155  1.00  0.00
I    H
ATOM   1759 HD12 LEU I 149       7.297 -28.725  17.952  1.00  0.00
I    H
ATOM   1760 HD13 LEU I 149       5.571 -28.310  17.637  1.00  0.00
I    H
ATOM   1761  CD2 LEU I 149       4.650 -30.958  17.701  1.00  0.00
I    C
ATOM   1762 HD21 LEU I 149       4.532 -31.091  18.798  1.00  0.00
I    H
ATOM   1763 HD22 LEU I 149       3.857 -30.263  17.359  1.00  0.00
I    H
ATOM   1764 HD23 LEU I 149       4.489 -31.942  17.212  1.00  0.00
I    H
ATOM   1765  C   LEU I 149       6.422 -31.268  13.610  1.00  0.00
I    C
ATOM   1766  O   LEU I 149       5.421 -31.348  12.904  1.00  0.00
I    O
ATOM   1767  N   VAL I 150       7.624 -30.971  13.061  1.00  0.00
I    N
ATOM   1768  HN  VAL I 150       8.465 -30.857  13.584  1.00  0.00
I    H
ATOM   1769  CA  VAL I 150       7.780 -30.789  11.618  1.00  0.00
I    C
ATOM   1770  HA  VAL I 150       7.050 -30.046  11.317  1.00  0.00
I    H
ATOM   1771  CB  VAL I 150       9.087 -30.136  11.146  1.00  0.00
I    C
ATOM   1772  HB  VAL I 150       8.954 -29.852  10.073  1.00  0.00
I    H
ATOM   1773  CG1 VAL I 150       9.371 -28.872  11.983  1.00  0.00
I    C
ATOM   1774 HG11 VAL I 150      10.336 -28.414  11.675  1.00  0.00
I    H
ATOM   1775 HG12 VAL I 150       8.582 -28.104  11.845  1.00  0.00
I    H
ATOM   1776 HG13 VAL I 150       9.440 -29.124  13.063  1.00  0.00
I    H
ATOM   1777  CG2 VAL I 150      10.297 -31.091  11.167  1.00  0.00
I    C
ATOM   1778 HG21 VAL I 150      11.221 -30.561  10.856  1.00  0.00
I    H
ATOM   1779 HG22 VAL I 150      10.454 -31.499  12.189  1.00  0.00
I    H
ATOM   1780 HG23 VAL I 150      10.149 -31.942  10.470  1.00  0.00
I    H
ATOM   1781  C   VAL I 150       7.488 -32.062  10.830  1.00  0.00
I    C
```

```
ATOM   1782  O   VAL I 150       6.906 -32.016   9.748  1.00  0.00
I    O
ATOM   1783  N   LYS I 151       7.847 -33.244  11.379  1.00  0.00
I    N
ATOM   1784  HN  LYS I 151       8.407 -33.279  12.205  1.00  0.00
I    H
ATOM   1785  CA  LYS I 151       7.465 -34.536  10.838  1.00  0.00
I    C
ATOM   1786  HA  LYS I 151       7.787 -34.529   9.804  1.00  0.00
I    H
ATOM   1787  CB  LYS I 151       8.198 -35.671  11.595  1.00  0.00
I    C
ATOM   1788  HB1 LYS I 151       9.284 -35.417  11.595  1.00  0.00
I    H
ATOM   1789  HB2 LYS I 151       7.876 -35.662  12.660  1.00  0.00
I    H
ATOM   1790  CG  LYS I 151       7.997 -37.059  10.965  1.00  0.00
I    C
ATOM   1791  HG1 LYS I 151       6.910 -37.299  10.953  1.00  0.00
I    H
ATOM   1792  HG2 LYS I 151       8.343 -37.030   9.906  1.00  0.00
I    H
ATOM   1793  CD  LYS I 151       8.736 -38.171  11.726  1.00  0.00
I    C
ATOM   1794  HD1 LYS I 151       9.823 -37.930  11.732  1.00  0.00
I    H
ATOM   1795  HD2 LYS I 151       8.385 -38.159  12.783  1.00  0.00
I    H
ATOM   1796  CE  LYS I 151       8.498 -39.553  11.108  1.00  0.00
I    C
ATOM   1797  HE1 LYS I 151       7.413 -39.800  11.114  1.00  0.00
I    H
ATOM   1798  HE2 LYS I 151       8.859 -39.578  10.056  1.00  0.00
I    H
ATOM   1799  NZ  LYS I 151       9.215 -40.599  11.871  1.00  0.00
I    N
ATOM   1800  HZ1 LYS I 151       9.045 -41.532  11.444  1.00  0.00
I    H
ATOM   1801  HZ2 LYS I 151      10.236 -40.396  11.863  1.00  0.00
I    H
ATOM   1802  HZ3 LYS I 151       8.876 -40.603  12.854  1.00  0.00
I    H
ATOM   1803  C   LYS I 151       5.954 -34.799  10.845  1.00  0.00
I    C
ATOM   1804  O   LYS I 151       5.405 -35.304   9.867  1.00  0.00
I    O
ATOM   1805  N   VAL I 152       5.234 -34.438  11.932  1.00  0.00
I    N
ATOM   1806  HN  VAL I 152       5.682 -34.123  12.765  1.00  0.00
I    H
ATOM   1807  CA  VAL I 152       3.770 -34.467  12.006  1.00  0.00
I    C
ATOM   1808  HA  VAL I 152       3.461 -35.476  11.764  1.00  0.00
I    H
```

```
ATOM   1809  CB  VAL I 152       3.261 -34.136  13.415  1.00  0.00
I    C
ATOM   1810  HB  VAL I 152       3.733 -33.171  13.727  1.00  0.00
I    H
ATOM   1811  CG1 VAL I 152       1.724 -33.963  13.462  1.00  0.00
I    C
ATOM   1812 HG11 VAL I 152       1.391 -33.763  14.503  1.00  0.00
I    H
ATOM   1813 HG12 VAL I 152       1.389 -33.110  12.838  1.00  0.00
I    H
ATOM   1814 HG13 VAL I 152       1.215 -34.885  13.109  1.00  0.00
I    H
ATOM   1815  CG2 VAL I 152       3.651 -35.281  14.373  1.00  0.00
I    C
ATOM   1816 HG21 VAL I 152       3.260 -35.087  15.395  1.00  0.00
I    H
ATOM   1817 HG22 VAL I 152       3.234 -36.247  14.016  1.00  0.00
I    H
ATOM   1818 HG23 VAL I 152       4.753 -35.383  14.449  1.00  0.00
I    H
ATOM   1819  C   VAL I 152       3.127 -33.534  10.983  1.00  0.00
I    C
ATOM   1820  O   VAL I 152       2.175 -33.901  10.294  1.00  0.00
I    O
ATOM   1821  N   LEU I 153       3.665 -32.306  10.813  1.00  0.00
I    N
ATOM   1822  HN  LEU I 153       4.394 -31.961  11.400  1.00  0.00
I    H
ATOM   1823  CA  LEU I 153       3.244 -31.377   9.773  1.00  0.00
I    C
ATOM   1824  HA  LEU I 153       2.179 -31.244   9.909  1.00  0.00
I    H
ATOM   1825  CB  LEU I 153       3.959 -30.008   9.913  1.00  0.00
I    C
ATOM   1826  HB1 LEU I 153       4.939 -30.197  10.409  1.00  0.00
I    H
ATOM   1827  HB2 LEU I 153       4.198 -29.619   8.899  1.00  0.00
I    H
ATOM   1828  CG  LEU I 153       3.166 -28.968  10.734  1.00  0.00
I    C
ATOM   1829  HG  LEU I 153       2.184 -28.889  10.209  1.00  0.00
I    H
ATOM   1830  CD1 LEU I 153       2.944 -29.359  12.204  1.00  0.00
I    C
ATOM   1831 HD11 LEU I 153       2.412 -28.549  12.747  1.00  0.00
I    H
ATOM   1832 HD12 LEU I 153       2.334 -30.285  12.274  1.00  0.00
I    H
ATOM   1833 HD13 LEU I 153       3.914 -29.538  12.713  1.00  0.00
I    H
ATOM   1834  CD2 LEU I 153       3.861 -27.604  10.649  1.00  0.00
I    C
ATOM   1835 HD21 LEU I 153       3.287 -26.843  11.222  1.00  0.00
I    H
```

```
ATOM   1836 HD22 LEU I 153       4.886 -27.639  11.070  1.00  0.00
I    H
ATOM   1837 HD23 LEU I 153       3.927 -27.265   9.593  1.00  0.00
I    H
ATOM   1838  C   LEU I 153       3.464 -31.918   8.369  1.00  0.00
I    C
ATOM   1839  O   LEU I 153       2.570 -31.843   7.528  1.00  0.00
I    O
ATOM   1840  N   PHE I 154       4.637 -32.532   8.090  1.00  0.00
I    N
ATOM   1841  HN  PHE I 154       5.388 -32.561   8.748  1.00  0.00
I    H
ATOM   1842  CA  PHE I 154       4.917 -33.199   6.825  1.00  0.00
I    C
ATOM   1843  HA  PHE I 154       4.767 -32.460   6.047  1.00  0.00
I    H
ATOM   1844  CB  PHE I 154       6.386 -33.744   6.828  1.00  0.00
I    C
ATOM   1845  HB1 PHE I 154       7.087 -32.899   7.007  1.00  0.00
I    H
ATOM   1846  HB2 PHE I 154       6.539 -34.478   7.648  1.00  0.00
I    H
ATOM   1847  CG  PHE I 154       6.824 -34.374   5.516  1.00  0.00
I    C
ATOM   1848  CD1 PHE I 154       6.416 -35.676   5.165  1.00  0.00
I    C
ATOM   1849  HD1 PHE I 154       5.821 -36.255   5.858  1.00  0.00
I    H
ATOM   1850  CE1 PHE I 154       6.775 -36.236   3.932  1.00  0.00
I    C
ATOM   1851  HE1 PHE I 154       6.461 -37.240   3.683  1.00  0.00
I    H
ATOM   1852  CZ  PHE I 154       7.533 -35.494   3.022  1.00  0.00
I    C
ATOM   1853  HZ  PHE I 154       7.765 -35.901   2.048  1.00  0.00
I    H
ATOM   1854  CD2 PHE I 154       7.658 -33.677   4.622  1.00  0.00
I    C
ATOM   1855  HD2 PHE I 154       8.073 -32.720   4.905  1.00  0.00
I    H
ATOM   1856  CE2 PHE I 154       7.985 -34.216   3.371  1.00  0.00
I    C
ATOM   1857  HE2 PHE I 154       8.586 -33.643   2.678  1.00  0.00
I    H
ATOM   1858  C   PHE I 154       3.915 -34.319   6.576  1.00  0.00
I    C
ATOM   1859  O   PHE I 154       3.305 -34.384   5.510  1.00  0.00
I    O
ATOM   1860  N   LEU I 155       3.661 -35.174   7.593  1.00  0.00
I    N
ATOM   1861  HN  LEU I 155       4.124 -35.098   8.474  1.00  0.00
I    H
ATOM   1862  CA  LEU I 155       2.710 -36.267   7.513  1.00  0.00
I    C
```

```
ATOM   1863  HA  LEU I 155       3.034 -36.887   6.688  1.00  0.00
I    H
ATOM   1864  CB  LEU I 155       2.694 -37.087   8.835  1.00  0.00
I    C
ATOM   1865  HB1 LEU I 155       3.688 -37.583   8.926  1.00  0.00
I    H
ATOM   1866  HB2 LEU I 155       2.620 -36.385   9.695  1.00  0.00
I    H
ATOM   1867  CG  LEU I 155       1.598 -38.177   8.947  1.00  0.00
I    C
ATOM   1868  HG  LEU I 155       0.632 -37.623   8.854  1.00  0.00
I    H
ATOM   1869  CD1 LEU I 155       1.693 -39.240   7.839  1.00  0.00
I    C
ATOM   1870 HD11 LEU I 155       0.922 -40.025   7.985  1.00  0.00
I    H
ATOM   1871 HD12 LEU I 155       1.538 -38.779   6.840  1.00  0.00
I    H
ATOM   1872 HD13 LEU I 155       2.691 -39.727   7.849  1.00  0.00
I    H
ATOM   1873  CD2 LEU I 155       1.634 -38.838  10.335  1.00  0.00
I    C
ATOM   1874 HD21 LEU I 155       0.834 -39.605  10.417  1.00  0.00
I    H
ATOM   1875 HD22 LEU I 155       2.603 -39.340  10.524  1.00  0.00
I    H
ATOM   1876 HD23 LEU I 155       1.471 -38.079  11.131  1.00  0.00
I    H
ATOM   1877  C   LEU I 155       1.307 -35.792   7.203  1.00  0.00
I    C
ATOM   1878  O   LEU I 155       0.657 -36.332   6.314  1.00  0.00
I    O
ATOM   1879  N   PHE I 156       0.808 -34.736   7.876  1.00  0.00
I    N
ATOM   1880  HN  PHE I 156       1.289 -34.275   8.619  1.00  0.00
I    H
ATOM   1881  CA  PHE I 156      -0.492 -34.177   7.565  1.00  0.00
I    C
ATOM   1882  HA  PHE I 156      -1.190 -35.002   7.617  1.00  0.00
I    H
ATOM   1883  CB  PHE I 156      -0.867 -33.105   8.627  1.00  0.00
I    C
ATOM   1884  HB1 PHE I 156      -0.929 -33.589   9.626  1.00  0.00
I    H
ATOM   1885  HB2 PHE I 156      -0.087 -32.316   8.692  1.00  0.00
I    H
ATOM   1886  CG  PHE I 156      -2.220 -32.477   8.360  1.00  0.00
I    C
ATOM   1887  CD1 PHE I 156      -2.316 -31.324   7.561  1.00  0.00
I    C
ATOM   1888  HD1 PHE I 156      -1.416 -30.862   7.178  1.00  0.00
I    H
ATOM   1889  CE1 PHE I 156      -3.561 -30.758   7.258  1.00  0.00
I    C
```

```
ATOM   1890  HE1 PHE I 156      -3.617 -29.871   6.643  1.00  0.00
I    H
ATOM   1891  CZ  PHE I 156      -4.731 -31.348   7.746  1.00  0.00
I    C
ATOM   1892  HZ  PHE I 156      -5.694 -30.908   7.527  1.00  0.00
I    H
ATOM   1893  CD2 PHE I 156      -3.406 -33.069   8.830  1.00  0.00
I    C
ATOM   1894  HD2 PHE I 156      -3.358 -33.946   9.461  1.00  0.00
I    H
ATOM   1895  CE2 PHE I 156      -4.655 -32.518   8.513  1.00  0.00
I    C
ATOM   1896  HE2 PHE I 156      -5.559 -32.997   8.861  1.00  0.00
I    H
ATOM   1897  C   PHE I 156      -0.550 -33.627   6.137  1.00  0.00
I    C
ATOM   1898  O   PHE I 156      -1.469 -33.976   5.397  1.00  0.00
I    O
ATOM   1899  N   SER I 157       0.458 -32.827   5.699  1.00  0.00
I    N
ATOM   1900  HN  SER I 157       1.243 -32.603   6.276  1.00  0.00
I    H
ATOM   1901  CA  SER I 157       0.501 -32.222   4.364  1.00  0.00
I    C
ATOM   1902  HA  SER I 157      -0.401 -31.630   4.277  1.00  0.00
I    H
ATOM   1903  CB  SER I 157       1.785 -31.377   4.104  1.00  0.00
I    C
ATOM   1904  HB1 SER I 157       2.670 -32.040   4.241  1.00  0.00
I    H
ATOM   1905  HB2 SER I 157       1.806 -31.016   3.051  1.00  0.00
I    H
ATOM   1906  OG  SER I 157       1.889 -30.284   5.021  1.00  0.00
I    O
ATOM   1907  HG1 SER I 157       2.791 -29.946   4.958  1.00  0.00
I    H
ATOM   1908  C   SER I 157       0.456 -33.270   3.280  1.00  0.00
I    C
ATOM   1909  O   SER I 157      -0.494 -33.341   2.502  1.00  0.00
I    O
ATOM   1910  N   ILE I 158       1.393 -34.245   3.310  1.00  0.00
I    N
ATOM   1911  HN  ILE I 158       2.057 -34.393   4.039  1.00  0.00
I    H
ATOM   1912  CA  ILE I 158       1.494 -35.190   2.212  1.00  0.00
I    C
ATOM   1913  HA  ILE I 158       1.191 -34.655   1.320  1.00  0.00
I    H
ATOM   1914  CB  ILE I 158       2.922 -35.656   1.934  1.00  0.00
I    C
ATOM   1915  HB  ILE I 158       3.535 -34.721   1.983  1.00  0.00
I    H
ATOM   1916  CG2 ILE I 158       3.402 -36.692   2.971  1.00  0.00
I    C
```

```
ATOM   1917 HG21 ILE I 158       4.486 -36.898   2.844  1.00  0.00
I    H
ATOM   1918 HG22 ILE I 158       3.250 -36.318   4.005  1.00  0.00
I    H
ATOM   1919 HG23 ILE I 158       2.852 -37.650   2.860  1.00  0.00
I    H
ATOM   1920  CG1 ILE I 158       3.074 -36.203   0.488  1.00  0.00
I    C
ATOM   1921 HG11 ILE I 158       2.897 -37.302   0.481  1.00  0.00
I    H
ATOM   1922 HG12 ILE I 158       2.291 -35.740  -0.156  1.00  0.00
I    H
ATOM   1923  CD  ILE I 158       4.445 -35.908  -0.129  1.00  0.00
I    C
ATOM   1924  HD1 ILE I 158       4.481 -36.268  -1.181  1.00  0.00
I    H
ATOM   1925  HD2 ILE I 158       4.648 -34.815  -0.129  1.00  0.00
I    H
ATOM   1926  HD3 ILE I 158       5.255 -36.416   0.436  1.00  0.00
I    H
ATOM   1927  C   ILE I 158       0.515 -36.345   2.386  1.00  0.00
I    C
ATOM   1928  O   ILE I 158       0.392 -37.225   1.548  1.00  0.00
I    O
ATOM   1929  N   SER I 159      -0.283 -36.338   3.472  1.00  0.00
I    N
ATOM   1930  HN  SER I 159      -0.097 -35.785   4.285  1.00  0.00
I    H
ATOM   1931  CA  SER I 159      -1.496 -37.139   3.561  1.00  0.00
I    C
ATOM   1932  HA  SER I 159      -1.303 -38.055   3.018  1.00  0.00
I    H
ATOM   1933  CB  SER I 159      -1.865 -37.428   5.047  1.00  0.00
I    C
ATOM   1934  HB1 SER I 159      -1.012 -37.970   5.517  1.00  0.00
I    H
ATOM   1935  HB2 SER I 159      -1.995 -36.475   5.607  1.00  0.00
I    H
ATOM   1936  OG  SER I 159      -3.028 -38.242   5.207  1.00  0.00
I    O
ATOM   1937  HG1 SER I 159      -3.040 -38.543   6.124  1.00  0.00
I    H
ATOM   1938  C   SER I 159      -2.635 -36.428   2.857  1.00  0.00
I    C
ATOM   1939  O   SER I 159      -3.200 -36.967   1.903  1.00  0.00
I    O
ATOM   1940  N   ILE I 160      -2.973 -35.167   3.216  1.00  0.00
I    N
ATOM   1941  HN  ILE I 160      -2.451 -34.616   3.864  1.00  0.00
I    H
ATOM   1942  CA  ILE I 160      -4.153 -34.497   2.674  1.00  0.00
I    C
ATOM   1943  HA  ILE I 160      -4.980 -35.168   2.870  1.00  0.00
I    H
```

```
ATOM   1944  CB  ILE I 160      -4.500 -33.198   3.420  1.00  0.00
I    C
ATOM   1945  HB  ILE I 160      -4.629 -33.513   4.486  1.00  0.00
I    H
ATOM   1946  CG2 ILE I 160      -3.384 -32.139   3.312  1.00  0.00
I    C
ATOM   1947 HG21 ILE I 160      -3.593 -31.280   3.984  1.00  0.00
I    H
ATOM   1948 HG22 ILE I 160      -2.403 -32.566   3.607  1.00  0.00
I    H
ATOM   1949 HG23 ILE I 160      -3.300 -31.754   2.274  1.00  0.00
I    H
ATOM   1950  CG1 ILE I 160      -5.860 -32.586   3.000  1.00  0.00
I    C
ATOM   1951 HG11 ILE I 160      -5.950 -31.553   3.405  1.00  0.00
I    H
ATOM   1952 HG12 ILE I 160      -5.893 -32.509   1.889  1.00  0.00
I    H
ATOM   1953  CD  ILE I 160      -7.068 -33.412   3.460  1.00  0.00
I    C
ATOM   1954  HD1 ILE I 160      -8.010 -32.960   3.083  1.00  0.00
I    H
ATOM   1955  HD2 ILE I 160      -7.002 -34.452   3.073  1.00  0.00
I    H
ATOM   1956  HD3 ILE I 160      -7.125 -33.451   4.568  1.00  0.00
I    H
ATOM   1957  C   ILE I 160      -4.094 -34.300   1.161  1.00  0.00
I    C
ATOM   1958  O   ILE I 160      -5.046 -34.599   0.437  1.00  0.00
I    O
ATOM   1959  N   GLU I 161      -2.936 -33.861   0.628  1.00  0.00
I    N
ATOM   1960  HN  GLU I 161      -2.139 -33.624   1.177  1.00  0.00
I    H
ATOM   1961  CA  GLU I 161      -2.740 -33.691  -0.796  1.00  0.00
I    C
ATOM   1962  HA  GLU I 161      -3.582 -33.124  -1.172  1.00  0.00
I    H
ATOM   1963  CB  GLU I 161      -1.534 -32.765  -1.078  1.00  0.00
I    C
ATOM   1964  HB1 GLU I 161      -1.549 -32.493  -2.159  1.00  0.00
I    H
ATOM   1965  HB2 GLU I 161      -1.655 -31.808  -0.520  1.00  0.00
I    H
ATOM   1966  CG  GLU I 161      -0.169 -33.388  -0.744  1.00  0.00
I    C
ATOM   1967  HG1 GLU I 161      -0.238 -33.939   0.212  1.00  0.00
I    H
ATOM   1968  HG2 GLU I 161       0.156 -34.084  -1.543  1.00  0.00
I    H
ATOM   1969  CD  GLU I 161       0.903 -32.324  -0.546  1.00  0.00
I    C
ATOM   1970  OE1 GLU I 161       1.090 -31.833   0.593  1.00  0.00
I    O
```

```
ATOM   1971  OE2 GLU I 161       1.574 -32.001  -1.550  1.00  0.00
I    O
ATOM   1972  C   GLU I 161      -2.710 -35.019  -1.558  1.00  0.00
I    C
ATOM   1973  O   GLU I 161      -3.285 -35.123  -2.639  1.00  0.00
I    O
ATOM   1974  N   LEU I 162      -2.115 -36.106  -1.000  1.00  0.00
I    N
ATOM   1975  HN  LEU I 162      -1.598 -36.065  -0.147  1.00  0.00
I    H
ATOM   1976  CA  LEU I 162      -2.177 -37.438  -1.601  1.00  0.00
I    C
ATOM   1977  HA  LEU I 162      -1.847 -37.305  -2.624  1.00  0.00
I    H
ATOM   1978  CB  LEU I 162      -1.218 -38.471  -0.955  1.00  0.00
I    C
ATOM   1979  HB1 LEU I 162      -1.058 -38.155   0.101  1.00  0.00
I    H
ATOM   1980  HB2 LEU I 162      -1.728 -39.458  -0.909  1.00  0.00
I    H
ATOM   1981  CG  LEU I 162       0.141 -38.569  -1.678  1.00  0.00
I    C
ATOM   1982  HG  LEU I 162       0.523 -37.528  -1.818  1.00  0.00
I    H
ATOM   1983  CD1 LEU I 162       1.141 -39.404  -0.866  1.00  0.00
I    C
ATOM   1984 HD11 LEU I 162       2.100 -39.509  -1.418  1.00  0.00
I    H
ATOM   1985 HD12 LEU I 162       1.352 -38.921   0.112  1.00  0.00
I    H
ATOM   1986 HD13 LEU I 162       0.739 -40.422  -0.676  1.00  0.00
I    H
ATOM   1987  CD2 LEU I 162      -0.020 -39.208  -3.069  1.00  0.00
I    C
ATOM   1988 HD21 LEU I 162       0.962 -39.257  -3.588  1.00  0.00
I    H
ATOM   1989 HD22 LEU I 162      -0.417 -40.241  -3.001  1.00  0.00
I    H
ATOM   1990 HD23 LEU I 162      -0.715 -38.607  -3.695  1.00  0.00
I    H
ATOM   1991  C   LEU I 162      -3.571 -38.039  -1.644  1.00  0.00
I    C
ATOM   1992  O   LEU I 162      -3.960 -38.605  -2.666  1.00  0.00
I    O
ATOM   1993  N   ILE I 163      -4.363 -37.906  -0.552  1.00  0.00
I    N
ATOM   1994  HN  ILE I 163      -4.041 -37.468   0.285  1.00  0.00
I    H
ATOM   1995  CA  ILE I 163      -5.745 -38.381  -0.487  1.00  0.00
I    C
ATOM   1996  HA  ILE I 163      -5.714 -39.433  -0.741  1.00  0.00
I    H
ATOM   1997  CB  ILE I 163      -6.355 -38.271   0.917  1.00  0.00
I    C
```

```
ATOM   1998  HB  ILE I 163       -6.134 -37.221   1.237  1.00  0.00
I    H
ATOM   1999  CG2 ILE I 163       -7.873 -38.584   0.909  1.00  0.00
I    C
ATOM   2000 HG21 ILE I 163       -8.325 -38.349   1.896  1.00  0.00
I    H
ATOM   2001 HG22 ILE I 163       -8.399 -37.975   0.144  1.00  0.00
I    H
ATOM   2002 HG23 ILE I 163       -8.057 -39.657   0.691  1.00  0.00
I    H
ATOM   2003  CG1 ILE I 163       -5.634 -39.199   1.933  1.00  0.00
I    C
ATOM   2004 HG11 ILE I 163       -4.572 -38.884   2.044  1.00  0.00
I    H
ATOM   2005 HG12 ILE I 163       -6.117 -39.086   2.931  1.00  0.00
I    H
ATOM   2006  CD  ILE I 163       -5.641 -40.702   1.604  1.00  0.00
I    C
ATOM   2007  HD1 ILE I 163       -5.153 -41.278   2.420  1.00  0.00
I    H
ATOM   2008  HD2 ILE I 163       -6.683 -41.074   1.492  1.00  0.00
I    H
ATOM   2009  HD3 ILE I 163       -5.091 -40.906   0.662  1.00  0.00
I    H
ATOM   2010  C   ILE I 163       -6.625 -37.702  -1.528  1.00  0.00
I    C
ATOM   2011  O   ILE I 163       -7.426 -38.329  -2.220  1.00  0.00
I    O
ATOM   2012  N   ALA I 164       -6.458 -36.384  -1.717  1.00  0.00
I    N
ATOM   2013  HN  ALA I 164       -5.895 -35.821  -1.113  1.00  0.00
I    H
ATOM   2014  CA  ALA I 164       -7.084 -35.684  -2.811  1.00  0.00
I    C
ATOM   2015  HA  ALA I 164       -8.147 -35.898  -2.805  1.00  0.00
I    H
ATOM   2016  CB  ALA I 164       -6.954 -34.182  -2.540  1.00  0.00
I    C
ATOM   2017  HB1 ALA I 164       -7.383 -33.945  -1.543  1.00  0.00
I    H
ATOM   2018  HB2 ALA I 164       -5.887 -33.868  -2.535  1.00  0.00
I    H
ATOM   2019  HB3 ALA I 164       -7.496 -33.580  -3.300  1.00  0.00
I    H
ATOM   2020  C   ALA I 164       -6.570 -36.085  -4.206  1.00  0.00
I    C
ATOM   2021  O   ALA I 164       -7.353 -36.210  -5.145  1.00  0.00
I    O
ATOM   2022  N   ALA I 165       -5.252 -36.320  -4.384  1.00  0.00
I    N
ATOM   2023  HN  ALA I 165       -4.621 -36.301  -3.608  1.00  0.00
I    H
ATOM   2024  CA  ALA I 165       -4.650 -36.613  -5.670  1.00  0.00
I    C
```

```
ATOM   2025  HA  ALA I 165      -5.023 -35.890  -6.387  1.00  0.00
I    H
ATOM   2026  CB  ALA I 165      -3.121 -36.502  -5.533  1.00  0.00
I    C
ATOM   2027  HB1 ALA I 165      -2.854 -35.493  -5.152  1.00  0.00
I    H
ATOM   2028  HB2 ALA I 165      -2.730 -37.254  -4.814  1.00  0.00
I    H
ATOM   2029  HB3 ALA I 165      -2.611 -36.647  -6.510  1.00  0.00
I    H
ATOM   2030  C   ALA I 165      -4.936 -37.986  -6.258  1.00  0.00
I    C
ATOM   2031  O   ALA I 165      -5.600 -38.129  -7.286  1.00  0.00
I    O
ATOM   2032  N   LEU I 166      -4.466 -39.078  -5.625  1.00  0.00
I    N
ATOM   2033  HN  LEU I 166      -4.149 -39.104  -4.679  1.00  0.00
I    H
ATOM   2034  CA  LEU I 166      -4.387 -40.359  -6.319  1.00  0.00
I    C
ATOM   2035  HA  LEU I 166      -4.248 -40.136  -7.368  1.00  0.00
I    H
ATOM   2036  CB  LEU I 166      -3.194 -41.216  -5.814  1.00  0.00
I    C
ATOM   2037  HB1 LEU I 166      -3.263 -41.255  -4.703  1.00  0.00
I    H
ATOM   2038  HB2 LEU I 166      -3.326 -42.263  -6.167  1.00  0.00
I    H
ATOM   2039  CG  LEU I 166      -1.785 -40.699  -6.198  1.00  0.00
I    C
ATOM   2040  HG  LEU I 166      -1.674 -39.687  -5.739  1.00  0.00
I    H
ATOM   2041  CD1 LEU I 166      -0.726 -41.663  -5.635  1.00  0.00
I    C
ATOM   2042 HD11 LEU I 166       0.293 -41.341  -5.937  1.00  0.00
I    H
ATOM   2043 HD12 LEU I 166      -0.770 -41.687  -4.525  1.00  0.00
I    H
ATOM   2044 HD13 LEU I 166      -0.892 -42.693  -6.016  1.00  0.00
I    H
ATOM   2045  CD2 LEU I 166      -1.578 -40.557  -7.719  1.00  0.00
I    C
ATOM   2046 HD21 LEU I 166      -0.560 -40.166  -7.935  1.00  0.00
I    H
ATOM   2047 HD22 LEU I 166      -1.682 -41.530  -8.239  1.00  0.00
I    H
ATOM   2048 HD23 LEU I 166      -2.320 -39.850  -8.148  1.00  0.00
I    H
ATOM   2049  C   LEU I 166      -5.687 -41.137  -6.182  1.00  0.00
I    C
ATOM   2050  O   LEU I 166      -5.807 -42.288  -6.597  1.00  0.00
I    O
ATOM   2051  N   ILE I 167      -6.716 -40.475  -5.629  1.00  0.00
I    N
```

```
ATOM   2052  HN  ILE I 167      -6.614 -39.529  -5.327  1.00  0.00
I    H
ATOM   2053  CA  ILE I 167      -8.031 -41.025  -5.415  1.00  0.00
I    C
ATOM   2054  HA  ILE I 167      -8.076 -41.970  -5.942  1.00  0.00
I    H
ATOM   2055  CB  ILE I 167      -8.330 -41.270  -3.941  1.00  0.00
I    C
ATOM   2056  HB  ILE I 167      -8.212 -40.266  -3.460  1.00  0.00
I    H
ATOM   2057  CG2 ILE I 167      -9.731 -41.914  -3.809  1.00  0.00
I    C
ATOM   2058 HG21 ILE I 167     -10.041 -41.960  -2.745  1.00  0.00
I    H
ATOM   2059 HG22 ILE I 167     -10.493 -41.319  -4.356  1.00  0.00
I    H
ATOM   2060 HG23 ILE I 167      -9.734 -42.947  -4.217  1.00  0.00
I    H
ATOM   2061  CG1 ILE I 167      -7.267 -42.196  -3.288  1.00  0.00
I    C
ATOM   2062 HG11 ILE I 167      -7.744 -42.820  -2.500  1.00  0.00
I    H
ATOM   2063 HG12 ILE I 167      -6.869 -42.891  -4.063  1.00  0.00
I    H
ATOM   2064  CD  ILE I 167      -6.073 -41.457  -2.669  1.00  0.00
I    C
ATOM   2065  HD1 ILE I 167      -5.331 -42.184  -2.275  1.00  0.00
I    H
ATOM   2066  HD2 ILE I 167      -5.569 -40.822  -3.428  1.00  0.00
I    H
ATOM   2067  HD3 ILE I 167      -6.398 -40.809  -1.827  1.00  0.00
I    H
ATOM   2068  C   ILE I 167      -9.050 -40.112  -6.063  1.00  0.00
I    C
ATOM   2069  O   ILE I 167      -9.546 -40.422  -7.145  1.00  0.00
I    O
ATOM   2070  N   LEU I 168      -9.414 -38.967  -5.442  1.00  0.00
I    N
ATOM   2071  HN  LEU I 168      -8.987 -38.616  -4.611  1.00  0.00
I    H
ATOM   2072  CA  LEU I 168     -10.490 -38.137  -5.956  1.00  0.00
I    C
ATOM   2073  HA  LEU I 168     -11.324 -38.807  -6.123  1.00  0.00
I    H
ATOM   2074  CB  LEU I 168     -10.866 -37.079  -4.885  1.00  0.00
I    C
ATOM   2075  HB1 LEU I 168     -10.739 -37.560  -3.889  1.00  0.00
I    H
ATOM   2076  HB2 LEU I 168     -10.127 -36.248  -4.922  1.00  0.00
I    H
ATOM   2077  CG  LEU I 168     -12.304 -36.499  -4.955  1.00  0.00
I    C
ATOM   2078  HG  LEU I 168     -12.234 -35.701  -4.357  1.00  0.00
I    H
```

```
ATOM   2079  CD1 LEU I 168      -13.369 -37.475  -4.434  1.00  0.00
I    C
ATOM   2080 HD11 LEU I 168      -14.375 -37.003  -4.458  1.00  0.00
I    H
ATOM   2081 HD12 LEU I 168      -13.400 -38.392  -5.061  1.00  0.00
I    H
ATOM   2082 HD13 LEU I 168      -13.150 -37.772  -3.387  1.00  0.00
I    H
ATOM   2083  CD2 LEU I 168      -12.754 -36.011  -6.337  1.00  0.00
I    C
ATOM   2084 HD21 LEU I 168      -13.798 -35.633  -6.292  1.00  0.00
I    H
ATOM   2085 HD22 LEU I 168      -12.113 -35.187  -6.709  1.00  0.00
I    H
ATOM   2086 HD23 LEU I 168      -12.717 -36.843  -7.074  1.00  0.00
I    H
ATOM   2087  C   LEU I 168      -10.157 -37.476  -7.289  1.00  0.00
I    C
ATOM   2088  O   LEU I 168      -10.835 -37.707  -8.291  1.00  0.00
I    O
ATOM   2089  N   SER I 169       -9.052 -36.701  -7.364  1.00  0.00
I    N
ATOM   2090  HN  SER I 169       -8.423 -36.583  -6.596  1.00  0.00
I    H
ATOM   2091  CA  SER I 169       -8.674 -35.967  -8.564  1.00  0.00
I    C
ATOM   2092  HA  SER I 169       -9.538 -35.372  -8.828  1.00  0.00
I    H
ATOM   2093  CB  SER I 169       -7.463 -35.022  -8.345  1.00  0.00
I    C
ATOM   2094  HB1 SER I 169       -6.692 -35.576  -7.762  1.00  0.00
I    H
ATOM   2095  HB2 SER I 169       -7.002 -34.750  -9.321  1.00  0.00
I    H
ATOM   2096  OG  SER I 169       -7.824 -33.852  -7.614  1.00  0.00
I    O
ATOM   2097  HG1 SER I 169       -7.003 -33.432  -7.326  1.00  0.00
I    H
ATOM   2098  C   SER I 169       -8.392 -36.896  -9.727  1.00  0.00
I    C
ATOM   2099  O   SER I 169       -8.778 -36.618 -10.857  1.00  0.00
I    O
ATOM   2100  N   ILE I 170       -7.765 -38.076  -9.494  1.00  0.00
I    N
ATOM   2101  HN  ILE I 170       -7.485 -38.409  -8.596  1.00  0.00
I    H
ATOM   2102  CA  ILE I 170       -7.443 -38.978 -10.591  1.00  0.00
I    C
ATOM   2103  HA  ILE I 170       -6.938 -38.361 -11.324  1.00  0.00
I    H
ATOM   2104  CB  ILE I 170       -6.439 -40.080 -10.227  1.00  0.00
I    C
ATOM   2105  HB  ILE I 170       -5.583 -39.534  -9.755  1.00  0.00
I    H
```

```
ATOM   2106  CG2 ILE I 170      -7.059 -41.166  -9.339  1.00  0.00
I    C
ATOM   2107 HG21 ILE I 170      -6.277 -41.860  -8.966  1.00  0.00
I    H
ATOM   2108 HG22 ILE I 170      -7.560 -40.717  -8.455  1.00  0.00
I    H
ATOM   2109 HG23 ILE I 170      -7.810 -41.760  -9.903  1.00  0.00
I    H
ATOM   2110  CG1 ILE I 170      -5.856 -40.789 -11.465  1.00  0.00
I    C
ATOM   2111 HG11 ILE I 170      -5.081 -41.523 -11.149  1.00  0.00
I    H
ATOM   2112 HG12 ILE I 170      -6.667 -41.363 -11.968  1.00  0.00
I    H
ATOM   2113  CD  ILE I 170      -5.253 -39.820 -12.474  1.00  0.00
I    C
ATOM   2114  HD1 ILE I 170      -4.899 -40.370 -13.373  1.00  0.00
I    H
ATOM   2115  HD2 ILE I 170      -6.007 -39.071 -12.798  1.00  0.00
I    H
ATOM   2116  HD3 ILE I 170      -4.386 -39.279 -12.037  1.00  0.00
I    H
ATOM   2117  C   ILE I 170      -8.661 -39.577 -11.285  1.00  0.00
I    C
ATOM   2118  O   ILE I 170      -8.619 -39.923 -12.463  1.00  0.00
I    O
ATOM   2119  N   ARG I 171      -9.822 -39.692 -10.600  1.00  0.00
I    N
ATOM   2120  HN  ARG I 171      -9.916 -39.396  -9.653  1.00  0.00
I    H
ATOM   2121  CA  ARG I 171     -11.005 -40.265 -11.227  1.00  0.00
I    C
ATOM   2122  HA  ARG I 171     -10.707 -41.213 -11.660  1.00  0.00
I    H
ATOM   2123  CB  ARG I 171     -12.152 -40.629 -10.242  1.00  0.00
I    C
ATOM   2124  HB1 ARG I 171     -12.442 -39.680  -9.731  1.00  0.00
I    H
ATOM   2125  HB2 ARG I 171     -13.047 -40.953 -10.817  1.00  0.00
I    H
ATOM   2126  CG  ARG I 171     -11.856 -41.669  -9.129  1.00  0.00
I    C
ATOM   2127  HG1 ARG I 171     -11.686 -41.126  -8.173  1.00  0.00
I    H
ATOM   2128  HG2 ARG I 171     -12.767 -42.290  -8.962  1.00  0.00
I    H
ATOM   2129  CD  ARG I 171     -10.714 -42.683  -9.335  1.00  0.00
I    C
ATOM   2130  HD1 ARG I 171      -9.743 -42.140  -9.390  1.00  0.00
I    H
ATOM   2131  HD2 ARG I 171     -10.664 -43.420  -8.500  1.00  0.00
I    H
ATOM   2132  NE  ARG I 171     -10.956 -43.448 -10.611  1.00  0.00
I    N
```

```
ATOM   2133  HE  ARG I 171     -11.927 -43.479 -10.875  1.00  0.00
I    H
ATOM   2134  CZ  ARG I 171      -9.988 -44.033 -11.332  1.00  0.00
I    C
ATOM   2135  NH1 ARG I 171      -8.761 -44.195 -10.849  1.00  0.00
I    N
ATOM   2136 HH11 ARG I 171      -8.051 -44.605 -11.404  1.00  0.00
I    H
ATOM   2137 HH12 ARG I 171      -8.570 -43.760  -9.967  1.00  0.00
I    H
ATOM   2138  NH2 ARG I 171     -10.243 -44.444 -12.574  1.00  0.00
I    N
ATOM   2139 HH21 ARG I 171      -9.558 -44.953 -13.076  1.00  0.00
I    H
ATOM   2140 HH22 ARG I 171     -11.187 -44.406 -12.874  1.00  0.00
I    H
ATOM   2141  C   ARG I 171     -11.575 -39.428 -12.363  1.00  0.00
I    C
ATOM   2142  O   ARG I 171     -12.154 -39.982 -13.293  1.00  0.00
I    O
ATOM   2143  N   LEU I 172     -11.402 -38.092 -12.335  1.00  0.00
I    N
ATOM   2144  HN  LEU I 172     -10.983 -37.599 -11.575  1.00  0.00
I    H
ATOM   2145  CA  LEU I 172     -11.825 -37.242 -13.436  1.00  0.00
I    C
ATOM   2146  HA  LEU I 172     -12.680 -37.729 -13.886  1.00  0.00
I    H
ATOM   2147  CB  LEU I 172     -12.271 -35.883 -12.837  1.00  0.00
I    C
ATOM   2148  HB1 LEU I 172     -12.841 -36.106 -11.905  1.00  0.00
I    H
ATOM   2149  HB2 LEU I 172     -11.369 -35.315 -12.517  1.00  0.00
I    H
ATOM   2150  CG  LEU I 172     -13.159 -34.965 -13.713  1.00  0.00
I    C
ATOM   2151  HG  LEU I 172     -13.629 -34.350 -12.907  1.00  0.00
I    H
ATOM   2152  CD1 LEU I 172     -12.372 -34.039 -14.653  1.00  0.00
I    C
ATOM   2153 HD11 LEU I 172     -13.065 -33.444 -15.285  1.00  0.00
I    H
ATOM   2154 HD12 LEU I 172     -11.740 -33.335 -14.070  1.00  0.00
I    H
ATOM   2155 HD13 LEU I 172     -11.712 -34.630 -15.324  1.00  0.00
I    H
ATOM   2156  CD2 LEU I 172     -14.281 -35.720 -14.452  1.00  0.00
I    C
ATOM   2157 HD21 LEU I 172     -14.895 -35.011 -15.048  1.00  0.00
I    H
ATOM   2158 HD22 LEU I 172     -13.873 -36.480 -15.148  1.00  0.00
I    H
ATOM   2159 HD23 LEU I 172     -14.948 -36.233 -13.726  1.00  0.00
I    H
```

```
ATOM   2160  C   LEU I 172     -10.743 -37.121 -14.513  1.00  0.00
I    C
ATOM   2161  O   LEU I 172     -11.029 -36.923 -15.696  1.00  0.00
I    O
ATOM   2162  N   VAL I 173      -9.460 -37.356 -14.159  1.00  0.00
I    N
ATOM   2163  HN  VAL I 173      -9.234 -37.682 -13.244  1.00  0.00
I    H
ATOM   2164  CA  VAL I 173      -8.310 -37.171 -15.034  1.00  0.00
I    C
ATOM   2165  HA  VAL I 173      -8.664 -36.567 -15.860  1.00  0.00
I    H
ATOM   2166  CB  VAL I 173      -7.249 -36.298 -14.328  1.00  0.00
I    C
ATOM   2167  HB  VAL I 173      -6.687 -36.955 -13.619  1.00  0.00
I    H
ATOM   2168  CG1 VAL I 173      -6.243 -35.684 -15.324  1.00  0.00
I    C
ATOM   2169 HG11 VAL I 173      -5.519 -35.032 -14.789  1.00  0.00
I    H
ATOM   2170 HG12 VAL I 173      -5.657 -36.466 -15.849  1.00  0.00
I    H
ATOM   2171 HG13 VAL I 173      -6.769 -35.064 -16.081  1.00  0.00
I    H
ATOM   2172  CG2 VAL I 173      -7.937 -35.087 -13.659  1.00  0.00
I    C
ATOM   2173 HG21 VAL I 173      -7.187 -34.424 -13.180  1.00  0.00
I    H
ATOM   2174 HG22 VAL I 173      -8.499 -34.492 -14.411  1.00  0.00
I    H
ATOM   2175 HG23 VAL I 173      -8.648 -35.414 -12.872  1.00  0.00
I    H
ATOM   2176  C   VAL I 173      -7.726 -38.490 -15.636  1.00  0.00
I    C
ATOM   2177  O   VAL I 173      -6.505 -38.568 -15.768  1.00  0.00
I    O
ATOM   2178  N   PRO I 174      -8.403 -39.586 -16.060  1.00  0.00
I    N
ATOM   2179  CD  PRO I 174      -9.800 -39.932 -15.772  1.00  0.00
I    C
ATOM   2180  HD1 PRO I 174      -9.939 -39.910 -14.667  1.00  0.00
I    H
ATOM   2181  HD2 PRO I 174     -10.510 -39.231 -16.268  1.00  0.00
I    H
ATOM   2182  CA  PRO I 174      -7.716 -40.710 -16.696  1.00  0.00
I    C
ATOM   2183  HA  PRO I 174      -6.703 -40.816 -16.328  1.00  0.00
I    H
ATOM   2184  CB  PRO I 174      -8.592 -41.914 -16.308  1.00  0.00
I    C
ATOM   2185  HB1 PRO I 174      -8.280 -42.280 -15.304  1.00  0.00
I    H
ATOM   2186  HB2 PRO I 174      -8.519 -42.749 -17.034  1.00  0.00
I    H
```

```
ATOM   2187  CG  PRO I 174     -10.014 -41.353 -16.298  1.00  0.00
I    C
ATOM   2188  HG1 PRO I 174     -10.706 -41.942 -15.664  1.00  0.00
I    H
ATOM   2189  HG2 PRO I 174     -10.403 -41.323 -17.342  1.00  0.00
I    H
ATOM   2190  C   PRO I 174      -7.680 -40.501 -18.199  1.00  0.00
I    C
ATOM   2191  O   PRO I 174      -7.034 -41.275 -18.896  1.00  0.00
I    O
ATOM   2192  N   GLN I 175      -8.375 -39.467 -18.721  1.00  0.00
I    N
ATOM   2193  HN  GLN I 175      -8.818 -38.818 -18.105  1.00  0.00
I    H
ATOM   2194  CA  GLN I 175      -8.559 -39.178 -20.136  1.00  0.00
I    C
ATOM   2195  HA  GLN I 175      -9.019 -40.065 -20.555  1.00  0.00
I    H
ATOM   2196  CB  GLN I 175      -9.469 -37.938 -20.293  1.00  0.00
I    C
ATOM   2197  HB1 GLN I 175      -8.976 -37.105 -19.741  1.00  0.00
I    H
ATOM   2198  HB2 GLN I 175      -9.492 -37.631 -21.363  1.00  0.00
I    H
ATOM   2199  CG  GLN I 175     -10.901 -38.147 -19.746  1.00  0.00
I    C
ATOM   2200  HG1 GLN I 175     -11.469 -38.844 -20.401  1.00  0.00
I    H
ATOM   2201  HG2 GLN I 175     -10.873 -38.585 -18.727  1.00  0.00
I    H
ATOM   2202  CD  GLN I 175     -11.661 -36.821 -19.721  1.00  0.00
I    C
ATOM   2203  OE1 GLN I 175     -11.101 -35.745 -19.922  1.00  0.00
I    O
ATOM   2204  NE2 GLN I 175     -12.984 -36.885 -19.453  1.00  0.00
I    N
ATOM   2205 HE21 GLN I 175     -13.483 -36.024 -19.440  1.00  0.00
I    H
ATOM   2206 HE22 GLN I 175     -13.409 -37.764 -19.264  1.00  0.00
I    H
ATOM   2207  C   GLN I 175      -7.266 -38.927 -20.899  1.00  0.00
I    C
ATOM   2208  O   GLN I 175      -7.101 -39.370 -22.030  1.00  0.00
I    O
ATOM   2209  N   TYR I 176      -6.303 -38.220 -20.272  1.00  0.00
I    N
ATOM   2210  HN  TYR I 176      -6.458 -37.803 -19.377  1.00  0.00
I    H
ATOM   2211  CA  TYR I 176      -4.972 -37.998 -20.821  1.00  0.00
I    C
ATOM   2212  HA  TYR I 176      -5.131 -37.636 -21.829  1.00  0.00
I    H
ATOM   2213  CB  TYR I 176      -4.154 -37.011 -19.945  1.00  0.00
I    C
```

ATOM 2214 HB1 TYR I 176 -4.135 -37.383 -18.897 1.00 0.00
I H
ATOM 2215 HB2 TYR I 176 -3.099 -36.957 -20.293 1.00 0.00
I H
ATOM 2216 CG TYR I 176 -4.777 -35.653 -19.939 1.00 0.00
I C
ATOM 2217 CD1 TYR I 176 -4.687 -34.820 -21.066 1.00 0.00
I C
ATOM 2218 HD1 TYR I 176 -4.135 -35.149 -21.935 1.00 0.00
I H
ATOM 2219 CE1 TYR I 176 -5.291 -33.555 -21.058 1.00 0.00
I C
ATOM 2220 HE1 TYR I 176 -5.214 -32.904 -21.916 1.00 0.00
I H
ATOM 2221 CZ TYR I 176 -5.995 -33.121 -19.932 1.00 0.00
I C
ATOM 2222 OH TYR I 176 -6.621 -31.860 -19.973 1.00 0.00
I O
ATOM 2223 HH TYR I 176 -7.112 -31.755 -19.155 1.00 0.00
I H
ATOM 2224 CD2 TYR I 176 -5.448 -35.194 -18.799 1.00 0.00
I C
ATOM 2225 HD2 TYR I 176 -5.464 -35.798 -17.902 1.00 0.00
I H
ATOM 2226 CE2 TYR I 176 -6.062 -33.933 -18.791 1.00 0.00
I C
ATOM 2227 HE2 TYR I 176 -6.563 -33.587 -17.899 1.00 0.00
I H
ATOM 2228 C TYR I 176 -4.138 -39.268 -20.927 1.00 0.00
I C
ATOM 2229 O TYR I 176 -3.214 -39.351 -21.732 1.00 0.00
I O
ATOM 2230 N GLY I 177 -4.433 -40.278 -20.087 1.00 0.00
I N
ATOM 2231 HN GLY I 177 -5.192 -40.183 -19.444 1.00 0.00
I H
ATOM 2232 CA GLY I 177 -3.718 -41.540 -20.031 1.00 0.00
I C
ATOM 2233 HA1 GLY I 177 -2.829 -41.460 -20.641 1.00 0.00
I H
ATOM 2234 HA2 GLY I 177 -4.390 -42.335 -20.327 1.00 0.00
I H
ATOM 2235 C GLY I 177 -3.289 -41.800 -18.618 1.00 0.00
I C
ATOM 2236 O GLY I 177 -3.279 -40.899 -17.787 1.00 0.00
I O
ATOM 2237 N TRP I 178 -2.909 -43.048 -18.283 1.00 0.00
I N
ATOM 2238 HN TRP I 178 -2.893 -43.795 -18.943 1.00 0.00
I H
ATOM 2239 CA TRP I 178 -2.490 -43.410 -16.935 1.00 0.00
I C
ATOM 2240 HA TRP I 178 -3.299 -43.113 -16.279 1.00 0.00
I H

ATOM 2241 CB TRP I 178 -2.286 -44.945 -16.827 1.00 0.00
I C
ATOM 2242 HB1 TRP I 178 -1.707 -45.275 -17.718 1.00 0.00
I H
ATOM 2243 HB2 TRP I 178 -1.668 -45.182 -15.934 1.00 0.00
I H
ATOM 2244 CG TRP I 178 -3.596 -45.725 -16.808 1.00 0.00
I C
ATOM 2245 CD1 TRP I 178 -4.360 -46.183 -17.849 1.00 0.00
I C
ATOM 2246 HD1 TRP I 178 -4.093 -46.083 -18.893 1.00 0.00
I H
ATOM 2247 NE1 TRP I 178 -5.484 -46.821 -17.368 1.00 0.00
I N
ATOM 2248 HE1 TRP I 178 -6.176 -47.257 -17.902 1.00 0.00
I H
ATOM 2249 CE2 TRP I 178 -5.459 -46.790 -15.993 1.00 0.00
I C
ATOM 2250 CD2 TRP I 178 -4.285 -46.103 -15.601 1.00 0.00
I C
ATOM 2251 CE3 TRP I 178 -3.981 -45.915 -14.254 1.00 0.00
I C
ATOM 2252 HE3 TRP I 178 -3.098 -45.376 -13.938 1.00 0.00
I H
ATOM 2253 CZ3 TRP I 178 -4.860 -46.440 -13.297 1.00 0.00
I C
ATOM 2254 HZ3 TRP I 178 -4.641 -46.311 -12.246 1.00 0.00
I H
ATOM 2255 CZ2 TRP I 178 -6.335 -47.299 -15.042 1.00 0.00
I C
ATOM 2256 HZ2 TRP I 178 -7.237 -47.818 -15.328 1.00 0.00
I H
ATOM 2257 CH2 TRP I 178 -6.023 -47.126 -13.683 1.00 0.00
I C
ATOM 2258 HH2 TRP I 178 -6.684 -47.524 -12.925 1.00 0.00
I H
ATOM 2259 C TRP I 178 -1.236 -42.680 -16.452 1.00 0.00
I C
ATOM 2260 O TRP I 178 -1.206 -42.140 -15.350 1.00 0.00
I O
ATOM 2261 N SER I 179 -0.174 -42.584 -17.277 1.00 0.00
I N
ATOM 2262 HN SER I 179 -0.149 -43.001 -18.186 1.00 0.00
I H
ATOM 2263 CA SER I 179 1.042 -41.859 -16.917 1.00 0.00
I C
ATOM 2264 HA SER I 179 1.334 -42.259 -15.955 1.00 0.00
I H
ATOM 2265 CB SER I 179 2.139 -42.069 -18.001 1.00 0.00
I C
ATOM 2266 HB1 SER I 179 2.956 -41.335 -17.813 1.00 0.00
I H
ATOM 2267 HB2 SER I 179 2.585 -43.086 -17.909 1.00 0.00
I H

```
ATOM   2268  OG  SER I 179       1.614 -41.850 -19.314  1.00  0.00
I    O
ATOM   2269  HG1 SER I 179       2.369 -41.773 -19.912  1.00  0.00
I    H
ATOM   2270  C   SER I 179       0.822 -40.361 -16.737  1.00  0.00
I    C
ATOM   2271  O   SER I 179       1.162 -39.779 -15.707  1.00  0.00
I    O
ATOM   2272  N   SER I 180       0.179 -39.720 -17.731  1.00  0.00
I    N
ATOM   2273  HN  SER I 180      -0.101 -40.211 -18.555  1.00  0.00
I    H
ATOM   2274  CA  SER I 180      -0.172 -38.307 -17.730  1.00  0.00
I    C
ATOM   2275  HA  SER I 180       0.753 -37.789 -17.515  1.00  0.00
I    H
ATOM   2276  CB  SER I 180      -0.791 -37.863 -19.075  1.00  0.00
I    C
ATOM   2277  HB1 SER I 180      -1.643 -38.543 -19.306  1.00  0.00
I    H
ATOM   2278  HB2 SER I 180      -1.207 -36.833 -18.990  1.00  0.00
I    H
ATOM   2279  OG  SER I 180       0.182 -37.957 -20.112  1.00  0.00
I    O
ATOM   2280  HG1 SER I 180      -0.290 -37.891 -20.951  1.00  0.00
I    H
ATOM   2281  C   SER I 180      -1.156 -37.934 -16.653  1.00  0.00
I    C
ATOM   2282  O   SER I 180      -1.012 -36.907 -15.993  1.00  0.00
I    O
ATOM   2283  N   GLY I 181      -2.188 -38.770 -16.428  1.00  0.00
I    N
ATOM   2284  HN  GLY I 181      -2.271 -39.633 -16.925  1.00  0.00
I    H
ATOM   2285  CA  GLY I 181      -3.242 -38.496 -15.469  1.00  0.00
I    C
ATOM   2286  HA1 GLY I 181      -3.991 -39.265 -15.599  1.00  0.00
I    H
ATOM   2287  HA2 GLY I 181      -3.597 -37.493 -15.665  1.00  0.00
I    H
ATOM   2288  C   GLY I 181      -2.764 -38.547 -14.052  1.00  0.00
I    C
ATOM   2289  O   GLY I 181      -3.050 -37.636 -13.279  1.00  0.00
I    O
ATOM   2290  N   LEU I 182      -1.987 -39.587 -13.662  1.00  0.00
I    N
ATOM   2291  HN  LEU I 182      -1.739 -40.328 -14.282  1.00  0.00
I    H
ATOM   2292  CA  LEU I 182      -1.442 -39.725 -12.314  1.00  0.00
I    C
ATOM   2293  HA  LEU I 182      -2.285 -39.700 -11.635  1.00  0.00
I    H
ATOM   2294  CB  LEU I 182      -0.667 -41.064 -12.130  1.00  0.00
I    C
```

```
ATOM   2295  HB1 LEU I 182      -0.222 -41.325 -13.118  1.00  0.00
I    H
ATOM   2296  HB2 LEU I 182       0.191 -40.893 -11.442  1.00  0.00
I    H
ATOM   2297  CG  LEU I 182      -1.505 -42.275 -11.640  1.00  0.00
I    C
ATOM   2298  HG  LEU I 182      -1.804 -42.008 -10.597  1.00  0.00
I    H
ATOM   2299  CD1 LEU I 182      -2.740 -42.599 -12.492  1.00  0.00
I    C
ATOM   2300 HD11 LEU I 182      -3.255 -43.504 -12.103  1.00  0.00
I    H
ATOM   2301 HD12 LEU I 182      -3.461 -41.754 -12.475  1.00  0.00
I    H
ATOM   2302 HD13 LEU I 182      -2.449 -42.792 -13.547  1.00  0.00
I    H
ATOM   2303  CD2 LEU I 182      -0.617 -43.526 -11.574  1.00  0.00
I    C
ATOM   2304 HD21 LEU I 182      -1.203 -44.396 -11.206  1.00  0.00
I    H
ATOM   2305 HD22 LEU I 182      -0.210 -43.792 -12.570  1.00  0.00
I    H
ATOM   2306 HD23 LEU I 182       0.235 -43.362 -10.879  1.00  0.00
I    H
ATOM   2307  C   LEU I 182      -0.535 -38.556 -11.962  1.00  0.00
I    C
ATOM   2308  O   LEU I 182      -0.651 -37.971 -10.886  1.00  0.00
I    O
ATOM   2309  N   PHE I 183       0.350 -38.153 -12.897  1.00  0.00
I    N
ATOM   2310  HN  PHE I 183       0.466 -38.629 -13.767  1.00  0.00
I    H
ATOM   2311  CA  PHE I 183       1.208 -36.999 -12.725  1.00  0.00
I    C
ATOM   2312  HA  PHE I 183       1.677 -37.134 -11.758  1.00  0.00
I    H
ATOM   2313  CB  PHE I 183       2.294 -36.972 -13.832  1.00  0.00
I    C
ATOM   2314  HB1 PHE I 183       2.717 -37.994 -13.952  1.00  0.00
I    H
ATOM   2315  HB2 PHE I 183       1.859 -36.681 -14.812  1.00  0.00
I    H
ATOM   2316  CG  PHE I 183       3.410 -36.047 -13.421  1.00  0.00
I    C
ATOM   2317  CD1 PHE I 183       4.331 -36.439 -12.433  1.00  0.00
I    C
ATOM   2318  HD1 PHE I 183       4.272 -37.432 -12.010  1.00  0.00
I    H
ATOM   2319  CE1 PHE I 183       5.320 -35.553 -11.986  1.00  0.00
I    C
ATOM   2320  HE1 PHE I 183       6.035 -35.872 -11.241  1.00  0.00
I    H
ATOM   2321  CZ  PHE I 183       5.383 -34.257 -12.512  1.00  0.00
I    C
```

ATOM 2322 HZ PHE I 183 6.152 -33.573 -12.182 1.00 0.00
I H
ATOM 2323 CD2 PHE I 183 3.484 -34.747 -13.941 1.00 0.00
I C
ATOM 2324 HD2 PHE I 183 2.777 -34.424 -14.691 1.00 0.00
I H
ATOM 2325 CE2 PHE I 183 4.453 -33.851 -13.476 1.00 0.00
I C
ATOM 2326 HE2 PHE I 183 4.483 -32.841 -13.862 1.00 0.00
I H
ATOM 2327 C PHE I 183 0.442 -35.676 -12.670 1.00 0.00
I C
ATOM 2328 O PHE I 183 0.679 -34.869 -11.779 1.00 0.00
I O
ATOM 2329 N ALA I 184 -0.540 -35.436 -13.573 1.00 0.00
I N
ATOM 2330 HN ALA I 184 -0.742 -36.080 -14.311 1.00 0.00
I H
ATOM 2331 CA ALA I 184 -1.369 -34.241 -13.545 1.00 0.00
I C
ATOM 2332 HA ALA I 184 -0.744 -33.357 -13.594 1.00 0.00
I H
ATOM 2333 CB ALA I 184 -2.328 -34.242 -14.760 1.00 0.00
I C
ATOM 2334 HB1 ALA I 184 -1.739 -34.278 -15.701 1.00 0.00
I H
ATOM 2335 HB2 ALA I 184 -2.996 -35.131 -14.741 1.00 0.00
I H
ATOM 2336 HB3 ALA I 184 -2.958 -33.327 -14.785 1.00 0.00
I H
ATOM 2337 C ALA I 184 -2.177 -34.124 -12.269 1.00 0.00
I C
ATOM 2338 O ALA I 184 -2.209 -33.102 -11.597 1.00 0.00
I O
ATOM 2339 N SER I 185 -2.799 -35.226 -11.847 1.00 0.00
I N
ATOM 2340 HN SER I 185 -2.796 -36.093 -12.346 1.00 0.00
I H
ATOM 2341 CA SER I 185 -3.543 -35.260 -10.609 1.00 0.00
I C
ATOM 2342 HA SER I 185 -4.255 -34.451 -10.695 1.00 0.00
I H
ATOM 2343 CB SER I 185 -4.213 -36.636 -10.483 1.00 0.00
I C
ATOM 2344 HB1 SER I 185 -4.752 -36.846 -11.435 1.00 0.00
I H
ATOM 2345 HB2 SER I 185 -3.445 -37.432 -10.359 1.00 0.00
I H
ATOM 2346 OG SER I 185 -5.134 -36.604 -9.408 1.00 0.00
I O
ATOM 2347 HG1 SER I 185 -5.693 -37.388 -9.490 1.00 0.00
I H
ATOM 2348 C SER I 185 -2.699 -34.992 -9.364 1.00 0.00
I C

```
ATOM   2349  O   SER I 185      -3.067 -34.209  -8.488  1.00  0.00
I    O
ATOM   2350  N   LEU I 186      -1.510 -35.626  -9.280  1.00  0.00
I    N
ATOM   2351  HN  LEU I 186      -1.205 -36.273  -9.975  1.00  0.00
I    H
ATOM   2352  CA  LEU I 186      -0.574 -35.433  -8.194  1.00  0.00
I    C
ATOM   2353  HA  LEU I 186      -1.144 -35.552  -7.281  1.00  0.00
I    H
ATOM   2354  CB  LEU I 186       0.450 -36.599  -8.201  1.00  0.00
I    C
ATOM   2355  HB1 LEU I 186      -0.126 -37.542  -8.342  1.00  0.00
I    H
ATOM   2356  HB2 LEU I 186       1.104 -36.500  -9.096  1.00  0.00
I    H
ATOM   2357  CG  LEU I 186       1.340 -36.781  -6.944  1.00  0.00
I    C
ATOM   2358  HG  LEU I 186       1.552 -37.876  -6.992  1.00  0.00
I    H
ATOM   2359  CD1 LEU I 186       2.655 -36.000  -7.054  1.00  0.00
I    C
ATOM   2360 HD11 LEU I 186       3.252 -36.108  -6.122  1.00  0.00
I    H
ATOM   2361 HD12 LEU I 186       3.264 -36.378  -7.904  1.00  0.00
I    H
ATOM   2362 HD13 LEU I 186       2.458 -34.919  -7.215  1.00  0.00
I    H
ATOM   2363  CD2 LEU I 186       0.633 -36.490  -5.604  1.00  0.00
I    C
ATOM   2364 HD21 LEU I 186       1.330 -36.661  -4.756  1.00  0.00
I    H
ATOM   2365 HD22 LEU I 186       0.283 -35.440  -5.546  1.00  0.00
I    H
ATOM   2366 HD23 LEU I 186      -0.244 -37.160  -5.473  1.00  0.00
I    H
ATOM   2367  C   LEU I 186       0.024 -34.035  -8.182  1.00  0.00
I    C
ATOM   2368  O   LEU I 186      -0.014 -33.366  -7.150  1.00  0.00
I    O
ATOM   2369  N   PHE I 187       0.480 -33.495  -9.333  1.00  0.00
I    N
ATOM   2370  HN  PHE I 187       0.443 -33.996 -10.196  1.00  0.00
I    H
ATOM   2371  CA  PHE I 187       1.057 -32.163  -9.434  1.00  0.00
I    C
ATOM   2372  HA  PHE I 187       1.897 -32.153  -8.751  1.00  0.00
I    H
ATOM   2373  CB  PHE I 187       1.491 -31.922 -10.918  1.00  0.00
I    C
ATOM   2374  HB1 PHE I 187       2.260 -32.674 -11.199  1.00  0.00
I    H
ATOM   2375  HB2 PHE I 187       0.634 -32.055 -11.613  1.00  0.00
I    H
```

```
ATOM   2376  CG  PHE I 187       2.123 -30.574 -11.176  1.00  0.00
I    C
ATOM   2377  CD1 PHE I 187       1.318 -29.445 -11.412  1.00  0.00
I    C
ATOM   2378  HD1 PHE I 187       0.242 -29.550 -11.429  1.00  0.00
I    H
ATOM   2379  CE1 PHE I 187       1.893 -28.190 -11.631  1.00  0.00
I    C
ATOM   2380  HE1 PHE I 187       1.264 -27.333 -11.824  1.00  0.00
I    H
ATOM   2381  CZ  PHE I 187       3.285 -28.041 -11.595  1.00  0.00
I    C
ATOM   2382  HZ  PHE I 187       3.730 -27.067 -11.734  1.00  0.00
I    H
ATOM   2383  CD2 PHE I 187       3.519 -30.419 -11.197  1.00  0.00
I    C
ATOM   2384  HD2 PHE I 187       4.159 -31.284 -11.103  1.00  0.00
I    H
ATOM   2385  CE2 PHE I 187       4.098 -29.155 -11.366  1.00  0.00
I    C
ATOM   2386  HE2 PHE I 187       5.173 -29.045 -11.320  1.00  0.00
I    H
ATOM   2387  C   PHE I 187       0.088 -31.080  -8.968  1.00  0.00
I    C
ATOM   2388  O   PHE I 187       0.427 -30.227  -8.148  1.00  0.00
I    O
ATOM   2389  N   HSE I 188      -1.169 -31.134  -9.449  1.00  0.00
I    N
ATOM   2390  HN  HSE I 188      -1.479 -31.863 -10.058  1.00  0.00
I    H
ATOM   2391  CA  HSE I 188      -2.184 -30.149  -9.132  1.00  0.00
I    C
ATOM   2392  HA  HSE I 188      -1.708 -29.187  -9.278  1.00  0.00
I    H
ATOM   2393  CB  HSE I 188      -3.386 -30.235 -10.092  1.00  0.00
I    C
ATOM   2394  HB1 HSE I 188      -3.634 -31.310 -10.234  1.00  0.00
I    H
ATOM   2395  HB2 HSE I 188      -4.271 -29.757  -9.621  1.00  0.00
I    H
ATOM   2396  ND1 HSE I 188      -2.245 -30.279 -12.307  1.00  0.00
I    N
ATOM   2397  CG  HSE I 188      -3.068 -29.626 -11.415  1.00  0.00
I    C
ATOM   2398  CE1 HSE I 188      -2.126 -29.472 -13.328  1.00  0.00
I    C
ATOM   2399  HE1 HSE I 188      -1.535 -29.685 -14.222  1.00  0.00
I    H
ATOM   2400  NE2 HSE I 188      -2.822 -28.330 -13.142  1.00  0.00
I    N
ATOM   2401  HE2 HSE I 188      -2.874 -27.551 -13.767  1.00  0.00
I    H
ATOM   2402  CD2 HSE I 188      -3.432 -28.419 -11.913  1.00  0.00
I    C
```

```
ATOM   2403  HD2 HSE I 188      -4.052 -27.648 -11.479  1.00  0.00
I    H
ATOM   2404  C   HSE I 188      -2.674 -30.191  -7.700  1.00  0.00
I    C
ATOM   2405  O   HSE I 188      -3.092 -29.176  -7.151  1.00  0.00
I    O
ATOM   2406  N   ALA I 189      -2.643 -31.356  -7.028  1.00  0.00
I    N
ATOM   2407  HN  ALA I 189      -2.412 -32.231  -7.454  1.00  0.00
I    H
ATOM   2408  CA  ALA I 189      -2.949 -31.415  -5.614  1.00  0.00
I    C
ATOM   2409  HA  ALA I 189      -3.868 -30.873  -5.422  1.00  0.00
I    H
ATOM   2410  CB  ALA I 189      -3.211 -32.866  -5.198  1.00  0.00
I    C
ATOM   2411  HB1 ALA I 189      -4.025 -33.291  -5.822  1.00  0.00
I    H
ATOM   2412  HB2 ALA I 189      -2.305 -33.494  -5.344  1.00  0.00
I    H
ATOM   2413  HB3 ALA I 189      -3.521 -32.937  -4.133  1.00  0.00
I    H
ATOM   2414  C   ALA I 189      -1.877 -30.777  -4.727  1.00  0.00
I    C
ATOM   2415  O   ALA I 189      -2.202 -30.016  -3.817  1.00  0.00
I    O
ATOM   2416  N   ILE I 190      -0.575 -31.029  -5.022  1.00  0.00
I    N
ATOM   2417  HN  ILE I 190      -0.318 -31.699  -5.716  1.00  0.00
I    H
ATOM   2418  CA  ILE I 190       0.562 -30.370  -4.376  1.00  0.00
I    C
ATOM   2419  HA  ILE I 190       0.438 -30.513  -3.311  1.00  0.00
I    H
ATOM   2420  CB  ILE I 190       1.908 -30.933  -4.868  1.00  0.00
I    C
ATOM   2421  HB  ILE I 190       1.892 -30.771  -5.975  1.00  0.00
I    H
ATOM   2422  CG2 ILE I 190       3.028 -30.173  -4.116  1.00  0.00
I    C
ATOM   2423 HG21 ILE I 190       4.023 -30.424  -4.539  1.00  0.00
I    H
ATOM   2424 HG22 ILE I 190       2.891 -29.075  -4.207  1.00  0.00
I    H
ATOM   2425 HG23 ILE I 190       3.033 -30.437  -3.037  1.00  0.00
I    H
ATOM   2426  CG1 ILE I 190       1.950 -32.469  -4.630  1.00  0.00
I    C
ATOM   2427 HG11 ILE I 190       1.386 -32.722  -3.705  1.00  0.00
I    H
ATOM   2428 HG12 ILE I 190       1.438 -32.979  -5.479  1.00  0.00
I    H
ATOM   2429  CD  ILE I 190       3.340 -33.110  -4.508  1.00  0.00
I    C
```

```
ATOM   2430  HD1 ILE I 190       3.247 -34.213  -4.399  1.00  0.00
I    H
ATOM   2431  HD2 ILE I 190       3.949 -32.900  -5.413  1.00  0.00
I    H
ATOM   2432  HD3 ILE I 190       3.882 -32.721  -3.620  1.00  0.00
I    H
ATOM   2433  C   ILE I 190       0.525 -28.868  -4.626  1.00  0.00
I    C
ATOM   2434  O   ILE I 190       0.648 -28.033  -3.728  1.00  0.00
I    O
ATOM   2435  N   SER I 191       0.279 -28.468  -5.891  1.00  0.00
I    N
ATOM   2436  HN  SER I 191       0.172 -29.101  -6.657  1.00  0.00
I    H
ATOM   2437  CA  SER I 191       0.145 -27.069  -6.255  1.00  0.00
I    C
ATOM   2438  HA  SER I 191       1.045 -26.591  -5.892  1.00  0.00
I    H
ATOM   2439  CB  SER I 191       0.223 -26.788  -7.793  1.00  0.00
I    C
ATOM   2440  HB1 SER I 191       0.496 -25.718  -7.936  1.00  0.00
I    H
ATOM   2441  HB2 SER I 191       1.031 -27.398  -8.258  1.00  0.00
I    H
ATOM   2442  OG  SER I 191      -0.995 -27.006  -8.501  1.00  0.00
I    O
ATOM   2443  HG1 SER I 191      -0.896 -26.596  -9.369  1.00  0.00
I    H
ATOM   2444  C   SER I 191      -1.033 -26.394  -5.570  1.00  0.00
I    C
ATOM   2445  O   SER I 191      -0.919 -25.259  -5.112  1.00  0.00
I    O
ATOM   2446  N   ALA I 192      -2.189 -27.067  -5.425  1.00  0.00
I    N
ATOM   2447  HN  ALA I 192      -2.345 -27.966  -5.834  1.00  0.00
I    H
ATOM   2448  CA  ALA I 192      -3.293 -26.531  -4.662  1.00  0.00
I    C
ATOM   2449  HA  ALA I 192      -3.498 -25.528  -5.019  1.00  0.00
I    H
ATOM   2450  CB  ALA I 192      -4.555 -27.364  -4.947  1.00  0.00
I    C
ATOM   2451  HB1 ALA I 192      -4.736 -27.403  -6.042  1.00  0.00
I    H
ATOM   2452  HB2 ALA I 192      -4.434 -28.408  -4.583  1.00  0.00
I    H
ATOM   2453  HB3 ALA I 192      -5.454 -26.923  -4.464  1.00  0.00
I    H
ATOM   2454  C   ALA I 192      -3.073 -26.415  -3.153  1.00  0.00
I    C
ATOM   2455  O   ALA I 192      -3.256 -25.341  -2.591  1.00  0.00
I    O
ATOM   2456  N   PHE I 193      -2.620 -27.480  -2.454  1.00  0.00
I    N
```

```
ATOM   2457  HN  PHE I 193      -2.368 -28.340  -2.894  1.00  0.00
I    H
ATOM   2458  CA  PHE I 193      -2.458 -27.473  -1.000  1.00  0.00
I    C
ATOM   2459  HA  PHE I 193      -3.417 -27.162  -0.602  1.00  0.00
I    H
ATOM   2460  CB  PHE I 193      -2.155 -28.907  -0.471  1.00  0.00
I    C
ATOM   2461  HB1 PHE I 193      -2.987 -29.586  -0.760  1.00  0.00
I    H
ATOM   2462  HB2 PHE I 193      -1.227 -29.315  -0.928  1.00  0.00
I    H
ATOM   2463  CG  PHE I 193      -2.059 -28.932   1.040  1.00  0.00
I    C
ATOM   2464  CD1 PHE I 193      -3.188 -28.725   1.857  1.00  0.00
I    C
ATOM   2465  HD1 PHE I 193      -4.164 -28.618   1.404  1.00  0.00
I    H
ATOM   2466  CE1 PHE I 193      -3.055 -28.649   3.252  1.00  0.00
I    C
ATOM   2467  HE1 PHE I 193      -3.929 -28.508   3.872  1.00  0.00
I    H
ATOM   2468  CZ  PHE I 193      -1.790 -28.764   3.841  1.00  0.00
I    C
ATOM   2469  HZ  PHE I 193      -1.683 -28.725   4.916  1.00  0.00
I    H
ATOM   2470  CD2 PHE I 193      -0.799 -29.051   1.646  1.00  0.00
I    C
ATOM   2471  HD2 PHE I 193       0.080 -29.208   1.037  1.00  0.00
I    H
ATOM   2472  CE2 PHE I 193      -0.662 -28.940   3.034  1.00  0.00
I    C
ATOM   2473  HE2 PHE I 193       0.320 -28.990   3.485  1.00  0.00
I    H
ATOM   2474  C   PHE I 193      -1.422 -26.448  -0.526  1.00  0.00
I    C
ATOM   2475  O   PHE I 193      -1.601 -25.745   0.468  1.00  0.00
I    O
ATOM   2476  N   ASN I 194      -0.318 -26.283  -1.272  1.00  0.00
I    N
ATOM   2477  HN  ASN I 194      -0.149 -26.789  -2.118  1.00  0.00
I    H
ATOM   2478  CA  ASN I 194       0.729 -25.352  -0.911  1.00  0.00
I    C
ATOM   2479  HA  ASN I 194       0.677 -25.270   0.169  1.00  0.00
I    H
ATOM   2480  CB  ASN I 194       2.110 -25.886  -1.361  1.00  0.00
I    C
ATOM   2481  HB1 ASN I 194       2.050 -26.114  -2.448  1.00  0.00
I    H
ATOM   2482  HB2 ASN I 194       2.888 -25.107  -1.226  1.00  0.00
I    H
ATOM   2483  CG  ASN I 194       2.416 -27.156  -0.579  1.00  0.00
I    C
```

ATOM   2484 OD1 ASN I 194   1.922 -28.233 -0.887 1.00 0.00
I   O
ATOM   2485 ND2 ASN I 194   3.252 -27.046   0.476 1.00 0.00
I   N
ATOM   2486 HD21 ASN I 194   3.463 -27.878   0.982 1.00 0.00
I   H
ATOM   2487 HD22 ASN I 194   3.655 -26.166   0.704 1.00 0.00
I   H
ATOM   2488 C   ASN I 194   0.502 -23.963 -1.491 1.00 0.00
I   C
ATOM   2489 O   ASN I 194   1.407 -23.135 -1.426 1.00 0.00
I   O
ATOM   2490 N   ASN I 195   -0.706 -23.666 -2.038 1.00 0.00
I   N
ATOM   2491 HN ASN I 195   -1.421 -24.359 -2.115 1.00 0.00
I   H
ATOM   2492 CA ASN I 195   -1.078 -22.353 -2.552 1.00 0.00
I   C
ATOM   2493 HA ASN I 195   -2.080 -22.483 -2.945 1.00 0.00
I   H
ATOM   2494 CB ASN I 195   -1.163 -21.295 -1.413 1.00 0.00
I   C
ATOM   2495 HB1 ASN I 195   -0.375 -21.535 -0.666 1.00 0.00
I   H
ATOM   2496 HB2 ASN I 195   -0.947 -20.282 -1.811 1.00 0.00
I   H
ATOM   2497 CG ASN I 195   -2.525 -21.358 -0.745 1.00 0.00
I   C
ATOM   2498 OD1 ASN I 195   -2.741 -22.022   0.263 1.00 0.00
I   O
ATOM   2499 ND2 ASN I 195   -3.503 -20.639 -1.341 1.00 0.00
I   N
ATOM   2500 HD21 ASN I 195   -4.405 -20.653 -0.917 1.00 0.00
I   H
ATOM   2501 HD22 ASN I 195   -3.301 -20.099 -2.152 1.00 0.00
I   H
ATOM   2502 C   ASN I 195   -0.192 -21.876 -3.695 1.00 0.00
I   C
ATOM   2503 O   ASN I 195   0.248 -20.735 -3.746 1.00 0.00
I   O
ATOM   2504 N   ALA I 196   0.098 -22.764 -4.661 1.00 0.00
I   N
ATOM   2505 HN ALA I 196   -0.323 -23.669 -4.714 1.00 0.00
I   H
ATOM   2506 CA ALA I 196   1.050 -22.464 -5.698 1.00 0.00
I   C
ATOM   2507 HA ALA I 196   1.693 -21.663 -5.352 1.00 0.00
I   H
ATOM   2508 CB ALA I 196   1.859 -23.741 -6.003 1.00 0.00
I   C
ATOM   2509 HB1 ALA I 196   2.357 -24.097 -5.076 1.00 0.00
I   H
ATOM   2510 HB2 ALA I 196   1.197 -24.555 -6.370 1.00 0.00
I   H

ATOM 2511 HB3 ALA I 196 2.646 -23.554 -6.766 1.00 0.00
I H
ATOM 2512 C ALA I 196 0.466 -22.005 -6.998 1.00 0.00
I C
ATOM 2513 O ALA I 196 1.115 -21.264 -7.721 1.00 0.00
I O
ATOM 2514 N GLY I 197 -0.720 -22.499 -7.405 1.00 0.00
I N
ATOM 2515 HN GLY I 197 -1.203 -23.224 -6.915 1.00 0.00
I H
ATOM 2516 CA GLY I 197 -1.385 -21.997 -8.611 1.00 0.00
I C
ATOM 2517 HA1 GLY I 197 -1.328 -20.918 -8.556 1.00 0.00
I H
ATOM 2518 HA2 GLY I 197 -2.392 -22.391 -8.589 1.00 0.00
I H
ATOM 2519 C GLY I 197 -0.744 -22.432 -9.901 1.00 0.00
I C
ATOM 2520 O GLY I 197 -1.228 -22.156 -10.992 1.00 0.00
I O
ATOM 2521 N PHE I 198 0.383 -23.159 -9.816 1.00 0.00
I N
ATOM 2522 HN PHE I 198 0.838 -23.301 -8.939 1.00 0.00
I H
ATOM 2523 CA PHE I 198 1.035 -23.788 -10.937 1.00 0.00
I C
ATOM 2524 HA PHE I 198 1.181 -23.008 -11.675 1.00 0.00
I H
ATOM 2525 CB PHE I 198 2.382 -24.454 -10.550 1.00 0.00
I C
ATOM 2526 HB1 PHE I 198 2.319 -24.819 -9.501 1.00 0.00
I H
ATOM 2527 HB2 PHE I 198 2.591 -25.336 -11.191 1.00 0.00
I H
ATOM 2528 CG PHE I 198 3.515 -23.476 -10.614 1.00 0.00
I C
ATOM 2529 CD1 PHE I 198 4.087 -23.153 -11.855 1.00 0.00
I C
ATOM 2530 HD1 PHE I 198 3.660 -23.558 -12.763 1.00 0.00
I H
ATOM 2531 CE1 PHE I 198 5.209 -22.316 -11.930 1.00 0.00
I C
ATOM 2532 HE1 PHE I 198 5.650 -22.089 -12.890 1.00 0.00
I H
ATOM 2533 CZ PHE I 198 5.748 -21.774 -10.757 1.00 0.00
I C
ATOM 2534 HZ PHE I 198 6.618 -21.134 -10.805 1.00 0.00
I H
ATOM 2535 CD2 PHE I 198 4.048 -22.905 -9.452 1.00 0.00
I C
ATOM 2536 HD2 PHE I 198 3.617 -23.140 -8.488 1.00 0.00
I H
ATOM 2537 CE2 PHE I 198 5.153 -22.053 -9.522 1.00 0.00
I C

```
ATOM   2538  HE2 PHE I 198       5.549 -21.610  -8.618  1.00  0.00
I    H
ATOM   2539  C   PHE I 198       0.160 -24.844 -11.575  1.00  0.00
I    C
ATOM   2540  O   PHE I 198      -0.499 -25.639 -10.903  1.00  0.00
I    O
ATOM   2541  N   SER I 199       0.194 -24.909 -12.911  1.00  0.00
I    N
ATOM   2542  HN  SER I 199       0.757 -24.311 -13.480  1.00  0.00
I    H
ATOM   2543  CA  SER I 199      -0.589 -25.860 -13.651  1.00  0.00
I    C
ATOM   2544  HA  SER I 199      -0.772 -26.709 -13.007  1.00  0.00
I    H
ATOM   2545  CB  SER I 199      -1.896 -25.224 -14.182  1.00  0.00
I    C
ATOM   2546  HB1 SER I 199      -2.484 -24.862 -13.308  1.00  0.00
I    H
ATOM   2547  HB2 SER I 199      -1.667 -24.335 -14.812  1.00  0.00
I    H
ATOM   2548  OG  SER I 199      -2.710 -26.149 -14.908  1.00  0.00
I    O
ATOM   2549  HG1 SER I 199      -3.581 -25.746 -15.003  1.00  0.00
I    H
ATOM   2550  C   SER I 199       0.256 -26.340 -14.793  1.00  0.00
I    C
ATOM   2551  O   SER I 199       1.118 -25.619 -15.288  1.00  0.00
I    O
ATOM   2552  N   LEU I 200       0.055 -27.608 -15.183  1.00  0.00
I    N
ATOM   2553  HN  LEU I 200      -0.546 -28.216 -14.668  1.00  0.00
I    H
ATOM   2554  CA  LEU I 200       0.660 -28.219 -16.347  1.00  0.00
I    C
ATOM   2555  HA  LEU I 200       1.675 -27.845 -16.385  1.00  0.00
I    H
ATOM   2556  CB  LEU I 200       0.529 -29.759 -16.255  1.00  0.00
I    C
ATOM   2557  HB1 LEU I 200      -0.559 -29.998 -16.275  1.00  0.00
I    H
ATOM   2558  HB2 LEU I 200       0.961 -30.214 -17.173  1.00  0.00
I    H
ATOM   2559  CG  LEU I 200       1.148 -30.399 -14.999  1.00  0.00
I    C
ATOM   2560  HG  LEU I 200       0.600 -29.991 -14.116  1.00  0.00
I    H
ATOM   2561  CD1 LEU I 200       0.967 -31.920 -15.071  1.00  0.00
I    C
ATOM   2562 HD11 LEU I 200       1.448 -32.412 -14.198  1.00  0.00
I    H
ATOM   2563 HD12 LEU I 200      -0.111 -32.187 -15.071  1.00  0.00
I    H
ATOM   2564 HD13 LEU I 200       1.428 -32.328 -15.996  1.00  0.00
I    H
```

```
ATOM   2565  CD2 LEU I 200       2.635 -30.057 -14.834  1.00  0.00
I    C
ATOM   2566 HD21 LEU I 200       3.037 -30.522 -13.908  1.00  0.00
I    H
ATOM   2567 HD22 LEU I 200       3.238 -30.428 -15.687  1.00  0.00
I    H
ATOM   2568 HD23 LEU I 200       2.774 -28.956 -14.759  1.00  0.00
I    H
ATOM   2569  C   LEU I 200      -0.025 -27.823 -17.643  1.00  0.00
I    C
ATOM   2570  O   LEU I 200       0.559 -27.894 -18.717  1.00  0.00
I    O
ATOM   2571  N   TRP I 201      -1.308 -27.429 -17.588  1.00  0.00
I    N
ATOM   2572  HN  TRP I 201      -1.810 -27.337 -16.731  1.00  0.00
I    H
ATOM   2573  CA  TRP I 201      -2.065 -27.104 -18.777  1.00  0.00
I    C
ATOM   2574  HA  TRP I 201      -1.730 -27.765 -19.568  1.00  0.00
I    H
ATOM   2575  CB  TRP I 201      -3.575 -27.331 -18.508  1.00  0.00
I    C
ATOM   2576  HB1 TRP I 201      -3.865 -26.701 -17.639  1.00  0.00
I    H
ATOM   2577  HB2 TRP I 201      -4.172 -26.977 -19.377  1.00  0.00
I    H
ATOM   2578  CG  TRP I 201      -3.902 -28.778 -18.170  1.00  0.00
I    C
ATOM   2579  CD1 TRP I 201      -3.697 -29.887 -18.940  1.00  0.00
I    C
ATOM   2580  HD1 TRP I 201      -3.289 -29.869 -19.942  1.00  0.00
I    H
ATOM   2581  NE1 TRP I 201      -4.044 -31.021 -18.239  1.00  0.00
I    N
ATOM   2582  HE1 TRP I 201      -3.960 -31.942 -18.553  1.00  0.00
I    H
ATOM   2583  CE2 TRP I 201      -4.487 -30.657 -16.992  1.00  0.00
I    C
ATOM   2584  CD2 TRP I 201      -4.430 -29.241 -16.917  1.00  0.00
I    C
ATOM   2585  CE3 TRP I 201      -4.844 -28.570 -15.773  1.00  0.00
I    C
ATOM   2586  HE3 TRP I 201      -4.826 -27.490 -15.708  1.00  0.00
I    H
ATOM   2587  CZ3 TRP I 201      -5.300 -29.332 -14.689  1.00  0.00
I    C
ATOM   2588  HZ3 TRP I 201      -5.635 -28.831 -13.791  1.00  0.00
I    H
ATOM   2589  CZ2 TRP I 201      -4.943 -31.407 -15.916  1.00  0.00
I    C
ATOM   2590  HZ2 TRP I 201      -5.000 -32.484 -15.963  1.00  0.00
I    H
ATOM   2591  CH2 TRP I 201      -5.333 -30.732 -14.749  1.00  0.00
I    C
```

```
ATOM   2592  HH2 TRP I 201       -5.664 -31.295 -13.888  1.00  0.00
I    H
ATOM   2593  C   TRP I 201       -1.776 -25.673 -19.241  1.00  0.00
I    C
ATOM   2594  O   TRP I 201       -1.447 -24.844 -18.390  1.00  0.00
I    O
ATOM   2595  N   PRO I 202       -1.872 -25.327 -20.543  1.00  0.00
I    N
ATOM   2596  CD  PRO I 202       -2.313 -26.234 -21.605  1.00  0.00
I    C
ATOM   2597  HD1 PRO I 202       -1.410 -26.486 -21.003  1.00  0.00
I    H
ATOM   2598  HD2 PRO I 202       -2.900 -27.154 -21.829  1.00  0.00
I    H
ATOM   2599  CA  PRO I 202       -1.626 -23.970 -21.057  1.00  0.00
I    C
ATOM   2600  HA  PRO I 202       -0.584 -23.700 -20.927  1.00  0.00
I    H
ATOM   2601  CB  PRO I 202       -1.974 -24.053 -22.563  1.00  0.00
I    C
ATOM   2602  HB1 PRO I 202       -1.085 -24.424 -23.121  1.00  0.00
I    H
ATOM   2603  HB2 PRO I 202       -2.283 -23.076 -22.986  1.00  0.00
I    H
ATOM   2604  CG  PRO I 202       -1.902 -25.546 -22.905  1.00  0.00
I    C
ATOM   2605  HG1 PRO I 202       -1.721 -25.734 -23.982  1.00  0.00
I    H
ATOM   2606  HG2 PRO I 202       -2.852 -26.039 -22.598  1.00  0.00
I    H
ATOM   2607  C   PRO I 202       -2.493 -22.943 -20.366  1.00  0.00
I    C
ATOM   2608  O   PRO I 202       -2.030 -21.893 -19.922  1.00  0.00
I    O
ATOM   2609  N   ASP I 203       -3.775 -23.297 -20.241  1.00  0.00
I    N
ATOM   2610  HN  ASP I 203       -4.143 -24.091 -20.718  1.00  0.00
I    H
ATOM   2611  CA  ASP I 203       -4.750 -22.623 -19.446  1.00  0.00
I    C
ATOM   2612  HA  ASP I 203       -4.448 -21.587 -19.344  1.00  0.00
I    H
ATOM   2613  CB  ASP I 203       -6.147 -22.960 -19.985  1.00  0.00
I    C
ATOM   2614  HB1 ASP I 203       -6.208 -24.048 -20.188  1.00  0.00
I    H
ATOM   2615  HB2 ASP I 203       -6.925 -22.699 -19.240  1.00  0.00
I    H
ATOM   2616  CG  ASP I 203       -6.480 -22.250 -21.279  1.00  0.00
I    C
ATOM   2617  OD1 ASP I 203       -5.749 -21.318 -21.678  1.00  0.00
I    O
ATOM   2618  OD2 ASP I 203       -7.531 -22.642 -21.846  1.00  0.00
I    O
```

```
ATOM   2619  C   ASP I 203      -4.726 -23.222 -18.062  1.00  0.00
I    C
ATOM   2620  O   ASP I 203      -4.761 -24.440 -17.905  1.00  0.00
I    O
ATOM   2621  N   ASN I 204      -4.702 -22.396 -17.001  1.00  0.00
I    N
ATOM   2622  HN  ASN I 204      -4.826 -21.406 -17.047  1.00  0.00
I    H
ATOM   2623  CA  ASN I 204      -4.484 -22.911 -15.665  1.00  0.00
I    C
ATOM   2624  HA  ASN I 204      -3.526 -23.415 -15.731  1.00  0.00
I    H
ATOM   2625  CB  ASN I 204      -4.369 -21.703 -14.707  1.00  0.00
I    C
ATOM   2626  HB1 ASN I 204      -3.955 -20.847 -15.285  1.00  0.00
I    H
ATOM   2627  HB2 ASN I 204      -5.373 -21.404 -14.342  1.00  0.00
I    H
ATOM   2628  CG  ASN I 204      -3.435 -22.032 -13.564  1.00  0.00
I    C
ATOM   2629  OD1 ASN I 204      -3.789 -22.922 -12.779  1.00  0.00
I    O
ATOM   2630  ND2 ASN I 204      -2.277 -21.365 -13.443  1.00  0.00
I    N
ATOM   2631 HD21 ASN I 204      -1.681 -21.617 -12.685  1.00  0.00
I    H
ATOM   2632 HD22 ASN I 204      -2.028 -20.673 -14.112  1.00  0.00
I    H
ATOM   2633  C   ASN I 204      -5.532 -23.950 -15.182  1.00  0.00
I    C
ATOM   2634  O   ASN I 204      -5.167 -25.063 -14.814  1.00  0.00
I    O
ATOM   2635  N   LEU I 205      -6.847 -23.633 -15.285  1.00  0.00
I    N
ATOM   2636  HN  LEU I 205      -7.130 -22.704 -15.516  1.00  0.00
I    H
ATOM   2637  CA  LEU I 205      -7.954 -24.564 -15.080  1.00  0.00
I    C
ATOM   2638  HA  LEU I 205      -7.601 -25.542 -15.381  1.00  0.00
I    H
ATOM   2639  CB  LEU I 205      -8.488 -24.575 -13.618  1.00  0.00
I    C
ATOM   2640  HB1 LEU I 205      -8.740 -23.522 -13.351  1.00  0.00
I    H
ATOM   2641  HB2 LEU I 205      -9.447 -25.138 -13.587  1.00  0.00
I    H
ATOM   2642  CG  LEU I 205      -7.517 -25.124 -12.557  1.00  0.00
I    C
ATOM   2643  HG  LEU I 205      -6.649 -24.636 -12.641  1.00  0.00
I    H
ATOM   2644  CD1 LEU I 205      -7.195 -26.616 -12.750  1.00  0.00
I    C
ATOM   2645 HD11 LEU I 205      -6.456 -26.957 -11.994  1.00  0.00
I    H
```

```
ATOM   2646 HD12 LEU I 205      -8.114 -27.232 -12.642  1.00  0.00
I    H
ATOM   2647 HD13 LEU I 205      -6.767 -26.797 -13.759  1.00  0.00
I    H
ATOM   2648  CD2 LEU I 205      -8.157 -24.910 -11.185  1.00  0.00
I    C
ATOM   2649 HD21 LEU I 205      -7.498 -25.310 -10.385  1.00  0.00
I    H
ATOM   2650 HD22 LEU I 205      -8.326 -23.835 -10.976  1.00  0.00
I    H
ATOM   2651 HD23 LEU I 205      -9.135 -25.436 -11.126  1.00  0.00
I    H
ATOM   2652  C   LEU I 205      -9.142 -24.205 -15.978  1.00  0.00
I    C
ATOM   2653  O   LEU I 205     -10.241 -24.705 -15.767  1.00  0.00
I    O
ATOM   2654  N   MET I 206      -8.970 -23.374 -17.042  1.00  0.00
I    N
ATOM   2655  HN  MET I 206      -8.057 -23.099 -17.335  1.00  0.00
I    H
ATOM   2656  CA  MET I 206     -10.063 -22.809 -17.847  1.00  0.00
I    C
ATOM   2657  HA  MET I 206     -10.660 -22.224 -17.158  1.00  0.00
I    H
ATOM   2658  CB  MET I 206      -9.525 -21.925 -18.995  1.00  0.00
I    C
ATOM   2659  HB1 MET I 206      -8.877 -22.576 -19.629  1.00  0.00
I    H
ATOM   2660  HB2 MET I 206     -10.371 -21.603 -19.642  1.00  0.00
I    H
ATOM   2661  CG  MET I 206      -8.700 -20.702 -18.543  1.00  0.00
I    C
ATOM   2662  HG1 MET I 206      -8.292 -20.867 -17.523  1.00  0.00
I    H
ATOM   2663  HG2 MET I 206      -7.838 -20.557 -19.230  1.00  0.00
I    H
ATOM   2664  SD  MET I 206      -9.630 -19.151 -18.491  1.00  0.00
I    S
ATOM   2665  CE  MET I 206      -9.608 -18.913 -20.291  1.00  0.00
I    C
ATOM   2666  HE1 MET I 206     -10.130 -17.974 -20.574  1.00  0.00
I    H
ATOM   2667  HE2 MET I 206      -8.566 -18.852 -20.673  1.00  0.00
I    H
ATOM   2668  HE3 MET I 206     -10.113 -19.755 -20.811  1.00  0.00
I    H
ATOM   2669  C   MET I 206     -10.979 -23.849 -18.483  1.00  0.00
I    C
ATOM   2670  O   MET I 206     -12.190 -23.671 -18.571  1.00  0.00
I    O
ATOM   2671  N   SER I 207     -10.398 -24.998 -18.895  1.00  0.00
I    N
ATOM   2672  HN  SER I 207      -9.402 -25.090 -18.873  1.00  0.00
I    H
```

ATOM 2673 CA SER I 207 −11.099 −26.181 −19.395 1.00 0.00
I C
ATOM 2674 HA SER I 207 −11.619 −25.855 −20.285 1.00 0.00
I H
ATOM 2675 CB SER I 207 −10.070 −27.331 −19.666 1.00 0.00
I C
ATOM 2676 HB1 SER I 207 −9.321 −26.959 −20.402 1.00 0.00
I H
ATOM 2677 HB2 SER I 207 −9.515 −27.583 −18.734 1.00 0.00
I H
ATOM 2678 OG SER I 207 −10.662 −28.511 −20.211 1.00 0.00
I O
ATOM 2679 HG1 SER I 207 −9.942 −29.060 −20.547 1.00 0.00
I H
ATOM 2680 C SER I 207 −12.156 −26.693 −18.412 1.00 0.00
I C
ATOM 2681 O SER I 207 −13.278 −27.024 −18.776 1.00 0.00
I O
ATOM 2682 N TYR I 208 −11.832 −26.699 −17.102 1.00 0.00
I N
ATOM 2683 HN TYR I 208 −10.976 −26.293 −16.785 1.00 0.00
I H
ATOM 2684 CA TYR I 208 −12.655 −27.269 −16.050 1.00 0.00
I C
ATOM 2685 HA TYR I 208 −13.352 −27.940 −16.536 1.00 0.00
I H
ATOM 2686 CB TYR I 208 −11.806 −28.031 −14.988 1.00 0.00
I C
ATOM 2687 HB1 TYR I 208 −11.266 −27.292 −14.356 1.00 0.00
I H
ATOM 2688 HB2 TYR I 208 −12.461 −28.624 −14.313 1.00 0.00
I H
ATOM 2689 CG TYR I 208 −10.785 −28.912 −15.647 1.00 0.00
I C
ATOM 2690 CD1 TYR I 208 −11.154 −30.068 −16.359 1.00 0.00
I C
ATOM 2691 HD1 TYR I 208 −12.195 −30.354 −16.416 1.00 0.00
I H
ATOM 2692 CE1 TYR I 208 −10.175 −30.866 −16.980 1.00 0.00
I C
ATOM 2693 HE1 TYR I 208 −10.455 −31.757 −17.520 1.00 0.00
I H
ATOM 2694 CZ TYR I 208 −8.825 −30.502 −16.887 1.00 0.00
I C
ATOM 2695 OH TYR I 208 −7.782 −31.259 −17.450 1.00 0.00
I O
ATOM 2696 HH TYR I 208 −6.966 −30.773 −17.316 1.00 0.00
I H
ATOM 2697 CD2 TYR I 208 −9.430 −28.559 −15.573 1.00 0.00
I C
ATOM 2698 HD2 TYR I 208 −9.128 −27.695 −14.998 1.00 0.00
I H
ATOM 2699 CE2 TYR I 208 −8.461 −29.346 −16.197 1.00 0.00
I C

ATOM 2700 HE2 TYR I 208 -7.419 -29.068 -16.126 1.00 0.00
I H
ATOM 2701 C TYR I 208 -13.479 -26.197 -15.348 1.00 0.00
I C
ATOM 2702 O TYR I 208 -13.783 -26.313 -14.167 1.00 0.00
I O
ATOM 2703 N VAL I 209 -13.890 -25.117 -16.062 1.00 0.00
I N
ATOM 2704 HN VAL I 209 -13.743 -25.054 -17.045 1.00 0.00
I H
ATOM 2705 CA VAL I 209 -14.573 -23.964 -15.478 1.00 0.00
I C
ATOM 2706 HA VAL I 209 -13.871 -23.528 -14.776 1.00 0.00
I H
ATOM 2707 CB VAL I 209 -14.843 -22.875 -16.527 1.00 0.00
I C
ATOM 2708 HB VAL I 209 -13.863 -22.606 -16.994 1.00 0.00
I H
ATOM 2709 CG1 VAL I 209 -15.797 -23.346 -17.649 1.00 0.00
I C
ATOM 2710 HG11 VAL I 209 -15.993 -22.516 -18.362 1.00 0.00
I H
ATOM 2711 HG12 VAL I 209 -15.362 -24.185 -18.230 1.00 0.00
I H
ATOM 2712 HG13 VAL I 209 -16.770 -23.673 -17.224 1.00 0.00
I H
ATOM 2713 CG2 VAL I 209 -15.389 -21.594 -15.865 1.00 0.00
I C
ATOM 2714 HG21 VAL I 209 -15.620 -20.824 -16.631 1.00 0.00
I H
ATOM 2715 HG22 VAL I 209 -16.319 -21.813 -15.297 1.00 0.00
I H
ATOM 2716 HG23 VAL I 209 -14.648 -21.157 -15.163 1.00 0.00
I H
ATOM 2717 C VAL I 209 -15.842 -24.310 -14.702 1.00 0.00
I C
ATOM 2718 O VAL I 209 -16.093 -23.759 -13.629 1.00 0.00
I O
ATOM 2719 N GLY I 210 -16.641 -25.269 -15.208 1.00 0.00
I N
ATOM 2720 HN GLY I 210 -16.393 -25.699 -16.076 1.00 0.00
I H
ATOM 2721 CA GLY I 210 -17.869 -25.753 -14.592 1.00 0.00
I C
ATOM 2722 HA1 GLY I 210 -18.618 -25.732 -15.372 1.00 0.00
I H
ATOM 2723 HA2 GLY I 210 -18.071 -25.088 -13.763 1.00 0.00
I H
ATOM 2724 C GLY I 210 -17.798 -27.154 -14.054 1.00 0.00
I C
ATOM 2725 O GLY I 210 -18.827 -27.768 -13.786 1.00 0.00
I O
ATOM 2726 N ASP I 211 -16.599 -27.739 -13.876 1.00 0.00
I N

```
ATOM   2727  HN  ASP I 211     -15.710 -27.326 -14.057  1.00  0.00
I    H
ATOM   2728  CA  ASP I 211     -16.501 -29.099 -13.378  1.00  0.00
I    C
ATOM   2729  HA  ASP I 211     -17.338 -29.656 -13.783  1.00  0.00
I    H
ATOM   2730  CB  ASP I 211     -15.156 -29.729 -13.826  1.00  0.00
I    C
ATOM   2731  HB1 ASP I 211     -15.141 -29.809 -14.931  1.00  0.00
I    H
ATOM   2732  HB2 ASP I 211     -14.303 -29.095 -13.511  1.00  0.00
I    H
ATOM   2733  CG  ASP I 211     -14.916 -31.132 -13.283  1.00  0.00
I    C
ATOM   2734  OD1 ASP I 211     -15.857 -31.788 -12.773  1.00  0.00
I    O
ATOM   2735  OD2 ASP I 211     -13.732 -31.536 -13.295  1.00  0.00
I    O
ATOM   2736  C   ASP I 211     -16.656 -29.117 -11.843  1.00  0.00
I    C
ATOM   2737  O   ASP I 211     -15.858 -28.471 -11.158  1.00  0.00
I    O
ATOM   2738  N   PRO I 212     -17.615 -29.812 -11.225  1.00  0.00
I    N
ATOM   2739  CD  PRO I 212     -18.655 -30.593 -11.897  1.00  0.00
I    C
ATOM   2740  HD1 PRO I 212     -19.474 -29.971 -12.325  1.00  0.00
I    H
ATOM   2741  HD2 PRO I 212     -18.165 -31.195 -12.696  1.00  0.00
I    H
ATOM   2742  CA  PRO I 212     -17.674 -29.958  -9.780  1.00  0.00
I    C
ATOM   2743  HA  PRO I 212     -17.748 -28.981  -9.318  1.00  0.00
I    H
ATOM   2744  CB  PRO I 212     -18.994 -30.713  -9.527  1.00  0.00
I    C
ATOM   2745  HB1 PRO I 212     -19.830 -29.979  -9.488  1.00  0.00
I    H
ATOM   2746  HB2 PRO I 212     -18.980 -31.295  -8.583  1.00  0.00
I    H
ATOM   2747  CG  PRO I 212     -19.212 -31.517 -10.812  1.00  0.00
I    C
ATOM   2748  HG1 PRO I 212     -20.273 -31.787 -10.978  1.00  0.00
I    H
ATOM   2749  HG2 PRO I 212     -18.596 -32.445 -10.773  1.00  0.00
I    H
ATOM   2750  C   PRO I 212     -16.479 -30.690  -9.204  1.00  0.00
I    C
ATOM   2751  O   PRO I 212     -16.074 -30.344  -8.099  1.00  0.00
I    O
ATOM   2752  N   THR I 213     -15.887 -31.696  -9.883  1.00  0.00
I    N
ATOM   2753  HN  THR I 213     -16.124 -31.973 -10.813  1.00  0.00
I    H
```

```
ATOM   2754  CA  THR I 213     -14.816 -32.481  -9.276  1.00  0.00
I    C
ATOM   2755  HA  THR I 213     -15.173 -32.755  -8.293  1.00  0.00
I    H
ATOM   2756  CB  THR I 213     -14.454 -33.698 -10.112  1.00  0.00
I    C
ATOM   2757  HB  THR I 213     -14.215 -33.312 -11.134  1.00  0.00
I    H
ATOM   2758  OG1 THR I 213     -15.578 -34.555 -10.220  1.00  0.00
I    O
ATOM   2759  HG1 THR I 213     -15.280 -35.304 -10.748  1.00  0.00
I    H
ATOM   2760  CG2 THR I 213     -13.382 -34.548  -9.420  1.00  0.00
I    C
ATOM   2761 HG21 THR I 213     -13.040 -35.370 -10.084  1.00  0.00
I    H
ATOM   2762 HG22 THR I 213     -12.498 -33.924  -9.166  1.00  0.00
I    H
ATOM   2763 HG23 THR I 213     -13.771 -34.998  -8.482  1.00  0.00
I    H
ATOM   2764  C   THR I 213     -13.574 -31.654  -9.067  1.00  0.00
I    C
ATOM   2765  O   THR I 213     -13.065 -31.530  -7.949  1.00  0.00
I    O
ATOM   2766  N   VAL I 214     -13.086 -30.976 -10.120  1.00  0.00
I    N
ATOM   2767  HN  VAL I 214     -13.488 -31.011 -11.031  1.00  0.00
I    H
ATOM   2768  CA  VAL I 214     -11.916 -30.123 -10.014  1.00  0.00
I    C
ATOM   2769  HA  VAL I 214     -11.144 -30.705  -9.525  1.00  0.00
I    H
ATOM   2770  CB  VAL I 214     -11.411 -29.749 -11.398  1.00  0.00
I    C
ATOM   2771  HB  VAL I 214     -12.254 -29.258 -11.944  1.00  0.00
I    H
ATOM   2772  CG1 VAL I 214     -10.230 -28.762 -11.341  1.00  0.00
I    C
ATOM   2773 HG11 VAL I 214      -9.862 -28.541 -12.366  1.00  0.00
I    H
ATOM   2774 HG12 VAL I 214     -10.525 -27.797 -10.880  1.00  0.00
I    H
ATOM   2775 HG13 VAL I 214      -9.387 -29.191 -10.759  1.00  0.00
I    H
ATOM   2776  CG2 VAL I 214     -10.925 -31.039 -12.088  1.00  0.00
I    C
ATOM   2777 HG21 VAL I 214     -10.507 -30.814 -13.092  1.00  0.00
I    H
ATOM   2778 HG22 VAL I 214     -10.133 -31.532 -11.482  1.00  0.00
I    H
ATOM   2779 HG23 VAL I 214     -11.758 -31.759 -12.225  1.00  0.00
I    H
ATOM   2780  C   VAL I 214     -12.177 -28.899  -9.152  1.00  0.00
I    C
```

```
ATOM   2781  O   VAL I 214     -11.395 -28.582  -8.253  1.00  0.00
I    O
ATOM   2782  N   ASN I 215     -13.324 -28.209  -9.345  1.00  0.00
I    N
ATOM   2783  HN  ASN I 215     -14.032 -28.474  -9.999  1.00  0.00
I    H
ATOM   2784  CA  ASN I 215     -13.619 -27.000  -8.596  1.00  0.00
I    C
ATOM   2785  HA  ASN I 215     -12.736 -26.381  -8.714  1.00  0.00
I    H
ATOM   2786  CB  ASN I 215     -14.839 -26.232  -9.149  1.00  0.00
I    C
ATOM   2787  HB1 ASN I 215     -15.654 -26.968  -9.324  1.00  0.00
I    H
ATOM   2788  HB2 ASN I 215     -15.207 -25.502  -8.399  1.00  0.00
I    H
ATOM   2789  CG  ASN I 215     -14.476 -25.556 -10.466  1.00  0.00
I    C
ATOM   2790  OD1 ASN I 215     -13.365 -25.061 -10.654  1.00  0.00
I    O
ATOM   2791  ND2 ASN I 215     -15.467 -25.452 -11.373  1.00  0.00
I    N
ATOM   2792 HD21 ASN I 215     -15.247 -25.023 -12.245  1.00  0.00
I    H
ATOM   2793 HD22 ASN I 215     -16.368 -25.822 -11.172  1.00  0.00
I    H
ATOM   2794  C   ASN I 215     -13.822 -27.234  -7.110  1.00  0.00
I    C
ATOM   2795  O   ASN I 215     -13.492 -26.364  -6.301  1.00  0.00
I    O
ATOM   2796  N   LEU I 216     -14.366 -28.382  -6.670  1.00  0.00
I    N
ATOM   2797  HN  LEU I 216     -14.738 -29.103  -7.251  1.00  0.00
I    H
ATOM   2798  CA  LEU I 216     -14.450 -28.658  -5.250  1.00  0.00
I    C
ATOM   2799  HA  LEU I 216     -14.730 -27.720  -4.787  1.00  0.00
I    H
ATOM   2800  CB  LEU I 216     -15.565 -29.683  -4.945  1.00  0.00
I    C
ATOM   2801  HB1 LEU I 216     -15.353 -30.590  -5.556  1.00  0.00
I    H
ATOM   2802  HB2 LEU I 216     -15.482 -30.002  -3.882  1.00  0.00
I    H
ATOM   2803  CG  LEU I 216     -16.979 -29.149  -5.272  1.00  0.00
I    C
ATOM   2804  HG  LEU I 216     -16.924 -28.828  -6.340  1.00  0.00
I    H
ATOM   2805  CD1 LEU I 216     -18.030 -30.258  -5.121  1.00  0.00
I    C
ATOM   2806 HD11 LEU I 216     -19.049 -29.858  -5.310  1.00  0.00
I    H
ATOM   2807 HD12 LEU I 216     -17.838 -31.081  -5.843  1.00  0.00
I    H
```

```
ATOM   2808 HD13 LEU I 216     -18.009 -30.682  -4.094  1.00  0.00
I    H
ATOM   2809  CD2 LEU I 216     -17.360 -27.922  -4.424  1.00  0.00
I    C
ATOM   2810 HD21 LEU I 216     -18.373 -27.560  -4.703  1.00  0.00
I    H
ATOM   2811 HD22 LEU I 216     -17.373 -28.160  -3.342  1.00  0.00
I    H
ATOM   2812 HD23 LEU I 216     -16.637 -27.094  -4.592  1.00  0.00
I    H
ATOM   2813  C   LEU I 216     -13.133 -29.097  -4.634  1.00  0.00
I    C
ATOM   2814  O   LEU I 216     -12.718 -28.555  -3.616  1.00  0.00
I    O
ATOM   2815  N   VAL I 217     -12.402 -30.056  -5.241  1.00  0.00
I    N
ATOM   2816  HN  VAL I 217     -12.657 -30.480  -6.106  1.00  0.00
I    H
ATOM   2817  CA  VAL I 217     -11.160 -30.558  -4.666  1.00  0.00
I    C
ATOM   2818  HA  VAL I 217     -11.390 -30.844  -3.647  1.00  0.00
I    H
ATOM   2819  CB  VAL I 217     -10.672 -31.775  -5.433  1.00  0.00
I    C
ATOM   2820  HB  VAL I 217     -10.468 -31.453  -6.484  1.00  0.00
I    H
ATOM   2821  CG1 VAL I 217      -9.374 -32.354  -4.838  1.00  0.00
I    C
ATOM   2822 HG11 VAL I 217      -9.066 -33.264  -5.396  1.00  0.00
I    H
ATOM   2823 HG12 VAL I 217      -8.536 -31.630  -4.900  1.00  0.00
I    H
ATOM   2824 HG13 VAL I 217      -9.521 -32.637  -3.774  1.00  0.00
I    H
ATOM   2825  CG2 VAL I 217     -11.743 -32.870  -5.341  1.00  0.00
I    C
ATOM   2826 HG21 VAL I 217     -11.406 -33.794  -5.857  1.00  0.00
I    H
ATOM   2827 HG22 VAL I 217     -11.955 -33.121  -4.280  1.00  0.00
I    H
ATOM   2828 HG23 VAL I 217     -12.692 -32.547  -5.819  1.00  0.00
I    H
ATOM   2829  C   VAL I 217     -10.052 -29.518  -4.609  1.00  0.00
I    C
ATOM   2830  O   VAL I 217      -9.408 -29.336  -3.576  1.00  0.00
I    O
ATOM   2831  N   ILE I 218      -9.820 -28.761  -5.704  1.00  0.00
I    N
ATOM   2832  HN  ILE I 218     -10.338 -28.841  -6.553  1.00  0.00
I    H
ATOM   2833  CA  ILE I 218      -8.776 -27.745  -5.736  1.00  0.00
I    C
ATOM   2834  HA  ILE I 218      -7.875 -28.233  -5.386  1.00  0.00
I    H
```

```
ATOM   2835  CB  ILE I 218      -8.493 -27.260  -7.157  1.00  0.00
I    C
ATOM   2836  HB  ILE I 218      -9.501 -26.998  -7.569  1.00  0.00
I    H
ATOM   2837  CG2 ILE I 218      -7.513 -26.057  -7.152  1.00  0.00
I    C
ATOM   2838 HG21 ILE I 218      -7.434 -25.613  -8.166  1.00  0.00
I    H
ATOM   2839 HG22 ILE I 218      -7.866 -25.261  -6.463  1.00  0.00
I    H
ATOM   2840 HG23 ILE I 218      -6.498 -26.372  -6.833  1.00  0.00
I    H
ATOM   2841  CG1 ILE I 218      -7.946 -28.469  -7.964  1.00  0.00
I    C
ATOM   2842 HG11 ILE I 218      -7.164 -28.995  -7.372  1.00  0.00
I    H
ATOM   2843 HG12 ILE I 218      -8.775 -29.195  -8.133  1.00  0.00
I    H
ATOM   2844  CD  ILE I 218      -7.363 -28.098  -9.329  1.00  0.00
I    C
ATOM   2845  HD1 ILE I 218      -7.045 -29.012  -9.875  1.00  0.00
I    H
ATOM   2846  HD2 ILE I 218      -8.119 -27.569  -9.948  1.00  0.00
I    H
ATOM   2847  HD3 ILE I 218      -6.476 -27.439  -9.218  1.00  0.00
I    H
ATOM   2848  C   ILE I 218      -9.060 -26.606  -4.774  1.00  0.00
I    C
ATOM   2849  O   ILE I 218      -8.177 -26.201  -4.017  1.00  0.00
I    O
ATOM   2850  N   THR I 219     -10.318 -26.100  -4.721  1.00  0.00
I    N
ATOM   2851  HN  THR I 219     -11.067 -26.399  -5.311  1.00  0.00
I    H
ATOM   2852  CA  THR I 219     -10.685 -25.047  -3.768  1.00  0.00
I    C
ATOM   2853  HA  THR I 219      -9.951 -24.266  -3.917  1.00  0.00
I    H
ATOM   2854  CB  THR I 219     -12.052 -24.392  -3.941  1.00  0.00
I    C
ATOM   2855  HB  THR I 219     -12.165 -23.681  -3.086  1.00  0.00
I    H
ATOM   2856  OG1 THR I 219     -13.151 -25.294  -3.847  1.00  0.00
I    O
ATOM   2857  HG1 THR I 219     -13.937 -24.755  -3.984  1.00  0.00
I    H
ATOM   2858  CG2 THR I 219     -12.125 -23.732  -5.315  1.00  0.00
I    C
ATOM   2859 HG21 THR I 219     -13.065 -23.151  -5.425  1.00  0.00
I    H
ATOM   2860 HG22 THR I 219     -11.273 -23.034  -5.455  1.00  0.00
I    H
ATOM   2861 HG23 THR I 219     -12.089 -24.491  -6.126  1.00  0.00
I    H
```

```
ATOM   2862  C   THR I 219     -10.565 -25.479  -2.332  1.00  0.00
I    C
ATOM   2863  O   THR I 219     -10.013 -24.755  -1.512  1.00  0.00
I    O
ATOM   2864  N   PHE I 220     -11.010 -26.706  -2.005  1.00  0.00
I    N
ATOM   2865  HN  PHE I 220     -11.491 -27.278  -2.668  1.00  0.00
I    H
ATOM   2866  CA  PHE I 220     -10.846 -27.311  -0.699  1.00  0.00
I    C
ATOM   2867  HA  PHE I 220     -11.329 -26.640   0.001  1.00  0.00
I    H
ATOM   2868  CB  PHE I 220     -11.519 -28.719  -0.721  1.00  0.00
I    C
ATOM   2869  HB1 PHE I 220     -12.615 -28.596  -0.860  1.00  0.00
I    H
ATOM   2870  HB2 PHE I 220     -11.147 -29.326  -1.573  1.00  0.00
I    H
ATOM   2871  CG  PHE I 220     -11.336 -29.483   0.564  1.00  0.00
I    C
ATOM   2872  CD1 PHE I 220     -10.192 -30.281   0.736  1.00  0.00
I    C
ATOM   2873  HD1 PHE I 220      -9.503 -30.414  -0.087  1.00  0.00
I    H
ATOM   2874  CE1 PHE I 220      -9.941 -30.908   1.959  1.00  0.00
I    C
ATOM   2875  HE1 PHE I 220      -9.038 -31.486   2.095  1.00  0.00
I    H
ATOM   2876  CZ  PHE I 220     -10.857 -30.784   3.009  1.00  0.00
I    C
ATOM   2877  HZ  PHE I 220     -10.670 -31.277   3.953  1.00  0.00
I    H
ATOM   2878  CD2 PHE I 220     -12.247 -29.357   1.623  1.00  0.00
I    C
ATOM   2879  HD2 PHE I 220     -13.146 -28.769   1.496  1.00  0.00
I    H
ATOM   2880  CE2 PHE I 220     -12.013 -30.012   2.840  1.00  0.00
I    C
ATOM   2881  HE2 PHE I 220     -12.728 -29.920   3.647  1.00  0.00
I    H
ATOM   2882  C   PHE I 220      -9.375 -27.399  -0.293  1.00  0.00
I    C
ATOM   2883  O   PHE I 220      -9.016 -27.016   0.821  1.00  0.00
I    O
ATOM   2884  N   LEU I 221      -8.471 -27.862  -1.181  1.00  0.00
I    N
ATOM   2885  HN  LEU I 221      -8.702 -28.152  -2.108  1.00  0.00
I    H
ATOM   2886  CA  LEU I 221      -7.060 -27.979  -0.855  1.00  0.00
I    C
ATOM   2887  HA  LEU I 221      -7.018 -28.593   0.036  1.00  0.00
I    H
ATOM   2888  CB  LEU I 221      -6.273 -28.674  -1.989  1.00  0.00
I    C
```

ATOM 2889 HB1 LEU I 221 -6.585 -28.196 -2.947 1.00 0.00
I H
ATOM 2890 HB2 LEU I 221 -5.190 -28.447 -1.870 1.00 0.00
I H
ATOM 2891 CG LEU I 221 -6.503 -30.195 -2.091 1.00 0.00
I C
ATOM 2892 HG LEU I 221 -7.602 -30.361 -2.203 1.00 0.00
I H
ATOM 2893 CD1 LEU I 221 -5.768 -30.720 -3.331 1.00 0.00
I C
ATOM 2894 HD11 LEU I 221 -5.873 -31.823 -3.409 1.00 0.00
I H
ATOM 2895 HD12 LEU I 221 -6.183 -30.264 -4.255 1.00 0.00
I H
ATOM 2896 HD13 LEU I 221 -4.685 -30.478 -3.277 1.00 0.00
I H
ATOM 2897 CD2 LEU I 221 -6.034 -30.949 -0.834 1.00 0.00
I C
ATOM 2898 HD21 LEU I 221 -6.234 -32.037 -0.939 1.00 0.00
I H
ATOM 2899 HD22 LEU I 221 -4.946 -30.822 -0.662 1.00 0.00
I H
ATOM 2900 HD23 LEU I 221 -6.574 -30.582 0.065 1.00 0.00
I H
ATOM 2901 C LEU I 221 -6.398 -26.653 -0.528 1.00 0.00
I C
ATOM 2902 O LEU I 221 -5.729 -26.539 0.498 1.00 0.00
I O
ATOM 2903 N PHE I 222 -6.617 -25.599 -1.345 1.00 0.00
I N
ATOM 2904 HN PHE I 222 -7.182 -25.614 -2.168 1.00 0.00
I H
ATOM 2905 CA PHE I 222 -6.010 -24.310 -1.054 1.00 0.00
I C
ATOM 2906 HA PHE I 222 -5.028 -24.544 -0.661 1.00 0.00
I H
ATOM 2907 CB PHE I 222 -5.731 -23.459 -2.331 1.00 0.00
I C
ATOM 2908 HB1 PHE I 222 -5.128 -22.569 -2.050 1.00 0.00
I H
ATOM 2909 HB2 PHE I 222 -5.140 -24.036 -3.074 1.00 0.00
I H
ATOM 2910 CG PHE I 222 -6.919 -22.891 -3.051 1.00 0.00
I C
ATOM 2911 CD1 PHE I 222 -7.675 -21.841 -2.503 1.00 0.00
I C
ATOM 2912 HD1 PHE I 222 -7.462 -21.493 -1.502 1.00 0.00
I H
ATOM 2913 CE1 PHE I 222 -8.704 -21.247 -3.238 1.00 0.00
I C
ATOM 2914 HE1 PHE I 222 -9.299 -20.460 -2.797 1.00 0.00
I H
ATOM 2915 CZ PHE I 222 -8.955 -21.674 -4.548 1.00 0.00
I C

```
ATOM   2916  HZ  PHE I 222      -9.739 -21.203  -5.122  1.00  0.00
I    H
ATOM   2917  CD2 PHE I 222      -7.186 -23.307  -4.364  1.00  0.00
I    C
ATOM   2918  HD2 PHE I 222      -6.625 -24.123  -4.797  1.00  0.00
I    H
ATOM   2919  CE2 PHE I 222      -8.191 -22.695  -5.117  1.00  0.00
I    C
ATOM   2920  HE2 PHE I 222      -8.373 -23.015  -6.133  1.00  0.00
I    H
ATOM   2921  C   PHE I 222      -6.742 -23.509   0.020  1.00  0.00
I    C
ATOM   2922  O   PHE I 222      -6.162 -22.624   0.642  1.00  0.00
I    O
ATOM   2923  N   ILE I 223      -8.041 -23.768   0.297  1.00  0.00
I    N
ATOM   2924  HN  ILE I 223      -8.622 -24.346  -0.272  1.00  0.00
I    H
ATOM   2925  CA  ILE I 223      -8.713 -23.215   1.472  1.00  0.00
I    C
ATOM   2926  HA  ILE I 223      -8.428 -22.172   1.521  1.00  0.00
I    H
ATOM   2927  CB  ILE I 223     -10.242 -23.247   1.364  1.00  0.00
I    C
ATOM   2928  HB  ILE I 223     -10.474 -24.273   0.983  1.00  0.00
I    H
ATOM   2929  CG2 ILE I 223     -10.926 -22.926   2.721  1.00  0.00
I    C
ATOM   2930 HG21 ILE I 223     -12.019 -23.107   2.660  1.00  0.00
I    H
ATOM   2931 HG22 ILE I 223     -10.522 -23.571   3.530  1.00  0.00
I    H
ATOM   2932 HG23 ILE I 223     -10.766 -21.865   3.006  1.00  0.00
I    H
ATOM   2933  CG1 ILE I 223     -10.691 -22.235   0.278  1.00  0.00
I    C
ATOM   2934 HG11 ILE I 223     -10.511 -21.196   0.634  1.00  0.00
I    H
ATOM   2935 HG12 ILE I 223     -10.066 -22.383  -0.632  1.00  0.00
I    H
ATOM   2936  CD  ILE I 223     -12.164 -22.378  -0.124  1.00  0.00
I    C
ATOM   2937  HD1 ILE I 223     -12.411 -21.669  -0.943  1.00  0.00
I    H
ATOM   2938  HD2 ILE I 223     -12.374 -23.410  -0.481  1.00  0.00
I    H
ATOM   2939  HD3 ILE I 223     -12.833 -22.159   0.735  1.00  0.00
I    H
ATOM   2940  C   ILE I 223      -8.217 -23.867   2.754  1.00  0.00
I    C
ATOM   2941  O   ILE I 223      -7.904 -23.177   3.716  1.00  0.00
I    O
ATOM   2942  N   THR I 224      -8.072 -25.214   2.785  1.00  0.00
I    N
```

```
ATOM   2943  HN  THR I 224       -8.336 -25.792   2.014  1.00  0.00
I    H
ATOM   2944  CA  THR I 224       -7.520 -25.954   3.933  1.00  0.00
I    C
ATOM   2945  HA  THR I 224       -8.110 -25.641   4.784  1.00  0.00
I    H
ATOM   2946  CB  THR I 224       -7.546 -27.472   3.749  1.00  0.00
I    C
ATOM   2947  HB  THR I 224       -7.071 -27.673   2.758  1.00  0.00
I    H
ATOM   2948  OG1 THR I 224       -8.885 -27.935   3.689  1.00  0.00
I    O
ATOM   2949  HG1 THR I 224       -8.821 -28.891   3.586  1.00  0.00
I    H
ATOM   2950  CG2 THR I 224       -6.926 -28.232   4.937  1.00  0.00
I    C
ATOM   2951 HG21 THR I 224       -6.860 -29.319   4.720  1.00  0.00
I    H
ATOM   2952 HG22 THR I 224       -5.899 -27.861   5.141  1.00  0.00
I    H
ATOM   2953 HG23 THR I 224       -7.534 -28.099   5.859  1.00  0.00
I    H
ATOM   2954  C   THR I 224       -6.092 -25.570   4.236  1.00  0.00
I    C
ATOM   2955  O   THR I 224       -5.722 -25.397   5.400  1.00  0.00
I    O
ATOM   2956  N   GLY I 225       -5.245 -25.388   3.204  1.00  0.00
I    N
ATOM   2957  HN  GLY I 225       -5.505 -25.622   2.268  1.00  0.00
I    H
ATOM   2958  CA  GLY I 225       -3.901 -24.843   3.368  1.00  0.00
I    C
ATOM   2959  HA1 GLY I 225       -3.363 -25.098   2.465  1.00  0.00
I    H
ATOM   2960  HA2 GLY I 225       -3.499 -25.292   4.267  1.00  0.00
I    H
ATOM   2961  C   GLY I 225       -3.852 -23.344   3.534  1.00  0.00
I    C
ATOM   2962  O   GLY I 225       -2.803 -22.793   3.824  1.00  0.00
I    O
ATOM   2963  N   GLY I 226       -4.993 -22.642   3.371  1.00  0.00
I    N
ATOM   2964  HN  GLY I 226       -5.845 -23.122   3.166  1.00  0.00
I    H
ATOM   2965  CA  GLY I 226       -5.096 -21.190   3.472  1.00  0.00
I    C
ATOM   2966  HA1 GLY I 226       -5.890 -20.884   2.805  1.00  0.00
I    H
ATOM   2967  HA2 GLY I 226       -4.125 -20.783   3.225  1.00  0.00
I    H
ATOM   2968  C   GLY I 226       -5.452 -20.733   4.860  1.00  0.00
I    C
ATOM   2969  O   GLY I 226       -4.986 -19.700   5.337  1.00  0.00
I    O
```

```
ATOM   2970  N   ILE I 227      -6.284 -21.489   5.596  1.00  0.00
I    N
ATOM   2971  HN  ILE I 227      -6.877 -22.194   5.214  1.00  0.00
I    H
ATOM   2972  CA  ILE I 227      -6.391 -21.337   7.042  1.00  0.00
I    C
ATOM   2973  HA  ILE I 227      -6.442 -20.273   7.238  1.00  0.00
I    H
ATOM   2974  CB  ILE I 227      -7.671 -21.904   7.660  1.00  0.00
I    C
ATOM   2975  HB  ILE I 227      -7.447 -21.974   8.755  1.00  0.00
I    H
ATOM   2976  CG2 ILE I 227      -8.817 -20.918   7.325  1.00  0.00
I    C
ATOM   2977 HG21 ILE I 227      -9.741 -21.191   7.877  1.00  0.00
I    H
ATOM   2978 HG22 ILE I 227      -8.546 -19.882   7.617  1.00  0.00
I    H
ATOM   2979 HG23 ILE I 227      -9.045 -20.928   6.239  1.00  0.00
I    H
ATOM   2980  CG1 ILE I 227      -7.957 -23.351   7.185  1.00  0.00
I    C
ATOM   2981 HG11 ILE I 227      -7.972 -23.386   6.073  1.00  0.00
I    H
ATOM   2982 HG12 ILE I 227      -7.128 -24.013   7.524  1.00  0.00
I    H
ATOM   2983  CD  ILE I 227      -9.274 -23.941   7.712  1.00  0.00
I    C
ATOM   2984  HD1 ILE I 227      -9.384 -24.996   7.379  1.00  0.00
I    H
ATOM   2985  HD2 ILE I 227      -9.295 -23.923   8.823  1.00  0.00
I    H
ATOM   2986  HD3 ILE I 227     -10.146 -23.368   7.333  1.00  0.00
I    H
ATOM   2987  C   ILE I 227      -5.132 -21.852   7.734  1.00  0.00
I    C
ATOM   2988  O   ILE I 227      -4.476 -22.786   7.290  1.00  0.00
I    O
ATOM   2989  N   GLY I 228      -4.703 -21.192   8.834  1.00  0.00
I    N
ATOM   2990  HN  GLY I 228      -5.199 -20.410   9.210  1.00  0.00
I    H
ATOM   2991  CA  GLY I 228      -3.486 -21.574   9.552  1.00  0.00
I    C
ATOM   2992  HA1 GLY I 228      -3.414 -20.888  10.385  1.00  0.00
I    H
ATOM   2993  HA2 GLY I 228      -2.682 -21.486   8.834  1.00  0.00
I    H
ATOM   2994  C   GLY I 228      -3.467 -22.974  10.100  1.00  0.00
I    C
ATOM   2995  O   GLY I 228      -4.498 -23.505  10.498  1.00  0.00
I    O
ATOM   2996  N   PHE I 229      -2.264 -23.574  10.240  1.00  0.00
I    N
```

```
ATOM   2997  HN  PHE I 229      -1.409 -23.149   9.949  1.00  0.00
I    H
ATOM   2998  CA  PHE I 229      -2.109 -24.898  10.830  1.00  0.00
I    C
ATOM   2999  HA  PHE I 229      -2.724 -25.562  10.234  1.00  0.00
I    H
ATOM   3000  CB  PHE I 229      -0.625 -25.337  10.775  1.00  0.00
I    C
ATOM   3001  HB1 PHE I 229      -0.126 -24.828   9.923  1.00  0.00
I    H
ATOM   3002  HB2 PHE I 229      -0.085 -25.036  11.699  1.00  0.00
I    H
ATOM   3003  CG  PHE I 229      -0.555 -26.818  10.547  1.00  0.00
I    C
ATOM   3004  CD1 PHE I 229      -0.521 -27.311   9.233  1.00  0.00
I    C
ATOM   3005  HD1 PHE I 229      -0.566 -26.621   8.401  1.00  0.00
I    H
ATOM   3006  CE1 PHE I 229      -0.434 -28.684   8.985  1.00  0.00
I    C
ATOM   3007  HE1 PHE I 229      -0.360 -29.046   7.969  1.00  0.00
I    H
ATOM   3008  CZ  PHE I 229      -0.439 -29.581  10.059  1.00  0.00
I    C
ATOM   3009  HZ  PHE I 229      -0.382 -30.643   9.870  1.00  0.00
I    H
ATOM   3010  CD2 PHE I 229      -0.556 -27.728  11.617  1.00  0.00
I    C
ATOM   3011  HD2 PHE I 229      -0.615 -27.365  12.634  1.00  0.00
I    H
ATOM   3012  CE2 PHE I 229      -0.508 -29.107  11.374  1.00  0.00
I    C
ATOM   3013  HE2 PHE I 229      -0.525 -29.801  12.204  1.00  0.00
I    H
ATOM   3014  C   PHE I 229      -2.646 -24.956  12.263  1.00  0.00
I    C
ATOM   3015  O   PHE I 229      -3.245 -25.930  12.702  1.00  0.00
I    O
ATOM   3016  N   THR I 230      -2.501 -23.826  12.984  1.00  0.00
I    N
ATOM   3017  HN  THR I 230      -1.890 -23.112  12.641  1.00  0.00
I    H
ATOM   3018  CA  THR I 230      -3.151 -23.503  14.255  1.00  0.00
I    C
ATOM   3019  HA  THR I 230      -2.781 -24.251  14.944  1.00  0.00
I    H
ATOM   3020  CB  THR I 230      -2.854 -22.059  14.651  1.00  0.00
I    C
ATOM   3021  HB  THR I 230      -3.112 -21.434  13.761  1.00  0.00
I    H
ATOM   3022  OG1 THR I 230      -1.467 -21.935  14.898  1.00  0.00
I    O
ATOM   3023  HG1 THR I 230      -1.333 -21.016  15.154  1.00  0.00
I    H
```

```
ATOM   3024  CG2 THR I 230      -3.603 -21.593  15.912  1.00  0.00
I    C
ATOM   3025 HG21 THR I 230      -3.442 -20.509  16.090  1.00  0.00
I    H
ATOM   3026 HG22 THR I 230      -4.695 -21.762  15.800  1.00  0.00
I    H
ATOM   3027 HG23 THR I 230      -3.256 -22.148  16.811  1.00  0.00
I    H
ATOM   3028  C   THR I 230      -4.661 -23.635  14.244  1.00  0.00
I    C
ATOM   3029  O   THR I 230      -5.233 -24.251  15.137  1.00  0.00
I    O
ATOM   3030  N   VAL I 231      -5.351 -23.097  13.216  1.00  0.00
I    N
ATOM   3031  HN  VAL I 231      -4.881 -22.618  12.479  1.00  0.00
I    H
ATOM   3032  CA  VAL I 231      -6.803 -23.153  13.070  1.00  0.00
I    C
ATOM   3033  HA  VAL I 231      -7.241 -22.782  13.988  1.00  0.00
I    H
ATOM   3034  CB  VAL I 231      -7.267 -22.303  11.884  1.00  0.00
I    C
ATOM   3035  HB  VAL I 231      -6.776 -22.709  10.965  1.00  0.00
I    H
ATOM   3036  CG1 VAL I 231      -8.800 -22.371  11.698  1.00  0.00
I    C
ATOM   3037 HG11 VAL I 231      -9.117 -21.717  10.857  1.00  0.00
I    H
ATOM   3038 HG12 VAL I 231      -9.139 -23.401  11.461  1.00  0.00
I    H
ATOM   3039 HG13 VAL I 231      -9.323 -22.026  12.616  1.00  0.00
I    H
ATOM   3040  CG2 VAL I 231      -6.833 -20.847  12.138  1.00  0.00
I    C
ATOM   3041 HG21 VAL I 231      -7.184 -20.184  11.318  1.00  0.00
I    H
ATOM   3042 HG22 VAL I 231      -7.257 -20.475  13.096  1.00  0.00
I    H
ATOM   3043 HG23 VAL I 231      -5.727 -20.760  12.188  1.00  0.00
I    H
ATOM   3044  C   VAL I 231      -7.263 -24.590  12.894  1.00  0.00
I    C
ATOM   3045  O   VAL I 231      -8.207 -25.051  13.532  1.00  0.00
I    O
ATOM   3046  N   LEU I 232      -6.528 -25.355  12.052  1.00  0.00
I    N
ATOM   3047  HN  LEU I 232      -5.791 -24.960  11.507  1.00  0.00
I    H
ATOM   3048  CA  LEU I 232      -6.719 -26.780  11.853  1.00  0.00
I    C
ATOM   3049  HA  LEU I 232      -7.755 -26.905  11.563  1.00  0.00
I    H
ATOM   3050  CB  LEU I 232      -5.795 -27.331  10.745  1.00  0.00
I    C
```

ATOM   3051 HB1 LEU I 232   -4.745 -27.150 11.072 1.00 0.00
I   H
ATOM   3052 HB2 LEU I 232   -5.913 -28.436 10.691 1.00 0.00
I   H
ATOM   3053 CG LEU I 232   -6.008 -26.689   9.362 1.00 0.00
I   C
ATOM   3054 HG LEU I 232   -5.833 -25.593   9.483 1.00 0.00
I   H
ATOM   3055 CD1 LEU I 232   -4.999 -27.276   8.367 1.00 0.00
I   C
ATOM   3056 HD11 LEU I 232   -5.167 -26.860   7.350 1.00 0.00
I   H
ATOM   3057 HD12 LEU I 232   -3.959 -27.033   8.676 1.00 0.00
I   H
ATOM   3058 HD13 LEU I 232   -5.099 -28.381   8.311 1.00 0.00
I   H
ATOM   3059 CD2 LEU I 232   -7.438 -26.888   8.830 1.00 0.00
I   C
ATOM   3060 HD21 LEU I 232   -7.553 -26.394   7.841 1.00 0.00
I   H
ATOM   3061 HD22 LEU I 232   -7.681 -27.962   8.702 1.00 0.00
I   H
ATOM   3062 HD23 LEU I 232   -8.177 -26.442   9.529 1.00 0.00
I   H
ATOM   3063 C   LEU I 232   -6.497 -27.585 13.124 1.00 0.00
I   C
ATOM   3064 O   LEU I 232   -7.267 -28.484 13.459 1.00 0.00
I   O
ATOM   3065 N   PHE I 233   -5.453 -27.254 13.903 1.00 0.00
I   N
ATOM   3066 HN PHE I 233   -4.785 -26.573 13.610 1.00 0.00
I   H
ATOM   3067 CA PHE I 233   -5.192 -27.828 15.207 1.00 0.00
I   C
ATOM   3068 HA PHE I 233   -5.138 -28.899 15.052 1.00 0.00
I   H
ATOM   3069 CB PHE I 233   -3.829 -27.257 15.711 1.00 0.00
I   C
ATOM   3070 HB1 PHE I 233   -3.081 -27.325 14.890 1.00 0.00
I   H
ATOM   3071 HB2 PHE I 233   -3.920 -26.184 15.983 1.00 0.00
I   H
ATOM   3072 CG PHE I 233   -3.206 -27.987 16.875 1.00 0.00
I   C
ATOM   3073 CD1 PHE I 233   -3.693 -27.801 18.178 1.00 0.00
I   C
ATOM   3074 HD1 PHE I 233   -4.582 -27.205 18.337 1.00 0.00
I   H
ATOM   3075 CE1 PHE I 233   -3.040 -28.372 19.277 1.00 0.00
I   C
ATOM   3076 HE1 PHE I 233   -3.434 -28.230 20.274 1.00 0.00
I   H
ATOM   3077 CZ PHE I 233   -1.880 -29.134 19.085 1.00 0.00
I   C

```
ATOM   3078  HZ  PHE I 233      -1.377 -29.579  19.931  1.00  0.00
I    H
ATOM   3079  CD2 PHE I 233      -2.041 -28.759  16.693  1.00  0.00
I    C
ATOM   3080  HD2 PHE I 233      -1.636 -28.901  15.702  1.00  0.00
I    H
ATOM   3081  CE2 PHE I 233      -1.379 -29.326  17.791  1.00  0.00
I    C
ATOM   3082  HE2 PHE I 233      -0.483 -29.911  17.636  1.00  0.00
I    H
ATOM   3083  C   PHE I 233      -6.337 -27.574  16.192  1.00  0.00
I    C
ATOM   3084  O   PHE I 233      -6.853 -28.519  16.789  1.00  0.00
I    O
ATOM   3085  N   ASP I 234      -6.813 -26.322  16.329  1.00  0.00
I    N
ATOM   3086  HN  ASP I 234      -6.444 -25.557  15.807  1.00  0.00
I    H
ATOM   3087  CA  ASP I 234      -7.886 -25.931  17.221  1.00  0.00
I    C
ATOM   3088  HA  ASP I 234      -7.597 -26.262  18.212  1.00  0.00
I    H
ATOM   3089  CB  ASP I 234      -7.968 -24.393  17.094  1.00  0.00
I    C
ATOM   3090  HB1 ASP I 234      -6.949 -23.984  16.946  1.00  0.00
I    H
ATOM   3091  HB2 ASP I 234      -8.588 -24.106  16.223  1.00  0.00
I    H
ATOM   3092  CG  ASP I 234      -8.537 -23.675  18.302  1.00  0.00
I    C
ATOM   3093  OD1 ASP I 234      -9.634 -24.012  18.799  1.00  0.00
I    O
ATOM   3094  OD2 ASP I 234      -7.904 -22.660  18.693  1.00  0.00
I    O
ATOM   3095  C   ASP I 234      -9.216 -26.632  16.889  1.00  0.00
I    C
ATOM   3096  O   ASP I 234      -9.866 -27.232  17.748  1.00  0.00
I    O
ATOM   3097  N   VAL I 235      -9.601 -26.663  15.589  1.00  0.00
I    N
ATOM   3098  HN  VAL I 235      -9.072 -26.216  14.873  1.00  0.00
I    H
ATOM   3099  CA  VAL I 235     -10.806 -27.340  15.115  1.00  0.00
I    C
ATOM   3100  HA  VAL I 235     -11.619 -26.950  15.715  1.00  0.00
I    H
ATOM   3101  CB  VAL I 235     -11.247 -26.908  13.711  1.00  0.00
I    C
ATOM   3102  HB  VAL I 235     -11.219 -25.791  13.677  1.00  0.00
I    H
ATOM   3103  CG1 VAL I 235     -10.316 -27.448  12.617  1.00  0.00
I    C
ATOM   3104 HG11 VAL I 235     -10.687 -27.149  11.613  1.00  0.00
I    H
```

```
ATOM   3105 HG12 VAL I 235      -9.286 -27.049  12.722  1.00  0.00
I    H
ATOM   3106 HG13 VAL I 235     -10.274 -28.558  12.650  1.00  0.00
I    H
ATOM   3107  CG2 VAL I 235     -12.699 -27.343  13.425  1.00  0.00
I    C
ATOM   3108 HG21 VAL I 235     -12.997 -27.063  12.393  1.00  0.00
I    H
ATOM   3109 HG22 VAL I 235     -12.804 -28.445  13.533  1.00  0.00
I    H
ATOM   3110 HG23 VAL I 235     -13.410 -26.855  14.124  1.00  0.00
I    H
ATOM   3111  C   VAL I 235     -10.795 -28.856  15.291  1.00  0.00
I    C
ATOM   3112  O   VAL I 235     -11.803 -29.440  15.676  1.00  0.00
I    O
ATOM   3113  N   MET I 236      -9.654 -29.552  15.064  1.00  0.00
I    N
ATOM   3114  HN  MET I 236      -8.816 -29.144  14.710  1.00  0.00
I    H
ATOM   3115  CA  MET I 236      -9.562 -30.985  15.327  1.00  0.00
I    C
ATOM   3116  HA  MET I 236     -10.365 -31.437  14.758  1.00  0.00
I    H
ATOM   3117  CB  MET I 236      -8.164 -31.537  14.944  1.00  0.00
I    C
ATOM   3118  HB1 MET I 236      -7.410 -30.872  15.428  1.00  0.00
I    H
ATOM   3119  HB2 MET I 236      -8.028 -32.543  15.398  1.00  0.00
I    H
ATOM   3120  CG  MET I 236      -7.858 -31.595  13.442  1.00  0.00
I    C
ATOM   3121  HG1 MET I 236      -8.519 -32.334  12.941  1.00  0.00
I    H
ATOM   3122  HG2 MET I 236      -8.053 -30.602  12.981  1.00  0.00
I    H
ATOM   3123  SD  MET I 236      -6.127 -32.071  13.140  1.00  0.00
I    S
ATOM   3124  CE  MET I 236      -6.098 -31.373  11.470  1.00  0.00
I    C
ATOM   3125  HE1 MET I 236      -5.107 -31.528  10.993  1.00  0.00
I    H
ATOM   3126  HE2 MET I 236      -6.867 -31.850  10.825  1.00  0.00
I    H
ATOM   3127  HE3 MET I 236      -6.299 -30.280  11.490  1.00  0.00
I    H
ATOM   3128  C   MET I 236      -9.759 -31.374  16.792  1.00  0.00
I    C
ATOM   3129  O   MET I 236     -10.456 -32.350  17.102  1.00  0.00
I    O
ATOM   3130  N   LYS I 237      -9.114 -30.627  17.717  1.00  0.00
I    N
ATOM   3131  HN  LYS I 237      -8.612 -29.803  17.457  1.00  0.00
I    H
```

ATOM 3132 CA LYS I 237 -9.089 -30.943 19.136 1.00 0.00
I C
ATOM 3133 HA LYS I 237 -9.028 -32.022 19.216 1.00 0.00
I H
ATOM 3134 CB LYS I 237 -7.880 -30.252 19.846 1.00 0.00
I C
ATOM 3135 HB1 LYS I 237 -7.732 -29.264 19.353 1.00 0.00
I H
ATOM 3136 HB2 LYS I 237 -8.155 -30.037 20.902 1.00 0.00
I H
ATOM 3137 CG LYS I 237 -6.537 -31.023 19.800 1.00 0.00
I C
ATOM 3138 HG1 LYS I 237 -5.790 -30.490 20.431 1.00 0.00
I H
ATOM 3139 HG2 LYS I 237 -6.685 -32.035 20.242 1.00 0.00
I H
ATOM 3140 CD LYS I 237 -5.937 -31.174 18.391 1.00 0.00
I C
ATOM 3141 HD1 LYS I 237 -6.607 -31.833 17.792 1.00 0.00
I H
ATOM 3142 HD2 LYS I 237 -5.934 -30.172 17.905 1.00 0.00
I H
ATOM 3143 CE LYS I 237 -4.515 -31.739 18.313 1.00 0.00
I C
ATOM 3144 HE1 LYS I 237 -3.832 -31.166 18.980 1.00 0.00
I H
ATOM 3145 HE2 LYS I 237 -4.501 -32.806 18.628 1.00 0.00
I H
ATOM 3146 NZ LYS I 237 -4.049 -31.627 16.912 1.00 0.00
I N
ATOM 3147 HZ1 LYS I 237 -3.082 -32.002 16.826 1.00 0.00
I H
ATOM 3148 HZ2 LYS I 237 -4.685 -32.166 16.290 1.00 0.00
I H
ATOM 3149 HZ3 LYS I 237 -4.056 -30.627 16.623 1.00 0.00
I H
ATOM 3150 C LYS I 237 -10.370 -30.509 19.831 1.00 0.00
I C
ATOM 3151 O LYS I 237 -11.082 -31.332 20.397 1.00 0.00
I O
ATOM 3152 N ASN I 238 -10.705 -29.204 19.777 1.00 0.00
I N
ATOM 3153 HN ASN I 238 -10.205 -28.541 19.221 1.00 0.00
I H
ATOM 3154 CA ASN I 238 -11.821 -28.647 20.522 1.00 0.00
I C
ATOM 3155 HA ASN I 238 -11.797 -29.157 21.479 1.00 0.00
I H
ATOM 3156 CB ASN I 238 -11.650 -27.109 20.627 1.00 0.00
I C
ATOM 3157 HB1 ASN I 238 -11.255 -26.744 19.654 1.00 0.00
I H
ATOM 3158 HB2 ASN I 238 -12.633 -26.623 20.790 1.00 0.00
I H

ATOM 3159 CG ASN I 238 -10.663 -26.757 21.730 1.00 0.00
I C
ATOM 3160 OD1 ASN I 238 -10.486 -27.502 22.695 1.00 0.00
I O
ATOM 3161 ND2 ASN I 238 -10.047 -25.560 21.637 1.00 0.00
I N
ATOM 3162 HD21 ASN I 238 -9.391 -25.327 22.349 1.00 0.00
I H
ATOM 3163 HD22 ASN I 238 -10.234 -24.963 20.863 1.00 0.00
I H
ATOM 3164 C ASN I 238 -13.175 -28.921 19.887 1.00 0.00
I C
ATOM 3165 O ASN I 238 -14.137 -29.274 20.564 1.00 0.00
I O
ATOM 3166 N ARG I 239 -13.268 -28.734 18.557 1.00 0.00
I N
ATOM 3167 HN ARG I 239 -12.499 -28.289 18.107 1.00 0.00
I H
ATOM 3168 CA ARG I 239 -14.389 -29.120 17.709 1.00 0.00
I C
ATOM 3169 HA ARG I 239 -14.200 -28.622 16.765 1.00 0.00
I H
ATOM 3170 CB ARG I 239 -14.278 -30.643 17.473 1.00 0.00
I C
ATOM 3171 HB1 ARG I 239 -13.195 -30.898 17.562 1.00 0.00
I H
ATOM 3172 HB2 ARG I 239 -14.790 -31.185 18.298 1.00 0.00
I H
ATOM 3173 CG ARG I 239 -14.782 -31.128 16.096 1.00 0.00
I C
ATOM 3174 HG1 ARG I 239 -15.731 -30.600 15.860 1.00 0.00
I H
ATOM 3175 HG2 ARG I 239 -14.058 -30.807 15.313 1.00 0.00
I H
ATOM 3176 CD ARG I 239 -14.941 -32.647 15.985 1.00 0.00
I C
ATOM 3177 HD1 ARG I 239 -15.768 -32.983 16.651 1.00 0.00
I H
ATOM 3178 HD2 ARG I 239 -15.175 -32.958 14.940 1.00 0.00
I H
ATOM 3179 NE ARG I 239 -13.623 -33.228 16.396 1.00 0.00
I N
ATOM 3180 HE ARG I 239 -12.933 -32.527 16.613 1.00 0.00
I H
ATOM 3181 CZ ARG I 239 -13.349 -34.534 16.479 1.00 0.00
I C
ATOM 3182 NH1 ARG I 239 -14.184 -35.444 15.991 1.00 0.00
I N
ATOM 3183 HH11 ARG I 239 -13.984 -36.411 16.080 1.00 0.00
I H
ATOM 3184 HH12 ARG I 239 -15.069 -35.105 15.662 1.00 0.00
I H
ATOM 3185 NH2 ARG I 239 -12.214 -34.916 17.059 1.00 0.00
I N

```
ATOM   3186 HH21 ARG I 239     -11.953 -35.871  17.053  1.00  0.00
I    H
ATOM   3187 HH22 ARG I 239     -11.568 -34.202  17.295  1.00  0.00
I    H
ATOM   3188  C   ARG I 239     -15.825 -28.737  18.131  1.00  0.00
I    C
ATOM   3189  O   ARG I 239     -16.780 -29.453  17.842  1.00  0.00
I    O
ATOM   3190  N   ARG I 240     -16.039 -27.578  18.796  1.00  0.00
I    N
ATOM   3191  HN  ARG I 240     -15.317 -26.903  18.932  1.00  0.00
I    H
ATOM   3192  CA  ARG I 240     -17.349 -27.255  19.352  1.00  0.00
I    C
ATOM   3193  HA  ARG I 240     -18.088 -27.827  18.805  1.00  0.00
I    H
ATOM   3194  CB  ARG I 240     -17.337 -27.616  20.870  1.00  0.00
I    C
ATOM   3195  HB1 ARG I 240     -16.910 -28.644  20.946  1.00  0.00
I    H
ATOM   3196  HB2 ARG I 240     -16.626 -26.949  21.406  1.00  0.00
I    H
ATOM   3197  CG  ARG I 240     -18.717 -27.616  21.570  1.00  0.00
I    C
ATOM   3198  HG1 ARG I 240     -19.217 -26.643  21.368  1.00  0.00
I    H
ATOM   3199  HG2 ARG I 240     -19.364 -28.389  21.095  1.00  0.00
I    H
ATOM   3200  CD  ARG I 240     -18.660 -27.918  23.070  1.00  0.00
I    C
ATOM   3201  HD1 ARG I 240     -18.300 -28.962  23.223  1.00  0.00
I    H
ATOM   3202  HD2 ARG I 240     -17.972 -27.220  23.602  1.00  0.00
I    H
ATOM   3203  NE  ARG I 240     -20.065 -27.727  23.577  1.00  0.00
I    N
ATOM   3204  HE  ARG I 240     -20.756 -27.750  22.846  1.00  0.00
I    H
ATOM   3205  CZ  ARG I 240     -20.387 -27.542  24.866  1.00  0.00
I    C
ATOM   3206  NH1 ARG I 240     -19.459 -27.554  25.814  1.00  0.00
I    N
ATOM   3207 HH11 ARG I 240     -19.708 -27.443  26.767  1.00  0.00
I    H
ATOM   3208 HH12 ARG I 240     -18.535 -27.816  25.527  1.00  0.00
I    H
ATOM   3209  NH2 ARG I 240     -21.659 -27.363  25.219  1.00  0.00
I    N
ATOM   3210 HH21 ARG I 240     -21.885 -27.141  26.156  1.00  0.00
I    H
ATOM   3211 HH22 ARG I 240     -22.316 -27.253  24.485  1.00  0.00
I    H
ATOM   3212  C   ARG I 240     -17.720 -25.782  19.178  1.00  0.00
I    C
```

```
ATOM   3213  O   ARG I 240     -18.826 -25.372  19.523  1.00  0.00
I    O
ATOM   3214  N   PHE I 241     -16.820 -24.921  18.645  1.00  0.00
I    N
ATOM   3215  HN  PHE I 241     -15.918 -25.236  18.353  1.00  0.00
I    H
ATOM   3216  CA  PHE I 241     -17.057 -23.489  18.443  1.00  0.00
I    C
ATOM   3217  HA  PHE I 241     -16.165 -23.145  17.934  1.00  0.00
I    H
ATOM   3218  CB  PHE I 241     -18.182 -23.271  17.378  1.00  0.00
I    C
ATOM   3219  HB1 PHE I 241     -17.953 -23.881  16.477  1.00  0.00
I    H
ATOM   3220  HB2 PHE I 241     -19.168 -23.611  17.762  1.00  0.00
I    H
ATOM   3221  CG  PHE I 241     -18.281 -21.839  16.915  1.00  0.00
I    C
ATOM   3222  CD1 PHE I 241     -17.402 -21.334  15.941  1.00  0.00
I    C
ATOM   3223  HD1 PHE I 241     -16.682 -21.995  15.476  1.00  0.00
I    H
ATOM   3224  CE1 PHE I 241     -17.442 -19.985  15.568  1.00  0.00
I    C
ATOM   3225  HE1 PHE I 241     -16.747 -19.606  14.832  1.00  0.00
I    H
ATOM   3226  CZ  PHE I 241     -18.389 -19.131  16.147  1.00  0.00
I    C
ATOM   3227  HZ  PHE I 241     -18.432 -18.092  15.853  1.00  0.00
I    H
ATOM   3228  CD2 PHE I 241     -19.231 -20.973  17.481  1.00  0.00
I    C
ATOM   3229  HD2 PHE I 241     -19.914 -21.339  18.234  1.00  0.00
I    H
ATOM   3230  CE2 PHE I 241     -19.287 -19.626  17.101  1.00  0.00
I    C
ATOM   3231  HE2 PHE I 241     -20.024 -18.972  17.546  1.00  0.00
I    H
ATOM   3232  C   PHE I 241     -17.205 -22.639  19.716  1.00  0.00
I    C
ATOM   3233  O   PHE I 241     -16.832 -21.468  19.750  1.00  0.00
I    O
ATOM   3234  N   LYS I 242     -17.650 -23.206  20.849  1.00  0.00
I    N
ATOM   3235  HN  LYS I 242     -17.950 -24.158  20.880  1.00  0.00
I    H
ATOM   3236  CA  LYS I 242     -17.731 -22.494  22.108  1.00  0.00
I    C
ATOM   3237  HA  LYS I 242     -18.093 -21.500  21.877  1.00  0.00
I    H
ATOM   3238  CB  LYS I 242     -18.747 -23.195  23.045  1.00  0.00
I    C
ATOM   3239  HB1 LYS I 242     -18.385 -24.238  23.198  1.00  0.00
I    H
```

ATOM 3240 HB2 LYS I 242 -18.717 -22.704 24.044 1.00 0.00
I H
ATOM 3241 CG LYS I 242 -20.161 -23.197 22.432 1.00 0.00
I C
ATOM 3242 HG1 LYS I 242 -20.463 -22.148 22.209 1.00 0.00
I H
ATOM 3243 HG2 LYS I 242 -20.135 -23.747 21.463 1.00 0.00
I H
ATOM 3244 CD LYS I 242 -21.217 -23.822 23.358 1.00 0.00
I C
ATOM 3245 HD1 LYS I 242 -20.893 -24.858 23.611 1.00 0.00
I H
ATOM 3246 HD2 LYS I 242 -21.236 -23.238 24.307 1.00 0.00
I H
ATOM 3247 CE LYS I 242 -22.638 -23.865 22.773 1.00 0.00
I C
ATOM 3248 HE1 LYS I 242 -22.635 -24.360 21.777 1.00 0.00
I H
ATOM 3249 HE2 LYS I 242 -23.320 -24.432 23.445 1.00 0.00
I H
ATOM 3250 NZ LYS I 242 -23.171 -22.495 22.613 1.00 0.00
I N
ATOM 3251 HZ1 LYS I 242 -24.132 -22.531 22.216 1.00 0.00
I H
ATOM 3252 HZ2 LYS I 242 -23.197 -22.022 23.538 1.00 0.00
I H
ATOM 3253 HZ3 LYS I 242 -22.554 -21.956 21.972 1.00 0.00
I H
ATOM 3254 C LYS I 242 -16.373 -22.339 22.779 1.00 0.00
I C
ATOM 3255 O LYS I 242 -16.162 -21.420 23.566 1.00 0.00
I O
ATOM 3256 N THR I 243 -15.415 -23.215 22.426 1.00 0.00
I N
ATOM 3257 HN THR I 243 -15.568 -23.864 21.682 1.00 0.00
I H
ATOM 3258 CA THR I 243 -14.105 -23.320 23.051 1.00 0.00
I C
ATOM 3259 HA THR I 243 -14.114 -22.590 23.849 1.00 0.00
I H
ATOM 3260 CB THR I 243 -13.839 -24.751 23.513 1.00 0.00
I C
ATOM 3261 HB THR I 243 -12.734 -24.830 23.658 1.00 0.00
I H
ATOM 3262 OG1 THR I 243 -14.199 -25.704 22.519 1.00 0.00
I O
ATOM 3263 HG1 THR I 243 -14.006 -26.564 22.906 1.00 0.00
I H
ATOM 3264 CG2 THR I 243 -14.734 -25.066 24.718 1.00 0.00
I C
ATOM 3265 HG21 THR I 243 -14.483 -26.059 25.148 1.00 0.00
I H
ATOM 3266 HG22 THR I 243 -14.593 -24.304 25.514 1.00 0.00
I H

```
ATOM   3267 HG23 THR I 243     -15.807 -25.073  24.429  1.00  0.00
I    H
ATOM   3268  C   THR I 243     -12.974 -22.930  22.121  1.00  0.00
I    C
ATOM   3269  O   THR I 243     -11.811 -23.000  22.504  1.00  0.00
I    O
ATOM   3270  N   PHE I 244     -13.270 -22.484  20.880  1.00  0.00
I    N
ATOM   3271  HN  PHE I 244     -14.204 -22.436  20.532  1.00  0.00
I    H
ATOM   3272  CA  PHE I 244     -12.251 -22.033  19.940  1.00  0.00
I    C
ATOM   3273  HA  PHE I 244     -11.544 -22.852  19.882  1.00  0.00
I    H
ATOM   3274  CB  PHE I 244     -12.834 -21.752  18.521  1.00  0.00
I    C
ATOM   3275  HB1 PHE I 244     -13.755 -21.136  18.621  1.00  0.00
I    H
ATOM   3276  HB2 PHE I 244     -12.118 -21.171  17.902  1.00  0.00
I    H
ATOM   3277  CG  PHE I 244     -13.253 -22.978  17.746  1.00  0.00
I    C
ATOM   3278  CD1 PHE I 244     -12.782 -24.279  18.014  1.00  0.00
I    C
ATOM   3279  HD1 PHE I 244     -12.065 -24.435  18.808  1.00  0.00
I    H
ATOM   3280  CE1 PHE I 244     -13.225 -25.370  17.262  1.00  0.00
I    C
ATOM   3281  HE1 PHE I 244     -12.849 -26.361  17.474  1.00  0.00
I    H
ATOM   3282  CZ  PHE I 244     -14.158 -25.185  16.239  1.00  0.00
I    C
ATOM   3283  HZ  PHE I 244     -14.548 -26.036  15.699  1.00  0.00
I    H
ATOM   3284  CD2 PHE I 244     -14.125 -22.799  16.658  1.00  0.00
I    C
ATOM   3285  HD2 PHE I 244     -14.426 -21.803  16.364  1.00  0.00
I    H
ATOM   3286  CE2 PHE I 244     -14.594 -23.896  15.921  1.00  0.00
I    C
ATOM   3287  HE2 PHE I 244     -15.292 -23.741  15.110  1.00  0.00
I    H
ATOM   3288  C   PHE I 244     -11.473 -20.804  20.398  1.00  0.00
I    C
ATOM   3289  O   PHE I 244     -12.010 -19.881  21.015  1.00  0.00
I    O
ATOM   3290  N   SER I 245     -10.165 -20.762  20.071  1.00  0.00
I    N
ATOM   3291  HN  SER I 245      -9.690 -21.547  19.673  1.00  0.00
I    H
ATOM   3292  CA  SER I 245      -9.321 -19.586  20.261  1.00  0.00
I    C
ATOM   3293  HA  SER I 245      -9.386 -19.365  21.318  1.00  0.00
I    H
```

```
ATOM   3294  CB  SER I 245      -7.850 -19.848  19.853  1.00  0.00
I    C
ATOM   3295  HB1 SER I 245      -7.505 -20.769  20.375  1.00  0.00
I    H
ATOM   3296  HB2 SER I 245      -7.780 -20.045  18.759  1.00  0.00
I    H
ATOM   3297  OG  SER I 245      -6.984 -18.770  20.227  1.00  0.00
I    O
ATOM   3298  HG1 SER I 245      -6.077 -19.094  20.149  1.00  0.00
I    H
ATOM   3299  C   SER I 245      -9.807 -18.353  19.510  1.00  0.00
I    C
ATOM   3300  O   SER I 245     -10.511 -18.464  18.503  1.00  0.00
I    O
ATOM   3301  N   LEU I 246      -9.444 -17.124  19.947  1.00  0.00
I    N
ATOM   3302  HN  LEU I 246      -8.880 -16.986  20.759  1.00  0.00
I    H
ATOM   3303  CA  LEU I 246      -9.840 -15.891  19.276  1.00  0.00
I    C
ATOM   3304  HA  LEU I 246     -10.922 -15.884  19.266  1.00  0.00
I    H
ATOM   3305  CB  LEU I 246      -9.323 -14.622  20.016  1.00  0.00
I    C
ATOM   3306  HB1 LEU I 246      -9.769 -14.633  21.037  1.00  0.00
I    H
ATOM   3307  HB2 LEU I 246      -8.222 -14.711  20.160  1.00  0.00
I    H
ATOM   3308  CG  LEU I 246      -9.676 -13.277  19.330  1.00  0.00
I    C
ATOM   3309  HG  LEU I 246      -9.294 -13.385  18.286  1.00  0.00
I    H
ATOM   3310  CD1 LEU I 246     -11.190 -13.030  19.305  1.00  0.00
I    C
ATOM   3311 HD11 LEU I 246     -11.416 -12.040  18.854  1.00  0.00
I    H
ATOM   3312 HD12 LEU I 246     -11.705 -13.813  18.707  1.00  0.00
I    H
ATOM   3313 HD13 LEU I 246     -11.607 -13.045  20.335  1.00  0.00
I    H
ATOM   3314  CD2 LEU I 246      -8.952 -12.088  19.981  1.00  0.00
I    C
ATOM   3315 HD21 LEU I 246      -9.211 -11.143  19.456  1.00  0.00
I    H
ATOM   3316 HD22 LEU I 246      -9.236 -11.970  21.046  1.00  0.00
I    H
ATOM   3317 HD23 LEU I 246      -7.850 -12.225  19.925  1.00  0.00
I    H
ATOM   3318  C   LEU I 246      -9.365 -15.851  17.827  1.00  0.00
I    C
ATOM   3319  O   LEU I 246     -10.131 -15.537  16.924  1.00  0.00
I    O
ATOM   3320  N   HSE I 247      -8.097 -16.257  17.573  1.00  0.00
I    N
```

```
ATOM   3321  HN  HSE I 247      -7.464 -16.505  18.306  1.00  0.00
I    H
ATOM   3322  CA  HSE I 247      -7.538 -16.370  16.232  1.00  0.00
I    C
ATOM   3323  HA  HSE I 247      -7.633 -15.391  15.777  1.00  0.00
I    H
ATOM   3324  CB  HSE I 247      -6.056 -16.831  16.333  1.00  0.00
I    C
ATOM   3325  HB1 HSE I 247      -5.511 -16.094  16.962  1.00  0.00
I    H
ATOM   3326  HB2 HSE I 247      -6.006 -17.806  16.860  1.00  0.00
I    H
ATOM   3327  ND1 HSE I 247      -5.397 -18.084  14.274  1.00  0.00
I    N
ATOM   3328  CG  HSE I 247      -5.331 -16.925  15.021  1.00  0.00
I    C
ATOM   3329  CE1 HSE I 247      -4.768 -17.812  13.153  1.00  0.00
I    C
ATOM   3330  HE1 HSE I 247      -4.638 -18.517  12.327  1.00  0.00
I    H
ATOM   3331  NE2 HSE I 247      -4.291 -16.548  13.141  1.00  0.00
I    N
ATOM   3332  HE2 HSE I 247      -3.758 -16.111  12.417  1.00  0.00
I    H
ATOM   3333  CD2 HSE I 247      -4.651 -15.972  14.337  1.00  0.00
I    C
ATOM   3334  HD2 HSE I 247      -4.401 -14.959  14.622  1.00  0.00
I    H
ATOM   3335  C   HSE I 247      -8.320 -17.346  15.362  1.00  0.00
I    C
ATOM   3336  O   HSE I 247      -8.714 -17.023  14.240  1.00  0.00
I    O
ATOM   3337  N   THR I 248      -8.635 -18.535  15.909  1.00  0.00
I    N
ATOM   3338  HN  THR I 248      -8.328 -18.757  16.834  1.00  0.00
I    H
ATOM   3339  CA  THR I 248      -9.416 -19.588  15.266  1.00  0.00
I    C
ATOM   3340  HA  THR I 248      -8.914 -19.779  14.327  1.00  0.00
I    H
ATOM   3341  CB  THR I 248      -9.464 -20.837  16.136  1.00  0.00
I    C
ATOM   3342  HB  THR I 248      -9.805 -20.499  17.146  1.00  0.00
I    H
ATOM   3343  OG1 THR I 248      -8.139 -21.335  16.242  1.00  0.00
I    O
ATOM   3344  HG1 THR I 248      -8.204 -22.127  16.786  1.00  0.00
I    H
ATOM   3345  CG2 THR I 248     -10.351 -21.956  15.556  1.00  0.00
I    C
ATOM   3346 HG21 THR I 248     -10.448 -22.797  16.275  1.00  0.00
I    H
ATOM   3347 HG22 THR I 248     -11.371 -21.571  15.343  1.00  0.00
I    H
```

ATOM 3348 HG23 THR I 248 -9.925 -22.355 14.611 1.00 0.00
I H
ATOM 3349 C THR I 248 -10.818 -19.151 14.923 1.00 0.00
I C
ATOM 3350 O THR I 248 -11.278 -19.337 13.798 1.00 0.00
I O
ATOM 3351 N LYS I 249 -11.521 -18.480 15.864 1.00 0.00
I N
ATOM 3352 HN LYS I 249 -11.178 -18.351 16.793 1.00 0.00
I H
ATOM 3353 CA LYS I 249 -12.825 -17.897 15.601 1.00 0.00
I C
ATOM 3354 HA LYS I 249 -13.452 -18.705 15.244 1.00 0.00
I H
ATOM 3355 CB LYS I 249 -13.432 -17.247 16.872 1.00 0.00
I C
ATOM 3356 HB1 LYS I 249 -12.650 -16.583 17.308 1.00 0.00
I H
ATOM 3357 HB2 LYS I 249 -14.274 -16.583 16.573 1.00 0.00
I H
ATOM 3358 CG LYS I 249 -13.884 -18.254 17.936 1.00 0.00
I C
ATOM 3359 HG1 LYS I 249 -14.559 -19.004 17.464 1.00 0.00
I H
ATOM 3360 HG2 LYS I 249 -12.993 -18.801 18.319 1.00 0.00
I H
ATOM 3361 CD LYS I 249 -14.629 -17.573 19.095 1.00 0.00
I C
ATOM 3362 HD1 LYS I 249 -13.966 -16.790 19.527 1.00 0.00
I H
ATOM 3363 HD2 LYS I 249 -15.525 -17.058 18.677 1.00 0.00
I H
ATOM 3364 CE LYS I 249 -15.053 -18.572 20.172 1.00 0.00
I C
ATOM 3365 HE1 LYS I 249 -15.692 -19.370 19.733 1.00 0.00
I H
ATOM 3366 HE2 LYS I 249 -14.161 -19.050 20.634 1.00 0.00
I H
ATOM 3367 NZ LYS I 249 -15.818 -17.888 21.231 1.00 0.00
I N
ATOM 3368 HZ1 LYS I 249 -16.105 -18.572 21.961 1.00 0.00
I H
ATOM 3369 HZ2 LYS I 249 -15.228 -17.148 21.663 1.00 0.00
I H
ATOM 3370 HZ3 LYS I 249 -16.665 -17.450 20.816 1.00 0.00
I H
ATOM 3371 C LYS I 249 -12.794 -16.831 14.515 1.00 0.00
I C
ATOM 3372 O LYS I 249 -13.620 -16.859 13.604 1.00 0.00
I O
ATOM 3373 N LEU I 250 -11.816 -15.899 14.566 1.00 0.00
I N
ATOM 3374 HN LEU I 250 -11.159 -15.880 15.317 1.00 0.00
I H

```
ATOM   3375  CA  LEU I 250     -11.619 -14.855  13.571  1.00  0.00
I    C
ATOM   3376  HA  LEU I 250     -12.565 -14.331  13.519  1.00  0.00
I    H
ATOM   3377  CB  LEU I 250     -10.454 -13.913  13.975  1.00  0.00
I    C
ATOM   3378  HB1 LEU I 250      -9.621 -14.557  14.341  1.00  0.00
I    H
ATOM   3379  HB2 LEU I 250     -10.069 -13.401  13.065  1.00  0.00
I    H
ATOM   3380  CG  LEU I 250     -10.799 -12.885  15.067  1.00  0.00
I    C
ATOM   3381  HG  LEU I 250     -11.129 -13.447  15.974  1.00  0.00
I    H
ATOM   3382  CD1 LEU I 250      -9.561 -12.037  15.396  1.00  0.00
I    C
ATOM   3383 HD11 LEU I 250      -9.811 -11.259  16.149  1.00  0.00
I    H
ATOM   3384 HD12 LEU I 250      -8.749 -12.673  15.808  1.00  0.00
I    H
ATOM   3385 HD13 LEU I 250      -9.180 -11.528  14.485  1.00  0.00
I    H
ATOM   3386  CD2 LEU I 250     -11.952 -11.975  14.625  1.00  0.00
I    C
ATOM   3387 HD21 LEU I 250     -12.203 -11.250  15.431  1.00  0.00
I    H
ATOM   3388 HD22 LEU I 250     -11.691 -11.393  13.719  1.00  0.00
I    H
ATOM   3389 HD23 LEU I 250     -12.860 -12.577  14.405  1.00  0.00
I    H
ATOM   3390  C   LEU I 250     -11.301 -15.364  12.178  1.00  0.00
I    C
ATOM   3391  O   LEU I 250     -11.835 -14.878  11.183  1.00  0.00
I    O
ATOM   3392  N   MET I 251     -10.409 -16.362  12.060  1.00  0.00
I    N
ATOM   3393  HN  MET I 251      -9.927 -16.767  12.834  1.00  0.00
I    H
ATOM   3394  CA  MET I 251     -10.070 -16.940  10.778  1.00  0.00
I    C
ATOM   3395  HA  MET I 251      -9.775 -16.107  10.151  1.00  0.00
I    H
ATOM   3396  CB  MET I 251      -8.885 -17.928  10.903  1.00  0.00
I    C
ATOM   3397  HB1 MET I 251      -9.037 -18.498  11.849  1.00  0.00
I    H
ATOM   3398  HB2 MET I 251      -8.937 -18.674  10.079  1.00  0.00
I    H
ATOM   3399  CG  MET I 251      -7.502 -17.246  10.943  1.00  0.00
I    C
ATOM   3400  HG1 MET I 251      -7.398 -16.639  11.867  1.00  0.00
I    H
ATOM   3401  HG2 MET I 251      -6.704 -18.021  10.957  1.00  0.00
I    H
```

```
ATOM   3402  SD  MET I 251      -7.141 -16.123   9.547  1.00  0.00
I    S
ATOM   3403  CE  MET I 251      -7.718 -17.118   8.135  1.00  0.00
I    C
ATOM   3404  HE1 MET I 251      -7.560 -16.577   7.177  1.00  0.00
I    H
ATOM   3405  HE2 MET I 251      -7.169 -18.083   8.076  1.00  0.00
I    H
ATOM   3406  HE3 MET I 251      -8.802 -17.346   8.223  1.00  0.00
I    H
ATOM   3407  C   MET I 251     -11.219 -17.648  10.087  1.00  0.00
I    C
ATOM   3408  O   MET I 251     -11.399 -17.501   8.878  1.00  0.00
I    O
ATOM   3409  N   LEU I 252     -12.026 -18.436  10.822  1.00  0.00
I    N
ATOM   3410  HN  LEU I 252     -11.888 -18.616  11.794  1.00  0.00
I    H
ATOM   3411  CA  LEU I 252     -13.183 -19.099  10.250  1.00  0.00
I    C
ATOM   3412  HA  LEU I 252     -12.818 -19.604   9.365  1.00  0.00
I    H
ATOM   3413  CB  LEU I 252     -13.766 -20.108  11.268  1.00  0.00
I    C
ATOM   3414  HB1 LEU I 252     -13.968 -19.547  12.209  1.00  0.00
I    H
ATOM   3415  HB2 LEU I 252     -14.756 -20.460  10.901  1.00  0.00
I    H
ATOM   3416  CG  LEU I 252     -12.836 -21.300  11.580  1.00  0.00
I    C
ATOM   3417  HG  LEU I 252     -11.858 -20.860  11.891  1.00  0.00
I    H
ATOM   3418  CD1 LEU I 252     -13.420 -22.132  12.734  1.00  0.00
I    C
ATOM   3419 HD11 LEU I 252     -12.783 -23.019  12.935  1.00  0.00
I    H
ATOM   3420 HD12 LEU I 252     -13.478 -21.525  13.664  1.00  0.00
I    H
ATOM   3421 HD13 LEU I 252     -14.442 -22.488  12.484  1.00  0.00
I    H
ATOM   3422  CD2 LEU I 252     -12.590 -22.185  10.346  1.00  0.00
I    C
ATOM   3423 HD21 LEU I 252     -11.903 -23.021  10.602  1.00  0.00
I    H
ATOM   3424 HD22 LEU I 252     -13.532 -22.629   9.965  1.00  0.00
I    H
ATOM   3425 HD23 LEU I 252     -12.126 -21.592   9.528  1.00  0.00
I    H
ATOM   3426  C   LEU I 252     -14.287 -18.146   9.814  1.00  0.00
I    C
ATOM   3427  O   LEU I 252     -14.775 -18.224   8.685  1.00  0.00
I    O
ATOM   3428  N   THR I 253     -14.682 -17.185  10.679  1.00  0.00
I    N
```

```
ATOM   3429  HN  THR I 253     -14.280 -17.076  11.588  1.00  0.00
I    H
ATOM   3430  CA  THR I 253     -15.732 -16.218  10.366  1.00  0.00
I    C
ATOM   3431  HA  THR I 253     -16.550 -16.805   9.969  1.00  0.00
I    H
ATOM   3432  CB  THR I 253     -16.253 -15.437  11.569  1.00  0.00
I    C
ATOM   3433  HB  THR I 253     -16.955 -14.671  11.160  1.00  0.00
I    H
ATOM   3434  OG1 THR I 253     -15.220 -14.732  12.243  1.00  0.00
I    O
ATOM   3435  HG1 THR I 253     -15.652 -14.290  12.981  1.00  0.00
I    H
ATOM   3436  CG2 THR I 253     -16.856 -16.439  12.565  1.00  0.00
I    C
ATOM   3437 HG21 THR I 253     -17.350 -15.910  13.406  1.00  0.00
I    H
ATOM   3438 HG22 THR I 253     -17.619 -17.072  12.063  1.00  0.00
I    H
ATOM   3439 HG23 THR I 253     -16.073 -17.105  12.985  1.00  0.00
I    H
ATOM   3440  C   THR I 253     -15.328 -15.253   9.277  1.00  0.00
I    C
ATOM   3441  O   THR I 253     -16.095 -14.986   8.353  1.00  0.00
I    O
ATOM   3442  N   GLY I 254     -14.076 -14.751   9.324  1.00  0.00
I    N
ATOM   3443  HN  GLY I 254     -13.442 -15.004  10.053  1.00  0.00
I    H
ATOM   3444  CA  GLY I 254     -13.558 -13.821   8.338  1.00  0.00
I    C
ATOM   3445  HA1 GLY I 254     -12.590 -13.494   8.695  1.00  0.00
I    H
ATOM   3446  HA2 GLY I 254     -14.285 -13.027   8.239  1.00  0.00
I    H
ATOM   3447  C   GLY I 254     -13.367 -14.446   6.987  1.00  0.00
I    C
ATOM   3448  O   GLY I 254     -13.696 -13.840   5.973  1.00  0.00
I    O
ATOM   3449  N   THR I 255     -12.849 -15.693   6.915  1.00  0.00
I    N
ATOM   3450  HN  THR I 255     -12.540 -16.209   7.714  1.00  0.00
I    H
ATOM   3451  CA  THR I 255     -12.692 -16.405   5.639  1.00  0.00
I    C
ATOM   3452  HA  THR I 255     -12.167 -15.721   4.986  1.00  0.00
I    H
ATOM   3453  CB  THR I 255     -11.906 -17.717   5.741  1.00  0.00
I    C
ATOM   3454  HB  THR I 255     -12.200 -18.180   6.715  1.00  0.00
I    H
ATOM   3455  OG1 THR I 255     -10.516 -17.433   5.789  1.00  0.00
I    O
```

ATOM 3456 HG1 THR I 255 -10.081 -18.291 5.841 1.00 0.00
I H
ATOM 3457 CG2 THR I 255 -12.060 -18.678 4.546 1.00 0.00
I C
ATOM 3458 HG21 THR I 255 -11.553 -19.646 4.750 1.00 0.00
I H
ATOM 3459 HG22 THR I 255 -13.133 -18.890 4.353 1.00 0.00
I H
ATOM 3460 HG23 THR I 255 -11.620 -18.243 3.623 1.00 0.00
I H
ATOM 3461 C THR I 255 -14.019 -16.685 4.987 1.00 0.00
I C
ATOM 3462 O THR I 255 -14.175 -16.468 3.787 1.00 0.00
I O
ATOM 3463 N LEU I 256 -15.027 -17.133 5.762 1.00 0.00
I N
ATOM 3464 HN LEU I 256 -14.927 -17.324 6.736 1.00 0.00
I H
ATOM 3465 CA LEU I 256 -16.359 -17.379 5.241 1.00 0.00
I C
ATOM 3466 HA LEU I 256 -16.231 -18.064 4.413 1.00 0.00
I H
ATOM 3467 CB LEU I 256 -17.248 -17.954 6.369 1.00 0.00
I C
ATOM 3468 HB1 LEU I 256 -16.664 -18.759 6.870 1.00 0.00
I H
ATOM 3469 HB2 LEU I 256 -17.411 -17.167 7.138 1.00 0.00
I H
ATOM 3470 CG LEU I 256 -18.592 -18.534 5.880 1.00 0.00
I C
ATOM 3471 HG LEU I 256 -18.980 -17.834 5.100 1.00 0.00
I H
ATOM 3472 CD1 LEU I 256 -18.394 -19.930 5.268 1.00 0.00
I C
ATOM 3473 HD11 LEU I 256 -19.369 -20.363 4.960 1.00 0.00
I H
ATOM 3474 HD12 LEU I 256 -17.738 -19.875 4.372 1.00 0.00
I H
ATOM 3475 HD13 LEU I 256 -17.925 -20.617 6.003 1.00 0.00
I H
ATOM 3476 CD2 LEU I 256 -19.614 -18.584 7.025 1.00 0.00
I C
ATOM 3477 HD21 LEU I 256 -20.584 -18.985 6.660 1.00 0.00
I H
ATOM 3478 HD22 LEU I 256 -19.270 -19.237 7.853 1.00 0.00
I H
ATOM 3479 HD23 LEU I 256 -19.791 -17.565 7.434 1.00 0.00
I H
ATOM 3480 C LEU I 256 -17.029 -16.121 4.699 1.00 0.00
I C
ATOM 3481 O LEU I 256 -17.536 -16.098 3.578 1.00 0.00
I O
ATOM 3482 N MET I 257 -16.992 -15.020 5.480 1.00 0.00
I N

```
ATOM   3483  HN  MET I 257     -16.549 -15.022   6.374  1.00  0.00
I    H
ATOM   3484  CA  MET I 257     -17.581 -13.751   5.107  1.00  0.00
I    C
ATOM   3485  HA  MET I 257     -18.603 -13.964   4.819  1.00  0.00
I    H
ATOM   3486  CB  MET I 257     -17.602 -12.814   6.339  1.00  0.00
I    C
ATOM   3487  HB1 MET I 257     -17.799 -13.455   7.230  1.00  0.00
I    H
ATOM   3488  HB2 MET I 257     -16.590 -12.375   6.488  1.00  0.00
I    H
ATOM   3489  CG  MET I 257     -18.672 -11.708   6.274  1.00  0.00
I    C
ATOM   3490  HG1 MET I 257     -18.316 -10.863   5.645  1.00  0.00
I    H
ATOM   3491  HG2 MET I 257     -19.597 -12.109   5.805  1.00  0.00
I    H
ATOM   3492  SD  MET I 257     -19.083 -11.050   7.921  1.00  0.00
I    S
ATOM   3493  CE  MET I 257     -20.600 -10.191   7.416  1.00  0.00
I    C
ATOM   3494  HE1 MET I 257     -21.067  -9.672   8.280  1.00  0.00
I    H
ATOM   3495  HE2 MET I 257     -20.386  -9.431   6.635  1.00  0.00
I    H
ATOM   3496  HE3 MET I 257     -21.345 -10.905   7.004  1.00  0.00
I    H
ATOM   3497  C   MET I 257     -16.893 -13.102   3.915  1.00  0.00
I    C
ATOM   3498  O   MET I 257     -17.541 -12.659   2.969  1.00  0.00
I    O
ATOM   3499  N   LEU I 258     -15.545 -13.092   3.893  1.00  0.00
I    N
ATOM   3500  HN  LEU I 258     -14.999 -13.472   4.637  1.00  0.00
I    H
ATOM   3501  CA  LEU I 258     -14.763 -12.535   2.804  1.00  0.00
I    C
ATOM   3502  HA  LEU I 258     -15.137 -11.529   2.663  1.00  0.00
I    H
ATOM   3503  CB  LEU I 258     -13.272 -12.457   3.210  1.00  0.00
I    C
ATOM   3504  HB1 LEU I 258     -13.233 -12.042   4.243  1.00  0.00
I    H
ATOM   3505  HB2 LEU I 258     -12.861 -13.489   3.278  1.00  0.00
I    H
ATOM   3506  CG  LEU I 258     -12.364 -11.590   2.310  1.00  0.00
I    C
ATOM   3507  HG  LEU I 258     -12.263 -12.115   1.330  1.00  0.00
I    H
ATOM   3508  CD1 LEU I 258     -12.912 -10.168   2.111  1.00  0.00
I    C
ATOM   3509 HD11 LEU I 258     -12.210  -9.562   1.500  1.00  0.00
I    H
```

```
ATOM   3510 HD12 LEU I 258     -13.893 -10.197   1.591  1.00  0.00
I    H
ATOM   3511 HD13 LEU I 258     -13.048  -9.659   3.089  1.00  0.00
I    H
ATOM   3512  CD2 LEU I 258     -10.968 -11.491   2.940  1.00  0.00
I    C
ATOM   3513 HD21 LEU I 258     -10.296 -10.885   2.295  1.00  0.00
I    H
ATOM   3514 HD22 LEU I 258     -11.002 -11.008   3.938  1.00  0.00
I    H
ATOM   3515 HD23 LEU I 258     -10.520 -12.502   3.056  1.00  0.00
I    H
ATOM   3516  C   LEU I 258     -14.935 -13.279   1.483  1.00  0.00
I    C
ATOM   3517  O   LEU I 258     -15.084 -12.664   0.428  1.00  0.00
I    O
ATOM   3518  N   ASN I 259     -14.975 -14.633   1.516  1.00  0.00
I    N
ATOM   3519  HN  ASN I 259     -14.797 -15.145   2.356  1.00  0.00
I    H
ATOM   3520  CA  ASN I 259     -15.276 -15.464   0.355  1.00  0.00
I    C
ATOM   3521  HA  ASN I 259     -14.570 -15.156  -0.409  1.00  0.00
I    H
ATOM   3522  CB  ASN I 259     -15.142 -16.985   0.666  1.00  0.00
I    C
ATOM   3523  HB1 ASN I 259     -15.746 -17.201   1.574  1.00  0.00
I    H
ATOM   3524  HB2 ASN I 259     -15.563 -17.587  -0.164  1.00  0.00
I    H
ATOM   3525  CG  ASN I 259     -13.694 -17.380   0.941  1.00  0.00
I    C
ATOM   3526  OD1 ASN I 259     -12.738 -16.608   0.835  1.00  0.00
I    O
ATOM   3527  ND2 ASN I 259     -13.484 -18.671   1.290  1.00  0.00
I    N
ATOM   3528 HD21 ASN I 259     -12.546 -18.946   1.485  1.00  0.00
I    H
ATOM   3529 HD22 ASN I 259     -14.254 -19.296   1.374  1.00  0.00
I    H
ATOM   3530  C   ASN I 259     -16.685 -15.218  -0.189  1.00  0.00
I    C
ATOM   3531  O   ASN I 259     -16.881 -15.113  -1.397  1.00  0.00
I    O
ATOM   3532  N   ALA I 260     -17.697 -15.094   0.699  1.00  0.00
I    N
ATOM   3533  HN  ALA I 260     -17.532 -15.211   1.678  1.00  0.00
I    H
ATOM   3534  CA  ALA I 260     -19.068 -14.791   0.330  1.00  0.00
I    C
ATOM   3535  HA  ALA I 260     -19.415 -15.527  -0.387  1.00  0.00
I    H
ATOM   3536  CB  ALA I 260     -19.959 -14.875   1.588  1.00  0.00
I    C
```

ATOM 3537 HB1 ALA I 260 -19.867 -15.883 2.043 1.00 0.00
I H
ATOM 3538 HB2 ALA I 260 -19.647 -14.126 2.349 1.00 0.00
I H
ATOM 3539 HB3 ALA I 260 -21.030 -14.705 1.343 1.00 0.00
I H
ATOM 3540 C ALA I 260 -19.250 -13.423 -0.332 1.00 0.00
I C
ATOM 3541 O ALA I 260 -19.893 -13.307 -1.377 1.00 0.00
I O
ATOM 3542 N ILE I 261 -18.637 -12.354 0.233 1.00 0.00
I N
ATOM 3543 HN ILE I 261 -18.140 -12.429 1.095 1.00 0.00
I H
ATOM 3544 CA ILE I 261 -18.638 -11.010 -0.345 1.00 0.00
I C
ATOM 3545 HA ILE I 261 -19.674 -10.757 -0.531 1.00 0.00
I H
ATOM 3546 CB ILE I 261 -18.003 -9.974 0.591 1.00 0.00
I C
ATOM 3547 HB ILE I 261 -17.005 -10.408 0.854 1.00 0.00
I H
ATOM 3548 CG2 ILE I 261 -17.935 -8.584 -0.096 1.00 0.00
I C
ATOM 3549 HG21 ILE I 261 -17.338 -7.875 0.516 1.00 0.00
I H
ATOM 3550 HG22 ILE I 261 -17.451 -8.658 -1.092 1.00 0.00
I H
ATOM 3551 HG23 ILE I 261 -18.951 -8.156 -0.229 1.00 0.00
I H
ATOM 3552 CG1 ILE I 261 -18.810 -9.870 1.910 1.00 0.00
I C
ATOM 3553 HG11 ILE I 261 -19.779 -9.356 1.719 1.00 0.00
I H
ATOM 3554 HG12 ILE I 261 -19.048 -10.898 2.269 1.00 0.00
I H
ATOM 3555 CD ILE I 261 -18.053 -9.131 3.021 1.00 0.00
I C
ATOM 3556 HD1 ILE I 261 -18.647 -9.133 3.961 1.00 0.00
I H
ATOM 3557 HD2 ILE I 261 -17.078 -9.622 3.225 1.00 0.00
I H
ATOM 3558 HD3 ILE I 261 -17.863 -8.074 2.738 1.00 0.00
I H
ATOM 3559 C ILE I 261 -17.934 -11.001 -1.692 1.00 0.00
I C
ATOM 3560 O ILE I 261 -18.431 -10.452 -2.672 1.00 0.00
I O
ATOM 3561 N ALA I 262 -16.770 -11.669 -1.799 1.00 0.00
I N
ATOM 3562 HN ALA I 262 -16.348 -12.114 -1.010 1.00 0.00
I H
ATOM 3563 CA ALA I 262 -16.046 -11.796 -3.044 1.00 0.00
I C

ATOM 3564 HA ALA I 262 -15.826 -10.806 -3.425 1.00 0.00
I H
ATOM 3565 CB ALA I 262 -14.706 -12.493 -2.765 1.00 0.00
I C
ATOM 3566 HB1 ALA I 262 -14.143 -11.924 -1.994 1.00 0.00
I H
ATOM 3567 HB2 ALA I 262 -14.865 -13.524 -2.380 1.00 0.00
I H
ATOM 3568 HB3 ALA I 262 -14.076 -12.549 -3.679 1.00 0.00
I H
ATOM 3569 C ALA I 262 -16.802 -12.524 -4.147 1.00 0.00
I C
ATOM 3570 O ALA I 262 -16.801 -12.089 -5.300 1.00 0.00
I O
ATOM 3571 N MET I 263 -17.502 -13.628 -3.820 1.00 0.00
I N
ATOM 3572 HN MET I 263 -17.482 -14.006 -2.897 1.00 0.00
I H
ATOM 3573 CA MET I 263 -18.337 -14.364 -4.751 1.00 0.00
I C
ATOM 3574 HA MET I 263 -17.702 -14.622 -5.590 1.00 0.00
I H
ATOM 3575 CB MET I 263 -18.842 -15.668 -4.096 1.00 0.00
I C
ATOM 3576 HB1 MET I 263 -17.941 -16.272 -3.838 1.00 0.00
I H
ATOM 3577 HB2 MET I 263 -19.342 -15.428 -3.132 1.00 0.00
I H
ATOM 3578 CG MET I 263 -19.764 -16.495 -5.009 1.00 0.00
I C
ATOM 3579 HG1 MET I 263 -20.740 -15.981 -5.139 1.00 0.00
I H
ATOM 3580 HG2 MET I 263 -19.301 -16.597 -6.014 1.00 0.00
I H
ATOM 3581 SD MET I 263 -20.116 -18.157 -4.383 1.00 0.00
I S
ATOM 3582 CE MET I 263 -21.192 -18.575 -5.783 1.00 0.00
I C
ATOM 3583 HE1 MET I 263 -21.600 -19.603 -5.676 1.00 0.00
I H
ATOM 3584 HE2 MET I 263 -22.050 -17.872 -5.850 1.00 0.00
I H
ATOM 3585 HE3 MET I 263 -20.635 -18.528 -6.743 1.00 0.00
I H
ATOM 3586 C MET I 263 -19.491 -13.542 -5.285 1.00 0.00
I C
ATOM 3587 O MET I 263 -19.768 -13.548 -6.484 1.00 0.00
I O
ATOM 3588 N LEU I 264 -20.158 -12.765 -4.406 1.00 0.00
I N
ATOM 3589 HN LEU I 264 -20.002 -12.807 -3.422 1.00 0.00
I H
ATOM 3590 CA LEU I 264 -21.160 -11.798 -4.810 1.00 0.00
I C

```
ATOM   3591  HA  LEU I 264     -21.895 -12.346  -5.387  1.00  0.00
I    H
ATOM   3592  CB  LEU I 264     -21.787 -11.138  -3.555  1.00  0.00
I    C
ATOM   3593  HB1 LEU I 264     -22.143 -11.961  -2.893  1.00  0.00
I    H
ATOM   3594  HB2 LEU I 264     -20.989 -10.609  -2.987  1.00  0.00
I    H
ATOM   3595  CG  LEU I 264     -22.970 -10.185  -3.837  1.00  0.00
I    C
ATOM   3596  HG  LEU I 264     -22.668  -9.566  -4.715  1.00  0.00
I    H
ATOM   3597  CD1 LEU I 264     -24.256 -10.960  -4.172  1.00  0.00
I    C
ATOM   3598 HD11 LEU I 264     -25.104 -10.259  -4.324  1.00  0.00
I    H
ATOM   3599 HD12 LEU I 264     -24.124 -11.554  -5.101  1.00  0.00
I    H
ATOM   3600 HD13 LEU I 264     -24.523 -11.653  -3.346  1.00  0.00
I    H
ATOM   3601  CD2 LEU I 264     -23.194  -9.242  -2.645  1.00  0.00
I    C
ATOM   3602 HD21 LEU I 264     -24.033  -8.544  -2.857  1.00  0.00
I    H
ATOM   3603 HD22 LEU I 264     -23.447  -9.801  -1.722  1.00  0.00
I    H
ATOM   3604 HD23 LEU I 264     -22.281  -8.638  -2.449  1.00  0.00
I    H
ATOM   3605  C   LEU I 264     -20.583 -10.711  -5.711  1.00  0.00
I    C
ATOM   3606  O   LEU I 264     -21.112 -10.447  -6.789  1.00  0.00
I    O
ATOM   3607  N   THR I 265     -19.438 -10.096  -5.336  1.00  0.00
I    N
ATOM   3608  HN  THR I 265     -18.953 -10.325  -4.492  1.00  0.00
I    H
ATOM   3609  CA  THR I 265     -18.802  -9.040  -6.127  1.00  0.00
I    C
ATOM   3610  HA  THR I 265     -19.571  -8.290  -6.261  1.00  0.00
I    H
ATOM   3611  CB  THR I 265     -17.566  -8.456  -5.453  1.00  0.00
I    C
ATOM   3612  HB  THR I 265     -16.922  -9.326  -5.179  1.00  0.00
I    H
ATOM   3613  OG1 THR I 265     -17.940  -7.796  -4.257  1.00  0.00
I    O
ATOM   3614  HG1 THR I 265     -17.122  -7.444  -3.891  1.00  0.00
I    H
ATOM   3615  CG2 THR I 265     -16.880  -7.376  -6.306  1.00  0.00
I    C
ATOM   3616 HG21 THR I 265     -15.924  -7.054  -5.841  1.00  0.00
I    H
ATOM   3617 HG22 THR I 265     -16.651  -7.771  -7.319  1.00  0.00
I    H
```

```
ATOM   3618 HG23 THR I 265     -17.530  -6.482  -6.419  1.00  0.00
I    H
ATOM   3619  C   THR I 265     -18.396  -9.492  -7.511  1.00  0.00
I    C
ATOM   3620  O   THR I 265     -18.739  -8.844  -8.497  1.00  0.00
I    O
ATOM   3621  N   VAL I 266     -17.717 -10.657  -7.641  1.00  0.00
I    N
ATOM   3622  HN  VAL I 266     -17.440 -11.207  -6.858  1.00  0.00
I    H
ATOM   3623  CA  VAL I 266     -17.329 -11.211  -8.936  1.00  0.00
I    C
ATOM   3624  HA  VAL I 266     -16.763 -10.447  -9.454  1.00  0.00
I    H
ATOM   3625  CB  VAL I 266     -16.418 -12.439  -8.784  1.00  0.00
I    C
ATOM   3626  HB  VAL I 266     -16.930 -13.156  -8.097  1.00  0.00
I    H
ATOM   3627  CG1 VAL I 266     -16.169 -13.148 -10.138  1.00  0.00
I    C
ATOM   3628 HG11 VAL I 266     -15.477 -14.007 -10.001  1.00  0.00
I    H
ATOM   3629 HG12 VAL I 266     -17.110 -13.547 -10.570  1.00  0.00
I    H
ATOM   3630 HG13 VAL I 266     -15.709 -12.449 -10.868  1.00  0.00
I    H
ATOM   3631  CG2 VAL I 266     -15.066 -11.983  -8.194  1.00  0.00
I    C
ATOM   3632 HG21 VAL I 266     -14.366 -12.842  -8.108  1.00  0.00
I    H
ATOM   3633 HG22 VAL I 266     -14.596 -11.213  -8.843  1.00  0.00
I    H
ATOM   3634 HG23 VAL I 266     -15.194 -11.553  -7.180  1.00  0.00
I    H
ATOM   3635  C   VAL I 266     -18.542 -11.541  -9.792  1.00  0.00
I    C
ATOM   3636  O   VAL I 266     -18.597 -11.182 -10.967  1.00  0.00
I    O
ATOM   3637  N   PHE I 267     -19.583 -12.180  -9.209  1.00  0.00
I    N
ATOM   3638  HN  PHE I 267     -19.593 -12.440  -8.245  1.00  0.00
I    H
ATOM   3639  CA  PHE I 267     -20.781 -12.542  -9.943  1.00  0.00
I    C
ATOM   3640  HA  PHE I 267     -20.433 -13.114 -10.794  1.00  0.00
I    H
ATOM   3641  CB  PHE I 267     -21.710 -13.402  -9.030  1.00  0.00
I    C
ATOM   3642  HB1 PHE I 267     -21.149 -14.295  -8.678  1.00  0.00
I    H
ATOM   3643  HB2 PHE I 267     -22.021 -12.834  -8.127  1.00  0.00
I    H
ATOM   3644  CG  PHE I 267     -22.933 -13.917  -9.759  1.00  0.00
I    C
```

ATOM 3645 CD1 PHE I 267 -22.876 -15.023 -10.630 1.00 0.00
I C
ATOM 3646 HD1 PHE I 267 -21.942 -15.551 -10.765 1.00 0.00
I H
ATOM 3647 CE1 PHE I 267 -24.018 -15.442 -11.328 1.00 0.00
I C
ATOM 3648 HE1 PHE I 267 -23.966 -16.298 -11.986 1.00 0.00
I H
ATOM 3649 CZ PHE I 267 -25.227 -14.755 -11.169 1.00 0.00
I C
ATOM 3650 HZ PHE I 267 -26.109 -15.081 -11.702 1.00 0.00
I H
ATOM 3651 CD2 PHE I 267 -24.156 -13.243 -9.607 1.00 0.00
I C
ATOM 3652 HD2 PHE I 267 -24.220 -12.382 -8.955 1.00 0.00
I H
ATOM 3653 CE2 PHE I 267 -25.296 -13.653 -10.311 1.00 0.00
I C
ATOM 3654 HE2 PHE I 267 -26.225 -13.115 -10.190 1.00 0.00
I H
ATOM 3655 C PHE I 267 -21.538 -11.339 -10.493 1.00 0.00
I C
ATOM 3656 O PHE I 267 -21.878 -11.317 -11.669 1.00 0.00
I O
ATOM 3657 N ILE I 268 -21.788 -10.298 -9.672 1.00 0.00
I N
ATOM 3658 HN ILE I 268 -21.490 -10.267 -8.721 1.00 0.00
I H
ATOM 3659 CA ILE I 268 -22.524 -9.114 -10.109 1.00 0.00
I C
ATOM 3660 HA ILE I 268 -23.421 -9.488 -10.586 1.00 0.00
I H
ATOM 3661 CB ILE I 268 -22.940 -8.242 -8.918 1.00 0.00
I C
ATOM 3662 HB ILE I 268 -21.983 -8.032 -8.376 1.00 0.00
I H
ATOM 3663 CG2 ILE I 268 -23.644 -6.944 -9.384 1.00 0.00
I C
ATOM 3664 HG21 ILE I 268 -23.798 -6.254 -8.528 1.00 0.00
I H
ATOM 3665 HG22 ILE I 268 -23.030 -6.410 -10.140 1.00 0.00
I H
ATOM 3666 HG23 ILE I 268 -24.635 -7.168 -9.832 1.00 0.00
I H
ATOM 3667 CG1 ILE I 268 -23.844 -9.024 -7.920 1.00 0.00
I C
ATOM 3668 HG11 ILE I 268 -23.294 -9.907 -7.526 1.00 0.00
I H
ATOM 3669 HG12 ILE I 268 -24.076 -8.366 -7.051 1.00 0.00
I H
ATOM 3670 CD ILE I 268 -25.186 -9.517 -8.483 1.00 0.00
I C
ATOM 3671 HD1 ILE I 268 -25.783 -10.010 -7.685 1.00 0.00
I H

```
ATOM   3672  HD2 ILE I 268     -25.779  -8.667  -8.885  1.00  0.00
I    H
ATOM   3673  HD3 ILE I 268     -25.029 -10.254  -9.299  1.00  0.00
I    H
ATOM   3674  C   ILE I 268     -21.770  -8.302 -11.160  1.00  0.00
I    C
ATOM   3675  O   ILE I 268     -22.339  -7.889 -12.169  1.00  0.00
I    O
ATOM   3676  N   LEU I 269     -20.453  -8.076 -10.971  1.00  0.00
I    N
ATOM   3677  HN  LEU I 269     -19.966  -8.415 -10.169  1.00  0.00
I    H
ATOM   3678  CA  LEU I 269     -19.622  -7.326 -11.902  1.00  0.00
I    C
ATOM   3679  HA  LEU I 269     -20.157  -6.400 -12.073  1.00  0.00
I    H
ATOM   3680  CB  LEU I 269     -18.252  -7.008 -11.254  1.00  0.00
I    C
ATOM   3681  HB1 LEU I 269     -18.022  -7.840 -10.549  1.00  0.00
I    H
ATOM   3682  HB2 LEU I 269     -17.463  -7.043 -12.038  1.00  0.00
I    H
ATOM   3683  CG  LEU I 269     -18.190  -5.667 -10.480  1.00  0.00
I    C
ATOM   3684  HG  LEU I 269     -18.249  -4.881 -11.271  1.00  0.00
I    H
ATOM   3685  CD1 LEU I 269     -19.298  -5.457  -9.435  1.00  0.00
I    C
ATOM   3686 HD11 LEU I 269     -19.150  -4.496  -8.898  1.00  0.00
I    H
ATOM   3687 HD12 LEU I 269     -20.296  -5.435  -9.921  1.00  0.00
I    H
ATOM   3688 HD13 LEU I 269     -19.291  -6.278  -8.686  1.00  0.00
I    H
ATOM   3689  CD2 LEU I 269     -16.824  -5.552  -9.796  1.00  0.00
I    C
ATOM   3690 HD21 LEU I 269     -16.748  -4.587  -9.248  1.00  0.00
I    H
ATOM   3691 HD22 LEU I 269     -16.662  -6.368  -9.064  1.00  0.00
I    H
ATOM   3692 HD23 LEU I 269     -16.007  -5.589 -10.548  1.00  0.00
I    H
ATOM   3693  C   LEU I 269     -19.416  -7.997 -13.261  1.00  0.00
I    C
ATOM   3694  O   LEU I 269     -19.487  -7.345 -14.306  1.00  0.00
I    O
ATOM   3695  N   GLU I 270     -19.167  -9.322 -13.288  1.00  0.00
I    N
ATOM   3696  HN  GLU I 270     -19.229  -9.916 -12.491  1.00  0.00
I    H
ATOM   3697  CA  GLU I 270     -18.780 -10.009 -14.508  1.00  0.00
I    C
ATOM   3698  HA  GLU I 270     -18.392  -9.262 -15.188  1.00  0.00
I    H
```

ATOM 3699 CB GLU I 270 -17.678 -11.062 -14.221 1.00 0.00
I C
ATOM 3700 HB1 GLU I 270 -18.058 -11.759 -13.439 1.00 0.00
I H
ATOM 3701 HB2 GLU I 270 -17.505 -11.685 -15.127 1.00 0.00
I H
ATOM 3702 CG GLU I 270 -16.334 -10.455 -13.740 1.00 0.00
I C
ATOM 3703 HG1 GLU I 270 -16.521 -9.772 -12.891 1.00 0.00
I H
ATOM 3704 HG2 GLU I 270 -15.631 -11.251 -13.420 1.00 0.00
I H
ATOM 3705 CD GLU I 270 -15.660 -9.613 -14.808 1.00 0.00
I C
ATOM 3706 OE1 GLU I 270 -15.599 -10.030 -15.986 1.00 0.00
I O
ATOM 3707 OE2 GLU I 270 -15.149 -8.520 -14.457 1.00 0.00
I O
ATOM 3708 C GLU I 270 -19.948 -10.694 -15.208 1.00 0.00
I C
ATOM 3709 O GLU I 270 -19.771 -11.275 -16.277 1.00 0.00
I O
ATOM 3710 N TYR I 271 -21.197 -10.600 -14.682 1.00 0.00
I N
ATOM 3711 HN TYR I 271 -21.368 -10.026 -13.883 1.00 0.00
I H
ATOM 3712 CA TYR I 271 -22.381 -11.295 -15.196 1.00 0.00
I C
ATOM 3713 HA TYR I 271 -22.178 -12.351 -15.060 1.00 0.00
I H
ATOM 3714 CB TYR I 271 -23.646 -10.880 -14.379 1.00 0.00
I C
ATOM 3715 HB1 TYR I 271 -23.444 -11.027 -13.296 1.00 0.00
I H
ATOM 3716 HB2 TYR I 271 -23.873 -9.803 -14.528 1.00 0.00
I H
ATOM 3717 CG TYR I 271 -24.863 -11.705 -14.715 1.00 0.00
I C
ATOM 3718 CD1 TYR I 271 -24.866 -13.099 -14.535 1.00 0.00
I C
ATOM 3719 HD1 TYR I 271 -23.989 -13.591 -14.135 1.00 0.00
I H
ATOM 3720 CE1 TYR I 271 -25.989 -13.858 -14.892 1.00 0.00
I C
ATOM 3721 HE1 TYR I 271 -25.995 -14.929 -14.752 1.00 0.00
I H
ATOM 3722 CZ TYR I 271 -27.112 -13.235 -15.440 1.00 0.00
I C
ATOM 3723 OH TYR I 271 -28.198 -14.032 -15.855 1.00 0.00
I O
ATOM 3724 HH TYR I 271 -28.899 -13.440 -16.135 1.00 0.00
I H
ATOM 3725 CD2 TYR I 271 -26.016 -11.082 -15.218 1.00 0.00
I C

```
ATOM   3726  HD2 TYR I 271     -26.031 -10.011 -15.367  1.00  0.00
I    H
ATOM   3727  CE2 TYR I 271     -27.140 -11.842 -15.580  1.00  0.00
I    C
ATOM   3728  HE2 TYR I 271     -28.013 -11.350 -15.984  1.00  0.00
I    H
ATOM   3729  C   TYR I 271     -22.624 -11.072 -16.689  1.00  0.00
I    C
ATOM   3730  O   TYR I 271     -22.963 -11.987 -17.440  1.00  0.00
I    O
ATOM   3731  N   SER I 272     -22.397  -9.826 -17.137  1.00  0.00
I    N
ATOM   3732  HN  SER I 272     -22.054  -9.112 -16.526  1.00  0.00
I    H
ATOM   3733  CA  SER I 272     -22.615  -9.385 -18.502  1.00  0.00
I    C
ATOM   3734  HA  SER I 272     -23.126 -10.202 -18.995  1.00  0.00
I    H
ATOM   3735  CB  SER I 272     -23.376  -8.036 -18.570  1.00  0.00
I    C
ATOM   3736  HB1 SER I 272     -22.692  -7.234 -18.208  1.00  0.00
I    H
ATOM   3737  HB2 SER I 272     -23.637  -7.791 -19.624  1.00  0.00
I    H
ATOM   3738  OG  SER I 272     -24.535  -8.066 -17.740  1.00  0.00
I    O
ATOM   3739  HG1 SER I 272     -24.839  -7.154 -17.643  1.00  0.00
I    H
ATOM   3740  C   SER I 272     -21.309  -9.155 -19.246  1.00  0.00
I    C
ATOM   3741  O   SER I 272     -21.264  -8.402 -20.218  1.00  0.00
I    O
ATOM   3742  N   ASN I 273     -20.174  -9.741 -18.805  1.00  0.00
I    N
ATOM   3743  HN  ASN I 273     -20.115 -10.285 -17.970  1.00  0.00
I    H
ATOM   3744  CA  ASN I 273     -18.922  -9.618 -19.535  1.00  0.00
I    C
ATOM   3745  HA  ASN I 273     -18.934  -8.639 -20.002  1.00  0.00
I    H
ATOM   3746  CB  ASN I 273     -17.705  -9.693 -18.566  1.00  0.00
I    C
ATOM   3747  HB1 ASN I 273     -17.912  -9.017 -17.707  1.00  0.00
I    H
ATOM   3748  HB2 ASN I 273     -17.596 -10.720 -18.162  1.00  0.00
I    H
ATOM   3749  CG  ASN I 273     -16.422  -9.232 -19.261  1.00  0.00
I    C
ATOM   3750  OD1 ASN I 273     -16.375  -9.049 -20.470  1.00  0.00
I    O
ATOM   3751  ND2 ASN I 273     -15.354  -9.026 -18.466  1.00  0.00
I    N
ATOM   3752 HD21 ASN I 273     -14.513  -8.715 -18.900  1.00  0.00
I    H
```

ATOM 3753 HD22 ASN I 273 −15.433 −9.155 −17.482 1.00 0.00
I H
ATOM 3754 C ASN I 273 −18.862 −10.671 −20.659 1.00 0.00
I C
ATOM 3755 O ASN I 273 −18.909 −11.862 −20.367 1.00 0.00
I O
ATOM 3756 N PRO I 274 −18.767 −10.361 −21.957 1.00 0.00
I N
ATOM 3757 CD PRO I 274 −18.798 −9.010 −22.520 1.00 0.00
I C
ATOM 3758 HD1 PRO I 274 −19.762 −8.467 −22.397 1.00 0.00
I H
ATOM 3759 HD2 PRO I 274 −17.968 −8.447 −22.033 1.00 0.00
I H
ATOM 3760 CA PRO I 274 −18.758 −11.384 −22.994 1.00 0.00
I C
ATOM 3761 HA PRO I 274 −19.518 −12.132 −22.799 1.00 0.00
I H
ATOM 3762 CB PRO I 274 −19.081 −10.596 −24.276 1.00 0.00
I C
ATOM 3763 HB1 PRO I 274 −20.186 −10.499 −24.373 1.00 0.00
I H
ATOM 3764 HB2 PRO I 274 −18.681 −11.081 −25.190 1.00 0.00
I H
ATOM 3765 CG PRO I 274 −18.494 −9.209 −24.005 1.00 0.00
I C
ATOM 3766 HG1 PRO I 274 −18.970 −8.413 −24.612 1.00 0.00
I H
ATOM 3767 HG2 PRO I 274 −17.400 −9.227 −24.210 1.00 0.00
I H
ATOM 3768 C PRO I 274 −17.404 −12.065 −23.090 1.00 0.00
I C
ATOM 3769 O PRO I 274 −17.308 −13.081 −23.766 1.00 0.00
I O
ATOM 3770 N GLY I 275 −16.342 −11.530 −22.449 1.00 0.00
I N
ATOM 3771 HN GLY I 275 −16.410 −10.689 −21.914 1.00 0.00
I H
ATOM 3772 CA GLY I 275 −15.022 −12.143 −22.494 1.00 0.00
I C
ATOM 3773 HA1 GLY I 275 −14.308 −11.367 −22.256 1.00 0.00
I H
ATOM 3774 HA2 GLY I 275 −14.902 −12.568 −23.481 1.00 0.00
I H
ATOM 3775 C GLY I 275 −14.857 −13.249 −21.490 1.00 0.00
I C
ATOM 3776 O GLY I 275 −14.174 −14.242 −21.736 1.00 0.00
I O
ATOM 3777 N THR I 276 −15.487 −13.110 −20.316 1.00 0.00
I N
ATOM 3778 HN THR I 276 −16.076 −12.327 −20.118 1.00 0.00
I H
ATOM 3779 CA THR I 276 −15.379 −14.065 −19.229 1.00 0.00
I C

ATOM 3780 HA THR I 276 -15.220 -15.038 -19.674 1.00 0.00
I H
ATOM 3781 CB THR I 276 -14.330 -13.710 -18.179 1.00 0.00
I C
ATOM 3782 HB THR I 276 -14.550 -14.354 -17.292 1.00 0.00
I H
ATOM 3783 OG1 THR I 276 -14.450 -12.363 -17.760 1.00 0.00
I O
ATOM 3784 HG1 THR I 276 -13.748 -12.231 -17.114 1.00 0.00
I H
ATOM 3785 CG2 THR I 276 -12.939 -13.824 -18.794 1.00 0.00
I C
ATOM 3786 HG21 THR I 276 -12.153 -13.682 -18.023 1.00 0.00
I H
ATOM 3787 HG22 THR I 276 -12.796 -14.829 -19.246 1.00 0.00
I H
ATOM 3788 HG23 THR I 276 -12.789 -13.061 -19.589 1.00 0.00
I H
ATOM 3789 C THR I 276 -16.705 -14.129 -18.529 1.00 0.00
I C
ATOM 3790 O THR I 276 -17.379 -13.124 -18.358 1.00 0.00
I O
ATOM 3791 N LEU I 277 -17.147 -15.334 -18.123 1.00 0.00
I N
ATOM 3792 HN LEU I 277 -16.658 -16.178 -18.329 1.00 0.00
I H
ATOM 3793 CA LEU I 277 -18.365 -15.522 -17.356 1.00 0.00
I C
ATOM 3794 HA LEU I 277 -18.345 -16.568 -17.077 1.00 0.00
I H
ATOM 3795 CB LEU I 277 -18.211 -14.866 -15.953 1.00 0.00
I C
ATOM 3796 HB1 LEU I 277 -17.198 -15.137 -15.573 1.00 0.00
I H
ATOM 3797 HB2 LEU I 277 -18.211 -13.760 -16.068 1.00 0.00
I H
ATOM 3798 CG LEU I 277 -19.247 -15.281 -14.893 1.00 0.00
I C
ATOM 3799 HG LEU I 277 -19.762 -16.196 -15.275 1.00 0.00
I H
ATOM 3800 CD1 LEU I 277 -18.593 -15.540 -13.534 1.00 0.00
I C
ATOM 3801 HD11 LEU I 277 -19.361 -15.791 -12.773 1.00 0.00
I H
ATOM 3802 HD12 LEU I 277 -17.878 -16.388 -13.601 1.00 0.00
I H
ATOM 3803 HD13 LEU I 277 -18.041 -14.641 -13.187 1.00 0.00
I H
ATOM 3804 CD2 LEU I 277 -20.302 -14.195 -14.688 1.00 0.00
I C
ATOM 3805 HD21 LEU I 277 -21.050 -14.522 -13.934 1.00 0.00
I H
ATOM 3806 HD22 LEU I 277 -19.851 -13.250 -14.327 1.00 0.00
I H

```
ATOM   3807 HD23 LEU I 277     -20.837 -13.987 -15.640  1.00  0.00
I    H
ATOM   3808  C   LEU I 277     -19.685 -15.266 -18.094  1.00  0.00
I    C
ATOM   3809  O   LEU I 277     -20.673 -15.922 -17.780  1.00  0.00
I    O
ATOM   3810  N   GLY I 278     -19.768 -14.431 -19.154  1.00  0.00
I    N
ATOM   3811  HN  GLY I 278     -18.952 -13.985 -19.521  1.00  0.00
I    H
ATOM   3812  CA  GLY I 278     -21.024 -14.116 -19.840  1.00  0.00
I    C
ATOM   3813  HA1 GLY I 278     -20.762 -13.331 -20.536  1.00  0.00
I    H
ATOM   3814  HA2 GLY I 278     -21.697 -13.805 -19.052  1.00  0.00
I    H
ATOM   3815  C   GLY I 278     -21.697 -15.226 -20.617  1.00  0.00
I    C
ATOM   3816  O   GLY I 278     -22.883 -15.491 -20.427  1.00  0.00
I    O
ATOM   3817  N   HSE I 279     -20.948 -15.929 -21.500  1.00  0.00
I    N
ATOM   3818  HN  HSE I 279     -19.969 -15.765 -21.611  1.00  0.00
I    H
ATOM   3819  CA  HSE I 279     -21.486 -16.984 -22.366  1.00  0.00
I    C
ATOM   3820  HA  HSE I 279     -22.439 -16.618 -22.728  1.00  0.00
I    H
ATOM   3821  CB  HSE I 279     -20.524 -17.335 -23.527  1.00  0.00
I    C
ATOM   3822  HB1 HSE I 279     -19.509 -17.477 -23.099  1.00  0.00
I    H
ATOM   3823  HB2 HSE I 279     -20.824 -18.304 -23.978  1.00  0.00
I    H
ATOM   3824  ND1 HSE I 279     -21.341 -16.297 -25.617  1.00  0.00
I    N
ATOM   3825  CG  HSE I 279     -20.456 -16.264 -24.561  1.00  0.00
I    C
ATOM   3826  CE1 HSE I 279     -21.034 -15.252 -26.355  1.00  0.00
I    C
ATOM   3827  HE1 HSE I 279     -21.538 -14.979 -27.285  1.00  0.00
I    H
ATOM   3828  NE2 HSE I 279     -20.003 -14.548 -25.825  1.00  0.00
I    N
ATOM   3829  HE2 HSE I 279     -19.573 -13.728 -26.200  1.00  0.00
I    H
ATOM   3830  CD2 HSE I 279     -19.628 -15.198 -24.669  1.00  0.00
I    C
ATOM   3831  HD2 HSE I 279     -18.821 -14.879 -24.025  1.00  0.00
I    H
ATOM   3832  C   HSE I 279     -21.759 -18.282 -21.625  1.00  0.00
I    C
ATOM   3833  O   HSE I 279     -22.451 -19.170 -22.110  1.00  0.00
I    O
```

```
ATOM   3834  N   LEU I 280     -21.192 -18.417 -20.416  1.00  0.00
I    N
ATOM   3835  HN  LEU I 280     -20.617 -17.686 -20.056  1.00  0.00
I    H
ATOM   3836  CA  LEU I 280     -21.325 -19.558 -19.536  1.00  0.00
I    C
ATOM   3837  HA  LEU I 280     -21.048 -20.425 -20.123  1.00  0.00
I    H
ATOM   3838  CB  LEU I 280     -20.350 -19.384 -18.344  1.00  0.00
I    C
ATOM   3839  HB1 LEU I 280     -20.347 -18.302 -18.076  1.00  0.00
I    H
ATOM   3840  HB2 LEU I 280     -20.760 -19.920 -17.459  1.00  0.00
I    H
ATOM   3841  CG  LEU I 280     -18.896 -19.835 -18.634  1.00  0.00
I    C
ATOM   3842  HG  LEU I 280     -18.993 -20.935 -18.796  1.00  0.00
I    H
ATOM   3843  CD1 LEU I 280     -18.233 -19.172 -19.859  1.00  0.00
I    C
ATOM   3844 HD11 LEU I 280     -17.181 -19.515 -19.965  1.00  0.00
I    H
ATOM   3845 HD12 LEU I 280     -18.781 -19.433 -20.790  1.00  0.00
I    H
ATOM   3846 HD13 LEU I 280     -18.228 -18.067 -19.752  1.00  0.00
I    H
ATOM   3847  CD2 LEU I 280     -18.018 -19.611 -17.397  1.00  0.00
I    C
ATOM   3848 HD21 LEU I 280     -16.979 -19.951 -17.595  1.00  0.00
I    H
ATOM   3849 HD22 LEU I 280     -17.974 -18.540 -17.114  1.00  0.00
I    H
ATOM   3850 HD23 LEU I 280     -18.413 -20.186 -16.531  1.00  0.00
I    H
ATOM   3851  C   LEU I 280     -22.759 -19.764 -19.059  1.00  0.00
I    C
ATOM   3852  O   LEU I 280     -23.495 -18.806 -18.802  1.00  0.00
I    O
ATOM   3853  N   HSE I 281     -23.212 -21.028 -18.904  1.00  0.00
I    N
ATOM   3854  HN  HSE I 281     -22.667 -21.835 -19.129  1.00  0.00
I    H
ATOM   3855  CA  HSE I 281     -24.542 -21.327 -18.394  1.00  0.00
I    C
ATOM   3856  HA  HSE I 281     -25.242 -20.762 -18.997  1.00  0.00
I    H
ATOM   3857  CB  HSE I 281     -24.907 -22.822 -18.507  1.00  0.00
I    C
ATOM   3858  HB1 HSE I 281     -24.211 -23.394 -17.855  1.00  0.00
I    H
ATOM   3859  HB2 HSE I 281     -25.932 -22.984 -18.111  1.00  0.00
I    H
ATOM   3860  ND1 HSE I 281     -25.699 -22.845 -20.828  1.00  0.00
I    N
```

ATOM 3861 CG HSE I 281 -24.767 -23.284 -19.910 1.00 0.00
I C
ATOM 3862 CE1 HSE I 281 -25.279 -23.312 -21.985 1.00 0.00
I C
ATOM 3863 HE1 HSE I 281 -25.788 -23.155 -22.939 1.00 0.00
I H
ATOM 3864 NE2 HSE I 281 -24.132 -24.025 -21.857 1.00 0.00
I N
ATOM 3865 HE2 HSE I 281 -23.628 -24.495 -22.582 1.00 0.00
I H
ATOM 3866 CD2 HSE I 281 -23.797 -24.009 -20.521 1.00 0.00
I C
ATOM 3867 HD2 HSE I 281 -22.931 -24.502 -20.101 1.00 0.00
I H
ATOM 3868 C HSE I 281 -24.709 -20.858 -16.959 1.00 0.00
I C
ATOM 3869 O HSE I 281 -23.737 -20.564 -16.271 1.00 0.00
I O
ATOM 3870 N ILE I 282 -25.950 -20.734 -16.446 1.00 0.00
I N
ATOM 3871 HN ILE I 282 -26.769 -21.017 -16.943 1.00 0.00
I H
ATOM 3872 CA ILE I 282 -26.216 -20.177 -15.118 1.00 0.00
I C
ATOM 3873 HA ILE I 282 -25.820 -19.169 -15.138 1.00 0.00
I H
ATOM 3874 CB ILE I 282 -27.723 -20.088 -14.853 1.00 0.00
I C
ATOM 3875 HB ILE I 282 -28.086 -21.143 -14.945 1.00 0.00
I H
ATOM 3876 CG2 ILE I 282 -27.977 -19.449 -13.465 1.00 0.00
I C
ATOM 3877 HG21 ILE I 282 -29.050 -19.520 -13.193 1.00 0.00
I H
ATOM 3878 HG22 ILE I 282 -27.395 -19.971 -12.676 1.00 0.00
I H
ATOM 3879 HG23 ILE I 282 -27.688 -18.376 -13.463 1.00 0.00
I H
ATOM 3880 CG1 ILE I 282 -28.419 -19.269 -15.978 1.00 0.00
I C
ATOM 3881 HG11 ILE I 282 -27.982 -18.247 -16.025 1.00 0.00
I H
ATOM 3882 HG12 ILE I 282 -28.217 -19.759 -16.958 1.00 0.00
I H
ATOM 3883 CD ILE I 282 -29.941 -19.150 -15.811 1.00 0.00
I C
ATOM 3884 HD1 ILE I 282 -30.383 -18.606 -16.674 1.00 0.00
I H
ATOM 3885 HD2 ILE I 282 -30.409 -20.156 -15.759 1.00 0.00
I H
ATOM 3886 HD3 ILE I 282 -30.198 -18.593 -14.886 1.00 0.00
I H
ATOM 3887 C ILE I 282 -25.472 -20.923 -14.004 1.00 0.00
I C

```
ATOM   3888  O   ILE I 282     -24.874 -20.308 -13.129  1.00  0.00
I    O
ATOM   3889  N   VAL I 283     -25.421 -22.271 -14.070  1.00  0.00
I    N
ATOM   3890  HN  VAL I 283     -25.960 -22.767 -14.746  1.00  0.00
I    H
ATOM   3891  CA  VAL I 283     -24.612 -23.119 -13.206  1.00  0.00
I    C
ATOM   3892  HA  VAL I 283     -24.897 -22.865 -12.193  1.00  0.00
I    H
ATOM   3893  CB  VAL I 283     -25.030 -24.583 -13.407  1.00  0.00
I    C
ATOM   3894  HB  VAL I 283     -26.088 -24.683 -13.061  1.00  0.00
I    H
ATOM   3895  CG1 VAL I 283     -24.963 -25.024 -14.890  1.00  0.00
I    C
ATOM   3896 HG11 VAL I 283     -25.236 -26.097 -14.986  1.00  0.00
I    H
ATOM   3897 HG12 VAL I 283     -25.670 -24.446 -15.521  1.00  0.00
I    H
ATOM   3898 HG13 VAL I 283     -23.936 -24.892 -15.292  1.00  0.00
I    H
ATOM   3899  CG2 VAL I 283     -24.200 -25.515 -12.502  1.00  0.00
I    C
ATOM   3900 HG21 VAL I 283     -24.475 -26.577 -12.675  1.00  0.00
I    H
ATOM   3901 HG22 VAL I 283     -23.115 -25.396 -12.709  1.00  0.00
I    H
ATOM   3902 HG23 VAL I 283     -24.377 -25.295 -11.429  1.00  0.00
I    H
ATOM   3903  C   VAL I 283     -23.090 -22.892 -13.330  1.00  0.00
I    C
ATOM   3904  O   VAL I 283     -22.404 -22.714 -12.322  1.00  0.00
I    O
ATOM   3905  N   ASP I 284     -22.526 -22.801 -14.561  1.00  0.00
I    N
ATOM   3906  HN  ASP I 284     -23.064 -22.915 -15.393  1.00  0.00
I    H
ATOM   3907  CA  ASP I 284     -21.116 -22.537 -14.828  1.00  0.00
I    C
ATOM   3908  HA  ASP I 284     -20.556 -23.290 -14.285  1.00  0.00
I    H
ATOM   3909  CB  ASP I 284     -20.820 -22.536 -16.343  1.00  0.00
I    C
ATOM   3910  HB1 ASP I 284     -21.456 -21.775 -16.838  1.00  0.00
I    H
ATOM   3911  HB2 ASP I 284     -19.759 -22.281 -16.534  1.00  0.00
I    H
ATOM   3912  CG  ASP I 284     -21.129 -23.877 -16.959  1.00  0.00
I    C
ATOM   3913  OD1 ASP I 284     -20.573 -24.893 -16.482  1.00  0.00
I    O
ATOM   3914  OD2 ASP I 284     -21.935 -23.877 -17.921  1.00  0.00
I    O
```

```
ATOM   3915  C   ASP I 284     -20.663 -21.187 -14.297  1.00  0.00
I    C
ATOM   3916  O   ASP I 284     -19.566 -21.036 -13.759  1.00  0.00
I    O
ATOM   3917  N   LYS I 285     -21.534 -20.160 -14.414  1.00  0.00
I    N
ATOM   3918  HN  LYS I 285     -22.404 -20.279 -14.888  1.00  0.00
I    H
ATOM   3919  CA  LYS I 285     -21.300 -18.831 -13.880  1.00  0.00
I    C
ATOM   3920  HA  LYS I 285     -20.397 -18.461 -14.351  1.00  0.00
I    H
ATOM   3921  CB  LYS I 285     -22.473 -17.858 -14.180  1.00  0.00
I    C
ATOM   3922  HB1 LYS I 285     -23.406 -18.345 -13.809  1.00  0.00
I    H
ATOM   3923  HB2 LYS I 285     -22.342 -16.934 -13.574  1.00  0.00
I    H
ATOM   3924  CG  LYS I 285     -22.666 -17.496 -15.668  1.00  0.00
I    C
ATOM   3925  HG1 LYS I 285     -21.675 -17.259 -16.117  1.00  0.00
I    H
ATOM   3926  HG2 LYS I 285     -23.071 -18.384 -16.208  1.00  0.00
I    H
ATOM   3927  CD  LYS I 285     -23.608 -16.283 -15.813  1.00  0.00
I    C
ATOM   3928  HD1 LYS I 285     -24.609 -16.564 -15.414  1.00  0.00
I    H
ATOM   3929  HD2 LYS I 285     -23.213 -15.459 -15.173  1.00  0.00
I    H
ATOM   3930  CE  LYS I 285     -23.841 -15.675 -17.214  1.00  0.00
I    C
ATOM   3931  HE1 LYS I 285     -24.333 -14.681 -17.131  1.00  0.00
I    H
ATOM   3932  HE2 LYS I 285     -22.875 -15.546 -17.750  1.00  0.00
I    H
ATOM   3933  NZ  LYS I 285     -24.703 -16.519 -18.063  1.00  0.00
I    N
ATOM   3934  HZ1 LYS I 285     -24.834 -16.072 -18.993  1.00  0.00
I    H
ATOM   3935  HZ2 LYS I 285     -24.261 -17.452 -18.188  1.00  0.00
I    H
ATOM   3936  HZ3 LYS I 285     -25.629 -16.637 -17.605  1.00  0.00
I    H
ATOM   3937  C   LYS I 285     -21.053 -18.850 -12.375  1.00  0.00
I    C
ATOM   3938  O   LYS I 285     -20.144 -18.195 -11.877  1.00  0.00
I    O
ATOM   3939  N   LEU I 286     -21.827 -19.644 -11.609  1.00  0.00
I    N
ATOM   3940  HN  LEU I 286     -22.589 -20.169 -11.985  1.00  0.00
I    H
ATOM   3941  CA  LEU I 286     -21.633 -19.815 -10.179  1.00  0.00
I    C
```

```
ATOM   3942  HA  LEU I 286     -21.585 -18.814  -9.769  1.00  0.00
I    H
ATOM   3943  CB  LEU I 286     -22.815 -20.571  -9.534  1.00  0.00
I    C
ATOM   3944  HB1 LEU I 286     -22.800 -21.610  -9.936  1.00  0.00
I    H
ATOM   3945  HB2 LEU I 286     -22.631 -20.663  -8.440  1.00  0.00
I    H
ATOM   3946  CG  LEU I 286     -24.183 -19.924  -9.823  1.00  0.00
I    C
ATOM   3947  HG  LEU I 286     -24.228 -19.828 -10.935  1.00  0.00
I    H
ATOM   3948  CD1 LEU I 286     -25.316 -20.842  -9.346  1.00  0.00
I    C
ATOM   3949 HD11 LEU I 286     -26.303 -20.359  -9.509  1.00  0.00
I    H
ATOM   3950 HD12 LEU I 286     -25.302 -21.803  -9.904  1.00  0.00
I    H
ATOM   3951 HD13 LEU I 286     -25.212 -21.062  -8.263  1.00  0.00
I    H
ATOM   3952  CD2 LEU I 286     -24.312 -18.515  -9.220  1.00  0.00
I    C
ATOM   3953 HD21 LEU I 286     -25.304 -18.079  -9.468  1.00  0.00
I    H
ATOM   3954 HD22 LEU I 286     -24.219 -18.533  -8.116  1.00  0.00
I    H
ATOM   3955 HD23 LEU I 286     -23.526 -17.844  -9.627  1.00  0.00
I    H
ATOM   3956  C   LEU I 286     -20.337 -20.517  -9.826  1.00  0.00
I    C
ATOM   3957  O   LEU I 286     -19.622 -20.077  -8.927  1.00  0.00
I    O
ATOM   3958  N   TRP I 287     -19.968 -21.593 -10.560  1.00  0.00
I    N
ATOM   3959  HN  TRP I 287     -20.567 -21.976 -11.259  1.00  0.00
I    H
ATOM   3960  CA  TRP I 287     -18.694 -22.275 -10.399  1.00  0.00
I    C
ATOM   3961  HA  TRP I 287     -18.629 -22.585  -9.363  1.00  0.00
I    H
ATOM   3962  CB  TRP I 287     -18.592 -23.502 -11.346  1.00  0.00
I    C
ATOM   3963  HB1 TRP I 287     -18.964 -23.192 -12.348  1.00  0.00
I    H
ATOM   3964  HB2 TRP I 287     -17.527 -23.793 -11.474  1.00  0.00
I    H
ATOM   3965  CG  TRP I 287     -19.417 -24.704 -10.917  1.00  0.00
I    C
ATOM   3966  CD1 TRP I 287     -20.440 -25.330 -11.572  1.00  0.00
I    C
ATOM   3967  HD1 TRP I 287     -20.791 -25.051 -12.557  1.00  0.00
I    H
ATOM   3968  NE1 TRP I 287     -20.922 -26.382 -10.817  1.00  0.00
I    N
```

ATOM   3969 HE1 TRP I 287   -21.634 -27.003 -11.064 1.00 0.00
I   H
ATOM   3970 CE2 TRP I 287   -20.189 -26.452 -9.652 1.00 0.00
I   C
ATOM   3971 CD2 TRP I 287   -19.223 -25.429 -9.684 1.00 0.00
I   C
ATOM   3972 CE3 TRP I 287   -18.313 -25.264 -8.639 1.00 0.00
I   C
ATOM   3973 HE3 TRP I 287   -17.564 -24.484 -8.652 1.00 0.00
I   H
ATOM   3974 CZ3 TRP I 287   -18.398 -26.144 -7.549 1.00 0.00
I   C
ATOM   3975 HZ3 TRP I 287   -17.706 -26.037 -6.724 1.00 0.00
I   H
ATOM   3976 CZ2 TRP I 287   -20.278 -27.320 -8.570 1.00 0.00
I   C
ATOM   3977 HZ2 TRP I 287   -21.017 -28.105 -8.529 1.00 0.00
I   H
ATOM   3978 CH2 TRP I 287   -19.368 -27.155 -7.513 1.00 0.00
I   C
ATOM   3979 HH2 TRP I 287   -19.418 -27.817 -6.659 1.00 0.00
I   H
ATOM   3980 C   TRP I 287   -17.512 -21.355 -10.661 1.00 0.00
I   C
ATOM   3981 O   TRP I 287   -16.578 -21.276 -9.863 1.00 0.00
I   O
ATOM   3982 N   ALA I 288   -17.569 -20.576 -11.756 1.00 0.00
I   N
ATOM   3983 HN ALA I 288   -18.319 -20.668 -12.410 1.00 0.00
I   H
ATOM   3984 CA ALA I 288   -16.586 -19.570 -12.078 1.00 0.00
I   C
ATOM   3985 HA ALA I 288   -15.603 -20.024 -12.097 1.00 0.00
I   H
ATOM   3986 CB ALA I 288   -16.893 -19.018 -13.481 1.00 0.00
I   C
ATOM   3987 HB1 ALA I 288   -16.895 -19.850 -14.217 1.00 0.00
I   H
ATOM   3988 HB2 ALA I 288   -17.894 -18.534 -13.510 1.00 0.00
I   H
ATOM   3989 HB3 ALA I 288   -16.132 -18.276 -13.804 1.00 0.00
I   H
ATOM   3990 C   ALA I 288   -16.513 -18.425 -11.067 1.00 0.00
I   C
ATOM   3991 O   ALA I 288   -15.423 -18.039 -10.642 1.00 0.00
I   O
ATOM   3992 N   SER I 289   -17.653 -17.864 -10.614 1.00 0.00
I   N
ATOM   3993 HN SER I 289   -18.554 -18.123 -10.960 1.00 0.00
I   H
ATOM   3994 CA SER I 289   -17.683 -16.828 -9.580 1.00 0.00
I   C
ATOM   3995 HA SER I 289   -17.011 -16.062 -9.945 1.00 0.00
I   H

ATOM 3996 CB SER I 289 -19.082 -16.260 -9.291 1.00 0.00
I C
ATOM 3997 HB1 SER I 289 -19.791 -17.115 -9.203 1.00 0.00
I H
ATOM 3998 HB2 SER I 289 -19.089 -15.725 -8.314 1.00 0.00
I H
ATOM 3999 OG SER I 289 -19.451 -15.427 -10.377 1.00 0.00
I O
ATOM 4000 HG1 SER I 289 -20.399 -15.260 -10.296 1.00 0.00
I H
ATOM 4001 C SER I 289 -17.174 -17.267 -8.239 1.00 0.00
I C
ATOM 4002 O SER I 289 -16.421 -16.543 -7.596 1.00 0.00
I O
ATOM 4003 N TYR I 290 -17.556 -18.480 -7.787 1.00 0.00
I N
ATOM 4004 HN TYR I 290 -18.227 -19.037 -8.274 1.00 0.00
I H
ATOM 4005 CA TYR I 290 -17.040 -19.085 -6.573 1.00 0.00
I C
ATOM 4006 HA TYR I 290 -17.250 -18.397 -5.762 1.00 0.00
I H
ATOM 4007 CB TYR I 290 -17.738 -20.473 -6.369 1.00 0.00
I C
ATOM 4008 HB1 TYR I 290 -18.782 -20.309 -6.024 1.00 0.00
I H
ATOM 4009 HB2 TYR I 290 -17.793 -21.029 -7.330 1.00 0.00
I H
ATOM 4010 CG TYR I 290 -17.096 -21.372 -5.334 1.00 0.00
I C
ATOM 4011 CD1 TYR I 290 -16.951 -20.970 -3.996 1.00 0.00
I C
ATOM 4012 HD1 TYR I 290 -17.349 -20.018 -3.675 1.00 0.00
I H
ATOM 4013 CE1 TYR I 290 -16.275 -21.788 -3.080 1.00 0.00
I C
ATOM 4014 HE1 TYR I 290 -16.161 -21.482 -2.051 1.00 0.00
I H
ATOM 4015 CZ TYR I 290 -15.732 -23.007 -3.484 1.00 0.00
I C
ATOM 4016 OH TYR I 290 -15.023 -23.747 -2.511 1.00 0.00
I O
ATOM 4017 HH TYR I 290 -14.774 -24.583 -2.910 1.00 0.00
I H
ATOM 4018 CD2 TYR I 290 -16.566 -22.614 -5.730 1.00 0.00
I C
ATOM 4019 HD2 TYR I 290 -16.646 -22.932 -6.760 1.00 0.00
I H
ATOM 4020 CE2 TYR I 290 -15.884 -23.431 -4.814 1.00 0.00
I C
ATOM 4021 HE2 TYR I 290 -15.462 -24.369 -5.144 1.00 0.00
I H
ATOM 4022 C TYR I 290 -15.529 -19.228 -6.660 1.00 0.00
I C

```
ATOM   4023  O   TYR I 290     -14.806 -18.722  -5.806  1.00  0.00
I    O
ATOM   4024  N   PHE I 291     -15.017 -19.830  -7.752  1.00  0.00
I    N
ATOM   4025  HN  PHE I 291     -15.601 -20.140  -8.500  1.00  0.00
I    H
ATOM   4026  CA  PHE I 291     -13.608 -20.082  -7.949  1.00  0.00
I    C
ATOM   4027  HA  PHE I 291     -13.289 -20.631  -7.071  1.00  0.00
I    H
ATOM   4028  CB  PHE I 291     -13.445 -20.942  -9.227  1.00  0.00
I    C
ATOM   4029  HB1 PHE I 291     -14.236 -21.724  -9.244  1.00  0.00
I    H
ATOM   4030  HB2 PHE I 291     -13.574 -20.326 -10.143  1.00  0.00
I    H
ATOM   4031  CG  PHE I 291     -12.118 -21.618  -9.198  1.00  0.00
I    C
ATOM   4032  CD1 PHE I 291     -11.976 -22.854  -8.551  1.00  0.00
I    C
ATOM   4033  HD1 PHE I 291     -12.847 -23.347  -8.143  1.00  0.00
I    H
ATOM   4034  CE1 PHE I 291     -10.716 -23.448  -8.427  1.00  0.00
I    C
ATOM   4035  HE1 PHE I 291     -10.616 -24.403  -7.931  1.00  0.00
I    H
ATOM   4036  CZ  PHE I 291      -9.594 -22.803  -8.952  1.00  0.00
I    C
ATOM   4037  HZ  PHE I 291      -8.613 -23.240  -8.830  1.00  0.00
I    H
ATOM   4038  CD2 PHE I 291     -10.996 -20.996  -9.757  1.00  0.00
I    C
ATOM   4039  HD2 PHE I 291     -11.099 -20.043 -10.256  1.00  0.00
I    H
ATOM   4040  CE2 PHE I 291      -9.741 -21.593  -9.646  1.00  0.00
I    C
ATOM   4041  HE2 PHE I 291      -8.878 -21.121 -10.097  1.00  0.00
I    H
ATOM   4042  C   PHE I 291     -12.766 -18.799  -8.008  1.00  0.00
I    C
ATOM   4043  O   PHE I 291     -11.730 -18.683  -7.356  1.00  0.00
I    O
ATOM   4044  N   GLN I 292     -13.243 -17.772  -8.745  1.00  0.00
I    N
ATOM   4045  HN  GLN I 292     -14.117 -17.834  -9.224  1.00  0.00
I    H
ATOM   4046  CA  GLN I 292     -12.551 -16.501  -8.915  1.00  0.00
I    C
ATOM   4047  HA  GLN I 292     -11.509 -16.747  -9.081  1.00  0.00
I    H
ATOM   4048  CB  GLN I 292     -13.027 -15.713 -10.169  1.00  0.00
I    C
ATOM   4049  HB1 GLN I 292     -14.040 -16.100 -10.428  1.00  0.00
I    H
```

```
ATOM   4050  HB2 GLN I 292     -13.163 -14.641  -9.901  1.00  0.00
I    H
ATOM   4051  CG  GLN I 292     -12.079 -15.870 -11.389  1.00  0.00
I    C
ATOM   4052  HG1 GLN I 292     -12.481 -15.318 -12.265  1.00  0.00
I    H
ATOM   4053  HG2 GLN I 292     -11.074 -15.461 -11.161  1.00  0.00
I    H
ATOM   4054  CD  GLN I 292     -11.873 -17.299 -11.892  1.00  0.00
I    C
ATOM   4055  OE1 GLN I 292     -10.773 -17.708 -12.255  1.00  0.00
I    O
ATOM   4056  NE2 GLN I 292     -12.966 -18.087 -11.952  1.00  0.00
I    N
ATOM   4057 HE21 GLN I 292     -12.831 -19.019 -12.275  1.00  0.00
I    H
ATOM   4058 HE22 GLN I 292     -13.854 -17.721 -11.695  1.00  0.00
I    H
ATOM   4059  C   GLN I 292     -12.621 -15.614  -7.684  1.00  0.00
I    C
ATOM   4060  O   GLN I 292     -11.845 -14.675  -7.556  1.00  0.00
I    O
ATOM   4061  N   ALA I 293     -13.530 -15.894  -6.739  1.00  0.00
I    N
ATOM   4062  HN  ALA I 293     -14.227 -16.595  -6.891  1.00  0.00
I    H
ATOM   4063  CA  ALA I 293     -13.585 -15.234  -5.460  1.00  0.00
I    C
ATOM   4064  HA  ALA I 293     -13.339 -14.187  -5.587  1.00  0.00
I    H
ATOM   4065  CB  ALA I 293     -15.005 -15.416  -4.927  1.00  0.00
I    C
ATOM   4066  HB1 ALA I 293     -15.730 -14.974  -5.642  1.00  0.00
I    H
ATOM   4067  HB2 ALA I 293     -15.253 -16.493  -4.811  1.00  0.00
I    H
ATOM   4068  HB3 ALA I 293     -15.142 -14.913  -3.946  1.00  0.00
I    H
ATOM   4069  C   ALA I 293     -12.602 -15.784  -4.437  1.00  0.00
I    C
ATOM   4070  O   ALA I 293     -12.071 -15.049  -3.607  1.00  0.00
I    O
ATOM   4071  N   VAL I 294     -12.342 -17.105  -4.467  1.00  0.00
I    N
ATOM   4072  HN  VAL I 294     -12.693 -17.716  -5.172  1.00  0.00
I    H
ATOM   4073  CA  VAL I 294     -11.518 -17.757  -3.463  1.00  0.00
I    C
ATOM   4074  HA  VAL I 294     -11.534 -17.125  -2.584  1.00  0.00
I    H
ATOM   4075  CB  VAL I 294     -12.090 -19.119  -3.069  1.00  0.00
I    C
ATOM   4076  HB  VAL I 294     -11.394 -19.575  -2.322  1.00  0.00
I    H
```

```
ATOM   4077  CG1 VAL I 294      -13.479 -18.901  -2.437  1.00  0.00
I    C
ATOM   4078 HG11 VAL I 294      -13.941 -19.877  -2.174  1.00  0.00
I    H
ATOM   4079 HG12 VAL I 294      -13.416 -18.303  -1.504  1.00  0.00
I    H
ATOM   4080 HG13 VAL I 294      -14.156 -18.381  -3.148  1.00  0.00
I    H
ATOM   4081  CG2 VAL I 294      -12.225 -20.070  -4.271  1.00  0.00
I    C
ATOM   4082 HG21 VAL I 294      -12.680 -21.034  -3.960  1.00  0.00
I    H
ATOM   4083 HG22 VAL I 294      -12.868 -19.616  -5.057  1.00  0.00
I    H
ATOM   4084 HG23 VAL I 294      -11.235 -20.294  -4.719  1.00  0.00
I    H
ATOM   4085  C   VAL I 294      -10.068 -17.886  -3.905  1.00  0.00
I    C
ATOM   4086  O   VAL I 294       -9.153 -17.831  -3.090  1.00  0.00
I    O
ATOM   4087  N   THR I 295       -9.807 -17.989  -5.226  1.00  0.00
I    N
ATOM   4088  HN  THR I 295      -10.543 -18.042  -5.900  1.00  0.00
I    H
ATOM   4089  CA  THR I 295       -8.459 -18.033  -5.801  1.00  0.00
I    C
ATOM   4090  HA  THR I 295       -7.991 -18.855  -5.276  1.00  0.00
I    H
ATOM   4091  CB  THR I 295       -8.454 -18.486  -7.269  1.00  0.00
I    C
ATOM   4092  HB  THR I 295       -9.111 -19.388  -7.317  1.00  0.00
I    H
ATOM   4093  OG1 THR I 295       -7.177 -18.894  -7.718  1.00  0.00
I    O
ATOM   4094  HG1 THR I 295       -7.297 -19.141  -8.641  1.00  0.00
I    H
ATOM   4095  CG2 THR I 295       -8.887 -17.391  -8.241  1.00  0.00
I    C
ATOM   4096 HG21 THR I 295       -8.978 -17.792  -9.272  1.00  0.00
I    H
ATOM   4097 HG22 THR I 295       -9.876 -16.980  -7.945  1.00  0.00
I    H
ATOM   4098 HG23 THR I 295       -8.154 -16.556  -8.256  1.00  0.00
I    H
ATOM   4099  C   THR I 295       -7.557 -16.801  -5.579  1.00  0.00
I    C
ATOM   4100  O   THR I 295       -6.351 -17.015  -5.420  1.00  0.00
I    O
ATOM   4101  N   PRO I 296       -7.968 -15.511  -5.521  1.00  0.00
I    N
ATOM   4102  CD  PRO I 296       -9.322 -15.016  -5.739  1.00  0.00
I    C
ATOM   4103  HD1 PRO I 296       -8.746 -15.747  -6.349  1.00  0.00
I    H
```

```
ATOM   4104  HD2 PRO I 296     -10.407 -15.269  -5.746  1.00  0.00
I    H
ATOM   4105  CA  PRO I 296      -7.065 -14.385  -5.298  1.00  0.00
I    C
ATOM   4106  HA  PRO I 296      -6.286 -14.382  -6.051  1.00  0.00
I    H
ATOM   4107  CB  PRO I 296      -7.951 -13.134  -5.412  1.00  0.00
I    C
ATOM   4108  HB1 PRO I 296      -8.037 -12.846  -6.485  1.00  0.00
I    H
ATOM   4109  HB2 PRO I 296      -7.552 -12.272  -4.840  1.00  0.00
I    H
ATOM   4110  CG  PRO I 296      -9.113 -13.608  -6.278  1.00  0.00
I    C
ATOM   4111  HG1 PRO I 296      -9.628 -12.779  -6.802  1.00  0.00
I    H
ATOM   4112  HG2 PRO I 296      -9.434 -13.880  -7.185  1.00  0.00
I    H
ATOM   4113  C   PRO I 296      -6.432 -14.412  -3.939  1.00  0.00
I    C
ATOM   4114  O   PRO I 296      -5.389 -13.800  -3.746  1.00  0.00
I    O
ATOM   4115  N   ARG I 297      -7.048 -15.101  -2.965  1.00  0.00
I    N
ATOM   4116  HN  ARG I 297      -7.915 -15.551  -3.162  1.00  0.00
I    H
ATOM   4117  CA  ARG I 297      -6.534 -15.242  -1.621  1.00  0.00
I    C
ATOM   4118  HA  ARG I 297      -6.099 -14.298  -1.312  1.00  0.00
I    H
ATOM   4119  CB  ARG I 297      -7.685 -15.579  -0.653  1.00  0.00
I    C
ATOM   4120  HB1 ARG I 297      -8.151 -16.519  -1.032  1.00  0.00
I    H
ATOM   4121  HB2 ARG I 297      -7.268 -15.827   0.349  1.00  0.00
I    H
ATOM   4122  CG  ARG I 297      -8.746 -14.452  -0.596  1.00  0.00
I    C
ATOM   4123  HG1 ARG I 297      -8.755 -13.924  -1.575  1.00  0.00
I    H
ATOM   4124  HG2 ARG I 297      -9.757 -14.911  -0.495  1.00  0.00
I    H
ATOM   4125  CD  ARG I 297      -8.608 -13.440   0.561  1.00  0.00
I    C
ATOM   4126  HD1 ARG I 297      -7.646 -12.887   0.457  1.00  0.00
I    H
ATOM   4127  HD2 ARG I 297      -9.445 -12.703   0.562  1.00  0.00
I    H
ATOM   4128  NE  ARG I 297      -8.642 -14.171   1.882  1.00  0.00
I    N
ATOM   4129  HE  ARG I 297      -7.802 -14.049   2.423  1.00  0.00
I    H
ATOM   4130  CZ  ARG I 297      -9.672 -14.915   2.321  1.00  0.00
I    C
```

```
ATOM   4131  NH1 ARG I 297     -10.855 -14.845   1.724  1.00  0.00
I    N
ATOM   4132 HH11 ARG I 297     -11.622 -15.373   2.062  1.00  0.00
I    H
ATOM   4133 HH12 ARG I 297     -10.955 -14.139   1.019  1.00  0.00
I    H
ATOM   4134  NH2 ARG I 297      -9.511 -15.733   3.357  1.00  0.00
I    N
ATOM   4135 HH21 ARG I 297     -10.243 -16.343   3.624  1.00  0.00
I    H
ATOM   4136 HH22 ARG I 297      -8.586 -15.851   3.695  1.00  0.00
I    H
ATOM   4137  C   ARG I 297      -5.410 -16.268  -1.593  1.00  0.00
I    C
ATOM   4138  O   ARG I 297      -5.465 -17.307  -0.940  1.00  0.00
I    O
ATOM   4139  N   THR I 298      -4.342 -15.918  -2.337  1.00  0.00
I    N
ATOM   4140  HN  THR I 298      -4.471 -15.104  -2.902  1.00  0.00
I    H
ATOM   4141  CA  THR I 298      -3.028 -16.524  -2.457  1.00  0.00
I    C
ATOM   4142  HA  THR I 298      -2.478 -15.821  -3.068  1.00  0.00
I    H
ATOM   4143  CB  THR I 298      -2.032 -16.250  -1.301  1.00  0.00
I    C
ATOM   4144  HB  THR I 298      -1.238 -15.589  -1.725  1.00  0.00
I    H
ATOM   4145  OG1 THR I 298      -1.346 -17.382  -0.798  1.00  0.00
I    O
ATOM   4146  HG1 THR I 298      -0.785 -17.048  -0.090  1.00  0.00
I    H
ATOM   4147  CG2 THR I 298      -2.750 -15.628  -0.100  1.00  0.00
I    C
ATOM   4148 HG21 THR I 298      -2.022 -15.327   0.684  1.00  0.00
I    H
ATOM   4149 HG22 THR I 298      -3.311 -14.721  -0.411  1.00  0.00
I    H
ATOM   4150 HG23 THR I 298      -3.469 -16.344   0.351  1.00  0.00
I    H
ATOM   4151  C   THR I 298      -2.963 -17.859  -3.171  1.00  0.00
I    C
ATOM   4152  O   THR I 298      -1.980 -18.579  -3.067  1.00  0.00
I    O
ATOM   4153  N   ALA I 299      -3.978 -18.236  -3.976  1.00  0.00
I    N
ATOM   4154  HN  ALA I 299      -4.810 -17.696  -4.100  1.00  0.00
I    H
ATOM   4155  CA  ALA I 299      -3.923 -19.478  -4.730  1.00  0.00
I    C
ATOM   4156  HA  ALA I 299      -3.210 -20.139  -4.252  1.00  0.00
I    H
ATOM   4157  CB  ALA I 299      -5.285 -20.181  -4.681  1.00  0.00
I    C
```

ATOM 4158 HB1 ALA I 299 -5.593 -20.325 -3.623 1.00 0.00
I H
ATOM 4159 HB2 ALA I 299 -6.068 -19.573 -5.185 1.00 0.00
I H
ATOM 4160 HB3 ALA I 299 -5.247 -21.181 -5.166 1.00 0.00
I H
ATOM 4161 C ALA I 299 -3.481 -19.348 -6.182 1.00 0.00
I C
ATOM 4162 O ALA I 299 -2.634 -20.110 -6.621 1.00 0.00
I O
ATOM 4163 N GLY I 300 -4.047 -18.407 -6.973 1.00 0.00
I N
ATOM 4164 HN GLY I 300 -4.832 -17.868 -6.669 1.00 0.00
I H
ATOM 4165 CA GLY I 300 -3.556 -18.109 -8.328 1.00 0.00
I C
ATOM 4166 HA1 GLY I 300 -2.483 -18.202 -8.235 1.00 0.00
I H
ATOM 4167 HA2 GLY I 300 -3.901 -17.100 -8.512 1.00 0.00
I H
ATOM 4168 C GLY I 300 -3.998 -18.966 -9.479 1.00 0.00
I C
ATOM 4169 O GLY I 300 -3.550 -18.791 -10.605 1.00 0.00
I O
ATOM 4170 N PHE I 301 -4.909 -19.912 -9.244 1.00 0.00
I N
ATOM 4171 HN PHE I 301 -5.197 -20.153 -8.318 1.00 0.00
I H
ATOM 4172 CA PHE I 301 -5.563 -20.674 -10.294 1.00 0.00
I C
ATOM 4173 HA PHE I 301 -4.774 -20.977 -10.971 1.00 0.00
I H
ATOM 4174 CB PHE I 301 -6.340 -21.873 -9.697 1.00 0.00
I C
ATOM 4175 HB1 PHE I 301 -6.997 -21.509 -8.877 1.00 0.00
I H
ATOM 4176 HB2 PHE I 301 -6.998 -22.343 -10.459 1.00 0.00
I H
ATOM 4177 CG PHE I 301 -5.440 -22.905 -9.098 1.00 0.00
I C
ATOM 4178 CD1 PHE I 301 -4.858 -23.874 -9.926 1.00 0.00
I C
ATOM 4179 HD1 PHE I 301 -5.091 -23.887 -10.982 1.00 0.00
I H
ATOM 4180 CE1 PHE I 301 -3.982 -24.829 -9.402 1.00 0.00
I C
ATOM 4181 HE1 PHE I 301 -3.491 -25.530 -10.061 1.00 0.00
I H
ATOM 4182 CZ PHE I 301 -3.742 -24.866 -8.025 1.00 0.00
I C
ATOM 4183 HZ PHE I 301 -3.081 -25.616 -7.612 1.00 0.00
I H
ATOM 4184 CD2 PHE I 301 -5.184 -22.941 -7.719 1.00 0.00
I C

```
ATOM   4185  HD2 PHE I 301      -5.648 -22.218  -7.064  1.00  0.00
I    H
ATOM   4186  CE2 PHE I 301      -4.349 -23.927  -7.181  1.00  0.00
I    C
ATOM   4187  HE2 PHE I 301      -4.175 -23.961  -6.114  1.00  0.00
I    H
ATOM   4188  C   PHE I 301      -6.552 -19.824 -11.106  1.00  0.00
I    C
ATOM   4189  O   PHE I 301      -7.205 -18.932 -10.556  1.00  0.00
I    O
ATOM   4190  N   ASN I 302      -6.730 -20.112 -12.416  1.00  0.00
I    N
ATOM   4191  HN  ASN I 302      -6.229 -20.871 -12.832  1.00  0.00
I    H
ATOM   4192  CA  ASN I 302      -7.618 -19.399 -13.331  1.00  0.00
I    C
ATOM   4193  HA  ASN I 302      -8.114 -18.642 -12.734  1.00  0.00
I    H
ATOM   4194  CB  ASN I 302      -6.930 -18.971 -14.666  1.00  0.00
I    C
ATOM   4195  HB1 ASN I 302      -6.252 -19.795 -14.979  1.00  0.00
I    H
ATOM   4196  HB2 ASN I 302      -7.687 -18.840 -15.466  1.00  0.00
I    H
ATOM   4197  CG  ASN I 302      -6.093 -17.713 -14.547  1.00  0.00
I    C
ATOM   4198  OD1 ASN I 302      -5.030 -17.691 -13.938  1.00  0.00
I    O
ATOM   4199  ND2 ASN I 302      -6.556 -16.637 -15.223  1.00  0.00
I    N
ATOM   4200 HD21 ASN I 302      -6.021 -15.799 -15.172  1.00  0.00
I    H
ATOM   4201 HD22 ASN I 302      -7.386 -16.711 -15.768  1.00  0.00
I    H
ATOM   4202  C   ASN I 302      -8.680 -20.313 -13.881  1.00  0.00
I    C
ATOM   4203  O   ASN I 302      -8.343 -21.238 -14.640  1.00  0.00
I    O
ATOM   4204  N   SER I 303      -9.961 -20.008 -13.654  1.00  0.00
I    N
ATOM   4205  HN  SER I 303     -10.255 -19.345 -12.966  1.00  0.00
I    H
ATOM   4206  CA  SER I 303     -11.054 -20.622 -14.398  1.00  0.00
I    C
ATOM   4207  HA  SER I 303     -10.607 -21.371 -15.038  1.00  0.00
I    H
ATOM   4208  CB  SER I 303     -12.152 -21.270 -13.510  1.00  0.00
I    C
ATOM   4209  HB1 SER I 303     -12.615 -20.468 -12.891  1.00  0.00
I    H
ATOM   4210  HB2 SER I 303     -12.959 -21.701 -14.144  1.00  0.00
I    H
ATOM   4211  OG  SER I 303     -11.598 -22.256 -12.643  1.00  0.00
I    O
```

```
ATOM   4212  HG1 SER I 303     -12.267 -22.457 -11.977  1.00  0.00
I    H
ATOM   4213  C   SER I 303     -11.720 -19.595 -15.300  1.00  0.00
I    C
ATOM   4214  O   SER I 303     -12.601 -19.926 -16.079  1.00  0.00
I    O
ATOM   4215  N   LEU I 304     -11.281 -18.318 -15.245  1.00  0.00
I    N
ATOM   4216  HN  LEU I 304     -10.648 -18.025 -14.531  1.00  0.00
I    H
ATOM   4217  CA  LEU I 304     -11.659 -17.268 -16.175  1.00  0.00
I    C
ATOM   4218  HA  LEU I 304     -12.023 -17.724 -17.088  1.00  0.00
I    H
ATOM   4219  CB  LEU I 304     -12.622 -16.222 -15.561  1.00  0.00
I    C
ATOM   4220  HB1 LEU I 304     -12.113 -15.790 -14.668  1.00  0.00
I    H
ATOM   4221  HB2 LEU I 304     -12.750 -15.382 -16.280  1.00  0.00
I    H
ATOM   4222  CG  LEU I 304     -13.998 -16.754 -15.122  1.00  0.00
I    C
ATOM   4223  HG  LEU I 304     -13.788 -17.579 -14.398  1.00  0.00
I    H
ATOM   4224  CD1 LEU I 304     -14.772 -15.632 -14.414  1.00  0.00
I    C
ATOM   4225 HD11 LEU I 304     -15.789 -15.979 -14.130  1.00  0.00
I    H
ATOM   4226 HD12 LEU I 304     -14.242 -15.313 -13.491  1.00  0.00
I    H
ATOM   4227 HD13 LEU I 304     -14.881 -14.749 -15.079  1.00  0.00
I    H
ATOM   4228  CD2 LEU I 304     -14.823 -17.339 -16.281  1.00  0.00
I    C
ATOM   4229 HD21 LEU I 304     -15.796 -17.724 -15.907  1.00  0.00
I    H
ATOM   4230 HD22 LEU I 304     -15.039 -16.575 -17.055  1.00  0.00
I    H
ATOM   4231 HD23 LEU I 304     -14.279 -18.179 -16.762  1.00  0.00
I    H
ATOM   4232  C   LEU I 304     -10.381 -16.520 -16.514  1.00  0.00
I    C
ATOM   4233  O   LEU I 304      -9.393 -16.618 -15.785  1.00  0.00
I    O
ATOM   4234  N   ASP I 305     -10.357 -15.749 -17.621  1.00  0.00
I    N
ATOM   4235  HN  ASP I 305     -11.112 -15.646 -18.264  1.00  0.00
I    H
ATOM   4236  CA  ASP I 305      -9.193 -14.973 -17.990  1.00  0.00
I    C
ATOM   4237  HA  ASP I 305      -8.325 -15.518 -17.636  1.00  0.00
I    H
ATOM   4238  CB  ASP I 305      -9.103 -14.795 -19.527  1.00  0.00
I    C
```

```
ATOM   4239  HB1 ASP I 305      -9.206 -15.784 -20.015  1.00  0.00
I    H
ATOM   4240  HB2 ASP I 305      -9.917 -14.139 -19.892  1.00  0.00
I    H
ATOM   4241  CG  ASP I 305      -7.731 -14.206 -19.788  1.00  0.00
I    C
ATOM   4242  OD1 ASP I 305      -6.737 -14.893 -19.460  1.00  0.00
I    O
ATOM   4243  OD2 ASP I 305      -7.682 -13.000 -20.135  1.00  0.00
I    O
ATOM   4244  C   ASP I 305      -9.183 -13.609 -17.276  1.00  0.00
I    C
ATOM   4245  O   ASP I 305     -10.119 -12.819 -17.371  1.00  0.00
I    O
ATOM   4246  N   PHE I 306      -8.097 -13.289 -16.548  1.00  0.00
I    N
ATOM   4247  HN  PHE I 306      -7.308 -13.897 -16.475  1.00  0.00
I    H
ATOM   4248  CA  PHE I 306      -7.970 -12.042 -15.807  1.00  0.00
I    C
ATOM   4249  HA  PHE I 306      -8.962 -11.827 -15.430  1.00  0.00
I    H
ATOM   4250  CB  PHE I 306      -6.964 -12.183 -14.632  1.00  0.00
I    C
ATOM   4251  HB1 PHE I 306      -6.180 -12.920 -14.911  1.00  0.00
I    H
ATOM   4252  HB2 PHE I 306      -6.449 -11.219 -14.429  1.00  0.00
I    H
ATOM   4253  CG  PHE I 306      -7.670 -12.698 -13.404  1.00  0.00
I    C
ATOM   4254  CD1 PHE I 306      -8.018 -14.051 -13.265  1.00  0.00
I    C
ATOM   4255  HD1 PHE I 306      -7.802 -14.744 -14.067  1.00  0.00
I    H
ATOM   4256  CE1 PHE I 306      -8.649 -14.520 -12.106  1.00  0.00
I    C
ATOM   4257  HE1 PHE I 306      -8.881 -15.571 -12.003  1.00  0.00
I    H
ATOM   4258  CZ  PHE I 306      -8.972 -13.624 -11.079  1.00  0.00
I    C
ATOM   4259  HZ  PHE I 306      -9.444 -13.980 -10.175  1.00  0.00
I    H
ATOM   4260  CD2 PHE I 306      -8.017 -11.809 -12.372  1.00  0.00
I    C
ATOM   4261  HD2 PHE I 306      -7.798 -10.755 -12.471  1.00  0.00
I    H
ATOM   4262  CE2 PHE I 306      -8.671 -12.265 -11.221  1.00  0.00
I    C
ATOM   4263  HE2 PHE I 306      -8.944 -11.565 -10.443  1.00  0.00
I    H
ATOM   4264  C   PHE I 306      -7.564 -10.868 -16.688  1.00  0.00
I    C
ATOM   4265  O   PHE I 306      -7.699  -9.716 -16.283  1.00  0.00
I    O
```

```
ATOM   4266  N   GLY I 307      -7.099 -11.097 -17.935  1.00  0.00
I    N
ATOM   4267  HN  GLY I 307      -6.934 -12.018 -18.286  1.00  0.00
I    H
ATOM   4268  CA  GLY I 307      -6.806 -10.006 -18.860  1.00  0.00
I    C
ATOM   4269  HA1 GLY I 307      -6.144 -10.409 -19.615  1.00  0.00
I    H
ATOM   4270  HA2 GLY I 307      -6.377  -9.203 -18.275  1.00  0.00
I    H
ATOM   4271  C   GLY I 307      -8.038  -9.471 -19.555  1.00  0.00
I    C
ATOM   4272  O   GLY I 307      -7.977  -8.481 -20.275  1.00  0.00
I    O
ATOM   4273  N   SER I 308      -9.191 -10.137 -19.344  1.00  0.00
I    N
ATOM   4274  HN  SER I 308      -9.234 -10.870 -18.665  1.00  0.00
I    H
ATOM   4275  CA  SER I 308     -10.451  -9.888 -20.036  1.00  0.00
I    C
ATOM   4276  HA  SER I 308     -10.263  -9.054 -20.698  1.00  0.00
I    H
ATOM   4277  CB  SER I 308     -10.799 -11.209 -20.792  1.00  0.00
I    C
ATOM   4278  HB1 SER I 308      -9.917 -11.498 -21.409  1.00  0.00
I    H
ATOM   4279  HB2 SER I 308     -10.977 -12.035 -20.067  1.00  0.00
I    H
ATOM   4280  OG  SER I 308     -11.911 -11.135 -21.683  1.00  0.00
I    O
ATOM   4281  HG1 SER I 308     -11.882 -11.924 -22.240  1.00  0.00
I    H
ATOM   4282  C   SER I 308     -11.557  -9.462 -19.067  1.00  0.00
I    C
ATOM   4283  O   SER I 308     -12.693  -9.192 -19.454  1.00  0.00
I    O
ATOM   4284  N   MET I 309     -11.246  -9.315 -17.761  1.00  0.00
I    N
ATOM   4285  HN  MET I 309     -10.328  -9.491 -17.411  1.00  0.00
I    H
ATOM   4286  CA  MET I 309     -12.205  -8.893 -16.751  1.00  0.00
I    C
ATOM   4287  HA  MET I 309     -13.132  -9.398 -16.991  1.00  0.00
I    H
ATOM   4288  CB  MET I 309     -11.755  -9.313 -15.334  1.00  0.00
I    C
ATOM   4289  HB1 MET I 309     -10.687  -9.011 -15.231  1.00  0.00
I    H
ATOM   4290  HB2 MET I 309     -12.320  -8.722 -14.577  1.00  0.00
I    H
ATOM   4291  CG  MET I 309     -11.892 -10.821 -15.094  1.00  0.00
I    C
ATOM   4292  HG1 MET I 309     -12.944 -11.142 -15.247  1.00  0.00
I    H
```

```
ATOM   4293  HG2 MET I 309      -11.263 -11.374 -15.824  1.00  0.00
I    H
ATOM   4294  SD  MET I 309      -11.397 -11.284 -13.412  1.00  0.00
I    S
ATOM   4295  CE  MET I 309      -12.675 -12.553 -13.235  1.00  0.00
I    C
ATOM   4296  HE1 MET I 309      -12.621 -13.034 -12.236  1.00  0.00
I    H
ATOM   4297  HE2 MET I 309      -13.690 -12.115 -13.349  1.00  0.00
I    H
ATOM   4298  HE3 MET I 309      -12.558 -13.348 -14.003  1.00  0.00
I    H
ATOM   4299  C   MET I 309      -12.480  -7.395 -16.760  1.00  0.00
I    C
ATOM   4300  O   MET I 309      -11.747  -6.586 -17.327  1.00  0.00
I    O
ATOM   4301  N   ARG I 310      -13.587  -6.969 -16.118  1.00  0.00
I    N
ATOM   4302  HN  ARG I 310      -14.266  -7.627 -15.800  1.00  0.00
I    H
ATOM   4303  CA  ARG I 310      -13.860  -5.571 -15.852  1.00  0.00
I    C
ATOM   4304  HA  ARG I 310      -13.755  -5.032 -16.785  1.00  0.00
I    H
ATOM   4305  CB  ARG I 310      -15.310  -5.374 -15.320  1.00  0.00
I    C
ATOM   4306  HB1 ARG I 310      -15.361  -5.906 -14.340  1.00  0.00
I    H
ATOM   4307  HB2 ARG I 310      -15.480  -4.299 -15.094  1.00  0.00
I    H
ATOM   4308  CG  ARG I 310      -16.420  -5.932 -16.241  1.00  0.00
I    C
ATOM   4309  HG1 ARG I 310      -16.378  -7.044 -16.213  1.00  0.00
I    H
ATOM   4310  HG2 ARG I 310      -17.414  -5.660 -15.817  1.00  0.00
I    H
ATOM   4311  CD  ARG I 310      -16.345  -5.387 -17.668  1.00  0.00
I    C
ATOM   4312  HD1 ARG I 310      -16.315  -4.274 -17.638  1.00  0.00
I    H
ATOM   4313  HD2 ARG I 310      -15.436  -5.755 -18.199  1.00  0.00
I    H
ATOM   4314  NE  ARG I 310      -17.554  -5.866 -18.414  1.00  0.00
I    N
ATOM   4315  HE  ARG I 310      -18.208  -6.362 -17.832  1.00  0.00
I    H
ATOM   4316  CZ  ARG I 310      -17.737  -5.650 -19.723  1.00  0.00
I    C
ATOM   4317  NH1 ARG I 310      -16.800  -5.050 -20.454  1.00  0.00
I    N
ATOM   4318 HH11 ARG I 310      -16.950  -4.870 -21.416  1.00  0.00
I    H
ATOM   4319 HH12 ARG I 310      -16.016  -4.679 -19.952  1.00  0.00
I    H
```

```
ATOM   4320  NH2 ARG I 310     -18.866  -6.026 -20.321  1.00  0.00
I    N
ATOM   4321 HH21 ARG I 310     -18.956  -5.941 -21.304  1.00  0.00
I    H
ATOM   4322 HH22 ARG I 310     -19.497  -6.572 -19.786  1.00  0.00
I    H
ATOM   4323  C   ARG I 310     -12.866  -4.949 -14.874  1.00  0.00
I    C
ATOM   4324  O   ARG I 310     -12.414  -5.586 -13.923  1.00  0.00
I    O
ATOM   4325  N   GLU I 311     -12.512  -3.656 -15.057  1.00  0.00
I    N
ATOM   4326  HN  GLU I 311     -12.883  -3.086 -15.785  1.00  0.00
I    H
ATOM   4327  CA  GLU I 311     -11.552  -2.975 -14.203  1.00  0.00
I    C
ATOM   4328  HA  GLU I 311     -10.626  -3.534 -14.264  1.00  0.00
I    H
ATOM   4329  CB  GLU I 311     -11.201  -1.556 -14.713  1.00  0.00
I    C
ATOM   4330  HB1 GLU I 311     -12.152  -1.027 -14.953  1.00  0.00
I    H
ATOM   4331  HB2 GLU I 311     -10.724  -0.969 -13.896  1.00  0.00
I    H
ATOM   4332  CG  GLU I 311     -10.289  -1.529 -15.970  1.00  0.00
I    C
ATOM   4333  HG1 GLU I 311      -9.556  -2.354 -15.912  1.00  0.00
I    H
ATOM   4334  HG2 GLU I 311     -10.888  -1.638 -16.897  1.00  0.00
I    H
ATOM   4335  CD  GLU I 311      -9.456  -0.242 -16.078  1.00  0.00
I    C
ATOM   4336  OE1 GLU I 311      -8.540  -0.210 -16.943  1.00  0.00
I    O
ATOM   4337  OE2 GLU I 311      -9.677   0.688 -15.261  1.00  0.00
I    O
ATOM   4338  C   GLU I 311     -11.955  -2.924 -12.731  1.00  0.00
I    C
ATOM   4339  O   GLU I 311     -11.123  -3.122 -11.852  1.00  0.00
I    O
ATOM   4340  N   GLY I 312     -13.257  -2.728 -12.410  1.00  0.00
I    N
ATOM   4341  HN  GLY I 312     -13.919  -2.444 -13.103  1.00  0.00
I    H
ATOM   4342  CA  GLY I 312     -13.796  -2.910 -11.055  1.00  0.00
I    C
ATOM   4343  HA1 GLY I 312     -14.871  -2.863 -11.156  1.00  0.00
I    H
ATOM   4344  HA2 GLY I 312     -13.371  -2.117 -10.455  1.00  0.00
I    H
ATOM   4345  C   GLY I 312     -13.444  -4.230 -10.401  1.00  0.00
I    C
ATOM   4346  O   GLY I 312     -13.013  -4.276  -9.254  1.00  0.00
I    O
```

```
ATOM   4347  N   THR I 313     -13.581  -5.355 -11.135  1.00  0.00
I    N
ATOM   4348  HN  THR I 313     -13.920  -5.315 -12.074  1.00  0.00
I    H
ATOM   4349  CA  THR I 313     -13.266  -6.705 -10.666  1.00  0.00
I    C
ATOM   4350  HA  THR I 313     -13.786  -6.822  -9.723  1.00  0.00
I    H
ATOM   4351  CB  THR I 313     -13.641  -7.777 -11.680  1.00  0.00
I    C
ATOM   4352  HB  THR I 313     -13.028  -7.569 -12.591  1.00  0.00
I    H
ATOM   4353  OG1 THR I 313     -15.005  -7.634 -12.023  1.00  0.00
I    O
ATOM   4354  HG1 THR I 313     -15.187  -8.339 -12.653  1.00  0.00
I    H
ATOM   4355  CG2 THR I 313     -13.498  -9.195 -11.111  1.00  0.00
I    C
ATOM   4356 HG21 THR I 313     -13.666  -9.957 -11.901  1.00  0.00
I    H
ATOM   4357 HG22 THR I 313     -12.476  -9.347 -10.702  1.00  0.00
I    H
ATOM   4358 HG23 THR I 313     -14.230  -9.375 -10.294  1.00  0.00
I    H
ATOM   4359  C   THR I 313     -11.801  -6.843 -10.366  1.00  0.00
I    C
ATOM   4360  O   THR I 313     -11.403  -7.348  -9.320  1.00  0.00
I    O
ATOM   4361  N   ILE I 314     -10.945  -6.306 -11.255  1.00  0.00
I    N
ATOM   4362  HN  ILE I 314     -11.260  -5.922 -12.120  1.00  0.00
I    H
ATOM   4363  CA  ILE I 314      -9.507  -6.230 -11.058  1.00  0.00
I    C
ATOM   4364  HA  ILE I 314      -9.177  -7.242 -10.864  1.00  0.00
I    H
ATOM   4365  CB  ILE I 314      -8.814  -5.707 -12.314  1.00  0.00
I    C
ATOM   4366  HB  ILE I 314      -9.311  -4.725 -12.520  1.00  0.00
I    H
ATOM   4367  CG2 ILE I 314      -7.295  -5.608 -12.079  1.00  0.00
I    C
ATOM   4368 HG21 ILE I 314      -6.803  -5.086 -12.926  1.00  0.00
I    H
ATOM   4369 HG22 ILE I 314      -7.074  -5.034 -11.155  1.00  0.00
I    H
ATOM   4370 HG23 ILE I 314      -6.841  -6.617 -11.983  1.00  0.00
I    H
ATOM   4371  CG1 ILE I 314      -9.107  -6.613 -13.542  1.00  0.00
I    C
ATOM   4372 HG11 ILE I 314     -10.206  -6.693 -13.698  1.00  0.00
I    H
ATOM   4373 HG12 ILE I 314      -8.678  -6.135 -14.453  1.00  0.00
I    H
```

```
ATOM   4374  CD  ILE I 314      -8.527  -8.034 -13.450  1.00  0.00
I    C
ATOM   4375  HD1 ILE I 314      -8.731  -8.595 -14.388  1.00  0.00
I    H
ATOM   4376  HD2 ILE I 314      -7.427  -8.000 -13.296  1.00  0.00
I    H
ATOM   4377  HD3 ILE I 314      -8.983  -8.597 -12.608  1.00  0.00
I    H
ATOM   4378  C   ILE I 314      -9.146  -5.402  -9.825  1.00  0.00
I    C
ATOM   4379  O   ILE I 314      -8.357  -5.850  -8.995  1.00  0.00
I    O
ATOM   4380  N   VAL I 315      -9.776  -4.224  -9.613  1.00  0.00
I    N
ATOM   4381  HN  VAL I 315     -10.406  -3.849 -10.289  1.00  0.00
I    H
ATOM   4382  CA  VAL I 315      -9.613  -3.397  -8.415  1.00  0.00
I    C
ATOM   4383  HA  VAL I 315      -8.557  -3.165  -8.342  1.00  0.00
I    H
ATOM   4384  CB  VAL I 315     -10.377  -2.072  -8.524  1.00  0.00
I    C
ATOM   4385  HB  VAL I 315     -11.430  -2.313  -8.815  1.00  0.00
I    H
ATOM   4386  CG1 VAL I 315     -10.410  -1.294  -7.187  1.00  0.00
I    C
ATOM   4387 HG11 VAL I 315     -10.937  -0.325  -7.317  1.00  0.00
I    H
ATOM   4388 HG12 VAL I 315     -10.946  -1.859  -6.398  1.00  0.00
I    H
ATOM   4389 HG13 VAL I 315      -9.379  -1.079  -6.833  1.00  0.00
I    H
ATOM   4390  CG2 VAL I 315      -9.695  -1.191  -9.586  1.00  0.00
I    C
ATOM   4391 HG21 VAL I 315     -10.200  -0.204  -9.662  1.00  0.00
I    H
ATOM   4392 HG22 VAL I 315      -8.629  -1.018  -9.323  1.00  0.00
I    H
ATOM   4393 HG23 VAL I 315      -9.734  -1.666 -10.587  1.00  0.00
I    H
ATOM   4394  C   VAL I 315     -10.012  -4.120  -7.134  1.00  0.00
I    C
ATOM   4395  O   VAL I 315      -9.302  -4.079  -6.130  1.00  0.00
I    O
ATOM   4396  N   PHE I 316     -11.143  -4.850  -7.139  1.00  0.00
I    N
ATOM   4397  HN  PHE I 316     -11.770  -4.870  -7.917  1.00  0.00
I    H
ATOM   4398  CA  PHE I 316     -11.551  -5.672  -6.016  1.00  0.00
I    C
ATOM   4399  HA  PHE I 316     -11.540  -5.016  -5.155  1.00  0.00
I    H
ATOM   4400  CB  PHE I 316     -12.975  -6.224  -6.268  1.00  0.00
I    C
```

```
ATOM   4401  HB1 PHE I 316     -13.615  -5.408  -6.669  1.00  0.00
I    H
ATOM   4402  HB2 PHE I 316     -12.961  -7.034  -7.029  1.00  0.00
I    H
ATOM   4403  CG  PHE I 316     -13.573  -6.693  -4.973  1.00  0.00
I    C
ATOM   4404  CD1 PHE I 316     -14.011  -5.759  -4.020  1.00  0.00
I    C
ATOM   4405  HD1 PHE I 316     -13.938  -4.701  -4.235  1.00  0.00
I    H
ATOM   4406  CE1 PHE I 316     -14.534  -6.187  -2.793  1.00  0.00
I    C
ATOM   4407  HE1 PHE I 316     -14.885  -5.462  -2.072  1.00  0.00
I    H
ATOM   4408  CZ  PHE I 316     -14.607  -7.556  -2.505  1.00  0.00
I    C
ATOM   4409  HZ  PHE I 316     -15.022  -7.892  -1.565  1.00  0.00
I    H
ATOM   4410  CD2 PHE I 316     -13.648  -8.062  -4.676  1.00  0.00
I    C
ATOM   4411  HD2 PHE I 316     -13.299  -8.791  -5.394  1.00  0.00
I    H
ATOM   4412  CE2 PHE I 316     -14.149  -8.491  -3.442  1.00  0.00
I    C
ATOM   4413  HE2 PHE I 316     -14.181  -9.547  -3.211  1.00  0.00
I    H
ATOM   4414  C   PHE I 316     -10.564  -6.805  -5.730  1.00  0.00
I    C
ATOM   4415  O   PHE I 316     -10.162  -7.024  -4.590  1.00  0.00
I    O
ATOM   4416  N   THR I 317     -10.085  -7.494  -6.791  1.00  0.00
I    N
ATOM   4417  HN  THR I 317     -10.430  -7.314  -7.712  1.00  0.00
I    H
ATOM   4418  CA  THR I 317      -9.056  -8.535  -6.726  1.00  0.00
I    C
ATOM   4419  HA  THR I 317      -9.444  -9.283  -6.048  1.00  0.00
I    H
ATOM   4420  CB  THR I 317      -8.751  -9.189  -8.072  1.00  0.00
I    C
ATOM   4421  HB  THR I 317      -8.459  -8.360  -8.761  1.00  0.00
I    H
ATOM   4422  OG1 THR I 317      -9.930  -9.791  -8.581  1.00  0.00
I    O
ATOM   4423  HG1 THR I 317      -9.673 -10.193  -9.418  1.00  0.00
I    H
ATOM   4424  CG2 THR I 317      -7.758 -10.353  -7.943  1.00  0.00
I    C
ATOM   4425 HG21 THR I 317      -7.466 -10.736  -8.944  1.00  0.00
I    H
ATOM   4426 HG22 THR I 317      -6.834 -10.018  -7.423  1.00  0.00
I    H
ATOM   4427 HG23 THR I 317      -8.198 -11.193  -7.364  1.00  0.00
I    H
```

ATOM   4428 C   THR I 317   -7.766 -8.025 -6.113 1.00 0.00
I   C
ATOM   4429 O   THR I 317   -7.175 -8.713 -5.287 1.00 0.00
I   O
ATOM   4430 N   LEU I 318   -7.321 -6.782 -6.420 1.00 0.00
I   N
ATOM   4431 HN   LEU I 318   -7.788 -6.214 -7.095 1.00 0.00
I   H
ATOM   4432 CA   LEU I 318   -6.145 -6.156 -5.816 1.00 0.00
I   C
ATOM   4433 HA   LEU I 318   -5.311 -6.793 -6.077 1.00 0.00
I   H
ATOM   4434 CB   LEU I 318   -5.936 -4.705 -6.335 1.00 0.00
I   C
ATOM   4435 HB1 LEU I 318   -6.900 -4.163 -6.195 1.00 0.00
I   H
ATOM   4436 HB2 LEU I 318   -5.196 -4.191 -5.683 1.00 0.00
I   H
ATOM   4437 CG   LEU I 318   -5.519 -4.576 -7.811 1.00 0.00
I   C
ATOM   4438 HG   LEU I 318   -6.244 -5.176 -8.412 1.00 0.00
I   H
ATOM   4439 CD1 LEU I 318   -5.543 -3.105 -8.254 1.00 0.00
I   C
ATOM   4440 HD11 LEU I 318   -5.195 -3.008 -9.305 1.00 0.00
I   H
ATOM   4441 HD12 LEU I 318   -6.573 -2.693 -8.187 1.00 0.00
I   H
ATOM   4442 HD13 LEU I 318   -4.877 -2.490 -7.611 1.00 0.00
I   H
ATOM   4443 CD2 LEU I 318   -4.122 -5.144 -8.052 1.00 0.00
I   C
ATOM   4444 HD21 LEU I 318   -3.851 -5.058 -9.127 1.00 0.00
I   H
ATOM   4445 HD22 LEU I 318   -3.352 -4.604 -7.466 1.00 0.00
I   H
ATOM   4446 HD23 LEU I 318   -4.086 -6.219 -7.770 1.00 0.00
I   H
ATOM   4447 C   LEU I 318   -6.205 -6.076 -4.293 1.00 0.00
I   C
ATOM   4448 O   LEU I 318   -5.251 -6.425 -3.605 1.00 0.00
I   O
ATOM   4449 N   LEU I 319   -7.360 -5.668 -3.720 1.00 0.00
I   N
ATOM   4450 HN   LEU I 319   -8.127 -5.308 -4.248 1.00 0.00
I   H
ATOM   4451 CA   LEU I 319   -7.593 -5.716 -2.281 1.00 0.00
I   C
ATOM   4452 HA   LEU I 319   -6.766 -5.192 -1.819 1.00 0.00
I   H
ATOM   4453 CB   LEU I 319   -8.960 -5.068 -1.938 1.00 0.00
I   C
ATOM   4454 HB1 LEU I 319   -8.905 -3.999 -2.250 1.00 0.00
I   H

```
ATOM   4455  HB2 LEU I 319      -9.749  -5.533  -2.570  1.00  0.00
I    H
ATOM   4456  CG  LEU I 319      -9.376  -5.117  -0.448  1.00  0.00
I    C
ATOM   4457  HG  LEU I 319      -9.391  -6.198  -0.166  1.00  0.00
I    H
ATOM   4458  CD1 LEU I 319      -8.395  -4.354   0.458  1.00  0.00
I    C
ATOM   4459 HD11 LEU I 319      -8.747  -4.368   1.511  1.00  0.00
I    H
ATOM   4460 HD12 LEU I 319      -7.387  -4.819   0.420  1.00  0.00
I    H
ATOM   4461 HD13 LEU I 319      -8.307  -3.295   0.135  1.00  0.00
I    H
ATOM   4462  CD2 LEU I 319     -10.799  -4.563  -0.276  1.00  0.00
I    C
ATOM   4463 HD21 LEU I 319     -11.107  -4.615   0.790  1.00  0.00
I    H
ATOM   4464 HD22 LEU I 319     -10.867  -3.504  -0.595  1.00  0.00
I    H
ATOM   4465 HD23 LEU I 319     -11.523  -5.156  -0.876  1.00  0.00
I    H
ATOM   4466  C   LEU I 319      -7.582  -7.145  -1.754  1.00  0.00
I    C
ATOM   4467  O   LEU I 319      -6.968  -7.463  -0.736  1.00  0.00
I    O
ATOM   4468  N   LEU I 320      -8.238  -8.052  -2.497  1.00  0.00
I    N
ATOM   4469  HN  LEU I 320      -8.636  -7.779  -3.371  1.00  0.00
I    H
ATOM   4470  CA  LEU I 320      -8.442  -9.443  -2.160  1.00  0.00
I    C
ATOM   4471  HA  LEU I 320      -8.841  -9.430  -1.154  1.00  0.00
I    H
ATOM   4472  CB  LEU I 320      -9.445 -10.025  -3.182  1.00  0.00
I    C
ATOM   4473  HB1 LEU I 320     -10.019  -9.167  -3.604  1.00  0.00
I    H
ATOM   4474  HB2 LEU I 320      -8.880 -10.465  -4.034  1.00  0.00
I    H
ATOM   4475  CG  LEU I 320     -10.454 -11.052  -2.647  1.00  0.00
I    C
ATOM   4476  HG  LEU I 320      -9.902 -11.971  -2.333  1.00  0.00
I    H
ATOM   4477  CD1 LEU I 320     -11.282 -10.504  -1.471  1.00  0.00
I    C
ATOM   4478 HD11 LEU I 320     -12.033 -11.253  -1.139  1.00  0.00
I    H
ATOM   4479 HD12 LEU I 320     -10.624 -10.264  -0.607  1.00  0.00
I    H
ATOM   4480 HD13 LEU I 320     -11.822  -9.580  -1.768  1.00  0.00
I    H
ATOM   4481  CD2 LEU I 320     -11.390 -11.433  -3.800  1.00  0.00
I    C
```

```
ATOM   4482 HD21 LEU I 320     -12.129 -12.192  -3.463  1.00  0.00
I    H
ATOM   4483 HD22 LEU I 320     -11.955 -10.556  -4.176  1.00  0.00
I    H
ATOM   4484 HD23 LEU I 320     -10.812 -11.864  -4.646  1.00  0.00
I    H
ATOM   4485  C   LEU I 320      -7.181 -10.295  -2.144  1.00  0.00
I    C
ATOM   4486  O   LEU I 320      -7.167 -11.355  -1.524  1.00  0.00
I    O
ATOM   4487  N   MET I 321      -6.105  -9.845  -2.826  1.00  0.00
I    N
ATOM   4488  HN  MET I 321      -6.166  -9.057  -3.437  1.00  0.00
I    H
ATOM   4489  CA  MET I 321      -4.786 -10.441  -2.746  1.00  0.00
I    C
ATOM   4490  HA  MET I 321      -4.924 -11.356  -2.184  1.00  0.00
I    H
ATOM   4491  CB  MET I 321      -4.266 -10.817  -4.146  1.00  0.00
I    C
ATOM   4492  HB1 MET I 321      -3.276 -11.310  -4.003  1.00  0.00
I    H
ATOM   4493  HB2 MET I 321      -4.935 -11.585  -4.594  1.00  0.00
I    H
ATOM   4494  CG  MET I 321      -4.043  -9.736  -5.211  1.00  0.00
I    C
ATOM   4495  HG1 MET I 321      -4.890  -9.017  -5.216  1.00  0.00
I    H
ATOM   4496  HG2 MET I 321      -3.118  -9.165  -4.975  1.00  0.00
I    H
ATOM   4497  SD  MET I 321      -3.903 -10.525  -6.845  1.00  0.00
I    S
ATOM   4498  CE  MET I 321      -3.577  -9.015  -7.778  1.00  0.00
I    C
ATOM   4499  HE1 MET I 321      -3.457  -9.236  -8.860  1.00  0.00
I    H
ATOM   4500  HE2 MET I 321      -4.412  -8.290  -7.667  1.00  0.00
I    H
ATOM   4501  HE3 MET I 321      -2.646  -8.521  -7.426  1.00  0.00
I    H
ATOM   4502  C   MET I 321      -3.738  -9.612  -2.022  1.00  0.00
I    C
ATOM   4503  O   MET I 321      -2.616 -10.066  -1.808  1.00  0.00
I    O
ATOM   4504  N   PHE I 322      -4.072  -8.393  -1.553  1.00  0.00
I    N
ATOM   4505  HN  PHE I 322      -4.860  -7.869  -1.872  1.00  0.00
I    H
ATOM   4506  CA  PHE I 322      -3.288  -7.734  -0.522  1.00  0.00
I    C
ATOM   4507  HA  PHE I 322      -2.246  -7.843  -0.795  1.00  0.00
I    H
ATOM   4508  CB  PHE I 322      -3.712  -6.243  -0.403  1.00  0.00
I    C
```

```
ATOM   4509  HB1 PHE I 322      -3.660  -5.769  -1.406  1.00  0.00
I    H
ATOM   4510  HB2 PHE I 322      -4.763  -6.152  -0.052  1.00  0.00
I    H
ATOM   4511  CG  PHE I 322      -2.783  -5.468   0.505  1.00  0.00
I    C
ATOM   4512  CD1 PHE I 322      -1.401  -5.413   0.245  1.00  0.00
I    C
ATOM   4513  HD1 PHE I 322      -0.997  -5.932  -0.613  1.00  0.00
I    H
ATOM   4514  CE1 PHE I 322      -0.540  -4.698   1.087  1.00  0.00
I    C
ATOM   4515  HE1 PHE I 322       0.517  -4.653   0.867  1.00  0.00
I    H
ATOM   4516  CZ  PHE I 322      -1.052  -4.038   2.210  1.00  0.00
I    C
ATOM   4517  HZ  PHE I 322      -0.391  -3.486   2.864  1.00  0.00
I    H
ATOM   4518  CD2 PHE I 322      -3.285  -4.799   1.635  1.00  0.00
I    C
ATOM   4519  HD2 PHE I 322      -4.338  -4.850   1.869  1.00  0.00
I    H
ATOM   4520  CE2 PHE I 322      -2.425  -4.084   2.481  1.00  0.00
I    C
ATOM   4521  HE2 PHE I 322      -2.825  -3.569   3.344  1.00  0.00
I    H
ATOM   4522  C   PHE I 322      -3.484  -8.470   0.804  1.00  0.00
I    C
ATOM   4523  O   PHE I 322      -2.541  -8.778   1.535  1.00  0.00
I    O
ATOM   4524  N   ILE I 323      -4.759  -8.818   1.084  1.00  0.00
I    N
ATOM   4525  HN  ILE I 323      -5.518  -8.512   0.514  1.00  0.00
I    H
ATOM   4526  CA  ILE I 323      -5.154  -9.645   2.203  1.00  0.00
I    C
ATOM   4527  HA  ILE I 323      -4.451  -9.464   3.005  1.00  0.00
I    H
ATOM   4528  CB  ILE I 323      -6.612  -9.440   2.634  1.00  0.00
I    C
ATOM   4529  HB  ILE I 323      -7.214  -9.906   1.813  1.00  0.00
I    H
ATOM   4530  CG2 ILE I 323      -6.818 -10.117   4.011  1.00  0.00
I    C
ATOM   4531 HG21 ILE I 323      -7.894 -10.126   4.285  1.00  0.00
I    H
ATOM   4532 HG22 ILE I 323      -6.467 -11.170   3.992  1.00  0.00
I    H
ATOM   4533 HG23 ILE I 323      -6.263  -9.577   4.807  1.00  0.00
I    H
ATOM   4534  CG1 ILE I 323      -7.063  -7.960   2.682  1.00  0.00
I    C
ATOM   4535 HG11 ILE I 323      -6.743  -7.499   3.643  1.00  0.00
I    H
```

ATOM 4536 HG12 ILE I 323 -6.553 -7.401 1.864 1.00 0.00
I H
ATOM 4537 CD ILE I 323 -8.586 -7.834 2.512 1.00 0.00
I C
ATOM 4538 HD1 ILE I 323 -8.884 -6.764 2.490 1.00 0.00
I H
ATOM 4539 HD2 ILE I 323 -8.914 -8.306 1.561 1.00 0.00
I H
ATOM 4540 HD3 ILE I 323 -9.122 -8.323 3.352 1.00 0.00
I H
ATOM 4541 C ILE I 323 -5.002 -11.105 1.807 1.00 0.00
I C
ATOM 4542 O ILE I 323 -5.653 -11.605 0.900 1.00 0.00
I O
ATOM 4543 N GLY I 324 -4.136 -11.847 2.508 1.00 0.00
I N
ATOM 4544 HN GLY I 324 -3.553 -11.430 3.204 1.00 0.00
I H
ATOM 4545 CA GLY I 324 -3.973 -13.273 2.327 1.00 0.00
I C
ATOM 4546 HA1 GLY I 324 -2.984 -13.529 2.681 1.00 0.00
I H
ATOM 4547 HA2 GLY I 324 -4.138 -13.488 1.279 1.00 0.00
I H
ATOM 4548 C GLY I 324 -4.971 -14.063 3.124 1.00 0.00
I C
ATOM 4549 O GLY I 324 -6.142 -13.716 3.294 1.00 0.00
I O
ATOM 4550 N ALA I 325 -4.540 -15.203 3.664 1.00 0.00
I N
ATOM 4551 HN ALA I 325 -3.637 -15.594 3.487 1.00 0.00
I H
ATOM 4552 CA ALA I 325 -5.371 -15.956 4.557 1.00 0.00
I C
ATOM 4553 HA ALA I 325 -6.019 -15.327 5.157 1.00 0.00
I H
ATOM 4554 CB ALA I 325 -6.189 -16.984 3.743 1.00 0.00
I C
ATOM 4555 HB1 ALA I 325 -6.899 -16.450 3.077 1.00 0.00
I H
ATOM 4556 HB2 ALA I 325 -5.525 -17.607 3.104 1.00 0.00
I H
ATOM 4557 HB3 ALA I 325 -6.776 -17.656 4.404 1.00 0.00
I H
ATOM 4558 C ALA I 325 -4.438 -16.657 5.526 1.00 0.00
I C
ATOM 4559 O ALA I 325 -3.429 -17.193 5.098 1.00 0.00
I O
ATOM 4560 N GLY I 326 -4.734 -16.658 6.846 1.00 0.00
I N
ATOM 4561 HN GLY I 326 -5.420 -16.035 7.221 1.00 0.00
I H
ATOM 4562 CA GLY I 326 -4.091 -17.551 7.816 1.00 0.00
I C

```
ATOM   4563  HA1 GLY I 326      -4.187 -18.531   7.368  1.00  0.00
I    H
ATOM   4564  HA2 GLY I 326      -4.670 -17.418   8.720  1.00  0.00
I    H
ATOM   4565  C   GLY I 326      -2.633 -17.334   8.158  1.00  0.00
I    C
ATOM   4566  O   GLY I 326      -1.732 -17.493   7.353  1.00  0.00
I    O
ATOM   4567  N   SER I 327      -2.313 -16.998   9.416  1.00  0.00
I    N
ATOM   4568  HN  SER I 327      -2.968 -16.970  10.172  1.00  0.00
I    H
ATOM   4569  CA  SER I 327      -0.949 -16.639   9.793  1.00  0.00
I    C
ATOM   4570  HA  SER I 327      -0.691 -15.853   9.095  1.00  0.00
I    H
ATOM   4571  CB  SER I 327      -0.898 -16.077  11.232  1.00  0.00
I    C
ATOM   4572  HB1 SER I 327      -0.684 -16.921  11.926  1.00  0.00
I    H
ATOM   4573  HB2 SER I 327      -0.062 -15.349  11.335  1.00  0.00
I    H
ATOM   4574  OG  SER I 327      -2.161 -15.493  11.566  1.00  0.00
I    O
ATOM   4575  HG1 SER I 327      -2.167 -15.359  12.522  1.00  0.00
I    H
ATOM   4576  C   SER I 327       0.102 -17.725   9.657  1.00  0.00
I    C
ATOM   4577  O   SER I 327       1.207 -17.493   9.177  1.00  0.00
I    O
ATOM   4578  N   ALA I 328      -0.253 -18.956  10.064  1.00  0.00
I    N
ATOM   4579  HN  ALA I 328      -1.181 -19.116  10.398  1.00  0.00
I    H
ATOM   4580  CA  ALA I 328       0.616 -20.115  10.061  1.00  0.00
I    C
ATOM   4581  HA  ALA I 328       1.645 -19.814   9.900  1.00  0.00
I    H
ATOM   4582  CB  ALA I 328       0.478 -20.856  11.407  1.00  0.00
I    C
ATOM   4583  HB1 ALA I 328       0.777 -20.180  12.237  1.00  0.00
I    H
ATOM   4584  HB2 ALA I 328      -0.574 -21.170  11.585  1.00  0.00
I    H
ATOM   4585  HB3 ALA I 328       1.127 -21.757  11.447  1.00  0.00
I    H
ATOM   4586  C   ALA I 328       0.266 -21.081   8.930  1.00  0.00
I    C
ATOM   4587  O   ALA I 328       0.314 -22.298   9.108  1.00  0.00
I    O
ATOM   4588  N   SER I 329      -0.199 -20.573   7.774  1.00  0.00
I    N
ATOM   4589  HN  SER I 329      -0.236 -19.586   7.619  1.00  0.00
I    H
```

ATOM 4590 CA SER I 329 -0.685 -21.369 6.657 1.00 0.00
I C
ATOM 4591 HA SER I 329 -0.817 -22.370 7.048 1.00 0.00
I H
ATOM 4592 CB SER I 329 -2.019 -20.770 6.143 1.00 0.00
I C
ATOM 4593 HB1 SER I 329 -2.501 -21.520 5.475 1.00 0.00
I H
ATOM 4594 HB2 SER I 329 -2.717 -20.592 6.993 1.00 0.00
I H
ATOM 4595 OG SER I 329 -1.831 -19.573 5.398 1.00 0.00
I O
ATOM 4596 HG1 SER I 329 -2.660 -19.395 4.935 1.00 0.00
I H
ATOM 4597 C SER I 329 0.339 -21.455 5.527 1.00 0.00
I C
ATOM 4598 O SER I 329 1.467 -20.966 5.629 1.00 0.00
I O
ATOM 4599 N THR I 330 -0.026 -22.087 4.390 1.00 0.00
I N
ATOM 4600 HN THR I 330 -0.879 -22.600 4.306 1.00 0.00
I H
ATOM 4601 CA THR I 330 0.791 -22.079 3.179 1.00 0.00
I C
ATOM 4602 HA THR I 330 1.818 -22.000 3.508 1.00 0.00
I H
ATOM 4603 CB THR I 330 0.640 -23.303 2.267 1.00 0.00
I C
ATOM 4604 HB THR I 330 0.922 -22.963 1.242 1.00 0.00
I H
ATOM 4605 OG1 THR I 330 -0.706 -23.738 2.212 1.00 0.00
I O
ATOM 4606 HG1 THR I 330 -0.704 -24.508 1.633 1.00 0.00
I H
ATOM 4607 CG2 THR I 330 1.432 -24.466 2.868 1.00 0.00
I C
ATOM 4608 HG21 THR I 330 1.440 -25.337 2.179 1.00 0.00
I H
ATOM 4609 HG22 THR I 330 2.486 -24.165 3.049 1.00 0.00
I H
ATOM 4610 HG23 THR I 330 0.993 -24.793 3.835 1.00 0.00
I H
ATOM 4611 C THR I 330 0.506 -20.842 2.357 1.00 0.00
I C
ATOM 4612 O THR I 330 1.265 -20.536 1.440 1.00 0.00
I O
ATOM 4613 N ALA I 331 -0.519 -20.043 2.691 1.00 0.00
I N
ATOM 4614 HN ALA I 331 -1.150 -20.309 3.419 1.00 0.00
I H
ATOM 4615 CA ALA I 331 -0.802 -18.772 2.058 1.00 0.00
I C
ATOM 4616 HA ALA I 331 -0.619 -18.854 0.992 1.00 0.00
I H

```
ATOM   4617  CB  ALA I 331      -2.304 -18.480   2.243  1.00  0.00
I    C
ATOM   4618  HB1 ALA I 331      -2.901 -19.317   1.821  1.00  0.00
I    H
ATOM   4619  HB2 ALA I 331      -2.563 -18.385   3.320  1.00  0.00
I    H
ATOM   4620  HB3 ALA I 331      -2.607 -17.546   1.722  1.00  0.00
I    H
ATOM   4621  C   ALA I 331       0.085 -17.610   2.566  1.00  0.00
I    C
ATOM   4622  O   ALA I 331       0.802 -17.729   3.560  1.00  0.00
I    O
ATOM   4623  N   SER I 332       0.113 -16.445   1.869  1.00  0.00
I    N
ATOM   4624  HN  SER I 332      -0.438 -16.287   1.049  1.00  0.00
I    H
ATOM   4625  CA  SER I 332       0.958 -15.303   2.256  1.00  0.00
I    C
ATOM   4626  HA  SER I 332       1.049 -15.379   3.331  1.00  0.00
I    H
ATOM   4627  CB  SER I 332       2.440 -15.391   1.779  1.00  0.00
I    C
ATOM   4628  HB1 SER I 332       2.770 -16.452   1.868  1.00  0.00
I    H
ATOM   4629  HB2 SER I 332       2.519 -15.114   0.704  1.00  0.00
I    H
ATOM   4630  OG  SER I 332       3.297 -14.573   2.588  1.00  0.00
I    O
ATOM   4631  HG1 SER I 332       4.206 -14.837   2.396  1.00  0.00
I    H
ATOM   4632  C   SER I 332       0.339 -13.935   1.946  1.00  0.00
I    C
ATOM   4633  O   SER I 332      -0.833 -13.815   1.607  1.00  0.00
I    O
ATOM   4634  N   GLY I 333       1.085 -12.838   2.175  1.00  0.00
I    N
ATOM   4635  HN  GLY I 333       2.033 -12.956   2.466  1.00  0.00
I    H
ATOM   4636  CA  GLY I 333       0.634 -11.453   2.036  1.00  0.00
I    C
ATOM   4637  HA1 GLY I 333      -0.329 -11.454   1.544  1.00  0.00
I    H
ATOM   4638  HA2 GLY I 333       1.398 -10.890   1.516  1.00  0.00
I    H
ATOM   4639  C   GLY I 333       0.460 -10.847   3.398  1.00  0.00
I    C
ATOM   4640  O   GLY I 333       1.101 -11.281   4.354  1.00  0.00
I    O
ATOM   4641  N   ILE I 334      -0.424  -9.838   3.576  1.00  0.00
I    N
ATOM   4642  HN  ILE I 334      -0.856  -9.306   2.850  1.00  0.00
I    H
ATOM   4643  CA  ILE I 334      -0.824  -9.444   4.925  1.00  0.00
I    C
```

```
ATOM   4644  HA  ILE I 334       0.017  -9.601   5.589  1.00  0.00
I    H
ATOM   4645  CB  ILE I 334      -1.281  -7.984   5.108  1.00  0.00
I    C
ATOM   4646  HB  ILE I 334      -1.124  -7.776   6.197  1.00  0.00
I    H
ATOM   4647  CG2 ILE I 334      -0.389  -7.097   4.216  1.00  0.00
I    C
ATOM   4648 HG21 ILE I 334      -0.571  -6.023   4.427  1.00  0.00
I    H
ATOM   4649 HG22 ILE I 334       0.686  -7.299   4.405  1.00  0.00
I    H
ATOM   4650 HG23 ILE I 334      -0.596  -7.280   3.140  1.00  0.00
I    H
ATOM   4651  CG1 ILE I 334      -2.792  -7.691   4.874  1.00  0.00
I    C
ATOM   4652 HG11 ILE I 334      -3.032  -7.789   3.792  1.00  0.00
I    H
ATOM   4653 HG12 ILE I 334      -3.396  -8.454   5.416  1.00  0.00
I    H
ATOM   4654  CD  ILE I 334      -3.225  -6.300   5.362  1.00  0.00
I    C
ATOM   4655  HD1 ILE I 334      -4.320  -6.167   5.224  1.00  0.00
I    H
ATOM   4656  HD2 ILE I 334      -2.993  -6.172   6.441  1.00  0.00
I    H
ATOM   4657  HD3 ILE I 334      -2.707  -5.499   4.793  1.00  0.00
I    H
ATOM   4658  C   ILE I 334      -1.902 -10.407   5.353  1.00  0.00
I    C
ATOM   4659  O   ILE I 334      -2.724 -10.821   4.540  1.00  0.00
I    O
ATOM   4660  N   LYS I 335      -1.958 -10.863   6.607  1.00  0.00
I    N
ATOM   4661  HN  LYS I 335      -1.393 -10.566   7.376  1.00  0.00
I    H
ATOM   4662  CA  LYS I 335      -2.923 -11.891   6.918  1.00  0.00
I    C
ATOM   4663  HA  LYS I 335      -2.989 -12.515   6.034  1.00  0.00
I    H
ATOM   4664  CB  LYS I 335      -2.415 -12.814   8.031  1.00  0.00
I    C
ATOM   4665  HB1 LYS I 335      -2.417 -12.221   8.975  1.00  0.00
I    H
ATOM   4666  HB2 LYS I 335      -3.147 -13.639   8.179  1.00  0.00
I    H
ATOM   4667  CG  LYS I 335      -0.999 -13.328   7.713  1.00  0.00
I    C
ATOM   4668  HG1 LYS I 335      -0.321 -12.458   7.561  1.00  0.00
I    H
ATOM   4669  HG2 LYS I 335      -0.616 -13.899   8.589  1.00  0.00
I    H
ATOM   4670  CD  LYS I 335      -0.856 -14.223   6.461  1.00  0.00
I    C
```

ATOM 4671 HD1 LYS I 335 -1.283 -15.225 6.695 1.00 0.00
I H
ATOM 4672 HD2 LYS I 335 -1.472 -13.780 5.645 1.00 0.00
I H
ATOM 4673 CE LYS I 335 0.581 -14.413 5.934 1.00 0.00
I C
ATOM 4674 HE1 LYS I 335 0.659 -15.353 5.343 1.00 0.00
I H
ATOM 4675 HE2 LYS I 335 0.873 -13.561 5.281 1.00 0.00
I H
ATOM 4676 NZ LYS I 335 1.588 -14.496 7.004 1.00 0.00
I N
ATOM 4677 HZ1 LYS I 335 2.535 -14.625 6.592 1.00 0.00
I H
ATOM 4678 HZ2 LYS I 335 1.571 -13.621 7.566 1.00 0.00
I H
ATOM 4679 HZ3 LYS I 335 1.369 -15.304 7.622 1.00 0.00
I H
ATOM 4680 C LYS I 335 -4.306 -11.361 7.198 1.00 0.00
I C
ATOM 4681 O LYS I 335 -4.540 -10.186 7.466 1.00 0.00
I O
ATOM 4682 N LEU I 336 -5.296 -12.261 7.117 1.00 0.00
I N
ATOM 4683 HN LEU I 336 -5.141 -13.224 6.902 1.00 0.00
I H
ATOM 4684 CA LEU I 336 -6.683 -11.918 7.334 1.00 0.00
I C
ATOM 4685 HA LEU I 336 -6.907 -11.127 6.631 1.00 0.00
I H
ATOM 4686 CB LEU I 336 -7.556 -13.143 7.023 1.00 0.00
I C
ATOM 4687 HB1 LEU I 336 -7.299 -13.478 5.991 1.00 0.00
I H
ATOM 4688 HB2 LEU I 336 -7.264 -13.978 7.698 1.00 0.00
I H
ATOM 4689 CG LEU I 336 -9.064 -12.874 7.103 1.00 0.00
I C
ATOM 4690 HG LEU I 336 -9.304 -12.634 8.167 1.00 0.00
I H
ATOM 4691 CD1 LEU I 336 -9.512 -11.723 6.188 1.00 0.00
I C
ATOM 4692 HD11 LEU I 336 -10.614 -11.591 6.238 1.00 0.00
I H
ATOM 4693 HD12 LEU I 336 -9.032 -10.769 6.496 1.00 0.00
I H
ATOM 4694 HD13 LEU I 336 -9.236 -11.930 5.133 1.00 0.00
I H
ATOM 4695 CD2 LEU I 336 -9.810 -14.152 6.728 1.00 0.00
I C
ATOM 4696 HD21 LEU I 336 -10.908 -13.991 6.796 1.00 0.00
I H
ATOM 4697 HD22 LEU I 336 -9.581 -14.472 5.692 1.00 0.00
I H

```
ATOM   4698 HD23 LEU I 336      -9.537 -14.979   7.418  1.00  0.00
I    H
ATOM   4699  C   LEU I 336      -6.943 -11.378   8.732  1.00  0.00
I    C
ATOM   4700  O   LEU I 336      -7.634 -10.379   8.916  1.00  0.00
I    O
ATOM   4701  N   THR I 337      -6.299 -11.968   9.754  1.00  0.00
I    N
ATOM   4702  HN  THR I 337      -5.784 -12.814   9.621  1.00  0.00
I    H
ATOM   4703  CA  THR I 337      -6.278 -11.464  11.116  1.00  0.00
I    C
ATOM   4704  HA  THR I 337      -7.318 -11.384  11.402  1.00  0.00
I    H
ATOM   4705  CB  THR I 337      -5.492 -12.372  12.043  1.00  0.00
I    C
ATOM   4706  HB  THR I 337      -5.113 -11.721  12.868  1.00  0.00
I    H
ATOM   4707  OG1 THR I 337      -4.359 -12.919  11.376  1.00  0.00
I    O
ATOM   4708  HG1 THR I 337      -3.928 -13.485  12.025  1.00  0.00
I    H
ATOM   4709  CG2 THR I 337      -6.407 -13.538  12.432  1.00  0.00
I    C
ATOM   4710 HG21 THR I 337      -5.926 -14.179  13.201  1.00  0.00
I    H
ATOM   4711 HG22 THR I 337      -7.361 -13.156  12.854  1.00  0.00
I    H
ATOM   4712 HG23 THR I 337      -6.645 -14.171  11.551  1.00  0.00
I    H
ATOM   4713  C   THR I 337      -5.693 -10.075  11.228  1.00  0.00
I    C
ATOM   4714  O   THR I 337      -6.231  -9.244  11.943  1.00  0.00
I    O
ATOM   4715  N   THR I 338      -4.610  -9.759  10.493  1.00  0.00
I    N
ATOM   4716  HN  THR I 338      -4.137 -10.447   9.944  1.00  0.00
I    H
ATOM   4717  CA  THR I 338      -4.020  -8.416  10.418  1.00  0.00
I    C
ATOM   4718  HA  THR I 338      -3.800  -8.151  11.443  1.00  0.00
I    H
ATOM   4719  CB  THR I 338      -2.802  -8.381   9.507  1.00  0.00
I    C
ATOM   4720  HB  THR I 338      -3.171  -8.638   8.484  1.00  0.00
I    H
ATOM   4721  OG1 THR I 338      -1.926  -9.400   9.955  1.00  0.00
I    O
ATOM   4722  HG1 THR I 338      -1.159  -9.353   9.375  1.00  0.00
I    H
ATOM   4723  CG2 THR I 338      -2.053  -7.039   9.558  1.00  0.00
I    C
ATOM   4724 HG21 THR I 338      -1.241  -7.010   8.801  1.00  0.00
I    H
```

```
ATOM   4725 HG22 THR I 338      -2.748  -6.199   9.345  1.00  0.00
I    H
ATOM   4726 HG23 THR I 338      -1.601  -6.870  10.559  1.00  0.00
I    H
ATOM   4727  C   THR I 338      -4.973  -7.371   9.900  1.00  0.00
I    C
ATOM   4728  O   THR I 338      -5.111  -6.292  10.480  1.00  0.00
I    O
ATOM   4729  N   PHE I 339      -5.697  -7.696   8.812  1.00  0.00
I    N
ATOM   4730  HN  PHE I 339      -5.540  -8.555   8.328  1.00  0.00
I    H
ATOM   4731  CA  PHE I 339      -6.743  -6.871   8.245  1.00  0.00
I    C
ATOM   4732  HA  PHE I 339      -6.291  -5.900   8.074  1.00  0.00
I    H
ATOM   4733  CB  PHE I 339      -7.207  -7.542   6.918  1.00  0.00
I    C
ATOM   4734  HB1 PHE I 339      -6.339  -7.623   6.228  1.00  0.00
I    H
ATOM   4735  HB2 PHE I 339      -7.577  -8.575   7.100  1.00  0.00
I    H
ATOM   4736  CG  PHE I 339      -8.260  -6.738   6.198  1.00  0.00
I    C
ATOM   4737  CD1 PHE I 339      -7.923  -5.536   5.556  1.00  0.00
I    C
ATOM   4738  HD1 PHE I 339      -6.894  -5.202   5.551  1.00  0.00
I    H
ATOM   4739  CE1 PHE I 339      -8.908  -4.762   4.928  1.00  0.00
I    C
ATOM   4740  HE1 PHE I 339      -8.637  -3.841   4.432  1.00  0.00
I    H
ATOM   4741  CZ  PHE I 339     -10.243  -5.187   4.937  1.00  0.00
I    C
ATOM   4742  HZ  PHE I 339     -11.002  -4.594   4.448  1.00  0.00
I    H
ATOM   4743  CD2 PHE I 339      -9.602  -7.157   6.199  1.00  0.00
I    C
ATOM   4744  HD2 PHE I 339      -9.883  -8.070   6.704  1.00  0.00
I    H
ATOM   4745  CE2 PHE I 339     -10.590  -6.385   5.573  1.00  0.00
I    C
ATOM   4746  HE2 PHE I 339     -11.620  -6.716   5.583  1.00  0.00
I    H
ATOM   4747  C   PHE I 339      -7.910  -6.659   9.219  1.00  0.00
I    C
ATOM   4748  O   PHE I 339      -8.332  -5.531   9.461  1.00  0.00
I    O
ATOM   4749  N   ILE I 340      -8.417  -7.740   9.860  1.00  0.00
I    N
ATOM   4750  HN  ILE I 340      -8.084  -8.666   9.692  1.00  0.00
I    H
ATOM   4751  CA  ILE I 340      -9.492  -7.661  10.849  1.00  0.00
I    C
```

```
ATOM   4752  HA  ILE I 340     -10.291  -7.111  10.369  1.00  0.00
I    H
ATOM   4753  CB  ILE I 340     -10.036  -9.041  11.239  1.00  0.00
I    C
ATOM   4754  HB  ILE I 340      -9.127  -9.629  11.523  1.00  0.00
I    H
ATOM   4755  CG2 ILE I 340     -11.093  -8.904  12.365  1.00  0.00
I    C
ATOM   4756 HG21 ILE I 340     -11.375  -9.903  12.760  1.00  0.00
I    H
ATOM   4757 HG22 ILE I 340     -10.694  -8.308  13.213  1.00  0.00
I    H
ATOM   4758 HG23 ILE I 340     -12.011  -8.407  11.990  1.00  0.00
I    H
ATOM   4759  CG1 ILE I 340     -10.656  -9.734   9.999  1.00  0.00
I    C
ATOM   4760 HG11 ILE I 340     -11.552  -9.167   9.659  1.00  0.00
I    H
ATOM   4761 HG12 ILE I 340      -9.918  -9.712   9.165  1.00  0.00
I    H
ATOM   4762  CD  ILE I 340     -11.053 -11.196  10.248  1.00  0.00
I    C
ATOM   4763  HD1 ILE I 340     -11.437 -11.657   9.313  1.00  0.00
I    H
ATOM   4764  HD2 ILE I 340     -10.177 -11.787  10.594  1.00  0.00
I    H
ATOM   4765  HD3 ILE I 340     -11.850 -11.266  11.018  1.00  0.00
I    H
ATOM   4766  C   ILE I 340      -9.094  -6.868  12.091  1.00  0.00
I    C
ATOM   4767  O   ILE I 340      -9.853  -6.009  12.548  1.00  0.00
I    O
ATOM   4768  N   VAL I 341      -7.879  -7.095  12.644  1.00  0.00
I    N
ATOM   4769  HN  VAL I 341      -7.277  -7.787  12.255  1.00  0.00
I    H
ATOM   4770  CA  VAL I 341      -7.328  -6.400  13.808  1.00  0.00
I    C
ATOM   4771  HA  VAL I 341      -8.014  -6.557  14.630  1.00  0.00
I    H
ATOM   4772  CB  VAL I 341      -5.946  -6.964  14.190  1.00  0.00
I    C
ATOM   4773  HB  VAL I 341      -5.373  -7.124  13.244  1.00  0.00
I    H
ATOM   4774  CG1 VAL I 341      -5.122  -6.011  15.087  1.00  0.00
I    C
ATOM   4775 HG11 VAL I 341      -4.155  -6.483  15.364  1.00  0.00
I    H
ATOM   4776 HG12 VAL I 341      -4.889  -5.058  14.568  1.00  0.00
I    H
ATOM   4777 HG13 VAL I 341      -5.672  -5.780  16.025  1.00  0.00
I    H
ATOM   4778  CG2 VAL I 341      -6.142  -8.305  14.930  1.00  0.00
I    C
```

```
ATOM   4779 HG21 VAL I 341      -5.164  -8.721  15.256  1.00  0.00
I    H
ATOM   4780 HG22 VAL I 341      -6.780  -8.166  15.829  1.00  0.00
I    H
ATOM   4781 HG23 VAL I 341      -6.624  -9.059  14.274  1.00  0.00
I    H
ATOM   4782  C   VAL I 341      -7.237  -4.904  13.571  1.00  0.00
I    C
ATOM   4783  O   VAL I 341      -7.681  -4.108  14.392  1.00  0.00
I    O
ATOM   4784  N   ILE I 342      -6.707  -4.474  12.405  1.00  0.00
I    N
ATOM   4785  HN  ILE I 342      -6.307  -5.081  11.722  1.00  0.00
I    H
ATOM   4786  CA  ILE I 342      -6.672  -3.064  12.044  1.00  0.00
I    C
ATOM   4787  HA  ILE I 342      -6.221  -2.553  12.885  1.00  0.00
I    H
ATOM   4788  CB  ILE I 342      -5.772  -2.834  10.827  1.00  0.00
I    C
ATOM   4789  HB  ILE I 342      -5.868  -3.773  10.226  1.00  0.00
I    H
ATOM   4790  CG2 ILE I 342      -6.152  -1.580   9.998  1.00  0.00
I    C
ATOM   4791 HG21 ILE I 342      -5.574  -1.548   9.051  1.00  0.00
I    H
ATOM   4792 HG22 ILE I 342      -7.230  -1.592   9.734  1.00  0.00
I    H
ATOM   4793 HG23 ILE I 342      -5.940  -0.649  10.565  1.00  0.00
I    H
ATOM   4794  CG1 ILE I 342      -4.319  -2.740  11.342  1.00  0.00
I    C
ATOM   4795 HG11 ILE I 342      -4.205  -1.841  11.987  1.00  0.00
I    H
ATOM   4796 HG12 ILE I 342      -4.103  -3.630  11.977  1.00  0.00
I    H
ATOM   4797  CD  ILE I 342      -3.299  -2.689  10.207  1.00  0.00
I    C
ATOM   4798  HD1 ILE I 342      -2.266  -2.681  10.617  1.00  0.00
I    H
ATOM   4799  HD2 ILE I 342      -3.405  -3.576   9.546  1.00  0.00
I    H
ATOM   4800  HD3 ILE I 342      -3.432  -1.774   9.593  1.00  0.00
I    H
ATOM   4801  C   ILE I 342      -8.060  -2.483  11.871  1.00  0.00
I    C
ATOM   4802  O   ILE I 342      -8.372  -1.436  12.441  1.00  0.00
I    O
ATOM   4803  N   LEU I 343      -8.959  -3.170  11.141  1.00  0.00
I    N
ATOM   4804  HN  LEU I 343      -8.763  -4.069  10.755  1.00  0.00
I    H
ATOM   4805  CA  LEU I 343     -10.287  -2.673  10.848  1.00  0.00
I    C
```

ATOM 4806 HA LEU I 343 -10.143 -1.700 10.397 1.00 0.00
I H
ATOM 4807 CB LEU I 343 -10.947 -3.627 9.823 1.00 0.00
I C
ATOM 4808 HB1 LEU I 343 -10.221 -3.766 8.989 1.00 0.00
I H
ATOM 4809 HB2 LEU I 343 -11.074 -4.629 10.290 1.00 0.00
I H
ATOM 4810 CG LEU I 343 -12.286 -3.147 9.226 1.00 0.00
I C
ATOM 4811 HG LEU I 343 -12.256 -2.031 9.234 1.00 0.00
I H
ATOM 4812 CD1 LEU I 343 -12.429 -3.666 7.788 1.00 0.00
I C
ATOM 4813 HD11 LEU I 343 -13.411 -3.368 7.362 1.00 0.00
I H
ATOM 4814 HD12 LEU I 343 -11.627 -3.250 7.140 1.00 0.00
I H
ATOM 4815 HD13 LEU I 343 -12.362 -4.774 7.761 1.00 0.00
I H
ATOM 4816 CD2 LEU I 343 -13.502 -3.597 10.052 1.00 0.00
I C
ATOM 4817 HD21 LEU I 343 -14.441 -3.214 9.597 1.00 0.00
I H
ATOM 4818 HD22 LEU I 343 -13.578 -4.702 10.100 1.00 0.00
I H
ATOM 4819 HD23 LEU I 343 -13.434 -3.205 11.090 1.00 0.00
I H
ATOM 4820 C LEU I 343 -11.150 -2.483 12.091 1.00 0.00
I C
ATOM 4821 O LEU I 343 -11.753 -1.430 12.296 1.00 0.00
I O
ATOM 4822 N THR I 344 -11.178 -3.479 13.005 1.00 0.00
I N
ATOM 4823 HN THR I 344 -10.696 -4.345 12.882 1.00 0.00
I H
ATOM 4824 CA THR I 344 -11.922 -3.378 14.262 1.00 0.00
I C
ATOM 4825 HA THR I 344 -12.909 -3.037 13.979 1.00 0.00
I H
ATOM 4826 CB THR I 344 -12.107 -4.672 15.045 1.00 0.00
I C
ATOM 4827 HB THR I 344 -12.580 -4.383 16.015 1.00 0.00
I H
ATOM 4828 OG1 THR I 344 -10.880 -5.306 15.366 1.00 0.00
I O
ATOM 4829 HG1 THR I 344 -11.123 -6.108 15.843 1.00 0.00
I H
ATOM 4830 CG2 THR I 344 -12.947 -5.636 14.200 1.00 0.00
I C
ATOM 4831 HG21 THR I 344 -13.202 -6.549 14.779 1.00 0.00
I H
ATOM 4832 HG22 THR I 344 -13.897 -5.152 13.890 1.00 0.00
I H

```
ATOM   4833 HG23 THR I 344     -12.400  -5.946  13.284  1.00  0.00
I    H
ATOM   4834  C   THR I 344     -11.360  -2.337  15.197  1.00  0.00
I    C
ATOM   4835  O   THR I 344     -12.115  -1.660  15.890  1.00  0.00
I    O
ATOM   4836  N   SER I 345     -10.021  -2.182  15.228  1.00  0.00
I    N
ATOM   4837  HN  SER I 345      -9.429  -2.811  14.726  1.00  0.00
I    H
ATOM   4838  CA  SER I 345      -9.305  -1.138  15.957  1.00  0.00
I    C
ATOM   4839  HA  SER I 345      -9.657  -1.216  16.977  1.00  0.00
I    H
ATOM   4840  CB  SER I 345      -7.774  -1.406  15.882  1.00  0.00
I    C
ATOM   4841  HB1 SER I 345      -7.593  -2.465  16.176  1.00  0.00
I    H
ATOM   4842  HB2 SER I 345      -7.410  -1.289  14.836  1.00  0.00
I    H
ATOM   4843  OG  SER I 345      -7.008  -0.581  16.755  1.00  0.00
I    O
ATOM   4844  HG1 SER I 345      -6.124  -0.965  16.804  1.00  0.00
I    H
ATOM   4845  C   SER I 345      -9.625   0.266  15.474  1.00  0.00
I    C
ATOM   4846  O   SER I 345      -9.957   1.154  16.258  1.00  0.00
I    O
ATOM   4847  N   VAL I 346      -9.629   0.493  14.145  1.00  0.00
I    N
ATOM   4848  HN  VAL I 346      -9.353  -0.198  13.483  1.00  0.00
I    H
ATOM   4849  CA  VAL I 346     -10.034   1.763  13.557  1.00  0.00
I    C
ATOM   4850  HA  VAL I 346      -9.431   2.534  14.019  1.00  0.00
I    H
ATOM   4851  CB  VAL I 346      -9.737   1.765  12.057  1.00  0.00
I    C
ATOM   4852  HB  VAL I 346     -10.140   0.812  11.632  1.00  0.00
I    H
ATOM   4853  CG1 VAL I 346     -10.407   2.942  11.315  1.00  0.00
I    C
ATOM   4854 HG11 VAL I 346     -10.132   2.925  10.238  1.00  0.00
I    H
ATOM   4855 HG12 VAL I 346     -11.514   2.888  11.374  1.00  0.00
I    H
ATOM   4856 HG13 VAL I 346     -10.074   3.913  11.739  1.00  0.00
I    H
ATOM   4857  CG2 VAL I 346      -8.207   1.849  11.905  1.00  0.00
I    C
ATOM   4858 HG21 VAL I 346      -7.922   1.894  10.832  1.00  0.00
I    H
ATOM   4859 HG22 VAL I 346      -7.814   2.757  12.411  1.00  0.00
I    H
```

```
ATOM   4860 HG23 VAL I 346      -7.709   0.960  12.346  1.00  0.00
I    H
ATOM   4861  C   VAL I 346     -11.491   2.087  13.849  1.00  0.00
I    C
ATOM   4862  O   VAL I 346     -11.814   3.179  14.317  1.00  0.00
I    O
ATOM   4863  N   ILE I 347     -12.416   1.122  13.673  1.00  0.00
I    N
ATOM   4864  HN  ILE I 347     -12.203   0.218  13.306  1.00  0.00
I    H
ATOM   4865  CA  ILE I 347     -13.822   1.314  14.006  1.00  0.00
I    C
ATOM   4866  HA  ILE I 347     -14.118   2.236  13.520  1.00  0.00
I    H
ATOM   4867  CB  ILE I 347     -14.733   0.254  13.382  1.00  0.00
I    C
ATOM   4868  HB  ILE I 347     -14.269  -0.721  13.676  1.00  0.00
I    H
ATOM   4869  CG2 ILE I 347     -16.197   0.449  13.852  1.00  0.00
I    C
ATOM   4870 HG21 ILE I 347     -16.828  -0.405  13.529  1.00  0.00
I    H
ATOM   4871 HG22 ILE I 347     -16.253   0.510  14.959  1.00  0.00
I    H
ATOM   4872 HG23 ILE I 347     -16.629   1.381  13.428  1.00  0.00
I    H
ATOM   4873  CG1 ILE I 347     -14.638   0.373  11.839  1.00  0.00
I    C
ATOM   4874 HG11 ILE I 347     -14.975   1.384  11.517  1.00  0.00
I    H
ATOM   4875 HG12 ILE I 347     -13.571   0.270  11.534  1.00  0.00
I    H
ATOM   4876  CD  ILE I 347     -15.450  -0.683  11.080  1.00  0.00
I    C
ATOM   4877  HD1 ILE I 347     -15.293  -0.577   9.984  1.00  0.00
I    H
ATOM   4878  HD2 ILE I 347     -15.138  -1.707  11.379  1.00  0.00
I    H
ATOM   4879  HD3 ILE I 347     -16.536  -0.572  11.283  1.00  0.00
I    H
ATOM   4880  C   ILE I 347     -14.041   1.515  15.506  1.00  0.00
I    C
ATOM   4881  O   ILE I 347     -14.880   2.311  15.916  1.00  0.00
I    O
ATOM   4882  N   ALA I 348     -13.260   0.853  16.387  1.00  0.00
I    N
ATOM   4883  HN  ALA I 348     -12.605   0.163  16.082  1.00  0.00
I    H
ATOM   4884  CA  ALA I 348     -13.303   1.083  17.822  1.00  0.00
I    C
ATOM   4885  HA  ALA I 348     -14.299   0.869  18.192  1.00  0.00
I    H
ATOM   4886  CB  ALA I 348     -12.302   0.146  18.538  1.00  0.00
I    C
```

```
ATOM    4887  HB1 ALA I 348     -12.544  -0.912  18.299  1.00  0.00
I    H
ATOM    4888  HB2 ALA I 348     -11.260   0.343  18.203  1.00  0.00
I    H
ATOM    4889  HB3 ALA I 348     -12.347   0.267  19.642  1.00  0.00
I    H
ATOM    4890  C   ALA I 348     -12.996   2.526  18.218  1.00  0.00
I    C
ATOM    4891  O   ALA I 348     -13.733   3.122  19.003  1.00  0.00
I    O
ATOM    4892  N   TYR I 349     -11.951   3.128  17.607  1.00  0.00
I    N
ATOM    4893  HN  TYR I 349     -11.333   2.597  17.029  1.00  0.00
I    H
ATOM    4894  CA  TYR I 349     -11.618   4.541  17.711  1.00  0.00
I    C
ATOM    4895  HA  TYR I 349     -11.507   4.748  18.768  1.00  0.00
I    H
ATOM    4896  CB  TYR I 349     -10.269   4.812  16.964  1.00  0.00
I    C
ATOM    4897  HB1 TYR I 349      -9.469   4.189  17.420  1.00  0.00
I    H
ATOM    4898  HB2 TYR I 349     -10.345   4.517  15.895  1.00  0.00
I    H
ATOM    4899  CG  TYR I 349      -9.848   6.255  17.082  1.00  0.00
I    C
ATOM    4900  CD1 TYR I 349      -9.124   6.718  18.194  1.00  0.00
I    C
ATOM    4901  HD1 TYR I 349      -8.787   6.018  18.946  1.00  0.00
I    H
ATOM    4902  CE1 TYR I 349      -8.854   8.091  18.348  1.00  0.00
I    C
ATOM    4903  HE1 TYR I 349      -8.279   8.447  19.190  1.00  0.00
I    H
ATOM    4904  CZ  TYR I 349      -9.340   9.005  17.405  1.00  0.00
I    C
ATOM    4905  OH  TYR I 349      -9.175  10.396  17.559  1.00  0.00
I    O
ATOM    4906  HH  TYR I 349      -9.519  10.816  16.768  1.00  0.00
I    H
ATOM    4907  CD2 TYR I 349     -10.295   7.182  16.127  1.00  0.00
I    C
ATOM    4908  HD2 TYR I 349     -10.850   6.843  15.264  1.00  0.00
I    H
ATOM    4909  CE2 TYR I 349     -10.071   8.550  16.306  1.00  0.00
I    C
ATOM    4910  HE2 TYR I 349     -10.484   9.257  15.601  1.00  0.00
I    H
ATOM    4911  C   TYR I 349     -12.750   5.440  17.189  1.00  0.00
I    C
ATOM    4912  O   TYR I 349     -13.133   6.413  17.838  1.00  0.00
I    O
ATOM    4913  N   LEU I 350     -13.356   5.109  16.026  1.00  0.00
I    N
```

```
ATOM   4914  HN  LEU I 350     -13.100   4.298  15.504  1.00  0.00
I    H
ATOM   4915  CA  LEU I 350     -14.432   5.893  15.427  1.00  0.00
I    C
ATOM   4916  HA  LEU I 350     -14.078   6.916  15.440  1.00  0.00
I    H
ATOM   4917  CB  LEU I 350     -14.701   5.462  13.957  1.00  0.00
I    C
ATOM   4918  HB1 LEU I 350     -14.905   4.366  13.968  1.00  0.00
I    H
ATOM   4919  HB2 LEU I 350     -15.639   5.945  13.604  1.00  0.00
I    H
ATOM   4920  CG  LEU I 350     -13.544   5.741  12.970  1.00  0.00
I    C
ATOM   4921  HG  LEU I 350     -12.665   5.183  13.373  1.00  0.00
I    H
ATOM   4922  CD1 LEU I 350     -13.889   5.233  11.558  1.00  0.00
I    C
ATOM   4923 HD11 LEU I 350     -13.074   5.480  10.844  1.00  0.00
I    H
ATOM   4924 HD12 LEU I 350     -14.027   4.130  11.562  1.00  0.00
I    H
ATOM   4925 HD13 LEU I 350     -14.826   5.702  11.190  1.00  0.00
I    H
ATOM   4926  CD2 LEU I 350     -13.175   7.232  12.909  1.00  0.00
I    C
ATOM   4927 HD21 LEU I 350     -12.329   7.391  12.205  1.00  0.00
I    H
ATOM   4928 HD22 LEU I 350     -14.025   7.850  12.559  1.00  0.00
I    H
ATOM   4929 HD23 LEU I 350     -12.865   7.597  13.912  1.00  0.00
I    H
ATOM   4930  C   LEU I 350     -15.755   5.853  16.196  1.00  0.00
I    C
ATOM   4931  O   LEU I 350     -16.601   6.728  16.030  1.00  0.00
I    O
ATOM   4932  N   ARG I 351     -15.964   4.848  17.073  1.00  0.00
I    N
ATOM   4933  HN  ARG I 351     -15.298   4.111  17.162  1.00  0.00
I    H
ATOM   4934  CA  ARG I 351     -17.142   4.773  17.924  1.00  0.00
I    C
ATOM   4935  HA  ARG I 351     -17.879   5.460  17.527  1.00  0.00
I    H
ATOM   4936  CB  ARG I 351     -17.654   3.317  18.001  1.00  0.00
I    C
ATOM   4937  HB1 ARG I 351     -16.814   2.709  18.412  1.00  0.00
I    H
ATOM   4938  HB2 ARG I 351     -18.475   3.250  18.748  1.00  0.00
I    H
ATOM   4939  CG  ARG I 351     -18.074   2.743  16.634  1.00  0.00
I    C
ATOM   4940  HG1 ARG I 351     -18.821   3.428  16.178  1.00  0.00
I    H
```

ATOM 4941 HG2 ARG I 351 -17.195 2.759 15.950 1.00 0.00
I H
ATOM 4942 CD ARG I 351 -18.580 1.306 16.734 1.00 0.00
I C
ATOM 4943 HD1 ARG I 351 -18.527 0.823 15.732 1.00 0.00
I H
ATOM 4944 HD2 ARG I 351 -17.972 0.707 17.452 1.00 0.00
I H
ATOM 4945 NE ARG I 351 -19.986 1.398 17.230 1.00 0.00
I N
ATOM 4946 HE ARG I 351 -20.384 2.316 17.123 1.00 0.00
I H
ATOM 4947 CZ ARG I 351 -20.671 0.386 17.765 1.00 0.00
I C
ATOM 4948 NH1 ARG I 351 -20.053 -0.702 18.196 1.00 0.00
I N
ATOM 4949 HH11 ARG I 351 -20.569 -1.461 18.569 1.00 0.00
I H
ATOM 4950 HH12 ARG I 351 -19.072 -0.764 17.996 1.00 0.00
I H
ATOM 4951 NH2 ARG I 351 -21.892 0.616 18.230 1.00 0.00
I N
ATOM 4952 HH21 ARG I 351 -22.381 -0.101 18.708 1.00 0.00
I H
ATOM 4953 HH22 ARG I 351 -22.189 1.561 18.266 1.00 0.00
I H
ATOM 4954 C ARG I 351 -16.864 5.210 19.351 1.00 0.00
I C
ATOM 4955 O ARG I 351 -17.787 5.379 20.145 1.00 0.00
I O
ATOM 4956 N GLY I 352 -15.595 5.432 19.741 1.00 0.00
I N
ATOM 4957 HN GLY I 352 -14.815 5.198 19.163 1.00 0.00
I H
ATOM 4958 CA GLY I 352 -15.268 6.027 21.033 1.00 0.00
I C
ATOM 4959 HA1 GLY I 352 -16.191 6.465 21.386 1.00 0.00
I H
ATOM 4960 HA2 GLY I 352 -14.486 6.746 20.830 1.00 0.00
I H
ATOM 4961 C GLY I 352 -14.767 5.084 22.089 1.00 0.00
I C
ATOM 4962 O GLY I 352 -14.367 5.539 23.167 1.00 0.00
I O
ATOM 4963 N LYS I 353 -14.738 3.762 21.811 1.00 0.00
I N
ATOM 4964 HN LYS I 353 -15.056 3.435 20.923 1.00 0.00
I H
ATOM 4965 CA LYS I 353 -14.267 2.725 22.720 1.00 0.00
I C
ATOM 4966 HA LYS I 353 -14.898 2.788 23.599 1.00 0.00
I H
ATOM 4967 CB LYS I 353 -14.371 1.296 22.113 1.00 0.00
I C

ATOM 4968 HB1 LYS I 353 -13.676 1.259 21.243 1.00 0.00
I H
ATOM 4969 HB2 LYS I 353 -13.985 0.559 22.853 1.00 0.00
I H
ATOM 4970 CG LYS I 353 -15.783 0.916 21.647 1.00 0.00
I C
ATOM 4971 HG1 LYS I 353 -16.524 1.277 22.396 1.00 0.00
I H
ATOM 4972 HG2 LYS I 353 -16.001 1.437 20.687 1.00 0.00
I H
ATOM 4973 CD LYS I 353 -16.029 -0.599 21.451 1.00 0.00
I C
ATOM 4974 HD1 LYS I 353 -16.168 -1.066 22.453 1.00 0.00
I H
ATOM 4975 HD2 LYS I 353 -16.982 -0.726 20.889 1.00 0.00
I H
ATOM 4976 CE LYS I 353 -14.924 -1.355 20.707 1.00 0.00
I C
ATOM 4977 HE1 LYS I 353 -14.532 -0.742 19.864 1.00 0.00
I H
ATOM 4978 HE2 LYS I 353 -14.081 -1.589 21.392 1.00 0.00
I H
ATOM 4979 NZ LYS I 353 -15.446 -2.629 20.153 1.00 0.00
I N
ATOM 4980 HZ1 LYS I 353 -14.689 -3.134 19.648 1.00 0.00
I H
ATOM 4981 HZ2 LYS I 353 -15.802 -3.225 20.928 1.00 0.00
I H
ATOM 4982 HZ3 LYS I 353 -16.224 -2.426 19.494 1.00 0.00
I H
ATOM 4983 C LYS I 353 -12.824 2.926 23.179 1.00 0.00
I C
ATOM 4984 O LYS I 353 -11.973 3.417 22.444 1.00 0.00
I O
ATOM 4985 N LYS I 354 -12.509 2.566 24.442 1.00 0.00
I N
ATOM 4986 HN LYS I 354 -13.164 2.149 25.069 1.00 0.00
I H
ATOM 4987 CA LYS I 354 -11.171 2.764 24.986 1.00 0.00
I C
ATOM 4988 HA LYS I 354 -10.756 3.621 24.469 1.00 0.00
I H
ATOM 4989 CB LYS I 354 -11.232 3.030 26.515 1.00 0.00
I C
ATOM 4990 HB1 LYS I 354 -11.482 2.061 27.007 1.00 0.00
I H
ATOM 4991 HB2 LYS I 354 -10.217 3.310 26.875 1.00 0.00
I H
ATOM 4992 CG LYS I 354 -12.272 4.089 26.934 1.00 0.00
I C
ATOM 4993 HG1 LYS I 354 -13.292 3.713 26.695 1.00 0.00
I H
ATOM 4994 HG2 LYS I 354 -12.220 4.232 28.037 1.00 0.00
I H

ATOM	4995	CD	LYS	I	354	-12.107	5.457	26.246	1.00	0.00	I C
ATOM	4996	HD1	LYS	I	354	-11.331	6.036	26.797	1.00	0.00	I H
ATOM	4997	HD2	LYS	I	354	-11.724	5.282	25.214	1.00	0.00	I H
ATOM	4998	CE	LYS	I	354	-13.425	6.236	26.194	1.00	0.00	I C
ATOM	4999	HE1	LYS	I	354	-14.276	5.550	25.986	1.00	0.00	I H
ATOM	5000	HE2	LYS	I	354	-13.615	6.748	27.163	1.00	0.00	I H
ATOM	5001	NZ	LYS	I	354	-13.350	7.246	25.123	1.00	0.00	I N
ATOM	5002	HZ1	LYS	I	354	-14.239	7.782	25.076	1.00	0.00	I H
ATOM	5003	HZ2	LYS	I	354	-12.563	7.899	25.316	1.00	0.00	I H
ATOM	5004	HZ3	LYS	I	354	-13.185	6.772	24.212	1.00	0.00	I H
ATOM	5005	C	LYS	I	354	-10.250	1.591	24.725	1.00	0.00	I C
ATOM	5006	O	LYS	I	354	-9.030	1.713	24.800	1.00	0.00	I O
ATOM	5007	N	GLU	I	355	-10.828	0.436	24.356	1.00	0.00	I N
ATOM	5008	HN	GLU	I	355	-11.815	0.303	24.341	1.00	0.00	I H
ATOM	5009	CA	GLU	I	355	-10.078	-0.720	23.948	1.00	0.00	I C
ATOM	5010	HA	GLU	I	355	-9.098	-0.407	23.612	1.00	0.00	I H
ATOM	5011	CB	GLU	I	355	-9.879	-1.765	25.089	1.00	0.00	I C
ATOM	5012	HB1	GLU	I	355	-9.311	-2.629	24.673	1.00	0.00	I H
ATOM	5013	HB2	GLU	I	355	-9.236	-1.333	25.888	1.00	0.00	I H
ATOM	5014	CG	GLU	I	355	-11.153	-2.348	25.771	1.00	0.00	I C
ATOM	5015	HG1	GLU	I	355	-11.642	-1.561	26.373	1.00	0.00	I H
ATOM	5016	HG2	GLU	I	355	-11.870	-2.727	25.015	1.00	0.00	I H
ATOM	5017	CD	GLU	I	355	-10.821	-3.484	26.750	1.00	0.00	I C
ATOM	5018	OE1	GLU	I	355	-9.664	-3.551	27.257	1.00	0.00	I O
ATOM	5019	OE2	GLU	I	355	-11.697	-4.359	26.970	1.00	0.00	I O
ATOM	5020	C	GLU	I	355	-10.769	-1.361	22.773	1.00	0.00	I C
ATOM	5021	O	GLU	I	355	-11.974	-1.222	22.548	1.00	0.00	I O

```
ATOM   5022  N   THR I 356      -9.983  -2.087  21.966  1.00  0.00
I    N
ATOM   5023  HN  THR I 356      -8.997  -2.169  22.113  1.00  0.00
I    H
ATOM   5024  CA  THR I 356     -10.476  -2.815  20.815  1.00  0.00
I    C
ATOM   5025  HA  THR I 356     -11.374  -2.312  20.485  1.00  0.00
I    H
ATOM   5026  CB  THR I 356      -9.446  -2.964  19.714  1.00  0.00
I    C
ATOM   5027  HB  THR I 356      -8.576  -3.495  20.172  1.00  0.00
I    H
ATOM   5028  OG1 THR I 356      -9.030  -1.669  19.319  1.00  0.00
I    O
ATOM   5029  HG1 THR I 356      -8.389  -1.809  18.614  1.00  0.00
I    H
ATOM   5030  CG2 THR I 356     -10.070  -3.618  18.473  1.00  0.00
I    C
ATOM   5031 HG21 THR I 356      -9.295  -3.833  17.707  1.00  0.00
I    H
ATOM   5032 HG22 THR I 356     -10.557  -4.579  18.745  1.00  0.00
I    H
ATOM   5033 HG23 THR I 356     -10.837  -2.958  18.015  1.00  0.00
I    H
ATOM   5034  C   THR I 356     -10.892  -4.188  21.260  1.00  0.00
I    C
ATOM   5035  O   THR I 356     -10.099  -4.965  21.775  1.00  0.00
I    O
ATOM   5036  N   VAL I 357     -12.174  -4.530  21.083  1.00  0.00
I    N
ATOM   5037  HN  VAL I 357     -12.834  -3.921  20.651  1.00  0.00
I    H
ATOM   5038  CA  VAL I 357     -12.717  -5.804  21.500  1.00  0.00
I    C
ATOM   5039  HA  VAL I 357     -11.899  -6.491  21.676  1.00  0.00
I    H
ATOM   5040  CB  VAL I 357     -13.515  -5.699  22.810  1.00  0.00
I    C
ATOM   5041  HB  VAL I 357     -12.785  -5.506  23.635  1.00  0.00
I    H
ATOM   5042  CG1 VAL I 357     -14.546  -4.550  22.811  1.00  0.00
I    C
ATOM   5043 HG11 VAL I 357     -15.118  -4.547  23.763  1.00  0.00
I    H
ATOM   5044 HG12 VAL I 357     -14.055  -3.560  22.716  1.00  0.00
I    H
ATOM   5045 HG13 VAL I 357     -15.269  -4.671  21.976  1.00  0.00
I    H
ATOM   5046  CG2 VAL I 357     -14.196  -7.035  23.158  1.00  0.00
I    C
ATOM   5047 HG21 VAL I 357     -14.802  -6.938  24.085  1.00  0.00
I    H
ATOM   5048 HG22 VAL I 357     -14.867  -7.358  22.334  1.00  0.00
I    H
```

```
ATOM   5049 HG23 VAL I 357      -13.446  -7.835  23.331  1.00  0.00
I    H
ATOM   5050  C   VAL I 357      -13.538  -6.360  20.358  1.00  0.00
I    C
ATOM   5051  O   VAL I 357      -14.381  -5.657  19.786  1.00  0.00
I    O
ATOM   5052  N   ILE I 358      -13.270  -7.634  19.999  1.00  0.00
I    N
ATOM   5053  HN  ILE I 358      -12.501  -8.104  20.426  1.00  0.00
I    H
ATOM   5054  CA  ILE I 358      -13.993  -8.435  19.030  1.00  0.00
I    C
ATOM   5055  HA  ILE I 358      -14.985  -8.025  18.893  1.00  0.00
I    H
ATOM   5056  CB  ILE I 358      -13.270  -8.567  17.682  1.00  0.00
I    C
ATOM   5057  HB  ILE I 358      -13.209  -7.518  17.294  1.00  0.00
I    H
ATOM   5058  CG2 ILE I 358      -11.875  -9.199  17.872  1.00  0.00
I    C
ATOM   5059 HG21 ILE I 358      -11.293  -9.151  16.928  1.00  0.00
I    H
ATOM   5060 HG22 ILE I 358      -11.297  -8.657  18.651  1.00  0.00
I    H
ATOM   5061 HG23 ILE I 358      -11.959 -10.264  18.174  1.00  0.00
I    H
ATOM   5062  CG1 ILE I 358      -14.060  -9.354  16.606  1.00  0.00
I    C
ATOM   5063 HG11 ILE I 358      -13.371  -9.675  15.793  1.00  0.00
I    H
ATOM   5064 HG12 ILE I 358      -14.476 -10.279  17.069  1.00  0.00
I    H
ATOM   5065  CD  ILE I 358      -15.226  -8.587  15.978  1.00  0.00
I    C
ATOM   5066  HD1 ILE I 358      -15.775  -9.237  15.263  1.00  0.00
I    H
ATOM   5067  HD2 ILE I 358      -15.941  -8.251  16.760  1.00  0.00
I    H
ATOM   5068  HD3 ILE I 358      -14.865  -7.696  15.424  1.00  0.00
I    H
ATOM   5069  C   ILE I 358      -14.165  -9.781  19.719  1.00  0.00
I    C
ATOM   5070  O   ILE I 358      -13.299 -10.213  20.478  1.00  0.00
I    O
ATOM   5071  N   PHE I 359      -15.342 -10.429  19.575  1.00  0.00
I    N
ATOM   5072  HN  PHE I 359      -16.055 -10.047  18.989  1.00  0.00
I    H
ATOM   5073  CA  PHE I 359      -15.700 -11.694  20.219  1.00  0.00
I    C
ATOM   5074  HA  PHE I 359      -16.748 -11.826  19.980  1.00  0.00
I    H
ATOM   5075  CB  PHE I 359      -14.973 -12.919  19.585  1.00  0.00
I    C
```

```
ATOM   5076  HB1 PHE I 359     -13.893 -12.681  19.469  1.00  0.00
I    H
ATOM   5077  HB2 PHE I 359     -15.043 -13.812  20.243  1.00  0.00
I    H
ATOM   5078  CG  PHE I 359     -15.478 -13.269  18.210  1.00  0.00
I    C
ATOM   5079  CD1 PHE I 359     -16.857 -13.342  17.929  1.00  0.00
I    C
ATOM   5080  HD1 PHE I 359     -17.575 -13.114  18.706  1.00  0.00
I    H
ATOM   5081  CE1 PHE I 359     -17.310 -13.711  16.657  1.00  0.00
I    C
ATOM   5082  HE1 PHE I 359     -18.370 -13.733  16.446  1.00  0.00
I    H
ATOM   5083  CZ  PHE I 359     -16.389 -14.045  15.661  1.00  0.00
I    C
ATOM   5084  HZ  PHE I 359     -16.739 -14.317  14.675  1.00  0.00
I    H
ATOM   5085  CD2 PHE I 359     -14.567 -13.624  17.199  1.00  0.00
I    C
ATOM   5086  HD2 PHE I 359     -13.505 -13.616  17.402  1.00  0.00
I    H
ATOM   5087  CE2 PHE I 359     -15.019 -14.018  15.933  1.00  0.00
I    C
ATOM   5088  HE2 PHE I 359     -14.305 -14.300  15.171  1.00  0.00
I    H
ATOM   5089  C   PHE I 359     -15.581 -11.724  21.743  1.00  0.00
I    C
ATOM   5090  O   PHE I 359     -15.442 -12.786  22.356  1.00  0.00
I    O
ATOM   5091  N   ARG I 360     -15.696 -10.539  22.384  1.00  0.00
I    N
ATOM   5092  HN  ARG I 360     -15.857  -9.730  21.823  1.00  0.00
I    H
ATOM   5093  CA  ARG I 360     -15.608 -10.330  23.820  1.00  0.00
I    C
ATOM   5094  HA  ARG I 360     -15.723  -9.270  24.012  1.00  0.00
I    H
ATOM   5095  CB  ARG I 360     -16.757 -11.031  24.573  1.00  0.00
I    C
ATOM   5096  HB1 ARG I 360     -16.575 -12.128  24.475  1.00  0.00
I    H
ATOM   5097  HB2 ARG I 360     -16.682 -10.805  25.659  1.00  0.00
I    H
ATOM   5098  CG  ARG I 360     -18.152 -10.685  23.996  1.00  0.00
I    C
ATOM   5099  HG1 ARG I 360     -18.275  -9.580  24.005  1.00  0.00
I    H
ATOM   5100  HG2 ARG I 360     -18.184 -10.981  22.922  1.00  0.00
I    H
ATOM   5101  CD  ARG I 360     -19.357 -11.362  24.669  1.00  0.00
I    C
ATOM   5102  HD1 ARG I 360     -19.649 -10.783  25.575  1.00  0.00
I    H
```

```
ATOM   5103  HD2 ARG I 360     -20.233 -11.414  23.980  1.00  0.00
I    H
ATOM   5104  NE  ARG I 360     -18.902 -12.753  25.013  1.00  0.00
I    N
ATOM   5105  HE  ARG I 360     -18.012 -12.774  25.480  1.00  0.00
I    H
ATOM   5106  CZ  ARG I 360     -19.533 -13.909  24.771  1.00  0.00
I    C
ATOM   5107  NH1 ARG I 360     -20.723 -13.952  24.190  1.00  0.00
I    N
ATOM   5108 HH11 ARG I 360     -21.182 -14.817  24.044  1.00  0.00
I    H
ATOM   5109 HH12 ARG I 360     -21.176 -13.071  24.040  1.00  0.00
I    H
ATOM   5110  NH2 ARG I 360     -18.956 -15.051  25.151  1.00  0.00
I    N
ATOM   5111 HH21 ARG I 360     -19.366 -15.917  24.904  1.00  0.00
I    H
ATOM   5112 HH22 ARG I 360     -18.025 -14.992  25.489  1.00  0.00
I    H
ATOM   5113  C   ARG I 360     -14.247 -10.726  24.369  1.00  0.00
I    C
ATOM   5114  O   ARG I 360     -14.098 -11.319  25.444  1.00  0.00
I    O
ATOM   5115  N   ARG I 361     -13.206 -10.393  23.597  1.00  0.00
I    N
ATOM   5116  HN  ARG I 361     -13.363  -9.962  22.712  1.00  0.00
I    H
ATOM   5117  CA  ARG I 361     -11.848 -10.621  23.966  1.00  0.00
I    C
ATOM   5118  HA  ARG I 361     -11.760 -10.703  25.042  1.00  0.00
I    H
ATOM   5119  CB  ARG I 361     -11.397 -11.929  23.266  1.00  0.00
I    C
ATOM   5120  HB1 ARG I 361     -12.305 -12.576  23.193  1.00  0.00
I    H
ATOM   5121  HB2 ARG I 361     -11.096 -11.706  22.219  1.00  0.00
I    H
ATOM   5122  CG  ARG I 361     -10.302 -12.672  24.040  1.00  0.00
I    C
ATOM   5123  HG1 ARG I 361      -9.878 -13.463  23.382  1.00  0.00
I    H
ATOM   5124  HG2 ARG I 361      -9.461 -11.969  24.241  1.00  0.00
I    H
ATOM   5125  CD  ARG I 361     -10.704 -13.265  25.394  1.00  0.00
I    C
ATOM   5126  HD1 ARG I 361      -9.788 -13.493  25.986  1.00  0.00
I    H
ATOM   5127  HD2 ARG I 361     -11.339 -12.558  25.977  1.00  0.00
I    H
ATOM   5128  NE  ARG I 361     -11.499 -14.516  25.139  1.00  0.00
I    N
ATOM   5129  HE  ARG I 361     -11.145 -15.060  24.370  1.00  0.00
I    H
```

```
ATOM   5130  CZ  ARG I 361     -12.570 -14.868  25.862  1.00  0.00
I    C
ATOM   5131  NH1 ARG I 361     -13.279 -13.968  26.532  1.00  0.00
I    N
ATOM   5132 HH11 ARG I 361     -14.054 -14.244  27.083  1.00  0.00
I    H
ATOM   5133 HH12 ARG I 361     -12.907 -13.038  26.555  1.00  0.00
I    H
ATOM   5134  NH2 ARG I 361     -12.925 -16.150  25.927  1.00  0.00
I    N
ATOM   5135 HH21 ARG I 361     -13.763 -16.408  26.387  1.00  0.00
I    H
ATOM   5136 HH22 ARG I 361     -12.444 -16.780  25.331  1.00  0.00
I    H
ATOM   5137  C   ARG I 361     -11.064  -9.373  23.557  1.00  0.00
I    C
ATOM   5138  O   ARG I 361     -11.163  -8.911  22.421  1.00  0.00
I    O
ATOM   5139  N   SER I 362     -10.334  -8.745  24.508  1.00  0.00
I    N
ATOM   5140  HN  SER I 362     -10.216  -9.144  25.417  1.00  0.00
I    H
ATOM   5141  CA  SER I 362      -9.657  -7.456  24.323  1.00  0.00
I    C
ATOM   5142  HA  SER I 362     -10.330  -6.850  23.731  1.00  0.00
I    H
ATOM   5143  CB  SER I 362      -9.344  -6.758  25.691  1.00  0.00
I    C
ATOM   5144  HB1 SER I 362     -10.310  -6.555  26.206  1.00  0.00
I    H
ATOM   5145  HB2 SER I 362      -8.759  -7.440  26.350  1.00  0.00
I    H
ATOM   5146  OG  SER I 362      -8.655  -5.502  25.592  1.00  0.00
I    O
ATOM   5147  HG1 SER I 362      -8.719  -5.074  26.456  1.00  0.00
I    H
ATOM   5148  C   SER I 362      -8.374  -7.608  23.532  1.00  0.00
I    C
ATOM   5149  O   SER I 362      -7.486  -8.385  23.875  1.00  0.00
I    O
ATOM   5150  N   ILE I 363      -8.235  -6.852  22.429  1.00  0.00
I    N
ATOM   5151  HN  ILE I 363      -8.943  -6.224  22.113  1.00  0.00
I    H
ATOM   5152  CA  ILE I 363      -7.050  -6.875  21.599  1.00  0.00
I    C
ATOM   5153  HA  ILE I 363      -6.568  -7.834  21.746  1.00  0.00
I    H
ATOM   5154  CB  ILE I 363      -7.320  -6.619  20.125  1.00  0.00
I    C
ATOM   5155  HB  ILE I 363      -7.709  -5.570  20.090  1.00  0.00
I    H
ATOM   5156  CG2 ILE I 363      -5.996  -6.856  19.355  1.00  0.00
I    C
```

```
ATOM   5157 HG21 ILE I 363      -6.095  -6.533  18.299  1.00  0.00
I    H
ATOM   5158 HG22 ILE I 363      -5.165  -6.276  19.809  1.00  0.00
I    H
ATOM   5159 HG23 ILE I 363      -5.717  -7.932  19.365  1.00  0.00
I    H
ATOM   5160  CG1 ILE I 363      -8.456  -7.533  19.610  1.00  0.00
I    C
ATOM   5161 HG11 ILE I 363      -8.214  -8.596  19.832  1.00  0.00
I    H
ATOM   5162 HG12 ILE I 363      -9.394  -7.288  20.160  1.00  0.00
I    H
ATOM   5163  CD  ILE I 363      -8.702  -7.360  18.108  1.00  0.00
I    C
ATOM   5164  HD1 ILE I 363      -9.561  -7.987  17.784  1.00  0.00
I    H
ATOM   5165  HD2 ILE I 363      -8.935  -6.300  17.868  1.00  0.00
I    H
ATOM   5166  HD3 ILE I 363      -7.812  -7.666  17.521  1.00  0.00
I    H
ATOM   5167  C   ILE I 363      -6.092  -5.823  22.105  1.00  0.00
I    C
ATOM   5168  O   ILE I 363      -6.383  -4.628  22.141  1.00  0.00
I    O
ATOM   5169  N   LYS I 364      -4.902  -6.252  22.553  1.00  0.00
I    N
ATOM   5170  HN  LYS I 364      -4.598  -7.202  22.483  1.00  0.00
I    H
ATOM   5171  CA  LYS I 364      -3.962  -5.353  23.179  1.00  0.00
I    C
ATOM   5172  HA  LYS I 364      -4.552  -4.648  23.753  1.00  0.00
I    H
ATOM   5173  CB  LYS I 364      -3.045  -6.090  24.190  1.00  0.00
I    C
ATOM   5174  HB1 LYS I 364      -2.335  -6.712  23.596  1.00  0.00
I    H
ATOM   5175  HB2 LYS I 364      -2.426  -5.340  24.732  1.00  0.00
I    H
ATOM   5176  CG  LYS I 364      -3.784  -7.001  25.190  1.00  0.00
I    C
ATOM   5177  HG1 LYS I 364      -4.005  -7.976  24.701  1.00  0.00
I    H
ATOM   5178  HG2 LYS I 364      -3.113  -7.209  26.055  1.00  0.00
I    H
ATOM   5179  CD  LYS I 364      -5.121  -6.471  25.744  1.00  0.00
I    C
ATOM   5180  HD1 LYS I 364      -5.795  -6.251  24.884  1.00  0.00
I    H
ATOM   5181  HD2 LYS I 364      -5.596  -7.285  26.341  1.00  0.00
I    H
ATOM   5182  CE  LYS I 364      -5.056  -5.229  26.623  1.00  0.00
I    C
ATOM   5183  HE1 LYS I 364      -4.379  -5.400  27.490  1.00  0.00
I    H
```

ATOM 5184 HE2 LYS I 364 -4.672 -4.360 26.044 1.00 0.00
I H
ATOM 5185 NZ LYS I 364 -6.426 -4.968 27.089 1.00 0.00
I N
ATOM 5186 HZ1 LYS I 364 -6.440 -4.125 27.699 1.00 0.00
I H
ATOM 5187 HZ2 LYS I 364 -7.048 -4.812 26.270 1.00 0.00
I H
ATOM 5188 HZ3 LYS I 364 -6.770 -5.788 27.628 1.00 0.00
I H
ATOM 5189 C LYS I 364 -3.149 -4.552 22.179 1.00 0.00
I C
ATOM 5190 O LYS I 364 -2.899 -4.979 21.055 1.00 0.00
I O
ATOM 5191 N TYR I 365 -2.672 -3.358 22.599 1.00 0.00
I N
ATOM 5192 HN TYR I 365 -2.879 -3.030 23.519 1.00 0.00
I H
ATOM 5193 CA TYR I 365 -1.846 -2.458 21.807 1.00 0.00
I C
ATOM 5194 HA TYR I 365 -2.504 -2.083 21.033 1.00 0.00
I H
ATOM 5195 CB TYR I 365 -1.442 -1.226 22.684 1.00 0.00
I C
ATOM 5196 HB1 TYR I 365 -2.342 -0.600 22.871 1.00 0.00
I H
ATOM 5197 HB2 TYR I 365 -1.061 -1.556 23.674 1.00 0.00
I H
ATOM 5198 CG TYR I 365 -0.418 -0.350 22.002 1.00 0.00
I C
ATOM 5199 CD1 TYR I 365 -0.802 0.545 20.994 1.00 0.00
I C
ATOM 5200 HD1 TYR I 365 -1.847 0.651 20.739 1.00 0.00
I H
ATOM 5201 CE1 TYR I 365 0.161 1.289 20.296 1.00 0.00
I C
ATOM 5202 HE1 TYR I 365 -0.134 1.983 19.524 1.00 0.00
I H
ATOM 5203 CZ TYR I 365 1.518 1.131 20.593 1.00 0.00
I C
ATOM 5204 OH TYR I 365 2.469 1.885 19.879 1.00 0.00
I O
ATOM 5205 HH TYR I 365 3.329 1.701 20.262 1.00 0.00
I H
ATOM 5206 CD2 TYR I 365 0.951 -0.514 22.281 1.00 0.00
I C
ATOM 5207 HD2 TYR I 365 1.269 -1.237 23.020 1.00 0.00
I H
ATOM 5208 CE2 TYR I 365 1.917 0.216 21.573 1.00 0.00
I C
ATOM 5209 HE2 TYR I 365 2.965 0.052 21.774 1.00 0.00
I H
ATOM 5210 C TYR I 365 -0.647 -3.135 21.099 1.00 0.00
I C

ATOM 5211 O TYR I 365 -0.462 -2.823 19.921 1.00 0.00
I O
ATOM 5212 N PRO I 366 0.157 -4.060 21.662 1.00 0.00
I N
ATOM 5213 CD PRO I 366 0.261 -4.324 23.099 1.00 0.00
I C
ATOM 5214 HD1 PRO I 366 0.382 -3.288 22.709 1.00 0.00
I H
ATOM 5215 HD2 PRO I 366 -0.292 -4.313 24.066 1.00 0.00
I H
ATOM 5216 CA PRO I 366 1.221 -4.747 20.931 1.00 0.00
I C
ATOM 5217 HA PRO I 366 1.988 -4.025 20.676 1.00 0.00
I H
ATOM 5218 CB PRO I 366 1.826 -5.714 21.961 1.00 0.00
I C
ATOM 5219 HB1 PRO I 366 2.590 -5.172 22.563 1.00 0.00
I H
ATOM 5220 HB2 PRO I 366 2.301 -6.598 21.491 1.00 0.00
I H
ATOM 5221 CG PRO I 366 1.631 -4.977 23.288 1.00 0.00
I C
ATOM 5222 HG1 PRO I 366 2.342 -5.303 24.071 1.00 0.00
I H
ATOM 5223 HG2 PRO I 366 1.902 -4.229 23.893 1.00 0.00
I H
ATOM 5224 C PRO I 366 0.774 -5.467 19.666 1.00 0.00
I C
ATOM 5225 O PRO I 366 1.544 -5.538 18.707 1.00 0.00
I O
ATOM 5226 N ILE I 367 -0.453 -6.015 19.639 1.00 0.00
I N
ATOM 5227 HN ILE I 367 -1.034 -6.024 20.450 1.00 0.00
I H
ATOM 5228 CA ILE I 367 -1.053 -6.632 18.469 1.00 0.00
I C
ATOM 5229 HA ILE I 367 -0.296 -7.281 18.048 1.00 0.00
I H
ATOM 5230 CB ILE I 367 -2.310 -7.402 18.862 1.00 0.00
I C
ATOM 5231 HB ILE I 367 -2.995 -6.623 19.283 1.00 0.00
I H
ATOM 5232 CG2 ILE I 367 -2.912 -8.109 17.631 1.00 0.00
I C
ATOM 5233 HG21 ILE I 367 -3.906 -8.539 17.875 1.00 0.00
I H
ATOM 5234 HG22 ILE I 367 -3.052 -7.395 16.793 1.00 0.00
I H
ATOM 5235 HG23 ILE I 367 -2.253 -8.933 17.285 1.00 0.00
I H
ATOM 5236 CG1 ILE I 367 -2.050 -8.413 20.013 1.00 0.00
I C
ATOM 5237 HG11 ILE I 367 -1.810 -7.863 20.951 1.00 0.00
I H

ATOM 5238 HG12 ILE I 367 -2.982 -8.993 20.201 1.00 0.00
I H
ATOM 5239 CD ILE I 367 -0.924 -9.422 19.742 1.00 0.00
I C
ATOM 5240 HD1 ILE I 367 -0.840 -10.142 20.584 1.00 0.00
I H
ATOM 5241 HD2 ILE I 367 -1.127 -9.996 18.812 1.00 0.00
I H
ATOM 5242 HD3 ILE I 367 0.054 -8.907 19.632 1.00 0.00
I H
ATOM 5243 C ILE I 367 -1.389 -5.595 17.402 1.00 0.00
I C
ATOM 5244 O ILE I 367 -1.109 -5.775 16.216 1.00 0.00
I O
ATOM 5245 N ILE I 368 -1.976 -4.449 17.822 1.00 0.00
I N
ATOM 5246 HN ILE I 368 -2.190 -4.304 18.786 1.00 0.00
I H
ATOM 5247 CA ILE I 368 -2.350 -3.337 16.954 1.00 0.00
I C
ATOM 5248 HA ILE I 368 -2.957 -3.764 16.166 1.00 0.00
I H
ATOM 5249 CB ILE I 368 -3.119 -2.231 17.694 1.00 0.00
I C
ATOM 5250 HB ILE I 368 -2.358 -1.768 18.373 1.00 0.00
I H
ATOM 5251 CG2 ILE I 368 -3.690 -1.223 16.665 1.00 0.00
I C
ATOM 5252 HG21 ILE I 368 -4.113 -0.336 17.181 1.00 0.00
I H
ATOM 5253 HG22 ILE I 368 -2.895 -0.865 15.978 1.00 0.00
I H
ATOM 5254 HG23 ILE I 368 -4.495 -1.687 16.058 1.00 0.00
I H
ATOM 5255 CG1 ILE I 368 -4.238 -2.767 18.627 1.00 0.00
I C
ATOM 5256 HG11 ILE I 368 -3.786 -3.342 19.466 1.00 0.00
I H
ATOM 5257 HG12 ILE I 368 -4.779 -1.902 19.075 1.00 0.00
I H
ATOM 5258 CD ILE I 368 -5.283 -3.655 17.947 1.00 0.00
I C
ATOM 5259 HD1 ILE I 368 -6.074 -3.945 18.671 1.00 0.00
I H
ATOM 5260 HD2 ILE I 368 -5.765 -3.117 17.103 1.00 0.00
I H
ATOM 5261 HD3 ILE I 368 -4.820 -4.585 17.554 1.00 0.00
I H
ATOM 5262 C ILE I 368 -1.137 -2.705 16.283 1.00 0.00
I C
ATOM 5263 O ILE I 368 -1.129 -2.489 15.072 1.00 0.00
I O
ATOM 5264 N ILE I 369 -0.054 -2.430 17.046 1.00 0.00
I N

ATOM 5265 HN ILE I 369 -0.037 -2.582 18.032 1.00 0.00
I H
ATOM 5266 CA ILE I 369 1.188 -1.886 16.503 1.00 0.00
I C
ATOM 5267 HA ILE I 369 0.893 -1.033 15.904 1.00 0.00
I H
ATOM 5268 CB ILE I 369 2.126 -1.303 17.566 1.00 0.00
I C
ATOM 5269 HB ILE I 369 1.481 -0.605 18.157 1.00 0.00
I H
ATOM 5270 CG2 ILE I 369 2.736 -2.400 18.457 1.00 0.00
I C
ATOM 5271 HG21 ILE I 369 3.283 -1.950 19.311 1.00 0.00
I H
ATOM 5272 HG22 ILE I 369 1.944 -3.058 18.874 1.00 0.00
I H
ATOM 5273 HG23 ILE I 369 3.448 -3.030 17.882 1.00 0.00
I H
ATOM 5274 CG1 ILE I 369 3.249 -0.431 16.960 1.00 0.00
I C
ATOM 5275 HG11 ILE I 369 3.967 -0.135 17.757 1.00 0.00
I H
ATOM 5276 HG12 ILE I 369 3.814 -1.036 16.216 1.00 0.00
I H
ATOM 5277 CD ILE I 369 2.742 0.831 16.258 1.00 0.00
I C
ATOM 5278 HD1 ILE I 369 3.589 1.382 15.796 1.00 0.00
I H
ATOM 5279 HD2 ILE I 369 2.018 0.569 15.456 1.00 0.00
I H
ATOM 5280 HD3 ILE I 369 2.240 1.513 16.976 1.00 0.00
I H
ATOM 5281 C ILE I 369 1.908 -2.851 15.571 1.00 0.00
I C
ATOM 5282 O ILE I 369 2.404 -2.459 14.515 1.00 0.00
I O
ATOM 5283 N LYS I 370 1.931 -4.166 15.892 1.00 0.00
I N
ATOM 5284 HN LYS I 370 1.578 -4.512 16.760 1.00 0.00
I H
ATOM 5285 CA LYS I 370 2.470 -5.175 15.000 1.00 0.00
I C
ATOM 5286 HA LYS I 370 3.482 -4.870 14.764 1.00 0.00
I H
ATOM 5287 CB LYS I 370 2.484 -6.572 15.662 1.00 0.00
I C
ATOM 5288 HB1 LYS I 370 3.055 -6.478 16.614 1.00 0.00
I H
ATOM 5289 HB2 LYS I 370 1.445 -6.849 15.946 1.00 0.00
I H
ATOM 5290 CG LYS I 370 3.135 -7.633 14.759 1.00 0.00
I C
ATOM 5291 HG1 LYS I 370 2.480 -7.812 13.876 1.00 0.00
I H

```
ATOM   5292  HG2 LYS I 370       4.106  -7.240  14.379  1.00  0.00
I    H
ATOM   5293  CD  LYS I 370       3.363  -8.972  15.468  1.00  0.00
I    C
ATOM   5294  HD1 LYS I 370       3.910  -8.778  16.419  1.00  0.00
I    H
ATOM   5295  HD2 LYS I 370       2.368  -9.396  15.736  1.00  0.00
I    H
ATOM   5296  CE  LYS I 370       4.140  -9.948  14.584  1.00  0.00
I    C
ATOM   5297  HE1 LYS I 370       3.533 -10.238  13.698  1.00  0.00
I    H
ATOM   5298  HE2 LYS I 370       5.086  -9.485  14.227  1.00  0.00
I    H
ATOM   5299  NZ  LYS I 370       4.466 -11.164  15.356  1.00  0.00
I    N
ATOM   5300  HZ1 LYS I 370       4.994 -11.833  14.761  1.00  0.00
I    H
ATOM   5301  HZ2 LYS I 370       5.044 -10.906  16.182  1.00  0.00
I    H
ATOM   5302  HZ3 LYS I 370       3.586 -11.613  15.682  1.00  0.00
I    H
ATOM   5303  C   LYS I 370       1.704  -5.257  13.690  1.00  0.00
I    C
ATOM   5304  O   LYS I 370       2.294  -5.294  12.612  1.00  0.00
I    O
ATOM   5305  N   ALA I 371       0.357  -5.226  13.745  1.00  0.00
I    N
ATOM   5306  HN  ALA I 371      -0.124  -5.235  14.622  1.00  0.00
I    H
ATOM   5307  CA  ALA I 371      -0.490  -5.179  12.573  1.00  0.00
I    C
ATOM   5308  HA  ALA I 371      -0.281  -6.030  11.934  1.00  0.00
I    H
ATOM   5309  CB  ALA I 371      -1.970  -5.286  13.001  1.00  0.00
I    C
ATOM   5310  HB1 ALA I 371      -2.125  -6.224  13.576  1.00  0.00
I    H
ATOM   5311  HB2 ALA I 371      -2.259  -4.434  13.654  1.00  0.00
I    H
ATOM   5312  HB3 ALA I 371      -2.650  -5.305  12.122  1.00  0.00
I    H
ATOM   5313  C   ALA I 371      -0.252  -3.930  11.720  1.00  0.00
I    C
ATOM   5314  O   ALA I 371      -0.183  -4.005  10.492  1.00  0.00
I    O
ATOM   5315  N   LEU I 372      -0.069  -2.756  12.370  1.00  0.00
I    N
ATOM   5316  HN  LEU I 372      -0.177  -2.711  13.361  1.00  0.00
I    H
ATOM   5317  CA  LEU I 372       0.286  -1.487  11.748  1.00  0.00
I    C
ATOM   5318  HA  LEU I 372      -0.494  -1.267  11.030  1.00  0.00
I    H
```

```
ATOM   5319  CB  LEU I 372       0.392  -0.398  12.854  1.00  0.00
I    C
ATOM   5320  HB1 LEU I 372      -0.293  -0.699  13.680  1.00  0.00
I    H
ATOM   5321  HB2 LEU I 372       1.419  -0.416  13.283  1.00  0.00
I    H
ATOM   5322  CG  LEU I 372       0.031   1.046  12.441  1.00  0.00
I    C
ATOM   5323  HG  LEU I 372      -1.018   0.976  12.063  1.00  0.00
I    H
ATOM   5324  CD1 LEU I 372       0.105   1.977  13.661  1.00  0.00
I    C
ATOM   5325 HD11 LEU I 372      -0.107   3.027  13.365  1.00  0.00
I    H
ATOM   5326 HD12 LEU I 372      -0.638   1.675  14.430  1.00  0.00
I    H
ATOM   5327 HD13 LEU I 372       1.116   1.943  14.119  1.00  0.00
I    H
ATOM   5328  CD2 LEU I 372       0.897   1.636  11.325  1.00  0.00
I    C
ATOM   5329 HD21 LEU I 372       0.560   2.665  11.077  1.00  0.00
I    H
ATOM   5330 HD22 LEU I 372       1.964   1.694  11.622  1.00  0.00
I    H
ATOM   5331 HD23 LEU I 372       0.824   1.015  10.406  1.00  0.00
I    H
ATOM   5332  C   LEU I 372       1.604  -1.589  10.981  1.00  0.00
I    C
ATOM   5333  O   LEU I 372       1.698  -1.233   9.807  1.00  0.00
I    O
ATOM   5334  N   ALA I 373       2.635  -2.173  11.628  1.00  0.00
I    N
ATOM   5335  HN  ALA I 373       2.512  -2.452  12.580  1.00  0.00
I    H
ATOM   5336  CA  ALA I 373       3.939  -2.443  11.065  1.00  0.00
I    C
ATOM   5337  HA  ALA I 373       4.361  -1.517  10.691  1.00  0.00
I    H
ATOM   5338  CB  ALA I 373       4.889  -2.932  12.179  1.00  0.00
I    C
ATOM   5339  HB1 ALA I 373       4.918  -2.183  13.000  1.00  0.00
I    H
ATOM   5340  HB2 ALA I 373       4.538  -3.896  12.609  1.00  0.00
I    H
ATOM   5341  HB3 ALA I 373       5.926  -3.069  11.803  1.00  0.00
I    H
ATOM   5342  C   ALA I 373       3.927  -3.419   9.888  1.00  0.00
I    C
ATOM   5343  O   ALA I 373       4.619  -3.194   8.892  1.00  0.00
I    O
ATOM   5344  N   VAL I 374       3.125  -4.513   9.948  1.00  0.00
I    N
ATOM   5345  HN  VAL I 374       2.625  -4.765  10.773  1.00  0.00
I    H
```

```
ATOM   5346  CA  VAL I 374       2.920  -5.421   8.815  1.00  0.00
I    C
ATOM   5347  HA  VAL I 374       3.898  -5.762   8.501  1.00  0.00
I    H
ATOM   5348  CB  VAL I 374       2.050  -6.634   9.174  1.00  0.00
I    C
ATOM   5349  HB  VAL I 374       1.091  -6.248   9.597  1.00  0.00
I    H
ATOM   5350  CG1 VAL I 374       1.722  -7.507   7.937  1.00  0.00
I    C
ATOM   5351 HG11 VAL I 374       1.125  -8.395   8.239  1.00  0.00
I    H
ATOM   5352 HG12 VAL I 374       1.125  -6.947   7.187  1.00  0.00
I    H
ATOM   5353 HG13 VAL I 374       2.654  -7.869   7.453  1.00  0.00
I    H
ATOM   5354  CG2 VAL I 374       2.803  -7.507  10.187  1.00  0.00
I    C
ATOM   5355 HG21 VAL I 374       2.215  -8.416  10.437  1.00  0.00
I    H
ATOM   5356 HG22 VAL I 374       3.784  -7.828   9.773  1.00  0.00
I    H
ATOM   5357 HG23 VAL I 374       2.988  -6.957  11.133  1.00  0.00
I    H
ATOM   5358  C   VAL I 374       2.283  -4.717   7.635  1.00  0.00
I    C
ATOM   5359  O   VAL I 374       2.807  -4.748   6.524  1.00  0.00
I    O
ATOM   5360  N   SER I 375       1.156  -4.011   7.850  1.00  0.00
I    N
ATOM   5361  HN  SER I 375       0.748  -3.889   8.755  1.00  0.00
I    H
ATOM   5362  CA  SER I 375       0.426  -3.355   6.772  1.00  0.00
I    C
ATOM   5363  HA  SER I 375       0.289  -4.128   6.027  1.00  0.00
I    H
ATOM   5364  CB  SER I 375      -0.943  -2.803   7.220  1.00  0.00
I    C
ATOM   5365  HB1 SER I 375      -0.777  -2.133   8.094  1.00  0.00
I    H
ATOM   5366  HB2 SER I 375      -1.394  -2.183   6.413  1.00  0.00
I    H
ATOM   5367  OG  SER I 375      -1.793  -3.881   7.601  1.00  0.00
I    O
ATOM   5368  HG1 SER I 375      -2.540  -3.499   8.077  1.00  0.00
I    H
ATOM   5369  C   SER I 375       1.173  -2.219   6.110  1.00  0.00
I    C
ATOM   5370  O   SER I 375       1.208  -2.139   4.884  1.00  0.00
I    O
ATOM   5371  N   VAL I 376       1.815  -1.325   6.895  1.00  0.00
I    N
ATOM   5372  HN  VAL I 376       1.784  -1.375   7.890  1.00  0.00
I    H
```

```
ATOM   5373  CA  VAL I 376       2.606  -0.214   6.371  1.00  0.00
I    C
ATOM   5374  HA  VAL I 376       1.966   0.328   5.686  1.00  0.00
I    H
ATOM   5375  CB  VAL I 376       3.032   0.744   7.488  1.00  0.00
I    C
ATOM   5376  HB  VAL I 376       3.375   0.124   8.352  1.00  0.00
I    H
ATOM   5377  CG1 VAL I 376       4.197   1.685   7.093  1.00  0.00
I    C
ATOM   5378 HG11 VAL I 376       4.432   2.380   7.929  1.00  0.00
I    H
ATOM   5379 HG12 VAL I 376       5.123   1.118   6.865  1.00  0.00
I    H
ATOM   5380 HG13 VAL I 376       3.926   2.296   6.206  1.00  0.00
I    H
ATOM   5381  CG2 VAL I 376       1.793   1.582   7.854  1.00  0.00
I    C
ATOM   5382 HG21 VAL I 376       2.042   2.330   8.636  1.00  0.00
I    H
ATOM   5383 HG22 VAL I 376       1.410   2.122   6.962  1.00  0.00
I    H
ATOM   5384 HG23 VAL I 376       0.978   0.941   8.251  1.00  0.00
I    H
ATOM   5385  C   VAL I 376       3.818  -0.679   5.591  1.00  0.00
I    C
ATOM   5386  O   VAL I 376       4.038  -0.241   4.464  1.00  0.00
I    O
ATOM   5387  N   THR I 377       4.612  -1.628   6.143  1.00  0.00
I    N
ATOM   5388  HN  THR I 377       4.441  -2.050   7.033  1.00  0.00
I    H
ATOM   5389  CA  THR I 377       5.812  -2.121   5.464  1.00  0.00
I    C
ATOM   5390  HA  THR I 377       6.372  -1.242   5.176  1.00  0.00
I    H
ATOM   5391  CB  THR I 377       6.727  -3.015   6.304  1.00  0.00
I    C
ATOM   5392  HB  THR I 377       6.183  -3.982   6.431  1.00  0.00
I    H
ATOM   5393  OG1 THR I 377       6.943  -2.487   7.605  1.00  0.00
I    O
ATOM   5394  HG1 THR I 377       7.534  -3.112   8.041  1.00  0.00
I    H
ATOM   5395  CG2 THR I 377       8.114  -3.094   5.637  1.00  0.00
I    C
ATOM   5396 HG21 THR I 377       8.764  -3.826   6.162  1.00  0.00
I    H
ATOM   5397 HG22 THR I 377       8.017  -3.420   4.579  1.00  0.00
I    H
ATOM   5398 HG23 THR I 377       8.621  -2.106   5.652  1.00  0.00
I    H
ATOM   5399  C   THR I 377       5.456  -2.849   4.176  1.00  0.00
I    C
```

```
ATOM   5400  O   THR I 377       6.054  -2.614   3.128  1.00  0.00
I    O
ATOM   5401  N   SER I 378       4.401  -3.698   4.198  1.00  0.00
I    N
ATOM   5402  HN  SER I 378       3.923  -3.916   5.049  1.00  0.00
I    H
ATOM   5403  CA  SER I 378       3.859  -4.369   3.016  1.00  0.00
I    C
ATOM   5404  HA  SER I 378       4.695  -4.923   2.608  1.00  0.00
I    H
ATOM   5405  CB  SER I 378       2.637  -5.250   3.380  1.00  0.00
I    C
ATOM   5406  HB1 SER I 378       1.971  -4.659   4.049  1.00  0.00
I    H
ATOM   5407  HB2 SER I 378       2.050  -5.496   2.466  1.00  0.00
I    H
ATOM   5408  OG  SER I 378       3.072  -6.425   4.063  1.00  0.00
I    O
ATOM   5409  HG1 SER I 378       2.290  -6.826   4.463  1.00  0.00
I    H
ATOM   5410  C   SER I 378       3.372  -3.431   1.937  1.00  0.00
I    C
ATOM   5411  O   SER I 378       3.697  -3.590   0.761  1.00  0.00
I    O
ATOM   5412  N   LEU I 379       2.596  -2.396   2.307  1.00  0.00
I    N
ATOM   5413  HN  LEU I 379       2.320  -2.236   3.252  1.00  0.00
I    H
ATOM   5414  CA  LEU I 379       2.091  -1.419   1.365  1.00  0.00
I    C
ATOM   5415  HA  LEU I 379       1.614  -1.989   0.578  1.00  0.00
I    H
ATOM   5416  CB  LEU I 379       1.034  -0.524   2.047  1.00  0.00
I    C
ATOM   5417  HB1 LEU I 379       0.461  -1.170   2.752  1.00  0.00
I    H
ATOM   5418  HB2 LEU I 379       1.553   0.233   2.676  1.00  0.00
I    H
ATOM   5419  CG  LEU I 379       0.051   0.154   1.074  1.00  0.00
I    C
ATOM   5420  HG  LEU I 379       0.661   0.744   0.348  1.00  0.00
I    H
ATOM   5421  CD1 LEU I 379      -0.827  -0.867   0.323  1.00  0.00
I    C
ATOM   5422 HD11 LEU I 379      -1.556  -0.346  -0.333  1.00  0.00
I    H
ATOM   5423 HD12 LEU I 379      -0.200  -1.532  -0.309  1.00  0.00
I    H
ATOM   5424 HD13 LEU I 379      -1.395  -1.498   1.040  1.00  0.00
I    H
ATOM   5425  CD2 LEU I 379      -0.844   1.130   1.845  1.00  0.00
I    C
ATOM   5426 HD21 LEU I 379      -1.546   1.641   1.151  1.00  0.00
I    H
```

```
ATOM   5427 HD22 LEU I 379      -1.449   0.611   2.614  1.00  0.00
I    H
ATOM   5428 HD23 LEU I 379      -0.228   1.907   2.350  1.00  0.00
I    H
ATOM   5429  C   LEU I 379       3.185  -0.574   0.724  1.00  0.00
I    C
ATOM   5430  O   LEU I 379       3.155  -0.286  -0.471  1.00  0.00
I    O
ATOM   5431  N   PHE I 380       4.213  -0.186   1.510  1.00  0.00
I    N
ATOM   5432  HN  PHE I 380       4.225  -0.383   2.489  1.00  0.00
I    H
ATOM   5433  CA  PHE I 380       5.374   0.538   1.029  1.00  0.00
I    C
ATOM   5434  HA  PHE I 380       4.979   1.405   0.514  1.00  0.00
I    H
ATOM   5435  CB  PHE I 380       6.246   0.994   2.233  1.00  0.00
I    C
ATOM   5436  HB1 PHE I 380       5.580   1.279   3.077  1.00  0.00
I    H
ATOM   5437  HB2 PHE I 380       6.896   0.167   2.593  1.00  0.00
I    H
ATOM   5438  CG  PHE I 380       7.069   2.205   1.880  1.00  0.00
I    C
ATOM   5439  CD1 PHE I 380       6.437   3.394   1.472  1.00  0.00
I    C
ATOM   5440  HD1 PHE I 380       5.358   3.431   1.406  1.00  0.00
I    H
ATOM   5441  CE1 PHE I 380       7.189   4.530   1.144  1.00  0.00
I    C
ATOM   5442  HE1 PHE I 380       6.690   5.434   0.826  1.00  0.00
I    H
ATOM   5443  CZ  PHE I 380       8.584   4.490   1.236  1.00  0.00
I    C
ATOM   5444  HZ  PHE I 380       9.168   5.368   0.997  1.00  0.00
I    H
ATOM   5445  CD2 PHE I 380       8.470   2.178   1.966  1.00  0.00
I    C
ATOM   5446  HD2 PHE I 380       8.976   1.268   2.258  1.00  0.00
I    H
ATOM   5447  CE2 PHE I 380       9.226   3.315   1.649  1.00  0.00
I    C
ATOM   5448  HE2 PHE I 380      10.304   3.283   1.723  1.00  0.00
I    H
ATOM   5449  C   PHE I 380       6.195  -0.254   0.010  1.00  0.00
I    C
ATOM   5450  O   PHE I 380       6.582   0.269  -1.031  1.00  0.00
I    O
ATOM   5451  N   ILE I 381       6.423  -1.564   0.249  1.00  0.00
I    N
ATOM   5452  HN  ILE I 381       6.146  -1.996   1.105  1.00  0.00
I    H
ATOM   5453  CA  ILE I 381       7.083  -2.468  -0.694  1.00  0.00
I    C
```

```
ATOM   5454  HA  ILE I 381       8.022  -1.994  -0.951  1.00  0.00
I    H
ATOM   5455  CB  ILE I 381       7.356  -3.827  -0.058  1.00  0.00
I    C
ATOM   5456  HB  ILE I 381       6.376  -4.128   0.391  1.00  0.00
I    H
ATOM   5457  CG2 ILE I 381       7.912  -4.845  -1.089  1.00  0.00
I    C
ATOM   5458 HG21 ILE I 381       7.956  -5.863  -0.649  1.00  0.00
I    H
ATOM   5459 HG22 ILE I 381       7.261  -4.896  -1.987  1.00  0.00
I    H
ATOM   5460 HG23 ILE I 381       8.936  -4.563  -1.414  1.00  0.00
I    H
ATOM   5461  CG1 ILE I 381       8.360  -3.653   1.103  1.00  0.00
I    C
ATOM   5462 HG11 ILE I 381       9.396  -3.598   0.700  1.00  0.00
I    H
ATOM   5463 HG12 ILE I 381       8.154  -2.686   1.616  1.00  0.00
I    H
ATOM   5464  CD  ILE I 381       8.248  -4.792   2.113  1.00  0.00
I    C
ATOM   5465  HD1 ILE I 381       8.936  -4.618   2.969  1.00  0.00
I    H
ATOM   5466  HD2 ILE I 381       7.212  -4.864   2.510  1.00  0.00
I    H
ATOM   5467  HD3 ILE I 381       8.515  -5.764   1.649  1.00  0.00
I    H
ATOM   5468  C   ILE I 381       6.298  -2.624  -1.991  1.00  0.00
I    C
ATOM   5469  O   ILE I 381       6.862  -2.573  -3.083  1.00  0.00
I    O
ATOM   5470  N   VAL I 382       4.955  -2.757  -1.910  1.00  0.00
I    N
ATOM   5471  HN  VAL I 382       4.481  -2.872  -1.040  1.00  0.00
I    H
ATOM   5472  CA  VAL I 382       4.077  -2.746  -3.077  1.00  0.00
I    C
ATOM   5473  HA  VAL I 382       4.416  -3.539  -3.733  1.00  0.00
I    H
ATOM   5474  CB  VAL I 382       2.628  -3.045  -2.688  1.00  0.00
I    C
ATOM   5475  HB  VAL I 382       2.356  -2.359  -1.847  1.00  0.00
I    H
ATOM   5476  CG1 VAL I 382       1.647  -2.799  -3.853  1.00  0.00
I    C
ATOM   5477 HG11 VAL I 382       0.611  -3.062  -3.548  1.00  0.00
I    H
ATOM   5478 HG12 VAL I 382       1.639  -1.733  -4.164  1.00  0.00
I    H
ATOM   5479 HG13 VAL I 382       1.916  -3.424  -4.731  1.00  0.00
I    H
ATOM   5480  CG2 VAL I 382       2.530  -4.520  -2.257  1.00  0.00
I    C
```

```
ATOM   5481 HG21 VAL I 382       1.482  -4.786  -2.004  1.00  0.00
I    H
ATOM   5482 HG22 VAL I 382       2.874  -5.190  -3.075  1.00  0.00
I    H
ATOM   5483 HG23 VAL I 382       3.153  -4.718  -1.360  1.00  0.00
I    H
ATOM   5484  C   VAL I 382       4.167  -1.436  -3.857  1.00  0.00
I    C
ATOM   5485  O   VAL I 382       4.327  -1.440  -5.075  1.00  0.00
I    O
ATOM   5486  N   PHE I 383       4.145  -0.271  -3.172  1.00  0.00
I    N
ATOM   5487  HN  PHE I 383       4.000  -0.250  -2.184  1.00  0.00
I    H
ATOM   5488  CA  PHE I 383       4.326   1.037  -3.783  1.00  0.00
I    C
ATOM   5489  HA  PHE I 383       3.561   1.111  -4.547  1.00  0.00
I    H
ATOM   5490  CB  PHE I 383       4.128   2.134  -2.688  1.00  0.00
I    C
ATOM   5491  HB1 PHE I 383       3.070   2.124  -2.350  1.00  0.00
I    H
ATOM   5492  HB2 PHE I 383       4.759   1.927  -1.796  1.00  0.00
I    H
ATOM   5493  CG  PHE I 383       4.406   3.525  -3.204  1.00  0.00
I    C
ATOM   5494  CD1 PHE I 383       3.450   4.221  -3.959  1.00  0.00
I    C
ATOM   5495  HD1 PHE I 383       2.471   3.787  -4.113  1.00  0.00
I    H
ATOM   5496  CE1 PHE I 383       3.756   5.467  -4.518  1.00  0.00
I    C
ATOM   5497  HE1 PHE I 383       3.010   6.004  -5.085  1.00  0.00
I    H
ATOM   5498  CZ  PHE I 383       5.030   6.023  -4.338  1.00  0.00
I    C
ATOM   5499  HZ  PHE I 383       5.265   6.987  -4.768  1.00  0.00
I    H
ATOM   5500  CD2 PHE I 383       5.678   4.098  -3.020  1.00  0.00
I    C
ATOM   5501  HD2 PHE I 383       6.431   3.571  -2.449  1.00  0.00
I    H
ATOM   5502  CE2 PHE I 383       5.994   5.335  -3.592  1.00  0.00
I    C
ATOM   5503  HE2 PHE I 383       6.981   5.755  -3.457  1.00  0.00
I    H
ATOM   5504  C   PHE I 383       5.682   1.172  -4.488  1.00  0.00
I    C
ATOM   5505  O   PHE I 383       5.749   1.579  -5.648  1.00  0.00
I    O
ATOM   5506  N   LEU I 384       6.789   0.764  -3.830  1.00  0.00
I    N
ATOM   5507  HN  LEU I 384       6.751   0.440  -2.887  1.00  0.00
I    H
```

```
ATOM   5508  CA  LEU I 384       8.124   0.753  -4.413  1.00  0.00
I    C
ATOM   5509  HA  LEU I 384       8.312   1.766  -4.743  1.00  0.00
I    H
ATOM   5510  CB  LEU I 384       9.207   0.350  -3.378  1.00  0.00
I    C
ATOM   5511  HB1 LEU I 384       8.859  -0.588  -2.886  1.00  0.00
I    H
ATOM   5512  HB2 LEU I 384      10.143   0.085  -3.918  1.00  0.00
I    H
ATOM   5513  CG  LEU I 384       9.489   1.409  -2.289  1.00  0.00
I    C
ATOM   5514  HG  LEU I 384       8.516   1.602  -1.776  1.00  0.00
I    H
ATOM   5515  CD1 LEU I 384      10.513   0.858  -1.285  1.00  0.00
I    C
ATOM   5516 HD11 LEU I 384      10.763   1.626  -0.521  1.00  0.00
I    H
ATOM   5517 HD12 LEU I 384      10.108  -0.034  -0.762  1.00  0.00
I    H
ATOM   5518 HD13 LEU I 384      11.452   0.566  -1.802  1.00  0.00
I    H
ATOM   5519  CD2 LEU I 384       9.994   2.743  -2.870  1.00  0.00
I    C
ATOM   5520 HD21 LEU I 384      10.164   3.479  -2.056  1.00  0.00
I    H
ATOM   5521 HD22 LEU I 384      10.950   2.616  -3.416  1.00  0.00
I    H
ATOM   5522 HD23 LEU I 384       9.245   3.170  -3.572  1.00  0.00
I    H
ATOM   5523  C   LEU I 384       8.229  -0.153  -5.629  1.00  0.00
I    C
ATOM   5524  O   LEU I 384       8.850   0.202  -6.630  1.00  0.00
I    O
ATOM   5525  N   GLY I 385       7.582  -1.336  -5.596  1.00  0.00
I    N
ATOM   5526  HN  GLY I 385       7.098  -1.646  -4.778  1.00  0.00
I    H
ATOM   5527  CA  GLY I 385       7.537  -2.244  -6.734  1.00  0.00
I    C
ATOM   5528  HA1 GLY I 385       7.057  -3.145  -6.380  1.00  0.00
I    H
ATOM   5529  HA2 GLY I 385       8.563  -2.383  -7.052  1.00  0.00
I    H
ATOM   5530  C   GLY I 385       6.751  -1.728  -7.914  1.00  0.00
I    C
ATOM   5531  O   GLY I 385       7.179  -1.866  -9.055  1.00  0.00
I    O
ATOM   5532  N   ILE I 386       5.594  -1.071  -7.678  1.00  0.00
I    N
ATOM   5533  HN  ILE I 386       5.189  -1.014  -6.768  1.00  0.00
I    H
ATOM   5534  CA  ILE I 386       4.830  -0.386  -8.720  1.00  0.00
I    C
```

```
ATOM   5535  HA  ILE I 386       4.683  -1.111  -9.510  1.00  0.00
I    H
ATOM   5536  CB  ILE I 386       3.454   0.077  -8.217  1.00  0.00
I    C
ATOM   5537  HB  ILE I 386       3.672   0.617  -7.261  1.00  0.00
I    H
ATOM   5538  CG2 ILE I 386       2.729   0.944  -9.278  1.00  0.00
I    C
ATOM   5539 HG21 ILE I 386       1.812   1.400  -8.850  1.00  0.00
I    H
ATOM   5540 HG22 ILE I 386       3.383   1.770  -9.628  1.00  0.00
I    H
ATOM   5541 HG23 ILE I 386       2.435   0.333 -10.157  1.00  0.00
I    H
ATOM   5542  CG1 ILE I 386       2.593  -1.159  -7.860  1.00  0.00
I    C
ATOM   5543 HG11 ILE I 386       2.332  -1.717  -8.787  1.00  0.00
I    H
ATOM   5544 HG12 ILE I 386       3.195  -1.846  -7.222  1.00  0.00
I    H
ATOM   5545  CD  ILE I 386       1.300  -0.829  -7.103  1.00  0.00
I    C
ATOM   5546  HD1 ILE I 386       0.763  -1.764  -6.830  1.00  0.00
I    H
ATOM   5547  HD2 ILE I 386       1.524  -0.270  -6.169  1.00  0.00
I    H
ATOM   5548  HD3 ILE I 386       0.620  -0.213  -7.728  1.00  0.00
I    H
ATOM   5549  C   ILE I 386       5.611   0.776  -9.328  1.00  0.00
I    C
ATOM   5550  O   ILE I 386       5.680   0.909 -10.547  1.00  0.00
I    O
ATOM   5551  N   PHE I 387       6.266   1.612  -8.491  1.00  0.00
I    N
ATOM   5552  HN  PHE I 387       6.190   1.513  -7.500  1.00  0.00
I    H
ATOM   5553  CA  PHE I 387       7.120   2.708  -8.924  1.00  0.00
I    C
ATOM   5554  HA  PHE I 387       6.505   3.310  -9.581  1.00  0.00
I    H
ATOM   5555  CB  PHE I 387       7.584   3.497  -7.660  1.00  0.00
I    C
ATOM   5556  HB1 PHE I 387       6.692   3.922  -7.149  1.00  0.00
I    H
ATOM   5557  HB2 PHE I 387       8.090   2.827  -6.932  1.00  0.00
I    H
ATOM   5558  CG  PHE I 387       8.487   4.668  -7.977  1.00  0.00
I    C
ATOM   5559  CD1 PHE I 387       7.970   5.880  -8.466  1.00  0.00
I    C
ATOM   5560  HD1 PHE I 387       6.904   5.989  -8.607  1.00  0.00
I    H
ATOM   5561  CE1 PHE I 387       8.828   6.944  -8.777  1.00  0.00
I    C
```

```
ATOM   5562  HE1 PHE I 387       8.422   7.879  -9.137  1.00  0.00
I    H
ATOM   5563  CZ  PHE I 387      10.210   6.808  -8.618  1.00  0.00
I    C
ATOM   5564  HZ  PHE I 387      10.868   7.633  -8.849  1.00  0.00
I    H
ATOM   5565  CD2 PHE I 387       9.881   4.542  -7.826  1.00  0.00
I    C
ATOM   5566  HD2 PHE I 387      10.304   3.611  -7.477  1.00  0.00
I    H
ATOM   5567  CE2 PHE I 387      10.739   5.602  -8.148  1.00  0.00
I    C
ATOM   5568  HE2 PHE I 387      11.808   5.483  -8.034  1.00  0.00
I    H
ATOM   5569  C   PHE I 387       8.325   2.240  -9.741  1.00  0.00
I    C
ATOM   5570  O   PHE I 387       8.627   2.791 -10.794  1.00  0.00
I    O
ATOM   5571  N   ALA I 388       9.047   1.191  -9.300  1.00  0.00
I    N
ATOM   5572  HN  ALA I 388       8.838   0.723  -8.442  1.00  0.00
I    H
ATOM   5573  CA  ALA I 388      10.178   0.665 -10.037  1.00  0.00
I    C
ATOM   5574  HA  ALA I 388      10.871   1.472 -10.250  1.00  0.00
I    H
ATOM   5575  CB  ALA I 388      10.964  -0.311  -9.137  1.00  0.00
I    C
ATOM   5576  HB1 ALA I 388      11.251   0.201  -8.193  1.00  0.00
I    H
ATOM   5577  HB2 ALA I 388      10.345  -1.196  -8.872  1.00  0.00
I    H
ATOM   5578  HB3 ALA I 388      11.894  -0.664  -9.631  1.00  0.00
I    H
ATOM   5579  C   ALA I 388       9.808   0.043 -11.391  1.00  0.00
I    C
ATOM   5580  O   ALA I 388      10.496   0.240 -12.390  1.00  0.00
I    O
ATOM   5581  N   LEU I 389       8.681  -0.698 -11.471  1.00  0.00
I    N
ATOM   5582  HN  LEU I 389       8.133  -0.916 -10.666  1.00  0.00
I    H
ATOM   5583  CA  LEU I 389       8.160  -1.244 -12.719  1.00  0.00
I    C
ATOM   5584  HA  LEU I 389       8.990  -1.784 -13.155  1.00  0.00
I    H
ATOM   5585  CB  LEU I 389       7.053  -2.291 -12.464  1.00  0.00
I    C
ATOM   5586  HB1 LEU I 389       6.341  -1.845 -11.732  1.00  0.00
I    H
ATOM   5587  HB2 LEU I 389       6.476  -2.443 -13.402  1.00  0.00
I    H
ATOM   5588  CG  LEU I 389       7.581  -3.632 -11.910  1.00  0.00
I    C
```

```
ATOM   5589  HG  LEU I 389       8.174  -3.393 -10.994  1.00  0.00
I    H
ATOM   5590  CD1 LEU I 389       6.401  -4.540 -11.559  1.00  0.00
I    C
ATOM   5591 HD11 LEU I 389       6.762  -5.529 -11.204  1.00  0.00
I    H
ATOM   5592 HD12 LEU I 389       5.783  -4.085 -10.756  1.00  0.00
I    H
ATOM   5593 HD13 LEU I 389       5.756  -4.706 -12.447  1.00  0.00
I    H
ATOM   5594  CD2 LEU I 389       8.502  -4.368 -12.896  1.00  0.00
I    C
ATOM   5595 HD21 LEU I 389       8.870  -5.316 -12.446  1.00  0.00
I    H
ATOM   5596 HD22 LEU I 389       7.975  -4.625 -13.836  1.00  0.00
I    H
ATOM   5597 HD23 LEU I 389       9.383  -3.739 -13.149  1.00  0.00
I    H
ATOM   5598  C   LEU I 389       7.701  -0.203 -13.736  1.00  0.00
I    C
ATOM   5599  O   LEU I 389       7.924  -0.366 -14.931  1.00  0.00
I    O
ATOM   5600  N   THR I 390       7.098   0.934 -13.325  1.00  0.00
I    N
ATOM   5601  HN  THR I 390       6.920   1.145 -12.365  1.00  0.00
I    H
ATOM   5602  CA  THR I 390       6.646   1.971 -14.267  1.00  0.00
I    C
ATOM   5603  HA  THR I 390       6.166   1.425 -15.067  1.00  0.00
I    H
ATOM   5604  CB  THR I 390       5.667   2.984 -13.686  1.00  0.00
I    C
ATOM   5605  HB  THR I 390       5.644   3.840 -14.403  1.00  0.00
I    H
ATOM   5606  OG1 THR I 390       6.096   3.508 -12.441  1.00  0.00
I    O
ATOM   5607  HG1 THR I 390       5.404   4.120 -12.168  1.00  0.00
I    H
ATOM   5608  CG2 THR I 390       4.311   2.322 -13.442  1.00  0.00
I    C
ATOM   5609 HG21 THR I 390       3.556   3.072 -13.127  1.00  0.00
I    H
ATOM   5610 HG22 THR I 390       3.943   1.838 -14.372  1.00  0.00
I    H
ATOM   5611 HG23 THR I 390       4.382   1.545 -12.651  1.00  0.00
I    H
ATOM   5612  C   THR I 390       7.778   2.762 -14.900  1.00  0.00
I    C
ATOM   5613  O   THR I 390       7.566   3.566 -15.808  1.00  0.00
I    O
ATOM   5614  N   ILE I 391       9.024   2.571 -14.431  1.00  0.00
I    N
ATOM   5615  HN  ILE I 391       9.215   2.019 -13.623  1.00  0.00
I    H
```

```
ATOM   5616  CA  ILE I 391      10.206   3.148 -15.052  1.00  0.00
I    C
ATOM   5617  HA  ILE I 391       9.862   3.990 -15.638  1.00  0.00
I    H
ATOM   5618  CB  ILE I 391      11.212   3.560 -13.973  1.00  0.00
I    C
ATOM   5619  HB  ILE I 391      11.426   2.614 -13.414  1.00  0.00
I    H
ATOM   5620  CG2 ILE I 391      12.476   4.195 -14.605  1.00  0.00
I    C
ATOM   5621 HG21 ILE I 391      13.262   4.348 -13.836  1.00  0.00
I    H
ATOM   5622 HG22 ILE I 391      12.899   3.536 -15.392  1.00  0.00
I    H
ATOM   5623 HG23 ILE I 391      12.240   5.180 -15.061  1.00  0.00
I    H
ATOM   5624  CG1 ILE I 391      10.525   4.537 -12.984  1.00  0.00
I    C
ATOM   5625 HG11 ILE I 391      10.173   5.440 -13.531  1.00  0.00
I    H
ATOM   5626 HG12 ILE I 391       9.625   4.041 -12.554  1.00  0.00
I    H
ATOM   5627  CD  ILE I 391      11.413   4.986 -11.818  1.00  0.00
I    C
ATOM   5628  HD1 ILE I 391      10.838   5.635 -11.123  1.00  0.00
I    H
ATOM   5629  HD2 ILE I 391      11.784   4.107 -11.247  1.00  0.00
I    H
ATOM   5630  HD3 ILE I 391      12.288   5.563 -12.183  1.00  0.00
I    H
ATOM   5631  C   ILE I 391      10.853   2.167 -16.026  1.00  0.00
I    C
ATOM   5632  O   ILE I 391      11.471   2.576 -17.012  1.00  0.00
I    O
ATOM   5633  N   THR I 392      10.699   0.852 -15.782  1.00  0.00
I    N
ATOM   5634  HN  THR I 392      10.074   0.522 -15.075  1.00  0.00
I    H
ATOM   5635  CA  THR I 392      11.404  -0.206 -16.501  1.00  0.00
I    C
ATOM   5636  HA  THR I 392      12.234   0.283 -16.992  1.00  0.00
I    H
ATOM   5637  CB  THR I 392      11.823  -1.300 -15.521  1.00  0.00
I    C
ATOM   5638  HB  THR I 392      10.894  -1.608 -14.983  1.00  0.00
I    H
ATOM   5639  OG1 THR I 392      12.728  -0.783 -14.555  1.00  0.00
I    O
ATOM   5640  HG1 THR I 392      12.952  -1.529 -13.988  1.00  0.00
I    H
ATOM   5641  CG2 THR I 392      12.576  -2.441 -16.200  1.00  0.00
I    C
ATOM   5642 HG21 THR I 392      12.766  -3.269 -15.485  1.00  0.00
I    H
```

ATOM   5643 HG22 THR I 392   11.981 -2.848 -17.045 1.00 0.00
I   H
ATOM   5644 HG23 THR I 392   13.553 -2.094 -16.598 1.00 0.00
I   H
ATOM   5645 C   THR I 392   10.552 -0.824 -17.602 1.00 0.00
I   C
ATOM   5646 O   THR I 392   11.055 -1.207 -18.661 1.00 0.00
I   O
ATOM   5647 N   GLU I 393   9.218 -0.881 -17.426 1.00 0.00
I   N
ATOM   5648 HN GLU I 393   8.769 -0.476 -16.634 1.00 0.00
I   H
ATOM   5649 CA GLU I 393   8.310 -1.525 -18.352 1.00 0.00
I   C
ATOM   5650 HA GLU I 393   8.902 -2.052 -19.089 1.00 0.00
I   H
ATOM   5651 CB GLU I 393   7.360 -2.508 -17.617 1.00 0.00
I   C
ATOM   5652 HB1 GLU I 393   7.220 -2.142 -16.574 1.00 0.00
I   H
ATOM   5653 HB2 GLU I 393   6.351 -2.481 -18.088 1.00 0.00
I   H
ATOM   5654 CG GLU I 393   7.862 -3.977 -17.561 1.00 0.00
I   C
ATOM   5655 HG1 GLU I 393   7.144 -4.590 -16.988 1.00 0.00
I   H
ATOM   5656 HG2 GLU I 393   7.970 -4.399 -18.580 1.00 0.00
I   H
ATOM   5657 CD GLU I 393   9.196 -4.206 -16.849 1.00 0.00
I   C
ATOM   5658 OE1 GLU I 393   10.016 -5.006 -17.380 1.00 0.00
I   O
ATOM   5659 OE2 GLU I 393   9.420 -3.641 -15.755 1.00 0.00
I   O
ATOM   5660 C   GLU I 393   7.464 -0.511 -19.095 1.00 0.00
I   C
ATOM   5661 O   GLU I 393   6.971   0.476 -18.558 1.00 0.00
I   O
ATOM   5662 N   GLN I 394   7.236 -0.771 -20.396 1.00 0.00
I   N
ATOM   5663 HN GLN I 394   7.663 -1.542 -20.864 1.00 0.00
I   H
ATOM   5664 CA GLN I 394   6.362   0.028 -21.231 1.00 0.00
I   C
ATOM   5665 HA GLN I 394   6.225   0.992 -20.757 1.00 0.00
I   H
ATOM   5666 CB GLN I 394   6.943   0.160 -22.664 1.00 0.00
I   C
ATOM   5667 HB1 GLN I 394   6.814 -0.832 -23.157 1.00 0.00
I   H
ATOM   5668 HB2 GLN I 394   6.324   0.880 -23.248 1.00 0.00
I   H
ATOM   5669 CG GLN I 394   8.435   0.564 -22.698 1.00 0.00
I   C

```
ATOM   5670  HG1 GLN I 394       8.572   1.582 -22.275  1.00  0.00
I    H
ATOM   5671  HG2 GLN I 394       9.046  -0.140 -22.095  1.00  0.00
I    H
ATOM   5672  CD  GLN I 394       8.941   0.591 -24.141  1.00  0.00
I    C
ATOM   5673  OE1 GLN I 394       8.533   1.414 -24.957  1.00  0.00
I    O
ATOM   5674  NE2 GLN I 394       9.856  -0.344 -24.485  1.00  0.00
I    N
ATOM   5675 HE21 GLN I 394      10.189  -0.324 -25.423  1.00  0.00
I    H
ATOM   5676 HE22 GLN I 394      10.151  -1.017 -23.815  1.00  0.00
I    H
ATOM   5677  C   GLN I 394       5.001  -0.655 -21.284  1.00  0.00
I    C
ATOM   5678  O   GLN I 394       4.503  -1.017 -22.346  1.00  0.00
I    O
ATOM   5679  N   ALA I 395       4.385  -0.899 -20.113  1.00  0.00
I    N
ATOM   5680  HN  ALA I 395       4.718  -0.504 -19.257  1.00  0.00
I    H
ATOM   5681  CA  ALA I 395       3.214  -1.736 -19.995  1.00  0.00
I    C
ATOM   5682  HA  ALA I 395       2.893  -2.103 -20.963  1.00  0.00
I    H
ATOM   5683  CB  ALA I 395       3.507  -2.906 -19.033  1.00  0.00
I    C
ATOM   5684  HB1 ALA I 395       4.317  -3.540 -19.452  1.00  0.00
I    H
ATOM   5685  HB2 ALA I 395       3.844  -2.533 -18.041  1.00  0.00
I    H
ATOM   5686  HB3 ALA I 395       2.612  -3.548 -18.884  1.00  0.00
I    H
ATOM   5687  C   ALA I 395       2.057  -0.917 -19.440  1.00  0.00
I    C
ATOM   5688  O   ALA I 395       2.317   0.008 -18.676  1.00  0.00
I    O
ATOM   5689  N   PRO I 396       0.788  -1.172 -19.767  1.00  0.00
I    N
ATOM   5690  CD  PRO I 396       0.372  -2.136 -20.786  1.00  0.00
I    C
ATOM   5691  HD1 PRO I 396       0.402  -1.765 -21.836  1.00  0.00
I    H
ATOM   5692  HD2 PRO I 396       1.034  -3.025 -20.669  1.00  0.00
I    H
ATOM   5693  CA  PRO I 396      -0.358  -0.536 -19.117  1.00  0.00
I    C
ATOM   5694  HA  PRO I 396      -0.394   0.502 -19.425  1.00  0.00
I    H
ATOM   5695  CB  PRO I 396      -1.596  -1.221 -19.737  1.00  0.00
I    C
ATOM   5696  HB1 PRO I 396      -1.855  -0.707 -20.689  1.00  0.00
I    H
```

```
ATOM   5697  HB2 PRO I 396      -2.478  -1.201 -19.065  1.00  0.00
I    H
ATOM   5698  CG  PRO I 396      -1.059  -2.496 -20.400  1.00  0.00
I    C
ATOM   5699  HG1 PRO I 396      -1.715  -2.871 -21.210  1.00  0.00
I    H
ATOM   5700  HG2 PRO I 396      -0.936  -3.288 -19.626  1.00  0.00
I    H
ATOM   5701  C   PRO I 396      -0.369  -0.605 -17.596  1.00  0.00
I    C
ATOM   5702  O   PRO I 396      -0.227  -1.691 -17.032  1.00  0.00
I    O
ATOM   5703  N   PHE I 397      -0.609   0.547 -16.924  1.00  0.00
I    N
ATOM   5704  HN  PHE I 397      -0.882   1.366 -17.428  1.00  0.00
I    H
ATOM   5705  CA  PHE I 397      -0.510   0.731 -15.487  1.00  0.00
I    C
ATOM   5706  HA  PHE I 397       0.545   0.621 -15.270  1.00  0.00
I    H
ATOM   5707  CB  PHE I 397      -1.011   2.163 -15.130  1.00  0.00
I    C
ATOM   5708  HB1 PHE I 397      -0.456   2.907 -15.743  1.00  0.00
I    H
ATOM   5709  HB2 PHE I 397      -2.090   2.282 -15.369  1.00  0.00
I    H
ATOM   5710  CG  PHE I 397      -0.752   2.498 -13.681  1.00  0.00
I    C
ATOM   5711  CD1 PHE I 397       0.564   2.515 -13.199  1.00  0.00
I    C
ATOM   5712  HD1 PHE I 397       1.385   2.335 -13.879  1.00  0.00
I    H
ATOM   5713  CE1 PHE I 397       0.826   2.756 -11.846  1.00  0.00
I    C
ATOM   5714  HE1 PHE I 397       1.844   2.757 -11.485  1.00  0.00
I    H
ATOM   5715  CZ  PHE I 397      -0.230   3.002 -10.961  1.00  0.00
I    C
ATOM   5716  HZ  PHE I 397      -0.029   3.207  -9.919  1.00  0.00
I    H
ATOM   5717  CD2 PHE I 397      -1.807   2.745 -12.786  1.00  0.00
I    C
ATOM   5718  HD2 PHE I 397      -2.829   2.723 -13.137  1.00  0.00
I    H
ATOM   5719  CE2 PHE I 397      -1.551   2.994 -11.429  1.00  0.00
I    C
ATOM   5720  HE2 PHE I 397      -2.373   3.177 -10.751  1.00  0.00
I    H
ATOM   5721  C   PHE I 397      -1.250  -0.316 -14.655  1.00  0.00
I    C
ATOM   5722  O   PHE I 397      -0.702  -0.866 -13.705  1.00  0.00
I    O
ATOM   5723  N   LEU I 398      -2.500  -0.680 -15.020  1.00  0.00
I    N
```

```
ATOM   5724  HN  LEU I 398      -2.966  -0.299 -15.816  1.00  0.00
I    H
ATOM   5725  CA  LEU I 398      -3.281  -1.664 -14.283  1.00  0.00
I    C
ATOM   5726  HA  LEU I 398      -3.364  -1.275 -13.276  1.00  0.00
I    H
ATOM   5727  CB  LEU I 398      -4.701  -1.804 -14.881  1.00  0.00
I    C
ATOM   5728  HB1 LEU I 398      -5.141  -0.781 -14.927  1.00  0.00
I    H
ATOM   5729  HB2 LEU I 398      -4.618  -2.151 -15.934  1.00  0.00
I    H
ATOM   5730  CG  LEU I 398      -5.666  -2.720 -14.088  1.00  0.00
I    C
ATOM   5731  HG  LEU I 398      -5.324  -3.768 -14.265  1.00  0.00
I    H
ATOM   5732  CD1 LEU I 398      -5.711  -2.414 -12.581  1.00  0.00
I    C
ATOM   5733 HD11 LEU I 398      -6.452  -3.067 -12.073  1.00  0.00
I    H
ATOM   5734 HD12 LEU I 398      -4.716  -2.589 -12.117  1.00  0.00
I    H
ATOM   5735 HD13 LEU I 398      -6.000  -1.356 -12.403  1.00  0.00
I    H
ATOM   5736  CD2 LEU I 398      -7.081  -2.581 -14.660  1.00  0.00
I    C
ATOM   5737 HD21 LEU I 398      -7.785  -3.246 -14.114  1.00  0.00
I    H
ATOM   5738 HD22 LEU I 398      -7.460  -1.542 -14.569  1.00  0.00
I    H
ATOM   5739 HD23 LEU I 398      -7.097  -2.865 -15.734  1.00  0.00
I    H
ATOM   5740  C   LEU I 398      -2.608  -3.032 -14.194  1.00  0.00
I    C
ATOM   5741  O   LEU I 398      -2.605  -3.676 -13.147  1.00  0.00
I    O
ATOM   5742  N   GLN I 399      -1.958  -3.479 -15.286  1.00  0.00
I    N
ATOM   5743  HN  GLN I 399      -1.972  -2.983 -16.152  1.00  0.00
I    H
ATOM   5744  CA  GLN I 399      -1.186  -4.705 -15.310  1.00  0.00
I    C
ATOM   5745  HA  GLN I 399      -1.840  -5.479 -14.929  1.00  0.00
I    H
ATOM   5746  CB  GLN I 399      -0.810  -5.090 -16.755  1.00  0.00
I    C
ATOM   5747  HB1 GLN I 399      -0.073  -4.334 -17.110  1.00  0.00
I    H
ATOM   5748  HB2 GLN I 399      -0.273  -6.066 -16.749  1.00  0.00
I    H
ATOM   5749  CG  GLN I 399      -2.057  -5.112 -17.670  1.00  0.00
I    C
ATOM   5750  HG1 GLN I 399      -2.913  -5.592 -17.148  1.00  0.00
I    H
```

```
ATOM   5751  HG2 GLN I 399      -2.361  -4.080 -17.943  1.00  0.00
I    H
ATOM   5752  CD  GLN I 399      -1.804  -5.917 -18.942  1.00  0.00
I    C
ATOM   5753  OE1 GLN I 399      -0.675  -6.280 -19.264  1.00  0.00
I    O
ATOM   5754  NE2 GLN I 399      -2.891  -6.222 -19.686  1.00  0.00
I    N
ATOM   5755 HE21 GLN I 399      -2.735  -6.751 -20.515  1.00  0.00
I    H
ATOM   5756 HE22 GLN I 399      -3.790  -5.904 -19.403  1.00  0.00
I    H
ATOM   5757  C   GLN I 399       0.043  -4.660 -14.412  1.00  0.00
I    C
ATOM   5758  O   GLN I 399       0.323  -5.616 -13.694  1.00  0.00
I    O
ATOM   5759  N   ILE I 400       0.773  -3.521 -14.384  1.00  0.00
I    N
ATOM   5760  HN  ILE I 400       0.592  -2.763 -15.008  1.00  0.00
I    H
ATOM   5761  CA  ILE I 400       1.878  -3.282 -13.458  1.00  0.00
I    C
ATOM   5762  HA  ILE I 400       2.564  -4.108 -13.595  1.00  0.00
I    H
ATOM   5763  CB  ILE I 400       2.603  -1.966 -13.769  1.00  0.00
I    C
ATOM   5764  HB  ILE I 400       1.779  -1.222 -13.921  1.00  0.00
I    H
ATOM   5765  CG2 ILE I 400       3.566  -1.583 -12.616  1.00  0.00
I    C
ATOM   5766 HG21 ILE I 400       3.970  -0.560 -12.767  1.00  0.00
I    H
ATOM   5767 HG22 ILE I 400       3.039  -1.595 -11.639  1.00  0.00
I    H
ATOM   5768 HG23 ILE I 400       4.422  -2.289 -12.562  1.00  0.00
I    H
ATOM   5769  CG1 ILE I 400       3.350  -2.112 -15.120  1.00  0.00
I    C
ATOM   5770 HG11 ILE I 400       4.103  -2.929 -15.047  1.00  0.00
I    H
ATOM   5771 HG12 ILE I 400       2.619  -2.408 -15.907  1.00  0.00
I    H
ATOM   5772  CD  ILE I 400       4.055  -0.834 -15.588  1.00  0.00
I    C
ATOM   5773  HD1 ILE I 400       4.520  -0.992 -16.585  1.00  0.00
I    H
ATOM   5774  HD2 ILE I 400       3.332   0.005 -15.670  1.00  0.00
I    H
ATOM   5775  HD3 ILE I 400       4.858  -0.539 -14.879  1.00  0.00
I    H
ATOM   5776  C   ILE I 400       1.426  -3.323 -12.003  1.00  0.00
I    C
ATOM   5777  O   ILE I 400       2.043  -3.982 -11.165  1.00  0.00
I    O
```

```
ATOM   5778  N   VAL I 401       0.292  -2.667 -11.665  1.00  0.00
I    N
ATOM   5779  HN  VAL I 401      -0.217  -2.107 -12.312  1.00  0.00
I    H
ATOM   5780  CA  VAL I 401      -0.286  -2.719 -10.327  1.00  0.00
I    C
ATOM   5781  HA  VAL I 401       0.476  -2.387  -9.634  1.00  0.00
I    H
ATOM   5782  CB  VAL I 401      -1.493  -1.783 -10.202  1.00  0.00
I    C
ATOM   5783  HB  VAL I 401      -2.251  -2.106 -10.958  1.00  0.00
I    H
ATOM   5784  CG1 VAL I 401      -2.130  -1.862  -8.798  1.00  0.00
I    C
ATOM   5785 HG11 VAL I 401      -2.978  -1.147  -8.719  1.00  0.00
I    H
ATOM   5786 HG12 VAL I 401      -2.531  -2.874  -8.587  1.00  0.00
I    H
ATOM   5787 HG13 VAL I 401      -1.388  -1.602  -8.015  1.00  0.00
I    H
ATOM   5788  CG2 VAL I 401      -1.037  -0.331 -10.442  1.00  0.00
I    C
ATOM   5789 HG21 VAL I 401      -1.886   0.373 -10.317  1.00  0.00
I    H
ATOM   5790 HG22 VAL I 401      -0.239  -0.049  -9.720  1.00  0.00
I    H
ATOM   5791 HG23 VAL I 401      -0.640  -0.199 -11.470  1.00  0.00
I    H
ATOM   5792  C   VAL I 401      -0.668  -4.145  -9.938  1.00  0.00
I    C
ATOM   5793  O   VAL I 401      -0.292  -4.640  -8.877  1.00  0.00
I    O
ATOM   5794  N   PHE I 402      -1.361  -4.872 -10.838  1.00  0.00
I    N
ATOM   5795  HN  PHE I 402      -1.622  -4.492 -11.724  1.00  0.00
I    H
ATOM   5796  CA  PHE I 402      -1.789  -6.240 -10.622  1.00  0.00
I    C
ATOM   5797  HA  PHE I 402      -2.355  -6.210  -9.698  1.00  0.00
I    H
ATOM   5798  CB  PHE I 402      -2.703  -6.662 -11.793  1.00  0.00
I    C
ATOM   5799  HB1 PHE I 402      -3.404  -5.831 -12.024  1.00  0.00
I    H
ATOM   5800  HB2 PHE I 402      -2.112  -6.857 -12.713  1.00  0.00
I    H
ATOM   5801  CG  PHE I 402      -3.537  -7.859 -11.443  1.00  0.00
I    C
ATOM   5802  CD1 PHE I 402      -4.778  -7.696 -10.807  1.00  0.00
I    C
ATOM   5803  HD1 PHE I 402      -5.089  -6.712 -10.485  1.00  0.00
I    H
ATOM   5804  CE1 PHE I 402      -5.614  -8.798 -10.580  1.00  0.00
I    C
```

```
ATOM   5805  HE1 PHE I 402      -6.580  -8.657 -10.116  1.00  0.00
I    H
ATOM   5806  CZ  PHE I 402      -5.198 -10.077 -10.963  1.00  0.00
I    C
ATOM   5807  HZ  PHE I 402      -5.838 -10.932 -10.798  1.00  0.00
I    H
ATOM   5808  CD2 PHE I 402      -3.109  -9.148 -11.783  1.00  0.00
I    C
ATOM   5809  HD2 PHE I 402      -2.119  -9.300 -12.190  1.00  0.00
I    H
ATOM   5810  CE2 PHE I 402      -3.950 -10.245 -11.571  1.00  0.00
I    C
ATOM   5811  HE2 PHE I 402      -3.635 -11.233 -11.878  1.00  0.00
I    H
ATOM   5812  C   PHE I 402      -0.652  -7.231 -10.416  1.00  0.00
I    C
ATOM   5813  O   PHE I 402      -0.664  -7.981  -9.442  1.00  0.00
I    O
ATOM   5814  N   GLU I 403       0.397  -7.208 -11.265  1.00  0.00
I    N
ATOM   5815  HN  GLU I 403       0.436  -6.601 -12.053  1.00  0.00
I    H
ATOM   5816  CA  GLU I 403       1.567  -8.062 -11.117  1.00  0.00
I    C
ATOM   5817  HA  GLU I 403       1.221  -9.088 -11.096  1.00  0.00
I    H
ATOM   5818  CB  GLU I 403       2.556  -7.900 -12.292  1.00  0.00
I    C
ATOM   5819  HB1 GLU I 403       2.729  -6.811 -12.453  1.00  0.00
I    H
ATOM   5820  HB2 GLU I 403       3.546  -8.328 -12.015  1.00  0.00
I    H
ATOM   5821  CG  GLU I 403       2.083  -8.524 -13.628  1.00  0.00
I    C
ATOM   5822  HG1 GLU I 403       1.032  -8.238 -13.817  1.00  0.00
I    H
ATOM   5823  HG2 GLU I 403       2.712  -8.180 -14.472  1.00  0.00
I    H
ATOM   5824  CD  GLU I 403       2.047 -10.041 -13.745  1.00  0.00
I    C
ATOM   5825  OE1 GLU I 403       0.938 -10.560 -14.028  1.00  0.00
I    O
ATOM   5826  OE2 GLU I 403       3.136 -10.671 -13.691  1.00  0.00
I    O
ATOM   5827  C   GLU I 403       2.312  -7.809  -9.814  1.00  0.00
I    C
ATOM   5828  O   GLU I 403       2.698  -8.748  -9.127  1.00  0.00
I    O
ATOM   5829  N   THR I 404       2.498  -6.535  -9.398  1.00  0.00
I    N
ATOM   5830  HN  THR I 404       2.227  -5.735  -9.931  1.00  0.00
I    H
ATOM   5831  CA  THR I 404       3.121  -6.218  -8.106  1.00  0.00
I    C
```

ATOM 5832 HA THR I 404 4.058 -6.758 -8.109 1.00 0.00
I H
ATOM 5833 CB THR I 404 3.373 -4.736 -7.889 1.00 0.00
I C
ATOM 5834 HB THR I 404 2.393 -4.227 -8.055 1.00 0.00
I H
ATOM 5835 OG1 THR I 404 4.282 -4.279 -8.870 1.00 0.00
I O
ATOM 5836 HG1 THR I 404 4.415 -3.344 -8.681 1.00 0.00
I H
ATOM 5837 CG2 THR I 404 4.079 -4.476 -6.550 1.00 0.00
I C
ATOM 5838 HG21 THR I 404 4.156 -3.386 -6.349 1.00 0.00
I H
ATOM 5839 HG22 THR I 404 3.509 -4.937 -5.716 1.00 0.00
I H
ATOM 5840 HG23 THR I 404 5.104 -4.903 -6.548 1.00 0.00
I H
ATOM 5841 C THR I 404 2.333 -6.727 -6.915 1.00 0.00
I C
ATOM 5842 O THR I 404 2.896 -7.344 -6.009 1.00 0.00
I O
ATOM 5843 N PHE I 405 0.996 -6.534 -6.896 1.00 0.00
I N
ATOM 5844 HN PHE I 405 0.523 -5.994 -7.590 1.00 0.00
I H
ATOM 5845 CA PHE I 405 0.126 -7.090 -5.866 1.00 0.00
I C
ATOM 5846 HA PHE I 405 0.554 -6.781 -4.921 1.00 0.00
I H
ATOM 5847 CB PHE I 405 -1.322 -6.526 -5.967 1.00 0.00
I C
ATOM 5848 HB1 PHE I 405 -1.545 -6.286 -7.030 1.00 0.00
I H
ATOM 5849 HB2 PHE I 405 -2.069 -7.279 -5.638 1.00 0.00
I H
ATOM 5850 CG PHE I 405 -1.481 -5.246 -5.180 1.00 0.00
I C
ATOM 5851 CD1 PHE I 405 -1.769 -5.299 -3.805 1.00 0.00
I C
ATOM 5852 HD1 PHE I 405 -1.809 -6.256 -3.303 1.00 0.00
I H
ATOM 5853 CE1 PHE I 405 -2.010 -4.123 -3.079 1.00 0.00
I C
ATOM 5854 HE1 PHE I 405 -2.221 -4.175 -2.021 1.00 0.00
I H
ATOM 5855 CZ PHE I 405 -1.974 -2.880 -3.720 1.00 0.00
I C
ATOM 5856 HZ PHE I 405 -2.172 -1.974 -3.164 1.00 0.00
I H
ATOM 5857 CD2 PHE I 405 -1.414 -3.987 -5.799 1.00 0.00
I C
ATOM 5858 HD2 PHE I 405 -1.181 -3.914 -6.852 1.00 0.00
I H

```
ATOM   5859  CE2 PHE I 405      -1.672  -2.811  -5.083  1.00  0.00
I    C
ATOM   5860  HE2 PHE I 405      -1.637  -1.855  -5.587  1.00  0.00
I    H
ATOM   5861  C   PHE I 405       0.118  -8.624  -5.876  1.00  0.00
I    C
ATOM   5862  O   PHE I 405       0.237  -9.252  -4.829  1.00  0.00
I    O
ATOM   5863  N   SER I 406       0.047  -9.265  -7.068  1.00  0.00
I    N
ATOM   5864  HN  SER I 406      -0.090  -8.765  -7.923  1.00  0.00
I    H
ATOM   5865  CA  SER I 406       0.161 -10.718  -7.235  1.00  0.00
I    C
ATOM   5866  HA  SER I 406      -0.599 -11.130  -6.585  1.00  0.00
I    H
ATOM   5867  CB  SER I 406       0.004 -11.201  -8.706  1.00  0.00
I    C
ATOM   5868  HB1 SER I 406       0.448 -10.427  -9.373  1.00  0.00
I    H
ATOM   5869  HB2 SER I 406       0.578 -12.141  -8.869  1.00  0.00
I    H
ATOM   5870  OG  SER I 406      -1.366 -11.368  -9.044  1.00  0.00
I    O
ATOM   5871  HG1 SER I 406      -1.413 -11.446 -10.005  1.00  0.00
I    H
ATOM   5872  C   SER I 406       1.488 -11.288  -6.792  1.00  0.00
I    C
ATOM   5873  O   SER I 406       1.530 -12.346  -6.164  1.00  0.00
I    O
ATOM   5874  N   ALA I 407       2.613 -10.632  -7.131  1.00  0.00
I    N
ATOM   5875  HN  ALA I 407       2.568  -9.827  -7.721  1.00  0.00
I    H
ATOM   5876  CA  ALA I 407       3.938 -11.011  -6.696  1.00  0.00
I    C
ATOM   5877  HA  ALA I 407       4.110 -12.056  -6.929  1.00  0.00
I    H
ATOM   5878  CB  ALA I 407       4.981 -10.165  -7.460  1.00  0.00
I    C
ATOM   5879  HB1 ALA I 407       4.854 -10.315  -8.553  1.00  0.00
I    H
ATOM   5880  HB2 ALA I 407       4.851  -9.082  -7.245  1.00  0.00
I    H
ATOM   5881  HB3 ALA I 407       6.020 -10.456  -7.191  1.00  0.00
I    H
ATOM   5882  C   ALA I 407       4.150 -10.865  -5.192  1.00  0.00
I    C
ATOM   5883  O   ALA I 407       4.629 -11.782  -4.536  1.00  0.00
I    O
ATOM   5884  N   PHE I 408       3.756  -9.716  -4.596  1.00  0.00
I    N
ATOM   5885  HN  PHE I 408       3.338  -8.972  -5.115  1.00  0.00
I    H
```

ATOM 5886 CA PHE I 408 3.898 -9.452 -3.173 1.00 0.00
I C
ATOM 5887 HA PHE I 408 4.917 -9.724 -2.926 1.00 0.00
I H
ATOM 5888 CB PHE I 408 3.682 -7.940 -2.883 1.00 0.00
I C
ATOM 5889 HB1 PHE I 408 4.362 -7.345 -3.531 1.00 0.00
I H
ATOM 5890 HB2 PHE I 408 2.644 -7.630 -3.126 1.00 0.00
I H
ATOM 5891 CG PHE I 408 4.026 -7.645 -1.450 1.00 0.00
I C
ATOM 5892 CD1 PHE I 408 5.363 -7.527 -1.033 1.00 0.00
I C
ATOM 5893 HD1 PHE I 408 6.156 -7.536 -1.769 1.00 0.00
I H
ATOM 5894 CE1 PHE I 408 5.674 -7.403 0.328 1.00 0.00
I C
ATOM 5895 HE1 PHE I 408 6.701 -7.284 0.639 1.00 0.00
I H
ATOM 5896 CZ PHE I 408 4.648 -7.429 1.280 1.00 0.00
I C
ATOM 5897 HZ PHE I 408 4.886 -7.339 2.330 1.00 0.00
I H
ATOM 5898 CD2 PHE I 408 3.006 -7.648 -0.487 1.00 0.00
I C
ATOM 5899 HD2 PHE I 408 1.973 -7.738 -0.794 1.00 0.00
I H
ATOM 5900 CE2 PHE I 408 3.316 -7.561 0.872 1.00 0.00
I C
ATOM 5901 HE2 PHE I 408 2.524 -7.598 1.608 1.00 0.00
I H
ATOM 5902 C PHE I 408 2.982 -10.325 -2.317 1.00 0.00
I C
ATOM 5903 O PHE I 408 3.387 -10.870 -1.293 1.00 0.00
I O
ATOM 5904 N GLY I 409 1.716 -10.525 -2.728 1.00 0.00
I N
ATOM 5905 HN GLY I 409 1.351 -10.108 -3.560 1.00 0.00
I H
ATOM 5906 CA GLY I 409 0.784 -11.368 -1.983 1.00 0.00
I C
ATOM 5907 HA1 GLY I 409 -0.206 -11.132 -2.350 1.00 0.00
I H
ATOM 5908 HA2 GLY I 409 0.934 -11.150 -0.934 1.00 0.00
I H
ATOM 5909 C GLY I 409 1.019 -12.850 -2.190 1.00 0.00
I C
ATOM 5910 O GLY I 409 0.326 -13.681 -1.616 1.00 0.00
I O
ATOM 5911 N THR I 410 1.995 -13.189 -3.063 1.00 0.00
I N
ATOM 5912 HN THR I 410 2.543 -12.439 -3.433 1.00 0.00
I H

```
ATOM   5913  CA  THR I 410       2.376 -14.513  -3.556  1.00  0.00
I    C
ATOM   5914  HA  THR I 410       3.028 -14.323  -4.399  1.00  0.00
I    H
ATOM   5915  CB  THR I 410       3.395 -15.322  -2.747  1.00  0.00
I    C
ATOM   5916  HB  THR I 410       3.811 -16.086  -3.447  1.00  0.00
I    H
ATOM   5917  OG1 THR I 410       2.813 -16.043  -1.665  1.00  0.00
I    O
ATOM   5918  HG1 THR I 410       3.546 -16.494  -1.234  1.00  0.00
I    H
ATOM   5919  CG2 THR I 410       4.489 -14.375  -2.219  1.00  0.00
I    C
ATOM   5920 HG21 THR I 410       5.302 -14.947  -1.725  1.00  0.00
I    H
ATOM   5921 HG22 THR I 410       4.937 -13.798  -3.056  1.00  0.00
I    H
ATOM   5922 HG23 THR I 410       4.073 -13.655  -1.482  1.00  0.00
I    H
ATOM   5923  C   THR I 410       1.209 -15.308  -4.086  1.00  0.00
I    C
ATOM   5924  O   THR I 410       0.954 -16.449  -3.709  1.00  0.00
I    O
ATOM   5925  N   VAL I 411       0.445 -14.660  -4.983  1.00  0.00
I    N
ATOM   5926  HN  VAL I 411       0.697 -13.757  -5.324  1.00  0.00
I    H
ATOM   5927  CA  VAL I 411      -0.788 -15.184  -5.533  1.00  0.00
I    C
ATOM   5928  HA  VAL I 411      -1.079 -16.024  -4.913  1.00  0.00
I    H
ATOM   5929  CB  VAL I 411      -1.867 -14.114  -5.604  1.00  0.00
I    C
ATOM   5930  HB  VAL I 411      -1.542 -13.357  -6.360  1.00  0.00
I    H
ATOM   5931  CG1 VAL I 411      -3.218 -14.723  -6.047  1.00  0.00
I    C
ATOM   5932 HG11 VAL I 411      -4.008 -13.941  -6.058  1.00  0.00
I    H
ATOM   5933 HG12 VAL I 411      -3.159 -15.149  -7.070  1.00  0.00
I    H
ATOM   5934 HG13 VAL I 411      -3.536 -15.524  -5.345  1.00  0.00
I    H
ATOM   5935  CG2 VAL I 411      -1.971 -13.466  -4.218  1.00  0.00
I    C
ATOM   5936 HG21 VAL I 411      -2.775 -12.700  -4.202  1.00  0.00
I    H
ATOM   5937 HG22 VAL I 411      -2.201 -14.232  -3.445  1.00  0.00
I    H
ATOM   5938 HG23 VAL I 411      -1.023 -12.961  -3.938  1.00  0.00
I    H
ATOM   5939  C   VAL I 411      -0.608 -15.695  -6.926  1.00  0.00
I    C
```

```
ATOM   5940  O   VAL I 411      -1.160 -16.717  -7.283  1.00  0.00
I    O
ATOM   5941  N   GLY I 412       0.154 -14.994  -7.780  1.00  0.00
I    N
ATOM   5942  HN  GLY I 412       0.559 -14.106  -7.566  1.00  0.00
I    H
ATOM   5943  CA  GLY I 412       0.438 -15.513  -9.112  1.00  0.00
I    C
ATOM   5944  HA1 GLY I 412       0.622 -16.566  -8.951  1.00  0.00
I    H
ATOM   5945  HA2 GLY I 412       1.308 -14.956  -9.436  1.00  0.00
I    H
ATOM   5946  C   GLY I 412      -0.613 -15.381 -10.168  1.00  0.00
I    C
ATOM   5947  O   GLY I 412      -0.431 -15.883 -11.270  1.00  0.00
I    O
ATOM   5948  N   LEU I 413      -1.729 -14.680  -9.897  1.00  0.00
I    N
ATOM   5949  HN  LEU I 413      -1.999 -14.385  -8.983  1.00  0.00
I    H
ATOM   5950  CA  LEU I 413      -2.652 -14.288 -10.948  1.00  0.00
I    C
ATOM   5951  HA  LEU I 413      -2.897 -15.195 -11.487  1.00  0.00
I    H
ATOM   5952  CB  LEU I 413      -3.931 -13.622 -10.382  1.00  0.00
I    C
ATOM   5953  HB1 LEU I 413      -3.601 -12.811  -9.693  1.00  0.00
I    H
ATOM   5954  HB2 LEU I 413      -4.474 -13.119 -11.211  1.00  0.00
I    H
ATOM   5955  CG  LEU I 413      -4.872 -14.564  -9.617  1.00  0.00
I    C
ATOM   5956  HG  LEU I 413      -4.257 -15.098  -8.853  1.00  0.00
I    H
ATOM   5957  CD1 LEU I 413      -5.969 -13.748  -8.918  1.00  0.00
I    C
ATOM   5958 HD11 LEU I 413      -6.686 -14.421  -8.400  1.00  0.00
I    H
ATOM   5959 HD12 LEU I 413      -5.526 -13.063  -8.163  1.00  0.00
I    H
ATOM   5960 HD13 LEU I 413      -6.535 -13.139  -9.654  1.00  0.00
I    H
ATOM   5961  CD2 LEU I 413      -5.492 -15.618 -10.550  1.00  0.00
I    C
ATOM   5962 HD21 LEU I 413      -6.155 -16.301  -9.975  1.00  0.00
I    H
ATOM   5963 HD22 LEU I 413      -6.102 -15.151 -11.349  1.00  0.00
I    H
ATOM   5964 HD23 LEU I 413      -4.697 -16.229 -11.030  1.00  0.00
I    H
ATOM   5965  C   LEU I 413      -2.022 -13.317 -11.942  1.00  0.00
I    C
ATOM   5966  O   LEU I 413      -1.375 -12.344 -11.555  1.00  0.00
I    O
```

```
ATOM   5967  N   THR I 414      -2.241 -13.543 -13.249  1.00  0.00
I    N
ATOM   5968  HN  THR I 414      -2.935 -14.183 -13.579  1.00  0.00
I    H
ATOM   5969  CA  THR I 414      -1.488 -12.877 -14.307  1.00  0.00
I    C
ATOM   5970  HA  THR I 414      -0.864 -12.141 -13.818  1.00  0.00
I    H
ATOM   5971  CB  THR I 414      -0.706 -13.877 -15.162  1.00  0.00
I    C
ATOM   5972  HB  THR I 414      -1.469 -14.540 -15.638  1.00  0.00
I    H
ATOM   5973  OG1 THR I 414       0.112 -14.681 -14.335  1.00  0.00
I    O
ATOM   5974  HG1 THR I 414       0.579 -15.274 -14.935  1.00  0.00
I    H
ATOM   5975  CG2 THR I 414       0.263 -13.185 -16.125  1.00  0.00
I    C
ATOM   5976 HG21 THR I 414       0.732 -13.922 -16.810  1.00  0.00
I    H
ATOM   5977 HG22 THR I 414      -0.275 -12.437 -16.745  1.00  0.00
I    H
ATOM   5978 HG23 THR I 414       1.071 -12.660 -15.571  1.00  0.00
I    H
ATOM   5979  C   THR I 414      -2.426 -12.108 -15.220  1.00  0.00
I    C
ATOM   5980  O   THR I 414      -3.436 -12.644 -15.668  1.00  0.00
I    O
ATOM   5981  N   MET I 415      -2.123 -10.827 -15.552  1.00  0.00
I    N
ATOM   5982  HN  MET I 415      -1.337 -10.347 -15.169  1.00  0.00
I    H
ATOM   5983  CA  MET I 415      -2.920 -10.030 -16.501  1.00  0.00
I    C
ATOM   5984  HA  MET I 415      -3.895 -10.500 -16.548  1.00  0.00
I    H
ATOM   5985  CB  MET I 415      -3.088  -8.554 -16.049  1.00  0.00
I    C
ATOM   5986  HB1 MET I 415      -2.138  -8.259 -15.546  1.00  0.00
I    H
ATOM   5987  HB2 MET I 415      -3.180  -7.901 -16.946  1.00  0.00
I    H
ATOM   5988  CG  MET I 415      -4.269  -8.360 -15.084  1.00  0.00
I    C
ATOM   5989  HG1 MET I 415      -5.170  -8.886 -15.465  1.00  0.00
I    H
ATOM   5990  HG2 MET I 415      -4.017  -8.799 -14.093  1.00  0.00
I    H
ATOM   5991  SD  MET I 415      -4.730  -6.619 -14.830  1.00  0.00
I    S
ATOM   5992  CE  MET I 415      -5.675  -6.451 -16.374  1.00  0.00
I    C
ATOM   5993  HE1 MET I 415      -6.082  -5.423 -16.481  1.00  0.00
I    H
```

```
ATOM   5994  HE2 MET I 415      -5.034  -6.658 -17.258  1.00  0.00
I    H
ATOM   5995  HE3 MET I 415      -6.530  -7.161 -16.398  1.00  0.00
I    H
ATOM   5996  C   MET I 415      -2.341 -10.026 -17.914  1.00  0.00
I    C
ATOM   5997  O   MET I 415      -2.760  -9.269 -18.785  1.00  0.00
I    O
ATOM   5998  N   GLY I 416      -1.357 -10.900 -18.166  1.00  0.00
I    N
ATOM   5999  HN  GLY I 416      -1.076 -11.529 -17.442  1.00  0.00
I    H
ATOM   6000  CA  GLY I 416      -0.636 -11.028 -19.428  1.00  0.00
I    C
ATOM   6001  HA1 GLY I 416      -1.233 -10.525 -20.176  1.00  0.00
I    H
ATOM   6002  HA2 GLY I 416      -0.502 -12.087 -19.604  1.00  0.00
I    H
ATOM   6003  C   GLY I 416       0.723 -10.386 -19.408  1.00  0.00
I    C
ATOM   6004  O   GLY I 416       1.488 -10.532 -20.351  1.00  0.00
I    O
ATOM   6005  N   LEU I 417       1.083  -9.668 -18.326  1.00  0.00
I    N
ATOM   6006  HN  LEU I 417       0.502  -9.560 -17.523  1.00  0.00
I    H
ATOM   6007  CA  LEU I 417       2.360  -8.983 -18.235  1.00  0.00
I    C
ATOM   6008  HA  LEU I 417       2.497  -8.519 -19.204  1.00  0.00
I    H
ATOM   6009  CB  LEU I 417       2.270  -7.822 -17.213  1.00  0.00
I    C
ATOM   6010  HB1 LEU I 417       1.469  -7.132 -17.568  1.00  0.00
I    H
ATOM   6011  HB2 LEU I 417       1.917  -8.225 -16.237  1.00  0.00
I    H
ATOM   6012  CG  LEU I 417       3.565  -6.994 -17.005  1.00  0.00
I    C
ATOM   6013  HG  LEU I 417       4.315  -7.732 -16.630  1.00  0.00
I    H
ATOM   6014  CD1 LEU I 417       4.051  -6.357 -18.316  1.00  0.00
I    C
ATOM   6015 HD11 LEU I 417       4.951  -5.731 -18.136  1.00  0.00
I    H
ATOM   6016 HD12 LEU I 417       4.315  -7.140 -19.058  1.00  0.00
I    H
ATOM   6017 HD13 LEU I 417       3.261  -5.710 -18.755  1.00  0.00
I    H
ATOM   6018  CD2 LEU I 417       3.407  -5.892 -15.940  1.00  0.00
I    C
ATOM   6019 HD21 LEU I 417       4.362  -5.340 -15.813  1.00  0.00
I    H
ATOM   6020 HD22 LEU I 417       2.628  -5.157 -16.224  1.00  0.00
I    H
```

```
ATOM   6021 HD23 LEU I 417       3.128  -6.336 -14.960  1.00  0.00
I    H
ATOM   6022  C   LEU I 417       3.565  -9.890 -17.984  1.00  0.00
I    C
ATOM   6023  O   LEU I 417       4.627  -9.644 -18.551  1.00  0.00
I    O
ATOM   6024  N   THR I 418       3.479 -10.976 -17.174  1.00  0.00
I    N
ATOM   6025  HN  THR I 418       2.622 -11.291 -16.769  1.00  0.00
I    H
ATOM   6026  CA  THR I 418       4.653 -11.792 -16.818  1.00  0.00
I    C
ATOM   6027  HA  THR I 418       5.278 -11.098 -16.273  1.00  0.00
I    H
ATOM   6028  CB  THR I 418       4.366 -12.980 -15.900  1.00  0.00
I    C
ATOM   6029  HB  THR I 418       4.182 -13.853 -16.572  1.00  0.00
I    H
ATOM   6030  OG1 THR I 418       3.190 -12.805 -15.130  1.00  0.00
I    O
ATOM   6031  HG1 THR I 418       3.117 -13.598 -14.589  1.00  0.00
I    H
ATOM   6032  CG2 THR I 418       5.559 -13.266 -14.969  1.00  0.00
I    C
ATOM   6033 HG21 THR I 418       5.390 -14.196 -14.386  1.00  0.00
I    H
ATOM   6034 HG22 THR I 418       6.490 -13.398 -15.560  1.00  0.00
I    H
ATOM   6035 HG23 THR I 418       5.716 -12.431 -14.252  1.00  0.00
I    H
ATOM   6036  C   THR I 418       5.510 -12.340 -17.971  1.00  0.00
I    C
ATOM   6037  O   THR I 418       6.735 -12.273 -17.846  1.00  0.00
I    O
ATOM   6038  N   PRO I 419       5.000 -12.857 -19.104  1.00  0.00
I    N
ATOM   6039  CD  PRO I 419       3.598 -13.217 -19.321  1.00  0.00
I    C
ATOM   6040  HD1 PRO I 419       4.207 -13.546 -18.449  1.00  0.00
I    H
ATOM   6041  HD2 PRO I 419       2.542 -13.039 -19.013  1.00  0.00
I    H
ATOM   6042  CA  PRO I 419       5.814 -13.183 -20.278  1.00  0.00
I    C
ATOM   6043  HA  PRO I 419       6.524 -13.957 -20.014  1.00  0.00
I    H
ATOM   6044  CB  PRO I 419       4.805 -13.767 -21.291  1.00  0.00
I    C
ATOM   6045  HB1 PRO I 419       4.670 -14.853 -21.083  1.00  0.00
I    H
ATOM   6046  HB2 PRO I 419       5.132 -13.640 -22.343  1.00  0.00
I    H
ATOM   6047  CG  PRO I 419       3.649 -14.270 -20.422  1.00  0.00
I    C
```

```
ATOM   6048  HG1 PRO I 419       3.049 -15.060 -20.915  1.00  0.00
I    H
ATOM   6049  HG2 PRO I 419       3.257 -14.938 -19.788  1.00  0.00
I    H
ATOM   6050  C   PRO I 419       6.567 -11.994 -20.865  1.00  0.00
I    C
ATOM   6051  O   PRO I 419       7.728 -12.149 -21.243  1.00  0.00
I    O
ATOM   6052  N   GLU I 420       5.928 -10.805 -20.915  1.00  0.00
I    N
ATOM   6053  HN  GLU I 420       5.077 -10.670 -20.416  1.00  0.00
I    H
ATOM   6054  CA  GLU I 420       6.368  -9.637 -21.658  1.00  0.00
I    C
ATOM   6055  HA  GLU I 420       7.029  -9.981 -22.444  1.00  0.00
I    H
ATOM   6056  CB  GLU I 420       5.145  -8.901 -22.275  1.00  0.00
I    C
ATOM   6057  HB1 GLU I 420       4.553  -8.454 -21.443  1.00  0.00
I    H
ATOM   6058  HB2 GLU I 420       5.493  -8.045 -22.895  1.00  0.00
I    H
ATOM   6059  CG  GLU I 420       4.202  -9.802 -23.121  1.00  0.00
I    C
ATOM   6060  HG1 GLU I 420       3.731 -10.559 -22.468  1.00  0.00
I    H
ATOM   6061  HG2 GLU I 420       3.411  -9.200 -23.611  1.00  0.00
I    H
ATOM   6062  CD  GLU I 420       4.919 -10.604 -24.207  1.00  0.00
I    C
ATOM   6063  OE1 GLU I 420       5.790 -10.026 -24.904  1.00  0.00
I    O
ATOM   6064  OE2 GLU I 420       4.573 -11.805 -24.354  1.00  0.00
I    O
ATOM   6065  C   GLU I 420       7.163  -8.658 -20.794  1.00  0.00
I    C
ATOM   6066  O   GLU I 420       7.489  -7.542 -21.201  1.00  0.00
I    O
ATOM   6067  N   LEU I 421       7.520  -9.052 -19.555  1.00  0.00
I    N
ATOM   6068  HN  LEU I 421       7.151  -9.879 -19.135  1.00  0.00
I    H
ATOM   6069  CA  LEU I 421       8.462  -8.326 -18.717  1.00  0.00
I    C
ATOM   6070  HA  LEU I 421       8.075  -7.317 -18.657  1.00  0.00
I    H
ATOM   6071  CB  LEU I 421       8.600  -8.942 -17.300  1.00  0.00
I    C
ATOM   6072  HB1 LEU I 421       8.773 -10.035 -17.432  1.00  0.00
I    H
ATOM   6073  HB2 LEU I 421       9.520  -8.542 -16.819  1.00  0.00
I    H
ATOM   6074  CG  LEU I 421       7.388  -8.747 -16.376  1.00  0.00
I    C
```

```
ATOM   6075  HG  LEU I 421       6.484  -9.053 -16.957  1.00  0.00
I    H
ATOM   6076  CD1 LEU I 421       7.533  -9.632 -15.130  1.00  0.00
I    C
ATOM   6077 HD11 LEU I 421       6.686  -9.463 -14.431  1.00  0.00
I    H
ATOM   6078 HD12 LEU I 421       7.545 -10.706 -15.412  1.00  0.00
I    H
ATOM   6079 HD13 LEU I 421       8.477  -9.400 -14.591  1.00  0.00
I    H
ATOM   6080  CD2 LEU I 421       7.211  -7.279 -15.962  1.00  0.00
I    C
ATOM   6081 HD21 LEU I 421       6.317  -7.167 -15.311  1.00  0.00
I    H
ATOM   6082 HD22 LEU I 421       8.088  -6.905 -15.397  1.00  0.00
I    H
ATOM   6083 HD23 LEU I 421       7.071  -6.637 -16.858  1.00  0.00
I    H
ATOM   6084  C   LEU I 421       9.867  -8.245 -19.297  1.00  0.00
I    C
ATOM   6085  O   LEU I 421      10.389  -9.180 -19.912  1.00  0.00
I    O
ATOM   6086  N   THR I 422      10.567  -7.123 -19.044  1.00  0.00
I    N
ATOM   6087  HN  THR I 422      10.168  -6.306 -18.628  1.00  0.00
I    H
ATOM   6088  CA  THR I 422      11.983  -7.003 -19.358  1.00  0.00
I    C
ATOM   6089  HA  THR I 422      12.119  -7.434 -20.341  1.00  0.00
I    H
ATOM   6090  CB  THR I 422      12.521  -5.562 -19.347  1.00  0.00
I    C
ATOM   6091  HB  THR I 422      13.547  -5.611 -19.787  1.00  0.00
I    H
ATOM   6092  OG1 THR I 422      12.659  -5.057 -18.025  1.00  0.00
I    O
ATOM   6093  HG1 THR I 422      12.988  -4.158 -18.127  1.00  0.00
I    H
ATOM   6094  CG2 THR I 422      11.592  -4.599 -20.109  1.00  0.00
I    C
ATOM   6095 HG21 THR I 422      12.050  -3.590 -20.189  1.00  0.00
I    H
ATOM   6096 HG22 THR I 422      11.407  -4.972 -21.139  1.00  0.00
I    H
ATOM   6097 HG23 THR I 422      10.612  -4.496 -19.595  1.00  0.00
I    H
ATOM   6098  C   THR I 422      12.795  -7.864 -18.390  1.00  0.00
I    C
ATOM   6099  O   THR I 422      12.299  -8.367 -17.382  1.00  0.00
I    O
ATOM   6100  N   THR I 423      14.100  -8.063 -18.654  1.00  0.00
I    N
ATOM   6101  HN  THR I 423      14.547  -7.752 -19.492  1.00  0.00
I    H
```

```
ATOM   6102  CA  THR I 423      14.989  -8.766 -17.721  1.00  0.00
I    C
ATOM   6103  HA  THR I 423      14.512  -9.720 -17.541  1.00  0.00
I    H
ATOM   6104  CB  THR I 423      16.386  -8.992 -18.288  1.00  0.00
I    C
ATOM   6105  HB  THR I 423      16.763  -7.986 -18.594  1.00  0.00
I    H
ATOM   6106  OG1 THR I 423      16.266  -9.802 -19.445  1.00  0.00
I    O
ATOM   6107  HG1 THR I 423      17.166  -9.930 -19.764  1.00  0.00
I    H
ATOM   6108  CG2 THR I 423      17.308  -9.752 -17.317  1.00  0.00
I    C
ATOM   6109 HG21 THR I 423      18.345  -9.800 -17.712  1.00  0.00
I    H
ATOM   6110 HG22 THR I 423      17.341  -9.240 -16.333  1.00  0.00
I    H
ATOM   6111 HG23 THR I 423      16.949 -10.792 -17.156  1.00  0.00
I    H
ATOM   6112  C   THR I 423      15.096  -8.076 -16.373  1.00  0.00
I    C
ATOM   6113  O   THR I 423      15.045  -8.717 -15.327  1.00  0.00
I    O
ATOM   6114  N   ALA I 424      15.190  -6.730 -16.358  1.00  0.00
I    N
ATOM   6115  HN  ALA I 424      15.259  -6.213 -17.211  1.00  0.00
I    H
ATOM   6116  CA  ALA I 424      15.200  -5.935 -15.148  1.00  0.00
I    C
ATOM   6117  HA  ALA I 424      15.977  -6.291 -14.481  1.00  0.00
I    H
ATOM   6118  CB  ALA I 424      15.562  -4.478 -15.507  1.00  0.00
I    C
ATOM   6119  HB1 ALA I 424      16.543  -4.456 -16.028  1.00  0.00
I    H
ATOM   6120  HB2 ALA I 424      14.803  -4.034 -16.187  1.00  0.00
I    H
ATOM   6121  HB3 ALA I 424      15.641  -3.841 -14.600  1.00  0.00
I    H
ATOM   6122  C   ALA I 424      13.884  -6.023 -14.365  1.00  0.00
I    C
ATOM   6123  O   ALA I 424      13.893  -6.164 -13.142  1.00  0.00
I    O
ATOM   6124  N   GLY I 425      12.716  -6.008 -15.054  1.00  0.00
I    N
ATOM   6125  HN  GLY I 425      12.705  -5.880 -16.045  1.00  0.00
I    H
ATOM   6126  CA  GLY I 425      11.404  -6.174 -14.423  1.00  0.00
I    C
ATOM   6127  HA1 GLY I 425      10.671  -6.047 -15.208  1.00  0.00
I    H
ATOM   6128  HA2 GLY I 425      11.344  -5.434 -13.635  1.00  0.00
I    H
```

```
ATOM   6129  C   GLY I 425      11.194  -7.530 -13.804  1.00  0.00
I    C
ATOM   6130  O   GLY I 425      10.590  -7.660 -12.740  1.00  0.00
I    O
ATOM   6131  N   LYS I 426      11.764  -8.594 -14.407  1.00  0.00
I    N
ATOM   6132  HN  LYS I 426      12.179  -8.518 -15.313  1.00  0.00
I    H
ATOM   6133  CA  LYS I 426      11.826  -9.919 -13.807  1.00  0.00
I    C
ATOM   6134  HA  LYS I 426      10.804 -10.199 -13.581  1.00  0.00
I    H
ATOM   6135  CB  LYS I 426      12.416 -10.958 -14.790  1.00  0.00
I    C
ATOM   6136  HB1 LYS I 426      13.244 -10.455 -15.341  1.00  0.00
I    H
ATOM   6137  HB2 LYS I 426      12.884 -11.783 -14.207  1.00  0.00
I    H
ATOM   6138  CG  LYS I 426      11.360 -11.475 -15.774  1.00  0.00
I    C
ATOM   6139  HG1 LYS I 426      10.821 -12.334 -15.313  1.00  0.00
I    H
ATOM   6140  HG2 LYS I 426      10.610 -10.671 -15.956  1.00  0.00
I    H
ATOM   6141  CD  LYS I 426      11.951 -11.940 -17.113  1.00  0.00
I    C
ATOM   6142  HD1 LYS I 426      12.688 -11.177 -17.457  1.00  0.00
I    H
ATOM   6143  HD2 LYS I 426      12.511 -12.887 -16.935  1.00  0.00
I    H
ATOM   6144  CE  LYS I 426      10.878 -12.163 -18.188  1.00  0.00
I    C
ATOM   6145  HE1 LYS I 426      10.133 -12.915 -17.845  1.00  0.00
I    H
ATOM   6146  HE2 LYS I 426      10.343 -11.214 -18.410  1.00  0.00
I    H
ATOM   6147  NZ  LYS I 426      11.486 -12.653 -19.439  1.00  0.00
I    N
ATOM   6148  HZ1 LYS I 426      10.749 -12.802 -20.158  1.00  0.00
I    H
ATOM   6149  HZ2 LYS I 426      12.173 -11.954 -19.787  1.00  0.00
I    H
ATOM   6150  HZ3 LYS I 426      11.976 -13.552 -19.256  1.00  0.00
I    H
ATOM   6151  C   LYS I 426      12.602  -9.943 -12.500  1.00  0.00
I    C
ATOM   6152  O   LYS I 426      12.142 -10.531 -11.526  1.00  0.00
I    O
ATOM   6153  N   CYS I 427      13.763  -9.250 -12.420  1.00  0.00
I    N
ATOM   6154  HN  CYS I 427      14.169  -8.802 -13.215  1.00  0.00
I    H
ATOM   6155  CA  CYS I 427      14.518  -9.099 -11.180  1.00  0.00
I    C
```

```
ATOM   6156  HA  CYS I 427      14.732 -10.104 -10.834  1.00  0.00
I    H
ATOM   6157  CB  CYS I 427      15.848  -8.310 -11.371  1.00  0.00
I    C
ATOM   6158  HB1 CYS I 427      15.577  -7.280 -11.695  1.00  0.00
I    H
ATOM   6159  HB2 CYS I 427      16.364  -8.198 -10.391  1.00  0.00
I    H
ATOM   6160  SG  CYS I 427      16.950  -9.043 -12.623  1.00  0.00
I    S
ATOM   6161  HG1 CYS I 427      17.904  -8.112 -12.571  1.00  0.00
I    H
ATOM   6162  C   CYS I 427      13.707  -8.400 -10.098  1.00  0.00
I    C
ATOM   6163  O   CYS I 427      13.657  -8.862  -8.960  1.00  0.00
I    O
ATOM   6164  N   ILE I 428      12.990  -7.303 -10.438  1.00  0.00
I    N
ATOM   6165  HN  ILE I 428      13.016  -6.919 -11.358  1.00  0.00
I    H
ATOM   6166  CA  ILE I 428      12.120  -6.581  -9.513  1.00  0.00
I    C
ATOM   6167  HA  ILE I 428      12.742  -6.298  -8.673  1.00  0.00
I    H
ATOM   6168  CB  ILE I 428      11.511  -5.341 -10.175  1.00  0.00
I    C
ATOM   6169  HB  ILE I 428      11.099  -5.715 -11.146  1.00  0.00
I    H
ATOM   6170  CG2 ILE I 428      10.433  -4.694  -9.268  1.00  0.00
I    C
ATOM   6171 HG21 ILE I 428       9.890  -3.895  -9.814  1.00  0.00
I    H
ATOM   6172 HG22 ILE I 428       9.686  -5.448  -8.943  1.00  0.00
I    H
ATOM   6173 HG23 ILE I 428      10.894  -4.244  -8.363  1.00  0.00
I    H
ATOM   6174  CG1 ILE I 428      12.620  -4.318 -10.522  1.00  0.00
I    C
ATOM   6175 HG11 ILE I 428      13.036  -3.883  -9.586  1.00  0.00
I    H
ATOM   6176 HG12 ILE I 428      13.452  -4.851 -11.036  1.00  0.00
I    H
ATOM   6177  CD  ILE I 428      12.136  -3.183 -11.436  1.00  0.00
I    C
ATOM   6178  HD1 ILE I 428      12.982  -2.513 -11.699  1.00  0.00
I    H
ATOM   6179  HD2 ILE I 428      11.709  -3.592 -12.377  1.00  0.00
I    H
ATOM   6180  HD3 ILE I 428      11.357  -2.571 -10.932  1.00  0.00
I    H
ATOM   6181  C   ILE I 428      11.018  -7.477  -8.961  1.00  0.00
I    C
ATOM   6182  O   ILE I 428      10.813  -7.551  -7.752  1.00  0.00
I    O
```

```
ATOM   6183  N   ILE I 429      10.334  -8.249  -9.835  1.00  0.00
I    N
ATOM   6184  HN  ILE I 429      10.483  -8.201 -10.820  1.00  0.00
I    H
ATOM   6185  CA  ILE I 429       9.322  -9.218  -9.429  1.00  0.00
I    C
ATOM   6186  HA  ILE I 429       8.603  -8.662  -8.841  1.00  0.00
I    H
ATOM   6187  CB  ILE I 429       8.585  -9.793 -10.641  1.00  0.00
I    C
ATOM   6188  HB  ILE I 429       9.400 -10.089 -11.350  1.00  0.00
I    H
ATOM   6189  CG2 ILE I 429       7.686 -10.995 -10.267  1.00  0.00
I    C
ATOM   6190 HG21 ILE I 429       7.289 -11.486 -11.180  1.00  0.00
I    H
ATOM   6191 HG22 ILE I 429       8.259 -11.756  -9.697  1.00  0.00
I    H
ATOM   6192 HG23 ILE I 429       6.824 -10.668  -9.647  1.00  0.00
I    H
ATOM   6193  CG1 ILE I 429       7.745  -8.710 -11.370  1.00  0.00
I    C
ATOM   6194 HG11 ILE I 429       8.390  -7.838 -11.620  1.00  0.00
I    H
ATOM   6195 HG12 ILE I 429       7.371  -9.131 -12.331  1.00  0.00
I    H
ATOM   6196  CD  ILE I 429       6.524  -8.192 -10.595  1.00  0.00
I    C
ATOM   6197  HD1 ILE I 429       5.953  -7.467 -11.214  1.00  0.00
I    H
ATOM   6198  HD2 ILE I 429       5.845  -9.031 -10.326  1.00  0.00
I    H
ATOM   6199  HD3 ILE I 429       6.834  -7.677  -9.662  1.00  0.00
I    H
ATOM   6200  C   ILE I 429       9.884 -10.311  -8.521  1.00  0.00
I    C
ATOM   6201  O   ILE I 429       9.290 -10.598  -7.482  1.00  0.00
I    O
ATOM   6202  N   ILE I 430      11.070 -10.896  -8.815  1.00  0.00
I    N
ATOM   6203  HN  ILE I 430      11.582 -10.668  -9.641  1.00  0.00
I    H
ATOM   6204  CA  ILE I 430      11.716 -11.905  -7.966  1.00  0.00
I    C
ATOM   6205  HA  ILE I 430      10.997 -12.708  -7.870  1.00  0.00
I    H
ATOM   6206  CB  ILE I 430      13.001 -12.447  -8.607  1.00  0.00
I    C
ATOM   6207  HB  ILE I 430      13.552 -11.533  -8.946  1.00  0.00
I    H
ATOM   6208  CG2 ILE I 430      13.808 -13.331  -7.621  1.00  0.00
I    C
ATOM   6209 HG21 ILE I 430      14.802 -13.583  -8.046  1.00  0.00
I    H
```

```
ATOM   6210 HG22 ILE I 430      13.978 -12.800  -6.662  1.00  0.00
I    H
ATOM   6211 HG23 ILE I 430      13.272 -14.279  -7.407  1.00  0.00
I    H
ATOM   6212  CG1 ILE I 430      12.657 -13.264  -9.875  1.00  0.00
I    C
ATOM   6213 HG11 ILE I 430      12.263 -14.264  -9.584  1.00  0.00
I    H
ATOM   6214 HG12 ILE I 430      11.848 -12.741 -10.434  1.00  0.00
I    H
ATOM   6215  CD  ILE I 430      13.861 -13.443 -10.810  1.00  0.00
I    C
ATOM   6216  HD1 ILE I 430      13.556 -13.983 -11.732  1.00  0.00
I    H
ATOM   6217  HD2 ILE I 430      14.276 -12.455 -11.107  1.00  0.00
I    H
ATOM   6218  HD3 ILE I 430      14.665 -14.029 -10.318  1.00  0.00
I    H
ATOM   6219  C   ILE I 430      12.002 -11.397  -6.554  1.00  0.00
I    C
ATOM   6220  O   ILE I 430      11.708 -12.074  -5.567  1.00  0.00
I    O
ATOM   6221  N   VAL I 431      12.532 -10.159  -6.410  1.00  0.00
I    N
ATOM   6222  HN  VAL I 431      12.806  -9.595  -7.185  1.00  0.00
I    H
ATOM   6223  CA  VAL I 431      12.747  -9.538  -5.105  1.00  0.00
I    C
ATOM   6224  HA  VAL I 431      13.341 -10.226  -4.517  1.00  0.00
I    H
ATOM   6225  CB  VAL I 431      13.533  -8.225  -5.188  1.00  0.00
I    C
ATOM   6226  HB  VAL I 431      12.905  -7.491  -5.751  1.00  0.00
I    H
ATOM   6227  CG1 VAL I 431      13.814  -7.672  -3.773  1.00  0.00
I    C
ATOM   6228 HG11 VAL I 431      14.418  -6.741  -3.835  1.00  0.00
I    H
ATOM   6229 HG12 VAL I 431      12.876  -7.423  -3.236  1.00  0.00
I    H
ATOM   6230 HG13 VAL I 431      14.384  -8.411  -3.169  1.00  0.00
I    H
ATOM   6231  CG2 VAL I 431      14.889  -8.469  -5.879  1.00  0.00
I    C
ATOM   6232 HG21 VAL I 431      15.488  -7.534  -5.911  1.00  0.00
I    H
ATOM   6233 HG22 VAL I 431      15.473  -9.240  -5.331  1.00  0.00
I    H
ATOM   6234 HG23 VAL I 431      14.751  -8.815  -6.924  1.00  0.00
I    H
ATOM   6235  C   VAL I 431      11.429  -9.320  -4.363  1.00  0.00
I    C
ATOM   6236  O   VAL I 431      11.302  -9.649  -3.184  1.00  0.00
I    O
```

```
ATOM   6237  N   ILE I 432      10.380  -8.826  -5.053  1.00  0.00
I    N
ATOM   6238  HN  ILE I 432      10.462  -8.552  -6.009  1.00  0.00
I    H
ATOM   6239  CA  ILE I 432       9.044  -8.642  -4.490  1.00  0.00
I    C
ATOM   6240  HA  ILE I 432       9.182  -8.017  -3.617  1.00  0.00
I    H
ATOM   6241  CB  ILE I 432       8.132  -7.924  -5.485  1.00  0.00
I    C
ATOM   6242  HB  ILE I 432       8.295  -8.463  -6.453  1.00  0.00
I    H
ATOM   6243  CG2 ILE I 432       6.658  -7.928  -5.023  1.00  0.00
I    C
ATOM   6244 HG21 ILE I 432       5.996  -7.544  -5.828  1.00  0.00
I    H
ATOM   6245 HG22 ILE I 432       6.324  -8.957  -4.776  1.00  0.00
I    H
ATOM   6246 HG23 ILE I 432       6.522  -7.288  -4.126  1.00  0.00
I    H
ATOM   6247  CG1 ILE I 432       8.613  -6.471  -5.692  1.00  0.00
I    C
ATOM   6248 HG11 ILE I 432       8.364  -5.862  -4.794  1.00  0.00
I    H
ATOM   6249 HG12 ILE I 432       9.722  -6.470  -5.794  1.00  0.00
I    H
ATOM   6250  CD  ILE I 432       7.993  -5.844  -6.943  1.00  0.00
I    C
ATOM   6251  HD1 ILE I 432       8.399  -4.821  -7.102  1.00  0.00
I    H
ATOM   6252  HD2 ILE I 432       8.225  -6.453  -7.843  1.00  0.00
I    H
ATOM   6253  HD3 ILE I 432       6.890  -5.765  -6.844  1.00  0.00
I    H
ATOM   6254  C   ILE I 432       8.411  -9.947  -4.003  1.00  0.00
I    C
ATOM   6255  O   ILE I 432       7.879 -10.004  -2.895  1.00  0.00
I    O
ATOM   6256  N   MET I 433       8.505 -11.048  -4.785  1.00  0.00
I    N
ATOM   6257  HN  MET I 433       8.908 -11.005  -5.698  1.00  0.00
I    H
ATOM   6258  CA  MET I 433       8.042 -12.376  -4.393  1.00  0.00
I    C
ATOM   6259  HA  MET I 433       6.994 -12.273  -4.140  1.00  0.00
I    H
ATOM   6260  CB  MET I 433       8.307 -13.426  -5.500  1.00  0.00
I    C
ATOM   6261  HB1 MET I 433       9.403 -13.410  -5.706  1.00  0.00
I    H
ATOM   6262  HB2 MET I 433       8.078 -14.442  -5.108  1.00  0.00
I    H
ATOM   6263  CG  MET I 433       7.576 -13.212  -6.831  1.00  0.00
I    C
```

```
ATOM   6264  HG1 MET I 433       6.563 -13.665  -6.792  1.00  0.00
I    H
ATOM   6265  HG2 MET I 433       7.450 -12.124  -7.018  1.00  0.00
I    H
ATOM   6266  SD  MET I 433       8.503 -13.968  -8.202  1.00  0.00
I    S
ATOM   6267  CE  MET I 433       7.210 -15.117  -8.697  1.00  0.00
I    C
ATOM   6268  HE1 MET I 433       7.542 -15.741  -9.556  1.00  0.00
I    H
ATOM   6269  HE2 MET I 433       6.943 -15.801  -7.862  1.00  0.00
I    H
ATOM   6270  HE3 MET I 433       6.290 -14.576  -9.004  1.00  0.00
I    H
ATOM   6271  C   MET I 433       8.759 -12.908  -3.167  1.00  0.00
I    C
ATOM   6272  O   MET I 433       8.163 -13.521  -2.281  1.00  0.00
I    O
ATOM   6273  N   PHE I 434      10.090 -12.696  -3.092  1.00  0.00
I    N
ATOM   6274  HN  PHE I 434      10.602 -12.277  -3.841  1.00  0.00
I    H
ATOM   6275  CA  PHE I 434      10.891 -13.049  -1.940  1.00  0.00
I    C
ATOM   6276  HA  PHE I 434      10.699 -14.100  -1.759  1.00  0.00
I    H
ATOM   6277  CB  PHE I 434      12.392 -12.815  -2.281  1.00  0.00
I    C
ATOM   6278  HB1 PHE I 434      12.663 -13.425  -3.169  1.00  0.00
I    H
ATOM   6279  HB2 PHE I 434      12.581 -11.752  -2.544  1.00  0.00
I    H
ATOM   6280  CG  PHE I 434      13.298 -13.244  -1.160  1.00  0.00
I    C
ATOM   6281  CD1 PHE I 434      13.376 -14.597  -0.807  1.00  0.00
I    C
ATOM   6282  HD1 PHE I 434      12.799 -15.327  -1.358  1.00  0.00
I    H
ATOM   6283  CE1 PHE I 434      14.186 -15.010   0.254  1.00  0.00
I    C
ATOM   6284  HE1 PHE I 434      14.257 -16.060   0.504  1.00  0.00
I    H
ATOM   6285  CZ  PHE I 434      14.914 -14.069   0.992  1.00  0.00
I    C
ATOM   6286  HZ  PHE I 434      15.547 -14.387   1.808  1.00  0.00
I    H
ATOM   6287  CD2 PHE I 434      14.027 -12.301  -0.413  1.00  0.00
I    C
ATOM   6288  HD2 PHE I 434      13.956 -11.249  -0.654  1.00  0.00
I    H
ATOM   6289  CE2 PHE I 434      14.827 -12.710   0.662  1.00  0.00
I    C
ATOM   6290  HE2 PHE I 434      15.375 -11.975   1.235  1.00  0.00
I    H
```

```
ATOM   6291  C   PHE I 434      10.465 -12.294  -0.687  1.00  0.00
I    C
ATOM   6292  O   PHE I 434      10.120 -12.904   0.323  1.00  0.00
I    O
ATOM   6293  N   ILE I 435      10.381 -10.953  -0.732  1.00  0.00
I    N
ATOM   6294  HN  ILE I 435      10.576 -10.416  -1.551  1.00  0.00
I    H
ATOM   6295  CA  ILE I 435       9.994 -10.157   0.425  1.00  0.00
I    C
ATOM   6296  HA  ILE I 435      10.641 -10.473   1.234  1.00  0.00
I    H
ATOM   6297  CB  ILE I 435      10.232  -8.667   0.199  1.00  0.00
I    C
ATOM   6298  HB  ILE I 435       9.628  -8.421  -0.711  1.00  0.00
I    H
ATOM   6299  CG2 ILE I 435       9.866  -7.902   1.489  1.00  0.00
I    C
ATOM   6300 HG21 ILE I 435       9.879  -6.806   1.310  1.00  0.00
I    H
ATOM   6301 HG22 ILE I 435       8.847  -8.175   1.835  1.00  0.00
I    H
ATOM   6302 HG23 ILE I 435      10.586  -8.129   2.303  1.00  0.00
I    H
ATOM   6303  CG1 ILE I 435      11.713  -8.379  -0.166  1.00  0.00
I    C
ATOM   6304 HG11 ILE I 435      12.365  -8.593   0.710  1.00  0.00
I    H
ATOM   6305 HG12 ILE I 435      12.024  -9.068  -0.984  1.00  0.00
I    H
ATOM   6306  CD  ILE I 435      11.953  -6.936  -0.633  1.00  0.00
I    C
ATOM   6307  HD1 ILE I 435      13.014  -6.800  -0.935  1.00  0.00
I    H
ATOM   6308  HD2 ILE I 435      11.308  -6.694  -1.505  1.00  0.00
I    H
ATOM   6309  HD3 ILE I 435      11.732  -6.213   0.180  1.00  0.00
I    H
ATOM   6310  C   ILE I 435       8.556 -10.447   0.869  1.00  0.00
I    C
ATOM   6311  O   ILE I 435       8.266 -10.575   2.057  1.00  0.00
I    O
ATOM   6312  N   GLY I 436       7.623 -10.614  -0.094  1.00  0.00
I    N
ATOM   6313  HN  GLY I 436       7.861 -10.498  -1.057  1.00  0.00
I    H
ATOM   6314  CA  GLY I 436       6.232 -10.968   0.175  1.00  0.00
I    C
ATOM   6315  HA1 GLY I 436       5.730 -10.904  -0.781  1.00  0.00
I    H
ATOM   6316  HA2 GLY I 436       5.886 -10.251   0.907  1.00  0.00
I    H
ATOM   6317  C   GLY I 436       6.001 -12.352   0.732  1.00  0.00
I    C
```

```
ATOM   6318  O   GLY I 436       5.088 -12.577   1.525  1.00  0.00
I    O
ATOM   6319  N   ARG I 437       6.827 -13.349   0.354  1.00  0.00
I    N
ATOM   6320  HN  ARG I 437       7.530 -13.224  -0.344  1.00  0.00
I    H
ATOM   6321  CA  ARG I 437       6.731 -14.668   0.953  1.00  0.00
I    C
ATOM   6322  HA  ARG I 437       5.677 -14.900   1.039  1.00  0.00
I    H
ATOM   6323  CB  ARG I 437       7.426 -15.765   0.109  1.00  0.00
I    C
ATOM   6324  HB1 ARG I 437       6.991 -15.695  -0.917  1.00  0.00
I    H
ATOM   6325  HB2 ARG I 437       8.507 -15.524   0.001  1.00  0.00
I    H
ATOM   6326  CG  ARG I 437       7.208 -17.197   0.656  1.00  0.00
I    C
ATOM   6327  HG1 ARG I 437       7.879 -17.892   0.105  1.00  0.00
I    H
ATOM   6328  HG2 ARG I 437       7.545 -17.234   1.717  1.00  0.00
I    H
ATOM   6329  CD  ARG I 437       5.737 -17.617   0.592  1.00  0.00
I    C
ATOM   6330  HD1 ARG I 437       5.094 -16.767   0.915  1.00  0.00
I    H
ATOM   6331  HD2 ARG I 437       5.441 -17.916  -0.440  1.00  0.00
I    H
ATOM   6332  NE  ARG I 437       5.538 -18.792   1.489  1.00  0.00
I    N
ATOM   6333  HE  ARG I 437       6.393 -19.123   1.902  1.00  0.00
I    H
ATOM   6334  CZ  ARG I 437       4.308 -19.284   1.663  1.00  0.00
I    C
ATOM   6335  NH1 ARG I 437       3.308 -18.947   0.855  1.00  0.00
I    N
ATOM   6336 HH11 ARG I 437       2.395 -19.298   1.010  1.00  0.00
I    H
ATOM   6337 HH12 ARG I 437       3.502 -18.227   0.186  1.00  0.00
I    H
ATOM   6338  NH2 ARG I 437       4.027 -20.083   2.684  1.00  0.00
I    N
ATOM   6339 HH21 ARG I 437       3.134 -20.507   2.748  1.00  0.00
I    H
ATOM   6340 HH22 ARG I 437       4.791 -20.393   3.233  1.00  0.00
I    H
ATOM   6341  C   ARG I 437       7.309 -14.760   2.348  1.00  0.00
I    C
ATOM   6342  O   ARG I 437       6.711 -15.359   3.237  1.00  0.00
I    O
ATOM   6343  N   ILE I 438       8.507 -14.185   2.560  1.00  0.00
I    N
ATOM   6344  HN  ILE I 438       9.003 -13.721   1.829  1.00  0.00
I    H
```

```
ATOM   6345  CA  ILE I 438       9.191 -14.177   3.845  1.00  0.00
I    C
ATOM   6346  HA  ILE I 438       9.193 -15.200   4.198  1.00  0.00
I    H
ATOM   6347  CB  ILE I 438      10.634 -13.697   3.677  1.00  0.00
I    C
ATOM   6348  HB  ILE I 438      10.540 -12.702   3.173  1.00  0.00
I    H
ATOM   6349  CG2 ILE I 438      11.345 -13.581   5.043  1.00  0.00
I    C
ATOM   6350 HG21 ILE I 438      12.332 -13.086   4.929  1.00  0.00
I    H
ATOM   6351 HG22 ILE I 438      10.744 -12.973   5.752  1.00  0.00
I    H
ATOM   6352 HG23 ILE I 438      11.509 -14.584   5.493  1.00  0.00
I    H
ATOM   6353  CG1 ILE I 438      11.423 -14.615   2.700  1.00  0.00
I    C
ATOM   6354 HG11 ILE I 438      11.072 -14.444   1.657  1.00  0.00
I    H
ATOM   6355 HG12 ILE I 438      12.501 -14.336   2.735  1.00  0.00
I    H
ATOM   6356  CD  ILE I 438      11.357 -16.122   2.959  1.00  0.00
I    C
ATOM   6357  HD1 ILE I 438      12.003 -16.666   2.237  1.00  0.00
I    H
ATOM   6358  HD2 ILE I 438      11.708 -16.358   3.986  1.00  0.00
I    H
ATOM   6359  HD3 ILE I 438      10.320 -16.500   2.846  1.00  0.00
I    H
ATOM   6360  C   ILE I 438       8.420 -13.362   4.866  1.00  0.00
I    C
ATOM   6361  O   ILE I 438       8.320 -13.709   6.045  1.00  0.00
I    O
ATOM   6362  N   GLY I 439       7.820 -12.259   4.391  1.00  0.00
I    N
ATOM   6363  HN  GLY I 439       7.913 -12.031   3.422  1.00  0.00
I    H
ATOM   6364  CA  GLY I 439       7.029 -11.341   5.174  1.00  0.00
I    C
ATOM   6365  HA1 GLY I 439       6.679 -11.848   6.063  1.00  0.00
I    H
ATOM   6366  HA2 GLY I 439       6.240 -10.934   4.555  1.00  0.00
I    H
ATOM   6367  C   GLY I 439       7.915 -10.200   5.606  1.00  0.00
I    C
ATOM   6368  O   GLY I 439       9.026 -10.443   6.083  1.00  0.00
I    O
ATOM   6369  N   PRO I 440       7.521  -8.938   5.494  1.00  0.00
I    N
ATOM   6370  CD  PRO I 440       6.227  -8.516   4.964  1.00  0.00
I    C
ATOM   6371  HD1 PRO I 440       6.024  -8.660   3.878  1.00  0.00
I    H
```

```
ATOM   6372  HD2 PRO I 440       5.489  -9.083   5.577  1.00  0.00
I    H
ATOM   6373  CA  PRO I 440       8.454  -7.825   5.606  1.00  0.00
I    C
ATOM   6374  HA  PRO I 440       9.264  -7.953   4.898  1.00  0.00
I    H
ATOM   6375  CB  PRO I 440       7.628  -6.602   5.188  1.00  0.00
I    C
ATOM   6376  HB1 PRO I 440       7.651  -6.511   4.079  1.00  0.00
I    H
ATOM   6377  HB2 PRO I 440       8.003  -5.659   5.634  1.00  0.00
I    H
ATOM   6378  CG  PRO I 440       6.165  -7.033   5.313  1.00  0.00
I    C
ATOM   6379  HG1 PRO I 440       5.482  -6.433   4.679  1.00  0.00
I    H
ATOM   6380  HG2 PRO I 440       5.848  -6.953   6.378  1.00  0.00
I    H
ATOM   6381  C   PRO I 440       9.030  -7.676   6.996  1.00  0.00
I    C
ATOM   6382  O   PRO I 440      10.206  -7.362   7.124  1.00  0.00
I    O
ATOM   6383  N   LEU I 441       8.234  -7.915   8.051  1.00  0.00
I    N
ATOM   6384  HN  LEU I 441       7.266  -8.137   7.945  1.00  0.00
I    H
ATOM   6385  CA  LEU I 441       8.690  -7.876   9.430  1.00  0.00
I    C
ATOM   6386  HA  LEU I 441       9.156  -6.908   9.561  1.00  0.00
I    H
ATOM   6387  CB  LEU I 441       7.480  -8.016  10.386  1.00  0.00
I    C
ATOM   6388  HB1 LEU I 441       6.632  -8.418   9.786  1.00  0.00
I    H
ATOM   6389  HB2 LEU I 441       7.711  -8.792  11.150  1.00  0.00
I    H
ATOM   6390  CG  LEU I 441       7.090  -6.674  11.024  1.00  0.00
I    C
ATOM   6391  HG  LEU I 441       7.991  -6.314  11.576  1.00  0.00
I    H
ATOM   6392  CD1 LEU I 441       6.674  -5.632   9.974  1.00  0.00
I    C
ATOM   6393 HD11 LEU I 441       6.351  -4.689  10.467  1.00  0.00
I    H
ATOM   6394 HD12 LEU I 441       7.523  -5.399   9.296  1.00  0.00
I    H
ATOM   6395 HD13 LEU I 441       5.829  -6.009   9.360  1.00  0.00
I    H
ATOM   6396  CD2 LEU I 441       5.965  -6.889  12.042  1.00  0.00
I    C
ATOM   6397 HD21 LEU I 441       5.689  -5.925  12.523  1.00  0.00
I    H
ATOM   6398 HD22 LEU I 441       5.053  -7.300  11.565  1.00  0.00
I    H
```

ATOM 6399 HD23 LEU I 441 6.291 -7.593 12.839 1.00 0.00
I H
ATOM 6400 C LEU I 441 9.735 -8.932 9.740 1.00 0.00
I C
ATOM 6401 O LEU I 441 10.781 -8.652 10.316 1.00 0.00
I O
ATOM 6402 N THR I 442 9.485 -10.174 9.287 1.00 0.00
I N
ATOM 6403 HN THR I 442 8.628 -10.378 8.815 1.00 0.00
I H
ATOM 6404 CA THR I 442 10.383 -11.316 9.419 1.00 0.00
I C
ATOM 6405 HA THR I 442 10.604 -11.392 10.475 1.00 0.00
I H
ATOM 6406 CB THR I 442 9.735 -12.576 8.858 1.00 0.00
I C
ATOM 6407 HB THR I 442 9.530 -12.365 7.780 1.00 0.00
I H
ATOM 6408 OG1 THR I 442 8.491 -12.805 9.501 1.00 0.00
I O
ATOM 6409 HG1 THR I 442 8.146 -13.614 9.110 1.00 0.00
I H
ATOM 6410 CG2 THR I 442 10.580 -13.835 9.098 1.00 0.00
I C
ATOM 6411 HG21 THR I 442 10.138 -14.713 8.580 1.00 0.00
I H
ATOM 6412 HG22 THR I 442 11.609 -13.692 8.706 1.00 0.00
I H
ATOM 6413 HG23 THR I 442 10.650 -14.070 10.182 1.00 0.00
I H
ATOM 6414 C THR I 442 11.707 -11.075 8.717 1.00 0.00
I C
ATOM 6415 O THR I 442 12.773 -11.366 9.245 1.00 0.00
I O
ATOM 6416 N PHE I 443 11.666 -10.483 7.505 1.00 0.00
I N
ATOM 6417 HN PHE I 443 10.796 -10.325 7.041 1.00 0.00
I H
ATOM 6418 CA PHE I 443 12.832 -10.029 6.768 1.00 0.00
I C
ATOM 6419 HA PHE I 443 13.495 -10.885 6.748 1.00 0.00
I H
ATOM 6420 CB PHE I 443 12.317 -9.699 5.329 1.00 0.00
I C
ATOM 6421 HB1 PHE I 443 12.145 -10.650 4.778 1.00 0.00
I H
ATOM 6422 HB2 PHE I 443 11.345 -9.161 5.366 1.00 0.00
I H
ATOM 6423 CG PHE I 443 13.267 -8.907 4.472 1.00 0.00
I C
ATOM 6424 CD1 PHE I 443 14.400 -9.498 3.892 1.00 0.00
I C
ATOM 6425 HD1 PHE I 443 14.590 -10.552 4.044 1.00 0.00
I H

```
ATOM   6426  CE1 PHE I 443      15.287  -8.729   3.125  1.00  0.00
I    C
ATOM   6427  HE1 PHE I 443      16.146  -9.195   2.664  1.00  0.00
I    H
ATOM   6428  CZ  PHE I 443      15.056  -7.358   2.952  1.00  0.00
I    C
ATOM   6429  HZ  PHE I 443      15.738  -6.766   2.359  1.00  0.00
I    H
ATOM   6430  CD2 PHE I 443      13.044  -7.532   4.288  1.00  0.00
I    C
ATOM   6431  HD2 PHE I 443      12.186  -7.058   4.744  1.00  0.00
I    H
ATOM   6432  CE2 PHE I 443      13.937  -6.757   3.539  1.00  0.00
I    C
ATOM   6433  HE2 PHE I 443      13.761  -5.698   3.418  1.00  0.00
I    H
ATOM   6434  C   PHE I 443      13.606  -8.871   7.440  1.00  0.00
I    C
ATOM   6435  O   PHE I 443      14.817  -8.977   7.639  1.00  0.00
I    O
ATOM   6436  N   VAL I 444      12.942  -7.760   7.851  1.00  0.00
I    N
ATOM   6437  HN  VAL I 444      11.949  -7.694   7.801  1.00  0.00
I    H
ATOM   6438  CA  VAL I 444      13.603  -6.567   8.398  1.00  0.00
I    C
ATOM   6439  HA  VAL I 444      14.356  -6.275   7.676  1.00  0.00
I    H
ATOM   6440  CB  VAL I 444      12.681  -5.346   8.519  1.00  0.00
I    C
ATOM   6441  HB  VAL I 444      11.803  -5.647   9.142  1.00  0.00
I    H
ATOM   6442  CG1 VAL I 444      13.392  -4.156   9.208  1.00  0.00
I    C
ATOM   6443 HG11 VAL I 444      12.717  -3.273   9.248  1.00  0.00
I    H
ATOM   6444 HG12 VAL I 444      13.680  -4.399  10.251  1.00  0.00
I    H
ATOM   6445 HG13 VAL I 444      14.305  -3.864   8.646  1.00  0.00
I    H
ATOM   6446  CG2 VAL I 444      12.263  -4.888   7.106  1.00  0.00
I    C
ATOM   6447 HG21 VAL I 444      11.626  -3.980   7.160  1.00  0.00
I    H
ATOM   6448 HG22 VAL I 444      13.159  -4.651   6.492  1.00  0.00
I    H
ATOM   6449 HG23 VAL I 444      11.680  -5.675   6.584  1.00  0.00
I    H
ATOM   6450  C   VAL I 444      14.313  -6.827   9.719  1.00  0.00
I    C
ATOM   6451  O   VAL I 444      15.383  -6.278   9.979  1.00  0.00
I    O
ATOM   6452  N   PHE I 445      13.773  -7.708  10.586  1.00  0.00
I    N
```

```
ATOM   6453  HN  PHE I 445      12.917  -8.189  10.401  1.00  0.00
I    H
ATOM   6454  CA  PHE I 445      14.386  -8.038  11.867  1.00  0.00
I    C
ATOM   6455  HA  PHE I 445      14.435  -7.093  12.394  1.00  0.00
I    H
ATOM   6456  CB  PHE I 445      13.514  -9.034  12.679  1.00  0.00
I    C
ATOM   6457  HB1 PHE I 445      13.061  -9.772  11.982  1.00  0.00
I    H
ATOM   6458  HB2 PHE I 445      14.131  -9.606  13.406  1.00  0.00
I    H
ATOM   6459  CG  PHE I 445      12.377  -8.340  13.389  1.00  0.00
I    C
ATOM   6460  CD1 PHE I 445      12.579  -7.165  14.140  1.00  0.00
I    C
ATOM   6461  HD1 PHE I 445      13.564  -6.721  14.185  1.00  0.00
I    H
ATOM   6462  CE1 PHE I 445      11.522  -6.566  14.834  1.00  0.00
I    C
ATOM   6463  HE1 PHE I 445      11.687  -5.649  15.383  1.00  0.00
I    H
ATOM   6464  CZ  PHE I 445      10.252  -7.151  14.812  1.00  0.00
I    C
ATOM   6465  HZ  PHE I 445       9.434  -6.690  15.346  1.00  0.00
I    H
ATOM   6466  CD2 PHE I 445      11.097  -8.918  13.383  1.00  0.00
I    C
ATOM   6467  HD2 PHE I 445      10.923  -9.841  12.846  1.00  0.00
I    H
ATOM   6468  CE2 PHE I 445      10.039  -8.330  14.087  1.00  0.00
I    C
ATOM   6469  HE2 PHE I 445       9.060  -8.789  14.070  1.00  0.00
I    H
ATOM   6470  C   PHE I 445      15.812  -8.579  11.783  1.00  0.00
I    C
ATOM   6471  O   PHE I 445      16.613  -8.366  12.687  1.00  0.00
I    O
ATOM   6472  N   SER I 446      16.189  -9.253  10.680  1.00  0.00
I    N
ATOM   6473  HN  SER I 446      15.542  -9.549   9.978  1.00  0.00
I    H
ATOM   6474  CA  SER I 446      17.579  -9.615  10.406  1.00  0.00
I    C
ATOM   6475  HA  SER I 446      17.895 -10.138  11.298  1.00  0.00
I    H
ATOM   6476  CB  SER I 446      17.735 -10.470   9.129  1.00  0.00
I    C
ATOM   6477  HB1 SER I 446      17.239  -9.932   8.290  1.00  0.00
I    H
ATOM   6478  HB2 SER I 446      18.811 -10.573   8.861  1.00  0.00
I    H
ATOM   6479  OG  SER I 446      17.109 -11.730   9.324  1.00  0.00
I    O
```

ATOM 6480 HG1 SER I 446 17.027 -12.143 8.456 1.00 0.00
I H
ATOM 6481 C SER I 446 18.536 -8.453 10.202 1.00 0.00
I C
ATOM 6482 O SER I 446 19.732 -8.584 10.459 1.00 0.00
I O
ATOM 6483 N PHE I 447 18.028 -7.323 9.673 1.00 0.00
I N
ATOM 6484 HN PHE I 447 17.041 -7.251 9.540 1.00 0.00
I H
ATOM 6485 CA PHE I 447 18.780 -6.150 9.256 1.00 0.00
I C
ATOM 6486 HA PHE I 447 19.782 -6.499 9.039 1.00 0.00
I H
ATOM 6487 CB PHE I 447 18.034 -5.419 8.090 1.00 0.00
I C
ATOM 6488 HB1 PHE I 447 16.947 -5.386 8.319 1.00 0.00
I H
ATOM 6489 HB2 PHE I 447 18.380 -4.368 7.991 1.00 0.00
I H
ATOM 6490 CG PHE I 447 18.168 -6.121 6.761 1.00 0.00
I C
ATOM 6491 CD1 PHE I 447 17.632 -7.400 6.530 1.00 0.00
I C
ATOM 6492 HD1 PHE I 447 17.107 -7.911 7.325 1.00 0.00
I H
ATOM 6493 CE1 PHE I 447 17.774 -8.025 5.285 1.00 0.00
I C
ATOM 6494 HE1 PHE I 447 17.386 -9.022 5.134 1.00 0.00
I H
ATOM 6495 CZ PHE I 447 18.413 -7.354 4.236 1.00 0.00
I C
ATOM 6496 HZ PHE I 447 18.512 -7.827 3.269 1.00 0.00
I H
ATOM 6497 CD2 PHE I 447 18.799 -5.458 5.694 1.00 0.00
I C
ATOM 6498 HD2 PHE I 447 19.223 -4.474 5.843 1.00 0.00
I H
ATOM 6499 CE2 PHE I 447 18.914 -6.063 4.437 1.00 0.00
I C
ATOM 6500 HE2 PHE I 447 19.392 -5.531 3.625 1.00 0.00
I H
ATOM 6501 C PHE I 447 18.895 -5.124 10.379 1.00 0.00
I C
ATOM 6502 O PHE I 447 19.746 -4.234 10.341 1.00 0.00
I O
ATOM 6503 N ALA I 448 18.028 -5.200 11.410 1.00 0.00
I N
ATOM 6504 HN ALA I 448 17.339 -5.922 11.457 1.00 0.00
I H
ATOM 6505 CA ALA I 448 18.020 -4.260 12.515 1.00 0.00
I C
ATOM 6506 HA ALA I 448 17.980 -3.251 12.122 1.00 0.00
I H

ATOM 6507 CB ALA I 448 16.732 -4.409 13.352 1.00 0.00
I C
ATOM 6508 HB1 ALA I 448 15.845 -4.307 12.691 1.00 0.00
I H
ATOM 6509 HB2 ALA I 448 16.684 -5.408 13.836 1.00 0.00
I H
ATOM 6510 HB3 ALA I 448 16.664 -3.629 14.141 1.00 0.00
I H
ATOM 6511 C ALA I 448 19.268 -4.329 13.407 1.00 0.00
I C
ATOM 6512 O ALA I 448 19.659 -5.374 13.914 1.00 0.00
I O
ATOM 6513 N LYS I 449 19.948 -3.177 13.600 1.00 0.00
I N
ATOM 6514 HN LYS I 449 19.601 -2.306 13.255 1.00 0.00
I H
ATOM 6515 CA LYS I 449 21.214 -3.100 14.309 1.00 0.00
I C
ATOM 6516 HA LYS I 449 21.828 -3.863 13.848 1.00 0.00
I H
ATOM 6517 CB LYS I 449 21.853 -1.702 14.077 1.00 0.00
I C
ATOM 6518 HB1 LYS I 449 21.108 -0.941 14.408 1.00 0.00
I H
ATOM 6519 HB2 LYS I 449 22.734 -1.590 14.748 1.00 0.00
I H
ATOM 6520 CG LYS I 449 22.241 -1.430 12.609 1.00 0.00
I C
ATOM 6521 HG1 LYS I 449 22.981 -2.195 12.280 1.00 0.00
I H
ATOM 6522 HG2 LYS I 449 21.336 -1.545 11.968 1.00 0.00
I H
ATOM 6523 CD LYS I 449 22.855 -0.031 12.407 1.00 0.00
I C
ATOM 6524 HD1 LYS I 449 22.092 0.732 12.684 1.00 0.00
I H
ATOM 6525 HD2 LYS I 449 23.708 0.080 13.117 1.00 0.00
I H
ATOM 6526 CE LYS I 449 23.344 0.210 10.970 1.00 0.00
I C
ATOM 6527 HE1 LYS I 449 24.122 -0.535 10.692 1.00 0.00
I H
ATOM 6528 HE2 LYS I 449 22.500 0.122 10.251 1.00 0.00
I H
ATOM 6529 NZ LYS I 449 23.932 1.565 10.826 1.00 0.00
I N
ATOM 6530 HZ1 LYS I 449 24.258 1.711 9.849 1.00 0.00
I H
ATOM 6531 HZ2 LYS I 449 23.216 2.281 11.061 1.00 0.00
I H
ATOM 6532 HZ3 LYS I 449 24.739 1.662 11.476 1.00 0.00
I H
ATOM 6533 C LYS I 449 21.188 -3.382 15.817 1.00 0.00
I C

```
ATOM   6534  O   LYS I 449      22.083  -4.045  16.334  1.00  0.00
I    O
ATOM   6535  N   THR I 450      20.213  -2.837  16.580  1.00  0.00
I    N
ATOM   6536  HN  THR I 450      19.451  -2.311  16.204  1.00  0.00
I    H
ATOM   6537  CA  THR I 450      20.188  -2.972  18.037  1.00  0.00
I    C
ATOM   6538  HA  THR I 450      20.223  -4.030  18.259  1.00  0.00
I    H
ATOM   6539  CB  THR I 450      21.337  -2.241  18.753  1.00  0.00
I    C
ATOM   6540  HB  THR I 450      22.283  -2.695  18.367  1.00  0.00
I    H
ATOM   6541  OG1 THR I 450      21.341  -2.442  20.162  1.00  0.00
I    O
ATOM   6542  HG1 THR I 450      22.089  -1.931  20.492  1.00  0.00
I    H
ATOM   6543  CG2 THR I 450      21.274  -0.727  18.492  1.00  0.00
I    C
ATOM   6544 HG21 THR I 450      22.164  -0.216  18.913  1.00  0.00
I    H
ATOM   6545 HG22 THR I 450      21.247  -0.524  17.399  1.00  0.00
I    H
ATOM   6546 HG23 THR I 450      20.366  -0.280  18.950  1.00  0.00
I    H
ATOM   6547  C   THR I 450      18.846  -2.469  18.542  1.00  0.00
I    C
ATOM   6548  O   THR I 450      18.171  -1.723  17.827  1.00  0.00
I    O
ATOM   6549  N   GLU I 451      18.453  -2.854  19.780  1.00  0.00
I    N
ATOM   6550  HN  GLU I 451      19.069  -3.368  20.371  1.00  0.00
I    H
ATOM   6551  CA  GLU I 451      17.154  -2.582  20.374  1.00  0.00
I    C
ATOM   6552  HA  GLU I 451      16.701  -1.818  19.756  1.00  0.00
I    H
ATOM   6553  CB  GLU I 451      16.301  -3.876  20.363  1.00  0.00
I    C
ATOM   6554  HB1 GLU I 451      15.311  -3.644  20.818  1.00  0.00
I    H
ATOM   6555  HB2 GLU I 451      16.090  -4.180  19.313  1.00  0.00
I    H
ATOM   6556  CG  GLU I 451      16.883  -5.100  21.115  1.00  0.00
I    C
ATOM   6557  HG1 GLU I 451      17.721  -5.526  20.533  1.00  0.00
I    H
ATOM   6558  HG2 GLU I 451      17.247  -4.812  22.121  1.00  0.00
I    H
ATOM   6559  CD  GLU I 451      15.862  -6.233  21.269  1.00  0.00
I    C
ATOM   6560  OE1 GLU I 451      14.641  -5.944  21.408  1.00  0.00
I    O
```

ATOM   6561 OE2 GLU I 451   16.321 -7.401 21.330 1.00 0.00
I   O
ATOM   6562 C   GLU I 451   17.167 -2.052 21.823 1.00 0.00
I   C
ATOM   6563 O   GLU I 451   16.149 -1.552 22.305 1.00 0.00
I   O
ATOM   6564 N   GLN I 452   18.288 -2.113 22.589 1.00 0.00
I   N
ATOM   6565 HN GLN I 452   19.194 -2.353 22.241 1.00 0.00
I   H
ATOM   6566 CA GLN I 452   18.247 -1.825 24.024 1.00 0.00
I   C
ATOM   6567 HA GLN I 452   17.236 -2.070 24.324 1.00 0.00
I   H
ATOM   6568 CB GLN I 452   19.147 -2.774 24.857 1.00 0.00
I   C
ATOM   6569 HB1 GLN I 452   19.992 -3.077 24.197 1.00 0.00
I   H
ATOM   6570 HB2 GLN I 452   19.600 -2.207 25.702 1.00 0.00
I   H
ATOM   6571 CG GLN I 452   18.354 -4.013 25.338 1.00 0.00
I   C
ATOM   6572 HG1 GLN I 452   17.398 -3.702 25.811 1.00 0.00
I   H
ATOM   6573 HG2 GLN I 452   18.112 -4.680 24.485 1.00 0.00
I   H
ATOM   6574 CD GLN I 452   19.141 -4.793 26.388 1.00 0.00
I   C
ATOM   6575 OE1 GLN I 452   20.370 -4.754 26.449 1.00 0.00
I   O
ATOM   6576 NE2 GLN I 452   18.418 -5.512 27.276 1.00 0.00
I   N
ATOM   6577 HE21 GLN I 452   18.928 -6.019 27.965 1.00 0.00
I   H
ATOM   6578 HE22 GLN I 452   17.426 -5.528 27.208 1.00 0.00
I   H
ATOM   6579 C   GLN I 452   18.509 -0.372 24.429 1.00 0.00
I   C
ATOM   6580 O   GLN I 452   19.342   0.330 23.862 1.00 0.00
I   O
ATOM   6581 N   SER I 453   17.765   0.086 25.462 1.00 0.00
I   N
ATOM   6582 HN SER I 453   17.032 -0.502 25.805 1.00 0.00
I   H
ATOM   6583 CA SER I 453   17.888   1.357 26.163 1.00 0.00
I   C
ATOM   6584 HA SER I 453   18.918   1.502 26.460 1.00 0.00
I   H
ATOM   6585 CB SER I 453   17.326   2.587 25.395 1.00 0.00
I   C
ATOM   6586 HB1 SER I 453   16.834   2.220 24.465 1.00 0.00
I   H
ATOM   6587 HB2 SER I 453   16.542   3.097 25.999 1.00 0.00
I   H

```
ATOM   6588  OG  SER I 453      18.378   3.481  25.043  1.00  0.00
I    O
ATOM   6589  HG1 SER I 453      18.022   4.096  24.390  1.00  0.00
I    H
ATOM   6590  C   SER I 453      17.079   1.149  27.427  1.00  0.00
I    C
ATOM   6591  O   SER I 453      16.473   0.090  27.580  1.00  0.00
I    O
ATOM   6592  N   ASN I 454      17.043   2.125  28.362  1.00  0.00
I    N
ATOM   6593  HN  ASN I 454      17.549   2.984  28.287  1.00  0.00
I    H
ATOM   6594  CA  ASN I 454      16.247   1.997  29.577  1.00  0.00
I    C
ATOM   6595  HA  ASN I 454      15.392   1.389  29.302  1.00  0.00
I    H
ATOM   6596  CB  ASN I 454      17.120   1.356  30.698  1.00  0.00
I    C
ATOM   6597  HB1 ASN I 454      17.882   0.710  30.208  1.00  0.00
I    H
ATOM   6598  HB2 ASN I 454      17.662   2.142  31.261  1.00  0.00
I    H
ATOM   6599  CG  ASN I 454      16.281   0.493  31.637  1.00  0.00
I    C
ATOM   6600  OD1 ASN I 454      15.074   0.316  31.483  1.00  0.00
I    O
ATOM   6601  ND2 ASN I 454      16.950  -0.116  32.643  1.00  0.00
I    N
ATOM   6602 HD21 ASN I 454      16.423  -0.693  33.261  1.00  0.00
I    H
ATOM   6603 HD22 ASN I 454      17.935  -0.003  32.727  1.00  0.00
I    H
ATOM   6604  C   ASN I 454      15.715   3.354  30.058  1.00  0.00
I    C
ATOM   6605  O   ASN I 454      15.337   3.518  31.214  1.00  0.00
I    O
ATOM   6606  N   ILE I 455      15.682   4.386  29.189  1.00  0.00
I    N
ATOM   6607  HN  ILE I 455      15.960   4.301  28.234  1.00  0.00
I    H
ATOM   6608  CA  ILE I 455      15.238   5.719  29.564  1.00  0.00
I    C
ATOM   6609  HA  ILE I 455      14.860   5.660  30.576  1.00  0.00
I    H
ATOM   6610  CB  ILE I 455      16.352   6.768  29.535  1.00  0.00
I    C
ATOM   6611  HB  ILE I 455      15.826   7.740  29.707  1.00  0.00
I    H
ATOM   6612  CG2 ILE I 455      17.355   6.407  30.658  1.00  0.00
I    C
ATOM   6613 HG21 ILE I 455      18.102   7.216  30.790  1.00  0.00
I    H
ATOM   6614 HG22 ILE I 455      16.831   6.271  31.628  1.00  0.00
I    H
```

ATOM 6615 HG23 ILE I 455 17.898 5.468 30.419 1.00 0.00
I H
ATOM 6616 CG1 ILE I 455 17.034 6.899 28.149 1.00 0.00
I C
ATOM 6617 HG11 ILE I 455 17.574 5.957 27.904 1.00 0.00
I H
ATOM 6618 HG12 ILE I 455 16.247 7.039 27.372 1.00 0.00
I H
ATOM 6619 CD ILE I 455 18.008 8.083 28.077 1.00 0.00
I C
ATOM 6620 HD1 ILE I 455 18.428 8.175 27.052 1.00 0.00
I H
ATOM 6621 HD2 ILE I 455 17.489 9.034 28.327 1.00 0.00
I H
ATOM 6622 HD3 ILE I 455 18.854 7.947 28.784 1.00 0.00
I H
ATOM 6623 C ILE I 455 14.073 6.117 28.683 1.00 0.00
I C
ATOM 6624 O ILE I 455 13.999 5.734 27.516 1.00 0.00
I O
ATOM 6625 N ARG I 456 13.112 6.863 29.260 1.00 0.00
I N
ATOM 6626 HN ARG I 456 13.208 7.089 30.227 1.00 0.00
I H
ATOM 6627 CA ARG I 456 11.934 7.372 28.586 1.00 0.00
I C
ATOM 6628 HA ARG I 456 11.871 6.926 27.601 1.00 0.00
I H
ATOM 6629 CB ARG I 456 10.656 7.159 29.441 1.00 0.00
I C
ATOM 6630 HB1 ARG I 456 10.835 7.682 30.411 1.00 0.00
I H
ATOM 6631 HB2 ARG I 456 9.798 7.682 28.965 1.00 0.00
I H
ATOM 6632 CG ARG I 456 10.324 5.680 29.729 1.00 0.00
I C
ATOM 6633 HG1 ARG I 456 9.994 5.202 28.781 1.00 0.00
I H
ATOM 6634 HG2 ARG I 456 11.259 5.149 30.023 1.00 0.00
I H
ATOM 6635 CD ARG I 456 9.300 5.485 30.854 1.00 0.00
I C
ATOM 6636 HD1 ARG I 456 9.155 4.395 31.036 1.00 0.00
I H
ATOM 6637 HD2 ARG I 456 9.638 5.964 31.803 1.00 0.00
I H
ATOM 6638 NE ARG I 456 8.030 6.139 30.400 1.00 0.00
I N
ATOM 6639 HE ARG I 456 7.991 6.278 29.404 1.00 0.00
I H
ATOM 6640 CZ ARG I 456 7.034 6.508 31.217 1.00 0.00
I C
ATOM 6641 NH1 ARG I 456 5.961 7.117 30.722 1.00 0.00
I N

```
ATOM   6642 HH11 ARG I 456       5.231   7.417  31.319  1.00  0.00
I    H
ATOM   6643 HH12 ARG I 456       6.002   7.371  29.753  1.00  0.00
I    H
ATOM   6644  NH2 ARG I 456       7.084   6.281  32.526  1.00  0.00
I    N
ATOM   6645 HH21 ARG I 456       6.298   6.486  33.093  1.00  0.00
I    H
ATOM   6646 HH22 ARG I 456       7.832   5.720  32.856  1.00  0.00
I    H
ATOM   6647  C   ARG I 456      12.067   8.868  28.377  1.00  0.00
I    C
ATOM   6648  O   ARG I 456      12.826   9.544  29.068  1.00  0.00
I    O
ATOM   6649  N   TYR I 457      11.298   9.420  27.425  1.00  0.00
I    N
ATOM   6650  HN  TYR I 457      10.678   8.875  26.863  1.00  0.00
I    H
ATOM   6651  CA  TYR I 457      11.291  10.837  27.125  1.00  0.00
I    C
ATOM   6652  HA  TYR I 457      12.073  11.282  27.729  1.00  0.00
I    H
ATOM   6653  CB  TYR I 457      11.525  11.033  25.605  1.00  0.00
I    C
ATOM   6654  HB1 TYR I 457      10.907  10.298  25.045  1.00  0.00
I    H
ATOM   6655  HB2 TYR I 457      11.201  12.047  25.286  1.00  0.00
I    H
ATOM   6656  CG  TYR I 457      12.970  10.789  25.290  1.00  0.00
I    C
ATOM   6657  CD1 TYR I 457      13.873  11.861  25.307  1.00  0.00
I    C
ATOM   6658  HD1 TYR I 457      13.520  12.858  25.533  1.00  0.00
I    H
ATOM   6659  CE1 TYR I 457      15.226  11.655  25.011  1.00  0.00
I    C
ATOM   6660  HE1 TYR I 457      15.922  12.481  25.017  1.00  0.00
I    H
ATOM   6661  CZ  TYR I 457      15.686  10.375  24.697  1.00  0.00
I    C
ATOM   6662  OH  TYR I 457      17.044  10.200  24.369  1.00  0.00
I    O
ATOM   6663  HH  TYR I 457      17.194   9.259  24.255  1.00  0.00
I    H
ATOM   6664  CD2 TYR I 457      13.445   9.498  25.001  1.00  0.00
I    C
ATOM   6665  HD2 TYR I 457      12.757   8.664  24.964  1.00  0.00
I    H
ATOM   6666  CE2 TYR I 457      14.802   9.289  24.709  1.00  0.00
I    C
ATOM   6667  HE2 TYR I 457      15.154   8.294  24.475  1.00  0.00
I    H
ATOM   6668  C   TYR I 457       9.973  11.493  27.540  1.00  0.00
I    C
```

```
ATOM   6669  O   TYR I 457       8.953  10.806  27.658  1.00  0.00
I    O
ATOM   6670  N   PRO I 458       9.926  12.811  27.792  1.00  0.00
I    N
ATOM   6671  CD  PRO I 458      11.082  13.714  27.798  1.00  0.00
I    C
ATOM   6672  HD1 PRO I 458      11.743  13.593  28.686  1.00  0.00
I    H
ATOM   6673  HD2 PRO I 458      11.653  13.535  26.859  1.00  0.00
I    H
ATOM   6674  CA  PRO I 458       8.682  13.527  28.056  1.00  0.00
I    C
ATOM   6675  HA  PRO I 458       8.117  13.035  28.839  1.00  0.00
I    H
ATOM   6676  CB  PRO I 458       9.136  14.933  28.496  1.00  0.00
I    C
ATOM   6677  HB1 PRO I 458       9.358  14.917  29.586  1.00  0.00
I    H
ATOM   6678  HB2 PRO I 458       8.375  15.712  28.295  1.00  0.00
I    H
ATOM   6679  CG  PRO I 458      10.477  15.119  27.783  1.00  0.00
I    C
ATOM   6680  HG1 PRO I 458      11.131  15.861  28.280  1.00  0.00
I    H
ATOM   6681  HG2 PRO I 458      10.292  15.433  26.729  1.00  0.00
I    H
ATOM   6682  C   PRO I 458       7.823  13.622  26.811  1.00  0.00
I    C
ATOM   6683  O   PRO I 458       8.349  13.650  25.698  1.00  0.00
I    O
ATOM   6684  N   ASP I 459       6.495  13.663  26.997  1.00  0.00
I    N
ATOM   6685  HN  ASP I 459       6.107  13.738  27.912  1.00  0.00
I    H
ATOM   6686  CA  ASP I 459       5.489  13.609  25.962  1.00  0.00
I    C
ATOM   6687  HA  ASP I 459       5.663  12.677  25.435  1.00  0.00
I    H
ATOM   6688  CB  ASP I 459       4.061  13.538  26.592  1.00  0.00
I    C
ATOM   6689  HB1 ASP I 459       3.908  14.418  27.250  1.00  0.00
I    H
ATOM   6690  HB2 ASP I 459       3.284  13.554  25.802  1.00  0.00
I    H
ATOM   6691  CG  ASP I 459       3.860  12.294  27.442  1.00  0.00
I    C
ATOM   6692  OD1 ASP I 459       4.838  11.531  27.649  1.00  0.00
I    O
ATOM   6693  OD2 ASP I 459       2.718  12.039  27.887  1.00  0.00
I    O
ATOM   6694  C   ASP I 459       5.589  14.729  24.923  1.00  0.00
I    C
ATOM   6695  O   ASP I 459       5.976  15.871  25.195  1.00  0.00
I    O
```

ATOM 6696 N GLY I 460 5.271 14.393 23.661 1.00 0.00
I N
ATOM 6697 HN GLY I 460 5.056 13.443 23.440 1.00 0.00
I H
ATOM 6698 CA GLY I 460 5.211 15.324 22.551 1.00 0.00
I C
ATOM 6699 HA1 GLY I 460 5.920 14.988 21.806 1.00 0.00
I H
ATOM 6700 HA2 GLY I 460 5.409 16.314 22.939 1.00 0.00
I H
ATOM 6701 C GLY I 460 3.850 15.337 21.923 1.00 0.00
I C
ATOM 6702 O GLY I 460 3.437 14.362 21.306 1.00 0.00
I O
ATOM 6703 N GLU I 461 3.133 16.474 22.034 1.00 0.00
I N
ATOM 6704 HN GLU I 461 3.453 17.266 22.546 1.00 0.00
I H
ATOM 6705 CA GLU I 461 1.829 16.653 21.420 1.00 0.00
I C
ATOM 6706 HA GLU I 461 1.301 15.708 21.460 1.00 0.00
I H
ATOM 6707 CB GLU I 461 0.976 17.701 22.186 1.00 0.00
I C
ATOM 6708 HB1 GLU I 461 0.940 17.404 23.259 1.00 0.00
I H
ATOM 6709 HB2 GLU I 461 1.483 18.692 22.160 1.00 0.00
I H
ATOM 6710 CG GLU I 461 -0.481 17.856 21.669 1.00 0.00
I C
ATOM 6711 HG1 GLU I 461 -1.066 18.455 22.390 1.00 0.00
I H
ATOM 6712 HG2 GLU I 461 -0.500 18.354 20.679 1.00 0.00
I H
ATOM 6713 CD GLU I 461 -1.208 16.517 21.553 1.00 0.00
I C
ATOM 6714 OE1 GLU I 461 -1.357 15.823 22.587 1.00 0.00
I O
ATOM 6715 OE2 GLU I 461 -1.602 16.176 20.407 1.00 0.00
I O
ATOM 6716 C GLU I 461 1.970 17.031 19.952 1.00 0.00
I C
ATOM 6717 O GLU I 461 2.753 17.908 19.575 1.00 0.00
I O
ATOM 6718 N VAL I 462 1.233 16.328 19.074 1.00 0.00
I N
ATOM 6719 HN VAL I 462 0.553 15.654 19.355 1.00 0.00
I H
ATOM 6720 CA VAL I 462 1.361 16.487 17.644 1.00 0.00
I C
ATOM 6721 HA VAL I 462 1.593 17.525 17.443 1.00 0.00
I H
ATOM 6722 CB VAL I 462 2.537 15.687 17.051 1.00 0.00
I C

```
ATOM   6723  HB  VAL I 462       3.483  16.153  17.423  1.00  0.00
I    H
ATOM   6724  CG1 VAL I 462       2.486  14.217  17.508  1.00  0.00
I    C
ATOM   6725 HG11 VAL I 462       3.315  13.640  17.045  1.00  0.00
I    H
ATOM   6726 HG12 VAL I 462       2.595  14.126  18.608  1.00  0.00
I    H
ATOM   6727 HG13 VAL I 462       1.527  13.745  17.205  1.00  0.00
I    H
ATOM   6728  CG2 VAL I 462       2.577  15.779  15.511  1.00  0.00
I    C
ATOM   6729 HG21 VAL I 462       3.407  15.165  15.103  1.00  0.00
I    H
ATOM   6730 HG22 VAL I 462       1.622  15.414  15.074  1.00  0.00
I    H
ATOM   6731 HG23 VAL I 462       2.736  16.824  15.175  1.00  0.00
I    H
ATOM   6732  C   VAL I 462       0.033  16.176  16.967  1.00  0.00
I    C
ATOM   6733  O   VAL I 462      -0.530  15.089  17.032  1.00  0.00
I    O
ATOM   6734  N   PHE I 463      -0.508  17.180  16.252  1.00  0.00
I    N
ATOM   6735  HN  PHE I 463      -0.023  18.041  16.110  1.00  0.00
I    H
ATOM   6736  CA  PHE I 463      -1.814  17.118  15.631  1.00  0.00
I    C
ATOM   6737  HA  PHE I 463      -2.455  16.599  16.332  1.00  0.00
I    H
ATOM   6738  CB  PHE I 463      -2.329  18.549  15.291  1.00  0.00
I    C
ATOM   6739  HB1 PHE I 463      -1.743  18.952  14.435  1.00  0.00
I    H
ATOM   6740  HB2 PHE I 463      -3.395  18.528  14.978  1.00  0.00
I    H
ATOM   6741  CG  PHE I 463      -2.127  19.472  16.458  1.00  0.00
I    C
ATOM   6742  CD1 PHE I 463      -2.765  19.207  17.677  1.00  0.00
I    C
ATOM   6743  HD1 PHE I 463      -3.474  18.394  17.743  1.00  0.00
I    H
ATOM   6744  CE1 PHE I 463      -2.496  19.988  18.806  1.00  0.00
I    C
ATOM   6745  HE1 PHE I 463      -2.987  19.770  19.744  1.00  0.00
I    H
ATOM   6746  CZ  PHE I 463      -1.585  21.047  18.721  1.00  0.00
I    C
ATOM   6747  HZ  PHE I 463      -1.379  21.653  19.591  1.00  0.00
I    H
ATOM   6748  CD2 PHE I 463      -1.217  20.544  16.378  1.00  0.00
I    C
ATOM   6749  HD2 PHE I 463      -0.749  20.786  15.434  1.00  0.00
I    H
```

```
ATOM   6750  CE2 PHE I 463      -0.935  21.315  17.513  1.00  0.00
I    C
ATOM   6751  HE2 PHE I 463      -0.214  22.118  17.452  1.00  0.00
I    H
ATOM   6752  C   PHE I 463      -1.789  16.288  14.348  1.00  0.00
I    C
ATOM   6753  O   PHE I 463      -1.132  16.640  13.369  1.00  0.00
I    O
ATOM   6754  N   THR I 464      -2.528  15.166  14.318  1.00  0.00
I    N
ATOM   6755  HN  THR I 464      -3.093  14.920  15.105  1.00  0.00
I    H
ATOM   6756  CA  THR I 464      -2.599  14.216  13.206  1.00  0.00
I    C
ATOM   6757  HA  THR I 464      -1.862  14.559  12.493  1.00  0.00
I    H
ATOM   6758  CB  THR I 464      -2.342  12.784  13.682  1.00  0.00
I    C
ATOM   6759  HB  THR I 464      -2.638  12.118  12.835  1.00  0.00
I    H
ATOM   6760  OG1 THR I 464      -3.165  12.438  14.790  1.00  0.00
I    O
ATOM   6761  HG1 THR I 464      -2.921  11.534  15.018  1.00  0.00
I    H
ATOM   6762  CG2 THR I 464      -0.893  12.677  14.175  1.00  0.00
I    C
ATOM   6763 HG21 THR I 464      -0.637  11.625  14.421  1.00  0.00
I    H
ATOM   6764 HG22 THR I 464      -0.190  13.024  13.389  1.00  0.00
I    H
ATOM   6765 HG23 THR I 464      -0.733  13.296  15.083  1.00  0.00
I    H
ATOM   6766  C   THR I 464      -3.946  14.247  12.488  1.00  0.00
I    C
ATOM   6767  O   THR I 464      -4.307  13.293  11.803  1.00  0.00
I    O
ATOM   6768  N   GLY I 465      -4.728  15.343  12.617  1.00  0.00
I    N
ATOM   6769  HN  GLY I 465      -4.439  16.097  13.205  1.00  0.00
I    H
ATOM   6770  CA  GLY I 465      -6.015  15.524  11.941  1.00  0.00
I    C
ATOM   6771  HA1 GLY I 465      -6.659  16.058  12.626  1.00  0.00
I    H
ATOM   6772  HA2 GLY I 465      -6.374  14.541  11.668  1.00  0.00
I    H
ATOM   6773  C   GLY I 465      -5.893  16.355  10.659  1.00  0.00
I    C
ATOM   6774  OT1 GLY I 465      -4.937  16.617  10.530  1.00  0.00
I    O
ATOM   6775  OT2 GLY I 465      -6.798  16.446  10.242  1.00  0.00
I    O
ATOM   6776  N   THR J  35     -23.054  21.030  18.759  1.00  0.00
J    N
```

ATOM 6777 HT1 THR J 35 −23.836 20.887 19.366 1.00 0.00
J H
ATOM 6778 HT2 THR J 35 −23.299 21.673 18.034 1.00 0.00
J H
ATOM 6779 HT3 THR J 35 −22.277 21.384 19.281 1.00 0.00
J H
ATOM 6780 CA THR J 35 −22.665 19.702 18.133 1.00 0.00
J C
ATOM 6781 HA THR J 35 −22.333 19.077 18.951 1.00 0.00
J H
ATOM 6782 CB THR J 35 −23.867 19.123 17.363 1.00 0.00
J C
ATOM 6783 HB THR J 35 −23.440 18.443 16.587 1.00 0.00
J H
ATOM 6784 OG1 THR J 35 −24.605 20.126 16.669 1.00 0.00
J O
ATOM 6785 HG1 THR J 35 −25.330 19.661 16.239 1.00 0.00
J H
ATOM 6786 CG2 THR J 35 −24.840 18.463 18.348 1.00 0.00
J C
ATOM 6787 HG21 THR J 35 −25.656 17.940 17.806 1.00 0.00
J H
ATOM 6788 HG22 THR J 35 −24.309 17.712 18.972 1.00 0.00
J H
ATOM 6789 HG23 THR J 35 −25.298 19.216 19.024 1.00 0.00
J H
ATOM 6790 C THR J 35 −21.476 19.856 17.194 1.00 0.00
J C
ATOM 6791 O THR J 35 −21.692 20.009 15.998 1.00 0.00
J O
ATOM 6792 N PRO J 36 −20.219 19.894 17.656 1.00 0.00
J N
ATOM 6793 CD PRO J 36 −19.792 19.439 18.983 1.00 0.00
J C
ATOM 6794 HD1 PRO J 36 −20.557 20.245 18.903 1.00 0.00
J H
ATOM 6795 HD2 PRO J 36 −20.031 18.758 19.832 1.00 0.00
J H
ATOM 6796 CA PRO J 36 −19.069 20.335 16.852 1.00 0.00
J C
ATOM 6797 HA PRO J 36 −19.140 21.414 16.790 1.00 0.00
J H
ATOM 6798 CB PRO J 36 −17.834 20.152 17.756 1.00 0.00
J C
ATOM 6799 HB1 PRO J 36 −17.718 21.054 18.399 1.00 0.00
J H
ATOM 6800 HB2 PRO J 36 −16.899 20.000 17.178 1.00 0.00
J H
ATOM 6801 CG PRO J 36 −18.401 20.052 19.177 1.00 0.00
J C
ATOM 6802 HG1 PRO J 36 −17.663 20.325 19.956 1.00 0.00
J H
ATOM 6803 HG2 PRO J 36 −18.761 19.013 19.356 1.00 0.00
J H

```
ATOM   6804  C   PRO J  36      -18.866  19.765  15.446  1.00  0.00
J    C
ATOM   6805  O   PRO J  36      -18.708  20.613  14.566  1.00  0.00
J    O
ATOM   6806  N   PRO J  37      -18.863  18.471  15.116  1.00  0.00
J    N
ATOM   6807  CD  PRO J  37      -18.929  17.342  16.052  1.00  0.00
J    C
ATOM   6808  HD1 PRO J  37      -18.080  17.388  16.771  1.00  0.00
J    H
ATOM   6809  HD2 PRO J  37      -19.902  17.340  16.597  1.00  0.00
J    H
ATOM   6810  CA  PRO J  37      -18.565  18.037  13.759  1.00  0.00
J    C
ATOM   6811  HA  PRO J  37      -17.777  18.634  13.315  1.00  0.00
J    H
ATOM   6812  CB  PRO J  37      -18.063  16.597  13.952  1.00  0.00
J    C
ATOM   6813  HB1 PRO J  37      -16.976  16.620  14.191  1.00  0.00
J    H
ATOM   6814  HB2 PRO J  37      -18.220  15.964  13.055  1.00  0.00
J    H
ATOM   6815  CG  PRO J  37      -18.813  16.088  15.185  1.00  0.00
J    C
ATOM   6816  HG1 PRO J  37      -18.292  15.254  15.694  1.00  0.00
J    H
ATOM   6817  HG2 PRO J  37      -19.834  15.758  14.883  1.00  0.00
J    H
ATOM   6818  C   PRO J  37      -19.818  18.150  12.901  1.00  0.00
J    C
ATOM   6819  O   PRO J  37      -19.697  18.369  11.695  1.00  0.00
J    O
ATOM   6820  N   GLN J  38      -21.037  18.042  13.486  1.00  0.00
J    N
ATOM   6821  HN  GLN J  38      -21.139  17.776  14.443  1.00  0.00
J    H
ATOM   6822  CA  GLN J  38      -22.290  18.297  12.795  1.00  0.00
J    C
ATOM   6823  HA  GLN J  38      -22.297  17.661  11.918  1.00  0.00
J    H
ATOM   6824  CB  GLN J  38      -23.554  18.038  13.668  1.00  0.00
J    C
ATOM   6825  HB1 GLN J  38      -23.402  18.589  14.625  1.00  0.00
J    H
ATOM   6826  HB2 GLN J  38      -24.441  18.501  13.179  1.00  0.00
J    H
ATOM   6827  CG  GLN J  38      -23.874  16.556  14.010  1.00  0.00
J    C
ATOM   6828  HG1 GLN J  38      -24.940  16.448  14.304  1.00  0.00
J    H
ATOM   6829  HG2 GLN J  38      -23.695  15.902  13.132  1.00  0.00
J    H
ATOM   6830  CD  GLN J  38      -23.079  15.969  15.182  1.00  0.00
J    C
```

ATOM 6831 OE1 GLN J 38 -22.231 16.623 15.797 1.00 0.00
J O
ATOM 6832 NE2 GLN J 38 -23.383 14.698 15.534 1.00 0.00
J N
ATOM 6833 HE21 GLN J 38 -22.875 14.309 16.297 1.00 0.00
J H
ATOM 6834 HE22 GLN J 38 -24.074 14.197 15.026 1.00 0.00
J H
ATOM 6835 C GLN J 38 -22.388 19.737 12.317 1.00 0.00
J C
ATOM 6836 O GLN J 38 -22.790 19.986 11.187 1.00 0.00
J O
ATOM 6837 N VAL J 39 -21.986 20.721 13.158 1.00 0.00
J N
ATOM 6838 HN VAL J 39 -21.655 20.517 14.075 1.00 0.00
J H
ATOM 6839 CA VAL J 39 -21.995 22.146 12.820 1.00 0.00
J C
ATOM 6840 HA VAL J 39 -23.019 22.415 12.593 1.00 0.00
J H
ATOM 6841 CB VAL J 39 -21.455 23.004 13.978 1.00 0.00
J C
ATOM 6842 HB VAL J 39 -20.523 22.515 14.354 1.00 0.00
J H
ATOM 6843 CG1 VAL J 39 -21.101 24.448 13.545 1.00 0.00
J C
ATOM 6844 HG11 VAL J 39 -20.753 25.039 14.421 1.00 0.00
J H
ATOM 6845 HG12 VAL J 39 -20.285 24.461 12.793 1.00 0.00
J H
ATOM 6846 HG13 VAL J 39 -21.989 24.960 13.117 1.00 0.00
J H
ATOM 6847 CG2 VAL J 39 -22.520 23.074 15.090 1.00 0.00
J C
ATOM 6848 HG21 VAL J 39 -22.174 23.720 15.924 1.00 0.00
J H
ATOM 6849 HG22 VAL J 39 -23.471 23.491 14.694 1.00 0.00
J H
ATOM 6850 HG23 VAL J 39 -22.729 22.068 15.510 1.00 0.00
J H
ATOM 6851 C VAL J 39 -21.166 22.429 11.585 1.00 0.00
J C
ATOM 6852 O VAL J 39 -21.631 23.061 10.637 1.00 0.00
J O
ATOM 6853 N LEU J 40 -19.925 21.900 11.546 1.00 0.00
J N
ATOM 6854 HN LEU J 40 -19.512 21.396 12.301 1.00 0.00
J H
ATOM 6855 CA LEU J 40 -19.080 22.027 10.384 1.00 0.00
J C
ATOM 6856 HA LEU J 40 -19.051 23.082 10.149 1.00 0.00
J H
ATOM 6857 CB LEU J 40 -17.648 21.552 10.719 1.00 0.00
J C

ATOM 6858 HB1 LEU J 40 -17.248 22.239 11.500 1.00 0.00
J H
ATOM 6859 HB2 LEU J 40 -17.702 20.546 11.191 1.00 0.00
J H
ATOM 6860 CG LEU J 40 -16.681 21.541 9.518 1.00 0.00
J C
ATOM 6861 HG LEU J 40 -17.154 22.161 8.718 1.00 0.00
J H
ATOM 6862 CD1 LEU J 40 -15.318 22.108 9.902 1.00 0.00
J C
ATOM 6863 HD11 LEU J 40 -14.616 22.051 9.043 1.00 0.00
J H
ATOM 6864 HD12 LEU J 40 -15.409 23.172 10.209 1.00 0.00
J H
ATOM 6865 HD13 LEU J 40 -14.879 21.536 10.747 1.00 0.00
J H
ATOM 6866 CD2 LEU J 40 -16.480 20.133 8.966 1.00 0.00
J C
ATOM 6867 HD21 LEU J 40 -15.795 20.155 8.091 1.00 0.00
J H
ATOM 6868 HD22 LEU J 40 -16.037 19.456 9.724 1.00 0.00
J H
ATOM 6869 HD23 LEU J 40 -17.451 19.702 8.637 1.00 0.00
J H
ATOM 6870 C LEU J 40 -19.638 21.303 9.169 1.00 0.00
J C
ATOM 6871 O LEU J 40 -19.780 21.911 8.114 1.00 0.00
J O
ATOM 6872 N ALA J 41 -20.036 20.018 9.273 1.00 0.00
J N
ATOM 6873 HN ALA J 41 -20.034 19.532 10.147 1.00 0.00
J H
ATOM 6874 CA ALA J 41 -20.496 19.246 8.131 1.00 0.00
J C
ATOM 6875 HA ALA J 41 -19.738 19.276 7.356 1.00 0.00
J H
ATOM 6876 CB ALA J 41 -20.664 17.768 8.544 1.00 0.00
J C
ATOM 6877 HB1 ALA J 41 -19.707 17.387 8.959 1.00 0.00
J H
ATOM 6878 HB2 ALA J 41 -21.445 17.659 9.328 1.00 0.00
J H
ATOM 6879 HB3 ALA J 41 -20.940 17.130 7.677 1.00 0.00
J H
ATOM 6880 C ALA J 41 -21.785 19.764 7.495 1.00 0.00
J C
ATOM 6881 O ALA J 41 -21.886 19.882 6.272 1.00 0.00
J O
ATOM 6882 N ILE J 42 -22.791 20.127 8.324 1.00 0.00
J N
ATOM 6883 HN ILE J 42 -22.728 20.001 9.311 1.00 0.00
J H
ATOM 6884 CA ILE J 42 -24.043 20.725 7.876 1.00 0.00
J C

ATOM 6885 HA ILE J 42 -24.422 20.094 7.083 1.00 0.00
J H
ATOM 6886 CB ILE J 42 -25.056 20.830 9.028 1.00 0.00
J C
ATOM 6887 HB ILE J 42 -24.505 21.374 9.837 1.00 0.00
J H
ATOM 6888 CG2 ILE J 42 -26.328 21.604 8.603 1.00 0.00
J C
ATOM 6889 HG21 ILE J 42 -26.976 21.806 9.481 1.00 0.00
J H
ATOM 6890 HG22 ILE J 42 -26.064 22.582 8.149 1.00 0.00
J H
ATOM 6891 HG23 ILE J 42 -26.918 21.022 7.863 1.00 0.00
J H
ATOM 6892 CG1 ILE J 42 -25.416 19.431 9.610 1.00 0.00
J C
ATOM 6893 HG11 ILE J 42 -24.487 18.841 9.775 1.00 0.00
J H
ATOM 6894 HG12 ILE J 42 -25.899 19.568 10.605 1.00 0.00
J H
ATOM 6895 CD ILE J 42 -26.369 18.591 8.752 1.00 0.00
J C
ATOM 6896 HD1 ILE J 42 -26.616 17.639 9.269 1.00 0.00
J H
ATOM 6897 HD2 ILE J 42 -27.317 19.142 8.567 1.00 0.00
J H
ATOM 6898 HD3 ILE J 42 -25.909 18.342 7.773 1.00 0.00
J H
ATOM 6899 C ILE J 42 -23.792 22.096 7.266 1.00 0.00
J C
ATOM 6900 O ILE J 42 -24.214 22.372 6.144 1.00 0.00
J O
ATOM 6901 N GLY J 43 -23.015 22.972 7.949 1.00 0.00
J N
ATOM 6902 HN GLY J 43 -22.596 22.739 8.826 1.00 0.00
J H
ATOM 6903 CA GLY J 43 -22.735 24.317 7.455 1.00 0.00
J C
ATOM 6904 HA1 GLY J 43 -22.156 24.811 8.225 1.00 0.00
J H
ATOM 6905 HA2 GLY J 43 -23.693 24.778 7.253 1.00 0.00
J H
ATOM 6906 C GLY J 43 -21.934 24.339 6.185 1.00 0.00
J C
ATOM 6907 O GLY J 43 -22.136 25.184 5.319 1.00 0.00
J O
ATOM 6908 N PHE J 44 -21.020 23.366 6.016 1.00 0.00
J N
ATOM 6909 HN PHE J 44 -20.815 22.690 6.723 1.00 0.00
J H
ATOM 6910 CA PHE J 44 -20.255 23.212 4.803 1.00 0.00
J C
ATOM 6911 HA PHE J 44 -19.822 24.184 4.601 1.00 0.00
J H

```
ATOM   6912  CB  PHE J  44      -19.150  22.145   5.017  1.00  0.00
J    C
ATOM   6913  HB1 PHE J  44      -18.886  22.105   6.096  1.00  0.00
J    H
ATOM   6914  HB2 PHE J  44      -19.510  21.133   4.730  1.00  0.00
J    H
ATOM   6915  CG  PHE J  44      -17.901  22.496   4.263  1.00  0.00
J    C
ATOM   6916  CD1 PHE J  44      -16.790  22.997   4.967  1.00  0.00
J    C
ATOM   6917  HD1 PHE J  44      -16.853  23.136   6.037  1.00  0.00
J    H
ATOM   6918  CE1 PHE J  44      -15.607  23.324   4.295  1.00  0.00
J    C
ATOM   6919  HE1 PHE J  44      -14.744  23.660   4.854  1.00  0.00
J    H
ATOM   6920  CZ  PHE J  44      -15.546  23.207   2.903  1.00  0.00
J    C
ATOM   6921  HZ  PHE J  44      -14.635  23.456   2.378  1.00  0.00
J    H
ATOM   6922  CD2 PHE J  44      -17.822  22.364   2.869  1.00  0.00
J    C
ATOM   6923  HD2 PHE J  44      -18.666  21.976   2.315  1.00  0.00
J    H
ATOM   6924  CE2 PHE J  44      -16.663  22.757   2.189  1.00  0.00
J    C
ATOM   6925  HE2 PHE J  44      -16.632  22.714   1.109  1.00  0.00
J    H
ATOM   6926  C   PHE J  44      -21.138  22.860   3.612  1.00  0.00
J    C
ATOM   6927  O   PHE J  44      -21.044  23.498   2.565  1.00  0.00
J    O
ATOM   6928  N   PHE J  45      -22.070  21.883   3.758  1.00  0.00
J    N
ATOM   6929  HN  PHE J  45      -22.219  21.415   4.627  1.00  0.00
J    H
ATOM   6930  CA  PHE J  45      -22.932  21.430   2.673  1.00  0.00
J    C
ATOM   6931  HA  PHE J  45      -22.267  21.138   1.870  1.00  0.00
J    H
ATOM   6932  CB  PHE J  45      -23.809  20.208   3.093  1.00  0.00
J    C
ATOM   6933  HB1 PHE J  45      -23.152  19.418   3.517  1.00  0.00
J    H
ATOM   6934  HB2 PHE J  45      -24.535  20.490   3.886  1.00  0.00
J    H
ATOM   6935  CG  PHE J  45      -24.577  19.529   1.959  1.00  0.00
J    C
ATOM   6936  CD1 PHE J  45      -24.256  19.657   0.588  1.00  0.00
J    C
ATOM   6937  HD1 PHE J  45      -23.414  20.267   0.290  1.00  0.00
J    H
ATOM   6938  CE1 PHE J  45      -25.018  19.010  -0.397  1.00  0.00
J    C
```

```
ATOM   6939  HE1 PHE J  45      -24.769  19.134  -1.442  1.00  0.00
J    H
ATOM   6940  CZ  PHE J  45      -26.096  18.199  -0.028  1.00  0.00
J    C
ATOM   6941  HZ  PHE J  45      -26.677  17.687  -0.782  1.00  0.00
J    H
ATOM   6942  CD2 PHE J  45      -25.665  18.708   2.304  1.00  0.00
J    C
ATOM   6943  HD2 PHE J  45      -25.950  18.596   3.341  1.00  0.00
J    H
ATOM   6944  CE2 PHE J  45      -26.415  18.043   1.326  1.00  0.00
J    C
ATOM   6945  HE2 PHE J  45      -27.242  17.411   1.618  1.00  0.00
J    H
ATOM   6946  C   PHE J  45      -23.816  22.551   2.135  1.00  0.00
J    C
ATOM   6947  O   PHE J  45      -23.914  22.754   0.925  1.00  0.00
J    O
ATOM   6948  N   LEU J  46      -24.421  23.361   3.030  1.00  0.00
J    N
ATOM   6949  HN  LEU J  46      -24.358  23.221   4.016  1.00  0.00
J    H
ATOM   6950  CA  LEU J  46      -25.219  24.512   2.641  1.00  0.00
J    C
ATOM   6951  HA  LEU J  46      -25.991  24.136   1.982  1.00  0.00
J    H
ATOM   6952  CB  LEU J  46      -25.951  25.161   3.850  1.00  0.00
J    C
ATOM   6953  HB1 LEU J  46      -25.235  25.163   4.704  1.00  0.00
J    H
ATOM   6954  HB2 LEU J  46      -26.157  26.230   3.618  1.00  0.00
J    H
ATOM   6955  CG  LEU J  46      -27.262  24.457   4.314  1.00  0.00
J    C
ATOM   6956  HG  LEU J  46      -27.603  25.162   5.111  1.00  0.00
J    H
ATOM   6957  CD1 LEU J  46      -28.320  24.348   3.205  1.00  0.00
J    C
ATOM   6958 HD11 LEU J  46      -29.214  23.798   3.571  1.00  0.00
J    H
ATOM   6959 HD12 LEU J  46      -28.645  25.358   2.872  1.00  0.00
J    H
ATOM   6960 HD13 LEU J  46      -27.914  23.802   2.327  1.00  0.00
J    H
ATOM   6961  CD2 LEU J  46      -27.065  23.075   4.956  1.00  0.00
J    C
ATOM   6962 HD21 LEU J  46      -28.044  22.655   5.273  1.00  0.00
J    H
ATOM   6963 HD22 LEU J  46      -26.604  22.356   4.248  1.00  0.00
J    H
ATOM   6964 HD23 LEU J  46      -26.413  23.151   5.852  1.00  0.00
J    H
ATOM   6965  C   LEU J  46      -24.407  25.546   1.858  1.00  0.00
J    C
```

```
ATOM   6966  O   LEU J  46      -24.859  26.046   0.832  1.00  0.00
J    O
ATOM   6967  N   THR J  47      -23.148  25.831   2.253  1.00  0.00
J    N
ATOM   6968  HN  THR J  47      -22.759  25.479   3.103  1.00  0.00
J    H
ATOM   6969  CA  THR J  47      -22.234  26.682   1.478  1.00  0.00
J    C
ATOM   6970  HA  THR J  47      -22.762  27.618   1.357  1.00  0.00
J    H
ATOM   6971  CB  THR J  47      -20.893  26.899   2.169  1.00  0.00
J    C
ATOM   6972  HB  THR J  47      -20.430  25.888   2.271  1.00  0.00
J    H
ATOM   6973  OG1 THR J  47      -21.113  27.415   3.469  1.00  0.00
J    O
ATOM   6974  HG1 THR J  47      -20.236  27.542   3.847  1.00  0.00
J    H
ATOM   6975  CG2 THR J  47      -20.040  27.965   1.464  1.00  0.00
J    C
ATOM   6976 HG21 THR J  47      -19.028  28.023   1.917  1.00  0.00
J    H
ATOM   6977 HG22 THR J  47      -19.920  27.715   0.388  1.00  0.00
J    H
ATOM   6978 HG23 THR J  47      -20.512  28.968   1.537  1.00  0.00
J    H
ATOM   6979  C   THR J  47      -21.953  26.158   0.070  1.00  0.00
J    C
ATOM   6980  O   THR J  47      -21.908  26.928  -0.890  1.00  0.00
J    O
ATOM   6981  N   ILE J  48      -21.787  24.822  -0.110  1.00  0.00
J    N
ATOM   6982  HN  ILE J  48      -21.768  24.179   0.652  1.00  0.00
J    H
ATOM   6983  CA  ILE J  48      -21.621  24.199  -1.428  1.00  0.00
J    C
ATOM   6984  HA  ILE J  48      -20.762  24.687  -1.872  1.00  0.00
J    H
ATOM   6985  CB  ILE J  48      -21.399  22.670  -1.358  1.00  0.00
J    C
ATOM   6986  HB  ILE J  48      -22.301  22.280  -0.823  1.00  0.00
J    H
ATOM   6987  CG2 ILE J  48      -21.260  22.098  -2.792  1.00  0.00
J    C
ATOM   6988 HG21 ILE J  48      -21.257  20.988  -2.771  1.00  0.00
J    H
ATOM   6989 HG22 ILE J  48      -22.108  22.420  -3.431  1.00  0.00
J    H
ATOM   6990 HG23 ILE J  48      -20.316  22.439  -3.265  1.00  0.00
J    H
ATOM   6991  CG1 ILE J  48      -20.174  22.329  -0.471  1.00  0.00
J    C
ATOM   6992 HG11 ILE J  48      -19.361  23.068  -0.651  1.00  0.00
J    H
```

```
ATOM   6993 HG12 ILE J  48      -20.469  22.418   0.601  1.00  0.00
J    H
ATOM   6994  CD  ILE J  48      -19.556  20.932  -0.631  1.00  0.00
J    C
ATOM   6995  HD1 ILE J  48      -18.728  20.790   0.096  1.00  0.00
J    H
ATOM   6996  HD2 ILE J  48      -20.317  20.143  -0.449  1.00  0.00
J    H
ATOM   6997  HD3 ILE J  48      -19.143  20.794  -1.653  1.00  0.00
J    H
ATOM   6998  C   ILE J  48      -22.801  24.448  -2.362  1.00  0.00
J    C
ATOM   6999  O   ILE J  48      -22.634  24.904  -3.495  1.00  0.00
J    O
ATOM   7000  N   ILE J  49      -24.043  24.183  -1.906  1.00  0.00
J    N
ATOM   7001  HN  ILE J  49      -24.245  23.863  -0.982  1.00  0.00
J    H
ATOM   7002  CA  ILE J  49      -25.219  24.350  -2.751  1.00  0.00
J    C
ATOM   7003  HA  ILE J  49      -24.927  23.986  -3.728  1.00  0.00
J    H
ATOM   7004  CB  ILE J  49      -26.392  23.431  -2.393  1.00  0.00
J    C
ATOM   7005  HB  ILE J  49      -27.265  23.862  -2.945  1.00  0.00
J    H
ATOM   7006  CG2 ILE J  49      -25.990  21.999  -2.821  1.00  0.00
J    C
ATOM   7007 HG21 ILE J  49      -26.854  21.308  -2.733  1.00  0.00
J    H
ATOM   7008 HG22 ILE J  49      -25.651  21.982  -3.878  1.00  0.00
J    H
ATOM   7009 HG23 ILE J  49      -25.168  21.609  -2.184  1.00  0.00
J    H
ATOM   7010  CG1 ILE J  49      -26.815  23.450  -0.905  1.00  0.00
J    C
ATOM   7011 HG11 ILE J  49      -27.356  22.510  -0.655  1.00  0.00
J    H
ATOM   7012 HG12 ILE J  49      -25.900  23.482  -0.270  1.00  0.00
J    H
ATOM   7013  CD  ILE J  49      -27.698  24.638  -0.506  1.00  0.00
J    C
ATOM   7014  HD1 ILE J  49      -27.911  24.612   0.585  1.00  0.00
J    H
ATOM   7015  HD2 ILE J  49      -27.191  25.599  -0.738  1.00  0.00
J    H
ATOM   7016  HD3 ILE J  49      -28.668  24.612  -1.045  1.00  0.00
J    H
ATOM   7017  C   ILE J  49      -25.629  25.805  -2.928  1.00  0.00
J    C
ATOM   7018  O   ILE J  49      -26.160  26.170  -3.973  1.00  0.00
J    O
ATOM   7019  N   ILE J  50      -25.318  26.708  -1.968  1.00  0.00
J    N
```

```
ATOM   7020  HN  ILE J  50      -24.963  26.441  -1.075  1.00  0.00
J    H
ATOM   7021  CA  ILE J  50      -25.472  28.152  -2.144  1.00  0.00
J    C
ATOM   7022  HA  ILE J  50      -26.493  28.313  -2.466  1.00  0.00
J    H
ATOM   7023  CB  ILE J  50      -25.233  28.905  -0.829  1.00  0.00
J    C
ATOM   7024  HB  ILE J  50      -24.380  28.363  -0.347  1.00  0.00
J    H
ATOM   7025  CG2 ILE J  50      -24.928  30.409  -1.052  1.00  0.00
J    C
ATOM   7026 HG21 ILE J  50      -24.608  30.888  -0.103  1.00  0.00
J    H
ATOM   7027 HG22 ILE J  50      -24.108  30.542  -1.788  1.00  0.00
J    H
ATOM   7028 HG23 ILE J  50      -25.825  30.946  -1.425  1.00  0.00
J    H
ATOM   7029  CG1 ILE J  50      -26.473  28.726   0.084  1.00  0.00
J    C
ATOM   7030 HG11 ILE J  50      -27.348  29.249  -0.362  1.00  0.00
J    H
ATOM   7031 HG12 ILE J  50      -26.728  27.643   0.137  1.00  0.00
J    H
ATOM   7032  CD  ILE J  50      -26.268  29.235   1.517  1.00  0.00
J    C
ATOM   7033  HD1 ILE J  50      -27.168  29.026   2.134  1.00  0.00
J    H
ATOM   7034  HD2 ILE J  50      -25.397  28.732   1.991  1.00  0.00
J    H
ATOM   7035  HD3 ILE J  50      -26.090  30.331   1.529  1.00  0.00
J    H
ATOM   7036  C   ILE J  50      -24.575  28.666  -3.256  1.00  0.00
J    C
ATOM   7037  O   ILE J  50      -25.016  29.396  -4.144  1.00  0.00
J    O
ATOM   7038  N   GLY J  51      -23.294  28.235  -3.297  1.00  0.00
J    N
ATOM   7039  HN  GLY J  51      -22.903  27.651  -2.587  1.00  0.00
J    H
ATOM   7040  CA  GLY J  51      -22.392  28.594  -4.387  1.00  0.00
J    C
ATOM   7041  HA1 GLY J  51      -21.434  28.154  -4.145  1.00  0.00
J    H
ATOM   7042  HA2 GLY J  51      -22.385  29.675  -4.437  1.00  0.00
J    H
ATOM   7043  C   GLY J  51      -22.817  28.072  -5.735  1.00  0.00
J    C
ATOM   7044  O   GLY J  51      -22.636  28.733  -6.749  1.00  0.00
J    O
ATOM   7045  N   ALA J  52      -23.445  26.880  -5.772  1.00  0.00
J    N
ATOM   7046  HN  ALA J  52      -23.538  26.334  -4.940  1.00  0.00
J    H
```

ATOM 7047 CA ALA J 52 -24.022 26.294 -6.968 1.00 0.00
J C
ATOM 7048 HA ALA J 52 -23.268 26.230 -7.744 1.00 0.00
J H
ATOM 7049 CB ALA J 52 -24.472 24.848 -6.671 1.00 0.00
J C
ATOM 7050 HB1 ALA J 52 -23.607 24.257 -6.300 1.00 0.00
J H
ATOM 7051 HB2 ALA J 52 -25.261 24.828 -5.888 1.00 0.00
J H
ATOM 7052 HB3 ALA J 52 -24.865 24.347 -7.582 1.00 0.00
J H
ATOM 7053 C ALA J 52 -25.167 27.127 -7.546 1.00 0.00
J C
ATOM 7054 O ALA J 52 -25.230 27.356 -8.752 1.00 0.00
J O
ATOM 7055 N VAL J 53 -26.068 27.653 -6.683 1.00 0.00
J N
ATOM 7056 HN VAL J 53 -26.059 27.418 -5.715 1.00 0.00
J H
ATOM 7057 CA VAL J 53 -27.121 28.594 -7.068 1.00 0.00
J C
ATOM 7058 HA VAL J 53 -27.689 28.126 -7.862 1.00 0.00
J H
ATOM 7059 CB VAL J 53 -28.075 28.891 -5.904 1.00 0.00
J C
ATOM 7060 HB VAL J 53 -27.462 29.287 -5.058 1.00 0.00
J H
ATOM 7061 CG1 VAL J 53 -29.129 29.953 -6.296 1.00 0.00
J C
ATOM 7062 HG11 VAL J 53 -29.834 30.126 -5.454 1.00 0.00
J H
ATOM 7063 HG12 VAL J 53 -28.657 30.928 -6.537 1.00 0.00
J H
ATOM 7064 HG13 VAL J 53 -29.719 29.615 -7.174 1.00 0.00
J H
ATOM 7065 CG2 VAL J 53 -28.802 27.589 -5.507 1.00 0.00
J C
ATOM 7066 HG21 VAL J 53 -29.532 27.780 -4.691 1.00 0.00
J H
ATOM 7067 HG22 VAL J 53 -29.353 27.169 -6.377 1.00 0.00
J H
ATOM 7068 HG23 VAL J 53 -28.086 26.824 -5.144 1.00 0.00
J H
ATOM 7069 C VAL J 53 -26.551 29.901 -7.620 1.00 0.00
J C
ATOM 7070 O VAL J 53 -26.961 30.371 -8.681 1.00 0.00
J O
ATOM 7071 N LEU J 54 -25.531 30.491 -6.953 1.00 0.00
J N
ATOM 7072 HN LEU J 54 -25.149 30.097 -6.120 1.00 0.00
J H
ATOM 7073 CA LEU J 54 -24.897 31.740 -7.374 1.00 0.00
J C

```
ATOM   7074  HA  LEU J  54      -25.706  32.449  -7.496  1.00  0.00
J    H
ATOM   7075  CB  LEU J  54      -23.935  32.308  -6.285  1.00  0.00
J    C
ATOM   7076  HB1 LEU J  54      -23.487  31.436  -5.755  1.00  0.00
J    H
ATOM   7077  HB2 LEU J  54      -23.091  32.833  -6.786  1.00  0.00
J    H
ATOM   7078  CG  LEU J  54      -24.598  33.235  -5.235  1.00  0.00
J    C
ATOM   7079  HG  LEU J  54      -25.229  33.945  -5.823  1.00  0.00
J    H
ATOM   7080  CD1 LEU J  54      -25.454  32.491  -4.201  1.00  0.00
J    C
ATOM   7081 HD11 LEU J  54      -25.863  33.201  -3.450  1.00  0.00
J    H
ATOM   7082 HD12 LEU J  54      -26.307  31.977  -4.695  1.00  0.00
J    H
ATOM   7083 HD13 LEU J  54      -24.849  31.729  -3.666  1.00  0.00
J    H
ATOM   7084  CD2 LEU J  54      -23.517  34.052  -4.505  1.00  0.00
J    C
ATOM   7085 HD21 LEU J  54      -23.985  34.734  -3.762  1.00  0.00
J    H
ATOM   7086 HD22 LEU J  54      -22.807  33.397  -3.961  1.00  0.00
J    H
ATOM   7087 HD23 LEU J  54      -22.941  34.668  -5.228  1.00  0.00
J    H
ATOM   7088  C   LEU J  54      -24.160  31.641  -8.719  1.00  0.00
J    C
ATOM   7089  O   LEU J  54      -23.999  32.625  -9.442  1.00  0.00
J    O
ATOM   7090  N   LEU J  55      -23.704  30.437  -9.108  1.00  0.00
J    N
ATOM   7091  HN  LEU J  55      -23.839  29.609  -8.567  1.00  0.00
J    H
ATOM   7092  CA  LEU J  55      -22.977  30.228 -10.347  1.00  0.00
J    C
ATOM   7093  HA  LEU J  55      -22.516  31.181 -10.572  1.00  0.00
J    H
ATOM   7094  CB  LEU J  55      -21.955  29.085 -10.167  1.00  0.00
J    C
ATOM   7095  HB1 LEU J  55      -22.500  28.223  -9.718  1.00  0.00
J    H
ATOM   7096  HB2 LEU J  55      -21.613  28.745 -11.170  1.00  0.00
J    H
ATOM   7097  CG  LEU J  55      -20.764  29.465  -9.269  1.00  0.00
J    C
ATOM   7098  HG  LEU J  55      -21.181  29.995  -8.378  1.00  0.00
J    H
ATOM   7099  CD1 LEU J  55      -20.050  28.186  -8.818  1.00  0.00
J    C
ATOM   7100 HD11 LEU J  55      -19.156  28.435  -8.206  1.00  0.00
J    H
```

ATOM 7101 HD12 LEU J 55 -20.728 27.555 -8.205 1.00 0.00
J H
ATOM 7102 HD13 LEU J 55 -19.715 27.594 -9.696 1.00 0.00
J H
ATOM 7103 CD2 LEU J 55 -19.782 30.416 -9.971 1.00 0.00
J C
ATOM 7104 HD21 LEU J 55 -18.947 30.682 -9.287 1.00 0.00
J H
ATOM 7105 HD22 LEU J 55 -19.341 29.953 -10.876 1.00 0.00
J H
ATOM 7106 HD23 LEU J 55 -20.297 31.353 -10.273 1.00 0.00
J H
ATOM 7107 C LEU J 55 -23.852 29.851 -11.528 1.00 0.00
J C
ATOM 7108 O LEU J 55 -23.374 29.867 -12.658 1.00 0.00
J O
ATOM 7109 N MET J 56 -25.146 29.528 -11.332 1.00 0.00
J N
ATOM 7110 HN MET J 56 -25.613 29.580 -10.452 1.00 0.00
J H
ATOM 7111 CA MET J 56 -25.983 29.065 -12.431 1.00 0.00
J C
ATOM 7112 HA MET J 56 -25.315 28.740 -13.219 1.00 0.00
J H
ATOM 7113 CB MET J 56 -26.876 27.907 -11.928 1.00 0.00
J C
ATOM 7114 HB1 MET J 56 -26.354 27.450 -11.056 1.00 0.00
J H
ATOM 7115 HB2 MET J 56 -27.832 28.322 -11.537 1.00 0.00
J H
ATOM 7116 CG MET J 56 -27.137 26.820 -12.990 1.00 0.00
J C
ATOM 7117 HG1 MET J 56 -27.535 27.276 -13.921 1.00 0.00
J H
ATOM 7118 HG2 MET J 56 -26.182 26.311 -13.246 1.00 0.00
J H
ATOM 7119 SD MET J 56 -28.329 25.529 -12.492 1.00 0.00
J S
ATOM 7120 CE MET J 56 -27.711 25.171 -10.819 1.00 0.00
J C
ATOM 7121 HE1 MET J 56 -28.318 24.376 -10.333 1.00 0.00
J H
ATOM 7122 HE2 MET J 56 -26.655 24.824 -10.849 1.00 0.00
J H
ATOM 7123 HE3 MET J 56 -27.756 26.074 -10.174 1.00 0.00
J H
ATOM 7124 C MET J 56 -26.847 30.186 -12.993 1.00 0.00
J C
ATOM 7125 O MET J 56 -27.578 30.027 -13.968 1.00 0.00
J O
ATOM 7126 N LEU J 57 -26.769 31.379 -12.375 1.00 0.00
J N
ATOM 7127 HN LEU J 57 -26.240 31.495 -11.537 1.00 0.00
J H

ATOM 7128 CA LEU J 57 -27.419 32.587 -12.839 1.00 0.00
J C
ATOM 7129 HA LEU J 57 -28.461 32.326 -12.975 1.00 0.00
J H
ATOM 7130 CB LEU J 57 -27.259 33.718 -11.795 1.00 0.00
J C
ATOM 7131 HB1 LEU J 57 -26.167 33.894 -11.666 1.00 0.00
J H
ATOM 7132 HB2 LEU J 57 -27.674 34.660 -12.217 1.00 0.00
J H
ATOM 7133 CG LEU J 57 -27.884 33.413 -10.419 1.00 0.00
J C
ATOM 7134 HG LEU J 57 -27.498 32.410 -10.115 1.00 0.00
J H
ATOM 7135 CD1 LEU J 57 -27.445 34.471 -9.394 1.00 0.00
J C
ATOM 7136 HD11 LEU J 57 -27.929 34.286 -8.411 1.00 0.00
J H
ATOM 7137 HD12 LEU J 57 -26.344 34.444 -9.251 1.00 0.00
J H
ATOM 7138 HD13 LEU J 57 -27.730 35.489 -9.734 1.00 0.00
J H
ATOM 7139 CD2 LEU J 57 -29.419 33.333 -10.482 1.00 0.00
J C
ATOM 7140 HD21 LEU J 57 -29.834 33.094 -9.479 1.00 0.00
J H
ATOM 7141 HD22 LEU J 57 -29.866 34.292 -10.812 1.00 0.00
J H
ATOM 7142 HD23 LEU J 57 -29.737 32.537 -11.188 1.00 0.00
J H
ATOM 7143 C LEU J 57 -26.885 33.082 -14.195 1.00 0.00
J C
ATOM 7144 O LEU J 57 -25.669 33.034 -14.389 1.00 0.00
J O
ATOM 7145 N PRO J 58 -27.680 33.616 -15.142 1.00 0.00
J N
ATOM 7146 CD PRO J 58 -29.143 33.656 -15.062 1.00 0.00
J C
ATOM 7147 HD1 PRO J 58 -28.614 32.702 -14.835 1.00 0.00
J H
ATOM 7148 HD2 PRO J 58 -30.010 33.793 -14.376 1.00 0.00
J H
ATOM 7149 CA PRO J 58 -27.210 33.923 -16.502 1.00 0.00
J C
ATOM 7150 HA PRO J 58 -26.809 33.020 -16.945 1.00 0.00
J H
ATOM 7151 CB PRO J 58 -28.480 34.356 -17.258 1.00 0.00
J C
ATOM 7152 HB1 PRO J 58 -29.000 33.451 -17.643 1.00 0.00
J H
ATOM 7153 HB2 PRO J 58 -28.262 35.035 -18.107 1.00 0.00
J H
ATOM 7154 CG PRO J 58 -29.620 33.659 -16.515 1.00 0.00
J C

ATOM 7155 HG1 PRO J 58 -30.525 33.524 -17.140 1.00 0.00
J H
ATOM 7156 HG2 PRO J 58 -29.882 34.249 -15.607 1.00 0.00
J H
ATOM 7157 C PRO J 58 -26.164 35.022 -16.584 1.00 0.00
J C
ATOM 7158 O PRO J 58 -25.518 35.170 -17.612 1.00 0.00
J O
ATOM 7159 N ILE J 59 -26.019 35.843 -15.527 1.00 0.00
J N
ATOM 7160 HN ILE J 59 -26.674 35.812 -14.775 1.00 0.00
J H
ATOM 7161 CA ILE J 59 -24.956 36.820 -15.352 1.00 0.00
J C
ATOM 7162 HA ILE J 59 -24.946 37.403 -16.263 1.00 0.00
J H
ATOM 7163 CB ILE J 59 -25.265 37.732 -14.152 1.00 0.00
J C
ATOM 7164 HB ILE J 59 -26.189 38.287 -14.454 1.00 0.00
J H
ATOM 7165 CG2 ILE J 59 -25.563 36.903 -12.874 1.00 0.00
J C
ATOM 7166 HG21 ILE J 59 -25.934 37.559 -12.060 1.00 0.00
J H
ATOM 7167 HG22 ILE J 59 -26.342 36.137 -13.071 1.00 0.00
J H
ATOM 7168 HG23 ILE J 59 -24.648 36.387 -12.512 1.00 0.00
J H
ATOM 7169 CG1 ILE J 59 -24.163 38.805 -13.942 1.00 0.00
J C
ATOM 7170 HG11 ILE J 59 -23.185 38.308 -13.749 1.00 0.00
J H
ATOM 7171 HG12 ILE J 59 -24.052 39.398 -14.879 1.00 0.00
J H
ATOM 7172 CD ILE J 59 -24.451 39.784 -12.797 1.00 0.00
J C
ATOM 7173 HD1 ILE J 59 -23.655 40.556 -12.740 1.00 0.00
J H
ATOM 7174 HD2 ILE J 59 -25.424 40.299 -12.957 1.00 0.00
J H
ATOM 7175 HD3 ILE J 59 -24.488 39.256 -11.821 1.00 0.00
J H
ATOM 7176 C ILE J 59 -23.570 36.182 -15.219 1.00 0.00
J C
ATOM 7177 O ILE J 59 -22.570 36.699 -15.714 1.00 0.00
J O
ATOM 7178 N SER J 60 -23.476 35.038 -14.512 1.00 0.00
J N
ATOM 7179 HN SER J 60 -24.291 34.569 -14.174 1.00 0.00
J H
ATOM 7180 CA SER J 60 -22.227 34.380 -14.174 1.00 0.00
J C
ATOM 7181 HA SER J 60 -21.557 35.178 -13.883 1.00 0.00
J H

```
ATOM   7182  CB  SER J  60      -22.395  33.360 -13.015  1.00  0.00
J    C
ATOM   7183  HB1 SER J  60      -23.194  32.639 -13.301  1.00  0.00
J    H
ATOM   7184  HB2 SER J  60      -21.458  32.773 -12.876  1.00  0.00
J    H
ATOM   7185  OG  SER J  60      -22.777  34.056 -11.826  1.00  0.00
J    O
ATOM   7186  HG1 SER J  60      -23.087  33.393 -11.197  1.00  0.00
J    H
ATOM   7187  C   SER J  60      -21.594  33.667 -15.341  1.00  0.00
J    C
ATOM   7188  O   SER J  60      -20.369  33.596 -15.429  1.00  0.00
J    O
ATOM   7189  N   THR J  61      -22.414  33.097 -16.249  1.00  0.00
J    N
ATOM   7190  HN  THR J  61      -23.393  33.300 -16.275  1.00  0.00
J    H
ATOM   7191  CA  THR J  61      -21.972  32.148 -17.265  1.00  0.00
J    C
ATOM   7192  HA  THR J  61      -20.906  32.041 -17.119  1.00  0.00
J    H
ATOM   7193  CB  THR J  61      -22.618  30.757 -17.138  1.00  0.00
J    C
ATOM   7194  HB  THR J  61      -22.104  30.107 -17.887  1.00  0.00
J    H
ATOM   7195  OG1 THR J  61      -23.997  30.691 -17.464  1.00  0.00
J    O
ATOM   7196  HG1 THR J  61      -24.251  29.773 -17.324  1.00  0.00
J    H
ATOM   7197  CG2 THR J  61      -22.540  30.311 -15.674  1.00  0.00
J    C
ATOM   7198 HG21 THR J  61      -22.892  29.264 -15.560  1.00  0.00
J    H
ATOM   7199 HG22 THR J  61      -21.491  30.359 -15.310  1.00  0.00
J    H
ATOM   7200 HG23 THR J  61      -23.164  30.961 -15.024  1.00  0.00
J    H
ATOM   7201  C   THR J  61      -22.163  32.695 -18.671  1.00  0.00
J    C
ATOM   7202  O   THR J  61      -23.204  33.240 -19.017  1.00  0.00
J    O
ATOM   7203  N   THR J  62      -21.134  32.605 -19.543  1.00  0.00
J    N
ATOM   7204  HN  THR J  62      -20.242  32.226 -19.299  1.00  0.00
J    H
ATOM   7205  CA  THR J  62      -21.235  33.060 -20.934  1.00  0.00
J    C
ATOM   7206  HA  THR J  62      -21.845  33.952 -20.891  1.00  0.00
J    H
ATOM   7207  CB  THR J  62      -19.865  33.299 -21.578  1.00  0.00
J    C
ATOM   7208  HB  THR J  62      -20.057  33.443 -22.669  1.00  0.00
J    H
```

ATOM 7209 OG1 THR J 62 -18.986 32.185 -21.469 1.00 0.00
J O
ATOM 7210 HG1 THR J 62 -18.167 32.462 -21.895 1.00 0.00
J H
ATOM 7211 CG2 THR J 62 -19.176 34.415 -20.810 1.00 0.00
J C
ATOM 7212 HG21 THR J 62 -18.226 34.707 -21.305 1.00 0.00
J H
ATOM 7213 HG22 THR J 62 -19.827 35.314 -20.764 1.00 0.00
J H
ATOM 7214 HG23 THR J 62 -18.944 34.101 -19.770 1.00 0.00
J H
ATOM 7215 C THR J 62 -21.957 32.090 -21.835 1.00 0.00
J C
ATOM 7216 O THR J 62 -22.850 32.429 -22.606 1.00 0.00
J O
ATOM 7217 N LYS J 63 -21.553 30.822 -21.735 1.00 0.00
J N
ATOM 7218 HN LYS J 63 -20.782 30.602 -21.140 1.00 0.00
J H
ATOM 7219 CA LYS J 63 -22.133 29.702 -22.419 1.00 0.00
J C
ATOM 7220 HA LYS J 63 -22.688 30.058 -23.279 1.00 0.00
J H
ATOM 7221 CB LYS J 63 -21.025 28.674 -22.784 1.00 0.00
J C
ATOM 7222 HB1 LYS J 63 -20.673 28.218 -21.830 1.00 0.00
J H
ATOM 7223 HB2 LYS J 63 -21.479 27.848 -23.377 1.00 0.00
J H
ATOM 7224 CG LYS J 63 -19.807 29.258 -23.528 1.00 0.00
J C
ATOM 7225 HG1 LYS J 63 -20.079 29.426 -24.596 1.00 0.00
J H
ATOM 7226 HG2 LYS J 63 -19.555 30.251 -23.089 1.00 0.00
J H
ATOM 7227 CD LYS J 63 -18.577 28.332 -23.475 1.00 0.00
J C
ATOM 7228 HD1 LYS J 63 -17.740 28.827 -24.020 1.00 0.00
J H
ATOM 7229 HD2 LYS J 63 -18.266 28.231 -22.409 1.00 0.00
J H
ATOM 7230 CE LYS J 63 -18.813 26.940 -24.060 1.00 0.00
J C
ATOM 7231 HE1 LYS J 63 -19.681 26.451 -23.566 1.00 0.00
J H
ATOM 7232 HE2 LYS J 63 -19.020 27.005 -25.151 1.00 0.00
J H
ATOM 7233 NZ LYS J 63 -17.610 26.111 -23.848 1.00 0.00
J N
ATOM 7234 HZ1 LYS J 63 -17.759 25.159 -24.242 1.00 0.00
J H
ATOM 7235 HZ2 LYS J 63 -16.795 26.555 -24.318 1.00 0.00
J H

ATOM 7236 HZ3 LYS J 63 -17.418 26.035 -22.828 1.00 0.00
J H
ATOM 7237 C LYS J 63 -23.137 29.079 -21.455 1.00 0.00
J C
ATOM 7238 O LYS J 63 -22.996 29.279 -20.242 1.00 0.00
J O
ATOM 7239 N PRO J 64 -24.153 28.326 -21.873 1.00 0.00
J N
ATOM 7240 CD PRO J 64 -24.421 27.987 -23.267 1.00 0.00
J C
ATOM 7241 HD1 PRO J 64 -24.409 29.031 -22.880 1.00 0.00
J H
ATOM 7242 HD2 PRO J 64 -24.023 27.952 -24.308 1.00 0.00
J H
ATOM 7243 CA PRO J 64 -24.957 27.516 -20.961 1.00 0.00
J C
ATOM 7244 HA PRO J 64 -25.586 28.160 -20.358 1.00 0.00
J H
ATOM 7245 CB PRO J 64 -25.905 26.712 -21.879 1.00 0.00
J C
ATOM 7246 HB1 PRO J 64 -26.799 27.334 -22.109 1.00 0.00
J H
ATOM 7247 HB2 PRO J 64 -26.237 25.759 -21.421 1.00 0.00
J H
ATOM 7248 CG PRO J 64 -25.844 27.433 -23.234 1.00 0.00
J C
ATOM 7249 HG1 PRO J 64 -26.740 27.252 -23.860 1.00 0.00
J H
ATOM 7250 HG2 PRO J 64 -24.933 27.104 -23.785 1.00 0.00
J H
ATOM 7251 C PRO J 64 -24.115 26.637 -20.041 1.00 0.00
J C
ATOM 7252 O PRO J 64 -23.164 26.007 -20.504 1.00 0.00
J O
ATOM 7253 N LEU J 65 -24.416 26.612 -18.732 1.00 0.00
J N
ATOM 7254 HN LEU J 65 -25.176 27.121 -18.335 1.00 0.00
J H
ATOM 7255 CA LEU J 65 -23.658 25.837 -17.778 1.00 0.00
J C
ATOM 7256 HA LEU J 65 -22.783 25.441 -18.276 1.00 0.00
J H
ATOM 7257 CB LEU J 65 -23.308 26.685 -16.532 1.00 0.00
J C
ATOM 7258 HB1 LEU J 65 -22.963 27.680 -16.897 1.00 0.00
J H
ATOM 7259 HB2 LEU J 65 -24.237 26.876 -15.952 1.00 0.00
J H
ATOM 7260 CG LEU J 65 -22.215 26.081 -15.626 1.00 0.00
J C
ATOM 7261 HG LEU J 65 -22.489 25.014 -15.443 1.00 0.00
J H
ATOM 7262 CD1 LEU J 65 -20.825 26.188 -16.261 1.00 0.00
J C

```
ATOM   7263 HD11 LEU J  65      -20.050  25.791 -15.572  1.00  0.00
J    H
ATOM   7264 HD12 LEU J  65      -20.781  25.607 -17.208  1.00  0.00
J    H
ATOM   7265 HD13 LEU J  65      -20.579  27.247 -16.488  1.00  0.00
J    H
ATOM   7266  CD2 LEU J  65      -22.173  26.799 -14.273  1.00  0.00
J    C
ATOM   7267 HD21 LEU J  65      -21.397  26.346 -13.619  1.00  0.00
J    H
ATOM   7268 HD22 LEU J  65      -21.931  27.875 -14.387  1.00  0.00
J    H
ATOM   7269 HD23 LEU J  65      -23.154  26.714 -13.757  1.00  0.00
J    H
ATOM   7270  C   LEU J  65      -24.517  24.666 -17.368  1.00  0.00
J    C
ATOM   7271  O   LEU J  65      -25.668  24.833 -16.969  1.00  0.00
J    O
ATOM   7272  N   SER J  66      -23.994  23.431 -17.480  1.00  0.00
J    N
ATOM   7273  HN  SER J  66      -23.101  23.257 -17.894  1.00  0.00
J    H
ATOM   7274  CA  SER J  66      -24.676  22.236 -17.013  1.00  0.00
J    C
ATOM   7275  HA  SER J  66      -25.637  22.234 -17.511  1.00  0.00
J    H
ATOM   7276  CB  SER J  66      -23.866  20.933 -17.250  1.00  0.00
J    C
ATOM   7277  HB1 SER J  66      -23.132  20.826 -16.419  1.00  0.00
J    H
ATOM   7278  HB2 SER J  66      -24.538  20.046 -17.211  1.00  0.00
J    H
ATOM   7279  OG  SER J  66      -23.146  20.960 -18.477  1.00  0.00
J    O
ATOM   7280  HG1 SER J  66      -22.507  20.236 -18.447  1.00  0.00
J    H
ATOM   7281  C   SER J  66      -24.944  22.276 -15.519  1.00  0.00
J    C
ATOM   7282  O   SER J  66      -24.130  22.779 -14.749  1.00  0.00
J    O
ATOM   7283  N   TRP J  67      -26.067  21.696 -15.047  1.00  0.00
J    N
ATOM   7284  HN  TRP J  67      -26.733  21.270 -15.654  1.00  0.00
J    H
ATOM   7285  CA  TRP J  67      -26.400  21.639 -13.628  1.00  0.00
J    C
ATOM   7286  HA  TRP J  67      -26.486  22.664 -13.288  1.00  0.00
J    H
ATOM   7287  CB  TRP J  67      -27.760  20.878 -13.494  1.00  0.00
J    C
ATOM   7288  HB1 TRP J  67      -28.572  21.574 -13.800  1.00  0.00
J    H
ATOM   7289  HB2 TRP J  67      -27.784  20.022 -14.202  1.00  0.00
J    H
```

ATOM 7290 CG TRP J 67 -28.113 20.391 -12.096 1.00 0.00
J C
ATOM 7291 CD1 TRP J 67 -28.419 21.115 -10.979 1.00 0.00
J C
ATOM 7292 HD1 TRP J 67 -28.608 22.181 -10.986 1.00 0.00
J H
ATOM 7293 NE1 TRP J 67 -28.494 20.293 -9.868 1.00 0.00
J N
ATOM 7294 HE1 TRP J 67 -28.733 20.548 -8.956 1.00 0.00
J H
ATOM 7295 CE2 TRP J 67 -28.215 19.001 -10.278 1.00 0.00
J C
ATOM 7296 CD2 TRP J 67 -27.974 19.018 -11.654 1.00 0.00
J C
ATOM 7297 CE3 TRP J 67 -27.645 17.852 -12.352 1.00 0.00
J C
ATOM 7298 HE3 TRP J 67 -27.439 17.862 -13.414 1.00 0.00
J H
ATOM 7299 CZ3 TRP J 67 -27.582 16.652 -11.622 1.00 0.00
J C
ATOM 7300 HZ3 TRP J 67 -27.337 15.731 -12.133 1.00 0.00
J H
ATOM 7301 CZ2 TRP J 67 -28.142 17.814 -9.546 1.00 0.00
J C
ATOM 7302 HZ2 TRP J 67 -28.318 17.782 -8.482 1.00 0.00
J H
ATOM 7303 CH2 TRP J 67 -27.829 16.633 -10.244 1.00 0.00
J C
ATOM 7304 HH2 TRP J 67 -27.777 15.696 -9.705 1.00 0.00
J H
ATOM 7305 C TRP J 67 -25.302 20.978 -12.782 1.00 0.00
J C
ATOM 7306 O TRP J 67 -24.841 21.526 -11.779 1.00 0.00
J O
ATOM 7307 N ILE J 68 -24.797 19.808 -13.224 1.00 0.00
J N
ATOM 7308 HN ILE J 68 -25.139 19.356 -14.045 1.00 0.00
J H
ATOM 7309 CA ILE J 68 -23.719 19.100 -12.559 1.00 0.00
J C
ATOM 7310 HA ILE J 68 -24.017 19.019 -11.522 1.00 0.00
J H
ATOM 7311 CB ILE J 68 -23.564 17.672 -13.083 1.00 0.00
J C
ATOM 7312 HB ILE J 68 -24.573 17.210 -12.937 1.00 0.00
J H
ATOM 7313 CG2 ILE J 68 -23.163 17.639 -14.581 1.00 0.00
J C
ATOM 7314 HG21 ILE J 68 -23.210 16.602 -14.975 1.00 0.00
J H
ATOM 7315 HG22 ILE J 68 -23.852 18.262 -15.189 1.00 0.00
J H
ATOM 7316 HG23 ILE J 68 -22.129 18.018 -14.724 1.00 0.00
J H

```
ATOM   7317  CG1 ILE J  68      -22.580  16.842 -12.225  1.00  0.00
J    C
ATOM   7318 HG11 ILE J  68      -22.451  15.831 -12.672  1.00  0.00
J    H
ATOM   7319 HG12 ILE J  68      -21.583  17.338 -12.239  1.00  0.00
J    H
ATOM   7320  CD  ILE J  68      -22.997  16.676 -10.756  1.00  0.00
J    C
ATOM   7321  HD1 ILE J  68      -22.215  16.120 -10.193  1.00  0.00
J    H
ATOM   7322  HD2 ILE J  68      -23.135  17.667 -10.273  1.00  0.00
J    H
ATOM   7323  HD3 ILE J  68      -23.948  16.108 -10.674  1.00  0.00
J    H
ATOM   7324  C   ILE J  68      -22.410  19.875 -12.580  1.00  0.00
J    C
ATOM   7325  O   ILE J  68      -21.684  19.924 -11.589  1.00  0.00
J    O
ATOM   7326  N   ASP J  69      -22.108  20.572 -13.695  1.00  0.00
J    N
ATOM   7327  HN  ASP J  69      -22.711  20.634 -14.486  1.00  0.00
J    H
ATOM   7328  CA  ASP J  69      -20.877  21.306 -13.868  1.00  0.00
J    C
ATOM   7329  HA  ASP J  69      -20.085  20.709 -13.432  1.00  0.00
J    H
ATOM   7330  CB  ASP J  69      -20.512  21.457 -15.383  1.00  0.00
J    C
ATOM   7331  HB1 ASP J  69      -21.298  22.052 -15.891  1.00  0.00
J    H
ATOM   7332  HB2 ASP J  69      -19.545  21.986 -15.501  1.00  0.00
J    H
ATOM   7333  CG  ASP J  69      -20.413  20.122 -16.134  1.00  0.00
J    C
ATOM   7334  OD1 ASP J  69      -19.907  19.102 -15.597  1.00  0.00
J    O
ATOM   7335  OD2 ASP J  69      -20.876  20.089 -17.303  1.00  0.00
J    O
ATOM   7336  C   ASP J  69      -20.914  22.638 -13.083  1.00  0.00
J    C
ATOM   7337  O   ASP J  69      -19.879  23.232 -12.786  1.00  0.00
J    O
ATOM   7338  N   ALA J  70      -22.107  23.111 -12.642  1.00  0.00
J    N
ATOM   7339  HN  ALA J  70      -22.965  22.728 -12.983  1.00  0.00
J    H
ATOM   7340  CA  ALA J  70      -22.244  24.178 -11.669  1.00  0.00
J    C
ATOM   7341  HA  ALA J  70      -21.525  24.962 -11.880  1.00  0.00
J    H
ATOM   7342  CB  ALA J  70      -23.672  24.760 -11.735  1.00  0.00
J    C
ATOM   7343  HB1 ALA J  70      -23.870  25.146 -12.757  1.00  0.00
J    H
```

ATOM 7344 HB2 ALA J 70 -24.432 23.981 -11.511 1.00 0.00
J H
ATOM 7345 HB3 ALA J 70 -23.805 25.600 -11.019 1.00 0.00
J H
ATOM 7346 C ALA J 70 -21.972 23.719 -10.243 1.00 0.00
J C
ATOM 7347 O ALA J 70 -21.178 24.318 -9.522 1.00 0.00
J O
ATOM 7348 N LEU J 71 -22.596 22.599 -9.813 1.00 0.00
J N
ATOM 7349 HN LEU J 71 -23.243 22.098 -10.383 1.00 0.00
J H
ATOM 7350 CA LEU J 71 -22.389 22.021 -8.495 1.00 0.00
J C
ATOM 7351 HA LEU J 71 -22.554 22.823 -7.786 1.00 0.00
J H
ATOM 7352 CB LEU J 71 -23.457 20.934 -8.208 1.00 0.00
J C
ATOM 7353 HB1 LEU J 71 -24.455 21.400 -8.380 1.00 0.00
J H
ATOM 7354 HB2 LEU J 71 -23.361 20.124 -8.964 1.00 0.00
J H
ATOM 7355 CG LEU J 71 -23.450 20.325 -6.782 1.00 0.00
J C
ATOM 7356 HG LEU J 71 -22.539 19.681 -6.740 1.00 0.00
J H
ATOM 7357 CD1 LEU J 71 -23.425 21.374 -5.658 1.00 0.00
J C
ATOM 7358 HD11 LEU J 71 -23.469 20.880 -4.663 1.00 0.00
J H
ATOM 7359 HD12 LEU J 71 -22.494 21.977 -5.706 1.00 0.00
J H
ATOM 7360 HD13 LEU J 71 -24.294 22.061 -5.743 1.00 0.00
J H
ATOM 7361 CD2 LEU J 71 -24.679 19.423 -6.584 1.00 0.00
J C
ATOM 7362 HD21 LEU J 71 -24.666 18.970 -5.569 1.00 0.00
J H
ATOM 7363 HD22 LEU J 71 -25.625 19.991 -6.686 1.00 0.00
J H
ATOM 7364 HD23 LEU J 71 -24.682 18.599 -7.330 1.00 0.00
J H
ATOM 7365 C LEU J 71 -20.964 21.514 -8.294 1.00 0.00
J C
ATOM 7366 O LEU J 71 -20.370 21.698 -7.237 1.00 0.00
J O
ATOM 7367 N PHE J 72 -20.352 20.918 -9.341 1.00 0.00
J N
ATOM 7368 HN PHE J 72 -20.837 20.704 -10.187 1.00 0.00
J H
ATOM 7369 CA PHE J 72 -18.951 20.537 -9.336 1.00 0.00
J C
ATOM 7370 HA PHE J 72 -18.829 19.898 -8.470 1.00 0.00
J H

ATOM 7371 CB PHE J 72 −18.612 19.832 −10.679 1.00 0.00
J C
ATOM 7372 HB1 PHE J 72 −19.388 19.066 −10.894 1.00 0.00
J H
ATOM 7373 HB2 PHE J 72 −18.621 20.554 −11.523 1.00 0.00
J H
ATOM 7374 CG PHE J 72 −17.291 19.113 −10.622 1.00 0.00
J C
ATOM 7375 CD1 PHE J 72 −16.072 19.801 −10.763 1.00 0.00
J C
ATOM 7376 HD1 PHE J 72 −16.077 20.872 −10.914 1.00 0.00
J H
ATOM 7377 CE1 PHE J 72 −14.857 19.111 −10.716 1.00 0.00
J C
ATOM 7378 HE1 PHE J 72 −13.925 19.649 −10.814 1.00 0.00
J H
ATOM 7379 CZ PHE J 72 −14.846 17.724 −10.536 1.00 0.00
J C
ATOM 7380 HZ PHE J 72 −13.908 17.189 −10.486 1.00 0.00
J H
ATOM 7381 CD2 PHE J 72 −17.267 17.723 −10.439 1.00 0.00
J C
ATOM 7382 HD2 PHE J 72 −18.194 17.175 −10.341 1.00 0.00
J H
ATOM 7383 CE2 PHE J 72 −16.054 17.030 −10.408 1.00 0.00
J C
ATOM 7384 HE2 PHE J 72 −16.052 15.956 −10.286 1.00 0.00
J H
ATOM 7385 C PHE J 72 −18.013 21.724 −9.151 1.00 0.00
J C
ATOM 7386 O PHE J 72 −17.136 21.704 −8.289 1.00 0.00
J O
ATOM 7387 N THR J 73 −18.192 22.810 −9.930 1.00 0.00
J N
ATOM 7388 HN THR J 73 −18.897 22.852 −10.637 1.00 0.00
J H
ATOM 7389 CA THR J 73 −17.381 24.024 −9.817 1.00 0.00
J C
ATOM 7390 HA THR J 73 −16.353 23.691 −9.862 1.00 0.00
J H
ATOM 7391 CB THR J 73 −17.635 25.013 −10.944 1.00 0.00
J C
ATOM 7392 HB THR J 73 −18.742 25.163 −10.980 1.00 0.00
J H
ATOM 7393 OG1 THR J 73 −17.221 24.419 −12.163 1.00 0.00
J O
ATOM 7394 HG1 THR J 73 −17.389 25.081 −12.841 1.00 0.00
J H
ATOM 7395 CG2 THR J 73 −16.804 26.295 −10.810 1.00 0.00
J C
ATOM 7396 HG21 THR J 73 −17.096 27.038 −11.581 1.00 0.00
J H
ATOM 7397 HG22 THR J 73 −16.963 26.758 −9.812 1.00 0.00
J H

```
ATOM   7398 HG23 THR J  73      -15.719  26.082 -10.926  1.00  0.00
J    H
ATOM   7399  C   THR J  73      -17.556  24.704  -8.476  1.00  0.00
J    C
ATOM   7400  O   THR J  73      -16.587  25.127  -7.854  1.00  0.00
J    O
ATOM   7401  N   ALA J  74      -18.803  24.773  -7.957  1.00  0.00
J    N
ATOM   7402  HN  ALA J  74      -19.603  24.478  -8.479  1.00  0.00
J    H
ATOM   7403  CA  ALA J  74      -19.080  25.273  -6.625  1.00  0.00
J    C
ATOM   7404  HA  ALA J  74      -18.705  26.287  -6.535  1.00  0.00
J    H
ATOM   7405  CB  ALA J  74      -20.600  25.247  -6.374  1.00  0.00
J    C
ATOM   7406  HB1 ALA J  74      -21.114  25.868  -7.136  1.00  0.00
J    H
ATOM   7407  HB2 ALA J  74      -20.999  24.212  -6.450  1.00  0.00
J    H
ATOM   7408  HB3 ALA J  74      -20.856  25.652  -5.371  1.00  0.00
J    H
ATOM   7409  C   ALA J  74      -18.410  24.468  -5.520  1.00  0.00
J    C
ATOM   7410  O   ALA J  74      -17.728  25.034  -4.660  1.00  0.00
J    O
ATOM   7411  N   ALA J  75      -18.530  23.120  -5.556  1.00  0.00
J    N
ATOM   7412  HN  ALA J  75      -19.075  22.692  -6.277  1.00  0.00
J    H
ATOM   7413  CA  ALA J  75      -17.920  22.208  -4.611  1.00  0.00
J    C
ATOM   7414  HA  ALA J  75      -18.221  22.457  -3.600  1.00  0.00
J    H
ATOM   7415  CB  ALA J  75      -18.384  20.755  -4.871  1.00  0.00
J    C
ATOM   7416  HB1 ALA J  75      -19.491  20.699  -4.795  1.00  0.00
J    H
ATOM   7417  HB2 ALA J  75      -18.093  20.419  -5.891  1.00  0.00
J    H
ATOM   7418  HB3 ALA J  75      -17.953  20.049  -4.130  1.00  0.00
J    H
ATOM   7419  C   ALA J  75      -16.408  22.296  -4.643  1.00  0.00
J    C
ATOM   7420  O   ALA J  75      -15.793  22.430  -3.597  1.00  0.00
J    O
ATOM   7421  N   SER J  76      -15.814  22.328  -5.857  1.00  0.00
J    N
ATOM   7422  HN  SER J  76      -16.382  22.267  -6.678  1.00  0.00
J    H
ATOM   7423  CA  SER J  76      -14.383  22.446  -6.128  1.00  0.00
J    C
ATOM   7424  HA  SER J  76      -13.924  21.677  -5.520  1.00  0.00
J    H
```

ATOM 7425 CB SER J 76 -14.091 22.296 -7.652 1.00 0.00
J C
ATOM 7426 HB1 SER J 76 -14.692 21.441 -8.037 1.00 0.00
J H
ATOM 7427 HB2 SER J 76 -14.425 23.205 -8.202 1.00 0.00
J H
ATOM 7428 OG SER J 76 -12.716 22.028 -7.927 1.00 0.00
J O
ATOM 7429 HG1 SER J 76 -12.663 21.732 -8.845 1.00 0.00
J H
ATOM 7430 C SER J 76 -13.781 23.765 -5.709 1.00 0.00
J C
ATOM 7431 O SER J 76 -12.575 23.863 -5.485 1.00 0.00
J O
ATOM 7432 N ALA J 77 -14.599 24.830 -5.624 1.00 0.00
J N
ATOM 7433 HN ALA J 77 -15.547 24.785 -5.940 1.00 0.00
J H
ATOM 7434 CA ALA J 77 -14.186 26.100 -5.083 1.00 0.00
J C
ATOM 7435 HA ALA J 77 -13.164 26.294 -5.387 1.00 0.00
J H
ATOM 7436 CB ALA J 77 -15.060 27.201 -5.705 1.00 0.00
J C
ATOM 7437 HB1 ALA J 77 -14.991 27.150 -6.813 1.00 0.00
J H
ATOM 7438 HB2 ALA J 77 -16.128 27.070 -5.424 1.00 0.00
J H
ATOM 7439 HB3 ALA J 77 -14.732 28.214 -5.387 1.00 0.00
J H
ATOM 7440 C ALA J 77 -14.206 26.186 -3.556 1.00 0.00
J C
ATOM 7441 O ALA J 77 -13.258 26.679 -2.963 1.00 0.00
J O
ATOM 7442 N THR J 78 -15.277 25.728 -2.867 1.00 0.00
J N
ATOM 7443 HN THR J 78 -16.078 25.329 -3.312 1.00 0.00
J H
ATOM 7444 CA THR J 78 -15.361 25.776 -1.394 1.00 0.00
J C
ATOM 7445 HA THR J 78 -14.848 26.685 -1.112 1.00 0.00
J H
ATOM 7446 CB THR J 78 -16.797 25.918 -0.890 1.00 0.00
J C
ATOM 7447 HB THR J 78 -17.169 26.895 -1.286 1.00 0.00
J H
ATOM 7448 OG1 THR J 78 -16.901 25.997 0.524 1.00 0.00
J O
ATOM 7449 HG1 THR J 78 -17.844 26.071 0.708 1.00 0.00
J H
ATOM 7450 CG2 THR J 78 -17.655 24.741 -1.328 1.00 0.00
J C
ATOM 7451 HG21 THR J 78 -18.719 24.906 -1.056 1.00 0.00
J H

```
ATOM   7452 HG22 THR J  78      -17.600  24.610  -2.430  1.00  0.00
J    H
ATOM   7453 HG23 THR J  78      -17.316  23.798  -0.848  1.00  0.00
J    H
ATOM   7454  C   THR J  78      -14.615  24.627  -0.714  1.00  0.00
J    C
ATOM   7455  O   THR J  78      -13.889  24.819   0.265  1.00  0.00
J    O
ATOM   7456  N   THR J  79      -14.679  23.386  -1.255  1.00  0.00
J    N
ATOM   7457  HN  THR J  79      -15.397  23.087  -1.882  1.00  0.00
J    H
ATOM   7458  CA  THR J  79      -13.678  22.363  -0.957  1.00  0.00
J    C
ATOM   7459  HA  THR J  79      -13.266  22.528   0.028  1.00  0.00
J    H
ATOM   7460  CB  THR J  79      -14.093  20.863  -1.057  1.00  0.00
J    C
ATOM   7461  HB  THR J  79      -13.282  20.283  -0.554  1.00  0.00
J    H
ATOM   7462  OG1 THR J  79      -14.186  20.295  -2.350  1.00  0.00
J    O
ATOM   7463  HG1 THR J  79      -14.461  19.382  -2.210  1.00  0.00
J    H
ATOM   7464  CG2 THR J  79      -15.493  20.647  -0.487  1.00  0.00
J    C
ATOM   7465 HG21 THR J  79      -15.743  19.565  -0.453  1.00  0.00
J    H
ATOM   7466 HG22 THR J  79      -15.556  21.045   0.548  1.00  0.00
J    H
ATOM   7467 HG23 THR J  79      -16.260  21.162  -1.104  1.00  0.00
J    H
ATOM   7468  C   THR J  79      -12.599  22.714  -1.934  1.00  0.00
J    C
ATOM   7469  O   THR J  79      -12.780  22.529  -3.129  1.00  0.00
J    O
ATOM   7470  N   VAL J  80      -11.497  23.351  -1.490  1.00  0.00
J    N
ATOM   7471  HN  VAL J  80      -11.255  23.441  -0.528  1.00  0.00
J    H
ATOM   7472  CA  VAL J  80      -10.555  23.972  -2.407  1.00  0.00
J    C
ATOM   7473  HA  VAL J  80      -11.139  24.491  -3.158  1.00  0.00
J    H
ATOM   7474  CB  VAL J  80       -9.715  25.062  -1.738  1.00  0.00
J    C
ATOM   7475  HB  VAL J  80       -8.891  25.333  -2.442  1.00  0.00
J    H
ATOM   7476  CG1 VAL J  80      -10.565  26.315  -1.453  1.00  0.00
J    C
ATOM   7477 HG11 VAL J  80       -9.955  27.085  -0.932  1.00  0.00
J    H
ATOM   7478 HG12 VAL J  80      -10.945  26.774  -2.389  1.00  0.00
J    H
```

```
ATOM   7479 HG13 VAL J  80     -11.430  26.064  -0.802  1.00  0.00
J    H
ATOM   7480  CG2 VAL J  80      -9.039  24.562  -0.463  1.00  0.00
J    C
ATOM   7481 HG21 VAL J  80      -8.468  25.381   0.025  1.00  0.00
J    H
ATOM   7482 HG22 VAL J  80      -9.795  24.184   0.258  1.00  0.00
J    H
ATOM   7483 HG23 VAL J  80      -8.325  23.741  -0.682  1.00  0.00
J    H
ATOM   7484  C   VAL J  80      -9.692  22.937  -3.132  1.00  0.00
J    C
ATOM   7485  O   VAL J  80      -8.490  22.821  -2.946  1.00  0.00
J    O
ATOM   7486  N   THR J  81     -10.319  22.107  -3.976  1.00  0.00
J    N
ATOM   7487  HN  THR J  81     -11.310  22.146  -4.105  1.00  0.00
J    H
ATOM   7488  CA  THR J  81      -9.658  21.095  -4.767  1.00  0.00
J    C
ATOM   7489  HA  THR J  81      -8.839  20.732  -4.161  1.00  0.00
J    H
ATOM   7490  CB  THR J  81     -10.589  19.974  -5.210  1.00  0.00
J    C
ATOM   7491  HB  THR J  81     -11.399  20.461  -5.807  1.00  0.00
J    H
ATOM   7492  OG1 THR J  81     -11.204  19.345  -4.100  1.00  0.00
J    O
ATOM   7493  HG1 THR J  81     -11.758  18.651  -4.473  1.00  0.00
J    H
ATOM   7494  CG2 THR J  81      -9.824  18.849  -5.913  1.00  0.00
J    C
ATOM   7495 HG21 THR J  81     -10.525  18.095  -6.330  1.00  0.00
J    H
ATOM   7496 HG22 THR J  81      -9.222  19.256  -6.753  1.00  0.00
J    H
ATOM   7497 HG23 THR J  81      -9.136  18.332  -5.210  1.00  0.00
J    H
ATOM   7498  C   THR J  81      -9.045  21.694  -6.001  1.00  0.00
J    C
ATOM   7499  O   THR J  81      -7.906  21.399  -6.313  1.00  0.00
J    O
ATOM   7500  N   GLY J  82      -9.762  22.548  -6.762  1.00  0.00
J    N
ATOM   7501  HN  GLY J  82     -10.688  22.842  -6.528  1.00  0.00
J    H
ATOM   7502  CA  GLY J  82      -9.215  23.105  -8.003  1.00  0.00
J    C
ATOM   7503  HA1 GLY J  82      -8.148  23.170  -7.839  1.00  0.00
J    H
ATOM   7504  HA2 GLY J  82      -9.703  24.062  -8.130  1.00  0.00
J    H
ATOM   7505  C   GLY J  82      -9.447  22.296  -9.253  1.00  0.00
J    C
```

ATOM 7506 O GLY J 82 -9.092 22.725 -10.346 1.00 0.00
J O
ATOM 7507 N LEU J 83 -10.104 21.118 -9.152 1.00 0.00
J N
ATOM 7508 HN LEU J 83 -10.317 20.698 -8.273 1.00 0.00
J H
ATOM 7509 CA LEU J 83 -10.562 20.355 -10.310 1.00 0.00
J C
ATOM 7510 HA LEU J 83 -9.690 20.184 -10.927 1.00 0.00
J H
ATOM 7511 CB LEU J 83 -11.238 19.006 -9.914 1.00 0.00
J C
ATOM 7512 HB1 LEU J 83 -11.988 19.235 -9.121 1.00 0.00
J H
ATOM 7513 HB2 LEU J 83 -11.816 18.623 -10.783 1.00 0.00
J H
ATOM 7514 CG LEU J 83 -10.277 17.925 -9.387 1.00 0.00
J C
ATOM 7515 HG LEU J 83 -9.685 18.444 -8.595 1.00 0.00
J H
ATOM 7516 CD1 LEU J 83 -10.995 16.691 -8.817 1.00 0.00
J C
ATOM 7517 HD11 LEU J 83 -10.257 15.924 -8.496 1.00 0.00
J H
ATOM 7518 HD12 LEU J 83 -11.614 16.969 -7.938 1.00 0.00
J H
ATOM 7519 HD13 LEU J 83 -11.658 16.234 -9.583 1.00 0.00
J H
ATOM 7520 CD2 LEU J 83 -9.306 17.446 -10.466 1.00 0.00
J C
ATOM 7521 HD21 LEU J 83 -8.615 16.681 -10.051 1.00 0.00
J H
ATOM 7522 HD22 LEU J 83 -9.839 16.988 -11.323 1.00 0.00
J H
ATOM 7523 HD23 LEU J 83 -8.697 18.294 -10.845 1.00 0.00
J H
ATOM 7524 C LEU J 83 -11.573 21.137 -11.145 1.00 0.00
J C
ATOM 7525 O LEU J 83 -12.477 21.779 -10.608 1.00 0.00
J O
ATOM 7526 N ALA J 84 -11.459 21.085 -12.483 1.00 0.00
J N
ATOM 7527 HN ALA J 84 -10.797 20.492 -12.941 1.00 0.00
J H
ATOM 7528 CA ALA J 84 -12.285 21.885 -13.357 1.00 0.00
J C
ATOM 7529 HA ALA J 84 -13.094 22.346 -12.803 1.00 0.00
J H
ATOM 7530 CB ALA J 84 -11.418 22.982 -14.006 1.00 0.00
J C
ATOM 7531 HB1 ALA J 84 -10.988 23.633 -13.214 1.00 0.00
J H
ATOM 7532 HB2 ALA J 84 -10.575 22.539 -14.579 1.00 0.00
J H

ATOM 7533 HB3 ALA J 84 -12.014 23.622 -14.691 1.00 0.00
J H
ATOM 7534 C ALA J 84 -12.937 21.025 -14.429 1.00 0.00
J C
ATOM 7535 O ALA J 84 -12.277 20.445 -15.289 1.00 0.00
J O
ATOM 7536 N VAL J 85 -14.280 20.927 -14.390 1.00 0.00
J N
ATOM 7537 HN VAL J 85 -14.782 21.254 -13.594 1.00 0.00
J H
ATOM 7538 CA VAL J 85 -15.115 20.366 -15.439 1.00 0.00
J C
ATOM 7539 HA VAL J 85 -14.633 19.454 -15.768 1.00 0.00
J H
ATOM 7540 CB VAL J 85 -16.507 20.040 -14.911 1.00 0.00
J C
ATOM 7541 HB VAL J 85 -17.178 19.898 -15.795 1.00 0.00
J H
ATOM 7542 CG1 VAL J 85 -16.408 18.741 -14.095 1.00 0.00
J C
ATOM 7543 HG11 VAL J 85 -17.395 18.484 -13.657 1.00 0.00
J H
ATOM 7544 HG12 VAL J 85 -16.093 17.884 -14.726 1.00 0.00
J H
ATOM 7545 HG13 VAL J 85 -15.681 18.855 -13.262 1.00 0.00
J H
ATOM 7546 CG2 VAL J 85 -17.097 21.187 -14.057 1.00 0.00
J C
ATOM 7547 HG21 VAL J 85 -18.093 20.905 -13.655 1.00 0.00
J H
ATOM 7548 HG22 VAL J 85 -16.427 21.421 -13.202 1.00 0.00
J H
ATOM 7549 HG23 VAL J 85 -17.228 22.111 -14.659 1.00 0.00
J H
ATOM 7550 C VAL J 85 -15.225 21.284 -16.644 1.00 0.00
J C
ATOM 7551 O VAL J 85 -15.267 20.839 -17.788 1.00 0.00
J O
ATOM 7552 N VAL J 86 -15.255 22.606 -16.403 1.00 0.00
J N
ATOM 7553 HN VAL J 86 -15.227 22.951 -15.469 1.00 0.00
J H
ATOM 7554 CA VAL J 86 -15.329 23.631 -17.416 1.00 0.00
J C
ATOM 7555 HA VAL J 86 -15.109 23.204 -18.387 1.00 0.00
J H
ATOM 7556 CB VAL J 86 -16.671 24.381 -17.433 1.00 0.00
J C
ATOM 7557 HB VAL J 86 -16.606 25.171 -18.222 1.00 0.00
J H
ATOM 7558 CG1 VAL J 86 -17.805 23.395 -17.777 1.00 0.00
J C
ATOM 7559 HG11 VAL J 86 -18.787 23.913 -17.755 1.00 0.00
J H

```
ATOM   7560 HG12 VAL J  86      -17.681  22.965 -18.793  1.00  0.00
J    H
ATOM   7561 HG13 VAL J  86      -17.839  22.563 -17.040  1.00  0.00
J    H
ATOM   7562  CG2 VAL J  86      -16.959  25.089 -16.087  1.00  0.00
J    C
ATOM   7563 HG21 VAL J  86      -17.951  25.590 -16.111  1.00  0.00
J    H
ATOM   7564 HG22 VAL J  86      -16.958  24.356 -15.253  1.00  0.00
J    H
ATOM   7565 HG23 VAL J  86      -16.196  25.866 -15.871  1.00  0.00
J    H
ATOM   7566  C   VAL J  86      -14.226  24.609 -17.091  1.00  0.00
J    C
ATOM   7567  O   VAL J  86      -13.762  24.670 -15.954  1.00  0.00
J    O
ATOM   7568  N   ASP J  87      -13.767  25.395 -18.083  1.00  0.00
J    N
ATOM   7569  HN  ASP J  87      -14.062  25.303 -19.031  1.00  0.00
J    H
ATOM   7570  CA  ASP J  87      -12.800  26.455 -17.895  1.00  0.00
J    C
ATOM   7571  HA  ASP J  87      -11.945  26.010 -17.399  1.00  0.00
J    H
ATOM   7572  CB  ASP J  87      -12.442  27.011 -19.295  1.00  0.00
J    C
ATOM   7573  HB1 ASP J  87      -12.333  26.168 -20.006  1.00  0.00
J    H
ATOM   7574  HB2 ASP J  87      -13.243  27.680 -19.666  1.00  0.00
J    H
ATOM   7575  CG  ASP J  87      -11.136  27.787 -19.339  1.00  0.00
J    C
ATOM   7576  OD1 ASP J  87      -10.904  28.624 -18.432  1.00  0.00
J    O
ATOM   7577  OD2 ASP J  87      -10.415  27.614 -20.349  1.00  0.00
J    O
ATOM   7578  C   ASP J  87      -13.323  27.562 -16.966  1.00  0.00
J    C
ATOM   7579  O   ASP J  87      -14.502  27.911 -16.975  1.00  0.00
J    O
ATOM   7580  N   THR J  88      -12.436  28.142 -16.146  1.00  0.00
J    N
ATOM   7581  HN  THR J  88      -11.486  27.837 -16.098  1.00  0.00
J    H
ATOM   7582  CA  THR J  88      -12.754  29.254 -15.258  1.00  0.00
J    C
ATOM   7583  HA  THR J  88      -13.783  29.107 -14.961  1.00  0.00
J    H
ATOM   7584  CB  THR J  88      -11.867  29.301 -14.018  1.00  0.00
J    C
ATOM   7585  HB  THR J  88      -10.824  29.119 -14.377  1.00  0.00
J    H
ATOM   7586  OG1 THR J  88      -12.242  28.234 -13.162  1.00  0.00
J    O
```

```
ATOM   7587  HG1 THR J  88      -11.666  28.309 -12.392  1.00  0.00
J    H
ATOM   7588  CG2 THR J  88      -12.050  30.577 -13.176  1.00  0.00
J    C
ATOM   7589 HG21 THR J  88      -11.307  30.617 -12.352  1.00  0.00
J    H
ATOM   7590 HG22 THR J  88      -11.911  31.480 -13.807  1.00  0.00
J    H
ATOM   7591 HG23 THR J  88      -13.067  30.618 -12.729  1.00  0.00
J    H
ATOM   7592  C   THR J  88      -12.703  30.576 -15.983  1.00  0.00
J    C
ATOM   7593  O   THR J  88      -13.511  31.466 -15.726  1.00  0.00
J    O
ATOM   7594  N   GLY J  89      -11.761  30.763 -16.931  1.00  0.00
J    N
ATOM   7595  HN  GLY J  89      -11.168  30.026 -17.253  1.00  0.00
J    H
ATOM   7596  CA  GLY J  89      -11.545  32.063 -17.551  1.00  0.00
J    C
ATOM   7597  HA1 GLY J  89      -10.536  32.050 -17.939  1.00  0.00
J    H
ATOM   7598  HA2 GLY J  89      -11.716  32.804 -16.782  1.00  0.00
J    H
ATOM   7599  C   GLY J  89      -12.477  32.356 -18.689  1.00  0.00
J    C
ATOM   7600  O   GLY J  89      -12.744  33.514 -19.003  1.00  0.00
J    O
ATOM   7601  N   THR J  90      -12.968  31.298 -19.365  1.00  0.00
J    N
ATOM   7602  HN  THR J  90      -12.800  30.353 -19.087  1.00  0.00
J    H
ATOM   7603  CA  THR J  90      -13.794  31.434 -20.570  1.00  0.00
J    C
ATOM   7604  HA  THR J  90      -13.595  32.435 -20.929  1.00  0.00
J    H
ATOM   7605  CB  THR J  90      -13.423  30.462 -21.705  1.00  0.00
J    C
ATOM   7606  HB  THR J  90      -13.947  30.837 -22.618  1.00  0.00
J    H
ATOM   7607  OG1 THR J  90      -13.866  29.121 -21.545  1.00  0.00
J    O
ATOM   7608  HG1 THR J  90      -13.547  28.651 -22.323  1.00  0.00
J    H
ATOM   7609  CG2 THR J  90      -11.900  30.352 -21.804  1.00  0.00
J    C
ATOM   7610 HG21 THR J  90      -11.604  29.746 -22.686  1.00  0.00
J    H
ATOM   7611 HG22 THR J  90      -11.446  31.359 -21.913  1.00  0.00
J    H
ATOM   7612 HG23 THR J  90      -11.474  29.875 -20.896  1.00  0.00
J    H
ATOM   7613  C   THR J  90      -15.284  31.361 -20.297  1.00  0.00
J    C
```

ATOM 7614 O THR J 90 -16.079 32.154 -20.805 1.00 0.00
J O
ATOM 7615 N GLN J 91 -15.697 30.373 -19.481 1.00 0.00
J N
ATOM 7616 HN GLN J 91 -15.033 29.756 -19.063 1.00 0.00
J H
ATOM 7617 CA GLN J 91 -17.076 30.098 -19.128 1.00 0.00
J C
ATOM 7618 HA GLN J 91 -17.637 30.123 -20.055 1.00 0.00
J H
ATOM 7619 CB GLN J 91 -17.160 28.715 -18.437 1.00 0.00
J C
ATOM 7620 HB1 GLN J 91 -16.608 27.997 -19.086 1.00 0.00
J H
ATOM 7621 HB2 GLN J 91 -16.605 28.751 -17.471 1.00 0.00
J H
ATOM 7622 CG GLN J 91 -18.588 28.171 -18.203 1.00 0.00
J C
ATOM 7623 HG1 GLN J 91 -18.580 27.393 -17.409 1.00 0.00
J H
ATOM 7624 HG2 GLN J 91 -19.269 28.983 -17.877 1.00 0.00
J H
ATOM 7625 CD GLN J 91 -19.216 27.499 -19.427 1.00 0.00
J C
ATOM 7626 OE1 GLN J 91 -18.563 26.975 -20.334 1.00 0.00
J O
ATOM 7627 NE2 GLN J 91 -20.571 27.473 -19.437 1.00 0.00
J N
ATOM 7628 HE21 GLN J 91 -21.007 27.038 -20.219 1.00 0.00
J H
ATOM 7629 HE22 GLN J 91 -21.079 27.891 -18.691 1.00 0.00
J H
ATOM 7630 C GLN J 91 -17.669 31.148 -18.207 1.00 0.00
J C
ATOM 7631 O GLN J 91 -18.834 31.525 -18.330 1.00 0.00
J O
ATOM 7632 N PHE J 92 -16.862 31.640 -17.253 1.00 0.00
J N
ATOM 7633 HN PHE J 92 -15.904 31.363 -17.192 1.00 0.00
J H
ATOM 7634 CA PHE J 92 -17.274 32.586 -16.249 1.00 0.00
J C
ATOM 7635 HA PHE J 92 -18.344 32.728 -16.346 1.00 0.00
J H
ATOM 7636 CB PHE J 92 -16.815 32.149 -14.831 1.00 0.00
J C
ATOM 7637 HB1 PHE J 92 -15.718 31.962 -14.845 1.00 0.00
J H
ATOM 7638 HB2 PHE J 92 -17.001 32.950 -14.084 1.00 0.00
J H
ATOM 7639 CG PHE J 92 -17.493 30.867 -14.433 1.00 0.00
J C
ATOM 7640 CD1 PHE J 92 -18.798 30.876 -13.916 1.00 0.00
J C

```
ATOM   7641  HD1 PHE J  92      -19.317  31.816 -13.789  1.00  0.00
J    H
ATOM   7642  CE1 PHE J  92      -19.434  29.680 -13.558  1.00  0.00
J    C
ATOM   7643  HE1 PHE J  92      -20.442  29.699 -13.170  1.00  0.00
J    H
ATOM   7644  CZ  PHE J  92      -18.763  28.459 -13.709  1.00  0.00
J    C
ATOM   7645  HZ  PHE J  92      -19.250  27.535 -13.433  1.00  0.00
J    H
ATOM   7646  CD2 PHE J  92      -16.838  29.635 -14.594  1.00  0.00
J    C
ATOM   7647  HD2 PHE J  92      -15.830  29.606 -14.986  1.00  0.00
J    H
ATOM   7648  CE2 PHE J  92      -17.463  28.437 -14.229  1.00  0.00
J    C
ATOM   7649  HE2 PHE J  92      -16.940  27.498 -14.348  1.00  0.00
J    H
ATOM   7650  C   PHE J  92      -16.621  33.910 -16.574  1.00  0.00
J    C
ATOM   7651  O   PHE J  92      -15.419  33.976 -16.817  1.00  0.00
J    O
ATOM   7652  N   THR J  93      -17.386  35.018 -16.579  1.00  0.00
J    N
ATOM   7653  HN  THR J  93      -18.381  34.994 -16.490  1.00  0.00
J    H
ATOM   7654  CA  THR J  93      -16.825  36.365 -16.715  1.00  0.00
J    C
ATOM   7655  HA  THR J  93      -15.879  36.263 -17.229  1.00  0.00
J    H
ATOM   7656  CB  THR J  93      -17.697  37.368 -17.478  1.00  0.00
J    C
ATOM   7657  HB  THR J  93      -17.143  38.338 -17.457  1.00  0.00
J    H
ATOM   7658  OG1 THR J  93      -18.944  37.593 -16.838  1.00  0.00
J    O
ATOM   7659  HG1 THR J  93      -19.411  38.221 -17.399  1.00  0.00
J    H
ATOM   7660  CG2 THR J  93      -17.998  36.779 -18.852  1.00  0.00
J    C
ATOM   7661 HG21 THR J  93      -18.532  37.515 -19.489  1.00  0.00
J    H
ATOM   7662 HG22 THR J  93      -17.055  36.501 -19.370  1.00  0.00
J    H
ATOM   7663 HG23 THR J  93      -18.629  35.868 -18.768  1.00  0.00
J    H
ATOM   7664  C   THR J  93      -16.511  36.895 -15.336  1.00  0.00
J    C
ATOM   7665  O   THR J  93      -16.524  36.151 -14.355  1.00  0.00
J    O
ATOM   7666  N   VAL J  94      -16.198  38.192 -15.187  1.00  0.00
J    N
ATOM   7667  HN  VAL J  94      -16.295  38.834 -15.943  1.00  0.00
J    H
```

ATOM 7668 CA VAL J 94 -15.699 38.785 -13.953 1.00 0.00
J C
ATOM 7669 HA VAL J 94 -14.761 38.286 -13.743 1.00 0.00
J H
ATOM 7670 CB VAL J 94 -15.368 40.267 -14.141 1.00 0.00
J C
ATOM 7671 HB VAL J 94 -16.322 40.841 -14.047 1.00 0.00
J H
ATOM 7672 CG1 VAL J 94 -14.380 40.693 -13.034 1.00 0.00
J C
ATOM 7673 HG11 VAL J 94 -14.089 41.757 -13.167 1.00 0.00
J H
ATOM 7674 HG12 VAL J 94 -14.830 40.596 -12.025 1.00 0.00
J H
ATOM 7675 HG13 VAL J 94 -13.458 40.076 -13.073 1.00 0.00
J H
ATOM 7676 CG2 VAL J 94 -14.727 40.509 -15.529 1.00 0.00
J C
ATOM 7677 HG21 VAL J 94 -14.449 41.577 -15.653 1.00 0.00
J H
ATOM 7678 HG22 VAL J 94 -13.811 39.892 -15.649 1.00 0.00
J H
ATOM 7679 HG23 VAL J 94 -15.431 40.249 -16.347 1.00 0.00
J H
ATOM 7680 C VAL J 94 -16.615 38.580 -12.731 1.00 0.00
J C
ATOM 7681 O VAL J 94 -16.148 38.278 -11.635 1.00 0.00
J O
ATOM 7682 N PHE J 95 -17.962 38.668 -12.905 1.00 0.00
J N
ATOM 7683 HN PHE J 95 -18.364 38.989 -13.761 1.00 0.00
J H
ATOM 7684 CA PHE J 95 -18.937 38.310 -11.876 1.00 0.00
J C
ATOM 7685 HA PHE J 95 -18.661 38.882 -10.998 1.00 0.00
J H
ATOM 7686 CB PHE J 95 -20.387 38.702 -12.298 1.00 0.00
J C
ATOM 7687 HB1 PHE J 95 -20.417 39.790 -12.524 1.00 0.00
J H
ATOM 7688 HB2 PHE J 95 -20.693 38.168 -13.222 1.00 0.00
J H
ATOM 7689 CG PHE J 95 -21.347 38.433 -11.159 1.00 0.00
J C
ATOM 7690 CD1 PHE J 95 -21.285 39.180 -9.968 1.00 0.00
J C
ATOM 7691 HD1 PHE J 95 -20.592 40.006 -9.889 1.00 0.00
J H
ATOM 7692 CE1 PHE J 95 -22.109 38.858 -8.881 1.00 0.00
J C
ATOM 7693 HE1 PHE J 95 -22.059 39.443 -7.973 1.00 0.00
J H
ATOM 7694 CZ PHE J 95 -23.002 37.784 -8.973 1.00 0.00
J C

ATOM 7695 HZ PHE J 95 −23.658 37.551 −8.146 1.00 0.00
J H
ATOM 7696 CD2 PHE J 95 −22.227 37.342 −11.225 1.00 0.00
J C
ATOM 7697 HD2 PHE J 95 −22.259 36.726 −12.113 1.00 0.00
J H
ATOM 7698 CE2 PHE J 95 −23.053 37.018 −10.142 1.00 0.00
J C
ATOM 7699 HE2 PHE J 95 −23.727 36.176 −10.212 1.00 0.00
J H
ATOM 7700 C PHE J 95 −18.861 36.824 −11.488 1.00 0.00
J C
ATOM 7701 O PHE J 95 −18.808 36.469 −10.311 1.00 0.00
J O
ATOM 7702 N GLY J 96 −18.776 35.904 −12.472 1.00 0.00
J N
ATOM 7703 HN GLY J 96 −18.777 36.148 −13.441 1.00 0.00
J H
ATOM 7704 CA GLY J 96 −18.676 34.479 −12.181 1.00 0.00
J C
ATOM 7705 HA1 GLY J 96 −18.714 33.961 −13.129 1.00 0.00
J H
ATOM 7706 HA2 GLY J 96 −19.486 34.233 −11.507 1.00 0.00
J H
ATOM 7707 C GLY J 96 −17.375 34.114 −11.498 1.00 0.00
J C
ATOM 7708 O GLY J 96 −17.341 33.284 −10.601 1.00 0.00
J O
ATOM 7709 N GLN J 97 −16.269 34.776 −11.885 1.00 0.00
J N
ATOM 7710 HN GLN J 97 −16.316 35.452 −12.619 1.00 0.00
J H
ATOM 7711 CA GLN J 97 −14.946 34.600 −11.313 1.00 0.00
J C
ATOM 7712 HA GLN J 97 −14.777 33.530 −11.297 1.00 0.00
J H
ATOM 7713 CB GLN J 97 −13.872 35.235 −12.231 1.00 0.00
J C
ATOM 7714 HB1 GLN J 97 −14.128 36.315 −12.327 1.00 0.00
J H
ATOM 7715 HB2 GLN J 97 −12.882 35.194 −11.723 1.00 0.00
J H
ATOM 7716 CG GLN J 97 −13.808 34.584 −13.639 1.00 0.00
J C
ATOM 7717 HG1 GLN J 97 −13.353 33.572 −13.577 1.00 0.00
J H
ATOM 7718 HG2 GLN J 97 −14.825 34.475 −14.068 1.00 0.00
J H
ATOM 7719 CD GLN J 97 −12.944 35.398 −14.604 1.00 0.00
J C
ATOM 7720 OE1 GLN J 97 −12.298 36.382 −14.251 1.00 0.00
J O
ATOM 7721 NE2 GLN J 97 −12.943 34.990 −15.891 1.00 0.00
J N

```
ATOM   7722 HE21 GLN J  97      -12.380  35.511 -16.528  1.00  0.00
J    H
ATOM   7723 HE22 GLN J  97      -13.501  34.215 -16.165  1.00  0.00
J    H
ATOM   7724  C   GLN J  97      -14.806  35.110  -9.872  1.00  0.00
J    C
ATOM   7725  O   GLN J  97      -14.086  34.536  -9.060  1.00  0.00
J    O
ATOM   7726  N   THR J  98      -15.498  36.211  -9.499  1.00  0.00
J    N
ATOM   7727  HN  THR J  98      -16.050  36.743 -10.140  1.00  0.00
J    H
ATOM   7728  CA  THR J  98      -15.504  36.730  -8.123  1.00  0.00
J    C
ATOM   7729  HA  THR J  98      -14.470  36.702  -7.806  1.00  0.00
J    H
ATOM   7730  CB  THR J  98      -15.878  38.212  -7.996  1.00  0.00
J    C
ATOM   7731  HB  THR J  98      -15.156  38.770  -8.642  1.00  0.00
J    H
ATOM   7732  OG1 THR J  98      -15.712  38.709  -6.677  1.00  0.00
J    O
ATOM   7733  HG1 THR J  98      -15.984  39.633  -6.716  1.00  0.00
J    H
ATOM   7734  CG2 THR J  98      -17.320  38.488  -8.413  1.00  0.00
J    C
ATOM   7735 HG21 THR J  98      -17.527  39.580  -8.421  1.00  0.00
J    H
ATOM   7736 HG22 THR J  98      -17.509  38.098  -9.436  1.00  0.00
J    H
ATOM   7737 HG23 THR J  98      -18.038  38.002  -7.717  1.00  0.00
J    H
ATOM   7738  C   THR J  98      -16.279  35.839  -7.153  1.00  0.00
J    C
ATOM   7739  O   THR J  98      -15.862  35.641  -6.015  1.00  0.00
J    O
ATOM   7740  N   VAL J  99      -17.393  35.189  -7.578  1.00  0.00
J    N
ATOM   7741  HN  VAL J  99      -17.817  35.355  -8.465  1.00  0.00
J    H
ATOM   7742  CA  VAL J  99      -18.074  34.180  -6.761  1.00  0.00
J    C
ATOM   7743  HA  VAL J  99      -18.322  34.645  -5.815  1.00  0.00
J    H
ATOM   7744  CB  VAL J  99      -19.393  33.712  -7.378  1.00  0.00
J    C
ATOM   7745  HB  VAL J  99      -19.165  33.312  -8.396  1.00  0.00
J    H
ATOM   7746  CG1 VAL J  99      -20.048  32.589  -6.538  1.00  0.00
J    C
ATOM   7747 HG11 VAL J  99      -21.022  32.293  -6.983  1.00  0.00
J    H
ATOM   7748 HG12 VAL J  99      -19.412  31.680  -6.502  1.00  0.00
J    H
```

```
ATOM   7749 HG13 VAL J  99      -20.236  32.935  -5.499  1.00  0.00
J    H
ATOM   7750  CG2 VAL J  99      -20.348  34.922  -7.445  1.00  0.00
J    C
ATOM   7751 HG21 VAL J  99      -21.337  34.619  -7.851  1.00  0.00
J    H
ATOM   7752 HG22 VAL J  99      -20.502  35.354  -6.433  1.00  0.00
J    H
ATOM   7753 HG23 VAL J  99      -19.942  35.716  -8.106  1.00  0.00
J    H
ATOM   7754  C   VAL J  99      -17.156  32.998  -6.465  1.00  0.00
J    C
ATOM   7755  O   VAL J  99      -17.112  32.494  -5.344  1.00  0.00
J    O
ATOM   7756  N   ILE J 100      -16.330  32.583  -7.451  1.00  0.00
J    N
ATOM   7757  HN  ILE J 100      -16.407  32.917  -8.388  1.00  0.00
J    H
ATOM   7758  CA  ILE J 100      -15.253  31.615  -7.247  1.00  0.00
J    C
ATOM   7759  HA  ILE J 100      -15.727  30.730  -6.842  1.00  0.00
J    H
ATOM   7760  CB  ILE J 100      -14.569  31.230  -8.564  1.00  0.00
J    C
ATOM   7761  HB  ILE J 100      -14.253  32.207  -9.011  1.00  0.00
J    H
ATOM   7762  CG2 ILE J 100      -13.359  30.290  -8.350  1.00  0.00
J    C
ATOM   7763 HG21 ILE J 100      -12.795  30.156  -9.297  1.00  0.00
J    H
ATOM   7764 HG22 ILE J 100      -12.659  30.710  -7.597  1.00  0.00
J    H
ATOM   7765 HG23 ILE J 100      -13.690  29.289  -8.000  1.00  0.00
J    H
ATOM   7766  CG1 ILE J 100      -15.577  30.598  -9.555  1.00  0.00
J    C
ATOM   7767 HG11 ILE J 100      -16.344  31.351  -9.842  1.00  0.00
J    H
ATOM   7768 HG12 ILE J 100      -15.035  30.308 -10.484  1.00  0.00
J    H
ATOM   7769  CD  ILE J 100      -16.304  29.348  -9.045  1.00  0.00
J    C
ATOM   7770  HD1 ILE J 100      -16.960  28.932  -9.839  1.00  0.00
J    H
ATOM   7771  HD2 ILE J 100      -15.574  28.564  -8.750  1.00  0.00
J    H
ATOM   7772  HD3 ILE J 100      -16.938  29.587  -8.166  1.00  0.00
J    H
ATOM   7773  C   ILE J 100      -14.232  32.081  -6.213  1.00  0.00
J    C
ATOM   7774  O   ILE J 100      -13.921  31.329  -5.294  1.00  0.00
J    O
ATOM   7775  N   MET J 101      -13.743  33.345  -6.247  1.00  0.00
J    N
```

ATOM 7776 HN MET J 101 -13.929 33.985 -6.989 1.00 0.00
J H
ATOM 7777 CA MET J 101 -12.890 33.881 -5.184 1.00 0.00
J C
ATOM 7778 HA MET J 101 -12.024 33.232 -5.142 1.00 0.00
J H
ATOM 7779 CB MET J 101 -12.513 35.357 -5.435 1.00 0.00
J C
ATOM 7780 HB1 MET J 101 -13.391 35.837 -5.928 1.00 0.00
J H
ATOM 7781 HB2 MET J 101 -12.382 35.876 -4.459 1.00 0.00
J H
ATOM 7782 CG MET J 101 -11.279 35.521 -6.323 1.00 0.00
J C
ATOM 7783 HG1 MET J 101 -10.355 35.331 -5.739 1.00 0.00
J H
ATOM 7784 HG2 MET J 101 -11.312 34.779 -7.150 1.00 0.00
J H
ATOM 7785 SD MET J 101 -11.106 37.179 -7.067 1.00 0.00
J S
ATOM 7786 CE MET J 101 -11.962 38.231 -5.852 1.00 0.00
J C
ATOM 7787 HE1 MET J 101 -11.953 39.295 -6.173 1.00 0.00
J H
ATOM 7788 HE2 MET J 101 -13.023 37.920 -5.731 1.00 0.00
J H
ATOM 7789 HE3 MET J 101 -11.473 38.172 -4.856 1.00 0.00
J H
ATOM 7790 C MET J 101 -13.540 33.867 -3.818 1.00 0.00
J C
ATOM 7791 O MET J 101 -12.922 33.468 -2.836 1.00 0.00
J O
ATOM 7792 N GLY J 102 -14.823 34.282 -3.749 1.00 0.00
J N
ATOM 7793 HN GLY J 102 -15.256 34.714 -4.539 1.00 0.00
J H
ATOM 7794 CA GLY J 102 -15.664 34.147 -2.567 1.00 0.00
J C
ATOM 7795 HA1 GLY J 102 -16.672 34.389 -2.872 1.00 0.00
J H
ATOM 7796 HA2 GLY J 102 -15.257 34.806 -1.812 1.00 0.00
J H
ATOM 7797 C GLY J 102 -15.667 32.751 -2.010 1.00 0.00
J C
ATOM 7798 O GLY J 102 -15.269 32.540 -0.870 1.00 0.00
J O
ATOM 7799 N LEU J 103 -16.039 31.742 -2.813 1.00 0.00
J N
ATOM 7800 HN LEU J 103 -16.352 31.896 -3.749 1.00 0.00
J H
ATOM 7801 CA LEU J 103 -16.023 30.348 -2.410 1.00 0.00
J C
ATOM 7802 HA LEU J 103 -16.665 30.299 -1.540 1.00 0.00
J H

ATOM 7803 CB LEU J 103 −16.581 29.464 −3.544 1.00 0.00
J C
ATOM 7804 HB1 LEU J 103 −16.034 29.740 −4.475 1.00 0.00
J H
ATOM 7805 HB2 LEU J 103 −16.321 28.403 −3.337 1.00 0.00
J H
ATOM 7806 CG LEU J 103 −18.095 29.623 −3.783 1.00 0.00
J C
ATOM 7807 HG LEU J 103 −18.329 30.711 −3.699 1.00 0.00
J H
ATOM 7808 CD1 LEU J 103 −18.452 29.126 −5.192 1.00 0.00
J C
ATOM 7809 HD11 LEU J 103 −19.549 29.190 −5.361 1.00 0.00
J H
ATOM 7810 HD12 LEU J 103 −17.944 29.741 −5.965 1.00 0.00
J H
ATOM 7811 HD13 LEU J 103 −18.142 28.069 −5.325 1.00 0.00
J H
ATOM 7812 CD2 LEU J 103 −18.917 28.875 −2.720 1.00 0.00
J C
ATOM 7813 HD21 LEU J 103 −20.005 29.019 −2.901 1.00 0.00
J H
ATOM 7814 HD22 LEU J 103 −18.714 27.786 −2.737 1.00 0.00
J H
ATOM 7815 HD23 LEU J 103 −18.681 29.260 −1.704 1.00 0.00
J H
ATOM 7816 C LEU J 103 −14.650 29.819 −2.001 1.00 0.00
J C
ATOM 7817 O LEU J 103 −14.536 29.140 −0.987 1.00 0.00
J O
ATOM 7818 N ILE J 104 −13.568 30.152 −2.740 1.00 0.00
J N
ATOM 7819 HN ILE J 104 −13.642 30.678 −3.584 1.00 0.00
J H
ATOM 7820 CA ILE J 104 −12.200 29.781 −2.384 1.00 0.00
J C
ATOM 7821 HA ILE J 104 −12.207 28.704 −2.269 1.00 0.00
J H
ATOM 7822 CB ILE J 104 −11.218 30.237 −3.473 1.00 0.00
J C
ATOM 7823 HB ILE J 104 −11.535 31.283 −3.715 1.00 0.00
J H
ATOM 7824 CG2 ILE J 104 −9.753 30.154 −2.978 1.00 0.00
J C
ATOM 7825 HG21 ILE J 104 −9.066 30.629 −3.708 1.00 0.00
J H
ATOM 7826 HG22 ILE J 104 −9.632 30.682 −2.009 1.00 0.00
J H
ATOM 7827 HG23 ILE J 104 −9.439 29.097 −2.843 1.00 0.00
J H
ATOM 7828 CG1 ILE J 104 −11.402 29.407 −4.767 1.00 0.00
J C
ATOM 7829 HG11 ILE J 104 −11.098 28.352 −4.584 1.00 0.00
J H

ATOM 7830 HG12 ILE J 104 -12.483 29.396 -5.037 1.00 0.00
J H
ATOM 7831 CD ILE J 104 -10.609 29.966 -5.957 1.00 0.00
J C
ATOM 7832 HD1 ILE J 104 -10.819 29.373 -6.873 1.00 0.00
J H
ATOM 7833 HD2 ILE J 104 -10.892 31.022 -6.155 1.00 0.00
J H
ATOM 7834 HD3 ILE J 104 -9.517 29.923 -5.763 1.00 0.00
J H
ATOM 7835 C ILE J 104 -11.767 30.363 -1.044 1.00 0.00
J C
ATOM 7836 O ILE J 104 -11.261 29.656 -0.163 1.00 0.00
J O
ATOM 7837 N GLN J 105 -11.981 31.681 -0.833 1.00 0.00
J N
ATOM 7838 HN GLN J 105 -12.412 32.285 -1.499 1.00 0.00
J H
ATOM 7839 CA GLN J 105 -11.592 32.330 0.401 1.00 0.00
J C
ATOM 7840 HA GLN J 105 -10.569 32.031 0.600 1.00 0.00
J H
ATOM 7841 CB GLN J 105 -11.609 33.878 0.274 1.00 0.00
J C
ATOM 7842 HB1 GLN J 105 -10.883 34.144 -0.529 1.00 0.00
J H
ATOM 7843 HB2 GLN J 105 -12.609 34.207 -0.090 1.00 0.00
J H
ATOM 7844 CG GLN J 105 -11.234 34.699 1.552 1.00 0.00
J C
ATOM 7845 HG1 GLN J 105 -11.260 35.788 1.335 1.00 0.00
J H
ATOM 7846 HG2 GLN J 105 -11.953 34.498 2.373 1.00 0.00
J H
ATOM 7847 CD GLN J 105 -9.822 34.439 2.111 1.00 0.00
J C
ATOM 7848 OE1 GLN J 105 -9.306 33.332 2.065 1.00 0.00
J O
ATOM 7849 NE2 GLN J 105 -9.188 35.473 2.705 1.00 0.00
J N
ATOM 7850 HE21 GLN J 105 -8.274 35.297 3.059 1.00 0.00
J H
ATOM 7851 HE22 GLN J 105 -9.645 36.353 2.785 1.00 0.00
J H
ATOM 7852 C GLN J 105 -12.438 31.873 1.575 1.00 0.00
J C
ATOM 7853 O GLN J 105 -11.918 31.481 2.609 1.00 0.00
J O
ATOM 7854 N ILE J 106 -13.779 31.829 1.415 1.00 0.00
J N
ATOM 7855 HN ILE J 106 -14.205 32.117 0.560 1.00 0.00
J H
ATOM 7856 CA ILE J 106 -14.722 31.375 2.433 1.00 0.00
J C

ATOM 7857 HA ILE J 106 −14.493 31.943 3.326 1.00 0.00
J H
ATOM 7858 CB ILE J 106 −16.172 31.667 2.032 1.00 0.00
J C
ATOM 7859 HB ILE J 106 −16.261 31.265 0.990 1.00 0.00
J H
ATOM 7860 CG2 ILE J 106 −17.185 31.021 3.012 1.00 0.00
J C
ATOM 7861 HG21 ILE J 106 −18.220 31.112 2.620 1.00 0.00
J H
ATOM 7862 HG22 ILE J 106 −16.970 29.941 3.150 1.00 0.00
J H
ATOM 7863 HG23 ILE J 106 −17.146 31.515 4.006 1.00 0.00
J H
ATOM 7864 CG1 ILE J 106 −16.392 33.200 1.950 1.00 0.00
J C
ATOM 7865 HG11 ILE J 106 −16.348 33.641 2.971 1.00 0.00
J H
ATOM 7866 HG12 ILE J 106 −15.563 33.653 1.359 1.00 0.00
J H
ATOM 7867 CD ILE J 106 −17.724 33.582 1.289 1.00 0.00
J C
ATOM 7868 HD1 ILE J 106 −17.809 34.687 1.202 1.00 0.00
J H
ATOM 7869 HD2 ILE J 106 −17.794 33.146 0.269 1.00 0.00
J H
ATOM 7870 HD3 ILE J 106 −18.584 33.218 1.888 1.00 0.00
J H
ATOM 7871 C ILE J 106 −14.530 29.907 2.783 1.00 0.00
J C
ATOM 7872 O ILE J 106 −14.558 29.533 3.952 1.00 0.00
J O
ATOM 7873 N GLY J 107 −14.287 29.027 1.792 1.00 0.00
J N
ATOM 7874 HN GLY J 107 −14.251 29.331 0.840 1.00 0.00
J H
ATOM 7875 CA GLY J 107 −14.062 27.601 2.009 1.00 0.00
J C
ATOM 7876 HA1 GLY J 107 −14.039 27.148 1.027 1.00 0.00
J H
ATOM 7877 HA2 GLY J 107 −14.873 27.258 2.638 1.00 0.00
J H
ATOM 7878 C GLY J 107 −12.768 27.275 2.702 1.00 0.00
J C
ATOM 7879 O GLY J 107 −12.694 26.377 3.540 1.00 0.00
J O
ATOM 7880 N GLY J 108 −11.697 28.043 2.413 1.00 0.00
J N
ATOM 7881 HN GLY J 108 −11.702 28.705 1.665 1.00 0.00
J H
ATOM 7882 CA GLY J 108 −10.457 27.961 3.175 1.00 0.00
J C
ATOM 7883 HA1 GLY J 108 −9.707 28.509 2.621 1.00 0.00
J H

ATOM 7884 HA2 GLY J 108 -10.230 26.913 3.312 1.00 0.00
J H
ATOM 7885 C GLY J 108 -10.584 28.590 4.533 1.00 0.00
J C
ATOM 7886 O GLY J 108 -10.077 28.044 5.510 1.00 0.00
J O
ATOM 7887 N LEU J 109 -11.296 29.725 4.648 1.00 0.00
J N
ATOM 7888 HN LEU J 109 -11.672 30.188 3.847 1.00 0.00
J H
ATOM 7889 CA LEU J 109 -11.589 30.382 5.908 1.00 0.00
J C
ATOM 7890 HA LEU J 109 -10.630 30.570 6.372 1.00 0.00
J H
ATOM 7891 CB LEU J 109 -12.361 31.696 5.634 1.00 0.00
J C
ATOM 7892 HB1 LEU J 109 -11.743 32.301 4.932 1.00 0.00
J H
ATOM 7893 HB2 LEU J 109 -13.301 31.457 5.088 1.00 0.00
J H
ATOM 7894 CG LEU J 109 -12.738 32.655 6.795 1.00 0.00
J C
ATOM 7895 HG LEU J 109 -11.888 32.780 7.306 1.00 0.00
J H
ATOM 7896 CD1 LEU J 109 -13.768 32.172 7.834 1.00 0.00
J C
ATOM 7897 HD11 LEU J 109 -13.906 32.934 8.629 1.00 0.00
J H
ATOM 7898 HD12 LEU J 109 -14.752 31.987 7.352 1.00 0.00
J H
ATOM 7899 HD13 LEU J 109 -13.429 31.229 8.312 1.00 0.00
J H
ATOM 7900 CD2 LEU J 109 -13.360 33.895 6.142 1.00 0.00
J C
ATOM 7901 HD21 LEU J 109 -13.673 34.624 6.920 1.00 0.00
J H
ATOM 7902 HD22 LEU J 109 -12.642 34.408 5.470 1.00 0.00
J H
ATOM 7903 HD23 LEU J 109 -14.256 33.614 5.548 1.00 0.00
J H
ATOM 7904 C LEU J 109 -12.394 29.525 6.856 1.00 0.00
J C
ATOM 7905 O LEU J 109 -12.009 29.328 8.002 1.00 0.00
J O
ATOM 7906 N GLY J 110 -13.517 28.943 6.390 1.00 0.00
J N
ATOM 7907 HN GLY J 110 -13.817 29.046 5.442 1.00 0.00
J H
ATOM 7908 CA GLY J 110 -14.372 28.122 7.235 1.00 0.00
J C
ATOM 7909 HA1 GLY J 110 -15.255 27.879 6.659 1.00 0.00
J H
ATOM 7910 HA2 GLY J 110 -14.572 28.682 8.138 1.00 0.00
J H

ATOM 7911 C GLY J 110 -13.705 26.842 7.625 1.00 0.00
J C
ATOM 7912 O GLY J 110 -13.858 26.345 8.733 1.00 0.00
J O
ATOM 7913 N PHE J 111 -12.881 26.271 6.739 1.00 0.00
J N
ATOM 7914 HN PHE J 111 -12.777 26.560 5.788 1.00 0.00
J H
ATOM 7915 CA PHE J 111 -12.054 25.154 7.118 1.00 0.00
J C
ATOM 7916 HA PHE J 111 -12.727 24.450 7.591 1.00 0.00
J H
ATOM 7917 CB PHE J 111 -11.445 24.552 5.831 1.00 0.00
J C
ATOM 7918 HB1 PHE J 111 -12.264 24.308 5.120 1.00 0.00
J H
ATOM 7919 HB2 PHE J 111 -10.777 25.282 5.324 1.00 0.00
J H
ATOM 7920 CG PHE J 111 -10.711 23.276 6.128 1.00 0.00
J C
ATOM 7921 CD1 PHE J 111 -11.390 22.177 6.685 1.00 0.00
J C
ATOM 7922 HD1 PHE J 111 -12.453 22.246 6.875 1.00 0.00
J H
ATOM 7923 CE1 PHE J 111 -10.698 21.002 6.999 1.00 0.00
J C
ATOM 7924 HE1 PHE J 111 -11.225 20.163 7.430 1.00 0.00
J H
ATOM 7925 CZ PHE J 111 -9.324 20.913 6.747 1.00 0.00
J C
ATOM 7926 HZ PHE J 111 -8.795 19.995 6.962 1.00 0.00
J H
ATOM 7927 CD2 PHE J 111 -9.328 23.186 5.910 1.00 0.00
J C
ATOM 7928 HD2 PHE J 111 -8.785 24.039 5.528 1.00 0.00
J H
ATOM 7929 CE2 PHE J 111 -8.638 22.007 6.208 1.00 0.00
J C
ATOM 7930 HE2 PHE J 111 -7.574 21.944 6.022 1.00 0.00
J H
ATOM 7931 C PHE J 111 -10.979 25.514 8.153 1.00 0.00
J C
ATOM 7932 O PHE J 111 -10.865 24.843 9.173 1.00 0.00
J O
ATOM 7933 N MET J 112 -10.198 26.605 7.957 1.00 0.00
J N
ATOM 7934 HN MET J 112 -10.288 27.210 7.168 1.00 0.00
J H
ATOM 7935 CA MET J 112 -9.149 26.989 8.893 1.00 0.00
J C
ATOM 7936 HA MET J 112 -8.542 26.104 9.036 1.00 0.00
J H
ATOM 7937 CB MET J 112 -8.135 28.017 8.298 1.00 0.00
J C

```
ATOM   7938  HB1 MET J 112      -7.243  28.017   8.967  1.00  0.00
J    H
ATOM   7939  HB2 MET J 112      -7.781  27.656   7.307  1.00  0.00
J    H
ATOM   7940  CG  MET J 112      -8.630  29.478   8.169  1.00  0.00
J    C
ATOM   7941  HG1 MET J 112      -9.697  29.498   7.863  1.00  0.00
J    H
ATOM   7942  HG2 MET J 112      -8.553  29.987   9.154  1.00  0.00
J    H
ATOM   7943  SD  MET J 112      -7.739  30.519   6.960  1.00  0.00
J    S
ATOM   7944  CE  MET J 112      -8.491  32.126   7.375  1.00  0.00
J    C
ATOM   7945  HE1 MET J 112      -8.086  32.932   6.727  1.00  0.00
J    H
ATOM   7946  HE2 MET J 112      -9.593  32.099   7.235  1.00  0.00
J    H
ATOM   7947  HE3 MET J 112      -8.287  32.402   8.432  1.00  0.00
J    H
ATOM   7948  C   MET J 112      -9.669  27.417  10.261  1.00  0.00
J    C
ATOM   7949  O   MET J 112      -9.115  27.010  11.280  1.00  0.00
J    O
ATOM   7950  N   THR J 113     -10.768  28.204  10.355  1.00  0.00
J    N
ATOM   7951  HN  THR J 113     -11.278  28.536   9.561  1.00  0.00
J    H
ATOM   7952  CA  THR J 113     -11.312  28.640  11.644  1.00  0.00
J    C
ATOM   7953  HA  THR J 113     -10.478  29.087  12.169  1.00  0.00
J    H
ATOM   7954  CB  THR J 113     -12.472  29.631  11.546  1.00  0.00
J    C
ATOM   7955  HB  THR J 113     -12.928  29.676  12.566  1.00  0.00
J    H
ATOM   7956  OG1 THR J 113     -13.488  29.182  10.659  1.00  0.00
J    O
ATOM   7957  HG1 THR J 113     -14.157  29.875  10.662  1.00  0.00
J    H
ATOM   7958  CG2 THR J 113     -11.936  30.949  10.985  1.00  0.00
J    C
ATOM   7959 HG21 THR J 113     -12.721  31.735  11.003  1.00  0.00
J    H
ATOM   7960 HG22 THR J 113     -11.079  31.309  11.593  1.00  0.00
J    H
ATOM   7961 HG23 THR J 113     -11.591  30.827   9.936  1.00  0.00
J    H
ATOM   7962  C   THR J 113     -11.785  27.491  12.492  1.00  0.00
J    C
ATOM   7963  O   THR J 113     -11.430  27.384  13.663  1.00  0.00
J    O
ATOM   7964  N   PHE J 114     -12.553  26.565  11.897  1.00  0.00
J    N
```

ATOM 7965 HN PHE J 114 -12.889 26.666 10.962 1.00 0.00
J H
ATOM 7966 CA PHE J 114 -12.962 25.346 12.550 1.00 0.00
J C
ATOM 7967 HA PHE J 114 -13.360 25.662 13.506 1.00 0.00
J H
ATOM 7968 CB PHE J 114 -14.084 24.646 11.760 1.00 0.00
J C
ATOM 7969 HB1 PHE J 114 -13.887 24.757 10.672 1.00 0.00
J H
ATOM 7970 HB2 PHE J 114 -14.108 23.557 11.978 1.00 0.00
J H
ATOM 7971 CG PHE J 114 -15.446 25.257 12.021 1.00 0.00
J C
ATOM 7972 CD1 PHE J 114 -16.164 24.889 13.172 1.00 0.00
J C
ATOM 7973 HD1 PHE J 114 -15.717 24.213 13.889 1.00 0.00
J H
ATOM 7974 CE1 PHE J 114 -17.456 25.381 13.402 1.00 0.00
J C
ATOM 7975 HE1 PHE J 114 -17.993 25.094 14.295 1.00 0.00
J H
ATOM 7976 CZ PHE J 114 -18.051 26.249 12.478 1.00 0.00
J C
ATOM 7977 HZ PHE J 114 -19.042 26.639 12.658 1.00 0.00
J H
ATOM 7978 CD2 PHE J 114 -16.064 26.125 11.105 1.00 0.00
J C
ATOM 7979 HD2 PHE J 114 -15.550 26.409 10.198 1.00 0.00
J H
ATOM 7980 CE2 PHE J 114 -17.354 26.619 11.324 1.00 0.00
J C
ATOM 7981 HE2 PHE J 114 -17.806 27.284 10.601 1.00 0.00
J H
ATOM 7982 C PHE J 114 -11.810 24.375 12.840 1.00 0.00
J C
ATOM 7983 O PHE J 114 -11.771 23.786 13.911 1.00 0.00
J O
ATOM 7984 N ALA J 115 -10.823 24.187 11.927 1.00 0.00
J N
ATOM 7985 HN ALA J 115 -10.860 24.615 11.025 1.00 0.00
J H
ATOM 7986 CA ALA J 115 -9.648 23.361 12.180 1.00 0.00
J C
ATOM 7987 HA ALA J 115 -9.972 22.362 12.446 1.00 0.00
J H
ATOM 7988 CB ALA J 115 -8.789 23.243 10.900 1.00 0.00
J C
ATOM 7989 HB1 ALA J 115 -9.410 22.844 10.070 1.00 0.00
J H
ATOM 7990 HB2 ALA J 115 -8.399 24.236 10.588 1.00 0.00
J H
ATOM 7991 HB3 ALA J 115 -7.929 22.555 11.046 1.00 0.00
J H

ATOM 7992 C ALA J 115 -8.760 23.846 13.329 1.00 0.00
J C
ATOM 7993 O ALA J 115 -8.339 23.059 14.178 1.00 0.00
J O
ATOM 7994 N VAL J 116 -8.483 25.166 13.429 1.00 0.00
J N
ATOM 7995 HN VAL J 116 -8.739 25.815 12.718 1.00 0.00
J H
ATOM 7996 CA VAL J 116 -7.791 25.765 14.572 1.00 0.00
J C
ATOM 7997 HA VAL J 116 -6.866 25.220 14.715 1.00 0.00
J H
ATOM 7998 CB VAL J 116 -7.429 27.234 14.328 1.00 0.00
J C
ATOM 7999 HB VAL J 116 -8.383 27.795 14.174 1.00 0.00
J H
ATOM 8000 CG1 VAL J 116 -6.684 27.836 15.541 1.00 0.00
J C
ATOM 8001 HG11 VAL J 116 -6.395 28.889 15.332 1.00 0.00
J H
ATOM 8002 HG12 VAL J 116 -7.320 27.842 16.450 1.00 0.00
J H
ATOM 8003 HG13 VAL J 116 -5.759 27.262 15.757 1.00 0.00
J H
ATOM 8004 CG2 VAL J 116 -6.501 27.350 13.099 1.00 0.00
J C
ATOM 8005 HG21 VAL J 116 -6.199 28.407 12.934 1.00 0.00
J H
ATOM 8006 HG22 VAL J 116 -5.583 26.742 13.245 1.00 0.00
J H
ATOM 8007 HG23 VAL J 116 -7.009 26.999 12.177 1.00 0.00
J H
ATOM 8008 C VAL J 116 -8.614 25.611 15.851 1.00 0.00
J C
ATOM 8009 O VAL J 116 -8.098 25.297 16.922 1.00 0.00
J O
ATOM 8010 N LEU J 117 -9.947 25.775 15.763 1.00 0.00
J N
ATOM 8011 HN LEU J 117 -10.388 26.072 14.918 1.00 0.00
J H
ATOM 8012 CA LEU J 117 -10.865 25.542 16.866 1.00 0.00
J C
ATOM 8013 HA LEU J 117 -10.509 26.171 17.672 1.00 0.00
J H
ATOM 8014 CB LEU J 117 -12.260 26.077 16.471 1.00 0.00
J C
ATOM 8015 HB1 LEU J 117 -12.112 27.089 16.029 1.00 0.00
J H
ATOM 8016 HB2 LEU J 117 -12.674 25.442 15.656 1.00 0.00
J H
ATOM 8017 CG LEU J 117 -13.326 26.217 17.580 1.00 0.00
J C
ATOM 8018 HG LEU J 117 -13.684 25.299 17.754 1.00 0.00
J H

```
ATOM   8019  CD1 LEU J 117      -14.422  27.181  17.091  1.00  0.00
J    C
ATOM   8020 HD11 LEU J 117      -15.231  27.268  17.848  1.00  0.00
J    H
ATOM   8021 HD12 LEU J 117      -14.001  28.194  16.912  1.00  0.00
J    H
ATOM   8022 HD13 LEU J 117      -14.871  26.815  16.143  1.00  0.00
J    H
ATOM   8023  CD2 LEU J 117      -12.773  26.726  18.923  1.00  0.00
J    C
ATOM   8024 HD21 LEU J 117      -13.592  26.822  19.667  1.00  0.00
J    H
ATOM   8025 HD22 LEU J 117      -12.016  26.034  19.343  1.00  0.00
J    H
ATOM   8026 HD23 LEU J 117      -12.301  27.725  18.796  1.00  0.00
J    H
ATOM   8027  C   LEU J 117      -10.878  24.100  17.376  1.00  0.00
J    C
ATOM   8028  O   LEU J 117      -10.875  23.871  18.586  1.00  0.00
J    O
ATOM   8029  N   ILE J 118      -10.793  23.081  16.489  1.00  0.00
J    N
ATOM   8030  HN  ILE J 118      -10.865  23.234  15.505  1.00  0.00
J    H
ATOM   8031  CA  ILE J 118      -10.594  21.680  16.865  1.00  0.00
J    C
ATOM   8032  HA  ILE J 118      -11.429  21.416  17.501  1.00  0.00
J    H
ATOM   8033  CB  ILE J 118      -10.573  20.759  15.633  1.00  0.00
J    C
ATOM   8034  HB  ILE J 118       -9.968  21.318  14.875  1.00  0.00
J    H
ATOM   8035  CG2 ILE J 118       -9.982  19.362  15.957  1.00  0.00
J    C
ATOM   8036 HG21 ILE J 118       -9.834  18.775  15.027  1.00  0.00
J    H
ATOM   8037 HG22 ILE J 118       -8.994  19.456  16.456  1.00  0.00
J    H
ATOM   8038 HG23 ILE J 118      -10.658  18.789  16.626  1.00  0.00
J    H
ATOM   8039  CG1 ILE J 118      -12.003  20.615  15.052  1.00  0.00
J    C
ATOM   8040 HG11 ILE J 118      -12.621  19.981  15.726  1.00  0.00
J    H
ATOM   8041 HG12 ILE J 118      -12.480  21.621  15.016  1.00  0.00
J    H
ATOM   8042  CD  ILE J 118      -12.025  20.019  13.636  1.00  0.00
J    C
ATOM   8043  HD1 ILE J 118      -13.066  19.992  13.246  1.00  0.00
J    H
ATOM   8044  HD2 ILE J 118      -11.410  20.632  12.943  1.00  0.00
J    H
ATOM   8045  HD3 ILE J 118      -11.631  18.982  13.634  1.00  0.00
J    H
```

```
ATOM   8046  C   ILE J 118      -9.328  21.499  17.701  1.00  0.00
J    C
ATOM   8047  O   ILE J 118      -9.353  20.840  18.739  1.00  0.00
J    O
ATOM   8048  N   VAL J 119      -8.203  22.138  17.319  1.00  0.00
J    N
ATOM   8049  HN  VAL J 119      -8.160  22.666  16.475  1.00  0.00
J    H
ATOM   8050  CA  VAL J 119      -6.958  22.124  18.086  1.00  0.00
J    C
ATOM   8051  HA  VAL J 119      -6.676  21.086  18.205  1.00  0.00
J    H
ATOM   8052  CB  VAL J 119      -5.838  22.844  17.332  1.00  0.00
J    C
ATOM   8053  HB  VAL J 119      -6.193  23.881  17.109  1.00  0.00
J    H
ATOM   8054  CG1 VAL J 119      -4.553  22.930  18.182  1.00  0.00
J    C
ATOM   8055 HG11 VAL J 119      -3.741  23.421  17.603  1.00  0.00
J    H
ATOM   8056 HG12 VAL J 119      -4.711  23.528  19.104  1.00  0.00
J    H
ATOM   8057 HG13 VAL J 119      -4.205  21.915  18.470  1.00  0.00
J    H
ATOM   8058  CG2 VAL J 119      -5.544  22.087  16.020  1.00  0.00
J    C
ATOM   8059 HG21 VAL J 119      -4.707  22.565  15.469  1.00  0.00
J    H
ATOM   8060 HG22 VAL J 119      -5.266  21.032  16.232  1.00  0.00
J    H
ATOM   8061 HG23 VAL J 119      -6.429  22.087  15.349  1.00  0.00
J    H
ATOM   8062  C   VAL J 119      -7.128  22.716  19.484  1.00  0.00
J    C
ATOM   8063  O   VAL J 119      -6.682  22.147  20.481  1.00  0.00
J    O
ATOM   8064  N   MET J 120      -7.847  23.849  19.604  1.00  0.00
J    N
ATOM   8065  HN  MET J 120      -8.192  24.348  18.811  1.00  0.00
J    H
ATOM   8066  CA  MET J 120      -8.186  24.449  20.885  1.00  0.00
J    C
ATOM   8067  HA  MET J 120      -7.244  24.610  21.395  1.00  0.00
J    H
ATOM   8068  CB  MET J 120      -8.919  25.798  20.691  1.00  0.00
J    C
ATOM   8069  HB1 MET J 120      -9.817  25.593  20.062  1.00  0.00
J    H
ATOM   8070  HB2 MET J 120      -9.301  26.156  21.673  1.00  0.00
J    H
ATOM   8071  CG  MET J 120      -8.064  26.882  20.006  1.00  0.00
J    C
ATOM   8072  HG1 MET J 120      -7.227  27.189  20.668  1.00  0.00
J    H
```

```
ATOM   8073  HG2 MET J 120      -7.622  26.474  19.071  1.00  0.00
J    H
ATOM   8074  SD  MET J 120      -8.994  28.391  19.579  1.00  0.00
J    S
ATOM   8075  CE  MET J 120      -9.225  29.011  21.271  1.00  0.00
J    C
ATOM   8076  HE1 MET J 120      -9.801  29.961  21.270  1.00  0.00
J    H
ATOM   8077  HE2 MET J 120      -9.780  28.276  21.893  1.00  0.00
J    H
ATOM   8078  HE3 MET J 120      -8.247  29.206  21.763  1.00  0.00
J    H
ATOM   8079  C   MET J 120      -9.047  23.554  21.775  1.00  0.00
J    C
ATOM   8080  O   MET J 120      -8.825  23.466  22.981  1.00  0.00
J    O
ATOM   8081  N   ILE J 121     -10.049  22.851  21.205  1.00  0.00
J    N
ATOM   8082  HN  ILE J 121     -10.292  22.975  20.246  1.00  0.00
J    H
ATOM   8083  CA  ILE J 121     -10.864  21.861  21.907  1.00  0.00
J    C
ATOM   8084  HA  ILE J 121     -11.258  22.365  22.780  1.00  0.00
J    H
ATOM   8085  CB  ILE J 121     -12.024  21.373  21.033  1.00  0.00
J    C
ATOM   8086  HB  ILE J 121     -11.543  21.094  20.061  1.00  0.00
J    H
ATOM   8087  CG2 ILE J 121     -12.785  20.201  21.703  1.00  0.00
J    C
ATOM   8088 HG21 ILE J 121     -13.517  19.757  20.998  1.00  0.00
J    H
ATOM   8089 HG22 ILE J 121     -12.083  19.396  22.010  1.00  0.00
J    H
ATOM   8090 HG23 ILE J 121     -13.335  20.548  22.603  1.00  0.00
J    H
ATOM   8091  CG1 ILE J 121     -12.998  22.541  20.736  1.00  0.00
J    C
ATOM   8092 HG11 ILE J 121     -13.569  22.797  21.656  1.00  0.00
J    H
ATOM   8093 HG12 ILE J 121     -12.405  23.441  20.454  1.00  0.00
J    H
ATOM   8094  CD  ILE J 121     -13.983  22.238  19.599  1.00  0.00
J    C
ATOM   8095  HD1 ILE J 121     -14.617  23.127  19.391  1.00  0.00
J    H
ATOM   8096  HD2 ILE J 121     -13.438  21.973  18.668  1.00  0.00
J    H
ATOM   8097  HD3 ILE J 121     -14.653  21.394  19.867  1.00  0.00
J    H
ATOM   8098  C   ILE J 121     -10.032  20.686  22.413  1.00  0.00
J    C
ATOM   8099  O   ILE J 121     -10.181  20.252  23.555  1.00  0.00
J    O
```

```
ATOM   8100  N   LEU J 122      -9.086  20.170  21.598  1.00  0.00
J    N
ATOM   8101  HN  LEU J 122      -8.957  20.477  20.657  1.00  0.00
J    H
ATOM   8102  CA  LEU J 122      -8.170  19.118  22.015  1.00  0.00
J    C
ATOM   8103  HA  LEU J 122      -8.795  18.298  22.344  1.00  0.00
J    H
ATOM   8104  CB  LEU J 122      -7.274  18.643  20.841  1.00  0.00
J    C
ATOM   8105  HB1 LEU J 122      -6.755  19.544  20.438  1.00  0.00
J    H
ATOM   8106  HB2 LEU J 122      -6.475  17.981  21.241  1.00  0.00
J    H
ATOM   8107  CG  LEU J 122      -8.036  17.947  19.691  1.00  0.00
J    C
ATOM   8108  HG  LEU J 122      -8.867  18.643  19.419  1.00  0.00
J    H
ATOM   8109  CD1 LEU J 122      -7.104  17.764  18.482  1.00  0.00
J    C
ATOM   8110 HD11 LEU J 122      -7.626  17.221  17.665  1.00  0.00
J    H
ATOM   8111 HD12 LEU J 122      -6.773  18.750  18.091  1.00  0.00
J    H
ATOM   8112 HD13 LEU J 122      -6.203  17.179  18.766  1.00  0.00
J    H
ATOM   8113  CD2 LEU J 122      -8.659  16.604  20.114  1.00  0.00
J    C
ATOM   8114 HD21 LEU J 122      -9.211  16.150  19.263  1.00  0.00
J    H
ATOM   8115 HD22 LEU J 122      -7.886  15.879  20.439  1.00  0.00
J    H
ATOM   8116 HD23 LEU J 122      -9.374  16.754  20.952  1.00  0.00
J    H
ATOM   8117  C   LEU J 122      -7.287  19.522  23.187  1.00  0.00
J    C
ATOM   8118  O   LEU J 122      -7.154  18.767  24.150  1.00  0.00
J    O
ATOM   8119  N   GLY J 123      -6.721  20.748  23.177  1.00  0.00
J    N
ATOM   8120  HN  GLY J 123      -6.817  21.365  22.397  1.00  0.00
J    H
ATOM   8121  CA  GLY J 123      -5.932  21.262  24.295  1.00  0.00
J    C
ATOM   8122  HA1 GLY J 123      -5.544  22.217  23.971  1.00  0.00
J    H
ATOM   8123  HA2 GLY J 123      -5.171  20.518  24.494  1.00  0.00
J    H
ATOM   8124  C   GLY J 123      -6.711  21.482  25.571  1.00  0.00
J    C
ATOM   8125  O   GLY J 123      -6.214  21.247  26.667  1.00  0.00
J    O
ATOM   8126  N   LYS J 124      -7.992  21.895  25.464  1.00  0.00
J    N
```

ATOM 8127 HN LYS J 124 -8.390 22.193 24.598 1.00 0.00
J H
ATOM 8128 CA LYS J 124 -8.895 21.938 26.607 1.00 0.00
J C
ATOM 8129 HA LYS J 124 -8.396 22.517 27.374 1.00 0.00
J H
ATOM 8130 CB LYS J 124 -10.249 22.600 26.236 1.00 0.00
J C
ATOM 8131 HB1 LYS J 124 -10.629 22.083 25.325 1.00 0.00
J H
ATOM 8132 HB2 LYS J 124 -10.986 22.394 27.043 1.00 0.00
J H
ATOM 8133 CG LYS J 124 -10.133 24.107 25.959 1.00 0.00
J C
ATOM 8134 HG1 LYS J 124 -9.822 24.626 26.894 1.00 0.00
J H
ATOM 8135 HG2 LYS J 124 -9.332 24.276 25.203 1.00 0.00
J H
ATOM 8136 CD LYS J 124 -11.459 24.716 25.472 1.00 0.00
J C
ATOM 8137 HD1 LYS J 124 -11.813 24.131 24.593 1.00 0.00
J H
ATOM 8138 HD2 LYS J 124 -12.216 24.593 26.281 1.00 0.00
J H
ATOM 8139 CE LYS J 124 -11.334 26.199 25.101 1.00 0.00
J C
ATOM 8140 HE1 LYS J 124 -10.976 26.790 25.973 1.00 0.00
J H
ATOM 8141 HE2 LYS J 124 -10.613 26.333 24.265 1.00 0.00
J H
ATOM 8142 NZ LYS J 124 -12.648 26.738 24.679 1.00 0.00
J N
ATOM 8143 HZ1 LYS J 124 -12.556 27.744 24.430 1.00 0.00
J H
ATOM 8144 HZ2 LYS J 124 -12.992 26.209 23.852 1.00 0.00
J H
ATOM 8145 HZ3 LYS J 124 -13.330 26.637 25.458 1.00 0.00
J H
ATOM 8146 C LYS J 124 -9.161 20.558 27.181 1.00 0.00
J C
ATOM 8147 O LYS J 124 -9.112 20.360 28.401 1.00 0.00
J O
ATOM 8148 N LYS J 125 -9.399 19.553 26.324 1.00 0.00
J N
ATOM 8149 HN LYS J 125 -9.459 19.727 25.343 1.00 0.00
J H
ATOM 8150 CA LYS J 125 -9.586 18.172 26.714 1.00 0.00
J C
ATOM 8151 HA LYS J 125 -10.397 18.176 27.431 1.00 0.00
J H
ATOM 8152 CB LYS J 125 -9.991 17.332 25.482 1.00 0.00
J C
ATOM 8153 HB1 LYS J 125 -10.855 17.848 25.002 1.00 0.00
J H

ATOM 8154 HB2 LYS J 125 -9.161 17.355 24.739 1.00 0.00
J H
ATOM 8155 CG LYS J 125 -10.389 15.883 25.811 1.00 0.00
J C
ATOM 8156 HG1 LYS J 125 -9.527 15.368 26.294 1.00 0.00
J H
ATOM 8157 HG2 LYS J 125 -11.225 15.896 26.548 1.00 0.00
J H
ATOM 8158 CD LYS J 125 -10.797 15.104 24.550 1.00 0.00
J C
ATOM 8159 HD1 LYS J 125 -11.609 15.668 24.036 1.00 0.00
J H
ATOM 8160 HD2 LYS J 125 -9.922 15.076 23.861 1.00 0.00
J H
ATOM 8161 CE LYS J 125 -11.265 13.672 24.822 1.00 0.00
J C
ATOM 8162 HE1 LYS J 125 -10.438 13.066 25.256 1.00 0.00
J H
ATOM 8163 HE2 LYS J 125 -12.116 13.668 25.538 1.00 0.00
J H
ATOM 8164 NZ LYS J 125 -11.706 13.037 23.557 1.00 0.00
J N
ATOM 8165 HZ1 LYS J 125 -12.023 12.063 23.738 1.00 0.00
J H
ATOM 8166 HZ2 LYS J 125 -12.489 13.586 23.147 1.00 0.00
J H
ATOM 8167 HZ3 LYS J 125 -10.913 13.021 22.884 1.00 0.00
J H
ATOM 8168 C LYS J 125 -8.374 17.548 27.400 1.00 0.00
J C
ATOM 8169 O LYS J 125 -8.520 16.790 28.355 1.00 0.00
J O
ATOM 8170 N ILE J 126 -7.138 17.850 26.937 1.00 0.00
J N
ATOM 8171 HN ILE J 126 -6.991 18.377 26.102 1.00 0.00
J H
ATOM 8172 CA ILE J 126 -5.906 17.439 27.610 1.00 0.00
J C
ATOM 8173 HA ILE J 126 -5.965 16.364 27.720 1.00 0.00
J H
ATOM 8174 CB ILE J 126 -4.671 17.817 26.789 1.00 0.00
J C
ATOM 8175 HB ILE J 126 -4.844 18.885 26.500 1.00 0.00
J H
ATOM 8176 CG2 ILE J 126 -3.367 17.599 27.599 1.00 0.00
J C
ATOM 8177 HG21 ILE J 126 -2.495 18.020 27.056 1.00 0.00
J H
ATOM 8178 HG22 ILE J 126 -3.424 18.103 28.586 1.00 0.00
J H
ATOM 8179 HG23 ILE J 126 -3.180 16.517 27.768 1.00 0.00
J H
ATOM 8180 CG1 ILE J 126 -4.646 16.986 25.483 1.00 0.00
J C

ATOM 8181 HG11 ILE J 126 -4.409 15.924 25.717 1.00 0.00
J H
ATOM 8182 HG12 ILE J 126 -5.661 17.004 25.025 1.00 0.00
J H
ATOM 8183 CD ILE J 126 -3.643 17.509 24.448 1.00 0.00
J C
ATOM 8184 HD1 ILE J 126 -3.709 16.912 23.513 1.00 0.00
J H
ATOM 8185 HD2 ILE J 126 -3.854 18.571 24.199 1.00 0.00
J H
ATOM 8186 HD3 ILE J 126 -2.603 17.436 24.829 1.00 0.00
J H
ATOM 8187 C ILE J 126 -5.819 18.019 29.005 1.00 0.00
J C
ATOM 8188 O ILE J 126 -5.667 17.268 29.969 1.00 0.00
J O
ATOM 8189 N GLY J 127 -6.041 19.345 29.157 1.00 0.00
J N
ATOM 8190 HN GLY J 127 -6.141 19.937 28.358 1.00 0.00
J H
ATOM 8191 CA GLY J 127 -6.150 20.009 30.452 1.00 0.00
J C
ATOM 8192 HA1 GLY J 127 -6.503 21.012 30.252 1.00 0.00
J H
ATOM 8193 HA2 GLY J 127 -5.168 19.966 30.906 1.00 0.00
J H
ATOM 8194 C GLY J 127 -7.126 19.351 31.394 1.00 0.00
J C
ATOM 8195 O GLY J 127 -6.806 19.049 32.534 1.00 0.00
J O
ATOM 8196 N LEU J 128 -8.362 19.063 30.927 1.00 0.00
J N
ATOM 8197 HN LEU J 128 -8.653 19.310 30.006 1.00 0.00
J H
ATOM 8198 CA LEU J 128 -9.380 18.372 31.713 1.00 0.00
J C
ATOM 8199 HA LEU J 128 -9.571 19.002 32.572 1.00 0.00
J H
ATOM 8200 CB LEU J 128 -10.698 18.165 30.912 1.00 0.00
J C
ATOM 8201 HB1 LEU J 128 -10.428 17.651 29.961 1.00 0.00
J H
ATOM 8202 HB2 LEU J 128 -11.353 17.459 31.469 1.00 0.00
J H
ATOM 8203 CG LEU J 128 -11.483 19.453 30.563 1.00 0.00
J C
ATOM 8204 HG LEU J 128 -10.745 20.130 30.069 1.00 0.00
J H
ATOM 8205 CD1 LEU J 128 -12.633 19.132 29.588 1.00 0.00
J C
ATOM 8206 HD11 LEU J 128 -13.228 20.045 29.372 1.00 0.00
J H
ATOM 8207 HD12 LEU J 128 -12.234 18.741 28.627 1.00 0.00
J H

```
ATOM   8208 HD13 LEU J 128     -13.314  18.369  30.021  1.00  0.00
J    H
ATOM   8209  CD2 LEU J 128     -12.029  20.172  31.809  1.00  0.00
J    C
ATOM   8210 HD21 LEU J 128     -12.569  21.098  31.514  1.00  0.00
J    H
ATOM   8211 HD22 LEU J 128     -12.739  19.534  32.372  1.00  0.00
J    H
ATOM   8212 HD23 LEU J 128     -11.198  20.455  32.490  1.00  0.00
J    H
ATOM   8213  C   LEU J 128      -8.909  17.018  32.232  1.00  0.00
J    C
ATOM   8214  O   LEU J 128      -9.111  16.699  33.404  1.00  0.00
J    O
ATOM   8215  N   LYS J 129      -8.215  16.211  31.409  1.00  0.00
J    N
ATOM   8216  HN  LYS J 129      -8.086  16.434  30.444  1.00  0.00
J    H
ATOM   8217  CA  LYS J 129      -7.604  14.967  31.841  1.00  0.00
J    C
ATOM   8218  HA  LYS J 129      -8.404  14.390  32.289  1.00  0.00
J    H
ATOM   8219  CB  LYS J 129      -6.960  14.233  30.654  1.00  0.00
J    C
ATOM   8220  HB1 LYS J 129      -6.265  14.953  30.161  1.00  0.00
J    H
ATOM   8221  HB2 LYS J 129      -6.329  13.403  31.040  1.00  0.00
J    H
ATOM   8222  CG  LYS J 129      -7.967  13.721  29.625  1.00  0.00
J    C
ATOM   8223  HG1 LYS J 129      -8.605  12.940  30.097  1.00  0.00
J    H
ATOM   8224  HG2 LYS J 129      -8.636  14.560  29.324  1.00  0.00
J    H
ATOM   8225  CD  LYS J 129      -7.202  13.143  28.432  1.00  0.00
J    C
ATOM   8226  HD1 LYS J 129      -6.530  13.934  28.027  1.00  0.00
J    H
ATOM   8227  HD2 LYS J 129      -6.555  12.314  28.804  1.00  0.00
J    H
ATOM   8228  CE  LYS J 129      -8.096  12.618  27.320  1.00  0.00
J    C
ATOM   8229  HE1 LYS J 129      -8.729  11.782  27.691  1.00  0.00
J    H
ATOM   8230  HE2 LYS J 129      -8.764  13.424  26.943  1.00  0.00
J    H
ATOM   8231  NZ  LYS J 129      -7.229  12.136  26.224  1.00  0.00
J    N
ATOM   8232  HZ1 LYS J 129      -7.813  11.768  25.445  1.00  0.00
J    H
ATOM   8233  HZ2 LYS J 129      -6.642  12.921  25.875  1.00  0.00
J    H
ATOM   8234  HZ3 LYS J 129      -6.612  11.379  26.580  1.00  0.00
J    H
```

ATOM 8235 C LYS J 129 -6.519 15.130 32.892 1.00 0.00
J C
ATOM 8236 O LYS J 129 -6.507 14.398 33.873 1.00 0.00
J O
ATOM 8237 N GLU J 130 -5.606 16.118 32.737 1.00 0.00
J N
ATOM 8238 HN GLU J 130 -5.572 16.711 31.938 1.00 0.00
J H
ATOM 8239 CA GLU J 130 -4.581 16.405 33.734 1.00 0.00
J C
ATOM 8240 HA GLU J 130 -3.976 15.515 33.857 1.00 0.00
J H
ATOM 8241 CB GLU J 130 -3.713 17.606 33.285 1.00 0.00
J C
ATOM 8242 HB1 GLU J 130 -4.393 18.454 33.040 1.00 0.00
J H
ATOM 8243 HB2 GLU J 130 -3.084 17.956 34.135 1.00 0.00
J H
ATOM 8244 CG GLU J 130 -2.819 17.310 32.058 1.00 0.00
J C
ATOM 8245 HG1 GLU J 130 -2.016 16.607 32.348 1.00 0.00
J H
ATOM 8246 HG2 GLU J 130 -3.411 16.867 31.233 1.00 0.00
J H
ATOM 8247 CD GLU J 130 -2.116 18.567 31.544 1.00 0.00
J C
ATOM 8248 OE1 GLU J 130 -2.517 19.689 31.942 1.00 0.00
J O
ATOM 8249 OE2 GLU J 130 -1.178 18.401 30.724 1.00 0.00
J O
ATOM 8250 C GLU J 130 -5.188 16.724 35.081 1.00 0.00
J C
ATOM 8251 O GLU J 130 -4.824 16.172 36.122 1.00 0.00
J O
ATOM 8252 N ARG J 131 -6.218 17.565 35.079 1.00 0.00
J N
ATOM 8253 HN ARG J 131 -6.495 17.963 34.207 1.00 0.00
J H
ATOM 8254 CA ARG J 131 -6.973 17.952 36.228 1.00 0.00
J C
ATOM 8255 HA ARG J 131 -6.264 18.310 36.965 1.00 0.00
J H
ATOM 8256 CB ARG J 131 -7.913 19.069 35.727 1.00 0.00
J C
ATOM 8257 HB1 ARG J 131 -8.500 18.632 34.883 1.00 0.00
J H
ATOM 8258 HB2 ARG J 131 -8.653 19.317 36.519 1.00 0.00
J H
ATOM 8259 CG ARG J 131 -7.127 20.325 35.227 1.00 0.00
J C
ATOM 8260 HG1 ARG J 131 -6.741 20.875 36.112 1.00 0.00
J H
ATOM 8261 HG2 ARG J 131 -6.225 19.987 34.667 1.00 0.00
J H

ATOM 8262 CD ARG J 131 -7.839 21.329 34.281 1.00 0.00
J C
ATOM 8263 HD1 ARG J 131 -7.624 21.057 33.221 1.00 0.00
J H
ATOM 8264 HD2 ARG J 131 -8.944 21.322 34.433 1.00 0.00
J H
ATOM 8265 NE ARG J 131 -7.437 22.746 34.465 1.00 0.00
J N
ATOM 8266 HE ARG J 131 -6.725 22.867 35.166 1.00 0.00
J H
ATOM 8267 CZ ARG J 131 -7.943 23.779 33.793 1.00 0.00
J C
ATOM 8268 NH1 ARG J 131 -7.314 24.916 33.907 1.00 0.00
J N
ATOM 8269 HH11 ARG J 131 -7.653 25.728 33.453 1.00 0.00
J H
ATOM 8270 HH12 ARG J 131 -6.569 24.946 34.577 1.00 0.00
J H
ATOM 8271 NH2 ARG J 131 -9.070 23.610 33.167 1.00 0.00
J N
ATOM 8272 HH21 ARG J 131 -9.426 24.331 32.589 1.00 0.00
J H
ATOM 8273 HH22 ARG J 131 -9.425 22.686 33.127 1.00 0.00
J H
ATOM 8274 C ARG J 131 -7.747 16.819 36.883 1.00 0.00
J C
ATOM 8275 O ARG J 131 -7.830 16.735 38.101 1.00 0.00
J O
ATOM 8276 N MET J 132 -8.304 15.880 36.094 1.00 0.00
J N
ATOM 8277 HN MET J 132 -8.373 15.954 35.101 1.00 0.00
J H
ATOM 8278 CA MET J 132 -8.862 14.658 36.644 1.00 0.00
J C
ATOM 8279 HA MET J 132 -9.602 14.964 37.373 1.00 0.00
J H
ATOM 8280 CB MET J 132 -9.519 13.801 35.538 1.00 0.00
J C
ATOM 8281 HB1 MET J 132 -8.773 13.705 34.715 1.00 0.00
J H
ATOM 8282 HB2 MET J 132 -9.692 12.771 35.923 1.00 0.00
J H
ATOM 8283 CG MET J 132 -10.819 14.404 34.974 1.00 0.00
J C
ATOM 8284 HG1 MET J 132 -11.637 14.323 35.720 1.00 0.00
J H
ATOM 8285 HG2 MET J 132 -10.666 15.483 34.757 1.00 0.00
J H
ATOM 8286 SD MET J 132 -11.390 13.588 33.449 1.00 0.00
J S
ATOM 8287 CE MET J 132 -12.015 12.063 34.216 1.00 0.00
J C
ATOM 8288 HE1 MET J 132 -12.424 11.370 33.448 1.00 0.00
J H

ATOM 8289 HE2 MET J 132 -11.206 11.529 34.760 1.00 0.00
J H
ATOM 8290 HE3 MET J 132 -12.827 12.284 34.942 1.00 0.00
J H
ATOM 8291 C MET J 132 -7.827 13.802 37.369 1.00 0.00
J C
ATOM 8292 O MET J 132 -8.094 13.302 38.462 1.00 0.00
J O
ATOM 8293 N LEU J 133 -6.605 13.655 36.804 1.00 0.00
J N
ATOM 8294 HN LEU J 133 -6.378 14.060 35.921 1.00 0.00
J H
ATOM 8295 CA LEU J 133 -5.514 12.904 37.415 1.00 0.00
J C
ATOM 8296 HA LEU J 133 -5.903 11.907 37.578 1.00 0.00
J H
ATOM 8297 CB LEU J 133 -4.248 12.878 36.512 1.00 0.00
J C
ATOM 8298 HB1 LEU J 133 -3.988 13.936 36.279 1.00 0.00
J H
ATOM 8299 HB2 LEU J 133 -3.393 12.473 37.099 1.00 0.00
J H
ATOM 8300 CG LEU J 133 -4.387 12.110 35.183 1.00 0.00
J C
ATOM 8301 HG LEU J 133 -5.350 12.444 34.726 1.00 0.00
J H
ATOM 8302 CD1 LEU J 133 -3.215 12.457 34.251 1.00 0.00
J C
ATOM 8303 HD11 LEU J 133 -3.280 11.873 33.309 1.00 0.00
J H
ATOM 8304 HD12 LEU J 133 -3.228 13.538 33.993 1.00 0.00
J H
ATOM 8305 HD13 LEU J 133 -2.245 12.224 34.738 1.00 0.00
J H
ATOM 8306 CD2 LEU J 133 -4.460 10.589 35.386 1.00 0.00
J C
ATOM 8307 HD21 LEU J 133 -4.578 10.074 34.408 1.00 0.00
J H
ATOM 8308 HD22 LEU J 133 -3.541 10.195 35.864 1.00 0.00
J H
ATOM 8309 HD23 LEU J 133 -5.328 10.323 36.026 1.00 0.00
J H
ATOM 8310 C LEU J 133 -5.077 13.456 38.768 1.00 0.00
J C
ATOM 8311 O LEU J 133 -4.868 12.701 39.714 1.00 0.00
J O
ATOM 8312 N VAL J 134 -4.930 14.794 38.893 1.00 0.00
J N
ATOM 8313 HN VAL J 134 -5.190 15.440 38.179 1.00 0.00
J H
ATOM 8314 CA VAL J 134 -4.373 15.412 40.094 1.00 0.00
J C
ATOM 8315 HA VAL J 134 -3.884 14.617 40.642 1.00 0.00
J H

ATOM 8316 CB VAL J 134 -3.333 16.488 39.762 1.00 0.00
J C
ATOM 8317 HB VAL J 134 -2.911 16.861 40.728 1.00 0.00
J H
ATOM 8318 CG1 VAL J 134 -2.217 15.851 38.909 1.00 0.00
J C
ATOM 8319 HG11 VAL J 134 -1.467 16.618 38.620 1.00 0.00
J H
ATOM 8320 HG12 VAL J 134 -1.679 15.056 39.465 1.00 0.00
J H
ATOM 8321 HG13 VAL J 134 -2.636 15.412 37.978 1.00 0.00
J H
ATOM 8322 CG2 VAL J 134 -3.962 17.709 39.053 1.00 0.00
J C
ATOM 8323 HG21 VAL J 134 -3.182 18.454 38.788 1.00 0.00
J H
ATOM 8324 HG22 VAL J 134 -4.477 17.395 38.120 1.00 0.00
J H
ATOM 8325 HG23 VAL J 134 -4.701 18.216 39.707 1.00 0.00
J H
ATOM 8326 C VAL J 134 -5.416 16.026 41.015 1.00 0.00
J C
ATOM 8327 O VAL J 134 -5.074 16.533 42.080 1.00 0.00
J O
ATOM 8328 N GLN J 135 -6.712 15.984 40.634 1.00 0.00
J N
ATOM 8329 HN GLN J 135 -6.967 15.522 39.787 1.00 0.00
J H
ATOM 8330 CA GLN J 135 -7.832 16.572 41.363 1.00 0.00
J C
ATOM 8331 HA GLN J 135 -8.712 16.362 40.766 1.00 0.00
J H
ATOM 8332 CB GLN J 135 -8.110 15.855 42.716 1.00 0.00
J C
ATOM 8333 HB1 GLN J 135 -7.201 15.992 43.345 1.00 0.00
J H
ATOM 8334 HB2 GLN J 135 -8.937 16.379 43.247 1.00 0.00
J H
ATOM 8335 CG GLN J 135 -8.420 14.341 42.596 1.00 0.00
J C
ATOM 8336 HG1 GLN J 135 -7.700 13.851 41.906 1.00 0.00
J H
ATOM 8337 HG2 GLN J 135 -8.336 13.844 43.585 1.00 0.00
J H
ATOM 8338 CD GLN J 135 -9.815 14.076 42.027 1.00 0.00
J C
ATOM 8339 OE1 GLN J 135 -10.828 14.350 42.670 1.00 0.00
J O
ATOM 8340 NE2 GLN J 135 -9.904 13.504 40.806 1.00 0.00
J N
ATOM 8341 HE21 GLN J 135 -10.820 13.344 40.450 1.00 0.00
J H
ATOM 8342 HE22 GLN J 135 -9.076 13.251 40.317 1.00 0.00
J H

ATOM 8343 C GLN J 135 -7.741 18.093 41.531 1.00 0.00
J C
ATOM 8344 O GLN J 135 -8.002 18.647 42.600 1.00 0.00
J O
ATOM 8345 N GLU J 136 -7.382 18.824 40.451 1.00 0.00
J N
ATOM 8346 HN GLU J 136 -7.109 18.393 39.596 1.00 0.00
J H
ATOM 8347 CA GLU J 136 -7.354 20.283 40.416 1.00 0.00
J C
ATOM 8348 HA GLU J 136 -6.827 20.626 41.298 1.00 0.00
J H
ATOM 8349 CB GLU J 136 -6.636 20.795 39.128 1.00 0.00
J C
ATOM 8350 HB1 GLU J 136 -5.632 20.315 39.076 1.00 0.00
J H
ATOM 8351 HB2 GLU J 136 -7.187 20.447 38.225 1.00 0.00
J H
ATOM 8352 CG GLU J 136 -6.405 22.335 38.967 1.00 0.00
J C
ATOM 8353 HG1 GLU J 136 -7.371 22.865 39.062 1.00 0.00
J H
ATOM 8354 HG2 GLU J 136 -5.703 22.714 39.735 1.00 0.00
J H
ATOM 8355 CD GLU J 136 -5.862 22.702 37.582 1.00 0.00
J C
ATOM 8356 OE1 GLU J 136 -6.471 23.541 36.849 1.00 0.00
J O
ATOM 8357 OE2 GLU J 136 -4.852 22.079 37.187 1.00 0.00
J O
ATOM 8358 C GLU J 136 -8.755 20.878 40.469 1.00 0.00
J C
ATOM 8359 O GLU J 136 -9.719 20.277 39.998 1.00 0.00
J O
ATOM 8360 N ALA J 137 -8.901 22.100 41.019 1.00 0.00
J N
ATOM 8361 HN ALA J 137 -8.149 22.543 41.508 1.00 0.00
J H
ATOM 8362 CA ALA J 137 -10.128 22.859 40.952 1.00 0.00
J C
ATOM 8363 HA ALA J 137 -10.961 22.254 40.614 1.00 0.00
J H
ATOM 8364 CB ALA J 137 -10.494 23.477 42.311 1.00 0.00
J C
ATOM 8365 HB1 ALA J 137 -10.672 22.669 43.051 1.00 0.00
J H
ATOM 8366 HB2 ALA J 137 -9.669 24.117 42.694 1.00 0.00
J H
ATOM 8367 HB3 ALA J 137 -11.417 24.093 42.245 1.00 0.00
J H
ATOM 8368 C ALA J 137 -9.950 23.956 39.920 1.00 0.00
J C
ATOM 8369 O ALA J 137 -8.996 24.734 39.948 1.00 0.00
J O

ATOM 8370 N LEU J 138 -10.860 23.981 38.939 1.00 0.00
J N
ATOM 8371 HN LEU J 138 -11.784 23.624 39.059 1.00 0.00
J H
ATOM 8372 CA LEU J 138 -10.599 24.522 37.622 1.00 0.00
J C
ATOM 8373 HA LEU J 138 -9.524 24.477 37.511 1.00 0.00
J H
ATOM 8374 CB LEU J 138 -11.431 23.772 36.542 1.00 0.00
J C
ATOM 8375 HB1 LEU J 138 -12.479 23.713 36.917 1.00 0.00
J H
ATOM 8376 HB2 LEU J 138 -11.469 24.393 35.620 1.00 0.00
J H
ATOM 8377 CG LEU J 138 -10.943 22.360 36.220 1.00 0.00
J C
ATOM 8378 HG LEU J 138 -9.930 22.669 35.865 1.00 0.00
J H
ATOM 8379 CD1 LEU J 138 -10.821 21.338 37.343 1.00 0.00
J C
ATOM 8380 HD11 LEU J 138 -10.502 20.353 36.940 1.00 0.00
J H
ATOM 8381 HD12 LEU J 138 -10.071 21.669 38.093 1.00 0.00
J H
ATOM 8382 HD13 LEU J 138 -11.796 21.203 37.858 1.00 0.00
J H
ATOM 8383 CD2 LEU J 138 -11.677 21.734 35.054 1.00 0.00
J C
ATOM 8384 HD21 LEU J 138 -11.275 20.719 34.846 1.00 0.00
J H
ATOM 8385 HD22 LEU J 138 -12.761 21.632 35.260 1.00 0.00
J H
ATOM 8386 HD23 LEU J 138 -11.551 22.352 34.138 1.00 0.00
J H
ATOM 8387 C LEU J 138 -11.016 25.951 37.429 1.00 0.00
J C
ATOM 8388 O LEU J 138 -12.207 26.239 37.285 1.00 0.00
J O
ATOM 8389 N ASN J 139 -10.058 26.871 37.272 1.00 0.00
J N
ATOM 8390 HN ASN J 139 -9.093 26.736 37.495 1.00 0.00
J H
ATOM 8391 CA ASN J 139 -10.373 28.180 36.742 1.00 0.00
J C
ATOM 8392 HA ASN J 139 -11.445 28.311 36.839 1.00 0.00
J H
ATOM 8393 CB ASN J 139 -9.615 29.305 37.481 1.00 0.00
J C
ATOM 8394 HB1 ASN J 139 -8.527 29.080 37.419 1.00 0.00
J H
ATOM 8395 HB2 ASN J 139 -9.783 30.279 36.977 1.00 0.00
J H
ATOM 8396 CG ASN J 139 -10.064 29.313 38.936 1.00 0.00
J C

```
ATOM   8397  OD1 ASN J 139     -11.257  29.361  39.233  1.00  0.00
J    O
ATOM   8398  ND2 ASN J 139      -9.105  29.289  39.885  1.00  0.00
J    N
ATOM   8399 HD21 ASN J 139      -9.403  29.283  40.836  1.00  0.00
J    H
ATOM   8400 HD22 ASN J 139      -8.145  29.248  39.624  1.00  0.00
J    H
ATOM   8401  C   ASN J 139     -10.039  28.200  35.256  1.00  0.00
J    C
ATOM   8402  O   ASN J 139      -9.075  27.572  34.804  1.00  0.00
J    O
ATOM   8403  N   GLN J 140     -10.844  28.915  34.447  1.00  0.00
J    N
ATOM   8404  HN  GLN J 140     -11.695  29.318  34.780  1.00  0.00
J    H
ATOM   8405  CA  GLN J 140     -10.569  29.166  33.047  1.00  0.00
J    C
ATOM   8406  HA  GLN J 140      -9.596  28.765  32.793  1.00  0.00
J    H
ATOM   8407  CB  GLN J 140     -11.676  28.618  32.104  1.00  0.00
J    C
ATOM   8408  HB1 GLN J 140     -12.645  29.033  32.468  1.00  0.00
J    H
ATOM   8409  HB2 GLN J 140     -11.527  29.031  31.080  1.00  0.00
J    H
ATOM   8410  CG  GLN J 140     -11.734  27.073  32.090  1.00  0.00
J    C
ATOM   8411  HG1 GLN J 140     -10.740  26.650  31.832  1.00  0.00
J    H
ATOM   8412  HG2 GLN J 140     -12.023  26.681  33.087  1.00  0.00
J    H
ATOM   8413  CD  GLN J 140     -12.719  26.541  31.044  1.00  0.00
J    C
ATOM   8414  OE1 GLN J 140     -13.183  27.222  30.137  1.00  0.00
J    O
ATOM   8415  NE2 GLN J 140     -13.036  25.227  31.154  1.00  0.00
J    N
ATOM   8416 HE21 GLN J 140     -13.672  24.860  30.481  1.00  0.00
J    H
ATOM   8417 HE22 GLN J 140     -12.650  24.685  31.892  1.00  0.00
J    H
ATOM   8418  C   GLN J 140     -10.490  30.680  32.912  1.00  0.00
J    C
ATOM   8419  O   GLN J 140     -11.329  31.351  33.515  1.00  0.00
J    O
ATOM   8420  N   PRO J 141      -9.537  31.297  32.212  1.00  0.00
J    N
ATOM   8421  CD  PRO J 141      -8.474  30.602  31.481  1.00  0.00
J    C
ATOM   8422  HD1 PRO J 141      -7.753  30.277  32.264  1.00  0.00
J    H
ATOM   8423  HD2 PRO J 141      -8.800  29.731  30.866  1.00  0.00
J    H
```

```
ATOM   8424  CA  PRO J 141      -9.652  32.692  31.799  1.00  0.00
J    C
ATOM   8425  HA  PRO J 141      -9.856  33.326  32.655  1.00  0.00
J    H
ATOM   8426  CB  PRO J 141      -8.257  32.988  31.224  1.00  0.00
J    C
ATOM   8427  HB1 PRO J 141      -7.576  33.278  32.056  1.00  0.00
J    H
ATOM   8428  HB2 PRO J 141      -8.268  33.799  30.468  1.00  0.00
J    H
ATOM   8429  CG  PRO J 141      -7.830  31.664  30.584  1.00  0.00
J    C
ATOM   8430  HG1 PRO J 141      -6.731  31.566  30.489  1.00  0.00
J    H
ATOM   8431  HG2 PRO J 141      -8.300  31.576  29.577  1.00  0.00
J    H
ATOM   8432  C   PRO J 141     -10.751  32.881  30.766  1.00  0.00
J    C
ATOM   8433  O   PRO J 141     -11.299  31.905  30.258  1.00  0.00
J    O
ATOM   8434  N   THR J 142     -11.104  34.144  30.452  1.00  0.00
J    N
ATOM   8435  HN  THR J 142     -10.629  34.920  30.865  1.00  0.00
J    H
ATOM   8436  CA  THR J 142     -12.164  34.516  29.523  1.00  0.00
J    C
ATOM   8437  HA  THR J 142     -13.073  34.187  30.008  1.00  0.00
J    H
ATOM   8438  CB  THR J 142     -12.171  36.020  29.264  1.00  0.00
J    C
ATOM   8439  HB  THR J 142     -11.189  36.257  28.788  1.00  0.00
J    H
ATOM   8440  OG1 THR J 142     -12.240  36.702  30.506  1.00  0.00
J    O
ATOM   8441  HG1 THR J 142     -12.252  37.639  30.284  1.00  0.00
J    H
ATOM   8442  CG2 THR J 142     -13.410  36.456  28.466  1.00  0.00
J    C
ATOM   8443 HG21 THR J 142     -13.343  37.529  28.188  1.00  0.00
J    H
ATOM   8444 HG22 THR J 142     -13.494  35.866  27.528  1.00  0.00
J    H
ATOM   8445 HG23 THR J 142     -14.340  36.305  29.056  1.00  0.00
J    H
ATOM   8446  C   THR J 142     -12.111  33.809  28.178  1.00  0.00
J    C
ATOM   8447  O   THR J 142     -11.275  34.096  27.324  1.00  0.00
J    O
ATOM   8448  N   ILE J 143     -13.061  32.883  27.913  1.00  0.00
J    N
ATOM   8449  HN  ILE J 143     -13.844  32.722  28.511  1.00  0.00
J    H
ATOM   8450  CA  ILE J 143     -13.030  32.034  26.727  1.00  0.00
J    C
```

```
ATOM   8451  HA  ILE J 143     -11.984  31.864  26.505  1.00  0.00
J    H
ATOM   8452  CB  ILE J 143     -13.776  30.715  26.948  1.00  0.00
J    C
ATOM   8453  HB  ILE J 143     -13.772  30.220  25.944  1.00  0.00
J    H
ATOM   8454  CG2 ILE J 143     -13.029  29.905  28.031  1.00  0.00
J    C
ATOM   8455 HG21 ILE J 143     -13.445  28.879  28.109  1.00  0.00
J    H
ATOM   8456 HG22 ILE J 143     -11.950  29.812  27.783  1.00  0.00
J    H
ATOM   8457 HG23 ILE J 143     -13.119  30.393  29.025  1.00  0.00
J    H
ATOM   8458  CG1 ILE J 143     -15.267  30.935  27.312  1.00  0.00
J    C
ATOM   8459 HG11 ILE J 143     -15.345  31.385  28.327  1.00  0.00
J    H
ATOM   8460 HG12 ILE J 143     -15.710  31.664  26.595  1.00  0.00
J    H
ATOM   8461  CD  ILE J 143     -16.091  29.643  27.264  1.00  0.00
J    C
ATOM   8462  HD1 ILE J 143     -17.160  29.860  27.475  1.00  0.00
J    H
ATOM   8463  HD2 ILE J 143     -16.022  29.172  26.259  1.00  0.00
J    H
ATOM   8464  HD3 ILE J 143     -15.732  28.913  28.020  1.00  0.00
J    H
ATOM   8465  C   ILE J 143     -13.628  32.738  25.516  1.00  0.00
J    C
ATOM   8466  O   ILE J 143     -13.687  32.201  24.412  1.00  0.00
J    O
ATOM   8467  N   GLY J 144     -14.021  34.015  25.699  1.00  0.00
J    N
ATOM   8468  HN  GLY J 144     -13.905  34.406  26.611  1.00  0.00
J    H
ATOM   8469  CA  GLY J 144     -14.605  34.903  24.702  1.00  0.00
J    C
ATOM   8470  HA1 GLY J 144     -15.048  35.723  25.248  1.00  0.00
J    H
ATOM   8471  HA2 GLY J 144     -15.314  34.310  24.139  1.00  0.00
J    H
ATOM   8472  C   GLY J 144     -13.606  35.473  23.733  1.00  0.00
J    C
ATOM   8473  O   GLY J 144     -13.968  36.204  22.821  1.00  0.00
J    O
ATOM   8474  N   GLY J 145     -12.309  35.131  23.867  1.00  0.00
J    N
ATOM   8475  HN  GLY J 145     -11.997  34.535  24.606  1.00  0.00
J    H
ATOM   8476  CA  GLY J 145     -11.269  35.601  22.953  1.00  0.00
J    C
ATOM   8477  HA1 GLY J 145     -10.321  35.371  23.420  1.00  0.00
J    H
```

ATOM 8478 HA2 GLY J 145 −11.443 36.657 22.797 1.00 0.00
J H
ATOM 8479 C GLY J 145 −11.302 34.919 21.603 1.00 0.00
J C
ATOM 8480 O GLY J 145 −10.580 35.307 20.691 1.00 0.00
J O
ATOM 8481 N VAL J 146 −12.185 33.912 21.421 1.00 0.00
J N
ATOM 8482 HN VAL J 146 −12.767 33.629 22.179 1.00 0.00
J H
ATOM 8483 CA VAL J 146 −12.394 33.159 20.186 1.00 0.00
J C
ATOM 8484 HA VAL J 146 −11.454 32.669 19.964 1.00 0.00
J H
ATOM 8485 CB VAL J 146 −13.505 32.127 20.396 1.00 0.00
J C
ATOM 8486 HB VAL J 146 −14.383 32.661 20.837 1.00 0.00
J H
ATOM 8487 CG1 VAL J 146 −13.966 31.456 19.080 1.00 0.00
J C
ATOM 8488 HG11 VAL J 146 −14.743 30.690 19.290 1.00 0.00
J H
ATOM 8489 HG12 VAL J 146 −14.408 32.192 18.378 1.00 0.00
J H
ATOM 8490 HG13 VAL J 146 −13.113 30.951 18.577 1.00 0.00
J H
ATOM 8491 CG2 VAL J 146 −12.989 31.047 21.368 1.00 0.00
J C
ATOM 8492 HG21 VAL J 146 −13.754 30.255 21.517 1.00 0.00
J H
ATOM 8493 HG22 VAL J 146 −12.066 30.573 20.970 1.00 0.00
J H
ATOM 8494 HG23 VAL J 146 −12.757 31.480 22.363 1.00 0.00
J H
ATOM 8495 C VAL J 146 −12.742 34.042 18.995 1.00 0.00
J C
ATOM 8496 O VAL J 146 −12.135 33.933 17.932 1.00 0.00
J O
ATOM 8497 N ILE J 147 −13.681 34.998 19.156 1.00 0.00
J N
ATOM 8498 HN ILE J 147 −14.191 35.091 20.009 1.00 0.00
J H
ATOM 8499 CA ILE J 147 −14.043 35.973 18.133 1.00 0.00
J C
ATOM 8500 HA ILE J 147 −14.302 35.395 17.255 1.00 0.00
J H
ATOM 8501 CB ILE J 147 −15.274 36.796 18.527 1.00 0.00
J C
ATOM 8502 HB ILE J 147 −15.288 37.645 17.798 1.00 0.00
J H
ATOM 8503 CG2 ILE J 147 −16.510 35.870 18.428 1.00 0.00
J C
ATOM 8504 HG21 ILE J 147 −17.446 36.453 18.551 1.00 0.00
J H

```
ATOM   8505 HG22 ILE J 147     -16.552  35.371  17.437  1.00  0.00
J    H
ATOM   8506 HG23 ILE J 147     -16.483  35.086  19.214  1.00  0.00
J    H
ATOM   8507  CG1 ILE J 147     -15.191  37.466  19.927  1.00  0.00
J    C
ATOM   8508 HG11 ILE J 147     -16.184  37.417  20.428  1.00  0.00
J    H
ATOM   8509 HG12 ILE J 147     -14.475  36.894  20.560  1.00  0.00
J    H
ATOM   8510  CD  ILE J 147     -14.728  38.932  19.917  1.00  0.00
J    C
ATOM   8511  HD1 ILE J 147     -14.640  39.316  20.956  1.00  0.00
J    H
ATOM   8512  HD2 ILE J 147     -13.735  39.025  19.427  1.00  0.00
J    H
ATOM   8513  HD3 ILE J 147     -15.452  39.574  19.373  1.00  0.00
J    H
ATOM   8514  C   ILE J 147     -12.878  36.871  17.741  1.00  0.00
J    C
ATOM   8515  O   ILE J 147     -12.703  37.232  16.576  1.00  0.00
J    O
ATOM   8516  N   GLY J 148     -12.005  37.199  18.718  1.00  0.00
J    N
ATOM   8517  HN  GLY J 148     -12.181  36.915  19.659  1.00  0.00
J    H
ATOM   8518  CA  GLY J 148     -10.785  37.961  18.508  1.00  0.00
J    C
ATOM   8519  HA1 GLY J 148     -10.372  38.170  19.485  1.00  0.00
J    H
ATOM   8520  HA2 GLY J 148     -11.051  38.842  17.940  1.00  0.00
J    H
ATOM   8521  C   GLY J 148      -9.755  37.195  17.719  1.00  0.00
J    C
ATOM   8522  O   GLY J 148      -9.149  37.736  16.799  1.00  0.00
J    O
ATOM   8523  N   LEU J 149      -9.565  35.900  18.030  1.00  0.00
J    N
ATOM   8524  HN  LEU J 149     -10.025  35.490  18.814  1.00  0.00
J    H
ATOM   8525  CA  LEU J 149      -8.706  34.983  17.295  1.00  0.00
J    C
ATOM   8526  HA  LEU J 149      -7.736  35.462  17.262  1.00  0.00
J    H
ATOM   8527  CB  LEU J 149      -8.596  33.621  18.027  1.00  0.00
J    C
ATOM   8528  HB1 LEU J 149      -9.635  33.251  18.192  1.00  0.00
J    H
ATOM   8529  HB2 LEU J 149      -8.112  32.884  17.349  1.00  0.00
J    H
ATOM   8530  CG  LEU J 149      -7.862  33.690  19.383  1.00  0.00
J    C
ATOM   8531  HG  LEU J 149      -8.359  34.501  19.967  1.00  0.00
J    H
```

ATOM 8532 CD1 LEU J 149 -7.998 32.351 20.126 1.00 0.00
J C
ATOM 8533 HD11 LEU J 149 -7.431 32.377 21.082 1.00 0.00
J H
ATOM 8534 HD12 LEU J 149 -9.064 32.139 20.357 1.00 0.00
J H
ATOM 8535 HD13 LEU J 149 -7.601 31.518 19.509 1.00 0.00
J H
ATOM 8536 CD2 LEU J 149 -6.376 34.058 19.225 1.00 0.00
J C
ATOM 8537 HD21 LEU J 149 -5.886 34.116 20.221 1.00 0.00
J H
ATOM 8538 HD22 LEU J 149 -5.829 33.304 18.623 1.00 0.00
J H
ATOM 8539 HD23 LEU J 149 -6.271 35.047 18.729 1.00 0.00
J H
ATOM 8540 C LEU J 149 -9.149 34.740 15.856 1.00 0.00
J C
ATOM 8541 O LEU J 149 -8.333 34.780 14.937 1.00 0.00
J O
ATOM 8542 N VAL J 150 -10.465 34.533 15.610 1.00 0.00
J N
ATOM 8543 HN VAL J 150 -11.136 34.477 16.345 1.00 0.00
J H
ATOM 8544 CA VAL J 150 -11.022 34.372 14.267 1.00 0.00
J C
ATOM 8545 HA VAL J 150 -10.495 33.548 13.804 1.00 0.00
J H
ATOM 8546 CB VAL J 150 -12.520 34.039 14.303 1.00 0.00
J C
ATOM 8547 HB VAL J 150 -13.027 34.832 14.906 1.00 0.00
J H
ATOM 8548 CG1 VAL J 150 -13.131 34.027 12.882 1.00 0.00
J C
ATOM 8549 HG11 VAL J 150 -14.206 33.746 12.927 1.00 0.00
J H
ATOM 8550 HG12 VAL J 150 -13.071 35.026 12.401 1.00 0.00
J H
ATOM 8551 HG13 VAL J 150 -12.610 33.289 12.237 1.00 0.00
J H
ATOM 8552 CG2 VAL J 150 -12.711 32.649 14.940 1.00 0.00
J C
ATOM 8553 HG21 VAL J 150 -13.784 32.362 14.942 1.00 0.00
J H
ATOM 8554 HG22 VAL J 150 -12.145 31.879 14.372 1.00 0.00
J H
ATOM 8555 HG23 VAL J 150 -12.358 32.637 15.991 1.00 0.00
J H
ATOM 8556 C VAL J 150 -10.798 35.604 13.400 1.00 0.00
J C
ATOM 8557 O VAL J 150 -10.337 35.502 12.261 1.00 0.00
J O
ATOM 8558 N LYS J 151 -11.059 36.822 13.926 1.00 0.00
J N

ATOM 8559 HN LYS J 151 -11.440 36.959 14.840 1.00 0.00
J H
ATOM 8560 CA LYS J 151 -10.794 38.040 13.179 1.00 0.00
J C
ATOM 8561 HA LYS J 151 -11.267 37.904 12.215 1.00 0.00
J H
ATOM 8562 CB LYS J 151 -11.486 39.301 13.768 1.00 0.00
J C
ATOM 8563 HB1 LYS J 151 -11.392 40.111 13.009 1.00 0.00
J H
ATOM 8564 HB2 LYS J 151 -12.575 39.099 13.876 1.00 0.00
J H
ATOM 8565 CG LYS J 151 -10.903 39.803 15.098 1.00 0.00
J C
ATOM 8566 HG1 LYS J 151 -11.298 39.176 15.929 1.00 0.00
J H
ATOM 8567 HG2 LYS J 151 -9.796 39.673 15.081 1.00 0.00
J H
ATOM 8568 CD LYS J 151 -11.239 41.272 15.409 1.00 0.00
J C
ATOM 8569 HD1 LYS J 151 -11.472 41.790 14.451 1.00 0.00
J H
ATOM 8570 HD2 LYS J 151 -12.163 41.291 16.032 1.00 0.00
J H
ATOM 8571 CE LYS J 151 -10.121 42.027 16.143 1.00 0.00
J C
ATOM 8572 HE1 LYS J 151 -10.449 43.060 16.395 1.00 0.00
J H
ATOM 8573 HE2 LYS J 151 -9.851 41.504 17.087 1.00 0.00
J H
ATOM 8574 NZ LYS J 151 -8.925 42.100 15.271 1.00 0.00
J N
ATOM 8575 HZ1 LYS J 151 -8.160 42.610 15.757 1.00 0.00
J H
ATOM 8576 HZ2 LYS J 151 -8.605 41.138 15.039 1.00 0.00
J H
ATOM 8577 HZ3 LYS J 151 -9.169 42.599 14.391 1.00 0.00
J H
ATOM 8578 C LYS J 151 -9.305 38.304 12.925 1.00 0.00
J C
ATOM 8579 O LYS J 151 -8.943 38.837 11.881 1.00 0.00
J O
ATOM 8580 N VAL J 152 -8.397 37.945 13.873 1.00 0.00
J N
ATOM 8581 HN VAL J 152 -8.683 37.616 14.769 1.00 0.00
J H
ATOM 8582 CA VAL J 152 -6.945 38.002 13.684 1.00 0.00
J C
ATOM 8583 HA VAL J 152 -6.701 39.009 13.368 1.00 0.00
J H
ATOM 8584 CB VAL J 152 -6.185 37.711 14.986 1.00 0.00
J C
ATOM 8585 HB VAL J 152 -6.626 36.787 15.435 1.00 0.00
J H

ATOM 8586 CG1 VAL J 152 -4.674 37.462 14.753 1.00 0.00
J C
ATOM 8587 HG11 VAL J 152 -4.157 37.295 15.723 1.00 0.00
J H
ATOM 8588 HG12 VAL J 152 -4.498 36.564 14.126 1.00 0.00
J H
ATOM 8589 HG13 VAL J 152 -4.203 38.339 14.259 1.00 0.00
J H
ATOM 8590 CG2 VAL J 152 -6.344 38.913 15.941 1.00 0.00
J C
ATOM 8591 HG21 VAL J 152 -5.771 38.749 16.878 1.00 0.00
J H
ATOM 8592 HG22 VAL J 152 -5.972 39.844 15.462 1.00 0.00
J H
ATOM 8593 HG23 VAL J 152 -7.407 39.065 16.221 1.00 0.00
J H
ATOM 8594 C VAL J 152 -6.490 37.057 12.584 1.00 0.00
J C
ATOM 8595 O VAL J 152 -5.736 37.454 11.695 1.00 0.00
J O
ATOM 8596 N LEU J 153 -6.984 35.801 12.572 1.00 0.00
J N
ATOM 8597 HN LEU J 153 -7.590 35.460 13.288 1.00 0.00
J H
ATOM 8598 CA LEU J 153 -6.688 34.833 11.531 1.00 0.00
J C
ATOM 8599 HA LEU J 153 -5.608 34.759 11.499 1.00 0.00
J H
ATOM 8600 CB LEU J 153 -7.312 33.455 11.864 1.00 0.00
J C
ATOM 8601 HB1 LEU J 153 -8.331 33.646 12.273 1.00 0.00
J H
ATOM 8602 HB2 LEU J 153 -7.461 32.887 10.919 1.00 0.00
J H
ATOM 8603 CG LEU J 153 -6.503 32.630 12.883 1.00 0.00
J C
ATOM 8604 HG LEU J 153 -6.367 33.241 13.807 1.00 0.00
J H
ATOM 8605 CD1 LEU J 153 -7.243 31.324 13.206 1.00 0.00
J C
ATOM 8606 HD11 LEU J 153 -6.646 30.699 13.903 1.00 0.00
J H
ATOM 8607 HD12 LEU J 153 -8.224 31.538 13.683 1.00 0.00
J H
ATOM 8608 HD13 LEU J 153 -7.424 30.736 12.282 1.00 0.00
J H
ATOM 8609 CD2 LEU J 153 -5.114 32.290 12.331 1.00 0.00
J C
ATOM 8610 HD21 LEU J 153 -4.532 31.711 13.081 1.00 0.00
J H
ATOM 8611 HD22 LEU J 153 -5.178 31.679 11.408 1.00 0.00
J H
ATOM 8612 HD23 LEU J 153 -4.551 33.219 12.095 1.00 0.00
J H

```
ATOM   8613  C   LEU J 153      -7.158  35.262  10.155  1.00  0.00
J    C
ATOM   8614  O   LEU J 153      -6.413  35.177   9.185  1.00  0.00
J    O
ATOM   8615  N   PHE J 154      -8.391  35.798  10.047  1.00  0.00
J    N
ATOM   8616  HN  PHE J 154      -9.037  35.831  10.808  1.00  0.00
J    H
ATOM   8617  CA  PHE J 154      -8.890  36.375   8.810  1.00  0.00
J    C
ATOM   8618  HA  PHE J 154      -8.806  35.591   8.068  1.00  0.00
J    H
ATOM   8619  CB  PHE J 154     -10.356  36.854   9.044  1.00  0.00
J    C
ATOM   8620  HB1 PHE J 154     -10.999  35.970   9.247  1.00  0.00
J    H
ATOM   8621  HB2 PHE J 154     -10.422  37.520   9.932  1.00  0.00
J    H
ATOM   8622  CG  PHE J 154     -10.946  37.550   7.834  1.00  0.00
J    C
ATOM   8623  CD1 PHE J 154     -10.817  38.939   7.621  1.00  0.00
J    C
ATOM   8624  HD1 PHE J 154     -10.367  39.559   8.386  1.00  0.00
J    H
ATOM   8625  CE1 PHE J 154     -11.275  39.522   6.429  1.00  0.00
J    C
ATOM   8626  HE1 PHE J 154     -11.122  40.577   6.250  1.00  0.00
J    H
ATOM   8627  CZ  PHE J 154     -11.923  38.732   5.471  1.00  0.00
J    C
ATOM   8628  HZ  PHE J 154     -12.300  39.183   4.564  1.00  0.00
J    H
ATOM   8629  CD2 PHE J 154     -11.554  36.774   6.844  1.00  0.00
J    C
ATOM   8630  HD2 PHE J 154     -11.648  35.705   6.977  1.00  0.00
J    H
ATOM   8631  CE2 PHE J 154     -12.072  37.358   5.684  1.00  0.00
J    C
ATOM   8632  HE2 PHE J 154     -12.587  36.746   4.956  1.00  0.00
J    H
ATOM   8633  C   PHE J 154      -8.038  37.545   8.324  1.00  0.00
J    C
ATOM   8634  O   PHE J 154      -7.644  37.604   7.160  1.00  0.00
J    O
ATOM   8635  N   LEU J 155      -7.714  38.488   9.230  1.00  0.00
J    N
ATOM   8636  HN  LEU J 155      -8.011  38.414  10.180  1.00  0.00
J    H
ATOM   8637  CA  LEU J 155      -6.930  39.674   8.942  1.00  0.00
J    C
ATOM   8638  HA  LEU J 155      -7.436  40.175   8.127  1.00  0.00
J    H
ATOM   8639  CB  LEU J 155      -6.895  40.595  10.186  1.00  0.00
J    C
```

```
ATOM   8640  HB1 LEU J 155      -7.908  41.050  10.288  1.00  0.00
J    H
ATOM   8641  HB2 LEU J 155      -6.747  39.970  11.095  1.00  0.00
J    H
ATOM   8642  CG  LEU J 155      -5.859  41.742  10.172  1.00  0.00
J    C
ATOM   8643  HG  LEU J 155      -4.864  41.238  10.107  1.00  0.00
J    H
ATOM   8644  CD1 LEU J 155      -6.028  42.711   8.987  1.00  0.00
J    C
ATOM   8645 HD11 LEU J 155      -5.291  43.540   9.053  1.00  0.00
J    H
ATOM   8646 HD12 LEU J 155      -5.873  42.181   8.023  1.00  0.00
J    H
ATOM   8647 HD13 LEU J 155      -7.046  43.153   8.985  1.00  0.00
J    H
ATOM   8648  CD2 LEU J 155      -5.918  42.506  11.501  1.00  0.00
J    C
ATOM   8649 HD21 LEU J 155      -5.164  43.322  11.512  1.00  0.00
J    H
ATOM   8650 HD22 LEU J 155      -6.913  42.965  11.666  1.00  0.00
J    H
ATOM   8651 HD23 LEU J 155      -5.703  41.823  12.353  1.00  0.00
J    H
ATOM   8652  C   LEU J 155      -5.520  39.351   8.477  1.00  0.00
J    C
ATOM   8653  O   LEU J 155      -5.031  39.922   7.506  1.00  0.00
J    O
ATOM   8654  N   PHE J 156      -4.839  38.390   9.130  1.00  0.00
J    N
ATOM   8655  HN  PHE J 156      -5.194  37.942   9.949  1.00  0.00
J    H
ATOM   8656  CA  PHE J 156      -3.534  37.920   8.708  1.00  0.00
J    C
ATOM   8657  HA  PHE J 156      -2.900  38.796   8.662  1.00  0.00
J    H
ATOM   8658  CB  PHE J 156      -3.011  36.892   9.762  1.00  0.00
J    C
ATOM   8659  HB1 PHE J 156      -2.762  37.431  10.702  1.00  0.00
J    H
ATOM   8660  HB2 PHE J 156      -3.792  36.142  10.011  1.00  0.00
J    H
ATOM   8661  CG  PHE J 156      -1.749  36.194   9.310  1.00  0.00
J    C
ATOM   8662  CD1 PHE J 156      -1.853  35.020   8.548  1.00  0.00
J    C
ATOM   8663  HD1 PHE J 156      -2.824  34.570   8.388  1.00  0.00
J    H
ATOM   8664  CE1 PHE J 156      -0.722  34.415   7.993  1.00  0.00
J    C
ATOM   8665  HE1 PHE J 156      -0.825  33.519   7.397  1.00  0.00
J    H
ATOM   8666  CZ  PHE J 156       0.541  34.979   8.207  1.00  0.00
J    C
```

```
ATOM   8667  HZ  PHE J 156       1.422  34.513   7.790  1.00  0.00
J    H
ATOM   8668  CD2 PHE J 156      -0.480  36.758   9.512  1.00  0.00
J    C
ATOM   8669  HD2 PHE J 156      -0.373  37.649  10.117  1.00  0.00
J    H
ATOM   8670  CE2 PHE J 156       0.661  36.158   8.957  1.00  0.00
J    C
ATOM   8671  HE2 PHE J 156       1.633  36.605   9.109  1.00  0.00
J    H
ATOM   8672  C   PHE J 156      -3.579  37.324   7.296  1.00  0.00
J    C
ATOM   8673  O   PHE J 156      -2.764  37.680   6.444  1.00  0.00
J    O
ATOM   8674  N   SER J 157      -4.564  36.440   7.019  1.00  0.00
J    N
ATOM   8675  HN  SER J 157      -5.244  36.171   7.700  1.00  0.00
J    H
ATOM   8676  CA  SER J 157      -4.733  35.798   5.719  1.00  0.00
J    C
ATOM   8677  HA  SER J 157      -3.781  35.332   5.505  1.00  0.00
J    H
ATOM   8678  CB  SER J 157      -5.895  34.785   5.728  1.00  0.00
J    C
ATOM   8679  HB1 SER J 157      -6.818  35.319   6.050  1.00  0.00
J    H
ATOM   8680  HB2 SER J 157      -6.083  34.400   4.699  1.00  0.00
J    H
ATOM   8681  OG  SER J 157      -5.584  33.743   6.654  1.00  0.00
J    O
ATOM   8682  HG1 SER J 157      -6.400  33.251   6.813  1.00  0.00
J    H
ATOM   8683  C   SER J 157      -5.010  36.777   4.613  1.00  0.00
J    C
ATOM   8684  O   SER J 157      -4.277  36.824   3.628  1.00  0.00
J    O
ATOM   8685  N   ILE J 158      -6.007  37.676   4.818  1.00  0.00
J    N
ATOM   8686  HN  ILE J 158      -6.572  37.696   5.640  1.00  0.00
J    H
ATOM   8687  CA  ILE J 158      -6.338  38.699   3.842  1.00  0.00
J    C
ATOM   8688  HA  ILE J 158      -6.495  38.180   2.905  1.00  0.00
J    H
ATOM   8689  CB  ILE J 158      -7.682  39.404   4.035  1.00  0.00
J    C
ATOM   8690  HB  ILE J 158      -8.399  38.579   4.275  1.00  0.00
J    H
ATOM   8691  CG2 ILE J 158      -7.624  40.428   5.188  1.00  0.00
J    C
ATOM   8692 HG21 ILE J 158      -8.639  40.816   5.418  1.00  0.00
J    H
ATOM   8693 HG22 ILE J 158      -7.226  39.960   6.113  1.00  0.00
J    H
```

```
ATOM   8694 HG23 ILE J 158      -6.976  41.289   4.923  1.00  0.00
J    H
ATOM   8695  CG1 ILE J 158      -8.131  40.028   2.686  1.00  0.00
J    C
ATOM   8696 HG11 ILE J 158      -7.373  40.767   2.343  1.00  0.00
J    H
ATOM   8697 HG12 ILE J 158      -8.179  39.224   1.916  1.00  0.00
J    H
ATOM   8698  CD  ILE J 158      -9.499  40.714   2.730  1.00  0.00
J    C
ATOM   8699  HD1 ILE J 158      -9.777  41.084   1.720  1.00  0.00
J    H
ATOM   8700  HD2 ILE J 158     -10.284  40.004   3.069  1.00  0.00
J    H
ATOM   8701  HD3 ILE J 158      -9.487  41.583   3.421  1.00  0.00
J    H
ATOM   8702  C   ILE J 158      -5.186  39.666   3.622  1.00  0.00
J    C
ATOM   8703  O   ILE J 158      -4.875  40.042   2.500  1.00  0.00
J    O
ATOM   8704  N   SER J 159      -4.447  40.055   4.684  1.00  0.00
J    N
ATOM   8705  HN  SER J 159      -4.658  39.821   5.632  1.00  0.00
J    H
ATOM   8706  CA  SER J 159      -3.250  40.870   4.523  1.00  0.00
J    C
ATOM   8707  HA  SER J 159      -3.575  41.753   3.988  1.00  0.00
J    H
ATOM   8708  CB  SER J 159      -2.644  41.267   5.901  1.00  0.00
J    C
ATOM   8709  HB1 SER J 159      -3.453  41.720   6.518  1.00  0.00
J    H
ATOM   8710  HB2 SER J 159      -2.287  40.361   6.443  1.00  0.00
J    H
ATOM   8711  OG  SER J 159      -1.587  42.224   5.782  1.00  0.00
J    O
ATOM   8712  HG1 SER J 159      -1.424  42.577   6.666  1.00  0.00
J    H
ATOM   8713  C   SER J 159      -2.184  40.192   3.672  1.00  0.00
J    C
ATOM   8714  O   SER J 159      -1.727  40.764   2.684  1.00  0.00
J    O
ATOM   8715  N   ILE J 160      -1.799  38.923   3.943  1.00  0.00
J    N
ATOM   8716  HN  ILE J 160      -2.175  38.353   4.670  1.00  0.00
J    H
ATOM   8717  CA  ILE J 160      -0.754  38.274   3.152  1.00  0.00
J    C
ATOM   8718  HA  ILE J 160       0.067  38.980   3.146  1.00  0.00
J    H
ATOM   8719  CB  ILE J 160      -0.117  37.053   3.825  1.00  0.00
J    C
ATOM   8720  HB  ILE J 160       0.197  37.422   4.834  1.00  0.00
J    H
```

```
ATOM   8721  CG2 ILE J 160      -1.122  35.889   3.950  1.00  0.00
J    C
ATOM   8722 HG21 ILE J 160      -0.700  35.074   4.575  1.00  0.00
J    H
ATOM   8723 HG22 ILE J 160      -2.063  36.229   4.432  1.00  0.00
J    H
ATOM   8724 HG23 ILE J 160      -1.370  35.469   2.952  1.00  0.00
J    H
ATOM   8725  CG1 ILE J 160       1.187  36.585   3.120  1.00  0.00
J    C
ATOM   8726 HG11 ILE J 160       1.611  35.707   3.657  1.00  0.00
J    H
ATOM   8727 HG12 ILE J 160       0.938  36.250   2.087  1.00  0.00
J    H
ATOM   8728  CD  ILE J 160       2.284  37.658   3.006  1.00  0.00
J    C
ATOM   8729  HD1 ILE J 160       3.154  37.261   2.441  1.00  0.00
J    H
ATOM   8730  HD2 ILE J 160       1.902  38.554   2.471  1.00  0.00
J    H
ATOM   8731  HD3 ILE J 160       2.640  37.972   4.010  1.00  0.00
J    H
ATOM   8732  C   ILE J 160      -1.127  38.016   1.688  1.00  0.00
J    C
ATOM   8733  O   ILE J 160      -0.317  38.254   0.790  1.00  0.00
J    O
ATOM   8734  N   GLU J 161      -2.377  37.583   1.388  1.00  0.00
J    N
ATOM   8735  HN  GLU J 161      -3.039  37.316   2.082  1.00  0.00
J    H
ATOM   8736  CA  GLU J 161      -2.872  37.469   0.020  1.00  0.00
J    C
ATOM   8737  HA  GLU J 161      -2.173  36.853  -0.531  1.00  0.00
J    H
ATOM   8738  CB  GLU J 161      -4.207  36.657  -0.065  1.00  0.00
J    C
ATOM   8739  HB1 GLU J 161      -4.408  36.430  -1.138  1.00  0.00
J    H
ATOM   8740  HB2 GLU J 161      -4.082  35.671   0.436  1.00  0.00
J    H
ATOM   8741  CG  GLU J 161      -5.428  37.399   0.529  1.00  0.00
J    C
ATOM   8742  HG1 GLU J 161      -5.130  37.911   1.462  1.00  0.00
J    H
ATOM   8743  HG2 GLU J 161      -5.826  38.146  -0.186  1.00  0.00
J    H
ATOM   8744  CD  GLU J 161      -6.632  36.552   0.961  1.00  0.00
J    C
ATOM   8745  OE1 GLU J 161      -6.482  35.699   1.870  1.00  0.00
J    O
ATOM   8746  OE2 GLU J 161      -7.754  36.855   0.478  1.00  0.00
J    O
ATOM   8747  C   GLU J 161      -2.942  38.831  -0.692  1.00  0.00
J    C
```

```
ATOM   8748  O   GLU J 161      -2.536  38.948  -1.848  1.00  0.00
J    O
ATOM   8749  N   LEU J 162      -3.373  39.929  -0.015  1.00  0.00
J    N
ATOM   8750  HN  LEU J 162      -3.757  39.877   0.905  1.00  0.00
J    H
ATOM   8751  CA  LEU J 162      -3.315  41.280  -0.566  1.00  0.00
J    C
ATOM   8752  HA  LEU J 162      -3.849  41.227  -1.506  1.00  0.00
J    H
ATOM   8753  CB  LEU J 162      -3.977  42.327   0.373  1.00  0.00
J    C
ATOM   8754  HB1 LEU J 162      -3.541  42.180   1.388  1.00  0.00
J    H
ATOM   8755  HB2 LEU J 162      -3.671  43.347   0.052  1.00  0.00
J    H
ATOM   8756  CG  LEU J 162      -5.516  42.246   0.487  1.00  0.00
J    C
ATOM   8757  HG  LEU J 162      -5.740  41.176   0.716  1.00  0.00
J    H
ATOM   8758  CD1 LEU J 162      -5.993  43.135   1.650  1.00  0.00
J    C
ATOM   8759 HD11 LEU J 162      -7.103  43.127   1.716  1.00  0.00
J    H
ATOM   8760 HD12 LEU J 162      -5.583  42.770   2.616  1.00  0.00
J    H
ATOM   8761 HD13 LEU J 162      -5.663  44.185   1.502  1.00  0.00
J    H
ATOM   8762  CD2 LEU J 162      -6.245  42.616  -0.811  1.00  0.00
J    C
ATOM   8763 HD21 LEU J 162      -7.345  42.522  -0.676  1.00  0.00
J    H
ATOM   8764 HD22 LEU J 162      -6.032  43.659  -1.118  1.00  0.00
J    H
ATOM   8765 HD23 LEU J 162      -5.938  41.938  -1.636  1.00  0.00
J    H
ATOM   8766  C   LEU J 162      -1.903  41.782  -0.866  1.00  0.00
J    C
ATOM   8767  O   LEU J 162      -1.651  42.292  -1.955  1.00  0.00
J    O
ATOM   8768  N   ILE J 163      -0.925  41.623   0.059  1.00  0.00
J    N
ATOM   8769  HN  ILE J 163      -1.092  41.214   0.954  1.00  0.00
J    H
ATOM   8770  CA  ILE J 163       0.467  42.033  -0.166  1.00  0.00
J    C
ATOM   8771  HA  ILE J 163       0.433  43.075  -0.461  1.00  0.00
J    H
ATOM   8772  CB  ILE J 163       1.348  41.885   1.087  1.00  0.00
J    C
ATOM   8773  HB  ILE J 163       1.218  40.816   1.396  1.00  0.00
J    H
ATOM   8774  CG2 ILE J 163       2.825  42.244   0.784  1.00  0.00
J    C
```

```
ATOM   8775 HG21 ILE J 163       3.474  41.985   1.647  1.00  0.00
J    H
ATOM   8776 HG22 ILE J 163       3.197  41.682  -0.097  1.00  0.00
J    H
ATOM   8777 HG23 ILE J 163       2.936  43.330   0.580  1.00  0.00
J    H
ATOM   8778  CG1 ILE J 163       0.850  42.735   2.288  1.00  0.00
J    C
ATOM   8779 HG11 ILE J 163      -0.133  42.349   2.640  1.00  0.00
J    H
ATOM   8780 HG12 ILE J 163       1.568  42.623   3.132  1.00  0.00
J    H
ATOM   8781  CD  ILE J 163       0.695  44.241   2.032  1.00  0.00
J    C
ATOM   8782  HD1 ILE J 163       0.391  44.762   2.966  1.00  0.00
J    H
ATOM   8783  HD2 ILE J 163       1.655  44.681   1.685  1.00  0.00
J    H
ATOM   8784  HD3 ILE J 163      -0.081  44.437   1.263  1.00  0.00
J    H
ATOM   8785  C   ILE J 163       1.088  41.266  -1.326  1.00  0.00
J    C
ATOM   8786  O   ILE J 163       1.736  41.834  -2.208  1.00  0.00
J    O
ATOM   8787  N   ALA J 164       0.852  39.943  -1.390  1.00  0.00
J    N
ATOM   8788  HN  ALA J 164       0.335  39.481  -0.671  1.00  0.00
J    H
ATOM   8789  CA  ALA J 164       1.310  39.095  -2.467  1.00  0.00
J    C
ATOM   8790  HA  ALA J 164       2.385  39.195  -2.564  1.00  0.00
J    H
ATOM   8791  CB  ALA J 164       0.970  37.651  -2.113  1.00  0.00
J    C
ATOM   8792  HB1 ALA J 164       1.442  37.383  -1.144  1.00  0.00
J    H
ATOM   8793  HB2 ALA J 164      -0.128  37.514  -2.009  1.00  0.00
J    H
ATOM   8794  HB3 ALA J 164       1.343  36.942  -2.884  1.00  0.00
J    H
ATOM   8795  C   ALA J 164       0.721  39.442  -3.828  1.00  0.00
J    C
ATOM   8796  O   ALA J 164       1.424  39.442  -4.837  1.00  0.00
J    O
ATOM   8797  N   ALA J 165      -0.585  39.803  -3.867  1.00  0.00
J    N
ATOM   8798  HN  ALA J 165      -1.157  39.708  -3.054  1.00  0.00
J    H
ATOM   8799  CA  ALA J 165      -1.252  40.336  -5.038  1.00  0.00
J    C
ATOM   8800  HA  ALA J 165      -1.208  39.624  -5.854  1.00  0.00
J    H
ATOM   8801  CB  ALA J 165      -2.745  40.621  -4.737  1.00  0.00
J    C
```

```
ATOM   8802  HB1 ALA J 165      -3.246  39.682  -4.417  1.00  0.00
J    H
ATOM   8803  HB2 ALA J 165      -2.853  41.362  -3.915  1.00  0.00
J    H
ATOM   8804  HB3 ALA J 165      -3.278  41.007  -5.633  1.00  0.00
J    H
ATOM   8805  C   ALA J 165      -0.579  41.608  -5.541  1.00  0.00
J    C
ATOM   8806  O   ALA J 165      -0.375  41.794  -6.737  1.00  0.00
J    O
ATOM   8807  N   LEU J 166      -0.145  42.531  -4.663  1.00  0.00
J    N
ATOM   8808  HN  LEU J 166      -0.283  42.492  -3.676  1.00  0.00
J    H
ATOM   8809  CA  LEU J 166       0.588  43.701  -5.119  1.00  0.00
J    C
ATOM   8810  HA  LEU J 166      -0.057  44.172  -5.850  1.00  0.00
J    H
ATOM   8811  CB  LEU J 166       0.802  44.736  -3.995  1.00  0.00
J    C
ATOM   8812  HB1 LEU J 166       1.448  44.255  -3.224  1.00  0.00
J    H
ATOM   8813  HB2 LEU J 166       1.389  45.591  -4.398  1.00  0.00
J    H
ATOM   8814  CG  LEU J 166      -0.508  45.219  -3.337  1.00  0.00
J    C
ATOM   8815  HG  LEU J 166      -0.959  44.293  -2.904  1.00  0.00
J    H
ATOM   8816  CD1 LEU J 166      -0.192  46.213  -2.214  1.00  0.00
J    C
ATOM   8817 HD11 LEU J 166      -1.130  46.604  -1.764  1.00  0.00
J    H
ATOM   8818 HD12 LEU J 166       0.402  45.722  -1.413  1.00  0.00
J    H
ATOM   8819 HD13 LEU J 166       0.392  47.074  -2.605  1.00  0.00
J    H
ATOM   8820  CD2 LEU J 166      -1.522  45.828  -4.325  1.00  0.00
J    C
ATOM   8821 HD21 LEU J 166      -2.445  46.137  -3.789  1.00  0.00
J    H
ATOM   8822 HD22 LEU J 166      -1.112  46.723  -4.833  1.00  0.00
J    H
ATOM   8823 HD23 LEU J 166      -1.804  45.084  -5.102  1.00  0.00
J    H
ATOM   8824  C   LEU J 166       1.917  43.392  -5.806  1.00  0.00
J    C
ATOM   8825  O   LEU J 166       2.210  43.957  -6.855  1.00  0.00
J    O
ATOM   8826  N   ILE J 167       2.722  42.444  -5.276  1.00  0.00
J    N
ATOM   8827  HN  ILE J 167       2.511  41.992  -4.412  1.00  0.00
J    H
ATOM   8828  CA  ILE J 167       3.965  41.988  -5.905  1.00  0.00
J    C
```

ATOM 8829 HA ILE J 167 4.541 42.884 -6.098 1.00 0.00
J H
ATOM 8830 CB ILE J 167 4.728 41.008 -5.006 1.00 0.00
J C
ATOM 8831 HB ILE J 167 4.011 40.164 -4.840 1.00 0.00
J H
ATOM 8832 CG2 ILE J 167 6.038 40.535 -5.687 1.00 0.00
J C
ATOM 8833 HG21 ILE J 167 6.506 39.713 -5.106 1.00 0.00
J H
ATOM 8834 HG22 ILE J 167 5.837 40.151 -6.710 1.00 0.00
J H
ATOM 8835 HG23 ILE J 167 6.770 41.366 -5.761 1.00 0.00
J H
ATOM 8836 CG1 ILE J 167 5.018 41.611 -3.607 1.00 0.00
J C
ATOM 8837 HG11 ILE J 167 4.061 41.863 -3.099 1.00 0.00
J H
ATOM 8838 HG12 ILE J 167 5.530 40.844 -2.982 1.00 0.00
J H
ATOM 8839 CD ILE J 167 5.905 42.865 -3.624 1.00 0.00
J C
ATOM 8840 HD1 ILE J 167 6.115 43.203 -2.586 1.00 0.00
J H
ATOM 8841 HD2 ILE J 167 6.875 42.653 -4.123 1.00 0.00
J H
ATOM 8842 HD3 ILE J 167 5.407 43.699 -4.161 1.00 0.00
J H
ATOM 8843 C ILE J 167 3.733 41.314 -7.251 1.00 0.00
J C
ATOM 8844 O ILE J 167 4.395 41.606 -8.248 1.00 0.00
J O
ATOM 8845 N LEU J 168 2.741 40.405 -7.324 1.00 0.00
J N
ATOM 8846 HN LEU J 168 2.190 40.169 -6.526 1.00 0.00
J H
ATOM 8847 CA LEU J 168 2.378 39.692 -8.531 1.00 0.00
J C
ATOM 8848 HA LEU J 168 3.292 39.258 -8.916 1.00 0.00
J H
ATOM 8849 CB LEU J 168 1.428 38.532 -8.132 1.00 0.00
J C
ATOM 8850 HB1 LEU J 168 1.760 38.164 -7.133 1.00 0.00
J H
ATOM 8851 HB2 LEU J 168 0.403 38.938 -7.985 1.00 0.00
J H
ATOM 8852 CG LEU J 168 1.375 37.326 -9.089 1.00 0.00
J C
ATOM 8853 HG LEU J 168 1.163 37.707 -10.118 1.00 0.00
J H
ATOM 8854 CD1 LEU J 168 2.696 36.539 -9.075 1.00 0.00
J C
ATOM 8855 HD11 LEU J 168 2.626 35.646 -9.733 1.00 0.00
J H

```
ATOM   8856 HD12 LEU J 168       3.534  37.173  -9.436  1.00  0.00
J    H
ATOM   8857 HD13 LEU J 168       2.935  36.193  -8.046  1.00  0.00
J    H
ATOM   8858  CD2 LEU J 168       0.223  36.391  -8.680  1.00  0.00
J    C
ATOM   8859 HD21 LEU J 168       0.163  35.526  -9.376  1.00  0.00
J    H
ATOM   8860 HD22 LEU J 168       0.364  35.991  -7.656  1.00  0.00
J    H
ATOM   8861 HD23 LEU J 168      -0.748  36.932  -8.714  1.00  0.00
J    H
ATOM   8862  C   LEU J 168       1.820  40.637  -9.612  1.00  0.00
J    C
ATOM   8863  O   LEU J 168       2.105  40.500 -10.802  1.00  0.00
J    O
ATOM   8864  N   SER J 169       1.079  41.696  -9.187  1.00  0.00
J    N
ATOM   8865  HN  SER J 169       0.889  41.792  -8.210  1.00  0.00
J    H
ATOM   8866  CA  SER J 169       0.499  42.760 -10.021  1.00  0.00
J    C
ATOM   8867  HA  SER J 169      -0.133  42.254 -10.739  1.00  0.00
J    H
ATOM   8868  CB  SER J 169      -0.323  43.846  -9.255  1.00  0.00
J    C
ATOM   8869  HB1 SER J 169       0.245  44.127  -8.338  1.00  0.00
J    H
ATOM   8870  HB2 SER J 169      -0.421  44.766  -9.874  1.00  0.00
J    H
ATOM   8871  OG  SER J 169      -1.607  43.366  -8.859  1.00  0.00
J    O
ATOM   8872  HG1 SER J 169      -1.958  43.993  -8.213  1.00  0.00
J    H
ATOM   8873  C   SER J 169       1.549  43.512 -10.799  1.00  0.00
J    C
ATOM   8874  O   SER J 169       1.330  43.847 -11.958  1.00  0.00
J    O
ATOM   8875  N   ILE J 170       2.740  43.766 -10.212  1.00  0.00
J    N
ATOM   8876  HN  ILE J 170       2.944  43.488  -9.276  1.00  0.00
J    H
ATOM   8877  CA  ILE J 170       3.837  44.466 -10.882  1.00  0.00
J    C
ATOM   8878  HA  ILE J 170       3.446  45.434 -11.168  1.00  0.00
J    H
ATOM   8879  CB  ILE J 170       5.056  44.647  -9.965  1.00  0.00
J    C
ATOM   8880  HB  ILE J 170       5.311  43.610  -9.630  1.00  0.00
J    H
ATOM   8881  CG2 ILE J 170       6.202  45.367 -10.722  1.00  0.00
J    C
ATOM   8882 HG21 ILE J 170       7.134  45.361 -10.119  1.00  0.00
J    H
```

```
ATOM   8883 HG22 ILE J 170       6.421  44.860 -11.685  1.00  0.00
J    H
ATOM   8884 HG23 ILE J 170       5.935  46.424 -10.936  1.00  0.00
J    H
ATOM   8885  CG1 ILE J 170       4.672  45.445  -8.695  1.00  0.00
J    C
ATOM   8886 HG11 ILE J 170       4.395  46.486  -8.974  1.00  0.00
J    H
ATOM   8887 HG12 ILE J 170       3.773  44.977  -8.233  1.00  0.00
J    H
ATOM   8888  CD  ILE J 170       5.785  45.489  -7.638  1.00  0.00
J    C
ATOM   8889  HD1 ILE J 170       5.433  46.024  -6.729  1.00  0.00
J    H
ATOM   8890  HD2 ILE J 170       6.086  44.461  -7.342  1.00  0.00
J    H
ATOM   8891  HD3 ILE J 170       6.681  46.021  -8.024  1.00  0.00
J    H
ATOM   8892  C   ILE J 170       4.254  43.760 -12.171  1.00  0.00
J    C
ATOM   8893  O   ILE J 170       4.502  44.390 -13.197  1.00  0.00
J    O
ATOM   8894  N   ARG J 171       4.310  42.413 -12.170  1.00  0.00
J    N
ATOM   8895  HN  ARG J 171       4.202  41.893 -11.326  1.00  0.00
J    H
ATOM   8896  CA  ARG J 171       4.528  41.646 -13.386  1.00  0.00
J    C
ATOM   8897  HA  ARG J 171       5.258  42.195 -13.969  1.00  0.00
J    H
ATOM   8898  CB  ARG J 171       5.168  40.273 -13.071  1.00  0.00
J    C
ATOM   8899  HB1 ARG J 171       4.518  39.790 -12.303  1.00  0.00
J    H
ATOM   8900  HB2 ARG J 171       5.114  39.622 -13.972  1.00  0.00
J    H
ATOM   8901  CG  ARG J 171       6.609  40.381 -12.522  1.00  0.00
J    C
ATOM   8902  HG1 ARG J 171       6.562  40.755 -11.476  1.00  0.00
J    H
ATOM   8903  HG2 ARG J 171       7.049  39.360 -12.456  1.00  0.00
J    H
ATOM   8904  CD  ARG J 171       7.543  41.236 -13.390  1.00  0.00
J    C
ATOM   8905  HD1 ARG J 171       7.523  40.859 -14.438  1.00  0.00
J    H
ATOM   8906  HD2 ARG J 171       7.234  42.308 -13.390  1.00  0.00
J    H
ATOM   8907  NE  ARG J 171       8.931  41.147 -12.821  1.00  0.00
J    N
ATOM   8908  HE  ARG J 171       9.016  41.576 -11.915  1.00  0.00
J    H
ATOM   8909  CZ  ARG J 171       9.953  40.554 -13.456  1.00  0.00
J    C
```

```
ATOM   8910  NH1 ARG J 171       9.759  39.706 -14.461  1.00  0.00
J    N
ATOM   8911 HH11 ARG J 171      10.527  39.260 -14.900  1.00  0.00
J    H
ATOM   8912 HH12 ARG J 171       8.808  39.448 -14.647  1.00  0.00
J    H
ATOM   8913  NH2 ARG J 171      11.197  40.821 -13.061  1.00  0.00
J    N
ATOM   8914 HH21 ARG J 171      11.965  40.463 -13.571  1.00  0.00
J    H
ATOM   8915 HH22 ARG J 171      11.308  41.542 -12.390  1.00  0.00
J    H
ATOM   8916  C   ARG J 171       3.303  41.474 -14.272  1.00  0.00
J    C
ATOM   8917  O   ARG J 171       3.414  41.521 -15.496  1.00  0.00
J    O
ATOM   8918  N   LEU J 172       2.108  41.267 -13.694  1.00  0.00
J    N
ATOM   8919  HN  LEU J 172       1.994  41.233 -12.704  1.00  0.00
J    H
ATOM   8920  CA  LEU J 172       0.877  41.074 -14.445  1.00  0.00
J    C
ATOM   8921  HA  LEU J 172       1.116  40.309 -15.172  1.00  0.00
J    H
ATOM   8922  CB  LEU J 172      -0.205  40.451 -13.533  1.00  0.00
J    C
ATOM   8923  HB1 LEU J 172       0.007  40.789 -12.492  1.00  0.00
J    H
ATOM   8924  HB2 LEU J 172      -1.196  40.880 -13.800  1.00  0.00
J    H
ATOM   8925  CG  LEU J 172      -0.222  38.900 -13.586  1.00  0.00
J    C
ATOM   8926  HG  LEU J 172      -0.558  38.657 -14.623  1.00  0.00
J    H
ATOM   8927  CD1 LEU J 172       1.129  38.226 -13.282  1.00  0.00
J    C
ATOM   8928 HD11 LEU J 172       1.024  37.120 -13.293  1.00  0.00
J    H
ATOM   8929 HD12 LEU J 172       1.888  38.512 -14.042  1.00  0.00
J    H
ATOM   8930 HD13 LEU J 172       1.502  38.530 -12.281  1.00  0.00
J    H
ATOM   8931  CD2 LEU J 172      -1.270  38.359 -12.611  1.00  0.00
J    C
ATOM   8932 HD21 LEU J 172      -1.302  37.248 -12.656  1.00  0.00
J    H
ATOM   8933 HD22 LEU J 172      -1.044  38.646 -11.565  1.00  0.00
J    H
ATOM   8934 HD23 LEU J 172      -2.279  38.746 -12.870  1.00  0.00
J    H
ATOM   8935  C   LEU J 172       0.368  42.301 -15.214  1.00  0.00
J    C
ATOM   8936  O   LEU J 172      -0.102  42.169 -16.344  1.00  0.00
J    O
```

```
ATOM   8937  N   VAL J 173       0.460  43.532 -14.659  1.00  0.00
J    N
ATOM   8938  HN  VAL J 173       0.851  43.675 -13.753  1.00  0.00
J    H
ATOM   8939  CA  VAL J 173       0.003  44.761 -15.325  1.00  0.00
J    C
ATOM   8940  HA  VAL J 173      -1.055  44.613 -15.498  1.00  0.00
J    H
ATOM   8941  CB  VAL J 173       0.086  46.001 -14.425  1.00  0.00
J    C
ATOM   8942  HB  VAL J 173       1.150  46.113 -14.102  1.00  0.00
J    H
ATOM   8943  CG1 VAL J 173      -0.338  47.301 -15.156  1.00  0.00
J    C
ATOM   8944 HG11 VAL J 173      -0.300  48.165 -14.458  1.00  0.00
J    H
ATOM   8945 HG12 VAL J 173       0.339  47.532 -16.005  1.00  0.00
J    H
ATOM   8946 HG13 VAL J 173      -1.376  47.215 -15.541  1.00  0.00
J    H
ATOM   8947  CG2 VAL J 173      -0.889  45.795 -13.259  1.00  0.00
J    C
ATOM   8948 HG21 VAL J 173      -0.899  46.684 -12.594  1.00  0.00
J    H
ATOM   8949 HG22 VAL J 173      -1.921  45.629 -13.638  1.00  0.00
J    H
ATOM   8950 HG23 VAL J 173      -0.598  44.919 -12.643  1.00  0.00
J    H
ATOM   8951  C   VAL J 173       0.652  45.069 -16.679  1.00  0.00
J    C
ATOM   8952  O   VAL J 173      -0.120  45.364 -17.593  1.00  0.00
J    O
ATOM   8953  N   PRO J 174       1.959  45.017 -16.975  1.00  0.00
J    N
ATOM   8954  CD  PRO J 174       3.050  44.861 -16.014  1.00  0.00
J    C
ATOM   8955  HD1 PRO J 174       3.281  45.699 -15.318  1.00  0.00
J    H
ATOM   8956  HD2 PRO J 174       2.754  43.950 -15.445  1.00  0.00
J    H
ATOM   8957  CA  PRO J 174       2.465  45.357 -18.306  1.00  0.00
J    C
ATOM   8958  HA  PRO J 174       2.117  46.341 -18.598  1.00  0.00
J    H
ATOM   8959  CB  PRO J 174       3.999  45.403 -18.140  1.00  0.00
J    C
ATOM   8960  HB1 PRO J 174       4.294  46.411 -17.771  1.00  0.00
J    H
ATOM   8961  HB2 PRO J 174       4.539  45.193 -19.085  1.00  0.00
J    H
ATOM   8962  CG  PRO J 174       4.266  44.558 -16.890  1.00  0.00
J    C
ATOM   8963  HG1 PRO J 174       5.226  44.808 -16.396  1.00  0.00
J    H
```

```
ATOM   8964  HG2 PRO J 174       4.262  43.479 -17.169  1.00  0.00
J    H
ATOM   8965  C   PRO J 174       2.039  44.349 -19.354  1.00  0.00
J    C
ATOM   8966  O   PRO J 174       1.880  44.722 -20.511  1.00  0.00
J    O
ATOM   8967  N   GLN J 175       1.882  43.067 -18.977  1.00  0.00
J    N
ATOM   8968  HN  GLN J 175       2.086  42.755 -18.051  1.00  0.00
J    H
ATOM   8969  CA  GLN J 175       1.407  42.030 -19.867  1.00  0.00
J    C
ATOM   8970  HA  GLN J 175       1.926  42.188 -20.805  1.00  0.00
J    H
ATOM   8971  CB  GLN J 175       1.791  40.638 -19.309  1.00  0.00
J    C
ATOM   8972  HB1 GLN J 175       1.289  40.540 -18.319  1.00  0.00
J    H
ATOM   8973  HB2 GLN J 175       1.354  39.846 -19.959  1.00  0.00
J    H
ATOM   8974  CG  GLN J 175       3.315  40.423 -19.135  1.00  0.00
J    C
ATOM   8975  HG1 GLN J 175       3.842  40.581 -20.102  1.00  0.00
J    H
ATOM   8976  HG2 GLN J 175       3.732  41.142 -18.399  1.00  0.00
J    H
ATOM   8977  CD  GLN J 175       3.598  38.989 -18.684  1.00  0.00
J    C
ATOM   8978  OE1 GLN J 175       2.838  38.060 -18.947  1.00  0.00
J    O
ATOM   8979  NE2 GLN J 175       4.738  38.784 -17.988  1.00  0.00
J    N
ATOM   8980 HE21 GLN J 175       4.926  37.849 -17.697  1.00  0.00
J    H
ATOM   8981 HE22 GLN J 175       5.334  39.551 -17.776  1.00  0.00
J    H
ATOM   8982  C   GLN J 175      -0.098  42.099 -20.155  1.00  0.00
J    C
ATOM   8983  O   GLN J 175      -0.521  41.908 -21.293  1.00  0.00
J    O
ATOM   8984  N   TYR J 176      -0.953  42.358 -19.134  1.00  0.00
J    N
ATOM   8985  HN  TYR J 176      -0.644  42.665 -18.236  1.00  0.00
J    H
ATOM   8986  CA  TYR J 176      -2.403  42.211 -19.261  1.00  0.00
J    C
ATOM   8987  HA  TYR J 176      -2.557  41.855 -20.272  1.00  0.00
J    H
ATOM   8988  CB  TYR J 176      -2.946  41.243 -18.168  1.00  0.00
J    C
ATOM   8989  HB1 TYR J 176      -2.473  41.492 -17.193  1.00  0.00
J    H
ATOM   8990  HB2 TYR J 176      -4.043  41.367 -18.043  1.00  0.00
J    H
```

ATOM 8991 CG TYR J 176 −2.593 39.831 −18.524 1.00 0.00
J C
ATOM 8992 CD1 TYR J 176 −1.320 39.324 −18.229 1.00 0.00
J C
ATOM 8993 HD1 TYR J 176 −0.631 39.912 −17.639 1.00 0.00
J H
ATOM 8994 CE1 TYR J 176 −0.926 38.071 −18.711 1.00 0.00
J C
ATOM 8995 HE1 TYR J 176 0.080 37.712 −18.550 1.00 0.00
J H
ATOM 8996 CZ TYR J 176 −1.836 37.275 −19.415 1.00 0.00
J C
ATOM 8997 OH TYR J 176 −1.441 36.006 −19.877 1.00 0.00
J O
ATOM 8998 HH TYR J 176 −2.212 35.588 −20.267 1.00 0.00
J H
ATOM 8999 CD2 TYR J 176 −3.509 39.018 −19.212 1.00 0.00
J C
ATOM 9000 HD2 TYR J 176 −4.494 39.396 −19.453 1.00 0.00
J H
ATOM 9001 CE2 TYR J 176 −3.141 37.732 −19.636 1.00 0.00
J C
ATOM 9002 HE2 TYR J 176 −3.860 37.112 −20.150 1.00 0.00
J H
ATOM 9003 C TYR J 176 −3.199 43.504 −19.113 1.00 0.00
J C
ATOM 9004 O TYR J 176 −4.417 43.510 −19.284 1.00 0.00
J O
ATOM 9005 N GLY J 177 −2.553 44.645 −18.798 1.00 0.00
J N
ATOM 9006 HN GLY J 177 −1.556 44.675 −18.751 1.00 0.00
J H
ATOM 9007 CA GLY J 177 −3.236 45.902 −18.505 1.00 0.00
J C
ATOM 9008 HA1 GLY J 177 −3.980 46.027 −19.279 1.00 0.00
J H
ATOM 9009 HA2 GLY J 177 −2.473 46.669 −18.495 1.00 0.00
J H
ATOM 9010 C GLY J 177 −3.940 45.928 −17.169 1.00 0.00
J C
ATOM 9011 O GLY J 177 −4.035 44.944 −16.445 1.00 0.00
J O
ATOM 9012 N TRP J 178 −4.450 47.104 −16.765 1.00 0.00
J N
ATOM 9013 HN TRP J 178 −4.458 47.912 −17.350 1.00 0.00
J H
ATOM 9014 CA TRP J 178 −5.032 47.295 −15.445 1.00 0.00
J C
ATOM 9015 HA TRP J 178 −4.288 46.940 −14.743 1.00 0.00
J H
ATOM 9016 CB TRP J 178 −5.288 48.799 −15.169 1.00 0.00
J C
ATOM 9017 HB1 TRP J 178 −5.999 49.170 −15.939 1.00 0.00
J H

```
ATOM   9018  HB2 TRP J 178      -5.787  48.922 -14.183  1.00  0.00
J    H
ATOM   9019  CG  TRP J 178      -4.019  49.638 -15.256  1.00  0.00
J    C
ATOM   9020  CD1 TRP J 178      -3.387  50.122 -16.371  1.00  0.00
J    C
ATOM   9021  HD1 TRP J 178      -3.779  50.045 -17.376  1.00  0.00
J    H
ATOM   9022  NE1 TRP J 178      -2.215  50.749 -16.018  1.00  0.00
J    N
ATOM   9023  HE1 TRP J 178      -1.587  51.187 -16.624  1.00  0.00
J    H
ATOM   9024  CE2 TRP J 178      -2.084  50.710 -14.650  1.00  0.00
J    C
ATOM   9025  CD2 TRP J 178      -3.208  50.021 -14.131  1.00  0.00
J    C
ATOM   9026  CE3 TRP J 178      -3.361  49.833 -12.760  1.00  0.00
J    C
ATOM   9027  HE3 TRP J 178      -4.208  49.302 -12.347  1.00  0.00
J    H
ATOM   9028  CZ3 TRP J 178      -2.373  50.346 -11.907  1.00  0.00
J    C
ATOM   9029  HZ3 TRP J 178      -2.473  50.214 -10.838  1.00  0.00
J    H
ATOM   9030  CZ2 TRP J 178      -1.104  51.209 -13.804  1.00  0.00
J    C
ATOM   9031  HZ2 TRP J 178      -0.242  51.732 -14.187  1.00  0.00
J    H
ATOM   9032  CH2 TRP J 178      -1.257  51.024 -12.422  1.00  0.00
J    C
ATOM   9033  HH2 TRP J 178      -0.506  51.407 -11.744  1.00  0.00
J    H
ATOM   9034  C   TRP J 178      -6.310  46.497 -15.183  1.00  0.00
J    C
ATOM   9035  O   TRP J 178      -6.464  45.883 -14.129  1.00  0.00
J    O
ATOM   9036  N   SER J 179      -7.240  46.462 -16.164  1.00  0.00
J    N
ATOM   9037  HN  SER J 179      -7.069  46.912 -17.040  1.00  0.00
J    H
ATOM   9038  CA  SER J 179      -8.538  45.798 -16.075  1.00  0.00
J    C
ATOM   9039  HA  SER J 179      -9.008  46.220 -15.196  1.00  0.00
J    H
ATOM   9040  CB  SER J 179      -9.387  46.063 -17.356  1.00  0.00
J    C
ATOM   9041  HB1 SER J 179     -10.252  45.360 -17.349  1.00  0.00
J    H
ATOM   9042  HB2 SER J 179      -9.804  47.095 -17.338  1.00  0.00
J    H
ATOM   9043  OG  SER J 179      -8.619  45.836 -18.541  1.00  0.00
J    O
ATOM   9044  HG1 SER J 179      -9.241  45.795 -19.278  1.00  0.00
J    H
```

```
ATOM   9045  C   SER J 179      -8.439  44.296 -15.858  1.00  0.00
J    C
ATOM   9046  O   SER J 179      -9.086  43.743 -14.972  1.00  0.00
J    O
ATOM   9047  N   SER J 180      -7.577  43.607 -16.630  1.00  0.00
J    N
ATOM   9048  HN  SER J 180      -6.972  44.040 -17.298  1.00  0.00
J    H
ATOM   9049  CA  SER J 180      -7.447  42.157 -16.560  1.00  0.00
J    C
ATOM   9050  HA  SER J 180      -8.326  41.813 -16.029  1.00  0.00
J    H
ATOM   9051  CB  SER J 180      -7.327  41.519 -17.970  1.00  0.00
J    C
ATOM   9052  HB1 SER J 180      -6.529  42.061 -18.527  1.00  0.00
J    H
ATOM   9053  HB2 SER J 180      -7.006  40.456 -17.888  1.00  0.00
J    H
ATOM   9054  OG  SER J 180      -8.568  41.653 -18.664  1.00  0.00
J    O
ATOM   9055  HG1 SER J 180      -8.395  41.456 -19.593  1.00  0.00
J    H
ATOM   9056  C   SER J 180      -6.240  41.725 -15.756  1.00  0.00
J    C
ATOM   9057  O   SER J 180      -6.071  40.543 -15.480  1.00  0.00
J    O
ATOM   9058  N   GLY J 181      -5.369  42.661 -15.317  1.00  0.00
J    N
ATOM   9059  HN  GLY J 181      -5.525  43.637 -15.463  1.00  0.00
J    H
ATOM   9060  CA  GLY J 181      -4.143  42.317 -14.602  1.00  0.00
J    C
ATOM   9061  HA1 GLY J 181      -3.399  43.038 -14.911  1.00  0.00
J    H
ATOM   9062  HA2 GLY J 181      -3.908  41.295 -14.868  1.00  0.00
J    H
ATOM   9063  C   GLY J 181      -4.269  42.389 -13.105  1.00  0.00
J    C
ATOM   9064  O   GLY J 181      -3.795  41.498 -12.406  1.00  0.00
J    O
ATOM   9065  N   LEU J 182      -4.933  43.432 -12.550  1.00  0.00
J    N
ATOM   9066  HN  LEU J 182      -5.326  44.171 -13.093  1.00  0.00
J    H
ATOM   9067  CA  LEU J 182      -5.134  43.573 -11.106  1.00  0.00
J    C
ATOM   9068  HA  LEU J 182      -4.163  43.447 -10.646  1.00  0.00
J    H
ATOM   9069  CB  LEU J 182      -5.737  44.956 -10.716  1.00  0.00
J    C
ATOM   9070  HB1 LEU J 182      -6.467  45.232 -11.512  1.00  0.00
J    H
ATOM   9071  HB2 LEU J 182      -6.329  44.842  -9.781  1.00  0.00
J    H
```

```
ATOM   9072  CG  LEU J 182      -4.720  46.110 -10.564  1.00  0.00
J    C
ATOM   9073  HG  LEU J 182      -4.100  45.852  -9.672  1.00  0.00
J    H
ATOM   9074  CD1 LEU J 182      -3.832  46.295 -11.793  1.00  0.00
J    C
ATOM   9075 HD11 LEU J 182      -3.153  47.164 -11.656  1.00  0.00
J    H
ATOM   9076 HD12 LEU J 182      -3.211  45.391 -11.967  1.00  0.00
J    H
ATOM   9077 HD13 LEU J 182      -4.449  46.477 -12.699  1.00  0.00
J    H
ATOM   9078  CD2 LEU J 182      -5.451  47.427 -10.268  1.00  0.00
J    C
ATOM   9079 HD21 LEU J 182      -4.720  48.254 -10.140  1.00  0.00
J    H
ATOM   9080 HD22 LEU J 182      -6.137  47.708 -11.092  1.00  0.00
J    H
ATOM   9081 HD23 LEU J 182      -6.045  47.341  -9.333  1.00  0.00
J    H
ATOM   9082  C   LEU J 182      -6.045  42.488 -10.560  1.00  0.00
J    C
ATOM   9083  O   LEU J 182      -5.759  41.864  -9.538  1.00  0.00
J    O
ATOM   9084  N   PHE J 183      -7.156  42.209 -11.275  1.00  0.00
J    N
ATOM   9085  HN  PHE J 183      -7.400  42.736 -12.087  1.00  0.00
J    H
ATOM   9086  CA  PHE J 183      -8.084  41.145 -10.947  1.00  0.00
J    C
ATOM   9087  HA  PHE J 183      -8.364  41.303  -9.913  1.00  0.00
J    H
ATOM   9088  CB  PHE J 183      -9.346  41.220 -11.854  1.00  0.00
J    C
ATOM   9089  HB1 PHE J 183      -9.804  42.228 -11.757  1.00  0.00
J    H
ATOM   9090  HB2 PHE J 183      -9.078  41.084 -12.924  1.00  0.00
J    H
ATOM   9091  CG  PHE J 183     -10.372  40.201 -11.413  1.00  0.00
J    C
ATOM   9092  CD1 PHE J 183     -11.171  40.432 -10.279  1.00  0.00
J    C
ATOM   9093  HD1 PHE J 183     -11.104  41.379  -9.762  1.00  0.00
J    H
ATOM   9094  CE1 PHE J 183     -12.049  39.444  -9.811  1.00  0.00
J    C
ATOM   9095  HE1 PHE J 183     -12.672  39.639  -8.950  1.00  0.00
J    H
ATOM   9096  CZ  PHE J 183     -12.123  38.208 -10.464  1.00  0.00
J    C
ATOM   9097  HZ  PHE J 183     -12.808  37.449 -10.114  1.00  0.00
J    H
ATOM   9098  CD2 PHE J 183     -10.457  38.956 -12.059  1.00  0.00
J    C
```

ATOM 9099 HD2 PHE J 183 -9.839 38.753 -12.923 1.00 0.00
J H
ATOM 9100 CE2 PHE J 183 -11.317 37.960 -11.580 1.00 0.00
J C
ATOM 9101 HE2 PHE J 183 -11.354 36.998 -12.072 1.00 0.00
J H
ATOM 9102 C PHE J 183 -7.426 39.771 -11.027 1.00 0.00
J C
ATOM 9103 O PHE J 183 -7.572 38.967 -10.111 1.00 0.00
J O
ATOM 9104 N ALA J 184 -6.632 39.486 -12.091 1.00 0.00
J N
ATOM 9105 HN ALA J 184 -6.507 40.126 -12.848 1.00 0.00
J H
ATOM 9106 CA ALA J 184 -5.909 38.234 -12.208 1.00 0.00
J C
ATOM 9107 HA ALA J 184 -6.597 37.397 -12.164 1.00 0.00
J H
ATOM 9108 CB ALA J 184 -5.173 38.181 -13.567 1.00 0.00
J C
ATOM 9109 HB1 ALA J 184 -5.910 38.275 -14.393 1.00 0.00
J H
ATOM 9110 HB2 ALA J 184 -4.446 39.017 -13.661 1.00 0.00
J H
ATOM 9111 HB3 ALA J 184 -4.628 37.221 -13.701 1.00 0.00
J H
ATOM 9112 C ALA J 184 -4.931 38.040 -11.073 1.00 0.00
J C
ATOM 9113 O ALA J 184 -4.943 37.029 -10.377 1.00 0.00
J O
ATOM 9114 N SER J 185 -4.137 39.068 -10.766 1.00 0.00
J N
ATOM 9115 HN SER J 185 -4.142 39.941 -11.255 1.00 0.00
J H
ATOM 9116 CA SER J 185 -3.184 39.005 -9.681 1.00 0.00
J C
ATOM 9117 HA SER J 185 -2.557 38.154 -9.915 1.00 0.00
J H
ATOM 9118 CB SER J 185 -2.442 40.342 -9.621 1.00 0.00
J C
ATOM 9119 HB1 SER J 185 -2.003 40.539 -10.626 1.00 0.00
J H
ATOM 9120 HB2 SER J 185 -3.154 41.171 -9.407 1.00 0.00
J H
ATOM 9121 OG SER J 185 -1.408 40.269 -8.660 1.00 0.00
J O
ATOM 9122 HG1 SER J 185 -0.832 41.029 -8.805 1.00 0.00
J H
ATOM 9123 C SER J 185 -3.809 38.745 -8.322 1.00 0.00
J C
ATOM 9124 O SER J 185 -3.370 37.886 -7.561 1.00 0.00
J O
ATOM 9125 N LEU J 186 -4.911 39.461 -8.021 1.00 0.00
J N

```
ATOM   9126  HN  LEU J 186      -5.267  40.179  -8.616  1.00  0.00
J    H
ATOM   9127  CA  LEU J 186      -5.675  39.257  -6.812  1.00  0.00
J    C
ATOM   9128  HA  LEU J 186      -4.977  39.354  -5.990  1.00  0.00
J    H
ATOM   9129  CB  LEU J 186      -6.739  40.383  -6.719  1.00  0.00
J    C
ATOM   9130  HB1 LEU J 186      -6.209  41.352  -6.876  1.00  0.00
J    H
ATOM   9131  HB2 LEU J 186      -7.450  40.281  -7.569  1.00  0.00
J    H
ATOM   9132  CG  LEU J 186      -7.546  40.508  -5.402  1.00  0.00
J    C
ATOM   9133  HG  LEU J 186      -7.979  41.532  -5.514  1.00  0.00
J    H
ATOM   9134  CD1 LEU J 186      -8.685  39.487  -5.285  1.00  0.00
J    C
ATOM   9135 HD11 LEU J 186      -9.200  39.587  -4.305  1.00  0.00
J    H
ATOM   9136 HD12 LEU J 186      -9.435  39.643  -6.090  1.00  0.00
J    H
ATOM   9137 HD13 LEU J 186      -8.294  38.451  -5.366  1.00  0.00
J    H
ATOM   9138  CD2 LEU J 186      -6.654  40.503  -4.146  1.00  0.00
J    C
ATOM   9139 HD21 LEU J 186      -7.276  40.613  -3.232  1.00  0.00
J    H
ATOM   9140 HD22 LEU J 186      -6.084  39.557  -4.054  1.00  0.00
J    H
ATOM   9141 HD23 LEU J 186      -5.932  41.347  -4.179  1.00  0.00
J    H
ATOM   9142  C   LEU J 186      -6.274  37.856  -6.744  1.00  0.00
J    C
ATOM   9143  O   LEU J 186      -6.116  37.159  -5.741  1.00  0.00
J    O
ATOM   9144  N   PHE J 187      -6.894  37.366  -7.843  1.00  0.00
J    N
ATOM   9145  HN  PHE J 187      -6.960  37.907  -8.680  1.00  0.00
J    H
ATOM   9146  CA  PHE J 187      -7.510  36.051  -7.927  1.00  0.00
J    C
ATOM   9147  HA  PHE J 187      -8.264  36.030  -7.149  1.00  0.00
J    H
ATOM   9148  CB  PHE J 187      -8.103  35.878  -9.369  1.00  0.00
J    C
ATOM   9149  HB1 PHE J 187      -8.889  36.647  -9.533  1.00  0.00
J    H
ATOM   9150  HB2 PHE J 187      -7.323  36.036 -10.144  1.00  0.00
J    H
ATOM   9151  CG  PHE J 187      -8.772  34.543  -9.604  1.00  0.00
J    C
ATOM   9152  CD1 PHE J 187     -10.158  34.399  -9.444  1.00  0.00
J    C
```

```
ATOM   9153  HD1 PHE J 187     -10.770  35.272  -9.268  1.00  0.00
J    H
ATOM   9154  CE1 PHE J 187     -10.759  33.135  -9.503  1.00  0.00
J    C
ATOM   9155  HE1 PHE J 187     -11.818  33.032  -9.312  1.00  0.00
J    H
ATOM   9156  CZ  PHE J 187      -9.988  32.011  -9.815  1.00  0.00
J    C
ATOM   9157  HZ  PHE J 187     -10.450  31.035  -9.875  1.00  0.00
J    H
ATOM   9158  CD2 PHE J 187      -8.012  33.408  -9.948  1.00  0.00
J    C
ATOM   9159  HD2 PHE J 187      -6.946  33.499 -10.108  1.00  0.00
J    H
ATOM   9160  CE2 PHE J 187      -8.617  32.152 -10.058  1.00  0.00
J    C
ATOM   9161  HE2 PHE J 187      -8.024  31.290 -10.331  1.00  0.00
J    H
ATOM   9162  C   PHE J 187      -6.524  34.929  -7.624  1.00  0.00
J    C
ATOM   9163  O   PHE J 187      -6.777  34.052  -6.796  1.00  0.00
J    O
ATOM   9164  N   HSE J 188      -5.344  34.962  -8.271  1.00  0.00
J    N
ATOM   9165  HN  HSE J 188      -5.093  35.675  -8.924  1.00  0.00
J    H
ATOM   9166  CA  HSE J 188      -4.328  33.950  -8.069  1.00  0.00
J    C
ATOM   9167  HA  HSE J 188      -4.852  33.003  -8.081  1.00  0.00
J    H
ATOM   9168  CB  HSE J 188      -3.277  33.956  -9.191  1.00  0.00
J    C
ATOM   9169  HB1 HSE J 188      -2.909  34.999  -9.308  1.00  0.00
J    H
ATOM   9170  HB2 HSE J 188      -2.408  33.337  -8.887  1.00  0.00
J    H
ATOM   9171  ND1 HSE J 188      -4.389  34.406 -11.365  1.00  0.00
J    N
ATOM   9172  CG  HSE J 188      -3.842  33.496 -10.492  1.00  0.00
J    C
ATOM   9173  CE1 HSE J 188      -4.770  33.715 -12.409  1.00  0.00
J    C
ATOM   9174  HE1 HSE J 188      -5.253  34.141 -13.292  1.00  0.00
J    H
ATOM   9175  NE2 HSE J 188      -4.497  32.402 -12.254  1.00  0.00
J    N
ATOM   9176  HE2 HSE J 188      -4.702  31.668 -12.900  1.00  0.00
J    H
ATOM   9177  CD2 HSE J 188      -3.905  32.250 -11.020  1.00  0.00
J    C
ATOM   9178  HD2 HSE J 188      -3.586  31.304 -10.605  1.00  0.00
J    H
ATOM   9179  C   HSE J 188      -3.616  34.049  -6.734  1.00  0.00
J    C
```

```
ATOM   9180  O   HSE J 188      -3.137  33.046  -6.213  1.00  0.00
J    O
ATOM   9181  N   ALA J 189      -3.537  35.244  -6.113  1.00  0.00
J    N
ATOM   9182  HN  ALA J 189      -3.811  36.096  -6.559  1.00  0.00
J    H
ATOM   9183  CA  ALA J 189      -3.048  35.383  -4.756  1.00  0.00
J    C
ATOM   9184  HA  ALA J 189      -2.068  34.927  -4.675  1.00  0.00
J    H
ATOM   9185  CB  ALA J 189      -2.869  36.875  -4.417  1.00  0.00
J    C
ATOM   9186  HB1 ALA J 189      -2.184  37.345  -5.156  1.00  0.00
J    H
ATOM   9187  HB2 ALA J 189      -3.841  37.413  -4.461  1.00  0.00
J    H
ATOM   9188  HB3 ALA J 189      -2.432  37.016  -3.405  1.00  0.00
J    H
ATOM   9189  C   ALA J 189      -3.935  34.687  -3.714  1.00  0.00
J    C
ATOM   9190  O   ALA J 189      -3.448  33.946  -2.861  1.00  0.00
J    O
ATOM   9191  N   ILE J 190      -5.274  34.857  -3.811  1.00  0.00
J    N
ATOM   9192  HN  ILE J 190      -5.680  35.493  -4.462  1.00  0.00
J    H
ATOM   9193  CA  ILE J 190      -6.246  34.140  -2.987  1.00  0.00
J    C
ATOM   9194  HA  ILE J 190      -5.929  34.273  -1.961  1.00  0.00
J    H
ATOM   9195  CB  ILE J 190      -7.661  34.684  -3.202  1.00  0.00
J    C
ATOM   9196  HB  ILE J 190      -7.834  34.588  -4.304  1.00  0.00
J    H
ATOM   9197  CG2 ILE J 190      -8.658  33.904  -2.308  1.00  0.00
J    C
ATOM   9198 HG21 ILE J 190      -9.704  34.174  -2.562  1.00  0.00
J    H
ATOM   9199 HG22 ILE J 190      -8.548  32.808  -2.456  1.00  0.00
J    H
ATOM   9200 HG23 ILE J 190      -8.491  34.131  -1.234  1.00  0.00
J    H
ATOM   9201  CG1 ILE J 190      -7.700  36.196  -2.866  1.00  0.00
J    C
ATOM   9202 HG11 ILE J 190      -7.329  36.361  -1.830  1.00  0.00
J    H
ATOM   9203 HG12 ILE J 190      -7.009  36.735  -3.552  1.00  0.00
J    H
ATOM   9204  CD  ILE J 190      -9.091  36.829  -3.000  1.00  0.00
J    C
ATOM   9205  HD1 ILE J 190      -9.038  37.921  -2.802  1.00  0.00
J    H
ATOM   9206  HD2 ILE J 190      -9.492  36.680  -4.025  1.00  0.00
J    H
```

ATOM 9207 HD3 ILE J 190 -9.803 36.383 -2.274 1.00 0.00
J H
ATOM 9208 C ILE J 190 -6.220 32.639 -3.265 1.00 0.00
J C
ATOM 9209 O ILE J 190 -6.143 31.809 -2.357 1.00 0.00
J O
ATOM 9210 N SER J 191 -6.223 32.218 -4.555 1.00 0.00
J N
ATOM 9211 HN SER J 191 -6.240 32.829 -5.346 1.00 0.00
J H
ATOM 9212 CA SER J 191 -6.201 30.797 -4.896 1.00 0.00
J C
ATOM 9213 HA SER J 191 -7.029 30.364 -4.349 1.00 0.00
J H
ATOM 9214 CB SER J 191 -6.536 30.442 -6.379 1.00 0.00
J C
ATOM 9215 HB1 SER J 191 -6.709 29.343 -6.442 1.00 0.00
J H
ATOM 9216 HB2 SER J 191 -7.481 30.940 -6.693 1.00 0.00
J H
ATOM 9217 OG SER J 191 -5.494 30.765 -7.298 1.00 0.00
J O
ATOM 9218 HG1 SER J 191 -5.696 30.314 -8.127 1.00 0.00
J H
ATOM 9219 C SER J 191 -4.941 30.087 -4.442 1.00 0.00
J C
ATOM 9220 O SER J 191 -5.003 28.949 -3.980 1.00 0.00
J O
ATOM 9221 N ALA J 192 -3.771 30.752 -4.528 1.00 0.00
J N
ATOM 9222 HN ALA J 192 -3.703 31.632 -4.996 1.00 0.00
J H
ATOM 9223 CA ALA J 192 -2.540 30.254 -3.960 1.00 0.00
J C
ATOM 9224 HA ALA J 192 -2.367 29.248 -4.322 1.00 0.00
J H
ATOM 9225 CB ALA J 192 -1.359 31.096 -4.484 1.00 0.00
J C
ATOM 9226 HB1 ALA J 192 -1.366 31.096 -5.595 1.00 0.00
J H
ATOM 9227 HB2 ALA J 192 -1.436 32.151 -4.141 1.00 0.00
J H
ATOM 9228 HB3 ALA J 192 -0.383 30.686 -4.147 1.00 0.00
J H
ATOM 9229 C ALA J 192 -2.531 30.170 -2.437 1.00 0.00
J C
ATOM 9230 O ALA J 192 -2.268 29.098 -1.899 1.00 0.00
J O
ATOM 9231 N PHE J 193 -2.890 31.228 -1.676 1.00 0.00
J N
ATOM 9232 HN PHE J 193 -3.213 32.094 -2.056 1.00 0.00
J H
ATOM 9233 CA PHE J 193 -2.835 31.191 -0.216 1.00 0.00
J C

```
ATOM   9234  HA  PHE J 193      -1.819  30.912   0.034  1.00  0.00
J    H
ATOM   9235  CB  PHE J 193      -3.123  32.605   0.365  1.00  0.00
J    C
ATOM   9236  HB1 PHE J 193      -2.380  33.325  -0.043  1.00  0.00
J    H
ATOM   9237  HB2 PHE J 193      -4.129  32.966   0.061  1.00  0.00
J    H
ATOM   9238  CG  PHE J 193      -2.978  32.598   1.868  1.00  0.00
J    C
ATOM   9239  CD1 PHE J 193      -1.724  32.464   2.494  1.00  0.00
J    C
ATOM   9240  HD1 PHE J 193      -0.824  32.464   1.893  1.00  0.00
J    H
ATOM   9241  CE1 PHE J 193      -1.637  32.324   3.888  1.00  0.00
J    C
ATOM   9242  HE1 PHE J 193      -0.670  32.262   4.366  1.00  0.00
J    H
ATOM   9243  CZ  PHE J 193      -2.806  32.271   4.659  1.00  0.00
J    C
ATOM   9244  HZ  PHE J 193      -2.745  32.173   5.733  1.00  0.00
J    H
ATOM   9245  CD2 PHE J 193      -4.136  32.570   2.656  1.00  0.00
J    C
ATOM   9246  HD2 PHE J 193      -5.106  32.694   2.196  1.00  0.00
J    H
ATOM   9247  CE2 PHE J 193      -4.054  32.354   4.034  1.00  0.00
J    C
ATOM   9248  HE2 PHE J 193      -4.959  32.251   4.617  1.00  0.00
J    H
ATOM   9249  C   PHE J 193      -3.755  30.112   0.372  1.00  0.00
J    C
ATOM   9250  O   PHE J 193      -3.415  29.374   1.301  1.00  0.00
J    O
ATOM   9251  N   ASN J 194      -4.945  29.939  -0.224  1.00  0.00
J    N
ATOM   9252  HN  ASN J 194      -5.222  30.418  -1.056  1.00  0.00
J    H
ATOM   9253  CA  ASN J 194      -5.933  29.030   0.300  1.00  0.00
J    C
ATOM   9254  HA  ASN J 194      -5.745  28.981   1.368  1.00  0.00
J    H
ATOM   9255  CB  ASN J 194      -7.366  29.520  -0.013  1.00  0.00
J    C
ATOM   9256  HB1 ASN J 194      -7.412  29.766  -1.097  1.00  0.00
J    H
ATOM   9257  HB2 ASN J 194      -8.100  28.710   0.177  1.00  0.00
J    H
ATOM   9258  CG  ASN J 194      -7.607  30.758   0.828  1.00  0.00
J    C
ATOM   9259  OD1 ASN J 194      -6.889  31.750   0.736  1.00  0.00
J    O
ATOM   9260  ND2 ASN J 194      -8.645  30.718   1.683  1.00  0.00
J    N
```

```
ATOM   9261 HD21 ASN J 194      -8.818  31.532   2.232  1.00  0.00
J    H
ATOM   9262 HD22 ASN J 194      -9.223  29.909   1.728  1.00  0.00
J    H
ATOM   9263  C   ASN J 194      -5.773  27.636  -0.256  1.00  0.00
J    C
ATOM   9264  O   ASN J 194      -6.580  26.777   0.081  1.00  0.00
J    O
ATOM   9265  N   ASN J 195      -4.727  27.369  -1.073  1.00  0.00
J    N
ATOM   9266  HN  ASN J 195      -4.067  28.074  -1.327  1.00  0.00
J    H
ATOM   9267  CA  ASN J 195      -4.463  26.058  -1.649  1.00  0.00
J    C
ATOM   9268  HA  ASN J 195      -3.581  26.194  -2.265  1.00  0.00
J    H
ATOM   9269  CB  ASN J 195      -4.137  24.994  -0.555  1.00  0.00
J    C
ATOM   9270  HB1 ASN J 195      -4.783  25.202   0.325  1.00  0.00
J    H
ATOM   9271  HB2 ASN J 195      -4.388  23.977  -0.920  1.00  0.00
J    H
ATOM   9272  CG  ASN J 195      -2.681  25.084  -0.132  1.00  0.00
J    C
ATOM   9273  OD1 ASN J 195      -1.800  24.619  -0.852  1.00  0.00
J    O
ATOM   9274  ND2 ASN J 195      -2.380  25.681   1.040  1.00  0.00
J    N
ATOM   9275 HD21 ASN J 195      -1.418  25.738   1.291  1.00  0.00
J    H
ATOM   9276 HD22 ASN J 195      -3.104  26.069   1.603  1.00  0.00
J    H
ATOM   9277  C   ASN J 195      -5.590  25.571  -2.555  1.00  0.00
J    C
ATOM   9278  O   ASN J 195      -6.024  24.431  -2.462  1.00  0.00
J    O
ATOM   9279  N   ALA J 196      -6.109  26.433  -3.451  1.00  0.00
J    N
ATOM   9280  HN  ALA J 196      -5.763  27.363  -3.572  1.00  0.00
J    H
ATOM   9281  CA  ALA J 196      -7.214  26.064  -4.310  1.00  0.00
J    C
ATOM   9282  HA  ALA J 196      -7.657  25.145  -3.942  1.00  0.00
J    H
ATOM   9283  CB  ALA J 196      -8.234  27.220  -4.332  1.00  0.00
J    C
ATOM   9284  HB1 ALA J 196      -8.592  27.421  -3.300  1.00  0.00
J    H
ATOM   9285  HB2 ALA J 196      -7.771  28.154  -4.721  1.00  0.00
J    H
ATOM   9286  HB3 ALA J 196      -9.117  26.975  -4.960  1.00  0.00
J    H
ATOM   9287  C   ALA J 196      -6.831  25.808  -5.737  1.00  0.00
J    C
```

```
ATOM   9288  O   ALA J 196      -7.624  25.272  -6.501  1.00  0.00
J    O
ATOM   9289  N   GLY J 197      -5.635  26.245  -6.170  1.00  0.00
J    N
ATOM   9290  HN  GLY J 197      -5.080  26.863  -5.615  1.00  0.00
J    H
ATOM   9291  CA  GLY J 197      -5.059  25.862  -7.458  1.00  0.00
J    C
ATOM   9292  HA1 GLY J 197      -5.155  24.786  -7.503  1.00  0.00
J    H
ATOM   9293  HA2 GLY J 197      -4.038  26.217  -7.433  1.00  0.00
J    H
ATOM   9294  C   GLY J 197      -5.721  26.440  -8.680  1.00  0.00
J    C
ATOM   9295  O   GLY J 197      -5.207  26.342  -9.786  1.00  0.00
J    O
ATOM   9296  N   PHE J 198      -6.888  27.095  -8.512  1.00  0.00
J    N
ATOM   9297  HN  PHE J 198      -7.292  27.191  -7.604  1.00  0.00
J    H
ATOM   9298  CA  PHE J 198      -7.652  27.707  -9.575  1.00  0.00
J    C
ATOM   9299  HA  PHE J 198      -7.860  26.910 -10.279  1.00  0.00
J    H
ATOM   9300  CB  PHE J 198      -8.966  28.377  -9.093  1.00  0.00
J    C
ATOM   9301  HB1 PHE J 198      -8.824  28.754  -8.056  1.00  0.00
J    H
ATOM   9302  HB2 PHE J 198      -9.224  29.252  -9.726  1.00  0.00
J    H
ATOM   9303  CG  PHE J 198     -10.083  27.387  -9.066  1.00  0.00
J    C
ATOM   9304  CD1 PHE J 198     -10.761  27.044 -10.250  1.00  0.00
J    C
ATOM   9305  HD1 PHE J 198     -10.437  27.463 -11.193  1.00  0.00
J    H
ATOM   9306  CE1 PHE J 198     -11.856  26.170 -10.218  1.00  0.00
J    C
ATOM   9307  HE1 PHE J 198     -12.376  25.921 -11.133  1.00  0.00
J    H
ATOM   9308  CZ  PHE J 198     -12.265  25.617  -8.999  1.00  0.00
J    C
ATOM   9309  HZ  PHE J 198     -13.107  24.940  -8.959  1.00  0.00
J    H
ATOM   9310  CD2 PHE J 198     -10.488  26.814  -7.860  1.00  0.00
J    C
ATOM   9311  HD2 PHE J 198      -9.973  27.065  -6.943  1.00  0.00
J    H
ATOM   9312  CE2 PHE J 198     -11.570  25.937  -7.829  1.00  0.00
J    C
ATOM   9313  HE2 PHE J 198     -11.881  25.499  -6.890  1.00  0.00
J    H
ATOM   9314  C   PHE J 198      -6.876  28.760 -10.316  1.00  0.00
J    C
```

```
ATOM   9315  O   PHE J 198      -6.119  29.548  -9.745  1.00  0.00
J    O
ATOM   9316  N   SER J 199      -7.100  28.820 -11.632  1.00  0.00
J    N
ATOM   9317  HN  SER J 199      -7.744  28.231 -12.121  1.00  0.00
J    H
ATOM   9318  CA  SER J 199      -6.412  29.760 -12.468  1.00  0.00
J    C
ATOM   9319  HA  SER J 199      -6.177  30.622 -11.858  1.00  0.00
J    H
ATOM   9320  CB  SER J 199      -5.139  29.126 -13.087  1.00  0.00
J    C
ATOM   9321  HB1 SER J 199      -4.489  28.771 -12.255  1.00  0.00
J    H
ATOM   9322  HB2 SER J 199      -5.406  28.233 -13.696  1.00  0.00
J    H
ATOM   9323  OG  SER J 199      -4.377  30.043 -13.873  1.00  0.00
J    O
ATOM   9324  HG1 SER J 199      -3.513  29.637 -14.025  1.00  0.00
J    H
ATOM   9325  C   SER J 199      -7.361  30.218 -13.542  1.00  0.00
J    C
ATOM   9326  O   SER J 199      -8.220  29.461 -13.986  1.00  0.00
J    O
ATOM   9327  N   LEU J 200      -7.256  31.505 -13.916  1.00  0.00
J    N
ATOM   9328  HN  LEU J 200      -6.625  32.115 -13.441  1.00  0.00
J    H
ATOM   9329  CA  LEU J 200      -7.999  32.135 -14.987  1.00  0.00
J    C
ATOM   9330  HA  LEU J 200      -9.010  31.760 -14.892  1.00  0.00
J    H
ATOM   9331  CB  LEU J 200      -7.900  33.672 -14.846  1.00  0.00
J    C
ATOM   9332  HB1 LEU J 200      -6.830  33.948 -14.994  1.00  0.00
J    H
ATOM   9333  HB2 LEU J 200      -8.459  34.148 -15.682  1.00  0.00
J    H
ATOM   9334  CG  LEU J 200      -8.375  34.228 -13.490  1.00  0.00
J    C
ATOM   9335  HG  LEU J 200      -7.748  33.749 -12.699  1.00  0.00
J    H
ATOM   9336  CD1 LEU J 200      -8.176  35.746 -13.473  1.00  0.00
J    C
ATOM   9337 HD11 LEU J 200      -8.555  36.178 -12.522  1.00  0.00
J    H
ATOM   9338 HD12 LEU J 200      -7.098  36.000 -13.566  1.00  0.00
J    H
ATOM   9339 HD13 LEU J 200      -8.723  36.222 -14.314  1.00  0.00
J    H
ATOM   9340  CD2 LEU J 200      -9.845  33.898 -13.194  1.00  0.00
J    C
ATOM   9341 HD21 LEU J 200     -10.139  34.301 -12.201  1.00  0.00
J    H
```

```
ATOM   9342 HD22 LEU J 200     -10.523  34.340 -13.952  1.00  0.00
J    H
ATOM   9343 HD23 LEU J 200     -10.002  32.798 -13.184  1.00  0.00
J    H
ATOM   9344  C   LEU J 200      -7.505  31.778 -16.383  1.00  0.00
J    C
ATOM   9345  O   LEU J 200      -8.273  31.799 -17.338  1.00  0.00
J    O
ATOM   9346  N   TRP J 201      -6.195  31.501 -16.563  1.00  0.00
J    N
ATOM   9347  HN  TRP J 201      -5.538  31.496 -15.814  1.00  0.00
J    H
ATOM   9348  CA  TRP J 201      -5.640  31.187 -17.873  1.00  0.00
J    C
ATOM   9349  HA  TRP J 201      -6.021  31.932 -18.561  1.00  0.00
J    H
ATOM   9350  CB  TRP J 201      -4.092  31.316 -17.863  1.00  0.00
J    C
ATOM   9351  HB1 TRP J 201      -3.697  30.625 -17.086  1.00  0.00
J    H
ATOM   9352  HB2 TRP J 201      -3.678  30.976 -18.837  1.00  0.00
J    H
ATOM   9353  CG  TRP J 201      -3.627  32.733 -17.523  1.00  0.00
J    C
ATOM   9354  CD1 TRP J 201      -3.775  33.870 -18.267  1.00  0.00
J    C
ATOM   9355  HD1 TRP J 201      -4.206  33.902 -19.260  1.00  0.00
J    H
ATOM   9356  NE1 TRP J 201      -3.335  34.965 -17.550  1.00  0.00
J    N
ATOM   9357  HE1 TRP J 201      -3.363  35.896 -17.844  1.00  0.00
J    H
ATOM   9358  CE2 TRP J 201      -2.892  34.548 -16.322  1.00  0.00
J    C
ATOM   9359  CD2 TRP J 201      -3.028  33.134 -16.281  1.00  0.00
J    C
ATOM   9360  CE3 TRP J 201      -2.613  32.415 -15.170  1.00  0.00
J    C
ATOM   9361  HE3 TRP J 201      -2.675  31.336 -15.134  1.00  0.00
J    H
ATOM   9362  CZ3 TRP J 201      -2.094  33.120 -14.077  1.00  0.00
J    C
ATOM   9363  HZ3 TRP J 201      -1.779  32.577 -13.196  1.00  0.00
J    H
ATOM   9364  CZ2 TRP J 201      -2.357  35.242 -15.246  1.00  0.00
J    C
ATOM   9365  HZ2 TRP J 201      -2.234  36.314 -15.271  1.00  0.00
J    H
ATOM   9366  CH2 TRP J 201      -1.975  34.517 -14.109  1.00  0.00
J    C
ATOM   9367  HH2 TRP J 201      -1.583  35.040 -13.248  1.00  0.00
J    H
ATOM   9368  C   TRP J 201      -6.098  29.816 -18.399  1.00  0.00
J    C
```

ATOM 9369 O TRP J 201 -6.263 28.918 -17.570 1.00 0.00
J O
ATOM 9370 N PRO J 202 -6.320 29.601 -19.717 1.00 0.00
J N
ATOM 9371 CD PRO J 202 -5.974 30.553 -20.772 1.00 0.00
J C
ATOM 9372 HD1 PRO J 202 -6.684 30.815 -19.955 1.00 0.00
J H
ATOM 9373 HD2 PRO J 202 -5.346 31.434 -21.040 1.00 0.00
J H
ATOM 9374 CA PRO J 202 -6.809 28.335 -20.285 1.00 0.00
J C
ATOM 9375 HA PRO J 202 -7.825 28.153 -19.958 1.00 0.00
J H
ATOM 9376 CB PRO J 202 -6.770 28.548 -21.818 1.00 0.00
J C
ATOM 9377 HB1 PRO J 202 -7.710 29.051 -22.138 1.00 0.00
J H
ATOM 9378 HB2 PRO J 202 -6.661 27.599 -22.380 1.00 0.00
J H
ATOM 9379 CG PRO J 202 -6.744 30.068 -22.000 1.00 0.00
J C
ATOM 9380 HG1 PRO J 202 -7.121 30.390 -22.990 1.00 0.00
J H
ATOM 9381 HG2 PRO J 202 -5.702 30.437 -21.861 1.00 0.00
J H
ATOM 9382 C PRO J 202 -5.945 27.149 -19.907 1.00 0.00
J C
ATOM 9383 O PRO J 202 -6.437 26.101 -19.490 1.00 0.00
J O
ATOM 9384 N ASP J 203 -4.620 27.324 -20.038 1.00 0.00
J N
ATOM 9385 HN ASP J 203 -4.248 28.067 -20.588 1.00 0.00
J H
ATOM 9386 CA ASP J 203 -3.607 26.515 -19.440 1.00 0.00
J C
ATOM 9387 HA ASP J 203 -3.989 25.508 -19.314 1.00 0.00
J H
ATOM 9388 CB ASP J 203 -2.327 26.507 -20.339 1.00 0.00
J C
ATOM 9389 HB1 ASP J 203 -1.516 25.964 -19.814 1.00 0.00
J H
ATOM 9390 HB2 ASP J 203 -2.528 25.993 -21.299 1.00 0.00
J H
ATOM 9391 CG ASP J 203 -1.754 27.885 -20.683 1.00 0.00
J C
ATOM 9392 OD1 ASP J 203 -2.532 28.774 -21.119 1.00 0.00
J O
ATOM 9393 OD2 ASP J 203 -0.517 28.059 -20.539 1.00 0.00
J O
ATOM 9394 C ASP J 203 -3.373 27.072 -18.046 1.00 0.00
J C
ATOM 9395 O ASP J 203 -3.045 28.240 -17.849 1.00 0.00
J O

```
ATOM   9396  N   ASN J 204      -3.576  26.248 -16.996  1.00  0.00
J    N
ATOM   9397  HN  ASN J 204      -3.872  25.299 -17.096  1.00  0.00
J    H
ATOM   9398  CA  ASN J 204      -3.380  26.675 -15.627  1.00  0.00
J    C
ATOM   9399  HA  ASN J 204      -3.971  27.576 -15.505  1.00  0.00
J    H
ATOM   9400  CB  ASN J 204      -3.877  25.533 -14.672  1.00  0.00
J    C
ATOM   9401  HB1 ASN J 204      -4.943  25.328 -14.912  1.00  0.00
J    H
ATOM   9402  HB2 ASN J 204      -3.308  24.600 -14.859  1.00  0.00
J    H
ATOM   9403  CG  ASN J 204      -3.810  25.873 -13.181  1.00  0.00
J    C
ATOM   9404  OD1 ASN J 204      -2.790  26.324 -12.662  1.00  0.00
J    O
ATOM   9405  ND2 ASN J 204      -4.929  25.671 -12.453  1.00  0.00
J    N
ATOM   9406 HD21 ASN J 204      -4.893  25.901 -11.485  1.00  0.00
J    H
ATOM   9407 HD22 ASN J 204      -5.755  25.333 -12.894  1.00  0.00
J    H
ATOM   9408  C   ASN J 204      -1.916  27.045 -15.383  1.00  0.00
J    C
ATOM   9409  O   ASN J 204      -1.125  26.168 -15.086  1.00  0.00
J    O
ATOM   9410  N   LEU J 205      -1.524  28.334 -15.547  1.00  0.00
J    N
ATOM   9411  HN  LEU J 205      -2.104  28.993 -16.023  1.00  0.00
J    H
ATOM   9412  CA  LEU J 205      -0.260  28.884 -15.067  1.00  0.00
J    C
ATOM   9413  HA  LEU J 205      -0.364  29.959 -15.153  1.00  0.00
J    H
ATOM   9414  CB  LEU J 205      -0.179  28.641 -13.531  1.00  0.00
J    C
ATOM   9415  HB1 LEU J 205      -1.128  29.023 -13.087  1.00  0.00
J    H
ATOM   9416  HB2 LEU J 205      -0.168  27.546 -13.338  1.00  0.00
J    H
ATOM   9417  CG  LEU J 205       0.978  29.290 -12.754  1.00  0.00
J    C
ATOM   9418  HG  LEU J 205       1.366  30.162 -13.332  1.00  0.00
J    H
ATOM   9419  CD1 LEU J 205       0.479  29.717 -11.373  1.00  0.00
J    C
ATOM   9420 HD11 LEU J 205       1.310  30.145 -10.773  1.00  0.00
J    H
ATOM   9421 HD12 LEU J 205      -0.318  30.486 -11.464  1.00  0.00
J    H
ATOM   9422 HD13 LEU J 205       0.065  28.848 -10.819  1.00  0.00
J    H
```

```
ATOM   9423  CD2 LEU J 205       2.137  28.312 -12.536  1.00  0.00
J    C
ATOM   9424 HD21 LEU J 205       2.965  28.812 -11.989  1.00  0.00
J    H
ATOM   9425 HD22 LEU J 205       1.824  27.431 -11.940  1.00  0.00
J    H
ATOM   9426 HD23 LEU J 205       2.531  27.953 -13.511  1.00  0.00
J    H
ATOM   9427  C   LEU J 205       0.963  28.468 -15.900  1.00  0.00
J    C
ATOM   9428  O   LEU J 205       2.067  28.991 -15.731  1.00  0.00
J    O
ATOM   9429  N   MET J 206       0.762  27.603 -16.920  1.00  0.00
J    N
ATOM   9430  HN  MET J 206      -0.168  27.409 -17.225  1.00  0.00
J    H
ATOM   9431  CA  MET J 206       1.792  26.894 -17.654  1.00  0.00
J    C
ATOM   9432  HA  MET J 206       2.423  26.426 -16.908  1.00  0.00
J    H
ATOM   9433  CB  MET J 206       1.135  25.771 -18.490  1.00  0.00
J    C
ATOM   9434  HB1 MET J 206       0.347  25.309 -17.850  1.00  0.00
J    H
ATOM   9435  HB2 MET J 206       0.605  26.220 -19.360  1.00  0.00
J    H
ATOM   9436  CG  MET J 206       2.109  24.673 -18.953  1.00  0.00
J    C
ATOM   9437  HG1 MET J 206       2.836  25.086 -19.684  1.00  0.00
J    H
ATOM   9438  HG2 MET J 206       2.686  24.294 -18.081  1.00  0.00
J    H
ATOM   9439  SD  MET J 206       1.268  23.271 -19.743  1.00  0.00
J    S
ATOM   9440  CE  MET J 206       2.762  22.561 -20.485  1.00  0.00
J    C
ATOM   9441  HE1 MET J 206       2.520  21.647 -21.068  1.00  0.00
J    H
ATOM   9442  HE2 MET J 206       3.248  23.286 -21.174  1.00  0.00
J    H
ATOM   9443  HE3 MET J 206       3.502  22.282 -19.705  1.00  0.00
J    H
ATOM   9444  C   MET J 206       2.675  27.805 -18.495  1.00  0.00
J    C
ATOM   9445  O   MET J 206       3.848  27.522 -18.740  1.00  0.00
J    O
ATOM   9446  N   SER J 207       2.138  28.987 -18.868  1.00  0.00
J    N
ATOM   9447  HN  SER J 207       1.171  29.164 -18.690  1.00  0.00
J    H
ATOM   9448  CA  SER J 207       2.834  30.082 -19.527  1.00  0.00
J    C
ATOM   9449  HA  SER J 207       3.169  29.677 -20.473  1.00  0.00
J    H
```

```
ATOM   9450  CB  SER J 207       1.948  31.364 -19.699  1.00  0.00
J    C
ATOM   9451  HB1 SER J 207       2.221  32.084 -18.894  1.00  0.00
J    H
ATOM   9452  HB2 SER J 207       2.167  31.860 -20.671  1.00  0.00
J    H
ATOM   9453  OG  SER J 207       0.544  31.110 -19.590  1.00  0.00
J    O
ATOM   9454  HG1 SER J 207       0.108  31.965 -19.484  1.00  0.00
J    H
ATOM   9455  C   SER J 207       4.072  30.542 -18.769  1.00  0.00
J    C
ATOM   9456  O   SER J 207       5.121  30.816 -19.349  1.00  0.00
J    O
ATOM   9457  N   TYR J 208       3.983  30.630 -17.425  1.00  0.00
J    N
ATOM   9458  HN  TYR J 208       3.200  30.253 -16.932  1.00  0.00
J    H
ATOM   9459  CA  TYR J 208       4.983  31.261 -16.577  1.00  0.00
J    C
ATOM   9460  HA  TYR J 208       5.562  31.911 -17.223  1.00  0.00
J    H
ATOM   9461  CB  TYR J 208       4.362  32.064 -15.401  1.00  0.00
J    C
ATOM   9462  HB1 TYR J 208       3.896  31.355 -14.683  1.00  0.00
J    H
ATOM   9463  HB2 TYR J 208       5.147  32.623 -14.848  1.00  0.00
J    H
ATOM   9464  CG  TYR J 208       3.305  32.982 -15.906  1.00  0.00
J    C
ATOM   9465  CD1 TYR J 208       3.616  34.078 -16.729  1.00  0.00
J    C
ATOM   9466  HD1 TYR J 208       4.646  34.282 -16.985  1.00  0.00
J    H
ATOM   9467  CE1 TYR J 208       2.592  34.916 -17.196  1.00  0.00
J    C
ATOM   9468  HE1 TYR J 208       2.820  35.764 -17.826  1.00  0.00
J    H
ATOM   9469  CZ  TYR J 208       1.266  34.656 -16.838  1.00  0.00
J    C
ATOM   9470  OH  TYR J 208       0.236  35.511 -17.245  1.00  0.00
J    O
ATOM   9471  HH  TYR J 208      -0.592  35.120 -16.960  1.00  0.00
J    H
ATOM   9472  CD2 TYR J 208       1.970  32.723 -15.571  1.00  0.00
J    C
ATOM   9473  HD2 TYR J 208       1.729  31.904 -14.906  1.00  0.00
J    H
ATOM   9474  CE2 TYR J 208       0.952  33.548 -16.051  1.00  0.00
J    C
ATOM   9475  HE2 TYR J 208      -0.076  33.338 -15.794  1.00  0.00
J    H
ATOM   9476  C   TYR J 208       5.942  30.246 -15.988  1.00  0.00
J    C
```

```
ATOM   9477  O   TYR J 208       6.459  30.434 -14.894  1.00  0.00
J    O
ATOM   9478  N   VAL J 209       6.228  29.148 -16.723  1.00  0.00
J    N
ATOM   9479  HN  VAL J 209       5.874  29.070 -17.652  1.00  0.00
J    H
ATOM   9480  CA  VAL J 209       7.035  28.013 -16.287  1.00  0.00
J    C
ATOM   9481  HA  VAL J 209       6.463  27.531 -15.504  1.00  0.00
J    H
ATOM   9482  CB  VAL J 209       7.182  26.991 -17.433  1.00  0.00
J    C
ATOM   9483  HB  VAL J 209       6.180  26.527 -17.602  1.00  0.00
J    H
ATOM   9484  CG1 VAL J 209       7.643  27.650 -18.757  1.00  0.00
J    C
ATOM   9485 HG11 VAL J 209       7.776  26.879 -19.546  1.00  0.00
J    H
ATOM   9486 HG12 VAL J 209       6.898  28.381 -19.131  1.00  0.00
J    H
ATOM   9487 HG13 VAL J 209       8.615  28.170 -18.618  1.00  0.00
J    H
ATOM   9488  CG2 VAL J 209       8.115  25.817 -17.051  1.00  0.00
J    C
ATOM   9489 HG21 VAL J 209       8.236  25.117 -17.905  1.00  0.00
J    H
ATOM   9490 HG22 VAL J 209       9.119  26.194 -16.763  1.00  0.00
J    H
ATOM   9491 HG23 VAL J 209       7.703  25.238 -16.199  1.00  0.00
J    H
ATOM   9492  C   VAL J 209       8.394  28.382 -15.699  1.00  0.00
J    C
ATOM   9493  O   VAL J 209       8.785  27.862 -14.653  1.00  0.00
J    O
ATOM   9494  N   GLY J 210       9.120  29.316 -16.344  1.00  0.00
J    N
ATOM   9495  HN  GLY J 210       8.773  29.698 -17.199  1.00  0.00
J    H
ATOM   9496  CA  GLY J 210      10.404  29.838 -15.897  1.00  0.00
J    C
ATOM   9497  HA1 GLY J 210      11.049  29.803 -16.764  1.00  0.00
J    H
ATOM   9498  HA2 GLY J 210      10.717  29.196 -15.084  1.00  0.00
J    H
ATOM   9499  C   GLY J 210      10.381  31.254 -15.389  1.00  0.00
J    C
ATOM   9500  O   GLY J 210      11.443  31.824 -15.156  1.00  0.00
J    O
ATOM   9501  N   ASP J 211       9.202  31.900 -15.198  1.00  0.00
J    N
ATOM   9502  HN  ASP J 211       8.291  31.550 -15.396  1.00  0.00
J    H
ATOM   9503  CA  ASP J 211       9.185  33.255 -14.650  1.00  0.00
J    C
```

```
ATOM   9504  HA  ASP J 211       9.947  33.832 -15.161  1.00  0.00
J    H
ATOM   9505  CB  ASP J 211       7.835  34.018 -14.868  1.00  0.00
J    C
ATOM   9506  HB1 ASP J 211       7.597  34.036 -15.951  1.00  0.00
J    H
ATOM   9507  HB2 ASP J 211       7.007  33.506 -14.339  1.00  0.00
J    H
ATOM   9508  CG  ASP J 211       7.896  35.470 -14.387  1.00  0.00
J    C
ATOM   9509  OD1 ASP J 211       7.844  35.676 -13.144  1.00  0.00
J    O
ATOM   9510  OD2 ASP J 211       8.022  36.400 -15.224  1.00  0.00
J    O
ATOM   9511  C   ASP J 211       9.591  33.200 -13.161  1.00  0.00
J    C
ATOM   9512  O   ASP J 211       8.965  32.471 -12.382  1.00  0.00
J    O
ATOM   9513  N   PRO J 212      10.627  33.907 -12.703  1.00  0.00
J    N
ATOM   9514  CD  PRO J 212      11.508  34.734 -13.528  1.00  0.00
J    C
ATOM   9515  HD1 PRO J 212      12.122  34.170 -14.267  1.00  0.00
J    H
ATOM   9516  HD2 PRO J 212      10.858  35.475 -14.048  1.00  0.00
J    H
ATOM   9517  CA  PRO J 212      11.120  33.759 -11.345  1.00  0.00
J    C
ATOM   9518  HA  PRO J 212      11.239  32.714 -11.084  1.00  0.00
J    H
ATOM   9519  CB  PRO J 212      12.505  34.437 -11.377  1.00  0.00
J    C
ATOM   9520  HB1 PRO J 212      13.261  33.702 -11.734  1.00  0.00
J    H
ATOM   9521  HB2 PRO J 212      12.818  34.821 -10.385  1.00  0.00
J    H
ATOM   9522  CG  PRO J 212      12.416  35.447 -12.528  1.00  0.00
J    C
ATOM   9523  HG1 PRO J 212      13.406  35.714 -12.947  1.00  0.00
J    H
ATOM   9524  HG2 PRO J 212      11.903  36.369 -12.169  1.00  0.00
J    H
ATOM   9525  C   PRO J 212      10.168  34.409 -10.362  1.00  0.00
J    C
ATOM   9526  O   PRO J 212      10.002  33.892  -9.259  1.00  0.00
J    O
ATOM   9527  N   THR J 213       9.530  35.539 -10.726  1.00  0.00
J    N
ATOM   9528  HN  THR J 213       9.631  35.939 -11.635  1.00  0.00
J    H
ATOM   9529  CA  THR J 213       8.641  36.280  -9.840  1.00  0.00
J    C
ATOM   9530  HA  THR J 213       9.162  36.358  -8.896  1.00  0.00
J    H
```

ATOM 9531 CB THR J 213 8.241 37.644 -10.397 1.00 0.00
J C
ATOM 9532 HB THR J 213 7.772 37.447 -11.393 1.00 0.00
J H
ATOM 9533 OG1 THR J 213 9.382 38.460 -10.619 1.00 0.00
J O
ATOM 9534 HG1 THR J 213 9.040 39.296 -10.956 1.00 0.00
J H
ATOM 9535 CG2 THR J 213 7.364 38.424 -9.401 1.00 0.00
J C
ATOM 9536 HG21 THR J 213 6.986 39.363 -9.858 1.00 0.00
J H
ATOM 9537 HG22 THR J 213 6.487 37.814 -9.096 1.00 0.00
J H
ATOM 9538 HG23 THR J 213 7.937 38.690 -8.486 1.00 0.00
J H
ATOM 9539 C THR J 213 7.369 35.508 -9.568 1.00 0.00
J C
ATOM 9540 O THR J 213 6.963 35.342 -8.418 1.00 0.00
J O
ATOM 9541 N VAL J 214 6.721 34.968 -10.627 1.00 0.00
J N
ATOM 9542 HN VAL J 214 7.059 35.052 -11.560 1.00 0.00
J H
ATOM 9543 CA VAL J 214 5.476 34.216 -10.501 1.00 0.00
J C
ATOM 9544 HA VAL J 214 4.778 34.842 -9.959 1.00 0.00
J H
ATOM 9545 CB VAL J 214 4.856 33.897 -11.865 1.00 0.00
J C
ATOM 9546 HB VAL J 214 5.634 33.376 -12.476 1.00 0.00
J H
ATOM 9547 CG1 VAL J 214 3.626 32.968 -11.732 1.00 0.00
J C
ATOM 9548 HG11 VAL J 214 3.173 32.784 -12.729 1.00 0.00
J H
ATOM 9549 HG12 VAL J 214 3.903 31.981 -11.308 1.00 0.00
J H
ATOM 9550 HG13 VAL J 214 2.853 33.432 -11.082 1.00 0.00
J H
ATOM 9551 CG2 VAL J 214 4.432 35.222 -12.536 1.00 0.00
J C
ATOM 9552 HG21 VAL J 214 3.941 35.028 -13.513 1.00 0.00
J H
ATOM 9553 HG22 VAL J 214 3.718 35.776 -11.889 1.00 0.00
J H
ATOM 9554 HG23 VAL J 214 5.309 35.875 -12.726 1.00 0.00
J H
ATOM 9555 C VAL J 214 5.670 32.948 -9.697 1.00 0.00
J C
ATOM 9556 O VAL J 214 4.935 32.690 -8.739 1.00 0.00
J O
ATOM 9557 N ASN J 215 6.723 32.160 -10.007 1.00 0.00
J N

ATOM 9558 HN ASN J 215 7.371 32.372 -10.737 1.00 0.00
J H
ATOM 9559 CA ASN J 215 7.001 30.927 -9.294 1.00 0.00
J C
ATOM 9560 HA ASN J 215 6.089 30.346 -9.371 1.00 0.00
J H
ATOM 9561 CB ASN J 215 8.196 30.179 -9.936 1.00 0.00
J C
ATOM 9562 HB1 ASN J 215 8.958 30.937 -10.222 1.00 0.00
J H
ATOM 9563 HB2 ASN J 215 8.665 29.497 -9.198 1.00 0.00
J H
ATOM 9564 CG ASN J 215 7.723 29.436 -11.180 1.00 0.00
J C
ATOM 9565 OD1 ASN J 215 6.546 29.121 -11.354 1.00 0.00
J O
ATOM 9566 ND2 ASN J 215 8.692 29.075 -12.042 1.00 0.00
J N
ATOM 9567 HD21 ASN J 215 8.414 28.598 -12.872 1.00 0.00
J H
ATOM 9568 HD22 ASN J 215 9.641 29.312 -11.854 1.00 0.00
J H
ATOM 9569 C ASN J 215 7.285 31.145 -7.814 1.00 0.00
J C
ATOM 9570 O ASN J 215 6.714 30.464 -6.959 1.00 0.00
J O
ATOM 9571 N LEU J 216 8.139 32.124 -7.453 1.00 0.00
J N
ATOM 9572 HN LEU J 216 8.613 32.716 -8.102 1.00 0.00
J H
ATOM 9573 CA LEU J 216 8.445 32.398 -6.060 1.00 0.00
J C
ATOM 9574 HA LEU J 216 8.739 31.446 -5.637 1.00 0.00
J H
ATOM 9575 CB LEU J 216 9.638 33.373 -5.925 1.00 0.00
J C
ATOM 9576 HB1 LEU J 216 9.394 34.277 -6.529 1.00 0.00
J H
ATOM 9577 HB2 LEU J 216 9.706 33.717 -4.868 1.00 0.00
J H
ATOM 9578 CG LEU J 216 10.985 32.789 -6.405 1.00 0.00
J C
ATOM 9579 HG LEU J 216 10.810 32.460 -7.458 1.00 0.00
J H
ATOM 9580 CD1 LEU J 216 12.076 33.872 -6.375 1.00 0.00
J C
ATOM 9581 HD11 LEU J 216 13.058 33.447 -6.674 1.00 0.00
J H
ATOM 9582 HD12 LEU J 216 11.827 34.698 -7.075 1.00 0.00
J H
ATOM 9583 HD13 LEU J 216 12.179 34.297 -5.355 1.00 0.00
J H
ATOM 9584 CD2 LEU J 216 11.429 31.560 -5.589 1.00 0.00
J C

ATOM 9585 HD21 LEU J 216 12.390 31.163 -5.981 1.00 0.00
J H
ATOM 9586 HD22 LEU J 216 11.579 31.811 -4.520 1.00 0.00
J H
ATOM 9587 HD23 LEU J 216 10.667 30.754 -5.656 1.00 0.00
J H
ATOM 9588 C LEU J 216 7.261 32.912 -5.255 1.00 0.00
J C
ATOM 9589 O LEU J 216 6.993 32.406 -4.168 1.00 0.00
J O
ATOM 9590 N VAL J 217 6.480 33.885 -5.778 1.00 0.00
J N
ATOM 9591 HN VAL J 217 6.641 34.305 -6.668 1.00 0.00
J H
ATOM 9592 CA VAL J 217 5.316 34.407 -5.068 1.00 0.00
J C
ATOM 9593 HA VAL J 217 5.656 34.706 -4.085 1.00 0.00
J H
ATOM 9594 CB VAL J 217 4.744 35.642 -5.771 1.00 0.00
J C
ATOM 9595 HB VAL J 217 4.506 35.351 -6.823 1.00 0.00
J H
ATOM 9596 CG1 VAL J 217 3.448 36.145 -5.094 1.00 0.00
J C
ATOM 9597 HG11 VAL J 217 3.079 37.062 -5.602 1.00 0.00
J H
ATOM 9598 HG12 VAL J 217 2.638 35.388 -5.148 1.00 0.00
J H
ATOM 9599 HG13 VAL J 217 3.632 36.392 -4.027 1.00 0.00
J H
ATOM 9600 CG2 VAL J 217 5.795 36.771 -5.739 1.00 0.00
J C
ATOM 9601 HG21 VAL J 217 5.394 37.695 -6.208 1.00 0.00
J H
ATOM 9602 HG22 VAL J 217 6.083 37.007 -4.692 1.00 0.00
J H
ATOM 9603 HG23 VAL J 217 6.712 36.484 -6.296 1.00 0.00
J H
ATOM 9604 C VAL J 217 4.232 33.351 -4.875 1.00 0.00
J C
ATOM 9605 O VAL J 217 3.736 33.145 -3.767 1.00 0.00
J O
ATOM 9606 N ILE J 218 3.866 32.599 -5.937 1.00 0.00
J N
ATOM 9607 HN ILE J 218 4.277 32.680 -6.842 1.00 0.00
J H
ATOM 9608 CA ILE J 218 2.821 31.588 -5.845 1.00 0.00
J C
ATOM 9609 HA ILE J 218 1.988 32.076 -5.356 1.00 0.00
J H
ATOM 9610 CB ILE J 218 2.277 31.207 -7.219 1.00 0.00
J C
ATOM 9611 HB ILE J 218 3.190 30.993 -7.832 1.00 0.00
J H

```
ATOM   9612  CG2 ILE J 218       1.322  29.987  -7.128  1.00  0.00
J    C
ATOM   9613 HG21 ILE J 218       1.056  29.623  -8.143  1.00  0.00
J    H
ATOM   9614 HG22 ILE J 218       1.802  29.147  -6.585  1.00  0.00
J    H
ATOM   9615 HG23 ILE J 218       0.383  30.256  -6.600  1.00  0.00
J    H
ATOM   9616  CG1 ILE J 218       1.595  32.489  -7.776  1.00  0.00
J    C
ATOM   9617 HG11 ILE J 218       0.971  32.959  -6.983  1.00  0.00
J    H
ATOM   9618 HG12 ILE J 218       2.384  33.226  -8.053  1.00  0.00
J    H
ATOM   9619  CD  ILE J 218       0.720  32.271  -9.009  1.00  0.00
J    C
ATOM   9620  HD1 ILE J 218       0.320  33.242  -9.375  1.00  0.00
J    H
ATOM   9621  HD2 ILE J 218       1.308  31.806  -9.830  1.00  0.00
J    H
ATOM   9622  HD3 ILE J 218      -0.141  31.611  -8.775  1.00  0.00
J    H
ATOM   9623  C   ILE J 218       3.209  30.405  -4.975  1.00  0.00
J    C
ATOM   9624  O   ILE J 218       2.421  29.985  -4.131  1.00  0.00
J    O
ATOM   9625  N   THR J 219       4.455  29.880  -5.095  1.00  0.00
J    N
ATOM   9626  HN  THR J 219       5.123  30.167  -5.781  1.00  0.00
J    H
ATOM   9627  CA  THR J 219       4.931  28.818  -4.198  1.00  0.00
J    C
ATOM   9628  HA  THR J 219       4.186  28.038  -4.265  1.00  0.00
J    H
ATOM   9629  CB  THR J 219       6.266  28.158  -4.546  1.00  0.00
J    C
ATOM   9630  HB  THR J 219       6.523  27.503  -3.678  1.00  0.00
J    H
ATOM   9631  OG1 THR J 219       7.348  29.074  -4.682  1.00  0.00
J    O
ATOM   9632  HG1 THR J 219       8.113  28.536  -4.909  1.00  0.00
J    H
ATOM   9633  CG2 THR J 219       6.127  27.391  -5.863  1.00  0.00
J    C
ATOM   9634 HG21 THR J 219       7.048  26.810  -6.081  1.00  0.00
J    H
ATOM   9635 HG22 THR J 219       5.277  26.679  -5.808  1.00  0.00
J    H
ATOM   9636 HG23 THR J 219       5.946  28.085  -6.712  1.00  0.00
J    H
ATOM   9637  C   THR J 219       4.977  29.243  -2.754  1.00  0.00
J    C
ATOM   9638  O   THR J 219       4.472  28.520  -1.893  1.00  0.00
J    O
```

ATOM 9639 N PHE J 220 5.481 30.452 -2.436 1.00 0.00
J N
ATOM 9640 HN PHE J 220 5.892 31.054 -3.118 1.00 0.00
J H
ATOM 9641 CA PHE J 220 5.474 30.989 -1.087 1.00 0.00
J C
ATOM 9642 HA PHE J 220 6.053 30.294 -0.490 1.00 0.00
J H
ATOM 9643 CB PHE J 220 6.116 32.415 -1.103 1.00 0.00
J C
ATOM 9644 HB1 PHE J 220 7.203 32.323 -1.315 1.00 0.00
J H
ATOM 9645 HB2 PHE J 220 5.677 33.042 -1.909 1.00 0.00
J H
ATOM 9646 CG PHE J 220 6.007 33.149 0.217 1.00 0.00
J C
ATOM 9647 CD1 PHE J 220 4.878 33.949 0.480 1.00 0.00
J C
ATOM 9648 HD1 PHE J 220 4.141 34.106 -0.296 1.00 0.00
J H
ATOM 9649 CE1 PHE J 220 4.702 34.548 1.732 1.00 0.00
J C
ATOM 9650 HE1 PHE J 220 3.814 35.129 1.933 1.00 0.00
J H
ATOM 9651 CZ PHE J 220 5.676 34.388 2.725 1.00 0.00
J C
ATOM 9652 HZ PHE J 220 5.548 34.855 3.691 1.00 0.00
J H
ATOM 9653 CD2 PHE J 220 6.973 32.990 1.222 1.00 0.00
J C
ATOM 9654 HD2 PHE J 220 7.859 32.400 1.031 1.00 0.00
J H
ATOM 9655 CE2 PHE J 220 6.814 33.613 2.468 1.00 0.00
J C
ATOM 9656 HE2 PHE J 220 7.572 33.494 3.230 1.00 0.00
J H
ATOM 9657 C PHE J 220 4.068 31.028 -0.491 1.00 0.00
J C
ATOM 9658 O PHE J 220 3.858 30.603 0.645 1.00 0.00
J O
ATOM 9659 N LEU J 221 3.059 31.503 -1.247 1.00 0.00
J N
ATOM 9660 HN LEU J 221 3.166 31.820 -2.188 1.00 0.00
J H
ATOM 9661 CA LEU J 221 1.706 31.590 -0.740 1.00 0.00
J C
ATOM 9662 HA LEU J 221 1.780 32.188 0.159 1.00 0.00
J H
ATOM 9663 CB LEU J 221 0.795 32.253 -1.793 1.00 0.00
J C
ATOM 9664 HB1 LEU J 221 1.051 31.803 -2.780 1.00 0.00
J H
ATOM 9665 HB2 LEU J 221 -0.261 31.966 -1.589 1.00 0.00
J H

ATOM 9666 CG LEU J 221 0.931 33.780 −1.894 1.00 0.00
J C
ATOM 9667 HG LEU J 221 1.986 34.044 −2.146 1.00 0.00
J H
ATOM 9668 CD1 LEU J 221 0.005 34.262 −3.019 1.00 0.00
J C
ATOM 9669 HD11 LEU J 221 0.033 35.370 −3.099 1.00 0.00
J H
ATOM 9670 HD12 LEU J 221 0.319 33.833 −3.995 1.00 0.00
J H
ATOM 9671 HD13 LEU J 221 −1.044 33.955 −2.822 1.00 0.00
J H
ATOM 9672 CD2 LEU J 221 0.578 34.454 −0.555 1.00 0.00
J C
ATOM 9673 HD21 LEU J 221 0.698 35.557 −0.635 1.00 0.00
J H
ATOM 9674 HD22 LEU J 221 −0.470 34.249 −0.258 1.00 0.00
J H
ATOM 9675 HD23 LEU J 221 1.248 34.089 0.252 1.00 0.00
J H
ATOM 9676 C LEU J 221 1.085 30.261 −0.356 1.00 0.00
J C
ATOM 9677 O LEU J 221 0.570 30.114 0.754 1.00 0.00
J O
ATOM 9678 N PHE J 222 1.153 29.242 −1.234 1.00 0.00
J N
ATOM 9679 HN PHE J 222 1.582 29.295 −2.134 1.00 0.00
J H
ATOM 9680 CA PHE J 222 0.585 27.947 −0.916 1.00 0.00
J C
ATOM 9681 HA PHE J 222 −0.384 28.164 −0.482 1.00 0.00
J H
ATOM 9682 CB PHE J 222 0.139 27.150 −2.171 1.00 0.00
J C
ATOM 9683 HB1 PHE J 222 −0.420 26.244 −1.848 1.00 0.00
J H
ATOM 9684 HB2 PHE J 222 −0.549 27.751 −2.803 1.00 0.00
J H
ATOM 9685 CG PHE J 222 1.217 26.630 −3.053 1.00 0.00
J C
ATOM 9686 CD1 PHE J 222 1.896 25.470 −2.671 1.00 0.00
J C
ATOM 9687 HD1 PHE J 222 1.763 25.076 −1.672 1.00 0.00
J H
ATOM 9688 CE1 PHE J 222 2.745 24.826 −3.569 1.00 0.00
J C
ATOM 9689 HE1 PHE J 222 3.229 23.902 −3.288 1.00 0.00
J H
ATOM 9690 CZ PHE J 222 2.972 25.367 −4.839 1.00 0.00
J C
ATOM 9691 HZ PHE J 222 3.673 24.897 −5.515 1.00 0.00
J H
ATOM 9692 CD2 PHE J 222 1.395 27.137 −4.349 1.00 0.00
J C

```
ATOM   9693  HD2 PHE J 222       0.861  28.025  -4.660  1.00  0.00
J    H
ATOM   9694  CE2 PHE J 222       2.281  26.516  -5.238  1.00  0.00
J    C
ATOM   9695  HE2 PHE J 222       2.427  26.927  -6.227  1.00  0.00
J    H
ATOM   9696  C   PHE J 222       1.379  27.161   0.130  1.00  0.00
J    C
ATOM   9697  O   PHE J 222       0.792  26.501   0.984  1.00  0.00
J    O
ATOM   9698  N   ILE J 223       2.735  27.250   0.149  1.00  0.00
J    N
ATOM   9699  HN  ILE J 223       3.260  27.718  -0.557  1.00  0.00
J    H
ATOM   9700  CA  ILE J 223       3.552  26.662   1.215  1.00  0.00
J    C
ATOM   9701  HA  ILE J 223       3.260  25.621   1.275  1.00  0.00
J    H
ATOM   9702  CB  ILE J 223       5.056  26.775   0.933  1.00  0.00
J    C
ATOM   9703  HB  ILE J 223       5.218  27.861   0.714  1.00  0.00
J    H
ATOM   9704  CG2 ILE J 223       5.889  26.247   2.131  1.00  0.00
J    C
ATOM   9705 HG21 ILE J 223       6.966  26.474   1.988  1.00  0.00
J    H
ATOM   9706 HG22 ILE J 223       5.567  26.727   3.079  1.00  0.00
J    H
ATOM   9707 HG23 ILE J 223       5.777  25.147   2.239  1.00  0.00
J    H
ATOM   9708  CG1 ILE J 223       5.455  26.008  -0.349  1.00  0.00
J    C
ATOM   9709 HG11 ILE J 223       5.356  24.912  -0.178  1.00  0.00
J    H
ATOM   9710 HG12 ILE J 223       4.747  26.277  -1.166  1.00  0.00
J    H
ATOM   9711  CD  ILE J 223       6.887  26.351  -0.785  1.00  0.00
J    C
ATOM   9712  HD1 ILE J 223       7.134  25.835  -1.738  1.00  0.00
J    H
ATOM   9713  HD2 ILE J 223       6.998  27.445  -0.943  1.00  0.00
J    H
ATOM   9714  HD3 ILE J 223       7.624  26.029  -0.019  1.00  0.00
J    H
ATOM   9715  C   ILE J 223       3.251  27.284   2.579  1.00  0.00
J    C
ATOM   9716  O   ILE J 223       3.032  26.573   3.559  1.00  0.00
J    O
ATOM   9717  N   THR J 224       3.174  28.633   2.668  1.00  0.00
J    N
ATOM   9718  HN  THR J 224       3.349  29.222   1.880  1.00  0.00
J    H
ATOM   9719  CA  THR J 224       2.833  29.358   3.900  1.00  0.00
J    C
```

```
ATOM   9720  HA  THR J 224       3.515  28.987   4.653  1.00  0.00
J    H
ATOM   9721  CB  THR J 224       2.954  30.875   3.762  1.00  0.00
J    C
ATOM   9722  HB  THR J 224       2.375  31.151   2.847  1.00  0.00
J    H
ATOM   9723  OG1 THR J 224       4.312  31.227   3.546  1.00  0.00
J    O
ATOM   9724  HG1 THR J 224       4.320  32.188   3.480  1.00  0.00
J    H
ATOM   9725  CG2 THR J 224       2.560  31.621   5.047  1.00  0.00
J    C
ATOM   9726 HG21 THR J 224       2.549  32.719   4.879  1.00  0.00
J    H
ATOM   9727 HG22 THR J 224       1.543  31.316   5.376  1.00  0.00
J    H
ATOM   9728 HG23 THR J 224       3.272  31.402   5.871  1.00  0.00
J    H
ATOM   9729  C   THR J 224       1.436  29.024   4.380  1.00  0.00
J    C
ATOM   9730  O   THR J 224       1.210  28.775   5.564  1.00  0.00
J    O
ATOM   9731  N   GLY J 225       0.450  28.950   3.460  1.00  0.00
J    N
ATOM   9732  HN  GLY J 225       0.597  29.204   2.505  1.00  0.00
J    H
ATOM   9733  CA  GLY J 225      -0.898  28.497   3.791  1.00  0.00
J    C
ATOM   9734  HA1 GLY J 225      -1.484  28.610   2.890  1.00  0.00
J    H
ATOM   9735  HA2 GLY J 225      -1.232  29.110   4.618  1.00  0.00
J    H
ATOM   9736  C   GLY J 225      -0.976  27.053   4.221  1.00  0.00
J    C
ATOM   9737  O   GLY J 225      -1.799  26.693   5.060  1.00  0.00
J    O
ATOM   9738  N   GLY J 226      -0.092  26.199   3.662  1.00  0.00
J    N
ATOM   9739  HN  GLY J 226       0.605  26.577   3.052  1.00  0.00
J    H
ATOM   9740  CA  GLY J 226      -0.043  24.755   3.856  1.00  0.00
J    C
ATOM   9741  HA1 GLY J 226       0.491  24.375   2.996  1.00  0.00
J    H
ATOM   9742  HA2 GLY J 226      -1.077  24.440   3.913  1.00  0.00
J    H
ATOM   9743  C   GLY J 226       0.662  24.264   5.097  1.00  0.00
J    C
ATOM   9744  O   GLY J 226       0.404  23.152   5.539  1.00  0.00
J    O
ATOM   9745  N   ILE J 227       1.557  25.023   5.751  1.00  0.00
J    N
ATOM   9746  HN  ILE J 227       1.905  25.898   5.419  1.00  0.00
J    H
```

```
ATOM   9747  CA  ILE J 227       2.107  24.609   7.049  1.00  0.00
J    C
ATOM   9748  HA  ILE J 227       2.349  23.560   6.933  1.00  0.00
J    H
ATOM   9749  CB  ILE J 227       3.415  25.295   7.417  1.00  0.00
J    C
ATOM   9750  HB  ILE J 227       3.477  25.200   8.531  1.00  0.00
J    H
ATOM   9751  CG2 ILE J 227       4.545  24.541   6.675  1.00  0.00
J    C
ATOM   9752 HG21 ILE J 227       5.540  24.890   7.021  1.00  0.00
J    H
ATOM   9753 HG22 ILE J 227       4.487  23.450   6.868  1.00  0.00
J    H
ATOM   9754 HG23 ILE J 227       4.481  24.708   5.579  1.00  0.00
J    H
ATOM   9755  CG1 ILE J 227       3.380  26.812   7.115  1.00  0.00
J    C
ATOM   9756 HG11 ILE J 227       3.117  26.977   6.046  1.00  0.00
J    H
ATOM   9757 HG12 ILE J 227       2.578  27.283   7.727  1.00  0.00
J    H
ATOM   9758  CD  ILE J 227       4.698  27.528   7.421  1.00  0.00
J    C
ATOM   9759  HD1 ILE J 227       4.595  28.620   7.240  1.00  0.00
J    H
ATOM   9760  HD2 ILE J 227       4.988  27.378   8.483  1.00  0.00
J    H
ATOM   9761  HD3 ILE J 227       5.518  27.149   6.774  1.00  0.00
J    H
ATOM   9762  C   ILE J 227       1.114  24.707   8.201  1.00  0.00
J    C
ATOM   9763  O   ILE J 227       1.102  23.876   9.110  1.00  0.00
J    O
ATOM   9764  N   GLY J 228       0.226  25.716   8.163  1.00  0.00
J    N
ATOM   9765  HN  GLY J 228       0.253  26.388   7.425  1.00  0.00
J    H
ATOM   9766  CA  GLY J 228      -0.818  25.914   9.154  1.00  0.00
J    C
ATOM   9767  HA1 GLY J 228      -0.946  24.959   9.646  1.00  0.00
J    H
ATOM   9768  HA2 GLY J 228      -1.689  26.254   8.611  1.00  0.00
J    H
ATOM   9769  C   GLY J 228      -0.502  26.929  10.195  1.00  0.00
J    C
ATOM   9770  O   GLY J 228       0.643  27.171  10.571  1.00  0.00
J    O
ATOM   9771  N   PHE J 229      -1.571  27.553  10.717  1.00  0.00
J    N
ATOM   9772  HN  PHE J 229      -2.502  27.261  10.505  1.00  0.00
J    H
ATOM   9773  CA  PHE J 229      -1.488  28.685  11.619  1.00  0.00
J    C
```

ATOM 9774 HA PHE J 229 -0.904 29.422 11.082 1.00 0.00
J H
ATOM 9775 CB PHE J 229 -2.868 29.288 11.932 1.00 0.00
J C
ATOM 9776 HB1 PHE J 229 -3.548 28.480 12.280 1.00 0.00
J H
ATOM 9777 HB2 PHE J 229 -2.800 30.035 12.752 1.00 0.00
J H
ATOM 9778 CG PHE J 229 -3.389 29.863 10.657 1.00 0.00
J C
ATOM 9779 CD1 PHE J 229 -2.886 31.083 10.171 1.00 0.00
J C
ATOM 9780 HD1 PHE J 229 -2.124 31.607 10.730 1.00 0.00
J H
ATOM 9781 CE1 PHE J 229 -3.374 31.616 8.974 1.00 0.00
J C
ATOM 9782 HE1 PHE J 229 -3.014 32.572 8.621 1.00 0.00
J H
ATOM 9783 CZ PHE J 229 -4.324 30.912 8.229 1.00 0.00
J C
ATOM 9784 HZ PHE J 229 -4.690 31.315 7.295 1.00 0.00
J H
ATOM 9785 CD2 PHE J 229 -4.350 29.169 9.911 1.00 0.00
J C
ATOM 9786 HD2 PHE J 229 -4.767 28.247 10.291 1.00 0.00
J H
ATOM 9787 CE2 PHE J 229 -4.793 29.678 8.688 1.00 0.00
J C
ATOM 9788 HE2 PHE J 229 -5.502 29.115 8.095 1.00 0.00
J H
ATOM 9789 C PHE J 229 -0.765 28.403 12.910 1.00 0.00
J C
ATOM 9790 O PHE J 229 -0.077 29.276 13.410 1.00 0.00
J O
ATOM 9791 N THR J 230 -0.857 27.170 13.452 1.00 0.00
J N
ATOM 9792 HN THR J 230 -1.491 26.483 13.099 1.00 0.00
J H
ATOM 9793 CA THR J 230 -0.052 26.718 14.594 1.00 0.00
J C
ATOM 9794 HA THR J 230 -0.317 27.382 15.405 1.00 0.00
J H
ATOM 9795 CB THR J 230 -0.314 25.248 14.927 1.00 0.00
J C
ATOM 9796 HB THR J 230 -0.277 24.698 13.954 1.00 0.00
J H
ATOM 9797 OG1 THR J 230 -1.624 25.124 15.457 1.00 0.00
J O
ATOM 9798 HG1 THR J 230 -1.732 24.188 15.659 1.00 0.00
J H
ATOM 9799 CG2 THR J 230 0.662 24.652 15.959 1.00 0.00
J C
ATOM 9800 HG21 THR J 230 0.496 23.560 16.075 1.00 0.00
J H

ATOM   9801 HG22 THR J 230   1.713 24.804 15.631 1.00 0.00
J   H
ATOM   9802 HG23 THR J 230   0.534 25.130 16.953 1.00 0.00
J   H
ATOM   9803 C   THR J 230   1.438 26.881 14.371 1.00 0.00
J   C
ATOM   9804 O   THR J 230   2.121 27.492 15.189 1.00 0.00
J   O
ATOM   9805 N   VAL J 231   1.968 26.417 13.219 1.00 0.00
J   N
ATOM   9806 HN VAL J 231   1.402 25.968 12.532 1.00 0.00
J   H
ATOM   9807 CA VAL J 231   3.378 26.518 12.865 1.00 0.00
J   C
ATOM   9808 HA VAL J 231   3.953 26.103 13.683 1.00 0.00
J   H
ATOM   9809 CB VAL J 231   3.664 25.755 11.570 1.00 0.00
J   C
ATOM   9810 HB VAL J 231   3.061 26.230 10.758 1.00 0.00
J   H
ATOM   9811 CG1 VAL J 231   5.159 25.831 11.189 1.00 0.00
J   C
ATOM   9812 HG11 VAL J 231   5.353 25.238 10.269 1.00 0.00
J   H
ATOM   9813 HG12 VAL J 231   5.477 26.874 10.983 1.00 0.00
J   H
ATOM   9814 HG13 VAL J 231   5.793 25.418 12.003 1.00 0.00
J   H
ATOM   9815 CG2 VAL J 231   3.251 24.281 11.763 1.00 0.00
J   C
ATOM   9816 HG21 VAL J 231   3.482 23.686 10.853 1.00 0.00
J   H
ATOM   9817 HG22 VAL J 231   3.795 23.832 12.622 1.00 0.00
J   H
ATOM   9818 HG23 VAL J 231   2.162 24.190 11.954 1.00 0.00
J   H
ATOM   9819 C   VAL J 231   3.790 27.974 12.711 1.00 0.00
J   C
ATOM   9820 O   VAL J 231   4.815 28.419 13.222 1.00 0.00
J   O
ATOM   9821 N   LEU J 232   2.932 28.770 12.031 1.00 0.00
J   N
ATOM   9822 HN LEU J 232   2.109 28.404 11.602 1.00 0.00
J   H
ATOM   9823 CA LEU J 232   3.111 30.200 11.856 1.00 0.00
J   C
ATOM   9824 HA LEU J 232   4.091 30.318 11.413 1.00 0.00
J   H
ATOM   9825 CB LEU J 232   2.040 30.792 10.915 1.00 0.00
J   C
ATOM   9826 HB1 LEU J 232   1.045 30.570 11.367 1.00 0.00
J   H
ATOM   9827 HB2 LEU J 232   2.135 31.901 10.911 1.00 0.00
J   H

ATOM   9828 CG LEU J 232   2.080 30.229   9.484 1.00 0.00
J   C
ATOM   9829 HG LEU J 232   1.974 29.120   9.568 1.00 0.00
J   H
ATOM   9830 CD1 LEU J 232   0.925 30.810   8.656 1.00 0.00
J   C
ATOM   9831 HD11 LEU J 232   0.975 30.445   7.608 1.00 0.00
J   H
ATOM   9832 HD12 LEU J 232   -0.055 30.507   9.084 1.00 0.00
J   H
ATOM   9833 HD13 LEU J 232   0.973 31.919   8.640 1.00 0.00
J   H
ATOM   9834 CD2 LEU J 232   3.415 30.528   8.786 1.00 0.00
J   C
ATOM   9835 HD21 LEU J 232   3.421 30.093   7.762 1.00 0.00
J   H
ATOM   9836 HD22 LEU J 232   3.592 31.619   8.689 1.00 0.00
J   H
ATOM   9837 HD23 LEU J 232   4.260 30.085   9.356 1.00 0.00
J   H
ATOM   9838 C   LEU J 232   3.099 30.984 13.159 1.00 0.00
J   C
ATOM   9839 O   LEU J 232   3.926 31.865 13.379 1.00 0.00
J   O
ATOM   9840 N   PHE J 233   2.174 30.663 14.080 1.00 0.00
J   N
ATOM   9841 HN PHE J 233   1.478 29.979 13.871 1.00 0.00
J   H
ATOM   9842 CA PHE J 233   2.075 31.234 15.407 1.00 0.00
J   C
ATOM   9843 HA PHE J 233   2.005 32.306 15.274 1.00 0.00
J   H
ATOM   9844 CB PHE J 233   0.809 30.633 16.093 1.00 0.00
J   C
ATOM   9845 HB1 PHE J 233   -0.053 30.710 15.394 1.00 0.00
J   H
ATOM   9846 HB2 PHE J 233   0.950 29.555 16.320 1.00 0.00
J   H
ATOM   9847 CG PHE J 233   0.362 31.329 17.354 1.00 0.00
J   C
ATOM   9848 CD1 PHE J 233   1.038 31.124 18.568 1.00 0.00
J   C
ATOM   9849 HD1 PHE J 233   1.932 30.516 18.586 1.00 0.00
J   H
ATOM   9850 CE1 PHE J 233   0.563 31.694 19.757 1.00 0.00
J   C
ATOM   9851 HE1 PHE J 233   1.103 31.543 20.681 1.00 0.00
J   H
ATOM   9852 CZ PHE J 233   -0.606 32.463 19.746 1.00 0.00
J   C
ATOM   9853 HZ PHE J 233   -0.971 32.907 20.661 1.00 0.00
J   H
ATOM   9854 CD2 PHE J 233   -0.812 32.105 17.356 1.00 0.00
J   C

ATOM   9855 HD2 PHE J 233   -1.362 32.257 16.438 1.00 0.00
J   H
ATOM   9856 CE2 PHE J 233   -1.299 32.667 18.545 1.00 0.00
J   C
ATOM   9857 HE2 PHE J 233   -2.206 33.253 18.532 1.00 0.00
J   H
ATOM   9858 C   PHE J 233   3.341 30.966 16.208 1.00 0.00
J   C
ATOM   9859 O   PHE J 233   3.955 31.904 16.717 1.00 0.00
J   O
ATOM   9860 N   ASP J 234   3.803 29.698 16.247 1.00 0.00
J   N
ATOM   9861 HN ASP J 234   3.336 28.948 15.785 1.00 0.00
J   H
ATOM   9862 CA ASP J 234   5.000 29.275 16.941 1.00 0.00
J   C
ATOM   9863 HA ASP J 234   4.887 29.571 17.978 1.00 0.00
J   H
ATOM   9864 CB ASP J 234   5.050 27.732 16.759 1.00 0.00
J   C
ATOM   9865 HB1 ASP J 234   4.016 27.332 16.751 1.00 0.00
J   H
ATOM   9866 HB2 ASP J 234   5.533 27.469 15.797 1.00 0.00
J   H
ATOM   9867 CG ASP J 234   5.779 26.948 17.835 1.00 0.00
J   C
ATOM   9868 OD1 ASP J 234   6.996 27.144 18.048 1.00 0.00
J   O
ATOM   9869 OD2 ASP J 234   5.141 26.011 18.381 1.00 0.00
J   O
ATOM   9870 C   ASP J 234   6.240 30.004 16.405 1.00 0.00
J   C
ATOM   9871 O   ASP J 234   6.958 30.673 17.150 1.00 0.00
J   O
ATOM   9872 N   VAL J 235   6.465 30.029 15.070 1.00 0.00
J   N
ATOM   9873 HN VAL J 235   5.869 29.584 14.408 1.00 0.00
J   H
ATOM   9874 CA VAL J 235   7.612 30.718 14.483 1.00 0.00
J   C
ATOM   9875 HA VAL J 235   8.485 30.311 14.979 1.00 0.00
J   H
ATOM   9876 CB VAL J 235   7.877 30.319 13.028 1.00 0.00
J   C
ATOM   9877 HB VAL J 235   7.865 29.202 12.976 1.00 0.00
J   H
ATOM   9878 CG1 VAL J 235   6.802 30.859 12.074 1.00 0.00
J   C
ATOM   9879 HG11 VAL J 235   7.045 30.585 11.024 1.00 0.00
J   H
ATOM   9880 HG12 VAL J 235   5.802 30.438 12.303 1.00 0.00
J   H
ATOM   9881 HG13 VAL J 235   6.744 31.967 12.136 1.00 0.00
J   H

```
ATOM   9882  CG2 VAL J 235       9.271  30.781  12.561  1.00  0.00
J    C
ATOM   9883 HG21 VAL J 235       9.434  30.521  11.493  1.00  0.00
J    H
ATOM   9884 HG22 VAL J 235       9.375  31.883  12.672  1.00  0.00
J    H
ATOM   9885 HG23 VAL J 235      10.075  30.295  13.152  1.00  0.00
J    H
ATOM   9886  C   VAL J 235       7.627  32.232  14.700  1.00  0.00
J    C
ATOM   9887  O   VAL J 235       8.672  32.813  14.989  1.00  0.00
J    O
ATOM   9888  N   MET J 236       6.463  32.921  14.618  1.00  0.00
J    N
ATOM   9889  HN  MET J 236       5.590  32.507  14.369  1.00  0.00
J    H
ATOM   9890  CA  MET J 236       6.393  34.349  14.894  1.00  0.00
J    C
ATOM   9891  HA  MET J 236       7.123  34.802  14.233  1.00  0.00
J    H
ATOM   9892  CB  MET J 236       4.955  34.899  14.704  1.00  0.00
J    C
ATOM   9893  HB1 MET J 236       4.270  34.213  15.256  1.00  0.00
J    H
ATOM   9894  HB2 MET J 236       4.870  35.890  15.203  1.00  0.00
J    H
ATOM   9895  CG  MET J 236       4.456  35.004  13.258  1.00  0.00
J    C
ATOM   9896  HG1 MET J 236       5.087  35.715  12.683  1.00  0.00
J    H
ATOM   9897  HG2 MET J 236       4.531  34.011  12.764  1.00  0.00
J    H
ATOM   9898  SD  MET J 236       2.732  35.582  13.204  1.00  0.00
J    S
ATOM   9899  CE  MET J 236       2.423  34.821  11.591  1.00  0.00
J    C
ATOM   9900  HE1 MET J 236       1.383  35.017  11.253  1.00  0.00
J    H
ATOM   9901  HE2 MET J 236       3.115  35.225  10.821  1.00  0.00
J    H
ATOM   9902  HE3 MET J 236       2.566  33.720  11.635  1.00  0.00
J    H
ATOM   9903  C   MET J 236       6.751  34.754  16.322  1.00  0.00
J    C
ATOM   9904  O   MET J 236       7.490  35.726  16.529  1.00  0.00
J    O
ATOM   9905  N   LYS J 237       6.186  34.061  17.338  1.00  0.00
J    N
ATOM   9906  HN  LYS J 237       5.632  33.243  17.195  1.00  0.00
J    H
ATOM   9907  CA  LYS J 237       6.349  34.463  18.730  1.00  0.00
J    C
ATOM   9908  HA  LYS J 237       6.429  35.543  18.749  1.00  0.00
J    H
```

ATOM 9909 CB LYS J 237 5.158 34.078 19.669 1.00 0.00
J C
ATOM 9910 HB1 LYS J 237 5.297 33.009 19.953 1.00 0.00
J H
ATOM 9911 HB2 LYS J 237 5.237 34.664 20.612 1.00 0.00
J H
ATOM 9912 CG LYS J 237 3.719 34.219 19.117 1.00 0.00
J C
ATOM 9913 HG1 LYS J 237 3.521 33.391 18.398 1.00 0.00
J H
ATOM 9914 HG2 LYS J 237 2.996 34.110 19.958 1.00 0.00
J H
ATOM 9915 CD LYS J 237 3.330 35.510 18.369 1.00 0.00
J C
ATOM 9916 HD1 LYS J 237 3.259 36.338 19.111 1.00 0.00
J H
ATOM 9917 HD2 LYS J 237 4.154 35.763 17.663 1.00 0.00
J H
ATOM 9918 CE LYS J 237 2.001 35.322 17.615 1.00 0.00
J C
ATOM 9919 HE1 LYS J 237 2.049 34.425 16.959 1.00 0.00
J H
ATOM 9920 HE2 LYS J 237 1.161 35.190 18.332 1.00 0.00
J H
ATOM 9921 NZ LYS J 237 1.708 36.500 16.767 1.00 0.00
J N
ATOM 9922 HZ1 LYS J 237 0.809 36.361 16.262 1.00 0.00
J H
ATOM 9923 HZ2 LYS J 237 1.641 37.350 17.365 1.00 0.00
J H
ATOM 9924 HZ3 LYS J 237 2.474 36.629 16.077 1.00 0.00
J H
ATOM 9925 C LYS J 237 7.644 33.902 19.298 1.00 0.00
J C
ATOM 9926 O LYS J 237 8.441 34.630 19.881 1.00 0.00
J O
ATOM 9927 N ASN J 238 7.892 32.586 19.107 1.00 0.00
J N
ATOM 9928 HN ASN J 238 7.312 32.019 18.523 1.00 0.00
J H
ATOM 9929 CA ASN J 238 9.004 31.878 19.718 1.00 0.00
J C
ATOM 9930 HA ASN J 238 9.026 32.222 20.747 1.00 0.00
J H
ATOM 9931 CB ASN J 238 8.827 30.337 19.617 1.00 0.00
J C
ATOM 9932 HB1 ASN J 238 8.640 30.086 18.550 1.00 0.00
J H
ATOM 9933 HB2 ASN J 238 9.761 29.821 19.921 1.00 0.00
J H
ATOM 9934 CG ASN J 238 7.643 29.845 20.444 1.00 0.00
J C
ATOM 9935 OD1 ASN J 238 6.706 30.569 20.780 1.00 0.00
J O

ATOM 9936 ND2 ASN J 238 7.692 28.548 20.821 1.00 0.00
J N
ATOM 9937 HD21 ASN J 238 6.927 28.196 21.352 1.00 0.00
J H
ATOM 9938 HD22 ASN J 238 8.457 27.976 20.541 1.00 0.00
J H
ATOM 9939 C ASN J 238 10.343 32.223 19.087 1.00 0.00
J C
ATOM 9940 O ASN J 238 11.326 32.468 19.786 1.00 0.00
J O
ATOM 9941 N ARG J 239 10.397 32.229 17.739 1.00 0.00
J N
ATOM 9942 HN ARG J 239 9.608 31.848 17.262 1.00 0.00
J H
ATOM 9943 CA ARG J 239 11.483 32.733 16.907 1.00 0.00
J C
ATOM 9944 HA ARG J 239 11.133 32.594 15.891 1.00 0.00
J H
ATOM 9945 CB ARG J 239 11.546 34.278 17.032 1.00 0.00
J C
ATOM 9946 HB1 ARG J 239 10.537 34.608 17.377 1.00 0.00
J H
ATOM 9947 HB2 ARG J 239 12.254 34.557 17.845 1.00 0.00
J H
ATOM 9948 CG ARG J 239 11.885 34.996 15.707 1.00 0.00
J C
ATOM 9949 HG1 ARG J 239 12.884 34.649 15.361 1.00 0.00
J H
ATOM 9950 HG2 ARG J 239 11.166 34.667 14.923 1.00 0.00
J H
ATOM 9951 CD ARG J 239 11.818 36.527 15.774 1.00 0.00
J C
ATOM 9952 HD1 ARG J 239 12.488 36.892 16.587 1.00 0.00
J H
ATOM 9953 HD2 ARG J 239 12.136 36.993 14.813 1.00 0.00
J H
ATOM 9954 NE ARG J 239 10.386 36.885 16.043 1.00 0.00
J N
ATOM 9955 HE ARG J 239 9.786 36.081 16.120 1.00 0.00
J H
ATOM 9956 CZ ARG J 239 9.915 38.129 16.173 1.00 0.00
J C
ATOM 9957 NH1 ARG J 239 10.702 39.192 16.048 1.00 0.00
J N
ATOM 9958 HH11 ARG J 239 10.339 40.105 16.173 1.00 0.00
J H
ATOM 9959 HH12 ARG J 239 11.683 39.010 15.962 1.00 0.00
J H
ATOM 9960 NH2 ARG J 239 8.624 38.302 16.441 1.00 0.00
J N
ATOM 9961 HH21 ARG J 239 8.241 39.215 16.455 1.00 0.00
J H
ATOM 9962 HH22 ARG J 239 8.049 37.496 16.411 1.00 0.00
J H

ATOM 9963 C ARG J 239 12.879 32.065 16.990 1.00 0.00
J C
ATOM 9964 O ARG J 239 13.706 32.231 16.098 1.00 0.00
J O
ATOM 9965 N ARG J 240 13.189 31.286 18.052 1.00 0.00
J N
ATOM 9966 HN ARG J 240 12.448 30.991 18.651 1.00 0.00
J H
ATOM 9967 CA ARG J 240 14.531 30.835 18.400 1.00 0.00
J C
ATOM 9968 HA ARG J 240 15.216 31.416 17.793 1.00 0.00
J H
ATOM 9969 CB ARG J 240 14.753 31.053 19.922 1.00 0.00
J C
ATOM 9970 HB1 ARG J 240 13.888 30.573 20.439 1.00 0.00
J H
ATOM 9971 HB2 ARG J 240 15.655 30.493 20.251 1.00 0.00
J H
ATOM 9972 CG ARG J 240 14.827 32.531 20.366 1.00 0.00
J C
ATOM 9973 HG1 ARG J 240 15.556 33.060 19.715 1.00 0.00
J H
ATOM 9974 HG2 ARG J 240 13.844 33.017 20.171 1.00 0.00
J H
ATOM 9975 CD ARG J 240 15.155 32.633 21.860 1.00 0.00
J C
ATOM 9976 HD1 ARG J 240 14.344 32.151 22.452 1.00 0.00
J H
ATOM 9977 HD2 ARG J 240 16.120 32.129 22.103 1.00 0.00
J H
ATOM 9978 NE ARG J 240 15.282 34.081 22.226 1.00 0.00
J N
ATOM 9979 HE ARG J 240 15.163 34.699 21.441 1.00 0.00
J H
ATOM 9980 CZ ARG J 240 15.528 34.519 23.470 1.00 0.00
J C
ATOM 9981 NH1 ARG J 240 15.634 35.824 23.709 1.00 0.00
J N
ATOM 9982 HH11 ARG J 240 15.841 36.154 24.620 1.00 0.00
J H
ATOM 9983 HH12 ARG J 240 15.631 36.424 22.907 1.00 0.00
J H
ATOM 9984 NH2 ARG J 240 15.662 33.676 24.489 1.00 0.00
J N
ATOM 9985 HH21 ARG J 240 15.756 34.026 25.410 1.00 0.00
J H
ATOM 9986 HH22 ARG J 240 15.452 32.722 24.312 1.00 0.00
J H
ATOM 9987 C ARG J 240 14.832 29.355 18.107 1.00 0.00
J C
ATOM 9988 O ARG J 240 15.872 28.847 18.533 1.00 0.00
J O
ATOM 9989 N PHE J 241 13.940 28.602 17.422 1.00 0.00
J N

ATOM 9990 HN PHE J 241 13.036 28.963 17.198 1.00 0.00
J H
ATOM 9991 CA PHE J 241 14.182 27.234 16.950 1.00 0.00
J C
ATOM 9992 HA PHE J 241 13.377 27.038 16.252 1.00 0.00
J H
ATOM 9993 CB PHE J 241 15.413 27.065 15.999 1.00 0.00
J C
ATOM 9994 HB1 PHE J 241 15.309 27.766 15.141 1.00 0.00
J H
ATOM 9995 HB2 PHE J 241 16.359 27.322 16.520 1.00 0.00
J H
ATOM 9996 CG PHE J 241 15.460 25.651 15.440 1.00 0.00
J C
ATOM 9997 CD1 PHE J 241 14.604 25.263 14.394 1.00 0.00
J C
ATOM 9998 HD1 PHE J 241 14.005 26.008 13.888 1.00 0.00
J H
ATOM 9999 CE1 PHE J 241 14.510 23.920 14.002 1.00 0.00
J C
ATOM 10000 HE1 PHE J 241 13.838 23.635 13.204 1.00 0.00
J H
ATOM 10001 CZ PHE J 241 15.292 22.950 14.640 1.00 0.00
J C
ATOM 10002 HZ PHE J 241 15.232 21.916 14.333 1.00 0.00
J H
ATOM 10003 CD2 PHE J 241 16.247 24.665 16.065 1.00 0.00
J C
ATOM 10004 HD2 PHE J 241 16.912 24.937 16.872 1.00 0.00
J H
ATOM 10005 CE2 PHE J 241 16.161 23.323 15.673 1.00 0.00
J C
ATOM 10006 HE2 PHE J 241 16.765 22.577 16.169 1.00 0.00
J H
ATOM 10007 C PHE J 241 14.105 26.161 18.035 1.00 0.00
J C
ATOM 10008 O PHE J 241 13.241 25.287 18.023 1.00 0.00
J O
ATOM 10009 N LYS J 242 15.012 26.185 19.030 1.00 0.00
J N
ATOM 10010 HN LYS J 242 15.698 26.908 19.093 1.00 0.00
J H
ATOM 10011 CA LYS J 242 15.075 25.180 20.084 1.00 0.00
J C
ATOM 10012 HA LYS J 242 15.176 24.223 19.587 1.00 0.00
J H
ATOM 10013 CB LYS J 242 16.327 25.313 20.995 1.00 0.00
J C
ATOM 10014 HB1 LYS J 242 16.055 24.893 21.990 1.00 0.00
J H
ATOM 10015 HB2 LYS J 242 17.138 24.665 20.593 1.00 0.00
J H
ATOM 10016 CG LYS J 242 16.852 26.743 21.201 1.00 0.00
J C

ATOM 10017 HG1 LYS J 242   17.136 27.173 20.214 1.00 0.00
J   H
ATOM 10018 HG2 LYS J 242   16.032 27.374 21.614 1.00 0.00
J   H
ATOM 10019 CD LYS J 242   18.074 26.757 22.136 1.00 0.00
J   C
ATOM 10020 HD1 LYS J 242   17.712 26.703 23.189 1.00 0.00
J   H
ATOM 10021 HD2 LYS J 242   18.671 25.836 21.941 1.00 0.00
J   H
ATOM 10022 CE LYS J 242   18.962 27.992 21.952 1.00 0.00
J   C
ATOM 10023 HE1 LYS J 242   19.311 28.069 20.899 1.00 0.00
J   H
ATOM 10024 HE2 LYS J 242   18.400 28.918 22.205 1.00 0.00
J   H
ATOM 10025 NZ LYS J 242   20.149 27.905 22.834 1.00 0.00
J   N
ATOM 10026 HZ1 LYS J 242   20.751 28.744 22.705 1.00 0.00
J   H
ATOM 10027 HZ2 LYS J 242   19.841 27.849 23.826 1.00 0.00
J   H
ATOM 10028 HZ3 LYS J 242   20.694 27.052 22.597 1.00 0.00
J   H
ATOM 10029 C   LYS J 242   13.809 25.126 20.922 1.00 0.00
J   C
ATOM 10030 O   LYS J 242   13.402 24.052 21.372 1.00 0.00
J   O
ATOM 10031 N   THR J 243   13.162 26.299 21.068 1.00 0.00
J   N
ATOM 10032 HN THR J 243   13.559 27.108 20.636 1.00 0.00
J   H
ATOM 10033 CA THR J 243   11.929 26.552 21.792 1.00 0.00
J   C
ATOM 10034 HA THR J 243   11.986 25.924 22.671 1.00 0.00
J   H
ATOM 10035 CB THR J 243   11.768 28.024 22.194 1.00 0.00
J   C
ATOM 10036 HB THR J 243   10.817 28.084 22.777 1.00 0.00
J   H
ATOM 10037 OG1 THR J 243   11.613 28.899 21.088 1.00 0.00
J   O
ATOM 10038 HG1 THR J 243   11.534 29.781 21.468 1.00 0.00
J   H
ATOM 10039 CG2 THR J 243   13.043 28.531 22.882 1.00 0.00
J   C
ATOM 10040 HG21 THR J 243   12.897 29.558 23.278 1.00 0.00
J   H
ATOM 10041 HG22 THR J 243   13.311 27.870 23.735 1.00 0.00
J   H
ATOM 10042 HG23 THR J 243   13.899 28.549 22.174 1.00 0.00
J   H
ATOM 10043 C   THR J 243   10.687 26.118 21.030 1.00 0.00
J   C

```
ATOM  10044  O   THR J 243       9.601  26.103  21.602  1.00  0.00
J    O
ATOM  10045  N   PHE J 244      10.801  25.758  19.727  1.00  0.00
J    N
ATOM  10046  HN  PHE J 244      11.670  25.805  19.239  1.00  0.00
J    H
ATOM  10047  CA  PHE J 244       9.684  25.277  18.925  1.00  0.00
J    C
ATOM  10048  HA  PHE J 244       8.939  26.062  18.980  1.00  0.00
J    H
ATOM  10049  CB  PHE J 244      10.061  25.015  17.432  1.00  0.00
J    C
ATOM  10050  HB1 PHE J 244      10.979  24.389  17.393  1.00  0.00
J    H
ATOM  10051  HB2 PHE J 244       9.258  24.451  16.910  1.00  0.00
J    H
ATOM  10052  CG  PHE J 244      10.382  26.255  16.624  1.00  0.00
J    C
ATOM  10053  CD1 PHE J 244       9.969  27.554  16.980  1.00  0.00
J    C
ATOM  10054  HD1 PHE J 244       9.377  27.702  17.873  1.00  0.00
J    H
ATOM  10055  CE1 PHE J 244      10.309  28.660  16.195  1.00  0.00
J    C
ATOM  10056  HE1 PHE J 244       9.993  29.650  16.491  1.00  0.00
J    H
ATOM  10057  CZ  PHE J 244      11.061  28.486  15.031  1.00  0.00
J    C
ATOM  10058  HZ  PHE J 244      11.363  29.344  14.446  1.00  0.00
J    H
ATOM  10059  CD2 PHE J 244      11.103  26.096  15.424  1.00  0.00
J    C
ATOM  10060  HD2 PHE J 244      11.391  25.107  15.095  1.00  0.00
J    H
ATOM  10061  CE2 PHE J 244      11.432  27.200  14.627  1.00  0.00
J    C
ATOM  10062  HE2 PHE J 244      11.972  27.055  13.702  1.00  0.00
J    H
ATOM  10063  C   PHE J 244       9.040  24.014  19.469  1.00  0.00
J    C
ATOM  10064  O   PHE J 244       9.703  23.062  19.898  1.00  0.00
J    O
ATOM  10065  N   SER J 245       7.689  24.000  19.416  1.00  0.00
J    N
ATOM  10066  HN  SER J 245       7.185  24.820  19.145  1.00  0.00
J    H
ATOM  10067  CA  SER J 245       6.845  22.858  19.728  1.00  0.00
J    C
ATOM  10068  HA  SER J 245       7.094  22.584  20.744  1.00  0.00
J    H
ATOM  10069  CB  SER J 245       5.340  23.183  19.555  1.00  0.00
J    C
ATOM  10070  HB1 SER J 245       5.111  24.092  20.157  1.00  0.00
J    H
```

ATOM 10071 HB2 SER J 245   5.115 23.429 18.493 1.00 0.00
J   H
ATOM 10072 OG SER J 245   4.501 22.116 20.015 1.00 0.00
J   O
ATOM 10073 HG1 SER J 245   3.605 22.469 20.086 1.00 0.00
J   H
ATOM 10074 C   SER J 245   7.146 21.658 18.851 1.00 0.00
J   C
ATOM 10075 O   SER J 245   7.607 21.823 17.711 1.00 0.00
J   O
ATOM 10076 N   LEU J 246   6.889 20.412 19.296 1.00 0.00
J   N
ATOM 10077 HN LEU J 246   6.469 20.239 20.185 1.00 0.00
J   H
ATOM 10078 CA LEU J 246   7.194 19.208 18.533 1.00 0.00
J   C
ATOM 10079 HA LEU J 246   8.263 19.226 18.367 1.00 0.00
J   H
ATOM 10080 CB LEU J 246   6.829 17.900 19.294 1.00 0.00
J   C
ATOM 10081 HB1 LEU J 246   7.424 17.891 20.236 1.00 0.00
J   H
ATOM 10082 HB2 LEU J 246   5.762 17.948 19.604 1.00 0.00
J   H
ATOM 10083 CG LEU J 246   7.118 16.598 18.500 1.00 0.00
J   C
ATOM 10084 HG LEU J 246   6.555 16.729 17.545 1.00 0.00
J   H
ATOM 10085 CD1 LEU J 246   8.615 16.438 18.204 1.00 0.00
J   C
ATOM 10086 HD11 LEU J 246   8.808 15.479 17.678 1.00 0.00
J   H
ATOM 10087 HD12 LEU J 246   8.976 17.268 17.560 1.00 0.00
J   H
ATOM 10088 HD13 LEU J 246   9.204 16.442 19.146 1.00 0.00
J   H
ATOM 10089 CD2 LEU J 246   6.578 15.344 19.202 1.00 0.00
J   C
ATOM 10090 HD21 LEU J 246   6.789 14.439 18.591 1.00 0.00
J   H
ATOM 10091 HD22 LEU J 246   7.047 15.196 20.195 1.00 0.00
J   H
ATOM 10092 HD23 LEU J 246   5.479 15.419 19.342 1.00 0.00
J   H
ATOM 10093 C   LEU J 246   6.515 19.181 17.164 1.00 0.00
J   C
ATOM 10094 O   LEU J 246   7.131 18.854 16.160 1.00 0.00
J   O
ATOM 10095 N   HSE J 247   5.228 19.592 17.098 1.00 0.00
J   N
ATOM 10096 HN HSE J 247   4.698 19.839 17.908 1.00 0.00
J   H
ATOM 10097 CA HSE J 247   4.499 19.711 15.842 1.00 0.00
J   C

ATOM 10098 HA HSE J 247   4.531 18.730 15.383 1.00 0.00
J   H
ATOM 10099 CB HSE J 247   3.047 20.171 16.142 1.00 0.00
J   C
ATOM 10100 HB1 HSE J 247   2.588 19.427 16.828 1.00 0.00
J   H
ATOM 10101 HB2 HSE J 247   3.070 21.141 16.682 1.00 0.00
J   H
ATOM 10102 ND1 HSE J 247   2.135 21.445 14.196 1.00 0.00
J   N
ATOM 10103 CG HSE J 247   2.164 20.279 14.933 1.00 0.00
J   C
ATOM 10104 CE1 HSE J 247   1.374 21.182 13.156 1.00 0.00
J   C
ATOM 10105 HE1 HSE J 247   1.144 21.895 12.360 1.00 0.00
J   H
ATOM 10106 NE2 HSE J 247   0.901 19.916 13.192 1.00 0.00
J   N
ATOM 10107 HE2 HSE J 247   0.284 19.482 12.536 1.00 0.00
J   H
ATOM 10108 CD2 HSE J 247   1.408 19.331 14.329 1.00 0.00
J   C
ATOM 10109 HD2 HSE J 247   1.199 18.315 14.632 1.00 0.00
J   H
ATOM 10110 C   HSE J 247   5.148 20.686 14.863 1.00 0.00
J   C
ATOM 10111 O   HSE J 247   5.374 20.359 13.698 1.00 0.00
J   O
ATOM 10112 N   THR J 248   5.538 21.876 15.356 1.00 0.00
J   N
ATOM 10113 HN THR J 248   5.335 22.104 16.308 1.00 0.00
J   H
ATOM 10114 CA THR J 248   6.258 22.919 14.629 1.00 0.00
J   C
ATOM 10115 HA THR J 248   5.662 23.119 13.749 1.00 0.00
J   H
ATOM 10116 CB THR J 248   6.424 24.163 15.499 1.00 0.00
J   C
ATOM 10117 HB THR J 248   6.918 23.818 16.440 1.00 0.00
J   H
ATOM 10118 OG1 THR J 248   5.126 24.648 15.814 1.00 0.00
J   O
ATOM 10119 HG1 THR J 248   5.270 25.434 16.351 1.00 0.00
J   H
ATOM 10120 CG2 THR J 248   7.196 25.299 14.804 1.00 0.00
J   C
ATOM 10121 HG21 THR J 248   7.391 26.134 15.510 1.00 0.00
J   H
ATOM 10122 HG22 THR J 248   8.176 24.931 14.433 1.00 0.00
J   H
ATOM 10123 HG23 THR J 248   6.625 25.701 13.939 1.00 0.00
J   H
ATOM 10124 C   THR J 248   7.614 22.447 14.135 1.00 0.00
J   C

```
ATOM  10125  O   THR J 248       7.977  22.641  12.979  1.00  0.00
J    O
ATOM  10126  N   LYS J 249       8.370  21.736  15.004  1.00  0.00
J    N
ATOM  10127  HN  LYS J 249       8.053  21.603  15.941  1.00  0.00
J    H
ATOM  10128  CA  LYS J 249       9.653  21.120  14.692  1.00  0.00
J    C
ATOM  10129  HA  LYS J 249      10.299  21.922  14.353  1.00  0.00
J    H
ATOM  10130  CB  LYS J 249      10.212  20.429  15.971  1.00  0.00
J    C
ATOM  10131  HB1 LYS J 249       9.499  20.647  16.800  1.00  0.00
J    H
ATOM  10132  HB2 LYS J 249      10.194  19.326  15.824  1.00  0.00
J    H
ATOM  10133  CG  LYS J 249      11.615  20.891  16.391  1.00  0.00
J    C
ATOM  10134  HG1 LYS J 249      12.375  20.314  15.818  1.00  0.00
J    H
ATOM  10135  HG2 LYS J 249      11.739  21.965  16.119  1.00  0.00
J    H
ATOM  10136  CD  LYS J 249      11.819  20.672  17.900  1.00  0.00
J    C
ATOM  10137  HD1 LYS J 249      10.914  21.040  18.435  1.00  0.00
J    H
ATOM  10138  HD2 LYS J 249      11.893  19.575  18.084  1.00  0.00
J    H
ATOM  10139  CE  LYS J 249      13.066  21.365  18.453  1.00  0.00
J    C
ATOM  10140  HE1 LYS J 249      13.984  20.815  18.151  1.00  0.00
J    H
ATOM  10141  HE2 LYS J 249      13.136  22.406  18.069  1.00  0.00
J    H
ATOM  10142  NZ  LYS J 249      13.001  21.407  19.931  1.00  0.00
J    N
ATOM  10143  HZ1 LYS J 249      13.847  21.877  20.312  1.00  0.00
J    H
ATOM  10144  HZ2 LYS J 249      12.152  21.931  20.227  1.00  0.00
J    H
ATOM  10145  HZ3 LYS J 249      12.949  20.437  20.303  1.00  0.00
J    H
ATOM  10146  C   LYS J 249       9.573  20.096  13.566  1.00  0.00
J    C
ATOM  10147  O   LYS J 249      10.387  20.099  12.640  1.00  0.00
J    O
ATOM  10148  N   LEU J 250       8.552  19.212  13.628  1.00  0.00
J    N
ATOM  10149  HN  LEU J 250       7.944  19.198  14.419  1.00  0.00
J    H
ATOM  10150  CA  LEU J 250       8.230  18.227  12.610  1.00  0.00
J    C
ATOM  10151  HA  LEU J 250       9.157  17.700  12.425  1.00  0.00
J    H
```

```
ATOM  10152  CB  LEU J 250       7.111  17.272  13.103  1.00  0.00
J    C
ATOM  10153  HB1 LEU J 250       6.318  17.905  13.565  1.00  0.00
J    H
ATOM  10154  HB2 LEU J 250       6.639  16.782  12.222  1.00  0.00
J    H
ATOM  10155  CG  LEU J 250       7.585  16.230  14.127  1.00  0.00
J    C
ATOM  10156  HG  LEU J 250       8.042  16.775  14.988  1.00  0.00
J    H
ATOM  10157  CD1 LEU J 250       6.400  15.380  14.608  1.00  0.00
J    C
ATOM  10158 HD11 LEU J 250       6.745  14.593  15.312  1.00  0.00
J    H
ATOM  10159 HD12 LEU J 250       5.652  16.014  15.132  1.00  0.00
J    H
ATOM  10160 HD13 LEU J 250       5.899  14.883  13.750  1.00  0.00
J    H
ATOM  10161  CD2 LEU J 250       8.657  15.318  13.520  1.00  0.00
J    C
ATOM  10162 HD21 LEU J 250       9.007  14.579  14.274  1.00  0.00
J    H
ATOM  10163 HD22 LEU J 250       8.269  14.752  12.650  1.00  0.00
J    H
ATOM  10164 HD23 LEU J 250       9.532  15.916  13.187  1.00  0.00
J    H
ATOM  10165  C   LEU J 250       7.764  18.807  11.292  1.00  0.00
J    C
ATOM  10166  O   LEU J 250       8.199  18.380  10.224  1.00  0.00
J    O
ATOM  10167  N   MET J 251       6.855  19.800  11.319  1.00  0.00
J    N
ATOM  10168  HN  MET J 251       6.486  20.189  12.160  1.00  0.00
J    H
ATOM  10169  CA  MET J 251       6.330  20.393  10.105  1.00  0.00
J    C
ATOM  10170  HA  MET J 251       5.949  19.568   9.516  1.00  0.00
J    H
ATOM  10171  CB  MET J 251       5.159  21.366  10.400  1.00  0.00
J    C
ATOM  10172  HB1 MET J 251       5.420  21.914  11.334  1.00  0.00
J    H
ATOM  10173  HB2 MET J 251       5.104  22.132   9.594  1.00  0.00
J    H
ATOM  10174  CG  MET J 251       3.793  20.672  10.591  1.00  0.00
J    C
ATOM  10175  HG1 MET J 251       3.797  20.059  11.517  1.00  0.00
J    H
ATOM  10176  HG2 MET J 251       2.996  21.441  10.701  1.00  0.00
J    H
ATOM  10177  SD  MET J 251       3.281  19.552   9.237  1.00  0.00
J    S
ATOM  10178  CE  MET J 251       3.615  20.614   7.797  1.00  0.00
J    C
```

ATOM 10179 HE1 MET J 251 3.349 20.095 6.851 1.00 0.00
J H
ATOM 10180 HE2 MET J 251 3.024 21.554 7.849 1.00 0.00
J H
ATOM 10181 HE3 MET J 251 4.690 20.889 7.744 1.00 0.00
J H
ATOM 10182 C MET J 251 7.369 21.112 9.267 1.00 0.00
J C
ATOM 10183 O MET J 251 7.381 20.981 8.043 1.00 0.00
J O
ATOM 10184 N LEU J 252 8.274 21.884 9.896 1.00 0.00
J N
ATOM 10185 HN LEU J 252 8.273 22.048 10.880 1.00 0.00
J H
ATOM 10186 CA LEU J 252 9.343 22.554 9.182 1.00 0.00
J C
ATOM 10187 HA LEU J 252 8.857 23.079 8.369 1.00 0.00
J H
ATOM 10188 CB LEU J 252 10.055 23.549 10.126 1.00 0.00
J C
ATOM 10189 HB1 LEU J 252 10.394 22.973 11.018 1.00 0.00
J H
ATOM 10190 HB2 LEU J 252 10.979 23.920 9.630 1.00 0.00
J H
ATOM 10191 CG LEU J 252 9.165 24.724 10.589 1.00 0.00
J C
ATOM 10192 HG LEU J 252 8.244 24.266 11.023 1.00 0.00
J H
ATOM 10193 CD1 LEU J 252 9.894 25.543 11.667 1.00 0.00
J C
ATOM 10194 HD11 LEU J 252 9.282 26.418 11.972 1.00 0.00
J H
ATOM 10195 HD12 LEU J 252 10.086 24.921 12.568 1.00 0.00
J H
ATOM 10196 HD13 LEU J 252 10.868 25.917 11.284 1.00 0.00
J H
ATOM 10197 CD2 LEU J 252 8.740 25.630 9.420 1.00 0.00
J C
ATOM 10198 HD21 LEU J 252 8.086 26.452 9.785 1.00 0.00
J H
ATOM 10199 HD22 LEU J 252 9.614 26.092 8.920 1.00 0.00
J H
ATOM 10200 HD23 LEU J 252 8.173 25.046 8.662 1.00 0.00
J H
ATOM 10201 C LEU J 252 10.378 21.614 8.570 1.00 0.00
J C
ATOM 10202 O LEU J 252 10.695 21.720 7.385 1.00 0.00
J O
ATOM 10203 N THR J 253 10.900 20.631 9.342 1.00 0.00
J N
ATOM 10204 HN THR J 253 10.657 20.505 10.304 1.00 0.00
J H
ATOM 10205 CA THR J 253 11.873 19.658 8.833 1.00 0.00
J C

```
ATOM  10206  HA  THR J 253      12.624  20.246   8.321  1.00  0.00
J    H
ATOM  10207  CB  THR J 253      12.583  18.822   9.894  1.00  0.00
J    C
ATOM  10208  HB  THR J 253      13.089  17.990   9.346  1.00  0.00
J    H
ATOM  10209  OG1 THR J 253      11.686  18.215  10.811  1.00  0.00
J    O
ATOM  10210  HG1 THR J 253      12.241  17.730  11.431  1.00  0.00
J    H
ATOM  10211  CG2 THR J 253      13.525  19.734  10.686  1.00  0.00
J    C
ATOM  10212 HG21 THR J 253      14.146  19.145  11.393  1.00  0.00
J    H
ATOM  10213 HG22 THR J 253      14.211  20.274   9.998  1.00  0.00
J    H
ATOM  10214 HG23 THR J 253      12.954  20.488  11.269  1.00  0.00
J    H
ATOM  10215  C   THR J 253      11.282  18.729   7.804  1.00  0.00
J    C
ATOM  10216  O   THR J 253      11.885  18.483   6.760  1.00  0.00
J    O
ATOM  10217  N   GLY J 254      10.050  18.231   8.040  1.00  0.00
J    N
ATOM  10218  HN  GLY J 254       9.537  18.468   8.864  1.00  0.00
J    H
ATOM  10219  CA  GLY J 254       9.382  17.326   7.125  1.00  0.00
J    C
ATOM  10220  HA1 GLY J 254       8.468  17.010   7.609  1.00  0.00
J    H
ATOM  10221  HA2 GLY J 254      10.074  16.522   6.913  1.00  0.00
J    H
ATOM  10222  C   GLY J 254       9.016  17.976   5.822  1.00  0.00
J    C
ATOM  10223  O   GLY J 254       9.208  17.394   4.764  1.00  0.00
J    O
ATOM  10224  N   THR J 255       8.490  19.223   5.846  1.00  0.00
J    N
ATOM  10225  HN  THR J 255       8.290  19.722   6.689  1.00  0.00
J    H
ATOM  10226  CA  THR J 255       8.166  19.959   4.617  1.00  0.00
J    C
ATOM  10227  HA  THR J 255       7.556  19.285   4.030  1.00  0.00
J    H
ATOM  10228  CB  THR J 255       7.398  21.263   4.855  1.00  0.00
J    C
ATOM  10229  HB  THR J 255       7.819  21.705   5.791  1.00  0.00
J    H
ATOM  10230  OG1 THR J 255       6.029  20.967   5.084  1.00  0.00
J    O
ATOM  10231  HG1 THR J 255       5.600  21.820   5.214  1.00  0.00
J    H
ATOM  10232  CG2 THR J 255       7.387  22.253   3.674  1.00  0.00
J    C
```

```
ATOM  10233 HG21 THR J 255       6.911  23.213   3.967  1.00  0.00
J    H
ATOM  10234 HG22 THR J 255       8.425  22.474   3.345  1.00  0.00
J    H
ATOM  10235 HG23 THR J 255       6.828  21.838   2.808  1.00  0.00
J    H
ATOM  10236  C   THR J 255       9.390  20.256   3.795  1.00  0.00
J    C
ATOM  10237  O   THR J 255       9.376  20.070   2.579  1.00  0.00
J    O
ATOM  10238  N   LEU J 256      10.498  20.690   4.430  1.00  0.00
J    N
ATOM  10239  HN  LEU J 256      10.533  20.859   5.413  1.00  0.00
J    H
ATOM  10240  CA  LEU J 256      11.744  20.950   3.736  1.00  0.00
J    C
ATOM  10241  HA  LEU J 256      11.503  21.652   2.949  1.00  0.00
J    H
ATOM  10242  CB  LEU J 256      12.785  21.502   4.739  1.00  0.00
J    C
ATOM  10243  HB1 LEU J 256      12.278  22.297   5.335  1.00  0.00
J    H
ATOM  10244  HB2 LEU J 256      13.053  20.700   5.462  1.00  0.00
J    H
ATOM  10245  CG  LEU J 256      14.048  22.094   4.079  1.00  0.00
J    C
ATOM  10246  HG  LEU J 256      14.323  21.411   3.240  1.00  0.00
J    H
ATOM  10247  CD1 LEU J 256      13.769  23.502   3.530  1.00  0.00
J    C
ATOM  10248 HD11 LEU J 256      14.693  23.943   3.099  1.00  0.00
J    H
ATOM  10249 HD12 LEU J 256      12.995  23.465   2.733  1.00  0.00
J    H
ATOM  10250 HD13 LEU J 256      13.409  24.174   4.338  1.00  0.00
J    H
ATOM  10251  CD2 LEU J 256      15.220  22.123   5.072  1.00  0.00
J    C
ATOM  10252 HD21 LEU J 256      16.131  22.532   4.583  1.00  0.00
J    H
ATOM  10253 HD22 LEU J 256      14.996  22.759   5.952  1.00  0.00
J    H
ATOM  10254 HD23 LEU J 256      15.451  21.097   5.431  1.00  0.00
J    H
ATOM  10255  C   LEU J 256      12.332  19.705   3.079  1.00  0.00
J    C
ATOM  10256  O   LEU J 256      12.678  19.707   1.896  1.00  0.00
J    O
ATOM  10257  N   MET J 257      12.404  18.587   3.833  1.00  0.00
J    N
ATOM  10258  HN  MET J 257      12.090  18.570   4.780  1.00  0.00
J    H
ATOM  10259  CA  MET J 257      12.933  17.327   3.355  1.00  0.00
J    C
```

```
ATOM  10260  HA  MET J 257      13.905  17.548   2.930  1.00  0.00
J    H
ATOM  10261  CB  MET J 257      13.127  16.365   4.552  1.00  0.00
J    C
ATOM  10262  HB1 MET J 257      13.448  16.988   5.420  1.00  0.00
J    H
ATOM  10263  HB2 MET J 257      12.144  15.922   4.832  1.00  0.00
J    H
ATOM  10264  CG  MET J 257      14.174  15.261   4.316  1.00  0.00
J    C
ATOM  10265  HG1 MET J 257      13.732  14.429   3.729  1.00  0.00
J    H
ATOM  10266  HG2 MET J 257      15.024  15.672   3.730  1.00  0.00
J    H
ATOM  10267  SD  MET J 257      14.810  14.573   5.876  1.00  0.00
J    S
ATOM  10268  CE  MET J 257      16.240  13.725   5.147  1.00  0.00
J    C
ATOM  10269  HE1 MET J 257      16.823  13.189   5.926  1.00  0.00
J    H
ATOM  10270  HE2 MET J 257      15.917  12.979   4.388  1.00  0.00
J    H
ATOM  10271  HE3 MET J 257      16.921  14.448   4.649  1.00  0.00
J    H
ATOM  10272  C   MET J 257      12.085  16.704   2.259  1.00  0.00
J    C
ATOM  10273  O   MET J 257      12.593  16.281   1.222  1.00  0.00
J    O
ATOM  10274  N   LEU J 258      10.747  16.691   2.426  1.00  0.00
J    N
ATOM  10275  HN  LEU J 258      10.312  17.056   3.247  1.00  0.00
J    H
ATOM  10276  CA  LEU J 258       9.819  16.155   1.447  1.00  0.00
J    C
ATOM  10277  HA  LEU J 258      10.168  15.153   1.236  1.00  0.00
J    H
ATOM  10278  CB  LEU J 258       8.402  16.067   2.062  1.00  0.00
J    C
ATOM  10279  HB1 LEU J 258       8.513  15.630   3.082  1.00  0.00
J    H
ATOM  10280  HB2 LEU J 258       8.008  17.097   2.211  1.00  0.00
J    H
ATOM  10281  CG  LEU J 258       7.370  15.219   1.287  1.00  0.00
J    C
ATOM  10282  HG  LEU J 258       7.122  15.764   0.344  1.00  0.00
J    H
ATOM  10283  CD1 LEU J 258       7.884  13.804   0.975  1.00  0.00
J    C
ATOM  10284 HD11 LEU J 258       7.100  13.208   0.461  1.00  0.00
J    H
ATOM  10285 HD12 LEU J 258       8.778  13.849   0.316  1.00  0.00
J    H
ATOM  10286 HD13 LEU J 258       8.165  13.274   1.910  1.00  0.00
J    H
```

ATOM 10287 CD2 LEU J 258   6.085 15.099   2.118 1.00 0.00
J   C
ATOM 10288 HD21 LEU J 258   5.322 14.506   1.567 1.00 0.00
J   H
ATOM 10289 HD22 LEU J 258   6.270 14.592   3.087 1.00 0.00
J   H
ATOM 10290 HD23 LEU J 258   5.660 16.105   2.325 1.00 0.00
J   H
ATOM 10291 C   LEU J 258   9.804 16.925   0.130 1.00 0.00
J   C
ATOM 10292 O   LEU J 258   9.804 16.330 -0.947 1.00 0.00
J   O
ATOM 10293 N   ASN J 259   9.847 18.279   0.180 1.00 0.00
J   N
ATOM 10294 HN ASN J 259   9.788 18.776   1.044 1.00 0.00
J   H
ATOM 10295 CA ASN J 259   9.982 19.128 -0.999 1.00 0.00
J   C
ATOM 10296 HA ASN J 259   9.179 18.827 -1.663 1.00 0.00
J   H
ATOM 10297 CB ASN J 259   9.884 20.642 -0.650 1.00 0.00
J   C
ATOM 10298 HB1 ASN J 259   10.601 20.848   0.175 1.00 0.00
J   H
ATOM 10299 HB2 ASN J 259   10.191 21.259 -1.520 1.00 0.00
J   H
ATOM 10300 CG ASN J 259   8.484 21.025 -0.181 1.00 0.00
J   C
ATOM 10301 OD1 ASN J 259   7.525 20.255 -0.177 1.00 0.00
J   O
ATOM 10302 ND2 ASN J 259   8.327 22.307   0.230 1.00 0.00
J   N
ATOM 10303 HD21 ASN J 259   7.424 22.578   0.552 1.00 0.00
J   H
ATOM 10304 HD22 ASN J 259   9.105 22.928   0.231 1.00 0.00
J   H
ATOM 10305 C   ASN J 259   11.303 18.898 -1.736 1.00 0.00
J   C
ATOM 10306 O   ASN J 259   11.332 18.819 -2.961 1.00 0.00
J   O
ATOM 10307 N   ALA J 260   12.429 18.760 -0.998 1.00 0.00
J   N
ATOM 10308 HN ALA J 260   12.402 18.858 -0.004 1.00 0.00
J   H
ATOM 10309 CA ALA J 260   13.735 18.468 -1.561 1.00 0.00
J   C
ATOM 10310 HA ALA J 260   13.978 19.219 -2.304 1.00 0.00
J   H
ATOM 10311 CB ALA J 260   14.793 18.527 -0.437 1.00 0.00
J   C
ATOM 10312 HB1 ALA J 260   14.765 19.527   0.048 1.00 0.00
J   H
ATOM 10313 HB2 ALA J 260   14.589 17.763   0.344 1.00 0.00
J   H

```
ATOM  10314  HB3 ALA J 260      15.819  18.366  -0.832  1.00  0.00
J    H
ATOM  10315  C   ALA J 260      13.824  17.114  -2.271  1.00  0.00
J    C
ATOM  10316  O   ALA J 260      14.315  17.022  -3.397  1.00  0.00
J    O
ATOM  10317  N   ILE J 261      13.295  16.032  -1.649  1.00  0.00
J    N
ATOM  10318  HN  ILE J 261      12.923  16.088  -0.725  1.00  0.00
J    H
ATOM  10319  CA  ILE J 261      13.214  14.700  -2.249  1.00  0.00
J    C
ATOM  10320  HA  ILE J 261      14.214  14.452  -2.582  1.00  0.00
J    H
ATOM  10321  CB  ILE J 261      12.714  13.645  -1.255  1.00  0.00
J    C
ATOM  10322  HB  ILE J 261      11.764  14.072  -0.846  1.00  0.00
J    H
ATOM  10323  CG2 ILE J 261      12.548  12.269  -1.951  1.00  0.00
J    C
ATOM  10324 HG21 ILE J 261      12.041  11.547  -1.276  1.00  0.00
J    H
ATOM  10325 HG22 ILE J 261      11.930  12.361  -2.869  1.00  0.00
J    H
ATOM  10326 HG23 ILE J 261      13.534  11.844  -2.234  1.00  0.00
J    H
ATOM  10327  CG1 ILE J 261      13.697  13.517  -0.064  1.00  0.00
J    C
ATOM  10328 HG11 ILE J 261      14.629  13.008  -0.398  1.00  0.00
J    H
ATOM  10329 HG12 ILE J 261      13.984  14.538   0.278  1.00  0.00
J    H
ATOM  10330  CD  ILE J 261      13.102  12.755   1.129  1.00  0.00
J    C
ATOM  10331  HD1 ILE J 261      13.821  12.741   1.976  1.00  0.00
J    H
ATOM  10332  HD2 ILE J 261      12.165  13.241   1.477  1.00  0.00
J    H
ATOM  10333  HD3 ILE J 261      12.873  11.703   0.856  1.00  0.00
J    H
ATOM  10334  C   ILE J 261      12.329  14.714  -3.485  1.00  0.00
J    C
ATOM  10335  O   ILE J 261      12.682  14.183  -4.534  1.00  0.00
J    O
ATOM  10336  N   ALA J 262      11.164  15.385  -3.416  1.00  0.00
J    N
ATOM  10337  HN  ALA J 262      10.857  15.813  -2.566  1.00  0.00
J    H
ATOM  10338  CA  ALA J 262      10.274  15.539  -4.545  1.00  0.00
J    C
ATOM  10339  HA  ALA J 262      10.000  14.558  -4.913  1.00  0.00
J    H
ATOM  10340  CB  ALA J 262       8.987  16.230  -4.068  1.00  0.00
J    C
```

ATOM 10341 HB1 ALA J 262 8.535 15.644 -3.240 1.00 0.00
J H
ATOM 10342 HB2 ALA J 262 9.199 17.253 -3.688 1.00 0.00
J H
ATOM 10343 HB3 ALA J 262 8.237 16.304 -4.885 1.00 0.00
J H
ATOM 10344 C ALA J 262 10.875 16.291 -5.727 1.00 0.00
J C
ATOM 10345 O ALA J 262 10.712 15.884 -6.876 1.00 0.00
J O
ATOM 10346 N MET J 263 11.617 17.387 -5.471 1.00 0.00
J N
ATOM 10347 HN MET J 263 11.726 17.744 -4.546 1.00 0.00
J H
ATOM 10348 CA MET J 263 12.319 18.145 -6.491 1.00 0.00
J C
ATOM 10349 HA MET J 263 11.577 18.418 -7.231 1.00 0.00
J H
ATOM 10350 CB MET J 263 12.905 19.440 -5.887 1.00 0.00
J C
ATOM 10351 HB1 MET J 263 12.046 20.033 -5.493 1.00 0.00
J H
ATOM 10352 HB2 MET J 263 13.536 19.185 -5.006 1.00 0.00
J H
ATOM 10353 CG MET J 263 13.687 20.289 -6.904 1.00 0.00
J C
ATOM 10354 HG1 MET J 263 14.636 19.782 -7.181 1.00 0.00
J H
ATOM 10355 HG2 MET J 263 13.085 20.408 -7.832 1.00 0.00
J H
ATOM 10356 SD MET J 263 14.120 21.941 -6.300 1.00 0.00
J S
ATOM 10357 CE MET J 263 14.995 22.386 -7.827 1.00 0.00
J C
ATOM 10358 HE1 MET J 263 15.415 23.412 -7.759 1.00 0.00
J H
ATOM 10359 HE2 MET J 263 15.835 21.685 -8.024 1.00 0.00
J H
ATOM 10360 HE3 MET J 263 14.312 22.354 -8.703 1.00 0.00
J H
ATOM 10361 C MET J 263 13.389 17.338 -7.193 1.00 0.00
J C
ATOM 10362 O MET J 263 13.502 17.373 -8.419 1.00 0.00
J O
ATOM 10363 N LEU J 264 14.165 16.541 -6.431 1.00 0.00
J N
ATOM 10364 HN LEU J 264 14.144 16.557 -5.433 1.00 0.00
J H
ATOM 10365 CA LEU J 264 15.103 15.590 -6.991 1.00 0.00
J C
ATOM 10366 HA LEU J 264 15.754 16.157 -7.646 1.00 0.00
J H
ATOM 10367 CB LEU J 264 15.890 14.897 -5.850 1.00 0.00
J C

ATOM 10368 HB1 LEU J 264 16.335 15.703 -5.221 1.00 0.00
J H
ATOM 10369 HB2 LEU J 264 15.175 14.354 -5.194 1.00 0.00
J H
ATOM 10370 CG LEU J 264 17.019 13.952 -6.318 1.00 0.00
J C
ATOM 10371 HG LEU J 264 16.596 13.352 -7.159 1.00 0.00
J H
ATOM 10372 CD1 LEU J 264 18.245 14.736 -6.815 1.00 0.00
J C
ATOM 10373 HD11 LEU J 264 19.063 14.041 -7.101 1.00 0.00
J H
ATOM 10374 HD12 LEU J 264 17.982 15.352 -7.703 1.00 0.00
J H
ATOM 10375 HD13 LEU J 264 18.627 15.412 -6.020 1.00 0.00
J H
ATOM 10376 CD2 LEU J 264 17.407 12.983 -5.190 1.00 0.00
J C
ATOM 10377 HD21 LEU J 264 18.206 12.291 -5.534 1.00 0.00
J H
ATOM 10378 HD22 LEU J 264 17.789 13.522 -4.300 1.00 0.00
J H
ATOM 10379 HD23 LEU J 264 16.529 12.375 -4.881 1.00 0.00
J H
ATOM 10380 C LEU J 264 14.412 14.526 -7.837 1.00 0.00
J C
ATOM 10381 O LEU J 264 14.792 14.296 -8.983 1.00 0.00
J O
ATOM 10382 N THR J 265 13.329 13.894 -7.330 1.00 0.00
J N
ATOM 10383 HN THR J 265 12.960 14.093 -6.423 1.00 0.00
J H
ATOM 10384 CA THR J 265 12.596 12.859 -8.064 1.00 0.00
J C
ATOM 10385 HA THR J 265 13.341 12.121 -8.326 1.00 0.00
J H
ATOM 10386 CB THR J 265 11.457 12.246 -7.255 1.00 0.00
J C
ATOM 10387 HB THR J 265 10.849 13.104 -6.875 1.00 0.00
J H
ATOM 10388 OG1 THR J 265 11.989 11.557 -6.139 1.00 0.00
J O
ATOM 10389 HG1 THR J 265 11.227 11.186 -5.680 1.00 0.00
J H
ATOM 10390 CG2 THR J 265 10.670 11.188 -8.044 1.00 0.00
J C
ATOM 10391 HG21 THR J 265 9.784 10.846 -7.470 1.00 0.00
J H
ATOM 10392 HG22 THR J 265 10.311 11.610 -9.007 1.00 0.00
J H
ATOM 10393 HG23 THR J 265 11.303 10.303 -8.266 1.00 0.00
J H
ATOM 10394 C THR J 265 12.004 13.355 -9.365 1.00 0.00
J C

ATOM 10395 O   THR J 265   12.203 12.741 -10.411 1.00 0.00
J   O
ATOM 10396 N   VAL J 266   11.314 14.518 -9.353 1.00 0.00
J   N
ATOM 10397 HN VAL J 266   11.152 15.030 -8.513 1.00 0.00
J   H
ATOM 10398 CA VAL J 266   10.747 15.130 -10.552 1.00 0.00
J   C
ATOM 10399 HA VAL J 266   10.116 14.384 -11.020 1.00 0.00
J   H
ATOM 10400 CB VAL J 266   9.844 16.323 -10.208 1.00 0.00
J   C
ATOM 10401 HB VAL J 266   10.422 16.999 -9.532 1.00 0.00
J   H
ATOM 10402 CG1 VAL J 266   9.432 17.123 -11.466 1.00 0.00
J   C
ATOM 10403 HG11 VAL J 266   8.749 17.954 -11.187 1.00 0.00
J   H
ATOM 10404 HG12 VAL J 266   10.311 17.573 -11.972 1.00 0.00
J   H
ATOM 10405 HG13 VAL J 266   8.900 16.469 -12.190 1.00 0.00
J   H
ATOM 10406 CG2 VAL J 266   8.576 15.783 -9.508 1.00 0.00
J   C
ATOM 10407 HG21 VAL J 266   7.873 16.612 -9.276 1.00 0.00
J   H
ATOM 10408 HG22 VAL J 266   8.052 15.051 -10.160 1.00 0.00
J   H
ATOM 10409 HG23 VAL J 266   8.829 15.280 -8.552 1.00 0.00
J   H
ATOM 10410 C   VAL J 266   11.822 15.515 -11.558 1.00 0.00
J   C
ATOM 10411 O   VAL J 266   11.696 15.229 -12.749 1.00 0.00
J   O
ATOM 10412 N   PHE J 267   12.945 16.124 -11.106 1.00 0.00
J   N
ATOM 10413 HN PHE J 267   13.093 16.358 -10.146 1.00 0.00
J   H
ATOM 10414 CA PHE J 267   14.039 16.488 -11.989 1.00 0.00
J   C
ATOM 10415 HA PHE J 267   13.588 17.083 -12.774 1.00 0.00
J   H
ATOM 10416 CB PHE J 267   15.100 17.327 -11.212 1.00 0.00
J   C
ATOM 10417 HB1 PHE J 267   14.599 18.202 -10.742 1.00 0.00
J   H
ATOM 10418 HB2 PHE J 267   15.559 16.733 -10.392 1.00 0.00
J   H
ATOM 10419 CG PHE J 267   16.183 17.880 -12.117 1.00 0.00
J   C
ATOM 10420 CD1 PHE J 267   15.958 18.998 -12.942 1.00 0.00
J   C
ATOM 10421 HD1 PHE J 267   14.997 19.494 -12.921 1.00 0.00
J   H

ATOM 10422 CE1 PHE J 267   16.964 19.473 -13.795 1.00 0.00
J   C
ATOM 10423 HE1 PHE J 267   16.784 20.339 -14.414 1.00 0.00
J   H
ATOM 10424 CZ PHE J 267   18.205 18.829 -13.840 1.00 0.00
J   C
ATOM 10425 HZ PHE J 267   18.983 19.196 -14.494 1.00 0.00
J   H
ATOM 10426 CD2 PHE J 267   17.437 17.246 -12.172 1.00 0.00
J   C
ATOM 10427 HD2 PHE J 267   17.629 16.374 -11.563 1.00 0.00
J   H
ATOM 10428 CE2 PHE J 267   18.441 17.713 -13.030 1.00 0.00
J   C
ATOM 10429 HE2 PHE J 267   19.397 17.209 -13.065 1.00 0.00
J   H
ATOM 10430 C   PHE J 267   14.693 15.285 -12.659 1.00 0.00
J   C
ATOM 10431 O   PHE J 267   14.857 15.279 -13.872 1.00 0.00
J   O
ATOM 10432 N   ILE J 268   15.047 14.221 -11.907 1.00 0.00
J   N
ATOM 10433 HN ILE J 268   14.887 14.166 -10.923 1.00 0.00
J   H
ATOM 10434 CA ILE J 268   15.703 13.044 -12.472 1.00 0.00
J   C
ATOM 10435 HA ILE J 268   16.524 13.426 -13.065 1.00 0.00
J   H
ATOM 10436 CB ILE J 268   16.279 12.137 -11.377 1.00 0.00
J   C
ATOM 10437 HB ILE J 268   15.409 11.917 -10.709 1.00 0.00
J   H
ATOM 10438 CG2 ILE J 268   16.899 10.848 -11.973 1.00 0.00
J   C
ATOM 10439 HG21 ILE J 268   17.169 10.135 -11.166 1.00 0.00
J   H
ATOM 10440 HG22 ILE J 268   16.180 10.337 -12.646 1.00 0.00
J   H
ATOM 10441 HG23 ILE J 268   17.818 11.080 -12.552 1.00 0.00
J   H
ATOM 10442 CG1 ILE J 268   17.326 12.886 -10.501 1.00 0.00
J   C
ATOM 10443 HG11 ILE J 268   16.844 13.754 -9.998 1.00 0.00
J   H
ATOM 10444 HG12 ILE J 268   17.684 12.199 -9.701 1.00 0.00
J   H
ATOM 10445 CD ILE J 268   18.568 13.401 -11.243 1.00 0.00
J   C
ATOM 10446 HD1 ILE J 268   19.279 13.867 -10.527 1.00 0.00
J   H
ATOM 10447 HD2 ILE J 268   19.091 12.566 -11.758 1.00 0.00
J   H
ATOM 10448 HD3 ILE J 268   18.293 14.165 -12.000 1.00 0.00
J   H

```
ATOM  10449  C   ILE J 268      14.802  12.255 -13.420  1.00  0.00
J    C
ATOM  10450  O   ILE J 268      15.220  11.855 -14.506  1.00  0.00
J    O
ATOM  10451  N   LEU J 269      13.526  12.024 -13.050  1.00  0.00
J    N
ATOM  10452  HN  LEU J 269      13.148  12.354 -12.187  1.00  0.00
J    H
ATOM  10453  CA  LEU J 269      12.585  11.277 -13.868  1.00  0.00
J    C
ATOM  10454  HA  LEU J 269      13.099  10.357 -14.115  1.00  0.00
J    H
ATOM  10455  CB  LEU J 269      11.310  10.936 -13.059  1.00  0.00
J    C
ATOM  10456  HB1 LEU J 269      11.176  11.743 -12.302  1.00  0.00
J    H
ATOM  10457  HB2 LEU J 269      10.427  10.995 -13.733  1.00  0.00
J    H
ATOM  10458  CG  LEU J 269      11.347   9.571 -12.331  1.00  0.00
J    C
ATOM  10459  HG  LEU J 269      11.306   8.805 -13.142  1.00  0.00
J    H
ATOM  10460  CD1 LEU J 269      12.581   9.339 -11.444  1.00  0.00
J    C
ATOM  10461 HD11 LEU J 269      12.508   8.362 -10.922  1.00  0.00
J    H
ATOM  10462 HD12 LEU J 269      13.509   9.340 -12.055  1.00  0.00
J    H
ATOM  10463 HD13 LEU J 269      12.667  10.138 -10.676  1.00  0.00
J    H
ATOM  10464  CD2 LEU J 269      10.080   9.432 -11.484  1.00  0.00
J    C
ATOM  10465 HD21 LEU J 269      10.070   8.450 -10.963  1.00  0.00
J    H
ATOM  10466 HD22 LEU J 269      10.014  10.223 -10.711  1.00  0.00
J    H
ATOM  10467 HD23 LEU J 269       9.174   9.493 -12.126  1.00  0.00
J    H
ATOM  10468  C   LEU J 269      12.196  11.953 -15.177  1.00  0.00
J    C
ATOM  10469  O   LEU J 269      12.153  11.305 -16.223  1.00  0.00
J    O
ATOM  10470  N   GLU J 270      11.914  13.269 -15.156  1.00  0.00
J    N
ATOM  10471  HN  GLU J 270      12.080  13.858 -14.371  1.00  0.00
J    H
ATOM  10472  CA  GLU J 270      11.334  13.953 -16.293  1.00  0.00
J    C
ATOM  10473  HA  GLU J 270      10.909  13.199 -16.944  1.00  0.00
J    H
ATOM  10474  CB  GLU J 270      10.222  14.919 -15.807  1.00  0.00
J    C
ATOM  10475  HB1 GLU J 270      10.665  15.604 -15.048  1.00  0.00
J    H
```

```
ATOM  10476  HB2 GLU J 270       9.892  15.569 -16.649  1.00  0.00
J    H
ATOM  10477  CG  GLU J 270       8.983  14.245 -15.180  1.00  0.00
J    C
ATOM  10478  HG1 GLU J 270       9.307  13.519 -14.412  1.00  0.00
J    H
ATOM  10479  HG2 GLU J 270       8.316  14.997 -14.714  1.00  0.00
J    H
ATOM  10480  CD  GLU J 270       8.196  13.455 -16.202  1.00  0.00
J    C
ATOM  10481  OE1 GLU J 270       7.790  14.013 -17.244  1.00  0.00
J    O
ATOM  10482  OE2 GLU J 270       7.941  12.256 -15.929  1.00  0.00
J    O
ATOM  10483  C   GLU J 270      12.373  14.723 -17.109  1.00  0.00
J    C
ATOM  10484  O   GLU J 270      12.037  15.367 -18.098  1.00  0.00
J    O
ATOM  10485  N   TYR J 271      13.686  14.645 -16.770  1.00  0.00
J    N
ATOM  10486  HN  TYR J 271      13.988  14.050 -16.027  1.00  0.00
J    H
ATOM  10487  CA  TYR J 271      14.764  15.393 -17.427  1.00  0.00
J    C
ATOM  10488  HA  TYR J 271      14.534  16.437 -17.248  1.00  0.00
J    H
ATOM  10489  CB  TYR J 271      16.146  14.977 -16.822  1.00  0.00
J    C
ATOM  10490  HB1 TYR J 271      16.116  15.110 -15.719  1.00  0.00
J    H
ATOM  10491  HB2 TYR J 271      16.353  13.903 -17.018  1.00  0.00
J    H
ATOM  10492  CG  TYR J 271      17.301  15.806 -17.327  1.00  0.00
J    C
ATOM  10493  CD1 TYR J 271      17.311  17.199 -17.150  1.00  0.00
J    C
ATOM  10494  HD1 TYR J 271      16.492  17.681 -16.633  1.00  0.00
J    H
ATOM  10495  CE1 TYR J 271      18.366  17.970 -17.659  1.00  0.00
J    C
ATOM  10496  HE1 TYR J 271      18.384  19.040 -17.516  1.00  0.00
J    H
ATOM  10497  CZ  TYR J 271      19.402  17.357 -18.366  1.00  0.00
J    C
ATOM  10498  OH  TYR J 271      20.390  18.164 -18.959  1.00  0.00
J    O
ATOM  10499  HH  TYR J 271      21.051  17.580 -19.335  1.00  0.00
J    H
ATOM  10500  CD2 TYR J 271      18.381  15.193 -17.983  1.00  0.00
J    C
ATOM  10501  HD2 TYR J 271      18.389  14.121 -18.127  1.00  0.00
J    H
ATOM  10502  CE2 TYR J 271      19.432  15.964 -18.501  1.00  0.00
J    C
```

```
ATOM  10503  HE2 TYR J 271      20.247  15.481 -19.019  1.00  0.00
J    H
ATOM  10504  C   TYR J 271      14.830  15.199 -18.944  1.00  0.00
J    C
ATOM  10505  O   TYR J 271      15.119  16.127 -19.701  1.00  0.00
J    O
ATOM  10506  N   SER J 272      14.563  13.960 -19.393  1.00  0.00
J    N
ATOM  10507  HN  SER J 272      14.290  13.228 -18.768  1.00  0.00
J    H
ATOM  10508  CA  SER J 272      14.637  13.557 -20.787  1.00  0.00
J    C
ATOM  10509  HA  SER J 272      15.134  14.372 -21.296  1.00  0.00
J    H
ATOM  10510  CB  SER J 272      15.331  12.182 -20.970  1.00  0.00
J    C
ATOM  10511  HB1 SER J 272      14.667  11.398 -20.538  1.00  0.00
J    H
ATOM  10512  HB2 SER J 272      15.451  11.949 -22.053  1.00  0.00
J    H
ATOM  10513  OG  SER J 272      16.585  12.156 -20.290  1.00  0.00
J    O
ATOM  10514  HG1 SER J 272      16.865  11.233 -20.243  1.00  0.00
J    H
ATOM  10515  C   SER J 272      13.270  13.396 -21.421  1.00  0.00
J    C
ATOM  10516  O   SER J 272      13.167  12.870 -22.526  1.00  0.00
J    O
ATOM  10517  N   ASN J 273      12.169  13.805 -20.752  1.00  0.00
J    N
ATOM  10518  HN  ASN J 273      12.195  14.216 -19.841  1.00  0.00
J    H
ATOM  10519  CA  ASN J 273      10.834  13.677 -21.316  1.00  0.00
J    C
ATOM  10520  HA  ASN J 273      10.796  12.707 -21.801  1.00  0.00
J    H
ATOM  10521  CB  ASN J 273       9.760  13.741 -20.187  1.00  0.00
J    C
ATOM  10522  HB1 ASN J 273      10.052  13.015 -19.397  1.00  0.00
J    H
ATOM  10523  HB2 ASN J 273       9.747  14.750 -19.724  1.00  0.00
J    H
ATOM  10524  CG  ASN J 273       8.370  13.366 -20.694  1.00  0.00
J    C
ATOM  10525  OD1 ASN J 273       8.096  13.366 -21.898  1.00  0.00
J    O
ATOM  10526  ND2 ASN J 273       7.461  13.023 -19.766  1.00  0.00
J    N
ATOM  10527 HD21 ASN J 273       6.553  12.766 -20.088  1.00  0.00
J    H
ATOM  10528 HD22 ASN J 273       7.713  13.006 -18.804  1.00  0.00
J    H
ATOM  10529  C   ASN J 273      10.624  14.751 -22.404  1.00  0.00
J    C
```

ATOM 10530 O   ASN J 273   10.746 15.933 -22.098 1.00 0.00
J   O
ATOM 10531 N   PRO J 274   10.330 14.486 -23.676 1.00 0.00
J   N
ATOM 10532 CD PRO J 274   10.249 13.151 -24.265 1.00 0.00
J   C
ATOM 10533 HD1 PRO J 274   11.211 12.590 -24.285 1.00 0.00
J   H
ATOM 10534 HD2 PRO J 274   9.482 12.587 -23.686 1.00 0.00
J   H
ATOM 10535 CA PRO J 274   10.220 15.542 -24.675 1.00 0.00
J   C
ATOM 10536 HA PRO J 274   11.037 16.247 -24.574 1.00 0.00
J   H
ATOM 10537 CB PRO J 274   10.323 14.783 -26.009 1.00 0.00
J   C
ATOM 10538 HB1 PRO J 274   11.397 14.645 -26.266 1.00 0.00
J   H
ATOM 10539 HB2 PRO J 274   9.819 15.313 -26.843 1.00 0.00
J   H
ATOM 10540 CG PRO J 274   9.755 13.396 -25.690 1.00 0.00
J   C
ATOM 10541 HG1 PRO J 274   10.114 12.612 -26.385 1.00 0.00
J   H
ATOM 10542 HG2 PRO J 274   8.642 13.439 -25.724 1.00 0.00
J   H
ATOM 10543 C   PRO J 274   8.903 16.290 -24.557 1.00 0.00
J   C
ATOM 10544 O   PRO J 274   8.787 17.366 -25.135 1.00 0.00
J   O
ATOM 10545 N   GLY J 275   7.895 15.739 -23.841 1.00 0.00
J   N
ATOM 10546 HN GLY J 275   8.013 14.869 -23.364 1.00 0.00
J   H
ATOM 10547 CA GLY J 275   6.583 16.356 -23.711 1.00 0.00
J   C
ATOM 10548 HA1 GLY J 275   5.895 15.569 -23.435 1.00 0.00
J   H
ATOM 10549 HA2 GLY J 275   6.361 16.828 -24.659 1.00 0.00
J   H
ATOM 10550 C   GLY J 275   6.533 17.413 -22.645 1.00 0.00
J   C
ATOM 10551 O   GLY J 275   5.804 18.396 -22.754 1.00 0.00
J   O
ATOM 10552 N   THR J 276   7.318 17.244 -21.574 1.00 0.00
J   N
ATOM 10553 HN THR J 276   7.921 16.453 -21.481 1.00 0.00
J   H
ATOM 10554 CA THR J 276   7.375 18.171 -20.461 1.00 0.00
J   C
ATOM 10555 HA THR J 276   7.183 19.164 -20.848 1.00 0.00
J   H
ATOM 10556 CB THR J 276   6.494 17.792 -19.271 1.00 0.00
J   C

```
ATOM  10557  HB  THR J 276       6.832  18.431 -18.419  1.00  0.00
J    H
ATOM  10558  OG1 THR J 276       6.692  16.443 -18.890  1.00  0.00
J    O
ATOM  10559  HG1 THR J 276       6.094  16.295 -18.151  1.00  0.00
J    H
ATOM  10560  CG2 THR J 276       5.026  17.894 -19.673  1.00  0.00
J    C
ATOM  10561 HG21 THR J 276       4.364  17.735 -18.796  1.00  0.00
J    H
ATOM  10562 HG22 THR J 276       4.807  18.902 -20.087  1.00  0.00
J    H
ATOM  10563 HG23 THR J 276       4.769  17.137 -20.446  1.00  0.00
J    H
ATOM  10564  C   THR J 276       8.795  18.171 -19.970  1.00  0.00
J    C
ATOM  10565  O   THR J 276       9.461  17.148 -19.997  1.00  0.00
J    O
ATOM  10566  N   LEU J 277       9.325  19.327 -19.534  1.00  0.00
J    N
ATOM  10567  HN  LEU J 277       8.839  20.194 -19.619  1.00  0.00
J    H
ATOM  10568  CA  LEU J 277      10.632  19.434 -18.907  1.00  0.00
J    C
ATOM  10569  HA  LEU J 277      10.664  20.450 -18.534  1.00  0.00
J    H
ATOM  10570  CB  LEU J 277      10.673  18.615 -17.578  1.00  0.00
J    C
ATOM  10571  HB1 LEU J 277       9.756  18.878 -17.001  1.00  0.00
J    H
ATOM  10572  HB2 LEU J 277      10.586  17.532 -17.817  1.00  0.00
J    H
ATOM  10573  CG  LEU J 277      11.895  18.857 -16.675  1.00  0.00
J    C
ATOM  10574  HG  LEU J 277      12.792  18.561 -17.270  1.00  0.00
J    H
ATOM  10575  CD1 LEU J 277      11.979  20.301 -16.211  1.00  0.00
J    C
ATOM  10576 HD11 LEU J 277      12.840  20.440 -15.523  1.00  0.00
J    H
ATOM  10577 HD12 LEU J 277      12.111  20.983 -17.078  1.00  0.00
J    H
ATOM  10578 HD13 LEU J 277      11.053  20.594 -15.672  1.00  0.00
J    H
ATOM  10579  CD2 LEU J 277      11.866  18.003 -15.407  1.00  0.00
J    C
ATOM  10580 HD21 LEU J 277      12.773  18.192 -14.793  1.00  0.00
J    H
ATOM  10581 HD22 LEU J 277      10.980  18.231 -14.780  1.00  0.00
J    H
ATOM  10582 HD23 LEU J 277      11.843  16.922 -15.667  1.00  0.00
J    H
ATOM  10583  C   LEU J 277      11.855  19.234 -19.817  1.00  0.00
J    C
```

ATOM 10584 O   LEU J 277   12.861 19.905 -19.625 1.00 0.00
J   O
ATOM 10585 N   GLY J 278   11.806 18.415 -20.891 1.00 0.00
J   N
ATOM 10586 HN GLY J 278   10.947 17.999 -21.185 1.00 0.00
J   H
ATOM 10587 CA GLY J 278   12.983 18.076 -21.696 1.00 0.00
J   C
ATOM 10588 HA1 GLY J 278   12.641 17.282 -22.347 1.00 0.00
J   H
ATOM 10589 HA2 GLY J 278   13.727 17.768 -20.974 1.00 0.00
J   H
ATOM 10590 C   GLY J 278   13.589 19.157 -22.556 1.00 0.00
J   C
ATOM 10591 O   GLY J 278   14.810 19.321 -22.579 1.00 0.00
J   O
ATOM 10592 N   HSE J 279   12.752 19.937 -23.269 1.00 0.00
J   N
ATOM 10593 HN HSE J 279   11.760 19.902 -23.152 1.00 0.00
J   H
ATOM 10594 CA HSE J 279   13.208 20.901 -24.272 1.00 0.00
J   C
ATOM 10595 HA HSE J 279   14.206 20.583 -24.549 1.00 0.00
J   H
ATOM 10596 CB HSE J 279   12.290 20.906 -25.521 1.00 0.00
J   C
ATOM 10597 HB1 HSE J 279   11.236 20.858 -25.172 1.00 0.00
J   H
ATOM 10598 HB2 HSE J 279   12.409 21.867 -26.065 1.00 0.00
J   H
ATOM 10599 ND1 HSE J 279   13.781 19.666 -27.033 1.00 0.00
J   N
ATOM 10600 CG HSE J 279   12.550 19.744 -26.417 1.00 0.00
J   C
ATOM 10601 CE1 HSE J 279   13.717 18.602 -27.800 1.00 0.00
J   C
ATOM 10602 HE1 HSE J 279   14.529 18.249 -28.442 1.00 0.00
J   H
ATOM 10603 NE2 HSE J 279   12.511 17.988 -27.707 1.00 0.00
J   N
ATOM 10604 HE2 HSE J 279   12.209 17.173 -28.202 1.00 0.00
J   H
ATOM 10605 CD2 HSE J 279   11.754 18.721 -26.817 1.00 0.00
J   C
ATOM 10606 HD2 HSE J 279   10.736 18.486 -26.541 1.00 0.00
J   H
ATOM 10607 C   HSE J 279   13.321 22.326 -23.754 1.00 0.00
J   C
ATOM 10608 O   HSE J 279   13.529 23.263 -24.519 1.00 0.00
J   O
ATOM 10609 N   LEU J 280   13.204 22.544 -22.433 1.00 0.00
J   N
ATOM 10610 HN LEU J 280   12.958 21.835 -21.775 1.00 0.00
J   H

```
ATOM  10611  CA  LEU J 280      13.428  23.853 -21.844  1.00  0.00
J    C
ATOM  10612  HA  LEU J 280      13.135  24.579 -22.592  1.00  0.00
J    H
ATOM  10613  CB  LEU J 280      12.563  24.057 -20.579  1.00  0.00
J    C
ATOM  10614  HB1 LEU J 280      12.818  23.235 -19.871  1.00  0.00
J    H
ATOM  10615  HB2 LEU J 280      12.872  25.002 -20.079  1.00  0.00
J    H
ATOM  10616  CG  LEU J 280      11.043  24.043 -20.842  1.00  0.00
J    C
ATOM  10617  HG  LEU J 280      10.909  23.993 -21.949  1.00  0.00
J    H
ATOM  10618  CD1 LEU J 280      10.370  22.835 -20.178  1.00  0.00
J    C
ATOM  10619 HD11 LEU J 280       9.271  22.865 -20.336  1.00  0.00
J    H
ATOM  10620 HD12 LEU J 280      10.759  21.886 -20.607  1.00  0.00
J    H
ATOM  10621 HD13 LEU J 280      10.563  22.830 -19.084  1.00  0.00
J    H
ATOM  10622  CD2 LEU J 280      10.395  25.344 -20.343  1.00  0.00
J    C
ATOM  10623 HD21 LEU J 280       9.303  25.336 -20.552  1.00  0.00
J    H
ATOM  10624 HD22 LEU J 280      10.527  25.475 -19.249  1.00  0.00
J    H
ATOM  10625 HD23 LEU J 280      10.841  26.223 -20.856  1.00  0.00
J    H
ATOM  10626  C   LEU J 280      14.907  24.089 -21.525  1.00  0.00
J    C
ATOM  10627  O   LEU J 280      15.741  23.186 -21.617  1.00  0.00
J    O
ATOM  10628  N   HSE J 281      15.303  25.320 -21.130  1.00  0.00
J    N
ATOM  10629  HN  HSE J 281      14.699  26.115 -21.095  1.00  0.00
J    H
ATOM  10630  CA  HSE J 281      16.674  25.586 -20.714  1.00  0.00
J    C
ATOM  10631  HA  HSE J 281      17.320  25.099 -21.435  1.00  0.00
J    H
ATOM  10632  CB  HSE J 281      16.977  27.096 -20.652  1.00  0.00
J    C
ATOM  10633  HB1 HSE J 281      16.313  27.545 -19.882  1.00  0.00
J    H
ATOM  10634  HB2 HSE J 281      18.022  27.250 -20.310  1.00  0.00
J    H
ATOM  10635  ND1 HSE J 281      17.735  27.746 -22.896  1.00  0.00
J    N
ATOM  10636  CG  HSE J 281      16.721  27.746 -21.962  1.00  0.00
J    C
ATOM  10637  CE1 HSE J 281      17.215  28.321 -23.960  1.00  0.00
J    C
```

```
ATOM  10638  HE1 HSE J 281      17.751  28.495 -24.895  1.00  0.00
J    H
ATOM  10639  NE2 HSE J 281      15.924  28.683 -23.760  1.00  0.00
J    N
ATOM  10640  HE2 HSE J 281      15.319  29.149 -24.406  1.00  0.00
J    H
ATOM  10641  CD2 HSE J 281      15.600  28.315 -22.474  1.00  0.00
J    C
ATOM  10642  HD2 HSE J 281      14.635  28.482 -22.015  1.00  0.00
J    H
ATOM  10643  C   HSE J 281      16.975  24.970 -19.356  1.00  0.00
J    C
ATOM  10644  O   HSE J 281      16.073  24.636 -18.595  1.00  0.00
J    O
ATOM  10645  N   ILE J 282      18.264  24.795 -18.982  1.00  0.00
J    N
ATOM  10646  HN  ILE J 282      19.033  25.074 -19.553  1.00  0.00
J    H
ATOM  10647  CA  ILE J 282      18.652  24.183 -17.707  1.00  0.00
J    C
ATOM  10648  HA  ILE J 282      18.211  23.195 -17.709  1.00  0.00
J    H
ATOM  10649  CB  ILE J 282      20.175  24.005 -17.603  1.00  0.00
J    C
ATOM  10650  HB  ILE J 282      20.589  25.033 -17.766  1.00  0.00
J    H
ATOM  10651  CG2 ILE J 282      20.555  23.386 -16.233  1.00  0.00
J    C
ATOM  10652 HG21 ILE J 282      21.656  23.400 -16.090  1.00  0.00
J    H
ATOM  10653 HG22 ILE J 282      20.099  23.961 -15.400  1.00  0.00
J    H
ATOM  10654 HG23 ILE J 282      20.210  22.334 -16.163  1.00  0.00
J    H
ATOM  10655  CG1 ILE J 282      20.690  23.118 -18.771  1.00  0.00
J    C
ATOM  10656 HG11 ILE J 282      20.190  22.124 -18.738  1.00  0.00
J    H
ATOM  10657 HG12 ILE J 282      20.409  23.596 -19.738  1.00  0.00
J    H
ATOM  10658  CD  ILE J 282      22.212  22.908 -18.770  1.00  0.00
J    C
ATOM  10659  HD1 ILE J 282      22.521  22.317 -19.659  1.00  0.00
J    H
ATOM  10660  HD2 ILE J 282      22.742  23.884 -18.801  1.00  0.00
J    H
ATOM  10661  HD3 ILE J 282      22.538  22.359 -17.861  1.00  0.00
J    H
ATOM  10662  C   ILE J 282      18.071  24.925 -16.499  1.00  0.00
J    C
ATOM  10663  O   ILE J 282      17.567  24.308 -15.565  1.00  0.00
J    O
ATOM  10664  N   VAL J 283      18.053  26.272 -16.528  1.00  0.00
J    N
```

ATOM 10665 HN VAL J 283   18.500 26.775 -17.263 1.00 0.00
J   H
ATOM 10666 CA VAL J 283   17.404 27.113 -15.532 1.00 0.00
J   C
ATOM 10667 HA VAL J 283   17.837 26.835 -14.580 1.00 0.00
J   H
ATOM 10668 CB VAL J 283   17.788 28.579 -15.772 1.00 0.00
J   C
ATOM 10669 HB VAL J 283   18.885 28.677 -15.578 1.00 0.00
J   H
ATOM 10670 CG1 VAL J 283   17.510 29.037 -17.224 1.00 0.00
J   C
ATOM 10671 HG11 VAL J 283   17.764 30.111 -17.345 1.00 0.00
J   H
ATOM 10672 HG12 VAL J 283   18.120 28.469 -17.956 1.00 0.00
J   H
ATOM 10673 HG13 VAL J 283   16.437 28.907 -17.478 1.00 0.00
J   H
ATOM 10674 CG2 VAL J 283   17.090 29.498 -14.748 1.00 0.00
J   C
ATOM 10675 HG21 VAL J 283   17.335 30.564 -14.945 1.00 0.00
J   H
ATOM 10676 HG22 VAL J 283   15.987 29.377 -14.803 1.00 0.00
J   H
ATOM 10677 HG23 VAL J 283   17.415 29.265 -13.712 1.00 0.00
J   H
ATOM 10678 C   VAL J 283   15.885 26.909 -15.424 1.00 0.00
J   C
ATOM 10679 O   VAL J 283   15.359 26.701 -14.330 1.00 0.00
J   O
ATOM 10680 N   ASP J 284   15.144 26.863 -16.556 1.00 0.00
J   N
ATOM 10681 HN ASP J 284   15.543 27.008 -17.458 1.00 0.00
J   H
ATOM 10682 CA ASP J 284   13.716 26.605 -16.605 1.00 0.00
J   C
ATOM 10683 HA ASP J 284   13.247 27.333 -15.951 1.00 0.00
J   H
ATOM 10684 CB ASP J 284   13.212 26.604 -18.053 1.00 0.00
J   C
ATOM 10685 HB1 ASP J 284   13.800 25.874 -18.643 1.00 0.00
J   H
ATOM 10686 HB2 ASP J 284   12.145 26.309 -18.095 1.00 0.00
J   H
ATOM 10687 CG ASP J 284   13.368 27.952 -18.706 1.00 0.00
J   C
ATOM 10688 OD1 ASP J 284   12.859 28.950 -18.149 1.00 0.00
J   O
ATOM 10689 OD2 ASP J 284   14.001 27.968 -19.791 1.00 0.00
J   O
ATOM 10690 C   ASP J 284   13.366 25.238 -16.058 1.00 0.00
J   C
ATOM 10691 O   ASP J 284   12.350 25.038 -15.393 1.00 0.00
J   O

ATOM 10692 N LYS J 285 14.238 24.243 -16.324 1.00 0.00
J N
ATOM 10693 HN LYS J 285 15.048 24.392 -16.889 1.00 0.00
J H
ATOM 10694 CA LYS J 285 14.078 22.899 -15.824 1.00 0.00
J C
ATOM 10695 HA LYS J 285 13.123 22.548 -16.195 1.00 0.00
J H
ATOM 10696 CB LYS J 285 15.181 21.940 -16.356 1.00 0.00
J C
ATOM 10697 HB1 LYS J 285 16.163 22.435 -16.170 1.00 0.00
J H
ATOM 10698 HB2 LYS J 285 15.180 21.011 -15.743 1.00 0.00
J H
ATOM 10699 CG LYS J 285 15.062 21.606 -17.850 1.00 0.00
J C
ATOM 10700 HG1 LYS J 285 14.068 21.141 -18.042 1.00 0.00
J H
ATOM 10701 HG2 LYS J 285 15.105 22.551 -18.438 1.00 0.00
J H
ATOM 10702 CD LYS J 285 16.153 20.638 -18.346 1.00 0.00
J C
ATOM 10703 HD1 LYS J 285 17.147 21.106 -18.163 1.00 0.00
J H
ATOM 10704 HD2 LYS J 285 16.101 19.711 -17.729 1.00 0.00
J H
ATOM 10705 CE LYS J 285 15.985 20.294 -19.831 1.00 0.00
J C
ATOM 10706 HE1 LYS J 285 15.107 19.627 -19.980 1.00 0.00
J H
ATOM 10707 HE2 LYS J 285 15.830 21.218 -20.431 1.00 0.00
J H
ATOM 10708 NZ LYS J 285 17.151 19.598 -20.415 1.00 0.00
J N
ATOM 10709 HZ1 LYS J 285 16.977 19.392 -21.419 1.00 0.00
J H
ATOM 10710 HZ2 LYS J 285 17.995 20.200 -20.327 1.00 0.00
J H
ATOM 10711 HZ3 LYS J 285 17.313 18.707 -19.903 1.00 0.00
J H
ATOM 10712 C LYS J 285 14.026 22.834 -14.302 1.00 0.00
J C
ATOM 10713 O LYS J 285 13.199 22.140 -13.719 1.00 0.00
J O
ATOM 10714 N LEU J 286 14.879 23.606 -13.611 1.00 0.00
J N
ATOM 10715 HN LEU J 286 15.587 24.151 -14.055 1.00 0.00
J H
ATOM 10716 CA LEU J 286 14.856 23.723 -12.165 1.00 0.00
J C
ATOM 10717 HA LEU J 286 14.859 22.709 -11.787 1.00 0.00
J H
ATOM 10718 CB LEU J 286 16.112 24.467 -11.669 1.00 0.00
J C

ATOM 10719 HB1 LEU J 286   16.037 25.518 -12.030 1.00 0.00
J   H
ATOM 10720 HB2 LEU J 286   16.086 24.520 -10.558 1.00 0.00
J   H
ATOM 10721 CG LEU J 286   17.421 23.831 -12.180 1.00 0.00
J   C
ATOM 10722 HG LEU J 286   17.293 23.769 -13.288 1.00 0.00
J   H
ATOM 10723 CD1 LEU J 286   18.617 24.736 -11.860 1.00 0.00
J   C
ATOM 10724 HD11 LEU J 286   19.566 24.258 -12.184 1.00 0.00
J   H
ATOM 10725 HD12 LEU J 286   18.521 25.711 -12.383 1.00 0.00
J   H
ATOM 10726 HD13 LEU J 286   18.681 24.929 -10.768 1.00 0.00
J   H
ATOM 10727 CD2 LEU J 286   17.633 22.405 -11.645 1.00 0.00
J   C
ATOM 10728 HD21 LEU J 286   18.575 21.977 -12.051 1.00 0.00
J   H
ATOM 10729 HD22 LEU J 286   17.705 22.389 -10.538 1.00 0.00
J   H
ATOM 10730 HD23 LEU J 286   16.792 21.746 -11.950 1.00 0.00
J   H
ATOM 10731 C   LEU J 286   13.607 24.405 -11.641 1.00 0.00
J   C
ATOM 10732 O   LEU J 286   13.000 23.937 -10.677 1.00 0.00
J   O
ATOM 10733 N   TRP J 287   13.155 25.496 -12.299 1.00 0.00
J   N
ATOM 10734 HN TRP J 287   13.667 25.886 -13.060 1.00 0.00
J   H
ATOM 10735 CA TRP J 287   11.917 26.185 -11.973 1.00 0.00
J   C
ATOM 10736 HA TRP J 287   11.991 26.482 -10.934 1.00 0.00
J   H
ATOM 10737 CB TRP J 287   11.729 27.418 -12.896 1.00 0.00
J   C
ATOM 10738 HB1 TRP J 287   12.017 27.118 -13.929 1.00 0.00
J   H
ATOM 10739 HB2 TRP J 287   10.656 27.703 -12.932 1.00 0.00
J   H
ATOM 10740 CG TRP J 287   12.580 28.619 -12.527 1.00 0.00
J   C
ATOM 10741 CD1 TRP J 287   13.499 29.282 -13.293 1.00 0.00
J   C
ATOM 10742 HD1 TRP J 287   13.718 29.040 -14.325 1.00 0.00
J   H
ATOM 10743 NE1 TRP J 287   14.065 30.317 -12.576 1.00 0.00
J   N
ATOM 10744 HE1 TRP J 287   14.731 30.955 -12.896 1.00 0.00
J   H
ATOM 10745 CE2 TRP J 287   13.493 30.343 -11.322 1.00 0.00
J   C

ATOM 10746 CD2 TRP J 287   12.540 29.307 -11.258 1.00 0.00
J   C
ATOM 10747 CE3 TRP J 287   11.775 29.103 -10.106 1.00 0.00
J   C
ATOM 10748 HE3 TRP J 287   11.038 28.314 -10.044 1.00 0.00
J   H
ATOM 10749 CZ3 TRP J 287   11.995 29.953 -9.011 1.00 0.00
J   C
ATOM 10750 HZ3 TRP J 287   11.418 29.816 -8.107 1.00 0.00
J   H
ATOM 10751 CZ2 TRP J 287   13.714 31.182 -10.234 1.00 0.00
J   C
ATOM 10752 HZ2 TRP J 287   14.444 31.976 -10.268 1.00 0.00
J   H
ATOM 10753 CH2 TRP J 287   12.954 30.973 -9.073 1.00 0.00
J   C
ATOM 10754 HH2 TRP J 287   13.113 31.609 -8.213 1.00 0.00
J   H
ATOM 10755 C   TRP J 287   10.691 25.292 -12.092 1.00 0.00
J   C
ATOM 10756 O   TRP J 287   9.868 25.214 -11.181 1.00 0.00
J   O
ATOM 10757 N   ALA J 288   10.587 24.533 -13.198 1.00 0.00
J   N
ATOM 10758 HN ALA J 288   11.245 24.624 -13.945 1.00 0.00
J   H
ATOM 10759 CA ALA J 288   9.549 23.552 -13.402 1.00 0.00
J   C
ATOM 10760 HA ALA J 288   8.580 24.020 -13.271 1.00 0.00
J   H
ATOM 10761 CB ALA J 288   9.648 23.019 -14.840 1.00 0.00
J   C
ATOM 10762 HB1 ALA J 288   9.558 23.863 -15.558 1.00 0.00
J   H
ATOM 10763 HB2 ALA J 288   10.628 22.524 -15.016 1.00 0.00
J   H
ATOM 10764 HB3 ALA J 288   8.839 22.291 -15.063 1.00 0.00
J   H
ATOM 10765 C   ALA J 288   9.604 22.389 -12.413 1.00 0.00
J   C
ATOM 10766 O   ALA J 288   8.582 22.004 -11.847 1.00 0.00
J   O
ATOM 10767 N   SER J 289   10.790 21.812 -12.141 1.00 0.00
J   N
ATOM 10768 HN SER J 289   11.640 22.075 -12.596 1.00 0.00
J   H
ATOM 10769 CA SER J 289   10.946 20.745 -11.153 1.00 0.00
J   C
ATOM 10770 HA SER J 289   10.213 20.003 -11.443 1.00 0.00
J   H
ATOM 10771 CB SER J 289   12.346 20.116 -11.149 1.00 0.00
J   C
ATOM 10772 HB1 SER J 289   13.095 20.938 -11.087 1.00 0.00
J   H

```
ATOM  10773  HB2 SER J 289      12.482  19.480 -10.246  1.00  0.00
J    H
ATOM  10774  OG  SER J 289      12.501  19.397 -12.365  1.00  0.00
J    O
ATOM  10775  HG1 SER J 289      13.441  19.196 -12.458  1.00  0.00
J    H
ATOM  10776  C   SER J 289      10.643  21.144  -9.734  1.00  0.00
J    C
ATOM  10777  O   SER J 289       9.983  20.404  -9.011  1.00  0.00
J    O
ATOM  10778  N   TYR J 290      11.083  22.342  -9.297  1.00  0.00
J    N
ATOM  10779  HN  TYR J 290      11.696  22.903  -9.850  1.00  0.00
J    H
ATOM  10780  CA  TYR J 290      10.722  22.924  -8.015  1.00  0.00
J    C
ATOM  10781  HA  TYR J 290      11.010  22.207  -7.257  1.00  0.00
J    H
ATOM  10782  CB  TYR J 290      11.484  24.285  -7.868  1.00  0.00
J    C
ATOM  10783  HB1 TYR J 290      12.558  24.081  -7.667  1.00  0.00
J    H
ATOM  10784  HB2 TYR J 290      11.431  24.869  -8.812  1.00  0.00
J    H
ATOM  10785  CG  TYR J 290      11.011  25.166  -6.734  1.00  0.00
J    C
ATOM  10786  CD1 TYR J 290      11.065  24.742  -5.396  1.00  0.00
J    C
ATOM  10787  HD1 TYR J 290      11.501  23.783  -5.154  1.00  0.00
J    H
ATOM  10788  CE1 TYR J 290      10.534  25.547  -4.377  1.00  0.00
J    C
ATOM  10789  HE1 TYR J 290      10.571  25.225  -3.347  1.00  0.00
J    H
ATOM  10790  CZ  TYR J 290       9.944  26.775  -4.680  1.00  0.00
J    C
ATOM  10791  OH  TYR J 290       9.379  27.506  -3.612  1.00  0.00
J    O
ATOM  10792  HH  TYR J 290       9.082  28.347  -3.965  1.00  0.00
J    H
ATOM  10793  CD2 TYR J 290      10.441  26.418  -7.028  1.00  0.00
J    C
ATOM  10794  HD2 TYR J 290      10.376  26.754  -8.054  1.00  0.00
J    H
ATOM  10795  CE2 TYR J 290       9.906  27.221  -6.009  1.00  0.00
J    C
ATOM  10796  HE2 TYR J 290       9.449  28.168  -6.257  1.00  0.00
J    H
ATOM  10797  C   TYR J 290       9.210  23.111  -7.907  1.00  0.00
J    C
ATOM  10798  O   TYR J 290       8.579  22.663  -6.955  1.00  0.00
J    O
ATOM  10799  N   PHE J 291       8.568  23.710  -8.927  1.00  0.00
J    N
```

ATOM 10800 HN PHE J 291   9.027 24.027 -9.755 1.00 0.00
J   H
ATOM 10801 CA PHE J 291   7.141 23.948 -8.905 1.00 0.00
J   C
ATOM 10802 HA PHE J 291   6.949 24.473 -7.977 1.00 0.00
J   H
ATOM 10803 CB PHE J 291   6.783 24.838 -10.118 1.00 0.00
J   C
ATOM 10804 HB1 PHE J 291   7.563 25.621 -10.237 1.00 0.00
J   H
ATOM 10805 HB2 PHE J 291   6.768 24.246 -11.058 1.00 0.00
J   H
ATOM 10806 CG PHE J 291   5.477 25.518 -9.877 1.00 0.00
J   C
ATOM 10807 CD1 PHE J 291   5.434 26.725 -9.161 1.00 0.00
J   C
ATOM 10808 HD1 PHE J 291   6.352 27.159 -8.789 1.00 0.00
J   H
ATOM 10809 CE1 PHE J 291   4.213 27.366 -8.919 1.00 0.00
J   C
ATOM 10810 HE1 PHE J 291   4.189 28.290 -8.361 1.00 0.00
J   H
ATOM 10811 CZ PHE J 291   3.029 26.806 -9.410 1.00 0.00
J   C
ATOM 10812 HZ PHE J 291   2.083 27.300 -9.236 1.00 0.00
J   H
ATOM 10813 CD2 PHE J 291   4.287 24.957 -10.351 1.00 0.00
J   C
ATOM 10814 HD2 PHE J 291   4.306 24.006 -10.866 1.00 0.00
J   H
ATOM 10815 CE2 PHE J 291   3.070 25.607 -10.137 1.00 0.00
J   C
ATOM 10816 HE2 PHE J 291   2.158 25.185 -10.532 1.00 0.00
J   H
ATOM 10817 C   PHE J 291   6.321 22.651 -8.867 1.00 0.00
J   C
ATOM 10818 O   PHE J 291   5.407 22.498 -8.059 1.00 0.00
J   O
ATOM 10819 N   GLN J 292   6.703 21.648 -9.690 1.00 0.00
J   N
ATOM 10820 HN GLN J 292   7.496 21.747 -10.288 1.00 0.00
J   H
ATOM 10821 CA GLN J 292   6.026 20.361 -9.794 1.00 0.00
J   C
ATOM 10822 HA GLN J 292   4.967 20.584 -9.800 1.00 0.00
J   H
ATOM 10823 CB GLN J 292   6.331 19.606 -11.118 1.00 0.00
J   C
ATOM 10824 HB1 GLN J 292   7.280 20.030 -11.520 1.00 0.00
J   H
ATOM 10825 HB2 GLN J 292   6.540 18.535 -10.893 1.00 0.00
J   H
ATOM 10826 CG GLN J 292   5.206 19.747 -12.176 1.00 0.00
J   C

ATOM 10827 HG1 GLN J 292 5.481 19.208 -13.108 1.00 0.00
J H
ATOM 10828 HG2 GLN J 292 4.257 19.313 -11.800 1.00 0.00
J H
ATOM 10829 CD GLN J 292 4.905 21.173 -12.621 1.00 0.00
J C
ATOM 10830 OE1 GLN J 292 3.754 21.551 -12.836 1.00 0.00
J O
ATOM 10831 NE2 GLN J 292 5.956 21.995 -12.803 1.00 0.00
J N
ATOM 10832 HE21 GLN J 292 5.754 22.927 -13.090 1.00 0.00
J H
ATOM 10833 HE22 GLN J 292 6.880 21.654 -12.667 1.00 0.00
J H
ATOM 10834 C GLN J 292 6.291 19.438 -8.620 1.00 0.00
J C
ATOM 10835 O GLN J 292 5.580 18.459 -8.433 1.00 0.00
J O
ATOM 10836 N ALA J 293 7.307 19.719 -7.792 1.00 0.00
J N
ATOM 10837 HN ALA J 293 7.952 20.455 -7.992 1.00 0.00
J H
ATOM 10838 CA ALA J 293 7.550 19.000 -6.567 1.00 0.00
J C
ATOM 10839 HA ALA J 293 7.289 17.958 -6.710 1.00 0.00
J H
ATOM 10840 CB ALA J 293 9.025 19.201 -6.236 1.00 0.00
J C
ATOM 10841 HB1 ALA J 293 9.650 18.805 -7.064 1.00 0.00
J H
ATOM 10842 HB2 ALA J 293 9.263 20.280 -6.113 1.00 0.00
J H
ATOM 10843 HB3 ALA J 293 9.311 18.666 -5.304 1.00 0.00
J H
ATOM 10844 C ALA J 293 6.732 19.487 -5.388 1.00 0.00
J C
ATOM 10845 O ALA J 293 6.395 18.726 -4.483 1.00 0.00
J O
ATOM 10846 N VAL J 294 6.389 20.786 -5.366 1.00 0.00
J N
ATOM 10847 HN VAL J 294 6.538 21.413 -6.126 1.00 0.00
J H
ATOM 10848 CA VAL J 294 5.762 21.387 -4.209 1.00 0.00
J C
ATOM 10849 HA VAL J 294 5.880 20.701 -3.379 1.00 0.00
J H
ATOM 10850 CB VAL J 294 6.451 22.707 -3.900 1.00 0.00
J C
ATOM 10851 HB VAL J 294 6.174 23.429 -4.707 1.00 0.00
J H
ATOM 10852 CG1 VAL J 294 5.964 23.233 -2.545 1.00 0.00
J C
ATOM 10853 HG11 VAL J 294 6.489 24.179 -2.288 1.00 0.00
J H

ATOM 10854 HG12 VAL J 294   4.877 23.451 -2.557 1.00 0.00
J   H
ATOM 10855 HG13 VAL J 294   6.171 22.496 -1.740 1.00 0.00
J   H
ATOM 10856 CG2 VAL J 294   7.981 22.510 -3.772 1.00 0.00
J   C
ATOM 10857 HG21 VAL J 294   8.479 23.468 -3.507 1.00 0.00
J   H
ATOM 10858 HG22 VAL J 294   8.214 21.765 -2.982 1.00 0.00
J   H
ATOM 10859 HG23 VAL J 294   8.422 22.156 -4.727 1.00 0.00
J   H
ATOM 10860 C   VAL J 294   4.264 21.574 -4.441 1.00 0.00
J   C
ATOM 10861 O   VAL J 294   3.455 21.419 -3.528 1.00 0.00
J   O
ATOM 10862 N   THR J 295   3.830 21.805 -5.702 1.00 0.00
J   N
ATOM 10863 HN THR J 295   4.470 21.963 -6.454 1.00 0.00
J   H
ATOM 10864 CA THR J 295   2.413 21.848 -6.096 1.00 0.00
J   C
ATOM 10865 HA THR J 295   2.003 22.633 -5.474 1.00 0.00
J   H
ATOM 10866 CB THR J 295   2.193 22.360 -7.531 1.00 0.00
J   C
ATOM 10867 HB THR J 295   2.824 23.276 -7.634 1.00 0.00
J   H
ATOM 10868 OG1 THR J 295   0.862 22.762 -7.781 1.00 0.00
J   O
ATOM 10869 HG1 THR J 295   0.848 23.046 -8.701 1.00 0.00
J   H
ATOM 10870 CG2 THR J 295   2.486 21.317 -8.607 1.00 0.00
J   C
ATOM 10871 HG21 THR J 295   2.422 21.766 -9.620 1.00 0.00
J   H
ATOM 10872 HG22 THR J 295   3.510 20.907 -8.479 1.00 0.00
J   H
ATOM 10873 HG23 THR J 295   1.763 20.474 -8.554 1.00 0.00
J   H
ATOM 10874 C   THR J 295   1.576 20.581 -5.813 1.00 0.00
J   C
ATOM 10875 O   THR J 295   0.391 20.750 -5.505 1.00 0.00
J   O
ATOM 10876 N   PRO J 296   2.028 19.306 -5.847 1.00 0.00
J   N
ATOM 10877 CD PRO J 296   3.349 18.859 -6.280 1.00 0.00
J   C
ATOM 10878 HD1 PRO J 296   2.645 19.547 -6.801 1.00 0.00
J   H
ATOM 10879 HD2 PRO J 296   4.404 19.162 -6.473 1.00 0.00
J   H
ATOM 10880 CA PRO J 296   1.216 18.150 -5.475 1.00 0.00
J   C

```
ATOM  10881  HA  PRO J 296       0.364  18.088  -6.143  1.00  0.00
J    H
ATOM  10882  CB  PRO J 296       2.146  16.941  -5.687  1.00  0.00
J    C
ATOM  10883  HB1 PRO J 296       2.097  16.629  -6.754  1.00  0.00
J    H
ATOM  10884  HB2 PRO J 296       1.877  16.077  -5.048  1.00  0.00
J    H
ATOM  10885  CG  PRO J 296       3.122  17.430  -6.749  1.00  0.00
J    C
ATOM  10886  HG1 PRO J 296       3.593  16.603  -7.317  1.00  0.00
J    H
ATOM  10887  HG2 PRO J 296       3.254  17.678  -7.709  1.00  0.00
J    H
ATOM  10888  C   PRO J 296       0.726  18.164  -4.052  1.00  0.00
J    C
ATOM  10889  O   PRO J 296      -0.291  17.541  -3.770  1.00  0.00
J    O
ATOM  10890  N   ARG J 297       1.431  18.840  -3.127  1.00  0.00
J    N
ATOM  10891  HN  ARG J 297       2.227  19.368  -3.414  1.00  0.00
J    H
ATOM  10892  CA  ARG J 297       1.113  18.861  -1.712  1.00  0.00
J    C
ATOM  10893  HA  ARG J 297       0.788  17.868  -1.424  1.00  0.00
J    H
ATOM  10894  CB  ARG J 297       2.385  19.221  -0.915  1.00  0.00
J    C
ATOM  10895  HB1 ARG J 297       2.772  20.173  -1.353  1.00  0.00
J    H
ATOM  10896  HB2 ARG J 297       2.111  19.458   0.137  1.00  0.00
J    H
ATOM  10897  CG  ARG J 297       3.469  18.122  -1.013  1.00  0.00
J    C
ATOM  10898  HG1 ARG J 297       3.376  17.619  -2.000  1.00  0.00
J    H
ATOM  10899  HG2 ARG J 297       4.474  18.604  -1.016  1.00  0.00
J    H
ATOM  10900  CD  ARG J 297       3.477  17.084   0.130  1.00  0.00
J    C
ATOM  10901  HD1 ARG J 297       2.517  16.519   0.122  1.00  0.00
J    H
ATOM  10902  HD2 ARG J 297       4.319  16.362   0.019  1.00  0.00
J    H
ATOM  10903  NE  ARG J 297       3.651  17.789   1.455  1.00  0.00
J    N
ATOM  10904  HE  ARG J 297       2.881  17.642   2.086  1.00  0.00
J    H
ATOM  10905  CZ  ARG J 297       4.710  18.545   1.800  1.00  0.00
J    C
ATOM  10906  NH1 ARG J 297       5.822  18.516   1.080  1.00  0.00
J    N
ATOM  10907 HH11 ARG J 297       6.610  19.052   1.348  1.00  0.00
J    H
```

```
ATOM  10908 HH12 ARG J 297       5.860  17.830   0.350  1.00  0.00
J    H
ATOM  10909  NH2 ARG J 297       4.641  19.331   2.869  1.00  0.00
J    N
ATOM  10910 HH21 ARG J 297       5.385  19.951   3.074  1.00  0.00
J    H
ATOM  10911 HH22 ARG J 297       3.756  19.418   3.306  1.00  0.00
J    H
ATOM  10912  C   ARG J 297      -0.048  19.803  -1.409  1.00  0.00
J    C
ATOM  10913  O   ARG J 297       0.044  20.729  -0.611  1.00  0.00
J    O
ATOM  10914  N   THR J 298      -1.180  19.509  -2.073  1.00  0.00
J    N
ATOM  10915  HN  THR J 298      -1.109  18.756  -2.727  1.00  0.00
J    H
ATOM  10916  CA  THR J 298      -2.502  20.110  -1.997  1.00  0.00
J    C
ATOM  10917  HA  THR J 298      -3.117  19.424  -2.564  1.00  0.00
J    H
ATOM  10918  CB  THR J 298      -3.401  19.749  -0.790  1.00  0.00
J    C
ATOM  10919  HB  THR J 298      -4.311  19.260  -1.216  1.00  0.00
J    H
ATOM  10920  OG1 THR J 298      -3.876  20.856  -0.045  1.00  0.00
J    O
ATOM  10921  HG1 THR J 298      -4.403  20.479   0.668  1.00  0.00
J    H
ATOM  10922  CG2 THR J 298      -2.681  18.872   0.236  1.00  0.00
J    C
ATOM  10923 HG21 THR J 298      -3.380  18.537   1.030  1.00  0.00
J    H
ATOM  10924 HG22 THR J 298      -2.261  17.967  -0.254  1.00  0.00
J    H
ATOM  10925 HG23 THR J 298      -1.846  19.425   0.719  1.00  0.00
J    H
ATOM  10926  C   THR J 298      -2.684  21.472  -2.626  1.00  0.00
J    C
ATOM  10927  O   THR J 298      -3.646  22.169  -2.311  1.00  0.00
J    O
ATOM  10928  N   ALA J 299      -1.827  21.869  -3.587  1.00  0.00
J    N
ATOM  10929  HN  ALA J 299      -1.052  21.312  -3.883  1.00  0.00
J    H
ATOM  10930  CA  ALA J 299      -1.971  23.146  -4.265  1.00  0.00
J    C
ATOM  10931  HA  ALA J 299      -2.584  23.799  -3.653  1.00  0.00
J    H
ATOM  10932  CB  ALA J 299      -0.594  23.799  -4.428  1.00  0.00
J    C
ATOM  10933  HB1 ALA J 299      -0.110  23.901  -3.434  1.00  0.00
J    H
ATOM  10934  HB2 ALA J 299       0.069  23.179  -5.069  1.00  0.00
J    H
```

ATOM 10935 HB3 ALA J 299 -0.672 24.813 -4.876 1.00 0.00
J H
ATOM 10936 C ALA J 299 -2.646 23.078 -5.628 1.00 0.00
J C
ATOM 10937 O ALA J 299 -3.626 23.772 -5.857 1.00 0.00
J O
ATOM 10938 N GLY J 300 -2.141 22.254 -6.575 1.00 0.00
J N
ATOM 10939 HN GLY J 300 -1.275 21.771 -6.450 1.00 0.00
J H
ATOM 10940 CA GLY J 300 -2.827 22.008 -7.850 1.00 0.00
J C
ATOM 10941 HA1 GLY J 300 -3.873 22.063 -7.582 1.00 0.00
J H
ATOM 10942 HA2 GLY J 300 -2.490 21.019 -8.131 1.00 0.00
J H
ATOM 10943 C GLY J 300 -2.599 22.921 -9.014 1.00 0.00
J C
ATOM 10944 O GLY J 300 -3.309 22.858 -10.011 1.00 0.00
J O
ATOM 10945 N PHE J 301 -1.591 23.791 -8.937 1.00 0.00
J N
ATOM 10946 HN PHE J 301 -1.085 23.948 -8.091 1.00 0.00
J H
ATOM 10947 CA PHE J 301 -1.138 24.585 -10.066 1.00 0.00
J C
ATOM 10948 HA PHE J 301 -2.031 24.852 -10.619 1.00 0.00
J H
ATOM 10949 CB PHE J 301 -0.313 25.798 -9.589 1.00 0.00
J C
ATOM 10950 HB1 PHE J 301 0.474 25.448 -8.886 1.00 0.00
J H
ATOM 10951 HB2 PHE J 301 0.205 26.290 -10.440 1.00 0.00
J H
ATOM 10952 CG PHE J 301 -1.164 26.773 -8.854 1.00 0.00
J C
ATOM 10953 CD1 PHE J 301 -2.007 27.634 -9.569 1.00 0.00
J C
ATOM 10954 HD1 PHE J 301 -2.064 27.555 -10.646 1.00 0.00
J H
ATOM 10955 CE1 PHE J 301 -2.768 28.597 -8.901 1.00 0.00
J C
ATOM 10956 HE1 PHE J 301 -3.413 29.260 -9.461 1.00 0.00
J H
ATOM 10957 CZ PHE J 301 -2.696 28.695 -7.507 1.00 0.00
J C
ATOM 10958 HZ PHE J 301 -3.277 29.444 -6.987 1.00 0.00
J H
ATOM 10959 CD2 PHE J 301 -1.111 26.861 -7.458 1.00 0.00
J C
ATOM 10960 HD2 PHE J 301 -0.460 26.203 -6.900 1.00 0.00
J H
ATOM 10961 CE2 PHE J 301 -1.877 27.816 -6.783 1.00 0.00
J C

```
ATOM  10962  HE2 PHE J 301      -1.835  27.876  -5.705  1.00  0.00
J    H
ATOM  10963  C   PHE J 301      -0.251  23.780 -11.012  1.00  0.00
J    C
ATOM  10964  O   PHE J 301       0.441  22.853 -10.589  1.00  0.00
J    O
ATOM  10965  N   ASN J 302      -0.209  24.153 -12.309  1.00  0.00
J    N
ATOM  10966  HN  ASN J 302      -0.726  24.946 -12.629  1.00  0.00
J    H
ATOM  10967  CA  ASN J 302       0.557  23.476 -13.343  1.00  0.00
J    C
ATOM  10968  HA  ASN J 302       1.046  22.639 -12.858  1.00  0.00
J    H
ATOM  10969  CB  ASN J 302      -0.371  23.136 -14.549  1.00  0.00
J    C
ATOM  10970  HB1 ASN J 302      -1.125  23.948 -14.639  1.00  0.00
J    H
ATOM  10971  HB2 ASN J 302       0.213  23.119 -15.491  1.00  0.00
J    H
ATOM  10972  CG  ASN J 302      -1.127  21.822 -14.389  1.00  0.00
J    C
ATOM  10973  OD1 ASN J 302      -2.088  21.687 -13.648  1.00  0.00
J    O
ATOM  10974  ND2 ASN J 302      -0.711  20.807 -15.185  1.00  0.00
J    N
ATOM  10975 HD21 ASN J 302      -1.195  19.939 -15.111  1.00  0.00
J    H
ATOM  10976 HD22 ASN J 302       0.030  20.953 -15.833  1.00  0.00
J    H
ATOM  10977  C   ASN J 302       1.648  24.399 -13.888  1.00  0.00
J    C
ATOM  10978  O   ASN J 302       1.396  25.533 -14.261  1.00  0.00
J    O
ATOM  10979  N   SER J 303       2.902  23.917 -14.006  1.00  0.00
J    N
ATOM  10980  HN  SER J 303       3.248  23.115 -13.518  1.00  0.00
J    H
ATOM  10981  CA  SER J 303       3.886  24.555 -14.888  1.00  0.00
J    C
ATOM  10982  HA  SER J 303       3.359  25.332 -15.426  1.00  0.00
J    H
ATOM  10983  CB  SER J 303       5.140  25.146 -14.191  1.00  0.00
J    C
ATOM  10984  HB1 SER J 303       5.663  24.319 -13.660  1.00  0.00
J    H
ATOM  10985  HB2 SER J 303       5.850  25.549 -14.948  1.00  0.00
J    H
ATOM  10986  OG  SER J 303       4.762  26.141 -13.247  1.00  0.00
J    O
ATOM  10987  HG1 SER J 303       5.533  26.313 -12.692  1.00  0.00
J    H
ATOM  10988  C   SER J 303       4.386  23.561 -15.911  1.00  0.00
J    C
```

ATOM 10989 O   SER J 303   5.215 23.887 -16.757 1.00 0.00
J   O
ATOM 10990 N   LEU J 304   3.880 22.316 -15.879 1.00 0.00
J   N
ATOM 10991 HN LEU J 304   3.277 22.036 -15.134 1.00 0.00
J   H
ATOM 10992 CA LEU J 304   4.129 21.283 -16.860 1.00 0.00
J   C
ATOM 10993 HA LEU J 304   4.394 21.746 -17.802 1.00 0.00
J   H
ATOM 10994 CB LEU J 304   5.148 20.216 -16.379 1.00 0.00
J   C
ATOM 10995 HB1 LEU J 304   4.751 19.786 -15.430 1.00 0.00
J   H
ATOM 10996 HB2 LEU J 304   5.167 19.378 -17.111 1.00 0.00
J   H
ATOM 10997 CG LEU J 304   6.580 20.727 -16.120 1.00 0.00
J   C
ATOM 10998 HG LEU J 304   6.467 21.540 -15.363 1.00 0.00
J   H
ATOM 10999 CD1 LEU J 304   7.445 19.595 -15.544 1.00 0.00
J   C
ATOM 11000 HD11 LEU J 304   8.492 19.939 -15.399 1.00 0.00
J   H
ATOM 11001 HD12 LEU J 304   7.048 19.259 -14.562 1.00 0.00
J   H
ATOM 11002 HD13 LEU J 304   7.460 18.722 -16.233 1.00 0.00
J   H
ATOM 11003 CD2 LEU J 304   7.249 21.328 -17.368 1.00 0.00
J   C
ATOM 11004 HD21 LEU J 304   8.269 21.696 -17.120 1.00 0.00
J   H
ATOM 11005 HD22 LEU J 304   7.351 20.580 -18.179 1.00 0.00
J   H
ATOM 11006 HD23 LEU J 304   6.655 22.185 -17.754 1.00 0.00
J   H
ATOM 11007 C   LEU J 304   2.805 20.573 -17.056 1.00 0.00
J   C
ATOM 11008 O   LEU J 304   1.912 20.703 -16.214 1.00 0.00
J   O
ATOM 11009 N   ASP J 305   2.625 19.791 -18.137 1.00 0.00
J   N
ATOM 11010 HN ASP J 305   3.288 19.667 -18.871 1.00 0.00
J   H
ATOM 11011 CA ASP J 305   1.411 19.032 -18.339 1.00 0.00
J   C
ATOM 11012 HA ASP J 305   0.610 19.578 -17.853 1.00 0.00
J   H
ATOM 11013 CB ASP J 305   1.107 18.874 -19.848 1.00 0.00
J   C
ATOM 11014 HB1 ASP J 305   1.184 19.864 -20.341 1.00 0.00
J   H
ATOM 11015 HB2 ASP J 305   1.837 18.189 -20.325 1.00 0.00
J   H

```
ATOM  11016  CG  ASP J 305      -0.307  18.343 -19.929  1.00  0.00
J    C
ATOM  11017  OD1 ASP J 305      -1.230  19.106 -19.565  1.00  0.00
J    O
ATOM  11018  OD2 ASP J 305      -0.449  17.122 -20.187  1.00  0.00
J    O
ATOM  11019  C   ASP J 305       1.494  17.657 -17.649  1.00  0.00
J    C
ATOM  11020  O   ASP J 305       2.411  16.873 -17.880  1.00  0.00
J    O
ATOM  11021  N   PHE J 306       0.517  17.319 -16.786  1.00  0.00
J    N
ATOM  11022  HN  PHE J 306      -0.256  17.921 -16.593  1.00  0.00
J    H
ATOM  11023  CA  PHE J 306       0.494  16.057 -16.058  1.00  0.00
J    C
ATOM  11024  HA  PHE J 306       1.529  15.837 -15.828  1.00  0.00
J    H
ATOM  11025  CB  PHE J 306      -0.339  16.169 -14.750  1.00  0.00
J    C
ATOM  11026  HB1 PHE J 306      -1.154  16.910 -14.899  1.00  0.00
J    H
ATOM  11027  HB2 PHE J 306      -0.819  15.200 -14.496  1.00  0.00
J    H
ATOM  11028  CG  PHE J 306       0.531  16.661 -13.620  1.00  0.00
J    C
ATOM  11029  CD1 PHE J 306       0.900  18.012 -13.505  1.00  0.00
J    C
ATOM  11030  HD1 PHE J 306       0.576  18.721 -14.254  1.00  0.00
J    H
ATOM  11031  CE1 PHE J 306       1.690  18.453 -12.434  1.00  0.00
J    C
ATOM  11032  HE1 PHE J 306       1.946  19.499 -12.346  1.00  0.00
J    H
ATOM  11033  CZ  PHE J 306       2.140  17.537 -11.476  1.00  0.00
J    C
ATOM  11034  HZ  PHE J 306       2.733  17.875 -10.638  1.00  0.00
J    H
ATOM  11035  CD2 PHE J 306       1.008  15.751 -12.661  1.00  0.00
J    C
ATOM  11036  HD2 PHE J 306       0.772  14.700 -12.750  1.00  0.00
J    H
ATOM  11037  CE2 PHE J 306       1.813  16.182 -11.599  1.00  0.00
J    C
ATOM  11038  HE2 PHE J 306       2.181  15.467 -10.877  1.00  0.00
J    H
ATOM  11039  C   PHE J 306      -0.034  14.899 -16.894  1.00  0.00
J    C
ATOM  11040  O   PHE J 306       0.150  13.740 -16.530  1.00  0.00
J    O
ATOM  11041  N   GLY J 307      -0.665  15.151 -18.062  1.00  0.00
J    N
ATOM  11042  HN  GLY J 307      -0.876  16.079 -18.366  1.00  0.00
J    H
```

```
ATOM  11043  CA  GLY J 307      -1.081  14.081 -18.963  1.00  0.00
J    C
ATOM  11044  HA1 GLY J 307      -1.845  14.495 -19.606  1.00  0.00
J    H
ATOM  11045  HA2 GLY J 307      -1.419  13.261 -18.343  1.00  0.00
J    H
ATOM  11046  C   GLY J 307       0.041  13.571 -19.838  1.00  0.00
J    C
ATOM  11047  O   GLY J 307      -0.119  12.599 -20.567  1.00  0.00
J    O
ATOM  11048  N   SER J 308       1.210  14.237 -19.771  1.00  0.00
J    N
ATOM  11049  HN  SER J 308       1.346  14.955 -19.089  1.00  0.00
J    H
ATOM  11050  CA  SER J 308       2.362  14.008 -20.636  1.00  0.00
J    C
ATOM  11051  HA  SER J 308       2.088  13.181 -21.279  1.00  0.00
J    H
ATOM  11052  CB  SER J 308       2.598  15.341 -21.415  1.00  0.00
J    C
ATOM  11053  HB1 SER J 308       1.636  15.638 -21.892  1.00  0.00
J    H
ATOM  11054  HB2 SER J 308       2.879  16.156 -20.711  1.00  0.00
J    H
ATOM  11055  OG  SER J 308       3.570  15.283 -22.459  1.00  0.00
J    O
ATOM  11056  HG1 SER J 308       3.460  16.079 -22.993  1.00  0.00
J    H
ATOM  11057  C   SER J 308       3.594  13.575 -19.837  1.00  0.00
J    C
ATOM  11058  O   SER J 308       4.672  13.350 -20.380  1.00  0.00
J    O
ATOM  11059  N   MET J 309       3.465  13.376 -18.507  1.00  0.00
J    N
ATOM  11060  HN  MET J 309       2.607  13.531 -18.022  1.00  0.00
J    H
ATOM  11061  CA  MET J 309       4.559  12.919 -17.663  1.00  0.00
J    C
ATOM  11062  HA  MET J 309       5.453  13.406 -18.033  1.00  0.00
J    H
ATOM  11063  CB  MET J 309       4.320  13.295 -16.184  1.00  0.00
J    C
ATOM  11064  HB1 MET J 309       3.279  12.982 -15.934  1.00  0.00
J    H
ATOM  11065  HB2 MET J 309       4.991  12.689 -15.535  1.00  0.00
J    H
ATOM  11066  CG  MET J 309       4.484  14.798 -15.921  1.00  0.00
J    C
ATOM  11067  HG1 MET J 309       5.504  15.131 -16.210  1.00  0.00
J    H
ATOM  11068  HG2 MET J 309       3.757  15.367 -16.540  1.00  0.00
J    H
ATOM  11069  SD  MET J 309       4.228  15.218 -14.175  1.00  0.00
J    S
```

```
ATOM  11070  CE  MET J 309       5.509  16.499 -14.146  1.00  0.00
J    C
ATOM  11071  HE1 MET J 309       5.590  16.955 -13.137  1.00  0.00
J    H
ATOM  11072  HE2 MET J 309       6.502  16.077 -14.412  1.00  0.00
J    H
ATOM  11073  HE3 MET J 309       5.278  17.311 -14.869  1.00  0.00
J    H
ATOM  11074  C   MET J 309       4.786  11.414 -17.748  1.00  0.00
J    C
ATOM  11075  O   MET J 309       3.910  10.648 -18.148  1.00  0.00
J    O
ATOM  11076  N   ARG J 310       5.983  10.931 -17.347  1.00  0.00
J    N
ATOM  11077  HN  ARG J 310       6.745  11.552 -17.178  1.00  0.00
J    H
ATOM  11078  CA  ARG J 310       6.237   9.515 -17.141  1.00  0.00
J    C
ATOM  11079  HA  ARG J 310       5.953   8.995 -18.048  1.00  0.00
J    H
ATOM  11080  CB  ARG J 310       7.717   9.201 -16.789  1.00  0.00
J    C
ATOM  11081  HB1 ARG J 310       8.030   9.963 -16.034  1.00  0.00
J    H
ATOM  11082  HB2 ARG J 310       7.778   8.217 -16.274  1.00  0.00
J    H
ATOM  11083  CG  ARG J 310       8.709   9.260 -17.968  1.00  0.00
J    C
ATOM  11084  HG1 ARG J 310       8.287   8.675 -18.815  1.00  0.00
J    H
ATOM  11085  HG2 ARG J 310       8.776  10.311 -18.334  1.00  0.00
J    H
ATOM  11086  CD  ARG J 310      10.109   8.786 -17.543  1.00  0.00
J    C
ATOM  11087  HD1 ARG J 310      10.684   9.650 -17.136  1.00  0.00
J    H
ATOM  11088  HD2 ARG J 310      10.052   7.993 -16.761  1.00  0.00
J    H
ATOM  11089  NE  ARG J 310      10.811   8.206 -18.746  1.00  0.00
J    N
ATOM  11090  HE  ARG J 310      10.537   8.640 -19.611  1.00  0.00
J    H
ATOM  11091  CZ  ARG J 310      11.712   7.213 -18.686  1.00  0.00
J    C
ATOM  11092  NH1 ARG J 310      12.145   6.738 -17.523  1.00  0.00
J    N
ATOM  11093 HH11 ARG J 310      12.829   6.022 -17.494  1.00  0.00
J    H
ATOM  11094 HH12 ARG J 310      11.848   7.232 -16.703  1.00  0.00
J    H
ATOM  11095  NH2 ARG J 310      12.194   6.670 -19.802  1.00  0.00
J    N
ATOM  11096 HH21 ARG J 310      12.791   5.883 -19.750  1.00  0.00
J    H
```

```
ATOM  11097 HH22 ARG J 310      11.770   6.946 -20.655  1.00  0.00
J    H
ATOM  11098  C   ARG J 310       5.393   8.923 -16.017  1.00  0.00
J    C
ATOM  11099  O   ARG J 310       5.138   9.555 -14.991  1.00  0.00
J    O
ATOM  11100  N   GLU J 311       4.992   7.640 -16.148  1.00  0.00
J    N
ATOM  11101  HN  GLU J 311       5.228   7.082 -16.938  1.00  0.00
J    H
ATOM  11102  CA  GLU J 311       4.185   6.952 -15.160  1.00  0.00
J    C
ATOM  11103  HA  GLU J 311       3.264   7.513 -15.066  1.00  0.00
J    H
ATOM  11104  CB  GLU J 311       3.779   5.540 -15.640  1.00  0.00
J    C
ATOM  11105  HB1 GLU J 311       4.695   5.023 -16.010  1.00  0.00
J    H
ATOM  11106  HB2 GLU J 311       3.417   4.938 -14.777  1.00  0.00
J    H
ATOM  11107  CG  GLU J 311       2.711   5.529 -16.768  1.00  0.00
J    C
ATOM  11108  HG1 GLU J 311       1.986   6.345 -16.597  1.00  0.00
J    H
ATOM  11109  HG2 GLU J 311       3.182   5.660 -17.762  1.00  0.00
J    H
ATOM  11110  CD  GLU J 311       1.882   4.237 -16.791  1.00  0.00
J    C
ATOM  11111  OE1 GLU J 311       0.841   4.222 -17.502  1.00  0.00
J    O
ATOM  11112  OE2 GLU J 311       2.237   3.282 -16.054  1.00  0.00
J    O
ATOM  11113  C   GLU J 311       4.815   6.879 -13.771  1.00  0.00
J    C
ATOM  11114  O   GLU J 311       4.128   7.046 -12.768  1.00  0.00
J    O
ATOM  11115  N   GLY J 312       6.152   6.687 -13.653  1.00  0.00
J    N
ATOM  11116  HN  GLY J 312       6.715   6.442 -14.442  1.00  0.00
J    H
ATOM  11117  CA  GLY J 312       6.870   6.822 -12.378  1.00  0.00
J    C
ATOM  11118  HA1 GLY J 312       7.921   6.756 -12.623  1.00  0.00
J    H
ATOM  11119  HA2 GLY J 312       6.510   6.021 -11.746  1.00  0.00
J    H
ATOM  11120  C   GLY J 312       6.637   8.129 -11.652  1.00  0.00
J    C
ATOM  11121  O   GLY J 312       6.389   8.153 -10.450  1.00  0.00
J    O
ATOM  11122  N   THR J 313       6.669   9.265 -12.376  1.00  0.00
J    N
ATOM  11123  HN  THR J 313       6.858   9.246 -13.357  1.00  0.00
J    H
```

ATOM 11124 CA THR J 313   6.440 10.606 -11.834 1.00 0.00
J   C
ATOM 11125 HA THR J 313   7.094 10.698 -10.977 1.00 0.00
J   H
ATOM 11126 CB THR J 313   6.672 11.694 -12.872 1.00 0.00
J   C
ATOM 11127 HB THR J 313   5.929 11.510 -13.686 1.00 0.00
J   H
ATOM 11128 OG1 THR J 313   7.971 11.538 -13.409 1.00 0.00
J   O
ATOM 11129 HG1 THR J 313   8.073 12.252 -14.047 1.00 0.00
J   H
ATOM 11130 CG2 THR J 313   6.629 13.099 -12.253 1.00 0.00
J   C
ATOM 11131 HG21 THR J 313   6.686 13.880 -13.039 1.00 0.00
J   H
ATOM 11132 HG22 THR J 313   5.681 13.247 -11.692 1.00 0.00
J   H
ATOM 11133 HG23 THR J 313   7.475 13.252 -11.549 1.00 0.00
J   H
ATOM 11134 C   THR J 313   5.037 10.741 -11.317 1.00 0.00
J   C
ATOM 11135 O   THR J 313   4.801 11.220 -10.211 1.00 0.00
J   O
ATOM 11136 N   ILE J 314   4.061 10.226 -12.083 1.00 0.00
J   N
ATOM 11137 HN ILE J 314   4.247   9.862 -12.994 1.00 0.00
J   H
ATOM 11138 CA ILE J 314   2.668 10.143 -11.683 1.00 0.00
J   C
ATOM 11139 HA ILE J 314   2.371 11.151 -11.423 1.00 0.00
J   H
ATOM 11140 CB ILE J 314   1.807   9.641 -12.837 1.00 0.00
J   C
ATOM 11141 HB ILE J 314   2.272   8.666 -13.129 1.00 0.00
J   H
ATOM 11142 CG2 ILE J 314   0.336   9.526 -12.394 1.00 0.00
J   C
ATOM 11143 HG21 ILE J 314   -0.268   9.016 -13.173 1.00 0.00
J   H
ATOM 11144 HG22 ILE J 314   0.249   8.933 -11.459 1.00 0.00
J   H
ATOM 11145 HG23 ILE J 314   -0.104 10.530 -12.214 1.00 0.00
J   H
ATOM 11146 CG1 ILE J 314   1.920 10.573 -14.076 1.00 0.00
J   C
ATOM 11147 HG11 ILE J 314   2.986 10.657 -14.387 1.00 0.00
J   H
ATOM 11148 HG12 ILE J 314   1.364 10.114 -14.924 1.00 0.00
J   H
ATOM 11149 CD ILE J 314   1.363 11.992 -13.872 1.00 0.00
J   C
ATOM 11150 HD1 ILE J 314   1.429 12.571 -14.818 1.00 0.00
J   H

```
ATOM  11151  HD2 ILE J 314       0.296  11.953 -13.562  1.00  0.00
J    H
ATOM  11152  HD3 ILE J 314       1.936  12.537 -13.094  1.00  0.00
J    H
ATOM  11153  C   ILE J 314       2.487   9.291 -10.427  1.00  0.00
J    C
ATOM  11154  O   ILE J 314       1.833   9.725  -9.481  1.00  0.00
J    O
ATOM  11155  N   VAL J 315       3.134   8.106 -10.335  1.00  0.00
J    N
ATOM  11156  HN  VAL J 315       3.659   7.746 -11.102  1.00  0.00
J    H
ATOM  11157  CA  VAL J 315       3.137   7.251  -9.146  1.00  0.00
J    C
ATOM  11158  HA  VAL J 315       2.102   7.019  -8.931  1.00  0.00
J    H
ATOM  11159  CB  VAL J 315       3.873   5.927  -9.393  1.00  0.00
J    C
ATOM  11160  HB  VAL J 315       4.871   6.173  -9.831  1.00  0.00
J    H
ATOM  11161  CG1 VAL J 315       4.099   5.122  -8.091  1.00  0.00
J    C
ATOM  11162 HG11 VAL J 315       4.598   4.156  -8.317  1.00  0.00
J    H
ATOM  11163 HG12 VAL J 315       4.748   5.671  -7.378  1.00  0.00
J    H
ATOM  11164 HG13 VAL J 315       3.131   4.901  -7.594  1.00  0.00
J    H
ATOM  11165  CG2 VAL J 315       3.043   5.067 -10.363  1.00  0.00
J    C
ATOM  11166 HG21 VAL J 315       3.530   4.083 -10.531  1.00  0.00
J    H
ATOM  11167 HG22 VAL J 315       2.025   4.888  -9.953  1.00  0.00
J    H
ATOM  11168 HG23 VAL J 315       2.938   5.563 -11.350  1.00  0.00
J    H
ATOM  11169  C   VAL J 315       3.716   7.943  -7.918  1.00  0.00
J    C
ATOM  11170  O   VAL J 315       3.151   7.884  -6.827  1.00  0.00
J    O
ATOM  11171  N   PHE J 316       4.840   8.672  -8.065  1.00  0.00
J    N
ATOM  11172  HN  PHE J 316       5.352   8.711  -8.922  1.00  0.00
J    H
ATOM  11173  CA  PHE J 316       5.407   9.465  -6.991  1.00  0.00
J    C
ATOM  11174  HA  PHE J 316       5.518   8.786  -6.153  1.00  0.00
J    H
ATOM  11175  CB  PHE J 316       6.781  10.024  -7.430  1.00  0.00
J    C
ATOM  11176  HB1 PHE J 316       7.358   9.218  -7.933  1.00  0.00
J    H
ATOM  11177  HB2 PHE J 316       6.661  10.849  -8.164  1.00  0.00
J    H
```

ATOM 11178 CG PHE J 316   7.560 10.469 -6.225 1.00 0.00
J   C
ATOM 11179 CD1 PHE J 316   8.120   9.517 -5.356 1.00 0.00
J   C
ATOM 11180 HD1 PHE J 316   8.015   8.464 -5.578 1.00 0.00
J   H
ATOM 11181 CE1 PHE J 316   8.806   9.922 -4.204 1.00 0.00
J   C
ATOM 11182 HE1 PHE J 316   9.250   9.184 -3.551 1.00 0.00
J   H
ATOM 11183 CZ PHE J 316   8.922 11.286 -3.904 1.00 0.00
J   C
ATOM 11184 HZ PHE J 316   9.462 11.604 -3.024 1.00 0.00
J   H
ATOM 11185 CD2 PHE J 316   7.681 11.832 -5.917 1.00 0.00
J   C
ATOM 11186 HD2 PHE J 316   7.243 12.575 -6.568 1.00 0.00
J   H
ATOM 11187 CE2 PHE J 316   8.345 12.238 -4.753 1.00 0.00
J   C
ATOM 11188 HE2 PHE J 316   8.412 13.289 -4.510 1.00 0.00
J   H
ATOM 11189 C   PHE J 316   4.471 10.587 -6.536 1.00 0.00
J   C
ATOM 11190 O   PHE J 316   4.234 10.775 -5.344 1.00 0.00
J   O
ATOM 11191 N   THR J 317   3.848 11.301 -7.499 1.00 0.00
J   N
ATOM 11192 HN THR J 317   4.061 11.148 -8.464 1.00 0.00
J   H
ATOM 11193 CA THR J 317   2.835 12.335 -7.265 1.00 0.00
J   C
ATOM 11194 HA THR J 317   3.314 13.066 -6.628 1.00 0.00
J   H
ATOM 11195 CB THR J 317   2.343 13.027 -8.534 1.00 0.00
J   C
ATOM 11196 HB THR J 317   1.929 12.221 -9.187 1.00 0.00
J   H
ATOM 11197 OG1 THR J 317   3.443 13.617 -9.207 1.00 0.00
J   O
ATOM 11198 HG1 THR J 317   3.069 14.043 -9.985 1.00 0.00
J   H
ATOM 11199 CG2 THR J 317   1.409 14.209 -8.229 1.00 0.00
J   C
ATOM 11200 HG21 THR J 317   0.976 14.623 -9.164 1.00 0.00
J   H
ATOM 11201 HG22 THR J 317   0.570 13.882 -7.580 1.00 0.00
J   H
ATOM 11202 HG23 THR J 317   1.954 15.026 -7.708 1.00 0.00
J   H
ATOM 11203 C   THR J 317   1.638 11.806 -6.492 1.00 0.00
J   C
ATOM 11204 O   THR J 317   1.157 12.480 -5.590 1.00 0.00
J   O

```
ATOM  11205  N   LEU J 318       1.157  10.566  -6.758  1.00  0.00
J    N
ATOM  11206  HN  LEU J 318       1.525  10.005  -7.498  1.00  0.00
J    H
ATOM  11207  CA  LEU J 318       0.073   9.932  -6.004  1.00  0.00
J    C
ATOM  11208  HA  LEU J 318      -0.786  10.577  -6.136  1.00  0.00
J    H
ATOM  11209  CB  LEU J 318      -0.210   8.484  -6.502  1.00  0.00
J    C
ATOM  11210  HB1 LEU J 318       0.757   7.931  -6.474  1.00  0.00
J    H
ATOM  11211  HB2 LEU J 318      -0.877   7.974  -5.771  1.00  0.00
J    H
ATOM  11212  CG  LEU J 318      -0.795   8.354  -7.920  1.00  0.00
J    C
ATOM  11213  HG  LEU J 318      -0.128   8.940  -8.597  1.00  0.00
J    H
ATOM  11214  CD1 LEU J 318      -0.836   6.879  -8.357  1.00  0.00
J    C
ATOM  11215 HD11 LEU J 318      -1.302   6.784  -9.361  1.00  0.00
J    H
ATOM  11216 HD12 LEU J 318       0.190   6.457  -8.408  1.00  0.00
J    H
ATOM  11217 HD13 LEU J 318      -1.430   6.274  -7.640  1.00  0.00
J    H
ATOM  11218  CD2 LEU J 318      -2.200   8.946  -8.010  1.00  0.00
J    C
ATOM  11219 HD21 LEU J 318      -2.590   8.856  -9.047  1.00  0.00
J    H
ATOM  11220 HD22 LEU J 318      -2.909   8.424  -7.337  1.00  0.00
J    H
ATOM  11221 HD23 LEU J 318      -2.186  10.023  -7.736  1.00  0.00
J    H
ATOM  11222  C   LEU J 318       0.342   9.823  -4.505  1.00  0.00
J    C
ATOM  11223  O   LEU J 318      -0.509  10.162  -3.684  1.00  0.00
J    O
ATOM  11224  N   LEU J 319       1.560   9.398  -4.103  1.00  0.00
J    N
ATOM  11225  HN  LEU J 319       2.241   9.042  -4.741  1.00  0.00
J    H
ATOM  11226  CA  LEU J 319       1.995   9.419  -2.712  1.00  0.00
J    C
ATOM  11227  HA  LEU J 319       1.240   8.889  -2.146  1.00  0.00
J    H
ATOM  11228  CB  LEU J 319       3.393   8.760  -2.587  1.00  0.00
J    C
ATOM  11229  HB1 LEU J 319       3.283   7.695  -2.896  1.00  0.00
J    H
ATOM  11230  HB2 LEU J 319       4.080   9.226  -3.326  1.00  0.00
J    H
ATOM  11231  CG  LEU J 319       4.035   8.790  -1.179  1.00  0.00
J    C
```

```
ATOM  11232  HG  LEU J 319       4.077   9.865  -0.880  1.00  0.00
J    H
ATOM  11233  CD1 LEU J 319       3.218   7.995  -0.147  1.00  0.00
J    C
ATOM  11234 HD11 LEU J 319       3.729   7.997   0.839  1.00  0.00
J    H
ATOM  11235 HD12 LEU J 319       2.209   8.442  -0.018  1.00  0.00
J    H
ATOM  11236 HD13 LEU J 319       3.095   6.941  -0.473  1.00  0.00
J    H
ATOM  11237  CD2 LEU J 319       5.474   8.258  -1.243  1.00  0.00
J    C
ATOM  11238 HD21 LEU J 319       5.946   8.296  -0.236  1.00  0.00
J    H
ATOM  11239 HD22 LEU J 319       5.507   7.206  -1.591  1.00  0.00
J    H
ATOM  11240 HD23 LEU J 319       6.086   8.875  -1.935  1.00  0.00
J    H
ATOM  11241  C   LEU J 319       2.064  10.841  -2.167  1.00  0.00
J    C
ATOM  11242  O   LEU J 319       1.601  11.144  -1.068  1.00  0.00
J    O
ATOM  11243  N   LEU J 320       2.610  11.759  -2.982  1.00  0.00
J    N
ATOM  11244  HN  LEU J 320       2.878  11.504  -3.908  1.00  0.00
J    H
ATOM  11245  CA  LEU J 320       2.864  13.145  -2.651  1.00  0.00
J    C
ATOM  11246  HA  LEU J 320       3.394  13.111  -1.709  1.00  0.00
J    H
ATOM  11247  CB  LEU J 320       3.721  13.743  -3.789  1.00  0.00
J    C
ATOM  11248  HB1 LEU J 320       4.234  12.892  -4.297  1.00  0.00
J    H
ATOM  11249  HB2 LEU J 320       3.048  14.195  -4.551  1.00  0.00
J    H
ATOM  11250  CG  LEU J 320       4.795  14.764  -3.382  1.00  0.00
J    C
ATOM  11251  HG  LEU J 320       4.291  15.675  -2.980  1.00  0.00
J    H
ATOM  11252  CD1 LEU J 320       5.774  14.198  -2.338  1.00  0.00
J    C
ATOM  11253 HD11 LEU J 320       6.565  14.942  -2.101  1.00  0.00
J    H
ATOM  11254 HD12 LEU J 320       5.240  13.944  -1.398  1.00  0.00
J    H
ATOM  11255 HD13 LEU J 320       6.267  13.279  -2.721  1.00  0.00
J    H
ATOM  11256  CD2 LEU J 320       5.565  15.164  -4.646  1.00  0.00
J    C
ATOM  11257 HD21 LEU J 320       6.343  15.919  -4.401  1.00  0.00
J    H
ATOM  11258 HD22 LEU J 320       6.073  14.295  -5.108  1.00  0.00
J    H
```

```
ATOM  11259 HD23 LEU J 320       4.875  15.607  -5.397  1.00  0.00
J    H
ATOM  11260  C   LEU J 320       1.622  14.004  -2.450  1.00  0.00
J    C
ATOM  11261  O   LEU J 320       1.704  15.058  -1.825  1.00  0.00
J    O
ATOM  11262  N   MET J 321       0.457  13.567  -2.978  1.00  0.00
J    N
ATOM  11263  HN  MET J 321       0.424  12.787  -3.600  1.00  0.00
J    H
ATOM  11264  CA  MET J 321      -0.834  14.170  -2.703  1.00  0.00
J    C
ATOM  11265  HA  MET J 321      -0.612  15.077  -2.154  1.00  0.00
J    H
ATOM  11266  CB  MET J 321      -1.543  14.570  -4.012  1.00  0.00
J    C
ATOM  11267  HB1 MET J 321      -2.500  15.063  -3.725  1.00  0.00
J    H
ATOM  11268  HB2 MET J 321      -0.939  15.343  -4.538  1.00  0.00
J    H
ATOM  11269  CG  MET J 321      -1.918  13.503  -5.050  1.00  0.00
J    C
ATOM  11270  HG1 MET J 321      -1.088  12.775  -5.172  1.00  0.00
J    H
ATOM  11271  HG2 MET J 321      -2.810  12.940  -4.701  1.00  0.00
J    H
ATOM  11272  SD  MET J 321      -2.260  14.313  -6.644  1.00  0.00
J    S
ATOM  11273  CE  MET J 321      -2.737  12.824  -7.544  1.00  0.00
J    C
ATOM  11274  HE1 MET J 321      -2.996  13.063  -8.598  1.00  0.00
J    H
ATOM  11275  HE2 MET J 321      -1.910  12.082  -7.556  1.00  0.00
J    H
ATOM  11276  HE3 MET J 321      -3.623  12.341  -7.078  1.00  0.00
J    H
ATOM  11277  C   MET J 321      -1.772  13.336  -1.850  1.00  0.00
J    C
ATOM  11278  O   MET J 321      -2.839  13.804  -1.449  1.00  0.00
J    O
ATOM  11279  N   PHE J 322      -1.394  12.098  -1.472  1.00  0.00
J    N
ATOM  11280  HN  PHE J 322      -0.676  11.573  -1.926  1.00  0.00
J    H
ATOM  11281  CA  PHE J 322      -2.018  11.418  -0.348  1.00  0.00
J    C
ATOM  11282  HA  PHE J 322      -3.089  11.535  -0.459  1.00  0.00
J    H
ATOM  11283  CB  PHE J 322      -1.588   9.924  -0.323  1.00  0.00
J    C
ATOM  11284  HB1 PHE J 322      -1.785   9.473  -1.320  1.00  0.00
J    H
ATOM  11285  HB2 PHE J 322      -0.499   9.824  -0.127  1.00  0.00
J    H
```

ATOM 11286 CG PHE J 322   -2.381   9.128   0.690 1.00 0.00
J   C
ATOM 11287 CD1 PHE J 322   -3.786   9.080   0.630 1.00 0.00
J   C
ATOM 11288 HD1 PHE J 322   -4.308   9.621 -0.147 1.00 0.00
J   H
ATOM 11289 CE1 PHE J 322   -4.521   8.344   1.569 1.00 0.00
J   C
ATOM 11290 HE1 PHE J 322   -5.598   8.306   1.502 1.00 0.00
J   H
ATOM 11291 CZ PHE J 322   -3.855   7.655   2.590 1.00 0.00
J   C
ATOM 11292 HZ PHE J 322   -4.417   7.092   3.321 1.00 0.00
J   H
ATOM 11293 CD2 PHE J 322   -1.726   8.426   1.715 1.00 0.00
J   C
ATOM 11294 HD2 PHE J 322   -0.648   8.469   1.796 1.00 0.00
J   H
ATOM 11295 CE2 PHE J 322   -2.456   7.687   2.656 1.00 0.00
J   C
ATOM 11296 HE2 PHE J 322   -1.938   7.144   3.434 1.00 0.00
J   H
ATOM 11297 C   PHE J 322   -1.621 12.124   0.948 1.00 0.00
J   C
ATOM 11298 O   PHE J 322   -2.447 12.447   1.804 1.00 0.00
J   O
ATOM 11299 N   ILE J 323   -0.313 12.443   1.054 1.00 0.00
J   N
ATOM 11300 HN ILE J 323   0.359 12.128   0.386 1.00 0.00
J   H
ATOM 11301 CA ILE J 323   0.242 13.254   2.115 1.00 0.00
J   C
ATOM 11302 HA ILE J 323   -0.344 13.068   3.007 1.00 0.00
J   H
ATOM 11303 CB ILE J 323   1.743 13.042   2.340 1.00 0.00
J   C
ATOM 11304 HB ILE J 323   2.230 13.522   1.453 1.00 0.00
J   H
ATOM 11305 CG2 ILE J 323   2.128 13.691   3.692 1.00 0.00
J   C
ATOM 11306 HG21 ILE J 323   3.231 13.698   3.821 1.00 0.00
J   H
ATOM 11307 HG22 ILE J 323   1.777 14.743   3.742 1.00 0.00
J   H
ATOM 11308 HG23 ILE J 323   1.683 13.132   4.543 1.00 0.00
J   H
ATOM 11309 CG1 ILE J 323   2.194 11.561   2.301 1.00 0.00
J   C
ATOM 11310 HG11 ILE J 323   2.011 11.084   3.290 1.00 0.00
J   H
ATOM 11311 HG12 ILE J 323   1.575 11.015   1.554 1.00 0.00
J   H
ATOM 11312 CD ILE J 323   3.678 11.440   1.918 1.00 0.00
J   C

```
ATOM  11313  HD1 ILE J 323       3.970  10.371   1.838  1.00  0.00
J    H
ATOM  11314  HD2 ILE J 323       3.871  11.928   0.938  1.00  0.00
J    H
ATOM  11315  HD3 ILE J 323       4.327  11.917   2.683  1.00  0.00
J    H
ATOM  11316  C   ILE J 323       0.046  14.719   1.759  1.00  0.00
J    C
ATOM  11317  O   ILE J 323       0.548  15.222   0.762  1.00  0.00
J    O
ATOM  11318  N   GLY J 324      -0.691  15.460   2.591  1.00  0.00
J    N
ATOM  11319  HN  GLY J 324      -1.159  15.037   3.365  1.00  0.00
J    H
ATOM  11320  CA  GLY J 324      -0.873  16.887   2.454  1.00  0.00
J    C
ATOM  11321  HA1 GLY J 324      -1.816  17.133   2.924  1.00  0.00
J    H
ATOM  11322  HA2 GLY J 324      -0.829  17.119   1.399  1.00  0.00
J    H
ATOM  11323  C   GLY J 324       0.206  17.667   3.144  1.00  0.00
J    C
ATOM  11324  O   GLY J 324       1.395  17.344   3.140  1.00  0.00
J    O
ATOM  11325  N   ALA J 325      -0.184  18.771   3.782  1.00  0.00
J    N
ATOM  11326  HN  ALA J 325      -1.119  19.123   3.745  1.00  0.00
J    H
ATOM  11327  CA  ALA J 325       0.731  19.538   4.576  1.00  0.00
J    C
ATOM  11328  HA  ALA J 325       1.474  18.921   5.067  1.00  0.00
J    H
ATOM  11329  CB  ALA J 325       1.419  20.605   3.694  1.00  0.00
J    C
ATOM  11330  HB1 ALA J 325       2.047  20.105   2.926  1.00  0.00
J    H
ATOM  11331  HB2 ALA J 325       0.667  21.231   3.166  1.00  0.00
J    H
ATOM  11332  HB3 ALA J 325       2.076  21.270   4.294  1.00  0.00
J    H
ATOM  11333  C   ALA J 325      -0.083  20.187   5.678  1.00  0.00
J    C
ATOM  11334  O   ALA J 325      -1.176  20.676   5.421  1.00  0.00
J    O
ATOM  11335  N   GLY J 326       0.434  20.176   6.929  1.00  0.00
J    N
ATOM  11336  HN  GLY J 326       1.182  19.549   7.138  1.00  0.00
J    H
ATOM  11337  CA  GLY J 326      -0.014  21.023   8.040  1.00  0.00
J    C
ATOM  11338  HA1 GLY J 326       0.041  22.028   7.645  1.00  0.00
J    H
ATOM  11339  HA2 GLY J 326       0.692  20.818   8.835  1.00  0.00
J    H
```

```
ATOM  11340  C   GLY J 326      -1.400  20.812   8.586  1.00  0.00
J    C
ATOM  11341  O   GLY J 326      -2.400  20.944   7.901  1.00  0.00
J    O
ATOM  11342  N   SER J 327      -1.551  20.512   9.880  1.00  0.00
J    N
ATOM  11343  HN  SER J 327      -0.813  20.519  10.556  1.00  0.00
J    H
ATOM  11344  CA  SER J 327      -2.853  20.140  10.421  1.00  0.00
J    C
ATOM  11345  HA  SER J 327      -3.199  19.376   9.738  1.00  0.00
J    H
ATOM  11346  CB  SER J 327      -2.714  19.512  11.820  1.00  0.00
J    C
ATOM  11347  HB1 SER J 327      -2.780  20.330  12.575  1.00  0.00
J    H
ATOM  11348  HB2 SER J 327      -3.560  18.817  12.018  1.00  0.00
J    H
ATOM  11349  OG  SER J 327      -1.444  18.864  11.921  1.00  0.00
J    O
ATOM  11350  HG1 SER J 327      -1.293  18.680  12.857  1.00  0.00
J    H
ATOM  11351  C   SER J 327      -3.913  21.220  10.465  1.00  0.00
J    C
ATOM  11352  O   SER J 327      -5.078  20.982  10.167  1.00  0.00
J    O
ATOM  11353  N   ALA J 328      -3.513  22.453  10.827  1.00  0.00
J    N
ATOM  11354  HN  ALA J 328      -2.545  22.625  11.008  1.00  0.00
J    H
ATOM  11355  CA  ALA J 328      -4.388  23.598  10.983  1.00  0.00
J    C
ATOM  11356  HA  ALA J 328      -5.429  23.292  10.985  1.00  0.00
J    H
ATOM  11357  CB  ALA J 328      -4.063  24.309  12.313  1.00  0.00
J    C
ATOM  11358  HB1 ALA J 328      -4.235  23.611  13.160  1.00  0.00
J    H
ATOM  11359  HB2 ALA J 328      -2.999  24.628  12.344  1.00  0.00
J    H
ATOM  11360  HB3 ALA J 328      -4.705  25.202  12.467  1.00  0.00
J    H
ATOM  11361  C   ALA J 328      -4.223  24.561   9.808  1.00  0.00
J    C
ATOM  11362  O   ALA J 328      -4.218  25.782   9.963  1.00  0.00
J    O
ATOM  11363  N   SER J 329      -3.974  24.005   8.609  1.00  0.00
J    N
ATOM  11364  HN  SER J 329      -3.973  23.009   8.521  1.00  0.00
J    H
ATOM  11365  CA  SER J 329      -3.694  24.711   7.373  1.00  0.00
J    C
ATOM  11366  HA  SER J 329      -3.308  25.669   7.696  1.00  0.00
J    H
```

ATOM 11367 CB SER J 329 -2.591 23.961 6.576 1.00 0.00
J C
ATOM 11368 HB1 SER J 329 -2.234 24.634 5.763 1.00 0.00
J H
ATOM 11369 HB2 SER J 329 -1.717 23.746 7.231 1.00 0.00
J H
ATOM 11370 OG SER J 329 -3.064 22.762 5.977 1.00 0.00
J O
ATOM 11371 HG1 SER J 329 -2.399 22.486 5.333 1.00 0.00
J H
ATOM 11372 C SER J 329 -4.899 24.983 6.498 1.00 0.00
J C
ATOM 11373 O SER J 329 -6.048 24.667 6.806 1.00 0.00
J O
ATOM 11374 N THR J 330 -4.646 25.625 5.345 1.00 0.00
J N
ATOM 11375 HN THR J 330 -3.771 26.065 5.149 1.00 0.00
J H
ATOM 11376 CA THR J 330 -5.625 25.737 4.275 1.00 0.00
J C
ATOM 11377 HA THR J 330 -6.592 25.748 4.759 1.00 0.00
J H
ATOM 11378 CB THR J 330 -5.432 26.998 3.430 1.00 0.00
J C
ATOM 11379 HB THR J 330 -6.127 26.898 2.561 1.00 0.00
J H
ATOM 11380 OG1 THR J 330 -4.117 27.099 2.896 1.00 0.00
J O
ATOM 11381 HG1 THR J 330 -4.104 27.923 2.398 1.00 0.00
J H
ATOM 11382 CG2 THR J 330 -5.641 28.237 4.313 1.00 0.00
J C
ATOM 11383 HG21 THR J 330 -5.620 29.166 3.704 1.00 0.00
J H
ATOM 11384 HG22 THR J 330 -6.626 28.188 4.824 1.00 0.00
J H
ATOM 11385 HG23 THR J 330 -4.850 28.314 5.090 1.00 0.00
J H
ATOM 11386 C THR J 330 -5.606 24.505 3.371 1.00 0.00
J C
ATOM 11387 O THR J 330 -6.512 24.309 2.555 1.00 0.00
J O
ATOM 11388 N ALA J 331 -4.594 23.622 3.535 1.00 0.00
J N
ATOM 11389 HN ALA J 331 -3.925 23.809 4.254 1.00 0.00
J H
ATOM 11390 CA ALA J 331 -4.366 22.413 2.774 1.00 0.00
J C
ATOM 11391 HA ALA J 331 -4.698 22.548 1.751 1.00 0.00
J H
ATOM 11392 CB ALA J 331 -2.855 22.093 2.755 1.00 0.00
J C
ATOM 11393 HB1 ALA J 331 -2.299 22.942 2.304 1.00 0.00
J H

ATOM 11394 HB2 ALA J 331 -2.466 21.940 3.786 1.00 0.00
J H
ATOM 11395 HB3 ALA J 331 -2.636 21.183 2.156 1.00 0.00
J H
ATOM 11396 C ALA J 331 -5.157 21.222 3.331 1.00 0.00
J C
ATOM 11397 O ALA J 331 -5.820 21.301 4.361 1.00 0.00
J O
ATOM 11398 N SER J 332 -5.150 20.077 2.619 1.00 0.00
J N
ATOM 11399 HN SER J 332 -4.569 19.937 1.817 1.00 0.00
J H
ATOM 11400 CA SER J 332 -5.992 18.934 2.955 1.00 0.00
J C
ATOM 11401 HA SER J 332 -6.338 19.121 3.963 1.00 0.00
J H
ATOM 11402 CB SER J 332 -7.138 18.858 1.932 1.00 0.00
J C
ATOM 11403 HB1 SER J 332 -7.425 19.899 1.657 1.00 0.00
J H
ATOM 11404 HB2 SER J 332 -6.794 18.355 1.000 1.00 0.00
J H
ATOM 11405 OG SER J 332 -8.284 18.205 2.459 1.00 0.00
J O
ATOM 11406 HG1 SER J 332 -9.018 18.392 1.858 1.00 0.00
J H
ATOM 11407 C SER J 332 -5.225 17.615 2.975 1.00 0.00
J C
ATOM 11408 O SER J 332 -4.069 17.565 3.385 1.00 0.00
J O
ATOM 11409 N GLY J 333 -5.874 16.504 2.551 1.00 0.00
J N
ATOM 11410 HN GLY J 333 -6.806 16.614 2.207 1.00 0.00
J H
ATOM 11411 CA GLY J 333 -5.352 15.135 2.539 1.00 0.00
J C
ATOM 11412 HA1 GLY J 333 -4.396 15.174 2.036 1.00 0.00
J H
ATOM 11413 HA2 GLY J 333 -6.090 14.522 2.041 1.00 0.00
J H
ATOM 11414 C GLY J 333 -5.131 14.569 3.914 1.00 0.00
J C
ATOM 11415 O GLY J 333 -5.802 14.947 4.870 1.00 0.00
J O
ATOM 11416 N ILE J 334 -4.181 13.625 4.069 1.00 0.00
J N
ATOM 11417 HN ILE J 334 -3.740 13.147 3.312 1.00 0.00
J H
ATOM 11418 CA ILE J 334 -3.713 13.217 5.388 1.00 0.00
J C
ATOM 11419 HA ILE J 334 -4.478 13.464 6.113 1.00 0.00
J H
ATOM 11420 CB ILE J 334 -3.371 11.724 5.521 1.00 0.00
J C

```
ATOM  11421  HB  ILE J 334      -3.434  11.519   6.620  1.00  0.00
J    H
ATOM  11422  CG2 ILE J 334      -4.417  10.935   4.702  1.00  0.00
J    C
ATOM  11423 HG21 ILE J 334      -4.316   9.845   4.886  1.00  0.00
J    H
ATOM  11424 HG22 ILE J 334      -5.449  11.230   4.987  1.00  0.00
J    H
ATOM  11425 HG23 ILE J 334      -4.290  11.116   3.614  1.00  0.00
J    H
ATOM  11426  CG1 ILE J 334      -1.917  11.334   5.127  1.00  0.00
J    C
ATOM  11427 HG11 ILE J 334      -1.752  11.542   4.046  1.00  0.00
J    H
ATOM  11428 HG12 ILE J 334      -1.205  11.974   5.697  1.00  0.00
J    H
ATOM  11429  CD  ILE J 334      -1.573   9.868   5.411  1.00  0.00
J    C
ATOM  11430  HD1 ILE J 334      -0.509   9.666   5.163  1.00  0.00
J    H
ATOM  11431  HD2 ILE J 334      -1.732   9.629   6.485  1.00  0.00
J    H
ATOM  11432  HD3 ILE J 334      -2.203   9.185   4.802  1.00  0.00
J    H
ATOM  11433  C   ILE J 334      -2.520  14.083   5.700  1.00  0.00
J    C
ATOM  11434  O   ILE J 334      -1.749  14.431   4.811  1.00  0.00
J    O
ATOM  11435  N   LYS J 335      -2.303  14.526   6.942  1.00  0.00
J    N
ATOM  11436  HN  LYS J 335      -2.799  14.260   7.767  1.00  0.00
J    H
ATOM  11437  CA  LYS J 335      -1.246  15.492   7.146  1.00  0.00
J    C
ATOM  11438  HA  LYS J 335      -1.270  16.141   6.279  1.00  0.00
J    H
ATOM  11439  CB  LYS J 335      -1.536  16.418   8.330  1.00  0.00
J    C
ATOM  11440  HB1 LYS J 335      -1.373  15.823   9.259  1.00  0.00
J    H
ATOM  11441  HB2 LYS J 335      -0.779  17.235   8.345  1.00  0.00
J    H
ATOM  11442  CG  LYS J 335      -2.980  16.942   8.258  1.00  0.00
J    C
ATOM  11443  HG1 LYS J 335      -3.683  16.079   8.289  1.00  0.00
J    H
ATOM  11444  HG2 LYS J 335      -3.183  17.569   9.156  1.00  0.00
J    H
ATOM  11445  CD  LYS J 335      -3.396  17.778   7.021  1.00  0.00
J    C
ATOM  11446  HD1 LYS J 335      -3.087  18.835   7.190  1.00  0.00
J    H
ATOM  11447  HD2 LYS J 335      -2.828  17.400   6.140  1.00  0.00
J    H
```

ATOM 11448 CE LYS J 335 -4.907 17.719 6.719 1.00 0.00
J C
ATOM 11449 HE1 LYS J 335 -5.183 18.489 5.963 1.00 0.00
J H
ATOM 11450 HE2 LYS J 335 -5.187 16.719 6.321 1.00 0.00
J H
ATOM 11451 NZ LYS J 335 -5.721 17.966 7.926 1.00 0.00
J N
ATOM 11452 HZ1 LYS J 335 -6.733 17.920 7.688 1.00 0.00
J H
ATOM 11453 HZ2 LYS J 335 -5.503 17.247 8.645 1.00 0.00
J H
ATOM 11454 HZ3 LYS J 335 -5.499 18.908 8.307 1.00 0.00
J H
ATOM 11455 C LYS J 335 0.134 14.894 7.222 1.00 0.00
J C
ATOM 11456 O LYS J 335 0.351 13.706 7.443 1.00 0.00
J O
ATOM 11457 N LEU J 336 1.134 15.758 7.016 1.00 0.00
J N
ATOM 11458 HN LEU J 336 0.979 16.725 6.825 1.00 0.00
J H
ATOM 11459 CA LEU J 336 2.523 15.376 7.050 1.00 0.00
J C
ATOM 11460 HA LEU J 336 2.635 14.581 6.325 1.00 0.00
J H
ATOM 11461 CB LEU J 336 3.382 16.573 6.616 1.00 0.00
J C
ATOM 11462 HB1 LEU J 336 2.995 16.914 5.628 1.00 0.00
J H
ATOM 11463 HB2 LEU J 336 3.210 17.416 7.322 1.00 0.00
J H
ATOM 11464 CG LEU J 336 4.878 16.255 6.491 1.00 0.00
J C
ATOM 11465 HG LEU J 336 5.283 16.118 7.523 1.00 0.00
J H
ATOM 11466 CD1 LEU J 336 5.172 15.010 5.634 1.00 0.00
J C
ATOM 11467 HD11 LEU J 336 6.268 14.854 5.536 1.00 0.00
J H
ATOM 11468 HD12 LEU J 336 4.729 14.103 6.098 1.00 0.00
J H
ATOM 11469 HD13 LEU J 336 4.746 15.127 4.614 1.00 0.00
J H
ATOM 11470 CD2 LEU J 336 5.564 17.467 5.872 1.00 0.00
J C
ATOM 11471 HD21 LEU J 336 6.657 17.287 5.779 1.00 0.00
J H
ATOM 11472 HD22 LEU J 336 5.172 17.686 4.858 1.00 0.00
J H
ATOM 11473 HD23 LEU J 336 5.413 18.366 6.508 1.00 0.00
J H
ATOM 11474 C LEU J 336 2.938 14.825 8.405 1.00 0.00
J C

```
ATOM  11475  O   LEU J 336       3.617  13.804   8.498  1.00  0.00
J    O
ATOM  11476  N   THR J 337       2.440  15.419   9.503  1.00  0.00
J    N
ATOM  11477  HN  THR J 337       1.943  16.283   9.441  1.00  0.00
J    H
ATOM  11478  CA  THR J 337       2.561  14.896  10.854  1.00  0.00
J    C
ATOM  11479  HA  THR J 337       3.625  14.787  11.018  1.00  0.00
J    H
ATOM  11480  CB  THR J 337       1.929  15.822  11.876  1.00  0.00
J    C
ATOM  11481  HB  THR J 337       1.643  15.177  12.743  1.00  0.00
J    H
ATOM  11482  OG1 THR J 337       0.735  16.400  11.358  1.00  0.00
J    O
ATOM  11483  HG1 THR J 337       0.404  16.976  12.055  1.00  0.00
J    H
ATOM  11484  CG2 THR J 337       2.924  16.959  12.142  1.00  0.00
J    C
ATOM  11485 HG21 THR J 337       2.568  17.611  12.968  1.00  0.00
J    H
ATOM  11486 HG22 THR J 337       3.913  16.548  12.434  1.00  0.00
J    H
ATOM  11487 HG23 THR J 337       3.062  17.589  11.238  1.00  0.00
J    H
ATOM  11488  C   THR J 337       1.956  13.516  11.015  1.00  0.00
J    C
ATOM  11489  O   THR J 337       2.559  12.661  11.643  1.00  0.00
J    O
ATOM  11490  N   THR J 338       0.784  13.232  10.411  1.00  0.00
J    N
ATOM  11491  HN  THR J 338       0.253  13.934   9.937  1.00  0.00
J    H
ATOM  11492  CA  THR J 338       0.175  11.895  10.385  1.00  0.00
J    C
ATOM  11493  HA  THR J 338       0.082  11.609  11.424  1.00  0.00
J    H
ATOM  11494  CB  THR J 338      -1.152  11.877   9.635  1.00  0.00
J    C
ATOM  11495  HB  THR J 338      -0.911  12.126   8.572  1.00  0.00
J    H
ATOM  11496  OG1 THR J 338      -1.965  12.908  10.173  1.00  0.00
J    O
ATOM  11497  HG1 THR J 338      -2.795  12.861   9.686  1.00  0.00
J    H
ATOM  11498  CG2 THR J 338      -1.891  10.536   9.787  1.00  0.00
J    C
ATOM  11499 HG21 THR J 338      -2.795  10.507   9.144  1.00  0.00
J    H
ATOM  11500 HG22 THR J 338      -1.231   9.694   9.488  1.00  0.00
J    H
ATOM  11501 HG23 THR J 338      -2.209  10.371  10.840  1.00  0.00
J    H
```

```
ATOM  11502  C   THR J 338       1.053  10.856   9.730  1.00  0.00
J    C
ATOM  11503  O   THR J 338       1.258   9.764  10.267  1.00  0.00
J    O
ATOM  11504  N   PHE J 339       1.636  11.201   8.566  1.00  0.00
J    N
ATOM  11505  HN  PHE J 339       1.433  12.075   8.126  1.00  0.00
J    H
ATOM  11506  CA  PHE J 339       2.589  10.377   7.849  1.00  0.00
J    C
ATOM  11507  HA  PHE J 339       2.103   9.418   7.718  1.00  0.00
J    H
ATOM  11508  CB  PHE J 339       2.890  11.071   6.487  1.00  0.00
J    C
ATOM  11509  HB1 PHE J 339       1.943  11.172   5.913  1.00  0.00
J    H
ATOM  11510  HB2 PHE J 339       3.292  12.096   6.641  1.00  0.00
J    H
ATOM  11511  CG  PHE J 339       3.835  10.278   5.619  1.00  0.00
J    C
ATOM  11512  CD1 PHE J 339       3.411   9.091   5.002  1.00  0.00
J    C
ATOM  11513  HD1 PHE J 339       2.392   8.752   5.135  1.00  0.00
J    H
ATOM  11514  CE1 PHE J 339       4.297   8.336   4.220  1.00  0.00
J    C
ATOM  11515  HE1 PHE J 339       3.959   7.426   3.744  1.00  0.00
J    H
ATOM  11516  CZ  PHE J 339       5.620   8.766   4.050  1.00  0.00
J    C
ATOM  11517  HZ  PHE J 339       6.303   8.191   3.441  1.00  0.00
J    H
ATOM  11518  CD2 PHE J 339       5.165  10.699   5.441  1.00  0.00
J    C
ATOM  11519  HD2 PHE J 339       5.517  11.600   5.925  1.00  0.00
J    H
ATOM  11520  CE2 PHE J 339       6.055   9.948   4.662  1.00  0.00
J    C
ATOM  11521  HE2 PHE J 339       7.075  10.282   4.536  1.00  0.00
J    H
ATOM  11522  C   PHE J 339       3.872  10.125   8.652  1.00  0.00
J    C
ATOM  11523  O   PHE J 339       4.303   8.987   8.810  1.00  0.00
J    O
ATOM  11524  N   ILE J 340       4.479  11.187   9.241  1.00  0.00
J    N
ATOM  11525  HN  ILE J 340       4.140  12.120   9.138  1.00  0.00
J    H
ATOM  11526  CA  ILE J 340       5.675  11.070  10.074  1.00  0.00
J    C
ATOM  11527  HA  ILE J 340       6.397  10.526   9.480  1.00  0.00
J    H
ATOM  11528  CB  ILE J 340       6.287  12.430  10.434  1.00  0.00
J    C
```

```
ATOM  11529  HB  ILE J 340       5.434  13.017  10.861  1.00  0.00
J    H
ATOM  11530  CG2 ILE J 340       7.490  12.239  11.394  1.00  0.00
J    C
ATOM  11531 HG21 ILE J 340       7.841  13.218  11.781  1.00  0.00
J    H
ATOM  11532 HG22 ILE J 340       7.206  11.613  12.266  1.00  0.00
J    H
ATOM  11533 HG23 ILE J 340       8.340  11.749  10.873  1.00  0.00
J    H
ATOM  11534  CG1 ILE J 340       6.744  13.170   9.152  1.00  0.00
J    C
ATOM  11535 HG11 ILE J 340       7.605  12.634   8.694  1.00  0.00
J    H
ATOM  11536 HG12 ILE J 340       5.914  13.155   8.410  1.00  0.00
J    H
ATOM  11537  CD  ILE J 340       7.139  14.634   9.401  1.00  0.00
J    C
ATOM  11538  HD1 ILE J 340       7.402  15.132   8.443  1.00  0.00
J    H
ATOM  11539  HD2 ILE J 340       6.297  15.193   9.864  1.00  0.00
J    H
ATOM  11540  HD3 ILE J 340       8.019  14.700  10.075  1.00  0.00
J    H
ATOM  11541  C   ILE J 340       5.426  10.245  11.330  1.00  0.00
J    C
ATOM  11542  O   ILE J 340       6.224   9.362  11.653  1.00  0.00
J    O
ATOM  11543  N   VAL J 341       4.294  10.463  12.040  1.00  0.00
J    N
ATOM  11544  HN  VAL J 341       3.649  11.168  11.757  1.00  0.00
J    H
ATOM  11545  CA  VAL J 341       3.898   9.731  13.243  1.00  0.00
J    C
ATOM  11546  HA  VAL J 341       4.687   9.870  13.972  1.00  0.00
J    H
ATOM  11547  CB  VAL J 341       2.575  10.279  13.811  1.00  0.00
J    C
ATOM  11548  HB  VAL J 341       1.884  10.451  12.950  1.00  0.00
J    H
ATOM  11549  CG1 VAL J 341       1.883   9.304  14.790  1.00  0.00
J    C
ATOM  11550 HG11 VAL J 341       0.957   9.762  15.199  1.00  0.00
J    H
ATOM  11551 HG12 VAL J 341       1.590   8.358  14.289  1.00  0.00
J    H
ATOM  11552 HG13 VAL J 341       2.553   9.062  15.644  1.00  0.00
J    H
ATOM  11553  CG2 VAL J 341       2.875  11.598  14.552  1.00  0.00
J    C
ATOM  11554 HG21 VAL J 341       1.951  12.008  15.013  1.00  0.00
J    H
ATOM  11555 HG22 VAL J 341       3.624  11.432  15.357  1.00  0.00
J    H
```

```
ATOM  11556 HG23 VAL J 341       3.273  12.367  13.859  1.00  0.00
J    H
ATOM  11557  C   VAL J 341       3.774   8.242  12.985  1.00  0.00
J    C
ATOM  11558  O   VAL J 341       4.322   7.426  13.719  1.00  0.00
J    O
ATOM  11559  N   ILE J 342       3.088   7.840  11.892  1.00  0.00
J    N
ATOM  11560  HN  ILE J 342       2.603   8.459  11.279  1.00  0.00
J    H
ATOM  11561  CA  ILE J 342       3.000   6.436  11.514  1.00  0.00
J    C
ATOM  11562  HA  ILE J 342       2.669   5.909  12.400  1.00  0.00
J    H
ATOM  11563  CB  ILE J 342       1.945   6.228  10.426  1.00  0.00
J    C
ATOM  11564  HB  ILE J 342       1.950   7.185   9.844  1.00  0.00
J    H
ATOM  11565  CG2 ILE J 342       2.219   5.002   9.515  1.00  0.00
J    C
ATOM  11566 HG21 ILE J 342       1.517   4.989   8.656  1.00  0.00
J    H
ATOM  11567 HG22 ILE J 342       3.251   5.036   9.106  1.00  0.00
J    H
ATOM  11568 HG23 ILE J 342       2.094   4.053  10.078  1.00  0.00
J    H
ATOM  11569  CG1 ILE J 342       0.578   6.104  11.135  1.00  0.00
J    C
ATOM  11570 HG11 ILE J 342       0.572   5.201  11.785  1.00  0.00
J    H
ATOM  11571 HG12 ILE J 342       0.434   6.989  11.797  1.00  0.00
J    H
ATOM  11572  CD  ILE J 342      -0.580   6.035  10.143  1.00  0.00
J    C
ATOM  11573  HD1 ILE J 342      -1.550   6.005  10.685  1.00  0.00
J    H
ATOM  11574  HD2 ILE J 342      -0.580   6.926   9.479  1.00  0.00
J    H
ATOM  11575  HD3 ILE J 342      -0.510   5.124   9.513  1.00  0.00
J    H
ATOM  11576  C   ILE J 342       4.349   5.861  11.140  1.00  0.00
J    C
ATOM  11577  O   ILE J 342       4.734   4.801  11.637  1.00  0.00
J    O
ATOM  11578  N   LEU J 343       5.138   6.570  10.313  1.00  0.00
J    N
ATOM  11579  HN  LEU J 343       4.893   7.476   9.974  1.00  0.00
J    H
ATOM  11580  CA  LEU J 343       6.414   6.085   9.840  1.00  0.00
J    C
ATOM  11581  HA  LEU J 343       6.214   5.122   9.390  1.00  0.00
J    H
ATOM  11582  CB  LEU J 343       6.964   7.073   8.786  1.00  0.00
J    C
```

ATOM 11583 HB1 LEU J 343   6.196   7.159   7.984 1.00 0.00
J   H
ATOM 11584 HB2 LEU J 343   7.044   8.083   9.245 1.00 0.00
J   H
ATOM 11585 CG LEU J 343   8.305   6.665   8.146 1.00 0.00
J   C
ATOM 11586 HG LEU J 343   9.030   6.564   8.989 1.00 0.00
J   H
ATOM 11587 CD1 LEU J 343   8.211   5.332   7.382 1.00 0.00
J   C
ATOM 11588 HD11 LEU J 343   9.180   5.092   6.893 1.00 0.00
J   H
ATOM 11589 HD12 LEU J 343   7.955   4.501   8.074 1.00 0.00
J   H
ATOM 11590 HD13 LEU J 343   7.430   5.385   6.593 1.00 0.00
J   H
ATOM 11591 CD2 LEU J 343   8.805   7.784   7.222 1.00 0.00
J   C
ATOM 11592 HD21 LEU J 343   9.782   7.504   6.771 1.00 0.00
J   H
ATOM 11593 HD22 LEU J 343   8.095   7.979   6.394 1.00 0.00
J   H
ATOM 11594 HD23 LEU J 343   8.944   8.727   7.794 1.00 0.00
J   H
ATOM 11595 C   LEU J 343   7.430   5.869 10.959 1.00 0.00
J   C
ATOM 11596 O   LEU J 343   8.022   4.800 11.078 1.00 0.00
J   O
ATOM 11597 N   THR J 344   7.606   6.850 11.873 1.00 0.00
J   N
ATOM 11598 HN THR J 344   7.125   7.725 11.830 1.00 0.00
J   H
ATOM 11599 CA THR J 344   8.518   6.720 13.011 1.00 0.00
J   C
ATOM 11600 HA THR J 344   9.453   6.378 12.587 1.00 0.00
J   H
ATOM 11601 CB THR J 344   8.800   8.000 13.788 1.00 0.00
J   C
ATOM 11602 HB THR J 344   9.388   7.694 14.688 1.00 0.00
J   H
ATOM 11603 OG1 THR J 344   7.609   8.608 14.260 1.00 0.00
J   O
ATOM 11604 HG1 THR J 344   7.892   9.405 14.721 1.00 0.00
J   H
ATOM 11605 CG2 THR J 344   9.525   8.984 12.863 1.00 0.00
J   C
ATOM 11606 HG21 THR J 344   9.850   9.886 13.423 1.00 0.00
J   H
ATOM 11607 HG22 THR J 344   10.429   8.510 12.425 1.00 0.00
J   H
ATOM 11608 HG23 THR J 344   8.866   9.310 12.030 1.00 0.00
J   H
ATOM 11609 C   THR J 344   8.083   5.665 13.998 1.00 0.00
J   C

```
ATOM  11610  O   THR J 344       8.917   4.982  14.588  1.00  0.00
J    O
ATOM  11611  N   SER J 345       6.759   5.505  14.193  1.00  0.00
J    N
ATOM  11612  HN  SER J 345       6.102   6.139  13.785  1.00  0.00
J    H
ATOM  11613  CA  SER J 345       6.151   4.440  14.982  1.00  0.00
J    C
ATOM  11614  HA  SER J 345       6.620   4.511  15.955  1.00  0.00
J    H
ATOM  11615  CB  SER J 345       4.618   4.681  15.099  1.00  0.00
J    C
ATOM  11616  HB1 SER J 345       4.458   5.724  15.454  1.00  0.00
J    H
ATOM  11617  HB2 SER J 345       4.135   4.597  14.098  1.00  0.00
J    H
ATOM  11618  OG  SER J 345       3.979   3.807  16.021  1.00  0.00
J    O
ATOM  11619  HG1 SER J 345       3.101   4.169  16.193  1.00  0.00
J    H
ATOM  11620  C   SER J 345       6.432   3.045  14.443  1.00  0.00
J    C
ATOM  11621  O   SER J 345       6.901   2.167  15.170  1.00  0.00
J    O
ATOM  11622  N   VAL J 346       6.254   2.827  13.125  1.00  0.00
J    N
ATOM  11623  HN  VAL J 346       5.868   3.521  12.523  1.00  0.00
J    H
ATOM  11624  CA  VAL J 346       6.601   1.577  12.457  1.00  0.00
J    C
ATOM  11625  HA  VAL J 346       6.093   0.780  12.985  1.00  0.00
J    H
ATOM  11626  CB  VAL J 346       6.092   1.596  11.013  1.00  0.00
J    C
ATOM  11627  HB  VAL J 346       6.427   2.557  10.550  1.00  0.00
J    H
ATOM  11628  CG1 VAL J 346       6.652   0.433  10.167  1.00  0.00
J    C
ATOM  11629 HG11 VAL J 346       6.226   0.464   9.140  1.00  0.00
J    H
ATOM  11630 HG12 VAL J 346       7.755   0.492  10.069  1.00  0.00
J    H
ATOM  11631 HG13 VAL J 346       6.385  -0.546  10.619  1.00  0.00
J    H
ATOM  11632  CG2 VAL J 346       4.554   1.508  11.068  1.00  0.00
J    C
ATOM  11633 HG21 VAL J 346       4.125   1.478  10.044  1.00  0.00
J    H
ATOM  11634 HG22 VAL J 346       4.235   0.591  11.608  1.00  0.00
J    H
ATOM  11635 HG23 VAL J 346       4.119   2.387  11.587  1.00  0.00
J    H
ATOM  11636  C   VAL J 346       8.097   1.297  12.538  1.00  0.00
J    C
```

```
ATOM  11637  O   VAL J 346       8.520   0.215  12.948  1.00  0.00
J    O
ATOM  11638  N   ILE J 347       8.950   2.303  12.252  1.00  0.00
J    N
ATOM  11639  HN  ILE J 347       8.625   3.196  11.950  1.00  0.00
J    H
ATOM  11640  CA  ILE J 347      10.404   2.198  12.351  1.00  0.00
J    C
ATOM  11641  HA  ILE J 347      10.680   1.347  11.740  1.00  0.00
J    H
ATOM  11642  CB  ILE J 347      11.093   3.448  11.792  1.00  0.00
J    C
ATOM  11643  HB  ILE J 347      10.546   4.304  12.262  1.00  0.00
J    H
ATOM  11644  CG2 ILE J 347      12.612   3.446  12.097  1.00  0.00
J    C
ATOM  11645 HG21 ILE J 347      13.066   4.423  11.826  1.00  0.00
J    H
ATOM  11646 HG22 ILE J 347      12.799   3.276  13.178  1.00  0.00
J    H
ATOM  11647 HG23 ILE J 347      13.132   2.651  11.522  1.00  0.00
J    H
ATOM  11648  CG1 ILE J 347      10.860   3.547  10.265  1.00  0.00
J    C
ATOM  11649 HG11 ILE J 347      11.431   2.745   9.744  1.00  0.00
J    H
ATOM  11650 HG12 ILE J 347       9.781   3.373  10.053  1.00  0.00
J    H
ATOM  11651  CD  ILE J 347      11.258   4.913   9.691  1.00  0.00
J    C
ATOM  11652  HD1 ILE J 347      11.021   4.959   8.606  1.00  0.00
J    H
ATOM  11653  HD2 ILE J 347      10.705   5.729  10.205  1.00  0.00
J    H
ATOM  11654  HD3 ILE J 347      12.346   5.094   9.813  1.00  0.00
J    H
ATOM  11655  C   ILE J 347      10.871   1.891  13.774  1.00  0.00
J    C
ATOM  11656  O   ILE J 347      11.803   1.118  13.991  1.00  0.00
J    O
ATOM  11657  N   ALA J 348      10.219   2.475  14.802  1.00  0.00
J    N
ATOM  11658  HN  ALA J 348       9.497   3.146  14.631  1.00  0.00
J    H
ATOM  11659  CA  ALA J 348      10.492   2.197  16.197  1.00  0.00
J    C
ATOM  11660  HA  ALA J 348      11.532   2.414  16.412  1.00  0.00
J    H
ATOM  11661  CB  ALA J 348       9.614   3.109  17.085  1.00  0.00
J    C
ATOM  11662  HB1 ALA J 348       9.813   4.173  16.839  1.00  0.00
J    H
ATOM  11663  HB2 ALA J 348       8.533   2.915  16.911  1.00  0.00
J    H
```

ATOM 11664 HB3 ALA J 348 9.831 2.960 18.165 1.00 0.00
J H
ATOM 11665 C ALA J 348 10.267 0.739 16.584 1.00 0.00
J C
ATOM 11666 O ALA J 348 11.138 0.130 17.211 1.00 0.00
J O
ATOM 11667 N TYR J 349 9.137 0.144 16.138 1.00 0.00
J N
ATOM 11668 HN TYR J 349 8.443 0.687 15.669 1.00 0.00
J H
ATOM 11669 CA TYR J 349 8.811 -1.268 16.273 1.00 0.00
J C
ATOM 11670 HA TYR J 349 8.861 -1.492 17.331 1.00 0.00
J H
ATOM 11671 CB TYR J 349 7.356 -1.510 15.737 1.00 0.00
J C
ATOM 11672 HB1 TYR J 349 6.639 -0.929 16.357 1.00 0.00
J H
ATOM 11673 HB2 TYR J 349 7.257 -1.147 14.692 1.00 0.00
J H
ATOM 11674 CG TYR J 349 6.941 -2.961 15.816 1.00 0.00
J C
ATOM 11675 CD1 TYR J 349 6.295 -3.488 16.949 1.00 0.00
J C
ATOM 11676 HD1 TYR J 349 6.015 -2.831 17.762 1.00 0.00
J H
ATOM 11677 CE1 TYR J 349 6.029 -4.867 17.043 1.00 0.00
J C
ATOM 11678 HE1 TYR J 349 5.513 -5.270 17.901 1.00 0.00
J H
ATOM 11679 CZ TYR J 349 6.445 -5.726 16.017 1.00 0.00
J C
ATOM 11680 OH TYR J 349 6.310 -7.126 16.115 1.00 0.00
J O
ATOM 11681 HH TYR J 349 6.590 -7.502 15.278 1.00 0.00
J H
ATOM 11682 CD2 TYR J 349 7.297 -3.831 14.773 1.00 0.00
J C
ATOM 11683 HD2 TYR J 349 7.775 -3.442 13.885 1.00 0.00
J H
ATOM 11684 CE2 TYR J 349 7.085 -5.207 14.890 1.00 0.00
J C
ATOM 11685 HE2 TYR J 349 7.439 -5.871 14.116 1.00 0.00
J H
ATOM 11686 C TYR J 349 9.849 -2.159 15.571 1.00 0.00
J C
ATOM 11687 O TYR J 349 10.321 -3.139 16.140 1.00 0.00
J O
ATOM 11688 N LEU J 350 10.270 -1.811 14.335 1.00 0.00
J N
ATOM 11689 HN LEU J 350 9.945 -0.990 13.871 1.00 0.00
J H
ATOM 11690 CA LEU J 350 11.232 -2.595 13.565 1.00 0.00
J C

ATOM 11691 HA LEU J 350   10.882 -3.618 13.625 1.00 0.00
J   H
ATOM 11692 CB LEU J 350   11.280 -2.116 12.089 1.00 0.00
J   C
ATOM 11693 HB1 LEU J 350   11.504 -1.025 12.105 1.00 0.00
J   H
ATOM 11694 HB2 LEU J 350   12.141 -2.600 11.579 1.00 0.00
J   H
ATOM 11695 CG LEU J 350   9.976 -2.346 11.295 1.00 0.00
J   C
ATOM 11696 HG LEU J 350   9.177 -1.803 11.856 1.00 0.00
J   H
ATOM 11697 CD1 LEU J 350   10.099 -1.771   9.874 1.00 0.00
J   C
ATOM 11698 HD11 LEU J 350   9.179 -1.979   9.288 1.00 0.00
J   H
ATOM 11699 HD12 LEU J 350   10.249 -0.670   9.908 1.00 0.00
J   H
ATOM 11700 HD13 LEU J 350   10.960 -2.225   9.340 1.00 0.00
J   H
ATOM 11701 CD2 LEU J 350   9.588 -3.832 11.233 1.00 0.00
J   C
ATOM 11702 HD21 LEU J 350   8.637 -3.959 10.671 1.00 0.00
J   H
ATOM 11703 HD22 LEU J 350   10.364 -4.437 10.723 1.00 0.00
J   H
ATOM 11704 HD23 LEU J 350   9.444 -4.239 12.257 1.00 0.00
J   H
ATOM 11705 C   LEU J 350   12.649 -2.561 14.120 1.00 0.00
J   C
ATOM 11706 O   LEU J 350   13.461 -3.441 13.854 1.00 0.00
J   O
ATOM 11707 N   ARG J 351   12.979 -1.532 14.916 1.00 0.00
J   N
ATOM 11708 HN ARG J 351   12.354 -0.763 15.023 1.00 0.00
J   H
ATOM 11709 CA ARG J 351   14.223 -1.466 15.651 1.00 0.00
J   C
ATOM 11710 HA ARG J 351   14.978 -1.968 15.057 1.00 0.00
J   H
ATOM 11711 CB ARG J 351   14.594   0.011 15.885 1.00 0.00
J   C
ATOM 11712 HB1 ARG J 351   13.651   0.529 16.183 1.00 0.00
J   H
ATOM 11713 HB2 ARG J 351   15.276   0.089 16.760 1.00 0.00
J   H
ATOM 11714 CG ARG J 351   15.170   0.647 14.610 1.00 0.00
J   C
ATOM 11715 HG1 ARG J 351   16.160   0.185 14.400 1.00 0.00
J   H
ATOM 11716 HG2 ARG J 351   14.524   0.374 13.744 1.00 0.00
J   H
ATOM 11717 CD ARG J 351   15.240   2.167 14.703 1.00 0.00
J   C

```
ATOM  11718  HD1 ARG J 351      14.327   2.608  14.240  1.00  0.00
J    H
ATOM  11719  HD2 ARG J 351      15.304   2.509  15.763  1.00  0.00
J    H
ATOM  11720  NE  ARG J 351      16.477   2.629  13.983  1.00  0.00
J    N
ATOM  11721  HE  ARG J 351      16.340   2.737  12.992  1.00  0.00
J    H
ATOM  11722  CZ  ARG J 351      17.650   2.876  14.585  1.00  0.00
J    C
ATOM  11723  NH1 ARG J 351      18.660   3.377  13.880  1.00  0.00
J    N
ATOM  11724 HH11 ARG J 351      19.539   3.541  14.304  1.00  0.00
J    H
ATOM  11725 HH12 ARG J 351      18.520   3.444  12.889  1.00  0.00
J    H
ATOM  11726  NH2 ARG J 351      17.835   2.640  15.880  1.00  0.00
J    N
ATOM  11727 HH21 ARG J 351      18.680   2.912  16.315  1.00  0.00
J    H
ATOM  11728 HH22 ARG J 351      17.035   2.387  16.408  1.00  0.00
J    H
ATOM  11729  C   ARG J 351      14.169  -2.186  16.980  1.00  0.00
J    C
ATOM  11730  O   ARG J 351      15.139  -2.824  17.370  1.00  0.00
J    O
ATOM  11731  N   GLY J 352      13.053  -2.068  17.726  1.00  0.00
J    N
ATOM  11732  HN  GLY J 352      12.285  -1.494  17.447  1.00  0.00
J    H
ATOM  11733  CA  GLY J 352      12.888  -2.771  18.993  1.00  0.00
J    C
ATOM  11734  HA1 GLY J 352      13.845  -3.210  19.235  1.00  0.00
J    H
ATOM  11735  HA2 GLY J 352      12.091  -3.490  18.857  1.00  0.00
J    H
ATOM  11736  C   GLY J 352      12.498  -1.857  20.118  1.00  0.00
J    C
ATOM  11737  O   GLY J 352      12.267  -2.308  21.240  1.00  0.00
J    O
ATOM  11738  N   LYS J 353      12.403  -0.533  19.855  1.00  0.00
J    N
ATOM  11739  HN  LYS J 353      12.544  -0.184  18.930  1.00  0.00
J    H
ATOM  11740  CA  LYS J 353      12.100   0.479  20.857  1.00  0.00
J    C
ATOM  11741  HA  LYS J 353      12.845   0.372  21.636  1.00  0.00
J    H
ATOM  11742  CB  LYS J 353      12.156   1.938  20.307  1.00  0.00
J    C
ATOM  11743  HB1 LYS J 353      11.335   2.039  19.560  1.00  0.00
J    H
ATOM  11744  HB2 LYS J 353      11.916   2.646  21.133  1.00  0.00
J    H
```

```
ATOM  11745  CG  LYS J 353      13.484   2.334  19.634  1.00  0.00
J    C
ATOM  11746  HG1 LYS J 353      14.332   1.957  20.251  1.00  0.00
J    H
ATOM  11747  HG2 LYS J 353      13.550   1.836  18.640  1.00  0.00
J    H
ATOM  11748  CD  LYS J 353      13.701   3.859  19.437  1.00  0.00
J    C
ATOM  11749  HD1 LYS J 353      14.038   4.295  20.406  1.00  0.00
J    H
ATOM  11750  HD2 LYS J 353      14.528   4.000  18.703  1.00  0.00
J    H
ATOM  11751  CE  LYS J 353      12.476   4.653  18.943  1.00  0.00
J    C
ATOM  11752  HE1 LYS J 353      11.936   4.084  18.154  1.00  0.00
J    H
ATOM  11753  HE2 LYS J 353      11.772   4.847  19.781  1.00  0.00
J    H
ATOM  11754  NZ  LYS J 353      12.864   5.971  18.364  1.00  0.00
J    N
ATOM  11755  HZ1 LYS J 353      12.017   6.480  18.039  1.00  0.00
J    H
ATOM  11756  HZ2 LYS J 353      13.351   6.537  19.087  1.00  0.00
J    H
ATOM  11757  HZ3 LYS J 353      13.505   5.819  17.559  1.00  0.00
J    H
ATOM  11758  C   LYS J 353      10.731   0.278  21.505  1.00  0.00
J    C
ATOM  11759  O   LYS J 353       9.755  -0.063  20.846  1.00  0.00
J    O
ATOM  11760  N   LYS J 354      10.624   0.490  22.835  1.00  0.00
J    N
ATOM  11761  HN  LYS J 354      11.391   0.785  23.402  1.00  0.00
J    H
ATOM  11762  CA  LYS J 354       9.366   0.301  23.546  1.00  0.00
J    C
ATOM  11763  HA  LYS J 354       8.890  -0.563  23.098  1.00  0.00
J    H
ATOM  11764  CB  LYS J 354       9.604   0.068  25.066  1.00  0.00
J    C
ATOM  11765  HB1 LYS J 354       9.933   1.042  25.499  1.00  0.00
J    H
ATOM  11766  HB2 LYS J 354       8.633  -0.178  25.552  1.00  0.00
J    H
ATOM  11767  CG  LYS J 354      10.658  -1.003  25.423  1.00  0.00
J    C
ATOM  11768  HG1 LYS J 354      11.643  -0.700  25.001  1.00  0.00
J    H
ATOM  11769  HG2 LYS J 354      10.769  -1.046  26.530  1.00  0.00
J    H
ATOM  11770  CD  LYS J 354      10.358  -2.428  24.904  1.00  0.00
J    C
ATOM  11771  HD1 LYS J 354       9.992  -3.043  25.757  1.00  0.00
J    H
```

ATOM 11772 HD2 LYS J 354 9.529 -2.359 24.162 1.00 0.00
J H
ATOM 11773 CE LYS J 354 11.602 -3.068 24.261 1.00 0.00
J C
ATOM 11774 HE1 LYS J 354 12.124 -2.335 23.607 1.00 0.00
J H
ATOM 11775 HE2 LYS J 354 12.315 -3.410 25.044 1.00 0.00
J H
ATOM 11776 NZ LYS J 354 11.278 -4.247 23.421 1.00 0.00
J N
ATOM 11777 HZ1 LYS J 354 12.148 -4.641 23.009 1.00 0.00
J H
ATOM 11778 HZ2 LYS J 354 10.812 -4.971 24.004 1.00 0.00
J H
ATOM 11779 HZ3 LYS J 354 10.635 -3.959 22.655 1.00 0.00
J H
ATOM 11780 C LYS J 354 8.410 1.478 23.390 1.00 0.00
J C
ATOM 11781 O LYS J 354 7.200 1.331 23.545 1.00 0.00
J O
ATOM 11782 N GLU J 355 8.944 2.665 23.044 1.00 0.00
J N
ATOM 11783 HN GLU J 355 9.926 2.790 22.939 1.00 0.00
J H
ATOM 11784 CA GLU J 355 8.170 3.859 22.792 1.00 0.00
J C
ATOM 11785 HA GLU J 355 7.136 3.586 22.622 1.00 0.00
J H
ATOM 11786 CB GLU J 355 8.217 4.874 23.974 1.00 0.00
J C
ATOM 11787 HB1 GLU J 355 7.529 5.718 23.735 1.00 0.00
J H
ATOM 11788 HB2 GLU J 355 7.809 4.400 24.895 1.00 0.00
J H
ATOM 11789 CG GLU J 355 9.597 5.502 24.336 1.00 0.00
J C
ATOM 11790 HG1 GLU J 355 10.249 4.728 24.782 1.00 0.00
J H
ATOM 11791 HG2 GLU J 355 10.091 5.921 23.438 1.00 0.00
J H
ATOM 11792 CD GLU J 355 9.481 6.607 25.392 1.00 0.00
J C
ATOM 11793 OE1 GLU J 355 8.373 6.793 25.970 1.00 0.00
J O
ATOM 11794 OE2 GLU J 355 10.476 7.346 25.596 1.00 0.00
J O
ATOM 11795 C GLU J 355 8.679 4.507 21.523 1.00 0.00
J C
ATOM 11796 O GLU J 355 9.785 4.235 21.048 1.00 0.00
J O
ATOM 11797 N THR J 356 7.863 5.385 20.904 1.00 0.00
J N
ATOM 11798 HN THR J 356 6.970 5.647 21.269 1.00 0.00
J H

ATOM 11799 CA THR J 356   8.210   6.030 19.649 1.00 0.00
J   C
ATOM 11800 HA THR J 356   9.087   5.533 19.258 1.00 0.00
J   H
ATOM 11801 CB THR J 356   7.168   5.896 18.515 1.00 0.00
J   C
ATOM 11802 HB THR J 356   7.737   5.991 17.559 1.00 0.00
J   H
ATOM 11803 OG1 THR J 356   6.180   6.906 18.464 1.00 0.00
J   O
ATOM 11804 HG1 THR J 356   5.613   6.672 17.721 1.00 0.00
J   H
ATOM 11805 CG2 THR J 356   6.397   4.577 18.681 1.00 0.00
J   C
ATOM 11806 HG21 THR J 356   5.722   4.403 17.816 1.00 0.00
J   H
ATOM 11807 HG22 THR J 356   7.102   3.721 18.742 1.00 0.00
J   H
ATOM 11808 HG23 THR J 356   5.781   4.587 19.606 1.00 0.00
J   H
ATOM 11809 C   THR J 356   8.629   7.450 19.976 1.00 0.00
J   C
ATOM 11810 O   THR J 356   7.866   8.278 20.455 1.00 0.00
J   O
ATOM 11811 N   VAL J 357   9.921   7.769 19.801 1.00 0.00
J   N
ATOM 11812 HN VAL J 357   10.601   7.128 19.454 1.00 0.00
J   H
ATOM 11813 CA VAL J 357   10.440   9.085 20.105 1.00 0.00
J   C
ATOM 11814 HA VAL J 357   9.614   9.713 20.414 1.00 0.00
J   H
ATOM 11815 CB VAL J 357   11.419   9.071 21.283 1.00 0.00
J   C
ATOM 11816 HB VAL J 357   10.838   8.806 22.200 1.00 0.00
J   H
ATOM 11817 CG1 VAL J 357   12.551   8.035 21.118 1.00 0.00
J   C
ATOM 11818 HG11 VAL J 357   13.253   8.091 21.977 1.00 0.00
J   H
ATOM 11819 HG12 VAL J 357   12.155   6.999 21.082 1.00 0.00
J   H
ATOM 11820 HG13 VAL J 357   13.127   8.228 20.189 1.00 0.00
J   H
ATOM 11821 CG2 VAL J 357   11.992 10.479 21.532 1.00 0.00
J   C
ATOM 11822 HG21 VAL J 357   12.732 10.461 22.361 1.00 0.00
J   H
ATOM 11823 HG22 VAL J 357   12.498 10.862 20.619 1.00 0.00
J   H
ATOM 11824 HG23 VAL J 357   11.192 11.195 21.813 1.00 0.00
J   H
ATOM 11825 C   VAL J 357   11.025   9.688 18.855 1.00 0.00
J   C

ATOM 11826 O   VAL J 357   11.810   9.052 18.140 1.00 0.00
J   O
ATOM 11827 N   ILE J 358   10.602 10.934 18.570 1.00 0.00
J   N
ATOM 11828 HN ILE J 358   9.864 11.335 19.108 1.00 0.00
J   H
ATOM 11829 CA ILE J 358   11.113 11.797 17.534 1.00 0.00
J   C
ATOM 11830 HA ILE J 358   12.047 11.385 17.170 1.00 0.00
J   H
ATOM 11831 CB ILE J 358   10.113 11.922 16.386 1.00 0.00
J   C
ATOM 11832 HB ILE J 358   9.925 10.867 16.063 1.00 0.00
J   H
ATOM 11833 CG2 ILE J 358   8.816 12.613 16.857 1.00 0.00
J   C
ATOM 11834 HG21 ILE J 358   8.038 12.562 16.067 1.00 0.00
J   H
ATOM 11835 HG22 ILE J 358   8.410 12.116 17.763 1.00 0.00
J   H
ATOM 11836 HG23 ILE J 358   9.000 13.681 17.095 1.00 0.00
J   H
ATOM 11837 CG1 ILE J 358   10.684 12.640 15.146 1.00 0.00
J   C
ATOM 11838 HG11 ILE J 358   9.885 12.764 14.381 1.00 0.00
J   H
ATOM 11839 HG12 ILE J 358   11.017 13.661 15.442 1.00 0.00
J   H
ATOM 11840 CD ILE J 358   11.877 11.943 14.486 1.00 0.00
J   C
ATOM 11841 HD1 ILE J 358   12.261 12.553 13.640 1.00 0.00
J   H
ATOM 11842 HD2 ILE J 358   12.702 11.802 15.217 1.00 0.00
J   H
ATOM 11843 HD3 ILE J 358   11.586 10.947 14.088 1.00 0.00
J   H
ATOM 11844 C   ILE J 358   11.451 13.118 18.220 1.00 0.00
J   C
ATOM 11845 O   ILE J 358   10.789 13.506 19.182 1.00 0.00
J   O
ATOM 11846 N   PHE J 359   12.564 13.788 17.833 1.00 0.00
J   N
ATOM 11847 HN PHE J 359   13.117 13.430 17.082 1.00 0.00
J   H
ATOM 11848 CA PHE J 359   13.069 15.037 18.419 1.00 0.00
J   C
ATOM 11849 HA PHE J 359   14.073 15.118 18.023 1.00 0.00
J   H
ATOM 11850 CB PHE J 359   12.284 16.276 17.899 1.00 0.00
J   C
ATOM 11851 HB1 PHE J 359   11.197 16.041 17.891 1.00 0.00
J   H
ATOM 11852 HB2 PHE J 359   12.424 17.149 18.572 1.00 0.00
J   H

ATOM 11853 CG PHE J 359 12.667 16.634 16.495 1.00 0.00
J C
ATOM 11854 CD1 PHE J 359 13.998 16.979 16.204 1.00 0.00
J C
ATOM 11855 HD1 PHE J 359 14.737 16.976 16.993 1.00 0.00
J H
ATOM 11856 CE1 PHE J 359 14.375 17.333 14.904 1.00 0.00
J C
ATOM 11857 HE1 PHE J 359 15.405 17.576 14.687 1.00 0.00
J H
ATOM 11858 CZ PHE J 359 13.414 17.363 13.887 1.00 0.00
J C
ATOM 11859 HZ PHE J 359 13.707 17.611 12.876 1.00 0.00
J H
ATOM 11860 CD2 PHE J 359 11.706 16.695 15.473 1.00 0.00
J C
ATOM 11861 HD2 PHE J 359 10.670 16.478 15.692 1.00 0.00
J H
ATOM 11862 CE2 PHE J 359 12.078 17.064 14.173 1.00 0.00
J C
ATOM 11863 HE2 PHE J 359 11.329 17.117 13.395 1.00 0.00
J H
ATOM 11864 C PHE J 359 13.183 15.088 19.948 1.00 0.00
J C
ATOM 11865 O PHE J 359 13.103 16.151 20.563 1.00 0.00
J O
ATOM 11866 N ARG J 360 13.422 13.920 20.582 1.00 0.00
J N
ATOM 11867 HN ARG J 360 13.560 13.109 20.017 1.00 0.00
J H
ATOM 11868 CA ARG J 360 13.497 13.733 22.024 1.00 0.00
J C
ATOM 11869 HA ARG J 360 13.677 12.678 22.195 1.00 0.00
J H
ATOM 11870 CB ARG J 360 14.720 14.438 22.662 1.00 0.00
J C
ATOM 11871 HB1 ARG J 360 14.535 15.535 22.572 1.00 0.00
J H
ATOM 11872 HB2 ARG J 360 14.748 14.221 23.753 1.00 0.00
J H
ATOM 11873 CG ARG J 360 16.056 14.090 21.969 1.00 0.00
J C
ATOM 11874 HG1 ARG J 360 16.043 13.011 21.699 1.00 0.00
J H
ATOM 11875 HG2 ARG J 360 16.116 14.639 21.002 1.00 0.00
J H
ATOM 11876 CD ARG J 360 17.319 14.444 22.767 1.00 0.00
J C
ATOM 11877 HD1 ARG J 360 17.429 13.734 23.618 1.00 0.00
J H
ATOM 11878 HD2 ARG J 360 18.233 14.385 22.132 1.00 0.00
J H
ATOM 11879 NE ARG J 360 17.170 15.859 23.261 1.00 0.00
J N

ATOM 11880 HE   ARG J 360   16.716 15.919 24.157 1.00 0.00
J   H
ATOM 11881 CZ   ARG J 360   17.577 16.967 22.627 1.00 0.00
J   C
ATOM 11882 NH1 ARG J 360   18.228 16.915 21.472 1.00 0.00
J   N
ATOM 11883 HH11 ARG J 360   18.542 17.744 21.032 1.00 0.00
J   H
ATOM 11884 HH12 ARG J 360   18.496 16.002 21.154 1.00 0.00
J   H
ATOM 11885 NH2 ARG J 360   17.346 18.158 23.180 1.00 0.00
J   N
ATOM 11886 HH21 ARG J 360   17.564 18.985 22.681 1.00 0.00
J   H
ATOM 11887 HH22 ARG J 360   16.770 18.173 23.987 1.00 0.00
J   H
ATOM 11888 C   ARG J 360   12.214 14.085 22.777 1.00 0.00
J   C
ATOM 11889 O   ARG J 360   12.248 14.485 23.939 1.00 0.00
J   O
ATOM 11890 N   ARG J 361   11.046 13.905 22.130 1.00 0.00
J   N
ATOM 11891 HN   ARG J 361   11.063 13.634 21.170 1.00 0.00
J   H
ATOM 11892 CA   ARG J 361   9.744 14.076 22.732 1.00 0.00
J   C
ATOM 11893 HA   ARG J 361   9.831 14.025 23.811 1.00 0.00
J   H
ATOM 11894 CB   ARG J 361   9.108 15.417 22.258 1.00 0.00
J   C
ATOM 11895 HB1 ARG J 361   9.244 15.456 21.151 1.00 0.00
J   H
ATOM 11896 HB2 ARG J 361   8.010 15.389 22.431 1.00 0.00
J   H
ATOM 11897 CG   ARG J 361   9.720 16.701 22.861 1.00 0.00
J   C
ATOM 11898 HG1 ARG J 361   10.746 16.830 22.452 1.00 0.00
J   H
ATOM 11899 HG2 ARG J 361   9.141 17.581 22.500 1.00 0.00
J   H
ATOM 11900 CD   ARG J 361   9.682 16.673 24.391 1.00 0.00
J   C
ATOM 11901 HD1 ARG J 361   8.751 16.165 24.729 1.00 0.00
J   H
ATOM 11902 HD2 ARG J 361   10.560 16.129 24.812 1.00 0.00
J   H
ATOM 11903 NE   ARG J 361   9.723 18.083 24.908 1.00 0.00
J   N
ATOM 11904 HE   ARG J 361   10.644 18.489 24.877 1.00 0.00
J   H
ATOM 11905 CZ   ARG J 361   8.657 18.752 25.369 1.00 0.00
J   C
ATOM 11906 NH1 ARG J 361   8.818 19.988 25.844 1.00 0.00
J   N

ATOM 11907 HH11 ARG J 361   8.049 20.490 26.213 1.00 0.00
J   H
ATOM 11908 HH12 ARG J 361   9.763 20.311 25.929 1.00 0.00
J   H
ATOM 11909 NH2 ARG J 361   7.435 18.232 25.395 1.00 0.00
J   N
ATOM 11910 HH21 ARG J 361   6.665 18.794 25.662 1.00 0.00
J   H
ATOM 11911 HH22 ARG J 361   7.309 17.358 24.943 1.00 0.00
J   H
ATOM 11912 C   ARG J 361   8.865 12.903 22.310 1.00 0.00
J   C
ATOM 11913 O   ARG J 361   8.256 12.915 21.243 1.00 0.00
J   O
ATOM 11914 N   SER J 362   8.816 11.843 23.152 1.00 0.00
J   N
ATOM 11915 HN SER J 362   9.369 11.876 23.983 1.00 0.00
J   H
ATOM 11916 CA SER J 362   8.032 10.611 22.999 1.00 0.00
J   C
ATOM 11917 HA SER J 362   8.509 10.096 22.176 1.00 0.00
J   H
ATOM 11918 CB SER J 362   8.177   9.685 24.250 1.00 0.00
J   C
ATOM 11919 HB1 SER J 362   9.237   9.349 24.310 1.00 0.00
J   H
ATOM 11920 HB2 SER J 362   7.960 10.254 25.181 1.00 0.00
J   H
ATOM 11921 OG SER J 362   7.366   8.506 24.222 1.00 0.00
J   O
ATOM 11922 HG1 SER J 362   7.698   7.918 24.912 1.00 0.00
J   H
ATOM 11923 C   SER J 362   6.567 10.806 22.623 1.00 0.00
J   C
ATOM 11924 O   SER J 362   5.825 11.596 23.208 1.00 0.00
J   O
ATOM 11925 N   ILE J 363   6.116 10.056 21.604 1.00 0.00
J   N
ATOM 11926 HN ILE J 363   6.711   9.401 21.142 1.00 0.00
J   H
ATOM 11927 CA ILE J 363   4.775 10.107 21.080 1.00 0.00
J   C
ATOM 11928 HA ILE J 363   4.288 10.981 21.493 1.00 0.00
J   H
ATOM 11929 CB ILE J 363   4.733 10.091 19.562 1.00 0.00
J   C
ATOM 11930 HB ILE J 363   5.199   9.113 19.279 1.00 0.00
J   H
ATOM 11931 CG2 ILE J 363   3.259 10.260 19.125 1.00 0.00
J   C
ATOM 11932 HG21 ILE J 363   3.154 10.100 18.031 1.00 0.00
J   H
ATOM 11933 HG22 ILE J 363   2.607   9.522 19.637 1.00 0.00
J   H

```
ATOM  11934 HG23 ILE J 363       2.888  11.280  19.361  1.00  0.00
J    H
ATOM  11935  CG1 ILE J 363       5.640  11.205  18.984  1.00  0.00
J    C
ATOM  11936 HG11 ILE J 363       5.338  12.191  19.401  1.00  0.00
J    H
ATOM  11937 HG12 ILE J 363       6.690  11.018  19.307  1.00  0.00
J    H
ATOM  11938  CD  ILE J 363       5.594  11.253  17.455  1.00  0.00
J    C
ATOM  11939  HD1 ILE J 363       6.298  12.022  17.072  1.00  0.00
J    H
ATOM  11940  HD2 ILE J 363       5.883  10.270  17.023  1.00  0.00
J    H
ATOM  11941  HD3 ILE J 363       4.575  11.510  17.095  1.00  0.00
J    H
ATOM  11942  C   ILE J 363       4.038   8.901  21.618  1.00  0.00
J    C
ATOM  11943  O   ILE J 363       4.370   7.745  21.358  1.00  0.00
J    O
ATOM  11944  N   LYS J 364       2.995   9.137  22.426  1.00  0.00
J    N
ATOM  11945  HN  LYS J 364       2.685  10.058  22.659  1.00  0.00
J    H
ATOM  11946  CA  LYS J 364       2.236   8.065  23.023  1.00  0.00
J    C
ATOM  11947  HA  LYS J 364       2.936   7.251  23.170  1.00  0.00
J    H
ATOM  11948  CB  LYS J 364       1.736   8.474  24.424  1.00  0.00
J    C
ATOM  11949  HB1 LYS J 364       1.161   9.421  24.304  1.00  0.00
J    H
ATOM  11950  HB2 LYS J 364       1.010   7.711  24.787  1.00  0.00
J    H
ATOM  11951  CG  LYS J 364       2.960   8.654  25.329  1.00  0.00
J    C
ATOM  11952  HG1 LYS J 364       3.643   7.785  25.194  1.00  0.00
J    H
ATOM  11953  HG2 LYS J 364       3.516   9.566  25.009  1.00  0.00
J    H
ATOM  11954  CD  LYS J 364       2.657   8.766  26.827  1.00  0.00
J    C
ATOM  11955  HD1 LYS J 364       2.321   9.806  27.041  1.00  0.00
J    H
ATOM  11956  HD2 LYS J 364       1.806   8.085  27.061  1.00  0.00
J    H
ATOM  11957  CE  LYS J 364       3.865   8.410  27.709  1.00  0.00
J    C
ATOM  11958  HE1 LYS J 364       3.710   8.774  28.749  1.00  0.00
J    H
ATOM  11959  HE2 LYS J 364       4.015   7.308  27.740  1.00  0.00
J    H
ATOM  11960  NZ  LYS J 364       5.085   9.040  27.161  1.00  0.00
J    N
```

ATOM 11961 HZ1 LYS J 364   5.905   8.803 27.755 1.00 0.00
J   H
ATOM 11962 HZ2 LYS J 364   5.248   8.697 26.193 1.00 0.00
J   H
ATOM 11963 HZ3 LYS J 364   4.961 10.073 27.142 1.00 0.00
J   H
ATOM 11964 C   LYS J 364   1.117   7.546 22.144 1.00 0.00
J   C
ATOM 11965 O   LYS J 364   0.641   8.211 21.225 1.00 0.00
J   O
ATOM 11966 N   TYR J 365   0.667   6.308 22.455 1.00 0.00
J   N
ATOM 11967 HN TYR J 365   1.104   5.844 23.223 1.00 0.00
J   H
ATOM 11968 CA TYR J 365   -0.393   5.542 21.808 1.00 0.00
J   C
ATOM 11969 HA TYR J 365   0.090   5.095 20.948 1.00 0.00
J   H
ATOM 11970 CB TYR J 365   -0.759   4.315 22.722 1.00 0.00
J   C
ATOM 11971 HB1 TYR J 365   -0.105   3.455 22.457 1.00 0.00
J   H
ATOM 11972 HB2 TYR J 365   -0.574   4.551 23.792 1.00 0.00
J   H
ATOM 11973 CG TYR J 365   -2.184   3.810 22.584 1.00 0.00
J   C
ATOM 11974 CD1 TYR J 365   -2.639   3.260 21.373 1.00 0.00
J   C
ATOM 11975 HD1 TYR J 365   -1.952   3.138 20.546 1.00 0.00
J   H
ATOM 11976 CE1 TYR J 365   -3.980   2.885 21.215 1.00 0.00
J   C
ATOM 11977 HE1 TYR J 365   -4.319   2.435 20.294 1.00 0.00
J   H
ATOM 11978 CZ TYR J 365   -4.895   3.097 22.249 1.00 0.00
J   C
ATOM 11979 OH TYR J 365   -6.249   2.774 22.032 1.00 0.00
J   O
ATOM 11980 HH TYR J 365   -6.715   2.913 22.860 1.00 0.00
J   H
ATOM 11981 CD2 TYR J 365   -3.110   3.993 23.627 1.00 0.00
J   C
ATOM 11982 HD2 TYR J 365   -2.791   4.435 24.561 1.00 0.00
J   H
ATOM 11983 CE2 TYR J 365   -4.463   3.656 23.456 1.00 0.00
J   C
ATOM 11984 HE2 TYR J 365   -5.168   3.851 24.250 1.00 0.00
J   H
ATOM 11985 C   TYR J 365   -1.598   6.354 21.284 1.00 0.00
J   C
ATOM 11986 O   TYR J 365   -1.930   6.125 20.120 1.00 0.00
J   O
ATOM 11987 N   PRO J 366   -2.274   7.291 21.981 1.00 0.00
J   N

ATOM 11988 CD PRO J 366   -2.105   7.559 23.412 1.00 0.00
J   C
ATOM 11989 HD1 PRO J 366   -2.263   6.526 23.028 1.00 0.00
J   H
ATOM 11990 HD2 PRO J 366   -1.387   7.552 24.265 1.00 0.00
J   H
ATOM 11991 CA PRO J 366   -3.456   7.976 21.460 1.00 0.00
J   C
ATOM 11992 HA PRO J 366   -4.250   7.252 21.313 1.00 0.00
J   H
ATOM 11993 CB PRO J 366   -3.889   8.914 22.599 1.00 0.00
J   C
ATOM 11994 HB1 PRO J 366   -4.530   8.348 23.311 1.00 0.00
J   H
ATOM 11995 HB2 PRO J 366   -4.449   9.799 22.236 1.00 0.00
J   H
ATOM 11996 CG PRO J 366   -3.439   8.163 23.855 1.00 0.00
J   C
ATOM 11997 HG1 PRO J 366   -4.003   8.459 24.761 1.00 0.00
J   H
ATOM 11998 HG2 PRO J 366   -3.583   7.393 24.476 1.00 0.00
J   H
ATOM 11999 C   PRO J 366   -3.219   8.726 20.160 1.00 0.00
J   C
ATOM 12000 O   PRO J 366   -4.123   8.809 19.328 1.00 0.00
J   O
ATOM 12001 N   ILE J 367   -2.010   9.280 19.966 1.00 0.00
J   N
ATOM 12002 HN ILE J 367   -1.317   9.275 20.684 1.00 0.00
J   H
ATOM 12003 CA ILE J 367   -1.589   9.923 18.736 1.00 0.00
J   C
ATOM 12004 HA ILE J 367   -2.401 10.577 18.446 1.00 0.00
J   H
ATOM 12005 CB ILE J 367   -0.289 10.678 18.972 1.00 0.00
J   C
ATOM 12006 HB ILE J 367   0.442   9.888 19.281 1.00 0.00
J   H
ATOM 12007 CG2 ILE J 367   0.150 11.412 17.688 1.00 0.00
J   C
ATOM 12008 HG21 ILE J 367   1.171 11.832 17.808 1.00 0.00
J   H
ATOM 12009 HG22 ILE J 367   0.172 10.717 16.823 1.00 0.00
J   H
ATOM 12010 HG23 ILE J 367   -0.544 12.245 17.450 1.00 0.00
J   H
ATOM 12011 CG1 ILE J 367   -0.386 11.665 20.167 1.00 0.00
J   C
ATOM 12012 HG11 ILE J 367   -0.499 11.096 21.117 1.00 0.00
J   H
ATOM 12013 HG12 ILE J 367   0.566 12.240 20.239 1.00 0.00
J   H
ATOM 12014 CD ILE J 367   -1.533 12.682 20.075 1.00 0.00
J   C

ATOM 12015 HD1 ILE J 367   -1.497 13.383 20.936 1.00 0.00
J   H
ATOM 12016 HD2 ILE J 367   -1.456 13.276 19.139 1.00 0.00
J   H
ATOM 12017 HD3 ILE J 367   -2.519 12.172 20.088 1.00 0.00
J   H
ATOM 12018 C   ILE J 367   -1.408   8.916 17.605 1.00 0.00
J   C
ATOM 12019 O   ILE J 367   -1.848   9.128 16.474 1.00 0.00
J   O
ATOM 12020 N   ILE J 368   -0.771   7.760 17.910 1.00 0.00
J   N
ATOM 12021 HN ILE J 368   -0.428   7.601 18.834 1.00 0.00
J   H
ATOM 12022 CA ILE J 368   -0.520   6.664 16.981 1.00 0.00
J   C
ATOM 12023 HA ILE J 368   -0.021   7.105 16.126 1.00 0.00
J   H
ATOM 12024 CB ILE J 368   0.334   5.547 17.601 1.00 0.00
J   C
ATOM 12025 HB ILE J 368   -0.337   5.068 18.357 1.00 0.00
J   H
ATOM 12026 CG2 ILE J 368   0.769   4.553 16.495 1.00 0.00
J   C
ATOM 12027 HG21 ILE J 368   1.252   3.658 16.941 1.00 0.00
J   H
ATOM 12028 HG22 ILE J 368   -0.107   4.205 15.908 1.00 0.00
J   H
ATOM 12029 HG23 ILE J 368   1.492   5.026 15.797 1.00 0.00
J   H
ATOM 12030 CG1 ILE J 368   1.560   6.067 18.403 1.00 0.00
J   C
ATOM 12031 HG11 ILE J 368   1.213   6.618 19.304 1.00 0.00
J   H
ATOM 12032 HG12 ILE J 368   2.154   5.195 18.758 1.00 0.00
J   H
ATOM 12033 CD ILE J 368   2.512   6.979 17.625 1.00 0.00
J   C
ATOM 12034 HD1 ILE J 368   3.384   7.254 18.257 1.00 0.00
J   H
ATOM 12035 HD2 ILE J 368   2.890   6.467 16.714 1.00 0.00
J   H
ATOM 12036 HD3 ILE J 368   2.002   7.916 17.317 1.00 0.00
J   H
ATOM 12037 C   ILE J 368   -1.814   6.046 16.466 1.00 0.00
J   C
ATOM 12038 O   ILE J 368   -1.985   5.852 15.264 1.00 0.00
J   O
ATOM 12039 N   ILE J 369   -2.787   5.756 17.362 1.00 0.00
J   N
ATOM 12040 HN ILE J 369   -2.669   5.891 18.344 1.00 0.00
J   H
ATOM 12041 CA ILE J 369   -4.092   5.222 16.982 1.00 0.00
J   C

```
ATOM  12042  HA  ILE J 369      -3.881   4.386  16.326  1.00  0.00
J    H
ATOM  12043  CB  ILE J 369      -4.868   4.617  18.153  1.00  0.00
J    C
ATOM  12044  HB  ILE J 369      -4.139   3.923  18.643  1.00  0.00
J    H
ATOM  12045  CG2 ILE J 369      -5.351   5.690  19.149  1.00  0.00
J    C
ATOM  12046 HG21 ILE J 369      -5.771   5.215  20.060  1.00  0.00
J    H
ATOM  12047 HG22 ILE J 369      -4.511   6.342  19.466  1.00  0.00
J    H
ATOM  12048 HG23 ILE J 369      -6.139   6.327  18.695  1.00  0.00
J    H
ATOM  12049  CG1 ILE J 369      -6.055   3.731  17.709  1.00  0.00
J    C
ATOM  12050 HG11 ILE J 369      -6.650   3.425  18.599  1.00  0.00
J    H
ATOM  12051 HG12 ILE J 369      -6.728   4.329  17.054  1.00  0.00
J    H
ATOM  12052  CD  ILE J 369      -5.647   2.472  16.937  1.00  0.00
J    C
ATOM  12053  HD1 ILE J 369      -6.549   1.915  16.601  1.00  0.00
J    H
ATOM  12054  HD2 ILE J 369      -5.049   2.740  16.039  1.00  0.00
J    H
ATOM  12055  HD3 ILE J 369      -5.041   1.793  17.574  1.00  0.00
J    H
ATOM  12056  C   ILE J 369      -4.935   6.203  16.186  1.00  0.00
J    C
ATOM  12057  O   ILE J 369      -5.574   5.834  15.203  1.00  0.00
J    O
ATOM  12058  N   LYS J 370      -4.909   7.508  16.542  1.00  0.00
J    N
ATOM  12059  HN  LYS J 370      -4.440   7.836  17.360  1.00  0.00
J    H
ATOM  12060  CA  LYS J 370      -5.564   8.535  15.756  1.00  0.00
J    C
ATOM  12061  HA  LYS J 370      -6.601   8.237  15.657  1.00  0.00
J    H
ATOM  12062  CB  LYS J 370      -5.478   9.917  16.442  1.00  0.00
J    C
ATOM  12063  HB1 LYS J 370      -5.912   9.804  17.462  1.00  0.00
J    H
ATOM  12064  HB2 LYS J 370      -4.406  10.183  16.587  1.00  0.00
J    H
ATOM  12065  CG  LYS J 370      -6.239  10.998  15.658  1.00  0.00
J    C
ATOM  12066  HG1 LYS J 370      -5.725  11.174  14.686  1.00  0.00
J    H
ATOM  12067  HG2 LYS J 370      -7.263  10.626  15.429  1.00  0.00
J    H
ATOM  12068  CD  LYS J 370      -6.331  12.333  16.404  1.00  0.00
J    C
```

ATOM 12069 HD1 LYS J 370   -6.716 12.137 17.430 1.00 0.00
J   H
ATOM 12070 HD2 LYS J 370   -5.300 12.744 16.507 1.00 0.00
J   H
ATOM 12071 CE LYS J 370   -7.230 13.326 15.666 1.00 0.00
J   C
ATOM 12072 HE1 LYS J 370   -6.770 13.623 14.697 1.00 0.00
J   H
ATOM 12073 HE2 LYS J 370   -8.225 12.873 15.459 1.00 0.00
J   H
ATOM 12074 NZ LYS J 370   -7.421 14.537 16.491 1.00 0.00
J   N
ATOM 12075 HZ1 LYS J 370   -8.032 15.215 15.992 1.00 0.00
J   H
ATOM 12076 HZ2 LYS J 370   -7.863 14.275 17.395 1.00 0.00
J   H
ATOM 12077 HZ3 LYS J 370   -6.498 14.977 16.677 1.00 0.00
J   H
ATOM 12078 C   LYS J 370   -4.986   8.639 14.356 1.00 0.00
J   C
ATOM 12079 O   LYS J 370   -5.720   8.690 13.369 1.00 0.00
J   O
ATOM 12080 N   ALA J 371   -3.645   8.605 14.223 1.00 0.00
J   N
ATOM 12081 HN ALA J 371   -3.046   8.595 15.024 1.00 0.00
J   H
ATOM 12082 CA ALA J 371   -2.969   8.580 12.944 1.00 0.00
J   C
ATOM 12083 HA ALA J 371   -3.262   9.444 12.358 1.00 0.00
J   H
ATOM 12084 CB ALA J 371   -1.444   8.676 13.160 1.00 0.00
J   C
ATOM 12085 HB1 ALA J 371   -1.205   9.601 13.726 1.00 0.00
J   H
ATOM 12086 HB2 ALA J 371   -1.068   7.810 13.748 1.00 0.00
J   H
ATOM 12087 HB3 ALA J 371   -0.894   8.713 12.194 1.00 0.00
J   H
ATOM 12088 C   ALA J 371   -3.326   7.348 12.107 1.00 0.00
J   C
ATOM 12089 O   ALA J 371   -3.564   7.447 10.903 1.00 0.00
J   O
ATOM 12090 N   LEU J 372   -3.419   6.160 12.754 1.00 0.00
J   N
ATOM 12091 HN LEU J 372   -3.174   6.095 13.719 1.00 0.00
J   H
ATOM 12092 CA LEU J 372   -3.862   4.904 12.164 1.00 0.00
J   C
ATOM 12093 HA LEU J 372   -3.190   4.698 11.341 1.00 0.00
J   H
ATOM 12094 CB LEU J 372   -3.814   3.793 13.254 1.00 0.00
J   C
ATOM 12095 HB1 LEU J 372   -3.014   4.075 13.976 1.00 0.00
J   H

ATOM 12096 HB2 LEU J 372 -4.767 3.808 13.829 1.00 0.00
J H
ATOM 12097 CG LEU J 372 -3.528 2.357 12.763 1.00 0.00
J C
ATOM 12098 HG LEU J 372 -2.550 2.432 12.230 1.00 0.00
J H
ATOM 12099 CD1 LEU J 372 -3.420 1.401 13.962 1.00 0.00
J C
ATOM 12100 HD11 LEU J 372 -3.262 0.357 13.616 1.00 0.00
J H
ATOM 12101 HD12 LEU J 372 -2.567 1.686 14.613 1.00 0.00
J H
ATOM 12102 HD13 LEU J 372 -4.350 1.428 14.570 1.00 0.00
J H
ATOM 12103 CD2 LEU J 372 -4.559 1.791 11.783 1.00 0.00
J C
ATOM 12104 HD21 LEU J 372 -4.269 0.766 11.468 1.00 0.00
J H
ATOM 12105 HD22 LEU J 372 -5.567 1.730 12.240 1.00 0.00
J H
ATOM 12106 HD23 LEU J 372 -4.624 2.429 10.876 1.00 0.00
J H
ATOM 12107 C LEU J 372 -5.274 5.017 11.590 1.00 0.00
J C
ATOM 12108 O LEU J 372 -5.528 4.684 10.433 1.00 0.00
J O
ATOM 12109 N ALA J 373 -6.203 5.585 12.386 1.00 0.00
J N
ATOM 12110 HN ALA J 373 -5.949 5.850 13.316 1.00 0.00
J H
ATOM 12111 CA ALA J 373 -7.574 5.857 12.016 1.00 0.00
J C
ATOM 12112 HA ALA J 373 -8.043 4.936 11.688 1.00 0.00
J H
ATOM 12113 CB ALA J 373 -8.354 6.331 13.260 1.00 0.00
J C
ATOM 12114 HB1 ALA J 373 -8.269 5.570 14.065 1.00 0.00
J H
ATOM 12115 HB2 ALA J 373 -7.941 7.287 13.650 1.00 0.00
J H
ATOM 12116 HB3 ALA J 373 -9.433 6.476 13.036 1.00 0.00
J H
ATOM 12117 C ALA J 373 -7.726 6.852 10.864 1.00 0.00
J C
ATOM 12118 O ALA J 373 -8.548 6.644 9.970 1.00 0.00
J O
ATOM 12119 N VAL J 374 -6.924 7.945 10.832 1.00 0.00
J N
ATOM 12120 HN VAL J 374 -6.313 8.183 11.582 1.00 0.00
J H
ATOM 12121 CA VAL J 374 -6.882 8.873 9.700 1.00 0.00
J C
ATOM 12122 HA VAL J 374 -7.896 9.211 9.529 1.00 0.00
J H

ATOM 12123 CB VAL J 374   -5.981 10.086   9.965 1.00 0.00
J   C
ATOM 12124 HB VAL J 374   -4.973   9.700 10.254 1.00 0.00
J   H
ATOM 12125 CG1 VAL J 374   -5.816 10.990   8.718 1.00 0.00
J   C
ATOM 12126 HG11 VAL J 374   -5.190 11.874   8.964 1.00 0.00
J   H
ATOM 12127 HG12 VAL J 374   -5.316 10.452   7.885 1.00 0.00
J   H
ATOM 12128 HG13 VAL J 374   -6.804 11.355   8.365 1.00 0.00
J   H
ATOM 12129 CG2 VAL J 374   -6.621 10.922 11.080 1.00 0.00
J   C
ATOM 12130 HG21 VAL J 374   -6.021 11.836 11.279 1.00 0.00
J   H
ATOM 12131 HG22 VAL J 374   -7.648 11.234 10.795 1.00 0.00
J   H
ATOM 12132 HG23 VAL J 374   -6.681 10.347 12.028 1.00 0.00
J   H
ATOM 12133 C   VAL J 374   -6.408   8.201   8.431 1.00 0.00
J   C
ATOM 12134 O   VAL J 374   -7.081   8.248   7.404 1.00 0.00
J   O
ATOM 12135 N   SER J 375   -5.258   7.502   8.475 1.00 0.00
J   N
ATOM 12136 HN SER J 375   -4.726   7.366   9.311 1.00 0.00
J   H
ATOM 12137 CA SER J 375   -4.683   6.874   7.293 1.00 0.00
J   C
ATOM 12138 HA SER J 375   -4.652   7.663   6.552 1.00 0.00
J   H
ATOM 12139 CB SER J 375   -3.263   6.322   7.533 1.00 0.00
J   C
ATOM 12140 HB1 SER J 375   -3.303   5.630   8.405 1.00 0.00
J   H
ATOM 12141 HB2 SER J 375   -2.926   5.724   6.656 1.00 0.00
J   H
ATOM 12142 OG SER J 375   -2.373   7.396   7.820 1.00 0.00
J   O
ATOM 12143 HG1 SER J 375   -1.565   7.008   8.179 1.00 0.00
J   H
ATOM 12144 C   SER J 375   -5.513   5.749   6.717 1.00 0.00
J   C
ATOM 12145 O   SER J 375   -5.718   5.693   5.508 1.00 0.00
J   O
ATOM 12146 N   VAL J 376   -6.042   4.838   7.566 1.00 0.00
J   N
ATOM 12147 HN VAL J 376   -5.872   4.863   8.547 1.00 0.00
J   H
ATOM 12148 CA VAL J 376   -6.903   3.741   7.131 1.00 0.00
J   C
ATOM 12149 HA VAL J 376   -6.370   3.218   6.347 1.00 0.00
J   H

ATOM 12150 CB VAL J 376 -7.173 2.755 8.269 1.00 0.00
J C
ATOM 12151 HB VAL J 376 -7.403 3.352 9.186 1.00 0.00
J H
ATOM 12152 CG1 VAL J 376 -8.377 1.815 8.002 1.00 0.00
J C
ATOM 12153 HG11 VAL J 376 -8.499 1.098 8.843 1.00 0.00
J H
ATOM 12154 HG12 VAL J 376 -9.326 2.382 7.910 1.00 0.00
J H
ATOM 12155 HG13 VAL J 376 -8.219 1.230 7.072 1.00 0.00
J H
ATOM 12156 CG2 VAL J 376 -5.894 1.917 8.450 1.00 0.00
J C
ATOM 12157 HG21 VAL J 376 -6.037 1.149 9.240 1.00 0.00
J H
ATOM 12158 HG22 VAL J 376 -5.629 1.400 7.503 1.00 0.00
J H
ATOM 12159 HG23 VAL J 376 -5.037 2.553 8.752 1.00 0.00
J H
ATOM 12160 C VAL J 376 -8.211 4.226 6.543 1.00 0.00
J C
ATOM 12161 O VAL J 376 -8.590 3.815 5.446 1.00 0.00
J O
ATOM 12162 N THR J 377 -8.918 5.160 7.222 1.00 0.00
J N
ATOM 12163 HN THR J 377 -8.623 5.562 8.089 1.00 0.00
J H
ATOM 12164 CA THR J 377 -10.202 5.665 6.731 1.00 0.00
J C
ATOM 12165 HA THR J 377 -10.799 4.792 6.506 1.00 0.00
J H
ATOM 12166 CB THR J 377 -10.991 6.539 7.708 1.00 0.00
J C
ATOM 12167 HB THR J 377 -10.438 7.507 7.775 1.00 0.00
J H
ATOM 12168 OG1 THR J 377 -11.018 5.986 9.017 1.00 0.00
J O
ATOM 12169 HG1 THR J 377 -11.542 6.599 9.544 1.00 0.00
J H
ATOM 12170 CG2 THR J 377 -12.458 6.622 7.245 1.00 0.00
J C
ATOM 12171 HG21 THR J 377 -13.029 7.340 7.871 1.00 0.00
J H
ATOM 12172 HG22 THR J 377 -12.512 6.968 6.191 1.00 0.00
J H
ATOM 12173 HG23 THR J 377 -12.956 5.631 7.312 1.00 0.00
J H
ATOM 12174 C THR J 377 -10.030 6.421 5.421 1.00 0.00
J C
ATOM 12175 O THR J 377 -10.769 6.209 4.464 1.00 0.00
J O
ATOM 12176 N SER J 378 -8.979 7.269 5.313 1.00 0.00
J N

ATOM 12177 HN SER J 378   -8.387   7.471   6.093 1.00 0.00
J   H
ATOM 12178 CA SER J 378   -8.605   7.964   4.081 1.00 0.00
J   C
ATOM 12179 HA SER J 378   -9.486   8.528   3.807 1.00 0.00
J   H
ATOM 12180 CB SER J 378   -7.337   8.831   4.289 1.00 0.00
J   C
ATOM 12181 HB1 SER J 378   -6.583   8.216   4.833 1.00 0.00
J   H
ATOM 12182 HB2 SER J 378   -6.889   9.105   3.308 1.00 0.00
J   H
ATOM 12183 OG SER J 378   -7.659   9.982   5.067 1.00 0.00
J   O
ATOM 12184 HG1 SER J 378   -6.824 10.368   5.360 1.00 0.00
J   H
ATOM 12185 C   SER J 378   -8.278   7.050   2.924 1.00 0.00
J   C
ATOM 12186 O   SER J 378   -8.765   7.233   1.811 1.00 0.00
J   O
ATOM 12187 N   LEU J 379   -7.460   6.010   3.162 1.00 0.00
J   N
ATOM 12188 HN LEU J 379   -7.055   5.832   4.057 1.00 0.00
J   H
ATOM 12189 CA LEU J 379   -7.091   5.051   2.140 1.00 0.00
J   C
ATOM 12190 HA LEU J 379   -6.728   5.638   1.306 1.00 0.00
J   H
ATOM 12191 CB LEU J 379   -5.952   4.146   2.653 1.00 0.00
J   C
ATOM 12192 HB1 LEU J 379   -5.287   4.780   3.283 1.00 0.00
J   H
ATOM 12193 HB2 LEU J 379   -6.380   3.377   3.335 1.00 0.00
J   H
ATOM 12194 CG LEU J 379   -5.111   3.486   1.544 1.00 0.00
J   C
ATOM 12195 HG LEU J 379   -5.814   2.906   0.899 1.00 0.00
J   H
ATOM 12196 CD1 LEU J 379   -4.345   4.521   0.697 1.00 0.00
J   C
ATOM 12197 HD11 LEU J 379   -3.712   4.013 -0.062 1.00 0.00
J   H
ATOM 12198 HD12 LEU J 379   -5.053   5.194   0.167 1.00 0.00
J   H
ATOM 12199 HD13 LEU J 379   -3.684   5.142   1.339 1.00 0.00
J   H
ATOM 12200 CD2 LEU J 379   -4.120   2.501   2.170 1.00 0.00
J   C
ATOM 12201 HD21 LEU J 379   -3.518   2.003   1.379 1.00 0.00
J   H
ATOM 12202 HD22 LEU J 379   -3.415   3.009   2.858 1.00 0.00
J   H
ATOM 12203 HD23 LEU J 379   -4.661   1.716   2.742 1.00 0.00
J   H

ATOM 12204 C   LEU J 379   -8.264   4.218   1.640 1.00 0.00
J   C
ATOM 12205 O   LEU J 379   -8.402   3.954   0.446 1.00 0.00
J   O
ATOM 12206 N   PHE J 380   -9.173   3.814   2.554 1.00 0.00
J   N
ATOM 12207 HN PHE J 380   -9.047   3.991   3.529 1.00 0.00
J   H
ATOM 12208 CA PHE J 380   -10.392   3.099   2.225 1.00 0.00
J   C
ATOM 12209 HA PHE J 380   -10.075   2.243   1.642 1.00 0.00
J   H
ATOM 12210 CB PHE J 380   -11.088   2.618   3.530 1.00 0.00
J   C
ATOM 12211 HB1 PHE J 380   -10.311   2.317   4.267 1.00 0.00
J   H
ATOM 12212 HB2 PHE J 380   -11.679   3.437   3.994 1.00 0.00
J   H
ATOM 12213 CG PHE J 380   -11.954   1.414   3.271 1.00 0.00
J   C
ATOM 12214 CD1 PHE J 380   -11.387   0.234   2.754 1.00 0.00
J   C
ATOM 12215 HD1 PHE J 380   -10.329   0.200   2.536 1.00 0.00
J   H
ATOM 12216 CE1 PHE J 380   -12.180 -0.895   2.512 1.00 0.00
J   C
ATOM 12217 HE1 PHE J 380   -11.733 -1.793   2.109 1.00 0.00
J   H
ATOM 12218 CZ PHE J 380   -13.549 -0.859   2.799 1.00 0.00
J   C
ATOM 12219 HZ PHE J 380   -14.160 -1.732   2.625 1.00 0.00
J   H
ATOM 12220 CD2 PHE J 380   -13.330   1.438   3.552 1.00 0.00
J   C
ATOM 12221 HD2 PHE J 380   -13.788   2.342   3.930 1.00 0.00
J   H
ATOM 12222 CE2 PHE J 380   -14.124   0.307   3.322 1.00 0.00
J   C
ATOM 12223 HE2 PHE J 380   -15.181   0.335   3.547 1.00 0.00
J   H
ATOM 12224 C   PHE J 380   -11.345   3.912   1.347 1.00 0.00
J   C
ATOM 12225 O   PHE J 380   -11.875   3.411   0.359 1.00 0.00
J   O
ATOM 12226 N   ILE J 381   -11.536   5.216   1.644 1.00 0.00
J   N
ATOM 12227 HN ILE J 381   -11.142   5.630   2.462 1.00 0.00
J   H
ATOM 12228 CA ILE J 381   -12.319   6.140   0.822 1.00 0.00
J   C
ATOM 12229 HA ILE J 381   -13.285   5.670   0.688 1.00 0.00
J   H
ATOM 12230 CB ILE J 381   -12.499   7.485   1.517 1.00 0.00
J   C

ATOM 12231 HB ILE J 381   -11.465   7.777   1.832 1.00 0.00
J   H
ATOM 12232 CG2 ILE J 381   -13.191   8.523   0.595 1.00 0.00
J   C
ATOM 12233 HG21 ILE J 381   -13.172   9.532   1.058 1.00 0.00
J   H
ATOM 12234 HG22 ILE J 381   -12.671   8.593 -0.384 1.00 0.00
J   H
ATOM 12235 HG23 ILE J 381   -14.250   8.246   0.410 1.00 0.00
J   H
ATOM 12236 CG1 ILE J 381   -13.332   7.286   2.803 1.00 0.00
J   C
ATOM 12237 HG11 ILE J 381   -14.414   7.236   2.546 1.00 0.00
J   H
ATOM 12238 HG12 ILE J 381   -13.058   6.309   3.262 1.00 0.00
J   H
ATOM 12239 CD ILE J 381   -13.081   8.404   3.810 1.00 0.00
J   C
ATOM 12240 HD1 ILE J 381   -13.645   8.210   4.748 1.00 0.00
J   H
ATOM 12241 HD2 ILE J 381   -12.001   8.471   4.061 1.00 0.00
J   H
ATOM 12242 HD3 ILE J 381   -13.411   9.385   3.407 1.00 0.00
J   H
ATOM 12243 C   ILE J 381   -11.721   6.323 -0.568 1.00 0.00
J   C
ATOM 12244 O   ILE J 381   -12.433   6.295 -1.572 1.00 0.00
J   O
ATOM 12245 N   VAL J 382   -10.380   6.457 -0.672 1.00 0.00
J   N
ATOM 12246 HN VAL J 382   -9.790   6.554   0.126 1.00 0.00
J   H
ATOM 12247 CA VAL J 382   -9.672   6.470 -1.950 1.00 0.00
J   C
ATOM 12248 HA VAL J 382   -10.098   7.274 -2.536 1.00 0.00
J   H
ATOM 12249 CB VAL J 382   -8.182   6.763 -1.761 1.00 0.00
J   C
ATOM 12250 HB VAL J 382   -7.797   6.062 -0.981 1.00 0.00
J   H
ATOM 12251 CG1 VAL J 382   -7.373   6.542 -3.057 1.00 0.00
J   C
ATOM 12252 HG11 VAL J 382   -6.305   6.801 -2.893 1.00 0.00
J   H
ATOM 12253 HG12 VAL J 382   -7.410   5.483 -3.387 1.00 0.00
J   H
ATOM 12254 HG13 VAL J 382   -7.760   7.184 -3.876 1.00 0.00
J   H
ATOM 12255 CG2 VAL J 382   -8.025   8.231 -1.318 1.00 0.00
J   C
ATOM 12256 HG21 VAL J 382   -6.952   8.495 -1.208 1.00 0.00
J   H
ATOM 12257 HG22 VAL J 382   -8.479   8.915 -2.067 1.00 0.00
J   H

```
ATOM  12258 HG23 VAL J 382      -8.517   8.410  -0.339  1.00  0.00
J    H
ATOM  12259  C   VAL J 382      -9.872   5.176  -2.736  1.00  0.00
J    C
ATOM  12260  O   VAL J 382     -10.200   5.205  -3.921  1.00  0.00
J    O
ATOM  12261  N   PHE J 383      -9.757   3.997  -2.086  1.00  0.00
J    N
ATOM  12262  HN  PHE J 383      -9.474   3.953  -1.129  1.00  0.00
J    H
ATOM  12263  CA  PHE J 383     -10.024   2.703  -2.695  1.00  0.00
J    C
ATOM  12264  HA  PHE J 383      -9.375   2.646  -3.559  1.00  0.00
J    H
ATOM  12265  CB  PHE J 383      -9.678   1.579  -1.666  1.00  0.00
J    C
ATOM  12266  HB1 PHE J 383      -8.582   1.580  -1.482  1.00  0.00
J    H
ATOM  12267  HB2 PHE J 383     -10.175   1.763  -0.690  1.00  0.00
J    H
ATOM  12268  CG  PHE J 383     -10.032   0.201  -2.175  1.00  0.00
J    C
ATOM  12269  CD1 PHE J 383      -9.198  -0.472  -3.082  1.00  0.00
J    C
ATOM  12270  HD1 PHE J 383      -8.247  -0.038  -3.358  1.00  0.00
J    H
ATOM  12271  CE1 PHE J 383      -9.593  -1.699  -3.633  1.00  0.00
J    C
ATOM  12272  HE1 PHE J 383      -8.940  -2.218  -4.320  1.00  0.00
J    H
ATOM  12273  CZ  PHE J 383     -10.830  -2.256  -3.289  1.00  0.00
J    C
ATOM  12274  HZ  PHE J 383     -11.133  -3.203  -3.712  1.00  0.00
J    H
ATOM  12275  CD2 PHE J 383     -11.268  -0.374  -1.827  1.00  0.00
J    C
ATOM  12276  HD2 PHE J 383     -11.925   0.134  -1.136  1.00  0.00
J    H
ATOM  12277  CE2 PHE J 383     -11.672  -1.591  -2.389  1.00  0.00
J    C
ATOM  12278  HE2 PHE J 383     -12.632  -2.012  -2.127  1.00  0.00
J    H
ATOM  12279  C   PHE J 383     -11.468   2.583  -3.206  1.00  0.00
J    C
ATOM  12280  O   PHE J 383     -11.697   2.201  -4.354  1.00  0.00
J    O
ATOM  12281  N   LEU J 384     -12.471   2.975  -2.391  1.00  0.00
J    N
ATOM  12282  HN  LEU J 384     -12.301   3.278  -1.455  1.00  0.00
J    H
ATOM  12283  CA  LEU J 384     -13.873   2.996  -2.781  1.00  0.00
J    C
ATOM  12284  HA  LEU J 384     -14.109   1.989  -3.101  1.00  0.00
J    H
```

ATOM 12285 CB LEU J 384 -14.802 3.375 -1.598 1.00 0.00
J C
ATOM 12286 HB1 LEU J 384 -14.389 4.304 -1.139 1.00 0.00
J H
ATOM 12287 HB2 LEU J 384 -15.804 3.649 -1.997 1.00 0.00
J H
ATOM 12288 CG LEU J 384 -14.930 2.293 -0.501 1.00 0.00
J C
ATOM 12289 HG LEU J 384 -13.895 2.090 -0.132 1.00 0.00
J H
ATOM 12290 CD1 LEU J 384 -15.804 2.822 0.647 1.00 0.00
J C
ATOM 12291 HD11 LEU J 384 -15.946 2.039 1.423 1.00 0.00
J H
ATOM 12292 HD12 LEU J 384 -15.328 3.706 1.126 1.00 0.00
J H
ATOM 12293 HD13 LEU J 384 -16.805 3.123 0.272 1.00 0.00
J H
ATOM 12294 CD2 LEU J 384 -15.513 0.971 -1.034 1.00 0.00
J C
ATOM 12295 HD21 LEU J 384 -15.570 0.218 -0.219 1.00 0.00
J H
ATOM 12296 HD22 LEU J 384 -16.537 1.108 -1.437 1.00 0.00
J H
ATOM 12297 HD23 LEU J 384 -14.871 0.560 -1.843 1.00 0.00
J H
ATOM 12298 C LEU J 384 -14.146 3.927 -3.952 1.00 0.00
J C
ATOM 12299 O LEU J 384 -14.900 3.592 -4.862 1.00 0.00
J O
ATOM 12300 N GLY J 385 -13.498 5.109 -3.984 1.00 0.00
J N
ATOM 12301 HN GLY J 385 -12.905 5.402 -3.235 1.00 0.00
J H
ATOM 12302 CA GLY J 385 -13.611 6.040 -5.098 1.00 0.00
J C
ATOM 12303 HA1 GLY J 385 -13.084 6.935 -4.795 1.00 0.00
J H
ATOM 12304 HA2 GLY J 385 -14.669 6.184 -5.268 1.00 0.00
J H
ATOM 12305 C GLY J 385 -12.997 5.551 -6.386 1.00 0.00
J C
ATOM 12306 O GLY J 385 -13.579 5.712 -7.453 1.00 0.00
J O
ATOM 12307 N ILE J 386 -11.819 4.890 -6.326 1.00 0.00
J N
ATOM 12308 HN ILE J 386 -11.290 4.813 -5.485 1.00 0.00
J H
ATOM 12309 CA ILE J 386 -11.210 4.228 -7.481 1.00 0.00
J C
ATOM 12310 HA ILE J 386 -11.174 4.971 -8.267 1.00 0.00
J H
ATOM 12311 CB ILE J 386 -9.778 3.756 -7.185 1.00 0.00
J C

ATOM 12312 HB ILE J 386   -9.862   3.196 -6.219 1.00 0.00
J   H
ATOM 12313 CG2 ILE J 386   -9.211   2.911 -8.354 1.00 0.00
J   C
ATOM 12314 HG21 ILE J 386   -8.244   2.448 -8.067 1.00 0.00
J   H
ATOM 12315 HG22 ILE J 386   -9.910   2.092 -8.626 1.00 0.00
J   H
ATOM 12316 HG23 ILE J 386   -9.042   3.540 -9.253 1.00 0.00
J   H
ATOM 12317 CG1 ILE J 386   -8.874   4.985 -6.925 1.00 0.00
J   C
ATOM 12318 HG11 ILE J 386   -8.743   5.563 -7.868 1.00 0.00
J   H
ATOM 12319 HG12 ILE J 386   -9.380   5.657 -6.195 1.00 0.00
J   H
ATOM 12320 CD ILE J 386   -7.489   4.640 -6.364 1.00 0.00
J   C
ATOM 12321 HD1 ILE J 386   -6.918   5.569 -6.149 1.00 0.00
J   H
ATOM 12322 HD2 ILE J 386   -7.582   4.061 -5.420 1.00 0.00
J   H
ATOM 12323 HD3 ILE J 386   -6.903   4.039 -7.090 1.00 0.00
J   H
ATOM 12324 C   ILE J 386   -12.069   3.080 -7.998 1.00 0.00
J   C
ATOM 12325 O   ILE J 386   -12.310   2.975 -9.198 1.00 0.00
J   O
ATOM 12326 N   PHE J 387   -12.603   2.224 -7.096 1.00 0.00
J   N
ATOM 12327 HN PHE J 387   -12.390   2.301 -6.123 1.00 0.00
J   H
ATOM 12328 CA PHE J 387   -13.512   1.138 -7.430 1.00 0.00
J   C
ATOM 12329 HA PHE J 387   -12.996   0.551 -8.181 1.00 0.00
J   H
ATOM 12330 CB PHE J 387   -13.790   0.315 -6.134 1.00 0.00
J   C
ATOM 12331 HB1 PHE J 387   -12.834 -0.117 -5.764 1.00 0.00
J   H
ATOM 12332 HB2 PHE J 387   -14.191   0.965 -5.326 1.00 0.00
J   H
ATOM 12333 CG PHE J 387   -14.728 -0.853 -6.354 1.00 0.00
J   C
ATOM 12334 CD1 PHE J 387   -14.284 -2.040 -6.961 1.00 0.00
J   C
ATOM 12335 HD1 PHE J 387   -13.248 -2.135 -7.257 1.00 0.00
J   H
ATOM 12336 CE1 PHE J 387   -15.175 -3.098 -7.189 1.00 0.00
J   C
ATOM 12337 HE1 PHE J 387   -14.823 -4.013 -7.646 1.00 0.00
J   H
ATOM 12338 CZ PHE J 387   -16.518 -2.980 -6.824 1.00 0.00
J   C

```
ATOM  12339  HZ  PHE J 387     -17.201  -3.801  -6.990  1.00  0.00
J    H
ATOM  12340  CD2 PHE J 387     -16.084  -0.744  -5.997  1.00  0.00
J    C
ATOM  12341  HD2 PHE J 387     -16.454   0.171  -5.555  1.00  0.00
J    H
ATOM  12342  CE2 PHE J 387     -16.976  -1.799  -6.231  1.00  0.00
J    C
ATOM  12343  HE2 PHE J 387     -18.016  -1.697  -5.954  1.00  0.00
J    H
ATOM  12344  C   PHE J 387     -14.819   1.622  -8.060  1.00  0.00
J    C
ATOM  12345  O   PHE J 387     -15.264   1.096  -9.074  1.00  0.00
J    O
ATOM  12346  N   ALA J 388     -15.469   2.659  -7.499  1.00  0.00
J    N
ATOM  12347  HN  ALA J 388     -15.141   3.108  -6.667  1.00  0.00
J    H
ATOM  12348  CA  ALA J 388     -16.691   3.202  -8.058  1.00  0.00
J    C
ATOM  12349  HA  ALA J 388     -17.408   2.401  -8.193  1.00  0.00
J    H
ATOM  12350  CB  ALA J 388     -17.345   4.152  -7.034  1.00  0.00
J    C
ATOM  12351  HB1 ALA J 388     -17.498   3.616  -6.074  1.00  0.00
J    H
ATOM  12352  HB2 ALA J 388     -16.697   5.033  -6.835  1.00  0.00
J    H
ATOM  12353  HB3 ALA J 388     -18.336   4.513  -7.387  1.00  0.00
J    H
ATOM  12354  C   ALA J 388     -16.511   3.859  -9.435  1.00  0.00
J    C
ATOM  12355  O   ALA J 388     -17.343   3.703 -10.327  1.00  0.00
J    O
ATOM  12356  N   LEU J 389     -15.398   4.588  -9.659  1.00  0.00
J    N
ATOM  12357  HN  LEU J 389     -14.734   4.779  -8.939  1.00  0.00
J    H
ATOM  12358  CA  LEU J 389     -15.058   5.162 -10.956  1.00  0.00
J    C
ATOM  12359  HA  LEU J 389     -15.944   5.703 -11.263  1.00  0.00
J    H
ATOM  12360  CB  LEU J 389     -13.929   6.208 -10.836  1.00  0.00
J    C
ATOM  12361  HB1 LEU J 389     -13.120   5.749 -10.222  1.00  0.00
J    H
ATOM  12362  HB2 LEU J 389     -13.489   6.382 -11.844  1.00  0.00
J    H
ATOM  12363  CG  LEU J 389     -14.374   7.535 -10.186  1.00  0.00
J    C
ATOM  12364  HG  LEU J 389     -14.835   7.277  -9.202  1.00  0.00
J    H
ATOM  12365  CD1 LEU J 389     -13.156   8.438  -9.984  1.00  0.00
J    C
```

ATOM 12366 HD11 LEU J 389   -13.462   9.418 -9.561 1.00 0.00
J   H
ATOM 12367 HD12 LEU J 389   -12.432   7.968 -9.285 1.00 0.00
J   H
ATOM 12368 HD13 LEU J 389   -12.640   8.624 -10.950 1.00 0.00
J   H
ATOM 12369 CD2 LEU J 389   -15.423   8.290 -11.018 1.00 0.00
J   C
ATOM 12370 HD21 LEU J 389   -15.724   9.227 -10.501 1.00 0.00
J   H
ATOM 12371 HD22 LEU J 389   -15.031   8.568 -12.017 1.00 0.00
J   H
ATOM 12372 HD23 LEU J 389   -16.330   7.663 -11.161 1.00 0.00
J   H
ATOM 12373 C   LEU J 389   -14.736   4.146 -12.049 1.00 0.00
J   C
ATOM 12374 O   LEU J 389   -15.102   4.344 -13.203 1.00 0.00
J   O
ATOM 12375 N   THR J 390   -14.097   2.994 -11.748 1.00 0.00
J   N
ATOM 12376 HN THR J 390   -13.802   2.753 -10.825 1.00 0.00
J   H
ATOM 12377 CA THR J 390   -13.775   1.985 -12.769 1.00 0.00
J   C
ATOM 12378 HA THR J 390   -13.406   2.553 -13.613 1.00 0.00
J   H
ATOM 12379 CB THR J 390   -12.729   0.956 -12.357 1.00 0.00
J   C
ATOM 12380 HB THR J 390   -12.820   0.113 -13.084 1.00 0.00
J   H
ATOM 12381 OG1 THR J 390   -12.973   0.412 -11.070 1.00 0.00
J   O
ATOM 12382 HG1 THR J 390   -12.253 -0.209 -10.914 1.00 0.00
J   H
ATOM 12383 CG2 THR J 390   -11.348   1.610 -12.304 1.00 0.00
J   C
ATOM 12384 HG21 THR J 390   -10.558   0.852 -12.117 1.00 0.00
J   H
ATOM 12385 HG22 THR J 390   -11.119   2.110 -13.269 1.00 0.00
J   H
ATOM 12386 HG23 THR J 390   -11.297   2.373 -11.497 1.00 0.00
J   H
ATOM 12387 C   THR J 390   -14.983   1.211 -13.266 1.00 0.00
J   C
ATOM 12388 O   THR J 390   -14.897   0.445 -14.226 1.00 0.00
J   O
ATOM 12389 N   ILE J 391   -16.149   1.376 -12.615 1.00 0.00
J   N
ATOM 12390 HN ILE J 391   -16.214   1.889 -11.761 1.00 0.00
J   H
ATOM 12391 CA ILE J 391   -17.414   0.832 -13.082 1.00 0.00
J   C
ATOM 12392 HA ILE J 391   -17.170   0.003 -13.734 1.00 0.00
J   H

```
ATOM  12393  CB  ILE J 391     -18.265   0.395 -11.886  1.00  0.00
J    C
ATOM  12394  HB  ILE J 391     -18.398   1.328 -11.281  1.00  0.00
J    H
ATOM  12395  CG2 ILE J 391     -19.599  -0.229 -12.364  1.00  0.00
J    C
ATOM  12396 HG21 ILE J 391     -20.280  -0.402 -11.504  1.00  0.00
J    H
ATOM  12397 HG22 ILE J 391     -20.121   0.446 -13.075  1.00  0.00
J    H
ATOM  12398 HG23 ILE J 391     -19.425  -1.202 -12.869  1.00  0.00
J    H
ATOM  12399  CG1 ILE J 391     -17.455  -0.605 -11.018  1.00  0.00
J    C
ATOM  12400 HG11 ILE J 391     -17.187  -1.498 -11.625  1.00  0.00
J    H
ATOM  12401 HG12 ILE J 391     -16.501  -0.120 -10.706  1.00  0.00
J    H
ATOM  12402  CD  ILE J 391     -18.176  -1.075  -9.751  1.00  0.00
J    C
ATOM  12403  HD1 ILE J 391     -17.514  -1.740  -9.154  1.00  0.00
J    H
ATOM  12404  HD2 ILE J 391     -18.459  -0.208  -9.116  1.00  0.00
J    H
ATOM  12405  HD3 ILE J 391     -19.096  -1.643 -10.003  1.00  0.00
J    H
ATOM  12406  C   ILE J 391     -18.169   1.853 -13.930  1.00  0.00
J    C
ATOM  12407  O   ILE J 391     -18.892   1.498 -14.860  1.00  0.00
J    O
ATOM  12408  N   THR J 392     -17.997   3.158 -13.647  1.00  0.00
J    N
ATOM  12409  HN  THR J 392     -17.304   3.477 -13.000  1.00  0.00
J    H
ATOM  12410  CA  THR J 392     -18.795   4.225 -14.248  1.00  0.00
J    C
ATOM  12411  HA  THR J 392     -19.685   3.740 -14.626  1.00  0.00
J    H
ATOM  12412  CB  THR J 392     -19.081   5.301 -13.204  1.00  0.00
J    C
ATOM  12413  HB  THR J 392     -18.088   5.606 -12.793  1.00  0.00
J    H
ATOM  12414  OG1 THR J 392     -19.843   4.768 -12.130  1.00  0.00
J    O
ATOM  12415  HG1 THR J 392     -19.988   5.506 -11.527  1.00  0.00
J    H
ATOM  12416  CG2 THR J 392     -19.923   6.447 -13.755  1.00  0.00
J    C
ATOM  12417 HG21 THR J 392     -20.017   7.263 -13.008  1.00  0.00
J    H
ATOM  12418 HG22 THR J 392     -19.450   6.871 -14.667  1.00  0.00
J    H
ATOM  12419 HG23 THR J 392     -20.945   6.100 -14.022  1.00  0.00
J    H
```

```
ATOM  12420  C   THR J 392      -18.105   4.863 -15.449  1.00  0.00
J    C
ATOM  12421  O   THR J 392      -18.754   5.289 -16.409  1.00  0.00
J    O
ATOM  12422  N   GLU J 393      -16.760   4.895 -15.475  1.00  0.00
J    N
ATOM  12423  HN  GLU J 393      -16.204   4.453 -14.776  1.00  0.00
J    H
ATOM  12424  CA  GLU J 393      -15.990   5.563 -16.504  1.00  0.00
J    C
ATOM  12425  HA  GLU J 393      -16.680   6.100 -17.142  1.00  0.00
J    H
ATOM  12426  CB  GLU J 393      -14.949   6.537 -15.890  1.00  0.00
J    C
ATOM  12427  HB1 GLU J 393      -14.669   6.154 -14.882  1.00  0.00
J    H
ATOM  12428  HB2 GLU J 393      -14.015   6.520 -16.494  1.00  0.00
J    H
ATOM  12429  CG  GLU J 393      -15.441   8.002 -15.741  1.00  0.00
J    C
ATOM  12430  HG1 GLU J 393      -14.651   8.609 -15.263  1.00  0.00
J    H
ATOM  12431  HG2 GLU J 393      -15.690   8.440 -16.728  1.00  0.00
J    H
ATOM  12432  CD  GLU J 393      -16.663   8.215 -14.847  1.00  0.00
J    C
ATOM  12433  OE1 GLU J 393      -17.553   9.015 -15.249  1.00  0.00
J    O
ATOM  12434  OE2 GLU J 393      -16.727   7.639 -13.738  1.00  0.00
J    O
ATOM  12435  C   GLU J 393      -15.250   4.570 -17.378  1.00  0.00
J    C
ATOM  12436  O   GLU J 393      -14.659   3.592 -16.931  1.00  0.00
J    O
ATOM  12437  N   GLN J 394      -15.224   4.845 -18.695  1.00  0.00
J    N
ATOM  12438  HN  GLN J 394      -15.730   5.613 -19.084  1.00  0.00
J    H
ATOM  12439  CA  GLN J 394      -14.474   4.070 -19.662  1.00  0.00
J    C
ATOM  12440  HA  GLN J 394      -14.276   3.095 -19.233  1.00  0.00
J    H
ATOM  12441  CB  GLN J 394      -15.249   3.966 -21.003  1.00  0.00
J    C
ATOM  12442  HB1 GLN J 394      -15.189   4.968 -21.487  1.00  0.00
J    H
ATOM  12443  HB2 GLN J 394      -14.718   3.260 -21.681  1.00  0.00
J    H
ATOM  12444  CG  GLN J 394      -16.732   3.561 -20.839  1.00  0.00
J    C
ATOM  12445  HG1 GLN J 394      -16.810   2.534 -20.423  1.00  0.00
J    H
ATOM  12446  HG2 GLN J 394      -17.253   4.251 -20.143  1.00  0.00
J    H
```

ATOM 12447 CD GLN J 394   -17.430   3.563 -22.199 1.00 0.00
J   C
ATOM 12448 OE1 GLN J 394   -17.133   2.765 -23.084 1.00 0.00
J   O
ATOM 12449 NE2 GLN J 394   -18.387   4.502 -22.390 1.00 0.00
J   N
ATOM 12450 HE21 GLN J 394   -18.846   4.502 -23.274 1.00 0.00
J   H
ATOM 12451 HE22 GLN J 394   -18.589   5.157 -21.670 1.00 0.00
J   H
ATOM 12452 C   GLN J 394   -13.132   4.753 -19.890 1.00 0.00
J   C
ATOM 12453 O   GLN J 394   -12.786   5.136 -21.005 1.00 0.00
J   O
ATOM 12454 N   ALA J 395   -12.359   4.977 -18.812 1.00 0.00
J   N
ATOM 12455 HN ALA J 395   -12.572   4.565 -17.927 1.00 0.00
J   H
ATOM 12456 CA ALA J 395   -11.183   5.814 -18.840 1.00 0.00
J   C
ATOM 12457 HA ALA J 395   -10.998   6.200 -19.836 1.00 0.00
J   H
ATOM 12458 CB ALA J 395   -11.341   6.962 -17.823 1.00 0.00
J   C
ATOM 12459 HB1 ALA J 395   -12.200   7.602 -18.113 1.00 0.00
J   H
ATOM 12460 HB2 ALA J 395   -11.538   6.567 -16.802 1.00 0.00
J   H
ATOM 12461 HB3 ALA J 395   -10.433   7.602 -17.785 1.00 0.00
J   H
ATOM 12462 C   ALA J 395   -9.960   4.988 -18.464 1.00 0.00
J   C
ATOM 12463 O   ALA J 395   -10.111   4.053 -17.685 1.00 0.00
J   O
ATOM 12464 N   PRO J 396   -8.749   5.249 -18.964 1.00 0.00
J   N
ATOM 12465 CD PRO J 396   -8.477   6.234 -20.012 1.00 0.00
J   C
ATOM 12466 HD1 PRO J 396   -8.654   5.884 -21.054 1.00 0.00
J   H
ATOM 12467 HD2 PRO J 396   -9.114   7.119 -19.784 1.00 0.00
J   H
ATOM 12468 CA PRO J 396   -7.524   4.599 -18.495 1.00 0.00
J   C
ATOM 12469 HA PRO J 396   -7.533   3.569 -18.830 1.00 0.00
J   H
ATOM 12470 CB PRO J 396   -6.384   5.298 -19.267 1.00 0.00
J   C
ATOM 12471 HB1 PRO J 396   -6.261   4.807 -20.257 1.00 0.00
J   H
ATOM 12472 HB2 PRO J 396   -5.416   5.265 -18.726 1.00 0.00
J   H
ATOM 12473 CG PRO J 396   -7.006   6.587 -19.822 1.00 0.00
J   C

```
ATOM  12474  HG1 PRO J 396      -6.467   6.980 -20.707  1.00  0.00
J    H
ATOM  12475  HG2 PRO J 396      -7.018   7.363 -19.021  1.00  0.00
J    H
ATOM  12476  C   PRO J 396      -7.299   4.631 -16.989  1.00  0.00
J    C
ATOM  12477  O   PRO J 396      -7.361   5.702 -16.384  1.00  0.00
J    O
ATOM  12478  N   PHE J 397      -6.969   3.463 -16.387  1.00  0.00
J    N
ATOM  12479  HN  PHE J 397      -6.773   2.656 -16.942  1.00  0.00
J    H
ATOM  12480  CA  PHE J 397      -6.864   3.247 -14.956  1.00  0.00
J    C
ATOM  12481  HA  PHE J 397      -7.878   3.352 -14.589  1.00  0.00
J    H
ATOM  12482  CB  PHE J 397      -6.327   1.804 -14.705  1.00  0.00
J    C
ATOM  12483  HB1 PHE J 397      -6.967   1.077 -15.249  1.00  0.00
J    H
ATOM  12484  HB2 PHE J 397      -5.294   1.688 -15.096  1.00  0.00
J    H
ATOM  12485  CG  PHE J 397      -6.381   1.440 -13.242  1.00  0.00
J    C
ATOM  12486  CD1 PHE J 397      -7.616   1.425 -12.579  1.00  0.00
J    C
ATOM  12487  HD1 PHE J 397      -8.523   1.624 -13.133  1.00  0.00
J    H
ATOM  12488  CE1 PHE J 397      -7.687   1.164 -11.207  1.00  0.00
J    C
ATOM  12489  HE1 PHE J 397      -8.644   1.171 -10.705  1.00  0.00
J    H
ATOM  12490  CZ  PHE J 397      -6.521   0.884 -10.485  1.00  0.00
J    C
ATOM  12491  HZ  PHE J 397      -6.576   0.660  -9.429  1.00  0.00
J    H
ATOM  12492  CD2 PHE J 397      -5.214   1.163 -12.508  1.00  0.00
J    C
ATOM  12493  HD2 PHE J 397      -4.250   1.185 -12.998  1.00  0.00
J    H
ATOM  12494  CE2 PHE J 397      -5.281   0.886 -11.135  1.00  0.00
J    C
ATOM  12495  HE2 PHE J 397      -4.374   0.677 -10.584  1.00  0.00
J    H
ATOM  12496  C   PHE J 397      -6.010   4.273 -14.212  1.00  0.00
J    C
ATOM  12497  O   PHE J 397      -6.417   4.801 -13.180  1.00  0.00
J    O
ATOM  12498  N   LEU J 398      -4.822   4.644 -14.740  1.00  0.00
J    N
ATOM  12499  HN  LEU J 398      -4.473   4.279 -15.601  1.00  0.00
J    H
ATOM  12500  CA  LEU J 398      -3.944   5.612 -14.099  1.00  0.00
J    C
```

```
ATOM  12501  HA  LEU J 398      -3.722   5.202 -13.122  1.00  0.00
J    H
ATOM  12502  CB  LEU J 398      -2.617   5.766 -14.882  1.00  0.00
J    C
ATOM  12503  HB1 LEU J 398      -2.190   4.745 -15.009  1.00  0.00
J    H
ATOM  12504  HB2 LEU J 398      -2.843   6.135 -15.907  1.00  0.00
J    H
ATOM  12505  CG  LEU J 398      -1.553   6.665 -14.210  1.00  0.00
J    C
ATOM  12506  HG  LEU J 398      -1.920   7.714 -14.321  1.00  0.00
J    H
ATOM  12507  CD1 LEU J 398      -1.300   6.333 -12.728  1.00  0.00
J    C
ATOM  12508 HD11 LEU J 398      -0.495   6.978 -12.317  1.00  0.00
J    H
ATOM  12509 HD12 LEU J 398      -2.220   6.498 -12.128  1.00  0.00
J    H
ATOM  12510 HD13 LEU J 398      -0.989   5.273 -12.611  1.00  0.00
J    H
ATOM  12511  CD2 LEU J 398      -0.230   6.544 -14.972  1.00  0.00
J    C
ATOM  12512 HD21 LEU J 398       0.541   7.199 -14.512  1.00  0.00
J    H
ATOM  12513 HD22 LEU J 398       0.157   5.506 -14.957  1.00  0.00
J    H
ATOM  12514 HD23 LEU J 398      -0.362   6.853 -16.032  1.00  0.00
J    H
ATOM  12515  C   LEU J 398      -4.591   6.977 -13.890  1.00  0.00
J    C
ATOM  12516  O   LEU J 398      -4.449   7.600 -12.840  1.00  0.00
J    O
ATOM  12517  N   GLN J 399      -5.387   7.447 -14.872  1.00  0.00
J    N
ATOM  12518  HN  GLN J 399      -5.495   6.974 -15.745  1.00  0.00
J    H
ATOM  12519  CA  GLN J 399      -6.154   8.670 -14.755  1.00  0.00
J    C
ATOM  12520  HA  GLN J 399      -5.452   9.438 -14.453  1.00  0.00
J    H
ATOM  12521  CB  GLN J 399      -6.729   9.099 -16.121  1.00  0.00
J    C
ATOM  12522  HB1 GLN J 399      -7.508   8.347 -16.394  1.00  0.00
J    H
ATOM  12523  HB2 GLN J 399      -7.262  10.070 -16.009  1.00  0.00
J    H
ATOM  12524  CG  GLN J 399      -5.619   9.156 -17.199  1.00  0.00
J    C
ATOM  12525  HG1 GLN J 399      -4.698   9.618 -16.782  1.00  0.00
J    H
ATOM  12526  HG2 GLN J 399      -5.359   8.136 -17.550  1.00  0.00
J    H
ATOM  12527  CD  GLN J 399      -6.040  10.007 -18.396  1.00  0.00
J    C
```

```
ATOM  12528  OE1 GLN J 399      -7.200  10.380 -18.547  1.00  0.00
J    O
ATOM  12529  NE2 GLN J 399      -5.063  10.340 -19.271  1.00  0.00
J    N
ATOM  12530 HE21 GLN J 399      -5.330  10.899 -20.051  1.00  0.00
J    H
ATOM  12531 HE22 GLN J 399      -4.136  10.014 -19.124  1.00  0.00
J    H
ATOM  12532  C   GLN J 399      -7.236   8.593 -13.687  1.00  0.00
J    C
ATOM  12533  O   GLN J 399      -7.381   9.512 -12.887  1.00  0.00
J    O
ATOM  12534  N   ILE J 400      -7.970   7.461 -13.593  1.00  0.00
J    N
ATOM  12535  HN  ILE J 400      -7.892   6.720 -14.257  1.00  0.00
J    H
ATOM  12536  CA  ILE J 400      -8.939   7.209 -12.526  1.00  0.00
J    C
ATOM  12537  HA  ILE J 400      -9.632   8.040 -12.553  1.00  0.00
J    H
ATOM  12538  CB  ILE J 400      -9.709   5.903 -12.760  1.00  0.00
J    C
ATOM  12539  HB  ILE J 400      -8.922   5.159 -13.040  1.00  0.00
J    H
ATOM  12540  CG2 ILE J 400     -10.505   5.500 -11.491  1.00  0.00
J    C
ATOM  12541 HG21 ILE J 400     -10.934   4.483 -11.605  1.00  0.00
J    H
ATOM  12542 HG22 ILE J 400      -9.846   5.489 -10.598  1.00  0.00
J    H
ATOM  12543 HG23 ILE J 400     -11.338   6.209 -11.303  1.00  0.00
J    H
ATOM  12544  CG1 ILE J 400     -10.638   6.081 -13.989  1.00  0.00
J    C
ATOM  12545 HG11 ILE J 400     -11.369   6.896 -13.793  1.00  0.00
J    H
ATOM  12546 HG12 ILE J 400     -10.023   6.394 -14.864  1.00  0.00
J    H
ATOM  12547  CD  ILE J 400     -11.406   4.814 -14.382  1.00  0.00
J    C
ATOM  12548  HD1 ILE J 400     -12.006   4.995 -15.301  1.00  0.00
J    H
ATOM  12549  HD2 ILE J 400     -10.704   3.976 -14.585  1.00  0.00
J    H
ATOM  12550  HD3 ILE J 400     -12.102   4.503 -13.575  1.00  0.00
J    H
ATOM  12551  C   ILE J 400      -8.290   7.218 -11.144  1.00  0.00
J    C
ATOM  12552  O   ILE J 400      -8.786   7.857 -10.216  1.00  0.00
J    O
ATOM  12553  N   VAL J 401      -7.122   6.559 -10.980  1.00  0.00
J    N
ATOM  12554  HN  VAL J 401      -6.706   6.013 -11.703  1.00  0.00
J    H
```

```
ATOM  12555  CA  VAL J 401      -6.364   6.586  -9.733  1.00  0.00
J    C
ATOM  12556  HA  VAL J 401      -7.026   6.238  -8.950  1.00  0.00
J    H
ATOM  12557  CB  VAL J 401      -5.151   5.653  -9.800  1.00  0.00
J    C
ATOM  12558  HB  VAL J 401      -4.509   5.994 -10.648  1.00  0.00
J    H
ATOM  12559  CG1 VAL J 401      -4.322   5.704  -8.499  1.00  0.00
J    C
ATOM  12560 HG11 VAL J 401      -3.471   4.991  -8.555  1.00  0.00
J    H
ATOM  12561 HG12 VAL J 401      -3.896   6.714  -8.324  1.00  0.00
J    H
ATOM  12562 HG13 VAL J 401      -4.947   5.425  -7.624  1.00  0.00
J    H
ATOM  12563  CG2 VAL J 401      -5.636   4.205 -10.004  1.00  0.00
J    C
ATOM  12564 HG21 VAL J 401      -4.777   3.500 -10.016  1.00  0.00
J    H
ATOM  12565 HG22 VAL J 401      -6.324   3.904  -9.184  1.00  0.00
J    H
ATOM  12566 HG23 VAL J 401      -6.174   4.094 -10.969  1.00  0.00
J    H
ATOM  12567  C   VAL J 401      -5.928   8.003  -9.369  1.00  0.00
J    C
ATOM  12568  O   VAL J 401      -6.156   8.473  -8.254  1.00  0.00
J    O
ATOM  12569  N   PHE J 402      -5.357   8.750 -10.335  1.00  0.00
J    N
ATOM  12570  HN  PHE J 402      -5.225   8.393 -11.258  1.00  0.00
J    H
ATOM  12571  CA  PHE J 402      -4.889  10.109 -10.144  1.00  0.00
J    C
ATOM  12572  HA  PHE J 402      -4.200  10.052  -9.311  1.00  0.00
J    H
ATOM  12573  CB  PHE J 402      -4.163  10.556 -11.433  1.00  0.00
J    C
ATOM  12574  HB1 PHE J 402      -3.512   9.728 -11.790  1.00  0.00
J    H
ATOM  12575  HB2 PHE J 402      -4.888  10.777 -12.245  1.00  0.00
J    H
ATOM  12576  CG  PHE J 402      -3.274  11.740 -11.193  1.00  0.00
J    C
ATOM  12577  CD1 PHE J 402      -1.963  11.556 -10.723  1.00  0.00
J    C
ATOM  12578  HD1 PHE J 402      -1.625  10.565 -10.456  1.00  0.00
J    H
ATOM  12579  CE1 PHE J 402      -1.090  12.644 -10.590  1.00  0.00
J    C
ATOM  12580  HE1 PHE J 402      -0.075  12.484 -10.254  1.00  0.00
J    H
ATOM  12581  CZ  PHE J 402      -1.533  13.932 -10.900  1.00  0.00
J    C
```

```
ATOM  12582  HZ  PHE J 402      -0.868  14.779 -10.805  1.00  0.00
J    H
ATOM  12583  CD2 PHE J 402      -3.720  13.039 -11.470  1.00  0.00
J    C
ATOM  12584  HD2 PHE J 402      -4.748  13.207 -11.758  1.00  0.00
J    H
ATOM  12585  CE2 PHE J 402      -2.844  14.121 -11.347  1.00  0.00
J    C
ATOM  12586  HE2 PHE J 402      -3.181  15.117 -11.598  1.00  0.00
J    H
ATOM  12587  C   PHE J 402      -5.980  11.094  -9.755  1.00  0.00
J    C
ATOM  12588  O   PHE J 402      -5.832  11.828  -8.781  1.00  0.00
J    O
ATOM  12589  N   GLU J 403      -7.135  11.079 -10.449  1.00  0.00
J    N
ATOM  12590  HN  GLU J 403      -7.282  10.478 -11.228  1.00  0.00
J    H
ATOM  12591  CA  GLU J 403      -8.271  11.930 -10.135  1.00  0.00
J    C
ATOM  12592  HA  GLU J 403      -7.921  12.955 -10.149  1.00  0.00
J    H
ATOM  12593  CB  GLU J 403      -9.393  11.764 -11.176  1.00  0.00
J    C
ATOM  12594  HB1 GLU J 403      -9.636  10.680 -11.254  1.00  0.00
J    H
ATOM  12595  HB2 GLU J 403     -10.320  12.260 -10.813  1.00  0.00
J    H
ATOM  12596  CG  GLU J 403      -9.023  12.300 -12.582  1.00  0.00
J    C
ATOM  12597  HG1 GLU J 403      -7.990  11.996 -12.833  1.00  0.00
J    H
ATOM  12598  HG2 GLU J 403      -9.716  11.907 -13.352  1.00  0.00
J    H
ATOM  12599  CD  GLU J 403      -8.988  13.805 -12.817  1.00  0.00
J    C
ATOM  12600  OE1 GLU J 403      -8.019  14.261 -13.480  1.00  0.00
J    O
ATOM  12601  OE2 GLU J 403      -9.976  14.503 -12.474  1.00  0.00
J    O
ATOM  12602  C   GLU J 403      -8.840  11.667  -8.753  1.00  0.00
J    C
ATOM  12603  O   GLU J 403      -9.126  12.599  -8.009  1.00  0.00
J    O
ATOM  12604  N   THR J 404      -8.976  10.388  -8.335  1.00  0.00
J    N
ATOM  12605  HN  THR J 404      -8.784   9.597  -8.914  1.00  0.00
J    H
ATOM  12606  CA  THR J 404      -9.423  10.049  -6.979  1.00  0.00
J    C
ATOM  12607  HA  THR J 404     -10.348  10.593  -6.843  1.00  0.00
J    H
ATOM  12608  CB  THR J 404      -9.647   8.563  -6.760  1.00  0.00
J    C
```

ATOM 12609 HB THR J 404 -8.699 8.058 -7.069 1.00 0.00
J H
ATOM 12610 OG1 THR J 404 -10.683 8.125 -7.616 1.00 0.00
J O
ATOM 12611 HG1 THR J 404 -10.790 7.186 -7.429 1.00 0.00
J H
ATOM 12612 CG2 THR J 404 -10.159 8.273 -5.341 1.00 0.00
J C
ATOM 12613 HG21 THR J 404 -10.211 7.179 -5.156 1.00 0.00
J H
ATOM 12614 HG22 THR J 404 -9.477 8.716 -4.584 1.00 0.00
J H
ATOM 12615 HG23 THR J 404 -11.174 8.700 -5.187 1.00 0.00
J H
ATOM 12616 C THR J 404 -8.475 10.535 -5.901 1.00 0.00
J C
ATOM 12617 O THR J 404 -8.901 11.142 -4.917 1.00 0.00
J O
ATOM 12618 N PHE J 405 -7.148 10.334 -6.074 1.00 0.00
J N
ATOM 12619 HN PHE J 405 -6.781 9.809 -6.841 1.00 0.00
J H
ATOM 12620 CA PHE J 405 -6.137 10.862 -5.165 1.00 0.00
J C
ATOM 12621 HA PHE J 405 -6.427 10.526 -4.176 1.00 0.00
J H
ATOM 12622 CB PHE J 405 -4.718 10.310 -5.493 1.00 0.00
J C
ATOM 12623 HB1 PHE J 405 -4.654 10.095 -6.582 1.00 0.00
J H
ATOM 12624 HB2 PHE J 405 -3.934 11.062 -5.261 1.00 0.00
J H
ATOM 12625 CG PHE J 405 -4.444 9.015 -4.764 1.00 0.00
J C
ATOM 12626 CD1 PHE J 405 -3.976 9.041 -3.438 1.00 0.00
J C
ATOM 12627 HD1 PHE J 405 -3.871 9.988 -2.927 1.00 0.00
J H
ATOM 12628 CE1 PHE J 405 -3.641 7.851 -2.775 1.00 0.00
J C
ATOM 12629 HE1 PHE J 405 -3.291 7.881 -1.753 1.00 0.00
J H
ATOM 12630 CZ PHE J 405 -3.764 6.621 -3.431 1.00 0.00
J C
ATOM 12631 HZ PHE J 405 -3.498 5.704 -2.924 1.00 0.00
J H
ATOM 12632 CD2 PHE J 405 -4.589 7.770 -5.396 1.00 0.00
J C
ATOM 12633 HD2 PHE J 405 -4.956 7.720 -6.412 1.00 0.00
J H
ATOM 12634 CE2 PHE J 405 -4.242 6.580 -4.745 1.00 0.00
J C
ATOM 12635 HE2 PHE J 405 -4.344 5.634 -5.260 1.00 0.00
J H

ATOM 12636 C PHE J 405 -6.127 12.394 -5.133 1.00 0.00
J C
ATOM 12637 O PHE J 405 -6.103 12.995 -4.062 1.00 0.00
J O
ATOM 12638 N SER J 406 -6.220 13.067 -6.303 1.00 0.00
J N
ATOM 12639 HN SER J 406 -6.201 12.591 -7.183 1.00 0.00
J H
ATOM 12640 CA SER J 406 -6.356 14.521 -6.413 1.00 0.00
J C
ATOM 12641 HA SER J 406 -5.518 14.917 -5.854 1.00 0.00
J H
ATOM 12642 CB SER J 406 -6.396 15.038 -7.876 1.00 0.00
J C
ATOM 12643 HB1 SER J 406 -6.924 14.278 -8.497 1.00 0.00
J H
ATOM 12644 HB2 SER J 406 -6.986 15.979 -7.938 1.00 0.00
J H
ATOM 12645 OG SER J 406 -5.084 15.216 -8.384 1.00 0.00
J O
ATOM 12646 HG1 SER J 406 -5.161 15.317 -9.341 1.00 0.00
J H
ATOM 12647 C SER J 406 -7.614 15.074 -5.788 1.00 0.00
J C
ATOM 12648 O SER J 406 -7.575 16.109 -5.127 1.00 0.00
J O
ATOM 12649 N ALA J 407 -8.776 14.428 -5.996 1.00 0.00
J N
ATOM 12650 HN ALA J 407 -8.814 13.636 -6.606 1.00 0.00
J H
ATOM 12651 CA ALA J 407 -10.031 14.800 -5.384 1.00 0.00
J C
ATOM 12652 HA ALA J 407 -10.231 15.848 -5.578 1.00 0.00
J H
ATOM 12653 CB ALA J 407 -11.167 13.963 -6.010 1.00 0.00
J C
ATOM 12654 HB1 ALA J 407 -11.188 14.125 -7.109 1.00 0.00
J H
ATOM 12655 HB2 ALA J 407 -11.009 12.877 -5.829 1.00 0.00
J H
ATOM 12656 HB3 ALA J 407 -12.160 14.249 -5.602 1.00 0.00
J H
ATOM 12657 C ALA J 407 -10.040 14.634 -3.867 1.00 0.00
J C
ATOM 12658 O ALA J 407 -10.418 15.548 -3.143 1.00 0.00
J O
ATOM 12659 N PHE J 408 -9.575 13.476 -3.348 1.00 0.00
J N
ATOM 12660 HN PHE J 408 -9.240 12.739 -3.933 1.00 0.00
J H
ATOM 12661 CA PHE J 408 -9.513 13.190 -1.923 1.00 0.00
J C
ATOM 12662 HA PHE J 408 -10.485 13.458 -1.528 1.00 0.00
J H

```
ATOM  12663  CB  PHE J 408      -9.241  11.676  -1.701  1.00  0.00
J    C
ATOM  12664  HB1 PHE J 408      -9.996  11.085  -2.261  1.00  0.00
J    H
ATOM  12665  HB2 PHE J 408      -8.242  11.387  -2.092  1.00  0.00
J    H
ATOM  12666  CG  PHE J 408      -9.378  11.348  -0.241  1.00  0.00
J    C
ATOM  12667  CD1 PHE J 408     -10.644  11.208   0.352  1.00  0.00
J    C
ATOM  12668  HD1 PHE J 408     -11.530  11.230  -0.266  1.00  0.00
J    H
ATOM  12669  CE1 PHE J 408     -10.762  11.045   1.739  1.00  0.00
J    C
ATOM  12670  HE1 PHE J 408     -11.736  10.906   2.186  1.00  0.00
J    H
ATOM  12671  CZ  PHE J 408      -9.616  11.056   2.540  1.00  0.00
J    C
ATOM  12672  HZ  PHE J 408      -9.706  10.937   3.611  1.00  0.00
J    H
ATOM  12673  CD2 PHE J 408      -8.234  11.336   0.570  1.00  0.00
J    C
ATOM  12674  HD2 PHE J 408      -7.255  11.446   0.126  1.00  0.00
J    H
ATOM  12675  CE2 PHE J 408      -8.352  11.210   1.957  1.00  0.00
J    C
ATOM  12676  HE2 PHE J 408      -7.468  11.233   2.577  1.00  0.00
J    H
ATOM  12677  C   PHE J 408      -8.483  14.054  -1.203  1.00  0.00
J    C
ATOM  12678  O   PHE J 408      -8.733  14.611  -0.136  1.00  0.00
J    O
ATOM  12679  N   GLY J 409      -7.291  14.246  -1.799  1.00  0.00
J    N
ATOM  12680  HN  GLY J 409      -7.064  13.841  -2.683  1.00  0.00
J    H
ATOM  12681  CA  GLY J 409      -6.247  15.062  -1.194  1.00  0.00
J    C
ATOM  12682  HA1 GLY J 409      -5.329  14.835  -1.719  1.00  0.00
J    H
ATOM  12683  HA2 GLY J 409      -6.226  14.821  -0.140  1.00  0.00
J    H
ATOM  12684  C   GLY J 409      -6.506  16.538  -1.327  1.00  0.00
J    C
ATOM  12685  O   GLY J 409      -5.746  17.354  -0.823  1.00  0.00
J    O
ATOM  12686  N   THR J 410      -7.597  16.896  -2.035  1.00  0.00
J    N
ATOM  12687  HN  THR J 410      -8.206  16.159  -2.326  1.00  0.00
J    H
ATOM  12688  CA  THR J 410      -8.027  18.227  -2.445  1.00  0.00
J    C
ATOM  12689  HA  THR J 410      -8.799  18.053  -3.182  1.00  0.00
J    H
```

ATOM 12690 CB THR J 410 -8.986 19.011 -1.531 1.00 0.00
J C
ATOM 12691 HB THR J 410 -9.660 19.588 -2.211 1.00 0.00
J H
ATOM 12692 OG1 THR J 410 -8.367 19.985 -0.692 1.00 0.00
J O
ATOM 12693 HG1 THR J 410 -9.084 20.376 -0.180 1.00 0.00
J H
ATOM 12694 CG2 THR J 410 -9.770 18.012 -0.660 1.00 0.00
J C
ATOM 12695 HG21 THR J 410 -10.552 18.532 -0.069 1.00 0.00
J H
ATOM 12696 HG22 THR J 410 -10.272 17.254 -1.298 1.00 0.00
J H
ATOM 12697 HG23 THR J 410 -9.096 17.481 0.046 1.00 0.00
J H
ATOM 12698 C THR J 410 -6.947 19.030 -3.136 1.00 0.00
J C
ATOM 12699 O THR J 410 -6.631 20.154 -2.761 1.00 0.00
J O
ATOM 12700 N VAL J 411 -6.340 18.417 -4.169 1.00 0.00
J N
ATOM 12701 HN VAL J 411 -6.658 17.540 -4.520 1.00 0.00
J H
ATOM 12702 CA VAL J 411 -5.187 18.957 -4.863 1.00 0.00
J C
ATOM 12703 HA VAL J 411 -4.845 19.811 -4.292 1.00 0.00
J H
ATOM 12704 CB VAL J 411 -4.126 17.884 -5.064 1.00 0.00
J C
ATOM 12705 HB VAL J 411 -4.543 17.126 -5.772 1.00 0.00
J H
ATOM 12706 CG1 VAL J 411 -2.839 18.491 -5.670 1.00 0.00
J C
ATOM 12707 HG11 VAL J 411 -2.057 17.709 -5.777 1.00 0.00
J H
ATOM 12708 HG12 VAL J 411 -3.022 18.915 -6.679 1.00 0.00
J H
ATOM 12709 HG13 VAL J 411 -2.436 19.292 -5.014 1.00 0.00
J H
ATOM 12710 CG2 VAL J 411 -3.849 17.212 -3.711 1.00 0.00
J C
ATOM 12711 HG21 VAL J 411 -3.049 16.448 -3.809 1.00 0.00
J H
ATOM 12712 HG22 VAL J 411 -3.523 17.965 -2.961 1.00 0.00
J H
ATOM 12713 HG23 VAL J 411 -4.754 16.702 -3.323 1.00 0.00
J H
ATOM 12714 C VAL J 411 -5.532 19.445 -6.241 1.00 0.00
J C
ATOM 12715 O VAL J 411 -5.027 20.458 -6.682 1.00 0.00
J O
ATOM 12716 N GLY J 412 -6.394 18.742 -6.992 1.00 0.00
J N

```
ATOM  12717  HN  GLY J 412      -6.745  17.843  -6.731  1.00  0.00
J    H
ATOM  12718  CA  GLY J 412      -6.884  19.267  -8.268  1.00  0.00
J    C
ATOM  12719  HA1 GLY J 412      -7.041  20.318  -8.069  1.00  0.00
J    H
ATOM  12720  HA2 GLY J 412      -7.793  18.709  -8.448  1.00  0.00
J    H
ATOM  12721  C   GLY J 412      -6.025  19.149  -9.480  1.00  0.00
J    C
ATOM  12722  O   GLY J 412      -6.406  19.600 -10.555  1.00  0.00
J    O
ATOM  12723  N   LEU J 413      -4.859  18.503  -9.368  1.00  0.00
J    N
ATOM  12724  HN  LEU J 413      -4.435  18.264  -8.497  1.00  0.00
J    H
ATOM  12725  CA  LEU J 413      -4.102  18.089 -10.531  1.00  0.00
J    C
ATOM  12726  HA  LEU J 413      -3.987  18.971 -11.147  1.00  0.00
J    H
ATOM  12727  CB  LEU J 413      -2.729  17.502 -10.141  1.00  0.00
J    C
ATOM  12728  HB1 LEU J 413      -2.916  16.696  -9.395  1.00  0.00
J    H
ATOM  12729  HB2 LEU J 413      -2.284  17.002 -11.030  1.00  0.00
J    H
ATOM  12730  CG  LEU J 413      -1.767  18.537  -9.543  1.00  0.00
J    C
ATOM  12731  HG  LEU J 413      -2.354  19.100  -8.779  1.00  0.00
J    H
ATOM  12732  CD1 LEU J 413      -0.574  17.845  -8.870  1.00  0.00
J    C
ATOM  12733 HD11 LEU J 413       0.145  18.598  -8.480  1.00  0.00
J    H
ATOM  12734 HD12 LEU J 413      -0.915  17.217  -8.020  1.00  0.00
J    H
ATOM  12735 HD13 LEU J 413      -0.037  17.195  -9.593  1.00  0.00
J    H
ATOM  12736  CD2 LEU J 413      -1.286  19.539 -10.607  1.00  0.00
J    C
ATOM  12737 HD21 LEU J 413      -0.606  20.290 -10.151  1.00  0.00
J    H
ATOM  12738 HD22 LEU J 413      -0.731  19.035 -11.424  1.00  0.00
J    H
ATOM  12739 HD23 LEU J 413      -2.150  20.078 -11.052  1.00  0.00
J    H
ATOM  12740  C   LEU J 413      -4.836  17.046 -11.356  1.00  0.00
J    C
ATOM  12741  O   LEU J 413      -5.470  16.134 -10.826  1.00  0.00
J    O
ATOM  12742  N   THR J 414      -4.715  17.121 -12.686  1.00  0.00
J    N
ATOM  12743  HN  THR J 414      -4.050  17.706 -13.150  1.00  0.00
J    H
```

```
ATOM  12744  CA  THR J 414      -5.556  16.344 -13.584  1.00  0.00
J    C
ATOM  12745  HA  THR J 414      -5.864  15.461 -13.041  1.00  0.00
J    H
ATOM  12746  CB  THR J 414      -6.717  17.200 -14.095  1.00  0.00
J    C
ATOM  12747  HB  THR J 414      -6.309  18.233 -14.221  1.00  0.00
J    H
ATOM  12748  OG1 THR J 414      -7.726  17.245 -13.115  1.00  0.00
J    O
ATOM  12749  HG1 THR J 414      -8.429  17.783 -13.495  1.00  0.00
J    H
ATOM  12750  CG2 THR J 414      -7.422  16.745 -15.379  1.00  0.00
J    C
ATOM  12751 HG21 THR J 414      -8.165  17.500 -15.711  1.00  0.00
J    H
ATOM  12752 HG22 THR J 414      -6.685  16.609 -16.198  1.00  0.00
J    H
ATOM  12753 HG23 THR J 414      -7.951  15.780 -15.224  1.00  0.00
J    H
ATOM  12754  C   THR J 414      -4.721  15.866 -14.738  1.00  0.00
J    C
ATOM  12755  O   THR J 414      -3.896  16.608 -15.265  1.00  0.00
J    O
ATOM  12756  N   MET J 415      -4.895  14.593 -15.170  1.00  0.00
J    N
ATOM  12757  HN  MET J 415      -5.531  13.957 -14.737  1.00  0.00
J    H
ATOM  12758  CA  MET J 415      -4.163  14.035 -16.311  1.00  0.00
J    C
ATOM  12759  HA  MET J 415      -3.297  14.667 -16.468  1.00  0.00
J    H
ATOM  12760  CB  MET J 415      -3.766  12.551 -16.120  1.00  0.00
J    C
ATOM  12761  HB1 MET J 415      -4.714  11.969 -16.045  1.00  0.00
J    H
ATOM  12762  HB2 MET J 415      -3.249  12.187 -17.035  1.00  0.00
J    H
ATOM  12763  CG  MET J 415      -2.917  12.245 -14.880  1.00  0.00
J    C
ATOM  12764  HG1 MET J 415      -1.939  12.768 -14.944  1.00  0.00
J    H
ATOM  12765  HG2 MET J 415      -3.437  12.615 -13.970  1.00  0.00
J    H
ATOM  12766  SD  MET J 415      -2.574  10.470 -14.666  1.00  0.00
J    S
ATOM  12767  CE  MET J 415      -1.715  10.142 -16.233  1.00  0.00
J    C
ATOM  12768  HE1 MET J 415      -1.420   9.074 -16.307  1.00  0.00
J    H
ATOM  12769  HE2 MET J 415      -2.366  10.378 -17.101  1.00  0.00
J    H
ATOM  12770  HE3 MET J 415      -0.794  10.757 -16.319  1.00  0.00
J    H
```

```
ATOM  12771  C   MET J 415      -4.994  14.068 -17.586  1.00  0.00
J    C
ATOM  12772  O   MET J 415      -4.596  13.550 -18.625  1.00  0.00
J    O
ATOM  12773  N   GLY J 416      -6.183  14.690 -17.506  1.00  0.00
J    N
ATOM  12774  HN  GLY J 416      -6.466  15.076 -16.630  1.00  0.00
J    H
ATOM  12775  CA  GLY J 416      -7.125  14.861 -18.603  1.00  0.00
J    C
ATOM  12776  HA1 GLY J 416      -6.633  14.446 -19.472  1.00  0.00
J    H
ATOM  12777  HA2 GLY J 416      -7.311  15.925 -18.664  1.00  0.00
J    H
ATOM  12778  C   GLY J 416      -8.452  14.167 -18.443  1.00  0.00
J    C
ATOM  12779  O   GLY J 416      -9.187  14.059 -19.416  1.00  0.00
J    O
ATOM  12780  N   LEU J 417      -8.844  13.703 -17.235  1.00  0.00
J    N
ATOM  12781  HN  LEU J 417      -8.331  13.798 -16.384  1.00  0.00
J    H
ATOM  12782  CA  LEU J 417     -10.110  12.996 -17.096  1.00  0.00
J    C
ATOM  12783  HA  LEU J 417     -10.356  12.641 -18.088  1.00  0.00
J    H
ATOM  12784  CB  LEU J 417      -9.936  11.798 -16.127  1.00  0.00
J    C
ATOM  12785  HB1 LEU J 417      -9.183  11.114 -16.581  1.00  0.00
J    H
ATOM  12786  HB2 LEU J 417      -9.485  12.163 -15.177  1.00  0.00
J    H
ATOM  12787  CG  LEU J 417     -11.218  10.976 -15.822  1.00  0.00
J    C
ATOM  12788  HG  LEU J 417     -11.919  11.716 -15.366  1.00  0.00
J    H
ATOM  12789  CD1 LEU J 417     -11.828  10.382 -17.102  1.00  0.00
J    C
ATOM  12790 HD11 LEU J 417     -12.715   9.759 -16.858  1.00  0.00
J    H
ATOM  12791 HD12 LEU J 417     -12.149  11.190 -17.794  1.00  0.00
J    H
ATOM  12792 HD13 LEU J 417     -11.089   9.742 -17.628  1.00  0.00
J    H
ATOM  12793  CD2 LEU J 417     -10.985   9.847 -14.802  1.00  0.00
J    C
ATOM  12794 HD21 LEU J 417     -11.935   9.303 -14.605  1.00  0.00
J    H
ATOM  12795 HD22 LEU J 417     -10.244   9.109 -15.169  1.00  0.00
J    H
ATOM  12796 HD23 LEU J 417     -10.617  10.263 -13.839  1.00  0.00
J    H
ATOM  12797  C   LEU J 417     -11.257  13.886 -16.626  1.00  0.00
J    C
```

ATOM 12798 O LEU J 417 -12.399 13.651 -17.007 1.00 0.00
J O
ATOM 12799 N THR J 418 -11.031 14.969 -15.842 1.00 0.00
J N
ATOM 12800 HN THR J 418 -10.121 15.286 -15.576 1.00 0.00
J H
ATOM 12801 CA THR J 418 -12.140 15.786 -15.305 1.00 0.00
J C
ATOM 12802 HA THR J 418 -12.687 15.083 -14.691 1.00 0.00
J H
ATOM 12803 CB THR J 418 -11.733 16.952 -14.418 1.00 0.00
J C
ATOM 12804 HB THR J 418 -11.575 17.822 -15.101 1.00 0.00
J H
ATOM 12805 OG1 THR J 418 -10.496 16.682 -13.776 1.00 0.00
J O
ATOM 12806 HG1 THR J 418 -10.316 17.456 -13.230 1.00 0.00
J H
ATOM 12807 CG2 THR J 418 -12.773 17.250 -13.331 1.00 0.00
J C
ATOM 12808 HG21 THR J 418 -12.508 18.172 -12.772 1.00 0.00
J H
ATOM 12809 HG22 THR J 418 -13.775 17.404 -13.786 1.00 0.00
J H
ATOM 12810 HG23 THR J 418 -12.845 16.412 -12.605 1.00 0.00
J H
ATOM 12811 C THR J 418 -13.144 16.363 -16.310 1.00 0.00
J C
ATOM 12812 O THR J 418 -14.339 16.300 -16.010 1.00 0.00
J O
ATOM 12813 N PRO J 419 -12.795 16.898 -17.495 1.00 0.00
J N
ATOM 12814 CD PRO J 419 -11.435 17.267 -17.894 1.00 0.00
J C
ATOM 12815 HD1 PRO J 419 -11.914 17.573 -16.937 1.00 0.00
J H
ATOM 12816 HD2 PRO J 419 -10.346 17.081 -17.744 1.00 0.00
J H
ATOM 12817 CA PRO J 419 -13.767 17.262 -18.531 1.00 0.00
J C
ATOM 12818 HA PRO J 419 -14.428 18.032 -18.150 1.00 0.00
J H
ATOM 12819 CB PRO J 419 -12.907 17.869 -19.661 1.00 0.00
J C
ATOM 12820 HB1 PRO J 419 -12.732 18.947 -19.445 1.00 0.00
J H
ATOM 12821 HB2 PRO J 419 -13.381 17.774 -20.659 1.00 0.00
J H
ATOM 12822 CG PRO J 419 -11.638 18.349 -18.950 1.00 0.00
J C
ATOM 12823 HG1 PRO J 419 -11.109 19.149 -19.503 1.00 0.00
J H
ATOM 12824 HG2 PRO J 419 -11.165 19.001 -18.358 1.00 0.00
J H

ATOM 12825 C PRO J 419 -14.604 16.087 -19.028 1.00 0.00
J C
ATOM 12826 O PRO J 419 -15.806 16.254 -19.238 1.00 0.00
J O
ATOM 12827 N GLU J 420 -13.985 14.897 -19.185 1.00 0.00
J N
ATOM 12828 HN GLU J 420 -13.076 14.748 -18.804 1.00 0.00
J H
ATOM 12829 CA GLU J 420 -14.528 13.744 -19.885 1.00 0.00
J C
ATOM 12830 HA GLU J 420 -15.289 14.104 -20.565 1.00 0.00
J H
ATOM 12831 CB GLU J 420 -13.402 13.020 -20.680 1.00 0.00
J C
ATOM 12832 HB1 GLU J 420 -12.704 12.553 -19.948 1.00 0.00
J H
ATOM 12833 HB2 GLU J 420 -13.835 12.180 -21.268 1.00 0.00
J H
ATOM 12834 CG GLU J 420 -12.578 13.939 -21.627 1.00 0.00
J C
ATOM 12835 HG1 GLU J 420 -12.011 14.674 -21.026 1.00 0.00
J H
ATOM 12836 HG2 GLU J 420 -11.870 13.346 -22.239 1.00 0.00
J H
ATOM 12837 CD GLU J 420 -13.434 14.776 -22.576 1.00 0.00
J C
ATOM 12838 OE1 GLU J 420 -14.393 14.222 -23.169 1.00 0.00
J O
ATOM 12839 OE2 GLU J 420 -13.109 15.984 -22.725 1.00 0.00
J O
ATOM 12840 C GLU J 420 -15.197 12.749 -18.938 1.00 0.00
J C
ATOM 12841 O GLU J 420 -15.585 11.644 -19.320 1.00 0.00
J O
ATOM 12842 N LEU J 421 -15.372 13.114 -17.651 1.00 0.00
J N
ATOM 12843 HN LEU J 421 -14.941 13.929 -17.268 1.00 0.00
J H
ATOM 12844 CA LEU J 421 -16.193 12.371 -16.706 1.00 0.00
J C
ATOM 12845 HA LEU J 421 -15.806 11.360 -16.720 1.00 0.00
J H
ATOM 12846 CB LEU J 421 -16.132 12.959 -15.271 1.00 0.00
J C
ATOM 12847 HB1 LEU J 421 -16.318 14.055 -15.357 1.00 0.00
J H
ATOM 12848 HB2 LEU J 421 -16.978 12.549 -14.676 1.00 0.00
J H
ATOM 12849 CG LEU J 421 -14.804 12.744 -14.528 1.00 0.00
J C
ATOM 12850 HG LEU J 421 -13.988 13.059 -15.222 1.00 0.00
J H
ATOM 12851 CD1 LEU J 421 -14.771 13.605 -13.258 1.00 0.00
J C

ATOM 12852 HD11 LEU J 421   -13.835 13.421 -12.687 1.00 0.00
J   H
ATOM 12853 HD12 LEU J 421   -14.816 14.685 -13.515 1.00 0.00
J   H
ATOM 12854 HD13 LEU J 421   -15.633 13.365 -12.598 1.00 0.00
J   H
ATOM 12855 CD2 LEU J 421   -14.581 11.269 -14.170 1.00 0.00
J   C
ATOM 12856 HD21 LEU J 421   -13.606 11.141 -13.651 1.00 0.00
J   H
ATOM 12857 HD22 LEU J 421   -15.373 10.886 -13.495 1.00 0.00
J   H
ATOM 12858 HD23 LEU J 421   -14.570 10.642 -15.088 1.00 0.00
J   H
ATOM 12859 C   LEU J 421   -17.665 12.306 -17.087 1.00 0.00
J   C
ATOM 12860 O   LEU J 421   -18.263 13.258 -17.598 1.00 0.00
J   O
ATOM 12861 N   THR J 422   -18.327 11.176 -16.768 1.00 0.00
J   N
ATOM 12862 HN THR J 422   -17.876 10.350 -16.435 1.00 0.00
J   H
ATOM 12863 CA THR J 422   -19.771 11.062 -16.884 1.00 0.00
J   C
ATOM 12864 HA THR J 422   -20.041 11.515 -17.829 1.00 0.00
J   H
ATOM 12865 CB THR J 422   -20.305   9.621 -16.829 1.00 0.00
J   C
ATOM 12866 HB THR J 422   -21.382   9.678 -17.119 1.00 0.00
J   H
ATOM 12867 OG1 THR J 422   -20.256   9.086 -15.513 1.00 0.00
J   O
ATOM 12868 HG1 THR J 422   -20.597   8.189 -15.588 1.00 0.00
J   H
ATOM 12869 CG2 THR J 422   -19.495   8.677 -17.739 1.00 0.00
J   C
ATOM 12870 HG21 THR J 422   -19.960   7.670 -17.777 1.00 0.00
J   H
ATOM 12871 HG22 THR J 422   -19.458   9.075 -18.775 1.00 0.00
J   H
ATOM 12872 HG23 THR J 422   -18.453   8.565 -17.372 1.00 0.00
J   H
ATOM 12873 C   THR J 422   -20.438 11.899 -15.793 1.00 0.00
J   C
ATOM 12874 O   THR J 422   -19.806 12.383 -14.854 1.00 0.00
J   O
ATOM 12875 N   THR J 423   -21.768 12.100 -15.865 1.00 0.00
J   N
ATOM 12876 HN THR J 423   -22.329 11.804 -16.637 1.00 0.00
J   H
ATOM 12877 CA THR J 423   -22.516 12.781 -14.801 1.00 0.00
J   C
ATOM 12878 HA THR J 423   -22.017 13.731 -14.669 1.00 0.00
J   H

ATOM 12879 CB THR J 423   -23.977 13.024 -15.161 1.00 0.00
J   C
ATOM 12880 HB THR J 423   -24.397 12.027 -15.443 1.00 0.00
J   H
ATOM 12881 OG1 THR J 423   -24.014 13.869 -16.300 1.00 0.00
J   O
ATOM 12882 HG1 THR J 423   -24.950 14.007 -16.487 1.00 0.00
J   H
ATOM 12883 CG2 THR J 423   -24.750 13.757 -14.051 1.00 0.00
J   C
ATOM 12884 HG21 THR J 423   -25.832 13.817 -14.296 1.00 0.00
J   H
ATOM 12885 HG22 THR J 423   -24.648 13.217 -13.087 1.00 0.00
J   H
ATOM 12886 HG23 THR J 423   -24.368 14.792 -13.913 1.00 0.00
J   H
ATOM 12887 C   THR J 423   -22.434 12.061 -13.467 1.00 0.00
J   C
ATOM 12888 O   THR J 423   -22.238 12.682 -12.424 1.00 0.00
J   O
ATOM 12889 N   ALA J 424   -22.525 10.715 -13.467 1.00 0.00
J   N
ATOM 12890 HN ALA J 424   -22.711 10.216 -14.312 1.00 0.00
J   H
ATOM 12891 CA ALA J 424   -22.368   9.896 -12.284 1.00 0.00
J   C
ATOM 12892 HA ALA J 424   -23.043 10.239 -11.508 1.00 0.00
J   H
ATOM 12893 CB ALA J 424   -22.780   8.447 -12.617 1.00 0.00
J   C
ATOM 12894 HB1 ALA J 424   -23.823   8.434 -12.996 1.00 0.00
J   H
ATOM 12895 HB2 ALA J 424   -22.125   8.017 -13.406 1.00 0.00
J   H
ATOM 12896 HB3 ALA J 424   -22.732   7.791 -11.721 1.00 0.00
J   H
ATOM 12897 C   ALA J 424   -20.955   9.970 -11.690 1.00 0.00
J   C
ATOM 12898 O   ALA J 424   -20.792 10.087 -10.476 1.00 0.00
J   O
ATOM 12899 N   GLY J 425   -19.895   9.971 -12.536 1.00 0.00
J   N
ATOM 12900 HN GLY J 425   -20.024   9.862 -13.521 1.00 0.00
J   H
ATOM 12901 CA GLY J 425   -18.507 10.127 -12.093 1.00 0.00
J   C
ATOM 12902 HA1 GLY J 425   -17.891 10.020 -12.975 1.00 0.00
J   H
ATOM 12903 HA2 GLY J 425   -18.337   9.371 -11.338 1.00 0.00
J   H
ATOM 12904 C   GLY J 425   -18.213 11.471 -11.478 1.00 0.00
J   C
ATOM 12905 O   GLY J 425   -17.464 11.579 -10.509 1.00 0.00
J   O

ATOM 12906 N   LYS J 426   -18.865 12.545 -11.971 1.00 0.00
J   N
ATOM 12907 HN LYS J 426   -19.404 12.488 -12.810 1.00 0.00
J   H
ATOM 12908 CA LYS J 426   -18.844 13.858 -11.338 1.00 0.00
J   C
ATOM 12909 HA LYS J 426   -17.801 14.138 -11.255 1.00 0.00
J   H
ATOM 12910 CB LYS J 426   -19.576 14.914 -12.200 1.00 0.00
J   C
ATOM 12911 HB1 LYS J 426   -20.464 14.412 -12.650 1.00 0.00
J   H
ATOM 12912 HB2 LYS J 426   -19.972 15.713 -11.535 1.00 0.00
J   H
ATOM 12913 CG LYS J 426   -18.670 15.476 -13.303 1.00 0.00
J   C
ATOM 12914 HG1 LYS J 426   -18.089 16.335 -12.895 1.00 0.00
J   H
ATOM 12915 HG2 LYS J 426   -17.936 14.693 -13.605 1.00 0.00
J   H
ATOM 12916 CD LYS J 426   -19.436 15.965 -14.543 1.00 0.00
J   C
ATOM 12917 HD1 LYS J 426   -20.217 15.211 -14.793 1.00 0.00
J   H
ATOM 12918 HD2 LYS J 426   -19.960 16.912 -14.277 1.00 0.00
J   H
ATOM 12919 CE LYS J 426   -18.515 16.197 -15.749 1.00 0.00
J   C
ATOM 12920 HE1 LYS J 426   -17.734 16.951 -15.503 1.00 0.00
J   H
ATOM 12921 HE2 LYS J 426   -18.011 15.251 -16.044 1.00 0.00
J   H
ATOM 12922 NZ LYS J 426   -19.273 16.692 -16.914 1.00 0.00
J   N
ATOM 12923 HZ1 LYS J 426   -18.630 16.845 -17.719 1.00 0.00
J   H
ATOM 12924 HZ2 LYS J 426   -19.998 15.995 -17.178 1.00 0.00
J   H
ATOM 12925 HZ3 LYS J 426   -19.736 17.590 -16.669 1.00 0.00
J   H
ATOM 12926 C   LYS J 426   -19.424 13.849 -9.933 1.00 0.00
J   C
ATOM 12927 O   LYS J 426   -18.828 14.415 -9.021 1.00 0.00
J   O
ATOM 12928 N   CYS J 427   -20.563 13.154 -9.707 1.00 0.00
J   N
ATOM 12929 HN CYS J 427   -21.077 12.723 -10.446 1.00 0.00
J   H
ATOM 12930 CA CYS J 427   -21.136 12.974 -8.377 1.00 0.00
J   C
ATOM 12931 HA CYS J 427   -21.298 13.970 -7.983 1.00 0.00
J   H
ATOM 12932 CB CYS J 427   -22.481 12.189 -8.398 1.00 0.00
J   C

ATOM 12933 HB1 CYS J 427   -22.260 11.166 -8.777 1.00 0.00
J   H
ATOM 12934 HB2 CYS J 427   -22.857 12.057 -7.359 1.00 0.00
J   H
ATOM 12935 SG CYS J 427   -23.744 12.947 -9.470 1.00 0.00
J   S
ATOM 12936 HG1 CYS J 427   -24.683 12.014 -9.305 1.00 0.00
J   H
ATOM 12937 C   CYS J 427   -20.184 12.252 -7.434 1.00 0.00
J   C
ATOM 12938 O   CYS J 427   -19.974 12.689 -6.305 1.00 0.00
J   O
ATOM 12939 N   ILE J 428   -19.525 11.163 -7.898 1.00 0.00
J   N
ATOM 12940 HN ILE J 428   -19.681 10.800 -8.814 1.00 0.00
J   H
ATOM 12941 CA ILE J 428   -18.536 10.421 -7.120 1.00 0.00
J   C
ATOM 12942 HA ILE J 428   -19.035 10.121 -6.207 1.00 0.00
J   H
ATOM 12943 CB ILE J 428   -18.025   9.198 -7.889 1.00 0.00
J   C
ATOM 12944 HB ILE J 428   -17.753   9.596 -8.899 1.00 0.00
J   H
ATOM 12945 CG2 ILE J 428   -16.831   8.536 -7.154 1.00 0.00
J   C
ATOM 12946 HG21 ILE J 428   -16.369   7.751 -7.789 1.00 0.00
J   H
ATOM 12947 HG22 ILE J 428   -16.046   9.284 -6.920 1.00 0.00
J   H
ATOM 12948 HG23 ILE J 428   -17.161   8.064 -6.204 1.00 0.00
J   H
ATOM 12949 CG1 ILE J 428   -19.172   8.182 -8.099 1.00 0.00
J   C
ATOM 12950 HG11 ILE J 428   -19.453   7.725 -7.123 1.00 0.00
J   H
ATOM 12951 HG12 ILE J 428   -20.068   8.723 -8.480 1.00 0.00
J   H
ATOM 12952 CD ILE J 428   -18.821   7.067 -9.095 1.00 0.00
J   C
ATOM 12953 HD1 ILE J 428   -19.697   6.401 -9.252 1.00 0.00
J   H
ATOM 12954 HD2 ILE J 428   -18.531   7.498 -10.077 1.00 0.00
J   H
ATOM 12955 HD3 ILE J 428   -17.981   6.447 -8.720 1.00 0.00
J   H
ATOM 12956 C   ILE J 428   -17.366 11.306 -6.711 1.00 0.00
J   C
ATOM 12957 O   ILE J 428   -16.991 11.355 -5.542 1.00 0.00
J   O
ATOM 12958 N   ILE J 429   -16.812 12.094 -7.659 1.00 0.00
J   N
ATOM 12959 HN ILE J 429   -17.097 12.066 -8.614 1.00 0.00
J   H

ATOM 12960 CA ILE J 429   -15.752 13.054 -7.379 1.00 0.00
J   C
ATOM 12961 HA ILE J 429   -14.957 12.488 -6.909 1.00 0.00
J   H
ATOM 12962 CB ILE J 429   -15.189 13.652 -8.671 1.00 0.00
J   C
ATOM 12963 HB ILE J 429   -16.094 13.958 -9.256 1.00 0.00
J   H
ATOM 12964 CG2 ILE J 429   -14.244 14.846 -8.403 1.00 0.00
J   C
ATOM 12965 HG21 ILE J 429   -13.977 15.354 -9.354 1.00 0.00
J   H
ATOM 12966 HG22 ILE J 429   -14.732 15.596 -7.746 1.00 0.00
J   H
ATOM 12967 HG23 ILE J 429   -13.306 14.509 -7.915 1.00 0.00
J   H
ATOM 12968 CG1 ILE J 429   -14.456 12.587 -9.530 1.00 0.00
J   C
ATOM 12969 HG11 ILE J 429   -15.127 11.717 -9.705 1.00 0.00
J   H
ATOM 12970 HG12 ILE J 429   -14.221 13.027 -10.526 1.00 0.00
J   H
ATOM 12971 CD ILE J 429   -13.137 12.062 -8.941 1.00 0.00
J   C
ATOM 12972 HD1 ILE J 429   -12.656 11.351 -9.646 1.00 0.00
J   H
ATOM 12973 HD2 ILE J 429   -12.431 12.900 -8.754 1.00 0.00
J   H
ATOM 12974 HD3 ILE J 429   -13.314 11.530 -7.982 1.00 0.00
J   H
ATOM 12975 C   ILE J 429   -16.183 14.126 -6.379 1.00 0.00
J   C
ATOM 12976 O   ILE J 429   -15.455 14.383 -5.422 1.00 0.00
J   O
ATOM 12977 N   ILE J 430   -17.395 14.720 -6.491 1.00 0.00
J   N
ATOM 12978 HN ILE J 430   -18.017 14.513 -7.243 1.00 0.00
J   H
ATOM 12979 CA ILE J 430   -17.913 15.710 -5.539 1.00 0.00
J   C
ATOM 12980 HA ILE J 430   -17.186 16.513 -5.532 1.00 0.00
J   H
ATOM 12981 CB ILE J 430   -19.271 16.270 -5.980 1.00 0.00
J   C
ATOM 12982 HB ILE J 430   -19.868 15.366 -6.262 1.00 0.00
J   H
ATOM 12983 CG2 ILE J 430   -19.931 17.131 -4.871 1.00 0.00
J   C
ATOM 12984 HG21 ILE J 430   -20.973 17.395 -5.149 1.00 0.00
J   H
ATOM 12985 HG22 ILE J 430   -19.968 16.578 -3.910 1.00 0.00
J   H
ATOM 12986 HG23 ILE J 430   -19.365 18.074 -4.712 1.00 0.00
J   H

ATOM 12987 CG1 ILE J 430 -19.102 17.117 -7.263 1.00 0.00
J C
ATOM 12988 HG11 ILE J 430 -18.669 18.109 -7.005 1.00 0.00
J H
ATOM 12989 HG12 ILE J 430 -18.378 16.607 -7.939 1.00 0.00
J H
ATOM 12990 CD ILE J 430 -20.423 17.316 -8.020 1.00 0.00
J C
ATOM 12991 HD1 ILE J 430 -20.247 17.878 -8.962 1.00 0.00
J H
ATOM 12992 HD2 ILE J 430 -20.877 16.335 -8.280 1.00 0.00
J H
ATOM 12993 HD3 ILE J 430 -21.150 17.890 -7.408 1.00 0.00
J H
ATOM 12994 C ILE J 430 -17.996 15.178 -4.109 1.00 0.00
J C
ATOM 12995 O ILE J 430 -17.562 15.838 -3.164 1.00 0.00
J O
ATOM 12996 N VAL J 431 -18.504 13.937 -3.915 1.00 0.00
J N
ATOM 12997 HN VAL J 431 -18.888 13.391 -4.654 1.00 0.00
J H
ATOM 12998 CA VAL J 431 -18.534 13.289 -2.605 1.00 0.00
J C
ATOM 12999 HA VAL J 431 -19.037 13.965 -1.924 1.00 0.00
J H
ATOM 13000 CB VAL J 431 -19.327 11.978 -2.604 1.00 0.00
J C
ATOM 13001 HB VAL J 431 -18.786 11.257 -3.266 1.00 0.00
J H
ATOM 13002 CG1 VAL J 431 -19.408 11.396 -1.176 1.00 0.00
J C
ATOM 13003 HG11 VAL J 431 -20.016 10.466 -1.173 1.00 0.00
J H
ATOM 13004 HG12 VAL J 431 -18.404 11.135 -0.782 1.00 0.00
J H
ATOM 13005 HG13 VAL J 431 -19.886 12.121 -0.483 1.00 0.00
J H
ATOM 13006 CG2 VAL J 431 -20.765 12.237 -3.093 1.00 0.00
J C
ATOM 13007 HG21 VAL J 431 -21.365 11.302 -3.061 1.00 0.00
J H
ATOM 13008 HG22 VAL J 431 -21.265 12.995 -2.452 1.00 0.00
J H
ATOM 13009 HG23 VAL J 431 -20.774 12.605 -4.140 1.00 0.00
J H
ATOM 13010 C VAL J 431 -17.127 13.056 -2.062 1.00 0.00
J C
ATOM 13011 O VAL J 431 -16.833 13.370 -0.908 1.00 0.00
J O
ATOM 13012 N ILE J 432 -16.187 12.569 -2.900 1.00 0.00
J N
ATOM 13013 HN ILE J 432 -16.405 12.313 -3.840 1.00 0.00
J H

ATOM 13014 CA ILE J 432 -14.786 12.370 -2.534 1.00 0.00
J C
ATOM 13015 HA ILE J 432 -14.802 11.728 -1.662 1.00 0.00
J H
ATOM 13016 CB ILE J 432 -14.020 11.677 -3.663 1.00 0.00
J C
ATOM 13017 HB ILE J 432 -14.315 12.238 -4.585 1.00 0.00
J H
ATOM 13018 CG2 ILE J 432 -12.496 11.676 -3.411 1.00 0.00
J C
ATOM 13019 HG21 ILE J 432 -11.953 11.312 -4.308 1.00 0.00
J H
ATOM 13020 HG22 ILE J 432 -12.131 12.700 -3.189 1.00 0.00
J H
ATOM 13021 HG23 ILE J 432 -12.236 11.017 -2.556 1.00 0.00
J H
ATOM 13022 CG1 ILE J 432 -14.527 10.229 -3.834 1.00 0.00
J C
ATOM 13023 HG11 ILE J 432 -14.155 9.599 -2.994 1.00 0.00
J H
ATOM 13024 HG12 ILE J 432 -15.639 10.227 -3.779 1.00 0.00
J H
ATOM 13025 CD ILE J 432 -14.087 9.635 -5.173 1.00 0.00
J C
ATOM 13026 HD1 ILE J 432 -14.510 8.615 -5.300 1.00 0.00
J H
ATOM 13027 HD2 ILE J 432 -14.440 10.266 -6.017 1.00 0.00
J H
ATOM 13028 HD3 ILE J 432 -12.980 9.557 -5.231 1.00 0.00
J H
ATOM 13029 C ILE J 432 -14.088 13.662 -2.117 1.00 0.00
J C
ATOM 13030 O ILE J 432 -13.396 13.699 -1.101 1.00 0.00
J O
ATOM 13031 N MET J 433 -14.293 14.774 -2.856 1.00 0.00
J N
ATOM 13032 HN MET J 433 -14.828 14.753 -3.698 1.00 0.00
J H
ATOM 13033 CA MET J 433 -13.767 16.088 -2.511 1.00 0.00
J C
ATOM 13034 HA MET J 433 -12.695 15.969 -2.412 1.00 0.00
J H
ATOM 13035 CB MET J 433 -14.172 17.144 -3.566 1.00 0.00
J C
ATOM 13036 HB1 MET J 433 -15.287 17.155 -3.603 1.00 0.00
J H
ATOM 13037 HB2 MET J 433 -13.862 18.153 -3.215 1.00 0.00
J H
ATOM 13038 CG MET J 433 -13.660 16.923 -4.996 1.00 0.00
J C
ATOM 13039 HG1 MET J 433 -12.647 17.362 -5.115 1.00 0.00
J H
ATOM 13040 HG2 MET J 433 -13.581 15.833 -5.200 1.00 0.00
J H

ATOM 13041 SD MET J 433   −14.775 17.692 −6.210 1.00 0.00
J   S
ATOM 13042 CE MET J 433   −13.636 18.935 −6.829 1.00 0.00
J   C
ATOM 13043 HE1 MET J 433   −14.119 19.562 −7.609 1.00 0.00
J   H
ATOM 13044 HE2 MET J 433   −13.297 19.608 −6.013 1.00 0.00
J   H
ATOM 13045 HE3 MET J 433   −12.736 18.464 −7.280 1.00 0.00
J   H
ATOM 13046 C   MET J 433   −14.296 16.610 −1.185 1.00 0.00
J   C
ATOM 13047 O   MET J 433   −13.566 17.185 −0.377 1.00 0.00
J   O
ATOM 13048 N   PHE J 434   −15.608 16.424 −0.929 1.00 0.00
J   N
ATOM 13049 HN PHE J 434   −16.227 16.034 −1.610 1.00 0.00
J   H
ATOM 13050 CA PHE J 434   −16.239 16.763   0.330 1.00 0.00
J   C
ATOM 13051 HA PHE J 434   −16.005 17.806   0.505 1.00 0.00
J   H
ATOM 13052 CB PHE J 434   −17.776 16.543   0.198 1.00 0.00
J   C
ATOM 13053 HB1 PHE J 434   −18.165 17.182 −0.624 1.00 0.00
J   H
ATOM 13054 HB2 PHE J 434   −18.008 15.489 −0.069 1.00 0.00
J   H
ATOM 13055 CG PHE J 434   −18.517 16.938   1.448 1.00 0.00
J   C
ATOM 13056 CD1 PHE J 434   −18.555 18.281   1.848 1.00 0.00
J   C
ATOM 13057 HD1 PHE J 434   −18.067 19.033   1.242 1.00 0.00
J   H
ATOM 13058 CE1 PHE J 434   −19.211 18.654   3.026 1.00 0.00
J   C
ATOM 13059 HE1 PHE J 434   −19.250 19.694   3.316 1.00 0.00
J   H
ATOM 13060 CZ PHE J 434   −19.823 17.684   3.828 1.00 0.00
J   C
ATOM 13061 HZ PHE J 434   −20.338 17.972   4.733 1.00 0.00
J   H
ATOM 13062 CD2 PHE J 434   −19.129 15.967   2.261 1.00 0.00
J   C
ATOM 13063 HD2 PHE J 434   −19.086 14.924   1.983 1.00 0.00
J   H
ATOM 13064 CE2 PHE J 434   −19.775 16.337   3.449 1.00 0.00
J   C
ATOM 13065 HE2 PHE J 434   −20.233 15.580   4.071 1.00 0.00
J   H
ATOM 13066 C   PHE J 434   −15.658 15.972   1.497 1.00 0.00
J   C
ATOM 13067 O   PHE J 434   −15.165 16.555   2.459 1.00 0.00
J   O

ATOM 13068 N ILE J 435 -15.606 14.628 1.416 1.00 0.00
J N
ATOM 13069 HN ILE J 435 -15.925 14.114 0.623 1.00 0.00
J H
ATOM 13070 CA ILE J 435 -15.072 13.799 2.490 1.00 0.00
J C
ATOM 13071 HA ILE J 435 -15.592 14.103 3.390 1.00 0.00
J H
ATOM 13072 CB ILE J 435 -15.338 12.312 2.267 1.00 0.00
J C
ATOM 13073 HB ILE J 435 -14.868 12.089 1.275 1.00 0.00
J H
ATOM 13074 CG2 ILE J 435 -14.787 11.518 3.473 1.00 0.00
J C
ATOM 13075 HG21 ILE J 435 -14.823 10.428 3.273 1.00 0.00
J H
ATOM 13076 HG22 ILE J 435 -13.730 11.788 3.676 1.00 0.00
J H
ATOM 13077 HG23 ILE J 435 -15.383 11.723 4.389 1.00 0.00
J H
ATOM 13078 CG1 ILE J 435 -16.853 12.024 2.110 1.00 0.00
J C
ATOM 13079 HG11 ILE J 435 -17.375 12.217 3.074 1.00 0.00
J H
ATOM 13080 HG12 ILE J 435 -17.277 12.729 1.359 1.00 0.00
J H
ATOM 13081 CD ILE J 435 -17.147 10.589 1.650 1.00 0.00
J C
ATOM 13082 HD1 ILE J 435 -18.239 10.449 1.497 1.00 0.00
J H
ATOM 13083 HD2 ILE J 435 -16.631 10.370 0.691 1.00 0.00
J H
ATOM 13084 HD3 ILE J 435 -16.811 9.851 2.409 1.00 0.00
J H
ATOM 13085 C ILE J 435 -13.586 14.063 2.728 1.00 0.00
J C
ATOM 13086 O ILE J 435 -13.127 14.155 3.866 1.00 0.00
J O
ATOM 13087 N GLY J 436 -12.805 14.243 1.641 1.00 0.00
J N
ATOM 13088 HN GLY J 436 -13.186 14.170 0.721 1.00 0.00
J H
ATOM 13089 CA GLY J 436 -11.385 14.550 1.711 1.00 0.00
J C
ATOM 13090 HA1 GLY J 436 -11.031 14.540 0.689 1.00 0.00
J H
ATOM 13091 HA2 GLY J 436 -10.946 13.788 2.341 1.00 0.00
J H
ATOM 13092 C GLY J 436 -11.052 15.888 2.304 1.00 0.00
J C
ATOM 13093 O GLY J 436 -10.073 16.011 3.030 1.00 0.00
J O
ATOM 13094 N ARG J 437 -11.862 16.937 2.043 1.00 0.00
J N

```
ATOM  13095  HN  ARG J 437     -12.602  16.871   1.379  1.00  0.00
J    H
ATOM  13096  CA  ARG J 437     -11.699  18.219   2.715  1.00  0.00
J    C
ATOM  13097  HA  ARG J 437     -10.640  18.450   2.705  1.00  0.00
J    H
ATOM  13098  CB  ARG J 437     -12.448  19.349   1.970  1.00  0.00
J    C
ATOM  13099  HB1 ARG J 437     -11.921  19.487   0.996  1.00  0.00
J    H
ATOM  13100  HB2 ARG J 437     -13.477  19.010   1.717  1.00  0.00
J    H
ATOM  13101  CG  ARG J 437     -12.518  20.741   2.652  1.00  0.00
J    C
ATOM  13102  HG1 ARG J 437     -12.954  21.467   1.930  1.00  0.00
J    H
ATOM  13103  HG2 ARG J 437     -13.238  20.693   3.500  1.00  0.00
J    H
ATOM  13104  CD  ARG J 437     -11.237  21.352   3.254  1.00  0.00
J    C
ATOM  13105  HD1 ARG J 437     -11.452  22.386   3.609  1.00  0.00
J    H
ATOM  13106  HD2 ARG J 437     -10.861  20.749   4.113  1.00  0.00
J    H
ATOM  13107  NE  ARG J 437     -10.156  21.380   2.212  1.00  0.00
J    N
ATOM  13108  HE  ARG J 437     -10.173  20.579   1.603  1.00  0.00
J    H
ATOM  13109  CZ  ARG J 437      -9.265  22.373   2.109  1.00  0.00
J    C
ATOM  13110  NH1 ARG J 437      -9.455  23.560   2.674  1.00  0.00
J    N
ATOM  13111 HH11 ARG J 437      -8.790  24.284   2.561  1.00  0.00
J    H
ATOM  13112 HH12 ARG J 437     -10.352  23.714   3.093  1.00  0.00
J    H
ATOM  13113  NH2 ARG J 437      -8.149  22.201   1.410  1.00  0.00
J    N
ATOM  13114 HH21 ARG J 437      -7.454  22.906   1.409  1.00  0.00
J    H
ATOM  13115 HH22 ARG J 437      -7.960  21.285   1.083  1.00  0.00
J    H
ATOM  13116  C   ARG J 437     -12.126  18.218   4.161  1.00  0.00
J    C
ATOM  13117  O   ARG J 437     -11.393  18.682   5.025  1.00  0.00
J    O
ATOM  13118  N   ILE J 438     -13.317  17.685   4.484  1.00  0.00
J    N
ATOM  13119  HN  ILE J 438     -13.938  17.268   3.822  1.00  0.00
J    H
ATOM  13120  CA  ILE J 438     -13.810  17.673   5.854  1.00  0.00
J    C
ATOM  13121  HA  ILE J 438     -13.747  18.694   6.208  1.00  0.00
J    H
```

ATOM 13122 CB ILE J 438 -15.268 17.223 5.882 1.00 0.00
J C
ATOM 13123 HB ILE J 438 -15.267 16.240 5.346 1.00 0.00
J H
ATOM 13124 CG2 ILE J 438 -15.773 17.074 7.332 1.00 0.00
J C
ATOM 13125 HG21 ILE J 438 -16.777 16.600 7.348 1.00 0.00
J H
ATOM 13126 HG22 ILE J 438 -15.089 16.433 7.926 1.00 0.00
J H
ATOM 13127 HG23 ILE J 438 -15.848 18.063 7.830 1.00 0.00
J H
ATOM 13128 CG1 ILE J 438 -16.168 18.180 5.049 1.00 0.00
J C
ATOM 13129 HG11 ILE J 438 -15.987 18.018 3.963 1.00 0.00
J H
ATOM 13130 HG12 ILE J 438 -17.236 17.932 5.246 1.00 0.00
J H
ATOM 13131 CD ILE J 438 -16.023 19.683 5.308 1.00 0.00
J C
ATOM 13132 HD1 ILE J 438 -16.756 20.253 4.697 1.00 0.00
J H
ATOM 13133 HD2 ILE J 438 -16.207 19.916 6.379 1.00 0.00
J H
ATOM 13134 HD3 ILE J 438 -15.005 20.037 5.040 1.00 0.00
J H
ATOM 13135 C ILE J 438 -12.920 16.841 6.757 1.00 0.00
J C
ATOM 13136 O ILE J 438 -12.666 17.171 7.918 1.00 0.00
J O
ATOM 13137 N GLY J 439 -12.379 15.753 6.188 1.00 0.00
J N
ATOM 13138 HN GLY J 439 -12.622 15.525 5.247 1.00 0.00
J H
ATOM 13139 CA GLY J 439 -11.454 14.853 6.828 1.00 0.00
J C
ATOM 13140 HA1 GLY J 439 -10.982 15.355 7.662 1.00 0.00
J H
ATOM 13141 HA2 GLY J 439 -10.760 14.463 6.096 1.00 0.00
J H
ATOM 13142 C GLY J 439 -12.251 13.700 7.370 1.00 0.00
J C
ATOM 13143 O GLY J 439 -13.272 13.926 8.023 1.00 0.00
J O
ATOM 13144 N PRO J 440 -11.878 12.447 7.164 1.00 0.00
J N
ATOM 13145 CD PRO J 440 -10.677 12.047 6.436 1.00 0.00
J C
ATOM 13146 HD1 PRO J 440 -10.634 12.216 5.336 1.00 0.00
J H
ATOM 13147 HD2 PRO J 440 -9.859 12.604 6.948 1.00 0.00
J H
ATOM 13148 CA PRO J 440 -12.788 11.330 7.379 1.00 0.00
J C

```
ATOM  13149  HA  PRO J 440     -13.688  11.472   6.792  1.00  0.00
J    H
ATOM  13150  CB  PRO J 440     -12.028  10.123   6.819  1.00  0.00
J    C
ATOM  13151  HB1 PRO J 440     -12.205  10.059   5.722  1.00  0.00
J    H
ATOM  13152  HB2 PRO J 440     -12.336   9.166   7.290  1.00  0.00
J    H
ATOM  13153  CG  PRO J 440     -10.563  10.559   6.739  1.00  0.00
J    C
ATOM  13154  HG1 PRO J 440      -9.978   9.977   6.001  1.00  0.00
J    H
ATOM  13155  HG2 PRO J 440     -10.094  10.458   7.745  1.00  0.00
J    H
ATOM  13156  C   PRO J 440     -13.167  11.152   8.833  1.00  0.00
J    C
ATOM  13157  O   PRO J 440     -14.312  10.826   9.119  1.00  0.00
J    O
ATOM  13158  N   LEU J 441     -12.233  11.383   9.770  1.00  0.00
J    N
ATOM  13159  HN  LEU J 441     -11.292  11.611   9.530  1.00  0.00
J    H
ATOM  13160  CA  LEU J 441     -12.490  11.329  11.199  1.00  0.00
J    C
ATOM  13161  HA  LEU J 441     -12.938  10.360  11.379  1.00  0.00
J    H
ATOM  13162  CB  LEU J 441     -11.153  11.455  11.967  1.00  0.00
J    C
ATOM  13163  HB1 LEU J 441     -10.404  11.875  11.256  1.00  0.00
J    H
ATOM  13164  HB2 LEU J 441     -11.269  12.213  12.774  1.00  0.00
J    H
ATOM  13165  CG  LEU J 441     -10.672  10.101  12.508  1.00  0.00
J    C
ATOM  13166  HG  LEU J 441     -11.479   9.721  13.180  1.00  0.00
J    H
ATOM  13167  CD1 LEU J 441     -10.412   9.083  11.387  1.00  0.00
J    C
ATOM  13168 HD11 LEU J 441     -10.020   8.132  11.807  1.00  0.00
J    H
ATOM  13169 HD12 LEU J 441     -11.351   8.861  10.835  1.00  0.00
J    H
ATOM  13170 HD13 LEU J 441      -9.666   9.477  10.664  1.00  0.00
J    H
ATOM  13171  CD2 LEU J 441      -9.411  10.307  13.354  1.00  0.00
J    C
ATOM  13172 HD21 LEU J 441      -9.061   9.337  13.769  1.00  0.00
J    H
ATOM  13173 HD22 LEU J 441      -8.582  10.736  12.756  1.00  0.00
J    H
ATOM  13174 HD23 LEU J 441      -9.620  10.993  14.203  1.00  0.00
J    H
ATOM  13175  C   LEU J 441     -13.471  12.382  11.675  1.00  0.00
J    C
```

ATOM 13176 O LEU J 441 -14.416 12.095 12.406 1.00 0.00
J O
ATOM 13177 N THR J 442 -13.290 13.630 11.208 1.00 0.00
J N
ATOM 13178 HN THR J 442 -12.510 13.841 10.620 1.00 0.00
J H
ATOM 13179 CA THR J 442 -14.159 14.769 11.482 1.00 0.00
J C
ATOM 13180 HA THR J 442 -14.236 14.826 12.560 1.00 0.00
J H
ATOM 13181 CB THR J 442 -13.595 16.042 10.864 1.00 0.00
J C
ATOM 13182 HB THR J 442 -13.550 15.859 9.763 1.00 0.00
J H
ATOM 13183 OG1 THR J 442 -12.269 16.260 11.324 1.00 0.00
J O
ATOM 13184 HG1 THR J 442 -11.984 17.079 10.906 1.00 0.00
J H
ATOM 13185 CG2 THR J 442 -14.393 17.297 11.250 1.00 0.00
J C
ATOM 13186 HG21 THR J 442 -14.026 18.185 10.694 1.00 0.00
J H
ATOM 13187 HG22 THR J 442 -15.469 17.162 11.006 1.00 0.00
J H
ATOM 13188 HG23 THR J 442 -14.307 17.507 12.337 1.00 0.00
J H
ATOM 13189 C THR J 442 -15.564 14.536 10.960 1.00 0.00
J C
ATOM 13190 O THR J 442 -16.550 14.821 11.625 1.00 0.00
J O
ATOM 13191 N PHE J 443 -15.684 13.962 9.744 1.00 0.00
J N
ATOM 13192 HN PHE J 443 -14.882 13.801 9.171 1.00 0.00
J H
ATOM 13193 CA PHE J 443 -16.939 13.531 9.154 1.00 0.00
J C
ATOM 13194 HA PHE J 443 -17.590 14.393 9.236 1.00 0.00
J H
ATOM 13195 CB PHE J 443 -16.614 13.236 7.653 1.00 0.00
J C
ATOM 13196 HB1 PHE J 443 -16.512 14.199 7.106 1.00 0.00
J H
ATOM 13197 HB2 PHE J 443 -15.647 12.698 7.550 1.00 0.00
J H
ATOM 13198 CG PHE J 443 -17.664 12.464 6.899 1.00 0.00
J C
ATOM 13199 CD1 PHE J 443 -18.832 13.081 6.425 1.00 0.00
J C
ATOM 13200 HD1 PHE J 443 -18.985 14.138 6.595 1.00 0.00
J H
ATOM 13201 CE1 PHE J 443 -19.801 12.333 5.739 1.00 0.00
J C
ATOM 13202 HE1 PHE J 443 -20.681 12.821 5.345 1.00 0.00
J H

ATOM 13203 CZ PHE J 443 -19.624 10.955 5.560 1.00 0.00
J C
ATOM 13204 HZ PHE J 443 -20.376 10.376 5.043 1.00 0.00
J H
ATOM 13205 CD2 PHE J 443 -17.489 11.086 6.694 1.00 0.00
J C
ATOM 13206 HD2 PHE J 443 -16.598 10.594 7.059 1.00 0.00
J H
ATOM 13207 CE2 PHE J 443 -18.468 10.329 6.042 1.00 0.00
J C
ATOM 13208 HE2 PHE J 443 -18.329 9.265 5.914 1.00 0.00
J H
ATOM 13209 C PHE J 443 -17.634 12.365 9.894 1.00 0.00
J C
ATOM 13210 O PHE J 443 -18.810 12.479 10.241 1.00 0.00
J O
ATOM 13211 N VAL J 444 -16.942 11.237 10.197 1.00 0.00
J N
ATOM 13212 HN VAL J 444 -15.961 11.163 10.040 1.00 0.00
J H
ATOM 13213 CA VAL J 444 -17.558 10.033 10.778 1.00 0.00
J C
ATOM 13214 HA VAL J 444 -18.375 9.761 10.122 1.00 0.00
J H
ATOM 13215 CB VAL J 444 -16.623 8.814 10.788 1.00 0.00
J C
ATOM 13216 HB VAL J 444 -15.679 9.118 11.303 1.00 0.00
J H
ATOM 13217 CG1 VAL J 444 -17.243 7.621 11.556 1.00 0.00
J C
ATOM 13218 HG11 VAL J 444 -16.565 6.742 11.515 1.00 0.00
J H
ATOM 13219 HG12 VAL J 444 -17.405 7.863 12.628 1.00 0.00
J H
ATOM 13220 HG13 VAL J 444 -18.215 7.327 11.106 1.00 0.00
J H
ATOM 13221 CG2 VAL J 444 -16.371 8.368 9.333 1.00 0.00
J C
ATOM 13222 HG21 VAL J 444 -15.729 7.462 9.305 1.00 0.00
J H
ATOM 13223 HG22 VAL J 444 -17.330 8.132 8.824 1.00 0.00
J H
ATOM 13224 HG23 VAL J 444 -15.856 9.161 8.753 1.00 0.00
J H
ATOM 13225 C VAL J 444 -18.144 10.262 12.166 1.00 0.00
J C
ATOM 13226 O VAL J 444 -19.223 9.759 12.487 1.00 0.00
J O
ATOM 13227 N PHE J 445 -17.470 11.073 13.011 1.00 0.00
J N
ATOM 13228 HN PHE J 445 -16.613 11.505 12.734 1.00 0.00
J H
ATOM 13229 CA PHE J 445 -17.898 11.397 14.365 1.00 0.00
J C

ATOM 13230 HA   PHE J 445   -17.995 10.437 14.859 1.00 0.00
J   H
ATOM 13231 CB   PHE J 445   -16.845 12.297 15.075 1.00 0.00
J   C
ATOM 13232 HB1 PHE J 445   -16.449 13.039 14.346 1.00 0.00
J   H
ATOM 13233 HB2 PHE J 445   -17.306 12.871 15.908 1.00 0.00
J   H
ATOM 13234 CG   PHE J 445   -15.634 11.555 15.604 1.00 0.00
J   C
ATOM 13235 CD1 PHE J 445   -15.694 10.250 16.141 1.00 0.00
J   C
ATOM 13236 HD1 PHE J 445   -16.626   9.702 16.113 1.00 0.00
J   H
ATOM 13237 CE1 PHE J 445   -14.562   9.657 16.716 1.00 0.00
J   C
ATOM 13238 HE1 PHE J 445   -14.619   8.651 17.109 1.00 0.00
J   H
ATOM 13239 CZ   PHE J 445   -13.356 10.361 16.779 1.00 0.00
J   C
ATOM 13240 HZ   PHE J 445   -12.483   9.904 17.223 1.00 0.00
J   H
ATOM 13241 CD2 PHE J 445   -14.410 12.244 15.673 1.00 0.00
J   C
ATOM 13242 HD2 PHE J 445   -14.336 13.253 15.291 1.00 0.00
J   H
ATOM 13243 CE2 PHE J 445   -13.279 11.658 16.257 1.00 0.00
J   C
ATOM 13244 HE2 PHE J 445   -12.350 12.209 16.303 1.00 0.00
J   H
ATOM 13245 C   PHE J 445   -19.257 12.088 14.458 1.00 0.00
J   C
ATOM 13246 O   PHE J 445   -19.912 12.053 15.496 1.00 0.00
J   O
ATOM 13247 N   SER J 446   -19.734 12.739 13.383 1.00 0.00
J   N
ATOM 13248 HN   SER J 446   -19.187 12.936 12.569 1.00 0.00
J   H
ATOM 13249 CA   SER J 446   -21.109 13.221 13.309 1.00 0.00
J   C
ATOM 13250 HA   SER J 446   -21.254 13.743 14.245 1.00 0.00
J   H
ATOM 13251 CB   SER J 446   -21.361 14.102 12.071 1.00 0.00
J   C
ATOM 13252 HB1 SER J 446   -21.144 13.494 11.162 1.00 0.00
J   H
ATOM 13253 HB2 SER J 446   -22.433 14.398 12.017 1.00 0.00
J   H
ATOM 13254 OG   SER J 446   -20.496 15.228 12.110 1.00 0.00
J   O
ATOM 13255 HG1 SER J 446   -20.515 15.631 11.233 1.00 0.00
J   H
ATOM 13256 C   SER J 446   -22.180 12.148 13.219 1.00 0.00
J   C

ATOM 13257 O   SER J 446   -23.299 12.335 13.699 1.00 0.00
J   O
ATOM 13258 N   PHE J 447   -21.868 11.046 12.511 1.00 0.00
J   N
ATOM 13259 HN PHE J 447   -20.937 10.927 12.171 1.00 0.00
J   H
ATOM 13260 CA PHE J 447   -22.775   9.960 12.172 1.00 0.00
J   C
ATOM 13261 HA PHE J 447   -23.760 10.405 12.095 1.00 0.00
J   H
ATOM 13262 CB PHE J 447   -22.287   9.191 10.904 1.00 0.00
J   C
ATOM 13263 HB1 PHE J 447   -21.189   9.034 10.974 1.00 0.00
J   H
ATOM 13264 HB2 PHE J 447   -22.758   8.186 10.841 1.00 0.00
J   H
ATOM 13265 CG PHE J 447   -22.537   9.957   9.636 1.00 0.00
J   C
ATOM 13266 CD1 PHE J 447   -21.822 11.129   9.344 1.00 0.00
J   C
ATOM 13267 HD1 PHE J 447   -21.086 11.496 10.045 1.00 0.00
J   H
ATOM 13268 CE1 PHE J 447   -22.054 11.833   8.159 1.00 0.00
J   C
ATOM 13269 HE1 PHE J 447   -21.528 12.758   7.969 1.00 0.00
J   H
ATOM 13270 CZ PHE J 447   -22.964 11.336   7.218 1.00 0.00
J   C
ATOM 13271 HZ PHE J 447   -23.115 11.856   6.284 1.00 0.00
J   H
ATOM 13272 CD2 PHE J 447   -23.467   9.480   8.695 1.00 0.00
J   C
ATOM 13273 HD2 PHE J 447   -24.048   8.595   8.911 1.00 0.00
J   H
ATOM 13274 CE2 PHE J 447   -23.670 10.157   7.485 1.00 0.00
J   C
ATOM 13275 HE2 PHE J 447   -24.373   9.768   6.761 1.00 0.00
J   H
ATOM 13276 C   PHE J 447   -22.839   8.928 13.277 1.00 0.00
J   C
ATOM 13277 O   PHE J 447   -23.912   8.516 13.720 1.00 0.00
J   O
ATOM 13278 N   ALA J 448   -21.663   8.467 13.742 1.00 0.00
J   N
ATOM 13279 HN ALA J 448   -20.790   8.809 13.398 1.00 0.00
J   H
ATOM 13280 CA ALA J 448   -21.572   7.454 14.764 1.00 0.00
J   C
ATOM 13281 HA ALA J 448   -22.366   6.724 14.655 1.00 0.00
J   H
ATOM 13282 CB ALA J 448   -20.233   6.691 14.660 1.00 0.00
J   C
ATOM 13283 HB1 ALA J 448   -20.155   6.209 13.662 1.00 0.00
J   H

ATOM 13284 HB2 ALA J 448   -19.369   7.381 14.775 1.00 0.00
J   H
ATOM 13285 HB3 ALA J 448   -20.154   5.895 15.432 1.00 0.00
J   H
ATOM 13286 C   ALA J 448   -21.740   8.069 16.143 1.00 0.00
J   C
ATOM 13287 O   ALA J 448   -21.282   9.170 16.430 1.00 0.00
J   O
ATOM 13288 N   LYS J 449   -22.428   7.362 17.055 1.00 0.00
J   N
ATOM 13289 HN LYS J 449   -22.815   6.461 16.867 1.00 0.00
J   H
ATOM 13290 CA LYS J 449   -22.660   7.863 18.390 1.00 0.00
J   C
ATOM 13291 HA LYS J 449   -22.486   8.932 18.385 1.00 0.00
J   H
ATOM 13292 CB LYS J 449   -24.088   7.543 18.875 1.00 0.00
J   C
ATOM 13293 HB1 LYS J 449   -24.240   6.446 18.749 1.00 0.00
J   H
ATOM 13294 HB2 LYS J 449   -24.152   7.743 19.969 1.00 0.00
J   H
ATOM 13295 CG LYS J 449   -25.138   8.326 18.078 1.00 0.00
J   C
ATOM 13296 HG1 LYS J 449   -24.947   9.417 18.195 1.00 0.00
J   H
ATOM 13297 HG2 LYS J 449   -25.027   8.083 16.996 1.00 0.00
J   H
ATOM 13298 CD LYS J 449   -26.566   8.017 18.547 1.00 0.00
J   C
ATOM 13299 HD1 LYS J 449   -26.751   6.926 18.419 1.00 0.00
J   H
ATOM 13300 HD2 LYS J 449   -26.629   8.240 19.638 1.00 0.00
J   H
ATOM 13301 CE LYS J 449   -27.621   8.827 17.788 1.00 0.00
J   C
ATOM 13302 HE1 LYS J 449   -27.467   9.917 17.952 1.00 0.00
J   H
ATOM 13303 HE2 LYS J 449   -27.559   8.623 16.696 1.00 0.00
J   H
ATOM 13304 NZ LYS J 449   -28.975   8.465 18.262 1.00 0.00
J   N
ATOM 13305 HZ1 LYS J 449   -29.692   9.016 17.747 1.00 0.00
J   H
ATOM 13306 HZ2 LYS J 449   -29.139   7.450 18.102 1.00 0.00
J   H
ATOM 13307 HZ3 LYS J 449   -29.051   8.666 19.280 1.00 0.00
J   H
ATOM 13308 C   LYS J 449   -21.648   7.247 19.319 1.00 0.00
J   C
ATOM 13309 O   LYS J 449   -21.391   6.048 19.264 1.00 0.00
J   O
ATOM 13310 N   THR J 450   -21.037   8.088 20.177 1.00 0.00
J   N

ATOM 13311 HN THR J 450 -21.226 9.069 20.167 1.00 0.00
J H
ATOM 13312 CA THR J 450 -20.062 7.675 21.182 1.00 0.00
J C
ATOM 13313 HA THR J 450 -19.301 7.136 20.633 1.00 0.00
J H
ATOM 13314 CB THR J 450 -19.363 8.869 21.856 1.00 0.00
J C
ATOM 13315 HB THR J 450 -18.795 9.392 21.048 1.00 0.00
J H
ATOM 13316 OG1 THR J 450 -18.402 8.501 22.838 1.00 0.00
J O
ATOM 13317 HG1 THR J 450 -18.056 9.332 23.182 1.00 0.00
J H
ATOM 13318 CG2 THR J 450 -20.368 9.818 22.531 1.00 0.00
J C
ATOM 13319 HG21 THR J 450 -19.858 10.728 22.911 1.00 0.00
J H
ATOM 13320 HG22 THR J 450 -21.144 10.140 21.805 1.00 0.00
J H
ATOM 13321 HG23 THR J 450 -20.875 9.321 23.386 1.00 0.00
J H
ATOM 13322 C THR J 450 -20.634 6.700 22.201 1.00 0.00
J C
ATOM 13323 O THR J 450 -21.726 6.889 22.738 1.00 0.00
J O
ATOM 13324 N GLU J 451 -19.886 5.616 22.480 1.00 0.00
J N
ATOM 13325 HN GLU J 451 -19.004 5.456 22.045 1.00 0.00
J H
ATOM 13326 CA GLU J 451 -20.272 4.590 23.419 1.00 0.00
J C
ATOM 13327 HA GLU J 451 -21.236 4.851 23.838 1.00 0.00
J H
ATOM 13328 CB GLU J 451 -20.495 3.217 22.720 1.00 0.00
J C
ATOM 13329 HB1 GLU J 451 -20.766 2.470 23.501 1.00 0.00
J H
ATOM 13330 HB2 GLU J 451 -21.374 3.278 22.040 1.00 0.00
J H
ATOM 13331 CG GLU J 451 -19.310 2.611 21.907 1.00 0.00
J C
ATOM 13332 HG1 GLU J 451 -18.849 3.399 21.285 1.00 0.00
J H
ATOM 13333 HG2 GLU J 451 -18.542 2.184 22.582 1.00 0.00
J H
ATOM 13334 CD GLU J 451 -19.753 1.519 20.926 1.00 0.00
J C
ATOM 13335 OE1 GLU J 451 -18.905 0.700 20.463 1.00 0.00
J O
ATOM 13336 OE2 GLU J 451 -20.957 1.490 20.560 1.00 0.00
J O
ATOM 13337 C GLU J 451 -19.284 4.530 24.567 1.00 0.00
J C

ATOM 13338 O   GLU J 451   -18.063   4.458 24.410 1.00 0.00
J   O
ATOM 13339 N   GLN J 452   -19.819   4.601 25.801 1.00 0.00
J   N
ATOM 13340 HN GLN J 452   -20.806   4.653 25.946 1.00 0.00
J   H
ATOM 13341 CA GLN J 452   -19.037   4.609 27.013 1.00 0.00
J   C
ATOM 13342 HA GLN J 452   -17.986   4.660 26.755 1.00 0.00
J   H
ATOM 13343 CB GLN J 452   -19.415   5.804 27.936 1.00 0.00
J   C
ATOM 13344 HB1 GLN J 452   -19.791   6.616 27.273 1.00 0.00
J   H
ATOM 13345 HB2 GLN J 452   -20.271   5.512 28.586 1.00 0.00
J   H
ATOM 13346 CG GLN J 452   -18.253   6.367 28.807 1.00 0.00
J   C
ATOM 13347 HG1 GLN J 452   -17.329   6.476 28.198 1.00 0.00
J   H
ATOM 13348 HG2 GLN J 452   -18.513   7.367 29.212 1.00 0.00
J   H
ATOM 13349 CD GLN J 452   -17.849   5.484 29.993 1.00 0.00
J   C
ATOM 13350 OE1 GLN J 452   -18.536   4.532 30.364 1.00 0.00
J   O
ATOM 13351 NE2 GLN J 452   -16.713   5.818 30.648 1.00 0.00
J   N
ATOM 13352 HE21 GLN J 452   -16.450   5.241 31.415 1.00 0.00
J   H
ATOM 13353 HE22 GLN J 452   -16.196   6.619 30.364 1.00 0.00
J   H
ATOM 13354 C   GLN J 452   -19.273   3.280 27.691 1.00 0.00
J   C
ATOM 13355 O   GLN J 452   -20.407   2.869 27.928 1.00 0.00
J   O
ATOM 13356 N   SER J 453   -18.187   2.551 27.977 1.00 0.00
J   N
ATOM 13357 HN SER J 453   -17.260   2.826 27.725 1.00 0.00
J   H
ATOM 13358 CA SER J 453   -18.259   1.294 28.681 1.00 0.00
J   C
ATOM 13359 HA SER J 453   -19.231   1.233 29.153 1.00 0.00
J   H
ATOM 13360 CB SER J 453   -17.952   0.086 27.766 1.00 0.00
J   C
ATOM 13361 HB1 SER J 453   -18.661   0.114 26.907 1.00 0.00
J   H
ATOM 13362 HB2 SER J 453   -16.925   0.170 27.345 1.00 0.00
J   H
ATOM 13363 OG SER J 453   -18.128 -1.141 28.473 1.00 0.00
J   O
ATOM 13364 HG1 SER J 453   -18.145 -1.847 27.815 1.00 0.00
J   H

ATOM 13365 C SER J 453 -17.230 1.345 29.781 1.00 0.00
J C
ATOM 13366 O SER J 453 -16.071 1.675 29.535 1.00 0.00
J O
ATOM 13367 N ASN J 454 -17.636 1.005 31.020 1.00 0.00
J N
ATOM 13368 HN ASN J 454 -18.587 0.781 31.225 1.00 0.00
J H
ATOM 13369 CA ASN J 454 -16.740 0.932 32.160 1.00 0.00
J C
ATOM 13370 HA ASN J 454 -15.876 1.535 31.904 1.00 0.00
J H
ATOM 13371 CB ASN J 454 -17.458 1.378 33.462 1.00 0.00
J C
ATOM 13372 HB1 ASN J 454 -18.382 0.768 33.567 1.00 0.00
J H
ATOM 13373 HB2 ASN J 454 -16.820 1.170 34.345 1.00 0.00
J H
ATOM 13374 CG ASN J 454 -17.830 2.853 33.363 1.00 0.00
J C
ATOM 13375 OD1 ASN J 454 -16.990 3.714 33.103 1.00 0.00
J O
ATOM 13376 ND2 ASN J 454 -19.122 3.174 33.607 1.00 0.00
J N
ATOM 13377 HD21 ASN J 454 -19.376 4.135 33.534 1.00 0.00
J H
ATOM 13378 HD22 ASN J 454 -19.781 2.460 33.821 1.00 0.00
J H
ATOM 13379 C ASN J 454 -16.258 -0.499 32.371 1.00 0.00
J C
ATOM 13380 O ASN J 454 -15.469 -0.769 33.274 1.00 0.00
J O
ATOM 13381 N ILE J 455 -16.711 -1.462 31.538 1.00 0.00
J N
ATOM 13382 HN ILE J 455 -17.338 -1.285 30.783 1.00 0.00
J H
ATOM 13383 CA ILE J 455 -16.318 -2.854 31.671 1.00 0.00
J C
ATOM 13384 HA ILE J 455 -15.890 -2.976 32.658 1.00 0.00
J H
ATOM 13385 CB ILE J 455 -17.473 -3.867 31.589 1.00 0.00
J C
ATOM 13386 HB ILE J 455 -17.032 -4.824 31.967 1.00 0.00
J H
ATOM 13387 CG2 ILE J 455 -18.616 -3.349 32.497 1.00 0.00
J C
ATOM 13388 HG21 ILE J 455 -19.411 -4.116 32.599 1.00 0.00
J H
ATOM 13389 HG22 ILE J 455 -18.238 -3.117 33.515 1.00 0.00
J H
ATOM 13390 HG23 ILE J 455 -19.073 -2.429 32.075 1.00 0.00
J H
ATOM 13391 CG1 ILE J 455 -17.945 -4.173 30.143 1.00 0.00
J C

```
ATOM  13392 HG11 ILE J 455     -18.188  -3.223  29.616  1.00  0.00
J    H
ATOM  13393 HG12 ILE J 455     -17.110  -4.655  29.585  1.00  0.00
J    H
ATOM  13394  CD  ILE J 455     -19.162  -5.104  30.047  1.00  0.00
J    C
ATOM  13395  HD1 ILE J 455     -19.400  -5.319  28.982  1.00  0.00
J    H
ATOM  13396  HD2 ILE J 455     -18.958  -6.068  30.559  1.00  0.00
J    H
ATOM  13397  HD3 ILE J 455     -20.057  -4.639  30.512  1.00  0.00
J    H
ATOM  13398  C   ILE J 455     -15.215  -3.149  30.675  1.00  0.00
J    C
ATOM  13399  O   ILE J 455     -15.204  -2.654  29.548  1.00  0.00
J    O
ATOM  13400  N   ARG J 456     -14.230  -3.949  31.108  1.00  0.00
J    N
ATOM  13401  HN  ARG J 456     -14.242  -4.259  32.056  1.00  0.00
J    H
ATOM  13402  CA  ARG J 456     -13.130  -4.406  30.291  1.00  0.00
J    C
ATOM  13403  HA  ARG J 456     -13.148  -3.883  29.342  1.00  0.00
J    H
ATOM  13404  CB  ARG J 456     -11.786  -4.288  31.047  1.00  0.00
J    C
ATOM  13405  HB1 ARG J 456     -11.868  -4.951  31.942  1.00  0.00
J    H
ATOM  13406  HB2 ARG J 456     -10.969  -4.714  30.425  1.00  0.00
J    H
ATOM  13407  CG  ARG J 456     -11.439  -2.860  31.523  1.00  0.00
J    C
ATOM  13408  HG1 ARG J 456     -12.352  -2.230  31.445  1.00  0.00
J    H
ATOM  13409  HG2 ARG J 456     -11.190  -2.890  32.609  1.00  0.00
J    H
ATOM  13410  CD  ARG J 456     -10.248  -2.205  30.803  1.00  0.00
J    C
ATOM  13411  HD1 ARG J 456     -10.526  -1.996  29.744  1.00  0.00
J    H
ATOM  13412  HD2 ARG J 456      -9.951  -1.248  31.291  1.00  0.00
J    H
ATOM  13413  NE  ARG J 456      -9.064  -3.140  30.857  1.00  0.00
J    N
ATOM  13414  HE  ARG J 456      -8.825  -3.528  29.959  1.00  0.00
J    H
ATOM  13415  CZ  ARG J 456      -8.357  -3.464  31.951  1.00  0.00
J    C
ATOM  13416  NH1 ARG J 456      -8.571  -2.868  33.118  1.00  0.00
J    N
ATOM  13417 HH11 ARG J 456      -8.024  -3.100  33.911  1.00  0.00
J    H
ATOM  13418 HH12 ARG J 456      -9.207  -2.094  33.111  1.00  0.00
J    H
```

ATOM 13419 NH2 ARG J 456   -7.438 -4.429 31.895 1.00 0.00
J   N
ATOM 13420 HH21 ARG J 456   -6.981 -4.724 32.722 1.00 0.00
J   H
ATOM 13421 HH22 ARG J 456   -7.397 -4.961 31.059 1.00 0.00
J   H
ATOM 13422 C   ARG J 456   -13.320 -5.872 29.979 1.00 0.00
J   C
ATOM 13423 O   ARG J 456   -14.082 -6.572 30.646 1.00 0.00
J   O
ATOM 13424 N   TYR J 457   -12.598 -6.375 28.970 1.00 0.00
J   N
ATOM 13425 HN TYR J 457   -11.992 -5.814 28.409 1.00 0.00
J   H
ATOM 13426 CA TYR J 457   -12.633 -7.772 28.603 1.00 0.00
J   C
ATOM 13427 HA TYR J 457   -13.334 -8.257 29.272 1.00 0.00
J   H
ATOM 13428 CB TYR J 457   -13.046 -7.895 27.117 1.00 0.00
J   C
ATOM 13429 HB1 TYR J 457   -12.500 -7.130 26.523 1.00 0.00
J   H
ATOM 13430 HB2 TYR J 457   -12.763 -8.890 26.712 1.00 0.00
J   H
ATOM 13431 CG TYR J 457   -14.522 -7.641 27.024 1.00 0.00
J   C
ATOM 13432 CD1 TYR J 457   -15.422 -8.703 27.201 1.00 0.00
J   C
ATOM 13433 HD1 TYR J 457   -15.046 -9.703 27.372 1.00 0.00
J   H
ATOM 13434 CE1 TYR J 457   -16.804 -8.484 27.136 1.00 0.00
J   C
ATOM 13435 HE1 TYR J 457   -17.497 -9.301 27.268 1.00 0.00
J   H
ATOM 13436 CZ TYR J 457   -17.297 -7.200 26.893 1.00 0.00
J   C
ATOM 13437 OH TYR J 457   -18.687 -7.014 26.779 1.00 0.00
J   O
ATOM 13438 HH TYR J 457   -18.845 -6.073 26.686 1.00 0.00
J   H
ATOM 13439 CD2 TYR J 457   -15.029 -6.344 26.825 1.00 0.00
J   C
ATOM 13440 HD2 TYR J 457   -14.350 -5.515 26.679 1.00 0.00
J   H
ATOM 13441 CE2 TYR J 457   -16.412 -6.122 26.761 1.00 0.00
J   C
ATOM 13442 HE2 TYR J 457   -16.787 -5.124 26.590 1.00 0.00
J   H
ATOM 13443 C   TYR J 457   -11.271 -8.413 28.837 1.00 0.00
J   C
ATOM 13444 O   TYR J 457   -10.259 -7.709 28.861 1.00 0.00
J   O
ATOM 13445 N   PRO J 458   -11.174 -9.734 29.041 1.00 0.00
J   N

ATOM 13446 CD PRO J 458   -12.303 -10.653 29.202 1.00 0.00
J   C
ATOM 13447 HD1 PRO J 458   -12.834 -10.555 30.176 1.00 0.00
J   H
ATOM 13448 HD2 PRO J 458   -13.004 -10.471 28.355 1.00 0.00
J   H
ATOM 13449 CA PRO J 458   -9.891 -10.420 29.147 1.00 0.00
J   C
ATOM 13450 HA PRO J 458   -9.256 -9.941 29.884 1.00 0.00
J   H
ATOM 13451 CB PRO J 458   -10.261 -11.849 29.601 1.00 0.00
J   C
ATOM 13452 HB1 PRO J 458   -10.362 -11.863 30.709 1.00 0.00
J   H
ATOM 13453 HB2 PRO J 458   -9.507 -12.604 29.298 1.00 0.00
J   H
ATOM 13454 CG PRO J 458   -11.687 -12.047 29.080 1.00 0.00
J   C
ATOM 13455 HG1 PRO J 458   -12.249 -12.818 29.642 1.00 0.00
J   H
ATOM 13456 HG2 PRO J 458   -11.648 -12.328 28.002 1.00 0.00
J   H
ATOM 13457 C   PRO J 458   -9.175 -10.435 27.808 1.00 0.00
J   C
ATOM 13458 O   PRO J 458   -9.831 -10.453 26.763 1.00 0.00
J   O
ATOM 13459 N   ASP J 459   -7.833 -10.393 27.836 1.00 0.00
J   N
ATOM 13460 HN ASP J 459   -7.350 -10.422 28.707 1.00 0.00
J   H
ATOM 13461 CA ASP J 459   -6.943 -10.307 26.701 1.00 0.00
J   C
ATOM 13462 HA ASP J 459   -7.211 -9.388 26.192 1.00 0.00
J   H
ATOM 13463 CB ASP J 459   -5.470 -10.236 27.200 1.00 0.00
J   C
ATOM 13464 HB1 ASP J 459   -5.255 -11.125 27.829 1.00 0.00
J   H
ATOM 13465 HB2 ASP J 459   -4.767 -10.237 26.344 1.00 0.00
J   H
ATOM 13466 CG ASP J 459   -5.154 -9.010 28.058 1.00 0.00
J   C
ATOM 13467 OD1 ASP J 459   -6.074 -8.229 28.427 1.00 0.00
J   O
ATOM 13468 OD2 ASP J 459   -3.939 -8.785 28.281 1.00 0.00
J   O
ATOM 13469 C   ASP J 459   -7.097 -11.439 25.679 1.00 0.00
J   C
ATOM 13470 O   ASP J 459   -7.367 -12.597 26.001 1.00 0.00
J   O
ATOM 13471 N   GLY J 460   -6.966 -11.103 24.382 1.00 0.00
J   N
ATOM 13472 HN GLY J 460   -6.773 -10.158 24.120 1.00 0.00
J   H

ATOM 13473 CA GLY J 460 -7.090 -12.046 23.284 1.00 0.00
J C
ATOM 13474 HA1 GLY J 460 -7.937 -11.727 22.691 1.00 0.00
J H
ATOM 13475 HA2 GLY J 460 -7.199 -13.029 23.722 1.00 0.00
J H
ATOM 13476 C GLY J 460 -5.896 -12.068 22.403 1.00 0.00
J C
ATOM 13477 O GLY J 460 -5.603 -11.093 21.718 1.00 0.00
J O
ATOM 13478 N GLU J 461 -5.204 -13.216 22.355 1.00 0.00
J N
ATOM 13479 HN GLU J 461 -5.385 -14.016 22.920 1.00 0.00
J H
ATOM 13480 CA GLU J 461 -4.099 -13.381 21.439 1.00 0.00
J C
ATOM 13481 HA GLU J 461 -3.555 -12.445 21.416 1.00 0.00
J H
ATOM 13482 CB GLU J 461 -3.128 -14.491 21.878 1.00 0.00
J C
ATOM 13483 HB1 GLU J 461 -2.815 -14.284 22.928 1.00 0.00
J H
ATOM 13484 HB2 GLU J 461 -3.658 -15.469 21.904 1.00 0.00
J H
ATOM 13485 CG GLU J 461 -1.877 -14.565 20.961 1.00 0.00
J C
ATOM 13486 HG1 GLU J 461 -1.218 -15.384 21.302 1.00 0.00
J H
ATOM 13487 HG2 GLU J 461 -2.167 -14.745 19.906 1.00 0.00
J H
ATOM 13488 CD GLU J 461 -1.011 -13.301 21.007 1.00 0.00
J C
ATOM 13489 OE1 GLU J 461 -0.906 -12.664 22.087 1.00 0.00
J O
ATOM 13490 OE2 GLU J 461 -0.441 -12.967 19.937 1.00 0.00
J O
ATOM 13491 C GLU J 461 -4.552 -13.675 20.019 1.00 0.00
J C
ATOM 13492 O GLU J 461 -5.443 -14.489 19.762 1.00 0.00
J O
ATOM 13493 N VAL J 462 -3.910 -13.000 19.051 1.00 0.00
J N
ATOM 13494 HN VAL J 462 -3.189 -12.340 19.240 1.00 0.00
J H
ATOM 13495 CA VAL J 462 -4.208 -13.172 17.654 1.00 0.00
J C
ATOM 13496 HA VAL J 462 -4.474 -14.209 17.493 1.00 0.00
J H
ATOM 13497 CB VAL J 462 -5.430 -12.343 17.239 1.00 0.00
J C
ATOM 13498 HB VAL J 462 -6.330 -12.812 17.708 1.00 0.00
J H
ATOM 13499 CG1 VAL J 462 -5.296 -10.891 17.738 1.00 0.00
J C

```
ATOM  13500 HG11 VAL J 462      -6.165 -10.286  17.402  1.00  0.00
J    H
ATOM  13501 HG12 VAL J 462      -5.268 -10.840  18.846  1.00  0.00
J    H
ATOM  13502 HG13 VAL J 462      -4.374 -10.421  17.336  1.00  0.00
J    H
ATOM  13503  CG2 VAL J 462      -5.659 -12.396  15.717  1.00  0.00
J    C
ATOM  13504 HG21 VAL J 462      -6.521 -11.760  15.427  1.00  0.00
J    H
ATOM  13505 HG22 VAL J 462      -4.759 -12.033  15.174  1.00  0.00
J    H
ATOM  13506 HG23 VAL J 462      -5.876 -13.430  15.379  1.00  0.00
J    H
ATOM  13507  C   VAL J 462      -2.961 -12.885  16.826  1.00  0.00
J    C
ATOM  13508  O   VAL J 462      -2.365 -11.813  16.848  1.00  0.00
J    O
ATOM  13509  N   PHE J 463      -2.524 -13.889  16.038  1.00  0.00
J    N
ATOM  13510  HN  PHE J 463      -3.043 -14.736  15.940  1.00  0.00
J    H
ATOM  13511  CA  PHE J 463      -1.292 -13.847  15.272  1.00  0.00
J    C
ATOM  13512  HA  PHE J 463      -0.574 -13.327  15.894  1.00  0.00
J    H
ATOM  13513  CB  PHE J 463      -0.815 -15.283  14.895  1.00  0.00
J    C
ATOM  13514  HB1 PHE J 463      -1.458 -15.678  14.078  1.00  0.00
J    H
ATOM  13515  HB2 PHE J 463       0.228 -15.271  14.513  1.00  0.00
J    H
ATOM  13516  CG  PHE J 463      -0.945 -16.218  16.066  1.00  0.00
J    C
ATOM  13517  CD1 PHE J 463      -0.182 -16.020  17.228  1.00  0.00
J    C
ATOM  13518  HD1 PHE J 463       0.550 -15.225  17.258  1.00  0.00
J    H
ATOM  13519  CE1 PHE J 463      -0.353 -16.846  18.345  1.00  0.00
J    C
ATOM  13520  HE1 PHE J 463       0.254 -16.697  19.227  1.00  0.00
J    H
ATOM  13521  CZ  PHE J 463      -1.317 -17.862  18.323  1.00  0.00
J    C
ATOM  13522  HZ  PHE J 463      -1.461 -18.492  19.188  1.00  0.00
J    H
ATOM  13523  CD2 PHE J 463      -1.897 -17.255  16.048  1.00  0.00
J    C
ATOM  13524  HD2 PHE J 463      -2.461 -17.454  15.147  1.00  0.00
J    H
ATOM  13525  CE2 PHE J 463      -2.102 -18.053  17.180  1.00  0.00
J    C
ATOM  13526  HE2 PHE J 463      -2.867 -18.816  17.169  1.00  0.00
J    H
```

```
ATOM  13527  C   PHE J 463      -1.446 -13.031  13.986  1.00  0.00
J    C
ATOM  13528  O   PHE J 463      -2.313 -13.307  13.158  1.00  0.00
J    O
ATOM  13529  N   THR J 464      -0.598 -12.002  13.790  1.00  0.00
J    N
ATOM  13530  HN  THR J 464       0.168 -11.846  14.414  1.00  0.00
J    H
ATOM  13531  CA  THR J 464      -0.689 -11.030  12.693  1.00  0.00
J    C
ATOM  13532  HA  THR J 464      -1.538 -11.367  12.113  1.00  0.00
J    H
ATOM  13533  CB  THR J 464      -0.828  -9.611  13.241  1.00  0.00
J    C
ATOM  13534  HB  THR J 464      -0.665  -8.926  12.374  1.00  0.00
J    H
ATOM  13535  OG1 THR J 464       0.181  -9.321  14.201  1.00  0.00
J    O
ATOM  13536  HG1 THR J 464       0.006  -8.420  14.494  1.00  0.00
J    H
ATOM  13537  CG2 THR J 464      -2.166  -9.482  13.978  1.00  0.00
J    C
ATOM  13538 HG21 THR J 464      -2.346  -8.431  14.289  1.00  0.00
J    H
ATOM  13539 HG22 THR J 464      -3.004  -9.791  13.318  1.00  0.00
J    H
ATOM  13540 HG23 THR J 464      -2.186 -10.122  14.887  1.00  0.00
J    H
ATOM  13541  C   THR J 464       0.504 -11.007  11.743  1.00  0.00
J    C
ATOM  13542  O   THR J 464       0.622 -10.104  10.920  1.00  0.00
J    O
ATOM  13543  N   GLY J 465       1.419 -11.996  11.810  1.00  0.00
J    N
ATOM  13544  HN  GLY J 465       1.375 -12.704  12.515  1.00  0.00
J    H
ATOM  13545  CA  GLY J 465       2.533 -12.109  10.869  1.00  0.00
J    C
ATOM  13546  HA1 GLY J 465       3.350 -12.574  11.403  1.00  0.00
J    H
ATOM  13547  HA2 GLY J 465       2.748 -11.112  10.508  1.00  0.00
J    H
ATOM  13548  C   GLY J 465       2.198 -12.985   9.662  1.00  0.00
J    C
ATOM  13549  OT1 GLY J 465       1.259 -13.318   9.753  1.00  0.00
J    O
ATOM  13550  OT2 GLY J 465       2.993 -13.025   9.058  1.00  0.00
J    O
ATOM  13551  N   GLN A   8     -30.063   8.471  84.614  1.00  0.00
A    N
ATOM  13552  HT1 GLN A   8     -30.528   8.479  85.499  1.00  0.00
A    H
ATOM  13553  HT2 GLN A   8     -29.342   9.163  84.601  1.00  0.00
A    H
```

ATOM 13554 HT3 GLN A   8   -29.670   7.567 84.442 1.00 0.00
A   H
ATOM 13555 CA GLN A   8   -31.052   8.782 83.542 1.00 0.00
A   C
ATOM 13556 HA GLN A   8   -31.785   7.984 83.572 1.00 0.00
A   H
ATOM 13557 CB GLN A   8   -31.742 10.110 83.844 1.00 0.00
A   C
ATOM 13558 HB1 GLN A   8   -31.650 10.280 84.941 1.00 0.00
A   H
ATOM 13559 HB2 GLN A   8   -31.179 10.938 83.354 1.00 0.00
A   H
ATOM 13560 CG GLN A   8   -33.209 10.152 83.451 1.00 0.00
A   C
ATOM 13561 HG1 GLN A   8   -33.313 10.190 82.345 1.00 0.00
A   H
ATOM 13562 HG2 GLN A   8   -33.739   9.246 83.812 1.00 0.00
A   H
ATOM 13563 CD GLN A   8   -33.920 11.377 83.989 1.00 0.00
A   C
ATOM 13564 OE1 GLN A   8   -33.351 12.171 84.743 1.00 0.00
A   O
ATOM 13565 NE2 GLN A   8   -35.180 11.536 83.603 1.00 0.00
A   N
ATOM 13566 HE21 GLN A   8   -35.658 12.339 83.946 1.00 0.00
A   H
ATOM 13567 HE22 GLN A   8   -35.606 10.858 83.013 1.00 0.00
A   H
ATOM 13568 C   GLN A   8   -30.439   8.797 82.126 1.00 0.00
A   C
ATOM 13569 O   GLN A   8   -31.080   8.362 81.163 1.00 0.00
A   O
ATOM 13570 N   PHE A   9   -29.211   9.299 82.000 1.00 0.00
A   N
ATOM 13571 HN PHE A   9   -28.674   9.664 82.759 1.00 0.00
A   H
ATOM 13572 CA PHE A   9   -28.538   9.352 80.703 1.00 0.00
A   C
ATOM 13573 HA PHE A   9   -29.091   8.686 80.051 1.00 0.00
A   H
ATOM 13574 CB PHE A   9   -28.466 10.782 80.165 1.00 0.00
A   C
ATOM 13575 HB1 PHE A   9   -27.819 11.390 80.834 1.00 0.00
A   H
ATOM 13576 HB2 PHE A   9   -28.007 10.807 79.154 1.00 0.00
A   H
ATOM 13577 CG PHE A   9   -29.783 11.489 80.118 1.00 0.00
A   C
ATOM 13578 CD1 PHE A   9   -30.611 11.362 79.015 1.00 0.00
A   C
ATOM 13579 HD1 PHE A   9   -30.302 10.746 78.182 1.00 0.00
A   H
ATOM 13580 CE1 PHE A   9   -31.830 12.021 78.967 1.00 0.00
A   C

```
ATOM  13581  HE1 PHE A   9      -32.474  11.904  78.108  1.00  0.00
A    H
ATOM  13582  CZ  PHE A   9      -32.226  12.824  80.024  1.00  0.00
A    C
ATOM  13583  HZ  PHE A   9      -33.177  13.334  79.988  1.00  0.00
A    H
ATOM  13584  CD2 PHE A   9      -30.190  12.304  81.173  1.00  0.00
A    C
ATOM  13585  HD2 PHE A   9      -29.543  12.444  82.028  1.00  0.00
A    H
ATOM  13586  CE2 PHE A   9      -31.406  12.964  81.132  1.00  0.00
A    C
ATOM  13587  HE2 PHE A   9      -31.710  13.586  81.963  1.00  0.00
A    H
ATOM  13588  C   PHE A   9      -27.124   8.818  80.784  1.00  0.00
A    C
ATOM  13589  O   PHE A   9      -26.395   9.111  81.738  1.00  0.00
A    O
ATOM  13590  N   ALA A  10      -26.746   8.037  79.773  1.00  0.00
A    N
ATOM  13591  HN  ALA A  10      -27.397   7.701  79.093  1.00  0.00
A    H
ATOM  13592  CA  ALA A  10      -25.365   7.615  79.580  1.00  0.00
A    C
ATOM  13593  HA  ALA A  10      -24.695   8.110  80.273  1.00  0.00
A    H
ATOM  13594  CB  ALA A  10      -25.228   6.116  79.774  1.00  0.00
A    C
ATOM  13595  HB1 ALA A  10      -25.513   5.847  80.813  1.00  0.00
A    H
ATOM  13596  HB2 ALA A  10      -25.897   5.560  79.081  1.00  0.00
A    H
ATOM  13597  HB3 ALA A  10      -24.183   5.775  79.606  1.00  0.00
A    H
ATOM  13598  C   ALA A  10      -24.915   8.021  78.185  1.00  0.00
A    C
ATOM  13599  O   ALA A  10      -25.663   7.873  77.212  1.00  0.00
A    O
ATOM  13600  N   VAL A  11      -23.707   8.559  78.092  1.00  0.00
A    N
ATOM  13601  HN  VAL A  11      -23.118   8.745  78.875  1.00  0.00
A    H
ATOM  13602  CA  VAL A  11      -23.131   8.931  76.802  1.00  0.00
A    C
ATOM  13603  HA  VAL A  11      -23.829   8.670  76.018  1.00  0.00
A    H
ATOM  13604  CB  VAL A  11      -22.805  10.438  76.725  1.00  0.00
A    C
ATOM  13605  HB  VAL A  11      -22.143  10.685  77.591  1.00  0.00
A    H
ATOM  13606  CG1 VAL A  11      -22.076  10.765  75.431  1.00  0.00
A    C
ATOM  13607 HG11 VAL A  11      -21.880  11.857  75.365  1.00  0.00
A    H
```

```
ATOM  13608 HG12 VAL A  11      -21.096  10.249  75.372  1.00  0.00
A    H
ATOM  13609 HG13 VAL A  11      -22.688  10.472  74.551  1.00  0.00
A    H
ATOM  13610  CG2 VAL A  11      -24.079  11.262  76.846  1.00  0.00
A    C
ATOM  13611 HG21 VAL A  11      -23.856  12.346  76.752  1.00  0.00
A    H
ATOM  13612 HG22 VAL A  11      -24.802  10.983  76.050  1.00  0.00
A    H
ATOM  13613 HG23 VAL A  11      -24.568  11.105  77.831  1.00  0.00
A    H
ATOM  13614  C   VAL A  11      -21.882   8.099  76.595  1.00  0.00
A    C
ATOM  13615  O   VAL A  11      -20.934   8.182  77.378  1.00  0.00
A    O
ATOM  13616  N   ILE A  12      -21.883   7.283  75.543  1.00  0.00
A    N
ATOM  13617  HN  ILE A  12      -22.573   7.267  74.823  1.00  0.00
A    H
ATOM  13618  CA  ILE A  12      -20.814   6.307  75.369  1.00  0.00
A    C
ATOM  13619  HA  ILE A  12      -20.473   6.095  76.374  1.00  0.00
A    H
ATOM  13620  CB  ILE A  12      -21.340   4.934  74.899  1.00  0.00
A    C
ATOM  13621  HB  ILE A  12      -22.128   5.180  74.144  1.00  0.00
A    H
ATOM  13622  CG2 ILE A  12      -20.207   4.051  74.395  1.00  0.00
A    C
ATOM  13623 HG21 ILE A  12      -20.609   3.133  73.918  1.00  0.00
A    H
ATOM  13624 HG22 ILE A  12      -19.597   4.585  73.637  1.00  0.00
A    H
ATOM  13625 HG23 ILE A  12      -19.541   3.745  75.229  1.00  0.00
A    H
ATOM  13626  CG1 ILE A  12      -22.031   4.233  76.065  1.00  0.00
A    C
ATOM  13627 HG11 ILE A  12      -21.324   4.134  76.919  1.00  0.00
A    H
ATOM  13628 HG12 ILE A  12      -22.880   4.866  76.413  1.00  0.00
A    H
ATOM  13629  CD  ILE A  12      -22.584   2.864  75.739  1.00  0.00
A    C
ATOM  13630  HD1 ILE A  12      -23.120   2.445  76.618  1.00  0.00
A    H
ATOM  13631  HD2 ILE A  12      -23.298   2.921  74.890  1.00  0.00
A    H
ATOM  13632  HD3 ILE A  12      -21.769   2.160  75.467  1.00  0.00
A    H
ATOM  13633  C   ILE A  12      -19.594   6.803  74.582  1.00  0.00
A    C
ATOM  13634  O   ILE A  12      -18.491   6.303  74.787  1.00  0.00
A    O
```

```
ATOM  13635  N   GLY A  13     -19.745   7.795  73.722  1.00  0.00
A    N
ATOM  13636  HN  GLY A  13     -20.607   8.221  73.450  1.00  0.00
A    H
ATOM  13637  CA  GLY A  13     -18.549   8.338  73.091  1.00  0.00
A    C
ATOM  13638  HA1 GLY A  13     -18.708   8.263  72.025  1.00  0.00
A    H
ATOM  13639  HA2 GLY A  13     -17.716   7.754  73.459  1.00  0.00
A    H
ATOM  13640  C   GLY A  13     -18.285   9.791  73.426  1.00  0.00
A    C
ATOM  13641  O   GLY A  13     -19.046  10.666  73.030  1.00  0.00
A    O
ATOM  13642  N   LEU A  14     -17.190  10.071  74.119  1.00  0.00
A    N
ATOM  13643  HN  LEU A  14     -16.514   9.409  74.435  1.00  0.00
A    H
ATOM  13644  CA  LEU A  14     -16.893  11.456  74.484  1.00  0.00
A    C
ATOM  13645  HA  LEU A  14     -17.832  11.993  74.461  1.00  0.00
A    H
ATOM  13646  CB  LEU A  14     -16.308  11.531  75.904  1.00  0.00
A    C
ATOM  13647  HB1 LEU A  14     -15.302  11.053  75.871  1.00  0.00
A    H
ATOM  13648  HB2 LEU A  14     -16.134  12.598  76.167  1.00  0.00
A    H
ATOM  13649  CG  LEU A  14     -17.134  10.844  76.997  1.00  0.00
A    C
ATOM  13650  HG  LEU A  14     -17.408   9.846  76.576  1.00  0.00
A    H
ATOM  13651  CD1 LEU A  14     -16.316  10.646  78.260  1.00  0.00
A    C
ATOM  13652 HD11 LEU A  14     -16.939  10.191  79.060  1.00  0.00
A    H
ATOM  13653 HD12 LEU A  14     -15.452   9.975  78.066  1.00  0.00
A    H
ATOM  13654 HD13 LEU A  14     -15.928  11.618  78.632  1.00  0.00
A    H
ATOM  13655  CD2 LEU A  14     -18.423  11.605  77.277  1.00  0.00
A    C
ATOM  13656 HD21 LEU A  14     -19.017  11.084  78.058  1.00  0.00
A    H
ATOM  13657 HD22 LEU A  14     -18.221  12.634  77.639  1.00  0.00
A    H
ATOM  13658 HD23 LEU A  14     -19.042  11.674  76.357  1.00  0.00
A    H
ATOM  13659  C   LEU A  14     -15.959  12.109  73.456  1.00  0.00
A    C
ATOM  13660  O   LEU A  14     -14.792  12.364  73.744  1.00  0.00
A    O
ATOM  13661  N   GLY A  15     -16.484  12.395  72.266  1.00  0.00
A    N
```

ATOM 13662 HN GLY A 15   -17.428 12.178 72.022 1.00 0.00
A   H
ATOM 13663 CA GLY A 15   -15.698 13.057 71.225 1.00 0.00
A   C
ATOM 13664 HA1 GLY A 15   -15.769 12.478 70.314 1.00 0.00
A   H
ATOM 13665 HA2 GLY A 15   -14.688 13.201 71.582 1.00 0.00
A   H
ATOM 13666 C   GLY A 15   -16.297 14.422 70.960 1.00 0.00
A   C
ATOM 13667 O   GLY A 15   -16.976 14.982 71.815 1.00 0.00
A   O
ATOM 13668 N   ARG A 16   -16.050 14.957 69.768 1.00 0.00
A   N
ATOM 13669 HN ARG A 16   -15.463 14.507 69.099 1.00 0.00
A   H
ATOM 13670 CA ARG A 16   -16.626 16.231 69.378 1.00 0.00
A   C
ATOM 13671 HA ARG A 16   -16.198 17.007 70.001 1.00 0.00
A   H
ATOM 13672 CB ARG A 16   -16.311 16.533 67.914 1.00 0.00
A   C
ATOM 13673 HB1 ARG A 16   -16.078 15.553 67.433 1.00 0.00
A   H
ATOM 13674 HB2 ARG A 16   -17.225 16.913 67.407 1.00 0.00
A   H
ATOM 13675 CG ARG A 16   -15.133 17.470 67.733 1.00 0.00
A   C
ATOM 13676 HG1 ARG A 16   -15.518 18.470 67.436 1.00 0.00
A   H
ATOM 13677 HG2 ARG A 16   -14.636 17.622 68.719 1.00 0.00
A   H
ATOM 13678 CD ARG A 16   -14.122 16.908 66.753 1.00 0.00
A   C
ATOM 13679 HD1 ARG A 16   -13.302 17.648 66.601 1.00 0.00
A   H
ATOM 13680 HD2 ARG A 16   -13.679 15.955 67.123 1.00 0.00
A   H
ATOM 13681 NE ARG A 16   -14.712 16.601 65.456 1.00 0.00
A   N
ATOM 13682 HE ARG A 16   -15.432 17.251 65.184 1.00 0.00
A   H
ATOM 13683 CZ ARG A 16   -14.347 15.572 64.702 1.00 0.00
A   C
ATOM 13684 NH1 ARG A 16   -13.401 14.738 65.119 1.00 0.00
A   N
ATOM 13685 HH11 ARG A 16   -13.107 13.984 64.549 1.00 0.00
A   H
ATOM 13686 HH12 ARG A 16   -12.921 14.997 65.960 1.00 0.00
A   H
ATOM 13687 NH2 ARG A 16   -14.930 15.369 63.529 1.00 0.00
A   N
ATOM 13688 HH21 ARG A 16   -14.719 14.554 63.008 1.00 0.00
A   H

ATOM 13689 HH22 ARG A 16 −15.713 15.937 63.313 1.00 0.00
A H
ATOM 13690 C ARG A 16 −18.129 16.252 69.628 1.00 0.00
A C
ATOM 13691 O ARG A 16 −18.665 17.223 70.164 1.00 0.00
A O
ATOM 13692 N PHE A 17 −18.792 15.158 69.272 1.00 0.00
A N
ATOM 13693 HN PHE A 17 −18.354 14.339 68.907 1.00 0.00
A H
ATOM 13694 CA PHE A 17 −20.241 15.067 69.388 1.00 0.00
A C
ATOM 13695 HA PHE A 17 −20.606 16.078 69.255 1.00 0.00
A H
ATOM 13696 CB PHE A 17 −20.793 14.148 68.295 1.00 0.00
A C
ATOM 13697 HB1 PHE A 17 −20.506 14.553 67.299 1.00 0.00
A H
ATOM 13698 HB2 PHE A 17 −20.356 13.128 68.371 1.00 0.00
A H
ATOM 13699 CG PHE A 17 −22.287 14.040 68.280 1.00 0.00
A C
ATOM 13700 CD1 PHE A 17 −23.082 15.149 68.014 1.00 0.00
A C
ATOM 13701 HD1 PHE A 17 −22.617 16.108 67.824 1.00 0.00
A H
ATOM 13702 CE1 PHE A 17 −24.467 15.044 67.994 1.00 0.00
A C
ATOM 13703 HE1 PHE A 17 −25.073 15.917 67.794 1.00 0.00
A H
ATOM 13704 CZ PHE A 17 −25.066 13.822 68.225 1.00 0.00
A C
ATOM 13705 HZ PHE A 17 −26.143 13.732 68.193 1.00 0.00
A H
ATOM 13706 CD2 PHE A 17 −22.902 12.820 68.513 1.00 0.00
A C
ATOM 13707 HD2 PHE A 17 −22.305 11.948 68.738 1.00 0.00
A H
ATOM 13708 CE2 PHE A 17 −24.285 12.708 68.488 1.00 0.00
A C
ATOM 13709 HE2 PHE A 17 −24.752 11.751 68.676 1.00 0.00
A H
ATOM 13710 C PHE A 17 −20.700 14.603 70.775 1.00 0.00
A C
ATOM 13711 O PHE A 17 −21.459 15.303 71.450 1.00 0.00
A O
ATOM 13712 N GLY A 18 −20.249 13.422 71.194 1.00 0.00
A N
ATOM 13713 HN GLY A 18 −19.592 12.874 70.677 1.00 0.00
A H
ATOM 13714 CA GLY A 18 −20.693 12.848 72.454 1.00 0.00
A C
ATOM 13715 HA1 GLY A 18 −20.198 11.891 72.555 1.00 0.00
A H

ATOM 13716 HA2 GLY A 18 −21.773 12.787 72.413 1.00 0.00
A H
ATOM 13717 C GLY A 18 −20.317 13.698 73.653 1.00 0.00
A C
ATOM 13718 O GLY A 18 −21.129 13.887 74.569 1.00 0.00
A O
ATOM 13719 N GLY A 19 −19.090 14.218 73.639 1.00 0.00
A N
ATOM 13720 HN GLY A 19 −18.430 14.021 72.915 1.00 0.00
A H
ATOM 13721 CA GLY A 19 −18.627 15.112 74.683 1.00 0.00
A C
ATOM 13722 HA1 GLY A 19 −17.645 15.461 74.392 1.00 0.00
A H
ATOM 13723 HA2 GLY A 19 −18.646 14.562 75.613 1.00 0.00
A H
ATOM 13724 C GLY A 19 −19.527 16.321 74.837 1.00 0.00
A C
ATOM 13725 O GLY A 19 −19.823 16.748 75.944 1.00 0.00
A O
ATOM 13726 N SER A 20 −19.979 16.861 73.715 1.00 0.00
A N
ATOM 13727 HN SER A 20 −19.772 16.520 72.798 1.00 0.00
A H
ATOM 13728 CA SER A 20 −20.838 18.027 73.734 1.00 0.00
A C
ATOM 13729 HA SER A 20 −20.352 18.741 74.384 1.00 0.00
A H
ATOM 13730 CB SER A 20 −21.005 18.592 72.333 1.00 0.00
A C
ATOM 13731 HB1 SER A 20 −20.008 18.943 71.981 1.00 0.00
A H
ATOM 13732 HB2 SER A 20 −21.339 17.795 71.631 1.00 0.00
A H
ATOM 13733 OG SER A 20 −21.901 19.677 72.361 1.00 0.00
A O
ATOM 13734 HG1 SER A 20 −21.810 20.141 71.520 1.00 0.00
A H
ATOM 13735 C SER A 20 −22.196 17.712 74.344 1.00 0.00
A C
ATOM 13736 O SER A 20 −22.704 18.508 75.133 1.00 0.00
A O
ATOM 13737 N ILE A 21 −22.777 16.565 73.981 1.00 0.00
A N
ATOM 13738 HN ILE A 21 −22.404 15.941 73.298 1.00 0.00
A H
ATOM 13739 CA ILE A 21 −24.041 16.122 74.572 1.00 0.00
A C
ATOM 13740 HA ILE A 21 −24.769 16.902 74.391 1.00 0.00
A H
ATOM 13741 CB ILE A 21 −24.499 14.758 74.024 1.00 0.00
A C
ATOM 13742 HB ILE A 21 −23.587 14.112 74.085 1.00 0.00
A H

ATOM 13743 CG2 ILE A 21 -25.704 14.246 74.804 1.00 0.00
A C
ATOM 13744 HG21 ILE A 21 -25.938 13.199 74.517 1.00 0.00
A H
ATOM 13745 HG22 ILE A 21 -25.503 14.258 75.896 1.00 0.00
A H
ATOM 13746 HG23 ILE A 21 -26.601 14.869 74.605 1.00 0.00
A H
ATOM 13747 CG1 ILE A 21 -24.863 14.864 72.554 1.00 0.00
A C
ATOM 13748 HG11 ILE A 21 -25.709 15.576 72.425 1.00 0.00
A H
ATOM 13749 HG12 ILE A 21 -23.993 15.281 71.996 1.00 0.00
A H
ATOM 13750 CD ILE A 21 -25.234 13.542 71.923 1.00 0.00
A C
ATOM 13751 HD1 ILE A 21 -25.433 13.675 70.838 1.00 0.00
A H
ATOM 13752 HD2 ILE A 21 -24.408 12.808 72.039 1.00 0.00
A H
ATOM 13753 HD3 ILE A 21 -26.148 13.121 72.391 1.00 0.00
A H
ATOM 13754 C ILE A 21 -23.879 16.012 76.087 1.00 0.00
A C
ATOM 13755 O ILE A 21 -24.698 16.526 76.852 1.00 0.00
A O
ATOM 13756 N CYS A 22 -22.806 15.345 76.502 1.00 0.00
A N
ATOM 13757 HN CYS A 22 -22.138 14.950 75.875 1.00 0.00
A H
ATOM 13758 CA CYS A 22 -22.519 15.136 77.908 1.00 0.00
A C
ATOM 13759 HA CYS A 22 -23.355 14.580 78.315 1.00 0.00
A H
ATOM 13760 CB CYS A 22 -21.217 14.357 78.059 1.00 0.00
A C
ATOM 13761 HB1 CYS A 22 -21.290 13.461 77.402 1.00 0.00
A H
ATOM 13762 HB2 CYS A 22 -20.369 14.959 77.664 1.00 0.00
A H
ATOM 13763 SG CYS A 22 -20.888 13.801 79.743 1.00 0.00
A S
ATOM 13764 HG1 CYS A 22 -19.764 13.132 79.480 1.00 0.00
A H
ATOM 13765 C CYS A 22 -22.436 16.463 78.654 1.00 0.00
A C
ATOM 13766 O CYS A 22 -23.213 16.713 79.581 1.00 0.00
A O
ATOM 13767 N LYS A 23 -21.513 17.324 78.227 1.00 0.00
A N
ATOM 13768 HN LYS A 23 -20.913 17.136 77.451 1.00 0.00
A H
ATOM 13769 CA LYS A 23 -21.310 18.618 78.868 1.00 0.00
A C

ATOM 13770 HA LYS A 23 −20.943 18.420 79.868 1.00 0.00
A H
ATOM 13771 CB LYS A 23 −20.267 19.456 78.118 1.00 0.00
A C
ATOM 13772 HB1 LYS A 23 −19.414 18.780 77.880 1.00 0.00
A H
ATOM 13773 HB2 LYS A 23 −20.694 19.777 77.142 1.00 0.00
A H
ATOM 13774 CG LYS A 23 −19.747 20.657 78.904 1.00 0.00
A C
ATOM 13775 HG1 LYS A 23 −20.591 21.355 79.105 1.00 0.00
A H
ATOM 13776 HG2 LYS A 23 −19.364 20.307 79.891 1.00 0.00
A H
ATOM 13777 CD LYS A 23 −18.652 21.425 78.171 1.00 0.00
A C
ATOM 13778 HD1 LYS A 23 −18.977 21.579 77.116 1.00 0.00
A H
ATOM 13779 HD2 LYS A 23 −18.559 22.431 78.641 1.00 0.00
A H
ATOM 13780 CE LYS A 23 −17.319 20.692 78.221 1.00 0.00
A C
ATOM 13781 HE1 LYS A 23 −17.070 20.407 79.268 1.00 0.00
A H
ATOM 13782 HE2 LYS A 23 −17.358 19.766 77.605 1.00 0.00
A H
ATOM 13783 NZ LYS A 23 −16.190 21.517 77.708 1.00 0.00
A N
ATOM 13784 HZ1 LYS A 23 −15.301 20.978 77.763 1.00 0.00
A H
ATOM 13785 HZ2 LYS A 23 −16.373 21.780 76.720 1.00 0.00
A H
ATOM 13786 HZ3 LYS A 23 −16.105 22.380 78.282 1.00 0.00
A H
ATOM 13787 C LYS A 23 −22.617 19.387 79.006 1.00 0.00
A C
ATOM 13788 O LYS A 23 −22.849 20.019 80.032 1.00 0.00
A O
ATOM 13789 N GLU A 24 −23.475 19.321 77.993 1.00 0.00
A N
ATOM 13790 HN GLU A 24 −23.355 18.770 77.173 1.00 0.00
A H
ATOM 13791 CA GLU A 24 −24.714 20.089 78.015 1.00 0.00
A C
ATOM 13792 HA GLU A 24 −24.462 21.082 78.366 1.00 0.00
A H
ATOM 13793 CB GLU A 24 −25.276 20.261 76.610 1.00 0.00
A C
ATOM 13794 HB1 GLU A 24 −24.478 20.699 75.968 1.00 0.00
A H
ATOM 13795 HB2 GLU A 24 −25.508 19.264 76.171 1.00 0.00
A H
ATOM 13796 CG GLU A 24 −26.512 21.154 76.532 1.00 0.00
A C

```
ATOM  13797  HG1 GLU A  24      -26.918  21.133  75.505  1.00  0.00
A    H
ATOM  13798  HG2 GLU A  24      -27.294  20.814  77.241  1.00  0.00
A    H
ATOM  13799  CD  GLU A  24      -26.238  22.621  76.817  1.00  0.00
A    C
ATOM  13800  OE1 GLU A  24      -27.219  23.393  76.872  1.00  0.00
A    O
ATOM  13801  OE2 GLU A  24      -25.061  23.010  76.976  1.00  0.00
A    O
ATOM  13802  C   GLU A  24      -25.775  19.526  78.967  1.00  0.00
A    C
ATOM  13803  O   GLU A  24      -26.384  20.284  79.719  1.00  0.00
A    O
ATOM  13804  N   LEU A  25      -25.996  18.211  78.936  1.00  0.00
A    N
ATOM  13805  HN  LEU A  25      -25.554  17.579  78.304  1.00  0.00
A    H
ATOM  13806  CA  LEU A  25      -26.938  17.578  79.860  1.00  0.00
A    C
ATOM  13807  HA  LEU A  25      -27.910  18.017  79.669  1.00  0.00
A    H
ATOM  13808  CB  LEU A  25      -26.978  16.063  79.650  1.00  0.00
A    C
ATOM  13809  HB1 LEU A  25      -25.922  15.708  79.609  1.00  0.00
A    H
ATOM  13810  HB2 LEU A  25      -27.428  15.585  80.548  1.00  0.00
A    H
ATOM  13811  CG  LEU A  25      -27.665  15.461  78.425  1.00  0.00
A    C
ATOM  13812  HG  LEU A  25      -27.269  15.992  77.527  1.00  0.00
A    H
ATOM  13813  CD1 LEU A  25      -27.337  13.978  78.337  1.00  0.00
A    C
ATOM  13814 HD11 LEU A  25      -27.867  13.512  77.478  1.00  0.00
A    H
ATOM  13815 HD12 LEU A  25      -26.246  13.826  78.197  1.00  0.00
A    H
ATOM  13816 HD13 LEU A  25      -27.649  13.451  79.264  1.00  0.00
A    H
ATOM  13817  CD2 LEU A  25      -29.171  15.662  78.464  1.00  0.00
A    C
ATOM  13818 HD21 LEU A  25      -29.641  15.232  77.554  1.00  0.00
A    H
ATOM  13819 HD22 LEU A  25      -29.627  15.168  79.344  1.00  0.00
A    H
ATOM  13820 HD23 LEU A  25      -29.418  16.745  78.504  1.00  0.00
A    H
ATOM  13821  C   LEU A  25      -26.566  17.891  81.312  1.00  0.00
A    C
ATOM  13822  O   LEU A  25      -27.436  18.138  82.153  1.00  0.00
A    O
ATOM  13823  N   HSE A  26      -25.266  17.876  81.584  1.00  0.00
A    N
```

ATOM 13824 HN HSE A 26 -24.576 17.668 80.892 1.00 0.00
A H
ATOM 13825 CA HSE A 26 -24.727 18.158 82.903 1.00 0.00
A C
ATOM 13826 HA HSE A 26 -25.277 17.548 83.611 1.00 0.00
A H
ATOM 13827 CB HSE A 26 -23.237 17.850 82.917 1.00 0.00
A C
ATOM 13828 HB1 HSE A 26 -23.105 16.786 82.621 1.00 0.00
A H
ATOM 13829 HB2 HSE A 26 -22.724 18.467 82.151 1.00 0.00
A H
ATOM 13830 ND1 HSE A 26 -22.904 17.281 85.323 1.00 0.00
A N
ATOM 13831 CG HSE A 26 -22.589 18.038 84.256 1.00 0.00
A C
ATOM 13832 CE1 HSE A 26 -22.167 17.669 86.380 1.00 0.00
A C
ATOM 13833 HE1 HSE A 26 -22.229 17.224 87.376 1.00 0.00
A H
ATOM 13834 NE2 HSE A 26 -21.374 18.679 85.985 1.00 0.00
A N
ATOM 13835 HE2 HSE A 26 -20.731 19.174 86.569 1.00 0.00
A H
ATOM 13836 CD2 HSE A 26 -21.612 18.931 84.680 1.00 0.00
A C
ATOM 13837 HD2 HSE A 26 -21.103 19.712 84.134 1.00 0.00
A H
ATOM 13838 C HSE A 26 -24.965 19.589 83.277 1.00 0.00
A C
ATOM 13839 O HSE A 26 -25.354 19.883 84.407 1.00 0.00
A O
ATOM 13840 N ARG A 27 -24.749 20.486 82.314 1.00 0.00
A N
ATOM 13841 HN ARG A 27 -24.435 20.192 81.414 1.00 0.00
A H
ATOM 13842 CA ARG A 27 -24.950 21.914 82.502 1.00 0.00
A C
ATOM 13843 HA ARG A 27 -24.419 22.221 83.394 1.00 0.00
A H
ATOM 13844 CB ARG A 27 -24.420 22.684 81.286 1.00 0.00
A C
ATOM 13845 HB1 ARG A 27 -23.462 22.192 80.993 1.00 0.00
A H
ATOM 13846 HB2 ARG A 27 -25.112 22.544 80.427 1.00 0.00
A H
ATOM 13847 CG ARG A 27 -24.152 24.158 81.548 1.00 0.00
A C
ATOM 13848 HG1 ARG A 27 -24.902 24.529 82.282 1.00 0.00
A H
ATOM 13849 HG2 ARG A 27 -23.164 24.262 82.052 1.00 0.00
A H
ATOM 13850 CD ARG A 27 -24.129 24.992 80.270 1.00 0.00
A C

ATOM 13851 HD1 ARG A 27 -24.021 26.069 80.533 1.00 0.00
A H
ATOM 13852 HD2 ARG A 27 -23.282 24.700 79.606 1.00 0.00
A H
ATOM 13853 NE ARG A 27 -25.349 24.825 79.473 1.00 0.00
A N
ATOM 13854 HE ARG A 27 -25.231 24.197 78.696 1.00 0.00
A H
ATOM 13855 CZ ARG A 27 -26.518 25.422 79.714 1.00 0.00
A C
ATOM 13856 NH1 ARG A 27 -26.663 26.251 80.736 1.00 0.00
A N
ATOM 13857 HH11 ARG A 27 -27.540 26.671 80.924 1.00 0.00
A H
ATOM 13858 HH12 ARG A 27 -25.889 26.314 81.370 1.00 0.00
A H
ATOM 13859 NH2 ARG A 27 -27.556 25.191 78.926 1.00 0.00
A N
ATOM 13860 HH21 ARG A 27 -28.395 25.699 79.054 1.00 0.00
A H
ATOM 13861 HH22 ARG A 27 -27.385 24.665 78.104 1.00 0.00
A H
ATOM 13862 C ARG A 27 -26.423 22.227 82.740 1.00 0.00
A C
ATOM 13863 O ARG A 27 -26.750 23.147 83.478 1.00 0.00
A O
ATOM 13864 N MET A 28 -27.306 21.458 82.114 1.00 0.00
A N
ATOM 13865 HN MET A 28 -27.046 20.741 81.470 1.00 0.00
A H
ATOM 13866 CA MET A 28 -28.750 21.595 82.316 1.00 0.00
A C
ATOM 13867 HA MET A 28 -28.956 22.658 82.312 1.00 0.00
A H
ATOM 13868 CB MET A 28 -29.516 20.902 81.191 1.00 0.00
A C
ATOM 13869 HB1 MET A 28 -29.108 19.868 81.110 1.00 0.00
A H
ATOM 13870 HB2 MET A 28 -30.584 20.793 81.484 1.00 0.00
A H
ATOM 13871 CG MET A 28 -29.404 21.576 79.840 1.00 0.00
A C
ATOM 13872 HG1 MET A 28 -29.818 22.606 79.886 1.00 0.00
A H
ATOM 13873 HG2 MET A 28 -28.333 21.656 79.550 1.00 0.00
A H
ATOM 13874 SD MET A 28 -30.302 20.655 78.567 1.00 0.00
A S
ATOM 13875 CE MET A 28 -31.929 21.392 78.710 1.00 0.00
A C
ATOM 13876 HE1 MET A 28 -32.638 20.930 77.990 1.00 0.00
A H
ATOM 13877 HE2 MET A 28 -32.341 21.253 79.733 1.00 0.00
A H

ATOM 13878 HE3 MET A 28   -31.894 22.483 78.501 1.00 0.00
A   H
ATOM 13879 C   MET A 28   -29.225 21.035 83.662 1.00 0.00
A   C
ATOM 13880 O   MET A 28   -30.323 21.357 84.116 1.00 0.00
A   O
ATOM 13881 N   GLY A 29   -28.414 20.180 84.277 1.00 0.00
A   N
ATOM 13882 HN GLY A 29   -27.559 19.852 83.878 1.00 0.00
A   H
ATOM 13883 CA GLY A 29   -28.730 19.663 85.590 1.00 0.00
A   C
ATOM 13884 HA1 GLY A 29   -29.548 20.288 85.922 1.00 0.00
A   H
ATOM 13885 HA2 GLY A 29   -27.814 19.796 86.150 1.00 0.00
A   H
ATOM 13886 C   GLY A 29   -29.155 18.226 85.730 1.00 0.00
A   C
ATOM 13887 O   GLY A 29   -29.511 17.787 86.825 1.00 0.00
A   O
ATOM 13888 N   HSE A 30   -29.122 17.495 84.626 1.00 0.00
A   N
ATOM 13889 HN HSE A 30   -28.891 17.856 83.724 1.00 0.00
A   H
ATOM 13890 CA HSE A 30   -29.428 16.076 84.642 1.00 0.00
A   C
ATOM 13891 HA HSE A 30   -30.194 15.906 85.390 1.00 0.00
A   H
ATOM 13892 CB HSE A 30   -29.842 15.616 83.255 1.00 0.00
A   C
ATOM 13893 HB1 HSE A 30   -28.960 15.713 82.585 1.00 0.00
A   H
ATOM 13894 HB2 HSE A 30   -30.111 14.540 83.286 1.00 0.00
A   H
ATOM 13895 ND1 HSE A 30   -32.261 16.118 82.870 1.00 0.00
A   N
ATOM 13896 CG HSE A 30   -30.969 16.423 82.662 1.00 0.00
A   C
ATOM 13897 CE1 HSE A 30   -33.041 17.009 82.230 1.00 0.00
A   C
ATOM 13898 HE1 HSE A 30   -34.133 16.990 82.231 1.00 0.00
A   H
ATOM 13899 NE2 HSE A 30   -32.240 17.888 81.608 1.00 0.00
A   N
ATOM 13900 HE2 HSE A 30   -32.550 18.650 81.038 1.00 0.00
A   H
ATOM 13901 CD2 HSE A 30   -30.957 17.556 81.854 1.00 0.00
A   C
ATOM 13902 HD2 HSE A 30   -30.122 18.112 81.451 1.00 0.00
A   H
ATOM 13903 C   HSE A 30   -28.214 15.334 85.083 1.00 0.00
A   C
ATOM 13904 O   HSE A 30   -27.100 15.860 85.002 1.00 0.00
A   O

ATOM 13905 N GLU A 31 −28.405 14.114 85.573 1.00 0.00
A N
ATOM 13906 HN GLU A 31 −29.288 13.692 85.756 1.00 0.00
A H
ATOM 13907 CA GLU A 31 −27.264 13.265 85.892 1.00 0.00
A C
ATOM 13908 HA GLU A 31 −26.418 13.902 86.122 1.00 0.00
A H
ATOM 13909 CB GLU A 31 −27.467 12.441 87.178 1.00 0.00
A C
ATOM 13910 HB1 GLU A 31 −26.670 11.664 87.222 1.00 0.00
A H
ATOM 13911 HB2 GLU A 31 −27.312 13.089 88.069 1.00 0.00
A H
ATOM 13912 CG GLU A 31 −28.805 11.732 87.331 1.00 0.00
A C
ATOM 13913 HG1 GLU A 31 −29.624 12.435 87.096 1.00 0.00
A H
ATOM 13914 HG2 GLU A 31 −28.869 10.856 86.655 1.00 0.00
A H
ATOM 13915 CD GLU A 31 −29.059 11.262 88.760 1.00 0.00
A C
ATOM 13916 OE1 GLU A 31 −28.088 11.126 89.543 1.00 0.00
A O
ATOM 13917 OE2 GLU A 31 −30.238 11.024 89.102 1.00 0.00
A O
ATOM 13918 C GLU A 31 −26.874 12.410 84.694 1.00 0.00
A C
ATOM 13919 O GLU A 31 −27.727 11.826 84.022 1.00 0.00
A O
ATOM 13920 N VAL A 32 −25.573 12.377 84.423 1.00 0.00
A N
ATOM 13921 HN VAL A 32 −24.882 12.762 85.029 1.00 0.00
A H
ATOM 13922 CA VAL A 32 −25.040 11.774 83.212 1.00 0.00
A C
ATOM 13923 HA VAL A 32 −25.818 11.156 82.781 1.00 0.00
A H
ATOM 13924 CB VAL A 32 −24.546 12.852 82.220 1.00 0.00
A C
ATOM 13925 HB VAL A 32 −23.607 13.292 82.639 1.00 0.00
A H
ATOM 13926 CG1 VAL A 32 −24.243 12.242 80.860 1.00 0.00
A C
ATOM 13927 HG11 VAL A 32 −23.926 13.031 80.144 1.00 0.00
A H
ATOM 13928 HG12 VAL A 32 −23.420 11.499 80.918 1.00 0.00
A H
ATOM 13929 HG13 VAL A 32 −25.144 11.742 80.444 1.00 0.00
A H
ATOM 13930 CG2 VAL A 32 −25.562 13.970 82.067 1.00 0.00
A C
ATOM 13931 HG21 VAL A 32 −25.215 14.718 81.322 1.00 0.00
A H

ATOM 13932 HG22 VAL A 32   -26.540 13.564 81.726 1.00 0.00
A   H
ATOM 13933 HG23 VAL A 32   -25.722 14.502 83.027 1.00 0.00
A   H
ATOM 13934 C   VAL A 32   -23.867 10.863 83.547 1.00 0.00
A   C
ATOM 13935 O   VAL A 32   -22.969 11.244 84.305 1.00 0.00
A   O
ATOM 13936 N   LEU A 33   -23.878   9.661 82.983 1.00 0.00
A   N
ATOM 13937 HN LEU A 33   -24.655   9.265 82.497 1.00 0.00
A   H
ATOM 13938 CA LEU A 33   -22.710   8.802 83.039 1.00 0.00
A   C
ATOM 13939 HA LEU A 33   -22.070   9.160 83.836 1.00 0.00
A   H
ATOM 13940 CB LEU A 33   -23.096   7.344 83.298 1.00 0.00
A   C
ATOM 13941 HB1 LEU A 33   -23.787   7.337 84.173 1.00 0.00
A   H
ATOM 13942 HB2 LEU A 33   -23.690   6.968 82.435 1.00 0.00
A   H
ATOM 13943 CG LEU A 33   -21.934   6.389 83.594 1.00 0.00
A   C
ATOM 13944 HG LEU A 33   -21.105   7.020 83.997 1.00 0.00
A   H
ATOM 13945 CD1 LEU A 33   -22.311   5.323 84.605 1.00 0.00
A   C
ATOM 13946 HD11 LEU A 33   -21.466   4.619 84.763 1.00 0.00
A   H
ATOM 13947 HD12 LEU A 33   -22.569   5.783 85.583 1.00 0.00
A   H
ATOM 13948 HD13 LEU A 33   -23.188   4.739 84.251 1.00 0.00
A   H
ATOM 13949 CD2 LEU A 33   -21.451   5.743 82.315 1.00 0.00
A   C
ATOM 13950 HD21 LEU A 33   -20.595   5.064 82.524 1.00 0.00
A   H
ATOM 13951 HD22 LEU A 33   -22.247   5.140 81.833 1.00 0.00
A   H
ATOM 13952 HD23 LEU A 33   -21.112   6.516 81.592 1.00 0.00
A   H
ATOM 13953 C   LEU A 33   -21.944   8.947 81.736 1.00 0.00
A   C
ATOM 13954 O   LEU A 33   -22.474   8.693 80.658 1.00 0.00
A   O
ATOM 13955 N   ALA A 34   -20.698   9.380 81.846 1.00 0.00
A   N
ATOM 13956 HN ALA A 34   -20.285   9.632 82.721 1.00 0.00
A   H
ATOM 13957 CA ALA A 34   -19.836   9.520 80.685 1.00 0.00
A   C
ATOM 13958 HA ALA A 34   -20.410   9.593 79.768 1.00 0.00
A   H

ATOM 13959 CB ALA A 34 −18.969 10.762 80.817 1.00 0.00
A C
ATOM 13960 HB1 ALA A 34 −19.616 11.665 80.859 1.00 0.00
A H
ATOM 13961 HB2 ALA A 34 −18.365 10.729 81.750 1.00 0.00
A H
ATOM 13962 HB3 ALA A 34 −18.280 10.874 79.953 1.00 0.00
A H
ATOM 13963 C ALA A 34 −18.977 8.272 80.556 1.00 0.00
A C
ATOM 13964 O ALA A 34 −18.318 7.866 81.514 1.00 0.00
A O
ATOM 13965 N VAL A 35 −19.005 7.658 79.378 1.00 0.00
A N
ATOM 13966 HN VAL A 35 −19.577 7.949 78.615 1.00 0.00
A H
ATOM 13967 CA VAL A 35 −18.186 6.485 79.102 1.00 0.00
A C
ATOM 13968 HA VAL A 35 −17.568 6.296 79.971 1.00 0.00
A H
ATOM 13969 CB VAL A 35 −19.042 5.254 78.751 1.00 0.00
A C
ATOM 13970 HB VAL A 35 −19.568 5.472 77.788 1.00 0.00
A H
ATOM 13971 CG1 VAL A 35 −18.170 4.025 78.574 1.00 0.00
A C
ATOM 13972 HG11 VAL A 35 −18.798 3.134 78.359 1.00 0.00
A H
ATOM 13973 HG12 VAL A 35 −17.463 4.143 77.727 1.00 0.00
A H
ATOM 13974 HG13 VAL A 35 −17.587 3.821 79.498 1.00 0.00
A H
ATOM 13975 CG2 VAL A 35 −20.076 4.991 79.825 1.00 0.00
A C
ATOM 13976 HG21 VAL A 35 −20.665 4.081 79.588 1.00 0.00
A H
ATOM 13977 HG22 VAL A 35 −19.585 4.844 80.810 1.00 0.00
A H
ATOM 13978 HG23 VAL A 35 −20.788 5.838 79.913 1.00 0.00
A H
ATOM 13979 C VAL A 35 −17.256 6.772 77.934 1.00 0.00
A C
ATOM 13980 O VAL A 35 −17.646 7.432 76.974 1.00 0.00
A O
ATOM 13981 N ASP A 36 −16.024 6.281 78.035 1.00 0.00
A N
ATOM 13982 HN ASP A 36 −15.649 5.850 78.851 1.00 0.00
A H
ATOM 13983 CA ASP A 36 −15.072 6.326 76.926 1.00 0.00
A C
ATOM 13984 HA ASP A 36 −15.607 6.042 76.027 1.00 0.00
A H
ATOM 13985 CB ASP A 36 −14.470 7.727 76.772 1.00 0.00
A C

ATOM 13986 HB1 ASP A 36 −15.255 8.485 76.968 1.00 0.00
A H
ATOM 13987 HB2 ASP A 36 −13.647 7.881 77.497 1.00 0.00
A H
ATOM 13988 CG ASP A 36 −13.934 7.981 75.377 1.00 0.00
A C
ATOM 13989 OD1 ASP A 36 −14.464 8.879 74.685 1.00 0.00
A O
ATOM 13990 OD2 ASP A 36 −12.984 7.281 74.972 1.00 0.00
A O
ATOM 13991 C ASP A 36 −13.971 5.290 77.131 1.00 0.00
A C
ATOM 13992 O ASP A 36 −13.636 4.937 78.266 1.00 0.00
A O
ATOM 13993 N ILE A 37 −13.422 4.796 76.026 1.00 0.00
A N
ATOM 13994 HN ILE A 37 −13.709 5.050 75.105 1.00 0.00
A H
ATOM 13995 CA ILE A 37 −12.335 3.823 76.067 1.00 0.00
A C
ATOM 13996 HA ILE A 37 −12.561 3.138 76.873 1.00 0.00
A H
ATOM 13997 CB ILE A 37 −12.208 3.044 74.730 1.00 0.00
A C
ATOM 13998 HB ILE A 37 −13.251 2.706 74.503 1.00 0.00
A H
ATOM 13999 CG2 ILE A 37 −11.673 3.935 73.606 1.00 0.00
A C
ATOM 14000 HG21 ILE A 37 −11.735 3.412 72.630 1.00 0.00
A H
ATOM 14001 HG22 ILE A 37 −12.266 4.869 73.526 1.00 0.00
A H
ATOM 14002 HG23 ILE A 37 −10.612 4.207 73.787 1.00 0.00
A H
ATOM 14003 CG1 ILE A 37 −11.365 1.774 74.917 1.00 0.00
A C
ATOM 14004 HG11 ILE A 37 −10.291 2.047 75.017 1.00 0.00
A H
ATOM 14005 HG12 ILE A 37 −11.668 1.277 75.866 1.00 0.00
A H
ATOM 14006 CD ILE A 37 −11.498 0.756 73.803 1.00 0.00
A C
ATOM 14007 HD1 ILE A 37 −10.905 −0.154 74.039 1.00 0.00
A H
ATOM 14008 HD2 ILE A 37 −12.560 0.454 73.671 1.00 0.00
A H
ATOM 14009 HD3 ILE A 37 −11.127 1.167 72.841 1.00 0.00
A H
ATOM 14010 C ILE A 37 −11.020 4.508 76.427 1.00 0.00
A C
ATOM 14011 O ILE A 37 −10.143 3.900 77.036 1.00 0.00
A O
ATOM 14012 N ASN A 38 −10.908 5.781 76.063 1.00 0.00
A N

```
ATOM  14013  HN  ASN A  38     -11.666   6.288  75.653  1.00  0.00
A    H
ATOM  14014  CA  ASN A  38      -9.678   6.556  76.227  1.00  0.00
A    C
ATOM  14015  HA  ASN A  38      -8.873   5.831  76.166  1.00  0.00
A    H
ATOM  14016  CB  ASN A  38      -9.585   7.592  75.106  1.00  0.00
A    C
ATOM  14017  HB1 ASN A  38      -9.631   7.050  74.136  1.00  0.00
A    H
ATOM  14018  HB2 ASN A  38     -10.454   8.280  75.143  1.00  0.00
A    H
ATOM  14019  CG  ASN A  38      -8.300   8.380  75.137  1.00  0.00
A    C
ATOM  14020  OD1 ASN A  38      -8.127   9.279  75.957  1.00  0.00
A    O
ATOM  14021  ND2 ASN A  38      -7.392   8.061  74.228  1.00  0.00
A    N
ATOM  14022 HD21 ASN A  38      -6.533   8.564  74.243  1.00  0.00
A    H
ATOM  14023 HD22 ASN A  38      -7.570   7.323  73.584  1.00  0.00
A    H
ATOM  14024  C   ASN A  38      -9.591   7.232  77.596  1.00  0.00
A    C
ATOM  14025  O   ASN A  38     -10.478   7.993  77.972  1.00  0.00
A    O
ATOM  14026  N   GLU A  39      -8.513   6.971  78.330  1.00  0.00
A    N
ATOM  14027  HN  GLU A  39      -7.725   6.447  78.017  1.00  0.00
A    H
ATOM  14028  CA  GLU A  39      -8.399   7.451  79.706  1.00  0.00
A    C
ATOM  14029  HA  GLU A  39      -9.283   7.114  80.231  1.00  0.00
A    H
ATOM  14030  CB  GLU A  39      -7.198   6.818  80.415  1.00  0.00
A    C
ATOM  14031  HB1 GLU A  39      -7.133   5.750  80.100  1.00  0.00
A    H
ATOM  14032  HB2 GLU A  39      -6.257   7.297  80.064  1.00  0.00
A    H
ATOM  14033  CG  GLU A  39      -7.290   6.888  81.934  1.00  0.00
A    C
ATOM  14034  HG1 GLU A  39      -7.399   7.942  82.249  1.00  0.00
A    H
ATOM  14035  HG2 GLU A  39      -8.156   6.305  82.307  1.00  0.00
A    H
ATOM  14036  CD  GLU A  39      -6.044   6.385  82.641  1.00  0.00
A    C
ATOM  14037  OE1 GLU A  39      -5.994   6.478  83.887  1.00  0.00
A    O
ATOM  14038  OE2 GLU A  39      -5.114   5.898  81.960  1.00  0.00
A    O
ATOM  14039  C   GLU A  39      -8.351   8.981  79.827  1.00  0.00
A    C
```

```
ATOM  14040  O   GLU A  39      -8.936   9.551  80.753  1.00  0.00
A    O
ATOM  14041  N   GLU A  40      -7.660   9.632  78.895  1.00  0.00
A    N
ATOM  14042  HN  GLU A  40      -7.153   9.189  78.161  1.00  0.00
A    H
ATOM  14043  CA  GLU A  40      -7.585  11.095  78.870  1.00  0.00
A    C
ATOM  14044  HA  GLU A  40      -7.174  11.422  79.817  1.00  0.00
A    H
ATOM  14045  CB  GLU A  40      -6.670  11.573  77.744  1.00  0.00
A    C
ATOM  14046  HB1 GLU A  40      -6.894  10.971  76.834  1.00  0.00
A    H
ATOM  14047  HB2 GLU A  40      -6.915  12.627  77.480  1.00  0.00
A    H
ATOM  14048  CG  GLU A  40      -5.186  11.457  78.053  1.00  0.00
A    C
ATOM  14049  HG1 GLU A  40      -4.845  12.369  78.575  1.00  0.00
A    H
ATOM  14050  HG2 GLU A  40      -4.981  10.573  78.688  1.00  0.00
A    H
ATOM  14051  CD  GLU A  40      -4.336  11.358  76.800  1.00  0.00
A    C
ATOM  14052  OE1 GLU A  40      -4.743  10.646  75.852  1.00  0.00
A    O
ATOM  14053  OE2 GLU A  40      -3.252  11.985  76.765  1.00  0.00
A    O
ATOM  14054  C   GLU A  40      -8.964  11.734  78.731  1.00  0.00
A    C
ATOM  14055  O   GLU A  40      -9.292  12.666  79.463  1.00  0.00
A    O
ATOM  14056  N   LYS A  41      -9.764  11.222  77.794  1.00  0.00
A    N
ATOM  14057  HN  LYS A  41      -9.467  10.498  77.173  1.00  0.00
A    H
ATOM  14058  CA  LYS A  41     -11.139  11.680  77.600  1.00  0.00
A    C
ATOM  14059  HA  LYS A  41     -11.092  12.733  77.349  1.00  0.00
A    H
ATOM  14060  CB  LYS A  41     -11.825  10.911  76.461  1.00  0.00
A    C
ATOM  14061  HB1 LYS A  41     -11.686   9.825  76.668  1.00  0.00
A    H
ATOM  14062  HB2 LYS A  41     -12.922  11.100  76.505  1.00  0.00
A    H
ATOM  14063  CG  LYS A  41     -11.290  11.217  75.068  1.00  0.00
A    C
ATOM  14064  HG1 LYS A  41     -10.179  11.288  75.112  1.00  0.00
A    H
ATOM  14065  HG2 LYS A  41     -11.541  10.369  74.388  1.00  0.00
A    H
ATOM  14066  CD  LYS A  41     -11.845  12.520  74.509  1.00  0.00
A    C
```

ATOM 14067 HD1 LYS A 41 -12.922 12.368 74.266 1.00 0.00
A H
ATOM 14068 HD2 LYS A 41 -11.790 13.293 75.310 1.00 0.00
A H
ATOM 14069 CE LYS A 41 -11.078 12.981 73.279 1.00 0.00
A C
ATOM 14070 HE1 LYS A 41 -11.536 13.902 72.856 1.00 0.00
A H
ATOM 14071 HE2 LYS A 41 -10.020 13.201 73.539 1.00 0.00
A H
ATOM 14072 NZ LYS A 41 -11.057 11.966 72.188 1.00 0.00
A N
ATOM 14073 HZ1 LYS A 41 -10.523 12.331 71.373 1.00 0.00
A H
ATOM 14074 HZ2 LYS A 41 -10.607 11.094 72.534 1.00 0.00
A H
ATOM 14075 HZ3 LYS A 41 -12.032 11.755 71.895 1.00 0.00
A H
ATOM 14076 C LYS A 41 -11.950 11.554 78.894 1.00 0.00
A C
ATOM 14077 O LYS A 41 -12.617 12.506 79.298 1.00 0.00
A O
ATOM 14078 N VAL A 42 -11.873 10.390 79.539 1.00 0.00
A N
ATOM 14079 HN VAL A 42 -11.337 9.619 79.204 1.00 0.00
A H
ATOM 14080 CA VAL A 42 -12.572 10.138 80.804 1.00 0.00
A C
ATOM 14081 HA VAL A 42 -13.628 10.303 80.627 1.00 0.00
A H
ATOM 14082 CB VAL A 42 -12.380 8.680 81.292 1.00 0.00
A C
ATOM 14083 HB VAL A 42 -11.297 8.545 81.534 1.00 0.00
A H
ATOM 14084 CG1 VAL A 42 -13.204 8.411 82.540 1.00 0.00
A C
ATOM 14085 HG11 VAL A 42 -13.089 7.353 82.858 1.00 0.00
A H
ATOM 14086 HG12 VAL A 42 -12.881 9.048 83.390 1.00 0.00
A H
ATOM 14087 HG13 VAL A 42 -14.282 8.596 82.347 1.00 0.00
A H
ATOM 14088 CG2 VAL A 42 -12.777 7.695 80.204 1.00 0.00
A C
ATOM 14089 HG21 VAL A 42 -12.678 6.649 80.565 1.00 0.00
A H
ATOM 14090 HG22 VAL A 42 -13.832 7.860 79.894 1.00 0.00
A H
ATOM 14091 HG23 VAL A 42 -12.130 7.803 79.309 1.00 0.00
A H
ATOM 14092 C VAL A 42 -12.128 11.124 81.890 1.00 0.00
A C
ATOM 14093 O VAL A 42 -12.957 11.643 82.643 1.00 0.00
A O

```
ATOM  14094  N   ASN A  43     -10.820  11.379  81.960  1.00  0.00
A    N
ATOM  14095  HN  ASN A  43     -10.138  10.912  81.399  1.00  0.00
A    H
ATOM  14096  CA  ASN A  43     -10.270  12.381  82.878  1.00  0.00
A    C
ATOM  14097  HA  ASN A  43     -10.628  12.097  83.861  1.00  0.00
A    H
ATOM  14098  CB  ASN A  43      -8.740  12.380  82.832  1.00  0.00
A    C
ATOM  14099  HB1 ASN A  43      -8.432  12.325  81.765  1.00  0.00
A    H
ATOM  14100  HB2 ASN A  43      -8.341  13.329  83.245  1.00  0.00
A    H
ATOM  14101  CG  ASN A  43      -8.130  11.194  83.558  1.00  0.00
A    C
ATOM  14102  OD1 ASN A  43      -8.818  10.462  84.274  1.00  0.00
A    O
ATOM  14103  ND2 ASN A  43      -6.828  11.003  83.381  1.00  0.00
A    N
ATOM  14104 HD21 ASN A  43      -6.413  10.222  83.841  1.00  0.00
A    H
ATOM  14105 HD22 ASN A  43      -6.312  11.609  82.784  1.00  0.00
A    H
ATOM  14106  C   ASN A  43     -10.791  13.780  82.585  1.00  0.00
A    C
ATOM  14107  O   ASN A  43     -11.161  14.512  83.503  1.00  0.00
A    O
ATOM  14108  N   ALA A  44     -10.831  14.135  81.302  1.00  0.00
A    N
ATOM  14109  HN  ALA A  44     -10.576  13.506  80.568  1.00  0.00
A    H
ATOM  14110  CA  ALA A  44     -11.250  15.465  80.870  1.00  0.00
A    C
ATOM  14111  HA  ALA A  44     -10.705  16.219  81.427  1.00  0.00
A    H
ATOM  14112  CB  ALA A  44     -10.905  15.677  79.403  1.00  0.00
A    C
ATOM  14113  HB1 ALA A  44      -9.815  15.527  79.250  1.00  0.00
A    H
ATOM  14114  HB2 ALA A  44     -11.444  14.950  78.757  1.00  0.00
A    H
ATOM  14115  HB3 ALA A  44     -11.160  16.706  79.068  1.00  0.00
A    H
ATOM  14116  C   ALA A  44     -12.735  15.733  81.120  1.00  0.00
A    C
ATOM  14117  O   ALA A  44     -13.148  16.892  81.216  1.00  0.00
A    O
ATOM  14118  N   TYR A  45     -13.531  14.673  81.231  1.00  0.00
A    N
ATOM  14119  HN  TYR A  45     -13.216  13.726  81.181  1.00  0.00
A    H
ATOM  14120  CA  TYR A  45     -14.972  14.823  81.441  1.00  0.00
A    C
```

ATOM 14121 HA TYR A 45 −15.182 15.868 81.245 1.00 0.00
A H
ATOM 14122 CB TYR A 45 −15.768 14.015 80.403 1.00 0.00
A C
ATOM 14123 HB1 TYR A 45 −15.274 13.031 80.251 1.00 0.00
A H
ATOM 14124 HB2 TYR A 45 −16.798 13.813 80.769 1.00 0.00
A H
ATOM 14125 CG TYR A 45 −15.814 14.717 79.068 1.00 0.00
A C
ATOM 14126 CD1 TYR A 45 −14.836 14.483 78.105 1.00 0.00
A C
ATOM 14127 HD1 TYR A 45 −14.036 13.786 78.311 1.00 0.00
A H
ATOM 14128 CE1 TYR A 45 −14.860 15.147 76.891 1.00 0.00
A C
ATOM 14129 HE1 TYR A 45 −14.100 14.960 76.147 1.00 0.00
A H
ATOM 14130 CZ TYR A 45 −15.860 16.061 76.632 1.00 0.00
A C
ATOM 14131 OH TYR A 45 −15.881 16.718 75.431 1.00 0.00
A O
ATOM 14132 HH TYR A 45 −16.679 17.250 75.407 1.00 0.00
A H
ATOM 14133 CD2 TYR A 45 −16.808 15.652 78.783 1.00 0.00
A C
ATOM 14134 HD2 TYR A 45 −17.553 15.893 79.529 1.00 0.00
A H
ATOM 14135 CE2 TYR A 45 −16.835 16.321 77.573 1.00 0.00
A C
ATOM 14136 HE2 TYR A 45 −17.603 17.055 77.384 1.00 0.00
A H
ATOM 14137 C TYR A 45 −15.422 14.533 82.876 1.00 0.00
A C
ATOM 14138 O TYR A 45 −16.617 14.587 83.190 1.00 0.00
A O
ATOM 14139 N ALA A 46 −14.458 14.256 83.748 1.00 0.00
A N
ATOM 14140 HN ALA A 46 −13.496 14.181 83.487 1.00 0.00
A H
ATOM 14141 CA ALA A 46 −14.744 14.039 85.168 1.00 0.00
A C
ATOM 14142 HA ALA A 46 −15.318 13.130 85.303 1.00 0.00
A H
ATOM 14143 CB ALA A 46 −13.450 13.854 85.958 1.00 0.00
A C
ATOM 14144 HB1 ALA A 46 −12.880 12.994 85.545 1.00 0.00
A H
ATOM 14145 HB2 ALA A 46 −12.806 14.758 85.886 1.00 0.00
A H
ATOM 14146 HB3 ALA A 46 −13.651 13.648 87.031 1.00 0.00
A H
ATOM 14147 C ALA A 46 −15.589 15.176 85.755 1.00 0.00
A C

ATOM 14148 O ALA A 46 −16.550 14.930 86.486 1.00 0.00
A O
ATOM 14149 N SER A 47 −15.238 16.412 85.402 1.00 0.00
A N
ATOM 14150 HN SER A 47 −14.477 16.602 84.782 1.00 0.00
A H
ATOM 14151 CA SER A 47 −15.931 17.609 85.888 1.00 0.00
A C
ATOM 14152 HA SER A 47 −16.155 17.389 86.924 1.00 0.00
A H
ATOM 14153 CB SER A 47 −15.027 18.830 85.735 1.00 0.00
A C
ATOM 14154 HB1 SER A 47 −14.702 18.888 84.671 1.00 0.00
A H
ATOM 14155 HB2 SER A 47 −15.595 19.762 85.956 1.00 0.00
A H
ATOM 14156 OG SER A 47 −13.886 18.712 86.559 1.00 0.00
A O
ATOM 14157 HG1 SER A 47 −13.254 19.380 86.264 1.00 0.00
A H
ATOM 14158 C SER A 47 −17.256 17.908 85.190 1.00 0.00
A C
ATOM 14159 O SER A 47 −18.049 18.714 85.686 1.00 0.00
A O
ATOM 14160 N TYR A 48 −17.489 17.278 84.042 1.00 0.00
A N
ATOM 14161 HN TYR A 48 −16.900 16.564 83.667 1.00 0.00
A H
ATOM 14162 CA TYR A 48 −18.659 17.595 83.221 1.00 0.00
A C
ATOM 14163 HA TYR A 48 −19.137 18.437 83.706 1.00 0.00
A H
ATOM 14164 CB TYR A 48 −18.233 17.995 81.803 1.00 0.00
A C
ATOM 14165 HB1 TYR A 48 −17.743 17.126 81.311 1.00 0.00
A H
ATOM 14166 HB2 TYR A 48 −19.119 18.265 81.188 1.00 0.00
A H
ATOM 14167 CG TYR A 48 −17.233 19.133 81.753 1.00 0.00
A C
ATOM 14168 CD1 TYR A 48 −15.879 18.892 81.530 1.00 0.00
A C
ATOM 14169 HD1 TYR A 48 −15.526 17.878 81.405 1.00 0.00
A H
ATOM 14170 CE1 TYR A 48 −14.964 19.930 81.485 1.00 0.00
A C
ATOM 14171 HE1 TYR A 48 −13.916 19.731 81.315 1.00 0.00
A H
ATOM 14172 CZ TYR A 48 −15.399 21.224 81.665 1.00 0.00
A C
ATOM 14173 OH TYR A 48 −14.504 22.260 81.620 1.00 0.00
A O
ATOM 14174 HH TYR A 48 −15.003 23.075 81.694 1.00 0.00
A H

ATOM 14175 CD2 TYR A 48 -17.640 20.449 81.933 1.00 0.00
A C
ATOM 14176 HD2 TYR A 48 -18.675 20.670 82.150 1.00 0.00
A H
ATOM 14177 CE2 TYR A 48 -16.731 21.491 81.888 1.00 0.00
A C
ATOM 14178 HE2 TYR A 48 -17.068 22.504 82.050 1.00 0.00
A H
ATOM 14179 C TYR A 48 -19.682 16.464 83.171 1.00 0.00
A C
ATOM 14180 O TYR A 48 -20.500 16.400 82.246 1.00 0.00
A O
ATOM 14181 N ALA A 49 -19.634 15.586 84.175 1.00 0.00
A N
ATOM 14182 HN ALA A 49 -18.970 15.677 84.917 1.00 0.00
A H
ATOM 14183 CA ALA A 49 -20.528 14.444 84.273 1.00 0.00
A C
ATOM 14184 HA ALA A 49 -21.521 14.701 83.924 1.00 0.00
A H
ATOM 14185 CB ALA A 49 -19.997 13.298 83.435 1.00 0.00
A C
ATOM 14186 HB1 ALA A 49 -19.935 13.612 82.371 1.00 0.00
A H
ATOM 14187 HB2 ALA A 49 -18.977 13.002 83.763 1.00 0.00
A H
ATOM 14188 HB3 ALA A 49 -20.660 12.407 83.492 1.00 0.00
A H
ATOM 14189 C ALA A 49 -20.682 14.007 85.726 1.00 0.00
A C
ATOM 14190 O ALA A 49 -19.769 14.202 86.535 1.00 0.00
A O
ATOM 14191 N THR A 50 -21.829 13.413 86.056 1.00 0.00
A N
ATOM 14192 HN THR A 50 -22.602 13.318 85.429 1.00 0.00
A H
ATOM 14193 CA THR A 50 -22.058 12.840 87.384 1.00 0.00
A C
ATOM 14194 HA THR A 50 -21.894 13.639 88.094 1.00 0.00
A H
ATOM 14195 CB THR A 50 -23.474 12.241 87.501 1.00 0.00
A C
ATOM 14196 HB THR A 50 -23.481 11.331 86.852 1.00 0.00
A H
ATOM 14197 OG1 THR A 50 -24.426 13.170 86.975 1.00 0.00
A O
ATOM 14198 HG1 THR A 50 -25.285 12.747 87.085 1.00 0.00
A H
ATOM 14199 CG2 THR A 50 -23.825 11.952 88.946 1.00 0.00
A C
ATOM 14200 HG21 THR A 50 -24.796 11.417 89.018 1.00 0.00
A H
ATOM 14201 HG22 THR A 50 -23.047 11.311 89.412 1.00 0.00
A H

ATOM 14202 HG23 THR A 50   -23.901 12.891 89.535 1.00 0.00
A   H
ATOM 14203 C   THR A 50   -21.005 11.770 87.681 1.00 0.00
A   C
ATOM 14204 O   THR A 50   -20.377 11.791 88.739 1.00 0.00
A   O
ATOM 14205 N   HSE A 51   -20.816 10.854 86.733 1.00 0.00
A   N
ATOM 14206 HN HSE A 51   -21.382 10.806 85.911 1.00 0.00
A   H
ATOM 14207 CA HSE A 51   -19.768   9.836 86.805 1.00 0.00
A   C
ATOM 14208 HA HSE A 51   -19.016 10.204 87.492 1.00 0.00
A   H
ATOM 14209 CB HSE A 51   -20.339   8.509 87.290 1.00 0.00
A   C
ATOM 14210 HB1 HSE A 51   -21.373   8.416 86.892 1.00 0.00
A   H
ATOM 14211 HB2 HSE A 51   -19.749   7.673 86.860 1.00 0.00
A   H
ATOM 14212 ND1 HSE A 51   -21.459   8.825 89.508 1.00 0.00
A   N
ATOM 14213 CG HSE A 51   -20.407   8.386 88.790 1.00 0.00
A   C
ATOM 14214 CE1 HSE A 51   -21.240   8.585 90.819 1.00 0.00
A   C
ATOM 14215 HE1 HSE A 51   -21.935   8.844 91.622 1.00 0.00
A   H
ATOM 14216 NE2 HSE A 51   -20.041   7.992 90.940 1.00 0.00
A   N
ATOM 14217 HE2 HSE A 51   -19.613   7.721 91.801 1.00 0.00
A   H
ATOM 14218 CD2 HSE A 51   -19.500   7.852 89.706 1.00 0.00
A   C
ATOM 14219 HD2 HSE A 51   -18.529   7.406 89.541 1.00 0.00
A   H
ATOM 14220 C   HSE A 51   -19.099   9.656 85.472 1.00 0.00
A   C
ATOM 14221 O   HSE A 51   -19.722   9.843 84.420 1.00 0.00
A   O
ATOM 14222 N   ALA A 52   -17.820   9.291 85.503 1.00 0.00
A   N
ATOM 14223 HN ALA A 52   -17.315   9.183 86.359 1.00 0.00
A   H
ATOM 14224 CA ALA A 52   -17.057   9.022 84.287 1.00 0.00
A   C
ATOM 14225 HA ALA A 52   -17.714   8.854 83.441 1.00 0.00
A   H
ATOM 14226 CB ALA A 52   -16.155 10.198 83.958 1.00 0.00
A   C
ATOM 14227 HB1 ALA A 52   -16.774 11.105 83.787 1.00 0.00
A   H
ATOM 14228 HB2 ALA A 52   -15.457 10.412 84.796 1.00 0.00
A   H

ATOM 14229 HB3 ALA A 52   -15.559 10.011 83.039 1.00 0.00
A   H
ATOM 14230 C   ALA A 52   -16.245   7.744 84.461 1.00 0.00
A   C
ATOM 14231 O   ALA A 52   -15.556   7.580 85.466 1.00 0.00
A   O
ATOM 14232 N   VAL A 53   -16.334   6.839 83.488 1.00 0.00
A   N
ATOM 14233 HN VAL A 53   -16.821   6.994 82.633 1.00 0.00
A   H
ATOM 14234 CA VAL A 53   -15.711   5.516 83.599 1.00 0.00
A   C
ATOM 14235 HA VAL A 53   -14.960   5.582 84.377 1.00 0.00
A   H
ATOM 14236 CB VAL A 53   -16.749   4.413 83.911 1.00 0.00
A   C
ATOM 14237 HB VAL A 53   -17.210   4.099 82.941 1.00 0.00
A   H
ATOM 14238 CG1 VAL A 53   -16.076   3.210 84.557 1.00 0.00
A   C
ATOM 14239 HG11 VAL A 53   -16.832   2.437 84.814 1.00 0.00
A   H
ATOM 14240 HG12 VAL A 53   -15.339   2.738 83.874 1.00 0.00
A   H
ATOM 14241 HG13 VAL A 53   -15.556   3.507 85.492 1.00 0.00
A   H
ATOM 14242 CG2 VAL A 53   -17.875   4.926 84.801 1.00 0.00
A   C
ATOM 14243 HG21 VAL A 53   -18.587   4.110 85.045 1.00 0.00
A   H
ATOM 14244 HG22 VAL A 53   -17.466   5.331 85.753 1.00 0.00
A   H
ATOM 14245 HG23 VAL A 53   -18.448   5.732 84.298 1.00 0.00
A   H
ATOM 14246 C   VAL A 53   -15.000   5.115 82.311 1.00 0.00
A   C
ATOM 14247 O   VAL A 53   -15.525   5.328 81.215 1.00 0.00
A   O
ATOM 14248 N   ILE A 54   -13.814   4.525 82.447 1.00 0.00
A   N
ATOM 14249 HN ILE A 54   -13.314   4.465 83.308 1.00 0.00
A   H
ATOM 14250 CA ILE A 54   -13.132   3.902 81.314 1.00 0.00
A   C
ATOM 14251 HA ILE A 54   -13.278   4.541 80.453 1.00 0.00
A   H
ATOM 14252 CB ILE A 54   -11.626   3.644 81.593 1.00 0.00
A   C
ATOM 14253 HB ILE A 54   -11.614   3.072 82.555 1.00 0.00
A   H
ATOM 14254 CG2 ILE A 54   -10.971   2.909 80.429 1.00 0.00
A   C
ATOM 14255 HG21 ILE A 54   -9.942   2.591 80.697 1.00 0.00
A   H

```
ATOM  14256 HG22 ILE A  54      -11.546   1.998  80.161  1.00  0.00
A    H
ATOM  14257 HG23 ILE A  54      -10.909   3.561  79.532  1.00  0.00
A    H
ATOM  14258  CG1 ILE A  54      -10.867   4.951  81.856  1.00  0.00
A    C
ATOM  14259 HG11 ILE A  54       -9.876   4.920  81.350  1.00  0.00
A    H
ATOM  14260 HG12 ILE A  54      -11.439   5.796  81.409  1.00  0.00
A    H
ATOM  14261  CD  ILE A  54      -10.654   5.260  83.320  1.00  0.00
A    C
ATOM  14262  HD1 ILE A  54      -10.152   6.244  83.438  1.00  0.00
A    H
ATOM  14263  HD2 ILE A  54      -11.625   5.298  83.859  1.00  0.00
A    H
ATOM  14264  HD3 ILE A  54      -10.015   4.489  83.801  1.00  0.00
A    H
ATOM  14265  C   ILE A  54      -13.824   2.580  81.006  1.00  0.00
A    C
ATOM  14266  O   ILE A  54      -13.992   1.749  81.898  1.00  0.00
A    O
ATOM  14267  N   ALA A  55      -14.234   2.395  79.752  1.00  0.00
A    N
ATOM  14268  HN  ALA A  55      -14.133   3.101  79.052  1.00  0.00
A    H
ATOM  14269  CA  ALA A  55      -14.861   1.149  79.304  1.00  0.00
A    C
ATOM  14270  HA  ALA A  55      -14.336   0.296  79.719  1.00  0.00
A    H
ATOM  14271  CB  ALA A  55      -16.300   1.060  79.792  1.00  0.00
A    C
ATOM  14272  HB1 ALA A  55      -16.322   1.124  80.901  1.00  0.00
A    H
ATOM  14273  HB2 ALA A  55      -16.910   1.897  79.389  1.00  0.00
A    H
ATOM  14274  HB3 ALA A  55      -16.774   0.100  79.494  1.00  0.00
A    H
ATOM  14275  C   ALA A  55      -14.803   0.995  77.787  1.00  0.00
A    C
ATOM  14276  O   ALA A  55      -14.903   1.974  77.048  1.00  0.00
A    O
ATOM  14277  N   ASN A  56      -14.623  -0.239  77.327  1.00  0.00
A    N
ATOM  14278  HN  ASN A  56      -14.365  -1.016  77.900  1.00  0.00
A    H
ATOM  14279  CA  ASN A  56      -14.790  -0.559  75.923  1.00  0.00
A    C
ATOM  14280  HA  ASN A  56      -14.613   0.350  75.357  1.00  0.00
A    H
ATOM  14281  CB  ASN A  56      -13.841  -1.686  75.498  1.00  0.00
A    C
ATOM  14282  HB1 ASN A  56      -12.801  -1.356  75.715  1.00  0.00
A    H
```

ATOM 14283 HB2 ASN A 56   -14.033 -2.599 76.099 1.00 0.00
A   H
ATOM 14284 CG ASN A 56   -13.930 -2.009 74.009 1.00 0.00
A   C
ATOM 14285 OD1 ASN A 56   -14.958 -1.787 73.362 1.00 0.00
A   O
ATOM 14286 ND2 ASN A 56   -12.850 -2.547 73.464 1.00 0.00
A   N
ATOM 14287 HD21 ASN A 56   -12.883 -2.755 72.490 1.00 0.00
A   H
ATOM 14288 HD22 ASN A 56   -12.036 -2.700 74.016 1.00 0.00
A   H
ATOM 14289 C   ASN A 56   -16.248 -0.955 75.716 1.00 0.00
A   C
ATOM 14290 O   ASN A 56   -16.663 -2.055 76.099 1.00 0.00
A   O
ATOM 14291 N   ALA A 57   -17.023 -0.055 75.109 1.00 0.00
A   N
ATOM 14292 HN ALA A 57   -16.665   0.799 74.732 1.00 0.00
A   H
ATOM 14293 CA ALA A 57   -18.463 -0.256 74.952 1.00 0.00
A   C
ATOM 14294 HA ALA A 57   -18.890 -0.475 75.924 1.00 0.00
A   H
ATOM 14295 CB ALA A 57   -19.130   1.028 74.507 1.00 0.00
A   C
ATOM 14296 HB1 ALA A 57   -18.869   1.847 75.212 1.00 0.00
A   H
ATOM 14297 HB2 ALA A 57   -18.785   1.327 73.494 1.00 0.00
A   H
ATOM 14298 HB3 ALA A 57   -20.237   0.930 74.490 1.00 0.00
A   H
ATOM 14299 C   ALA A 57   -18.864 -1.416 74.032 1.00 0.00
A   C
ATOM 14300 O   ALA A 57   -20.053 -1.715 73.892 1.00 0.00
A   O
ATOM 14301 N   THR A 58   -17.881 -2.065 73.412 1.00 0.00
A   N
ATOM 14302 HN THR A 58   -16.915 -1.816 73.477 1.00 0.00
A   H
ATOM 14303 CA THR A 58   -18.148 -3.228 72.564 1.00 0.00
A   C
ATOM 14304 HA THR A 58   -19.197 -3.170 72.307 1.00 0.00
A   H
ATOM 14305 CB THR A 58   -17.243 -3.241 71.322 1.00 0.00
A   C
ATOM 14306 HB THR A 58   -17.568 -4.119 70.712 1.00 0.00
A   H
ATOM 14307 OG1 THR A 58   -15.887 -3.456 71.723 1.00 0.00
A   O
ATOM 14308 HG1 THR A 58   -15.374 -3.442 70.908 1.00 0.00
A   H
ATOM 14309 CG2 THR A 58   -17.338 -1.929 70.575 1.00 0.00
A   C

ATOM 14310 HG21 THR A 58 -16.776 -1.977 69.619 1.00 0.00
A H
ATOM 14311 HG22 THR A 58 -18.398 -1.694 70.336 1.00 0.00
A H
ATOM 14312 HG23 THR A 58 -16.926 -1.094 71.179 1.00 0.00
A H
ATOM 14313 C THR A 58 -17.960 -4.541 73.338 1.00 0.00
A C
ATOM 14314 O THR A 58 -18.101 -5.632 72.777 1.00 0.00
A O
ATOM 14315 N GLU A 59 -17.648 -4.419 74.625 1.00 0.00
A N
ATOM 14316 HN GLU A 59 -17.564 -3.539 75.084 1.00 0.00
A H
ATOM 14317 CA GLU A 59 -17.400 -5.562 75.489 1.00 0.00
A C
ATOM 14318 HA GLU A 59 -17.352 -6.454 74.878 1.00 0.00
A H
ATOM 14319 CB GLU A 59 -16.084 -5.349 76.243 1.00 0.00
A C
ATOM 14320 HB1 GLU A 59 -15.366 -4.850 75.553 1.00 0.00
A H
ATOM 14321 HB2 GLU A 59 -16.242 -4.638 77.085 1.00 0.00
A H
ATOM 14322 CG GLU A 59 -15.444 -6.623 76.770 1.00 0.00
A C
ATOM 14323 HG1 GLU A 59 -14.513 -6.371 77.310 1.00 0.00
A H
ATOM 14324 HG2 GLU A 59 -16.131 -7.158 77.455 1.00 0.00
A H
ATOM 14325 CD GLU A 59 -15.030 -7.577 75.663 1.00 0.00
A C
ATOM 14326 OE1 GLU A 59 -15.495 -8.737 75.677 1.00 0.00
A O
ATOM 14327 OE2 GLU A 59 -14.250 -7.167 74.773 1.00 0.00
A O
ATOM 14328 C GLU A 59 -18.565 -5.742 76.464 1.00 0.00
A C
ATOM 14329 O GLU A 59 -18.764 -4.917 77.362 1.00 0.00
A O
ATOM 14330 N GLU A 60 -19.320 -6.829 76.287 1.00 0.00
A N
ATOM 14331 HN GLU A 60 -19.078 -7.563 75.659 1.00 0.00
A H
ATOM 14332 CA GLU A 60 -20.588 -7.050 77.006 1.00 0.00
A C
ATOM 14333 HA GLU A 60 -21.249 -6.247 76.706 1.00 0.00
A H
ATOM 14334 CB GLU A 60 -21.264 -8.349 76.556 1.00 0.00
A C
ATOM 14335 HB1 GLU A 60 -21.217 -8.397 75.444 1.00 0.00
A H
ATOM 14336 HB2 GLU A 60 -20.681 -9.224 76.923 1.00 0.00
A H

ATOM 14337 CG GLU A 60   -22.716 -8.457 77.004 1.00 0.00
A   C
ATOM 14338 HG1 GLU A 60   -22.795 -8.171 78.069 1.00 0.00
A   H
ATOM 14339 HG2 GLU A 60   -23.368 -7.795 76.400 1.00 0.00
A   H
ATOM 14340 CD GLU A 60   -23.270 -9.865 76.923 1.00 0.00
A   C
ATOM 14341 OE1 GLU A 60   -24.025 -10.255 77.840 1.00 0.00
A   O
ATOM 14342 OE2 GLU A 60   -22.956 -10.580 75.945 1.00 0.00
A   O
ATOM 14343 C   GLU A 60   -20.495 -7.014 78.537 1.00 0.00
A   C
ATOM 14344 O   GLU A 60   -21.349 -6.410 79.196 1.00 0.00
A   O
ATOM 14345 N   ASN A 61   -19.479 -7.663 79.099 1.00 0.00
A   N
ATOM 14346 HN ASN A 61   -18.792 -8.189 78.599 1.00 0.00
A   H
ATOM 14347 CA ASN A 61   -19.292 -7.646 80.553 1.00 0.00
A   C
ATOM 14348 HA ASN A 61   -20.257 -7.925 80.965 1.00 0.00
A   H
ATOM 14349 CB ASN A 61   -18.278 -8.709 81.013 1.00 0.00
A   C
ATOM 14350 HB1 ASN A 61   -18.063 -8.531 82.089 1.00 0.00
A   H
ATOM 14351 HB2 ASN A 61   -18.716 -9.724 80.924 1.00 0.00
A   H
ATOM 14352 CG ASN A 61   -16.957 -8.646 80.257 1.00 0.00
A   C
ATOM 14353 OD1 ASN A 61   -16.511 -7.586 79.815 1.00 0.00
A   O
ATOM 14354 ND2 ASN A 61   -16.315 -9.799 80.118 1.00 0.00
A   N
ATOM 14355 HD21 ASN A 61   -15.443 -9.782 79.637 1.00 0.00
A   H
ATOM 14356 HD22 ASN A 61   -16.693 -10.631 80.512 1.00 0.00
A   H
ATOM 14357 C   ASN A 61   -18.941 -6.255 81.094 1.00 0.00
A   C
ATOM 14358 O   ASN A 61   -19.345 -5.894 82.203 1.00 0.00
A   O
ATOM 14359 N   GLU A 62   -18.206 -5.478 80.297 1.00 0.00
A   N
ATOM 14360 HN GLU A 62   -17.853 -5.762 79.410 1.00 0.00
A   H
ATOM 14361 CA GLU A 62   -17.849 -4.110 80.668 1.00 0.00
A   C
ATOM 14362 HA GLU A 62   -17.413 -4.126 81.660 1.00 0.00
A   H
ATOM 14363 CB GLU A 62   -16.825 -3.517 79.699 1.00 0.00
A   C

ATOM 14364 HB1 GLU A 62   -17.210 -3.653 78.661 1.00 0.00
A   H
ATOM 14365 HB2 GLU A 62   -16.751 -2.417 79.855 1.00 0.00
A   H
ATOM 14366 CG GLU A 62   -15.443 -4.146 79.798 1.00 0.00
A   C
ATOM 14367 HG1 GLU A 62   -15.185 -4.303 80.862 1.00 0.00
A   H
ATOM 14368 HG2 GLU A 62   -15.410 -5.119 79.268 1.00 0.00
A   H
ATOM 14369 CD GLU A 62   -14.340 -3.264 79.241 1.00 0.00
A   C
ATOM 14370 OE1 GLU A 62   -14.373 -2.040 79.477 1.00 0.00
A   O
ATOM 14371 OE2 GLU A 62   -13.425 -3.796 78.578 1.00 0.00
A   O
ATOM 14372 C   GLU A 62   -19.094 -3.238 80.737 1.00 0.00
A   C
ATOM 14373 O   GLU A 62   -19.217 -2.404 81.632 1.00 0.00
A   O
ATOM 14374 N   LEU A 63   -20.018 -3.451 79.803 1.00 0.00
A   N
ATOM 14375 HN LEU A 63   -19.901 -4.089 79.045 1.00 0.00
A   H
ATOM 14376 CA LEU A 63   -21.306 -2.755 79.809 1.00 0.00
A   C
ATOM 14377 HA LEU A 63   -21.085 -1.696 79.845 1.00 0.00
A   H
ATOM 14378 CB LEU A 63   -22.114 -3.074 78.547 1.00 0.00
A   C
ATOM 14379 HB1 LEU A 63   -21.794 -4.083 78.197 1.00 0.00
A   H
ATOM 14380 HB2 LEU A 63   -23.189 -3.170 78.819 1.00 0.00
A   H
ATOM 14381 CG LEU A 63   -21.975 -2.109 77.368 1.00 0.00
A   C
ATOM 14382 HG LEU A 63   -22.279 -1.097 77.729 1.00 0.00
A   H
ATOM 14383 CD1 LEU A 63   -20.538 -2.072 76.888 1.00 0.00
A   C
ATOM 14384 HD11 LEU A 63   -20.439 -1.409 76.001 1.00 0.00
A   H
ATOM 14385 HD12 LEU A 63   -19.869 -1.687 77.687 1.00 0.00
A   H
ATOM 14386 HD13 LEU A 63   -20.193 -3.088 76.601 1.00 0.00
A   H
ATOM 14387 CD2 LEU A 63   -22.902 -2.500 76.229 1.00 0.00
A   C
ATOM 14388 HD21 LEU A 63   -22.804 -1.780 75.387 1.00 0.00
A   H
ATOM 14389 HD22 LEU A 63   -22.667 -3.510 75.837 1.00 0.00
A   H
ATOM 14390 HD23 LEU A 63   -23.960 -2.494 76.569 1.00 0.00
A   H

```
ATOM  14391  C   LEU A  63      -22.118  -3.097  81.050  1.00  0.00
A    C
ATOM  14392  O   LEU A  63      -22.668  -2.205  81.699  1.00  0.00
A    O
ATOM  14393  N   LEU A  64      -22.181  -4.389  81.373  1.00  0.00
A    N
ATOM  14394  HN  LEU A  64      -21.768  -5.121  80.834  1.00  0.00
A    H
ATOM  14395  CA  LEU A  64      -22.874  -4.844  82.569  1.00  0.00
A    C
ATOM  14396  HA  LEU A  64      -23.903  -4.518  82.484  1.00  0.00
A    H
ATOM  14397  CB  LEU A  64      -22.810  -6.369  82.685  1.00  0.00
A    C
ATOM  14398  HB1 LEU A  64      -21.747  -6.667  82.529  1.00  0.00
A    H
ATOM  14399  HB2 LEU A  64      -23.061  -6.663  83.729  1.00  0.00
A    H
ATOM  14400  CG  LEU A  64      -23.649  -7.233  81.734  1.00  0.00
A    C
ATOM  14401  HG  LEU A  64      -23.379  -6.928  80.694  1.00  0.00
A    H
ATOM  14402  CD1 LEU A  64      -23.317  -8.706  81.940  1.00  0.00
A    C
ATOM  14403 HD11 LEU A  64      -23.954  -9.344  81.290  1.00  0.00
A    H
ATOM  14404 HD12 LEU A  64      -22.253  -8.907  81.692  1.00  0.00
A    H
ATOM  14405 HD13 LEU A  64      -23.493  -9.004  82.996  1.00  0.00
A    H
ATOM  14406  CD2 LEU A  64      -25.140  -6.990  81.925  1.00  0.00
A    C
ATOM  14407 HD21 LEU A  64      -25.727  -7.612  81.213  1.00  0.00
A    H
ATOM  14408 HD22 LEU A  64      -25.470  -7.251  82.951  1.00  0.00
A    H
ATOM  14409 HD23 LEU A  64      -25.387  -5.923  81.738  1.00  0.00
A    H
ATOM  14410  C   LEU A  64      -22.283  -4.181  83.808  1.00  0.00
A    C
ATOM  14411  O   LEU A  64      -23.021  -3.713  84.673  1.00  0.00
A    O
ATOM  14412  N   SER A  65      -20.951  -4.121  83.860  1.00  0.00
A    N
ATOM  14413  HN  SER A  65      -20.370  -4.483  83.130  1.00  0.00
A    H
ATOM  14414  CA  SER A  65      -20.209  -3.525  84.980  1.00  0.00
A    C
ATOM  14415  HA  SER A  65      -20.560  -4.054  85.856  1.00  0.00
A    H
ATOM  14416  CB  SER A  65      -18.703  -3.743  84.809  1.00  0.00
A    C
ATOM  14417  HB1 SER A  65      -18.374  -3.189  83.900  1.00  0.00
A    H
```

ATOM 14418 HB2 SER A 65   -18.148 -3.313 85.674 1.00 0.00
A   H
ATOM 14419 OG SER A 65   -18.398 -5.116 84.639 1.00 0.00
A   O
ATOM 14420 HG1 SER A 65   -17.489 -5.167 84.318 1.00 0.00
A   H
ATOM 14421 C   SER A 65   -20.498 -2.039 85.199 1.00 0.00
A   C
ATOM 14422 O   SER A 65   -20.430 -1.547 86.328 1.00 0.00
A   O
ATOM 14423 N   LEU A 66   -20.815 -1.334 84.117 1.00 0.00
A   N
ATOM 14424 HN LEU A 66   -20.811 -1.709 83.192 1.00 0.00
A   H
ATOM 14425 CA LEU A 66   -21.200   0.071 84.192 1.00 0.00
A   C
ATOM 14426 HA LEU A 66   -20.481   0.555 84.839 1.00 0.00
A   H
ATOM 14427 CB LEU A 66   -21.220   0.695 82.790 1.00 0.00
A   C
ATOM 14428 HB1 LEU A 66   -21.978   0.138 82.192 1.00 0.00
A   H
ATOM 14429 HB2 LEU A 66   -21.596   1.740 82.865 1.00 0.00
A   H
ATOM 14430 CG LEU A 66   -19.926   0.702 81.973 1.00 0.00
A   C
ATOM 14431 HG LEU A 66   -19.432 -0.280 82.173 1.00 0.00
A   H
ATOM 14432 CD1 LEU A 66   -20.224   0.829 80.489 1.00 0.00
A   C
ATOM 14433 HD11 LEU A 66   -19.280   0.882 79.905 1.00 0.00
A   H
ATOM 14434 HD12 LEU A 66   -20.809 -0.045 80.130 1.00 0.00
A   H
ATOM 14435 HD13 LEU A 66   -20.810   1.750 80.284 1.00 0.00
A   H
ATOM 14436 CD2 LEU A 66   -18.984   1.802 82.428 1.00 0.00
A   C
ATOM 14437 HD21 LEU A 66   -18.044   1.776 81.835 1.00 0.00
A   H
ATOM 14438 HD22 LEU A 66   -19.435   2.807 82.301 1.00 0.00
A   H
ATOM 14439 HD23 LEU A 66   -18.722   1.671 83.501 1.00 0.00
A   H
ATOM 14440 C   LEU A 66   -22.578   0.235 84.830 1.00 0.00
A   C
ATOM 14441 O   LEU A 66   -22.929   1.321 85.297 1.00 0.00
A   O
ATOM 14442 N   GLY A 67   -23.352 -0.851 84.846 1.00 0.00
A   N
ATOM 14443 HN GLY A 67   -23.009 -1.748 84.570 1.00 0.00
A   H
ATOM 14444 CA GLY A 67   -24.748 -0.811 85.264 1.00 0.00
A   C

ATOM 14445 HA1 GLY A 67 -24.819 -0.214 86.163 1.00 0.00
A H
ATOM 14446 HA2 GLY A 67 -25.107 -1.826 85.374 1.00 0.00
A H
ATOM 14447 C GLY A 67 -25.575 -0.139 84.189 1.00 0.00
A C
ATOM 14448 O GLY A 67 -26.462 0.663 84.480 1.00 0.00
A O
ATOM 14449 N ILE A 68 -25.271 -0.475 82.938 1.00 0.00
A N
ATOM 14450 HN ILE A 68 -24.632 -1.207 82.710 1.00 0.00
A H
ATOM 14451 CA ILE A 68 -25.847 0.196 81.778 1.00 0.00
A C
ATOM 14452 HA ILE A 68 -25.620 1.246 81.916 1.00 0.00
A H
ATOM 14453 CB ILE A 68 -25.181 -0.289 80.468 1.00 0.00
A C
ATOM 14454 HB ILE A 68 -24.083 -0.239 80.677 1.00 0.00
A H
ATOM 14455 CG2 ILE A 68 -25.619 -1.706 80.123 1.00 0.00
A C
ATOM 14456 HG21 ILE A 68 -25.024 -2.103 79.275 1.00 0.00
A H
ATOM 14457 HG22 ILE A 68 -25.471 -2.387 80.987 1.00 0.00
A H
ATOM 14458 HG23 ILE A 68 -26.691 -1.731 79.836 1.00 0.00
A H
ATOM 14459 CG1 ILE A 68 -25.452 0.685 79.319 1.00 0.00
A C
ATOM 14460 HG11 ILE A 68 -25.125 0.232 78.356 1.00 0.00
A H
ATOM 14461 HG12 ILE A 68 -26.550 0.861 79.245 1.00 0.00
A H
ATOM 14462 CD ILE A 68 -24.779 2.032 79.472 1.00 0.00
A C
ATOM 14463 HD1 ILE A 68 -25.065 2.704 78.635 1.00 0.00
A H
ATOM 14464 HD2 ILE A 68 -25.082 2.515 80.425 1.00 0.00
A H
ATOM 14465 HD3 ILE A 68 -23.673 1.928 79.466 1.00 0.00
A H
ATOM 14466 C ILE A 68 -27.372 0.079 81.695 1.00 0.00
A C
ATOM 14467 O ILE A 68 -28.029 0.923 81.087 1.00 0.00
A O
ATOM 14468 N ARG A 69 -27.925 -0.951 82.325 1.00 0.00
A N
ATOM 14469 HN ARG A 69 -27.376 -1.603 82.843 1.00 0.00
A H
ATOM 14470 CA ARG A 69 -29.366 -1.185 82.295 1.00 0.00
A C
ATOM 14471 HA ARG A 69 -29.704 -1.009 81.281 1.00 0.00
A H

ATOM 14472 CB ARG A 69   -29.679 -2.604 82.716 1.00 0.00
A   C
ATOM 14473 HB1 ARG A 69   -29.207 -2.747 83.718 1.00 0.00
A   H
ATOM 14474 HB2 ARG A 69   -30.774 -2.713 82.875 1.00 0.00
A   H
ATOM 14475 CG ARG A 69   -29.143 -3.638 81.764 1.00 0.00
A   C
ATOM 14476 HG1 ARG A 69   -29.258 -3.255 80.726 1.00 0.00
A   H
ATOM 14477 HG2 ARG A 69   -28.045 -3.743 81.924 1.00 0.00
A   H
ATOM 14478 CD ARG A 69   -29.832 -4.953 82.021 1.00 0.00
A   C
ATOM 14479 HD1 ARG A 69   -30.223 -4.968 83.064 1.00 0.00
A   H
ATOM 14480 HD2 ARG A 69   -30.683 -5.113 81.318 1.00 0.00
A   H
ATOM 14481 NE ARG A 69   -28.892 -6.040 81.826 1.00 0.00
A   N
ATOM 14482 HE ARG A 69   -27.946 -5.793 82.066 1.00 0.00
A   H
ATOM 14483 CZ ARG A 69   -29.223 -7.242 81.380 1.00 0.00
A   C
ATOM 14484 NH1 ARG A 69   -30.483 -7.528 81.077 1.00 0.00
A   N
ATOM 14485 HH11 ARG A 69   -30.734 -8.435 80.767 1.00 0.00
A   H
ATOM 14486 HH12 ARG A 69   -31.165 -6.831 81.307 1.00 0.00
A   H
ATOM 14487 NH2 ARG A 69   -28.282 -8.161 81.231 1.00 0.00
A   N
ATOM 14488 HH21 ARG A 69   -28.506 -9.032 80.817 1.00 0.00
A   H
ATOM 14489 HH22 ARG A 69   -27.343 -7.865 81.349 1.00 0.00
A   H
ATOM 14490 C   ARG A 69   -30.139 -0.231 83.182 1.00 0.00
A   C
ATOM 14491 O   ARG A 69   -31.336 -0.013 82.980 1.00 0.00
A   O
ATOM 14492 N   ASN A 70   -29.451   0.319 84.176 1.00 0.00
A   N
ATOM 14493 HN ASN A 70   -28.498   0.095 84.375 1.00 0.00
A   H
ATOM 14494 CA ASN A 70   -30.038   1.309 85.069 1.00 0.00
A   C
ATOM 14495 HA ASN A 70   -30.988   0.890 85.385 1.00 0.00
A   H
ATOM 14496 CB ASN A 70   -29.116   1.556 86.269 1.00 0.00
A   C
ATOM 14497 HB1 ASN A 70   -28.137   1.912 85.878 1.00 0.00
A   H
ATOM 14498 HB2 ASN A 70   -29.528   2.358 86.913 1.00 0.00
A   H

ATOM 14499 CG ASN A 70 -28.868 0.300 87.083 1.00 0.00
A C
ATOM 14500 OD1 ASN A 70 -29.589 -0.691 86.961 1.00 0.00
A O
ATOM 14501 ND2 ASN A 70 -27.842 0.338 87.928 1.00 0.00
A N
ATOM 14502 HD21 ASN A 70 -27.659 -0.482 88.463 1.00 0.00
A H
ATOM 14503 HD22 ASN A 70 -27.274 1.153 87.985 1.00 0.00
A H
ATOM 14504 C ASN A 70 -30.329 2.622 84.346 1.00 0.00
A C
ATOM 14505 O ASN A 70 -30.817 3.579 84.955 1.00 0.00
A O
ATOM 14506 N PHE A 71 -30.039 2.656 83.047 1.00 0.00
A N
ATOM 14507 HN PHE A 71 -29.692 1.868 82.540 1.00 0.00
A H
ATOM 14508 CA PHE A 71 -30.205 3.863 82.248 1.00 0.00
A C
ATOM 14509 HA PHE A 71 -30.447 4.646 82.957 1.00 0.00
A H
ATOM 14510 CB PHE A 71 -28.902 4.211 81.537 1.00 0.00
A C
ATOM 14511 HB1 PHE A 71 -28.501 3.298 81.044 1.00 0.00
A H
ATOM 14512 HB2 PHE A 71 -29.070 4.966 80.739 1.00 0.00
A H
ATOM 14513 CG PHE A 71 -27.833 4.693 82.469 1.00 0.00
A C
ATOM 14514 CD1 PHE A 71 -26.995 3.789 83.115 1.00 0.00
A C
ATOM 14515 HD1 PHE A 71 -27.112 2.729 82.937 1.00 0.00
A H
ATOM 14516 CE1 PHE A 71 -26.019 4.237 83.989 1.00 0.00
A C
ATOM 14517 HE1 PHE A 71 -25.367 3.529 84.480 1.00 0.00
A H
ATOM 14518 CZ PHE A 71 -25.876 5.593 84.228 1.00 0.00
A C
ATOM 14519 HZ PHE A 71 -25.110 5.943 84.905 1.00 0.00
A H
ATOM 14520 CD2 PHE A 71 -27.682 6.048 82.727 1.00 0.00
A C
ATOM 14521 HD2 PHE A 71 -28.348 6.764 82.265 1.00 0.00
A H
ATOM 14522 CE2 PHE A 71 -26.705 6.500 83.594 1.00 0.00
A C
ATOM 14523 HE2 PHE A 71 -26.592 7.560 83.774 1.00 0.00
A H
ATOM 14524 C PHE A 71 -31.362 3.773 81.272 1.00 0.00
A C
ATOM 14525 O PHE A 71 -31.614 2.730 80.674 1.00 0.00
A O

ATOM 14526 N GLU A 72 -32.064 4.889 81.136 1.00 0.00
A N
ATOM 14527 HN GLU A 72 -31.830 5.738 81.600 1.00 0.00
A H
ATOM 14528 CA GLU A 72 -33.242 4.981 80.298 1.00 0.00
A C
ATOM 14529 HA GLU A 72 -33.677 3.993 80.201 1.00 0.00
A H
ATOM 14530 CB GLU A 72 -34.231 5.964 80.915 1.00 0.00
A C
ATOM 14531 HB1 GLU A 72 -34.114 5.925 82.022 1.00 0.00
A H
ATOM 14532 HB2 GLU A 72 -33.968 7.002 80.616 1.00 0.00
A H
ATOM 14533 CG GLU A 72 -35.682 5.689 80.571 1.00 0.00
A C
ATOM 14534 HG1 GLU A 72 -35.771 5.477 79.490 1.00 0.00
A H
ATOM 14535 HG2 GLU A 72 -36.069 4.824 81.145 1.00 0.00
A H
ATOM 14536 CD GLU A 72 -36.582 6.884 80.833 1.00 0.00
A C
ATOM 14537 OE1 GLU A 72 -36.388 7.591 81.851 1.00 0.00
A O
ATOM 14538 OE2 GLU A 72 -37.497 7.114 80.017 1.00 0.00
A O
ATOM 14539 C GLU A 72 -32.854 5.444 78.897 1.00 0.00
A C
ATOM 14540 O GLU A 72 -33.550 5.149 77.917 1.00 0.00
A O
ATOM 14541 N TYR A 73 -31.749 6.186 78.820 1.00 0.00
A N
ATOM 14542 HN TYR A 73 -31.218 6.444 79.626 1.00 0.00
A H
ATOM 14543 CA TYR A 73 -31.219 6.686 77.560 1.00 0.00
A C
ATOM 14544 HA TYR A 73 -31.705 6.126 76.771 1.00 0.00
A H
ATOM 14545 CB TYR A 73 -31.420 8.190 77.447 1.00 0.00
A C
ATOM 14546 HB1 TYR A 73 -31.080 8.675 78.389 1.00 0.00
A H
ATOM 14547 HB2 TYR A 73 -30.804 8.608 76.621 1.00 0.00
A H
ATOM 14548 CG TYR A 73 -32.846 8.610 77.247 1.00 0.00
A C
ATOM 14549 CD1 TYR A 73 -33.722 8.696 78.323 1.00 0.00
A C
ATOM 14550 HD1 TYR A 73 -33.380 8.437 79.316 1.00 0.00
A H
ATOM 14551 CE1 TYR A 73 -35.033 9.085 78.142 1.00 0.00
A C
ATOM 14552 HE1 TYR A 73 -35.712 9.133 78.980 1.00 0.00
A H

ATOM	14553	CZ	TYR	A	73	-35.473	9.409	76.873	1.00	0.00	A	C
ATOM	14554	OH	TYR	A	73	-36.773	9.801	76.679	1.00	0.00	A	O
ATOM	14555	HH	TYR	A	73	-36.864	10.049	75.757	1.00	0.00	A	H
ATOM	14556	CD2	TYR	A	73	-33.314	8.943	75.983	1.00	0.00	A	C
ATOM	14557	HD2	TYR	A	73	-32.661	8.860	75.124	1.00	0.00	A	H
ATOM	14558	CE2	TYR	A	73	-34.622	9.336	75.791	1.00	0.00	A	C
ATOM	14559	HE2	TYR	A	73	-34.973	9.562	74.795	1.00	0.00	A	H
ATOM	14560	C	TYR	A	73	-29.738	6.406	77.459	1.00	0.00	A	C
ATOM	14561	O	TYR	A	73	-28.955	6.820	78.317	1.00	0.00	A	O
ATOM	14562	N	VAL	A	74	-29.351	5.696	76.408	1.00	0.00	A	N
ATOM	14563	HN	VAL	A	74	-29.962	5.235	75.771	1.00	0.00	A	H
ATOM	14564	CA	VAL	A	74	-27.942	5.539	76.107	1.00	0.00	A	C
ATOM	14565	HA	VAL	A	74	-27.372	6.120	76.821	1.00	0.00	A	H
ATOM	14566	CB	VAL	A	74	-27.467	4.079	76.231	1.00	0.00	A	C
ATOM	14567	HB	VAL	A	74	-28.011	3.481	75.459	1.00	0.00	A	H
ATOM	14568	CG1	VAL	A	74	-25.971	3.988	75.978	1.00	0.00	A	C
ATOM	14569	HG11	VAL	A	74	-25.622	2.941	76.105	1.00	0.00	A	H
ATOM	14570	HG12	VAL	A	74	-25.711	4.300	74.945	1.00	0.00	A	H
ATOM	14571	HG13	VAL	A	74	-25.412	4.626	76.694	1.00	0.00	A	H
ATOM	14572	CG2	VAL	A	74	-27.783	3.530	77.617	1.00	0.00	A	C
ATOM	14573	HG21	VAL	A	74	-27.407	2.490	77.723	1.00	0.00	A	H
ATOM	14574	HG22	VAL	A	74	-27.307	4.156	78.402	1.00	0.00	A	H
ATOM	14575	HG23	VAL	A	74	-28.877	3.511	77.802	1.00	0.00	A	H
ATOM	14576	C	VAL	A	74	-27.669	6.116	74.726	1.00	0.00	A	C
ATOM	14577	O	VAL	A	74	-28.361	5.799	73.758	1.00	0.00	A	O
ATOM	14578	N	ILE	A	75	-26.678	6.999	74.663	1.00	0.00	A	N
ATOM	14579	HN	ILE	A	75	-26.162	7.296	75.463	1.00	0.00	A	H

```
ATOM  14580  CA  ILE A  75     -26.254   7.615  73.418  1.00  0.00
A    C
ATOM  14581  HA  ILE A  75     -27.006   7.396  72.670  1.00  0.00
A    H
ATOM  14582  CB  ILE A  75     -25.950   9.112  73.609  1.00  0.00
A    C
ATOM  14583  HB  ILE A  75     -25.037   9.131  74.257  1.00  0.00
A    H
ATOM  14584  CG2 ILE A  75     -25.647   9.780  72.273  1.00  0.00
A    C
ATOM  14585 HG21 ILE A  75     -25.277  10.814  72.428  1.00  0.00
A    H
ATOM  14586 HG22 ILE A  75     -24.863   9.222  71.719  1.00  0.00
A    H
ATOM  14587 HG23 ILE A  75     -26.556   9.829  71.637  1.00  0.00
A    H
ATOM  14588  CG1 ILE A  75     -27.068   9.806  74.404  1.00  0.00
A    C
ATOM  14589 HG11 ILE A  75     -27.261   9.248  75.347  1.00  0.00
A    H
ATOM  14590 HG12 ILE A  75     -26.726  10.827  74.691  1.00  0.00
A    H
ATOM  14591  CD  ILE A  75     -28.375   9.957  73.666  1.00  0.00
A    C
ATOM  14592  HD1 ILE A  75     -29.106  10.518  74.287  1.00  0.00
A    H
ATOM  14593  HD2 ILE A  75     -28.227  10.514  72.716  1.00  0.00
A    H
ATOM  14594  HD3 ILE A  75     -28.815   8.965  73.429  1.00  0.00
A    H
ATOM  14595  C   ILE A  75     -24.979   6.942  72.966  1.00  0.00
A    C
ATOM  14596  O   ILE A  75     -23.991   6.911  73.701  1.00  0.00
A    O
ATOM  14597  N   VAL A  76     -25.000   6.394  71.759  1.00  0.00
A    N
ATOM  14598  HN  VAL A  76     -25.796   6.356  71.161  1.00  0.00
A    H
ATOM  14599  CA  VAL A  76     -23.801   5.782  71.205  1.00  0.00
A    C
ATOM  14600  HA  VAL A  76     -23.099   5.594  72.008  1.00  0.00
A    H
ATOM  14601  CB  VAL A  76     -24.083   4.459  70.474  1.00  0.00
A    C
ATOM  14602  HB  VAL A  76     -24.633   4.705  69.532  1.00  0.00
A    H
ATOM  14603  CG1 VAL A  76     -22.775   3.771  70.133  1.00  0.00
A    C
ATOM  14604 HG11 VAL A  76     -22.969   2.795  69.639  1.00  0.00
A    H
ATOM  14605 HG12 VAL A  76     -22.161   4.378  69.436  1.00  0.00
A    H
ATOM  14606 HG13 VAL A  76     -22.181   3.578  71.053  1.00  0.00
A    H
```

```
ATOM  14607  CG2 VAL A  76     -24.926   3.548  71.342  1.00  0.00
A    C
ATOM  14608 HG21 VAL A  76     -25.100   2.575  70.837  1.00  0.00
A    H
ATOM  14609 HG22 VAL A  76     -24.417   3.352  72.311  1.00  0.00
A    H
ATOM  14610 HG23 VAL A  76     -25.918   3.998  71.554  1.00  0.00
A    H
ATOM  14611  C   VAL A  76     -23.162   6.783  70.269  1.00  0.00
A    C
ATOM  14612  O   VAL A  76     -23.632   7.012  69.149  1.00  0.00
A    O
ATOM  14613  N   ALA A  77     -22.093   7.398  70.752  1.00  0.00
A    N
ATOM  14614  HN  ALA A  77     -21.690   7.188  71.642  1.00  0.00
A    H
ATOM  14615  CA  ALA A  77     -21.425   8.438  69.998  1.00  0.00
A    C
ATOM  14616  HA  ALA A  77     -21.750   8.523  68.967  1.00  0.00
A    H
ATOM  14617  CB  ALA A  77     -21.635   9.801  70.645  1.00  0.00
A    C
ATOM  14618  HB1 ALA A  77     -22.708  10.083  70.579  1.00  0.00
A    H
ATOM  14619  HB2 ALA A  77     -21.352   9.780  71.720  1.00  0.00
A    H
ATOM  14620  HB3 ALA A  77     -21.040  10.591  70.138  1.00  0.00
A    H
ATOM  14621  C   ALA A  77     -19.991   7.948  69.954  1.00  0.00
A    C
ATOM  14622  O   ALA A  77     -19.136   8.376  70.735  1.00  0.00
A    O
ATOM  14623  N   ILE A  78     -19.743   7.018  69.046  1.00  0.00
A    N
ATOM  14624  HN  ILE A  78     -20.429   6.426  68.629  1.00  0.00
A    H
ATOM  14625  CA  ILE A  78     -18.403   6.790  68.577  1.00  0.00
A    C
ATOM  14626  HA  ILE A  78     -17.727   7.577  68.888  1.00  0.00
A    H
ATOM  14627  CB  ILE A  78     -17.868   5.380  68.793  1.00  0.00
A    C
ATOM  14628  HB  ILE A  78     -18.583   4.722  68.238  1.00  0.00
A    H
ATOM  14629  CG2 ILE A  78     -16.441   5.301  68.273  1.00  0.00
A    C
ATOM  14630 HG21 ILE A  78     -16.083   4.251  68.274  1.00  0.00
A    H
ATOM  14631 HG22 ILE A  78     -16.379   5.677  67.230  1.00  0.00
A    H
ATOM  14632 HG23 ILE A  78     -15.754   5.903  68.904  1.00  0.00
A    H
ATOM  14633  CG1 ILE A  78     -17.968   5.006  70.267  1.00  0.00
A    C
```

ATOM 14634 HG11 ILE A 78 -16.975 5.127 70.755 1.00 0.00
A H
ATOM 14635 HG12 ILE A 78 -18.670 5.710 70.771 1.00 0.00
A H
ATOM 14636 CD ILE A 78 -18.465 3.597 70.483 1.00 0.00
A C
ATOM 14637 HD1 ILE A 78 -18.577 3.389 71.569 1.00 0.00
A H
ATOM 14638 HD2 ILE A 78 -19.454 3.451 69.998 1.00 0.00
A H
ATOM 14639 HD3 ILE A 78 -17.754 2.855 70.064 1.00 0.00
A H
ATOM 14640 C ILE A 78 -18.671 7.001 67.104 1.00 0.00
A C
ATOM 14641 O ILE A 78 -19.526 6.325 66.499 1.00 0.00
A O
ATOM 14642 N GLY A 79 -17.965 7.964 66.538 1.00 0.00
A N
ATOM 14643 HN GLY A 79 -17.263 8.496 67.009 1.00 0.00
A H
ATOM 14644 CA GLY A 79 -18.183 8.306 65.161 1.00 0.00
A C
ATOM 14645 HA1 GLY A 79 -17.741 9.279 64.990 1.00 0.00
A H
ATOM 14646 HA2 GLY A 79 -19.247 8.263 64.970 1.00 0.00
A H
ATOM 14647 C GLY A 79 -17.504 7.316 64.243 1.00 0.00
A C
ATOM 14648 O GLY A 79 -18.153 6.462 63.635 1.00 0.00
A O
ATOM 14649 N ALA A 80 -16.183 7.422 64.184 1.00 0.00
A N
ATOM 14650 HN ALA A 80 -15.651 7.874 64.900 1.00 0.00
A H
ATOM 14651 CA ALA A 80 -15.409 6.890 63.073 1.00 0.00
A C
ATOM 14652 HA ALA A 80 -15.738 7.355 62.151 1.00 0.00
A H
ATOM 14653 CB ALA A 80 -13.945 7.263 63.244 1.00 0.00
A C
ATOM 14654 HB1 ALA A 80 -13.850 8.366 63.335 1.00 0.00
A H
ATOM 14655 HB2 ALA A 80 -13.521 6.807 64.165 1.00 0.00
A H
ATOM 14656 HB3 ALA A 80 -13.335 6.937 62.374 1.00 0.00
A H
ATOM 14657 C ALA A 80 -15.568 5.389 62.878 1.00 0.00
A C
ATOM 14658 O ALA A 80 -15.834 4.921 61.772 1.00 0.00
A O
ATOM 14659 N ASN A 81 -15.416 4.647 63.965 1.00 0.00
A N
ATOM 14660 HN ASN A 81 -15.285 5.039 64.874 1.00 0.00
A H

ATOM 14661 CA ASN A 81   -15.425   3.197 63.931 1.00 0.00
A   C
ATOM 14662 HA ASN A 81   -14.845   2.922 63.056 1.00 0.00
A   H
ATOM 14663 CB ASN A 81   -14.811   2.676 65.228 1.00 0.00
A   C
ATOM 14664 HB1 ASN A 81   -13.938   3.319 65.474 1.00 0.00
A   H
ATOM 14665 HB2 ASN A 81   -15.537   2.768 66.062 1.00 0.00
A   H
ATOM 14666 CG ASN A 81   -14.321   1.246 65.117 1.00 0.00
A   C
ATOM 14667 OD1 ASN A 81   -14.808   0.466 64.298 1.00 0.00
A   O
ATOM 14668 ND2 ASN A 81   -13.351   0.889 65.967 1.00 0.00
A   N
ATOM 14669 HD21 ASN A 81   -13.007 -0.044 65.903 1.00 0.00
A   H
ATOM 14670 HD22 ASN A 81   -12.982   1.553 66.610 1.00 0.00
A   H
ATOM 14671 C   ASN A 81   -16.836   2.643 63.745 1.00 0.00
A   C
ATOM 14672 O   ASN A 81   -17.558   2.424 64.716 1.00 0.00
A   O
ATOM 14673 N   ILE A 82   -17.223   2.413 62.494 1.00 0.00
A   N
ATOM 14674 HN ILE A 82   -16.653   2.574 61.691 1.00 0.00
A   H
ATOM 14675 CA ILE A 82   -18.561   1.887 62.187 1.00 0.00
A   C
ATOM 14676 HA ILE A 82   -19.260   2.589 62.627 1.00 0.00
A   H
ATOM 14677 CB ILE A 82   -18.836   1.825 60.666 1.00 0.00
A   C
ATOM 14678 HB ILE A 82   -17.943   1.300 60.241 1.00 0.00
A   H
ATOM 14679 CG2 ILE A 82   -20.161   1.139 60.378 1.00 0.00
A   C
ATOM 14680 HG21 ILE A 82   -20.279   0.958 59.289 1.00 0.00
A   H
ATOM 14681 HG22 ILE A 82   -20.219   0.156 60.891 1.00 0.00
A   H
ATOM 14682 HG23 ILE A 82   -21.014   1.763 60.720 1.00 0.00
A   H
ATOM 14683 CG1 ILE A 82   -18.847   3.234 60.072 1.00 0.00
A   C
ATOM 14684 HG11 ILE A 82   -19.698   3.813 60.495 1.00 0.00
A   H
ATOM 14685 HG12 ILE A 82   -17.911   3.759 60.370 1.00 0.00
A   H
ATOM 14686 CD ILE A 82   -18.939   3.268 58.563 1.00 0.00
A   C
ATOM 14687 HD1 ILE A 82   -18.884   4.316 58.197 1.00 0.00
A   H

ATOM 14688 HD2 ILE A 82 −18.102 2.696 58.106 1.00 0.00
A H
ATOM 14689 HD3 ILE A 82 −19.897 2.831 58.212 1.00 0.00
A H
ATOM 14690 C ILE A 82 −18.827 0.528 62.842 1.00 0.00
A C
ATOM 14691 O ILE A 82 −19.931 0.279 63.321 1.00 0.00
A O
ATOM 14692 N GLN A 83 −17.812 −0.334 62.866 1.00 0.00
A N
ATOM 14693 HN GLN A 83 −16.917 −0.142 62.468 1.00 0.00
A H
ATOM 14694 CA GLN A 83 −17.927 −1.652 63.486 1.00 0.00
A C
ATOM 14695 HA GLN A 83 −18.757 −2.159 63.008 1.00 0.00
A H
ATOM 14696 CB GLN A 83 −16.632 −2.446 63.311 1.00 0.00
A C
ATOM 14697 HB1 GLN A 83 −16.550 −2.693 62.227 1.00 0.00
A H
ATOM 14698 HB2 GLN A 83 −15.762 −1.791 63.547 1.00 0.00
A H
ATOM 14699 CG GLN A 83 −16.579 −3.733 64.130 1.00 0.00
A C
ATOM 14700 HG1 GLN A 83 −16.997 −3.564 65.145 1.00 0.00
A H
ATOM 14701 HG2 GLN A 83 −17.176 −4.533 63.646 1.00 0.00
A H
ATOM 14702 CD GLN A 83 −15.168 −4.259 64.344 1.00 0.00
A C
ATOM 14703 OE1 GLN A 83 −14.251 −3.950 63.579 1.00 0.00
A O
ATOM 14704 NE2 GLN A 83 −14.992 −5.074 65.388 1.00 0.00
A N
ATOM 14705 HE21 GLN A 83 −14.070 −5.420 65.538 1.00 0.00
A H
ATOM 14706 HE22 GLN A 83 −15.766 −5.319 65.961 1.00 0.00
A H
ATOM 14707 C GLN A 83 −18.257 −1.527 64.965 1.00 0.00
A C
ATOM 14708 O GLN A 83 −19.235 −2.109 65.437 1.00 0.00
A O
ATOM 14709 N ALA A 84 −17.432 −0.763 65.683 1.00 0.00
A N
ATOM 14710 HN ALA A 84 −16.649 −0.295 65.274 1.00 0.00
A H
ATOM 14711 CA ALA A 84 −17.608 −0.554 67.115 1.00 0.00
A C
ATOM 14712 HA ALA A 84 −17.544 −1.493 67.653 1.00 0.00
A H
ATOM 14713 CB ALA A 84 −16.528 0.370 67.654 1.00 0.00
A C
ATOM 14714 HB1 ALA A 84 −15.529 −0.082 67.476 1.00 0.00
A H

ATOM 14715 HB2 ALA A 84 -16.553 1.355 67.141 1.00 0.00
A H
ATOM 14716 HB3 ALA A 84 -16.640 0.537 68.747 1.00 0.00
A H
ATOM 14717 C ALA A 84 -18.989 0.014 67.407 1.00 0.00
A C
ATOM 14718 O ALA A 84 -19.681 -0.460 68.312 1.00 0.00
A O
ATOM 14719 N SER A 85 -19.387 1.010 66.615 1.00 0.00
A N
ATOM 14720 HN SER A 85 -18.845 1.352 65.848 1.00 0.00
A H
ATOM 14721 CA SER A 85 -20.655 1.693 66.798 1.00 0.00
A C
ATOM 14722 HA SER A 85 -20.626 2.104 67.799 1.00 0.00
A H
ATOM 14723 CB SER A 85 -20.816 2.811 65.764 1.00 0.00
A C
ATOM 14724 HB1 SER A 85 -19.985 3.537 65.909 1.00 0.00
A H
ATOM 14725 HB2 SER A 85 -20.719 2.402 64.734 1.00 0.00
A H
ATOM 14726 OG SER A 85 -22.047 3.490 65.929 1.00 0.00
A O
ATOM 14727 HG1 SER A 85 -21.992 4.305 65.412 1.00 0.00
A H
ATOM 14728 C SER A 85 -21.831 0.727 66.739 1.00 0.00
A C
ATOM 14729 O SER A 85 -22.700 0.750 67.605 1.00 0.00
A O
ATOM 14730 N THR A 86 -21.851 -0.132 65.728 1.00 0.00
A N
ATOM 14731 HN THR A 86 -21.125 -0.243 65.050 1.00 0.00
A H
ATOM 14732 CA THR A 86 -22.995 -1.016 65.533 1.00 0.00
A C
ATOM 14733 HA THR A 86 -23.872 -0.435 65.785 1.00 0.00
A H
ATOM 14734 CB THR A 86 -23.115 -1.541 64.084 1.00 0.00
A C
ATOM 14735 HB THR A 86 -24.121 -2.021 64.011 1.00 0.00
A H
ATOM 14736 OG1 THR A 86 -22.128 -2.549 63.851 1.00 0.00
A O
ATOM 14737 HG1 THR A 86 -22.251 -2.820 62.934 1.00 0.00
A H
ATOM 14738 CG2 THR A 86 -22.929 -0.424 63.077 1.00 0.00
A C
ATOM 14739 HG21 THR A 86 -23.129 -0.784 62.046 1.00 0.00
A H
ATOM 14740 HG22 THR A 86 -23.632 0.410 63.290 1.00 0.00
A H
ATOM 14741 HG23 THR A 86 -21.893 -0.023 63.111 1.00 0.00
A H

ATOM 14742 C THR A 86 −22.946 −2.178 66.521 1.00 0.00
A C
ATOM 14743 O THR A 86 −23.982 −2.626 67.014 1.00 0.00
A O
ATOM 14744 N LEU A 87 −21.740 −2.656 66.806 1.00 0.00
A N
ATOM 14745 HN LEU A 87 −20.892 −2.339 66.388 1.00 0.00
A H
ATOM 14746 CA LEU A 87 −21.563 −3.719 67.790 1.00 0.00
A C
ATOM 14747 HA LEU A 87 −22.174 −4.553 67.472 1.00 0.00
A H
ATOM 14748 CB LEU A 87 −20.095 −4.164 67.858 1.00 0.00
A C
ATOM 14749 HB1 LEU A 87 −19.807 −4.504 66.836 1.00 0.00
A H
ATOM 14750 HB2 LEU A 87 −19.458 −3.279 68.079 1.00 0.00
A H
ATOM 14751 CG LEU A 87 −19.668 −5.289 68.812 1.00 0.00
A C
ATOM 14752 HG LEU A 87 −19.687 −4.832 69.832 1.00 0.00
A H
ATOM 14753 CD1 LEU A 87 −20.598 −6.488 68.770 1.00 0.00
A C
ATOM 14754 HD11 LEU A 87 −20.225 −7.294 69.438 1.00 0.00
A H
ATOM 14755 HD12 LEU A 87 −21.619 −6.203 69.102 1.00 0.00
A H
ATOM 14756 HD13 LEU A 87 −20.665 −6.896 67.739 1.00 0.00
A H
ATOM 14757 CD2 LEU A 87 −18.238 −5.716 68.516 1.00 0.00
A C
ATOM 14758 HD21 LEU A 87 −17.920 −6.517 69.218 1.00 0.00
A H
ATOM 14759 HD22 LEU A 87 −18.134 −6.111 67.485 1.00 0.00
A H
ATOM 14760 HD23 LEU A 87 −17.543 −4.856 68.632 1.00 0.00
A H
ATOM 14761 C LEU A 87 −22.078 −3.264 69.157 1.00 0.00
A C
ATOM 14762 O LEU A 87 −22.791 −4.013 69.831 1.00 0.00
A O
ATOM 14763 N THR A 88 −21.731 −2.033 69.543 1.00 0.00
A N
ATOM 14764 HN THR A 88 −21.115 −1.446 69.018 1.00 0.00
A H
ATOM 14765 CA THR A 88 −22.227 −1.429 70.780 1.00 0.00
A C
ATOM 14766 HA THR A 88 −21.970 −2.114 71.576 1.00 0.00
A H
ATOM 14767 CB THR A 88 −21.624 −0.031 71.030 1.00 0.00
A C
ATOM 14768 HB THR A 88 −21.801 0.554 70.095 1.00 0.00
A H

ATOM 14769 OG1 THR A 88 -20.209 -0.146 71.221 1.00 0.00
A O
ATOM 14770 HG1 THR A 88 -19.899 0.751 71.382 1.00 0.00
A H
ATOM 14771 CG2 THR A 88 -22.239 0.608 72.264 1.00 0.00
A C
ATOM 14772 HG21 THR A 88 -21.891 1.657 72.381 1.00 0.00
A H
ATOM 14773 HG22 THR A 88 -23.347 0.623 72.182 1.00 0.00
A H
ATOM 14774 HG23 THR A 88 -21.966 0.047 73.184 1.00 0.00
A H
ATOM 14775 C THR A 88 -23.751 -1.347 70.753 1.00 0.00
A C
ATOM 14776 O THR A 88 -24.421 -1.852 71.661 1.00 0.00
A O
ATOM 14777 N THR A 89 -24.289 -0.729 69.704 1.00 0.00
A N
ATOM 14778 HN THR A 89 -23.761 -0.312 68.965 1.00 0.00
A H
ATOM 14779 CA THR A 89 -25.737 -0.611 69.547 1.00 0.00
A C
ATOM 14780 HA THR A 89 -26.087 -0.018 70.381 1.00 0.00
A H
ATOM 14781 CB THR A 89 -26.122 0.008 68.189 1.00 0.00
A C
ATOM 14782 HB THR A 89 -25.709 -0.676 67.409 1.00 0.00
A H
ATOM 14783 OG1 THR A 89 -25.486 1.277 68.048 1.00 0.00
A O
ATOM 14784 HG1 THR A 89 -25.773 1.613 67.192 1.00 0.00
A H
ATOM 14785 CG2 THR A 89 -27.622 0.203 68.090 1.00 0.00
A C
ATOM 14786 HG21 THR A 89 -27.910 0.541 67.073 1.00 0.00
A H
ATOM 14787 HG22 THR A 89 -28.151 -0.753 68.295 1.00 0.00
A H
ATOM 14788 HG23 THR A 89 -27.976 0.960 68.822 1.00 0.00
A H
ATOM 14789 C THR A 89 -26.389 -1.983 69.687 1.00 0.00
A C
ATOM 14790 O THR A 89 -27.389 -2.132 70.388 1.00 0.00
A O
ATOM 14791 N LEU A 90 -25.796 -2.977 69.030 1.00 0.00
A N
ATOM 14792 HN LEU A 90 -24.967 -2.856 68.487 1.00 0.00
A H
ATOM 14793 CA LEU A 90 -26.304 -4.337 69.047 1.00 0.00
A C
ATOM 14794 HA LEU A 90 -27.305 -4.301 68.636 1.00 0.00
A H
ATOM 14795 CB LEU A 90 -25.415 -5.241 68.200 1.00 0.00
A C

ATOM 14796 HB1 LEU A 90   -25.132 -4.664 67.289 1.00 0.00
A   H
ATOM 14797 HB2 LEU A 90   -24.466 -5.432 68.749 1.00 0.00
A   H
ATOM 14798 CG LEU A 90   -26.024 -6.566 67.749 1.00 0.00
A   C
ATOM 14799 HG LEU A 90   -25.903 -7.298 68.585 1.00 0.00
A   H
ATOM 14800 CD1 LEU A 90   -27.493 -6.431 67.387 1.00 0.00
A   C
ATOM 14801 HD11 LEU A 90   -27.895 -7.399 67.018 1.00 0.00
A   H
ATOM 14802 HD12 LEU A 90   -28.088 -6.122 68.274 1.00 0.00
A   H
ATOM 14803 HD13 LEU A 90   -27.632 -5.671 66.589 1.00 0.00
A   H
ATOM 14804 CD2 LEU A 90   -25.246 -7.082 66.559 1.00 0.00
A   C
ATOM 14805 HD21 LEU A 90   -25.659 -8.057 66.222 1.00 0.00
A   H
ATOM 14806 HD22 LEU A 90   -25.296 -6.380 65.702 1.00 0.00
A   H
ATOM 14807 HD23 LEU A 90   -24.178 -7.230 66.827 1.00 0.00
A   H
ATOM 14808 C   LEU A 90   -26.411 -4.863 70.475 1.00 0.00
A   C
ATOM 14809 O   LEU A 90   -27.464 -5.362 70.883 1.00 0.00
A   O
ATOM 14810 N   LEU A 91   -25.322 -4.724 71.230 1.00 0.00
A   N
ATOM 14811 HN LEU A 91   -24.464 -4.346 70.887 1.00 0.00
A   H
ATOM 14812 CA LEU A 91   -25.285 -5.114 72.635 1.00 0.00
A   C
ATOM 14813 HA LEU A 91   -25.540 -6.166 72.662 1.00 0.00
A   H
ATOM 14814 CB LEU A 91   -23.892 -4.890 73.216 1.00 0.00
A   C
ATOM 14815 HB1 LEU A 91   -23.653 -3.809 73.089 1.00 0.00
A   H
ATOM 14816 HB2 LEU A 91   -23.924 -5.071 74.313 1.00 0.00
A   H
ATOM 14817 CG LEU A 91   -22.724 -5.682 72.622 1.00 0.00
A   C
ATOM 14818 HG LEU A 91   -22.733 -5.495 71.522 1.00 0.00
A   H
ATOM 14819 CD1 LEU A 91   -21.418 -5.181 73.213 1.00 0.00
A   C
ATOM 14820 HD11 LEU A 91   -20.562 -5.777 72.829 1.00 0.00
A   H
ATOM 14821 HD12 LEU A 91   -21.250 -4.116 72.944 1.00 0.00
A   H
ATOM 14822 HD13 LEU A 91   -21.431 -5.266 74.320 1.00 0.00
A   H

ATOM 14823 CD2 LEU A 91   -22.877 -7.180 72.847 1.00 0.00
A   C
ATOM 14824 HD21 LEU A 91   -22.025 -7.728 72.390 1.00 0.00
A   H
ATOM 14825 HD22 LEU A 91   -22.898 -7.431 73.927 1.00 0.00
A   H
ATOM 14826 HD23 LEU A 91   -23.817 -7.549 72.383 1.00 0.00
A   H
ATOM 14827 C   LEU A 91   -26.317 -4.369 73.475 1.00 0.00
A   C
ATOM 14828 O   LEU A 91   -26.985 -4.970 74.313 1.00 0.00
A   O
ATOM 14829 N   LEU A 92   -26.444 -3.062 73.248 1.00 0.00
A   N
ATOM 14830 HN LEU A 92   -25.907 -2.552 72.579 1.00 0.00
A   H
ATOM 14831 CA LEU A 92   -27.410 -2.252 73.983 1.00 0.00
A   C
ATOM 14832 HA LEU A 92   -27.182 -2.393 75.031 1.00 0.00
A   H
ATOM 14833 CB LEU A 92   -27.259 -0.772 73.635 1.00 0.00
A   C
ATOM 14834 HB1 LEU A 92   -27.314 -0.688 72.525 1.00 0.00
A   H
ATOM 14835 HB2 LEU A 92   -28.138 -0.216 74.029 1.00 0.00
A   H
ATOM 14836 CG LEU A 92   -25.972 -0.086 74.094 1.00 0.00
A   C
ATOM 14837 HG LEU A 92   -25.115 -0.623 73.621 1.00 0.00
A   H
ATOM 14838 CD1 LEU A 92   -25.987   1.356 73.631 1.00 0.00
A   C
ATOM 14839 HD11 LEU A 92   -25.084   1.892 73.993 1.00 0.00
A   H
ATOM 14840 HD12 LEU A 92   -26.004   1.411 72.521 1.00 0.00
A   H
ATOM 14841 HD13 LEU A 92   -26.883   1.884 74.022 1.00 0.00
A   H
ATOM 14842 CD2 LEU A 92   -25.798 -0.160 75.602 1.00 0.00
A   C
ATOM 14843 HD21 LEU A 92   -24.847   0.329 75.905 1.00 0.00
A   H
ATOM 14844 HD22 LEU A 92   -26.625   0.352 76.135 1.00 0.00
A   H
ATOM 14845 HD23 LEU A 92   -25.766 -1.219 75.938 1.00 0.00
A   H
ATOM 14846 C   LEU A 92   -28.848 -2.711 73.758 1.00 0.00
A   C
ATOM 14847 O   LEU A 92   -29.656 -2.700 74.685 1.00 0.00
A   O
ATOM 14848 N   LYS A 93   -29.158 -3.124 72.533 1.00 0.00
A   N
ATOM 14849 HN LYS A 93   -28.508 -3.132 71.774 1.00 0.00
A   H

ATOM 14850 CA LYS A 93 -30.492 -3.601 72.208 1.00 0.00
A C
ATOM 14851 HA LYS A 93 -31.201 -2.888 72.610 1.00 0.00
A H
ATOM 14852 CB LYS A 93 -30.681 -3.674 70.688 1.00 0.00
A C
ATOM 14853 HB1 LYS A 93 -30.424 -2.671 70.276 1.00 0.00
A H
ATOM 14854 HB2 LYS A 93 -29.937 -4.386 70.264 1.00 0.00
A H
ATOM 14855 CG LYS A 93 -32.083 -4.042 70.222 1.00 0.00
A C
ATOM 14856 HG1 LYS A 93 -32.080 -4.161 69.115 1.00 0.00
A H
ATOM 14857 HG2 LYS A 93 -32.364 -5.028 70.661 1.00 0.00
A H
ATOM 14858 CD LYS A 93 -33.135 -3.004 70.587 1.00 0.00
A C
ATOM 14859 HD1 LYS A 93 -33.052 -2.787 71.677 1.00 0.00
A H
ATOM 14860 HD2 LYS A 93 -32.897 -2.063 70.040 1.00 0.00
A H
ATOM 14861 CE LYS A 93 -34.523 -3.513 70.225 1.00 0.00
A C
ATOM 14862 HE1 LYS A 93 -34.653 -3.539 69.120 1.00 0.00
A H
ATOM 14863 HE2 LYS A 93 -34.679 -4.541 70.621 1.00 0.00
A H
ATOM 14864 NZ LYS A 93 -35.611 -2.663 70.774 1.00 0.00
A N
ATOM 14865 HZ1 LYS A 93 -36.538 -3.048 70.500 1.00 0.00
A H
ATOM 14866 HZ2 LYS A 93 -35.542 -2.636 71.811 1.00 0.00
A H
ATOM 14867 HZ3 LYS A 93 -35.516 -1.696 70.401 1.00 0.00
A H
ATOM 14868 C LYS A 93 -30.756 -4.946 72.895 1.00 0.00
A C
ATOM 14869 O LYS A 93 -31.889 -5.233 73.293 1.00 0.00
A O
ATOM 14870 N GLU A 94 -29.704 -5.751 73.054 1.00 0.00
A N
ATOM 14871 HN GLU A 94 -28.796 -5.551 72.694 1.00 0.00
A H
ATOM 14872 CA GLU A 94 -29.791 -7.020 73.783 1.00 0.00
A C
ATOM 14873 HA GLU A 94 -30.605 -7.589 73.353 1.00 0.00
A H
ATOM 14874 CB GLU A 94 -28.494 -7.811 73.654 1.00 0.00
A C
ATOM 14875 HB1 GLU A 94 -27.643 -7.093 73.694 1.00 0.00
A H
ATOM 14876 HB2 GLU A 94 -28.370 -8.479 74.535 1.00 0.00
A H

ATOM 14877 CG GLU A 94 -28.393 -8.622 72.376 1.00 0.00
A C
ATOM 14878 HG1 GLU A 94 -29.231 -9.342 72.329 1.00 0.00
A H
ATOM 14879 HG2 GLU A 94 -28.427 -7.965 71.485 1.00 0.00
A H
ATOM 14880 CD GLU A 94 -27.127 -9.456 72.318 1.00 0.00
A C
ATOM 14881 OE1 GLU A 94 -26.345 -9.273 71.356 1.00 0.00
A O
ATOM 14882 OE2 GLU A 94 -26.906 -10.291 73.230 1.00 0.00
A O
ATOM 14883 C GLU A 94 -30.109 -6.821 75.260 1.00 0.00
A C
ATOM 14884 O GLU A 94 -30.842 -7.612 75.851 1.00 0.00
A O
ATOM 14885 N LEU A 95 -29.561 -5.758 75.846 1.00 0.00
A N
ATOM 14886 HN LEU A 95 -28.997 -5.094 75.362 1.00 0.00
A H
ATOM 14887 CA LEU A 95 -29.743 -5.469 77.267 1.00 0.00
A C
ATOM 14888 HA LEU A 95 -29.807 -6.430 77.761 1.00 0.00
A H
ATOM 14889 CB LEU A 95 -28.527 -4.717 77.824 1.00 0.00
A C
ATOM 14890 HB1 LEU A 95 -28.462 -3.746 77.280 1.00 0.00
A H
ATOM 14891 HB2 LEU A 95 -28.717 -4.460 78.889 1.00 0.00
A H
ATOM 14892 CG LEU A 95 -27.174 -5.438 77.689 1.00 0.00
A C
ATOM 14893 HG LEU A 95 -27.107 -5.733 76.614 1.00 0.00
A H
ATOM 14894 CD1 LEU A 95 -26.023 -4.510 78.042 1.00 0.00
A C
ATOM 14895 HD11 LEU A 95 -25.054 -5.053 77.988 1.00 0.00
A H
ATOM 14896 HD12 LEU A 95 -25.981 -3.652 77.337 1.00 0.00
A H
ATOM 14897 HD13 LEU A 95 -26.141 -4.113 79.072 1.00 0.00
A H
ATOM 14898 CD2 LEU A 95 -27.109 -6.704 78.528 1.00 0.00
A C
ATOM 14899 HD21 LEU A 95 -26.127 -7.207 78.391 1.00 0.00
A H
ATOM 14900 HD22 LEU A 95 -27.228 -6.485 79.608 1.00 0.00
A H
ATOM 14901 HD23 LEU A 95 -27.908 -7.413 78.225 1.00 0.00
A H
ATOM 14902 C LEU A 95 -31.037 -4.715 77.559 1.00 0.00
A C
ATOM 14903 O LEU A 95 -31.246 -4.242 78.674 1.00 0.00
A O

```
ATOM  14904  N   ASP A  96     -31.897  -4.617  76.548  1.00  0.00
A    N
ATOM  14905  HN  ASP A  96     -31.690  -4.934  75.626  1.00  0.00
A    H
ATOM  14906  CA  ASP A  96     -33.243  -4.035  76.665  1.00  0.00
A    C
ATOM  14907  HA  ASP A  96     -33.683  -4.165  75.682  1.00  0.00
A    H
ATOM  14908  CB  ASP A  96     -34.110  -4.837  77.648  1.00  0.00
A    C
ATOM  14909  HB1 ASP A  96     -33.459  -5.287  78.425  1.00  0.00
A    H
ATOM  14910  HB2 ASP A  96     -34.844  -4.177  78.149  1.00  0.00
A    H
ATOM  14911  CG  ASP A  96     -34.857  -5.962  76.976  1.00  0.00
A    C
ATOM  14912  OD1 ASP A  96     -34.611  -7.131  77.338  1.00  0.00
A    O
ATOM  14913  OD2 ASP A  96     -35.687  -5.683  76.084  1.00  0.00
A    O
ATOM  14914  C   ASP A  96     -33.317  -2.538  76.989  1.00  0.00
A    C
ATOM  14915  O   ASP A  96     -34.328  -2.071  77.531  1.00  0.00
A    O
ATOM  14916  N   ILE A  97     -32.267  -1.790  76.646  1.00  0.00
A    N
ATOM  14917  HN  ILE A  97     -31.426  -2.146  76.241  1.00  0.00
A    H
ATOM  14918  CA  ILE A  97     -32.268  -0.337  76.846  1.00  0.00
A    C
ATOM  14919  HA  ILE A  97     -32.405  -0.165  77.905  1.00  0.00
A    H
ATOM  14920  CB  ILE A  97     -30.949   0.309  76.359  1.00  0.00
A    C
ATOM  14921  HB  ILE A  97     -30.891   0.043  75.274  1.00  0.00
A    H
ATOM  14922  CG2 ILE A  97     -30.972   1.827  76.547  1.00  0.00
A    C
ATOM  14923 HG21 ILE A  97     -30.093   2.294  76.055  1.00  0.00
A    H
ATOM  14924 HG22 ILE A  97     -31.885   2.268  76.095  1.00  0.00
A    H
ATOM  14925 HG23 ILE A  97     -30.949   2.094  77.624  1.00  0.00
A    H
ATOM  14926  CG1 ILE A  97     -29.729  -0.341  77.038  1.00  0.00
A    C
ATOM  14927 HG11 ILE A  97     -29.640  -1.402  76.716  1.00  0.00
A    H
ATOM  14928 HG12 ILE A  97     -28.806   0.184  76.699  1.00  0.00
A    H
ATOM  14929  CD  ILE A  97     -29.740  -0.303  78.552  1.00  0.00
A    C
ATOM  14930  HD1 ILE A  97     -28.801  -0.739  78.956  1.00  0.00
A    H
```

ATOM 14931 HD2 ILE A 97 -29.824 0.743 78.918 1.00 0.00
A H
ATOM 14932 HD3 ILE A 97 -30.592 -0.888 78.959 1.00 0.00
A H
ATOM 14933 C ILE A 97 -33.479 0.288 76.134 1.00 0.00
A C
ATOM 14934 O ILE A 97 -33.712 0.025 74.956 1.00 0.00
A O
ATOM 14935 N PRO A 98 -34.271 1.088 76.865 1.00 0.00
A N
ATOM 14936 CD PRO A 98 -34.130 1.365 78.304 1.00 0.00
A C
ATOM 14937 HD1 PRO A 98 -34.127 0.320 77.920 1.00 0.00
A H
ATOM 14938 HD2 PRO A 98 -33.438 1.464 79.171 1.00 0.00
A H
ATOM 14939 CA PRO A 98 -35.479 1.695 76.304 1.00 0.00
A C
ATOM 14940 HA PRO A 98 -36.140 0.910 75.954 1.00 0.00
A H
ATOM 14941 CB PRO A 98 -36.095 2.429 77.507 1.00 0.00
A C
ATOM 14942 HB1 PRO A 98 -36.718 1.713 78.090 1.00 0.00
A H
ATOM 14943 HB2 PRO A 98 -36.725 3.291 77.207 1.00 0.00
A H
ATOM 14944 CG PRO A 98 -35.517 1.759 78.708 1.00 0.00
A C
ATOM 14945 HG1 PRO A 98 -36.144 1.881 79.613 1.00 0.00
A H
ATOM 14946 HG2 PRO A 98 -35.449 1.007 79.363 1.00 0.00
A H
ATOM 14947 C PRO A 98 -35.218 2.679 75.158 1.00 0.00
A C
ATOM 14948 O PRO A 98 -35.957 2.672 74.174 1.00 0.00
A O
ATOM 14949 N ASN A 99 -34.191 3.520 75.286 1.00 0.00
A N
ATOM 14950 HN ASN A 99 -33.587 3.554 76.081 1.00 0.00
A H
ATOM 14951 CA ASN A 99 -33.867 4.484 74.234 1.00 0.00
A C
ATOM 14952 HA ASN A 99 -34.434 4.157 73.370 1.00 0.00
A H
ATOM 14953 CB ASN A 99 -34.296 5.898 74.619 1.00 0.00
A C
ATOM 14954 HB1 ASN A 99 -33.746 6.181 75.543 1.00 0.00
A H
ATOM 14955 HB2 ASN A 99 -34.008 6.617 73.826 1.00 0.00
A H
ATOM 14956 CG ASN A 99 -35.777 5.997 74.904 1.00 0.00
A C
ATOM 14957 OD1 ASN A 99 -36.218 5.700 76.011 1.00 0.00
A O

ATOM 14958 ND2 ASN A 99   -36.553   6.421 73.912 1.00 0.00
A   N
ATOM 14959 HD21 ASN A 99   -37.529   6.500 74.096 1.00 0.00
A   H
ATOM 14960 HD22 ASN A 99   -36.150   6.673 73.037 1.00 0.00
A   H
ATOM 14961 C   ASN A 99   -32.397   4.480 73.848 1.00 0.00
A   C
ATOM 14962 O   ASN A 99   -31.532   4.843 74.656 1.00 0.00
A   O
ATOM 14963 N   ILE A 100   -32.131   4.071 72.607 1.00 0.00
A   N
ATOM 14964 HN ILE A 100   -32.832   3.761 71.969 1.00 0.00
A   H
ATOM 14965 CA ILE A 100   -30.781   4.042 72.069 1.00 0.00
A   C
ATOM 14966 HA ILE A 100   -30.125   4.402 72.850 1.00 0.00
A   H
ATOM 14967 CB ILE A 100   -30.382   2.626 71.628 1.00 0.00
A   C
ATOM 14968 HB ILE A 100   -31.170   2.329 70.891 1.00 0.00
A   H
ATOM 14969 CG2 ILE A 100   -28.966   2.622 71.071 1.00 0.00
A   C
ATOM 14970 HG21 ILE A 100   -28.730   1.640 70.609 1.00 0.00
A   H
ATOM 14971 HG22 ILE A 100   -28.845   3.400 70.288 1.00 0.00
A   H
ATOM 14972 HG23 ILE A 100   -28.223   2.815 71.874 1.00 0.00
A   H
ATOM 14973 CG1 ILE A 100   -30.489   1.658 72.809 1.00 0.00
A   C
ATOM 14974 HG11 ILE A 100   -29.588   1.752 73.456 1.00 0.00
A   H
ATOM 14975 HG12 ILE A 100   -31.370   1.942 73.430 1.00 0.00
A   H
ATOM 14976 CD ILE A 100   -30.651   0.201 72.414 1.00 0.00
A   C
ATOM 14977 HD1 ILE A 100   -30.780 -0.432 73.319 1.00 0.00
A   H
ATOM 14978 HD2 ILE A 100   -31.544   0.069 71.766 1.00 0.00
A   H
ATOM 14979 HD3 ILE A 100   -29.759 -0.164 71.864 1.00 0.00
A   H
ATOM 14980 C   ILE A 100   -30.656   5.019 70.903 1.00 0.00
A   C
ATOM 14981 O   ILE A 100   -31.285   4.840 69.852 1.00 0.00
A   O
ATOM 14982 N   TRP A 101   -29.854   6.061 71.110 1.00 0.00
A   N
ATOM 14983 HN TRP A 101   -29.384   6.200 71.979 1.00 0.00
A   H
ATOM 14984 CA TRP A 101   -29.593   7.083 70.093 1.00 0.00
A   C

ATOM 14985 HA TRP A 101 -30.239 6.890 69.246 1.00 0.00
A H
ATOM 14986 CB TRP A 101 -29.808 8.476 70.662 1.00 0.00
A C
ATOM 14987 HB1 TRP A 101 -29.203 8.562 71.592 1.00 0.00
A H
ATOM 14988 HB2 TRP A 101 -29.419 9.238 69.952 1.00 0.00
A H
ATOM 14989 CG TRP A 101 -31.241 8.782 71.030 1.00 0.00
A C
ATOM 14990 CD1 TRP A 101 -32.246 7.880 71.376 1.00 0.00
A C
ATOM 14991 HD1 TRP A 101 -32.092 6.811 71.444 1.00 0.00
A H
ATOM 14992 NE1 TRP A 101 -33.417 8.531 71.652 1.00 0.00
A N
ATOM 14993 HE1 TRP A 101 -34.242 8.093 71.935 1.00 0.00
A H
ATOM 14994 CE2 TRP A 101 -33.261 9.867 71.524 1.00 0.00
A C
ATOM 14995 CD2 TRP A 101 -31.869 10.104 71.124 1.00 0.00
A C
ATOM 14996 CE3 TRP A 101 -31.437 11.407 70.927 1.00 0.00
A C
ATOM 14997 HE3 TRP A 101 -30.422 11.632 70.630 1.00 0.00
A H
ATOM 14998 CZ3 TRP A 101 -32.356 12.449 71.116 1.00 0.00
A C
ATOM 14999 HZ3 TRP A 101 -32.040 13.471 70.962 1.00 0.00
A H
ATOM 15000 CZ2 TRP A 101 -34.160 10.906 71.711 1.00 0.00
A C
ATOM 15001 HZ2 TRP A 101 -35.185 10.749 72.009 1.00 0.00
A H
ATOM 15002 CH2 TRP A 101 -33.683 12.200 71.496 1.00 0.00
A C
ATOM 15003 HH2 TRP A 101 -34.364 13.029 71.629 1.00 0.00
A H
ATOM 15004 C TRP A 101 -28.189 6.956 69.606 1.00 0.00
A C
ATOM 15005 O TRP A 101 -27.249 6.930 70.402 1.00 0.00
A O
ATOM 15006 N VAL A 102 -28.028 6.870 68.289 1.00 0.00
A N
ATOM 15007 HN VAL A 102 -28.767 6.968 67.627 1.00 0.00
A H
ATOM 15008 CA VAL A 102 -26.719 6.619 67.699 1.00 0.00
A C
ATOM 15009 HA VAL A 102 -25.986 6.717 68.489 1.00 0.00
A H
ATOM 15010 CB VAL A 102 -26.637 5.212 67.065 1.00 0.00
A C
ATOM 15011 HB VAL A 102 -27.204 5.242 66.102 1.00 0.00
A H

```
ATOM  15012  CG1 VAL A 102     -25.195   4.841  66.784  1.00  0.00
A    C
ATOM  15013 HG11 VAL A 102     -25.136   3.813  66.364  1.00  0.00
A    H
ATOM  15014 HG12 VAL A 102     -24.732   5.527  66.045  1.00  0.00
A    H
ATOM  15015 HG13 VAL A 102     -24.593   4.866  67.718  1.00  0.00
A    H
ATOM  15016  CG2 VAL A 102     -27.271   4.168  67.973  1.00  0.00
A    C
ATOM  15017 HG21 VAL A 102     -27.175   3.153  67.532  1.00  0.00
A    H
ATOM  15018 HG22 VAL A 102     -26.778   4.164  68.968  1.00  0.00
A    H
ATOM  15019 HG23 VAL A 102     -28.353   4.367  68.121  1.00  0.00
A    H
ATOM  15020  C   VAL A 102     -26.383   7.654  66.634  1.00  0.00
A    C
ATOM  15021  O   VAL A 102     -27.248   8.060  65.853  1.00  0.00
A    O
ATOM  15022  N   LYS A 103     -25.124   8.085  66.618  1.00  0.00
A    N
ATOM  15023  HN  LYS A 103     -24.461   7.870  67.334  1.00  0.00
A    H
ATOM  15024  CA  LYS A 103     -24.617   8.913  65.536  1.00  0.00
A    C
ATOM  15025  HA  LYS A 103     -25.416   9.574  65.223  1.00  0.00
A    H
ATOM  15026  CB  LYS A 103     -23.407   9.724  66.003  1.00  0.00
A    C
ATOM  15027  HB1 LYS A 103     -23.767  10.428  66.788  1.00  0.00
A    H
ATOM  15028  HB2 LYS A 103     -22.684   9.041  66.504  1.00  0.00
A    H
ATOM  15029  CG  LYS A 103     -22.721  10.519  64.901  1.00  0.00
A    C
ATOM  15030  HG1 LYS A 103     -22.414   9.823  64.087  1.00  0.00
A    H
ATOM  15031  HG2 LYS A 103     -23.451  11.239  64.465  1.00  0.00
A    H
ATOM  15032  CD  LYS A 103     -21.503  11.237  65.449  1.00  0.00
A    C
ATOM  15033  HD1 LYS A 103     -21.809  12.263  65.758  1.00  0.00
A    H
ATOM  15034  HD2 LYS A 103     -21.167  10.699  66.365  1.00  0.00
A    H
ATOM  15035  CE  LYS A 103     -20.379  11.300  64.436  1.00  0.00
A    C
ATOM  15036  HE1 LYS A 103     -19.396  11.134  64.933  1.00  0.00
A    H
ATOM  15037  HE2 LYS A 103     -20.514  10.521  63.654  1.00  0.00
A    H
ATOM  15038  NZ  LYS A 103     -20.351  12.622  63.777  1.00  0.00
A    N
```

ATOM 15039 HZ1 LYS A 103 -19.576 12.658 63.084 1.00 0.00
A H
ATOM 15040 HZ2 LYS A 103 -21.256 12.789 63.292 1.00 0.00
A H
ATOM 15041 HZ3 LYS A 103 -20.207 13.363 64.493 1.00 0.00
A H
ATOM 15042 C LYS A 103 -24.256 8.031 64.336 1.00 0.00
A C
ATOM 15043 O LYS A 103 -23.478 7.075 64.462 1.00 0.00
A O
ATOM 15044 N ALA A 104 -24.829 8.350 63.182 1.00 0.00
A N
ATOM 15045 HN ALA A 104 -25.482 9.102 63.087 1.00 0.00
A H
ATOM 15046 CA ALA A 104 -24.541 7.613 61.956 1.00 0.00
A C
ATOM 15047 HA ALA A 104 -24.239 6.597 62.184 1.00 0.00
A H
ATOM 15048 CB ALA A 104 -25.773 7.545 61.075 1.00 0.00
A C
ATOM 15049 HB1 ALA A 104 -26.595 7.038 61.623 1.00 0.00
A H
ATOM 15050 HB2 ALA A 104 -26.123 8.563 60.799 1.00 0.00
A H
ATOM 15051 HB3 ALA A 104 -25.578 6.973 60.143 1.00 0.00
A H
ATOM 15052 C ALA A 104 -23.382 8.244 61.196 1.00 0.00
A C
ATOM 15053 O ALA A 104 -23.287 9.473 61.105 1.00 0.00
A O
ATOM 15054 N GLN A 105 -22.513 7.403 60.647 1.00 0.00
A N
ATOM 15055 HN GLN A 105 -22.593 6.410 60.705 1.00 0.00
A H
ATOM 15056 CA GLN A 105 -21.354 7.872 59.902 1.00 0.00
A C
ATOM 15057 HA GLN A 105 -21.055 8.807 60.359 1.00 0.00
A H
ATOM 15058 CB GLN A 105 -20.213 6.875 60.005 1.00 0.00
A C
ATOM 15059 HB1 GLN A 105 -20.629 5.879 59.728 1.00 0.00
A H
ATOM 15060 HB2 GLN A 105 -19.444 7.114 59.235 1.00 0.00
A H
ATOM 15061 CG GLN A 105 -19.615 6.823 61.391 1.00 0.00
A C
ATOM 15062 HG1 GLN A 105 -20.366 6.453 62.123 1.00 0.00
A H
ATOM 15063 HG2 GLN A 105 -18.746 6.134 61.418 1.00 0.00
A H
ATOM 15064 CD GLN A 105 -19.176 8.195 61.871 1.00 0.00
A C
ATOM 15065 OE1 GLN A 105 -18.444 8.910 61.182 1.00 0.00
A O

ATOM 15066 NE2 GLN A 105 -19.623 8.570 63.059 1.00 0.00
A N
ATOM 15067 HE21 GLN A 105 -19.345 9.470 63.381 1.00 0.00
A H
ATOM 15068 HE22 GLN A 105 -20.195 7.953 63.588 1.00 0.00
A H
ATOM 15069 C GLN A 105 -21.674 8.155 58.450 1.00 0.00
A C
ATOM 15070 O GLN A 105 -21.202 9.142 57.884 1.00 0.00
A O
ATOM 15071 N ASN A 106 -22.486 7.292 57.853 1.00 0.00
A N
ATOM 15072 HN ASN A 106 -22.888 6.507 58.321 1.00 0.00
A H
ATOM 15073 CA ASN A 106 -22.863 7.419 56.455 1.00 0.00
A C
ATOM 15074 HA ASN A 106 -22.994 8.480 56.273 1.00 0.00
A H
ATOM 15075 CB ASN A 106 -21.772 6.824 55.558 1.00 0.00
A C
ATOM 15076 HB1 ASN A 106 -22.148 6.837 54.511 1.00 0.00
A H
ATOM 15077 HB2 ASN A 106 -20.860 7.453 55.592 1.00 0.00
A H
ATOM 15078 CG ASN A 106 -21.427 5.381 55.912 1.00 0.00
A C
ATOM 15079 OD1 ASN A 106 -22.287 4.594 56.308 1.00 0.00
A O
ATOM 15080 ND2 ASN A 106 -20.156 5.028 55.759 1.00 0.00
A N
ATOM 15081 HD21 ASN A 106 -19.913 4.086 55.975 1.00 0.00
A H
ATOM 15082 HD22 ASN A 106 -19.495 5.685 55.412 1.00 0.00
A H
ATOM 15083 C ASN A 106 -24.200 6.739 56.199 1.00 0.00
A C
ATOM 15084 O ASN A 106 -24.849 6.264 57.132 1.00 0.00
A O
ATOM 15085 N TYR A 107 -24.602 6.683 54.934 1.00 0.00
A N
ATOM 15086 HN TYR A 107 -24.089 7.072 54.171 1.00 0.00
A H
ATOM 15087 CA TYR A 107 -25.854 6.033 54.554 1.00 0.00
A C
ATOM 15088 HA TYR A 107 -26.638 6.564 55.081 1.00 0.00
A H
ATOM 15089 CB TYR A 107 -26.081 6.127 53.042 1.00 0.00
A C
ATOM 15090 HB1 TYR A 107 -26.248 7.191 52.764 1.00 0.00
A H
ATOM 15091 HB2 TYR A 107 -25.182 5.779 52.488 1.00 0.00
A H
ATOM 15092 CG TYR A 107 -27.293 5.354 52.569 1.00 0.00
A C

ATOM 15093 CD1 TYR A 107 -28.578 5.878 52.711 1.00 0.00
A C
ATOM 15094 HD1 TYR A 107 -28.715 6.846 53.174 1.00 0.00
A H
ATOM 15095 CE1 TYR A 107 -29.693 5.172 52.288 1.00 0.00
A C
ATOM 15096 HE1 TYR A 107 -30.683 5.586 52.405 1.00 0.00
A H
ATOM 15097 CZ TYR A 107 -29.529 3.918 51.717 1.00 0.00
A C
ATOM 15098 OH TYR A 107 -30.630 3.206 51.294 1.00 0.00
A O
ATOM 15099 HH TYR A 107 -30.312 2.408 50.868 1.00 0.00
A H
ATOM 15100 CD2 TYR A 107 -27.156 4.088 51.991 1.00 0.00
A C
ATOM 15101 HD2 TYR A 107 -26.177 3.637 51.904 1.00 0.00
A H
ATOM 15102 CE2 TYR A 107 -28.264 3.369 51.566 1.00 0.00
A C
ATOM 15103 HE2 TYR A 107 -28.134 2.383 51.145 1.00 0.00
A H
ATOM 15104 C TYR A 107 -25.913 4.572 55.009 1.00 0.00
A C
ATOM 15105 O TYR A 107 -26.911 4.131 55.581 1.00 0.00
A O
ATOM 15106 N TYR A 108 -24.840 3.833 54.739 1.00 0.00
A N
ATOM 15107 HN TYR A 108 -24.034 4.201 54.275 1.00 0.00
A H
ATOM 15108 CA TYR A 108 -24.742 2.421 55.096 1.00 0.00
A C
ATOM 15109 HA TYR A 108 -25.567 1.926 54.598 1.00 0.00
A H
ATOM 15110 CB TYR A 108 -23.385 1.862 54.655 1.00 0.00
A C
ATOM 15111 HB1 TYR A 108 -23.465 1.496 53.607 1.00 0.00
A H
ATOM 15112 HB2 TYR A 108 -22.609 2.658 54.668 1.00 0.00
A H
ATOM 15113 CG TYR A 108 -22.897 0.684 55.484 1.00 0.00
A C
ATOM 15114 CD1 TYR A 108 -23.528 -0.562 55.399 1.00 0.00
A C
ATOM 15115 HD1 TYR A 108 -24.383 -0.689 54.749 1.00 0.00
A H
ATOM 15116 CE1 TYR A 108 -23.090 -1.642 56.152 1.00 0.00
A C
ATOM 15117 HE1 TYR A 108 -23.590 -2.597 56.085 1.00 0.00
A H
ATOM 15118 CZ TYR A 108 -22.007 -1.492 57.003 1.00 0.00
A C
ATOM 15119 OH TYR A 108 -21.578 -2.569 57.746 1.00 0.00
A O

ATOM 15120 HH TYR A 108 -20.785 -2.299 58.214 1.00 0.00
A H
ATOM 15121 CD2 TYR A 108 -21.807 0.813 56.358 1.00 0.00
A C
ATOM 15122 HD2 TYR A 108 -21.324 1.773 56.481 1.00 0.00
A H
ATOM 15123 CE2 TYR A 108 -21.362 -0.267 57.113 1.00 0.00
A C
ATOM 15124 HE2 TYR A 108 -20.533 -0.141 57.793 1.00 0.00
A H
ATOM 15125 C TYR A 108 -24.919 2.196 56.593 1.00 0.00
A C
ATOM 15126 O TYR A 108 -25.635 1.283 57.013 1.00 0.00
A O
ATOM 15127 N HSE A 109 -24.243 3.025 57.381 1.00 0.00
A N
ATOM 15128 HN HSE A 109 -23.676 3.771 57.032 1.00 0.00
A H
ATOM 15129 CA HSE A 109 -24.267 2.919 58.821 1.00 0.00
A C
ATOM 15130 HA HSE A 109 -23.989 1.907 59.087 1.00 0.00
A H
ATOM 15131 CB HSE A 109 -23.301 3.932 59.419 1.00 0.00
A C
ATOM 15132 HB1 HSE A 109 -22.334 3.841 58.878 1.00 0.00
A H
ATOM 15133 HB2 HSE A 109 -23.680 4.959 59.237 1.00 0.00
A H
ATOM 15134 ND1 HSE A 109 -22.549 4.717 61.664 1.00 0.00
A N
ATOM 15135 CG HSE A 109 -23.026 3.734 60.884 1.00 0.00
A C
ATOM 15136 CE1 HSE A 109 -22.403 4.268 62.918 1.00 0.00
A C
ATOM 15137 HE1 HSE A 109 -22.018 4.863 63.749 1.00 0.00
A H
ATOM 15138 NE2 HSE A 109 -22.800 2.991 62.948 1.00 0.00
A N
ATOM 15139 HE2 HSE A 109 -22.786 2.399 63.754 1.00 0.00
A H
ATOM 15140 CD2 HSE A 109 -23.193 2.630 61.708 1.00 0.00
A C
ATOM 15141 HD2 HSE A 109 -23.552 1.638 61.473 1.00 0.00
A H
ATOM 15142 C HSE A 109 -25.671 3.128 59.298 1.00 0.00
A C
ATOM 15143 O HSE A 109 -26.164 2.382 60.140 1.00 0.00
A O
ATOM 15144 N HSE A 110 -26.344 4.125 58.725 1.00 0.00
A N
ATOM 15145 HN HSE A 110 -25.944 4.743 58.050 1.00 0.00
A H
ATOM 15146 CA HSE A 110 -27.745 4.399 59.040 1.00 0.00
A C

ATOM 15147 HA HSE A 110   -27.782   4.684 60.084 1.00 0.00
A   H
ATOM 15148 CB HSE A 110   -28.274   5.528 58.154 1.00 0.00
A   C
ATOM 15149 HB1 HSE A 110   -27.585   6.395 58.256 1.00 0.00
A   H
ATOM 15150 HB2 HSE A 110   -28.241   5.212 57.090 1.00 0.00
A   H
ATOM 15151 ND1 HSE A 110   -29.883   7.119 59.205 1.00 0.00
A   N
ATOM 15152 CG HSE A 110   -29.660   5.990 58.521 1.00 0.00
A   C
ATOM 15153 CE1 HSE A 110   -31.204   7.278 59.396 1.00 0.00
A   C
ATOM 15154 HE1 HSE A 110   -31.657   8.117 59.930 1.00 0.00
A   H
ATOM 15155 NE2 HSE A 110   -31.832   6.241 58.832 1.00 0.00
A   N
ATOM 15156 HE2 HSE A 110   -32.820   6.087 58.838 1.00 0.00
A   H
ATOM 15157 CD2 HSE A 110   -30.909   5.425 58.279 1.00 0.00
A   C
ATOM 15158 HD2 HSE A 110   -31.169   4.509 57.767 1.00 0.00
A   H
ATOM 15159 C   HSE A 110   -28.593   3.165 58.886 1.00 0.00
A   C
ATOM 15160 O   HSE A 110   -29.388   2.833 59.770 1.00 0.00
A   O
ATOM 15161 N   LYS A 111   -28.405   2.472 57.767 1.00 0.00
A   N
ATOM 15162 HN LYS A 111   -27.696   2.722 57.109 1.00 0.00
A   H
ATOM 15163 CA LYS A 111   -29.206   1.299 57.402 1.00 0.00
A   C
ATOM 15164 HA LYS A 111   -30.230   1.651 57.376 1.00 0.00
A   H
ATOM 15165 CB LYS A 111   -28.823   0.824 56.001 1.00 0.00
A   C
ATOM 15166 HB1 LYS A 111   -27.721   0.652 56.003 1.00 0.00
A   H
ATOM 15167 HB2 LYS A 111   -29.294 -0.167 55.813 1.00 0.00
A   H
ATOM 15168 CG LYS A 111   -29.169   1.803 54.890 1.00 0.00
A   C
ATOM 15169 HG1 LYS A 111   -29.400   2.796 55.337 1.00 0.00
A   H
ATOM 15170 HG2 LYS A 111   -28.280   1.933 54.232 1.00 0.00
A   H
ATOM 15171 CD LYS A 111   -30.365   1.341 54.072 1.00 0.00
A   C
ATOM 15172 HD1 LYS A 111   -30.286   1.783 53.052 1.00 0.00
A   H
ATOM 15173 HD2 LYS A 111   -30.303   0.234 53.962 1.00 0.00
A   H

ATOM 15174 CE LYS A 111   -31.695   1.723 54.709 1.00 0.00
A   C
ATOM 15175 HE1 LYS A 111   -31.691   1.478 55.794 1.00 0.00
A   H
ATOM 15176 HE2 LYS A 111   -31.885   2.812 54.594 1.00 0.00
A   H
ATOM 15177 NZ LYS A 111   -32.813   0.978 54.061 1.00 0.00
A   N
ATOM 15178 HZ1 LYS A 111   -33.718   1.241 54.501 1.00 0.00
A   H
ATOM 15179 HZ2 LYS A 111   -32.841   1.207 53.047 1.00 0.00
A   H
ATOM 15180 HZ3 LYS A 111   -32.658 -0.045 54.176 1.00 0.00
A   H
ATOM 15181 C   LYS A 111   -29.148   0.129 58.400 1.00 0.00
A   C
ATOM 15182 O   LYS A 111   -30.191 -0.422 58.749 1.00 0.00
A   O
ATOM 15183 N   VAL A 112   -27.948 -0.253 58.847 1.00 0.00
A   N
ATOM 15184 HN VAL A 112   -27.083   0.145 58.554 1.00 0.00
A   H
ATOM 15185 CA VAL A 112   -27.816 -1.331 59.843 1.00 0.00
A   C
ATOM 15186 HA VAL A 112   -28.420 -2.159 59.496 1.00 0.00
A   H
ATOM 15187 CB VAL A 112   -26.380 -1.904 59.996 1.00 0.00
A   C
ATOM 15188 HB VAL A 112   -26.376 -2.570 60.893 1.00 0.00
A   H
ATOM 15189 CG1 VAL A 112   -25.991 -2.712 58.778 1.00 0.00
A   C
ATOM 15190 HG11 VAL A 112   -24.949 -3.084 58.880 1.00 0.00
A   H
ATOM 15191 HG12 VAL A 112   -26.646 -3.597 58.648 1.00 0.00
A   H
ATOM 15192 HG13 VAL A 112   -26.045 -2.088 57.860 1.00 0.00
A   H
ATOM 15193 CG2 VAL A 112   -25.351 -0.812 60.251 1.00 0.00
A   C
ATOM 15194 HG21 VAL A 112   -24.329 -1.243 60.317 1.00 0.00
A   H
ATOM 15195 HG22 VAL A 112   -25.364 -0.064 59.428 1.00 0.00
A   H
ATOM 15196 HG23 VAL A 112   -25.554 -0.284 61.205 1.00 0.00
A   H
ATOM 15197 C   VAL A 112   -28.350 -0.915 61.204 1.00 0.00
A   C
ATOM 15198 O   VAL A 112   -28.985 -1.720 61.878 1.00 0.00
A   O
ATOM 15199 N   LEU A 113   -28.094   0.334 61.601 1.00 0.00
A   N
ATOM 15200 HN LEU A 113   -27.554   0.983 61.069 1.00 0.00
A   H

```
ATOM  15201  CA  LEU A 113     -28.591   0.865  62.872  1.00  0.00
A    C
ATOM  15202  HA  LEU A 113     -28.244   0.202  63.654  1.00  0.00
A    H
ATOM  15203  CB  LEU A 113     -28.066   2.284  63.113  1.00  0.00
A    C
ATOM  15204  HB1 LEU A 113     -28.322   2.887  62.212  1.00  0.00
A    H
ATOM  15205  HB2 LEU A 113     -28.629   2.740  63.958  1.00  0.00
A    H
ATOM  15206  CG  LEU A 113     -26.559   2.425  63.363  1.00  0.00
A    C
ATOM  15207  HG  LEU A 113     -26.067   1.801  62.578  1.00  0.00
A    H
ATOM  15208  CD1 LEU A 113     -26.112   3.873  63.206  1.00  0.00
A    C
ATOM  15209 HD11 LEU A 113     -25.030   3.973  63.436  1.00  0.00
A    H
ATOM  15210 HD12 LEU A 113     -26.283   4.224  62.166  1.00  0.00
A    H
ATOM  15211 HD13 LEU A 113     -26.676   4.534  63.898  1.00  0.00
A    H
ATOM  15212  CD2 LEU A 113     -26.165   1.867  64.724  1.00  0.00
A    C
ATOM  15213 HD21 LEU A 113     -25.068   1.961  64.878  1.00  0.00
A    H
ATOM  15214 HD22 LEU A 113     -26.669   2.408  65.550  1.00  0.00
A    H
ATOM  15215 HD23 LEU A 113     -26.435   0.790  64.796  1.00  0.00
A    H
ATOM  15216  C   LEU A 113     -30.121   0.835  62.886  1.00  0.00
A    C
ATOM  15217  O   LEU A 113     -30.740   0.458  63.881  1.00  0.00
A    O
ATOM  15218  N   GLU A 114     -30.717   1.209  61.763  1.00  0.00
A    N
ATOM  15219  HN  GLU A 114     -30.221   1.577  60.982  1.00  0.00
A    H
ATOM  15220  CA  GLU A 114     -32.154   1.118  61.560  1.00  0.00
A    C
ATOM  15221  HA  GLU A 114     -32.636   1.781  62.268  1.00  0.00
A    H
ATOM  15222  CB  GLU A 114     -32.471   1.520  60.120  1.00  0.00
A    C
ATOM  15223  HB1 GLU A 114     -31.749   2.312  59.814  1.00  0.00
A    H
ATOM  15224  HB2 GLU A 114     -32.285   0.659  59.439  1.00  0.00
A    H
ATOM  15225  CG  GLU A 114     -33.867   2.045  59.858  1.00  0.00
A    C
ATOM  15226  HG1 GLU A 114     -34.576   1.199  59.812  1.00  0.00
A    H
ATOM  15227  HG2 GLU A 114     -34.184   2.746  60.656  1.00  0.00
A    H
```

ATOM 15228 CD GLU A 114 -33.952 2.749 58.513 1.00 0.00
A C
ATOM 15229 OE1 GLU A 114 -33.746 2.087 57.469 1.00 0.00
A O
ATOM 15230 OE2 GLU A 114 -34.215 3.973 58.498 1.00 0.00
A O
ATOM 15231 C GLU A 114 -32.668 -0.302 61.843 1.00 0.00
A C
ATOM 15232 O GLU A 114 -33.637 -0.476 62.575 1.00 0.00
A O
ATOM 15233 N LYS A 115 -31.997 -1.304 61.277 1.00 0.00
A N
ATOM 15234 HN LYS A 115 -31.170 -1.154 60.737 1.00 0.00
A H
ATOM 15235 CA LYS A 115 -32.412 -2.701 61.401 1.00 0.00
A C
ATOM 15236 HA LYS A 115 -33.490 -2.692 61.293 1.00 0.00
A H
ATOM 15237 CB LYS A 115 -31.819 -3.538 60.264 1.00 0.00
A C
ATOM 15238 HB1 LYS A 115 -30.724 -3.326 60.237 1.00 0.00
A H
ATOM 15239 HB2 LYS A 115 -31.922 -4.618 60.517 1.00 0.00
A H
ATOM 15240 CG LYS A 115 -32.408 -3.255 58.890 1.00 0.00
A C
ATOM 15241 HG1 LYS A 115 -32.398 -2.155 58.709 1.00 0.00
A H
ATOM 15242 HG2 LYS A 115 -31.761 -3.725 58.114 1.00 0.00
A H
ATOM 15243 CD LYS A 115 -33.834 -3.768 58.777 1.00 0.00
A C
ATOM 15244 HD1 LYS A 115 -33.804 -4.879 58.706 1.00 0.00
A H
ATOM 15245 HD2 LYS A 115 -34.372 -3.509 59.718 1.00 0.00
A H
ATOM 15246 CE LYS A 115 -34.554 -3.173 57.578 1.00 0.00
A C
ATOM 15247 HE1 LYS A 115 -35.645 -3.391 57.630 1.00 0.00
A H
ATOM 15248 HE2 LYS A 115 -34.417 -2.069 57.549 1.00 0.00
A H
ATOM 15249 NZ LYS A 115 -34.049 -3.723 56.294 1.00 0.00
A N
ATOM 15250 HZ1 LYS A 115 -34.563 -3.296 55.497 1.00 0.00
A H
ATOM 15251 HZ2 LYS A 115 -33.034 -3.513 56.202 1.00 0.00
A H
ATOM 15252 HZ3 LYS A 115 -34.188 -4.753 56.281 1.00 0.00
A H
ATOM 15253 C LYS A 115 -32.092 -3.349 62.751 1.00 0.00
A C
ATOM 15254 O LYS A 115 -32.826 -4.228 63.197 1.00 0.00
A O

ATOM 15255 N   ILE A 116   -31.005 -2.928 63.398 1.00 0.00
A   N
ATOM 15256 HN ILE A 116   -30.367 -2.234 63.070 1.00 0.00
A   H
ATOM 15257 CA ILE A 116   -30.659 -3.490 64.709 1.00 0.00
A   C
ATOM 15258 HA ILE A 116   -31.017 -4.511 64.701 1.00 0.00
A   H
ATOM 15259 CB ILE A 116   -29.129 -3.587 64.959 1.00 0.00
A   C
ATOM 15260 HB ILE A 116   -29.040 -4.054 65.972 1.00 0.00
A   H
ATOM 15261 CG2 ILE A 116   -28.468 -4.418 63.873 1.00 0.00
A   C
ATOM 15262 HG21 ILE A 116   -27.407 -4.621 64.129 1.00 0.00
A   H
ATOM 15263 HG22 ILE A 116   -28.979 -5.396 63.757 1.00 0.00
A   H
ATOM 15264 HG23 ILE A 116   -28.494 -3.890 62.897 1.00 0.00
A   H
ATOM 15265 CG1 ILE A 116   -28.478 -2.210 65.065 1.00 0.00
A   C
ATOM 15266 HG11 ILE A 116   -28.705 -1.615 64.152 1.00 0.00
A   H
ATOM 15267 HG12 ILE A 116   -28.920 -1.668 65.931 1.00 0.00
A   H
ATOM 15268 CD ILE A 116   -26.974 -2.263 65.259 1.00 0.00
A   C
ATOM 15269 HD1 ILE A 116   -26.566 -1.237 65.387 1.00 0.00
A   H
ATOM 15270 HD2 ILE A 116   -26.717 -2.855 66.163 1.00 0.00
A   H
ATOM 15271 HD3 ILE A 116   -26.475 -2.723 64.381 1.00 0.00
A   H
ATOM 15272 C   ILE A 116   -31.390 -2.775 65.846 1.00 0.00
A   C
ATOM 15273 O   ILE A 116   -31.078 -2.981 67.022 1.00 0.00
A   O
ATOM 15274 N   GLY A 117   -32.366 -1.946 65.479 1.00 0.00
A   N
ATOM 15275 HN GLY A 117   -32.531 -1.725 64.519 1.00 0.00
A   H
ATOM 15276 CA GLY A 117   -33.262 -1.303 66.437 1.00 0.00
A   C
ATOM 15277 HA1 GLY A 117   -33.498 -2.080 67.152 1.00 0.00
A   H
ATOM 15278 HA2 GLY A 117   -34.105 -0.972 65.846 1.00 0.00
A   H
ATOM 15279 C   GLY A 117   -32.727 -0.104 67.205 1.00 0.00
A   C
ATOM 15280 O   GLY A 117   -32.899 -0.021 68.426 1.00 0.00
A   O
ATOM 15281 N   ALA A 118   -32.079   0.820 66.499 1.00 0.00
A   N

ATOM 15282 HN ALA A 118 -31.807 0.688 65.546 1.00 0.00
A H
ATOM 15283 CA ALA A 118 -31.716 2.110 67.080 1.00 0.00
A C
ATOM 15284 HA ALA A 118 -31.388 2.023 68.110 1.00 0.00
A H
ATOM 15285 CB ALA A 118 -30.626 2.784 66.262 1.00 0.00
A C
ATOM 15286 HB1 ALA A 118 -29.699 2.172 66.300 1.00 0.00
A H
ATOM 15287 HB2 ALA A 118 -30.929 2.880 65.197 1.00 0.00
A H
ATOM 15288 HB3 ALA A 118 -30.386 3.795 66.655 1.00 0.00
A H
ATOM 15289 C ALA A 118 -32.974 2.958 67.102 1.00 0.00
A C
ATOM 15290 O ALA A 118 -33.708 3.006 66.110 1.00 0.00
A O
ATOM 15291 N ASP A 119 -33.237 3.609 68.235 1.00 0.00
A N
ATOM 15292 HN ASP A 119 -32.651 3.639 69.041 1.00 0.00
A H
ATOM 15293 CA ASP A 119 -34.475 4.375 68.399 1.00 0.00
A C
ATOM 15294 HA ASP A 119 -35.254 3.804 67.907 1.00 0.00
A H
ATOM 15295 CB ASP A 119 -34.813 4.528 69.868 1.00 0.00
A C
ATOM 15296 HB1 ASP A 119 -33.962 5.007 70.392 1.00 0.00
A H
ATOM 15297 HB2 ASP A 119 -35.707 5.170 69.998 1.00 0.00
A H
ATOM 15298 CG ASP A 119 -35.068 3.203 70.531 1.00 0.00
A C
ATOM 15299 OD1 ASP A 119 -36.060 2.541 70.168 1.00 0.00
A O
ATOM 15300 OD2 ASP A 119 -34.273 2.820 71.416 1.00 0.00
A O
ATOM 15301 C ASP A 119 -34.423 5.732 67.709 1.00 0.00
A C
ATOM 15302 O ASP A 119 -35.438 6.216 67.204 1.00 0.00
A O
ATOM 15303 N ARG A 120 -33.240 6.342 67.696 1.00 0.00
A N
ATOM 15304 HN ARG A 120 -32.462 5.988 68.212 1.00 0.00
A H
ATOM 15305 CA ARG A 120 -33.017 7.554 66.930 1.00 0.00
A C
ATOM 15306 HA ARG A 120 -33.708 7.569 66.095 1.00 0.00
A H
ATOM 15307 CB ARG A 120 -33.258 8.800 67.789 1.00 0.00
A C
ATOM 15308 HB1 ARG A 120 -34.278 8.688 68.227 1.00 0.00
A H

ATOM 15309 HB2 ARG A 120   -32.553   8.799 68.651 1.00 0.00
A   H
ATOM 15310 CG ARG A 120   -33.187 10.090 66.991 1.00 0.00
A   C
ATOM 15311 HG1 ARG A 120   -32.168 10.181 66.556 1.00 0.00
A   H
ATOM 15312 HG2 ARG A 120   -33.879 10.014 66.121 1.00 0.00
A   H
ATOM 15313 CD ARG A 120   -33.575 11.311 67.794 1.00 0.00
A   C
ATOM 15314 HD1 ARG A 120   -34.682 11.334 67.922 1.00 0.00
A   H
ATOM 15315 HD2 ARG A 120   -33.099 11.306 68.802 1.00 0.00
A   H
ATOM 15316 NE ARG A 120   -33.131 12.524 67.108 1.00 0.00
A   N
ATOM 15317 HE ARG A 120   -32.567 12.336 66.295 1.00 0.00
A   H
ATOM 15318 CZ ARG A 120   -33.418 13.770 67.483 1.00 0.00
A   C
ATOM 15319 NH1 ARG A 120   -34.170 14.008 68.558 1.00 0.00
A   N
ATOM 15320 HH11 ARG A 120   -34.400 14.937 68.816 1.00 0.00
A   H
ATOM 15321 HH12 ARG A 120   -34.590 13.208 68.992 1.00 0.00
A   H
ATOM 15322 NH2 ARG A 120   -32.948 14.785 66.775 1.00 0.00
A   N
ATOM 15323 HH21 ARG A 120   -33.082 15.713 67.093 1.00 0.00
A   H
ATOM 15324 HH22 ARG A 120   -32.297 14.570 66.059 1.00 0.00
A   H
ATOM 15325 C   ARG A 120   -31.615   7.560 66.336 1.00 0.00
A   C
ATOM 15326 O   ARG A 120   -30.640   7.256 67.020 1.00 0.00
A   O
ATOM 15327 N   ILE A 121   -31.525   7.907 65.056 1.00 0.00
A   N
ATOM 15328 HN ILE A 121   -32.307   8.161 64.491 1.00 0.00
A   H
ATOM 15329 CA ILE A 121   -30.242   7.948 64.357 1.00 0.00
A   C
ATOM 15330 HA ILE A 121   -29.487   7.659 65.077 1.00 0.00
A   H
ATOM 15331 CB ILE A 121   -30.240   7.004 63.141 1.00 0.00
A   C
ATOM 15332 HB ILE A 121   -31.093   7.360 62.509 1.00 0.00
A   H
ATOM 15333 CG2 ILE A 121   -28.894   7.055 62.436 1.00 0.00
A   C
ATOM 15334 HG21 ILE A 121   -28.935   6.501 61.475 1.00 0.00
A   H
ATOM 15335 HG22 ILE A 121   -28.606   8.103 62.208 1.00 0.00
A   H

```
ATOM  15336 HG23 ILE A 121     -28.099   6.601  63.064  1.00  0.00
A    H
ATOM  15337  CG1 ILE A 121     -30.575   5.581  63.581  1.00  0.00
A    C
ATOM  15338 HG11 ILE A 121     -29.683   5.115  64.057  1.00  0.00
A    H
ATOM  15339 HG12 ILE A 121     -31.377   5.621  64.352  1.00  0.00
A    H
ATOM  15340  CD  ILE A 121     -31.049   4.696  62.460  1.00  0.00
A    C
ATOM  15341  HD1 ILE A 121     -31.330   3.694  62.852  1.00  0.00
A    H
ATOM  15342  HD2 ILE A 121     -31.938   5.137  61.961  1.00  0.00
A    H
ATOM  15343  HD3 ILE A 121     -30.252   4.555  61.699  1.00  0.00
A    H
ATOM  15344  C   ILE A 121     -29.915   9.373  63.921  1.00  0.00
A    C
ATOM  15345  O   ILE A 121     -30.668   9.986  63.175  1.00  0.00
A    O
ATOM  15346  N   ILE A 122     -28.787   9.898  64.393  1.00  0.00
A    N
ATOM  15347  HN  ILE A 122     -28.106   9.394  64.920  1.00  0.00
A    H
ATOM  15348  CA  ILE A 122     -28.452  11.297  64.165  1.00  0.00
A    C
ATOM  15349  HA  ILE A 122     -29.338  11.749  63.739  1.00  0.00
A    H
ATOM  15350  CB  ILE A 122     -28.061  12.019  65.480  1.00  0.00
A    C
ATOM  15351  HB  ILE A 122     -27.191  11.434  65.873  1.00  0.00
A    H
ATOM  15352  CG2 ILE A 122     -27.776  13.494  65.240  1.00  0.00
A    C
ATOM  15353 HG21 ILE A 122     -27.349  13.962  66.151  1.00  0.00
A    H
ATOM  15354 HG22 ILE A 122     -27.042  13.627  64.417  1.00  0.00
A    H
ATOM  15355 HG23 ILE A 122     -28.705  14.040  64.973  1.00  0.00
A    H
ATOM  15356  CG1 ILE A 122     -29.169  11.902  66.522  1.00  0.00
A    C
ATOM  15357 HG11 ILE A 122     -29.508  12.917  66.828  1.00  0.00
A    H
ATOM  15358 HG12 ILE A 122     -30.045  11.389  66.061  1.00  0.00
A    H
ATOM  15359  CD  ILE A 122     -28.743  11.124  67.743  1.00  0.00
A    C
ATOM  15360  HD1 ILE A 122     -29.596  11.014  68.446  1.00  0.00
A    H
ATOM  15361  HD2 ILE A 122     -28.394  10.108  67.458  1.00  0.00
A    H
ATOM  15362  HD3 ILE A 122     -27.921  11.642  68.279  1.00  0.00
A    H
```

ATOM 15363 C   ILE A 122   -27.336 11.466 63.142 1.00 0.00
A   C
ATOM 15364 O   ILE A 122   -26.290 10.836 63.244 1.00 0.00
A   O
ATOM 15365 N   HSE A 123   -27.585 12.328 62.160 1.00 0.00
A   N
ATOM 15366 HN HSE A 123   -28.497 12.698 61.988 1.00 0.00
A   H
ATOM 15367 CA HSE A 123   -26.557 12.813 61.246 1.00 0.00
A   C
ATOM 15368 HA HSE A 123   -25.659 12.235 61.426 1.00 0.00
A   H
ATOM 15369 CB HSE A 123   -27.044 12.704 59.819 1.00 0.00
A   C
ATOM 15370 HB1 HSE A 123   -27.981 13.296 59.732 1.00 0.00
A   H
ATOM 15371 HB2 HSE A 123   -26.302 13.170 59.138 1.00 0.00
A   H
ATOM 15372 ND1 HSE A 123   -26.505 10.582 58.604 1.00 0.00
A   N
ATOM 15373 CG HSE A 123   -27.343 11.294 59.386 1.00 0.00
A   C
ATOM 15374 CE1 HSE A 123   -27.033   9.363 58.379 1.00 0.00
A   C
ATOM 15375 HE1 HSE A 123   -26.563   8.586 57.771 1.00 0.00
A   H
ATOM 15376 NE2 HSE A 123   -28.213   9.294 59.019 1.00 0.00
A   N
ATOM 15377 HE2 HSE A 123   -28.831   8.507 59.008 1.00 0.00
A   H
ATOM 15378 CD2 HSE A 123   -28.436 10.465 59.650 1.00 0.00
A   C
ATOM 15379 HD2 HSE A 123   -29.329 10.658 60.228 1.00 0.00
A   H
ATOM 15380 C   HSE A 123   -26.231 14.251 61.565 1.00 0.00
A   C
ATOM 15381 O   HSE A 123   -26.833 15.164 60.985 1.00 0.00
A   O
ATOM 15382 N   PRO A 124   -25.273 14.480 62.489 1.00 0.00
A   N
ATOM 15383 CD PRO A 124   -24.392 13.467 63.087 1.00 0.00
A   C
ATOM 15384 HD1 PRO A 124   -25.497 13.380 63.189 1.00 0.00
A   H
ATOM 15385 HD2 PRO A 124   -23.929 12.459 62.986 1.00 0.00
A   H
ATOM 15386 CA PRO A 124   -24.962 15.818 62.975 1.00 0.00
A   C
ATOM 15387 HA PRO A 124   -25.806 16.204 63.535 1.00 0.00
A   H
ATOM 15388 CB PRO A 124   -23.754 15.597 63.884 1.00 0.00
A   C
ATOM 15389 HB1 PRO A 124   -24.111 15.306 64.898 1.00 0.00
A   H

ATOM 15390 HB2 PRO A 124 -23.114 16.498 63.973 1.00 0.00
A H
ATOM 15391 CG PRO A 124 -23.824 14.173 64.272 1.00 0.00
A C
ATOM 15392 HG1 PRO A 124 -23.290 13.955 65.218 1.00 0.00
A H
ATOM 15393 HG2 PRO A 124 -24.209 13.412 64.796 1.00 0.00
A H
ATOM 15394 C PRO A 124 -24.608 16.800 61.868 1.00 0.00
A C
ATOM 15395 O PRO A 124 -25.225 17.862 61.790 1.00 0.00
A O
ATOM 15396 N GLU A 125 -23.641 16.458 61.022 1.00 0.00
A N
ATOM 15397 HN GLU A 125 -23.152 15.590 61.029 1.00 0.00
A H
ATOM 15398 CA GLU A 125 -23.209 17.375 59.972 1.00 0.00
A C
ATOM 15399 HA GLU A 125 -22.916 18.303 60.446 1.00 0.00
A H
ATOM 15400 CB GLU A 125 -21.990 16.846 59.217 1.00 0.00
A C
ATOM 15401 HB1 GLU A 125 -22.226 15.821 58.850 1.00 0.00
A H
ATOM 15402 HB2 GLU A 125 -21.814 17.462 58.307 1.00 0.00
A H
ATOM 15403 CG GLU A 125 -20.709 16.778 60.028 1.00 0.00
A C
ATOM 15404 HG1 GLU A 125 -19.875 17.196 59.435 1.00 0.00
A H
ATOM 15405 HG2 GLU A 125 -20.806 17.348 60.973 1.00 0.00
A H
ATOM 15406 CD GLU A 125 -20.315 15.348 60.357 1.00 0.00
A C
ATOM 15407 OE1 GLU A 125 -19.155 15.004 60.062 1.00 0.00
A O
ATOM 15408 OE2 GLU A 125 -21.152 14.566 60.886 1.00 0.00
A O
ATOM 15409 C GLU A 125 -24.333 17.702 58.991 1.00 0.00
A C
ATOM 15410 O GLU A 125 -24.546 18.869 58.662 1.00 0.00
A O
ATOM 15411 N LYS A 126 -25.047 16.675 58.532 1.00 0.00
A N
ATOM 15412 HN LYS A 126 -24.845 15.727 58.772 1.00 0.00
A H
ATOM 15413 CA LYS A 126 -26.184 16.857 57.642 1.00 0.00
A C
ATOM 15414 HA LYS A 126 -25.817 17.276 56.713 1.00 0.00
A H
ATOM 15415 CB LYS A 126 -26.895 15.527 57.405 1.00 0.00
A C
ATOM 15416 HB1 LYS A 126 -26.107 14.768 57.191 1.00 0.00
A H

ATOM 15417 HB2 LYS A 126 -27.388 15.207 58.350 1.00 0.00
A H
ATOM 15418 CG LYS A 126 -27.895 15.535 56.257 1.00 0.00
A C
ATOM 15419 HG1 LYS A 126 -28.290 16.568 56.125 1.00 0.00
A H
ATOM 15420 HG2 LYS A 126 -27.368 15.258 55.315 1.00 0.00
A H
ATOM 15421 CD LYS A 126 -29.059 14.587 56.512 1.00 0.00
A C
ATOM 15422 HD1 LYS A 126 -28.652 13.624 56.899 1.00 0.00
A H
ATOM 15423 HD2 LYS A 126 -29.695 15.027 57.315 1.00 0.00
A H
ATOM 15424 CE LYS A 126 -29.877 14.360 55.247 1.00 0.00
A C
ATOM 15425 HE1 LYS A 126 -30.859 13.899 55.494 1.00 0.00
A H
ATOM 15426 HE2 LYS A 126 -30.063 15.325 54.724 1.00 0.00
A H
ATOM 15427 NZ LYS A 126 -29.184 13.453 54.284 1.00 0.00
A N
ATOM 15428 HZ1 LYS A 126 -29.770 13.321 53.434 1.00 0.00
A H
ATOM 15429 HZ2 LYS A 126 -28.270 13.868 54.012 1.00 0.00
A H
ATOM 15430 HZ3 LYS A 126 -29.019 12.531 54.736 1.00 0.00
A H
ATOM 15431 C LYS A 126 -27.148 17.876 58.252 1.00 0.00
A C
ATOM 15432 O LYS A 126 -27.499 18.860 57.611 1.00 0.00
A O
ATOM 15433 N ASP A 127 -27.539 17.648 59.505 1.00 0.00
A N
ATOM 15434 HN ASP A 127 -27.229 16.885 60.065 1.00 0.00
A H
ATOM 15435 CA ASP A 127 -28.488 18.519 60.201 1.00 0.00
A C
ATOM 15436 HA ASP A 127 -29.362 18.588 59.565 1.00 0.00
A H
ATOM 15437 CB ASP A 127 -28.879 17.915 61.549 1.00 0.00
A C
ATOM 15438 HB1 ASP A 127 -27.963 17.602 62.088 1.00 0.00
A H
ATOM 15439 HB2 ASP A 127 -29.411 18.662 62.171 1.00 0.00
A H
ATOM 15440 CG ASP A 127 -29.767 16.684 61.407 1.00 0.00
A C
ATOM 15441 OD1 ASP A 127 -30.232 16.382 60.289 1.00 0.00
A O
ATOM 15442 OD2 ASP A 127 -30.012 16.011 62.427 1.00 0.00
A O
ATOM 15443 C ASP A 127 -27.961 19.939 60.382 1.00 0.00
A C

```
ATOM  15444  O   ASP A 127     -28.694  20.906  60.195  1.00  0.00
A    O
ATOM  15445  N   MET A 128     -26.689  20.070  60.739  1.00  0.00
A    N
ATOM  15446  HN  MET A 128     -26.074  19.312  60.944  1.00  0.00
A    H
ATOM  15447  CA  MET A 128     -26.077  21.398  60.861  1.00  0.00
A    C
ATOM  15448  HA  MET A 128     -26.737  21.982  61.489  1.00  0.00
A    H
ATOM  15449  CB  MET A 128     -24.712  21.336  61.539  1.00  0.00
A    C
ATOM  15450  HB1 MET A 128     -24.191  20.435  61.137  1.00  0.00
A    H
ATOM  15451  HB2 MET A 128     -24.104  22.213  61.226  1.00  0.00
A    H
ATOM  15452  CG  MET A 128     -24.788  21.236  63.052  1.00  0.00
A    C
ATOM  15453  HG1 MET A 128     -25.256  20.274  63.353  1.00  0.00
A    H
ATOM  15454  HG2 MET A 128     -23.762  21.259  63.480  1.00  0.00
A    H
ATOM  15455  SD  MET A 128     -25.731  22.526  63.897  1.00  0.00
A    S
ATOM  15456  CE  MET A 128     -24.770  23.974  63.451  1.00  0.00
A    C
ATOM  15457  HE1 MET A 128     -25.204  24.892  63.901  1.00  0.00
A    H
ATOM  15458  HE2 MET A 128     -23.721  23.881  63.808  1.00  0.00
A    H
ATOM  15459  HE3 MET A 128     -24.745  24.114  62.348  1.00  0.00
A    H
ATOM  15460  C   MET A 128     -25.958  22.115  59.525  1.00  0.00
A    C
ATOM  15461  O   MET A 128     -25.998  23.346  59.489  1.00  0.00
A    O
ATOM  15462  N   GLY A 129     -25.809  21.347  58.441  1.00  0.00
A    N
ATOM  15463  HN  GLY A 129     -25.651  20.362  58.498  1.00  0.00
A    H
ATOM  15464  CA  GLY A 129     -25.866  21.900  57.087  1.00  0.00
A    C
ATOM  15465  HA1 GLY A 129     -25.863  21.071  56.393  1.00  0.00
A    H
ATOM  15466  HA2 GLY A 129     -25.033  22.580  56.970  1.00  0.00
A    H
ATOM  15467  C   GLY A 129     -27.151  22.690  56.872  1.00  0.00
A    C
ATOM  15468  O   GLY A 129     -27.118  23.872  56.527  1.00  0.00
A    O
ATOM  15469  N   VAL A 130     -28.288  22.040  57.112  1.00  0.00
A    N
ATOM  15470  HN  VAL A 130     -28.348  21.090  57.408  1.00  0.00
A    H
```

```
ATOM  15471  CA  VAL A 130     -29.576  22.704  56.950  1.00  0.00
A    C
ATOM  15472  HA  VAL A 130     -29.688  22.959  55.903  1.00  0.00
A    H
ATOM  15473  CB  VAL A 130     -30.800  21.792  57.268  1.00  0.00
A    C
ATOM  15474  HB  VAL A 130     -31.724  22.400  57.110  1.00  0.00
A    H
ATOM  15475  CG1 VAL A 130     -30.827  20.591  56.338  1.00  0.00
A    C
ATOM  15476 HG11 VAL A 130     -31.678  19.924  56.593  1.00  0.00
A    H
ATOM  15477 HG12 VAL A 130     -30.953  20.896  55.278  1.00  0.00
A    H
ATOM  15478 HG13 VAL A 130     -29.889  20.002  56.429  1.00  0.00
A    H
ATOM  15479  CG2 VAL A 130     -30.814  21.328  58.720  1.00  0.00
A    C
ATOM  15480 HG21 VAL A 130     -31.673  20.649  58.907  1.00  0.00
A    H
ATOM  15481 HG22 VAL A 130     -29.876  20.783  58.965  1.00  0.00
A    H
ATOM  15482 HG23 VAL A 130     -30.908  22.188  59.416  1.00  0.00
A    H
ATOM  15483  C   VAL A 130     -29.603  24.006  57.744  1.00  0.00
A    C
ATOM  15484  O   VAL A 130     -29.939  25.052  57.196  1.00  0.00
A    O
ATOM  15485  N   LYS A 131     -29.204  23.938  59.013  1.00  0.00
A    N
ATOM  15486  HN  LYS A 131     -28.850  23.096  59.417  1.00  0.00
A    H
ATOM  15487  CA  LYS A 131     -29.250  25.093  59.920  1.00  0.00
A    C
ATOM  15488  HA  LYS A 131     -30.273  25.449  59.911  1.00  0.00
A    H
ATOM  15489  CB  LYS A 131     -28.886  24.700  61.357  1.00  0.00
A    C
ATOM  15490  HB1 LYS A 131     -27.888  24.204  61.319  1.00  0.00
A    H
ATOM  15491  HB2 LYS A 131     -28.752  25.625  61.962  1.00  0.00
A    H
ATOM  15492  CG  LYS A 131     -29.885  23.761  62.017  1.00  0.00
A    C
ATOM  15493  HG1 LYS A 131     -30.775  23.654  61.356  1.00  0.00
A    H
ATOM  15494  HG2 LYS A 131     -29.423  22.753  62.117  1.00  0.00
A    H
ATOM  15495  CD  LYS A 131     -30.324  24.291  63.374  1.00  0.00
A    C
ATOM  15496  HD1 LYS A 131     -29.613  23.914  64.145  1.00  0.00
A    H
ATOM  15497  HD2 LYS A 131     -30.240  25.402  63.358  1.00  0.00
A    H
```

ATOM 15498 CE LYS A 131   -31.756 23.864 63.695 1.00 0.00
A   C
ATOM 15499 HE1 LYS A 131   -32.325 23.675 62.758 1.00 0.00
A   H
ATOM 15500 HE2 LYS A 131   -31.759 22.932 64.303 1.00 0.00
A   H
ATOM 15501 NZ LYS A 131   -32.513 24.897 64.468 1.00 0.00
A   N
ATOM 15502 HZ1 LYS A 131   -33.478 24.561 64.659 1.00 0.00
A   H
ATOM 15503 HZ2 LYS A 131   -32.027 25.082 65.368 1.00 0.00
A   H
ATOM 15504 HZ3 LYS A 131   -32.557 25.777 63.916 1.00 0.00
A   H
ATOM 15505 C   LYS A 131   -28.364 26.243 59.453 1.00 0.00
A   C
ATOM 15506 O   LYS A 131   -28.739 27.402 59.560 1.00 0.00
A   O
ATOM 15507 N   ILE A 132   -27.195 25.914 58.924 1.00 0.00
A   N
ATOM 15508 HN ILE A 132   -26.857 24.978 58.850 1.00 0.00
A   H
ATOM 15509 CA ILE A 132   -26.287 26.920 58.393 1.00 0.00
A   C
ATOM 15510 HA ILE A 132   -26.211 27.694 59.146 1.00 0.00
A   H
ATOM 15511 CB ILE A 132   -24.889 26.320 58.112 1.00 0.00
A   C
ATOM 15512 HB ILE A 132   -25.099 25.351 57.592 1.00 0.00
A   H
ATOM 15513 CG2 ILE A 132   -24.029 27.266 57.293 1.00 0.00
A   C
ATOM 15514 HG21 ILE A 132   -23.094 26.762 56.969 1.00 0.00
A   H
ATOM 15515 HG22 ILE A 132   -24.565 27.598 56.379 1.00 0.00
A   H
ATOM 15516 HG23 ILE A 132   -23.752 28.164 57.884 1.00 0.00
A   H
ATOM 15517 CG1 ILE A 132   -24.167 25.970 59.419 1.00 0.00
A   C
ATOM 15518 HG11 ILE A 132   -24.667 25.102 59.905 1.00 0.00
A   H
ATOM 15519 HG12 ILE A 132   -23.124 25.659 59.183 1.00 0.00
A   H
ATOM 15520 CD ILE A 132   -24.082 27.091 60.432 1.00 0.00
A   C
ATOM 15521 HD1 ILE A 132   -23.496 26.766 61.318 1.00 0.00
A   H
ATOM 15522 HD2 ILE A 132   -23.583 27.980 59.991 1.00 0.00
A   H
ATOM 15523 HD3 ILE A 132   -25.093 27.391 60.781 1.00 0.00
A   H
ATOM 15524 C   ILE A 132   -26.870 27.576 57.144 1.00 0.00
A   C

ATOM 15525 O ILE A 132 -26.811 28.793 56.998 1.00 0.00
A O
ATOM 15526 N ALA A 133 -27.441 26.766 56.255 1.00 0.00
A N
ATOM 15527 HN ALA A 133 -27.472 25.772 56.359 1.00 0.00
A H
ATOM 15528 CA ALA A 133 -28.068 27.286 55.050 1.00 0.00
A C
ATOM 15529 HA ALA A 133 -27.364 27.878 54.477 1.00 0.00
A H
ATOM 15530 CB ALA A 133 -28.524 26.157 54.148 1.00 0.00
A C
ATOM 15531 HB1 ALA A 133 -27.652 25.525 53.873 1.00 0.00
A H
ATOM 15532 HB2 ALA A 133 -29.266 25.509 54.663 1.00 0.00
A H
ATOM 15533 HB3 ALA A 133 -28.980 26.541 53.210 1.00 0.00
A H
ATOM 15534 C ALA A 133 -29.233 28.216 55.395 1.00 0.00
A C
ATOM 15535 O ALA A 133 -29.419 29.232 54.726 1.00 0.00
A O
ATOM 15536 N GLN A 134 -29.992 27.873 56.441 1.00 0.00
A N
ATOM 15537 HN GLN A 134 -29.872 27.021 56.948 1.00 0.00
A H
ATOM 15538 CA GLN A 134 -31.077 28.727 56.939 1.00 0.00
A C
ATOM 15539 HA GLN A 134 -31.762 28.896 56.116 1.00 0.00
A H
ATOM 15540 CB GLN A 134 -31.782 28.085 58.138 1.00 0.00
A C
ATOM 15541 HB1 GLN A 134 -31.008 27.938 58.927 1.00 0.00
A H
ATOM 15542 HB2 GLN A 134 -32.523 28.802 58.559 1.00 0.00
A H
ATOM 15543 CG GLN A 134 -32.456 26.746 57.864 1.00 0.00
A C
ATOM 15544 HG1 GLN A 134 -31.743 26.046 57.378 1.00 0.00
A H
ATOM 15545 HG2 GLN A 134 -32.801 26.279 58.809 1.00 0.00
A H
ATOM 15546 CD GLN A 134 -33.652 26.833 56.920 1.00 0.00
A C
ATOM 15547 OE1 GLN A 134 -34.333 27.863 56.833 1.00 0.00
A O
ATOM 15548 NE2 GLN A 134 -33.923 25.735 56.221 1.00 0.00
A N
ATOM 15549 HE21 GLN A 134 -34.698 25.779 55.597 1.00 0.00
A H
ATOM 15550 HE22 GLN A 134 -33.370 24.919 56.348 1.00 0.00
A H
ATOM 15551 C GLN A 134 -30.525 30.084 57.362 1.00 0.00
A C

ATOM 15552 O   GLN A 134   -31.104 31.139 57.079 1.00 0.00
A   O
ATOM 15553 N   SER A 135   -29.386 30.029 58.034 1.00 0.00
A   N
ATOM 15554 HN SER A 135   -28.934 29.157 58.221 1.00 0.00
A   H
ATOM 15555 CA SER A 135   -28.690 31.180 58.559 1.00 0.00
A   C
ATOM 15556 HA SER A 135   -29.434 31.770 59.077 1.00 0.00
A   H
ATOM 15557 CB SER A 135   -27.545 30.626 59.395 1.00 0.00
A   C
ATOM 15558 HB1 SER A 135   -27.758 29.552 59.604 1.00 0.00
A   H
ATOM 15559 HB2 SER A 135   -26.592 30.664 58.820 1.00 0.00
A   H
ATOM 15560 OG SER A 135   -27.444 31.359 60.669 1.00 0.00
A   O
ATOM 15561 HG1 SER A 135   -26.870 30.842 61.249 1.00 0.00
A   H
ATOM 15562 C   SER A 135   -28.129 32.049 57.429 1.00 0.00
A   C
ATOM 15563 O   SER A 135   -28.169 33.276 57.494 1.00 0.00
A   O
ATOM 15564 N   LEU A 136   -27.609 31.396 56.392 1.00 0.00
A   N
ATOM 15565 HN LEU A 136   -27.606 30.401 56.323 1.00 0.00
A   H
ATOM 15566 CA LEU A 136   -26.996 32.076 55.259 1.00 0.00
A   C
ATOM 15567 HA LEU A 136   -26.299 32.791 55.676 1.00 0.00
A   H
ATOM 15568 CB LEU A 136   -26.327 31.042 54.355 1.00 0.00
A   C
ATOM 15569 HB1 LEU A 136   -27.044 30.198 54.235 1.00 0.00
A   H
ATOM 15570 HB2 LEU A 136   -26.194 31.481 53.342 1.00 0.00
A   H
ATOM 15571 CG LEU A 136   -24.996 30.469 54.850 1.00 0.00
A   C
ATOM 15572 HG LEU A 136   -25.120 30.437 55.959 1.00 0.00
A   H
ATOM 15573 CD1 LEU A 136   -24.734 29.060 54.338 1.00 0.00
A   C
ATOM 15574 HD11 LEU A 136   -23.740 28.700 54.679 1.00 0.00
A   H
ATOM 15575 HD12 LEU A 136   -25.507 28.356 54.713 1.00 0.00
A   H
ATOM 15576 HD13 LEU A 136   -24.748 29.038 53.227 1.00 0.00
A   H
ATOM 15577 CD2 LEU A 136   -23.845 31.406 54.533 1.00 0.00
A   C
ATOM 15578 HD21 LEU A 136   -22.888 30.985 54.914 1.00 0.00
A   H

ATOM 15579 HD22 LEU A 136   -23.732 31.562 53.441 1.00 0.00
A   H
ATOM 15580 HD23 LEU A 136   -24.005 32.396 55.013 1.00 0.00
A   H
ATOM 15581 C   LEU A 136   -28.010 32.851 54.438 1.00 0.00
A   C
ATOM 15582 O   LEU A 136   -27.710 33.890 53.858 1.00 0.00
A   O
ATOM 15583 N   SER A 137   -29.217 32.321 54.407 1.00 0.00
A   N
ATOM 15584 HN SER A 137   -29.482 31.586 55.031 1.00 0.00
A   H
ATOM 15585 CA SER A 137   -30.258 32.727 53.502 1.00 0.00
A   C
ATOM 15586 HA SER A 137   -29.772 32.971 52.567 1.00 0.00
A   H
ATOM 15587 CB SER A 137   -31.193 31.541 53.486 1.00 0.00
A   C
ATOM 15588 HB1 SER A 137   -30.626 30.659 53.109 1.00 0.00
A   H
ATOM 15589 HB2 SER A 137   -31.529 31.297 54.519 1.00 0.00
A   H
ATOM 15590 OG SER A 137   -32.292 31.672 52.685 1.00 0.00
A   O
ATOM 15591 HG1 SER A 137   -32.678 30.792 52.586 1.00 0.00
A   H
ATOM 15592 C   SER A 137   -30.937 34.000 54.006 1.00 0.00
A   C
ATOM 15593 O   SER A 137   -31.327 34.872 53.223 1.00 0.00
A   O
ATOM 15594 N   ASP A 138   -31.059 34.091 55.326 1.00 0.00
A   N
ATOM 15595 HN ASP A 138   -30.851 33.352 55.962 1.00 0.00
A   H
ATOM 15596 CA ASP A 138   -31.517 35.297 55.990 1.00 0.00
A   C
ATOM 15597 HA ASP A 138   -31.332 36.146 55.342 1.00 0.00
A   H
ATOM 15598 CB ASP A 138   -32.992 35.167 56.379 1.00 0.00
A   C
ATOM 15599 HB1 ASP A 138   -33.517 34.557 55.618 1.00 0.00
A   H
ATOM 15600 HB2 ASP A 138   -33.094 34.664 57.360 1.00 0.00
A   H
ATOM 15601 CG ASP A 138   -33.706 36.507 56.452 1.00 0.00
A   C
ATOM 15602 OD1 ASP A 138   -33.042 37.566 56.407 1.00 0.00
A   O
ATOM 15603 OD2 ASP A 138   -34.949 36.489 56.547 1.00 0.00
A   O
ATOM 15604 C   ASP A 138   -30.641 35.481 57.218 1.00 0.00
A   C
ATOM 15605 O   ASP A 138   -30.790 34.773 58.215 1.00 0.00
A   O

ATOM 15606 N GLU A 139 -29.709 36.421 57.121 1.00 0.00
A N
ATOM 15607 HN GLU A 139 -29.619 37.025 56.334 1.00 0.00
A H
ATOM 15608 CA GLU A 139 -28.722 36.659 58.167 1.00 0.00
A C
ATOM 15609 HA GLU A 139 -28.331 35.702 58.489 1.00 0.00
A H
ATOM 15610 CB GLU A 139 -27.613 37.551 57.616 1.00 0.00
A C
ATOM 15611 HB1 GLU A 139 -27.596 37.440 56.507 1.00 0.00
A H
ATOM 15612 HB2 GLU A 139 -27.857 38.619 57.812 1.00 0.00
A H
ATOM 15613 CG GLU A 139 -26.231 37.235 58.159 1.00 0.00
A C
ATOM 15614 HG1 GLU A 139 -26.098 37.728 59.139 1.00 0.00
A H
ATOM 15615 HG2 GLU A 139 -26.090 36.142 58.277 1.00 0.00
A H
ATOM 15616 CD GLU A 139 -25.131 37.774 57.270 1.00 0.00
A C
ATOM 15617 OE1 GLU A 139 -25.108 38.997 56.995 1.00 0.00
A O
ATOM 15618 OE2 GLU A 139 -24.284 36.966 56.847 1.00 0.00
A O
ATOM 15619 C GLU A 139 -29.364 37.316 59.384 1.00 0.00
A C
ATOM 15620 O GLU A 139 -28.887 37.175 60.512 1.00 0.00
A O
ATOM 15621 N ASN A 140 -30.460 38.020 59.131 1.00 0.00
A N
ATOM 15622 HN ASN A 140 -30.862 38.056 58.217 1.00 0.00
A H
ATOM 15623 CA ASN A 140 -31.176 38.796 60.138 1.00 0.00
A C
ATOM 15624 HA ASN A 140 -30.400 39.459 60.508 1.00 0.00
A H
ATOM 15625 CB ASN A 140 -32.257 39.647 59.457 1.00 0.00
A C
ATOM 15626 HB1 ASN A 140 -32.622 39.085 58.569 1.00 0.00
A H
ATOM 15627 HB2 ASN A 140 -33.121 39.790 60.138 1.00 0.00
A H
ATOM 15628 CG ASN A 140 -31.749 40.991 58.978 1.00 0.00
A C
ATOM 15629 OD1 ASN A 140 -31.071 41.690 59.669 1.00 0.00
A O
ATOM 15630 ND2 ASN A 140 -32.137 41.362 57.788 1.00 0.00
A N
ATOM 15631 HD21 ASN A 140 -31.805 42.238 57.450 1.00 0.00
A H
ATOM 15632 HD22 ASN A 140 -32.718 40.764 57.244 1.00 0.00
A H

```
ATOM  15633  C   ASN A 140     -31.798  38.062  61.369  1.00  0.00
A    C
ATOM  15634  O   ASN A 140     -31.756  38.604  62.455  1.00  0.00
A    O
ATOM  15635  N   VAL A 141     -32.379  36.864  61.215  1.00  0.00
A    N
ATOM  15636  HN  VAL A 141     -32.367  36.448  60.309  1.00  0.00
A    H
ATOM  15637  CA  VAL A 141     -33.067  36.045  62.262  1.00  0.00
A    C
ATOM  15638  HA  VAL A 141     -33.745  36.717  62.774  1.00  0.00
A    H
ATOM  15639  CB  VAL A 141     -33.736  34.860  61.511  1.00  0.00
A    C
ATOM  15640  HB  VAL A 141     -33.000  34.477  60.761  1.00  0.00
A    H
ATOM  15641  CG1 VAL A 141     -34.127  33.685  62.402  1.00  0.00
A    C
ATOM  15642 HG11 VAL A 141     -34.633  32.897  61.803  1.00  0.00
A    H
ATOM  15643 HG12 VAL A 141     -33.240  33.219  62.880  1.00  0.00
A    H
ATOM  15644 HG13 VAL A 141     -34.829  34.013  63.199  1.00  0.00
A    H
ATOM  15645  CG2 VAL A 141     -34.854  35.342  60.582  1.00  0.00
A    C
ATOM  15646 HG21 VAL A 141     -35.348  34.481  60.083  1.00  0.00
A    H
ATOM  15647 HG22 VAL A 141     -35.623  35.903  61.155  1.00  0.00
A    H
ATOM  15648 HG23 VAL A 141     -34.457  36.007  59.787  1.00  0.00
A    H
ATOM  15649  C   VAL A 141     -32.109  35.457  63.322  1.00  0.00
A    C
ATOM  15650  O   VAL A 141     -31.050  34.939  62.960  1.00  0.00
A    O
ATOM  15651  N   LEU A 142     -32.467  35.522  64.609  1.00  0.00
A    N
ATOM  15652  HN  LEU A 142     -33.296  35.970  64.939  1.00  0.00
A    H
ATOM  15653  CA  LEU A 142     -31.640  34.917  65.672  1.00  0.00
A    C
ATOM  15654  HA  LEU A 142     -30.634  34.847  65.280  1.00  0.00
A    H
ATOM  15655  CB  LEU A 142     -31.658  35.761  66.950  1.00  0.00
A    C
ATOM  15656  HB1 LEU A 142     -32.703  35.750  67.337  1.00  0.00
A    H
ATOM  15657  HB2 LEU A 142     -31.037  35.257  67.724  1.00  0.00
A    H
ATOM  15658  CG  LEU A 142     -31.232  37.230  66.878  1.00  0.00
A    C
ATOM  15659  HG  LEU A 142     -31.789  37.676  66.019  1.00  0.00
A    H
```

ATOM 15660 CD1 LEU A 142   -31.618 37.947 68.163 1.00 0.00
A   C
ATOM 15661 HD11 LEU A 142   -31.270 39.002 68.140 1.00 0.00
A   H
ATOM 15662 HD12 LEU A 142   -32.721 37.946 68.296 1.00 0.00
A   H
ATOM 15663 HD13 LEU A 142   -31.158 37.450 69.043 1.00 0.00
A   H
ATOM 15664 CD2 LEU A 142   -29.742 37.380 66.601 1.00 0.00
A   C
ATOM 15665 HD21 LEU A 142   -29.468 38.455 66.536 1.00 0.00
A   H
ATOM 15666 HD22 LEU A 142   -29.128 36.924 67.403 1.00 0.00
A   H
ATOM 15667 HD23 LEU A 142   -29.474 36.894 65.638 1.00 0.00
A   H
ATOM 15668 C   LEU A 142   -32.103 33.506 66.001 1.00 0.00
A   C
ATOM 15669 O   LEU A 142   -31.293 32.623 66.269 1.00 0.00
A   O
ATOM 15670 N   ASN A 143   -33.417 33.315 65.992 1.00 0.00
A   N
ATOM 15671 HN ASN A 143   -34.060 34.051 65.783 1.00 0.00
A   H
ATOM 15672 CA ASN A 143   -34.043 32.032 66.281 1.00 0.00
A   C
ATOM 15673 HA ASN A 143   -33.385 31.273 65.870 1.00 0.00
A   H
ATOM 15674 CB ASN A 143   -34.266 31.866 67.787 1.00 0.00
A   C
ATOM 15675 HB1 ASN A 143   -34.018 32.833 68.277 1.00 0.00
A   H
ATOM 15676 HB2 ASN A 143   -35.333 31.649 67.998 1.00 0.00
A   H
ATOM 15677 CG ASN A 143   -33.384 30.792 68.400 1.00 0.00
A   C
ATOM 15678 OD1 ASN A 143   -32.838 29.941 67.699 1.00 0.00
A   O
ATOM 15679 ND2 ASN A 143   -33.256 30.816 69.721 1.00 0.00
A   N
ATOM 15680 HD21 ASN A 143   -32.672 30.126 70.137 1.00 0.00
A   H
ATOM 15681 HD22 ASN A 143   -33.711 31.524 70.252 1.00 0.00
A   H
ATOM 15682 C   ASN A 143   -35.371 31.923 65.564 1.00 0.00
A   C
ATOM 15683 O   ASN A 143   -36.003 32.932 65.261 1.00 0.00
A   O
ATOM 15684 N   TYR A 144   -35.798 30.697 65.296 1.00 0.00
A   N
ATOM 15685 HN TYR A 144   -35.297 29.867 65.536 1.00 0.00
A   H
ATOM 15686 CA TYR A 144   -37.063 30.464 64.611 1.00 0.00
A   C

ATOM 15687 HA TYR A 144   -37.744 31.237 64.948 1.00 0.00
A   H
ATOM 15688 CB TYR A 144   -36.870 30.510 63.087 1.00 0.00
A   C
ATOM 15689 HB1 TYR A 144   -37.867 30.507 62.594 1.00 0.00
A   H
ATOM 15690 HB2 TYR A 144   -36.356 31.448 62.783 1.00 0.00
A   H
ATOM 15691 CG TYR A 144   -36.109 29.321 62.546 1.00 0.00
A   C
ATOM 15692 CD1 TYR A 144   -36.783 28.237 61.982 1.00 0.00
A   C
ATOM 15693 HD1 TYR A 144   -37.863 28.241 61.934 1.00 0.00
A   H
ATOM 15694 CE1 TYR A 144   -36.091 27.138 61.499 1.00 0.00
A   C
ATOM 15695 HE1 TYR A 144   -36.619 26.302 61.066 1.00 0.00
A   H
ATOM 15696 CZ TYR A 144   -34.704 27.112 61.582 1.00 0.00
A   C
ATOM 15697 OH TYR A 144   -34.007 26.028 61.105 1.00 0.00
A   O
ATOM 15698 HH TYR A 144   -33.072 26.230 61.186 1.00 0.00
A   H
ATOM 15699 CD2 TYR A 144   -34.716 29.266 62.619 1.00 0.00
A   C
ATOM 15700 HD2 TYR A 144   -34.168 30.070 63.091 1.00 0.00
A   H
ATOM 15701 CE2 TYR A 144   -34.015 28.172 62.141 1.00 0.00
A   C
ATOM 15702 HE2 TYR A 144   -32.939 28.145 62.227 1.00 0.00
A   H
ATOM 15703 C   TYR A 144   -37.666 29.126 65.018 1.00 0.00
A   C
ATOM 15704 O   TYR A 144   -36.957 28.235 65.486 1.00 0.00
A   O
ATOM 15705 N   ILE A 145   -38.979 29.002 64.841 1.00 0.00
A   N
ATOM 15706 HN ILE A 145   -39.568 29.773 64.609 1.00 0.00
A   H
ATOM 15707 CA ILE A 145   -39.683 27.725 64.969 1.00 0.00
A   C
ATOM 15708 HA ILE A 145   -38.942 26.938 64.904 1.00 0.00
A   H
ATOM 15709 CB ILE A 145   -40.497 27.624 66.286 1.00 0.00
A   C
ATOM 15710 HB ILE A 145   -41.022 28.610 66.367 1.00 0.00
A   H
ATOM 15711 CG2 ILE A 145   -41.427 26.421 66.258 1.00 0.00
A   C
ATOM 15712 HG21 ILE A 145   -42.116 26.440 67.128 1.00 0.00
A   H
ATOM 15713 HG22 ILE A 145   -42.049 26.421 65.338 1.00 0.00
A   H

ATOM 15714 HG23 ILE A 145   -40.851 25.472 66.293 1.00 0.00
A   H
ATOM 15715 CG1 ILE A 145   -39.575 27.522 67.502 1.00 0.00
A   C
ATOM 15716 HG11 ILE A 145   -39.010 26.564 67.467 1.00 0.00
A   H
ATOM 15717 HG12 ILE A 145   -38.828 28.348 67.459 1.00 0.00
A   H
ATOM 15718 CD ILE A 145   -40.313 27.616 68.819 1.00 0.00
A   C
ATOM 15719 HD1 ILE A 145   -39.593 27.595 69.666 1.00 0.00
A   H
ATOM 15720 HD2 ILE A 145   -40.891 28.564 68.878 1.00 0.00
A   H
ATOM 15721 HD3 ILE A 145   -41.016 26.766 68.944 1.00 0.00
A   H
ATOM 15722 C   ILE A 145   -40.613 27.556 63.768 1.00 0.00
A   C
ATOM 15723 O   ILE A 145   -41.477 28.401 63.518 1.00 0.00
A   O
ATOM 15724 N   ASP A 146   -40.421 26.467 63.025 1.00 0.00
A   N
ATOM 15725 HN ASP A 146   -39.703 25.793 63.180 1.00 0.00
A   H
ATOM 15726 CA ASP A 146   -41.267 26.144 61.876 1.00 0.00
A   C
ATOM 15727 HA ASP A 146   -41.548 27.087 61.421 1.00 0.00
A   H
ATOM 15728 CB ASP A 146   -40.515 25.272 60.867 1.00 0.00
A   C
ATOM 15729 HB1 ASP A 146   -39.690 24.742 61.385 1.00 0.00
A   H
ATOM 15730 HB2 ASP A 146   -41.192 24.514 60.425 1.00 0.00
A   H
ATOM 15731 CG ASP A 146   -39.897 26.079 59.742 1.00 0.00
A   C
ATOM 15732 OD1 ASP A 146   -40.428 27.159 59.409 1.00 0.00
A   O
ATOM 15733 OD2 ASP A 146   -38.883 25.623 59.177 1.00 0.00
A   O
ATOM 15734 C   ASP A 146   -42.561 25.457 62.299 1.00 0.00
A   C
ATOM 15735 O   ASP A 146   -42.544 24.350 62.838 1.00 0.00
A   O
ATOM 15736 N   LEU A 147   -43.679 26.129 62.041 1.00 0.00
A   N
ATOM 15737 HN LEU A 147   -43.689 27.021 61.593 1.00 0.00
A   H
ATOM 15738 CA LEU A 147   -44.997 25.621 62.390 1.00 0.00
A   C
ATOM 15739 HA LEU A 147   -44.869 24.987 63.257 1.00 0.00
A   H
ATOM 15740 CB LEU A 147   -45.940 26.789 62.671 1.00 0.00
A   C

ATOM 15741 HB1 LEU A 147   -45.674 27.606 61.961 1.00 0.00
A   H
ATOM 15742 HB2 LEU A 147   -46.979 26.485 62.415 1.00 0.00
A   H
ATOM 15743 CG LEU A 147   -45.969 27.428 64.068 1.00 0.00
A   C
ATOM 15744 HG LEU A 147   -46.581 26.635 64.562 1.00 0.00
A   H
ATOM 15745 CD1 LEU A 147   -44.639 27.558 64.806 1.00 0.00
A   C
ATOM 15746 HD11 LEU A 147   -44.782 28.076 65.780 1.00 0.00
A   H
ATOM 15747 HD12 LEU A 147   -44.202 26.556 65.004 1.00 0.00
A   H
ATOM 15748 HD13 LEU A 147   -43.912 28.145 64.205 1.00 0.00
A   H
ATOM 15749 CD2 LEU A 147   -46.768 28.722 64.094 1.00 0.00
A   C
ATOM 15750 HD21 LEU A 147   -46.784 29.146 65.121 1.00 0.00
A   H
ATOM 15751 HD22 LEU A 147   -46.331 29.487 63.421 1.00 0.00
A   H
ATOM 15752 HD23 LEU A 147   -47.818 28.538 63.777 1.00 0.00
A   H
ATOM 15753 C   LEU A 147   -45.558 24.758 61.269 1.00 0.00
A   C
ATOM 15754 O   LEU A 147   -46.088 23.677 61.514 1.00 0.00
A   O
ATOM 15755 N   SER A 152   -44.214 30.350 60.365 1.00 0.00
A   N
ATOM 15756 HN SER A 152   -44.451 31.219 59.931 1.00 0.00
A   H
ATOM 15757 CA SER A 152   -42.991 30.392 61.155 1.00 0.00
A   C
ATOM 15758 HA SER A 152   -42.919 29.427 61.639 1.00 0.00
A   H
ATOM 15759 CB SER A 152   -41.784 30.666 60.251 1.00 0.00
A   C
ATOM 15760 HB1 SER A 152   -41.665 29.800 59.561 1.00 0.00
A   H
ATOM 15761 HB2 SER A 152   -41.969 31.565 59.620 1.00 0.00
A   H
ATOM 15762 OG SER A 152   -40.593 30.810 61.008 1.00 0.00
A   O
ATOM 15763 HG1 SER A 152   -39.855 30.756 60.388 1.00 0.00
A   H
ATOM 15764 C   SER A 152   -43.064 31.448 62.256 1.00 0.00
A   C
ATOM 15765 O   SER A 152   -43.558 32.556 62.032 1.00 0.00
A   O
ATOM 15766 N   ILE A 153   -42.586 31.091 63.448 1.00 0.00
A   N
ATOM 15767 HN ILE A 153   -42.371 30.149 63.700 1.00 0.00
A   H

ATOM 15768 CA ILE A 153   -42.334 32.066 64.507 1.00 0.00
A   C
ATOM 15769 HA ILE A 153   -42.905 32.959 64.285 1.00 0.00
A   H
ATOM 15770 CB ILE A 153   -42.652 31.521 65.918 1.00 0.00
A   C
ATOM 15771 HB ILE A 153   -42.031 30.593 66.006 1.00 0.00
A   H
ATOM 15772 CG2 ILE A 153   -42.343 32.567 66.984 1.00 0.00
A   C
ATOM 15773 HG21 ILE A 153   -42.421 32.125 67.999 1.00 0.00
A   H
ATOM 15774 HG22 ILE A 153   -41.311 32.960 66.867 1.00 0.00
A   H
ATOM 15775 HG23 ILE A 153   -43.052 33.419 66.921 1.00 0.00
A   H
ATOM 15776 CG1 ILE A 153   -44.116 31.083 66.007 1.00 0.00
A   C
ATOM 15777 HG11 ILE A 153   -44.781 31.967 65.887 1.00 0.00
A   H
ATOM 15778 HG12 ILE A 153   -44.337 30.388 65.164 1.00 0.00
A   H
ATOM 15779 CD ILE A 153   -44.480 30.375 67.296 1.00 0.00
A   C
ATOM 15780 HD1 ILE A 153   -45.538 30.037 67.265 1.00 0.00
A   H
ATOM 15781 HD2 ILE A 153   -43.835 29.484 67.449 1.00 0.00
A   H
ATOM 15782 HD3 ILE A 153   -44.361 31.051 68.168 1.00 0.00
A   H
ATOM 15783 C   ILE A 153   -40.864 32.450 64.418 1.00 0.00
A   C
ATOM 15784 O   ILE A 153   -39.995 31.582 64.361 1.00 0.00
A   O
ATOM 15785 N   VAL A 154   -40.590 33.749 64.397 1.00 0.00
A   N
ATOM 15786 HN VAL A 154   -41.264 34.472 64.523 1.00 0.00
A   H
ATOM 15787 CA VAL A 154   -39.234 34.223 64.179 1.00 0.00
A   C
ATOM 15788 HA VAL A 154   -38.582 33.363 64.267 1.00 0.00
A   H
ATOM 15789 CB VAL A 154   -39.078 34.832 62.769 1.00 0.00
A   C
ATOM 15790 HB VAL A 154   -39.750 35.724 62.711 1.00 0.00
A   H
ATOM 15791 CG1 VAL A 154   -37.647 35.266 62.542 1.00 0.00
A   C
ATOM 15792 HG11 VAL A 154   -37.524 35.669 61.514 1.00 0.00
A   H
ATOM 15793 HG12 VAL A 154   -37.344 36.068 63.249 1.00 0.00
A   H
ATOM 15794 HG13 VAL A 154   -36.952 34.407 62.660 1.00 0.00
A   H

ATOM 15795 CG2 VAL A 154   -39.482 33.837 61.693 1.00 0.00
A   C
ATOM 15796 HG21 VAL A 154   -39.329 34.269 60.681 1.00 0.00
A   H
ATOM 15797 HG22 VAL A 154   -38.876 32.909 61.773 1.00 0.00
A   H
ATOM 15798 HG23 VAL A 154   -40.554 33.563 61.782 1.00 0.00
A   H
ATOM 15799 C   VAL A 154   -38.803 35.236 65.234 1.00 0.00
A   C
ATOM 15800 O   VAL A 154   -39.490 36.225 65.472 1.00 0.00
A   O
ATOM 15801 N   GLU A 155   -37.665 34.964 65.868 1.00 0.00
A   N
ATOM 15802 HN GLU A 155   -37.188 34.092 65.796 1.00 0.00
A   H
ATOM 15803 CA GLU A 155   -37.006 35.930 66.736 1.00 0.00
A   C
ATOM 15804 HA GLU A 155   -37.744 36.602 67.157 1.00 0.00
A   H
ATOM 15805 CB GLU A 155   -36.239 35.220 67.859 1.00 0.00
A   C
ATOM 15806 HB1 GLU A 155   -36.957 34.578 68.418 1.00 0.00
A   H
ATOM 15807 HB2 GLU A 155   -35.486 34.526 67.422 1.00 0.00
A   H
ATOM 15808 CG GLU A 155   -35.554 36.156 68.848 1.00 0.00
A   C
ATOM 15809 HG1 GLU A 155   -34.892 36.851 68.299 1.00 0.00
A   H
ATOM 15810 HG2 GLU A 155   -36.299 36.739 69.424 1.00 0.00
A   H
ATOM 15811 CD GLU A 155   -34.652 35.435 69.836 1.00 0.00
A   C
ATOM 15812 OE1 GLU A 155   -33.767 36.092 70.422 1.00 0.00
A   O
ATOM 15813 OE2 GLU A 155   -34.822 34.213 70.033 1.00 0.00
A   O
ATOM 15814 C   GLU A 155   -36.062 36.771 65.881 1.00 0.00
A   C
ATOM 15815 O   GLU A 155   -35.124 36.246 65.285 1.00 0.00
A   O
ATOM 15816 N   LEU A 156   -36.325 38.073 65.814 1.00 0.00
A   N
ATOM 15817 HN LEU A 156   -37.075 38.511 66.304 1.00 0.00
A   H
ATOM 15818 CA LEU A 156   -35.522 38.988 65.003 1.00 0.00
A   C
ATOM 15819 HA LEU A 156   -34.782 38.384 64.495 1.00 0.00
A   H
ATOM 15820 CB LEU A 156   -36.403 39.764 64.023 1.00 0.00
A   C
ATOM 15821 HB1 LEU A 156   -37.351 40.008 64.554 1.00 0.00
A   H

ATOM 15822 HB2 LEU A 156   -35.918 40.740 63.796 1.00 0.00
A   H
ATOM 15823 CG LEU A 156   -36.808 39.127 62.697 1.00 0.00
A   C
ATOM 15824 HG LEU A 156   -35.885 38.801 62.160 1.00 0.00
A   H
ATOM 15825 CD1 LEU A 156   -37.726 37.953 62.938 1.00 0.00
A   C
ATOM 15826 HD11 LEU A 156   -38.062 37.517 61.972 1.00 0.00
A   H
ATOM 15827 HD12 LEU A 156   -37.204 37.161 63.516 1.00 0.00
A   H
ATOM 15828 HD13 LEU A 156   -38.626 38.268 63.508 1.00 0.00
A   H
ATOM 15829 CD2 LEU A 156   -37.529 40.132 61.822 1.00 0.00
A   C
ATOM 15830 HD21 LEU A 156   -37.805 39.669 60.850 1.00 0.00
A   H
ATOM 15831 HD22 LEU A 156   -38.461 40.496 62.299 1.00 0.00
A   H
ATOM 15832 HD23 LEU A 156   -36.875 41.007 61.616 1.00 0.00
A   H
ATOM 15833 C   LEU A 156   -34.786 39.983 65.865 1.00 0.00
A   C
ATOM 15834 O   LEU A 156   -35.319 40.482 66.857 1.00 0.00
A   O
ATOM 15835 N   LEU A 157   -33.556 40.280 65.475 1.00 0.00
A   N
ATOM 15836 HN LEU A 157   -33.055 39.810 64.750 1.00 0.00
A   H
ATOM 15837 CA LEU A 157   -32.822 41.355 66.102 1.00 0.00
A   C
ATOM 15838 HA LEU A 157   -33.223 41.493 67.097 1.00 0.00
A   H
ATOM 15839 CB LEU A 157   -31.334 41.039 66.135 1.00 0.00
A   C
ATOM 15840 HB1 LEU A 157   -31.221 40.034 66.603 1.00 0.00
A   H
ATOM 15841 HB2 LEU A 157   -30.963 40.935 65.092 1.00 0.00
A   H
ATOM 15842 CG LEU A 157   -30.439 42.016 66.901 1.00 0.00
A   C
ATOM 15843 HG LEU A 157   -30.686 43.043 66.538 1.00 0.00
A   H
ATOM 15844 CD1 LEU A 157   -30.664 41.942 68.408 1.00 0.00
A   C
ATOM 15845 HD11 LEU A 157   -29.969 42.626 68.940 1.00 0.00
A   H
ATOM 15846 HD12 LEU A 157   -31.705 42.235 68.663 1.00 0.00
A   H
ATOM 15847 HD13 LEU A 157   -30.488 40.910 68.781 1.00 0.00
A   H
ATOM 15848 CD2 LEU A 157   -28.988 41.731 66.555 1.00 0.00
A   C

ATOM 15849 HD21 LEU A 157   -28.318 42.441 67.085 1.00 0.00
A   H
ATOM 15850 HD22 LEU A 157   -28.691 40.704 66.849 1.00 0.00
A   H
ATOM 15851 HD23 LEU A 157   -28.820 41.844 65.463 1.00 0.00
A   H
ATOM 15852 C   LEU A 157   -33.075 42.634 65.323 1.00 0.00
A   C
ATOM 15853 O   LEU A 157   -32.926 42.660 64.105 1.00 0.00
A   O
ATOM 15854 N   LEU A 162   -36.319 50.541 62.699 1.00 0.00
A   N
ATOM 15855 HN LEU A 162   -36.314 51.386 62.169 1.00 0.00
A   H
ATOM 15856 CA LEU A 162   -37.461 50.403 63.597 1.00 0.00
A   C
ATOM 15857 HA LEU A 162   -38.283 50.928 63.127 1.00 0.00
A   H
ATOM 15858 CB LEU A 162   -37.807 48.925 63.794 1.00 0.00
A   C
ATOM 15859 HB1 LEU A 162   -36.887 48.420 64.171 1.00 0.00
A   H
ATOM 15860 HB2 LEU A 162   -38.561 48.835 64.606 1.00 0.00
A   H
ATOM 15861 CG LEU A 162   -38.289 48.172 62.552 1.00 0.00
A   C
ATOM 15862 HG LEU A 162   -37.508 48.309 61.765 1.00 0.00
A   H
ATOM 15863 CD1 LEU A 162   -38.453 46.690 62.853 1.00 0.00
A   C
ATOM 15864 HD11 LEU A 162   -38.849 46.153 61.965 1.00 0.00
A   H
ATOM 15865 HD12 LEU A 162   -37.477 46.237 63.130 1.00 0.00
A   H
ATOM 15866 HD13 LEU A 162   -39.161 46.537 63.695 1.00 0.00
A   H
ATOM 15867 CD2 LEU A 162   -39.589 48.766 62.033 1.00 0.00
A   C
ATOM 15868 HD21 LEU A 162   -39.923 48.226 61.120 1.00 0.00
A   H
ATOM 15869 HD22 LEU A 162   -40.401 48.693 62.783 1.00 0.00
A   H
ATOM 15870 HD23 LEU A 162   -39.449 49.838 61.771 1.00 0.00
A   H
ATOM 15871 C   LEU A 162   -37.208 51.064 64.956 1.00 0.00
A   C
ATOM 15872 O   LEU A 162   -38.077 51.046 65.834 1.00 0.00
A   O
ATOM 15873 N   ASP A 163   -36.016 51.648 65.111 1.00 0.00
A   N
ATOM 15874 HN ASP A 163   -35.335 51.712 64.387 1.00 0.00
A   H
ATOM 15875 CA ASP A 163   -35.576 52.263 66.369 1.00 0.00
A   C

ATOM 15876 HA ASP A 163 -35.564 51.470 67.107 1.00 0.00
A H
ATOM 15877 CB ASP A 163 -34.183 52.874 66.191 1.00 0.00
A C
ATOM 15878 HB1 ASP A 163 -33.470 52.078 65.898 1.00 0.00
A H
ATOM 15879 HB2 ASP A 163 -34.193 53.645 65.395 1.00 0.00
A H
ATOM 15880 CG ASP A 163 -33.658 53.506 67.464 1.00 0.00
A C
ATOM 15881 OD1 ASP A 163 -33.429 52.770 68.445 1.00 0.00
A O
ATOM 15882 OD2 ASP A 163 -33.445 54.732 67.577 1.00 0.00
A O
ATOM 15883 C ASP A 163 -36.543 53.329 66.881 1.00 0.00
A C
ATOM 15884 O ASP A 163 -36.948 54.222 66.133 1.00 0.00
A O
ATOM 15885 N SER A 164 -36.907 53.209 68.159 1.00 0.00
A N
ATOM 15886 HN SER A 164 -36.558 52.469 68.733 1.00 0.00
A H
ATOM 15887 CA SER A 164 -37.833 54.121 68.848 1.00 0.00
A C
ATOM 15888 HA SER A 164 -37.946 53.689 69.834 1.00 0.00
A H
ATOM 15889 CB SER A 164 -37.218 55.520 68.913 1.00 0.00
A C
ATOM 15890 HB1 SER A 164 -36.945 55.826 67.877 1.00 0.00
A H
ATOM 15891 HB2 SER A 164 -37.966 56.255 69.287 1.00 0.00
A H
ATOM 15892 OG SER A 164 -36.049 55.526 69.713 1.00 0.00
A O
ATOM 15893 HG1 SER A 164 -35.590 56.357 69.538 1.00 0.00
A H
ATOM 15894 C SER A 164 -39.239 54.224 68.230 1.00 0.00
A C
ATOM 15895 O SER A 164 -39.879 55.277 68.300 1.00 0.00
A O
ATOM 15896 N LYS A 165 -39.722 53.131 67.638 1.00 0.00
A N
ATOM 15897 HN LYS A 165 -39.206 52.278 67.569 1.00 0.00
A H
ATOM 15898 CA LYS A 165 -41.060 53.100 67.038 1.00 0.00
A C
ATOM 15899 HA LYS A 165 -41.458 54.104 67.116 1.00 0.00
A H
ATOM 15900 CB LYS A 165 -40.973 52.670 65.571 1.00 0.00
A C
ATOM 15901 HB1 LYS A 165 -40.418 51.704 65.545 1.00 0.00
A H
ATOM 15902 HB2 LYS A 165 -41.997 52.452 65.196 1.00 0.00
A H

ATOM 15903 CG LYS A 165   -40.268 53.672 64.671 1.00 0.00
A   C
ATOM 15904 HG1 LYS A 165   -40.822 54.637 64.697 1.00 0.00
A   H
ATOM 15905 HG2 LYS A 165   -39.247 53.863 65.071 1.00 0.00
A   H
ATOM 15906 CD LYS A 165   -40.197 53.172 63.238 1.00 0.00
A   C
ATOM 15907 HD1 LYS A 165   -39.651 52.201 63.230 1.00 0.00
A   H
ATOM 15908 HD2 LYS A 165   -41.236 52.972 62.887 1.00 0.00
A   H
ATOM 15909 CE LYS A 165   -39.515 54.184 62.333 1.00 0.00
A   C
ATOM 15910 HE1 LYS A 165   -40.086 55.139 62.318 1.00 0.00
A   H
ATOM 15911 HE2 LYS A 165   -38.484 54.396 62.691 1.00 0.00
A   H
ATOM 15912 NZ LYS A 165   -39.415 53.696 60.930 1.00 0.00
A   N
ATOM 15913 HZ1 LYS A 165   -38.945 54.412 60.337 1.00 0.00
A   H
ATOM 15914 HZ2 LYS A 165   -38.865 52.814 60.907 1.00 0.00
A   H
ATOM 15915 HZ3 LYS A 165   -40.369 53.513 60.558 1.00 0.00
A   H
ATOM 15916 C   LYS A 165   -42.012 52.171 67.795 1.00 0.00
A   C
ATOM 15917 O   LYS A 165   -41.614 51.097 68.248 1.00 0.00
A   O
ATOM 15918 N   SER A 166   -43.264 52.601 67.925 1.00 0.00
A   N
ATOM 15919 HN SER A 166   -43.575 53.480 67.564 1.00 0.00
A   H
ATOM 15920 CA SER A 166   -44.304 51.831 68.607 1.00 0.00
A   C
ATOM 15921 HA SER A 166   -43.822 51.380 69.464 1.00 0.00
A   H
ATOM 15922 CB SER A 166   -45.458 52.745 69.026 1.00 0.00
A   C
ATOM 15923 HB1 SER A 166   -45.813 53.291 68.122 1.00 0.00
A   H
ATOM 15924 HB2 SER A 166   -46.315 52.140 69.400 1.00 0.00
A   H
ATOM 15925 OG SER A 166   -45.035 53.689 69.995 1.00 0.00
A   O
ATOM 15926 HG1 SER A 166   -45.723 54.364 70.052 1.00 0.00
A   H
ATOM 15927 C   SER A 166   -44.836 50.692 67.732 1.00 0.00
A   C
ATOM 15928 O   SER A 166   -44.539 50.633 66.535 1.00 0.00
A   O
ATOM 15929 N   ILE A 167   -45.615 49.791 68.334 1.00 0.00
A   N

ATOM 15930 HN ILE A 167   -45.794 49.789 69.316 1.00 0.00
A   H
ATOM 15931 CA ILE A 167   -46.284 48.714 67.598 1.00 0.00
A   C
ATOM 15932 HA ILE A 167   -45.516 48.220 67.017 1.00 0.00
A   H
ATOM 15933 CB ILE A 167   -46.977 47.743 68.573 1.00 0.00
A   C
ATOM 15934 HB ILE A 167   -47.681 48.388 69.157 1.00 0.00
A   H
ATOM 15935 CG2 ILE A 167   -47.666 46.622 67.812 1.00 0.00
A   C
ATOM 15936 HG21 ILE A 167   -48.293 46.013 68.497 1.00 0.00
A   H
ATOM 15937 HG22 ILE A 167   -48.329 47.030 67.022 1.00 0.00
A   H
ATOM 15938 HG23 ILE A 167   -46.921 45.951 67.335 1.00 0.00
A   H
ATOM 15939 CG1 ILE A 167   -45.963 47.175 69.571 1.00 0.00
A   C
ATOM 15940 HG11 ILE A 167   -45.548 47.998 70.196 1.00 0.00
A   H
ATOM 15941 HG12 ILE A 167   -46.488 46.473 70.258 1.00 0.00
A   H
ATOM 15942 CD ILE A 167   -44.820 46.428 68.922 1.00 0.00
A   C
ATOM 15943 HD1 ILE A 167   -44.153 45.993 69.697 1.00 0.00
A   H
ATOM 15944 HD2 ILE A 167   -45.204 45.600 68.287 1.00 0.00
A   H
ATOM 15945 HD3 ILE A 167   -44.212 47.106 68.286 1.00 0.00
A   H
ATOM 15946 C   ILE A 167   -47.303 49.272 66.601 1.00 0.00
A   C
ATOM 15947 O   ILE A 167   -47.511 48.696 65.528 1.00 0.00
A   O
ATOM 15948 N   ILE A 168   -47.928 50.393 66.967 1.00 0.00
A   N
ATOM 15949 HN ILE A 168   -47.807 50.813 67.864 1.00 0.00
A   H
ATOM 15950 CA ILE A 168   -48.851 51.114 66.086 1.00 0.00
A   C
ATOM 15951 HA ILE A 168   -49.388 50.360 65.525 1.00 0.00
A   H
ATOM 15952 CB ILE A 168   -49.802 52.001 66.912 1.00 0.00
A   C
ATOM 15953 HB ILE A 168   -50.397 52.561 66.148 1.00 0.00
A   H
ATOM 15954 CG2 ILE A 168   -50.653 51.147 67.843 1.00 0.00
A   C
ATOM 15955 HG21 ILE A 168   -51.443 51.762 68.323 1.00 0.00
A   H
ATOM 15956 HG22 ILE A 168   -51.155 50.331 67.283 1.00 0.00
A   H

```
ATOM  15957 HG23 ILE A 168     -50.032  50.693  68.644  1.00  0.00
A    H
ATOM  15958  CG1 ILE A 168     -49.010  53.039  67.706  1.00  0.00
A    C
ATOM  15959 HG11 ILE A 168     -48.394  52.531  68.481  1.00  0.00
A    H
ATOM  15960 HG12 ILE A 168     -48.308  53.562  67.018  1.00  0.00
A    H
ATOM  15961  CD  ILE A 168     -49.874  54.085  68.376  1.00  0.00
A    C
ATOM  15962  HD1 ILE A 168     -49.239  54.841  68.886  1.00  0.00
A    H
ATOM  15963  HD2 ILE A 168     -50.505  54.610  67.627  1.00  0.00
A    H
ATOM  15964  HD3 ILE A 168     -50.539  53.625  69.137  1.00  0.00
A    H
ATOM  15965  C   ILE A 168     -48.096  51.965  65.071  1.00  0.00
A    C
ATOM  15966  O   ILE A 168     -48.646  52.301  64.019  1.00  0.00
A    O
ATOM  15967  N   ASP A 169     -46.846  52.309  65.388  1.00  0.00
A    N
ATOM  15968  HN  ASP A 169     -46.399  52.092  66.252  1.00  0.00
A    H
ATOM  15969  CA  ASP A 169     -45.984  53.062  64.471  1.00  0.00
A    C
ATOM  15970  HA  ASP A 169     -46.529  53.959  64.198  1.00  0.00
A    H
ATOM  15971  CB  ASP A 169     -44.659  53.408  65.155  1.00  0.00
A    C
ATOM  15972  HB1 ASP A 169     -44.866  53.975  66.085  1.00  0.00
A    H
ATOM  15973  HB2 ASP A 169     -44.106  52.487  65.424  1.00  0.00
A    H
ATOM  15974  CG  ASP A 169     -43.765  54.271  64.288  1.00  0.00
A    C
ATOM  15975  OD1 ASP A 169     -44.179  54.619  63.163  1.00  0.00
A    O
ATOM  15976  OD2 ASP A 169     -42.629  54.651  64.648  1.00  0.00
A    O
ATOM  15977  C   ASP A 169     -45.716  52.296  63.174  1.00  0.00
A    C
ATOM  15978  O   ASP A 169     -45.556  52.901  62.111  1.00  0.00
A    O
ATOM  15979  N   LEU A 170     -45.660  50.968  63.273  1.00  0.00
A    N
ATOM  15980  HN  LEU A 170     -45.611  50.482  64.144  1.00  0.00
A    H
ATOM  15981  CA  LEU A 170     -45.665  50.099  62.100  1.00  0.00
A    C
ATOM  15982  HA  LEU A 170     -46.019  50.647  61.237  1.00  0.00
A    H
ATOM  15983  CB  LEU A 170     -44.291  49.483  61.823  1.00  0.00
A    C
```

```
ATOM  15984  HB1 LEU A 170     -44.381  48.885  60.888  1.00  0.00
A    H
ATOM  15985  HB2 LEU A 170     -43.567  50.298  61.599  1.00  0.00
A    H
ATOM  15986  CG  LEU A 170     -43.724  48.573  62.916  1.00  0.00
A    C
ATOM  15987  HG  LEU A 170     -43.983  49.041  63.896  1.00  0.00
A    H
ATOM  15988  CD1 LEU A 170     -44.344  47.186  62.834  1.00  0.00
A    C
ATOM  15989 HD11 LEU A 170     -43.897  46.514  63.596  1.00  0.00
A    H
ATOM  15990 HD12 LEU A 170     -45.441  47.236  63.011  1.00  0.00
A    H
ATOM  15991 HD13 LEU A 170     -44.171  46.738  61.832  1.00  0.00
A    H
ATOM  15992  CD2 LEU A 170     -42.210  48.495  62.818  1.00  0.00
A    C
ATOM  15993 HD21 LEU A 170     -41.804  47.848  63.626  1.00  0.00
A    H
ATOM  15994 HD22 LEU A 170     -41.883  48.067  61.849  1.00  0.00
A    H
ATOM  15995 HD23 LEU A 170     -41.759  49.505  62.924  1.00  0.00
A    H
ATOM  15996  C   LEU A 170     -46.714  49.035  62.433  1.00  0.00
A    C
ATOM  15997  O   LEU A 170     -46.503  48.195  63.313  1.00  0.00
A    O
ATOM  15998  N   ASN A 171     -47.847  49.085  61.730  1.00  0.00
A    N
ATOM  15999  HN  ASN A 171     -48.000  49.750  61.000  1.00  0.00
A    H
ATOM  16000  CA  ASN A 171     -48.971  48.171  61.965  1.00  0.00
A    C
ATOM  16001  HA  ASN A 171     -48.981  47.935  63.024  1.00  0.00
A    H
ATOM  16002  CB  ASN A 171     -50.302  48.788  61.535  1.00  0.00
A    C
ATOM  16003  HB1 ASN A 171     -50.158  49.242  60.530  1.00  0.00
A    H
ATOM  16004  HB2 ASN A 171     -51.078  48.001  61.436  1.00  0.00
A    H
ATOM  16005  CG  ASN A 171     -50.773  49.877  62.480  1.00  0.00
A    C
ATOM  16006  OD1 ASN A 171     -50.381  49.914  63.647  1.00  0.00
A    O
ATOM  16007  ND2 ASN A 171     -51.619  50.769  61.981  1.00  0.00
A    N
ATOM  16008 HD21 ASN A 171     -51.928  51.497  62.588  1.00  0.00
A    H
ATOM  16009 HD22 ASN A 171     -51.896  50.714  61.028  1.00  0.00
A    H
ATOM  16010  C   ASN A 171     -48.688  46.877  61.204  1.00  0.00
A    C
```

ATOM 16011 O   ASN A 171   -48.945 46.770 60.000 1.00 0.00
A   O
ATOM 16012 N   VAL A 172   -48.155 45.900 61.930 1.00 0.00
A   N
ATOM 16013 HN VAL A 172   -48.005 45.968 62.914 1.00 0.00
A   H
ATOM 16014 CA VAL A 172   -47.741 44.632 61.345 1.00 0.00
A   C
ATOM 16015 HA VAL A 172   -47.231 44.874 60.420 1.00 0.00
A   H
ATOM 16016 CB VAL A 172   -46.792 43.875 62.289 1.00 0.00
A   C
ATOM 16017 HB VAL A 172   -47.322 43.738 63.262 1.00 0.00
A   H
ATOM 16018 CG1 VAL A 172   -46.443 42.510 61.713 1.00 0.00
A   C
ATOM 16019 HG11 VAL A 172   -45.727 41.983 62.380 1.00 0.00
A   H
ATOM 16020 HG12 VAL A 172   -47.341 41.866 61.616 1.00 0.00
A   H
ATOM 16021 HG13 VAL A 172   -45.970 42.615 60.714 1.00 0.00
A   H
ATOM 16022 CG2 VAL A 172   -45.532 44.688 62.545 1.00 0.00
A   C
ATOM 16023 HG21 VAL A 172   -44.827 44.127 63.194 1.00 0.00
A   H
ATOM 16024 HG22 VAL A 172   -45.017 44.921 61.588 1.00 0.00
A   H
ATOM 16025 HG23 VAL A 172   -45.769 45.645 63.055 1.00 0.00
A   H
ATOM 16026 C   VAL A 172   -48.915 43.714 60.996 1.00 0.00
A   C
ATOM 16027 O   VAL A 172   -48.805 42.898 60.082 1.00 0.00
A   O
ATOM 16028 N   ARG A 173   -50.028 43.846 61.717 1.00 0.00
A   N
ATOM 16029 HN ARG A 173   -50.085 44.484 62.481 1.00 0.00
A   H
ATOM 16030 CA ARG A 173   -51.227 43.060 61.426 1.00 0.00
A   C
ATOM 16031 HA ARG A 173   -50.934 42.026 61.284 1.00 0.00
A   H
ATOM 16032 CB ARG A 173   -52.232 43.166 62.574 1.00 0.00
A   C
ATOM 16033 HB1 ARG A 173   -52.363 44.255 62.778 1.00 0.00
A   H
ATOM 16034 HB2 ARG A 173   -53.225 42.797 62.234 1.00 0.00
A   H
ATOM 16035 CG ARG A 173   -51.787 42.475 63.853 1.00 0.00
A   C
ATOM 16036 HG1 ARG A 173   -51.324 41.500 63.587 1.00 0.00
A   H
ATOM 16037 HG2 ARG A 173   -50.973 43.074 64.324 1.00 0.00
A   H

ATOM 16038 CD ARG A 173 -52.891 42.308 64.886 1.00 0.00
A C
ATOM 16039 HD1 ARG A 173 -53.732 41.731 64.439 1.00 0.00
A H
ATOM 16040 HD2 ARG A 173 -52.527 41.766 65.790 1.00 0.00
A H
ATOM 16041 NE ARG A 173 -53.401 43.591 65.356 1.00 0.00
A N
ATOM 16042 HE ARG A 173 -54.211 43.906 64.846 1.00 0.00
A H
ATOM 16043 CZ ARG A 173 -52.867 44.286 66.349 1.00 0.00
A C
ATOM 16044 NH1 ARG A 173 -51.795 43.827 66.983 1.00 0.00
A N
ATOM 16045 HH11 ARG A 173 -51.405 44.328 67.743 1.00 0.00
A H
ATOM 16046 HH12 ARG A 173 -51.499 42.900 66.741 1.00 0.00
A H
ATOM 16047 NH2 ARG A 173 -53.398 45.448 66.711 1.00 0.00
A N
ATOM 16048 HH21 ARG A 173 -52.947 46.006 67.393 1.00 0.00
A H
ATOM 16049 HH22 ARG A 173 -54.116 45.812 66.132 1.00 0.00
A H
ATOM 16050 C ARG A 173 -51.873 43.514 60.117 1.00 0.00
A C
ATOM 16051 O ARG A 173 -52.365 42.693 59.342 1.00 0.00
A O
ATOM 16052 N ALA A 174 -51.860 44.824 59.878 1.00 0.00
A N
ATOM 16053 HN ALA A 174 -51.469 45.484 60.519 1.00 0.00
A H
ATOM 16054 CA ALA A 174 -52.418 45.392 58.658 1.00 0.00
A C
ATOM 16055 HA ALA A 174 -53.312 44.858 58.358 1.00 0.00
A H
ATOM 16056 CB ALA A 174 -52.796 46.848 58.875 1.00 0.00
A C
ATOM 16057 HB1 ALA A 174 -53.552 46.919 59.686 1.00 0.00
A H
ATOM 16058 HB2 ALA A 174 -51.911 47.449 59.179 1.00 0.00
A H
ATOM 16059 HB3 ALA A 174 -53.233 47.298 57.958 1.00 0.00
A H
ATOM 16060 C ALA A 174 -51.431 45.261 57.504 1.00 0.00
A C
ATOM 16061 O ALA A 174 -51.718 44.601 56.508 1.00 0.00
A O
ATOM 16062 N GLY A 177 -49.288 41.682 57.726 1.00 0.00
A N
ATOM 16063 HN GLY A 177 -48.368 41.353 57.516 1.00 0.00
A H
ATOM 16064 CA GLY A 177 -50.203 40.690 58.267 1.00 0.00
A C

ATOM 16065 HA1 GLY A 177 -50.570 40.123 57.423 1.00 0.00
A H
ATOM 16066 HA2 GLY A 177 -50.968 41.235 58.803 1.00 0.00
A H
ATOM 16067 C GLY A 177 -49.555 39.721 59.236 1.00 0.00
A C
ATOM 16068 O GLY A 177 -49.916 38.542 59.270 1.00 0.00
A O
ATOM 16069 N CYS A 178 -48.605 40.211 60.034 1.00 0.00
A N
ATOM 16070 HN CYS A 178 -48.280 41.153 59.984 1.00 0.00
A H
ATOM 16071 CA CYS A 178 -47.955 39.392 61.059 1.00 0.00
A C
ATOM 16072 HA CYS A 178 -48.416 38.413 61.006 1.00 0.00
A H
ATOM 16073 CB CYS A 178 -46.456 39.278 60.790 1.00 0.00
A C
ATOM 16074 HB1 CYS A 178 -46.053 40.314 60.722 1.00 0.00
A H
ATOM 16075 HB2 CYS A 178 -45.951 38.809 61.662 1.00 0.00
A H
ATOM 16076 SG CYS A 178 -46.091 38.403 59.255 1.00 0.00
A S
ATOM 16077 HG1 CYS A 178 -44.766 38.544 59.295 1.00 0.00
A H
ATOM 16078 C CYS A 178 -48.204 39.946 62.455 1.00 0.00
A C
ATOM 16079 O CYS A 178 -48.414 41.147 62.626 1.00 0.00
A O
ATOM 16080 N THR A 179 -48.191 39.062 63.447 1.00 0.00
A N
ATOM 16081 HN THR A 179 -48.019 38.085 63.323 1.00 0.00
A H
ATOM 16082 CA THR A 179 -48.433 39.457 64.831 1.00 0.00
A C
ATOM 16083 HA THR A 179 -48.763 40.486 64.790 1.00 0.00
A H
ATOM 16084 CB THR A 179 -49.480 38.546 65.508 1.00 0.00
A C
ATOM 16085 HB THR A 179 -49.375 38.718 66.607 1.00 0.00
A H
ATOM 16086 OG1 THR A 179 -49.162 37.175 65.240 1.00 0.00
A O
ATOM 16087 HG1 THR A 179 -49.852 36.662 65.676 1.00 0.00
A H
ATOM 16088 CG2 THR A 179 -50.881 38.850 64.982 1.00 0.00
A C
ATOM 16089 HG21 THR A 179 -51.648 38.278 65.547 1.00 0.00
A H
ATOM 16090 HG22 THR A 179 -51.111 39.931 65.093 1.00 0.00
A H
ATOM 16091 HG23 THR A 179 -50.971 38.583 63.907 1.00 0.00
A H

ATOM 16092 C   THR A 179   -47.145 39.446 65.651 1.00 0.00
A   C
ATOM 16093 O   THR A 179   -46.523 38.397 65.831 1.00 0.00
A   O
ATOM 16094 N   ILE A 180   -46.744 40.624 66.132 1.00 0.00
A   N
ATOM 16095 HN ILE A 180   -47.199 41.489 65.932 1.00 0.00
A   H
ATOM 16096 CA ILE A 180   -45.580 40.747 67.010 1.00 0.00
A   C
ATOM 16097 HA ILE A 180   -44.794 40.133 66.589 1.00 0.00
A   H
ATOM 16098 CB ILE A 180   -45.100 42.211 67.162 1.00 0.00
A   C
ATOM 16099 HB ILE A 180   -46.027 42.784 67.416 1.00 0.00
A   H
ATOM 16100 CG2 ILE A 180   -44.005 42.314 68.222 1.00 0.00
A   C
ATOM 16101 HG21 ILE A 180   -43.773 43.377 68.441 1.00 0.00
A   H
ATOM 16102 HG22 ILE A 180   -44.327 41.837 69.171 1.00 0.00
A   H
ATOM 16103 HG23 ILE A 180   -43.072 41.819 67.879 1.00 0.00
A   H
ATOM 16104 CG1 ILE A 180   -44.598 42.753 65.818 1.00 0.00
A   C
ATOM 16105 HG11 ILE A 180   -43.703 42.177 65.491 1.00 0.00
A   H
ATOM 16106 HG12 ILE A 180   -45.388 42.598 65.049 1.00 0.00
A   H
ATOM 16107 CD ILE A 180   -44.245 44.225 65.825 1.00 0.00
A   C
ATOM 16108 HD1 ILE A 180   -43.947 44.555 64.807 1.00 0.00
A   H
ATOM 16109 HD2 ILE A 180   -45.117 44.835 66.147 1.00 0.00
A   H
ATOM 16110 HD3 ILE A 180   -43.398 44.428 66.514 1.00 0.00
A   H
ATOM 16111 C   ILE A 180   -45.920 40.156 68.372 1.00 0.00
A   C
ATOM 16112 O   ILE A 180   -46.819 40.636 69.069 1.00 0.00
A   O
ATOM 16113 N   LEU A 181   -45.197 39.104 68.740 1.00 0.00
A   N
ATOM 16114 HN LEU A 181   -44.431 38.739 68.216 1.00 0.00
A   H
ATOM 16115 CA LEU A 181   -45.477 38.382 69.972 1.00 0.00
A   C
ATOM 16116 HA LEU A 181   -46.544 38.469 70.125 1.00 0.00
A   H
ATOM 16117 CB LEU A 181   -45.145 36.890 69.833 1.00 0.00
A   C
ATOM 16118 HB1 LEU A 181   -44.102 36.820 69.447 1.00 0.00
A   H

ATOM 16119 HB2 LEU A 181 -45.131 36.427 70.844 1.00 0.00
A H
ATOM 16120 CG LEU A 181 -46.037 36.052 68.908 1.00 0.00
A C
ATOM 16121 HG LEU A 181 -46.034 36.570 67.919 1.00 0.00
A H
ATOM 16122 CD1 LEU A 181 -45.466 34.653 68.757 1.00 0.00
A C
ATOM 16123 HD11 LEU A 181 -46.132 34.026 68.127 1.00 0.00
A H
ATOM 16124 HD12 LEU A 181 -44.464 34.689 68.277 1.00 0.00
A H
ATOM 16125 HD13 LEU A 181 -45.362 34.162 69.748 1.00 0.00
A H
ATOM 16126 CD2 LEU A 181 -47.475 35.986 69.400 1.00 0.00
A C
ATOM 16127 HD21 LEU A 181 -48.096 35.388 68.698 1.00 0.00
A H
ATOM 16128 HD22 LEU A 181 -47.546 35.512 70.400 1.00 0.00
A H
ATOM 16129 HD23 LEU A 181 -47.912 37.007 69.466 1.00 0.00
A H
ATOM 16130 C LEU A 181 -44.788 38.975 71.190 1.00 0.00
A C
ATOM 16131 O LEU A 181 -45.369 38.956 72.264 1.00 0.00
A O
ATOM 16132 N ALA A 182 -43.572 39.501 71.032 1.00 0.00
A N
ATOM 16133 HN ALA A 182 -43.102 39.537 70.151 1.00 0.00
A H
ATOM 16134 CA ALA A 182 -42.828 40.074 72.170 1.00 0.00
A C
ATOM 16135 HA ALA A 182 -43.524 40.604 72.810 1.00 0.00
A H
ATOM 16136 CB ALA A 182 -42.234 38.966 73.030 1.00 0.00
A C
ATOM 16137 HB1 ALA A 182 -43.038 38.266 73.343 1.00 0.00
A H
ATOM 16138 HB2 ALA A 182 -41.476 38.383 72.463 1.00 0.00
A H
ATOM 16139 HB3 ALA A 182 -41.756 39.371 73.947 1.00 0.00
A H
ATOM 16140 C ALA A 182 -41.739 41.080 71.790 1.00 0.00
A C
ATOM 16141 O ALA A 182 -41.312 41.146 70.639 1.00 0.00
A O
ATOM 16142 N ILE A 183 -41.311 41.865 72.775 1.00 0.00
A N
ATOM 16143 HN ILE A 183 -41.765 41.908 73.662 1.00 0.00
A H
ATOM 16144 CA ILE A 183 -40.142 42.737 72.657 1.00 0.00
A C
ATOM 16145 HA ILE A 183 -39.633 42.488 71.734 1.00 0.00
A H

ATOM 16146 CB ILE A 183   -40.504 44.244 72.762 1.00 0.00
A   C
ATOM 16147 HB ILE A 183   -40.856 44.374 73.817 1.00 0.00
A   H
ATOM 16148 CG2 ILE A 183   -39.304 45.118 72.413 1.00 0.00
A   C
ATOM 16149 HG21 ILE A 183   -39.519 46.183 72.639 1.00 0.00
A   H
ATOM 16150 HG22 ILE A 183   -38.413 44.821 73.005 1.00 0.00
A   H
ATOM 16151 HG23 ILE A 183   -39.053 45.036 71.335 1.00 0.00
A   H
ATOM 16152 CG1 ILE A 183   -41.693 44.601 71.862 1.00 0.00
A   C
ATOM 16153 HG11 ILE A 183   -41.366 44.631 70.799 1.00 0.00
A   H
ATOM 16154 HG12 ILE A 183   -42.464 43.800 71.947 1.00 0.00
A   H
ATOM 16155 CD ILE A 183   -42.352 45.923 72.207 1.00 0.00
A   C
ATOM 16156 HD1 ILE A 183   -43.238 46.093 71.557 1.00 0.00
A   H
ATOM 16157 HD2 ILE A 183   -42.691 45.927 73.266 1.00 0.00
A   H
ATOM 16158 HD3 ILE A 183   -41.649 46.769 72.058 1.00 0.00
A   H
ATOM 16159 C   ILE A 183   -39.186 42.386 73.796 1.00 0.00
A   C
ATOM 16160 O   ILE A 183   -39.622 42.067 74.905 1.00 0.00
A   O
ATOM 16161 N   LYS A 184   -37.886 42.439 73.524 1.00 0.00
A   N
ATOM 16162 HN LYS A 184   -37.513 42.603 72.612 1.00 0.00
A   H
ATOM 16163 CA LYS A 184   -36.891 42.252 74.575 1.00 0.00
A   C
ATOM 16164 HA LYS A 184   -37.434 42.115 75.503 1.00 0.00
A   H
ATOM 16165 CB LYS A 184   -36.029 41.013 74.316 1.00 0.00
A   C
ATOM 16166 HB1 LYS A 184   -36.723 40.149 74.201 1.00 0.00
A   H
ATOM 16167 HB2 LYS A 184   -35.511 41.130 73.338 1.00 0.00
A   H
ATOM 16168 CG LYS A 184   -35.034 40.715 75.427 1.00 0.00
A   C
ATOM 16169 HG1 LYS A 184   -34.440 41.633 75.639 1.00 0.00
A   H
ATOM 16170 HG2 LYS A 184   -35.594 40.460 76.356 1.00 0.00
A   H
ATOM 16171 CD LYS A 184   -34.084 39.586 75.062 1.00 0.00
A   C
ATOM 16172 HD1 LYS A 184   -34.670 38.642 74.986 1.00 0.00
A   H

ATOM 16173 HD2 LYS A 184 −33.663 39.801 74.052 1.00 0.00
A H
ATOM 16174 CE LYS A 184 −32.977 39.467 76.096 1.00 0.00
A C
ATOM 16175 HE1 LYS A 184 −32.163 40.195 75.882 1.00 0.00
A H
ATOM 16176 HE2 LYS A 184 −33.372 39.671 77.116 1.00 0.00
A H
ATOM 16177 NZ LYS A 184 −32.372 38.109 76.111 1.00 0.00
A N
ATOM 16178 HZ1 LYS A 184 −31.619 38.062 76.827 1.00 0.00
A H
ATOM 16179 HZ2 LYS A 184 −33.105 37.405 76.334 1.00 0.00
A H
ATOM 16180 HZ3 LYS A 184 −31.970 37.899 75.175 1.00 0.00
A H
ATOM 16181 C LYS A 184 −36.010 43.486 74.741 1.00 0.00
A C
ATOM 16182 O LYS A 184 −35.162 43.770 73.896 1.00 0.00
A O
ATOM 16183 N HSE A 185 −36.219 44.216 75.834 1.00 0.00
A N
ATOM 16184 HN HSE A 185 −36.979 44.074 76.466 1.00 0.00
A H
ATOM 16185 CA HSE A 185 −35.328 45.308 76.201 1.00 0.00
A C
ATOM 16186 HA HSE A 185 −34.556 45.362 75.443 1.00 0.00
A H
ATOM 16187 CB HSE A 185 −36.085 46.629 76.305 1.00 0.00
A C
ATOM 16188 HB1 HSE A 185 −36.587 46.813 75.330 1.00 0.00
A H
ATOM 16189 HB2 HSE A 185 −36.884 46.538 77.070 1.00 0.00
A H
ATOM 16190 ND1 HSE A 185 −34.467 48.432 75.657 1.00 0.00
A N
ATOM 16191 CG HSE A 185 −35.195 47.812 76.606 1.00 0.00
A C
ATOM 16192 CE1 HSE A 185 −33.766 49.441 76.214 1.00 0.00
A C
ATOM 16193 HE1 HSE A 185 −33.090 50.104 75.667 1.00 0.00
A H
ATOM 16194 NE2 HSE A 185 −34.046 49.467 77.529 1.00 0.00
A N
ATOM 16195 HE2 HSE A 185 −33.653 50.103 78.192 1.00 0.00
A H
ATOM 16196 CD2 HSE A 185 −34.924 48.475 77.805 1.00 0.00
A C
ATOM 16197 HD2 HSE A 185 −35.295 48.284 78.802 1.00 0.00
A H
ATOM 16198 C HSE A 185 −34.648 45.015 77.500 1.00 0.00
A C
ATOM 16199 O HSE A 185 −35.315 44.809 78.515 1.00 0.00
A O

ATOM 16200 N   HSE A 186   -33.316 45.000 77.496 1.00 0.00
A   N
ATOM 16201 HN HSE A 186   -32.749 45.028 76.674 1.00 0.00
A   H
ATOM 16202 CA HSE A 186   -32.559 44.937 78.749 1.00 0.00
A   C
ATOM 16203 HA HSE A 186   -31.510 44.918 78.480 1.00 0.00
A   H
ATOM 16204 CB HSE A 186   -32.866 46.227 79.556 1.00 0.00
A   C
ATOM 16205 HB1 HSE A 186   -32.651 47.099 78.901 1.00 0.00
A   H
ATOM 16206 HB2 HSE A 186   -33.949 46.262 79.800 1.00 0.00
A   H
ATOM 16207 ND1 HSE A 186   -30.723 46.608 80.812 1.00 0.00
A   N
ATOM 16208 CG HSE A 186   -32.052 46.402 80.823 1.00 0.00
A   C
ATOM 16209 CE1 HSE A 186   -30.281 46.743 82.079 1.00 0.00
A   C
ATOM 16210 HE1 HSE A 186   -29.237 46.903 82.359 1.00 0.00
A   H
ATOM 16211 NE2 HSE A 186   -31.336 46.639 82.902 1.00 0.00
A   N
ATOM 16212 HE2 HSE A 186   -31.298 46.681 83.900 1.00 0.00
A   H
ATOM 16213 CD2 HSE A 186   -32.446 46.440 82.162 1.00 0.00
A   C
ATOM 16214 HD2 HSE A 186   -33.427 46.327 82.598 1.00 0.00
A   H
ATOM 16215 C   HSE A 186   -32.850 43.646 79.517 1.00 0.00
A   C
ATOM 16216 O   HSE A 186   -32.952 43.670 80.748 1.00 0.00
A   O
ATOM 16217 N   GLY A 187   -32.983 42.503 78.809 1.00 0.00
A   N
ATOM 16218 HN GLY A 187   -32.856 42.484 77.818 1.00 0.00
A   H
ATOM 16219 CA GLY A 187   -33.322 41.204 79.435 1.00 0.00
A   C
ATOM 16220 HA1 GLY A 187   -32.499 40.942 80.087 1.00 0.00
A   H
ATOM 16221 HA2 GLY A 187   -33.497 40.497 78.635 1.00 0.00
A   H
ATOM 16222 C   GLY A 187   -34.589 41.278 80.282 1.00 0.00
A   C
ATOM 16223 O   GLY A 187   -34.778 40.469 81.201 1.00 0.00
A   O
ATOM 16224 N   ASP A 188   -35.423 42.292 79.994 1.00 0.00
A   N
ATOM 16225 HN ASP A 188   -35.165 43.004 79.346 1.00 0.00
A   H
ATOM 16226 CA ASP A 188   -36.767 42.492 80.561 1.00 0.00
A   C

ATOM 16227 HA ASP A 188 -37.000 41.644 81.195 1.00 0.00
A H
ATOM 16228 CB ASP A 188 -36.883 43.857 81.260 1.00 0.00
A C
ATOM 16229 HB1 ASP A 188 -36.377 44.627 80.643 1.00 0.00
A H
ATOM 16230 HB2 ASP A 188 -37.945 44.147 81.377 1.00 0.00
A H
ATOM 16231 CG ASP A 188 -36.233 43.885 82.624 1.00 0.00
A C
ATOM 16232 OD1 ASP A 188 -35.657 42.864 83.047 1.00 0.00
A O
ATOM 16233 OD2 ASP A 188 -36.301 44.945 83.279 1.00 0.00
A O
ATOM 16234 C ASP A 188 -37.771 42.471 79.413 1.00 0.00
A C
ATOM 16235 O ASP A 188 -37.815 43.409 78.615 1.00 0.00
A O
ATOM 16236 N ILE A 189 -38.583 41.424 79.326 1.00 0.00
A N
ATOM 16237 HN ILE A 189 -38.663 40.685 79.991 1.00 0.00
A H
ATOM 16238 CA ILE A 189 -39.465 41.288 78.169 1.00 0.00
A C
ATOM 16239 HA ILE A 189 -38.978 41.854 77.386 1.00 0.00
A H
ATOM 16240 CB ILE A 189 -39.567 39.834 77.654 1.00 0.00
A C
ATOM 16241 HB ILE A 189 -40.264 39.895 76.780 1.00 0.00
A H
ATOM 16242 CG2 ILE A 189 -38.197 39.322 77.247 1.00 0.00
A C
ATOM 16243 HG21 ILE A 189 -38.282 38.341 76.733 1.00 0.00
A H
ATOM 16244 HG22 ILE A 189 -37.702 40.027 76.546 1.00 0.00
A H
ATOM 16245 HG23 ILE A 189 -37.541 39.192 78.133 1.00 0.00
A H
ATOM 16246 CG1 ILE A 189 -40.242 38.923 78.685 1.00 0.00
A C
ATOM 16247 HG11 ILE A 189 -39.467 38.409 79.297 1.00 0.00
A H
ATOM 16248 HG12 ILE A 189 -40.847 39.550 79.378 1.00 0.00
A H
ATOM 16249 CD ILE A 189 -41.149 37.891 78.052 1.00 0.00
A C
ATOM 16250 HD1 ILE A 189 -41.660 37.293 78.837 1.00 0.00
A H
ATOM 16251 HD2 ILE A 189 -41.927 38.383 77.429 1.00 0.00
A H
ATOM 16252 HD3 ILE A 189 -40.572 37.193 77.409 1.00 0.00
A H
ATOM 16253 C ILE A 189 -40.860 41.886 78.343 1.00 0.00
A C

ATOM 16254 O   ILE A 189   −41.428 41.874 79.438 1.00 0.00
A   O
ATOM 16255 N   CYS A 190   −41.382 42.418 77.242 1.00 0.00
A   N
ATOM 16256 HN CYS A 190   −40.843 42.522 76.408 1.00 0.00
A   H
ATOM 16257 CA CYS A 190   −42.752 42.888 77.143 1.00 0.00
A   C
ATOM 16258 HA CYS A 190   −43.166 42.960 78.142 1.00 0.00
A   H
ATOM 16259 CB CYS A 190   −42.780 44.207 76.369 1.00 0.00
A   C
ATOM 16260 HB1 CYS A 190   −41.827 44.739 76.587 1.00 0.00
A   H
ATOM 16261 HB2 CYS A 190   −42.774 44.002 75.275 1.00 0.00
A   H
ATOM 16262 SG CYS A 190   −44.137 45.322 76.787 1.00 0.00
A   S
ATOM 16263 HG1 CYS A 190   −43.777 46.329 75.988 1.00 0.00
A   H
ATOM 16264 C   CYS A 190   −43.525 41.804 76.395 1.00 0.00
A   C
ATOM 16265 O   CYS A 190   −43.507 41.768 75.164 1.00 0.00
A   O
ATOM 16266 N   LEU A 191   −44.190 40.921 77.147 1.00 0.00
A   N
ATOM 16267 HN LEU A 191   −44.369 41.061 78.119 1.00 0.00
A   H
ATOM 16268 CA LEU A 191   −44.720 39.654 76.609 1.00 0.00
A   C
ATOM 16269 HA LEU A 191   −43.850 39.110 76.264 1.00 0.00
A   H
ATOM 16270 CB LEU A 191   −45.340 38.775 77.708 1.00 0.00
A   C
ATOM 16271 HB1 LEU A 191   −44.601 38.718 78.540 1.00 0.00
A   H
ATOM 16272 HB2 LEU A 191   −46.233 39.292 78.125 1.00 0.00
A   H
ATOM 16273 CG LEU A 191   −45.720 37.333 77.318 1.00 0.00
A   C
ATOM 16274 HG LEU A 191   −46.222 37.421 76.325 1.00 0.00
A   H
ATOM 16275 CD1 LEU A 191   −44.498 36.429 77.200 1.00 0.00
A   C
ATOM 16276 HD11 LEU A 191   −44.806 35.387 76.966 1.00 0.00
A   H
ATOM 16277 HD12 LEU A 191   −43.825 36.785 76.391 1.00 0.00
A   H
ATOM 16278 HD13 LEU A 191   −43.925 36.415 78.152 1.00 0.00
A   H
ATOM 16279 CD2 LEU A 191   −46.724 36.745 78.304 1.00 0.00
A   C
ATOM 16280 HD21 LEU A 191   −47.003 35.713 78.001 1.00 0.00
A   H

ATOM 16281 HD22 LEU A 191   -46.309 36.693 79.330 1.00 0.00
A   H
ATOM 16282 HD23 LEU A 191   -47.649 37.360 78.332 1.00 0.00
A   H
ATOM 16283 C   LEU A 191   -45.674 39.809 75.417 1.00 0.00
A   C
ATOM 16284 O   LEU A 191   -45.384 39.300 74.348 1.00 0.00
A   O
ATOM 16285 N   SER A 192   -46.802 40.482 75.612 1.00 0.00
A   N
ATOM 16286 HN SER A 192   -47.123 40.788 76.508 1.00 0.00
A   H
ATOM 16287 CA SER A 192   -47.691 40.840 74.502 1.00 0.00
A   C
ATOM 16288 HA SER A 192   -47.189 40.564 73.584 1.00 0.00
A   H
ATOM 16289 CB SER A 192   -49.050 40.146 74.622 1.00 0.00
A   C
ATOM 16290 HB1 SER A 192   -49.298 40.056 75.704 1.00 0.00
A   H
ATOM 16291 HB2 SER A 192   -49.845 40.768 74.155 1.00 0.00
A   H
ATOM 16292 OG SER A 192   -49.017 38.849 74.055 1.00 0.00
A   O
ATOM 16293 HG1 SER A 192   -49.811 38.388 74.354 1.00 0.00
A   H
ATOM 16294 C   SER A 192   -47.849 42.356 74.495 1.00 0.00
A   C
ATOM 16295 O   SER A 192   -48.590 42.907 75.314 1.00 0.00
A   O
ATOM 16296 N   PRO A 193   -47.148 43.035 73.569 1.00 0.00
A   N
ATOM 16297 CD PRO A 193   -46.578 42.503 72.321 1.00 0.00
A   C
ATOM 16298 HD1 PRO A 193   -46.375 42.001 73.294 1.00 0.00
A   H
ATOM 16299 HD2 PRO A 193   -46.651 41.751 71.502 1.00 0.00
A   H
ATOM 16300 CA PRO A 193   -46.994 44.486 73.646 1.00 0.00
A   C
ATOM 16301 HA PRO A 193   -46.610 44.761 74.621 1.00 0.00
A   H
ATOM 16302 CB PRO A 193   -45.973 44.803 72.538 1.00 0.00
A   C
ATOM 16303 HB1 PRO A 193   -44.945 44.668 72.943 1.00 0.00
A   H
ATOM 16304 HB2 PRO A 193   -46.072 45.835 72.146 1.00 0.00
A   H
ATOM 16305 CG PRO A 193   -45.495 43.488 72.017 1.00 0.00
A   C
ATOM 16306 HG1 PRO A 193   -44.501 43.550 71.534 1.00 0.00
A   H
ATOM 16307 HG2 PRO A 193   -46.238 43.091 71.288 1.00 0.00
A   H

ATOM 16308 C PRO A 193 -48.293 45.244 73.390 1.00 0.00
A C
ATOM 16309 O PRO A 193 -49.083 44.855 72.525 1.00 0.00
A O
ATOM 16310 N ALA A 194 -48.504 46.310 74.162 1.00 0.00
A N
ATOM 16311 HN ALA A 194 -47.923 46.516 74.949 1.00 0.00
A H
ATOM 16312 CA ALA A 194 -49.592 47.254 73.920 1.00 0.00
A C
ATOM 16313 HA ALA A 194 -50.530 46.742 73.741 1.00 0.00
A H
ATOM 16314 CB ALA A 194 -49.770 48.178 75.118 1.00 0.00
A C
ATOM 16315 HB1 ALA A 194 -50.040 47.579 76.014 1.00 0.00
A H
ATOM 16316 HB2 ALA A 194 -48.829 48.725 75.344 1.00 0.00
A H
ATOM 16317 HB3 ALA A 194 -50.579 48.920 74.946 1.00 0.00
A H
ATOM 16318 C ALA A 194 -49.284 48.055 72.647 1.00 0.00
A C
ATOM 16319 O ALA A 194 -48.115 48.191 72.272 1.00 0.00
A O
ATOM 16320 N PRO A 195 -50.327 48.579 71.972 1.00 0.00
A N
ATOM 16321 CD PRO A 195 -51.762 48.338 72.225 1.00 0.00
A C
ATOM 16322 HD1 PRO A 195 -51.090 47.464 72.066 1.00 0.00
A H
ATOM 16323 HD2 PRO A 195 -52.540 48.098 72.985 1.00 0.00
A H
ATOM 16324 CA PRO A 195 -50.122 49.306 70.715 1.00 0.00
A C
ATOM 16325 HA PRO A 195 -49.803 48.580 69.977 1.00 0.00
A H
ATOM 16326 CB PRO A 195 -51.532 49.782 70.360 1.00 0.00
A C
ATOM 16327 HB1 PRO A 195 -52.062 48.970 69.814 1.00 0.00
A H
ATOM 16328 HB2 PRO A 195 -51.530 50.696 69.733 1.00 0.00
A H
ATOM 16329 CG PRO A 195 -52.414 48.728 70.928 1.00 0.00
A C
ATOM 16330 HG1 PRO A 195 -53.404 48.681 70.434 1.00 0.00
A H
ATOM 16331 HG2 PRO A 195 -52.723 47.781 70.846 1.00 0.00
A H
ATOM 16332 C PRO A 195 -49.136 50.492 70.717 1.00 0.00
A C
ATOM 16333 O PRO A 195 -48.778 50.958 69.634 1.00 0.00
A O
ATOM 16334 N GLU A 196 -48.700 50.935 71.895 1.00 0.00
A N

ATOM 16335 HN GLU A 196 -48.949 50.532 72.771 1.00 0.00
A H
ATOM 16336 CA GLU A 196 -47.795 52.080 72.003 1.00 0.00
A C
ATOM 16337 HA GLU A 196 -47.294 52.304 71.071 1.00 0.00
A H
ATOM 16338 CB GLU A 196 -48.524 53.275 72.624 1.00 0.00
A C
ATOM 16339 HB1 GLU A 196 -47.780 54.088 72.788 1.00 0.00
A H
ATOM 16340 HB2 GLU A 196 -49.266 53.681 71.901 1.00 0.00
A H
ATOM 16341 CG GLU A 196 -49.211 52.966 73.945 1.00 0.00
A C
ATOM 16342 HG1 GLU A 196 -48.764 52.059 74.390 1.00 0.00
A H
ATOM 16343 HG2 GLU A 196 -49.105 53.811 74.655 1.00 0.00
A H
ATOM 16344 CD GLU A 196 -50.685 52.659 73.776 1.00 0.00
A C
ATOM 16345 OE1 GLU A 196 -51.296 52.129 74.729 1.00 0.00
A O
ATOM 16346 OE2 GLU A 196 -51.234 52.950 72.693 1.00 0.00
A O
ATOM 16347 C GLU A 196 -46.738 51.528 72.968 1.00 0.00
A C
ATOM 16348 O GLU A 196 -46.758 51.835 74.160 1.00 0.00
A O
ATOM 16349 N ASP A 197 -45.817 50.726 72.445 1.00 0.00
A N
ATOM 16350 HN ASP A 197 -45.812 50.413 71.499 1.00 0.00
A H
ATOM 16351 CA ASP A 197 -44.688 50.205 73.226 1.00 0.00
A C
ATOM 16352 HA ASP A 197 -44.509 50.840 74.087 1.00 0.00
A H
ATOM 16353 CB ASP A 197 -44.750 48.790 73.808 1.00 0.00
A C
ATOM 16354 HB1 ASP A 197 -45.300 48.129 73.107 1.00 0.00
A H
ATOM 16355 HB2 ASP A 197 -43.730 48.379 73.946 1.00 0.00
A H
ATOM 16356 CG ASP A 197 -45.475 48.740 75.145 1.00 0.00
A C
ATOM 16357 OD1 ASP A 197 -45.679 49.808 75.762 1.00 0.00
A O
ATOM 16358 OD2 ASP A 197 -45.844 47.634 75.584 1.00 0.00
A O
ATOM 16359 C ASP A 197 -43.485 50.369 72.298 1.00 0.00
A C
ATOM 16360 O ASP A 197 -43.460 49.833 71.185 1.00 0.00
A O
ATOM 16361 N ILE A 198 -42.492 51.111 72.785 1.00 0.00
A N

ATOM 16362 HN ILE A 198   -42.498 51.448 73.724 1.00 0.00
A   H
ATOM 16363 CA ILE A 198   -41.311 51.505 72.012 1.00 0.00
A   C
ATOM 16364 HA ILE A 198   -41.679 51.818 71.043 1.00 0.00
A   H
ATOM 16365 CB ILE A 198   -40.545 52.661 72.709 1.00 0.00
A   C
ATOM 16366 HB ILE A 198   -40.338 52.278 73.740 1.00 0.00
A   H
ATOM 16367 CG2 ILE A 198   -39.282 53.036 71.942 1.00 0.00
A   C
ATOM 16368 HG21 ILE A 198   -38.666 53.749 72.528 1.00 0.00
A   H
ATOM 16369 HG22 ILE A 198   -38.658 52.138 71.743 1.00 0.00
A   H
ATOM 16370 HG23 ILE A 198   -39.533 53.510 70.970 1.00 0.00
A   H
ATOM 16371 CG1 ILE A 198   -41.450 53.891 72.869 1.00 0.00
A   C
ATOM 16372 HG11 ILE A 198   -41.496 54.451 71.909 1.00 0.00
A   H
ATOM 16373 HG12 ILE A 198   -42.484 53.550 73.101 1.00 0.00
A   H
ATOM 16374 CD ILE A 198   -41.019 54.841 73.965 1.00 0.00
A   C
ATOM 16375 HD1 ILE A 198   -41.748 55.674 74.062 1.00 0.00
A   H
ATOM 16376 HD2 ILE A 198   -40.962 54.313 74.941 1.00 0.00
A   H
ATOM 16377 HD3 ILE A 198   -40.024 55.280 73.743 1.00 0.00
A   H
ATOM 16378 C   ILE A 198   -40.369 50.327 71.767 1.00 0.00
A   C
ATOM 16379 O   ILE A 198   -40.024 49.593 72.698 1.00 0.00
A   O
ATOM 16380 N   ILE A 199   -39.976 50.149 70.506 1.00 0.00
A   N
ATOM 16381 HN ILE A 199   -40.363 50.656 69.738 1.00 0.00
A   H
ATOM 16382 CA ILE A 199   -38.938 49.192 70.129 1.00 0.00
A   C
ATOM 16383 HA ILE A 199   -38.760 48.555 70.987 1.00 0.00
A   H
ATOM 16384 CB ILE A 199   -39.332 48.350 68.892 1.00 0.00
A   C
ATOM 16385 HB ILE A 199   -39.804 49.085 68.192 1.00 0.00
A   H
ATOM 16386 CG2 ILE A 199   -38.116 47.625 68.316 1.00 0.00
A   C
ATOM 16387 HG21 ILE A 199   -38.368 47.151 67.345 1.00 0.00
A   H
ATOM 16388 HG22 ILE A 199   -37.280 48.333 68.138 1.00 0.00
A   H

```
ATOM  16389 HG23 ILE A 199     -37.762  46.831  69.007  1.00  0.00
A    H
ATOM  16390  CG1 ILE A 199     -40.415  47.331  69.263  1.00  0.00
A    C
ATOM  16391 HG11 ILE A 199     -39.954  46.467  69.791  1.00  0.00
A    H
ATOM  16392 HG12 ILE A 199     -41.130  47.807  69.973  1.00  0.00
A    H
ATOM  16393  CD  ILE A 199     -41.210  46.811  68.084  1.00  0.00
A    C
ATOM  16394  HD1 ILE A 199     -42.012  46.125  68.432  1.00  0.00
A    H
ATOM  16395  HD2 ILE A 199     -41.686  47.650  67.532  1.00  0.00
A    H
ATOM  16396  HD3 ILE A 199     -40.560  46.250  67.381  1.00  0.00
A    H
ATOM  16397  C   ILE A 199     -37.644  49.945  69.864  1.00  0.00
A    C
ATOM  16398  O   ILE A 199     -37.578  50.800  68.977  1.00  0.00
A    O
ATOM  16399  N   ARG A 200     -36.577  49.591  70.576  1.00  0.00
A    N
ATOM  16400  HN  ARG A 200     -36.620  48.908  71.302  1.00  0.00
A    H
ATOM  16401  CA  ARG A 200     -35.267  50.200  70.321  1.00  0.00
A    C
ATOM  16402  HA  ARG A 200     -35.432  51.153  69.833  1.00  0.00
A    H
ATOM  16403  CB  ARG A 200     -34.488  50.390  71.618  1.00  0.00
A    C
ATOM  16404  HB1 ARG A 200     -35.092  49.903  72.422  1.00  0.00
A    H
ATOM  16405  HB2 ARG A 200     -33.533  49.821  71.566  1.00  0.00
A    H
ATOM  16406  CG  ARG A 200     -34.253  51.839  71.990  1.00  0.00
A    C
ATOM  16407  HG1 ARG A 200     -33.346  52.199  71.456  1.00  0.00
A    H
ATOM  16408  HG2 ARG A 200     -35.095  52.454  71.599  1.00  0.00
A    H
ATOM  16409  CD  ARG A 200     -34.170  52.002  73.493  1.00  0.00
A    C
ATOM  16410  HD1 ARG A 200     -34.023  51.005  73.967  1.00  0.00
A    H
ATOM  16411  HD2 ARG A 200     -33.323  52.665  73.786  1.00  0.00
A    H
ATOM  16412  NE  ARG A 200     -35.376  52.609  74.038  1.00  0.00
A    N
ATOM  16413  HE  ARG A 200     -36.175  52.511  73.435  1.00  0.00
A    H
ATOM  16414  CZ  ARG A 200     -35.427  53.228  75.208  1.00  0.00
A    C
ATOM  16415  NH1 ARG A 200     -34.338  53.322  75.953  1.00  0.00
A    N
```

ATOM 16416 HH11 ARG A 200 −34.370 53.761 76.840 1.00 0.00
A H
ATOM 16417 HH12 ARG A 200 −33.536 52.813 75.632 1.00 0.00
A H
ATOM 16418 NH2 ARG A 200 −36.563 53.754 75.629 1.00 0.00
A N
ATOM 16419 HH21 ARG A 200 −36.581 54.291 76.461 1.00 0.00
A H
ATOM 16420 HH22 ARG A 200 −37.317 53.767 74.985 1.00 0.00
A H
ATOM 16421 C ARG A 200 −34.421 49.365 69.370 1.00 0.00
A C
ATOM 16422 O ARG A 200 −34.886 48.373 68.828 1.00 0.00
A O
ATOM 16423 N GLU A 201 −33.177 49.772 69.161 1.00 0.00
A N
ATOM 16424 HN GLU A 201 −32.746 50.567 69.580 1.00 0.00
A H
ATOM 16425 CA GLU A 201 −32.309 49.039 68.251 1.00 0.00
A C
ATOM 16426 HA GLU A 201 −32.918 48.944 67.361 1.00 0.00
A H
ATOM 16427 CB GLU A 201 −30.962 49.744 68.087 1.00 0.00
A C
ATOM 16428 HB1 GLU A 201 −30.891 50.543 68.861 1.00 0.00
A H
ATOM 16429 HB2 GLU A 201 −30.135 49.033 68.307 1.00 0.00
A H
ATOM 16430 CG GLU A 201 −30.761 50.368 66.711 1.00 0.00
A C
ATOM 16431 HG1 GLU A 201 −31.320 49.785 65.956 1.00 0.00
A H
ATOM 16432 HG2 GLU A 201 −31.114 51.418 66.696 1.00 0.00
A H
ATOM 16433 CD GLU A 201 −29.319 50.346 66.251 1.00 0.00
A C
ATOM 16434 OE1 GLU A 201 −28.421 50.420 67.111 1.00 0.00
A O
ATOM 16435 OE2 GLU A 201 −29.081 50.264 65.028 1.00 0.00
A O
ATOM 16436 C GLU A 201 −31.780 47.670 68.578 1.00 0.00
A C
ATOM 16437 O GLU A 201 −31.844 46.759 67.767 1.00 0.00
A O
ATOM 16438 N GLN A 202 −31.268 47.529 69.788 1.00 0.00
A N
ATOM 16439 HN GLN A 202 −31.135 48.288 70.423 1.00 0.00
A H
ATOM 16440 CA GLN A 202 −30.852 46.241 70.298 1.00 0.00
A C
ATOM 16441 HA GLN A 202 −30.582 45.571 69.491 1.00 0.00
A H
ATOM 16442 CB GLN A 202 −29.875 46.492 71.438 1.00 0.00
A C

ATOM 16443 HB1 GLN A 202 −29.537 47.549 71.346 1.00 0.00
A H
ATOM 16444 HB2 GLN A 202 −30.415 46.422 72.409 1.00 0.00
A H
ATOM 16445 CG GLN A 202 −28.668 45.589 71.443 1.00 0.00
A C
ATOM 16446 HG1 GLN A 202 −28.199 45.574 72.450 1.00 0.00
A H
ATOM 16447 HG2 GLN A 202 −28.955 44.549 71.185 1.00 0.00
A H
ATOM 16448 CD GLN A 202 −27.630 46.078 70.478 1.00 0.00
A C
ATOM 16449 OE1 GLN A 202 −27.956 46.694 69.466 1.00 0.00
A O
ATOM 16450 NE2 GLN A 202 −26.371 45.814 70.782 1.00 0.00
A N
ATOM 16451 HE21 GLN A 202 −25.677 46.140 70.146 1.00 0.00
A H
ATOM 16452 HE22 GLN A 202 −26.159 45.297 71.604 1.00 0.00
A H
ATOM 16453 C GLN A 202 −32.136 45.721 70.912 1.00 0.00
A C
ATOM 16454 O GLN A 202 −32.314 45.816 72.119 1.00 0.00
A O
ATOM 16455 N ASP A 203 −33.034 45.166 70.119 1.00 0.00
A N
ATOM 16456 HN ASP A 203 −33.009 45.065 69.128 1.00 0.00
A H
ATOM 16457 CA ASP A 203 −34.241 44.610 70.715 1.00 0.00
A C
ATOM 16458 HA ASP A 203 −33.977 44.313 71.724 1.00 0.00
A H
ATOM 16459 CB ASP A 203 −35.346 45.653 70.795 1.00 0.00
A C
ATOM 16460 HB1 ASP A 203 −35.042 46.552 70.221 1.00 0.00
A H
ATOM 16461 HB2 ASP A 203 −36.285 45.261 70.359 1.00 0.00
A H
ATOM 16462 CG ASP A 203 −35.617 46.089 72.214 1.00 0.00
A C
ATOM 16463 OD1 ASP A 203 −35.445 45.261 73.133 1.00 0.00
A O
ATOM 16464 OD2 ASP A 203 −35.995 47.258 72.413 1.00 0.00
A O
ATOM 16465 C ASP A 203 −34.712 43.368 70.005 1.00 0.00
A C
ATOM 16466 O ASP A 203 −34.468 43.205 68.822 1.00 0.00
A O
ATOM 16467 N CYS A 204 −35.375 42.480 70.728 1.00 0.00
A N
ATOM 16468 HN CYS A 204 −35.746 42.632 71.641 1.00 0.00
A H
ATOM 16469 CA CYS A 204 −35.609 41.148 70.187 1.00 0.00
A C

ATOM 16470 HA CYS A 204   -34.996 41.064 69.298 1.00 0.00
A   H
ATOM 16471 CB CYS A 204   -35.225 40.078 71.212 1.00 0.00
A   C
ATOM 16472 HB1 CYS A 204   -35.238 40.562 72.215 1.00 0.00
A   H
ATOM 16473 HB2 CYS A 204   -36.007 39.287 71.243 1.00 0.00
A   H
ATOM 16474 SG CYS A 204   -33.589 39.353 70.958 1.00 0.00
A   S
ATOM 16475 HG1 CYS A 204   -33.589 38.562 72.031 1.00 0.00
A   H
ATOM 16476 C   CYS A 204   -37.064 40.971 69.767 1.00 0.00
A   C
ATOM 16477 O   CYS A 204   -37.912 40.588 70.575 1.00 0.00
A   O
ATOM 16478 N   LEU A 205   -37.348 41.253 68.500 1.00 0.00
A   N
ATOM 16479 HN LEU A 205   -36.675 41.568 67.834 1.00 0.00
A   H
ATOM 16480 CA LEU A 205   -38.695 41.123 67.973 1.00 0.00
A   C
ATOM 16481 HA LEU A 205   -39.376 41.548 68.700 1.00 0.00
A   H
ATOM 16482 CB LEU A 205   -38.805 41.789 66.600 1.00 0.00
A   C
ATOM 16483 HB1 LEU A 205   -38.009 41.348 65.957 1.00 0.00
A   H
ATOM 16484 HB2 LEU A 205   -39.773 41.500 66.134 1.00 0.00
A   H
ATOM 16485 CG LEU A 205   -38.654 43.305 66.551 1.00 0.00
A   C
ATOM 16486 HG LEU A 205   -37.687 43.575 67.040 1.00 0.00
A   H
ATOM 16487 CD1 LEU A 205   -38.650 43.789 65.112 1.00 0.00
A   C
ATOM 16488 HD11 LEU A 205   -38.588 44.897 65.074 1.00 0.00
A   H
ATOM 16489 HD12 LEU A 205   -37.779 43.372 64.562 1.00 0.00
A   H
ATOM 16490 HD13 LEU A 205   -39.578 43.475 64.590 1.00 0.00
A   H
ATOM 16491 CD2 LEU A 205   -39.776 43.963 67.332 1.00 0.00
A   C
ATOM 16492 HD21 LEU A 205   -39.661 45.069 67.317 1.00 0.00
A   H
ATOM 16493 HD22 LEU A 205   -40.770 43.721 66.904 1.00 0.00
A   H
ATOM 16494 HD23 LEU A 205   -39.760 43.627 68.392 1.00 0.00
A   H
ATOM 16495 C   LEU A 205   -39.029 39.658 67.837 1.00 0.00
A   C
ATOM 16496 O   LEU A 205   -38.229 38.888 67.321 1.00 0.00
A   O

ATOM 16497 N VAL A 206 -40.199 39.264 68.317 1.00 0.00
A N
ATOM 16498 HN VAL A 206 -40.817 39.830 68.858 1.00 0.00
A H
ATOM 16499 CA VAL A 206 -40.680 37.911 68.073 1.00 0.00
A C
ATOM 16500 HA VAL A 206 -39.951 37.389 67.466 1.00 0.00
A H
ATOM 16501 CB VAL A 206 -40.855 37.084 69.361 1.00 0.00
A C
ATOM 16502 HB VAL A 206 -41.635 37.589 69.983 1.00 0.00
A H
ATOM 16503 CG1 VAL A 206 -41.314 35.673 69.024 1.00 0.00
A C
ATOM 16504 HG11 VAL A 206 -41.402 35.065 69.951 1.00 0.00
A H
ATOM 16505 HG12 VAL A 206 -42.309 35.672 68.532 1.00 0.00
A H
ATOM 16506 HG13 VAL A 206 -40.584 35.173 68.353 1.00 0.00
A H
ATOM 16507 CG2 VAL A 206 -39.550 37.034 70.143 1.00 0.00
A C
ATOM 16508 HG21 VAL A 206 -39.661 36.405 71.052 1.00 0.00
A H
ATOM 16509 HG22 VAL A 206 -38.737 36.605 69.518 1.00 0.00
A H
ATOM 16510 HG23 VAL A 206 -39.239 38.047 70.471 1.00 0.00
A H
ATOM 16511 C VAL A 206 -41.966 38.002 67.267 1.00 0.00
A C
ATOM 16512 O VAL A 206 -42.987 38.498 67.746 1.00 0.00
A O
ATOM 16513 N ILE A 207 -41.897 37.507 66.039 1.00 0.00
A N
ATOM 16514 HN ILE A 207 -41.151 36.923 65.726 1.00 0.00
A H
ATOM 16515 CA ILE A 207 -42.903 37.772 65.037 1.00 0.00
A C
ATOM 16516 HA ILE A 207 -43.709 38.320 65.508 1.00 0.00
A H
ATOM 16517 CB ILE A 207 -42.197 38.584 63.914 1.00 0.00
A C
ATOM 16518 HB ILE A 207 -41.255 38.958 64.389 1.00 0.00
A H
ATOM 16519 CG2 ILE A 207 -42.236 37.721 62.564 1.00 0.00
A C
ATOM 16520 HG21 ILE A 207 -41.609 38.194 61.780 1.00 0.00
A H
ATOM 16521 HG22 ILE A 207 -41.843 36.697 62.738 1.00 0.00
A H
ATOM 16522 HG23 ILE A 207 -43.273 37.638 62.175 1.00 0.00
A H
ATOM 16523 CG1 ILE A 207 -43.067 39.869 63.530 1.00 0.00
A C

ATOM 16524 HG11 ILE A 207   -44.072 39.551 63.173 1.00 0.00
A   H
ATOM 16525 HG12 ILE A 207   -43.223 40.485 64.445 1.00 0.00
A   H
ATOM 16526 CD ILE A 207   -42.258 40.600 62.475 1.00 0.00
A   C
ATOM 16527 HD1 ILE A 207   -42.765 41.547 62.188 1.00 0.00
A   H
ATOM 16528 HD2 ILE A 207   -41.246 40.850 62.861 1.00 0.00
A   H
ATOM 16529 HD3 ILE A 207   -42.144 39.981 61.561 1.00 0.00
A   H
ATOM 16530 C   ILE A 207   -43.482 36.420 64.600 1.00 0.00
A   C
ATOM 16531 O   ILE A 207   -42.793 35.396 64.653 1.00 0.00
A   O
ATOM 16532 N   MET A 208   -44.753 36.408 64.204 1.00 0.00
A   N
ATOM 16533 HN MET A 208   -45.303 37.233 64.094 1.00 0.00
A   H
ATOM 16534 CA MET A 208   -45.459 35.163 63.890 1.00 0.00
A   C
ATOM 16535 HA MET A 208   -44.707 34.434 63.617 1.00 0.00
A   H
ATOM 16536 CB MET A 208   -46.252 34.664 65.106 1.00 0.00
A   C
ATOM 16537 HB1 MET A 208   -45.548 34.655 65.971 1.00 0.00
A   H
ATOM 16538 HB2 MET A 208   -47.046 35.402 65.357 1.00 0.00
A   H
ATOM 16539 CG MET A 208   -46.858 33.272 64.969 1.00 0.00
A   C
ATOM 16540 HG1 MET A 208   -47.356 33.163 63.983 1.00 0.00
A   H
ATOM 16541 HG2 MET A 208   -46.054 32.507 65.026 1.00 0.00
A   H
ATOM 16542 SD MET A 208   -48.078 32.953 66.261 1.00 0.00
A   S
ATOM 16543 CE MET A 208   -48.209 31.170 66.196 1.00 0.00
A   C
ATOM 16544 HE1 MET A 208   -48.946 30.798 66.940 1.00 0.00
A   H
ATOM 16545 HE2 MET A 208   -48.538 30.831 65.190 1.00 0.00
A   H
ATOM 16546 HE3 MET A 208   -47.232 30.689 66.415 1.00 0.00
A   H
ATOM 16547 C   MET A 208   -46.395 35.332 62.694 1.00 0.00
A   C
ATOM 16548 O   MET A 208   -47.007 36.388 62.513 1.00 0.00
A   O
ATOM 16549 N   GLY A 209   -46.506 34.281 61.886 1.00 0.00
A   N
ATOM 16550 HN GLY A 209   -45.974 33.445 62.019 1.00 0.00
A   H

ATOM 16551 CA GLY A 209 -47.413 34.276 60.749 1.00 0.00
A C
ATOM 16552 HA1 GLY A 209 -47.633 35.310 60.521 1.00 0.00
A H
ATOM 16553 HA2 GLY A 209 -48.277 33.690 61.035 1.00 0.00
A H
ATOM 16554 C GLY A 209 -46.808 33.641 59.514 1.00 0.00
A C
ATOM 16555 O GLY A 209 -46.059 32.663 59.604 1.00 0.00
A O
ATOM 16556 N HSE A 210 -47.135 34.210 58.358 1.00 0.00
A N
ATOM 16557 HN HSE A 210 -47.704 35.029 58.290 1.00 0.00
A H
ATOM 16558 CA HSE A 210 -46.687 33.682 57.078 1.00 0.00
A C
ATOM 16559 HA HSE A 210 -46.749 32.602 57.144 1.00 0.00
A H
ATOM 16560 CB HSE A 210 -47.580 34.198 55.958 1.00 0.00
A C
ATOM 16561 HB1 HSE A 210 -48.578 34.420 56.392 1.00 0.00
A H
ATOM 16562 HB2 HSE A 210 -47.173 35.155 55.571 1.00 0.00
A H
ATOM 16563 ND1 HSE A 210 -48.463 32.071 54.979 1.00 0.00
A N
ATOM 16564 CG HSE A 210 -47.773 33.216 54.828 1.00 0.00
A C
ATOM 16565 CE1 HSE A 210 -48.478 31.402 53.810 1.00 0.00
A C
ATOM 16566 HE1 HSE A 210 -48.960 30.433 53.656 1.00 0.00
A H
ATOM 16567 NE2 HSE A 210 -47.802 32.129 52.904 1.00 0.00
A N
ATOM 16568 HE2 HSE A 210 -47.641 31.866 51.953 1.00 0.00
A H
ATOM 16569 CD2 HSE A 210 -47.355 33.255 53.500 1.00 0.00
A C
ATOM 16570 HD2 HSE A 210 -46.773 34.002 52.977 1.00 0.00
A H
ATOM 16571 C HSE A 210 -45.249 34.025 56.819 1.00 0.00
A C
ATOM 16572 O HSE A 210 -44.827 35.173 56.992 1.00 0.00
A O
ATOM 16573 N LYS A 211 -44.496 33.012 56.402 1.00 0.00
A N
ATOM 16574 HN LYS A 211 -44.904 32.117 56.223 1.00 0.00
A H
ATOM 16575 CA LYS A 211 -43.054 33.094 56.169 1.00 0.00
A C
ATOM 16576 HA LYS A 211 -42.605 33.324 57.128 1.00 0.00
A H
ATOM 16577 CB LYS A 211 -42.570 31.753 55.621 1.00 0.00
A C

ATOM 16578 HB1 LYS A 211 -43.291 30.977 55.969 1.00 0.00
A H
ATOM 16579 HB2 LYS A 211 -42.640 31.768 54.511 1.00 0.00
A H
ATOM 16580 CG LYS A 211 -41.176 31.346 56.053 1.00 0.00
A C
ATOM 16581 HG1 LYS A 211 -40.450 32.119 55.712 1.00 0.00
A H
ATOM 16582 HG2 LYS A 211 -41.135 31.318 57.166 1.00 0.00
A H
ATOM 16583 CD LYS A 211 -40.832 29.998 55.447 1.00 0.00
A C
ATOM 16584 HD1 LYS A 211 -41.726 29.338 55.526 1.00 0.00
A H
ATOM 16585 HD2 LYS A 211 -40.623 30.148 54.362 1.00 0.00
A H
ATOM 16586 CE LYS A 211 -39.636 29.366 56.132 1.00 0.00
A C
ATOM 16587 HE1 LYS A 211 -38.746 30.029 56.053 1.00 0.00
A H
ATOM 16588 HE2 LYS A 211 -39.849 29.191 57.210 1.00 0.00
A H
ATOM 16589 NZ LYS A 211 -39.295 28.058 55.510 1.00 0.00
A N
ATOM 16590 HZ1 LYS A 211 -38.473 27.639 55.990 1.00 0.00
A H
ATOM 16591 HZ2 LYS A 211 -40.108 27.413 55.590 1.00 0.00
A H
ATOM 16592 HZ3 LYS A 211 -39.071 28.202 54.504 1.00 0.00
A H
ATOM 16593 C LYS A 211 -42.665 34.214 55.203 1.00 0.00
A C
ATOM 16594 O LYS A 211 -41.681 34.924 55.421 1.00 0.00
A O
ATOM 16595 N LYS A 212 -43.454 34.361 54.141 1.00 0.00
A N
ATOM 16596 HN LYS A 212 -44.267 33.798 54.002 1.00 0.00
A H
ATOM 16597 CA LYS A 212 -43.198 35.348 53.099 1.00 0.00
A C
ATOM 16598 HA LYS A 212 -42.137 35.310 52.879 1.00 0.00
A H
ATOM 16599 CB LYS A 212 -44.019 35.021 51.852 1.00 0.00
A C
ATOM 16600 HB1 LYS A 212 -45.055 35.389 52.036 1.00 0.00
A H
ATOM 16601 HB2 LYS A 212 -43.627 35.612 50.994 1.00 0.00
A H
ATOM 16602 CG LYS A 212 -44.063 33.540 51.518 1.00 0.00
A C
ATOM 16603 HG1 LYS A 212 -43.051 33.101 51.677 1.00 0.00
A H
ATOM 16604 HG2 LYS A 212 -44.761 33.030 52.222 1.00 0.00
A H

ATOM 16605 CD LYS A 212 -44.489 33.309 50.084 1.00 0.00
A C
ATOM 16606 HD1 LYS A 212 -45.381 33.943 49.876 1.00 0.00
A H
ATOM 16607 HD2 LYS A 212 -43.668 33.661 49.418 1.00 0.00
A H
ATOM 16608 CE LYS A 212 -44.789 31.843 49.843 1.00 0.00
A C
ATOM 16609 HE1 LYS A 212 -43.922 31.213 50.144 1.00 0.00
A H
ATOM 16610 HE2 LYS A 212 -45.677 31.524 50.431 1.00 0.00
A H
ATOM 16611 NZ LYS A 212 -45.066 31.602 48.404 1.00 0.00
A N
ATOM 16612 HZ1 LYS A 212 -45.270 30.595 48.244 1.00 0.00
A H
ATOM 16613 HZ2 LYS A 212 -45.885 32.171 48.107 1.00 0.00
A H
ATOM 16614 HZ3 LYS A 212 -44.237 31.879 47.841 1.00 0.00
A H
ATOM 16615 C LYS A 212 -43.511 36.760 53.580 1.00 0.00
A C
ATOM 16616 O LYS A 212 -42.838 37.719 53.195 1.00 0.00
A O
ATOM 16617 N ASP A 213 -44.533 36.873 54.425 1.00 0.00
A N
ATOM 16618 HN ASP A 213 -45.103 36.115 54.727 1.00 0.00
A H
ATOM 16619 CA ASP A 213 -44.930 38.150 55.007 1.00 0.00
A C
ATOM 16620 HA ASP A 213 -45.029 38.855 54.190 1.00 0.00
A H
ATOM 16621 CB ASP A 213 -46.256 38.006 55.758 1.00 0.00
A C
ATOM 16622 HB1 ASP A 213 -46.206 37.119 56.420 1.00 0.00
A H
ATOM 16623 HB2 ASP A 213 -46.449 38.899 56.385 1.00 0.00
A H
ATOM 16624 CG ASP A 213 -47.442 37.810 54.829 1.00 0.00
A C
ATOM 16625 OD1 ASP A 213 -48.377 37.076 55.213 1.00 0.00
A O
ATOM 16626 OD2 ASP A 213 -47.453 38.386 53.721 1.00 0.00
A O
ATOM 16627 C ASP A 213 -43.842 38.695 55.930 1.00 0.00
A C
ATOM 16628 O ASP A 213 -43.517 39.885 55.881 1.00 0.00
A O
ATOM 16629 N ILE A 214 -43.284 37.814 56.759 1.00 0.00
A N
ATOM 16630 HN ILE A 214 -43.589 36.867 56.830 1.00 0.00
A H
ATOM 16631 CA ILE A 214 -42.168 38.154 57.645 1.00 0.00
A C

ATOM 16632 HA ILE A 214 -42.485 39.000 58.242 1.00 0.00
A H
ATOM 16633 CB ILE A 214 -41.762 36.960 58.540 1.00 0.00
A C
ATOM 16634 HB ILE A 214 -41.556 36.128 57.821 1.00 0.00
A H
ATOM 16635 CG2 ILE A 214 -40.561 37.321 59.407 1.00 0.00
A C
ATOM 16636 HG21 ILE A 214 -40.173 36.423 59.932 1.00 0.00
A H
ATOM 16637 HG22 ILE A 214 -39.736 37.732 58.788 1.00 0.00
A H
ATOM 16638 HG23 ILE A 214 -40.835 38.079 60.171 1.00 0.00
A H
ATOM 16639 CG1 ILE A 214 -42.947 36.517 59.406 1.00 0.00
A C
ATOM 16640 HG11 ILE A 214 -43.086 37.231 60.248 1.00 0.00
A H
ATOM 16641 HG12 ILE A 214 -43.876 36.548 58.791 1.00 0.00
A H
ATOM 16642 CD ILE A 214 -42.820 35.120 59.977 1.00 0.00
A C
ATOM 16643 HD1 ILE A 214 -43.738 34.850 60.543 1.00 0.00
A H
ATOM 16644 HD2 ILE A 214 -42.681 34.375 59.163 1.00 0.00
A H
ATOM 16645 HD3 ILE A 214 -41.956 35.050 60.671 1.00 0.00
A H
ATOM 16646 C ILE A 214 -40.974 38.622 56.817 1.00 0.00
A C
ATOM 16647 O ILE A 214 -40.432 39.701 57.053 1.00 0.00
A O
ATOM 16648 N LYS A 215 -40.592 37.811 55.835 1.00 0.00
A N
ATOM 16649 HN LYS A 215 -41.026 36.929 55.656 1.00 0.00
A H
ATOM 16650 CA LYS A 215 -39.498 38.153 54.936 1.00 0.00
A C
ATOM 16651 HA LYS A 215 -38.596 38.209 55.536 1.00 0.00
A H
ATOM 16652 CB LYS A 215 -39.353 37.092 53.844 1.00 0.00
A C
ATOM 16653 HB1 LYS A 215 -40.076 36.277 54.081 1.00 0.00
A H
ATOM 16654 HB2 LYS A 215 -39.678 37.526 52.872 1.00 0.00
A H
ATOM 16655 CG LYS A 215 -37.955 36.504 53.734 1.00 0.00
A C
ATOM 16656 HG1 LYS A 215 -37.216 37.333 53.648 1.00 0.00
A H
ATOM 16657 HG2 LYS A 215 -37.721 35.947 54.669 1.00 0.00
A H
ATOM 16658 CD LYS A 215 -37.835 35.593 52.522 1.00 0.00
A C

ATOM 16659 HD1 LYS A 215 −38.786 35.649 51.944 1.00 0.00
A H
ATOM 16660 HD2 LYS A 215 −37.024 35.991 51.868 1.00 0.00
A H
ATOM 16661 CE LYS A 215 −37.533 34.162 52.933 1.00 0.00
A C
ATOM 16662 HE1 LYS A 215 −36.510 34.088 53.364 1.00 0.00
A H
ATOM 16663 HE2 LYS A 215 −38.263 33.813 53.697 1.00 0.00
A H
ATOM 16664 NZ LYS A 215 −37.605 33.225 51.776 1.00 0.00
A N
ATOM 16665 HZ1 LYS A 215 −37.393 32.256 52.090 1.00 0.00
A H
ATOM 16666 HZ2 LYS A 215 −38.561 33.254 51.365 1.00 0.00
A H
ATOM 16667 HZ3 LYS A 215 −36.914 33.513 51.054 1.00 0.00
A H
ATOM 16668 C LYS A 215 −39.713 39.527 54.308 1.00 0.00
A C
ATOM 16669 O LYS A 215 −38.775 40.317 54.196 1.00 0.00
A O
ATOM 16670 N ARG A 216 −40.948 39.809 53.901 1.00 0.00
A N
ATOM 16671 HN ARG A 216 −41.702 39.164 53.992 1.00 0.00
A H
ATOM 16672 CA ARG A 216 −41.257 41.093 53.294 1.00 0.00
A C
ATOM 16673 HA ARG A 216 −40.557 41.280 52.489 1.00 0.00
A H
ATOM 16674 CB ARG A 216 −42.683 41.123 52.748 1.00 0.00
A C
ATOM 16675 HB1 ARG A 216 −42.820 40.176 52.172 1.00 0.00
A H
ATOM 16676 HB2 ARG A 216 −43.406 41.074 53.592 1.00 0.00
A H
ATOM 16677 CG ARG A 216 −42.957 42.312 51.832 1.00 0.00
A C
ATOM 16678 HG1 ARG A 216 −42.671 43.244 52.368 1.00 0.00
A H
ATOM 16679 HG2 ARG A 216 −42.277 42.253 50.950 1.00 0.00
A H
ATOM 16680 CD ARG A 216 −44.391 42.359 51.322 1.00 0.00
A C
ATOM 16681 HD1 ARG A 216 −45.068 42.672 52.150 1.00 0.00
A H
ATOM 16682 HD2 ARG A 216 −44.502 43.078 50.478 1.00 0.00
A H
ATOM 16683 NE ARG A 216 −44.839 41.063 50.817 1.00 0.00
A N
ATOM 16684 HE ARG A 216 −44.382 40.792 49.962 1.00 0.00
A H
ATOM 16685 CZ ARG A 216 −45.756 40.300 51.408 1.00 0.00
A C

ATOM 16686 NH1 ARG A 216   -46.348 40.701 52.526 1.00 0.00
A   N
ATOM 16687 HH11 ARG A 216   -47.012 40.124 52.980 1.00 0.00
A   H
ATOM 16688 HH12 ARG A 216   -45.988 41.538 52.944 1.00 0.00
A   H
ATOM 16689 NH2 ARG A 216   -46.087 39.131 50.877 1.00 0.00
A   N
ATOM 16690 HH21 ARG A 216   -46.830 38.604 51.266 1.00 0.00
A   H
ATOM 16691 HH22 ARG A 216   -45.717 38.924 49.981 1.00 0.00
A   H
ATOM 16692 C   ARG A 216   -41.058 42.207 54.310 1.00 0.00
A   C
ATOM 16693 O   ARG A 216   -40.474 43.243 53.989 1.00 0.00
A   O
ATOM 16694 N   PHE A 217   -41.544 41.982 55.532 1.00 0.00
A   N
ATOM 16695 HN PHE A 217   -42.044 41.154 55.784 1.00 0.00
A   H
ATOM 16696 CA PHE A 217   -41.379 42.944 56.620 1.00 0.00
A   C
ATOM 16697 HA PHE A 217   -41.823 43.870 56.275 1.00 0.00
A   H
ATOM 16698 CB PHE A 217   -42.083 42.459 57.891 1.00 0.00
A   C
ATOM 16699 HB1 PHE A 217   -43.172 42.667 57.806 1.00 0.00
A   H
ATOM 16700 HB2 PHE A 217   -41.969 41.361 58.018 1.00 0.00
A   H
ATOM 16701 CG PHE A 217   -41.623 43.154 59.144 1.00 0.00
A   C
ATOM 16702 CD1 PHE A 217   -41.903 44.502 59.356 1.00 0.00
A   C
ATOM 16703 HD1 PHE A 217   -42.460 45.055 58.613 1.00 0.00
A   H
ATOM 16704 CE1 PHE A 217   -41.470 45.145 60.507 1.00 0.00
A   C
ATOM 16705 HE1 PHE A 217   -41.696 46.191 60.659 1.00 0.00
A   H
ATOM 16706 CZ PHE A 217   -40.753 44.443 61.463 1.00 0.00
A   C
ATOM 16707 HZ PHE A 217   -40.424 44.937 62.366 1.00 0.00
A   H
ATOM 16708 CD2 PHE A 217   -40.899 42.464 60.109 1.00 0.00
A   C
ATOM 16709 HD2 PHE A 217   -40.648 41.424 59.955 1.00 0.00
A   H
ATOM 16710 CE2 PHE A 217   -40.468 43.103 61.264 1.00 0.00
A   C
ATOM 16711 HE2 PHE A 217   -39.908 42.552 62.007 1.00 0.00
A   H
ATOM 16712 C   PHE A 217   -39.900 43.228 56.888 1.00 0.00
A   C

ATOM 16713 O PHE A 217 -39.519 44.369 57.161 1.00 0.00
A O
ATOM 16714 N GLU A 218 -39.081 42.183 56.805 1.00 0.00
A N
ATOM 16715 HN GLU A 218 -39.393 41.250 56.649 1.00 0.00
A H
ATOM 16716 CA GLU A 218 -37.633 42.310 56.937 1.00 0.00
A C
ATOM 16717 HA GLU A 218 -37.415 42.788 57.884 1.00 0.00
A H
ATOM 16718 CB GLU A 218 -36.959 40.934 56.894 1.00 0.00
A C
ATOM 16719 HB1 GLU A 218 -37.244 40.437 55.938 1.00 0.00
A H
ATOM 16720 HB2 GLU A 218 -35.854 41.055 56.857 1.00 0.00
A H
ATOM 16721 CG GLU A 218 -37.333 40.015 58.051 1.00 0.00
A C
ATOM 16722 HG1 GLU A 218 -37.086 40.509 59.009 1.00 0.00
A H
ATOM 16723 HG2 GLU A 218 -38.415 39.773 58.033 1.00 0.00
A H
ATOM 16724 CD GLU A 218 -36.553 38.715 58.050 1.00 0.00
A C
ATOM 16725 OE1 GLU A 218 -35.306 38.766 58.139 1.00 0.00
A O
ATOM 16726 OE2 GLU A 218 -37.192 37.642 57.970 1.00 0.00
A O
ATOM 16727 C GLU A 218 -37.075 43.209 55.837 1.00 0.00
A C
ATOM 16728 O GLU A 218 -36.350 44.163 56.120 1.00 0.00
A O
ATOM 16729 N ASN A 219 -37.442 42.907 54.589 1.00 0.00
A N
ATOM 16730 HN ASN A 219 -38.030 42.126 54.380 1.00 0.00
A H
ATOM 16731 CA ASN A 219 -37.023 43.684 53.417 1.00 0.00
A C
ATOM 16732 HA ASN A 219 -35.945 43.570 53.393 1.00 0.00
A H
ATOM 16733 CB ASN A 219 -37.656 43.116 52.139 1.00 0.00
A C
ATOM 16734 HB1 ASN A 219 -38.603 42.606 52.425 1.00 0.00
A H
ATOM 16735 HB2 ASN A 219 -37.919 43.937 51.441 1.00 0.00
A H
ATOM 16736 CG ASN A 219 -36.771 42.099 51.440 1.00 0.00
A C
ATOM 16737 OD1 ASN A 219 -35.566 42.298 51.288 1.00 0.00
A O
ATOM 16738 ND2 ASN A 219 -37.374 41.006 50.990 1.00 0.00
A N
ATOM 16739 HD21 ASN A 219 -36.807 40.322 50.539 1.00 0.00
A H

ATOM 16740 HD22 ASN A 219   -38.350 40.880 51.136 1.00 0.00
A   H
ATOM 16741 C   ASN A 219   -37.329 45.179 53.495 1.00 0.00
A   C
ATOM 16742 O   ASN A 219   -36.535 46.002 53.037 1.00 0.00
A   O
ATOM 16743 N   GLU A 220   -38.475 45.522 54.079 1.00 0.00
A   N
ATOM 16744 HN GLU A 220   -39.054 44.893 54.590 1.00 0.00
A   H
ATOM 16745 CA GLU A 220   -38.993 46.889 54.019 1.00 0.00
A   C
ATOM 16746 HA GLU A 220   -38.426 47.396 53.246 1.00 0.00
A   H
ATOM 16747 CB GLU A 220   -40.454 46.883 53.567 1.00 0.00
A   C
ATOM 16748 HB1 GLU A 220   -41.001 46.125 54.175 1.00 0.00
A   H
ATOM 16749 HB2 GLU A 220   -40.926 47.863 53.804 1.00 0.00
A   H
ATOM 16750 CG GLU A 220   -40.628 46.560 52.090 1.00 0.00
A   C
ATOM 16751 HG1 GLU A 220   -40.244 47.400 51.481 1.00 0.00
A   H
ATOM 16752 HG2 GLU A 220   -40.082 45.634 51.818 1.00 0.00
A   H
ATOM 16753 CD GLU A 220   -42.080 46.393 51.679 1.00 0.00
A   C
ATOM 16754 OE1 GLU A 220   -42.933 46.111 52.548 1.00 0.00
A   O
ATOM 16755 OE2 GLU A 220   -42.370 46.532 50.471 1.00 0.00
A   O
ATOM 16756 C   GLU A 220   -38.833 47.702 55.306 1.00 0.00
A   C
ATOM 16757 O   GLU A 220   -38.444 48.869 55.256 1.00 0.00
A   O
ATOM 16758 N   GLY A 221   -39.139 47.085 56.444 1.00 0.00
A   N
ATOM 16759 HN GLY A 221   -39.462 46.139 56.472 1.00 0.00
A   H
ATOM 16760 CA GLY A 221   -39.025 47.752 57.742 1.00 0.00
A   C
ATOM 16761 HA1 GLY A 221   -39.656 47.201 58.425 1.00 0.00
A   H
ATOM 16762 HA2 GLY A 221   -39.327 48.779 57.589 1.00 0.00
A   H
ATOM 16763 C   GLY A 221   -37.612 47.757 58.304 1.00 0.00
A   C
ATOM 16764 O   GLY A 221   -37.333 48.446 59.289 1.00 0.00
A   O
ATOM 16765 N   MET A 222   -36.727 46.983 57.676 1.00 0.00
A   N
ATOM 16766 HN MET A 222   -36.952 46.452 56.862 1.00 0.00
A   H

ATOM 16767 CA MET A 222 -35.342 46.844 58.123 1.00 0.00
A C
ATOM 16768 HA MET A 222 -35.150 47.645 58.827 1.00 0.00
A H
ATOM 16769 CB MET A 222 -35.127 45.482 58.781 1.00 0.00
A C
ATOM 16770 HB1 MET A 222 -35.446 44.711 58.041 1.00 0.00
A H
ATOM 16771 HB2 MET A 222 -34.039 45.321 58.950 1.00 0.00
A H
ATOM 16772 CG MET A 222 -35.908 45.282 60.069 1.00 0.00
A C
ATOM 16773 HG1 MET A 222 -35.380 45.763 60.919 1.00 0.00
A H
ATOM 16774 HG2 MET A 222 -36.907 45.762 59.979 1.00 0.00
A H
ATOM 16775 SD MET A 222 -36.134 43.541 60.469 1.00 0.00
A S
ATOM 16776 CE MET A 222 -36.961 43.693 62.048 1.00 0.00
A C
ATOM 16777 HE1 MET A 222 -37.183 42.692 62.477 1.00 0.00
A H
ATOM 16778 HE2 MET A 222 -36.330 44.245 62.778 1.00 0.00
A H
ATOM 16779 HE3 MET A 222 -37.922 44.241 61.946 1.00 0.00
A H
ATOM 16780 C MET A 222 -34.376 47.014 56.961 1.00 0.00
A C
ATOM 16781 O MET A 222 -33.296 47.576 57.122 1.00 0.00
A O
ATOM 16782 N GLN B 8 -23.907 34.539 44.701 1.00 0.00
B N
ATOM 16783 HT1 GLN B 8 -24.387 35.185 44.107 1.00 0.00
B H
ATOM 16784 HT2 GLN B 8 -24.293 33.623 44.592 1.00 0.00
B H
ATOM 16785 HT3 GLN B 8 -22.933 34.528 44.475 1.00 0.00
B H
ATOM 16786 CA GLN B 8 -24.072 34.969 46.118 1.00 0.00
B C
ATOM 16787 HA GLN B 8 -23.604 35.944 46.186 1.00 0.00
B H
ATOM 16788 CB GLN B 8 -25.556 35.118 46.441 1.00 0.00
B C
ATOM 16789 HB1 GLN B 8 -26.086 35.250 45.469 1.00 0.00
B H
ATOM 16790 HB2 GLN B 8 -25.937 34.165 46.874 1.00 0.00
B H
ATOM 16791 CG GLN B 8 -25.866 36.292 47.353 1.00 0.00
B C
ATOM 16792 HG1 GLN B 8 -25.523 36.079 48.388 1.00 0.00
B H
ATOM 16793 HG2 GLN B 8 -25.347 37.209 47.004 1.00 0.00
B H

ATOM 16794 CD GLN B   8   -27.351 36.583 47.449 1.00 0.00
B   C
ATOM 16795 OE1 GLN B   8   -28.169 35.983 46.740 1.00 0.00
B   O
ATOM 16796 NE2 GLN B   8   -27.711 37.508 48.328 1.00 0.00
B   N
ATOM 16797 HE21 GLN B   8   -28.685 37.704 48.402 1.00 0.00
B   H
ATOM 16798 HE22 GLN B   8   -27.017 37.979 48.862 1.00 0.00
B   H
ATOM 16799 C   GLN B   8   -23.366 34.041 47.138 1.00 0.00
B   C
ATOM 16800 O   GLN B   8   -22.791 34.525 48.122 1.00 0.00
B   O
ATOM 16801 N   PHE B   9   -23.406 32.725 46.906 1.00 0.00
B   N
ATOM 16802 HN PHE B   9   -23.851 32.318 46.110 1.00 0.00
B   H
ATOM 16803 CA PHE B   9   -22.785 31.751 47.823 1.00 0.00
B   C
ATOM 16804 HA PHE B   9   -22.126 32.328 48.460 1.00 0.00
B   H
ATOM 16805 CB PHE B   9   -23.834 30.971 48.630 1.00 0.00
B   C
ATOM 16806 HB1 PHE B   9   -24.430 30.339 47.936 1.00 0.00
B   H
ATOM 16807 HB2 PHE B   9   -23.351 30.289 49.361 1.00 0.00
B   H
ATOM 16808 CG PHE B   9   -24.824 31.834 49.350 1.00 0.00
B   C
ATOM 16809 CD1 PHE B   9   -24.548 32.314 50.621 1.00 0.00
B   C
ATOM 16810 HD1 PHE B   9   -23.612 32.060 51.097 1.00 0.00
B   H
ATOM 16811 CE1 PHE B   9   -25.456 33.110 51.291 1.00 0.00
B   C
ATOM 16812 HE1 PHE B   9   -25.224 33.479 52.281 1.00 0.00
B   H
ATOM 16813 CZ PHE B   9   -26.657 33.436 50.690 1.00 0.00
B   C
ATOM 16814 HZ PHE B   9   -27.368 34.066 51.206 1.00 0.00
B   H
ATOM 16815 CD2 PHE B   9   -26.035 32.162 48.758 1.00 0.00
B   C
ATOM 16816 HD2 PHE B   9   -26.282 31.771 47.780 1.00 0.00
B   H
ATOM 16817 CE2 PHE B   9   -26.946 32.964 49.417 1.00 0.00
B   C
ATOM 16818 HE2 PHE B   9   -27.882 33.220 48.940 1.00 0.00
B   H
ATOM 16819 C   PHE B   9   -21.924 30.735 47.096 1.00 0.00
B   C
ATOM 16820 O   PHE B   9   -22.313 30.209 46.063 1.00 0.00
B   O

ATOM 16821 N ALA B 10 -20.751 30.463 47.646 1.00 0.00
B N
ATOM 16822 HN ALA B 10 -20.382 30.986 48.413 1.00 0.00
B H
ATOM 16823 CA ALA B 10 -19.914 29.380 47.172 1.00 0.00
B C
ATOM 16824 HA ALA B 10 -20.409 28.785 46.413 1.00 0.00
B H
ATOM 16825 CB ALA B 10 -18.612 29.915 46.600 1.00 0.00
B C
ATOM 16826 HB1 ALA B 10 -18.827 30.553 45.716 1.00 0.00
B H
ATOM 16827 HB2 ALA B 10 -18.073 30.537 47.347 1.00 0.00
B H
ATOM 16828 HB3 ALA B 10 -17.939 29.093 46.273 1.00 0.00
B H
ATOM 16829 C ALA B 10 -19.648 28.451 48.338 1.00 0.00
B C
ATOM 16830 O ALA B 10 -19.348 28.908 49.446 1.00 0.00
B O
ATOM 16831 N VAL B 11 -19.779 27.148 48.098 1.00 0.00
B N
ATOM 16832 HN VAL B 11 -20.111 26.776 47.235 1.00 0.00
B H
ATOM 16833 CA VAL B 11 -19.439 26.137 49.103 1.00 0.00
B C
ATOM 16834 HA VAL B 11 -19.124 26.647 50.006 1.00 0.00
B H
ATOM 16835 CB VAL B 11 -20.640 25.225 49.423 1.00 0.00
B C
ATOM 16836 HB VAL B 11 -20.979 24.764 48.463 1.00 0.00
B H
ATOM 16837 CG1 VAL B 11 -20.237 24.123 50.391 1.00 0.00
B C
ATOM 16838 HG11 VAL B 11 -21.116 23.494 50.647 1.00 0.00
B H
ATOM 16839 HG12 VAL B 11 -19.467 23.454 49.953 1.00 0.00
B H
ATOM 16840 HG13 VAL B 11 -19.837 24.556 51.333 1.00 0.00
B H
ATOM 16841 CG2 VAL B 11 -21.784 26.049 49.999 1.00 0.00
B C
ATOM 16842 HG21 VAL B 11 -22.640 25.396 50.270 1.00 0.00
B H
ATOM 16843 HG22 VAL B 11 -21.454 26.591 50.912 1.00 0.00
B H
ATOM 16844 HG23 VAL B 11 -22.150 26.795 49.264 1.00 0.00
B H
ATOM 16845 C VAL B 11 -18.255 25.315 48.604 1.00 0.00
B C
ATOM 16846 O VAL B 11 -18.346 24.650 47.566 1.00 0.00
B O
ATOM 16847 N ILE B 12 -17.147 25.364 49.341 1.00 0.00
B N

ATOM 16848 HN ILE B 12 -17.071 25.792 50.239 1.00 0.00
B H
ATOM 16849 CA ILE B 12 -15.908 24.766 48.858 1.00 0.00
B C
ATOM 16850 HA ILE B 12 -15.987 24.815 47.780 1.00 0.00
B H
ATOM 16851 CB ILE B 12 -14.676 25.638 49.156 1.00 0.00
B C
ATOM 16852 HB ILE B 12 -14.846 26.015 50.196 1.00 0.00
B H
ATOM 16853 CG2 ILE B 12 -13.399 24.849 48.949 1.00 0.00
B C
ATOM 16854 HG21 ILE B 12 -12.522 25.425 49.312 1.00 0.00
B H
ATOM 16855 HG22 ILE B 12 -13.429 23.892 49.510 1.00 0.00
B H
ATOM 16856 HG23 ILE B 12 -13.243 24.620 47.873 1.00 0.00
B H
ATOM 16857 CG1 ILE B 12 -14.694 26.849 48.218 1.00 0.00
B C
ATOM 16858 HG11 ILE B 12 -14.712 26.505 47.160 1.00 0.00
B H
ATOM 16859 HG12 ILE B 12 -15.629 27.427 48.393 1.00 0.00
B H
ATOM 16860 CD ILE B 12 -13.537 27.809 48.380 1.00 0.00
B C
ATOM 16861 HD1 ILE B 12 -13.662 28.682 47.702 1.00 0.00
B H
ATOM 16862 HD2 ILE B 12 -13.482 28.184 49.424 1.00 0.00
B H
ATOM 16863 HD3 ILE B 12 -12.573 27.317 48.131 1.00 0.00
B H
ATOM 16864 C ILE B 12 -15.702 23.292 49.221 1.00 0.00
B C
ATOM 16865 O ILE B 12 -15.012 22.579 48.502 1.00 0.00
B O
ATOM 16866 N GLY B 13 -16.314 22.801 50.281 1.00 0.00
B N
ATOM 16867 HN GLY B 13 -16.845 23.316 50.952 1.00 0.00
B H
ATOM 16868 CA GLY B 13 -16.224 21.361 50.519 1.00 0.00
B C
ATOM 16869 HA1 GLY B 13 -15.820 21.246 51.516 1.00 0.00
B H
ATOM 16870 HA2 GLY B 13 -15.592 20.962 49.736 1.00 0.00
B H
ATOM 16871 C GLY B 13 -17.552 20.633 50.464 1.00 0.00
B C
ATOM 16872 O GLY B 13 -18.420 20.857 51.307 1.00 0.00
B O
ATOM 16873 N LEU B 14 -17.721 19.742 49.498 1.00 0.00
B N
ATOM 16874 HN LEU B 14 -17.044 19.484 48.813 1.00 0.00
B H

ATOM 16875 CA LEU B 14 −19.002 19.039 49.373 1.00 0.00
B C
ATOM 16876 HA LEU B 14 −19.756 19.596 49.915 1.00 0.00
B H
ATOM 16877 CB LEU B 14 −19.411 18.918 47.900 1.00 0.00
B C
ATOM 16878 HB1 LEU B 14 −18.709 18.196 47.422 1.00 0.00
B H
ATOM 16879 HB2 LEU B 14 −20.419 18.450 47.842 1.00 0.00
B H
ATOM 16880 CG LEU B 14 −19.394 20.207 47.080 1.00 0.00
B C
ATOM 16881 HG LEU B 14 −18.429 20.724 47.302 1.00 0.00
B H
ATOM 16882 CD1 LEU B 14 −19.459 19.884 45.599 1.00 0.00
B C
ATOM 16883 HD11 LEU B 14 −19.495 20.818 44.997 1.00 0.00
B H
ATOM 16884 HD12 LEU B 14 −18.566 19.301 45.285 1.00 0.00
B H
ATOM 16885 HD13 LEU B 14 −20.366 19.287 45.367 1.00 0.00
B H
ATOM 16886 CD2 LEU B 14 −20.527 21.129 47.498 1.00 0.00
B C
ATOM 16887 HD21 LEU B 14 −20.495 22.072 46.911 1.00 0.00
B H
ATOM 16888 HD22 LEU B 14 −21.518 20.661 47.331 1.00 0.00
B H
ATOM 16889 HD23 LEU B 14 −20.439 21.388 48.575 1.00 0.00
B H
ATOM 16890 C LEU B 14 −18.836 17.679 50.076 1.00 0.00
B C
ATOM 16891 O LEU B 14 −18.613 16.656 49.425 1.00 0.00
B O
ATOM 16892 N GLY B 15 −18.955 17.675 51.403 1.00 0.00
B N
ATOM 16893 HN GLY B 15 −19.111 18.507 51.933 1.00 0.00
B H
ATOM 16894 CA GLY B 15 −18.865 16.439 52.178 1.00 0.00
B C
ATOM 16895 HA1 GLY B 15 −18.064 16.513 52.902 1.00 0.00
B H
ATOM 16896 HA2 GLY B 15 −18.790 15.586 51.518 1.00 0.00
B H
ATOM 16897 C GLY B 15 −20.172 16.331 52.936 1.00 0.00
B C
ATOM 16898 O GLY B 15 −21.173 16.937 52.546 1.00 0.00
B O
ATOM 16899 N ARG B 16 −20.168 15.564 54.024 1.00 0.00
B N
ATOM 16900 HN ARG B 16 −19.372 15.016 54.271 1.00 0.00
B H
ATOM 16901 CA ARG B 16 −21.324 15.482 54.917 1.00 0.00
B C

```
ATOM  16902  HA  ARG B  16     -22.118  14.964  54.390  1.00  0.00
B    H
ATOM  16903  CB  ARG B  16     -20.964  14.693  56.178  1.00  0.00
B    C
ATOM  16904  HB1 ARG B  16     -19.854  14.751  56.279  1.00  0.00
B    H
ATOM  16905  HB2 ARG B  16     -21.380  15.208  57.071  1.00  0.00
B    H
ATOM  16906  CG  ARG B  16     -21.381  13.232  56.130  1.00  0.00
B    C
ATOM  16907  HG1 ARG B  16     -22.260  13.092  56.797  1.00  0.00
B    H
ATOM  16908  HG2 ARG B  16     -21.743  12.993  55.103  1.00  0.00
B    H
ATOM  16909  CD  ARG B  16     -20.230  12.310  56.470  1.00  0.00
B    C
ATOM  16910  HD1 ARG B  16     -20.588  11.255  56.466  1.00  0.00
B    H
ATOM  16911  HD2 ARG B  16     -19.397  12.408  55.735  1.00  0.00
B    H
ATOM  16912  NE  ARG B  16     -19.650  12.597  57.774  1.00  0.00
B    N
ATOM  16913  HE  ARG B  16     -20.332  12.892  58.453  1.00  0.00
B    H
ATOM  16914  CZ  ARG B  16     -18.353  12.487  58.059  1.00  0.00
B    C
ATOM  16915  NH1 ARG B  16     -17.486  12.112  57.118  1.00  0.00
B    N
ATOM  16916 HH11 ARG B  16     -16.525  12.005  57.333  1.00  0.00
B    H
ATOM  16917 HH12 ARG B  16     -17.878  11.820  56.244  1.00  0.00
B    H
ATOM  16918  NH2 ARG B  16     -17.916  12.754  59.284  1.00  0.00
B    N
ATOM  16919 HH21 ARG B  16     -16.943  12.756  59.472  1.00  0.00
B    H
ATOM  16920 HH22 ARG B  16     -18.569  13.147  59.917  1.00  0.00
B    H
ATOM  16921  C   ARG B  16     -21.870  16.866  55.279  1.00  0.00
B    C
ATOM  16922  O   ARG B  16     -23.075  17.104  55.202  1.00  0.00
B    O
ATOM  16923  N   PHE B  17     -20.972  17.779  55.638  1.00  0.00
B    N
ATOM  16924  HN  PHE B  17     -19.989  17.602  55.653  1.00  0.00
B    H
ATOM  16925  CA  PHE B  17     -21.349  19.128  56.043  1.00  0.00
B    C
ATOM  16926  HA  PHE B  17     -22.313  19.019  56.525  1.00  0.00
B    H
ATOM  16927  CB  PHE B  17     -20.308  19.684  57.012  1.00  0.00
B    C
ATOM  16928  HB1 PHE B  17     -20.232  19.009  57.892  1.00  0.00
B    H
```

ATOM 16929 HB2 PHE B 17 -19.302 19.721 56.540 1.00 0.00
B H
ATOM 16930 CG PHE B 17 -20.642 21.038 57.558 1.00 0.00
B C
ATOM 16931 CD1 PHE B 17 -21.770 21.221 58.360 1.00 0.00
B C
ATOM 16932 HD1 PHE B 17 -22.417 20.382 58.572 1.00 0.00
B H
ATOM 16933 CE1 PHE B 17 -22.074 22.474 58.885 1.00 0.00
B C
ATOM 16934 HE1 PHE B 17 -22.949 22.602 59.505 1.00 0.00
B H
ATOM 16935 CZ PHE B 17 -21.243 23.555 58.611 1.00 0.00
B C
ATOM 16936 HZ PHE B 17 -21.472 24.528 59.021 1.00 0.00
B H
ATOM 16937 CD2 PHE B 17 -19.812 22.126 57.303 1.00 0.00
B C
ATOM 16938 HD2 PHE B 17 -18.937 22.003 56.680 1.00 0.00
B H
ATOM 16939 CE2 PHE B 17 -20.110 23.381 57.819 1.00 0.00
B C
ATOM 16940 HE2 PHE B 17 -19.462 24.219 57.604 1.00 0.00
B H
ATOM 16941 C PHE B 17 -21.541 20.094 54.874 1.00 0.00
B C
ATOM 16942 O PHE B 17 -22.605 20.687 54.726 1.00 0.00
B O
ATOM 16943 N GLY B 18 -20.510 20.270 54.054 1.00 0.00
B N
ATOM 16944 HN GLY B 18 -19.647 19.773 54.143 1.00 0.00
B H
ATOM 16945 CA GLY B 18 -20.579 21.219 52.950 1.00 0.00
B C
ATOM 16946 HA1 GLY B 18 -19.631 21.163 52.432 1.00 0.00
B H
ATOM 16947 HA2 GLY B 18 -20.793 22.189 53.380 1.00 0.00
B H
ATOM 16948 C GLY B 18 -21.676 20.894 51.959 1.00 0.00
B C
ATOM 16949 O GLY B 18 -22.428 21.777 51.548 1.00 0.00
B O
ATOM 16950 N GLY B 19 -21.772 19.624 51.579 1.00 0.00
B N
ATOM 16951 HN GLY B 19 -21.125 18.925 51.879 1.00 0.00
B H
ATOM 16952 CA GLY B 19 -22.835 19.160 50.693 1.00 0.00
B C
ATOM 16953 HA1 GLY B 19 -22.752 18.083 50.648 1.00 0.00
B H
ATOM 16954 HA2 GLY B 19 -22.691 19.657 49.744 1.00 0.00
B H
ATOM 16955 C GLY B 19 -24.230 19.504 51.196 1.00 0.00
B C

ATOM 16956 O   GLY B 19   -25.105 19.874 50.418 1.00 0.00
B   O
ATOM 16957 N   SER B 20   -24.430 19.397 52.504 1.00 0.00
B   N
ATOM 16958 HN SER B 20   -23.726 19.121 53.159 1.00 0.00
B   H
ATOM 16959 CA SER B 20   -25.724 19.677 53.105 1.00 0.00
B   C
ATOM 16960 HA SER B 20   -26.450 19.138 52.511 1.00 0.00
B   H
ATOM 16961 CB SER B 20   -25.767 19.218 54.561 1.00 0.00
B   C
ATOM 16962 HB1 SER B 20   -25.608 18.115 54.580 1.00 0.00
B   H
ATOM 16963 HB2 SER B 20   -24.934 19.679 55.137 1.00 0.00
B   H
ATOM 16964 OG SER B 20   -27.025 19.516 55.132 1.00 0.00
B   O
ATOM 16965 HG1 SER B 20   -27.087 19.019 55.958 1.00 0.00
B   H
ATOM 16966 C   SER B 20   -26.071 21.150 53.014 1.00 0.00
B   C
ATOM 16967 O   SER B 20   -27.207 21.494 52.702 1.00 0.00
B   O
ATOM 16968 N   ILE B 21   -25.101 22.018 53.302 1.00 0.00
B   N
ATOM 16969 HN ILE B 21   -24.196 21.754 53.629 1.00 0.00
B   H
ATOM 16970 CA ILE B 21   -25.289 23.461 53.160 1.00 0.00
B   C
ATOM 16971 HA ILE B 21   -26.132 23.739 53.781 1.00 0.00
B   H
ATOM 16972 CB ILE B 21   -24.008 24.247 53.488 1.00 0.00
B   C
ATOM 16973 HB ILE B 21   -23.203 23.723 52.912 1.00 0.00
B   H
ATOM 16974 CG2 ILE B 21   -24.169 25.707 53.083 1.00 0.00
B   C
ATOM 16975 HG21 ILE B 21   -23.202 26.245 53.170 1.00 0.00
B   H
ATOM 16976 HG22 ILE B 21   -24.507 25.791 52.029 1.00 0.00
B   H
ATOM 16977 HG23 ILE B 21   -24.911 26.220 53.731 1.00 0.00
B   H
ATOM 16978 CG1 ILE B 21   -23.653 24.108 54.965 1.00 0.00
B   C
ATOM 16979 HG11 ILE B 21   -24.456 24.557 55.589 1.00 0.00
B   H
ATOM 16980 HG12 ILE B 21   -23.601 23.025 55.223 1.00 0.00
B   H
ATOM 16981 CD ILE B 21   -22.329 24.744 55.328 1.00 0.00
B   C
ATOM 16982 HD1 ILE B 21   -22.094 24.563 56.400 1.00 0.00
B   H

ATOM 16983 HD2 ILE B 21 −21.508 24.317 54.715 1.00 0.00
B H
ATOM 16984 HD3 ILE B 21 −22.357 25.842 55.164 1.00 0.00
B H
ATOM 16985 C ILE B 21 −25.681 23.771 51.721 1.00 0.00
B C
ATOM 16986 O ILE B 21 −26.663 24.478 51.471 1.00 0.00
B O
ATOM 16987 N CYS B 22 −24.909 23.221 50.782 1.00 0.00
B N
ATOM 16988 HN CYS B 22 −24.131 22.638 51.007 1.00 0.00
B H
ATOM 16989 CA CYS B 22 −25.129 23.420 49.355 1.00 0.00
B C
ATOM 16990 HA CYS B 22 −25.020 24.482 49.171 1.00 0.00
B H
ATOM 16991 CB CYS B 22 −24.112 22.617 48.553 1.00 0.00
B C
ATOM 16992 HB1 CYS B 22 −23.106 22.857 48.963 1.00 0.00
B H
ATOM 16993 HB2 CYS B 22 −24.265 21.529 48.731 1.00 0.00
B H
ATOM 16994 SG CYS B 22 −24.142 23.017 46.802 1.00 0.00
B S
ATOM 16995 HG1 CYS B 22 −23.132 22.227 46.435 1.00 0.00
B H
ATOM 16996 C CYS B 22 −26.545 23.024 48.956 1.00 0.00
B C
ATOM 16997 O CYS B 22 −27.310 23.854 48.466 1.00 0.00
B O
ATOM 16998 N LYS B 23 −26.892 21.762 49.199 1.00 0.00
B N
ATOM 16999 HN LYS B 23 −26.271 21.103 49.620 1.00 0.00
B H
ATOM 17000 CA LYS B 23 −28.215 21.240 48.865 1.00 0.00
B C
ATOM 17001 HA LYS B 23 −28.291 21.256 47.785 1.00 0.00
B H
ATOM 17002 CB LYS B 23 −28.369 19.795 49.347 1.00 0.00
B C
ATOM 17003 HB1 LYS B 23 −27.434 19.254 49.066 1.00 0.00
B H
ATOM 17004 HB2 LYS B 23 −28.412 19.786 50.459 1.00 0.00
B H
ATOM 17005 CG LYS B 23 −29.564 19.057 48.755 1.00 0.00
B C
ATOM 17006 HG1 LYS B 23 −30.502 19.561 49.085 1.00 0.00
B H
ATOM 17007 HG2 LYS B 23 −29.521 19.128 47.644 1.00 0.00
B H
ATOM 17008 CD LYS B 23 −29.610 17.596 49.188 1.00 0.00
B C
ATOM 17009 HD1 LYS B 23 −29.421 17.547 50.286 1.00 0.00
B H

```
ATOM  17010  HD2 LYS B  23      -30.642  17.214  49.011  1.00  0.00
B    H
ATOM  17011  CE  LYS B  23      -28.595  16.749  48.430  1.00  0.00
B    C
ATOM  17012  HE1 LYS B  23      -28.658  16.950  47.337  1.00  0.00
B    H
ATOM  17013  HE2 LYS B  23      -27.562  16.979  48.771  1.00  0.00
B    H
ATOM  17014  NZ  LYS B  23      -28.812  15.286  48.625  1.00  0.00
B    N
ATOM  17015  HZ1 LYS B  23      -28.101  14.746  48.091  1.00  0.00
B    H
ATOM  17016  HZ2 LYS B  23      -28.733  15.055  49.637  1.00  0.00
B    H
ATOM  17017  HZ3 LYS B  23      -29.762  15.029  48.289  1.00  0.00
B    H
ATOM  17018  C   LYS B  23      -29.341  22.118  49.415  1.00  0.00
B    C
ATOM  17019  O   LYS B  23      -30.339  22.347  48.732  1.00  0.00
B    O
ATOM  17020  N   GLU B  24      -29.163  22.624  50.634  1.00  0.00
B    N
ATOM  17021  HN  GLU B  24      -28.336  22.516  51.179  1.00  0.00
B    H
ATOM  17022  CA  GLU B  24      -30.208  23.405  51.289  1.00  0.00
B    C
ATOM  17023  HA  GLU B  24      -31.146  22.893  51.115  1.00  0.00
B    H
ATOM  17024  CB  GLU B  24      -30.016  23.437  52.805  1.00  0.00
B    C
ATOM  17025  HB1 GLU B  24      -29.954  22.385  53.170  1.00  0.00
B    H
ATOM  17026  HB2 GLU B  24      -29.036  23.903  53.051  1.00  0.00
B    H
ATOM  17027  CG  GLU B  24      -31.123  24.151  53.570  1.00  0.00
B    C
ATOM  17028  HG1 GLU B  24      -30.851  24.215  54.639  1.00  0.00
B    H
ATOM  17029  HG2 GLU B  24      -31.284  25.173  53.174  1.00  0.00
B    H
ATOM  17030  CD  GLU B  24      -32.453  23.428  53.543  1.00  0.00
B    C
ATOM  17031  OE1 GLU B  24      -33.439  23.997  54.058  1.00  0.00
B    O
ATOM  17032  OE2 GLU B  24      -32.520  22.296  53.013  1.00  0.00
B    O
ATOM  17033  C   GLU B  24      -30.349  24.811  50.727  1.00  0.00
B    C
ATOM  17034  O   GLU B  24      -31.462  25.254  50.485  1.00  0.00
B    O
ATOM  17035  N   LEU B  25      -29.232  25.507  50.518  1.00  0.00
B    N
ATOM  17036  HN  LEU B  25      -28.315  25.185  50.747  1.00  0.00
B    H
```

ATOM 17037 CA LEU B 25 -29.267 26.845 49.913 1.00 0.00
B C
ATOM 17038 HA LEU B 25 -29.861 27.470 50.568 1.00 0.00
B H
ATOM 17039 CB LEU B 25 -27.856 27.410 49.753 1.00 0.00
B C
ATOM 17040 HB1 LEU B 25 -27.229 26.606 49.303 1.00 0.00
B H
ATOM 17041 HB2 LEU B 25 -27.879 28.235 49.008 1.00 0.00
B H
ATOM 17042 CG LEU B 25 -27.085 27.912 50.973 1.00 0.00
B C
ATOM 17043 HG LEU B 25 -27.088 27.097 51.737 1.00 0.00
B H
ATOM 17044 CD1 LEU B 25 -25.658 28.232 50.569 1.00 0.00
B C
ATOM 17045 HD11 LEU B 25 -25.092 28.645 51.432 1.00 0.00
B H
ATOM 17046 HD12 LEU B 25 -25.136 27.316 50.217 1.00 0.00
B H
ATOM 17047 HD13 LEU B 25 -25.639 28.983 49.752 1.00 0.00
B H
ATOM 17048 CD2 LEU B 25 -27.748 29.137 51.588 1.00 0.00
B C
ATOM 17049 HD21 LEU B 25 -27.182 29.474 52.483 1.00 0.00
B H
ATOM 17050 HD22 LEU B 25 -27.785 29.984 50.874 1.00 0.00
B H
ATOM 17051 HD23 LEU B 25 -28.787 28.899 51.902 1.00 0.00
B H
ATOM 17052 C LEU B 25 -29.961 26.810 48.556 1.00 0.00
B C
ATOM 17053 O LEU B 25 -30.723 27.715 48.206 1.00 0.00
B O
ATOM 17054 N HSE B 26 -29.685 25.740 47.808 1.00 0.00
B N
ATOM 17055 HN HSE B 26 -29.050 25.030 48.110 1.00 0.00
B H
ATOM 17056 CA HSE B 26 -30.271 25.503 46.499 1.00 0.00
B C
ATOM 17057 HA HSE B 26 -30.150 26.421 45.937 1.00 0.00
B H
ATOM 17058 CB HSE B 26 -29.558 24.345 45.835 1.00 0.00
B C
ATOM 17059 HB1 HSE B 26 -28.474 24.587 45.798 1.00 0.00
B H
ATOM 17060 HB2 HSE B 26 -29.667 23.435 46.461 1.00 0.00
B H
ATOM 17061 ND1 HSE B 26 -29.795 24.926 43.413 1.00 0.00
B N
ATOM 17062 CG HSE B 26 -30.022 24.071 44.428 1.00 0.00
B C
ATOM 17063 CE1 HSE B 26 -30.310 24.421 42.280 1.00 0.00
B C

ATOM 17064 HE1 HSE B 26 -30.275 24.918 41.308 1.00 0.00
B H
ATOM 17065 NE2 HSE B 26 -30.862 23.229 42.569 1.00 0.00
B N
ATOM 17066 HE2 HSE B 26 -31.331 22.635 41.916 1.00 0.00
B H
ATOM 17067 CD2 HSE B 26 -30.704 22.985 43.886 1.00 0.00
B C
ATOM 17068 HD2 HSE B 26 -31.076 22.091 44.365 1.00 0.00
B H
ATOM 17069 C HSE B 26 -31.747 25.227 46.581 1.00 0.00
B C
ATOM 17070 O HSE B 26 -32.522 25.750 45.790 1.00 0.00
B O
ATOM 17071 N ARG B 27 -32.139 24.400 47.545 1.00 0.00
B N
ATOM 17072 HN ARG B 27 -31.471 23.959 48.139 1.00 0.00
B H
ATOM 17073 CA ARG B 27 -33.541 24.091 47.794 1.00 0.00
B C
ATOM 17074 HA ARG B 27 -33.990 23.770 46.862 1.00 0.00
B H
ATOM 17075 CB ARG B 27 -33.652 22.991 48.845 1.00 0.00
B C
ATOM 17076 HB1 ARG B 27 -32.858 22.245 48.605 1.00 0.00
B H
ATOM 17077 HB2 ARG B 27 -33.393 23.404 49.845 1.00 0.00
B H
ATOM 17078 CG ARG B 27 -34.992 22.278 48.878 1.00 0.00
B C
ATOM 17079 HG1 ARG B 27 -35.791 23.013 48.637 1.00 0.00
B H
ATOM 17080 HG2 ARG B 27 -35.021 21.524 48.058 1.00 0.00
B H
ATOM 17081 CD ARG B 27 -35.239 21.560 50.203 1.00 0.00
B C
ATOM 17082 HD1 ARG B 27 -36.259 21.110 50.196 1.00 0.00
B H
ATOM 17083 HD2 ARG B 27 -34.493 20.751 50.374 1.00 0.00
B H
ATOM 17084 NE ARG B 27 -35.136 22.459 51.358 1.00 0.00
B N
ATOM 17085 HE ARG B 27 -34.259 22.378 51.844 1.00 0.00
B H
ATOM 17086 CZ ARG B 27 -36.082 23.309 51.752 1.00 0.00
B C
ATOM 17087 NH1 ARG B 27 -37.230 23.398 51.095 1.00 0.00
B N
ATOM 17088 HH11 ARG B 27 -37.922 24.049 51.374 1.00 0.00
B H
ATOM 17089 HH12 ARG B 27 -37.292 22.880 50.239 1.00 0.00
B H
ATOM 17090 NH2 ARG B 27 -35.879 24.079 52.809 1.00 0.00
B N

```
ATOM  17091 HH21 ARG B  27      -36.614  24.646  53.154  1.00  0.00
B    H
ATOM  17092 HH22 ARG B  27      -35.063  23.901  53.343  1.00  0.00
B    H
ATOM  17093  C   ARG B  27      -34.310  25.336  48.245  1.00  0.00
B    C
ATOM  17094  O   ARG B  27      -35.494  25.456  47.965  1.00  0.00
B    O
ATOM  17095  N   MET B  28      -33.633  26.253  48.935  1.00  0.00
B    N
ATOM  17096  HN  MET B  28      -32.676  26.146  49.197  1.00  0.00
B    H
ATOM  17097  CA  MET B  28      -34.244  27.513  49.374  1.00  0.00
B    C
ATOM  17098  HA  MET B  28      -35.242  27.269  49.719  1.00  0.00
B    H
ATOM  17099  CB  MET B  28      -33.449  28.150  50.514  1.00  0.00
B    C
ATOM  17100  HB1 MET B  28      -32.382  28.181  50.188  1.00  0.00
B    H
ATOM  17101  HB2 MET B  28      -33.767  29.209  50.640  1.00  0.00
B    H
ATOM  17102  CG  MET B  28      -33.533  27.409  51.832  1.00  0.00
B    C
ATOM  17103  HG1 MET B  28      -34.579  27.400  52.203  1.00  0.00
B    H
ATOM  17104  HG2 MET B  28      -33.218  26.353  51.687  1.00  0.00
B    H
ATOM  17105  SD  MET B  28      -32.487  28.185  53.077  1.00  0.00
B    S
ATOM  17106  CE  MET B  28      -33.686  29.308  53.807  1.00  0.00
B    C
ATOM  17107  HE1 MET B  28      -33.224  29.909  54.619  1.00  0.00
B    H
ATOM  17108  HE2 MET B  28      -34.088  30.012  53.047  1.00  0.00
B    H
ATOM  17109  HE3 MET B  28      -34.543  28.750  54.242  1.00  0.00
B    H
ATOM  17110  C   MET B  28      -34.385  28.519  48.230  1.00  0.00
B    C
ATOM  17111  O   MET B  28      -35.128  29.494  48.354  1.00  0.00
B    O
ATOM  17112  N   GLY B  29      -33.657  28.293  47.138  1.00  0.00
B    N
ATOM  17113  HN  GLY B  29      -32.992  27.549  47.080  1.00  0.00
B    H
ATOM  17114  CA  GLY B  29      -33.781  29.124  45.944  1.00  0.00
B    C
ATOM  17115  HA1 GLY B  29      -34.698  29.680  46.087  1.00  0.00
B    H
ATOM  17116  HA2 GLY B  29      -33.808  28.427  45.117  1.00  0.00
B    H
ATOM  17117  C   GLY B  29      -32.664  30.119  45.681  1.00  0.00
B    C
```

ATOM 17118 O GLY B 29 -32.756 30.915 44.745 1.00 0.00
B O
ATOM 17119 N HSE B 30 -31.615 30.089 46.498 1.00 0.00
B N
ATOM 17120 HN HSE B 30 -31.552 29.507 47.308 1.00 0.00
B H
ATOM 17121 CA HSE B 30 -30.441 30.928 46.265 1.00 0.00
B C
ATOM 17122 HA HSE B 30 -30.785 31.867 45.850 1.00 0.00
B H
ATOM 17123 CB HSE B 30 -29.653 31.112 47.546 1.00 0.00
B C
ATOM 17124 HB1 HSE B 30 -29.234 30.124 47.835 1.00 0.00
B H
ATOM 17125 HB2 HSE B 30 -28.792 31.787 47.358 1.00 0.00
B H
ATOM 17126 ND1 HSE B 30 -30.658 32.931 48.928 1.00 0.00
B N
ATOM 17127 CG HSE B 30 -30.476 31.620 48.695 1.00 0.00
B C
ATOM 17128 CE1 HSE B 30 -31.436 33.086 50.010 1.00 0.00
B C
ATOM 17129 HE1 HSE B 30 -31.735 34.052 50.425 1.00 0.00
B H
ATOM 17130 NE2 HSE B 30 -31.757 31.866 50.469 1.00 0.00
B N
ATOM 17131 HE2 HSE B 30 -32.315 31.682 51.278 1.00 0.00
B H
ATOM 17132 CD2 HSE B 30 -31.182 30.942 49.682 1.00 0.00
B C
ATOM 17133 HD2 HSE B 30 -31.287 29.880 49.854 1.00 0.00
B H
ATOM 17134 C HSE B 30 -29.569 30.289 45.236 1.00 0.00
B C
ATOM 17135 O HSE B 30 -29.654 29.081 45.018 1.00 0.00
B O
ATOM 17136 N GLU B 31 -28.725 31.081 44.586 1.00 0.00
B N
ATOM 17137 HN GLU B 31 -28.679 32.073 44.670 1.00 0.00
B H
ATOM 17138 CA GLU B 31 -27.754 30.519 43.649 1.00 0.00
B C
ATOM 17139 HA GLU B 31 -28.155 29.589 43.266 1.00 0.00
B H
ATOM 17140 CB GLU B 31 -27.557 31.385 42.388 1.00 0.00
B C
ATOM 17141 HB1 GLU B 31 -26.662 31.005 41.842 1.00 0.00
B H
ATOM 17142 HB2 GLU B 31 -28.418 31.246 41.697 1.00 0.00
B H
ATOM 17143 CG GLU B 31 -27.356 32.881 42.599 1.00 0.00
B C
ATOM 17144 HG1 GLU B 31 -28.119 33.260 43.303 1.00 0.00
B H

ATOM 17145 HG2 GLU B 31 -26.348 33.093 43.005 1.00 0.00
B H
ATOM 17146 CD GLU B 31 -27.540 33.687 41.315 1.00 0.00
B C
ATOM 17147 OE1 GLU B 31 -27.424 33.113 40.208 1.00 0.00
B O
ATOM 17148 OE2 GLU B 31 -27.803 34.908 41.409 1.00 0.00
B O
ATOM 17149 C GLU B 31 -26.438 30.191 44.344 1.00 0.00
B C
ATOM 17150 O GLU B 31 -25.915 30.990 45.125 1.00 0.00
B O
ATOM 17151 N VAL B 32 -25.930 28.995 44.066 1.00 0.00
B N
ATOM 17152 HN VAL B 32 -26.283 28.413 43.339 1.00 0.00
B H
ATOM 17153 CA VAL B 32 -24.811 28.427 44.801 1.00 0.00
B C
ATOM 17154 HA VAL B 32 -24.321 29.236 45.330 1.00 0.00
B H
ATOM 17155 CB VAL B 32 -25.273 27.301 45.750 1.00 0.00
B C
ATOM 17156 HB VAL B 32 -25.551 26.421 45.119 1.00 0.00
B H
ATOM 17157 CG1 VAL B 32 -24.150 26.900 46.698 1.00 0.00
B C
ATOM 17158 HG11 VAL B 32 -24.503 26.116 47.403 1.00 0.00
B H
ATOM 17159 HG12 VAL B 32 -23.280 26.484 46.150 1.00 0.00
B H
ATOM 17160 HG13 VAL B 32 -23.810 27.773 47.296 1.00 0.00
B H
ATOM 17161 CG2 VAL B 32 -26.509 27.708 46.535 1.00 0.00
B C
ATOM 17162 HG21 VAL B 32 -26.808 26.905 47.243 1.00 0.00
B H
ATOM 17163 HG22 VAL B 32 -26.313 28.633 47.119 1.00 0.00
B H
ATOM 17164 HG23 VAL B 32 -27.369 27.897 45.860 1.00 0.00
B H
ATOM 17165 C VAL B 32 -23.793 27.827 43.844 1.00 0.00
B C
ATOM 17166 O VAL B 32 -24.142 27.054 42.951 1.00 0.00
B O
ATOM 17167 N LEU B 33 -22.530 28.187 44.038 1.00 0.00
B N
ATOM 17168 HN LEU B 33 -22.234 28.911 44.658 1.00 0.00
B H
ATOM 17169 CA LEU B 33 -21.449 27.527 43.336 1.00 0.00
B C
ATOM 17170 HA LEU B 33 -21.869 27.038 42.467 1.00 0.00
B H
ATOM 17171 CB LEU B 33 -20.374 28.526 42.916 1.00 0.00
B C

ATOM 17172 HB1 LEU B 33   -20.890 29.363 42.391 1.00 0.00
B   H
ATOM 17173 HB2 LEU B 33   -19.916 28.970 43.828 1.00 0.00
B   H
ATOM 17174 CG LEU B 33   -19.285 27.979 41.995 1.00 0.00
B   C
ATOM 17175 HG LEU B 33   -19.726 27.106 41.455 1.00 0.00
B   H
ATOM 17176 CD1 LEU B 33   -18.798 29.018 41.008 1.00 0.00
B   C
ATOM 17177 HD11 LEU B 33   -17.975 28.608 40.385 1.00 0.00
B   H
ATOM 17178 HD12 LEU B 33   -19.623 29.334 40.333 1.00 0.00
B   H
ATOM 17179 HD13 LEU B 33   -18.416 29.915 41.540 1.00 0.00
B   H
ATOM 17180 CD2 LEU B 33   -18.116 27.483 42.805 1.00 0.00
B   C
ATOM 17181 HD21 LEU B 33   -17.332 27.068 42.136 1.00 0.00
B   H
ATOM 17182 HD22 LEU B 33   -17.656 28.297 43.400 1.00 0.00
B   H
ATOM 17183 HD23 LEU B 33   -18.439 26.680 43.502 1.00 0.00
B   H
ATOM 17184 C   LEU B 33   -20.867 26.442 44.237 1.00 0.00
B   C
ATOM 17185 O   LEU B 33   -20.348 26.722 45.319 1.00 0.00
B   O
ATOM 17186 N   ALA B 34   -20.965 25.203 43.781 1.00 0.00
B   N
ATOM 17187 HN ALA B 34   -21.421 24.981 42.919 1.00 0.00
B   H
ATOM 17188 CA ALA B 34   -20.417 24.074 44.503 1.00 0.00
B   C
ATOM 17189 HA ALA B 34   -20.320 24.290 45.561 1.00 0.00
B   H
ATOM 17190 CB ALA B 34   -21.317 22.864 44.333 1.00 0.00
B   C
ATOM 17191 HB1 ALA B 34   -22.320 23.084 44.756 1.00 0.00
B   H
ATOM 17192 HB2 ALA B 34   -21.446 22.611 43.258 1.00 0.00
B   H
ATOM 17193 HB3 ALA B 34   -20.909 21.974 44.860 1.00 0.00
B   H
ATOM 17194 C   ALA B 34   -19.010 23.783 43.994 1.00 0.00
B   C
ATOM 17195 O   ALA B 34   -18.799 23.646 42.785 1.00 0.00
B   O
ATOM 17196 N   VAL B 35   -18.050 23.718 44.914 1.00 0.00
B   N
ATOM 17197 HN VAL B 35   -18.206 23.866 45.888 1.00 0.00
B   H
ATOM 17198 CA VAL B 35   -16.664 23.419 44.565 1.00 0.00
B   C

ATOM 17199 HA VAL B 35 −16.625 23.194 43.507 1.00 0.00
B H
ATOM 17200 CB VAL B 35 −15.698 24.573 44.916 1.00 0.00
B C
ATOM 17201 HB VAL B 35 −15.706 24.694 46.027 1.00 0.00
B H
ATOM 17202 CG1 VAL B 35 −14.285 24.249 44.459 1.00 0.00
B C
ATOM 17203 HG11 VAL B 35 −13.604 25.100 44.680 1.00 0.00
B H
ATOM 17204 HG12 VAL B 35 −13.880 23.358 44.981 1.00 0.00
B H
ATOM 17205 HG13 VAL B 35 −14.260 24.064 43.363 1.00 0.00
B H
ATOM 17206 CG2 VAL B 35 −16.139 25.870 44.268 1.00 0.00
B C
ATOM 17207 HG21 VAL B 35 −15.419 26.686 44.492 1.00 0.00
B H
ATOM 17208 HG22 VAL B 35 −16.203 25.752 43.164 1.00 0.00
B H
ATOM 17209 HG23 VAL B 35 −17.134 26.188 44.643 1.00 0.00
B H
ATOM 17210 C VAL B 35 −16.209 22.182 45.310 1.00 0.00
B C
ATOM 17211 O VAL B 35 −16.543 22.008 46.479 1.00 0.00
B O
ATOM 17212 N ASP B 36 −15.461 21.319 44.627 1.00 0.00
B N
ATOM 17213 HN ASP B 36 −15.270 21.378 43.651 1.00 0.00
B H
ATOM 17214 CA ASP B 36 −14.827 20.169 45.262 1.00 0.00
B C
ATOM 17215 HA ASP B 36 −14.405 20.504 46.202 1.00 0.00
B H
ATOM 17216 CB ASP B 36 −15.841 19.045 45.495 1.00 0.00
B C
ATOM 17217 HB1 ASP B 36 −16.808 19.486 45.810 1.00 0.00
B H
ATOM 17218 HB2 ASP B 36 −16.007 18.471 44.563 1.00 0.00
B H
ATOM 17219 CG ASP B 36 −15.405 18.080 46.584 1.00 0.00
B C
ATOM 17220 OD1 ASP B 36 −16.119 17.974 47.605 1.00 0.00
B O
ATOM 17221 OD2 ASP B 36 −14.348 17.428 46.429 1.00 0.00
B O
ATOM 17222 C ASP B 36 −13.662 19.685 44.404 1.00 0.00
B C
ATOM 17223 O ASP B 36 −13.670 19.852 43.189 1.00 0.00
B O
ATOM 17224 N ILE B 37 −12.657 19.105 45.051 1.00 0.00
B N
ATOM 17225 HN ILE B 37 −12.628 19.011 46.044 1.00 0.00
B H

ATOM 17226 CA ILE B 37   -11.496 18.548 44.364 1.00 0.00
B   C
ATOM 17227 HA ILE B 37   -11.253 19.241 43.567 1.00 0.00
B   H
ATOM 17228 CB ILE B 37   -10.287 18.400 45.328 1.00 0.00
B   C
ATOM 17229 HB ILE B 37   -10.194 19.403 45.816 1.00 0.00
B   H
ATOM 17230 CG2 ILE B 37   -10.524 17.301 46.363 1.00 0.00
B   C
ATOM 17231 HG21 ILE B 37   -9.727 17.312 47.135 1.00 0.00
B   H
ATOM 17232 HG22 ILE B 37   -11.495 17.449 46.881 1.00 0.00
B   H
ATOM 17233 HG23 ILE B 37   -10.530 16.299 45.885 1.00 0.00
B   H
ATOM 17234 CG1 ILE B 37   -8.986 18.168 44.546 1.00 0.00
B   C
ATOM 17235 HG11 ILE B 37   -8.964 17.128 44.148 1.00 0.00
B   H
ATOM 17236 HG12 ILE B 37   -8.964 18.856 43.669 1.00 0.00
B   H
ATOM 17237 CD ILE B 37   -7.727 18.406 45.354 1.00 0.00
B   C
ATOM 17238 HD1 ILE B 37   -6.827 18.278 44.714 1.00 0.00
B   H
ATOM 17239 HD2 ILE B 37   -7.716 19.438 45.767 1.00 0.00
B   H
ATOM 17240 HD3 ILE B 37   -7.653 17.688 46.197 1.00 0.00
B   H
ATOM 17241 C   ILE B 37   -11.834 17.207 43.698 1.00 0.00
B   C
ATOM 17242 O   ILE B 37   -11.246 16.848 42.683 1.00 0.00
B   O
ATOM 17243 N   ASN B 38   -12.799 16.497 44.273 1.00 0.00
B   N
ATOM 17244 HN ASN B 38   -13.314 16.839 45.058 1.00 0.00
B   H
ATOM 17245 CA ASN B 38   -13.194 15.170 43.824 1.00 0.00
B   C
ATOM 17246 HA ASN B 38   -12.300 14.745 43.382 1.00 0.00
B   H
ATOM 17247 CB ASN B 38   -13.682 14.373 45.027 1.00 0.00
B   C
ATOM 17248 HB1 ASN B 38   -12.863 14.357 45.780 1.00 0.00
B   H
ATOM 17249 HB2 ASN B 38   -14.554 14.874 45.495 1.00 0.00
B   H
ATOM 17250 CG ASN B 38   -14.025 12.945 44.680 1.00 0.00
B   C
ATOM 17251 OD1 ASN B 38   -15.075 12.670 44.105 1.00 0.00
B   O
ATOM 17252 ND2 ASN B 38   -13.146 12.024 45.039 1.00 0.00
B   N

ATOM 17253 HD21 ASN B 38   -13.354 11.078 44.805 1.00 0.00
B   H
ATOM 17254 HD22 ASN B 38   -12.303 12.292 45.496 1.00 0.00
B   H
ATOM 17255 C   ASN B 38   -14.275 15.203 42.745 1.00 0.00
B   C
ATOM 17256 O   ASN B 38   -15.354 15.759 42.956 1.00 0.00
B   O
ATOM 17257 N   GLU B 39   -13.992 14.579 41.603 1.00 0.00
B   N
ATOM 17258 HN GLU B 39   -13.175 14.032 41.444 1.00 0.00
B   H
ATOM 17259 CA GLU B 39   -14.894 14.651 40.449 1.00 0.00
B   C
ATOM 17260 HA GLU B 39   -15.049 15.702 40.236 1.00 0.00
B   H
ATOM 17261 CB GLU B 39   -14.249 14.042 39.197 1.00 0.00
B   C
ATOM 17262 HB1 GLU B 39   -13.169 14.321 39.190 1.00 0.00
B   H
ATOM 17263 HB2 GLU B 39   -14.275 12.931 39.261 1.00 0.00
B   H
ATOM 17264 CG GLU B 39   -14.897 14.511 37.897 1.00 0.00
B   C
ATOM 17265 HG1 GLU B 39   -15.975 14.265 37.913 1.00 0.00
B   H
ATOM 17266 HG2 GLU B 39   -14.775 15.604 37.766 1.00 0.00
B   H
ATOM 17267 CD GLU B 39   -14.343 13.822 36.670 1.00 0.00
B   C
ATOM 17268 OE1 GLU B 39   -14.849 14.097 35.561 1.00 0.00
B   O
ATOM 17269 OE2 GLU B 39   -13.406 13.009 36.807 1.00 0.00
B   O
ATOM 17270 C   GLU B 39   -16.269 14.031 40.697 1.00 0.00
B   C
ATOM 17271 O   GLU B 39   -17.278 14.561 40.223 1.00 0.00
B   O
ATOM 17272 N   GLU B 40   -16.303 12.918 41.427 1.00 0.00
B   N
ATOM 17273 HN GLU B 40   -15.498 12.465 41.798 1.00 0.00
B   H
ATOM 17274 CA GLU B 40   -17.565 12.249 41.754 1.00 0.00
B   C
ATOM 17275 HA GLU B 40   -18.073 12.019 40.826 1.00 0.00
B   H
ATOM 17276 CB GLU B 40   -17.321 10.947 42.515 1.00 0.00
B   C
ATOM 17277 HB1 GLU B 40   -16.521 11.128 43.268 1.00 0.00
B   H
ATOM 17278 HB2 GLU B 40   -18.231 10.673 43.096 1.00 0.00
B   H
ATOM 17279 CG GLU B 40   -16.907   9.784 41.630 1.00 0.00
B   C

ATOM 17280 HG1 GLU B 40 −17.809 9.266 41.254 1.00 0.00
B H
ATOM 17281 HG2 GLU B 40 −16.304 10.135 40.769 1.00 0.00
B H
ATOM 17282 CD GLU B 40 −16.112 8.734 42.379 1.00 0.00
B C
ATOM 17283 OE1 GLU B 40 −15.258 9.117 43.212 1.00 0.00
B O
ATOM 17284 OE2 GLU B 40 −16.331 7.527 42.130 1.00 0.00
B O
ATOM 17285 C GLU B 40 −18.483 13.159 42.558 1.00 0.00
B C
ATOM 17286 O GLU B 40 −19.680 13.249 42.263 1.00 0.00
B O
ATOM 17287 N LYS B 41 −17.913 13.830 43.561 1.00 0.00
B N
ATOM 17288 HN LYS B 41 −16.950 13.717 43.801 1.00 0.00
B H
ATOM 17289 CA LYS B 41 −18.651 14.787 44.390 1.00 0.00
B C
ATOM 17290 HA LYS B 41 −19.475 14.247 44.842 1.00 0.00
B H
ATOM 17291 CB LYS B 41 −17.753 15.365 45.489 1.00 0.00
B C
ATOM 17292 HB1 LYS B 41 −16.823 15.729 44.995 1.00 0.00
B H
ATOM 17293 HB2 LYS B 41 −18.247 16.261 45.926 1.00 0.00
B H
ATOM 17294 CG LYS B 41 −17.371 14.379 46.587 1.00 0.00
B C
ATOM 17295 HG1 LYS B 41 −17.141 13.392 46.126 1.00 0.00
B H
ATOM 17296 HG2 LYS B 41 −16.442 14.736 47.087 1.00 0.00
B H
ATOM 17297 CD LYS B 41 −18.484 14.199 47.611 1.00 0.00
B C
ATOM 17298 HD1 LYS B 41 −18.573 15.139 48.203 1.00 0.00
B H
ATOM 17299 HD2 LYS B 41 −19.443 14.060 47.060 1.00 0.00
B H
ATOM 17300 CE LYS B 41 −18.222 13.013 48.522 1.00 0.00
B C
ATOM 17301 HE1 LYS B 41 −19.010 12.940 49.304 1.00 0.00
B H
ATOM 17302 HE2 LYS B 41 −18.217 12.065 47.938 1.00 0.00
B H
ATOM 17303 NZ LYS B 41 −16.912 13.107 49.223 1.00 0.00
B N
ATOM 17304 HZ1 LYS B 41 −16.775 12.277 49.835 1.00 0.00
B H
ATOM 17305 HZ2 LYS B 41 −16.145 13.150 48.521 1.00 0.00
B H
ATOM 17306 HZ3 LYS B 41 −16.891 13.970 49.803 1.00 0.00
B H

ATOM 17307 C   LYS B 41   -19.253 15.911 43.539 1.00 0.00
B   C
ATOM 17308 O   LYS B 41   -20.432 16.238 43.680 1.00 0.00
B   O
ATOM 17309 N   VAL B 42   -18.441 16.477 42.648 1.00 0.00
B   N
ATOM 17310 HN VAL B 42   -17.482 16.222 42.548 1.00 0.00
B   H
ATOM 17311 CA VAL B 42   -18.886 17.529 41.733 1.00 0.00
B   C
ATOM 17312 HA VAL B 42   -19.281 18.335 42.338 1.00 0.00
B   H
ATOM 17313 CB VAL B 42   -17.718 18.084 40.887 1.00 0.00
B   C
ATOM 17314 HB VAL B 42   -17.370 17.263 40.213 1.00 0.00
B   H
ATOM 17315 CG1 VAL B 42   -18.176 19.260 40.043 1.00 0.00
B   C
ATOM 17316 HG11 VAL B 42   -17.322 19.678 39.467 1.00 0.00
B   H
ATOM 17317 HG12 VAL B 42   -18.953 18.960 39.310 1.00 0.00
B   H
ATOM 17318 HG13 VAL B 42   -18.586 20.069 40.686 1.00 0.00
B   H
ATOM 17319 CG2 VAL B 42   -16.575 18.526 41.784 1.00 0.00
B   C
ATOM 17320 HG21 VAL B 42   -15.749 18.963 41.182 1.00 0.00
B   H
ATOM 17321 HG22 VAL B 42   -16.923 19.294 42.509 1.00 0.00
B   H
ATOM 17322 HG23 VAL B 42   -16.158 17.671 42.355 1.00 0.00
B   H
ATOM 17323 C   VAL B 42   -20.019 17.040 40.822 1.00 0.00
B   C
ATOM 17324 O   VAL B 42   -20.980 17.771 40.576 1.00 0.00
B   O
ATOM 17325 N   ASN B 43   -19.901 15.806 40.329 1.00 0.00
B   N
ATOM 17326 HN ASN B 43   -19.093 15.235 40.470 1.00 0.00
B   H
ATOM 17327 CA ASN B 43   -20.959 15.178 39.531 1.00 0.00
B   C
ATOM 17328 HA ASN B 43   -21.167 15.877 38.728 1.00 0.00
B   H
ATOM 17329 CB ASN B 43   -20.494 13.831 38.973 1.00 0.00
B   C
ATOM 17330 HB1 ASN B 43   -19.988 13.279 39.794 1.00 0.00
B   H
ATOM 17331 HB2 ASN B 43   -21.367 13.225 38.653 1.00 0.00
B   H
ATOM 17332 CG ASN B 43   -19.507 13.972 37.828 1.00 0.00
B   C
ATOM 17333 OD1 ASN B 43   -19.309 15.062 37.286 1.00 0.00
B   O

ATOM 17334 ND2 ASN B 43 −18.887 12.861 37.447 1.00 0.00
B N
ATOM 17335 HD21 ASN B 43 −18.227 12.937 36.704 1.00 0.00
B H
ATOM 17336 HD22 ASN B 43 −19.068 12.003 37.918 1.00 0.00
B H
ATOM 17337 C ASN B 43 −22.248 14.991 40.326 1.00 0.00
B C
ATOM 17338 O ASN B 43 −23.337 15.271 39.834 1.00 0.00
B O
ATOM 17339 N ALA B 44 −22.106 14.531 41.567 1.00 0.00
B N
ATOM 17340 HN ALA B 44 −21.208 14.350 41.967 1.00 0.00
B H
ATOM 17341 CA ALA B 44 −23.245 14.255 42.436 1.00 0.00
B C
ATOM 17342 HA ALA B 44 −23.960 13.631 41.913 1.00 0.00
B H
ATOM 17343 CB ALA B 44 −22.794 13.478 43.666 1.00 0.00
B C
ATOM 17344 HB1 ALA B 44 −22.292 12.539 43.350 1.00 0.00
B H
ATOM 17345 HB2 ALA B 44 −22.068 14.069 44.268 1.00 0.00
B H
ATOM 17346 HB3 ALA B 44 −23.653 13.206 44.316 1.00 0.00
B H
ATOM 17347 C ALA B 44 −23.996 15.514 42.853 1.00 0.00
B C
ATOM 17348 O ALA B 44 −25.173 15.444 43.205 1.00 0.00
B O
ATOM 17349 N TYR B 45 −23.323 16.660 42.818 1.00 0.00
B N
ATOM 17350 HN TYR B 45 −22.372 16.748 42.524 1.00 0.00
B H
ATOM 17351 CA TYR B 45 −23.952 17.914 43.220 1.00 0.00
B C
ATOM 17352 HA TYR B 45 −24.866 17.625 43.724 1.00 0.00
B H
ATOM 17353 CB TYR B 45 −23.091 18.652 44.255 1.00 0.00
B C
ATOM 17354 HB1 TYR B 45 −22.021 18.573 43.963 1.00 0.00
B H
ATOM 17355 HB2 TYR B 45 −23.346 19.734 44.279 1.00 0.00
B H
ATOM 17356 CG TYR B 45 −23.233 18.051 45.627 1.00 0.00
B C
ATOM 17357 CD1 TYR B 45 −22.375 17.046 46.059 1.00 0.00
B C
ATOM 17358 HD1 TYR B 45 −21.589 16.692 45.406 1.00 0.00
B H
ATOM 17359 CE1 TYR B 45 −22.521 16.470 47.307 1.00 0.00
B C
ATOM 17360 HE1 TYR B 45 −21.849 15.691 47.635 1.00 0.00
B H

ATOM 17361 CZ TYR B 45 -23.542 16.895 48.138 1.00 0.00
B C
ATOM 17362 OH TYR B 45 -23.683 16.329 49.380 1.00 0.00
B O
ATOM 17363 HH TYR B 45 -24.389 16.796 49.831 1.00 0.00
B H
ATOM 17364 CD2 TYR B 45 -24.259 18.454 46.480 1.00 0.00
B C
ATOM 17365 HD2 TYR B 45 -24.973 19.196 46.151 1.00 0.00
B H
ATOM 17366 CE2 TYR B 45 -24.413 17.885 47.732 1.00 0.00
B C
ATOM 17367 HE2 TYR B 45 -25.225 18.204 48.369 1.00 0.00
B H
ATOM 17368 C TYR B 45 -24.347 18.815 42.052 1.00 0.00
B C
ATOM 17369 O TYR B 45 -24.843 19.927 42.254 1.00 0.00
B O
ATOM 17370 N ALA B 46 -24.153 18.319 40.834 1.00 0.00
B N
ATOM 17371 HN ALA B 46 -23.735 17.426 40.673 1.00 0.00
B H
ATOM 17372 CA ALA B 46 -24.544 19.055 39.632 1.00 0.00
B C
ATOM 17373 HA ALA B 46 -23.954 19.958 39.532 1.00 0.00
B H
ATOM 17374 CB ALA B 46 -24.279 18.218 38.388 1.00 0.00
B C
ATOM 17375 HB1 ALA B 46 -23.202 17.952 38.341 1.00 0.00
B H
ATOM 17376 HB2 ALA B 46 -24.864 17.273 38.412 1.00 0.00
B H
ATOM 17377 HB3 ALA B 46 -24.537 18.772 37.460 1.00 0.00
B H
ATOM 17378 C ALA B 46 -26.011 19.501 39.701 1.00 0.00
B C
ATOM 17379 O ALA B 46 -26.335 20.642 39.362 1.00 0.00
B O
ATOM 17380 N SER B 47 -26.878 18.608 40.179 1.00 0.00
B N
ATOM 17381 HN SER B 47 -26.601 17.701 40.497 1.00 0.00
B H
ATOM 17382 CA SER B 47 -28.313 18.867 40.284 1.00 0.00
B C
ATOM 17383 HA SER B 47 -28.553 19.434 39.394 1.00 0.00
B H
ATOM 17384 CB SER B 47 -29.076 17.548 40.359 1.00 0.00
B C
ATOM 17385 HB1 SER B 47 -28.673 16.964 41.218 1.00 0.00
B H
ATOM 17386 HB2 SER B 47 -30.154 17.734 40.567 1.00 0.00
B H
ATOM 17387 OG SER B 47 -28.903 16.800 39.175 1.00 0.00
B O

ATOM 17388 HG1 SER B 47 −29.208 15.903 39.358 1.00 0.00
B H
ATOM 17389 C SER B 47 −28.713 19.716 41.484 1.00 0.00
B C
ATOM 17390 O SER B 47 −29.836 20.216 41.536 1.00 0.00
B O
ATOM 17391 N TYR B 48 −27.810 19.859 42.450 1.00 0.00
B N
ATOM 17392 HN TYR B 48 −26.870 19.528 42.388 1.00 0.00
B H
ATOM 17393 CA TYR B 48 −28.138 20.535 43.713 1.00 0.00
B C
ATOM 17394 HA TYR B 48 −29.197 20.755 43.663 1.00 0.00
B H
ATOM 17395 CB TYR B 48 −27.854 19.615 44.907 1.00 0.00
B C
ATOM 17396 HB1 TYR B 48 −26.765 19.396 44.948 1.00 0.00
B H
ATOM 17397 HB2 TYR B 48 −28.126 20.116 45.862 1.00 0.00
B H
ATOM 17398 CG TYR B 48 −28.557 18.275 44.839 1.00 0.00
B C
ATOM 17399 CD1 TYR B 48 −27.866 17.121 44.462 1.00 0.00
B C
ATOM 17400 HD1 TYR B 48 −26.819 17.185 44.197 1.00 0.00
B H
ATOM 17401 CE1 TYR B 48 −28.504 15.893 44.398 1.00 0.00
B C
ATOM 17402 HE1 TYR B 48 −27.963 15.008 44.099 1.00 0.00
B H
ATOM 17403 CZ TYR B 48 −29.851 15.805 44.714 1.00 0.00
B C
ATOM 17404 OH TYR B 48 −30.489 14.587 44.654 1.00 0.00
B O
ATOM 17405 HH TYR B 48 −31.384 14.714 44.973 1.00 0.00
B H
ATOM 17406 CD2 TYR B 48 −29.914 18.157 45.151 1.00 0.00
B C
ATOM 17407 HD2 TYR B 48 −30.488 19.037 45.408 1.00 0.00
B H
ATOM 17408 CE2 TYR B 48 −30.558 16.933 45.091 1.00 0.00
B C
ATOM 17409 HE2 TYR B 48 −31.612 16.870 45.317 1.00 0.00
B H
ATOM 17410 C TYR B 48 −27.408 21.866 43.881 1.00 0.00
B C
ATOM 17411 O TYR B 48 −27.238 22.354 44.999 1.00 0.00
B O
ATOM 17412 N ALA B 49 −26.984 22.447 42.763 1.00 0.00
B N
ATOM 17413 HN ALA B 49 −27.142 22.035 41.866 1.00 0.00
B H
ATOM 17414 CA ALA B 49 −26.264 23.715 42.751 1.00 0.00
B C

ATOM 17415 HA ALA B 49 -26.649 24.386 43.509 1.00 0.00
B H
ATOM 17416 CB ALA B 49 -24.784 23.481 43.014 1.00 0.00
B C
ATOM 17417 HB1 ALA B 49 -24.653 23.010 44.012 1.00 0.00
B H
ATOM 17418 HB2 ALA B 49 -24.346 22.798 42.254 1.00 0.00
B H
ATOM 17419 HB3 ALA B 49 -24.211 24.434 43.006 1.00 0.00
B H
ATOM 17420 C ALA B 49 -26.454 24.424 41.412 1.00 0.00
B C
ATOM 17421 O ALA B 49 -26.675 23.776 40.389 1.00 0.00
B O
ATOM 17422 N THR B 50 -26.375 25.750 41.424 1.00 0.00
B N
ATOM 17423 HN THR B 50 -26.283 26.294 42.258 1.00 0.00
B H
ATOM 17424 CA THR B 50 -26.416 26.538 40.194 1.00 0.00
B C
ATOM 17425 HA THR B 50 -27.332 26.262 39.689 1.00 0.00
B H
ATOM 17426 CB THR B 50 -26.343 28.044 40.497 1.00 0.00
B C
ATOM 17427 HB THR B 50 -25.298 28.241 40.838 1.00 0.00
B H
ATOM 17428 OG1 THR B 50 -27.232 28.353 41.577 1.00 0.00
B O
ATOM 17429 HG1 THR B 50 -27.152 29.303 41.711 1.00 0.00
B H
ATOM 17430 CG2 THR B 50 -26.733 28.859 39.276 1.00 0.00
B C
ATOM 17431 HG21 THR B 50 -26.572 29.943 39.456 1.00 0.00
B H
ATOM 17432 HG22 THR B 50 -26.117 28.564 38.400 1.00 0.00
B H
ATOM 17433 HG23 THR B 50 -27.803 28.704 39.016 1.00 0.00
B H
ATOM 17434 C THR B 50 -25.267 26.139 39.262 1.00 0.00
B C
ATOM 17435 O THR B 50 -25.479 25.900 38.073 1.00 0.00
B O
ATOM 17436 N HSE B 51 -24.060 26.064 39.814 1.00 0.00
B N
ATOM 17437 HN HSE B 51 -23.882 26.335 40.758 1.00 0.00
B H
ATOM 17438 CA HSE B 51 -22.884 25.579 39.094 1.00 0.00
B C
ATOM 17439 HA HSE B 51 -23.236 24.959 38.279 1.00 0.00
B H
ATOM 17440 CB HSE B 51 -22.046 26.746 38.585 1.00 0.00
B C
ATOM 17441 HB1 HSE B 51 -22.121 27.570 39.327 1.00 0.00
B H

ATOM 17442 HB2 HSE B 51   -20.978 26.445 38.541 1.00 0.00
B   H
ATOM 17443 ND1 HSE B 51   -23.452 28.218 37.128 1.00 0.00
B   N
ATOM 17444 CG HSE B 51   -22.497 27.277 37.250 1.00 0.00
B   C
ATOM 17445 CE1 HSE B 51   -23.654 28.485 35.820 1.00 0.00
B   C
ATOM 17446 HE1 HSE B 51   -24.379 29.208 35.439 1.00 0.00
B   H
ATOM 17447 NE2 HSE B 51   -22.822 27.709 35.105 1.00 0.00
B   N
ATOM 17448 HE2 HSE B 51   -22.770 27.684 34.107 1.00 0.00
B   H
ATOM 17449 CD2 HSE B 51   -22.094 26.953 35.956 1.00 0.00
B   C
ATOM 17450 HD2 HSE B 51   -21.357 26.237 35.618 1.00 0.00
B   H
ATOM 17451 C   HSE B 51   -22.055 24.705 39.988 1.00 0.00
B   C
ATOM 17452 O   HSE B 51   -22.036 24.889 41.210 1.00 0.00
B   O
ATOM 17453 N   ALA B 52   -21.364 23.741 39.388 1.00 0.00
B   N
ATOM 17454 HN ALA B 52   -21.420 23.577 38.403 1.00 0.00
B   H
ATOM 17455 CA ALA B 52   -20.468 22.848 40.122 1.00 0.00
B   C
ATOM 17456 HA ALA B 52   -20.205 23.261 41.089 1.00 0.00
B   H
ATOM 17457 CB ALA B 52   -21.131 21.497 40.349 1.00 0.00
B   C
ATOM 17458 HB1 ALA B 52   -22.054 21.632 40.953 1.00 0.00
B   H
ATOM 17459 HB2 ALA B 52   -21.420 21.027 39.384 1.00 0.00
B   H
ATOM 17460 HB3 ALA B 52   -20.462 20.800 40.896 1.00 0.00
B   H
ATOM 17461 C   ALA B 52   -19.163 22.688 39.354 1.00 0.00
B   C
ATOM 17462 O   ALA B 52   -19.179 22.437 38.151 1.00 0.00
B   O
ATOM 17463 N   VAL B 53   -18.037 22.841 40.046 1.00 0.00
B   N
ATOM 17464 HN VAL B 53   -18.001 22.948 41.037 1.00 0.00
B   H
ATOM 17465 CA VAL B 53   -16.728 22.865 39.387 1.00 0.00
B   C
ATOM 17466 HA VAL B 53   -16.847 22.398 38.417 1.00 0.00
B   H
ATOM 17467 CB VAL B 53   -16.169 24.305 39.249 1.00 0.00
B   C
ATOM 17468 HB VAL B 53   -15.689 24.576 40.222 1.00 0.00
B   H

ATOM 17469 CG1 VAL B 53   -15.130 24.372 38.143 1.00 0.00
B   C
ATOM 17470 HG11 VAL B 53   -14.762 25.413 38.022 1.00 0.00
B   H
ATOM 17471 HG12 VAL B 53   -14.250 23.735 38.369 1.00 0.00
B   H
ATOM 17472 HG13 VAL B 53   -15.565 24.048 37.174 1.00 0.00
B   H
ATOM 17473 CG2 VAL B 53   -17.280 25.310 38.978 1.00 0.00
B   C
ATOM 17474 HG21 VAL B 53   -16.863 26.330 38.842 1.00 0.00
B   H
ATOM 17475 HG22 VAL B 53   -17.840 25.035 38.058 1.00 0.00
B   H
ATOM 17476 HG23 VAL B 53   -17.998 25.352 39.824 1.00 0.00
B   H
ATOM 17477 C   VAL B 53   -15.704 22.050 40.162 1.00 0.00
B   C
ATOM 17478 O   VAL B 53   -15.643 22.136 41.387 1.00 0.00
B   O
ATOM 17479 N   ILE B 54   -14.897 21.272 39.446 1.00 0.00
B   N
ATOM 17480 HN ILE B 54   -15.030 21.072 38.477 1.00 0.00
B   H
ATOM 17481 CA ILE B 54   -13.721 20.630 40.033 1.00 0.00
B   C
ATOM 17482 HA ILE B 54   -14.005 20.268 41.013 1.00 0.00
B   H
ATOM 17483 CB ILE B 54   -13.156 19.498 39.142 1.00 0.00
B   C
ATOM 17484 HB ILE B 54   -12.989 19.982 38.146 1.00 0.00
B   H
ATOM 17485 CG2 ILE B 54   -11.899 18.895 39.750 1.00 0.00
B   C
ATOM 17486 HG21 ILE B 54   -11.415 18.197 39.036 1.00 0.00
B   H
ATOM 17487 HG22 ILE B 54   -11.162 19.687 40.000 1.00 0.00
B   H
ATOM 17488 HG23 ILE B 54   -12.136 18.333 40.678 1.00 0.00
B   H
ATOM 17489 CG1 ILE B 54   -14.192 18.397 38.921 1.00 0.00
B   C
ATOM 17490 HG11 ILE B 54   -13.702 17.400 38.989 1.00 0.00
B   H
ATOM 17491 HG12 ILE B 54   -14.950 18.447 39.736 1.00 0.00
B   H
ATOM 17492 CD ILE B 54   -14.925 18.490 37.598 1.00 0.00
B   C
ATOM 17493 HD1 ILE B 54   -15.700 17.696 37.530 1.00 0.00
B   H
ATOM 17494 HD2 ILE B 54   -15.427 19.476 37.496 1.00 0.00
B   H
ATOM 17495 HD3 ILE B 54   -14.225 18.361 36.745 1.00 0.00
B   H

```
ATOM  17496  C   ILE B  54     -12.640  21.691  40.250  1.00  0.00
B    C
ATOM  17497  O   ILE B  54     -12.283  22.424  39.320  1.00  0.00
B    O
ATOM  17498  N   ALA B  55     -12.138  21.780  41.478  1.00  0.00
B    N
ATOM  17499  HN  ALA B  55     -12.481  21.224  42.234  1.00  0.00
B    H
ATOM  17500  CA  ALA B  55     -11.046  22.699  41.817  1.00  0.00
B    C
ATOM  17501  HA  ALA B  55     -10.299  22.695  41.031  1.00  0.00
B    H
ATOM  17502  CB  ALA B  55     -11.563  24.121  41.938  1.00  0.00
B    C
ATOM  17503  HB1 ALA B  55     -12.041  24.426  40.983  1.00  0.00
B    H
ATOM  17504  HB2 ALA B  55     -12.325  24.202  42.743  1.00  0.00
B    H
ATOM  17505  HB3 ALA B  55     -10.742  24.838  42.155  1.00  0.00
B    H
ATOM  17506  C   ALA B  55     -10.324  22.289  43.103  1.00  0.00
B    C
ATOM  17507  O   ALA B  55     -10.934  21.762  44.030  1.00  0.00
B    O
ATOM  17508  N   ASN B  56      -9.019  22.515  43.142  1.00  0.00
B    N
ATOM  17509  HN  ASN B  56      -8.472  22.774  42.346  1.00  0.00
B    H
ATOM  17510  CA  ASN B  56      -8.266  22.400  44.376  1.00  0.00
B    C
ATOM  17511  HA  ASN B  56      -8.809  21.718  45.022  1.00  0.00
B    H
ATOM  17512  CB  ASN B  56      -6.841  21.929  44.093  1.00  0.00
B    C
ATOM  17513  HB1 ASN B  56      -6.904  20.948  43.573  1.00  0.00
B    H
ATOM  17514  HB2 ASN B  56      -6.330  22.637  43.408  1.00  0.00
B    H
ATOM  17515  CG  ASN B  56      -6.022  21.739  45.359  1.00  0.00
B    C
ATOM  17516  OD1 ASN B  56      -6.281  22.372  46.386  1.00  0.00
B    O
ATOM  17517  ND2 ASN B  56      -5.028  20.858  45.295  1.00  0.00
B    N
ATOM  17518 HD21 ASN B  56      -4.490  20.714  46.122  1.00  0.00
B    H
ATOM  17519 HD22 ASN B  56      -4.864  20.352  44.454  1.00  0.00
B    H
ATOM  17520  C   ASN B  56      -8.259  23.769  45.050  1.00  0.00
B    C
ATOM  17521  O   ASN B  56      -7.521  24.670  44.638  1.00  0.00
B    O
ATOM  17522  N   ALA B  57      -9.081  23.928  46.082  1.00  0.00
B    N
```

ATOM 17523 HN ALA B 57 -9.615 23.179 46.473 1.00 0.00
B H
ATOM 17524 CA ALA B 57 -9.262 25.233 46.726 1.00 0.00
B C
ATOM 17525 HA ALA B 57 -9.549 25.956 45.970 1.00 0.00
B H
ATOM 17526 CB ALA B 57 -10.426 25.194 47.696 1.00 0.00
B C
ATOM 17527 HB1 ALA B 57 -11.335 24.828 47.173 1.00 0.00
B H
ATOM 17528 HB2 ALA B 57 -10.217 24.504 48.542 1.00 0.00
B H
ATOM 17529 HB3 ALA B 57 -10.649 26.201 48.108 1.00 0.00
B H
ATOM 17530 C ALA B 57 -8.017 25.801 47.414 1.00 0.00
B C
ATOM 17531 O ALA B 57 -8.050 26.918 47.929 1.00 0.00
B O
ATOM 17532 N THR B 58 -6.923 25.043 47.414 1.00 0.00
B N
ATOM 17533 HN THR B 58 -6.866 24.131 47.007 1.00 0.00
B H
ATOM 17534 CA THR B 58 -5.686 25.503 48.029 1.00 0.00
B C
ATOM 17535 HA THR B 58 -5.960 26.311 48.694 1.00 0.00
B H
ATOM 17536 CB THR B 58 -4.950 24.370 48.772 1.00 0.00
B C
ATOM 17537 HB THR B 58 -4.050 24.839 49.240 1.00 0.00
B H
ATOM 17538 OG1 THR B 58 -4.499 23.391 47.830 1.00 0.00
B O
ATOM 17539 HG1 THR B 58 -4.062 22.712 48.355 1.00 0.00
B H
ATOM 17540 CG2 THR B 58 -5.860 23.714 49.798 1.00 0.00
B C
ATOM 17541 HG21 THR B 58 -5.298 22.977 50.410 1.00 0.00
B H
ATOM 17542 HG22 THR B 58 -6.282 24.478 50.485 1.00 0.00
B H
ATOM 17543 HG23 THR B 58 -6.704 23.186 49.306 1.00 0.00
B H
ATOM 17544 C THR B 58 -4.764 26.102 46.978 1.00 0.00
B C
ATOM 17545 O THR B 58 -3.662 26.556 47.293 1.00 0.00
B O
ATOM 17546 N GLU B 59 -5.236 26.110 45.732 1.00 0.00
B N
ATOM 17547 HN GLU B 59 -6.154 25.801 45.501 1.00 0.00
B H
ATOM 17548 CA GLU B 59 -4.460 26.570 44.589 1.00 0.00
B C
ATOM 17549 HA GLU B 59 -3.431 26.713 44.895 1.00 0.00
B H

ATOM 17550 CB GLU B 59 -4.552 25.537 43.468 1.00 0.00
B C
ATOM 17551 HB1 GLU B 59 -4.533 24.522 43.928 1.00 0.00
B H
ATOM 17552 HB2 GLU B 59 -5.536 25.622 42.956 1.00 0.00
B H
ATOM 17553 CG GLU B 59 -3.438 25.621 42.438 1.00 0.00
B C
ATOM 17554 HG1 GLU B 59 -3.589 24.844 41.666 1.00 0.00
B H
ATOM 17555 HG2 GLU B 59 -3.419 26.618 41.954 1.00 0.00
B H
ATOM 17556 CD GLU B 59 -2.069 25.349 43.028 1.00 0.00
B C
ATOM 17557 OE1 GLU B 59 -1.187 26.230 42.910 1.00 0.00
B O
ATOM 17558 OE2 GLU B 59 -1.882 24.262 43.617 1.00 0.00
B O
ATOM 17559 C GLU B 59 -4.990 27.928 44.134 1.00 0.00
B C
ATOM 17560 O GLU B 59 -6.106 28.024 43.623 1.00 0.00
B O
ATOM 17561 N GLU B 60 -4.180 28.971 44.318 1.00 0.00
B N
ATOM 17562 HN GLU B 60 -3.231 28.880 44.606 1.00 0.00
B H
ATOM 17563 CA GLU B 60 -4.612 30.361 44.110 1.00 0.00
B C
ATOM 17564 HA GLU B 60 -5.403 30.535 44.830 1.00 0.00
B H
ATOM 17565 CB GLU B 60 -3.493 31.347 44.467 1.00 0.00
B C
ATOM 17566 HB1 GLU B 60 -3.045 31.029 45.436 1.00 0.00
B H
ATOM 17567 HB2 GLU B 60 -2.676 31.277 43.715 1.00 0.00
B H
ATOM 17568 CG GLU B 60 -3.989 32.788 44.584 1.00 0.00
B C
ATOM 17569 HG1 GLU B 60 -4.639 33.026 43.722 1.00 0.00
B H
ATOM 17570 HG2 GLU B 60 -4.557 32.937 45.523 1.00 0.00
B H
ATOM 17571 CD GLU B 60 -2.877 33.817 44.541 1.00 0.00
B C
ATOM 17572 OE1 GLU B 60 -3.085 34.878 43.919 1.00 0.00
B O
ATOM 17573 OE2 GLU B 60 -1.797 33.564 45.124 1.00 0.00
B O
ATOM 17574 C GLU B 60 -5.179 30.695 42.722 1.00 0.00
B C
ATOM 17575 O GLU B 60 -6.185 31.399 42.621 1.00 0.00
B O
ATOM 17576 N ASN B 61 -4.525 30.217 41.665 1.00 0.00
B N

ATOM 17577 HN ASN B 61   -3.685 29.678 41.721 1.00 0.00
B   H
ATOM 17578 CA ASN B 61   -5.004 30.453 40.304 1.00 0.00
B   C
ATOM 17579 HA ASN B 61   -5.201 31.518 40.251 1.00 0.00
B   H
ATOM 17580 CB ASN B 61   -3.930 30.126 39.251 1.00 0.00
B   C
ATOM 17581 HB1 ASN B 61   -4.410 30.173 38.249 1.00 0.00
B   H
ATOM 17582 HB2 ASN B 61   -3.125 30.890 39.271 1.00 0.00
B   H
ATOM 17583 CG ASN B 61   -3.329 28.732 39.416 1.00 0.00
B   C
ATOM 17584 OD1 ASN B 61   -3.982 27.799 39.895 1.00 0.00
B   O
ATOM 17585 ND2 ASN B 61   -2.073 28.586 39.010 1.00 0.00
B   N
ATOM 17586 HD21 ASN B 61   -1.666 27.680 39.099 1.00 0.00
B   H
ATOM 17587 HD22 ASN B 61   -1.587 29.355 38.608 1.00 0.00
B   H
ATOM 17588 C   ASN B 61   -6.321 29.722 40.012 1.00 0.00
B   C
ATOM 17589 O   ASN B 61   -7.149 30.215 39.245 1.00 0.00
B   O
ATOM 17590 N   GLU B 62   -6.515 28.561 40.638 1.00 0.00
B   N
ATOM 17591 HN GLU B 62   -5.848 28.132 41.240 1.00 0.00
B   H
ATOM 17592 CA GLU B 62   -7.754 27.805 40.489 1.00 0.00
B   C
ATOM 17593 HA GLU B 62   -7.974 27.710 39.433 1.00 0.00
B   H
ATOM 17594 CB GLU B 62   -7.634 26.404 41.088 1.00 0.00
B   C
ATOM 17595 HB1 GLU B 62   -7.250 26.503 42.129 1.00 0.00
B   H
ATOM 17596 HB2 GLU B 62   -8.644 25.945 41.178 1.00 0.00
B   H
ATOM 17597 CG GLU B 62   -6.714 25.474 40.310 1.00 0.00
B   C
ATOM 17598 HG1 GLU B 62   -6.892 25.599 39.227 1.00 0.00
B   H
ATOM 17599 HG2 GLU B 62   -5.650 25.692 40.532 1.00 0.00
B   H
ATOM 17600 CD GLU B 62   -6.974 24.010 40.601 1.00 0.00
B   C
ATOM 17601 OE1 GLU B 62   -8.158 23.624 40.706 1.00 0.00
B   O
ATOM 17602 OE2 GLU B 62   -5.995 23.246 40.711 1.00 0.00
B   O
ATOM 17603 C   GLU B 62   -8.910 28.558 41.124 1.00 0.00
B   C

ATOM 17604 O GLU B 62 -10.016 28.559 40.586 1.00 0.00
B O
ATOM 17605 N LEU B 63 -8.642 29.204 42.259 1.00 0.00
B N
ATOM 17606 HN LEU B 63 -7.762 29.160 42.726 1.00 0.00
B H
ATOM 17607 CA LEU B 63 -9.634 30.048 42.925 1.00 0.00
B C
ATOM 17608 HA LEU B 63 -10.517 29.436 43.057 1.00 0.00
B H
ATOM 17609 CB LEU B 63 -9.127 30.524 44.287 1.00 0.00
B C
ATOM 17610 HB1 LEU B 63 -8.014 30.525 44.242 1.00 0.00
B H
ATOM 17611 HB2 LEU B 63 -9.430 31.584 44.436 1.00 0.00
B H
ATOM 17612 CG LEU B 63 -9.540 29.704 45.510 1.00 0.00
B C
ATOM 17613 HG LEU B 63 -10.656 29.676 45.554 1.00 0.00
B H
ATOM 17614 CD1 LEU B 63 -8.992 28.301 45.391 1.00 0.00
B C
ATOM 17615 HD11 LEU B 63 -9.243 27.706 46.295 1.00 0.00
B H
ATOM 17616 HD12 LEU B 63 -9.422 27.786 44.505 1.00 0.00
B H
ATOM 17617 HD13 LEU B 63 -7.886 28.319 45.285 1.00 0.00
B H
ATOM 17618 CD2 LEU B 63 -9.036 30.349 46.790 1.00 0.00
B C
ATOM 17619 HD21 LEU B 63 -9.358 29.756 47.674 1.00 0.00
B H
ATOM 17620 HD22 LEU B 63 -7.929 30.409 46.809 1.00 0.00
B H
ATOM 17621 HD23 LEU B 63 -9.444 31.378 46.895 1.00 0.00
B H
ATOM 17622 C LEU B 63 -10.025 31.240 42.066 1.00 0.00
B C
ATOM 17623 O LEU B 63 -11.214 31.541 41.925 1.00 0.00
B O
ATOM 17624 N LEU B 64 -9.024 31.908 41.493 1.00 0.00
B N
ATOM 17625 HN LEU B 64 -8.059 31.701 41.643 1.00 0.00
B H
ATOM 17626 CA LEU B 64 -9.263 33.018 40.584 1.00 0.00
B C
ATOM 17627 HA LEU B 64 -9.812 33.770 41.135 1.00 0.00
B H
ATOM 17628 CB LEU B 64 -7.942 33.601 40.072 1.00 0.00
B C
ATOM 17629 HB1 LEU B 64 -7.320 32.746 39.719 1.00 0.00
B H
ATOM 17630 HB2 LEU B 64 -8.146 34.225 39.173 1.00 0.00
B H

ATOM 17631 CG LEU B 64 -7.071 34.415 41.036 1.00 0.00
B C
ATOM 17632 HG LEU B 64 -6.886 33.764 41.924 1.00 0.00
B H
ATOM 17633 CD1 LEU B 64 -5.740 34.780 40.382 1.00 0.00
B C
ATOM 17634 HD11 LEU B 64 -5.130 35.410 41.065 1.00 0.00
B H
ATOM 17635 HD12 LEU B 64 -5.158 33.865 40.140 1.00 0.00
B H
ATOM 17636 HD13 LEU B 64 -5.907 35.348 39.442 1.00 0.00
B H
ATOM 17637 CD2 LEU B 64 -7.800 35.665 41.511 1.00 0.00
B C
ATOM 17638 HD21 LEU B 64 -7.164 36.235 42.222 1.00 0.00
B H
ATOM 17639 HD22 LEU B 64 -8.049 36.339 40.667 1.00 0.00
B H
ATOM 17640 HD23 LEU B 64 -8.743 35.392 42.030 1.00 0.00
B H
ATOM 17641 C LEU B 64 -10.142 32.565 39.422 1.00 0.00
B C
ATOM 17642 O LEU B 64 -11.116 33.236 39.098 1.00 0.00
B O
ATOM 17643 N SER B 65 -9.812 31.411 38.837 1.00 0.00
B N
ATOM 17644 HN SER B 65 -9.041 30.855 39.148 1.00 0.00
B H
ATOM 17645 CA SER B 65 -10.539 30.846 37.691 1.00 0.00
B C
ATOM 17646 HA SER B 65 -10.509 31.618 36.933 1.00 0.00
B H
ATOM 17647 CB SER B 65 -9.848 29.575 37.189 1.00 0.00
B C
ATOM 17648 HB1 SER B 65 -9.917 28.804 37.990 1.00 0.00
B H
ATOM 17649 HB2 SER B 65 -10.380 29.170 36.298 1.00 0.00
B H
ATOM 17650 OG SER B 65 -8.482 29.816 36.905 1.00 0.00
B O
ATOM 17651 HG1 SER B 65 -8.055 28.954 36.813 1.00 0.00
B H
ATOM 17652 C SER B 65 -12.015 30.553 37.985 1.00 0.00
B C
ATOM 17653 O SER B 65 -12.851 30.614 37.089 1.00 0.00
B O
ATOM 17654 N LEU B 66 -12.329 30.240 39.238 1.00 0.00
B N
ATOM 17655 HN LEU B 66 -11.661 30.142 39.973 1.00 0.00
B H
ATOM 17656 CA LEU B 66 -13.707 30.011 39.647 1.00 0.00
B C
ATOM 17657 HA LEU B 66 -14.157 29.377 38.895 1.00 0.00
B H

ATOM 17658 CB LEU B 66 -13.745 29.399 41.051 1.00 0.00
B C
ATOM 17659 HB1 LEU B 66 -13.265 30.131 41.742 1.00 0.00
B H
ATOM 17660 HB2 LEU B 66 -14.805 29.314 41.380 1.00 0.00
B H
ATOM 17661 CG LEU B 66 -13.053 28.057 41.262 1.00 0.00
B C
ATOM 17662 HG LEU B 66 -12.109 28.077 40.666 1.00 0.00
B H
ATOM 17663 CD1 LEU B 66 -12.714 27.873 42.732 1.00 0.00
B C
ATOM 17664 HD11 LEU B 66 -12.253 26.877 42.904 1.00 0.00
B H
ATOM 17665 HD12 LEU B 66 -11.997 28.653 43.069 1.00 0.00
B H
ATOM 17666 HD13 LEU B 66 -13.629 27.945 43.357 1.00 0.00
B H
ATOM 17667 CD2 LEU B 66 -13.913 26.913 40.744 1.00 0.00
B C
ATOM 17668 HD21 LEU B 66 -13.390 25.942 40.885 1.00 0.00
B H
ATOM 17669 HD22 LEU B 66 -14.882 26.855 41.279 1.00 0.00
B H
ATOM 17670 HD23 LEU B 66 -14.119 27.041 39.659 1.00 0.00
B H
ATOM 17671 C LEU B 66 -14.486 31.315 39.674 1.00 0.00
B C
ATOM 17672 O LEU B 66 -15.723 31.314 39.667 1.00 0.00
B O
ATOM 17673 N GLY B 67 -13.754 32.426 39.713 1.00 0.00
B N
ATOM 17674 HN GLY B 67 -12.759 32.418 39.622 1.00 0.00
B H
ATOM 17675 CA GLY B 67 -14.359 33.736 39.890 1.00 0.00
B C
ATOM 17676 HA1 GLY B 67 -15.238 33.793 39.262 1.00 0.00
B H
ATOM 17677 HA2 GLY B 67 -13.614 34.491 39.681 1.00 0.00
B H
ATOM 17678 C GLY B 67 -14.798 33.902 41.322 1.00 0.00
B C
ATOM 17679 O GLY B 67 -15.871 34.453 41.595 1.00 0.00
B O
ATOM 17680 N ILE B 68 -13.954 33.434 42.234 1.00 0.00
B N
ATOM 17681 HN ILE B 68 -13.039 33.102 42.013 1.00 0.00
B H
ATOM 17682 CA ILE B 68 -14.287 33.364 43.647 1.00 0.00
B C
ATOM 17683 HA ILE B 68 -15.198 32.781 43.699 1.00 0.00
B H
ATOM 17684 CB ILE B 68 -13.188 32.618 44.451 1.00 0.00
B C

ATOM 17685 HB ILE B 68   -12.995 31.685 43.862 1.00 0.00
B   H
ATOM 17686 CG2 ILE B 68   -11.926 33.455 44.580 1.00 0.00
B   C
ATOM 17687 HG21 ILE B 68   -11.101 32.854 45.018 1.00 0.00
B   H
ATOM 17688 HG22 ILE B 68   -11.588 33.817 43.587 1.00 0.00
B   H
ATOM 17689 HG23 ILE B 68   -12.098 34.335 45.235 1.00 0.00
B   H
ATOM 17690 CG1 ILE B 68   -13.702 32.163 45.827 1.00 0.00
B   C
ATOM 17691 HG11 ILE B 68   -12.856 31.775 46.436 1.00 0.00
B   H
ATOM 17692 HG12 ILE B 68   -14.118 33.045 46.366 1.00 0.00
B   H
ATOM 17693 CD ILE B 68   -14.781 31.107 45.753 1.00 0.00
B   C
ATOM 17694 HD1 ILE B 68   -15.151 30.862 46.772 1.00 0.00
B   H
ATOM 17695 HD2 ILE B 68   -15.640 31.466 45.147 1.00 0.00
B   H
ATOM 17696 HD3 ILE B 68   -14.392 30.172 45.296 1.00 0.00
B   H
ATOM 17697 C   ILE B 68   -14.600 34.730 44.264 1.00 0.00
B   C
ATOM 17698 O   ILE B 68   -15.348 34.813 45.241 1.00 0.00
B   O
ATOM 17699 N   ARG B 69   -14.052 35.795 43.684 1.00 0.00
B   N
ATOM 17700 HN ARG B 69   -13.475 35.716 42.874 1.00 0.00
B   H
ATOM 17701 CA ARG B 69   -14.267 37.142 44.209 1.00 0.00
B   C
ATOM 17702 HA ARG B 69   -14.152 37.097 45.285 1.00 0.00
B   H
ATOM 17703 CB ARG B 69   -13.264 38.110 43.623 1.00 0.00
B   C
ATOM 17704 HB1 ARG B 69   -13.381 38.053 42.514 1.00 0.00
B   H
ATOM 17705 HB2 ARG B 69   -13.540 39.150 43.906 1.00 0.00
B   H
ATOM 17706 CG ARG B 69   -11.852 37.772 43.999 1.00 0.00
B   C
ATOM 17707 HG1 ARG B 69   -11.850 37.367 45.034 1.00 0.00
B   H
ATOM 17708 HG2 ARG B 69   -11.494 36.940 43.350 1.00 0.00
B   H
ATOM 17709 CD ARG B 69   -10.975 38.974 43.812 1.00 0.00
B   C
ATOM 17710 HD1 ARG B 69   -11.447 39.666 43.076 1.00 0.00
B   H
ATOM 17711 HD2 ARG B 69   -10.823 39.523 44.771 1.00 0.00
B   H

```
ATOM  17712  NE  ARG B  69      -9.679  38.538  43.335  1.00  0.00
B    N
ATOM  17713  HE  ARG B  69      -9.719  37.677  42.815  1.00  0.00
B    H
ATOM  17714  CZ  ARG B  69      -8.544  39.196  43.555  1.00  0.00
B    C
ATOM  17715  NH1 ARG B  69      -8.552  40.330  44.255  1.00  0.00
B    N
ATOM  17716 HH11 ARG B  69      -7.715  40.839  44.401  1.00  0.00
B    H
ATOM  17717 HH12 ARG B  69      -9.453  40.697  44.497  1.00  0.00
B    H
ATOM  17718  NH2 ARG B  69      -7.394  38.721  43.074  1.00  0.00
B    N
ATOM  17719 HH21 ARG B  69      -6.540  39.163  43.311  1.00  0.00
B    H
ATOM  17720 HH22 ARG B  69      -7.417  37.817  42.668  1.00  0.00
B    H
ATOM  17721  C   ARG B  69     -15.661  37.661  43.943  1.00  0.00
B    C
ATOM  17722  O   ARG B  69     -16.143  38.546  44.648  1.00  0.00
B    O
ATOM  17723  N   ASN B  70     -16.306  37.101  42.925  1.00  0.00
B    N
ATOM  17724  HN  ASN B  70     -15.903  36.411  42.326  1.00  0.00
B    H
ATOM  17725  CA  ASN B  70     -17.675  37.457  42.599  1.00  0.00
B    C
ATOM  17726  HA  ASN B  70     -17.692  38.542  42.578  1.00  0.00
B    H
ATOM  17727  CB  ASN B  70     -18.053  36.873  41.241  1.00  0.00
B    C
ATOM  17728  HB1 ASN B  70     -17.909  35.772  41.292  1.00  0.00
B    H
ATOM  17729  HB2 ASN B  70     -19.125  37.058  41.027  1.00  0.00
B    H
ATOM  17730  CG  ASN B  70     -17.191  37.410  40.113  1.00  0.00
B    C
ATOM  17731  OD1 ASN B  70     -16.502  38.432  40.252  1.00  0.00
B    O
ATOM  17732  ND2 ASN B  70     -17.225  36.722  38.975  1.00  0.00
B    N
ATOM  17733 HD21 ASN B  70     -16.658  37.050  38.224  1.00  0.00
B    H
ATOM  17734 HD22 ASN B  70     -17.779  35.898  38.907  1.00  0.00
B    H
ATOM  17735  C   ASN B  70     -18.658  37.002  43.665  1.00  0.00
B    C
ATOM  17736  O   ASN B  70     -19.864  37.225  43.543  1.00  0.00
B    O
ATOM  17737  N   PHE B  71     -18.133  36.382  44.722  1.00  0.00
B    N
ATOM  17738  HN  PHE B  71     -17.150  36.256  44.849  1.00  0.00
B    H
```

ATOM 17739 CA PHE B 71 −18.956 35.831 45.791 1.00 0.00
B C
ATOM 17740 HA PHE B 71 −19.976 35.915 45.437 1.00 0.00
B H
ATOM 17741 CB PHE B 71 −18.628 34.357 46.009 1.00 0.00
B C
ATOM 17742 HB1 PHE B 71 −17.525 34.234 46.071 1.00 0.00
B H
ATOM 17743 HB2 PHE B 71 −19.053 33.990 46.967 1.00 0.00
B H
ATOM 17744 CG PHE B 71 −19.092 33.483 44.882 1.00 0.00
B C
ATOM 17745 CD1 PHE B 71 −18.304 33.313 43.742 1.00 0.00
B C
ATOM 17746 HD1 PHE B 71 −17.344 33.806 43.676 1.00 0.00
B H
ATOM 17747 CE1 PHE B 71 −18.739 32.524 42.690 1.00 0.00
B C
ATOM 17748 HE1 PHE B 71 −18.112 32.389 41.820 1.00 0.00
B H
ATOM 17749 CZ PHE B 71 −19.976 31.909 42.761 1.00 0.00
B C
ATOM 17750 HZ PHE B 71 −20.317 31.291 41.943 1.00 0.00
B H
ATOM 17751 CD2 PHE B 71 −20.336 32.862 44.936 1.00 0.00
B C
ATOM 17752 HD2 PHE B 71 −20.976 33.008 45.794 1.00 0.00
B H
ATOM 17753 CE2 PHE B 71 −20.775 32.075 43.886 1.00 0.00
B C
ATOM 17754 HE2 PHE B 71 −21.740 31.592 43.942 1.00 0.00
B H
ATOM 17755 C PHE B 71 −18.868 36.618 47.083 1.00 0.00
B C
ATOM 17756 O PHE B 71 −17.800 37.090 47.466 1.00 0.00
B O
ATOM 17757 N GLU B 72 −20.019 36.757 47.735 1.00 0.00
B N
ATOM 17758 HN GLU B 72 −20.872 36.345 47.426 1.00 0.00
B H
ATOM 17759 CA GLU B 72 −20.143 37.523 48.960 1.00 0.00
B C
ATOM 17760 HA GLU B 72 −19.369 38.281 48.974 1.00 0.00
B H
ATOM 17761 CB GLU B 72 −21.528 38.162 49.030 1.00 0.00
B C
ATOM 17762 HB1 GLU B 72 −21.872 38.360 47.989 1.00 0.00
B H
ATOM 17763 HB2 GLU B 72 −22.255 37.437 49.460 1.00 0.00
B H
ATOM 17764 CG GLU B 72 −21.575 39.462 49.817 1.00 0.00
B C
ATOM 17765 HG1 GLU B 72 −21.014 39.341 50.763 1.00 0.00
B H

ATOM 17766 HG2 GLU B 72 −21.133 40.295 49.235 1.00 0.00
B H
ATOM 17767 CD GLU B 72 −22.990 39.854 50.223 1.00 0.00
B C
ATOM 17768 OE1 GLU B 72 −23.927 39.665 49.413 1.00 0.00
B O
ATOM 17769 OE2 GLU B 72 −23.167 40.356 51.360 1.00 0.00
B O
ATOM 17770 C GLU B 72 −19.914 36.619 50.171 1.00 0.00
B C
ATOM 17771 O GLU B 72 −19.470 37.077 51.229 1.00 0.00
B O
ATOM 17772 N TYR B 73 −20.230 35.338 49.998 1.00 0.00
B N
ATOM 17773 HN TYR B 73 −20.603 34.989 49.140 1.00 0.00
B H
ATOM 17774 CA TYR B 73 −20.062 34.329 51.042 1.00 0.00
B C
ATOM 17775 HA TYR B 73 −19.423 34.762 51.802 1.00 0.00
B H
ATOM 17776 CB TYR B 73 −21.416 33.869 51.566 1.00 0.00
B C
ATOM 17777 HB1 TYR B 73 −22.080 33.638 50.705 1.00 0.00
B H
ATOM 17778 HB2 TYR B 73 −21.310 32.936 52.161 1.00 0.00
B H
ATOM 17779 CG TYR B 73 −22.118 34.899 52.385 1.00 0.00
B C
ATOM 17780 CD1 TYR B 73 −22.830 35.927 51.783 1.00 0.00
B C
ATOM 17781 HD1 TYR B 73 −22.866 35.996 50.705 1.00 0.00
B H
ATOM 17782 CE1 TYR B 73 −23.479 36.883 52.537 1.00 0.00
B C
ATOM 17783 HE1 TYR B 73 −24.022 37.684 52.059 1.00 0.00
B H
ATOM 17784 CZ TYR B 73 −23.422 36.813 53.914 1.00 0.00
B C
ATOM 17785 OH TYR B 73 −24.074 37.764 54.656 1.00 0.00
B O
ATOM 17786 HH TYR B 73 −23.987 37.519 55.579 1.00 0.00
B H
ATOM 17787 CD2 TYR B 73 −22.078 34.848 53.772 1.00 0.00
B C
ATOM 17788 HD2 TYR B 73 −21.502 34.079 54.269 1.00 0.00
B H
ATOM 17789 CE2 TYR B 73 −22.725 35.798 54.541 1.00 0.00
B C
ATOM 17790 HE2 TYR B 73 −22.661 35.750 55.617 1.00 0.00
B H
ATOM 17791 C TYR B 73 −19.342 33.123 50.488 1.00 0.00
B C
ATOM 17792 O TYR B 73 −19.787 32.517 49.513 1.00 0.00
B O

ATOM 17793 N   VAL B 74   -18.223 32.777 51.108 1.00 0.00
B   N
ATOM 17794 HN VAL B 74   -17.761 33.314 51.809 1.00 0.00
B   H
ATOM 17795 CA VAL B 74   -17.569 31.527 50.783 1.00 0.00
B   C
ATOM 17796 HA VAL B 74   -18.174 30.992 50.063 1.00 0.00
B   H
ATOM 17797 CB VAL B 74   -16.170 31.730 50.162 1.00 0.00
B   C
ATOM 17798 HB VAL B 74   -15.526 32.218 50.934 1.00 0.00
B   H
ATOM 17799 CG1 VAL B 74   -15.566 30.388 49.776 1.00 0.00
B   C
ATOM 17800 HG11 VAL B 74   -14.576 30.536 49.294 1.00 0.00
B   H
ATOM 17801 HG12 VAL B 74   -15.408 29.739 50.663 1.00 0.00
B   H
ATOM 17802 HG13 VAL B 74   -16.223 29.854 49.057 1.00 0.00
B   H
ATOM 17803 CG2 VAL B 74   -16.264 32.624 48.931 1.00 0.00
B   C
ATOM 17804 HG21 VAL B 74   -15.268 32.742 48.455 1.00 0.00
B   H
ATOM 17805 HG22 VAL B 74   -16.960 32.186 48.183 1.00 0.00
B   H
ATOM 17806 HG23 VAL B 74   -16.631 33.636 49.196 1.00 0.00
B   H
ATOM 17807 C   VAL B 74   -17.514 30.687 52.046 1.00 0.00
B   C
ATOM 17808 O   VAL B 74   -17.108 31.168 53.103 1.00 0.00
B   O
ATOM 17809 N   ILE B 75   -17.959 29.443 51.925 1.00 0.00
B   N
ATOM 17810 HN ILE B 75   -18.337 29.079 51.077 1.00 0.00
B   H
ATOM 17811 CA ILE B 75   -17.936 28.487 53.029 1.00 0.00
B   C
ATOM 17812 HA ILE B 75   -17.671 29.032 53.926 1.00 0.00
B   H
ATOM 17813 CB ILE B 75   -19.240 27.677 53.104 1.00 0.00
B   C
ATOM 17814 HB ILE B 75   -19.229 27.041 52.183 1.00 0.00
B   H
ATOM 17815 CG2 ILE B 75   -19.245 26.763 54.319 1.00 0.00
B   C
ATOM 17816 HG21 ILE B 75   -20.112 26.071 54.285 1.00 0.00
B   H
ATOM 17817 HG22 ILE B 75   -18.323 26.145 54.352 1.00 0.00
B   H
ATOM 17818 HG23 ILE B 75   -19.307 27.351 55.259 1.00 0.00
B   H
ATOM 17819 CG1 ILE B 75   -20.473 28.595 52.989 1.00 0.00
B   C

ATOM 17820 HG11 ILE B 75 −20.386 29.231 52.080 1.00 0.00
B H
ATOM 17821 HG12 ILE B 75 −21.383 27.964 52.864 1.00 0.00
B H
ATOM 17822 CD ILE B 75 −20.704 29.493 54.175 1.00 0.00
B C
ATOM 17823 HD1 ILE B 75 −21.640 30.078 54.041 1.00 0.00
B H
ATOM 17824 HD2 ILE B 75 −20.800 28.894 55.106 1.00 0.00
B H
ATOM 17825 HD3 ILE B 75 −19.866 30.210 54.302 1.00 0.00
B H
ATOM 17826 C ILE B 75 −16.831 27.493 52.781 1.00 0.00
B C
ATOM 17827 O ILE B 75 −16.814 26.823 51.744 1.00 0.00
B O
ATOM 17828 N VAL B 76 −15.903 27.403 53.725 1.00 0.00
B N
ATOM 17829 HN VAL B 76 −15.874 27.952 54.556 1.00 0.00
B H
ATOM 17830 CA VAL B 76 −14.820 26.452 53.599 1.00 0.00
B C
ATOM 17831 HA VAL B 76 −14.743 26.131 52.567 1.00 0.00
B H
ATOM 17832 CB VAL B 76 −13.464 27.029 54.034 1.00 0.00
B C
ATOM 17833 HB VAL B 76 −13.504 27.186 55.140 1.00 0.00
B H
ATOM 17834 CG1 VAL B 76 −12.345 26.046 53.702 1.00 0.00
B C
ATOM 17835 HG11 VAL B 76 −11.359 26.477 53.979 1.00 0.00
B H
ATOM 17836 HG12 VAL B 76 −12.458 25.093 54.259 1.00 0.00
B H
ATOM 17837 HG13 VAL B 76 −12.329 25.823 52.613 1.00 0.00
B H
ATOM 17838 CG2 VAL B 76 −13.221 28.360 53.338 1.00 0.00
B C
ATOM 17839 HG21 VAL B 76 −12.226 28.770 53.611 1.00 0.00
B H
ATOM 17840 HG22 VAL B 76 −13.256 28.234 52.234 1.00 0.00
B H
ATOM 17841 HG23 VAL B 76 −13.985 29.110 53.627 1.00 0.00
B H
ATOM 17842 C VAL B 76 −15.196 25.246 54.420 1.00 0.00
B C
ATOM 17843 O VAL B 76 −15.151 25.275 55.644 1.00 0.00
B O
ATOM 17844 N ALA B 77 −15.605 24.198 53.723 1.00 0.00
B N
ATOM 17845 HN ALA B 77 −15.621 24.172 52.724 1.00 0.00
B H
ATOM 17846 CA ALA B 77 −16.062 22.996 54.381 1.00 0.00
B C

ATOM 17847 HA ALA B 77 -15.883 23.000 55.450 1.00 0.00
B H
ATOM 17848 CB ALA B 77 -17.558 22.823 54.180 1.00 0.00
B C
ATOM 17849 HB1 ALA B 77 -18.099 23.659 54.671 1.00 0.00
B H
ATOM 17850 HB2 ALA B 77 -17.820 22.834 53.099 1.00 0.00
B H
ATOM 17851 HB3 ALA B 77 -17.923 21.870 54.622 1.00 0.00
B H
ATOM 17852 C ALA B 77 -15.207 21.865 53.804 1.00 0.00
B C
ATOM 17853 O ALA B 77 -15.647 21.076 52.959 1.00 0.00
B O
ATOM 17854 N ILE B 78 -13.959 21.820 54.246 1.00 0.00
B N
ATOM 17855 HN ILE B 78 -13.480 22.573 54.692 1.00 0.00
B H
ATOM 17856 CA ILE B 78 -13.171 20.620 54.106 1.00 0.00
B C
ATOM 17857 HA ILE B 78 -13.768 19.794 53.742 1.00 0.00
B H
ATOM 17858 CB ILE B 78 -11.852 20.780 53.343 1.00 0.00
B C
ATOM 17859 HB ILE B 78 -11.266 21.518 53.947 1.00 0.00
B H
ATOM 17860 CG2 ILE B 78 -11.179 19.422 53.211 1.00 0.00
B C
ATOM 17861 HG21 ILE B 78 -10.153 19.532 52.801 1.00 0.00
B H
ATOM 17862 HG22 ILE B 78 -11.095 18.923 54.199 1.00 0.00
B H
ATOM 17863 HG23 ILE B 78 -11.754 18.758 52.531 1.00 0.00
B H
ATOM 17864 CG1 ILE B 78 -12.107 21.424 51.980 1.00 0.00
B C
ATOM 17865 HG11 ILE B 78 -12.161 20.636 51.195 1.00 0.00
B H
ATOM 17866 HG12 ILE B 78 -13.095 21.937 52.002 1.00 0.00
B H
ATOM 17867 CD ILE B 78 -11.054 22.435 51.600 1.00 0.00
B C
ATOM 17868 HD1 ILE B 78 -11.313 22.916 50.632 1.00 0.00
B H
ATOM 17869 HD2 ILE B 78 -10.977 23.229 52.374 1.00 0.00
B H
ATOM 17870 HD3 ILE B 78 -10.060 21.953 51.489 1.00 0.00
B H
ATOM 17871 C ILE B 78 -12.902 20.354 55.574 1.00 0.00
B C
ATOM 17872 O ILE B 78 -12.333 21.198 56.290 1.00 0.00
B O
ATOM 17873 N GLY B 79 -13.350 19.194 56.024 1.00 0.00
B N

ATOM 17874 HN   GLY B 79   -13.789 18.500 55.456 1.00 0.00
B   H
ATOM 17875 CA   GLY B 79   -13.218 18.866 57.408 1.00 0.00
B   C
ATOM 17876 HA1 GLY B 79   -13.892 18.046 57.616 1.00 0.00
B   H
ATOM 17877 HA2 GLY B 79   -13.417 19.761 57.982 1.00 0.00
B   H
ATOM 17878 C   GLY B 79   -11.813 18.410 57.727 1.00 0.00
B   C
ATOM 17879 O   GLY B 79   -11.022 19.148 58.324 1.00 0.00
B   O
ATOM 17880 N   ALA B 80   -11.503 17.194 57.295 1.00 0.00
B   N
ATOM 17881 HN   ALA B 80   -12.022 16.737 56.573 1.00 0.00
B   H
ATOM 17882 CA   ALA B 80   -10.380 16.429 57.842 1.00 0.00
B   C
ATOM 17883 HA   ALA B 80   -10.542 16.271 58.902 1.00 0.00
B   H
ATOM 17884 CB   ALA B 80   -10.336 15.038 57.223 1.00 0.00
B   C
ATOM 17885 HB1 ALA B 80   -11.314 14.533 57.372 1.00 0.00
B   H
ATOM 17886 HB2 ALA B 80   -10.142 15.093 56.130 1.00 0.00
B   H
ATOM 17887 HB3 ALA B 80   -9.549 14.407 57.690 1.00 0.00
B   H
ATOM 17888 C   ALA B 80   -9.026 17.120 57.711 1.00 0.00
B   C
ATOM 17889 O   ALA B 80   -8.282 17.226 58.688 1.00 0.00
B   O
ATOM 17890 N   ASN B 81   -8.725 17.591 56.505 1.00 0.00
B   N
ATOM 17891 HN   ASN B 81   -9.368 17.572 55.740 1.00 0.00
B   H
ATOM 17892 CA   ASN B 81   -7.442 18.172 56.193 1.00 0.00
B   C
ATOM 17893 HA   ASN B 81   -6.704 17.545 56.681 1.00 0.00
B   H
ATOM 17894 CB   ASN B 81   -7.281 18.202 54.675 1.00 0.00
B   C
ATOM 17895 HB1 ASN B 81   -7.693 17.252 54.271 1.00 0.00
B   H
ATOM 17896 HB2 ASN B 81   -7.873 19.034 54.241 1.00 0.00
B   H
ATOM 17897 CG   ASN B 81   -5.839 18.307 54.237 1.00 0.00
B   C
ATOM 17898 OD1 ASN B 81   -4.982 18.837 54.952 1.00 0.00
B   O
ATOM 17899 ND2 ASN B 81   -5.563 17.810 53.035 1.00 0.00
B   N
ATOM 17900 HD21 ASN B 81   -4.617 17.857 52.727 1.00 0.00
B   H

ATOM 17901 HD22 ASN B 81 -6.283 17.385 52.495 1.00 0.00
B H
ATOM 17902 C ASN B 81 -7.316 19.577 56.782 1.00 0.00
B C
ATOM 17903 O ASN B 81 -7.713 20.561 56.158 1.00 0.00
B O
ATOM 17904 N ILE B 82 -6.769 19.674 57.989 1.00 0.00
B N
ATOM 17905 HN ILE B 82 -6.450 18.893 58.522 1.00 0.00
B H
ATOM 17906 CA ILE B 82 -6.584 20.975 58.653 1.00 0.00
B C
ATOM 17907 HA ILE B 82 -7.565 21.430 58.699 1.00 0.00
B H
ATOM 17908 CB ILE B 82 -6.040 20.816 60.096 1.00 0.00
B C
ATOM 17909 HB ILE B 82 -5.174 20.114 59.992 1.00 0.00
B H
ATOM 17910 CG2 ILE B 82 -5.695 22.158 60.716 1.00 0.00
B C
ATOM 17911 HG21 ILE B 82 -5.165 22.017 61.681 1.00 0.00
B H
ATOM 17912 HG22 ILE B 82 -5.029 22.742 60.048 1.00 0.00
B H
ATOM 17913 HG23 ILE B 82 -6.611 22.754 60.908 1.00 0.00
B H
ATOM 17914 CG1 ILE B 82 -7.072 20.120 60.972 1.00 0.00
B C
ATOM 17915 HG11 ILE B 82 -7.971 20.767 61.082 1.00 0.00
B H
ATOM 17916 HG12 ILE B 82 -7.401 19.182 60.468 1.00 0.00
B H
ATOM 17917 CD ILE B 82 -6.566 19.754 62.347 1.00 0.00
B C
ATOM 17918 HD1 ILE B 82 -7.348 19.200 62.912 1.00 0.00
B H
ATOM 17919 HD2 ILE B 82 -5.665 19.107 62.274 1.00 0.00
B H
ATOM 17920 HD3 ILE B 82 -6.301 20.662 62.929 1.00 0.00
B H
ATOM 17921 C ILE B 82 -5.696 21.918 57.829 1.00 0.00
B C
ATOM 17922 O ILE B 82 -5.988 23.115 57.746 1.00 0.00
B O
ATOM 17923 N GLN B 83 -4.636 21.376 57.219 1.00 0.00
B N
ATOM 17924 HN GLN B 83 -4.403 20.407 57.282 1.00 0.00
B H
ATOM 17925 CA GLN B 83 -3.719 22.167 56.397 1.00 0.00
B C
ATOM 17926 HA GLN B 83 -3.335 22.968 57.017 1.00 0.00
B H
ATOM 17927 CB GLN B 83 -2.560 21.313 55.868 1.00 0.00
B C

ATOM 17928 HB1 GLN B 83   -1.901 21.090 56.739 1.00 0.00
B   H
ATOM 17929 HB2 GLN B 83   -2.954 20.332 55.518 1.00 0.00
B   H
ATOM 17930 CG GLN B 83   -1.745 21.993 54.773 1.00 0.00
B   C
ATOM 17931 HG1 GLN B 83   -2.416 22.520 54.061 1.00 0.00
B   H
ATOM 17932 HG2 GLN B 83   -1.052 22.744 55.207 1.00 0.00
B   H
ATOM 17933 CD GLN B 83   -0.925 21.038 53.928 1.00 0.00
B   C
ATOM 17934 OE1 GLN B 83   -0.650 19.909 54.336 1.00 0.00
B   O
ATOM 17935 NE2 GLN B 83   -0.523 21.489 52.740 1.00 0.00
B   N
ATOM 17936 HE21 GLN B 83   0.012 20.864 52.178 1.00 0.00
B   H
ATOM 17937 HE22 GLN B 83   -0.749 22.416 52.461 1.00 0.00
B   H
ATOM 17938 C   GLN B 83   -4.473 22.805 55.236 1.00 0.00
B   C
ATOM 17939 O   GLN B 83   -4.420 24.015 55.050 1.00 0.00
B   O
ATOM 17940 N   ALA B 84   -5.180 21.977 54.477 1.00 0.00
B   N
ATOM 17941 HN ALA B 84   -5.229 20.995 54.659 1.00 0.00
B   H
ATOM 17942 CA ALA B 84   -5.936 22.436 53.325 1.00 0.00
B   C
ATOM 17943 HA ALA B 84   -5.281 22.895 52.593 1.00 0.00
B   H
ATOM 17944 CB ALA B 84   -6.649 21.265 52.671 1.00 0.00
B   C
ATOM 17945 HB1 ALA B 84   -5.902 20.515 52.334 1.00 0.00
B   H
ATOM 17946 HB2 ALA B 84   -7.337 20.766 53.387 1.00 0.00
B   H
ATOM 17947 HB3 ALA B 84   -7.236 21.588 51.784 1.00 0.00
B   H
ATOM 17948 C   ALA B 84   -6.943 23.507 53.733 1.00 0.00
B   C
ATOM 17949 O   ALA B 84   -7.062 24.545 53.086 1.00 0.00
B   O
ATOM 17950 N   SER B 85   -7.654 23.247 54.822 1.00 0.00
B   N
ATOM 17951 HN SER B 85   -7.521 22.434 55.388 1.00 0.00
B   H
ATOM 17952 CA SER B 85   -8.699 24.131 55.290 1.00 0.00
B   C
ATOM 17953 HA SER B 85   -9.393 24.233 54.466 1.00 0.00
B   H
ATOM 17954 CB SER B 85   -9.375 23.532 56.525 1.00 0.00
B   C

ATOM 17955 HB1 SER B 85   -9.867 22.579 56.222 1.00 0.00
B   H
ATOM 17956 HB2 SER B 85   -8.615 23.276 57.298 1.00 0.00
B   H
ATOM 17957 OG SER B 85   -10.364 24.399 57.055 1.00 0.00
B   O
ATOM 17958 HG1 SER B 85   -10.893 23.881 57.674 1.00 0.00
B   H
ATOM 17959 C   SER B 85   -8.158 25.523 55.602 1.00 0.00
B   C
ATOM 17960 O   SER B 85   -8.758 26.528 55.197 1.00 0.00
B   O
ATOM 17961 N   THR B 86   -7.025 25.591 56.304 1.00 0.00
B   N
ATOM 17962 HN THR B 86   -6.481 24.810 56.607 1.00 0.00
B   H
ATOM 17963 CA THR B 86   -6.482 26.890 56.695 1.00 0.00
B   C
ATOM 17964 HA THR B 86   -7.336 27.501 56.956 1.00 0.00
B   H
ATOM 17965 CB THR B 86   -5.540 26.818 57.920 1.00 0.00
B   C
ATOM 17966 HB THR B 86   -5.410 27.870 58.273 1.00 0.00
B   H
ATOM 17967 OG1 THR B 86   -4.260 26.329 57.526 1.00 0.00
B   O
ATOM 17968 HG1 THR B 86   -3.737 26.300 58.334 1.00 0.00
B   H
ATOM 17969 CG2 THR B 86   -6.102 25.908 58.982 1.00 0.00
B   C
ATOM 17970 HG21 THR B 86   -5.484 25.947 59.903 1.00 0.00
B   H
ATOM 17971 HG22 THR B 86   -7.135 26.218 59.250 1.00 0.00
B   H
ATOM 17972 HG23 THR B 86   -6.135 24.855 58.629 1.00 0.00
B   H
ATOM 17973 C   THR B 86   -5.796 27.582 55.519 1.00 0.00
B   C
ATOM 17974 O   THR B 86   -5.876 28.798 55.378 1.00 0.00
B   O
ATOM 17975 N   LEU B 87   -5.144 26.798 54.667 1.00 0.00
B   N
ATOM 17976 HN LEU B 87   -5.044 25.810 54.771 1.00 0.00
B   H
ATOM 17977 CA LEU B 87   -4.506 27.339 53.481 1.00 0.00
B   C
ATOM 17978 HA LEU B 87   -3.837 28.124 53.810 1.00 0.00
B   H
ATOM 17979 CB LEU B 87   -3.694 26.255 52.766 1.00 0.00
B   C
ATOM 17980 HB1 LEU B 87   -2.955 25.860 53.501 1.00 0.00
B   H
ATOM 17981 HB2 LEU B 87   -4.366 25.404 52.517 1.00 0.00
B   H

ATOM 17982 CG LEU B 87 -2.903 26.614 51.507 1.00 0.00
B C
ATOM 17983 HG LEU B 87 -3.665 26.783 50.709 1.00 0.00
B H
ATOM 17984 CD1 LEU B 87 -2.063 27.873 51.689 1.00 0.00
B C
ATOM 17985 HD11 LEU B 87 -1.465 28.079 50.775 1.00 0.00
B H
ATOM 17986 HD12 LEU B 87 -2.714 28.753 51.886 1.00 0.00
B H
ATOM 17987 HD13 LEU B 87 -1.362 27.757 52.543 1.00 0.00
B H
ATOM 17988 CD2 LEU B 87 -2.029 25.448 51.082 1.00 0.00
B C
ATOM 17989 HD21 LEU B 87 -1.468 25.703 50.157 1.00 0.00
B H
ATOM 17990 HD22 LEU B 87 -1.287 25.187 51.864 1.00 0.00
B H
ATOM 17991 HD23 LEU B 87 -2.651 24.551 50.876 1.00 0.00
B H
ATOM 17992 C LEU B 87 -5.551 27.977 52.557 1.00 0.00
B C
ATOM 17993 O LEU B 87 -5.333 29.068 52.023 1.00 0.00
B O
ATOM 17994 N THR B 88 -6.683 27.300 52.386 1.00 0.00
B N
ATOM 17995 HN THR B 88 -6.845 26.388 52.762 1.00 0.00
B H
ATOM 17996 CA THR B 88 -7.803 27.842 51.623 1.00 0.00
B C
ATOM 17997 HA THR B 88 -7.412 28.108 50.650 1.00 0.00
B H
ATOM 17998 CB THR B 88 -8.959 26.827 51.522 1.00 0.00
B C
ATOM 17999 HB THR B 88 -9.172 26.494 52.567 1.00 0.00
B H
ATOM 18000 OG1 THR B 88 -8.517 25.695 50.771 1.00 0.00
B O
ATOM 18001 HG1 THR B 88 -9.276 25.103 50.729 1.00 0.00
B H
ATOM 18002 CG2 THR B 88 -10.165 27.423 50.825 1.00 0.00
B C
ATOM 18003 HG21 THR B 88 -11.027 26.724 50.860 1.00 0.00
B H
ATOM 18004 HG22 THR B 88 -10.471 28.368 51.323 1.00 0.00
B H
ATOM 18005 HG23 THR B 88 -9.941 27.647 49.760 1.00 0.00
B H
ATOM 18006 C THR B 88 -8.285 29.138 52.266 1.00 0.00
B C
ATOM 18007 O THR B 88 -8.373 30.179 51.595 1.00 0.00
B O
ATOM 18008 N THR B 89 -8.573 29.077 53.569 1.00 0.00
B N

ATOM 18009 HN THR B 89 -8.470 28.259 54.133 1.00 0.00
B H
ATOM 18010 CA THR B 89 -9.077 30.244 54.289 1.00 0.00
B C
ATOM 18011 HA THR B 89 -10.006 30.529 53.812 1.00 0.00
B H
ATOM 18012 CB THR B 89 -9.278 29.953 55.794 1.00 0.00
B C
ATOM 18013 HB THR B 89 -8.283 29.626 56.182 1.00 0.00
B H
ATOM 18014 OG1 THR B 89 -10.194 28.866 55.941 1.00 0.00
B O
ATOM 18015 HG1 THR B 89 -10.289 28.737 56.891 1.00 0.00
B H
ATOM 18016 CG2 THR B 89 -9.864 31.145 56.507 1.00 0.00
B C
ATOM 18017 HG21 THR B 89 -9.899 30.971 57.603 1.00 0.00
B H
ATOM 18018 HG22 THR B 89 -9.244 32.048 56.324 1.00 0.00
B H
ATOM 18019 HG23 THR B 89 -10.896 31.354 56.154 1.00 0.00
B H
ATOM 18020 C THR B 89 -8.116 31.401 54.089 1.00 0.00
B C
ATOM 18021 O THR B 89 -8.524 32.516 53.807 1.00 0.00
B O
ATOM 18022 N LEU B 90 -6.832 31.105 54.210 1.00 0.00
B N
ATOM 18023 HN LEU B 90 -6.505 30.183 54.405 1.00 0.00
B H
ATOM 18024 CA LEU B 90 -5.779 32.096 54.069 1.00 0.00
B C
ATOM 18025 HA LEU B 90 -5.944 32.837 54.840 1.00 0.00
B H
ATOM 18026 CB LEU B 90 -4.420 31.419 54.249 1.00 0.00
B C
ATOM 18027 HB1 LEU B 90 -4.531 30.668 55.065 1.00 0.00
B H
ATOM 18028 HB2 LEU B 90 -4.179 30.840 53.329 1.00 0.00
B H
ATOM 18029 CG LEU B 90 -3.254 32.321 54.601 1.00 0.00
B C
ATOM 18030 HG LEU B 90 -2.838 32.769 53.666 1.00 0.00
B H
ATOM 18031 CD1 LEU B 90 -3.728 33.433 55.523 1.00 0.00
B C
ATOM 18032 HD11 LEU B 90 -2.875 34.076 55.830 1.00 0.00
B H
ATOM 18033 HD12 LEU B 90 -4.480 34.072 55.010 1.00 0.00
B H
ATOM 18034 HD13 LEU B 90 -4.192 33.012 56.440 1.00 0.00
B H
ATOM 18035 CD2 LEU B 90 -2.159 31.491 55.253 1.00 0.00
B C

ATOM 18036 HD21 LEU B 90 −1.285 32.132 55.502 1.00 0.00
B H
ATOM 18037 HD22 LEU B 90 −2.507 31.017 56.192 1.00 0.00
B H
ATOM 18038 HD23 LEU B 90 −1.816 30.690 54.563 1.00 0.00
B H
ATOM 18039 C LEU B 90 −5.865 32.804 52.723 1.00 0.00
B C
ATOM 18040 O LEU B 90 −5.854 34.036 52.670 1.00 0.00
B O
ATOM 18041 N LEU B 91 −5.983 32.010 51.656 1.00 0.00
B N
ATOM 18042 HN LEU B 91 −5.977 31.015 51.726 1.00 0.00
B H
ATOM 18043 CA LEU B 91 −6.130 32.515 50.293 1.00 0.00
B C
ATOM 18044 HA LEU B 91 −5.290 33.177 50.120 1.00 0.00
B H
ATOM 18045 CB LEU B 91 −6.119 31.369 49.280 1.00 0.00
B C
ATOM 18046 HB1 LEU B 91 −6.956 30.684 49.549 1.00 0.00
B H
ATOM 18047 HB2 LEU B 91 −6.364 31.774 48.273 1.00 0.00
B H
ATOM 18048 CG LEU B 91 −4.862 30.507 49.162 1.00 0.00
B C
ATOM 18049 HG LEU B 91 −4.631 30.136 50.189 1.00 0.00
B H
ATOM 18050 CD1 LEU B 91 −5.150 29.325 48.246 1.00 0.00
B C
ATOM 18051 HD11 LEU B 91 −4.235 28.709 48.107 1.00 0.00
B H
ATOM 18052 HD12 LEU B 91 −5.944 28.678 48.679 1.00 0.00
B H
ATOM 18053 HD13 LEU B 91 −5.488 29.674 47.248 1.00 0.00
B H
ATOM 18054 CD2 LEU B 91 −3.661 31.296 48.666 1.00 0.00
B C
ATOM 18055 HD21 LEU B 91 −2.765 30.642 48.611 1.00 0.00
B H
ATOM 18056 HD22 LEU B 91 −3.835 31.715 47.654 1.00 0.00
B H
ATOM 18057 HD23 LEU B 91 −3.435 32.137 49.358 1.00 0.00
B H
ATOM 18058 C LEU B 91 −7.407 33.337 50.124 1.00 0.00
B C
ATOM 18059 O LEU B 91 −7.396 34.392 49.472 1.00 0.00
B O
ATOM 18060 N LEU B 92 −8.503 32.860 50.711 1.00 0.00
B N
ATOM 18061 HN LEU B 92 −8.542 32.008 51.230 1.00 0.00
B H
ATOM 18062 CA LEU B 92 −9.770 33.574 50.637 1.00 0.00
B C

ATOM 18063 HA LEU B 92   -9.983 33.713 49.586 1.00 0.00
B   H
ATOM 18064 CB LEU B 92   -10.903 32.770 51.270 1.00 0.00
B   C
ATOM 18065 HB1 LEU B 92   -10.563 32.466 52.287 1.00 0.00
B   H
ATOM 18066 HB2 LEU B 92   -11.778 33.439 51.426 1.00 0.00
B   H
ATOM 18067 CG LEU B 92   -11.314 31.521 50.495 1.00 0.00
B   C
ATOM 18068 HG LEU B 92   -10.419 30.854 50.453 1.00 0.00
B   H
ATOM 18069 CD1 LEU B 92   -12.438 30.828 51.225 1.00 0.00
B   C
ATOM 18070 HD11 LEU B 92   -12.786 29.941 50.652 1.00 0.00
B   H
ATOM 18071 HD12 LEU B 92   -12.102 30.486 52.227 1.00 0.00
B   H
ATOM 18072 HD13 LEU B 92   -13.301 31.514 51.358 1.00 0.00
B   H
ATOM 18073 CD2 LEU B 92   -11.735 31.834 49.065 1.00 0.00
B   C
ATOM 18074 HD21 LEU B 92   -12.007 30.899 48.530 1.00 0.00
B   H
ATOM 18075 HD22 LEU B 92   -12.614 32.507 49.036 1.00 0.00
B   H
ATOM 18076 HD23 LEU B 92   -10.902 32.321 48.513 1.00 0.00
B   H
ATOM 18077 C   LEU B 92   -9.674 34.948 51.270 1.00 0.00
B   C
ATOM 18078 O   LEU B 92   -10.235 35.917 50.755 1.00 0.00
B   O
ATOM 18079 N   LYS B 93   -8.949 35.042 52.376 1.00 0.00
B   N
ATOM 18080 HN LYS B 93   -8.485 34.272 52.811 1.00 0.00
B   H
ATOM 18081 CA LYS B 93   -8.781 36.323 53.046 1.00 0.00
B   C
ATOM 18082 HA LYS B 93   -9.765 36.766 53.139 1.00 0.00
B   H
ATOM 18083 CB LYS B 93   -8.215 36.120 54.457 1.00 0.00
B   C
ATOM 18084 HB1 LYS B 93   -8.873 35.382 54.972 1.00 0.00
B   H
ATOM 18085 HB2 LYS B 93   -7.212 35.645 54.380 1.00 0.00
B   H
ATOM 18086 CG LYS B 93   -8.139 37.377 55.307 1.00 0.00
B   C
ATOM 18087 HG1 LYS B 93   -7.613 37.142 56.260 1.00 0.00
B   H
ATOM 18088 HG2 LYS B 93   -7.530 38.141 54.771 1.00 0.00
B   H
ATOM 18089 CD LYS B 93   -9.505 37.956 55.645 1.00 0.00
B   C

ATOM 18090 HD1 LYS B 93 -10.072 38.097 54.696 1.00 0.00
B H
ATOM 18091 HD2 LYS B 93 -10.058 37.207 56.256 1.00 0.00
B H
ATOM 18092 CE LYS B 93 -9.340 39.267 56.398 1.00 0.00
B C
ATOM 18093 HE1 LYS B 93 -8.955 39.081 57.425 1.00 0.00
B H
ATOM 18094 HE2 LYS B 93 -8.623 39.934 55.869 1.00 0.00
B H
ATOM 18095 NZ LYS B 93 -10.609 40.026 56.544 1.00 0.00
B N
ATOM 18096 HZ1 LYS B 93 -10.437 40.910 57.066 1.00 0.00
B H
ATOM 18097 HZ2 LYS B 93 -10.990 40.250 55.602 1.00 0.00
B H
ATOM 18098 HZ3 LYS B 93 -11.300 39.447 57.064 1.00 0.00
B H
ATOM 18099 C LYS B 93 -7.925 37.281 52.205 1.00 0.00
B C
ATOM 18100 O LYS B 93 -8.145 38.487 52.214 1.00 0.00
B O
ATOM 18101 N GLU B 94 -6.963 36.730 51.468 1.00 0.00
B N
ATOM 18102 HN GLU B 94 -6.740 35.759 51.487 1.00 0.00
B H
ATOM 18103 CA GLU B 94 -6.135 37.512 50.552 1.00 0.00
B C
ATOM 18104 HA GLU B 94 -5.713 38.336 51.112 1.00 0.00
B H
ATOM 18105 CB GLU B 94 -5.010 36.660 49.969 1.00 0.00
B C
ATOM 18106 HB1 GLU B 94 -5.417 35.644 49.760 1.00 0.00
B H
ATOM 18107 HB2 GLU B 94 -4.696 37.072 48.984 1.00 0.00
B H
ATOM 18108 CG GLU B 94 -3.808 36.530 50.880 1.00 0.00
B C
ATOM 18109 HG1 GLU B 94 -3.391 37.533 51.085 1.00 0.00
B H
ATOM 18110 HG2 GLU B 94 -4.086 36.048 51.839 1.00 0.00
B H
ATOM 18111 CD GLU B 94 -2.677 35.739 50.260 1.00 0.00
B C
ATOM 18112 OE1 GLU B 94 -2.234 34.750 50.876 1.00 0.00
B O
ATOM 18113 OE2 GLU B 94 -2.223 36.100 49.154 1.00 0.00
B O
ATOM 18114 C GLU B 94 -6.945 38.108 49.414 1.00 0.00
B C
ATOM 18115 O GLU B 94 -6.670 39.218 48.976 1.00 0.00
B O
ATOM 18116 N LEU B 95 -7.942 37.368 48.941 1.00 0.00
B N

ATOM 18117 HN LEU B 95   -8.194 36.476 49.312 1.00 0.00
B   H
ATOM 18118 CA LEU B 95   -8.765 37.807 47.820 1.00 0.00
B   C
ATOM 18119 HA LEU B 95   -8.114 38.388 47.179 1.00 0.00
B   H
ATOM 18120 CB LEU B 95   -9.279 36.597 47.030 1.00 0.00
B   C
ATOM 18121 HB1 LEU B 95   -9.914 35.998 47.722 1.00 0.00
B   H
ATOM 18122 HB2 LEU B 95   -9.956 36.954 46.223 1.00 0.00
B   H
ATOM 18123 CG LEU B 95   -8.202 35.674 46.444 1.00 0.00
B   C
ATOM 18124 HG LEU B 95   -7.529 35.433 47.303 1.00 0.00
B   H
ATOM 18125 CD1 LEU B 95   -8.810 34.394 45.895 1.00 0.00
B   C
ATOM 18126 HD11 LEU B 95   -8.025 33.758 45.431 1.00 0.00
B   H
ATOM 18127 HD12 LEU B 95   -9.296 33.813 46.707 1.00 0.00
B   H
ATOM 18128 HD13 LEU B 95   -9.573 34.623 45.121 1.00 0.00
B   H
ATOM 18129 CD2 LEU B 95   -7.370 36.366 45.376 1.00 0.00
B   C
ATOM 18130 HD21 LEU B 95   -6.589 35.677 44.987 1.00 0.00
B   H
ATOM 18131 HD22 LEU B 95   -7.992 36.689 44.517 1.00 0.00
B   H
ATOM 18132 HD23 LEU B 95   -6.864 37.262 45.796 1.00 0.00
B   H
ATOM 18133 C   LEU B 95   -9.917 38.717 48.255 1.00 0.00
B   C
ATOM 18134 O   LEU B 95   -10.805 39.023 47.455 1.00 0.00
B   O
ATOM 18135 N   ASP B 96   -9.884 39.147 49.516 1.00 0.00
B   N
ATOM 18136 HN ASP B 96   -9.209 38.838 50.181 1.00 0.00
B   H
ATOM 18137 CA ASP B 96   -10.832 40.122 50.072 1.00 0.00
B   C
ATOM 18138 HA ASP B 96   -10.497 40.278 51.091 1.00 0.00
B   H
ATOM 18139 CB ASP B 96   -10.732 41.462 49.336 1.00 0.00
B   C
ATOM 18140 HB1 ASP B 96   -10.435 41.281 48.284 1.00 0.00
B   H
ATOM 18141 HB2 ASP B 96   -11.711 41.982 49.338 1.00 0.00
B   H
ATOM 18142 CG ASP B 96   -9.705 42.376 49.943 1.00 0.00
B   C
ATOM 18143 OD1 ASP B 96   -8.736 42.727 49.229 1.00 0.00
B   O

ATOM 18144 OD2 ASP B 96   -9.863 42.742 51.133 1.00 0.00
B   O
ATOM 18145 C   ASP B 96   -12.298 39.682 50.150 1.00 0.00
B   C
ATOM 18146 O   ASP B 96   -13.198 40.528 50.138 1.00 0.00
B   O
ATOM 18147 N   ILE B 97   -12.537 38.375 50.243 1.00 0.00
B   N
ATOM 18148 HN ILE B 97   -11.828 37.675 50.213 1.00 0.00
B   H
ATOM 18149 CA ILE B 97   -13.896 37.847 50.400 1.00 0.00
B   C
ATOM 18150 HA ILE B 97   -14.469 38.181 49.544 1.00 0.00
B   H
ATOM 18151 CB ILE B 97   -13.906 36.300 50.480 1.00 0.00
B   C
ATOM 18152 HB ILE B 97   -13.257 36.061 51.361 1.00 0.00
B   H
ATOM 18153 CG2 ILE B 97   -15.317 35.751 50.665 1.00 0.00
B   C
ATOM 18154 HG21 ILE B 97   -15.288 34.662 50.876 1.00 0.00
B   H
ATOM 18155 HG22 ILE B 97   -15.826 36.246 51.519 1.00 0.00
B   H
ATOM 18156 HG23 ILE B 97   -15.929 35.912 49.751 1.00 0.00
B   H
ATOM 18157 CG1 ILE B 97   -13.222 35.679 49.249 1.00 0.00
B   C
ATOM 18158 HG11 ILE B 97   -12.139 35.933 49.253 1.00 0.00
B   H
ATOM 18159 HG12 ILE B 97   -13.300 34.569 49.315 1.00 0.00
B   H
ATOM 18160 CD ILE B 97   -13.794 36.088 47.906 1.00 0.00
B   C
ATOM 18161 HD1 ILE B 97   -13.276 35.547 47.085 1.00 0.00
B   H
ATOM 18162 HD2 ILE B 97   -14.877 35.848 47.852 1.00 0.00
B   H
ATOM 18163 HD3 ILE B 97   -13.666 37.177 47.734 1.00 0.00
B   H
ATOM 18164 C   ILE B 97   -14.543 38.472 51.635 1.00 0.00
B   C
ATOM 18165 O   ILE B 97   -13.928 38.511 52.695 1.00 0.00
B   O
ATOM 18166 N   PRO B 98   -15.766 39.005 51.492 1.00 0.00
B   N
ATOM 18167 CD PRO B 98   -16.493 39.185 50.221 1.00 0.00
B   C
ATOM 18168 HD1 PRO B 98   -15.422 39.471 50.332 1.00 0.00
B   H
ATOM 18169 HD2 PRO B 98   -16.689 38.797 49.195 1.00 0.00
B   H
ATOM 18170 CA PRO B 98   -16.446 39.680 52.604 1.00 0.00
B   C

ATOM 18171 HA PRO B 98 −15.813 40.477 52.978 1.00 0.00
B H
ATOM 18172 CB PRO B 98 −17.712 40.254 51.949 1.00 0.00
B C
ATOM 18173 HB1 PRO B 98 −17.470 41.239 51.491 1.00 0.00
B H
ATOM 18174 HB2 PRO B 98 −18.544 40.387 52.669 1.00 0.00
B H
ATOM 18175 CG PRO B 98 −17.373 40.371 50.499 1.00 0.00
B C
ATOM 18176 HG1 PRO B 98 −17.992 41.121 49.971 1.00 0.00
B H
ATOM 18177 HG2 PRO B 98 −16.887 40.832 49.756 1.00 0.00
B H
ATOM 18178 C PRO B 98 −16.820 38.753 53.761 1.00 0.00
B C
ATOM 18179 O PRO B 98 −16.670 39.133 54.924 1.00 0.00
B O
ATOM 18180 N ASN B 99 −17.306 37.554 53.448 1.00 0.00
B N
ATOM 18181 HN ASN B 99 −17.453 37.220 52.518 1.00 0.00
B H
ATOM 18182 CA ASN B 99 −17.672 36.605 54.495 1.00 0.00
B C
ATOM 18183 HA ASN B 99 −17.227 36.998 55.404 1.00 0.00
B H
ATOM 18184 CB ASN B 99 −19.183 36.510 54.654 1.00 0.00
B C
ATOM 18185 HB1 ASN B 99 −19.605 36.154 53.688 1.00 0.00
B H
ATOM 18186 HB2 ASN B 99 −19.443 35.760 55.429 1.00 0.00
B H
ATOM 18187 CG ASN B 99 −19.817 37.844 54.980 1.00 0.00
B C
ATOM 18188 OD1 ASN B 99 −20.105 38.637 54.087 1.00 0.00
B O
ATOM 18189 ND2 ASN B 99 −20.028 38.101 56.267 1.00 0.00
B N
ATOM 18190 HD21 ASN B 99 −20.455 38.971 56.496 1.00 0.00
B H
ATOM 18191 HD22 ASN B 99 −19.790 37.424 56.957 1.00 0.00
B H
ATOM 18192 C ASN B 99 −17.095 35.228 54.262 1.00 0.00
B C
ATOM 18193 O ASN B 99 −17.430 34.570 53.270 1.00 0.00
B O
ATOM 18194 N ILE B 100 −16.228 34.805 55.180 1.00 0.00
B N
ATOM 18195 HN ILE B 100 −15.949 35.353 55.966 1.00 0.00
B H
ATOM 18196 CA ILE B 100 −15.606 33.495 55.117 1.00 0.00
B C
ATOM 18197 HA ILE B 100 −16.015 32.990 54.251 1.00 0.00
B H

ATOM 18198 CB ILE B 100 -14.074 33.597 55.024 1.00 0.00
B C
ATOM 18199 HB ILE B 100 -13.777 34.176 55.935 1.00 0.00
B H
ATOM 18200 CG2 ILE B 100 -13.458 32.209 54.934 1.00 0.00
B C
ATOM 18201 HG21 ILE B 100 -12.353 32.266 55.025 1.00 0.00
B H
ATOM 18202 HG22 ILE B 100 -13.828 31.557 55.752 1.00 0.00
B H
ATOM 18203 HG23 ILE B 100 -13.702 31.727 53.963 1.00 0.00
B H
ATOM 18204 CG1 ILE B 100 -13.675 34.440 53.808 1.00 0.00
B C
ATOM 18205 HG11 ILE B 100 -13.737 33.822 52.884 1.00 0.00
B H
ATOM 18206 HG12 ILE B 100 -14.403 35.275 53.693 1.00 0.00
B H
ATOM 18207 CD ILE B 100 -12.287 35.053 53.876 1.00 0.00
B C
ATOM 18208 HD1 ILE B 100 -12.102 35.691 52.985 1.00 0.00
B H
ATOM 18209 HD2 ILE B 100 -12.181 35.685 54.784 1.00 0.00
B H
ATOM 18210 HD3 ILE B 100 -11.505 34.265 53.902 1.00 0.00
B H
ATOM 18211 C ILE B 100 -16.012 32.678 56.337 1.00 0.00
B C
ATOM 18212 O ILE B 100 -15.657 33.013 57.471 1.00 0.00
B O
ATOM 18213 N TRP B 101 -16.776 31.614 56.092 1.00 0.00
B N
ATOM 18214 HN TRP B 101 -17.087 31.377 55.175 1.00 0.00
B H
ATOM 18215 CA TRP B 101 -17.218 30.711 57.146 1.00 0.00
B C
ATOM 18216 HA TRP B 101 -16.913 31.120 58.102 1.00 0.00
B H
ATOM 18217 CB TRP B 101 -18.713 30.487 57.060 1.00 0.00
B C
ATOM 18218 HB1 TRP B 101 -18.951 30.166 56.022 1.00 0.00
B H
ATOM 18219 HB2 TRP B 101 -19.009 29.652 57.731 1.00 0.00
B H
ATOM 18220 CG TRP B 101 -19.536 31.717 57.352 1.00 0.00
B C
ATOM 18221 CD1 TRP B 101 -19.164 33.049 57.211 1.00 0.00
B C
ATOM 18222 HD1 TRP B 101 -18.199 33.362 56.835 1.00 0.00
B H
ATOM 18223 NE1 TRP B 101 -20.189 33.890 57.572 1.00 0.00
B N
ATOM 18224 HE1 TRP B 101 -20.145 34.864 57.528 1.00 0.00
B H

ATOM 18225 CE2 TRP B 101   -21.274 33.189 57.946 1.00 0.00
B   C
ATOM 18226 CD2 TRP B 101   -20.932 31.765 57.822 1.00 0.00
B   C
ATOM 18227 CE3 TRP B 101   -21.890 30.808 58.149 1.00 0.00
B   C
ATOM 18228 HE3 TRP B 101   -21.686 29.749 58.078 1.00 0.00
B   H
ATOM 18229 CZ3 TRP B 101   -23.147 31.251 58.588 1.00 0.00
B   C
ATOM 18230 HZ3 TRP B 101   -23.904 30.526 58.852 1.00 0.00
B   H
ATOM 18231 CZ2 TRP B 101   -22.521 33.609 58.374 1.00 0.00
B   C
ATOM 18232 HZ2 TRP B 101   -22.791 34.650 58.461 1.00 0.00
B   H
ATOM 18233 CH2 TRP B 101   -23.450 32.615 58.697 1.00 0.00
B   C
ATOM 18234 HH2 TRP B 101   -24.428 32.919 59.042 1.00 0.00
B   H
ATOM 18235 C   TRP B 101   -16.511 29.411 56.987 1.00 0.00
B   C
ATOM 18236 O   TRP B 101   -16.487 28.853 55.894 1.00 0.00
B   O
ATOM 18237 N   VAL B 102   -15.927 28.916 58.074 1.00 0.00
B   N
ATOM 18238 HN VAL B 102   -16.005 29.327 58.978 1.00 0.00
B   H
ATOM 18239 CA VAL B 102   -15.115 27.715 58.029 1.00 0.00
B   C
ATOM 18240 HA VAL B 102   -15.284 27.247 57.067 1.00 0.00
B   H
ATOM 18241 CB VAL B 102   -13.617 28.053 58.211 1.00 0.00
B   C
ATOM 18242 HB VAL B 102   -13.456 28.305 59.288 1.00 0.00
B   H
ATOM 18243 CG1 VAL B 102   -12.743 26.865 57.845 1.00 0.00
B   C
ATOM 18244 HG11 VAL B 102   -11.669 27.132 57.942 1.00 0.00
B   H
ATOM 18245 HG12 VAL B 102   -12.930 25.997 58.512 1.00 0.00
B   H
ATOM 18246 HG13 VAL B 102   -12.927 26.552 56.795 1.00 0.00
B   H
ATOM 18247 CG2 VAL B 102   -13.227 29.259 57.376 1.00 0.00
B   C
ATOM 18248 HG21 VAL B 102   -12.143 29.474 57.478 1.00 0.00
B   H
ATOM 18249 HG22 VAL B 102   -13.448 29.078 56.301 1.00 0.00
B   H
ATOM 18250 HG23 VAL B 102   -13.781 30.166 57.696 1.00 0.00
B   H
ATOM 18251 C   VAL B 102   -15.539 26.716 59.105 1.00 0.00
B   C

ATOM 18252 O VAL B 102 -15.865 27.099 60.230 1.00 0.00
B O
ATOM 18253 N LYS B 103 -15.543 25.436 58.746 1.00 0.00
B N
ATOM 18254 HN LYS B 103 -15.412 25.139 57.801 1.00 0.00
B H
ATOM 18255 CA LYS B 103 -15.739 24.363 59.710 1.00 0.00
B C
ATOM 18256 HA LYS B 103 -16.442 24.706 60.458 1.00 0.00
B H
ATOM 18257 CB LYS B 103 -16.253 23.105 59.014 1.00 0.00
B C
ATOM 18258 HB1 LYS B 103 -17.276 23.334 58.637 1.00 0.00
B H
ATOM 18259 HB2 LYS B 103 -15.626 22.906 58.115 1.00 0.00
B H
ATOM 18260 CG LYS B 103 -16.321 21.871 59.898 1.00 0.00
B C
ATOM 18261 HG1 LYS B 103 -15.307 21.656 60.303 1.00 0.00
B H
ATOM 18262 HG2 LYS B 103 -16.988 22.083 60.766 1.00 0.00
B H
ATOM 18263 CD LYS B 103 -16.821 20.681 59.098 1.00 0.00
B C
ATOM 18264 HD1 LYS B 103 -17.930 20.633 59.196 1.00 0.00
B H
ATOM 18265 HD2 LYS B 103 -16.588 20.863 58.024 1.00 0.00
B H
ATOM 18266 CE LYS B 103 -16.184 19.388 59.566 1.00 0.00
B C
ATOM 18267 HE1 LYS B 103 -15.914 18.749 58.695 1.00 0.00
B H
ATOM 18268 HE2 LYS B 103 -15.260 19.596 60.150 1.00 0.00
B H
ATOM 18269 NZ LYS B 103 -17.109 18.615 60.431 1.00 0.00
B N
ATOM 18270 HZ1 LYS B 103 -16.653 17.732 60.739 1.00 0.00
B H
ATOM 18271 HZ2 LYS B 103 -17.361 19.183 61.264 1.00 0.00
B H
ATOM 18272 HZ3 LYS B 103 -17.973 18.388 59.897 1.00 0.00
B H
ATOM 18273 C LYS B 103 -14.422 24.088 60.417 1.00 0.00
B C
ATOM 18274 O LYS B 103 -13.394 23.899 59.780 1.00 0.00
B O
ATOM 18275 N ALA B 104 -14.455 24.085 61.739 1.00 0.00
B N
ATOM 18276 HN ALA B 104 -15.286 24.272 62.262 1.00 0.00
B H
ATOM 18277 CA ALA B 104 -13.270 23.806 62.528 1.00 0.00
B C
ATOM 18278 HA ALA B 104 -12.368 23.998 61.959 1.00 0.00
B H

ATOM 18279 CB ALA B 104   -13.253 24.674 63.769 1.00 0.00
B   C
ATOM 18280 HB1 ALA B 104   -13.231 25.746 63.474 1.00 0.00
B   H
ATOM 18281 HB2 ALA B 104   -14.163 24.508 64.386 1.00 0.00
B   H
ATOM 18282 HB3 ALA B 104   -12.358 24.473 64.396 1.00 0.00
B   H
ATOM 18283 C   ALA B 104   -13.232 22.335 62.910 1.00 0.00
B   C
ATOM 18284 O   ALA B 104   -14.255 21.750 63.263 1.00 0.00
B   O
ATOM 18285 N   GLN B 105   -12.043 21.747 62.844 1.00 0.00
B   N
ATOM 18286 HN GLN B 105   -11.214 22.221 62.552 1.00 0.00
B   H
ATOM 18287 CA GLN B 105   -11.846 20.348 63.194 1.00 0.00
B   C
ATOM 18288 HA GLN B 105   -12.747 19.826 62.896 1.00 0.00
B   H
ATOM 18289 CB GLN B 105   -10.643 19.772 62.453 1.00 0.00
B   C
ATOM 18290 HB1 GLN B 105   -9.791 20.469 62.631 1.00 0.00
B   H
ATOM 18291 HB2 GLN B 105   -10.354 18.802 62.917 1.00 0.00
B   H
ATOM 18292 CG GLN B 105   -10.894 19.627 60.972 1.00 0.00
B   C
ATOM 18293 HG1 GLN B 105   -11.025 20.625 60.501 1.00 0.00
B   H
ATOM 18294 HG2 GLN B 105   -10.039 19.126 60.476 1.00 0.00
B   H
ATOM 18295 CD GLN B 105   -12.160 18.842 60.696 1.00 0.00
B   C
ATOM 18296 OE1 GLN B 105   -12.337 17.726 61.186 1.00 0.00
B   O
ATOM 18297 NE2 GLN B 105   -13.058 19.429 59.912 1.00 0.00
B   N
ATOM 18298 HE21 GLN B 105   -13.895 18.922 59.728 1.00 0.00
B   H
ATOM 18299 HE22 GLN B 105   -12.867 20.326 59.527 1.00 0.00
B   H
ATOM 18300 C   GLN B 105   -11.678 20.151 64.686 1.00 0.00
B   C
ATOM 18301 O   GLN B 105   -12.225 19.206 65.250 1.00 0.00
B   O
ATOM 18302 N   ASN B 106   -10.930 21.046 65.320 1.00 0.00
B   N
ATOM 18303 HN ASN B 106   -10.539 21.849 64.873 1.00 0.00
B   H
ATOM 18304 CA ASN B 106   -10.616 20.929 66.733 1.00 0.00
B   C
ATOM 18305 HA ASN B 106   -11.520 20.569 67.214 1.00 0.00
B   H

ATOM 18306 CB ASN B 106   -9.457 19.950 66.930 1.00 0.00
B   C
ATOM 18307 HB1 ASN B 106   -9.185 19.957 68.009 1.00 0.00
B   H
ATOM 18308 HB2 ASN B 106   -9.775 18.919 66.674 1.00 0.00
B   H
ATOM 18309 CG ASN B 106   -8.217 20.330 66.132 1.00 0.00
B   C
ATOM 18310 OD1 ASN B 106   -7.927 21.506 65.916 1.00 0.00
B   O
ATOM 18311 ND2 ASN B 106   -7.475 19.324 65.700 1.00 0.00
B   N
ATOM 18312 HD21 ASN B 106   -6.653 19.551 65.185 1.00 0.00
B   H
ATOM 18313 HD22 ASN B 106   -7.730 18.388 65.918 1.00 0.00
B   H
ATOM 18314 C   ASN B 106   -10.288 22.300 67.329 1.00 0.00
B   C
ATOM 18315 O   ASN B 106   -10.389 23.315 66.644 1.00 0.00
B   O
ATOM 18316 N   TYR B 107   -9.896 22.328 68.598 1.00 0.00
B   N
ATOM 18317 HN TYR B 107   -9.822 21.519 69.178 1.00 0.00
B   H
ATOM 18318 CA TYR B 107   -9.533 23.581 69.255 1.00 0.00
B   C
ATOM 18319 HA TYR B 107   -10.417 24.205 69.214 1.00 0.00
B   H
ATOM 18320 CB TYR B 107   -9.116 23.332 70.706 1.00 0.00
B   C
ATOM 18321 HB1 TYR B 107   -9.998 22.975 71.283 1.00 0.00
B   H
ATOM 18322 HB2 TYR B 107   -8.344 22.535 70.762 1.00 0.00
B   H
ATOM 18323 CG TYR B 107   -8.610 24.571 71.406 1.00 0.00
B   C
ATOM 18324 CD1 TYR B 107   -9.499 25.535 71.880 1.00 0.00
B   C
ATOM 18325 HD1 TYR B 107   -10.563 25.405 71.732 1.00 0.00
B   H
ATOM 18326 CE1 TYR B 107   -9.045 26.677 72.523 1.00 0.00
B   C
ATOM 18327 HE1 TYR B 107   -9.742 27.418 72.886 1.00 0.00
B   H
ATOM 18328 CZ TYR B 107   -7.683 26.867 72.694 1.00 0.00
B   C
ATOM 18329 OH TYR B 107   -7.226 28.006 73.331 1.00 0.00
B   O
ATOM 18330 HH TYR B 107   -6.273 27.924 73.417 1.00 0.00
B   H
ATOM 18331 CD2 TYR B 107   -7.247 24.787 71.587 1.00 0.00
B   C
ATOM 18332 HD2 TYR B 107   -6.530 24.079 71.193 1.00 0.00
B   H

ATOM 18333 CE2 TYR B 107 -6.781 25.927 72.226 1.00 0.00
B C
ATOM 18334 HE2 TYR B 107 -5.718 26.082 72.336 1.00 0.00
B H
ATOM 18335 C TYR B 107 -8.421 24.328 68.521 1.00 0.00
B C
ATOM 18336 O TYR B 107 -8.503 25.543 68.314 1.00 0.00
B O
ATOM 18337 N TYR B 108 -7.378 23.599 68.139 1.00 0.00
B N
ATOM 18338 HN TYR B 108 -7.313 22.615 68.296 1.00 0.00
B H
ATOM 18339 CA TYR B 108 -6.227 24.181 67.453 1.00 0.00
B C
ATOM 18340 HA TYR B 108 -5.855 24.964 68.102 1.00 0.00
B H
ATOM 18341 CB TYR B 108 -5.180 23.103 67.191 1.00 0.00
B C
ATOM 18342 HB1 TYR B 108 -4.509 23.026 68.075 1.00 0.00
B H
ATOM 18343 HB2 TYR B 108 -5.663 22.111 67.057 1.00 0.00
B H
ATOM 18344 CG TYR B 108 -4.296 23.387 66.003 1.00 0.00
B C
ATOM 18345 CD1 TYR B 108 -3.330 24.397 66.049 1.00 0.00
B C
ATOM 18346 HD1 TYR B 108 -3.225 24.999 66.940 1.00 0.00
B H
ATOM 18347 CE1 TYR B 108 -2.516 24.658 64.961 1.00 0.00
B C
ATOM 18348 HE1 TYR B 108 -1.783 25.450 65.001 1.00 0.00
B H
ATOM 18349 CZ TYR B 108 -2.655 23.900 63.816 1.00 0.00
B C
ATOM 18350 OH TYR B 108 -1.852 24.153 62.737 1.00 0.00
B O
ATOM 18351 HH TYR B 108 -2.043 23.488 62.073 1.00 0.00
B H
ATOM 18352 CD2 TYR B 108 -4.414 22.645 64.835 1.00 0.00
B C
ATOM 18353 HD2 TYR B 108 -5.178 21.883 64.753 1.00 0.00
B H
ATOM 18354 CE2 TYR B 108 -3.599 22.894 63.742 1.00 0.00
B C
ATOM 18355 HE2 TYR B 108 -3.723 22.316 62.838 1.00 0.00
B H
ATOM 18356 C TYR B 108 -6.614 24.837 66.137 1.00 0.00
B C
ATOM 18357 O TYR B 108 -6.162 25.931 65.812 1.00 0.00
B O
ATOM 18358 N HSE B 109 -7.446 24.141 65.380 1.00 0.00
B N
ATOM 18359 HN HSE B 109 -7.817 23.255 65.655 1.00 0.00
B H

ATOM 18360 CA HSE B 109 -7.895 24.600 64.090 1.00 0.00
B C
ATOM 18361 HA HSE B 109 -7.020 24.840 63.499 1.00 0.00
B H
ATOM 18362 CB HSE B 109 -8.717 23.495 63.419 1.00 0.00
B C
ATOM 18363 HB1 HSE B 109 -8.141 22.548 63.493 1.00 0.00
B H
ATOM 18364 HB2 HSE B 109 -9.663 23.345 63.979 1.00 0.00
B H
ATOM 18365 ND1 HSE B 109 -10.044 23.151 61.319 1.00 0.00
B N
ATOM 18366 CG HSE B 109 -9.014 23.739 61.955 1.00 0.00
B C
ATOM 18367 CE1 HSE B 109 -10.080 23.557 60.044 1.00 0.00
B C
ATOM 18368 HE1 HSE B 109 -10.813 23.223 59.307 1.00 0.00
B H
ATOM 18369 NE2 HSE B 109 -9.071 24.418 59.857 1.00 0.00
B N
ATOM 18370 HE2 HSE B 109 -8.849 24.867 58.992 1.00 0.00
B H
ATOM 18371 CD2 HSE B 109 -8.393 24.554 61.017 1.00 0.00
B C
ATOM 18372 HD2 HSE B 109 -7.527 25.192 61.119 1.00 0.00
B H
ATOM 18373 C HSE B 109 -8.670 25.873 64.269 1.00 0.00
B C
ATOM 18374 O HSE B 109 -8.478 26.832 63.523 1.00 0.00
B O
ATOM 18375 N HSE B 110 -9.522 25.913 65.289 1.00 0.00
B N
ATOM 18376 HN HSE B 110 -9.698 25.135 65.890 1.00 0.00
B H
ATOM 18377 CA HSE B 110 -10.285 27.116 65.621 1.00 0.00
B C
ATOM 18378 HA HSE B 110 -10.928 27.327 64.776 1.00 0.00
B H
ATOM 18379 CB HSE B 110 -11.106 26.891 66.885 1.00 0.00
B C
ATOM 18380 HB1 HSE B 110 -11.713 25.971 66.739 1.00 0.00
B H
ATOM 18381 HB2 HSE B 110 -10.425 26.698 67.740 1.00 0.00
B H
ATOM 18382 ND1 HSE B 110 -13.366 27.951 66.974 1.00 0.00
B N
ATOM 18383 CG HSE B 110 -12.049 28.022 67.211 1.00 0.00
B C
ATOM 18384 CE1 HSE B 110 -13.956 29.096 67.356 1.00 0.00
B C
ATOM 18385 HE1 HSE B 110 -15.025 29.302 67.266 1.00 0.00
B H
ATOM 18386 NE2 HSE B 110 -13.005 29.909 67.844 1.00 0.00
B N

ATOM 18387 HE2 HSE B 110   -13.154 30.838 68.183 1.00 0.00
B   H
ATOM 18388 CD2 HSE B 110   -11.817 29.277 67.772 1.00 0.00
B   C
ATOM 18389 HD2 HSE B 110   -10.891 29.729 68.100 1.00 0.00
B   H
ATOM 18390 C   HSE B 110   -9.377 28.300 65.792 1.00 0.00
B   C
ATOM 18391 O   HSE B 110   -9.630 29.372 65.241 1.00 0.00
B   O
ATOM 18392 N   LYS B 111   -8.296 28.100 66.542 1.00 0.00
B   N
ATOM 18393 HN LYS B 111   -8.068 27.198 66.905 1.00 0.00
B   H
ATOM 18394 CA LYS B 111   -7.362 29.166 66.895 1.00 0.00
B   C
ATOM 18395 HA LYS B 111   -7.958 29.914 67.403 1.00 0.00
B   H
ATOM 18396 CB LYS B 111   -6.311 28.645 67.878 1.00 0.00
B   C
ATOM 18397 HB1 LYS B 111   -5.845 27.745 67.413 1.00 0.00
B   H
ATOM 18398 HB2 LYS B 111   -5.500 29.401 67.977 1.00 0.00
B   H
ATOM 18399 CG LYS B 111   -6.858 28.264 69.249 1.00 0.00
B   C
ATOM 18400 HG1 LYS B 111   -7.970 28.204 69.194 1.00 0.00
B   H
ATOM 18401 HG2 LYS B 111   -6.484 27.250 69.521 1.00 0.00
B   H
ATOM 18402 CD LYS B 111   -6.476 29.268 70.328 1.00 0.00
B   C
ATOM 18403 HD1 LYS B 111   -6.459 28.740 71.310 1.00 0.00
B   H
ATOM 18404 HD2 LYS B 111   -5.439 29.621 70.121 1.00 0.00
B   H
ATOM 18405 CE LYS B 111   -7.423 30.456 70.387 1.00 0.00
B   C
ATOM 18406 HE1 LYS B 111   -7.623 30.846 69.364 1.00 0.00
B   H
ATOM 18407 HE2 LYS B 111   -8.392 30.161 70.847 1.00 0.00
B   H
ATOM 18408 NZ LYS B 111   -6.828 31.553 71.202 1.00 0.00
B   N
ATOM 18409 HZ1 LYS B 111   -7.479 32.364 71.237 1.00 0.00
B   H
ATOM 18410 HZ2 LYS B 111   -6.649 31.211 72.168 1.00 0.00
B   H
ATOM 18411 HZ3 LYS B 111   -5.928 31.853 70.774 1.00 0.00
B   H
ATOM 18412 C   LYS B 111   -6.683 29.853 65.709 1.00 0.00
B   C
ATOM 18413 O   LYS B 111   -6.621 31.082 65.682 1.00 0.00
B   O

ATOM 18414 N VAL B 112 -6.170 29.085 64.744 1.00 0.00
B N
ATOM 18415 HN VAL B 112 -6.170 28.088 64.750 1.00 0.00
B H
ATOM 18416 CA VAL B 112 -5.541 29.686 63.553 1.00 0.00
B C
ATOM 18417 HA VAL B 112 -4.869 30.461 63.903 1.00 0.00
B H
ATOM 18418 CB VAL B 112 -4.668 28.719 62.718 1.00 0.00
B C
ATOM 18419 HB VAL B 112 -4.432 29.227 61.750 1.00 0.00
B H
ATOM 18420 CG1 VAL B 112 -3.378 28.408 63.445 1.00 0.00
B C
ATOM 18421 HG11 VAL B 112 -2.771 27.683 62.860 1.00 0.00
B H
ATOM 18422 HG12 VAL B 112 -2.758 29.317 63.588 1.00 0.00
B H
ATOM 18423 HG13 VAL B 112 -3.586 27.958 64.439 1.00 0.00
B H
ATOM 18424 CG2 VAL B 112 -5.417 27.445 62.377 1.00 0.00
B C
ATOM 18425 HG21 VAL B 112 -4.765 26.746 61.813 1.00 0.00
B H
ATOM 18426 HG22 VAL B 112 -5.760 26.935 63.302 1.00 0.00
B H
ATOM 18427 HG23 VAL B 112 -6.305 27.658 61.747 1.00 0.00
B H
ATOM 18428 C VAL B 112 -6.570 30.348 62.648 1.00 0.00
B C
ATOM 18429 O VAL B 112 -6.300 31.411 62.093 1.00 0.00
B O
ATOM 18430 N LEU B 113 -7.741 29.724 62.508 1.00 0.00
B N
ATOM 18431 HN LEU B 113 -7.960 28.845 62.924 1.00 0.00
B H
ATOM 18432 CA LEU B 113 -8.824 30.298 61.708 1.00 0.00
B C
ATOM 18433 HA LEU B 113 -8.432 30.452 60.711 1.00 0.00
B H
ATOM 18434 CB LEU B 113 -10.019 29.347 61.642 1.00 0.00
B C
ATOM 18435 HB1 LEU B 113 -10.285 29.079 62.691 1.00 0.00
B H
ATOM 18436 HB2 LEU B 113 -10.896 29.896 61.234 1.00 0.00
B H
ATOM 18437 CG LEU B 113 -9.795 28.046 60.853 1.00 0.00
B C
ATOM 18438 HG LEU B 113 -8.825 27.642 61.227 1.00 0.00
B H
ATOM 18439 CD1 LEU B 113 -10.910 27.061 61.150 1.00 0.00
B C
ATOM 18440 HD11 LEU B 113 -10.783 26.136 60.548 1.00 0.00
B H

```
ATOM  18441 HD12 LEU B 113     -10.907  26.781  62.225  1.00  0.00
B    H
ATOM  18442 HD13 LEU B 113     -11.899  27.502  60.905  1.00  0.00
B    H
ATOM  18443  CD2 LEU B 113      -9.667  28.264  59.350  1.00  0.00
B    C
ATOM  18444 HD21 LEU B 113      -9.489  27.296  58.833  1.00  0.00
B    H
ATOM  18445 HD22 LEU B 113     -10.587  28.710  58.921  1.00  0.00
B    H
ATOM  18446 HD23 LEU B 113      -8.814  28.940  59.127  1.00  0.00
B    H
ATOM  18447  C   LEU B 113      -9.234  31.657  62.257  1.00  0.00
B    C
ATOM  18448  O   LEU B 113      -9.458  32.604  61.503  1.00  0.00
B    O
ATOM  18449  N   GLU B 114      -9.295  31.741  63.584  1.00  0.00
B    N
ATOM  18450  HN  GLU B 114      -9.160  30.950  64.173  1.00  0.00
B    H
ATOM  18451  CA  GLU B 114      -9.562  32.978  64.305  1.00  0.00
B    C
ATOM  18452  HA  GLU B 114     -10.546  33.330  64.022  1.00  0.00
B    H
ATOM  18453  CB  GLU B 114      -9.493  32.701  65.804  1.00  0.00
B    C
ATOM  18454  HB1 GLU B 114      -9.859  31.664  65.983  1.00  0.00
B    H
ATOM  18455  HB2 GLU B 114      -8.432  32.713  66.141  1.00  0.00
B    H
ATOM  18456  CG  GLU B 114     -10.291  33.630  66.703  1.00  0.00
B    C
ATOM  18457  HG1 GLU B 114      -9.752  34.589  66.817  1.00  0.00
B    H
ATOM  18458  HG2 GLU B 114     -11.297  33.825  66.281  1.00  0.00
B    H
ATOM  18459  CD  GLU B 114     -10.439  33.049  68.109  1.00  0.00
B    C
ATOM  18460  OE1 GLU B 114      -9.407  32.787  68.777  1.00  0.00
B    O
ATOM  18461  OE2 GLU B 114     -11.592  32.833  68.549  1.00  0.00
B    O
ATOM  18462  C   GLU B 114      -8.562  34.065  63.916  1.00  0.00
B    C
ATOM  18463  O   GLU B 114      -8.956  35.190  63.627  1.00  0.00
B    O
ATOM  18464  N   LYS B 115      -7.279  33.716  63.881  1.00  0.00
B    N
ATOM  18465  HN  LYS B 115      -6.966  32.782  64.047  1.00  0.00
B    H
ATOM  18466  CA  LYS B 115      -6.217  34.688  63.594  1.00  0.00
B    C
ATOM  18467  HA  LYS B 115      -6.510  35.596  64.107  1.00  0.00
B    H
```

ATOM 18468 CB LYS B 115 -4.879 34.234 64.181 1.00 0.00
B C
ATOM 18469 HB1 LYS B 115 -4.706 33.189 63.833 1.00 0.00
B H
ATOM 18470 HB2 LYS B 115 -4.063 34.850 63.740 1.00 0.00
B H
ATOM 18471 CG LYS B 115 -4.840 34.276 65.695 1.00 0.00
B C
ATOM 18472 HG1 LYS B 115 -5.762 33.797 66.096 1.00 0.00
B H
ATOM 18473 HG2 LYS B 115 -3.972 33.676 66.052 1.00 0.00
B H
ATOM 18474 CD LYS B 115 -4.751 35.701 66.231 1.00 0.00
B C
ATOM 18475 HD1 LYS B 115 -3.738 36.101 66.000 1.00 0.00
B H
ATOM 18476 HD2 LYS B 115 -5.490 36.326 65.679 1.00 0.00
B H
ATOM 18477 CE LYS B 115 -5.025 35.760 67.733 1.00 0.00
B C
ATOM 18478 HE1 LYS B 115 -5.140 36.814 68.067 1.00 0.00
B H
ATOM 18479 HE2 LYS B 115 -5.959 35.208 67.981 1.00 0.00
B H
ATOM 18480 NZ LYS B 115 -3.936 35.161 68.561 1.00 0.00
B N
ATOM 18481 HZ1 LYS B 115 -4.177 35.231 69.571 1.00 0.00
B H
ATOM 18482 HZ2 LYS B 115 -3.814 34.161 68.303 1.00 0.00
B H
ATOM 18483 HZ3 LYS B 115 -3.047 35.670 68.383 1.00 0.00
B H
ATOM 18484 C LYS B 115 -6.048 35.033 62.119 1.00 0.00
B C
ATOM 18485 O LYS B 115 -5.658 36.149 61.789 1.00 0.00
B O
ATOM 18486 N ILE B 116 -6.333 34.089 61.229 1.00 0.00
B N
ATOM 18487 HN ILE B 116 -6.638 33.164 61.447 1.00 0.00
B H
ATOM 18488 CA ILE B 116 -6.214 34.357 59.796 1.00 0.00
B C
ATOM 18489 HA ILE B 116 -5.428 35.094 59.696 1.00 0.00
B H
ATOM 18490 CB ILE B 116 -5.765 33.120 58.979 1.00 0.00
B C
ATOM 18491 HB ILE B 116 -5.671 33.496 57.929 1.00 0.00
B H
ATOM 18492 CG2 ILE B 116 -4.439 32.578 59.503 1.00 0.00
B C
ATOM 18493 HG21 ILE B 116 -4.042 31.794 58.826 1.00 0.00
B H
ATOM 18494 HG22 ILE B 116 -3.679 33.385 59.567 1.00 0.00
B H

```
ATOM  18495 HG23 ILE B 116      -4.563  32.133  60.513  1.00  0.00
B    H
ATOM  18496  CG1 ILE B 116      -6.847  32.038  58.953  1.00  0.00
B    C
ATOM  18497 HG11 ILE B 116      -7.123  31.757  59.994  1.00  0.00
B    H
ATOM  18498 HG12 ILE B 116      -7.761  32.453  58.471  1.00  0.00
B    H
ATOM  18499  CD  ILE B 116      -6.441  30.788  58.196  1.00  0.00
B    C
ATOM  18500  HD1 ILE B 116      -7.285  30.065  58.163  1.00  0.00
B    H
ATOM  18501  HD2 ILE B 116      -6.156  31.038  57.152  1.00  0.00
B    H
ATOM  18502  HD3 ILE B 116      -5.579  30.287  58.687  1.00  0.00
B    H
ATOM  18503  C   ILE B 116      -7.489  34.987  59.220  1.00  0.00
B    C
ATOM  18504  O   ILE B 116      -7.668  35.060  57.997  1.00  0.00
B    O
ATOM  18505  N   GLY B 117      -8.360  35.443  60.121  1.00  0.00
B    N
ATOM  18506  HN  GLY B 117      -8.248  35.263  61.097  1.00  0.00
B    H
ATOM  18507  CA  GLY B 117      -9.534  36.232  59.756  1.00  0.00
B    C
ATOM  18508  HA1 GLY B 117      -9.174  36.939  59.021  1.00  0.00
B    H
ATOM  18509  HA2 GLY B 117      -9.867  36.683  60.680  1.00  0.00
B    H
ATOM  18510  C   GLY B 117     -10.707  35.481  59.151  1.00  0.00
B    C
ATOM  18511  O   GLY B 117     -11.284  35.932  58.159  1.00  0.00
B    O
ATOM  18512  N   ALA B 118     -11.066  34.340  59.738  1.00  0.00
B    N
ATOM  18513  HN  ALA B 118     -10.498  33.865  60.408  1.00  0.00
B    H
ATOM  18514  CA  ALA B 118     -12.339  33.702  59.433  1.00  0.00
B    C
ATOM  18515  HA  ALA B 118     -12.556  33.723  58.371  1.00  0.00
B    H
ATOM  18516  CB  ALA B 118     -12.341  32.257  59.893  1.00  0.00
B    C
ATOM  18517  HB1 ALA B 118     -11.555  31.692  59.347  1.00  0.00
B    H
ATOM  18518  HB2 ALA B 118     -12.122  32.185  60.981  1.00  0.00
B    H
ATOM  18519  HB3 ALA B 118     -13.318  31.766  59.695  1.00  0.00
B    H
ATOM  18520  C   ALA B 118     -13.456  34.493  60.117  1.00  0.00
B    C
ATOM  18521  O   ALA B 118     -13.363  34.832  61.290  1.00  0.00
B    O
```

ATOM 18522 N ASP B 119 −14.503 34.803 59.369 1.00 0.00
B N
ATOM 18523 HN ASP B 119 −14.633 34.539 58.417 1.00 0.00
B H
ATOM 18524 CA ASP B 119 −15.607 35.593 59.894 1.00 0.00
B C
ATOM 18525 HA ASP B 119 −15.156 36.377 60.493 1.00 0.00
B H
ATOM 18526 CB ASP B 119 −16.388 36.230 58.744 1.00 0.00
B C
ATOM 18527 HB1 ASP B 119 −16.743 35.433 58.059 1.00 0.00
B H
ATOM 18528 HB2 ASP B 119 −17.271 36.778 59.128 1.00 0.00
B H
ATOM 18529 CG ASP B 119 −15.538 37.185 57.934 1.00 0.00
B C
ATOM 18530 OD1 ASP B 119 −15.113 38.214 58.492 1.00 0.00
B O
ATOM 18531 OD2 ASP B 119 −15.295 36.910 56.742 1.00 0.00
B O
ATOM 18532 C ASP B 119 −16.532 34.804 60.822 1.00 0.00
B C
ATOM 18533 O ASP B 119 −17.062 35.352 61.788 1.00 0.00
B O
ATOM 18534 N ARG B 120 −16.733 33.524 60.514 1.00 0.00
B N
ATOM 18535 HN ARG B 120 −16.384 33.125 59.669 1.00 0.00
B H
ATOM 18536 CA ARG B 120 −17.474 32.637 61.399 1.00 0.00
B C
ATOM 18537 HA ARG B 120 −17.381 33.002 62.415 1.00 0.00
B H
ATOM 18538 CB ARG B 120 −18.961 32.597 61.035 1.00 0.00
B C
ATOM 18539 HB1 ARG B 120 −19.316 33.656 61.041 1.00 0.00
B H
ATOM 18540 HB2 ARG B 120 −19.078 32.240 59.988 1.00 0.00
B H
ATOM 18541 CG ARG B 120 −19.787 31.782 62.015 1.00 0.00
B C
ATOM 18542 HG1 ARG B 120 −19.417 30.734 62.002 1.00 0.00
B H
ATOM 18543 HG2 ARG B 120 −19.595 32.155 63.047 1.00 0.00
B H
ATOM 18544 CD ARG B 120 −21.276 31.869 61.750 1.00 0.00
B C
ATOM 18545 HD1 ARG B 120 −21.656 32.859 62.095 1.00 0.00
B H
ATOM 18546 HD2 ARG B 120 −21.505 31.755 60.665 1.00 0.00
B H
ATOM 18547 NE ARG B 120 −21.969 30.795 62.461 1.00 0.00
B N
ATOM 18548 HE ARG B 120 −21.341 30.148 62.909 1.00 0.00
B H

ATOM 18549 CZ ARG B 120 -23.290 30.650 62.532 1.00 0.00
B C
ATOM 18550 NH1 ARG B 120 -24.096 31.525 61.935 1.00 0.00
B N
ATOM 18551 HH11 ARG B 120 -25.080 31.435 62.007 1.00 0.00
B H
ATOM 18552 HH12 ARG B 120 -23.658 32.334 61.538 1.00 0.00
B H
ATOM 18553 NH2 ARG B 120 -23.804 29.624 63.211 1.00 0.00
B N
ATOM 18554 HH21 ARG B 120 -24.780 29.461 63.196 1.00 0.00
B H
ATOM 18555 HH22 ARG B 120 -23.167 28.938 63.537 1.00 0.00
B H
ATOM 18556 C ARG B 120 -16.865 31.242 61.380 1.00 0.00
B C
ATOM 18557 O ARG B 120 -16.538 30.711 60.327 1.00 0.00
B O
ATOM 18558 N ILE B 121 -16.706 30.658 62.560 1.00 0.00
B N
ATOM 18559 HN ILE B 121 -16.990 31.068 63.424 1.00 0.00
B H
ATOM 18560 CA ILE B 121 -16.093 29.349 62.696 1.00 0.00
B C
ATOM 18561 HA ILE B 121 -15.858 28.991 61.702 1.00 0.00
B H
ATOM 18562 CB ILE B 121 -14.829 29.416 63.583 1.00 0.00
B C
ATOM 18563 HB ILE B 121 -15.183 29.877 64.540 1.00 0.00
B H
ATOM 18564 CG2 ILE B 121 -14.209 28.036 63.752 1.00 0.00
B C
ATOM 18565 HG21 ILE B 121 -13.399 28.063 64.510 1.00 0.00
B H
ATOM 18566 HG22 ILE B 121 -14.966 27.301 64.094 1.00 0.00
B H
ATOM 18567 HG23 ILE B 121 -13.776 27.675 62.795 1.00 0.00
B H
ATOM 18568 CG1 ILE B 121 -13.818 30.381 62.966 1.00 0.00
B C
ATOM 18569 HG11 ILE B 121 -13.376 29.928 62.050 1.00 0.00
B H
ATOM 18570 HG12 ILE B 121 -14.350 31.308 62.652 1.00 0.00
B H
ATOM 18571 CD ILE B 121 -12.696 30.784 63.899 1.00 0.00
B C
ATOM 18572 HD1 ILE B 121 -12.034 31.528 63.406 1.00 0.00
B H
ATOM 18573 HD2 ILE B 121 -13.102 31.240 64.827 1.00 0.00
B H
ATOM 18574 HD3 ILE B 121 -12.077 29.906 64.180 1.00 0.00
B H
ATOM 18575 C ILE B 121 -17.136 28.395 63.274 1.00 0.00
B C

ATOM 18576 O   ILE B 121   -17.671 28.642 64.351 1.00 0.00
B   O
ATOM 18577 N   ILE B 122   -17.424 27.315 62.550 1.00 0.00
B   N
ATOM 18578 HN ILE B 122   -16.968 27.057 61.700 1.00 0.00
B   H
ATOM 18579 CA ILE B 122   -18.475 26.393 62.967 1.00 0.00
B   C
ATOM 18580 HA ILE B 122   -18.965 26.876 63.802 1.00 0.00
B   H
ATOM 18581 CB ILE B 122   -19.490 26.114 61.840 1.00 0.00
B   C
ATOM 18582 HB ILE B 122   -18.876 25.675 61.013 1.00 0.00
B   H
ATOM 18583 CG2 ILE B 122   -20.617 25.225 62.335 1.00 0.00
B   C
ATOM 18584 HG21 ILE B 122   -21.258 24.899 61.490 1.00 0.00
B   H
ATOM 18585 HG22 ILE B 122   -20.217 24.313 62.824 1.00 0.00
B   H
ATOM 18586 HG23 ILE B 122   -21.256 25.764 63.067 1.00 0.00
B   H
ATOM 18587 CG1 ILE B 122   -20.083 27.411 61.302 1.00 0.00
B   C
ATOM 18588 HG11 ILE B 122   -21.190 27.392 61.409 1.00 0.00
B   H
ATOM 18589 HG12 ILE B 122   -19.707 28.262 61.915 1.00 0.00
B   H
ATOM 18590 CD ILE B 122   -19.726 27.668 59.859 1.00 0.00
B   C
ATOM 18591 HD1 ILE B 122   -20.131 28.650 59.530 1.00 0.00
B   H
ATOM 18592 HD2 ILE B 122   -18.622 27.685 59.725 1.00 0.00
B   H
ATOM 18593 HD3 ILE B 122   -20.150 26.883 59.198 1.00 0.00
B   H
ATOM 18594 C   ILE B 122   -17.943 25.061 63.478 1.00 0.00
B   C
ATOM 18595 O   ILE B 122   -17.121 24.417 62.819 1.00 0.00
B   O
ATOM 18596 N   HSE B 123   -18.428 24.652 64.646 1.00 0.00
B   N
ATOM 18597 HN HSE B 123   -18.980 25.231 65.243 1.00 0.00
B   H
ATOM 18598 CA HSE B 123   -18.193 23.313 65.159 1.00 0.00
B   C
ATOM 18599 HA HSE B 123   -17.506 22.807 64.492 1.00 0.00
B   H
ATOM 18600 CB HSE B 123   -17.700 23.378 66.593 1.00 0.00
B   C
ATOM 18601 HB1 HSE B 123   -18.460 23.925 67.192 1.00 0.00
B   H
ATOM 18602 HB2 HSE B 123   -17.634 22.352 67.010 1.00 0.00
B   H

ATOM 18603 ND1 HSE B 123   -15.235 23.432 66.973 1.00 0.00
B   N
ATOM 18604 CG HSE B 123   -16.391 24.089 66.754 1.00 0.00
B   C
ATOM 18605 CE1 HSE B 123   -14.232 24.320 67.091 1.00 0.00
B   C
ATOM 18606 HE1 HSE B 123   -13.189 24.055 67.285 1.00 0.00
B   H
ATOM 18607 NE2 HSE B 123   -14.746 25.553 66.936 1.00 0.00
B   N
ATOM 18608 HE2 HSE B 123   -14.229 26.407 66.991 1.00 0.00
B   H
ATOM 18609 CD2 HSE B 123   -16.075 25.445 66.726 1.00 0.00
B   C
ATOM 18610 HD2 HSE B 123   -16.717 26.302 66.580 1.00 0.00
B   H
ATOM 18611 C   HSE B 123   -19.489 22.566 65.106 1.00 0.00
B   C
ATOM 18612 O   HSE B 123   -20.253 22.579 66.074 1.00 0.00
B   O
ATOM 18613 N   PRO B 124   -19.775 21.915 63.977 1.00 0.00
B   N
ATOM 18614 CD PRO B 124   -18.842 21.724 62.858 1.00 0.00
B   C
ATOM 18615 HD1 PRO B 124   -19.111 22.765 63.147 1.00 0.00
B   H
ATOM 18616 HD2 PRO B 124   -17.774 21.670 62.541 1.00 0.00
B   H
ATOM 18617 CA PRO B 124   -21.049 21.223 63.754 1.00 0.00
B   C
ATOM 18618 HA PRO B 124   -21.842 21.959 63.697 1.00 0.00
B   H
ATOM 18619 CB PRO B 124   -20.857 20.543 62.398 1.00 0.00
B   C
ATOM 18620 HB1 PRO B 124   -21.104 21.268 61.591 1.00 0.00
B   H
ATOM 18621 HB2 PRO B 124   -21.490 19.640 62.278 1.00 0.00
B   H
ATOM 18622 CG PRO B 124   -19.728 21.254 61.751 1.00 0.00
B   C
ATOM 18623 HG1 PRO B 124   -19.735 21.163 60.647 1.00 0.00
B   H
ATOM 18624 HG2 PRO B 124   -18.767 20.855 62.150 1.00 0.00
B   H
ATOM 18625 C   PRO B 124   -21.412 20.176 64.825 1.00 0.00
B   C
ATOM 18626 O   PRO B 124   -22.516 20.227 65.371 1.00 0.00
B   O
ATOM 18627 N   GLU B 125   -20.504 19.244 65.124 1.00 0.00
B   N
ATOM 18628 HN GLU B 125   -19.596 19.176 64.721 1.00 0.00
B   H
ATOM 18629 CA GLU B 125   -20.793 18.209 66.109 1.00 0.00
B   C

ATOM 18630 HA GLU B 125 -21.705 17.708 65.808 1.00 0.00
B H
ATOM 18631 CB GLU B 125 -19.680 17.166 66.202 1.00 0.00
B C
ATOM 18632 HB1 GLU B 125 -18.711 17.702 66.326 1.00 0.00
B H
ATOM 18633 HB2 GLU B 125 -19.811 16.555 67.123 1.00 0.00
B H
ATOM 18634 CG GLU B 125 -19.558 16.242 65.008 1.00 0.00
B C
ATOM 18635 HG1 GLU B 125 -19.433 15.201 65.359 1.00 0.00
B H
ATOM 18636 HG2 GLU B 125 -20.458 16.304 64.364 1.00 0.00
B H
ATOM 18637 CD GLU B 125 -18.335 16.554 64.176 1.00 0.00
B C
ATOM 18638 OE1 GLU B 125 -17.581 15.598 63.907 1.00 0.00
B O
ATOM 18639 OE2 GLU B 125 -18.118 17.746 63.814 1.00 0.00
B O
ATOM 18640 C GLU B 125 -21.038 18.797 67.488 1.00 0.00
B C
ATOM 18641 O GLU B 125 -21.998 18.404 68.147 1.00 0.00
B O
ATOM 18642 N LYS B 126 -20.173 19.726 67.912 1.00 0.00
B N
ATOM 18643 HN LYS B 126 -19.376 20.004 67.377 1.00 0.00
B H
ATOM 18644 CA LYS B 126 -20.328 20.407 69.191 1.00 0.00
B C
ATOM 18645 HA LYS B 126 -20.247 19.664 69.975 1.00 0.00
B H
ATOM 18646 CB LYS B 126 -19.261 21.486 69.366 1.00 0.00
B C
ATOM 18647 HB1 LYS B 126 -18.286 21.034 69.069 1.00 0.00
B H
ATOM 18648 HB2 LYS B 126 -19.456 22.309 68.643 1.00 0.00
B H
ATOM 18649 CG LYS B 126 -19.145 22.030 70.784 1.00 0.00
B C
ATOM 18650 HG1 LYS B 126 -20.124 21.912 71.301 1.00 0.00
B H
ATOM 18651 HG2 LYS B 126 -18.398 21.423 71.345 1.00 0.00
B H
ATOM 18652 CD LYS B 126 -18.750 23.501 70.789 1.00 0.00
B C
ATOM 18653 HD1 LYS B 126 -17.939 23.650 70.040 1.00 0.00
B H
ATOM 18654 HD2 LYS B 126 -19.629 24.097 70.451 1.00 0.00
B H
ATOM 18655 CE LYS B 126 -18.295 23.975 72.168 1.00 0.00
B C
ATOM 18656 HE1 LYS B 126 -18.258 25.087 72.205 1.00 0.00
B H

ATOM 18657 HE2 LYS B 126 −18.997 23.619 72.954 1.00 0.00
B H
ATOM 18658 NZ LYS B 126 −16.933 23.474 72.532 1.00 0.00
B N
ATOM 18659 HZ1 LYS B 126 −16.666 23.822 73.476 1.00 0.00
B H
ATOM 18660 HZ2 LYS B 126 −16.935 22.434 72.535 1.00 0.00
B H
ATOM 18661 HZ3 LYS B 126 −16.242 23.813 71.832 1.00 0.00
B H
ATOM 18662 C LYS B 126 −21.723 21.014 69.281 1.00 0.00
B C
ATOM 18663 O LYS B 126 −22.450 20.765 70.233 1.00 0.00
B O
ATOM 18664 N ASP B 127 −22.104 21.780 68.262 1.00 0.00
B N
ATOM 18665 HN ASP B 127 −21.550 21.965 67.454 1.00 0.00
B H
ATOM 18666 CA ASP B 127 −23.407 22.442 68.223 1.00 0.00
B C
ATOM 18667 HA ASP B 127 −23.486 23.009 69.145 1.00 0.00
B H
ATOM 18668 CB ASP B 127 −23.491 23.408 67.041 1.00 0.00
B C
ATOM 18669 HB1 ASP B 127 −23.164 22.885 66.120 1.00 0.00
B H
ATOM 18670 HB2 ASP B 127 −24.532 23.756 66.894 1.00 0.00
B H
ATOM 18671 CG ASP B 127 −22.592 24.629 67.215 1.00 0.00
B C
ATOM 18672 OD1 ASP B 127 −22.027 24.833 68.322 1.00 0.00
B O
ATOM 18673 OD2 ASP B 127 −22.460 25.393 66.235 1.00 0.00
B O
ATOM 18674 C ASP B 127 −24.574 21.463 68.209 1.00 0.00
B C
ATOM 18675 O ASP B 127 −25.563 21.658 68.914 1.00 0.00
B O
ATOM 18676 N MET B 128 −24.465 20.401 67.416 1.00 0.00
B N
ATOM 18677 HN MET B 128 −23.704 20.227 66.794 1.00 0.00
B H
ATOM 18678 CA MET B 128 −25.500 19.369 67.405 1.00 0.00
B C
ATOM 18679 HA MET B 128 −26.439 19.883 67.239 1.00 0.00
B H
ATOM 18680 CB MET B 128 −25.283 18.388 66.263 1.00 0.00
B C
ATOM 18681 HB1 MET B 128 −24.184 18.206 66.197 1.00 0.00
B H
ATOM 18682 HB2 MET B 128 −25.745 17.410 66.523 1.00 0.00
B H
ATOM 18683 CG MET B 128 −25.792 18.908 64.937 1.00 0.00
B C

ATOM 18684 HG1 MET B 128   -25.166 19.757 64.589 1.00 0.00
B   H
ATOM 18685 HG2 MET B 128   -25.725 18.105 64.170 1.00 0.00
B   H
ATOM 18686 SD MET B 128   -27.502 19.485 65.018 1.00 0.00
B   S
ATOM 18687 CE MET B 128   -28.358 17.920 65.267 1.00 0.00
B   C
ATOM 18688 HE1 MET B 128   -29.456 18.073 65.333 1.00 0.00
B   H
ATOM 18689 HE2 MET B 128   -28.161 17.220 64.426 1.00 0.00
B   H
ATOM 18690 HE3 MET B 128   -28.026 17.428 66.206 1.00 0.00
B   H
ATOM 18691 C   MET B 128   -25.613 18.622 68.727 1.00 0.00
B   C
ATOM 18692 O   MET B 128   -26.696 18.158 69.081 1.00 0.00
B   O
ATOM 18693 N   GLY B 129   -24.496 18.505 69.442 1.00 0.00
B   N
ATOM 18694 HN GLY B 129   -23.607 18.784 69.079 1.00 0.00
B   H
ATOM 18695 CA GLY B 129   -24.482 17.965 70.799 1.00 0.00
B   C
ATOM 18696 HA1 GLY B 129   -23.489 18.114 71.199 1.00 0.00
B   H
ATOM 18697 HA2 GLY B 129   -24.789 16.929 70.745 1.00 0.00
B   H
ATOM 18698 C   GLY B 129   -25.463 18.695 71.694 1.00 0.00
B   C
ATOM 18699 O   GLY B 129   -26.337 18.068 72.305 1.00 0.00
B   O
ATOM 18700 N   VAL B 130   -25.336 20.021 71.751 1.00 0.00
B   N
ATOM 18701 HN VAL B 130   -24.637 20.541 71.267 1.00 0.00
B   H
ATOM 18702 CA VAL B 130   -26.245 20.836 72.556 1.00 0.00
B   C
ATOM 18703 HA VAL B 130   -26.122 20.540 73.590 1.00 0.00
B   H
ATOM 18704 CB VAL B 130   -25.915 22.361 72.540 1.00 0.00
B   C
ATOM 18705 HB VAL B 130   -26.653 22.873 73.206 1.00 0.00
B   H
ATOM 18706 CG1 VAL B 130   -24.514 22.608 73.063 1.00 0.00
B   C
ATOM 18707 HG11 VAL B 130   -24.269 23.691 73.013 1.00 0.00
B   H
ATOM 18708 HG12 VAL B 130   -24.410 22.294 74.122 1.00 0.00
B   H
ATOM 18709 HG13 VAL B 130   -23.764 22.058 72.454 1.00 0.00
B   H
ATOM 18710 CG2 VAL B 130   -26.075 22.976 71.158 1.00 0.00
B   C

ATOM 18711 HG21 VAL B 130 −25.796 24.051 71.171 1.00 0.00
B H
ATOM 18712 HG22 VAL B 130 −25.426 22.455 70.421 1.00 0.00
B H
ATOM 18713 HG23 VAL B 130 −27.126 22.906 70.807 1.00 0.00
B H
ATOM 18714 C VAL B 130 −27.700 20.562 72.170 1.00 0.00
B C
ATOM 18715 O VAL B 130 −28.517 20.272 73.035 1.00 0.00
B O
ATOM 18716 N LYS B 131 −27.991 20.603 70.872 1.00 0.00
B N
ATOM 18717 HN LYS B 131 −27.294 20.759 70.173 1.00 0.00
B H
ATOM 18718 CA LYS B 131 −29.354 20.423 70.363 1.00 0.00
B C
ATOM 18719 HA LYS B 131 −29.963 21.177 70.846 1.00 0.00
B H
ATOM 18720 CB LYS B 131 −29.416 20.649 68.844 1.00 0.00
B C
ATOM 18721 HB1 LYS B 131 −28.668 19.964 68.380 1.00 0.00
B H
ATOM 18722 HB2 LYS B 131 −30.412 20.324 68.470 1.00 0.00
B H
ATOM 18723 CG LYS B 131 −29.113 22.086 68.431 1.00 0.00
B C
ATOM 18724 HG1 LYS B 131 −29.043 22.720 69.343 1.00 0.00
B H
ATOM 18725 HG2 LYS B 131 −28.120 22.117 67.927 1.00 0.00
B H
ATOM 18726 CD LYS B 131 −30.194 22.639 67.517 1.00 0.00
B C
ATOM 18727 HD1 LYS B 131 −29.934 22.378 66.466 1.00 0.00
B H
ATOM 18728 HD2 LYS B 131 −31.152 22.124 67.760 1.00 0.00
B H
ATOM 18729 CE LYS B 131 −30.332 24.148 67.691 1.00 0.00
B C
ATOM 18730 HE1 LYS B 131 −29.987 24.457 68.703 1.00 0.00
B H
ATOM 18731 HE2 LYS B 131 −29.722 24.686 66.932 1.00 0.00
B H
ATOM 18732 NZ LYS B 131 −31.741 24.626 67.542 1.00 0.00
B N
ATOM 18733 HZ1 LYS B 131 −31.783 25.657 67.670 1.00 0.00
B H
ATOM 18734 HZ2 LYS B 131 −32.092 24.381 66.594 1.00 0.00
B H
ATOM 18735 HZ3 LYS B 131 −32.339 24.165 68.258 1.00 0.00
B H
ATOM 18736 C LYS B 131 −29.942 19.065 70.722 1.00 0.00
B C
ATOM 18737 O LYS B 131 −31.127 18.975 71.059 1.00 0.00
B O

ATOM 18738 N ILE B 132 -29.116 18.021 70.651 1.00 0.00
B N
ATOM 18739 HN ILE B 132 -28.168 18.084 70.343 1.00 0.00
B H
ATOM 18740 CA ILE B 132 -29.532 16.682 71.023 1.00 0.00
B C
ATOM 18741 HA ILE B 132 -30.468 16.498 70.511 1.00 0.00
B H
ATOM 18742 CB ILE B 132 -28.502 15.619 70.585 1.00 0.00
B C
ATOM 18743 HB ILE B 132 -27.514 16.040 70.899 1.00 0.00
B H
ATOM 18744 CG2 ILE B 132 -28.798 14.257 71.203 1.00 0.00
B C
ATOM 18745 HG21 ILE B 132 -27.966 13.549 71.006 1.00 0.00
B H
ATOM 18746 HG22 ILE B 132 -28.916 14.339 72.304 1.00 0.00
B H
ATOM 18747 HG23 ILE B 132 -29.729 13.823 70.783 1.00 0.00
B H
ATOM 18748 CG1 ILE B 132 -28.464 15.497 69.052 1.00 0.00
B C
ATOM 18749 HG11 ILE B 132 -28.024 16.420 68.613 1.00 0.00
B H
ATOM 18750 HG12 ILE B 132 -27.797 14.649 68.774 1.00 0.00
B H
ATOM 18751 CD ILE B 132 -29.805 15.253 68.389 1.00 0.00
B C
ATOM 18752 HD1 ILE B 132 -29.675 15.121 67.293 1.00 0.00
B H
ATOM 18753 HD2 ILE B 132 -30.282 14.334 68.795 1.00 0.00
B H
ATOM 18754 HD3 ILE B 132 -30.493 16.109 68.553 1.00 0.00
B H
ATOM 18755 C ILE B 132 -29.823 16.602 72.512 1.00 0.00
B C
ATOM 18756 O ILE B 132 -30.841 16.043 72.913 1.00 0.00
B O
ATOM 18757 N ALA B 133 -28.950 17.183 73.332 1.00 0.00
B N
ATOM 18758 HN ALA B 133 -28.117 17.629 73.008 1.00 0.00
B H
ATOM 18759 CA ALA B 133 -29.154 17.209 74.788 1.00 0.00
B C
ATOM 18760 HA ALA B 133 -29.244 16.200 75.174 1.00 0.00
B H
ATOM 18761 CB ALA B 133 -27.955 17.824 75.491 1.00 0.00
B C
ATOM 18762 HB1 ALA B 133 -27.040 17.248 75.239 1.00 0.00
B H
ATOM 18763 HB2 ALA B 133 -27.798 18.876 75.168 1.00 0.00
B H
ATOM 18764 HB3 ALA B 133 -28.077 17.807 76.596 1.00 0.00
B H

ATOM 18765 C   ALA B 133   -30.451 17.935 75.166 1.00 0.00
B   C
ATOM 18766 O   ALA B 133   -31.158 17.506 76.075 1.00 0.00
B   O
ATOM 18767 N   GLN B 134   -30.756 19.018 74.451 1.00 0.00
B   N
ATOM 18768 HN GLN B 134   -30.142 19.403 73.764 1.00 0.00
B   H
ATOM 18769 CA GLN B 134   -32.019 19.740 74.608 1.00 0.00
B   C
ATOM 18770 HA GLN B 134   -32.091 20.046 75.645 1.00 0.00
B   H
ATOM 18771 CB GLN B 134   -32.093 20.943 73.660 1.00 0.00
B   C
ATOM 18772 HB1 GLN B 134   -32.040 20.539 72.622 1.00 0.00
B   H
ATOM 18773 HB2 GLN B 134   -33.093 21.426 73.754 1.00 0.00
B   H
ATOM 18774 CG GLN B 134   -31.005 21.997 73.816 1.00 0.00
B   C
ATOM 18775 HG1 GLN B 134   -30.000 21.524 73.782 1.00 0.00
B   H
ATOM 18776 HG2 GLN B 134   -31.058 22.741 72.995 1.00 0.00
B   H
ATOM 18777 CD GLN B 134   -31.076 22.736 75.136 1.00 0.00
B   C
ATOM 18778 OE1 GLN B 134   -32.151 22.880 75.734 1.00 0.00
B   O
ATOM 18779 NE2 GLN B 134   -29.924 23.220 75.603 1.00 0.00
B   N
ATOM 18780 HE21 GLN B 134   -29.960 23.704 76.473 1.00 0.00
B   H
ATOM 18781 HE22 GLN B 134   -29.088 23.102 75.078 1.00 0.00
B   H
ATOM 18782 C   GLN B 134   -33.195 18.812 74.319 1.00 0.00
B   C
ATOM 18783 O   GLN B 134   -34.185 18.811 75.045 1.00 0.00
B   O
ATOM 18784 N   SER B 135   -33.071 18.024 73.250 1.00 0.00
B   N
ATOM 18785 HN SER B 135   -32.268 18.046 72.655 1.00 0.00
B   H
ATOM 18786 CA SER B 135   -34.091 17.056 72.846 1.00 0.00
B   C
ATOM 18787 HA SER B 135   -35.020 17.610 72.802 1.00 0.00
B   H
ATOM 18788 CB SER B 135   -33.682 16.362 71.561 1.00 0.00
B   C
ATOM 18789 HB1 SER B 135   -32.603 16.574 71.383 1.00 0.00
B   H
ATOM 18790 HB2 SER B 135   -33.785 15.258 71.667 1.00 0.00
B   H
ATOM 18791 OG SER B 135   -34.483 16.887 70.419 1.00 0.00
B   O

ATOM 18792 HG1 SER B 135 -34.048 16.594 69.609 1.00 0.00
B H
ATOM 18793 C SER B 135 -34.269 15.965 73.867 1.00 0.00
B C
ATOM 18794 O SER B 135 -35.392 15.520 74.126 1.00 0.00
B O
ATOM 18795 N LEU B 136 -33.148 15.510 74.412 1.00 0.00
B N
ATOM 18796 HN LEU B 136 -32.248 15.894 74.213 1.00 0.00
B H
ATOM 18797 CA LEU B 136 -33.132 14.404 75.354 1.00 0.00
B C
ATOM 18798 HA LEU B 136 -33.709 13.609 74.898 1.00 0.00
B H
ATOM 18799 CB LEU B 136 -31.697 13.971 75.648 1.00 0.00
B C
ATOM 18800 HB1 LEU B 136 -31.104 14.897 75.834 1.00 0.00
B H
ATOM 18801 HB2 LEU B 136 -31.679 13.399 76.602 1.00 0.00
B H
ATOM 18802 CG LEU B 136 -30.996 13.173 74.555 1.00 0.00
B C
ATOM 18803 HG LEU B 136 -31.411 13.604 73.612 1.00 0.00
B H
ATOM 18804 CD1 LEU B 136 -29.489 13.393 74.571 1.00 0.00
B C
ATOM 18805 HD11 LEU B 136 -28.995 12.766 73.799 1.00 0.00
B H
ATOM 18806 HD12 LEU B 136 -29.246 14.458 74.364 1.00 0.00
B H
ATOM 18807 HD13 LEU B 136 -29.064 13.123 75.561 1.00 0.00
B H
ATOM 18808 CD2 LEU B 136 -31.370 11.699 74.604 1.00 0.00
B C
ATOM 18809 HD21 LEU B 136 -30.862 11.145 73.786 1.00 0.00
B H
ATOM 18810 HD22 LEU B 136 -31.072 11.233 75.563 1.00 0.00
B H
ATOM 18811 HD23 LEU B 136 -32.468 11.572 74.479 1.00 0.00
B H
ATOM 18812 C LEU B 136 -33.799 14.760 76.658 1.00 0.00
B C
ATOM 18813 O LEU B 136 -34.412 13.912 77.299 1.00 0.00
B O
ATOM 18814 N SER B 137 -33.663 16.013 77.067 1.00 0.00
B N
ATOM 18815 HN SER B 137 -33.273 16.775 76.552 1.00 0.00
B H
ATOM 18816 CA SER B 137 -34.113 16.365 78.394 1.00 0.00
B C
ATOM 18817 HA SER B 137 -33.936 15.483 78.996 1.00 0.00
B H
ATOM 18818 CB SER B 137 -33.211 17.413 79.040 1.00 0.00
B C

ATOM 18819 HB1 SER B 137 -33.297 17.309 80.146 1.00 0.00
B H
ATOM 18820 HB2 SER B 137 -32.146 17.225 78.775 1.00 0.00
B H
ATOM 18821 OG SER B 137 -33.497 18.767 78.756 1.00 0.00
B O
ATOM 18822 HG1 SER B 137 -33.005 19.300 79.394 1.00 0.00
B H
ATOM 18823 C SER B 137 -35.609 16.667 78.460 1.00 0.00
B C
ATOM 18824 O SER B 137 -36.242 16.451 79.491 1.00 0.00
B O
ATOM 18825 N ASP B 138 -36.167 17.118 77.343 1.00 0.00
B N
ATOM 18826 HN ASP B 138 -35.664 17.415 76.535 1.00 0.00
B H
ATOM 18827 CA ASP B 138 -37.604 17.226 77.184 1.00 0.00
B C
ATOM 18828 HA ASP B 138 -38.082 16.552 77.886 1.00 0.00
B H
ATOM 18829 CB ASP B 138 -38.073 18.666 77.371 1.00 0.00
B C
ATOM 18830 HB1 ASP B 138 -37.410 19.175 78.099 1.00 0.00
B H
ATOM 18831 HB2 ASP B 138 -38.027 19.220 76.411 1.00 0.00
B H
ATOM 18832 CG ASP B 138 -39.489 18.755 77.909 1.00 0.00
B C
ATOM 18833 OD1 ASP B 138 -40.193 17.715 77.962 1.00 0.00
B O
ATOM 18834 OD2 ASP B 138 -39.894 19.876 78.294 1.00 0.00
B O
ATOM 18835 C ASP B 138 -37.945 16.725 75.796 1.00 0.00
B C
ATOM 18836 O ASP B 138 -37.724 17.422 74.798 1.00 0.00
B O
ATOM 18837 N GLU B 139 -38.466 15.502 75.742 1.00 0.00
B N
ATOM 18838 HN GLU B 139 -38.686 14.967 76.553 1.00 0.00
B H
ATOM 18839 CA GLU B 139 -38.764 14.827 74.488 1.00 0.00
B C
ATOM 18840 HA GLU B 139 -37.926 14.963 73.818 1.00 0.00
B H
ATOM 18841 CB GLU B 139 -39.042 13.358 74.759 1.00 0.00
B C
ATOM 18842 HB1 GLU B 139 -38.543 13.080 75.716 1.00 0.00
B H
ATOM 18843 HB2 GLU B 139 -40.132 13.205 74.929 1.00 0.00
B H
ATOM 18844 CG GLU B 139 -38.559 12.424 73.666 1.00 0.00
B C
ATOM 18845 HG1 GLU B 139 -39.315 12.375 72.861 1.00 0.00
B H

ATOM 18846 HG2 GLU B 139 -37.597 12.773 73.242 1.00 0.00
B H
ATOM 18847 CD GLU B 139 -38.389 11.005 74.167 1.00 0.00
B C
ATOM 18848 OE1 GLU B 139 -39.378 10.420 74.671 1.00 0.00
B O
ATOM 18849 OE2 GLU B 139 -37.265 10.479 74.063 1.00 0.00
B O
ATOM 18850 C GLU B 139 -39.973 15.445 73.827 1.00 0.00
B C
ATOM 18851 O GLU B 139 -40.085 15.446 72.602 1.00 0.00
B O
ATOM 18852 N ASN B 140 -40.870 15.960 74.664 1.00 0.00
B N
ATOM 18853 HN ASN B 140 -40.731 15.948 75.653 1.00 0.00
B H
ATOM 18854 CA ASN B 140 -42.113 16.577 74.239 1.00 0.00
B C
ATOM 18855 HA ASN B 140 -42.605 15.827 73.630 1.00 0.00
B H
ATOM 18856 CB ASN B 140 -42.910 17.017 75.458 1.00 0.00
B C
ATOM 18857 HB1 ASN B 140 -42.186 17.323 76.245 1.00 0.00
B H
ATOM 18858 HB2 ASN B 140 -43.530 17.903 75.214 1.00 0.00
B H
ATOM 18859 CG ASN B 140 -43.768 15.925 76.019 1.00 0.00
B C
ATOM 18860 OD1 ASN B 140 -44.199 15.055 75.348 1.00 0.00
B O
ATOM 18861 ND2 ASN B 140 -44.055 16.016 77.234 1.00 0.00
B N
ATOM 18862 HD21 ASN B 140 -44.616 15.293 77.628 1.00 0.00
B H
ATOM 18863 HD22 ASN B 140 -43.701 16.773 77.774 1.00 0.00
B H
ATOM 18864 C ASN B 140 -41.900 17.801 73.376 1.00 0.00
B C
ATOM 18865 O ASN B 140 -42.699 18.052 72.465 1.00 0.00
B O
ATOM 18866 N VAL B 141 -40.835 18.555 73.699 1.00 0.00
B N
ATOM 18867 HN VAL B 141 -40.206 18.304 74.430 1.00 0.00
B H
ATOM 18868 CA VAL B 141 -40.501 19.807 73.016 1.00 0.00
B C
ATOM 18869 HA VAL B 141 -41.426 20.303 72.748 1.00 0.00
B H
ATOM 18870 CB VAL B 141 -39.616 20.727 73.902 1.00 0.00
B C
ATOM 18871 HB VAL B 141 -38.775 20.106 74.298 1.00 0.00
B H
ATOM 18872 CG1 VAL B 141 -39.020 21.896 73.118 1.00 0.00
B C

ATOM 18873 HG11 VAL B 141 −38.428 22.550 73.792 1.00 0.00
B H
ATOM 18874 HG12 VAL B 141 −38.339 21.546 72.315 1.00 0.00
B H
ATOM 18875 HG13 VAL B 141 −39.824 22.512 72.661 1.00 0.00
B H
ATOM 18876 CG2 VAL B 141 −40.355 21.175 75.158 1.00 0.00
B C
ATOM 18877 HG21 VAL B 141 −39.723 21.863 75.760 1.00 0.00
B H
ATOM 18878 HG22 VAL B 141 −41.294 21.706 74.891 1.00 0.00
B H
ATOM 18879 HG23 VAL B 141 −40.614 20.309 75.802 1.00 0.00
B H
ATOM 18880 C VAL B 141 −39.759 19.498 71.726 1.00 0.00
B C
ATOM 18881 O VAL B 141 −38.704 18.853 71.746 1.00 0.00
B O
ATOM 18882 N LEU B 142 −40.315 19.963 70.610 1.00 0.00
B N
ATOM 18883 HN LEU B 142 −41.191 20.441 70.584 1.00 0.00
B H
ATOM 18884 CA LEU B 142 −39.677 19.809 69.308 1.00 0.00
B C
ATOM 18885 HA LEU B 142 −39.003 18.965 69.380 1.00 0.00
B H
ATOM 18886 CB LEU B 142 −40.722 19.599 68.211 1.00 0.00
B C
ATOM 18887 HB1 LEU B 142 −41.322 20.536 68.145 1.00 0.00
B H
ATOM 18888 HB2 LEU B 142 −40.203 19.496 67.232 1.00 0.00
B H
ATOM 18889 CG LEU B 142 −41.712 18.442 68.366 1.00 0.00
B C
ATOM 18890 HG LEU B 142 −42.119 18.506 69.404 1.00 0.00
B H
ATOM 18891 CD1 LEU B 142 −42.848 18.599 67.367 1.00 0.00
B C
ATOM 18892 HD11 LEU B 142 −43.549 17.739 67.436 1.00 0.00
B H
ATOM 18893 HD12 LEU B 142 −43.418 19.532 67.566 1.00 0.00
B H
ATOM 18894 HD13 LEU B 142 −42.454 18.642 66.329 1.00 0.00
B H
ATOM 18895 CD2 LEU B 142 −41.030 17.089 68.209 1.00 0.00
B C
ATOM 18896 HD21 LEU B 142 −41.766 16.267 68.344 1.00 0.00
B H
ATOM 18897 HD22 LEU B 142 −40.577 16.972 67.205 1.00 0.00
B H
ATOM 18898 HD23 LEU B 142 −40.229 16.966 68.971 1.00 0.00
B H
ATOM 18899 C LEU B 142 −38.843 21.034 68.984 1.00 0.00
B C

```
ATOM  18900  O   LEU B 142      -37.776  20.930  68.387  1.00  0.00
B    O
ATOM  18901  N   ASN B 143      -39.351  22.196  69.374  1.00  0.00
B    N
ATOM  18902  HN  ASN B 143      -40.223  22.263  69.856  1.00  0.00
B    H
ATOM  18903  CA  ASN B 143      -38.693  23.472  69.140  1.00  0.00
B    C
ATOM  18904  HA  ASN B 143      -37.628  23.286  69.234  1.00  0.00
B    H
ATOM  18905  CB  ASN B 143      -39.064  24.027  67.766  1.00  0.00
B    C
ATOM  18906  HB1 ASN B 143      -39.861  23.380  67.341  1.00  0.00
B    H
ATOM  18907  HB2 ASN B 143      -39.483  25.050  67.864  1.00  0.00
B    H
ATOM  18908  CG  ASN B 143      -37.898  24.030  66.800  1.00  0.00
B    C
ATOM  18909  OD1 ASN B 143      -36.736  23.934  67.204  1.00  0.00
B    O
ATOM  18910  ND2 ASN B 143      -38.201  24.154  65.511  1.00  0.00
B    N
ATOM  18911 HD21 ASN B 143      -37.446  24.146  64.861  1.00  0.00
B    H
ATOM  18912 HD22 ASN B 143      -39.153  24.220  65.228  1.00  0.00
B    H
ATOM  18913  C   ASN B 143      -39.082  24.471  70.214  1.00  0.00
B    C
ATOM  18914  O   ASN B 143      -40.151  24.356  70.814  1.00  0.00
B    O
ATOM  18915  N   TYR B 144      -38.216  25.449  70.448  1.00  0.00
B    N
ATOM  18916  HN  TYR B 144      -37.350  25.548  69.962  1.00  0.00
B    H
ATOM  18917  CA  TYR B 144      -38.469  26.477  71.454  1.00  0.00
B    C
ATOM  18918  HA  TYR B 144      -39.537  26.661  71.446  1.00  0.00
B    H
ATOM  18919  CB  TYR B 144      -38.002  26.011  72.843  1.00  0.00
B    C
ATOM  18920  HB1 TYR B 144      -38.371  26.727  73.610  1.00  0.00
B    H
ATOM  18921  HB2 TYR B 144      -38.431  25.016  73.089  1.00  0.00
B    H
ATOM  18922  CG  TYR B 144      -36.498  25.959  72.979  1.00  0.00
B    C
ATOM  18923  CD1 TYR B 144      -35.795  27.002  73.580  1.00  0.00
B    C
ATOM  18924  HD1 TYR B 144      -36.329  27.866  73.953  1.00  0.00
B    H
ATOM  18925  CE1 TYR B 144      -34.414  26.964  73.690  1.00  0.00
B    C
ATOM  18926  HE1 TYR B 144      -33.877  27.777  74.155  1.00  0.00
B    H
```

ATOM 18927 CZ TYR B 144 −33.717 25.875 73.190 1.00 0.00
B C
ATOM 18928 OH TYR B 144 −32.346 25.828 73.292 1.00 0.00
B O
ATOM 18929 HH TYR B 144 −32.061 24.974 72.959 1.00 0.00
B H
ATOM 18930 CD2 TYR B 144 −35.771 24.875 72.480 1.00 0.00
B C
ATOM 18931 HD2 TYR B 144 −36.282 24.071 71.968 1.00 0.00
B H
ATOM 18932 CE2 TYR B 144 −34.391 24.828 72.583 1.00 0.00
B C
ATOM 18933 HE2 TYR B 144 −33.851 23.989 72.172 1.00 0.00
B H
ATOM 18934 C TYR B 144 −37.783 27.791 71.082 1.00 0.00
B C
ATOM 18935 O TYR B 144 −36.817 27.796 70.319 1.00 0.00
B O
ATOM 18936 N ILE B 145 −38.304 28.891 71.615 1.00 0.00
B N
ATOM 18937 HN ILE B 145 −39.182 28.904 72.090 1.00 0.00
B H
ATOM 18938 CA ILE B 145 −37.635 30.193 71.555 1.00 0.00
B C
ATOM 18939 HA ILE B 145 −36.599 30.013 71.300 1.00 0.00
B H
ATOM 18940 CB ILE B 145 −38.294 31.159 70.536 1.00 0.00
B C
ATOM 18941 HB ILE B 145 −39.393 31.067 70.732 1.00 0.00
B H
ATOM 18942 CG2 ILE B 145 −37.740 32.570 70.695 1.00 0.00
B C
ATOM 18943 HG21 ILE B 145 −38.318 33.289 70.078 1.00 0.00
B H
ATOM 18944 HG22 ILE B 145 −37.805 32.904 71.752 1.00 0.00
B H
ATOM 18945 HG23 ILE B 145 −36.676 32.618 70.378 1.00 0.00
B H
ATOM 18946 CG1 ILE B 145 −38.068 30.685 69.096 1.00 0.00
B C
ATOM 18947 HG11 ILE B 145 −36.985 30.745 68.845 1.00 0.00
B H
ATOM 18948 HG12 ILE B 145 −38.364 29.614 69.017 1.00 0.00
B H
ATOM 18949 CD ILE B 145 −38.850 31.461 68.057 1.00 0.00
B C
ATOM 18950 HD1 ILE B 145 −38.684 31.029 67.046 1.00 0.00
B H
ATOM 18951 HD2 ILE B 145 −39.939 31.423 68.278 1.00 0.00
B H
ATOM 18952 HD3 ILE B 145 −38.533 32.525 68.032 1.00 0.00
B H
ATOM 18953 C ILE B 145 −37.646 30.813 72.955 1.00 0.00
B C

ATOM 18954 O ILE B 145 −38.713 30.993 73.553 1.00 0.00
B O
ATOM 18955 N ASP B 146 −36.458 31.120 73.472 1.00 0.00
B N
ATOM 18956 HN ASP B 146 −35.583 30.937 73.031 1.00 0.00
B H
ATOM 18957 CA ASP B 146 −36.314 31.775 74.774 1.00 0.00
B C
ATOM 18958 HA ASP B 146 −37.109 31.395 75.406 1.00 0.00
B H
ATOM 18959 CB ASP B 146 −34.946 31.471 75.393 1.00 0.00
B C
ATOM 18960 HB1 ASP B 146 −34.235 31.194 74.588 1.00 0.00
B H
ATOM 18961 HB2 ASP B 146 −34.547 32.361 75.918 1.00 0.00
B H
ATOM 18962 CG ASP B 146 −34.987 30.316 76.376 1.00 0.00
B C
ATOM 18963 OD1 ASP B 146 −36.054 30.075 76.976 1.00 0.00
B O
ATOM 18964 OD2 ASP B 146 −33.945 29.655 76.568 1.00 0.00
B O
ATOM 18965 C ASP B 146 −36.518 33.283 74.669 1.00 0.00
B C
ATOM 18966 O ASP B 146 −35.729 33.981 74.024 1.00 0.00
B O
ATOM 18967 N LEU B 147 −37.579 33.781 75.305 1.00 0.00
B N
ATOM 18968 HN LEU B 147 −38.233 33.231 75.820 1.00 0.00
B H
ATOM 18969 CA LEU B 147 −37.872 35.214 75.296 1.00 0.00
B C
ATOM 18970 HA LEU B 147 −37.490 35.610 74.364 1.00 0.00
B H
ATOM 18971 CB LEU B 147 −39.373 35.484 75.404 1.00 0.00
B C
ATOM 18972 HB1 LEU B 147 −39.676 35.246 76.449 1.00 0.00
B H
ATOM 18973 HB2 LEU B 147 −39.555 36.573 75.270 1.00 0.00
B H
ATOM 18974 CG LEU B 147 −40.369 34.741 74.502 1.00 0.00
B C
ATOM 18975 HG LEU B 147 −40.378 33.776 75.063 1.00 0.00
B H
ATOM 18976 CD1 LEU B 147 −41.762 35.348 74.553 1.00 0.00
B C
ATOM 18977 HD11 LEU B 147 −42.444 34.817 73.854 1.00 0.00
B H
ATOM 18978 HD12 LEU B 147 −42.185 35.272 75.577 1.00 0.00
B H
ATOM 18979 HD13 LEU B 147 −41.734 36.420 74.263 1.00 0.00
B H
ATOM 18980 CD2 LEU B 147 −39.911 34.454 73.082 1.00 0.00
B C

ATOM 18981 HD21 LEU B 147   -40.698 33.900 72.527 1.00 0.00
B   H
ATOM 18982 HD22 LEU B 147   -39.698 35.387 72.524 1.00 0.00
B   H
ATOM 18983 HD23 LEU B 147   -38.988 33.834 73.089 1.00 0.00
B   H
ATOM 18984 C   LEU B 147   -37.137 35.915 76.428 1.00 0.00
B   C
ATOM 18985 O   LEU B 147   -36.551 36.980 76.235 1.00 0.00
B   O
ATOM 18986 N   SER B 148   -37.180 35.311 77.606 1.00 0.00
B   N
ATOM 18987 HN SER B 148   -37.734 34.499 77.790 1.00 0.00
B   H
ATOM 18988 CA SER B 148   -36.416 35.781 78.748 1.00 0.00
B   C
ATOM 18989 HA SER B 148   -35.544 36.290 78.357 1.00 0.00
B   H
ATOM 18990 CB SER B 148   -37.272 36.684 79.640 1.00 0.00
B   C
ATOM 18991 HB1 SER B 148   -36.639 37.035 80.487 1.00 0.00
B   H
ATOM 18992 HB2 SER B 148   -37.597 37.588 79.078 1.00 0.00
B   H
ATOM 18993 OG SER B 148   -38.396 35.976 80.170 1.00 0.00
B   O
ATOM 18994 HG1 SER B 148   -38.760 36.515 80.883 1.00 0.00
B   H
ATOM 18995 C   SER B 148   -35.924 34.586 79.542 1.00 0.00
B   C
ATOM 18996 O   SER B 148   -36.063 33.437 79.111 1.00 0.00
B   O
ATOM 18997 N   ASP B 149   -35.343 34.866 80.703 1.00 0.00
B   N
ATOM 18998 HN ASP B 149   -35.108 35.782 81.018 1.00 0.00
B   H
ATOM 18999 CA ASP B 149   -34.983 33.833 81.651 1.00 0.00
B   C
ATOM 19000 HA ASP B 149   -34.306 33.144 81.159 1.00 0.00
B   H
ATOM 19001 CB ASP B 149   -34.397 34.468 82.915 1.00 0.00
B   C
ATOM 19002 HB1 ASP B 149   -35.184 35.057 83.427 1.00 0.00
B   H
ATOM 19003 HB2 ASP B 149   -34.036 33.687 83.613 1.00 0.00
B   H
ATOM 19004 CG ASP B 149   -33.243 35.416 82.618 1.00 0.00
B   C
ATOM 19005 OD1 ASP B 149   -32.118 34.936 82.353 1.00 0.00
B   O
ATOM 19006 OD2 ASP B 149   -33.461 36.644 82.680 1.00 0.00
B   O
ATOM 19007 C   ASP B 149   -36.239 33.039 82.006 1.00 0.00
B   C

ATOM 19008 O ASP B 149 -36.214 31.807 82.089 1.00 0.00
B O
ATOM 19009 N GLU B 150 -37.342 33.765 82.168 1.00 0.00
B N
ATOM 19010 HN GLU B 150 -37.385 34.738 81.956 1.00 0.00
B H
ATOM 19011 CA GLU B 150 -38.593 33.211 82.669 1.00 0.00
B C
ATOM 19012 HA GLU B 150 -38.324 32.334 83.245 1.00 0.00
B H
ATOM 19013 CB GLU B 150 -39.279 34.237 83.564 1.00 0.00
B C
ATOM 19014 HB1 GLU B 150 -39.385 35.185 82.986 1.00 0.00
B H
ATOM 19015 HB2 GLU B 150 -40.315 33.903 83.796 1.00 0.00
B H
ATOM 19016 CG GLU B 150 -38.545 34.540 84.857 1.00 0.00
B C
ATOM 19017 HG1 GLU B 150 -38.525 33.635 85.491 1.00 0.00
B H
ATOM 19018 HG2 GLU B 150 -37.505 34.866 84.656 1.00 0.00
B H
ATOM 19019 CD GLU B 150 -39.242 35.611 85.677 1.00 0.00
B C
ATOM 19020 OE1 GLU B 150 -40.208 36.220 85.167 1.00 0.00
B O
ATOM 19021 OE2 GLU B 150 -38.819 35.848 86.830 1.00 0.00
B O
ATOM 19022 C GLU B 150 -39.590 32.765 81.603 1.00 0.00
B C
ATOM 19023 O GLU B 150 -40.421 31.893 81.862 1.00 0.00
B O
ATOM 19024 N TYR B 151 -39.524 33.373 80.420 1.00 0.00
B N
ATOM 19025 HN TYR B 151 -38.805 34.014 80.157 1.00 0.00
B H
ATOM 19026 CA TYR B 151 -40.538 33.137 79.387 1.00 0.00
B C
ATOM 19027 HA TYR B 151 -41.265 32.480 79.848 1.00 0.00
B H
ATOM 19028 CB TYR B 151 -41.230 34.448 79.006 1.00 0.00
B C
ATOM 19029 HB1 TYR B 151 -40.458 35.205 78.749 1.00 0.00
B H
ATOM 19030 HB2 TYR B 151 -41.867 34.309 78.107 1.00 0.00
B H
ATOM 19031 CG TYR B 151 -42.067 35.045 80.122 1.00 0.00
B C
ATOM 19032 CD1 TYR B 151 -43.414 34.714 80.261 1.00 0.00
B C
ATOM 19033 HD1 TYR B 151 -43.877 34.036 79.558 1.00 0.00
B H
ATOM 19034 CE1 TYR B 151 -44.189 35.255 81.277 1.00 0.00
B C

ATOM 19035 HE1 TYR B 151   -45.233 34.997 81.368 1.00 0.00
B   H
ATOM 19036 CZ TYR B 151   -43.618 36.137 82.172 1.00 0.00
B   C
ATOM 19037 OH TYR B 151   -44.392 36.667 83.173 1.00 0.00
B   O
ATOM 19038 HH TYR B 151   -43.817 37.193 83.733 1.00 0.00
B   H
ATOM 19039 CD2 TYR B 151   -41.514 35.938 81.036 1.00 0.00
B   C
ATOM 19040 HD2 TYR B 151   -40.483 36.247 80.933 1.00 0.00
B   H
ATOM 19041 CE2 TYR B 151   -42.281 36.484 82.058 1.00 0.00
B   C
ATOM 19042 HE2 TYR B 151   -41.837 37.190 82.743 1.00 0.00
B   H
ATOM 19043 C   TYR B 151   -40.009 32.422 78.147 1.00 0.00
B   C
ATOM 19044 O   TYR B 151   -38.892 32.680 77.692 1.00 0.00
B   O
ATOM 19045 N   SER B 152   -40.841 31.534 77.604 1.00 0.00
B   N
ATOM 19046 HN SER B 152   -41.773 31.393 77.936 1.00 0.00
B   H
ATOM 19047 CA SER B 152   -40.474 30.684 76.478 1.00 0.00
B   C
ATOM 19048 HA SER B 152   -39.779 31.263 75.884 1.00 0.00
B   H
ATOM 19049 CB SER B 152   -39.864 29.379 76.998 1.00 0.00
B   C
ATOM 19050 HB1 SER B 152   -38.906 29.625 77.511 1.00 0.00
B   H
ATOM 19051 HB2 SER B 152   -40.532 28.915 77.758 1.00 0.00
B   H
ATOM 19052 OG SER B 152   -39.593 28.477 75.942 1.00 0.00
B   O
ATOM 19053 HG1 SER B 152   -39.018 27.788 76.298 1.00 0.00
B   H
ATOM 19054 C   SER B 152   -41.678 30.371 75.591 1.00 0.00
B   C
ATOM 19055 O   SER B 152   -42.775 30.112 76.094 1.00 0.00
B   O
ATOM 19056 N   ILE B 153   -41.467 30.412 74.274 1.00 0.00
B   N
ATOM 19057 HN ILE B 153   -40.660 30.820 73.855 1.00 0.00
B   H
ATOM 19058 CA ILE B 153   -42.422 29.850 73.316 1.00 0.00
B   C
ATOM 19059 HA ILE B 153   -43.383 29.770 73.809 1.00 0.00
B   H
ATOM 19060 CB ILE B 153   -42.512 30.669 72.013 1.00 0.00
B   C
ATOM 19061 HB ILE B 153   -41.463 30.691 71.624 1.00 0.00
B   H

ATOM 19062 CG2 ILE B 153   -43.511 30.031 71.053 1.00 0.00
B   C
ATOM 19063 HG21 ILE B 153   -43.465 30.523 70.058 1.00 0.00
B   H
ATOM 19064 HG22 ILE B 153   -43.286 28.954 70.906 1.00 0.00
B   H
ATOM 19065 HG23 ILE B 153   -44.548 30.124 71.439 1.00 0.00
B   H
ATOM 19066 CG1 ILE B 153   -42.902 32.116 72.319 1.00 0.00
B   C
ATOM 19067 HG11 ILE B 153   -43.930 32.147 72.746 1.00 0.00
B   H
ATOM 19068 HG12 ILE B 153   -42.212 32.520 73.095 1.00 0.00
B   H
ATOM 19069 CD ILE B 153   -42.849 33.048 71.128 1.00 0.00
B   C
ATOM 19070 HD1 ILE B 153   -43.088 34.088 71.441 1.00 0.00
B   H
ATOM 19071 HD2 ILE B 153   -41.835 33.046 70.674 1.00 0.00
B   H
ATOM 19072 HD3 ILE B 153   -43.583 32.745 70.353 1.00 0.00
B   H
ATOM 19073 C   ILE B 153   -41.969 28.432 73.003 1.00 0.00
B   C
ATOM 19074 O   ILE B 153   -40.794 28.203 72.704 1.00 0.00
B   O
ATOM 19075 N   VAL B 154   -42.893 27.482 73.087 1.00 0.00
B   N
ATOM 19076 HN VAL B 154   -43.863 27.652 73.240 1.00 0.00
B   H
ATOM 19077 CA VAL B 154   -42.542 26.074 72.955 1.00 0.00
B   C
ATOM 19078 HA VAL B 154   -41.529 26.032 72.576 1.00 0.00
B   H
ATOM 19079 CB VAL B 154   -42.591 25.343 74.321 1.00 0.00
B   C
ATOM 19080 HB VAL B 154   -43.647 25.377 74.684 1.00 0.00
B   H
ATOM 19081 CG1 VAL B 154   -42.164 23.897 74.165 1.00 0.00
B   C
ATOM 19082 HG11 VAL B 154   -42.163 23.387 75.153 1.00 0.00
B   H
ATOM 19083 HG12 VAL B 154   -42.854 23.335 73.503 1.00 0.00
B   H
ATOM 19084 HG13 VAL B 154   -41.138 23.834 73.744 1.00 0.00
B   H
ATOM 19085 CG2 VAL B 154   -41.690 26.020 75.341 1.00 0.00
B   C
ATOM 19086 HG21 VAL B 154   -41.701 25.466 76.304 1.00 0.00
B   H
ATOM 19087 HG22 VAL B 154   -40.643 26.059 74.971 1.00 0.00
B   H
ATOM 19088 HG23 VAL B 154   -42.026 27.057 75.547 1.00 0.00
B   H

ATOM 19089 C VAL B 154 -43.438 25.358 71.948 1.00 0.00
B C
ATOM 19090 O VAL B 154 -44.665 25.417 72.044 1.00 0.00
B O
ATOM 19091 N GLU B 155 -42.808 24.701 70.978 1.00 0.00
B N
ATOM 19092 HN GLU B 155 -41.830 24.781 70.803 1.00 0.00
B H
ATOM 19093 CA GLU B 155 -43.498 23.794 70.072 1.00 0.00
B C
ATOM 19094 HA GLU B 155 -44.536 24.091 69.981 1.00 0.00
B H
ATOM 19095 CB GLU B 155 -42.831 23.782 68.695 1.00 0.00
B C
ATOM 19096 HB1 GLU B 155 -42.824 24.825 68.304 1.00 0.00
B H
ATOM 19097 HB2 GLU B 155 -41.762 23.487 68.797 1.00 0.00
B H
ATOM 19098 CG GLU B 155 -43.518 22.881 67.676 1.00 0.00
B C
ATOM 19099 HG1 GLU B 155 -43.610 21.860 68.090 1.00 0.00
B H
ATOM 19100 HG2 GLU B 155 -44.527 23.263 67.424 1.00 0.00
B H
ATOM 19101 CD GLU B 155 -42.733 22.725 66.384 1.00 0.00
B C
ATOM 19102 OE1 GLU B 155 -43.002 21.757 65.643 1.00 0.00
B O
ATOM 19103 OE2 GLU B 155 -41.849 23.562 66.104 1.00 0.00
B O
ATOM 19104 C GLU B 155 -43.480 22.403 70.693 1.00 0.00
B C
ATOM 19105 O GLU B 155 -42.410 21.819 70.897 1.00 0.00
B O
ATOM 19106 N LEU B 156 -44.666 21.892 71.008 1.00 0.00
B N
ATOM 19107 HN LEU B 156 -45.527 22.365 70.831 1.00 0.00
B H
ATOM 19108 CA LEU B 156 -44.819 20.585 71.645 1.00 0.00
B C
ATOM 19109 HA LEU B 156 -43.820 20.223 71.851 1.00 0.00
B H
ATOM 19110 CB LEU B 156 -45.653 20.685 72.929 1.00 0.00
B C
ATOM 19111 HB1 LEU B 156 -46.456 21.436 72.742 1.00 0.00
B H
ATOM 19112 HB2 LEU B 156 -46.172 19.715 73.099 1.00 0.00
B H
ATOM 19113 CG LEU B 156 -44.969 21.091 74.227 1.00 0.00
B C
ATOM 19114 HG LEU B 156 -44.086 20.429 74.402 1.00 0.00
B H
ATOM 19115 CD1 LEU B 156 -44.535 22.536 74.155 1.00 0.00
B C

ATOM 19116 HD11 LEU B 156 -44.078 22.854 75.117 1.00 0.00
B H
ATOM 19117 HD12 LEU B 156 -43.785 22.679 73.349 1.00 0.00
B H
ATOM 19118 HD13 LEU B 156 -45.403 23.197 73.949 1.00 0.00
B H
ATOM 19119 CD2 LEU B 156 -45.928 20.915 75.396 1.00 0.00
B C
ATOM 19120 HD21 LEU B 156 -45.430 21.193 76.350 1.00 0.00
B H
ATOM 19121 HD22 LEU B 156 -46.828 21.555 75.287 1.00 0.00
B H
ATOM 19122 HD23 LEU B 156 -46.259 19.856 75.472 1.00 0.00
B H
ATOM 19123 C LEU B 156 -45.485 19.589 70.716 1.00 0.00
B C
ATOM 19124 O LEU B 156 -46.431 19.923 69.997 1.00 0.00
B O
ATOM 19125 N LEU B 157 -44.987 18.360 70.738 1.00 0.00
B N
ATOM 19126 HN LEU B 157 -44.144 18.090 71.200 1.00 0.00
B H
ATOM 19127 CA LEU B 157 -45.664 17.280 70.061 1.00 0.00
B C
ATOM 19128 HA LEU B 157 -46.235 17.706 69.246 1.00 0.00
B H
ATOM 19129 CB LEU B 157 -44.669 16.246 69.554 1.00 0.00
B C
ATOM 19130 HB1 LEU B 157 -43.911 16.790 68.945 1.00 0.00
B H
ATOM 19131 HB2 LEU B 157 -44.118 15.818 70.421 1.00 0.00
B H
ATOM 19132 CG LEU B 157 -45.245 15.115 68.697 1.00 0.00
B C
ATOM 19133 HG LEU B 157 -46.111 14.674 69.246 1.00 0.00
B H
ATOM 19134 CD1 LEU B 157 -45.712 15.615 67.333 1.00 0.00
B C
ATOM 19135 HD11 LEU B 157 -46.080 14.770 66.713 1.00 0.00
B H
ATOM 19136 HD12 LEU B 157 -46.537 16.351 67.447 1.00 0.00
B H
ATOM 19137 HD13 LEU B 157 -44.878 16.107 66.788 1.00 0.00
B H
ATOM 19138 CD2 LEU B 157 -44.183 14.037 68.551 1.00 0.00
B C
ATOM 19139 HD21 LEU B 157 -44.575 13.188 67.948 1.00 0.00
B H
ATOM 19140 HD22 LEU B 157 -43.276 14.421 68.042 1.00 0.00
B H
ATOM 19141 HD23 LEU B 157 -43.885 13.646 69.547 1.00 0.00
B H
ATOM 19142 C LEU B 157 -46.647 16.642 71.025 1.00 0.00
B C

ATOM 19143 O LEU B 157 -46.280 16.251 72.141 1.00 0.00
B O
ATOM 19144 N ALA B 158 -47.903 16.583 70.595 1.00 0.00
B N
ATOM 19145 HN ALA B 158 -48.157 16.867 69.671 1.00 0.00
B H
ATOM 19146 CA ALA B 158 -48.998 16.105 71.426 1.00 0.00
B C
ATOM 19147 HA ALA B 158 -49.053 16.679 72.343 1.00 0.00
B H
ATOM 19148 CB ALA B 158 -50.325 16.276 70.702 1.00 0.00
B C
ATOM 19149 HB1 ALA B 158 -50.478 17.347 70.451 1.00 0.00
B H
ATOM 19150 HB2 ALA B 158 -50.340 15.697 69.753 1.00 0.00
B H
ATOM 19151 HB3 ALA B 158 -51.180 15.946 71.331 1.00 0.00
B H
ATOM 19152 C ALA B 158 -48.801 14.655 71.838 1.00 0.00
B C
ATOM 19153 O ALA B 158 -48.710 13.761 70.996 1.00 0.00
B O
ATOM 19154 N THR B 159 -48.741 14.432 73.145 1.00 0.00
B N
ATOM 19155 HN THR B 159 -48.789 15.150 73.839 1.00 0.00
B H
ATOM 19156 CA THR B 159 -48.595 13.084 73.684 1.00 0.00
B C
ATOM 19157 HA THR B 159 -48.157 12.419 72.951 1.00 0.00
B H
ATOM 19158 CB THR B 159 -47.902 13.062 75.068 1.00 0.00
B C
ATOM 19159 HB THR B 159 -48.417 13.838 75.685 1.00 0.00
B H
ATOM 19160 OG1 THR B 159 -46.527 13.451 74.923 1.00 0.00
B O
ATOM 19161 HG1 THR B 159 -46.153 13.406 75.810 1.00 0.00
B H
ATOM 19162 CG2 THR B 159 -47.956 11.673 75.712 1.00 0.00
B C
ATOM 19163 HG21 THR B 159 -47.557 11.702 76.748 1.00 0.00
B H
ATOM 19164 HG22 THR B 159 -49.004 11.308 75.760 1.00 0.00
B H
ATOM 19165 HG23 THR B 159 -47.358 10.939 75.130 1.00 0.00
B H
ATOM 19166 C THR B 159 -50.079 12.713 73.776 1.00 0.00
B C
ATOM 19167 O THR B 159 -50.945 13.584 73.677 1.00 0.00
B O
ATOM 19168 N ARG B 160 -50.361 11.430 73.977 1.00 0.00
B N
ATOM 19169 HN ARG B 160 -49.657 10.814 74.324 1.00 0.00
B H

ATOM 19170 CA ARG B 160   -51.683 10.840 73.711 1.00 0.00
B   C
ATOM 19171 HA ARG B 160   -51.853 11.007 72.654 1.00 0.00
B   H
ATOM 19172 CB ARG B 160   -51.634   9.327 73.932 1.00 0.00
B   C
ATOM 19173 HB1 ARG B 160   -51.690   9.167 75.035 1.00 0.00
B   H
ATOM 19174 HB2 ARG B 160   -52.549   8.859 73.508 1.00 0.00
B   H
ATOM 19175 CG ARG B 160   -50.374   8.674 73.402 1.00 0.00
B   C
ATOM 19176 HG1 ARG B 160   -50.145   9.107 72.404 1.00 0.00
B   H
ATOM 19177 HG2 ARG B 160   -49.518   8.957 74.056 1.00 0.00
B   H
ATOM 19178 CD ARG B 160   -50.508   7.166 73.383 1.00 0.00
B   C
ATOM 19179 HD1 ARG B 160   -50.294   6.762 74.399 1.00 0.00
B   H
ATOM 19180 HD2 ARG B 160   -51.535   6.850 73.083 1.00 0.00
B   H
ATOM 19181 NE ARG B 160   -49.588   6.554 72.426 1.00 0.00
B   N
ATOM 19182 HE ARG B 160   -48.992   7.223 71.967 1.00 0.00
B   H
ATOM 19183 CZ ARG B 160   -49.523   5.247 72.168 1.00 0.00
B   C
ATOM 19184 NH1 ARG B 160   -50.325   4.389 72.794 1.00 0.00
B   N
ATOM 19185 HH11 ARG B 160   -50.258   3.416 72.620 1.00 0.00
B   H
ATOM 19186 HH12 ARG B 160   -50.887   4.765 73.533 1.00 0.00
B   H
ATOM 19187 NH2 ARG B 160   -48.655   4.787 71.276 1.00 0.00
B   N
ATOM 19188 HH21 ARG B 160   -48.667   3.830 71.022 1.00 0.00
B   H
ATOM 19189 HH22 ARG B 160   -48.163   5.461 70.740 1.00 0.00
B   H
ATOM 19190 C   ARG B 160   -52.898 11.402 74.444 1.00 0.00
B   C
ATOM 19191 O   ARG B 160   -53.905 11.692 73.811 1.00 0.00
B   O
ATOM 19192 N   LYS B 161   -52.808 11.521 75.768 1.00 0.00
B   N
ATOM 19193 HN LYS B 161   -51.954 11.345 76.255 1.00 0.00
B   H
ATOM 19194 CA LYS B 161   -53.947 11.921 76.624 1.00 0.00
B   C
ATOM 19195 HA LYS B 161   -54.702 11.152 76.511 1.00 0.00
B   H
ATOM 19196 CB LYS B 161   -53.499 12.033 78.084 1.00 0.00
B   C

ATOM 19197 HB1 LYS B 161 -53.310 13.112 78.287 1.00 0.00
B H
ATOM 19198 HB2 LYS B 161 -54.343 11.734 78.746 1.00 0.00
B H
ATOM 19199 CG LYS B 161 -52.247 11.229 78.385 1.00 0.00
B C
ATOM 19200 HG1 LYS B 161 -52.324 10.234 77.890 1.00 0.00
B H
ATOM 19201 HG2 LYS B 161 -51.366 11.751 77.945 1.00 0.00
B H
ATOM 19202 CD LYS B 161 -52.036 11.017 79.874 1.00 0.00
B C
ATOM 19203 HD1 LYS B 161 -51.831 12.005 80.345 1.00 0.00
B H
ATOM 19204 HD2 LYS B 161 -52.988 10.632 80.308 1.00 0.00
B H
ATOM 19205 CE LYS B 161 -50.891 10.039 80.084 1.00 0.00
B C
ATOM 19206 HE1 LYS B 161 -51.130 9.054 79.626 1.00 0.00
B H
ATOM 19207 HE2 LYS B 161 -49.956 10.425 79.620 1.00 0.00
B H
ATOM 19208 NZ LYS B 161 -50.597 9.792 81.521 1.00 0.00
B N
ATOM 19209 HZ1 LYS B 161 -49.810 9.118 81.614 1.00 0.00
B H
ATOM 19210 HZ2 LYS B 161 -50.340 10.689 81.981 1.00 0.00
B H
ATOM 19211 HZ3 LYS B 161 -51.442 9.401 81.984 1.00 0.00
B H
ATOM 19212 C LYS B 161 -54.573 13.240 76.184 1.00 0.00
B C
ATOM 19213 O LYS B 161 -55.720 13.551 76.522 1.00 0.00
B O
ATOM 19214 N LEU B 162 -53.792 14.002 75.425 1.00 0.00
B N
ATOM 19215 HN LEU B 162 -52.857 13.736 75.199 1.00 0.00
B H
ATOM 19216 CA LEU B 162 -54.205 15.271 74.856 1.00 0.00
B C
ATOM 19217 HA LEU B 162 -54.818 15.758 75.602 1.00 0.00
B H
ATOM 19218 CB LEU B 162 -52.938 16.077 74.557 1.00 0.00
B C
ATOM 19219 HB1 LEU B 162 -52.292 16.021 75.462 1.00 0.00
B H
ATOM 19220 HB2 LEU B 162 -52.372 15.572 73.743 1.00 0.00
B H
ATOM 19221 CG LEU B 162 -53.017 17.554 74.199 1.00 0.00
B C
ATOM 19222 HG LEU B 162 -54.040 17.933 74.440 1.00 0.00
B H
ATOM 19223 CD1 LEU B 162 -51.992 18.325 75.016 1.00 0.00
B C

ATOM 19224 HD11 LEU B 162   -51.999 19.400 74.736 1.00 0.00
B   H
ATOM 19225 HD12 LEU B 162   -52.216 18.246 76.101 1.00 0.00
B   H
ATOM 19226 HD13 LEU B 162   -50.969 17.927 74.839 1.00 0.00
B   H
ATOM 19227 CD2 LEU B 162   -52.789 17.734 72.706 1.00 0.00
B   C
ATOM 19228 HD21 LEU B 162   -52.864 18.808 72.431 1.00 0.00
B   H
ATOM 19229 HD22 LEU B 162   -51.786 17.377 72.400 1.00 0.00
B   H
ATOM 19230 HD23 LEU B 162   -53.553 17.173 72.126 1.00 0.00
B   H
ATOM 19231 C   LEU B 162   -55.074 15.073 73.596 1.00 0.00
B   C
ATOM 19232 O   LEU B 162   -55.510 16.043 72.962 1.00 0.00
B   O
ATOM 19233 N   ASP B 163   -55.344 13.809 73.267 1.00 0.00
B   N
ATOM 19234 HN ASP B 163   -55.090 13.028 73.831 1.00 0.00
B   H
ATOM 19235 CA ASP B 163   -56.046 13.425 72.043 1.00 0.00
B   C
ATOM 19236 HA ASP B 163   -55.604 14.015 71.250 1.00 0.00
B   H
ATOM 19237 CB ASP B 163   -55.856 11.927 71.774 1.00 0.00
B   C
ATOM 19238 HB1 ASP B 163   -54.785 11.667 71.897 1.00 0.00
B   H
ATOM 19239 HB2 ASP B 163   -56.446 11.323 72.491 1.00 0.00
B   H
ATOM 19240 CG ASP B 163   -56.259 11.522 70.360 1.00 0.00
B   C
ATOM 19241 OD1 ASP B 163   -55.913 12.246 69.402 1.00 0.00
B   O
ATOM 19242 OD2 ASP B 163   -56.920 10.472 70.204 1.00 0.00
B   O
ATOM 19243 C   ASP B 163   -57.538 13.757 72.062 1.00 0.00
B   C
ATOM 19244 O   ASP B 163   -58.236 13.482 73.044 1.00 0.00
B   O
ATOM 19245 N   SER B 164   -57.998 14.339 70.952 1.00 0.00
B   N
ATOM 19246 HN SER B 164   -57.382 14.585 70.203 1.00 0.00
B   H
ATOM 19247 CA SER B 164   -59.408 14.674 70.706 1.00 0.00
B   C
ATOM 19248 HA SER B 164   -59.408 15.182 69.751 1.00 0.00
B   H
ATOM 19249 CB SER B 164   -60.263 13.403 70.561 1.00 0.00
B   C
ATOM 19250 HB1 SER B 164   -60.192 12.828 71.512 1.00 0.00
B   H

ATOM 19251 HB2 SER B 164 -61.335 13.671 70.420 1.00 0.00
B H
ATOM 19252 OG SER B 164 -59.792 12.592 69.498 1.00 0.00
B O
ATOM 19253 HG1 SER B 164 -60.210 11.727 69.594 1.00 0.00
B H
ATOM 19254 C SER B 164 -60.007 15.636 71.735 1.00 0.00
B C
ATOM 19255 O SER B 164 -61.195 15.557 72.059 1.00 0.00
B O
ATOM 19256 N LYS B 165 -59.176 16.547 72.238 1.00 0.00
B N
ATOM 19257 HN LYS B 165 -58.212 16.602 71.983 1.00 0.00
B H
ATOM 19258 CA LYS B 165 -59.617 17.543 73.210 1.00 0.00
B C
ATOM 19259 HA LYS B 165 -60.654 17.329 73.437 1.00 0.00
B H
ATOM 19260 CB LYS B 165 -58.771 17.478 74.485 1.00 0.00
B C
ATOM 19261 HB1 LYS B 165 -57.710 17.346 74.169 1.00 0.00
B H
ATOM 19262 HB2 LYS B 165 -58.822 18.461 75.005 1.00 0.00
B H
ATOM 19263 CG LYS B 165 -59.145 16.352 75.433 1.00 0.00
B C
ATOM 19264 HG1 LYS B 165 -60.179 16.010 75.202 1.00 0.00
B H
ATOM 19265 HG2 LYS B 165 -58.462 15.489 75.258 1.00 0.00
B H
ATOM 19266 CD LYS B 165 -59.067 16.833 76.875 1.00 0.00
B C
ATOM 19267 HD1 LYS B 165 -58.068 17.298 77.038 1.00 0.00
B H
ATOM 19268 HD2 LYS B 165 -59.833 17.630 77.016 1.00 0.00
B H
ATOM 19269 CE LYS B 165 -59.297 15.702 77.865 1.00 0.00
B C
ATOM 19270 HE1 LYS B 165 -59.548 16.109 78.870 1.00 0.00
B H
ATOM 19271 HE2 LYS B 165 -60.136 15.053 77.529 1.00 0.00
B H
ATOM 19272 NZ LYS B 165 -58.089 14.843 78.013 1.00 0.00
B N
ATOM 19273 HZ1 LYS B 165 -58.276 14.081 78.696 1.00 0.00
B H
ATOM 19274 HZ2 LYS B 165 -57.842 14.429 77.092 1.00 0.00
B H
ATOM 19275 HZ3 LYS B 165 -57.293 15.421 78.351 1.00 0.00
B H
ATOM 19276 C LYS B 165 -59.560 18.948 72.629 1.00 0.00
B C
ATOM 19277 O LYS B 165 -58.625 19.289 71.901 1.00 0.00
B O

ATOM 19278 N SER B 166 −60.561 19.761 72.957 1.00 0.00
B N
ATOM 19279 HN SER B 166 −61.339 19.494 73.527 1.00 0.00
B H
ATOM 19280 CA SER B 166 −60.603 21.145 72.497 1.00 0.00
B C
ATOM 19281 HA SER B 166 −60.145 21.147 71.516 1.00 0.00
B H
ATOM 19282 CB SER B 166 −62.049 21.649 72.399 1.00 0.00
B C
ATOM 19283 HB1 SER B 166 −62.067 22.517 71.700 1.00 0.00
B H
ATOM 19284 HB2 SER B 166 −62.706 20.863 71.961 1.00 0.00
B H
ATOM 19285 OG SER B 166 −62.549 22.077 73.657 1.00 0.00
B O
ATOM 19286 HG1 SER B 166 −63.356 22.578 73.486 1.00 0.00
B H
ATOM 19287 C SER B 166 −59.765 22.058 73.392 1.00 0.00
B C
ATOM 19288 O SER B 166 −59.267 21.638 74.442 1.00 0.00
B O
ATOM 19289 N ILE B 167 −59.606 23.306 72.959 1.00 0.00
B N
ATOM 19290 HN ILE B 167 −59.973 23.633 72.090 1.00 0.00
B H
ATOM 19291 CA ILE B 167 −58.877 24.317 73.719 1.00 0.00
B C
ATOM 19292 HA ILE B 167 −57.874 23.931 73.853 1.00 0.00
B H
ATOM 19293 CB ILE B 167 −58.844 25.670 72.967 1.00 0.00
B C
ATOM 19294 HB ILE B 167 −59.921 25.945 72.838 1.00 0.00
B H
ATOM 19295 CG2 ILE B 167 −58.091 26.733 73.763 1.00 0.00
B C
ATOM 19296 HG21 ILE B 167 −58.207 27.730 73.291 1.00 0.00
B H
ATOM 19297 HG22 ILE B 167 −58.483 26.803 74.799 1.00 0.00
B H
ATOM 19298 HG23 ILE B 167 −57.006 26.497 73.813 1.00 0.00
B H
ATOM 19299 CG1 ILE B 167 −58.262 25.502 71.555 1.00 0.00
B C
ATOM 19300 HG11 ILE B 167 −58.916 24.828 70.956 1.00 0.00
B H
ATOM 19301 HG12 ILE B 167 −58.257 26.493 71.046 1.00 0.00
B H
ATOM 19302 CD ILE B 167 −56.848 24.958 71.501 1.00 0.00
B C
ATOM 19303 HD1 ILE B 167 −56.488 24.923 70.450 1.00 0.00
B H
ATOM 19304 HD2 ILE B 167 −56.157 25.604 72.084 1.00 0.00
B H

ATOM 19305 HD3 ILE B 167 -56.801 23.929 71.914 1.00 0.00
B H
ATOM 19306 C ILE B 167 -59.470 24.501 75.114 1.00 0.00
B C
ATOM 19307 O ILE B 167 -58.745 24.473 76.105 1.00 0.00
B O
ATOM 19308 N ILE B 168 -60.789 24.665 75.186 1.00 0.00
B N
ATOM 19309 HN ILE B 168 -61.393 24.647 74.392 1.00 0.00
B H
ATOM 19310 CA ILE B 168 -61.478 24.891 76.459 1.00 0.00
B C
ATOM 19311 HA ILE B 168 -60.861 25.598 76.999 1.00 0.00
B H
ATOM 19312 CB ILE B 168 -62.863 25.577 76.252 1.00 0.00
B C
ATOM 19313 HB ILE B 168 -62.678 26.349 75.462 1.00 0.00
B H
ATOM 19314 CG2 ILE B 168 -63.920 24.583 75.775 1.00 0.00
B C
ATOM 19315 HG21 ILE B 168 -64.852 25.113 75.485 1.00 0.00
B H
ATOM 19316 HG22 ILE B 168 -63.563 24.020 74.888 1.00 0.00
B H
ATOM 19317 HG23 ILE B 168 -64.171 23.855 76.576 1.00 0.00
B H
ATOM 19318 CG1 ILE B 168 -63.308 26.340 77.514 1.00 0.00
B C
ATOM 19319 HG11 ILE B 168 -64.405 26.525 77.476 1.00 0.00
B H
ATOM 19320 HG12 ILE B 168 -63.112 25.704 78.408 1.00 0.00
B H
ATOM 19321 CD ILE B 168 -62.606 27.666 77.732 1.00 0.00
B C
ATOM 19322 HD1 ILE B 168 -62.938 28.125 78.688 1.00 0.00
B H
ATOM 19323 HD2 ILE B 168 -61.505 27.524 77.778 1.00 0.00
B H
ATOM 19324 HD3 ILE B 168 -62.834 28.378 76.911 1.00 0.00
B H
ATOM 19325 C ILE B 168 -61.572 23.623 77.333 1.00 0.00
B C
ATOM 19326 O ILE B 168 -61.795 23.718 78.545 1.00 0.00
B O
ATOM 19327 N ASP B 169 -61.388 22.450 76.718 1.00 0.00
B N
ATOM 19328 HN ASP B 169 -61.229 22.343 75.740 1.00 0.00
B H
ATOM 19329 CA ASP B 169 -61.401 21.169 77.440 1.00 0.00
B C
ATOM 19330 HA ASP B 169 -62.359 21.094 77.942 1.00 0.00
B H
ATOM 19331 CB ASP B 169 -61.226 19.988 76.479 1.00 0.00
B C

ATOM 19332 HB1 ASP B 169 -60.435 20.231 75.742 1.00 0.00
B H
ATOM 19333 HB2 ASP B 169 -60.920 19.078 77.032 1.00 0.00
B H
ATOM 19334 CG ASP B 169 -62.488 19.673 75.697 1.00 0.00
B C
ATOM 19335 OD1 ASP B 169 -63.423 20.507 75.682 1.00 0.00
B O
ATOM 19336 OD2 ASP B 169 -62.540 18.581 75.093 1.00 0.00
B O
ATOM 19337 C ASP B 169 -60.330 21.148 78.527 1.00 0.00
B C
ATOM 19338 O ASP B 169 -60.606 20.810 79.677 1.00 0.00
B O
ATOM 19339 N LEU B 170 -59.108 21.501 78.140 1.00 0.00
B N
ATOM 19340 HN LEU B 170 -58.806 21.514 77.189 1.00 0.00
B H
ATOM 19341 CA LEU B 170 -58.091 21.905 79.096 1.00 0.00
B C
ATOM 19342 HA LEU B 170 -58.499 21.829 80.096 1.00 0.00
B H
ATOM 19343 CB LEU B 170 -56.799 21.103 78.920 1.00 0.00
B C
ATOM 19344 HB1 LEU B 170 -56.520 21.160 77.842 1.00 0.00
B H
ATOM 19345 HB2 LEU B 170 -55.980 21.610 79.476 1.00 0.00
B H
ATOM 19346 CG LEU B 170 -56.707 19.616 79.275 1.00 0.00
B C
ATOM 19347 HG LEU B 170 -57.503 19.078 78.706 1.00 0.00
B H
ATOM 19348 CD1 LEU B 170 -55.340 19.101 78.851 1.00 0.00
B C
ATOM 19349 HD11 LEU B 170 -55.220 18.034 79.137 1.00 0.00
B H
ATOM 19350 HD12 LEU B 170 -55.215 19.183 77.750 1.00 0.00
B H
ATOM 19351 HD13 LEU B 170 -54.531 19.685 79.340 1.00 0.00
B H
ATOM 19352 CD2 LEU B 170 -56.939 19.366 80.762 1.00 0.00
B C
ATOM 19353 HD21 LEU B 170 -56.885 18.278 80.984 1.00 0.00
B H
ATOM 19354 HD22 LEU B 170 -56.178 19.876 81.386 1.00 0.00
B H
ATOM 19355 HD23 LEU B 170 -57.944 19.733 81.065 1.00 0.00
B H
ATOM 19356 C LEU B 170 -57.816 23.373 78.824 1.00 0.00
B C
ATOM 19357 O LEU B 170 -56.960 23.697 77.994 1.00 0.00
B O
ATOM 19358 N ASN B 171 -58.557 24.263 79.488 1.00 0.00
B N

ATOM 19359 HN ASN B 171 -59.317 24.044 80.099 1.00 0.00
B H
ATOM 19360 CA ASN B 171 -58.283 25.693 79.349 1.00 0.00
B C
ATOM 19361 HA ASN B 171 -58.238 25.893 78.284 1.00 0.00
B H
ATOM 19362 CB ASN B 171 -59.404 26.589 79.909 1.00 0.00
B C
ATOM 19363 HB1 ASN B 171 -59.040 27.640 79.891 1.00 0.00
B H
ATOM 19364 HB2 ASN B 171 -60.301 26.535 79.259 1.00 0.00
B H
ATOM 19365 CG ASN B 171 -59.786 26.258 81.344 1.00 0.00
B C
ATOM 19366 OD1 ASN B 171 -58.990 25.732 82.122 1.00 0.00
B O
ATOM 19367 ND2 ASN B 171 -61.022 26.581 81.700 1.00 0.00
B N
ATOM 19368 HD21 ASN B 171 -61.294 26.384 82.639 1.00 0.00
B H
ATOM 19369 HD22 ASN B 171 -61.628 27.028 81.050 1.00 0.00
B H
ATOM 19370 C ASN B 171 -56.921 26.024 79.938 1.00 0.00
B C
ATOM 19371 O ASN B 171 -56.685 25.864 81.135 1.00 0.00
B O
ATOM 19372 N VAL B 172 -56.022 26.454 79.068 1.00 0.00
B N
ATOM 19373 HN VAL B 172 -56.233 26.720 78.131 1.00 0.00
B H
ATOM 19374 CA VAL B 172 -54.621 26.576 79.423 1.00 0.00
B C
ATOM 19375 HA VAL B 172 -54.427 25.872 80.224 1.00 0.00
B H
ATOM 19376 CB VAL B 172 -53.722 26.142 78.241 1.00 0.00
B C
ATOM 19377 HB VAL B 172 -53.935 26.829 77.385 1.00 0.00
B H
ATOM 19378 CG1 VAL B 172 -52.251 26.242 78.589 1.00 0.00
B C
ATOM 19379 HG11 VAL B 172 -51.629 25.888 77.739 1.00 0.00
B H
ATOM 19380 HG12 VAL B 172 -51.952 27.288 78.806 1.00 0.00
B H
ATOM 19381 HG13 VAL B 172 -52.014 25.613 79.473 1.00 0.00
B H
ATOM 19382 CG2 VAL B 172 -54.028 24.701 77.867 1.00 0.00
B C
ATOM 19383 HG21 VAL B 172 -53.364 24.358 77.045 1.00 0.00
B H
ATOM 19384 HG22 VAL B 172 -53.877 24.031 78.741 1.00 0.00
B H
ATOM 19385 HG23 VAL B 172 -55.076 24.590 77.519 1.00 0.00
B H

ATOM 19386 C   VAL B 172   -54.309 27.977 79.956 1.00 0.00
B   C
ATOM 19387 O   VAL B 172   -53.270 28.182 80.591 1.00 0.00
B   O
ATOM 19388 N   ARG B 173   -55.213 28.927 79.722 1.00 0.00
B   N
ATOM 19389 HN ARG B 173   -56.026 28.761 79.170 1.00 0.00
B   H
ATOM 19390 CA ARG B 173   -55.055 30.272 80.269 1.00 0.00
B   C
ATOM 19391 HA ARG B 173   -54.011 30.550 80.194 1.00 0.00
B   H
ATOM 19392 CB ARG B 173   -55.887 31.289 79.501 1.00 0.00
B   C
ATOM 19393 HB1 ARG B 173   -56.857 30.790 79.266 1.00 0.00
B   H
ATOM 19394 HB2 ARG B 173   -56.136 32.145 80.167 1.00 0.00
B   H
ATOM 19395 CG ARG B 173   -55.247 31.754 78.210 1.00 0.00
B   C
ATOM 19396 HG1 ARG B 173   -54.144 31.764 78.347 1.00 0.00
B   H
ATOM 19397 HG2 ARG B 173   -55.444 30.997 77.416 1.00 0.00
B   H
ATOM 19398 CD ARG B 173   -55.830 33.091 77.801 1.00 0.00
B   C
ATOM 19399 HD1 ARG B 173   -56.689 33.340 78.465 1.00 0.00
B   H
ATOM 19400 HD2 ARG B 173   -55.073 33.908 77.876 1.00 0.00
B   H
ATOM 19401 NE ARG B 173   -56.282 33.052 76.413 1.00 0.00
B   N
ATOM 19402 HE ARG B 173   -56.974 32.341 76.243 1.00 0.00
B   H
ATOM 19403 CZ ARG B 173   -55.850 33.853 75.443 1.00 0.00
B   C
ATOM 19404 NH1 ARG B 173   -56.337 33.708 74.218 1.00 0.00
B   N
ATOM 19405 HH11 ARG B 173   -56.011 34.277 73.476 1.00 0.00
B   H
ATOM 19406 HH12 ARG B 173   -56.935 32.917 74.069 1.00 0.00
B   H
ATOM 19407 NH2 ARG B 173   -54.947 34.801 75.690 1.00 0.00
B   N
ATOM 19408 HH21 ARG B 173   -54.701 35.444 74.979 1.00 0.00
B   H
ATOM 19409 HH22 ARG B 173   -54.710 34.957 76.639 1.00 0.00
B   H
ATOM 19410 C   ARG B 173   -55.410 30.310 81.745 1.00 0.00
B   C
ATOM 19411 O   ARG B 173   -54.731 30.965 82.529 1.00 0.00
B   O
ATOM 19412 N   ALA B 174   -56.471 29.605 82.116 1.00 0.00
B   N

ATOM 19413 HN ALA B 174 -57.050 29.107 81.470 1.00 0.00
B H
ATOM 19414 CA ALA B 174 -56.866 29.507 83.510 1.00 0.00
B C
ATOM 19415 HA ALA B 174 -56.804 30.475 83.993 1.00 0.00
B H
ATOM 19416 CB ALA B 174 -58.313 29.048 83.622 1.00 0.00
B C
ATOM 19417 HB1 ALA B 174 -58.974 29.768 83.093 1.00 0.00
B H
ATOM 19418 HB2 ALA B 174 -58.452 28.049 83.153 1.00 0.00
B H
ATOM 19419 HB3 ALA B 174 -58.644 28.991 84.681 1.00 0.00
B H
ATOM 19420 C ALA B 174 -55.927 28.576 84.285 1.00 0.00
B C
ATOM 19421 O ALA B 174 -55.333 28.985 85.277 1.00 0.00
B O
ATOM 19422 N LYS B 175 -55.756 27.348 83.798 1.00 0.00
B N
ATOM 19423 HN LYS B 175 -56.102 27.081 82.900 1.00 0.00
B H
ATOM 19424 CA LYS B 175 -55.048 26.286 84.536 1.00 0.00
B C
ATOM 19425 HA LYS B 175 -55.539 26.252 85.501 1.00 0.00
B H
ATOM 19426 CB LYS B 175 -55.236 24.926 83.848 1.00 0.00
B C
ATOM 19427 HB1 LYS B 175 -55.338 25.123 82.755 1.00 0.00
B H
ATOM 19428 HB2 LYS B 175 -54.307 24.326 83.972 1.00 0.00
B H
ATOM 19429 CG LYS B 175 -56.447 24.135 84.319 1.00 0.00
B C
ATOM 19430 HG1 LYS B 175 -56.510 24.191 85.430 1.00 0.00
B H
ATOM 19431 HG2 LYS B 175 -57.370 24.607 83.911 1.00 0.00
B H
ATOM 19432 CD LYS B 175 -56.381 22.669 83.906 1.00 0.00
B C
ATOM 19433 HD1 LYS B 175 -57.422 22.284 83.807 1.00 0.00
B H
ATOM 19434 HD2 LYS B 175 -55.908 22.614 82.898 1.00 0.00
B H
ATOM 19435 CE LYS B 175 -55.591 21.828 84.903 1.00 0.00
B C
ATOM 19436 HE1 LYS B 175 -54.498 21.930 84.720 1.00 0.00
B H
ATOM 19437 HE2 LYS B 175 -55.805 22.153 85.945 1.00 0.00
B H
ATOM 19438 NZ LYS B 175 -55.950 20.384 84.781 1.00 0.00
B N
ATOM 19439 HZ1 LYS B 175 -55.405 19.821 85.465 1.00 0.00
B H

ATOM 19440 HZ2 LYS B 175 -56.967 20.262 84.966 1.00 0.00
B H
ATOM 19441 HZ3 LYS B 175 -55.738 20.055 83.817 1.00 0.00
B H
ATOM 19442 C LYS B 175 -53.555 26.502 84.791 1.00 0.00
B C
ATOM 19443 O LYS B 175 -53.077 26.292 85.903 1.00 0.00
B O
ATOM 19444 N TYR B 176 -52.823 26.884 83.752 1.00 0.00
B N
ATOM 19445 HN TYR B 176 -53.220 27.130 82.869 1.00 0.00
B H
ATOM 19446 CA TYR B 176 -51.359 26.977 83.808 1.00 0.00
B C
ATOM 19447 HA TYR B 176 -51.047 26.828 84.834 1.00 0.00
B H
ATOM 19448 CB TYR B 176 -50.739 26.010 82.807 1.00 0.00
B C
ATOM 19449 HB1 TYR B 176 -51.117 26.246 81.789 1.00 0.00
B H
ATOM 19450 HB2 TYR B 176 -49.633 26.125 82.782 1.00 0.00
B H
ATOM 19451 CG TYR B 176 -51.115 24.604 83.122 1.00 0.00
B C
ATOM 19452 CD1 TYR B 176 -50.481 23.921 84.149 1.00 0.00
B C
ATOM 19453 HD1 TYR B 176 -49.680 24.397 84.697 1.00 0.00
B H
ATOM 19454 CE1 TYR B 176 -50.838 22.631 84.474 1.00 0.00
B C
ATOM 19455 HE1 TYR B 176 -50.329 22.103 85.267 1.00 0.00
B H
ATOM 19456 CZ TYR B 176 -51.853 22.015 83.765 1.00 0.00
B C
ATOM 19457 OH TYR B 176 -52.220 20.731 84.068 1.00 0.00
B O
ATOM 19458 HH TYR B 176 -52.977 20.512 83.522 1.00 0.00
B H
ATOM 19459 CD2 TYR B 176 -52.142 23.972 82.435 1.00 0.00
B C
ATOM 19460 HD2 TYR B 176 -52.637 24.479 81.619 1.00 0.00
B H
ATOM 19461 CE2 TYR B 176 -52.510 22.679 82.746 1.00 0.00
B C
ATOM 19462 HE2 TYR B 176 -53.290 22.193 82.180 1.00 0.00
B H
ATOM 19463 C TYR B 176 -50.997 28.372 83.438 1.00 0.00
B C
ATOM 19464 O TYR B 176 -49.847 28.801 83.516 1.00 0.00
B O
ATOM 19465 N GLY B 177 -52.017 29.082 83.009 1.00 0.00
B N
ATOM 19466 HN GLY B 177 -52.951 28.733 82.946 1.00 0.00
B H

ATOM 19467 CA GLY B 177 -51.835 30.441 82.595 1.00 0.00
B C
ATOM 19468 HA1 GLY B 177 -51.375 30.967 83.421 1.00 0.00
B H
ATOM 19469 HA2 GLY B 177 -52.804 30.825 82.307 1.00 0.00
B H
ATOM 19470 C GLY B 177 -50.917 30.553 81.400 1.00 0.00
B C
ATOM 19471 O GLY B 177 -50.084 31.460 81.356 1.00 0.00
B O
ATOM 19472 N CYS B 178 -51.065 29.635 80.440 1.00 0.00
B N
ATOM 19473 HN CYS B 178 -51.672 28.847 80.527 1.00 0.00
B H
ATOM 19474 CA CYS B 178 -50.342 29.714 79.172 1.00 0.00
B C
ATOM 19475 HA CYS B 178 -49.729 30.607 79.220 1.00 0.00
B H
ATOM 19476 CB CYS B 178 -49.451 28.492 78.973 1.00 0.00
B C
ATOM 19477 HB1 CYS B 178 -50.104 27.593 79.041 1.00 0.00
B H
ATOM 19478 HB2 CYS B 178 -49.035 28.492 77.941 1.00 0.00
B H
ATOM 19479 SG CYS B 178 -48.153 28.364 80.210 1.00 0.00
B S
ATOM 19480 HG1 CYS B 178 -47.641 27.205 79.794 1.00 0.00
B H
ATOM 19481 C CYS B 178 -51.306 29.876 78.002 1.00 0.00
B C
ATOM 19482 O CYS B 178 -52.452 29.438 78.061 1.00 0.00
B O
ATOM 19483 N THR B 179 -50.833 30.520 76.941 1.00 0.00
B N
ATOM 19484 HN THR B 179 -49.907 30.889 76.876 1.00 0.00
B H
ATOM 19485 CA THR B 179 -51.653 30.745 75.754 1.00 0.00
B C
ATOM 19486 HA THR B 179 -52.653 30.432 76.024 1.00 0.00
B H
ATOM 19487 CB THR B 179 -51.612 32.218 75.311 1.00 0.00
B C
ATOM 19488 HB THR B 179 -52.020 32.240 74.271 1.00 0.00
B H
ATOM 19489 OG1 THR B 179 -50.251 32.663 75.265 1.00 0.00
B O
ATOM 19490 HG1 THR B 179 -50.293 33.586 74.996 1.00 0.00
B H
ATOM 19491 CG2 THR B 179 -52.384 33.086 76.288 1.00 0.00
B C
ATOM 19492 HG21 THR B 179 -52.456 34.130 75.916 1.00 0.00
B H
ATOM 19493 HG22 THR B 179 -53.417 32.698 76.418 1.00 0.00
B H

ATOM 19494 HG23 THR B 179   -51.891 33.103 77.283 1.00 0.00
B   H
ATOM 19495 C   THR B 179   -51.215 29.851 74.593 1.00 0.00
B   C
ATOM 19496 O   THR B 179   -50.080 29.953 74.112 1.00 0.00
B   O
ATOM 19497 N   ILE B 180   -52.116 28.971 74.158 1.00 0.00
B   N
ATOM 19498 HN ILE B 180   -53.008 28.827 74.582 1.00 0.00
B   H
ATOM 19499 CA ILE B 180   -51.862 28.120 72.993 1.00 0.00
B   C
ATOM 19500 HA ILE B 180   -50.850 27.746 73.081 1.00 0.00
B   H
ATOM 19501 CB ILE B 180   -52.854 26.942 72.887 1.00 0.00
B   C
ATOM 19502 HB ILE B 180   -53.863 27.413 73.008 1.00 0.00
B   H
ATOM 19503 CG2 ILE B 180   -52.663 26.205 71.567 1.00 0.00
B   C
ATOM 19504 HG21 ILE B 180   -53.470 25.459 71.415 1.00 0.00
B   H
ATOM 19505 HG22 ILE B 180   -52.692 26.912 70.710 1.00 0.00
B   H
ATOM 19506 HG23 ILE B 180   -51.690 25.671 71.547 1.00 0.00
B   H
ATOM 19507 CG1 ILE B 180   -52.668 25.981 74.066 1.00 0.00
B   C
ATOM 19508 HG11 ILE B 180   -51.657 25.519 74.020 1.00 0.00
B   H
ATOM 19509 HG12 ILE B 180   -52.722 26.560 75.016 1.00 0.00
B   H
ATOM 19510 CD ILE B 180   -53.701 24.876 74.140 1.00 0.00
B   C
ATOM 19511 HD1 ILE B 180   -53.538 24.256 75.049 1.00 0.00
B   H
ATOM 19512 HD2 ILE B 180   -54.727 25.300 74.187 1.00 0.00
B   H
ATOM 19513 HD3 ILE B 180   -53.636 24.209 73.253 1.00 0.00
B   H
ATOM 19514 C   ILE B 180   -51.906 28.980 71.733 1.00 0.00
B   C
ATOM 19515 O   ILE B 180   -52.936 29.576 71.406 1.00 0.00
B   O
ATOM 19516 N   LEU B 181   -50.774 29.045 71.041 1.00 0.00
B   N
ATOM 19517 HN LEU B 181   -49.949 28.529 71.262 1.00 0.00
B   H
ATOM 19518 CA LEU B 181   -50.648 29.900 69.878 1.00 0.00
B   C
ATOM 19519 HA LEU B 181   -51.292 30.750 70.066 1.00 0.00
B   H
ATOM 19520 CB LEU B 181   -49.218 30.423 69.726 1.00 0.00
B   C

ATOM 19521 HB1 LEU B 181 -48.537 29.541 69.780 1.00 0.00
B H
ATOM 19522 HB2 LEU B 181 -49.094 30.845 68.705 1.00 0.00
B H
ATOM 19523 CG LEU B 181 -48.737 31.446 70.760 1.00 0.00
B C
ATOM 19524 HG LEU B 181 -48.895 30.984 71.765 1.00 0.00
B H
ATOM 19525 CD1 LEU B 181 -47.256 31.735 70.557 1.00 0.00
B C
ATOM 19526 HD11 LEU B 181 -46.909 32.512 71.272 1.00 0.00
B H
ATOM 19527 HD12 LEU B 181 -46.653 30.816 70.720 1.00 0.00
B H
ATOM 19528 HD13 LEU B 181 -47.066 32.102 69.527 1.00 0.00
B H
ATOM 19529 CD2 LEU B 181 -49.544 32.735 70.703 1.00 0.00
B C
ATOM 19530 HD21 LEU B 181 -49.185 33.450 71.475 1.00 0.00
B H
ATOM 19531 HD22 LEU B 181 -49.453 33.232 69.716 1.00 0.00
B H
ATOM 19532 HD23 LEU B 181 -50.620 32.529 70.893 1.00 0.00
B H
ATOM 19533 C LEU B 181 -51.126 29.237 68.594 1.00 0.00
B C
ATOM 19534 O LEU B 181 -51.716 29.910 67.758 1.00 0.00
B O
ATOM 19535 N ALA B 182 -50.889 27.932 68.436 1.00 0.00
B N
ATOM 19536 HN ALA B 182 -50.436 27.371 69.129 1.00 0.00
B H
ATOM 19537 CA ALA B 182 -51.287 27.223 67.210 1.00 0.00
B C
ATOM 19538 HA ALA B 182 -52.239 27.617 66.872 1.00 0.00
B H
ATOM 19539 CB ALA B 182 -50.297 27.510 66.082 1.00 0.00
B C
ATOM 19540 HB1 ALA B 182 -50.202 28.608 65.940 1.00 0.00
B H
ATOM 19541 HB2 ALA B 182 -49.289 27.109 66.324 1.00 0.00
B H
ATOM 19542 HB3 ALA B 182 -50.632 27.068 65.120 1.00 0.00
B H
ATOM 19543 C ALA B 182 -51.479 25.711 67.369 1.00 0.00
B C
ATOM 19544 O ALA B 182 -51.017 25.115 68.347 1.00 0.00
B O
ATOM 19545 N ILE B 183 -52.175 25.113 66.402 1.00 0.00
B N
ATOM 19546 HN ILE B 183 -52.681 25.627 65.712 1.00 0.00
B H
ATOM 19547 CA ILE B 183 -52.267 23.658 66.255 1.00 0.00
B C

ATOM 19548 HA ILE B 183   -51.567 23.210 66.948 1.00 0.00
B   H
ATOM 19549 CB ILE B 183   -53.712 23.129 66.441 1.00 0.00
B   C
ATOM 19550 HB ILE B 183   -54.274 23.532 65.561 1.00 0.00
B   H
ATOM 19551 CG2 ILE B 183   -53.732 21.605 66.526 1.00 0.00
B   C
ATOM 19552 HG21 ILE B 183   -54.774 21.225 66.502 1.00 0.00
B   H
ATOM 19553 HG22 ILE B 183   -53.190 21.154 65.668 1.00 0.00
B   H
ATOM 19554 HG23 ILE B 183   -53.256 21.253 67.465 1.00 0.00
B   H
ATOM 19555 CG1 ILE B 183   -54.369 23.732 67.687 1.00 0.00
B   C
ATOM 19556 HG11 ILE B 183   -53.946 23.259 68.602 1.00 0.00
B   H
ATOM 19557 HG12 ILE B 183   -54.124 24.818 67.735 1.00 0.00
B   H
ATOM 19558 CD ILE B 183   -55.877 23.592 67.715 1.00 0.00
B   C
ATOM 19559 HD1 ILE B 183   -56.295 24.098 68.612 1.00 0.00
B   H
ATOM 19560 HD2 ILE B 183   -56.330 24.054 66.812 1.00 0.00
B   H
ATOM 19561 HD3 ILE B 183   -56.176 22.523 67.752 1.00 0.00
B   H
ATOM 19562 C   ILE B 183   -51.788 23.298 64.849 1.00 0.00
B   C
ATOM 19563 O   ILE B 183   -52.021 24.052 63.898 1.00 0.00
B   O
ATOM 19564 N   LYS B 184   -51.110 22.159 64.718 1.00 0.00
B   N
ATOM 19565 HN LYS B 184   -50.822 21.596 65.490 1.00 0.00
B   H
ATOM 19566 CA LYS B 184   -50.732 21.646 63.403 1.00 0.00
B   C
ATOM 19567 HA LYS B 184   -51.138 22.333 62.670 1.00 0.00
B   H
ATOM 19568 CB LYS B 184   -49.209 21.589 63.248 1.00 0.00
B   C
ATOM 19569 HB1 LYS B 184   -48.818 22.610 63.467 1.00 0.00
B   H
ATOM 19570 HB2 LYS B 184   -48.790 20.919 64.032 1.00 0.00
B   H
ATOM 19571 CG LYS B 184   -48.753 21.161 61.860 1.00 0.00
B   C
ATOM 19572 HG1 LYS B 184   -49.280 20.221 61.578 1.00 0.00
B   H
ATOM 19573 HG2 LYS B 184   -49.051 21.943 61.124 1.00 0.00
B   H
ATOM 19574 CD LYS B 184   -47.256 20.922 61.793 1.00 0.00
B   C

ATOM 19575 HD1 LYS B 184 -46.737 21.894 61.963 1.00 0.00
B H
ATOM 19576 HD2 LYS B 184 -46.974 20.243 62.631 1.00 0.00
B H
ATOM 19577 CE LYS B 184 -46.879 20.320 60.451 1.00 0.00
B C
ATOM 19578 HE1 LYS B 184 -47.000 19.213 60.474 1.00 0.00
B H
ATOM 19579 HE2 LYS B 184 -47.526 20.728 59.643 1.00 0.00
B H
ATOM 19580 NZ LYS B 184 -45.468 20.608 60.085 1.00 0.00
B N
ATOM 19581 HZ1 LYS B 184 -45.245 20.181 59.164 1.00 0.00
B H
ATOM 19582 HZ2 LYS B 184 -45.327 21.637 60.032 1.00 0.00
B H
ATOM 19583 HZ3 LYS B 184 -44.836 20.214 60.811 1.00 0.00
B H
ATOM 19584 C LYS B 184 -51.345 20.274 63.132 1.00 0.00
B C
ATOM 19585 O LYS B 184 -50.937 19.281 63.730 1.00 0.00
B O
ATOM 19586 N HSE B 185 -52.316 20.234 62.222 1.00 0.00
B N
ATOM 19587 HN HSE B 185 -52.733 21.052 61.829 1.00 0.00
B H
ATOM 19588 CA HSE B 185 -52.863 18.974 61.714 1.00 0.00
B C
ATOM 19589 HA HSE B 185 -52.317 18.172 62.192 1.00 0.00
B H
ATOM 19590 CB HSE B 185 -54.360 18.872 62.031 1.00 0.00
B C
ATOM 19591 HB1 HSE B 185 -54.484 18.990 63.130 1.00 0.00
B H
ATOM 19592 HB2 HSE B 185 -54.899 19.715 61.552 1.00 0.00
B H
ATOM 19593 ND1 HSE B 185 -54.878 16.445 62.389 1.00 0.00
B N
ATOM 19594 CG HSE B 185 -54.980 17.554 61.629 1.00 0.00
B C
ATOM 19595 CE1 HSE B 185 -55.515 15.425 61.781 1.00 0.00
B C
ATOM 19596 HE1 HSE B 185 -55.584 14.410 62.181 1.00 0.00
B H
ATOM 19597 NE2 HSE B 185 -56.027 15.880 60.622 1.00 0.00
B N
ATOM 19598 HE2 HSE B 185 -56.533 15.333 59.956 1.00 0.00
B H
ATOM 19599 CD2 HSE B 185 -55.717 17.188 60.498 1.00 0.00
B C
ATOM 19600 HD2 HSE B 185 -56.013 17.780 59.643 1.00 0.00
B H
ATOM 19601 C HSE B 185 -52.623 18.848 60.222 1.00 0.00
B C

ATOM 19602 O HSE B 185 -53.084 19.687 59.463 1.00 0.00
B O
ATOM 19603 N HSE B 186 -51.901 17.808 59.780 1.00 0.00
B N
ATOM 19604 HN HSE B 186 -51.466 17.166 60.410 1.00 0.00
B H
ATOM 19605 CA HSE B 186 -51.675 17.501 58.333 1.00 0.00
B C
ATOM 19606 HA HSE B 186 -51.163 16.546 58.330 1.00 0.00
B H
ATOM 19607 CB HSE B 186 -53.017 17.259 57.595 1.00 0.00
B C
ATOM 19608 HB1 HSE B 186 -53.537 16.415 58.098 1.00 0.00
B H
ATOM 19609 HB2 HSE B 186 -53.666 18.152 57.706 1.00 0.00
B H
ATOM 19610 ND1 HSE B 186 -52.345 15.733 55.691 1.00 0.00
B N
ATOM 19611 CG HSE B 186 -52.884 16.896 56.112 1.00 0.00
B C
ATOM 19612 CE1 HSE B 186 -52.373 15.685 54.341 1.00 0.00
B C
ATOM 19613 HE1 HSE B 186 -51.985 14.858 53.742 1.00 0.00
B H
ATOM 19614 NE2 HSE B 186 -52.943 16.816 53.895 1.00 0.00
B N
ATOM 19615 HE2 HSE B 186 -53.077 17.057 52.934 1.00 0.00
B H
ATOM 19616 CD2 HSE B 186 -53.278 17.583 54.958 1.00 0.00
B C
ATOM 19617 HD2 HSE B 186 -53.753 18.548 54.855 1.00 0.00
B H
ATOM 19618 C HSE B 186 -50.794 18.483 57.573 1.00 0.00
B C
ATOM 19619 O HSE B 186 -50.865 18.563 56.344 1.00 0.00
B O
ATOM 19620 N GLY B 187 -49.935 19.225 58.270 1.00 0.00
B N
ATOM 19621 HN GLY B 187 -49.757 19.117 59.248 1.00 0.00
B H
ATOM 19622 CA GLY B 187 -49.175 20.288 57.601 1.00 0.00
B C
ATOM 19623 HA1 GLY B 187 -48.722 19.880 56.708 1.00 0.00
B H
ATOM 19624 HA2 GLY B 187 -48.471 20.712 58.303 1.00 0.00
B H
ATOM 19625 C GLY B 187 -50.134 21.384 57.181 1.00 0.00
B C
ATOM 19626 O GLY B 187 -49.876 22.149 56.246 1.00 0.00
B O
ATOM 19627 N ASP B 188 -51.264 21.411 57.875 1.00 0.00
B N
ATOM 19628 HN ASP B 188 -51.506 20.712 58.542 1.00 0.00
B H

ATOM 19629 CA ASP B 188   -52.267 22.442 57.750 1.00 0.00
B   C
ATOM 19630 HA ASP B 188   -51.883 23.264 57.158 1.00 0.00
B   H
ATOM 19631 CB ASP B 188   -53.574 21.847 57.207 1.00 0.00
B   C
ATOM 19632 HB1 ASP B 188   -53.381 21.380 56.220 1.00 0.00
B   H
ATOM 19633 HB2 ASP B 188   -53.962 21.067 57.892 1.00 0.00
B   H
ATOM 19634 CG ASP B 188   -54.658 22.888 57.007 1.00 0.00
B   C
ATOM 19635 OD1 ASP B 188   -54.476 23.796 56.167 1.00 0.00
B   O
ATOM 19636 OD2 ASP B 188   -55.706 22.791 57.679 1.00 0.00
B   O
ATOM 19637 C   ASP B 188   -52.440 22.965 59.166 1.00 0.00
B   C
ATOM 19638 O   ASP B 188   -52.777 22.211 60.083 1.00 0.00
B   O
ATOM 19639 N   ILE B 189   -52.177 24.250 59.358 1.00 0.00
B   N
ATOM 19640 HN ILE B 189   -51.971 24.912 58.640 1.00 0.00
B   H
ATOM 19641 CA ILE B 189   -52.170 24.810 60.705 1.00 0.00
B   C
ATOM 19642 HA ILE B 189   -52.114 23.956 61.367 1.00 0.00
B   H
ATOM 19643 CB ILE B 189   -50.911 25.673 60.983 1.00 0.00
B   C
ATOM 19644 HB ILE B 189   -51.141 26.205 61.941 1.00 0.00
B   H
ATOM 19645 CG2 ILE B 189   -49.671 24.790 61.068 1.00 0.00
B   C
ATOM 19646 HG21 ILE B 189   -48.796 25.376 61.419 1.00 0.00
B   H
ATOM 19647 HG22 ILE B 189   -49.826 23.956 61.784 1.00 0.00
B   H
ATOM 19648 HG23 ILE B 189   -49.422 24.357 60.075 1.00 0.00
B   H
ATOM 19649 CG1 ILE B 189   -50.732 26.761 59.913 1.00 0.00
B   C
ATOM 19650 HG11 ILE B 189   -50.319 26.311 58.983 1.00 0.00
B   H
ATOM 19651 HG12 ILE B 189   -51.731 27.185 59.659 1.00 0.00
B   H
ATOM 19652 CD ILE B 189   -49.833 27.915 60.332 1.00 0.00
B   C
ATOM 19653 HD1 ILE B 189   -49.799 28.687 59.532 1.00 0.00
B   H
ATOM 19654 HD2 ILE B 189   -50.215 28.392 61.260 1.00 0.00
B   H
ATOM 19655 HD3 ILE B 189   -48.796 27.565 60.516 1.00 0.00
B   H

ATOM 19656 C ILE B 189 -53.448 25.565 61.065 1.00 0.00
B C
ATOM 19657 O ILE B 189 -54.097 26.177 60.211 1.00 0.00
B O
ATOM 19658 N CYS B 190 -53.810 25.469 62.341 1.00 0.00
B N
ATOM 19659 HN CYS B 190 -53.363 24.827 62.961 1.00 0.00
B H
ATOM 19660 CA CYS B 190 -54.862 26.266 62.944 1.00 0.00
B C
ATOM 19661 HA CYS B 190 -55.456 26.728 62.164 1.00 0.00
B H
ATOM 19662 CB CYS B 190 -55.673 25.392 63.909 1.00 0.00
B C
ATOM 19663 HB1 CYS B 190 -55.999 24.490 63.343 1.00 0.00
B H
ATOM 19664 HB2 CYS B 190 -55.015 25.021 64.726 1.00 0.00
B H
ATOM 19665 SG CYS B 190 -57.158 26.143 64.615 1.00 0.00
B S
ATOM 19666 HG1 CYS B 190 -57.574 25.076 65.298 1.00 0.00
B H
ATOM 19667 C CYS B 190 -54.125 27.376 63.685 1.00 0.00
B C
ATOM 19668 O CYS B 190 -53.646 27.164 64.800 1.00 0.00
B O
ATOM 19669 N LEU B 191 -54.006 28.544 63.049 1.00 0.00
B N
ATOM 19670 HN LEU B 191 -54.495 28.772 62.209 1.00 0.00
B H
ATOM 19671 CA LEU B 191 -53.126 29.611 63.545 1.00 0.00
B C
ATOM 19672 HA LEU B 191 -52.143 29.162 63.606 1.00 0.00
B H
ATOM 19673 CB LEU B 191 -53.011 30.773 62.546 1.00 0.00
B C
ATOM 19674 HB1 LEU B 191 -52.685 30.339 61.573 1.00 0.00
B H
ATOM 19675 HB2 LEU B 191 -54.023 31.200 62.369 1.00 0.00
B H
ATOM 19676 CG LEU B 191 -52.023 31.883 62.944 1.00 0.00
B C
ATOM 19677 HG LEU B 191 -52.222 32.087 64.024 1.00 0.00
B H
ATOM 19678 CD1 LEU B 191 -50.571 31.445 62.767 1.00 0.00
B C
ATOM 19679 HD11 LEU B 191 -49.879 32.279 63.015 1.00 0.00
B H
ATOM 19680 HD12 LEU B 191 -50.336 30.588 63.433 1.00 0.00
B H
ATOM 19681 HD13 LEU B 191 -50.379 31.137 61.717 1.00 0.00
B H
ATOM 19682 CD2 LEU B 191 -52.304 33.159 62.164 1.00 0.00
B C

ATOM 19683 HD21 LEU B 191 -51.600 33.962 62.470 1.00 0.00
B H
ATOM 19684 HD22 LEU B 191 -52.187 33.006 61.073 1.00 0.00
B H
ATOM 19685 HD23 LEU B 191 -53.340 33.514 62.358 1.00 0.00
B H
ATOM 19686 C LEU B 191 -53.483 30.131 64.933 1.00 0.00
B C
ATOM 19687 O LEU B 191 -52.638 30.127 65.818 1.00 0.00
B O
ATOM 19688 N SER B 192 -54.718 30.587 65.125 1.00 0.00
B N
ATOM 19689 HN SER B 192 -55.416 30.647 64.412 1.00 0.00
B H
ATOM 19690 CA SER B 192 -55.162 31.049 66.439 1.00 0.00
B C
ATOM 19691 HA SER B 192 -54.419 30.726 67.156 1.00 0.00
B H
ATOM 19692 CB SER B 192 -55.317 32.572 66.456 1.00 0.00
B C
ATOM 19693 HB1 SER B 192 -56.129 32.846 65.743 1.00 0.00
B H
ATOM 19694 HB2 SER B 192 -55.638 32.917 67.465 1.00 0.00
B H
ATOM 19695 OG SER B 192 -54.117 33.210 66.052 1.00 0.00
B O
ATOM 19696 HG1 SER B 192 -54.334 34.132 65.867 1.00 0.00
B H
ATOM 19697 C SER B 192 -56.470 30.350 66.796 1.00 0.00
B C
ATOM 19698 O SER B 192 -57.550 30.815 66.415 1.00 0.00
B O
ATOM 19699 N PRO B 193 -56.376 29.230 67.537 1.00 0.00
B N
ATOM 19700 CD PRO B 193 -55.230 28.831 68.372 1.00 0.00
B C
ATOM 19701 HD1 PRO B 193 -55.096 29.070 67.292 1.00 0.00
B H
ATOM 19702 HD2 PRO B 193 -54.341 29.158 68.957 1.00 0.00
B H
ATOM 19703 CA PRO B 193 -57.501 28.304 67.686 1.00 0.00
B C
ATOM 19704 HA PRO B 193 -57.786 27.932 66.709 1.00 0.00
B H
ATOM 19705 CB PRO B 193 -56.927 27.170 68.550 1.00 0.00
B C
ATOM 19706 HB1 PRO B 193 -56.408 26.439 67.891 1.00 0.00
B H
ATOM 19707 HB2 PRO B 193 -57.706 26.638 69.132 1.00 0.00
B H
ATOM 19708 CG PRO B 193 -55.447 27.360 68.548 1.00 0.00
B C
ATOM 19709 HG1 PRO B 193 -54.892 26.427 68.769 1.00 0.00
B H

ATOM 19710 HG2 PRO B 193 -55.176 28.139 69.297 1.00 0.00
B H
ATOM 19711 C PRO B 193 -58.720 28.908 68.364 1.00 0.00
B C
ATOM 19712 O PRO B 193 -58.591 29.682 69.313 1.00 0.00
B O
ATOM 19713 N ALA B 194 -59.895 28.554 67.856 1.00 0.00
B N
ATOM 19714 HN ALA B 194 -59.976 28.065 66.988 1.00 0.00
B H
ATOM 19715 CA ALA B 194 -61.153 28.852 68.524 1.00 0.00
B C
ATOM 19716 HA ALA B 194 -61.179 29.865 68.909 1.00 0.00
B H
ATOM 19717 CB ALA B 194 -62.320 28.648 67.568 1.00 0.00
B C
ATOM 19718 HB1 ALA B 194 -62.237 29.363 66.721 1.00 0.00
B H
ATOM 19719 HB2 ALA B 194 -62.317 27.618 67.148 1.00 0.00
B H
ATOM 19720 HB3 ALA B 194 -63.296 28.824 68.070 1.00 0.00
B H
ATOM 19721 C ALA B 194 -61.276 27.928 69.736 1.00 0.00
B C
ATOM 19722 O ALA B 194 -60.667 26.855 69.755 1.00 0.00
B O
ATOM 19723 N PRO B 195 -62.058 28.339 70.754 1.00 0.00
B N
ATOM 19724 CD PRO B 195 -62.747 29.641 70.866 1.00 0.00
B C
ATOM 19725 HD1 PRO B 195 -61.641 29.645 70.744 1.00 0.00
B H
ATOM 19726 HD2 PRO B 195 -63.205 30.493 70.313 1.00 0.00
B H
ATOM 19727 CA PRO B 195 -62.212 27.542 71.978 1.00 0.00
B C
ATOM 19728 HA PRO B 195 -61.265 27.541 72.507 1.00 0.00
B H
ATOM 19729 CB PRO B 195 -63.268 28.315 72.770 1.00 0.00
B C
ATOM 19730 HB1 PRO B 195 -62.768 29.122 73.350 1.00 0.00
B H
ATOM 19731 HB2 PRO B 195 -63.837 27.670 73.469 1.00 0.00
B H
ATOM 19732 CG PRO B 195 -63.103 29.724 72.322 1.00 0.00
B C
ATOM 19733 HG1 PRO B 195 -63.482 30.459 73.059 1.00 0.00
B H
ATOM 19734 HG2 PRO B 195 -62.566 30.567 72.331 1.00 0.00
B H
ATOM 19735 C PRO B 195 -62.672 26.093 71.772 1.00 0.00
B C
ATOM 19736 O PRO B 195 -62.585 25.297 72.711 1.00 0.00
B O

```
ATOM  19737  N   GLU B 196     -63.152  25.748  70.572  1.00  0.00
B    N
ATOM  19738  HN  GLU B 196     -63.219  26.345  69.778  1.00  0.00
B    H
ATOM  19739  CA  GLU B 196     -63.641  24.379  70.335  1.00  0.00
B    C
ATOM  19740  HA  GLU B 196     -63.636  23.888  71.301  1.00  0.00
B    H
ATOM  19741  CB  GLU B 196     -65.067  24.358  69.781  1.00  0.00
B    C
ATOM  19742  HB1 GLU B 196     -65.031  24.692  68.718  1.00  0.00
B    H
ATOM  19743  HB2 GLU B 196     -65.448  23.312  69.760  1.00  0.00
B    H
ATOM  19744  CG  GLU B 196     -66.084  25.209  70.481  1.00  0.00
B    C
ATOM  19745  HG1 GLU B 196     -67.097  24.813  70.282  1.00  0.00
B    H
ATOM  19746  HG2 GLU B 196     -65.907  25.223  71.575  1.00  0.00
B    H
ATOM  19747  CD  GLU B 196     -66.133  26.648  70.011  1.00  0.00
B    C
ATOM  19748  OE1 GLU B 196     -66.001  26.932  68.801  1.00  0.00
B    O
ATOM  19749  OE2 GLU B 196     -66.346  27.495  70.899  1.00  0.00
B    O
ATOM  19750  C   GLU B 196     -62.797  23.529  69.391  1.00  0.00
B    C
ATOM  19751  O   GLU B 196     -63.232  22.445  68.997  1.00  0.00
B    O
ATOM  19752  N   ASP B 197     -61.614  24.004  69.014  1.00  0.00
B    N
ATOM  19753  HN  ASP B 197     -61.171  24.834  69.346  1.00  0.00
B    H
ATOM  19754  CA  ASP B 197     -60.817  23.295  68.011  1.00  0.00
B    C
ATOM  19755  HA  ASP B 197     -61.521  22.955  67.258  1.00  0.00
B    H
ATOM  19756  CB  ASP B 197     -59.841  24.241  67.305  1.00  0.00
B    C
ATOM  19757  HB1 ASP B 197     -59.382  24.918  68.054  1.00  0.00
B    H
ATOM  19758  HB2 ASP B 197     -59.031  23.669  66.810  1.00  0.00
B    H
ATOM  19759  CG  ASP B 197     -60.525  25.111  66.264  1.00  0.00
B    C
ATOM  19760  OD1 ASP B 197     -61.668  24.796  65.864  1.00  0.00
B    O
ATOM  19761  OD2 ASP B 197     -59.919  26.113  65.837  1.00  0.00
B    O
ATOM  19762  C   ASP B 197     -60.114  22.049  68.551  1.00  0.00
B    C
ATOM  19763  O   ASP B 197     -59.382  22.108  69.540  1.00  0.00
B    O
```

ATOM 19764 N ILE B 198 -60.358 20.926 67.877 1.00 0.00
B N
ATOM 19765 HN ILE B 198 -60.888 20.920 67.033 1.00 0.00
B H
ATOM 19766 CA ILE B 198 -59.883 19.606 68.297 1.00 0.00
B C
ATOM 19767 HA ILE B 198 -60.068 19.546 69.362 1.00 0.00
B H
ATOM 19768 CB ILE B 198 -60.637 18.474 67.545 1.00 0.00
B C
ATOM 19769 HB ILE B 198 -60.485 18.710 66.461 1.00 0.00
B H
ATOM 19770 CG2 ILE B 198 -60.106 17.098 67.935 1.00 0.00
B C
ATOM 19771 HG21 ILE B 198 -60.540 16.312 67.282 1.00 0.00
B H
ATOM 19772 HG22 ILE B 198 -59.002 17.054 67.827 1.00 0.00
B H
ATOM 19773 HG23 ILE B 198 -60.366 16.857 68.988 1.00 0.00
B H
ATOM 19774 CG1 ILE B 198 -62.148 18.558 67.809 1.00 0.00
B C
ATOM 19775 HG11 ILE B 198 -62.381 18.117 68.804 1.00 0.00
B H
ATOM 19776 HG12 ILE B 198 -62.450 19.630 67.844 1.00 0.00
B H
ATOM 19777 CD ILE B 198 -63.008 17.871 66.765 1.00 0.00
B C
ATOM 19778 HD1 ILE B 198 -64.085 18.023 66.992 1.00 0.00
B H
ATOM 19779 HD2 ILE B 198 -62.802 18.285 65.754 1.00 0.00
B H
ATOM 19780 HD3 ILE B 198 -62.812 16.778 66.744 1.00 0.00
B H
ATOM 19781 C ILE B 198 -58.372 19.456 68.102 1.00 0.00
B C
ATOM 19782 O ILE B 198 -57.838 19.772 67.034 1.00 0.00
B O
ATOM 19783 N ILE B 199 -57.697 18.992 69.152 1.00 0.00
B N
ATOM 19784 HN ILE B 199 -58.100 18.872 70.056 1.00 0.00
B H
ATOM 19785 CA ILE B 199 -56.291 18.613 69.069 1.00 0.00
B C
ATOM 19786 HA ILE B 199 -55.916 18.973 68.120 1.00 0.00
B H
ATOM 19787 CB ILE B 199 -55.458 19.176 70.243 1.00 0.00
B C
ATOM 19788 HB ILE B 199 -56.104 19.021 71.144 1.00 0.00
B H
ATOM 19789 CG2 ILE B 199 -54.092 18.511 70.288 1.00 0.00
B C
ATOM 19790 HG21 ILE B 199 -53.549 18.793 71.215 1.00 0.00
B H

```
ATOM  19791 HG22 ILE B 199     -54.189  17.405  70.280  1.00  0.00
B    H
ATOM  19792 HG23 ILE B 199     -53.472  18.815  69.418  1.00  0.00
B    H
ATOM  19793  CG1 ILE B 199     -55.268  20.683  70.090  1.00  0.00
B    C
ATOM  19794 HG11 ILE B 199     -54.470  20.888  69.341  1.00  0.00
B    H
ATOM  19795 HG12 ILE B 199     -56.210  21.128  69.696  1.00  0.00
B    H
ATOM  19796  CD  ILE B 199     -54.921  21.396  71.379  1.00  0.00
B    C
ATOM  19797  HD1 ILE B 199     -54.853  22.492  71.208  1.00  0.00
B    H
ATOM  19798  HD2 ILE B 199     -55.698  21.211  72.151  1.00  0.00
B    H
ATOM  19799  HD3 ILE B 199     -53.942  21.050  71.775  1.00  0.00
B    H
ATOM  19800  C   ILE B 199     -56.185  17.092  69.036  1.00  0.00
B    C
ATOM  19801  O   ILE B 199     -56.631  16.407  69.959  1.00  0.00
B    O
ATOM  19802  N   ARG B 200     -55.603  16.581  67.955  1.00  0.00
B    N
ATOM  19803  HN  ARG B 200     -55.298  17.168  67.208  1.00  0.00
B    H
ATOM  19804  CA  ARG B 200     -55.379  15.156  67.797  1.00  0.00
B    C
ATOM  19805  HA  ARG B 200     -55.994  14.615  68.506  1.00  0.00
B    H
ATOM  19806  CB  ARG B 200     -55.721  14.728  66.367  1.00  0.00
B    C
ATOM  19807  HB1 ARG B 200     -56.229  15.599  65.890  1.00  0.00
B    H
ATOM  19808  HB2 ARG B 200     -54.782  14.568  65.791  1.00  0.00
B    H
ATOM  19809  CG  ARG B 200     -56.639  13.516  66.279  1.00  0.00
B    C
ATOM  19810  HG1 ARG B 200     -56.052  12.607  66.534  1.00  0.00
B    H
ATOM  19811  HG2 ARG B 200     -57.422  13.596  67.068  1.00  0.00
B    H
ATOM  19812  CD  ARG B 200     -57.314  13.430  64.916  1.00  0.00
B    C
ATOM  19813  HD1 ARG B 200     -56.558  13.601  64.117  1.00  0.00
B    H
ATOM  19814  HD2 ARG B 200     -57.784  12.431  64.755  1.00  0.00
B    H
ATOM  19815  NE  ARG B 200     -58.387  14.414  64.765  1.00  0.00
B    N
ATOM  19816  HE  ARG B 200     -58.614  14.887  65.623  1.00  0.00
B    H
ATOM  19817  CZ  ARG B 200     -59.016  14.681  63.621  1.00  0.00
B    C
```

ATOM 19818 NH1 ARG B 200 −58.689 14.042 62.501 1.00 0.00
B N
ATOM 19819 HH11 ARG B 200 −59.141 14.259 61.647 1.00 0.00
B H
ATOM 19820 HH12 ARG B 200 −57.885 13.446 62.548 1.00 0.00
B H
ATOM 19821 NH2 ARG B 200 −59.978 15.592 63.594 1.00 0.00
B N
ATOM 19822 HH21 ARG B 200 −60.505 15.729 62.768 1.00 0.00
B H
ATOM 19823 HH22 ARG B 200 −60.274 15.958 64.467 1.00 0.00
B H
ATOM 19824 C ARG B 200 −53.922 14.839 68.150 1.00 0.00
B C
ATOM 19825 O ARG B 200 −53.087 15.736 68.227 1.00 0.00
B O
ATOM 19826 N GLU B 201 −53.643 13.563 68.391 1.00 0.00
B N
ATOM 19827 HN GLU B 201 −54.339 12.854 68.319 1.00 0.00
B H
ATOM 19828 CA GLU B 201 −52.322 13.062 68.779 1.00 0.00
B C
ATOM 19829 HA GLU B 201 −52.004 13.567 69.684 1.00 0.00
B H
ATOM 19830 CB GLU B 201 −52.436 11.553 68.827 1.00 0.00
B C
ATOM 19831 HB1 GLU B 201 −53.477 11.293 69.127 1.00 0.00
B H
ATOM 19832 HB2 GLU B 201 −52.299 11.131 67.806 1.00 0.00
B H
ATOM 19833 CG GLU B 201 −51.495 10.851 69.770 1.00 0.00
B C
ATOM 19834 HG1 GLU B 201 −50.452 11.094 69.499 1.00 0.00
B H
ATOM 19835 HG2 GLU B 201 −51.681 11.158 70.819 1.00 0.00
B H
ATOM 19836 CD GLU B 201 −51.634 9.353 69.651 1.00 0.00
B C
ATOM 19837 OE1 GLU B 201 −52.190 8.883 68.633 1.00 0.00
B O
ATOM 19838 OE2 GLU B 201 −51.193 8.641 70.568 1.00 0.00
B O
ATOM 19839 C GLU B 201 −51.318 13.407 67.694 1.00 0.00
B C
ATOM 19840 O GLU B 201 −51.605 13.191 66.588 1.00 0.00
B O
ATOM 19841 N GLN B 202 −50.136 13.920 67.962 1.00 0.00
B N
ATOM 19842 HN GLN B 202 −49.782 14.142 68.869 1.00 0.00
B H
ATOM 19843 CA GLN B 202 −49.209 14.215 66.858 1.00 0.00
B C
ATOM 19844 HA GLN B 202 −48.238 14.389 67.303 1.00 0.00
B H

ATOM 19845 CB GLN B 202   -49.134 13.020 65.911 1.00 0.00
B   C
ATOM 19846 HB1 GLN B 202   -48.885 12.129 66.534 1.00 0.00
B   H
ATOM 19847 HB2 GLN B 202   -50.143 12.825 65.481 1.00 0.00
B   H
ATOM 19848 CG GLN B 202   -48.132 13.125 64.816 1.00 0.00
B   C
ATOM 19849 HG1 GLN B 202   -48.372 13.985 64.153 1.00 0.00
B   H
ATOM 19850 HG2 GLN B 202   -47.115 13.286 65.228 1.00 0.00
B   H
ATOM 19851 CD GLN B 202   -48.101 11.902 63.926 1.00 0.00
B   C
ATOM 19852 OE1 GLN B 202   -48.682 10.894 64.214 1.00 0.00
B   O
ATOM 19853 NE2 GLN B 202   -47.427 12.001 62.842 1.00 0.00
B   N
ATOM 19854 HE21 GLN B 202   -47.407 11.200 62.250 1.00 0.00
B   H
ATOM 19855 HE22 GLN B 202   -46.943 12.845 62.635 1.00 0.00
B   H
ATOM 19856 C   GLN B 202   -49.608 15.490 66.108 1.00 0.00
B   C
ATOM 19857 O   GLN B 202   -49.214 15.720 64.956 1.00 0.00
B   O
ATOM 19858 N   ASP B 203   -50.414 16.310 66.770 1.00 0.00
B   N
ATOM 19859 HN ASP B 203   -50.943 16.090 67.587 1.00 0.00
B   H
ATOM 19860 CA ASP B 203   -50.599 17.674 66.330 1.00 0.00
B   C
ATOM 19861 HA ASP B 203   -50.385 17.742 65.269 1.00 0.00
B   H
ATOM 19862 CB ASP B 203   -52.007 18.185 66.651 1.00 0.00
B   C
ATOM 19863 HB1 ASP B 203   -52.314 17.808 67.647 1.00 0.00
B   H
ATOM 19864 HB2 ASP B 203   -52.024 19.293 66.678 1.00 0.00
B   H
ATOM 19865 CG ASP B 203   -53.055 17.712 65.644 1.00 0.00
B   C
ATOM 19866 OD1 ASP B 203   -52.688 17.198 64.565 1.00 0.00
B   O
ATOM 19867 OD2 ASP B 203   -54.259 17.859 65.925 1.00 0.00
B   O
ATOM 19868 C   ASP B 203   -49.531 18.481 67.044 1.00 0.00
B   C
ATOM 19869 O   ASP B 203   -49.105 18.121 68.146 1.00 0.00
B   O
ATOM 19870 N   CYS B 204   -49.065 19.548 66.405 1.00 0.00
B   N
ATOM 19871 HN CYS B 204   -49.353 19.838 65.495 1.00 0.00
B   H

ATOM 19872 CA CYS B 204 -48.057 20.395 67.025 1.00 0.00
B C
ATOM 19873 HA CYS B 204 -47.511 19.762 67.714 1.00 0.00
B H
ATOM 19874 CB CYS B 204 -47.077 20.944 65.989 1.00 0.00
B C
ATOM 19875 HB1 CYS B 204 -47.669 21.210 65.083 1.00 0.00
B H
ATOM 19876 HB2 CYS B 204 -46.634 21.895 66.356 1.00 0.00
B H
ATOM 19877 SG CYS B 204 -45.795 19.768 65.509 1.00 0.00
B S
ATOM 19878 HG1 CYS B 204 -45.205 20.533 64.588 1.00 0.00
B H
ATOM 19879 C CYS B 204 -48.688 21.522 67.834 1.00 0.00
B C
ATOM 19880 O CYS B 204 -49.324 22.423 67.281 1.00 0.00
B O
ATOM 19881 N LEU B 205 -48.520 21.451 69.152 1.00 0.00
B N
ATOM 19882 HN LEU B 205 -48.094 20.675 69.612 1.00 0.00
B H
ATOM 19883 CA LEU B 205 -48.950 22.513 70.047 1.00 0.00
B C
ATOM 19884 HA LEU B 205 -49.840 22.958 69.623 1.00 0.00
B H
ATOM 19885 CB LEU B 205 -49.188 21.972 71.456 1.00 0.00
B C
ATOM 19886 HB1 LEU B 205 -48.259 21.441 71.766 1.00 0.00
B H
ATOM 19887 HB2 LEU B 205 -49.301 22.829 72.158 1.00 0.00
B H
ATOM 19888 CG LEU B 205 -50.356 21.009 71.642 1.00 0.00
B C
ATOM 19889 HG LEU B 205 -50.248 20.163 70.921 1.00 0.00
B H
ATOM 19890 CD1 LEU B 205 -50.354 20.475 73.059 1.00 0.00
B C
ATOM 19891 HD11 LEU B 205 -51.227 19.808 73.226 1.00 0.00
B H
ATOM 19892 HD12 LEU B 205 -49.427 19.893 73.256 1.00 0.00
B H
ATOM 19893 HD13 LEU B 205 -50.408 21.308 73.792 1.00 0.00
B H
ATOM 19894 CD2 LEU B 205 -51.664 21.714 71.345 1.00 0.00
B C
ATOM 19895 HD21 LEU B 205 -52.517 21.010 71.461 1.00 0.00
B H
ATOM 19896 HD22 LEU B 205 -51.835 22.566 72.033 1.00 0.00
B H
ATOM 19897 HD23 LEU B 205 -51.670 22.099 70.302 1.00 0.00
B H
ATOM 19898 C LEU B 205 -47.884 23.584 70.097 1.00 0.00
B C

ATOM 19899 O LEU B 205 −46.705 23.289 70.275 1.00 0.00
B O
ATOM 19900 N VAL B 206 −48.295 24.831 69.909 1.00 0.00
B N
ATOM 19901 HN VAL B 206 −49.216 25.082 69.621 1.00 0.00
B H
ATOM 19902 CA VAL B 206 −47.399 25.961 70.118 1.00 0.00
B C
ATOM 19903 HA VAL B 206 −46.411 25.592 70.360 1.00 0.00
B H
ATOM 19904 CB VAL B 206 −47.321 26.906 68.900 1.00 0.00
B C
ATOM 19905 HB VAL B 206 −48.327 27.376 68.772 1.00 0.00
B H
ATOM 19906 CG1 VAL B 206 −46.281 27.990 69.152 1.00 0.00
B C
ATOM 19907 HG11 VAL B 206 −46.190 28.652 68.263 1.00 0.00
B H
ATOM 19908 HG12 VAL B 206 −46.557 28.631 70.014 1.00 0.00
B H
ATOM 19909 HG13 VAL B 206 −45.284 27.540 69.347 1.00 0.00
B H
ATOM 19910 CG2 VAL B 206 −46.995 26.132 67.630 1.00 0.00
B C
ATOM 19911 HG21 VAL B 206 −46.900 26.821 66.763 1.00 0.00
B H
ATOM 19912 HG22 VAL B 206 −46.037 25.580 67.744 1.00 0.00
B H
ATOM 19913 HG23 VAL B 206 −47.793 25.399 67.389 1.00 0.00
B H
ATOM 19914 C VAL B 206 −47.913 26.718 71.330 1.00 0.00
B C
ATOM 19915 O VAL B 206 −49.019 27.262 71.309 1.00 0.00
B O
ATOM 19916 N ILE B 207 −47.097 26.749 72.379 1.00 0.00
B N
ATOM 19917 HN ILE B 207 −46.147 26.447 72.344 1.00 0.00
B H
ATOM 19918 CA ILE B 207 −47.524 27.228 73.685 1.00 0.00
B C
ATOM 19919 HA ILE B 207 −48.515 27.650 73.574 1.00 0.00
B H
ATOM 19920 CB ILE B 207 −47.553 26.016 74.645 1.00 0.00
B C
ATOM 19921 HB ILE B 207 −47.506 25.124 73.970 1.00 0.00
B H
ATOM 19922 CG2 ILE B 207 −46.503 26.210 75.820 1.00 0.00
B C
ATOM 19923 HG21 ILE B 207 −46.410 25.279 76.418 1.00 0.00
B H
ATOM 19924 HG22 ILE B 207 −45.498 26.452 75.416 1.00 0.00
B H
ATOM 19925 HG23 ILE B 207 −46.814 27.030 76.500 1.00 0.00
B H

ATOM 19926 CG1 ILE B 207   -48.940 25.932 75.414 1.00 0.00
B   C
ATOM 19927 HG11 ILE B 207   -49.093 26.853 76.020 1.00 0.00
B   H
ATOM 19928 HG12 ILE B 207   -49.765 25.890 74.667 1.00 0.00
B   H
ATOM 19929 CD ILE B 207   -48.850 24.659 76.253 1.00 0.00
B   C
ATOM 19930 HD1 ILE B 207   -49.807 24.481 76.789 1.00 0.00
B   H
ATOM 19931 HD2 ILE B 207   -48.644 23.778 75.608 1.00 0.00
B   H
ATOM 19932 HD3 ILE B 207   -48.042 24.738 77.010 1.00 0.00
B   H
ATOM 19933 C   ILE B 207   -46.594 28.382 74.094 1.00 0.00
B   C
ATOM 19934 O   ILE B 207   -45.429 28.424 73.680 1.00 0.00
B   O
ATOM 19935 N   MET B 208   -47.114 29.327 74.872 1.00 0.00
B   N
ATOM 19936 HN MET B 208   -48.031 29.284 75.261 1.00 0.00
B   H
ATOM 19937 CA MET B 208   -46.360 30.534 75.227 1.00 0.00
B   C
ATOM 19938 HA MET B 208   -45.308 30.280 75.175 1.00 0.00
B   H
ATOM 19939 CB MET B 208   -46.650 31.688 74.261 1.00 0.00
B   C
ATOM 19940 HB1 MET B 208   -46.519 31.287 73.229 1.00 0.00
B   H
ATOM 19941 HB2 MET B 208   -47.721 31.981 74.347 1.00 0.00
B   H
ATOM 19942 CG MET B 208   -45.756 32.910 74.420 1.00 0.00
B   C
ATOM 19943 HG1 MET B 208   -45.631 33.159 75.495 1.00 0.00
B   H
ATOM 19944 HG2 MET B 208   -44.748 32.691 74.007 1.00 0.00
B   H
ATOM 19945 SD MET B 208   -46.456 34.336 73.576 1.00 0.00
B   S
ATOM 19946 CE MET B 208   -45.036 35.406 73.405 1.00 0.00
B   C
ATOM 19947 HE1 MET B 208   -45.314 36.356 72.900 1.00 0.00
B   H
ATOM 19948 HE2 MET B 208   -44.607 35.661 74.398 1.00 0.00
B   H
ATOM 19949 HE3 MET B 208   -44.239 34.920 72.803 1.00 0.00
B   H
ATOM 19950 C   MET B 208   -46.659 30.971 76.656 1.00 0.00
B   C
ATOM 19951 O   MET B 208   -47.786 30.844 77.135 1.00 0.00
B   O
ATOM 19952 N   GLY B 209   -45.637 31.491 77.325 1.00 0.00
B   N

ATOM 19953 HN GLY B 209 −44.717 31.558 76.941 1.00 0.00
B H
ATOM 19954 CA GLY B 209 −45.794 32.005 78.678 1.00 0.00
B C
ATOM 19955 HA1 GLY B 209 −46.694 31.560 79.079 1.00 0.00
B H
ATOM 19956 HA2 GLY B 209 −45.826 33.084 78.606 1.00 0.00
B H
ATOM 19957 C GLY B 209 −44.643 31.633 79.585 1.00 0.00
B C
ATOM 19958 O GLY B 209 −43.489 31.591 79.162 1.00 0.00
B O
ATOM 19959 N HSE B 210 −44.971 31.363 80.842 1.00 0.00
B N
ATOM 19960 HN HSE B 210 −45.911 31.328 81.178 1.00 0.00
B H
ATOM 19961 CA HSE B 210 −43.964 31.089 81.852 1.00 0.00
B C
ATOM 19962 HA HSE B 210 −43.132 31.758 81.666 1.00 0.00
B H
ATOM 19963 CB HSE B 210 −44.542 31.326 83.239 1.00 0.00
B C
ATOM 19964 HB1 HSE B 210 −45.341 32.093 83.149 1.00 0.00
B H
ATOM 19965 HB2 HSE B 210 −45.025 30.394 83.603 1.00 0.00
B H
ATOM 19966 ND1 HSE B 210 −42.993 33.050 84.185 1.00 0.00
B N
ATOM 19967 CG HSE B 210 −43.536 31.821 84.248 1.00 0.00
B C
ATOM 19968 CE1 HSE B 210 −42.136 33.216 85.216 1.00 0.00
B C
ATOM 19969 HE1 HSE B 210 −41.548 34.120 85.395 1.00 0.00
B H
ATOM 19970 NE2 HSE B 210 −42.143 32.089 85.949 1.00 0.00
B N
ATOM 19971 HE2 HSE B 210 −41.589 31.926 86.765 1.00 0.00
B H
ATOM 19972 CD2 HSE B 210 −42.998 31.208 85.384 1.00 0.00
B C
ATOM 19973 HD2 HSE B 210 −43.174 30.221 85.788 1.00 0.00
B H
ATOM 19974 C HSE B 210 −43.445 29.688 81.703 1.00 0.00
B C
ATOM 19975 O HSE B 210 −44.215 28.741 81.543 1.00 0.00
B O
ATOM 19976 N LYS B 211 −42.122 29.568 81.752 1.00 0.00
B N
ATOM 19977 HN LYS B 211 −41.545 30.361 81.941 1.00 0.00
B H
ATOM 19978 CA LYS B 211 −41.401 28.314 81.543 1.00 0.00
B C
ATOM 19979 HA LYS B 211 −41.618 28.005 80.528 1.00 0.00
B H

```
ATOM  19980  CB  LYS B 211     -39.911  28.570  81.756  1.00  0.00
B    C
ATOM  19981  HB1 LYS B 211     -39.732  29.651  81.554  1.00  0.00
B    H
ATOM  19982  HB2 LYS B 211     -39.666  28.407  82.829  1.00  0.00
B    H
ATOM  19983  CG  LYS B 211     -38.987  27.755  80.877  1.00  0.00
B    C
ATOM  19984  HG1 LYS B 211     -39.159  26.672  81.073  1.00  0.00
B    H
ATOM  19985  HG2 LYS B 211     -39.241  27.944  79.809  1.00  0.00
B    H
ATOM  19986  CD  LYS B 211     -37.541  28.116  81.176  1.00  0.00
B    C
ATOM  19987  HD1 LYS B 211     -37.467  29.224  81.270  1.00  0.00
B    H
ATOM  19988  HD2 LYS B 211     -37.271  27.679  82.165  1.00  0.00
B    H
ATOM  19989  CE  LYS B 211     -36.597  27.606  80.099  1.00  0.00
B    C
ATOM  19990  HE1 LYS B 211     -36.681  26.500  79.999  1.00  0.00
B    H
ATOM  19991  HE2 LYS B 211     -36.839  28.068  79.117  1.00  0.00
B    H
ATOM  19992  NZ  LYS B 211     -35.182  27.933  80.428  1.00  0.00
B    N
ATOM  19993  HZ1 LYS B 211     -34.550  27.576  79.682  1.00  0.00
B    H
ATOM  19994  HZ2 LYS B 211     -35.074  28.965  80.507  1.00  0.00
B    H
ATOM  19995  HZ3 LYS B 211     -34.927  27.493  81.335  1.00  0.00
B    H
ATOM  19996  C   LYS B 211     -41.865  27.192  82.471  1.00  0.00
B    C
ATOM  19997  O   LYS B 211     -42.009  26.045  82.048  1.00  0.00
B    O
ATOM  19998  N   LYS B 212     -42.093  27.539  83.735  1.00  0.00
B    N
ATOM  19999  HN  LYS B 212     -42.000  28.483  84.050  1.00  0.00
B    H
ATOM  20000  CA  LYS B 212     -42.495  26.579  84.763  1.00  0.00
B    C
ATOM  20001  HA  LYS B 212     -41.865  25.706  84.646  1.00  0.00
B    H
ATOM  20002  CB  LYS B 212     -42.317  27.190  86.157  1.00  0.00
B    C
ATOM  20003  HB1 LYS B 212     -43.188  27.862  86.334  1.00  0.00
B    H
ATOM  20004  HB2 LYS B 212     -42.386  26.381  86.918  1.00  0.00
B    H
ATOM  20005  CG  LYS B 212     -41.031  27.985  86.324  1.00  0.00
B    C
ATOM  20006  HG1 LYS B 212     -40.197  27.439  85.825  1.00  0.00
B    H
```

ATOM 20007 HG2 LYS B 212 -41.141 28.967 85.809 1.00 0.00
B H
ATOM 20008 CD LYS B 212 -40.677 28.195 87.787 1.00 0.00
B C
ATOM 20009 HD1 LYS B 212 -41.592 28.534 88.325 1.00 0.00
B H
ATOM 20010 HD2 LYS B 212 -40.382 27.211 88.217 1.00 0.00
B H
ATOM 20011 CE LYS B 212 -39.552 29.206 87.927 1.00 0.00
B C
ATOM 20012 HE1 LYS B 212 -38.687 28.917 87.290 1.00 0.00
B H
ATOM 20013 HE2 LYS B 212 -39.895 30.217 87.617 1.00 0.00
B H
ATOM 20014 NZ LYS B 212 -39.069 29.300 89.331 1.00 0.00
B N
ATOM 20015 HZ1 LYS B 212 -38.299 29.996 89.398 1.00 0.00
B H
ATOM 20016 HZ2 LYS B 212 -39.854 29.592 89.950 1.00 0.00
B H
ATOM 20017 HZ3 LYS B 212 -38.720 28.370 89.639 1.00 0.00
B H
ATOM 20018 C LYS B 212 -43.940 26.129 84.565 1.00 0.00
B C
ATOM 20019 O LYS B 212 -44.283 24.975 84.833 1.00 0.00
B O
ATOM 20020 N ASP B 213 -44.774 27.050 84.090 1.00 0.00
B N
ATOM 20021 HN ASP B 213 -44.514 27.990 83.881 1.00 0.00
B H
ATOM 20022 CA ASP B 213 -46.178 26.777 83.821 1.00 0.00
B C
ATOM 20023 HA ASP B 213 -46.589 26.311 84.710 1.00 0.00
B H
ATOM 20024 CB ASP B 213 -46.925 28.080 83.507 1.00 0.00
B C
ATOM 20025 HB1 ASP B 213 -46.351 28.658 82.755 1.00 0.00
B H
ATOM 20026 HB2 ASP B 213 -47.928 27.864 83.090 1.00 0.00
B H
ATOM 20027 CG ASP B 213 -47.094 28.971 84.730 1.00 0.00
B C
ATOM 20028 OD1 ASP B 213 -47.044 30.204 84.575 1.00 0.00
B O
ATOM 20029 OD2 ASP B 213 -47.284 28.447 85.844 1.00 0.00
B O
ATOM 20030 C ASP B 213 -46.327 25.770 82.688 1.00 0.00
B C
ATOM 20031 O ASP B 213 -47.099 24.820 82.797 1.00 0.00
B O
ATOM 20032 N ILE B 214 -45.571 25.975 81.614 1.00 0.00
B N
ATOM 20033 HN ILE B 214 -44.982 26.773 81.504 1.00 0.00
B H

ATOM 20034 CA ILE B 214   -45.535 25.042 80.487 1.00 0.00
B   C
ATOM 20035 HA ILE B 214   -46.552 24.935 80.133 1.00 0.00
B   H
ATOM 20036 CB ILE B 214   -44.585 25.539 79.380 1.00 0.00
B   C
ATOM 20037 HB ILE B 214   -43.606 25.696 79.900 1.00 0.00
B   H
ATOM 20038 CG2 ILE B 214   -44.517 24.530 78.238 1.00 0.00
B   C
ATOM 20039 HG21 ILE B 214   -43.721 24.809 77.517 1.00 0.00
B   H
ATOM 20040 HG22 ILE B 214   -44.284 23.514 78.621 1.00 0.00
B   H
ATOM 20041 HG23 ILE B 214   -45.482 24.485 77.690 1.00 0.00
B   H
ATOM 20042 CG1 ILE B 214   -45.035 26.910 78.863 1.00 0.00
B   C
ATOM 20043 HG11 ILE B 214   -45.921 26.790 78.200 1.00 0.00
B   H
ATOM 20044 HG12 ILE B 214   -45.355 27.534 79.728 1.00 0.00
B   H
ATOM 20045 CD ILE B 214   -43.971 27.680 78.109 1.00 0.00
B   C
ATOM 20046 HD1 ILE B 214   -44.354 28.682 77.818 1.00 0.00
B   H
ATOM 20047 HD2 ILE B 214   -43.069 27.822 78.741 1.00 0.00
B   H
ATOM 20048 HD3 ILE B 214   -43.672 27.145 77.183 1.00 0.00
B   H
ATOM 20049 C   ILE B 214   -45.090 23.670 80.972 1.00 0.00
B   C
ATOM 20050 O   ILE B 214   -45.761 22.672 80.738 1.00 0.00
B   O
ATOM 20051 N   LYS B 215   -43.965 23.645 81.671 1.00 0.00
B   N
ATOM 20052 HN LYS B 215   -43.440 24.478 81.835 1.00 0.00
B   H
ATOM 20053 CA LYS B 215   -43.403 22.436 82.249 1.00 0.00
B   C
ATOM 20054 HA LYS B 215   -43.179 21.777 81.418 1.00 0.00
B   H
ATOM 20055 CB LYS B 215   -42.172 22.842 83.034 1.00 0.00
B   C
ATOM 20056 HB1 LYS B 215   -41.774 23.770 82.562 1.00 0.00
B   H
ATOM 20057 HB2 LYS B 215   -42.477 23.123 84.067 1.00 0.00
B   H
ATOM 20058 CG LYS B 215   -41.057 21.798 83.076 1.00 0.00
B   C
ATOM 20059 HG1 LYS B 215   -41.416 20.904 83.636 1.00 0.00
B   H
ATOM 20060 HG2 LYS B 215   -40.829 21.468 82.036 1.00 0.00
B   H

ATOM 20061 CD LYS B 215 −39.899 22.489 83.768 1.00 0.00
B C
ATOM 20062 HD1 LYS B 215 −39.586 23.359 83.147 1.00 0.00
B H
ATOM 20063 HD2 LYS B 215 −40.266 22.889 84.742 1.00 0.00
B H
ATOM 20064 CE LYS B 215 −38.744 21.512 83.979 1.00 0.00
B C
ATOM 20065 HE1 LYS B 215 −39.075 20.643 84.590 1.00 0.00
B H
ATOM 20066 HE2 LYS B 215 −38.370 21.133 83.002 1.00 0.00
B H
ATOM 20067 NZ LYS B 215 −37.612 22.167 84.684 1.00 0.00
B N
ATOM 20068 HZ1 LYS B 215 −36.836 21.487 84.820 1.00 0.00
B H
ATOM 20069 HZ2 LYS B 215 −37.272 22.973 84.122 1.00 0.00
B H
ATOM 20070 HZ3 LYS B 215 −37.935 22.510 85.611 1.00 0.00
B H
ATOM 20071 C LYS B 215 −44.384 21.700 83.164 1.00 0.00
B C
ATOM 20072 O LYS B 215 −44.357 20.472 83.252 1.00 0.00
B O
ATOM 20073 N ARG B 216 −45.238 22.456 83.852 1.00 0.00
B N
ATOM 20074 HN ARG B 216 −45.193 23.452 83.829 1.00 0.00
B H
ATOM 20075 CA ARG B 216 −46.287 21.866 84.680 1.00 0.00
B C
ATOM 20076 HA ARG B 216 −45.871 21.042 85.248 1.00 0.00
B H
ATOM 20077 CB ARG B 216 −46.894 22.897 85.638 1.00 0.00
B C
ATOM 20078 HB1 ARG B 216 −46.035 23.433 86.109 1.00 0.00
B H
ATOM 20079 HB2 ARG B 216 −47.451 23.663 85.055 1.00 0.00
B H
ATOM 20080 CG ARG B 216 −47.759 22.281 86.733 1.00 0.00
B C
ATOM 20081 HG1 ARG B 216 −48.523 21.630 86.256 1.00 0.00
B H
ATOM 20082 HG2 ARG B 216 −47.129 21.599 87.351 1.00 0.00
B H
ATOM 20083 CD ARG B 216 −48.383 23.320 87.659 1.00 0.00
B C
ATOM 20084 HD1 ARG B 216 −49.211 23.842 87.125 1.00 0.00
B H
ATOM 20085 HD2 ARG B 216 −48.794 22.850 88.582 1.00 0.00
B H
ATOM 20086 NE ARG B 216 −47.407 24.312 88.100 1.00 0.00
B N
ATOM 20087 HE ARG B 216 −46.723 23.938 88.736 1.00 0.00
B H

ATOM 20088 CZ ARG B 216 -47.405 25.585 87.712 1.00 0.00
B C
ATOM 20089 NH1 ARG B 216 -48.344 26.039 86.886 1.00 0.00
B N
ATOM 20090 HH11 ARG B 216 -48.330 26.979 86.575 1.00 0.00
B H
ATOM 20091 HH12 ARG B 216 -48.965 25.353 86.500 1.00 0.00
B H
ATOM 20092 NH2 ARG B 216 -46.463 26.408 88.160 1.00 0.00
B N
ATOM 20093 HH21 ARG B 216 -46.507 27.374 87.944 1.00 0.00
B H
ATOM 20094 HH22 ARG B 216 -45.864 26.060 88.868 1.00 0.00
B H
ATOM 20095 C ARG B 216 -47.371 21.276 83.786 1.00 0.00
B C
ATOM 20096 O ARG B 216 -47.854 20.167 84.034 1.00 0.00
B O
ATOM 20097 N PHE B 217 -47.741 22.016 82.746 1.00 0.00
B N
ATOM 20098 HN PHE B 217 -47.361 22.916 82.541 1.00 0.00
B H
ATOM 20099 CA PHE B 217 -48.755 21.561 81.805 1.00 0.00
B C
ATOM 20100 HA PHE B 217 -49.641 21.344 82.388 1.00 0.00
B H
ATOM 20101 CB PHE B 217 -49.048 22.632 80.754 1.00 0.00
B C
ATOM 20102 HB1 PHE B 217 -49.737 23.388 81.188 1.00 0.00
B H
ATOM 20103 HB2 PHE B 217 -48.120 23.167 80.459 1.00 0.00
B H
ATOM 20104 CG PHE B 217 -49.728 22.103 79.525 1.00 0.00
B C
ATOM 20105 CD1 PHE B 217 -51.036 21.621 79.578 1.00 0.00
B C
ATOM 20106 HD1 PHE B 217 -51.570 21.626 80.518 1.00 0.00
B H
ATOM 20107 CE1 PHE B 217 -51.661 21.127 78.441 1.00 0.00
B C
ATOM 20108 HE1 PHE B 217 -52.673 20.754 78.499 1.00 0.00
B H
ATOM 20109 CZ PHE B 217 -50.982 21.122 77.229 1.00 0.00
B C
ATOM 20110 HZ PHE B 217 -51.466 20.753 76.336 1.00 0.00
B H
ATOM 20111 CD2 PHE B 217 -49.061 22.087 78.308 1.00 0.00
B C
ATOM 20112 HD2 PHE B 217 -48.039 22.434 78.247 1.00 0.00
B H
ATOM 20113 CE2 PHE B 217 -49.682 21.602 77.164 1.00 0.00
B C
ATOM 20114 HE2 PHE B 217 -49.150 21.599 76.223 1.00 0.00
B H

ATOM 20115 C PHE B 217 −48.324 20.257 81.146 1.00 0.00
B C
ATOM 20116 O PHE B 217 −49.148 19.368 80.917 1.00 0.00
B O
ATOM 20117 N GLU B 218 −47.032 20.158 80.842 1.00 0.00
B N
ATOM 20118 HN GLU B 218 −46.380 20.902 80.954 1.00 0.00
B H
ATOM 20119 CA GLU B 218 −46.447 18.933 80.310 1.00 0.00
B C
ATOM 20120 HA GLU B 218 −46.981 18.662 79.408 1.00 0.00
B H
ATOM 20121 CB GLU B 218 −44.969 19.144 79.974 1.00 0.00
B C
ATOM 20122 HB1 GLU B 218 −44.443 19.454 80.906 1.00 0.00
B H
ATOM 20123 HB2 GLU B 218 −44.509 18.176 79.673 1.00 0.00
B H
ATOM 20124 CG GLU B 218 −44.716 20.189 78.896 1.00 0.00
B C
ATOM 20125 HG1 GLU B 218 −45.265 19.912 77.978 1.00 0.00
B H
ATOM 20126 HG2 GLU B 218 −45.047 21.192 79.230 1.00 0.00
B H
ATOM 20127 CD GLU B 218 −43.258 20.277 78.488 1.00 0.00
B C
ATOM 20128 OE1 GLU B 218 −42.708 19.252 78.039 1.00 0.00
B O
ATOM 20129 OE2 GLU B 218 −42.662 21.370 78.606 1.00 0.00
B O
ATOM 20130 C GLU B 218 −46.620 17.782 81.302 1.00 0.00
B C
ATOM 20131 O GLU B 218 −47.133 16.726 80.944 1.00 0.00
B O
ATOM 20132 N ASN B 219 −46.208 18.011 82.548 1.00 0.00
B N
ATOM 20133 HN ASN B 219 −45.789 18.873 82.829 1.00 0.00
B H
ATOM 20134 CA ASN B 219 −46.332 17.019 83.620 1.00 0.00
B C
ATOM 20135 HA ASN B 219 −45.689 16.206 83.301 1.00 0.00
B H
ATOM 20136 CB ASN B 219 −45.849 17.606 84.945 1.00 0.00
B C
ATOM 20137 HB1 ASN B 219 −45.997 18.707 84.903 1.00 0.00
B H
ATOM 20138 HB2 ASN B 219 −46.462 17.219 85.785 1.00 0.00
B H
ATOM 20139 CG ASN B 219 −44.381 17.337 85.199 1.00 0.00
B C
ATOM 20140 OD1 ASN B 219 −43.901 16.223 84.998 1.00 0.00
B O
ATOM 20141 ND2 ASN B 219 −43.661 18.356 85.656 1.00 0.00
B N

ATOM 20142 HD21 ASN B 219 −42.691 18.196 85.817 1.00 0.00
B H
ATOM 20143 HD22 ASN B 219 −44.088 19.244 85.796 1.00 0.00
B H
ATOM 20144 C ASN B 219 −47.732 16.450 83.817 1.00 0.00
B C
ATOM 20145 O ASN B 219 −47.885 15.268 84.111 1.00 0.00
B O
ATOM 20146 N GLU B 220 −48.745 17.292 83.643 1.00 0.00
B N
ATOM 20147 HN GLU B 220 −48.658 18.201 83.248 1.00 0.00
B H
ATOM 20148 CA GLU B 220 −50.111 16.939 84.031 1.00 0.00
B C
ATOM 20149 HA GLU B 220 −50.037 16.052 84.647 1.00 0.00
B H
ATOM 20150 CB GLU B 220 −50.717 18.042 84.905 1.00 0.00
B C
ATOM 20151 HB1 GLU B 220 −50.538 19.021 84.404 1.00 0.00
B H
ATOM 20152 HB2 GLU B 220 −51.822 17.921 84.950 1.00 0.00
B H
ATOM 20153 CG GLU B 220 −50.143 18.095 86.314 1.00 0.00
B C
ATOM 20154 HG1 GLU B 220 −50.471 17.204 86.880 1.00 0.00
B H
ATOM 20155 HG2 GLU B 220 −49.035 18.124 86.291 1.00 0.00
B H
ATOM 20156 CD GLU B 220 −50.629 19.287 87.121 1.00 0.00
B C
ATOM 20157 OE1 GLU B 220 −51.054 20.299 86.522 1.00 0.00
B O
ATOM 20158 OE2 GLU B 220 −50.575 19.216 88.369 1.00 0.00
B O
ATOM 20159 C GLU B 220 −51.036 16.605 82.866 1.00 0.00
B C
ATOM 20160 O GLU B 220 −51.776 15.620 82.931 1.00 0.00
B O
ATOM 20161 N GLY B 221 −51.000 17.418 81.812 1.00 0.00
B N
ATOM 20162 HN GLY B 221 −50.401 18.217 81.775 1.00 0.00
B H
ATOM 20163 CA GLY B 221 −51.838 17.199 80.631 1.00 0.00
B C
ATOM 20164 HA1 GLY B 221 −51.879 18.146 80.112 1.00 0.00
B H
ATOM 20165 HA2 GLY B 221 −52.795 16.850 80.996 1.00 0.00
B H
ATOM 20166 C GLY B 221 −51.297 16.157 79.665 1.00 0.00
B C
ATOM 20167 O GLY B 221 −51.991 15.750 78.724 1.00 0.00
B O
ATOM 20168 N MET B 222 −50.056 15.728 79.900 1.00 0.00
B N

ATOM 20169 HN MET B 222 -49.516 16.041 80.679 1.00 0.00
B H
ATOM 20170 CA MET B 222 -49.372 14.763 79.041 1.00 0.00
B C
ATOM 20171 HA MET B 222 -50.126 14.289 78.423 1.00 0.00
B H
ATOM 20172 CB MET B 222 -48.324 15.467 78.170 1.00 0.00
B C
ATOM 20173 HB1 MET B 222 -47.634 16.004 78.864 1.00 0.00
B H
ATOM 20174 HB2 MET B 222 -47.707 14.704 77.646 1.00 0.00
B H
ATOM 20175 CG MET B 222 -48.887 16.469 77.176 1.00 0.00
B C
ATOM 20176 HG1 MET B 222 -49.257 15.946 76.269 1.00 0.00
B H
ATOM 20177 HG2 MET B 222 -49.749 17.003 77.632 1.00 0.00
B H
ATOM 20178 SD MET B 222 -47.662 17.683 76.653 1.00 0.00
B S
ATOM 20179 CE MET B 222 -48.602 18.592 75.440 1.00 0.00
B C
ATOM 20180 HE1 MET B 222 -47.988 19.401 74.989 1.00 0.00
B H
ATOM 20181 HE2 MET B 222 -48.945 17.926 74.620 1.00 0.00
B H
ATOM 20182 HE3 MET B 222 -49.499 19.061 75.899 1.00 0.00
B H
ATOM 20183 C MET B 222 -48.714 13.674 79.880 1.00 0.00
B C
ATOM 20184 O MET B 222 -48.682 12.510 79.485 1.00 0.00
B O
ATOM 20185 N GLN C 8 28.365 19.502 34.204 1.00 0.00
C N
ATOM 20186 HT1 GLN C 8 28.768 19.741 33.320 1.00 0.00
C H
ATOM 20187 HT2 GLN C 8 27.488 19.969 34.317 1.00 0.00
C H
ATOM 20188 HT3 GLN C 8 28.236 18.513 34.263 1.00 0.00
C H
ATOM 20189 CA GLN C 8 29.288 19.935 35.291 1.00 0.00
C C
ATOM 20190 HA GLN C 8 30.202 19.373 35.150 1.00 0.00
C H
ATOM 20191 CB GLN C 8 29.595 21.426 35.160 1.00 0.00
C C
ATOM 20192 HB1 GLN C 8 29.391 21.705 34.100 1.00 0.00
C H
ATOM 20193 HB2 GLN C 8 28.875 22.007 35.779 1.00 0.00
C H
ATOM 20194 CG GLN C 8 31.029 21.794 35.511 1.00 0.00
C C
ATOM 20195 HG1 GLN C 8 31.192 21.716 36.608 1.00 0.00
C H

ATOM 20196 HG2 GLN C   8   31.744 21.109 35.011 1.00 0.00
C   H
ATOM 20197 CD GLN C   8   31.377 23.220 35.131 1.00 0.00
C   C
ATOM 20198 OE1 GLN C   8   30.594 23.916 34.478 1.00 0.00
C   O
ATOM 20199 NE2 GLN C   8   32.560 23.662 35.539 1.00 0.00
C   N
ATOM 20200 HE21 GLN C   8   32.798 24.598 35.298 1.00 0.00
C   H
ATOM 20201 HE22 GLN C   8   33.167 23.057 36.044 1.00 0.00
C   H
ATOM 20202 C   GLN C   8   28.756 19.610 36.698 1.00 0.00
C   C
ATOM 20203 O   GLN C   8   29.534 19.234 37.579 1.00 0.00
C   O
ATOM 20204 N   PHE C   9   27.444 19.754 36.909 1.00 0.00
C   N
ATOM 20205 HN PHE C   9   26.803 20.069 36.211 1.00 0.00
C   H
ATOM 20206 CA PHE C   9   26.832 19.456 38.209 1.00 0.00
C   C
ATOM 20207 HA PHE C   9   27.571 18.887 38.758 1.00 0.00
C   H
ATOM 20208 CB PHE C   9   26.426 20.731 38.945 1.00 0.00
C   C
ATOM 20209 HB1 PHE C   9   25.605 21.229 38.385 1.00 0.00
C   H
ATOM 20210 HB2 PHE C   9   26.035 20.499 39.960 1.00 0.00
C   H
ATOM 20211 CG PHE C   9   27.517 21.745 39.062 1.00 0.00
C   C
ATOM 20212 CD1 PHE C   9   28.385 21.718 40.136 1.00 0.00
C   C
ATOM 20213 HD1 PHE C   9   28.271 20.958 40.897 1.00 0.00
C   H
ATOM 20214 CE1 PHE C   9   29.396 22.658 40.251 1.00 0.00
C   C
ATOM 20215 HE1 PHE C   9   30.077 22.618 41.088 1.00 0.00
C   H
ATOM 20216 CZ PHE C   9   29.538 23.646 39.288 1.00 0.00
C   C
ATOM 20217 HZ PHE C   9   30.328 24.378 39.372 1.00 0.00
C   H
ATOM 20218 CD2 PHE C   9   27.662 22.745 38.103 1.00 0.00
C   C
ATOM 20219 HD2 PHE C   9   26.970 22.804 37.274 1.00 0.00
C   H
ATOM 20220 CE2 PHE C   9   28.668 23.689 38.209 1.00 0.00
C   C
ATOM 20221 HE2 PHE C   9   28.771 24.454 37.452 1.00 0.00
C   H
ATOM 20222 C   PHE C   9   25.596 18.576 38.078 1.00 0.00
C   C

```
ATOM  20223  O   PHE C   9      24.763  18.779  37.194  1.00  0.00
C    O
ATOM  20224  N   ALA C  10      25.484  17.597  38.972  1.00  0.00
C    N
ATOM  20225  HN  ALA C  10      26.229  17.346  39.590  1.00  0.00
C    H
ATOM  20226  CA  ALA C  10      24.262  16.815  39.116  1.00  0.00
C    C
ATOM  20227  HA  ALA C  10      23.459  17.209  38.503  1.00  0.00
C    H
ATOM  20228  CB  ALA C  10      24.491  15.371  38.715  1.00  0.00
C    C
ATOM  20229  HB1 ALA C  10      24.787  15.323  37.646  1.00  0.00
C    H
ATOM  20230  HB2 ALA C  10      25.307  14.914  39.317  1.00  0.00
C    H
ATOM  20231  HB3 ALA C  10      23.571  14.759  38.844  1.00  0.00
C    H
ATOM  20232  C   ALA C  10      23.788  16.910  40.559  1.00  0.00
C    C
ATOM  20233  O   ALA C  10      24.591  16.846  41.490  1.00  0.00
C    O
ATOM  20234  N   VAL C  11      22.489  17.102  40.741  1.00  0.00
C    N
ATOM  20235  HN  VAL C  11      21.837  17.220  39.998  1.00  0.00
C    H
ATOM  20236  CA  VAL C  11      21.903  17.154  42.068  1.00  0.00
C    C
ATOM  20237  HA  VAL C  11      22.681  16.988  42.803  1.00  0.00
C    H
ATOM  20238  CB  VAL C  11      21.198  18.497  42.337  1.00  0.00
C    C
ATOM  20239  HB  VAL C  11      20.457  18.660  41.515  1.00  0.00
C    H
ATOM  20240  CG1 VAL C  11      20.465  18.460  43.671  1.00  0.00
C    C
ATOM  20241 HG11 VAL C  11      19.991  19.444  43.879  1.00  0.00
C    H
ATOM  20242 HG12 VAL C  11      19.659  17.698  43.675  1.00  0.00
C    H
ATOM  20243 HG13 VAL C  11      21.170  18.238  44.501  1.00  0.00
C    H
ATOM  20244  CG2 VAL C  11      22.200  19.640  42.315  1.00  0.00
C    C
ATOM  20245 HG21 VAL C  11      21.701  20.604  42.547  1.00  0.00
C    H
ATOM  20246 HG22 VAL C  11      23.001  19.470  43.065  1.00  0.00
C    H
ATOM  20247 HG23 VAL C  11      22.673  19.740  41.316  1.00  0.00
C    H
ATOM  20248  C   VAL C  11      20.916  16.005  42.181  1.00  0.00
C    C
ATOM  20249  O   VAL C  11      19.938  15.945  41.432  1.00  0.00
C    O
```

ATOM 20250 N ILE C 12 21.177 15.096 43.117 1.00 0.00
C N
ATOM 20251 HN ILE C 12 21.883 15.181 43.818 1.00 0.00
C H
ATOM 20252 CA ILE C 12 20.412 13.855 43.186 1.00 0.00
C C
ATOM 20253 HA ILE C 12 20.122 13.661 42.160 1.00 0.00
C H
ATOM 20254 CB ILE C 12 21.300 12.632 43.488 1.00 0.00
C C
ATOM 20255 HB ILE C 12 22.030 13.000 44.253 1.00 0.00
C H
ATOM 20256 CG2 ILE C 12 20.456 11.444 43.913 1.00 0.00
C C
ATOM 20257 HG21 ILE C 12 21.101 10.618 44.279 1.00 0.00
C H
ATOM 20258 HG22 ILE C 12 19.767 11.723 44.737 1.00 0.00
C H
ATOM 20259 HG23 ILE C 12 19.851 11.062 43.064 1.00 0.00
C H
ATOM 20260 CG1 ILE C 12 22.093 12.279 42.224 1.00 0.00
C C
ATOM 20261 HG11 ILE C 12 21.392 12.082 41.382 1.00 0.00
C H
ATOM 20262 HG12 ILE C 12 22.718 13.155 41.934 1.00 0.00
C H
ATOM 20263 CD ILE C 12 23.018 11.093 42.350 1.00 0.00
C C
ATOM 20264 HD1 ILE C 12 23.590 10.949 41.408 1.00 0.00
C H
ATOM 20265 HD2 ILE C 12 23.743 11.248 43.178 1.00 0.00
C H
ATOM 20266 HD3 ILE C 12 22.447 10.163 42.551 1.00 0.00
C H
ATOM 20267 C ILE C 12 19.123 13.916 44.005 1.00 0.00
C C
ATOM 20268 O ILE C 12 18.178 13.188 43.716 1.00 0.00
C O
ATOM 20269 N GLY C 13 19.050 14.784 45.005 1.00 0.00
C N
ATOM 20270 HN GLY C 13 19.796 15.367 45.325 1.00 0.00
C H
ATOM 20271 CA GLY C 13 17.786 14.926 45.720 1.00 0.00
C C
ATOM 20272 HA1 GLY C 13 18.026 14.742 46.758 1.00 0.00
C H
ATOM 20273 HA2 GLY C 13 17.122 14.195 45.280 1.00 0.00
C H
ATOM 20274 C GLY C 13 17.116 16.280 45.627 1.00 0.00
C C
ATOM 20275 O GLY C 13 17.581 17.249 46.229 1.00 0.00
C O
ATOM 20276 N LEU C 14 16.038 16.362 44.855 1.00 0.00
C N

ATOM 20277 HN LEU C 14   15.682 15.607 44.307 1.00 0.00
C   H
ATOM 20278 CA LEU C 14   15.271 17.597 44.745 1.00 0.00
C   C
ATOM 20279 HA LEU C 14   15.956 18.429 44.844 1.00 0.00
C   H
ATOM 20280 CB LEU C 14   14.507 17.643 43.421 1.00 0.00
C   C
ATOM 20281 HB1 LEU C 14   13.839 16.751 43.395 1.00 0.00
C   H
ATOM 20282 HB2 LEU C 14   13.836 18.530 43.422 1.00 0.00
C   H
ATOM 20283 CG LEU C 14   15.355 17.637 42.149 1.00 0.00
C   C
ATOM 20284 HG LEU C 14   15.941 16.686 42.153 1.00 0.00
C   H
ATOM 20285 CD1 LEU C 14   14.470 17.684 40.913 1.00 0.00
C   C
ATOM 20286 HD11 LEU C 14   15.089 17.728 39.992 1.00 0.00
C   H
ATOM 20287 HD12 LEU C 14   13.826 16.781 40.859 1.00 0.00
C   H
ATOM 20288 HD13 LEU C 14   13.815 18.580 40.934 1.00 0.00
C   H
ATOM 20289 CD2 LEU C 14   16.339 18.795 42.152 1.00 0.00
C   C
ATOM 20290 HD21 LEU C 14   16.964 18.773 41.233 1.00 0.00
C   H
ATOM 20291 HD22 LEU C 14   15.819 19.774 42.182 1.00 0.00
C   H
ATOM 20292 HD23 LEU C 14   17.013 18.730 43.032 1.00 0.00
C   H
ATOM 20293 C   LEU C 14   14.305 17.656 45.916 1.00 0.00
C   C
ATOM 20294 O   LEU C 14   13.284 16.969 45.921 1.00 0.00
C   O
ATOM 20295 N   GLY C 15   14.633 18.463 46.918 1.00 0.00
C   N
ATOM 20296 HN GLY C 15   15.389 19.116 46.884 1.00 0.00
C   H
ATOM 20297 CA GLY C 15   13.876 18.437 48.171 1.00 0.00
C   C
ATOM 20298 HA1 GLY C 15   14.316 17.794 48.920 1.00 0.00
C   H
ATOM 20299 HA2 GLY C 15   12.813 18.291 48.028 1.00 0.00
C   H
ATOM 20300 C   GLY C 15   14.098 19.870 48.596 1.00 0.00
C   C
ATOM 20301 O   GLY C 15   14.591 20.680 47.812 1.00 0.00
C   O
ATOM 20302 N   ARG C 16   13.737 20.190 49.836 1.00 0.00
C   N
ATOM 20303 HN ARG C 16   13.281 19.537 50.435 1.00 0.00
C   H

ATOM 20304 CA ARG C 16   13.991 21.520 50.387 1.00 0.00
C   C
ATOM 20305 HA ARG C 16   13.349 22.226 49.874 1.00 0.00
C   H
ATOM 20306 CB ARG C 16   13.666 21.553 51.881 1.00 0.00
C   C
ATOM 20307 HB1 ARG C 16   13.729 20.498 52.240 1.00 0.00
C   H
ATOM 20308 HB2 ARG C 16   14.462 22.110 52.423 1.00 0.00
C   H
ATOM 20309 CG ARG C 16   12.285 22.101 52.196 1.00 0.00
C   C
ATOM 20310 HG1 ARG C 16   12.398 23.128 52.609 1.00 0.00
C   H
ATOM 20311 HG2 ARG C 16   11.718 22.223 51.245 1.00 0.00
C   H
ATOM 20312 CD ARG C 16   11.503 21.182 53.114 1.00 0.00
C   C
ATOM 20313 HD1 ARG C 16   10.530 21.658 53.374 1.00 0.00
C   H
ATOM 20314 HD2 ARG C 16   11.301 20.197 52.631 1.00 0.00
C   H
ATOM 20315 NE ARG C 16   12.220 20.886 54.350 1.00 0.00
C   N
ATOM 20316 HE ARG C 16   12.754 21.666 54.694 1.00 0.00
C   H
ATOM 20317 CZ ARG C 16   12.181 19.708 54.969 1.00 0.00
C   C
ATOM 20318 NH1 ARG C 16   11.467 18.706 54.459 1.00 0.00
C   N
ATOM 20319 HH11 ARG C 16   11.414 17.836 54.929 1.00 0.00
C   H
ATOM 20320 HH12 ARG C 16   10.892 18.927 53.668 1.00 0.00
C   H
ATOM 20321 NH2 ARG C 16   12.859 19.522 56.092 1.00 0.00
C   N
ATOM 20322 HH21 ARG C 16   12.898 18.622 56.502 1.00 0.00
C   H
ATOM 20323 HH22 ARG C 16   13.477 20.247 56.367 1.00 0.00
C   H
ATOM 20324 C   ARG C 16   15.431 21.972 50.130 1.00 0.00
C   C
ATOM 20325 O   ARG C 16   15.676 23.104 49.690 1.00 0.00
C   O
ATOM 20326 N   PHE C 17   16.375 21.065 50.366 1.00 0.00
C   N
ATOM 20327 HN PHE C 17   16.176 20.131 50.658 1.00 0.00
C   H
ATOM 20328 CA PHE C 17   17.794 21.362 50.216 1.00 0.00
C   C
ATOM 20329 HA PHE C 17   17.894 22.410 50.471 1.00 0.00
C   H
ATOM 20330 CB PHE C 17   18.611 20.500 51.175 1.00 0.00
C   C

ATOM 20331 HB1 PHE C 17   18.275 20.696 52.217 1.00 0.00
C   H
ATOM 20332 HB2 PHE C 17   18.449 19.418 50.977 1.00 0.00
C   H
ATOM 20333 CG PHE C 17   20.085 20.789 51.154 1.00 0.00
C   C
ATOM 20334 CD1 PHE C 17   20.570 22.038 51.532 1.00 0.00
C   C
ATOM 20335 HD1 PHE C 17   19.878 22.812 51.834 1.00 0.00
C   H
ATOM 20336 CE1 PHE C 17   21.935 22.306 51.520 1.00 0.00
C   C
ATOM 20337 HE1 PHE C 17   22.298 23.281 51.814 1.00 0.00
C   H
ATOM 20338 CZ PHE C 17   22.826 21.317 51.135 1.00 0.00
C   C
ATOM 20339 HZ PHE C 17   23.888 21.515 51.133 1.00 0.00
C   H
ATOM 20340 CD2 PHE C 17   20.991 19.807 50.774 1.00 0.00
C   C
ATOM 20341 HD2 PHE C 17   20.635 18.835 50.464 1.00 0.00
C   H
ATOM 20342 CE2 PHE C 17   22.352 20.067 50.762 1.00 0.00
C   C
ATOM 20343 HE2 PHE C 17   23.045 19.293 50.461 1.00 0.00
C   H
ATOM 20344 C   PHE C 17   18.299 21.199 48.783 1.00 0.00
C   C
ATOM 20345 O   PHE C 17   18.808 22.150 48.193 1.00 0.00
C   O
ATOM 20346 N   GLY C 18   18.165 19.998 48.227 1.00 0.00
C   N
ATOM 20347 HN GLY C 18   17.718 19.231 48.684 1.00 0.00
C   H
ATOM 20348 CA GLY C 18   18.676 19.724 46.882 1.00 0.00
C   C
ATOM 20349 HA1 GLY C 18   18.421 18.698 46.655 1.00 0.00
C   H
ATOM 20350 HA2 GLY C 18   19.739 19.924 46.900 1.00 0.00
C   H
ATOM 20351 C   GLY C 18   18.051 20.610 45.818 1.00 0.00
C   C
ATOM 20352 O   GLY C 18   18.745 21.143 44.945 1.00 0.00
C   O
ATOM 20353 N   GLY C 19   16.733 20.771 45.894 1.00 0.00
C   N
ATOM 20354 HN GLY C 19   16.173 20.318 46.586 1.00 0.00
C   H
ATOM 20355 CA GLY C 19   16.014 21.627 44.963 1.00 0.00
C   C
ATOM 20356 HA1 GLY C 19   14.985 21.663 45.293 1.00 0.00
C   H
ATOM 20357 HA2 GLY C 19   16.150 21.215 43.973 1.00 0.00
C   H

ATOM 20358 C   GLY C 19   16.555 23.045 44.957 1.00 0.00
C   C
ATOM 20359 O   GLY C 19   16.682 23.669 43.905 1.00 0.00
C   O
ATOM 20360 N   SER C 20   16.894 23.544 46.142 1.00 0.00
C   N
ATOM 20361 HN SER C 20   16.819 23.049 47.008 1.00 0.00
C   H
ATOM 20362 CA SER C 20   17.417 24.890 46.271 1.00 0.00
C   C
ATOM 20363 HA SER C 20   16.737 25.529 45.722 1.00 0.00
C   H
ATOM 20364 CB SER C 20   17.491 25.318 47.734 1.00 0.00
C   C
ATOM 20365 HB1 SER C 20   16.459 25.299 48.153 1.00 0.00
C   H
ATOM 20366 HB2 SER C 20   18.095 24.590 48.322 1.00 0.00
C   H
ATOM 20367 OG SER C 20   18.011 26.633 47.829 1.00 0.00
C   O
ATOM 20368 HG1 SER C 20   17.835 26.944 48.726 1.00 0.00
C   H
ATOM 20369 C   SER C 20   18.779 25.016 45.615 1.00 0.00
C   C
ATOM 20370 O   SER C 20   19.027 26.002 44.930 1.00 0.00
C   O
ATOM 20371 N   ILE C 21   19.653 24.032 45.825 1.00 0.00
C   N
ATOM 20372 HN ILE C 21   19.483 23.250 46.421 1.00 0.00
C   H
ATOM 20373 CA ILE C 21   20.967 24.018 45.177 1.00 0.00
C   C
ATOM 20374 HA ILE C 21   21.461 24.944 45.442 1.00 0.00
C   H
ATOM 20375 CB ILE C 21   21.799 22.776 45.536 1.00 0.00
C   C
ATOM 20376 HB ILE C 21   21.093 21.918 45.400 1.00 0.00
C   H
ATOM 20377 CG2 ILE C 21   23.045 22.699 44.665 1.00 0.00
C   C
ATOM 20378 HG21 ILE C 21   23.564 21.729 44.811 1.00 0.00
C   H
ATOM 20379 HG22 ILE C 21   22.782 22.781 43.589 1.00 0.00
C   H
ATOM 20380 HG23 ILE C 21   23.756 23.514 44.917 1.00 0.00
C   H
ATOM 20381 CG1 ILE C 21   22.188 22.793 47.010 1.00 0.00
C   C
ATOM 20382 HG11 ILE C 21   22.841 23.670 47.218 1.00 0.00
C   H
ATOM 20383 HG12 ILE C 21   21.268 22.917 47.626 1.00 0.00
C   H
ATOM 20384 CD ILE C 21   22.895 21.531 47.466 1.00 0.00
C   C

ATOM 20385 HD1 ILE C 21 23.102 21.577 48.557 1.00 0.00
C H
ATOM 20386 HD2 ILE C 21 22.266 20.637 47.268 1.00 0.00
C H
ATOM 20387 HD3 ILE C 21 23.864 21.405 46.938 1.00 0.00
C H
ATOM 20388 C ILE C 21 20.791 24.031 43.666 1.00 0.00
C C
ATOM 20389 O ILE C 21 21.454 24.799 42.966 1.00 0.00
C O
ATOM 20390 N CYS C 22 19.888 23.179 43.184 1.00 0.00
C N
ATOM 20391 HN CYS C 22 19.352 22.573 43.770 1.00 0.00
C H
ATOM 20392 CA CYS C 22 19.606 23.058 41.758 1.00 0.00
C C
ATOM 20393 HA CYS C 22 20.542 22.791 41.280 1.00 0.00
C H
ATOM 20394 CB CYS C 22 18.549 21.978 41.516 1.00 0.00
C C
ATOM 20395 HB1 CYS C 22 18.909 21.048 42.012 1.00 0.00
C H
ATOM 20396 HB2 CYS C 22 17.603 22.254 42.032 1.00 0.00
C H
ATOM 20397 SG CYS C 22 18.251 21.611 39.770 1.00 0.00
C S
ATOM 20398 HG1 CYS C 22 17.371 20.626 39.955 1.00 0.00
C H
ATOM 20399 C CYS C 22 19.151 24.395 41.174 1.00 0.00
C C
ATOM 20400 O CYS C 22 19.795 24.943 40.269 1.00 0.00
C O
ATOM 20401 N LYS C 23 18.060 24.924 41.719 1.00 0.00
C N
ATOM 20402 HN LYS C 23 17.566 24.490 42.471 1.00 0.00
C H
ATOM 20403 CA LYS C 23 17.499 26.187 41.258 1.00 0.00
C C
ATOM 20404 HA LYS C 23 17.144 26.022 40.248 1.00 0.00
C H
ATOM 20405 CB LYS C 23 16.312 26.617 42.129 1.00 0.00
C C
ATOM 20406 HB1 LYS C 23 15.689 25.710 42.306 1.00 0.00
C H
ATOM 20407 HB2 LYS C 23 16.690 26.935 43.126 1.00 0.00
C H
ATOM 20408 CG LYS C 23 15.452 27.705 41.497 1.00 0.00
C C
ATOM 20409 HG1 LYS C 23 16.063 28.629 41.379 1.00 0.00
C H
ATOM 20410 HG2 LYS C 23 15.144 27.377 40.478 1.00 0.00
C H
ATOM 20411 CD LYS C 23 14.223 28.037 42.332 1.00 0.00
C C

ATOM 20412 HD1 LYS C 23   14.535 28.128 43.398 1.00 0.00
C   H
ATOM 20413 HD2 LYS C 23   13.843 29.034 42.010 1.00 0.00
C   H
ATOM 20414 CE LYS C 23   13.125 26.989 42.186 1.00 0.00
C   C
ATOM 20415 HE1 LYS C 23   12.931 26.775 41.112 1.00 0.00
C   H
ATOM 20416 HE2 LYS C 23   13.423 26.040 42.686 1.00 0.00
C   H
ATOM 20417 NZ LYS C 23   11.843 27.442 42.797 1.00 0.00
C   N
ATOM 20418 HZ1 LYS C 23   11.114 26.708 42.681 1.00 0.00
C   H
ATOM 20419 HZ2 LYS C 23   11.988 27.629 43.811 1.00 0.00
C   H
ATOM 20420 HZ3 LYS C 23   11.527 28.316 42.331 1.00 0.00
C   H
ATOM 20421 C   LYS C 23   18.554 27.292 41.194 1.00 0.00
C   C
ATOM 20422 O   LYS C 23   18.551 28.101 40.263 1.00 0.00
C   O
ATOM 20423 N   GLU C 24   19.458 27.313 42.171 1.00 0.00
C   N
ATOM 20424 HN GLU C 24   19.531 26.637 42.898 1.00 0.00
C   H
ATOM 20425 CA GLU C 24   20.452 28.378 42.249 1.00 0.00
C   C
ATOM 20426 HA GLU C 24   19.939 29.307 42.037 1.00 0.00
C   H
ATOM 20427 CB GLU C 24   21.014 28.520 43.667 1.00 0.00
C   C
ATOM 20428 HB1 GLU C 24   20.158 28.644 44.369 1.00 0.00
C   H
ATOM 20429 HB2 GLU C 24   21.522 27.577 43.968 1.00 0.00
C   H
ATOM 20430 CG GLU C 24   21.972 29.698 43.854 1.00 0.00
C   C
ATOM 20431 HG1 GLU C 24   22.383 29.681 44.879 1.00 0.00
C   H
ATOM 20432 HG2 GLU C 24   22.806 29.651 43.125 1.00 0.00
C   H
ATOM 20433 CD GLU C 24   21.313 31.072 43.716 1.00 0.00
C   C
ATOM 20434 OE1 GLU C 24   22.049 32.083 43.765 1.00 0.00
C   O
ATOM 20435 OE2 GLU C 24   20.071 31.160 43.565 1.00 0.00
C   O
ATOM 20436 C   GLU C 24   21.579 28.238 41.230 1.00 0.00
C   C
ATOM 20437 O   GLU C 24   21.944 29.216 40.586 1.00 0.00
C   O
ATOM 20438 N   LEU C 25   22.120 27.035 41.081 1.00 0.00
C   N

```
ATOM  20439  HN  LEU C  25      21.865  26.233  41.617  1.00  0.00
C    H
ATOM  20440  CA  LEU C  25      23.164  26.788  40.090  1.00  0.00
C    C
ATOM  20441  HA  LEU C  25      23.985  27.453  40.323  1.00  0.00
C    H
ATOM  20442  CB  LEU C  25      23.606  25.324  40.122  1.00  0.00
C    C
ATOM  20443  HB1 LEU C  25      22.682  24.702  40.125  1.00  0.00
C    H
ATOM  20444  HB2 LEU C  25      24.136  25.086  39.174  1.00  0.00
C    H
ATOM  20445  CG  LEU C  25      24.470  24.783  41.266  1.00  0.00
C    C
ATOM  20446  HG  LEU C  25      23.965  25.067  42.220  1.00  0.00
C    H
ATOM  20447  CD1 LEU C  25      24.561  23.270  41.161  1.00  0.00
C    C
ATOM  20448 HD11 LEU C  25      25.223  22.864  41.954  1.00  0.00
C    H
ATOM  20449 HD12 LEU C  25      23.557  22.809  41.274  1.00  0.00
C    H
ATOM  20450 HD13 LEU C  25      24.976  22.970  40.175  1.00  0.00
C    H
ATOM  20451  CD2 LEU C  25      25.865  25.390  41.271  1.00  0.00
C    C
ATOM  20452 HD21 LEU C  25      26.457  24.990  42.122  1.00  0.00
C    H
ATOM  20453 HD22 LEU C  25      26.417  25.157  40.338  1.00  0.00
C    H
ATOM  20454 HD23 LEU C  25      25.808  26.496  41.376  1.00  0.00
C    H
ATOM  20455  C   LEU C  25      22.672  27.139  38.693  1.00  0.00
C    C
ATOM  20456  O   LEU C  25      23.409  27.687  37.869  1.00  0.00
C    O
ATOM  20457  N   HSE C  26      21.411  26.807  38.442  1.00  0.00
C    N
ATOM  20458  HN  HSE C  26      20.833  26.321  39.096  1.00  0.00
C    H
ATOM  20459  CA  HSE C  26      20.756  27.124  37.185  1.00  0.00
C    C
ATOM  20460  HA  HSE C  26      21.407  26.762  36.397  1.00  0.00
C    H
ATOM  20461  CB  HSE C  26      19.405  26.441  37.137  1.00  0.00
C    C
ATOM  20462  HB1 HSE C  26      19.566  25.354  37.304  1.00  0.00
C    H
ATOM  20463  HB2 HSE C  26      18.774  26.808  37.973  1.00  0.00
C    H
ATOM  20464  ND1 HSE C  26      19.134  26.063  34.678  1.00  0.00
C    N
ATOM  20465  CG  HSE C  26      18.683  26.607  35.825  1.00  0.00
C    C
```

ATOM 20466 CE1 HSE C 26   18.283 26.364 33.680 1.00 0.00
C   C
ATOM 20467 HE1 HSE C 26   18.414 26.055 32.640 1.00 0.00
C   H
ATOM 20468 NE2 HSE C 26   17.282 27.099 34.194 1.00 0.00
C   N
ATOM 20469 HE2 HSE C 26   16.515 27.473 33.673 1.00 0.00
C   H
ATOM 20470 CD2 HSE C 26   17.498 27.269 35.510 1.00 0.00
C   C
ATOM 20471 HD2 HSE C 26   16.833 27.839 36.144 1.00 0.00
C   H
ATOM 20472 C   HSE C 26   20.605 28.606 36.987 1.00 0.00
C   C
ATOM 20473 O   HSE C 26   20.882 29.119 35.908 1.00 0.00
C   O
ATOM 20474 N   ARG C 27   20.171 29.298 38.040 1.00 0.00
C   N
ATOM 20475 HN ARG C 27   19.942 28.826 38.888 1.00 0.00
C   H
ATOM 20476 CA ARG C 27   20.003 30.746 38.030 1.00 0.00
C   C
ATOM 20477 HA ARG C 27   19.387 31.013 37.179 1.00 0.00
C   H
ATOM 20478 CB ARG C 27   19.336 31.213 39.325 1.00 0.00
C   C
ATOM 20479 HB1 ARG C 27   18.547 30.461 39.563 1.00 0.00
C   H
ATOM 20480 HB2 ARG C 27   20.069 31.162 40.161 1.00 0.00
C   H
ATOM 20481 CG ARG C 27   18.687 32.588 39.247 1.00 0.00
C   C
ATOM 20482 HG1 ARG C 27   19.297 33.229 38.574 1.00 0.00
C   H
ATOM 20483 HG2 ARG C 27   17.694 32.492 38.750 1.00 0.00
C   H
ATOM 20484 CD ARG C 27   18.483 33.208 40.625 1.00 0.00
C   C
ATOM 20485 HD1 ARG C 27   18.084 34.242 40.512 1.00 0.00
C   H
ATOM 20486 HD2 ARG C 27   17.765 32.614 41.239 1.00 0.00
C   H
ATOM 20487 NE ARG C 27   19.729 33.267 41.395 1.00 0.00
C   N
ATOM 20488 HE ARG C 27   19.802 32.538 42.085 1.00 0.00
C   H
ATOM 20489 CZ ARG C 27   20.689 34.176 41.229 1.00 0.00
C   C
ATOM 20490 NH1 ARG C 27   20.571 35.132 40.315 1.00 0.00
C   N
ATOM 20491 HH11 ARG C 27   21.297 35.792 40.178 1.00 0.00
C   H
ATOM 20492 HH12 ARG C 27   19.784 35.061 39.697 1.00 0.00
C   H

ATOM 20493 NH2 ARG C 27   21.779 34.131 41.986 1.00 0.00
C   N
ATOM 20494 HH21 ARG C 27   22.452 34.853 41.925 1.00 0.00
C   H
ATOM 20495 HH22 ARG C 27   21.777 33.482 42.736 1.00 0.00
C   H
ATOM 20496 C   ARG C 27   21.342 31.452 37.838 1.00 0.00
C   C
ATOM 20497 O   ARG C 27   21.395 32.528 37.245 1.00 0.00
C   O
ATOM 20498 N   MET C 28   22.418 30.841 38.331 1.00 0.00
C   N
ATOM 20499 HN MET C 28   22.376 29.989 38.847 1.00 0.00
C   H
ATOM 20500 CA MET C 28   23.773 31.377 38.153 1.00 0.00
C   C
ATOM 20501 HA MET C 28   23.698 32.449 38.287 1.00 0.00
C   H
ATOM 20502 CB MET C 28   24.734 30.792 39.181 1.00 0.00
C   C
ATOM 20503 HB1 MET C 28   24.619 29.684 39.140 1.00 0.00
C   H
ATOM 20504 HB2 MET C 28   25.781 31.008 38.872 1.00 0.00
C   H
ATOM 20505 CG MET C 28   24.498 31.251 40.606 1.00 0.00
C   C
ATOM 20506 HG1 MET C 28   24.645 32.348 40.687 1.00 0.00
C   H
ATOM 20507 HG2 MET C 28   23.450 31.028 40.900 1.00 0.00
C   H
ATOM 20508 SD MET C 28   25.637 30.426 41.734 1.00 0.00
C   S
ATOM 20509 CE MET C 28   27.008 31.583 41.716 1.00 0.00
C   C
ATOM 20510 HE1 MET C 28   27.835 31.227 42.366 1.00 0.00
C   H
ATOM 20511 HE2 MET C 28   27.410 31.707 40.687 1.00 0.00
C   H
ATOM 20512 HE3 MET C 28   26.691 32.583 42.082 1.00 0.00
C   H
ATOM 20513 C   MET C 28   24.330 31.125 36.753 1.00 0.00
C   C
ATOM 20514 O   MET C 28   25.313 31.749 36.355 1.00 0.00
C   O
ATOM 20515 N   GLY C 29   23.718 30.198 36.018 1.00 0.00
C   N
ATOM 20516 HN GLY C 29   22.991 29.616 36.380 1.00 0.00
C   H
ATOM 20517 CA GLY C 29   24.073 29.969 34.621 1.00 0.00
C   C
ATOM 20518 HA1 GLY C 29   24.673 30.824 34.338 1.00 0.00
C   H
ATOM 20519 HA2 GLY C 29   23.132 29.892 34.094 1.00 0.00
C   H

```
ATOM  20520  C   GLY C  29      24.875  28.718  34.333  1.00  0.00
C    C
ATOM  20521  O   GLY C  29      25.281  28.489  33.197  1.00  0.00
C    O
ATOM  20522  N   HSE C  30      25.109  27.903  35.357  1.00  0.00
C    N
ATOM  20523  HN  HSE C  30      24.866  28.107  36.303  1.00  0.00
C    H
ATOM  20524  CA  HSE C  30      25.761  26.606  35.167  1.00  0.00
C    C
ATOM  20525  HA  HSE C  30      26.518  26.729  34.402  1.00  0.00
C    H
ATOM  20526  CB  HSE C  30      26.341  26.104  36.478  1.00  0.00
C    C
ATOM  20527  HB1 HSE C  30      25.495  25.885  37.165  1.00  0.00
C    H
ATOM  20528  HB2 HSE C  30      26.874  25.147  36.304  1.00  0.00
C    H
ATOM  20529  ND1 HSE C  30      28.560  27.174  36.889  1.00  0.00
C    N
ATOM  20530  CG  HSE C  30      27.244  27.095  37.154  1.00  0.00
C    C
ATOM  20531  CE1 HSE C  30      29.111  28.152  37.634  1.00  0.00
C    C
ATOM  20532  HE1 HSE C  30      30.170  28.422  37.618  1.00  0.00
C    H
ATOM  20533  NE2 HSE C  30      28.137  28.704  38.377  1.00  0.00
C    N
ATOM  20534  HE2 HSE C  30      28.263  29.444  39.038  1.00  0.00
C    H
ATOM  20535  CD2 HSE C  30      26.975  28.075  38.105  1.00  0.00
C    C
ATOM  20536  HD2 HSE C  30      26.043  28.335  38.587  1.00  0.00
C    H
ATOM  20537  C   HSE C  30      24.769  25.614  34.647  1.00  0.00
C    C
ATOM  20538  O   HSE C  30      23.560  25.789  34.824  1.00  0.00
C    O
ATOM  20539  N   GLU C  31      25.257  24.567  33.987  1.00  0.00
C    N
ATOM  20540  HN  GLU C  31      26.211  24.433  33.736  1.00  0.00
C    H
ATOM  20541  CA  GLU C  31      24.373  23.483  33.564  1.00  0.00
C    C
ATOM  20542  HA  GLU C  31      23.379  23.890  33.423  1.00  0.00
C    H
ATOM  20543  CB  GLU C  31      24.739  22.902  32.190  1.00  0.00
C    C
ATOM  20544  HB1 GLU C  31      24.168  21.957  32.049  1.00  0.00
C    H
ATOM  20545  HB2 GLU C  31      24.395  23.590  31.385  1.00  0.00
C    H
ATOM  20546  CG  GLU C  31      26.207  22.586  31.949  1.00  0.00
C    C
```

ATOM 20547 HG1 GLU C 31   26.828 23.438 32.281 1.00 0.00
C   H
ATOM 20548 HG2 GLU C 31   26.513 21.676 32.501 1.00 0.00
C   H
ATOM 20549 CD GLU C 31   26.526 22.390 30.471 1.00 0.00
C   C
ATOM 20550 OE1 GLU C 31   25.596 22.115 29.677 1.00 0.00
C   O
ATOM 20551 OE2 GLU C 31   27.715 22.512 30.100 1.00 0.00
C   O
ATOM 20552 C   GLU C 31   24.272 22.409 34.642 1.00 0.00
C   C
ATOM 20553 O   GLU C 31   25.276 21.990 35.223 1.00 0.00
C   O
ATOM 20554 N   VAL C 32   23.038 21.996 34.915 1.00 0.00
C   N
ATOM 20555 HN VAL C 32   22.251 22.245 34.356 1.00 0.00
C   H
ATOM 20556 CA VAL C 32   22.721 21.143 36.054 1.00 0.00
C   C
ATOM 20557 HA VAL C 32   23.646 20.701 36.401 1.00 0.00
C   H
ATOM 20558 CB VAL C 32   22.004 21.929 37.180 1.00 0.00
C   C
ATOM 20559 HB VAL C 32   20.971 22.161 36.823 1.00 0.00
C   H
ATOM 20560 CG1 VAL C 32   21.921 21.094 38.445 1.00 0.00
C   C
ATOM 20561 HG11 VAL C 32   21.441 21.677 39.261 1.00 0.00
C   H
ATOM 20562 HG12 VAL C 32   21.314 20.177 38.291 1.00 0.00
C   H
ATOM 20563 HG13 VAL C 32   22.936 20.796 38.786 1.00 0.00
C   H
ATOM 20564 CG2 VAL C 32   22.712 23.238 37.483 1.00 0.00
C   C
ATOM 20565 HG21 VAL C 32   22.211 23.771 38.319 1.00 0.00
C   H
ATOM 20566 HG22 VAL C 32   23.770 23.052 37.772 1.00 0.00
C   H
ATOM 20567 HG23 VAL C 32   22.705 23.913 36.602 1.00 0.00
C   H
ATOM 20568 C   VAL C 32   21.804 20.007 35.625 1.00 0.00
C   C
ATOM 20569 O   VAL C 32   20.789 20.232 34.958 1.00 0.00
C   O
ATOM 20570 N   LEU C 33   22.168 18.787 36.009 1.00 0.00
C   N
ATOM 20571 HN LEU C 33   23.049 18.558 36.417 1.00 0.00
C   H
ATOM 20572 CA LEU C 33   21.269 17.653 35.854 1.00 0.00
C   C
ATOM 20573 HA LEU C 33   20.526 17.921 35.114 1.00 0.00
C   H

```
ATOM  20574  CB  LEU C  33      22.023  16.401  35.409  1.00  0.00
C    C
ATOM  20575  HB1 LEU C  33      22.648  16.687  34.532  1.00  0.00
C    H
ATOM  20576  HB2 LEU C  33      22.735  16.104  36.210  1.00  0.00
C    H
ATOM  20577  CG  LEU C  33      21.148  15.209  35.013  1.00  0.00
C    C
ATOM  20578  HG  LEU C  33      20.165  15.633  34.697  1.00  0.00
C    H
ATOM  20579  CD1 LEU C  33      21.755  14.402  33.879  1.00  0.00
C    C
ATOM  20580 HD11 LEU C  33      21.122  13.519  33.646  1.00  0.00
C    H
ATOM  20581 HD12 LEU C  33      21.840  15.021  32.960  1.00  0.00
C    H
ATOM  20582 HD13 LEU C  33      22.768  14.039  34.154  1.00  0.00
C    H
ATOM  20583  CD2 LEU C  33      20.910  14.316  36.209  1.00  0.00
C    C
ATOM  20584 HD21 LEU C  33      20.259  13.460  35.928  1.00  0.00
C    H
ATOM  20585 HD22 LEU C  33      21.859  13.902  36.606  1.00  0.00
C    H
ATOM  20586 HD23 LEU C  33      20.407  14.882  37.024  1.00  0.00
C    H
ATOM  20587  C   LEU C  33      20.541  17.416  37.171  1.00  0.00
C    C
ATOM  20588  O   LEU C  33      21.163  17.135  38.193  1.00  0.00
C    O
ATOM  20589  N   ALA C  34      19.222  17.545  37.136  1.00  0.00
C    N
ATOM  20590  HN  ALA C  34      18.723  17.805  36.309  1.00  0.00
C    H
ATOM  20591  CA  ALA C  34      18.403  17.317  38.311  1.00  0.00
C    C
ATOM  20592  HA  ALA C  34      18.978  17.419  39.225  1.00  0.00
C    H
ATOM  20593  CB  ALA C  34      17.245  18.295  38.339  1.00  0.00
C    C
ATOM  20594  HB1 ALA C  34      17.635  19.333  38.409  1.00  0.00
C    H
ATOM  20595  HB2 ALA C  34      16.637  18.221  37.411  1.00  0.00
C    H
ATOM  20596  HB3 ALA C  34      16.580  18.118  39.212  1.00  0.00
C    H
ATOM  20597  C   ALA C  34      17.897  15.890  38.283  1.00  0.00
C    C
ATOM  20598  O   ALA C  34      17.330  15.452  37.281  1.00  0.00
C    O
ATOM  20599  N   VAL C  35      18.123  15.166  39.374  1.00  0.00
C    N
ATOM  20600  HN  VAL C  35      18.609  15.515  40.171  1.00  0.00
C    H
```

ATOM 20601 CA VAL C 35   17.672 13.778 39.499 1.00 0.00
C   C
ATOM 20602 HA VAL C 35   17.092 13.532 38.618 1.00 0.00
C   H
ATOM 20603 CB VAL C 35   18.847 12.792 39.684 1.00 0.00
C   C
ATOM 20604 HB VAL C 35   19.341 13.035 40.657 1.00 0.00
C   H
ATOM 20605 CG1 VAL C 35   18.347 11.358 39.726 1.00 0.00
C   C
ATOM 20606 HG11 VAL C 35   19.202 10.654 39.819 1.00 0.00
C   H
ATOM 20607 HG12 VAL C 35   17.678 11.183 40.593 1.00 0.00
C   H
ATOM 20608 HG13 VAL C 35   17.797 11.107 38.794 1.00 0.00
C   H
ATOM 20609 CG2 VAL C 35   19.866 12.940 38.571 1.00 0.00
C   C
ATOM 20610 HG21 VAL C 35   20.686 12.201 38.689 1.00 0.00
C   H
ATOM 20611 HG22 VAL C 35   19.389 12.777 37.580 1.00 0.00
C   H
ATOM 20612 HG23 VAL C 35   20.323 13.951 38.575 1.00 0.00
C   H
ATOM 20613 C   VAL C 35   16.752 13.646 40.702 1.00 0.00
C   C
ATOM 20614 O   VAL C 35   17.002 14.248 41.745 1.00 0.00
C   O
ATOM 20615 N   ASP C 36   15.687 12.868 40.548 1.00 0.00
C   N
ATOM 20616 HN ASP C 36   15.399 12.457 39.687 1.00 0.00
C   H
ATOM 20617 CA ASP C 36   14.809 12.531 41.662 1.00 0.00
C   C
ATOM 20618 HA ASP C 36   15.436 12.295 42.514 1.00 0.00
C   H
ATOM 20619 CB ASP C 36   13.867 13.694 41.996 1.00 0.00
C   C
ATOM 20620 HB1 ASP C 36   14.418 14.650 41.897 1.00 0.00
C   H
ATOM 20621 HB2 ASP C 36   13.007 13.714 41.298 1.00 0.00
C   H
ATOM 20622 CG ASP C 36   13.331 13.627 43.415 1.00 0.00
C   C
ATOM 20623 OD1 ASP C 36   13.644 14.540 44.217 1.00 0.00
C   O
ATOM 20624 OD2 ASP C 36   12.597 12.665 43.732 1.00 0.00
C   O
ATOM 20625 C   ASP C 36   14.020 11.268 41.334 1.00 0.00
C   C
ATOM 20626 O   ASP C 36   13.744 10.981 40.170 1.00 0.00
C   O
ATOM 20627 N   ILE C 37   13.673 10.510 42.369 1.00 0.00
C   N

```
ATOM  20628  HN  ILE C  37      13.929  10.710  43.313  1.00  0.00
C    H
ATOM  20629  CA  ILE C  37      12.873   9.298  42.209  1.00  0.00
C    C
ATOM  20630  HA  ILE C  37      13.244   8.802  41.321  1.00  0.00
C    H
ATOM  20631  CB  ILE C  37      13.023   8.364  43.433  1.00  0.00
C    C
ATOM  20632  HB  ILE C  37      14.129   8.281  43.588  1.00  0.00
C    H
ATOM  20633  CG2 ILE C  37      12.336   8.946  44.668  1.00  0.00
C    C
ATOM  20634 HG21 ILE C  37      12.582   8.348  45.570  1.00  0.00
C    H
ATOM  20635 HG22 ILE C  37      12.671   9.988  44.853  1.00  0.00
C    H
ATOM  20636 HG23 ILE C  37      11.233   8.950  44.545  1.00  0.00
C    H
ATOM  20637  CG1 ILE C  37      12.517   6.953  43.110  1.00  0.00
C    C
ATOM  20638 HG11 ILE C  37      11.406   6.955  43.052  1.00  0.00
C    H
ATOM  20639 HG12 ILE C  37      12.898   6.654  42.106  1.00  0.00
C    H
ATOM  20640  CD  ILE C  37      12.951   5.896  44.104  1.00  0.00
C    C
ATOM  20641  HD1 ILE C  37      12.600   4.893  43.779  1.00  0.00
C    H
ATOM  20642  HD2 ILE C  37      14.059   5.867  44.187  1.00  0.00
C    H
ATOM  20643  HD3 ILE C  37      12.527   6.098  45.110  1.00  0.00
C    H
ATOM  20644  C   ILE C  37      11.401   9.638  41.945  1.00  0.00
C    C
ATOM  20645  O   ILE C  37      10.690   8.880  41.285  1.00  0.00
C    O
ATOM  20646  N   ASN C  38      10.968  10.789  42.456  1.00  0.00
C    N
ATOM  20647  HN  ASN C  38      11.582  11.435  42.907  1.00  0.00
C    H
ATOM  20648  CA  ASN C  38       9.576  11.214  42.406  1.00  0.00
C    C
ATOM  20649  HA  ASN C  38       8.991  10.301  42.371  1.00  0.00
C    H
ATOM  20650  CB  ASN C  38       9.262  12.045  43.652  1.00  0.00
C    C
ATOM  20651  HB1 ASN C  38       9.480  11.417  44.544  1.00  0.00
C    H
ATOM  20652  HB2 ASN C  38       9.924  12.933  43.700  1.00  0.00
C    H
ATOM  20653  CG  ASN C  38       7.814  12.472  43.728  1.00  0.00
C    C
ATOM  20654  OD1 ASN C  38       7.391  13.403  43.048  1.00  0.00
C    O
```

ATOM 20655 ND2 ASN C 38   7.049 11.811 44.581 1.00 0.00
C   N
ATOM 20656 HD21 ASN C 38   6.089 12.074 44.634 1.00 0.00
C   H
ATOM 20657 HD22 ASN C 38   7.430 11.064 45.118 1.00 0.00
C   H
ATOM 20658 C   ASN C 38   9.279 12.003 41.135 1.00 0.00
C   C
ATOM 20659 O   ASN C 38   9.925 13.017 40.866 1.00 0.00
C   O
ATOM 20660 N   GLU C 39   8.295 11.542 40.365 1.00 0.00
C   N
ATOM 20661 HN GLU C 39   7.710 10.770 40.599 1.00 0.00
C   H
ATOM 20662 CA GLU C 39   7.982 12.154 39.068 1.00 0.00
C   C
ATOM 20663 HA GLU C 39   8.896 12.135 38.488 1.00 0.00
C   H
ATOM 20664 CB GLU C 39   6.960 11.319 38.291 1.00 0.00
C   C
ATOM 20665 HB1 GLU C 39   7.193 10.242 38.457 1.00 0.00
C   H
ATOM 20666 HB2 GLU C 39   5.942 11.479 38.712 1.00 0.00
C   H
ATOM 20667 CG GLU C 39   6.964 11.610 36.798 1.00 0.00
C   C
ATOM 20668 HG1 GLU C 39   6.765 12.684 36.631 1.00 0.00
C   H
ATOM 20669 HG2 GLU C 39   7.940 11.344 36.344 1.00 0.00
C   H
ATOM 20670 CD GLU C 39   5.876 10.876 36.042 1.00 0.00
C   C
ATOM 20671 OE1 GLU C 39   5.751 11.104 34.819 1.00 0.00
C   O
ATOM 20672 OE2 GLU C 39   5.141 10.074 36.664 1.00 0.00
C   O
ATOM 20673 C   GLU C 39   7.524 13.621 39.152 1.00 0.00
C   C
ATOM 20674 O   GLU C 39   7.891 14.433 38.293 1.00 0.00
C   O
ATOM 20675 N   GLU C 40   6.732 13.945 40.176 1.00 0.00
C   N
ATOM 20676 HN GLU C 40   6.402 13.294 40.853 1.00 0.00
C   H
ATOM 20677 CA GLU C 40   6.272 15.320 40.396 1.00 0.00
C   C
ATOM 20678 HA GLU C 40   5.749 15.641 39.505 1.00 0.00
C   H
ATOM 20679 CB GLU C 40   5.303 15.394 41.578 1.00 0.00
C   C
ATOM 20680 HB1 GLU C 40   5.708 14.759 42.399 1.00 0.00
C   H
ATOM 20681 HB2 GLU C 40   5.276 16.431 41.980 1.00 0.00
C   H

ATOM 20682 CG GLU C 40   3.889 14.934 41.256 1.00 0.00
C   C
ATOM 20683 HG1 GLU C 40   3.307 15.784 40.857 1.00 0.00
C   H
ATOM 20684 HG2 GLU C 40   3.898 14.115 40.510 1.00 0.00
C   H
ATOM 20685 CD GLU C 40   3.133 14.458 42.482 1.00 0.00
C   C
ATOM 20686 OE1 GLU C 40   3.748 13.788 43.346 1.00 0.00
C   O
ATOM 20687 OE2 GLU C 40   1.919 14.742 42.579 1.00 0.00
C   O
ATOM 20688 C   GLU C 40   7.439 16.285 40.609 1.00 0.00
C   C
ATOM 20689 O   GLU C 40   7.477 17.367 40.011 1.00 0.00
C   O
ATOM 20690 N   LYS C 41   8.388 15.882 41.453 1.00 0.00
C   N
ATOM 20691 HN LYS C 41   8.330 15.023 41.957 1.00 0.00
C   H
ATOM 20692 CA LYS C 41   9.589 16.670 41.707 1.00 0.00
C   C
ATOM 20693 HA LYS C 41   9.264 17.631 42.086 1.00 0.00
C   H
ATOM 20694 CB LYS C 41   10.481 15.974 42.740 1.00 0.00
C   C
ATOM 20695 HB1 LYS C 41   10.627 14.924 42.395 1.00 0.00
C   H
ATOM 20696 HB2 LYS C 41   11.488 16.450 42.728 1.00 0.00
C   H
ATOM 20697 CG LYS C 41   9.927 15.947 44.159 1.00 0.00
C   C
ATOM 20698 HG1 LYS C 41   8.836 15.730 44.119 1.00 0.00
C   H
ATOM 20699 HG2 LYS C 41   10.412 15.116 44.722 1.00 0.00
C   H
ATOM 20700 CD LYS C 41   10.144 17.271 44.879 1.00 0.00
C   C
ATOM 20701 HD1 LYS C 41   11.230 17.373 45.108 1.00 0.00
C   H
ATOM 20702 HD2 LYS C 41   9.872 18.095 44.180 1.00 0.00
C   H
ATOM 20703 CE LYS C 41   9.317 17.363 46.150 1.00 0.00
C   C
ATOM 20704 HE1 LYS C 41   9.516 18.323 46.677 1.00 0.00
C   H
ATOM 20705 HE2 LYS C 41   8.231 17.310 45.914 1.00 0.00
C   H
ATOM 20706 NZ LYS C 41   9.619 16.263 47.104 1.00 0.00
C   N
ATOM 20707 HZ1 LYS C 41   9.034 16.363 47.959 1.00 0.00
C   H
ATOM 20708 HZ2 LYS C 41   9.418 15.348 46.653 1.00 0.00
C   H

ATOM 20709 HZ3 LYS C 41   10.624 16.301 47.367 1.00 0.00
C   H
ATOM 20710 C   LYS C 41   10.370 16.928 40.416 1.00 0.00
C   C
ATOM 20711 O   LYS C 41   10.752 18.070 40.145 1.00 0.00
C   O
ATOM 20712 N   VAL C 42   10.585 15.870 39.628 1.00 0.00
C   N
ATOM 20713 HN VAL C 42   10.307 14.946 39.877 1.00 0.00
C   H
ATOM 20714 CA VAL C 42   11.252 15.972 38.321 1.00 0.00
C   C
ATOM 20715 HA VAL C 42   12.236 16.390 38.497 1.00 0.00
C   H
ATOM 20716 CB VAL C 42   11.434 14.590 37.654 1.00 0.00
C   C
ATOM 20717 HB VAL C 42   10.417 14.197 37.409 1.00 0.00
C   H
ATOM 20718 CG1 VAL C 42   12.242 14.705 36.371 1.00 0.00
C   C
ATOM 20719 HG11 VAL C 42   12.398 13.700 35.922 1.00 0.00
C   H
ATOM 20720 HG12 VAL C 42   11.722 15.328 35.613 1.00 0.00
C   H
ATOM 20721 HG13 VAL C 42   13.239 15.151 36.574 1.00 0.00
C   H
ATOM 20722 CG2 VAL C 42   12.127 13.632 38.604 1.00 0.00
C   C
ATOM 20723 HG21 VAL C 42   12.296 12.648 38.117 1.00 0.00
C   H
ATOM 20724 HG22 VAL C 42   13.112 14.040 38.920 1.00 0.00
C   H
ATOM 20725 HG23 VAL C 42   11.516 13.455 39.514 1.00 0.00
C   H
ATOM 20726 C   VAL C 42   10.509 16.927 37.375 1.00 0.00
C   C
ATOM 20727 O   VAL C 42   11.139 17.745 36.704 1.00 0.00
C   O
ATOM 20728 N   ASN C 43   9.180 16.826 37.335 1.00 0.00
C   N
ATOM 20729 HN ASN C 43   8.666 16.124 37.825 1.00 0.00
C   H
ATOM 20730 CA ASN C 43   8.354 17.753 36.556 1.00 0.00
C   C
ATOM 20731 HA ASN C 43   8.733 17.691 35.542 1.00 0.00
C   H
ATOM 20732 CB ASN C 43   6.879 17.342 36.596 1.00 0.00
C   C
ATOM 20733 HB1 ASN C 43   6.625 17.090 37.649 1.00 0.00
C   H
ATOM 20734 HB2 ASN C 43   6.235 18.191 36.291 1.00 0.00
C   H
ATOM 20735 CG ASN C 43   6.581 16.123 35.738 1.00 0.00
C   C

ATOM 20736 OD1 ASN C 43   7.412 15.684 34.942 1.00 0.00
C   O
ATOM 20737 ND2 ASN C 43   5.384 15.574 35.896 1.00 0.00
C   N
ATOM 20738 HD21 ASN C 43   5.175 14.768 35.349 1.00 0.00
C   H
ATOM 20739 HD22 ASN C 43   4.746 15.951 36.561 1.00 0.00
C   H
ATOM 20740 C   ASN C 43   8.503 19.198 37.017 1.00 0.00
C   C
ATOM 20741 O   ASN C 43   8.637 20.104 36.195 1.00 0.00
C   O
ATOM 20742 N   ALA C 44   8.502 19.396 38.337 1.00 0.00
C   N
ATOM 20743 HN ALA C 44   8.450 18.635 38.983 1.00 0.00
C   H
ATOM 20744 CA ALA C 44   8.574 20.724 38.937 1.00 0.00
C   C
ATOM 20745 HA ALA C 44   7.834 21.370 38.481 1.00 0.00
C   H
ATOM 20746 CB ALA C 44   8.243 20.656 40.419 1.00 0.00
C   C
ATOM 20747 HB1 ALA C 44   7.237 20.206 40.555 1.00 0.00
C   H
ATOM 20748 HB2 ALA C 44   8.977 20.023 40.963 1.00 0.00
C   H
ATOM 20749 HB3 ALA C 44   8.233 21.666 40.883 1.00 0.00
C   H
ATOM 20750 C   ALA C 44   9.926 21.403 38.728 1.00 0.00
C   C
ATOM 20751 O   ALA C 44   10.026 22.627 38.802 1.00 0.00
C   O
ATOM 20752 N   TYR C 45   10.962 20.613 38.467 1.00 0.00
C   N
ATOM 20753 HN TYR C 45   10.915 19.619 38.391 1.00 0.00
C   H
ATOM 20754 CA TYR C 45   12.296 21.175 38.266 1.00 0.00
C   C
ATOM 20755 HA TYR C 45   12.221 22.206 38.589 1.00 0.00
C   H
ATOM 20756 CB TYR C 45   13.315 20.494 39.188 1.00 0.00
C   C
ATOM 20757 HB1 TYR C 45   13.109 19.403 39.216 1.00 0.00
C   H
ATOM 20758 HB2 TYR C 45   14.347 20.624 38.796 1.00 0.00
C   H
ATOM 20759 CG TYR C 45   13.222 21.014 40.601 1.00 0.00
C   C
ATOM 20760 CD1 TYR C 45   12.396 20.399 41.538 1.00 0.00
C   C
ATOM 20761 HD1 TYR C 45   11.814 19.533 41.256 1.00 0.00
C   H
ATOM 20762 CE1 TYR C 45   12.289 20.891 42.829 1.00 0.00
C   C

ATOM 20763 HE1 TYR C 45   11.650 20.405 43.551 1.00 0.00
C   H
ATOM 20764 CZ TYR C 45   13.004 22.018 43.192 1.00 0.00
C   C
ATOM 20765 OH TYR C 45   12.902 22.513 44.467 1.00 0.00
C   O
ATOM 20766 HH TYR C 45   13.519 23.243 44.543 1.00 0.00
C   H
ATOM 20767 CD2 TYR C 45   13.926 22.152 40.990 1.00 0.00
C   C
ATOM 20768 HD2 TYR C 45   14.534 22.684 40.271 1.00 0.00
C   H
ATOM 20769 CE2 TYR C 45   13.825 22.651 42.280 1.00 0.00
C   C
ATOM 20770 HE2 TYR C 45   14.373 23.541 42.555 1.00 0.00
C   H
ATOM 20771 C   TYR C 45   12.755 21.190 36.809 1.00 0.00
C   C
ATOM 20772 O   TYR C 45   13.876 21.624 36.502 1.00 0.00
C   O
ATOM 20773 N   ALA C 46   11.872 20.754 35.912 1.00 0.00
C   N
ATOM 20774 HN ALA C 46   10.982 20.376 36.167 1.00 0.00
C   H
ATOM 20775 CA ALA C 46   12.146 20.800 34.478 1.00 0.00
C   C
ATOM 20776 HA ALA C 46   12.935 20.102 34.221 1.00 0.00
C   H
ATOM 20777 CB ALA C 46   10.911 20.381 33.689 1.00 0.00
C   C
ATOM 20778 HB1 ALA C 46   10.608 19.355 33.987 1.00 0.00
C   H
ATOM 20779 HB2 ALA C 46   10.056 21.063 33.893 1.00 0.00
C   H
ATOM 20780 HB3 ALA C 46   11.106 20.378 32.594 1.00 0.00
C   H
ATOM 20781 C   ALA C 46   12.636 22.192 34.042 1.00 0.00
C   C
ATOM 20782 O   ALA C 46   13.622 22.309 33.309 1.00 0.00
C   O
ATOM 20783 N   SER C 47   11.968 23.233 34.532 1.00 0.00
C   N
ATOM 20784 HN SER C 47   11.198 23.137 35.163 1.00 0.00
C   H
ATOM 20785 CA SER C 47   12.294 24.617 34.194 1.00 0.00
C   C
ATOM 20786 HA SER C 47   12.520 24.592 33.136 1.00 0.00
C   H
ATOM 20787 CB SER C 47   11.102 25.523 34.498 1.00 0.00
C   C
ATOM 20788 HB1 SER C 47   10.823 25.381 35.567 1.00 0.00
C   H
ATOM 20789 HB2 SER C 47   11.386 26.593 34.372 1.00 0.00
C   H

ATOM 20790 OG SER C 47 9.994 25.187 33.688 1.00 0.00
C O
ATOM 20791 HG1 SER C 47 9.222 25.625 34.069 1.00 0.00
C H
ATOM 20792 C SER C 47 13.525 25.173 34.907 1.00 0.00
C C
ATOM 20793 O SER C 47 14.046 26.220 34.509 1.00 0.00
C O
ATOM 20794 N TYR C 48 13.982 24.492 35.955 1.00 0.00
C N
ATOM 20795 HN TYR C 48 13.643 23.596 36.237 1.00 0.00
C H
ATOM 20796 CA TYR C 48 15.053 25.030 36.797 1.00 0.00
C C
ATOM 20797 HA TYR C 48 15.269 26.017 36.406 1.00 0.00
C H
ATOM 20798 CB TYR C 48 14.590 25.147 38.252 1.00 0.00
C C
ATOM 20799 HB1 TYR C 48 14.367 24.132 38.647 1.00 0.00
C H
ATOM 20800 HB2 TYR C 48 15.395 25.578 38.887 1.00 0.00
C H
ATOM 20801 CG TYR C 48 13.331 25.963 38.434 1.00 0.00
C C
ATOM 20802 CD1 TYR C 48 12.096 25.343 38.629 1.00 0.00
C C
ATOM 20803 HD1 TYR C 48 12.032 24.263 38.633 1.00 0.00
C H
ATOM 20804 CE1 TYR C 48 10.941 26.087 38.797 1.00 0.00
C C
ATOM 20805 HE1 TYR C 48 9.990 25.598 38.944 1.00 0.00
C H
ATOM 20806 CZ TYR C 48 11.009 27.472 38.769 1.00 0.00
C C
ATOM 20807 OH TYR C 48 9.868 28.224 38.933 1.00 0.00
C O
ATOM 20808 HH TYR C 48 10.132 29.147 38.954 1.00 0.00
C H
ATOM 20809 CD2 TYR C 48 13.370 27.354 38.412 1.00 0.00
C C
ATOM 20810 HD2 TYR C 48 14.304 27.866 38.225 1.00 0.00
C H
ATOM 20811 CE2 TYR C 48 12.220 28.108 38.577 1.00 0.00
C C
ATOM 20812 HE2 TYR C 48 12.275 29.186 38.534 1.00 0.00
C H
ATOM 20813 C TYR C 48 16.339 24.217 36.703 1.00 0.00
C C
ATOM 20814 O TYR C 48 17.186 24.266 37.600 1.00 0.00
C O
ATOM 20815 N ALA C 49 16.480 23.478 35.605 1.00 0.00
C N
ATOM 20816 HN ALA C 49 15.784 23.459 34.887 1.00 0.00
C H

```
ATOM  20817  CA  ALA C  49      17.653  22.641  35.363  1.00  0.00
C    C
ATOM  20818  HA  ALA C  49      18.553  23.122  35.730  1.00  0.00
C    H
ATOM  20819  CB  ALA C  49      17.493  21.301  36.061  1.00  0.00
C    C
ATOM  20820  HB1 ALA C  49      17.393  21.461  37.156  1.00  0.00
C    H
ATOM  20821  HB2 ALA C  49      16.580  20.773  35.707  1.00  0.00
C    H
ATOM  20822  HB3 ALA C  49      18.371  20.643  35.889  1.00  0.00
C    H
ATOM  20823  C   ALA C  49      17.859  22.429  33.871  1.00  0.00
C    C
ATOM  20824  O   ALA C  49      16.901  22.477  33.096  1.00  0.00
C    O
ATOM  20825  N   THR C  50      19.104  22.194  33.470  1.00  0.00
C    N
ATOM  20826  HN  THR C  50      19.900  22.223  34.074  1.00  0.00
C    H
ATOM  20827  CA  THR C  50      19.415  21.865  32.082  1.00  0.00
C    C
ATOM  20828  HA  THR C  50      19.014  22.673  31.484  1.00  0.00
C    H
ATOM  20829  CB  THR C  50      20.930  21.694  31.880  1.00  0.00
C    C
ATOM  20830  HB  THR C  50      21.209  20.750  32.410  1.00  0.00
C    H
ATOM  20831  OG1 THR C  50      21.612  22.781  32.513  1.00  0.00
C    O
ATOM  20832  HG1 THR C  50      22.548  22.630  32.345  1.00  0.00
C    H
ATOM  20833  CG2 THR C  50      21.285  21.669  30.399  1.00  0.00
C    C
ATOM  20834 HG21 THR C  50      22.361  21.431  30.254  1.00  0.00
C    H
ATOM  20835 HG22 THR C  50      20.690  20.894  29.871  1.00  0.00
C    H
ATOM  20836 HG23 THR C  50      21.078  22.651  29.920  1.00  0.00
C    H
ATOM  20837  C   THR C  50      18.678  20.595  31.650  1.00  0.00
C    C
ATOM  20838  O   THR C  50      18.049  20.561  30.593  1.00  0.00
C    O
ATOM  20839  N   HSE C  51      18.759  19.564  32.483  1.00  0.00
C    N
ATOM  20840  HN  HSE C  51      19.331  19.570  33.302  1.00  0.00
C    H
ATOM  20841  CA  HSE C  51      18.018  18.328  32.277  1.00  0.00
C    C
ATOM  20842  HA  HSE C  51      17.164  18.566  31.654  1.00  0.00
C    H
ATOM  20843  CB  HSE C  51      18.902  17.272  31.629  1.00  0.00
C    C
```

ATOM 20844 HB1 HSE C 51   19.937 17.414 32.007 1.00 0.00
C   H
ATOM 20845 HB2 HSE C 51   18.572 16.263 31.954 1.00 0.00
C   H
ATOM 20846 ND1 HSE C 51   19.819 18.127 29.460 1.00 0.00
C   N
ATOM 20847 CG HSE C 51   18.943 17.350 30.124 1.00 0.00
C   C
ATOM 20848 CE1 HSE C 51   19.622 17.994 28.131 1.00 0.00
C   C
ATOM 20849 HE1 HSE C 51   20.195 18.519 27.364 1.00 0.00
C   H
ATOM 20850 NE2 HSE C 51   18.613 17.125 27.946 1.00 0.00
C   N
ATOM 20851 HE2 HSE C 51   18.240 16.852 27.060 1.00 0.00
C   H
ATOM 20852 CD2 HSE C 51   18.173 16.707 29.154 1.00 0.00
C   C
ATOM 20853 HD2 HSE C 51   17.357 16.006 29.267 1.00 0.00
C   H
ATOM 20854 C   HSE C 51   17.482 17.813 33.581 1.00 0.00
C   C
ATOM 20855 O   HSE C 51   18.086 18.017 34.638 1.00 0.00
C   O
ATOM 20856 N   ALA C 52   16.340 17.137 33.517 1.00 0.00
C   N
ATOM 20857 HN ALA C 52   15.843 17.010 32.659 1.00 0.00
C   H
ATOM 20858 CA ALA C 52   15.729 16.532 34.687 1.00 0.00
C   C
ATOM 20859 HA ALA C 52   16.438 16.445 35.502 1.00 0.00
C   H
ATOM 20860 CB ALA C 52   14.560 17.374 35.161 1.00 0.00
C   C
ATOM 20861 HB1 ALA C 52   14.920 18.385 35.445 1.00 0.00
C   H
ATOM 20862 HB2 ALA C 52   13.801 17.493 34.357 1.00 0.00
C   H
ATOM 20863 HB3 ALA C 52   14.065 16.924 36.050 1.00 0.00
C   H
ATOM 20864 C   ALA C 52   15.281 15.115 34.355 1.00 0.00
C   C
ATOM 20865 O   ALA C 52   14.634 14.891 33.330 1.00 0.00
C   O
ATOM 20866 N   VAL C 53   15.635 14.159 35.212 1.00 0.00
C   N
ATOM 20867 HN VAL C 53   16.089 14.338 36.080 1.00 0.00
C   H
ATOM 20868 CA VAL C 53   15.379 12.740 34.944 1.00 0.00
C   C
ATOM 20869 HA VAL C 53   14.608 12.693 34.185 1.00 0.00
C   H
ATOM 20870 CB VAL C 53   16.658 11.998 34.485 1.00 0.00
C   C

```
ATOM  20871  HB  VAL C  53      17.217  11.690  35.402  1.00  0.00
C    H
ATOM  20872  CG1 VAL C  53      16.296  10.757  33.679  1.00  0.00
C    C
ATOM  20873 HG11 VAL C  53      17.216  10.250  33.318  1.00  0.00
C    H
ATOM  20874 HG12 VAL C  53      15.730  10.022  34.288  1.00  0.00
C    H
ATOM  20875 HG13 VAL C  53      15.684  11.029  32.793  1.00  0.00
C    H
ATOM  20876  CG2 VAL C  53      17.569  12.903  33.667  1.00  0.00
C    C
ATOM  20877 HG21 VAL C  53      18.462  12.344  33.312  1.00  0.00
C    H
ATOM  20878 HG22 VAL C  53      17.030  13.301  32.779  1.00  0.00
C    H
ATOM  20879 HG23 VAL C  53      17.930  13.762  34.270  1.00  0.00
C    H
ATOM  20880  C   VAL C  53      14.851  12.016  36.178  1.00  0.00
C    C
ATOM  20881  O   VAL C  53      15.342  12.234  37.284  1.00  0.00
C    O
ATOM  20882  N   ILE C  54      13.858  11.152  35.974  1.00  0.00
C    N
ATOM  20883  HN  ILE C  54      13.358  11.074  35.114  1.00  0.00
C    H
ATOM  20884  CA  ILE C  54      13.409  10.225  37.013  1.00  0.00
C    C
ATOM  20885  HA  ILE C  54      13.415  10.763  37.953  1.00  0.00
C    H
ATOM  20886  CB  ILE C  54      12.016   9.617  36.711  1.00  0.00
C    C
ATOM  20887  HB  ILE C  54      12.117   9.182  35.685  1.00  0.00
C    H
ATOM  20888  CG2 ILE C  54      11.620   8.606  37.783  1.00  0.00
C    C
ATOM  20889 HG21 ILE C  54      10.701   8.061  37.484  1.00  0.00
C    H
ATOM  20890 HG22 ILE C  54      12.423   7.855  37.933  1.00  0.00
C    H
ATOM  20891 HG23 ILE C  54      11.425   9.110  38.752  1.00  0.00
C    H
ATOM  20892  CG1 ILE C  54      10.937  10.701  36.617  1.00  0.00
C    C
ATOM  20893 HG11 ILE C  54      10.016  10.363  37.141  1.00  0.00
C    H
ATOM  20894 HG12 ILE C  54      11.298  11.615  37.143  1.00  0.00
C    H
ATOM  20895  CD  ILE C  54      10.573  11.093  35.200  1.00  0.00
C    C
ATOM  20896  HD1 ILE C  54       9.831  11.921  35.208  1.00  0.00
C    H
ATOM  20897  HD2 ILE C  54      11.473  11.437  34.645  1.00  0.00
C    H
```

```
ATOM  20898  HD3 ILE C  54      10.127  10.237  34.652  1.00  0.00
C    H
ATOM  20899  C   ILE C  54      14.435   9.101  37.141  1.00  0.00
C    C
ATOM  20900  O   ILE C  54      14.786   8.460  36.145  1.00  0.00
C    O
ATOM  20901  N   ALA C  55      14.922   8.878  38.362  1.00  0.00
C    N
ATOM  20902  HN  ALA C  55      14.659   9.435  39.149  1.00  0.00
C    H
ATOM  20903  CA  ALA C  55      15.876   7.805  38.643  1.00  0.00
C    C
ATOM  20904  HA  ALA C  55      15.581   6.898  38.128  1.00  0.00
C    H
ATOM  20905  CB  ALA C  55      17.267   8.175  38.145  1.00  0.00
C    C
ATOM  20906  HB1 ALA C  55      17.230   8.376  37.054  1.00  0.00
C    H
ATOM  20907  HB2 ALA C  55      17.641   9.093  38.650  1.00  0.00
C    H
ATOM  20908  HB3 ALA C  55      17.996   7.355  38.319  1.00  0.00
C    H
ATOM  20909  C   ALA C  55      15.925   7.461  40.123  1.00  0.00
C    C
ATOM  20910  O   ALA C  55      15.795   8.337  40.980  1.00  0.00
C    O
ATOM  20911  N   ASN C  56      16.103   6.176  40.415  1.00  0.00
C    N
ATOM  20912  HN  ASN C  56      16.043   5.441  39.741  1.00  0.00
C    H
ATOM  20913  CA  ASN C  56      16.406   5.733  41.763  1.00  0.00
C    C
ATOM  20914  HA  ASN C  56      16.013   6.480  42.444  1.00  0.00
C    H
ATOM  20915  CB  ASN C  56      15.811   4.349  42.034  1.00  0.00
C    C
ATOM  20916  HB1 ASN C  56      14.717   4.406  41.844  1.00  0.00
C    H
ATOM  20917  HB2 ASN C  56      16.233   3.603  41.329  1.00  0.00
C    H
ATOM  20918  CG  ASN C  56      16.015   3.890  43.466  1.00  0.00
C    C
ATOM  20919  OD1 ASN C  56      16.953   4.308  44.144  1.00  0.00
C    O
ATOM  20920  ND2 ASN C  56      15.134   3.017  43.932  1.00  0.00
C    N
ATOM  20921 HD21 ASN C  56      15.245   2.712  44.874  1.00  0.00
C    H
ATOM  20922 HD22 ASN C  56      14.381   2.713  43.357  1.00  0.00
C    H
ATOM  20923  C   ASN C  56      17.920   5.716  41.921  1.00  0.00
C    C
ATOM  20924  O   ASN C  56      18.594   4.810  41.433  1.00  0.00
C    O
```

ATOM 20925 N ALA C 57 18.451 6.719 42.614 1.00 0.00
C N
ATOM 20926 HN ALA C 57 17.889 7.403 43.079 1.00 0.00
C H
ATOM 20927 CA ALA C 57 19.895 6.898 42.746 1.00 0.00
C C
ATOM 20928 HA ALA C 57 20.334 6.919 41.755 1.00 0.00
C H
ATOM 20929 CB ALA C 57 20.200 8.255 43.348 1.00 0.00
C C
ATOM 20930 HB1 ALA C 57 19.706 9.048 42.748 1.00 0.00
C H
ATOM 20931 HB2 ALA C 57 19.817 8.326 44.390 1.00 0.00
C H
ATOM 20932 HB3 ALA C 57 21.292 8.463 43.358 1.00 0.00
C H
ATOM 20933 C ALA C 57 20.624 5.786 43.520 1.00 0.00
C C
ATOM 20934 O ALA C 57 21.855 5.805 43.629 1.00 0.00
C O
ATOM 20935 N THR C 58 19.871 4.825 44.045 1.00 0.00
C N
ATOM 20936 HN THR C 58 18.873 4.812 44.002 1.00 0.00
C H
ATOM 20937 CA THR C 58 20.459 3.681 44.746 1.00 0.00
C C
ATOM 20938 HA THR C 58 21.465 3.980 45.012 1.00 0.00
C H
ATOM 20939 CB THR C 58 19.632 3.277 45.983 1.00 0.00
C C
ATOM 20940 HB THR C 58 20.200 2.453 46.480 1.00 0.00
C H
ATOM 20941 OG1 THR C 58 18.364 2.753 45.566 1.00 0.00
C O
ATOM 20942 HG1 THR C 58 17.897 2.533 46.379 1.00 0.00
C H
ATOM 20943 CG2 THR C 58 19.414 4.471 46.905 1.00 0.00
C C
ATOM 20944 HG21 THR C 58 18.918 4.156 47.847 1.00 0.00
C H
ATOM 20945 HG22 THR C 58 20.386 4.939 47.169 1.00 0.00
C H
ATOM 20946 HG23 THR C 58 18.778 5.241 46.417 1.00 0.00
C H
ATOM 20947 C THR C 58 20.595 2.476 43.816 1.00 0.00
C C
ATOM 20948 O THR C 58 21.045 1.409 44.228 1.00 0.00
C O
ATOM 20949 N GLU C 59 20.217 2.664 42.556 1.00 0.00
C N
ATOM 20950 HN GLU C 59 19.892 3.539 42.210 1.00 0.00
C H
ATOM 20951 CA GLU C 59 20.241 1.605 41.558 1.00 0.00
C C

ATOM 20952 HA   GLU C 59   20.452   0.667 42.056 1.00 0.00
C   H
ATOM 20953 CB   GLU C 59   18.888   1.545 40.853 1.00 0.00
C   C
ATOM 20954 HB1 GLU C 59   18.093   1.735 41.611 1.00 0.00
C   H
ATOM 20955 HB2 GLU C 59   18.810   2.374 40.114 1.00 0.00
C   H
ATOM 20956 CG   GLU C 59   18.590   0.226 40.164 1.00 0.00
C   C
ATOM 20957 HG1 GLU C 59   17.611   0.289 39.655 1.00 0.00
C   H
ATOM 20958 HG2 GLU C 59   19.373 -0.020 39.419 1.00 0.00
C   H
ATOM 20959 CD   GLU C 59   18.471 -0.935 41.136 1.00 0.00
C   C
ATOM 20960 OE1 GLU C 59   19.225 -1.918 40.971 1.00 0.00
C   O
ATOM 20961 OE2 GLU C 59   17.635 -0.864 42.063 1.00 0.00
C   O
ATOM 20962 C   GLU C 59   21.369   1.847 40.550 1.00 0.00
C   C
ATOM 20963 O   GLU C 59   21.308   2.790 39.758 1.00 0.00
C   O
ATOM 20964 N   GLU C 60   22.382   0.983 40.577 1.00 0.00
C   N
ATOM 20965 HN   GLU C 60   22.363   0.135 41.098 1.00 0.00
C   H
ATOM 20966 CA   GLU C 60   23.634   1.202 39.835 1.00 0.00
C   C
ATOM 20967 HA   GLU C 60   24.069   2.108 40.236 1.00 0.00
C   H
ATOM 20968 CB   GLU C 60   24.642   0.082 40.104 1.00 0.00
C   C
ATOM 20969 HB1 GLU C 60   24.650 -0.123 41.200 1.00 0.00
C   H
ATOM 20970 HB2 GLU C 60   24.299 -0.860 39.620 1.00 0.00
C   H
ATOM 20971 CG   GLU C 60   26.056   0.423 39.647 1.00 0.00
C   C
ATOM 20972 HG1 GLU C 60   26.021   0.847 38.626 1.00 0.00
C   H
ATOM 20973 HG2 GLU C 60   26.529   1.157 40.330 1.00 0.00
C   H
ATOM 20974 CD   GLU C 60   26.967 -0.784 39.550 1.00 0.00
C   C
ATOM 20975 OE1 GLU C 60   27.760 -0.843 38.586 1.00 0.00
C   O
ATOM 20976 OE2 GLU C 60   26.895 -1.670 40.431 1.00 0.00
C   O
ATOM 20977 C   GLU C 60   23.477   1.403 38.331 1.00 0.00
C   C
ATOM 20978 O   GLU C 60   24.114   2.287 37.752 1.00 0.00
C   O

ATOM 20979 N   ASN C 61   22.647   0.580 37.696 1.00 0.00
C   N
ATOM 20980 HN ASN C 61   22.139 -0.166 38.125 1.00 0.00
C   H
ATOM 20981 CA ASN C 61   22.414   0.722 36.259 1.00 0.00
C   C
ATOM 20982 HA ASN C 61   23.404   0.763 35.815 1.00 0.00
C   H
ATOM 20983 CB ASN C 61   21.691 -0.499 35.671 1.00 0.00
C   C
ATOM 20984 HB1 ASN C 61   21.399 -0.250 34.627 1.00 0.00
C   H
ATOM 20985 HB2 ASN C 61   22.377 -1.368 35.626 1.00 0.00
C   H
ATOM 20986 CG ASN C 61   20.427 -0.872 36.432 1.00 0.00
C   C
ATOM 20987 OD1 ASN C 61   19.738 -0.021 37.001 1.00 0.00
C   O
ATOM 20988 ND2 ASN C 61   20.109 -2.162 36.430 1.00 0.00
C   N
ATOM 20989 HD21 ASN C 61   19.279 -2.430 36.912 1.00 0.00
C   H
ATOM 20990 HD22 ASN C 61   20.674 -2.815 35.934 1.00 0.00
C   H
ATOM 20991 C   ASN C 61   21.692   2.021 35.901 1.00 0.00
C   C
ATOM 20992 O   ASN C 61   21.941   2.606 34.842 1.00 0.00
C   O
ATOM 20993 N   GLU C 62   20.812   2.472 36.793 1.00 0.00
C   N
ATOM 20994 HN GLU C 62   20.582   2.000 37.639 1.00 0.00
C   H
ATOM 20995 CA GLU C 62   20.095   3.727 36.600 1.00 0.00
C   C
ATOM 20996 HA GLU C 62   19.638   3.718 35.618 1.00 0.00
C   H
ATOM 20997 CB GLU C 62   18.985   3.902 37.639 1.00 0.00
C   C
ATOM 20998 HB1 GLU C 62   19.426   3.736 38.649 1.00 0.00
C   H
ATOM 20999 HB2 GLU C 62   18.626   4.955 37.633 1.00 0.00
C   H
ATOM 21000 CG GLU C 62   17.807   2.954 37.455 1.00 0.00
C   C
ATOM 21001 HG1 GLU C 62   17.551   2.887 36.381 1.00 0.00
C   H
ATOM 21002 HG2 GLU C 62   18.049   1.942 37.836 1.00 0.00
C   H
ATOM 21003 CD GLU C 62   16.545   3.434 38.144 1.00 0.00
C   C
ATOM 21004 OE1 GLU C 62   16.250   4.645 38.080 1.00 0.00
C   O
ATOM 21005 OE2 GLU C 62   15.836   2.598 38.742 1.00 0.00
C   O

ATOM 21006 C   GLU C 62   21.070   4.896 36.633 1.00 0.00
C   C
ATOM 21007 O   GLU C 62   20.936   5.838 35.851 1.00 0.00
C   O
ATOM 21008 N   LEU C 63   22.058   4.816 37.526 1.00 0.00
C   N
ATOM 21009 HN LEU C 63   22.145   4.076 38.189 1.00 0.00
C   H
ATOM 21010 CA LEU C 63   23.115   5.826 37.608 1.00 0.00
C   C
ATOM 21011 HA LEU C 63   22.620   6.783 37.712 1.00 0.00
C   H
ATOM 21012 CB LEU C 63   24.023   5.582 38.819 1.00 0.00
C   C
ATOM 21013 HB1 LEU C 63   23.985   4.491 39.043 1.00 0.00
C   H
ATOM 21014 HB2 LEU C 63   25.075   5.800 38.532 1.00 0.00
C   H
ATOM 21015 CG LEU C 63   23.681   6.333 40.105 1.00 0.00
C   C
ATOM 21016 HG LEU C 63   23.697   7.425 39.874 1.00 0.00
C   H
ATOM 21017 CD1 LEU C 63   22.304   5.929 40.588 1.00 0.00
C   C
ATOM 21018 HD11 LEU C 63   22.065   6.433 41.550 1.00 0.00
C   H
ATOM 21019 HD12 LEU C 63   21.529   6.213 39.844 1.00 0.00
C   H
ATOM 21020 HD13 LEU C 63   22.250   4.831 40.751 1.00 0.00
C   H
ATOM 21021 CD2 LEU C 63   24.716   6.067 41.183 1.00 0.00
C   C
ATOM 21022 HD21 LEU C 63   24.466   6.634 42.106 1.00 0.00
C   H
ATOM 21023 HD22 LEU C 63   24.762   4.992 41.451 1.00 0.00
C   H
ATOM 21024 HD23 LEU C 63   25.725   6.386 40.843 1.00 0.00
C   H
ATOM 21025 C   LEU C 63   23.944   5.873 36.334 1.00 0.00
C   C
ATOM 21026 O   LEU C 63   24.233   6.954 35.815 1.00 0.00
C   O
ATOM 21027 N   LEU C 64   24.315   4.697 35.832 1.00 0.00
C   N
ATOM 21028 HN LEU C 64   24.123   3.820 36.268 1.00 0.00
C   H
ATOM 21029 CA LEU C 64   25.048   4.594 34.575 1.00 0.00
C   C
ATOM 21030 HA LEU C 64   25.960   5.165 34.695 1.00 0.00
C   H
ATOM 21031 CB LEU C 64   25.379   3.132 34.252 1.00 0.00
C   C
ATOM 21032 HB1 LEU C 64   24.436   2.549 34.366 1.00 0.00
C   H

ATOM 21033 HB2 LEU C 64   25.662   3.054 33.179 1.00 0.00
C   H
ATOM 21034 CG LEU C 64   26.446   2.417 35.087 1.00 0.00
C   C
ATOM 21035 HG LEU C 64   26.141   2.520 36.156 1.00 0.00
C   H
ATOM 21036 CD1 LEU C 64   26.506   0.945 34.712 1.00 0.00
C   C
ATOM 21037 HD11 LEU C 64   27.311   0.430 35.280 1.00 0.00
C   H
ATOM 21038 HD12 LEU C 64   25.542   0.443 34.941 1.00 0.00
C   H
ATOM 21039 HD13 LEU C 64   26.716   0.824 33.628 1.00 0.00
C   H
ATOM 21040 CD2 LEU C 64   27.814   3.067 34.922 1.00 0.00
C   C
ATOM 21041 HD21 LEU C 64   28.568   2.544 35.550 1.00 0.00
C   H
ATOM 21042 HD22 LEU C 64   28.163   3.025 33.871 1.00 0.00
C   H
ATOM 21043 HD23 LEU C 64   27.778   4.133 35.236 1.00 0.00
C   H
ATOM 21044 C   LEU C 64   24.256   5.224 33.435 1.00 0.00
C   C
ATOM 21045 O   LEU C 64   24.804   5.984 32.638 1.00 0.00
C   O
ATOM 21046 N   SER C 65   22.959   4.922 33.387 1.00 0.00
C   N
ATOM 21047 HN SER C 65   22.523   4.321 34.056 1.00 0.00
C   H
ATOM 21048 CA SER C 65   22.050   5.435 32.358 1.00 0.00
C   C
ATOM 21049 HA SER C 65   22.499   5.138 31.419 1.00 0.00
C   H
ATOM 21050 CB SER C 65   20.662   4.815 32.519 1.00 0.00
C   C
ATOM 21051 HB1 SER C 65   20.235   5.167 33.486 1.00 0.00
C   H
ATOM 21052 HB2 SER C 65   19.982   5.170 31.712 1.00 0.00
C   H
ATOM 21053 OG SER C 65   20.740   3.401 32.540 1.00 0.00
C   O
ATOM 21054 HG1 SER C 65   19.891   3.075 32.863 1.00 0.00
C   H
ATOM 21055 C   SER C 65   21.931   6.963 32.343 1.00 0.00
C   C
ATOM 21056 O   SER C 65   21.698   7.561 31.293 1.00 0.00
C   O
ATOM 21057 N   LEU C 66   22.086   7.584 33.512 1.00 0.00
C   N
ATOM 21058 HN LEU C 66   22.214   7.105 34.378 1.00 0.00
C   H
ATOM 21059 CA LEU C 66   22.081   9.042 33.627 1.00 0.00
C   C

```
ATOM  21060  HA  LEU C  66      21.230   9.396  33.059  1.00  0.00
C    H
ATOM  21061  CB  LEU C  66      21.990   9.462  35.098  1.00  0.00
C    C
ATOM  21062  HB1 LEU C  66      22.886   9.045  35.614  1.00  0.00
C    H
ATOM  21063  HB2 LEU C  66      22.083  10.568  35.164  1.00  0.00
C    H
ATOM  21064  CG  LEU C  66      20.770   9.028  35.907  1.00  0.00
C    C
ATOM  21065  HG  LEU C  66      20.552   7.973  35.611  1.00  0.00
C    H
ATOM  21066  CD1 LEU C  66      21.083   9.084  37.393  1.00  0.00
C    C
ATOM  21067 HD11 LEU C  66      20.186   8.816  37.991  1.00  0.00
C    H
ATOM  21068 HD12 LEU C  66      21.898   8.372  37.648  1.00  0.00
C    H
ATOM  21069 HD13 LEU C  66      21.405  10.105  37.689  1.00  0.00
C    H
ATOM  21070  CD2 LEU C  66      19.548   9.870  35.579  1.00  0.00
C    C
ATOM  21071 HD21 LEU C  66      18.671   9.523  36.166  1.00  0.00
C    H
ATOM  21072 HD22 LEU C  66      19.712  10.940  35.819  1.00  0.00
C    H
ATOM  21073 HD23 LEU C  66      19.298   9.788  34.499  1.00  0.00
C    H
ATOM  21074  C   LEU C  66      23.334   9.652  33.006  1.00  0.00
C    C
ATOM  21075  O   LEU C  66      23.359  10.843  32.676  1.00  0.00
C    O
ATOM  21076  N   GLY C  67      24.369   8.825  32.845  1.00  0.00
C    N
ATOM  21077  HN  GLY C  67      24.288   7.842  33.006  1.00  0.00
C    H
ATOM  21078  CA  GLY C  67      25.688   9.289  32.423  1.00  0.00
C    C
ATOM  21079  HA1 GLY C  67      25.561   9.986  31.606  1.00  0.00
C    H
ATOM  21080  HA2 GLY C  67      26.299   8.429  32.183  1.00  0.00
C    H
ATOM  21081  C   GLY C  67      26.347  10.026  33.571  1.00  0.00
C    C
ATOM  21082  O   GLY C  67      26.977  11.066  33.379  1.00  0.00
C    O
ATOM  21083  N   ILE C  68      26.196   9.470  34.770  1.00  0.00
C    N
ATOM  21084  HN  ILE C  68      25.784   8.572  34.904  1.00  0.00
C    H
ATOM  21085  CA  ILE C  68      26.620  10.125  36.005  1.00  0.00
C    C
ATOM  21086  HA  ILE C  68      26.115  11.083  36.008  1.00  0.00
C    H
```

```
ATOM  21087  CB  ILE C  68      26.154   9.324  37.246  1.00  0.00
C    C
ATOM  21088  HB  ILE C  68      25.073   9.108  37.051  1.00  0.00
C    H
ATOM  21089  CG2 ILE C  68      26.956   8.040  37.407  1.00  0.00
C    C
ATOM  21090 HG21 ILE C  68      26.517   7.401  38.200  1.00  0.00
C    H
ATOM  21091 HG22 ILE C  68      26.958   7.454  36.464  1.00  0.00
C    H
ATOM  21092 HG23 ILE C  68      28.009   8.261  37.684  1.00  0.00
C    H
ATOM  21093  CG1 ILE C  68      26.205  10.184  38.514  1.00  0.00
C    C
ATOM  21094 HG11 ILE C  68      26.050   9.543  39.410  1.00  0.00
C    H
ATOM  21095 HG12 ILE C  68      27.219  10.636  38.603  1.00  0.00
C    H
ATOM  21096  CD  ILE C  68      25.193  11.308  38.542  1.00  0.00
C    C
ATOM  21097  HD1 ILE C  68      25.325  11.922  39.459  1.00  0.00
C    H
ATOM  21098  HD2 ILE C  68      25.318  11.969  37.658  1.00  0.00
C    H
ATOM  21099  HD3 ILE C  68      24.157  10.910  38.541  1.00  0.00
C    H
ATOM  21100  C   ILE C  68      28.127  10.417  36.060  1.00  0.00
C    C
ATOM  21101  O   ILE C  68      28.556  11.341  36.751  1.00  0.00
C    O
ATOM  21102  N   ARG C  69      28.914   9.646  35.316  1.00  0.00
C    N
ATOM  21103  HN  ARG C  69      28.536   8.927  34.737  1.00  0.00
C    H
ATOM  21104  CA  ARG C  69      30.367   9.803  35.301  1.00  0.00
C    C
ATOM  21105  HA  ARG C  69      30.689   9.941  36.327  1.00  0.00
C    H
ATOM  21106  CB  ARG C  69      31.023   8.579  34.697  1.00  0.00
C    C
ATOM  21107  HB1 ARG C  69      30.583   8.456  33.678  1.00  0.00
C    H
ATOM  21108  HB2 ARG C  69      32.107   8.776  34.541  1.00  0.00
C    H
ATOM  21109  CG  ARG C  69      30.783   7.328  35.500  1.00  0.00
C    C
ATOM  21110  HG1 ARG C  69      30.787   7.595  36.580  1.00  0.00
C    H
ATOM  21111  HG2 ARG C  69      29.755   6.955  35.288  1.00  0.00
C    H
ATOM  21112  CD  ARG C  69      31.799   6.283  35.120  1.00  0.00
C    C
ATOM  21113  HD1 ARG C  69      32.190   6.500  34.099  1.00  0.00
C    H
```

ATOM 21114 HD2 ARG C 69   32.656   6.266 35.835 1.00 0.00
C   H
ATOM 21115 NE ARG C 69   31.198   4.959 35.140 1.00 0.00
C   N
ATOM 21116 HE ARG C 69   30.213   4.969 34.936 1.00 0.00
C   H
ATOM 21117 CZ ARG C 69   31.859   3.838 35.397 1.00 0.00
C   C
ATOM 21118 NH1 ARG C 69   33.157   3.869 35.673 1.00 0.00
C   N
ATOM 21119 HH11 ARG C 69   33.657   3.031 35.841 1.00 0.00
C   H
ATOM 21120 HH12 ARG C 69   33.613   4.755 35.566 1.00 0.00
C   H
ATOM 21121 NH2 ARG C 69   31.217   2.678 35.386 1.00 0.00
C   N
ATOM 21122 HH21 ARG C 69   31.687   1.851 35.663 1.00 0.00
C   H
ATOM 21123 HH22 ARG C 69   30.231   2.713 35.302 1.00 0.00
C   H
ATOM 21124 C   ARG C 69   30.826 11.026 34.535 1.00 0.00
C   C
ATOM 21125 O   ARG C 69   31.931 11.527 34.757 1.00 0.00
C   O
ATOM 21126 N   ASN C 70   29.978 11.490 33.622 1.00 0.00
C   N
ATOM 21127 HN ASN C 70   29.111 11.042 33.405 1.00 0.00
C   H
ATOM 21128 CA ASN C 70   30.240 12.699 32.856 1.00 0.00
C   C
ATOM 21129 HA ASN C 70   31.250 12.592 32.477 1.00 0.00
C   H
ATOM 21130 CB ASN C 70   29.235 12.833 31.705 1.00 0.00
C   C
ATOM 21131 HB1 ASN C 70   28.214 12.856 32.145 1.00 0.00
C   H
ATOM 21132 HB2 ASN C 70   29.388 13.792 31.169 1.00 0.00
C   H
ATOM 21133 CG ASN C 70   29.299 11.667 30.726 1.00 0.00
C   C
ATOM 21134 OD1 ASN C 70   30.260 10.893 30.710 1.00 0.00
C   O
ATOM 21135 ND2 ASN C 70   28.268 11.540 29.899 1.00 0.00
C   N
ATOM 21136 HD21 ASN C 70   28.286 10.777 29.259 1.00 0.00
C   H
ATOM 21137 HD22 ASN C 70   27.503 12.174 29.955 1.00 0.00
C   H
ATOM 21138 C   ASN C 70   30.208 13.945 33.742 1.00 0.00
C   C
ATOM 21139 O   ASN C 70   30.414 15.063 33.262 1.00 0.00
C   O
ATOM 21140 N   PHE C 71   29.962 13.740 35.035 1.00 0.00
C   N

ATOM 21141 HN PHE C 71 29.855 12.832 35.437 1.00 0.00
C H
ATOM 21142 CA PHE C 71 29.828 14.841 35.982 1.00 0.00
C C
ATOM 21143 HA PHE C 71 29.823 15.741 35.379 1.00 0.00
C H
ATOM 21144 CB PHE C 71 28.502 14.741 36.741 1.00 0.00
C C
ATOM 21145 HB1 PHE C 71 28.373 13.701 37.113 1.00 0.00
C H
ATOM 21146 HB2 PHE C 71 28.497 15.409 37.628 1.00 0.00
C H
ATOM 21147 CG PHE C 71 27.307 15.034 35.885 1.00 0.00
C C
ATOM 21148 CD1 PHE C 71 26.718 14.032 35.118 1.00 0.00
C C
ATOM 21149 HD1 PHE C 71 27.122 13.029 35.147 1.00 0.00
C H
ATOM 21150 CE1 PHE C 71 25.626 14.307 34.313 1.00 0.00
C C
ATOM 21151 HE1 PHE C 71 25.170 13.518 33.732 1.00 0.00
C H
ATOM 21152 CZ PHE C 71 25.117 15.594 34.261 1.00 0.00
C C
ATOM 21153 HZ PHE C 71 24.261 15.809 33.640 1.00 0.00
C H
ATOM 21154 CD2 PHE C 71 26.791 16.322 35.816 1.00 0.00
C C
ATOM 21155 HD2 PHE C 71 27.257 17.122 36.376 1.00 0.00
C H
ATOM 21156 CE2 PHE C 71 25.699 16.601 35.013 1.00 0.00
C C
ATOM 21157 HE2 PHE C 71 25.302 17.607 34.974 1.00 0.00
C H
ATOM 21158 C PHE C 71 31.001 14.951 36.936 1.00 0.00
C C
ATOM 21159 O PHE C 71 31.535 13.951 37.410 1.00 0.00
C O
ATOM 21160 N GLU C 72 31.392 16.192 37.195 1.00 0.00
C N
ATOM 21161 HN GLU C 72 30.927 16.997 36.838 1.00 0.00
C H
ATOM 21162 CA GLU C 72 32.539 16.494 38.028 1.00 0.00
C C
ATOM 21163 HA GLU C 72 33.220 15.652 38.001 1.00 0.00
C H
ATOM 21164 CB GLU C 72 33.219 17.764 37.518 1.00 0.00
C C
ATOM 21165 HB1 GLU C 72 33.068 17.821 36.415 1.00 0.00
C H
ATOM 21166 HB2 GLU C 72 32.711 18.659 37.942 1.00 0.00
C H
ATOM 21167 CG GLU C 72 34.710 17.846 37.806 1.00 0.00
C C

ATOM 21168 HG1 GLU C 72 34.902 17.535 38.849 1.00 0.00
C H
ATOM 21169 HG2 GLU C 72 35.283 17.192 37.119 1.00 0.00
C H
ATOM 21170 CD GLU C 72 35.258 19.260 37.692 1.00 0.00
C C
ATOM 21171 OE1 GLU C 72 34.850 20.001 36.770 1.00 0.00
C O
ATOM 21172 OE2 GLU C 72 36.106 19.629 38.530 1.00 0.00
C O
ATOM 21173 C GLU C 72 32.097 16.671 39.475 1.00 0.00
C C
ATOM 21174 O GLU C 72 32.881 16.455 40.408 1.00 0.00
C O
ATOM 21175 N TYR C 73 30.841 17.073 39.652 1.00 0.00
C N
ATOM 21176 HN TYR C 73 30.225 17.278 38.894 1.00 0.00
C H
ATOM 21177 CA TYR C 73 30.254 17.250 40.973 1.00 0.00
C C
ATOM 21178 HA TYR C 73 30.906 16.750 41.680 1.00 0.00
C H
ATOM 21179 CB TYR C 73 30.051 18.724 41.293 1.00 0.00
C C
ATOM 21180 HB1 TYR C 73 29.551 19.219 40.431 1.00 0.00
C H
ATOM 21181 HB2 TYR C 73 29.385 18.847 42.175 1.00 0.00
C H
ATOM 21182 CG TYR C 73 31.318 19.480 41.534 1.00 0.00
C C
ATOM 21183 CD1 TYR C 73 32.100 19.924 40.466 1.00 0.00
C C
ATOM 21184 HD1 TYR C 73 31.798 19.704 39.451 1.00 0.00
C H
ATOM 21185 CE1 TYR C 73 33.271 20.624 40.678 1.00 0.00
C C
ATOM 21186 HE1 TYR C 73 33.877 20.948 39.845 1.00 0.00
C H
ATOM 21187 CZ TYR C 73 33.665 20.900 41.977 1.00 0.00
C C
ATOM 21188 OH TYR C 73 34.827 21.602 42.194 1.00 0.00
C O
ATOM 21189 HH TYR C 73 34.898 21.758 43.138 1.00 0.00
C H
ATOM 21190 CD2 TYR C 73 31.734 19.771 42.825 1.00 0.00
C C
ATOM 21191 HD2 TYR C 73 31.163 19.413 43.670 1.00 0.00
C H
ATOM 21192 CE2 TYR C 73 32.903 20.476 43.054 1.00 0.00
C C
ATOM 21193 HE2 TYR C 73 33.222 20.669 44.068 1.00 0.00
C H
ATOM 21194 C TYR C 73 28.912 16.563 41.043 1.00 0.00
C C

ATOM 21195 O   TYR C 73   28.018 16.840 40.235 1.00 0.00
C   O
ATOM 21196 N   VAL C 74   28.765 15.658 42.006 1.00 0.00
C   N
ATOM 21197 HN VAL C 74   29.499 15.307 42.582 1.00 0.00
C   H
ATOM 21198 CA VAL C 74   27.460 15.098 42.290 1.00 0.00
C   C
ATOM 21199 HA VAL C 74   26.726 15.594 41.668 1.00 0.00
C   H
ATOM 21200 CB VAL C 74   27.379 13.592 41.980 1.00 0.00
C   C
ATOM 21201 HB VAL C 74   28.090 13.068 42.665 1.00 0.00
C   H
ATOM 21202 CG1 VAL C 74   25.975 13.076 42.226 1.00 0.00
C   C
ATOM 21203 HG11 VAL C 74   25.909 11.998 41.965 1.00 0.00
C   H
ATOM 21204 HG12 VAL C 74   25.683 13.176 43.292 1.00 0.00
C   H
ATOM 21205 HG13 VAL C 74   25.239 13.627 41.603 1.00 0.00
C   H
ATOM 21206 CG2 VAL C 74   27.756 13.330 40.528 1.00 0.00
C   C
ATOM 21207 HG21 VAL C 74   27.661 12.250 40.288 1.00 0.00
C   H
ATOM 21208 HG22 VAL C 74   27.092 13.900 39.843 1.00 0.00
C   H
ATOM 21209 HG23 VAL C 74   28.805 13.627 40.325 1.00 0.00
C   H
ATOM 21210 C   VAL C 74   27.113 15.406 43.735 1.00 0.00
C   C
ATOM 21211 O   VAL C 74   27.920 15.190 44.636 1.00 0.00
C   O
ATOM 21212 N   ILE C 75   25.922 15.960 43.925 1.00 0.00
C   N
ATOM 21213 HN ILE C 75   25.306 16.170 43.167 1.00 0.00
C   H
ATOM 21214 CA ILE C 75   25.398 16.312 45.232 1.00 0.00
C   C
ATOM 21215 HA ILE C 75   26.199 16.217 45.953 1.00 0.00
C   H
ATOM 21216 CB ILE C 75   24.694 17.692 45.216 1.00 0.00
C   C
ATOM 21217 HB ILE C 75   23.794 17.539 44.568 1.00 0.00
C   H
ATOM 21218 CG2 ILE C 75   24.274 18.111 46.622 1.00 0.00
C   C
ATOM 21219 HG21 ILE C 75   23.637 19.019 46.584 1.00 0.00
C   H
ATOM 21220 HG22 ILE C 75   23.688 17.309 47.116 1.00 0.00
C   H
ATOM 21221 HG23 ILE C 75   25.161 18.336 47.251 1.00 0.00
C   H

ATOM 21222 CG1 ILE C 75   25.555 18.755 44.518 1.00 0.00
C   C
ATOM 21223 HG11 ILE C 75   25.850 18.396 43.507 1.00 0.00
C   H
ATOM 21224 HG12 ILE C 75   24.945 19.676 44.375 1.00 0.00
C   H
ATOM 21225 CD ILE C 75   26.803 19.143 45.274 1.00 0.00
C   C
ATOM 21226 HD1 ILE C 75   27.342 19.954 44.738 1.00 0.00
C   H
ATOM 21227 HD2 ILE C 75   26.547 19.509 46.292 1.00 0.00
C   H
ATOM 21228 HD3 ILE C 75   27.494 18.280 45.372 1.00 0.00
C   H
ATOM 21229 C   ILE C 75   24.364 15.271 45.587 1.00 0.00
C   C
ATOM 21230 O   ILE C 75   23.389 15.077 44.855 1.00 0.00
C   O
ATOM 21231 N   VAL C 76   24.570 14.595 46.706 1.00 0.00
C   N
ATOM 21232 HN VAL C 76   25.380 14.673 47.281 1.00 0.00
C   H
ATOM 21233 CA VAL C 76   23.584 13.653 47.188 1.00 0.00
C   C
ATOM 21234 HA VAL C 76   22.916 13.396 46.374 1.00 0.00
C   H
ATOM 21235 CB VAL C 76   24.221 12.367 47.735 1.00 0.00
C   C
ATOM 21236 HB VAL C 76   24.731 12.626 48.695 1.00 0.00
C   H
ATOM 21237 CG1 VAL C 76   23.147 11.325 47.997 1.00 0.00
C   C
ATOM 21238 HG11 VAL C 76   23.608 10.379 48.356 1.00 0.00
C   H
ATOM 21239 HG12 VAL C 76   22.433 11.661 48.777 1.00 0.00
C   H
ATOM 21240 HG13 VAL C 76   22.581 11.101 47.068 1.00 0.00
C   H
ATOM 21241 CG2 VAL C 76   25.244 11.827 46.753 1.00 0.00
C   C
ATOM 21242 HG21 VAL C 76   25.680 10.875 47.124 1.00 0.00
C   H
ATOM 21243 HG22 VAL C 76   24.773 11.635 45.764 1.00 0.00
C   H
ATOM 21244 HG23 VAL C 76   26.079 12.544 46.607 1.00 0.00
C   H
ATOM 21245 C   VAL C 76   22.760 14.350 48.246 1.00 0.00
C   C
ATOM 21246 O   VAL C 76   23.211 14.559 49.376 1.00 0.00
C   O
ATOM 21247 N   ALA C 77   21.552 14.729 47.857 1.00 0.00
C   N
ATOM 21248 HN ALA C 77   21.187 14.551 46.943 1.00 0.00
C   H

ATOM 21249 CA ALA C 77   20.666 15.439 48.752 1.00 0.00
C   C
ATOM 21250 HA ALA C 77   21.012 15.487 49.778 1.00 0.00
C   H
ATOM 21251 CB ALA C 77   20.465 16.866 48.278 1.00 0.00
C   C
ATOM 21252 HB1 ALA C 77   21.421 17.425 48.362 1.00 0.00
C   H
ATOM 21253 HB2 ALA C 77   20.147 16.891 47.213 1.00 0.00
C   H
ATOM 21254 HB3 ALA C 77   19.703 17.396 48.889 1.00 0.00
C   H
ATOM 21255 C   ALA C 77   19.428 14.563 48.758 1.00 0.00
C   C
ATOM 21256 O   ALA C 77   18.485 14.792 48.009 1.00 0.00
C   O
ATOM 21257 N   ILE C 78   19.452 13.536 49.590 1.00 0.00
C   N
ATOM 21258 HN ILE C 78   20.277 13.146 49.995 1.00 0.00
C   H
ATOM 21259 CA ILE C 78   18.231 12.877 49.986 1.00 0.00
C   C
ATOM 21260 HA ILE C 78   17.352 13.475 49.781 1.00 0.00
C   H
ATOM 21261 CB ILE C 78   18.080 11.411 49.583 1.00 0.00
C   C
ATOM 21262 HB ILE C 78   18.965 10.902 50.042 1.00 0.00
C   H
ATOM 21263 CG2 ILE C 78   16.747 10.876 50.087 1.00 0.00
C   C
ATOM 21264 HG21 ILE C 78   16.687   9.778 49.942 1.00 0.00
C   H
ATOM 21265 HG22 ILE C 78   16.624 11.079 51.172 1.00 0.00
C   H
ATOM 21266 HG23 ILE C 78   15.900 11.345 49.544 1.00 0.00
C   H
ATOM 21267 CG1 ILE C 78   18.218 11.265 48.068 1.00 0.00
C   C
ATOM 21268 HG11 ILE C 78   17.209 11.202 47.602 1.00 0.00
C   H
ATOM 21269 HG12 ILE C 78   18.712 12.177 47.662 1.00 0.00
C   H
ATOM 21270 CD ILE C 78   19.032 10.061 47.661 1.00 0.00
C   C
ATOM 21271 HD1 ILE C 78   19.152 10.031 46.556 1.00 0.00
C   H
ATOM 21272 HD2 ILE C 78   20.043 10.098 48.120 1.00 0.00
C   H
ATOM 21273 HD3 ILE C 78   18.535   9.119 47.978 1.00 0.00
C   H
ATOM 21274 C   ILE C 78   18.487 12.970 51.465 1.00 0.00
C   C
ATOM 21275 O   ILE C 78   19.513 12.487 51.972 1.00 0.00
C   O

ATOM 21276 N   GLY C 79   17.570 13.632 52.153 1.00 0.00
C   N
ATOM 21277 HN GLY C 79   16.733 14.003 51.753 1.00 0.00
C   H
ATOM 21278 CA GLY C 79   17.739 13.858 53.565 1.00 0.00
C   C
ATOM 21279 HA1 GLY C 79   17.063 14.652 53.851 1.00 0.00
C   H
ATOM 21280 HA2 GLY C 79   18.784 14.077 53.741 1.00 0.00
C   H
ATOM 21281 C   GLY C 79   17.381 12.628 54.365 1.00 0.00
C   C
ATOM 21282 O   GLY C 79   18.254 11.917 54.865 1.00 0.00
C   O
ATOM 21283 N   ALA C 80   16.083 12.370 54.447 1.00 0.00
C   N
ATOM 21284 HN ALA C 80   15.424 12.767 53.809 1.00 0.00
C   H
ATOM 21285 CA ALA C 80   15.518 11.493 55.469 1.00 0.00
C   C
ATOM 21286 HA ALA C 80   15.737 11.908 56.446 1.00 0.00
C   H
ATOM 21287 CB ALA C 80   14.002 11.483 55.353 1.00 0.00
C   C
ATOM 21288 HB1 ALA C 80   13.617 12.524 55.402 1.00 0.00
C   H
ATOM 21289 HB2 ALA C 80   13.678 11.044 54.384 1.00 0.00
C   H
ATOM 21290 HB3 ALA C 80   13.532 10.905 56.178 1.00 0.00
C   H
ATOM 21291 C   ALA C 80   16.071 10.065 55.474 1.00 0.00
C   C
ATOM 21292 O   ALA C 80   16.514   9.561 56.508 1.00 0.00
C   O
ATOM 21293 N   ASN C 81   16.049   9.435 54.306 1.00 0.00
C   N
ATOM 21294 HN ASN C 81   15.740   9.873 53.463 1.00 0.00
C   H
ATOM 21295 CA ASN C 81   16.471   8.057 54.148 1.00 0.00
C   C
ATOM 21296 HA ASN C 81   16.025   7.523 54.980 1.00 0.00
C   H
ATOM 21297 CB ASN C 81   15.972   7.555 52.794 1.00 0.00
C   C
ATOM 21298 HB1 ASN C 81   14.957   7.978 52.627 1.00 0.00
C   H
ATOM 21299 HB2 ASN C 81   16.625   7.927 51.979 1.00 0.00
C   H
ATOM 21300 CG ASN C 81   15.865   6.047 52.726 1.00 0.00
C   C
ATOM 21301 OD1 ASN C 81   16.554   5.320 53.445 1.00 0.00
C   O
ATOM 21302 ND2 ASN C 81   15.000   5.565 51.842 1.00 0.00
C   N

ATOM 21303 HD21 ASN C 81   14.905   4.574 51.788 1.00 0.00
C   H
ATOM 21304 HD22 ASN C 81   14.456   6.186 51.287 1.00 0.00
C   H
ATOM 21305 C   ASN C 81   17.994   7.898 54.256 1.00 0.00
C   C
ATOM 21306 O   ASN C 81   18.713   8.021 53.263 1.00 0.00
C   O
ATOM 21307 N   ILE C 82   18.480   7.618 55.462 1.00 0.00
C   N
ATOM 21308 HN ILE C 82   17.924   7.507 56.284 1.00 0.00
C   H
ATOM 21309 CA ILE C 82   19.918   7.445 55.683 1.00 0.00
C   C
ATOM 21310 HA ILE C 82   20.384   8.357 55.331 1.00 0.00
C   H
ATOM 21311 CB ILE C 82   20.254   7.266 57.181 1.00 0.00
C   C
ATOM 21312 HB ILE C 82   19.545   6.476 57.534 1.00 0.00
C   H
ATOM 21313 CG2 ILE C 82   21.723   6.910 57.372 1.00 0.00
C   C
ATOM 21314 HG21 ILE C 82   21.924   6.630 58.427 1.00 0.00
C   H
ATOM 21315 HG22 ILE C 82   22.008   6.046 56.734 1.00 0.00
C   H
ATOM 21316 HG23 ILE C 82   22.377   7.769 57.110 1.00 0.00
C   H
ATOM 21317 CG1 ILE C 82   19.919   8.546 57.954 1.00 0.00
C   C
ATOM 21318 HG11 ILE C 82   20.565   9.380 57.600 1.00 0.00
C   H
ATOM 21319 HG12 ILE C 82   18.864   8.832 57.737 1.00 0.00
C   H
ATOM 21320 CD ILE C 82   20.064   8.428 59.457 1.00 0.00
C   C
ATOM 21321 HD1 ILE C 82   19.749   9.373 59.950 1.00 0.00
C   H
ATOM 21322 HD2 ILE C 82   19.430   7.604 59.849 1.00 0.00
C   H
ATOM 21323 HD3 ILE C 82   21.119   8.227 59.740 1.00 0.00
C   H
ATOM 21324 C   ILE C 82   20.497   6.300 54.848 1.00 0.00
C   C
ATOM 21325 O   ILE C 82   21.591   6.429 54.295 1.00 0.00
C   O
ATOM 21326 N   GLN C 83   19.755   5.196 54.745 1.00 0.00
C   N
ATOM 21327 HN GLN C 83   18.872   5.080 55.196 1.00 0.00
C   H
ATOM 21328 CA GLN C 83   20.184   4.045 53.949 1.00 0.00
C   C
ATOM 21329 HA GLN C 83   21.136   3.717 54.350 1.00 0.00
C   H

ATOM 21330 CB GLN C 83   19.153   2.920 54.023 1.00 0.00
C   C
ATOM 21331 HB1 GLN C 83   19.171   2.535 55.069 1.00 0.00
C   H
ATOM 21332 HB2 GLN C 83   18.135   3.342 53.863 1.00 0.00
C   H
ATOM 21333 CG GLN C 83   19.420   1.770 53.056 1.00 0.00
C   C
ATOM 21334 HG1 GLN C 83   19.743   2.162 52.067 1.00 0.00
C   H
ATOM 21335 HG2 GLN C 83   20.226   1.112 53.441 1.00 0.00
C   H
ATOM 21336 CD GLN C 83   18.193   0.910 52.783 1.00 0.00
C   C
ATOM 21337 OE1 GLN C 83   17.260   0.850 53.591 1.00 0.00
C   O
ATOM 21338 NE2 GLN C 83   18.192   0.230 51.640 1.00 0.00
C   N
ATOM 21339 HE21 GLN C 83   17.392 -0.332 51.450 1.00 0.00
C   H
ATOM 21340 HE22 GLN C 83   18.976   0.286 51.030 1.00 0.00
C   H
ATOM 21341 C   GLN C 83   20.411   4.446 52.496 1.00 0.00
C   C
ATOM 21342 O   GLN C 83   21.483   4.217 51.942 1.00 0.00
C   O
ATOM 21343 N   ALA C 84   19.394   5.058 51.894 1.00 0.00
C   N
ATOM 21344 HN ALA C 84   18.541   5.270 52.369 1.00 0.00
C   H
ATOM 21345 CA ALA C 84   19.449   5.464 50.497 1.00 0.00
C   C
ATOM 21346 HA ALA C 84   19.607   4.609 49.849 1.00 0.00
C   H
ATOM 21347 CB ALA C 84   18.156   6.140 50.085 1.00 0.00
C   C
ATOM 21348 HB1 ALA C 84   17.309   5.431 50.211 1.00 0.00
C   H
ATOM 21349 HB2 ALA C 84   17.952   7.031 50.717 1.00 0.00
C   H
ATOM 21350 HB3 ALA C 84   18.181   6.460 49.021 1.00 0.00
C   H
ATOM 21351 C   ALA C 84   20.620   6.394 50.268 1.00 0.00
C   C
ATOM 21352 O   ALA C 84   21.374   6.228 49.307 1.00 0.00
C   O
ATOM 21353 N   SER C 85   20.770   7.359 51.173 1.00 0.00
C   N
ATOM 21354 HN SER C 85   20.182   7.448 51.977 1.00 0.00
C   H
ATOM 21355 CA SER C 85   21.805   8.377 51.077 1.00 0.00
C   C
ATOM 21356 HA SER C 85   21.631   8.884 50.138 1.00 0.00
C   H

ATOM 21357 CB SER C 85   21.701   9.348 52.251 1.00 0.00
C   C
ATOM 21358 HB1 SER C 85   20.711   9.856 52.192 1.00 0.00
C   H
ATOM 21359 HB2 SER C 85   21.729   8.794 53.216 1.00 0.00
C   H
ATOM 21360 OG SER C 85   22.718 10.326 52.193 1.00 0.00
C   O
ATOM 21361 HG1 SER C 85   22.475 11.026 52.812 1.00 0.00
C   H
ATOM 21362 C   SER C 85   23.203   7.765 51.009 1.00 0.00
C   C
ATOM 21363 O   SER C 85   24.001   8.134 50.140 1.00 0.00
C   O
ATOM 21364 N   THR C 86   23.492   6.825 51.909 1.00 0.00
C   N
ATOM 21365 HN THR C 86   22.845   6.461 52.578 1.00 0.00
C   H
ATOM 21366 CA THR C 86   24.830   6.234 51.981 1.00 0.00
C   C
ATOM 21367 HA THR C 86   25.518   7.047 51.796 1.00 0.00
C   H
ATOM 21368 CB THR C 86   25.139   5.594 53.357 1.00 0.00
C   C
ATOM 21369 HB THR C 86   26.245   5.435 53.384 1.00 0.00
C   H
ATOM 21370 OG1 THR C 86   24.513   4.316 53.456 1.00 0.00
C   O
ATOM 21371 HG1 THR C 86   24.738   3.988 54.333 1.00 0.00
C   H
ATOM 21372 CG2 THR C 86   24.659   6.470 54.494 1.00 0.00
C   C
ATOM 21373 HG21 THR C 86   24.984   6.061 55.473 1.00 0.00
C   H
ATOM 21374 HG22 THR C 86   25.077   7.495 54.396 1.00 0.00
C   H
ATOM 21375 HG23 THR C 86   23.550   6.546 54.499 1.00 0.00
C   H
ATOM 21376 C   THR C 86   25.056   5.212 50.869 1.00 0.00
C   C
ATOM 21377 O   THR C 86   26.163   5.097 50.337 1.00 0.00
C   O
ATOM 21378 N   LEU C 87   24.003   4.479 50.521 1.00 0.00
C   N
ATOM 21379 HN LEU C 87   23.118   4.509 50.980 1.00 0.00
C   H
ATOM 21380 CA LEU C 87   24.067   3.547 49.406 1.00 0.00
C   C
ATOM 21381 HA LEU C 87   24.891   2.873 49.604 1.00 0.00
C   H
ATOM 21382 CB LEU C 87   22.773   2.731 49.303 1.00 0.00
C   C
ATOM 21383 HB1 LEU C 87   22.629   2.218 50.283 1.00 0.00
C   H

```
ATOM  21384  HB2 LEU C  87      21.914   3.428  49.192  1.00  0.00
C    H
ATOM  21385  CG  LEU C  87      22.631   1.644  48.229  1.00  0.00
C    C
ATOM  21386  HG  LEU C  87      22.512   2.194  47.264  1.00  0.00
C    H
ATOM  21387  CD1 LEU C  87      23.839   0.725  48.149  1.00  0.00
C    C
ATOM  21388 HD11 LEU C  87      23.673  -0.072  47.394  1.00  0.00
C    H
ATOM  21389 HD12 LEU C  87      24.746   1.297  47.859  1.00  0.00
C    H
ATOM  21390 HD13 LEU C  87      24.029   0.241  49.130  1.00  0.00
C    H
ATOM  21391  CD2 LEU C  87      21.364   0.837  48.469  1.00  0.00
C    C
ATOM  21392 HD21 LEU C  87      21.245   0.060  47.683  1.00  0.00
C    H
ATOM  21393 HD22 LEU C  87      21.386   0.322  49.451  1.00  0.00
C    H
ATOM  21394 HD23 LEU C  87      20.470   1.497  48.442  1.00  0.00
C    H
ATOM  21395  C   LEU C  87      24.377   4.285  48.102  1.00  0.00
C    C
ATOM  21396  O   LEU C  87      25.230   3.848  47.336  1.00  0.00
C    O
ATOM  21397  N   THR C  88      23.695   5.408  47.869  1.00  0.00
C    N
ATOM  21398  HN  THR C  88      22.953   5.735  48.453  1.00  0.00
C    H
ATOM  21399  CA  THR C  88      23.982   6.270  46.720  1.00  0.00
C    C
ATOM  21400  HA  THR C  88      23.882   5.640  45.846  1.00  0.00
C    H
ATOM  21401  CB  THR C  88      23.026   7.481  46.656  1.00  0.00
C    C
ATOM  21402  HB  THR C  88      23.087   7.977  47.655  1.00  0.00
C    H
ATOM  21403  OG1 THR C  88      21.683   7.020  46.469  1.00  0.00
C    O
ATOM  21404  HG1 THR C  88      21.143   7.817  46.425  1.00  0.00
C    H
ATOM  21405  CG2 THR C  88      23.399   8.408  45.506  1.00  0.00
C    C
ATOM  21406 HG21 THR C  88      22.784   9.333  45.529  1.00  0.00
C    H
ATOM  21407 HG22 THR C  88      24.467   8.705  45.578  1.00  0.00
C    H
ATOM  21408 HG23 THR C  88      23.241   7.911  44.524  1.00  0.00
C    H
ATOM  21409  C   THR C  88      25.428   6.757  46.739  1.00  0.00
C    C
ATOM  21410  O   THR C  88      26.147   6.606  45.747  1.00  0.00
C    O
```

ATOM 21411 N THR C 89 25.847 7.330 47.869 1.00 0.00
C N
ATOM 21412 HN THR C 89 25.267 7.455 48.674 1.00 0.00
C H
ATOM 21413 CA THR C 89 27.217 7.834 48.028 1.00 0.00
C C
ATOM 21414 HA THR C 89 27.358 8.593 47.270 1.00 0.00
C H
ATOM 21415 CB THR C 89 27.478 8.369 49.451 1.00 0.00
C C
ATOM 21416 HB THR C 89 27.281 7.516 50.145 1.00 0.00
C H
ATOM 21417 OG1 THR C 89 26.545 9.406 49.751 1.00 0.00
C O
ATOM 21418 HG1 THR C 89 26.769 9.703 50.640 1.00 0.00
C H
ATOM 21419 CG2 THR C 89 28.879 8.935 49.573 1.00 0.00
C C
ATOM 21420 HG21 THR C 89 29.102 9.215 50.624 1.00 0.00
C H
ATOM 21421 HG22 THR C 89 29.630 8.182 49.253 1.00 0.00
C H
ATOM 21422 HG23 THR C 89 29.002 9.839 48.939 1.00 0.00
C H
ATOM 21423 C THR C 89 28.202 6.720 47.714 1.00 0.00
C C
ATOM 21424 O THR C 89 29.173 6.928 46.990 1.00 0.00
C O
ATOM 21425 N LEU C 90 27.922 5.536 48.254 1.00 0.00
C N
ATOM 21426 HN LEU C 90 27.121 5.376 48.828 1.00 0.00
C H
ATOM 21427 CA LEU C 90 28.757 4.366 48.059 1.00 0.00
C C
ATOM 21428 HA LEU C 90 29.730 4.612 48.466 1.00 0.00
C H
ATOM 21429 CB LEU C 90 28.155 3.168 48.786 1.00 0.00
C C
ATOM 21430 HB1 LEU C 90 27.766 3.538 49.763 1.00 0.00
C H
ATOM 21431 HB2 LEU C 90 27.268 2.808 48.219 1.00 0.00
C H
ATOM 21432 CG LEU C 90 29.096 1.996 49.066 1.00 0.00
C C
ATOM 21433 HG LEU C 90 29.166 1.387 48.134 1.00 0.00
C H
ATOM 21434 CD1 LEU C 90 30.483 2.480 49.466 1.00 0.00
C C
ATOM 21435 HD11 LEU C 90 31.141 1.618 49.712 1.00 0.00
C H
ATOM 21436 HD12 LEU C 90 30.953 3.051 48.636 1.00 0.00
C H
ATOM 21437 HD13 LEU C 90 30.428 3.138 50.358 1.00 0.00
C H

ATOM 21438 CD2 LEU C 90   28.497   1.126 50.149 1.00 0.00
C   C
ATOM 21439 HD21 LEU C 90   29.158   0.257 50.355 1.00 0.00
C   H
ATOM 21440 HD22 LEU C 90   28.369   1.685 51.099 1.00 0.00
C   H
ATOM 21441 HD23 LEU C 90   27.503   0.741 49.834 1.00 0.00
C   H
ATOM 21442 C   LEU C 90   28.945   4.050 46.582 1.00 0.00
C   C
ATOM 21443 O   LEU C 90   30.071   3.857 46.124 1.00 0.00
C   O
ATOM 21444 N   LEU C 91   27.837   4.011 45.848 1.00 0.00
C   N
ATOM 21445 HN LEU C 91   26.925   4.128 46.237 1.00 0.00
C   H
ATOM 21446 CA LEU C 91   27.850   3.792 44.404 1.00 0.00
C   C
ATOM 21447 HA LEU C 91   28.384   2.864 44.245 1.00 0.00
C   H
ATOM 21448 CB LEU C 91   26.423   3.688 43.862 1.00 0.00
C   C
ATOM 21449 HB1 LEU C 91   25.902   4.637 44.123 1.00 0.00
C   H
ATOM 21450 HB2 LEU C 91   26.460   3.652 42.750 1.00 0.00
C   H
ATOM 21451 CG LEU C 91   25.531   2.542 44.353 1.00 0.00
C   C
ATOM 21452 HG LEU C 91   25.546   2.584 45.469 1.00 0.00
C   H
ATOM 21453 CD1 LEU C 91   24.108   2.745 43.852 1.00 0.00
C   C
ATOM 21454 HD11 LEU C 91   23.462   1.895 44.161 1.00 0.00
C   H
ATOM 21455 HD12 LEU C 91   23.677   3.681 44.267 1.00 0.00
C   H
ATOM 21456 HD13 LEU C 91   24.088   2.811 42.743 1.00 0.00
C   H
ATOM 21457 CD2 LEU C 91   26.058   1.178 43.926 1.00 0.00
C   C
ATOM 21458 HD21 LEU C 91   25.400   0.371 44.313 1.00 0.00
C   H
ATOM 21459 HD22 LEU C 91   26.097   1.081 42.822 1.00 0.00
C   H
ATOM 21460 HD23 LEU C 91   27.082   1.014 44.327 1.00 0.00
C   H
ATOM 21461 C   LEU C 91   28.604   4.892 43.657 1.00 0.00
C   C
ATOM 21462 O   LEU C 91   29.363   4.604 42.732 1.00 0.00
C   O
ATOM 21463 N   LEU C 92   28.397   6.144 44.062 1.00 0.00
C   N
ATOM 21464 HN LEU C 92   27.781   6.405 44.802 1.00 0.00
C   H

ATOM 21465 CA LEU C 92   29.080   7.267 43.426 1.00 0.00
C   C
ATOM 21466 HA LEU C 92   28.853   7.204 42.370 1.00 0.00
C   H
ATOM 21467 CB LEU C 92   28.572   8.604 43.963 1.00 0.00
C   C
ATOM 21468 HB1 LEU C 92   28.653   8.564 45.074 1.00 0.00
C   H
ATOM 21469 HB2 LEU C 92   29.262   9.412 43.634 1.00 0.00
C   H
ATOM 21470 CG LEU C 92   27.131   8.964 43.599 1.00 0.00
C   C
ATOM 21471 HG LEU C 92   26.480   8.148 43.997 1.00 0.00
C   H
ATOM 21472 CD1 LEU C 92   26.756 10.279 44.251 1.00 0.00
C   C
ATOM 21473 HD11 LEU C 92   25.727 10.580 43.959 1.00 0.00
C   H
ATOM 21474 HD12 LEU C 92   26.791 10.190 45.358 1.00 0.00
C   H
ATOM 21475 HD13 LEU C 92   27.455 11.084 43.941 1.00 0.00
C   H
ATOM 21476 CD2 LEU C 92   26.927   9.037 42.095 1.00 0.00
C   C
ATOM 21477 HD21 LEU C 92   25.869   9.282 41.859 1.00 0.00
C   H
ATOM 21478 HD22 LEU C 92   27.564   9.818 41.632 1.00 0.00
C   H
ATOM 21479 HD23 LEU C 92   27.171   8.061 41.621 1.00 0.00
C   H
ATOM 21480 C   LEU C 92   30.593   7.174 43.577 1.00 0.00
C   C
ATOM 21481 O   LEU C 92   31.336   7.495 42.649 1.00 0.00
C   O
ATOM 21482 N   LYS C 93   31.046   6.718 44.742 1.00 0.00
C   N
ATOM 21483 HN LYS C 93   30.454   6.455 45.503 1.00 0.00
C   H
ATOM 21484 CA LYS C 93   32.469   6.570 44.990 1.00 0.00
C   C
ATOM 21485 HA LYS C 93   32.949   7.492 44.683 1.00 0.00
C   H
ATOM 21486 CB LYS C 93   32.734   6.359 46.483 1.00 0.00
C   C
ATOM 21487 HB1 LYS C 93   32.238   7.196 47.025 1.00 0.00
C   H
ATOM 21488 HB2 LYS C 93   32.225   5.427 46.813 1.00 0.00
C   H
ATOM 21489 CG LYS C 93   34.204   6.328 46.877 1.00 0.00
C   C
ATOM 21490 HG1 LYS C 93   34.286   6.086 47.961 1.00 0.00
C   H
ATOM 21491 HG2 LYS C 93   34.712   5.511 46.315 1.00 0.00
C   H

```
ATOM  21492  CD  LYS C  93      34.928   7.645  46.627  1.00  0.00
C    C
ATOM  21493  HD1 LYS C  93      34.756   7.946  45.567  1.00  0.00
C    H
ATOM  21494  HD2 LYS C  93      34.465   8.424  47.274  1.00  0.00
C    H
ATOM  21495  CE  LYS C  93      36.411   7.495  46.928  1.00  0.00
C    C
ATOM  21496  HE1 LYS C  93      36.577   7.376  48.022  1.00  0.00
C    H
ATOM  21497  HE2 LYS C  93      36.826   6.602  46.411  1.00  0.00
C    H
ATOM  21498  NZ  LYS C  93      37.207   8.671  46.487  1.00  0.00
C    N
ATOM  21499  HZ1 LYS C  93      38.212   8.527  46.713  1.00  0.00
C    H
ATOM  21500  HZ2 LYS C  93      37.099   8.799  45.460  1.00  0.00
C    H
ATOM  21501  HZ3 LYS C  93      36.863   9.524  46.974  1.00  0.00
C    H
ATOM  21502  C   LYS C  93      33.052   5.445  44.132  1.00  0.00
C    C
ATOM  21503  O   LYS C  93      34.199   5.525  43.693  1.00  0.00
C    O
ATOM  21504  N   GLU C  94      32.245   4.415  43.881  1.00  0.00
C    N
ATOM  21505  HN  GLU C  94      31.332   4.322  44.271  1.00  0.00
C    H
ATOM  21506  CA  GLU C  94      32.630   3.312  42.999  1.00  0.00
C    C
ATOM  21507  HA  GLU C  94      33.580   2.928  43.348  1.00  0.00
C    H
ATOM  21508  CB  GLU C  94      31.590   2.192  43.037  1.00  0.00
C    C
ATOM  21509  HB1 GLU C  94      30.580   2.658  43.100  1.00  0.00
C    H
ATOM  21510  HB2 GLU C  94      31.607   1.629  42.077  1.00  0.00
C    H
ATOM  21511  CG  GLU C  94      31.759   1.230  44.201  1.00  0.00
C    C
ATOM  21512  HG1 GLU C  94      32.757   0.757  44.146  1.00  0.00
C    H
ATOM  21513  HG2 GLU C  94      31.657   1.757  45.170  1.00  0.00
C    H
ATOM  21514  CD  GLU C  94      30.762   0.088  44.167  1.00  0.00
C    C
ATOM  21515  OE1 GLU C  94      30.027  -0.078  45.161  1.00  0.00
C    O
ATOM  21516  OE2 GLU C  94      30.705  -0.644  43.153  1.00  0.00
C    O
ATOM  21517  C   GLU C  94      32.827   3.768  41.561  1.00  0.00
C    C
ATOM  21518  O   GLU C  94      33.722   3.281  40.869  1.00  0.00
C    O
```

ATOM 21519 N LEU C 95 32.001 4.710 41.119 1.00 0.00
C N
ATOM 21520 HN LEU C 95 31.301 5.144 41.683 1.00 0.00
C H
ATOM 21521 CA LEU C 95 32.049 5.197 39.740 1.00 0.00
C C
ATOM 21522 HA LEU C 95 32.342 4.349 39.135 1.00 0.00
C H
ATOM 21523 CB LEU C 95 30.655 5.645 39.281 1.00 0.00
C C
ATOM 21524 HB1 LEU C 95 30.350 6.494 39.935 1.00 0.00
C H
ATOM 21525 HB2 LEU C 95 30.729 6.061 38.251 1.00 0.00
C H
ATOM 21526 CG LEU C 95 29.547 4.580 39.333 1.00 0.00
C C
ATOM 21527 HG LEU C 95 29.619 4.152 40.362 1.00 0.00
C H
ATOM 21528 CD1 LEU C 95 28.170 5.184 39.123 1.00 0.00
C C
ATOM 21529 HD11 LEU C 95 27.392 4.390 39.118 1.00 0.00
C H
ATOM 21530 HD12 LEU C 95 27.929 5.902 39.937 1.00 0.00
C H
ATOM 21531 HD13 LEU C 95 28.121 5.724 38.154 1.00 0.00
C H
ATOM 21532 CD2 LEU C 95 29.788 3.458 38.340 1.00 0.00
C C
ATOM 21533 HD21 LEU C 95 28.982 2.696 38.415 1.00 0.00
C H
ATOM 21534 HD22 LEU C 95 29.802 3.831 37.296 1.00 0.00
C H
ATOM 21535 HD23 LEU C 95 30.759 2.957 38.546 1.00 0.00
C H
ATOM 21536 C LEU C 95 33.100 6.299 39.536 1.00 0.00
C C
ATOM 21537 O LEU C 95 33.148 6.934 38.481 1.00 0.00
C O
ATOM 21538 N ASP C 96 33.937 6.504 40.554 1.00 0.00
C N
ATOM 21539 HN ASP C 96 33.853 6.038 41.431 1.00 0.00
C H
ATOM 21540 CA ASP C 96 35.074 7.433 40.506 1.00 0.00
C C
ATOM 21541 HA ASP C 96 35.562 7.318 41.468 1.00 0.00
C H
ATOM 21542 CB ASP C 96 36.067 7.013 39.419 1.00 0.00
C C
ATOM 21543 HB1 ASP C 96 35.518 6.514 38.595 1.00 0.00
C H
ATOM 21544 HB2 ASP C 96 36.589 7.898 39.003 1.00 0.00
C H
ATOM 21545 CG ASP C 96 37.102 6.041 39.928 1.00 0.00
C C

ATOM 21546 OD1 ASP C 96   37.153   4.905 39.411 1.00 0.00
C   O
ATOM 21547 OD2 ASP C 96   37.865   6.408 40.851 1.00 0.00
C   O
ATOM 21548 C   ASP C 96   34.734   8.919 40.358 1.00 0.00
C   C
ATOM 21549 O   ASP C 96   35.556   9.693 39.864 1.00 0.00
C   O
ATOM 21550 N   ILE C 97   33.539   9.319 40.791 1.00 0.00
C   N
ATOM 21551 HN ILE C 97   32.839   8.706 41.152 1.00 0.00
C   H
ATOM 21552 CA ILE C 97   33.146 10.729 40.772 1.00 0.00
C   C
ATOM 21553 HA ILE C 97   33.194 11.063 39.743 1.00 0.00
C   H
ATOM 21554 CB ILE C 97   31.722 10.937 41.348 1.00 0.00
C   C
ATOM 21555 HB ILE C 97   31.780 10.534 42.391 1.00 0.00
C   H
ATOM 21556 CG2 ILE C 97   31.327 12.412 41.356 1.00 0.00
C   C
ATOM 21557 HG21 ILE C 97   30.377 12.560 41.911 1.00 0.00
C   H
ATOM 21558 HG22 ILE C 97   32.106 13.028 41.853 1.00 0.00
C   H
ATOM 21559 HG23 ILE C 97   31.187 12.791 40.322 1.00 0.00
C   H
ATOM 21560 CG1 ILE C 97   30.685 10.076 40.603 1.00 0.00
C   C
ATOM 21561 HG11 ILE C 97   30.885   8.998 40.791 1.00 0.00
C   H
ATOM 21562 HG12 ILE C 97   29.672 10.298 41.010 1.00 0.00
C   H
ATOM 21563 CD ILE C 97   30.622 10.285 39.107 1.00 0.00
C   C
ATOM 21564 HD1 ILE C 97   29.811   9.668 38.665 1.00 0.00
C   H
ATOM 21565 HD2 ILE C 97   30.417 11.351 38.868 1.00 0.00
C   H
ATOM 21566 HD3 ILE C 97   31.578   9.994 38.624 1.00 0.00
C   H
ATOM 21567 C   ILE C 97   34.187 11.547 41.553 1.00 0.00
C   C
ATOM 21568 O   ILE C 97   34.549 11.178 42.677 1.00 0.00
C   O
ATOM 21569 N   PRO C 98   34.695 12.636 40.941 1.00 0.00
C   N
ATOM 21570 CD PRO C 98   34.425 13.049 39.551 1.00 0.00
C   C
ATOM 21571 HD1 PRO C 98   34.695 11.999 39.808 1.00 0.00
C   H
ATOM 21572 HD2 PRO C 98   33.698 13.069 38.707 1.00 0.00
C   H

ATOM 21573 CA PRO C 98   35.732 13.471 41.564 1.00 0.00
C   C
ATOM 21574 HA PRO C 98   36.587 12.852 41.810 1.00 0.00
C   H
ATOM 21575 CB PRO C 98   36.092 14.474 40.458 1.00 0.00
C   C
ATOM 21576 HB1 PRO C 98   36.854 14.017 39.787 1.00 0.00
C   H
ATOM 21577 HB2 PRO C 98   36.491 15.428 40.858 1.00 0.00
C   H
ATOM 21578 CG PRO C 98   35.659 13.824 39.185 1.00 0.00
C   C
ATOM 21579 HG1 PRO C 98   36.199 14.210 38.298 1.00 0.00
C   H
ATOM 21580 HG2 PRO C 98   35.770 13.156 38.450 1.00 0.00
C   H
ATOM 21581 C   PRO C 98   35.271 14.209 42.821 1.00 0.00
C   C
ATOM 21582 O   PRO C 98   36.035 14.293 43.783 1.00 0.00
C   O
ATOM 21583 N   ASN C 99   34.049 14.743 42.812 1.00 0.00
C   N
ATOM 21584 HN ASN C 99   33.425 14.704 42.034 1.00 0.00
C   H
ATOM 21585 CA ASN C 99   33.520 15.447 43.979 1.00 0.00
C   C
ATOM 21586 HA ASN C 99   34.186 15.181 44.793 1.00 0.00
C   H
ATOM 21587 CB ASN C 99   33.536 16.958 43.771 1.00 0.00
C   C
ATOM 21588 HB1 ASN C 99   32.901 17.187 42.888 1.00 0.00
C   H
ATOM 21589 HB2 ASN C 99   33.088 17.472 44.646 1.00 0.00
C   H
ATOM 21590 CG ASN C 99   34.927 17.499 43.501 1.00 0.00
C   C
ATOM 21591 OD1 ASN C 99   35.393 17.515 42.355 1.00 0.00
C   O
ATOM 21592 ND2 ASN C 99   35.599 17.956 44.557 1.00 0.00
C   N
ATOM 21593 HD21 ASN C 99   36.510 18.325 44.395 1.00 0.00
C   H
ATOM 21594 HD22 ASN C 99   35.177 17.948 45.458 1.00 0.00
C   H
ATOM 21595 C   ASN C 99   32.121 14.996 44.362 1.00 0.00
C   C
ATOM 21596 O   ASN C 99   31.166 15.176 43.597 1.00 0.00
C   O
ATOM 21597 N   ILE C 100   32.014 14.407 45.554 1.00 0.00
C   N
ATOM 21598 HN ILE C 100   32.797 14.243 46.150 1.00 0.00
C   H
ATOM 21599 CA ILE C 100   30.741 13.946 46.099 1.00 0.00
C   C

```
ATOM  21600  HA  ILE C 100      29.978  14.209  45.377  1.00  0.00
C    H
ATOM  21601  CB  ILE C 100      30.747  12.426  46.361  1.00  0.00
C    C
ATOM  21602  HB  ILE C 100      31.619  12.263  47.045  1.00  0.00
C    H
ATOM  21603  CG2 ILE C 100      29.411  11.976  46.930  1.00  0.00
C    C
ATOM  21604 HG21 ILE C 100      29.465  10.918  47.264  1.00  0.00
C    H
ATOM  21605 HG22 ILE C 100      29.128  12.592  47.809  1.00  0.00
C    H
ATOM  21606 HG23 ILE C 100      28.606  12.059  46.169  1.00  0.00
C    H
ATOM  21607  CG1 ILE C 100      31.048  11.667  45.066  1.00  0.00
C    C
ATOM  21608 HG11 ILE C 100      30.126  11.598  44.447  1.00  0.00
C    H
ATOM  21609 HG12 ILE C 100      31.798  12.242  44.476  1.00  0.00
C    H
ATOM  21610  CD  ILE C 100      31.599  10.266  45.267  1.00  0.00
C    C
ATOM  21611  HD1 ILE C 100      31.845   9.804  44.287  1.00  0.00
C    H
ATOM  21612  HD2 ILE C 100      32.525  10.294  45.881  1.00  0.00
C    H
ATOM  21613  HD3 ILE C 100      30.857   9.615  45.776  1.00  0.00
C    H
ATOM  21614  C   ILE C 100      30.415  14.707  47.384  1.00  0.00
C    C
ATOM  21615  O   ILE C 100      31.112  14.581  48.400  1.00  0.00
C    O
ATOM  21616  N   TRP C 101      29.357  15.510  47.316  1.00  0.00
C    N
ATOM  21617  HN  TRP C 101      28.824  15.624  46.481  1.00  0.00
C    H
ATOM  21618  CA  TRP C 101      28.891  16.289  48.455  1.00  0.00
C    C
ATOM  21619  HA  TRP C 101      29.591  16.164  49.272  1.00  0.00
C    H
ATOM  21620  CB  TRP C 101      28.704  17.751  48.074  1.00  0.00
C    C
ATOM  21621  HB1 TRP C 101      28.048  17.788  47.176  1.00  0.00
C    H
ATOM  21622  HB2 TRP C 101      28.168  18.287  48.887  1.00  0.00
C    H
ATOM  21623  CG  TRP C 101      29.984  18.474  47.722  1.00  0.00
C    C
ATOM  21624  CD1 TRP C 101      31.169  17.926  47.234  1.00  0.00
C    C
ATOM  21625  HD1 TRP C 101      31.298  16.872  47.024  1.00  0.00
C    H
ATOM  21626  NE1 TRP C 101      32.110  18.897  47.025  1.00  0.00
C    N
```

ATOM 21627 HE1 TRP C 101   33.004 18.731 46.670 1.00 0.00
C   H
ATOM 21628 CE2 TRP C 101   31.620 20.115 47.330 1.00 0.00
C   C
ATOM 21629 CD2 TRP C 101   30.237 19.919 47.786 1.00 0.00
C   C
ATOM 21630 CE3 TRP C 101   29.490 21.032 48.171 1.00 0.00
C   C
ATOM 21631 HE3 TRP C 101   28.470 20.943 48.518 1.00 0.00
C   H
ATOM 21632 CZ3 TRP C 101   30.096 22.294 48.106 1.00 0.00
C   C
ATOM 21633 HZ3 TRP C 101   29.535 23.168 48.407 1.00 0.00
C   H
ATOM 21634 CZ2 TRP C 101   32.205 21.373 47.271 1.00 0.00
C   C
ATOM 21635 HZ2 TRP C 101   33.219 21.533 46.937 1.00 0.00
C   H
ATOM 21636 CH2 TRP C 101   31.417 22.457 47.663 1.00 0.00
C   C
ATOM 21637 HH2 TRP C 101   31.848 23.448 47.623 1.00 0.00
C   H
ATOM 21638 C   TRP C 101   27.596 15.724 48.907 1.00 0.00
C   C
ATOM 21639 O   TRP C 101   26.692 15.516 48.100 1.00 0.00
C   O
ATOM 21640 N   VAL C 102   27.491 15.467 50.205 1.00 0.00
C   N
ATOM 21641 HN VAL C 102   28.189 15.697 50.878 1.00 0.00
C   H
ATOM 21642 CA VAL C 102   26.318 14.813 50.756 1.00 0.00
C   C
ATOM 21643 HA VAL C 102   25.555 14.813 49.988 1.00 0.00
C   H
ATOM 21644 CB VAL C 102   26.641 13.368 51.195 1.00 0.00
C   C
ATOM 21645 HB VAL C 102   27.220 13.428 52.150 1.00 0.00
C   H
ATOM 21646 CG1 VAL C 102   25.357 12.582 51.435 1.00 0.00
C   C
ATOM 21647 HG11 VAL C 102   25.594 11.533 51.713 1.00 0.00
C   H
ATOM 21648 HG12 VAL C 102   24.759 13.016 52.263 1.00 0.00
C   H
ATOM 21649 HG13 VAL C 102   24.731 12.562 50.517 1.00 0.00
C   H
ATOM 21650 CG2 VAL C 102   27.494 12.660 50.155 1.00 0.00
C   C
ATOM 21651 HG21 VAL C 102   27.690 11.609 50.457 1.00 0.00
C   H
ATOM 21652 HG22 VAL C 102   26.981 12.651 49.169 1.00 0.00
C   H
ATOM 21653 HG23 VAL C 102   28.476 13.162 50.032 1.00 0.00
C   H

ATOM 21654 C VAL C 102 25.768 15.589 51.947 1.00 0.00
C C
ATOM 21655 O VAL C 102 26.532 16.122 52.762 1.00 0.00
C O
ATOM 21656 N LYS C 103 24.440 15.655 52.034 1.00 0.00
C N
ATOM 21657 HN LYS C 103 23.835 15.358 51.298 1.00 0.00
C H
ATOM 21658 CA LYS C 103 23.768 16.169 53.217 1.00 0.00
C C
ATOM 21659 HA LYS C 103 24.367 16.982 53.611 1.00 0.00
C H
ATOM 21660 CB LYS C 103 22.364 16.653 52.861 1.00 0.00
C C
ATOM 21661 HB1 LYS C 103 22.481 17.496 52.142 1.00 0.00
C H
ATOM 21662 HB2 LYS C 103 21.831 15.845 52.311 1.00 0.00
C H
ATOM 21663 CG LYS C 103 21.535 17.128 54.043 1.00 0.00
C C
ATOM 21664 HG1 LYS C 103 21.472 16.310 54.797 1.00 0.00
C H
ATOM 21665 HG2 LYS C 103 22.054 17.987 54.528 1.00 0.00
C H
ATOM 21666 CD LYS C 103 20.139 17.517 53.586 1.00 0.00
C C
ATOM 21667 HD1 LYS C 103 20.134 18.607 53.357 1.00 0.00
C H
ATOM 21668 HD2 LYS C 103 19.923 16.975 52.636 1.00 0.00
C H
ATOM 21669 CE LYS C 103 19.094 17.183 54.634 1.00 0.00
C C
ATOM 21670 HE1 LYS C 103 18.152 16.839 54.150 1.00 0.00
C H
ATOM 21671 HE2 LYS C 103 19.458 16.376 55.307 1.00 0.00
C H
ATOM 21672 NZ LYS C 103 18.757 18.356 55.480 1.00 0.00
C N
ATOM 21673 HZ1 LYS C 103 18.039 18.092 56.184 1.00 0.00
C H
ATOM 21674 HZ2 LYS C 103 19.613 18.688 55.969 1.00 0.00
C H
ATOM 21675 HZ3 LYS C 103 18.387 19.121 54.880 1.00 0.00
C H
ATOM 21676 C LYS C 103 23.712 15.082 54.294 1.00 0.00
C C
ATOM 21677 O LYS C 103 23.243 13.964 54.046 1.00 0.00
C O
ATOM 21678 N ALA C 104 24.192 15.417 55.486 1.00 0.00
C N
ATOM 21679 HN ALA C 104 24.595 16.311 55.680 1.00 0.00
C H
ATOM 21680 CA ALA C 104 24.164 14.488 56.608 1.00 0.00
C C

ATOM 21681 HA ALA C 104   24.127 13.463 56.257 1.00 0.00
C   H
ATOM 21682 CB ALA C 104   25.411 14.645 57.456 1.00 0.00
C   C
ATOM 21683 HB1 ALA C 104   26.311 14.442 56.837 1.00 0.00
C   H
ATOM 21684 HB2 ALA C 104   25.497 15.681 57.852 1.00 0.00
C   H
ATOM 21685 HB3 ALA C 104   25.416 13.937 58.313 1.00 0.00
C   H
ATOM 21686 C   ALA C 104   22.917 14.696 57.452 1.00 0.00
C   C
ATOM 21687 O   ALA C 104   22.502 15.828 57.692 1.00 0.00
C   O
ATOM 21688 N   GLN C 105   22.327 13.596 57.902 1.00 0.00
C   N
ATOM 21689 HN GLN C 105   22.668 12.675 57.719 1.00 0.00
C   H
ATOM 21690 CA GLN C 105   21.114 13.641 58.713 1.00 0.00
C   C
ATOM 21691 HA GLN C 105   20.559 14.510 58.383 1.00 0.00
C   H
ATOM 21692 CB GLN C 105   20.276 12.388 58.499 1.00 0.00
C   C
ATOM 21693 HB1 GLN C 105   20.960 11.518 58.628 1.00 0.00
C   H
ATOM 21694 HB2 GLN C 105   19.517 12.310 59.311 1.00 0.00
C   H
ATOM 21695 CG GLN C 105   19.630 12.339 57.133 1.00 0.00
C   C
ATOM 21696 HG1 GLN C 105   20.404 12.246 56.342 1.00 0.00
C   H
ATOM 21697 HG2 GLN C 105   18.952 11.465 57.051 1.00 0.00
C   H
ATOM 21698 CD GLN C 105   18.844 13.605 56.830 1.00 0.00
C   C
ATOM 21699 OE1 GLN C 105   17.968 14.007 57.597 1.00 0.00
C   O
ATOM 21700 NE2 GLN C 105   19.157 14.239 55.703 1.00 0.00
C   N
ATOM 21701 HE21 GLN C 105   18.649 15.071 55.500 1.00 0.00
C   H
ATOM 21702 HE22 GLN C 105   19.857 13.866 55.104 1.00 0.00
C   H
ATOM 21703 C   GLN C 105   21.407 13.820 60.188 1.00 0.00
C   C
ATOM 21704 O   GLN C 105   20.709 14.562 60.881 1.00 0.00
C   O
ATOM 21705 N   ASN C 106   22.445 13.140 60.658 1.00 0.00
C   N
ATOM 21706 HN ASN C 106   23.019 12.552 60.088 1.00 0.00
C   H
ATOM 21707 CA ASN C 106   22.839 13.193 62.053 1.00 0.00
C   C

```
ATOM  21708  HA  ASN C 106      22.704  14.222  62.366  1.00  0.00
C    H
ATOM  21709  CB  ASN C 106      21.971  12.238  62.877  1.00  0.00
C    C
ATOM  21710  HB1 ASN C 106      22.363  12.233  63.917  1.00  0.00
C    H
ATOM  21711  HB2 ASN C 106      20.926  12.606  62.918  1.00  0.00
C    H
ATOM  21712  CG  ASN C 106      22.006  10.814  62.356  1.00  0.00
C    C
ATOM  21713  OD1 ASN C 106      23.034  10.343  61.868  1.00  0.00
C    O
ATOM  21714  ND2 ASN C 106      20.880  10.117  62.467  1.00  0.00
C    N
ATOM  21715 HD21 ASN C 106      20.891   9.176  62.140  1.00  0.00
C    H
ATOM  21716 HD22 ASN C 106      20.080  10.527  62.891  1.00  0.00
C    H
ATOM  21717  C   ASN C 106      24.316  12.854  62.205  1.00  0.00
C    C
ATOM  21718  O   ASN C 106      25.027  12.673  61.211  1.00  0.00
C    O
ATOM  21719  N   TYR C 107      24.777  12.765  63.450  1.00  0.00
C    N
ATOM  21720  HN  TYR C 107      24.215  12.926  64.261  1.00  0.00
C    H
ATOM  21721  CA  TYR C 107      26.166  12.420  63.735  1.00  0.00
C    C
ATOM  21722  HA  TYR C 107      26.767  13.195  63.275  1.00  0.00
C    H
ATOM  21723  CB  TYR C 107      26.413  12.374  65.244  1.00  0.00
C    C
ATOM  21724  HB1 TYR C 107      26.296  13.397  65.664  1.00  0.00
C    H
ATOM  21725  HB2 TYR C 107      25.663  11.725  65.746  1.00  0.00
C    H
ATOM  21726  CG  TYR C 107      27.804  11.908  65.605  1.00  0.00
C    C
ATOM  21727  CD1 TYR C 107      28.896  12.774  65.516  1.00  0.00
C    C
ATOM  21728  HD1 TYR C 107      28.751  13.789  65.173  1.00  0.00
C    H
ATOM  21729  CE1 TYR C 107      30.176  12.351  65.839  1.00  0.00
C    C
ATOM  21730  HE1 TYR C 107      31.014  13.027  65.762  1.00  0.00
C    H
ATOM  21731  CZ  TYR C 107      30.379  11.046  66.259  1.00  0.00
C    C
ATOM  21732  OH  TYR C 107      31.652  10.636  66.583  1.00  0.00
C    O
ATOM  21733  HH  TYR C 107      31.589   9.738  66.916  1.00  0.00
C    H
ATOM  21734  CD2 TYR C 107      28.036  10.594  66.026  1.00  0.00
C    C
```

ATOM 21735 HD2 TYR C 107   27.220   9.886 66.063 1.00 0.00
C   H
ATOM 21736 CE2 TYR C 107   29.312 10.161 66.353 1.00 0.00
C   C
ATOM 21737 HE2 TYR C 107   29.469   9.136 66.656 1.00 0.00
C   H
ATOM 21738 C   TYR C 107   26.576 11.092 63.094 1.00 0.00
C   C
ATOM 21739 O   TYR C 107   27.623 11.001 62.453 1.00 0.00
C   O
ATOM 21740 N   TYR C 108   25.744 10.070 63.278 1.00 0.00
C   N
ATOM 21741 HN TYR C 108   24.888 10.150 63.786 1.00 0.00
C   H
ATOM 21742 CA TYR C 108   26.014   8.738 62.751 1.00 0.00
C   C
ATOM 21743 HA TYR C 108   26.956   8.426 63.184 1.00 0.00
C   H
ATOM 21744 CB TYR C 108   24.874   7.784 63.126 1.00 0.00
C   C
ATOM 21745 HB1 TYR C 108   25.095   7.324 64.114 1.00 0.00
C   H
ATOM 21746 HB2 TYR C 108   23.915   8.337 63.228 1.00 0.00
C   H
ATOM 21747 CG TYR C 108   24.678   6.635 62.153 1.00 0.00
C   C
ATOM 21748 CD1 TYR C 108   25.599   5.591 62.087 1.00 0.00
C   C
ATOM 21749 HD1 TYR C 108   26.474   5.606 62.722 1.00 0.00
C   H
ATOM 21750 CE1 TYR C 108   25.430   4.536 61.202 1.00 0.00
C   C
ATOM 21751 HE1 TYR C 108   26.155   3.737 61.156 1.00 0.00
C   H
ATOM 21752 CZ TYR C 108   24.327   4.514 60.366 1.00 0.00
C   C
ATOM 21753 OH TYR C 108   24.159   3.468 59.489 1.00 0.00
C   O
ATOM 21754 HH TYR C 108   23.314   3.591 59.053 1.00 0.00
C   H
ATOM 21755 CD2 TYR C 108   23.569   6.588 61.297 1.00 0.00
C   C
ATOM 21756 HD2 TYR C 108   22.853   7.398 61.296 1.00 0.00
C   H
ATOM 21757 CE2 TYR C 108   23.395   5.536 60.406 1.00 0.00
C   C
ATOM 21758 HE2 TYR C 108   22.547   5.534 59.738 1.00 0.00
C   H
ATOM 21759 C   TYR C 108   26.199   8.756 61.240 1.00 0.00
C   C
ATOM 21760 O   TYR C 108   27.127   8.145 60.709 1.00 0.00
C   O
ATOM 21761 N   HSE C 109   25.294   9.455 60.566 1.00 0.00
C   N

ATOM 21762 HN HSE C 109   24.560   9.961 61.018 1.00 0.00
C   H
ATOM 21763 CA HSE C 109   25.283   9.553 59.116 1.00 0.00
C   C
ATOM 21764 HA HSE C 109   25.271   8.545 58.719 1.00 0.00
C   H
ATOM 21765 CB HSE C 109   24.048 10.327 58.666 1.00 0.00
C   C
ATOM 21766 HB1 HSE C 109   23.167   9.903 59.195 1.00 0.00
C   H
ATOM 21767 HB2 HSE C 109   24.139 11.386 58.982 1.00 0.00
C   H
ATOM 21768 ND1 HSE C 109   23.042 11.174 56.540 1.00 0.00
C   N
ATOM 21769 CG HSE C 109   23.777 10.249 57.185 1.00 0.00
C   C
ATOM 21770 CE1 HSE C 109   22.962 10.860 55.237 1.00 0.00
C   C
ATOM 21771 HE1 HSE C 109   22.409 11.438 54.492 1.00 0.00
C   H
ATOM 21772 NE2 HSE C 109   23.661   9.735 55.041 1.00 0.00
C   N
ATOM 21773 HE2 HSE C 109   23.765   9.264 54.166 1.00 0.00
C   H
ATOM 21774 CD2 HSE C 109   24.178   9.332 56.224 1.00 0.00
C   C
ATOM 21775 HD2 HSE C 109   24.777   8.438 56.326 1.00 0.00
C   H
ATOM 21776 C   HSE C 109   26.552 10.197 58.645 1.00 0.00
C   C
ATOM 21777 O   HSE C 109   27.175   9.737 57.690 1.00 0.00
C   O
ATOM 21778 N   HSE C 110   26.970 11.248 59.343 1.00 0.00
C   N
ATOM 21779 HN HSE C 110   26.455 11.652 60.098 1.00 0.00
C   H
ATOM 21780 CA HSE C 110   28.234 11.909 59.058 1.00 0.00
C   C
ATOM 21781 HA HSE C 110   28.157 12.313 58.056 1.00 0.00
C   H
ATOM 21782 CB HSE C 110   28.479 13.008 60.081 1.00 0.00
C   C
ATOM 21783 HB1 HSE C 110   27.582 13.664 60.100 1.00 0.00
C   H
ATOM 21784 HB2 HSE C 110   28.574 12.560 61.092 1.00 0.00
C   H
ATOM 21785 ND1 HSE C 110   29.564 15.088 59.236 1.00 0.00
C   N
ATOM 21786 CG HSE C 110   29.676 13.868 59.775 1.00 0.00
C   C
ATOM 21787 CE1 HSE C 110   30.786 15.621 59.068 1.00 0.00
C   C
ATOM 21788 HE1 HSE C 110   30.979 16.609 58.643 1.00 0.00
C   H

ATOM 21789 NE2 HSE C 110   31.688 14.731 59.503 1.00 0.00
C   N
ATOM 21790 HE2 HSE C 110   32.680 14.854 59.482 1.00 0.00
C   H
ATOM 21791 CD2 HSE C 110   31.037 13.635 59.946 1.00 0.00
C   C
ATOM 21792 HD2 HSE C 110   31.548 12.767 60.339 1.00 0.00
C   H
ATOM 21793 C   HSE C 110   29.380 10.930 59.054 1.00 0.00
C   C
ATOM 21794 O   HSE C 110   30.206 10.926 58.140 1.00 0.00
C   O
ATOM 21795 N   LYS C 111   29.416 10.076 60.074 1.00 0.00
C   N
ATOM 21796 HN LYS C 111   28.684 10.035 60.753 1.00 0.00
C   H
ATOM 21797 CA LYS C 111   30.512   9.137 60.287 1.00 0.00
C   C
ATOM 21798 HA LYS C 111   31.408   9.743 60.348 1.00 0.00
C   H
ATOM 21799 CB LYS C 111   30.333   8.418 61.623 1.00 0.00
C   C
ATOM 21800 HB1 LYS C 111   29.316   7.961 61.616 1.00 0.00
C   H
ATOM 21801 HB2 LYS C 111   31.055   7.573 61.680 1.00 0.00
C   H
ATOM 21802 CG LYS C 111   30.465   9.328 62.842 1.00 0.00
C   C
ATOM 21803 HG1 LYS C 111   30.421 10.390 62.509 1.00 0.00
C   H
ATOM 21804 HG2 LYS C 111   29.596   9.157 63.519 1.00 0.00
C   H
ATOM 21805 CD LYS C 111   31.766   9.103 63.594 1.00 0.00
C   C
ATOM 21806 HD1 LYS C 111   31.609   9.383 64.661 1.00 0.00
C   H
ATOM 21807 HD2 LYS C 111   31.999   8.013 63.566 1.00 0.00
C   H
ATOM 21808 CE LYS C 111   32.932   9.887 63.020 1.00 0.00
C   C
ATOM 21809 HE1 LYS C 111   32.954   9.797 61.911 1.00 0.00
C   H
ATOM 21810 HE2 LYS C 111   32.846 10.964 63.284 1.00 0.00
C   H
ATOM 21811 NZ LYS C 111   34.210   9.354 63.572 1.00 0.00
C   N
ATOM 21812 HZ1 LYS C 111   35.015   9.884 63.182 1.00 0.00
C   H
ATOM 21813 HZ2 LYS C 111   34.206   9.444 64.608 1.00 0.00
C   H
ATOM 21814 HZ3 LYS C 111   34.304   8.350 63.318 1.00 0.00
C   H
ATOM 21815 C   LYS C 111   30.718   8.133 59.153 1.00 0.00
C   C

ATOM 21816 O   LYS C 111   31.852   7.935 58.719 1.00 0.00
C   O
ATOM 21817 N   VAL C 112   29.640   7.506 58.673 1.00 0.00
C   N
ATOM 21818 HN VAL C 112   28.712   7.623 59.017 1.00 0.00
C   H
ATOM 21819 CA VAL C 112   29.759   6.556 57.551 1.00 0.00
C   C
ATOM 21820 HA VAL C 112   30.574   5.884 57.790 1.00 0.00
C   H
ATOM 21821 CB VAL C 112   28.518   5.649 57.337 1.00 0.00
C   C
ATOM 21822 HB VAL C 112   28.645   5.129 56.356 1.00 0.00
C   H
ATOM 21823 CG1 VAL C 112   28.411   4.615 58.431 1.00 0.00
C   C
ATOM 21824 HG11 VAL C 112   27.499   3.997 58.290 1.00 0.00
C   H
ATOM 21825 HG12 VAL C 112   29.281   3.926 58.430 1.00 0.00
C   H
ATOM 21826 HG13 VAL C 112   28.345   5.103 59.427 1.00 0.00
C   H
ATOM 21827 CG2 VAL C 112   27.237   6.450 57.248 1.00 0.00
C   C
ATOM 21828 HG21 VAL C 112   26.360   5.777 57.135 1.00 0.00
C   H
ATOM 21829 HG22 VAL C 112   27.093   7.060 58.167 1.00 0.00
C   H
ATOM 21830 HG23 VAL C 112   27.250   7.134 56.374 1.00 0.00
C   H
ATOM 21831 C   VAL C 112   30.114   7.259 56.244 1.00 0.00
C   C
ATOM 21832 O   VAL C 112   30.924   6.748 55.465 1.00 0.00
C   O
ATOM 21833 N   LEU C 113   29.510   8.419 56.009 1.00 0.00
C   N
ATOM 21834 HN LEU C 113   28.826   8.827 56.611 1.00 0.00
C   H
ATOM 21835 CA LEU C 113   29.809   9.210 54.819 1.00 0.00
C   C
ATOM 21836 HA LEU C 113   29.612   8.582 53.960 1.00 0.00
C   H
ATOM 21837 CB LEU C 113   28.929 10.461 54.765 1.00 0.00
C   C
ATOM 21838 HB1 LEU C 113   29.064 10.999 55.732 1.00 0.00
C   H
ATOM 21839 HB2 LEU C 113   29.318 11.143 53.977 1.00 0.00
C   H
ATOM 21840 CG LEU C 113   27.427 10.224 54.555 1.00 0.00
C   C
ATOM 21841 HG LEU C 113   27.146   9.404 55.259 1.00 0.00
C   H
ATOM 21842 CD1 LEU C 113   26.635 11.477 54.893 1.00 0.00
C   C

ATOM 21843 HD11 LEU C 113   25.554 11.319 54.694 1.00 0.00
C   H
ATOM 21844 HD12 LEU C 113   26.759 11.740 55.965 1.00 0.00
C   H
ATOM 21845 HD13 LEU C 113   26.980 12.336 54.278 1.00 0.00
C   H
ATOM 21846 CD2 LEU C 113   27.123   9.755 53.141 1.00 0.00
C   C
ATOM 21847 HD21 LEU C 113   26.033   9.573 53.021 1.00 0.00
C   H
ATOM 21848 HD22 LEU C 113   27.423 10.508 52.386 1.00 0.00
C   H
ATOM 21849 HD23 LEU C 113   27.660   8.806 52.922 1.00 0.00
C   H
ATOM 21850 C   LEU C 113   31.291   9.576 54.791 1.00 0.00
C   C
ATOM 21851 O   LEU C 113   31.938   9.489 53.746 1.00 0.00
C   O
ATOM 21852 N   GLU C 114   31.818   9.958 55.953 1.00 0.00
C   N
ATOM 21853 HN GLU C 114   31.270 10.081 56.776 1.00 0.00
C   H
ATOM 21854 CA GLU C 114   33.237 10.234 56.139 1.00 0.00
C   C
ATOM 21855 HA GLU C 114   33.500 11.087 55.526 1.00 0.00
C   H
ATOM 21856 CB GLU C 114   33.495 10.527 57.618 1.00 0.00
C   C
ATOM 21857 HB1 GLU C 114   32.595 11.038 58.033 1.00 0.00
C   H
ATOM 21858 HB2 GLU C 114   33.588   9.571 58.181 1.00 0.00
C   H
ATOM 21859 CG GLU C 114   34.704 11.393 57.927 1.00 0.00
C   C
ATOM 21860 HG1 GLU C 114   35.624 10.786 57.844 1.00 0.00
C   H
ATOM 21861 HG2 GLU C 114   34.768 12.250 57.227 1.00 0.00
C   H
ATOM 21862 CD GLU C 114   34.665 11.916 59.352 1.00 0.00
C   C
ATOM 21863 OE1 GLU C 114   34.646 11.098 60.302 1.00 0.00
C   O
ATOM 21864 OE2 GLU C 114   34.642 13.152 59.526 1.00 0.00
C   O
ATOM 21865 C   GLU C 114   34.091   9.050 55.662 1.00 0.00
C   C
ATOM 21866 O   GLU C 114   35.047   9.229 54.901 1.00 0.00
C   O
ATOM 21867 N   LYS C 115   33.719   7.844 56.093 1.00 0.00
C   N
ATOM 21868 HN LYS C 115   32.895   7.702 56.639 1.00 0.00
C   H
ATOM 21869 CA LYS C 115   34.487   6.635 55.800 1.00 0.00
C   C

ATOM 21870 HA LYS C 115   35.527   6.919 55.903 1.00 0.00
C   H
ATOM 21871 CB LYS C 115   34.187   5.543 56.831 1.00 0.00
C   C
ATOM 21872 HB1 LYS C 115   33.078   5.450 56.896 1.00 0.00
C   H
ATOM 21873 HB2 LYS C 115   34.560   4.568 56.445 1.00 0.00
C   H
ATOM 21874 CG LYS C 115   34.746   5.824 58.220 1.00 0.00
C   C
ATOM 21875 HG1 LYS C 115   34.446   6.851 58.533 1.00 0.00
C   H
ATOM 21876 HG2 LYS C 115   34.291   5.111 58.945 1.00 0.00
C   H
ATOM 21877 CD LYS C 115   36.266   5.719 58.254 1.00 0.00
C   C
ATOM 21878 HD1 LYS C 115   36.545   4.644 58.168 1.00 0.00
C   H
ATOM 21879 HD2 LYS C 115   36.670   6.241 57.357 1.00 0.00
C   H
ATOM 21880 CE LYS C 115   36.848   6.325 59.524 1.00 0.00
C   C
ATOM 21881 HE1 LYS C 115   37.954   6.415 59.441 1.00 0.00
C   H
ATOM 21882 HE2 LYS C 115   36.425   7.337 59.704 1.00 0.00
C   H
ATOM 21883 NZ LYS C 115   36.565   5.506 60.740 1.00 0.00
C   N
ATOM 21884 HZ1 LYS C 115   36.982   5.958 61.578 1.00 0.00
C   H
ATOM 21885 HZ2 LYS C 115   35.536   5.422 60.869 1.00 0.00
C   H
ATOM 21886 HZ3 LYS C 115   36.973   4.557 60.620 1.00 0.00
C   H
ATOM 21887 C   LYS C 115   34.300   6.095 54.385 1.00 0.00
C   C
ATOM 21888 O   LYS C 115   35.216   5.495 53.828 1.00 0.00
C   O
ATOM 21889 N   ILE C 116   33.124   6.297 53.801 1.00 0.00
C   N
ATOM 21890 HN ILE C 116   32.345   6.765 54.211 1.00 0.00
C   H
ATOM 21891 CA ILE C 116   32.885   5.815 52.434 1.00 0.00
C   C
ATOM 21892 HA ILE C 116   33.500   4.932 52.317 1.00 0.00
C   H
ATOM 21893 CB ILE C 116   31.427   5.343 52.194 1.00 0.00
C   C
ATOM 21894 HB ILE C 116   31.416   4.997 51.130 1.00 0.00
C   H
ATOM 21895 CG2 ILE C 116   31.054   4.239 53.176 1.00 0.00
C   C
ATOM 21896 HG21 ILE C 116   30.073   3.793 52.909 1.00 0.00
C   H

```
ATOM  21897 HG22 ILE C 116      31.808   3.425  53.162  1.00  0.00
C    H
ATOM  21898 HG23 ILE C 116      30.984   4.634  54.211  1.00  0.00
C    H
ATOM  21899  CG1 ILE C 116      30.428   6.495  52.276  1.00  0.00
C    C
ATOM  21900 HG11 ILE C 116      30.526   7.008  53.258  1.00  0.00
C    H
ATOM  21901 HG12 ILE C 116      30.675   7.242  51.487  1.00  0.00
C    H
ATOM  21902  CD  ILE C 116      28.986   6.059  52.084  1.00  0.00
C    C
ATOM  21903  HD1 ILE C 116      28.311   6.942  52.096  1.00  0.00
C    H
ATOM  21904  HD2 ILE C 116      28.860   5.538  51.110  1.00  0.00
C    H
ATOM  21905  HD3 ILE C 116      28.666   5.372  52.895  1.00  0.00
C    H
ATOM  21906  C   ILE C 116      33.351   6.820  51.384  1.00  0.00
C    C
ATOM  21907  O   ILE C 116      33.054   6.670  50.195  1.00  0.00
C    O
ATOM  21908  N   GLY C 117      34.087   7.837  51.837  1.00  0.00
C    N
ATOM  21909  HN  GLY C 117      34.222   7.989  52.815  1.00  0.00
C    H
ATOM  21910  CA  GLY C 117      34.745   8.795  50.951  1.00  0.00
C    C
ATOM  21911  HA1 GLY C 117      35.131   8.194  50.139  1.00  0.00
C    H
ATOM  21912  HA2 GLY C 117      35.509   9.257  51.562  1.00  0.00
C    H
ATOM  21913  C   GLY C 117      33.889   9.902  50.352  1.00  0.00
C    C
ATOM  21914  O   GLY C 117      33.993  10.185  49.155  1.00  0.00
C    O
ATOM  21915  N   ALA C 118      33.045  10.526  51.175  1.00  0.00
C    N
ATOM  21916  HN  ALA C 118      32.846  10.201  52.099  1.00  0.00
C    H
ATOM  21917  CA  ALA C 118      32.348  11.741  50.775  1.00  0.00
C    C
ATOM  21918  HA  ALA C 118      32.016  11.706  49.744  1.00  0.00
C    H
ATOM  21919  CB  ALA C 118      31.146  11.998  51.668  1.00  0.00
C    C
ATOM  21920  HB1 ALA C 118      30.409  11.176  51.548  1.00  0.00
C    H
ATOM  21921  HB2 ALA C 118      31.444  12.035  52.738  1.00  0.00
C    H
ATOM  21922  HB3 ALA C 118      30.640  12.953  51.408  1.00  0.00
C    H
ATOM  21923  C   ALA C 118      33.346  12.883  50.866  1.00  0.00
C    C
```

ATOM 21924 O ALA C 118 34.064 13.010 51.858 1.00 0.00
C O
ATOM 21925 N ASP C 119 33.397 13.706 49.826 1.00 0.00
C N
ATOM 21926 HN ASP C 119 32.802 13.673 49.027 1.00 0.00
C H
ATOM 21927 CA ASP C 119 34.378 14.785 49.759 1.00 0.00
C C
ATOM 21928 HA ASP C 119 35.301 14.386 50.164 1.00 0.00
C H
ATOM 21929 CB ASP C 119 34.600 15.208 48.309 1.00 0.00
C C
ATOM 21930 HB1 ASP C 119 33.628 15.495 47.859 1.00 0.00
C H
ATOM 21931 HB2 ASP C 119 35.279 16.082 48.257 1.00 0.00
C H
ATOM 21932 CG ASP C 119 35.182 14.092 47.464 1.00 0.00
C C
ATOM 21933 OD1 ASP C 119 36.342 13.697 47.706 1.00 0.00
C O
ATOM 21934 OD2 ASP C 119 34.480 13.607 46.554 1.00 0.00
C O
ATOM 21935 C ASP C 119 33.995 15.977 50.630 1.00 0.00
C C
ATOM 21936 O ASP C 119 34.865 16.635 51.203 1.00 0.00
C O
ATOM 21937 N ARG C 120 32.696 16.250 50.726 1.00 0.00
C N
ATOM 21938 HN ARG C 120 32.016 15.769 50.177 1.00 0.00
C H
ATOM 21939 CA ARG C 120 32.196 17.269 51.641 1.00 0.00
C C
ATOM 21940 HA ARG C 120 32.887 17.367 52.470 1.00 0.00
C H
ATOM 21941 CB ARG C 120 32.055 18.631 50.941 1.00 0.00
C C
ATOM 21942 HB1 ARG C 120 33.049 18.857 50.487 1.00 0.00
C H
ATOM 21943 HB2 ARG C 120 31.343 18.541 50.091 1.00 0.00
C H
ATOM 21944 CG ARG C 120 31.669 19.755 51.893 1.00 0.00
C C
ATOM 21945 HG1 ARG C 120 30.674 19.520 52.333 1.00 0.00
C H
ATOM 21946 HG2 ARG C 120 32.380 19.764 52.749 1.00 0.00
C H
ATOM 21947 CD ARG C 120 31.697 21.121 51.246 1.00 0.00
C C
ATOM 21948 HD1 ARG C 120 32.754 21.453 51.129 1.00 0.00
C H
ATOM 21949 HD2 ARG C 120 31.213 21.108 50.242 1.00 0.00
C H
ATOM 21950 NE ARG C 120 30.964 22.078 52.073 1.00 0.00
C N

```
ATOM  21951  HE  ARG C 120      30.491  21.648  52.850  1.00  0.00
C    H
ATOM  21952  CZ  ARG C 120      30.899  23.390  51.859  1.00  0.00
C    C
ATOM  21953  NH1 ARG C 120      31.530  23.948  50.827  1.00  0.00
C    N
ATOM  21954 HH11 ARG C 120      31.499  24.927  50.687  1.00  0.00
C    H
ATOM  21955 HH12 ARG C 120      32.135  23.348  50.300  1.00  0.00
C    H
ATOM  21956  NH2 ARG C 120      30.197  24.152  52.687  1.00  0.00
C    N
ATOM  21957 HH21 ARG C 120      30.072  25.114  52.487  1.00  0.00
C    H
ATOM  21958 HH22 ARG C 120      29.648  23.687  53.368  1.00  0.00
C    H
ATOM  21959  C   ARG C 120      30.870  16.823  52.231  1.00  0.00
C    C
ATOM  21960  O   ARG C 120      30.007  16.324  51.520  1.00  0.00
C    O
ATOM  21961  N   ILE C 121      30.719  17.007  53.537  1.00  0.00
C    N
ATOM  21962  HN  ILE C 121      31.411  17.418  54.126  1.00  0.00
C    H
ATOM  21963  CA  ILE C 121      29.497  16.610  54.236  1.00  0.00
C    C
ATOM  21964  HA  ILE C 121      28.816  16.232  53.484  1.00  0.00
C    H
ATOM  21965  CB  ILE C 121      29.791  15.550  55.312  1.00  0.00
C    C
ATOM  21966  HB  ILE C 121      30.550  16.033  55.979  1.00  0.00
C    H
ATOM  21967  CG2 ILE C 121      28.515  15.145  56.030  1.00  0.00
C    C
ATOM  21968 HG21 ILE C 121      28.747  14.502  56.905  1.00  0.00
C    H
ATOM  21969 HG22 ILE C 121      27.970  16.038  56.403  1.00  0.00
C    H
ATOM  21970 HG23 ILE C 121      27.841  14.580  55.353  1.00  0.00
C    H
ATOM  21971  CG1 ILE C 121      30.464  14.328  54.684  1.00  0.00
C    C
ATOM  21972 HG11 ILE C 121      29.703  13.705  54.162  1.00  0.00
C    H
ATOM  21973 HG12 ILE C 121      31.192  14.672  53.915  1.00  0.00
C    H
ATOM  21974  CD  ILE C 121      31.206  13.465  55.680  1.00  0.00
C    C
ATOM  21975  HD1 ILE C 121      31.720  12.628  55.158  1.00  0.00
C    H
ATOM  21976  HD2 ILE C 121      31.973  14.061  56.220  1.00  0.00
C    H
ATOM  21977  HD3 ILE C 121      30.510  13.028  56.425  1.00  0.00
C    H
```

```
ATOM  21978  C   ILE C 121      28.828  17.830  54.865  1.00  0.00
C    C
ATOM  21979  O   ILE C 121      29.430  18.516  55.689  1.00  0.00
C    O
ATOM  21980  N   ILE C 122      27.585  18.091  54.469  1.00  0.00
C    N
ATOM  21981  HN  ILE C 122      27.044  17.491  53.884  1.00  0.00
C    H
ATOM  21982  CA  ILE C 122      26.896  19.315  54.872  1.00  0.00
C    C
ATOM  21983  HA  ILE C 122      27.647  19.937  55.341  1.00  0.00
C    H
ATOM  21984  CB  ILE C 122      26.268  20.035  53.661  1.00  0.00
C    C
ATOM  21985  HB  ILE C 122      25.567  19.282  53.219  1.00  0.00
C    H
ATOM  21986  CG2 ILE C 122      25.615  21.342  54.084  1.00  0.00
C    C
ATOM  21987 HG21 ILE C 122      25.032  21.777  53.245  1.00  0.00
C    H
ATOM  21988 HG22 ILE C 122      24.915  21.178  54.931  1.00  0.00
C    H
ATOM  21989 HG23 ILE C 122      26.378  22.084  54.398  1.00  0.00
C    H
ATOM  21990  CG1 ILE C 122      27.333  20.323  52.608  1.00  0.00
C    C
ATOM  21991 HG11 ILE C 122      27.476  21.422  52.507  1.00  0.00
C    H
ATOM  21992 HG12 ILE C 122      28.302  19.895  52.950  1.00  0.00
C    H
ATOM  21993  CD  ILE C 122      27.005  19.738  51.255  1.00  0.00
C    C
ATOM  21994  HD1 ILE C 122      27.838  19.925  50.543  1.00  0.00
C    H
ATOM  21995  HD2 ILE C 122      26.852  18.639  51.330  1.00  0.00
C    H
ATOM  21996  HD3 ILE C 122      26.084  20.194  50.836  1.00  0.00
C    H
ATOM  21997  C   ILE C 122      25.825  19.052  55.919  1.00  0.00
C    C
ATOM  21998  O   ILE C 122      25.006  18.153  55.773  1.00  0.00
C    O
ATOM  21999  N   HSE C 123      25.858  19.847  56.979  1.00  0.00
C    N
ATOM  22000  HN  HSE C 123      26.624  20.457  57.175  1.00  0.00
C    H
ATOM  22001  CA  HSE C 123      24.781  19.905  57.953  1.00  0.00
C    C
ATOM  22002  HA  HSE C 123      24.048  19.151  57.696  1.00  0.00
C    H
ATOM  22003  CB  HSE C 123      25.334  19.757  59.358  1.00  0.00
C    C
ATOM  22004  HB1 HSE C 123      26.077  20.568  59.520  1.00  0.00
C    H
```

ATOM 22005 HB2 HSE C 123   24.519 19.910 60.096 1.00 0.00
C   H
ATOM 22006 ND1 HSE C 123   25.447 17.443 60.313 1.00 0.00
C   N
ATOM 22007 CG HSE C 123   26.026 18.441 59.610 1.00 0.00
C   C
ATOM 22008 CE1 HSE C 123   26.290 16.400 60.380 1.00 0.00
C   C
ATOM 22009 HE1 HSE C 123   26.074 15.460 60.894 1.00 0.00
C   H
ATOM 22010 NE2 HSE C 123   27.415 16.731 59.720 1.00 0.00
C   N
ATOM 22011 HE2 HSE C 123   28.221 16.146 59.623 1.00 0.00
C   H
ATOM 22012 CD2 HSE C 123   27.286 17.984 59.239 1.00 0.00
C   C
ATOM 22013 HD2 HSE C 123   28.068 18.482 58.683 1.00 0.00
C   H
ATOM 22014 C   HSE C 123   24.105 21.240 57.823 1.00 0.00
C   C
ATOM 22015 O   HSE C 123   24.509 22.205 58.480 1.00 0.00
C   O
ATOM 22016 N   PRO C 124   23.064 21.317 56.966 1.00 0.00
C   N
ATOM 22017 CD PRO C 124   22.443 20.183 56.263 1.00 0.00
C   C
ATOM 22018 HD1 PRO C 124   23.533 20.403 56.195 1.00 0.00
C   H
ATOM 22019 HD2 PRO C 124   22.265 19.083 56.231 1.00 0.00
C   H
ATOM 22020 CA PRO C 124   22.393 22.573 56.651 1.00 0.00
C   C
ATOM 22021 HA PRO C 124   23.088 23.237 56.152 1.00 0.00
C   H
ATOM 22022 CB PRO C 124   21.271 22.149 55.708 1.00 0.00
C   C
ATOM 22023 HB1 PRO C 124   21.669 22.095 54.671 1.00 0.00
C   H
ATOM 22024 HB2 PRO C 124   20.408 22.846 55.727 1.00 0.00
C   H
ATOM 22025 CG PRO C 124   21.714 20.851 55.144 1.00 0.00
C   C
ATOM 22026 HG1 PRO C 124   21.237 20.617 54.173 1.00 0.00
C   H
ATOM 22027 HG2 PRO C 124   22.303 20.327 54.530 1.00 0.00
C   H
ATOM 22028 C   PRO C 124   21.815 23.282 57.872 1.00 0.00
C   C
ATOM 22029 O   PRO C 124   22.088 24.466 58.066 1.00 0.00
C   O
ATOM 22030 N   GLU C 125   21.038 22.574 58.693 1.00 0.00
C   N
ATOM 22031 HN GLU C 125   20.828 21.605 58.591 1.00 0.00
C   H

ATOM 22032 CA GLU C 125   20.409 23.202 59.860 1.00 0.00
C   C
ATOM 22033 HA GLU C 125   19.869 24.075 59.514 1.00 0.00
C   H
ATOM 22034 CB GLU C 125   19.409 22.275 60.553 1.00 0.00
C   C
ATOM 22035 HB1 GLU C 125   19.924 21.313 60.776 1.00 0.00
C   H
ATOM 22036 HB2 GLU C 125   19.119 22.703 61.539 1.00 0.00
C   H
ATOM 22037 CG GLU C 125   18.158 21.974 59.751 1.00 0.00
C   C
ATOM 22038 HG1 GLU C 125   17.271 22.055 60.406 1.00 0.00
C   H
ATOM 22039 HG2 GLU C 125   18.050 22.678 58.902 1.00 0.00
C   H
ATOM 22040 CD GLU C 125   18.151 20.556 59.214 1.00 0.00
C   C
ATOM 22041 OE1 GLU C 125   17.135 19.866 59.441 1.00 0.00
C   O
ATOM 22042 OE2 GLU C 125   19.155 20.128 58.591 1.00 0.00
C   O
ATOM 22043 C   GLU C 125   21.431 23.677 60.872 1.00 0.00
C   C
ATOM 22044 O   GLU C 125   21.302 24.773 61.420 1.00 0.00
C   O
ATOM 22045 N   LYS C 126   22.440 22.847 61.122 1.00 0.00
C   N
ATOM 22046 HN LYS C 126   22.513 21.941 60.708 1.00 0.00
C   H
ATOM 22047 CA LYS C 126   23.523 23.202 62.026 1.00 0.00
C   C
ATOM 22048 HA LYS C 126   23.092 23.378 63.004 1.00 0.00
C   H
ATOM 22049 CB LYS C 126   24.571 22.090 62.088 1.00 0.00
C   C
ATOM 22050 HB1 LYS C 126   24.020 21.127 62.191 1.00 0.00
C   H
ATOM 22051 HB2 LYS C 126   25.105 22.042 61.113 1.00 0.00
C   H
ATOM 22052 CG LYS C 126   25.557 22.228 63.242 1.00 0.00
C   C
ATOM 22053 HG1 LYS C 126   25.662 23.305 63.505 1.00 0.00
C   H
ATOM 22054 HG2 LYS C 126   25.145 21.706 64.136 1.00 0.00
C   H
ATOM 22055 CD LYS C 126   26.922 21.665 62.871 1.00 0.00
C   C
ATOM 22056 HD1 LYS C 126   26.772 20.686 62.361 1.00 0.00
C   H
ATOM 22057 HD2 LYS C 126   27.394 22.356 62.136 1.00 0.00
C   H
ATOM 22058 CE LYS C 126   27.814 21.502 64.090 1.00 0.00
C   C

ATOM 22059 HE1 LYS C 126 28.876 21.374 63.781 1.00 0.00
C H
ATOM 22060 HE2 LYS C 126 27.745 22.396 64.747 1.00 0.00
C H
ATOM 22061 NZ LYS C 126 27.423 20.309 64.894 1.00 0.00
C N
ATOM 22062 HZ1 LYS C 126 28.047 20.217 65.721 1.00 0.00
C H
ATOM 22063 HZ2 LYS C 126 26.439 20.414 65.214 1.00 0.00
C H
ATOM 22064 HZ3 LYS C 126 27.502 19.455 64.306 1.00 0.00
C H
ATOM 22065 C LYS C 126 24.169 24.501 61.576 1.00 0.00
C C
ATOM 22066 O LYS C 126 24.283 25.439 62.361 1.00 0.00
C O
ATOM 22067 N ASP C 127 24.560 24.556 60.300 1.00 0.00
C N
ATOM 22068 HN ASP C 127 24.452 23.817 59.641 1.00 0.00
C H
ATOM 22069 CA ASP C 127 25.203 25.739 59.730 1.00 0.00
C C
ATOM 22070 HA ASP C 127 26.042 25.970 60.377 1.00 0.00
C H
ATOM 22071 CB ASP C 127 25.711 25.453 58.319 1.00 0.00
C C
ATOM 22072 HB1 ASP C 127 24.894 25.002 57.721 1.00 0.00
C H
ATOM 22073 HB2 ASP C 127 26.031 26.390 57.821 1.00 0.00
C H
ATOM 22074 CG ASP C 127 26.883 24.473 58.297 1.00 0.00
C C
ATOM 22075 OD1 ASP C 127 27.438 24.143 59.372 1.00 0.00
C O
ATOM 22076 OD2 ASP C 127 27.254 24.036 57.185 1.00 0.00
C O
ATOM 22077 C ASP C 127 24.294 26.969 59.731 1.00 0.00
C C
ATOM 22078 O ASP C 127 24.749 28.078 60.029 1.00 0.00
C O
ATOM 22079 N MET C 128 23.016 26.785 59.410 1.00 0.00
C N
ATOM 22080 HN MET C 128 22.618 25.917 59.124 1.00 0.00
C H
ATOM 22081 CA MET C 128 22.069 27.898 59.457 1.00 0.00
C C
ATOM 22082 HA MET C 128 22.518 28.709 58.897 1.00 0.00
C H
ATOM 22083 CB MET C 128 20.751 27.547 58.779 1.00 0.00
C C
ATOM 22084 HB1 MET C 128 20.524 26.487 59.039 1.00 0.00
C H
ATOM 22085 HB2 MET C 128 19.933 28.158 59.222 1.00 0.00
C H

```
ATOM  22086  CG  MET C 128      20.796  27.697  57.271  1.00  0.00
C    C
ATOM  22087  HG1 MET C 128      21.459  26.925  56.827  1.00  0.00
C    H
ATOM  22088  HG2 MET C 128      19.777  27.549  56.850  1.00  0.00
C    H
ATOM  22089  SD  MET C 128      21.401  29.297  56.690  1.00  0.00
C    S
ATOM  22090  CE  MET C 128      20.080  30.380  57.249  1.00  0.00
C    C
ATOM  22091  HE1 MET C 128      20.283  31.434  56.962  1.00  0.00
C    H
ATOM  22092  HE2 MET C 128      19.106  30.085  56.801  1.00  0.00
C    H
ATOM  22093  HE3 MET C 128      19.973  30.342  58.354  1.00  0.00
C    H
ATOM  22094  C   MET C 128      21.827  28.398  60.876  1.00  0.00
C    C
ATOM  22095  O   MET C 128      21.549  29.582  61.075  1.00  0.00
C    O
ATOM  22096  N   GLY C 129      21.940  27.494  61.849  1.00  0.00
C    N
ATOM  22097  HN  GLY C 129      22.055  26.519  61.664  1.00  0.00
C    H
ATOM  22098  CA  GLY C 129      21.900  27.868  63.259  1.00  0.00
C    C
ATOM  22099  HA1 GLY C 129      22.142  26.984  63.833  1.00  0.00
C    H
ATOM  22100  HA2 GLY C 129      20.919  28.275  63.462  1.00  0.00
C    H
ATOM  22101  C   GLY C 129      22.927  28.937  63.587  1.00  0.00
C    C
ATOM  22102  O   GLY C 129      22.584  29.985  64.134  1.00  0.00
C    O
ATOM  22103  N   VAL C 130      24.185  28.681  63.229  1.00  0.00
C    N
ATOM  22104  HN  VAL C 130      24.474  27.839  62.781  1.00  0.00
C    H
ATOM  22105  CA  VAL C 130      25.264  29.637  63.474  1.00  0.00
C    C
ATOM  22106  HA  VAL C 130      25.335  29.785  64.545  1.00  0.00
C    H
ATOM  22107  CB  VAL C 130      26.664  29.122  63.024  1.00  0.00
C    C
ATOM  22108  HB  VAL C 130      27.409  29.920  63.261  1.00  0.00
C    H
ATOM  22109  CG1 VAL C 130      27.038  27.851  63.774  1.00  0.00
C    C
ATOM  22110 HG11 VAL C 130      28.019  27.468  63.419  1.00  0.00
C    H
ATOM  22111 HG12 VAL C 130      27.128  28.031  64.866  1.00  0.00
C    H
ATOM  22112 HG13 VAL C 130      26.279  27.057  63.605  1.00  0.00
C    H
```

ATOM 22113 CG2 VAL C 130   26.741 28.888 61.519 1.00 0.00
C   C
ATOM 22114 HG21 VAL C 130   27.736 28.484 61.235 1.00 0.00
C   H
ATOM 22115 HG22 VAL C 130   25.964 28.162 61.196 1.00 0.00
C   H
ATOM 22116 HG23 VAL C 130   26.590 29.833 60.958 1.00 0.00
C   H
ATOM 22117 C   VAL C 130   24.937 30.989 62.845 1.00 0.00
C   C
ATOM 22118 O   VAL C 130   25.025 32.016 63.517 1.00 0.00
C   O
ATOM 22119 N   LYS C 131   24.515 30.969 61.579 1.00 0.00
C   N
ATOM 22120 HN LYS C 131   24.376 30.118 61.075 1.00 0.00
C   H
ATOM 22121 CA LYS C 131   24.223 32.189 60.828 1.00 0.00
C   C
ATOM 22122 HA LYS C 131   25.116 32.801 60.869 1.00 0.00
C   H
ATOM 22123 CB LYS C 131   23.910 31.879 59.360 1.00 0.00
C   C
ATOM 22124 HB1 LYS C 131   23.090 31.125 59.352 1.00 0.00
C   H
ATOM 22125 HB2 LYS C 131   23.498 32.793 58.877 1.00 0.00
C   H
ATOM 22126 CG LYS C 131   25.096 31.340 58.582 1.00 0.00
C   C
ATOM 22127 HG1 LYS C 131   26.008 31.400 59.219 1.00 0.00
C   H
ATOM 22128 HG2 LYS C 131   24.920 30.263 58.354 1.00 0.00
C   H
ATOM 22129 CD LYS C 131   25.316 32.134 57.306 1.00 0.00
C   C
ATOM 22130 HD1 LYS C 131   24.693 31.684 56.500 1.00 0.00
C   H
ATOM 22131 HD2 LYS C 131   24.944 33.171 57.475 1.00 0.00
C   H
ATOM 22132 CE LYS C 131   26.790 32.143 56.922 1.00 0.00
C   C
ATOM 22133 HE1 LYS C 131   27.427 31.984 57.820 1.00 0.00
C   H
ATOM 22134 HE2 LYS C 131   27.008 31.335 56.189 1.00 0.00
C   H
ATOM 22135 NZ LYS C 131   27.219 33.429 56.300 1.00 0.00
C   N
ATOM 22136 HZ1 LYS C 131   28.230 33.387 56.058 1.00 0.00
C   H
ATOM 22137 HZ2 LYS C 131   26.664 33.600 55.437 1.00 0.00
C   H
ATOM 22138 HZ3 LYS C 131   27.057 34.207 56.970 1.00 0.00
C   H
ATOM 22139 C   LYS C 131   23.092 32.994 61.454 1.00 0.00
C   C

ATOM 22140 O   LYS C 131   23.152 34.219 61.495 1.00 0.00
C   O
ATOM 22141 N   ILE C 132   22.071 32.302 61.942 1.00 0.00
C   N
ATOM 22142 HN ILE C 132   21.992 31.308 61.891 1.00 0.00
C   H
ATOM 22143 CA ILE C 132   20.948 32.958 62.609 1.00 0.00
C   C
ATOM 22144 HA ILE C 132   20.634 33.768 61.962 1.00 0.00
C   H
ATOM 22145 CB ILE C 132   19.759 31.988 62.812 1.00 0.00
C   C
ATOM 22146 HB ILE C 132   20.227 31.052 63.211 1.00 0.00
C   H
ATOM 22147 CG2 ILE C 132   18.708 32.561 63.745 1.00 0.00
C   C
ATOM 22148 HG21 ILE C 132   17.949 31.792 63.998 1.00 0.00
C   H
ATOM 22149 HG22 ILE C 132   19.167 32.905 64.695 1.00 0.00
C   H
ATOM 22150 HG23 ILE C 132   18.187 33.422 63.275 1.00 0.00
C   H
ATOM 22151 CG1 ILE C 132   19.120 31.622 61.465 1.00 0.00
C   C
ATOM 22152 HG11 ILE C 132   19.824 30.997 60.871 1.00 0.00
C   H
ATOM 22153 HG12 ILE C 132   18.210 31.007 61.653 1.00 0.00
C   H
ATOM 22154 CD ILE C 132   18.697 32.797 60.607 1.00 0.00
C   C
ATOM 22155 HD1 ILE C 132   18.195 32.439 59.683 1.00 0.00
C   H
ATOM 22156 HD2 ILE C 132   17.987 33.450 61.158 1.00 0.00
C   H
ATOM 22157 HD3 ILE C 132   19.575 33.406 60.306 1.00 0.00
C   H
ATOM 22158 C   ILE C 132   21.402 33.594 63.928 1.00 0.00
C   C
ATOM 22159 O   ILE C 132   21.052 34.735 64.223 1.00 0.00
C   O
ATOM 22160 N   ALA C 133   22.192 32.861 64.706 1.00 0.00
C   N
ATOM 22161 HN ALA C 133   22.469 31.930 64.471 1.00 0.00
C   H
ATOM 22162 CA ALA C 133   22.718 33.373 65.971 1.00 0.00
C   C
ATOM 22163 HA ALA C 133   21.903 33.671 66.622 1.00 0.00
C   H
ATOM 22164 CB ALA C 133   23.484 32.292 66.706 1.00 0.00
C   C
ATOM 22165 HB1 ALA C 133   22.819 31.420 66.885 1.00 0.00
C   H
ATOM 22166 HB2 ALA C 133   24.354 31.941 66.107 1.00 0.00
C   H

ATOM 22167 HB3 ALA C 133   23.854 32.650 67.691 1.00 0.00
C   H
ATOM 22168 C   ALA C 133   23.588 34.617 65.769 1.00 0.00
C   C
ATOM 22169 O   ALA C 133   23.543 35.552 66.568 1.00 0.00
C   O
ATOM 22170 N   GLN C 134   24.364 34.621 64.689 1.00 0.00
C   N
ATOM 22171 HN GLN C 134   24.477 33.828 64.094 1.00 0.00
C   H
ATOM 22172 CA GLN C 134   25.122 35.798 64.277 1.00 0.00
C   C
ATOM 22173 HA GLN C 134   25.766 36.065 65.106 1.00 0.00
C   H
ATOM 22174 CB GLN C 134   25.930 35.494 63.019 1.00 0.00
C   C
ATOM 22175 HB1 GLN C 134   25.195 35.257 62.215 1.00 0.00
C   H
ATOM 22176 HB2 GLN C 134   26.462 36.416 62.691 1.00 0.00
C   H
ATOM 22177 CG GLN C 134   26.924 34.342 63.107 1.00 0.00
C   C
ATOM 22178 HG1 GLN C 134   26.440 33.446 63.551 1.00 0.00
C   H
ATOM 22179 HG2 GLN C 134   27.294 34.064 62.098 1.00 0.00
C   H
ATOM 22180 CD GLN C 134   28.116 34.646 63.980 1.00 0.00
C   C
ATOM 22181 OE1 GLN C 134   28.486 35.808 64.181 1.00 0.00
C   O
ATOM 22182 NE2 GLN C 134   28.729 33.598 64.513 1.00 0.00
C   N
ATOM 22183 HE21 GLN C 134   29.515 33.785 65.095 1.00 0.00
C   H
ATOM 22184 HE22 GLN C 134   28.406 32.681 64.308 1.00 0.00
C   H
ATOM 22185 C   GLN C 134   24.196 36.994 64.008 1.00 0.00
C   C
ATOM 22186 O   GLN C 134   24.467 38.114 64.448 1.00 0.00
C   O
ATOM 22187 N   SER C 135   23.108 36.739 63.280 1.00 0.00
C   N
ATOM 22188 HN SER C 135   22.923 35.836 62.891 1.00 0.00
C   H
ATOM 22189 CA SER C 135   22.089 37.740 62.980 1.00 0.00
C   C
ATOM 22190 HA SER C 135   22.615 38.566 62.522 1.00 0.00
C   H
ATOM 22191 CB SER C 135   20.978 37.117 62.151 1.00 0.00
C   C
ATOM 22192 HB1 SER C 135   21.102 36.011 62.181 1.00 0.00
C   H
ATOM 22193 HB2 SER C 135   19.985 37.348 62.597 1.00 0.00
C   H

ATOM 22194 OG SER C 135   21.085 37.600 60.723 1.00 0.00
C   O
ATOM 22195 HG1 SER C 135   20.522 37.026 60.187 1.00 0.00
C   H
ATOM 22196 C   SER C 135   21.431 38.265 64.233 1.00 0.00
C   C
ATOM 22197 O   SER C 135   21.146 39.456 64.335 1.00 0.00
C   O
ATOM 22198 N   LEU C 136   21.148 37.355 65.161 1.00 0.00
C   N
ATOM 22199 HN LEU C 136   21.404 36.395 65.072 1.00 0.00
C   H
ATOM 22200 CA LEU C 136   20.442 37.671 66.390 1.00 0.00
C   C
ATOM 22201 HA LEU C 136   19.549 38.209 66.097 1.00 0.00
C   H
ATOM 22202 CB LEU C 136   20.158 36.395 67.164 1.00 0.00
C   C
ATOM 22203 HB1 LEU C 136   20.938 35.653 66.874 1.00 0.00
C   H
ATOM 22204 HB2 LEU C 136   20.308 36.588 68.249 1.00 0.00
C   H
ATOM 22205 CG LEU C 136   18.906 35.497 67.182 1.00 0.00
C   C
ATOM 22206 HG LEU C 136   18.932 35.342 68.288 1.00 0.00
C   H
ATOM 22207 CD1 LEU C 136   17.574 36.226 66.831 1.00 0.00
C   C
ATOM 22208 HD11 LEU C 136   16.728 35.505 66.818 1.00 0.00
C   H
ATOM 22209 HD12 LEU C 136   17.350 37.014 67.581 1.00 0.00
C   H
ATOM 22210 HD13 LEU C 136   17.640 36.702 65.829 1.00 0.00
C   H
ATOM 22211 CD2 LEU C 136   19.077 34.049 66.467 1.00 0.00
C   C
ATOM 22212 HD21 LEU C 136   18.133 33.466 66.545 1.00 0.00
C   H
ATOM 22213 HD22 LEU C 136   19.323 34.149 65.391 1.00 0.00
C   H
ATOM 22214 HD23 LEU C 136   19.886 33.465 66.958 1.00 0.00
C   H
ATOM 22215 C   LEU C 136   21.255 38.588 67.296 1.00 0.00
C   C
ATOM 22216 O   LEU C 136   20.695 39.432 68.000 1.00 0.00
C   O
ATOM 22217 N   SER C 137   22.571 38.394 67.282 1.00 0.00
C   N
ATOM 22218 HN SER C 137   23.002 37.744 66.655 1.00 0.00
C   H
ATOM 22219 CA SER C 137   23.517 39.095 68.165 1.00 0.00
C   C
ATOM 22220 HA SER C 137   23.085 39.033 69.156 1.00 0.00
C   H

ATOM 22221 CB SER C 137   24.918 38.601 67.897 1.00 0.00
C   C
ATOM 22222 HB1 SER C 137   24.928 38.116 66.894 1.00 0.00
C   H
ATOM 22223 HB2 SER C 137   25.629 39.457 67.853 1.00 0.00
C   H
ATOM 22224 OG SER C 137   25.364 37.628 68.868 1.00 0.00
C   O
ATOM 22225 HG1 SER C 137   26.154 37.207 68.506 1.00 0.00
C   H
ATOM 22226 C   SER C 137   23.651 40.546 67.830 1.00 0.00
C   C
ATOM 22227 O   SER C 137   23.793 41.397 68.706 1.00 0.00
C   O
ATOM 22228 N   ASP C 138   23.728 40.795 66.532 1.00 0.00
C   N
ATOM 22229 HN ASP C 138   23.721 40.098 65.820 1.00 0.00
C   H
ATOM 22230 CA ASP C 138   23.834 42.127 66.009 1.00 0.00
C   C
ATOM 22231 HA ASP C 138   23.444 42.826 66.740 1.00 0.00
C   H
ATOM 22232 CB ASP C 138   25.274 42.433 65.621 1.00 0.00
C   C
ATOM 22233 HB1 ASP C 138   25.959 41.869 66.287 1.00 0.00
C   H
ATOM 22234 HB2 ASP C 138   25.472 42.123 64.576 1.00 0.00
C   H
ATOM 22235 CG ASP C 138   25.613 43.899 65.761 1.00 0.00
C   C
ATOM 22236 OD1 ASP C 138   24.692 44.721 65.977 1.00 0.00
C   O
ATOM 22237 OD2 ASP C 138   26.809 44.232 65.648 1.00 0.00
C   O
ATOM 22238 C   ASP C 138   22.914 42.191 64.808 1.00 0.00
C   C
ATOM 22239 O   ASP C 138   23.223 41.667 63.732 1.00 0.00
C   O
ATOM 22240 N   GLU C 139   21.765 42.818 65.021 1.00 0.00
C   N
ATOM 22241 HN GLU C 139   21.563 43.309 65.864 1.00 0.00
C   H
ATOM 22242 CA GLU C 139   20.687 42.852 64.043 1.00 0.00
C   C
ATOM 22243 HA GLU C 139   20.599 41.870 63.595 1.00 0.00
C   H
ATOM 22244 CB GLU C 139   19.426 43.349 64.732 1.00 0.00
C   C
ATOM 22245 HB1 GLU C 139   19.477 43.053 65.805 1.00 0.00
C   H
ATOM 22246 HB2 GLU C 139   19.405 44.462 64.723 1.00 0.00
C   H
ATOM 22247 CG GLU C 139   18.143 42.808 64.137 1.00 0.00
C   C

ATOM 22248 HG1 GLU C 139   17.866 43.407 63.251 1.00 0.00
C   H
ATOM 22249 HG2 GLU C 139   18.260 41.748 63.838 1.00 0.00
C   H
ATOM 22250 CD GLU C 139   16.983 42.922 65.101 1.00 0.00
C   C
ATOM 22251 OE1 GLU C 139   16.651 44.053 65.518 1.00 0.00
C   O
ATOM 22252 OE2 GLU C 139   16.403 41.872 65.440 1.00 0.00
C   O
ATOM 22253 C   GLU C 139   21.010 43.820 62.936 1.00 0.00
C   C
ATOM 22254 O   GLU C 139   20.586 43.676 61.789 1.00 0.00
C   O
ATOM 22255 N   ASN C 140   21.625 44.890 63.436 1.00 0.00
C   N
ATOM 22256 HN ASN C 140   22.104 44.812 64.309 1.00 0.00
C   H
ATOM 22257 CA ASN C 140   21.682 46.202 62.839 1.00 0.00
C   C
ATOM 22258 HA ASN C 140   20.786 46.305 62.237 1.00 0.00
C   H
ATOM 22259 CB ASN C 140   22.010 47.259 63.894 1.00 0.00
C   C
ATOM 22260 HB1 ASN C 140   21.329 47.099 64.759 1.00 0.00
C   H
ATOM 22261 HB2 ASN C 140   23.049 47.129 64.260 1.00 0.00
C   H
ATOM 22262 CG ASN C 140   21.804 48.674 63.388 1.00 0.00
C   C
ATOM 22263 OD1 ASN C 140   20.951 48.921 62.536 1.00 0.00
C   O
ATOM 22264 ND2 ASN C 140   22.586 49.610 63.912 1.00 0.00
C   N
ATOM 22265 HD21 ASN C 140   22.453 50.546 63.597 1.00 0.00
C   H
ATOM 22266 HD22 ASN C 140   23.250 49.370 64.613 1.00 0.00
C   H
ATOM 22267 C   ASN C 140   22.853 46.292 61.925 1.00 0.00
C   C
ATOM 22268 O   ASN C 140   22.947 47.225 61.125 1.00 0.00
C   O
ATOM 22269 N   VAL C 141   23.703 45.273 61.934 1.00 0.00
C   N
ATOM 22270 HN VAL C 141   23.715 44.547 62.616 1.00 0.00
C   H
ATOM 22271 CA VAL C 141   24.722 45.143 60.897 1.00 0.00
C   C
ATOM 22272 HA VAL C 141   25.000 46.114 60.506 1.00 0.00
C   H
ATOM 22273 CB VAL C 141   25.918 44.305 61.383 1.00 0.00
C   C
ATOM 22274 HB VAL C 141   26.817 44.632 60.804 1.00 0.00
C   H

ATOM 22275 CG1 VAL C 141   26.163 44.541 62.866 1.00 0.00
C   C
ATOM 22276 HG11 VAL C 141   27.001 43.906 63.225 1.00 0.00
C   H
ATOM 22277 HG12 VAL C 141   26.436 45.597 63.074 1.00 0.00
C   H
ATOM 22278 HG13 VAL C 141   25.259 44.283 63.460 1.00 0.00
C   H
ATOM 22279 CG2 VAL C 141   25.680 42.829 61.104 1.00 0.00
C   C
ATOM 22280 HG21 VAL C 141   26.523 42.216 61.489 1.00 0.00
C   H
ATOM 22281 HG22 VAL C 141   24.744 42.485 61.595 1.00 0.00
C   H
ATOM 22282 HG23 VAL C 141   25.594 42.635 60.015 1.00 0.00
C   H
ATOM 22283 C   VAL C 141   23.995 44.388 59.779 1.00 0.00
C   C
ATOM 22284 O   VAL C 141   23.742 43.188 59.890 1.00 0.00
C   O
ATOM 22285 N   LEU C 142   23.657 45.103 58.710 1.00 0.00
C   N
ATOM 22286 HN LEU C 142   23.922 46.058 58.597 1.00 0.00
C   H
ATOM 22287 CA LEU C 142   22.881 44.570 57.617 1.00 0.00
C   C
ATOM 22288 HA LEU C 142   21.912 44.271 57.997 1.00 0.00
C   H
ATOM 22289 CB LEU C 142   22.790 45.597 56.488 1.00 0.00
C   C
ATOM 22290 HB1 LEU C 142   23.828 45.785 56.129 1.00 0.00
C   H
ATOM 22291 HB2 LEU C 142   22.246 45.144 55.629 1.00 0.00
C   H
ATOM 22292 CG LEU C 142   22.168 46.961 56.798 1.00 0.00
C   C
ATOM 22293 HG LEU C 142   22.639 47.327 57.741 1.00 0.00
C   H
ATOM 22294 CD1 LEU C 142   22.467 47.931 55.671 1.00 0.00
C   C
ATOM 22295 HD11 LEU C 142   21.979 48.911 55.860 1.00 0.00
C   H
ATOM 22296 HD12 LEU C 142   23.561 48.097 55.578 1.00 0.00
C   H
ATOM 22297 HD13 LEU C 142   22.088 47.538 54.704 1.00 0.00
C   H
ATOM 22298 CD2 LEU C 142   20.667 46.851 57.033 1.00 0.00
C   C
ATOM 22299 HD21 LEU C 142   20.239 47.848 57.274 1.00 0.00
C   H
ATOM 22300 HD22 LEU C 142   20.139 46.467 56.137 1.00 0.00
C   H
ATOM 22301 HD23 LEU C 142   20.455 46.167 57.883 1.00 0.00
C   H

ATOM 22302 C   LEU C 142   23.589 43.318 57.130 1.00 0.00
C   C
ATOM 22303 O   LEU C 142   22.951 42.355 56.707 1.00 0.00
C   O
ATOM 22304 N   ASN C 143   24.918 43.353 57.208 1.00 0.00
C   N
ATOM 22305 HN ASN C 143   25.403 44.150 57.567 1.00 0.00
C   H
ATOM 22306 CA ASN C 143   25.782 42.259 56.789 1.00 0.00
C   C
ATOM 22307 HA ASN C 143   25.251 41.347 57.041 1.00 0.00
C   H
ATOM 22308 CB ASN C 143   26.080 42.349 55.292 1.00 0.00
C   C
ATOM 22309 HB1 ASN C 143   25.693 43.325 54.927 1.00 0.00
C   H
ATOM 22310 HB2 ASN C 143   27.175 42.339 55.116 1.00 0.00
C   H
ATOM 22311 CG ASN C 143   25.411 41.251 54.489 1.00 0.00
C   C
ATOM 22312 OD1 ASN C 143   25.001 40.221 55.032 1.00 0.00
C   O
ATOM 22313 ND2 ASN C 143   25.312 41.457 53.180 1.00 0.00
C   N
ATOM 22314 HD21 ASN C 143   24.865 40.751 52.639 1.00 0.00
C   H
ATOM 22315 HD22 ASN C 143   25.650 42.304 52.782 1.00 0.00
C   H
ATOM 22316 C   ASN C 143   27.085 42.262 57.570 1.00 0.00
C   C
ATOM 22317 O   ASN C 143   27.525 43.304 58.053 1.00 0.00
C   O
ATOM 22318 N   TYR C 144   27.702 41.092 57.684 1.00 0.00
C   N
ATOM 22319 HN TYR C 144   27.351 40.242 57.294 1.00 0.00
C   H
ATOM 22320 CA TYR C 144   28.963 40.953 58.403 1.00 0.00
C   C
ATOM 22321 HA TYR C 144   29.521 41.866 58.231 1.00 0.00
C   H
ATOM 22322 CB TYR C 144   28.704 40.731 59.903 1.00 0.00
C   C
ATOM 22323 HB1 TYR C 144   29.667 40.812 60.454 1.00 0.00
C   H
ATOM 22324 HB2 TYR C 144   28.028 41.516 60.305 1.00 0.00
C   H
ATOM 22325 CG TYR C 144   28.134 39.365 60.194 1.00 0.00
C   C
ATOM 22326 CD1 TYR C 144   28.960 38.323 60.623 1.00 0.00
C   C
ATOM 22327 HD1 TYR C 144   30.020 38.496 60.754 1.00 0.00
C   H
ATOM 22328 CE1 TYR C 144   28.446 37.062 60.864 1.00 0.00
C   C

```
ATOM  22329  HE1 TYR C 144      29.088  36.261  61.198  1.00  0.00
C    H
ATOM  22330  CZ  TYR C 144      27.092  36.831  60.666  1.00  0.00
C    C
ATOM  22331  OH  TYR C 144      26.571  35.585  60.894  1.00  0.00
C    O
ATOM  22332  HH  TYR C 144      25.621  35.643  60.773  1.00  0.00
C    H
ATOM  22333  CD2 TYR C 144      26.776  39.098  60.001  1.00  0.00
C    C
ATOM  22334  HD2 TYR C 144      26.121  39.871  59.624  1.00  0.00
C    H
ATOM  22335  CE2 TYR C 144      26.254  37.840  60.239  1.00  0.00
C    C
ATOM  22336  HE2 TYR C 144      25.204  37.655  60.069  1.00  0.00
C    H
ATOM  22337  C   TYR C 144      29.793  39.801  57.829  1.00  0.00
C    C
ATOM  22338  O   TYR C 144      29.256  38.896  57.185  1.00  0.00
C    O
ATOM  22339  N   ILE C 145      31.104  39.857  58.053  1.00  0.00
C    N
ATOM  22340  HN  ILE C 145      31.556  40.667  58.421  1.00  0.00
C    H
ATOM  22341  CA  ILE C 145      32.006  38.737  57.782  1.00  0.00
C    C
ATOM  22342  HA  ILE C 145      31.401  37.845  57.691  1.00  0.00
C    H
ATOM  22343  CB  ILE C 145      32.877  38.960  56.519  1.00  0.00
C    C
ATOM  22344  HB  ILE C 145      33.218  40.023  56.598  1.00  0.00
C    H
ATOM  22345  CG2 ILE C 145      33.990  37.921  56.437  1.00  0.00
C    C
ATOM  22346 HG21 ILE C 145      34.701  38.174  55.623  1.00  0.00
C    H
ATOM  22347 HG22 ILE C 145      34.564  37.881  57.386  1.00  0.00
C    H
ATOM  22348 HG23 ILE C 145      33.576  36.910  56.233  1.00  0.00
C    H
ATOM  22349  CG1 ILE C 145      32.041  38.867  55.244  1.00  0.00
C    C
ATOM  22350 HG11 ILE C 145      31.667  37.827  55.114  1.00  0.00
C    H
ATOM  22351 HG12 ILE C 145      31.149  39.526  55.349  1.00  0.00
C    H
ATOM  22352  CD  ILE C 145      32.786  39.283  53.987  1.00  0.00
C    C
ATOM  22353  HD1 ILE C 145      32.107  39.248  53.108  1.00  0.00
C    H
ATOM  22354  HD2 ILE C 145      33.174  40.319  54.086  1.00  0.00
C    H
ATOM  22355  HD3 ILE C 145      33.641  38.603  53.788  1.00  0.00
C    H
```

```
ATOM  22356  C   ILE C 145      32.905  38.538  59.001  1.00  0.00
C    C
ATOM  22357  O   ILE C 145      33.602  39.468  59.426  1.00  0.00
C    O
ATOM  22358  N   ASP C 146      32.876  37.334  59.565  1.00  0.00
C    N
ATOM  22359  HN  ASP C 146      32.286  36.584  59.278  1.00  0.00
C    H
ATOM  22360  CA  ASP C 146      33.734  36.983  60.695  1.00  0.00
C    C
ATOM  22361  HA  ASP C 146      33.833  37.879  61.298  1.00  0.00
C    H
ATOM  22362  CB  ASP C 146      33.108  35.858  61.528  1.00  0.00
C    C
ATOM  22363  HB1 ASP C 146      32.409  35.279  60.893  1.00  0.00
C    H
ATOM  22364  HB2 ASP C 146      33.889  35.169  61.905  1.00  0.00
C    H
ATOM  22365  CG  ASP C 146      32.314  36.377  62.716  1.00  0.00
C    C
ATOM  22366  OD1 ASP C 146      32.631  37.468  63.235  1.00  0.00
C    O
ATOM  22367  OD2 ASP C 146      31.378  35.682  63.148  1.00  0.00
C    O
ATOM  22368  C   ASP C 146      35.143  36.593  60.250  1.00  0.00
C    C
ATOM  22369  O   ASP C 146      35.332  35.590  59.560  1.00  0.00
C    O
ATOM  22370  N   LEU C 147      36.129  37.390  60.647  1.00  0.00
C    N
ATOM  22371  HN  LEU C 147      35.999  38.211  61.200  1.00  0.00
C    H
ATOM  22372  CA  LEU C 147      37.520  37.117  60.292  1.00  0.00
C    C
ATOM  22373  HA  LEU C 147      37.511  36.593  59.345  1.00  0.00
C    H
ATOM  22374  CB  LEU C 147      38.336  38.410  60.165  1.00  0.00
C    C
ATOM  22375  HB1 LEU C 147      38.497  38.800  61.196  1.00  0.00
C    H
ATOM  22376  HB2 LEU C 147      39.345  38.163  59.767  1.00  0.00
C    H
ATOM  22377  CG  LEU C 147      37.810  39.599  59.352  1.00  0.00
C    C
ATOM  22378  HG  LEU C 147      37.079  39.990  60.100  1.00  0.00
C    H
ATOM  22379  CD1 LEU C 147      38.881  40.647  59.113  1.00  0.00
C    C
ATOM  22380 HD11 LEU C 147      38.485  41.472  58.482  1.00  0.00
C    H
ATOM  22381 HD12 LEU C 147      39.226  41.079  60.077  1.00  0.00
C    H
ATOM  22382 HD13 LEU C 147      39.756  40.202  58.593  1.00  0.00
C    H
```

ATOM 22383 CD2 LEU C 147   37.045 39.265 58.077 1.00 0.00
C   C
ATOM 22384 HD21 LEU C 147   36.697 40.196 57.581 1.00 0.00
C   H
ATOM 22385 HD22 LEU C 147   37.678 38.713 57.353 1.00 0.00
C   H
ATOM 22386 HD23 LEU C 147   36.156 38.641 58.310 1.00 0.00
C   H
ATOM 22387 C   LEU C 147   38.154 36.194 61.322 1.00 0.00
C   C
ATOM 22388 O   LEU C 147   38.862 35.245 60.975 1.00 0.00
C   O
ATOM 22389 N   SER C 148   37.889 36.491 62.591 1.00 0.00
C   N
ATOM 22390 HN SER C 148   37.363 37.295 62.866 1.00 0.00
C   H
ATOM 22391 CA SER C 148   38.342 35.672 63.698 1.00 0.00
C   C
ATOM 22392 HA SER C 148   38.481 34.667 63.320 1.00 0.00
C   H
ATOM 22393 CB SER C 148   39.625 36.251 64.314 1.00 0.00
C   C
ATOM 22394 HB1 SER C 148   39.453 37.333 64.518 1.00 0.00
C   H
ATOM 22395 HB2 SER C 148   39.841 35.764 65.292 1.00 0.00
C   H
ATOM 22396 OG SER C 148   40.733 36.130 63.433 1.00 0.00
C   O
ATOM 22397 HG1 SER C 148   41.432 36.700 63.776 1.00 0.00
C   H
ATOM 22398 C   SER C 148   37.235 35.614 64.738 1.00 0.00
C   C
ATOM 22399 O   SER C 148   36.105 36.045 64.486 1.00 0.00
C   O
ATOM 22400 N   ASP C 149   37.562 35.067 65.902 1.00 0.00
C   N
ATOM 22401 HN ASP C 149   38.418 34.590 66.086 1.00 0.00
C   H
ATOM 22402 CA ASP C 149   36.680 35.113 67.053 1.00 0.00
C   C
ATOM 22403 HA ASP C 149   35.697 34.784 66.736 1.00 0.00
C   H
ATOM 22404 CB ASP C 149   37.253 34.255 68.185 1.00 0.00
C   C
ATOM 22405 HB1 ASP C 149   38.099 34.793 68.659 1.00 0.00
C   H
ATOM 22406 HB2 ASP C 149   36.484 34.065 68.960 1.00 0.00
C   H
ATOM 22407 CG ASP C 149   37.787 32.911 67.698 1.00 0.00
C   C
ATOM 22408 OD1 ASP C 149   36.970 32.044 67.315 1.00 0.00
C   O
ATOM 22409 OD2 ASP C 149   39.025 32.725 67.709 1.00 0.00
C   O

ATOM 22410 C ASP C 149 36.535 36.570 67.502 1.00 0.00
C C
ATOM 22411 O ASP C 149 35.456 36.998 67.919 1.00 0.00
C O
ATOM 22412 N GLU C 150 37.628 37.323 67.379 1.00 0.00
C N
ATOM 22413 HN GLU C 150 38.474 36.982 66.979 1.00 0.00
C H
ATOM 22414 CA GLU C 150 37.696 38.715 67.816 1.00 0.00
C C
ATOM 22415 HA GLU C 150 36.967 38.826 68.609 1.00 0.00
C H
ATOM 22416 CB GLU C 150 39.093 39.023 68.368 1.00 0.00
C C
ATOM 22417 HB1 GLU C 150 39.843 38.724 67.600 1.00 0.00
C H
ATOM 22418 HB2 GLU C 150 39.213 40.122 68.501 1.00 0.00
C H
ATOM 22419 CG GLU C 150 39.437 38.318 69.673 1.00 0.00
C C
ATOM 22420 HG1 GLU C 150 38.756 38.668 70.472 1.00 0.00
C H
ATOM 22421 HG2 GLU C 150 39.343 37.219 69.569 1.00 0.00
C H
ATOM 22422 CD GLU C 150 40.841 38.633 70.169 1.00 0.00
C C
ATOM 22423 OE1 GLU C 150 41.649 39.215 69.408 1.00 0.00
C O
ATOM 22424 OE2 GLU C 150 41.143 38.286 71.331 1.00 0.00
C O
ATOM 22425 C GLU C 150 37.346 39.743 66.730 1.00 0.00
C C
ATOM 22426 O GLU C 150 36.757 40.781 67.035 1.00 0.00
C O
ATOM 22427 N TYR C 151 37.712 39.460 65.479 1.00 0.00
C N
ATOM 22428 HN TYR C 151 38.061 38.570 65.191 1.00 0.00
C H
ATOM 22429 CA TYR C 151 37.629 40.463 64.405 1.00 0.00
C C
ATOM 22430 HA TYR C 151 37.422 41.399 64.911 1.00 0.00
C H
ATOM 22431 CB TYR C 151 38.976 40.597 63.682 1.00 0.00
C C
ATOM 22432 HB1 TYR C 151 39.311 39.591 63.347 1.00 0.00
C H
ATOM 22433 HB2 TYR C 151 38.872 41.229 62.773 1.00 0.00
C H
ATOM 22434 CG TYR C 151 40.074 41.155 64.560 1.00 0.00
C C
ATOM 22435 CD1 TYR C 151 40.307 42.530 64.627 1.00 0.00
C C
ATOM 22436 HD1 TYR C 151 39.711 43.206 64.030 1.00 0.00
C H

ATOM 22437 CE1 TYR C 151   41.307 43.050 65.434 1.00 0.00
C   C
ATOM 22438 HE1 TYR C 151   41.482 44.115 65.475 1.00 0.00
C   H
ATOM 22439 CZ TYR C 151   42.092 42.189 66.187 1.00 0.00
C   C
ATOM 22440 OH TYR C 151   43.084 42.704 66.988 1.00 0.00
C   O
ATOM 22441 HH TYR C 151   43.465 41.974 67.479 1.00 0.00
C   H
ATOM 22442 CD2 TYR C 151   40.878 40.312 65.331 1.00 0.00
C   C
ATOM 22443 HD2 TYR C 151   40.749 39.240 65.272 1.00 0.00
C   H
ATOM 22444 CE2 TYR C 151   41.880 40.821 66.141 1.00 0.00
C   C
ATOM 22445 HE2 TYR C 151   42.500 40.147 66.713 1.00 0.00
C   H
ATOM 22446 C   TYR C 151   36.498 40.231 63.404 1.00 0.00
C   C
ATOM 22447 O   TYR C 151   36.220 39.096 63.010 1.00 0.00
C   O
ATOM 22448 N   SER C 152   35.868 41.329 62.994 1.00 0.00
C   N
ATOM 22449 HN SER C 152   36.157 42.251 63.251 1.00 0.00
C   H
ATOM 22450 CA SER C 152   34.694 41.292 62.129 1.00 0.00
C   C
ATOM 22451 HA SER C 152   34.821 40.417 61.504 1.00 0.00
C   H
ATOM 22452 CB SER C 152   33.428 41.228 62.983 1.00 0.00
C   C
ATOM 22453 HB1 SER C 152   33.421 40.252 63.519 1.00 0.00
C   H
ATOM 22454 HB2 SER C 152   33.444 42.026 63.759 1.00 0.00
C   H
ATOM 22455 OG SER C 152   32.267 41.316 62.179 1.00 0.00
C   O
ATOM 22456 HG1 SER C 152   31.520 41.050 62.730 1.00 0.00
C   H
ATOM 22457 C   SER C 152   34.617 42.502 61.205 1.00 0.00
C   C
ATOM 22458 O   SER C 152   34.889 43.629 61.622 1.00 0.00
C   O
ATOM 22459 N   ILE C 153   34.255 42.257 59.949 1.00 0.00
C   N
ATOM 22460 HN ILE C 153   34.215 41.344 59.549 1.00 0.00
C   H
ATOM 22461 CA ILE C 153   33.878 43.329 59.032 1.00 0.00
C   C
ATOM 22462 HA ILE C 153   34.285 44.257 59.415 1.00 0.00
C   H
ATOM 22463 CB ILE C 153   34.327 43.060 57.578 1.00 0.00
C   C

ATOM 22464 HB ILE C 153   33.870 42.071 57.322 1.00 0.00
C   H
ATOM 22465 CG2 ILE C 153   33.878 44.194 56.660 1.00 0.00
C   C
ATOM 22466 HG21 ILE C 153   34.060 43.932 55.597 1.00 0.00
C   H
ATOM 22467 HG22 ILE C 153   32.792 44.391 56.776 1.00 0.00
C   H
ATOM 22468 HG23 ILE C 153   34.431 45.131 56.885 1.00 0.00
C   H
ATOM 22469 CG1 ILE C 153   35.847 42.876 57.509 1.00 0.00
C   C
ATOM 22470 HG11 ILE C 153   36.354 43.826 57.790 1.00 0.00
C   H
ATOM 22471 HG12 ILE C 153   36.152 42.107 58.255 1.00 0.00
C   H
ATOM 22472 CD ILE C 153   36.367 42.437 56.156 1.00 0.00
C   C
ATOM 22473 HD1 ILE C 153   37.465 42.269 56.200 1.00 0.00
C   H
ATOM 22474 HD2 ILE C 153   35.883 41.488 55.839 1.00 0.00
C   H
ATOM 22475 HD3 ILE C 153   36.168 43.209 55.383 1.00 0.00
C   H
ATOM 22476 C   ILE C 153   32.365 43.456 59.090 1.00 0.00
C   C
ATOM 22477 O   ILE C 153   31.650 42.464 58.963 1.00 0.00
C   O
ATOM 22478 N   VAL C 154   31.877 44.673 59.293 1.00 0.00
C   N
ATOM 22479 HN VAL C 154   32.427 45.504 59.314 1.00 0.00
C   H
ATOM 22480 CA VAL C 154   30.454 44.886 59.505 1.00 0.00
C   C
ATOM 22481 HA VAL C 154   29.955 43.956 59.267 1.00 0.00
C   H
ATOM 22482 CB VAL C 154   30.164 45.254 60.976 1.00 0.00
C   C
ATOM 22483 HB VAL C 154   30.690 46.216 61.194 1.00 0.00
C   H
ATOM 22484 CG1 VAL C 154   28.679 45.439 61.190 1.00 0.00
C   C
ATOM 22485 HG11 VAL C 154   28.468 45.666 62.257 1.00 0.00
C   H
ATOM 22486 HG12 VAL C 154   28.280 46.282 60.588 1.00 0.00
C   H
ATOM 22487 HG13 VAL C 154   28.125 44.514 60.918 1.00 0.00
C   H
ATOM 22488 CG2 VAL C 154   30.672 44.178 61.921 1.00 0.00
C   C
ATOM 22489 HG21 VAL C 154   30.425 44.431 62.974 1.00 0.00
C   H
ATOM 22490 HG22 VAL C 154   30.210 43.196 61.679 1.00 0.00
C   H

ATOM 22491 HG23 VAL C 154   31.775 44.071 61.853 1.00 0.00
C   H
ATOM 22492 C   VAL C 154   29.893 45.959 58.574 1.00 0.00
C   C
ATOM 22493 O   VAL C 154   30.419 47.071 58.507 1.00 0.00
C   O
ATOM 22494 N   GLU C 155   28.836 45.606 57.846 1.00 0.00
C   N
ATOM 22495 HN GLU C 155   28.507 44.669 57.766 1.00 0.00
C   H
ATOM 22496 CA GLU C 155   28.054 46.576 57.088 1.00 0.00
C   C
ATOM 22497 HA GLU C 155   28.684 47.414 56.815 1.00 0.00
C   H
ATOM 22498 CB GLU C 155   27.457 45.936 55.833 1.00 0.00
C   C
ATOM 22499 HB1 GLU C 155   28.292 45.512 55.229 1.00 0.00
C   H
ATOM 22500 HB2 GLU C 155   26.817 45.072 56.118 1.00 0.00
C   H
ATOM 22501 CG GLU C 155   26.659 46.894 54.957 1.00 0.00
C   C
ATOM 22502 HG1 GLU C 155   25.878 47.386 55.565 1.00 0.00
C   H
ATOM 22503 HG2 GLU C 155   27.317 47.666 54.512 1.00 0.00
C   H
ATOM 22504 CD GLU C 155   25.914 46.194 53.830 1.00 0.00
C   C
ATOM 22505 OE1 GLU C 155   24.947 46.780 53.302 1.00 0.00
C   O
ATOM 22506 OE2 GLU C 155   26.291 45.061 53.464 1.00 0.00
C   O
ATOM 22507 C   GLU C 155   26.950 47.116 57.996 1.00 0.00
C   C
ATOM 22508 O   GLU C 155   26.090 46.364 58.449 1.00 0.00
C   O
ATOM 22509 N   LEU C 156   26.994 48.418 58.268 1.00 0.00
C   N
ATOM 22510 HN LEU C 156   27.679 49.032 57.880 1.00 0.00
C   H
ATOM 22511 CA LEU C 156   26.039 49.072 59.164 1.00 0.00
C   C
ATOM 22512 HA LEU C 156   25.386 48.296 59.541 1.00 0.00
C   H
ATOM 22513 CB LEU C 156   26.759 49.816 60.298 1.00 0.00
C   C
ATOM 22514 HB1 LEU C 156   27.679 50.269 59.861 1.00 0.00
C   H
ATOM 22515 HB2 LEU C 156   26.125 50.668 60.631 1.00 0.00
C   H
ATOM 22516 CG LEU C 156   27.215 49.068 61.551 1.00 0.00
C   C
ATOM 22517 HG LEU C 156   26.334 48.537 61.987 1.00 0.00
C   H

ATOM 22518 CD1 LEU C 156   28.315 48.094 61.211 1.00 0.00
C   C
ATOM 22519 HD11 LEU C 156   28.681 47.584 62.127 1.00 0.00
C   H
ATOM 22520 HD12 LEU C 156   27.947 47.320 60.504 1.00 0.00
C   H
ATOM 22521 HD13 LEU C 156   29.173 48.620 60.740 1.00 0.00
C   H
ATOM 22522 CD2 LEU C 156   27.742 50.032 62.592 1.00 0.00
C   C
ATOM 22523 HD21 LEU C 156   28.053 49.480 63.506 1.00 0.00
C   H
ATOM 22524 HD22 LEU C 156   28.623 50.593 62.222 1.00 0.00
C   H
ATOM 22525 HD23 LEU C 156   26.956 50.764 62.879 1.00 0.00
C   H
ATOM 22526 C   LEU C 156   25.180 50.069 58.417 1.00 0.00
C   C
ATOM 22527 O   LEU C 156   25.657 50.798 57.549 1.00 0.00
C   O
ATOM 22528 N   LEU C 157   23.908 50.108 58.778 1.00 0.00
C   N
ATOM 22529 HN LEU C 157   23.465 49.466 59.401 1.00 0.00
C   H
ATOM 22530 CA LEU C 157   23.034 51.136 58.267 1.00 0.00
C   C
ATOM 22531 HA LEU C 157   23.450 51.483 57.330 1.00 0.00
C   H
ATOM 22532 CB LEU C 157   21.633 50.583 58.082 1.00 0.00
C   C
ATOM 22533 HB1 LEU C 157   21.724 49.657 57.468 1.00 0.00
C   H
ATOM 22534 HB2 LEU C 157   21.239 50.259 59.071 1.00 0.00
C   H
ATOM 22535 CG LEU C 157   20.635 51.504 57.404 1.00 0.00
C   C
ATOM 22536 HG LEU C 157   20.564 52.484 57.936 1.00 0.00
C   H
ATOM 22537 CD1 LEU C 157   20.930 51.721 55.932 1.00 0.00
C   C
ATOM 22538 HD11 LEU C 157   20.150 52.360 55.468 1.00 0.00
C   H
ATOM 22539 HD12 LEU C 157   21.914 52.219 55.800 1.00 0.00
C   H
ATOM 22540 HD13 LEU C 157   20.952 50.752 55.389 1.00 0.00
C   H
ATOM 22541 CD2 LEU C 157   19.314 50.805 57.540 1.00 0.00
C   C
ATOM 22542 HD21 LEU C 157   18.505 51.417 57.085 1.00 0.00
C   H
ATOM 22543 HD22 LEU C 157   19.318 49.821 57.031 1.00 0.00
C   H
ATOM 22544 HD23 LEU C 157   19.067 50.644 58.612 1.00 0.00
C   H

ATOM 22545 C LEU C 157 23.026 52.312 59.231 1.00 0.00
C C
ATOM 22546 O LEU C 157 22.853 52.136 60.435 1.00 0.00
C O
ATOM 22547 N LYS C 161 22.150 60.614 64.034 1.00 0.00
C N
ATOM 22548 HN LYS C 161 21.342 61.195 64.112 1.00 0.00
C H
ATOM 22549 CA LYS C 161 23.333 61.098 64.742 1.00 0.00
C C
ATOM 22550 HA LYS C 161 23.153 62.139 64.982 1.00 0.00
C H
ATOM 22551 CB LYS C 161 23.557 60.284 66.018 1.00 0.00
C C
ATOM 22552 HB1 LYS C 161 23.698 59.222 65.710 1.00 0.00
C H
ATOM 22553 HB2 LYS C 161 24.515 60.603 66.488 1.00 0.00
C H
ATOM 22554 CG LYS C 161 22.413 60.366 67.017 1.00 0.00
C C
ATOM 22555 HG1 LYS C 161 21.471 60.039 66.519 1.00 0.00
C H
ATOM 22556 HG2 LYS C 161 22.610 59.658 67.854 1.00 0.00
C H
ATOM 22557 CD LYS C 161 22.237 61.781 67.543 1.00 0.00
C C
ATOM 22558 HD1 LYS C 161 23.211 62.125 67.962 1.00 0.00
C H
ATOM 22559 HD2 LYS C 161 21.992 62.445 66.682 1.00 0.00
C H
ATOM 22560 CE LYS C 161 21.142 61.847 68.594 1.00 0.00
C C
ATOM 22561 HE1 LYS C 161 20.173 61.501 68.170 1.00 0.00
C H
ATOM 22562 HE2 LYS C 161 21.395 61.203 69.464 1.00 0.00
C H
ATOM 22563 NZ LYS C 161 20.944 63.233 69.100 1.00 0.00
C N
ATOM 22564 HZ1 LYS C 161 20.189 63.246 69.815 1.00 0.00
C H
ATOM 22565 HZ2 LYS C 161 21.829 63.577 69.524 1.00 0.00
C H
ATOM 22566 HZ3 LYS C 161 20.679 63.854 68.309 1.00 0.00
C H
ATOM 22567 C LYS C 161 24.578 61.042 63.864 1.00 0.00
C C
ATOM 22568 O LYS C 161 25.389 61.970 63.869 1.00 0.00
C O
ATOM 22569 N LEU C 162 24.724 59.944 63.124 1.00 0.00
C N
ATOM 22570 HN LEU C 162 24.092 59.173 63.165 1.00 0.00
C H
ATOM 22571 CA LEU C 162 25.826 59.767 62.183 1.00 0.00
C C

ATOM 22572 HA LEU C 162   26.727 60.077 62.695 1.00 0.00
C   H
ATOM 22573 CB LEU C 162   25.929 58.302 61.747 1.00 0.00
C   C
ATOM 22574 HB1 LEU C 162   24.890 57.906 61.677 1.00 0.00
C   H
ATOM 22575 HB2 LEU C 162   26.343 58.260 60.715 1.00 0.00
C   H
ATOM 22576 CG LEU C 162   26.730 57.377 62.664 1.00 0.00
C   C
ATOM 22577 HG LEU C 162   27.730 57.851 62.813 1.00 0.00
C   H
ATOM 22578 CD1 LEU C 162   26.035 57.222 64.008 1.00 0.00
C   C
ATOM 22579 HD11 LEU C 162   26.592 56.512 64.656 1.00 0.00
C   H
ATOM 22580 HD12 LEU C 162   25.974 58.200 64.533 1.00 0.00
C   H
ATOM 22581 HD13 LEU C 162   25.004 56.831 63.874 1.00 0.00
C   H
ATOM 22582 CD2 LEU C 162   26.944 56.022 62.008 1.00 0.00
C   C
ATOM 22583 HD21 LEU C 162   27.543 55.362 62.673 1.00 0.00
C   H
ATOM 22584 HD22 LEU C 162   25.983 55.509 61.803 1.00 0.00
C   H
ATOM 22585 HD23 LEU C 162   27.492 56.136 61.048 1.00 0.00
C   H
ATOM 22586 C   LEU C 162   25.656 60.670 60.959 1.00 0.00
C   C
ATOM 22587 O   LEU C 162   26.645 61.067 60.332 1.00 0.00
C   O
ATOM 22588 N   ASP C 163   24.397 60.993 60.647 1.00 0.00
C   N
ATOM 22589 HN ASP C 163   23.617 60.717 61.203 1.00 0.00
C   H
ATOM 22590 CA ASP C 163   24.009 61.782 59.467 1.00 0.00
C   C
ATOM 22591 HA ASP C 163   24.395 61.236 58.614 1.00 0.00
C   H
ATOM 22592 CB ASP C 163   22.484 61.897 59.408 1.00 0.00
C   C
ATOM 22593 HB1 ASP C 163   22.042 60.880 59.395 1.00 0.00
C   H
ATOM 22594 HB2 ASP C 163   22.099 62.434 60.296 1.00 0.00
C   H
ATOM 22595 CG ASP C 163   22.004 62.616 58.163 1.00 0.00
C   C
ATOM 22596 OD1 ASP C 163   22.852 63.009 57.336 1.00 0.00
C   O
ATOM 22597 OD2 ASP C 163   20.796 62.833 57.927 1.00 0.00
C   O
ATOM 22598 C   ASP C 163   24.624 63.187 59.409 1.00 0.00
C   C

ATOM 22599 O   ASP C 163   24.657 63.902 60.415 1.00 0.00
C   O
ATOM 22600 N   SER C 164   25.097 63.562 58.216 1.00 0.00
C   N
ATOM 22601 HN SER C 164   25.030 62.962 57.419 1.00 0.00
C   H
ATOM 22602 CA SER C 164   25.747 64.857 57.945 1.00 0.00
C   C
ATOM 22603 HA SER C 164   25.974 64.822 56.888 1.00 0.00
C   H
ATOM 22604 CB SER C 164   24.777 65.992 58.275 1.00 0.00
C   C
ATOM 22605 HB1 SER C 164   24.428 65.855 59.324 1.00 0.00
C   H
ATOM 22606 HB2 SER C 164   25.300 66.974 58.229 1.00 0.00
C   H
ATOM 22607 OG SER C 164   23.654 65.969 57.412 1.00 0.00
C   O
ATOM 22608 HG1 SER C 164   22.986 66.549 57.800 1.00 0.00
C   H
ATOM 22609 C   SER C 164   27.073 65.106 58.690 1.00 0.00
C   C
ATOM 22610 O   SER C 164   27.427 66.250 58.975 1.00 0.00
C   O
ATOM 22611 N   LYS C 165   27.801 64.033 58.993 1.00 0.00
C   N
ATOM 22612 HN LYS C 165   27.502 63.101 58.799 1.00 0.00
C   H
ATOM 22613 CA LYS C 165   29.110 64.144 59.641 1.00 0.00
C   C
ATOM 22614 HA LYS C 165   29.349 65.198 59.699 1.00 0.00
C   H
ATOM 22615 CB LYS C 165   29.067 63.529 61.040 1.00 0.00
C   C
ATOM 22616 HB1 LYS C 165   28.681 62.489 60.929 1.00 0.00
C   H
ATOM 22617 HB2 LYS C 165   30.107 63.436 61.426 1.00 0.00
C   H
ATOM 22618 CG LYS C 165   28.189 64.283 62.027 1.00 0.00
C   C
ATOM 22619 HG1 LYS C 165   28.575 65.322 62.138 1.00 0.00
C   H
ATOM 22620 HG2 LYS C 165   27.157 64.355 61.612 1.00 0.00
C   H
ATOM 22621 CD LYS C 165   28.174 63.604 63.385 1.00 0.00
C   C
ATOM 22622 HD1 LYS C 165   27.796 62.564 63.255 1.00 0.00
C   H
ATOM 22623 HD2 LYS C 165   29.225 63.534 63.749 1.00 0.00
C   H
ATOM 22624 CE LYS C 165   27.316 64.373 64.377 1.00 0.00
C   C
ATOM 22625 HE1 LYS C 165   27.718 65.399 64.528 1.00 0.00
C   H

ATOM 22626 HE2 LYS C 165 26.270 64.456 64.007 1.00 0.00
C H
ATOM 22627 NZ LYS C 165 27.272 63.705 65.707 1.00 0.00
C N
ATOM 22628 HZ1 LYS C 165 26.679 64.254 66.362 1.00 0.00
C H
ATOM 22629 HZ2 LYS C 165 26.875 62.748 65.602 1.00 0.00
C H
ATOM 22630 HZ3 LYS C 165 28.236 63.634 66.091 1.00 0.00
C H
ATOM 22631 C LYS C 165 30.198 63.475 58.807 1.00 0.00
C C
ATOM 22632 O LYS C 165 29.965 62.443 58.173 1.00 0.00
C O
ATOM 22633 N SER C 166 31.385 64.073 58.812 1.00 0.00
C N
ATOM 22634 HN SER C 166 31.586 64.895 59.347 1.00 0.00
C H
ATOM 22635 CA SER C 166 32.515 63.578 58.028 1.00 0.00
C C
ATOM 22636 HA SER C 166 32.078 63.090 57.167 1.00 0.00
C H
ATOM 22637 CB SER C 166 33.425 64.740 57.619 1.00 0.00
C C
ATOM 22638 HB1 SER C 166 33.714 65.295 58.540 1.00 0.00
C H
ATOM 22639 HB2 SER C 166 34.364 64.354 57.161 1.00 0.00
C H
ATOM 22640 OG SER C 166 32.754 65.627 56.741 1.00 0.00
C O
ATOM 22641 HG1 SER C 166 33.281 66.435 56.698 1.00 0.00
C H
ATOM 22642 C SER C 166 33.334 62.524 58.783 1.00 0.00
C C
ATOM 22643 O SER C 166 33.040 62.216 59.941 1.00 0.00
C O
ATOM 22644 N ILE C 167 34.351 61.972 58.117 1.00 0.00
C N
ATOM 22645 HN ILE C 167 34.551 62.173 57.160 1.00 0.00
C H
ATOM 22646 CA ILE C 167 35.274 61.015 58.735 1.00 0.00
C C
ATOM 22647 HA ILE C 167 34.660 60.333 59.309 1.00 0.00
C H
ATOM 22648 CB ILE C 167 36.090 60.281 57.653 1.00 0.00
C C
ATOM 22649 HB ILE C 167 36.605 61.098 57.087 1.00 0.00
C H
ATOM 22650 CG2 ILE C 167 37.046 59.283 58.291 1.00 0.00
C C
ATOM 22651 HG21 ILE C 167 37.740 58.867 57.532 1.00 0.00
C H
ATOM 22652 HG22 ILE C 167 37.659 59.769 59.078 1.00 0.00
C H

ATOM 22653 HG23 ILE C 167   36.490 58.439 58.751 1.00 0.00
C   H
ATOM 22654 CG1 ILE C 167   35.154 59.579 56.667 1.00 0.00
C   C
ATOM 22655 HG11 ILE C 167   34.537 60.334 56.132 1.00 0.00
C   H
ATOM 22656 HG12 ILE C 167   35.768 59.051 55.901 1.00 0.00
C   H
ATOM 22657 CD ILE C 167   34.237 58.565 57.312 1.00 0.00
C   C
ATOM 22658 HD1 ILE C 167   33.626 58.050 56.539 1.00 0.00
C   H
ATOM 22659 HD2 ILE C 167   34.824 57.797 57.859 1.00 0.00
C   H
ATOM 22660 HD3 ILE C 167   33.543 59.053 58.028 1.00 0.00
C   H
ATOM 22661 C   ILE C 167   36.222 61.699 59.727 1.00 0.00
C   C
ATOM 22662 O   ILE C 167   36.664 61.082 60.699 1.00 0.00
C   O
ATOM 22663 N   ILE C 168   36.532 62.971 59.469 1.00 0.00
C   N
ATOM 22664 HN ILE C 168   36.223 63.452 58.651 1.00 0.00
C   H
ATOM 22665 CA ILE C 168   37.358 63.783 60.367 1.00 0.00
C   C
ATOM 22666 HA ILE C 168   38.128 63.126 60.750 1.00 0.00
C   H
ATOM 22667 CB ILE C 168   37.958 64.985 59.611 1.00 0.00
C   C
ATOM 22668 HB ILE C 168   38.562 64.525 58.789 1.00 0.00
C   H
ATOM 22669 CG2 ILE C 168   36.854 65.908 59.118 1.00 0.00
C   C
ATOM 22670 HG21 ILE C 168   37.268 66.686 58.444 1.00 0.00
C   H
ATOM 22671 HG22 ILE C 168   36.089 65.341 58.547 1.00 0.00
C   H
ATOM 22672 HG23 ILE C 168   36.352 66.417 59.967 1.00 0.00
C   H
ATOM 22673 CG1 ILE C 168   38.937 65.744 60.509 1.00 0.00
C   C
ATOM 22674 HG11 ILE C 168   38.377 66.277 61.310 1.00 0.00
C   H
ATOM 22675 HG12 ILE C 168   39.611 65.011 61.009 1.00 0.00
C   H
ATOM 22676 CD ILE C 168   39.797 66.740 59.765 1.00 0.00
C   C
ATOM 22677 HD1 ILE C 168   40.524 67.216 60.459 1.00 0.00
C   H
ATOM 22678 HD2 ILE C 168   40.367 66.237 58.954 1.00 0.00
C   H
ATOM 22679 HD3 ILE C 168   39.177 67.543 59.314 1.00 0.00
C   H

```
ATOM  22680  C   ILE C 168      36.553  64.268  61.568  1.00  0.00
C    C
ATOM  22681  O   ILE C 168      37.073  64.315  62.687  1.00  0.00
C    O
ATOM  22682  N   ASP C 169      35.294  64.635  61.325  1.00  0.00
C    N
ATOM  22683  HN  ASP C 169      34.877  64.640  60.419  1.00  0.00
C    H
ATOM  22684  CA  ASP C 169      34.375  65.076  62.381  1.00  0.00
C    C
ATOM  22685  HA  ASP C 169      34.917  65.793  62.986  1.00  0.00
C    H
ATOM  22686  CB  ASP C 169      33.123  65.706  61.765  1.00  0.00
C    C
ATOM  22687  HB1 ASP C 169      32.728  65.037  60.975  1.00  0.00
C    H
ATOM  22688  HB2 ASP C 169      32.336  65.842  62.533  1.00  0.00
C    H
ATOM  22689  CG  ASP C 169      33.403  67.050  61.122  1.00  0.00
C    C
ATOM  22690  OD1 ASP C 169      34.477  67.627  61.390  1.00  0.00
C    O
ATOM  22691  OD2 ASP C 169      32.607  67.607  60.335  1.00  0.00
C    O
ATOM  22692  C   ASP C 169      33.977  63.924  63.311  1.00  0.00
C    C
ATOM  22693  O   ASP C 169      33.733  64.138  64.501  1.00  0.00
C    O
ATOM  22694  N   LEU C 170      33.908  62.715  62.751  1.00  0.00
C    N
ATOM  22695  HN  LEU C 170      33.985  62.569  61.767  1.00  0.00
C    H
ATOM  22696  CA  LEU C 170      33.713  61.492  63.529  1.00  0.00
C    C
ATOM  22697  HA  LEU C 170      33.745  61.767  64.575  1.00  0.00
C    H
ATOM  22698  CB  LEU C 170      32.373  60.835  63.181  1.00  0.00
C    C
ATOM  22699  HB1 LEU C 170      31.571  61.558  63.457  1.00  0.00
C    H
ATOM  22700  HB2 LEU C 170      32.308  60.710  62.077  1.00  0.00
C    H
ATOM  22701  CG  LEU C 170      32.074  59.503  63.873  1.00  0.00
C    C
ATOM  22702  HG  LEU C 170      32.919  58.814  63.633  1.00  0.00
C    H
ATOM  22703  CD1 LEU C 170      31.983  59.690  65.380  1.00  0.00
C    C
ATOM  22704 HD11 LEU C 170      31.719  58.732  65.877  1.00  0.00
C    H
ATOM  22705 HD12 LEU C 170      32.956  60.039  65.790  1.00  0.00
C    H
ATOM  22706 HD13 LEU C 170      31.205  60.440  65.636  1.00  0.00
C    H
```

ATOM 22707 CD2 LEU C 170   30.795 58.888 63.326 1.00 0.00
C   C
ATOM 22708 HD21 LEU C 170   30.594 57.913 63.820 1.00 0.00
C   H
ATOM 22709 HD22 LEU C 170   29.917 59.542 63.503 1.00 0.00
C   H
ATOM 22710 HD23 LEU C 170   30.884 58.712 62.233 1.00 0.00
C   H
ATOM 22711 C   LEU C 170   34.872 60.517 63.270 1.00 0.00
C   C
ATOM 22712 O   LEU C 170   34.805 59.679 62.364 1.00 0.00
C   O
ATOM 22713 N   ASN C 171   35.936 60.650 64.065 1.00 0.00
C   N
ATOM 22714 HN ASN C 171   35.980 61.316 64.809 1.00 0.00
C   H
ATOM 22715 CA ASN C 171   37.138 59.827 63.913 1.00 0.00
C   C
ATOM 22716 HA ASN C 171   37.225 59.615 62.853 1.00 0.00
C   H
ATOM 22717 CB ASN C 171   38.373 60.570 64.421 1.00 0.00
C   C
ATOM 22718 HB1 ASN C 171   39.259 59.926 64.230 1.00 0.00
C   H
ATOM 22719 HB2 ASN C 171   38.517 61.512 63.853 1.00 0.00
C   H
ATOM 22720 CG ASN C 171   38.313 60.859 65.908 1.00 0.00
C   C
ATOM 22721 OD1 ASN C 171   37.770 60.070 66.683 1.00 0.00
C   O
ATOM 22722 ND2 ASN C 171   38.870 61.992 66.316 1.00 0.00
C   N
ATOM 22723 HD21 ASN C 171   38.847 62.188 67.292 1.00 0.00
C   H
ATOM 22724 HD22 ASN C 171   39.314 62.593 65.659 1.00 0.00
C   H
ATOM 22725 C   ASN C 171   36.977 58.497 64.639 1.00 0.00
C   C
ATOM 22726 O   ASN C 171   36.670 58.466 65.832 1.00 0.00
C   O
ATOM 22727 N   VAL C 172   37.190 57.406 63.911 1.00 0.00
C   N
ATOM 22728 HN VAL C 172   37.505 57.428 62.966 1.00 0.00
C   H
ATOM 22729 CA VAL C 172   36.975 56.065 64.443 1.00 0.00
C   C
ATOM 22730 HA VAL C 172   36.512 56.193 65.413 1.00 0.00
C   H
ATOM 22731 CB VAL C 172   36.051 55.249 63.519 1.00 0.00
C   C
ATOM 22732 HB VAL C 172   36.520 55.231 62.504 1.00 0.00
C   H
ATOM 22733 CG1 VAL C 172   35.908 53.824 64.030 1.00 0.00
C   C

ATOM 22734 HG11 VAL C 172   35.210 53.250 63.382 1.00 0.00
C   H
ATOM 22735 HG12 VAL C 172   36.880 53.288 64.024 1.00 0.00
C   H
ATOM 22736 HG13 VAL C 172   35.503 53.816 65.064 1.00 0.00
C   H
ATOM 22737 CG2 VAL C 172   34.689 55.918 63.401 1.00 0.00
C   C
ATOM 22738 HG21 VAL C 172   34.007 55.312 62.767 1.00 0.00
C   H
ATOM 22739 HG22 VAL C 172   34.224 56.035 64.404 1.00 0.00
C   H
ATOM 22740 HG23 VAL C 172   34.774 56.922 62.937 1.00 0.00
C   H
ATOM 22741 C   VAL C 172   38.269 55.275 64.652 1.00 0.00
C   C
ATOM 22742 O   VAL C 172   38.276 54.296 65.398 1.00 0.00
C   O
ATOM 22743 N   ARG C 173   39.351 55.698 63.996 1.00 0.00
C   N
ATOM 22744 HN ARG C 173   39.304 56.464 63.360 1.00 0.00
C   H
ATOM 22745 CA ARG C 173   40.662 55.069 64.172 1.00 0.00
C   C
ATOM 22746 HA ARG C 173   40.539 53.993 64.119 1.00 0.00
C   H
ATOM 22747 CB ARG C 173   41.636 55.539 63.089 1.00 0.00
C   C
ATOM 22748 HB1 ARG C 173   41.617 56.655 63.111 1.00 0.00
C   H
ATOM 22749 HB2 ARG C 173   42.672 55.244 63.367 1.00 0.00
C   H
ATOM 22750 CG ARG C 173   41.278 55.069 61.687 1.00 0.00
C   C
ATOM 22751 HG1 ARG C 173   41.146 53.965 61.707 1.00 0.00
C   H
ATOM 22752 HG2 ARG C 173   40.283 55.484 61.409 1.00 0.00
C   H
ATOM 22753 CD ARG C 173   42.272 55.496 60.617 1.00 0.00
C   C
ATOM 22754 HD1 ARG C 173   42.377 56.605 60.631 1.00 0.00
C   H
ATOM 22755 HD2 ARG C 173   43.275 55.040 60.785 1.00 0.00
C   H
ATOM 22756 NE ARG C 173   41.851 55.080 59.283 1.00 0.00
C   N
ATOM 22757 HE ARG C 173   41.029 54.499 59.291 1.00 0.00
C   H
ATOM 22758 CZ ARG C 173   42.474 55.422 58.165 1.00 0.00
C   C
ATOM 22759 NH1 ARG C 173   43.556 56.189 58.214 1.00 0.00
C   N
ATOM 22760 HH11 ARG C 173   44.013 56.470 57.381 1.00 0.00
C   H

```
ATOM  22761 HH12 ARG C 173      43.791  56.568  59.111  1.00  0.00
C    H
ATOM  22762  NH2 ARG C 173      42.021  54.998  56.992  1.00  0.00
C    N
ATOM  22763 HH21 ARG C 173      42.536  55.182  56.168  1.00  0.00
C    H
ATOM  22764 HH22 ARG C 173      41.287  54.330  57.010  1.00  0.00
C    H
ATOM  22765  C   ARG C 173      41.225  55.375  65.557  1.00  0.00
C    C
ATOM  22766  O   ARG C 173      41.862  54.526  66.188  1.00  0.00
C    O
ATOM  22767  N   ALA C 174      40.974  56.598  66.021  1.00  0.00
C    N
ATOM  22768  HN  ALA C 174      40.484  57.280  65.479  1.00  0.00
C    H
ATOM  22769  CA  ALA C 174      41.389  57.034  67.346  1.00  0.00
C    C
ATOM  22770  HA  ALA C 174      42.342  56.591  67.609  1.00  0.00
C    H
ATOM  22771  CB  ALA C 174      41.559  58.545  67.374  1.00  0.00
C    C
ATOM  22772  HB1 ALA C 174      42.320  58.848  66.623  1.00  0.00
C    H
ATOM  22773  HB2 ALA C 174      40.606  59.058  67.122  1.00  0.00
C    H
ATOM  22774  HB3 ALA C 174      41.900  58.899  68.370  1.00  0.00
C    H
ATOM  22775  C   ALA C 174      40.393  56.589  68.422  1.00  0.00
C    C
ATOM  22776  O   ALA C 174      40.753  55.839  69.335  1.00  0.00
C    O
ATOM  22777  N   LYS C 175      39.140  57.035  68.290  1.00  0.00
C    N
ATOM  22778  HN  LYS C 175      38.826  57.467  67.446  1.00  0.00
C    H
ATOM  22779  CA  LYS C 175      38.129  56.934  69.353  1.00  0.00
C    C
ATOM  22780  HA  LYS C 175      38.612  57.332  70.237  1.00  0.00
C    H
ATOM  22781  CB  LYS C 175      36.915  57.784  68.975  1.00  0.00
C    C
ATOM  22782  HB1 LYS C 175      37.283  58.821  68.797  1.00  0.00
C    H
ATOM  22783  HB2 LYS C 175      36.510  57.425  68.003  1.00  0.00
C    H
ATOM  22784  CG  LYS C 175      35.825  57.819  70.036  1.00  0.00
C    C
ATOM  22785  HG1 LYS C 175      35.443  56.786  70.199  1.00  0.00
C    H
ATOM  22786  HG2 LYS C 175      36.267  58.166  70.998  1.00  0.00
C    H
ATOM  22787  CD  LYS C 175      34.676  58.719  69.616  1.00  0.00
C    C
```

ATOM 22788 HD1 LYS C 175   35.077 59.745 69.446 1.00 0.00
C   H
ATOM 22789 HD2 LYS C 175   34.283 58.348 68.641 1.00 0.00
C   H
ATOM 22790 CE LYS C 175   33.574 58.733 70.665 1.00 0.00
C   C
ATOM 22791 HE1 LYS C 175   33.168 57.708 70.817 1.00 0.00
C   H
ATOM 22792 HE2 LYS C 175   33.966 59.104 71.638 1.00 0.00
C   H
ATOM 22793 NZ LYS C 175   32.441 59.613 70.267 1.00 0.00
C   N
ATOM 22794 HZ1 LYS C 175   31.705 59.601 71.002 1.00 0.00
C   H
ATOM 22795 HZ2 LYS C 175   32.785 60.586 70.140 1.00 0.00
C   H
ATOM 22796 HZ3 LYS C 175   32.038 59.273 69.371 1.00 0.00
C   H
ATOM 22797 C   LYS C 175   37.651 55.514 69.692 1.00 0.00
C   C
ATOM 22798 O   LYS C 175   37.507 55.168 70.868 1.00 0.00
C   O
ATOM 22799 N   TYR C 176   37.394 54.712 68.662 1.00 0.00
C   N
ATOM 22800 HN TYR C 176   37.492 54.997 67.709 1.00 0.00
C   H
ATOM 22801 CA TYR C 176   36.941 53.334 68.839 1.00 0.00
C   C
ATOM 22802 HA TYR C 176   36.835 53.178 69.905 1.00 0.00
C   H
ATOM 22803 CB TYR C 176   35.616 53.105 68.111 1.00 0.00
C   C
ATOM 22804 HB1 TYR C 176   35.727 53.411 67.048 1.00 0.00
C   H
ATOM 22805 HB2 TYR C 176   35.345 52.027 68.117 1.00 0.00
C   H
ATOM 22806 CG TYR C 176   34.463 53.907 68.672 1.00 0.00
C   C
ATOM 22807 CD1 TYR C 176   33.701 53.419 69.726 1.00 0.00
C   C
ATOM 22808 HD1 TYR C 176   33.923 52.446 70.143 1.00 0.00
C   H
ATOM 22809 CE1 TYR C 176   32.646 54.147 70.242 1.00 0.00
C   C
ATOM 22810 HE1 TYR C 176   32.056 53.756 71.058 1.00 0.00
C   H
ATOM 22811 CZ TYR C 176   32.342 55.381 69.704 1.00 0.00
C   C
ATOM 22812 OH TYR C 176   31.291 56.108 70.215 1.00 0.00
C   O
ATOM 22813 HH TYR C 176   31.282 56.957 69.766 1.00 0.00
C   H
ATOM 22814 CD2 TYR C 176   34.138 55.151 68.148 1.00 0.00
C   C

ATOM 22815 HD2 TYR C 176   34.688 55.541 67.302 1.00 0.00
C   H
ATOM 22816 CE2 TYR C 176   33.084 55.886 68.658 1.00 0.00
C   C
ATOM 22817 HE2 TYR C 176   32.835 56.841 68.221 1.00 0.00
C   H
ATOM 22818 C   TYR C 176   37.992 52.341 68.341 1.00 0.00
C   C
ATOM 22819 O   TYR C 176   37.977 51.170 68.713 1.00 0.00
C   O
ATOM 22820 N   GLY C 177   38.908 52.829 67.507 1.00 0.00
C   N
ATOM 22821 HN GLY C 177   38.898 53.791 67.237 1.00 0.00
C   H
ATOM 22822 CA GLY C 177   39.980 52.022 66.930 1.00 0.00
C   C
ATOM 22823 HA1 GLY C 177   40.341 51.386 67.725 1.00 0.00
C   H
ATOM 22824 HA2 GLY C 177   40.715 52.717 66.547 1.00 0.00
C   H
ATOM 22825 C   GLY C 177   39.536 51.132 65.782 1.00 0.00
C   C
ATOM 22826 O   GLY C 177   40.086 50.046 65.595 1.00 0.00
C   O
ATOM 22827 N   CYS C 178   38.548 51.590 65.014 1.00 0.00
C   N
ATOM 22828 HN CYS C 178   38.076 52.453 65.184 1.00 0.00
C   H
ATOM 22829 CA CYS C 178   38.072 50.853 63.844 1.00 0.00
C   C
ATOM 22830 HA CYS C 178   38.681 49.959 63.780 1.00 0.00
C   H
ATOM 22831 CB CYS C 178   36.603 50.472 64.010 1.00 0.00
C   C
ATOM 22832 HB1 CYS C 178   36.036 51.415 64.184 1.00 0.00
C   H
ATOM 22833 HB2 CYS C 178   36.213 50.050 63.057 1.00 0.00
C   H
ATOM 22834 SG CYS C 178   36.321 49.357 65.398 1.00 0.00
C   S
ATOM 22835 HG1 CYS C 178   34.992 49.298 65.295 1.00 0.00
C   H
ATOM 22836 C   CYS C 178   38.274 51.644 62.556 1.00 0.00
C   C
ATOM 22837 O   CYS C 178   38.277 52.877 62.568 1.00 0.00
C   O
ATOM 22838 N   THR C 179   38.450 50.928 61.448 1.00 0.00
C   N
ATOM 22839 HN THR C 179   38.442 49.928 61.419 1.00 0.00
C   H
ATOM 22840 CA THR C 179   38.673 51.563 60.154 1.00 0.00
C   C
ATOM 22841 HA THR C 179   38.832 52.613 60.362 1.00 0.00
C   H

ATOM 22842 CB THR C 179   39.878 50.944 59.416 1.00 0.00
C   C
ATOM 22843 HB THR C 179   39.788 51.267 58.350 1.00 0.00
C   H
ATOM 22844 OG1 THR C 179   39.782 49.518 59.455 1.00 0.00
C   O
ATOM 22845 HG1 THR C 179   40.563 49.195 58.992 1.00 0.00
C   H
ATOM 22846 CG2 THR C 179   41.189 51.371 60.063 1.00 0.00
C   C
ATOM 22847 HG21 THR C 179   42.058 51.020 59.467 1.00 0.00
C   H
ATOM 22848 HG22 THR C 179   41.246 52.479 60.131 1.00 0.00
C   H
ATOM 22849 HG23 THR C 179   41.283 50.956 61.090 1.00 0.00
C   H
ATOM 22850 C   THR C 179   37.427 51.478 59.277 1.00 0.00
C   C
ATOM 22851 O   THR C 179   36.990 50.382 58.913 1.00 0.00
C   O
ATOM 22852 N   ILE C 180   36.850 52.637 58.954 1.00 0.00
C   N
ATOM 22853 HN ILE C 180   37.145 53.522 59.307 1.00 0.00
C   H
ATOM 22854 CA ILE C 180   35.713 52.706 58.033 1.00 0.00
C   C
ATOM 22855 HA ILE C 180   35.025 51.919 58.314 1.00 0.00
C   H
ATOM 22856 CB ILE C 180   34.995 54.076 58.073 1.00 0.00
C   C
ATOM 22857 HB ILE C 180   35.818 54.827 57.956 1.00 0.00
C   H
ATOM 22858 CG2 ILE C 180   33.931 54.162 56.981 1.00 0.00
C   C
ATOM 22859 HG21 ILE C 180   33.527 55.193 56.908 1.00 0.00
C   H
ATOM 22860 HG22 ILE C 180   34.358 53.898 55.991 1.00 0.00
C   H
ATOM 22861 HG23 ILE C 180   33.087 53.473 57.194 1.00 0.00
C   H
ATOM 22862 CG1 ILE C 180   34.370 54.312 59.454 1.00 0.00
C   C
ATOM 22863 HG11 ILE C 180   33.576 53.554 59.643 1.00 0.00
C   H
ATOM 22864 HG12 ILE C 180   35.153 54.170 60.233 1.00 0.00
C   H
ATOM 22865 CD ILE C 180   33.773 55.691 59.650 1.00 0.00
C   C
ATOM 22866 HD1 ILE C 180   33.393 55.803 60.689 1.00 0.00
C   H
ATOM 22867 HD2 ILE C 180   34.537 56.477 59.471 1.00 0.00
C   H
ATOM 22868 HD3 ILE C 180   32.924 55.859 58.954 1.00 0.00
C   H

ATOM 22869 C   ILE C 180   36.189 52.391 56.617 1.00 0.00
C   C
ATOM 22870 O   ILE C 180   37.017 53.108 56.051 1.00 0.00
C   O
ATOM 22871 N   LEU C 181   35.662 51.309 56.058 1.00 0.00
C   N
ATOM 22872 HN LEU C 181   34.947 50.749 56.472 1.00 0.00
C   H
ATOM 22873 CA LEU C 181   36.105 50.843 54.756 1.00 0.00
C   C
ATOM 22874 HA LEU C 181   37.148 51.127 54.682 1.00 0.00
C   H
ATOM 22875 CB LEU C 181   36.034 49.319 54.659 1.00 0.00
C   C
ATOM 22876 HB1 LEU C 181   35.003 49.018 54.957 1.00 0.00
C   H
ATOM 22877 HB2 LEU C 181   36.145 49.019 53.593 1.00 0.00
C   H
ATOM 22878 CG LEU C 181   37.015 48.508 55.514 1.00 0.00
C   C
ATOM 22879 HG LEU C 181   36.889 48.865 56.564 1.00 0.00
C   H
ATOM 22880 CD1 LEU C 181   36.679 47.026 55.433 1.00 0.00
C   C
ATOM 22881 HD11 LEU C 181   37.416 46.427 56.012 1.00 0.00
C   H
ATOM 22882 HD12 LEU C 181   35.668 46.831 55.849 1.00 0.00
C   H
ATOM 22883 HD13 LEU C 181   36.699 46.676 54.379 1.00 0.00
C   H
ATOM 22884 CD2 LEU C 181   38.460 48.754 55.104 1.00 0.00
C   C
ATOM 22885 HD21 LEU C 181   39.149 48.169 55.750 1.00 0.00
C   H
ATOM 22886 HD22 LEU C 181   38.645 48.451 54.053 1.00 0.00
C   H
ATOM 22887 HD23 LEU C 181   38.716 49.830 55.207 1.00 0.00
C   H
ATOM 22888 C   LEU C 181   35.367 51.501 53.599 1.00 0.00
C   C
ATOM 22889 O   LEU C 181   35.979 51.751 52.568 1.00 0.00
C   O
ATOM 22890 N   ALA C 182   34.073 51.786 53.763 1.00 0.00
C   N
ATOM 22891 HN ALA C 182   33.578 51.604 54.612 1.00 0.00
C   H
ATOM 22892 CA ALA C 182   33.276 52.396 52.685 1.00 0.00
C   C
ATOM 22893 HA ALA C 182   33.893 53.119 52.163 1.00 0.00
C   H
ATOM 22894 CB ALA C 182   32.902 51.348 51.640 1.00 0.00
C   C
ATOM 22895 HB1 ALA C 182   33.821 50.842 51.274 1.00 0.00
C   H

ATOM 22896 HB2 ALA C 182 32.235 50.572 52.075 1.00 0.00
C H
ATOM 22897 HB3 ALA C 182 32.391 51.805 50.766 1.00 0.00
C H
ATOM 22898 C ALA C 182 32.028 53.148 53.148 1.00 0.00
C C
ATOM 22899 O ALA C 182 31.563 52.969 54.276 1.00 0.00
C O
ATOM 22900 N ILE C 183 31.512 54.003 52.263 1.00 0.00
C N
ATOM 22901 HN ILE C 183 31.992 54.256 51.425 1.00 0.00
C H
ATOM 22902 CA ILE C 183 30.207 54.649 52.427 1.00 0.00
C C
ATOM 22903 HA ILE C 183 29.714 54.189 53.273 1.00 0.00
C H
ATOM 22904 CB ILE C 183 30.316 56.192 52.562 1.00 0.00
C C
ATOM 22905 HB ILE C 183 30.672 56.536 51.557 1.00 0.00
C H
ATOM 22906 CG2 ILE C 183 28.973 56.803 52.962 1.00 0.00
C C
ATOM 22907 HG21 ILE C 183 29.016 57.911 52.905 1.00 0.00
C H
ATOM 22908 HG22 ILE C 183 28.165 56.462 52.282 1.00 0.00
C H
ATOM 22909 HG23 ILE C 183 28.701 56.519 54.001 1.00 0.00
C H
ATOM 22910 CG1 ILE C 183 31.403 56.593 53.567 1.00 0.00
C C
ATOM 22911 HG11 ILE C 183 31.049 56.395 54.604 1.00 0.00
C H
ATOM 22912 HG12 ILE C 183 32.302 55.956 53.399 1.00 0.00
C H
ATOM 22913 CD ILE C 183 31.833 58.042 53.467 1.00 0.00
C C
ATOM 22914 HD1 ILE C 183 32.659 58.249 54.181 1.00 0.00
C H
ATOM 22915 HD2 ILE C 183 32.193 58.274 52.442 1.00 0.00
C H
ATOM 22916 HD3 ILE C 183 30.991 58.724 53.709 1.00 0.00
C H
ATOM 22917 C ILE C 183 29.366 54.321 51.191 1.00 0.00
C C
ATOM 22918 O ILE C 183 29.895 54.238 50.079 1.00 0.00
C O
ATOM 22919 N LYS C 184 28.065 54.125 51.386 1.00 0.00
C N
ATOM 22920 HN LYS C 184 27.639 54.090 52.289 1.00 0.00
C H
ATOM 22921 CA LYS C 184 27.153 53.942 50.265 1.00 0.00
C C
ATOM 22922 HA LYS C 184 27.739 54.037 49.358 1.00 0.00
C H

ATOM 22923 CB LYS C 184   26.500 52.557 50.298 1.00 0.00
C   C
ATOM 22924 HB1 LYS C 184   27.325 51.807 50.324 1.00 0.00
C   H
ATOM 22925 HB2 LYS C 184   25.943 52.442 51.255 1.00 0.00
C   H
ATOM 22926 CG LYS C 184   25.601 52.278 49.104 1.00 0.00
C   C
ATOM 22927 HG1 LYS C 184   24.866 53.108 48.999 1.00 0.00
C   H
ATOM 22928 HG2 LYS C 184   26.222 52.267 48.178 1.00 0.00
C   H
ATOM 22929 CD LYS C 184   24.846 50.968 49.246 1.00 0.00
C   C
ATOM 22930 HD1 LYS C 184   25.584 50.133 49.224 1.00 0.00
C   H
ATOM 22931 HD2 LYS C 184   24.358 50.957 50.247 1.00 0.00
C   H
ATOM 22932 CE LYS C 184   23.818 50.829 48.136 1.00 0.00
C   C
ATOM 22933 HE1 LYS C 184   22.882 51.368 48.401 1.00 0.00
C   H
ATOM 22934 HE2 LYS C 184   24.209 51.252 47.185 1.00 0.00
C   H
ATOM 22935 NZ LYS C 184   23.452 49.410 47.874 1.00 0.00
C   N
ATOM 22936 HZ1 LYS C 184   22.747 49.359 47.110 1.00 0.00
C   H
ATOM 22937 HZ2 LYS C 184   24.301 48.879 47.595 1.00 0.00
C   H
ATOM 22938 HZ3 LYS C 184   23.054 48.991 48.739 1.00 0.00
C   H
ATOM 22939 C   LYS C 184   26.091 55.035 50.236 1.00 0.00
C   C
ATOM 22940 O   LYS C 184   25.174 55.045 51.058 1.00 0.00
C   O
ATOM 22941 N   HSE C 185   26.228 55.958 49.291 1.00 0.00
C   N
ATOM 22942 HN HSE C 185   27.029 56.053 48.700 1.00 0.00
C   H
ATOM 22943 CA HSE C 185   25.181 56.934 49.042 1.00 0.00
C   C
ATOM 22944 HA HSE C 185   24.412 56.791 49.792 1.00 0.00
C   H
ATOM 22945 CB HSE C 185   25.699 58.364 49.141 1.00 0.00
C   C
ATOM 22946 HB1 HSE C 185   26.157 58.493 50.145 1.00 0.00
C   H
ATOM 22947 HB2 HSE C 185   26.505 58.519 48.393 1.00 0.00
C   H
ATOM 22948 ND1 HSE C 185   23.779 59.751 49.974 1.00 0.00
C   N
ATOM 22949 CG HSE C 185   24.621 59.415 48.979 1.00 0.00
C   C

ATOM 22950 CE1 HSE C 185   22.931 60.702 49.540 1.00 0.00
C   C
ATOM 22951 HE1 HSE C 185   22.140 61.151 50.146 1.00 0.00
C   H
ATOM 22952 NE2 HSE C 185   23.233 60.976 48.262 1.00 0.00
C   N
ATOM 22953 HE2 HSE C 185   22.752 61.633 47.683 1.00 0.00
C   H
ATOM 22954 CD2 HSE C 185   24.269 60.201 47.884 1.00 0.00
C   C
ATOM 22955 HD2 HSE C 185   24.691 60.232 46.889 1.00 0.00
C   H
ATOM 22956 C   HSE C 185   24.554 56.661 47.714 1.00 0.00
C   C
ATOM 22957 O   HSE C 185   25.183 56.819 46.663 1.00 0.00
C   O
ATOM 22958 N   HSE C 186   23.308 56.200 47.787 1.00 0.00
C   N
ATOM 22959 HN HSE C 186   22.878 56.020 48.671 1.00 0.00
C   H
ATOM 22960 CA HSE C 186   22.458 55.915 46.633 1.00 0.00
C   C
ATOM 22961 HA HSE C 186   21.623 55.361 47.042 1.00 0.00
C   H
ATOM 22962 CB HSE C 186   21.841 57.184 46.086 1.00 0.00
C   C
ATOM 22963 HB1 HSE C 186   22.567 58.011 46.241 1.00 0.00
C   H
ATOM 22964 HB2 HSE C 186   21.693 57.082 44.991 1.00 0.00
C   H
ATOM 22965 ND1 HSE C 186   19.485 56.736 46.727 1.00 0.00
C   N
ATOM 22966 CG HSE C 186   20.564 57.539 46.770 1.00 0.00
C   C
ATOM 22967 CE1 HSE C 186   18.491 57.288 47.440 1.00 0.00
C   C
ATOM 22968 HE1 HSE C 186   17.497 56.850 47.561 1.00 0.00
C   H
ATOM 22969 NE2 HSE C 186   18.939 58.446 47.951 1.00 0.00
C   N
ATOM 22970 HE2 HSE C 186   18.403 59.077 48.510 1.00 0.00
C   H
ATOM 22971 CD2 HSE C 186   20.217 58.627 47.561 1.00 0.00
C   C
ATOM 22972 HD2 HSE C 186   20.799 59.492 47.844 1.00 0.00
C   H
ATOM 22973 C   HSE C 186   23.053 55.077 45.557 1.00 0.00
C   C
ATOM 22974 O   HSE C 186   23.151 55.488 44.394 1.00 0.00
C   O
ATOM 22975 N   GLY C 187   23.443 53.877 45.952 1.00 0.00
C   N
ATOM 22976 HN GLY C 187   23.404 53.599 46.912 1.00 0.00
C   H

```
ATOM  22977  CA  GLY C 187      23.955  52.878  45.029  1.00  0.00
C    C
ATOM  22978  HA1 GLY C 187      23.189  52.708  44.285  1.00  0.00
C    H
ATOM  22979  HA2 GLY C 187      24.213  52.002  45.609  1.00  0.00
C    H
ATOM  22980  C   GLY C 187      25.202  53.347  44.317  1.00  0.00
C    C
ATOM  22981  O   GLY C 187      25.555  52.839  43.253  1.00  0.00
C    O
ATOM  22982  N   ASP C 188      25.866  54.319  44.934  1.00  0.00
C    N
ATOM  22983  HN  ASP C 188      25.595  54.675  45.824  1.00  0.00
C    H
ATOM  22984  CA  ASP C 188      27.043  54.971  44.400  1.00  0.00
C    C
ATOM  22985  HA  ASP C 188      27.510  54.338  43.653  1.00  0.00
C    H
ATOM  22986  CB  ASP C 188      26.654  56.368  43.895  1.00  0.00
C    C
ATOM  22987  HB1 ASP C 188      25.869  56.270  43.118  1.00  0.00
C    H
ATOM  22988  HB2 ASP C 188      26.249  56.984  44.722  1.00  0.00
C    H
ATOM  22989  CG  ASP C 188      27.816  57.132  43.262  1.00  0.00
C    C
ATOM  22990  OD1 ASP C 188      27.554  58.218  42.703  1.00  0.00
C    O
ATOM  22991  OD2 ASP C 188      28.982  56.668  43.308  1.00  0.00
C    O
ATOM  22992  C   ASP C 188      28.007  55.041  45.582  1.00  0.00
C    C
ATOM  22993  O   ASP C 188      27.844  55.873  46.478  1.00  0.00
C    O
ATOM  22994  N   ILE C 189      28.999  54.153  45.587  1.00  0.00
C    N
ATOM  22995  HN  ILE C 189      29.243  53.570  44.816  1.00  0.00
C    H
ATOM  22996  CA  ILE C 189      29.835  53.961  46.773  1.00  0.00
C    C
ATOM  22997  HA  ILE C 189      29.224  54.317  47.593  1.00  0.00
C    H
ATOM  22998  CB  ILE C 189      30.161  52.470  47.038  1.00  0.00
C    C
ATOM  22999  HB  ILE C 189      30.863  52.487  47.910  1.00  0.00
C    H
ATOM  23000  CG2 ILE C 189      28.889  51.690  47.343  1.00  0.00
C    C
ATOM  23001 HG21 ILE C 189      29.133  50.663  47.685  1.00  0.00
C    H
ATOM  23002 HG22 ILE C 189      28.305  52.183  48.149  1.00  0.00
C    H
ATOM  23003 HG23 ILE C 189      28.246  51.611  46.442  1.00  0.00
C    H
```

ATOM 23004 CG1 ILE C 189   30.935 51.848 45.870 1.00 0.00
C   C
ATOM 23005 HG11 ILE C 189   30.222 51.394 45.145 1.00 0.00
C   H
ATOM 23006 HG12 ILE C 189   31.483 52.654 45.331 1.00 0.00
C   H
ATOM 23007 CD ILE C 189   31.937 50.800 46.302 1.00 0.00
C   C
ATOM 23008 HD1 ILE C 189   32.507 50.425 45.425 1.00 0.00
C   H
ATOM 23009 HD2 ILE C 189   32.659 51.225 47.031 1.00 0.00
C   H
ATOM 23010 HD3 ILE C 189   31.428 49.933 46.775 1.00 0.00
C   H
ATOM 23011 C   ILE C 189   31.129 54.768 46.804 1.00 0.00
C   C
ATOM 23012 O   ILE C 189   31.822 54.898 45.792 1.00 0.00
C   O
ATOM 23013 N   CYS C 190   31.431 55.310 47.985 1.00 0.00
C   N
ATOM 23014 HN CYS C 190   30.779 55.339 48.739 1.00 0.00
C   H
ATOM 23015 CA CYS C 190   32.733 55.905 48.277 1.00 0.00
C   C
ATOM 23016 HA CYS C 190   33.182 56.219 47.341 1.00 0.00
C   H
ATOM 23017 CB CYS C 190   32.604 57.079 49.261 1.00 0.00
C   C
ATOM 23018 HB1 CYS C 190   31.974 56.730 50.110 1.00 0.00
C   H
ATOM 23019 HB2 CYS C 190   33.600 57.319 49.695 1.00 0.00
C   H
ATOM 23020 SG CYS C 190   31.838 58.594 48.611 1.00 0.00
C   S
ATOM 23021 HG1 CYS C 190   31.857 59.290 49.749 1.00 0.00
C   H
ATOM 23022 C   CYS C 190   33.614 54.803 48.867 1.00 0.00
C   C
ATOM 23023 O   CYS C 190   33.526 54.498 50.060 1.00 0.00
C   O
ATOM 23024 N   LEU C 191   34.450 54.205 48.020 1.00 0.00
C   N
ATOM 23025 HN LEU C 191   34.607 54.530 47.089 1.00 0.00
C   H
ATOM 23026 CA LEU C 191   35.220 53.011 48.378 1.00 0.00
C   C
ATOM 23027 HA LEU C 191   34.481 52.255 48.613 1.00 0.00
C   H
ATOM 23028 CB LEU C 191   36.032 52.495 47.184 1.00 0.00
C   C
ATOM 23029 HB1 LEU C 191   35.357 52.507 46.297 1.00 0.00
C   H
ATOM 23030 HB2 LEU C 191   36.847 53.219 46.961 1.00 0.00
C   H

ATOM 23031 CG LEU C 191   36.618 51.084 47.310 1.00 0.00
C   C
ATOM 23032 HG LEU C 191   37.166 51.051 48.283 1.00 0.00
C   H
ATOM 23033 CD1 LEU C 191   35.527 50.021 47.302 1.00 0.00
C   C
ATOM 23034 HD11 LEU C 191   35.972 49.005 47.346 1.00 0.00
C   H
ATOM 23035 HD12 LEU C 191   34.854 50.145 48.178 1.00 0.00
C   H
ATOM 23036 HD13 LEU C 191   34.916 50.093 46.378 1.00 0.00
C   H
ATOM 23037 CD2 LEU C 191   37.623 50.830 46.196 1.00 0.00
C   C
ATOM 23038 HD21 LEU C 191   38.063 49.814 46.296 1.00 0.00
C   H
ATOM 23039 HD22 LEU C 191   37.150 50.894 45.196 1.00 0.00
C   H
ATOM 23040 HD23 LEU C 191   38.449 51.573 46.240 1.00 0.00
C   H
ATOM 23041 C   LEU C 191   36.110 53.185 49.611 1.00 0.00
C   C
ATOM 23042 O   LEU C 191   35.906 52.505 50.602 1.00 0.00
C   O
ATOM 23043 N   SER C 192   37.098 54.073 49.541 1.00 0.00
C   N
ATOM 23044 HN SER C 192   37.358 54.559 48.707 1.00 0.00
C   H
ATOM 23045 CA SER C 192   37.905 54.419 50.712 1.00 0.00
C   C
ATOM 23046 HA SER C 192   37.464 53.913 51.560 1.00 0.00
C   H
ATOM 23047 CB SER C 192   39.368 53.996 50.530 1.00 0.00
C   C
ATOM 23048 HB1 SER C 192   39.627 54.101 49.452 1.00 0.00
C   H
ATOM 23049 HB2 SER C 192   40.042 54.678 51.096 1.00 0.00
C   H
ATOM 23050 OG SER C 192   39.561 52.650 50.917 1.00 0.00
C   O
ATOM 23051 HG1 SER C 192   40.423 52.378 50.577 1.00 0.00
C   H
ATOM 23052 C   SER C 192   37.795 55.920 50.972 1.00 0.00
C   C
ATOM 23053 O   SER C 192   38.438 56.720 50.280 1.00 0.00
C   O
ATOM 23054 N   PRO C 193   36.972 56.307 51.968 1.00 0.00
C   N
ATOM 23055 CD PRO C 193   36.406 55.432 53.008 1.00 0.00
C   C
ATOM 23056 HD1 PRO C 193   36.338 55.183 51.925 1.00 0.00
C   H
ATOM 23057 HD2 PRO C 193   36.518 54.504 53.616 1.00 0.00
C   H

ATOM 23058 CA PRO C 193   36.575 57.704 52.170 1.00 0.00
C   C
ATOM 23059 HA PRO C 193   36.119 58.077 51.259 1.00 0.00
C   H
ATOM 23060 CB PRO C 193   35.542 57.626 53.309 1.00 0.00
C   C
ATOM 23061 HB1 PRO C 193   34.541 57.399 52.876 1.00 0.00
C   H
ATOM 23062 HB2 PRO C 193   35.476 58.566 53.893 1.00 0.00
C   H
ATOM 23063 CG PRO C 193   35.184 56.181 53.439 1.00 0.00
C   C
ATOM 23064 HG1 PRO C 193   34.193 56.022 53.907 1.00 0.00
C   H
ATOM 23065 HG2 PRO C 193   35.966 55.663 54.040 1.00 0.00
C   H
ATOM 23066 C   PRO C 193   37.720 58.633 52.572 1.00 0.00
C   C
ATOM 23067 O   PRO C 193   38.546 58.276 53.417 1.00 0.00
C   O
ATOM 23068 N   ALA C 194   37.760 59.810 51.953 1.00 0.00
C   N
ATOM 23069 HN ALA C 194   37.176 60.017 51.167 1.00 0.00
C   H
ATOM 23070 CA ALA C 194   38.654 60.885 52.369 1.00 0.00
C   C
ATOM 23071 HA ALA C 194   39.668 60.532 52.517 1.00 0.00
C   H
ATOM 23072 CB ALA C 194   38.661 61.996 51.331 1.00 0.00
C   C
ATOM 23073 HB1 ALA C 194   39.056 61.604 50.369 1.00 0.00
C   H
ATOM 23074 HB2 ALA C 194   37.632 62.377 51.147 1.00 0.00
C   H
ATOM 23075 HB3 ALA C 194   39.303 62.846 51.645 1.00 0.00
C   H
ATOM 23076 C   ALA C 194   38.176 61.414 53.726 1.00 0.00
C   C
ATOM 23077 O   ALA C 194   36.978 61.342 54.021 1.00 0.00
C   O
ATOM 23078 N   PRO C 195   39.102 61.937 54.557 1.00 0.00
C   N
ATOM 23079 CD PRO C 195   40.563 61.993 54.346 1.00 0.00
C   C
ATOM 23080 HD1 PRO C 195   40.104 60.978 54.382 1.00 0.00
C   H
ATOM 23081 HD2 PRO C 195   41.381 62.024 53.589 1.00 0.00
C   H
ATOM 23082 CA PRO C 195   38.736 62.411 55.897 1.00 0.00
C   C
ATOM 23083 HA PRO C 195   38.548 61.546 56.521 1.00 0.00
C   H
ATOM 23084 CB PRO C 195   40.008 63.122 56.368 1.00 0.00
C   C

ATOM 23085 HB1 PRO C 195 40.700 62.371 56.811 1.00 0.00
C H
ATOM 23086 HB2 PRO C 195 39.804 63.913 57.118 1.00 0.00
C H
ATOM 23087 CG PRO C 195 41.102 62.362 55.702 1.00 0.00
C C
ATOM 23088 HG1 PRO C 195 42.074 62.458 56.225 1.00 0.00
C H
ATOM 23089 HG2 PRO C 195 41.625 61.511 55.659 1.00 0.00
C H
ATOM 23090 C PRO C 195 37.528 63.358 55.973 1.00 0.00
C C
ATOM 23091 O PRO C 195 36.933 63.483 57.041 1.00 0.00
C O
ATOM 23092 N GLU C 196 37.153 63.999 54.870 1.00 0.00
C N
ATOM 23093 HN GLU C 196 37.562 63.895 53.967 1.00 0.00
C H
ATOM 23094 CA GLU C 196 36.039 64.963 54.916 1.00 0.00
C C
ATOM 23095 HA GLU C 196 35.846 65.147 55.965 1.00 0.00
C H
ATOM 23096 CB GLU C 196 36.399 66.287 54.239 1.00 0.00
C C
ATOM 23097 HB1 GLU C 196 36.417 66.120 53.137 1.00 0.00
C H
ATOM 23098 HB2 GLU C 196 35.595 67.034 54.421 1.00 0.00
C H
ATOM 23099 CG GLU C 196 37.708 66.918 54.611 1.00 0.00
C C
ATOM 23100 HG1 GLU C 196 37.676 67.999 54.381 1.00 0.00
C H
ATOM 23101 HG2 GLU C 196 37.922 66.781 55.690 1.00 0.00
C H
ATOM 23102 CD GLU C 196 38.907 66.397 53.844 1.00 0.00
C C
ATOM 23103 OE1 GLU C 196 38.826 66.143 52.624 1.00 0.00
C O
ATOM 23104 OE2 GLU C 196 39.957 66.285 54.504 1.00 0.00
C O
ATOM 23105 C GLU C 196 34.729 64.489 54.290 1.00 0.00
C C
ATOM 23106 O GLU C 196 33.817 65.300 54.105 1.00 0.00
C O
ATOM 23107 N ASP C 197 34.623 63.209 53.955 1.00 0.00
C N
ATOM 23108 HN ASP C 197 35.284 62.488 54.147 1.00 0.00
C H
ATOM 23109 CA ASP C 197 33.448 62.726 53.227 1.00 0.00
C C
ATOM 23110 HA ASP C 197 33.217 63.494 52.496 1.00 0.00
C H
ATOM 23111 CB ASP C 197 33.765 61.451 52.444 1.00 0.00
C C

ATOM 23112 HB1 ASP C 197   34.390 60.784 53.072 1.00 0.00
C   H
ATOM 23113 HB2 ASP C 197   32.833 60.912 52.182 1.00 0.00
C   H
ATOM 23114 CG ASP C 197   34.539 61.730 51.165 1.00 0.00
C   C
ATOM 23115 OD1 ASP C 197   34.590 62.901 50.728 1.00 0.00
C   O
ATOM 23116 OD2 ASP C 197   35.098 60.773 50.593 1.00 0.00
C   O
ATOM 23117 C   ASP C 197   32.205 62.562 54.099 1.00 0.00
C   C
ATOM 23118 O   ASP C 197   32.233 61.873 55.122 1.00 0.00
C   O
ATOM 23119 N   ILE C 198   31.122 63.208 53.669 1.00 0.00
C   N
ATOM 23120 HN ILE C 198   31.106 63.689 52.795 1.00 0.00
C   H
ATOM 23121 CA ILE C 198   29.866 63.278 54.417 1.00 0.00
C   C
ATOM 23122 HA ILE C 198   30.141 63.490 55.442 1.00 0.00
C   H
ATOM 23123 CB ILE C 198   28.943 64.393 53.856 1.00 0.00
C   C
ATOM 23124 HB ILE C 198   28.829 64.142 52.771 1.00 0.00
C   H
ATOM 23125 CG2 ILE C 198   27.612 64.440 54.602 1.00 0.00
C   C
ATOM 23126 HG21 ILE C 198   26.905 65.130 54.095 1.00 0.00
C   H
ATOM 23127 HG22 ILE C 198   27.139 63.436 54.632 1.00 0.00
C   H
ATOM 23128 HG23 ILE C 198   27.753 64.793 55.645 1.00 0.00
C   H
ATOM 23129 CG1 ILE C 198   29.643 65.755 53.924 1.00 0.00
C   C
ATOM 23130 HG11 ILE C 198   29.570 66.166 54.956 1.00 0.00
C   H
ATOM 23131 HG12 ILE C 198   30.726 65.617 53.701 1.00 0.00
C   H
ATOM 23132 CD ILE C 198   29.103 66.789 52.955 1.00 0.00
C   C
ATOM 23133 HD1 ILE C 198   29.695 67.727 53.022 1.00 0.00
C   H
ATOM 23134 HD2 ILE C 198   29.160 66.415 51.910 1.00 0.00
C   H
ATOM 23135 HD3 ILE C 198   28.044 67.034 53.184 1.00 0.00
C   H
ATOM 23136 C   ILE C 198   29.125 61.938 54.427 1.00 0.00
C   C
ATOM 23137 O   ILE C 198   28.937 61.312 53.380 1.00 0.00
C   O
ATOM 23138 N   ILE C 199   28.727 61.504 55.623 1.00 0.00
C   N

ATOM 23139 HN ILE C 199 28.998 61.937 56.480 1.00 0.00
C H
ATOM 23140 CA ILE C 199 27.852 60.347 55.788 1.00 0.00
C C
ATOM 23141 HA ILE C 199 27.805 59.836 54.834 1.00 0.00
C H
ATOM 23142 CB ILE C 199 28.339 59.399 56.911 1.00 0.00
C C
ATOM 23143 HB ILE C 199 28.648 60.084 57.741 1.00 0.00
C H
ATOM 23144 CG2 ILE C 199 27.250 58.405 57.298 1.00 0.00
C C
ATOM 23145 HG21 ILE C 199 27.545 57.835 58.203 1.00 0.00
C H
ATOM 23146 HG22 ILE C 199 26.298 58.929 57.526 1.00 0.00
C H
ATOM 23147 HG23 ILE C 199 27.064 57.680 56.477 1.00 0.00
C H
ATOM 23148 CG1 ILE C 199 29.595 58.649 56.461 1.00 0.00
C C
ATOM 23149 HG11 ILE C 199 29.311 57.810 55.787 1.00 0.00
C H
ATOM 23150 HG12 ILE C 199 30.238 59.342 55.872 1.00 0.00
C H
ATOM 23151 CD ILE C 199 30.430 58.096 57.597 1.00 0.00
C C
ATOM 23152 HD1 ILE C 199 31.351 57.616 57.201 1.00 0.00
C H
ATOM 23153 HD2 ILE C 199 30.732 58.908 58.292 1.00 0.00
C H
ATOM 23154 HD3 ILE C 199 29.865 57.331 58.171 1.00 0.00
C H
ATOM 23155 C ILE C 199 26.446 60.843 56.090 1.00 0.00
C C
ATOM 23156 O ILE C 199 26.222 61.546 57.075 1.00 0.00
C O
ATOM 23157 N ARG C 200 25.508 60.486 55.220 1.00 0.00
C N
ATOM 23158 HN ARG C 200 25.736 59.971 54.396 1.00 0.00
C H
ATOM 23159 CA ARG C 200 24.109 60.822 55.413 1.00 0.00
C C
ATOM 23160 HA ARG C 200 24.028 61.614 56.148 1.00 0.00
C H
ATOM 23161 CB ARG C 200 23.491 61.271 54.086 1.00 0.00
C C
ATOM 23162 HB1 ARG C 200 24.340 61.393 53.369 1.00 0.00
C H
ATOM 23163 HB2 ARG C 200 22.859 60.454 53.674 1.00 0.00
C H
ATOM 23164 CG ARG C 200 22.725 62.582 54.167 1.00 0.00
C C
ATOM 23165 HG1 ARG C 200 21.757 62.393 54.681 1.00 0.00
C H

ATOM 23166 HG2 ARG C 200   23.281 63.286 54.826 1.00 0.00
C   H
ATOM 23167 CD ARG C 200   22.562 63.208 52.791 1.00 0.00
C   C
ATOM 23168 HD1 ARG C 200   22.245 62.425 52.063 1.00 0.00
C   H
ATOM 23169 HD2 ARG C 200   21.798 64.019 52.797 1.00 0.00
C   H
ATOM 23170 NE ARG C 200   23.801 63.819 52.309 1.00 0.00
C   N
ATOM 23171 HE ARG C 200   24.518 63.869 53.014 1.00 0.00
C   H
ATOM 23172 CZ ARG C 200   23.994 64.273 51.071 1.00 0.00
C   C
ATOM 23173 NH1 ARG C 200   23.030 64.188 50.162 1.00 0.00
C   N
ATOM 23174 HH11 ARG C 200   23.182 64.503 49.235 1.00 0.00
C   H
ATOM 23175 HH12 ARG C 200   22.213 63.673 50.428 1.00 0.00
C   H
ATOM 23176 NH2 ARG C 200   25.159 64.813 50.739 1.00 0.00
C   N
ATOM 23177 HH21 ARG C 200   25.275 65.229 49.849 1.00 0.00
C   H
ATOM 23178 HH22 ARG C 200   25.807 64.966 51.474 1.00 0.00
C   H
ATOM 23179 C   ARG C 200   23.379 59.606 55.998 1.00 0.00
C   C
ATOM 23180 O   ARG C 200   23.912 58.496 56.007 1.00 0.00
C   O
ATOM 23181 N   GLU C 201   22.176 59.849 56.510 1.00 0.00
C   N
ATOM 23182 HN GLU C 201   21.736 60.743 56.490 1.00 0.00
C   H
ATOM 23183 CA GLU C 201   21.381 58.814 57.155 1.00 0.00
C   C
ATOM 23184 HA GLU C 201   21.933 58.358 57.968 1.00 0.00
C   H
ATOM 23185 CB GLU C 201   20.150 59.424 57.827 1.00 0.00
C   C
ATOM 23186 HB1 GLU C 201   20.494 60.223 58.524 1.00 0.00
C   H
ATOM 23187 HB2 GLU C 201   19.519 59.935 57.066 1.00 0.00
C   H
ATOM 23188 CG GLU C 201   19.309 58.424 58.604 1.00 0.00
C   C
ATOM 23189 HG1 GLU C 201   18.965 57.624 57.925 1.00 0.00
C   H
ATOM 23190 HG2 GLU C 201   19.892 57.975 59.433 1.00 0.00
C   H
ATOM 23191 CD GLU C 201   18.051 59.044 59.180 1.00 0.00
C   C
ATOM 23192 OE1 GLU C 201   17.379 59.808 58.456 1.00 0.00
C   O

ATOM 23193 OE2 GLU C 201   17.734 58.768 60.356 1.00 0.00
C   O
ATOM 23194 C   GLU C 201   21.173 57.743 56.095 1.00 0.00
C   C
ATOM 23195 O   GLU C 201   21.116 58.038 54.902 1.00 0.00
C   O
ATOM 23196 N   GLN C 202   21.062 56.498 56.540 1.00 0.00
C   N
ATOM 23197 HN GLN C 202   21.103 56.243 57.505 1.00 0.00
C   H
ATOM 23198 CA GLN C 202   20.867 55.382 55.630 1.00 0.00
C   C
ATOM 23199 HA GLN C 202   20.829 54.511 56.272 1.00 0.00
C   H
ATOM 23200 CB GLN C 202   19.720 55.772 54.696 1.00 0.00
C   C
ATOM 23201 HB1 GLN C 202   18.912 55.019 54.847 1.00 0.00
C   H
ATOM 23202 HB2 GLN C 202   19.297 56.751 55.020 1.00 0.00
C   H
ATOM 23203 CG GLN C 202   20.104 55.812 53.227 1.00 0.00
C   C
ATOM 23204 HG1 GLN C 202   20.765 56.682 53.022 1.00 0.00
C   H
ATOM 23205 HG2 GLN C 202   20.655 54.892 52.939 1.00 0.00
C   H
ATOM 23206 CD GLN C 202   18.904 55.964 52.313 1.00 0.00
C   C
ATOM 23207 OE1 GLN C 202   17.758 55.903 52.761 1.00 0.00
C   O
ATOM 23208 NE2 GLN C 202   19.160 56.165 51.026 1.00 0.00
C   N
ATOM 23209 HE21 GLN C 202   18.375 56.270 50.422 1.00 0.00
C   H
ATOM 23210 HE22 GLN C 202   20.101 56.189 50.707 1.00 0.00
C   H
ATOM 23211 C   GLN C 202   21.967 55.146 54.602 1.00 0.00
C   C
ATOM 23212 O   GLN C 202   21.718 54.560 53.549 1.00 0.00
C   O
ATOM 23213 N   ASP C 203   23.179 55.600 54.906 1.00 0.00
C   N
ATOM 23214 HN ASP C 203   23.447 56.081 55.736 1.00 0.00
C   H
ATOM 23215 CA ASP C 203   24.271 55.414 53.986 1.00 0.00
C   C
ATOM 23216 HA ASP C 203   23.939 55.101 53.003 1.00 0.00
C   H
ATOM 23217 CB ASP C 203   25.320 56.527 54.025 1.00 0.00
C   C
ATOM 23218 HB1 ASP C 203   25.339 56.976 55.039 1.00 0.00
C   H
ATOM 23219 HB2 ASP C 203   26.328 56.122 53.805 1.00 0.00
C   H

ATOM 23220 CG ASP C 203 25.025 57.655 53.034 1.00 0.00
C C
ATOM 23221 OD1 ASP C 203 24.155 57.489 52.152 1.00 0.00
C O
ATOM 23222 OD2 ASP C 203 25.678 58.715 53.131 1.00 0.00
C O
ATOM 23223 C ASP C 203 24.888 54.195 54.647 1.00 0.00
C C
ATOM 23224 O ASP C 203 24.943 54.106 55.879 1.00 0.00
C O
ATOM 23225 N CYS C 204 25.318 53.239 53.837 1.00 0.00
C N
ATOM 23226 HN CYS C 204 25.257 53.271 52.841 1.00 0.00
C H
ATOM 23227 CA CYS C 204 25.931 52.034 54.372 1.00 0.00
C C
ATOM 23228 HA CYS C 204 25.332 51.774 55.236 1.00 0.00
C H
ATOM 23229 CB CYS C 204 25.821 50.898 53.354 1.00 0.00
C C
ATOM 23230 HB1 CYS C 204 26.064 51.325 52.355 1.00 0.00
C H
ATOM 23231 HB2 CYS C 204 26.602 50.131 53.559 1.00 0.00
C H
ATOM 23232 SG CYS C 204 24.178 50.147 53.270 1.00 0.00
C S
ATOM 23233 HG1 CYS C 204 24.437 49.293 52.278 1.00 0.00
C H
ATOM 23234 C CYS C 204 27.364 52.210 54.840 1.00 0.00
C C
ATOM 23235 O CYS C 204 28.273 52.439 54.039 1.00 0.00
C O
ATOM 23236 N LEU C 205 27.548 52.121 56.150 1.00 0.00
C N
ATOM 23237 HN LEU C 205 26.805 52.042 56.812 1.00 0.00
C H
ATOM 23238 CA LEU C 205 28.872 52.130 56.740 1.00 0.00
C C
ATOM 23239 HA LEU C 205 29.492 52.780 56.137 1.00 0.00
C H
ATOM 23240 CB LEU C 205 28.814 52.576 58.200 1.00 0.00
C C
ATOM 23241 HB1 LEU C 205 28.072 51.924 58.714 1.00 0.00
C H
ATOM 23242 HB2 LEU C 205 29.794 52.364 58.681 1.00 0.00
C H
ATOM 23243 CG LEU C 205 28.421 54.028 58.453 1.00 0.00
C C
ATOM 23244 HG LEU C 205 27.444 54.218 57.949 1.00 0.00
C H
ATOM 23245 CD1 LEU C 205 28.282 54.282 59.942 1.00 0.00
C C
ATOM 23246 HD11 LEU C 205 28.042 55.350 60.135 1.00 0.00
C H

ATOM 23247 HD12 LEU C 205   27.468 53.659 60.371 1.00 0.00
C   H
ATOM 23248 HD13 LEU C 205   29.227 54.039 60.472 1.00 0.00
C   H
ATOM 23249 CD2 LEU C 205   29.453 54.957 57.844 1.00 0.00
C   C
ATOM 23250 HD21 LEU C 205   29.163 56.017 58.009 1.00 0.00
C   H
ATOM 23251 HD22 LEU C 205   30.454 54.808 58.295 1.00 0.00
C   H
ATOM 23252 HD23 LEU C 205   29.533 54.785 56.749 1.00 0.00
C   H
ATOM 23253 C   LEU C 205   29.460 50.743 56.668 1.00 0.00
C   C
ATOM 23254 O   LEU C 205   28.802 49.761 57.007 1.00 0.00
C   O
ATOM 23255 N   VAL C 206   30.696 50.666 56.201 1.00 0.00
C   N
ATOM 23256 HN VAL C 206   31.178 51.409 55.745 1.00 0.00
C   H
ATOM 23257 CA VAL C 206   31.460 49.447 56.329 1.00 0.00
C   C
ATOM 23258 HA VAL C 206   30.826 48.657 56.711 1.00 0.00
C   H
ATOM 23259 CB VAL C 206   32.084 48.994 54.994 1.00 0.00
C   C
ATOM 23260 HB VAL C 206   32.844 49.758 54.700 1.00 0.00
C   H
ATOM 23261 CG1 VAL C 206   32.769 47.642 55.147 1.00 0.00
C   C
ATOM 23262 HG11 VAL C 206   33.181 47.306 54.171 1.00 0.00
C   H
ATOM 23263 HG12 VAL C 206   33.615 47.691 55.864 1.00 0.00
C   H
ATOM 23264 HG13 VAL C 206   32.048 46.873 55.498 1.00 0.00
C   H
ATOM 23265 CG2 VAL C 206   31.013 48.912 53.912 1.00 0.00
C   C
ATOM 23266 HG21 VAL C 206   31.447 48.548 52.957 1.00 0.00
C   H
ATOM 23267 HG22 VAL C 206   30.203 48.215 54.217 1.00 0.00
C   H
ATOM 23268 HG23 VAL C 206   30.562 49.907 53.718 1.00 0.00
C   H
ATOM 23269 C   VAL C 206   32.519 49.728 57.377 1.00 0.00
C   C
ATOM 23270 O   VAL C 206   33.210 50.749 57.330 1.00 0.00
C   O
ATOM 23271 N   ILE C 207   32.619 48.826 58.335 1.00 0.00
C   N
ATOM 23272 HN ILE C 207   32.101 47.972 58.362 1.00 0.00
C   H
ATOM 23273 CA ILE C 207   33.510 49.010 59.449 1.00 0.00
C   C

ATOM 23274 HA ILE C 207   34.218 49.783 59.179 1.00 0.00
C   H
ATOM 23275 CB ILE C 207   32.669 49.394 60.667 1.00 0.00
C   C
ATOM 23276 HB ILE C 207   31.610 49.284 60.321 1.00 0.00
C   H
ATOM 23277 CG2 ILE C 207   33.152 48.668 61.946 1.00 0.00
C   C
ATOM 23278 HG21 ILE C 207   32.441 48.834 62.782 1.00 0.00
C   H
ATOM 23279 HG22 ILE C 207   33.222 47.573 61.777 1.00 0.00
C   H
ATOM 23280 HG23 ILE C 207   34.150 49.040 62.261 1.00 0.00
C   H
ATOM 23281 CG1 ILE C 207   32.876 50.904 61.020 1.00 0.00
C   C
ATOM 23282 HG11 ILE C 207   33.944 51.088 61.274 1.00 0.00
C   H
ATOM 23283 HG12 ILE C 207   32.641 51.520 60.122 1.00 0.00
C   H
ATOM 23284 CD ILE C 207   31.859 51.175 62.245 1.00 0.00
C   C
ATOM 23285 HD1 ILE C 207   31.885 52.247 62.539 1.00 0.00
C   H
ATOM 23286 HD2 ILE C 207   30.817 50.920 61.954 1.00 0.00
C   H
ATOM 23287 HD3 ILE C 207   32.135 50.569 63.134 1.00 0.00
C   H
ATOM 23288 C   ILE C 207   34.310 47.727 59.637 1.00 0.00
C   C
ATOM 23289 O   ILE C 207   33.816 46.637 59.348 1.00 0.00
C   O
ATOM 23290 N   MET C 208   35.542 47.865 60.114 1.00 0.00
C   N
ATOM 23291 HN MET C 208   35.936 48.738 60.397 1.00 0.00
C   H
ATOM 23292 CA MET C 208   36.434 46.728 60.264 1.00 0.00
C   C
ATOM 23293 HA MET C 208   35.817 45.845 60.380 1.00 0.00
C   H
ATOM 23294 CB MET C 208   37.342 46.573 59.046 1.00 0.00
C   C
ATOM 23295 HB1 MET C 208   36.683 46.589 58.146 1.00 0.00
C   H
ATOM 23296 HB2 MET C 208   38.004 47.463 58.963 1.00 0.00
C   H
ATOM 23297 CG MET C 208   38.167 45.297 59.019 1.00 0.00
C   C
ATOM 23298 HG1 MET C 208   38.631 45.117 60.012 1.00 0.00
C   H
ATOM 23299 HG2 MET C 208   37.507 44.431 58.794 1.00 0.00
C   H
ATOM 23300 SD MET C 208   39.473 45.398 57.783 1.00 0.00
C   S

```
ATOM  23301  CE  MET C 208      39.895  43.670  57.581  1.00  0.00
C    C
ATOM  23302  HE1 MET C 208      40.715  43.546  56.841  1.00  0.00
C    H
ATOM  23303  HE2 MET C 208      40.232  43.227  58.542  1.00  0.00
C    H
ATOM  23304  HE3 MET C 208      39.021  43.084  57.223  1.00  0.00
C    H
ATOM  23305  C   MET C 208      37.271  46.872  61.523  1.00  0.00
C    C
ATOM  23306  O   MET C 208      37.674  47.978  61.891  1.00  0.00
C    O
ATOM  23307  N   GLY C 209      37.528  45.744  62.179  1.00  0.00
C    N
ATOM  23308  HN  GLY C 209      37.150  44.863  61.900  1.00  0.00
C    H
ATOM  23309  CA  GLY C 209      38.383  45.718  63.356  1.00  0.00
C    C
ATOM  23310  HA1 GLY C 209      38.419  46.728  63.740  1.00  0.00
C    H
ATOM  23311  HA2 GLY C 209      39.342  45.326  63.045  1.00  0.00
C    H
ATOM  23312  C   GLY C 209      37.846  44.821  64.448  1.00  0.00
C    C
ATOM  23313  O   GLY C 209      37.267  43.768  64.171  1.00  0.00
C    O
ATOM  23314  N   HSE C 210      38.042  45.246  65.692  1.00  0.00
C    N
ATOM  23315  HN  HSE C 210      38.467  46.124  65.909  1.00  0.00
C    H
ATOM  23316  CA  HSE C 210      37.652  44.462  66.858  1.00  0.00
C    C
ATOM  23317  HA  HSE C 210      37.897  43.429  66.640  1.00  0.00
C    H
ATOM  23318  CB  HSE C 210      38.409  44.936  68.093  1.00  0.00
C    C
ATOM  23319  HB1 HSE C 210      39.372  45.377  67.756  1.00  0.00
C    H
ATOM  23320  HB2 HSE C 210      37.836  45.747  68.591  1.00  0.00
C    H
ATOM  23321  ND1 HSE C 210      39.605  42.856  68.811  1.00  0.00
C    N
ATOM  23322  CG  HSE C 210      38.724  43.837  69.081  1.00  0.00
C    C
ATOM  23323  CE1 HSE C 210      39.690  42.028  69.871  1.00  0.00
C    C
ATOM  23324  HE1 HSE C 210      40.327  41.140  69.916  1.00  0.00
C    H
ATOM  23325  NE2 HSE C 210      38.864  42.487  70.826  1.00  0.00
C    N
ATOM  23326  HE2 HSE C 210      38.713  42.058  71.716  1.00  0.00
C    H
ATOM  23327  CD2 HSE C 210      38.256  43.602  70.373  1.00  0.00
C    C
```

ATOM 23328 HD2 HSE C 210   37.538 44.155 70.961 1.00 0.00
C   H
ATOM 23329 C   HSE C 210   36.171 44.525 67.081 1.00 0.00
C   C
ATOM 23330 O   HSE C 210   35.567 45.599 67.047 1.00 0.00
C   O
ATOM 23331 N   LYS C 211   35.585 43.354 67.310 1.00 0.00
C   N
ATOM 23332 HN LYS C 211   36.129 42.520 67.389 1.00 0.00
C   H
ATOM 23333 CA LYS C 211   34.142 43.173 67.465 1.00 0.00
C   C
ATOM 23334 HA LYS C 211   33.694 43.476 66.527 1.00 0.00
C   H
ATOM 23335 CB LYS C 211   33.860 41.705 67.786 1.00 0.00
C   C
ATOM 23336 HB1 LYS C 211   34.710 41.110 67.379 1.00 0.00
C   H
ATOM 23337 HB2 LYS C 211   33.881 41.565 68.891 1.00 0.00
C   H
ATOM 23338 CG LYS C 211   32.570 41.148 67.217 1.00 0.00
C   C
ATOM 23339 HG1 LYS C 211   31.711 41.725 67.629 1.00 0.00
C   H
ATOM 23340 HG2 LYS C 211   32.568 41.295 66.112 1.00 0.00
C   H
ATOM 23341 CD LYS C 211   32.442 39.680 67.586 1.00 0.00
C   C
ATOM 23342 HD1 LYS C 211   33.437 39.196 67.454 1.00 0.00
C   H
ATOM 23343 HD2 LYS C 211   32.181 39.616 68.668 1.00 0.00
C   H
ATOM 23344 CE LYS C 211   31.389 38.981 66.745 1.00 0.00
C   C
ATOM 23345 HE1 LYS C 211   30.401 39.477 66.867 1.00 0.00
C   H
ATOM 23346 HE2 LYS C 211   31.667 39.010 65.669 1.00 0.00
C   H
ATOM 23347 NZ LYS C 211   31.248 37.557 67.152 1.00 0.00
C   N
ATOM 23348 HZ1 LYS C 211   30.523 37.091 66.569 1.00 0.00
C   H
ATOM 23349 HZ2 LYS C 211   32.158 37.068 67.028 1.00 0.00
C   H
ATOM 23350 HZ3 LYS C 211   30.968 37.509 68.153 1.00 0.00
C   H
ATOM 23351 C   LYS C 211   33.545 44.056 68.561 1.00 0.00
C   C
ATOM 23352 O   LYS C 211   32.461 44.621 68.395 1.00 0.00
C   O
ATOM 23353 N   LYS C 212   34.264 44.163 69.678 1.00 0.00
C   N
ATOM 23354 HN LYS C 212   35.156 43.727 69.775 1.00 0.00
C   H

ATOM 23355 CA LYS C 212 33.813 44.921 70.842 1.00 0.00
C C
ATOM 23356 HA LYS C 212 32.769 44.676 70.995 1.00 0.00
C H
ATOM 23357 CB LYS C 212 34.637 44.544 72.075 1.00 0.00
C C
ATOM 23358 HB1 LYS C 212 35.604 45.096 72.004 1.00 0.00
C H
ATOM 23359 HB2 LYS C 212 34.125 44.929 72.985 1.00 0.00
C H
ATOM 23360 CG LYS C 212 34.916 43.055 72.195 1.00 0.00
C C
ATOM 23361 HG1 LYS C 212 34.000 42.489 71.913 1.00 0.00
C H
ATOM 23362 HG2 LYS C 212 35.716 42.776 71.470 1.00 0.00
C H
ATOM 23363 CD LYS C 212 35.322 42.677 73.606 1.00 0.00
C C
ATOM 23364 HD1 LYS C 212 36.094 43.400 73.956 1.00 0.00
C H
ATOM 23365 HD2 LYS C 212 34.431 42.793 74.266 1.00 0.00
C H
ATOM 23366 CE LYS C 212 35.845 41.251 73.650 1.00 0.00
C C
ATOM 23367 HE1 LYS C 212 35.110 40.552 73.193 1.00 0.00
C H
ATOM 23368 HE2 LYS C 212 36.803 41.169 73.092 1.00 0.00
C H
ATOM 23369 NZ LYS C 212 36.092 40.803 75.048 1.00 0.00
C N
ATOM 23370 HZ1 LYS C 212 36.447 39.825 75.051 1.00 0.00
C H
ATOM 23371 HZ2 LYS C 212 36.794 41.427 75.494 1.00 0.00
C H
ATOM 23372 HZ3 LYS C 212 35.203 40.848 75.586 1.00 0.00
C H
ATOM 23373 C LYS C 212 33.897 46.420 70.598 1.00 0.00
C C
ATOM 23374 O LYS C 212 33.065 47.188 71.087 1.00 0.00
C O
ATOM 23375 N ASP C 213 34.911 46.827 69.838 1.00 0.00
C N
ATOM 23376 HN ASP C 213 35.612 46.225 69.463 1.00 0.00
C H
ATOM 23377 CA ASP C 213 35.104 48.223 69.469 1.00 0.00
C C
ATOM 23378 HA ASP C 213 35.055 48.801 70.384 1.00 0.00
C H
ATOM 23379 CB ASP C 213 36.462 48.406 68.785 1.00 0.00
C C
ATOM 23380 HB1 ASP C 213 36.581 47.634 67.999 1.00 0.00
C H
ATOM 23381 HB2 ASP C 213 36.528 49.403 68.307 1.00 0.00
C H

ATOM 23382 CG ASP C 213   37.631 48.256 69.748 1.00 0.00
C   C
ATOM 23383 OD1 ASP C 213   38.685 47.737 69.327 1.00 0.00
C   O
ATOM 23384 OD2 ASP C 213   37.504 48.661 70.923 1.00 0.00
C   O
ATOM 23385 C   ASP C 213   33.974 48.725 68.569 1.00 0.00
C   C
ATOM 23386 O   ASP C 213   33.452 49.823 68.780 1.00 0.00
C   O
ATOM 23387 N   ILE C 214   33.604 47.910 67.580 1.00 0.00
C   N
ATOM 23388 HN ILE C 214   34.069 47.049 67.384 1.00 0.00
C   H
ATOM 23389 CA ILE C 214   32.479 48.202 66.687 1.00 0.00
C   C
ATOM 23390 HA ILE C 214   32.671 49.171 66.244 1.00 0.00
C   H
ATOM 23391 CB ILE C 214   32.301 47.104 65.609 1.00 0.00
C   C
ATOM 23392 HB ILE C 214   32.197 46.154 66.191 1.00 0.00
C   H
ATOM 23393 CG2 ILE C 214   31.091 47.400 64.727 1.00 0.00
C   C
ATOM 23394 HG21 ILE C 214   30.873 46.538 64.061 1.00 0.00
C   H
ATOM 23395 HG22 ILE C 214   30.187 47.585 65.344 1.00 0.00
C   H
ATOM 23396 HG23 ILE C 214   31.271 48.292 64.092 1.00 0.00
C   H
ATOM 23397 CG1 ILE C 214   33.573 46.970 64.766 1.00 0.00
C   C
ATOM 23398 HG11 ILE C 214   33.631 47.808 64.036 1.00 0.00
C   H
ATOM 23399 HG12 ILE C 214   34.459 47.055 65.435 1.00 0.00
C   H
ATOM 23400 CD ILE C 214   33.699 45.667 64.006 1.00 0.00
C   C
ATOM 23401 HD1 ILE C 214   34.671 45.626 63.469 1.00 0.00
C   H
ATOM 23402 HD2 ILE C 214   33.649 44.801 64.701 1.00 0.00
C   H
ATOM 23403 HD3 ILE C 214   32.887 45.565 63.255 1.00 0.00
C   H
ATOM 23404 C   ILE C 214   31.197 48.337 67.505 1.00 0.00
C   C
ATOM 23405 O   ILE C 214   30.479 49.327 67.397 1.00 0.00
C   O
ATOM 23406 N   LYS C 215   30.948 47.332 68.337 1.00 0.00
C   N
ATOM 23407 HN LYS C 215   31.562 46.547 68.393 1.00 0.00
C   H
ATOM 23408 CA LYS C 215   29.794 47.277 69.220 1.00 0.00
C   C

ATOM 23409 HA LYS C 215   28.918 47.302 68.582 1.00 0.00
C   H
ATOM 23410 CB LYS C 215   29.918 46.008 70.035 1.00 0.00
C   C
ATOM 23411 HB1 LYS C 215   30.495 45.279 69.420 1.00 0.00
C   H
ATOM 23412 HB2 LYS C 215   30.537 46.215 70.936 1.00 0.00
C   H
ATOM 23413 CG LYS C 215   28.594 45.371 70.436 1.00 0.00
C   C
ATOM 23414 HG1 LYS C 215   28.069 46.042 71.153 1.00 0.00
C   H
ATOM 23415 HG2 LYS C 215   27.947 45.280 69.532 1.00 0.00
C   H
ATOM 23416 CD LYS C 215   28.970 44.045 71.054 1.00 0.00
C   C
ATOM 23417 HD1 LYS C 215   29.484 43.430 70.280 1.00 0.00
C   H
ATOM 23418 HD2 LYS C 215   29.705 44.239 71.869 1.00 0.00
C   H
ATOM 23419 CE LYS C 215   27.703 43.339 71.596 1.00 0.00
C   C
ATOM 23420 HE1 LYS C 215   27.158 44.004 72.302 1.00 0.00
C   H
ATOM 23421 HE2 LYS C 215   27.017 43.073 70.762 1.00 0.00
C   H
ATOM 23422 NZ LYS C 215   27.938 42.139 72.300 1.00 0.00
C   N
ATOM 23423 HZ1 LYS C 215   27.036 41.735 72.628 1.00 0.00
C   H
ATOM 23424 HZ2 LYS C 215   28.414 41.457 71.676 1.00 0.00
C   H
ATOM 23425 HZ3 LYS C 215   28.546 42.333 73.121 1.00 0.00
C   H
ATOM 23426 C   LYS C 215   29.711 48.481 70.157 1.00 0.00
C   C
ATOM 23427 O   LYS C 215   28.616 48.934 70.499 1.00 0.00
C   O
ATOM 23428 N   ARG C 216   30.871 48.990 70.573 1.00 0.00
C   N
ATOM 23429 HN ARG C 216   31.739 48.549 70.354 1.00 0.00
C   H
ATOM 23430 CA ARG C 216   30.939 50.214 71.367 1.00 0.00
C   C
ATOM 23431 HA ARG C 216   30.188 50.161 72.147 1.00 0.00
C   H
ATOM 23432 CB ARG C 216   32.321 50.392 72.004 1.00 0.00
C   C
ATOM 23433 HB1 ARG C 216   32.591 49.406 72.453 1.00 0.00
C   H
ATOM 23434 HB2 ARG C 216   33.075 50.583 71.210 1.00 0.00
C   H
ATOM 23435 CG ARG C 216   32.357 51.462 73.091 1.00 0.00
C   C

ATOM 23436 HG1 ARG C 216   31.932 52.402 72.676 1.00 0.00
C   H
ATOM 23437 HG2 ARG C 216   31.671 51.164 73.916 1.00 0.00
C   H
ATOM 23438 CD ARG C 216   33.747 51.667 73.676 1.00 0.00
C   C
ATOM 23439 HD1 ARG C 216   34.383 52.210 72.939 1.00 0.00
C   H
ATOM 23440 HD2 ARG C 216   33.710 52.259 74.620 1.00 0.00
C   H
ATOM 23441 NE ARG C 216   34.392 50.398 74.011 1.00 0.00
C   N
ATOM 23442 HE ARG C 216   33.964 49.930 74.793 1.00 0.00
C   H
ATOM 23443 CZ ARG C 216   35.439 49.892 73.365 1.00 0.00
C   C
ATOM 23444 NH1 ARG C 216   35.988 50.552 72.352 1.00 0.00
C   N
ATOM 23445 HH11 ARG C 216   36.751 50.166 71.852 1.00 0.00
C   H
ATOM 23446 HH12 ARG C 216   35.505 51.374 72.045 1.00 0.00
C   H
ATOM 23447 NH2 ARG C 216   35.944 48.723 73.741 1.00 0.00
C   N
ATOM 23448 HH21 ARG C 216   36.774 48.385 73.320 1.00 0.00
C   H
ATOM 23449 HH22 ARG C 216   35.588 48.329 74.578 1.00 0.00
C   H
ATOM 23450 C   ARG C 216   30.598 51.431 70.516 1.00 0.00
C   C
ATOM 23451 O   ARG C 216   29.845 52.305 70.951 1.00 0.00
C   O
ATOM 23452 N   PHE C 217   31.159 51.485 69.306 1.00 0.00
C   N
ATOM 23453 HN PHE C 217   31.804 50.801 68.968 1.00 0.00
C   H
ATOM 23454 CA PHE C 217   30.870 52.564 68.362 1.00 0.00
C   C
ATOM 23455 HA PHE C 217   31.143 53.484 68.864 1.00 0.00
C   H
ATOM 23456 CB PHE C 217   31.691 52.407 67.074 1.00 0.00
C   C
ATOM 23457 HB1 PHE C 217   32.725 52.770 67.255 1.00 0.00
C   H
ATOM 23458 HB2 PHE C 217   31.767 51.339 66.776 1.00 0.00
C   H
ATOM 23459 CG PHE C 217   31.162 53.208 65.917 1.00 0.00
C   C
ATOM 23460 CD1 PHE C 217   31.232 54.599 65.919 1.00 0.00
C   C
ATOM 23461 HD1 PHE C 217   31.674 55.111 66.762 1.00 0.00
C   H
ATOM 23462 CE1 PHE C 217   30.735 55.336 64.856 1.00 0.00
C   C

```
ATOM  23463  HE1 PHE C 217      30.797  56.415  64.870  1.00  0.00
C    H
ATOM  23464  CZ  PHE C 217      30.165  54.688  63.771  1.00  0.00
C    C
ATOM  23465  HZ  PHE C 217      29.787  55.256  62.933  1.00  0.00
C    H
ATOM  23466  CD2 PHE C 217      30.586  52.574  64.829  1.00  0.00
C    C
ATOM  23467  HD2 PHE C 217      30.499  51.496  64.816  1.00  0.00
C    H
ATOM  23468  CE2 PHE C 217      30.090  53.307  63.760  1.00  0.00
C    C
ATOM  23469  HE2 PHE C 217      29.644  52.796  62.918  1.00  0.00
C    H
ATOM  23470  C   PHE C 217      29.374  52.647  68.053  1.00  0.00
C    C
ATOM  23471  O   PHE C 217      28.820  53.743  67.936  1.00  0.00
C    O
ATOM  23472  N   GLU C 218      28.737  51.484  67.928  1.00  0.00
C    N
ATOM  23473  HN  GLU C 218      29.198  50.600  67.953  1.00  0.00
C    H
ATOM  23474  CA  GLU C 218      27.288  51.394  67.743  1.00  0.00
C    C
ATOM  23475  HA  GLU C 218      27.020  51.970  66.866  1.00  0.00
C    H
ATOM  23476  CB  GLU C 218      26.850  49.936  67.545  1.00  0.00
C    C
ATOM  23477  HB1 GLU C 218      27.190  49.348  68.428  1.00  0.00
C    H
ATOM  23478  HB2 GLU C 218      25.739  49.873  67.546  1.00  0.00
C    H
ATOM  23479  CG  GLU C 218      27.396  49.282  66.286  1.00  0.00
C    C
ATOM  23480  HG1 GLU C 218      27.088  49.869  65.402  1.00  0.00
C    H
ATOM  23481  HG2 GLU C 218      28.503  49.222  66.317  1.00  0.00
C    H
ATOM  23482  CD  GLU C 218      26.847  47.884  66.062  1.00  0.00
C    C
ATOM  23483  OE1 GLU C 218      25.614  47.736  65.919  1.00  0.00
C    O
ATOM  23484  OE2 GLU C 218      27.655  46.936  66.015  1.00  0.00
C    O
ATOM  23485  C   GLU C 218      26.550  52.010  68.933  1.00  0.00
C    C
ATOM  23486  O   GLU C 218      25.677  52.860  68.755  1.00  0.00
C    O
ATOM  23487  N   ASN C 219      26.925  51.585  70.141  1.00  0.00
C    N
ATOM  23488  HN  ASN C 219      27.632  50.890  70.269  1.00  0.00
C    H
ATOM  23489  CA  ASN C 219      26.342  52.093  71.385  1.00  0.00
C    C
```

ATOM 23490 HA ASN C 219   25.298 51.803 71.329 1.00 0.00
C   H
ATOM 23491 CB ASN C 219   27.020 51.447 72.600 1.00 0.00
C   C
ATOM 23492 HB1 ASN C 219   28.049 51.155 72.299 1.00 0.00
C   H
ATOM 23493 HB2 ASN C 219   27.110 52.181 73.427 1.00 0.00
C   H
ATOM 23494 CG ASN C 219   26.301 50.203 73.080 1.00 0.00
C   C
ATOM 23495 OD1 ASN C 219   25.074 50.180 73.185 1.00 0.00
C   O
ATOM 23496 ND2 ASN C 219   27.066 49.165 73.392 1.00 0.00
C   N
ATOM 23497 HD21 ASN C 219   26.608 48.334 73.699 1.00 0.00
C   H
ATOM 23498 HD22 ASN C 219   28.053 49.224 73.287 1.00 0.00
C   H
ATOM 23499 C   ASN C 219   26.392 53.613 71.540 1.00 0.00
C   C
ATOM 23500 O   ASN C 219   25.453 54.217 72.063 1.00 0.00
C   O
ATOM 23501 N   GLU C 220   27.484 54.221 71.078 1.00 0.00
C   N
ATOM 23502 HN GLU C 220   28.176 53.775 70.516 1.00 0.00
C   H
ATOM 23503 CA GLU C 220   27.767 55.630 71.363 1.00 0.00
C   C
ATOM 23504 HA GLU C 220   27.102 55.917 72.168 1.00 0.00
C   H
ATOM 23505 CB GLU C 220   29.197 55.788 71.900 1.00 0.00
C   C
ATOM 23506 HB1 GLU C 220   29.886 55.233 71.221 1.00 0.00
C   H
ATOM 23507 HB2 GLU C 220   29.507 56.855 71.841 1.00 0.00
C   H
ATOM 23508 CG GLU C 220   29.373 55.270 73.323 1.00 0.00
C   C
ATOM 23509 HG1 GLU C 220   28.832 55.930 74.025 1.00 0.00
C   H
ATOM 23510 HG2 GLU C 220   28.984 54.237 73.420 1.00 0.00
C   H
ATOM 23511 CD GLU C 220   30.817 55.277 73.794 1.00 0.00
C   C
ATOM 23512 OE1 GLU C 220   31.733 55.286 72.945 1.00 0.00
C   O
ATOM 23513 OE2 GLU C 220   31.036 55.264 75.022 1.00 0.00
C   O
ATOM 23514 C   GLU C 220   27.515 56.593 70.200 1.00 0.00
C   C
ATOM 23515 O   GLU C 220   26.917 57.656 70.393 1.00 0.00
C   O
ATOM 23516 N   GLY C 221   27.965 56.226 69.004 1.00 0.00
C   N

ATOM 23517 HN GLY C 221   28.451 55.366 68.854 1.00 0.00
C   H
ATOM 23518 CA GLY C 221   27.776 57.062 67.819 1.00 0.00
C   C
ATOM 23519 HA1 GLY C 221   28.509 56.734 67.095 1.00 0.00
C   H
ATOM 23520 HA2 GLY C 221   27.899 58.087 68.142 1.00 0.00
C   H
ATOM 23521 C   GLY C 221   26.401 56.930 67.190 1.00 0.00
C   C
ATOM 23522 O   GLY C 221   26.044 57.706 66.304 1.00 0.00
C   O
ATOM 23523 N   MET C 222   25.634 55.944 67.645 1.00 0.00
C   N
ATOM 23524 HN MET C 222   25.914 55.339 68.388 1.00 0.00
C   H
ATOM 23525 CA MET C 222   24.311 55.656 67.099 1.00 0.00
C   C
ATOM 23526 HA MET C 222   24.009 56.519 66.518 1.00 0.00
C   H
ATOM 23527 CB MET C 222   24.353 54.399 66.226 1.00 0.00
C   C
ATOM 23528 HB1 MET C 222   24.765 53.579 66.858 1.00 0.00
C   H
ATOM 23529 HB2 MET C 222   23.314 54.097 65.963 1.00 0.00
C   H
ATOM 23530 CG MET C 222   25.205 54.530 64.975 1.00 0.00
C   C
ATOM 23531 HG1 MET C 222   24.632 55.037 64.170 1.00 0.00
C   H
ATOM 23532 HG2 MET C 222   26.099 55.153 65.196 1.00 0.00
C   H
ATOM 23533 SD MET C 222   25.753 52.936 64.341 1.00 0.00
C   S
ATOM 23534 CE MET C 222   26.583 53.450 62.843 1.00 0.00
C   C
ATOM 23535 HE1 MET C 222   26.990 52.574 62.295 1.00 0.00
C   H
ATOM 23536 HE2 MET C 222   25.883 53.983 62.164 1.00 0.00
C   H
ATOM 23537 HE3 MET C 222   27.428 54.134 63.069 1.00 0.00
C   H
ATOM 23538 C   MET C 222   23.294 55.475 68.217 1.00 0.00
C   C
ATOM 23539 O   MET C 222   22.138 55.875 68.085 1.00 0.00
C   O
ATOM 23540 N   GLN D   8   17.433 37.941 77.332 1.00 0.00
D   N
ATOM 23541 HT1 GLN D   8   17.730 38.604 78.019 1.00 0.00
D   H
ATOM 23542 HT2 GLN D   8   18.060 37.163 77.314 1.00 0.00
D   H
ATOM 23543 HT3 GLN D   8   16.505 37.627 77.540 1.00 0.00
D   H

ATOM 23544 CA GLN D   8   17.435 38.595 75.993 1.00 0.00
D   C
ATOM 23545 HA GLN D   8   16.713 39.401 76.050 1.00 0.00
D   H
ATOM 23546 CB GLN D   8   18.821 39.172 75.702 1.00 0.00
D   C
ATOM 23547 HB1 GLN D   8   19.315 39.336 76.688 1.00 0.00
D   H
ATOM 23548 HB2 GLN D   8   19.434 38.409 75.171 1.00 0.00
D   H
ATOM 23549 CG GLN D   8   18.802 40.479 74.928 1.00 0.00
D   C
ATOM 23550 HG1 GLN D   8   18.499 40.300 73.873 1.00 0.00
D   H
ATOM 23551 HG2 GLN D   8   18.077 41.189 75.375 1.00 0.00
D   H
ATOM 23552 CD GLN D   8   20.165 41.147 74.872 1.00 0.00
D   C
ATOM 23553 OE1 GLN D   8   21.127 40.691 75.491 1.00 0.00
D   O
ATOM 23554 NE2 GLN D   8   20.249 42.242 74.126 1.00 0.00
D   N
ATOM 23555 HE21 GLN D   8   21.139 42.687 74.078 1.00 0.00
D   H
ATOM 23556 HE22 GLN D   8   19.440 42.587 73.662 1.00 0.00
D   H
ATOM 23557 C   GLN D   8   16.988 37.650 74.858 1.00 0.00
D   C
ATOM 23558 O   GLN D   8   16.301 38.078 73.925 1.00 0.00
D   O
ATOM 23559 N   PHE D   9   17.377 36.375 74.941 1.00 0.00
D   N
ATOM 23560 HN PHE D   9   17.945 36.021 75.681 1.00 0.00
D   H
ATOM 23561 CA PHE D   9   16.996 35.375 73.936 1.00 0.00
D   C
ATOM 23562 HA PHE D   9   16.190 35.826 73.370 1.00 0.00
D   H
ATOM 23563 CB PHE D   9   18.180 35.002 73.036 1.00 0.00
D   C
ATOM 23564 HB1 PHE D   9   18.948 34.478 73.645 1.00 0.00
D   H
ATOM 23565 HB2 PHE D   9   17.866 34.302 72.231 1.00 0.00
D   H
ATOM 23566 CG PHE D   9   18.874 36.174 72.419 1.00 0.00
D   C
ATOM 23567 CD1 PHE D   9   18.423 36.717 71.227 1.00 0.00
D   C
ATOM 23568 HD1 PHE D   9   17.561 36.286 70.736 1.00 0.00
D   H
ATOM 23569 CE1 PHE D   9   19.068 37.802 70.655 1.00 0.00
D   C
ATOM 23570 HE1 PHE D   9   18.701 38.223 69.731 1.00 0.00
D   H

```
ATOM  23571  CZ  PHE D   9      20.180  38.348  71.275  1.00  0.00
D    C
ATOM  23572  HZ  PHE D   9      20.682  39.197  70.833  1.00  0.00
D    H
ATOM  23573  CD2 PHE D   9      19.991  36.730  73.028  1.00  0.00
D    C
ATOM  23574  HD2 PHE D   9      20.375  36.304  73.944  1.00  0.00
D    H
ATOM  23575  CE2 PHE D   9      20.643  37.813  72.465  1.00  0.00
D    C
ATOM  23576  HE2 PHE D   9      21.510  38.234  72.956  1.00  0.00
D    H
ATOM  23577  C   PHE D   9      16.445  34.095  74.555  1.00  0.00
D    C
ATOM  23578  O   PHE D   9      16.989  33.574  75.527  1.00  0.00
D    O
ATOM  23579  N   ALA D  10      15.363  33.592  73.971  1.00  0.00
D    N
ATOM  23580  HN  ALA D  10      14.841  34.097  73.284  1.00  0.00
D    H
ATOM  23581  CA  ALA D  10      14.858  32.268  74.291  1.00  0.00
D    C
ATOM  23582  HA  ALA D  10      15.527  31.731  74.955  1.00  0.00
D    H
ATOM  23583  CB  ALA D  10      13.496  32.360  74.953  1.00  0.00
D    C
ATOM  23584  HB1 ALA D  10      13.584  32.914  75.912  1.00  0.00
D    H
ATOM  23585  HB2 ALA D  10      12.774  32.906  74.308  1.00  0.00
D    H
ATOM  23586  HB3 ALA D  10      13.080  31.354  75.176  1.00  0.00
D    H
ATOM  23587  C   ALA D  10      14.784  31.453  73.005  1.00  0.00
D    C
ATOM  23588  O   ALA D  10      14.355  31.959  71.964  1.00  0.00
D    O
ATOM  23589  N   VAL D  11      15.233  30.203  73.076  1.00  0.00
D    N
ATOM  23590  HN  VAL D  11      15.656  29.820  73.893  1.00  0.00
D    H
ATOM  23591  CA  VAL D  11      15.142  29.274  71.948  1.00  0.00
D    C
ATOM  23592  HA  VAL D  11      14.679  29.786  71.114  1.00  0.00
D    H
ATOM  23593  CB  VAL D  11      16.531  28.744  71.524  1.00  0.00
D    C
ATOM  23594  HB  VAL D  11      17.004  28.278  72.424  1.00  0.00
D    H
ATOM  23595  CG1 VAL D  11      16.404  27.692  70.433  1.00  0.00
D    C
ATOM  23596 HG11 VAL D  11      17.409  27.347  70.110  1.00  0.00
D    H
ATOM  23597 HG12 VAL D  11      15.847  26.800  70.787  1.00  0.00
D    H
```

```
ATOM  23598 HG13 VAL D  11      15.882  28.109  69.545  1.00  0.00
D    H
ATOM  23599  CG2 VAL D  11      17.419  29.887  71.051  1.00  0.00
D    C
ATOM  23600 HG21 VAL D  11      18.404  29.504  70.708  1.00  0.00
D    H
ATOM  23601 HG22 VAL D  11      16.941  30.429  70.207  1.00  0.00
D    H
ATOM  23602 HG23 VAL D  11      17.609  30.614  71.869  1.00  0.00
D    H
ATOM  23603  C   VAL D  11      14.223  28.123  72.344  1.00  0.00
D    C
ATOM  23604  O   VAL D  11      14.503  27.392  73.303  1.00  0.00
D    O
ATOM  23605  N   ILE D  12      13.125  27.963  71.611  1.00  0.00
D    N
ATOM  23606  HN  ILE D  12      12.917  28.449  70.765  1.00  0.00
D    H
ATOM  23607  CA  ILE D  12      12.098  27.016  72.021  1.00  0.00
D    C
ATOM  23608  HA  ILE D  12      12.188  26.978  73.099  1.00  0.00
D    H
ATOM  23609  CB  ILE D  12      10.672  27.576  71.845  1.00  0.00
D    C
ATOM  23610  HB  ILE D  12      10.699  28.106  70.860  1.00  0.00
D    H
ATOM  23611  CG2 ILE D  12       9.641  26.465  71.944  1.00  0.00
D    C
ATOM  23612 HG21 ILE D  12       8.634  26.839  71.664  1.00  0.00
D    H
ATOM  23613 HG22 ILE D  12       9.892  25.629  71.257  1.00  0.00
D    H
ATOM  23614 HG23 ILE D  12       9.586  26.064  72.978  1.00  0.00
D    H
ATOM  23615  CG1 ILE D  12      10.415  28.634  72.929  1.00  0.00
D    C
ATOM  23616 HG11 ILE D  12      10.579  28.189  73.936  1.00  0.00
D    H
ATOM  23617 HG12 ILE D  12      11.155  29.459  72.810  1.00  0.00
D    H
ATOM  23618  CD  ILE D  12       9.036  29.262  72.911  1.00  0.00
D    C
ATOM  23619  HD1 ILE D  12       8.961  30.053  73.690  1.00  0.00
D    H
ATOM  23620  HD2 ILE D  12       8.829  29.726  71.922  1.00  0.00
D    H
ATOM  23621  HD3 ILE D  12       8.251  28.504  73.115  1.00  0.00
D    H
ATOM  23622  C   ILE D  12      12.263  25.585  71.513  1.00  0.00
D    C
ATOM  23623  O   ILE D  12      11.814  24.651  72.168  1.00  0.00
D    O
ATOM  23624  N   GLY D  13      12.915  25.389  70.378  1.00  0.00
D    N
```

ATOM 23625 HN GLY D 13 13.239 26.104 69.759 1.00 0.00
D H
ATOM 23626 CA GLY D 13 13.203 24.012 69.963 1.00 0.00
D C
ATOM 23627 HA1 GLY D 13 12.772 23.923 68.975 1.00 0.00
D H
ATOM 23628 HA2 GLY D 13 12.734 23.386 70.711 1.00 0.00
D H
ATOM 23629 C GLY D 13 14.670 23.616 69.873 1.00 0.00
D C
ATOM 23630 O GLY D 13 15.376 24.047 68.961 1.00 0.00
D O
ATOM 23631 N LEU D 14 15.140 22.778 70.797 1.00 0.00
D N
ATOM 23632 HN LEU D 14 14.605 22.364 71.530 1.00 0.00
D H
ATOM 23633 CA LEU D 14 16.557 22.391 70.791 1.00 0.00
D C
ATOM 23634 HA LEU D 14 17.103 23.211 70.342 1.00 0.00
D H
ATOM 23635 CB LEU D 14 17.041 22.178 72.229 1.00 0.00
D C
ATOM 23636 HB1 LEU D 14 16.545 21.255 72.609 1.00 0.00
D H
ATOM 23637 HB2 LEU D 14 18.132 21.958 72.215 1.00 0.00
D H
ATOM 23638 CG LEU D 14 16.753 23.317 73.212 1.00 0.00
D C
ATOM 23639 HG LEU D 14 15.680 23.585 73.054 1.00 0.00
D H
ATOM 23640 CD1 LEU D 14 16.951 22.863 74.650 1.00 0.00
D C
ATOM 23641 HD11 LEU D 14 16.786 23.709 75.351 1.00 0.00
D H
ATOM 23642 HD12 LEU D 14 16.236 22.052 74.907 1.00 0.00
D H
ATOM 23643 HD13 LEU D 14 17.983 22.483 74.803 1.00 0.00
D H
ATOM 23644 CD2 LEU D 14 17.598 24.545 72.895 1.00 0.00
D C
ATOM 23645 HD21 LEU D 14 17.363 25.369 73.605 1.00 0.00
D H
ATOM 23646 HD22 LEU D 14 18.682 24.329 72.978 1.00 0.00
D H
ATOM 23647 HD23 LEU D 14 17.390 24.905 71.864 1.00 0.00
D H
ATOM 23648 C LEU D 14 16.785 21.152 69.920 1.00 0.00
D C
ATOM 23649 O LEU D 14 17.045 20.070 70.430 1.00 0.00
D O
ATOM 23650 N GLY D 15 16.695 21.321 68.603 1.00 0.00
D N
ATOM 23651 HN GLY D 15 16.452 22.198 68.190 1.00 0.00
D H

ATOM 23652 CA GLY D 15 16.948 20.229 67.665 1.00 0.00
D C
ATOM 23653 HA1 GLY D 15 16.122 20.158 66.971 1.00 0.00
D H
ATOM 23654 HA2 GLY D 15 17.145 19.319 68.217 1.00 0.00
D H
ATOM 23655 C GLY D 15 18.197 20.574 66.874 1.00 0.00
D C
ATOM 23656 O GLY D 15 19.016 21.392 67.314 1.00 0.00
D O
ATOM 23657 N ARG D 16 18.348 19.956 65.704 1.00 0.00
D N
ATOM 23658 HN ARG D 16 17.703 19.262 65.392 1.00 0.00
D H
ATOM 23659 CA ARG D 16 19.464 20.260 64.813 1.00 0.00
D C
ATOM 23660 HA ARG D 16 20.379 19.916 65.282 1.00 0.00
D H
ATOM 23661 CB ARG D 16 19.290 19.561 63.470 1.00 0.00
D C
ATOM 23662 HB1 ARG D 16 18.200 19.344 63.367 1.00 0.00
D H
ATOM 23663 HB2 ARG D 16 19.541 20.267 62.648 1.00 0.00
D H
ATOM 23664 CG ARG D 16 20.063 18.260 63.342 1.00 0.00
D C
ATOM 23665 HG1 ARG D 16 20.941 18.433 62.682 1.00 0.00
D H
ATOM 23666 HG2 ARG D 16 20.486 17.991 64.337 1.00 0.00
D H
ATOM 23667 CD ARG D 16 19.185 17.115 62.862 1.00 0.00
D C
ATOM 23668 HD1 ARG D 16 19.812 16.206 62.712 1.00 0.00
D H
ATOM 23669 HD2 ARG D 16 18.383 16.878 63.599 1.00 0.00
D H
ATOM 23670 NE ARG D 16 18.493 17.401 61.606 1.00 0.00
D N
ATOM 23671 HE ARG D 16 19.048 17.942 60.964 1.00 0.00
D H
ATOM 23672 CZ ARG D 16 17.257 16.996 61.325 1.00 0.00
D C
ATOM 23673 NH1 ARG D 16 16.563 16.289 62.215 1.00 0.00
D N
ATOM 23674 HH11 ARG D 16 15.652 15.963 62.001 1.00 0.00
D H
ATOM 23675 HH12 ARG D 16 17.060 15.997 63.035 1.00 0.00
D H
ATOM 23676 NH2 ARG D 16 16.705 17.304 60.160 1.00 0.00
D N
ATOM 23677 HH21 ARG D 16 15.757 17.078 59.987 1.00 0.00
D H
ATOM 23678 HH22 ARG D 16 17.203 17.938 59.583 1.00 0.00
D H

ATOM 23679 C   ARG D 16   19.613 21.761 64.618 1.00 0.00
D   C
ATOM 23680 O   ARG D 16   20.726 22.285 64.692 1.00 0.00
D   O
ATOM 23681 N   PHE D 17   18.490 22.448 64.410 1.00 0.00
D   N
ATOM 23682 HN PHE D 17   17.583 22.030 64.421 1.00 0.00
D   H
ATOM 23683 CA PHE D 17   18.491 23.882 64.149 1.00 0.00
D   C
ATOM 23684 HA PHE D 17   19.426 24.077 63.637 1.00 0.00
D   H
ATOM 23685 CB PHE D 17   17.313 24.255 63.252 1.00 0.00
D   C
ATOM 23686 HB1 PHE D 17   17.395 23.698 62.293 1.00 0.00
D   H
ATOM 23687 HB2 PHE D 17   16.347 23.963 63.719 1.00 0.00
D   H
ATOM 23688 CG PHE D 17   17.265 25.710 62.887 1.00 0.00
D   C
ATOM 23689 CD1 PHE D 17   18.284 26.285 62.134 1.00 0.00
D   C
ATOM 23690 HD1 PHE D 17   19.120 25.679 61.814 1.00 0.00
D   H
ATOM 23691 CE1 PHE D 17   18.242 27.629 61.794 1.00 0.00
D   C
ATOM 23692 HE1 PHE D 17   19.041 28.063 61.209 1.00 0.00
D   H
ATOM 23693 CZ PHE D 17   17.172 28.408 62.202 1.00 0.00
D   C
ATOM 23694 HZ PHE D 17   17.130 29.453 61.929 1.00 0.00
D   H
ATOM 23695 CD2 PHE D 17   16.197 26.505 63.291 1.00 0.00
D   C
ATOM 23696 HD2 PHE D 17   15.406 26.082 63.893 1.00 0.00
D   H
ATOM 23697 CE2 PHE D 17   16.149 27.845 62.952 1.00 0.00
D   C
ATOM 23698 HE2 PHE D 17   15.314 28.453 63.272 1.00 0.00
D   H
ATOM 23699 C   PHE D 17   18.490 24.732 65.420 1.00 0.00
D   C
ATOM 23700 O   PHE D 17   19.380 25.561 65.619 1.00 0.00
D   O
ATOM 23701 N   GLY D 18   17.491 24.536 66.274 1.00 0.00
D   N
ATOM 23702 HN GLY D 18   16.778 23.849 66.138 1.00 0.00
D   H
ATOM 23703 CA GLY D 18   17.375 25.336 67.491 1.00 0.00
D   C
ATOM 23704 HA1 GLY D 18   16.503 24.975 68.020 1.00 0.00
D   H
ATOM 23705 HA2 GLY D 18   17.311 26.372 67.185 1.00 0.00
D   H

ATOM 23706 C   GLY D 18   18.572 25.195 68.408 1.00 0.00
D   C
ATOM 23707 O   GLY D 18   19.101 26.187 68.911 1.00 0.00
D   O
ATOM 23708 N   GLY D 19   19.003 23.955 68.617 1.00 0.00
D   N
ATOM 23709 HN GLY D 19   18.559 23.154 68.216 1.00 0.00
D   H
ATOM 23710 CA GLY D 19   20.162 23.681 69.450 1.00 0.00
D   C
ATOM 23711 HA1 GLY D 19   20.357 22.619 69.380 1.00 0.00
D   H
ATOM 23712 HA2 GLY D 19   19.927 24.024 70.448 1.00 0.00
D   H
ATOM 23713 C   GLY D 19   21.404 24.418 68.982 1.00 0.00
D   C
ATOM 23714 O   GLY D 19   22.192 24.908 69.794 1.00 0.00
D   O
ATOM 23715 N   SER D 20   21.572 24.497 67.667 1.00 0.00
D   N
ATOM 23716 HN SER D 20   20.952 24.101 66.990 1.00 0.00
D   H
ATOM 23717 CA SER D 20   22.710 25.191 67.095 1.00 0.00
D   C
ATOM 23718 HA SER D 20   23.584 24.801 67.600 1.00 0.00
D   H
ATOM 23719 CB SER D 20   22.805 24.938 65.592 1.00 0.00
D   C
ATOM 23720 HB1 SER D 20   22.906 23.840 65.431 1.00 0.00
D   H
ATOM 23721 HB2 SER D 20   21.866 25.257 65.085 1.00 0.00
D   H
ATOM 23722 OG SER D 20   23.936 25.589 65.036 1.00 0.00
D   O
ATOM 23723 HG1 SER D 20   24.072 25.219 64.155 1.00 0.00
D   H
ATOM 23724 C   SER D 20   22.648 26.689 67.386 1.00 0.00
D   C
ATOM 23725 O   SER D 20   23.662 27.289 67.747 1.00 0.00
D   O
ATOM 23726 N   ILE D 21   21.465 27.289 67.240 1.00 0.00
D   N
ATOM 23727 HN ILE D 21   20.634 26.841 66.918 1.00 0.00
D   H
ATOM 23728 CA ILE D 21   21.298 28.704 67.553 1.00 0.00
D   C
ATOM 23729 HA ILE D 21   22.013 29.252 66.954 1.00 0.00
D   H
ATOM 23730 CB ILE D 21   19.858 29.187 67.322 1.00 0.00
D   C
ATOM 23731 HB ILE D 21   19.220 28.420 67.828 1.00 0.00
D   H
ATOM 23732 CG2 ILE D 21   19.679 30.599 67.876 1.00 0.00
D   C

ATOM 23733 HG21 ILE D 21   18.610 30.894 67.854 1.00 0.00
D   H
ATOM 23734 HG22 ILE D 21   20.024 30.658 68.930 1.00 0.00
D   H
ATOM 23735 HG23 ILE D 21   20.255 31.337 67.277 1.00 0.00
D   H
ATOM 23736 CG1 ILE D 21   19.509 29.140 65.833 1.00 0.00
D   C
ATOM 23737 HG11 ILE D 21   20.161 29.847 65.272 1.00 0.00
D   H
ATOM 23738 HG12 ILE D 21   19.722 28.118 65.446 1.00 0.00
D   H
ATOM 23739 CD ILE D 21   18.062 29.464 65.531 1.00 0.00
D   C
ATOM 23740 HD1 ILE D 21   17.861 29.355 64.443 1.00 0.00
D   H
ATOM 23741 HD2 ILE D 21   17.383 28.775 66.079 1.00 0.00
D   H
ATOM 23742 HD3 ILE D 21   17.816 30.506 65.823 1.00 0.00
D   H
ATOM 23743 C   ILE D 21   21.672 28.938 69.008 1.00 0.00
D   C
ATOM 23744 O   ILE D 21   22.442 29.849 69.322 1.00 0.00
D   O
ATOM 23745 N   CYS D 22   21.125 28.094 69.879 1.00 0.00
D   N
ATOM 23746 HN CYS D 22   20.528 27.350 69.586 1.00 0.00
D   H
ATOM 23747 CA CYS D 22   21.341 28.172 71.318 1.00 0.00
D   C
ATOM 23748 HA CYS D 22   20.971 29.141 71.632 1.00 0.00
D   H
ATOM 23749 CB CYS D 22   20.587 27.040 72.018 1.00 0.00
D   C
ATOM 23750 HB1 CYS D 22   19.527 27.094 71.683 1.00 0.00
D   H
ATOM 23751 HB2 CYS D 22   20.970 26.057 71.666 1.00 0.00
D   H
ATOM 23752 SG CYS D 22   20.602 27.115 73.821 1.00 0.00
D   S
ATOM 23753 HG1 CYS D 22   19.820 26.056 74.038 1.00 0.00
D   H
ATOM 23754 C   CYS D 22   22.822 28.110 71.661 1.00 0.00
D   C
ATOM 23755 O   CYS D 22   23.363 29.043 72.261 1.00 0.00
D   O
ATOM 23756 N   LYS D 23   23.474 27.020 71.255 1.00 0.00
D   N
ATOM 23757 HN LYS D 23   23.026 26.275 70.762 1.00 0.00
D   H
ATOM 23758 CA LYS D 23   24.900 26.830 71.502 1.00 0.00
D   C
ATOM 23759 HA LYS D 23   25.015 26.740 72.575 1.00 0.00
D   H

ATOM 23760 CB LYS D 23 25.399 25.546 70.838 1.00 0.00
D C
ATOM 23761 HB1 LYS D 23 24.641 24.754 71.045 1.00 0.00
D H
ATOM 23762 HB2 LYS D 23 25.407 25.688 69.734 1.00 0.00
D H
ATOM 23763 CG LYS D 23 26.762 25.086 71.343 1.00 0.00
D C
ATOM 23764 HG1 LYS D 23 27.525 25.856 71.086 1.00 0.00
D H
ATOM 23765 HG2 LYS D 23 26.730 25.008 72.454 1.00 0.00
D H
ATOM 23766 CD LYS D 23 27.188 23.755 70.738 1.00 0.00
D C
ATOM 23767 HD1 LYS D 23 26.991 23.788 69.642 1.00 0.00
D H
ATOM 23768 HD2 LYS D 23 28.289 23.648 70.877 1.00 0.00
D H
ATOM 23769 CE LYS D 23 26.457 22.580 71.379 1.00 0.00
D C
ATOM 23770 HE1 LYS D 23 26.515 22.645 72.489 1.00 0.00
D H
ATOM 23771 HE2 LYS D 23 25.385 22.577 71.082 1.00 0.00
D H
ATOM 23772 NZ LYS D 23 27.040 21.269 70.975 1.00 0.00
D N
ATOM 23773 HZ1 LYS D 23 26.520 20.492 71.431 1.00 0.00
D H
ATOM 23774 HZ2 LYS D 23 26.979 21.166 69.942 1.00 0.00
D H
ATOM 23775 HZ3 LYS D 23 28.038 21.231 71.264 1.00 0.00
D H
ATOM 23776 C LYS D 23 25.744 28.025 71.050 1.00 0.00
D C
ATOM 23777 O LYS D 23 26.679 28.422 71.744 1.00 0.00
D O
ATOM 23778 N GLU D 24 25.400 28.606 69.904 1.00 0.00
D N
ATOM 23779 HN GLU D 24 24.621 28.339 69.344 1.00 0.00
D H
ATOM 23780 CA GLU D 24 26.167 29.719 69.360 1.00 0.00
D C
ATOM 23781 HA GLU D 24 27.212 29.453 69.454 1.00 0.00
D H
ATOM 23782 CB GLU D 24 25.909 29.889 67.857 1.00 0.00
D C
ATOM 23783 HB1 GLU D 24 26.094 28.909 67.360 1.00 0.00
D H
ATOM 23784 HB2 GLU D 24 24.835 30.122 67.683 1.00 0.00
D H
ATOM 23785 CG GLU D 24 26.773 30.949 67.172 1.00 0.00
D C
ATOM 23786 HG1 GLU D 24 26.454 31.062 66.120 1.00 0.00
D H

ATOM 23787 HG2 GLU D 24   26.685 31.927 67.686 1.00 0.00
D   H
ATOM 23788 CD GLU D 24   28.255 30.595 67.099 1.00 0.00
D   C
ATOM 23789 OE1 GLU D 24   29.045 31.452 66.640 1.00 0.00
D   O
ATOM 23790 OE2 GLU D 24   28.643 29.472 67.492 1.00 0.00
D   O
ATOM 23791 C   GLU D 24   25.964 31.041 70.104 1.00 0.00
D   C
ATOM 23792 O   GLU D 24   26.939 31.732 70.394 1.00 0.00
D   O
ATOM 23793 N   LEU D 25   24.715 31.391 70.420 1.00 0.00
D   N
ATOM 23794 HN LEU D 25   23.902 30.866 70.177 1.00 0.00
D   H
ATOM 23795 CA LEU D 25   24.437 32.616 71.175 1.00 0.00
D   C
ATOM 23796 HA LEU D 25   24.834 33.441 70.598 1.00 0.00
D   H
ATOM 23797 CB LEU D 25   22.939 32.784 71.414 1.00 0.00
D   C
ATOM 23798 HB1 LEU D 25   22.551 31.802 71.771 1.00 0.00
D   H
ATOM 23799 HB2 LEU D 25   22.784 33.500 72.252 1.00 0.00
D   H
ATOM 23800 CG LEU D 25   22.014 33.209 70.273 1.00 0.00
D   C
ATOM 23801 HG LEU D 25   22.202 32.517 69.417 1.00 0.00
D   H
ATOM 23802 CD1 LEU D 25   20.567 33.075 70.729 1.00 0.00
D   C
ATOM 23803 HD11 LEU D 25   19.874 33.424 69.935 1.00 0.00
D   H
ATOM 23804 HD12 LEU D 25   20.327 32.016 70.961 1.00 0.00
D   H
ATOM 23805 HD13 LEU D 25   20.385 33.685 71.640 1.00 0.00
D   H
ATOM 23806 CD2 LEU D 25   22.302 34.630 69.802 1.00 0.00
D   C
ATOM 23807 HD21 LEU D 25   21.628 34.902 68.961 1.00 0.00
D   H
ATOM 23808 HD22 LEU D 25   22.146 35.370 70.613 1.00 0.00
D   H
ATOM 23809 HD23 LEU D 25   23.352 34.716 69.447 1.00 0.00
D   H
ATOM 23810 C   LEU D 25   25.172 32.593 72.514 1.00 0.00
D   C
ATOM 23811 O   LEU D 25   25.698 33.611 72.975 1.00 0.00
D   O
ATOM 23812 N   HSE D 26   25.202 31.411 73.119 1.00 0.00
D   N
ATOM 23813 HN HSE D 26   24.749 30.605 72.738 1.00 0.00
D   H

ATOM 23814 CA HSE D 26 25.888 31.174 74.381 1.00 0.00
D C
ATOM 23815 HA HSE D 26 25.561 31.952 75.060 1.00 0.00
D H
ATOM 23816 CB HSE D 26 25.531 29.790 74.887 1.00 0.00
D C
ATOM 23817 HB1 HSE D 26 24.424 29.729 74.961 1.00 0.00
D H
ATOM 23818 HB2 HSE D 26 25.850 29.030 74.144 1.00 0.00
D H
ATOM 23819 ND1 HSE D 26 25.709 30.111 77.351 1.00 0.00
D N
ATOM 23820 CG HSE D 26 26.103 29.474 76.236 1.00 0.00
D C
ATOM 23821 CE1 HSE D 26 26.383 29.621 78.407 1.00 0.00
D C
ATOM 23822 HE1 HSE D 26 26.264 29.970 79.435 1.00 0.00
D H
ATOM 23823 NE2 HSE D 26 27.207 28.658 77.964 1.00 0.00
D N
ATOM 23824 HE2 HSE D 26 27.839 28.129 78.531 1.00 0.00
D H
ATOM 23825 CD2 HSE D 26 27.058 28.543 76.628 1.00 0.00
D C
ATOM 23826 HD2 HSE D 26 27.624 27.839 76.036 1.00 0.00
D H
ATOM 23827 C HSE D 26 27.379 31.303 74.247 1.00 0.00
D C
ATOM 23828 O HSE D 26 28.036 31.903 75.101 1.00 0.00
D O
ATOM 23829 N ARG D 27 27.924 30.734 73.174 1.00 0.00
D N
ATOM 23830 HN ARG D 27 27.363 30.219 72.531 1.00 0.00
D H
ATOM 23831 CA ARG D 27 29.347 30.823 72.875 1.00 0.00
D C
ATOM 23832 HA ARG D 27 29.901 30.510 73.753 1.00 0.00
D H
ATOM 23833 CB ARG D 27 29.694 29.915 71.690 1.00 0.00
D C
ATOM 23834 HB1 ARG D 27 29.122 28.968 71.841 1.00 0.00
D H
ATOM 23835 HB2 ARG D 27 29.302 30.364 70.751 1.00 0.00
D H
ATOM 23836 CG ARG D 27 31.171 29.572 71.559 1.00 0.00
D C
ATOM 23837 HG1 ARG D 27 31.769 30.446 71.898 1.00 0.00
D H
ATOM 23838 HG2 ARG D 27 31.415 28.748 72.268 1.00 0.00
D H
ATOM 23839 CD ARG D 27 31.542 29.121 70.148 1.00 0.00
D C
ATOM 23840 HD1 ARG D 27 32.642 28.959 70.089 1.00 0.00
D H

ATOM 23841 HD2 ARG D 27 31.026 28.171 69.874 1.00 0.00
D H
ATOM 23842 NE ARG D 27 31.161 30.097 69.124 1.00 0.00
D N
ATOM 23843 HE ARG D 27 30.313 29.851 68.639 1.00 0.00
D H
ATOM 23844 CZ ARG D 27 31.840 31.208 68.835 1.00 0.00
D C
ATOM 23845 NH1 ARG D 27 32.956 31.506 69.488 1.00 0.00
D N
ATOM 23846 HH11 ARG D 27 33.447 32.343 69.292 1.00 0.00
D H
ATOM 23847 HH12 ARG D 27 33.185 30.917 70.267 1.00 0.00
D H
ATOM 23848 NH2 ARG D 27 31.398 32.031 67.890 1.00 0.00
D N
ATOM 23849 HH21 ARG D 27 31.946 32.809 67.616 1.00 0.00
D H
ATOM 23850 HH22 ARG D 27 30.630 31.718 67.347 1.00 0.00
D H
ATOM 23851 C ARG D 27 29.760 32.272 72.588 1.00 0.00
D C
ATOM 23852 O ARG D 27 30.882 32.665 72.885 1.00 0.00
D O
ATOM 23853 N MET D 28 28.847 33.060 72.021 1.00 0.00
D N
ATOM 23854 HN MET D 28 27.943 32.736 71.749 1.00 0.00
D H
ATOM 23855 CA MET D 28 29.090 34.483 71.746 1.00 0.00
D C
ATOM 23856 HA MET D 28 30.100 34.554 71.362 1.00 0.00
D H
ATOM 23857 CB MET D 28 28.108 35.022 70.702 1.00 0.00
D C
ATOM 23858 HB1 MET D 28 27.085 34.737 71.041 1.00 0.00
D H
ATOM 23859 HB2 MET D 28 28.139 36.134 70.706 1.00 0.00
D H
ATOM 23860 CG MET D 28 28.313 34.492 69.289 1.00 0.00
D C
ATOM 23861 HG1 MET D 28 29.321 34.773 68.914 1.00 0.00
D H
ATOM 23862 HG2 MET D 28 28.254 33.382 69.294 1.00 0.00
D H
ATOM 23863 SD MET D 28 27.087 35.137 68.129 1.00 0.00
D S
ATOM 23864 CE MET D 28 27.899 36.641 67.582 1.00 0.00
D C
ATOM 23865 HE1 MET D 28 27.267 37.189 66.851 1.00 0.00
D H
ATOM 23866 HE2 MET D 28 28.099 37.320 68.438 1.00 0.00
D H
ATOM 23867 HE3 MET D 28 28.870 36.415 67.090 1.00 0.00
D H

ATOM 23868 C MET D 28 29.015 35.344 73.009 1.00 0.00
D C
ATOM 23869 O MET D 28 29.477 36.483 73.010 1.00 0.00
D O
ATOM 23870 N GLY D 29 28.414 34.808 74.069 1.00 0.00
D N
ATOM 23871 HN GLY D 29 27.962 33.916 74.037 1.00 0.00
D H
ATOM 23872 CA GLY D 29 28.373 35.490 75.361 1.00 0.00
D C
ATOM 23873 HA1 GLY D 29 29.103 36.283 75.279 1.00 0.00
D H
ATOM 23874 HA2 GLY D 29 28.622 34.731 76.089 1.00 0.00
D H
ATOM 23875 C GLY D 29 27.048 36.111 75.765 1.00 0.00
D C
ATOM 23876 O GLY D 29 26.972 36.772 76.801 1.00 0.00
D O
ATOM 23877 N HSE D 30 26.008 35.909 74.957 1.00 0.00
D N
ATOM 23878 HN HSE D 30 26.064 35.435 74.079 1.00 0.00
D H
ATOM 23879 CA HSE D 30 24.669 36.380 75.302 1.00 0.00
D C
ATOM 23880 HA HSE D 30 24.774 37.320 75.829 1.00 0.00
D H
ATOM 23881 CB HSE D 30 23.812 36.509 74.057 1.00 0.00
D C
ATOM 23882 HB1 HSE D 30 23.648 35.489 73.647 1.00 0.00
D H
ATOM 23883 HB2 HSE D 30 22.815 36.912 74.334 1.00 0.00
D H
ATOM 23884 ND1 HSE D 30 24.271 38.686 72.911 1.00 0.00
D N
ATOM 23885 CG HSE D 30 24.439 37.352 72.977 1.00 0.00
D C
ATOM 23886 CE1 HSE D 30 24.947 39.166 71.852 1.00 0.00
D C
ATOM 23887 HE1 HSE D 30 24.973 40.219 71.558 1.00 0.00
D H
ATOM 23888 NE2 HSE D 30 25.555 38.137 71.242 1.00 0.00
D N
ATOM 23889 HE2 HSE D 30 26.114 38.202 70.417 1.00 0.00
D H
ATOM 23890 CD2 HSE D 30 25.262 37.003 71.910 1.00 0.00
D C
ATOM 23891 HD2 HSE D 30 25.628 36.032 71.606 1.00 0.00
D H
ATOM 23892 C HSE D 30 24.039 35.415 76.250 1.00 0.00
D C
ATOM 23893 O HSE D 30 24.441 34.253 76.312 1.00 0.00
D O
ATOM 23894 N GLU D 31 23.055 35.882 77.014 1.00 0.00
D N

ATOM 23895 HN GLU D 31 22.766 36.834 77.068 1.00 0.00
D H
ATOM 23896 CA GLU D 31 22.292 34.979 77.871 1.00 0.00
D C
ATOM 23897 HA GLU D 31 22.929 34.144 78.133 1.00 0.00
D H
ATOM 23898 CB GLU D 31 21.927 35.605 79.231 1.00 0.00
D C
ATOM 23899 HB1 GLU D 31 21.176 34.946 79.726 1.00 0.00
D H
ATOM 23900 HB2 GLU D 31 22.819 35.605 79.897 1.00 0.00
D H
ATOM 23901 CG GLU D 31 21.351 37.013 79.209 1.00 0.00
D C
ATOM 23902 HG1 GLU D 31 21.974 37.657 78.562 1.00 0.00
D H
ATOM 23903 HG2 GLU D 31 20.311 37.011 78.827 1.00 0.00
D H
ATOM 23904 CD GLU D 31 21.361 37.673 80.583 1.00 0.00
D C
ATOM 23905 OE1 GLU D 31 21.458 36.956 81.605 1.00 0.00
D O
ATOM 23906 OE2 GLU D 31 21.271 38.917 80.645 1.00 0.00
D O
ATOM 23907 C GLU D 31 21.080 34.411 77.134 1.00 0.00
D C
ATOM 23908 O GLU D 31 20.341 35.138 76.466 1.00 0.00
D O
ATOM 23909 N VAL D 32 20.912 33.095 77.250 1.00 0.00
D N
ATOM 23910 HN VAL D 32 21.442 32.538 77.884 1.00 0.00
D H
ATOM 23911 CA VAL D 32 19.939 32.349 76.463 1.00 0.00
D C
ATOM 23912 HA VAL D 32 19.241 33.062 76.042 1.00 0.00
D H
ATOM 23913 CB VAL D 32 20.627 31.493 75.373 1.00 0.00
D C
ATOM 23914 HB VAL D 32 21.139 30.643 75.886 1.00 0.00
D H
ATOM 23915 CG1 VAL D 32 19.601 30.941 74.396 1.00 0.00
D C
ATOM 23916 HG11 VAL D 32 20.106 30.360 73.595 1.00 0.00
D H
ATOM 23917 HG12 VAL D 32 18.883 30.258 74.897 1.00 0.00
D H
ATOM 23918 HG13 VAL D 32 19.032 31.765 73.916 1.00 0.00
D H
ATOM 23919 CG2 VAL D 32 21.675 32.298 74.625 1.00 0.00
D C
ATOM 23920 HG21 VAL D 32 22.136 31.690 73.818 1.00 0.00
D H
ATOM 23921 HG22 VAL D 32 21.219 33.201 74.164 1.00 0.00
D H

ATOM 23922 HG23 VAL D 32 22.490 32.627 75.303 1.00 0.00
D H
ATOM 23923 C VAL D 32 19.147 31.404 77.350 1.00 0.00
D C
ATOM 23924 O VAL D 32 19.722 30.656 78.143 1.00 0.00
D O
ATOM 23925 N LEU D 33 17.828 31.441 77.206 1.00 0.00
D N
ATOM 23926 HN LEU D 33 17.331 32.130 76.681 1.00 0.00
D H
ATOM 23927 CA LEU D 33 16.981 30.441 77.829 1.00 0.00
D C
ATOM 23928 HA LEU D 33 17.543 29.979 78.630 1.00 0.00
D H
ATOM 23929 CB LEU D 33 15.692 31.057 78.379 1.00 0.00
D C
ATOM 23930 HB1 LEU D 33 15.985 31.942 78.990 1.00 0.00
D H
ATOM 23931 HB2 LEU D 33 15.090 31.454 77.532 1.00 0.00
D H
ATOM 23932 CG LEU D 33 14.822 30.141 79.252 1.00 0.00
D C
ATOM 23933 HG LEU D 33 15.513 29.368 79.669 1.00 0.00
D H
ATOM 23934 CD1 LEU D 33 14.141 30.894 80.381 1.00 0.00
D C
ATOM 23935 HD11 LEU D 33 13.483 30.213 80.962 1.00 0.00
D H
ATOM 23936 HD12 LEU D 33 14.894 31.327 81.073 1.00 0.00
D H
ATOM 23937 HD13 LEU D 33 13.516 31.721 79.981 1.00 0.00
D H
ATOM 23938 CD2 LEU D 33 13.785 29.434 78.409 1.00 0.00
D C
ATOM 23939 HD21 LEU D 33 13.170 28.758 79.040 1.00 0.00
D H
ATOM 23940 HD22 LEU D 33 13.099 30.153 77.917 1.00 0.00
D H
ATOM 23941 HD23 LEU D 33 14.275 28.823 77.621 1.00 0.00
D H
ATOM 23942 C LEU D 33 16.682 29.366 76.801 1.00 0.00
D C
ATOM 23943 O LEU D 33 16.120 29.647 75.741 1.00 0.00
D O
ATOM 23944 N ALA D 34 17.083 28.138 77.115 1.00 0.00
D N
ATOM 23945 HN ALA D 34 17.588 27.935 77.953 1.00 0.00
D H
ATOM 23946 CA ALA D 34 16.814 26.997 76.256 1.00 0.00
D C
ATOM 23947 HA ALA D 34 16.622 27.306 75.236 1.00 0.00
D H
ATOM 23948 CB ALA D 34 17.995 26.047 76.262 1.00 0.00
D C

ATOM 23949 HB1 ALA D 34   18.893 26.566 75.864 1.00 0.00
D   H
ATOM 23950 HB2 ALA D 34   18.227 25.705 77.294 1.00 0.00
D   H
ATOM 23951 HB3 ALA D 34   17.805 25.154 75.628 1.00 0.00
D   H
ATOM 23952 C   ALA D 34   15.555 26.288 76.734 1.00 0.00
D   C
ATOM 23953 O   ALA D 34   15.451 25.930 77.910 1.00 0.00
D   O
ATOM 23954 N   VAL D 35   14.597 26.109 75.825 1.00 0.00
D   N
ATOM 23955 HN VAL D 35   14.662 26.440 74.887 1.00 0.00
D   H
ATOM 23956 CA VAL D 35   13.350 25.401 76.131 1.00 0.00
D   C
ATOM 23957 HA VAL D 35   13.414 25.042 77.151 1.00 0.00
D   H
ATOM 23958 CB VAL D 35   12.103 26.296 75.950 1.00 0.00
D   C
ATOM 23959 HB VAL D 35   12.022 26.546 74.864 1.00 0.00
D   H
ATOM 23960 CG1 VAL D 35   10.842 25.562 76.386 1.00 0.00
D   C
ATOM 23961 HG11 VAL D 35   9.956 26.226 76.289 1.00 0.00
D   H
ATOM 23962 HG12 VAL D 35   10.653 24.665 75.761 1.00 0.00
D   H
ATOM 23963 HG13 VAL D 35   10.920 25.246 77.449 1.00 0.00
D   H
ATOM 23964 CG2 VAL D 35   12.236 27.581 76.740 1.00 0.00
D   C
ATOM 23965 HG21 VAL D 35   11.320 28.202 76.637 1.00 0.00
D   H
ATOM 23966 HG22 VAL D 35   12.391 27.361 77.818 1.00 0.00
D   H
ATOM 23967 HG23 VAL D 35   13.093 28.187 76.380 1.00 0.00
D   H
ATOM 23968 C   VAL D 35   13.205 24.186 75.228 1.00 0.00
D   C
ATOM 23969 O   VAL D 35   13.536 24.245 74.045 1.00 0.00
D   O
ATOM 23970 N   ASP D 36   12.725 23.086 75.804 1.00 0.00
D   N
ATOM 23971 HN ASP D 36   12.564 22.982 76.782 1.00 0.00
D   H
ATOM 23972 CA ASP D 36   12.379 21.894 75.042 1.00 0.00
D   C
ATOM 23973 HA ASP D 36   11.841 22.218 74.158 1.00 0.00
D   H
ATOM 23974 CB ASP D 36   13.632 21.109 74.654 1.00 0.00
D   C
ATOM 23975 HB1 ASP D 36   14.443 21.819 74.395 1.00 0.00
D   H

ATOM 23976 HB2 ASP D 36   13.976 20.480 75.499 1.00 0.00
D   H
ATOM 23977 CG ASP D 36   13.410 20.216 73.448 1.00 0.00
D   C
ATOM 23978 OD1 ASP D 36   14.072 20.445 72.410 1.00 0.00
D   O
ATOM 23979 OD2 ASP D 36   12.572 19.294 73.534 1.00 0.00
D   O
ATOM 23980 C   ASP D 36   11.423 21.017 75.841 1.00 0.00
D   C
ATOM 23981 O   ASP D 36   11.431 21.029 77.068 1.00 0.00
D   O
ATOM 23982 N   ILE D 37   10.594 20.264 75.131 1.00 0.00
D   N
ATOM 23983 HN ILE D 37   10.566 20.264 74.133 1.00 0.00
D   H
ATOM 23984 CA ILE D 37   9.638 19.357 75.756 1.00 0.00
D   C
ATOM 23985 HA ILE D 37   9.259 19.866 76.633 1.00 0.00
D   H
ATOM 23986 CB ILE D 37   8.458 19.039 74.796 1.00 0.00
D   C
ATOM 23987 HB ILE D 37   8.108 20.041 74.440 1.00 0.00
D   H
ATOM 23988 CG2 ILE D 37   8.911 18.175 73.622 1.00 0.00
D   C
ATOM 23989 HG21 ILE D 37   8.102 18.085 72.867 1.00 0.00
D   H
ATOM 23990 HG22 ILE D 37   9.793 18.621 73.118 1.00 0.00
D   H
ATOM 23991 HG23 ILE D 37   9.182 17.153 73.962 1.00 0.00
D   H
ATOM 23992 CG1 ILE D 37   7.286 18.410 75.562 1.00 0.00
D   C
ATOM 23993 HG11 ILE D 37   7.549 17.371 75.867 1.00 0.00
D   H
ATOM 23994 HG12 ILE D 37   7.112 18.991 76.496 1.00 0.00
D   H
ATOM 23995 CD ILE D 37   5.982 18.371 74.795 1.00 0.00
D   C
ATOM 23996 HD1 ILE D 37   5.171 17.956 75.433 1.00 0.00
D   H
ATOM 23997 HD2 ILE D 37   5.685 19.392 74.474 1.00 0.00
D   H
ATOM 23998 HD3 ILE D 37   6.070 17.730 73.892 1.00 0.00
D   H
ATOM 23999 C   ILE D 37   10.337 18.085 76.248 1.00 0.00
D   C
ATOM 24000 O   ILE D 37   9.908 17.462 77.216 1.00 0.00
D   O
ATOM 24001 N   ASN D 38   11.430 17.733 75.585 1.00 0.00
D   N
ATOM 24002 HN ASN D 38   11.821 18.297 74.858 1.00 0.00
D   H

```
ATOM  24003  CA  ASN D  38      12.161  16.500  75.851  1.00  0.00
D    C
ATOM  24004  HA  ASN D  38      11.426  15.803  76.239  1.00  0.00
D    H
ATOM  24005  CB  ASN D  38      12.788  16.005  74.544  1.00  0.00
D    C
ATOM  24006  HB1 ASN D  38      11.970  15.876  73.802  1.00  0.00
D    H
ATOM  24007  HB2 ASN D  38      13.487  16.765  74.139  1.00  0.00
D    H
ATOM  24008  CG  ASN D  38      13.495  14.677  74.691  1.00  0.00
D    C
ATOM  24009  OD1 ASN D  38      14.613  14.603  75.207  1.00  0.00
D    O
ATOM  24010  ND2 ASN D  38      12.857  13.619  74.208  1.00  0.00
D    N
ATOM  24011 HD21 ASN D  38      13.302  12.733  74.305  1.00  0.00
D    H
ATOM  24012 HD22 ASN D  38      11.958  13.726  73.796  1.00  0.00
D    H
ATOM  24013  C   ASN D  38      13.228  16.697  76.929  1.00  0.00
D    C
ATOM  24014  O   ASN D  38      14.094  17.561  76.798  1.00  0.00
D    O
ATOM  24015  N   GLU D  39      13.169  15.886  77.982  1.00  0.00
D    N
ATOM  24016  HN  GLU D  39      12.522  15.134  78.075  1.00  0.00
D    H
ATOM  24017  CA  GLU D  39      14.077  16.035  79.124  1.00  0.00
D    C
ATOM  24018  HA  GLU D  39      13.973  17.056  79.471  1.00  0.00
D    H
ATOM  24019  CB  GLU D  39      13.651  15.138  80.288  1.00  0.00
D    C
ATOM  24020  HB1 GLU D  39      12.537  15.140  80.338  1.00  0.00
D    H
ATOM  24021  HB2 GLU D  39      13.945  14.085  80.080  1.00  0.00
D    H
ATOM  24022  CG  GLU D  39      14.216  15.589  81.628  1.00  0.00
D    C
ATOM  24023  HG1 GLU D  39      15.320  15.622  81.570  1.00  0.00
D    H
ATOM  24024  HG2 GLU D  39      13.835  16.593  81.903  1.00  0.00
D    H
ATOM  24025  CD  GLU D  39      13.905  14.641  82.775  1.00  0.00
D    C
ATOM  24026  OE1 GLU D  39      14.383  14.901  83.903  1.00  0.00
D    O
ATOM  24027  OE2 GLU D  39      13.187  13.638  82.557  1.00  0.00
D    O
ATOM  24028  C   GLU D  39      15.557  15.816  78.794  1.00  0.00
D    C
ATOM  24029  O   GLU D  39      16.419  16.527  79.314  1.00  0.00
D    O
```

ATOM 24030 N GLU D 40 15.850 14.845 77.929 1.00 0.00
D N
ATOM 24031 HN GLU D 40 15.176 14.230 77.528 1.00 0.00
D H
ATOM 24032 CA GLU D 40 17.227 14.594 77.484 1.00 0.00
D C
ATOM 24033 HA GLU D 40 17.825 14.390 78.363 1.00 0.00
D H
ATOM 24034 CB GLU D 40 17.293 13.375 76.562 1.00 0.00
D C
ATOM 24035 HB1 GLU D 40 16.437 13.429 75.851 1.00 0.00
D H
ATOM 24036 HB2 GLU D 40 18.213 13.425 75.936 1.00 0.00
D H
ATOM 24037 CG GLU D 40 17.242 12.038 77.281 1.00 0.00
D C
ATOM 24038 HG1 GLU D 40 18.264 11.734 77.569 1.00 0.00
D H
ATOM 24039 HG2 GLU D 40 16.609 12.100 78.189 1.00 0.00
D H
ATOM 24040 CD GLU D 40 16.712 10.916 76.401 1.00 0.00
D C
ATOM 24041 OE1 GLU D 40 15.757 11.157 75.627 1.00 0.00
D O
ATOM 24042 OE2 GLU D 40 17.241 9.788 76.493 1.00 0.00
D O
ATOM 24043 C GLU D 40 17.838 15.816 76.789 1.00 0.00
D C
ATOM 24044 O GLU D 40 18.972 16.203 77.082 1.00 0.00
D O
ATOM 24045 N LYS D 41 17.075 16.414 75.873 1.00 0.00
D N
ATOM 24046 HN LYS D 41 16.176 16.066 75.613 1.00 0.00
D H
ATOM 24047 CA LYS D 41 17.496 17.629 75.176 1.00 0.00
D C
ATOM 24048 HA LYS D 41 18.407 17.387 74.641 1.00 0.00
D H
ATOM 24049 CB LYS D 41 16.424 18.084 74.172 1.00 0.00
D C
ATOM 24050 HB1 LYS D 41 15.457 18.132 74.724 1.00 0.00
D H
ATOM 24051 HB2 LYS D 41 16.651 19.125 73.847 1.00 0.00
D H
ATOM 24052 CG LYS D 41 16.249 17.178 72.959 1.00 0.00
D C
ATOM 24053 HG1 LYS D 41 16.293 16.115 73.289 1.00 0.00
D H
ATOM 24054 HG2 LYS D 41 15.239 17.348 72.522 1.00 0.00
D H
ATOM 24055 CD LYS D 41 17.326 17.413 71.906 1.00 0.00
D C
ATOM 24056 HD1 LYS D 41 17.140 18.402 71.428 1.00 0.00
D H

ATOM 24057 HD2 LYS D 41   18.311 17.474 72.425 1.00 0.00
D   H
ATOM 24058 CE LYS D 41   17.349 16.309 70.861 1.00 0.00
D   C
ATOM 24059 HE1 LYS D 41   18.101 16.535 70.072 1.00 0.00
D   H
ATOM 24060 HE2 LYS D 41   17.612 15.334 71.327 1.00 0.00
D   H
ATOM 24061 NZ LYS D 41   16.037 16.128 70.175 1.00 0.00
D   N
ATOM 24062 HZ1 LYS D 41   16.106 15.364 69.471 1.00 0.00
D   H
ATOM 24063 HZ2 LYS D 41   15.309 15.885 70.877 1.00 0.00
D   H
ATOM 24064 HZ3 LYS D 41   15.771 17.013 69.698 1.00 0.00
D   H
ATOM 24065 C   LYS D 41   17.828 18.752 76.158 1.00 0.00
D   C
ATOM 24066 O   LYS D 41   18.883 19.384 76.052 1.00 0.00
D   O
ATOM 24067 N   VAL D 42   16.933 18.979 77.120 1.00 0.00
D   N
ATOM 24068 HN VAL D 42   16.075 18.477 77.188 1.00 0.00
D   H
ATOM 24069 CA VAL D 42   17.130 19.988 78.166 1.00 0.00
D   C
ATOM 24070 HA VAL D 42   17.279 20.939 77.670 1.00 0.00
D   H
ATOM 24071 CB VAL D 42   15.894 20.113 79.087 1.00 0.00
D   C
ATOM 24072 HB VAL D 42   15.785 19.146 79.638 1.00 0.00
D   H
ATOM 24073 CG1 VAL D 42   16.076 21.239 80.093 1.00 0.00
D   C
ATOM 24074 HG11 VAL D 42   15.167 21.344 80.724 1.00 0.00
D   H
ATOM 24075 HG12 VAL D 42   16.930 21.044 80.775 1.00 0.00
D   H
ATOM 24076 HG13 VAL D 42   16.248 22.206 79.574 1.00 0.00
D   H
ATOM 24077 CG2 VAL D 42   14.636 20.359 78.266 1.00 0.00
D   C
ATOM 24078 HG21 VAL D 42   13.754 20.489 78.928 1.00 0.00
D   H
ATOM 24079 HG22 VAL D 42   14.747 21.276 77.648 1.00 0.00
D   H
ATOM 24080 HG23 VAL D 42   14.425 19.506 77.589 1.00 0.00
D   H
ATOM 24081 C   VAL D 42   18.387 19.699 78.994 1.00 0.00
D   C
ATOM 24082 O   VAL D 42   19.155 20.615 79.302 1.00 0.00
D   O
ATOM 24083 N   ASN D 43   18.600 18.430 79.341 1.00 0.00
D   N

ATOM 24084 HN ASN D 43 17.954 17.690 79.157 1.00 0.00
D H
ATOM 24085 CA ASN D 43 19.820 18.012 80.034 1.00 0.00
D C
ATOM 24086 HA ASN D 43 19.879 18.638 80.918 1.00 0.00
D H
ATOM 24087 CB ASN D 43 19.752 16.527 80.426 1.00 0.00
D C
ATOM 24088 HB1 ASN D 43 19.358 15.963 79.553 1.00 0.00
D H
ATOM 24089 HB2 ASN D 43 20.768 16.139 80.642 1.00 0.00
D H
ATOM 24090 CG ASN D 43 18.828 16.265 81.609 1.00 0.00
D C
ATOM 24091 OD1 ASN D 43 18.382 17.192 82.290 1.00 0.00
D O
ATOM 24092 ND2 ASN D 43 18.540 14.991 81.859 1.00 0.00
D N
ATOM 24093 HD21 ASN D 43 17.929 14.801 82.622 1.00 0.00
D H
ATOM 24094 HD22 ASN D 43 18.908 14.273 81.276 1.00 0.00
D H
ATOM 24095 C ASN D 43 21.073 18.269 79.200 1.00 0.00
D C
ATOM 24096 O ASN D 43 22.069 18.778 79.711 1.00 0.00
D O
ATOM 24097 N ALA D 44 21.004 17.930 77.916 1.00 0.00
D N
ATOM 24098 HN ALA D 44 20.169 17.560 77.509 1.00 0.00
D H
ATOM 24099 CA ALA D 44 22.138 18.070 77.004 1.00 0.00
D C
ATOM 24100 HA ALA D 44 23.010 17.594 77.434 1.00 0.00
D H
ATOM 24101 CB ALA D 44 21.856 17.353 75.691 1.00 0.00
D C
ATOM 24102 HB1 ALA D 44 21.624 16.286 75.893 1.00 0.00
D H
ATOM 24103 HB2 ALA D 44 20.982 17.801 75.171 1.00 0.00
D H
ATOM 24104 HB3 ALA D 44 22.733 17.391 75.008 1.00 0.00
D H
ATOM 24105 C ALA D 44 22.526 19.527 76.739 1.00 0.00
D C
ATOM 24106 O ALA D 44 23.662 19.806 76.349 1.00 0.00
D O
ATOM 24107 N TYR D 45 21.592 20.450 76.952 1.00 0.00
D N
ATOM 24108 HN TYR D 45 20.670 20.259 77.285 1.00 0.00
D H
ATOM 24109 CA TYR D 45 21.864 21.859 76.703 1.00 0.00
D C
ATOM 24110 HA TYR D 45 22.799 21.876 76.156 1.00 0.00
D H

ATOM 24111 CB TYR D 45   20.801 22.465 75.777 1.00 0.00
D   C
ATOM 24112 HB1 TYR D 45   19.802 22.070 76.062 1.00 0.00
D   H
ATOM 24113 HB2 TYR D 45   20.763 23.570 75.893 1.00 0.00
D   H
ATOM 24114 CG TYR D 45   21.048 22.095 74.339 1.00 0.00
D   C
ATOM 24115 CD1 TYR D 45   20.465 20.962 73.787 1.00 0.00
D   C
ATOM 24116 HD1 TYR D 45   19.824 20.338 74.394 1.00 0.00
D   H
ATOM 24117 CE1 TYR D 45   20.706 20.599 72.473 1.00 0.00
D   C
ATOM 24118 HE1 TYR D 45   20.243 19.719 72.052 1.00 0.00
D   H
ATOM 24119 CZ TYR D 45   21.556 21.372 71.699 1.00 0.00
D   C
ATOM 24120 OH TYR D 45   21.800 21.017 70.391 1.00 0.00
D   O
ATOM 24121 HH TYR D 45   22.347 21.703 70.004 1.00 0.00
D   H
ATOM 24122 CD2 TYR D 45   21.902 22.849 73.541 1.00 0.00
D   C
ATOM 24123 HD2 TYR D 45   22.413 23.707 73.957 1.00 0.00
D   H
ATOM 24124 CE2 TYR D 45   22.156 22.498 72.229 1.00 0.00
D   C
ATOM 24125 HE2 TYR D 45   22.835 23.091 71.637 1.00 0.00
D   H
ATOM 24126 C   TYR D 45   22.067 22.682 77.961 1.00 0.00
D   C
ATOM 24127 O   TYR D 45   22.274 23.893 77.887 1.00 0.00
D   O
ATOM 24128 N   ALA D 46   22.039 22.018 79.112 1.00 0.00
D   N
ATOM 24129 HN ALA D 46   21.852 21.037 79.161 1.00 0.00
D   H
ATOM 24130 CA ALA D 46   22.278 22.676 80.396 1.00 0.00
D   C
ATOM 24131 HA ALA D 46   21.484 23.379 80.621 1.00 0.00
D   H
ATOM 24132 CB ALA D 46   22.300 21.656 81.526 1.00 0.00
D   C
ATOM 24133 HB1 ALA D 46   21.330 21.116 81.559 1.00 0.00
D   H
ATOM 24134 HB2 ALA D 46   23.105 20.904 81.369 1.00 0.00
D   H
ATOM 24135 HB3 ALA D 46   22.458 22.141 82.514 1.00 0.00
D   H
ATOM 24136 C   ALA D 46   23.579 23.492 80.360 1.00 0.00
D   C
ATOM 24137 O   ALA D 46   23.612 24.638 80.818 1.00 0.00
D   O

ATOM 24138 N   SER D 47   24.627 22.905 79.779 1.00 0.00
D   N
ATOM 24139 HN SER D 47   24.580 21.994 79.369 1.00 0.00
D   H
ATOM 24140 CA SER D 47   25.947 23.537 79.686 1.00 0.00
D   C
ATOM 24141 HA SER D 47   26.082 24.029 80.640 1.00 0.00
D   H
ATOM 24142 CB SER D 47   27.021 22.482 79.430 1.00 0.00
D   C
ATOM 24143 HB1 SER D 47   26.739 21.915 78.513 1.00 0.00
D   H
ATOM 24144 HB2 SER D 47   28.002 22.968 79.224 1.00 0.00
D   H
ATOM 24145 OG SER D 47   27.117 21.580 80.508 1.00 0.00
D   O
ATOM 24146 HG1 SER D 47   27.635 20.825 80.201 1.00 0.00
D   H
ATOM 24147 C   SER D 47   26.058 24.605 78.606 1.00 0.00
D   C
ATOM 24148 O   SER D 47   27.006 25.397 78.612 1.00 0.00
D   O
ATOM 24149 N   TYR D 48   25.107 24.618 77.675 1.00 0.00
D   N
ATOM 24150 HN TYR D 48   24.292 24.042 77.704 1.00 0.00
D   H
ATOM 24151 CA TYR D 48   25.188 25.501 76.511 1.00 0.00
D   C
ATOM 24152 HA TYR D 48   26.155 25.984 76.578 1.00 0.00
D   H
ATOM 24153 CB TYR D 48   25.106 24.686 75.220 1.00 0.00
D   C
ATOM 24154 HB1 TYR D 48   24.111 24.191 75.164 1.00 0.00
D   H
ATOM 24155 HB2 TYR D 48   25.195 25.347 74.331 1.00 0.00
D   H
ATOM 24156 CG TYR D 48   26.139 23.584 75.117 1.00 0.00
D   C
ATOM 24157 CD1 TYR D 48   25.790 22.255 75.358 1.00 0.00
D   C
ATOM 24158 HD1 TYR D 48   24.776 22.008 75.641 1.00 0.00
D   H
ATOM 24159 CE1 TYR D 48   26.726 21.241 75.264 1.00 0.00
D   C
ATOM 24160 HE1 TYR D 48   26.446 20.215 75.456 1.00 0.00
D   H
ATOM 24161 CZ TYR D 48   28.033 21.549 74.926 1.00 0.00
D   C
ATOM 24162 OH TYR D 48   28.967 20.542 74.832 1.00 0.00
D   O
ATOM 24163 HH TYR D 48   29.785 20.937 74.522 1.00 0.00
D   H
ATOM 24164 CD2 TYR D 48   27.464 23.868 74.777 1.00 0.00
D   C

ATOM 24165 HD2 TYR D 48   27.775 24.892 74.623 1.00 0.00
D   H
ATOM 24166 CE2 TYR D 48   28.408 22.858 74.681 1.00 0.00
D   C
ATOM 24167 HE2 TYR D 48   29.431 23.102 74.436 1.00 0.00
D   H
ATOM 24168 C   TYR D 48   24.131 26.605 76.517 1.00 0.00
D   C
ATOM 24169 O   TYR D 48   23.789 27.154 75.470 1.00 0.00
D   O
ATOM 24170 N   ALA D 49   23.629 26.925 77.706 1.00 0.00
D   N
ATOM 24171 HN ALA D 49   23.923 26.460 78.541 1.00 0.00
D   H
ATOM 24172 CA ALA D 49   22.623 27.965 77.889 1.00 0.00
D   C
ATOM 24173 HA ALA D 49   22.811 28.803 77.228 1.00 0.00
D   H
ATOM 24174 CB ALA D 49   21.236 27.415 77.591 1.00 0.00
D   C
ATOM 24175 HB1 ALA D 49   21.195 27.059 76.539 1.00 0.00
D   H
ATOM 24176 HB2 ALA D 49   20.997 26.554 78.253 1.00 0.00
D   H
ATOM 24177 HB3 ALA D 49   20.452 28.191 77.722 1.00 0.00
D   H
ATOM 24178 C   ALA D 49   22.672 28.519 79.311 1.00 0.00
D   C
ATOM 24179 O   ALA D 49   23.079 27.817 80.245 1.00 0.00
D   O
ATOM 24180 N   THR D 50   22.257 29.774 79.470 1.00 0.00
D   N
ATOM 24181 HN THR D 50   21.992 30.370 78.712 1.00 0.00
D   H
ATOM 24182 CA THR D 50   22.151 30.396 80.789 1.00 0.00
D   C
ATOM 24183 HA THR D 50   23.127 30.307 81.248 1.00 0.00
D   H
ATOM 24184 CB THR D 50   21.689 31.858 80.686 1.00 0.00
D   C
ATOM 24185 HB THR D 50   20.623 31.823 80.352 1.00 0.00
D   H
ATOM 24186 OG1 THR D 50   22.454 32.520 79.676 1.00 0.00
D   O
ATOM 24187 HG1 THR D 50   22.132 33.427 79.664 1.00 0.00
D   H
ATOM 24188 CG2 THR D 50   21.883 32.582 82.005 1.00 0.00
D   C
ATOM 24189 HG21 THR D 50   21.445 33.602 81.964 1.00 0.00
D   H
ATOM 24190 HG22 THR D 50   21.383 32.028 82.829 1.00 0.00
D   H
ATOM 24191 HG23 THR D 50   22.961 32.677 82.256 1.00 0.00
D   H

ATOM 24192 C   THR D 50   21.186 29.598 81.664 1.00 0.00
D   C
ATOM 24193 O   THR D 50   21.500 29.277 82.811 1.00 0.00
D   O
ATOM 24194 N   HSE D 51   20.021 29.278 81.108 1.00 0.00
D   N
ATOM 24195 HN HSE D 51   19.747 29.602 80.203 1.00 0.00
D   H
ATOM 24196 CA HSE D 51   19.034 28.425 81.767 1.00 0.00
D   C
ATOM 24197 HA HSE D 51   19.565 27.824 82.496 1.00 0.00
D   H
ATOM 24198 CB HSE D 51   17.950 29.261 82.439 1.00 0.00
D   C
ATOM 24199 HB1 HSE D 51   17.782 30.166 81.817 1.00 0.00
D   H
ATOM 24200 HB2 HSE D 51   16.996 28.692 82.449 1.00 0.00
D   H
ATOM 24201 ND1 HSE D 51   18.998 30.845 84.075 1.00 0.00
D   N
ATOM 24202 CG HSE D 51   18.306 29.714 83.834 1.00 0.00
D   C
ATOM 24203 CE1 HSE D 51   19.171 30.993 85.404 1.00 0.00
D   C
ATOM 24204 HE1 HSE D 51   19.704 31.824 85.871 1.00 0.00
D   H
ATOM 24205 NE2 HSE D 51   18.587 29.949 86.017 1.00 0.00
D   N
ATOM 24206 HE2 HSE D 51   18.576 29.791 87.004 1.00 0.00
D   H
ATOM 24207 CD2 HSE D 51   18.045 29.140 85.077 1.00 0.00
D   C
ATOM 24208 HD2 HSE D 51   17.526 28.223 85.322 1.00 0.00
D   H
ATOM 24209 C   HSE D 51   18.416 27.477 80.781 1.00 0.00
D   C
ATOM 24210 O   HSE D 51   18.289 27.796 79.598 1.00 0.00
D   O
ATOM 24211 N   ALA D 52   18.035 26.301 81.266 1.00 0.00
D   N
ATOM 24212 HN ALA D 52   18.191 26.041 82.219 1.00 0.00
D   H
ATOM 24213 CA ALA D 52   17.362 25.301 80.447 1.00 0.00
D   C
ATOM 24214 HA ALA D 52   16.962 25.740 79.540 1.00 0.00
D   H
ATOM 24215 CB ALA D 52   18.332 24.202 80.044 1.00 0.00
D   C
ATOM 24216 HB1 ALA D 52   19.163 24.637 79.449 1.00 0.00
D   H
ATOM 24217 HB2 ALA D 52   18.774 23.711 80.939 1.00 0.00
D   H
ATOM 24218 HB3 ALA D 52   17.835 23.425 79.423 1.00 0.00
D   H

```
ATOM  24219  C   ALA D  52      16.171  24.730  81.210  1.00  0.00
D    C
ATOM  24220  O   ALA D  52      16.296  24.354  82.378  1.00  0.00
D    O
ATOM  24221  N   VAL D  53      15.012  24.681  80.552  1.00  0.00
D    N
ATOM  24222  HN  VAL D  53      14.916  24.911  79.587  1.00  0.00
D    H
ATOM  24223  CA  VAL D  53      13.761  24.276  81.206  1.00  0.00
D    C
ATOM  24224  HA  VAL D  53      14.033  23.748  82.112  1.00  0.00
D    H
ATOM  24225  CB  VAL D  53      12.858  25.490  81.537  1.00  0.00
D    C
ATOM  24226  HB  VAL D  53      12.268  25.732  80.619  1.00  0.00
D    H
ATOM  24227  CG1 VAL D  53      11.899  25.157  82.672  1.00  0.00
D    C
ATOM  24228 HG11 VAL D  53      11.282  26.045  82.929  1.00  0.00
D    H
ATOM  24229 HG12 VAL D  53      11.202  24.339  82.393  1.00  0.00
D    H
ATOM  24230 HG13 VAL D  53      12.459  24.853  83.582  1.00  0.00
D    H
ATOM  24231  CG2 VAL D  53      13.679  26.720  81.901  1.00  0.00
D    C
ATOM  24232 HG21 VAL D  53      13.016  27.568  82.173  1.00  0.00
D    H
ATOM  24233 HG22 VAL D  53      14.344  26.505  82.765  1.00  0.00
D    H
ATOM  24234 HG23 VAL D  53      14.310  27.048  81.049  1.00  0.00
D    H
ATOM  24235  C   VAL D  53      12.950  23.311  80.338  1.00  0.00
D    C
ATOM  24236  O   VAL D  53      12.839  23.504  79.128  1.00  0.00
D    O
ATOM  24237  N   ILE D  54      12.388  22.279  80.967  1.00  0.00
D    N
ATOM  24238  HN  ILE D  54      12.590  22.025  81.911  1.00  0.00
D    H
ATOM  24239  CA  ILE D  54      11.408  21.416  80.312  1.00  0.00
D    C
ATOM  24240  HA  ILE D  54      11.734  21.264  79.291  1.00  0.00
D    H
ATOM  24241  CB  ILE D  54      11.195  20.075  81.062  1.00  0.00
D    C
ATOM  24242  HB  ILE D  54      10.950  20.377  82.112  1.00  0.00
D    H
ATOM  24243  CG2 ILE D  54      10.107  19.242  80.396  1.00  0.00
D    C
ATOM  24244 HG21 ILE D  54       9.851  18.361  81.021  1.00  0.00
D    H
ATOM  24245 HG22 ILE D  54       9.181  19.838  80.258  1.00  0.00
D    H
```

ATOM 24246 HG23 ILE D 54   10.440 18.876 79.401 1.00 0.00
D   H
ATOM 24247 CG1 ILE D 54   12.494 19.262 81.137 1.00 0.00
D   C
ATOM 24248 HG11 ILE D 54   12.275 18.185 80.957 1.00 0.00
D   H
ATOM 24249 HG12 ILE D 54   13.178 19.599 80.325 1.00 0.00
D   H
ATOM 24250 CD ILE D 54   13.235 19.393 82.452 1.00 0.00
D   C
ATOM 24251 HD1 ILE D 54   14.191 18.826 82.415 1.00 0.00
D   H
ATOM 24252 HD2 ILE D 54   13.470 20.458 82.666 1.00 0.00
D   H
ATOM 24253 HD3 ILE D 54   12.630 18.991 83.293 1.00 0.00
D   H
ATOM 24254 C   ILE D 54   10.085 22.170 80.245 1.00 0.00
D   C
ATOM 24255 O   ILE D 54   9.592 22.661 81.266 1.00 0.00
D   O
ATOM 24256 N   ALA D 55   9.526 22.275 79.036 1.00 0.00
D   N
ATOM 24257 HN ALA D 55   9.969 21.913 78.216 1.00 0.00
D   H
ATOM 24258 CA ALA D 55   8.230 22.923 78.816 1.00 0.00
D   C
ATOM 24259 HA ALA D 55   7.530 22.628 79.591 1.00 0.00
D   H
ATOM 24260 CB ALA D 55   8.363 24.439 78.881 1.00 0.00
D   C
ATOM 24261 HB1 ALA D 55   8.778 24.734 79.869 1.00 0.00
D   H
ATOM 24262 HB2 ALA D 55   9.054 24.813 78.095 1.00 0.00
D   H
ATOM 24263 HB3 ALA D 55   7.380 24.942 78.757 1.00 0.00
D   H
ATOM 24264 C   ALA D 55   7.600 22.518 77.493 1.00 0.00
D   C
ATOM 24265 O   ALA D 55   8.298 22.321 76.498 1.00 0.00
D   O
ATOM 24266 N   ASN D 56   6.278 22.385 77.494 1.00 0.00
D   N
ATOM 24267 HN ASN D 56   5.721 22.385 78.323 1.00 0.00
D   H
ATOM 24268 CA ASN D 56   5.526 22.225 76.262 1.00 0.00
D   C
ATOM 24269 HA ASN D 56   6.196 21.799 75.524 1.00 0.00
D   H
ATOM 24270 CB ASN D 56   4.288 21.347 76.484 1.00 0.00
D   C
ATOM 24271 HB1 ASN D 56   4.633 20.364 76.871 1.00 0.00
D   H
ATOM 24272 HB2 ASN D 56   3.630 21.796 77.256 1.00 0.00
D   H

ATOM 24273 CG ASN D 56   3.502 21.100 75.203 1.00 0.00
D   C
ATOM 24274 OD1 ASN D 56   3.534 21.903 74.269 1.00 0.00
D   O
ATOM 24275 ND2 ASN D 56   2.784 19.985 75.162 1.00 0.00
D   N
ATOM 24276 HD21 ASN D 56   2.272 19.800 74.328 1.00 0.00
D   H
ATOM 24277 HD22 ASN D 56   2.796 19.357 75.933 1.00 0.00
D   H
ATOM 24278 C   ASN D 56   5.133 23.619 75.780 1.00 0.00
D   C
ATOM 24279 O   ASN D 56   4.212 24.233 76.317 1.00 0.00
D   O
ATOM 24280 N   ALA D 57   5.838 24.113 74.769 1.00 0.00
D   N
ATOM 24281 HN ALA D 57   6.529 23.580 74.281 1.00 0.00
D   H
ATOM 24282 CA ALA D 57   5.651 25.486 74.293 1.00 0.00
D   C
ATOM 24283 HA ALA D 57   5.768 26.163 75.132 1.00 0.00
D   H
ATOM 24284 CB ALA D 57   6.757 25.865 73.324 1.00 0.00
D   C
ATOM 24285 HB1 ALA D 57   7.745 25.689 73.799 1.00 0.00
D   H
ATOM 24286 HB2 ALA D 57   6.708 25.249 72.401 1.00 0.00
D   H
ATOM 24287 HB3 ALA D 57   6.700 26.937 73.038 1.00 0.00
D   H
ATOM 24288 C   ALA D 57   4.269 25.784 73.684 1.00 0.00
D   C
ATOM 24289 O   ALA D 57   3.987 26.924 73.317 1.00 0.00
D   O
ATOM 24290 N   THR D 58   3.417 24.767 73.582 1.00 0.00
D   N
ATOM 24291 HN THR D 58   3.628 23.828 73.852 1.00 0.00
D   H
ATOM 24292 CA THR D 58   2.070 24.949 73.051 1.00 0.00
D   C
ATOM 24293 HA THR D 58   2.088 25.879 72.498 1.00 0.00
D   H
ATOM 24294 CB THR D 58   1.629 23.761 72.173 1.00 0.00
D   C
ATOM 24295 HB THR D 58   0.614 24.021 71.785 1.00 0.00
D   H
ATOM 24296 OG1 THR D 58   1.506 22.584 72.979 1.00 0.00
D   O
ATOM 24297 HG1 THR D 58   1.242 21.883 72.373 1.00 0.00
D   H
ATOM 24298 CG2 THR D 58   2.636 23.510 71.068 1.00 0.00
D   C
ATOM 24299 HG21 THR D 58   2.267 22.731 70.367 1.00 0.00
D   H

ATOM 24300 HG22 THR D 58   2.811 24.439 70.485 1.00 0.00
D   H
ATOM 24301 HG23 THR D 58   3.610 23.172 71.483 1.00 0.00
D   H
ATOM 24302 C   THR D 58   1.064 25.139 74.184 1.00 0.00
D   C
ATOM 24303 O   THR D 58   -0.137 25.265 73.942 1.00 0.00
D   O
ATOM 24304 N   GLU D 59   1.573 25.159 75.413 1.00 0.00
D   N
ATOM 24305 HN GLU D 59   2.551 25.114 75.596 1.00 0.00
D   H
ATOM 24306 CA GLU D 59   0.750 25.248 76.611 1.00 0.00
D   C
ATOM 24307 HA GLU D 59   -0.291 25.157 76.329 1.00 0.00
D   H
ATOM 24308 CB GLU D 59   1.141 24.141 77.589 1.00 0.00
D   C
ATOM 24309 HB1 GLU D 59   1.359 23.219 77.002 1.00 0.00
D   H
ATOM 24310 HB2 GLU D 59   2.092 24.409 78.102 1.00 0.00
D   H
ATOM 24311 CG GLU D 59   0.083 23.816 78.624 1.00 0.00
D   C
ATOM 24312 HG1 GLU D 59   0.458 23.028 79.301 1.00 0.00
D   H
ATOM 24313 HG2 GLU D 59   -0.179 24.714 79.219 1.00 0.00
D   H
ATOM 24314 CD GLU D 59   -1.185 23.260 78.004 1.00 0.00
D   C
ATOM 24315 OE1 GLU D 59   -2.250 23.861 78.230 1.00 0.00
D   O
ATOM 24316 OE2 GLU D 59   -1.119 22.236 77.290 1.00 0.00
D   O
ATOM 24317 C   GLU D 59   0.928 26.621 77.247 1.00 0.00
D   C
ATOM 24318 O   GLU D 59   1.996 26.935 77.776 1.00 0.00
D   O
ATOM 24319 N   GLU D 60   -0.126 27.430 77.195 1.00 0.00
D   N
ATOM 24320 HN GLU D 60   -1.031 27.136 76.899 1.00 0.00
D   H
ATOM 24321 CA GLU D 60   -0.052 28.847 77.574 1.00 0.00
D   C
ATOM 24322 HA GLU D 60   0.637 29.306 76.876 1.00 0.00
D   H
ATOM 24323 CB GLU D 60   -1.400 29.545 77.370 1.00 0.00
D   C
ATOM 24324 HB1 GLU D 60   -1.803 29.237 76.377 1.00 0.00
D   H
ATOM 24325 HB2 GLU D 60   -2.132 29.179 78.123 1.00 0.00
D   H
ATOM 24326 CG GLU D 60   -1.306 31.065 77.423 1.00 0.00
D   C

ATOM 24327 HG1 GLU D 60   -0.703 31.367 78.299 1.00 0.00
D   H
ATOM 24328 HG2 GLU D 60   -0.837 31.467 76.503 1.00 0.00
D   H
ATOM 24329 CD GLU D 60   -2.650 31.745 77.606 1.00 0.00
D   C
ATOM 24330 OE1 GLU D 60   -2.706 32.741 78.360 1.00 0.00
D   O
ATOM 24331 OE2 GLU D 60   -3.647 31.292 76.998 1.00 0.00
D   O
ATOM 24332 C   GLU D 60   0.471 29.135 78.985 1.00 0.00
D   C
ATOM 24333 O   GLU D 60   1.282 30.047 79.167 1.00 0.00
D   O
ATOM 24334 N   ASN D 61   -0.004 28.383 79.974 1.00 0.00
D   N
ATOM 24335 HN ASN D 61   -0.688 27.662 79.864 1.00 0.00
D   H
ATOM 24336 CA ASN D 61   0.464 28.568 81.349 1.00 0.00
D   C
ATOM 24337 HA ASN D 61   0.367 29.632 81.542 1.00 0.00
D   H
ATOM 24338 CB ASN D 61   -0.428 27.838 82.370 1.00 0.00
D   C
ATOM 24339 HB1 ASN D 61   0.078 27.886 83.360 1.00 0.00
D   H
ATOM 24340 HB2 ASN D 61   -1.404 28.355 82.469 1.00 0.00
D   H
ATOM 24341 CG ASN D 61   -0.651 26.369 82.035 1.00 0.00
D   C
ATOM 24342 OD1 ASN D 61   0.197 25.707 81.435 1.00 0.00
D   O
ATOM 24343 ND2 ASN D 61   -1.803 25.851 82.436 1.00 0.00
D   N
ATOM 24344 HD21 ASN D 61   -1.965 24.888 82.237 1.00 0.00
D   H
ATOM 24345 HD22 ASN D 61   -2.452 26.410 82.941 1.00 0.00
D   H
ATOM 24346 C   ASN D 61   1.939 28.198 81.522 1.00 0.00
D   C
ATOM 24347 O   ASN D 61   2.649 28.821 82.317 1.00 0.00
D   O
ATOM 24348 N   GLU D 62   2.393 27.203 80.757 1.00 0.00
D   N
ATOM 24349 HN GLU D 62   1.830 26.686 80.119 1.00 0.00
D   H
ATOM 24350 CA GLU D 62   3.789 26.780 80.796 1.00 0.00
D   C
ATOM 24351 HA GLU D 62   4.069 26.597 81.826 1.00 0.00
D   H
ATOM 24352 CB GLU D 62   4.010 25.483 80.013 1.00 0.00
D   C
ATOM 24353 HB1 GLU D 62   3.567 25.610 78.998 1.00 0.00
D   H

ATOM 24354 HB2 GLU D 62   5.099 25.319 79.853 1.00 0.00
D   H
ATOM 24355 CG GLU D 62   3.391 24.254 80.672 1.00 0.00
D   C
ATOM 24356 HG1 GLU D 62   3.571 24.290 81.762 1.00 0.00
D   H
ATOM 24357 HG2 GLU D 62   2.300 24.208 80.484 1.00 0.00
D   H
ATOM 24358 CD GLU D 62   4.008 22.946 80.204 1.00 0.00
D   C
ATOM 24359 OE1 GLU D 62   5.246 22.896 80.034 1.00 0.00
D   O
ATOM 24360 OE2 GLU D 62   3.261 21.960 80.022 1.00 0.00
D   O
ATOM 24361 C   GLU D 62   4.684 27.892 80.277 1.00 0.00
D   C
ATOM 24362 O   GLU D 62   5.759 28.135 80.825 1.00 0.00
D   O
ATOM 24363 N   LEU D 63   4.215 28.578 79.236 1.00 0.00
D   N
ATOM 24364 HN LEU D 63   3.359 28.358 78.772 1.00 0.00
D   H
ATOM 24365 CA LEU D 63   4.924 29.727 78.676 1.00 0.00
D   C
ATOM 24366 HA LEU D 63   5.929 29.391 78.455 1.00 0.00
D   H
ATOM 24367 CB LEU D 63   4.254 30.215 77.388 1.00 0.00
D   C
ATOM 24368 HB1 LEU D 63   3.177 29.938 77.452 1.00 0.00
D   H
ATOM 24369 HB2 LEU D 63   4.284 31.327 77.362 1.00 0.00
D   H
ATOM 24370 CG LEU D 63   4.789 29.672 76.063 1.00 0.00
D   C
ATOM 24371 HG LEU D 63   5.872 29.934 75.999 1.00 0.00
D   H
ATOM 24372 CD1 LEU D 63   4.612 28.173 76.001 1.00 0.00
D   C
ATOM 24373 HD11 LEU D 63   4.955 27.781 75.020 1.00 0.00
D   H
ATOM 24374 HD12 LEU D 63   5.202 27.677 76.802 1.00 0.00
D   H
ATOM 24375 HD13 LEU D 63   3.544 27.899 76.130 1.00 0.00
D   H
ATOM 24376 CD2 LEU D 63   4.079 30.317 74.889 1.00 0.00
D   C
ATOM 24377 HD21 LEU D 63   4.487 29.929 73.931 1.00 0.00
D   H
ATOM 24378 HD22 LEU D 63   2.991 30.106 74.902 1.00 0.00
D   H
ATOM 24379 HD23 LEU D 63   4.222 31.419 74.906 1.00 0.00
D   H
ATOM 24380 C   LEU D 63   5.031 30.866 79.684 1.00 0.00
D   C

```
ATOM  24381  O   LEU D  63       6.103  31.450  79.861  1.00  0.00
D    O
ATOM  24382  N   LEU D  64       3.918  31.169  80.348  1.00  0.00
D    N
ATOM  24383  HN  LEU D  64       3.033  30.739  80.178  1.00  0.00
D    H
ATOM  24384  CA  LEU D  64       3.900  32.179  81.401  1.00  0.00
D    C
ATOM  24385  HA  LEU D  64       4.215  33.112  80.949  1.00  0.00
D    H
ATOM  24386  CB  LEU D  64       2.497  32.327  81.997  1.00  0.00
D    C
ATOM  24387  HB1 LEU D  64       2.134  31.302  82.243  1.00  0.00
D    H
ATOM  24388  HB2 LEU D  64       2.570  32.867  82.966  1.00  0.00
D    H
ATOM  24389  CG  LEU D  64       1.407  32.994  81.149  1.00  0.00
D    C
ATOM  24390  HG  LEU D  64       1.356  32.426  80.188  1.00  0.00
D    H
ATOM  24391  CD1 LEU D  64       0.063  32.919  81.861  1.00  0.00
D    C
ATOM  24392 HD11 LEU D  64      -0.719  33.444  81.272  1.00  0.00
D    H
ATOM  24393 HD12 LEU D  64      -0.250  31.861  81.995  1.00  0.00
D    H
ATOM  24394 HD13 LEU D  64       0.123  33.397  82.862  1.00  0.00
D    H
ATOM  24395  CD2 LEU D  64       1.758  34.439  80.824  1.00  0.00
D    C
ATOM  24396 HD21 LEU D  64       0.965  34.896  80.194  1.00  0.00
D    H
ATOM  24397 HD22 LEU D  64       1.856  35.054  81.741  1.00  0.00
D    H
ATOM  24398 HD23 LEU D  64       2.718  34.490  80.265  1.00  0.00
D    H
ATOM  24399  C   LEU D  64       4.908  31.834  82.493  1.00  0.00
D    C
ATOM  24400  O   LEU D  64       5.680  32.691  82.921  1.00  0.00
D    O
ATOM  24401  N   SER D  65       4.909  30.566  82.905  1.00  0.00
D    N
ATOM  24402  HN  SER D  65       4.294  29.875  82.526  1.00  0.00
D    H
ATOM  24403  CA  SER D  65       5.806  30.062  83.947  1.00  0.00
D    C
ATOM  24404  HA  SER D  65       5.614  30.685  84.811  1.00  0.00
D    H
ATOM  24405  CB  SER D  65       5.499  28.596  84.263  1.00  0.00
D    C
ATOM  24406  HB1 SER D  65       5.721  27.992  83.353  1.00  0.00
D    H
ATOM  24407  HB2 SER D  65       6.168  28.226  85.072  1.00  0.00
D    H
```

ATOM 24408 OG SER D 65   4.136 28.418 84.606 1.00 0.00
D   O
ATOM 24409 HG1 SER D 65   3.957 27.470 84.573 1.00 0.00
D   H
ATOM 24410 C   SER D 65   7.285 30.218 83.594 1.00 0.00
D   C
ATOM 24411 O   SER D 65   8.120 30.381 84.482 1.00 0.00
D   O
ATOM 24412 N   LEU D 66   7.609 30.169 82.305 1.00 0.00
D   N
ATOM 24413 HN LEU D 66   6.955 30.001 81.569 1.00 0.00
D   H
ATOM 24414 CA LEU D 66   8.985 30.364 81.852 1.00 0.00
D   C
ATOM 24415 HA LEU D 66   9.615 29.773 82.503 1.00 0.00
D   H
ATOM 24416 CB LEU D 66   9.136 29.947 80.387 1.00 0.00
D   C
ATOM 24417 HB1 LEU D 66   8.447 30.588 79.789 1.00 0.00
D   H
ATOM 24418 HB2 LEU D 66   10.164 30.197 80.044 1.00 0.00
D   H
ATOM 24419 CG LEU D 66   8.838 28.498 80.006 1.00 0.00
D   C
ATOM 24420 HG LEU D 66   7.946 28.194 80.605 1.00 0.00
D   H
ATOM 24421 CD1 LEU D 66   8.511 28.397 78.524 1.00 0.00
D   C
ATOM 24422 HD11 LEU D 66   8.341 27.339 78.234 1.00 0.00
D   H
ATOM 24423 HD12 LEU D 66   7.594 28.976 78.285 1.00 0.00
D   H
ATOM 24424 HD13 LEU D 66   9.348 28.795 77.910 1.00 0.00
D   H
ATOM 24425 CD2 LEU D 66   9.990 27.574 80.375 1.00 0.00
D   C
ATOM 24426 HD21 LEU D 66   9.743 26.524 80.107 1.00 0.00
D   H
ATOM 24427 HD22 LEU D 66   10.922 27.849 79.841 1.00 0.00
D   H
ATOM 24428 HD23 LEU D 66   10.187 27.615 81.468 1.00 0.00
D   H
ATOM 24429 C   LEU D 66   9.406 31.819 82.001 1.00 0.00
D   C
ATOM 24430 O   LEU D 66   10.599 32.131 81.988 1.00 0.00
D   O
ATOM 24431 N   GLY D 67   8.415 32.698 82.142 1.00 0.00
D   N
ATOM 24432 HN GLY D 67   7.465 32.410 82.255 1.00 0.00
D   H
ATOM 24433 CA GLY D 67   8.640 34.139 82.143 1.00 0.00
D   C
ATOM 24434 HA1 GLY D 67   9.492 34.353 82.775 1.00 0.00
D   H

ATOM 24435 HA2 GLY D 67   7.727 34.634 82.444 1.00 0.00
D   H
ATOM 24436 C   GLY D 67   8.975 34.590 80.737 1.00 0.00
D   C
ATOM 24437 O   GLY D 67   9.859 35.421 80.534 1.00 0.00
D   O
ATOM 24438 N   ILE D 68   8.247 34.033 79.771 1.00 0.00
D   N
ATOM 24439 HN ILE D 68   7.451 33.459 79.955 1.00 0.00
D   H
ATOM 24440 CA ILE D 68   8.543 34.208 78.350 1.00 0.00
D   C
ATOM 24441 HA ILE D 68   9.573 33.894 78.233 1.00 0.00
D   H
ATOM 24442 CB ILE D 68   7.645 33.301 77.473 1.00 0.00
D   C
ATOM 24443 HB ILE D 68   7.720 32.290 77.948 1.00 0.00
D   H
ATOM 24444 CG2 ILE D 68   6.206 33.802 77.458 1.00 0.00
D   C
ATOM 24445 HG21 ILE D 68   5.540 33.066 76.960 1.00 0.00
D   H
ATOM 24446 HG22 ILE D 68   5.828 33.953 78.491 1.00 0.00
D   H
ATOM 24447 HG23 ILE D 68   6.127 34.766 76.912 1.00 0.00
D   H
ATOM 24448 CG1 ILE D 68   8.211 33.158 76.053 1.00 0.00
D   C
ATOM 24449 HG11 ILE D 68   7.463 32.659 75.398 1.00 0.00
D   H
ATOM 24450 HG12 ILE D 68   8.391 34.173 75.632 1.00 0.00
D   H
ATOM 24451 CD ILE D 68   9.513 32.388 75.972 1.00 0.00
D   C
ATOM 24452 HD1 ILE D 68   9.890 32.375 74.927 1.00 0.00
D   H
ATOM 24453 HD2 ILE D 68   10.287 32.858 76.615 1.00 0.00
D   H
ATOM 24454 HD3 ILE D 68   9.374 31.336 76.300 1.00 0.00
D   H
ATOM 24455 C   ILE D 68   8.468 35.664 77.880 1.00 0.00
D   C
ATOM 24456 O   ILE D 68   9.105 36.033 76.893 1.00 0.00
D   O
ATOM 24457 N   ARG D 69   7.707 36.487 78.599 1.00 0.00
D   N
ATOM 24458 HN ARG D 69   7.224 36.169 79.412 1.00 0.00
D   H
ATOM 24459 CA ARG D 69   7.536 37.894 78.243 1.00 0.00
D   C
ATOM 24460 HA ARG D 69   7.401 37.946 77.169 1.00 0.00
D   H
ATOM 24461 CB ARG D 69   6.338 38.481 78.960 1.00 0.00
D   C

```
ATOM  24462  HB1 ARG D  69       6.509  38.313  80.050  1.00  0.00
D    H
ATOM  24463  HB2 ARG D  69       6.323  39.585  78.818  1.00  0.00
D    H
ATOM  24464  CG  ARG D  69       5.036  37.844  78.558  1.00  0.00
D    C
ATOM  24465  HG1 ARG D  69       5.069  37.626  77.468  1.00  0.00
D    H
ATOM  24466  HG2 ARG D  69       4.949  36.853  79.059  1.00  0.00
D    H
ATOM  24467  CD  ARG D  69       3.889  38.733  78.974  1.00  0.00
D    C
ATOM  24468  HD1 ARG D  69       4.220  39.401  79.803  1.00  0.00
D    H
ATOM  24469  HD2 ARG D  69       3.532  39.364  78.127  1.00  0.00
D    H
ATOM  24470  NE  ARG D  69       2.760  37.928  79.417  1.00  0.00
D    N
ATOM  24471  HE  ARG D  69       3.036  37.055  79.835  1.00  0.00
D    H
ATOM  24472  CZ  ARG D  69       1.485  38.286  79.294  1.00  0.00
D    C
ATOM  24473  NH1 ARG D  69       1.168  39.440  78.722  1.00  0.00
D    N
ATOM  24474 HH11 ARG D  69       0.221  39.723  78.651  1.00  0.00
D    H
ATOM  24475 HH12 ARG D  69       1.931  40.050  78.501  1.00  0.00
D    H
ATOM  24476  NH2 ARG D  69       0.525  37.481  79.732  1.00  0.00
D    N
ATOM  24477 HH21 ARG D  69      -0.423  37.706  79.562  1.00  0.00
D    H
ATOM  24478 HH22 ARG D  69       0.801  36.576  80.030  1.00  0.00
D    H
ATOM  24479  C   ARG D  69       8.754  38.736  78.569  1.00  0.00
D    C
ATOM  24480  O   ARG D  69       8.952  39.798  77.983  1.00  0.00
D    O
ATOM  24481  N   ASN D  70       9.556  38.263  79.515  1.00  0.00
D    N
ATOM  24482  HN  ASN D  70       9.361  37.435  80.040  1.00  0.00
D    H
ATOM  24483  CA  ASN D  70      10.809  38.924  79.871  1.00  0.00
D    C
ATOM  24484  HA  ASN D  70      10.551  39.965  80.028  1.00  0.00
D    H
ATOM  24485  CB  ASN D  70      11.389  38.310  81.152  1.00  0.00
D    C
ATOM  24486  HB1 ASN D  70      11.572  37.231  80.960  1.00  0.00
D    H
ATOM  24487  HB2 ASN D  70      12.366  38.776  81.395  1.00  0.00
D    H
ATOM  24488  CG  ASN D  70      10.446  38.422  82.338  1.00  0.00
D    C
```

ATOM 24489 OD1 ASN D 70 9.485 39.195 82.314 1.00 0.00
D O
ATOM 24490 ND2 ASN D 70 10.719 37.650 83.387 1.00 0.00
D N
ATOM 24491 HD21 ASN D 70 10.103 37.704 84.168 1.00 0.00
D H
ATOM 24492 HD22 ASN D 70 11.495 37.027 83.358 1.00 0.00
D H
ATOM 24493 C ASN D 70 11.835 38.866 78.735 1.00 0.00
D C
ATOM 24494 O ASN D 70 12.949 39.376 78.869 1.00 0.00
D O
ATOM 24495 N PHE D 71 11.447 38.250 77.621 1.00 0.00
D N
ATOM 24496 HN PHE D 71 10.528 37.886 77.485 1.00 0.00
D H
ATOM 24497 CA PHE D 71 12.347 38.055 76.491 1.00 0.00
D C
ATOM 24498 HA PHE D 71 13.322 38.375 76.840 1.00 0.00
D H
ATOM 24499 CB PHE D 71 12.404 36.581 76.098 1.00 0.00
D C
ATOM 24500 HB1 PHE D 71 11.368 36.184 76.029 1.00 0.00
D H
ATOM 24501 HB2 PHE D 71 12.871 36.451 75.098 1.00 0.00
D H
ATOM 24502 CG PHE D 71 13.119 35.728 77.102 1.00 0.00
D C
ATOM 24503 CD1 PHE D 71 12.440 35.200 78.198 1.00 0.00
D C
ATOM 24504 HD1 PHE D 71 11.386 35.405 78.324 1.00 0.00
D H
ATOM 24505 CE1 PHE D 71 13.105 34.423 79.129 1.00 0.00
D C
ATOM 24506 HE1 PHE D 71 12.567 34.007 79.969 1.00 0.00
D H
ATOM 24507 CZ PHE D 71 14.459 34.175 78.980 1.00 0.00
D C
ATOM 24508 HZ PHE D 71 14.977 33.566 79.707 1.00 0.00
D H
ATOM 24509 CD2 PHE D 71 14.479 35.471 76.966 1.00 0.00
D C
ATOM 24510 HD2 PHE D 71 15.029 35.897 76.138 1.00 0.00
D H
ATOM 24511 CE2 PHE D 71 15.147 34.695 77.898 1.00 0.00
D C
ATOM 24512 HE2 PHE D 71 16.204 34.500 77.777 1.00 0.00
D H
ATOM 24513 C PHE D 71 11.997 38.923 75.299 1.00 0.00
D C
ATOM 24514 O PHE D 71 10.824 39.129 74.974 1.00 0.00
D O
ATOM 24515 N GLU D 72 13.042 39.431 74.661 1.00 0.00
D N

ATOM 24516 HN GLU D 72 13.985 39.222 74.906 1.00 0.00
D H
ATOM 24517 CA GLU D 72 12.913 40.343 73.541 1.00 0.00
D C
ATOM 24518 HA GLU D 72 11.966 40.861 73.625 1.00 0.00
D H
ATOM 24519 CB GLU D 72 14.078 41.328 73.546 1.00 0.00
D C
ATOM 24520 HB1 GLU D 72 14.399 41.481 74.602 1.00 0.00
D H
ATOM 24521 HB2 GLU D 72 14.953 40.880 73.023 1.00 0.00
D H
ATOM 24522 CG GLU D 72 13.756 42.678 72.935 1.00 0.00
D C
ATOM 24523 HG1 GLU D 72 13.210 42.532 71.985 1.00 0.00
D H
ATOM 24524 HG2 GLU D 72 13.136 43.287 73.622 1.00 0.00
D H
ATOM 24525 CD GLU D 72 14.999 43.471 72.577 1.00 0.00
D C
ATOM 24526 OE1 GLU D 72 15.980 43.452 73.357 1.00 0.00
D O
ATOM 24527 OE2 GLU D 72 14.993 44.120 71.511 1.00 0.00
D O
ATOM 24528 C GLU D 72 12.890 39.568 72.230 1.00 0.00
D C
ATOM 24529 O GLU D 72 12.317 40.023 71.235 1.00 0.00
D O
ATOM 24530 N TYR D 73 13.530 38.401 72.242 1.00 0.00
D N
ATOM 24531 HN TYR D 73 14.018 38.064 73.045 1.00 0.00
D H
ATOM 24532 CA TYR D 73 13.577 37.512 71.088 1.00 0.00
D C
ATOM 24533 HA TYR D 73 12.814 37.854 70.399 1.00 0.00
D H
ATOM 24534 CB TYR D 73 14.977 37.485 70.486 1.00 0.00
D C
ATOM 24535 HB1 TYR D 73 15.720 37.329 71.298 1.00 0.00
D H
ATOM 24536 HB2 TYR D 73 15.083 36.635 69.776 1.00 0.00
D H
ATOM 24537 CG TYR D 73 15.366 38.753 69.791 1.00 0.00
D C
ATOM 24538 CD1 TYR D 73 15.809 39.857 70.514 1.00 0.00
D C
ATOM 24539 HD1 TYR D 73 15.858 39.808 71.593 1.00 0.00
D H
ATOM 24540 CE1 TYR D 73 16.168 41.028 69.879 1.00 0.00
D C
ATOM 24541 HE1 TYR D 73 16.497 41.883 70.449 1.00 0.00
D H
ATOM 24542 CZ TYR D 73 16.095 41.098 68.504 1.00 0.00
D C

```
ATOM  24543  OH  TYR D  73      16.455  42.263  67.880  1.00  0.00
D    O
ATOM  24544  HH  TYR D  73      16.412  42.110  66.934  1.00  0.00
D    H
ATOM  24545  CD2 TYR D  73      15.306  38.851  68.407  1.00  0.00
D    C
ATOM  24546  HD2 TYR D  73      14.937  38.019  67.823  1.00  0.00
D    H
ATOM  24547  CE2 TYR D  73      15.664  40.017  67.761  1.00  0.00
D    C
ATOM  24548  HE2 TYR D  73      15.583  40.081  66.686  1.00  0.00
D    H
ATOM  24549  C   TYR D  73      13.215  36.101  71.488  1.00  0.00
D    C
ATOM  24550  O   TYR D  73      13.851  35.509  72.366  1.00  0.00
D    O
ATOM  24551  N   VAL D  74      12.191  35.556  70.844  1.00  0.00
D    N
ATOM  24552  HN  VAL D  74      11.572  36.040  70.232  1.00  0.00
D    H
ATOM  24553  CA  VAL D  74      11.895  34.147  71.003  1.00  0.00
D    C
ATOM  24554  HA  VAL D  74      12.651  33.704  71.640  1.00  0.00
D    H
ATOM  24555  CB  VAL D  74      10.529  33.900  71.669  1.00  0.00
D    C
ATOM  24556  HB  VAL D  74       9.742  34.303  70.985  1.00  0.00
D    H
ATOM  24557  CG1 VAL D  74      10.302  32.410  71.875  1.00  0.00
D    C
ATOM  24558 HG11 VAL D  74       9.334  32.235  72.391  1.00  0.00
D    H
ATOM  24559 HG12 VAL D  74      10.266  31.864  70.909  1.00  0.00
D    H
ATOM  24560 HG13 VAL D  74      11.109  31.973  72.501  1.00  0.00
D    H
ATOM  24561  CG2 VAL D  74      10.457  34.620  73.007  1.00  0.00
D    C
ATOM  24562 HG21 VAL D  74       9.489  34.414  73.512  1.00  0.00
D    H
ATOM  24563 HG22 VAL D  74      11.278  34.283  73.676  1.00  0.00
D    H
ATOM  24564 HG23 VAL D  74      10.540  35.719  72.878  1.00  0.00
D    H
ATOM  24565  C   VAL D  74      11.998  33.478  69.641  1.00  0.00
D    C
ATOM  24566  O   VAL D  74      11.434  33.950  68.657  1.00  0.00
D    O
ATOM  24567  N   ILE D  75      12.763  32.395  69.599  1.00  0.00
D    N
ATOM  24568  HN  ILE D  75      13.267  32.056  70.391  1.00  0.00
D    H
ATOM  24569  CA  ILE D  75      12.942  31.601  68.396  1.00  0.00
D    C
```

```
ATOM  24570  HA  ILE D  75      12.533  32.169  67.569  1.00  0.00
D    H
ATOM  24571  CB  ILE D  75      14.416  31.169  68.221  1.00  0.00
D    C
ATOM  24572  HB  ILE D  75      14.601  30.457  69.065  1.00  0.00
D    H
ATOM  24573  CG2 ILE D  75      14.619  30.451  66.886  1.00  0.00
D    C
ATOM  24574 HG21 ILE D  75      15.634  30.006  66.832  1.00  0.00
D    H
ATOM  24575 HG22 ILE D  75      13.884  29.627  66.764  1.00  0.00
D    H
ATOM  24576 HG23 ILE D  75      14.502  31.154  66.034  1.00  0.00
D    H
ATOM  24577  CG1 ILE D  75      15.375  32.353  68.432  1.00  0.00
D    C
ATOM  24578 HG11 ILE D  75      15.177  32.827  69.419  1.00  0.00
D    H
ATOM  24579 HG12 ILE D  75      16.421  31.970  68.454  1.00  0.00
D    H
ATOM  24580  CD  ILE D  75      15.299  33.419  67.362  1.00  0.00
D    C
ATOM  24581  HD1 ILE D  75      16.057  34.209  67.551  1.00  0.00
D    H
ATOM  24582  HD2 ILE D  75      15.493  32.983  66.359  1.00  0.00
D    H
ATOM  24583  HD3 ILE D  75      14.298  33.901  67.350  1.00  0.00
D    H
ATOM  24584  C   ILE D  75      12.109  30.342  68.517  1.00  0.00
D    C
ATOM  24585  O   ILE D  75      12.291  29.557  69.451  1.00  0.00
D    O
ATOM  24586  N   VAL D  76      11.190  30.149  67.581  1.00  0.00
D    N
ATOM  24587  HN  VAL D  76      10.963  30.785  66.848  1.00  0.00
D    H
ATOM  24588  CA  VAL D  76      10.413  28.920  67.572  1.00  0.00
D    C
ATOM  24589  HA  VAL D  76      10.462  28.467  68.556  1.00  0.00
D    H
ATOM  24590  CB  VAL D  76       8.936  29.153  67.234  1.00  0.00
D    C
ATOM  24591  HB  VAL D  76       8.876  29.421  66.150  1.00  0.00
D    H
ATOM  24592  CG1 VAL D  76       8.139  27.880  67.486  1.00  0.00
D    C
ATOM  24593 HG11 VAL D  76       7.061  28.053  67.280  1.00  0.00
D    H
ATOM  24594 HG12 VAL D  76       8.472  27.050  66.828  1.00  0.00
D    H
ATOM  24595 HG13 VAL D  76       8.239  27.557  68.545  1.00  0.00
D    H
ATOM  24596  CG2 VAL D  76       8.383  30.293  68.071  1.00  0.00
D    C
```

```
ATOM  24597 HG21 VAL D  76       7.303  30.446  67.863  1.00  0.00
D    H
ATOM  24598 HG22 VAL D  76       8.504  30.076  69.155  1.00  0.00
D    H
ATOM  24599 HG23 VAL D  76       8.906  31.246  67.847  1.00  0.00
D    H
ATOM  24600  C   VAL D  76      11.067  27.960  66.598  1.00  0.00
D    C
ATOM  24601  O   VAL D  76      10.992  28.128  65.379  1.00  0.00
D    O
ATOM  24602  N   ALA D  77      11.745  26.970  67.153  1.00  0.00
D    N
ATOM  24603  HN  ALA D  77      11.801  26.819  68.139  1.00  0.00
D    H
ATOM  24604  CA  ALA D  77      12.467  26.019  66.337  1.00  0.00
D    C
ATOM  24605  HA  ALA D  77      12.250  26.052  65.276  1.00  0.00
D    H
ATOM  24606  CB  ALA D  77      13.966  26.214  66.492  1.00  0.00
D    C
ATOM  24607  HB1 ALA D  77      14.267  27.175  66.024  1.00  0.00
D    H
ATOM  24608  HB2 ALA D  77      14.255  26.251  67.565  1.00  0.00
D    H
ATOM  24609  HB3 ALA D  77      14.538  25.398  66.000  1.00  0.00
D    H
ATOM  24610  C   ALA D  77      11.862  24.724  66.859  1.00  0.00
D    C
ATOM  24611  O   ALA D  77      12.364  24.119  67.809  1.00  0.00
D    O
ATOM  24612  N   ILE D  78      10.796  24.297  66.189  1.00  0.00
D    N
ATOM  24613  HN  ILE D  78      10.335  24.874  65.518  1.00  0.00
D    H
ATOM  24614  CA  ILE D  78      10.199  22.978  66.354  1.00  0.00
D    C
ATOM  24615  HA  ILE D  78      10.894  22.197  66.633  1.00  0.00
D    H
ATOM  24616  CB  ILE D  78       8.945  23.025  67.242  1.00  0.00
D    C
ATOM  24617  HB  ILE D  78       8.285  23.786  66.754  1.00  0.00
D    H
ATOM  24618  CG2 ILE D  78       8.315  21.645  67.346  1.00  0.00
D    C
ATOM  24619 HG21 ILE D  78       7.330  21.702  67.853  1.00  0.00
D    H
ATOM  24620 HG22 ILE D  78       8.150  21.208  66.339  1.00  0.00
D    H
ATOM  24621 HG23 ILE D  78       8.963  20.954  67.925  1.00  0.00
D    H
ATOM  24622  CG1 ILE D  78       9.296  23.566  68.630  1.00  0.00
D    C
ATOM  24623 HG11 ILE D  78       9.848  22.790  69.208  1.00  0.00
D    H
```

ATOM 24624 HG12 ILE D 78   9.979 24.439 68.516 1.00 0.00
D   H
ATOM 24625 CD ILE D 78   8.094 24.013 69.431 1.00 0.00
D   C
ATOM 24626 HD1 ILE D 78   8.416 24.447 70.403 1.00 0.00
D   H
ATOM 24627 HD2 ILE D 78   7.519 24.787 68.878 1.00 0.00
D   H
ATOM 24628 HD3 ILE D 78   7.418 23.158 69.645 1.00 0.00
D   H
ATOM 24629 C   ILE D 78   10.028 22.896 64.841 1.00 0.00
D   C
ATOM 24630 O   ILE D 78   9.532 23.831 64.211 1.00 0.00
D   O
ATOM 24631 N   GLY D 79   10.460 21.781 64.259 1.00 0.00
D   N
ATOM 24632 HN GLY D 79   10.657 20.951 64.779 1.00 0.00
D   H
ATOM 24633 CA GLY D 79   10.674 21.692 62.844 1.00 0.00
D   C
ATOM 24634 HA1 GLY D 79   11.530 21.084 62.581 1.00 0.00
D   H
ATOM 24635 HA2 GLY D 79   10.639 22.650 62.343 1.00 0.00
D   H
ATOM 24636 C   GLY D 79   9.425 20.926 62.473 1.00 0.00
D   C
ATOM 24637 O   GLY D 79   8.454 21.496 61.964 1.00 0.00
D   O
ATOM 24638 N   ALA D 80   9.445 19.632 62.762 1.00 0.00
D   N
ATOM 24639 HN ALA D 80   10.077 19.240 63.429 1.00 0.00
D   H
ATOM 24640 CA ALA D 80   8.539 18.682 62.124 1.00 0.00
D   C
ATOM 24641 HA ALA D 80   8.696 18.712 61.051 1.00 0.00
D   H
ATOM 24642 CB ALA D 80   8.888 17.268 62.557 1.00 0.00
D   C
ATOM 24643 HB1 ALA D 80   9.957 17.064 62.338 1.00 0.00
D   H
ATOM 24644 HB2 ALA D 80   8.731 17.136 63.650 1.00 0.00
D   H
ATOM 24645 HB3 ALA D 80   8.277 16.512 62.018 1.00 0.00
D   H
ATOM 24646 C   ALA D 80   7.060 18.965 62.351 1.00 0.00
D   C
ATOM 24647 O   ALA D 80   6.270 19.000 61.405 1.00 0.00
D   O
ATOM 24648 N   ASN D 81   6.701 19.173 63.612 1.00 0.00
D   N
ATOM 24649 HN ASN D 81   7.360 19.197 64.364 1.00 0.00
D   H
ATOM 24650 CA ASN D 81   5.320 19.384 64.014 1.00 0.00
D   C

ATOM 24651 HA ASN D 81 4.746 18.642 63.468 1.00 0.00
D H
ATOM 24652 CB ASN D 81 5.210 19.203 65.526 1.00 0.00
D C
ATOM 24653 HB1 ASN D 81 5.885 18.369 65.819 1.00 0.00
D H
ATOM 24654 HB2 ASN D 81 5.562 20.115 66.050 1.00 0.00
D H
ATOM 24655 CG ASN D 81 3.809 18.850 65.976 1.00 0.00
D C
ATOM 24656 OD1 ASN D 81 2.823 19.177 65.319 1.00 0.00
D O
ATOM 24657 ND2 ASN D 81 3.717 18.179 67.116 1.00 0.00
D N
ATOM 24658 HD21 ASN D 81 2.804 17.926 67.425 1.00 0.00
D H
ATOM 24659 HD22 ASN D 81 4.541 17.922 67.611 1.00 0.00
D H
ATOM 24660 C ASN D 81 4.800 20.764 63.606 1.00 0.00
D C
ATOM 24661 O ASN D 81 4.942 21.737 64.350 1.00 0.00
D O
ATOM 24662 N ILE D 82 4.192 20.850 62.425 1.00 0.00
D N
ATOM 24663 HN ILE D 82 4.051 20.084 61.801 1.00 0.00
D H
ATOM 24664 CA ILE D 82 3.666 22.126 61.939 1.00 0.00
D C
ATOM 24665 HA ILE D 82 4.499 22.817 61.950 1.00 0.00
D H
ATOM 24666 CB ILE D 82 3.136 22.021 60.490 1.00 0.00
D C
ATOM 24667 HB ILE D 82 2.465 21.124 60.502 1.00 0.00
D H
ATOM 24668 CG2 ILE D 82 2.451 23.312 60.066 1.00 0.00
D C
ATOM 24669 HG21 ILE D 82 1.938 23.180 59.090 1.00 0.00
D H
ATOM 24670 HG22 ILE D 82 1.685 23.617 60.810 1.00 0.00
D H
ATOM 24671 HG23 ILE D 82 3.187 24.137 59.962 1.00 0.00
D H
ATOM 24672 CG1 ILE D 82 4.279 21.707 59.526 1.00 0.00
D C
ATOM 24673 HG11 ILE D 82 4.995 22.559 59.501 1.00 0.00
D H
ATOM 24674 HG12 ILE D 82 4.837 20.819 59.905 1.00 0.00
D H
ATOM 24675 CD ILE D 82 3.832 21.406 58.113 1.00 0.00
D C
ATOM 24676 HD1 ILE D 82 4.705 21.131 57.482 1.00 0.00
D H
ATOM 24677 HD2 ILE D 82 3.113 20.558 58.101 1.00 0.00
D H

ATOM 24678 HD3 ILE D 82   3.341 22.290 57.654 1.00 0.00
D   H
ATOM 24679 C   ILE D 82   2.601 22.700 62.877 1.00 0.00
D   C
ATOM 24680 O   ILE D 82   2.581 23.907 63.117 1.00 0.00
D   O
ATOM 24681 N   GLN D 83   1.733 21.839 63.413 1.00 0.00
D   N
ATOM 24682 HN GLN D 83   1.738 20.861 63.214 1.00 0.00
D   H
ATOM 24683 CA GLN D 83   0.689 22.263 64.350 1.00 0.00
D   C
ATOM 24684 HA GLN D 83   0.077 22.995 63.837 1.00 0.00
D   H
ATOM 24685 CB GLN D 83   -0.163 21.070 64.796 1.00 0.00
D   C
ATOM 24686 HB1 GLN D 83   -0.753 20.746 63.909 1.00 0.00
D   H
ATOM 24687 HB2 GLN D 83   0.504 20.216 65.055 1.00 0.00
D   H
ATOM 24688 CG GLN D 83   -1.116 21.383 65.953 1.00 0.00
D   C
ATOM 24689 HG1 GLN D 83   -0.598 21.986 66.730 1.00 0.00
D   H
ATOM 24690 HG2 GLN D 83   -1.990 21.967 65.596 1.00 0.00
D   H
ATOM 24691 CD GLN D 83   -1.642 20.150 66.675 1.00 0.00
D   C
ATOM 24692 OE1 GLN D 83   -1.640 19.049 66.130 1.00 0.00
D   O
ATOM 24693 NE2 GLN D 83   -2.104 20.339 67.908 1.00 0.00
D   N
ATOM 24694 HE21 GLN D 83   -2.445 19.536 68.389 1.00 0.00
D   H
ATOM 24695 HE22 GLN D 83   -2.110 21.253 68.299 1.00 0.00
D   H
ATOM 24696 C   GLN D 83   1.299 22.946 65.568 1.00 0.00
D   C
ATOM 24697 O   GLN D 83   0.942 24.079 65.901 1.00 0.00
D   O
ATOM 24698 N   ALA D 84   2.221 22.245 66.222 1.00 0.00
D   N
ATOM 24699 HN ALA D 84   2.517 21.340 65.916 1.00 0.00
D   H
ATOM 24700 CA ALA D 84   2.867 22.738 67.424 1.00 0.00
D   C
ATOM 24701 HA ALA D 84   2.139 22.931 68.205 1.00 0.00
D   H
ATOM 24702 CB ALA D 84   3.865 21.721 67.944 1.00 0.00
D   C
ATOM 24703 HB1 ALA D 84   3.338 20.775 68.191 1.00 0.00
D   H
ATOM 24704 HB2 ALA D 84   4.637 21.493 67.178 1.00 0.00
D   H

ATOM 24705 HB3 ALA D 84   4.374 22.081 68.864 1.00 0.00
D   H
ATOM 24706 C   ALA D 84   3.555 24.058 67.145 1.00 0.00
D   C
ATOM 24707 O   ALA D 84   3.442 24.997 67.933 1.00 0.00
D   O
ATOM 24708 N   SER D 85   4.256 24.125 66.016 1.00 0.00
D   N
ATOM 24709 HN SER D 85   4.302 23.382 65.348 1.00 0.00
D   H
ATOM 24710 CA SER D 85   5.027 25.303 65.651 1.00 0.00
D   C
ATOM 24711 HA SER D 85   5.714 25.471 66.469 1.00 0.00
D   H
ATOM 24712 CB SER D 85   5.774 25.058 64.338 1.00 0.00
D   C
ATOM 24713 HB1 SER D 85   6.507 24.237 64.505 1.00 0.00
D   H
ATOM 24714 HB2 SER D 85   5.069 24.711 63.549 1.00 0.00
D   H
ATOM 24715 OG SER D 85   6.484 26.210 63.906 1.00 0.00
D   O
ATOM 24716 HG1 SER D 85   7.096 25.925 63.216 1.00 0.00
D   H
ATOM 24717 C   SER D 85   4.144 26.545 65.536 1.00 0.00
D   C
ATOM 24718 O   SER D 85   4.482 27.597 66.086 1.00 0.00
D   O
ATOM 24719 N   THR D 86   3.012 26.420 64.839 1.00 0.00
D   N
ATOM 24720 HN THR D 86   2.687 25.569 64.430 1.00 0.00
D   H
ATOM 24721 CA THR D 86   2.137 27.563 64.617 1.00 0.00
D   C
ATOM 24722 HA THR D 86   2.790 28.405 64.434 1.00 0.00
D   H
ATOM 24723 CB THR D 86   1.193 27.388 63.403 1.00 0.00
D   C
ATOM 24724 HB THR D 86   0.767 28.400 63.192 1.00 0.00
D   H
ATOM 24725 OG1 THR D 86   0.107 26.521 63.735 1.00 0.00
D   O
ATOM 24726 HG1 THR D 86   -0.423 26.455 62.933 1.00 0.00
D   H
ATOM 24727 CG2 THR D 86   1.932 26.811 62.225 1.00 0.00
D   C
ATOM 24728 HG21 THR D 86   1.285 26.791 61.323 1.00 0.00
D   H
ATOM 24729 HG22 THR D 86   2.826 27.426 61.990 1.00 0.00
D   H
ATOM 24730 HG23 THR D 86   2.269 25.772 62.433 1.00 0.00
D   H
ATOM 24731 C   THR D 86   1.341 27.897 65.870 1.00 0.00
D   C

ATOM 24732 O   THR D 86   1.109 29.075 66.158 1.00 0.00
D   O
ATOM 24733 N   LEU D 87   0.936 26.868 66.613 1.00 0.00
D   N
ATOM 24734 HN LEU D 87   1.073 25.911 66.366 1.00 0.00
D   H
ATOM 24735 CA LEU D 87   0.236 27.067 67.885 1.00 0.00
D   C
ATOM 24736 HA LEU D 87   -0.630 27.680 67.671 1.00 0.00
D   H
ATOM 24737 CB LEU D 87   -0.252 25.726 68.451 1.00 0.00
D   C
ATOM 24738 HB1 LEU D 87   -0.895 25.255 67.673 1.00 0.00
D   H
ATOM 24739 HB2 LEU D 87   0.621 25.049 68.578 1.00 0.00
D   H
ATOM 24740 CG LEU D 87   -1.062 25.710 69.755 1.00 0.00
D   C
ATOM 24741 HG LEU D 87   -0.329 25.963 70.558 1.00 0.00
D   H
ATOM 24742 CD1 LEU D 87   -2.195 26.736 69.756 1.00 0.00
D   C
ATOM 24743 HD11 LEU D 87   -2.787 26.663 70.694 1.00 0.00
D   H
ATOM 24744 HD12 LEU D 87   -1.788 27.768 69.681 1.00 0.00
D   H
ATOM 24745 HD13 LEU D 87   -2.880 26.565 68.898 1.00 0.00
D   H
ATOM 24746 CD2 LEU D 87   -1.597 24.308 70.029 1.00 0.00
D   C
ATOM 24747 HD21 LEU D 87   -2.167 24.292 70.983 1.00 0.00
D   H
ATOM 24748 HD22 LEU D 87   -2.277 23.965 69.224 1.00 0.00
D   H
ATOM 24749 HD23 LEU D 87   -0.760 23.581 70.112 1.00 0.00
D   H
ATOM 24750 C   LEU D 87   1.119 27.841 68.890 1.00 0.00
D   C
ATOM 24751 O   LEU D 87   0.657 28.781 69.545 1.00 0.00
D   O
ATOM 24752 N   THR D 88   2.388 27.452 68.978 1.00 0.00
D   N
ATOM 24753 HN THR D 88   2.754 26.650 68.508 1.00 0.00
D   H
ATOM 24754 CA THR D 88   3.363 28.172 69.777 1.00 0.00
D   C
ATOM 24755 HA THR D 88   2.962 28.209 70.781 1.00 0.00
D   H
ATOM 24756 CB THR D 88   4.744 27.492 69.729 1.00 0.00
D   C
ATOM 24757 HB THR D 88   4.994 27.375 68.646 1.00 0.00
D   H
ATOM 24758 OG1 THR D 88   4.651 26.182 70.309 1.00 0.00
D   O

ATOM 24759 HG1 THR D 88   5.540 25.817 70.260 1.00 0.00
D   H
ATOM 24760 CG2 THR D 88   5.780 28.309 70.494 1.00 0.00
D   C
ATOM 24761 HG21 THR D 88   6.796 27.880 70.364 1.00 0.00
D   H
ATOM 24762 HG22 THR D 88   5.801 29.355 70.121 1.00 0.00
D   H
ATOM 24763 HG23 THR D 88   5.549 28.332 71.581 1.00 0.00
D   H
ATOM 24764 C   THR D 88   3.477 29.616 69.301 1.00 0.00
D   C
ATOM 24765 O   THR D 88   3.336 30.549 70.101 1.00 0.00
D   O
ATOM 24766 N   THR D 89   3.709 29.796 67.999 1.00 0.00
D   N
ATOM 24767 HN THR D 89   3.792 29.053 67.336 1.00 0.00
D   H
ATOM 24768 CA THR D 89   3.864 31.135 67.425 1.00 0.00
D   C
ATOM 24769 HA THR D 89   4.700 31.594 67.936 1.00 0.00
D   H
ATOM 24770 CB THR D 89   4.080 31.093 65.902 1.00 0.00
D   C
ATOM 24771 HB THR D 89   3.185 30.578 65.476 1.00 0.00
D   H
ATOM 24772 OG1 THR D 89   5.235 30.295 65.618 1.00 0.00
D   O
ATOM 24773 HG1 THR D 89   5.328 30.312 64.660 1.00 0.00
D   H
ATOM 24774 CG2 THR D 89   4.293 32.495 65.335 1.00 0.00
D   C
ATOM 24775 HG21 THR D 89   4.335 32.471 64.225 1.00 0.00
D   H
ATOM 24776 HG22 THR D 89   3.456 33.163 65.630 1.00 0.00
D   H
ATOM 24777 HG23 THR D 89   5.241 32.938 65.708 1.00 0.00
D   H
ATOM 24778 C   THR D 89   2.645 31.972 67.754 1.00 0.00
D   C
ATOM 24779 O   THR D 89   2.763 33.124 68.160 1.00 0.00
D   O
ATOM 24780 N   LEU D 90   1.475 31.366 67.600 1.00 0.00
D   N
ATOM 24781 HN LEU D 90   1.380 30.428 67.275 1.00 0.00
D   H
ATOM 24782 CA LEU D 90   0.216 32.026 67.895 1.00 0.00
D   C
ATOM 24783 HA LEU D 90   0.157 32.878 67.230 1.00 0.00
D   H
ATOM 24784 CB LEU D 90   -0.932 31.060 67.639 1.00 0.00
D   C
ATOM 24785 HB1 LEU D 90   -0.680 30.485 66.718 1.00 0.00
D   H

```
ATOM  24786  HB2 LEU D  90      -0.970  30.316  68.465  1.00  0.00
D    H
ATOM  24787  CG  LEU D  90      -2.308  31.671  67.444  1.00  0.00
D    C
ATOM  24788  HG  LEU D  90      -2.771  31.874  68.439  1.00  0.00
D    H
ATOM  24789  CD1 LEU D  90      -2.186  32.970  66.670  1.00  0.00
D    C
ATOM  24790 HD11 LEU D  90      -3.190  33.405  66.478  1.00  0.00
D    H
ATOM  24791 HD12 LEU D  90      -1.588  33.712  67.243  1.00  0.00
D    H
ATOM  24792 HD13 LEU D  90      -1.689  32.799  65.692  1.00  0.00
D    H
ATOM  24793  CD2 LEU D  90      -3.192  30.678  66.708  1.00  0.00
D    C
ATOM  24794 HD21 LEU D  90      -4.213  31.098  66.571  1.00  0.00
D    H
ATOM  24795 HD22 LEU D  90      -2.789  30.435  65.704  1.00  0.00
D    H
ATOM  24796 HD23 LEU D  90      -3.281  29.733  67.287  1.00  0.00
D    H
ATOM  24797  C   LEU D  90       0.166  32.556  69.330  1.00  0.00
D    C
ATOM  24798  O   LEU D  90      -0.188  33.715  69.554  1.00  0.00
D    O
ATOM  24799  N   LEU D  91       0.537  31.697  70.281  1.00  0.00
D    N
ATOM  24800  HN  LEU D  91       0.791  30.753  70.083  1.00  0.00
D    H
ATOM  24801  CA  LEU D  91       0.606  32.055  71.699  1.00  0.00
D    C
ATOM  24802  HA  LEU D  91      -0.371  32.442  71.959  1.00  0.00
D    H
ATOM  24803  CB  LEU D  91       0.938  30.826  72.553  1.00  0.00
D    C
ATOM  24804  HB1 LEU D  91       1.909  30.421  72.184  1.00  0.00
D    H
ATOM  24805  HB2 LEU D  91       1.120  31.153  73.602  1.00  0.00
D    H
ATOM  24806  CG  LEU D  91      -0.059  29.663  72.568  1.00  0.00
D    C
ATOM  24807  HG  LEU D  91      -0.219  29.373  71.502  1.00  0.00
D    H
ATOM  24808  CD1 LEU D  91       0.540  28.492  73.327  1.00  0.00
D    C
ATOM  24809 HD11 LEU D  91      -0.187  27.655  73.390  1.00  0.00
D    H
ATOM  24810 HD12 LEU D  91       1.455  28.122  72.816  1.00  0.00
D    H
ATOM  24811 HD13 LEU D  91       0.813  28.793  74.361  1.00  0.00
D    H
ATOM  24812  CD2 LEU D  91      -1.409  30.061  73.158  1.00  0.00
D    C
```

ATOM 24813 HD21 LEU D 91   −2.110 29.199 73.135 1.00 0.00
D   H
ATOM 24814 HD22 LEU D 91   −1.315 30.389 74.212 1.00 0.00
D   H
ATOM 24815 HD23 LEU D 91   −1.860 30.891 72.572 1.00 0.00
D   H
ATOM 24816 C   LEU D 91   1.627 33.156 71.973 1.00 0.00
D   C
ATOM 24817 O   LEU D 91   1.365 34.076 72.749 1.00 0.00
D   O
ATOM 24818 N   LEU D 92   2.786 33.058 71.337 1.00 0.00
D   N
ATOM 24819 HN LEU D 92   3.032 32.316 70.716 1.00 0.00
D   H
ATOM 24820 CA LEU D 92   3.824 34.063 71.506 1.00 0.00
D   C
ATOM 24821 HA LEU D 92   4.023 34.123 72.568 1.00 0.00
D   H
ATOM 24822 CB LEU D 92   5.104 33.655 70.775 1.00 0.00
D   C
ATOM 24823 HB1 LEU D 92   4.820 33.415 69.724 1.00 0.00
D   H
ATOM 24824 HB2 LEU D 92   5.783 34.535 70.718 1.00 0.00
D   H
ATOM 24825 CG LEU D 92   5.858 32.453 71.350 1.00 0.00
D   C
ATOM 24826 HG LEU D 92   5.165 31.579 71.310 1.00 0.00
D   H
ATOM 24827 CD1 LEU D 92   7.090 32.171 70.514 1.00 0.00
D   C
ATOM 24828 HD11 LEU D 92   7.673 31.330 70.949 1.00 0.00
D   H
ATOM 24829 HD12 LEU D 92   6.804 31.897 69.476 1.00 0.00
D   H
ATOM 24830 HD13 LEU D 92   7.749 33.064 70.472 1.00 0.00
D   H
ATOM 24831 CD2 LEU D 92   6.248 32.678 72.800 1.00 0.00
D   C
ATOM 24832 HD21 LEU D 92   6.777 31.786 73.200 1.00 0.00
D   H
ATOM 24833 HD22 LEU D 92   6.925 33.549 72.913 1.00 0.00
D   H
ATOM 24834 HD23 LEU D 92   5.345 32.855 73.424 1.00 0.00
D   H
ATOM 24835 C   LEU D 92   3.354 35.440 71.047 1.00 0.00
D   C
ATOM 24836 O   LEU D 92   3.671 36.444 71.680 1.00 0.00
D   O
ATOM 24837 N   LYS D 93   2.584 35.480 69.963 1.00 0.00
D   N
ATOM 24838 HN LYS D 93   2.312 34.668 69.450 1.00 0.00
D   H
ATOM 24839 CA LYS D 93   2.076 36.742 69.440 1.00 0.00
D   C

ATOM 24840 HA LYS D 93 2.905 37.437 69.399 1.00 0.00
D H
ATOM 24841 CB LYS D 93 1.514 36.561 68.028 1.00 0.00
D C
ATOM 24842 HB1 LYS D 93 2.314 36.087 67.415 1.00 0.00
D H
ATOM 24843 HB2 LYS D 93 0.674 35.831 68.063 1.00 0.00
D H
ATOM 24844 CG LYS D 93 1.069 37.841 67.338 1.00 0.00
D C
ATOM 24845 HG1 LYS D 93 0.581 37.583 66.371 1.00 0.00
D H
ATOM 24846 HG2 LYS D 93 0.303 38.347 67.972 1.00 0.00
D H
ATOM 24847 CD LYS D 93 2.218 38.799 67.056 1.00 0.00
D C
ATOM 24848 HD1 LYS D 93 2.781 38.963 68.004 1.00 0.00
D H
ATOM 24849 HD2 LYS D 93 2.913 38.306 66.337 1.00 0.00
D H
ATOM 24850 CE LYS D 93 1.689 40.111 66.491 1.00 0.00
D C
ATOM 24851 HE1 LYS D 93 1.307 39.964 65.456 1.00 0.00
D H
ATOM 24852 HE2 LYS D 93 0.859 40.500 67.121 1.00 0.00
D H
ATOM 24853 NZ LYS D 93 2.726 41.181 66.424 1.00 0.00
D N
ATOM 24854 HZ1 LYS D 93 2.315 42.052 66.032 1.00 0.00
D H
ATOM 24855 HZ2 LYS D 93 3.091 41.371 67.380 1.00 0.00
D H
ATOM 24856 HZ3 LYS D 93 3.509 40.865 65.817 1.00 0.00
D H
ATOM 24857 C LYS D 93 1.038 37.327 70.395 1.00 0.00
D C
ATOM 24858 O LYS D 93 0.937 38.549 70.537 1.00 0.00
D O
ATOM 24859 N GLU D 94 0.291 36.451 71.063 1.00 0.00
D N
ATOM 24860 HN GLU D 94 0.329 35.465 70.928 1.00 0.00
D H
ATOM 24861 CA GLU D 94 -0.671 36.873 72.075 1.00 0.00
D C
ATOM 24862 HA GLU D 94 -1.323 37.612 71.627 1.00 0.00
D H
ATOM 24863 CB GLU D 94 -1.511 35.690 72.553 1.00 0.00
D C
ATOM 24864 HB1 GLU D 94 -0.847 34.799 72.626 1.00 0.00
D H
ATOM 24865 HB2 GLU D 94 -1.879 35.882 73.587 1.00 0.00
D H
ATOM 24866 CG GLU D 94 -2.678 35.361 71.645 1.00 0.00
D C

ATOM 24867 HG1 GLU D 94 -3.343 36.241 71.565 1.00 0.00
D H
ATOM 24868 HG2 GLU D 94 -2.326 35.076 70.634 1.00 0.00
D H
ATOM 24869 CD GLU D 94 -3.550 34.239 72.181 1.00 0.00
D C
ATOM 24870 OE1 GLU D 94 -3.758 33.251 71.444 1.00 0.00
D O
ATOM 24871 OE2 GLU D 94 -4.027 34.338 73.333 1.00 0.00
D O
ATOM 24872 C GLU D 94 0.014 37.536 73.265 1.00 0.00
D C
ATOM 24873 O GLU D 94 -0.508 38.496 73.833 1.00 0.00
D O
ATOM 24874 N LEU D 95 1.187 37.029 73.627 1.00 0.00
D N
ATOM 24875 HN LEU D 95 1.651 36.295 73.134 1.00 0.00
D H
ATOM 24876 CA LEU D 95 1.908 37.512 74.799 1.00 0.00
D C
ATOM 24877 HA LEU D 95 1.153 37.815 75.514 1.00 0.00
D H
ATOM 24878 CB LEU D 95 2.738 36.385 75.424 1.00 0.00
D C
ATOM 24879 HB1 LEU D 95 3.465 36.044 74.650 1.00 0.00
D H
ATOM 24880 HB2 LEU D 95 3.348 36.803 76.255 1.00 0.00
D H
ATOM 24881 CG LEU D 95 1.965 35.153 75.921 1.00 0.00
D C
ATOM 24882 HG LEU D 95 1.335 34.851 75.049 1.00 0.00
D H
ATOM 24883 CD1 LEU D 95 2.911 34.022 76.287 1.00 0.00
D C
ATOM 24884 HD11 LEU D 95 2.345 33.156 76.689 1.00 0.00
D H
ATOM 24885 HD12 LEU D 95 3.480 33.684 75.395 1.00 0.00
D H
ATOM 24886 HD13 LEU D 95 3.636 34.352 77.060 1.00 0.00
D H
ATOM 24887 CD2 LEU D 95 1.037 35.468 77.085 1.00 0.00
D C
ATOM 24888 HD21 LEU D 95 0.486 34.554 77.398 1.00 0.00
D H
ATOM 24889 HD22 LEU D 95 1.597 35.841 77.965 1.00 0.00
D H
ATOM 24890 HD23 LEU D 95 0.291 36.239 76.791 1.00 0.00
D H
ATOM 24891 C LEU D 95 2.768 38.737 74.489 1.00 0.00
D C
ATOM 24892 O LEU D 95 3.573 39.169 75.321 1.00 0.00
D O
ATOM 24893 N ASP D 96 2.580 39.293 73.292 1.00 0.00
D N

ATOM 24894 HN ASP D 96 1.991 38.896 72.593 1.00 0.00
D H
ATOM 24895 CA ASP D 96 3.213 40.550 72.863 1.00 0.00
D C
ATOM 24896 HA ASP D 96 2.800 40.745 71.880 1.00 0.00
D H
ATOM 24897 CB ASP D 96 2.793 41.715 73.773 1.00 0.00
D C
ATOM 24898 HB1 ASP D 96 2.585 41.326 74.791 1.00 0.00
D H
ATOM 24899 HB2 ASP D 96 3.604 42.466 73.846 1.00 0.00
D H
ATOM 24900 CG ASP D 96 1.540 42.409 73.291 1.00 0.00
D C
ATOM 24901 OD1 ASP D 96 0.542 42.429 74.046 1.00 0.00
D O
ATOM 24902 OD2 ASP D 96 1.553 42.932 72.153 1.00 0.00
D O
ATOM 24903 C ASP D 96 4.740 40.523 72.705 1.00 0.00
D C
ATOM 24904 O ASP D 96 5.393 41.566 72.791 1.00 0.00
D O
ATOM 24905 N ILE D 97 5.305 39.341 72.469 1.00 0.00
D N
ATOM 24906 HN ILE D 97 4.806 38.478 72.441 1.00 0.00
D H
ATOM 24907 CA ILE D 97 6.738 39.212 72.228 1.00 0.00
D C
ATOM 24908 HA ILE D 97 7.245 39.594 73.105 1.00 0.00
D H
ATOM 24909 CB ILE D 97 7.141 37.748 71.948 1.00 0.00
D C
ATOM 24910 HB ILE D 97 6.540 37.460 71.049 1.00 0.00
D H
ATOM 24911 CG2 ILE D 97 8.638 37.630 71.684 1.00 0.00
D C
ATOM 24912 HG21 ILE D 97 8.893 36.609 71.332 1.00 0.00
D H
ATOM 24913 HG22 ILE D 97 8.960 38.346 70.899 1.00 0.00
D H
ATOM 24914 HG23 ILE D 97 9.222 37.836 72.606 1.00 0.00
D H
ATOM 24915 CG1 ILE D 97 6.691 36.823 73.091 1.00 0.00
D C
ATOM 24916 HG11 ILE D 97 5.579 36.813 73.152 1.00 0.00
D H
ATOM 24917 HG12 ILE D 97 7.017 35.783 72.863 1.00 0.00
D H
ATOM 24918 CD ILE D 97 7.232 37.174 74.460 1.00 0.00
D C
ATOM 24919 HD1 ILE D 97 6.903 36.421 75.209 1.00 0.00
D H
ATOM 24920 HD2 ILE D 97 8.343 37.191 74.449 1.00 0.00
D H

ATOM 24921 HD3 ILE D 97 6.868 38.169 74.789 1.00 0.00
D H
ATOM 24922 C ILE D 97 7.141 40.114 71.057 1.00 0.00
D C
ATOM 24923 O ILE D 97 6.506 40.077 70.001 1.00 0.00
D O
ATOM 24924 N PRO D 98 8.176 40.953 71.254 1.00 0.00
D N
ATOM 24925 CD PRO D 98 8.904 41.139 72.522 1.00 0.00
D C
ATOM 24926 HD1 PRO D 98 7.792 41.142 72.455 1.00 0.00
D H
ATOM 24927 HD2 PRO D 98 9.236 40.692 73.487 1.00 0.00
D H
ATOM 24928 CA PRO D 98 8.625 41.908 70.233 1.00 0.00
D C
ATOM 24929 HA PRO D 98 7.798 42.556 69.965 1.00 0.00
D H
ATOM 24930 CB PRO D 98 9.728 42.698 70.950 1.00 0.00
D C
ATOM 24931 HB1 PRO D 98 9.262 43.526 71.530 1.00 0.00
D H
ATOM 24932 HB2 PRO D 98 10.474 43.125 70.249 1.00 0.00
D H
ATOM 24933 CG PRO D 98 9.429 42.538 72.398 1.00 0.00
D C
ATOM 24934 HG1 PRO D 98 9.858 43.347 73.020 1.00 0.00
D H
ATOM 24935 HG2 PRO D 98 8.857 42.764 73.187 1.00 0.00
D H
ATOM 24936 C PRO D 98 9.182 41.256 68.964 1.00 0.00
D C
ATOM 24937 O PRO D 98 8.902 41.725 67.863 1.00 0.00
D O
ATOM 24938 N ASN D 99 9.973 40.197 69.114 1.00 0.00
D N
ATOM 24939 HN ASN D 99 10.235 39.812 69.998 1.00 0.00
D H
ATOM 24940 CA ASN D 99 10.532 39.495 67.956 1.00 0.00
D C
ATOM 24941 HA ASN D 99 9.969 39.870 67.108 1.00 0.00
D H
ATOM 24942 CB ASN D 99 12.011 39.821 67.772 1.00 0.00
D C
ATOM 24943 HB1 ASN D 99 12.549 39.483 68.684 1.00 0.00
D H
ATOM 24944 HB2 ASN D 99 12.425 39.257 66.912 1.00 0.00
D H
ATOM 24945 CG ASN D 99 12.260 41.298 67.601 1.00 0.00
D C
ATOM 24946 OD1 ASN D 99 12.364 42.035 68.581 1.00 0.00
D O
ATOM 24947 ND2 ASN D 99 12.367 41.738 66.354 1.00 0.00
D N

```
ATOM  24948 HD21 ASN D  99      12.543  42.711  66.226  1.00  0.00
D    H
ATOM  24949 HD22 ASN D  99      12.302  41.101  65.593  1.00  0.00
D    H
ATOM  24950  C   ASN D  99      10.342  37.986  68.006  1.00  0.00
D    C
ATOM  24951  O   ASN D  99      10.889  37.304  68.878  1.00  0.00
D    O
ATOM  24952  N   ILE D 100       9.561  37.479  67.057  1.00  0.00
D    N
ATOM  24953  HN  ILE D 100       9.107  38.030  66.361  1.00  0.00
D    H
ATOM  24954  CA  ILE D 100       9.299  36.055  66.954  1.00  0.00
D    C
ATOM  24955  HA  ILE D 100       9.865  35.570  67.739  1.00  0.00
D    H
ATOM  24956  CB  ILE D 100       7.801  35.733  67.092  1.00  0.00
D    C
ATOM  24957  HB  ILE D 100       7.308  36.343  66.294  1.00  0.00
D    H
ATOM  24958  CG2 ILE D 100       7.572  34.234  66.971  1.00  0.00
D    C
ATOM  24959 HG21 ILE D 100       6.487  34.007  66.913  1.00  0.00
D    H
ATOM  24960 HG22 ILE D 100       8.048  33.834  66.051  1.00  0.00
D    H
ATOM  24961 HG23 ILE D 100       7.992  33.695  67.846  1.00  0.00
D    H
ATOM  24962  CG1 ILE D 100       7.271  36.246  68.435  1.00  0.00
D    C
ATOM  24963 HG11 ILE D 100       7.522  35.521  69.241  1.00  0.00
D    H
ATOM  24964 HG12 ILE D 100       7.784  37.203  68.682  1.00  0.00
D    H
ATOM  24965  CD  ILE D 100       5.778  36.499  68.459  1.00  0.00
D    C
ATOM  24966  HD1 ILE D 100       5.475  36.922  69.441  1.00  0.00
D    H
ATOM  24967  HD2 ILE D 100       5.491  37.221  67.666  1.00  0.00
D    H
ATOM  24968  HD3 ILE D 100       5.212  35.557  68.302  1.00  0.00
D    H
ATOM  24969  C   ILE D 100       9.843  35.532  65.629  1.00  0.00
D    C
ATOM  24970  O   ILE D 100       9.369  35.907  64.555  1.00  0.00
D    O
ATOM  24971  N   TRP D 101      10.863  34.680  65.732  1.00  0.00
D    N
ATOM  24972  HN  TRP D 101      11.250  34.428  66.616  1.00  0.00
D    H
ATOM  24973  CA  TRP D 101      11.495  34.048  64.580  1.00  0.00
D    C
ATOM  24974  HA  TRP D 101      11.059  34.469  63.682  1.00  0.00
D    H
```

ATOM 24975 CB TRP D 101   12.999 34.231 64.630 1.00 0.00
D   C
ATOM 24976 HB1 TRP D 101   13.353 33.876 65.623 1.00 0.00
D   H
ATOM 24977 HB2 TRP D 101   13.484 33.586 63.865 1.00 0.00
D   H
ATOM 24978 CG TRP D 101   13.462 35.657 64.478 1.00 0.00
D   C
ATOM 24979 CD1 TRP D 101   12.762 36.818 64.784 1.00 0.00
D   C
ATOM 24980 HD1 TRP D 101   11.762 36.821 65.200 1.00 0.00
D   H
ATOM 24981 NE1 TRP D 101   13.520 37.933 64.529 1.00 0.00
D   N
ATOM 24982 HE1 TRP D 101   13.228 38.851 64.692 1.00 0.00
D   H
ATOM 24983 CE2 TRP D 101   14.740 37.586 64.068 1.00 0.00
D   C
ATOM 24984 CD2 TRP D 101   14.777 36.119 64.012 1.00 0.00
D   C
ATOM 24985 CE3 TRP D 101   15.938 35.492 63.565 1.00 0.00
D   C
ATOM 24986 HE3 TRP D 101   16.015 34.415 63.502 1.00 0.00
D   H
ATOM 24987 CZ3 TRP D 101   17.022 36.291 63.183 1.00 0.00
D   C
ATOM 24988 HZ3 TRP D 101   17.930 35.821 62.830 1.00 0.00
D   H
ATOM 24989 CZ2 TRP D 101   15.823 38.364 63.689 1.00 0.00
D   C
ATOM 24990 HZ2 TRP D 101   15.812 39.442 63.729 1.00 0.00
D   H
ATOM 24991 CH2 TRP D 101   16.962 37.692 63.243 1.00 0.00
D   C
ATOM 24992 HH2 TRP D 101   17.818 38.277 62.935 1.00 0.00
D   H
ATOM 24993 C   TRP D 101   11.175 32.587 64.576 1.00 0.00
D   C
ATOM 24994 O   TRP D 101   11.363 31.896 65.585 1.00 0.00
D   O
ATOM 24995 N   VAL D 102   10.688 32.104 63.438 1.00 0.00
D   N
ATOM 24996 HN VAL D 102   10.612 32.633 62.597 1.00 0.00
D   H
ATOM 24997 CA VAL D 102   10.220 30.735 63.331 1.00 0.00
D   C
ATOM 24998 HA VAL D 102   10.538 30.216 64.227 1.00 0.00
D   H
ATOM 24999 CB VAL D 102   8.687 30.675 63.204 1.00 0.00
D   C
ATOM 25000 HB VAL D 102   8.423 30.979 62.161 1.00 0.00
D   H
ATOM 25001 CG1 VAL D 102   8.188 29.261 63.455 1.00 0.00
D   C

ATOM 25002 HG11 VAL D 102   7.078 29.229 63.401 1.00 0.00
D   H
ATOM 25003 HG12 VAL D 102   8.577 28.549 62.697 1.00 0.00
D   H
ATOM 25004 HG13 VAL D 102   8.494 28.910 64.464 1.00 0.00
D   H
ATOM 25005 CG2 VAL D 102   8.028 31.647 64.174 1.00 0.00
D   C
ATOM 25006 HG21 VAL D 102   6.922 31.575 64.110 1.00 0.00
D   H
ATOM 25007 HG22 VAL D 102   8.334 31.422 65.219 1.00 0.00
D   H
ATOM 25008 HG23 VAL D 102   8.310 32.695 63.946 1.00 0.00
D   H
ATOM 25009 C   VAL D 102   10.849 30.020 62.140 1.00 0.00
D   C
ATOM 25010 O   VAL D 102   11.033 30.607 61.067 1.00 0.00
D   O
ATOM 25011 N   LYS D 103   11.186 28.749 62.343 1.00 0.00
D   N
ATOM 25012 HN LYS D 103   11.169 28.320 63.245 1.00 0.00
D   H
ATOM 25013 CA LYS D 103   11.610 27.881 61.255 1.00 0.00
D   C
ATOM 25014 HA LYS D 103   12.173 28.479 60.549 1.00 0.00
D   H
ATOM 25015 CB LYS D 103   12.461 26.732 61.785 1.00 0.00
D   C
ATOM 25016 HB1 LYS D 103   13.387 27.179 62.214 1.00 0.00
D   H
ATOM 25017 HB2 LYS D 103   11.926 26.246 62.632 1.00 0.00
D   H
ATOM 25018 CG LYS D 103   12.852 25.701 60.739 1.00 0.00
D   C
ATOM 25019 HG1 LYS D 103   11.931 25.312 60.249 1.00 0.00
D   H
ATOM 25020 HG2 LYS D 103   13.462 26.197 59.951 1.00 0.00
D   H
ATOM 25021 CD LYS D 103   13.612 24.565 61.398 1.00 0.00
D   C
ATOM 25022 HD1 LYS D 103   14.701 24.800 61.356 1.00 0.00
D   H
ATOM 25023 HD2 LYS D 103   13.320 24.530 62.473 1.00 0.00
D   H
ATOM 25024 CE LYS D 103   13.334 23.229 60.739 1.00 0.00
D   C
ATOM 25025 HE1 LYS D 103   13.286 22.421 61.502 1.00 0.00
D   H
ATOM 25026 HE2 LYS D 103   12.364 23.257 60.195 1.00 0.00
D   H
ATOM 25027 NZ LYS D 103   14.398 22.870 59.761 1.00 0.00
D   N
ATOM 25028 HZ1 LYS D 103   14.185 21.950 59.324 1.00 0.00
D   H

ATOM 25029 HZ2 LYS D 103 14.450 23.600 59.022 1.00 0.00
D H
ATOM 25030 HZ3 LYS D 103 15.314 22.816 60.251 1.00 0.00
D H
ATOM 25031 C LYS D 103 10.379 27.356 60.525 1.00 0.00
D C
ATOM 25032 O LYS D 103 9.464 26.821 61.142 1.00 0.00
D O
ATOM 25033 N ALA D 104 10.355 27.532 59.211 1.00 0.00
D N
ATOM 25034 HN ALA D 104 11.089 27.992 58.712 1.00 0.00
D H
ATOM 25035 CA ALA D 104 9.243 27.061 58.396 1.00 0.00
D C
ATOM 25036 HA ALA D 104 8.344 26.949 58.992 1.00 0.00
D H
ATOM 25037 CB ALA D 104 8.948 28.045 57.280 1.00 0.00
D C
ATOM 25038 HB1 ALA D 104 8.674 29.030 57.715 1.00 0.00
D H
ATOM 25039 HB2 ALA D 104 9.839 28.193 56.632 1.00 0.00
D H
ATOM 25040 HB3 ALA D 104 8.102 27.702 56.646 1.00 0.00
D H
ATOM 25041 C ALA D 104 9.556 25.683 57.826 1.00 0.00
D C
ATOM 25042 O ALA D 104 10.682 25.414 57.399 1.00 0.00
D O
ATOM 25043 N GLN D 105 8.551 24.815 57.816 1.00 0.00
D N
ATOM 25044 HN GLN D 105 7.634 25.021 58.153 1.00 0.00
D H
ATOM 25045 CA GLN D 105 8.715 23.463 57.299 1.00 0.00
D C
ATOM 25046 HA GLN D 105 9.734 23.169 57.521 1.00 0.00
D H
ATOM 25047 CB GLN D 105 7.744 22.510 57.976 1.00 0.00
D C
ATOM 25048 HB1 GLN D 105 6.731 22.969 57.893 1.00 0.00
D H
ATOM 25049 HB2 GLN D 105 7.701 21.557 57.402 1.00 0.00
D H
ATOM 25050 CG GLN D 105 8.094 22.275 59.425 1.00 0.00
D C
ATOM 25051 HG1 GLN D 105 7.993 23.216 60.006 1.00 0.00
D H
ATOM 25052 HG2 GLN D 105 7.414 21.523 59.876 1.00 0.00
D H
ATOM 25053 CD GLN D 105 9.523 21.812 59.577 1.00 0.00
D C
ATOM 25054 OE1 GLN D 105 9.937 20.856 58.929 1.00 0.00
D O
ATOM 25055 NE2 GLN D 105 10.288 22.497 60.426 1.00 0.00
D N

ATOM 25056 HE21 GLN D 105 11.232 22.196 60.524 1.00 0.00
D H
ATOM 25057 HE22 GLN D 105 9.901 23.258 60.937 1.00 0.00
D H
ATOM 25058 C GLN D 105 8.540 23.402 55.795 1.00 0.00
D C
ATOM 25059 O GLN D 105 9.285 22.701 55.106 1.00 0.00
D O
ATOM 25060 N ASN D 106 7.558 24.141 55.291 1.00 0.00
D N
ATOM 25061 HN ASN D 106 6.983 24.748 55.839 1.00 0.00
D H
ATOM 25062 CA ASN D 106 7.238 24.125 53.879 1.00 0.00
D C
ATOM 25063 HA ASN D 106 8.187 24.081 53.356 1.00 0.00
D H
ATOM 25064 CB ASN D 106 6.371 22.906 53.565 1.00 0.00
D C
ATOM 25065 HB1 ASN D 106 6.061 22.974 52.499 1.00 0.00
D H
ATOM 25066 HB2 ASN D 106 6.960 21.974 53.681 1.00 0.00
D H
ATOM 25067 CG ASN D 106 5.119 22.842 54.411 1.00 0.00
D C
ATOM 25068 OD1 ASN D 106 4.541 23.863 54.783 1.00 0.00
D O
ATOM 25069 ND2 ASN D 106 4.687 21.635 54.713 1.00 0.00
D N
ATOM 25070 HD21 ASN D 106 3.857 21.570 55.261 1.00 0.00
D H
ATOM 25071 HD22 ASN D 106 5.167 20.833 54.373 1.00 0.00
D H
ATOM 25072 C ASN D 106 6.543 25.424 53.469 1.00 0.00
D C
ATOM 25073 O ASN D 106 6.379 26.324 54.290 1.00 0.00
D O
ATOM 25074 N TYR D 107 6.135 25.513 52.206 1.00 0.00
D N
ATOM 25075 HN TYR D 107 6.282 24.799 51.524 1.00 0.00
D H
ATOM 25076 CA TYR D 107 5.424 26.686 51.710 1.00 0.00
D C
ATOM 25077 HA TYR D 107 6.113 27.517 51.804 1.00 0.00
D H
ATOM 25078 CB TYR D 107 5.009 26.503 50.244 1.00 0.00
D C
ATOM 25079 HB1 TYR D 107 5.921 26.445 49.612 1.00 0.00
D H
ATOM 25080 HB2 TYR D 107 4.454 25.550 50.110 1.00 0.00
D H
ATOM 25081 CG TYR D 107 4.178 27.648 49.709 1.00 0.00
D C
ATOM 25082 CD1 TYR D 107 4.778 28.855 49.334 1.00 0.00
D C

ATOM 25083 HD1 TYR D 107   5.847 28.979 49.436 1.00 0.00
D   H
ATOM 25084 CE1 TYR D 107   4.021 29.913 48.851 1.00 0.00
D   C
ATOM 25085 HE1 TYR D 107   4.493 30.842 48.566 1.00 0.00
D   H
ATOM 25086 CZ TYR D 107   2.644 29.775 48.743 1.00 0.00
D   C
ATOM 25087 OH TYR D 107   1.876 30.823 48.262 1.00 0.00
D   O
ATOM 25088 HH TYR D 107   0.971 30.511 48.196 1.00 0.00
D   H
ATOM 25089 CD2 TYR D 107   2.798 27.535 49.593 1.00 0.00
D   C
ATOM 25090 HD2 TYR D 107   2.301 26.630 49.914 1.00 0.00
D   H
ATOM 25091 CE2 TYR D 107   2.030 28.588 49.114 1.00 0.00
D   C
ATOM 25092 HE2 TYR D 107   0.957 28.485 49.052 1.00 0.00
D   H
ATOM 25093 C   TYR D 107   4.200 27.018 52.563 1.00 0.00
D   C
ATOM 25094 O   TYR D 107   4.005 28.170 52.951 1.00 0.00
D   O
ATOM 25095 N   TYR D 108   3.386 26.003 52.844 1.00 0.00
D   N
ATOM 25096 HN TYR D 108   3.564 25.065 52.548 1.00 0.00
D   H
ATOM 25097 CA TYR D 108   2.158 26.173 53.614 1.00 0.00
D   C
ATOM 25098 HA TYR D 108   1.564 26.898 53.072 1.00 0.00
D   H
ATOM 25099 CB TYR D 108   1.429 24.834 53.741 1.00 0.00
D   C
ATOM 25100 HB1 TYR D 108   0.765 24.696 52.861 1.00 0.00
D   H
ATOM 25101 HB2 TYR D 108   2.153 23.990 53.743 1.00 0.00
D   H
ATOM 25102 CG TYR D 108   0.553 24.738 54.962 1.00 0.00
D   C
ATOM 25103 CD1 TYR D 108   -0.640 25.460 55.049 1.00 0.00
D   C
ATOM 25104 HD1 TYR D 108   -0.938 26.107 54.235 1.00 0.00
D   H
ATOM 25105 CE1 TYR D 108   -1.443 25.379 56.175 1.00 0.00
D   C
ATOM 25106 HE1 TYR D 108   -2.358 25.948 56.240 1.00 0.00
D   H
ATOM 25107 CZ TYR D 108   -1.061 24.563 57.228 1.00 0.00
D   C
ATOM 25108 OH TYR D 108   -1.867 24.478 58.338 1.00 0.00
D   O
ATOM 25109 HH TYR D 108   -1.483 23.820 58.922 1.00 0.00
D   H

ATOM 25110 CD2 TYR D 108 0.914 23.928 56.037 1.00 0.00
D C
ATOM 25111 HD2 TYR D 108 1.851 23.389 56.018 1.00 0.00
D H
ATOM 25112 CE2 TYR D 108 0.113 23.834 57.168 1.00 0.00
D C
ATOM 25113 HE2 TYR D 108 0.423 23.213 57.995 1.00 0.00
D H
ATOM 25114 C TYR D 108 2.418 26.753 55.001 1.00 0.00
D C
ATOM 25115 O TYR D 108 1.699 27.643 55.474 1.00 0.00
D O
ATOM 25116 N HSE D 109 3.446 26.222 55.647 1.00 0.00
D N
ATOM 25117 HN HSE D 109 4.035 25.519 55.250 1.00 0.00
D H
ATOM 25118 CA HSE D 109 3.810 26.616 56.991 1.00 0.00
D C
ATOM 25119 HA HSE D 109 2.925 26.532 57.611 1.00 0.00
D H
ATOM 25120 CB HSE D 109 4.930 25.711 57.496 1.00 0.00
D C
ATOM 25121 HB1 HSE D 109 4.636 24.658 57.295 1.00 0.00
D H
ATOM 25122 HB2 HSE D 109 5.855 25.906 56.915 1.00 0.00
D H
ATOM 25123 ND1 HSE D 109 6.379 25.467 59.517 1.00 0.00
D N
ATOM 25124 CG HSE D 109 5.210 25.836 58.973 1.00 0.00
D C
ATOM 25125 CE1 HSE D 109 6.357 25.693 60.847 1.00 0.00
D C
ATOM 25126 HE1 HSE D 109 7.180 25.467 61.528 1.00 0.00
D H
ATOM 25127 NE2 HSE D 109 5.170 26.222 61.154 1.00 0.00
D N
ATOM 25128 HE2 HSE D 109 4.869 26.477 62.072 1.00 0.00
D H
ATOM 25129 CD2 HSE D 109 4.431 26.327 60.017 1.00 0.00
D C
ATOM 25130 HD2 HSE D 109 3.425 26.721 60.006 1.00 0.00
D H
ATOM 25131 C HSE D 109 4.213 28.056 56.993 1.00 0.00
D C
ATOM 25132 O HSE D 109 3.797 28.820 57.862 1.00 0.00
D O
ATOM 25133 N HSE D 110 5.000 28.441 55.992 1.00 0.00
D N
ATOM 25134 HN HSE D 110 5.368 27.811 55.310 1.00 0.00
D H
ATOM 25135 CA HSE D 110 5.397 29.832 55.802 1.00 0.00
D C
ATOM 25136 HA HSE D 110 5.994 30.108 56.662 1.00 0.00
D H

ATOM 25137 CB HSE D 110 6.197 29.979 54.517 1.00 0.00
D C
ATOM 25138 HB1 HSE D 110 7.030 29.243 54.546 1.00 0.00
D H
ATOM 25139 HB2 HSE D 110 5.558 29.712 53.650 1.00 0.00
D H
ATOM 25140 ND1 HSE D 110 8.093 31.602 54.551 1.00 0.00
D N
ATOM 25141 CG HSE D 110 6.796 31.348 54.322 1.00 0.00
D C
ATOM 25142 CE1 HSE D 110 8.350 32.894 54.293 1.00 0.00
D C
ATOM 25143 HE1 HSE D 110 9.330 33.366 54.402 1.00 0.00
D H
ATOM 25144 NE2 HSE D 110 7.207 33.473 53.897 1.00 0.00
D N
ATOM 25145 HE2 HSE D 110 7.098 34.438 53.658 1.00 0.00
D H
ATOM 25146 CD2 HSE D 110 6.228 32.546 53.902 1.00 0.00
D C
ATOM 25147 HD2 HSE D 110 5.206 32.770 53.627 1.00 0.00
D H
ATOM 25148 C HSE D 110 4.203 30.749 55.781 1.00 0.00
D C
ATOM 25149 O HSE D 110 4.200 31.772 56.468 1.00 0.00
D O
ATOM 25150 N LYS D 111 3.176 30.369 55.019 1.00 0.00
D N
ATOM 25151 HN LYS D 111 3.171 29.487 54.550 1.00 0.00
D H
ATOM 25152 CA LYS D 111 1.988 31.192 54.805 1.00 0.00
D C
ATOM 25153 HA LYS D 111 2.351 32.129 54.401 1.00 0.00
D H
ATOM 25154 CB LYS D 111 1.070 30.549 53.762 1.00 0.00
D C
ATOM 25155 HB1 LYS D 111 0.856 29.512 54.109 1.00 0.00
D H
ATOM 25156 HB2 LYS D 111 0.095 31.086 53.759 1.00 0.00
D H
ATOM 25157 CG LYS D 111 1.653 30.488 52.353 1.00 0.00
D C
ATOM 25158 HG1 LYS D 111 2.742 30.715 52.398 1.00 0.00
D H
ATOM 25159 HG2 LYS D 111 1.545 29.451 51.961 1.00 0.00
D H
ATOM 25160 CD LYS D 111 0.983 31.477 51.412 1.00 0.00
D C
ATOM 25161 HD1 LYS D 111 1.057 31.081 50.374 1.00 0.00
D H
ATOM 25162 HD2 LYS D 111 -0.100 31.526 51.674 1.00 0.00
D H
ATOM 25163 CE LYS D 111 1.594 32.867 51.488 1.00 0.00
D C

ATOM 25164 HE1 LYS D 111   1.738 33.173 52.549 1.00 0.00
D   H
ATOM 25165 HE2 LYS D 111   2.584 32.887 50.979 1.00 0.00
D   H
ATOM 25166 NZ LYS D 111   0.699 33.860 50.825 1.00 0.00
D   N
ATOM 25167 HZ1 LYS D 111   1.119 34.810 50.879 1.00 0.00
D   H
ATOM 25168 HZ2 LYS D 111   0.567 33.597 49.828 1.00 0.00
D   H
ATOM 25169 HZ3 LYS D 111   -0.225 33.864 51.303 1.00 0.00
D   H
ATOM 25170 C   LYS D 111   1.207 31.516 56.078 1.00 0.00
D   C
ATOM 25171 O   LYS D 111   0.824 32.671 56.272 1.00 0.00
D   O
ATOM 25172 N   VAL D 112   0.965 30.520 56.940 1.00 0.00
D   N
ATOM 25173 HN VAL D 112   1.234 29.570 56.802 1.00 0.00
D   H
ATOM 25174 CA VAL D 112   0.250 30.776 58.213 1.00 0.00
D   C
ATOM 25175 HA VAL D 112   -0.616 31.379 57.969 1.00 0.00
D   H
ATOM 25176 CB VAL D 112   -0.310 29.512 58.913 1.00 0.00
D   C
ATOM 25177 HB VAL D 112   -0.631 29.810 59.941 1.00 0.00
D   H
ATOM 25178 CG1 VAL D 112   -1.506 28.959 58.165 1.00 0.00
D   C
ATOM 25179 HG11 VAL D 112   -1.873 28.032 58.655 1.00 0.00
D   H
ATOM 25180 HG12 VAL D 112   -2.349 29.680 58.149 1.00 0.00
D   H
ATOM 25181 HG13 VAL D 112   -1.233 28.708 57.117 1.00 0.00
D   H
ATOM 25182 CG2 VAL D 112   0.756 28.449 59.085 1.00 0.00
D   C
ATOM 25183 HG21 VAL D 112   0.329 27.536 59.552 1.00 0.00
D   H
ATOM 25184 HG22 VAL D 112   1.190 28.168 58.102 1.00 0.00
D   H
ATOM 25185 HG23 VAL D 112   1.578 28.806 59.740 1.00 0.00
D   H
ATOM 25186 C   VAL D 112   1.092 31.564 59.206 1.00 0.00
D   C
ATOM 25187 O   VAL D 112   0.569 32.423 59.920 1.00 0.00
D   O
ATOM 25188 N   LEU D 113   2.389 31.272 59.245 1.00 0.00
D   N
ATOM 25189 HN LEU D 113   2.814 30.547 58.709 1.00 0.00
D   H
ATOM 25190 CA LEU D 113   3.311 32.006 60.095 1.00 0.00
D   C

ATOM 25191 HA LEU D 113   2.926 31.941 61.105 1.00 0.00
D   H
ATOM 25192 CB LEU D 113   4.702 31.391 60.018 1.00 0.00
D   C
ATOM 25193 HB1 LEU D 113   4.967 31.311 58.938 1.00 0.00
D   H
ATOM 25194 HB2 LEU D 113   5.436 32.100 60.462 1.00 0.00
D   H
ATOM 25195 CG LEU D 113   4.877 30.002 60.652 1.00 0.00
D   C
ATOM 25196 HG LEU D 113   4.018 29.401 60.268 1.00 0.00
D   H
ATOM 25197 CD1 LEU D 113   6.184 29.368 60.219 1.00 0.00
D   C
ATOM 25198 HD11 LEU D 113   6.325 28.385 60.719 1.00 0.00
D   H
ATOM 25199 HD12 LEU D 113   6.193 29.204 59.120 1.00 0.00
D   H
ATOM 25200 HD13 LEU D 113   7.044 30.019 60.484 1.00 0.00
D   H
ATOM 25201 CD2 LEU D 113   4.785 30.037 62.170 1.00 0.00
D   C
ATOM 25202 HD21 LEU D 113   4.897 29.013 62.587 1.00 0.00
D   H
ATOM 25203 HD22 LEU D 113   5.580 30.671 62.615 1.00 0.00
D   H
ATOM 25204 HD23 LEU D 113   3.798 30.437 62.492 1.00 0.00
D   H
ATOM 25205 C   LEU D 113   3.348 33.483 59.710 1.00 0.00
D   C
ATOM 25206 O   LEU D 113   3.383 34.360 60.579 1.00 0.00
D   O
ATOM 25207 N   GLU D 114   3.317 33.743 58.402 1.00 0.00
D   N
ATOM 25208 HN GLU D 114   3.350 33.022 57.716 1.00 0.00
D   H
ATOM 25209 CA GLU D 114   3.234 35.088 57.846 1.00 0.00
D   C
ATOM 25210 HA GLU D 114   4.108 35.642 58.162 1.00 0.00
D   H
ATOM 25211 CB GLU D 114   3.159 34.989 56.328 1.00 0.00
D   C
ATOM 25212 HB1 GLU D 114   3.740 34.094 56.008 1.00 0.00
D   H
ATOM 25213 HB2 GLU D 114   2.108 34.798 56.013 1.00 0.00
D   H
ATOM 25214 CG GLU D 114   3.684 36.180 55.550 1.00 0.00
D   C
ATOM 25215 HG1 GLU D 114   2.930 36.989 55.558 1.00 0.00
D   H
ATOM 25216 HG2 GLU D 114   4.628 36.558 55.991 1.00 0.00
D   H
ATOM 25217 CD GLU D 114   3.921 35.831 54.089 1.00 0.00
D   C

ATOM 25218 OE1 GLU D 114   2.957 35.423 53.393 1.00 0.00
D   O
ATOM 25219 OE2 GLU D 114   5.080 35.958 53.634 1.00 0.00
D   O
ATOM 25220 C   GLU D 114   2.014 35.822 58.389 1.00 0.00
D   C
ATOM 25221 O   GLU D 114   2.125 36.965 58.827 1.00 0.00
D   O
ATOM 25222 N   LYS D 115   0.863 35.153 58.386 1.00 0.00
D   N
ATOM 25223 HN LYS D 115   0.795 34.196 58.109 1.00 0.00
D   H
ATOM 25224 CA LYS D 115   -0.399 35.774 58.791 1.00 0.00
D   C
ATOM 25225 HA LYS D 115   -0.365 36.784 58.401 1.00 0.00
D   H
ATOM 25226 CB LYS D 115   -1.597 35.066 58.153 1.00 0.00
D   C
ATOM 25227 HB1 LYS D 115   -1.483 33.976 58.361 1.00 0.00
D   H
ATOM 25228 HB2 LYS D 115   -2.526 35.387 58.674 1.00 0.00
D   H
ATOM 25229 CG LYS D 115   -1.699 35.288 56.657 1.00 0.00
D   C
ATOM 25230 HG1 LYS D 115   -0.702 35.111 56.192 1.00 0.00
D   H
ATOM 25231 HG2 LYS D 115   -2.403 34.540 56.226 1.00 0.00
D   H
ATOM 25232 CD LYS D 115   -2.158 36.697 56.305 1.00 0.00
D   C
ATOM 25233 HD1 LYS D 115   -3.226 36.804 56.604 1.00 0.00
D   H
ATOM 25234 HD2 LYS D 115   -1.567 37.417 56.916 1.00 0.00
D   H
ATOM 25235 CE LYS D 115   -1.977 36.981 54.819 1.00 0.00
D   C
ATOM 25236 HE1 LYS D 115   -2.139 38.062 54.607 1.00 0.00
D   H
ATOM 25237 HE2 LYS D 115   -0.950 36.708 54.491 1.00 0.00
D   H
ATOM 25238 NZ LYS D 115   -2.933 36.216 53.965 1.00 0.00
D   N
ATOM 25239 HZ1 LYS D 115   -2.775 36.443 52.962 1.00 0.00
D   H
ATOM 25240 HZ2 LYS D 115   -2.794 35.197 54.115 1.00 0.00
D   H
ATOM 25241 HZ3 LYS D 115   -3.908 36.470 54.225 1.00 0.00
D   H
ATOM 25242 C   LYS D 115   -0.599 35.875 60.296 1.00 0.00
D   C
ATOM 25243 O   LYS D 115   -1.271 36.794 60.762 1.00 0.00
D   O
ATOM 25244 N   ILE D 116   -0.036 34.944 61.060 1.00 0.00
D   N

ATOM 25245 HN ILE D 116   0.496 34.167 60.730 1.00 0.00
D   H
ATOM 25246 CA ILE D 116   -0.171 35.004 62.517 1.00 0.00
D   C
ATOM 25247 HA ILE D 116   -1.117 35.493 62.712 1.00 0.00
D   H
ATOM 25248 CB ILE D 116   -0.247 33.607 63.183 1.00 0.00
D   C
ATOM 25249 HB ILE D 116   -0.374 33.825 64.274 1.00 0.00
D   H
ATOM 25250 CG2 ILE D 116   -1.422 32.808 62.633 1.00 0.00
D   C
ATOM 25251 HG21 ILE D 116   -1.570 31.876 63.219 1.00 0.00
D   H
ATOM 25252 HG22 ILE D 116   -2.363 33.395 62.693 1.00 0.00
D   H
ATOM 25253 HG23 ILE D 116   -1.250 32.527 61.573 1.00 0.00
D   H
ATOM 25254 CG1 ILE D 116   1.074 32.845 63.045 1.00 0.00
D   C
ATOM 25255 HG11 ILE D 116   1.354 32.769 61.970 1.00 0.00
D   H
ATOM 25256 HG12 ILE D 116   1.879 33.423 63.554 1.00 0.00
D   H
ATOM 25257 CD ILE D 116   1.034 31.456 63.646 1.00 0.00
D   C
ATOM 25258 HD1 ILE D 116   2.033 30.974 63.569 1.00 0.00
D   H
ATOM 25259 HD2 ILE D 116   0.753 31.503 64.720 1.00 0.00
D   H
ATOM 25260 HD3 ILE D 116   0.300 30.813 63.117 1.00 0.00
D   H
ATOM 25261 C   ILE D 116   0.920 35.877 63.152 1.00 0.00
D   C
ATOM 25262 O   ILE D 116   1.123 35.859 64.374 1.00 0.00
D   O
ATOM 25263 N   GLY D 117   1.612 36.639 62.306 1.00 0.00
D   N
ATOM 25264 HN GLY D 117   1.512 36.542 61.316 1.00 0.00
D   H
ATOM 25265 CA GLY D 117   2.556 37.659 62.747 1.00 0.00
D   C
ATOM 25266 HA1 GLY D 117   2.055 38.177 63.553 1.00 0.00
D   H
ATOM 25267 HA2 GLY D 117   2.744 38.272 61.875 1.00 0.00
D   H
ATOM 25268 C   GLY D 117   3.894 37.161 63.267 1.00 0.00
D   C
ATOM 25269 O   GLY D 117   4.343 37.590 64.330 1.00 0.00
D   O
ATOM 25270 N   ALA D 118   4.526 36.255 62.525 1.00 0.00
D   N
ATOM 25271 HN ALA D 118   4.082 35.760 61.778 1.00 0.00
D   H

ATOM 25272 CA ALA D 118   5.928 35.917 62.754 1.00 0.00
D   C
ATOM 25273 HA ALA D 118   6.174 35.877 63.809 1.00 0.00
D   H
ATOM 25274 CB ALA D 118   6.270 34.585 62.113 1.00 0.00
D   C
ATOM 25275 HB1 ALA D 118   5.681 33.777 62.596 1.00 0.00
D   H
ATOM 25276 HB2 ALA D 118   6.023 34.589 61.029 1.00 0.00
D   H
ATOM 25277 HB3 ALA D 118   7.348 34.340 62.230 1.00 0.00
D   H
ATOM 25278 C   ALA D 118   6.774 37.043 62.158 1.00 0.00
D   C
ATOM 25279 O   ALA D 118   6.527 37.496 61.036 1.00 0.00
D   O
ATOM 25280 N   ASP D 119   7.758 37.506 62.916 1.00 0.00
D   N
ATOM 25281 HN ASP D 119   8.026 37.147 63.806 1.00 0.00
D   H
ATOM 25282 CA ASP D 119   8.577 38.642 62.489 1.00 0.00
D   C
ATOM 25283 HA ASP D 119   7.905 39.334 61.995 1.00 0.00
D   H
ATOM 25284 CB ASP D 119   9.206 39.326 63.696 1.00 0.00
D   C
ATOM 25285 HB1 ASP D 119   9.797 38.585 64.270 1.00 0.00
D   H
ATOM 25286 HB2 ASP D 119   9.886 40.140 63.373 1.00 0.00
D   H
ATOM 25287 CG ASP D 119   8.170 39.903 64.629 1.00 0.00
D   C
ATOM 25288 OD1 ASP D 119   7.467 40.849 64.228 1.00 0.00
D   O
ATOM 25289 OD2 ASP D 119   8.048 39.406 65.765 1.00 0.00
D   O
ATOM 25290 C   ASP D 119   9.633 38.249 61.464 1.00 0.00
D   C
ATOM 25291 O   ASP D 119   9.953 39.029 60.569 1.00 0.00
D   O
ATOM 25292 N   ARG D 120   10.171 37.041 61.600 1.00 0.00
D   N
ATOM 25293 HN ARG D 120   9.981 36.467 62.393 1.00 0.00
D   H
ATOM 25294 CA ARG D 120   11.068 36.490 60.594 1.00 0.00
D   C
ATOM 25295 HA ARG D 120   10.827 36.931 59.634 1.00 0.00
D   H
ATOM 25296 CB ARG D 120   12.529 36.806 60.924 1.00 0.00
D   C
ATOM 25297 HB1 ARG D 120   12.590 37.914 61.052 1.00 0.00
D   H
ATOM 25298 HB2 ARG D 120   12.790 36.368 61.912 1.00 0.00
D   H

ATOM 25299 CG ARG D 120   13.494 36.373 59.836 1.00 0.00
D   C
ATOM 25300 HG1 ARG D 120   13.374 35.280 59.671 1.00 0.00
D   H
ATOM 25301 HG2 ARG D 120   13.197 36.855 58.875 1.00 0.00
D   H
ATOM 25302 CD ARG D 120   14.929 36.758 60.137 1.00 0.00
D   C
ATOM 25303 HD1 ARG D 120   15.068 37.848 59.949 1.00 0.00
D   H
ATOM 25304 HD2 ARG D 120   15.197 36.541 61.198 1.00 0.00
D   H
ATOM 25305 NE ARG D 120   15.844 35.993 59.294 1.00 0.00
D   N
ATOM 25306 HE ARG D 120   15.385 35.270 58.766 1.00 0.00
D   H
ATOM 25307 CZ ARG D 120   17.155 36.200 59.204 1.00 0.00
D   C
ATOM 25308 NH1 ARG D 120   17.736 37.164 59.911 1.00 0.00
D   N
ATOM 25309 HH11 ARG D 120   18.708 37.336 59.827 1.00 0.00
D   H
ATOM 25310 HH12 ARG D 120   17.124 37.778 60.414 1.00 0.00
D   H
ATOM 25311 NH2 ARG D 120   17.884 35.441 58.396 1.00 0.00
D   N
ATOM 25312 HH21 ARG D 120   18.870 35.532 58.389 1.00 0.00
D   H
ATOM 25313 HH22 ARG D 120   17.429 34.665 57.979 1.00 0.00
D   H
ATOM 25314 C   ARG D 120   10.847 34.984 60.451 1.00 0.00
D   C
ATOM 25315 O   ARG D 120   10.734 34.262 61.439 1.00 0.00
D   O
ATOM 25316 N   ILE D 121   10.780 34.525 59.208 1.00 0.00
D   N
ATOM 25317 HN ILE D 121   10.894 35.098 58.398 1.00 0.00
D   H
ATOM 25318 CA ILE D 121   10.531 33.122 58.915 1.00 0.00
D   C
ATOM 25319 HA ILE D 121   10.448 32.609 59.865 1.00 0.00
D   H
ATOM 25320 CB ILE D 121   9.259 32.958 58.063 1.00 0.00
D   C
ATOM 25321 HB ILE D 121   9.445 33.589 57.158 1.00 0.00
D   H
ATOM 25322 CG2 ILE D 121   9.023 31.492 57.730 1.00 0.00
D   C
ATOM 25323 HG21 ILE D 121   8.202 31.386 56.992 1.00 0.00
D   H
ATOM 25324 HG22 ILE D 121   9.934 31.036 57.288 1.00 0.00
D   H
ATOM 25325 HG23 ILE D 121   8.749 30.917 58.639 1.00 0.00
D   H

ATOM 25326 CG1 ILE D 121   8.055 33.567 58.796 1.00 0.00
D   C
ATOM 25327 HG11 ILE D 121   7.770 32.920 59.655 1.00 0.00
D   H
ATOM 25328 HG12 ILE D 121   8.349 34.557 59.212 1.00 0.00
D   H
ATOM 25329 CD ILE D 121   6.838 33.777 57.925 1.00 0.00
D   C
ATOM 25330 HD1 ILE D 121   6.029 34.272 58.505 1.00 0.00
D   H
ATOM 25331 HD2 ILE D 121   7.085 34.421 57.054 1.00 0.00
D   H
ATOM 25332 HD3 ILE D 121   6.446 32.809 57.548 1.00 0.00
D   H
ATOM 25333 C   ILE D 121   11.739 32.528 58.196 1.00 0.00
D   C
ATOM 25334 O   ILE D 121   12.120 33.000 57.125 1.00 0.00
D   O
ATOM 25335 N   ILE D 122   12.341 31.500 58.787 1.00 0.00
D   N
ATOM 25336 HN ILE D 122   12.009 31.042 59.609 1.00 0.00
D   H
ATOM 25337 CA ILE D 122   13.583 30.946 58.254 1.00 0.00
D   C
ATOM 25338 HA ILE D 122   13.906 31.638 57.486 1.00 0.00
D   H
ATOM 25339 CB ILE D 122   14.677 30.806 59.343 1.00 0.00
D   C
ATOM 25340 HB ILE D 122   14.219 30.139 60.116 1.00 0.00
D   H
ATOM 25341 CG2 ILE D 122   15.980 30.286 58.755 1.00 0.00
D   C
ATOM 25342 HG21 ILE D 122   16.703 30.043 59.560 1.00 0.00
D   H
ATOM 25343 HG22 ILE D 122   15.806 29.360 58.167 1.00 0.00
D   H
ATOM 25344 HG23 ILE D 122   16.445 31.042 58.088 1.00 0.00
D   H
ATOM 25345 CG1 ILE D 122   14.947 32.141 60.028 1.00 0.00
D   C
ATOM 25346 HG11 ILE D 122   16.019 32.418 59.905 1.00 0.00
D   H
ATOM 25347 HG12 ILE D 122   14.343 32.932 59.528 1.00 0.00
D   H
ATOM 25348 CD ILE D 122   14.601 32.136 61.495 1.00 0.00
D   C
ATOM 25349 HD1 ILE D 122   14.761 33.145 61.933 1.00 0.00
D   H
ATOM 25350 HD2 ILE D 122   13.536 31.856 61.646 1.00 0.00
D   H
ATOM 25351 HD3 ILE D 122   15.236 31.416 62.053 1.00 0.00
D   H
ATOM 25352 C   ILE D 122   13.379 29.595 57.573 1.00 0.00
D   C

ATOM 25353 O   ILE D 122   12.747 28.696 58.127 1.00 0.00
D   O
ATOM 25354 N   HSE D 123   13.923 29.477 56.365 1.00 0.00
D   N
ATOM 25355 HN HSE D 123   14.302 30.251 55.859 1.00 0.00
D   H
ATOM 25356 CA HSE D 123   14.016 28.200 55.667 1.00 0.00
D   C
ATOM 25357 HA HSE D 123   13.504 27.451 56.259 1.00 0.00
D   H
ATOM 25358 CB HSE D 123   13.467 28.321 54.253 1.00 0.00
D   C
ATOM 25359 HB1 HSE D 123   14.041 29.115 53.729 1.00 0.00
D   H
ATOM 25360 HB2 HSE D 123   13.648 27.373 53.703 1.00 0.00
D   H
ATOM 25361 ND1 HSE D 123   11.056 27.790 53.893 1.00 0.00
D   N
ATOM 25362 CG HSE D 123   12.013 28.687 54.190 1.00 0.00
D   C
ATOM 25363 CE1 HSE D 123   9.854 28.398 53.898 1.00 0.00
D   C
ATOM 25364 HE1 HSE D 123   8.906 27.899 53.683 1.00 0.00
D   H
ATOM 25365 NE2 HSE D 123   10.047 29.694 54.201 1.00 0.00
D   N
ATOM 25366 HE2 HSE D 123   9.328 30.387 54.256 1.00 0.00
D   H
ATOM 25367 CD2 HSE D 123   11.365 29.907 54.387 1.00 0.00
D   C
ATOM 25368 HD2 HSE D 123   11.773 30.877 54.633 1.00 0.00
D   H
ATOM 25369 C   HSE D 123   15.463 27.802 55.609 1.00 0.00
D   C
ATOM 25370 O   HSE D 123   16.159 28.132 54.646 1.00 0.00
D   O
ATOM 25371 N   PRO D 124   15.938 27.080 56.637 1.00 0.00
D   N
ATOM 25372 CD PRO D 124   15.136 26.492 57.722 1.00 0.00
D   C
ATOM 25373 HD1 PRO D 124   15.110 27.585 57.510 1.00 0.00
D   H
ATOM 25374 HD2 PRO D 124   14.134 26.137 58.057 1.00 0.00
D   H
ATOM 25375 CA PRO D 124   17.352 26.737 56.766 1.00 0.00
D   C
ATOM 25376 HA PRO D 124   17.928 27.644 56.911 1.00 0.00
D   H
ATOM 25377 CB PRO D 124   17.395 25.862 58.018 1.00 0.00
D   C
ATOM 25378 HB1 PRO D 124   17.465 26.516 58.916 1.00 0.00
D   H
ATOM 25379 HB2 PRO D 124   18.250 25.156 58.018 1.00 0.00
D   H

ATOM 25380 CG PRO D 124 16.160 26.198 58.772 1.00 0.00
D C
ATOM 25381 HG1 PRO D 124 16.239 25.972 59.853 1.00 0.00
D H
ATOM 25382 HG2 PRO D 124 15.609 26.788 59.361 1.00 0.00
D H
ATOM 25383 C PRO D 124 17.913 25.976 55.571 1.00 0.00
D C
ATOM 25384 O PRO D 124 18.950 26.369 55.038 1.00 0.00
D O
ATOM 25385 N GLU D 125 17.240 24.911 55.143 1.00 0.00
D N
ATOM 25386 HN GLU D 125 16.376 24.583 55.514 1.00 0.00
D H
ATOM 25387 CA GLU D 125 17.754 24.108 54.038 1.00 0.00
D C
ATOM 25388 HA GLU D 125 18.773 23.829 54.279 1.00 0.00
D H
ATOM 25389 CB GLU D 125 16.948 22.826 53.836 1.00 0.00
D C
ATOM 25390 HB1 GLU D 125 15.869 23.101 53.776 1.00 0.00
D H
ATOM 25391 HB2 GLU D 125 17.201 22.372 52.853 1.00 0.00
D H
ATOM 25392 CG GLU D 125 17.118 21.785 54.931 1.00 0.00
D C
ATOM 25393 HG1 GLU D 125 17.258 20.789 54.473 1.00 0.00
D H
ATOM 25394 HG2 GLU D 125 17.993 22.019 55.569 1.00 0.00
D H
ATOM 25395 CD GLU D 125 15.891 21.669 55.811 1.00 0.00
D C
ATOM 25396 OE1 GLU D 125 15.403 20.535 55.967 1.00 0.00
D O
ATOM 25397 OE2 GLU D 125 15.400 22.699 56.329 1.00 0.00
D O
ATOM 25398 C GLU D 125 17.797 24.889 52.736 1.00 0.00
D C
ATOM 25399 O GLU D 125 18.790 24.825 51.998 1.00 0.00
D O
ATOM 25400 N LYS D 126 16.719 25.622 52.457 1.00 0.00
D N
ATOM 25401 HN LYS D 126 15.905 25.634 53.036 1.00 0.00
D H
ATOM 25402 CA LYS D 126 16.650 26.468 51.276 1.00 0.00
D C
ATOM 25403 HA LYS D 126 16.737 25.828 50.405 1.00 0.00
D H
ATOM 25404 CB LYS D 126 15.332 27.241 51.247 1.00 0.00
D C
ATOM 25405 HB1 LYS D 126 14.523 26.519 51.500 1.00 0.00
D H
ATOM 25406 HB2 LYS D 126 15.340 28.000 52.061 1.00 0.00
D H

ATOM 25407 CG LYS D 126 15.006 27.897 49.911 1.00 0.00
D C
ATOM 25408 HG1 LYS D 126 15.954 28.110 49.369 1.00 0.00
D H
ATOM 25409 HG2 LYS D 126 14.421 27.181 49.289 1.00 0.00
D H
ATOM 25410 CD LYS D 126 14.234 29.194 50.107 1.00 0.00
D C
ATOM 25411 HD1 LYS D 126 13.421 29.012 50.848 1.00 0.00
D H
ATOM 25412 HD2 LYS D 126 14.926 29.946 50.551 1.00 0.00
D H
ATOM 25413 CE LYS D 126 13.658 29.716 48.799 1.00 0.00
D C
ATOM 25414 HE1 LYS D 126 13.342 30.777 48.908 1.00 0.00
D H
ATOM 25415 HE2 LYS D 126 14.415 29.656 47.987 1.00 0.00
D H
ATOM 25416 NZ LYS D 126 12.470 28.922 48.382 1.00 0.00
D N
ATOM 25417 HZ1 LYS D 126 12.089 29.293 47.488 1.00 0.00
D H
ATOM 25418 HZ2 LYS D 126 12.745 27.927 48.254 1.00 0.00
D H
ATOM 25419 HZ3 LYS D 126 11.738 28.982 49.119 1.00 0.00
D H
ATOM 25420 C LYS D 126 17.840 27.426 51.257 1.00 0.00
D C
ATOM 25421 O LYS D 126 18.561 27.499 50.269 1.00 0.00
D O
ATOM 25422 N ASP D 127 18.056 28.134 52.363 1.00 0.00
D N
ATOM 25423 HN ASP D 127 17.511 28.072 53.196 1.00 0.00
D H
ATOM 25424 CA ASP D 127 19.147 29.102 52.468 1.00 0.00
D C
ATOM 25425 HA ASP D 127 19.048 29.766 51.617 1.00 0.00
D H
ATOM 25426 CB ASP D 127 19.037 29.909 53.764 1.00 0.00
D C
ATOM 25427 HB1 ASP D 127 18.885 29.215 54.615 1.00 0.00
D H
ATOM 25428 HB2 ASP D 127 19.967 30.485 53.945 1.00 0.00
D H
ATOM 25429 CG ASP D 127 17.863 30.881 53.757 1.00 0.00
D C
ATOM 25430 OD1 ASP D 127 17.239 31.086 52.689 1.00 0.00
D O
ATOM 25431 OD2 ASP D 127 17.564 31.448 54.829 1.00 0.00
D O
ATOM 25432 C ASP D 127 20.526 28.451 52.361 1.00 0.00
D C
ATOM 25433 O ASP D 127 21.419 28.987 51.715 1.00 0.00
D O

ATOM 25434 N MET D 128 20.703 27.293 52.988 1.00 0.00
D N
ATOM 25435 HN MET D 128 20.021 26.839 53.558 1.00 0.00
D H
ATOM 25436 CA MET D 128 21.971 26.574 52.875 1.00 0.00
D C
ATOM 25437 HA MET D 128 22.750 27.291 53.100 1.00 0.00
D H
ATOM 25438 CB MET D 128 22.059 25.443 53.891 1.00 0.00
D C
ATOM 25439 HB1 MET D 128 21.047 24.977 53.946 1.00 0.00
D H
ATOM 25440 HB2 MET D 128 22.743 24.654 53.507 1.00 0.00
D H
ATOM 25441 CG MET D 128 22.475 25.905 55.274 1.00 0.00
D C
ATOM 25442 HG1 MET D 128 21.675 26.524 55.732 1.00 0.00
D H
ATOM 25443 HG2 MET D 128 22.635 25.021 55.931 1.00 0.00
D H
ATOM 25444 SD MET D 128 23.982 26.895 55.320 1.00 0.00
D S
ATOM 25445 CE MET D 128 25.211 25.685 54.810 1.00 0.00
D C
ATOM 25446 HE1 MET D 128 26.227 26.135 54.793 1.00 0.00
D H
ATOM 25447 HE2 MET D 128 25.232 24.820 55.506 1.00 0.00
D H
ATOM 25448 HE3 MET D 128 24.993 25.298 53.791 1.00 0.00
D H
ATOM 25449 C MET D 128 22.226 26.042 51.470 1.00 0.00
D C
ATOM 25450 O MET D 128 23.377 25.922 51.051 1.00 0.00
D O
ATOM 25451 N GLY D 129 21.155 25.722 50.750 1.00 0.00
D N
ATOM 25452 HN GLY D 129 20.234 25.704 51.137 1.00 0.00
D H
ATOM 25453 CA GLY D 129 21.253 25.376 49.339 1.00 0.00
D C
ATOM 25454 HA1 GLY D 129 20.245 25.290 48.958 1.00 0.00
D H
ATOM 25455 HA2 GLY D 129 21.835 24.466 49.266 1.00 0.00
D H
ATOM 25456 C GLY D 129 21.968 26.456 48.541 1.00 0.00
D C
ATOM 25457 O GLY D 129 22.945 26.170 47.845 1.00 0.00
D O
ATOM 25458 N VAL D 130 21.491 27.697 48.654 1.00 0.00
D N
ATOM 25459 HN VAL D 130 20.698 27.943 49.206 1.00 0.00
D H
ATOM 25460 CA VAL D 130 22.114 28.822 47.955 1.00 0.00
D C

ATOM 25461 HA VAL D 130 22.026 28.638 46.892 1.00 0.00
D H
ATOM 25462 CB VAL D 130 21.395 30.193 48.185 1.00 0.00
D C
ATOM 25463 HB VAL D 130 21.947 30.966 47.595 1.00 0.00
D H
ATOM 25464 CG1 VAL D 130 19.955 30.138 47.706 1.00 0.00
D C
ATOM 25465 HG11 VAL D 130 19.444 31.104 47.910 1.00 0.00
D H
ATOM 25466 HG12 VAL D 130 19.893 29.956 46.613 1.00 0.00
D H
ATOM 25467 HG13 VAL D 130 19.396 29.336 48.233 1.00 0.00
D H
ATOM 25468 CG2 VAL D 130 21.443 30.643 49.637 1.00 0.00
D C
ATOM 25469 HG21 VAL D 130 20.890 31.598 49.771 1.00 0.00
D H
ATOM 25470 HG22 VAL D 130 20.984 29.877 50.298 1.00 0.00
D H
ATOM 25471 HG23 VAL D 130 22.488 30.811 49.971 1.00 0.00
D H
ATOM 25472 C VAL D 130 23.607 28.902 48.285 1.00 0.00
D C
ATOM 25473 O VAL D 130 24.435 28.948 47.378 1.00 0.00
D O
ATOM 25474 N LYS D 131 23.931 28.871 49.579 1.00 0.00
D N
ATOM 25475 HN LYS D 131 23.245 28.769 50.297 1.00 0.00
D H
ATOM 25476 CA LYS D 131 25.314 28.982 50.052 1.00 0.00
D C
ATOM 25477 HA LYS D 131 25.693 29.922 49.671 1.00 0.00
D H
ATOM 25478 CB LYS D 131 25.379 29.012 51.583 1.00 0.00
D C
ATOM 25479 HB1 LYS D 131 24.850 28.104 51.952 1.00 0.00
D H
ATOM 25480 HB2 LYS D 131 26.440 28.907 51.902 1.00 0.00
D H
ATOM 25481 CG LYS D 131 24.745 30.249 52.198 1.00 0.00
D C
ATOM 25482 HG1 LYS D 131 24.454 30.952 51.385 1.00 0.00
D H
ATOM 25483 HG2 LYS D 131 23.812 29.952 52.730 1.00 0.00
D H
ATOM 25484 CD LYS D 131 25.708 30.946 53.149 1.00 0.00
D C
ATOM 25485 HD1 LYS D 131 25.592 30.494 54.161 1.00 0.00
D H
ATOM 25486 HD2 LYS D 131 26.748 30.735 52.807 1.00 0.00
D H
ATOM 25487 CE LYS D 131 25.441 32.450 53.181 1.00 0.00
D C

```
ATOM  25488  HE1 LYS D 131      24.961  32.781  52.233  1.00  0.00
D    H
ATOM  25489  HE2 LYS D 131      24.766  32.709  54.026  1.00  0.00
D    H
ATOM  25490  NZ  LYS D 131      26.690  33.249  53.347  1.00  0.00
D    N
ATOM  25491  HZ1 LYS D 131      26.463  34.264  53.362  1.00  0.00
D    H
ATOM  25492  HZ2 LYS D 131      27.154  32.985  54.240  1.00  0.00
D    H
ATOM  25493  HZ3 LYS D 131      27.334  33.051  52.555  1.00  0.00
D    H
ATOM  25494  C   LYS D 131      26.210  27.876  49.511  1.00  0.00
D    C
ATOM  25495  O   LYS D 131      27.364  28.133  49.148  1.00  0.00
D    O
ATOM  25496  N   ILE D 132      25.675  26.656  49.450  1.00  0.00
D    N
ATOM  25497  HN  ILE D 132      24.753  26.437  49.762  1.00  0.00
D    H
ATOM  25498  CA  ILE D 132      26.411  25.517  48.909  1.00  0.00
D    C
ATOM  25499  HA  ILE D 132      27.380  25.516  49.392  1.00  0.00
D    H
ATOM  25500  CB  ILE D 132      25.708  24.177  49.219  1.00  0.00
D    C
ATOM  25501  HB  ILE D 132      24.631  24.363  48.976  1.00  0.00
D    H
ATOM  25502  CG2 ILE D 132      26.312  23.029  48.424  1.00  0.00
D    C
ATOM  25503 HG21 ILE D 132      25.700  22.110  48.538  1.00  0.00
D    H
ATOM  25504 HG22 ILE D 132      26.352  23.274  47.342  1.00  0.00
D    H
ATOM  25505 HG23 ILE D 132      27.343  22.805  48.770  1.00  0.00
D    H
ATOM  25506  CG1 ILE D 132      25.778  23.869  50.719  1.00  0.00
D    C
ATOM  25507 HG11 ILE D 132      25.131  24.580  51.280  1.00  0.00
D    H
ATOM  25508 HG12 ILE D 132      25.374  22.845  50.897  1.00  0.00
D    H
ATOM  25509  CD  ILE D 132      27.173  23.920  51.313  1.00  0.00
D    C
ATOM  25510  HD1 ILE D 132      27.147  23.630  52.386  1.00  0.00
D    H
ATOM  25511  HD2 ILE D 132      27.853  23.221  50.780  1.00  0.00
D    H
ATOM  25512  HD3 ILE D 132      27.597  24.945  51.245  1.00  0.00
D    H
ATOM  25513  C   ILE D 132      26.662  25.702  47.412  1.00  0.00
D    C
ATOM  25514  O   ILE D 132      27.776  25.483  46.937  1.00  0.00
D    O
```

ATOM 25515 N   ALA D 133   25.633 26.135 46.682 1.00 0.00
D   N
ATOM 25516 HN ALA D 133   24.727 26.309 47.065 1.00 0.00
D   H
ATOM 25517 CA ALA D 133   25.769 26.388 45.246 1.00 0.00
D   C
ATOM 25518 HA ALA D 133   26.117 25.496 44.738 1.00 0.00
D   H
ATOM 25519 CB ALA D 133   24.431 26.735 44.626 1.00 0.00
D   C
ATOM 25520 HB1 ALA D 133   23.711 25.908 44.806 1.00 0.00
D   H
ATOM 25521 HB2 ALA D 133   24.009 27.660 45.078 1.00 0.00
D   H
ATOM 25522 HB3 ALA D 133   24.514 26.884 43.528 1.00 0.00
D   H
ATOM 25523 C   ALA D 133   26.795 27.475 44.955 1.00 0.00
D   C
ATOM 25524 O   ALA D 133   27.530 27.381 43.979 1.00 0.00
D   O
ATOM 25525 N   GLN D 134   26.847 28.494 45.809 1.00 0.00
D   N
ATOM 25526 HN GLN D 134   26.196 28.616 46.557 1.00 0.00
D   H
ATOM 25527 CA GLN D 134   27.874 29.536 45.724 1.00 0.00
D   C
ATOM 25528 HA GLN D 134   27.813 29.974 44.735 1.00 0.00
D   H
ATOM 25529 CB GLN D 134   27.685 30.585 46.823 1.00 0.00
D   C
ATOM 25530 HB1 GLN D 134   27.770 30.050 47.797 1.00 0.00
D   H
ATOM 25531 HB2 GLN D 134   28.530 31.309 46.792 1.00 0.00
D   H
ATOM 25532 CG GLN D 134   26.361 31.337 46.799 1.00 0.00
D   C
ATOM 25533 HG1 GLN D 134   25.510 30.624 46.770 1.00 0.00
D   H
ATOM 25534 HG2 GLN D 134   26.249 31.963 47.709 1.00 0.00
D   H
ATOM 25535 CD GLN D 134   26.192 32.237 45.580 1.00 0.00
D   C
ATOM 25536 OE1 GLN D 134   27.169 32.728 45.003 1.00 0.00
D   O
ATOM 25537 NE2 GLN D 134   24.938 32.467 45.186 1.00 0.00
D   N
ATOM 25538 HE21 GLN D 134   24.817 33.049 44.388 1.00 0.00
D   H
ATOM 25539 HE22 GLN D 134   24.180 32.075 45.696 1.00 0.00
D   H
ATOM 25540 C   GLN D 134   29.261 28.917 45.867 1.00 0.00
D   C
ATOM 25541 O   GLN D 134   30.192 29.244 45.130 1.00 0.00
D   O

```
ATOM  25542  N   SER D 135      29.367  28.007  46.825  1.00  0.00
D    N
ATOM  25543  HN  SER D 135      28.583  27.742  47.385  1.00  0.00
D    H
ATOM  25544  CA  SER D 135      30.595  27.316  47.160  1.00  0.00
D    C
ATOM  25545  HA  SER D 135      31.349  28.083  47.271  1.00  0.00
D    H
ATOM  25546  CB  SER D 135      30.307  26.490  48.406  1.00  0.00
D    C
ATOM  25547  HB1 SER D 135      29.367  26.874  48.865  1.00  0.00
D    H
ATOM  25548  HB2 SER D 135      30.127  25.425  48.133  1.00  0.00
D    H
ATOM  25549  OG  SER D 135      31.431  26.638  49.372  1.00  0.00
D    O
ATOM  25550  HG1 SER D 135      31.122  26.296  50.220  1.00  0.00
D    H
ATOM  25551  C   SER D 135      31.043  26.398  46.022  1.00  0.00
D    C
ATOM  25552  O   SER D 135      32.232  26.307  45.722  1.00  0.00
D    O
ATOM  25553  N   LEU D 136      30.074  25.735  45.393  1.00  0.00
D    N
ATOM  25554  HN  LEU D 136      29.112  25.849  45.630  1.00  0.00
D    H
ATOM  25555  CA  LEU D 136      30.319  24.785  44.303  1.00  0.00
D    C
ATOM  25556  HA  LEU D 136      31.091  24.115  44.658  1.00  0.00
D    H
ATOM  25557  CB  LEU D 136      29.025  24.058  43.938  1.00  0.00
D    C
ATOM  25558  HB1 LEU D 136      28.221  24.828  43.874  1.00  0.00
D    H
ATOM  25559  HB2 LEU D 136      29.125  23.632  42.915  1.00  0.00
D    H
ATOM  25560  CG  LEU D 136      28.581  22.975  44.921  1.00  0.00
D    C
ATOM  25561  HG  LEU D 136      28.931  23.391  45.896  1.00  0.00
D    H
ATOM  25562  CD1 LEU D 136      27.067  22.806  44.973  1.00  0.00
D    C
ATOM  25563 HD11 LEU D 136      26.790  21.979  45.662  1.00  0.00
D    H
ATOM  25564 HD12 LEU D 136      26.582  23.739  45.335  1.00  0.00
D    H
ATOM  25565 HD13 LEU D 136      26.663  22.569  43.966  1.00  0.00
D    H
ATOM  25566  CD2 LEU D 136      29.305  21.651  44.686  1.00  0.00
D    C
ATOM  25567 HD21 LEU D 136      28.974  20.892  45.428  1.00  0.00
D    H
ATOM  25568 HD22 LEU D 136      29.099  21.245  43.675  1.00  0.00
D    H
```

ATOM 25569 HD23 LEU D 136   30.404 21.784 44.793 1.00 0.00
D   H
ATOM 25570 C   LEU D 136   30.837 25.458 43.051 1.00 0.00
D   C
ATOM 25571 O   LEU D 136   31.603 24.886 42.277 1.00 0.00
D   O
ATOM 25572 N   SER D 137   30.398 26.689 42.870 1.00 0.00
D   N
ATOM 25573 HN SER D 137   29.930 27.186 43.599 1.00 0.00
D   H
ATOM 25574 CA SER D 137   30.538 27.431 41.645 1.00 0.00
D   C
ATOM 25575 HA SER D 137   30.439 26.715 40.840 1.00 0.00
D   H
ATOM 25576 CB SER D 137   29.456 28.488 41.743 1.00 0.00
D   C
ATOM 25577 HB1 SER D 137   28.478 27.971 41.879 1.00 0.00
D   H
ATOM 25578 HB2 SER D 137   29.617 29.126 42.641 1.00 0.00
D   H
ATOM 25579 OG SER D 137   29.324 29.296 40.646 1.00 0.00
D   O
ATOM 25580 HG1 SER D 137   28.483 29.761 40.735 1.00 0.00
D   H
ATOM 25581 C   SER D 137   31.942 28.037 41.546 1.00 0.00
D   C
ATOM 25582 O   SER D 137   32.547 28.099 40.464 1.00 0.00
D   O
ATOM 25583 N   ASP D 138   32.445 28.474 42.695 1.00 0.00
D   N
ATOM 25584 HN ASP D 138   31.928 28.537 43.546 1.00 0.00
D   H
ATOM 25585 CA ASP D 138   33.821 28.912 42.837 1.00 0.00
D   C
ATOM 25586 HA ASP D 138   34.411 28.461 42.047 1.00 0.00
D   H
ATOM 25587 CB ASP D 138   33.902 30.433 42.833 1.00 0.00
D   C
ATOM 25588 HB1 ASP D 138   33.098 30.840 42.187 1.00 0.00
D   H
ATOM 25589 HB2 ASP D 138   33.763 30.833 43.857 1.00 0.00
D   H
ATOM 25590 CG ASP D 138   35.224 30.952 42.291 1.00 0.00
D   C
ATOM 25591 OD1 ASP D 138   36.177 30.164 42.102 1.00 0.00
D   O
ATOM 25592 OD2 ASP D 138   35.300 32.172 42.044 1.00 0.00
D   O
ATOM 25593 C   ASP D 138   34.343 28.352 44.148 1.00 0.00
D   C
ATOM 25594 O   ASP D 138   34.030 28.864 45.222 1.00 0.00
D   O
ATOM 25595 N   GLU D 139   35.129 27.285 44.042 1.00 0.00
D   N

ATOM 25596 HN GLU D 139 35.416 26.909 43.165 1.00 0.00
D H
ATOM 25597 CA GLU D 139 35.647 26.562 45.194 1.00 0.00
D C
ATOM 25598 HA GLU D 139 34.831 26.363 45.876 1.00 0.00
D H
ATOM 25599 CB GLU D 139 36.305 25.273 44.727 1.00 0.00
D C
ATOM 25600 HB1 GLU D 139 35.872 25.000 43.737 1.00 0.00
D H
ATOM 25601 HB2 GLU D 139 37.390 25.446 44.547 1.00 0.00
D H
ATOM 25602 CG GLU D 139 36.122 24.109 45.681 1.00 0.00
D C
ATOM 25603 HG1 GLU D 139 36.894 24.153 46.470 1.00 0.00
D H
ATOM 25604 HG2 GLU D 139 35.119 24.135 46.151 1.00 0.00
D H
ATOM 25605 CD GLU D 139 36.304 22.770 44.995 1.00 0.00
D C
ATOM 25606 OE1 GLU D 139 37.387 22.528 44.406 1.00 0.00
D O
ATOM 25607 OE2 GLU D 139 35.356 21.956 45.043 1.00 0.00
D O
ATOM 25608 C GLU D 139 36.652 27.423 45.935 1.00 0.00
D C
ATOM 25609 O GLU D 139 36.835 27.286 47.141 1.00 0.00
D O
ATOM 25610 N ASN D 140 37.197 28.449 45.293 1.00 0.00
D N
ATOM 25611 HN ASN D 140 37.075 28.654 44.322 1.00 0.00
D H
ATOM 25612 CA ASN D 140 38.062 29.400 46.015 1.00 0.00
D C
ATOM 25613 HA ASN D 140 38.854 28.766 46.400 1.00 0.00
D H
ATOM 25614 CB ASN D 140 38.680 30.401 45.035 1.00 0.00
D C
ATOM 25615 HB1 ASN D 140 37.863 30.791 44.389 1.00 0.00
D H
ATOM 25616 HB2 ASN D 140 39.111 31.263 45.584 1.00 0.00
D H
ATOM 25617 CG ASN D 140 39.728 29.769 44.140 1.00 0.00
D C
ATOM 25618 OD1 ASN D 140 39.723 29.965 42.923 1.00 0.00
D O
ATOM 25619 ND2 ASN D 140 40.636 29.007 44.739 1.00 0.00
D N
ATOM 25620 HD21 ASN D 140 41.338 28.595 44.165 1.00 0.00
D H
ATOM 25621 HD22 ASN D 140 40.616 28.891 45.727 1.00 0.00
D H
ATOM 25622 C ASN D 140 37.440 30.161 47.220 1.00 0.00
D C

ATOM 25623 O   ASN D 140   38.034 30.228 48.295 1.00 0.00
D   O
ATOM 25624 N   VAL D 141   36.251 30.734 47.060 1.00 0.00
D   N
ATOM 25625 HN VAL D 141   35.678 30.635 46.251 1.00 0.00
D   H
ATOM 25626 CA VAL D 141   35.675 31.591 48.112 1.00 0.00
D   C
ATOM 25627 HA VAL D 141   36.499 32.204 48.455 1.00 0.00
D   H
ATOM 25628 CB VAL D 141   34.594 32.528 47.538 1.00 0.00
D   C
ATOM 25629 HB VAL D 141   33.867 31.897 46.971 1.00 0.00
D   H
ATOM 25630 CG1 VAL D 141   33.863 33.249 48.662 1.00 0.00
D   C
ATOM 25631 HG11 VAL D 141   33.111 33.952 48.245 1.00 0.00
D   H
ATOM 25632 HG12 VAL D 141   33.322 32.538 49.320 1.00 0.00
D   H
ATOM 25633 HG13 VAL D 141   34.576 33.835 49.281 1.00 0.00
D   H
ATOM 25634 CG2 VAL D 141   35.216 33.526 46.572 1.00 0.00
D   C
ATOM 25635 HG21 VAL D 141   34.448 34.229 46.186 1.00 0.00
D   H
ATOM 25636 HG22 VAL D 141   36.009 34.118 47.079 1.00 0.00
D   H
ATOM 25637 HG23 VAL D 141   35.668 33.011 45.700 1.00 0.00
D   H
ATOM 25638 C   VAL D 141   35.092 30.870 49.344 1.00 0.00
D   C
ATOM 25639 O   VAL D 141   34.698 29.707 49.258 1.00 0.00
D   O
ATOM 25640 N   LEU D 142   35.035 31.574 50.480 1.00 0.00
D   N
ATOM 25641 HN LEU D 142   35.421 32.489 50.581 1.00 0.00
D   H
ATOM 25642 CA LEU D 142   34.385 31.051 51.700 1.00 0.00
D   C
ATOM 25643 HA LEU D 142   33.974 30.089 51.421 1.00 0.00
D   H
ATOM 25644 CB LEU D 142   35.403 30.895 52.829 1.00 0.00
D   C
ATOM 25645 HB1 LEU D 142   35.747 31.918 53.107 1.00 0.00
D   H
ATOM 25646 HB2 LEU D 142   34.888 30.489 53.729 1.00 0.00
D   H
ATOM 25647 CG LEU D 142   36.651 30.058 52.557 1.00 0.00
D   C
ATOM 25648 HG LEU D 142   37.068 30.410 51.583 1.00 0.00
D   H
ATOM 25649 CD1 LEU D 142   37.668 30.299 53.660 1.00 0.00
D   C

ATOM 25650 HD11 LEU D 142   38.566 29.663 53.508 1.00 0.00
D   H
ATOM 25651 HD12 LEU D 142   37.990 31.362 53.668 1.00 0.00
D   H
ATOM 25652 HD13 LEU D 142   37.235 30.056 54.654 1.00 0.00
D   H
ATOM 25653 CD2 LEU D 142   36.330 28.573 52.424 1.00 0.00
D   C
ATOM 25654 HD21 LEU D 142   37.256 27.996 52.208 1.00 0.00
D   H
ATOM 25655 HD22 LEU D 142   35.888 28.164 53.354 1.00 0.00
D   H
ATOM 25656 HD23 LEU D 142   35.613 28.404 51.592 1.00 0.00
D   H
ATOM 25657 C   LEU D 142   33.231 31.916 52.201 1.00 0.00
D   C
ATOM 25658 O   LEU D 142   32.221 31.407 52.700 1.00 0.00
D   O
ATOM 25659 N   ASN D 143   33.410 33.227 52.079 1.00 0.00
D   N
ATOM 25660 HN ASN D 143   34.244 33.614 51.688 1.00 0.00
D   H
ATOM 25661 CA ASN D 143   32.423 34.215 52.498 1.00 0.00
D   C
ATOM 25662 HA ASN D 143   31.451 33.780 52.290 1.00 0.00
D   H
ATOM 25663 CB ASN D 143   32.601 34.564 53.977 1.00 0.00
D   C
ATOM 25664 HB1 ASN D 143   33.537 34.078 54.330 1.00 0.00
D   H
ATOM 25665 HB2 ASN D 143   32.727 35.659 54.102 1.00 0.00
D   H
ATOM 25666 CG ASN D 143   31.455 34.063 54.842 1.00 0.00
D   C
ATOM 25667 OD1 ASN D 143   30.374 33.736 54.348 1.00 0.00
D   O
ATOM 25668 ND2 ASN D 143   31.683 34.019 56.150 1.00 0.00
D   N
ATOM 25669 HD21 ASN D 143   30.947 33.682 56.732 1.00 0.00
D   H
ATOM 25670 HD22 ASN D 143   32.571 34.287 56.510 1.00 0.00
D   H
ATOM 25671 C   ASN D 143   32.551 35.473 51.662 1.00 0.00
D   C
ATOM 25672 O   ASN D 143   33.626 35.771 51.148 1.00 0.00
D   O
ATOM 25673 N   TYR D 144   31.455 36.210 51.529 1.00 0.00
D   N
ATOM 25674 HN TYR D 144   30.575 35.983 51.943 1.00 0.00
D   H
ATOM 25675 CA TYR D 144   31.456 37.441 50.744 1.00 0.00
D   C
ATOM 25676 HA TYR D 144   32.436 37.886 50.869 1.00 0.00
D   H

ATOM 25677 CB TYR D 144   31.166 37.141 49.263 1.00 0.00
D   C
ATOM 25678 HB1 TYR D 144   31.350 38.059 48.663 1.00 0.00
D   H
ATOM 25679 HB2 TYR D 144   31.851 36.354 48.880 1.00 0.00
D   H
ATOM 25680 CG TYR D 144   29.736 36.731 49.008 1.00 0.00
D   C
ATOM 25681 CD1 TYR D 144   28.799 37.653 48.546 1.00 0.00
D   C
ATOM 25682 HD1 TYR D 144   29.096 38.677 48.370 1.00 0.00
D   H
ATOM 25683 CE1 TYR D 144   27.482 37.284 48.327 1.00 0.00
D   C
ATOM 25684 HE1 TYR D 144   26.762 38.005 47.970 1.00 0.00
D   H
ATOM 25685 CZ TYR D 144   27.090 35.980 48.578 1.00 0.00
D   C
ATOM 25686 OH TYR D 144   25.788 35.597 48.367 1.00 0.00
D   O
ATOM 25687 HH TYR D 144   25.736 34.653 48.527 1.00 0.00
D   H
ATOM 25688 CD2 TYR D 144   29.312 35.427 49.257 1.00 0.00
D   C
ATOM 25689 HD2 TYR D 144   30.004 34.702 49.661 1.00 0.00
D   H
ATOM 25690 CE2 TYR D 144   27.999 35.048 49.042 1.00 0.00
D   C
ATOM 25691 HE2 TYR D 144   27.690 34.036 49.258 1.00 0.00
D   H
ATOM 25692 C   TYR D 144   30.436 38.440 51.285 1.00 0.00
D   C
ATOM 25693 O   TYR D 144   29.478 38.055 51.958 1.00 0.00
D   O
ATOM 25694 N   ILE D 145   30.661 39.721 50.995 1.00 0.00
D   N
ATOM 25695 HN ILE D 145   31.515 40.045 50.594 1.00 0.00
D   H
ATOM 25696 CA ILE D 145   29.673 40.776 51.236 1.00 0.00
D   C
ATOM 25697 HA ILE D 145   28.714 40.294 51.381 1.00 0.00
D   H
ATOM 25698 CB ILE D 145   30.037 41.676 52.440 1.00 0.00
D   C
ATOM 25699 HB ILE D 145   31.132 41.877 52.318 1.00 0.00
D   H
ATOM 25700 CG2 ILE D 145   29.142 42.908 52.484 1.00 0.00
D   C
ATOM 25701 HG21 ILE D 145   29.503 43.626 53.249 1.00 0.00
D   H
ATOM 25702 HG22 ILE D 145   29.141 43.432 51.505 1.00 0.00
D   H
ATOM 25703 HG23 ILE D 145   28.096 42.631 52.734 1.00 0.00
D   H

ATOM 25704 CG1 ILE D 145   29.876 40.921 53.760 1.00 0.00
D   C
ATOM 25705 HG11 ILE D 145   28.803 40.677 53.927 1.00 0.00
D   H
ATOM 25706 HG12 ILE D 145   30.428 39.955 53.692 1.00 0.00
D   H
ATOM 25707 CD ILE D 145   30.403 41.683 54.966 1.00 0.00
D   C
ATOM 25708 HD1 ILE D 145   30.312 41.062 55.884 1.00 0.00
D   H
ATOM 25709 HD2 ILE D 145   31.474 41.944 54.828 1.00 0.00
D   H
ATOM 25710 HD3 ILE D 145   29.829 42.620 55.127 1.00 0.00
D   H
ATOM 25711 C   ILE D 145   29.552 41.631 49.977 1.00 0.00
D   C
ATOM 25712 O   ILE D 145   30.543 42.189 49.501 1.00 0.00
D   O
ATOM 25713 N   ASP D 146   28.337 41.714 49.441 1.00 0.00
D   N
ATOM 25714 HN ASP D 146   27.534 41.231 49.780 1.00 0.00
D   H
ATOM 25715 CA ASP D 146   28.052 42.543 48.270 1.00 0.00
D   C
ATOM 25716 HA ASP D 146   28.934 42.508 47.642 1.00 0.00
D   H
ATOM 25717 CB ASP D 146   26.835 42.010 47.501 1.00 0.00
D   C
ATOM 25718 HB1 ASP D 146   26.207 41.404 48.186 1.00 0.00
D   H
ATOM 25719 HB2 ASP D 146   26.221 42.846 47.113 1.00 0.00
D   H
ATOM 25720 CG ASP D 146   27.220 41.116 46.332 1.00 0.00
D   C
ATOM 25721 OD1 ASP D 146   28.327 41.282 45.773 1.00 0.00
D   O
ATOM 25722 OD2 ASP D 146   26.400 40.251 45.961 1.00 0.00
D   O
ATOM 25723 C   ASP D 146   27.835 44.007 48.643 1.00 0.00
D   C
ATOM 25724 O   ASP D 146   26.877 44.350 49.339 1.00 0.00
D   O
ATOM 25725 N   LEU D 147   28.735 44.863 48.165 1.00 0.00
D   N
ATOM 25726 HN LEU D 147   29.499 44.578 47.591 1.00 0.00
D   H
ATOM 25727 CA LEU D 147   28.689 46.296 48.436 1.00 0.00
D   C
ATOM 25728 HA LEU D 147   28.214 46.423 49.400 1.00 0.00
D   H
ATOM 25729 CB LEU D 147   30.106 46.865 48.414 1.00 0.00
D   C
ATOM 25730 HB1 LEU D 147   30.665 46.323 47.617 1.00 0.00
D   H

ATOM 25731 HB2 LEU D 147 30.063 47.929 48.090 1.00 0.00
D H
ATOM 25732 CG LEU D 147 30.976 46.794 49.676 1.00 0.00
D C
ATOM 25733 HG LEU D 147 30.501 47.639 50.229 1.00 0.00
D H
ATOM 25734 CD1 LEU D 147 30.862 45.528 50.519 1.00 0.00
D C
ATOM 25735 HD11 LEU D 147 31.568 45.566 51.376 1.00 0.00
D H
ATOM 25736 HD12 LEU D 147 29.832 45.416 50.919 1.00 0.00
D H
ATOM 25737 HD13 LEU D 147 31.102 44.630 49.911 1.00 0.00
D H
ATOM 25738 CD2 LEU D 147 32.422 47.170 49.393 1.00 0.00
D C
ATOM 25739 HD21 LEU D 147 33.023 47.127 50.328 1.00 0.00
D H
ATOM 25740 HD22 LEU D 147 32.889 46.480 48.662 1.00 0.00
D H
ATOM 25741 HD23 LEU D 147 32.484 48.203 48.988 1.00 0.00
D H
ATOM 25742 C LEU D 147 27.827 47.003 47.403 1.00 0.00
D C
ATOM 25743 O LEU D 147 26.972 47.821 47.744 1.00 0.00
D O
ATOM 25744 N SER D 148 28.076 46.683 46.138 1.00 0.00
D N
ATOM 25745 HN SER D 148 28.827 46.080 45.869 1.00 0.00
D H
ATOM 25746 CA SER D 148 27.283 47.178 45.025 1.00 0.00
D C
ATOM 25747 HA SER D 148 26.320 47.468 45.425 1.00 0.00
D H
ATOM 25748 CB SER D 148 28.008 48.320 44.311 1.00 0.00
D C
ATOM 25749 HB1 SER D 148 29.060 48.001 44.126 1.00 0.00
D H
ATOM 25750 HB2 SER D 148 27.545 48.512 43.316 1.00 0.00
D H
ATOM 25751 OG SER D 148 28.022 49.493 45.100 1.00 0.00
D O
ATOM 25752 HG1 SER D 148 28.671 50.091 44.708 1.00 0.00
D H
ATOM 25753 C SER D 148 27.043 46.037 44.052 1.00 0.00
D C
ATOM 25754 O SER D 148 27.337 44.873 44.351 1.00 0.00
D O
ATOM 25755 N ASP D 149 26.500 46.382 42.886 1.00 0.00
D N
ATOM 25756 HN ASP D 149 26.118 47.279 42.681 1.00 0.00
D H
ATOM 25757 CA ASP D 149 26.406 45.456 41.764 1.00 0.00
D C

ATOM 25758 HA ASP D 149 25.985 44.527 42.134 1.00 0.00
D H
ATOM 25759 CB ASP D 149 25.586 46.076 40.626 1.00 0.00
D C
ATOM 25760 HB1 ASP D 149 26.188 46.865 40.131 1.00 0.00
D H
ATOM 25761 HB2 ASP D 149 25.326 45.310 39.869 1.00 0.00
D H
ATOM 25762 CG ASP D 149 24.301 46.727 41.110 1.00 0.00
D C
ATOM 25763 OD1 ASP D 149 23.412 46.006 41.614 1.00 0.00
D O
ATOM 25764 OD2 ASP D 149 24.173 47.965 40.970 1.00 0.00
D O
ATOM 25765 C ASP D 149 27.813 45.133 41.260 1.00 0.00
D C
ATOM 25766 O ASP D 149 28.062 44.050 40.723 1.00 0.00
D O
ATOM 25767 N GLU D 150 28.729 46.082 41.459 1.00 0.00
D N
ATOM 25768 HN GLU D 150 28.547 46.913 41.978 1.00 0.00
D H
ATOM 25769 CA GLU D 150 30.090 45.995 40.937 1.00 0.00
D C
ATOM 25770 HA GLU D 150 30.085 45.219 40.181 1.00 0.00
D H
ATOM 25771 CB GLU D 150 30.496 47.331 40.322 1.00 0.00
D C
ATOM 25772 HB1 GLU D 150 30.286 48.134 41.066 1.00 0.00
D H
ATOM 25773 HB2 GLU D 150 31.596 47.351 40.155 1.00 0.00
D H
ATOM 25774 CG GLU D 150 29.781 47.674 39.027 1.00 0.00
D C
ATOM 25775 HG1 GLU D 150 30.039 46.927 38.255 1.00 0.00
D H
ATOM 25776 HG2 GLU D 150 28.683 47.687 39.171 1.00 0.00
D H
ATOM 25777 CD GLU D 150 30.209 49.014 38.462 1.00 0.00
D C
ATOM 25778 OE1 GLU D 150 30.939 49.757 39.153 1.00 0.00
D O
ATOM 25779 OE2 GLU D 150 29.813 49.328 37.321 1.00 0.00
D O
ATOM 25780 C GLU D 150 31.132 45.607 41.976 1.00 0.00
D C
ATOM 25781 O GLU D 150 32.158 45.008 41.638 1.00 0.00
D O
ATOM 25782 N TYR D 151 30.880 45.960 43.234 1.00 0.00
D N
ATOM 25783 HN TYR D 151 30.015 46.354 43.542 1.00 0.00
D H
ATOM 25784 CA TYR D 151 31.879 45.791 44.287 1.00 0.00
D C

ATOM 25785 HA TYR D 151   32.779 45.468 43.778 1.00 0.00
D   H
ATOM 25786 CB TYR D 151   32.171 47.134 44.966 1.00 0.00
D   C
ATOM 25787 HB1 TYR D 151   31.213 47.587 45.303 1.00 0.00
D   H
ATOM 25788 HB2 TYR D 151   32.801 46.988 45.869 1.00 0.00
D   H
ATOM 25789 CG TYR D 151   32.832 48.129 44.035 1.00 0.00
D   C
ATOM 25790 CD1 TYR D 151   34.224 48.210 43.948 1.00 0.00
D   C
ATOM 25791 HD1 TYR D 151   34.840 47.573 44.567 1.00 0.00
D   H
ATOM 25792 CE1 TYR D 151   34.838 49.111 43.093 1.00 0.00
D   C
ATOM 25793 HE1 TYR D 151   35.915 49.170 43.039 1.00 0.00
D   H
ATOM 25794 CZ TYR D 151   34.059 49.946 42.309 1.00 0.00
D   C
ATOM 25795 OH TYR D 151   34.668 50.843 41.460 1.00 0.00
D   O
ATOM 25796 HH TYR D 151   33.978 51.277 40.954 1.00 0.00
D   H
ATOM 25797 CD2 TYR D 151   32.071 48.979 43.231 1.00 0.00
D   C
ATOM 25798 HD2 TYR D 151   30.992 48.963 43.298 1.00 0.00
D   H
ATOM 25799 CE2 TYR D 151   32.675 49.886 42.374 1.00 0.00
D   C
ATOM 25800 HE2 TYR D 151   32.065 50.551 41.781 1.00 0.00
D   H
ATOM 25801 C   TYR D 151   31.527 44.711 45.306 1.00 0.00
D   C
ATOM 25802 O   TYR D 151   30.356 44.511 45.642 1.00 0.00
D   O
ATOM 25803 N   SER D 152   32.561 44.018 45.779 1.00 0.00
D   N
ATOM 25804 HN SER D 152   33.509 44.221 45.535 1.00 0.00
D   H
ATOM 25805 CA SER D 152   32.406 42.896 46.700 1.00 0.00
D   C
ATOM 25806 HA SER D 152   31.567 43.148 47.335 1.00 0.00
D   H
ATOM 25807 CB SER D 152   32.166 41.602 45.913 1.00 0.00
D   C
ATOM 25808 HB1 SER D 152   31.191 41.696 45.382 1.00 0.00
D   H
ATOM 25809 HB2 SER D 152   32.951 41.471 45.134 1.00 0.00
D   H
ATOM 25810 OG SER D 152   32.107 40.474 46.767 1.00 0.00
D   O
ATOM 25811 HG1 SER D 152   31.738 39.746 46.252 1.00 0.00
D   H

ATOM 25812 C SER D 152 33.627 42.738 47.603 1.00 0.00
D C
ATOM 25813 O SER D 152 34.765 42.882 47.151 1.00 0.00
D O
ATOM 25814 N ILE D 153 33.381 42.459 48.881 1.00 0.00
D N
ATOM 25815 HN ILE D 153 32.480 42.541 49.303 1.00 0.00
D H
ATOM 25816 CA ILE D 153 34.428 42.001 49.791 1.00 0.00
D C
ATOM 25817 HA ILE D 153 35.386 42.267 49.364 1.00 0.00
D H
ATOM 25818 CB ILE D 153 34.258 42.551 51.223 1.00 0.00
D C
ATOM 25819 HB ILE D 153 33.227 42.234 51.524 1.00 0.00
D H
ATOM 25820 CG2 ILE D 153 35.354 42.010 52.135 1.00 0.00
D C
ATOM 25821 HG21 ILE D 153 35.148 42.275 53.194 1.00 0.00
D H
ATOM 25822 HG22 ILE D 153 35.413 40.904 52.070 1.00 0.00
D H
ATOM 25823 HG23 ILE D 153 36.344 42.431 51.859 1.00 0.00
D H
ATOM 25824 CG1 ILE D 153 34.264 44.084 51.212 1.00 0.00
D C
ATOM 25825 HG11 ILE D 153 35.262 44.455 50.888 1.00 0.00
D H
ATOM 25826 HG12 ILE D 153 33.521 44.443 50.464 1.00 0.00
D H
ATOM 25827 CD ILE D 153 33.923 44.724 52.543 1.00 0.00
D C
ATOM 25828 HD1 ILE D 153 33.891 45.831 52.442 1.00 0.00
D H
ATOM 25829 HD2 ILE D 153 32.929 44.380 52.901 1.00 0.00
D H
ATOM 25830 HD3 ILE D 153 34.682 44.472 53.313 1.00 0.00
D H
ATOM 25831 C ILE D 153 34.362 40.481 49.815 1.00 0.00
D C
ATOM 25832 O ILE D 153 33.290 39.909 50.000 1.00 0.00
D O
ATOM 25833 N VAL D 154 35.505 39.832 49.617 1.00 0.00
D N
ATOM 25834 HN VAL D 154 36.397 40.271 49.553 1.00 0.00
D H
ATOM 25835 CA VAL D 154 35.534 38.382 49.476 1.00 0.00
D C
ATOM 25836 HA VAL D 154 34.558 38.016 49.768 1.00 0.00
D H
ATOM 25837 CB VAL D 154 35.816 37.963 48.017 1.00 0.00
D C
ATOM 25838 HB VAL D 154 36.833 38.338 47.748 1.00 0.00
D H

ATOM 25839 CG1 VAL D 154   35.789 36.454 47.887 1.00 0.00
D   C
ATOM 25840 HG11 VAL D 154   35.953 36.156 46.829 1.00 0.00
D   H
ATOM 25841 HG12 VAL D 154   36.586 35.976 48.492 1.00 0.00
D   H
ATOM 25842 HG13 VAL D 154   34.806 36.049 48.210 1.00 0.00
D   H
ATOM 25843 CG2 VAL D 154   34.799 38.570 47.064 1.00 0.00
D   C
ATOM 25844 HG21 VAL D 154   34.985 38.231 46.023 1.00 0.00
D   H
ATOM 25845 HG22 VAL D 154   33.767 38.270 47.350 1.00 0.00
D   H
ATOM 25846 HG23 VAL D 154   34.854 39.678 47.070 1.00 0.00
D   H
ATOM 25847 C   VAL D 154   36.561 37.737 50.402 1.00 0.00
D   C
ATOM 25848 O   VAL D 154   37.732 38.116 50.405 1.00 0.00
D   O
ATOM 25849 N   GLU D 155   36.101 36.772 51.191 1.00 0.00
D   N
ATOM 25850 HN GLU D 155   35.133 36.566 51.305 1.00 0.00
D   H
ATOM 25851 CA GLU D 155   36.984 35.915 51.971 1.00 0.00
D   C
ATOM 25852 HA GLU D 155   37.901 36.446 52.193 1.00 0.00
D   H
ATOM 25853 CB GLU D 155   36.307 35.467 53.271 1.00 0.00
D   C
ATOM 25854 HB1 GLU D 155   36.005 36.378 53.838 1.00 0.00
D   H
ATOM 25855 HB2 GLU D 155   35.363 34.926 53.038 1.00 0.00
D   H
ATOM 25856 CG GLU D 155   37.181 34.601 54.171 1.00 0.00
D   C
ATOM 25857 HG1 GLU D 155   37.546 33.727 53.600 1.00 0.00
D   H
ATOM 25858 HG2 GLU D 155   38.049 35.175 54.553 1.00 0.00
D   H
ATOM 25859 CD GLU D 155   36.432 34.025 55.360 1.00 0.00
D   C
ATOM 25860 OE1 GLU D 155   36.915 33.026 55.935 1.00 0.00
D   O
ATOM 25861 OE2 GLU D 155   35.364 34.564 55.726 1.00 0.00
D   O
ATOM 25862 C   GLU D 155   37.356 34.713 51.112 1.00 0.00
D   C
ATOM 25863 O   GLU D 155   36.489 33.936 50.705 1.00 0.00
D   O
ATOM 25864 N   LEU D 156   38.648 34.579 50.824 1.00 0.00
D   N
ATOM 25865 HN LEU D 156   39.353 35.197 51.167 1.00 0.00
D   H

ATOM 25866 CA LEU D 156   39.153 33.506 49.974 1.00 0.00
D   C
ATOM 25867 HA LEU D 156   38.289 32.946 49.639 1.00 0.00
D   H
ATOM 25868 CB LEU D 156   39.950 34.070 48.792 1.00 0.00
D   C
ATOM 25869 HB1 LEU D 156   40.520 34.950 49.169 1.00 0.00
D   H
ATOM 25870 HB2 LEU D 156   40.713 33.322 48.479 1.00 0.00
D   H
ATOM 25871 CG LEU D 156   39.216 34.540 47.538 1.00 0.00
D   C
ATOM 25872 HG LEU D 156   38.552 33.714 47.187 1.00 0.00
D   H
ATOM 25873 CD1 LEU D 156   38.406 35.778 47.838 1.00 0.00
D   C
ATOM 25874 HD11 LEU D 156   37.908 36.152 46.918 1.00 0.00
D   H
ATOM 25875 HD12 LEU D 156   37.622 35.560 48.595 1.00 0.00
D   H
ATOM 25876 HD13 LEU D 156   39.058 36.587 48.232 1.00 0.00
D   H
ATOM 25877 CD2 LEU D 156   40.197 34.858 46.425 1.00 0.00
D   C
ATOM 25878 HD21 LEU D 156   39.652 35.181 45.511 1.00 0.00
D   H
ATOM 25879 HD22 LEU D 156   40.889 35.675 46.711 1.00 0.00
D   H
ATOM 25880 HD23 LEU D 156   40.801 33.960 46.170 1.00 0.00
D   H
ATOM 25881 C   LEU D 156   40.041 32.556 50.747 1.00 0.00
D   C
ATOM 25882 O   LEU D 156   40.845 32.972 51.583 1.00 0.00
D   O
ATOM 25883 N   LEU D 157   39.902 31.274 50.452 1.00 0.00
D   N
ATOM 25884 HN LEU D 157   39.184 30.895 49.872 1.00 0.00
D   H
ATOM 25885 CA LEU D 157   40.822 30.292 50.974 1.00 0.00
D   C
ATOM 25886 HA LEU D 157   41.240 30.679 51.894 1.00 0.00
D   H
ATOM 25887 CB LEU D 157   40.115 28.961 51.216 1.00 0.00
D   C
ATOM 25888 HB1 LEU D 157   39.221 29.174 51.846 1.00 0.00
D   H
ATOM 25889 HB2 LEU D 157   39.723 28.578 50.247 1.00 0.00
D   H
ATOM 25890 CG LEU D 157   40.937 27.875 51.908 1.00 0.00
D   C
ATOM 25891 HG LEU D 157   41.902 27.778 51.355 1.00 0.00
D   H
ATOM 25892 CD1 LEU D 157   41.218 28.211 53.370 1.00 0.00
D   C

ATOM 25893 HD11 LEU D 157   41.775 27.384 53.860 1.00 0.00
D   H
ATOM 25894 HD12 LEU D 157   41.828 29.136 53.448 1.00 0.00
D   H
ATOM 25895 HD13 LEU D 157   40.269 28.368 53.926 1.00 0.00
D   H
ATOM 25896 CD2 LEU D 157   40.196 26.557 51.793 1.00 0.00
D   C
ATOM 25897 HD21 LEU D 157   40.780 25.742 52.273 1.00 0.00
D   H
ATOM 25898 HD22 LEU D 157   39.206 26.599 52.290 1.00 0.00
D   H
ATOM 25899 HD23 LEU D 157   40.039 26.291 50.726 1.00 0.00
D   H
ATOM 25900 C   LEU D 157   41.962 30.137 49.982 1.00 0.00
D   C
ATOM 25901 O   LEU D 157   41.738 29.880 48.796 1.00 0.00
D   O
ATOM 25902 N   ALA D 158   43.182 30.302 50.487 1.00 0.00
D   N
ATOM 25903 HN ALA D 158   43.326 30.404 51.471 1.00 0.00
D   H
ATOM 25904 CA ALA D 158   44.392 30.346 49.669 1.00 0.00
D   C
ATOM 25905 HA ALA D 158   44.245 31.038 48.847 1.00 0.00
D   H
ATOM 25906 CB ALA D 158   45.553 30.864 50.502 1.00 0.00
D   C
ATOM 25907 HB1 ALA D 158   45.299 31.863 50.915 1.00 0.00
D   H
ATOM 25908 HB2 ALA D 158   45.765 30.184 51.356 1.00 0.00
D   H
ATOM 25909 HB3 ALA D 158   46.479 30.968 49.894 1.00 0.00
D   H
ATOM 25910 C   ALA D 158   44.755 29.005 49.041 1.00 0.00
D   C
ATOM 25911 O   ALA D 158   44.675 27.962 49.692 1.00 0.00
D   O
ATOM 25912 N   THR D 159   45.166 29.046 47.776 1.00 0.00
D   N
ATOM 25913 HN THR D 159   45.190 29.882 47.229 1.00 0.00
D   H
ATOM 25914 CA THR D 159   45.622 27.851 47.067 1.00 0.00
D   C
ATOM 25915 HA THR D 159   45.102 27.021 47.526 1.00 0.00
D   H
ATOM 25916 CB THR D 159   45.346 27.928 45.546 1.00 0.00
D   C
ATOM 25917 HB THR D 159   45.821 27.021 45.098 1.00 0.00
D   H
ATOM 25918 OG1 THR D 159   45.991 29.082 44.991 1.00 0.00
D   O
ATOM 25919 HG1 THR D 159   45.770 29.074 44.054 1.00 0.00
D   H

ATOM 25920 CG2 THR D 159   43.855 27.986 45.268 1.00 0.00
D   C
ATOM 25921 HG21 THR D 159   43.654 27.926 44.177 1.00 0.00
D   H
ATOM 25922 HG22 THR D 159   43.337 27.134 45.758 1.00 0.00
D   H
ATOM 25923 HG23 THR D 159   43.413 28.930 45.652 1.00 0.00
D   H
ATOM 25924 C   THR D 159   47.114 27.604 47.286 1.00 0.00
D   C
ATOM 25925 O   THR D 159   47.761 28.284 48.089 1.00 0.00
D   O
ATOM 25926 N   ARG D 160   47.641 26.615 46.565 1.00 0.00
D   N
ATOM 25927 HN ARG D 160   47.051 26.044 46.000 1.00 0.00
D   H
ATOM 25928 CA ARG D 160   49.068 26.303 46.548 1.00 0.00
D   C
ATOM 25929 HA ARG D 160   49.411 26.257 47.575 1.00 0.00
D   H
ATOM 25930 CB ARG D 160   49.297 24.996 45.799 1.00 0.00
D   C
ATOM 25931 HB1 ARG D 160   48.509 24.944 45.010 1.00 0.00
D   H
ATOM 25932 HB2 ARG D 160   50.269 25.042 45.258 1.00 0.00
D   H
ATOM 25933 CG ARG D 160   49.205 23.756 46.657 1.00 0.00
D   C
ATOM 25934 HG1 ARG D 160   49.963 23.830 47.467 1.00 0.00
D   H
ATOM 25935 HG2 ARG D 160   48.217 23.746 47.173 1.00 0.00
D   H
ATOM 25936 CD ARG D 160   49.343 22.512 45.808 1.00 0.00
D   C
ATOM 25937 HD1 ARG D 160   48.346 22.228 45.400 1.00 0.00
D   H
ATOM 25938 HD2 ARG D 160   50.044 22.674 44.955 1.00 0.00
D   H
ATOM 25939 NE ARG D 160   49.877 21.399 46.585 1.00 0.00
D   N
ATOM 25940 HE ARG D 160   50.013 21.625 47.556 1.00 0.00
D   H
ATOM 25941 CZ ARG D 160   50.163 20.201 46.086 1.00 0.00
D   C
ATOM 25942 NH1 ARG D 160   49.966 19.943 44.798 1.00 0.00
D   N
ATOM 25943 HH11 ARG D 160   50.154 19.044 44.430 1.00 0.00
D   H
ATOM 25944 HH12 ARG D 160   49.497 20.654 44.270 1.00 0.00
D   H
ATOM 25945 NH2 ARG D 160   50.649 19.253 46.877 1.00 0.00
D   N
ATOM 25946 HH21 ARG D 160   50.942 18.390 46.490 1.00 0.00
D   H

ATOM 25947 HH22 ARG D 160 50.896 19.521 47.799 1.00 0.00
D H
ATOM 25948 C ARG D 160 49.889 27.387 45.863 1.00 0.00
D C
ATOM 25949 O ARG D 160 51.057 27.593 46.191 1.00 0.00
D O
ATOM 25950 N LYS D 161 49.266 28.067 44.905 1.00 0.00
D N
ATOM 25951 HN LYS D 161 48.281 28.003 44.751 1.00 0.00
D H
ATOM 25952 CA LYS D 161 49.975 28.965 44.000 1.00 0.00
D C
ATOM 25953 HA LYS D 161 50.835 28.405 43.654 1.00 0.00
D H
ATOM 25954 CB LYS D 161 49.112 29.280 42.779 1.00 0.00
D C
ATOM 25955 HB1 LYS D 161 48.124 29.633 43.158 1.00 0.00
D H
ATOM 25956 HB2 LYS D 161 49.560 30.138 42.230 1.00 0.00
D H
ATOM 25957 CG LYS D 161 48.901 28.091 41.856 1.00 0.00
D C
ATOM 25958 HG1 LYS D 161 49.858 27.529 41.762 1.00 0.00
D H
ATOM 25959 HG2 LYS D 161 48.155 27.402 42.316 1.00 0.00
D H
ATOM 25960 CD LYS D 161 48.442 28.552 40.487 1.00 0.00
D C
ATOM 25961 HD1 LYS D 161 47.341 28.718 40.523 1.00 0.00
D H
ATOM 25962 HD2 LYS D 161 48.921 29.536 40.274 1.00 0.00
D H
ATOM 25963 CE LYS D 161 48.807 27.538 39.421 1.00 0.00
D C
ATOM 25964 HE1 LYS D 161 49.728 26.983 39.709 1.00 0.00
D H
ATOM 25965 HE2 LYS D 161 47.984 26.803 39.282 1.00 0.00
D H
ATOM 25966 NZ LYS D 161 49.046 28.254 38.143 1.00 0.00
D N
ATOM 25967 HZ1 LYS D 161 49.299 27.576 37.396 1.00 0.00
D H
ATOM 25968 HZ2 LYS D 161 48.184 28.767 37.866 1.00 0.00
D H
ATOM 25969 HZ3 LYS D 161 49.822 28.934 38.269 1.00 0.00
D H
ATOM 25970 C LYS D 161 50.496 30.248 44.644 1.00 0.00
D C
ATOM 25971 O LYS D 161 51.512 30.789 44.205 1.00 0.00
D O
ATOM 25972 N LEU D 162 49.809 30.735 45.676 1.00 0.00
D N
ATOM 25973 HN LEU D 162 48.944 30.366 46.007 1.00 0.00
D H

ATOM 25974 CA LEU D 162   50.296 31.897 46.429 1.00 0.00
D   C
ATOM 25975 HA LEU D 162   51.129 32.292 45.861 1.00 0.00
D   H
ATOM 25976 CB LEU D 162   49.245 33.016 46.484 1.00 0.00
D   C
ATOM 25977 HB1 LEU D 162   49.640 33.805 47.164 1.00 0.00
D   H
ATOM 25978 HB2 LEU D 162   49.164 33.486 45.479 1.00 0.00
D   H
ATOM 25979 CG LEU D 162   47.816 32.753 46.962 1.00 0.00
D   C
ATOM 25980 HG LEU D 162   47.503 31.749 46.588 1.00 0.00
D   H
ATOM 25981 CD1 LEU D 162   47.700 32.803 48.475 1.00 0.00
D   C
ATOM 25982 HD11 LEU D 162   46.645 32.657 48.790 1.00 0.00
D   H
ATOM 25983 HD12 LEU D 162   48.318 32.006 48.941 1.00 0.00
D   H
ATOM 25984 HD13 LEU D 162   48.045 33.785 48.862 1.00 0.00
D   H
ATOM 25985 CD2 LEU D 162   46.902 33.791 46.341 1.00 0.00
D   C
ATOM 25986 HD21 LEU D 162   45.850 33.616 46.655 1.00 0.00
D   H
ATOM 25987 HD22 LEU D 162   47.180 34.819 46.651 1.00 0.00
D   H
ATOM 25988 HD23 LEU D 162   46.948 33.736 45.232 1.00 0.00
D   H
ATOM 25989 C   LEU D 162   50.819 31.524 47.818 1.00 0.00
D   C
ATOM 25990 O   LEU D 162   50.885 32.364 48.725 1.00 0.00
D   O
ATOM 25991 N   ASP D 163   51.210 30.259 47.967 1.00 0.00
D   N
ATOM 25992 HN ASP D 163   51.135 29.558 47.263 1.00 0.00
D   H
ATOM 25993 CA ASP D 163   51.802 29.761 49.204 1.00 0.00
D   C
ATOM 25994 HA ASP D 163   51.173 30.101 50.019 1.00 0.00
D   H
ATOM 25995 CB ASP D 163   51.888 28.231 49.183 1.00 0.00
D   C
ATOM 25996 HB1 ASP D 163   50.865 27.809 49.114 1.00 0.00
D   H
ATOM 25997 HB2 ASP D 163   52.468 27.885 48.304 1.00 0.00
D   H
ATOM 25998 CG ASP D 163   52.531 27.656 50.438 1.00 0.00
D   C
ATOM 25999 OD1 ASP D 163   52.280 28.183 51.543 1.00 0.00
D   O
ATOM 26000 OD2 ASP D 163   53.289 26.672 50.316 1.00 0.00
D   O

ATOM 26001 C   ASP D 163   53.184 30.370 49.432 1.00 0.00
D   C
ATOM 26002 O   ASP D 163   54.015 30.410 48.521 1.00 0.00
D   O
ATOM 26003 N   SER D 164   53.403 30.844 50.659 1.00 0.00
D   N
ATOM 26004 HN SER D 164   52.702 30.792 51.369 1.00 0.00
D   H
ATOM 26005 CA SER D 164   54.659 31.475 51.096 1.00 0.00
D   C
ATOM 26006 HA SER D 164   54.486 31.712 52.138 1.00 0.00
D   H
ATOM 26007 CB SER D 164   55.825 30.474 51.076 1.00 0.00
D   C
ATOM 26008 HB1 SER D 164   55.918 30.073 50.040 1.00 0.00
D   H
ATOM 26009 HB2 SER D 164   56.783 30.989 51.315 1.00 0.00
D   H
ATOM 26010 OG SER D 164   55.577 29.399 51.966 1.00 0.00
D   O
ATOM 26011 HG1 SER D 164   56.211 28.703 51.754 1.00 0.00
D   H
ATOM 26012 C   SER D 164   55.022 32.788 50.383 1.00 0.00
D   C
ATOM 26013 O   SER D 164   56.177 33.221 50.417 1.00 0.00
D   O
ATOM 26014 N   LYS D 165   54.025 33.418 49.761 1.00 0.00
D   N
ATOM 26015 HN LYS D 165   53.096 33.052 49.748 1.00 0.00
D   H
ATOM 26016 CA LYS D 165   54.204 34.687 49.049 1.00 0.00
D   C
ATOM 26017 HA LYS D 165   55.255 34.775 48.801 1.00 0.00
D   H
ATOM 26018 CB LYS D 165   53.306 34.733 47.809 1.00 0.00
D   C
ATOM 26019 HB1 LYS D 165   52.282 34.442 48.135 1.00 0.00
D   H
ATOM 26020 HB2 LYS D 165   53.233 35.785 47.453 1.00 0.00
D   H
ATOM 26021 CG LYS D 165   53.720 33.825 46.664 1.00 0.00
D   C
ATOM 26022 HG1 LYS D 165   54.293 32.961 47.072 1.00 0.00
D   H
ATOM 26023 HG2 LYS D 165   52.806 33.416 46.176 1.00 0.00
D   H
ATOM 26024 CD LYS D 165   54.582 34.562 45.648 1.00 0.00
D   C
ATOM 26025 HD1 LYS D 165   54.409 35.656 45.769 1.00 0.00
D   H
ATOM 26026 HD2 LYS D 165   55.651 34.364 45.893 1.00 0.00
D   H
ATOM 26027 CE LYS D 165   54.268 34.117 44.229 1.00 0.00
D   C

ATOM 26028 HE1 LYS D 165   53.239 34.427 43.941 1.00 0.00
D   H
ATOM 26029 HE2 LYS D 165   54.985 34.570 43.509 1.00 0.00
D   H
ATOM 26030 NZ LYS D 165   54.346 32.635 44.059 1.00 0.00
D   N
ATOM 26031 HZ1 LYS D 165   54.123 32.377 43.076 1.00 0.00
D   H
ATOM 26032 HZ2 LYS D 165   55.306 32.309 44.292 1.00 0.00
D   H
ATOM 26033 HZ3 LYS D 165   53.665 32.178 44.698 1.00 0.00
D   H
ATOM 26034 C   LYS D 165   53.852 35.862 49.956 1.00 0.00
D   C
ATOM 26035 O   LYS D 165   52.982 35.745 50.818 1.00 0.00
D   O
ATOM 26036 N   SER D 166   54.522 36.995 49.752 1.00 0.00
D   N
ATOM 26037 HN SER D 166   55.270 37.096 49.098 1.00 0.00
D   H
ATOM 26038 CA SER D 166   54.203 38.215 50.493 1.00 0.00
D   C
ATOM 26039 HA SER D 166   53.775 37.893 51.434 1.00 0.00
D   H
ATOM 26040 CB SER D 166   55.465 39.049 50.753 1.00 0.00
D   C
ATOM 26041 HB1 SER D 166   55.304 39.638 51.685 1.00 0.00
D   H
ATOM 26042 HB2 SER D 166   56.339 38.384 50.932 1.00 0.00
D   H
ATOM 26043 OG SER D 166   55.733 39.954 49.695 1.00 0.00
D   O
ATOM 26044 HG1 SER D 166   56.388 40.584 50.020 1.00 0.00
D   H
ATOM 26045 C   SER D 166   53.135 39.038 49.764 1.00 0.00
D   C
ATOM 26046 O   SER D 166   52.655 38.640 48.697 1.00 0.00
D   O
ATOM 26047 N   ILE D 167   52.766 40.174 50.351 1.00 0.00
D   N
ATOM 26048 HN ILE D 167   53.145 40.490 51.218 1.00 0.00
D   H
ATOM 26049 CA ILE D 167   51.764 41.072 49.773 1.00 0.00
D   C
ATOM 26050 HA ILE D 167   50.915 40.454 49.509 1.00 0.00
D   H
ATOM 26051 CB ILE D 167   51.351 42.190 50.767 1.00 0.00
D   C
ATOM 26052 HB ILE D 167   52.278 42.805 50.897 1.00 0.00
D   H
ATOM 26053 CG2 ILE D 167   50.213 43.040 50.201 1.00 0.00
D   C
ATOM 26054 HG21 ILE D 167   50.031 43.926 50.844 1.00 0.00
D   H

ATOM 26055 HG22 ILE D 167   50.462 43.407 49.183 1.00 0.00
D   H
ATOM 26056 HG23 ILE D 167   49.271 42.454 50.142 1.00 0.00
D   H
ATOM 26057 CG1 ILE D 167   51.009 41.617 52.159 1.00 0.00
D   C
ATOM 26058 HG11 ILE D 167   51.875 41.037 52.550 1.00 0.00
D   H
ATOM 26059 HG12 ILE D 167   50.833 42.462 52.864 1.00 0.00
D   H
ATOM 26060 CD ILE D 167   49.783 40.721 52.228 1.00 0.00
D   C
ATOM 26061 HD1 ILE D 167   49.591 40.408 53.277 1.00 0.00
D   H
ATOM 26062 HD2 ILE D 167   48.884 41.257 51.854 1.00 0.00
D   H
ATOM 26063 HD3 ILE D 167   49.925 39.804 51.617 1.00 0.00
D   H
ATOM 26064 C   ILE D 167   52.274 41.699 48.476 1.00 0.00
D   C
ATOM 26065 O   ILE D 167   51.530 41.806 47.497 1.00 0.00
D   O
ATOM 26066 N   ILE D 168   53.547 42.094 48.477 1.00 0.00
D   N
ATOM 26067 HN ILE D 168   54.156 41.968 49.258 1.00 0.00
D   H
ATOM 26068 CA ILE D 168   54.172 42.751 47.328 1.00 0.00
D   C
ATOM 26069 HA ILE D 168   53.378 43.303 46.841 1.00 0.00
D   H
ATOM 26070 CB ILE D 168   55.245 43.781 47.787 1.00 0.00
D   C
ATOM 26071 HB ILE D 168   54.764 44.326 48.639 1.00 0.00
D   H
ATOM 26072 CG2 ILE D 168   56.507 43.090 48.303 1.00 0.00
D   C
ATOM 26073 HG21 ILE D 168   57.194 43.827 48.767 1.00 0.00
D   H
ATOM 26074 HG22 ILE D 168   56.256 42.331 49.074 1.00 0.00
D   H
ATOM 26075 HG23 ILE D 168   57.049 42.584 47.477 1.00 0.00
D   H
ATOM 26076 CG1 ILE D 168   55.525 44.836 46.702 1.00 0.00
D   C
ATOM 26077 HG11 ILE D 168   56.513 45.313 46.887 1.00 0.00
D   H
ATOM 26078 HG12 ILE D 168   55.585 44.327 45.713 1.00 0.00
D   H
ATOM 26079 CD ILE D 168   54.475 45.925 46.598 1.00 0.00
D   C
ATOM 26080 HD1 ILE D 168   54.715 46.613 45.759 1.00 0.00
D   H
ATOM 26081 HD2 ILE D 168   53.471 45.485 46.412 1.00 0.00
D   H

ATOM 26082 HD3 ILE D 168   54.429 46.525 47.532 1.00 0.00
D   H
ATOM 26083 C   ILE D 168   54.722 41.751 46.291 1.00 0.00
D   C
ATOM 26084 O   ILE D 168   55.145 42.147 45.200 1.00 0.00
D   O
ATOM 26085 N   ASP D 169   54.701 40.462 46.635 1.00 0.00
D   N
ATOM 26086 HN ASP D 169   54.438 40.117 47.532 1.00 0.00
D   H
ATOM 26087 CA ASP D 169   55.077 39.393 45.699 1.00 0.00
D   C
ATOM 26088 HA ASP D 169   56.028 39.672 45.259 1.00 0.00
D   H
ATOM 26089 CB ASP D 169   55.200 38.051 46.424 1.00 0.00
D   C
ATOM 26090 HB1 ASP D 169   54.340 37.927 47.112 1.00 0.00
D   H
ATOM 26091 HB2 ASP D 169   55.191 37.214 45.698 1.00 0.00
D   H
ATOM 26092 CG ASP D 169   56.467 37.942 47.263 1.00 0.00
D   C
ATOM 26093 OD1 ASP D 169   57.205 38.943 47.400 1.00 0.00
D   O
ATOM 26094 OD2 ASP D 169   56.722 36.841 47.792 1.00 0.00
D   O
ATOM 26095 C   ASP D 169   54.070 39.299 44.550 1.00 0.00
D   C
ATOM 26096 O   ASP D 169   54.454 39.216 43.382 1.00 0.00
D   O
ATOM 26097 N   LEU D 170   52.784 39.295 44.900 1.00 0.00
D   N
ATOM 26098 HN LEU D 170   52.471 39.109 45.830 1.00 0.00
D   H
ATOM 26099 CA LEU D 170   51.712 39.563 43.950 1.00 0.00
D   C
ATOM 26100 HA LEU D 170   52.150 39.751 42.978 1.00 0.00
D   H
ATOM 26101 CB LEU D 170   50.689 38.416 43.910 1.00 0.00
D   C
ATOM 26102 HB1 LEU D 170   50.319 38.268 44.951 1.00 0.00
D   H
ATOM 26103 HB2 LEU D 170   49.807 38.738 43.313 1.00 0.00
D   H
ATOM 26104 CG LEU D 170   51.046 37.007 43.417 1.00 0.00
D   C
ATOM 26105 HG LEU D 170   51.971 36.716 43.970 1.00 0.00
D   H
ATOM 26106 CD1 LEU D 170   49.921 36.027 43.744 1.00 0.00
D   C
ATOM 26107 HD11 LEU D 170   50.156 35.016 43.346 1.00 0.00
D   H
ATOM 26108 HD12 LEU D 170   49.781 35.945 44.843 1.00 0.00
D   H

ATOM 26109 HD13 LEU D 170   48.964 36.363 43.292 1.00 0.00
D   H
ATOM 26110 CD2 LEU D 170   51.359 36.979 41.926 1.00 0.00
D   C
ATOM 26111 HD21 LEU D 170   51.630 35.948 41.607 1.00 0.00
D   H
ATOM 26112 HD22 LEU D 170   50.489 37.300 41.318 1.00 0.00
D   H
ATOM 26113 HD23 LEU D 170   52.214 37.651 41.695 1.00 0.00
D   H
ATOM 26114 C   LEU D 170   51.030 40.850 44.405 1.00 0.00
D   C
ATOM 26115 O   LEU D 170   50.035 40.796 45.138 1.00 0.00
D   O
ATOM 26116 N   ASN D 171   51.568 42.003 44.000 1.00 0.00
D   N
ATOM 26117 HN ASN D 171   52.398 42.092 43.450 1.00 0.00
D   H
ATOM 26118 CA ASN D 171   50.938 43.276 44.360 1.00 0.00
D   C
ATOM 26119 HA ASN D 171   50.784 43.241 45.433 1.00 0.00
D   H
ATOM 26120 CB ASN D 171   51.845 44.504 44.128 1.00 0.00
D   C
ATOM 26121 HB1 ASN D 171   51.249 45.415 44.353 1.00 0.00
D   H
ATOM 26122 HB2 ASN D 171   52.704 44.487 44.831 1.00 0.00
D   H
ATOM 26123 CG ASN D 171   52.353 44.626 42.699 1.00 0.00
D   C
ATOM 26124 OD1 ASN D 171   51.645 44.347 41.732 1.00 0.00
D   O
ATOM 26125 ND2 ASN D 171   53.593 45.076 42.563 1.00 0.00
D   N
ATOM 26126 HD21 ASN D 171   53.944 45.175 41.636 1.00 0.00
D   H
ATOM 26127 HD22 ASN D 171   54.122 45.327 43.367 1.00 0.00
D   H
ATOM 26128 C   ASN D 171   49.570 43.411 43.701 1.00 0.00
D   C
ATOM 26129 O   ASN D 171   49.447 43.510 42.479 1.00 0.00
D   O
ATOM 26130 N   VAL D 172   48.542 43.379 44.533 1.00 0.00
D   N
ATOM 26131 HN VAL D 172   48.626 43.428 45.525 1.00 0.00
D   H
ATOM 26132 CA VAL D 172   47.174 43.268 44.049 1.00 0.00
D   C
ATOM 26133 HA VAL D 172   47.215 42.717 43.118 1.00 0.00
D   H
ATOM 26134 CB VAL D 172   46.307 42.455 45.030 1.00 0.00
D   C
ATOM 26135 HB VAL D 172   45.237 42.635 44.762 1.00 0.00
D   H

ATOM 26136 CG1 VAL D 172   46.618 40.969 44.899 1.00 0.00
D   C
ATOM 26137 HG11 VAL D 172   46.021 40.385 45.633 1.00 0.00
D   H
ATOM 26138 HG12 VAL D 172   46.366 40.585 43.889 1.00 0.00
D   H
ATOM 26139 HG13 VAL D 172   47.694 40.774 45.097 1.00 0.00
D   H
ATOM 26140 CG2 VAL D 172   46.538 42.929 46.457 1.00 0.00
D   C
ATOM 26141 HG21 VAL D 172   45.945 42.321 47.174 1.00 0.00
D   H
ATOM 26142 HG22 VAL D 172   47.612 42.842 46.729 1.00 0.00
D   H
ATOM 26143 HG23 VAL D 172   46.232 43.988 46.584 1.00 0.00
D   H
ATOM 26144 C   VAL D 172   46.551 44.630 43.748 1.00 0.00
D   C
ATOM 26145 O   VAL D 172   45.554 44.712 43.034 1.00 0.00
D   O
ATOM 26146 N   ARG D 173   47.149 45.686 44.296 1.00 0.00
D   N
ATOM 26147 HN ARG D 173   47.920 45.570 44.919 1.00 0.00
D   H
ATOM 26148 CA ARG D 173   46.726 47.060 44.025 1.00 0.00
D   C
ATOM 26149 HA ARG D 173   45.645 47.094 44.081 1.00 0.00
D   H
ATOM 26150 CB ARG D 173   47.326 48.022 45.051 1.00 0.00
D   C
ATOM 26151 HB1 ARG D 173   48.263 47.541 45.423 1.00 0.00
D   H
ATOM 26152 HB2 ARG D 173   47.644 48.959 44.543 1.00 0.00
D   H
ATOM 26153 CG ARG D 173   46.408 48.298 46.223 1.00 0.00
D   C
ATOM 26154 HG1 ARG D 173   45.469 48.753 45.838 1.00 0.00
D   H
ATOM 26155 HG2 ARG D 173   46.105 47.329 46.681 1.00 0.00
D   H
ATOM 26156 CD ARG D 173   47.084 49.149 47.282 1.00 0.00
D   C
ATOM 26157 HD1 ARG D 173   47.776 48.514 47.882 1.00 0.00
D   H
ATOM 26158 HD2 ARG D 173   47.669 49.982 46.826 1.00 0.00
D   H
ATOM 26159 NE ARG D 173   46.094 49.757 48.167 1.00 0.00
D   N
ATOM 26160 HE ARG D 173   45.260 50.048 47.685 1.00 0.00
D   H
ATOM 26161 CZ ARG D 173   46.247 49.923 49.479 1.00 0.00
D   C
ATOM 26162 NH1 ARG D 173   47.355 49.516 50.085 1.00 0.00
D   N

ATOM 26163 HH11 ARG D 173   47.459 49.616 51.065 1.00 0.00
D   H
ATOM 26164 HH12 ARG D 173   48.000 48.990 49.527 1.00 0.00
D   H
ATOM 26165 NH2 ARG D 173   45.279 50.485 50.186 1.00 0.00
D   N
ATOM 26166 HH21 ARG D 173   45.423 50.689 51.145 1.00 0.00
D   H
ATOM 26167 HH22 ARG D 173   44.519 50.871 49.679 1.00 0.00
D   H
ATOM 26168 C   ARG D 173   47.103 47.506 42.617 1.00 0.00
D   C
ATOM 26169 O   ARG D 173   46.373 48.272 41.984 1.00 0.00
D   O
ATOM 26170 N   ALA D 174   48.249 47.029 42.140 1.00 0.00
D   N
ATOM 26171 HN ALA D 174   48.861 46.450 42.678 1.00 0.00
D   H
ATOM 26172 CA ALA D 174   48.695 47.321 40.789 1.00 0.00
D   C
ATOM 26173 HA ALA D 174   48.369 48.308 40.480 1.00 0.00
D   H
ATOM 26174 CB ALA D 174   50.217 47.300 40.712 1.00 0.00
D   C
ATOM 26175 HB1 ALA D 174   50.635 48.060 41.408 1.00 0.00
D   H
ATOM 26176 HB2 ALA D 174   50.618 46.307 41.009 1.00 0.00
D   H
ATOM 26177 HB3 ALA D 174   50.578 47.536 39.688 1.00 0.00
D   H
ATOM 26178 C   ALA D 174   48.085 46.335 39.795 1.00 0.00
D   C
ATOM 26179 O   ALA D 174   47.355 46.734 38.887 1.00 0.00
D   O
ATOM 26180 N   LYS D 175   48.356 45.049 39.995 1.00 0.00
D   N
ATOM 26181 HN LYS D 175   48.781 44.711 40.834 1.00 0.00
D   H
ATOM 26182 CA LYS D 175   48.052 44.021 38.993 1.00 0.00
D   C
ATOM 26183 HA LYS D 175   48.607 44.321 38.113 1.00 0.00
D   H
ATOM 26184 CB LYS D 175   48.598 42.660 39.429 1.00 0.00
D   C
ATOM 26185 HB1 LYS D 175   48.369 42.548 40.514 1.00 0.00
D   H
ATOM 26186 HB2 LYS D 175   48.030 41.858 38.906 1.00 0.00
D   H
ATOM 26187 CG LYS D 175   50.094 42.483 39.218 1.00 0.00
D   C
ATOM 26188 HG1 LYS D 175   50.332 42.641 38.142 1.00 0.00
D   H
ATOM 26189 HG2 LYS D 175   50.637 43.266 39.796 1.00 0.00
D   H

ATOM 26190 CD LYS D 175 50.549 41.094 39.636 1.00 0.00
D C
ATOM 26191 HD1 LYS D 175 51.659 41.102 39.736 1.00 0.00
D H
ATOM 26192 HD2 LYS D 175 50.125 40.878 40.643 1.00 0.00
D H
ATOM 26193 CE LYS D 175 50.107 40.038 38.637 1.00 0.00
D C
ATOM 26194 HE1 LYS D 175 49.038 40.185 38.362 1.00 0.00
D H
ATOM 26195 HE2 LYS D 175 50.719 40.096 37.710 1.00 0.00
D H
ATOM 26196 NZ LYS D 175 50.249 38.664 39.191 1.00 0.00
D N
ATOM 26197 HZ1 LYS D 175 49.939 37.962 38.489 1.00 0.00
D H
ATOM 26198 HZ2 LYS D 175 51.245 38.489 39.437 1.00 0.00
D H
ATOM 26199 HZ3 LYS D 175 49.666 38.575 40.048 1.00 0.00
D H
ATOM 26200 C LYS D 175 46.582 43.886 38.582 1.00 0.00
D C
ATOM 26201 O LYS D 175 46.276 43.801 37.394 1.00 0.00
D O
ATOM 26202 N TYR D 176 45.682 43.839 39.562 1.00 0.00
D N
ATOM 26203 HN TYR D 176 45.917 43.928 40.528 1.00 0.00
D H
ATOM 26204 CA TYR D 176 44.249 43.650 39.296 1.00 0.00
D C
ATOM 26205 HA TYR D 176 44.102 43.761 38.229 1.00 0.00
D H
ATOM 26206 CB TYR D 176 43.790 42.277 39.784 1.00 0.00
D C
ATOM 26207 HB1 TYR D 176 43.805 42.262 40.896 1.00 0.00
D H
ATOM 26208 HB2 TYR D 176 42.743 42.078 39.468 1.00 0.00
D H
ATOM 26209 CG TYR D 176 44.703 41.195 39.296 1.00 0.00
D C
ATOM 26210 CD1 TYR D 176 44.650 40.764 37.974 1.00 0.00
D C
ATOM 26211 HD1 TYR D 176 43.915 41.184 37.301 1.00 0.00
D H
ATOM 26212 CE1 TYR D 176 45.509 39.788 37.512 1.00 0.00
D C
ATOM 26213 HE1 TYR D 176 45.454 39.448 36.488 1.00 0.00
D H
ATOM 26214 CZ TYR D 176 46.444 39.236 38.372 1.00 0.00
D C
ATOM 26215 OH TYR D 176 47.300 38.259 37.921 1.00 0.00
D O
ATOM 26216 HH TYR D 176 47.910 38.060 38.634 1.00 0.00
D H

ATOM 26217 CD2 TYR D 176 45.660 40.637 40.139 1.00 0.00
D C
ATOM 26218 HD2 TYR D 176 45.709 40.940 41.176 1.00 0.00
D H
ATOM 26219 CE2 TYR D 176 46.525 39.658 39.686 1.00 0.00
D C
ATOM 26220 HE2 TYR D 176 47.239 39.218 40.366 1.00 0.00
D H
ATOM 26221 C TYR D 176 43.464 44.745 39.969 1.00 0.00
D C
ATOM 26222 O TYR D 176 42.278 44.943 39.706 1.00 0.00
D O
ATOM 26223 N GLY D 177 44.155 45.457 40.844 1.00 0.00
D N
ATOM 26224 HN GLY D 177 45.085 45.223 41.122 1.00 0.00
D H
ATOM 26225 CA GLY D 177 43.596 46.636 41.461 1.00 0.00
D C
ATOM 26226 HA1 GLY D 177 43.025 47.146 40.698 1.00 0.00
D H
ATOM 26227 HA2 GLY D 177 44.426 47.210 41.852 1.00 0.00
D H
ATOM 26228 C GLY D 177 42.667 46.320 42.609 1.00 0.00
D C
ATOM 26229 O GLY D 177 41.637 46.983 42.779 1.00 0.00
D O
ATOM 26230 N CYS D 178 43.015 45.302 43.396 1.00 0.00
D N
ATOM 26231 HN CYS D 178 43.801 44.711 43.223 1.00 0.00
D H
ATOM 26232 CA CYS D 178 42.254 44.970 44.597 1.00 0.00
D C
ATOM 26233 HA CYS D 178 41.436 45.679 44.652 1.00 0.00
D H
ATOM 26234 CB CYS D 178 41.682 43.550 44.514 1.00 0.00
D C
ATOM 26235 HB1 CYS D 178 42.535 42.861 44.322 1.00 0.00
D H
ATOM 26236 HB2 CYS D 178 41.264 43.253 45.501 1.00 0.00
D H
ATOM 26237 SG CYS D 178 40.465 43.347 43.193 1.00 0.00
D S
ATOM 26238 HG1 CYS D 178 40.250 42.041 43.365 1.00 0.00
D H
ATOM 26239 C CYS D 178 43.108 45.149 45.848 1.00 0.00
D C
ATOM 26240 O CYS D 178 44.330 45.031 45.797 1.00 0.00
D O
ATOM 26241 N THR D 179 42.459 45.453 46.966 1.00 0.00
D N
ATOM 26242 HN THR D 179 41.466 45.560 47.019 1.00 0.00
D H
ATOM 26243 CA THR D 179 43.152 45.661 48.232 1.00 0.00
D C

ATOM 26244 HA THR D 179 44.207 45.672 47.994 1.00 0.00
D H
ATOM 26245 CB THR D 179 42.703 46.965 48.918 1.00 0.00
D C
ATOM 26246 HB THR D 179 43.047 46.893 49.978 1.00 0.00
D H
ATOM 26247 OG1 THR D 179 41.271 47.033 48.919 1.00 0.00
D O
ATOM 26248 HG1 THR D 179 41.054 47.869 49.346 1.00 0.00
D H
ATOM 26249 CG2 THR D 179 43.256 48.177 48.188 1.00 0.00
D C
ATOM 26250 HG21 THR D 179 43.028 49.111 48.744 1.00 0.00
D H
ATOM 26251 HG22 THR D 179 44.360 48.098 48.089 1.00 0.00
D H
ATOM 26252 HG23 THR D 179 42.820 48.264 47.170 1.00 0.00
D H
ATOM 26253 C THR D 179 42.933 44.484 49.179 1.00 0.00
D C
ATOM 26254 O THR D 179 41.799 44.200 49.585 1.00 0.00
D O
ATOM 26255 N ILE D 180 44.023 43.794 49.515 1.00 0.00
D N
ATOM 26256 HN ILE D 180 44.930 43.963 49.133 1.00 0.00
D H
ATOM 26257 CA ILE D 180 43.978 42.708 50.494 1.00 0.00
D C
ATOM 26258 HA ILE D 180 43.105 42.108 50.269 1.00 0.00
D H
ATOM 26259 CB ILE D 180 45.250 41.827 50.465 1.00 0.00
D C
ATOM 26260 HB ILE D 180 46.097 42.558 50.510 1.00 0.00
D H
ATOM 26261 CG2 ILE D 180 45.244 40.822 51.616 1.00 0.00
D C
ATOM 26262 HG21 ILE D 180 46.222 40.302 51.686 1.00 0.00
D H
ATOM 26263 HG22 ILE D 180 45.064 41.332 52.585 1.00 0.00
D H
ATOM 26264 HG23 ILE D 180 44.453 40.056 51.470 1.00 0.00
D H
ATOM 26265 CG1 ILE D 180 45.365 41.096 49.123 1.00 0.00
D C
ATOM 26266 HG11 ILE D 180 44.509 40.395 49.001 1.00 0.00
D H
ATOM 26267 HG12 ILE D 180 45.299 41.843 48.299 1.00 0.00
D H
ATOM 26268 CD ILE D 180 46.653 40.320 48.936 1.00 0.00
D C
ATOM 26269 HD1 ILE D 180 46.686 39.868 47.921 1.00 0.00
D H
ATOM 26270 HD2 ILE D 180 47.533 40.988 49.049 1.00 0.00
D H

ATOM 26271 HD3 ILE D 180 46.734 39.500 49.680 1.00 0.00
D H
ATOM 26272 C ILE D 180 43.760 43.300 51.885 1.00 0.00
D C
ATOM 26273 O ILE D 180 44.587 44.065 52.386 1.00 0.00
D O
ATOM 26274 N LEU D 181 42.635 42.946 52.494 1.00 0.00
D N
ATOM 26275 HN LEU D 181 41.980 42.293 52.120 1.00 0.00
D H
ATOM 26276 CA LEU D 181 42.257 43.496 53.788 1.00 0.00
D C
ATOM 26277 HA LEU D 181 42.660 44.501 53.806 1.00 0.00
D H
ATOM 26278 CB LEU D 181 40.734 43.607 53.918 1.00 0.00
D C
ATOM 26279 HB1 LEU D 181 40.309 42.605 53.678 1.00 0.00
D H
ATOM 26280 HB2 LEU D 181 40.476 43.805 54.982 1.00 0.00
D H
ATOM 26281 CG LEU D 181 40.027 44.636 53.026 1.00 0.00
D C
ATOM 26282 HG LEU D 181 40.334 44.413 51.975 1.00 0.00
D H
ATOM 26283 CD1 LEU D 181 38.522 44.499 53.161 1.00 0.00
D C
ATOM 26284 HD11 LEU D 181 38.003 45.271 52.555 1.00 0.00
D H
ATOM 26285 HD12 LEU D 181 38.187 43.498 52.811 1.00 0.00
D H
ATOM 26286 HD13 LEU D 181 38.210 44.622 54.220 1.00 0.00
D H
ATOM 26287 CD2 LEU D 181 40.458 46.059 53.350 1.00 0.00
D C
ATOM 26288 HD21 LEU D 181 39.945 46.781 52.677 1.00 0.00
D H
ATOM 26289 HD22 LEU D 181 40.207 46.336 54.393 1.00 0.00
D H
ATOM 26290 HD23 LEU D 181 41.555 46.175 53.211 1.00 0.00
D H
ATOM 26291 C LEU D 181 42.853 42.740 54.973 1.00 0.00
D C
ATOM 26292 O LEU D 181 43.205 43.365 55.965 1.00 0.00
D O
ATOM 26293 N ALA D 182 42.972 41.415 54.877 1.00 0.00
D N
ATOM 26294 HN ALA D 182 42.696 40.896 54.069 1.00 0.00
D H
ATOM 26295 CA ALA D 182 43.529 40.616 55.981 1.00 0.00
D C
ATOM 26296 HA ALA D 182 44.346 41.166 56.434 1.00 0.00
D H
ATOM 26297 CB ALA D 182 42.493 40.442 57.086 1.00 0.00
D C

ATOM 26298 HB1 ALA D 182 42.123 41.438 57.411 1.00 0.00
D H
ATOM 26299 HB2 ALA D 182 41.620 39.854 56.726 1.00 0.00
D H
ATOM 26300 HB3 ALA D 182 42.923 39.929 57.973 1.00 0.00
D H
ATOM 26301 C ALA D 182 44.101 39.257 55.567 1.00 0.00
D C
ATOM 26302 O ALA D 182 43.829 38.760 54.473 1.00 0.00
D O
ATOM 26303 N ILE D 183 44.911 38.679 56.457 1.00 0.00
D N
ATOM 26304 HN ILE D 183 45.246 39.161 57.264 1.00 0.00
D H
ATOM 26305 CA ILE D 183 45.385 37.296 56.343 1.00 0.00
D C
ATOM 26306 HA ILE D 183 44.839 36.824 55.537 1.00 0.00
D H
ATOM 26307 CB ILE D 183 46.922 37.204 56.167 1.00 0.00
D C
ATOM 26308 HB ILE D 183 47.338 37.566 57.141 1.00 0.00
D H
ATOM 26309 CG2 ILE D 183 47.345 35.779 55.821 1.00 0.00
D C
ATOM 26310 HG21 ILE D 183 48.450 35.682 55.850 1.00 0.00
D H
ATOM 26311 HG22 ILE D 183 46.926 35.053 56.550 1.00 0.00
D H
ATOM 26312 HG23 ILE D 183 46.998 35.496 54.804 1.00 0.00
D H
ATOM 26313 CG1 ILE D 183 47.423 38.175 55.093 1.00 0.00
D C
ATOM 26314 HG11 ILE D 183 47.160 37.789 54.084 1.00 0.00
D H
ATOM 26315 HG12 ILE D 183 46.901 39.152 55.218 1.00 0.00
D H
ATOM 26316 CD ILE D 183 48.915 38.437 55.140 1.00 0.00
D C
ATOM 26317 HD1 ILE D 183 49.199 39.191 54.375 1.00 0.00
D H
ATOM 26318 HD2 ILE D 183 49.214 38.823 56.139 1.00 0.00
D H
ATOM 26319 HD3 ILE D 183 49.489 37.508 54.936 1.00 0.00
D H
ATOM 26320 C ILE D 183 44.996 36.566 57.628 1.00 0.00
D C
ATOM 26321 O ILE D 183 45.016 37.156 58.710 1.00 0.00
D O
ATOM 26322 N LYS D 184 44.628 35.292 57.507 1.00 0.00
D N
ATOM 26323 HN LYS D 184 44.512 34.829 56.630 1.00 0.00
D H
ATOM 26324 CA LYS D 184 44.363 34.470 58.681 1.00 0.00
D C

ATOM 26325 HA LYS D 184 44.557 35.084 59.552 1.00 0.00
D H
ATOM 26326 CB LYS D 184 42.906 33.997 58.714 1.00 0.00
D C
ATOM 26327 HB1 LYS D 184 42.265 34.907 58.653 1.00 0.00
D H
ATOM 26328 HB2 LYS D 184 42.697 33.404 57.796 1.00 0.00
D H
ATOM 26329 CG LYS D 184 42.548 33.211 59.967 1.00 0.00
D C
ATOM 26330 HG1 LYS D 184 43.286 32.387 60.103 1.00 0.00
D H
ATOM 26331 HG2 LYS D 184 42.633 33.882 60.853 1.00 0.00
D H
ATOM 26332 CD LYS D 184 41.153 32.614 59.892 1.00 0.00
D C
ATOM 26333 HD1 LYS D 184 40.414 33.448 59.895 1.00 0.00
D H
ATOM 26334 HD2 LYS D 184 41.055 32.084 58.917 1.00 0.00
D H
ATOM 26335 CE LYS D 184 40.923 31.664 61.055 1.00 0.00
D C
ATOM 26336 HE1 LYS D 184 41.337 30.658 60.825 1.00 0.00
D H
ATOM 26337 HE2 LYS D 184 41.418 32.046 61.975 1.00 0.00
D H
ATOM 26338 NZ LYS D 184 39.477 31.491 61.365 1.00 0.00
D N
ATOM 26339 HZ1 LYS D 184 39.360 30.834 62.163 1.00 0.00
D H
ATOM 26340 HZ2 LYS D 184 39.061 32.412 61.611 1.00 0.00
D H
ATOM 26341 HZ3 LYS D 184 38.988 31.108 60.531 1.00 0.00
D H
ATOM 26342 C LYS D 184 45.313 33.280 58.744 1.00 0.00
D C
ATOM 26343 O LYS D 184 45.191 32.333 57.966 1.00 0.00
D O
ATOM 26344 N HSE D 185 46.267 33.345 59.667 1.00 0.00
D N
ATOM 26345 HN HSE D 185 46.469 34.156 60.215 1.00 0.00
D H
ATOM 26346 CA HSE D 185 47.113 32.199 59.957 1.00 0.00
D C
ATOM 26347 HA HSE D 185 46.854 31.419 59.250 1.00 0.00
D H
ATOM 26348 CB HSE D 185 48.593 32.534 59.788 1.00 0.00
D C
ATOM 26349 HB1 HSE D 185 48.741 32.914 58.755 1.00 0.00
D H
ATOM 26350 HB2 HSE D 185 48.867 33.358 60.480 1.00 0.00
D H
ATOM 26351 ND1 HSE D 185 49.708 30.417 59.037 1.00 0.00
D N

ATOM 26352 CG HSE D 185 49.509 31.347 59.989 1.00 0.00
D C
ATOM 26353 CE1 HSE D 185 50.563 29.483 59.496 1.00 0.00
D C
ATOM 26354 HE1 HSE D 185 50.893 28.611 58.926 1.00 0.00
D H
ATOM 26355 NE2 HSE D 185 50.911 29.818 60.749 1.00 0.00
D N
ATOM 26356 HE2 HSE D 185 51.523 29.292 61.339 1.00 0.00
D H
ATOM 26357 CD2 HSE D 185 50.278 30.961 61.085 1.00 0.00
D C
ATOM 26358 HD2 HSE D 185 50.388 31.427 62.053 1.00 0.00
D H
ATOM 26359 C HSE D 185 46.815 31.668 61.329 1.00 0.00
D C
ATOM 26360 O HSE D 185 47.052 32.343 62.337 1.00 0.00
D O
ATOM 26361 N HSE D 186 46.246 30.461 61.346 1.00 0.00
D N
ATOM 26362 HN HSE D 186 45.948 30.023 60.499 1.00 0.00
D H
ATOM 26363 CA HSE D 186 46.002 29.667 62.557 1.00 0.00
D C
ATOM 26364 HA HSE D 186 45.475 28.784 62.214 1.00 0.00
D H
ATOM 26365 CB HSE D 186 47.341 29.289 63.196 1.00 0.00
D C
ATOM 26366 HB1 HSE D 186 47.931 28.723 62.444 1.00 0.00
D H
ATOM 26367 HB2 HSE D 186 47.913 30.212 63.430 1.00 0.00
D H
ATOM 26368 ND1 HSE D 186 46.641 27.177 64.388 1.00 0.00
D N
ATOM 26369 CG HSE D 186 47.207 28.435 64.417 1.00 0.00
D C
ATOM 26370 CE1 HSE D 186 46.659 26.661 65.604 1.00 0.00
D C
ATOM 26371 HE1 HSE D 186 46.268 25.675 65.865 1.00 0.00
D H
ATOM 26372 NE2 HSE D 186 47.215 27.536 66.421 1.00 0.00
D N
ATOM 26373 HE2 HSE D 186 47.338 27.410 67.404 1.00 0.00
D H
ATOM 26374 CD2 HSE D 186 47.569 28.654 65.702 1.00 0.00
D C
ATOM 26375 HD2 HSE D 186 48.038 29.517 66.153 1.00 0.00
D H
ATOM 26376 C HSE D 186 45.116 30.339 63.612 1.00 0.00
D C
ATOM 26377 O HSE D 186 45.382 30.236 64.814 1.00 0.00
D O
ATOM 26378 N GLY D 187 44.061 31.011 63.161 1.00 0.00
D N

ATOM 26379 HN GLY D 187   43.868 31.106 62.185 1.00 0.00
D   H
ATOM 26380 CA GLY D 187   43.108 31.658 64.061 1.00 0.00
D   C
ATOM 26381 HA1 GLY D 187   42.858 30.944 64.834 1.00 0.00
D   H
ATOM 26382 HA2 GLY D 187   42.265 31.983 63.467 1.00 0.00
D   H
ATOM 26383 C   GLY D 187   43.708 32.876 64.726 1.00 0.00
D   C
ATOM 26384 O   GLY D 187   43.221 33.349 65.752 1.00 0.00
D   O
ATOM 26385 N   ASP D 188   44.791 33.363 64.141 1.00 0.00
D   N
ATOM 26386 HN ASP D 188   45.273 32.922 63.388 1.00 0.00
D   H
ATOM 26387 CA ASP D 188   45.400 34.614 64.544 1.00 0.00
D   C
ATOM 26388 HA ASP D 188   44.725 35.153 65.199 1.00 0.00
D   H
ATOM 26389 CB ASP D 188   46.763 34.403 65.205 1.00 0.00
D   C
ATOM 26390 HB1 ASP D 188   47.352 33.680 64.605 1.00 0.00
D   H
ATOM 26391 HB2 ASP D 188   47.326 35.355 65.254 1.00 0.00
D   H
ATOM 26392 CG ASP D 188   46.649 33.838 66.608 1.00 0.00
D   C
ATOM 26393 OD1 ASP D 188   45.538 33.875 67.175 1.00 0.00
D   O
ATOM 26394 OD2 ASP D 188   47.616 33.339 67.221 1.00 0.00
D   O
ATOM 26395 C   ASP D 188   45.515 35.427 63.267 1.00 0.00
D   C
ATOM 26396 O   ASP D 188   46.180 35.020 62.311 1.00 0.00
D   O
ATOM 26397 N   ILE D 189   44.833 36.568 63.247 1.00 0.00
D   N
ATOM 26398 HN ILE D 189   44.377 36.960 64.043 1.00 0.00
D   H
ATOM 26399 CA ILE D 189   44.693 37.357 62.029 1.00 0.00
D   C
ATOM 26400 HA ILE D 189   44.930 36.676 61.222 1.00 0.00
D   H
ATOM 26401 CB ILE D 189   43.252 37.895 61.920 1.00 0.00
D   C
ATOM 26402 HB ILE D 189   42.611 36.976 61.907 1.00 0.00
D   H
ATOM 26403 CG2 ILE D 189   42.946 38.836 63.074 1.00 0.00
D   C
ATOM 26404 HG21 ILE D 189   41.868 39.101 63.087 1.00 0.00
D   H
ATOM 26405 HG22 ILE D 189   43.187 38.362 64.048 1.00 0.00
D   H

```
ATOM  26406 HG23 ILE D 189      43.532  39.775  62.986  1.00  0.00
D    H
ATOM  26407  CG1 ILE D 189      43.048  38.599  60.577  1.00  0.00
D    C
ATOM  26408 HG11 ILE D 189      43.615  39.556  60.564  1.00  0.00
D    H
ATOM  26409 HG12 ILE D 189      43.464  37.957  59.767  1.00  0.00
D    H
ATOM  26410  CD  ILE D 189      41.597  38.882  60.252  1.00  0.00
D    C
ATOM  26411  HD1 ILE D 189      41.511  39.341  59.244  1.00  0.00
D    H
ATOM  26412  HD2 ILE D 189      41.003  37.944  60.261  1.00  0.00
D    H
ATOM  26413  HD3 ILE D 189      41.154  39.587  60.988  1.00  0.00
D    H
ATOM  26414  C   ILE D 189      45.677  38.524  61.928  1.00  0.00
D    C
ATOM  26415  O   ILE D 189      45.886  39.263  62.897  1.00  0.00
D    O
ATOM  26416  N   CYS D 190      46.282  38.666  60.749  1.00  0.00
D    N
ATOM  26417  HN  CYS D 190      46.252  37.970  60.036  1.00  0.00
D    H
ATOM  26418  CA  CYS D 190      47.041  39.862  60.404  1.00  0.00
D    C
ATOM  26419  HA  CYS D 190      47.441  40.291  61.314  1.00  0.00
D    H
ATOM  26420  CB  CYS D 190      48.163  39.554  59.410  1.00  0.00
D    C
ATOM  26421  HB1 CYS D 190      47.703  39.017  58.549  1.00  0.00
D    H
ATOM  26422  HB2 CYS D 190      48.573  40.502  58.998  1.00  0.00
D    H
ATOM  26423  SG  CYS D 190      49.486  38.519  60.077  1.00  0.00
D    S
ATOM  26424  HG1 CYS D 190      50.199  38.417  58.954  1.00  0.00
D    H
ATOM  26425  C   CYS D 190      46.043  40.858  59.821  1.00  0.00
D    C
ATOM  26426  O   CYS D 190      45.624  40.735  58.666  1.00  0.00
D    O
ATOM  26427  N   LEU D 191      45.655  41.828  60.649  1.00  0.00
D    N
ATOM  26428  HN  LEU D 191      46.103  41.998  61.524  1.00  0.00
D    H
ATOM  26429  CA  LEU D 191      44.542  42.732  60.356  1.00  0.00
D    C
ATOM  26430  HA  LEU D 191      43.673  42.093  60.261  1.00  0.00
D    H
ATOM  26431  CB  LEU D 191      44.363  43.720  61.512  1.00  0.00
D    C
ATOM  26432  HB1 LEU D 191      44.314  43.122  62.451  1.00  0.00
D    H
```

ATOM 26433 HB2 LEU D 191   45.277 44.349 61.594 1.00 0.00
D   H
ATOM 26434 CG LEU D 191   43.122 44.615 61.454 1.00 0.00
D   C
ATOM 26435 HG LEU D 191   42.344 44.057 60.881 1.00 0.00
D   H
ATOM 26436 CD1 LEU D 191   42.609 44.909 62.855 1.00 0.00
D   C
ATOM 26437 HD11 LEU D 191   41.737 45.597 62.815 1.00 0.00
D   H
ATOM 26438 HD12 LEU D 191   42.290 43.973 63.360 1.00 0.00
D   H
ATOM 26439 HD13 LEU D 191   43.400 45.388 63.471 1.00 0.00
D   H
ATOM 26440 CD2 LEU D 191   43.423 45.905 60.707 1.00 0.00
D   C
ATOM 26441 HD21 LEU D 191   42.513 46.541 60.653 1.00 0.00
D   H
ATOM 26442 HD22 LEU D 191   44.216 46.495 61.209 1.00 0.00
D   H
ATOM 26443 HD23 LEU D 191   43.755 45.685 59.669 1.00 0.00
D   H
ATOM 26444 C   LEU D 191   44.689 43.508 59.050 1.00 0.00
D   C
ATOM 26445 O   LEU D 191   43.775 43.492 58.227 1.00 0.00
D   O
ATOM 26446 N   SER D 192   45.830 44.180 58.869 1.00 0.00
D   N
ATOM 26447 HN SER D 192   46.586 44.177 59.524 1.00 0.00
D   H
ATOM 26448 CA SER D 192   46.072 44.991 57.675 1.00 0.00
D   C
ATOM 26449 HA SER D 192   45.399 44.608 56.920 1.00 0.00
D   H
ATOM 26450 CB SER D 192   45.804 46.467 57.971 1.00 0.00
D   C
ATOM 26451 HB1 SER D 192   45.933 47.040 57.025 1.00 0.00
D   H
ATOM 26452 HB2 SER D 192   44.750 46.611 58.300 1.00 0.00
D   H
ATOM 26453 OG SER D 192   46.714 46.968 58.934 1.00 0.00
D   O
ATOM 26454 HG1 SER D 192   46.647 47.931 58.915 1.00 0.00
D   H
ATOM 26455 C   SER D 192   47.496 44.821 57.142 1.00 0.00
D   C
ATOM 26456 O   SER D 192   48.476 44.959 57.884 1.00 0.00
D   O
ATOM 26457 N   GLU D 196   55.390 42.883 53.339 1.00 0.00
D   N
ATOM 26458 HN GLU D 196   55.784 43.664 52.863 1.00 0.00
D   H
ATOM 26459 CA GLU D 196   56.212 41.667 53.273 1.00 0.00
D   C

ATOM 26460 HA GLU D 196 56.289 41.400 52.226 1.00 0.00
D H
ATOM 26461 CB GLU D 196 57.606 41.891 53.875 1.00 0.00
D C
ATOM 26462 HB1 GLU D 196 57.491 42.043 54.973 1.00 0.00
D H
ATOM 26463 HB2 GLU D 196 58.220 40.970 53.757 1.00 0.00
D H
ATOM 26464 CG GLU D 196 58.397 43.105 53.297 1.00 0.00
D C
ATOM 26465 HG1 GLU D 196 59.478 42.962 53.481 1.00 0.00
D H
ATOM 26466 HG2 GLU D 196 58.223 43.210 52.207 1.00 0.00
D H
ATOM 26467 CD GLU D 196 58.059 44.403 53.943 1.00 0.00
D C
ATOM 26468 OE1 GLU D 196 57.827 44.407 55.071 1.00 0.00
D O
ATOM 26469 OE2 GLU D 196 58.093 45.457 53.318 1.00 0.00
D O
ATOM 26470 C GLU D 196 55.621 40.471 54.002 1.00 0.00
D C
ATOM 26471 O GLU D 196 56.328 39.486 54.230 1.00 0.00
D O
ATOM 26472 N ASP D 197 54.346 40.538 54.372 1.00 0.00
D N
ATOM 26473 HN ASP D 197 53.700 41.265 54.158 1.00 0.00
D H
ATOM 26474 CA ASP D 197 53.756 39.472 55.179 1.00 0.00
D C
ATOM 26475 HA ASP D 197 54.514 39.191 55.901 1.00 0.00
D H
ATOM 26476 CB ASP D 197 52.550 39.980 55.972 1.00 0.00
D C
ATOM 26477 HB1 ASP D 197 51.943 40.648 55.327 1.00 0.00
D H
ATOM 26478 HB2 ASP D 197 51.912 39.135 56.297 1.00 0.00
D H
ATOM 26479 CG ASP D 197 52.960 40.780 57.201 1.00 0.00
D C
ATOM 26480 OD1 ASP D 197 54.143 40.712 57.605 1.00 0.00
D O
ATOM 26481 OD2 ASP D 197 52.095 41.479 57.765 1.00 0.00
D O
ATOM 26482 C ASP D 197 53.420 38.215 54.382 1.00 0.00
D C
ATOM 26483 O ASP D 197 52.710 38.273 53.376 1.00 0.00
D O
ATOM 26484 N ILE D 198 53.948 37.087 54.854 1.00 0.00
D N
ATOM 26485 HN ILE D 198 54.454 37.059 55.713 1.00 0.00
D H
ATOM 26486 CA ILE D 198 53.844 35.791 54.176 1.00 0.00
D C

ATOM 26487 HA ILE D 198   54.057 35.983 53.132 1.00 0.00
D   H
ATOM 26488 CB ILE D 198   54.854 34.768 54.764 1.00 0.00
D   C
ATOM 26489 HB ILE D 198   54.627 34.749 55.860 1.00 0.00
D   H
ATOM 26490 CG2 ILE D 198   54.714 33.405 54.098 1.00 0.00
D   C
ATOM 26491 HG21 ILE D 198   55.327 32.646 54.628 1.00 0.00
D   H
ATOM 26492 HG22 ILE D 198   53.659 33.060 54.118 1.00 0.00
D   H
ATOM 26493 HG23 ILE D 198   55.049 33.443 53.039 1.00 0.00
D   H
ATOM 26494 CG1 ILE D 198   56.294 35.282 54.620 1.00 0.00
D   C
ATOM 26495 HG11 ILE D 198   56.655 35.107 53.582 1.00 0.00
D   H
ATOM 26496 HG12 ILE D 198   56.303 36.383 54.791 1.00 0.00
D   H
ATOM 26497 CD ILE D 198   57.282 34.657 55.586 1.00 0.00
D   C
ATOM 26498 HD1 ILE D 198   58.286 35.118 55.463 1.00 0.00
D   H
ATOM 26499 HD2 ILE D 198   56.954 34.813 56.636 1.00 0.00
D   H
ATOM 26500 HD3 ILE D 198   57.380 33.566 55.406 1.00 0.00
D   H
ATOM 26501 C   ILE D 198   52.426 35.221 54.242 1.00 0.00
D   C
ATOM 26502 O   ILE D 198   51.811 35.171 55.313 1.00 0.00
D   O
ATOM 26503 N   ILE D 199   51.912 34.812 53.083 1.00 0.00
D   N
ATOM 26504 HN ILE D 199   52.353 34.977 52.203 1.00 0.00
D   H
ATOM 26505 CA ILE D 199   50.650 34.080 52.992 1.00 0.00
D   C
ATOM 26506 HA ILE D 199   50.167 34.145 53.959 1.00 0.00
D   H
ATOM 26507 CB ILE D 199   49.728 34.620 51.873 1.00 0.00
D   C
ATOM 26508 HB ILE D 199   50.415 34.807 51.009 1.00 0.00
D   H
ATOM 26509 CG2 ILE D 199   48.596 33.642 51.588 1.00 0.00
D   C
ATOM 26510 HG21 ILE D 199   48.029 33.953 50.685 1.00 0.00
D   H
ATOM 26511 HG22 ILE D 199   48.992 32.622 51.403 1.00 0.00
D   H
ATOM 26512 HG23 ILE D 199   47.889 33.593 52.442 1.00 0.00
D   H
ATOM 26513 CG1 ILE D 199   49.138 35.976 52.271 1.00 0.00
D   C

ATOM 26514 HG11 ILE D 199   48.289 35.826 52.974 1.00 0.00
D   H
ATOM 26515 HG12 ILE D 199   49.915 36.562 52.813 1.00 0.00
D   H
ATOM 26516 CD ILE D 199   48.659 36.811 51.101 1.00 0.00
D   C
ATOM 26517 HD1 ILE D 199   48.299 37.801 51.456 1.00 0.00
D   H
ATOM 26518 HD2 ILE D 199   49.485 36.980 50.377 1.00 0.00
D   H
ATOM 26519 HD3 ILE D 199   47.822 36.311 50.571 1.00 0.00
D   H
ATOM 26520 C   ILE D 199   50.954 32.606 52.750 1.00 0.00
D   C
ATOM 26521 O   ILE D 199   51.584 32.244 51.753 1.00 0.00
D   O
ATOM 26522 N   ARG D 200   50.512 31.769 53.683 1.00 0.00
D   N
ATOM 26523 HN ARG D 200   50.059 32.104 54.506 1.00 0.00
D   H
ATOM 26524 CA ARG D 200   50.663 30.330 53.561 1.00 0.00
D   C
ATOM 26525 HA ARG D 200   51.423 30.110 52.820 1.00 0.00
D   H
ATOM 26526 CB ARG D 200   51.070 29.728 54.911 1.00 0.00
D   C
ATOM 26527 HB1 ARG D 200   51.311 30.589 55.579 1.00 0.00
D   H
ATOM 26528 HB2 ARG D 200   50.194 29.220 55.370 1.00 0.00
D   H
ATOM 26529 CG ARG D 200   52.277 28.806 54.845 1.00 0.00
D   C
ATOM 26530 HG1 ARG D 200   51.961 27.842 54.390 1.00 0.00
D   H
ATOM 26531 HG2 ARG D 200   53.025 29.238 54.141 1.00 0.00
D   H
ATOM 26532 CD ARG D 200   52.917 28.643 56.215 1.00 0.00
D   C
ATOM 26533 HD1 ARG D 200   52.123 28.469 56.977 1.00 0.00
D   H
ATOM 26534 HD2 ARG D 200   53.626 27.782 56.236 1.00 0.00
D   H
ATOM 26535 NE ARG D 200   53.693 29.818 56.611 1.00 0.00
D   N
ATOM 26536 HE ARG D 200   53.810 30.485 55.866 1.00 0.00
D   H
ATOM 26537 CZ ARG D 200   54.206 30.016 57.825 1.00 0.00
D   C
ATOM 26538 NH1 ARG D 200   54.029 29.116 58.786 1.00 0.00
D   N
ATOM 26539 HH11 ARG D 200   54.388 29.272 59.696 1.00 0.00
D   H
ATOM 26540 HH12 ARG D 200   53.408 28.358 58.576 1.00 0.00
D   H

```
ATOM  26541  NH2 ARG D 200      54.898  31.117  58.081  1.00  0.00
D    N
ATOM  26542 HH21 ARG D 200      55.351  31.223  58.954  1.00  0.00
D    H
ATOM  26543 HH22 ARG D 200      55.110  31.703  57.310  1.00  0.00
D    H
ATOM  26544  C   ARG D 200      49.353  29.732  53.035  1.00  0.00
D    C
ATOM  26545  O   ARG D 200      48.316  30.395  53.028  1.00  0.00
D    O
ATOM  26546  N   GLU D 201      49.424  28.489  52.570  1.00  0.00
D    N
ATOM  26547  HN  GLU D 201      50.277  27.974  52.592  1.00  0.00
D    H
ATOM  26548  CA  GLU D 201      48.299  27.753  51.990  1.00  0.00
D    C
ATOM  26549  HA  GLU D 201      47.897  28.283  51.136  1.00  0.00
D    H
ATOM  26550  CB  GLU D 201      48.815  26.359  51.681  1.00  0.00
D    C
ATOM  26551  HB1 GLU D 201      49.894  26.439  51.416  1.00  0.00
D    H
ATOM  26552  HB2 GLU D 201      48.771  25.728  52.597  1.00  0.00
D    H
ATOM  26553  CG  GLU D 201      48.116  25.631  50.560  1.00  0.00
D    C
ATOM  26554  HG1 GLU D 201      47.036  25.559  50.784  1.00  0.00
D    H
ATOM  26555  HG2 GLU D 201      48.254  26.160  49.596  1.00  0.00
D    H
ATOM  26556  CD  GLU D 201      48.624  24.213  50.431  1.00  0.00
D    C
ATOM  26557  OE1 GLU D 201      49.261  23.714  51.386  1.00  0.00
D    O
ATOM  26558  OE2 GLU D 201      48.386  23.594  49.379  1.00  0.00
D    O
ATOM  26559  C   GLU D 201      47.220  27.737  53.057  1.00  0.00
D    C
ATOM  26560  O   GLU D 201      47.552  27.726  54.176  1.00  0.00
D    O
ATOM  26561  N   GLN D 202      45.936  27.722  52.764  1.00  0.00
D    N
ATOM  26562  HN  GLN D 202      45.543  27.732  51.846  1.00  0.00
D    H
ATOM  26563  CA  GLN D 202      44.944  27.690  53.850  1.00  0.00
D    C
ATOM  26564  HA  GLN D 202      43.982  27.751  53.355  1.00  0.00
D    H
ATOM  26565  CB  GLN D 202      45.167  26.361  54.566  1.00  0.00
D    C
ATOM  26566  HB1 GLN D 202      45.177  25.570  53.780  1.00  0.00
D    H
ATOM  26567  HB2 GLN D 202      46.182  26.356  55.025  1.00  0.00
D    H
```

ATOM 26568 CG GLN D 202 44.147 26.009 55.584 1.00 0.00
D C
ATOM 26569 HG1 GLN D 202 44.139 26.761 56.401 1.00 0.00
D H
ATOM 26570 HG2 GLN D 202 43.133 25.985 55.133 1.00 0.00
D H
ATOM 26571 CD GLN D 202 44.415 24.680 56.236 1.00 0.00
D C
ATOM 26572 OE1 GLN D 202 45.240 23.921 55.804 1.00 0.00
D O
ATOM 26573 NE2 GLN D 202 43.724 24.400 57.273 1.00 0.00
D N
ATOM 26574 HE21 GLN D 202 43.903 23.523 57.710 1.00 0.00
D H
ATOM 26575 HE22 GLN D 202 43.036 25.041 57.597 1.00 0.00
D H
ATOM 26576 C GLN D 202 45.025 28.851 54.846 1.00 0.00
D C
ATOM 26577 O GLN D 202 44.667 28.727 56.029 1.00 0.00
D O
ATOM 26578 N ASP D 203 45.521 29.979 54.361 1.00 0.00
D N
ATOM 26579 HN ASP D 203 46.026 30.087 53.509 1.00 0.00
D H
ATOM 26580 CA ASP D 203 45.355 31.221 55.079 1.00 0.00
D C
ATOM 26581 HA ASP D 203 45.097 31.022 56.113 1.00 0.00
D H
ATOM 26582 CB ASP D 203 46.590 32.124 54.963 1.00 0.00
D C
ATOM 26583 HB1 ASP D 203 47.013 32.035 53.941 1.00 0.00
D H
ATOM 26584 HB2 ASP D 203 46.318 33.183 55.137 1.00 0.00
D H
ATOM 26585 CG ASP D 203 47.699 31.746 55.945 1.00 0.00
D C
ATOM 26586 OD1 ASP D 203 47.452 30.953 56.876 1.00 0.00
D O
ATOM 26587 OD2 ASP D 203 48.827 32.254 55.784 1.00 0.00
D O
ATOM 26588 C ASP D 203 44.135 31.855 54.436 1.00 0.00
D C
ATOM 26589 O ASP D 203 43.859 31.629 53.257 1.00 0.00
D O
ATOM 26590 N CYS D 204 43.383 32.616 55.219 1.00 0.00
D N
ATOM 26591 HN CYS D 204 43.557 32.788 56.186 1.00 0.00
D H
ATOM 26592 CA CYS D 204 42.202 33.276 54.684 1.00 0.00
D C
ATOM 26593 HA CYS D 204 41.861 32.666 53.856 1.00 0.00
D H
ATOM 26594 CB CYS D 204 41.090 33.357 55.730 1.00 0.00
D C

ATOM 26595 HB1 CYS D 204   41.572 33.585 56.707 1.00 0.00
D   H
ATOM 26596 HB2 CYS D 204   40.424 34.221 55.508 1.00 0.00
D   H
ATOM 26597 SG CYS D 204   40.132 31.836 55.912 1.00 0.00
D   S
ATOM 26598 HG1 CYS D 204   39.349 32.250 56.910 1.00 0.00
D   H
ATOM 26599 C   CYS D 204   42.534 34.655 54.137 1.00 0.00
D   C
ATOM 26600 O   CYS D 204   42.888 35.569 54.884 1.00 0.00
D   O
ATOM 26601 N   LEU D 205   42.430 34.786 52.819 1.00 0.00
D   N
ATOM 26602 HN LEU D 205   42.246 34.024 52.202 1.00 0.00
D   H
ATOM 26603 CA LEU D 205   42.579 36.074 52.154 1.00 0.00
D   C
ATOM 26604 HA LEU D 205   43.322 36.635 52.707 1.00 0.00
D   H
ATOM 26605 CB LEU D 205   42.980 35.881 50.693 1.00 0.00
D   C
ATOM 26606 HB1 LEU D 205   42.230 35.197 50.233 1.00 0.00
D   H
ATOM 26607 HB2 LEU D 205   42.880 36.852 50.158 1.00 0.00
D   H
ATOM 26608 CG LEU D 205   44.365 35.297 50.434 1.00 0.00
D   C
ATOM 26609 HG LEU D 205   44.446 34.330 50.986 1.00 0.00
D   H
ATOM 26610 CD1 LEU D 205   44.558 35.059 48.949 1.00 0.00
D   C
ATOM 26611 HD11 LEU D 205   45.582 34.679 48.744 1.00 0.00
D   H
ATOM 26612 HD12 LEU D 205   43.827 34.312 48.574 1.00 0.00
D   H
ATOM 26613 HD13 LEU D 205   44.419 36.003 48.378 1.00 0.00
D   H
ATOM 26614 CD2 LEU D 205   45.441 36.231 50.963 1.00 0.00
D   C
ATOM 26615 HD21 LEU D 205   46.449 35.796 50.790 1.00 0.00
D   H
ATOM 26616 HD22 LEU D 205   45.410 37.218 50.460 1.00 0.00
D   H
ATOM 26617 HD23 LEU D 205   45.314 36.391 52.056 1.00 0.00
D   H
ATOM 26618 C   LEU D 205   41.279 36.849 52.218 1.00 0.00
D   C
ATOM 26619 O   LEU D 205   40.213 36.304 51.938 1.00 0.00
D   O
ATOM 26620 N   VAL D 206   41.378 38.121 52.584 1.00 0.00
D   N
ATOM 26621 HN VAL D 206   42.241 38.546 52.846 1.00 0.00
D   H

ATOM 26622 CA VAL D 206   40.227 39.015 52.636 1.00 0.00
D   C
ATOM 26623 HA VAL D 206   39.345 38.463 52.334 1.00 0.00
D   H
ATOM 26624 CB VAL D 206   40.017 39.618 54.036 1.00 0.00
D   C
ATOM 26625 HB VAL D 206   40.975 40.109 54.339 1.00 0.00
D   H
ATOM 26626 CG1 VAL D 206   38.908 40.659 54.008 1.00 0.00
D   C
ATOM 26627 HG11 VAL D 206   38.733 41.064 55.028 1.00 0.00
D   H
ATOM 26628 HG12 VAL D 206   39.167 41.516 53.352 1.00 0.00
D   H
ATOM 26629 HG13 VAL D 206   37.957 40.210 53.649 1.00 0.00
D   H
ATOM 26630 CG2 VAL D 206   39.708 38.525 55.048 1.00 0.00
D   C
ATOM 26631 HG21 VAL D 206   39.517 38.961 56.051 1.00 0.00
D   H
ATOM 26632 HG22 VAL D 206   38.808 37.951 54.738 1.00 0.00
D   H
ATOM 26633 HG23 VAL D 206   40.557 37.816 55.146 1.00 0.00
D   H
ATOM 26634 C   VAL D 206   40.441 40.128 51.616 1.00 0.00
D   C
ATOM 26635 O   VAL D 206   41.215 41.049 51.847 1.00 0.00
D   O
ATOM 26636 N   ILE D 207   39.793 39.992 50.466 1.00 0.00
D   N
ATOM 26637 HN ILE D 207   39.127 39.272 50.279 1.00 0.00
D   H
ATOM 26638 CA ILE D 207   40.009 40.908 49.356 1.00 0.00
D   C
ATOM 26639 HA ILE D 207   40.876 41.501 49.618 1.00 0.00
D   H
ATOM 26640 CB ILE D 207   40.215 40.149 48.032 1.00 0.00
D   C
ATOM 26641 HB ILE D 207   39.767 39.139 48.216 1.00 0.00
D   H
ATOM 26642 CG2 ILE D 207   39.570 40.903 46.880 1.00 0.00
D   C
ATOM 26643 HG21 ILE D 207   39.579 40.288 45.955 1.00 0.00
D   H
ATOM 26644 HG22 ILE D 207   38.511 41.148 47.110 1.00 0.00
D   H
ATOM 26645 HG23 ILE D 207   40.112 41.850 46.672 1.00 0.00
D   H
ATOM 26646 CG1 ILE D 207   41.706 39.931 47.768 1.00 0.00
D   C
ATOM 26647 HG11 ILE D 207   42.221 40.915 47.686 1.00 0.00
D   H
ATOM 26648 HG12 ILE D 207   42.152 39.398 48.639 1.00 0.00
D   H

ATOM 26649 CD ILE D 207 41.994 39.121 46.524 1.00 0.00
D C
ATOM 26650 HD1 ILE D 207 43.087 38.950 46.419 1.00 0.00
D H
ATOM 26651 HD2 ILE D 207 41.490 38.132 46.575 1.00 0.00
D H
ATOM 26652 HD3 ILE D 207 41.641 39.650 45.614 1.00 0.00
D H
ATOM 26653 C ILE D 207 38.846 41.874 49.198 1.00 0.00
D C
ATOM 26654 O ILE D 207 37.729 41.598 49.636 1.00 0.00
D O
ATOM 26655 N MET D 208 39.122 43.008 48.569 1.00 0.00
D N
ATOM 26656 HN MET D 208 40.024 43.247 48.214 1.00 0.00
D H
ATOM 26657 CA MET D 208 38.092 44.028 48.343 1.00 0.00
D C
ATOM 26658 HA MET D 208 37.148 43.501 48.279 1.00 0.00
D H
ATOM 26659 CB MET D 208 38.078 45.015 49.512 1.00 0.00
D C
ATOM 26660 HB1 MET D 208 38.045 44.409 50.448 1.00 0.00
D H
ATOM 26661 HB2 MET D 208 39.041 45.572 49.536 1.00 0.00
D H
ATOM 26662 CG MET D 208 36.907 45.989 49.529 1.00 0.00
D C
ATOM 26663 HG1 MET D 208 36.756 46.430 48.520 1.00 0.00
D H
ATOM 26664 HG2 MET D 208 35.975 45.448 49.802 1.00 0.00
D H
ATOM 26665 SD MET D 208 37.185 47.330 50.702 1.00 0.00
D S
ATOM 26666 CE MET D 208 35.525 47.971 50.903 1.00 0.00
D C
ATOM 26667 HE1 MET D 208 35.515 48.832 51.605 1.00 0.00
D H
ATOM 26668 HE2 MET D 208 35.110 48.317 49.932 1.00 0.00
D H
ATOM 26669 HE3 MET D 208 34.843 47.193 51.309 1.00 0.00
D H
ATOM 26670 C MET D 208 38.291 44.780 47.028 1.00 0.00
D C
ATOM 26671 O MET D 208 39.421 45.032 46.611 1.00 0.00
D O
ATOM 26672 N GLY D 209 37.186 45.145 46.387 1.00 0.00
D N
ATOM 26673 HN GLY D 209 36.271 44.904 46.708 1.00 0.00
D H
ATOM 26674 CA GLY D 209 37.236 45.928 45.163 1.00 0.00
D C
ATOM 26675 HA1 GLY D 209 38.229 45.808 44.754 1.00 0.00
D H

ATOM 26676 HA2 GLY D 209 36.984 46.947 45.426 1.00 0.00
D H
ATOM 26677 C GLY D 209 36.242 45.454 44.127 1.00 0.00
D C
ATOM 26678 O GLY D 209 35.132 45.030 44.459 1.00 0.00
D O
ATOM 26679 N HSE D 210 36.651 45.533 42.865 1.00 0.00
D N
ATOM 26680 HN HSE D 210 37.569 45.826 42.599 1.00 0.00
D H
ATOM 26681 CA HSE D 210 35.783 45.193 41.749 1.00 0.00
D C
ATOM 26682 HA HSE D 210 34.798 45.576 41.984 1.00 0.00
D H
ATOM 26683 CB HSE D 210 36.306 45.823 40.466 1.00 0.00
D C
ATOM 26684 HB1 HSE D 210 36.873 46.740 40.741 1.00 0.00
D H
ATOM 26685 HB2 HSE D 210 37.025 45.132 39.979 1.00 0.00
D H
ATOM 26686 ND1 HSE D 210 34.367 47.231 39.732 1.00 0.00
D N
ATOM 26687 CG HSE D 210 35.224 46.223 39.490 1.00 0.00
D C
ATOM 26688 CE1 HSE D 210 33.525 47.365 38.689 1.00 0.00
D C
ATOM 26689 HE1 HSE D 210 32.723 48.105 38.630 1.00 0.00
D H
ATOM 26690 NE2 HSE D 210 33.850 46.440 37.770 1.00 0.00
D N
ATOM 26691 HE2 HSE D 210 33.378 46.296 36.901 1.00 0.00
D H
ATOM 26692 CD2 HSE D 210 34.896 45.721 38.230 1.00 0.00
D C
ATOM 26693 HD2 HSE D 210 35.339 44.910 37.670 1.00 0.00
D H
ATOM 26694 C HSE D 210 35.655 43.706 41.603 1.00 0.00
D C
ATOM 26695 O HSE D 210 36.650 42.976 41.635 1.00 0.00
D O
ATOM 26696 N LYS D 211 34.412 43.258 41.442 1.00 0.00
D N
ATOM 26697 HN LYS D 211 33.649 43.897 41.362 1.00 0.00
D H
ATOM 26698 CA LYS D 211 34.047 41.845 41.366 1.00 0.00
D C
ATOM 26699 HA LYS D 211 34.315 41.410 42.321 1.00 0.00
D H
ATOM 26700 CB LYS D 211 32.547 41.736 41.094 1.00 0.00
D C
ATOM 26701 HB1 LYS D 211 32.076 42.672 41.476 1.00 0.00
D H
ATOM 26702 HB2 LYS D 211 32.378 41.728 39.994 1.00 0.00
D H

ATOM 26703 CG LYS D 211 31.857 40.551 41.739 1.00 0.00
D C
ATOM 26704 HG1 LYS D 211 32.320 39.610 41.361 1.00 0.00
D H
ATOM 26705 HG2 LYS D 211 32.027 40.587 42.839 1.00 0.00
D H
ATOM 26706 CD LYS D 211 30.375 40.576 41.403 1.00 0.00
D C
ATOM 26707 HD1 LYS D 211 30.011 41.624 41.495 1.00 0.00
D H
ATOM 26708 HD2 LYS D 211 30.255 40.272 40.337 1.00 0.00
D H
ATOM 26709 CE LYS D 211 29.578 39.650 42.308 1.00 0.00
D C
ATOM 26710 HE1 LYS D 211 29.942 38.603 42.213 1.00 0.00
D H
ATOM 26711 HE2 LYS D 211 29.677 39.962 43.371 1.00 0.00
D H
ATOM 26712 NZ LYS D 211 28.133 39.668 41.955 1.00 0.00
D N
ATOM 26713 HZ1 LYS D 211 27.605 39.028 42.584 1.00 0.00
D H
ATOM 26714 HZ2 LYS D 211 27.763 40.635 42.056 1.00 0.00
D H
ATOM 26715 HZ3 LYS D 211 28.014 39.358 40.970 1.00 0.00
D H
ATOM 26716 C LYS D 211 34.821 41.085 40.285 1.00 0.00
D C
ATOM 26717 O LYS D 211 35.257 39.951 40.502 1.00 0.00
D O
ATOM 26718 N LYS D 212 34.981 41.723 39.131 1.00 0.00
D N
ATOM 26719 HN LYS D 212 34.656 42.657 38.992 1.00 0.00
D H
ATOM 26720 CA LYS D 212 35.642 41.118 37.978 1.00 0.00
D C
ATOM 26721 HA LYS D 212 35.269 40.105 37.891 1.00 0.00
D H
ATOM 26722 CB LYS D 212 35.335 41.917 36.710 1.00 0.00
D C
ATOM 26723 HB1 LYS D 212 36.000 42.811 36.717 1.00 0.00
D H
ATOM 26724 HB2 LYS D 212 35.630 41.315 35.822 1.00 0.00
D H
ATOM 26725 CG LYS D 212 33.888 42.370 36.605 1.00 0.00
D C
ATOM 26726 HG1 LYS D 212 33.223 41.545 36.948 1.00 0.00
D H
ATOM 26727 HG2 LYS D 212 33.728 43.234 37.290 1.00 0.00
D H
ATOM 26728 CD LYS D 212 33.524 42.745 35.180 1.00 0.00
D C
ATOM 26729 HD1 LYS D 212 34.329 43.400 34.773 1.00 0.00
D H

ATOM 26730 HD2 LYS D 212 33.512 41.813 34.569 1.00 0.00
D H
ATOM 26731 CE LYS D 212 32.174 43.439 35.135 1.00 0.00
D C
ATOM 26732 HE1 LYS D 212 31.402 42.823 35.649 1.00 0.00
D H
ATOM 26733 HE2 LYS D 212 32.227 44.429 35.638 1.00 0.00
D H
ATOM 26734 NZ LYS D 212 31.725 43.659 33.736 1.00 0.00
D N
ATOM 26735 HZ1 LYS D 212 30.799 44.133 33.730 1.00 0.00
D H
ATOM 26736 HZ2 LYS D 212 32.420 44.251 33.237 1.00 0.00
D H
ATOM 26737 HZ3 LYS D 212 31.646 42.743 33.249 1.00 0.00
D H
ATOM 26738 C LYS D 212 37.148 41.031 38.190 1.00 0.00
D C
ATOM 26739 O LYS D 212 37.794 40.088 37.727 1.00 0.00
D O
ATOM 26740 N ASP D 213 37.692 42.022 38.892 1.00 0.00
D N
ATOM 26741 HN ASP D 213 37.188 42.805 39.251 1.00 0.00
D H
ATOM 26742 CA ASP D 213 39.114 42.064 39.215 1.00 0.00
D C
ATOM 26743 HA ASP D 213 39.653 41.901 38.289 1.00 0.00
D H
ATOM 26744 CB ASP D 213 39.483 43.423 39.816 1.00 0.00
D C
ATOM 26745 HB1 ASP D 213 38.754 43.681 40.610 1.00 0.00
D H
ATOM 26746 HB2 ASP D 213 40.494 43.388 40.268 1.00 0.00
D H
ATOM 26747 CG ASP D 213 39.454 44.551 38.791 1.00 0.00
D C
ATOM 26748 OD1 ASP D 213 39.094 45.683 39.166 1.00 0.00
D O
ATOM 26749 OD2 ASP D 213 39.803 44.315 37.613 1.00 0.00
D O
ATOM 26750 C ASP D 213 39.503 40.927 40.157 1.00 0.00
D C
ATOM 26751 O ASP D 213 40.517 40.258 39.946 1.00 0.00
D O
ATOM 26752 N ILE D 214 38.682 40.709 41.183 1.00 0.00
D N
ATOM 26753 HN ILE D 214 37.891 41.284 41.381 1.00 0.00
D H
ATOM 26754 CA ILE D 214 38.866 39.600 42.119 1.00 0.00
D C
ATOM 26755 HA ILE D 214 39.866 39.692 42.524 1.00 0.00
D H
ATOM 26756 CB ILE D 214 37.790 39.616 43.228 1.00 0.00
D C

ATOM 26757 HB ILE D 214 36.817 39.599 42.675 1.00 0.00
D H
ATOM 26758 CG2 ILE D 214 37.970 38.435 44.177 1.00 0.00
D C
ATOM 26759 HG21 ILE D 214 37.110 38.360 44.875 1.00 0.00
D H
ATOM 26760 HG22 ILE D 214 38.031 37.481 43.614 1.00 0.00
D H
ATOM 26761 HG23 ILE D 214 38.897 38.546 44.779 1.00 0.00
D H
ATOM 26762 CG1 ILE D 214 37.841 40.937 43.997 1.00 0.00
D C
ATOM 26763 HG11 ILE D 214 38.713 40.938 44.689 1.00 0.00
D H
ATOM 26764 HG12 ILE D 214 37.995 41.770 43.274 1.00 0.00
D H
ATOM 26765 CD ILE D 214 36.592 41.246 44.796 1.00 0.00
D C
ATOM 26766 HD1 ILE D 214 36.678 42.244 45.279 1.00 0.00
D H
ATOM 26767 HD2 ILE D 214 35.697 41.255 44.136 1.00 0.00
D H
ATOM 26768 HD3 ILE D 214 36.433 40.491 45.594 1.00 0.00
D H
ATOM 26769 C ILE D 214 38.814 38.271 41.368 1.00 0.00
D C
ATOM 26770 O ILE D 214 39.722 37.445 41.472 1.00 0.00
D O
ATOM 26771 N LYS D 215 37.753 38.096 40.592 1.00 0.00
D N
ATOM 26772 HN LYS D 215 37.034 38.786 40.540 1.00 0.00
D H
ATOM 26773 CA LYS D 215 37.538 36.920 39.768 1.00 0.00
D C
ATOM 26774 HA LYS D 215 37.467 36.078 40.446 1.00 0.00
D H
ATOM 26775 CB LYS D 215 36.275 37.158 38.967 1.00 0.00
D C
ATOM 26776 HB1 LYS D 215 35.643 37.862 39.555 1.00 0.00
D H
ATOM 26777 HB2 LYS D 215 36.539 37.687 38.024 1.00 0.00
D H
ATOM 26778 CG LYS D 215 35.461 35.899 38.663 1.00 0.00
D C
ATOM 26779 HG1 LYS D 215 36.041 35.248 37.970 1.00 0.00
D H
ATOM 26780 HG2 LYS D 215 35.311 35.328 39.608 1.00 0.00
D H
ATOM 26781 CD LYS D 215 34.176 36.404 38.044 1.00 0.00
D C
ATOM 26782 HD1 LYS D 215 33.649 37.037 38.793 1.00 0.00
D H
ATOM 26783 HD2 LYS D 215 34.441 37.059 37.181 1.00 0.00
D H

ATOM 26784 CE LYS D 215 33.309 35.222 37.594 1.00 0.00
D C
ATOM 26785 HE1 LYS D 215 33.852 34.602 36.847 1.00 0.00
D H
ATOM 26786 HE2 LYS D 215 33.046 34.578 38.463 1.00 0.00
D H
ATOM 26787 NZ LYS D 215 32.027 35.658 36.960 1.00 0.00
D N
ATOM 26788 HZ1 LYS D 215 31.472 34.828 36.670 1.00 0.00
D H
ATOM 26789 HZ2 LYS D 215 31.477 36.221 37.641 1.00 0.00
D H
ATOM 26790 HZ3 LYS D 215 32.236 36.242 36.125 1.00 0.00
D H
ATOM 26791 C LYS D 215 38.707 36.645 38.821 1.00 0.00
D C
ATOM 26792 O LYS D 215 39.028 35.487 38.545 1.00 0.00
D O
ATOM 26793 N ARG D 216 39.333 37.711 38.322 1.00 0.00
D N
ATOM 26794 HN ARG D 216 39.007 38.636 38.497 1.00 0.00
D H
ATOM 26795 CA ARG D 216 40.529 37.583 37.494 1.00 0.00
D C
ATOM 26796 HA ARG D 216 40.363 36.804 36.759 1.00 0.00
D H
ATOM 26797 CB ARG D 216 40.859 38.893 36.774 1.00 0.00
D C
ATOM 26798 HB1 ARG D 216 39.904 39.255 36.325 1.00 0.00
D H
ATOM 26799 HB2 ARG D 216 41.158 39.663 37.519 1.00 0.00
D H
ATOM 26800 CG ARG D 216 41.904 38.743 35.666 1.00 0.00
D C
ATOM 26801 HG1 ARG D 216 42.803 38.246 36.092 1.00 0.00
D H
ATOM 26802 HG2 ARG D 216 41.513 38.043 34.893 1.00 0.00
D H
ATOM 26803 CD ARG D 216 42.246 40.062 34.980 1.00 0.00
D C
ATOM 26804 HD1 ARG D 216 42.904 40.666 35.647 1.00 0.00
D H
ATOM 26805 HD2 ARG D 216 42.775 39.895 34.013 1.00 0.00
D H
ATOM 26806 NE ARG D 216 41.042 40.827 34.674 1.00 0.00
D N
ATOM 26807 HE ARG D 216 40.487 40.415 33.942 1.00 0.00
D H
ATOM 26808 CZ ARG D 216 40.687 41.955 35.282 1.00 0.00
D C
ATOM 26809 NH1 ARG D 216 41.458 42.494 36.220 1.00 0.00
D N
ATOM 26810 HH11 ARG D 216 41.179 43.320 36.689 1.00 0.00
D H

ATOM 26811 HH12 ARG D 216 42.249 41.950 36.511 1.00 0.00
D H
ATOM 26812 NH2 ARG D 216 39.555 42.551 34.943 1.00 0.00
D N
ATOM 26813 HH21 ARG D 216 39.327 43.434 35.328 1.00 0.00
D H
ATOM 26814 HH22 ARG D 216 39.076 42.187 34.155 1.00 0.00
D H
ATOM 26815 C ARG D 216 41.713 37.137 38.341 1.00 0.00
D C
ATOM 26816 O ARG D 216 42.472 36.254 37.933 1.00 0.00
D O
ATOM 26817 N PHE D 217 41.864 37.746 39.516 1.00 0.00
D N
ATOM 26818 HN PHE D 217 41.266 38.477 39.841 1.00 0.00
D H
ATOM 26819 CA PHE D 217 42.935 37.386 40.441 1.00 0.00
D C
ATOM 26820 HA PHE D 217 43.864 37.513 39.897 1.00 0.00
D H
ATOM 26821 CB PHE D 217 42.917 38.288 41.681 1.00 0.00
D C
ATOM 26822 HB1 PHE D 217 43.403 39.257 41.433 1.00 0.00
D H
ATOM 26823 HB2 PHE D 217 41.875 38.515 41.996 1.00 0.00
D H
ATOM 26824 CG PHE D 217 43.678 37.726 42.849 1.00 0.00
D C
ATOM 26825 CD1 PHE D 217 45.064 37.615 42.811 1.00 0.00
D C
ATOM 26826 HD1 PHE D 217 45.603 37.941 41.931 1.00 0.00
D H
ATOM 26827 CE1 PHE D 217 45.763 37.088 43.884 1.00 0.00
D C
ATOM 26828 HE1 PHE D 217 46.839 37.010 43.843 1.00 0.00
D H
ATOM 26829 CZ PHE D 217 45.079 36.666 45.013 1.00 0.00
D C
ATOM 26830 HZ PHE D 217 45.620 36.267 45.859 1.00 0.00
D H
ATOM 26831 CD2 PHE D 217 43.005 37.292 43.981 1.00 0.00
D C
ATOM 26832 HD2 PHE D 217 41.926 37.343 44.021 1.00 0.00
D H
ATOM 26833 CE2 PHE D 217 43.699 36.765 45.059 1.00 0.00
D C
ATOM 26834 HE2 PHE D 217 43.160 36.432 45.935 1.00 0.00
D H
ATOM 26835 C PHE D 217 42.843 35.911 40.833 1.00 0.00
D C
ATOM 26836 O PHE D 217 43.866 35.231 40.966 1.00 0.00
D O
ATOM 26837 N GLU D 218 41.613 35.430 41.007 1.00 0.00
D N

ATOM 26838 HN GLU D 218   40.789 35.988 40.963 1.00 0.00
D   H
ATOM 26839 CA GLU D 218   41.359 34.020 41.280 1.00 0.00
D   C
ATOM 26840 HA GLU D 218   41.928 33.733 42.156 1.00 0.00
D   H
ATOM 26841 CB GLU D 218   39.870 33.774 41.538 1.00 0.00
D   C
ATOM 26842 HB1 GLU D 218   39.300 34.141 40.654 1.00 0.00
D   H
ATOM 26843 HB2 GLU D 218   39.673 32.680 41.594 1.00 0.00
D   H
ATOM 26844 CG GLU D 218   39.328 34.456 42.786 1.00 0.00
D   C
ATOM 26845 HG1 GLU D 218   39.899 34.116 43.669 1.00 0.00
D   H
ATOM 26846 HG2 GLU D 218   39.407 35.558 42.702 1.00 0.00
D   H
ATOM 26847 CD GLU D 218   37.884 34.100 43.072 1.00 0.00
D   C
ATOM 26848 OE1 GLU D 218   37.600 32.905 43.298 1.00 0.00
D   O
ATOM 26849 OE2 GLU D 218   37.034 35.014 43.085 1.00 0.00
D   O
ATOM 26850 C   GLU D 218   41.846 33.156 40.123 1.00 0.00
D   C
ATOM 26851 O   GLU D 218   42.596 32.204 40.333 1.00 0.00
D   O
ATOM 26852 N   ASN D 219   41.430 33.512 38.907 1.00 0.00
D   N
ATOM 26853 HN ASN D 219   40.813 34.282 38.756 1.00 0.00
D   H
ATOM 26854 CA ASN D 219   41.836 32.809 37.686 1.00 0.00
D   C
ATOM 26855 HA ASN D 219   41.422 31.814 37.806 1.00 0.00
D   H
ATOM 26856 CB ASN D 219   41.239 33.493 36.453 1.00 0.00
D   C
ATOM 26857 HB1 ASN D 219   41.079 34.564 36.702 1.00 0.00
D   H
ATOM 26858 HB2 ASN D 219   41.954 33.451 35.606 1.00 0.00
D   H
ATOM 26859 CG ASN D 219   39.905 32.904 36.039 1.00 0.00
D   C
ATOM 26860 OD1 ASN D 219   39.736 31.686 35.991 1.00 0.00
D   O
ATOM 26861 ND2 ASN D 219   38.953 33.771 35.721 1.00 0.00
D   N
ATOM 26862 HD21 ASN D 219   38.067 33.400 35.458 1.00 0.00
D   H
ATOM 26863 HD22 ASN D 219   39.130 34.749 35.780 1.00 0.00
D   H
ATOM 26864 C   ASN D 219   43.342 32.664 37.487 1.00 0.00
D   C

ATOM 26865 O ASN D 219 43.812 31.633 36.998 1.00 0.00
D O
ATOM 26866 N GLU D 220 44.092 33.691 37.875 1.00 0.00
D N
ATOM 26867 HN GLU D 220 43.754 34.464 38.406 1.00 0.00
D H
ATOM 26868 CA GLU D 220 45.514 33.775 37.546 1.00 0.00
D C
ATOM 26869 HA GLU D 220 45.691 33.034 36.776 1.00 0.00
D H
ATOM 26870 CB GLU D 220 45.831 35.136 36.925 1.00 0.00
D C
ATOM 26871 HB1 GLU D 220 45.384 35.926 37.571 1.00 0.00
D H
ATOM 26872 HB2 GLU D 220 46.930 35.314 36.944 1.00 0.00
D H
ATOM 26873 CG GLU D 220 45.300 35.290 35.505 1.00 0.00
D C
ATOM 26874 HG1 GLU D 220 45.873 34.635 34.824 1.00 0.00
D H
ATOM 26875 HG2 GLU D 220 44.226 35.023 35.452 1.00 0.00
D H
ATOM 26876 CD GLU D 220 45.468 36.694 34.952 1.00 0.00
D C
ATOM 26877 OE1 GLU D 220 45.600 37.643 35.746 1.00 0.00
D O
ATOM 26878 OE2 GLU D 220 45.459 36.848 33.712 1.00 0.00
D O
ATOM 26879 C GLU D 220 46.467 33.468 38.698 1.00 0.00
D C
ATOM 26880 O GLU D 220 47.443 32.736 38.516 1.00 0.00
D O
ATOM 26881 N GLY D 221 46.188 34.024 39.875 1.00 0.00
D N
ATOM 26882 HN GLY D 221 45.396 34.618 40.012 1.00 0.00
D H
ATOM 26883 CA GLY D 221 47.024 33.804 41.052 1.00 0.00
D C
ATOM 26884 HA1 GLY D 221 46.806 34.612 41.736 1.00 0.00
D H
ATOM 26885 HA2 GLY D 221 48.048 33.782 40.704 1.00 0.00
D H
ATOM 26886 C GLY D 221 46.745 32.493 41.768 1.00 0.00
D C
ATOM 26887 O GLY D 221 47.492 32.097 42.667 1.00 0.00
D O
ATOM 26888 N MET D 222 45.663 31.823 41.367 1.00 0.00
D N
ATOM 26889 HN MET D 222 45.080 32.131 40.617 1.00 0.00
D H
ATOM 26890 CA MET D 222 45.235 30.574 41.991 1.00 0.00
D C
ATOM 26891 HA MET D 222 46.067 30.205 42.577 1.00 0.00
D H

ATOM 26892 CB MET D 222   44.012 30.816 42.877 1.00 0.00
D   C
ATOM 26893 HB1 MET D 222   43.233 31.290 42.233 1.00 0.00
D   H
ATOM 26894 HB2 MET D 222   43.592 29.838 43.204 1.00 0.00
D   H
ATOM 26895 CG MET D 222   44.271 31.715 44.075 1.00 0.00
D   C
ATOM 26896 HG1 MET D 222   44.726 31.132 44.903 1.00 0.00
D   H
ATOM 26897 HG2 MET D 222   44.989 32.515 43.793 1.00 0.00
D   H
ATOM 26898 SD MET D 222   42.767 32.482 44.702 1.00 0.00
D   S
ATOM 26899 CE MET D 222   43.408 33.297 46.156 1.00 0.00
D   C
ATOM 26900 HE1 MET D 222   42.600 33.834 46.698 1.00 0.00
D   H
ATOM 26901 HE2 MET D 222   43.862 32.563 46.857 1.00 0.00
D   H
ATOM 26902 HE3 MET D 222   44.189 34.041 45.885 1.00 0.00
D   H
ATOM 26903 C   MET D 222   44.914 29.521 40.935 1.00 0.00
D   C
ATOM 26904 O   MET D 222   45.173 28.334 41.128 1.00 0.00
D   O
ATOM 26905 N   GLN E   8   36.830 -11.263 77.757 1.00 0.00
E   N
ATOM 26906 HT1 GLN E   8   37.455 -11.308 78.537 1.00 0.00
E   H
ATOM 26907 HT2 GLN E   8   36.117 -11.958 77.851 1.00 0.00
E   H
ATOM 26908 HT3 GLN E   8   36.416 -10.354 77.704 1.00 0.00
E   H
ATOM 26909 CA GLN E   8   37.598 -11.521 76.504 1.00 0.00
E   C
ATOM 26910 HA GLN E   8   38.326 -10.723 76.431 1.00 0.00
E   H
ATOM 26911 CB GLN E   8   38.327 -12.860 76.608 1.00 0.00
E   C
ATOM 26912 HB1 GLN E   8   38.444 -13.080 77.695 1.00 0.00
E   H
ATOM 26913 HB2 GLN E   8   37.678 -13.667 76.196 1.00 0.00
E   H
ATOM 26914 CG GLN E   8   39.692 -12.877 75.942 1.00 0.00
E   C
ATOM 26915 HG1 GLN E   8   39.584 -12.865 74.836 1.00 0.00
E   H
ATOM 26916 HG2 GLN E   8   40.283 -11.986 76.236 1.00 0.00
E   H
ATOM 26917 CD GLN E   8   40.488 -14.121 76.279 1.00 0.00
E   C
ATOM 26918 OE1 GLN E   8   40.068 -14.951 77.092 1.00 0.00
E   O

ATOM 26919 NE2 GLN E 8 41.650 -14.258 75.656 1.00 0.00
E N
ATOM 26920 HE21 GLN E 8 42.180 -15.075 75.867 1.00 0.00
E H
ATOM 26921 HE22 GLN E 8 41.959 -13.554 75.026 1.00 0.00
E H
ATOM 26922 C GLN E 8 36.727 -11.476 75.231 1.00 0.00
E C
ATOM 26923 O GLN E 8 37.176 -10.994 74.184 1.00 0.00
E O
ATOM 26924 N PHE E 9 35.495 -11.976 75.317 1.00 0.00
E N
ATOM 26925 HN PHE E 9 35.109 -12.377 76.147 1.00 0.00
E H
ATOM 26926 CA PHE E 9 34.588 -11.973 74.168 1.00 0.00
E C
ATOM 26927 HA PHE E 9 35.010 -11.277 73.454 1.00 0.00
E H
ATOM 26928 CB PHE E 9 34.409 -13.378 73.592 1.00 0.00
E C
ATOM 26929 HB1 PHE E 9 33.899 -14.018 74.345 1.00 0.00
E H
ATOM 26930 HB2 PHE E 9 33.765 -13.360 72.687 1.00 0.00
E H
ATOM 26931 CG PHE E 9 35.691 -14.076 73.264 1.00 0.00
E C
ATOM 26932 CD1 PHE E 9 36.295 -13.896 72.030 1.00 0.00
E C
ATOM 26933 HD1 PHE E 9 35.835 -13.245 71.299 1.00 0.00
E H
ATOM 26934 CE1 PHE E 9 37.479 -14.546 71.721 1.00 0.00
E C
ATOM 26935 HE1 PHE E 9 37.949 -14.387 70.761 1.00 0.00
E H
ATOM 26936 CZ PHE E 9 38.066 -15.394 72.647 1.00 0.00
E C
ATOM 26937 HZ PHE E 9 38.992 -15.898 72.409 1.00 0.00
E H
ATOM 26938 CD2 PHE E 9 36.287 -14.937 74.184 1.00 0.00
E C
ATOM 26939 HD2 PHE E 9 35.814 -15.118 75.140 1.00 0.00
E H
ATOM 26940 CE2 PHE E 9 37.470 -15.590 73.883 1.00 0.00
E C
ATOM 26941 HE2 PHE E 9 37.921 -16.250 74.612 1.00 0.00
E H
ATOM 26942 C PHE E 9 33.218 -11.449 74.542 1.00 0.00
E C
ATOM 26943 O PHE E 9 32.681 -11.789 75.602 1.00 0.00
E O
ATOM 26944 N ALA E 10 32.658 -10.624 73.658 1.00 0.00
E N
ATOM 26945 HN ALA E 10 33.169 -10.254 72.883 1.00 0.00
E H

```
ATOM  26946  CA  ALA E  10      31.267 -10.200  73.750  1.00  0.00
E    C
ATOM  26947  HA  ALA E  10      30.739 -10.730  74.534  1.00  0.00
E    H
ATOM  26948  CB  ALA E  10      31.176  -8.711  74.034  1.00  0.00
E    C
ATOM  26949  HB1 ALA E  10      31.654  -8.488  75.011  1.00  0.00
E    H
ATOM  26950  HB2 ALA E  10      31.705  -8.123  73.252  1.00  0.00
E    H
ATOM  26951  HB3 ALA E  10      30.120  -8.368  74.082  1.00  0.00
E    H
ATOM  26952  C   ALA E  10      30.561 -10.545  72.448  1.00  0.00
E    C
ATOM  26953  O   ALA E  10      31.111 -10.349  71.359  1.00  0.00
E    O
ATOM  26954  N   VAL E  11      29.352 -11.083  72.562  1.00  0.00
E    N
ATOM  26955  HN  VAL E  11      28.921 -11.304  73.433  1.00  0.00
E    H
ATOM  26956  CA  VAL E  11      28.541 -11.401  71.388  1.00  0.00
E    C
ATOM  26957  HA  VAL E  11      29.079 -11.102  70.497  1.00  0.00
E    H
ATOM  26958  CB  VAL E  11      28.200 -12.902  71.306  1.00  0.00
E    C
ATOM  26959  HB  VAL E  11      27.712 -13.190  72.270  1.00  0.00
E    H
ATOM  26960  CG1 VAL E  11      27.239 -13.174  70.161  1.00  0.00
E    C
ATOM  26961 HG11 VAL E  11      27.029 -14.262  70.084  1.00  0.00
E    H
ATOM  26962 HG12 VAL E  11      26.267 -12.659  70.311  1.00  0.00
E    H
ATOM  26963 HG13 VAL E  11      27.673 -12.838  69.195  1.00  0.00
E    H
ATOM  26964  CG2 VAL E  11      29.471 -13.725  71.145  1.00  0.00
E    C
ATOM  26965 HG21 VAL E  11      29.230 -14.804  71.045  1.00  0.00
E    H
ATOM  26966 HG22 VAL E  11      30.030 -13.405  70.239  1.00  0.00
E    H
ATOM  26967 HG23 VAL E  11      30.138 -13.610  72.024  1.00  0.00
E    H
ATOM  26968  C   VAL E  11      27.280 -10.562  71.459  1.00  0.00
E    C
ATOM  26969  O   VAL E  11      26.496 -10.687  72.403  1.00  0.00
E    O
ATOM  26970  N   ILE E  12      27.085  -9.700  70.465  1.00  0.00
E    N
ATOM  26971  HN  ILE E  12      27.627  -9.647  69.629  1.00  0.00
E    H
ATOM  26972  CA  ILE E  12      26.005  -8.722  70.541  1.00  0.00
E    C
```

```
ATOM  26973  HA  ILE E  12      25.862  -8.557  71.601  1.00  0.00
E    H
ATOM  26974  CB  ILE E  12      26.439  -7.326  70.043  1.00  0.00
E    C
ATOM  26975  HB  ILE E  12      27.068  -7.534  69.141  1.00  0.00
E    H
ATOM  26976  CG2 ILE E  12      25.236  -6.428  69.804  1.00  0.00
E    C
ATOM  26977 HG21 ILE E  12      25.544  -5.487  69.301  1.00  0.00
E    H
ATOM  26978 HG22 ILE E  12      24.491  -6.929  69.152  1.00  0.00
E    H
ATOM  26979 HG23 ILE E  12      24.741  -6.163  70.762  1.00  0.00
E    H
ATOM  26980  CG1 ILE E  12      27.343  -6.677  71.089  1.00  0.00
E    C
ATOM  26981 HG11 ILE E  12      26.811  -6.621  72.064  1.00  0.00
E    H
ATOM  26982 HG12 ILE E  12      28.240  -7.321  71.240  1.00  0.00
E    H
ATOM  26983  CD  ILE E  12      27.830  -5.292  70.726  1.00  0.00
E    C
ATOM  26984  HD1 ILE E  12      28.526  -4.911  71.504  1.00  0.00
E    H
ATOM  26985  HD2 ILE E  12      28.369  -5.308  69.755  1.00  0.00
E    H
ATOM  26986  HD3 ILE E  12      26.981  -4.580  70.646  1.00  0.00
E    H
ATOM  26987  C   ILE E  12      24.656  -9.188  69.978  1.00  0.00
E    C
ATOM  26988  O   ILE E  12      23.616  -8.701  70.412  1.00  0.00
E    O
ATOM  26989  N   GLY E  13      24.638 -10.139  69.062  1.00  0.00
E    N
ATOM  26990  HN  GLY E  13      25.431 -10.549  68.614  1.00  0.00
E    H
ATOM  26991  CA  GLY E  13      23.343 -10.657  68.645  1.00  0.00
E    C
ATOM  26992  HA1 GLY E  13      23.299 -10.534  67.572  1.00  0.00
E    H
ATOM  26993  HA2 GLY E  13      22.597 -10.095  69.189  1.00  0.00
E    H
ATOM  26994  C   GLY E  13      23.139 -12.125  68.957  1.00  0.00
E    C
ATOM  26995  O   GLY E  13      23.809 -12.978  68.384  1.00  0.00
E    O
ATOM  26996  N   LEU E  14      22.195 -12.441  69.832  1.00  0.00
E    N
ATOM  26997  HN  LEU E  14      21.593 -11.798  70.300  1.00  0.00
E    H
ATOM  26998  CA  LEU E  14      21.966 -13.842  70.184  1.00  0.00
E    C
ATOM  26999  HA  LEU E  14      22.881 -14.374  69.959  1.00  0.00
E    H
```

ATOM 27000 CB LEU E 14   21.661 -13.985 71.683 1.00 0.00
E   C
ATOM 27001 HB1 LEU E 14   20.668 -13.511 71.864 1.00 0.00
E   H
ATOM 27002 HB2 LEU E 14   21.536 -15.063 71.927 1.00 0.00
E   H
ATOM 27003 CG LEU E 14   22.682 -13.345 72.630 1.00 0.00
E   C
ATOM 27004 HG LEU E 14   22.874 -12.327 72.211 1.00 0.00
E   H
ATOM 27005 CD1 LEU E 14   22.120 -13.207 74.033 1.00 0.00
E   C
ATOM 27006 HD11 LEU E 14   22.885 -12.787 74.720 1.00 0.00
E   H
ATOM 27007 HD12 LEU E 14   21.237 -12.531 74.037 1.00 0.00
E   H
ATOM 27008 HD13 LEU E 14   21.805 -14.197 74.428 1.00 0.00
E   H
ATOM 27009 CD2 LEU E 14   23.997 -14.112 72.625 1.00 0.00
E   C
ATOM 27010 HD21 LEU E 14   24.732 -13.624 73.302 1.00 0.00
E   H
ATOM 27011 HD22 LEU E 14   23.863 -15.157 72.971 1.00 0.00
E   H
ATOM 27012 HD23 LEU E 14   24.430 -14.135 71.602 1.00 0.00
E   H
ATOM 27013 C   LEU E 14   20.851 -14.453 69.322 1.00 0.00
E   C
ATOM 27014 O   LEU E 14   19.759 -14.725 69.814 1.00 0.00
E   O
ATOM 27015 N   GLY E 15   21.139 -14.683 68.043 1.00 0.00
E   N
ATOM 27016 HN GLY E 15   22.021 -14.450 67.635 1.00 0.00
E   H
ATOM 27017 CA GLY E 15   20.168 -15.300 67.140 1.00 0.00
E   C
ATOM 27018 HA1 GLY E 15   20.069 -14.680 66.260 1.00 0.00
E   H
ATOM 27019 HA2 GLY E 15   19.242 -15.463 67.675 1.00 0.00
E   H
ATOM 27020 C   GLY E 15   20.698 -16.649 66.705 1.00 0.00
E   C
ATOM 27021 O   GLY E 15   21.527 -17.243 67.389 1.00 0.00
E   O
ATOM 27022 N   ARG E 16   20.230 -17.129 65.558 1.00 0.00
E   N
ATOM 27023 HN ARG E 16   19.531 -16.650 65.032 1.00 0.00
E   H
ATOM 27024 CA ARG E 16   20.715 -18.383 65.008 1.00 0.00
E   C
ATOM 27025 HA ARG E 16   20.410 -19.188 65.666 1.00 0.00
E   H
ATOM 27026 CB ARG E 16   20.128 -18.619 63.619 1.00 0.00
E   C

ATOM 27027 HB1 ARG E 16   19.813 -17.619 63.237 1.00 0.00
E   H
ATOM 27028 HB2 ARG E 16   20.927 -18.971 62.931 1.00 0.00
E   H
ATOM 27029 CG ARG E 16   18.931 -19.552 63.623 1.00 0.00
E   C
ATOM 27030 HG1 ARG E 16   19.249 -20.535 63.213 1.00 0.00
E   H
ATOM 27031 HG2 ARG E 16   18.630 -19.750 64.677 1.00 0.00
E   H
ATOM 27032 CD ARG E 16   17.755 -18.949 62.879 1.00 0.00
E   C
ATOM 27033 HD1 ARG E 16   16.917 -19.684 62.853 1.00 0.00
E   H
ATOM 27034 HD2 ARG E 16   17.395 -18.015 63.371 1.00 0.00
E   H
ATOM 27035 NE ARG E 16   18.091 -18.582 61.509 1.00 0.00
E   N
ATOM 27036 HE ARG E 16   18.743 -19.216 61.078 1.00 0.00
E   H
ATOM 27037 CZ ARG E 16   17.594 -17.521 60.885 1.00 0.00
E   C
ATOM 27038 NH1 ARG E 16   16.748 -16.711 61.511 1.00 0.00
E   N
ATOM 27039 HH11 ARG E 16   16.354 -15.932 61.043 1.00 0.00
E   H
ATOM 27040 HH12 ARG E 16   16.435 -17.010 62.416 1.00 0.00
E   H
ATOM 27041 NH2 ARG E 16   17.945 -17.263 59.633 1.00 0.00
E   N
ATOM 27042 HH21 ARG E 16   17.643 -16.426 59.198 1.00 0.00
E   H
ATOM 27043 HH22 ARG E 16   18.671 -17.818 59.247 1.00 0.00
E   H
ATOM 27044 C   ARG E 16   22.239 -18.407 64.969 1.00 0.00
E   C
ATOM 27045 O   ARG E 16   22.861 -19.400 65.348 1.00 0.00
E   O
ATOM 27046 N   PHE E 17   22.827 -17.297 64.543 1.00 0.00
E   N
ATOM 27047 HN PHE E 17   22.330 -16.464 64.303 1.00 0.00
E   H
ATOM 27048 CA PHE E 17   24.272 -17.204 64.386 1.00 0.00
E   C
ATOM 27049 HA PHE E 17   24.601 -18.207 64.140 1.00 0.00
E   H
ATOM 27050 CB PHE E 17   24.609 -16.234 63.250 1.00 0.00
E   C
ATOM 27051 HB1 PHE E 17   24.138 -16.596 62.310 1.00 0.00
E   H
ATOM 27052 HB2 PHE E 17   24.198 -15.221 63.454 1.00 0.00
E   H
ATOM 27053 CG PHE E 17   26.074 -16.117 62.958 1.00 0.00
E   C

```
ATOM  27054  CD1 PHE E  17      26.800 -17.212  62.496  1.00  0.00
E    C
ATOM  27055  HD1 PHE E  17      26.304 -18.163  62.357  1.00  0.00
E    H
ATOM  27056  CE1 PHE E  17      28.156 -17.099  62.218  1.00  0.00
E    C
ATOM  27057  HE1 PHE E  17      28.709 -17.958  61.865  1.00  0.00
E    H
ATOM  27058  CZ  PHE E  17      28.795 -15.887  62.387  1.00  0.00
E    C
ATOM  27059  HZ  PHE E  17      29.847 -15.790  62.157  1.00  0.00
E    H
ATOM  27060  CD2 PHE E  17      26.729 -14.907  63.124  1.00  0.00
E    C
ATOM  27061  HD2 PHE E  17      26.189 -14.047  63.495  1.00  0.00
E    H
ATOM  27062  CE2 PHE E  17      28.082 -14.789  62.844  1.00  0.00
E    C
ATOM  27063  HE2 PHE E  17      28.580 -13.840  62.984  1.00  0.00
E    H
ATOM  27064  C   PHE E  17      24.987 -16.801  65.681  1.00  0.00
E    C
ATOM  27065  O   PHE E  17      25.857 -17.529  66.165  1.00  0.00
E    O
ATOM  27066  N   GLY E  18      24.629 -15.642  66.230  1.00  0.00
E    N
ATOM  27067  HN  GLY E  18      23.890 -15.073  65.872  1.00  0.00
E    H
ATOM  27068  CA  GLY E  18      25.306 -15.124  67.409  1.00  0.00
E    C
ATOM  27069  HA1 GLY E  18      24.842 -14.176  67.645  1.00  0.00
E    H
ATOM  27070  HA2 GLY E  18      26.357 -15.056  67.167  1.00  0.00
E    H
ATOM  27071  C   GLY E  18      25.161 -16.031  68.616  1.00  0.00
E    C
ATOM  27072  O   GLY E  18      26.130 -16.256  69.352  1.00  0.00
E    O
ATOM  27073  N   GLY E  19      23.952 -16.553  68.812  1.00  0.00
E    N
ATOM  27074  HN  GLY E  19      23.167 -16.326  68.236  1.00  0.00
E    H
ATOM  27075  CA  GLY E  19      23.690 -17.496  69.884  1.00  0.00
E    C
ATOM  27076  HA1 GLY E  19      22.669 -17.836  69.771  1.00  0.00
E    H
ATOM  27077  HA2 GLY E  19      23.889 -16.990  70.818  1.00  0.00
E    H
ATOM  27078  C   GLY E  19      24.598 -18.707  69.807  1.00  0.00
E    C
ATOM  27079  O   GLY E  19      25.097 -19.183  70.820  1.00  0.00
E    O
ATOM  27080  N   SER E  20      24.827 -19.193  68.598  1.00  0.00
E    N
```

ATOM 27081 HN SER E 20 24.450 -18.812 67.754 1.00 0.00
E H
ATOM 27082 CA SER E 20 25.669 -20.356 68.401 1.00 0.00
E C
ATOM 27083 HA SER E 20 25.311 -21.102 69.098 1.00 0.00
E H
ATOM 27084 CB SER E 20 25.564 -20.856 66.969 1.00 0.00
E C
ATOM 27085 HB1 SER E 20 24.517 -21.195 66.797 1.00 0.00
E H
ATOM 27086 HB2 SER E 20 25.762 -20.026 66.253 1.00 0.00
E H
ATOM 27087 OG SER E 20 26.446 -21.937 66.779 1.00 0.00
E O
ATOM 27088 HG1 SER E 20 26.195 -22.364 65.949 1.00 0.00
E H
ATOM 27089 C SER E 20 27.118 -20.063 68.755 1.00 0.00
E C
ATOM 27090 O SER E 20 27.764 -20.891 69.397 1.00 0.00
E O
ATOM 27091 N ILE E 21 27.626 -18.898 68.342 1.00 0.00
E N
ATOM 27092 HN ILE E 21 27.132 -18.245 67.771 1.00 0.00
E H
ATOM 27093 CA ILE E 21 28.981 -18.477 68.703 1.00 0.00
E C
ATOM 27094 HA ILE E 21 29.658 -19.245 68.352 1.00 0.00
E H
ATOM 27095 CB ILE E 21 29.332 -17.088 68.140 1.00 0.00
E C
ATOM 27096 HB ILE E 21 28.451 -16.449 68.403 1.00 0.00
E H
ATOM 27097 CG2 ILE E 21 30.665 -16.605 68.699 1.00 0.00
E C
ATOM 27098 HG21 ILE E 21 30.845 -15.546 68.421 1.00 0.00
E H
ATOM 27099 HG22 ILE E 21 30.675 -16.668 69.807 1.00 0.00
E H
ATOM 27100 HG23 ILE E 21 31.505 -17.215 68.305 1.00 0.00
E H
ATOM 27101 CG1 ILE E 21 29.410 -17.125 66.623 1.00 0.00
E C
ATOM 27102 HG11 ILE E 21 30.212 -17.827 66.304 1.00 0.00
E H
ATOM 27103 HG12 ILE E 21 28.448 -17.520 66.222 1.00 0.00
E H
ATOM 27104 CD ILE E 21 29.662 -15.775 65.996 1.00 0.00
E C
ATOM 27105 HD1 ILE E 21 29.650 -15.856 64.887 1.00 0.00
E H
ATOM 27106 HD2 ILE E 21 28.877 -15.049 66.300 1.00 0.00
E H
ATOM 27107 HD3 ILE E 21 30.651 -15.372 66.300 1.00 0.00
E H

ATOM 27108 C ILE E 21 29.110 -18.436 70.223 1.00 0.00
E C
ATOM 27109 O ILE E 21 30.056 -18.981 70.796 1.00 0.00
E O
ATOM 27110 N CYS E 22 28.138 -17.793 70.864 1.00 0.00
E N
ATOM 27111 HN CYS E 22 27.364 -17.373 70.395 1.00 0.00
E H
ATOM 27112 CA CYS E 22 28.123 -17.651 72.306 1.00 0.00
E C
ATOM 27113 HA CYS E 22 29.024 -17.111 72.572 1.00 0.00
E H
ATOM 27114 CB CYS E 22 26.876 -16.885 72.738 1.00 0.00
E C
ATOM 27115 HB1 CYS E 22 26.826 -15.961 72.119 1.00 0.00
E H
ATOM 27116 HB2 CYS E 22 25.967 -17.473 72.485 1.00 0.00
E H
ATOM 27117 SG CYS E 22 26.876 -16.406 74.477 1.00 0.00
E S
ATOM 27118 HG1 CYS E 22 25.724 -15.732 74.462 1.00 0.00
E H
ATOM 27119 C CYS E 22 28.178 -19.012 72.995 1.00 0.00
E C
ATOM 27120 O CYS E 22 29.115 -19.298 73.745 1.00 0.00
E O
ATOM 27121 N LYS E 23 27.187 -19.854 72.712 1.00 0.00
E N
ATOM 27122 HN LYS E 23 26.450 -19.633 72.075 1.00 0.00
E H
ATOM 27123 CA LYS E 23 27.104 -21.178 73.319 1.00 0.00
E C
ATOM 27124 HA LYS E 23 26.933 -21.028 74.379 1.00 0.00
E H
ATOM 27125 CB LYS E 23 25.933 -21.984 72.744 1.00 0.00
E C
ATOM 27126 HB1 LYS E 23 25.054 -21.301 72.703 1.00 0.00
E H
ATOM 27127 HB2 LYS E 23 26.166 -22.259 71.690 1.00 0.00
E H
ATOM 27128 CG LYS E 23 25.566 -23.223 73.560 1.00 0.00
E C
ATOM 27129 HG1 LYS E 23 26.431 -23.925 73.565 1.00 0.00
E H
ATOM 27130 HG2 LYS E 23 25.377 -22.920 74.615 1.00 0.00
E H
ATOM 27131 CD LYS E 23 24.350 -23.962 73.012 1.00 0.00
E C
ATOM 27132 HD1 LYS E 23 24.469 -24.067 71.909 1.00 0.00
E H
ATOM 27133 HD2 LYS E 23 24.343 -24.989 73.446 1.00 0.00
E H
ATOM 27134 CE LYS E 23 23.054 -23.236 73.348 1.00 0.00
E C

ATOM 27135 HE1 LYS E 23   23.010 -23.000 74.434 1.00 0.00
E   H
ATOM 27136 HE2 LYS E 23   22.981 -22.284 72.777 1.00 0.00
E   H
ATOM 27137 NZ LYS E 23   21.844 -24.043 73.021 1.00 0.00
E   N
ATOM 27138 HZ1 LYS E 23   20.984 -23.511 73.267 1.00 0.00
E   H
ATOM 27139 HZ2 LYS E 23   21.836 -24.261 72.004 1.00 0.00
E   H
ATOM 27140 HZ3 LYS E 23   21.865 -24.932 73.561 1.00 0.00
E   H
ATOM 27141 C   LYS E 23   28.411 -21.947 73.174 1.00 0.00
E   C
ATOM 27142 O   LYS E 23   28.830 -22.623 74.107 1.00 0.00
E   O
ATOM 27143 N   GLU E 24   29.060 -21.830 72.020 1.00 0.00
E   N
ATOM 27144 HN GLU E 24   28.788 -21.243 71.264 1.00 0.00
E   H
ATOM 27145 CA GLU E 24   30.277 -22.592 71.771 1.00 0.00
E   C
ATOM 27146 HA GLU E 24   30.093 -23.602 72.116 1.00 0.00
E   H
ATOM 27147 CB GLU E 24   30.562 -22.699 70.279 1.00 0.00
E   C
ATOM 27148 HB1 GLU E 24   29.654 -23.111 69.781 1.00 0.00
E   H
ATOM 27149 HB2 GLU E 24   30.709 -21.682 69.850 1.00 0.00
E   H
ATOM 27150 CG GLU E 24   31.756 -23.581 69.928 1.00 0.00
E   C
ATOM 27151 HG1 GLU E 24   31.961 -23.511 68.844 1.00 0.00
E   H
ATOM 27152 HG2 GLU E 24   32.659 -23.269 70.490 1.00 0.00
E   H
ATOM 27153 CD GLU E 24   31.535 -25.062 70.192 1.00 0.00
E   C
ATOM 27154 OE1 GLU E 24   32.506 -25.831 70.024 1.00 0.00
E   O
ATOM 27155 OE2 GLU E 24   30.408 -25.463 70.554 1.00 0.00
E   O
ATOM 27156 C   GLU E 24   31.502 -22.069 72.529 1.00 0.00
E   C
ATOM 27157 O   GLU E 24   32.240 -22.859 73.116 1.00 0.00
E   O
ATOM 27158 N   LEU E 25   31.719 -20.753 72.517 1.00 0.00
E   N
ATOM 27159 HN LEU E 25   31.167 -20.093 72.011 1.00 0.00
E   H
ATOM 27160 CA LEU E 25   32.822 -20.161 73.273 1.00 0.00
E   C
ATOM 27161 HA LEU E 25   33.736 -20.587 72.881 1.00 0.00
E   H

```
ATOM  27162  CB  LEU E  25      32.828 -18.635  73.128  1.00  0.00
E    C
ATOM  27163  HB1 LEU E  25      31.784 -18.284  73.303  1.00  0.00
E    H
ATOM  27164  HB2 LEU E  25      33.441 -18.195  73.945  1.00  0.00
E    H
ATOM  27165  CG  LEU E  25      33.274 -17.976  71.823  1.00  0.00
E    C
ATOM  27166  HG  LEU E  25      32.711 -18.467  70.993  1.00  0.00
E    H
ATOM  27167  CD1 LEU E  25      32.941 -16.492  71.866  1.00  0.00
E    C
ATOM  27168 HD11 LEU E  25      33.301 -15.985  70.945  1.00  0.00
E    H
ATOM  27169 HD12 LEU E  25      31.843 -16.339  71.942  1.00  0.00
E    H
ATOM  27170 HD13 LEU E  25      33.423 -16.007  72.741  1.00  0.00
E    H
ATOM  27171  CD2 LEU E  25      34.757 -18.173  71.567  1.00  0.00
E    C
ATOM  27172 HD21 LEU E  25      35.048 -17.700  70.604  1.00  0.00
E    H
ATOM  27173 HD22 LEU E  25      35.375 -17.716  72.366  1.00  0.00
E    H
ATOM  27174 HD23 LEU E  25      35.002 -19.255  71.510  1.00  0.00
E    H
ATOM  27175  C   LEU E  25      32.733 -20.539  74.754  1.00  0.00
E    C
ATOM  27176  O   LEU E  25      33.743 -20.820  75.401  1.00  0.00
E    O
ATOM  27177  N   HSE E  26      31.506 -20.543  75.267  1.00  0.00
E    N
ATOM  27178  HN  HSE E  26      30.699 -20.308  74.728  1.00  0.00
E    H
ATOM  27179  CA  HSE E  26      31.224 -20.886  76.650  1.00  0.00
E    C
ATOM  27180  HA  HSE E  26      31.899 -20.307  77.268  1.00  0.00
E    H
ATOM  27181  CB  HSE E  26      29.767 -20.586  76.960  1.00  0.00
E    C
ATOM  27182  HB1 HSE E  26      29.586 -19.511  76.742  1.00  0.00
E    H
ATOM  27183  HB2 HSE E  26      29.115 -21.170  76.278  1.00  0.00
E    H
ATOM  27184  ND1 HSE E  26      29.898 -20.129  79.408  1.00  0.00
E    N
ATOM  27185  CG  HSE E  26      29.384 -20.838  78.388  1.00  0.00
E    C
ATOM  27186  CE1 HSE E  26      29.373 -20.567  80.568  1.00  0.00
E    C
ATOM  27187  HE1 HSE E  26      29.624 -20.166  81.553  1.00  0.00
E    H
ATOM  27188  NE2 HSE E  26      28.516 -21.561  80.286  1.00  0.00
E    N
```

```
ATOM  27189  HE2 HSE E  26      27.995 -22.085  80.958  1.00  0.00
E    H
ATOM  27190  CD2 HSE E  26      28.500 -21.753  78.948  1.00  0.00
E    C
ATOM  27191  HD2 HSE E  26      27.892 -22.509  78.473  1.00  0.00
E    H
ATOM  27192  C   HSE E  26      31.524 -22.332  76.907  1.00  0.00
E    C
ATOM  27193  O   HSE E  26      32.118 -22.676  77.928  1.00  0.00
E    O
ATOM  27194  N   ARG E  27      31.126 -23.185  75.963  1.00  0.00
E    N
ATOM  27195  HN  ARG E  27      30.648 -22.851  75.154  1.00  0.00
E    H
ATOM  27196  CA  ARG E  27      31.352 -24.618  76.043  1.00  0.00
E    C
ATOM  27197  HA  ARG E  27      30.997 -24.969  77.004  1.00  0.00
E    H
ATOM  27198  CB  ARG E  27      30.599 -25.336  74.917  1.00  0.00
E    C
ATOM  27199  HB1 ARG E  27      29.606 -24.835  74.832  1.00  0.00
E    H
ATOM  27200  HB2 ARG E  27      31.117 -25.156  73.949  1.00  0.00
E    H
ATOM  27201  CG  ARG E  27      30.378 -26.821  75.158  1.00  0.00
E    C
ATOM  27202  HG1 ARG E  27      31.250 -27.223  75.718  1.00  0.00
E    H
ATOM  27203  HG2 ARG E  27      29.502 -26.951  75.834  1.00  0.00
E    H
ATOM  27204  CD  ARG E  27      30.108 -27.596  73.870  1.00  0.00
E    C
ATOM  27205  HD1 ARG E  27      30.045 -28.684  74.100  1.00  0.00
E    H
ATOM  27206  HD2 ARG E  27      29.153 -27.276  73.393  1.00  0.00
E    H
ATOM  27207  NE  ARG E  27      31.157 -27.389  72.866  1.00  0.00
E    N
ATOM  27208  HE  ARG E  27      30.897 -26.726  72.154  1.00  0.00
E    H
ATOM  27209  CZ  ARG E  27      32.346 -27.991  72.854  1.00  0.00
E    C
ATOM  27210  NH1 ARG E  27      32.680 -28.865  73.790  1.00  0.00
E    N
ATOM  27211 HH11 ARG E  27      33.575 -29.289  73.788  1.00  0.00
E    H
ATOM  27212 HH12 ARG E  27      32.041 -28.961  74.556  1.00  0.00
E    H
ATOM  27213  NH2 ARG E  27      33.219 -27.718  71.894  1.00  0.00
E    N
ATOM  27214 HH21 ARG E  27      34.065 -28.229  71.836  1.00  0.00
E    H
ATOM  27215 HH22 ARG E  27      32.898 -27.155  71.143  1.00  0.00
E    H
```

```
ATOM  27216  C   ARG E  27      32.842 -24.937  75.985  1.00  0.00
E    C
ATOM  27217  O   ARG E  27      33.298 -25.888  76.603  1.00  0.00
E    O
ATOM  27218  N   MET E  28      33.593 -24.135  75.237  1.00  0.00
E    N
ATOM  27219  HN  MET E  28      33.218 -23.392  74.687  1.00  0.00
E    H
ATOM  27220  CA  MET E  28      35.049 -24.275  75.155  1.00  0.00
E    C
ATOM  27221  HA  MET E  28      35.247 -25.336  75.063  1.00  0.00
E    H
ATOM  27222  CB  MET E  28      35.591 -23.529  73.940  1.00  0.00
E    C
ATOM  27223  HB1 MET E  28      35.179 -22.493  73.985  1.00  0.00
E    H
ATOM  27224  HB2 MET E  28      36.696 -23.428  74.031  1.00  0.00
E    H
ATOM  27225  CG  MET E  28      35.222 -24.141  72.603  1.00  0.00
E    C
ATOM  27226  HG1 MET E  28      35.632 -25.170  72.523  1.00  0.00
E    H
ATOM  27227  HG2 MET E  28      34.115 -24.213  72.518  1.00  0.00
E    H
ATOM  27228  SD  MET E  28      35.866 -23.159  71.227  1.00  0.00
E    S
ATOM  27229  CE  MET E  28      37.487 -23.896  71.026  1.00  0.00
E    C
ATOM  27230  HE1 MET E  28      38.049 -23.398  70.207  1.00  0.00
E    H
ATOM  27231  HE2 MET E  28      38.086 -23.803  71.958  1.00  0.00
E    H
ATOM  27232  HE3 MET E  28      37.407 -24.976  70.777  1.00  0.00
E    H
ATOM  27233  C   MET E  28      35.774 -23.776  76.412  1.00  0.00
E    C
ATOM  27234  O   MET E  28      36.936 -24.113  76.634  1.00  0.00
E    O
ATOM  27235  N   GLY E  29      35.097 -22.953  77.206  1.00  0.00
E    N
ATOM  27236  HN  GLY E  29      34.184 -22.611  76.991  1.00  0.00
E    H
ATOM  27237  CA  GLY E  29      35.659 -22.494  78.457  1.00  0.00
E    C
ATOM  27238  HA1 GLY E  29      36.522 -23.130  78.598  1.00  0.00
E    H
ATOM  27239  HA2 GLY E  29      34.865 -22.657  79.173  1.00  0.00
E    H
ATOM  27240  C   GLY E  29      36.108 -21.064  78.581  1.00  0.00
E    C
ATOM  27241  O   GLY E  29      36.669 -20.674  79.606  1.00  0.00
E    O
ATOM  27242  N   HSE E  30      35.871 -20.284  77.537  1.00  0.00
E    N
```

```
ATOM  27243  HN  HSE E  30      35.473 -20.606  76.680  1.00  0.00
E    H
ATOM  27244  CA  HSE E  30      36.181 -18.865  77.559  1.00  0.00
E    C
ATOM  27245  HA  HSE E  30      37.075 -18.725  78.154  1.00  0.00
E    H
ATOM  27246  CB  HSE E  30      36.325 -18.341  76.140  1.00  0.00
E    C
ATOM  27247  HB1 HSE E  30      35.332 -18.412  75.647  1.00  0.00
E    H
ATOM  27248  HB2 HSE E  30      36.598 -17.266  76.168  1.00  0.00
E    H
ATOM  27249  ND1 HSE E  30      38.626 -18.815  75.282  1.00  0.00
E    N
ATOM  27250  CG  HSE E  30      37.316 -19.115  75.310  1.00  0.00
E    C
ATOM  27251  CE1 HSE E  30      39.266 -19.671  74.465  1.00  0.00
E    C
ATOM  27252  HE1 HSE E  30      40.340 -19.646  74.259  1.00  0.00
E    H
ATOM  27253  NE2 HSE E  30      38.357 -20.526  73.967  1.00  0.00
E    N
ATOM  27254  HE2 HSE E  30      38.549 -21.261  73.317  1.00  0.00
E    H
ATOM  27255  CD2 HSE E  30      37.146 -20.210  74.466  1.00  0.00
E    C
ATOM  27256  HD2 HSE E  30      36.247 -20.750  74.204  1.00  0.00
E    H
ATOM  27257  C   HSE E  30      35.074 -18.150  78.256  1.00  0.00
E    C
ATOM  27258  O   HSE E  30      33.962 -18.676  78.363  1.00  0.00
E    O
ATOM  27259  N   GLU E  31      35.361 -16.952  78.756  1.00  0.00
E    N
ATOM  27260  HN  GLU E  31      36.265 -16.534  78.788  1.00  0.00
E    H
ATOM  27261  CA  GLU E  31      34.304 -16.123  79.324  1.00  0.00
E    C
ATOM  27262  HA  GLU E  31      33.515 -16.773  79.679  1.00  0.00
E    H
ATOM  27263  CB  GLU E  31      34.752 -15.359  80.583  1.00  0.00
E    C
ATOM  27264  HB1 GLU E  31      33.981 -14.589  80.815  1.00  0.00
E    H
ATOM  27265  HB2 GLU E  31      34.767 -16.048  81.457  1.00  0.00
E    H
ATOM  27266  CG  GLU E  31      36.098 -14.649  80.512  1.00  0.00
E    C
ATOM  27267  HG1 GLU E  31      36.855 -15.337  80.093  1.00  0.00
E    H
ATOM  27268  HG2 GLU E  31      36.036 -13.743  79.877  1.00  0.00
E    H
ATOM  27269  CD  GLU E  31      36.619 -14.245  81.887  1.00  0.00
E    C
```

```
ATOM  27270  OE1 GLU E  31      35.815 -14.150  82.845  1.00  0.00
E    O
ATOM  27271  OE2 GLU E  31      37.842 -14.018  82.009  1.00  0.00
E    O
ATOM  27272  C   GLU E  31      33.698 -15.216  78.261  1.00  0.00
E    C
ATOM  27273  O   GLU E  31      34.411 -14.599  77.468  1.00  0.00
E    O
ATOM  27274  N   VAL E  32      32.370 -15.177  78.244  1.00  0.00
E    N
ATOM  27275  HN  VAL E  32      31.806 -15.592  78.953  1.00  0.00
E    H
ATOM  27276  CA  VAL E  32      31.619 -14.521  77.184  1.00  0.00
E    C
ATOM  27277  HA  VAL E  32      32.303 -13.880  76.642  1.00  0.00
E    H
ATOM  27278  CB  VAL E  32      30.940 -15.556  76.256  1.00  0.00
E    C
ATOM  27279  HB  VAL E  32      30.096 -16.017  76.826  1.00  0.00
E    H
ATOM  27280  CG1 VAL E  32      30.388 -14.885  75.006  1.00  0.00
E    C
ATOM  27281 HG11 VAL E  32      29.938 -15.642  74.329  1.00  0.00
E    H
ATOM  27282 HG12 VAL E  32      29.596 -14.148  75.253  1.00  0.00
E    H
ATOM  27283 HG13 VAL E  32      31.197 -14.365  74.450  1.00  0.00
E    H
ATOM  27284  CG2 VAL E  32      31.905 -16.661  75.863  1.00  0.00
E    C
ATOM  27285 HG21 VAL E  32      31.420 -17.376  75.165  1.00  0.00
E    H
ATOM  27286 HG22 VAL E  32      32.801 -16.236  75.360  1.00  0.00
E    H
ATOM  27287 HG23 VAL E  32      32.242 -17.235  76.750  1.00  0.00
E    H
ATOM  27288  C   VAL E  32      30.535 -13.633  77.777  1.00  0.00
E    C
ATOM  27289  O   VAL E  32      29.794 -14.050  78.673  1.00  0.00
E    O
ATOM  27290  N   LEU E  33      30.444 -12.404  77.276  1.00  0.00
E    N
ATOM  27291  HN  LEU E  33      31.116 -11.984  76.670  1.00  0.00
E    H
ATOM  27292  CA  LEU E  33      29.310 -11.554  77.591  1.00  0.00
E    C
ATOM  27293  HA  LEU E  33      28.832 -11.952  78.477  1.00  0.00
E    H
ATOM  27294  CB  LEU E  33      29.746 -10.109  77.838  1.00  0.00
E    C
ATOM  27295  HB1 LEU E  33      30.591 -10.139  78.565  1.00  0.00
E    H
ATOM  27296  HB2 LEU E  33      30.166  -9.692  76.896  1.00  0.00
E    H
```

ATOM 27297 CG LEU E 33 28.666 -9.174 78.392 1.00 0.00
E C
ATOM 27298 HG LEU E 33 27.926 -9.825 78.915 1.00 0.00
E H
ATOM 27299 CD1 LEU E 33 29.233 -8.152 79.361 1.00 0.00
E C
ATOM 27300 HD11 LEU E 33 28.435 -7.460 79.707 1.00 0.00
E H
ATOM 27301 HD12 LEU E 33 29.669 -8.656 80.250 1.00 0.00
E H
ATOM 27302 HD13 LEU E 33 30.028 -7.549 78.874 1.00 0.00
E H
ATOM 27303 CD2 LEU E 33 27.952 -8.471 77.259 1.00 0.00
E C
ATOM 27304 HD21 LEU E 33 27.156 -7.806 77.658 1.00 0.00
E H
ATOM 27305 HD22 LEU E 33 28.646 -7.844 76.663 1.00 0.00
E H
ATOM 27306 HD23 LEU E 33 27.479 -9.212 76.579 1.00 0.00
E H
ATOM 27307 C LEU E 33 28.312 -11.643 76.452 1.00 0.00
E C
ATOM 27308 O LEU E 33 28.629 -11.339 75.306 1.00 0.00
E O
ATOM 27309 N ALA E 34 27.108 -12.087 76.776 1.00 0.00
E N
ATOM 27310 HN ALA E 34 26.868 -12.382 77.700 1.00 0.00
E H
ATOM 27311 CA ALA E 34 26.040 -12.178 75.796 1.00 0.00
E C
ATOM 27312 HA ALA E 34 26.428 -12.207 74.784 1.00 0.00
E H
ATOM 27313 CB ALA E 34 25.209 -13.428 76.034 1.00 0.00
E C
ATOM 27314 HB1 ALA E 34 25.848 -14.329 75.912 1.00 0.00
E H
ATOM 27315 HB2 ALA E 34 24.793 -13.441 77.065 1.00 0.00
E H
ATOM 27316 HB3 ALA E 34 24.368 -13.504 75.312 1.00 0.00
E H
ATOM 27317 C ALA E 34 25.178 -10.930 75.889 1.00 0.00
E C
ATOM 27318 O ALA E 34 24.714 -10.571 76.972 1.00 0.00
E O
ATOM 27319 N VAL E 35 24.985 -10.262 74.754 1.00 0.00
E N
ATOM 27320 HN VAL E 35 25.401 -10.516 73.884 1.00 0.00
E H
ATOM 27321 CA VAL E 35 24.132 -9.081 74.692 1.00 0.00
E C
ATOM 27322 HA VAL E 35 23.691 -8.933 75.671 1.00 0.00
E H
ATOM 27323 CB VAL E 35 24.913 -7.832 74.242 1.00 0.00
E C

ATOM 27324 HB VAL E 35   25.246 -8.004 73.188 1.00 0.00
E   H
ATOM 27325 CG1 VAL E 35   24.028 -6.601 74.290 1.00 0.00
E   C
ATOM 27326 HG11 VAL E 35   24.608 -5.698 74.001 1.00 0.00
E   H
ATOM 27327 HG12 VAL E 35   23.173 -6.683 73.587 1.00 0.00
E   H
ATOM 27328 HG13 VAL E 35   23.632 -6.442 75.316 1.00 0.00
E   H
ATOM 27329 CG2 VAL E 35   26.131 -7.614 75.111 1.00 0.00
E   C
ATOM 27330 HG21 VAL E 35   26.669 -6.690 74.808 1.00 0.00
E   H
ATOM 27331 HG22 VAL E 35   25.837 -7.513 76.178 1.00 0.00
E   H
ATOM 27332 HG23 VAL E 35   26.844 -8.461 75.025 1.00 0.00
E   H
ATOM 27333 C   VAL E 35   22.996 -9.319 73.711 1.00 0.00
E   C
ATOM 27334 O   VAL E 35   23.195 -9.933 72.666 1.00 0.00
E   O
ATOM 27335 N   ASP E 36   21.808 -8.838 74.066 1.00 0.00
E   N
ATOM 27336 HN ASP E 36   21.596 -8.446 74.957 1.00 0.00
E   H
ATOM 27337 CA ASP E 36   20.664 -8.837 73.157 1.00 0.00
E   C
ATOM 27338 HA ASP E 36   21.020 -8.509 72.187 1.00 0.00
E   H
ATOM 27339 CB ASP E 36   20.039 -10.233 73.056 1.00 0.00
E   C
ATOM 27340 HB1 ASP E 36   20.844 -10.996 73.064 1.00 0.00
E   H
ATOM 27341 HB2 ASP E 36   19.369 -10.424 73.917 1.00 0.00
E   H
ATOM 27342 CG ASP E 36   19.246 -10.426 71.779 1.00 0.00
E   C
ATOM 27343 OD1 ASP E 36   19.631 -11.289 70.957 1.00 0.00
E   O
ATOM 27344 OD2 ASP E 36   18.239 -9.712 71.592 1.00 0.00
E   O
ATOM 27345 C   ASP E 36   19.626 -7.816 73.614 1.00 0.00
E   C
ATOM 27346 O   ASP E 36   19.514 -7.516 74.806 1.00 0.00
E   O
ATOM 27347 N   ILE E 37   18.880 -7.275 72.656 1.00 0.00
E   N
ATOM 27348 HN ILE E 37   18.985 -7.486 71.686 1.00 0.00
E   H
ATOM 27349 CA ILE E 37   17.825 -6.309 72.946 1.00 0.00
E   C
ATOM 27350 HA ILE E 37   18.203 -5.661 73.726 1.00 0.00
E   H

```
ATOM  27351  CB  ILE E  37      17.449  -5.471  71.696  1.00  0.00
E    C
ATOM  27352  HB  ILE E  37      18.431  -5.119  71.290  1.00  0.00
E    H
ATOM  27353  CG2 ILE E  37      16.707  -6.314  70.655  1.00  0.00
E    C
ATOM  27354 HG21 ILE E  37      16.584  -5.747  69.708  1.00  0.00
E    H
ATOM  27355 HG22 ILE E  37      17.272  -7.241  70.422  1.00  0.00
E    H
ATOM  27356 HG23 ILE E  37      15.698  -6.600  71.022  1.00  0.00
E    H
ATOM  27357  CG1 ILE E  37      16.662  -4.215  72.096  1.00  0.00
E    C
ATOM  27358 HG11 ILE E  37      15.625  -4.496  72.385  1.00  0.00
E    H
ATOM  27359 HG12 ILE E  37      17.141  -3.760  72.993  1.00  0.00
E    H
ATOM  27360  CD  ILE E  37      16.588  -3.146  71.024  1.00  0.00
E    C
ATOM  27361  HD1 ILE E  37      16.054  -2.250  71.410  1.00  0.00
E    H
ATOM  27362  HD2 ILE E  37      17.607  -2.835  70.710  1.00  0.00
E    H
ATOM  27363  HD3 ILE E  37      16.040  -3.515  70.131  1.00  0.00
E    H
ATOM  27364  C   ILE E  37      16.597  -7.017  73.517  1.00  0.00
E    C
ATOM  27365  O   ILE E  37      15.857  -6.440  74.311  1.00  0.00
E    O
ATOM  27366  N   ASN E  38      16.414  -8.273  73.125  1.00  0.00
E    N
ATOM  27367  HN  ASN E  38      17.078  -8.759  72.558  1.00  0.00
E    H
ATOM  27368  CA  ASN E  38      15.234  -9.059  73.483  1.00  0.00
E    C
ATOM  27369  HA  ASN E  38      14.436  -8.336  73.610  1.00  0.00
E    H
ATOM  27370  CB  ASN E  38      14.927 -10.043  72.353  1.00  0.00
E    C
ATOM  27371  HB1 ASN E  38      14.790  -9.457  71.418  1.00  0.00
E    H
ATOM  27372  HB2 ASN E  38      15.786 -10.728  72.193  1.00  0.00
E    H
ATOM  27373  CG  ASN E  38      13.666 -10.838  72.593  1.00  0.00
E    C
ATOM  27374  OD1 ASN E  38      13.648 -11.774  73.390  1.00  0.00
E    O
ATOM  27375  ND2 ASN E  38      12.605 -10.481  71.888  1.00  0.00
E    N
ATOM  27376 HD21 ASN E  38      11.761 -10.987  72.042  1.00  0.00
E    H
ATOM  27377 HD22 ASN E  38      12.661  -9.714  71.256  1.00  0.00
E    H
```

```
ATOM  27378  C   ASN E  38      15.406  -9.797  74.813  1.00  0.00
E    C
ATOM  27379  O   ASN E  38      16.345 -10.571  74.977  1.00  0.00
E    O
ATOM  27380  N   GLU E  39      14.486  -9.572  75.748  1.00  0.00
E    N
ATOM  27381  HN  GLU E  39      13.656  -9.038  75.616  1.00  0.00
E    H
ATOM  27382  CA  GLU E  39      14.634 -10.118  77.097  1.00  0.00
E    C
ATOM  27383  HA  GLU E  39      15.605  -9.802  77.459  1.00  0.00
E    H
ATOM  27384  CB  GLU E  39      13.593  -9.521  78.050  1.00  0.00
E    C
ATOM  27385  HB1 GLU E  39      13.474  -8.441  77.803  1.00  0.00
E    H
ATOM  27386  HB2 GLU E  39      12.599  -9.988  77.864  1.00  0.00
E    H
ATOM  27387  CG  GLU E  39      13.971  -9.661  79.520  1.00  0.00
E    C
ATOM  27388  HG1 GLU E  39      14.133 -10.728  79.759  1.00  0.00
E    H
ATOM  27389  HG2 GLU E  39      14.895  -9.092  79.748  1.00  0.00
E    H
ATOM  27390  CD  GLU E  39      12.883  -9.195  80.471  1.00  0.00
E    C
ATOM  27391  OE1 GLU E  39      13.070  -9.344  81.698  1.00  0.00
E    O
ATOM  27392  OE2 GLU E  39      11.843  -8.682  80.001  1.00  0.00
E    O
ATOM  27393  C   GLU E  39      14.604 -11.650  77.156  1.00  0.00
E    C
ATOM  27394  O   GLU E  39      15.351 -12.259  77.927  1.00  0.00
E    O
ATOM  27395  N   GLU E  40      13.746 -12.261  76.344  1.00  0.00
E    N
ATOM  27396  HN  GLU E  40      13.111 -11.787  75.741  1.00  0.00
E    H
ATOM  27397  CA  GLU E  40      13.661 -13.721  76.267  1.00  0.00
E    C
ATOM  27398  HA  GLU E  40      13.435 -14.093  77.259  1.00  0.00
E    H
ATOM  27399  CB  GLU E  40      12.547 -14.153  75.314  1.00  0.00
E    C
ATOM  27400  HB1 GLU E  40      12.597 -13.508  74.406  1.00  0.00
E    H
ATOM  27401  HB2 GLU E  40      12.734 -15.192  74.960  1.00  0.00
E    H
ATOM  27402  CG  GLU E  40      11.149 -14.057  75.904  1.00  0.00
E    C
ATOM  27403  HG1 GLU E  40      10.911 -14.994  76.440  1.00  0.00
E    H
ATOM  27404  HG2 GLU E  40      11.073 -13.204  76.607  1.00  0.00
E    H
```

ATOM 27405 CD GLU E 40 10.076 -13.906 74.841 1.00 0.00
E C
ATOM 27406 OE1 GLU E 40 10.299 -13.150 73.867 1.00 0.00
E O
ATOM 27407 OE2 GLU E 40 9.004 -14.535 74.982 1.00 0.00
E O
ATOM 27408 C GLU E 40 14.986 -14.348 75.839 1.00 0.00
E C
ATOM 27409 O GLU E 40 15.442 -15.312 76.454 1.00 0.00
E O
ATOM 27410 N LYS E 41 15.595 -13.790 74.794 1.00 0.00
E N
ATOM 27411 HN LYS E 41 15.188 -13.039 74.276 1.00 0.00
E H
ATOM 27412 CA LYS E 41 16.907 -14.233 74.320 1.00 0.00
E C
ATOM 27413 HA LYS E 41 16.809 -15.273 74.033 1.00 0.00
E H
ATOM 27414 CB LYS E 41 17.368 -13.410 73.108 1.00 0.00
E C
ATOM 27415 HB1 LYS E 41 17.275 -12.335 73.387 1.00 0.00
E H
ATOM 27416 HB2 LYS E 41 18.452 -13.595 72.935 1.00 0.00
E H
ATOM 27417 CG LYS E 41 16.577 -13.655 71.829 1.00 0.00
E C
ATOM 27418 HG1 LYS E 41 15.494 -13.734 72.080 1.00 0.00
E H
ATOM 27419 HG2 LYS E 41 16.698 -12.778 71.154 1.00 0.00
E H
ATOM 27420 CD LYS E 41 17.010 -14.929 71.116 1.00 0.00
E C
ATOM 27421 HD1 LYS E 41 18.022 -14.762 70.681 1.00 0.00
E H
ATOM 27422 HD2 LYS E 41 17.104 -15.738 71.878 1.00 0.00
E H
ATOM 27423 CE LYS E 41 16.022 -15.337 70.034 1.00 0.00
E C
ATOM 27424 HE1 LYS E 41 16.389 -16.236 69.490 1.00 0.00
E H
ATOM 27425 HE2 LYS E 41 15.031 -15.575 70.479 1.00 0.00
E H
ATOM 27426 NZ LYS E 41 15.798 -14.274 69.015 1.00 0.00
E N
ATOM 27427 HZ1 LYS E 41 15.117 -14.603 68.300 1.00 0.00
E H
ATOM 27428 HZ2 LYS E 41 15.425 -13.421 69.479 1.00 0.00
E H
ATOM 27429 HZ3 LYS E 41 16.700 -14.043 68.552 1.00 0.00
E H
ATOM 27430 C LYS E 41 17.948 -14.164 75.440 1.00 0.00
E C
ATOM 27431 O LYS E 41 18.677 -15.129 75.668 1.00 0.00
E O

```
ATOM  27432  N   VAL E  42      18.000 -13.031  76.140  1.00  0.00
E    N
ATOM  27433  HN  VAL E  42      17.414 -12.248  75.947  1.00  0.00
E    H
ATOM  27434  CA  VAL E  42      18.928 -12.832  77.262  1.00  0.00
E    C
ATOM  27435  HA  VAL E  42      19.931 -12.983  76.883  1.00  0.00
E    H
ATOM  27436  CB  VAL E  42      18.839 -11.399  77.842  1.00  0.00
E    C
ATOM  27437  HB  VAL E  42      17.822 -11.280  78.291  1.00  0.00
E    H
ATOM  27438  CG1 VAL E  42      19.886 -11.183  78.923  1.00  0.00
E    C
ATOM  27439 HG11 VAL E  42      19.838 -10.140  79.304  1.00  0.00
E    H
ATOM  27440 HG12 VAL E  42      19.727 -11.859  79.789  1.00  0.00
E    H
ATOM  27441 HG13 VAL E  42      20.908 -11.354  78.520  1.00  0.00
E    H
ATOM  27442  CG2 VAL E  42      19.025 -10.364  76.745  1.00  0.00
E    C
ATOM  27443 HG21 VAL E  42      19.001  -9.336  77.166  1.00  0.00
E    H
ATOM  27444 HG22 VAL E  42      20.000 -10.509  76.232  1.00  0.00
E    H
ATOM  27445 HG23 VAL E  42      18.219 -10.433  75.986  1.00  0.00
E    H
ATOM  27446  C   VAL E  42      18.693 -13.868  78.366  1.00  0.00
E    C
ATOM  27447  O   VAL E  42      19.649 -14.418  78.924  1.00  0.00
E    O
ATOM  27448  N   ASN E  43      17.422 -14.134  78.670  1.00  0.00
E    N
ATOM  27449  HN  ASN E  43      16.647 -13.646  78.269  1.00  0.00
E    H
ATOM  27450  CA  ASN E  43      17.051 -15.177  79.630  1.00  0.00
E    C
ATOM  27451  HA  ASN E  43      17.590 -14.936  80.540  1.00  0.00
E    H
ATOM  27452  CB  ASN E  43      15.541 -15.180  79.875  1.00  0.00
E    C
ATOM  27453  HB1 ASN E  43      15.036 -15.077  78.889  1.00  0.00
E    H
ATOM  27454  HB2 ASN E  43      15.223 -16.149  80.312  1.00  0.00
E    H
ATOM  27455  CG  ASN E  43      15.085 -14.032  80.757  1.00  0.00
E    C
ATOM  27456  OD1 ASN E  43      15.898 -13.330  81.362  1.00  0.00
E    O
ATOM  27457  ND2 ASN E  43      13.774 -13.838  80.840  1.00  0.00
E    N
ATOM  27458 HD21 ASN E  43      13.457 -13.081  81.405  1.00  0.00
E    H
```

```
ATOM  27459 HD22 ASN E  43      13.151 -14.418  80.325  1.00  0.00
E    H
ATOM  27460  C   ASN E  43      17.502 -16.559  79.181  1.00  0.00
E    C
ATOM  27461  O   ASN E  43      18.035 -17.331  79.976  1.00  0.00
E    O
ATOM  27462  N   ALA E  44      17.296 -16.856  77.898  1.00  0.00
E    N
ATOM  27463  HN  ALA E  44      16.908 -16.195  77.255  1.00  0.00
E    H
ATOM  27464  CA  ALA E  44      17.620 -18.163  77.335  1.00  0.00
E    C
ATOM  27465  HA  ALA E  44      17.187 -18.944  77.950  1.00  0.00
E    H
ATOM  27466  CB  ALA E  44      17.001 -18.309  75.952  1.00  0.00
E    C
ATOM  27467  HB1 ALA E  44      15.903 -18.156  76.017  1.00  0.00
E    H
ATOM  27468  HB2 ALA E  44      17.410 -17.552  75.248  1.00  0.00
E    H
ATOM  27469  HB3 ALA E  44      17.183 -19.320  75.527  1.00  0.00
E    H
ATOM  27470  C   ALA E  44      19.124 -18.436  77.285  1.00  0.00
E    C
ATOM  27471  O   ALA E  44      19.544 -19.594  77.249  1.00  0.00
E    O
ATOM  27472  N   TYR E  45      19.931 -17.377  77.292  1.00  0.00
E    N
ATOM  27473  HN  TYR E  45      19.617 -16.430  77.347  1.00  0.00
E    H
ATOM  27474  CA  TYR E  45      21.385 -17.533  77.217  1.00  0.00
E    C
ATOM  27475  HA  TYR E  45      21.549 -18.567  76.940  1.00  0.00
E    H
ATOM  27476  CB  TYR E  45      21.974 -16.674  76.086  1.00  0.00
E    C
ATOM  27477  HB1 TYR E  45      21.464 -15.686  76.076  1.00  0.00
E    H
ATOM  27478  HB2 TYR E  45      23.055 -16.484  76.259  1.00  0.00
E    H
ATOM  27479  CG  TYR E  45      21.761 -17.314  74.735  1.00  0.00
E    C
ATOM  27480  CD1 TYR E  45      20.622 -17.041  73.987  1.00  0.00
E    C
ATOM  27481  HD1 TYR E  45      19.878 -16.357  74.370  1.00  0.00
E    H
ATOM  27482  CE1 TYR E  45      20.411 -17.648  72.761  1.00  0.00
E    C
ATOM  27483  HE1 TYR E  45      19.525 -17.428  72.185  1.00  0.00
E    H
ATOM  27484  CZ  TYR E  45      21.339 -18.546  72.277  1.00  0.00
E    C
ATOM  27485  OH  TYR E  45      21.130 -19.147  71.065  1.00  0.00
E    O
```

```
ATOM  27486  HH  TYR E  45      21.906 -19.675  70.868  1.00  0.00
E    H
ATOM  27487  CD2 TYR E  45      22.679 -18.230  74.225  1.00  0.00
E    C
ATOM  27488  HD2 TYR E  45      23.551 -18.502  74.804  1.00  0.00
E    H
ATOM  27489  CE2 TYR E  45      22.473 -18.843  73.002  1.00  0.00
E    C
ATOM  27490  HE2 TYR E  45      23.189 -19.564  72.636  1.00  0.00
E    H
ATOM  27491  C   TYR E  45      22.100 -17.304  78.553  1.00  0.00
E    C
ATOM  27492  O   TYR E  45      23.331 -17.367  78.632  1.00  0.00
E    O
ATOM  27493  N   ALA E  46      21.320 -17.073  79.604  1.00  0.00
E    N
ATOM  27494  HN  ALA E  46      20.327 -16.991  79.534  1.00  0.00
E    H
ATOM  27495  CA  ALA E  46      21.871 -16.919  80.953  1.00  0.00
E    C
ATOM  27496  HA  ALA E  46      22.464 -16.014  81.018  1.00  0.00
E    H
ATOM  27497  CB  ALA E  46      20.752 -16.775  81.982  1.00  0.00
E    C
ATOM  27498  HB1 ALA E  46      20.118 -15.901  81.723  1.00  0.00
E    H
ATOM  27499  HB2 ALA E  46      20.102 -17.678  81.994  1.00  0.00
E    H
ATOM  27500  HB3 ALA E  46      21.154 -16.617  83.005  1.00  0.00
E    H
ATOM  27501  C   ALA E  46      22.808 -18.077  81.316  1.00  0.00
E    C
ATOM  27502  O   ALA E  46      23.891 -17.860  81.861  1.00  0.00
E    O
ATOM  27503  N   SER E  47      22.391 -19.296  80.980  1.00  0.00
E    N
ATOM  27504  HN  SER E  47      21.526 -19.460  80.506  1.00  0.00
E    H
ATOM  27505  CA  SER E  47      23.158 -20.513  81.271  1.00  0.00
E    C
ATOM  27506  HA  SER E  47      23.574 -20.341  82.254  1.00  0.00
E    H
ATOM  27507  CB  SER E  47      22.236 -21.729  81.237  1.00  0.00
E    C
ATOM  27508  HB1 SER E  47      21.715 -21.740  80.252  1.00  0.00
E    H
ATOM  27509  HB2 SER E  47      22.831 -22.668  81.304  1.00  0.00
E    H
ATOM  27510  OG  SER E  47      21.271 -21.654  82.266  1.00  0.00
E    O
ATOM  27511  HG1 SER E  47      20.592 -22.310  82.066  1.00  0.00
E    H
ATOM  27512  C   SER E  47      24.326 -20.773  80.322  1.00  0.00
E    C
```

```
ATOM  27513  O   SER E  47      25.193 -21.597  80.622  1.00  0.00
E    O
ATOM  27514  N   TYR E  48      24.340 -20.091  79.180  1.00  0.00
E    N
ATOM  27515  HN  TYR E  48      23.694 -19.363  78.956  1.00  0.00
E    H
ATOM  27516  CA  TYR E  48      25.331 -20.365  78.138  1.00  0.00
E    C
ATOM  27517  HA  TYR E  48      25.889 -21.226  78.485  1.00  0.00
E    H
ATOM  27518  CB  TYR E  48      24.641 -20.702  76.812  1.00  0.00
E    C
ATOM  27519  HB1 TYR E  48      24.070 -19.814  76.462  1.00  0.00
E    H
ATOM  27520  HB2 TYR E  48      25.392 -20.939  76.027  1.00  0.00
E    H
ATOM  27521  CG  TYR E  48      23.646 -21.840  76.899  1.00  0.00
E    C
ATOM  27522  CD1 TYR E  48      22.275 -21.596  76.948  1.00  0.00
E    C
ATOM  27523  HD1 TYR E  48      21.909 -20.578  76.939  1.00  0.00
E    H
ATOM  27524  CE1 TYR E  48      21.362 -22.634  77.031  1.00  0.00
E    C
ATOM  27525  HE1 TYR E  48      20.302 -22.432  77.074  1.00  0.00
E    H
ATOM  27526  CZ  TYR E  48      21.819 -23.933  77.065  1.00  0.00
E    C
ATOM  27527  OH  TYR E  48      20.927 -24.971  77.144  1.00  0.00
E    O
ATOM  27528  HH  TYR E  48      21.429 -25.787  77.084  1.00  0.00
E    H
ATOM  27529  CD2 TYR E  48      24.073 -23.163  76.938  1.00  0.00
E    C
ATOM  27530  HD2 TYR E  48      25.130 -23.390  76.944  1.00  0.00
E    H
ATOM  27531  CE2 TYR E  48      23.167 -24.205  77.020  1.00  0.00
E    C
ATOM  27532  HE2 TYR E  48      23.524 -25.223  77.070  1.00  0.00
E    H
ATOM  27533  C   TYR E  48      26.331 -19.228  77.947  1.00  0.00
E    C
ATOM  27534  O   TYR E  48      26.959 -19.118  76.888  1.00  0.00
E    O
ATOM  27535  N   ALA E  49      26.477 -18.399  78.979  1.00  0.00
E    N
ATOM  27536  HN  ALA E  49      25.965 -18.526  79.829  1.00  0.00
E    H
ATOM  27537  CA  ALA E  49      27.379 -17.258  78.959  1.00  0.00
E    C
ATOM  27538  HA  ALA E  49      28.288 -17.494  78.417  1.00  0.00
E    H
ATOM  27539  CB  ALA E  49      26.702 -16.076  78.289  1.00  0.00
E    C
```

ATOM 27540 HB1 ALA E 49 26.439 -16.341 77.243 1.00 0.00
E H
ATOM 27541 HB2 ALA E 49 25.763 -15.802 78.818 1.00 0.00
E H
ATOM 27542 HB3 ALA E 49 27.366 -15.186 78.261 1.00 0.00
E H
ATOM 27543 C ALA E 49 27.807 -16.886 80.376 1.00 0.00
E C
ATOM 27544 O ALA E 49 27.063 -17.120 81.333 1.00 0.00
E O
ATOM 27545 N THR E 50 28.999 -16.303 80.509 1.00 0.00
E N
ATOM 27546 HN THR E 50 29.639 -16.177 79.751 1.00 0.00
E H
ATOM 27547 CA THR E 50 29.478 -15.789 81.793 1.00 0.00
E C
ATOM 27548 HA THR E 50 29.448 -16.620 82.484 1.00 0.00
E H
ATOM 27549 CB THR E 50 30.892 -15.189 81.666 1.00 0.00
E C
ATOM 27550 HB THR E 50 30.780 -14.252 81.069 1.00 0.00
E H
ATOM 27551 OG1 THR E 50 31.724 -16.090 80.928 1.00 0.00
E O
ATOM 27552 HG1 THR E 50 32.590 -15.669 80.893 1.00 0.00
E H
ATOM 27553 CG2 THR E 50 31.513 -14.965 83.030 1.00 0.00
E C
ATOM 27554 HG21 THR E 50 32.481 -14.429 82.940 1.00 0.00
E H
ATOM 27555 HG22 THR E 50 30.841 -14.351 83.666 1.00 0.00
E H
ATOM 27556 HG23 THR E 50 31.696 -15.930 83.550 1.00 0.00
E H
ATOM 27557 C THR E 50 28.505 -14.739 82.332 1.00 0.00
E C
ATOM 27558 O THR E 50 28.089 -14.811 83.487 1.00 0.00
E O
ATOM 27559 N HSE E 51 28.144 -13.782 81.481 1.00 0.00
E N
ATOM 27560 HN HSE E 51 28.543 -13.693 80.569 1.00 0.00
E H
ATOM 27561 CA HSE E 51 27.134 -12.772 81.797 1.00 0.00
E C
ATOM 27562 HA HSE E 51 26.524 -13.174 82.597 1.00 0.00
E H
ATOM 27563 CB HSE E 51 27.792 -11.466 82.223 1.00 0.00
E C
ATOM 27564 HB1 HSE E 51 28.732 -11.351 81.640 1.00 0.00
E H
ATOM 27565 HB2 HSE E 51 27.135 -10.614 81.950 1.00 0.00
E H
ATOM 27566 ND1 HSE E 51 29.310 -11.878 84.171 1.00 0.00
E N

ATOM 27567 CG HSE E 51   28.143 -11.412 83.688 1.00 0.00
E   C
ATOM 27568 CE1 HSE E 51   29.344 -11.698 85.510 1.00 0.00
E   C
ATOM 27569 HE1 HSE E 51   30.177 -11.991 86.154 1.00 0.00
E   H
ATOM 27570 NE2 HSE E 51   28.191 -11.116 85.883 1.00 0.00
E   N
ATOM 27571 HE2 HSE E 51   27.935 -10.885 86.821 1.00 0.00
E   H
ATOM 27572 CD2 HSE E 51   27.428 -10.924 84.783 1.00 0.00
E   C
ATOM 27573 HD2 HSE E 51   26.446 -10.475 84.825 1.00 0.00
E   H
ATOM 27574 C   HSE E 51   26.225 -12.535 80.623 1.00 0.00
E   C
ATOM 27575 O   HSE E 51   26.637 -12.671 79.464 1.00 0.00
E   O
ATOM 27576 N   ALA E 52   24.976 -12.177 80.914 1.00 0.00
E   N
ATOM 27577 HN ALA E 52   24.643 -12.109 81.854 1.00 0.00
E   H
ATOM 27578 CA ALA E 52   23.998 -11.856 79.877 1.00 0.00
E   C
ATOM 27579 HA ALA E 52   24.483 -11.647 78.931 1.00 0.00
E   H
ATOM 27580 CB ALA E 52   23.044 -13.019 79.672 1.00 0.00
E   C
ATOM 27581 HB1 ALA E 52   23.614 -13.915 79.346 1.00 0.00
E   H
ATOM 27582 HB2 ALA E 52   22.517 -13.275 80.617 1.00 0.00
E   H
ATOM 27583 HB3 ALA E 52   22.285 -12.793 78.892 1.00 0.00
E   H
ATOM 27584 C   ALA E 52   23.239 -10.592 80.260 1.00 0.00
E   C
ATOM 27585 O   ALA E 52   22.754 -10.476 81.385 1.00 0.00
E   O
ATOM 27586 N   VAL E 53   23.145 -9.641 79.331 1.00 0.00
E   N
ATOM 27587 HN VAL E 53   23.462 -9.756 78.394 1.00 0.00
E   H
ATOM 27588 CA VAL E 53   22.559 -8.329 79.617 1.00 0.00
E   C
ATOM 27589 HA VAL E 53   21.969 -8.435 80.519 1.00 0.00
E   H
ATOM 27590 CB VAL E 53   23.644 -7.235 79.775 1.00 0.00
E   C
ATOM 27591 HB VAL E 53   23.912 -6.876 78.752 1.00 0.00
E   H
ATOM 27592 CG1 VAL E 53   23.110 -6.068 80.592 1.00 0.00
E   C
ATOM 27593 HG11 VAL E 53   23.904 -5.303 80.736 1.00 0.00
E   H

ATOM 27594 HG12 VAL E 53 22.258 -5.569 80.086 1.00 0.00
E H
ATOM 27595 HG13 VAL E 53 22.777 -6.411 81.595 1.00 0.00
E H
ATOM 27596 CG2 VAL E 53 24.916 -7.785 80.413 1.00 0.00
E C
ATOM 27597 HG21 VAL E 53 25.665 -6.979 80.554 1.00 0.00
E H
ATOM 27598 HG22 VAL E 53 24.693 -8.233 81.406 1.00 0.00
E H
ATOM 27599 HG23 VAL E 53 25.379 -8.567 79.776 1.00 0.00
E H
ATOM 27600 C VAL E 53 21.620 -7.872 78.508 1.00 0.00
E C
ATOM 27601 O VAL E 53 21.927 -8.033 77.322 1.00 0.00
E O
ATOM 27602 N ILE E 54 20.484 -7.295 78.892 1.00 0.00
E N
ATOM 27603 HN ILE E 54 20.157 -7.277 79.835 1.00 0.00
E H
ATOM 27604 CA ILE E 54 19.601 -6.623 77.939 1.00 0.00
E C
ATOM 27605 HA ILE E 54 19.577 -7.221 77.037 1.00 0.00
E H
ATOM 27606 CB ILE E 54 18.178 -6.385 78.509 1.00 0.00
E C
ATOM 27607 HB ILE E 54 18.350 -5.858 79.481 1.00 0.00
E H
ATOM 27608 CG2 ILE E 54 17.316 -5.601 77.524 1.00 0.00
E C
ATOM 27609 HG21 ILE E 54 16.358 -5.301 77.998 1.00 0.00
E H
ATOM 27610 HG22 ILE E 54 17.833 -4.675 77.195 1.00 0.00
E H
ATOM 27611 HG23 ILE E 54 17.082 -6.211 76.626 1.00 0.00
E H
ATOM 27612 CG1 ILE E 54 17.476 -7.706 78.853 1.00 0.00
E C
ATOM 27613 HG11 ILE E 54 16.408 -7.655 78.546 1.00 0.00
E H
ATOM 27614 HG12 ILE E 54 17.949 -8.527 78.268 1.00 0.00
E H
ATOM 27615 CD ILE E 54 17.542 -8.083 80.315 1.00 0.00
E C
ATOM 27616 HD1 ILE E 54 17.066 -9.075 80.480 1.00 0.00
E H
ATOM 27617 HD2 ILE E 54 18.598 -8.144 80.657 1.00 0.00
E H
ATOM 27618 HD3 ILE E 54 17.010 -7.339 80.943 1.00 0.00
E H
ATOM 27619 C ILE E 54 20.229 -5.286 77.566 1.00 0.00
E C
ATOM 27620 O ILE E 54 20.567 -4.496 78.446 1.00 0.00
E O

ATOM 27621 N ALA E 55 20.393 -5.043 76.267 1.00 0.00
E N
ATOM 27622 HN ALA E 55 20.159 -5.717 75.568 1.00 0.00
E H
ATOM 27623 CA ALA E 55 20.930 -3.775 75.764 1.00 0.00
E C
ATOM 27624 HA ALA E 55 20.498 -2.944 76.310 1.00 0.00
E H
ATOM 27625 CB ALA E 55 22.436 -3.702 75.974 1.00 0.00
E C
ATOM 27626 HB1 ALA E 55 22.669 -3.816 77.054 1.00 0.00
E H
ATOM 27627 HB2 ALA E 55 22.956 -4.516 75.424 1.00 0.00
E H
ATOM 27628 HB3 ALA E 55 22.849 -2.727 75.636 1.00 0.00
E H
ATOM 27629 C ALA E 55 20.586 -3.552 74.294 1.00 0.00
E C
ATOM 27630 O ALA E 55 20.540 -4.496 73.507 1.00 0.00
E O
ATOM 27631 N ASN E 56 20.327 -2.299 73.934 1.00 0.00
E N
ATOM 27632 HN ASN E 56 20.187 -1.549 74.581 1.00 0.00
E H
ATOM 27633 CA ASN E 56 20.227 -1.916 72.539 1.00 0.00
E C
ATOM 27634 HA ASN E 56 19.942 -2.799 71.977 1.00 0.00
E H
ATOM 27635 CB ASN E 56 19.219 -0.773 72.353 1.00 0.00
E C
ATOM 27636 HB1 ASN E 56 18.238 -1.118 72.748 1.00 0.00
E H
ATOM 27637 HB2 ASN E 56 19.525 0.111 72.948 1.00 0.00
E H
ATOM 27638 CG ASN E 56 19.027 -0.383 70.892 1.00 0.00
E C
ATOM 27639 OD1 ASN E 56 19.910 -0.572 70.053 1.00 0.00
E O
ATOM 27640 ND2 ASN E 56 17.864 0.173 70.586 1.00 0.00
E N
ATOM 27641 HD21 ASN E 56 17.714 0.426 69.634 1.00 0.00
E H
ATOM 27642 HD22 ASN E 56 17.171 0.297 71.289 1.00 0.00
E H
ATOM 27643 C ASN E 56 21.621 -1.505 72.078 1.00 0.00
E C
ATOM 27644 O ASN E 56 22.106 -0.421 72.425 1.00 0.00
E O
ATOM 27645 N ALA E 57 22.262 -2.371 71.295 1.00 0.00
E N
ATOM 27646 HN ALA E 57 21.836 -3.208 70.953 1.00 0.00
E H
ATOM 27647 CA ALA E 57 23.647 -2.157 70.877 1.00 0.00
E C

```
ATOM  27648  HA  ALA E  57      24.253  -1.982  71.759  1.00  0.00
E    H
ATOM  27649  CB  ALA E  57      24.212  -3.418  70.255  1.00  0.00
E    C
ATOM  27650  HB1 ALA E  57      24.085  -4.268  70.959  1.00  0.00
E    H
ATOM  27651  HB2 ALA E  57      23.679  -3.671  69.313  1.00  0.00
E    H
ATOM  27652  HB3 ALA E  57      25.296  -3.314  70.032  1.00  0.00
E    H
ATOM  27653  C   ALA E  57      23.872  -0.955  69.952  1.00  0.00
E    C
ATOM  27654  O   ALA E  57      25.014  -0.646  69.602  1.00  0.00
E    O
ATOM  27655  N   THR E  58      22.793  -0.283  69.560  1.00  0.00
E    N
ATOM  27656  HN  THR E  58      21.856  -0.541  69.795  1.00  0.00
E    H
ATOM  27657  CA  THR E  58      22.897   0.919  68.730  1.00  0.00
E    C
ATOM  27658  HA  THR E  58      23.876   0.877  68.273  1.00  0.00
E    H
ATOM  27659  CB  THR E  58      21.775   0.984  67.683  1.00  0.00
E    C
ATOM  27660  HB  THR E  58      21.982   1.889  67.062  1.00  0.00
E    H
ATOM  27661  OG1 THR E  58      20.520   1.175  68.343  1.00  0.00
E    O
ATOM  27662  HG1 THR E  58      19.861   1.195  67.640  1.00  0.00
E    H
ATOM  27663  CG2 THR E  58      21.721  -0.292  66.873  1.00  0.00
E    C
ATOM  27664 HG21 THR E  58      20.988  -0.203  66.044  1.00  0.00
E    H
ATOM  27665 HG22 THR E  58      22.715  -0.511  66.428  1.00  0.00
E    H
ATOM  27666 HG23 THR E  58      21.425  -1.156  67.506  1.00  0.00
E    H
ATOM  27667  C   THR E  58      22.867   2.194  69.583  1.00  0.00
E    C
ATOM  27668  O   THR E  58      22.903   3.309  69.057  1.00  0.00
E    O
ATOM  27669  N   GLU E  59      22.803   2.012  70.901  1.00  0.00
E    N
ATOM  27670  HN  GLU E  59      22.803   1.111  71.327  1.00  0.00
E    H
ATOM  27671  CA  GLU E  59      22.728   3.113  71.846  1.00  0.00
E    C
ATOM  27672  HA  GLU E  59      22.567   4.032  71.296  1.00  0.00
E    H
ATOM  27673  CB  GLU E  59      21.579   2.861  72.825  1.00  0.00
E    C
ATOM  27674  HB1 GLU E  59      20.739   2.391  72.263  1.00  0.00
E    H
```

ATOM 27675 HB2 GLU E 59 21.891 2.114 73.589 1.00 0.00
E H
ATOM 27676 CG GLU E 59 21.055 4.106 73.521 1.00 0.00
E C
ATOM 27677 HG1 GLU E 59 20.243 3.826 74.216 1.00 0.00
E H
ATOM 27678 HG2 GLU E 59 21.862 4.614 74.088 1.00 0.00
E H
ATOM 27679 CD GLU E 59 20.444 5.108 72.558 1.00 0.00
E C
ATOM 27680 OE1 GLU E 59 20.909 6.268 72.537 1.00 0.00
E O
ATOM 27681 OE2 GLU E 59 19.506 4.736 71.816 1.00 0.00
E O
ATOM 27682 C GLU E 59 24.058 3.255 72.590 1.00 0.00
E C
ATOM 27683 O GLU E 59 24.421 2.391 73.395 1.00 0.00
E O
ATOM 27684 N GLU E 60 24.771 4.351 72.323 1.00 0.00
E N
ATOM 27685 HN GLU E 60 24.417 5.112 71.786 1.00 0.00
E H
ATOM 27686 CA GLU E 60 26.153 4.545 72.798 1.00 0.00
E C
ATOM 27687 HA GLU E 60 26.741 3.759 72.341 1.00 0.00
E H
ATOM 27688 CB GLU E 60 26.738 5.867 72.288 1.00 0.00
E C
ATOM 27689 HB1 GLU E 60 26.481 5.966 71.208 1.00 0.00
E H
ATOM 27690 HB2 GLU E 60 26.240 6.722 72.799 1.00 0.00
E H
ATOM 27691 CG GLU E 60 28.248 5.959 72.456 1.00 0.00
E C
ATOM 27692 HG1 GLU E 60 28.527 5.626 73.473 1.00 0.00
E H
ATOM 27693 HG2 GLU E 60 28.770 5.329 71.709 1.00 0.00
E H
ATOM 27694 CD GLU E 60 28.783 7.373 72.336 1.00 0.00
E C
ATOM 27695 OE1 GLU E 60 29.701 7.725 73.109 1.00 0.00
E O
ATOM 27696 OE2 GLU E 60 28.292 8.130 71.469 1.00 0.00
E O
ATOM 27697 C GLU E 60 26.351 4.438 74.315 1.00 0.00
E C
ATOM 27698 O GLU E 60 27.312 3.809 74.771 1.00 0.00
E O
ATOM 27699 N ASN E 61 25.464 5.056 75.089 1.00 0.00
E N
ATOM 27700 HN ASN E 61 24.698 5.602 74.751 1.00 0.00
E H
ATOM 27701 CA ASN E 61 25.555 4.974 76.549 1.00 0.00
E C

```
ATOM  27702  HA  ASN E  61      26.580   5.240  76.784  1.00  0.00
E    H
ATOM  27703  CB  ASN E  61      24.651   6.009  77.241  1.00  0.00
E    C
ATOM  27704  HB1 ASN E  61      24.642   5.779  78.330  1.00  0.00
E    H
ATOM  27705  HB2 ASN E  61      25.069   7.029  77.120  1.00  0.00
E    H
ATOM  27706  CG  ASN E  61      23.211   5.976  76.747  1.00  0.00
E    C
ATOM  27707  OD1 ASN E  61      22.685   4.934  76.351  1.00  0.00
E    O
ATOM  27708  ND2 ASN E  61      22.560   7.132  76.785  1.00  0.00
E    N
ATOM  27709 HD21 ASN E  61      21.612   7.133  76.477  1.00  0.00
E    H
ATOM  27710 HD22 ASN E  61      23.011   7.946  77.139  1.00  0.00
E    H
ATOM  27711  C   ASN E  61      25.308   3.557  77.083  1.00  0.00
E    C
ATOM  27712  O   ASN E  61      25.912   3.148  78.078  1.00  0.00
E    O
ATOM  27713  N   GLU E  62      24.432   2.815  76.406  1.00  0.00
E    N
ATOM  27714  HN  GLU E  62      23.918   3.138  75.617  1.00  0.00
E    H
ATOM  27715  CA  GLU E  62      24.146   1.429  76.774  1.00  0.00
E    C
ATOM  27716  HA  GLU E  62      23.903   1.396  77.829  1.00  0.00
E    H
ATOM  27717  CB  GLU E  62      22.954   0.877  75.992  1.00  0.00
E    C
ATOM  27718  HB1 GLU E  62      23.135   1.060  74.908  1.00  0.00
E    H
ATOM  27719  HB2 GLU E  62      22.905  -0.230  76.111  1.00  0.00
E    H
ATOM  27720  CG  GLU E  62      21.619   1.496  76.379  1.00  0.00
E    C
ATOM  27721  HG1 GLU E  62      21.567   1.605  77.478  1.00  0.00
E    H
ATOM  27722  HG2 GLU E  62      21.491   2.490  75.908  1.00  0.00
E    H
ATOM  27723  CD  GLU E  62      20.426   0.634  76.002  1.00  0.00
E    C
ATOM  27724  OE1 GLU E  62      20.497  -0.599  76.171  1.00  0.00
E    O
ATOM  27725  OE2 GLU E  62      19.404   1.191  75.550  1.00  0.00
E    O
ATOM  27726  C   GLU E  62      25.377   0.561  76.567  1.00  0.00
E    C
ATOM  27727  O   GLU E  62      25.663  -0.311  77.383  1.00  0.00
E    O
ATOM  27728  N   LEU E  63      26.109   0.821  75.484  1.00  0.00
E    N
```

```
ATOM  27729  HN  LEU E  63      25.853   1.493  74.792  1.00  0.00
E    H
ATOM  27730  CA  LEU E  63      27.371   0.131  75.214  1.00  0.00
E    C
ATOM  27731  HA  LEU E  63      27.156  -0.929  75.243  1.00  0.00
E    H
ATOM  27732  CB  LEU E  63      27.926   0.510  73.839  1.00  0.00
E    C
ATOM  27733  HB1 LEU E  63      27.550   1.532  73.603  1.00  0.00
E    H
ATOM  27734  HB2 LEU E  63      29.033   0.598  73.907  1.00  0.00
E    H
ATOM  27735  CG  LEU E  63      27.561  -0.401  72.665  1.00  0.00
E    C
ATOM  27736  HG  LEU E  63      27.924  -1.428  72.916  1.00  0.00
E    H
ATOM  27737  CD1 LEU E  63      26.060  -0.422  72.464  1.00  0.00
E    C
ATOM  27738 HD11 LEU E  63      25.793  -1.045  71.583  1.00  0.00
E    H
ATOM  27739 HD12 LEU E  63      25.551  -0.845  73.356  1.00  0.00
E    H
ATOM  27740 HD13 LEU E  63      25.673   0.605  72.293  1.00  0.00
E    H
ATOM  27741  CD2 LEU E  63      28.258   0.045  71.388  1.00  0.00
E    C
ATOM  27742 HD21 LEU E  63      27.999  -0.636  70.549  1.00  0.00
E    H
ATOM  27743 HD22 LEU E  63      27.956   1.071  71.094  1.00  0.00
E    H
ATOM  27744 HD23 LEU E  63      29.361   0.029  71.521  1.00  0.00
E    H
ATOM  27745  C   LEU E  63      28.405   0.420  76.293  1.00  0.00
E    C
ATOM  27746  O   LEU E  63      29.065  -0.498  76.785  1.00  0.00
E    O
ATOM  27747  N   LEU E  64      28.533   1.694  76.658  1.00  0.00
E    N
ATOM  27748  HN  LEU E  64      28.029   2.448  76.242  1.00  0.00
E    H
ATOM  27749  CA  LEU E  64      29.443   2.098  77.720  1.00  0.00
E    C
ATOM  27750  HA  LEU E  64      30.435   1.779  77.426  1.00  0.00
E    H
ATOM  27751  CB  LEU E  64      29.409   3.617  77.915  1.00  0.00
E    C
ATOM  27752  HB1 LEU E  64      28.337   3.916  77.976  1.00  0.00
E    H
ATOM  27753  HB2 LEU E  64      29.855   3.864  78.904  1.00  0.00
E    H
ATOM  27754  CG  LEU E  64      30.055   4.526  76.862  1.00  0.00
E    C
ATOM  27755  HG  LEU E  64      29.593   4.268  75.880  1.00  0.00
E    H
```

ATOM 27756 CD1 LEU E 64 29.776 5.987 77.194 1.00 0.00
E C
ATOM 27757 HD11 LEU E 64 30.280 6.656 76.464 1.00 0.00
E H
ATOM 27758 HD12 LEU E 64 28.684 6.194 77.161 1.00 0.00
E H
ATOM 27759 HD13 LEU E 64 30.149 6.238 78.209 1.00 0.00
E H
ATOM 27760 CD2 LEU E 64 31.554 4.282 76.756 1.00 0.00
E C
ATOM 27761 HD21 LEU E 64 31.997 4.937 75.975 1.00 0.00
E H
ATOM 27762 HD22 LEU E 64 32.073 4.497 77.711 1.00 0.00
E H
ATOM 27763 HD23 LEU E 64 31.757 3.225 76.477 1.00 0.00
E H
ATOM 27764 C LEU E 64 29.094 1.378 79.018 1.00 0.00
E C
ATOM 27765 O LEU E 64 29.981 0.873 79.704 1.00 0.00
E O
ATOM 27766 N SER E 65 27.796 1.310 79.317 1.00 0.00
E N
ATOM 27767 HN SER E 65 27.090 1.702 78.727 1.00 0.00
E H
ATOM 27768 CA SER E 65 27.277 0.660 80.529 1.00 0.00
E C
ATOM 27769 HA SER E 65 27.789 1.151 81.346 1.00 0.00
E H
ATOM 27770 CB SER E 65 25.767 0.878 80.658 1.00 0.00
E C
ATOM 27771 HB1 SER E 65 25.269 0.363 79.805 1.00 0.00
E H
ATOM 27772 HB2 SER E 65 25.386 0.407 81.593 1.00 0.00
E H
ATOM 27773 OG SER E 65 25.442 2.255 80.611 1.00 0.00
E O
ATOM 27774 HG1 SER E 65 24.488 2.316 80.472 1.00 0.00
E H
ATOM 27775 C SER E 65 27.597 -0.833 80.622 1.00 0.00
E C
ATOM 27776 O SER E 65 27.743 -1.376 81.719 1.00 0.00
E O
ATOM 27777 N LEU E 66 27.700 -1.487 79.469 1.00 0.00
E N
ATOM 27778 HN LEU E 66 27.522 -1.070 78.580 1.00 0.00
E H
ATOM 27779 CA LEU E 66 28.087 -2.893 79.404 1.00 0.00
E C
ATOM 27780 HA LEU E 66 27.501 -3.411 80.153 1.00 0.00
E H
ATOM 27781 CB LEU E 66 27.838 -3.451 77.998 1.00 0.00
E C
ATOM 27782 HB1 LEU E 66 28.470 -2.862 77.294 1.00 0.00
E H

ATOM 27783 HB2 LEU E 66   28.217 -4.495 77.951 1.00 0.00
E   H
ATOM 27784 CG LEU E 66   26.412 -3.427 77.442 1.00 0.00
E   C
ATOM 27785 HG LEU E 66   25.971 -2.457 77.776 1.00 0.00
E   H
ATOM 27786 CD1 LEU E 66   26.422 -3.486 75.925 1.00 0.00
E   C
ATOM 27787 HD11 LEU E 66   25.385 -3.516 75.528 1.00 0.00
E   H
ATOM 27788 HD12 LEU E 66   26.931 -2.593 75.502 1.00 0.00
E   H
ATOM 27789 HD13 LEU E 66   26.956 -4.394 75.571 1.00 0.00
E   H
ATOM 27790 CD2 LEU E 66   25.567 -4.551 78.018 1.00 0.00
E   C
ATOM 27791 HD21 LEU E 66   24.532 -4.502 77.614 1.00 0.00
E   H
ATOM 27792 HD22 LEU E 66   25.981 -5.547 77.762 1.00 0.00
E   H
ATOM 27793 HD23 LEU E 66   25.513 -4.468 79.125 1.00 0.00
E   H
ATOM 27794 C   LEU E 66   29.560 -3.078 79.762 1.00 0.00
E   C
ATOM 27795 O   LEU E 66   29.986 -4.184 80.105 1.00 0.00
E   O
ATOM 27796 N   GLY E 67   30.326 -1.992 79.682 1.00 0.00
E   N
ATOM 27797 HN GLY E 67   29.941 -1.084 79.518 1.00 0.00
E   H
ATOM 27798 CA GLY E 67   31.775 -2.045 79.825 1.00 0.00
E   C
ATOM 27799 HA1 GLY E 67   32.013 -2.683 80.665 1.00 0.00
E   H
ATOM 27800 HA2 GLY E 67   32.153 -1.035 79.913 1.00 0.00
E   H
ATOM 27801 C   GLY E 67   32.381 -2.664 78.581 1.00 0.00
E   C
ATOM 27802 O   GLY E 67   33.304 -3.474 78.663 1.00 0.00
E   O
ATOM 27803 N   ILE E 68   31.847 -2.272 77.428 1.00 0.00
E   N
ATOM 27804 HN ILE E 68   31.180 -1.534 77.360 1.00 0.00
E   H
ATOM 27805 CA ILE E 68   32.190 -2.887 76.151 1.00 0.00
E   C
ATOM 27806 HA ILE E 68   31.988 -3.943 76.279 1.00 0.00
E   H
ATOM 27807 CB ILE E 68   31.290 -2.346 75.016 1.00 0.00
E   C
ATOM 27808 HB ILE E 68   30.250 -2.411 75.427 1.00 0.00
E   H
ATOM 27809 CG2 ILE E 68   31.660 -0.914 74.657 1.00 0.00
E   C

ATOM 27810 HG21 ILE E 68   30.916 -0.481 73.957 1.00 0.00
E   H
ATOM 27811 HG22 ILE E 68   31.683 -0.272 75.563 1.00 0.00
E   H
ATOM 27812 HG23 ILE E 68   32.658 -0.871 74.171 1.00 0.00
E   H
ATOM 27813 CG1 ILE E 68   31.334 -3.266 73.791 1.00 0.00
E   C
ATOM 27814 HG11 ILE E 68   30.834 -2.771 72.930 1.00 0.00
E   H
ATOM 27815 HG12 ILE E 68   32.397 -3.433 73.503 1.00 0.00
E   H
ATOM 27816 CD ILE E 68   30.695 -4.622 74.007 1.00 0.00
E   C
ATOM 27817 HD1 ILE E 68   30.815 -5.253 73.101 1.00 0.00
E   H
ATOM 27818 HD2 ILE E 68   31.172 -5.147 74.863 1.00 0.00
E   H
ATOM 27819 HD3 ILE E 68   29.609 -4.522 74.215 1.00 0.00
E   H
ATOM 27820 C   ILE E 68   33.671 -2.761 75.786 1.00 0.00
E   C
ATOM 27821 O   ILE E 68   34.198 -3.572 75.025 1.00 0.00
E   O
ATOM 27822 N   ARG E 69   34.339 -1.757 76.347 1.00 0.00
E   N
ATOM 27823 HN ARG E 69   33.902 -1.132 76.989 1.00 0.00
E   H
ATOM 27824 CA ARG E 69   35.749 -1.516 76.053 1.00 0.00
E   C
ATOM 27825 HA ARG E 69   35.888 -1.646 74.986 1.00 0.00
E   H
ATOM 27826 CB ARG E 69   36.142 -0.116 76.472 1.00 0.00
E   C
ATOM 27827 HB1 ARG E 69   35.869 -0.020 77.550 1.00 0.00
E   H
ATOM 27828 HB2 ARG E 69   37.248 -0.010 76.424 1.00 0.00
E   H
ATOM 27829 CG ARG E 69   35.439   0.958 75.685 1.00 0.00
E   C
ATOM 27830 HG1 ARG E 69   35.354   0.623 74.628 1.00 0.00
E   H
ATOM 27831 HG2 ARG E 69   34.392   1.050 76.054 1.00 0.00
E   H
ATOM 27832 CD ARG E 69   36.171   2.264 75.867 1.00 0.00
E   C
ATOM 27833 HD1 ARG E 69   36.752   2.233 76.817 1.00 0.00
E   H
ATOM 27834 HD2 ARG E 69   36.875   2.458 75.025 1.00 0.00
E   H
ATOM 27835 NE ARG E 69   35.217   3.354 75.903 1.00 0.00
E   N
ATOM 27836 HE ARG E 69   34.332   3.092 76.304 1.00 0.00
E   H

```
ATOM  27837  CZ  ARG E  69      35.462   4.576  75.459  1.00  0.00
E    C
ATOM  27838  NH1 ARG E  69      36.643   4.882  74.935  1.00  0.00
E    N
ATOM  27839 HH11 ARG E  69      36.834   5.803  74.626  1.00  0.00
E    H
ATOM  27840 HH12 ARG E  69      37.353   4.178  74.999  1.00  0.00
E    H
ATOM  27841  NH2 ARG E  69      34.514   5.496  75.531  1.00  0.00
E    N
ATOM  27842 HH21 ARG E  69      34.658   6.386  75.124  1.00  0.00
E    H
ATOM  27843 HH22 ARG E  69      33.612   5.190  75.809  1.00  0.00
E    H
ATOM  27844  C   ARG E  69      36.672  -2.505  76.733  1.00  0.00
E    C
ATOM  27845  O   ARG E  69      37.808  -2.709  76.298  1.00  0.00
E    O
ATOM  27846  N   ASN E  70      36.184  -3.104  77.815  1.00  0.00
E    N
ATOM  27847  HN  ASN E  70      35.288  -2.891  78.203  1.00  0.00
E    H
ATOM  27848  CA  ASN E  70      36.924  -4.131  78.533  1.00  0.00
E    C
ATOM  27849  HA  ASN E  70      37.919  -3.723  78.683  1.00  0.00
E    H
ATOM  27850  CB  ASN E  70      36.246  -4.435  79.874  1.00  0.00
E    C
ATOM  27851  HB1 ASN E  70      35.210  -4.778  79.661  1.00  0.00
E    H
ATOM  27852  HB2 ASN E  70      36.771  -5.263  80.393  1.00  0.00
E    H
ATOM  27853  CG  ASN E  70      36.162  -3.219  80.777  1.00  0.00
E    C
ATOM  27854  OD1 ASN E  70      36.851  -2.220  80.565  1.00  0.00
E    O
ATOM  27855  ND2 ASN E  70      35.315  -3.299  81.797  1.00  0.00
E    N
ATOM  27856 HD21 ASN E  70      35.240  -2.505  82.393  1.00  0.00
E    H
ATOM  27857 HD22 ASN E  70      34.764  -4.118  81.925  1.00  0.00
E    H
ATOM  27858  C   ASN E  70      37.067  -5.408  77.709  1.00  0.00
E    C
ATOM  27859  O   ASN E  70      37.658  -6.389  78.170  1.00  0.00
E    O
ATOM  27860  N   PHE E  71      36.536  -5.384  76.489  1.00  0.00
E    N
ATOM  27861  HN  PHE E  71      36.104  -4.575  76.093  1.00  0.00
E    H
ATOM  27862  CA  PHE E  71      36.541  -6.553  75.619  1.00  0.00
E    C
ATOM  27863  HA  PHE E  71      36.909  -7.367  76.232  1.00  0.00
E    H
```

```
ATOM  27864  CB  PHE E  71      35.126  -6.874  75.153  1.00  0.00
E    C
ATOM  27865  HB1 PHE E  71      34.644  -5.941  74.787  1.00  0.00
E    H
ATOM  27866  HB2 PHE E  71      35.137  -7.592  74.305  1.00  0.00
E    H
ATOM  27867  CG  PHE E  71      34.251  -7.401  76.247  1.00  0.00
E    C
ATOM  27868  CD1 PHE E  71      33.556  -6.534  77.083  1.00  0.00
E    C
ATOM  27869  HD1 PHE E  71      33.642  -5.466  76.936  1.00  0.00
E    H
ATOM  27870  CE1 PHE E  71      32.760  -7.024  78.105  1.00  0.00
E    C
ATOM  27871  HE1 PHE E  71      32.216  -6.342  78.742  1.00  0.00
E    H
ATOM  27872  CZ  PHE E  71      32.660  -8.389  78.303  1.00  0.00
E    C
ATOM  27873  HZ  PHE E  71      32.034  -8.773  79.096  1.00  0.00
E    H
ATOM  27874  CD2 PHE E  71      34.147  -8.768  76.467  1.00  0.00
E    C
ATOM  27875  HD2 PHE E  71      34.711  -9.458  75.855  1.00  0.00
E    H
ATOM  27876  CE2 PHE E  71      33.349  -9.263  77.482  1.00  0.00
E    C
ATOM  27877  HE2 PHE E  71      33.268 -10.330  77.632  1.00  0.00
E    H
ATOM  27878  C   PHE E  71      37.493  -6.413  74.446  1.00  0.00
E    C
ATOM  27879  O   PHE E  71      37.632  -5.342  73.860  1.00  0.00
E    O
ATOM  27880  N   GLU E  72      38.152  -7.519  74.130  1.00  0.00
E    N
ATOM  27881  HN  GLU E  72      38.008  -8.391  74.590  1.00  0.00
E    H
ATOM  27882  CA  GLU E  72      39.149  -7.567  73.079  1.00  0.00
E    C
ATOM  27883  HA  GLU E  72      39.559  -6.573  72.946  1.00  0.00
E    H
ATOM  27884  CB  GLU E  72      40.233  -8.573  73.453  1.00  0.00
E    C
ATOM  27885  HB1 GLU E  72      40.328  -8.585  74.563  1.00  0.00
E    H
ATOM  27886  HB2 GLU E  72      39.911  -9.599  73.164  1.00  0.00
E    H
ATOM  27887  CG  GLU E  72      41.594  -8.278  72.852  1.00  0.00
E    C
ATOM  27888  HG1 GLU E  72      41.477  -8.018  71.783  1.00  0.00
E    H
ATOM  27889  HG2 GLU E  72      42.087  -7.437  73.379  1.00  0.00
E    H
ATOM  27890  CD  GLU E  72      42.522  -9.478  72.885  1.00  0.00
E    C
```

ATOM 27891 OE1 GLU E 72 42.522 -10.232 73.887 1.00 0.00
E O
ATOM 27892 OE2 GLU E 72 43.264 -9.667 71.900 1.00 0.00
E O
ATOM 27893 C GLU E 72 38.501 -7.968 71.758 1.00 0.00
E C
ATOM 27894 O GLU E 72 38.999 -7.626 70.678 1.00 0.00
E O
ATOM 27895 N TYR E 73 37.399 -8.710 71.858 1.00 0.00
E N
ATOM 27896 HN TYR E 73 37.029 -9.005 72.737 1.00 0.00
E H
ATOM 27897 CA TYR E 73 36.637 -9.157 70.700 1.00 0.00
E C
ATOM 27898 HA TYR E 73 36.967 -8.560 69.859 1.00 0.00
E H
ATOM 27899 CB TYR E 73 36.805 -10.652 70.483 1.00 0.00
E C
ATOM 27900 HB1 TYR E 73 36.649 -11.180 71.449 1.00 0.00
E H
ATOM 27901 HB2 TYR E 73 36.041 -11.034 69.772 1.00 0.00
E H
ATOM 27902 CG TYR E 73 38.165 -11.055 69.996 1.00 0.00
E C
ATOM 27903 CD1 TYR E 73 39.229 -11.188 70.883 1.00 0.00
E C
ATOM 27904 HD1 TYR E 73 39.082 -10.979 71.934 1.00 0.00
E H
ATOM 27905 CE1 TYR E 73 40.481 -11.562 70.440 1.00 0.00
E C
ATOM 27906 HE1 TYR E 73 41.307 -11.644 71.132 1.00 0.00
E H
ATOM 27907 CZ TYR E 73 40.670 -11.825 69.096 1.00 0.00
E C
ATOM 27908 OH TYR E 73 41.908 -12.203 68.641 1.00 0.00
E O
ATOM 27909 HH TYR E 73 41.823 -12.408 67.708 1.00 0.00
E H
ATOM 27910 CD2 TYR E 73 38.385 -11.329 68.653 1.00 0.00
E C
ATOM 27911 HD2 TYR E 73 37.583 -11.209 67.938 1.00 0.00
E H
ATOM 27912 CE2 TYR E 73 39.632 -11.708 68.199 1.00 0.00
E C
ATOM 27913 HE2 TYR E 73 39.787 -11.888 67.145 1.00 0.00
E H
ATOM 27914 C TYR E 73 35.165 -8.877 70.895 1.00 0.00
E C
ATOM 27915 O TYR E 73 34.557 -9.333 71.866 1.00 0.00
E O
ATOM 27916 N VAL E 74 34.589 -8.121 69.970 1.00 0.00
E N
ATOM 27917 HN VAL E 74 35.070 -7.627 69.251 1.00 0.00
E H

ATOM 27918 CA VAL E 74   33.148 -7.958 69.949 1.00 0.00
E   C
ATOM 27919 HA VAL E 74   32.721 -8.574 70.730 1.00 0.00
E   H
ATOM 27920 CB VAL E 74   32.712 -6.506 70.227 1.00 0.00
E   C
ATOM 27921 HB VAL E 74   33.101 -5.872 69.393 1.00 0.00
E   H
ATOM 27922 CG1 VAL E 74   31.195 -6.411 70.267 1.00 0.00
E   C
ATOM 27923 HG11 VAL E 74   30.879 -5.372 70.505 1.00 0.00
E   H
ATOM 27924 HG12 VAL E 74   30.741 -6.678 69.289 1.00 0.00
E   H
ATOM 27925 HG13 VAL E 74   30.780 -7.084 71.046 1.00 0.00
E   H
ATOM 27926 CG2 VAL E 74   33.286 -6.021 71.549 1.00 0.00
E   C
ATOM 27927 HG21 VAL E 74   32.942 -4.989 71.773 1.00 0.00
E   H
ATOM 27928 HG22 VAL E 74   32.965 -6.685 72.381 1.00 0.00
E   H
ATOM 27929 HG23 VAL E 74   34.396 -6.006 71.524 1.00 0.00
E   H
ATOM 27930 C   VAL E 74   32.616 -8.473 68.620 1.00 0.00
E   C
ATOM 27931 O   VAL E 74   33.114 -8.109 67.553 1.00 0.00
E   O
ATOM 27932 N   ILE E 75   31.627 -9.357 68.707 1.00 0.00
E   N
ATOM 27933 HN ILE E 75   31.270 -9.693 69.575 1.00 0.00
E   H
ATOM 27934 CA ILE E 75   30.971 -9.917 67.537 1.00 0.00
E   C
ATOM 27935 HA ILE E 75   31.569 -9.661 66.672 1.00 0.00
E   H
ATOM 27936 CB ILE E 75   30.702 -11.422 67.713 1.00 0.00
E   C
ATOM 27937 HB ILE E 75   29.928 -11.475 68.520 1.00 0.00
E   H
ATOM 27938 CG2 ILE E 75   30.149 -12.030 66.431 1.00 0.00
E   C
ATOM 27939 HG21 ILE E 75   29.812 -13.073 66.607 1.00 0.00
E   H
ATOM 27940 HG22 ILE E 75   29.277 -11.452 66.063 1.00 0.00
E   H
ATOM 27941 HG23 ILE E 75   30.922 -12.046 65.634 1.00 0.00
E   H
ATOM 27942 CG1 ILE E 75   31.947 -12.147 68.252 1.00 0.00
E   C
ATOM 27943 HG11 ILE E 75   32.318 -11.630 69.165 1.00 0.00
E   H
ATOM 27944 HG12 ILE E 75   31.661 -13.181 68.552 1.00 0.00
E   H

ATOM 27945 CD ILE E 75   33.091 -12.259 67.271 1.00 0.00
E   C
ATOM 27946 HD1 ILE E 75   33.926 -12.842 67.716 1.00 0.00
E   H
ATOM 27947 HD2 ILE E 75   32.763 -12.773 66.342 1.00 0.00
E   H
ATOM 27948 HD3 ILE E 75   33.482 -11.255 66.998 1.00 0.00
E   H
ATOM 27949 C   ILE E 75   29.638 -9.229 67.365 1.00 0.00
E   C
ATOM 27950 O   ILE E 75   28.806 -9.237 68.275 1.00 0.00
E   O
ATOM 27951 N   VAL E 76   29.431 -8.627 66.205 1.00 0.00
E   N
ATOM 27952 HN VAL E 76   30.100 -8.559 65.469 1.00 0.00
E   H
ATOM 27953 CA VAL E 76   28.152 -7.996 65.916 1.00 0.00
E   C
ATOM 27954 HA VAL E 76   27.616 -7.848 66.845 1.00 0.00
E   H
ATOM 27955 CB VAL E 76   28.296 -6.641 65.206 1.00 0.00
E   C
ATOM 27956 HB VAL E 76   28.656 -6.841 64.167 1.00 0.00
E   H
ATOM 27957 CG1 VAL E 76   26.949 -5.942 65.150 1.00 0.00
E   C
ATOM 27958 HG11 VAL E 76   27.053 -4.944 64.673 1.00 0.00
E   H
ATOM 27959 HG12 VAL E 76   26.212 -6.518 64.555 1.00 0.00
E   H
ATOM 27960 HG13 VAL E 76   26.542 -5.793 66.174 1.00 0.00
E   H
ATOM 27961 CG2 VAL E 76   29.291 -5.765 65.937 1.00 0.00
E   C
ATOM 27962 HG21 VAL E 76   29.370 -4.770 65.451 1.00 0.00
E   H
ATOM 27963 HG22 VAL E 76   28.978 -5.616 66.993 1.00 0.00
E   H
ATOM 27964 HG23 VAL E 76   30.304 -6.220 65.935 1.00 0.00
E   H
ATOM 27965 C   VAL E 76   27.342 -8.956 65.072 1.00 0.00
E   C
ATOM 27966 O   VAL E 76   27.591 -9.130 63.874 1.00 0.00
E   O
ATOM 27967 N   ALA E 77   26.381 -9.597 65.720 1.00 0.00
E   N
ATOM 27968 HN ALA E 77   26.157 -9.428 66.679 1.00 0.00
E   H
ATOM 27969 CA ALA E 77   25.577 -10.604 65.061 1.00 0.00
E   C
ATOM 27970 HA ALA E 77   25.702 -10.642 63.985 1.00 0.00
E   H
ATOM 27971 CB ALA E 77   25.901 -11.994 65.594 1.00 0.00
E   C

```
ATOM  27972  HB1 ALA E  77      26.941 -12.267  65.314  1.00  0.00
E    H
ATOM  27973  HB2 ALA E  77      25.827 -12.023  66.703  1.00  0.00
E    H
ATOM  27974  HB3 ALA E  77      25.219 -12.763  65.173  1.00  0.00
E    H
ATOM  27975  C   ALA E  77      24.163 -10.119  65.312  1.00  0.00
E    C
ATOM  27976  O   ALA E  77      23.470 -10.587  66.221  1.00  0.00
E    O
ATOM  27977  N   ILE E  78      23.753  -9.150  64.511  1.00  0.00
E    N
ATOM  27978  HN  ILE E  78      24.350  -8.534  64.000  1.00  0.00
E    H
ATOM  27979  CA  ILE E  78      22.350  -8.907  64.315  1.00  0.00
E    C
ATOM  27980  HA  ILE E  78      21.742  -9.710  64.712  1.00  0.00
E    H
ATOM  27981  CB  ILE E  78      21.870  -7.511  64.692  1.00  0.00
E    C
ATOM  27982  HB  ILE E  78      22.470  -6.824  64.042  1.00  0.00
E    H
ATOM  27983  CG2 ILE E  78      20.373  -7.413  64.456  1.00  0.00
E    C
ATOM  27984 HG21 ILE E  78      20.027  -6.365  64.573  1.00  0.00
E    H
ATOM  27985 HG22 ILE E  78      20.112  -7.740  63.428  1.00  0.00
E    H
ATOM  27986 HG23 ILE E  78      19.815  -8.046  65.178  1.00  0.00
E    H
ATOM  27987  CG1 ILE E  78      22.250  -7.203  66.137  1.00  0.00
E    C
ATOM  27988 HG11 ILE E  78      21.367  -7.350  66.798  1.00  0.00
E    H
ATOM  27989 HG12 ILE E  78      23.031  -7.926  66.466  1.00  0.00
E    H
ATOM  27990  CD  ILE E  78      22.785  -5.804  66.318  1.00  0.00
E    C
ATOM  27991  HD1 ILE E  78      23.101  -5.646  67.372  1.00  0.00
E    H
ATOM  27992  HD2 ILE E  78      23.664  -5.632  65.662  1.00  0.00
E    H
ATOM  27993  HD3 ILE E  78      22.009  -5.046  66.075  1.00  0.00
E    H
ATOM  27994  C   ILE E  78      22.332  -9.048  62.810  1.00  0.00
E    C
ATOM  27995  O   ILE E  78      23.060  -8.343  62.087  1.00  0.00
E    O
ATOM  27996  N   GLY E  79      21.528  -9.988  62.345  1.00  0.00
E    N
ATOM  27997  HN  GLY E  79      20.926 -10.543  62.917  1.00  0.00
E    H
ATOM  27998  CA  GLY E  79      21.479 -10.267  60.938  1.00  0.00
E    C
```

```
ATOM  27999  HA1 GLY E  79      21.008 -11.232  60.811  1.00  0.00
E    H
ATOM  28000  HA2 GLY E  79      22.488 -10.210  60.552  1.00  0.00
E    H
ATOM  28001  C   GLY E  79      20.643  -9.239  60.212  1.00  0.00
E    C
ATOM  28002  O   GLY E  79      21.168  -8.356  59.530  1.00  0.00
E    O
ATOM  28003  N   ALA E  80      19.334  -9.349  60.398  1.00  0.00
E    N
ATOM  28004  HN  ALA E  80      18.946  -9.836  61.180  1.00  0.00
E    H
ATOM  28005  CA  ALA E  80      18.365  -8.770  59.481  1.00  0.00
E    C
ATOM  28006  HA  ALA E  80      18.509  -9.192  58.493  1.00  0.00
E    H
ATOM  28007  CB  ALA E  80      16.959  -9.157  59.908  1.00  0.00
E    C
ATOM  28008  HB1 ALA E  80      16.879 -10.263  59.965  1.00  0.00
E    H
ATOM  28009  HB2 ALA E  80      16.719  -8.743  60.911  1.00  0.00
E    H
ATOM  28010  HB3 ALA E  80      16.198  -8.794  59.184  1.00  0.00
E    H
ATOM  28011  C   ALA E  80      18.491  -7.261  59.326  1.00  0.00
E    C
ATOM  28012  O   ALA E  80      18.544  -6.743  58.214  1.00  0.00
E    O
ATOM  28013  N   ASN E  81      18.551  -6.569  60.454  1.00  0.00
E    N
ATOM  28014  HN  ASN E  81      18.593  -7.004  61.353  1.00  0.00
E    H
ATOM  28015  CA  ASN E  81      18.561  -5.120  60.487  1.00  0.00
E    C
ATOM  28016  HA  ASN E  81      17.827  -4.807  59.752  1.00  0.00
E    H
ATOM  28017  CB  ASN E  81      18.205  -4.661  61.899  1.00  0.00
E    C
ATOM  28018  HB1 ASN E  81      17.392  -5.318  62.277  1.00  0.00
E    H
ATOM  28019  HB2 ASN E  81      19.075  -4.786  62.576  1.00  0.00
E    H
ATOM  28020  CG  ASN E  81      17.709  -3.230  61.949  1.00  0.00
E    C
ATOM  28021  OD1 ASN E  81      18.037  -2.410  61.089  1.00  0.00
E    O
ATOM  28022  ND2 ASN E  81      16.920  -2.915  62.981  1.00  0.00
E    N
ATOM  28023 HD21 ASN E  81      16.574  -1.982  63.025  1.00  0.00
E    H
ATOM  28024 HD22 ASN E  81      16.675  -3.608  63.651  1.00  0.00
E    H
ATOM  28025  C   ASN E  81      19.912  -4.551  60.061  1.00  0.00
E    C
```

ATOM 28026 O ASN E 81 20.805 -4.373 60.887 1.00 0.00
E O
ATOM 28027 N ILE E 82 20.056 -4.263 58.771 1.00 0.00
E N
ATOM 28028 HN ILE E 82 19.343 -4.390 58.085 1.00 0.00
E H
ATOM 28029 CA ILE E 82 21.315 -3.718 58.240 1.00 0.00
E C
ATOM 28030 HA ILE E 82 22.081 -4.435 58.507 1.00 0.00
E H
ATOM 28031 CB ILE E 82 21.295 -3.586 56.700 1.00 0.00
E C
ATOM 28032 HB ILE E 82 20.339 -3.047 56.477 1.00 0.00
E H
ATOM 28033 CG2 ILE E 82 22.545 -2.882 56.196 1.00 0.00
E C
ATOM 28034 HG21 ILE E 82 22.455 -2.651 55.115 1.00 0.00
E H
ATOM 28035 HG22 ILE E 82 22.702 -1.923 56.733 1.00 0.00
E H
ATOM 28036 HG23 ILE E 82 23.445 -3.516 56.342 1.00 0.00
E H
ATOM 28037 CG1 ILE E 82 21.188 -4.966 56.051 1.00 0.00
E C
ATOM 28038 HG11 ILE E 82 22.101 -5.561 56.279 1.00 0.00
E H
ATOM 28039 HG12 ILE E 82 20.323 -5.508 56.497 1.00 0.00
E H
ATOM 28040 CD ILE E 82 20.993 -4.931 54.551 1.00 0.00
E C
ATOM 28041 HD1 ILE E 82 20.865 -5.961 54.154 1.00 0.00
E H
ATOM 28042 HD2 ILE E 82 20.087 -4.342 54.288 1.00 0.00
E H
ATOM 28043 HD3 ILE E 82 21.869 -4.474 54.046 1.00 0.00
E H
ATOM 28044 C ILE E 82 21.704 -2.390 58.896 1.00 0.00
E C
ATOM 28045 O ILE E 82 22.881 -2.157 59.167 1.00 0.00
E O
ATOM 28046 N GLN E 83 20.718 -1.532 59.150 1.00 0.00
E N
ATOM 28047 HN GLN E 83 19.763 -1.711 58.920 1.00 0.00
E H
ATOM 28048 CA GLN E 83 20.953 -0.244 59.796 1.00 0.00
E C
ATOM 28049 HA GLN E 83 21.680 0.288 59.194 1.00 0.00
E H
ATOM 28050 CB GLN E 83 19.652 0.551 59.906 1.00 0.00
E C
ATOM 28051 HB1 GLN E 83 19.366 0.847 58.869 1.00 0.00
E H
ATOM 28052 HB2 GLN E 83 18.840 -0.119 60.272 1.00 0.00
E H

```
ATOM  28053  CG  GLN E  83      19.761   1.799  60.778  1.00  0.00
E    C
ATOM  28054  HG1 GLN E  83      20.363   1.585  61.687  1.00  0.00
E    H
ATOM  28055  HG2 GLN E  83      20.259   2.622  60.226  1.00  0.00
E    H
ATOM  28056  CD  GLN E  83      18.419   2.310  61.280  1.00  0.00
E    C
ATOM  28057  OE1 GLN E  83      17.372   2.032  60.688  1.00  0.00
E    O
ATOM  28058  NE2 GLN E  83      18.447   3.075  62.374  1.00  0.00
E    N
ATOM  28059 HE21 GLN E  83      17.572   3.410  62.711  1.00  0.00
E    H
ATOM  28060 HE22 GLN E  83      19.317   3.297  62.801  1.00  0.00
E    H
ATOM  28061  C   GLN E  83      21.556  -0.436  61.179  1.00  0.00
E    C
ATOM  28062  O   GLN E  83      22.609   0.129  61.482  1.00  0.00
E    O
ATOM  28063  N   ALA E  84      20.881  -1.233  62.004  1.00  0.00
E    N
ATOM  28064  HN  ALA E  84      20.033  -1.686  61.728  1.00  0.00
E    H
ATOM  28065  CA  ALA E  84      21.322  -1.507  63.366  1.00  0.00
E    C
ATOM  28066  HA  ALA E  84      21.364  -0.594  63.949  1.00  0.00
E    H
ATOM  28067  CB  ALA E  84      20.360  -2.460  64.057  1.00  0.00
E    C
ATOM  28068  HB1 ALA E  84      19.347  -2.005  64.094  1.00  0.00
E    H
ATOM  28069  HB2 ALA E  84      20.283  -3.421  63.503  1.00  0.00
E    H
ATOM  28070  HB3 ALA E  84      20.677  -2.677  65.099  1.00  0.00
E    H
ATOM  28071  C   ALA E  84      22.732  -2.082  63.364  1.00  0.00
E    C
ATOM  28072  O   ALA E  84      23.585  -1.646  64.143  1.00  0.00
E    O
ATOM  28073  N   SER E  85      22.969  -3.039  62.468  1.00  0.00
E    N
ATOM  28074  HN  SER E  85      22.290  -3.348  61.802  1.00  0.00
E    H
ATOM  28075  CA  SER E  85      24.245  -3.724  62.375  1.00  0.00
E    C
ATOM  28076  HA  SER E  85      24.405  -4.181  63.343  1.00  0.00
E    H
ATOM  28077  CB  SER E  85      24.203  -4.793  61.280  1.00  0.00
E    C
ATOM  28078  HB1 SER E  85      23.411  -5.530  61.546  1.00  0.00
E    H
ATOM  28079  HB2 SER E  85      23.913  -4.339  60.306  1.00  0.00
E    H
```

ATOM 28080 OG   SER E 85   25.439 -5.474 61.177 1.00 0.00
E   O
ATOM 28081 HG1 SER E 85   25.285 -6.264 60.645 1.00 0.00
E   H
ATOM 28082 C   SER E 85   25.392 -2.752 62.139 1.00 0.00
E   C
ATOM 28083 O   SER E 85   26.410 -2.810 62.821 1.00 0.00
E   O
ATOM 28084 N   THR E 86   25.225 -1.847 61.183 1.00 0.00
E   N
ATOM 28085 HN THR E 86   24.384 -1.707 60.660 1.00 0.00
E   H
ATOM 28086 CA THR E 86   26.314 -0.951 60.815 1.00 0.00
E   C
ATOM 28087 HA THR E 86   27.220 -1.540 60.869 1.00 0.00
E   H
ATOM 28088 CB THR E 86   26.160 -0.361 59.393 1.00 0.00
E   C
ATOM 28089 HB THR E 86   27.136   0.126 59.152 1.00 0.00
E   H
ATOM 28090 OG1 THR E 86   25.151   0.652 59.398 1.00 0.00
E   O
ATOM 28091 HG1 THR E 86   25.098   0.966 58.488 1.00 0.00
E   H
ATOM 28092 CG2 THR E 86   25.782 -1.430 58.392 1.00 0.00
E   C
ATOM 28093 HG21 THR E 86   25.784 -1.023 57.359 1.00 0.00
E   H
ATOM 28094 HG22 THR E 86   26.508 -2.270 58.429 1.00 0.00
E   H
ATOM 28095 HG23 THR E 86   24.769 -1.835 58.602 1.00 0.00
E   H
ATOM 28096 C   THR E 86   26.459   0.165 61.844 1.00 0.00
E   C
ATOM 28097 O   THR E 86   27.572   0.594 62.153 1.00 0.00
E   O
ATOM 28098 N   LEU E 87   25.331   0.625 62.375 1.00 0.00
E   N
ATOM 28099 HN LEU E 87   24.417   0.324 62.112 1.00 0.00
E   H
ATOM 28100 CA LEU E 87   25.348   1.640 63.422 1.00 0.00
E   C
ATOM 28101 HA LEU E 87   25.892   2.492 63.032 1.00 0.00
E   H
ATOM 28102 CB LEU E 87   23.922   2.076 63.787 1.00 0.00
E   C
ATOM 28103 HB1 LEU E 87   23.447   2.461 62.855 1.00 0.00
E   H
ATOM 28104 HB2 LEU E 87   23.335   1.179 64.085 1.00 0.00
E   H
ATOM 28105 CG LEU E 87   23.688   3.155 64.856 1.00 0.00
E   C
ATOM 28106 HG LEU E 87   23.898   2.653 65.831 1.00 0.00
E   H

```
ATOM  28107  CD1 LEU E  87      24.597   4.357  64.693  1.00  0.00
E    C
ATOM  28108 HD11 LEU E  87      24.362   5.131  65.455  1.00  0.00
E    H
ATOM  28109 HD12 LEU E  87      25.662   4.061  64.812  1.00  0.00
E    H
ATOM  28110 HD13 LEU E  87      24.470   4.812  63.687  1.00  0.00
E    H
ATOM  28111  CD2 LEU E  87      22.230   3.588  64.855  1.00  0.00
E    C
ATOM  28112 HD21 LEU E  87      22.054   4.355  65.640  1.00  0.00
E    H
ATOM  28113 HD22 LEU E  87      21.935   4.029  63.881  1.00  0.00
E    H
ATOM  28114 HD23 LEU E  87      21.566   2.720  65.061  1.00  0.00
E    H
ATOM  28115  C   LEU E  87      26.111   1.126  64.645  1.00  0.00
E    C
ATOM  28116  O   LEU E  87      26.942   1.847  65.204  1.00  0.00
E    O
ATOM  28117  N   THR E  88      25.838  -0.123  65.031  1.00  0.00
E    N
ATOM  28118  HN  THR E  88      25.132  -0.689  64.606  1.00  0.00
E    H
ATOM  28119  CA  THR E  88      26.558  -0.780  66.125  1.00  0.00
E    C
ATOM  28120  HA  THR E  88      26.460  -0.131  66.985  1.00  0.00
E    H
ATOM  28121  CB  THR E  88      26.007  -2.190  66.421  1.00  0.00
E    C
ATOM  28122  HB  THR E  88      26.001  -2.732  65.443  1.00  0.00
E    H
ATOM  28123  OG1 THR E  88      24.655  -2.090  66.881  1.00  0.00
E    O
ATOM  28124  HG1 THR E  88      24.377  -2.996  67.057  1.00  0.00
E    H
ATOM  28125  CG2 THR E  88      26.841  -2.882  67.486  1.00  0.00
E    C
ATOM  28126 HG21 THR E  88      26.518  -3.936  67.619  1.00  0.00
E    H
ATOM  28127 HG22 THR E  88      27.914  -2.888  67.195  1.00  0.00
E    H
ATOM  28128 HG23 THR E  88      26.750  -2.364  68.465  1.00  0.00
E    H
ATOM  28129  C   THR E  88      28.048  -0.855  65.805  1.00  0.00
E    C
ATOM  28130  O   THR E  88      28.881  -0.388  66.592  1.00  0.00
E    O
ATOM  28131  N   THR E  89      28.375  -1.421  64.646  1.00  0.00
E    N
ATOM  28132  HN  THR E  89      27.714  -1.806  64.003  1.00  0.00
E    H
ATOM  28133  CA  THR E  89      29.766  -1.525  64.212  1.00  0.00
E    C
```

ATOM 28134 HA THR E 89   30.265 -2.155 64.936 1.00 0.00
E   H
ATOM 28135 CB THR E 89   29.884 -2.080 62.779 1.00 0.00
E   C
ATOM 28136 HB THR E 89   29.334 -1.362 62.123 1.00 0.00
E   H
ATOM 28137 OG1 THR E 89   29.227 -3.344 62.703 1.00 0.00
E   O
ATOM 28138 HG1 THR E 89   29.345 -3.641 61.794 1.00 0.00
E   H
ATOM 28139 CG2 THR E 89   31.337 -2.264 62.389 1.00 0.00
E   C
ATOM 28140 HG21 THR E 89   31.426 -2.555 61.321 1.00 0.00
E   H
ATOM 28141 HG22 THR E 89   31.900 -1.318 62.532 1.00 0.00
E   H
ATOM 28142 HG23 THR E 89   31.820 -3.053 63.004 1.00 0.00
E   H
ATOM 28143 C   THR E 89   30.439 -0.158 64.289 1.00 0.00
E   C
ATOM 28144 O   THR E 89   31.554 -0.038 64.792 1.00 0.00
E   O
ATOM 28145 N   LEU E 90   29.734   0.863 63.802 1.00 0.00
E   N
ATOM 28146 HN LEU E 90   28.817   0.764 63.421 1.00 0.00
E   H
ATOM 28147 CA LEU E 90   30.245   2.222 63.784 1.00 0.00
E   C
ATOM 28148 HA LEU E 90   31.151   2.208 63.190 1.00 0.00
E   H
ATOM 28149 CB LEU E 90   29.214   3.159 63.164 1.00 0.00
E   C
ATOM 28150 HB1 LEU E 90   28.760   2.623 62.298 1.00 0.00
E   H
ATOM 28151 HB2 LEU E 90   28.388   3.321 63.892 1.00 0.00
E   H
ATOM 28152 CG LEU E 90   29.732   4.506 62.665 1.00 0.00
E   C
ATOM 28153 HG LEU E 90   29.774   5.198 63.541 1.00 0.00
E   H
ATOM 28154 CD1 LEU E 90   31.107   4.393 62.026 1.00 0.00
E   C
ATOM 28155 HD11 LEU E 90   31.436   5.378 61.631 1.00 0.00
E   H
ATOM 28156 HD12 LEU E 90   31.858   4.048 62.769 1.00 0.00
E   H
ATOM 28157 HD13 LEU E 90   31.090   3.670 61.183 1.00 0.00
E   H
ATOM 28158 CD2 LEU E 90   28.747   5.072 61.668 1.00 0.00
E   C
ATOM 28159 HD21 LEU E 90   29.093   6.063 61.304 1.00 0.00
E   H
ATOM 28160 HD22 LEU E 90   28.631   4.411 60.786 1.00 0.00
E   H

```
ATOM  28161 HD23 LEU E  90      27.749   5.205  62.140  1.00  0.00
E    H
ATOM  28162  C   LEU E  90      30.623   2.682  65.188  1.00  0.00
E    C
ATOM  28163  O   LEU E  90      31.736   3.166  65.412  1.00  0.00
E    O
ATOM  28164  N   LEU E  91      29.695   2.504  66.128  1.00  0.00
E    N
ATOM  28165  HN  LEU E  91      28.787   2.138  65.938  1.00  0.00
E    H
ATOM  28166  CA  LEU E  91      29.928   2.831  67.531  1.00  0.00
E    C
ATOM  28167  HA  LEU E  91      30.189   3.880  67.556  1.00  0.00
E    H
ATOM  28168  CB  LEU E  91      28.668   2.574  68.355  1.00  0.00
E    C
ATOM  28169  HB1 LEU E  91      28.404   1.499  68.225  1.00  0.00
E    H
ATOM  28170  HB2 LEU E  91      28.908   2.704  69.433  1.00  0.00
E    H
ATOM  28171  CG  LEU E  91      27.414   3.387  68.031  1.00  0.00
E    C
ATOM  28172  HG  LEU E  91      27.214   3.251  66.941  1.00  0.00
E    H
ATOM  28173  CD1 LEU E  91      26.241   2.854  68.834  1.00  0.00
E    C
ATOM  28174 HD11 LEU E  91      25.331   3.464  68.648  1.00  0.00
E    H
ATOM  28175 HD12 LEU E  91      26.020   1.802  68.552  1.00  0.00
E    H
ATOM  28176 HD13 LEU E  91      26.464   2.886  69.922  1.00  0.00
E    H
ATOM  28177  CD2 LEU E  91      27.612   4.874  68.290  1.00  0.00
E    C
ATOM  28178 HD21 LEU E  91      26.691   5.437  68.029  1.00  0.00
E    H
ATOM  28179 HD22 LEU E  91      27.840   5.077  69.356  1.00  0.00
E    H
ATOM  28180 HD23 LEU E  91      28.448   5.268  67.672  1.00  0.00
E    H
ATOM  28181  C   LEU E  91      31.096   2.052  68.125  1.00  0.00
E    C
ATOM  28182  O   LEU E  91      31.913   2.617  68.849  1.00  0.00
E    O
ATOM  28183  N   LEU E  92      31.173   0.758  67.820  1.00  0.00
E    N
ATOM  28184  HN  LEU E  92      30.515   0.278  67.243  1.00  0.00
E    H
ATOM  28185  CA  LEU E  92      32.256  -0.080  68.320  1.00  0.00
E    C
ATOM  28186  HA  LEU E  92      32.232   0.011  69.399  1.00  0.00
E    H
ATOM  28187  CB  LEU E  92      32.036  -1.543  67.940  1.00  0.00
E    C
```

ATOM 28188 HB1 LEU E 92   31.880 -1.577 66.837 1.00 0.00
E   H
ATOM 28189 HB2 LEU E 92   32.972 -2.113 68.135 1.00 0.00
E   H
ATOM 28190 CG LEU E 92   30.857 -2.256 68.603 1.00 0.00
E   C
ATOM 28191 HG LEU E 92   29.929 -1.701 68.325 1.00 0.00
E   H
ATOM 28192 CD1 LEU E 92   30.777 -3.675 68.080 1.00 0.00
E   C
ATOM 28193 HD11 LEU E 92   29.956 -4.230 68.583 1.00 0.00
E   H
ATOM 28194 HD12 LEU E 92   30.582 -3.679 66.987 1.00 0.00
E   H
ATOM 28195 HD13 LEU E 92   31.728 -4.217 68.269 1.00 0.00
E   H
ATOM 28196 CD2 LEU E 92   30.972 -2.251 70.118 1.00 0.00
E   C
ATOM 28197 HD21 LEU E 92   30.095 -2.758 70.574 1.00 0.00
E   H
ATOM 28198 HD22 LEU E 92   31.883 -2.781 70.461 1.00 0.00
E   H
ATOM 28199 HD23 LEU E 92   31.009 -1.209 70.501 1.00 0.00
E   H
ATOM 28200 C   LEU E 92   33.629   0.394 67.848 1.00 0.00
E   C
ATOM 28201 O   LEU E 92   34.598   0.346 68.604 1.00 0.00
E   O
ATOM 28202 N   LYS E 93   33.702   0.863 66.606 1.00 0.00
E   N
ATOM 28203 HN LYS E 93   32.920   0.903 65.985 1.00 0.00
E   H
ATOM 28204 CA LYS E 93   34.953   1.362 66.057 1.00 0.00
E   C
ATOM 28205 HA LYS E 93   35.723   0.634 66.285 1.00 0.00
E   H
ATOM 28206 CB LYS E 93   34.850   1.504 64.533 1.00 0.00
E   C
ATOM 28207 HB1 LYS E 93   34.515   0.519 64.133 1.00 0.00
E   H
ATOM 28208 HB2 LYS E 93   34.043   2.231 64.291 1.00 0.00
E   H
ATOM 28209 CG LYS E 93   36.141   1.899 63.828 1.00 0.00
E   C
ATOM 28210 HG1 LYS E 93   35.929   2.068 62.748 1.00 0.00
E   H
ATOM 28211 HG2 LYS E 93   36.504   2.864 64.251 1.00 0.00
E   H
ATOM 28212 CD LYS E 93   37.237   0.851 63.938 1.00 0.00
E   C
ATOM 28213 HD1 LYS E 93   37.362   0.584 65.013 1.00 0.00
E   H
ATOM 28214 HD2 LYS E 93   36.895 -0.066 63.404 1.00 0.00
E   H

```
ATOM  28215  CE  LYS E  93      38.534   1.380  63.342  1.00  0.00
E    C
ATOM  28216  HE1 LYS E  93      38.453   1.456  62.235  1.00  0.00
E    H
ATOM  28217  HE2 LYS E  93      38.767   2.389  63.747  1.00  0.00
E    H
ATOM  28218  NZ  LYS E  93      39.702   0.510  63.637  1.00  0.00
E    N
ATOM  28219  HZ1 LYS E  93      40.562   0.910  63.210  1.00  0.00
E    H
ATOM  28220  HZ2 LYS E  93      39.830   0.436  64.667  1.00  0.00
E    H
ATOM  28221  HZ3 LYS E  93      39.533  -0.440  63.245  1.00  0.00
E    H
ATOM  28222  C   LYS E  93      35.348   2.674  66.741  1.00  0.00
E    C
ATOM  28223  O   LYS E  93      36.538   2.949  66.929  1.00  0.00
E    O
ATOM  28224  N   GLU E  94      34.348   3.466  67.133  1.00  0.00
E    N
ATOM  28225  HN  GLU E  94      33.388   3.280  66.945  1.00  0.00
E    H
ATOM  28226  CA  GLU E  94      34.578   4.702  67.889  1.00  0.00
E    C
ATOM  28227  HA  GLU E  94      35.299   5.293  67.338  1.00  0.00
E    H
ATOM  28228  CB  GLU E  94      33.284   5.493  68.046  1.00  0.00
E    C
ATOM  28229  HB1 GLU E  94      32.453   4.769  68.214  1.00  0.00
E    H
ATOM  28230  HB2 GLU E  94      33.334   6.120  68.964  1.00  0.00
E    H
ATOM  28231  CG  GLU E  94      32.945   6.360  66.848  1.00  0.00
E    C
ATOM  28232  HG1 GLU E  94      33.761   7.085  66.675  1.00  0.00
E    H
ATOM  28233  HG2 GLU E  94      32.804   5.743  65.939  1.00  0.00
E    H
ATOM  28234  CD  GLU E  94      31.694   7.191  67.067  1.00  0.00
E    C
ATOM  28235  OE1 GLU E  94      30.743   7.047  66.266  1.00  0.00
E    O
ATOM  28236  OE2 GLU E  94      31.655   7.981  68.043  1.00  0.00
E    O
ATOM  28237  C   GLU E  94      35.170   4.438  69.268  1.00  0.00
E    C
ATOM  28238  O   GLU E  94      36.005   5.205  69.746  1.00  0.00
E    O
ATOM  28239  N   LEU E  95      34.737   3.346  69.899  1.00  0.00
E    N
ATOM  28240  HN  LEU E  95      34.087   2.702  69.501  1.00  0.00
E    H
ATOM  28241  CA  LEU E  95      35.185   2.994  71.246  1.00  0.00
E    C
```

ATOM 28242 HA LEU E 95 35.345 3.933 71.760 1.00 0.00
E H
ATOM 28243 CB LEU E 95 34.093 2.212 71.987 1.00 0.00
E C
ATOM 28244 HB1 LEU E 95 33.922 1.268 71.420 1.00 0.00
E H
ATOM 28245 HB2 LEU E 95 34.481 1.906 72.984 1.00 0.00
E H
ATOM 28246 CG LEU E 95 32.743 2.932 72.145 1.00 0.00
E C
ATOM 28247 HG LEU E 95 32.475 3.275 71.117 1.00 0.00
E H
ATOM 28248 CD1 LEU E 95 31.675 1.986 72.668 1.00 0.00
E C
ATOM 28249 HD11 LEU E 95 30.717 2.527 72.823 1.00 0.00
E H
ATOM 28250 HD12 LEU E 95 31.496 1.161 71.946 1.00 0.00
E H
ATOM 28251 HD13 LEU E 95 31.985 1.541 73.638 1.00 0.00
E H
ATOM 28252 CD2 LEU E 95 32.842 4.157 73.038 1.00 0.00
E C
ATOM 28253 HD21 LEU E 95 31.854 4.661 73.113 1.00 0.00
E H
ATOM 28254 HD22 LEU E 95 33.163 3.890 74.065 1.00 0.00
E H
ATOM 28255 HD23 LEU E 95 33.572 4.884 72.621 1.00 0.00
E H
ATOM 28256 C LEU E 95 36.508 2.233 71.253 1.00 0.00
E C
ATOM 28257 O LEU E 95 36.922 1.712 72.284 1.00 0.00
E O
ATOM 28258 N ASP E 96 37.160 2.185 70.092 1.00 0.00
E N
ATOM 28259 HN ASP E 96 36.783 2.544 69.242 1.00 0.00
E H
ATOM 28260 CA ASP E 96 38.501 1.604 69.926 1.00 0.00
E C
ATOM 28261 HA ASP E 96 38.749 1.780 68.886 1.00 0.00
E H
ATOM 28262 CB ASP E 96 39.542 2.364 70.762 1.00 0.00
E C
ATOM 28263 HB1 ASP E 96 39.052 2.776 71.667 1.00 0.00
E H
ATOM 28264 HB2 ASP E 96 40.356 1.686 71.084 1.00 0.00
E H
ATOM 28265 CG ASP E 96 40.152 3.522 70.012 1.00 0.00
E C
ATOM 28266 OD1 ASP E 96 39.985 4.672 70.467 1.00 0.00
E O
ATOM 28267 OD2 ASP E 96 40.796 3.288 68.968 1.00 0.00
E O
ATOM 28268 C ASP E 96 38.628 0.094 70.161 1.00 0.00
E C

ATOM 28269 O ASP E 96 39.721 -0.393 70.481 1.00 0.00
E O
ATOM 28270 N ILE E 97 37.528 -0.643 69.991 1.00 0.00
E N
ATOM 28271 HN ILE E 97 36.627 -0.273 69.771 1.00 0.00
E H
ATOM 28272 CA ILE E 97 37.562 -2.102 70.120 1.00 0.00
E C
ATOM 28273 HA ILE E 97 37.897 -2.320 71.126 1.00 0.00
E H
ATOM 28274 CB ILE E 97 36.172 -2.731 69.863 1.00 0.00
E C
ATOM 28275 HB ILE E 97 35.910 -2.415 68.822 1.00 0.00
E H
ATOM 28276 CG2 ILE E 97 36.222 -4.257 69.975 1.00 0.00
E C
ATOM 28277 HG21 ILE E 97 35.264 -4.705 69.637 1.00 0.00
E H
ATOM 28278 HG22 ILE E 97 37.031 -4.674 69.340 1.00 0.00
E H
ATOM 28279 HG23 ILE E 97 36.402 -4.573 71.024 1.00 0.00
E H
ATOM 28280 CG1 ILE E 97 35.105 -2.119 70.790 1.00 0.00
E C
ATOM 28281 HG11 ILE E 97 34.962 -1.044 70.540 1.00 0.00
E H
ATOM 28282 HG12 ILE E 97 34.133 -2.631 70.607 1.00 0.00
E H
ATOM 28283 CD ILE E 97 35.404 -2.226 72.272 1.00 0.00
E C
ATOM 28284 HD1 ILE E 97 34.560 -1.814 72.866 1.00 0.00
E H
ATOM 28285 HD2 ILE E 97 35.551 -3.288 72.566 1.00 0.00
E H
ATOM 28286 HD3 ILE E 97 36.321 -1.657 72.536 1.00 0.00
E H
ATOM 28287 C ILE E 97 38.612 -2.690 69.165 1.00 0.00
E C
ATOM 28288 O ILE E 97 38.619 -2.373 67.977 1.00 0.00
E O
ATOM 28289 N PRO E 98 39.525 -3.517 69.692 1.00 0.00
E N
ATOM 28290 CD PRO E 98 39.659 -3.861 71.120 1.00 0.00
E C
ATOM 28291 HD1 PRO E 98 39.587 -2.799 70.793 1.00 0.00
E H
ATOM 28292 HD2 PRO E 98 39.144 -4.002 72.099 1.00 0.00
E H
ATOM 28293 CA PRO E 98 40.604 -4.095 68.888 1.00 0.00
E C
ATOM 28294 HA PRO E 98 41.192 -3.294 68.454 1.00 0.00
E H
ATOM 28295 CB PRO E 98 41.433 -4.880 69.916 1.00 0.00
E C

ATOM 28296 HB1 PRO E 98 42.157 -4.189 70.402 1.00 0.00
E H
ATOM 28297 HB2 PRO E 98 41.990 -5.725 69.463 1.00 0.00
E H
ATOM 28298 CG PRO E 98 41.096 -4.267 71.234 1.00 0.00
E C
ATOM 28299 HG1 PRO E 98 41.881 -4.427 71.999 1.00 0.00
E H
ATOM 28300 HG2 PRO E 98 41.157 -3.544 71.922 1.00 0.00
E H
ATOM 28301 C PRO E 98 40.124 -5.026 67.768 1.00 0.00
E C
ATOM 28302 O PRO E 98 40.664 -4.971 66.664 1.00 0.00
E O
ATOM 28303 N ASN E 99 39.137 -5.876 68.050 1.00 0.00
E N
ATOM 28304 HN ASN E 99 38.695 -5.950 68.942 1.00 0.00
E H
ATOM 28305 CA ASN E 99 38.615 -6.793 67.036 1.00 0.00
E C
ATOM 28306 HA ASN E 99 39.009 -6.424 66.095 1.00 0.00
E H
ATOM 28307 CB ASN E 99 39.103 -8.221 67.267 1.00 0.00
E C
ATOM 28308 HB1 ASN E 99 38.737 -8.548 68.265 1.00 0.00
E H
ATOM 28309 HB2 ASN E 99 38.667 -8.905 66.511 1.00 0.00
E H
ATOM 28310 CG ASN E 99 40.611 -8.327 67.263 1.00 0.00
E C
ATOM 28311 OD1 ASN E 99 41.255 -8.078 68.278 1.00 0.00
E O
ATOM 28312 ND2 ASN E 99 41.182 -8.700 66.123 1.00 0.00
E N
ATOM 28313 HD21 ASN E 99 42.175 -8.784 66.114 1.00 0.00
E H
ATOM 28314 HD22 ASN E 99 40.620 -8.912 65.329 1.00 0.00
E H
ATOM 28315 C ASN E 99 37.099 -6.778 66.936 1.00 0.00
E C
ATOM 28316 O ASN E 99 36.401 -7.181 67.876 1.00 0.00
E O
ATOM 28317 N ILE E 100 36.604 -6.314 65.788 1.00 0.00
E N
ATOM 28318 HN ILE E 100 37.173 -5.971 65.044 1.00 0.00
E H
ATOM 28319 CA ILE E 100 35.176 -6.266 65.518 1.00 0.00
E C
ATOM 28320 HA ILE E 100 34.679 -6.665 66.393 1.00 0.00
E H
ATOM 28321 CB ILE E 100 34.707 -4.833 65.225 1.00 0.00
E C
ATOM 28322 HB ILE E 100 35.342 -4.501 64.364 1.00 0.00
E H

```
ATOM  28323  CG2 ILE E 100      33.210  -4.810  64.948  1.00  0.00
E    C
ATOM  28324 HG21 ILE E 100      32.896  -3.809  64.585  1.00  0.00
E    H
ATOM  28325 HG22 ILE E 100      32.940  -5.552  64.168  1.00  0.00
E    H
ATOM  28326 HG23 ILE E 100      32.633  -5.041  65.868  1.00  0.00
E    H
ATOM  28327  CG1 ILE E 100      35.041  -3.919  66.407  1.00  0.00
E    C
ATOM  28328 HG11 ILE E 100      34.278  -4.046  67.208  1.00  0.00
E    H
ATOM  28329 HG12 ILE E 100      36.022  -4.227  66.835  1.00  0.00
E    H
ATOM  28330  CD  ILE E 100      35.132  -2.447  66.056  1.00  0.00
E    C
ATOM  28331  HD1 ILE E 100      35.433  -1.856  66.947  1.00  0.00
E    H
ATOM  28332  HD2 ILE E 100      35.886  -2.281  65.257  1.00  0.00
E    H
ATOM  28333  HD3 ILE E 100      34.153  -2.061  65.702  1.00  0.00
E    H
ATOM  28334  C   ILE E 100      34.829  -7.189  64.353  1.00  0.00
E    C
ATOM  28335  O   ILE E 100      35.248  -6.961  63.212  1.00  0.00
E    O
ATOM  28336  N   TRP E 101      34.076  -8.244  64.662  1.00  0.00
E    N
ATOM  28337  HN  TRP E 101      33.780  -8.425  65.596  1.00  0.00
E    H
ATOM  28338  CA  TRP E 101      33.621  -9.219  63.667  1.00  0.00
E    C
ATOM  28339  HA  TRP E 101      34.095  -8.984  62.723  1.00  0.00
E    H
ATOM  28340  CB  TRP E 101      33.934 -10.636  64.121  1.00  0.00
E    C
ATOM  28341  HB1 TRP E 101      33.516 -10.767  65.144  1.00  0.00
E    H
ATOM  28342  HB2 TRP E 101      33.414 -11.366  63.464  1.00  0.00
E    H
ATOM  28343  CG  TRP E 101      35.410 -10.951  64.195  1.00  0.00
E    C
ATOM  28344  CD1 TRP E 101      36.466 -10.063  64.386  1.00  0.00
E    C
ATOM  28345  HD1 TRP E 101      36.331  -8.999  64.530  1.00  0.00
E    H
ATOM  28346  NE1 TRP E 101      37.666 -10.720  64.405  1.00  0.00
E    N
ATOM  28347  HE1 TRP E 101      38.531 -10.291  64.548  1.00  0.00
E    H
ATOM  28348  CE2 TRP E 101      37.483 -12.049  64.247  1.00  0.00
E    C
ATOM  28349  CD2 TRP E 101      36.039 -12.275  64.110  1.00  0.00
E    C
```

```
ATOM  28350  CE3 TRP E 101      35.572 -13.569  63.939  1.00  0.00
E    C
ATOM  28351  HE3 TRP E 101      34.518 -13.786  63.829  1.00  0.00
E    H
ATOM  28352  CZ3 TRP E 101      36.505 -14.614  63.902  1.00  0.00
E    C
ATOM  28353  HZ3 TRP E 101      36.160 -15.630  63.766  1.00  0.00
E    H
ATOM  28354  CZ2 TRP E 101      38.397 -13.091  64.214  1.00  0.00
E    C
ATOM  28355  HZ2 TRP E 101      39.461 -12.945  64.320  1.00  0.00
E    H
ATOM  28356  CH2 TRP E 101      37.882 -14.378  64.034  1.00  0.00
E    C
ATOM  28357  HH2 TRP E 101      38.572 -15.209  63.996  1.00  0.00
E    H
ATOM  28358  C   TRP E 101      32.151  -9.076  63.461  1.00  0.00
E    C
ATOM  28359  O   TRP E 101      31.380  -9.092  64.422  1.00  0.00
E    O
ATOM  28360  N   VAL E 102      31.744  -8.931  62.204  1.00  0.00
E    N
ATOM  28361  HN  VAL E 102      32.344  -8.995  61.411  1.00  0.00
E    H
ATOM  28362  CA  VAL E 102      30.348  -8.660  61.885  1.00  0.00
E    C
ATOM  28363  HA  VAL E 102      29.776  -8.797  62.795  1.00  0.00
E    H
ATOM  28364  CB  VAL E 102      30.154  -7.226  61.342  1.00  0.00
E    C
ATOM  28365  HB  VAL E 102      30.528  -7.210  60.289  1.00  0.00
E    H
ATOM  28366  CG1 VAL E 102      28.685  -6.848  61.357  1.00  0.00
E    C
ATOM  28367 HG11 VAL E 102      28.552  -5.802  61.003  1.00  0.00
E    H
ATOM  28368 HG12 VAL E 102      28.086  -7.502  60.689  1.00  0.00
E    H
ATOM  28369 HG13 VAL E 102      28.272  -6.918  62.386  1.00  0.00
E    H
ATOM  28370  CG2 VAL E 102      30.952  -6.222  62.159  1.00  0.00
E    C
ATOM  28371 HG21 VAL E 102      30.779  -5.189  61.791  1.00  0.00
E    H
ATOM  28372 HG22 VAL E 102      30.656  -6.267  63.230  1.00  0.00
E    H
ATOM  28373 HG23 VAL E 102      32.041  -6.424  62.090  1.00  0.00
E    H
ATOM  28374  C   VAL E 102      29.812  -9.647  60.857  1.00  0.00
E    C
ATOM  28375  O   VAL E 102      30.511 -10.013  59.908  1.00  0.00
E    O
ATOM  28376  N   LYS E 103      28.571 -10.082  61.060  1.00  0.00
E    N
```

ATOM 28377 HN LYS E 103   28.057 -9.902 61.897 1.00 0.00
E   H
ATOM 28378 CA LYS E 103   27.865 -10.863 60.058 1.00 0.00
E   C
ATOM 28379 HA LYS E 103   28.587 -11.505 59.568 1.00 0.00
E   H
ATOM 28380 CB LYS E 103   26.761 -11.699 60.708 1.00 0.00
E   C
ATOM 28381 HB1 LYS E 103   27.260 -12.437 61.377 1.00 0.00
E   H
ATOM 28382 HB2 LYS E 103   26.150 -11.043 61.367 1.00 0.00
E   H
ATOM 28383 CG LYS E 103   25.874 -12.446 59.722 1.00 0.00
E   C
ATOM 28384 HG1 LYS E 103   25.421 -11.716 59.013 1.00 0.00
E   H
ATOM 28385 HG2 LYS E 103   26.505 -13.143 59.123 1.00 0.00
E   H
ATOM 28386 CD LYS E 103   24.781 -13.193 60.458 1.00 0.00
E   C
ATOM 28387 HD1 LYS E 103   25.136 -14.230 60.657 1.00 0.00
E   H
ATOM 28388 HD2 LYS E 103   24.626 -12.698 61.444 1.00 0.00
E   H
ATOM 28389 CE LYS E 103   23.483 -13.216 59.675 1.00 0.00
E   C
ATOM 28390 HE1 LYS E 103   22.614 -13.080 60.357 1.00 0.00
E   H
ATOM 28391 HE2 LYS E 103   23.468 -12.400 58.919 1.00 0.00
E   H
ATOM 28392 NZ LYS E 103   23.326 -14.506 58.972 1.00 0.00
E   N
ATOM 28393 HZ1 LYS E 103   22.433 -14.515 58.438 1.00 0.00
E   H
ATOM 28394 HZ2 LYS E 103   24.121 -14.644 58.316 1.00 0.00
E   H
ATOM 28395 HZ3 LYS E 103   23.319 -15.279 59.668 1.00 0.00
E   H
ATOM 28396 C   LYS E 103   27.286 -9.928 58.990 1.00 0.00
E   C
ATOM 28397 O   LYS E 103   26.550 -8.984 59.305 1.00 0.00
E   O
ATOM 28398 N   ALA E 104   27.628 -10.193 57.734 1.00 0.00
E   N
ATOM 28399 HN ALA E 104   28.246 -10.937 57.483 1.00 0.00
E   H
ATOM 28400 CA ALA E 104   27.116 -9.400 56.619 1.00 0.00
E   C
ATOM 28401 HA ALA E 104   26.867 -8.397 56.946 1.00 0.00
E   H
ATOM 28402 CB ALA E 104   28.159 -9.287 55.525 1.00 0.00
E   C
ATOM 28403 HB1 ALA E 104   29.072 -8.801 55.929 1.00 0.00
E   H

ATOM 28404 HB2 ALA E 104 28.446 -10.291 55.142 1.00 0.00
E H
ATOM 28405 HB3 ALA E 104 27.794 -8.675 54.673 1.00 0.00
E H
ATOM 28406 C ALA E 104 25.832 -10.002 56.065 1.00 0.00
E C
ATOM 28407 O ALA E 104 25.715 -11.227 55.939 1.00 0.00
E O
ATOM 28408 N GLN E 105 24.877 -9.141 55.730 1.00 0.00
E N
ATOM 28409 HN GLN E 105 24.970 -8.151 55.818 1.00 0.00
E H
ATOM 28410 CA GLN E 105 23.596 -9.580 55.197 1.00 0.00
E C
ATOM 28411 HA GLN E 105 23.385 -10.537 55.659 1.00 0.00
E H
ATOM 28412 CB GLN E 105 22.500 -8.595 55.561 1.00 0.00
E C
ATOM 28413 HB1 GLN E 105 22.860 -7.585 55.256 1.00 0.00
E H
ATOM 28414 HB2 GLN E 105 21.597 -8.803 54.942 1.00 0.00
E H
ATOM 28415 CG GLN E 105 22.175 -8.607 57.037 1.00 0.00
E C
ATOM 28416 HG1 GLN E 105 23.052 -8.267 57.629 1.00 0.00
E H
ATOM 28417 HG2 GLN E 105 21.330 -7.923 57.259 1.00 0.00
E H
ATOM 28418 CD GLN E 105 21.830 -10.003 57.527 1.00 0.00
E C
ATOM 28419 OE1 GLN E 105 20.978 -10.688 56.959 1.00 0.00
E O
ATOM 28420 NE2 GLN E 105 22.494 -10.429 58.591 1.00 0.00
E N
ATOM 28421 HE21 GLN E 105 22.278 -11.345 58.919 1.00 0.00
E H
ATOM 28422 HE22 GLN E 105 23.158 -9.834 59.031 1.00 0.00
E H
ATOM 28423 C GLN E 105 23.633 -9.795 53.700 1.00 0.00
E C
ATOM 28424 O GLN E 105 23.058 -10.758 53.190 1.00 0.00
E O
ATOM 28425 N ASN E 106 24.322 -8.904 52.999 1.00 0.00
E N
ATOM 28426 HN ASN E 106 24.809 -8.139 53.418 1.00 0.00
E H
ATOM 28427 CA ASN E 106 24.426 -8.963 51.552 1.00 0.00
E C
ATOM 28428 HA ASN E 106 24.514 -10.014 51.300 1.00 0.00
E H
ATOM 28429 CB ASN E 106 23.188 -8.334 50.905 1.00 0.00
E C
ATOM 28430 HB1 ASN E 106 23.360 -8.299 49.806 1.00 0.00
E H

ATOM 28431 HB2 ASN E 106   22.296 -8.969 51.083 1.00 0.00
E   H
ATOM 28432 CG ASN E 106   22.923 -6.910 51.384 1.00 0.00
E   C
ATOM 28433 OD1 ASN E 106   23.845 -6.138 51.645 1.00 0.00
E   O
ATOM 28434 ND2 ASN E 106   21.646 -6.557 51.492 1.00 0.00
E   N
ATOM 28435 HD21 ASN E 106   21.453 -5.628 51.793 1.00 0.00
E   H
ATOM 28436 HD22 ASN E 106   20.930 -7.202 51.247 1.00 0.00
E   H
ATOM 28437 C   ASN E 106   25.694 -8.268 51.078 1.00 0.00
E   C
ATOM 28438 O   ASN E 106   26.511 -7.833 51.892 1.00 0.00
E   O
ATOM 28439 N   TYR E 107   25.848 -8.152 49.764 1.00 0.00
E   N
ATOM 28440 HN TYR E 107   25.197 -8.507 49.095 1.00 0.00
E   H
ATOM 28441 CA TYR E 107   27.009 -7.481 49.183 1.00 0.00
E   C
ATOM 28442 HA TYR E 107   27.877 -8.031 49.526 1.00 0.00
E   H
ATOM 28443 CB TYR E 107   26.945 -7.504 47.652 1.00 0.00
E   C
ATOM 28444 HB1 TYR E 107   27.051 -8.553 47.300 1.00 0.00
E   H
ATOM 28445 HB2 TYR E 107   25.959 -7.135 47.295 1.00 0.00
E   H
ATOM 28446 CG TYR E 107   28.049 -6.706 46.994 1.00 0.00
E   C
ATOM 28447 CD1 TYR E 107   29.334 -7.231 46.865 1.00 0.00
E   C
ATOM 28448 HD1 TYR E 107   29.553 -8.218 47.248 1.00 0.00
E   H
ATOM 28449 CE1 TYR E 107   30.351 -6.500 46.273 1.00 0.00
E   C
ATOM 28450 HE1 TYR E 107   31.345 -6.913 46.182 1.00 0.00
E   H
ATOM 28451 CZ TYR E 107   30.088 -5.222 45.799 1.00 0.00
E   C
ATOM 28452 OH TYR E 107   31.092 -4.487 45.209 1.00 0.00
E   O
ATOM 28453 HH TYR E 107   30.702 -3.672 44.886 1.00 0.00
E   H
ATOM 28454 CD2 TYR E 107   27.810 -5.415 46.510 1.00 0.00
E   C
ATOM 28455 HD2 TYR E 107   26.835 -4.965 46.632 1.00 0.00
E   H
ATOM 28456 CE2 TYR E 107   28.821 -4.673 45.916 1.00 0.00
E   C
ATOM 28457 HE2 TYR E 107   28.618 -3.670 45.571 1.00 0.00
E   H

ATOM 28458 C TYR E 107 27.159 -6.042 49.685 1.00 0.00
E C
ATOM 28459 O TYR E 107 28.250 -5.624 50.076 1.00 0.00
E O
ATOM 28460 N TYR E 108 26.058 -5.295 49.656 1.00 0.00
E N
ATOM 28461 HN TYR E 108 25.179 -5.644 49.336 1.00 0.00
E H
ATOM 28462 CA TYR E 108 26.035 -3.901 50.090 1.00 0.00
E C
ATOM 28463 HA TYR E 108 26.754 -3.380 49.469 1.00 0.00
E H
ATOM 28464 CB TYR E 108 24.622 -3.329 49.939 1.00 0.00
E C
ATOM 28465 HB1 TYR E 108 24.504 -2.916 48.913 1.00 0.00
E H
ATOM 28466 HB2 TYR E 108 23.860 -4.128 50.061 1.00 0.00
E H
ATOM 28467 CG TYR E 108 24.306 -2.192 50.899 1.00 0.00
E C
ATOM 28468 CD1 TYR E 108 24.913 -0.941 50.751 1.00 0.00
E C
ATOM 28469 HD1 TYR E 108 25.629 -0.782 49.957 1.00 0.00
E H
ATOM 28470 CE1 TYR E 108 24.631 0.103 51.623 1.00 0.00
E C
ATOM 28471 HE1 TYR E 108 25.113 1.063 51.504 1.00 0.00
E H
ATOM 28472 CZ TYR E 108 23.729 -0.093 52.658 1.00 0.00
E C
ATOM 28473 OH TYR E 108 23.454 0.948 53.516 1.00 0.00
E O
ATOM 28474 HH TYR E 108 22.764 0.654 54.114 1.00 0.00
E H
ATOM 28475 CD2 TYR E 108 23.398 -2.367 51.957 1.00 0.00
E C
ATOM 28476 HD2 TYR E 108 22.943 -3.333 52.124 1.00 0.00
E H
ATOM 28477 CE2 TYR E 108 23.111 -1.323 52.830 1.00 0.00
E C
ATOM 28478 HE2 TYR E 108 22.426 -1.482 53.649 1.00 0.00
E H
ATOM 28479 C TYR E 108 26.494 -3.744 51.534 1.00 0.00
E C
ATOM 28480 O TYR E 108 27.281 -2.848 51.853 1.00 0.00
E O
ATOM 28481 N HSE E 109 25.976 -4.612 52.398 1.00 0.00
E N
ATOM 28482 HN HSE E 109 25.352 -5.345 52.129 1.00 0.00
E H
ATOM 28483 CA HSE E 109 26.274 -4.571 53.811 1.00 0.00
E C
ATOM 28484 HA HSE E 109 26.055 -3.572 54.169 1.00 0.00
E H

ATOM 28485 CB HSE E 109 25.433 -5.614 54.534 1.00 0.00
E C
ATOM 28486 HB1 HSE E 109 24.381 -5.502 54.191 1.00 0.00
E H
ATOM 28487 HB2 HSE E 109 25.766 -6.630 54.237 1.00 0.00
E H
ATOM 28488 ND1 HSE E 109 25.117 -6.504 56.842 1.00 0.00
E N
ATOM 28489 CG HSE E 109 25.443 -5.483 56.032 1.00 0.00
E C
ATOM 28490 CE1 HSE E 109 25.213 -6.113 58.122 1.00 0.00
E C
ATOM 28491 HE1 HSE E 109 24.990 -6.748 58.982 1.00 0.00
E H
ATOM 28492 NE2 HSE E 109 25.616 -4.836 58.133 1.00 0.00
E N
ATOM 28493 HE2 HSE E 109 25.757 -4.281 58.952 1.00 0.00
E H
ATOM 28494 CD2 HSE E 109 25.768 -4.418 56.859 1.00 0.00
E C
ATOM 28495 HD2 HSE E 109 26.080 -3.415 56.606 1.00 0.00
E H
ATOM 28496 C HSE E 109 27.742 -4.796 54.003 1.00 0.00
E C
ATOM 28497 O HSE E 109 28.388 -4.085 54.769 1.00 0.00
E O
ATOM 28498 N HSE E 110 28.289 -5.761 53.267 1.00 0.00
E N
ATOM 28499 HN HSE E 110 27.765 -6.350 52.652 1.00 0.00
E H
ATOM 28500 CA HSE E 110 29.724 -6.045 53.297 1.00 0.00
E C
ATOM 28501 HA HSE E 110 29.959 -6.377 54.301 1.00 0.00
E H
ATOM 28502 CB HSE E 110 30.068 -7.130 52.277 1.00 0.00
E C
ATOM 28503 HB1 HSE E 110 29.407 -8.002 52.470 1.00 0.00
E H
ATOM 28504 HB2 HSE E 110 29.836 -6.764 51.255 1.00 0.00
E H
ATOM 28505 ND1 HSE E 110 31.842 -8.760 52.930 1.00 0.00
E N
ATOM 28506 CG HSE E 110 31.497 -7.601 52.355 1.00 0.00
E C
ATOM 28507 CE1 HSE E 110 33.174 -8.920 52.860 1.00 0.00
E C
ATOM 28508 HE1 HSE E 110 33.717 -9.781 53.259 1.00 0.00
E H
ATOM 28509 NE2 HSE E 110 33.688 -7.857 52.235 1.00 0.00
E N
ATOM 28510 HE2 HSE E 110 34.660 -7.700 52.061 1.00 0.00
E H
ATOM 28511 CD2 HSE E 110 32.681 -7.021 51.905 1.00 0.00
E C

ATOM 28512 HD2 HSE E 110 32.843 -6.082 51.394 1.00 0.00
E H
ATOM 28513 C HSE E 110 30.532 -4.800 53.043 1.00 0.00
E C
ATOM 28514 O HSE E 110 31.483 -4.505 53.774 1.00 0.00
E O
ATOM 28515 N LYS E 111 30.139 -4.059 52.011 1.00 0.00
E N
ATOM 28516 HN LYS E 111 29.318 -4.283 51.488 1.00 0.00
E H
ATOM 28517 CA LYS E 111 30.861 -2.867 51.556 1.00 0.00
E C
ATOM 28518 HA LYS E 111 31.860 -3.212 51.320 1.00 0.00
E H
ATOM 28519 CB LYS E 111 30.220 -2.331 50.276 1.00 0.00
E C
ATOM 28520 HB1 LYS E 111 29.140 -2.166 50.495 1.00 0.00
E H
ATOM 28521 HB2 LYS E 111 30.649 -1.330 50.048 1.00 0.00
E H
ATOM 28522 CG LYS E 111 30.346 -3.256 49.075 1.00 0.00
E C
ATOM 28523 HG1 LYS E 111 30.653 -4.268 49.425 1.00 0.00
E H
ATOM 28524 HG2 LYS E 111 29.348 -3.360 48.591 1.00 0.00
E H
ATOM 28525 CD LYS E 111 31.368 -2.753 48.069 1.00 0.00
E C
ATOM 28526 HD1 LYS E 111 31.095 -3.148 47.064 1.00 0.00
E H
ATOM 28527 HD2 LYS E 111 31.292 -1.642 48.023 1.00 0.00
E H
ATOM 28528 CE LYS E 111 32.792 -3.157 48.422 1.00 0.00
E C
ATOM 28529 HE1 LYS E 111 32.996 -2.964 49.498 1.00 0.00
E H
ATOM 28530 HE2 LYS E 111 32.952 -4.240 48.223 1.00 0.00
E H
ATOM 28531 NZ LYS E 111 33.769 -2.378 47.610 1.00 0.00
E N
ATOM 28532 HZ1 LYS E 111 34.740 -2.657 47.857 1.00 0.00
E H
ATOM 28533 HZ2 LYS E 111 33.604 -2.558 46.599 1.00 0.00
E H
ATOM 28534 HZ3 LYS E 111 33.644 -1.362 47.799 1.00 0.00
E H
ATOM 28535 C LYS E 111 30.998 -1.743 52.599 1.00 0.00
E C
ATOM 28536 O LYS E 111 32.091 -1.203 52.768 1.00 0.00
E O
ATOM 28537 N VAL E 112 29.905 -1.387 53.283 1.00 0.00
E N
ATOM 28538 HN VAL E 112 28.998 -1.774 53.142 1.00 0.00
E H

```
ATOM  28539  CA  VAL E 112      29.970  -0.356  54.333  1.00  0.00
E    C
ATOM  28540  HA  VAL E 112      30.502   0.489  53.915  1.00  0.00
E    H
ATOM  28541  CB  VAL E 112      28.591   0.202  54.782  1.00  0.00
E    C
ATOM  28542  HB  VAL E 112      28.761   0.827  55.694  1.00  0.00
E    H
ATOM  28543  CG1 VAL E 112      27.982   1.063  53.698  1.00  0.00
E    C
ATOM  28544 HG11 VAL E 112      26.980   1.426  54.012  1.00  0.00
E    H
ATOM  28545 HG12 VAL E 112      28.606   1.957  53.485  1.00  0.00
E    H
ATOM  28546 HG13 VAL E 112      27.859   0.484  52.759  1.00  0.00
E    H
ATOM  28547  CG2 VAL E 112      27.625  -0.903  55.177  1.00  0.00
E    C
ATOM  28548 HG21 VAL E 112      26.637  -0.480  55.455  1.00  0.00
E    H
ATOM  28549 HG22 VAL E 112      27.478  -1.612  54.334  1.00  0.00
E    H
ATOM  28550 HG23 VAL E 112      28.003  -1.474  56.049  1.00  0.00
E    H
ATOM  28551  C   VAL E 112      30.752  -0.831  55.549  1.00  0.00
E    C
ATOM  28552  O   VAL E 112      31.506  -0.055  56.126  1.00  0.00
E    O
ATOM  28553  N   LEU E 113      30.568  -2.099  55.929  1.00  0.00
E    N
ATOM  28554  HN  LEU E 113      29.935  -2.727  55.480  1.00  0.00
E    H
ATOM  28555  CA  LEU E 113      31.296  -2.685  57.057  1.00  0.00
E    C
ATOM  28556  HA  LEU E 113      31.106  -2.061  57.920  1.00  0.00
E    H
ATOM  28557  CB  LEU E 113      30.820  -4.116  57.329  1.00  0.00
E    C
ATOM  28558  HB1 LEU E 113      30.898  -4.677  56.370  1.00  0.00
E    H
ATOM  28559  HB2 LEU E 113      31.531  -4.605  58.031  1.00  0.00
E    H
ATOM  28560  CG  LEU E 113      29.387  -4.275  57.854  1.00  0.00
E    C
ATOM  28561  HG  LEU E 113      28.758  -3.618  57.205  1.00  0.00
E    H
ATOM  28562  CD1 LEU E 113      28.913  -5.715  57.718  1.00  0.00
E    C
ATOM  28563 HD11 LEU E 113      27.894  -5.832  58.143  1.00  0.00
E    H
ATOM  28564 HD12 LEU E 113      28.882  -6.017  56.649  1.00  0.00
E    H
ATOM  28565 HD13 LEU E 113      29.596  -6.405  58.258  1.00  0.00
E    H
```

ATOM 28566 CD2 LEU E 113   29.262 -3.780 59.290 1.00 0.00
E   C
ATOM 28567 HD21 LEU E 113   28.213 -3.887 59.645 1.00 0.00
E   H
ATOM 28568 HD22 LEU E 113   29.910 -4.356 59.980 1.00 0.00
E   H
ATOM 28569 HD23 LEU E 113   29.544 -2.708 59.357 1.00 0.00
E   H
ATOM 28570 C   LEU E 113   32.801 -2.649 56.782 1.00 0.00
E   C
ATOM 28571 O   LEU E 113   33.599 -2.315 57.658 1.00 0.00
E   O
ATOM 28572 N   GLU E 114   33.171 -2.968 55.551 1.00 0.00
E   N
ATOM 28573 HN GLU E 114   32.533 -3.301 54.861 1.00 0.00
E   H
ATOM 28574 CA GLU E 114   34.545 -2.862 55.084 1.00 0.00
E   C
ATOM 28575 HA GLU E 114   35.150 -3.554 55.657 1.00 0.00
E   H
ATOM 28576 CB GLU E 114   34.580 -3.197 53.594 1.00 0.00
E   C
ATOM 28577 HB1 GLU E 114   33.811 -3.980 53.396 1.00 0.00
E   H
ATOM 28578 HB2 GLU E 114   34.271 -2.308 53.000 1.00 0.00
E   H
ATOM 28579 CG GLU E 114   35.900 -3.703 53.046 1.00 0.00
E   C
ATOM 28580 HG1 GLU E 114   36.590 -2.852 52.903 1.00 0.00
E   H
ATOM 28581 HG2 GLU E 114   36.362 -4.436 53.736 1.00 0.00
E   H
ATOM 28582 CD GLU E 114   35.724 -4.345 51.680 1.00 0.00
E   C
ATOM 28583 OE1 GLU E 114   35.327 -3.636 50.724 1.00 0.00
E   O
ATOM 28584 OE2 GLU E 114   35.975 -5.566 51.560 1.00 0.00
E   O
ATOM 28585 C   GLU E 114   35.109 -1.455 55.326 1.00 0.00
E   C
ATOM 28586 O   GLU E 114   36.199 -1.309 55.871 1.00 0.00
E   O
ATOM 28587 N   LYS E 115   34.347 -0.430 54.946 1.00 0.00
E   N
ATOM 28588 HN LYS E 115   33.432 -0.559 54.566 1.00 0.00
E   H
ATOM 28589 CA LYS E 115   34.784   0.962 55.052 1.00 0.00
E   C
ATOM 28590 HA LYS E 115   35.822   0.963 54.743 1.00 0.00
E   H
ATOM 28591 CB LYS E 115   33.989   1.847 54.088 1.00 0.00
E   C
ATOM 28592 HB1 LYS E 115   32.909   1.631 54.259 1.00 0.00
E   H

ATOM 28593 HB2 LYS E 115   34.143   2.914 54.366 1.00 0.00
E   H
ATOM 28594 CG LYS E 115   34.306   1.629 52.615 1.00 0.00
E   C
ATOM 28595 HG1 LYS E 115   34.257   0.539 52.389 1.00 0.00
E   H
ATOM 28596 HG2 LYS E 115   33.526   2.132 51.998 1.00 0.00
E   H
ATOM 28597 CD LYS E 115   35.688   2.153 52.257 1.00 0.00
E   C
ATOM 28598 HD1 LYS E 115   35.650   3.266 52.243 1.00 0.00
E   H
ATOM 28599 HD2 LYS E 115   36.393   1.853 53.066 1.00 0.00
E   H
ATOM 28600 CE LYS E 115   36.165   1.616 50.917 1.00 0.00
E   C
ATOM 28601 HE1 LYS E 115   37.246   1.835 50.772 1.00 0.00
E   H
ATOM 28602 HE2 LYS E 115   36.018   0.514 50.863 1.00 0.00
E   H
ATOM 28603 NZ LYS E 115   35.428   2.222 49.778 1.00 0.00
E   N
ATOM 28604 HZ1 LYS E 115   35.779   1.834 48.879 1.00 0.00
E   H
ATOM 28605 HZ2 LYS E 115   34.413   2.013 49.871 1.00 0.00
E   H
ATOM 28606 HZ3 LYS E 115   35.567   3.253 49.786 1.00 0.00
E   H
ATOM 28607 C   LYS E 115   34.728   1.545 56.466 1.00 0.00
E   C
ATOM 28608 O   LYS E 115   35.537   2.406 56.805 1.00 0.00
E   O
ATOM 28609 N   ILE E 116   33.783   1.092 57.287 1.00 0.00
E   N
ATOM 28610 HN ILE E 116   33.091   0.412 57.053 1.00 0.00
E   H
ATOM 28611 CA ILE E 116   33.695   1.592 58.666 1.00 0.00
E   C
ATOM 28612 HA ILE E 116   34.049   2.615 58.636 1.00 0.00
E   H
ATOM 28613 CB ILE E 116   32.240   1.671 59.203 1.00 0.00
E   C
ATOM 28614 HB ILE E 116   32.345   2.092 60.235 1.00 0.00
E   H
ATOM 28615 CG2 ILE E 116   31.387   2.547 58.302 1.00 0.00
E   C
ATOM 28616 HG21 ILE E 116   30.395   2.733 58.763 1.00 0.00
E   H
ATOM 28617 HG22 ILE E 116   31.870   3.532 58.136 1.00 0.00
E   H
ATOM 28618 HG23 ILE E 116   31.227   2.064 57.315 1.00 0.00
E   H
ATOM 28619 CG1 ILE E 116   31.614   0.287 59.369 1.00 0.00
E   C

ATOM 28620 HG11 ILE E 116 31.662 -0.266 58.404 1.00 0.00
E H
ATOM 28621 HG12 ILE E 116 32.211 -0.292 60.110 1.00 0.00
E H
ATOM 28622 CD ILE E 116 30.174 0.324 59.847 1.00 0.00
E C
ATOM 28623 HD1 ILE E 116 29.794 -0.709 60.004 1.00 0.00
E H
ATOM 28624 HD2 ILE E 116 30.095 0.874 60.810 1.00 0.00
E H
ATOM 28625 HD3 ILE E 116 29.520 0.821 59.101 1.00 0.00
E H
ATOM 28626 C ILE E 116 34.623 0.830 59.609 1.00 0.00
E C
ATOM 28627 O ILE E 116 34.540 0.980 60.831 1.00 0.00
E O
ATOM 28628 N GLY E 117 35.510 0.022 59.026 1.00 0.00
E N
ATOM 28629 HN GLY E 117 35.488 -0.154 58.043 1.00 0.00
E H
ATOM 28630 CA GLY E 117 36.567 -0.660 59.766 1.00 0.00
E C
ATOM 28631 HA1 GLY E 117 36.937 0.084 60.458 1.00 0.00
E H
ATOM 28632 HA2 GLY E 117 37.281 -0.960 59.012 1.00 0.00
E H
ATOM 28633 C GLY E 117 36.183 -1.895 60.566 1.00 0.00
E C
ATOM 28634 O GLY E 117 36.583 -2.033 61.728 1.00 0.00
E O
ATOM 28635 N ALA E 118 35.408 -2.790 59.956 1.00 0.00
E N
ATOM 28636 HN ALA E 118 34.962 -2.613 59.079 1.00 0.00
E H
ATOM 28637 CA ALA E 118 35.156 -4.105 60.534 1.00 0.00
E C
ATOM 28638 HA ALA E 118 35.027 -4.067 61.609 1.00 0.00
E H
ATOM 28639 CB ALA E 118 33.929 -4.746 59.909 1.00 0.00
E C
ATOM 28640 HB1 ALA E 118 33.029 -4.140 60.149 1.00 0.00
E H
ATOM 28641 HB2 ALA E 118 34.023 -4.795 58.803 1.00 0.00
E H
ATOM 28642 HB3 ALA E 118 33.763 -5.775 60.296 1.00 0.00
E H
ATOM 28643 C ALA E 118 36.393 -4.948 60.281 1.00 0.00
E C
ATOM 28644 O ALA E 118 36.925 -4.948 59.165 1.00 0.00
E O
ATOM 28645 N ASP E 119 36.863 -5.649 61.311 1.00 0.00
E N
ATOM 28646 HN ASP E 119 36.441 -5.719 62.211 1.00 0.00
E H

ATOM 28647 CA ASP E 119 38.105 -6.417 61.203 1.00 0.00
E C
ATOM 28648 HA ASP E 119 38.780 -5.821 60.600 1.00 0.00
E H
ATOM 28649 CB ASP E 119 38.716 -6.634 62.573 1.00 0.00
E C
ATOM 28650 HB1 ASP E 119 37.978 -7.141 63.226 1.00 0.00
E H
ATOM 28651 HB2 ASP E 119 39.617 -7.276 62.501 1.00 0.00
E H
ATOM 28652 CG ASP E 119 39.097 -5.340 63.235 1.00 0.00
E C
ATOM 28653 OD1 ASP E 119 40.006 -4.657 62.720 1.00 0.00
E O
ATOM 28654 OD2 ASP E 119 38.485 -5.001 64.270 1.00 0.00
E O
ATOM 28655 C ASP E 119 37.918 -7.740 60.475 1.00 0.00
E C
ATOM 28656 O ASP E 119 38.817 -8.197 59.764 1.00 0.00
E O
ATOM 28657 N ARG E 120 36.752 -8.354 60.660 1.00 0.00
E N
ATOM 28658 HN ARG E 120 36.088 -8.028 61.329 1.00 0.00
E H
ATOM 28659 CA ARG E 120 36.383 -9.530 59.895 1.00 0.00
E C
ATOM 28660 HA ARG E 120 36.903 -9.503 58.944 1.00 0.00
E H
ATOM 28661 CB ARG E 120 36.775 -10.814 60.635 1.00 0.00
E C
ATOM 28662 HB1 ARG E 120 37.860 -10.719 60.879 1.00 0.00
E H
ATOM 28663 HB2 ARG E 120 36.245 -10.856 61.612 1.00 0.00
E H
ATOM 28664 CG ARG E 120 36.551 -12.067 59.806 1.00 0.00
E C
ATOM 28665 HG1 ARG E 120 35.467 -12.144 59.566 1.00 0.00
E H
ATOM 28666 HG2 ARG E 120 37.066 -11.949 58.825 1.00 0.00
E H
ATOM 28667 CD ARG E 120 37.077 -13.322 60.464 1.00 0.00
E C
ATOM 28668 HD1 ARG E 120 38.188 -13.347 60.377 1.00 0.00
E H
ATOM 28669 HD2 ARG E 120 36.802 -13.364 61.543 1.00 0.00
E H
ATOM 28670 NE ARG E 120 36.505 -14.503 59.821 1.00 0.00
E N
ATOM 28671 HE ARG E 120 35.799 -14.280 59.140 1.00 0.00
E H
ATOM 28672 CZ ARG E 120 36.853 -15.764 60.078 1.00 0.00
E C
ATOM 28673 NH1 ARG E 120 37.794 -16.049 60.978 1.00 0.00
E N

```
ATOM  28674 HH11 ARG E 120      38.065 -16.987  61.145  1.00  0.00
E    H
ATOM  28675 HH12 ARG E 120      38.294 -15.268  61.361  1.00  0.00
E    H
ATOM  28676  NH2 ARG E 120      36.254 -16.748  59.426  1.00  0.00
E    N
ATOM  28677 HH21 ARG E 120      36.442 -17.688  59.672  1.00  0.00
E    H
ATOM  28678 HH22 ARG E 120      35.480 -16.503  58.856  1.00  0.00
E    H
ATOM  28679  C   ARG E 120      34.893  -9.517  59.576  1.00  0.00
E    C
ATOM  28680  O   ARG E 120      34.066  -9.248  60.447  1.00  0.00
E    O
ATOM  28681  N   ILE E 121      34.561  -9.805  58.323  1.00  0.00
E    N
ATOM  28682  HN  ILE E 121      35.221 -10.028  57.608  1.00  0.00
E    H
ATOM  28683  CA  ILE E 121      33.168  -9.820  57.878  1.00  0.00
E    C
ATOM  28684  HA  ILE E 121      32.565  -9.568  58.741  1.00  0.00
E    H
ATOM  28685  CB  ILE E 121      32.939  -8.823  56.729  1.00  0.00
E    C
ATOM  28686  HB  ILE E 121      33.655  -9.146  55.930  1.00  0.00
E    H
ATOM  28687  CG2 ILE E 121      31.484  -8.847  56.290  1.00  0.00
E    C
ATOM  28688 HG21 ILE E 121      31.345  -8.250  55.365  1.00  0.00
E    H
ATOM  28689 HG22 ILE E 121      31.153  -9.884  56.074  1.00  0.00
E    H
ATOM  28690 HG23 ILE E 121      30.824  -8.425  57.078  1.00  0.00
E    H
ATOM  28691  CG1 ILE E 121      33.358  -7.419  57.162  1.00  0.00
E    C
ATOM  28692 HG11 ILE E 121      32.575  -6.979  57.820  1.00  0.00
E    H
ATOM  28693 HG12 ILE E 121      34.292  -7.491  57.765  1.00  0.00
E    H
ATOM  28694  CD  ILE E 121      33.613  -6.482  56.013  1.00  0.00
E    C
ATOM  28695  HD1 ILE E 121      33.969  -5.498  56.388  1.00  0.00
E    H
ATOM  28696  HD2 ILE E 121      34.390  -6.897  55.334  1.00  0.00
E    H
ATOM  28697  HD3 ILE E 121      32.687  -6.310  55.425  1.00  0.00
E    H
ATOM  28698  C   ILE E 121      32.757 -11.226  57.448  1.00  0.00
E    C
ATOM  28699  O   ILE E 121      33.353 -11.801  56.544  1.00  0.00
E    O
ATOM  28700  N   ILE E 122      31.739 -11.776  58.100  1.00  0.00
E    N
```

```
ATOM  28701  HN  ILE E 122      31.173 -11.299  58.770  1.00  0.00
E    H
ATOM  28702  CA  ILE E 122      31.358 -13.165  57.878  1.00  0.00
E    C
ATOM  28703  HA  ILE E 122      32.145 -13.594  57.272  1.00  0.00
E    H
ATOM  28704  CB  ILE E 122      31.223 -13.949  59.209  1.00  0.00
E    C
ATOM  28705  HB  ILE E 122      30.445 -13.388  59.786  1.00  0.00
E    H
ATOM  28706  CG2 ILE E 122      30.890 -15.411  58.961  1.00  0.00
E    C
ATOM  28707 HG21 ILE E 122      30.643 -15.923  59.915  1.00  0.00
E    H
ATOM  28708 HG22 ILE E 122      30.011 -15.510  58.290  1.00  0.00
E    H
ATOM  28709 HG23 ILE E 122      31.747 -15.941  58.495  1.00  0.00
E    H
ATOM  28710  CG1 ILE E 122      32.508 -13.872  60.025  1.00  0.00
E    C
ATOM  28711 HG11 ILE E 122      32.895 -14.898  60.215  1.00  0.00
E    H
ATOM  28712 HG12 ILE E 122      33.281 -13.333  59.431  1.00  0.00
E    H
ATOM  28713  CD  ILE E 122      32.325 -13.154  61.338  1.00  0.00
E    C
ATOM  28714  HD1 ILE E 122      33.297 -13.071  61.871  1.00  0.00
E    H
ATOM  28715  HD2 ILE E 122      31.932 -12.128  61.173  1.00  0.00
E    H
ATOM  28716  HD3 ILE E 122      31.618 -13.701  61.996  1.00  0.00
E    H
ATOM  28717  C   ILE E 122      30.069 -13.292  57.079  1.00  0.00
E    C
ATOM  28718  O   ILE E 122      29.063 -12.672  57.406  1.00  0.00
E    O
ATOM  28719  N   HSE E 123      30.124 -14.108  56.027  1.00  0.00
E    N
ATOM  28720  HN  HSE E 123      30.985 -14.465  55.667  1.00  0.00
E    H
ATOM  28721  CA  HSE E 123      28.939 -14.554  55.306  1.00  0.00
E    C
ATOM  28722  HA  HSE E 123      28.094 -13.989  55.682  1.00  0.00
E    H
ATOM  28723  CB  HSE E 123      29.146 -14.378  53.818  1.00  0.00
E    C
ATOM  28724  HB1 HSE E 123      30.046 -14.961  53.527  1.00  0.00
E    H
ATOM  28725  HB2 HSE E 123      28.285 -14.814  53.269  1.00  0.00
E    H
ATOM  28726  ND1 HSE E 123      28.395 -12.206  52.824  1.00  0.00
E    N
ATOM  28727  CG  HSE E 123      29.364 -12.950  53.400  1.00  0.00
E    C
```

```
ATOM  28728  CE1 HSE E 123      28.877 -10.976  52.558  1.00  0.00
E    C
ATOM  28729  HE1 HSE E 123      28.304 -10.175  52.085  1.00  0.00
E    H
ATOM  28730  NE2 HSE E 123      30.157 -10.931  52.968  1.00  0.00
E    N
ATOM  28731  HE2 HSE E 123      30.765 -10.141  52.878  1.00  0.00
E    H
ATOM  28732  CD2 HSE E 123      30.491 -12.130  53.491  1.00  0.00
E    C
ATOM  28733  HD2 HSE E 123      31.476 -12.345  53.881  1.00  0.00
E    H
ATOM  28734  C   HSE E 123      28.672 -16.006  55.614  1.00  0.00
E    C
ATOM  28735  O   HSE E 123      29.150 -16.890  54.890  1.00  0.00
E    O
ATOM  28736  N   PRO E 124      27.906 -16.282  56.691  1.00  0.00
E    N
ATOM  28737  CD  PRO E 124      27.158 -15.301  57.493  1.00  0.00
E    C
ATOM  28738  HD1 PRO E 124      28.263 -15.212  57.386  1.00  0.00
E    H
ATOM  28739  HD2 PRO E 124      26.688 -14.291  57.529  1.00  0.00
E    H
ATOM  28740  CA  PRO E 124      27.687 -17.641  57.167  1.00  0.00
E    C
ATOM  28741  HA  PRO E 124      28.621 -18.048  57.539  1.00  0.00
E    H
ATOM  28742  CB  PRO E 124      26.675 -17.469  58.298  1.00  0.00
E    C
ATOM  28743  HB1 PRO E 124      27.220 -17.223  59.237  1.00  0.00
E    H
ATOM  28744  HB2 PRO E 124      26.061 -18.376  58.465  1.00  0.00
E    H
ATOM  28745  CG  PRO E 124      26.824 -16.063  58.729  1.00  0.00
E    C
ATOM  28746  HG1 PRO E 124      26.480 -15.892  59.769  1.00  0.00
E    H
ATOM  28747  HG2 PRO E 124      27.307 -15.327  59.203  1.00  0.00
E    H
ATOM  28748  C   PRO E 124      27.126 -18.574  56.104  1.00  0.00
E    C
ATOM  28749  O   PRO E 124      27.711 -19.630  55.862  1.00  0.00
E    O
ATOM  28750  N   GLU E 125      26.018 -18.198  55.472  1.00  0.00
E    N
ATOM  28751  HN  GLU E 125      25.544 -17.333  55.609  1.00  0.00
E    H
ATOM  28752  CA  GLU E 125      25.390 -19.069  54.483  1.00  0.00
E    C
ATOM  28753  HA  GLU E 125      25.189 -20.019  54.963  1.00  0.00
E    H
ATOM  28754  CB  GLU E 125      24.053 -18.511  53.998  1.00  0.00
E    C
```

ATOM 28755 HB1 GLU E 125   24.220 -17.469 53.641 1.00 0.00
E   H
ATOM 28756 HB2 GLU E 125   23.704 -19.085 53.110 1.00 0.00
E   H
ATOM 28757 CG GLU E 125   22.948 -18.485 55.039 1.00 0.00
E   C
ATOM 28758 HG1 GLU E 125   22.015 -18.878 54.596 1.00 0.00
E   H
ATOM 28759 HG2 GLU E 125   23.220 -19.098 55.921 1.00 0.00
E   H
ATOM 28760 CD GLU E 125   22.630 -17.074 55.502 1.00 0.00
E   C
ATOM 28761 OE1 GLU E 125   21.437 -16.722 55.447 1.00 0.00
E   O
ATOM 28762 OE2 GLU E 125   23.555 -16.314 55.897 1.00 0.00
E   O
ATOM 28763 C   GLU E 125   26.305 -19.346 53.292 1.00 0.00
E   C
ATOM 28764 O   GLU E 125   26.447 -20.496 52.876 1.00 0.00
E   O
ATOM 28765 N   LYS E 126   26.924 -18.296 52.753 1.00 0.00
E   N
ATOM 28766 HN LYS E 126   26.773 -17.361 53.069 1.00 0.00
E   H
ATOM 28767 CA LYS E 126   27.873 -18.433 51.657 1.00 0.00
E   C
ATOM 28768 HA LYS E 126   27.335 -18.810 50.795 1.00 0.00
E   H
ATOM 28769 CB LYS E 126   28.529 -17.090 51.350 1.00 0.00
E   C
ATOM 28770 HB1 LYS E 126   27.718 -16.326 51.325 1.00 0.00
E   H
ATOM 28771 HB2 LYS E 126   29.195 -16.810 52.198 1.00 0.00
E   H
ATOM 28772 CG LYS E 126   29.293 -17.041 50.033 1.00 0.00
E   C
ATOM 28773 HG1 LYS E 126   29.649 -18.065 49.780 1.00 0.00
E   H
ATOM 28774 HG2 LYS E 126   28.598 -16.722 49.223 1.00 0.00
E   H
ATOM 28775 CD LYS E 126   30.491 -16.101 50.107 1.00 0.00
E   C
ATOM 28776 HD1 LYS E 126   30.169 -15.160 50.609 1.00 0.00
E   H
ATOM 28777 HD2 LYS E 126   31.265 -16.576 50.753 1.00 0.00
E   H
ATOM 28778 CE LYS E 126   31.054 -15.813 48.722 1.00 0.00
E   C
ATOM 28779 HE1 LYS E 126   32.067 -15.359 48.799 1.00 0.00
E   H
ATOM 28780 HE2 LYS E 126   31.134 -16.752 48.129 1.00 0.00
E   H
ATOM 28781 NZ LYS E 126   30.194 -14.866 47.950 1.00 0.00
E   N

ATOM 28782 HZ1 LYS E 126 30.609 -14.691 47.011 1.00 0.00
E H
ATOM 28783 HZ2 LYS E 126 29.243 -15.273 47.837 1.00 0.00
E H
ATOM 28784 HZ3 LYS E 126 30.120 -13.967 48.467 1.00 0.00
E H
ATOM 28785 C LYS E 126 28.928 -19.473 52.027 1.00 0.00
E C
ATOM 28786 O LYS E 126 29.148 -20.427 51.286 1.00 0.00
E O
ATOM 28787 N ASP E 127 29.552 -19.302 53.191 1.00 0.00
E N
ATOM 28788 HN ASP E 127 29.357 -18.566 53.835 1.00 0.00
E H
ATOM 28789 CA ASP E 127 30.611 -20.200 53.655 1.00 0.00
E C
ATOM 28790 HA ASP E 127 31.349 -20.237 52.862 1.00 0.00
E H
ATOM 28791 CB ASP E 127 31.254 -19.655 54.930 1.00 0.00
E C
ATOM 28792 HB1 ASP E 127 30.458 -19.369 55.647 1.00 0.00
E H
ATOM 28793 HB2 ASP E 127 31.891 -20.426 55.406 1.00 0.00
E H
ATOM 28794 CG ASP E 127 32.105 -18.416 54.679 1.00 0.00
E C
ATOM 28795 OD1 ASP E 127 32.350 -18.061 53.507 1.00 0.00
E O
ATOM 28796 OD2 ASP E 127 32.541 -17.787 55.662 1.00 0.00
E O
ATOM 28797 C ASP E 127 30.122 -21.628 53.866 1.00 0.00
E C
ATOM 28798 O ASP E 127 30.803 -22.582 53.501 1.00 0.00
E O
ATOM 28799 N MET E 128 28.940 -21.779 54.453 1.00 0.00
E N
ATOM 28800 HN MET E 128 28.378 -21.035 54.806 1.00 0.00
E H
ATOM 28801 CA MET E 128 28.357 -23.115 54.627 1.00 0.00
E C
ATOM 28802 HA MET E 128 29.123 -23.724 55.091 1.00 0.00
E H
ATOM 28803 CB MET E 128 27.145 -23.089 55.554 1.00 0.00
E C
ATOM 28804 HB1 MET E 128 26.562 -22.173 55.300 1.00 0.00
E H
ATOM 28805 HB2 MET E 128 26.484 -23.954 55.322 1.00 0.00
E H
ATOM 28806 CG MET E 128 27.507 -23.058 57.029 1.00 0.00
E C
ATOM 28807 HG1 MET E 128 28.028 -22.110 57.279 1.00 0.00
E H
ATOM 28808 HG2 MET E 128 26.581 -23.104 57.642 1.00 0.00
E H

ATOM 28809 SD MET E 128 28.588 -24.383 57.620 1.00 0.00
E S
ATOM 28810 CE MET E 128 27.553 -25.811 57.299 1.00 0.00
E C
ATOM 28811 HE1 MET E 128 28.061 -26.748 57.617 1.00 0.00
E H
ATOM 28812 HE2 MET E 128 26.592 -25.740 57.852 1.00 0.00
E H
ATOM 28813 HE3 MET E 128 27.320 -25.900 56.216 1.00 0.00
E H
ATOM 28814 C MET E 128 27.986 -23.771 53.307 1.00 0.00
E C
ATOM 28815 O MET E 128 28.011 -25.001 53.209 1.00 0.00
E O
ATOM 28816 N GLY E 129 27.635 -22.955 52.308 1.00 0.00
E N
ATOM 28817 HN GLY E 129 27.496 -21.975 52.440 1.00 0.00
E H
ATOM 28818 CA GLY E 129 27.432 -23.447 50.943 1.00 0.00
E C
ATOM 28819 HA1 GLY E 129 27.301 -22.586 50.301 1.00 0.00
E H
ATOM 28820 HA2 GLY E 129 26.589 -24.125 50.956 1.00 0.00
E H
[truncated: 449,933 more chars]
