# Supplementary material for: Structural basis and synergism of ATP and Na+ activation in bacterial K+ uptake system KtrAB
Source: Nat Commun. 2024 May 8;15:3850. doi: 10.1038/s41467-024-48057-y (PMC11078986; doi:10.1038/s41467-024-48057-y)
Supplement: Supplementary file 7 — Supplementary Data 4 [file 41467_2024_48057_MOESM7_ESM.pdf]

```
CRYST1    0.000    0.000    0.000  90.00  90.00  90.00 P 1
1
ATOM      1  N   THR I  35      20.254 -15.848  13.949  0.00  0.00
I
ATOM      2  HT1 THR I  35      20.463 -16.738  13.455  0.00  0.00
I
ATOM      3  HT2 THR I  35      20.964 -15.658  14.686  0.00  0.00
I
ATOM      4  HT3 THR I  35      20.232 -15.002  13.345  0.00  0.00
I
ATOM      5  CA  THR I  35      18.842 -15.916  14.475  0.00  0.00
I
ATOM      6  HA  THR I  35      18.877 -16.552  15.347  0.00  0.00
I
ATOM      7  CB  THR I  35      18.295 -14.530  14.845  0.00  0.00
I
ATOM      8  HB  THR I  35      17.303 -14.729  15.305  0.00  0.00
I
ATOM      9  OG1 THR I  35      18.145 -13.755  13.613  0.00  0.00
I
ATOM     10  HG1 THR I  35      17.465 -13.133  13.883  0.00  0.00
I
ATOM     11  CG2 THR I  35      19.165 -13.663  15.708  0.00  0.00
I
ATOM     12 HG21 THR I  35      20.034 -13.162  15.231  0.00  0.00
I
ATOM     13 HG22 THR I  35      18.626 -12.787  16.127  0.00  0.00
I
ATOM     14 HG23 THR I  35      19.483 -14.214  16.619  0.00  0.00
I
ATOM     15  C   THR I  35      17.874 -16.582  13.465  0.00  0.00
I
ATOM     16  O   THR I  35      18.190 -16.769  12.268  0.00  0.00
I
ATOM     17  N   PRO I  36      16.697 -17.184  13.914  0.00  0.00
I
ATOM     18  CD  PRO I  36      16.489 -17.629  15.292  0.00  0.00
I
ATOM     19  HD1 PRO I  36      17.378 -18.218  15.603  0.00  0.00
I
ATOM     20  HD2 PRO I  36      16.390 -16.681  15.862  0.00  0.00
I
ATOM     21  CA  PRO I  36      15.962 -18.062  12.984  0.00  0.00
I
ATOM     22  HA  PRO I  36      16.566 -18.815  12.500  0.00  0.00
I
ATOM     23  CB  PRO I  36      15.002 -18.927  13.833  0.00  0.00
I
ATOM     24  HB1 PRO I  36      15.288 -19.999  13.790  0.00  0.00
I
ATOM     25  HB2 PRO I  36      13.939 -18.815  13.531  0.00  0.00
I
ATOM     26  CG  PRO I  36      15.128 -18.391  15.274  0.00  0.00
I
```

```
ATOM     27  HG1 PRO I  36      15.191 -19.147  16.086  0.00  0.00
I
ATOM     28  HG2 PRO I  36      14.255 -17.727  15.449  0.00  0.00
I
ATOM     29  C   PRO I  36      15.327 -17.347  11.877  0.00  0.00
I
ATOM     30  O   PRO I  36      15.296 -17.934  10.802  0.00  0.00
I
ATOM     31  N   PRO I  37      14.877 -16.028  11.854  0.00  0.00
I
ATOM     32  CD  PRO I  37      14.495 -15.262  13.042  0.00  0.00
I
ATOM     33  HD1 PRO I  37      13.799 -15.863  13.665  0.00  0.00
I
ATOM     34  HD2 PRO I  37      15.428 -14.938  13.551  0.00  0.00
I
ATOM     35  CA  PRO I  37      14.571 -15.341  10.606  0.00  0.00
I
ATOM     36  HA  PRO I  37      13.773 -15.902  10.142  0.00  0.00
I
ATOM     37  CB  PRO I  37      14.187 -13.970  11.081  0.00  0.00
I
ATOM     38  HB1 PRO I  37      13.404 -13.520  10.434  0.00  0.00
I
ATOM     39  HB2 PRO I  37      15.108 -13.353  11.150  0.00  0.00
I
ATOM     40  CG  PRO I  37      13.713 -14.052  12.570  0.00  0.00
I
ATOM     41  HG1 PRO I  37      12.630 -14.221  12.750  0.00  0.00
I
ATOM     42  HG2 PRO I  37      14.094 -13.175  13.136  0.00  0.00
I
ATOM     43  C   PRO I  37      15.665 -15.275   9.586  0.00  0.00
I
ATOM     44  O   PRO I  37      15.459 -15.348   8.376  0.00  0.00
I
ATOM     45  N   GLN I  38      16.960 -15.101   9.993  0.00  0.00
I
ATOM     46  HN  GLN I  38      17.198 -14.913  10.942  0.00  0.00
I
ATOM     47  CA  GLN I  38      18.110 -15.092   9.111  0.00  0.00
I
ATOM     48  HA  GLN I  38      17.903 -14.386   8.321  0.00  0.00
I
ATOM     49  CB  GLN I  38      19.309 -14.554   9.920  0.00  0.00
I
ATOM     50  HB1 GLN I  38      19.518 -15.263  10.749  0.00  0.00
I
ATOM     51  HB2 GLN I  38      20.124 -14.578   9.165  0.00  0.00
I
ATOM     52  CG  GLN I  38      19.138 -13.143  10.471  0.00  0.00
I
ATOM     53  HG1 GLN I  38      19.162 -12.395   9.650  0.00  0.00
I
```

```
ATOM     54  HG2 GLN I  38      18.276 -13.032  11.163  0.00  0.00
I
ATOM     55  CD  GLN I  38      20.396 -12.824  11.347  0.00  0.00
I
ATOM     56  OE1 GLN I  38      20.816 -13.557  12.246  0.00  0.00
I
ATOM     57  NE2 GLN I  38      21.022 -11.694  10.964  0.00  0.00
I
ATOM     58 HE21 GLN I  38      21.776 -11.397  11.550  0.00  0.00
I
ATOM     59 HE22 GLN I  38      20.603 -11.037  10.338  0.00  0.00
I
ATOM     60  C   GLN I  38      18.365 -16.483   8.618  0.00  0.00
I
ATOM     61  O   GLN I  38      18.820 -16.669   7.436  0.00  0.00
I
ATOM     62  N   VAL I  39      18.044 -17.498   9.450  0.00  0.00
I
ATOM     63  HN  VAL I  39      17.799 -17.441  10.414  0.00  0.00
I
ATOM     64  CA  VAL I  39      18.209 -18.786   8.889  0.00  0.00
I
ATOM     65  HA  VAL I  39      19.146 -18.718   8.356  0.00  0.00
I
ATOM     66  CB  VAL I  39      18.288 -19.942   9.943  0.00  0.00
I
ATOM     67  HB  VAL I  39      17.396 -19.918  10.605  0.00  0.00
I
ATOM     68  CG1 VAL I  39      18.474 -21.300   9.288  0.00  0.00
I
ATOM     69 HG11 VAL I  39      17.606 -21.735   8.748  0.00  0.00
I
ATOM     70 HG12 VAL I  39      19.456 -21.323   8.770  0.00  0.00
I
ATOM     71 HG13 VAL I  39      18.634 -22.031  10.109  0.00  0.00
I
ATOM     72  CG2 VAL I  39      19.429 -19.758  11.004  0.00  0.00
I
ATOM     73 HG21 VAL I  39      19.097 -20.119  12.001  0.00  0.00
I
ATOM     74 HG22 VAL I  39      20.370 -20.149  10.561  0.00  0.00
I
ATOM     75 HG23 VAL I  39      19.678 -18.677  11.056  0.00  0.00
I
ATOM     76  C   VAL I  39      17.252 -19.317   7.805  0.00  0.00
I
ATOM     77  O   VAL I  39      17.639 -20.093   6.894  0.00  0.00
I
ATOM     78  N   LEU I  40      16.007 -18.843   7.905  0.00  0.00
I
ATOM     79  HN  LEU I  40      15.826 -18.352   8.753  0.00  0.00
I
ATOM     80  CA  LEU I  40      14.925 -18.938   7.043  0.00  0.00
I
```

```
ATOM     81  HA  LEU I  40      14.551 -19.923   6.805  0.00  0.00
I
ATOM     82  CB  LEU I  40      13.653 -18.169   7.526  0.00  0.00
I
ATOM     83  HB1 LEU I  40      13.364 -18.503   8.545  0.00  0.00
I
ATOM     84  HB2 LEU I  40      13.979 -17.120   7.695  0.00  0.00
I
ATOM     85  CG  LEU I  40      12.377 -18.359   6.602  0.00  0.00
I
ATOM     86  HG  LEU I  40      12.768 -18.331   5.563  0.00  0.00
I
ATOM     87  CD1 LEU I  40      11.628 -19.691   6.931  0.00  0.00
I
ATOM     88 HD11 LEU I  40      10.690 -19.665   6.336  0.00  0.00
I
ATOM     89 HD12 LEU I  40      12.349 -20.527   6.803  0.00  0.00
I
ATOM     90 HD13 LEU I  40      11.487 -19.587   8.028  0.00  0.00
I
ATOM     91  CD2 LEU I  40      11.454 -17.170   6.780  0.00  0.00
I
ATOM     92 HD21 LEU I  40      11.981 -16.197   6.681  0.00  0.00
I
ATOM     93 HD22 LEU I  40      10.654 -17.204   6.010  0.00  0.00
I
ATOM     94 HD23 LEU I  40      10.998 -17.354   7.776  0.00  0.00
I
ATOM     95  C   LEU I  40      15.400 -18.323   5.718  0.00  0.00
I
ATOM     96  O   LEU I  40      15.282 -18.917   4.620  0.00  0.00
I
ATOM     97  N   ALA I  41      16.109 -17.178   5.759  0.00  0.00
I
ATOM     98  HN  ALA I  41      16.097 -16.785   6.676  0.00  0.00
I
ATOM     99  CA  ALA I  41      16.559 -16.455   4.657  0.00  0.00
I
ATOM    100  HA  ALA I  41      15.780 -16.397   3.911  0.00  0.00
I
ATOM    101  CB  ALA I  41      16.817 -15.006   5.061  0.00  0.00
I
ATOM    102  HB1 ALA I  41      17.584 -14.897   5.858  0.00  0.00
I
ATOM    103  HB2 ALA I  41      17.109 -14.468   4.134  0.00  0.00
I
ATOM    104  HB3 ALA I  41      15.925 -14.438   5.404  0.00  0.00
I
ATOM    105  C   ALA I  41      17.840 -17.000   3.939  0.00  0.00
I
ATOM    106  O   ALA I  41      17.906 -16.956   2.703  0.00  0.00
I
ATOM    107  N   ILE I  42      18.819 -17.514   4.707  0.00  0.00
I
```

ATOM 108 HN ILE I 42 18.715 -17.354 5.685 0.00 0.00
I
ATOM 109 CA ILE I 42 19.902 -18.315 4.132 0.00 0.00
I
ATOM 110 HA ILE I 42 20.321 -17.815 3.271 0.00 0.00
I
ATOM 111 CB ILE I 42 21.086 -18.500 5.044 0.00 0.00
I
ATOM 112 HB ILE I 42 21.175 -17.531 5.579 0.00 0.00
I
ATOM 113 CG2 ILE I 42 21.034 -19.579 6.129 0.00 0.00
I
ATOM 114 HG21 ILE I 42 20.041 -19.450 6.610 0.00 0.00
I
ATOM 115 HG22 ILE I 42 20.977 -20.594 5.680 0.00 0.00
I
ATOM 116 HG23 ILE I 42 21.810 -19.399 6.904 0.00 0.00
I
ATOM 117 CG1 ILE I 42 22.415 -18.734 4.196 0.00 0.00
I
ATOM 118 HG11 ILE I 42 23.291 -19.036 4.809 0.00 0.00
I
ATOM 119 HG12 ILE I 42 22.256 -19.671 3.620 0.00 0.00
I
ATOM 120 CD ILE I 42 22.861 -17.573 3.353 0.00 0.00
I
ATOM 121 HD1 ILE I 42 22.659 -17.622 2.262 0.00 0.00
I
ATOM 122 HD2 ILE I 42 22.682 -16.537 3.712 0.00 0.00
I
ATOM 123 HD3 ILE I 42 23.959 -17.731 3.289 0.00 0.00
I
ATOM 124 C ILE I 42 19.507 -19.654 3.454 0.00 0.00
I
ATOM 125 O ILE I 42 19.971 -19.928 2.328 0.00 0.00
I
ATOM 126 N GLY I 43 18.648 -20.507 4.157 0.00 0.00
I
ATOM 127 HN GLY I 43 18.386 -20.377 5.110 0.00 0.00
I
ATOM 128 CA GLY I 43 18.180 -21.816 3.638 0.00 0.00
I
ATOM 129 HA1 GLY I 43 17.639 -22.252 4.464 0.00 0.00
I
ATOM 130 HA2 GLY I 43 19.082 -22.315 3.317 0.00 0.00
I
ATOM 131 C GLY I 43 17.252 -21.701 2.454 0.00 0.00
I
ATOM 132 O GLY I 43 17.202 -22.550 1.567 0.00 0.00
I
ATOM 133 N PHE I 44 16.377 -20.613 2.328 0.00 0.00
I
ATOM 134 HN PHE I 44 16.332 -19.907 3.031 0.00 0.00
I

ATOM	135	CA	PHE	I	44	15.529	-20.391	1.095	0.00	0.00	I
ATOM	136	HA	PHE	I	44	15.091	-21.345	0.841	0.00	0.00	I
ATOM	137	CB	PHE	I	44	14.360	-19.397	1.307	0.00	0.00	I
ATOM	138	HB1	PHE	I	44	14.694	-18.467	1.816	0.00	0.00	I
ATOM	139	HB2	PHE	I	44	13.766	-19.160	0.399	0.00	0.00	I
ATOM	140	CG	PHE	I	44	13.338	-19.862	2.217	0.00	0.00	I
ATOM	141	CD1	PHE	I	44	13.237	-21.231	2.523	0.00	0.00	I
ATOM	142	HD1	PHE	I	44	13.921	-22.009	2.218	0.00	0.00	I
ATOM	143	CE1	PHE	I	44	12.166	-21.642	3.337	0.00	0.00	I
ATOM	144	HE1	PHE	I	44	11.998	-22.693	3.520	0.00	0.00	I
ATOM	145	CZ	PHE	I	44	11.218	-20.691	3.729	0.00	0.00	I
ATOM	146	HZ	PHE	I	44	10.455	-21.051	4.404	0.00	0.00	I
ATOM	147	CD2	PHE	I	44	12.403	-18.924	2.666	0.00	0.00	I
ATOM	148	HD2	PHE	I	44	12.334	-17.925	2.264	0.00	0.00	I
ATOM	149	CE2	PHE	I	44	11.310	-19.351	3.382	0.00	0.00	I
ATOM	150	HE2	PHE	I	44	10.618	-18.604	3.743	0.00	0.00	I
ATOM	151	C	PHE	I	44	16.376	-19.960	-0.135	0.00	0.00	I
ATOM	152	O	PHE	I	44	16.117	-20.408	-1.277	0.00	0.00	I
ATOM	153	N	PHE	I	45	17.410	-19.081	0.156	0.00	0.00	I
ATOM	154	HN	PHE	I	45	17.369	-18.730	1.088	0.00	0.00	I
ATOM	155	CA	PHE	I	45	18.390	-18.619	-0.915	0.00	0.00	I
ATOM	156	HA	PHE	I	45	17.824	-18.237	-1.752	0.00	0.00	I
ATOM	157	CB	PHE	I	45	19.239	-17.425	-0.483	0.00	0.00	I
ATOM	158	HB1	PHE	I	45	18.652	-16.553	-0.125	0.00	0.00	I
ATOM	159	HB2	PHE	I	45	19.869	-17.716	0.384	0.00	0.00	I
ATOM	160	CG	PHE	I	45	20.195	-16.751	-1.479	0.00	0.00	I
ATOM	161	CD1	PHE	I	45	21.453	-16.280	-1.065	0.00	0.00	I

ATOM 162 HD1 PHE I 45 21.705 −16.365 −0.019 0.00 0.00
I
ATOM 163 CE1 PHE I 45 22.267 −15.597 −1.981 0.00 0.00
I
ATOM 164 HE1 PHE I 45 23.176 −15.128 −1.634 0.00 0.00
I
ATOM 165 CZ PHE I 45 21.923 −15.522 −3.350 0.00 0.00
I
ATOM 166 HZ PHE I 45 22.644 −15.239 −4.103 0.00 0.00
I
ATOM 167 CD2 PHE I 45 19.702 −16.425 −2.754 0.00 0.00
I
ATOM 168 HD2 PHE I 45 18.693 −16.655 −3.063 0.00 0.00
I
ATOM 169 CE2 PHE I 45 20.587 −15.918 −3.693 0.00 0.00
I
ATOM 170 HE2 PHE I 45 20.326 −15.963 −4.740 0.00 0.00
I
ATOM 171 C PHE I 45 19.287 −19.760 −1.447 0.00 0.00
I
ATOM 172 O PHE I 45 19.527 −19.865 −2.606 0.00 0.00
I
ATOM 173 N LEU I 46 19.768 −20.628 −0.509 0.00 0.00
I
ATOM 174 HN LEU I 46 19.637 −20.432 0.460 0.00 0.00
I
ATOM 175 CA LEU I 46 20.576 −21.788 −0.786 0.00 0.00
I
ATOM 176 HA LEU I 46 21.395 −21.507 −1.432 0.00 0.00
I
ATOM 177 CB LEU I 46 20.919 −22.416 0.570 0.00 0.00
I
ATOM 178 HB1 LEU I 46 21.534 −21.622 1.046 0.00 0.00
I
ATOM 179 HB2 LEU I 46 20.060 −22.734 1.198 0.00 0.00
I
ATOM 180 CG LEU I 46 21.805 −23.648 0.530 0.00 0.00
I
ATOM 181 HG LEU I 46 21.146 −24.350 −0.025 0.00 0.00
I
ATOM 182 CD1 LEU I 46 23.102 −23.269 −0.246 0.00 0.00
I
ATOM 183 HD11 LEU I 46 23.944 −23.982 −0.115 0.00 0.00
I
ATOM 184 HD12 LEU I 46 22.843 −23.349 −1.324 0.00 0.00
I
ATOM 185 HD13 LEU I 46 23.523 −22.292 0.074 0.00 0.00
I
ATOM 186 CD2 LEU I 46 22.235 −24.298 1.865 0.00 0.00
I
ATOM 187 HD21 LEU I 46 22.756 −23.537 2.485 0.00 0.00
I
ATOM 188 HD22 LEU I 46 21.393 −24.818 2.371 0.00 0.00
I

```
ATOM    189 HD23 LEU I  46      23.029 -25.050   1.669  0.00  0.00
I
ATOM    190  C   LEU I  46      19.839 -22.703  -1.700  0.00  0.00
I
ATOM    191  O   LEU I  46      20.511 -23.045  -2.667  0.00  0.00
I
ATOM    192  N   THR I  47      18.522 -22.875  -1.420  0.00  0.00
I
ATOM    193  HN  THR I  47      18.116 -22.509  -0.587  0.00  0.00
I
ATOM    194  CA  THR I  47      17.530 -23.722  -2.149  0.00  0.00
I
ATOM    195  HA  THR I  47      17.961 -24.708  -2.239  0.00  0.00
I
ATOM    196  CB  THR I  47      16.173 -23.772  -1.513  0.00  0.00
I
ATOM    197  HB  THR I  47      15.627 -22.806  -1.566  0.00  0.00
I
ATOM    198  OG1 THR I  47      16.262 -24.381  -0.225  0.00  0.00
I
ATOM    199  HG1 THR I  47      16.568 -23.767   0.446  0.00  0.00
I
ATOM    200  CG2 THR I  47      15.227 -24.704  -2.289  0.00  0.00
I
ATOM    201 HG21 THR I  47      15.789 -25.655  -2.404  0.00  0.00
I
ATOM    202 HG22 THR I  47      14.340 -24.888  -1.645  0.00  0.00
I
ATOM    203 HG23 THR I  47      14.802 -24.182  -3.173  0.00  0.00
I
ATOM    204  C   THR I  47      17.335 -23.170  -3.536  0.00  0.00
I
ATOM    205  O   THR I  47      17.486 -23.845  -4.561  0.00  0.00
I
ATOM    206  N   ILE I  48      17.107 -21.863  -3.670  0.00  0.00
I
ATOM    207  HN  ILE I  48      17.009 -21.304  -2.850  0.00  0.00
I
ATOM    208  CA  ILE I  48      16.793 -21.098  -4.838  0.00  0.00
I
ATOM    209  HA  ILE I  48      15.965 -21.590  -5.328  0.00  0.00
I
ATOM    210  CB  ILE I  48      16.331 -19.651  -4.432  0.00  0.00
I
ATOM    211  HB  ILE I  48      16.952 -19.218  -3.619  0.00  0.00
I
ATOM    212  CG2 ILE I  48      16.328 -18.684  -5.650  0.00  0.00
I
ATOM    213 HG21 ILE I  48      15.690 -19.039  -6.488  0.00  0.00
I
ATOM    214 HG22 ILE I  48      15.797 -17.774  -5.297  0.00  0.00
I
ATOM    215 HG23 ILE I  48      17.348 -18.331  -5.913  0.00  0.00
I
```

ATOM 216 CG1 ILE I 48 14.879 −19.800 −4.009 0.00 0.00
I
ATOM 217 HG11 ILE I 48 14.304 −19.888 −4.955 0.00 0.00
I
ATOM 218 HG12 ILE I 48 14.810 −20.685 −3.341 0.00 0.00
I
ATOM 219 CD ILE I 48 14.339 −18.553 −3.254 0.00 0.00
I
ATOM 220 HD1 ILE I 48 15.086 −18.296 −2.473 0.00 0.00
I
ATOM 221 HD2 ILE I 48 14.359 −17.646 −3.895 0.00 0.00
I
ATOM 222 HD3 ILE I 48 13.321 −18.700 −2.833 0.00 0.00
I
ATOM 223 C ILE I 48 17.952 −21.103 −5.810 0.00 0.00
I
ATOM 224 O ILE I 48 17.819 −21.433 −6.991 0.00 0.00
I
ATOM 225 N ILE I 49 19.219 −20.824 −5.332 0.00 0.00
I
ATOM 226 HN ILE I 49 19.360 −20.573 −4.377 0.00 0.00
I
ATOM 227 CA ILE I 49 20.467 −20.821 −6.151 0.00 0.00
I
ATOM 228 HA ILE I 49 20.313 −20.202 −7.022 0.00 0.00
I
ATOM 229 CB ILE I 49 21.483 −20.245 −5.306 0.00 0.00
I
ATOM 230 HB ILE I 49 21.524 −20.818 −4.355 0.00 0.00
I
ATOM 231 CG2 ILE I 49 22.982 −20.531 −5.814 0.00 0.00
I
ATOM 232 HG21 ILE I 49 23.041 −20.313 −6.902 0.00 0.00
I
ATOM 233 HG22 ILE I 49 23.766 −19.966 −5.267 0.00 0.00
I
ATOM 234 HG23 ILE I 49 23.088 −21.606 −5.552 0.00 0.00
I
ATOM 235 CG1 ILE I 49 21.369 −18.766 −5.160 0.00 0.00
I
ATOM 236 HG11 ILE I 49 20.384 −18.470 −4.741 0.00 0.00
I
ATOM 237 HG12 ILE I 49 22.117 −18.364 −4.443 0.00 0.00
I
ATOM 238 CD ILE I 49 21.408 −17.964 −6.469 0.00 0.00
I
ATOM 239 HD1 ILE I 49 20.670 −18.159 −7.275 0.00 0.00
I
ATOM 240 HD2 ILE I 49 21.320 −16.912 −6.123 0.00 0.00
I
ATOM 241 HD3 ILE I 49 22.376 −18.052 −7.008 0.00 0.00
I
ATOM 242 C ILE I 49 20.777 −22.200 −6.781 0.00 0.00
I

```
ATOM    243  O   ILE I  49      20.939 -22.408  -7.974  0.00  0.00
I
ATOM    244  N   ILE I  50      20.628 -23.274  -5.961  0.00  0.00
I
ATOM    245  HN  ILE I  50      20.469 -23.142  -4.985  0.00  0.00
I
ATOM    246  CA  ILE I  50      20.791 -24.616  -6.416  0.00  0.00
I
ATOM    247  HA  ILE I  50      21.683 -24.822  -6.989  0.00  0.00
I
ATOM    248  CB  ILE I  50      20.876 -25.627  -5.264  0.00  0.00
I
ATOM    249  HB  ILE I  50      20.043 -25.410  -4.562  0.00  0.00
I
ATOM    250  CG2 ILE I  50      20.694 -27.060  -5.788  0.00  0.00
I
ATOM    251 HG21 ILE I  50      19.630 -27.243  -6.052  0.00  0.00
I
ATOM    252 HG22 ILE I  50      21.419 -27.346  -6.579  0.00  0.00
I
ATOM    253 HG23 ILE I  50      20.875 -27.715  -4.909  0.00  0.00
I
ATOM    254  CG1 ILE I  50      22.226 -25.525  -4.541  0.00  0.00
I
ATOM    255 HG11 ILE I  50      22.934 -25.967  -5.274  0.00  0.00
I
ATOM    256 HG12 ILE I  50      22.580 -24.476  -4.456  0.00  0.00
I
ATOM    257  CD  ILE I  50      22.293 -26.188  -3.177  0.00  0.00
I
ATOM    258  HD1 ILE I  50      21.760 -25.440  -2.552  0.00  0.00
I
ATOM    259  HD2 ILE I  50      21.846 -27.186  -2.983  0.00  0.00
I
ATOM    260  HD3 ILE I  50      23.364 -26.220  -2.883  0.00  0.00
I
ATOM    261  C   ILE I  50      19.736 -24.978  -7.430  0.00  0.00
I
ATOM    262  O   ILE I  50      19.972 -25.520  -8.550  0.00  0.00
I
ATOM    263  N   GLY I  51      18.416 -24.698  -7.126  0.00  0.00
I
ATOM    264  HN  GLY I  51      18.243 -24.237  -6.259  0.00  0.00
I
ATOM    265  CA  GLY I  51      17.275 -25.057  -7.969  0.00  0.00
I
ATOM    266  HA1 GLY I  51      16.408 -24.893  -7.347  0.00  0.00
I
ATOM    267  HA2 GLY I  51      17.293 -26.092  -8.278  0.00  0.00
I
ATOM    268  C   GLY I  51      17.117 -24.239  -9.238  0.00  0.00
I
ATOM    269  O   GLY I  51      16.461 -24.729 -10.108  0.00  0.00
I
```

ATOM 270 N ALA I 52 17.752 -23.086 -9.428 0.00 0.00
I
ATOM 271 HN ALA I 52 18.030 -22.550 -8.635 0.00 0.00
I
ATOM 272 CA ALA I 52 18.038 -22.466 -10.696 0.00 0.00
I
ATOM 273 HA ALA I 52 17.143 -22.477 -11.301 0.00 0.00
I
ATOM 274 CB ALA I 52 18.484 -21.008 -10.517 0.00 0.00
I
ATOM 275 HB1 ALA I 52 19.310 -20.833 -9.795 0.00 0.00
I
ATOM 276 HB2 ALA I 52 18.869 -20.650 -11.496 0.00 0.00
I
ATOM 277 HB3 ALA I 52 17.685 -20.277 -10.267 0.00 0.00
I
ATOM 278 C ALA I 52 19.119 -23.240 -11.505 0.00 0.00
I
ATOM 279 O ALA I 52 19.021 -23.504 -12.707 0.00 0.00
I
ATOM 280 N VAL I 53 20.210 -23.703 -10.851 0.00 0.00
I
ATOM 281 HN VAL I 53 20.150 -23.678 -9.856 0.00 0.00
I
ATOM 282 CA VAL I 53 21.349 -24.450 -11.522 0.00 0.00
I
ATOM 283 HA VAL I 53 21.602 -23.901 -12.417 0.00 0.00
I
ATOM 284 CB VAL I 53 22.618 -24.386 -10.745 0.00 0.00
I
ATOM 285 HB VAL I 53 22.471 -24.794 -9.723 0.00 0.00
I
ATOM 286 CG1 VAL I 53 23.778 -25.212 -11.395 0.00 0.00
I
ATOM 287 HG11 VAL I 53 24.751 -24.997 -10.903 0.00 0.00
I
ATOM 288 HG12 VAL I 53 23.790 -26.322 -11.365 0.00 0.00
I
ATOM 289 HG13 VAL I 53 23.897 -24.873 -12.446 0.00 0.00
I
ATOM 290 CG2 VAL I 53 23.101 -22.965 -10.522 0.00 0.00
I
ATOM 291 HG21 VAL I 53 22.334 -22.369 -9.984 0.00 0.00
I
ATOM 292 HG22 VAL I 53 24.068 -22.981 -9.974 0.00 0.00
I
ATOM 293 HG23 VAL I 53 23.218 -22.495 -11.521 0.00 0.00
I
ATOM 294 C VAL I 53 20.887 -25.833 -11.984 0.00 0.00
I
ATOM 295 O VAL I 53 21.307 -26.264 -13.073 0.00 0.00
I
ATOM 296 N LEU I 54 20.030 -26.537 -11.273 0.00 0.00
I

```
ATOM    297  HN  LEU I  54      19.712 -26.105 -10.433  0.00  0.00
I
ATOM    298  CA  LEU I  54      19.314 -27.772 -11.542  0.00  0.00
I
ATOM    299  HA  LEU I  54      20.113 -28.415 -11.883  0.00  0.00
I
ATOM    300  CB  LEU I  54      18.823 -28.203 -10.169  0.00  0.00
I
ATOM    301  HB1 LEU I  54      19.587 -28.079  -9.372  0.00  0.00
I
ATOM    302  HB2 LEU I  54      17.961 -27.580  -9.844  0.00  0.00
I
ATOM    303  CG  LEU I  54      18.468 -29.744 -10.122  0.00  0.00
I
ATOM    304  HG  LEU I  54      17.850 -30.094 -10.976  0.00  0.00
I
ATOM    305  CD1 LEU I  54      19.689 -30.633 -10.170  0.00  0.00
I
ATOM    306 HD11 LEU I  54      20.551 -30.168  -9.645  0.00  0.00
I
ATOM    307 HD12 LEU I  54      19.594 -31.579  -9.596  0.00  0.00
I
ATOM    308 HD13 LEU I  54      19.969 -30.814 -11.230  0.00  0.00
I
ATOM    309  CD2 LEU I  54      17.738 -29.954  -8.844  0.00  0.00
I
ATOM    310 HD21 LEU I  54      17.198 -30.925  -8.844  0.00  0.00
I
ATOM    311 HD22 LEU I  54      18.498 -30.057  -8.041  0.00  0.00
I
ATOM    312 HD23 LEU I  54      16.953 -29.206  -8.600  0.00  0.00
I
ATOM    313  C   LEU I  54      18.313 -27.669 -12.629  0.00  0.00
I
ATOM    314  O   LEU I  54      18.157 -28.520 -13.481  0.00  0.00
I
ATOM    315  N   LEU I  55      17.576 -26.545 -12.760  0.00  0.00
I
ATOM    316  HN  LEU I  55      17.600 -25.867 -12.029  0.00  0.00
I
ATOM    317  CA  LEU I  55      16.695 -26.326 -13.910  0.00  0.00
I
ATOM    318  HA  LEU I  55      16.139 -27.230 -14.111  0.00  0.00
I
ATOM    319  CB  LEU I  55      15.664 -25.232 -13.651  0.00  0.00
I
ATOM    320  HB1 LEU I  55      16.147 -24.320 -13.238  0.00  0.00
I
ATOM    321  HB2 LEU I  55      15.000 -24.979 -14.505  0.00  0.00
I
ATOM    322  CG  LEU I  55      14.613 -25.575 -12.578  0.00  0.00
I
ATOM    323  HG  LEU I  55      15.080 -25.898 -11.622  0.00  0.00
I
```

```
ATOM    324  CD1 LEU I  55      13.798 -24.226 -12.452  0.00  0.00
I
ATOM    325 HD11 LEU I  55      13.615 -23.747 -13.438  0.00  0.00
I
ATOM    326 HD12 LEU I  55      12.818 -24.488 -11.998  0.00  0.00
I
ATOM    327 HD13 LEU I  55      14.410 -23.480 -11.901  0.00  0.00
I
ATOM    328  CD2 LEU I  55      13.726 -26.737 -13.004  0.00  0.00
I
ATOM    329 HD21 LEU I  55      13.011 -26.333 -13.753  0.00  0.00
I
ATOM    330 HD22 LEU I  55      14.296 -27.657 -13.254  0.00  0.00
I
ATOM    331 HD23 LEU I  55      12.966 -27.046 -12.254  0.00  0.00
I
ATOM    332  C   LEU I  55      17.328 -25.756 -15.188  0.00  0.00
I
ATOM    333  O   LEU I  55      16.707 -25.899 -16.220  0.00  0.00
I
ATOM    334  N   MET I  56      18.574 -25.229 -15.175  0.00  0.00
I
ATOM    335  HN  MET I  56      19.039 -25.206 -14.293  0.00  0.00
I
ATOM    336  CA  MET I  56      19.336 -24.820 -16.335  0.00  0.00
I
ATOM    337  HA  MET I  56      18.679 -24.419 -17.093  0.00  0.00
I
ATOM    338  CB  MET I  56      20.416 -23.797 -15.997  0.00  0.00
I
ATOM    339  HB1 MET I  56      20.047 -22.994 -15.324  0.00  0.00
I
ATOM    340  HB2 MET I  56      21.279 -24.241 -15.457  0.00  0.00
I
ATOM    341  CG  MET I  56      20.890 -22.861 -17.149  0.00  0.00
I
ATOM    342  HG1 MET I  56      21.347 -23.498 -17.935  0.00  0.00
I
ATOM    343  HG2 MET I  56      20.001 -22.393 -17.624  0.00  0.00
I
ATOM    344  SD  MET I  56      22.126 -21.646 -16.608  0.00  0.00
I
ATOM    345  CE  MET I  56      22.850 -21.212 -18.204  0.00  0.00
I
ATOM    346  HE1 MET I  56      22.016 -20.659 -18.687  0.00  0.00
I
ATOM    347  HE2 MET I  56      23.728 -20.536 -18.121  0.00  0.00
I
ATOM    348  HE3 MET I  56      23.288 -22.104 -18.702  0.00  0.00
I
ATOM    349  C   MET I  56      20.158 -25.882 -17.021  0.00  0.00
I
ATOM    350  O   MET I  56      20.525 -25.885 -18.204  0.00  0.00
I
```

ATOM 351 N LEU I 57 20.430 -26.925 -16.232 0.00 0.00
I
ATOM 352 HN LEU I 57 20.216 -26.898 -15.259 0.00 0.00
I
ATOM 353 CA LEU I 57 21.090 -28.141 -16.772 0.00 0.00
I
ATOM 354 HA LEU I 57 22.019 -27.835 -17.230 0.00 0.00
I
ATOM 355 CB LEU I 57 21.317 -29.099 -15.596 0.00 0.00
I
ATOM 356 HB1 LEU I 57 21.660 -28.451 -14.761 0.00 0.00
I
ATOM 357 HB2 LEU I 57 20.332 -29.524 -15.308 0.00 0.00
I
ATOM 358 CG LEU I 57 22.205 -30.458 -15.638 0.00 0.00
I
ATOM 359 HG LEU I 57 21.714 -31.065 -16.428 0.00 0.00
I
ATOM 360 CD1 LEU I 57 23.688 -30.338 -15.877 0.00 0.00
I
ATOM 361 HD11 LEU I 57 24.133 -31.356 -15.871 0.00 0.00
I
ATOM 362 HD12 LEU I 57 24.003 -29.822 -16.809 0.00 0.00
I
ATOM 363 HD13 LEU I 57 24.241 -29.828 -15.059 0.00 0.00
I
ATOM 364 CD2 LEU I 57 21.994 -31.104 -14.216 0.00 0.00
I
ATOM 365 HD21 LEU I 57 21.019 -31.580 -13.976 0.00 0.00
I
ATOM 366 HD22 LEU I 57 22.800 -31.864 -14.134 0.00 0.00
I
ATOM 367 HD23 LEU I 57 22.231 -30.332 -13.454 0.00 0.00
I
ATOM 368 C LEU I 57 20.356 -28.824 -17.944 0.00 0.00
I
ATOM 369 O LEU I 57 19.164 -29.139 -17.822 0.00 0.00
I
ATOM 370 N PRO I 58 21.015 -29.216 -19.138 0.00 0.00
I
ATOM 371 CD PRO I 58 22.417 -28.916 -19.365 0.00 0.00
I
ATOM 372 HD1 PRO I 58 22.613 -27.860 -19.080 0.00 0.00
I
ATOM 373 HD2 PRO I 58 23.063 -29.676 -18.878 0.00 0.00
I
ATOM 374 CA PRO I 58 20.363 -29.925 -20.306 0.00 0.00
I
ATOM 375 HA PRO I 58 19.768 -29.088 -20.641 0.00 0.00
I
ATOM 376 CB PRO I 58 21.543 -30.174 -21.176 0.00 0.00
I
ATOM 377 HB1 PRO I 58 21.152 -30.132 -22.215 0.00 0.00
I

ATOM	378	HB2	PRO	I	58	21.911	-31.222	-21.200	0.00	0.00	I
ATOM	379	CG	PRO	I	58	22.626	-29.196	-20.857	0.00	0.00	I
ATOM	380	HG1	PRO	I	58	22.568	-28.217	-21.379	0.00	0.00	I
ATOM	381	HG2	PRO	I	58	23.666	-29.473	-21.133	0.00	0.00	I
ATOM	382	C	PRO	I	58	19.484	-31.095	-20.181	0.00	0.00	I
ATOM	383	O	PRO	I	58	18.721	-31.358	-21.123	0.00	0.00	I
ATOM	384	N	ILE	I	59	19.566	-31.807	-19.053	0.00	0.00	I
ATOM	385	HN	ILE	I	59	20.258	-31.584	-18.371	0.00	0.00	I
ATOM	386	CA	ILE	I	59	18.643	-32.936	-18.847	0.00	0.00	I
ATOM	387	HA	ILE	I	59	18.333	-33.501	-19.714	0.00	0.00	I
ATOM	388	CB	ILE	I	59	19.314	-33.870	-17.863	0.00	0.00	I
ATOM	389	HB	ILE	I	59	20.387	-33.926	-18.145	0.00	0.00	I
ATOM	390	CG2	ILE	I	59	19.280	-33.242	-16.452	0.00	0.00	I
ATOM	391	HG21	ILE	I	59	19.889	-33.864	-15.761	0.00	0.00	I
ATOM	392	HG22	ILE	I	59	19.586	-32.174	-16.412	0.00	0.00	I
ATOM	393	HG23	ILE	I	59	18.280	-33.266	-15.968	0.00	0.00	I
ATOM	394	CG1	ILE	I	59	18.795	-35.273	-17.920	0.00	0.00	I
ATOM	395	HG11	ILE	I	59	19.253	-35.807	-17.060	0.00	0.00	I
ATOM	396	HG12	ILE	I	59	17.689	-35.308	-17.812	0.00	0.00	I
ATOM	397	CD	ILE	I	59	19.160	-36.039	-19.151	0.00	0.00	I
ATOM	398	HD1	ILE	I	59	20.252	-35.896	-19.296	0.00	0.00	I
ATOM	399	HD2	ILE	I	59	18.968	-37.132	-19.105	0.00	0.00	I
ATOM	400	HD3	ILE	I	59	18.700	-35.677	-20.096	0.00	0.00	I
ATOM	401	C	ILE	I	59	17.239	-32.488	-18.275	0.00	0.00	I
ATOM	402	O	ILE	I	59	16.317	-33.289	-18.259	0.00	0.00	I
ATOM	403	N	SER	I	60	17.115	-31.231	-17.827	0.00	0.00	I
ATOM	404	HN	SER	I	60	17.915	-30.647	-17.941	0.00	0.00	I

ATOM 405 CA SER I 60 15.941 -30.440 -17.345 0.00 0.00
I
ATOM 406 HA SER I 60 15.220 -31.124 -16.921 0.00 0.00
I
ATOM 407 CB SER I 60 16.347 -29.399 -16.336 0.00 0.00
I
ATOM 408 HB1 SER I 60 17.249 -28.794 -16.568 0.00 0.00
I
ATOM 409 HB2 SER I 60 15.601 -28.583 -16.228 0.00 0.00
I
ATOM 410 OG SER I 60 16.481 -29.952 -15.048 0.00 0.00
I
ATOM 411 HG1 SER I 60 17.195 -29.470 -14.624 0.00 0.00
I
ATOM 412 C SER I 60 15.247 -29.705 -18.446 0.00 0.00
I
ATOM 413 O SER I 60 14.094 -29.394 -18.259 0.00 0.00
I
ATOM 414 N THR I 61 15.912 -29.444 -19.646 0.00 0.00
I
ATOM 415 HN THR I 61 16.732 -29.862 -20.031 0.00 0.00
I
ATOM 416 CA THR I 61 15.396 -28.411 -20.543 0.00 0.00
I
ATOM 417 HA THR I 61 14.514 -27.953 -20.121 0.00 0.00
I
ATOM 418 CB THR I 61 16.372 -27.154 -20.655 0.00 0.00
I
ATOM 419 HB THR I 61 15.858 -26.339 -21.208 0.00 0.00
I
ATOM 420 OG1 THR I 61 17.637 -27.354 -21.189 0.00 0.00
I
ATOM 421 HG1 THR I 61 18.127 -26.543 -21.036 0.00 0.00
I
ATOM 422 CG2 THR I 61 16.752 -26.776 -19.260 0.00 0.00
I
ATOM 423 HG21 THR I 61 17.421 -27.487 -18.730 0.00 0.00
I
ATOM 424 HG22 THR I 61 17.345 -25.838 -19.200 0.00 0.00
I
ATOM 425 HG23 THR I 61 15.874 -26.610 -18.601 0.00 0.00
I
ATOM 426 C THR I 61 15.139 -28.954 -21.905 0.00 0.00
I
ATOM 427 O THR I 61 15.808 -29.878 -22.416 0.00 0.00
I
ATOM 428 N THR I 62 14.039 -28.432 -22.515 0.00 0.00
I
ATOM 429 HN THR I 62 13.346 -27.792 -22.191 0.00 0.00
I
ATOM 430 CA THR I 62 13.584 -29.097 -23.801 0.00 0.00
I
ATOM 431 HA THR I 62 14.047 -30.072 -23.793 0.00 0.00
I

```
ATOM    432  CB  THR I  62      12.074 -29.225 -23.842  0.00  0.00
I
ATOM    433  HB  THR I  62      11.737 -29.800 -24.731  0.00  0.00
I
ATOM    434  OG1 THR I  62      11.436 -27.930 -23.787  0.00  0.00
I
ATOM    435  HG1 THR I  62      10.590 -27.989 -24.236  0.00  0.00
I
ATOM    436  CG2 THR I  62      11.596 -30.014 -22.656  0.00  0.00
I
ATOM    437 HG21 THR I  62      10.491 -30.121 -22.688  0.00  0.00
I
ATOM    438 HG22 THR I  62      12.006 -31.046 -22.624  0.00  0.00
I
ATOM    439 HG23 THR I  62      11.767 -29.397 -21.748  0.00  0.00
I
ATOM    440  C   THR I  62      14.104 -28.316 -25.017  0.00  0.00
I
ATOM    441  O   THR I  62      14.669 -28.732 -26.049  0.00  0.00
I
ATOM    442  N   LYS I  63      14.143 -26.971 -24.862  0.00  0.00
I
ATOM    443  HN  LYS I  63      13.940 -26.589 -23.963  0.00  0.00
I
ATOM    444  CA  LYS I  63      14.719 -25.917 -25.741  0.00  0.00
I
ATOM    445  HA  LYS I  63      15.341 -26.250 -26.558  0.00  0.00
I
ATOM    446  CB  LYS I  63      13.529 -25.091 -26.268  0.00  0.00
I
ATOM    447  HB1 LYS I  63      12.790 -24.654 -25.563  0.00  0.00
I
ATOM    448  HB2 LYS I  63      13.962 -24.304 -26.922  0.00  0.00
I
ATOM    449  CG  LYS I  63      12.684 -25.995 -27.282  0.00  0.00
I
ATOM    450  HG1 LYS I  63      13.423 -26.306 -28.051  0.00  0.00
I
ATOM    451  HG2 LYS I  63      12.171 -26.897 -26.886  0.00  0.00
I
ATOM    452  CD  LYS I  63      11.600 -25.084 -28.040  0.00  0.00
I
ATOM    453  HD1 LYS I  63      10.866 -24.690 -27.305  0.00  0.00
I
ATOM    454  HD2 LYS I  63      12.097 -24.275 -28.618  0.00  0.00
I
ATOM    455  CE  LYS I  63      10.848 -26.004 -28.976  0.00  0.00
I
ATOM    456  HE1 LYS I  63      11.498 -26.253 -29.842  0.00  0.00
I
ATOM    457  HE2 LYS I  63      10.561 -26.948 -28.465  0.00  0.00
I
ATOM    458  NZ  LYS I  63       9.680 -25.307 -29.506  0.00  0.00
I
```

ATOM 459 HZ1 LYS I 63 8.943 -25.223 -28.778 0.00 0.00
I
ATOM 460 HZ2 LYS I 63 9.981 -24.348 -29.775 0.00 0.00
I
ATOM 461 HZ3 LYS I 63 9.243 -25.741 -30.344 0.00 0.00
I
ATOM 462 C LYS I 63 15.565 -25.068 -24.896 0.00 0.00
I
ATOM 463 O LYS I 63 15.350 -24.973 -23.679 0.00 0.00
I
ATOM 464 N PRO I 64 16.637 -24.465 -25.255 0.00 0.00
I
ATOM 465 CD PRO I 64 16.961 -24.199 -26.675 0.00 0.00
I
ATOM 466 HD1 PRO I 64 17.113 -25.152 -27.225 0.00 0.00
I
ATOM 467 HD2 PRO I 64 16.093 -23.668 -27.121 0.00 0.00
I
ATOM 468 CA PRO I 64 17.630 -23.965 -24.306 0.00 0.00
I
ATOM 469 HA PRO I 64 17.854 -24.768 -23.620 0.00 0.00
I
ATOM 470 CB PRO I 64 18.869 -23.640 -25.233 0.00 0.00
I
ATOM 471 HB1 PRO I 64 19.339 -24.618 -25.471 0.00 0.00
I
ATOM 472 HB2 PRO I 64 19.636 -22.947 -24.828 0.00 0.00
I
ATOM 473 CG PRO I 64 18.193 -23.280 -26.567 0.00 0.00
I
ATOM 474 HG1 PRO I 64 17.860 -22.222 -26.499 0.00 0.00
I
ATOM 475 HG2 PRO I 64 18.988 -23.344 -27.340 0.00 0.00
I
ATOM 476 C PRO I 64 17.301 -22.820 -23.396 0.00 0.00
I
ATOM 477 O PRO I 64 16.650 -21.926 -23.800 0.00 0.00
I
ATOM 478 N LEU I 65 17.617 -22.946 -22.046 0.00 0.00
I
ATOM 479 HN LEU I 65 18.166 -23.668 -21.632 0.00 0.00
I
ATOM 480 CA LEU I 65 17.122 -22.037 -21.012 0.00 0.00
I
ATOM 481 HA LEU I 65 16.390 -21.325 -21.365 0.00 0.00
I
ATOM 482 CB LEU I 65 16.499 -22.947 -19.954 0.00 0.00
I
ATOM 483 HB1 LEU I 65 15.903 -23.683 -20.536 0.00 0.00
I
ATOM 484 HB2 LEU I 65 17.314 -23.420 -19.366 0.00 0.00
I
ATOM 485 CG LEU I 65 15.636 -22.193 -18.852 0.00 0.00
I

ATOM 486 HG LEU I 65 16.209 −21.274 −18.602 0.00 0.00
I
ATOM 487 CD1 LEU I 65 14.264 −21.637 −19.380 0.00 0.00
I
ATOM 488 HD11 LEU I 65 14.423 −20.841 −20.138 0.00 0.00
I
ATOM 489 HD12 LEU I 65 13.779 −22.440 −19.974 0.00 0.00
I
ATOM 490 HD13 LEU I 65 13.608 −21.212 −18.590 0.00 0.00
I
ATOM 491 CD2 LEU I 65 15.416 −23.077 −17.665 0.00 0.00
I
ATOM 492 HD21 LEU I 65 14.811 −22.617 −16.854 0.00 0.00
I
ATOM 493 HD22 LEU I 65 14.885 −24.039 −17.828 0.00 0.00
I
ATOM 494 HD23 LEU I 65 16.310 −23.404 −17.091 0.00 0.00
I
ATOM 495 C LEU I 65 18.252 −21.133 −20.416 0.00 0.00
I
ATOM 496 O LEU I 65 19.302 −21.647 −20.008 0.00 0.00
I
ATOM 497 N SER I 66 17.973 −19.804 −20.337 0.00 0.00
I
ATOM 498 HN SER I 66 17.137 −19.360 −20.649 0.00 0.00
I
ATOM 499 CA SER I 66 18.994 −18.848 −19.727 0.00 0.00
I
ATOM 500 HA SER I 66 19.951 −19.225 −20.057 0.00 0.00
I
ATOM 501 CB SER I 66 18.721 −17.394 −20.151 0.00 0.00
I
ATOM 502 HB1 SER I 66 18.313 −17.288 −21.178 0.00 0.00
I
ATOM 503 HB2 SER I 66 17.942 −16.855 −19.571 0.00 0.00
I
ATOM 504 OG SER I 66 19.900 −16.641 −20.210 0.00 0.00
I
ATOM 505 HG1 SER I 66 19.609 −15.731 −20.302 0.00 0.00
I
ATOM 506 C SER I 66 18.947 −18.895 −18.133 0.00 0.00
I
ATOM 507 O SER I 66 17.856 −19.219 −17.647 0.00 0.00
I
ATOM 508 N TRP I 67 19.977 −18.608 −17.335 0.00 0.00
I
ATOM 509 HN TRP I 67 20.846 −18.309 −17.720 0.00 0.00
I
ATOM 510 CA TRP I 67 20.001 −18.861 −15.899 0.00 0.00
I
ATOM 511 HA TRP I 67 19.782 −19.909 −15.755 0.00 0.00
I
ATOM 512 CB TRP I 67 21.376 −18.627 −15.276 0.00 0.00
I

ATOM 513 HB1 TRP I 67 22.170 -18.865 -16.015 0.00 0.00
I
ATOM 514 HB2 TRP I 67 21.518 -17.525 -15.263 0.00 0.00
I
ATOM 515 CG TRP I 67 21.615 -19.165 -13.810 0.00 0.00
I
ATOM 516 CD1 TRP I 67 21.434 -20.472 -13.369 0.00 0.00
I
ATOM 517 HD1 TRP I 67 20.980 -21.210 -14.014 0.00 0.00
I
ATOM 518 NE1 TRP I 67 21.471 -20.459 -11.995 0.00 0.00
I
ATOM 519 HE1 TRP I 67 21.351 -21.222 -11.398 0.00 0.00
I
ATOM 520 CE2 TRP I 67 21.604 -19.123 -11.545 0.00 0.00
I
ATOM 521 CD2 TRP I 67 21.601 -18.311 -12.666 0.00 0.00
I
ATOM 522 CE3 TRP I 67 21.632 -16.905 -12.533 0.00 0.00
I
ATOM 523 HE3 TRP I 67 21.394 -16.264 -13.370 0.00 0.00
I
ATOM 524 CZ3 TRP I 67 21.490 -16.406 -11.221 0.00 0.00
I
ATOM 525 HZ3 TRP I 67 21.465 -15.340 -11.047 0.00 0.00
I
ATOM 526 CZ2 TRP I 67 21.490 -18.586 -10.285 0.00 0.00
I
ATOM 527 HZ2 TRP I 67 21.284 -19.237 -9.448 0.00 0.00
I
ATOM 528 CH2 TRP I 67 21.478 -17.157 -10.081 0.00 0.00
I
ATOM 529 HH2 TRP I 67 21.389 -16.628 -9.143 0.00 0.00
I
ATOM 530 C TRP I 67 18.960 -18.014 -15.136 0.00 0.00
I
ATOM 531 O TRP I 67 18.316 -18.427 -14.216 0.00 0.00
I
ATOM 532 N ILE I 68 18.830 -16.727 -15.505 0.00 0.00
I
ATOM 533 HN ILE I 68 19.531 -16.385 -16.126 0.00 0.00
I
ATOM 534 CA ILE I 68 17.756 -15.866 -15.145 0.00 0.00
I
ATOM 535 HA ILE I 68 17.873 -15.754 -14.078 0.00 0.00
I
ATOM 536 CB ILE I 68 17.967 -14.423 -15.768 0.00 0.00
I
ATOM 537 HB ILE I 68 18.908 -13.982 -15.375 0.00 0.00
I
ATOM 538 CG2 ILE I 68 17.988 -14.507 -17.303 0.00 0.00
I
ATOM 539 HG21 ILE I 68 18.789 -15.202 -17.634 0.00 0.00
I

ATOM 540 HG22 ILE I 68 17.070 -14.883 -17.802 0.00 0.00
I
ATOM 541 HG23 ILE I 68 18.203 -13.492 -17.702 0.00 0.00
I
ATOM 542 CG1 ILE I 68 16.974 -13.355 -15.246 0.00 0.00
I
ATOM 543 HG11 ILE I 68 17.155 -12.426 -15.829 0.00 0.00
I
ATOM 544 HG12 ILE I 68 15.948 -13.682 -15.519 0.00 0.00
I
ATOM 545 CD ILE I 68 17.022 -13.112 -13.714 0.00 0.00
I
ATOM 546 HD1 ILE I 68 16.410 -13.914 -13.249 0.00 0.00
I
ATOM 547 HD2 ILE I 68 17.993 -13.050 -13.179 0.00 0.00
I
ATOM 548 HD3 ILE I 68 16.524 -12.129 -13.571 0.00 0.00
I
ATOM 549 C ILE I 68 16.311 -16.331 -15.265 0.00 0.00
I
ATOM 550 O ILE I 68 15.508 -16.148 -14.340 0.00 0.00
I
ATOM 551 N ASP I 69 15.895 -17.043 -16.357 0.00 0.00
I
ATOM 552 HN ASP I 69 16.336 -17.075 -17.251 0.00 0.00
I
ATOM 553 CA ASP I 69 14.527 -17.679 -16.489 0.00 0.00
I
ATOM 554 HA ASP I 69 13.768 -16.981 -16.166 0.00 0.00
I
ATOM 555 CB ASP I 69 14.342 -18.058 -18.000 0.00 0.00
I
ATOM 556 HB1 ASP I 69 15.140 -18.790 -18.249 0.00 0.00
I
ATOM 557 HB2 ASP I 69 13.355 -18.561 -18.083 0.00 0.00
I
ATOM 558 CG ASP I 69 14.493 -16.808 -18.736 0.00 0.00
I
ATOM 559 OD1 ASP I 69 13.655 -15.858 -18.649 0.00 0.00
I
ATOM 560 OD2 ASP I 69 15.481 -16.694 -19.567 0.00 0.00
I
ATOM 561 C ASP I 69 14.429 -18.793 -15.397 0.00 0.00
I
ATOM 562 O ASP I 69 13.370 -19.011 -14.781 0.00 0.00
I
ATOM 563 N ALA I 70 15.501 -19.532 -15.095 0.00 0.00
I
ATOM 564 HN ALA I 70 16.369 -19.371 -15.559 0.00 0.00
I
ATOM 565 CA ALA I 70 15.489 -20.629 -14.147 0.00 0.00
I
ATOM 566 HA ALA I 70 14.548 -21.157 -14.198 0.00 0.00
I

```
ATOM    567  CB  ALA I  70      16.726 -21.508 -14.574  0.00  0.00
I
ATOM    568  HB1 ALA I  70      17.647 -20.920 -14.770  0.00  0.00
I
ATOM    569  HB2 ALA I  70      16.864 -22.301 -13.808  0.00  0.00
I
ATOM    570  HB3 ALA I  70      16.574 -22.035 -15.540  0.00  0.00
I
ATOM    571  C   ALA I  70      15.585 -20.129 -12.768  0.00  0.00
I
ATOM    572  O   ALA I  70      14.976 -20.745 -11.903  0.00  0.00
I
ATOM    573  N   LEU I  71      16.218 -18.926 -12.561  0.00  0.00
I
ATOM    574  HN  LEU I  71      16.863 -18.587 -13.241  0.00  0.00
I
ATOM    575  CA  LEU I  71      16.136 -18.172 -11.357  0.00  0.00
I
ATOM    576  HA  LEU I  71      16.477 -18.907 -10.643  0.00  0.00
I
ATOM    577  CB  LEU I  71      17.030 -16.934 -11.362  0.00  0.00
I
ATOM    578  HB1 LEU I  71      17.980 -17.396 -11.708  0.00  0.00
I
ATOM    579  HB2 LEU I  71      16.687 -16.228 -12.148  0.00  0.00
I
ATOM    580  CG  LEU I  71      17.239 -16.215 -10.024  0.00  0.00
I
ATOM    581  HG  LEU I  71      16.218 -15.988  -9.648  0.00  0.00
I
ATOM    582  CD1 LEU I  71      17.909 -17.094  -8.934  0.00  0.00
I
ATOM    583 HD11 LEU I  71      17.146 -17.823  -8.587  0.00  0.00
I
ATOM    584 HD12 LEU I  71      18.863 -17.557  -9.265  0.00  0.00
I
ATOM    585 HD13 LEU I  71      18.102 -16.312  -8.169  0.00  0.00
I
ATOM    586  CD2 LEU I  71      18.048 -14.867 -10.263  0.00  0.00
I
ATOM    587 HD21 LEU I  71      19.099 -15.053 -10.572  0.00  0.00
I
ATOM    588 HD22 LEU I  71      17.620 -14.279 -11.104  0.00  0.00
I
ATOM    589 HD23 LEU I  71      17.978 -14.168  -9.402  0.00  0.00
I
ATOM    590  C   LEU I  71      14.646 -17.796 -10.983  0.00  0.00
I
ATOM    591  O   LEU I  71      14.144 -18.129  -9.903  0.00  0.00
I
ATOM    592  N   PHE I  72      13.924 -17.247 -11.976  0.00  0.00
I
ATOM    593  HN  PHE I  72      14.391 -16.909 -12.789  0.00  0.00
I
```

ATOM 594 CA PHE I 72 12.464 -17.030 -11.962 0.00 0.00
I
ATOM 595 HA PHE I 72 12.272 -16.469 -11.059 0.00 0.00
I
ATOM 596 CB PHE I 72 11.912 -16.189 -13.195 0.00 0.00
I
ATOM 597 HB1 PHE I 72 12.589 -15.403 -13.593 0.00 0.00
I
ATOM 598 HB2 PHE I 72 11.833 -16.888 -14.055 0.00 0.00
I
ATOM 599 CG PHE I 72 10.610 -15.495 -12.982 0.00 0.00
I
ATOM 600 CD1 PHE I 72 9.398 -16.223 -13.014 0.00 0.00
I
ATOM 601 HD1 PHE I 72 9.513 -17.219 -13.413 0.00 0.00
I
ATOM 602 CE1 PHE I 72 8.228 -15.616 -12.595 0.00 0.00
I
ATOM 603 HE1 PHE I 72 7.348 -16.237 -12.519 0.00 0.00
I
ATOM 604 CZ PHE I 72 8.219 -14.299 -12.106 0.00 0.00
I
ATOM 605 HZ PHE I 72 7.363 -13.750 -11.742 0.00 0.00
I
ATOM 606 CD2 PHE I 72 10.584 -14.135 -12.548 0.00 0.00
I
ATOM 607 HD2 PHE I 72 11.541 -13.634 -12.516 0.00 0.00
I
ATOM 608 CE2 PHE I 72 9.384 -13.566 -12.186 0.00 0.00
I
ATOM 609 HE2 PHE I 72 9.463 -12.509 -11.978 0.00 0.00
I
ATOM 610 C PHE I 72 11.584 -18.220 -11.636 0.00 0.00
I
ATOM 611 O PHE I 72 10.759 -18.168 -10.668 0.00 0.00
I
ATOM 612 N THR I 73 11.722 -19.352 -12.368 0.00 0.00
I
ATOM 613 HN THR I 73 12.296 -19.359 -13.183 0.00 0.00
I
ATOM 614 CA THR I 73 10.960 -20.538 -12.028 0.00 0.00
I
ATOM 615 HA THR I 73 9.965 -20.212 -11.763 0.00 0.00
I
ATOM 616 CB THR I 73 11.008 -21.607 -13.159 0.00 0.00
I
ATOM 617 HB THR I 73 11.960 -22.180 -13.150 0.00 0.00
I
ATOM 618 OG1 THR I 73 10.814 -21.032 -14.452 0.00 0.00
I
ATOM 619 HG1 THR I 73 11.252 -21.653 -15.040 0.00 0.00
I
ATOM 620 CG2 THR I 73 9.771 -22.496 -12.858 0.00 0.00
I

```
ATOM    621 HG21 THR I  73       9.266 -22.841 -13.785  0.00  0.00
I
ATOM    622 HG22 THR I  73      10.039 -23.479 -12.414  0.00  0.00
I
ATOM    623 HG23 THR I  73       9.031 -21.919 -12.265  0.00  0.00
I
ATOM    624  C   THR I  73      11.367 -21.063 -10.628  0.00  0.00
I
ATOM    625  O   THR I  73      10.561 -21.309  -9.734  0.00  0.00
I
ATOM    626  N   ALA I  74      12.658 -21.127 -10.324  0.00  0.00
I
ATOM    627  HN  ALA I  74      13.373 -20.883 -10.975  0.00  0.00
I
ATOM    628  CA  ALA I  74      13.196 -21.668  -9.036  0.00  0.00
I
ATOM    629  HA  ALA I  74      12.801 -22.669  -8.945  0.00  0.00
I
ATOM    630  CB  ALA I  74      14.775 -21.765  -9.022  0.00  0.00
I
ATOM    631  HB1 ALA I  74      15.059 -20.728  -9.302  0.00  0.00
I
ATOM    632  HB2 ALA I  74      15.038 -22.086  -7.992  0.00  0.00
I
ATOM    633  HB3 ALA I  74      15.155 -22.410  -9.843  0.00  0.00
I
ATOM    634  C   ALA I  74      12.790 -20.862  -7.808  0.00  0.00
I
ATOM    635  O   ALA I  74      12.518 -21.374  -6.720  0.00  0.00
I
ATOM    636  N   ALA I  75      12.835 -19.537  -7.951  0.00  0.00
I
ATOM    637  HN  ALA I  75      12.875 -19.123  -8.857  0.00  0.00
I
ATOM    638  CA  ALA I  75      12.305 -18.645  -6.962  0.00  0.00
I
ATOM    639  HA  ALA I  75      12.681 -18.836  -5.968  0.00  0.00
I
ATOM    640  CB  ALA I  75      12.700 -17.209  -7.384  0.00  0.00
I
ATOM    641  HB1 ALA I  75      12.209 -16.967  -8.351  0.00  0.00
I
ATOM    642  HB2 ALA I  75      12.445 -16.568  -6.513  0.00  0.00
I
ATOM    643  HB3 ALA I  75      13.809 -17.163  -7.416  0.00  0.00
I
ATOM    644  C   ALA I  75      10.780 -18.661  -6.776  0.00  0.00
I
ATOM    645  O   ALA I  75      10.178 -18.837  -5.683  0.00  0.00
I
ATOM    646  N   SER I  76      10.060 -18.691  -7.928  0.00  0.00
I
ATOM    647  HN  SER I  76      10.467 -18.606  -8.834  0.00  0.00
I
```

ATOM 648 CA SER I 76 8.569 −18.887 −7.854 0.00 0.00
I
ATOM 649 HA SER I 76 8.090 −18.150 −7.227 0.00 0.00
I
ATOM 650 CB SER I 76 7.919 −18.620 −9.273 0.00 0.00
I
ATOM 651 HB1 SER I 76 8.562 −17.791 −9.638 0.00 0.00
I
ATOM 652 HB2 SER I 76 8.101 −19.480 −9.952 0.00 0.00
I
ATOM 653 OG SER I 76 6.602 −18.278 −9.321 0.00 0.00
I
ATOM 654 HG1 SER I 76 6.318 −18.272 −10.238 0.00 0.00
I
ATOM 655 C SER I 76 8.127 −20.207 −7.327 0.00 0.00
I
ATOM 656 O SER I 76 7.144 −20.305 −6.631 0.00 0.00
I
ATOM 657 N ALA I 77 8.876 −21.273 −7.627 0.00 0.00
I
ATOM 658 HN ALA I 77 9.639 −21.066 −8.234 0.00 0.00
I
ATOM 659 CA ALA I 77 8.754 −22.652 −7.218 0.00 0.00
I
ATOM 660 HA ALA I 77 7.744 −22.961 −7.447 0.00 0.00
I
ATOM 661 CB ALA I 77 9.622 −23.573 −8.044 0.00 0.00
I
ATOM 662 HB1 ALA I 77 9.335 −23.420 −9.106 0.00 0.00
I
ATOM 663 HB2 ALA I 77 10.674 −23.228 −7.949 0.00 0.00
I
ATOM 664 HB3 ALA I 77 9.601 −24.674 −7.894 0.00 0.00
I
ATOM 665 C ALA I 77 9.080 −22.828 −5.776 0.00 0.00
I
ATOM 666 O ALA I 77 8.303 −23.577 −5.186 0.00 0.00
I
ATOM 667 N THR I 78 10.134 −22.169 −5.153 0.00 0.00
I
ATOM 668 HN THR I 78 10.856 −21.671 −5.626 0.00 0.00
I
ATOM 669 CA THR I 78 10.410 −22.262 −3.661 0.00 0.00
I
ATOM 670 HA THR I 78 10.307 −23.269 −3.285 0.00 0.00
I
ATOM 671 CB THR I 78 11.826 −21.804 −3.367 0.00 0.00
I
ATOM 672 HB THR I 78 11.958 −20.730 −3.619 0.00 0.00
I
ATOM 673 OG1 THR I 78 12.795 −22.388 −4.258 0.00 0.00
I
ATOM 674 HG1 THR I 78 12.799 −21.927 −5.100 0.00 0.00
I

```
ATOM    675  CG2 THR I  78      12.258 -22.127  -1.925  0.00  0.00
I
ATOM    676 HG21 THR I  78      11.388 -21.960  -1.254  0.00  0.00
I
ATOM    677 HG22 THR I  78      12.434 -23.216  -1.789  0.00  0.00
I
ATOM    678 HG23 THR I  78      13.152 -21.536  -1.632  0.00  0.00
I
ATOM    679  C   THR I  78       9.430 -21.368  -2.862  0.00  0.00
I
ATOM    680  O   THR I  78       8.948 -21.807  -1.809  0.00  0.00
I
ATOM    681  N   THR I  79       9.065 -20.222  -3.443  0.00  0.00
I
ATOM    682  HN  THR I  79       9.447 -19.978  -4.331  0.00  0.00
I
ATOM    683  CA  THR I  79       8.013 -19.438  -2.939  0.00  0.00
I
ATOM    684  HA  THR I  79       8.122 -19.590  -1.876  0.00  0.00
I
ATOM    685  CB  THR I  79       8.010 -17.932  -3.278  0.00  0.00
I
ATOM    686  HB  THR I  79       7.221 -17.356  -2.749  0.00  0.00
I
ATOM    687  OG1 THR I  79       7.881 -17.624  -4.687  0.00  0.00
I
ATOM    688  HG1 THR I  79       8.626 -17.978  -5.178  0.00  0.00
I
ATOM    689  CG2 THR I  79       9.445 -17.298  -2.874  0.00  0.00
I
ATOM    690 HG21 THR I  79       9.623 -17.377  -1.781  0.00  0.00
I
ATOM    691 HG22 THR I  79      10.260 -17.781  -3.454  0.00  0.00
I
ATOM    692 HG23 THR I  79       9.372 -16.236  -3.194  0.00  0.00
I
ATOM    693  C   THR I  79       6.619 -19.891  -3.131  0.00  0.00
I
ATOM    694  O   THR I  79       5.724 -19.293  -2.441  0.00  0.00
I
ATOM    695  N   VAL I  80       6.322 -20.873  -3.994  0.00  0.00
I
ATOM    696  HN  VAL I  80       6.929 -21.305  -4.656  0.00  0.00
I
ATOM    697  CA  VAL I  80       4.983 -21.448  -4.191  0.00  0.00
I
ATOM    698  HA  VAL I  80       5.064 -22.107  -5.043  0.00  0.00
I
ATOM    699  CB  VAL I  80       4.600 -22.283  -3.003  0.00  0.00
I
ATOM    700  HB  VAL I  80       4.578 -21.625  -2.108  0.00  0.00
I
ATOM    701  CG1 VAL I  80       3.222 -23.007  -3.056  0.00  0.00
I
```

```
ATOM    702 HG11 VAL I  80       3.274 -23.743  -3.887  0.00  0.00
I
ATOM    703 HG12 VAL I  80       3.281 -23.655  -2.155  0.00  0.00
I
ATOM    704 HG13 VAL I  80       2.327 -22.350  -3.026  0.00  0.00
I
ATOM    705  CG2 VAL I  80       5.728 -23.382  -2.881  0.00  0.00
I
ATOM    706 HG21 VAL I  80       6.766 -22.987  -2.920  0.00  0.00
I
ATOM    707 HG22 VAL I  80       5.645 -23.876  -1.889  0.00  0.00
I
ATOM    708 HG23 VAL I  80       5.569 -24.169  -3.648  0.00  0.00
I
ATOM    709  C   VAL I  80       4.036 -20.348  -4.678  0.00  0.00
I
ATOM    710  O   VAL I  80       2.875 -20.267  -4.343  0.00  0.00
I
ATOM    711  N   THR I  81       4.560 -19.358  -5.511  0.00  0.00
I
ATOM    712  HN  THR I  81       5.470 -19.459  -5.907  0.00  0.00
I
ATOM    713  CA  THR I  81       3.790 -18.234  -5.992  0.00  0.00
I
ATOM    714  HA  THR I  81       3.006 -18.085  -5.265  0.00  0.00
I
ATOM    715  CB  THR I  81       4.649 -16.921  -6.204  0.00  0.00
I
ATOM    716  HB  THR I  81       5.488 -17.205  -6.876  0.00  0.00
I
ATOM    717  OG1 THR I  81       5.186 -16.429  -4.991  0.00  0.00
I
ATOM    718  HG1 THR I  81       6.023 -16.873  -4.833  0.00  0.00
I
ATOM    719  CG2 THR I  81       3.857 -15.783  -6.787  0.00  0.00
I
ATOM    720 HG21 THR I  81       3.518 -15.995  -7.823  0.00  0.00
I
ATOM    721 HG22 THR I  81       2.895 -15.555  -6.280  0.00  0.00
I
ATOM    722 HG23 THR I  81       4.500 -14.881  -6.869  0.00  0.00
I
ATOM    723  C   THR I  81       3.071 -18.601  -7.199  0.00  0.00
I
ATOM    724  O   THR I  81       1.906 -18.569  -7.228  0.00  0.00
I
ATOM    725  N   GLY I  82       3.720 -19.024  -8.270  0.00  0.00
I
ATOM    726  HN  GLY I  82       4.716 -18.980  -8.291  0.00  0.00
I
ATOM    727  CA  GLY I  82       3.131 -19.756  -9.337  0.00  0.00
I
ATOM    728  HA1 GLY I  82       2.090 -19.970  -9.143  0.00  0.00
I
```

ATOM 729 HA2 GLY I 82 3.777 −20.600 −9.529 0.00 0.00
I
ATOM 730 C GLY I 82 3.003 −19.026 −10.623 0.00 0.00
I
ATOM 731 O GLY I 82 2.615 −19.443 −11.741 0.00 0.00
I
ATOM 732 N LEU I 83 3.431 −17.758 −10.564 0.00 0.00
I
ATOM 733 HN LEU I 83 3.740 −17.363 −9.702 0.00 0.00
I
ATOM 734 CA LEU I 83 3.648 −16.964 −11.726 0.00 0.00
I
ATOM 735 HA LEU I 83 2.810 −16.858 −12.398 0.00 0.00
I
ATOM 736 CB LEU I 83 4.210 −15.584 −11.205 0.00 0.00
I
ATOM 737 HB1 LEU I 83 5.014 −15.782 −10.463 0.00 0.00
I
ATOM 738 HB2 LEU I 83 4.681 −15.080 −12.076 0.00 0.00
I
ATOM 739 CG LEU I 83 3.171 −14.612 −10.574 0.00 0.00
I
ATOM 740 HG LEU I 83 2.537 −15.186 −9.864 0.00 0.00
I
ATOM 741 CD1 LEU I 83 3.801 −13.471 −9.832 0.00 0.00
I
ATOM 742 HD11 LEU I 83 3.110 −12.652 −9.538 0.00 0.00
I
ATOM 743 HD12 LEU I 83 4.270 −13.700 −8.852 0.00 0.00
I
ATOM 744 HD13 LEU I 83 4.610 −13.005 −10.436 0.00 0.00
I
ATOM 745 CD2 LEU I 83 2.297 −14.116 −11.682 0.00 0.00
I
ATOM 746 HD21 LEU I 83 2.864 −13.623 −12.501 0.00 0.00
I
ATOM 747 HD22 LEU I 83 1.756 −14.964 −12.153 0.00 0.00
I
ATOM 748 HD23 LEU I 83 1.472 −13.410 −11.447 0.00 0.00
I
ATOM 749 C LEU I 83 4.835 −17.644 −12.573 0.00 0.00
I
ATOM 750 O LEU I 83 5.700 −18.321 −12.042 0.00 0.00
I
ATOM 751 N ALA I 84 4.762 −17.416 −13.930 0.00 0.00
I
ATOM 752 HN ALA I 84 4.002 −16.884 −14.295 0.00 0.00
I
ATOM 753 CA ALA I 84 5.624 −18.096 −14.793 0.00 0.00
I
ATOM 754 HA ALA I 84 6.530 −18.381 −14.280 0.00 0.00
I
ATOM 755 CB ALA I 84 5.044 −19.403 −15.330 0.00 0.00
I

ATOM 756 HB1 ALA I 84 4.092 −19.141 −15.839 0.00 0.00
I
ATOM 757 HB2 ALA I 84 5.730 −19.760 −16.128 0.00 0.00
I
ATOM 758 HB3 ALA I 84 4.842 −20.161 −14.544 0.00 0.00
I
ATOM 759 C ALA I 84 6.002 −17.116 −15.988 0.00 0.00
I
ATOM 760 O ALA I 84 5.149 −16.359 −16.507 0.00 0.00
I
ATOM 761 N VAL I 85 7.249 −17.167 −16.480 0.00 0.00
I
ATOM 762 HN VAL I 85 7.927 −17.726 −16.011 0.00 0.00
I
ATOM 763 CA VAL I 85 7.795 −16.512 −17.649 0.00 0.00
I
ATOM 764 HA VAL I 85 7.213 −15.646 −17.926 0.00 0.00
I
ATOM 765 CB VAL I 85 9.185 −15.905 −17.326 0.00 0.00
I
ATOM 766 HB VAL I 85 9.533 −15.469 −18.287 0.00 0.00
I
ATOM 767 CG1 VAL I 85 9.006 −14.773 −16.282 0.00 0.00
I
ATOM 768 HG11 VAL I 85 8.208 −14.022 −16.466 0.00 0.00
I
ATOM 769 HG12 VAL I 85 8.780 −15.364 −15.369 0.00 0.00
I
ATOM 770 HG13 VAL I 85 9.939 −14.205 −16.079 0.00 0.00
I
ATOM 771 CG2 VAL I 85 10.188 −17.021 −16.926 0.00 0.00
I
ATOM 772 HG21 VAL I 85 10.056 −17.877 −17.621 0.00 0.00
I
ATOM 773 HG22 VAL I 85 11.211 −16.587 −16.954 0.00 0.00
I
ATOM 774 HG23 VAL I 85 10.049 −17.342 −15.871 0.00 0.00
I
ATOM 775 C VAL I 85 7.836 −17.506 −18.823 0.00 0.00
I
ATOM 776 O VAL I 85 7.904 −17.076 −19.966 0.00 0.00
I
ATOM 777 N VAL I 86 7.882 −18.855 −18.552 0.00 0.00
I
ATOM 778 HN VAL I 86 7.678 −19.176 −17.630 0.00 0.00
I
ATOM 779 CA VAL I 86 8.050 −19.970 −19.485 0.00 0.00
I
ATOM 780 HA VAL I 86 7.621 −19.684 −20.434 0.00 0.00
I
ATOM 781 CB VAL I 86 9.489 −20.527 −19.608 0.00 0.00
I
ATOM 782 HB VAL I 86 9.387 −21.422 −20.259 0.00 0.00
I

```
ATOM    783  CG1 VAL I  86      10.254 -19.455 -20.435  0.00  0.00
I
ATOM    784 HG11 VAL I  86       9.574 -19.243 -21.288  0.00  0.00
I
ATOM    785 HG12 VAL I  86      10.574 -18.641 -19.750  0.00  0.00
I
ATOM    786 HG13 VAL I  86      11.119 -19.976 -20.899  0.00  0.00
I
ATOM    787  CG2 VAL I  86      10.164 -20.997 -18.315  0.00  0.00
I
ATOM    788 HG21 VAL I  86       9.654 -21.961 -18.106  0.00  0.00
I
ATOM    789 HG22 VAL I  86      11.233 -21.254 -18.476  0.00  0.00
I
ATOM    790 HG23 VAL I  86      10.124 -20.234 -17.508  0.00  0.00
I
ATOM    791  C   VAL I  86       7.081 -20.994 -19.102  0.00  0.00
I
ATOM    792  O   VAL I  86       6.741 -21.071 -17.914  0.00  0.00
I
ATOM    793  N   ASP I  87       6.590 -21.825 -20.016  0.00  0.00
I
ATOM    794  HN  ASP I  87       6.828 -21.821 -20.984  0.00  0.00
I
ATOM    795  CA  ASP I  87       5.460 -22.726 -19.745  0.00  0.00
I
ATOM    796  HA  ASP I  87       4.766 -22.288 -19.043  0.00  0.00
I
ATOM    797  CB  ASP I  87       4.727 -23.085 -21.062  0.00  0.00
I
ATOM    798  HB1 ASP I  87       5.331 -23.648 -21.806  0.00  0.00
I
ATOM    799  HB2 ASP I  87       3.804 -23.538 -20.641  0.00  0.00
I
ATOM    800  CG  ASP I  87       4.199 -21.788 -21.658  0.00  0.00
I
ATOM    801  OD1 ASP I  87       3.404 -21.102 -21.011  0.00  0.00
I
ATOM    802  OD2 ASP I  87       4.607 -21.413 -22.766  0.00  0.00
I
ATOM    803  C   ASP I  87       5.923 -24.023 -19.094  0.00  0.00
I
ATOM    804  O   ASP I  87       6.814 -24.740 -19.522  0.00  0.00
I
ATOM    805  N   THR I  88       5.366 -24.270 -17.898  0.00  0.00
I
ATOM    806  HN  THR I  88       4.789 -23.567 -17.490  0.00  0.00
I
ATOM    807  CA  THR I  88       5.574 -25.431 -17.001  0.00  0.00
I
ATOM    808  HA  THR I  88       6.543 -25.291 -16.544  0.00  0.00
I
ATOM    809  CB  THR I  88       4.640 -25.420 -15.781  0.00  0.00
I
```

ATOM 810 HB THR I 88 3.571 -25.582 -16.037 0.00 0.00
I
ATOM 811 OG1 THR I 88 4.788 -24.163 -15.153 0.00 0.00
I
ATOM 812 HG1 THR I 88 4.329 -23.529 -15.707 0.00 0.00
I
ATOM 813 CG2 THR I 88 5.234 -26.403 -14.643 0.00 0.00
I
ATOM 814 HG21 THR I 88 4.677 -26.214 -13.700 0.00 0.00
I
ATOM 815 HG22 THR I 88 5.121 -27.448 -15.003 0.00 0.00
I
ATOM 816 HG23 THR I 88 6.274 -26.123 -14.366 0.00 0.00
I
ATOM 817 C THR I 88 5.518 -26.742 -17.702 0.00 0.00
I
ATOM 818 O THR I 88 6.434 -27.487 -17.618 0.00 0.00
I
ATOM 819 N GLY I 89 4.541 -27.077 -18.430 0.00 0.00
I
ATOM 820 HN GLY I 89 3.721 -26.512 -18.412 0.00 0.00
I
ATOM 821 CA GLY I 89 4.464 -28.393 -19.021 0.00 0.00
I
ATOM 822 HA1 GLY I 89 3.451 -28.631 -19.311 0.00 0.00
I
ATOM 823 HA2 GLY I 89 4.930 -29.150 -18.408 0.00 0.00
I
ATOM 824 C GLY I 89 5.273 -28.538 -20.320 0.00 0.00
I
ATOM 825 O GLY I 89 5.333 -29.616 -20.856 0.00 0.00
I
ATOM 826 N THR I 90 5.957 -27.493 -20.851 0.00 0.00
I
ATOM 827 HN THR I 90 5.992 -26.560 -20.501 0.00 0.00
I
ATOM 828 CA THR I 90 6.525 -27.830 -22.187 0.00 0.00
I
ATOM 829 HA THR I 90 6.910 -28.839 -22.171 0.00 0.00
I
ATOM 830 CB THR I 90 5.535 -27.748 -23.427 0.00 0.00
I
ATOM 831 HB THR I 90 4.666 -28.228 -22.929 0.00 0.00
I
ATOM 832 OG1 THR I 90 5.982 -28.522 -24.455 0.00 0.00
I
ATOM 833 HG1 THR I 90 5.178 -28.737 -24.934 0.00 0.00
I
ATOM 834 CG2 THR I 90 5.199 -26.294 -23.899 0.00 0.00
I
ATOM 835 HG21 THR I 90 6.109 -25.659 -23.866 0.00 0.00
I
ATOM 836 HG22 THR I 90 4.823 -26.246 -24.944 0.00 0.00
I

ATOM 837 HG23 THR I 90 4.505 -25.861 -23.147 0.00 0.00
I
ATOM 838 C THR I 90 7.759 -27.052 -22.485 0.00 0.00
I
ATOM 839 O THR I 90 8.424 -27.311 -23.521 0.00 0.00
I
ATOM 840 N GLN I 91 8.205 -26.330 -21.481 0.00 0.00
I
ATOM 841 HN GLN I 91 7.760 -26.109 -20.617 0.00 0.00
I
ATOM 842 CA GLN I 91 9.602 -26.008 -21.424 0.00 0.00
I
ATOM 843 HA GLN I 91 10.048 -26.004 -22.407 0.00 0.00
I
ATOM 844 CB GLN I 91 9.681 -24.606 -20.694 0.00 0.00
I
ATOM 845 HB1 GLN I 91 9.003 -23.892 -21.210 0.00 0.00
I
ATOM 846 HB2 GLN I 91 9.231 -24.741 -19.687 0.00 0.00
I
ATOM 847 CG GLN I 91 11.100 -24.156 -20.402 0.00 0.00
I
ATOM 848 HG1 GLN I 91 11.071 -23.304 -19.690 0.00 0.00
I
ATOM 849 HG2 GLN I 91 11.644 -25.015 -19.952 0.00 0.00
I
ATOM 850 CD GLN I 91 11.917 -23.750 -21.597 0.00 0.00
I
ATOM 851 OE1 GLN I 91 11.686 -22.669 -22.180 0.00 0.00
I
ATOM 852 NE2 GLN I 91 12.703 -24.805 -22.081 0.00 0.00
I
ATOM 853 HE21 GLN I 91 13.503 -24.599 -22.644 0.00 0.00
I
ATOM 854 HE22 GLN I 91 12.556 -25.709 -21.680 0.00 0.00
I
ATOM 855 C GLN I 91 10.504 -26.945 -20.681 0.00 0.00
I
ATOM 856 O GLN I 91 11.720 -27.052 -20.872 0.00 0.00
I
ATOM 857 N PHE I 92 9.973 -27.756 -19.762 0.00 0.00
I
ATOM 858 HN PHE I 92 9.042 -27.695 -19.409 0.00 0.00
I
ATOM 859 CA PHE I 92 10.723 -28.612 -18.888 0.00 0.00
I
ATOM 860 HA PHE I 92 11.781 -28.486 -19.065 0.00 0.00
I
ATOM 861 CB PHE I 92 10.439 -28.176 -17.441 0.00 0.00
I
ATOM 862 HB1 PHE I 92 9.349 -28.285 -17.256 0.00 0.00
I
ATOM 863 HB2 PHE I 92 10.989 -28.826 -16.727 0.00 0.00
I

ATOM 864 CG PHE I 92 10.756 −26.779 −17.087 0.00 0.00
I
ATOM 865 CD1 PHE I 92 12.033 −26.330 −16.745 0.00 0.00
I
ATOM 866 HD1 PHE I 92 12.852 −27.033 −16.767 0.00 0.00
I
ATOM 867 CE1 PHE I 92 12.281 −25.005 −16.246 0.00 0.00
I
ATOM 868 HE1 PHE I 92 13.253 −24.719 −15.871 0.00 0.00
I
ATOM 869 CZ PHE I 92 11.223 −24.103 −16.216 0.00 0.00
I
ATOM 870 HZ PHE I 92 11.383 −23.059 −15.994 0.00 0.00
I
ATOM 871 CD2 PHE I 92 9.702 −25.834 −16.934 0.00 0.00
I
ATOM 872 HD2 PHE I 92 8.707 −26.176 −17.180 0.00 0.00
I
ATOM 873 CE2 PHE I 92 9.965 −24.486 −16.701 0.00 0.00
I
ATOM 874 HE2 PHE I 92 9.133 −23.805 −16.592 0.00 0.00
I
ATOM 875 C PHE I 92 10.377 −30.081 −19.157 0.00 0.00
I
ATOM 876 O PHE I 92 9.232 −30.474 −19.357 0.00 0.00
I
ATOM 877 N THR I 93 11.435 −30.936 −19.044 0.00 0.00
I
ATOM 878 HN THR I 93 12.288 −30.568 −18.680 0.00 0.00
I
ATOM 879 CA THR I 93 11.448 −32.388 −18.924 0.00 0.00
I
ATOM 880 HA THR I 93 10.922 −32.673 −19.823 0.00 0.00
I
ATOM 881 CB THR I 93 12.833 −33.091 −18.983 0.00 0.00
I
ATOM 882 HB THR I 93 12.768 −34.184 −19.171 0.00 0.00
I
ATOM 883 OG1 THR I 93 13.625 −32.841 −17.816 0.00 0.00
I
ATOM 884 HG1 THR I 93 14.527 −33.033 −18.082 0.00 0.00
I
ATOM 885 CG2 THR I 93 13.548 −32.492 −20.226 0.00 0.00
I
ATOM 886 HG21 THR I 93 13.496 −31.383 −20.256 0.00 0.00
I
ATOM 887 HG22 THR I 93 14.583 −32.892 −20.287 0.00 0.00
I
ATOM 888 HG23 THR I 93 13.084 −32.799 −21.188 0.00 0.00
I
ATOM 889 C THR I 93 10.743 −32.902 −17.760 0.00 0.00
I
ATOM 890 O THR I 93 10.498 −32.185 −16.813 0.00 0.00
I

ATOM 891 N VAL I 94 10.556 -34.263 -17.717 0.00 0.00
I
ATOM 892 HN VAL I 94 10.658 -34.829 -18.532 0.00 0.00
I
ATOM 893 CA VAL I 94 10.142 -34.977 -16.533 0.00 0.00
I
ATOM 894 HA VAL I 94 9.276 -34.429 -16.194 0.00 0.00
I
ATOM 895 CB VAL I 94 9.956 -36.527 -16.703 0.00 0.00
I
ATOM 896 HB VAL I 94 10.938 -36.985 -16.945 0.00 0.00
I
ATOM 897 CG1 VAL I 94 9.304 -37.035 -15.465 0.00 0.00
I
ATOM 898 HG11 VAL I 94 8.862 -38.051 -15.548 0.00 0.00
I
ATOM 899 HG12 VAL I 94 10.095 -37.176 -14.697 0.00 0.00
I
ATOM 900 HG13 VAL I 94 8.530 -36.368 -15.030 0.00 0.00
I
ATOM 901 CG2 VAL I 94 9.039 -36.691 -17.898 0.00 0.00
I
ATOM 902 HG21 VAL I 94 8.011 -36.329 -17.679 0.00 0.00
I
ATOM 903 HG22 VAL I 94 9.424 -36.186 -18.809 0.00 0.00
I
ATOM 904 HG23 VAL I 94 8.917 -37.777 -18.099 0.00 0.00
I
ATOM 905 C VAL I 94 11.132 -34.747 -15.349 0.00 0.00
I
ATOM 906 O VAL I 94 10.702 -34.347 -14.257 0.00 0.00
I
ATOM 907 N PHE I 95 12.469 -34.820 -15.543 0.00 0.00
I
ATOM 908 HN PHE I 95 12.865 -35.249 -16.352 0.00 0.00
I
ATOM 909 CA PHE I 95 13.469 -34.516 -14.600 0.00 0.00
I
ATOM 910 HA PHE I 95 13.268 -35.154 -13.752 0.00 0.00
I
ATOM 911 CB PHE I 95 14.895 -34.903 -15.077 0.00 0.00
I
ATOM 912 HB1 PHE I 95 14.971 -35.942 -15.463 0.00 0.00
I
ATOM 913 HB2 PHE I 95 15.301 -34.240 -15.871 0.00 0.00
I
ATOM 914 CG PHE I 95 15.836 -34.822 -13.870 0.00 0.00
I
ATOM 915 CD1 PHE I 95 16.536 -33.586 -13.598 0.00 0.00
I
ATOM 916 HD1 PHE I 95 16.552 -32.673 -14.175 0.00 0.00
I
ATOM 917 CE1 PHE I 95 17.317 -33.497 -12.438 0.00 0.00
I

```
ATOM    918  HE1 PHE I  95      17.911 -32.602 -12.321  0.00  0.00
I
ATOM    919  CZ  PHE I  95      17.386 -34.588 -11.615  0.00  0.00
I
ATOM    920  HZ  PHE I  95      18.035 -34.514 -10.756  0.00  0.00
I
ATOM    921  CD2 PHE I  95      15.968 -35.913 -12.990  0.00  0.00
I
ATOM    922  HD2 PHE I  95      15.537 -36.893 -13.131  0.00  0.00
I
ATOM    923  CE2 PHE I  95      16.805 -35.813 -11.885  0.00  0.00
I
ATOM    924  HE2 PHE I  95      16.950 -36.747 -11.362  0.00  0.00
I
ATOM    925  C   PHE I  95      13.330 -33.091 -14.144  0.00  0.00
I
ATOM    926  O   PHE I  95      13.322 -32.824 -12.927  0.00  0.00
I
ATOM    927  N   GLY I  96      13.204 -32.096 -15.094  0.00  0.00
I
ATOM    928  HN  GLY I  96      13.384 -32.369 -16.036  0.00  0.00
I
ATOM    929  CA  GLY I  96      12.874 -30.669 -14.856  0.00  0.00
I
ATOM    930  HA1 GLY I  96      12.825 -30.189 -15.822  0.00  0.00
I
ATOM    931  HA2 GLY I  96      13.689 -30.226 -14.302  0.00  0.00
I
ATOM    932  C   GLY I  96      11.564 -30.385 -14.140  0.00  0.00
I
ATOM    933  O   GLY I  96      11.449 -29.620 -13.244  0.00  0.00
I
ATOM    934  N   GLN I  97      10.433 -31.020 -14.502  0.00  0.00
I
ATOM    935  HN  GLN I  97      10.450 -31.521 -15.363  0.00  0.00
I
ATOM    936  CA  GLN I  97       9.113 -30.997 -13.864  0.00  0.00
I
ATOM    937  HA  GLN I  97       8.834 -29.964 -13.719  0.00  0.00
I
ATOM    938  CB  GLN I  97       8.095 -31.701 -14.793  0.00  0.00
I
ATOM    939  HB1 GLN I  97       8.377 -32.729 -15.108  0.00  0.00
I
ATOM    940  HB2 GLN I  97       7.167 -31.760 -14.186  0.00  0.00
I
ATOM    941  CG  GLN I  97       7.676 -30.921 -16.128  0.00  0.00
I
ATOM    942  HG1 GLN I  97       7.026 -30.042 -15.932  0.00  0.00
I
ATOM    943  HG2 GLN I  97       8.631 -30.703 -16.652  0.00  0.00
I
ATOM    944  CD  GLN I  97       6.879 -31.941 -16.973  0.00  0.00
I
```

```
ATOM    945  OE1 GLN I  97       6.480 -33.050 -16.518  0.00  0.00
I
ATOM    946  NE2 GLN I  97       6.583 -31.596 -18.276  0.00  0.00
I
ATOM    947 HE21 GLN I  97       5.982 -32.228 -18.766  0.00  0.00
I
ATOM    948 HE22 GLN I  97       6.990 -30.782 -18.689  0.00  0.00
I
ATOM    949  C   GLN I  97       9.217 -31.615 -12.421  0.00  0.00
I
ATOM    950  O   GLN I  97       8.562 -31.023 -11.547  0.00  0.00
I
ATOM    951  N   THR I  98       9.979 -32.688 -12.157  0.00  0.00
I
ATOM    952  HN  THR I  98      10.285 -33.175 -12.972  0.00  0.00
I
ATOM    953  CA  THR I  98      10.218 -33.283 -10.793  0.00  0.00
I
ATOM    954  HA  THR I  98       9.252 -33.284 -10.312  0.00  0.00
I
ATOM    955  CB  THR I  98      11.003 -34.574 -10.778  0.00  0.00
I
ATOM    956  HB  THR I  98      12.050 -34.310 -11.039  0.00  0.00
I
ATOM    957  OG1 THR I  98      10.560 -35.475 -11.771  0.00  0.00
I
ATOM    958  HG1 THR I  98      10.951 -35.121 -12.574  0.00  0.00
I
ATOM    959  CG2 THR I  98      10.593 -35.132  -9.442  0.00  0.00
I
ATOM    960 HG21 THR I  98       9.490 -35.118  -9.311  0.00  0.00
I
ATOM    961 HG22 THR I  98      11.014 -36.132  -9.204  0.00  0.00
I
ATOM    962 HG23 THR I  98      10.985 -34.485  -8.629  0.00  0.00
I
ATOM    963  C   THR I  98      11.033 -32.247  -9.973  0.00  0.00
I
ATOM    964  O   THR I  98      10.753 -32.050  -8.831  0.00  0.00
I
ATOM    965  N   VAL I  99      11.987 -31.539 -10.558  0.00  0.00
I
ATOM    966  HN  VAL I  99      12.350 -31.914 -11.408  0.00  0.00
I
ATOM    967  CA  VAL I  99      12.680 -30.448  -9.934  0.00  0.00
I
ATOM    968  HA  VAL I  99      13.032 -30.891  -9.014  0.00  0.00
I
ATOM    969  CB  VAL I  99      13.873 -29.921 -10.805  0.00  0.00
I
ATOM    970  HB  VAL I  99      13.456 -29.526 -11.756  0.00  0.00
I
ATOM    971  CG1 VAL I  99      14.493 -28.663 -10.168  0.00  0.00
I
```

```
ATOM    972 HG11 VAL I  99      15.390 -28.363 -10.751  0.00  0.00
I
ATOM    973 HG12 VAL I  99      13.813 -27.785 -10.134  0.00  0.00
I
ATOM    974 HG13 VAL I  99      14.872 -28.863  -9.143  0.00  0.00
I
ATOM    975  CG2 VAL I  99      14.934 -30.952 -11.122  0.00  0.00
I
ATOM    976 HG21 VAL I  99      15.688 -31.010 -10.309  0.00  0.00
I
ATOM    977 HG22 VAL I  99      14.526 -31.977 -11.259  0.00  0.00
I
ATOM    978 HG23 VAL I  99      15.365 -30.649 -12.100  0.00  0.00
I
ATOM    979  C   VAL I  99      11.729 -29.335  -9.472  0.00  0.00
I
ATOM    980  O   VAL I  99      11.943 -28.812  -8.388  0.00  0.00
I
ATOM    981  N   ILE I 100      10.720 -28.945 -10.207  0.00  0.00
I
ATOM    982  HN  ILE I 100      10.574 -29.381 -11.092  0.00  0.00
I
ATOM    983  CA  ILE I 100       9.716 -27.914  -9.729  0.00  0.00
I
ATOM    984  HA  ILE I 100      10.282 -27.077  -9.347  0.00  0.00
I
ATOM    985  CB  ILE I 100       8.808 -27.498 -10.875  0.00  0.00
I
ATOM    986  HB  ILE I 100       8.254 -28.333 -11.354  0.00  0.00
I
ATOM    987  CG2 ILE I 100       7.811 -26.420 -10.418  0.00  0.00
I
ATOM    988 HG21 ILE I 100       8.409 -25.555 -10.059  0.00  0.00
I
ATOM    989 HG22 ILE I 100       7.239 -26.158 -11.334  0.00  0.00
I
ATOM    990 HG23 ILE I 100       7.075 -26.833  -9.695  0.00  0.00
I
ATOM    991  CG1 ILE I 100       9.673 -26.841 -11.964  0.00  0.00
I
ATOM    992 HG11 ILE I 100      10.146 -25.935 -11.528  0.00  0.00
I
ATOM    993 HG12 ILE I 100      10.436 -27.566 -12.320  0.00  0.00
I
ATOM    994  CD  ILE I 100       8.877 -26.489 -13.277  0.00  0.00
I
ATOM    995  HD1 ILE I 100       8.163 -27.291 -13.566  0.00  0.00
I
ATOM    996  HD2 ILE I 100       8.261 -25.574 -13.141  0.00  0.00
I
ATOM    997  HD3 ILE I 100       9.686 -26.219 -13.989  0.00  0.00
I
ATOM    998  C   ILE I 100       8.950 -28.365  -8.466  0.00  0.00
I
```

```
ATOM    999  O   ILE I 100       8.858 -27.532  -7.535  0.00  0.00
I
ATOM   1000  N   MET I 101       8.427 -29.613  -8.477  0.00  0.00
I
ATOM   1001  HN  MET I 101       8.373 -30.040  -9.376  0.00  0.00
I
ATOM   1002  CA  MET I 101       7.777 -30.254  -7.392  0.00  0.00
I
ATOM   1003  HA  MET I 101       6.962 -29.659  -7.007  0.00  0.00
I
ATOM   1004  CB  MET I 101       7.211 -31.659  -7.676  0.00  0.00
I
ATOM   1005  HB1 MET I 101       7.904 -32.488  -7.933  0.00  0.00
I
ATOM   1006  HB2 MET I 101       6.658 -31.999  -6.774  0.00  0.00
I
ATOM   1007  CG  MET I 101       6.243 -31.660  -8.893  0.00  0.00
I
ATOM   1008  HG1 MET I 101       5.360 -31.115  -8.497  0.00  0.00
I
ATOM   1009  HG2 MET I 101       6.756 -31.022  -9.644  0.00  0.00
I
ATOM   1010  SD  MET I 101       5.913 -33.300  -9.616  0.00  0.00
I
ATOM   1011  CE  MET I 101       4.731 -33.709  -8.308  0.00  0.00
I
ATOM   1012  HE1 MET I 101       4.270 -34.712  -8.430  0.00  0.00
I
ATOM   1013  HE2 MET I 101       5.137 -33.689  -7.274  0.00  0.00
I
ATOM   1014  HE3 MET I 101       3.827 -33.064  -8.249  0.00  0.00
I
ATOM   1015  C   MET I 101       8.645 -30.388  -6.189  0.00  0.00
I
ATOM   1016  O   MET I 101       8.234 -30.262  -5.027  0.00  0.00
I
ATOM   1017  N   GLY I 102       9.931 -30.549  -6.397  0.00  0.00
I
ATOM   1018  HN  GLY I 102      10.238 -30.595  -7.344  0.00  0.00
I
ATOM   1019  CA  GLY I 102      10.967 -30.693  -5.399  0.00  0.00
I
ATOM   1020  HA1 GLY I 102      11.886 -30.758  -5.963  0.00  0.00
I
ATOM   1021  HA2 GLY I 102      10.713 -31.518  -4.750  0.00  0.00
I
ATOM   1022  C   GLY I 102      11.127 -29.416  -4.626  0.00  0.00
I
ATOM   1023  O   GLY I 102      11.035 -29.340  -3.373  0.00  0.00
I
ATOM   1024  N   LEU I 103      11.174 -28.347  -5.345  0.00  0.00
I
ATOM   1025  HN  LEU I 103      11.117 -28.462  -6.333  0.00  0.00
I
```

ATOM 1026 CA LEU I 103 11.157 -27.012 -4.770 0.00 0.00
I
ATOM 1027 HA LEU I 103 11.891 -26.799 -4.007 0.00 0.00
I
ATOM 1028 CB LEU I 103 11.548 -26.051 -5.887 0.00 0.00
I
ATOM 1029 HB1 LEU I 103 10.815 -26.179 -6.712 0.00 0.00
I
ATOM 1030 HB2 LEU I 103 11.289 -25.040 -5.506 0.00 0.00
I
ATOM 1031 CG LEU I 103 13.034 -26.005 -6.442 0.00 0.00
I
ATOM 1032 HG LEU I 103 13.277 -27.039 -6.766 0.00 0.00
I
ATOM 1033 CD1 LEU I 103 13.151 -25.082 -7.660 0.00 0.00
I
ATOM 1034 HD11 LEU I 103 14.152 -24.902 -8.109 0.00 0.00
I
ATOM 1035 HD12 LEU I 103 12.441 -25.345 -8.473 0.00 0.00
I
ATOM 1036 HD13 LEU I 103 12.803 -24.074 -7.351 0.00 0.00
I
ATOM 1037 CD2 LEU I 103 13.948 -25.527 -5.324 0.00 0.00
I
ATOM 1038 HD21 LEU I 103 13.597 -24.530 -4.982 0.00 0.00
I
ATOM 1039 HD22 LEU I 103 13.977 -26.211 -4.448 0.00 0.00
I
ATOM 1040 HD23 LEU I 103 15.024 -25.428 -5.580 0.00 0.00
I
ATOM 1041 C LEU I 103 9.866 -26.626 -4.045 0.00 0.00
I
ATOM 1042 O LEU I 103 9.915 -26.134 -2.917 0.00 0.00
I
ATOM 1043 N ILE I 104 8.628 -26.928 -4.519 0.00 0.00
I
ATOM 1044 HN ILE I 104 8.505 -27.307 -5.433 0.00 0.00
I
ATOM 1045 CA ILE I 104 7.349 -26.653 -3.827 0.00 0.00
I
ATOM 1046 HA ILE I 104 7.259 -25.606 -3.577 0.00 0.00
I
ATOM 1047 CB ILE I 104 6.236 -27.019 -4.751 0.00 0.00
I
ATOM 1048 HB ILE I 104 6.497 -28.019 -5.157 0.00 0.00
I
ATOM 1049 CG2 ILE I 104 4.879 -26.975 -4.012 0.00 0.00
I
ATOM 1050 HG21 ILE I 104 4.791 -27.836 -3.315 0.00 0.00
I
ATOM 1051 HG22 ILE I 104 4.670 -26.021 -3.483 0.00 0.00
I
ATOM 1052 HG23 ILE I 104 4.040 -27.072 -4.734 0.00 0.00
I

ATOM   1053 CG1 ILE I 104   6.093 -25.954 -5.865 0.00 0.00
I
ATOM   1054 HG11 ILE I 104   5.898 -24.997 -5.335 0.00 0.00
I
ATOM   1055 HG12 ILE I 104   7.084 -25.719 -6.310 0.00 0.00
I
ATOM   1056 CD ILE I 104   5.134 -26.256 -7.045 0.00 0.00
I
ATOM   1057 HD1 ILE I 104   5.177 -27.288 -7.454 0.00 0.00
I
ATOM   1058 HD2 ILE I 104   4.137 -25.947 -6.664 0.00 0.00
I
ATOM   1059 HD3 ILE I 104   5.418 -25.632 -7.919 0.00 0.00
I
ATOM   1060 C   ILE I 104   7.288 -27.427 -2.489 0.00 0.00
I
ATOM   1061 O   ILE I 104   6.891 -26.905 -1.428 0.00 0.00
I
ATOM   1062 N   GLN I 105   7.727 -28.697 -2.526 0.00 0.00
I
ATOM   1063 HN GLN I 105   8.018 -28.998 -3.431 0.00 0.00
I
ATOM   1064 CA GLN I 105   7.896 -29.627 -1.373 0.00 0.00
I
ATOM   1065 HA GLN I 105   6.951 -29.597 -0.851 0.00 0.00
I
ATOM   1066 CB GLN I 105   8.112 -31.052 -1.848 0.00 0.00
I
ATOM   1067 HB1 GLN I 105   7.442 -31.249 -2.712 0.00 0.00
I
ATOM   1068 HB2 GLN I 105   9.186 -31.084 -2.129 0.00 0.00
I
ATOM   1069 CG GLN I 105   8.058 -32.133 -0.750 0.00 0.00
I
ATOM   1070 HG1 GLN I 105   8.291 -33.121 -1.201 0.00 0.00
I
ATOM   1071 HG2 GLN I 105   8.846 -32.032   0.027 0.00 0.00
I
ATOM   1072 CD GLN I 105   6.723 -32.292 -0.037 0.00 0.00
I
ATOM   1073 OE1 GLN I 105   6.496 -31.764   1.086 0.00 0.00
I
ATOM   1074 NE2 GLN I 105   5.705 -32.965 -0.666 0.00 0.00
I
ATOM   1075 HE21 GLN I 105   4.754 -33.001 -0.360 0.00 0.00
I
ATOM   1076 HE22 GLN I 105   5.866 -33.390 -1.557 0.00 0.00
I
ATOM   1077 C   GLN I 105   8.851 -29.132 -0.261 0.00 0.00
I
ATOM   1078 O   GLN I 105   8.461 -28.950   0.844 0.00 0.00
I
ATOM   1079 N   ILE I 106   10.092 -28.784 -0.565 0.00 0.00
I

ATOM 1080 HN ILE I 106 10.524 -29.043 -1.425 0.00 0.00
I
ATOM 1081 CA ILE I 106 11.143 -28.125 0.289 0.00 0.00
I
ATOM 1082 HA ILE I 106 11.255 -28.705 1.193 0.00 0.00
I
ATOM 1083 CB ILE I 106 12.506 -28.101 -0.454 0.00 0.00
I
ATOM 1084 HB ILE I 106 12.418 -27.729 -1.497 0.00 0.00
I
ATOM 1085 CG2 ILE I 106 13.515 -27.112 0.371 0.00 0.00
I
ATOM 1086 HG21 ILE I 106 13.693 -27.580 1.363 0.00 0.00
I
ATOM 1087 HG22 ILE I 106 14.475 -27.063 -0.187 0.00 0.00
I
ATOM 1088 HG23 ILE I 106 13.069 -26.099 0.470 0.00 0.00
I
ATOM 1089 CG1 ILE I 106 13.055 -29.528 -0.571 0.00 0.00
I
ATOM 1090 HG11 ILE I 106 13.325 -29.807 0.470 0.00 0.00
I
ATOM 1091 HG12 ILE I 106 12.222 -30.164 -0.939 0.00 0.00
I
ATOM 1092 CD ILE I 106 14.204 -29.625 -1.532 0.00 0.00
I
ATOM 1093 HD1 ILE I 106 14.019 -28.999 -2.431 0.00 0.00
I
ATOM 1094 HD2 ILE I 106 15.142 -29.337 -1.011 0.00 0.00
I
ATOM 1095 HD3 ILE I 106 14.307 -30.706 -1.769 0.00 0.00
I
ATOM 1096 C ILE I 106 10.664 -26.690 0.786 0.00 0.00
I
ATOM 1097 O ILE I 106 10.978 -26.383 1.964 0.00 0.00
I
ATOM 1098 N GLY I 107 9.938 -25.901 -0.068 0.00 0.00
I
ATOM 1099 HN GLY I 107 9.944 -26.171 -1.028 0.00 0.00
I
ATOM 1100 CA GLY I 107 9.655 -24.527 0.297 0.00 0.00
I
ATOM 1101 HA1 GLY I 107 9.145 -24.099 -0.553 0.00 0.00
I
ATOM 1102 HA2 GLY I 107 10.592 -24.015 0.457 0.00 0.00
I
ATOM 1103 C GLY I 107 8.565 -24.384 1.395 0.00 0.00
I
ATOM 1104 O GLY I 107 8.667 -23.472 2.241 0.00 0.00
I
ATOM 1105 N GLY I 108 7.519 -25.214 1.276 0.00 0.00
I
ATOM 1106 HN GLY I 108 7.539 -25.847 0.506 0.00 0.00
I

ATOM	1107	CA	GLY	I	108	6.406	-25.211	2.115	0.00	0.00	I
ATOM	1108	HA1	GLY	I	108	5.498	-25.546	1.634	0.00	0.00	I
ATOM	1109	HA2	GLY	I	108	6.235	-24.242	2.560	0.00	0.00	I
ATOM	1110	C	GLY	I	108	6.795	-25.870	3.419	0.00	0.00	I
ATOM	1111	O	GLY	I	108	6.497	-25.318	4.505	0.00	0.00	I
ATOM	1112	N	LEU	I	109	7.492	-27.029	3.339	0.00	0.00	I
ATOM	1113	HN	LEU	I	109	7.592	-27.565	2.505	0.00	0.00	I
ATOM	1114	CA	LEU	I	109	7.935	-27.717	4.579	0.00	0.00	I
ATOM	1115	HA	LEU	I	109	7.016	-27.813	5.139	0.00	0.00	I
ATOM	1116	CB	LEU	I	109	8.407	-29.243	4.426	0.00	0.00	I
ATOM	1117	HB1	LEU	I	109	7.664	-29.764	3.786	0.00	0.00	I
ATOM	1118	HB2	LEU	I	109	9.424	-29.234	3.980	0.00	0.00	I
ATOM	1119	CG	LEU	I	109	8.472	-30.100	5.710	0.00	0.00	I
ATOM	1120	HG	LEU	I	109	8.316	-31.163	5.427	0.00	0.00	I
ATOM	1121	CD1	LEU	I	109	9.857	-29.978	6.328	0.00	0.00	I
ATOM	1122	HD11	LEU	I	109	10.662	-30.002	5.562	0.00	0.00	I
ATOM	1123	HD12	LEU	I	109	9.894	-28.935	6.709	0.00	0.00	I
ATOM	1124	HD13	LEU	I	109	10.046	-30.749	7.105	0.00	0.00	I
ATOM	1125	CD2	LEU	I	109	7.359	-29.801	6.648	0.00	0.00	I
ATOM	1126	HD21	LEU	I	109	7.552	-28.856	7.199	0.00	0.00	I
ATOM	1127	HD22	LEU	I	109	6.359	-29.783	6.164	0.00	0.00	I
ATOM	1128	HD23	LEU	I	109	7.371	-30.537	7.480	0.00	0.00	I
ATOM	1129	C	LEU	I	109	8.915	-26.874	5.375	0.00	0.00	I
ATOM	1130	O	LEU	I	109	8.831	-26.776	6.623	0.00	0.00	I
ATOM	1131	N	GLY	I	110	9.944	-26.176	4.783	0.00	0.00	I
ATOM	1132	HN	GLY	I	110	10.017	-26.393	3.812	0.00	0.00	I
ATOM	1133	CA	GLY	I	110	10.788	-25.138	5.368	0.00	0.00	I

ATOM 1134 HA1 GLY I 110 11.347 -24.697 4.557 0.00 0.00
I
ATOM 1135 HA2 GLY I 110 11.308 -25.583 6.204 0.00 0.00
I
ATOM 1136 C GLY I 110 9.984 -24.074 5.981 0.00 0.00
I
ATOM 1137 O GLY I 110 10.201 -23.755 7.119 0.00 0.00
I
ATOM 1138 N PHE I 111 9.021 -23.523 5.267 0.00 0.00
I
ATOM 1139 HN PHE I 111 8.931 -23.859 4.333 0.00 0.00
I
ATOM 1140 CA PHE I 111 8.172 -22.521 5.798 0.00 0.00
I
ATOM 1141 HA PHE I 111 8.780 -21.696 6.140 0.00 0.00
I
ATOM 1142 CB PHE I 111 7.226 -21.976 4.704 0.00 0.00
I
ATOM 1143 HB1 PHE I 111 7.877 -21.630 3.873 0.00 0.00
I
ATOM 1144 HB2 PHE I 111 6.481 -22.650 4.229 0.00 0.00
I
ATOM 1145 CG PHE I 111 6.544 -20.704 5.186 0.00 0.00
I
ATOM 1146 CD1 PHE I 111 5.326 -20.734 5.881 0.00 0.00
I
ATOM 1147 HD1 PHE I 111 4.828 -21.676 6.057 0.00 0.00
I
ATOM 1148 CE1 PHE I 111 4.777 -19.488 6.391 0.00 0.00
I
ATOM 1149 HE1 PHE I 111 3.843 -19.410 6.929 0.00 0.00
I
ATOM 1150 CZ PHE I 111 5.485 -18.324 6.270 0.00 0.00
I
ATOM 1151 HZ PHE I 111 5.069 -17.444 6.739 0.00 0.00
I
ATOM 1152 CD2 PHE I 111 7.222 -19.445 5.124 0.00 0.00
I
ATOM 1153 HD2 PHE I 111 8.142 -19.300 4.577 0.00 0.00
I
ATOM 1154 CE2 PHE I 111 6.678 -18.252 5.577 0.00 0.00
I
ATOM 1155 HE2 PHE I 111 7.246 -17.344 5.444 0.00 0.00
I
ATOM 1156 C PHE I 111 7.352 -22.895 6.980 0.00 0.00
I
ATOM 1157 O PHE I 111 7.245 -22.245 7.989 0.00 0.00
I
ATOM 1158 N MET I 112 6.689 -24.079 6.886 0.00 0.00
I
ATOM 1159 HN MET I 112 6.661 -24.595 6.033 0.00 0.00
I
ATOM 1160 CA MET I 112 6.033 -24.755 7.976 0.00 0.00
I

ATOM 1161 HA MET I 112 5.233 -24.144 8.367 0.00 0.00
I
ATOM 1162 CB MET I 112 5.272 -25.965 7.403 0.00 0.00
I
ATOM 1163 HB1 MET I 112 5.981 -26.438 6.690 0.00 0.00
I
ATOM 1164 HB2 MET I 112 5.030 -26.672 8.225 0.00 0.00
I
ATOM 1165 CG MET I 112 4.021 -25.487 6.659 0.00 0.00
I
ATOM 1166 HG1 MET I 112 4.233 -25.057 5.657 0.00 0.00
I
ATOM 1167 HG2 MET I 112 3.288 -26.298 6.457 0.00 0.00
I
ATOM 1168 SD MET I 112 2.942 -24.259 7.481 0.00 0.00
I
ATOM 1169 CE MET I 112 2.603 -25.338 8.897 0.00 0.00
I
ATOM 1170 HE1 MET I 112 1.929 -26.185 8.647 0.00 0.00
I
ATOM 1171 HE2 MET I 112 3.484 -25.758 9.427 0.00 0.00
I
ATOM 1172 HE3 MET I 112 1.951 -24.867 9.663 0.00 0.00
I
ATOM 1173 C MET I 112 6.874 -25.163 9.171 0.00 0.00
I
ATOM 1174 O MET I 112 6.400 -25.219 10.304 0.00 0.00
I
ATOM 1175 N THR I 113 8.169 -25.509 9.035 0.00 0.00
I
ATOM 1176 HN THR I 113 8.489 -25.529 8.091 0.00 0.00
I
ATOM 1177 CA THR I 113 9.103 -25.624 10.219 0.00 0.00
I
ATOM 1178 HA THR I 113 8.823 -26.460 10.842 0.00 0.00
I
ATOM 1179 CB THR I 113 10.538 -26.111 9.779 0.00 0.00
I
ATOM 1180 HB THR I 113 10.987 -25.285 9.187 0.00 0.00
I
ATOM 1181 OG1 THR I 113 10.497 -27.337 9.010 0.00 0.00
I
ATOM 1182 HG1 THR I 113 10.196 -27.211 8.108 0.00 0.00
I
ATOM 1183 CG2 THR I 113 11.263 -26.431 11.044 0.00 0.00
I
ATOM 1184 HG21 THR I 113 11.509 -25.490 11.581 0.00 0.00
I
ATOM 1185 HG22 THR I 113 10.784 -27.126 11.766 0.00 0.00
I
ATOM 1186 HG23 THR I 113 12.270 -26.794 10.747 0.00 0.00
I
ATOM 1187 C THR I 113 9.187 -24.340 11.080 0.00 0.00
I

ATOM 1188 O THR I 113 9.167 -24.283 12.334 0.00 0.00
I
ATOM 1189 N PHE I 114 9.390 -23.248 10.355 0.00 0.00
I
ATOM 1190 HN PHE I 114 9.192 -23.309 9.380 0.00 0.00
I
ATOM 1191 CA PHE I 114 9.399 -21.871 10.814 0.00 0.00
I
ATOM 1192 HA PHE I 114 10.158 -21.853 11.582 0.00 0.00
I
ATOM 1193 CB PHE I 114 9.580 -20.845 9.666 0.00 0.00
I
ATOM 1194 HB1 PHE I 114 10.297 -21.152 8.875 0.00 0.00
I
ATOM 1195 HB2 PHE I 114 8.659 -20.691 9.064 0.00 0.00
I
ATOM 1196 CG PHE I 114 9.914 -19.470 10.183 0.00 0.00
I
ATOM 1197 CD1 PHE I 114 9.187 -18.300 9.789 0.00 0.00
I
ATOM 1198 HD1 PHE I 114 8.328 -18.425 9.147 0.00 0.00
I
ATOM 1199 CE1 PHE I 114 9.537 -17.081 10.373 0.00 0.00
I
ATOM 1200 HE1 PHE I 114 8.968 -16.187 10.165 0.00 0.00
I
ATOM 1201 CZ PHE I 114 10.604 -16.969 11.311 0.00 0.00
I
ATOM 1202 HZ PHE I 114 10.904 -16.035 11.763 0.00 0.00
I
ATOM 1203 CD2 PHE I 114 11.027 -19.313 11.038 0.00 0.00
I
ATOM 1204 HD2 PHE I 114 11.493 -20.201 11.437 0.00 0.00
I
ATOM 1205 CE2 PHE I 114 11.385 -18.112 11.594 0.00 0.00
I
ATOM 1206 HE2 PHE I 114 12.281 -18.070 12.196 0.00 0.00
I
ATOM 1207 C PHE I 114 8.171 -21.476 11.585 0.00 0.00
I
ATOM 1208 O PHE I 114 8.188 -21.096 12.725 0.00 0.00
I
ATOM 1209 N ALA I 115 7.016 -21.768 10.984 0.00 0.00
I
ATOM 1210 HN ALA I 115 7.005 -22.137 10.058 0.00 0.00
I
ATOM 1211 CA ALA I 115 5.789 -21.368 11.421 0.00 0.00
I
ATOM 1212 HA ALA I 115 5.786 -20.329 11.716 0.00 0.00
I
ATOM 1213 CB ALA I 115 4.725 -21.522 10.334 0.00 0.00
I
ATOM 1214 HB1 ALA I 115 5.057 -20.742 9.616 0.00 0.00
I

ATOM 1215 HB2 ALA I 115 4.684 −22.581 10.000 0.00 0.00
I
ATOM 1216 HB3 ALA I 115 3.718 −21.314 10.755 0.00 0.00
I
ATOM 1217 C ALA I 115 5.327 −22.138 12.711 0.00 0.00
I
ATOM 1218 O ALA I 115 4.509 −21.606 13.419 0.00 0.00
I
ATOM 1219 N VAL I 116 5.833 −23.362 13.035 0.00 0.00
I
ATOM 1220 HN VAL I 116 6.394 −23.848 12.369 0.00 0.00
I
ATOM 1221 CA VAL I 116 5.567 −23.989 14.292 0.00 0.00
I
ATOM 1222 HA VAL I 116 4.577 −23.698 14.609 0.00 0.00
I
ATOM 1223 CB VAL I 116 5.618 −25.529 14.260 0.00 0.00
I
ATOM 1224 HB VAL I 116 5.411 −25.964 15.261 0.00 0.00
I
ATOM 1225 CG1 VAL I 116 4.491 −26.016 13.303 0.00 0.00
I
ATOM 1226 HG11 VAL I 116 4.614 −25.720 12.239 0.00 0.00
I
ATOM 1227 HG12 VAL I 116 4.288 −27.108 13.345 0.00 0.00
I
ATOM 1228 HG13 VAL I 116 3.545 −25.638 13.745 0.00 0.00
I
ATOM 1229 CG2 VAL I 116 6.976 −26.089 13.841 0.00 0.00
I
ATOM 1230 HG21 VAL I 116 7.785 −25.738 14.516 0.00 0.00
I
ATOM 1231 HG22 VAL I 116 7.004 −27.180 14.052 0.00 0.00
I
ATOM 1232 HG23 VAL I 116 7.170 −25.812 12.783 0.00 0.00
I
ATOM 1233 C VAL I 116 6.496 −23.519 15.347 0.00 0.00
I
ATOM 1234 O VAL I 116 6.223 −23.618 16.537 0.00 0.00
I
ATOM 1235 N LEU I 117 7.683 −23.025 14.968 0.00 0.00
I
ATOM 1236 HN LEU I 117 7.916 −23.031 13.999 0.00 0.00
I
ATOM 1237 CA LEU I 117 8.662 −22.426 15.842 0.00 0.00
I
ATOM 1238 HA LEU I 117 8.851 −23.100 16.664 0.00 0.00
I
ATOM 1239 CB LEU I 117 10.032 −22.248 15.105 0.00 0.00
I
ATOM 1240 HB1 LEU I 117 10.437 −23.262 14.900 0.00 0.00
I
ATOM 1241 HB2 LEU I 117 9.904 −21.782 14.105 0.00 0.00
I

```
ATOM   1242  CG  LEU I 117      11.077 -21.310  15.831  0.00  0.00
I
ATOM   1243  HG  LEU I 117      10.497 -20.462  16.255  0.00  0.00
I
ATOM   1244  CD1 LEU I 117      11.673 -22.045  17.063  0.00  0.00
I
ATOM   1245 HD11 LEU I 117      12.249 -22.953  16.787  0.00  0.00
I
ATOM   1246 HD12 LEU I 117      12.392 -21.420  17.634  0.00  0.00
I
ATOM   1247 HD13 LEU I 117      10.896 -22.377  17.785  0.00  0.00
I
ATOM   1248  CD2 LEU I 117      12.235 -20.787  14.964  0.00  0.00
I
ATOM   1249 HD21 LEU I 117      12.833 -20.112  15.613  0.00  0.00
I
ATOM   1250 HD22 LEU I 117      12.838 -21.616  14.536  0.00  0.00
I
ATOM   1251 HD23 LEU I 117      11.694 -20.246  14.158  0.00  0.00
I
ATOM   1252  C   LEU I 117       8.105 -21.086  16.447  0.00  0.00
I
ATOM   1253  O   LEU I 117       8.038 -20.919  17.674  0.00  0.00
I
ATOM   1254  N   ILE I 118       7.724 -20.077  15.582  0.00  0.00
I
ATOM   1255  HN  ILE I 118       7.759 -20.307  14.612  0.00  0.00
I
ATOM   1256  CA  ILE I 118       7.428 -18.756  16.090  0.00  0.00
I
ATOM   1257  HA  ILE I 118       8.195 -18.461  16.792  0.00  0.00
I
ATOM   1258  CB  ILE I 118       7.192 -17.660  14.989  0.00  0.00
I
ATOM   1259  HB  ILE I 118       7.055 -16.668  15.470  0.00  0.00
I
ATOM   1260  CG2 ILE I 118       8.574 -17.635  14.379  0.00  0.00
I
ATOM   1261 HG21 ILE I 118       9.281 -17.765  15.226  0.00  0.00
I
ATOM   1262 HG22 ILE I 118       8.734 -18.439  13.629  0.00  0.00
I
ATOM   1263 HG23 ILE I 118       8.719 -16.706  13.786  0.00  0.00
I
ATOM   1264  CG1 ILE I 118       6.077 -17.964  13.964  0.00  0.00
I
ATOM   1265 HG11 ILE I 118       6.259 -18.907  13.406  0.00  0.00
I
ATOM   1266 HG12 ILE I 118       5.134 -18.107  14.533  0.00  0.00
I
ATOM   1267  CD  ILE I 118       5.821 -16.894  12.922  0.00  0.00
I
ATOM   1268  HD1 ILE I 118       6.670 -16.700  12.232  0.00  0.00
I
```

ATOM 1269 HD2 ILE I 118 4.937 -17.152 12.301 0.00 0.00
I
ATOM 1270 HD3 ILE I 118 5.461 -15.998 13.472 0.00 0.00
I
ATOM 1271 C ILE I 118 6.211 -18.805 17.051 0.00 0.00
I
ATOM 1272 O ILE I 118 6.188 -18.011 17.977 0.00 0.00
I
ATOM 1273 N VAL I 119 5.239 -19.722 16.866 0.00 0.00
I
ATOM 1274 HN VAL I 119 5.253 -20.236 16.012 0.00 0.00
I
ATOM 1275 CA VAL I 119 4.154 -19.794 17.800 0.00 0.00
I
ATOM 1276 HA VAL I 119 3.864 -18.838 18.210 0.00 0.00
I
ATOM 1277 CB VAL I 119 2.958 -20.543 17.117 0.00 0.00
I
ATOM 1278 HB VAL I 119 2.111 -20.546 17.837 0.00 0.00
I
ATOM 1279 CG1 VAL I 119 2.490 -19.788 15.915 0.00 0.00
I
ATOM 1280 HG11 VAL I 119 1.994 -18.843 16.224 0.00 0.00
I
ATOM 1281 HG12 VAL I 119 3.287 -19.622 15.159 0.00 0.00
I
ATOM 1282 HG13 VAL I 119 1.656 -20.351 15.443 0.00 0.00
I
ATOM 1283 CG2 VAL I 119 3.335 -22.004 16.751 0.00 0.00
I
ATOM 1284 HG21 VAL I 119 2.684 -22.502 16.001 0.00 0.00
I
ATOM 1285 HG22 VAL I 119 4.344 -22.048 16.287 0.00 0.00
I
ATOM 1286 HG23 VAL I 119 3.225 -22.575 17.698 0.00 0.00
I
ATOM 1287 C VAL I 119 4.461 -20.462 19.115 0.00 0.00
I
ATOM 1288 O VAL I 119 3.711 -20.306 20.069 0.00 0.00
I
ATOM 1289 N MET I 120 5.577 -21.095 19.315 0.00 0.00
I
ATOM 1290 HN MET I 120 6.263 -20.970 18.603 0.00 0.00
I
ATOM 1291 CA MET I 120 6.105 -21.430 20.655 0.00 0.00
I
ATOM 1292 HA MET I 120 5.386 -21.913 21.300 0.00 0.00
I
ATOM 1293 CB MET I 120 7.284 -22.422 20.521 0.00 0.00
I
ATOM 1294 HB1 MET I 120 8.114 -21.920 19.979 0.00 0.00
I
ATOM 1295 HB2 MET I 120 7.670 -22.562 21.553 0.00 0.00
I

```
ATOM   1296  CG  MET I 120       7.006 -23.773  19.891  0.00  0.00
I
ATOM   1297  HG1 MET I 120       6.107 -24.217  20.370  0.00  0.00
I
ATOM   1298  HG2 MET I 120       6.752 -23.576  18.828  0.00  0.00
I
ATOM   1299  SD  MET I 120       8.423 -24.908  19.957  0.00  0.00
I
ATOM   1300  CE  MET I 120       7.337 -26.333  19.554  0.00  0.00
I
ATOM   1301  HE1 MET I 120       6.845 -26.842  20.411  0.00  0.00
I
ATOM   1302  HE2 MET I 120       6.470 -25.998  18.945  0.00  0.00
I
ATOM   1303  HE3 MET I 120       7.868 -27.184  19.076  0.00  0.00
I
ATOM   1304  C   MET I 120       6.651 -20.222  21.381  0.00  0.00
I
ATOM   1305  O   MET I 120       6.340 -19.898  22.517  0.00  0.00
I
ATOM   1306  N   ILE I 121       7.465 -19.395  20.562  0.00  0.00
I
ATOM   1307  HN  ILE I 121       7.597 -19.719  19.628  0.00  0.00
I
ATOM   1308  CA  ILE I 121       8.117 -18.229  21.032  0.00  0.00
I
ATOM   1309  HA  ILE I 121       8.852 -18.527  21.765  0.00  0.00
I
ATOM   1310  CB  ILE I 121       8.932 -17.563  19.910  0.00  0.00
I
ATOM   1311  HB  ILE I 121       8.269 -17.303  19.057  0.00  0.00
I
ATOM   1312  CG2 ILE I 121       9.494 -16.243  20.397  0.00  0.00
I
ATOM   1313 HG21 ILE I 121       8.720 -15.529  20.750  0.00  0.00
I
ATOM   1314 HG22 ILE I 121      10.175 -16.412  21.258  0.00  0.00
I
ATOM   1315 HG23 ILE I 121      10.053 -15.736  19.582  0.00  0.00
I
ATOM   1316  CG1 ILE I 121       9.951 -18.536  19.344  0.00  0.00
I
ATOM   1317 HG11 ILE I 121      10.646 -18.802  20.169  0.00  0.00
I
ATOM   1318 HG12 ILE I 121       9.595 -19.524  18.981  0.00  0.00
I
ATOM   1319  CD  ILE I 121      10.865 -17.968  18.262  0.00  0.00
I
ATOM   1320  HD1 ILE I 121      11.751 -17.537  18.775  0.00  0.00
I
ATOM   1321  HD2 ILE I 121      11.146 -18.835  17.627  0.00  0.00
I
ATOM   1322  HD3 ILE I 121      10.341 -17.214  17.636  0.00  0.00
I
```

ATOM   1323 C   ILE I 121   7.158 -17.177 21.571 0.00 0.00
I
ATOM   1324 O   ILE I 121   7.301 -16.592 22.730 0.00 0.00
I
ATOM   1325 N   LEU I 122   6.157 -16.841 20.733 0.00 0.00
I
ATOM   1326 HN LEU I 122   6.052 -17.239 19.825 0.00 0.00
I
ATOM   1327 CA LEU I 122   5.047 -15.912 21.108 0.00 0.00
I
ATOM   1328 HA LEU I 122   5.478 -14.970 21.412 0.00 0.00
I
ATOM   1329 CB LEU I 122   4.183 -15.567 19.870 0.00 0.00
I
ATOM   1330 HB1 LEU I 122   3.711 -16.478 19.445 0.00 0.00
I
ATOM   1331 HB2 LEU I 122   3.310 -14.901 20.039 0.00 0.00
I
ATOM   1332 CG LEU I 122   4.859 -14.968 18.607 0.00 0.00
I
ATOM   1333 HG LEU I 122   5.729 -15.647 18.485 0.00 0.00
I
ATOM   1334 CD1 LEU I 122   4.086 -15.042 17.302 0.00 0.00
I
ATOM   1335 HD11 LEU I 122   3.224 -15.740 17.234 0.00 0.00
I
ATOM   1336 HD12 LEU I 122   3.634 -14.039 17.150 0.00 0.00
I
ATOM   1337 HD13 LEU I 122   4.795 -15.154 16.454 0.00 0.00
I
ATOM   1338 CD2 LEU I 122   5.284 -13.471 18.870 0.00 0.00
I
ATOM   1339 HD21 LEU I 122   6.053 -12.995 18.224 0.00 0.00
I
ATOM   1340 HD22 LEU I 122   4.307 -12.948 18.783 0.00 0.00
I
ATOM   1341 HD23 LEU I 122   5.671 -13.393 19.908 0.00 0.00
I
ATOM   1342 C   LEU I 122   4.156 -16.412 22.181 0.00 0.00
I
ATOM   1343 O   LEU I 122   3.513 -15.625 22.958 0.00 0.00
I
ATOM   1344 N   GLY I 123   3.923 -17.690 22.280 0.00 0.00
I
ATOM   1345 HN GLY I 123   4.356 -18.345 21.666 0.00 0.00
I
ATOM   1346 CA GLY I 123   2.973 -18.277 23.256 0.00 0.00
I
ATOM   1347 HA1 GLY I 123   2.638 -19.258 22.952 0.00 0.00
I
ATOM   1348 HA2 GLY I 123   2.084 -17.665 23.300 0.00 0.00
I
ATOM   1349 C   GLY I 123   3.461 -18.325 24.643 0.00 0.00
I

```
ATOM   1350  O   GLY I 123       2.726 -18.583  25.604  0.00  0.00
I
ATOM   1351  N   LYS I 124       4.772 -17.995  24.925  0.00  0.00
I
ATOM   1352  HN  LYS I 124       5.465 -17.826  24.228  0.00  0.00
I
ATOM   1353  CA  LYS I 124       5.288 -17.965  26.311  0.00  0.00
I
ATOM   1354  HA  LYS I 124       5.027 -18.895  26.795  0.00  0.00
I
ATOM   1355  CB  LYS I 124       6.834 -17.809  26.312  0.00  0.00
I
ATOM   1356  HB1 LYS I 124       7.081 -17.192  25.422  0.00  0.00
I
ATOM   1357  HB2 LYS I 124       7.059 -17.200  27.213  0.00  0.00
I
ATOM   1358  CG  LYS I 124       7.452 -19.182  26.170  0.00  0.00
I
ATOM   1359  HG1 LYS I 124       7.122 -19.878  26.970  0.00  0.00
I
ATOM   1360  HG2 LYS I 124       7.149 -19.507  25.151  0.00  0.00
I
ATOM   1361  CD  LYS I 124       9.011 -19.077  26.258  0.00  0.00
I
ATOM   1362  HD1 LYS I 124       9.355 -18.446  25.411  0.00  0.00
I
ATOM   1363  HD2 LYS I 124       9.153 -18.470  27.178  0.00  0.00
I
ATOM   1364  CE  LYS I 124       9.731 -20.429  26.233  0.00  0.00
I
ATOM   1365  HE1 LYS I 124       9.540 -21.078  27.114  0.00  0.00
I
ATOM   1366  HE2 LYS I 124       9.542 -20.978  25.286  0.00  0.00
I
ATOM   1367  NZ  LYS I 124      11.155 -20.119  26.196  0.00  0.00
I
ATOM   1368  HZ1 LYS I 124      11.810 -20.911  26.357  0.00  0.00
I
ATOM   1369  HZ2 LYS I 124      11.305 -19.766  25.229  0.00  0.00
I
ATOM   1370  HZ3 LYS I 124      11.499 -19.448  26.911  0.00  0.00
I
ATOM   1371  C   LYS I 124       4.689 -16.902  27.139  0.00  0.00
I
ATOM   1372  O   LYS I 124       4.503 -17.103  28.311  0.00  0.00
I
ATOM   1373  N   LYS I 125       4.319 -15.745  26.457  0.00  0.00
I
ATOM   1374  HN  LYS I 125       4.540 -15.826  25.489  0.00  0.00
I
ATOM   1375  CA  LYS I 125       3.778 -14.551  27.121  0.00  0.00
I
ATOM   1376  HA  LYS I 125       4.490 -14.367  27.912  0.00  0.00
I
```

ATOM 1377 CB LYS I 125 3.625 −13.399 26.116 0.00 0.00
I
ATOM 1378 HB1 LYS I 125 4.528 −13.487 25.474 0.00 0.00
I
ATOM 1379 HB2 LYS I 125 2.812 −13.794 25.469 0.00 0.00
I
ATOM 1380 CG LYS I 125 3.414 −11.979 26.638 0.00 0.00
I
ATOM 1381 HG1 LYS I 125 3.342 −11.488 25.644 0.00 0.00
I
ATOM 1382 HG2 LYS I 125 2.405 −11.942 27.100 0.00 0.00
I
ATOM 1383 CD LYS I 125 4.522 −11.477 27.581 0.00 0.00
I
ATOM 1384 HD1 LYS I 125 4.315 −10.397 27.740 0.00 0.00
I
ATOM 1385 HD2 LYS I 125 4.346 −11.985 28.553 0.00 0.00
I
ATOM 1386 CE LYS I 125 6.005 −11.526 27.045 0.00 0.00
I
ATOM 1387 HE1 LYS I 125 6.346 −12.573 27.192 0.00 0.00
I
ATOM 1388 HE2 LYS I 125 5.970 −11.387 25.943 0.00 0.00
I
ATOM 1389 NZ LYS I 125 6.913 −10.577 27.673 0.00 0.00
I
ATOM 1390 HZ1 LYS I 125 7.831 −10.624 27.187 0.00 0.00
I
ATOM 1391 HZ2 LYS I 125 6.616 −9.611 27.429 0.00 0.00
I
ATOM 1392 HZ3 LYS I 125 7.108 −10.642 28.693 0.00 0.00
I
ATOM 1393 C LYS I 125 2.477 −14.808 27.885 0.00 0.00
I
ATOM 1394 O LYS I 125 2.201 −14.241 28.941 0.00 0.00
I
ATOM 1395 N ILE I 126 1.568 −15.603 27.292 0.00 0.00
I
ATOM 1396 HN ILE I 126 1.817 −16.138 26.488 0.00 0.00
I
ATOM 1397 CA ILE I 126 0.237 −15.856 27.808 0.00 0.00
I
ATOM 1398 HA ILE I 126 −0.026 −15.183 28.611 0.00 0.00
I
ATOM 1399 CB ILE I 126 −0.721 −15.603 26.579 0.00 0.00
I
ATOM 1400 HB ILE I 126 −0.396 −14.602 26.221 0.00 0.00
I
ATOM 1401 CG2 ILE I 126 −0.580 −16.690 25.460 0.00 0.00
I
ATOM 1402 HG21 ILE I 126 −1.148 −16.345 24.570 0.00 0.00
I
ATOM 1403 HG22 ILE I 126 0.496 −16.859 25.236 0.00 0.00
I

```
ATOM   1404 HG23 ILE I 126      -0.945 -17.726  25.627  0.00  0.00
I
ATOM   1405  CG1 ILE I 126      -2.281 -15.482  26.965  0.00  0.00
I
ATOM   1406 HG11 ILE I 126      -2.843 -15.358  26.015  0.00  0.00
I
ATOM   1407 HG12 ILE I 126      -2.550 -16.333  27.627  0.00  0.00
I
ATOM   1408  CD  ILE I 126      -2.640 -14.245  27.807  0.00  0.00
I
ATOM   1409  HD1 ILE I 126      -3.698 -14.331  28.137  0.00  0.00
I
ATOM   1410  HD2 ILE I 126      -2.042 -14.163  28.740  0.00  0.00
I
ATOM   1411  HD3 ILE I 126      -2.519 -13.373  27.130  0.00  0.00
I
ATOM   1412  C   ILE I 126       0.154 -17.276  28.352  0.00  0.00
I
ATOM   1413  O   ILE I 126      -0.854 -17.744  28.758  0.00  0.00
I
ATOM   1414  N   GLY I 127       1.318 -17.935  28.463  0.00  0.00
I
ATOM   1415  HN  GLY I 127       2.109 -17.460  28.085  0.00  0.00
I
ATOM   1416  CA  GLY I 127       1.538 -19.299  28.849  0.00  0.00
I
ATOM   1417  HA1 GLY I 127       2.612 -19.407  28.848  0.00  0.00
I
ATOM   1418  HA2 GLY I 127       0.957 -20.000  28.267  0.00  0.00
I
ATOM   1419  C   GLY I 127       1.044 -19.644  30.199  0.00  0.00
I
ATOM   1420  O   GLY I 127       0.180 -20.540  30.319  0.00  0.00
I
ATOM   1421  N   LEU I 128       1.499 -18.893  31.232  0.00  0.00
I
ATOM   1422  HN  LEU I 128       2.094 -18.119  31.028  0.00  0.00
I
ATOM   1423  CA  LEU I 128       0.958 -19.069  32.550  0.00  0.00
I
ATOM   1424  HA  LEU I 128       1.176 -20.092  32.819  0.00  0.00
I
ATOM   1425  CB  LEU I 128       1.757 -18.291  33.574  0.00  0.00
I
ATOM   1426  HB1 LEU I 128       2.784 -18.711  33.530  0.00  0.00
I
ATOM   1427  HB2 LEU I 128       1.852 -17.255  33.184  0.00  0.00
I
ATOM   1428  CG  LEU I 128       1.394 -18.398  35.081  0.00  0.00
I
ATOM   1429  HG  LEU I 128       0.372 -18.017  35.293  0.00  0.00
I
ATOM   1430  CD1 LEU I 128       1.368 -19.913  35.507  0.00  0.00
I
```

```
ATOM   1431 HD11 LEU I 128       2.094 -20.542  34.948  0.00  0.00
I
ATOM   1432 HD12 LEU I 128       1.460 -19.999  36.611  0.00  0.00
I
ATOM   1433 HD13 LEU I 128       0.417 -20.432  35.259  0.00  0.00
I
ATOM   1434  CD2 LEU I 128       2.346 -17.458  35.842  0.00  0.00
I
ATOM   1435 HD21 LEU I 128       3.383 -17.773  35.598  0.00  0.00
I
ATOM   1436 HD22 LEU I 128       2.188 -16.389  35.583  0.00  0.00
I
ATOM   1437 HD23 LEU I 128       2.127 -17.561  36.927  0.00  0.00
I
ATOM   1438  C   LEU I 128      -0.537 -18.847  32.642  0.00  0.00
I
ATOM   1439  O   LEU I 128      -1.236 -19.710  33.164  0.00  0.00
I
ATOM   1440  N   LYS I 129      -1.081 -17.875  31.940  0.00  0.00
I
ATOM   1441  HN  LYS I 129      -0.608 -17.117  31.499  0.00  0.00
I
ATOM   1442  CA  LYS I 129      -2.508 -17.730  32.086  0.00  0.00
I
ATOM   1443  HA  LYS I 129      -2.843 -17.850  33.106  0.00  0.00
I
ATOM   1444  CB  LYS I 129      -2.877 -16.264  31.788  0.00  0.00
I
ATOM   1445  HB1 LYS I 129      -2.222 -15.801  31.019  0.00  0.00
I
ATOM   1446  HB2 LYS I 129      -3.924 -16.138  31.439  0.00  0.00
I
ATOM   1447  CG  LYS I 129      -2.623 -15.279  32.938  0.00  0.00
I
ATOM   1448  HG1 LYS I 129      -3.161 -15.622  33.847  0.00  0.00
I
ATOM   1449  HG2 LYS I 129      -1.541 -15.276  33.193  0.00  0.00
I
ATOM   1450  CD  LYS I 129      -3.098 -13.909  32.376  0.00  0.00
I
ATOM   1451  HD1 LYS I 129      -2.328 -13.685  31.606  0.00  0.00
I
ATOM   1452  HD2 LYS I 129      -4.109 -13.837  31.923  0.00  0.00
I
ATOM   1453  CE  LYS I 129      -2.997 -12.970  33.601  0.00  0.00
I
ATOM   1454  HE1 LYS I 129      -3.780 -12.981  34.389  0.00  0.00
I
ATOM   1455  HE2 LYS I 129      -2.023 -13.070  34.125  0.00  0.00
I
ATOM   1456  NZ  LYS I 129      -3.009 -11.496  33.145  0.00  0.00
I
ATOM   1457  HZ1 LYS I 129      -2.189 -11.457  32.508  0.00  0.00
I
```

ATOM 1458 HZ2 LYS I 129 −3.859 −11.217 32.614 0.00 0.00
I
ATOM 1459 HZ3 LYS I 129 −2.846 −10.893 33.977 0.00 0.00
I
ATOM 1460 C LYS I 129 −3.366 −18.749 31.243 0.00 0.00
I
ATOM 1461 O LYS I 129 −4.483 −19.193 31.596 0.00 0.00
I
ATOM 1462 N GLU I 130 −2.815 −19.112 30.046 0.00 0.00
I
ATOM 1463 HN GLU I 130 −1.949 −18.752 29.707 0.00 0.00
I
ATOM 1464 CA GLU I 130 −3.458 −19.962 29.034 0.00 0.00
I
ATOM 1465 HA GLU I 130 −4.389 −19.544 28.681 0.00 0.00
I
ATOM 1466 CB GLU I 130 −2.828 −20.055 27.659 0.00 0.00
I
ATOM 1467 HB1 GLU I 130 −2.592 −19.044 27.263 0.00 0.00
I
ATOM 1468 HB2 GLU I 130 −1.837 −20.541 27.789 0.00 0.00
I
ATOM 1469 CG GLU I 130 −3.484 −21.049 26.675 0.00 0.00
I
ATOM 1470 HG1 GLU I 130 −3.140 −22.070 26.946 0.00 0.00
I
ATOM 1471 HG2 GLU I 130 −4.586 −21.140 26.791 0.00 0.00
I
ATOM 1472 CD GLU I 130 −3.245 −20.724 25.214 0.00 0.00
I
ATOM 1473 OE1 GLU I 130 −2.552 −19.732 24.832 0.00 0.00
I
ATOM 1474 OE2 GLU I 130 −3.790 −21.540 24.372 0.00 0.00
I
ATOM 1475 C GLU I 130 −3.590 −21.381 29.625 0.00 0.00
I
ATOM 1476 O GLU I 130 −4.644 −22.059 29.496 0.00 0.00
I
ATOM 1477 N ARG I 131 −2.558 −21.863 30.344 0.00 0.00
I
ATOM 1478 HN ARG I 131 −1.833 −21.178 30.324 0.00 0.00
I
ATOM 1479 CA ARG I 131 −2.554 −23.069 31.109 0.00 0.00
I
ATOM 1480 HA ARG I 131 −2.631 −23.841 30.358 0.00 0.00
I
ATOM 1481 CB ARG I 131 −1.296 −23.228 31.926 0.00 0.00
I
ATOM 1482 HB1 ARG I 131 −0.403 −23.009 31.302 0.00 0.00
I
ATOM 1483 HB2 ARG I 131 −1.267 −22.724 32.916 0.00 0.00
I
ATOM 1484 CG ARG I 131 −0.961 −24.694 32.330 0.00 0.00
I

```
ATOM   1485  HG1 ARG I 131      -1.839 -25.264  32.700  0.00  0.00
I
ATOM   1486  HG2 ARG I 131      -0.773 -25.318  31.430  0.00  0.00
I
ATOM   1487  CD  ARG I 131       0.057 -24.882  33.376  0.00  0.00
I
ATOM   1488  HD1 ARG I 131      -0.191 -24.195  34.213  0.00  0.00
I
ATOM   1489  HD2 ARG I 131       0.172 -25.909  33.783  0.00  0.00
I
ATOM   1490  NE  ARG I 131       1.379 -24.555  32.729  0.00  0.00
I
ATOM   1491  HE  ARG I 131       1.656 -25.083  31.926  0.00  0.00
I
ATOM   1492  CZ  ARG I 131       2.313 -23.760  33.290  0.00  0.00
I
ATOM   1493  NH1 ARG I 131       3.267 -23.406  32.489  0.00  0.00
I
ATOM   1494 HH11 ARG I 131       3.858 -22.654  32.781  0.00  0.00
I
ATOM   1495 HH12 ARG I 131       2.941 -23.328  31.546  0.00  0.00
I
ATOM   1496  NH2 ARG I 131       2.250 -23.320  34.525  0.00  0.00
I
ATOM   1497 HH21 ARG I 131       3.024 -22.791  34.873  0.00  0.00
I
ATOM   1498 HH22 ARG I 131       1.655 -23.857  35.122  0.00  0.00
I
ATOM   1499  C   ARG I 131      -3.774 -23.227  32.029  0.00  0.00
I
ATOM   1500  O   ARG I 131      -4.364 -24.352  32.006  0.00  0.00
I
ATOM   1501  N   MET I 132      -4.093 -22.251  32.858  0.00  0.00
I
ATOM   1502  HN  MET I 132      -3.589 -21.391  32.882  0.00  0.00
I
ATOM   1503  CA  MET I 132      -5.218 -22.296  33.734  0.00  0.00
I
ATOM   1504  HA  MET I 132      -5.194 -23.111  34.443  0.00  0.00
I
ATOM   1505  CB  MET I 132      -5.231 -20.852  34.426  0.00  0.00
I
ATOM   1506  HB1 MET I 132      -5.470 -20.099  33.646  0.00  0.00
I
ATOM   1507  HB2 MET I 132      -5.988 -20.943  35.235  0.00  0.00
I
ATOM   1508  CG  MET I 132      -3.991 -20.439  35.245  0.00  0.00
I
ATOM   1509  HG1 MET I 132      -4.013 -20.943  36.235  0.00  0.00
I
ATOM   1510  HG2 MET I 132      -3.095 -20.680  34.634  0.00  0.00
I
ATOM   1511  SD  MET I 132      -3.986 -18.667  35.655  0.00  0.00
I
```

ATOM 1512 CE MET I 132 -2.402 -18.834 36.459 0.00 0.00
I
ATOM 1513 HE1 MET I 132 -2.509 -19.387 37.416 0.00 0.00
I
ATOM 1514 HE2 MET I 132 -1.662 -19.342 35.804 0.00 0.00
I
ATOM 1515 HE3 MET I 132 -2.030 -17.866 36.857 0.00 0.00
I
ATOM 1516 C MET I 132 -6.547 -22.460 32.977 0.00 0.00
I
ATOM 1517 O MET I 132 -7.412 -23.224 33.416 0.00 0.00
I
ATOM 1518 N LEU I 133 -6.688 -21.745 31.812 0.00 0.00
I
ATOM 1519 HN LEU I 133 -5.988 -21.189 31.369 0.00 0.00
I
ATOM 1520 CA LEU I 133 -7.876 -21.895 31.000 0.00 0.00
I
ATOM 1521 HA LEU I 133 -8.700 -21.727 31.678 0.00 0.00
I
ATOM 1522 CB LEU I 133 -7.929 -20.737 29.971 0.00 0.00
I
ATOM 1523 HB1 LEU I 133 -8.004 -19.786 30.540 0.00 0.00
I
ATOM 1524 HB2 LEU I 133 -6.871 -20.711 29.632 0.00 0.00
I
ATOM 1525 CG LEU I 133 -8.869 -20.835 28.743 0.00 0.00
I
ATOM 1526 HG LEU I 133 -8.673 -21.774 28.184 0.00 0.00
I
ATOM 1527 CD1 LEU I 133 -10.411 -21.024 28.996 0.00 0.00
I
ATOM 1528 HD11 LEU I 133 -10.885 -20.178 29.538 0.00 0.00
I
ATOM 1529 HD12 LEU I 133 -10.859 -21.148 27.986 0.00 0.00
I
ATOM 1530 HD13 LEU I 133 -10.517 -22.001 29.513 0.00 0.00
I
ATOM 1531 CD2 LEU I 133 -8.530 -19.609 27.883 0.00 0.00
I
ATOM 1532 HD21 LEU I 133 -7.434 -19.428 27.878 0.00 0.00
I
ATOM 1533 HD22 LEU I 133 -8.917 -19.605 26.841 0.00 0.00
I
ATOM 1534 HD23 LEU I 133 -8.926 -18.659 28.300 0.00 0.00
I
ATOM 1535 C LEU I 133 -8.039 -23.240 30.267 0.00 0.00
I
ATOM 1536 O LEU I 133 -9.069 -23.849 30.359 0.00 0.00
I
ATOM 1537 N VAL I 134 -7.009 -23.792 29.533 0.00 0.00
I
ATOM 1538 HN VAL I 134 -6.093 -23.409 29.617 0.00 0.00
I

```
ATOM   1539  CA  VAL I 134      -7.164 -25.065  28.822  0.00  0.00
I
ATOM   1540  HA  VAL I 134      -8.213 -25.264  28.659  0.00  0.00
I
ATOM   1541  CB  VAL I 134      -6.480 -25.015  27.488  0.00  0.00
I
ATOM   1542  HB  VAL I 134      -6.448 -25.969  26.919  0.00  0.00
I
ATOM   1543  CG1 VAL I 134      -7.220 -24.019  26.567  0.00  0.00
I
ATOM   1544 HG11 VAL I 134      -7.087 -23.012  27.016  0.00  0.00
I
ATOM   1545 HG12 VAL I 134      -6.682 -24.025  25.595  0.00  0.00
I
ATOM   1546 HG13 VAL I 134      -8.300 -24.275  26.537  0.00  0.00
I
ATOM   1547  CG2 VAL I 134      -4.991 -24.550  27.682  0.00  0.00
I
ATOM   1548 HG21 VAL I 134      -5.013 -23.450  27.830  0.00  0.00
I
ATOM   1549 HG22 VAL I 134      -4.509 -24.831  28.643  0.00  0.00
I
ATOM   1550 HG23 VAL I 134      -4.284 -24.789  26.858  0.00  0.00
I
ATOM   1551  C   VAL I 134      -6.668 -26.269  29.542  0.00  0.00
I
ATOM   1552  O   VAL I 134      -6.881 -27.428  29.161  0.00  0.00
I
ATOM   1553  N   GLN I 135      -6.116 -26.072  30.704  0.00  0.00
I
ATOM   1554  HN  GLN I 135      -5.764 -25.223  31.089  0.00  0.00
I
ATOM   1555  CA  GLN I 135      -5.758 -27.159  31.575  0.00  0.00
I
ATOM   1556  HA  GLN I 135      -5.079 -26.697  32.277  0.00  0.00
I
ATOM   1557  CB  GLN I 135      -7.057 -27.582  32.344  0.00  0.00
I
ATOM   1558  HB1 GLN I 135      -7.682 -28.321  31.798  0.00  0.00
I
ATOM   1559  HB2 GLN I 135      -6.626 -28.078  33.239  0.00  0.00
I
ATOM   1560  CG  GLN I 135      -7.846 -26.363  32.884  0.00  0.00
I
ATOM   1561  HG1 GLN I 135      -7.264 -25.777  33.627  0.00  0.00
I
ATOM   1562  HG2 GLN I 135      -8.010 -25.683  32.021  0.00  0.00
I
ATOM   1563  CD  GLN I 135      -9.192 -26.741  33.467  0.00  0.00
I
ATOM   1564  OE1 GLN I 135     -10.097 -27.301  32.751  0.00  0.00
I
ATOM   1565  NE2 GLN I 135      -9.453 -26.458  34.732  0.00  0.00
I
```

ATOM 1566 HE21 GLN I 135 -10.446 -26.485 34.848 0.00 0.00
I
ATOM 1567 HE22 GLN I 135 -8.815 -26.010 35.358 0.00 0.00
I
ATOM 1568 C GLN I 135 -5.008 -28.290 31.027 0.00 0.00
I
ATOM 1569 O GLN I 135 -5.363 -29.437 31.261 0.00 0.00
I
ATOM 1570 N GLU I 136 -3.908 -27.958 30.391 0.00 0.00
I
ATOM 1571 HN GLU I 136 -3.613 -27.026 30.192 0.00 0.00
I
ATOM 1572 CA GLU I 136 -3.058 -28.899 29.677 0.00 0.00
I
ATOM 1573 HA GLU I 136 -2.843 -29.778 30.267 0.00 0.00
I
ATOM 1574 CB GLU I 136 -3.618 -29.235 28.310 0.00 0.00
I
ATOM 1575 HB1 GLU I 136 -3.067 -29.986 27.705 0.00 0.00
I
ATOM 1576 HB2 GLU I 136 -4.576 -29.727 28.584 0.00 0.00
I
ATOM 1577 CG GLU I 136 -4.000 -28.060 27.362 0.00 0.00
I
ATOM 1578 HG1 GLU I 136 -4.782 -27.358 27.722 0.00 0.00
I
ATOM 1579 HG2 GLU I 136 -3.037 -27.520 27.238 0.00 0.00
I
ATOM 1580 CD GLU I 136 -4.293 -28.500 25.927 0.00 0.00
I
ATOM 1581 OE1 GLU I 136 -5.243 -29.254 25.692 0.00 0.00
I
ATOM 1582 OE2 GLU I 136 -3.542 -28.169 24.992 0.00 0.00
I
ATOM 1583 C GLU I 136 -1.729 -28.168 29.559 0.00 0.00
I
ATOM 1584 O GLU I 136 -1.534 -27.093 30.132 0.00 0.00
I
ATOM 1585 N ALA I 137 -0.657 -28.800 28.919 0.00 0.00
I
ATOM 1586 HN ALA I 137 -0.655 -29.725 28.548 0.00 0.00
I
ATOM 1587 CA ALA I 137 0.697 -28.258 29.054 0.00 0.00
I
ATOM 1588 HA ALA I 137 0.858 -27.651 29.933 0.00 0.00
I
ATOM 1589 CB ALA I 137 1.627 -29.422 29.296 0.00 0.00
I
ATOM 1590 HB1 ALA I 137 2.711 -29.192 29.211 0.00 0.00
I
ATOM 1591 HB2 ALA I 137 1.357 -29.932 30.246 0.00 0.00
I
ATOM 1592 HB3 ALA I 137 1.637 -30.123 28.434 0.00 0.00
I

ATOM 1593 C ALA I 137 1.168 -27.367 27.908 0.00 0.00
I
ATOM 1594 O ALA I 137 2.310 -26.876 27.971 0.00 0.00
I
ATOM 1595 N LEU I 138 0.317 -27.163 27.001 0.00 0.00
I
ATOM 1596 HN LEU I 138 -0.598 -27.550 27.079 0.00 0.00
I
ATOM 1597 CA LEU I 138 0.373 -26.263 25.892 0.00 0.00
I
ATOM 1598 HA LEU I 138 -0.575 -26.215 25.378 0.00 0.00
I
ATOM 1599 CB LEU I 138 0.625 -24.807 26.314 0.00 0.00
I
ATOM 1600 HB1 LEU I 138 1.724 -24.671 26.404 0.00 0.00
I
ATOM 1601 HB2 LEU I 138 0.284 -24.227 25.430 0.00 0.00
I
ATOM 1602 CG LEU I 138 -0.204 -24.343 27.581 0.00 0.00
I
ATOM 1603 HG LEU I 138 0.046 -25.133 28.322 0.00 0.00
I
ATOM 1604 CD1 LEU I 138 0.305 -23.015 28.134 0.00 0.00
I
ATOM 1605 HD11 LEU I 138 -0.279 -22.230 27.608 0.00 0.00
I
ATOM 1606 HD12 LEU I 138 -0.101 -22.907 29.163 0.00 0.00
I
ATOM 1607 HD13 LEU I 138 1.389 -22.830 27.973 0.00 0.00
I
ATOM 1608 CD2 LEU I 138 -1.720 -24.339 27.370 0.00 0.00
I
ATOM 1609 HD21 LEU I 138 -2.203 -24.091 28.339 0.00 0.00
I
ATOM 1610 HD22 LEU I 138 -2.139 -23.557 26.702 0.00 0.00
I
ATOM 1611 HD23 LEU I 138 -2.082 -25.346 27.072 0.00 0.00
I
ATOM 1612 C LEU I 138 1.263 -26.587 24.696 0.00 0.00
I
ATOM 1613 O LEU I 138 1.463 -25.654 23.909 0.00 0.00
I
ATOM 1614 N ASN I 139 1.808 -27.792 24.559 0.00 0.00
I
ATOM 1615 HN ASN I 139 1.609 -28.566 25.154 0.00 0.00
I
ATOM 1616 CA ASN I 139 2.663 -28.119 23.425 0.00 0.00
I
ATOM 1617 HA ASN I 139 2.832 -29.185 23.399 0.00 0.00
I
ATOM 1618 CB ASN I 139 2.060 -27.843 22.019 0.00 0.00
I
ATOM 1619 HB1 ASN I 139 1.938 -26.761 21.795 0.00 0.00
I

ATOM	1620	HB2	ASN	I	139	2.726	-28.219	21.213	0.00	0.00	I
ATOM	1621	CG	ASN	I	139	0.687	-28.588	21.700	0.00	0.00	I
ATOM	1622	OD1	ASN	I	139	0.700	-29.713	21.176	0.00	0.00	I
ATOM	1623	ND2	ASN	I	139	-0.396	-27.808	21.915	0.00	0.00	I
ATOM	1624	HD21	ASN	I	139	-1.222	-28.199	21.511	0.00	0.00	I
ATOM	1625	HD22	ASN	I	139	-0.257	-26.853	22.180	0.00	0.00	I
ATOM	1626	C	ASN	I	139	4.027	-27.480	23.516	0.00	0.00	I
ATOM	1627	O	ASN	I	139	4.378	-26.621	22.718	0.00	0.00	I
ATOM	1628	N	GLN	I	140	4.821	-27.855	24.533	0.00	0.00	I
ATOM	1629	HN	GLN	I	140	4.522	-28.617	25.102	0.00	0.00	I
ATOM	1630	CA	GLN	I	140	6.166	-27.312	24.663	0.00	0.00	I
ATOM	1631	HA	GLN	I	140	6.269	-26.445	24.027	0.00	0.00	I
ATOM	1632	CB	GLN	I	140	6.378	-26.989	26.148	0.00	0.00	I
ATOM	1633	HB1	GLN	I	140	6.213	-27.941	26.696	0.00	0.00	I
ATOM	1634	HB2	GLN	I	140	7.451	-26.718	26.239	0.00	0.00	I
ATOM	1635	CG	GLN	I	140	5.472	-25.874	26.708	0.00	0.00	I
ATOM	1636	HG1	GLN	I	140	5.599	-24.904	26.181	0.00	0.00	I
ATOM	1637	HG2	GLN	I	140	4.398	-26.152	26.652	0.00	0.00	I
ATOM	1638	CD	GLN	I	140	5.816	-25.493	28.209	0.00	0.00	I
ATOM	1639	OE1	GLN	I	140	6.309	-24.385	28.436	0.00	0.00	I
ATOM	1640	NE2	GLN	I	140	5.427	-26.349	29.169	0.00	0.00	I
ATOM	1641	HE21	GLN	I	140	5.452	-25.960	30.090	0.00	0.00	I
ATOM	1642	HE22	GLN	I	140	4.918	-27.162	28.885	0.00	0.00	I
ATOM	1643	C	GLN	I	140	7.186	-28.300	24.261	0.00	0.00	I
ATOM	1644	O	GLN	I	140	6.913	-29.490	24.288	0.00	0.00	I
ATOM	1645	N	PRO	I	141	8.356	-27.883	23.635	0.00	0.00	I
ATOM	1646	CD	PRO	I	141	8.976	-26.610	23.936	0.00	0.00	I

ATOM 1647 HD1 PRO I 141 8.366 −25.829 23.433 0.00 0.00
I
ATOM 1648 HD2 PRO I 141 9.121 −26.543 25.035 0.00 0.00
I
ATOM 1649 CA PRO I 141 9.326 −28.857 23.085 0.00 0.00
I
ATOM 1650 HA PRO I 141 8.733 −29.584 22.552 0.00 0.00
I
ATOM 1651 CB PRO I 141 10.106 −27.875 22.237 0.00 0.00
I
ATOM 1652 HB1 PRO I 141 9.475 −27.492 21.407 0.00 0.00
I
ATOM 1653 HB2 PRO I 141 11.094 −28.106 21.783 0.00 0.00
I
ATOM 1654 CG PRO I 141 10.262 −26.631 23.162 0.00 0.00
I
ATOM 1655 HG1 PRO I 141 10.382 −25.731 22.522 0.00 0.00
I
ATOM 1656 HG2 PRO I 141 11.225 −26.781 23.695 0.00 0.00
I
ATOM 1657 C PRO I 141 10.253 −29.569 24.100 0.00 0.00
I
ATOM 1658 O PRO I 141 11.384 −29.929 23.843 0.00 0.00
I
ATOM 1659 N THR I 142 9.679 −29.979 25.222 0.00 0.00
I
ATOM 1660 HN THR I 142 8.759 −29.741 25.525 0.00 0.00
I
ATOM 1661 CA THR I 142 10.375 −30.679 26.343 0.00 0.00
I
ATOM 1662 HA THR I 142 11.249 −30.093 26.582 0.00 0.00
I
ATOM 1663 CB THR I 142 9.598 −30.749 27.672 0.00 0.00
I
ATOM 1664 HB THR I 142 8.704 −31.375 27.465 0.00 0.00
I
ATOM 1665 OG1 THR I 142 9.114 −29.453 27.939 0.00 0.00
I
ATOM 1666 HG1 THR I 142 8.614 −29.654 28.734 0.00 0.00
I
ATOM 1667 CG2 THR I 142 10.571 −31.278 28.790 0.00 0.00
I
ATOM 1668 HG21 THR I 142 10.737 −32.373 28.698 0.00 0.00
I
ATOM 1669 HG22 THR I 142 11.494 −30.661 28.747 0.00 0.00
I
ATOM 1670 HG23 THR I 142 10.148 −31.137 29.808 0.00 0.00
I
ATOM 1671 C THR I 142 10.788 −32.081 25.988 0.00 0.00
I
ATOM 1672 O THR I 142 11.886 −32.543 26.344 0.00 0.00
I
ATOM 1673 N ILE I 143 9.953 −32.849 25.274 0.00 0.00
I

ATOM   1674 HN ILE I 143   9.060 -32.409 25.215 0.00 0.00
I
ATOM   1675 CA ILE I 143   10.209 -34.179 24.838 0.00 0.00
I
ATOM   1676 HA ILE I 143   11.223 -34.432 25.114 0.00 0.00
I
ATOM   1677 CB ILE I 143   9.380 -35.242 25.455 0.00 0.00
I
ATOM   1678 HB ILE I 143   8.313 -34.979 25.293 0.00 0.00
I
ATOM   1679 CG2 ILE I 143   9.695 -36.616 24.797 0.00 0.00
I
ATOM   1680 HG21 ILE I 143   10.688 -37.001 25.114 0.00 0.00
I
ATOM   1681 HG22 ILE I 143   9.023 -37.329 25.320 0.00 0.00
I
ATOM   1682 HG23 ILE I 143   9.541 -36.883 23.729 0.00 0.00
I
ATOM   1683 CG1 ILE I 143   9.604 -35.324 26.946 0.00 0.00
I
ATOM   1684 HG11 ILE I 143   10.684 -35.509 27.129 0.00 0.00
I
ATOM   1685 HG12 ILE I 143   9.304 -34.350 27.388 0.00 0.00
I
ATOM   1686 CD ILE I 143   8.837 -36.489 27.621 0.00 0.00
I
ATOM   1687 HD1 ILE I 143   9.270 -37.492 27.422 0.00 0.00
I
ATOM   1688 HD2 ILE I 143   8.763 -36.272 28.708 0.00 0.00
I
ATOM   1689 HD3 ILE I 143   7.833 -36.522 27.145 0.00 0.00
I
ATOM   1690 C   ILE I 143   10.134 -34.247 23.318 0.00 0.00
I
ATOM   1691 O   ILE I 143   9.091 -33.881 22.743 0.00 0.00
I
ATOM   1692 N   GLY I 144   11.244 -34.613 22.667 0.00 0.00
I
ATOM   1693 HN GLY I 144   12.142 -34.855 23.028 0.00 0.00
I
ATOM   1694 CA GLY I 144   11.293 -34.809 21.223 0.00 0.00
I
ATOM   1695 HA1 GLY I 144   10.460 -35.369 20.824 0.00 0.00
I
ATOM   1696 HA2 GLY I 144   12.232 -35.313 21.048 0.00 0.00
I
ATOM   1697 C   GLY I 144   11.330 -33.514 20.394 0.00 0.00
I
ATOM   1698 O   GLY I 144   11.163 -33.558 19.161 0.00 0.00
I
ATOM   1699 N   GLY I 145   11.464 -32.377 21.026 0.00 0.00
I
ATOM   1700 HN GLY I 145   11.678 -32.455 21.996 0.00 0.00
I

ATOM 1701 CA GLY I 145 11.347 -31.036 20.508 0.00 0.00
I
ATOM 1702 HA1 GLY I 145 12.321 -30.878 20.069 0.00 0.00
I
ATOM 1703 HA2 GLY I 145 11.193 -30.368 21.343 0.00 0.00
I
ATOM 1704 C GLY I 145 10.345 -30.558 19.438 0.00 0.00
I
ATOM 1705 O GLY I 145 9.225 -31.025 19.197 0.00 0.00
I
ATOM 1706 N VAL I 146 10.787 -29.580 18.588 0.00 0.00
I
ATOM 1707 HN VAL I 146 11.700 -29.190 18.677 0.00 0.00
I
ATOM 1708 CA VAL I 146 10.121 -29.026 17.426 0.00 0.00
I
ATOM 1709 HA VAL I 146 9.104 -28.701 17.591 0.00 0.00
I
ATOM 1710 CB VAL I 146 10.961 -27.832 16.926 0.00 0.00
I
ATOM 1711 HB VAL I 146 11.979 -28.238 16.743 0.00 0.00
I
ATOM 1712 CG1 VAL I 146 10.535 -27.306 15.535 0.00 0.00
I
ATOM 1713 HG11 VAL I 146 10.629 -28.194 14.875 0.00 0.00
I
ATOM 1714 HG12 VAL I 146 9.459 -27.035 15.590 0.00 0.00
I
ATOM 1715 HG13 VAL I 146 11.158 -26.438 15.229 0.00 0.00
I
ATOM 1716 CG2 VAL I 146 11.056 -26.613 17.914 0.00 0.00
I
ATOM 1717 HG21 VAL I 146 11.709 -25.812 17.506 0.00 0.00
I
ATOM 1718 HG22 VAL I 146 10.057 -26.189 18.152 0.00 0.00
I
ATOM 1719 HG23 VAL I 146 11.497 -26.959 18.873 0.00 0.00
I
ATOM 1720 C VAL I 146 10.088 -30.041 16.332 0.00 0.00
I
ATOM 1721 O VAL I 146 9.115 -30.277 15.622 0.00 0.00
I
ATOM 1722 N ILE I 147 11.243 -30.848 16.222 0.00 0.00
I
ATOM 1723 HN ILE I 147 11.996 -30.798 16.874 0.00 0.00
I
ATOM 1724 CA ILE I 147 11.444 -31.835 15.178 0.00 0.00
I
ATOM 1725 HA ILE I 147 11.177 -31.313 14.272 0.00 0.00
I
ATOM 1726 CB ILE I 147 12.918 -32.290 15.219 0.00 0.00
I
ATOM 1727 HB ILE I 147 13.472 -31.328 15.244 0.00 0.00
I

ATOM 1728 CG2 ILE I 147 13.143 -33.111 16.541 0.00 0.00
I
ATOM 1729 HG21 ILE I 147 12.982 -32.433 17.406 0.00 0.00
I
ATOM 1730 HG22 ILE I 147 12.433 -33.933 16.776 0.00 0.00
I
ATOM 1731 HG23 ILE I 147 14.174 -33.515 16.635 0.00 0.00
I
ATOM 1732 CG1 ILE I 147 13.308 -32.938 13.869 0.00 0.00
I
ATOM 1733 HG11 ILE I 147 14.338 -33.293 14.084 0.00 0.00
I
ATOM 1734 HG12 ILE I 147 12.623 -33.785 13.650 0.00 0.00
I
ATOM 1735 CD ILE I 147 13.422 -31.946 12.759 0.00 0.00
I
ATOM 1736 HD1 ILE I 147 12.497 -31.402 12.469 0.00 0.00
I
ATOM 1737 HD2 ILE I 147 14.121 -31.108 12.969 0.00 0.00
I
ATOM 1738 HD3 ILE I 147 13.690 -32.375 11.771 0.00 0.00
I
ATOM 1739 C ILE I 147 10.508 -33.052 15.086 0.00 0.00
I
ATOM 1740 O ILE I 147 9.992 -33.443 14.023 0.00 0.00
I
ATOM 1741 N GLY I 148 10.144 -33.622 16.246 0.00 0.00
I
ATOM 1742 HN GLY I 148 10.774 -33.508 17.010 0.00 0.00
I
ATOM 1743 CA GLY I 148 9.049 -34.612 16.379 0.00 0.00
I
ATOM 1744 HA1 GLY I 148 9.044 -34.976 17.396 0.00 0.00
I
ATOM 1745 HA2 GLY I 148 9.258 -35.347 15.616 0.00 0.00
I
ATOM 1746 C GLY I 148 7.698 -34.062 16.028 0.00 0.00
I
ATOM 1747 O GLY I 148 6.875 -34.760 15.518 0.00 0.00
I
ATOM 1748 N LEU I 149 7.489 -32.813 16.317 0.00 0.00
I
ATOM 1749 HN LEU I 149 8.150 -32.280 16.840 0.00 0.00
I
ATOM 1750 CA LEU I 149 6.271 -32.096 15.874 0.00 0.00
I
ATOM 1751 HA LEU I 149 5.414 -32.682 16.173 0.00 0.00
I
ATOM 1752 CB LEU I 149 6.040 -30.710 16.685 0.00 0.00
I
ATOM 1753 HB1 LEU I 149 6.004 -30.862 17.785 0.00 0.00
I
ATOM 1754 HB2 LEU I 149 6.838 -29.995 16.391 0.00 0.00
I

ATOM 1755 CG LEU I 149 4.746 -29.955 16.413 0.00 0.00
I
ATOM 1756 HG LEU I 149 4.911 -29.544 15.394 0.00 0.00
I
ATOM 1757 CD1 LEU I 149 3.406 -30.752 16.289 0.00 0.00
I
ATOM 1758 HD11 LEU I 149 3.241 -31.254 17.267 0.00 0.00
I
ATOM 1759 HD12 LEU I 149 2.652 -29.972 16.050 0.00 0.00
I
ATOM 1760 HD13 LEU I 149 3.357 -31.564 15.532 0.00 0.00
I
ATOM 1761 CD2 LEU I 149 4.544 -28.781 17.420 0.00 0.00
I
ATOM 1762 HD21 LEU I 149 5.416 -28.094 17.383 0.00 0.00
I
ATOM 1763 HD22 LEU I 149 3.573 -28.259 17.285 0.00 0.00
I
ATOM 1764 HD23 LEU I 149 4.409 -29.226 18.429 0.00 0.00
I
ATOM 1765 C LEU I 149 6.201 -31.906 14.372 0.00 0.00
I
ATOM 1766 O LEU I 149 5.207 -32.141 13.701 0.00 0.00
I
ATOM 1767 N VAL I 150 7.335 -31.532 13.683 0.00 0.00
I
ATOM 1768 HN VAL I 150 8.122 -31.239 14.221 0.00 0.00
I
ATOM 1769 CA VAL I 150 7.455 -31.463 12.238 0.00 0.00
I
ATOM 1770 HA VAL I 150 6.543 -30.914 12.054 0.00 0.00
I
ATOM 1771 CB VAL I 150 8.726 -30.764 11.853 0.00 0.00
I
ATOM 1772 HB VAL I 150 9.534 -31.362 12.327 0.00 0.00
I
ATOM 1773 CG1 VAL I 150 8.935 -30.638 10.338 0.00 0.00
I
ATOM 1774 HG11 VAL I 150 8.109 -30.114 9.811 0.00 0.00
I
ATOM 1775 HG12 VAL I 150 9.885 -30.101 10.129 0.00 0.00
I
ATOM 1776 HG13 VAL I 150 9.092 -31.662 9.937 0.00 0.00
I
ATOM 1777 CG2 VAL I 150 8.670 -29.266 12.377 0.00 0.00
I
ATOM 1778 HG21 VAL I 150 8.438 -29.367 13.458 0.00 0.00
I
ATOM 1779 HG22 VAL I 150 9.599 -28.705 12.140 0.00 0.00
I
ATOM 1780 HG23 VAL I 150 7.814 -28.854 11.801 0.00 0.00
I
ATOM 1781 C VAL I 150 7.160 -32.796 11.507 0.00 0.00
I

```
ATOM   1782  O   VAL I 150       6.385 -32.775  10.502  0.00  0.00
I
ATOM   1783  N   LYS I 151       7.649 -33.994  12.007  0.00  0.00
I
ATOM   1784  HN  LYS I 151       8.120 -33.923  12.883  0.00  0.00
I
ATOM   1785  CA  LYS I 151       7.302 -35.318  11.413  0.00  0.00
I
ATOM   1786  HA  LYS I 151       7.670 -35.298  10.398  0.00  0.00
I
ATOM   1787  CB  LYS I 151       8.058 -36.455  12.164  0.00  0.00
I
ATOM   1788  HB1 LYS I 151       7.610 -36.505  13.180  0.00  0.00
I
ATOM   1789  HB2 LYS I 151       7.853 -37.480  11.789  0.00  0.00
I
ATOM   1790  CG  LYS I 151       9.564 -36.254  12.302  0.00  0.00
I
ATOM   1791  HG1 LYS I 151      10.143 -36.378  11.362  0.00  0.00
I
ATOM   1792  HG2 LYS I 151       9.631 -35.178  12.573  0.00  0.00
I
ATOM   1793  CD  LYS I 151      10.115 -37.129  13.460  0.00  0.00
I
ATOM   1794  HD1 LYS I 151       9.410 -37.094  14.318  0.00  0.00
I
ATOM   1795  HD2 LYS I 151      10.114 -38.121  12.960  0.00  0.00
I
ATOM   1796  CE  LYS I 151      11.538 -36.994  14.003  0.00  0.00
I
ATOM   1797  HE1 LYS I 151      12.249 -37.455  13.285  0.00  0.00
I
ATOM   1798  HE2 LYS I 151      11.742 -35.927  14.233  0.00  0.00
I
ATOM   1799  NZ  LYS I 151      11.741 -37.746  15.353  0.00  0.00
I
ATOM   1800  HZ1 LYS I 151      12.695 -37.545  15.715  0.00  0.00
I
ATOM   1801  HZ2 LYS I 151      11.099 -37.447  16.115  0.00  0.00
I
ATOM   1802  HZ3 LYS I 151      11.477 -38.741  15.203  0.00  0.00
I
ATOM   1803  C   LYS I 151       5.772 -35.619  11.406  0.00  0.00
I
ATOM   1804  O   LYS I 151       5.278 -36.068  10.335  0.00  0.00
I
ATOM   1805  N   VAL I 152       4.946 -35.378  12.532  0.00  0.00
I
ATOM   1806  HN  VAL I 152       5.266 -34.975  13.387  0.00  0.00
I
ATOM   1807  CA  VAL I 152       3.484 -35.695  12.569  0.00  0.00
I
ATOM   1808  HA  VAL I 152       3.383 -36.676  12.130  0.00  0.00
I
```

ATOM	1809	CB	VAL	I	152	2.968	-35.789	14.025	0.00	0.00	I
ATOM	1810	HB	VAL	I	152	3.100	-34.851	14.606	0.00	0.00	I
ATOM	1811	CG1	VAL	I	152	1.530	-36.352	13.879	0.00	0.00	I
ATOM	1812	HG11	VAL	I	152	0.857	-35.597	13.418	0.00	0.00	I
ATOM	1813	HG12	VAL	I	152	1.580	-37.253	13.230	0.00	0.00	I
ATOM	1814	HG13	VAL	I	152	1.162	-36.648	14.884	0.00	0.00	I
ATOM	1815	CG2	VAL	I	152	3.863	-36.761	14.907	0.00	0.00	I
ATOM	1816	HG21	VAL	I	152	3.402	-36.907	15.908	0.00	0.00	I
ATOM	1817	HG22	VAL	I	152	3.774	-37.751	14.411	0.00	0.00	I
ATOM	1818	HG23	VAL	I	152	4.928	-36.446	14.932	0.00	0.00	I
ATOM	1819	C	VAL	I	152	2.659	-34.644	11.721	0.00	0.00	I
ATOM	1820	O	VAL	I	152	1.763	-34.984	10.971	0.00	0.00	I
ATOM	1821	N	LEU	I	153	3.129	-33.319	11.797	0.00	0.00	I
ATOM	1822	HN	LEU	I	153	3.965	-33.201	12.327	0.00	0.00	I
ATOM	1823	CA	LEU	I	153	2.646	-32.344	10.797	0.00	0.00	I
ATOM	1824	HA	LEU	I	153	1.596	-32.243	11.030	0.00	0.00	I
ATOM	1825	CB	LEU	I	153	3.359	-31.060	11.072	0.00	0.00	I
ATOM	1826	HB1	LEU	I	153	3.474	-31.037	12.177	0.00	0.00	I
ATOM	1827	HB2	LEU	I	153	4.444	-31.101	10.836	0.00	0.00	I
ATOM	1828	CG	LEU	I	153	2.731	-29.823	10.448	0.00	0.00	I
ATOM	1829	HG	LEU	I	153	1.654	-29.958	10.214	0.00	0.00	I
ATOM	1830	CD1	LEU	I	153	3.013	-28.634	11.348	0.00	0.00	I
ATOM	1831	HD11	LEU	I	153	2.405	-28.808	12.262	0.00	0.00	I
ATOM	1832	HD12	LEU	I	153	4.055	-28.648	11.733	0.00	0.00	I
ATOM	1833	HD13	LEU	I	153	2.735	-27.653	10.907	0.00	0.00	I
ATOM	1834	CD2	LEU	I	153	3.461	-29.455	9.103	0.00	0.00	I
ATOM	1835	HD21	LEU	I	153	3.320	-30.398	8.534	0.00	0.00	I

ATOM 1836 HD22 LEU I 153 2.901 -28.566 8.742 0.00 0.00
I
ATOM 1837 HD23 LEU I 153 4.495 -29.053 9.167 0.00 0.00
I
ATOM 1838 C LEU I 153 2.739 -32.771 9.376 0.00 0.00
I
ATOM 1839 O LEU I 153 1.797 -32.792 8.568 0.00 0.00
I
ATOM 1840 N PHE I 154 3.975 -33.237 9.101 0.00 0.00
I
ATOM 1841 HN PHE I 154 4.727 -33.291 9.753 0.00 0.00
I
ATOM 1842 CA PHE I 154 4.428 -33.623 7.719 0.00 0.00
I
ATOM 1843 HA PHE I 154 4.139 -32.861 7.011 0.00 0.00
I
ATOM 1844 CB PHE I 154 5.989 -33.832 7.559 0.00 0.00
I
ATOM 1845 HB1 PHE I 154 6.414 -32.812 7.681 0.00 0.00
I
ATOM 1846 HB2 PHE I 154 6.300 -34.390 8.468 0.00 0.00
I
ATOM 1847 CG PHE I 154 6.372 -34.556 6.338 0.00 0.00
I
ATOM 1848 CD1 PHE I 154 6.555 -33.787 5.156 0.00 0.00
I
ATOM 1849 HD1 PHE I 154 6.122 -32.805 5.036 0.00 0.00
I
ATOM 1850 CE1 PHE I 154 6.994 -34.467 3.969 0.00 0.00
I
ATOM 1851 HE1 PHE I 154 6.986 -33.941 3.026 0.00 0.00
I
ATOM 1852 CZ PHE I 154 7.290 -35.802 4.067 0.00 0.00
I
ATOM 1853 HZ PHE I 154 7.569 -36.294 3.147 0.00 0.00
I
ATOM 1854 CD2 PHE I 154 6.726 -35.907 6.425 0.00 0.00
I
ATOM 1855 HD2 PHE I 154 6.639 -36.468 7.344 0.00 0.00
I
ATOM 1856 CE2 PHE I 154 7.211 -36.554 5.289 0.00 0.00
I
ATOM 1857 HE2 PHE I 154 7.495 -37.595 5.252 0.00 0.00
I
ATOM 1858 C PHE I 154 3.662 -34.834 7.229 0.00 0.00
I
ATOM 1859 O PHE I 154 3.134 -34.697 6.114 0.00 0.00
I
ATOM 1860 N LEU I 155 3.495 -35.843 8.042 0.00 0.00
I
ATOM 1861 HN LEU I 155 3.983 -35.784 8.909 0.00 0.00
I
ATOM 1862 CA LEU I 155 2.529 -36.930 7.874 0.00 0.00
I

ATOM 1863 HA LEU I 155 2.883 -37.590 7.097 0.00 0.00
I
ATOM 1864 CB LEU I 155 2.465 -37.802 9.126 0.00 0.00
I
ATOM 1865 HB1 LEU I 155 3.495 -38.203 9.244 0.00 0.00
I
ATOM 1866 HB2 LEU I 155 2.265 -37.217 10.049 0.00 0.00
I
ATOM 1867 CG LEU I 155 1.385 -38.875 9.163 0.00 0.00
I
ATOM 1868 HG LEU I 155 0.409 -38.459 8.833 0.00 0.00
I
ATOM 1869 CD1 LEU I 155 1.859 -40.048 8.273 0.00 0.00
I
ATOM 1870 HD11 LEU I 155 1.768 -39.770 7.201 0.00 0.00
I
ATOM 1871 HD12 LEU I 155 2.927 -40.320 8.412 0.00 0.00
I
ATOM 1872 HD13 LEU I 155 1.179 -40.923 8.351 0.00 0.00
I
ATOM 1873 CD2 LEU I 155 1.309 -39.391 10.536 0.00 0.00
I
ATOM 1874 HD21 LEU I 155 0.670 -40.299 10.544 0.00 0.00
I
ATOM 1875 HD22 LEU I 155 2.282 -39.770 10.914 0.00 0.00
I
ATOM 1876 HD23 LEU I 155 0.964 -38.671 11.309 0.00 0.00
I
ATOM 1877 C LEU I 155 1.069 -36.476 7.412 0.00 0.00
I
ATOM 1878 O LEU I 155 0.525 -37.140 6.507 0.00 0.00
I
ATOM 1879 N PHE I 156 0.447 -35.460 8.083 0.00 0.00
I
ATOM 1880 HN PHE I 156 1.009 -35.012 8.775 0.00 0.00
I
ATOM 1881 CA PHE I 156 -0.878 -34.931 7.612 0.00 0.00
I
ATOM 1882 HA PHE I 156 -1.583 -35.746 7.679 0.00 0.00
I
ATOM 1883 CB PHE I 156 -1.226 -33.826 8.657 0.00 0.00
I
ATOM 1884 HB1 PHE I 156 -1.232 -34.263 9.679 0.00 0.00
I
ATOM 1885 HB2 PHE I 156 -0.498 -32.992 8.752 0.00 0.00
I
ATOM 1886 CG PHE I 156 -2.599 -33.325 8.510 0.00 0.00
I
ATOM 1887 CD1 PHE I 156 -2.782 -32.015 8.087 0.00 0.00
I
ATOM 1888 HD1 PHE I 156 -1.871 -31.452 7.942 0.00 0.00
I
ATOM 1889 CE1 PHE I 156 -4.070 -31.495 7.875 0.00 0.00
I

```
ATOM   1890  HE1 PHE I 156      -4.418 -30.476   7.958  0.00  0.00
I
ATOM   1891  CZ  PHE I 156      -5.123 -32.328   8.207  0.00  0.00
I
ATOM   1892  HZ  PHE I 156      -6.124 -31.923   8.195  0.00  0.00
I
ATOM   1893  CD2 PHE I 156      -3.664 -34.207   8.643  0.00  0.00
I
ATOM   1894  HD2 PHE I 156      -3.442 -35.259   8.752  0.00  0.00
I
ATOM   1895  CE2 PHE I 156      -4.891 -33.744   8.447  0.00  0.00
I
ATOM   1896  HE2 PHE I 156      -5.739 -34.384   8.644  0.00  0.00
I
ATOM   1897  C   PHE I 156      -0.749 -34.262   6.167  0.00  0.00
I
ATOM   1898  O   PHE I 156      -1.625 -34.419   5.317  0.00  0.00
I
ATOM   1899  N   SER I 157       0.286 -33.411   5.965  0.00  0.00
I
ATOM   1900  HN  SER I 157       1.040 -33.415   6.617  0.00  0.00
I
ATOM   1901  CA  SER I 157       0.384 -32.769   4.710  0.00  0.00
I
ATOM   1902  HA  SER I 157      -0.575 -32.273   4.666  0.00  0.00
I
ATOM   1903  CB  SER I 157       1.485 -31.737   4.629  0.00  0.00
I
ATOM   1904  HB1 SER I 157       2.446 -32.238   4.870  0.00  0.00
I
ATOM   1905  HB2 SER I 157       1.588 -31.200   3.662  0.00  0.00
I
ATOM   1906  OG  SER I 157       1.437 -30.704   5.660  0.00  0.00
I
ATOM   1907  HG1 SER I 157       2.276 -30.266   5.502  0.00  0.00
I
ATOM   1908  C   SER I 157       0.659 -33.617   3.492  0.00  0.00
I
ATOM   1909  O   SER I 157       0.012 -33.468   2.516  0.00  0.00
I
ATOM   1910  N   ILE I 158       1.544 -34.614   3.625  0.00  0.00
I
ATOM   1911  HN  ILE I 158       2.128 -34.669   4.432  0.00  0.00
I
ATOM   1912  CA  ILE I 158       1.731 -35.665   2.587  0.00  0.00
I
ATOM   1913  HA  ILE I 158       1.886 -35.210   1.620  0.00  0.00
I
ATOM   1914  CB  ILE I 158       2.953 -36.561   2.705  0.00  0.00
I
ATOM   1915  HB  ILE I 158       3.156 -37.224   1.836  0.00  0.00
I
ATOM   1916  CG2 ILE I 158       4.176 -35.726   2.732  0.00  0.00
I
```

ATOM   1917 HG21 ILE I 158   5.125 -36.294   2.624 0.00 0.00
I
ATOM   1918 HG22 ILE I 158   4.162 -34.864   2.032 0.00 0.00
I
ATOM   1919 HG23 ILE I 158   4.325 -35.271   3.734 0.00 0.00
I
ATOM   1920 CG1 ILE I 158   2.985 -37.417   3.995 0.00 0.00
I
ATOM   1921 HG11 ILE I 158   4.044 -37.572   4.292 0.00 0.00
I
ATOM   1922 HG12 ILE I 158   2.550 -36.831   4.833 0.00 0.00
I
ATOM   1923 CD ILE I 158   2.326 -38.760   3.873 0.00 0.00
I
ATOM   1924 HD1 ILE I 158   1.305 -38.846   3.445 0.00 0.00
I
ATOM   1925 HD2 ILE I 158   2.945 -39.469   3.283 0.00 0.00
I
ATOM   1926 HD3 ILE I 158   2.300 -39.162   4.908 0.00 0.00
I
ATOM   1927 C   ILE I 158   0.471 -36.573   2.322 0.00 0.00
I
ATOM   1928 O   ILE I 158   0.235 -37.072   1.213 0.00 0.00
I
ATOM   1929 N   SER I 159   -0.361 -36.866   3.327 0.00 0.00
I
ATOM   1930 HN SER I 159   -0.077 -36.455   4.190 0.00 0.00
I
ATOM   1931 CA SER I 159   -1.497 -37.755   3.226 0.00 0.00
I
ATOM   1932 HA SER I 159   -1.258 -38.665   2.696 0.00 0.00
I
ATOM   1933 CB SER I 159   -2.100 -38.114   4.632 0.00 0.00
I
ATOM   1934 HB1 SER I 159   -2.173 -37.165   5.205 0.00 0.00
I
ATOM   1935 HB2 SER I 159   -3.179 -38.357   4.527 0.00 0.00
I
ATOM   1936 OG SER I 159   -1.200 -38.967   5.377 0.00 0.00
I
ATOM   1937 HG1 SER I 159   -0.538 -38.313   5.617 0.00 0.00
I
ATOM   1938 C   SER I 159   -2.650 -37.171   2.458 0.00 0.00
I
ATOM   1939 O   SER I 159   -3.211 -37.868   1.602 0.00 0.00
I
ATOM   1940 N   ILE I 160   -2.971 -35.848   2.598 0.00 0.00
I
ATOM   1941 HN ILE I 160   -2.553 -35.271   3.296 0.00 0.00
I
ATOM   1942 CA ILE I 160   -3.933 -35.118   1.724 0.00 0.00
I
ATOM   1943 HA ILE I 160   -4.847 -35.693   1.723 0.00 0.00
I

```
ATOM   1944  CB  ILE I 160      -4.136 -33.689   2.261  0.00  0.00
I
ATOM   1945  HB  ILE I 160      -3.179 -33.126   2.225  0.00  0.00
I
ATOM   1946  CG2 ILE I 160      -5.174 -32.898   1.548  0.00  0.00
I
ATOM   1947 HG21 ILE I 160      -4.983 -32.826   0.456  0.00  0.00
I
ATOM   1948 HG22 ILE I 160      -6.192 -33.334   1.635  0.00  0.00
I
ATOM   1949 HG23 ILE I 160      -5.278 -31.898   2.020  0.00  0.00
I
ATOM   1950  CG1 ILE I 160      -4.573 -33.875   3.678  0.00  0.00
I
ATOM   1951 HG11 ILE I 160      -5.662 -34.088   3.742  0.00  0.00
I
ATOM   1952 HG12 ILE I 160      -4.042 -34.685   4.221  0.00  0.00
I
ATOM   1953  CD  ILE I 160      -4.408 -32.546   4.458  0.00  0.00
I
ATOM   1954  HD1 ILE I 160      -3.339 -32.252   4.524  0.00  0.00
I
ATOM   1955  HD2 ILE I 160      -4.911 -31.660   4.016  0.00  0.00
I
ATOM   1956  HD3 ILE I 160      -4.665 -32.606   5.537  0.00  0.00
I
ATOM   1957  C   ILE I 160      -3.429 -35.109   0.291  0.00  0.00
I
ATOM   1958  O   ILE I 160      -4.256 -35.465  -0.562  0.00  0.00
I
ATOM   1959  N   GLU I 161      -2.114 -34.892   0.093  0.00  0.00
I
ATOM   1960  HN  GLU I 161      -1.504 -34.634   0.839  0.00  0.00
I
ATOM   1961  CA  GLU I 161      -1.549 -34.939  -1.291  0.00  0.00
I
ATOM   1962  HA  GLU I 161      -2.017 -34.236  -1.963  0.00  0.00
I
ATOM   1963  CB  GLU I 161      -0.032 -34.657  -1.202  0.00  0.00
I
ATOM   1964  HB1 GLU I 161       0.311 -35.320  -0.379  0.00  0.00
I
ATOM   1965  HB2 GLU I 161       0.504 -35.062  -2.086  0.00  0.00
I
ATOM   1966  CG  GLU I 161       0.365 -33.207  -0.976  0.00  0.00
I
ATOM   1967  HG1 GLU I 161      -0.072 -32.540  -1.750  0.00  0.00
I
ATOM   1968  HG2 GLU I 161      -0.040 -32.820  -0.016  0.00  0.00
I
ATOM   1969  CD  GLU I 161       1.882 -33.071  -0.914  0.00  0.00
I
ATOM   1970  OE1 GLU I 161       2.610 -34.120  -0.988  0.00  0.00
I
```

ATOM 1971 OE2 GLU I 161 2.334 -31.926 -0.746 0.00 0.00
I
ATOM 1972 C GLU I 161 -1.714 -36.283 -1.950 0.00 0.00
I
ATOM 1973 O GLU I 161 -2.125 -36.310 -3.102 0.00 0.00
I
ATOM 1974 N LEU I 162 -1.583 -37.479 -1.247 0.00 0.00
I
ATOM 1975 HN LEU I 162 -1.362 -37.429 -0.277 0.00 0.00
I
ATOM 1976 CA LEU I 162 -1.642 -38.841 -1.803 0.00 0.00
I
ATOM 1977 HA LEU I 162 -1.125 -38.768 -2.748 0.00 0.00
I
ATOM 1978 CB LEU I 162 -1.098 -39.890 -0.891 0.00 0.00
I
ATOM 1979 HB1 LEU I 162 -1.648 -39.809 0.071 0.00 0.00
I
ATOM 1980 HB2 LEU I 162 -1.306 -40.851 -1.408 0.00 0.00
I
ATOM 1981 CG LEU I 162 0.448 -39.881 -0.642 0.00 0.00
I
ATOM 1982 HG LEU I 162 0.789 -38.910 -0.223 0.00 0.00
I
ATOM 1983 CD1 LEU I 162 0.834 -41.001 0.307 0.00 0.00
I
ATOM 1984 HD11 LEU I 162 0.521 -41.966 -0.146 0.00 0.00
I
ATOM 1985 HD12 LEU I 162 1.940 -40.976 0.409 0.00 0.00
I
ATOM 1986 HD13 LEU I 162 0.395 -40.895 1.322 0.00 0.00
I
ATOM 1987 CD2 LEU I 162 1.075 -40.026 -2.059 0.00 0.00
I
ATOM 1988 HD21 LEU I 162 2.172 -40.102 -1.905 0.00 0.00
I
ATOM 1989 HD22 LEU I 162 0.593 -40.890 -2.564 0.00 0.00
I
ATOM 1990 HD23 LEU I 162 0.859 -39.093 -2.623 0.00 0.00
I
ATOM 1991 C LEU I 162 -3.067 -39.243 -2.048 0.00 0.00
I
ATOM 1992 O LEU I 162 -3.335 -39.761 -3.124 0.00 0.00
I
ATOM 1993 N ILE I 163 -3.938 -39.018 -1.111 0.00 0.00
I
ATOM 1994 HN ILE I 163 -3.740 -38.604 -0.226 0.00 0.00
I
ATOM 1995 CA ILE I 163 -5.333 -39.275 -1.303 0.00 0.00
I
ATOM 1996 HA ILE I 163 -5.442 -40.302 -1.617 0.00 0.00
I
ATOM 1997 CB ILE I 163 -6.143 -38.972 0.011 0.00 0.00
I

ATOM 1998 HB ILE I 163 -5.941 -37.926 0.328 0.00 0.00
I
ATOM 1999 CG2 ILE I 163 -7.615 -39.023 -0.240 0.00 0.00
I
ATOM 2000 HG21 ILE I 163 -7.888 -38.119 -0.826 0.00 0.00
I
ATOM 2001 HG22 ILE I 163 -7.896 -39.940 -0.801 0.00 0.00
I
ATOM 2002 HG23 ILE I 163 -8.060 -38.906 0.771 0.00 0.00
I
ATOM 2003 CG1 ILE I 163 -5.707 -39.901 1.146 0.00 0.00
I
ATOM 2004 HG11 ILE I 163 -6.011 -40.936 0.880 0.00 0.00
I
ATOM 2005 HG12 ILE I 163 -4.598 -39.906 1.203 0.00 0.00
I
ATOM 2006 CD ILE I 163 -6.185 -39.454 2.518 0.00 0.00
I
ATOM 2007 HD1 ILE I 163 -5.799 -40.051 3.372 0.00 0.00
I
ATOM 2008 HD2 ILE I 163 -5.922 -38.398 2.742 0.00 0.00
I
ATOM 2009 HD3 ILE I 163 -7.295 -39.474 2.571 0.00 0.00
I
ATOM 2010 C ILE I 163 -5.913 -38.475 -2.490 0.00 0.00
I
ATOM 2011 O ILE I 163 -6.729 -38.968 -3.297 0.00 0.00
I
ATOM 2012 N ALA I 164 -5.480 -37.236 -2.668 0.00 0.00
I
ATOM 2013 HN ALA I 164 -4.834 -36.893 -1.989 0.00 0.00
I
ATOM 2014 CA ALA I 164 -5.930 -36.256 -3.692 0.00 0.00
I
ATOM 2015 HA ALA I 164 -7.010 -36.272 -3.669 0.00 0.00
I
ATOM 2016 CB ALA I 164 -5.474 -34.777 -3.480 0.00 0.00
I
ATOM 2017 HB1 ALA I 164 -5.596 -34.384 -2.448 0.00 0.00
I
ATOM 2018 HB2 ALA I 164 -4.380 -34.656 -3.628 0.00 0.00
I
ATOM 2019 HB3 ALA I 164 -5.924 -34.075 -4.214 0.00 0.00
I
ATOM 2020 C ALA I 164 -5.676 -36.774 -5.056 0.00 0.00
I
ATOM 2021 O ALA I 164 -6.495 -36.673 -5.964 0.00 0.00
I
ATOM 2022 N ALA I 165 -4.500 -37.313 -5.206 0.00 0.00
I
ATOM 2023 HN ALA I 165 -3.835 -37.305 -4.462 0.00 0.00
I
ATOM 2024 CA ALA I 165 -3.918 -37.963 -6.389 0.00 0.00
I

ATOM 2025 HA ALA I 165 −4.017 −37.280 −7.219 0.00 0.00
I
ATOM 2026 CB ALA I 165 −2.403 −38.027 −6.260 0.00 0.00
I
ATOM 2027 HB1 ALA I 165 −2.085 −36.964 −6.197 0.00 0.00
I
ATOM 2028 HB2 ALA I 165 −2.235 −38.447 −5.245 0.00 0.00
I
ATOM 2029 HB3 ALA I 165 −1.980 −38.635 −7.087 0.00 0.00
I
ATOM 2030 C ALA I 165 −4.689 −39.250 −6.911 0.00 0.00
I
ATOM 2031 O ALA I 165 −4.859 −39.513 −8.065 0.00 0.00
I
ATOM 2032 N LEU I 166 −5.306 −39.922 −5.920 0.00 0.00
I
ATOM 2033 HN LEU I 166 −5.139 −39.774 −4.949 0.00 0.00
I
ATOM 2034 CA LEU I 166 −6.192 −41.032 −6.215 0.00 0.00
I
ATOM 2035 HA LEU I 166 −5.683 −41.688 −6.905 0.00 0.00
I
ATOM 2036 CB LEU I 166 −6.538 −41.767 −4.906 0.00 0.00
I
ATOM 2037 HB1 LEU I 166 −7.217 −41.173 −4.258 0.00 0.00
I
ATOM 2038 HB2 LEU I 166 −7.172 −42.588 −5.303 0.00 0.00
I
ATOM 2039 CG LEU I 166 −5.457 −42.417 −4.011 0.00 0.00
I
ATOM 2040 HG LEU I 166 −4.714 −41.634 −3.752 0.00 0.00
I
ATOM 2041 CD1 LEU I 166 −6.033 −43.024 −2.748 0.00 0.00
I
ATOM 2042 HD11 LEU I 166 −5.180 −43.507 −2.224 0.00 0.00
I
ATOM 2043 HD12 LEU I 166 −6.363 −42.188 −2.095 0.00 0.00
I
ATOM 2044 HD13 LEU I 166 −6.935 −43.619 −3.005 0.00 0.00
I
ATOM 2045 CD2 LEU I 166 −4.668 −43.597 −4.708 0.00 0.00
I
ATOM 2046 HD21 LEU I 166 −3.699 −43.744 −4.185 0.00 0.00
I
ATOM 2047 HD22 LEU I 166 −5.239 −44.549 −4.737 0.00 0.00
I
ATOM 2048 HD23 LEU I 166 −4.515 −43.424 −5.795 0.00 0.00
I
ATOM 2049 C LEU I 166 −7.500 −40.611 −6.825 0.00 0.00
I
ATOM 2050 O LEU I 166 −8.103 −41.303 −7.676 0.00 0.00
I
ATOM 2051 N ILE I 167 −8.032 −39.476 −6.332 0.00 0.00
I

ATOM 2052 HN ILE I 167 -7.513 -38.991 -5.632 0.00 0.00
I
ATOM 2053 CA ILE I 167 -9.415 -39.159 -6.660 0.00 0.00
I
ATOM 2054 HA ILE I 167 -9.907 -40.051 -7.019 0.00 0.00
I
ATOM 2055 CB ILE I 167 -10.135 -38.854 -5.426 0.00 0.00
I
ATOM 2056 HB ILE I 167 -11.185 -38.598 -5.684 0.00 0.00
I
ATOM 2057 CG2 ILE I 167 -10.218 -40.170 -4.649 0.00 0.00
I
ATOM 2058 HG21 ILE I 167 -11.001 -39.983 -3.884 0.00 0.00
I
ATOM 2059 HG22 ILE I 167 -10.531 -41.026 -5.284 0.00 0.00
I
ATOM 2060 HG23 ILE I 167 -9.281 -40.434 -4.113 0.00 0.00
I
ATOM 2061 CG1 ILE I 167 -9.697 -37.587 -4.620 0.00 0.00
I
ATOM 2062 HG11 ILE I 167 -8.616 -37.612 -4.363 0.00 0.00
I
ATOM 2063 HG12 ILE I 167 -9.703 -36.671 -5.248 0.00 0.00
I
ATOM 2064 CD ILE I 167 -10.552 -37.167 -3.419 0.00 0.00
I
ATOM 2065 HD1 ILE I 167 -11.573 -37.501 -3.698 0.00 0.00
I
ATOM 2066 HD2 ILE I 167 -10.363 -37.798 -2.524 0.00 0.00
I
ATOM 2067 HD3 ILE I 167 -10.561 -36.088 -3.153 0.00 0.00
I
ATOM 2068 C ILE I 167 -9.626 -38.010 -7.712 0.00 0.00
I
ATOM 2069 O ILE I 167 -10.744 -37.668 -8.009 0.00 0.00
I
ATOM 2070 N LEU I 168 -8.563 -37.470 -8.251 0.00 0.00
I
ATOM 2071 HN LEU I 168 -7.695 -37.796 -7.886 0.00 0.00
I
ATOM 2072 CA LEU I 168 -8.521 -36.340 -9.202 0.00 0.00
I
ATOM 2073 HA LEU I 168 -9.545 -36.004 -9.127 0.00 0.00
I
ATOM 2074 CB LEU I 168 -7.384 -35.281 -8.834 0.00 0.00
I
ATOM 2075 HB1 LEU I 168 -7.448 -35.123 -7.736 0.00 0.00
I
ATOM 2076 HB2 LEU I 168 -6.424 -35.826 -8.952 0.00 0.00
I
ATOM 2077 CG LEU I 168 -7.457 -33.880 -9.514 0.00 0.00
I
ATOM 2078 HG LEU I 168 -7.191 -34.152 -10.558 0.00 0.00
I

```
ATOM   2079  CD1 LEU I 168      -6.510 -32.872  -8.882  0.00  0.00
I
ATOM   2080 HD11 LEU I 168      -5.500 -33.313  -8.743  0.00  0.00
I
ATOM   2081 HD12 LEU I 168      -6.350 -31.953  -9.485  0.00  0.00
I
ATOM   2082 HD13 LEU I 168      -6.913 -32.535  -7.903  0.00  0.00
I
ATOM   2083  CD2 LEU I 168      -8.920 -33.346  -9.502  0.00  0.00
I
ATOM   2084 HD21 LEU I 168      -9.567 -33.863 -10.243  0.00  0.00
I
ATOM   2085 HD22 LEU I 168      -9.440 -33.549  -8.541  0.00  0.00
I
ATOM   2086 HD23 LEU I 168      -9.052 -32.272  -9.755  0.00  0.00
I
ATOM   2087  C   LEU I 168      -8.371 -36.722 -10.687  0.00  0.00
I
ATOM   2088  O   LEU I 168      -9.340 -36.906 -11.400  0.00  0.00
I
ATOM   2089  N   SER I 169      -7.138 -36.746 -11.212  0.00  0.00
I
ATOM   2090  HN  SER I 169      -6.270 -36.516 -10.779  0.00  0.00
I
ATOM   2091  CA  SER I 169      -7.035 -36.698 -12.659  0.00  0.00
I
ATOM   2092  HA  SER I 169      -7.891 -36.372 -13.232  0.00  0.00
I
ATOM   2093  CB  SER I 169      -6.092 -35.629 -13.141  0.00  0.00
I
ATOM   2094  HB1 SER I 169      -5.133 -35.741 -12.591  0.00  0.00
I
ATOM   2095  HB2 SER I 169      -5.945 -35.598 -14.241  0.00  0.00
I
ATOM   2096  OG  SER I 169      -6.600 -34.304 -12.989  0.00  0.00
I
ATOM   2097  HG1 SER I 169      -5.911 -33.640 -12.913  0.00  0.00
I
ATOM   2098  C   SER I 169      -6.493 -37.975 -13.295  0.00  0.00
I
ATOM   2099  O   SER I 169      -6.132 -37.924 -14.468  0.00  0.00
I
ATOM   2100  N   ILE I 170      -6.387 -39.060 -12.540  0.00  0.00
I
ATOM   2101  HN  ILE I 170      -6.710 -39.073 -11.597  0.00  0.00
I
ATOM   2102  CA  ILE I 170      -5.810 -40.392 -12.925  0.00  0.00
I
ATOM   2103  HA  ILE I 170      -4.967 -40.146 -13.553  0.00  0.00
I
ATOM   2104  CB  ILE I 170      -5.257 -41.173 -11.775  0.00  0.00
I
ATOM   2105  HB  ILE I 170      -4.551 -40.477 -11.272  0.00  0.00
I
```

ATOM   2106 CG2 ILE I 170   -6.369 -41.364 -10.701 0.00 0.00
I
ATOM   2107 HG21 ILE I 170   -6.721 -40.377 -10.334 0.00 0.00
I
ATOM   2108 HG22 ILE I 170   -7.203 -42.024 -11.023 0.00 0.00
I
ATOM   2109 HG23 ILE I 170   -5.987 -42.014 -9.884 0.00 0.00
I
ATOM   2110 CG1 ILE I 170   -4.685 -42.567 -12.225 0.00 0.00
I
ATOM   2111 HG11 ILE I 170   -5.503 -43.251 -12.541 0.00 0.00
I
ATOM   2112 HG12 ILE I 170   -4.062 -42.427 -13.134 0.00 0.00
I
ATOM   2113 CD ILE I 170   -3.762 -43.353 -11.274 0.00 0.00
I
ATOM   2114 HD1 ILE I 170   -2.883 -42.750 -10.960 0.00 0.00
I
ATOM   2115 HD2 ILE I 170   -4.276 -43.609 -10.322 0.00 0.00
I
ATOM   2116 HD3 ILE I 170   -3.227 -44.168 -11.807 0.00 0.00
I
ATOM   2117 C   ILE I 170   -6.710 -41.197 -13.832 0.00 0.00
I
ATOM   2118 O   ILE I 170   -6.297 -41.786 -14.852 0.00 0.00
I
ATOM   2119 N   ARG I 171   -8.060 -41.176 -13.578 0.00 0.00
I
ATOM   2120 HN ARG I 171   -8.466 -40.724 -12.788 0.00 0.00
I
ATOM   2121 CA ARG I 171   -9.085 -41.879 -14.327 0.00 0.00
I
ATOM   2122 HA ARG I 171   -8.509 -42.396 -15.080 0.00 0.00
I
ATOM   2123 CB ARG I 171   -9.885 -42.943 -13.528 0.00 0.00
I
ATOM   2124 HB1 ARG I 171   -10.519 -42.379 -12.812 0.00 0.00
I
ATOM   2125 HB2 ARG I 171   -10.560 -43.528 -14.188 0.00 0.00
I
ATOM   2126 CG ARG I 171   -9.093 -43.957 -12.812 0.00 0.00
I
ATOM   2127 HG1 ARG I 171   -8.284 -44.335 -13.474 0.00 0.00
I
ATOM   2128 HG2 ARG I 171   -8.603 -43.479 -11.937 0.00 0.00
I
ATOM   2129 CD ARG I 171   -9.906 -45.161 -12.265 0.00 0.00
I
ATOM   2130 HD1 ARG I 171   -10.582 -45.530 -13.066 0.00 0.00
I
ATOM   2131 HD2 ARG I 171   -9.199 -45.891 -11.815 0.00 0.00
I
ATOM   2132 NE ARG I 171   -10.760 -44.694 -11.125 0.00 0.00
I

```
ATOM   2133  HE  ARG I 171     -10.965 -43.725 -10.987  0.00  0.00
I
ATOM   2134  CZ  ARG I 171     -11.704 -45.460 -10.579  0.00  0.00
I
ATOM   2135  NH1 ARG I 171     -11.726 -46.782 -10.848  0.00  0.00
I
ATOM   2136 HH11 ARG I 171     -12.376 -47.339 -10.331  0.00  0.00
I
ATOM   2137 HH12 ARG I 171     -11.023 -47.197 -11.425  0.00  0.00
I
ATOM   2138  NH2 ARG I 171     -12.649 -44.884  -9.873  0.00  0.00
I
ATOM   2139 HH21 ARG I 171     -13.384 -45.372  -9.402  0.00  0.00
I
ATOM   2140 HH22 ARG I 171     -12.678 -43.885  -9.903  0.00  0.00
I
ATOM   2141  C   ARG I 171     -10.040 -40.882 -15.016  0.00  0.00
I
ATOM   2142  O   ARG I 171     -11.231 -41.068 -15.374  0.00  0.00
I
ATOM   2143  N   LEU I 172      -9.608 -39.656 -15.213  0.00  0.00
I
ATOM   2144  HN  LEU I 172      -8.640 -39.511 -15.401  0.00  0.00
I
ATOM   2145  CA  LEU I 172     -10.506 -38.585 -15.542  0.00  0.00
I
ATOM   2146  HA  LEU I 172     -11.524 -38.805 -15.257  0.00  0.00
I
ATOM   2147  CB  LEU I 172     -10.048 -37.353 -14.837  0.00  0.00
I
ATOM   2148  HB1 LEU I 172     -10.175 -37.429 -13.736  0.00  0.00
I
ATOM   2149  HB2 LEU I 172      -8.952 -37.283 -15.005  0.00  0.00
I
ATOM   2150  CG  LEU I 172     -10.680 -36.004 -15.255  0.00  0.00
I
ATOM   2151  HG  LEU I 172     -10.618 -35.889 -16.359  0.00  0.00
I
ATOM   2152  CD1 LEU I 172     -12.222 -36.141 -14.980  0.00  0.00
I
ATOM   2153 HD11 LEU I 172     -12.718 -36.889 -15.635  0.00  0.00
I
ATOM   2154 HD12 LEU I 172     -12.422 -36.267 -13.894  0.00  0.00
I
ATOM   2155 HD13 LEU I 172     -12.763 -35.202 -15.222  0.00  0.00
I
ATOM   2156  CD2 LEU I 172      -9.930 -34.927 -14.473  0.00  0.00
I
ATOM   2157 HD21 LEU I 172      -8.860 -34.937 -14.772  0.00  0.00
I
ATOM   2158 HD22 LEU I 172     -10.148 -33.848 -14.624  0.00  0.00
I
ATOM   2159 HD23 LEU I 172     -10.032 -35.204 -13.402  0.00  0.00
I
```

ATOM   2160 C   LEU I 172   -10.537 -38.363 -17.092 0.00 0.00
I
ATOM   2161 O   LEU I 172   -11.557 -38.260 -17.804 0.00 0.00
I
ATOM   2162 N   VAL I 173   -9.372 -38.315 -17.734 0.00 0.00
I
ATOM   2163 HN   VAL I 173   -8.533 -38.403 -17.202 0.00 0.00
I
ATOM   2164 CA   VAL I 173   -9.175 -37.975 -19.142 0.00 0.00
I
ATOM   2165 HA   VAL I 173   -10.009 -37.388 -19.498 0.00 0.00
I
ATOM   2166 CB   VAL I 173   -7.989 -37.053 -19.291 0.00 0.00
I
ATOM   2167 HB   VAL I 173   -7.111 -37.583 -18.864 0.00 0.00
I
ATOM   2168 CG1 VAL I 173   -7.726 -36.630 -20.758 0.00 0.00
I
ATOM   2169 HG11 VAL I 173   -8.677 -36.339 -21.253 0.00 0.00
I
ATOM   2170 HG12 VAL I 173   -7.043 -35.769 -20.915 0.00 0.00
I
ATOM   2171 HG13 VAL I 173   -7.461 -37.489 -21.412 0.00 0.00
I
ATOM   2172 CG2 VAL I 173   -8.229 -35.808 -18.438 0.00 0.00
I
ATOM   2173 HG21 VAL I 173   -8.275 -35.947 -17.336 0.00 0.00
I
ATOM   2174 HG22 VAL I 173   -7.357 -35.129 -18.551 0.00 0.00
I
ATOM   2175 HG23 VAL I 173   -9.037 -35.262 -18.969 0.00 0.00
I
ATOM   2176 C   VAL I 173   -8.835 -39.165 -20.043 0.00 0.00
I
ATOM   2177 O   VAL I 173   -7.918 -39.907 -19.685 0.00 0.00
I
ATOM   2178 N   PRO I 174   -9.718 -39.417 -21.122 0.00 0.00
I
ATOM   2179 CD   PRO I 174   -10.519 -38.468 -21.885 0.00 0.00
I
ATOM   2180 HD1 PRO I 174   -10.845 -37.632 -21.229 0.00 0.00
I
ATOM   2181 HD2 PRO I 174   -9.787 -38.026 -22.595 0.00 0.00
I
ATOM   2182 CA   PRO I 174   -9.920 -40.804 -21.592 0.00 0.00
I
ATOM   2183 HA   PRO I 174   -10.231 -41.256 -20.661 0.00 0.00
I
ATOM   2184 CB   PRO I 174   -11.022 -40.694 -22.779 0.00 0.00
I
ATOM   2185 HB1 PRO I 174   -11.915 -41.333 -22.613 0.00 0.00
I
ATOM   2186 HB2 PRO I 174   -10.592 -40.847 -23.792 0.00 0.00
I

```
ATOM   2187  CG  PRO I 174     -11.580 -39.246 -22.623  0.00  0.00
I
ATOM   2188  HG1 PRO I 174     -12.459 -39.166 -21.949  0.00  0.00
I
ATOM   2189  HG2 PRO I 174     -12.007 -38.804 -23.549  0.00  0.00
I
ATOM   2190  C   PRO I 174      -8.673 -41.570 -22.049  0.00  0.00
I
ATOM   2191  O   PRO I 174      -8.432 -42.685 -21.601  0.00  0.00
I
ATOM   2192  N   GLN I 175      -7.846 -40.924 -22.915  0.00  0.00
I
ATOM   2193  HN  GLN I 175      -8.027 -40.025 -23.308  0.00  0.00
I
ATOM   2194  CA  GLN I 175      -6.696 -41.597 -23.591  0.00  0.00
I
ATOM   2195  HA  GLN I 175      -6.759 -42.657 -23.394  0.00  0.00
I
ATOM   2196  CB  GLN I 175      -6.962 -41.357 -25.087  0.00  0.00
I
ATOM   2197  HB1 GLN I 175      -8.063 -41.365 -25.237  0.00  0.00
I
ATOM   2198  HB2 GLN I 175      -6.643 -40.320 -25.325  0.00  0.00
I
ATOM   2199  CG  GLN I 175      -6.256 -42.254 -26.115  0.00  0.00
I
ATOM   2200  HG1 GLN I 175      -5.160 -42.085 -26.051  0.00  0.00
I
ATOM   2201  HG2 GLN I 175      -6.581 -43.291 -25.886  0.00  0.00
I
ATOM   2202  CD  GLN I 175      -6.728 -41.951 -27.547  0.00  0.00
I
ATOM   2203  OE1 GLN I 175      -6.504 -40.838 -27.949  0.00  0.00
I
ATOM   2204  NE2 GLN I 175      -7.336 -42.874 -28.252  0.00  0.00
I
ATOM   2205 HE21 GLN I 175      -7.670 -42.648 -29.167  0.00  0.00
I
ATOM   2206 HE22 GLN I 175      -7.578 -43.775 -27.892  0.00  0.00
I
ATOM   2207  C   GLN I 175      -5.320 -41.224 -23.045  0.00  0.00
I
ATOM   2208  O   GLN I 175      -4.259 -41.446 -23.635  0.00  0.00
I
ATOM   2209  N   TYR I 176      -5.338 -40.551 -21.841  0.00  0.00
I
ATOM   2210  HN  TYR I 176      -6.229 -40.225 -21.534  0.00  0.00
I
ATOM   2211  CA  TYR I 176      -4.201 -39.931 -21.274  0.00  0.00
I
ATOM   2212  HA  TYR I 176      -3.699 -39.295 -21.989  0.00  0.00
I
ATOM   2213  CB  TYR I 176      -4.654 -39.065 -20.049  0.00  0.00
I
```

```
ATOM   2214  HB1 TYR I 176      -5.679 -38.675 -20.226  0.00  0.00
I
ATOM   2215  HB2 TYR I 176      -4.824 -39.688 -19.145  0.00  0.00
I
ATOM   2216  CG  TYR I 176      -3.682 -37.940 -19.675  0.00  0.00
I
ATOM   2217  CD1 TYR I 176      -4.091 -36.665 -20.089  0.00  0.00
I
ATOM   2218  HD1 TYR I 176      -5.070 -36.557 -20.533  0.00  0.00
I
ATOM   2219  CE1 TYR I 176      -3.365 -35.557 -19.932  0.00  0.00
I
ATOM   2220  HE1 TYR I 176      -3.606 -34.576 -20.312  0.00  0.00
I
ATOM   2221  CZ  TYR I 176      -2.080 -35.755 -19.331  0.00  0.00
I
ATOM   2222  OH  TYR I 176      -1.311 -34.585 -18.903  0.00  0.00
I
ATOM   2223  HH  TYR I 176      -0.366 -34.750 -18.924  0.00  0.00
I
ATOM   2224  CD2 TYR I 176      -2.431 -38.184 -19.012  0.00  0.00
I
ATOM   2225  HD2 TYR I 176      -2.072 -39.159 -18.718  0.00  0.00
I
ATOM   2226  CE2 TYR I 176      -1.685 -37.028 -18.842  0.00  0.00
I
ATOM   2227  HE2 TYR I 176      -0.758 -37.073 -18.289  0.00  0.00
I
ATOM   2228  C   TYR I 176      -3.067 -40.835 -20.768  0.00  0.00
I
ATOM   2229  O   TYR I 176      -1.893 -40.515 -20.931  0.00  0.00
I
ATOM   2230  N   GLY I 177      -3.426 -41.991 -20.275  0.00  0.00
I
ATOM   2231  HN  GLY I 177      -4.384 -42.205 -20.100  0.00  0.00
I
ATOM   2232  CA  GLY I 177      -2.575 -43.054 -19.821  0.00  0.00
I
ATOM   2233  HA1 GLY I 177      -1.618 -42.699 -20.173  0.00  0.00
I
ATOM   2234  HA2 GLY I 177      -2.823 -43.963 -20.350  0.00  0.00
I
ATOM   2235  C   GLY I 177      -2.551 -43.189 -18.340  0.00  0.00
I
ATOM   2236  O   GLY I 177      -2.709 -42.214 -17.568  0.00  0.00
I
ATOM   2237  N   TRP I 178      -2.115 -44.354 -17.848  0.00  0.00
I
ATOM   2238  HN  TRP I 178      -1.742 -45.112 -18.377  0.00  0.00
I
ATOM   2239  CA  TRP I 178      -2.046 -44.531 -16.422  0.00  0.00
I
ATOM   2240  HA  TRP I 178      -2.842 -44.080 -15.849  0.00  0.00
I
```

ATOM 2241 CB TRP I 178 −2.072 −46.064 −16.153 0.00 0.00
I
ATOM 2242 HB1 TRP I 178 −2.850 −46.529 −16.795 0.00 0.00
I
ATOM 2243 HB2 TRP I 178 −1.190 −46.583 −16.585 0.00 0.00
I
ATOM 2244 CG TRP I 178 −2.431 −46.514 −14.744 0.00 0.00
I
ATOM 2245 CD1 TRP I 178 −1.704 −46.425 −13.576 0.00 0.00
I
ATOM 2246 HD1 TRP I 178 −0.650 −46.203 −13.499 0.00 0.00
I
ATOM 2247 NE1 TRP I 178 −2.511 −46.787 −12.543 0.00 0.00
I
ATOM 2248 HE1 TRP I 178 −2.285 −46.778 −11.593 0.00 0.00
I
ATOM 2249 CE2 TRP I 178 −3.742 −47.224 −13.032 0.00 0.00
I
ATOM 2250 CD2 TRP I 178 −3.716 −47.038 −14.386 0.00 0.00
I
ATOM 2251 CE3 TRP I 178 −4.819 −47.464 −15.145 0.00 0.00
I
ATOM 2252 HE3 TRP I 178 −4.831 −47.317 −16.215 0.00 0.00
I
ATOM 2253 CZ3 TRP I 178 −5.918 −48.057 −14.598 0.00 0.00
I
ATOM 2254 HZ3 TRP I 178 −6.741 −48.433 −15.188 0.00 0.00
I
ATOM 2255 CZ2 TRP I 178 −4.860 −47.796 −12.421 0.00 0.00
I
ATOM 2256 HZ2 TRP I 178 −4.842 −47.740 −11.343 0.00 0.00
I
ATOM 2257 CH2 TRP I 178 −5.994 −48.189 −13.177 0.00 0.00
I
ATOM 2258 HH2 TRP I 178 −6.910 −48.534 −12.721 0.00 0.00
I
ATOM 2259 C TRP I 178 −0.774 −43.883 −15.748 0.00 0.00
I
ATOM 2260 O TRP I 178 −0.893 −42.981 −14.942 0.00 0.00
I
ATOM 2261 N SER I 179 0.441 −44.236 −16.231 0.00 0.00
I
ATOM 2262 HN SER I 179 0.606 −44.893 −16.963 0.00 0.00
I
ATOM 2263 CA SER I 179 1.700 −43.820 −15.591 0.00 0.00
I
ATOM 2264 HA SER I 179 1.644 −43.953 −14.520 0.00 0.00
I
ATOM 2265 CB SER I 179 2.933 −44.689 −15.946 0.00 0.00
I
ATOM 2266 HB1 SER I 179 3.840 −44.509 −15.330 0.00 0.00
I
ATOM 2267 HB2 SER I 179 2.615 −45.752 −15.882 0.00 0.00
I

ATOM 2268 OG SER I 179 3.276 -44.527 -17.327 0.00 0.00
I
ATOM 2269 HG1 SER I 179 3.886 -45.216 -17.600 0.00 0.00
I
ATOM 2270 C SER I 179 1.931 -42.342 -15.736 0.00 0.00
I
ATOM 2271 O SER I 179 2.196 -41.625 -14.813 0.00 0.00
I
ATOM 2272 N SER I 180 1.570 -41.737 -16.896 0.00 0.00
I
ATOM 2273 HN SER I 180 1.187 -42.220 -17.680 0.00 0.00
I
ATOM 2274 CA SER I 180 1.404 -40.278 -17.218 0.00 0.00
I
ATOM 2275 HA SER I 180 2.309 -39.760 -16.936 0.00 0.00
I
ATOM 2276 CB SER I 180 1.030 -40.226 -18.743 0.00 0.00
I
ATOM 2277 HB1 SER I 180 0.744 -39.206 -19.079 0.00 0.00
I
ATOM 2278 HB2 SER I 180 2.014 -40.413 -19.223 0.00 0.00
I
ATOM 2279 OG SER I 180 -0.011 -41.135 -19.028 0.00 0.00
I
ATOM 2280 HG1 SER I 180 -0.416 -41.026 -19.891 0.00 0.00
I
ATOM 2281 C SER I 180 0.291 -39.527 -16.383 0.00 0.00
I
ATOM 2282 O SER I 180 0.562 -38.493 -15.821 0.00 0.00
I
ATOM 2283 N GLY I 181 -0.918 -40.123 -16.342 0.00 0.00
I
ATOM 2284 HN GLY I 181 -0.997 -40.962 -16.875 0.00 0.00
I
ATOM 2285 CA GLY I 181 -2.064 -39.582 -15.664 0.00 0.00
I
ATOM 2286 HA1 GLY I 181 -2.885 -40.273 -15.789 0.00 0.00
I
ATOM 2287 HA2 GLY I 181 -2.384 -38.626 -16.053 0.00 0.00
I
ATOM 2288 C GLY I 181 -1.811 -39.544 -14.210 0.00 0.00
I
ATOM 2289 O GLY I 181 -2.225 -38.690 -13.399 0.00 0.00
I
ATOM 2290 N LEU I 182 -1.015 -40.594 -13.672 0.00 0.00
I
ATOM 2291 HN LEU I 182 -0.805 -41.328 -14.313 0.00 0.00
I
ATOM 2292 CA LEU I 182 -0.533 -40.675 -12.296 0.00 0.00
I
ATOM 2293 HA LEU I 182 -1.312 -40.782 -11.556 0.00 0.00
I
ATOM 2294 CB LEU I 182 0.296 -42.028 -12.154 0.00 0.00
I

```
ATOM   2295  HB1 LEU I 182      -0.226 -42.807 -12.749  0.00  0.00
I
ATOM   2296  HB2 LEU I 182       1.227 -42.014 -12.760  0.00  0.00
I
ATOM   2297  CG  LEU I 182       0.442 -42.728 -10.786  0.00  0.00
I
ATOM   2298  HG  LEU I 182      -0.601 -42.632 -10.417  0.00  0.00
I
ATOM   2299  CD1 LEU I 182       0.913 -44.167 -10.890  0.00  0.00
I
ATOM   2300 HD11 LEU I 182       1.887 -44.158 -11.424  0.00  0.00
I
ATOM   2301 HD12 LEU I 182       1.057 -44.630  -9.891  0.00  0.00
I
ATOM   2302 HD13 LEU I 182       0.185 -44.748 -11.496  0.00  0.00
I
ATOM   2303  CD2 LEU I 182       1.410 -41.827  -9.902  0.00  0.00
I
ATOM   2304 HD21 LEU I 182       1.650 -42.265  -8.910  0.00  0.00
I
ATOM   2305 HD22 LEU I 182       2.289 -41.621 -10.550  0.00  0.00
I
ATOM   2306 HD23 LEU I 182       1.050 -40.797  -9.692  0.00  0.00
I
ATOM   2307  C   LEU I 182       0.268 -39.561 -11.870  0.00  0.00
I
ATOM   2308  O   LEU I 182      -0.023 -38.909 -10.902  0.00  0.00
I
ATOM   2309  N   PHE I 183       1.299 -39.279 -12.660  0.00  0.00
I
ATOM   2310  HN  PHE I 183       1.447 -39.920 -13.409  0.00  0.00
I
ATOM   2311  CA  PHE I 183       2.298 -38.186 -12.540  0.00  0.00
I
ATOM   2312  HA  PHE I 183       2.654 -38.295 -11.526  0.00  0.00
I
ATOM   2313  CB  PHE I 183       3.437 -38.428 -13.589  0.00  0.00
I
ATOM   2314  HB1 PHE I 183       3.872 -39.440 -13.445  0.00  0.00
I
ATOM   2315  HB2 PHE I 183       3.167 -38.202 -14.643  0.00  0.00
I
ATOM   2316  CG  PHE I 183       4.558 -37.522 -13.274  0.00  0.00
I
ATOM   2317  CD1 PHE I 183       5.159 -36.694 -14.236  0.00  0.00
I
ATOM   2318  HD1 PHE I 183       4.890 -36.741 -15.280  0.00  0.00
I
ATOM   2319  CE1 PHE I 183       6.151 -35.834 -13.985  0.00  0.00
I
ATOM   2320  HE1 PHE I 183       6.474 -35.145 -14.751  0.00  0.00
I
ATOM   2321  CZ  PHE I 183       6.580 -35.629 -12.683  0.00  0.00
I
```

ATOM 2322 HZ PHE I 183 7.355 -34.909 -12.466 0.00 0.00
I
ATOM 2323 CD2 PHE I 183 5.147 -37.439 -11.958 0.00 0.00
I
ATOM 2324 HD2 PHE I 183 4.944 -38.159 -11.178 0.00 0.00
I
ATOM 2325 CE2 PHE I 183 6.121 -36.450 -11.686 0.00 0.00
I
ATOM 2326 HE2 PHE I 183 6.534 -36.313 -10.698 0.00 0.00
I
ATOM 2327 C PHE I 183 1.624 -36.819 -12.767 0.00 0.00
I
ATOM 2328 O PHE I 183 1.906 -35.888 -12.020 0.00 0.00
I
ATOM 2329 N ALA I 184 0.647 -36.640 -13.687 0.00 0.00
I
ATOM 2330 HN ALA I 184 0.384 -37.339 -14.348 0.00 0.00
I
ATOM 2331 CA ALA I 184 -0.175 -35.450 -13.795 0.00 0.00
I
ATOM 2332 HA ALA I 184 0.514 -34.623 -13.887 0.00 0.00
I
ATOM 2333 CB ALA I 184 -1.153 -35.519 -15.003 0.00 0.00
I
ATOM 2334 HB1 ALA I 184 -1.914 -34.710 -14.967 0.00 0.00
I
ATOM 2335 HB2 ALA I 184 -0.689 -35.377 -16.003 0.00 0.00
I
ATOM 2336 HB3 ALA I 184 -1.739 -36.462 -15.023 0.00 0.00
I
ATOM 2337 C ALA I 184 -1.092 -35.319 -12.572 0.00 0.00
I
ATOM 2338 O ALA I 184 -1.202 -34.249 -12.011 0.00 0.00
I
ATOM 2339 N SER I 185 -1.702 -36.377 -12.059 0.00 0.00
I
ATOM 2340 HN SER I 185 -1.663 -37.219 -12.591 0.00 0.00
I
ATOM 2341 CA SER I 185 -2.601 -36.306 -10.884 0.00 0.00
I
ATOM 2342 HA SER I 185 -3.358 -35.559 -11.070 0.00 0.00
I
ATOM 2343 CB SER I 185 -3.380 -37.626 -10.751 0.00 0.00
I
ATOM 2344 HB1 SER I 185 -3.765 -37.869 -11.764 0.00 0.00
I
ATOM 2345 HB2 SER I 185 -2.799 -38.444 -10.275 0.00 0.00
I
ATOM 2346 OG SER I 185 -4.517 -37.421 -9.937 0.00 0.00
I
ATOM 2347 HG1 SER I 185 -4.685 -38.290 -9.566 0.00 0.00
I
ATOM 2348 C SER I 185 -1.830 -35.845 -9.579 0.00 0.00
I

```
ATOM   2349  O   SER I 185      -2.329 -35.265  -8.652  0.00  0.00
I
ATOM   2350  N   LEU I 186      -0.550 -36.243  -9.506  0.00  0.00
I
ATOM   2351  HN  LEU I 186      -0.315 -36.915 -10.205  0.00  0.00
I
ATOM   2352  CA  LEU I 186       0.288 -36.039  -8.387  0.00  0.00
I
ATOM   2353  HA  LEU I 186      -0.209 -36.282  -7.459  0.00  0.00
I
ATOM   2354  CB  LEU I 186       1.580 -36.966  -8.444  0.00  0.00
I
ATOM   2355  HB1 LEU I 186       1.237 -38.012  -8.596  0.00  0.00
I
ATOM   2356  HB2 LEU I 186       2.131 -36.745  -9.383  0.00  0.00
I
ATOM   2357  CG  LEU I 186       2.420 -37.104  -7.149  0.00  0.00
I
ATOM   2358  HG  LEU I 186       2.764 -36.081  -6.885  0.00  0.00
I
ATOM   2359  CD1 LEU I 186       1.716 -37.684  -5.933  0.00  0.00
I
ATOM   2360 HD11 LEU I 186       1.500 -38.758  -6.115  0.00  0.00
I
ATOM   2361 HD12 LEU I 186       2.408 -37.438  -5.100  0.00  0.00
I
ATOM   2362 HD13 LEU I 186       0.802 -37.062  -5.828  0.00  0.00
I
ATOM   2363  CD2 LEU I 186       3.668 -37.845  -7.536  0.00  0.00
I
ATOM   2364 HD21 LEU I 186       4.211 -37.124  -8.184  0.00  0.00
I
ATOM   2365 HD22 LEU I 186       4.337 -38.121  -6.692  0.00  0.00
I
ATOM   2366 HD23 LEU I 186       3.562 -38.743  -8.181  0.00  0.00
I
ATOM   2367  C   LEU I 186       0.842 -34.587  -8.346  0.00  0.00
I
ATOM   2368  O   LEU I 186       0.935 -33.914  -7.308  0.00  0.00
I
ATOM   2369  N   PHE I 187       1.118 -34.107  -9.587  0.00  0.00
I
ATOM   2370  HN  PHE I 187       1.148 -34.729 -10.365  0.00  0.00
I
ATOM   2371  CA  PHE I 187       1.408 -32.748  -9.945  0.00  0.00
I
ATOM   2372  HA  PHE I 187       2.185 -32.540  -9.224  0.00  0.00
I
ATOM   2373  CB  PHE I 187       1.873 -32.765 -11.416  0.00  0.00
I
ATOM   2374  HB1 PHE I 187       2.565 -33.631 -11.503  0.00  0.00
I
ATOM   2375  HB2 PHE I 187       1.003 -32.942 -12.084  0.00  0.00
I
```

ATOM 2376 CG PHE I 187 2.679 −31.551 −11.832 0.00 0.00
I
ATOM 2377 CD1 PHE I 187 2.044 −30.275 −12.043 0.00 0.00
I
ATOM 2378 HD1 PHE I 187 0.978 −30.150 −11.924 0.00 0.00
I
ATOM 2379 CE1 PHE I 187 2.705 −29.058 −12.283 0.00 0.00
I
ATOM 2380 HE1 PHE I 187 2.198 −28.105 −12.329 0.00 0.00
I
ATOM 2381 CZ PHE I 187 4.085 −29.160 −12.184 0.00 0.00
I
ATOM 2382 HZ PHE I 187 4.705 −28.280 −12.272 0.00 0.00
I
ATOM 2383 CD2 PHE I 187 4.025 −31.572 −12.010 0.00 0.00
I
ATOM 2384 HD2 PHE I 187 4.558 −32.510 −11.975 0.00 0.00
I
ATOM 2385 CE2 PHE I 187 4.777 −30.345 −12.048 0.00 0.00
I
ATOM 2386 HE2 PHE I 187 5.849 −30.325 −12.178 0.00 0.00
I
ATOM 2387 C PHE I 187 0.379 −31.738 −9.791 0.00 0.00
I
ATOM 2388 O PHE I 187 0.587 −30.670 −9.308 0.00 0.00
I
ATOM 2389 N HSE I 188 −0.871 −32.070 −10.048 0.00 0.00
I
ATOM 2390 HN HSE I 188 −0.923 −32.947 −10.518 0.00 0.00
I
ATOM 2391 CA HSE I 188 −2.017 −31.270 −9.675 0.00 0.00
I
ATOM 2392 HA HSE I 188 −1.861 −30.233 −9.935 0.00 0.00
I
ATOM 2393 CB HSE I 188 −3.344 −31.733 −10.333 0.00 0.00
I
ATOM 2394 HB1 HSE I 188 −3.690 −32.703 −9.916 0.00 0.00
I
ATOM 2395 HB2 HSE I 188 −4.037 −30.882 −10.157 0.00 0.00
I
ATOM 2396 ND1 HSE I 188 −4.260 −32.276 −12.633 0.00 0.00
I
ATOM 2397 CG HSE I 188 −3.261 −31.776 −11.879 0.00 0.00
I
ATOM 2398 CE1 HSE I 188 −3.857 −32.194 −13.861 0.00 0.00
I
ATOM 2399 HE1 HSE I 188 −4.356 −32.431 −14.801 0.00 0.00
I
ATOM 2400 NE2 HSE I 188 −2.660 −31.699 −13.943 0.00 0.00
I
ATOM 2401 HE2 HSE I 188 −2.043 −31.628 −14.726 0.00 0.00
I
ATOM 2402 CD2 HSE I 188 −2.230 −31.374 −12.677 0.00 0.00
I

```
ATOM   2403  HD2 HSE I 188      -1.219 -31.015 -12.524  0.00  0.00
I
ATOM   2404  C   HSE I 188      -2.216 -31.283  -8.109  0.00  0.00
I
ATOM   2405  O   HSE I 188      -2.475 -30.229  -7.503  0.00  0.00
I
ATOM   2406  N   ALA I 189      -2.094 -32.407  -7.453  0.00  0.00
I
ATOM   2407  HN  ALA I 189      -1.817 -33.263  -7.884  0.00  0.00
I
ATOM   2408  CA  ALA I 189      -2.455 -32.538  -5.992  0.00  0.00
I
ATOM   2409  HA  ALA I 189      -3.428 -32.074  -5.924  0.00  0.00
I
ATOM   2410  CB  ALA I 189      -2.545 -34.035  -5.682  0.00  0.00
I
ATOM   2411  HB1 ALA I 189      -1.615 -34.610  -5.882  0.00  0.00
I
ATOM   2412  HB2 ALA I 189      -2.840 -34.180  -4.620  0.00  0.00
I
ATOM   2413  HB3 ALA I 189      -3.290 -34.473  -6.379  0.00  0.00
I
ATOM   2414  C   ALA I 189      -1.438 -31.804  -5.015  0.00  0.00
I
ATOM   2415  O   ALA I 189      -1.908 -30.932  -4.241  0.00  0.00
I
ATOM   2416  N   ILE I 190      -0.085 -31.906  -5.233  0.00  0.00
I
ATOM   2417  HN  ILE I 190       0.152 -32.607  -5.902  0.00  0.00
I
ATOM   2418  CA  ILE I 190       0.964 -31.003  -4.703  0.00  0.00
I
ATOM   2419  HA  ILE I 190       0.842 -31.167  -3.643  0.00  0.00
I
ATOM   2420  CB  ILE I 190       2.464 -31.333  -4.977  0.00  0.00
I
ATOM   2421  HB  ILE I 190       2.741 -32.405  -4.884  0.00  0.00
I
ATOM   2422  CG2 ILE I 190       2.930 -30.911  -6.408  0.00  0.00
I
ATOM   2423 HG21 ILE I 190       2.312 -31.436  -7.168  0.00  0.00
I
ATOM   2424 HG22 ILE I 190       2.990 -29.819  -6.600  0.00  0.00
I
ATOM   2425 HG23 ILE I 190       3.964 -31.312  -6.468  0.00  0.00
I
ATOM   2426  CG1 ILE I 190       3.388 -30.645  -3.935  0.00  0.00
I
ATOM   2427 HG11 ILE I 190       3.191 -29.552  -3.930  0.00  0.00
I
ATOM   2428 HG12 ILE I 190       3.083 -31.006  -2.930  0.00  0.00
I
ATOM   2429  CD  ILE I 190       4.886 -30.999  -4.168  0.00  0.00
I
```

```
ATOM   2430  HD1 ILE I 190       4.990 -32.097  -4.036  0.00  0.00
I
ATOM   2431  HD2 ILE I 190       5.369 -30.613  -5.092  0.00  0.00
I
ATOM   2432  HD3 ILE I 190       5.486 -30.497  -3.379  0.00  0.00
I
ATOM   2433  C   ILE I 190       0.658 -29.608  -5.043  0.00  0.00
I
ATOM   2434  O   ILE I 190       0.674 -28.807  -4.153  0.00  0.00
I
ATOM   2435  N   SER I 191       0.281 -29.271  -6.324  0.00  0.00
I
ATOM   2436  HN  SER I 191       0.351 -30.008  -6.991  0.00  0.00
I
ATOM   2437  CA  SER I 191      -0.071 -27.949  -6.683  0.00  0.00
I
ATOM   2438  HA  SER I 191       0.721 -27.332  -6.287  0.00  0.00
I
ATOM   2439  CB  SER I 191      -0.108 -27.816  -8.206  0.00  0.00
I
ATOM   2440  HB1 SER I 191       0.492 -28.479  -8.865  0.00  0.00
I
ATOM   2441  HB2 SER I 191      -1.097 -28.206  -8.528  0.00  0.00
I
ATOM   2442  OG  SER I 191      -0.050 -26.407  -8.614  0.00  0.00
I
ATOM   2443  HG1 SER I 191      -0.147 -26.418  -9.569  0.00  0.00
I
ATOM   2444  C   SER I 191      -1.260 -27.283  -5.978  0.00  0.00
I
ATOM   2445  O   SER I 191      -1.234 -26.081  -5.548  0.00  0.00
I
ATOM   2446  N   ALA I 192      -2.313 -28.059  -5.739  0.00  0.00
I
ATOM   2447  HN  ALA I 192      -2.370 -28.985  -6.103  0.00  0.00
I
ATOM   2448  CA  ALA I 192      -3.610 -27.547  -5.312  0.00  0.00
I
ATOM   2449  HA  ALA I 192      -3.664 -26.482  -5.479  0.00  0.00
I
ATOM   2450  CB  ALA I 192      -4.709 -28.364  -6.078  0.00  0.00
I
ATOM   2451  HB1 ALA I 192      -4.524 -28.349  -7.174  0.00  0.00
I
ATOM   2452  HB2 ALA I 192      -4.577 -29.421  -5.765  0.00  0.00
I
ATOM   2453  HB3 ALA I 192      -5.735 -27.975  -5.902  0.00  0.00
I
ATOM   2454  C   ALA I 192      -3.742 -27.703  -3.867  0.00  0.00
I
ATOM   2455  O   ALA I 192      -4.385 -26.819  -3.302  0.00  0.00
I
ATOM   2456  N   PHE I 193      -3.133 -28.658  -3.194  0.00  0.00
I
```

```
ATOM   2457  HN  PHE I 193      -2.783 -29.483  -3.631  0.00  0.00
I
ATOM   2458  CA  PHE I 193      -2.993 -28.694  -1.760  0.00  0.00
I
ATOM   2459  HA  PHE I 193      -3.998 -28.616  -1.373  0.00  0.00
I
ATOM   2460  CB  PHE I 193      -2.471 -30.125  -1.331  0.00  0.00
I
ATOM   2461  HB1 PHE I 193      -3.159 -30.877  -1.775  0.00  0.00
I
ATOM   2462  HB2 PHE I 193      -1.459 -30.412  -1.688  0.00  0.00
I
ATOM   2463  CG  PHE I 193      -2.469 -30.234   0.193  0.00  0.00
I
ATOM   2464  CD1 PHE I 193      -3.541 -29.899   0.980  0.00  0.00
I
ATOM   2465  HD1 PHE I 193      -4.480 -29.706   0.483  0.00  0.00
I
ATOM   2466  CE1 PHE I 193      -3.401 -29.775   2.374  0.00  0.00
I
ATOM   2467  HE1 PHE I 193      -4.231 -29.549   3.027  0.00  0.00
I
ATOM   2468  CZ  PHE I 193      -2.174 -29.864   2.981  0.00  0.00
I
ATOM   2469  HZ  PHE I 193      -1.957 -29.846   4.038  0.00  0.00
I
ATOM   2470  CD2 PHE I 193      -1.199 -30.441   0.834  0.00  0.00
I
ATOM   2471  HD2 PHE I 193      -0.363 -30.728   0.214  0.00  0.00
I
ATOM   2472  CE2 PHE I 193      -1.057 -30.250   2.203  0.00  0.00
I
ATOM   2473  HE2 PHE I 193      -0.043 -30.322   2.567  0.00  0.00
I
ATOM   2474  C   PHE I 193      -2.139 -27.585  -1.244  0.00  0.00
I
ATOM   2475  O   PHE I 193      -2.511 -26.942  -0.238  0.00  0.00
I
ATOM   2476  N   ASN I 194      -0.955 -27.361  -1.785  0.00  0.00
I
ATOM   2477  HN  ASN I 194      -0.760 -27.912  -2.592  0.00  0.00
I
ATOM   2478  CA  ASN I 194      -0.019 -26.424  -1.220  0.00  0.00
I
ATOM   2479  HA  ASN I 194      -0.237 -26.472  -0.164  0.00  0.00
I
ATOM   2480  CB  ASN I 194       1.456 -26.822  -1.381  0.00  0.00
I
ATOM   2481  HB1 ASN I 194       1.664 -27.041  -2.450  0.00  0.00
I
ATOM   2482  HB2 ASN I 194       2.099 -26.017  -0.965  0.00  0.00
I
ATOM   2483  CG  ASN I 194       1.684 -28.101  -0.637  0.00  0.00
I
```

ATOM	2484	OD1	ASN	I	194	1.917	-28.047	0.591	0.00	0.00	I
ATOM	2485	ND2	ASN	I	194	1.785	-29.175	-1.378	0.00	0.00	I
ATOM	2486	HD21	ASN	I	194	1.935	-30.091	-1.004	0.00	0.00	I
ATOM	2487	HD22	ASN	I	194	1.614	-29.252	-2.361	0.00	0.00	I
ATOM	2488	C	ASN	I	194	-0.188	-25.074	-1.758	0.00	0.00	I
ATOM	2489	O	ASN	I	194	0.514	-24.150	-1.476	0.00	0.00	I
ATOM	2490	N	ASN	I	195	-1.248	-24.944	-2.561	0.00	0.00	I
ATOM	2491	HN	ASN	I	195	-1.833	-25.746	-2.655	0.00	0.00	I
ATOM	2492	CA	ASN	I	195	-1.701	-23.692	-3.176	0.00	0.00	I
ATOM	2493	HA	ASN	I	195	-2.449	-23.995	-3.893	0.00	0.00	I
ATOM	2494	CB	ASN	I	195	-2.341	-22.779	-2.194	0.00	0.00	I
ATOM	2495	HB1	ASN	I	195	-1.702	-22.409	-1.363	0.00	0.00	I
ATOM	2496	HB2	ASN	I	195	-2.669	-21.873	-2.748	0.00	0.00	I
ATOM	2497	CG	ASN	I	195	-3.608	-23.367	-1.701	0.00	0.00	I
ATOM	2498	OD1	ASN	I	195	-4.632	-22.843	-2.183	0.00	0.00	I
ATOM	2499	ND2	ASN	I	195	-3.606	-24.337	-0.766	0.00	0.00	I
ATOM	2500	HD21	ASN	I	195	-4.413	-24.789	-0.385	0.00	0.00	I
ATOM	2501	HD22	ASN	I	195	-2.725	-24.709	-0.474	0.00	0.00	I
ATOM	2502	C	ASN	I	195	-0.701	-22.993	-4.052	0.00	0.00	I
ATOM	2503	O	ASN	I	195	-0.575	-21.794	-4.003	0.00	0.00	I
ATOM	2504	N	ALA	I	196	-0.094	-23.687	-5.044	0.00	0.00	I
ATOM	2505	HN	ALA	I	196	-0.256	-24.656	-5.215	0.00	0.00	I
ATOM	2506	CA	ALA	I	196	1.030	-23.291	-5.764	0.00	0.00	I
ATOM	2507	HA	ALA	I	196	1.621	-22.607	-5.173	0.00	0.00	I
ATOM	2508	CB	ALA	I	196	1.943	-24.586	-5.985	0.00	0.00	I
ATOM	2509	HB1	ALA	I	196	2.988	-24.276	-6.200	0.00	0.00	I
ATOM	2510	HB2	ALA	I	196	1.988	-25.246	-5.092	0.00	0.00	I

ATOM	2511	HB3	ALA	I	196	1.548	-25.307	-6.733	0.00	0.00	I
ATOM	2512	C	ALA	I	196	0.772	-22.621	-7.060	0.00	0.00	I
ATOM	2513	O	ALA	I	196	1.380	-21.564	-7.173	0.00	0.00	I
ATOM	2514	N	GLY	I	197	-0.011	-23.203	-8.012	0.00	0.00	I
ATOM	2515	HN	GLY	I	197	-0.465	-24.092	-8.009	0.00	0.00	I
ATOM	2516	CA	GLY	I	197	-0.413	-22.357	-9.141	0.00	0.00	I
ATOM	2517	HA1	GLY	I	197	-0.115	-21.341	-8.926	0.00	0.00	I
ATOM	2518	HA2	GLY	I	197	-1.486	-22.426	-9.237	0.00	0.00	I
ATOM	2519	C	GLY	I	197	0.204	-22.663	-10.454	0.00	0.00	I
ATOM	2520	O	GLY	I	197	0.154	-21.845	-11.320	0.00	0.00	I
ATOM	2521	N	PHE	I	198	0.973	-23.792	-10.551	0.00	0.00	I
ATOM	2522	HN	PHE	I	198	1.174	-24.352	-9.751	0.00	0.00	I
ATOM	2523	CA	PHE	I	198	1.499	-24.324	-11.832	0.00	0.00	I
ATOM	2524	HA	PHE	I	198	1.486	-23.523	-12.557	0.00	0.00	I
ATOM	2525	CB	PHE	I	198	2.954	-24.822	-11.564	0.00	0.00	I
ATOM	2526	HB1	PHE	I	198	2.788	-25.542	-10.734	0.00	0.00	I
ATOM	2527	HB2	PHE	I	198	3.317	-25.335	-12.480	0.00	0.00	I
ATOM	2528	CG	PHE	I	198	3.928	-23.755	-11.137	0.00	0.00	I
ATOM	2529	CD1	PHE	I	198	4.391	-23.669	-9.828	0.00	0.00	I
ATOM	2530	HD1	PHE	I	198	3.977	-24.228	-9.002	0.00	0.00	I
ATOM	2531	CE1	PHE	I	198	5.467	-22.816	-9.537	0.00	0.00	I
ATOM	2532	HE1	PHE	I	198	5.793	-22.743	-8.510	0.00	0.00	I
ATOM	2533	CZ	PHE	I	198	6.054	-22.062	-10.545	0.00	0.00	I
ATOM	2534	HZ	PHE	I	198	6.890	-21.420	-10.305	0.00	0.00	I
ATOM	2535	CD2	PHE	I	198	4.453	-22.933	-12.154	0.00	0.00	I
ATOM	2536	HD2	PHE	I	198	4.213	-22.848	-13.203	0.00	0.00	I
ATOM	2537	CE2	PHE	I	198	5.571	-22.191	-11.844	0.00	0.00	I

ATOM 2538 HE2 PHE I 198 6.132 -21.699 -12.625 0.00 0.00
I
ATOM 2539 C PHE I 198 0.621 -25.453 -12.290 0.00 0.00
I
ATOM 2540 O PHE I 198 0.017 -26.073 -11.412 0.00 0.00
I
ATOM 2541 N SER I 199 0.586 -25.819 -13.582 0.00 0.00
I
ATOM 2542 HN SER I 199 0.949 -25.231 -14.301 0.00 0.00
I
ATOM 2543 CA SER I 199 -0.102 -27.032 -14.052 0.00 0.00
I
ATOM 2544 HA SER I 199 -0.057 -27.842 -13.339 0.00 0.00
I
ATOM 2545 CB SER I 199 -1.601 -26.878 -14.413 0.00 0.00
I
ATOM 2546 HB1 SER I 199 -2.174 -26.619 -13.496 0.00 0.00
I
ATOM 2547 HB2 SER I 199 -1.742 -25.989 -15.064 0.00 0.00
I
ATOM 2548 OG SER I 199 -2.124 -28.057 -14.883 0.00 0.00
I
ATOM 2549 HG1 SER I 199 -3.083 -28.044 -14.836 0.00 0.00
I
ATOM 2550 C SER I 199 0.570 -27.431 -15.309 0.00 0.00
I
ATOM 2551 O SER I 199 1.082 -26.641 -16.101 0.00 0.00
I
ATOM 2552 N LEU I 200 0.596 -28.733 -15.583 0.00 0.00
I
ATOM 2553 HN LEU I 200 -0.105 -29.312 -15.177 0.00 0.00
I
ATOM 2554 CA LEU I 200 1.166 -29.307 -16.752 0.00 0.00
I
ATOM 2555 HA LEU I 200 2.098 -28.819 -16.996 0.00 0.00
I
ATOM 2556 CB LEU I 200 1.431 -30.789 -16.466 0.00 0.00
I
ATOM 2557 HB1 LEU I 200 0.577 -31.304 -15.976 0.00 0.00
I
ATOM 2558 HB2 LEU I 200 1.694 -31.254 -17.440 0.00 0.00
I
ATOM 2559 CG LEU I 200 2.567 -31.131 -15.564 0.00 0.00
I
ATOM 2560 HG LEU I 200 2.369 -30.556 -14.634 0.00 0.00
I
ATOM 2561 CD1 LEU I 200 2.527 -32.652 -15.306 0.00 0.00
I
ATOM 2562 HD11 LEU I 200 3.349 -32.916 -14.607 0.00 0.00
I
ATOM 2563 HD12 LEU I 200 1.572 -33.028 -14.882 0.00 0.00
I
ATOM 2564 HD13 LEU I 200 2.712 -33.137 -16.288 0.00 0.00
I

ATOM 2565 CD2 LEU I 200 3.965 -30.604 -16.092 0.00 0.00
I
ATOM 2566 HD21 LEU I 200 4.834 -31.074 -15.582 0.00 0.00
I
ATOM 2567 HD22 LEU I 200 4.147 -30.873 -17.155 0.00 0.00
I
ATOM 2568 HD23 LEU I 200 4.114 -29.513 -15.951 0.00 0.00
I
ATOM 2569 C LEU I 200 0.182 -29.226 -17.936 0.00 0.00
I
ATOM 2570 O LEU I 200 0.549 -29.453 -19.141 0.00 0.00
I
ATOM 2571 N TRP I 201 -1.077 -28.800 -17.770 0.00 0.00
I
ATOM 2572 HN TRP I 201 -1.329 -28.245 -16.981 0.00 0.00
I
ATOM 2573 CA TRP I 201 -2.054 -28.730 -18.833 0.00 0.00
I
ATOM 2574 HA TRP I 201 -1.869 -29.417 -19.645 0.00 0.00
I
ATOM 2575 CB TRP I 201 -3.394 -29.193 -18.102 0.00 0.00
I
ATOM 2576 HB1 TRP I 201 -3.620 -28.558 -17.219 0.00 0.00
I
ATOM 2577 HB2 TRP I 201 -4.183 -28.936 -18.841 0.00 0.00
I
ATOM 2578 CG TRP I 201 -3.452 -30.680 -17.758 0.00 0.00
I
ATOM 2579 CD1 TRP I 201 -2.626 -31.735 -18.115 0.00 0.00
I
ATOM 2580 HD1 TRP I 201 -1.645 -31.468 -18.479 0.00 0.00
I
ATOM 2581 NE1 TRP I 201 -3.196 -32.896 -17.710 0.00 0.00
I
ATOM 2582 HE1 TRP I 201 -2.790 -33.737 -17.993 0.00 0.00
I
ATOM 2583 CE2 TRP I 201 -4.452 -32.595 -17.318 0.00 0.00
I
ATOM 2584 CD2 TRP I 201 -4.655 -31.245 -17.178 0.00 0.00
I
ATOM 2585 CE3 TRP I 201 -5.856 -30.693 -16.744 0.00 0.00
I
ATOM 2586 HE3 TRP I 201 -6.006 -29.625 -16.800 0.00 0.00
I
ATOM 2587 CZ3 TRP I 201 -6.801 -31.580 -16.352 0.00 0.00
I
ATOM 2588 HZ3 TRP I 201 -7.774 -31.220 -16.053 0.00 0.00
I
ATOM 2589 CZ2 TRP I 201 -5.427 -33.502 -16.867 0.00 0.00
I
ATOM 2590 HZ2 TRP I 201 -5.252 -34.567 -16.903 0.00 0.00
I
ATOM 2591 CH2 TRP I 201 -6.614 -32.974 -16.303 0.00 0.00
I

ATOM	2592	HH2	TRP	I	201	−7.462	−33.594	−16.052	0.00	0.00	I
ATOM	2593	C	TRP	I	201	−2.112	−27.248	−19.227	0.00	0.00	I
ATOM	2594	O	TRP	I	201	−2.181	−26.458	−18.319	0.00	0.00	I
ATOM	2595	N	PRO	I	202	−2.018	−26.761	−20.526	0.00	0.00	I
ATOM	2596	CD	PRO	I	202	−1.698	−27.526	−21.705	0.00	0.00	I
ATOM	2597	HD1	PRO	I	202	−0.676	−27.950	−21.598	0.00	0.00	I
ATOM	2598	HD2	PRO	I	202	−2.330	−28.411	−21.933	0.00	0.00	I
ATOM	2599	CA	PRO	I	202	−2.000	−25.303	−20.693	0.00	0.00	I
ATOM	2600	HA	PRO	I	202	−1.428	−24.917	−19.863	0.00	0.00	I
ATOM	2601	CB	PRO	I	202	−1.458	−25.060	−22.095	0.00	0.00	I
ATOM	2602	HB1	PRO	I	202	−0.349	−25.064	−22.150	0.00	0.00	I
ATOM	2603	HB2	PRO	I	202	−1.754	−24.110	−22.590	0.00	0.00	I
ATOM	2604	CG	PRO	I	202	−1.816	−26.413	−22.771	0.00	0.00	I
ATOM	2605	HG1	PRO	I	202	−1.142	−26.689	−23.610	0.00	0.00	I
ATOM	2606	HG2	PRO	I	202	−2.888	−26.616	−22.982	0.00	0.00	I
ATOM	2607	C	PRO	I	202	−3.276	−24.592	−20.480	0.00	0.00	I
ATOM	2608	O	PRO	I	202	−3.286	−23.353	−20.383	0.00	0.00	I
ATOM	2609	N	ASP	I	203	−4.378	−25.276	−20.370	0.00	0.00	I
ATOM	2610	HN	ASP	I	203	−4.292	−26.269	−20.361	0.00	0.00	I
ATOM	2611	CA	ASP	I	203	−5.729	−24.785	−20.403	0.00	0.00	I
ATOM	2612	HA	ASP	I	203	−5.843	−23.803	−20.837	0.00	0.00	I
ATOM	2613	CB	ASP	I	203	−6.471	−25.771	−21.342	0.00	0.00	I
ATOM	2614	HB1	ASP	I	203	−7.544	−25.490	−21.409	0.00	0.00	I
ATOM	2615	HB2	ASP	I	203	−6.015	−25.675	−22.350	0.00	0.00	I
ATOM	2616	CG	ASP	I	203	−6.459	−27.228	−20.897	0.00	0.00	I
ATOM	2617	OD1	ASP	I	203	−7.508	−27.782	−20.545	0.00	0.00	I
ATOM	2618	OD2	ASP	I	203	−5.351	−27.810	−20.793	0.00	0.00	I

ATOM 2619 C ASP I 203 -6.332 -24.695 -19.017 0.00 0.00
I
ATOM 2620 O ASP I 203 -7.505 -24.367 -18.799 0.00 0.00
I
ATOM 2621 N ASN I 204 -5.497 -24.841 -17.947 0.00 0.00
I
ATOM 2622 HN ASN I 204 -4.579 -25.185 -18.123 0.00 0.00
I
ATOM 2623 CA ASN I 204 -5.898 -24.733 -16.567 0.00 0.00
I
ATOM 2624 HA ASN I 204 -5.136 -25.299 -16.050 0.00 0.00
I
ATOM 2625 CB ASN I 204 -5.850 -23.162 -16.104 0.00 0.00
I
ATOM 2626 HB1 ASN I 204 -5.148 -22.600 -16.758 0.00 0.00
I
ATOM 2627 HB2 ASN I 204 -6.882 -22.751 -16.109 0.00 0.00
I
ATOM 2628 CG ASN I 204 -5.341 -22.982 -14.704 0.00 0.00
I
ATOM 2629 OD1 ASN I 204 -6.020 -22.904 -13.681 0.00 0.00
I
ATOM 2630 ND2 ASN I 204 -3.980 -23.066 -14.581 0.00 0.00
I
ATOM 2631 HD21 ASN I 204 -3.637 -23.052 -13.642 0.00 0.00
I
ATOM 2632 HD22 ASN I 204 -3.353 -23.441 -15.265 0.00 0.00
I
ATOM 2633 C ASN I 204 -7.072 -25.511 -16.054 0.00 0.00
I
ATOM 2634 O ASN I 204 -7.128 -26.681 -16.187 0.00 0.00
I
ATOM 2635 N LEU I 205 -8.093 -24.824 -15.505 0.00 0.00
I
ATOM 2636 HN LEU I 205 -7.870 -23.907 -15.183 0.00 0.00
I
ATOM 2637 CA LEU I 205 -9.340 -25.520 -15.160 0.00 0.00
I
ATOM 2638 HA LEU I 205 -9.291 -26.509 -15.591 0.00 0.00
I
ATOM 2639 CB LEU I 205 -9.630 -25.619 -13.614 0.00 0.00
I
ATOM 2640 HB1 LEU I 205 -8.684 -25.727 -13.041 0.00 0.00
I
ATOM 2641 HB2 LEU I 205 -10.219 -24.770 -13.206 0.00 0.00
I
ATOM 2642 CG LEU I 205 -10.425 -26.944 -13.245 0.00 0.00
I
ATOM 2643 HG LEU I 205 -11.017 -27.236 -14.139 0.00 0.00
I
ATOM 2644 CD1 LEU I 205 -11.441 -26.712 -12.034 0.00 0.00
I
ATOM 2645 HD11 LEU I 205 -12.144 -25.897 -12.311 0.00 0.00
I

```
ATOM   2646 HD12 LEU I 205     -12.038 -27.617 -11.793  0.00  0.00
I
ATOM   2647 HD13 LEU I 205     -10.819 -26.269 -11.227  0.00  0.00
I
ATOM   2648  CD2 LEU I 205      -9.367 -28.120 -12.987  0.00  0.00
I
ATOM   2649 HD21 LEU I 205      -9.852 -29.049 -12.617  0.00  0.00
I
ATOM   2650 HD22 LEU I 205      -8.767 -28.325 -13.899  0.00  0.00
I
ATOM   2651 HD23 LEU I 205      -8.630 -27.720 -12.258  0.00  0.00
I
ATOM   2652  C   LEU I 205     -10.486 -25.048 -15.990  0.00  0.00
I
ATOM   2653  O   LEU I 205     -11.619 -25.236 -15.675  0.00  0.00
I
ATOM   2654  N   MET I 206     -10.239 -24.443 -17.157  0.00  0.00
I
ATOM   2655  HN  MET I 206      -9.325 -24.400 -17.554  0.00  0.00
I
ATOM   2656  CA  MET I 206     -11.286 -23.862 -17.978  0.00  0.00
I
ATOM   2657  HA  MET I 206     -11.913 -23.193 -17.408  0.00  0.00
I
ATOM   2658  CB  MET I 206     -10.480 -23.134 -19.125  0.00  0.00
I
ATOM   2659  HB1 MET I 206      -9.802 -23.832 -19.661  0.00  0.00
I
ATOM   2660  HB2 MET I 206     -11.254 -22.900 -19.887  0.00  0.00
I
ATOM   2661  CG  MET I 206      -9.780 -21.841 -18.633  0.00  0.00
I
ATOM   2662  HG1 MET I 206     -10.532 -21.127 -18.234  0.00  0.00
I
ATOM   2663  HG2 MET I 206      -9.077 -22.042 -17.796  0.00  0.00
I
ATOM   2664  SD  MET I 206      -8.815 -20.983 -19.913  0.00  0.00
I
ATOM   2665  CE  MET I 206      -8.832 -19.349 -19.092  0.00  0.00
I
ATOM   2666  HE1 MET I 206      -8.225 -19.371 -18.162  0.00  0.00
I
ATOM   2667  HE2 MET I 206      -8.469 -18.543 -19.765  0.00  0.00
I
ATOM   2668  HE3 MET I 206      -9.907 -19.167 -18.882  0.00  0.00
I
ATOM   2669  C   MET I 206     -12.185 -24.926 -18.583  0.00  0.00
I
ATOM   2670  O   MET I 206     -13.364 -24.721 -18.654  0.00  0.00
I
ATOM   2671  N   SER I 207     -11.701 -26.157 -18.892  0.00  0.00
I
ATOM   2672  HN  SER I 207     -10.743 -26.414 -18.788  0.00  0.00
I
```

```
ATOM   2673  CA  SER I 207      -12.532 -27.155 -19.498  0.00  0.00
I
ATOM   2674  HA  SER I 207      -13.019 -26.759 -20.377  0.00  0.00
I
ATOM   2675  CB  SER I 207      -11.654 -28.272 -19.998  0.00  0.00
I
ATOM   2676  HB1 SER I 207      -11.107 -28.868 -19.237  0.00  0.00
I
ATOM   2677  HB2 SER I 207      -12.254 -28.975 -20.616  0.00  0.00
I
ATOM   2678  OG  SER I 207      -10.627 -27.684 -20.805  0.00  0.00
I
ATOM   2679  HG1 SER I 207      -10.051 -28.398 -21.089  0.00  0.00
I
ATOM   2680  C   SER I 207      -13.461 -27.745 -18.546  0.00  0.00
I
ATOM   2681  O   SER I 207      -14.706 -27.876 -18.780  0.00  0.00
I
ATOM   2682  N   TYR I 208      -12.985 -28.067 -17.330  0.00  0.00
I
ATOM   2683  HN  TYR I 208      -12.086 -27.733 -17.058  0.00  0.00
I
ATOM   2684  CA  TYR I 208      -13.685 -28.756 -16.294  0.00  0.00
I
ATOM   2685  HA  TYR I 208      -14.412 -29.402 -16.764  0.00  0.00
I
ATOM   2686  CB  TYR I 208      -12.691 -29.584 -15.405  0.00  0.00
I
ATOM   2687  HB1 TYR I 208      -12.053 -28.808 -14.931  0.00  0.00
I
ATOM   2688  HB2 TYR I 208      -13.223 -30.102 -14.578  0.00  0.00
I
ATOM   2689  CG  TYR I 208      -11.964 -30.605 -16.222  0.00  0.00
I
ATOM   2690  CD1 TYR I 208      -12.736 -31.728 -16.455  0.00  0.00
I
ATOM   2691  HD1 TYR I 208      -13.712 -31.906 -16.027  0.00  0.00
I
ATOM   2692  CE1 TYR I 208      -12.224 -32.713 -17.359  0.00  0.00
I
ATOM   2693  HE1 TYR I 208      -12.718 -33.655 -17.545  0.00  0.00
I
ATOM   2694  CZ  TYR I 208      -11.158 -32.407 -18.180  0.00  0.00
I
ATOM   2695  OH  TYR I 208      -10.712 -33.308 -19.180  0.00  0.00
I
ATOM   2696  HH  TYR I 208       -9.868 -32.995 -19.513  0.00  0.00
I
ATOM   2697  CD2 TYR I 208      -10.754 -30.402 -16.873  0.00  0.00
I
ATOM   2698  HD2 TYR I 208      -10.317 -29.436 -16.667  0.00  0.00
I
ATOM   2699  CE2 TYR I 208      -10.355 -31.242 -17.929  0.00  0.00
I
```

ATOM 2700 HE2 TYR I 208 −9.436 −31.025 −18.452 0.00 0.00
I
ATOM 2701 C TYR I 208 −14.537 −27.788 −15.415 0.00 0.00
I
ATOM 2702 O TYR I 208 −15.133 −28.234 −14.396 0.00 0.00
I
ATOM 2703 N VAL I 209 −14.778 −26.539 −15.861 0.00 0.00
I
ATOM 2704 HN VAL I 209 −14.243 −26.138 −16.601 0.00 0.00
I
ATOM 2705 CA VAL I 209 −15.421 −25.482 −15.114 0.00 0.00
I
ATOM 2706 HA VAL I 209 −14.769 −25.265 −14.281 0.00 0.00
I
ATOM 2707 CB VAL I 209 −15.636 −24.337 −16.116 0.00 0.00
I
ATOM 2708 HB VAL I 209 −14.579 −24.107 −16.373 0.00 0.00
I
ATOM 2709 CG1 VAL I 209 −16.409 −24.819 −17.367 0.00 0.00
I
ATOM 2710 HG11 VAL I 209 −15.836 −25.578 −17.941 0.00 0.00
I
ATOM 2711 HG12 VAL I 209 −17.437 −25.139 −17.093 0.00 0.00
I
ATOM 2712 HG13 VAL I 209 −16.495 −24.006 −18.120 0.00 0.00
I
ATOM 2713 CG2 VAL I 209 −16.266 −23.108 −15.441 0.00 0.00
I
ATOM 2714 HG21 VAL I 209 −15.635 −22.949 −14.540 0.00 0.00
I
ATOM 2715 HG22 VAL I 209 −16.258 −22.176 −16.046 0.00 0.00
I
ATOM 2716 HG23 VAL I 209 −17.298 −23.433 −15.191 0.00 0.00
I
ATOM 2717 C VAL I 209 −16.767 −25.919 −14.374 0.00 0.00
I
ATOM 2718 O VAL I 209 −17.040 −25.462 −13.246 0.00 0.00
I
ATOM 2719 N GLY I 210 −17.610 −26.708 −15.016 0.00 0.00
I
ATOM 2720 HN GLY I 210 −17.315 −27.077 −15.894 0.00 0.00
I
ATOM 2721 CA GLY I 210 −18.826 −27.264 −14.414 0.00 0.00
I
ATOM 2722 HA1 GLY I 210 −19.670 −27.231 −15.087 0.00 0.00
I
ATOM 2723 HA2 GLY I 210 −19.023 −26.632 −13.561 0.00 0.00
I
ATOM 2724 C GLY I 210 −18.829 −28.627 −13.786 0.00 0.00
I
ATOM 2725 O GLY I 210 −19.815 −29.047 −13.182 0.00 0.00
I
ATOM 2726 N ASP I 211 −17.784 −29.460 −13.890 0.00 0.00
I

ATOM 2727 HN ASP I 211 −16.975 −29.057 −14.311 0.00 0.00
I
ATOM 2728 CA ASP I 211 −17.606 −30.813 −13.391 0.00 0.00
I
ATOM 2729 HA ASP I 211 −18.430 −31.349 −13.838 0.00 0.00
I
ATOM 2730 CB ASP I 211 −16.336 −31.549 −13.986 0.00 0.00
I
ATOM 2731 HB1 ASP I 211 −15.447 −30.934 −13.725 0.00 0.00
I
ATOM 2732 HB2 ASP I 211 −16.157 −32.493 −13.429 0.00 0.00
I
ATOM 2733 CG ASP I 211 −16.359 −31.809 −15.525 0.00 0.00
I
ATOM 2734 OD1 ASP I 211 −16.330 −33.038 −15.830 0.00 0.00
I
ATOM 2735 OD2 ASP I 211 −16.402 −30.890 −16.376 0.00 0.00
I
ATOM 2736 C ASP I 211 −17.730 −30.868 −11.829 0.00 0.00
I
ATOM 2737 O ASP I 211 −16.933 −30.241 −11.093 0.00 0.00
I
ATOM 2738 N PRO I 212 −18.598 −31.703 −11.184 0.00 0.00
I
ATOM 2739 CD PRO I 212 −19.767 −32.344 −11.810 0.00 0.00
I
ATOM 2740 HD1 PRO I 212 −20.156 −31.740 −12.657 0.00 0.00
I
ATOM 2741 HD2 PRO I 212 −19.435 −33.356 −12.126 0.00 0.00
I
ATOM 2742 CA PRO I 212 −18.721 −31.577 −9.722 0.00 0.00
I
ATOM 2743 HA PRO I 212 −18.948 −30.545 −9.499 0.00 0.00
I
ATOM 2744 CB PRO I 212 −19.919 −32.560 −9.424 0.00 0.00
I
ATOM 2745 HB1 PRO I 212 −20.414 −32.185 −8.504 0.00 0.00
I
ATOM 2746 HB2 PRO I 212 −19.573 −33.615 −9.387 0.00 0.00
I
ATOM 2747 CG PRO I 212 −20.752 −32.381 −10.705 0.00 0.00
I
ATOM 2748 HG1 PRO I 212 −21.228 −31.377 −10.671 0.00 0.00
I
ATOM 2749 HG2 PRO I 212 −21.509 −33.161 −10.933 0.00 0.00
I
ATOM 2750 C PRO I 212 −17.602 −32.126 −8.890 0.00 0.00
I
ATOM 2751 O PRO I 212 −17.113 −31.475 −7.939 0.00 0.00
I
ATOM 2752 N THR I 213 −17.087 −33.234 −9.291 0.00 0.00
I
ATOM 2753 HN THR I 213 −17.471 −33.678 −10.096 0.00 0.00
I

```
ATOM   2754  CA  THR I 213     -16.009 -33.898  -8.479  0.00  0.00
I
ATOM   2755  HA  THR I 213     -16.157 -33.866  -7.409  0.00  0.00
I
ATOM   2756  CB  THR I 213     -15.790 -35.395  -8.835  0.00  0.00
I
ATOM   2757  HB  THR I 213     -14.980 -35.811  -8.198  0.00  0.00
I
ATOM   2758  OG1 THR I 213     -15.459 -35.606 -10.198  0.00  0.00
I
ATOM   2759  HG1 THR I 213     -14.527 -35.818 -10.105  0.00  0.00
I
ATOM   2760  CG2 THR I 213     -17.048 -36.214  -8.486  0.00  0.00
I
ATOM   2761 HG21 THR I 213     -16.767 -37.283  -8.373  0.00  0.00
I
ATOM   2762 HG22 THR I 213     -17.504 -35.900  -7.523  0.00  0.00
I
ATOM   2763 HG23 THR I 213     -17.744 -36.217  -9.352  0.00  0.00
I
ATOM   2764  C   THR I 213     -14.650 -33.184  -8.662  0.00  0.00
I
ATOM   2765  O   THR I 213     -13.889 -32.814  -7.767  0.00  0.00
I
ATOM   2766  N   VAL I 214     -14.468 -32.565  -9.868  0.00  0.00
I
ATOM   2767  HN  VAL I 214     -15.028 -32.788 -10.662  0.00  0.00
I
ATOM   2768  CA  VAL I 214     -13.364 -31.670 -10.151  0.00  0.00
I
ATOM   2769  HA  VAL I 214     -12.382 -32.086  -9.977  0.00  0.00
I
ATOM   2770  CB  VAL I 214     -13.300 -31.467 -11.690  0.00  0.00
I
ATOM   2771  HB  VAL I 214     -13.994 -30.705 -12.106  0.00  0.00
I
ATOM   2772  CG1 VAL I 214     -12.007 -30.789 -11.955  0.00  0.00
I
ATOM   2773 HG11 VAL I 214     -11.931 -30.510 -13.028  0.00  0.00
I
ATOM   2774 HG12 VAL I 214     -11.823 -29.869 -11.361  0.00  0.00
I
ATOM   2775 HG13 VAL I 214     -11.193 -31.501 -11.703  0.00  0.00
I
ATOM   2776  CG2 VAL I 214     -13.236 -32.871 -12.307  0.00  0.00
I
ATOM   2777 HG21 VAL I 214     -12.574 -33.629 -11.836  0.00  0.00
I
ATOM   2778 HG22 VAL I 214     -14.315 -33.129 -12.349  0.00  0.00
I
ATOM   2779 HG23 VAL I 214     -12.834 -32.778 -13.339  0.00  0.00
I
ATOM   2780  C   VAL I 214     -13.473 -30.285  -9.480  0.00  0.00
I
```

ATOM 2781 O VAL I 214 −12.487 −29.795 −8.943 0.00 0.00
I
ATOM 2782 N ASN I 215 −14.653 −29.641 −9.321 0.00 0.00
I
ATOM 2783 HN ASN I 215 −15.487 −29.972 −9.755 0.00 0.00
I
ATOM 2784 CA ASN I 215 −14.787 −28.520 −8.425 0.00 0.00
I
ATOM 2785 HA ASN I 215 −13.984 −27.814 −8.576 0.00 0.00
I
ATOM 2786 CB ASN I 215 −16.070 −27.752 −8.554 0.00 0.00
I
ATOM 2787 HB1 ASN I 215 −17.028 −28.313 −8.510 0.00 0.00
I
ATOM 2788 HB2 ASN I 215 −16.075 −27.035 −7.705 0.00 0.00
I
ATOM 2789 CG ASN I 215 −16.009 −26.918 −9.850 0.00 0.00
I
ATOM 2790 OD1 ASN I 215 −15.644 −25.756 −9.765 0.00 0.00
I
ATOM 2791 ND2 ASN I 215 −16.516 −27.463 −10.954 0.00 0.00
I
ATOM 2792 HD21 ASN I 215 −16.651 −26.947 −11.800 0.00 0.00
I
ATOM 2793 HD22 ASN I 215 −16.845 −28.406 −11.004 0.00 0.00
I
ATOM 2794 C ASN I 215 −14.605 −28.906 −6.871 0.00 0.00
I
ATOM 2795 O ASN I 215 −13.848 −28.285 −6.087 0.00 0.00
I
ATOM 2796 N LEU I 216 −15.348 −29.986 −6.368 0.00 0.00
I
ATOM 2797 HN LEU I 216 −15.907 −30.522 −6.995 0.00 0.00
I
ATOM 2798 CA LEU I 216 −15.229 −30.453 −4.978 0.00 0.00
I
ATOM 2799 HA LEU I 216 −15.672 −29.638 −4.425 0.00 0.00
I
ATOM 2800 CB LEU I 216 −15.966 −31.808 −4.714 0.00 0.00
I
ATOM 2801 HB1 LEU I 216 −15.988 −32.442 −5.626 0.00 0.00
I
ATOM 2802 HB2 LEU I 216 −15.470 −32.490 −3.991 0.00 0.00
I
ATOM 2803 CG LEU I 216 −17.332 −31.556 −4.085 0.00 0.00
I
ATOM 2804 HG LEU I 216 −17.802 −30.701 −4.617 0.00 0.00
I
ATOM 2805 CD1 LEU I 216 −18.265 −32.824 −4.169 0.00 0.00
I
ATOM 2806 HD11 LEU I 216 −17.801 −33.654 −3.594 0.00 0.00
I
ATOM 2807 HD12 LEU I 216 −19.209 −32.448 −3.721 0.00 0.00
I

ATOM 2808 HD13 LEU I 216 -18.615 -33.195 -5.156 0.00 0.00
I
ATOM 2809 CD2 LEU I 216 -17.231 -31.173 -2.571 0.00 0.00
I
ATOM 2810 HD21 LEU I 216 -18.237 -30.964 -2.146 0.00 0.00
I
ATOM 2811 HD22 LEU I 216 -16.792 -31.931 -1.887 0.00 0.00
I
ATOM 2812 HD23 LEU I 216 -16.598 -30.283 -2.370 0.00 0.00
I
ATOM 2813 C LEU I 216 -13.857 -30.772 -4.416 0.00 0.00
I
ATOM 2814 O LEU I 216 -13.513 -30.414 -3.331 0.00 0.00
I
ATOM 2815 N VAL I 217 -13.023 -31.503 -5.204 0.00 0.00
I
ATOM 2816 HN VAL I 217 -13.333 -31.900 -6.065 0.00 0.00
I
ATOM 2817 CA VAL I 217 -11.748 -31.883 -4.783 0.00 0.00
I
ATOM 2818 HA VAL I 217 -11.768 -32.267 -3.773 0.00 0.00
I
ATOM 2819 CB VAL I 217 -11.069 -32.893 -5.786 0.00 0.00
I
ATOM 2820 HB VAL I 217 -10.983 -32.444 -6.799 0.00 0.00
I
ATOM 2821 CG1 VAL I 217 -9.563 -33.420 -5.403 0.00 0.00
I
ATOM 2822 HG11 VAL I 217 -9.598 -33.899 -4.401 0.00 0.00
I
ATOM 2823 HG12 VAL I 217 -9.299 -34.139 -6.208 0.00 0.00
I
ATOM 2824 HG13 VAL I 217 -8.825 -32.591 -5.364 0.00 0.00
I
ATOM 2825 CG2 VAL I 217 -11.885 -34.141 -5.846 0.00 0.00
I
ATOM 2826 HG21 VAL I 217 -11.558 -34.893 -6.596 0.00 0.00
I
ATOM 2827 HG22 VAL I 217 -11.871 -34.556 -4.815 0.00 0.00
I
ATOM 2828 HG23 VAL I 217 -12.963 -33.951 -6.038 0.00 0.00
I
ATOM 2829 C VAL I 217 -10.787 -30.686 -4.691 0.00 0.00
I
ATOM 2830 O VAL I 217 -10.096 -30.512 -3.729 0.00 0.00
I
ATOM 2831 N ILE I 218 -10.798 -29.865 -5.756 0.00 0.00
I
ATOM 2832 HN ILE I 218 -11.348 -30.164 -6.532 0.00 0.00
I
ATOM 2833 CA ILE I 218 -9.892 -28.747 -5.915 0.00 0.00
I
ATOM 2834 HA ILE I 218 -8.855 -29.011 -5.772 0.00 0.00
I

ATOM 2835 CB ILE I 218 -9.988 -28.197 -7.251 0.00 0.00
I
ATOM 2836 HB ILE I 218 -11.016 -27.885 -7.535 0.00 0.00
I
ATOM 2837 CG2 ILE I 218 -9.169 -26.940 -7.328 0.00 0.00
I
ATOM 2838 HG21 ILE I 218 -9.597 -26.115 -6.720 0.00 0.00
I
ATOM 2839 HG22 ILE I 218 -8.134 -27.048 -6.937 0.00 0.00
I
ATOM 2840 HG23 ILE I 218 -8.956 -26.616 -8.369 0.00 0.00
I
ATOM 2841 CG1 ILE I 218 -9.466 -29.292 -8.222 0.00 0.00
I
ATOM 2842 HG11 ILE I 218 -10.094 -30.205 -8.143 0.00 0.00
I
ATOM 2843 HG12 ILE I 218 -9.581 -28.872 -9.244 0.00 0.00
I
ATOM 2844 CD ILE I 218 -7.945 -29.573 -8.206 0.00 0.00
I
ATOM 2845 HD1 ILE I 218 -7.341 -28.676 -8.461 0.00 0.00
I
ATOM 2846 HD2 ILE I 218 -7.620 -29.882 -7.190 0.00 0.00
I
ATOM 2847 HD3 ILE I 218 -7.729 -30.327 -8.994 0.00 0.00
I
ATOM 2848 C ILE I 218 -10.142 -27.719 -4.838 0.00 0.00
I
ATOM 2849 O ILE I 218 -9.263 -27.053 -4.226 0.00 0.00
I
ATOM 2850 N THR I 219 -11.442 -27.465 -4.569 0.00 0.00
I
ATOM 2851 HN THR I 219 -12.168 -27.898 -5.098 0.00 0.00
I
ATOM 2852 CA THR I 219 -11.827 -26.485 -3.597 0.00 0.00
I
ATOM 2853 HA THR I 219 -11.146 -25.647 -3.582 0.00 0.00
I
ATOM 2854 CB THR I 219 -13.101 -25.852 -3.790 0.00 0.00
I
ATOM 2855 HB THR I 219 -13.419 -25.116 -3.021 0.00 0.00
I
ATOM 2856 OG1 THR I 219 -14.147 -26.759 -3.880 0.00 0.00
I
ATOM 2857 HG1 THR I 219 -13.990 -27.298 -4.659 0.00 0.00
I
ATOM 2858 CG2 THR I 219 -13.203 -25.042 -5.127 0.00 0.00
I
ATOM 2859 HG21 THR I 219 -12.504 -24.179 -5.123 0.00 0.00
I
ATOM 2860 HG22 THR I 219 -12.943 -25.709 -5.976 0.00 0.00
I
ATOM 2861 HG23 THR I 219 -14.241 -24.737 -5.380 0.00 0.00
I

ATOM 2862 C THR I 219 −11.643 −27.000 −2.155 0.00 0.00
I
ATOM 2863 O THR I 219 −11.470 −26.191 −1.195 0.00 0.00
I
ATOM 2864 N PHE I 220 −11.693 −28.293 −1.909 0.00 0.00
I
ATOM 2865 HN PHE I 220 −11.969 −28.859 −2.681 0.00 0.00
I
ATOM 2866 CA PHE I 220 −11.391 −28.878 −0.619 0.00 0.00
I
ATOM 2867 HA PHE I 220 −12.006 −28.352 0.097 0.00 0.00
I
ATOM 2868 CB PHE I 220 −11.857 −30.378 −0.523 0.00 0.00
I
ATOM 2869 HB1 PHE I 220 −12.954 −30.465 −0.676 0.00 0.00
I
ATOM 2870 HB2 PHE I 220 −11.405 −30.956 −1.358 0.00 0.00
I
ATOM 2871 CG PHE I 220 −11.526 −31.034 0.751 0.00 0.00
I
ATOM 2872 CD1 PHE I 220 −12.296 −30.967 1.910 0.00 0.00
I
ATOM 2873 HD1 PHE I 220 −13.141 −30.296 1.936 0.00 0.00
I
ATOM 2874 CE1 PHE I 220 −11.879 −31.452 3.115 0.00 0.00
I
ATOM 2875 HE1 PHE I 220 −12.384 −31.163 4.025 0.00 0.00
I
ATOM 2876 CZ PHE I 220 −10.820 −32.322 3.128 0.00 0.00
I
ATOM 2877 HZ PHE I 220 −10.588 −32.898 4.011 0.00 0.00
I
ATOM 2878 CD2 PHE I 220 −10.404 −31.885 0.782 0.00 0.00
I
ATOM 2879 HD2 PHE I 220 −9.726 −32.067 −0.038 0.00 0.00
I
ATOM 2880 CE2 PHE I 220 −10.136 −32.560 1.971 0.00 0.00
I
ATOM 2881 HE2 PHE I 220 −9.298 −33.239 2.023 0.00 0.00
I
ATOM 2882 C PHE I 220 −9.904 −28.758 −0.292 0.00 0.00
I
ATOM 2883 O PHE I 220 −9.526 −28.483 0.884 0.00 0.00
I
ATOM 2884 N LEU I 221 −9.028 −28.925 −1.240 0.00 0.00
I
ATOM 2885 HN LEU I 221 −9.296 −29.291 −2.127 0.00 0.00
I
ATOM 2886 CA LEU I 221 −7.596 −28.793 −1.082 0.00 0.00
I
ATOM 2887 HA LEU I 221 −7.252 −29.416 −0.269 0.00 0.00
I
ATOM 2888 CB LEU I 221 −6.858 −29.252 −2.390 0.00 0.00
I

ATOM 2889 HB1 LEU I 221 -7.317 -28.617 -3.178 0.00 0.00
I
ATOM 2890 HB2 LEU I 221 -5.759 -29.143 -2.266 0.00 0.00
I
ATOM 2891 CG LEU I 221 -7.056 -30.713 -2.779 0.00 0.00
I
ATOM 2892 HG LEU I 221 -8.149 -30.905 -2.725 0.00 0.00
I
ATOM 2893 CD1 LEU I 221 -6.420 -31.041 -4.111 0.00 0.00
I
ATOM 2894 HD11 LEU I 221 -5.318 -30.904 -4.112 0.00 0.00
I
ATOM 2895 HD12 LEU I 221 -6.760 -32.047 -4.440 0.00 0.00
I
ATOM 2896 HD13 LEU I 221 -6.836 -30.353 -4.879 0.00 0.00
I
ATOM 2897 CD2 LEU I 221 -6.443 -31.686 -1.745 0.00 0.00
I
ATOM 2898 HD21 LEU I 221 -6.431 -32.727 -2.133 0.00 0.00
I
ATOM 2899 HD22 LEU I 221 -5.408 -31.355 -1.513 0.00 0.00
I
ATOM 2900 HD23 LEU I 221 -7.022 -31.732 -0.798 0.00 0.00
I
ATOM 2901 C LEU I 221 -7.082 -27.403 -0.756 0.00 0.00
I
ATOM 2902 O LEU I 221 -6.259 -27.191 0.159 0.00 0.00
I
ATOM 2903 N PHE I 222 -7.628 -26.471 -1.508 0.00 0.00
I
ATOM 2904 HN PHE I 222 -8.172 -26.646 -2.325 0.00 0.00
I
ATOM 2905 CA PHE I 222 -7.277 -25.045 -1.345 0.00 0.00
I
ATOM 2906 HA PHE I 222 -6.210 -24.958 -1.484 0.00 0.00
I
ATOM 2907 CB PHE I 222 -8.106 -24.129 -2.271 0.00 0.00
I
ATOM 2908 HB1 PHE I 222 -9.177 -24.401 -2.161 0.00 0.00
I
ATOM 2909 HB2 PHE I 222 -7.965 -23.070 -1.964 0.00 0.00
I
ATOM 2910 CG PHE I 222 -7.883 -24.166 -3.761 0.00 0.00
I
ATOM 2911 CD1 PHE I 222 -8.955 -23.703 -4.457 0.00 0.00
I
ATOM 2912 HD1 PHE I 222 -9.827 -23.450 -3.873 0.00 0.00
I
ATOM 2913 CE1 PHE I 222 -8.810 -23.611 -5.815 0.00 0.00
I
ATOM 2914 HE1 PHE I 222 -9.617 -23.325 -6.473 0.00 0.00
I
ATOM 2915 CZ PHE I 222 -7.647 -24.110 -6.493 0.00 0.00
I

ATOM 2916 HZ PHE I 222 -7.508 -24.109 -7.564 0.00 0.00
I
ATOM 2917 CD2 PHE I 222 -6.660 -24.566 -4.358 0.00 0.00
I
ATOM 2918 HD2 PHE I 222 -5.854 -24.901 -3.723 0.00 0.00
I
ATOM 2919 CE2 PHE I 222 -6.550 -24.543 -5.687 0.00 0.00
I
ATOM 2920 HE2 PHE I 222 -5.640 -24.776 -6.220 0.00 0.00
I
ATOM 2921 C PHE I 222 -7.582 -24.579 0.040 0.00 0.00
I
ATOM 2922 O PHE I 222 -6.750 -23.876 0.642 0.00 0.00
I
ATOM 2923 N ILE I 223 -8.820 -24.823 0.512 0.00 0.00
I
ATOM 2924 HN ILE I 223 -9.410 -25.396 -0.051 0.00 0.00
I
ATOM 2925 CA ILE I 223 -9.420 -24.373 1.844 0.00 0.00
I
ATOM 2926 HA ILE I 223 -9.063 -23.360 1.960 0.00 0.00
I
ATOM 2927 CB ILE I 223 -10.946 -24.420 1.957 0.00 0.00
I
ATOM 2928 HB ILE I 223 -11.266 -25.484 1.927 0.00 0.00
I
ATOM 2929 CG2 ILE I 223 -11.465 -23.722 3.230 0.00 0.00
I
ATOM 2930 HG21 ILE I 223 -11.168 -22.651 3.227 0.00 0.00
I
ATOM 2931 HG22 ILE I 223 -12.574 -23.752 3.174 0.00 0.00
I
ATOM 2932 HG23 ILE I 223 -11.196 -24.230 4.180 0.00 0.00
I
ATOM 2933 CG1 ILE I 223 -11.509 -23.628 0.740 0.00 0.00
I
ATOM 2934 HG11 ILE I 223 -11.213 -24.240 -0.139 0.00 0.00
I
ATOM 2935 HG12 ILE I 223 -12.610 -23.650 0.886 0.00 0.00
I
ATOM 2936 CD ILE I 223 -11.052 -22.190 0.588 0.00 0.00
I
ATOM 2937 HD1 ILE I 223 -9.948 -22.066 0.622 0.00 0.00
I
ATOM 2938 HD2 ILE I 223 -11.435 -21.626 -0.289 0.00 0.00
I
ATOM 2939 HD3 ILE I 223 -11.445 -21.653 1.478 0.00 0.00
I
ATOM 2940 C ILE I 223 -8.733 -25.055 2.985 0.00 0.00
I
ATOM 2941 O ILE I 223 -8.287 -24.453 4.024 0.00 0.00
I
ATOM 2942 N THR I 224 -8.452 -26.367 2.821 0.00 0.00
I

ATOM 2943 HN THR I 224 −8.803 −26.849 2.022 0.00 0.00
I
ATOM 2944 CA THR I 224 −7.693 −27.108 3.795 0.00 0.00
I
ATOM 2945 HA THR I 224 −8.156 −26.917 4.752 0.00 0.00
I
ATOM 2946 CB THR I 224 −7.710 −28.658 3.590 0.00 0.00
I
ATOM 2947 HB THR I 224 −7.116 −29.007 2.718 0.00 0.00
I
ATOM 2948 OG1 THR I 224 −9.046 −29.177 3.613 0.00 0.00
I
ATOM 2949 HG1 THR I 224 −9.417 −29.051 2.737 0.00 0.00
I
ATOM 2950 CG2 THR I 224 −6.989 −29.304 4.795 0.00 0.00
I
ATOM 2951 HG21 THR I 224 −5.891 −29.447 4.707 0.00 0.00
I
ATOM 2952 HG22 THR I 224 −7.252 −28.856 5.776 0.00 0.00
I
ATOM 2953 HG23 THR I 224 −7.464 −30.307 4.842 0.00 0.00
I
ATOM 2954 C THR I 224 −6.227 −26.729 4.005 0.00 0.00
I
ATOM 2955 O THR I 224 −5.701 −26.577 5.075 0.00 0.00
I
ATOM 2956 N GLY I 225 −5.464 −26.567 2.911 0.00 0.00
I
ATOM 2957 HN GLY I 225 −5.801 −26.695 1.982 0.00 0.00
I
ATOM 2958 CA GLY I 225 −4.040 −26.097 2.928 0.00 0.00
I
ATOM 2959 HA1 GLY I 225 −3.740 −26.152 1.892 0.00 0.00
I
ATOM 2960 HA2 GLY I 225 −3.456 −26.751 3.558 0.00 0.00
I
ATOM 2961 C GLY I 225 −3.922 −24.624 3.424 0.00 0.00
I
ATOM 2962 O GLY I 225 −2.808 −24.169 3.710 0.00 0.00
I
ATOM 2963 N GLY I 226 −5.021 −23.769 3.451 0.00 0.00
I
ATOM 2964 HN GLY I 226 −5.927 −24.022 3.119 0.00 0.00
I
ATOM 2965 CA GLY I 226 −5.007 −22.305 3.872 0.00 0.00
I
ATOM 2966 HA1 GLY I 226 −5.818 −21.789 3.381 0.00 0.00
I
ATOM 2967 HA2 GLY I 226 −4.003 −21.909 3.818 0.00 0.00
I
ATOM 2968 C GLY I 226 −5.346 −22.076 5.281 0.00 0.00
I
ATOM 2969 O GLY I 226 −4.658 −21.303 6.016 0.00 0.00
I

ATOM 2970 N ILE I 227 -6.315 -22.827 5.833 0.00 0.00
I
ATOM 2971 HN ILE I 227 -6.902 -23.333 5.206 0.00 0.00
I
ATOM 2972 CA ILE I 227 -6.612 -22.830 7.253 0.00 0.00
I
ATOM 2973 HA ILE I 227 -6.402 -21.840 7.632 0.00 0.00
I
ATOM 2974 CB ILE I 227 -8.003 -23.096 7.611 0.00 0.00
I
ATOM 2975 HB ILE I 227 -8.211 -22.744 8.644 0.00 0.00
I
ATOM 2976 CG2 ILE I 227 -8.920 -22.294 6.586 0.00 0.00
I
ATOM 2977 HG21 ILE I 227 -8.791 -22.635 5.537 0.00 0.00
I
ATOM 2978 HG22 ILE I 227 -9.929 -22.595 6.941 0.00 0.00
I
ATOM 2979 HG23 ILE I 227 -8.629 -21.222 6.631 0.00 0.00
I
ATOM 2980 CG1 ILE I 227 -8.343 -24.637 7.553 0.00 0.00
I
ATOM 2981 HG11 ILE I 227 -8.382 -24.949 6.488 0.00 0.00
I
ATOM 2982 HG12 ILE I 227 -7.615 -25.244 8.133 0.00 0.00
I
ATOM 2983 CD ILE I 227 -9.778 -24.974 8.100 0.00 0.00
I
ATOM 2984 HD1 ILE I 227 -10.567 -24.579 7.425 0.00 0.00
I
ATOM 2985 HD2 ILE I 227 -9.943 -26.063 8.247 0.00 0.00
I
ATOM 2986 HD3 ILE I 227 -9.760 -24.397 9.049 0.00 0.00
I
ATOM 2987 C ILE I 227 -5.647 -23.660 8.150 0.00 0.00
I
ATOM 2988 O ILE I 227 -5.204 -23.262 9.251 0.00 0.00
I
ATOM 2989 N GLY I 228 -5.126 -24.876 7.658 0.00 0.00
I
ATOM 2990 HN GLY I 228 -5.589 -25.132 6.813 0.00 0.00
I
ATOM 2991 CA GLY I 228 -3.949 -25.675 8.189 0.00 0.00
I
ATOM 2992 HA1 GLY I 228 -3.252 -24.897 8.464 0.00 0.00
I
ATOM 2993 HA2 GLY I 228 -3.616 -26.325 7.394 0.00 0.00
I
ATOM 2994 C GLY I 228 -4.190 -26.580 9.308 0.00 0.00
I
ATOM 2995 O GLY I 228 -5.245 -26.477 9.970 0.00 0.00
I
ATOM 2996 N PHE I 229 -3.251 -27.440 9.707 0.00 0.00
I

ATOM 2997 HN PHE I 229 -2.371 -27.438 9.239 0.00 0.00
I
ATOM 2998 CA PHE I 229 -3.215 -28.454 10.845 0.00 0.00
I
ATOM 2999 HA PHE I 229 -3.741 -29.343 10.530 0.00 0.00
I
ATOM 3000 CB PHE I 229 -1.684 -28.870 11.169 0.00 0.00
I
ATOM 3001 HB1 PHE I 229 -1.375 -29.276 10.183 0.00 0.00
I
ATOM 3002 HB2 PHE I 229 -1.127 -27.937 11.399 0.00 0.00
I
ATOM 3003 CG PHE I 229 -1.511 -30.100 12.041 0.00 0.00
I
ATOM 3004 CD1 PHE I 229 -0.975 -29.945 13.334 0.00 0.00
I
ATOM 3005 HD1 PHE I 229 -0.775 -28.990 13.796 0.00 0.00
I
ATOM 3006 CE1 PHE I 229 -0.497 -31.023 14.076 0.00 0.00
I
ATOM 3007 HE1 PHE I 229 -0.042 -30.822 15.035 0.00 0.00
I
ATOM 3008 CZ PHE I 229 -0.765 -32.274 13.576 0.00 0.00
I
ATOM 3009 HZ PHE I 229 -0.380 -33.177 14.026 0.00 0.00
I
ATOM 3010 CD2 PHE I 229 -1.677 -31.392 11.517 0.00 0.00
I
ATOM 3011 HD2 PHE I 229 -2.192 -31.618 10.595 0.00 0.00
I
ATOM 3012 CE2 PHE I 229 -1.246 -32.454 12.298 0.00 0.00
I
ATOM 3013 HE2 PHE I 229 -1.391 -33.462 11.939 0.00 0.00
I
ATOM 3014 C PHE I 229 -3.868 -28.053 12.134 0.00 0.00
I
ATOM 3015 O PHE I 229 -4.742 -28.750 12.633 0.00 0.00
I
ATOM 3016 N THR I 230 -3.541 -26.915 12.706 0.00 0.00
I
ATOM 3017 HN THR I 230 -3.052 -26.298 12.095 0.00 0.00
I
ATOM 3018 CA THR I 230 -3.704 -26.573 14.121 0.00 0.00
I
ATOM 3019 HA THR I 230 -3.506 -27.420 14.761 0.00 0.00
I
ATOM 3020 CB THR I 230 -2.777 -25.421 14.580 0.00 0.00
I
ATOM 3021 HB THR I 230 -3.048 -25.048 15.591 0.00 0.00
I
ATOM 3022 OG1 THR I 230 -2.918 -24.314 13.654 0.00 0.00
I
ATOM 3023 HG1 THR I 230 -2.334 -23.637 14.003 0.00 0.00
I

ATOM 3024 CG2 THR I 230 -1.278 -25.864 14.537 0.00 0.00
I
ATOM 3025 HG21 THR I 230 -1.108 -26.531 15.409 0.00 0.00
I
ATOM 3026 HG22 THR I 230 -1.151 -26.422 13.585 0.00 0.00
I
ATOM 3027 HG23 THR I 230 -0.581 -25.011 14.687 0.00 0.00
I
ATOM 3028 C THR I 230 -5.157 -26.260 14.313 0.00 0.00
I
ATOM 3029 O THR I 230 -5.731 -26.675 15.311 0.00 0.00
I
ATOM 3030 N VAL I 231 -5.755 -25.500 13.365 0.00 0.00
I
ATOM 3031 HN VAL I 231 -5.262 -25.166 12.566 0.00 0.00
I
ATOM 3032 CA VAL I 231 -7.162 -25.161 13.319 0.00 0.00
I
ATOM 3033 HA VAL I 231 -7.369 -24.653 14.249 0.00 0.00
I
ATOM 3034 CB VAL I 231 -7.494 -24.178 12.251 0.00 0.00
I
ATOM 3035 HB VAL I 231 -7.126 -24.668 11.324 0.00 0.00
I
ATOM 3036 CG1 VAL I 231 -9.002 -23.586 12.263 0.00 0.00
I
ATOM 3037 HG11 VAL I 231 -9.091 -22.905 13.136 0.00 0.00
I
ATOM 3038 HG12 VAL I 231 -9.214 -22.998 11.344 0.00 0.00
I
ATOM 3039 HG13 VAL I 231 -9.796 -24.360 12.327 0.00 0.00
I
ATOM 3040 CG2 VAL I 231 -6.519 -23.041 12.347 0.00 0.00
I
ATOM 3041 HG21 VAL I 231 -6.754 -22.260 11.591 0.00 0.00
I
ATOM 3042 HG22 VAL I 231 -6.483 -22.464 13.295 0.00 0.00
I
ATOM 3043 HG23 VAL I 231 -5.518 -23.311 11.947 0.00 0.00
I
ATOM 3044 C VAL I 231 -8.074 -26.354 13.230 0.00 0.00
I
ATOM 3045 O VAL I 231 -8.985 -26.527 13.981 0.00 0.00
I
ATOM 3046 N LEU I 232 -7.783 -27.294 12.381 0.00 0.00
I
ATOM 3047 HN LEU I 232 -7.143 -27.136 11.633 0.00 0.00
I
ATOM 3048 CA LEU I 232 -8.494 -28.528 12.325 0.00 0.00
I
ATOM 3049 HA LEU I 232 -9.548 -28.318 12.217 0.00 0.00
I
ATOM 3050 CB LEU I 232 -7.987 -29.373 11.142 0.00 0.00
I

ATOM 3051 HB1 LEU I 232 -6.923 -29.656 11.293 0.00 0.00
I
ATOM 3052 HB2 LEU I 232 -8.566 -30.319 11.071 0.00 0.00
I
ATOM 3053 CG LEU I 232 -8.134 -28.729 9.765 0.00 0.00
I
ATOM 3054 HG LEU I 232 -8.100 -27.623 9.868 0.00 0.00
I
ATOM 3055 CD1 LEU I 232 -7.087 -29.223 8.764 0.00 0.00
I
ATOM 3056 HD11 LEU I 232 -7.427 -30.258 8.546 0.00 0.00
I
ATOM 3057 HD12 LEU I 232 -7.188 -28.536 7.897 0.00 0.00
I
ATOM 3058 HD13 LEU I 232 -6.075 -29.191 9.222 0.00 0.00
I
ATOM 3059 CD2 LEU I 232 -9.582 -28.921 9.184 0.00 0.00
I
ATOM 3060 HD21 LEU I 232 -10.074 -27.987 9.529 0.00 0.00
I
ATOM 3061 HD22 LEU I 232 -9.576 -29.015 8.077 0.00 0.00
I
ATOM 3062 HD23 LEU I 232 -10.133 -29.800 9.581 0.00 0.00
I
ATOM 3063 C LEU I 232 -8.317 -29.423 13.524 0.00 0.00
I
ATOM 3064 O LEU I 232 -9.229 -30.096 13.960 0.00 0.00
I
ATOM 3065 N PHE I 233 -7.084 -29.570 14.121 0.00 0.00
I
ATOM 3066 HN PHE I 233 -6.298 -29.190 13.639 0.00 0.00
I
ATOM 3067 CA PHE I 233 -6.835 -30.252 15.379 0.00 0.00
I
ATOM 3068 HA PHE I 233 -7.243 -31.251 15.328 0.00 0.00
I
ATOM 3069 CB PHE I 233 -5.306 -30.336 15.629 0.00 0.00
I
ATOM 3070 HB1 PHE I 233 -4.878 -30.570 14.631 0.00 0.00
I
ATOM 3071 HB2 PHE I 233 -4.949 -29.321 15.904 0.00 0.00
I
ATOM 3072 CG PHE I 233 -4.717 -31.211 16.785 0.00 0.00
I
ATOM 3073 CD1 PHE I 233 -5.261 -32.449 17.212 0.00 0.00
I
ATOM 3074 HD1 PHE I 233 -6.120 -32.890 16.728 0.00 0.00
I
ATOM 3075 CE1 PHE I 233 -4.808 -33.037 18.381 0.00 0.00
I
ATOM 3076 HE1 PHE I 233 -5.314 -33.922 18.736 0.00 0.00
I
ATOM 3077 CZ PHE I 233 -3.766 -32.422 19.126 0.00 0.00
I

ATOM 3078 HZ PHE I 233 -3.362 -32.904 20.004 0.00 0.00
I
ATOM 3079 CD2 PHE I 233 -3.674 -30.631 17.524 0.00 0.00
I
ATOM 3080 HD2 PHE I 233 -3.195 -29.720 17.197 0.00 0.00
I
ATOM 3081 CE2 PHE I 233 -3.200 -31.214 18.694 0.00 0.00
I
ATOM 3082 HE2 PHE I 233 -2.466 -30.730 19.322 0.00 0.00
I
ATOM 3083 C PHE I 233 -7.553 -29.610 16.584 0.00 0.00
I
ATOM 3084 O PHE I 233 -8.000 -30.400 17.410 0.00 0.00
I
ATOM 3085 N ASP I 234 -7.608 -28.240 16.632 0.00 0.00
I
ATOM 3086 HN ASP I 234 -7.038 -27.686 16.030 0.00 0.00
I
ATOM 3087 CA ASP I 234 -8.229 -27.525 17.751 0.00 0.00
I
ATOM 3088 HA ASP I 234 -7.731 -27.848 18.653 0.00 0.00
I
ATOM 3089 CB ASP I 234 -7.909 -26.068 17.590 0.00 0.00
I
ATOM 3090 HB1 ASP I 234 -6.954 -25.913 17.043 0.00 0.00
I
ATOM 3091 HB2 ASP I 234 -8.692 -25.593 16.961 0.00 0.00
I
ATOM 3092 CG ASP I 234 -7.701 -25.274 18.964 0.00 0.00
I
ATOM 3093 OD1 ASP I 234 -6.747 -25.579 19.723 0.00 0.00
I
ATOM 3094 OD2 ASP I 234 -8.492 -24.292 19.214 0.00 0.00
I
ATOM 3095 C ASP I 234 -9.797 -27.757 17.755 0.00 0.00
I
ATOM 3096 O ASP I 234 -10.374 -28.021 18.824 0.00 0.00
I
ATOM 3097 N VAL I 235 -10.465 -27.862 16.574 0.00 0.00
I
ATOM 3098 HN VAL I 235 -10.017 -27.570 15.733 0.00 0.00
I
ATOM 3099 CA VAL I 235 -11.845 -28.249 16.332 0.00 0.00
I
ATOM 3100 HA VAL I 235 -12.491 -27.542 16.832 0.00 0.00
I
ATOM 3101 CB VAL I 235 -12.139 -28.264 14.802 0.00 0.00
I
ATOM 3102 HB VAL I 235 -11.430 -28.964 14.309 0.00 0.00
I
ATOM 3103 CG1 VAL I 235 -13.512 -28.916 14.426 0.00 0.00
I
ATOM 3104 HG11 VAL I 235 -13.592 -29.136 13.340 0.00 0.00
I

ATOM   3105 HG12 VAL I 235   -13.685 -29.867 14.974 0.00 0.00
I
ATOM   3106 HG13 VAL I 235   -14.305 -28.204 14.740 0.00 0.00
I
ATOM   3107 CG2 VAL I 235   -12.163 -26.829 14.252 0.00 0.00
I
ATOM   3108 HG21 VAL I 235   -11.179 -26.327 14.367 0.00 0.00
I
ATOM   3109 HG22 VAL I 235   -12.444 -26.863 13.178 0.00 0.00
I
ATOM   3110 HG23 VAL I 235   -13.059 -26.290 14.627 0.00 0.00
I
ATOM   3111 C   VAL I 235   -12.105 -29.647 16.942 0.00 0.00
I
ATOM   3112 O   VAL I 235   -12.998 -29.905 17.824 0.00 0.00
I
ATOM   3113 N   MET I 236   -11.286 -30.711 16.650 0.00 0.00
I
ATOM   3114 HN MET I 236   -10.614 -30.571 15.927 0.00 0.00
I
ATOM   3115 CA MET I 236   -11.263 -32.054 17.156 0.00 0.00
I
ATOM   3116 HA MET I 236   -12.223 -32.515 16.976 0.00 0.00
I
ATOM   3117 CB MET I 236   -10.117 -32.782 16.340 0.00 0.00
I
ATOM   3118 HB1 MET I 236   -10.282 -32.761 15.242 0.00 0.00
I
ATOM   3119 HB2 MET I 236   -9.095 -32.378 16.504 0.00 0.00
I
ATOM   3120 CG MET I 236   -10.134 -34.295 16.671 0.00 0.00
I
ATOM   3121 HG1 MET I 236   -9.681 -34.370 17.683 0.00 0.00
I
ATOM   3122 HG2 MET I 236   -11.197 -34.616 16.696 0.00 0.00
I
ATOM   3123 SD MET I 236   -9.202 -35.321 15.545 0.00 0.00
I
ATOM   3124 CE MET I 236   -9.744 -36.801 16.328 0.00 0.00
I
ATOM   3125 HE1 MET I 236   -8.943 -37.561 16.214 0.00 0.00
I
ATOM   3126 HE2 MET I 236   -9.777 -36.719 17.436 0.00 0.00
I
ATOM   3127 HE3 MET I 236   -10.739 -37.178 16.007 0.00 0.00
I
ATOM   3128 C   MET I 236   -10.954 -32.241 18.643 0.00 0.00
I
ATOM   3129 O   MET I 236   -11.466 -33.094 19.375 0.00 0.00
I
ATOM   3130 N   LYS I 237   -9.932 -31.443 19.174 0.00 0.00
I
ATOM   3131 HN LYS I 237   -9.351 -30.888 18.585 0.00 0.00
I

ATOM 3132 CA LYS I 237 -9.414 -31.889 20.516 0.00 0.00
I
ATOM 3133 HA LYS I 237 -9.604 -32.947 20.614 0.00 0.00
I
ATOM 3134 CB LYS I 237 -7.923 -31.312 20.555 0.00 0.00
I
ATOM 3135 HB1 LYS I 237 -7.402 -31.631 19.627 0.00 0.00
I
ATOM 3136 HB2 LYS I 237 -8.009 -30.213 20.695 0.00 0.00
I
ATOM 3137 CG LYS I 237 -7.123 -31.775 21.785 0.00 0.00
I
ATOM 3138 HG1 LYS I 237 -7.741 -31.656 22.700 0.00 0.00
I
ATOM 3139 HG2 LYS I 237 -6.834 -32.841 21.668 0.00 0.00
I
ATOM 3140 CD LYS I 237 -5.961 -30.899 21.981 0.00 0.00
I
ATOM 3141 HD1 LYS I 237 -5.194 -31.415 22.596 0.00 0.00
I
ATOM 3142 HD2 LYS I 237 -5.453 -30.729 21.008 0.00 0.00
I
ATOM 3143 CE LYS I 237 -6.235 -29.485 22.529 0.00 0.00
I
ATOM 3144 HE1 LYS I 237 -6.797 -28.989 21.710 0.00 0.00
I
ATOM 3145 HE2 LYS I 237 -6.871 -29.483 23.440 0.00 0.00
I
ATOM 3146 NZ LYS I 237 -5.094 -28.590 22.790 0.00 0.00
I
ATOM 3147 HZ1 LYS I 237 -5.454 -27.621 22.682 0.00 0.00
I
ATOM 3148 HZ2 LYS I 237 -4.659 -28.727 23.725 0.00 0.00
I
ATOM 3149 HZ3 LYS I 237 -4.402 -28.772 22.035 0.00 0.00
I
ATOM 3150 C LYS I 237 -10.163 -31.217 21.646 0.00 0.00
I
ATOM 3151 O LYS I 237 -10.428 -31.839 22.694 0.00 0.00
I
ATOM 3152 N ASN I 238 -10.665 -29.961 21.522 0.00 0.00
I
ATOM 3153 HN ASN I 238 -10.465 -29.352 20.757 0.00 0.00
I
ATOM 3154 CA ASN I 238 -11.401 -29.249 22.526 0.00 0.00
I
ATOM 3155 HA ASN I 238 -10.994 -29.553 23.479 0.00 0.00
I
ATOM 3156 CB ASN I 238 -11.179 -27.670 22.313 0.00 0.00
I
ATOM 3157 HB1 ASN I 238 -11.431 -27.515 21.242 0.00 0.00
I
ATOM 3158 HB2 ASN I 238 -11.962 -27.092 22.848 0.00 0.00
I

ATOM 3159 CG ASN I 238 -9.680 -27.410 22.461 0.00 0.00
I
ATOM 3160 OD1 ASN I 238 -9.040 -27.918 23.379 0.00 0.00
I
ATOM 3161 ND2 ASN I 238 -9.030 -26.607 21.584 0.00 0.00
I
ATOM 3162 HD21 ASN I 238 -8.066 -26.370 21.703 0.00 0.00
I
ATOM 3163 HD22 ASN I 238 -9.583 -26.163 20.879 0.00 0.00
I
ATOM 3164 C ASN I 238 -12.893 -29.534 22.468 0.00 0.00
I
ATOM 3165 O ASN I 238 -13.592 -29.643 23.515 0.00 0.00
I
ATOM 3166 N ARG I 239 -13.427 -29.664 21.223 0.00 0.00
I
ATOM 3167 HN ARG I 239 -12.779 -29.576 20.471 0.00 0.00
I
ATOM 3168 CA ARG I 239 -14.746 -30.172 20.926 0.00 0.00
I
ATOM 3169 HA ARG I 239 -14.756 -30.110 19.848 0.00 0.00
I
ATOM 3170 CB ARG I 239 -15.018 -31.549 21.495 0.00 0.00
I
ATOM 3171 HB1 ARG I 239 -15.141 -31.580 22.599 0.00 0.00
I
ATOM 3172 HB2 ARG I 239 -15.961 -32.055 21.198 0.00 0.00
I
ATOM 3173 CG ARG I 239 -13.878 -32.544 21.177 0.00 0.00
I
ATOM 3174 HG1 ARG I 239 -13.598 -32.433 20.108 0.00 0.00
I
ATOM 3175 HG2 ARG I 239 -12.932 -32.280 21.695 0.00 0.00
I
ATOM 3176 CD ARG I 239 -14.135 -34.013 21.533 0.00 0.00
I
ATOM 3177 HD1 ARG I 239 -14.300 -34.342 22.581 0.00 0.00
I
ATOM 3178 HD2 ARG I 239 -15.043 -34.432 21.048 0.00 0.00
I
ATOM 3179 NE ARG I 239 -12.974 -34.788 20.954 0.00 0.00
I
ATOM 3180 HE ARG I 239 -12.220 -34.271 20.549 0.00 0.00
I
ATOM 3181 CZ ARG I 239 -13.045 -36.022 20.492 0.00 0.00
I
ATOM 3182 NH1 ARG I 239 -14.054 -36.855 20.471 0.00 0.00
I
ATOM 3183 HH11 ARG I 239 -13.952 -37.834 20.296 0.00 0.00
I
ATOM 3184 HH12 ARG I 239 -14.842 -36.535 20.997 0.00 0.00
I
ATOM 3185 NH2 ARG I 239 -11.857 -36.471 19.985 0.00 0.00
I

```
ATOM   3186 HH21 ARG I 239     −11.880 −37.408  19.638  0.00  0.00
I
ATOM   3187 HH22 ARG I 239     −11.024 −35.945  20.158  0.00  0.00
I
ATOM   3188  C   ARG I 239     −15.933 −29.199  21.287  0.00  0.00
I
ATOM   3189  O   ARG I 239     −17.093 −29.471  20.948  0.00  0.00
I
ATOM   3190  N   ARG I 240     −15.675 −28.114  22.083  0.00  0.00
I
ATOM   3191  HN  ARG I 240     −14.750 −27.902  22.388  0.00  0.00
I
ATOM   3192  CA  ARG I 240     −16.604 −27.229  22.618  0.00  0.00
I
ATOM   3193  HA  ARG I 240     −17.614 −27.404  22.277  0.00  0.00
I
ATOM   3194  CB  ARG I 240     −16.600 −27.207  24.220  0.00  0.00
I
ATOM   3195  HB1 ARG I 240     −17.003 −28.222  24.422  0.00  0.00
I
ATOM   3196  HB2 ARG I 240     −15.547 −27.191  24.573  0.00  0.00
I
ATOM   3197  CG  ARG I 240     −17.530 −26.243  24.929  0.00  0.00
I
ATOM   3198  HG1 ARG I 240     −17.204 −25.181  24.930  0.00  0.00
I
ATOM   3199  HG2 ARG I 240     −18.432 −26.254  24.282  0.00  0.00
I
ATOM   3200  CD  ARG I 240     −17.922 −26.804  26.247  0.00  0.00
I
ATOM   3201  HD1 ARG I 240     −18.313 −27.828  26.063  0.00  0.00
I
ATOM   3202  HD2 ARG I 240     −17.063 −26.880  26.948  0.00  0.00
I
ATOM   3203  NE  ARG I 240     −18.909 −25.920  26.939  0.00  0.00
I
ATOM   3204  HE  ARG I 240     −19.201 −25.098  26.449  0.00  0.00
I
ATOM   3205  CZ  ARG I 240     −19.298 −26.178  28.154  0.00  0.00
I
ATOM   3206  NH1 ARG I 240     −18.707 −27.095  28.904  0.00  0.00
I
ATOM   3207 HH11 ARG I 240     −19.068 −27.353  29.801  0.00  0.00
I
ATOM   3208 HH12 ARG I 240     −17.842 −27.421  28.523  0.00  0.00
I
ATOM   3209  NH2 ARG I 240     −20.170 −25.324  28.622  0.00  0.00
I
ATOM   3210 HH21 ARG I 240     −20.365 −25.361  29.603  0.00  0.00
I
ATOM   3211 HH22 ARG I 240     −20.287 −24.478  28.103  0.00  0.00
I
ATOM   3212  C   ARG I 240     −16.279 −25.840  22.194  0.00  0.00
I
```

ATOM 3213 O ARG I 240 −15.096 −25.468 22.107 0.00 0.00
I
ATOM 3214 N PHE I 241 −17.391 −25.042 21.845 0.00 0.00
I
ATOM 3215 HN PHE I 241 −18.313 −25.392 21.990 0.00 0.00
I
ATOM 3216 CA PHE I 241 −17.241 −23.808 21.127 0.00 0.00
I
ATOM 3217 HA PHE I 241 −16.617 −24.089 20.291 0.00 0.00
I
ATOM 3218 CB PHE I 241 −18.614 −23.253 20.769 0.00 0.00
I
ATOM 3219 HB1 PHE I 241 −19.349 −23.976 20.354 0.00 0.00
I
ATOM 3220 HB2 PHE I 241 −19.000 −22.790 21.702 0.00 0.00
I
ATOM 3221 CG PHE I 241 −18.521 −22.152 19.702 0.00 0.00
I
ATOM 3222 CD1 PHE I 241 −18.059 −22.540 18.465 0.00 0.00
I
ATOM 3223 HD1 PHE I 241 −17.772 −23.553 18.228 0.00 0.00
I
ATOM 3224 CE1 PHE I 241 −17.939 −21.631 17.388 0.00 0.00
I
ATOM 3225 HE1 PHE I 241 −17.642 −21.941 16.397 0.00 0.00
I
ATOM 3226 CZ PHE I 241 −18.291 −20.281 17.588 0.00 0.00
I
ATOM 3227 HZ PHE I 241 −18.024 −19.566 16.824 0.00 0.00
I
ATOM 3228 CD2 PHE I 241 −18.929 −20.820 19.848 0.00 0.00
I
ATOM 3229 HD2 PHE I 241 −19.333 −20.466 20.784 0.00 0.00
I
ATOM 3230 CE2 PHE I 241 −18.788 −19.865 18.823 0.00 0.00
I
ATOM 3231 HE2 PHE I 241 −19.107 −18.844 18.969 0.00 0.00
I
ATOM 3232 C PHE I 241 −16.462 −22.752 21.874 0.00 0.00
I
ATOM 3233 O PHE I 241 −15.666 −21.932 21.335 0.00 0.00
I
ATOM 3234 N LYS I 242 −16.597 −22.691 23.242 0.00 0.00
I
ATOM 3235 HN LYS I 242 −17.125 −23.414 23.680 0.00 0.00
I
ATOM 3236 CA LYS I 242 −15.975 −21.625 24.050 0.00 0.00
I
ATOM 3237 HA LYS I 242 −16.092 −20.677 23.547 0.00 0.00
I
ATOM 3238 CB LYS I 242 −16.768 −21.373 25.379 0.00 0.00
I
ATOM 3239 HB1 LYS I 242 −16.309 −20.479 25.853 0.00 0.00
I

ATOM 3240 HB2 LYS I 242 -17.830 -21.160 25.133 0.00 0.00
I
ATOM 3241 CG LYS I 242 -16.573 -22.433 26.490 0.00 0.00
I
ATOM 3242 HG1 LYS I 242 -16.944 -23.438 26.197 0.00 0.00
I
ATOM 3243 HG2 LYS I 242 -15.476 -22.463 26.663 0.00 0.00
I
ATOM 3244 CD LYS I 242 -17.199 -21.884 27.771 0.00 0.00
I
ATOM 3245 HD1 LYS I 242 -16.823 -20.842 27.853 0.00 0.00
I
ATOM 3246 HD2 LYS I 242 -18.298 -21.775 27.643 0.00 0.00
I
ATOM 3247 CE LYS I 242 -17.013 -22.681 29.060 0.00 0.00
I
ATOM 3248 HE1 LYS I 242 -17.746 -22.302 29.804 0.00 0.00
I
ATOM 3249 HE2 LYS I 242 -17.214 -23.763 28.905 0.00 0.00
I
ATOM 3250 NZ LYS I 242 -15.671 -22.559 29.567 0.00 0.00
I
ATOM 3251 HZ1 LYS I 242 -15.398 -21.574 29.759 0.00 0.00
I
ATOM 3252 HZ2 LYS I 242 -15.515 -23.030 30.481 0.00 0.00
I
ATOM 3253 HZ3 LYS I 242 -14.946 -22.980 28.951 0.00 0.00
I
ATOM 3254 C LYS I 242 -14.558 -21.900 24.287 0.00 0.00
I
ATOM 3255 O LYS I 242 -13.902 -20.987 24.762 0.00 0.00
I
ATOM 3256 N THR I 243 -14.111 -23.076 23.962 0.00 0.00
I
ATOM 3257 HN THR I 243 -14.736 -23.808 23.701 0.00 0.00
I
ATOM 3258 CA THR I 243 -12.805 -23.504 24.422 0.00 0.00
I
ATOM 3259 HA THR I 243 -12.379 -22.797 25.118 0.00 0.00
I
ATOM 3260 CB THR I 243 -12.973 -24.790 25.190 0.00 0.00
I
ATOM 3261 HB THR I 243 -13.237 -25.603 24.480 0.00 0.00
I
ATOM 3262 OG1 THR I 243 -13.978 -24.663 26.137 0.00 0.00
I
ATOM 3263 HG1 THR I 243 -14.123 -25.555 26.460 0.00 0.00
I
ATOM 3264 CG2 THR I 243 -11.693 -25.077 25.989 0.00 0.00
I
ATOM 3265 HG21 THR I 243 -11.723 -26.076 26.476 0.00 0.00
I
ATOM 3266 HG22 THR I 243 -10.889 -25.261 25.244 0.00 0.00
I

ATOM   3267 HG23 THR I 243   -11.355 -24.247 26.645 0.00 0.00
I
ATOM   3268 C   THR I 243   -11.822 -23.658 23.266 0.00 0.00
I
ATOM   3269 O   THR I 243   -10.626 -23.817 23.526 0.00 0.00
I
ATOM   3270 N   PHE I 244   -12.275 -23.529 21.959 0.00 0.00
I
ATOM   3271 HN PHE I 244   -13.225 -23.531 21.657 0.00 0.00
I
ATOM   3272 CA PHE I 244   -11.382 -23.328 20.834 0.00 0.00
I
ATOM   3273 HA PHE I 244   -10.673 -24.142 20.823 0.00 0.00
I
ATOM   3274 CB PHE I 244   -12.263 -23.247 19.516 0.00 0.00
I
ATOM   3275 HB1 PHE I 244   -13.040 -22.466 19.663 0.00 0.00
I
ATOM   3276 HB2 PHE I 244   -11.560 -23.031 18.683 0.00 0.00
I
ATOM   3277 CG PHE I 244   -13.073 -24.476 19.224 0.00 0.00
I
ATOM   3278 CD1 PHE I 244   -12.521 -25.775 19.236 0.00 0.00
I
ATOM   3279 HD1 PHE I 244   -11.504 -25.849 19.591 0.00 0.00
I
ATOM   3280 CE1 PHE I 244   -13.383 -26.916 18.952 0.00 0.00
I
ATOM   3281 HE1 PHE I 244   -12.951 -27.901 19.046 0.00 0.00
I
ATOM   3282 CZ PHE I 244   -14.680 -26.652 18.449 0.00 0.00
I
ATOM   3283 HZ PHE I 244   -15.368 -27.419 18.126 0.00 0.00
I
ATOM   3284 CD2 PHE I 244   -14.333 -24.224 18.763 0.00 0.00
I
ATOM   3285 HD2 PHE I 244   -14.793 -23.254 18.646 0.00 0.00
I
ATOM   3286 CE2 PHE I 244   -15.105 -25.321 18.305 0.00 0.00
I
ATOM   3287 HE2 PHE I 244   -16.042 -25.132 17.801 0.00 0.00
I
ATOM   3288 C   PHE I 244   -10.689 -22.046 20.855 0.00 0.00
I
ATOM   3289 O   PHE I 244   -11.264 -21.020 21.113 0.00 0.00
I
ATOM   3290 N   SER I 245   -9.361 -22.080 20.603 0.00 0.00
I
ATOM   3291 HN SER I 245   -8.926 -22.928 20.310 0.00 0.00
I
ATOM   3292 CA SER I 245   -8.449 -20.931 20.656 0.00 0.00
I
ATOM   3293 HA SER I 245   -8.289 -20.799 21.716 0.00 0.00
I

```
ATOM   3294  CB  SER I 245      -7.033 -21.345  20.118  0.00  0.00
I
ATOM   3295  HB1 SER I 245      -6.724 -22.326  20.538  0.00  0.00
I
ATOM   3296  HB2 SER I 245      -7.204 -21.365  19.020  0.00  0.00
I
ATOM   3297  OG  SER I 245      -5.893 -20.403  20.299  0.00  0.00
I
ATOM   3298  HG1 SER I 245      -5.192 -21.016  20.532  0.00  0.00
I
ATOM   3299  C   SER I 245      -9.026 -19.754  19.842  0.00  0.00
I
ATOM   3300  O   SER I 245      -9.887 -19.848  18.998  0.00  0.00
I
ATOM   3301  N   LEU I 246      -8.524 -18.566  20.084  0.00  0.00
I
ATOM   3302  HN  LEU I 246      -7.746 -18.529  20.706  0.00  0.00
I
ATOM   3303  CA  LEU I 246      -9.021 -17.350  19.453  0.00  0.00
I
ATOM   3304  HA  LEU I 246     -10.097 -17.371  19.542  0.00  0.00
I
ATOM   3305  CB  LEU I 246      -8.469 -16.005  20.061  0.00  0.00
I
ATOM   3306  HB1 LEU I 246      -7.381 -16.094  20.272  0.00  0.00
I
ATOM   3307  HB2 LEU I 246      -8.552 -15.181  19.321  0.00  0.00
I
ATOM   3308  CG  LEU I 246      -9.223 -15.505  21.297  0.00  0.00
I
ATOM   3309  HG  LEU I 246      -9.537 -16.307  21.999  0.00  0.00
I
ATOM   3310  CD1 LEU I 246      -8.388 -14.496  22.084  0.00  0.00
I
ATOM   3311 HD11 LEU I 246      -9.063 -13.760  22.572  0.00  0.00
I
ATOM   3312 HD12 LEU I 246      -7.672 -14.946  22.804  0.00  0.00
I
ATOM   3313 HD13 LEU I 246      -7.715 -14.081  21.303  0.00  0.00
I
ATOM   3314  CD2 LEU I 246     -10.566 -14.780  20.961  0.00  0.00
I
ATOM   3315 HD21 LEU I 246     -11.139 -14.401  21.834  0.00  0.00
I
ATOM   3316 HD22 LEU I 246     -10.259 -13.955  20.284  0.00  0.00
I
ATOM   3317 HD23 LEU I 246     -11.240 -15.422  20.353  0.00  0.00
I
ATOM   3318  C   LEU I 246      -8.745 -17.354  17.948  0.00  0.00
I
ATOM   3319  O   LEU I 246      -9.607 -17.143  17.090  0.00  0.00
I
ATOM   3320  N   HSE I 247      -7.495 -17.702  17.593  0.00  0.00
I
```

ATOM 3321 HN HSE I 247 -6.827 -17.886 18.310 0.00 0.00
I
ATOM 3322 CA HSE I 247 -7.113 -18.057 16.237 0.00 0.00
I
ATOM 3323 HA HSE I 247 -7.256 -17.162 15.650 0.00 0.00
I
ATOM 3324 CB HSE I 247 -5.714 -18.678 16.145 0.00 0.00
I
ATOM 3325 HB1 HSE I 247 -4.978 -17.850 16.226 0.00 0.00
I
ATOM 3326 HB2 HSE I 247 -5.729 -19.416 16.976 0.00 0.00
I
ATOM 3327 ND1 HSE I 247 -4.863 -20.653 14.776 0.00 0.00
I
ATOM 3328 CG HSE I 247 -5.312 -19.320 14.826 0.00 0.00
I
ATOM 3329 CE1 HSE I 247 -4.572 -20.860 13.553 0.00 0.00
I
ATOM 3330 HE1 HSE I 247 -4.056 -21.784 13.292 0.00 0.00
I
ATOM 3331 NE2 HSE I 247 -4.721 -19.770 12.750 0.00 0.00
I
ATOM 3332 HE2 HSE I 247 -4.554 -19.595 11.779 0.00 0.00
I
ATOM 3333 CD2 HSE I 247 -5.256 -18.824 13.546 0.00 0.00
I
ATOM 3334 HD2 HSE I 247 -5.421 -17.809 13.205 0.00 0.00
I
ATOM 3335 C HSE I 247 -8.062 -18.998 15.541 0.00 0.00
I
ATOM 3336 O HSE I 247 -8.394 -18.758 14.382 0.00 0.00
I
ATOM 3337 N THR I 248 -8.561 -20.105 16.207 0.00 0.00
I
ATOM 3338 HN THR I 248 -8.408 -20.273 17.178 0.00 0.00
I
ATOM 3339 CA THR I 248 -9.400 -21.091 15.707 0.00 0.00
I
ATOM 3340 HA THR I 248 -8.894 -21.407 14.807 0.00 0.00
I
ATOM 3341 CB THR I 248 -9.627 -22.212 16.621 0.00 0.00
I
ATOM 3342 HB THR I 248 -10.119 -21.786 17.521 0.00 0.00
I
ATOM 3343 OG1 THR I 248 -8.422 -22.723 17.153 0.00 0.00
I
ATOM 3344 HG1 THR I 248 -8.596 -23.371 17.840 0.00 0.00
I
ATOM 3345 CG2 THR I 248 -10.517 -23.410 16.160 0.00 0.00
I
ATOM 3346 HG21 THR I 248 -10.557 -24.262 16.872 0.00 0.00
I
ATOM 3347 HG22 THR I 248 -11.496 -23.057 15.772 0.00 0.00
I

ATOM 3348 HG23 THR I 248 -9.928 -23.805 15.305 0.00 0.00
I
ATOM 3349 C THR I 248 -10.735 -20.441 15.420 0.00 0.00
I
ATOM 3350 O THR I 248 -11.335 -20.568 14.348 0.00 0.00
I
ATOM 3351 N LYS I 249 -11.285 -19.640 16.294 0.00 0.00
I
ATOM 3352 HN LYS I 249 -10.805 -19.379 17.128 0.00 0.00
I
ATOM 3353 CA LYS I 249 -12.667 -19.215 16.261 0.00 0.00
I
ATOM 3354 HA LYS I 249 -13.388 -20.014 16.170 0.00 0.00
I
ATOM 3355 CB LYS I 249 -13.082 -18.538 17.566 0.00 0.00
I
ATOM 3356 HB1 LYS I 249 -12.799 -19.180 18.428 0.00 0.00
I
ATOM 3357 HB2 LYS I 249 -12.548 -17.578 17.730 0.00 0.00
I
ATOM 3358 CG LYS I 249 -14.614 -18.305 17.819 0.00 0.00
I
ATOM 3359 HG1 LYS I 249 -15.131 -17.674 17.065 0.00 0.00
I
ATOM 3360 HG2 LYS I 249 -15.087 -19.308 17.755 0.00 0.00
I
ATOM 3361 CD LYS I 249 -14.939 -17.765 19.193 0.00 0.00
I
ATOM 3362 HD1 LYS I 249 -14.062 -17.254 19.645 0.00 0.00
I
ATOM 3363 HD2 LYS I 249 -15.751 -17.032 18.998 0.00 0.00
I
ATOM 3364 CE LYS I 249 -15.431 -18.864 20.092 0.00 0.00
I
ATOM 3365 HE1 LYS I 249 -16.348 -19.296 19.638 0.00 0.00
I
ATOM 3366 HE2 LYS I 249 -14.630 -19.613 20.273 0.00 0.00
I
ATOM 3367 NZ LYS I 249 -15.741 -18.217 21.455 0.00 0.00
I
ATOM 3368 HZ1 LYS I 249 -16.124 -18.950 22.086 0.00 0.00
I
ATOM 3369 HZ2 LYS I 249 -14.880 -17.777 21.836 0.00 0.00
I
ATOM 3370 HZ3 LYS I 249 -16.437 -17.459 21.306 0.00 0.00
I
ATOM 3371 C LYS I 249 -12.816 -18.221 15.107 0.00 0.00
I
ATOM 3372 O LYS I 249 -13.758 -18.316 14.314 0.00 0.00
I
ATOM 3373 N LEU I 250 -11.807 -17.281 14.920 0.00 0.00
I
ATOM 3374 HN LEU I 250 -11.065 -17.216 15.582 0.00 0.00
I

ATOM   3375 CA LEU I 250   -11.763 -16.236 13.902 0.00 0.00
I
ATOM   3376 HA LEU I 250   -12.767 -15.851 13.798 0.00 0.00
I
ATOM   3377 CB LEU I 250   -10.619 -15.186 14.026 0.00 0.00
I
ATOM   3378 HB1 LEU I 250   -9.609 -15.641 13.946 0.00 0.00
I
ATOM   3379 HB2 LEU I 250   -10.614 -14.609 13.077 0.00 0.00
I
ATOM   3380 CG LEU I 250   -10.652 -14.109 15.144 0.00 0.00
I
ATOM   3381 HG LEU I 250   -11.124 -14.539 16.053 0.00 0.00
I
ATOM   3382 CD1 LEU I 250   -9.265 -13.643 15.603 0.00 0.00
I
ATOM   3383 HD11 LEU I 250   -8.623 -14.547 15.675 0.00 0.00
I
ATOM   3384 HD12 LEU I 250   -8.814 -12.995 14.821 0.00 0.00
I
ATOM   3385 HD13 LEU I 250   -9.177 -12.973 16.485 0.00 0.00
I
ATOM   3386 CD2 LEU I 250   -11.457 -12.809 14.727 0.00 0.00
I
ATOM   3387 HD21 LEU I 250   -12.474 -13.167 14.997 0.00 0.00
I
ATOM   3388 HD22 LEU I 250   -11.169 -11.896 15.290 0.00 0.00
I
ATOM   3389 HD23 LEU I 250   -11.274 -12.701 13.637 0.00 0.00
I
ATOM   3390 C   LEU I 250   -11.489 -16.920 12.525 0.00 0.00
I
ATOM   3391 O   LEU I 250   -12.157 -16.580 11.539 0.00 0.00
I
ATOM   3392 N   MET I 251   -10.499 -17.801 12.445 0.00 0.00
I
ATOM   3393 HN MET I 251   -9.985 -18.166 13.217 0.00 0.00
I
ATOM   3394 CA MET I 251   -10.146 -18.386 11.103 0.00 0.00
I
ATOM   3395 HA MET I 251   -9.986 -17.530 10.464 0.00 0.00
I
ATOM   3396 CB MET I 251   -8.822 -19.090 11.194 0.00 0.00
I
ATOM   3397 HB1 MET I 251   -8.084 -18.334 11.538 0.00 0.00
I
ATOM   3398 HB2 MET I 251   -8.830 -19.829 12.023 0.00 0.00
I
ATOM   3399 CG MET I 251   -8.363 -19.746   9.820 0.00 0.00
I
ATOM   3400 HG1 MET I 251   -8.984 -20.663   9.733 0.00 0.00
I
ATOM   3401 HG2 MET I 251   -8.769 -19.239   8.919 0.00 0.00
I

```
ATOM   3402  SD  MET I 251      -6.623 -20.194   9.796  0.00  0.00
I
ATOM   3403  CE  MET I 251      -6.096 -18.552   9.639  0.00  0.00
I
ATOM   3404  HE1 MET I 251      -5.005 -18.643   9.451  0.00  0.00
I
ATOM   3405  HE2 MET I 251      -6.656 -18.064   8.813  0.00  0.00
I
ATOM   3406  HE3 MET I 251      -6.222 -17.936  10.555  0.00  0.00
I
ATOM   3407  C   MET I 251     -11.255 -19.233  10.585  0.00  0.00
I
ATOM   3408  O   MET I 251     -11.591 -19.105   9.391  0.00  0.00
I
ATOM   3409  N   LEU I 252     -11.910 -20.021  11.431  0.00  0.00
I
ATOM   3410  HN  LEU I 252     -11.696 -20.086  12.402  0.00  0.00
I
ATOM   3411  CA  LEU I 252     -13.012 -20.859  11.046  0.00  0.00
I
ATOM   3412  HA  LEU I 252     -12.703 -21.470  10.211  0.00  0.00
I
ATOM   3413  CB  LEU I 252     -13.416 -21.839  12.277  0.00  0.00
I
ATOM   3414  HB1 LEU I 252     -12.566 -22.554  12.282  0.00  0.00
I
ATOM   3415  HB2 LEU I 252     -13.324 -21.388  13.289  0.00  0.00
I
ATOM   3416  CG  LEU I 252     -14.725 -22.528  12.089  0.00  0.00
I
ATOM   3417  HG  LEU I 252     -15.503 -21.741  11.992  0.00  0.00
I
ATOM   3418  CD1 LEU I 252     -14.691 -23.467  10.871  0.00  0.00
I
ATOM   3419 HD11 LEU I 252     -15.564 -24.153  10.905  0.00  0.00
I
ATOM   3420 HD12 LEU I 252     -14.848 -22.873   9.946  0.00  0.00
I
ATOM   3421 HD13 LEU I 252     -13.764 -24.075  10.793  0.00  0.00
I
ATOM   3422  CD2 LEU I 252     -15.096 -23.263  13.424  0.00  0.00
I
ATOM   3423 HD21 LEU I 252     -15.313 -22.468  14.169  0.00  0.00
I
ATOM   3424 HD22 LEU I 252     -16.026 -23.853  13.275  0.00  0.00
I
ATOM   3425 HD23 LEU I 252     -14.338 -23.986  13.795  0.00  0.00
I
ATOM   3426  C   LEU I 252     -14.196 -20.035  10.577  0.00  0.00
I
ATOM   3427  O   LEU I 252     -14.499 -20.084   9.411  0.00  0.00
I
ATOM   3428  N   THR I 253     -14.798 -19.136  11.477  0.00  0.00
I
```

ATOM	3429	HN	THR	I	253	−14.389	−18.917	12.360	0.00	0.00	I
ATOM	3430	CA	THR	I	253	−16.059	−18.439	11.178	0.00	0.00	I
ATOM	3431	HA	THR	I	253	−16.774	−19.165	10.821	0.00	0.00	I
ATOM	3432	CB	THR	I	253	−16.605	−17.678	12.371	0.00	0.00	I
ATOM	3433	HB	THR	I	253	−17.482	−17.126	11.971	0.00	0.00	I
ATOM	3434	OG1	THR	I	253	−15.637	−16.819	12.985	0.00	0.00	I
ATOM	3435	HG1	THR	I	253	−15.061	−17.398	13.489	0.00	0.00	I
ATOM	3436	CG2	THR	I	253	−17.110	−18.626	13.495	0.00	0.00	I
ATOM	3437	HG21	THR	I	253	−17.889	−19.347	13.167	0.00	0.00	I
ATOM	3438	HG22	THR	I	253	−16.277	−19.257	13.872	0.00	0.00	I
ATOM	3439	HG23	THR	I	253	−17.511	−18.075	14.373	0.00	0.00	I
ATOM	3440	C	THR	I	253	−15.826	−17.352	10.061	0.00	0.00	I
ATOM	3441	O	THR	I	253	−16.711	−17.194	9.206	0.00	0.00	I
ATOM	3442	N	GLY	I	254	−14.632	−16.666	10.029	0.00	0.00	I
ATOM	3443	HN	GLY	I	254	−13.923	−16.841	10.708	0.00	0.00	I
ATOM	3444	CA	GLY	I	254	−14.276	−15.679	9.097	0.00	0.00	I
ATOM	3445	HA1	GLY	I	254	−13.532	−15.102	9.626	0.00	0.00	I
ATOM	3446	HA2	GLY	I	254	−15.185	−15.141	8.871	0.00	0.00	I
ATOM	3447	C	GLY	I	254	−13.854	−16.245	7.824	0.00	0.00	I
ATOM	3448	O	GLY	I	254	−13.976	−15.517	6.815	0.00	0.00	I
ATOM	3449	N	THR	I	255	−13.376	−17.499	7.641	0.00	0.00	I
ATOM	3450	HN	THR	I	255	−13.205	−18.056	8.451	0.00	0.00	I
ATOM	3451	CA	THR	I	255	−13.336	−18.201	6.325	0.00	0.00	I
ATOM	3452	HA	THR	I	255	−12.793	−17.526	5.680	0.00	0.00	I
ATOM	3453	CB	THR	I	255	−12.569	−19.536	6.364	0.00	0.00	I
ATOM	3454	HB	THR	I	255	−13.049	−20.279	7.036	0.00	0.00	I
ATOM	3455	OG1	THR	I	255	−11.197	−19.295	6.816	0.00	0.00	I

```
ATOM   3456  HG1 THR I 255     -11.379 -19.103   7.739  0.00  0.00
I
ATOM   3457  CG2 THR I 255     -12.636 -20.093   4.882  0.00  0.00
I
ATOM   3458 HG21 THR I 255     -13.607 -20.470   4.496  0.00  0.00
I
ATOM   3459 HG22 THR I 255     -12.327 -19.367   4.100  0.00  0.00
I
ATOM   3460 HG23 THR I 255     -11.810 -20.831   4.795  0.00  0.00
I
ATOM   3461  C   THR I 255     -14.738 -18.366   5.699  0.00  0.00
I
ATOM   3462  O   THR I 255     -14.926 -18.053   4.533  0.00  0.00
I
ATOM   3463  N   LEU I 256     -15.713 -18.897   6.420  0.00  0.00
I
ATOM   3464  HN  LEU I 256     -15.491 -19.313   7.298  0.00  0.00
I
ATOM   3465  CA  LEU I 256     -17.081 -19.210   5.942  0.00  0.00
I
ATOM   3466  HA  LEU I 256     -17.021 -19.674   4.969  0.00  0.00
I
ATOM   3467  CB  LEU I 256     -17.689 -20.172   7.071  0.00  0.00
I
ATOM   3468  HB1 LEU I 256     -17.423 -19.723   8.052  0.00  0.00
I
ATOM   3469  HB2 LEU I 256     -18.795 -20.249   6.995  0.00  0.00
I
ATOM   3470  CG  LEU I 256     -17.211 -21.589   6.966  0.00  0.00
I
ATOM   3471  HG  LEU I 256     -16.107 -21.514   7.056  0.00  0.00
I
ATOM   3472  CD1 LEU I 256     -17.596 -22.409   8.164  0.00  0.00
I
ATOM   3473 HD11 LEU I 256     -17.154 -23.425   8.086  0.00  0.00
I
ATOM   3474 HD12 LEU I 256     -17.235 -21.935   9.102  0.00  0.00
I
ATOM   3475 HD13 LEU I 256     -18.673 -22.657   8.272  0.00  0.00
I
ATOM   3476  CD2 LEU I 256     -17.716 -22.203   5.723  0.00  0.00
I
ATOM   3477 HD21 LEU I 256     -17.628 -23.311   5.718  0.00  0.00
I
ATOM   3478 HD22 LEU I 256     -18.790 -21.958   5.574  0.00  0.00
I
ATOM   3479 HD23 LEU I 256     -17.155 -21.870   4.824  0.00  0.00
I
ATOM   3480  C   LEU I 256     -17.871 -17.979   5.653  0.00  0.00
I
ATOM   3481  O   LEU I 256     -18.556 -17.925   4.658  0.00  0.00
I
ATOM   3482  N   MET I 257     -17.859 -16.941   6.514  0.00  0.00
I
```

ATOM 3483 HN MET I 257 -17.315 -17.026 7.345 0.00 0.00
I
ATOM 3484 CA MET I 257 -18.398 -15.596 6.276 0.00 0.00
I
ATOM 3485 HA MET I 257 -19.453 -15.683 6.061 0.00 0.00
I
ATOM 3486 CB MET I 257 -18.121 -14.734 7.496 0.00 0.00
I
ATOM 3487 HB1 MET I 257 -18.406 -15.460 8.288 0.00 0.00
I
ATOM 3488 HB2 MET I 257 -17.043 -14.469 7.461 0.00 0.00
I
ATOM 3489 CG MET I 257 -19.054 -13.536 7.836 0.00 0.00
I
ATOM 3490 HG1 MET I 257 -18.698 -12.894 7.002 0.00 0.00
I
ATOM 3491 HG2 MET I 257 -20.105 -13.836 7.636 0.00 0.00
I
ATOM 3492 SD MET I 257 -18.883 -12.725 9.490 0.00 0.00
I
ATOM 3493 CE MET I 257 -17.545 -11.666 8.900 0.00 0.00
I
ATOM 3494 HE1 MET I 257 -17.055 -11.033 9.670 0.00 0.00
I
ATOM 3495 HE2 MET I 257 -16.732 -12.264 8.436 0.00 0.00
I
ATOM 3496 HE3 MET I 257 -17.807 -10.903 8.136 0.00 0.00
I
ATOM 3497 C MET I 257 -17.813 -14.913 5.124 0.00 0.00
I
ATOM 3498 O MET I 257 -18.550 -14.336 4.391 0.00 0.00
I
ATOM 3499 N LEU I 258 -16.473 -14.957 4.930 0.00 0.00
I
ATOM 3500 HN LEU I 258 -15.872 -15.425 5.573 0.00 0.00
I
ATOM 3501 CA LEU I 258 -15.860 -14.330 3.774 0.00 0.00
I
ATOM 3502 HA LEU I 258 -16.147 -13.293 3.869 0.00 0.00
I
ATOM 3503 CB LEU I 258 -14.410 -14.516 3.953 0.00 0.00
I
ATOM 3504 HB1 LEU I 258 -14.223 -14.157 4.987 0.00 0.00
I
ATOM 3505 HB2 LEU I 258 -14.104 -15.583 4.008 0.00 0.00
I
ATOM 3506 CG LEU I 258 -13.314 -13.708 3.090 0.00 0.00
I
ATOM 3507 HG LEU I 258 -13.544 -14.079 2.068 0.00 0.00
I
ATOM 3508 CD1 LEU I 258 -13.650 -12.210 2.994 0.00 0.00
I
ATOM 3509 HD11 LEU I 258 -12.785 -11.622 2.618 0.00 0.00
I

ATOM	3510	HD12	LEU	I	258	−14.562	−12.100	2.369	0.00	0.00	I
ATOM	3511	HD13	LEU	I	258	−13.950	−11.822	3.991	0.00	0.00	I
ATOM	3512	CD2	LEU	I	258	−11.898	−13.995	3.587	0.00	0.00	I
ATOM	3513	HD21	LEU	I	258	−11.736	−15.084	3.733	0.00	0.00	I
ATOM	3514	HD22	LEU	I	258	−11.139	−13.765	2.810	0.00	0.00	I
ATOM	3515	HD23	LEU	I	258	−11.535	−13.367	4.429	0.00	0.00	I
ATOM	3516	C	LEU	I	258	−16.233	−14.995	2.456	0.00	0.00	I
ATOM	3517	O	LEU	I	258	−16.573	−14.309	1.540	0.00	0.00	I
ATOM	3518	N	ASN	I	259	−16.165	−16.277	2.330	0.00	0.00	I
ATOM	3519	HN	ASN	I	259	−15.811	−16.764	3.125	0.00	0.00	I
ATOM	3520	CA	ASN	I	259	−16.667	−17.125	1.189	0.00	0.00	I
ATOM	3521	HA	ASN	I	259	−16.003	−16.712	0.443	0.00	0.00	I
ATOM	3522	CB	ASN	I	259	−16.437	−18.667	1.329	0.00	0.00	I
ATOM	3523	HB1	ASN	I	259	−17.010	−19.066	2.193	0.00	0.00	I
ATOM	3524	HB2	ASN	I	259	−16.848	−19.120	0.402	0.00	0.00	I
ATOM	3525	CG	ASN	I	259	−15.034	−19.161	1.384	0.00	0.00	I
ATOM	3526	OD1	ASN	I	259	−14.020	−18.479	1.114	0.00	0.00	I
ATOM	3527	ND2	ASN	I	259	−15.005	−20.491	1.726	0.00	0.00	I
ATOM	3528	HD21	ASN	I	259	−14.139	−20.989	1.719	0.00	0.00	I
ATOM	3529	HD22	ASN	I	259	−15.901	−20.890	1.921	0.00	0.00	I
ATOM	3530	C	ASN	I	259	−18.127	−16.831	0.911	0.00	0.00	I
ATOM	3531	O	ASN	I	259	−18.501	−16.696	−0.268	0.00	0.00	I
ATOM	3532	N	ALA	I	260	−18.981	−16.605	1.964	0.00	0.00	I
ATOM	3533	HN	ALA	I	260	−18.524	−16.457	2.838	0.00	0.00	I
ATOM	3534	CA	ALA	I	260	−20.398	−16.328	1.899	0.00	0.00	I
ATOM	3535	HA	ALA	I	260	−20.747	−17.111	1.241	0.00	0.00	I
ATOM	3536	CB	ALA	I	260	−21.082	−16.233	3.293	0.00	0.00	I

ATOM   3537 HB1 ALA I 260   -20.707 -15.420   3.951 0.00   0.00
I
ATOM   3538 HB2 ALA I 260   -22.169 -16.079   3.126 0.00   0.00
I
ATOM   3539 HB3 ALA I 260   -20.971 -17.171   3.879 0.00   0.00
I
ATOM   3540 C   ALA I 260   -20.720 -15.079   1.127 0.00   0.00
I
ATOM   3541 O   ALA I 260   -21.419 -15.089   0.128 0.00   0.00
I
ATOM   3542 N   ILE I 261   -20.040 -13.968   1.511 0.00   0.00
I
ATOM   3543 HN ILE I 261   -19.512 -14.204   2.322 0.00   0.00
I
ATOM   3544 CA ILE I 261   -20.008 -12.683   0.943 0.00   0.00
I
ATOM   3545 HA ILE I 261   -21.024 -12.317   0.909 0.00   0.00
I
ATOM   3546 CB ILE I 261   -19.203 -11.667   1.847 0.00   0.00
I
ATOM   3547 HB ILE I 261   -18.254 -12.190   2.094 0.00   0.00
I
ATOM   3548 CG2 ILE I 261   -18.898 -10.224   1.151 0.00   0.00
I
ATOM   3549 HG21 ILE I 261   -19.788 -9.945   0.549 0.00   0.00
I
ATOM   3550 HG22 ILE I 261   -18.780 -9.380   1.864 0.00   0.00
I
ATOM   3551 HG23 ILE I 261   -18.053 -10.373   0.444 0.00   0.00
I
ATOM   3552 CG1 ILE I 261   -19.918 -11.428   3.218 0.00   0.00
I
ATOM   3553 HG11 ILE I 261   -20.692 -10.656   3.025 0.00   0.00
I
ATOM   3554 HG12 ILE I 261   -20.381 -12.359   3.611 0.00   0.00
I
ATOM   3555 CD ILE I 261   -18.992 -11.069   4.394 0.00   0.00
I
ATOM   3556 HD1 ILE I 261   -18.098 -11.727   4.440 0.00   0.00
I
ATOM   3557 HD2 ILE I 261   -18.723 -9.991   4.419 0.00   0.00
I
ATOM   3558 HD3 ILE I 261   -19.614 -11.151   5.311 0.00   0.00
I
ATOM   3559 C   ILE I 261   -19.466 -12.705 -0.464 0.00   0.00
I
ATOM   3560 O   ILE I 261   -20.267 -12.264 -1.350 0.00   0.00
I
ATOM   3561 N   ALA I 262   -18.182 -13.207 -0.669 0.00   0.00
I
ATOM   3562 HN ALA I 262   -17.646 -13.422   0.144 0.00   0.00
I
ATOM   3563 CA ALA I 262   -17.514 -13.182 -1.906 0.00   0.00
I

ATOM 3564 HA ALA I 262 −17.488 −12.184 −2.318 0.00 0.00
I
ATOM 3565 CB ALA I 262 −16.118 −13.754 −1.856 0.00 0.00
I
ATOM 3566 HB1 ALA I 262 −15.461 −13.478 −1.003 0.00 0.00
I
ATOM 3567 HB2 ALA I 262 −16.248 −14.854 −1.782 0.00 0.00
I
ATOM 3568 HB3 ALA I 262 −15.629 −13.644 −2.848 0.00 0.00
I
ATOM 3569 C ALA I 262 −18.216 −13.965 −3.074 0.00 0.00
I
ATOM 3570 O ALA I 262 −18.329 −13.383 −4.155 0.00 0.00
I
ATOM 3571 N MET I 263 −18.718 −15.254 −2.866 0.00 0.00
I
ATOM 3572 HN MET I 263 −18.524 −15.630 −1.963 0.00 0.00
I
ATOM 3573 CA MET I 263 −19.516 −15.979 −3.857 0.00 0.00
I
ATOM 3574 HA MET I 263 −18.879 −16.226 −4.694 0.00 0.00
I
ATOM 3575 CB MET I 263 −20.043 −17.318 −3.333 0.00 0.00
I
ATOM 3576 HB1 MET I 263 −19.221 −17.964 −2.956 0.00 0.00
I
ATOM 3577 HB2 MET I 263 −20.673 −17.051 −2.458 0.00 0.00
I
ATOM 3578 CG MET I 263 −20.960 −18.124 −4.330 0.00 0.00
I
ATOM 3579 HG1 MET I 263 −22.030 −17.830 −4.375 0.00 0.00
I
ATOM 3580 HG2 MET I 263 −20.455 −18.032 −5.316 0.00 0.00
I
ATOM 3581 SD MET I 263 −20.993 −19.895 −3.795 0.00 0.00
I
ATOM 3582 CE MET I 263 −22.556 −20.311 −4.540 0.00 0.00
I
ATOM 3583 HE1 MET I 263 −22.606 −21.421 −4.583 0.00 0.00
I
ATOM 3584 HE2 MET I 263 −23.496 −20.036 −4.017 0.00 0.00
I
ATOM 3585 HE3 MET I 263 −22.572 −19.870 −5.560 0.00 0.00
I
ATOM 3586 C MET I 263 −20.721 −15.209 −4.358 0.00 0.00
I
ATOM 3587 O MET I 263 −20.983 −15.126 −5.527 0.00 0.00
I
ATOM 3588 N LEU I 264 −21.510 −14.513 −3.446 0.00 0.00
I
ATOM 3589 HN LEU I 264 −21.281 −14.732 −2.501 0.00 0.00
I
ATOM 3590 CA LEU I 264 −22.695 −13.708 −3.734 0.00 0.00
I

ATOM 3591 HA LEU I 264 -23.362 -14.394 -4.236 0.00 0.00
I
ATOM 3592 CB LEU I 264 -23.344 -13.203 -2.384 0.00 0.00
I
ATOM 3593 HB1 LEU I 264 -23.466 -14.095 -1.733 0.00 0.00
I
ATOM 3594 HB2 LEU I 264 -22.670 -12.651 -1.695 0.00 0.00
I
ATOM 3595 CG LEU I 264 -24.658 -12.377 -2.553 0.00 0.00
I
ATOM 3596 HG LEU I 264 -24.347 -11.402 -2.986 0.00 0.00
I
ATOM 3597 CD1 LEU I 264 -25.682 -12.951 -3.522 0.00 0.00
I
ATOM 3598 HD11 LEU I 264 -26.483 -12.240 -3.820 0.00 0.00
I
ATOM 3599 HD12 LEU I 264 -25.289 -13.196 -4.532 0.00 0.00
I
ATOM 3600 HD13 LEU I 264 -26.197 -13.809 -3.041 0.00 0.00
I
ATOM 3601 CD2 LEU I 264 -25.370 -12.153 -1.215 0.00 0.00
I
ATOM 3602 HD21 LEU I 264 -26.115 -11.363 -1.450 0.00 0.00
I
ATOM 3603 HD22 LEU I 264 -25.809 -13.047 -0.722 0.00 0.00
I
ATOM 3604 HD23 LEU I 264 -24.548 -11.788 -0.563 0.00 0.00
I
ATOM 3605 C LEU I 264 -22.372 -12.504 -4.572 0.00 0.00
I
ATOM 3606 O LEU I 264 -23.033 -12.197 -5.592 0.00 0.00
I
ATOM 3607 N THR I 265 -21.253 -11.808 -4.221 0.00 0.00
I
ATOM 3608 HN THR I 265 -20.698 -12.176 -3.479 0.00 0.00
I
ATOM 3609 CA THR I 265 -20.731 -10.538 -4.845 0.00 0.00
I
ATOM 3610 HA THR I 265 -21.501 -9.784 -4.915 0.00 0.00
I
ATOM 3611 CB THR I 265 -19.616 -10.067 -3.970 0.00 0.00
I
ATOM 3612 HB THR I 265 -18.710 -10.710 -3.965 0.00 0.00
I
ATOM 3613 OG1 THR I 265 -20.093 -9.839 -2.571 0.00 0.00
I
ATOM 3614 HG1 THR I 265 -20.289 -10.688 -2.169 0.00 0.00
I
ATOM 3615 CG2 THR I 265 -19.087 -8.690 -4.543 0.00 0.00
I
ATOM 3616 HG21 THR I 265 -19.901 -7.958 -4.733 0.00 0.00
I
ATOM 3617 HG22 THR I 265 -18.323 -8.425 -3.781 0.00 0.00
I

ATOM 3618 HG23 THR I 265 -18.563 -8.857 -5.508 0.00 0.00
I
ATOM 3619 C THR I 265 -20.259 -10.736 -6.262 0.00 0.00
I
ATOM 3620 O THR I 265 -20.520 -9.998 -7.146 0.00 0.00
I
ATOM 3621 N VAL I 266 -19.545 -11.918 -6.556 0.00 0.00
I
ATOM 3622 HN VAL I 266 -19.107 -12.461 -5.844 0.00 0.00
I
ATOM 3623 CA VAL I 266 -19.217 -12.362 -7.934 0.00 0.00
I
ATOM 3624 HA VAL I 266 -18.680 -11.532 -8.368 0.00 0.00
I
ATOM 3625 CB VAL I 266 -18.151 -13.502 -8.024 0.00 0.00
I
ATOM 3626 HB VAL I 266 -18.714 -14.286 -7.475 0.00 0.00
I
ATOM 3627 CG1 VAL I 266 -17.736 -13.994 -9.451 0.00 0.00
I
ATOM 3628 HG11 VAL I 266 -18.548 -14.588 -9.921 0.00 0.00
I
ATOM 3629 HG12 VAL I 266 -17.595 -13.116 -10.116 0.00 0.00
I
ATOM 3630 HG13 VAL I 266 -16.791 -14.567 -9.336 0.00 0.00
I
ATOM 3631 CG2 VAL I 266 -16.864 -13.133 -7.281 0.00 0.00
I
ATOM 3632 HG21 VAL I 266 -17.181 -13.044 -6.220 0.00 0.00
I
ATOM 3633 HG22 VAL I 266 -16.094 -13.923 -7.415 0.00 0.00
I
ATOM 3634 HG23 VAL I 266 -16.406 -12.266 -7.803 0.00 0.00
I
ATOM 3635 C VAL I 266 -20.470 -12.623 -8.778 0.00 0.00
I
ATOM 3636 O VAL I 266 -20.585 -12.140 -9.978 0.00 0.00
I
ATOM 3637 N PHE I 267 -21.451 -13.334 -8.180 0.00 0.00
I
ATOM 3638 HN PHE I 267 -21.486 -13.748 -7.273 0.00 0.00
I
ATOM 3639 CA PHE I 267 -22.685 -13.712 -8.881 0.00 0.00
I
ATOM 3640 HA PHE I 267 -22.351 -14.166 -9.802 0.00 0.00
I
ATOM 3641 CB PHE I 267 -23.551 -14.676 -8.039 0.00 0.00
I
ATOM 3642 HB1 PHE I 267 -22.824 -15.387 -7.591 0.00 0.00
I
ATOM 3643 HB2 PHE I 267 -24.050 -14.271 -7.133 0.00 0.00
I
ATOM 3644 CG PHE I 267 -24.480 -15.495 -8.799 0.00 0.00
I

```
ATOM   3645  CD1 PHE I 267     -25.803 -15.038  -9.118  0.00  0.00
I
ATOM   3646  HD1 PHE I 267     -26.184 -14.052  -8.897  0.00  0.00
I
ATOM   3647  CE1 PHE I 267     -26.692 -15.872  -9.843  0.00  0.00
I
ATOM   3648  HE1 PHE I 267     -27.607 -15.418 -10.195  0.00  0.00
I
ATOM   3649  CZ  PHE I 267     -26.145 -17.042 -10.404  0.00  0.00
I
ATOM   3650  HZ  PHE I 267     -26.777 -17.630 -11.053  0.00  0.00
I
ATOM   3651  CD2 PHE I 267     -24.048 -16.798  -9.302  0.00  0.00
I
ATOM   3652  HD2 PHE I 267     -23.084 -17.106  -8.924  0.00  0.00
I
ATOM   3653  CE2 PHE I 267     -24.897 -17.537 -10.034  0.00  0.00
I
ATOM   3654  HE2 PHE I 267     -24.665 -18.478 -10.512  0.00  0.00
I
ATOM   3655  C   PHE I 267     -23.547 -12.518  -9.338  0.00  0.00
I
ATOM   3656  O   PHE I 267     -24.083 -12.568 -10.465  0.00  0.00
I
ATOM   3657  N   ILE I 268     -23.761 -11.509  -8.497  0.00  0.00
I
ATOM   3658  HN  ILE I 268     -23.308 -11.561  -7.610  0.00  0.00
I
ATOM   3659  CA  ILE I 268     -24.556 -10.307  -8.762  0.00  0.00
I
ATOM   3660  HA  ILE I 268     -25.362 -10.672  -9.382  0.00  0.00
I
ATOM   3661  CB  ILE I 268     -25.292  -9.713  -7.550  0.00  0.00
I
ATOM   3662  HB  ILE I 268     -25.493 -10.598  -6.909  0.00  0.00
I
ATOM   3663  CG2 ILE I 268     -24.545  -8.653  -6.751  0.00  0.00
I
ATOM   3664 HG21 ILE I 268     -24.181  -7.794  -7.356  0.00  0.00
I
ATOM   3665 HG22 ILE I 268     -25.109  -8.266  -5.876  0.00  0.00
I
ATOM   3666 HG23 ILE I 268     -23.605  -9.090  -6.351  0.00  0.00
I
ATOM   3667  CG1 ILE I 268     -26.715  -9.257  -7.904  0.00  0.00
I
ATOM   3668 HG11 ILE I 268     -26.584  -8.324  -8.492  0.00  0.00
I
ATOM   3669 HG12 ILE I 268     -27.285  -9.969  -8.538  0.00  0.00
I
ATOM   3670  CD  ILE I 268     -27.678  -8.794  -6.688  0.00  0.00
I
ATOM   3671  HD1 ILE I 268     -27.638  -9.468  -5.805  0.00  0.00
I
```

ATOM 3672 HD2 ILE I 268 -27.216 -7.815 -6.437 0.00 0.00
I
ATOM 3673 HD3 ILE I 268 -28.711 -8.702 -7.087 0.00 0.00
I
ATOM 3674 C ILE I 268 -23.797 -9.256 -9.583 0.00 0.00
I
ATOM 3675 O ILE I 268 -24.492 -8.581 -10.398 0.00 0.00
I
ATOM 3676 N LEU I 269 -22.468 -9.049 -9.335 0.00 0.00
I
ATOM 3677 HN LEU I 269 -22.024 -9.541 -8.591 0.00 0.00
I
ATOM 3678 CA LEU I 269 -21.627 -8.098 -10.017 0.00 0.00
I
ATOM 3679 HA LEU I 269 -22.121 -7.153 -9.846 0.00 0.00
I
ATOM 3680 CB LEU I 269 -20.216 -8.118 -9.359 0.00 0.00
I
ATOM 3681 HB1 LEU I 269 -20.494 -7.956 -8.296 0.00 0.00
I
ATOM 3682 HB2 LEU I 269 -19.783 -9.141 -9.377 0.00 0.00
I
ATOM 3683 CG LEU I 269 -19.265 -7.066 -9.872 0.00 0.00
I
ATOM 3684 HG LEU I 269 -19.092 -7.128 -10.967 0.00 0.00
I
ATOM 3685 CD1 LEU I 269 -19.730 -5.594 -9.593 0.00 0.00
I
ATOM 3686 HD11 LEU I 269 -20.582 -5.296 -10.242 0.00 0.00
I
ATOM 3687 HD12 LEU I 269 -19.972 -5.476 -8.516 0.00 0.00
I
ATOM 3688 HD13 LEU I 269 -18.860 -4.921 -9.749 0.00 0.00
I
ATOM 3689 CD2 LEU I 269 -17.873 -7.388 -9.226 0.00 0.00
I
ATOM 3690 HD21 LEU I 269 -17.171 -6.691 -9.732 0.00 0.00
I
ATOM 3691 HD22 LEU I 269 -18.041 -7.207 -8.143 0.00 0.00
I
ATOM 3692 HD23 LEU I 269 -17.518 -8.439 -9.280 0.00 0.00
I
ATOM 3693 C LEU I 269 -21.536 -8.248 -11.554 0.00 0.00
I
ATOM 3694 O LEU I 269 -21.960 -7.469 -12.355 0.00 0.00
I
ATOM 3695 N GLU I 270 -21.280 -9.519 -11.942 0.00 0.00
I
ATOM 3696 HN GLU I 270 -21.154 -10.166 -11.194 0.00 0.00
I
ATOM 3697 CA GLU I 270 -21.022 -9.935 -13.276 0.00 0.00
I
ATOM 3698 HA GLU I 270 -20.793 -9.019 -13.800 0.00 0.00
I

```
ATOM   3699  CB  GLU I 270     -19.809 -10.888 -13.397  0.00  0.00
I
ATOM   3700  HB1 GLU I 270     -20.044 -11.740 -12.724  0.00  0.00
I
ATOM   3701  HB2 GLU I 270     -19.677 -11.247 -14.440  0.00  0.00
I
ATOM   3702  CG  GLU I 270     -18.520 -10.162 -12.872  0.00  0.00
I
ATOM   3703  HG1 GLU I 270     -18.589  -9.899 -11.795  0.00  0.00
I
ATOM   3704  HG2 GLU I 270     -17.699 -10.891 -13.035  0.00  0.00
I
ATOM   3705  CD  GLU I 270     -18.019  -8.974 -13.783  0.00  0.00
I
ATOM   3706  OE1 GLU I 270     -18.787  -7.998 -14.068  0.00  0.00
I
ATOM   3707  OE2 GLU I 270     -16.901  -9.102 -14.336  0.00  0.00
I
ATOM   3708  C   GLU I 270     -22.317 -10.475 -13.915  0.00  0.00
I
ATOM   3709  O   GLU I 270     -22.239 -10.914 -15.026  0.00  0.00
I
ATOM   3710  N   TYR I 271     -23.505 -10.365 -13.170  0.00  0.00
I
ATOM   3711  HN  TYR I 271     -23.475  -9.988 -12.248  0.00  0.00
I
ATOM   3712  CA  TYR I 271     -24.842 -10.654 -13.700  0.00  0.00
I
ATOM   3713  HA  TYR I 271     -24.939 -11.717 -13.864  0.00  0.00
I
ATOM   3714  CB  TYR I 271     -25.967 -10.285 -12.669  0.00  0.00
I
ATOM   3715  HB1 TYR I 271     -25.485 -10.265 -11.668  0.00  0.00
I
ATOM   3716  HB2 TYR I 271     -26.531  -9.361 -12.920  0.00  0.00
I
ATOM   3717  CG  TYR I 271     -27.026 -11.353 -12.503  0.00  0.00
I
ATOM   3718  CD1 TYR I 271     -27.554 -11.821 -11.206  0.00  0.00
I
ATOM   3719  HD1 TYR I 271     -27.009 -11.431 -10.359  0.00  0.00
I
ATOM   3720  CE1 TYR I 271     -28.589 -12.671 -10.981  0.00  0.00
I
ATOM   3721  HE1 TYR I 271     -28.872 -12.976  -9.984  0.00  0.00
I
ATOM   3722  CZ  TYR I 271     -29.212 -13.205 -12.036  0.00  0.00
I
ATOM   3723  OH  TYR I 271     -30.374 -13.995 -11.930  0.00  0.00
I
ATOM   3724  HH  TYR I 271     -30.791 -14.369 -12.710  0.00  0.00
I
ATOM   3725  CD2 TYR I 271     -27.695 -11.931 -13.561  0.00  0.00
I
```

ATOM 3726 HD2 TYR I 271 -27.418 -11.630 -14.560 0.00 0.00
I
ATOM 3727 CE2 TYR I 271 -28.708 -12.874 -13.386 0.00 0.00
I
ATOM 3728 HE2 TYR I 271 -29.065 -13.444 -14.232 0.00 0.00
I
ATOM 3729 C TYR I 271 -25.159 -10.010 -15.101 0.00 0.00
I
ATOM 3730 O TYR I 271 -25.674 -10.643 -16.002 0.00 0.00
I
ATOM 3731 N SER I 272 -24.653 -8.780 -15.159 0.00 0.00
I
ATOM 3732 HN SER I 272 -24.306 -8.342 -14.333 0.00 0.00
I
ATOM 3733 CA SER I 272 -24.781 -7.931 -16.314 0.00 0.00
I
ATOM 3734 HA SER I 272 -25.653 -8.167 -16.907 0.00 0.00
I
ATOM 3735 CB SER I 272 -24.909 -6.477 -15.863 0.00 0.00
I
ATOM 3736 HB1 SER I 272 -25.231 -5.803 -16.685 0.00 0.00
I
ATOM 3737 HB2 SER I 272 -25.802 -6.368 -15.210 0.00 0.00
I
ATOM 3738 OG SER I 272 -23.749 -5.964 -15.274 0.00 0.00
I
ATOM 3739 HG1 SER I 272 -23.995 -5.359 -14.570 0.00 0.00
I
ATOM 3740 C SER I 272 -23.585 -8.042 -17.268 0.00 0.00
I
ATOM 3741 O SER I 272 -23.452 -7.252 -18.167 0.00 0.00
I
ATOM 3742 N ASN I 273 -22.633 -8.973 -17.019 0.00 0.00
I
ATOM 3743 HN ASN I 273 -22.699 -9.595 -16.243 0.00 0.00
I
ATOM 3744 CA ASN I 273 -21.538 -9.059 -17.921 0.00 0.00
I
ATOM 3745 HA ASN I 273 -21.271 -8.087 -18.309 0.00 0.00
I
ATOM 3746 CB ASN I 273 -20.284 -9.422 -17.092 0.00 0.00
I
ATOM 3747 HB1 ASN I 273 -20.205 -8.695 -16.256 0.00 0.00
I
ATOM 3748 HB2 ASN I 273 -20.516 -10.369 -16.561 0.00 0.00
I
ATOM 3749 CG ASN I 273 -18.907 -9.347 -17.836 0.00 0.00
I
ATOM 3750 OD1 ASN I 273 -18.862 -9.119 -19.051 0.00 0.00
I
ATOM 3751 ND2 ASN I 273 -17.856 -9.435 -17.025 0.00 0.00
I
ATOM 3752 HD21 ASN I 273 -17.973 -9.384 -16.033 0.00 0.00
I

ATOM 3753 HD22 ASN I 273 -16.955 -9.527 -17.449 0.00 0.00
I
ATOM 3754 C ASN I 273 -21.781 -10.026 -19.105 0.00 0.00
I
ATOM 3755 O ASN I 273 -22.122 -11.196 -18.913 0.00 0.00
I
ATOM 3756 N PRO I 274 -21.706 -9.695 -20.417 0.00 0.00
I
ATOM 3757 CD PRO I 274 -21.625 -8.339 -20.887 0.00 0.00
I
ATOM 3758 HD1 PRO I 274 -22.309 -7.624 -20.381 0.00 0.00
I
ATOM 3759 HD2 PRO I 274 -20.611 -7.935 -20.678 0.00 0.00
I
ATOM 3760 CA PRO I 274 -21.876 -10.660 -21.546 0.00 0.00
I
ATOM 3761 HA PRO I 274 -22.569 -11.425 -21.228 0.00 0.00
I
ATOM 3762 CB PRO I 274 -22.351 -9.680 -22.646 0.00 0.00
I
ATOM 3763 HB1 PRO I 274 -23.445 -9.516 -22.541 0.00 0.00
I
ATOM 3764 HB2 PRO I 274 -22.073 -10.096 -23.638 0.00 0.00
I
ATOM 3765 CG PRO I 274 -21.769 -8.387 -22.389 0.00 0.00
I
ATOM 3766 HG1 PRO I 274 -22.404 -7.558 -22.766 0.00 0.00
I
ATOM 3767 HG2 PRO I 274 -20.744 -8.197 -22.775 0.00 0.00
I
ATOM 3768 C PRO I 274 -20.452 -11.317 -21.785 0.00 0.00
I
ATOM 3769 O PRO I 274 -20.310 -12.303 -22.500 0.00 0.00
I
ATOM 3770 N GLY I 275 -19.345 -10.690 -21.158 0.00 0.00
I
ATOM 3771 HN GLY I 275 -19.443 -9.844 -20.640 0.00 0.00
I
ATOM 3772 CA GLY I 275 -17.969 -11.059 -21.346 0.00 0.00
I
ATOM 3773 HA1 GLY I 275 -17.508 -10.191 -20.898 0.00 0.00
I
ATOM 3774 HA2 GLY I 275 -17.928 -11.214 -22.414 0.00 0.00
I
ATOM 3775 C GLY I 275 -17.536 -12.338 -20.627 0.00 0.00
I
ATOM 3776 O GLY I 275 -16.800 -13.141 -21.239 0.00 0.00
I
ATOM 3777 N THR I 276 -17.959 -12.570 -19.386 0.00 0.00
I
ATOM 3778 HN THR I 276 -18.648 -11.935 -19.045 0.00 0.00
I
ATOM 3779 CA THR I 276 -17.656 -13.729 -18.603 0.00 0.00
I

ATOM 3780 HA THR I 276 −17.720 −14.572 −19.274 0.00 0.00
I
ATOM 3781 CB THR I 276 −16.283 −13.917 −17.938 0.00 0.00
I
ATOM 3782 HB THR I 276 −15.517 −13.649 −18.697 0.00 0.00
I
ATOM 3783 OG1 THR I 276 −15.908 −15.299 −17.532 0.00 0.00
I
ATOM 3784 HG1 THR I 276 −15.354 −15.673 −18.220 0.00 0.00
I
ATOM 3785 CG2 THR I 276 −16.253 −12.987 −16.814 0.00 0.00
I
ATOM 3786 HG21 THR I 276 −16.592 −12.013 −17.228 0.00 0.00
I
ATOM 3787 HG22 THR I 276 −16.971 −13.364 −16.054 0.00 0.00
I
ATOM 3788 HG23 THR I 276 −15.287 −12.845 −16.285 0.00 0.00
I
ATOM 3789 C THR I 276 −18.746 −13.918 −17.610 0.00 0.00
I
ATOM 3790 O THR I 276 −19.576 −13.086 −17.342 0.00 0.00
I
ATOM 3791 N LEU I 277 −18.857 −15.142 −17.094 0.00 0.00
I
ATOM 3792 HN LEU I 277 −18.207 −15.821 −17.426 0.00 0.00
I
ATOM 3793 CA LEU I 277 −19.723 −15.696 −16.048 0.00 0.00
I
ATOM 3794 HA LEU I 277 −19.388 −16.705 −15.855 0.00 0.00
I
ATOM 3795 CB LEU I 277 −19.813 −14.900 −14.738 0.00 0.00
I
ATOM 3796 HB1 LEU I 277 −18.733 −14.843 −14.484 0.00 0.00
I
ATOM 3797 HB2 LEU I 277 −20.068 −13.827 −14.874 0.00 0.00
I
ATOM 3798 CG LEU I 277 −20.562 −15.501 −13.558 0.00 0.00
I
ATOM 3799 HG LEU I 277 −21.562 −15.845 −13.896 0.00 0.00
I
ATOM 3800 CD1 LEU I 277 −19.745 −16.722 −13.010 0.00 0.00
I
ATOM 3801 HD11 LEU I 277 −19.784 −17.511 −13.791 0.00 0.00
I
ATOM 3802 HD12 LEU I 277 −18.721 −16.421 −12.702 0.00 0.00
I
ATOM 3803 HD13 LEU I 277 −20.198 −17.137 −12.084 0.00 0.00
I
ATOM 3804 CD2 LEU I 277 −20.841 −14.621 −12.379 0.00 0.00
I
ATOM 3805 HD21 LEU I 277 −21.386 −13.710 −12.707 0.00 0.00
I
ATOM 3806 HD22 LEU I 277 −21.524 −15.129 −11.665 0.00 0.00
I

ATOM 3807 HD23 LEU I 277 −19.888 −14.390 −11.857 0.00 0.00
I
ATOM 3808 C LEU I 277 −21.072 −16.056 −16.733 0.00 0.00
I
ATOM 3809 O LEU I 277 −21.634 −17.175 −16.714 0.00 0.00
I
ATOM 3810 N GLY I 278 −21.699 −15.039 −17.430 0.00 0.00
I
ATOM 3811 HN GLY I 278 −21.194 −14.180 −17.396 0.00 0.00
I
ATOM 3812 CA GLY I 278 −22.938 −15.236 −18.148 0.00 0.00
I
ATOM 3813 HA1 GLY I 278 −23.402 −14.316 −18.473 0.00 0.00
I
ATOM 3814 HA2 GLY I 278 −23.689 −15.641 −17.486 0.00 0.00
I
ATOM 3815 C GLY I 278 −22.906 −16.166 −19.376 0.00 0.00
I
ATOM 3816 O GLY I 278 −23.983 −16.430 −19.943 0.00 0.00
I
ATOM 3817 N HSE I 279 −21.709 −16.594 −19.950 0.00 0.00
I
ATOM 3818 HN HSE I 279 −20.778 −16.334 −19.707 0.00 0.00
I
ATOM 3819 CA HSE I 279 −21.688 −17.610 −21.048 0.00 0.00
I
ATOM 3820 HA HSE I 279 −22.472 −17.365 −21.749 0.00 0.00
I
ATOM 3821 CB HSE I 279 −20.249 −17.485 −21.631 0.00 0.00
I
ATOM 3822 HB1 HSE I 279 −20.180 −18.021 −22.602 0.00 0.00
I
ATOM 3823 HB2 HSE I 279 −20.161 −16.439 −21.996 0.00 0.00
I
ATOM 3824 ND1 HSE I 279 −18.646 −16.885 −19.819 0.00 0.00
I
ATOM 3825 CG HSE I 279 −19.040 −17.784 −20.814 0.00 0.00
I
ATOM 3826 CE1 HSE I 279 −17.518 −17.369 −19.340 0.00 0.00
I
ATOM 3827 HE1 HSE I 279 −16.865 −16.941 −18.578 0.00 0.00
I
ATOM 3828 NE2 HSE I 279 −17.194 −18.526 −20.000 0.00 0.00
I
ATOM 3829 HE2 HSE I 279 −16.347 −19.053 −19.929 0.00 0.00
I
ATOM 3830 CD2 HSE I 279 −18.155 −18.766 −20.980 0.00 0.00
I
ATOM 3831 HD2 HSE I 279 −18.181 −19.681 −21.559 0.00 0.00
I
ATOM 3832 C HSE I 279 −21.813 −19.039 −20.581 0.00 0.00
I
ATOM 3833 O HSE I 279 −21.710 −19.981 −21.312 0.00 0.00
I

ATOM 3834 N LEU I 280 -22.044 -19.203 -19.298 0.00 0.00
I
ATOM 3835 HN LEU I 280 -22.180 -18.352 -18.797 0.00 0.00
I
ATOM 3836 CA LEU I 280 -22.186 -20.486 -18.607 0.00 0.00
I
ATOM 3837 HA LEU I 280 -22.060 -21.309 -19.295 0.00 0.00
I
ATOM 3838 CB LEU I 280 -21.220 -20.718 -17.446 0.00 0.00
I
ATOM 3839 HB1 LEU I 280 -21.391 -19.851 -16.773 0.00 0.00
I
ATOM 3840 HB2 LEU I 280 -21.585 -21.632 -16.930 0.00 0.00
I
ATOM 3841 CG LEU I 280 -19.807 -20.754 -17.841 0.00 0.00
I
ATOM 3842 HG LEU I 280 -19.489 -19.728 -18.124 0.00 0.00
I
ATOM 3843 CD1 LEU I 280 -18.885 -20.986 -16.576 0.00 0.00
I
ATOM 3844 HD11 LEU I 280 -19.012 -22.057 -16.312 0.00 0.00
I
ATOM 3845 HD12 LEU I 280 -17.830 -20.858 -16.901 0.00 0.00
I
ATOM 3846 HD13 LEU I 280 -19.007 -20.316 -15.698 0.00 0.00
I
ATOM 3847 CD2 LEU I 280 -19.542 -21.791 -18.953 0.00 0.00
I
ATOM 3848 HD21 LEU I 280 -18.464 -21.939 -19.178 0.00 0.00
I
ATOM 3849 HD22 LEU I 280 -20.200 -22.669 -18.778 0.00 0.00
I
ATOM 3850 HD23 LEU I 280 -19.966 -21.352 -19.882 0.00 0.00
I
ATOM 3851 C LEU I 280 -23.609 -20.623 -18.090 0.00 0.00
I
ATOM 3852 O LEU I 280 -24.432 -19.688 -18.064 0.00 0.00
I
ATOM 3853 N HSE I 281 -23.954 -21.851 -17.955 0.00 0.00
I
ATOM 3854 HN HSE I 281 -23.425 -22.667 -18.175 0.00 0.00
I
ATOM 3855 CA HSE I 281 -25.356 -22.254 -17.662 0.00 0.00
I
ATOM 3856 HA HSE I 281 -26.062 -21.515 -18.011 0.00 0.00
I
ATOM 3857 CB HSE I 281 -25.827 -23.561 -18.405 0.00 0.00
I
ATOM 3858 HB1 HSE I 281 -25.271 -24.466 -18.079 0.00 0.00
I
ATOM 3859 HB2 HSE I 281 -26.923 -23.726 -18.325 0.00 0.00
I
ATOM 3860 ND1 HSE I 281 -24.396 -23.505 -20.434 0.00 0.00
I

ATOM 3861 CG HSE I 281 -25.701 -23.570 -19.862 0.00 0.00
I
ATOM 3862 CE1 HSE I 281 -24.556 -23.417 -21.711 0.00 0.00
I
ATOM 3863 HE1 HSE I 281 -23.807 -23.582 -22.486 0.00 0.00
I
ATOM 3864 NE2 HSE I 281 -25.872 -23.307 -22.017 0.00 0.00
I
ATOM 3865 HE2 HSE I 281 -26.155 -23.679 -22.901 0.00 0.00
I
ATOM 3866 CD2 HSE I 281 -26.583 -23.504 -20.801 0.00 0.00
I
ATOM 3867 HD2 HSE I 281 -27.661 -23.406 -20.771 0.00 0.00
I
ATOM 3868 C HSE I 281 -25.497 -22.436 -16.171 0.00 0.00
I
ATOM 3869 O HSE I 281 -24.513 -22.312 -15.491 0.00 0.00
I
ATOM 3870 N ILE I 282 -26.690 -22.607 -15.589 0.00 0.00
I
ATOM 3871 HN ILE I 282 -27.533 -22.799 -16.086 0.00 0.00
I
ATOM 3872 CA ILE I 282 -26.930 -22.237 -14.253 0.00 0.00
I
ATOM 3873 HA ILE I 282 -26.609 -21.246 -13.968 0.00 0.00
I
ATOM 3874 CB ILE I 282 -28.436 -22.360 -13.840 0.00 0.00
I
ATOM 3875 HB ILE I 282 -28.946 -21.587 -14.454 0.00 0.00
I
ATOM 3876 CG2 ILE I 282 -28.948 -23.843 -14.120 0.00 0.00
I
ATOM 3877 HG21 ILE I 282 -28.605 -24.500 -13.292 0.00 0.00
I
ATOM 3878 HG22 ILE I 282 -30.058 -23.844 -14.073 0.00 0.00
I
ATOM 3879 HG23 ILE I 282 -28.597 -24.251 -15.092 0.00 0.00
I
ATOM 3880 CG1 ILE I 282 -28.746 -21.868 -12.411 0.00 0.00
I
ATOM 3881 HG11 ILE I 282 -29.846 -21.989 -12.310 0.00 0.00
I
ATOM 3882 HG12 ILE I 282 -28.259 -22.561 -11.692 0.00 0.00
I
ATOM 3883 CD ILE I 282 -28.397 -20.380 -12.201 0.00 0.00
I
ATOM 3884 HD1 ILE I 282 -28.836 -19.841 -13.068 0.00 0.00
I
ATOM 3885 HD2 ILE I 282 -28.790 -19.796 -11.342 0.00 0.00
I
ATOM 3886 HD3 ILE I 282 -27.295 -20.240 -12.156 0.00 0.00
I
ATOM 3887 C ILE I 282 -26.122 -22.988 -13.215 0.00 0.00
I

ATOM 3888 O ILE I 282 -25.477 -22.327 -12.430 0.00 0.00
I
ATOM 3889 N VAL I 283 -25.933 -24.266 -13.271 0.00 0.00
I
ATOM 3890 HN VAL I 283 -26.383 -24.873 -13.921 0.00 0.00
I
ATOM 3891 CA VAL I 283 -25.284 -25.000 -12.157 0.00 0.00
I
ATOM 3892 HA VAL I 283 -25.587 -24.604 -11.199 0.00 0.00
I
ATOM 3893 CB VAL I 283 -25.659 -26.435 -12.168 0.00 0.00
I
ATOM 3894 HB VAL I 283 -25.245 -26.954 -11.278 0.00 0.00
I
ATOM 3895 CG1 VAL I 283 -27.204 -26.481 -11.789 0.00 0.00
I
ATOM 3896 HG11 VAL I 283 -27.342 -27.536 -11.470 0.00 0.00
I
ATOM 3897 HG12 VAL I 283 -27.356 -25.734 -10.981 0.00 0.00
I
ATOM 3898 HG13 VAL I 283 -27.963 -26.336 -12.587 0.00 0.00
I
ATOM 3899 CG2 VAL I 283 -25.314 -27.245 -13.432 0.00 0.00
I
ATOM 3900 HG21 VAL I 283 -25.818 -26.969 -14.382 0.00 0.00
I
ATOM 3901 HG22 VAL I 283 -24.219 -27.264 -13.618 0.00 0.00
I
ATOM 3902 HG23 VAL I 283 -25.603 -28.306 -13.268 0.00 0.00
I
ATOM 3903 C VAL I 283 -23.768 -24.995 -12.146 0.00 0.00
I
ATOM 3904 O VAL I 283 -23.140 -25.012 -11.079 0.00 0.00
I
ATOM 3905 N ASP I 284 -23.240 -24.994 -13.450 0.00 0.00
I
ATOM 3906 HN ASP I 284 -23.903 -25.177 -14.171 0.00 0.00
I
ATOM 3907 CA ASP I 284 -21.959 -24.647 -13.804 0.00 0.00
I
ATOM 3908 HA ASP I 284 -21.213 -25.345 -13.452 0.00 0.00
I
ATOM 3909 CB ASP I 284 -21.866 -24.620 -15.340 0.00 0.00
I
ATOM 3910 HB1 ASP I 284 -22.411 -23.719 -15.693 0.00 0.00
I
ATOM 3911 HB2 ASP I 284 -20.847 -24.637 -15.782 0.00 0.00
I
ATOM 3912 CG ASP I 284 -22.549 -25.924 -15.957 0.00 0.00
I
ATOM 3913 OD1 ASP I 284 -23.754 -26.034 -16.043 0.00 0.00
I
ATOM 3914 OD2 ASP I 284 -21.783 -26.890 -16.212 0.00 0.00
I

ATOM	3915	C	ASP	I	284	-21.501	-23.245	-13.289	0.00	0.00	I
ATOM	3916	O	ASP	I	284	-20.402	-23.035	-12.695	0.00	0.00	I
ATOM	3917	N	LYS	I	285	-22.423	-22.233	-13.600	0.00	0.00	I
ATOM	3918	HN	LYS	I	285	-23.181	-22.373	-14.232	0.00	0.00	I
ATOM	3919	CA	LYS	I	285	-22.181	-20.813	-13.141	0.00	0.00	I
ATOM	3920	HA	LYS	I	285	-21.191	-20.536	-13.471	0.00	0.00	I
ATOM	3921	CB	LYS	I	285	-23.331	-19.879	-13.855	0.00	0.00	I
ATOM	3922	HB1	LYS	I	285	-23.349	-20.059	-14.951	0.00	0.00	I
ATOM	3923	HB2	LYS	I	285	-24.363	-20.069	-13.490	0.00	0.00	I
ATOM	3924	CG	LYS	I	285	-23.164	-18.351	-13.631	0.00	0.00	I
ATOM	3925	HG1	LYS	I	285	-23.086	-18.001	-12.579	0.00	0.00	I
ATOM	3926	HG2	LYS	I	285	-22.156	-18.257	-14.089	0.00	0.00	I
ATOM	3927	CD	LYS	I	285	-24.117	-17.419	-14.447	0.00	0.00	I
ATOM	3928	HD1	LYS	I	285	-23.585	-16.443	-14.442	0.00	0.00	I
ATOM	3929	HD2	LYS	I	285	-24.132	-17.830	-15.479	0.00	0.00	I
ATOM	3930	CE	LYS	I	285	-25.484	-17.323	-13.892	0.00	0.00	I
ATOM	3931	HE1	LYS	I	285	-25.926	-18.341	-13.835	0.00	0.00	I
ATOM	3932	HE2	LYS	I	285	-25.424	-16.902	-12.866	0.00	0.00	I
ATOM	3933	NZ	LYS	I	285	-26.402	-16.527	-14.700	0.00	0.00	I
ATOM	3934	HZ1	LYS	I	285	-26.119	-15.563	-14.969	0.00	0.00	I
ATOM	3935	HZ2	LYS	I	285	-26.352	-17.119	-15.553	0.00	0.00	I
ATOM	3936	HZ3	LYS	I	285	-27.409	-16.547	-14.440	0.00	0.00	I
ATOM	3937	C	LYS	I	285	-22.086	-20.811	-11.559	0.00	0.00	I
ATOM	3938	O	LYS	I	285	-21.278	-20.095	-10.972	0.00	0.00	I
ATOM	3939	N	LEU	I	286	-22.969	-21.646	-10.889	0.00	0.00	I
ATOM	3940	HN	LEU	I	286	-23.702	-22.168	-11.319	0.00	0.00	I
ATOM	3941	CA	LEU	I	286	-22.973	-21.749	-9.471	0.00	0.00	I

ATOM	3942	HA	LEU	I	286	-23.075	-20.759	-9.052	0.00	0.00	I
ATOM	3943	CB	LEU	I	286	-24.248	-22.540	-9.017	0.00	0.00	I
ATOM	3944	HB1	LEU	I	286	-25.048	-22.506	-9.787	0.00	0.00	I
ATOM	3945	HB2	LEU	I	286	-23.951	-23.610	-9.028	0.00	0.00	I
ATOM	3946	CG	LEU	I	286	-24.868	-22.119	-7.663	0.00	0.00	I
ATOM	3947	HG	LEU	I	286	-24.059	-22.115	-6.902	0.00	0.00	I
ATOM	3948	CD1	LEU	I	286	-25.338	-20.725	-7.677	0.00	0.00	I
ATOM	3949	HD11	LEU	I	286	-25.841	-20.463	-6.722	0.00	0.00	I
ATOM	3950	HD12	LEU	I	286	-24.513	-19.993	-7.812	0.00	0.00	I
ATOM	3951	HD13	LEU	I	286	-26.079	-20.551	-8.487	0.00	0.00	I
ATOM	3952	CD2	LEU	I	286	-25.998	-23.028	-7.162	0.00	0.00	I
ATOM	3953	HD21	LEU	I	286	-26.812	-22.922	-7.911	0.00	0.00	I
ATOM	3954	HD22	LEU	I	286	-25.764	-24.104	-7.015	0.00	0.00	I
ATOM	3955	HD23	LEU	I	286	-26.386	-22.627	-6.201	0.00	0.00	I
ATOM	3956	C	LEU	I	286	-21.594	-22.329	-8.892	0.00	0.00	I
ATOM	3957	O	LEU	I	286	-21.100	-21.831	-7.895	0.00	0.00	I
ATOM	3958	N	TRP	I	287	-21.046	-23.457	-9.539	0.00	0.00	I
ATOM	3959	HN	TRP	I	287	-21.417	-23.772	-10.409	0.00	0.00	I
ATOM	3960	CA	TRP	I	287	-19.754	-24.002	-9.189	0.00	0.00	I
ATOM	3961	HA	TRP	I	287	-19.716	-24.270	-8.144	0.00	0.00	I
ATOM	3962	CB	TRP	I	287	-19.313	-25.203	-10.071	0.00	0.00	I
ATOM	3963	HB1	TRP	I	287	-19.644	-24.959	-11.104	0.00	0.00	I
ATOM	3964	HB2	TRP	I	287	-18.254	-25.504	-9.927	0.00	0.00	I
ATOM	3965	CG	TRP	I	287	-20.010	-26.482	-9.619	0.00	0.00	I
ATOM	3966	CD1	TRP	I	287	-20.739	-27.252	-10.447	0.00	0.00	I
ATOM	3967	HD1	TRP	I	287	-21.220	-26.935	-11.360	0.00	0.00	I
ATOM	3968	NE1	TRP	I	287	-21.229	-28.357	-9.725	0.00	0.00	I

ATOM 3969 HE1 TRP I 287 −21.511 −29.154 −10.213 0.00 0.00
I
ATOM 3970 CE2 TRP I 287 −20.647 −28.328 −8.438 0.00 0.00
I
ATOM 3971 CD2 TRP I 287 −19.874 −27.173 −8.337 0.00 0.00
I
ATOM 3972 CE3 TRP I 287 −19.145 −26.823 −7.186 0.00 0.00
I
ATOM 3973 HE3 TRP I 287 −18.473 −25.978 −7.172 0.00 0.00
I
ATOM 3974 CZ3 TRP I 287 −19.120 −27.719 −6.138 0.00 0.00
I
ATOM 3975 HZ3 TRP I 287 −18.545 −27.473 −5.258 0.00 0.00
I
ATOM 3976 CZ2 TRP I 287 −20.743 −29.163 −7.290 0.00 0.00
I
ATOM 3977 HZ2 TRP I 287 −21.370 −30.036 −7.403 0.00 0.00
I
ATOM 3978 CH2 TRP I 287 −19.954 −28.885 −6.162 0.00 0.00
I
ATOM 3979 HH2 TRP I 287 −19.870 −29.571 −5.332 0.00 0.00
I
ATOM 3980 C TRP I 287 −18.605 −22.876 −9.379 0.00 0.00
I
ATOM 3981 O TRP I 287 −17.781 −22.655 −8.463 0.00 0.00
I
ATOM 3982 N ALA I 288 −18.528 −22.228 −10.513 0.00 0.00
I
ATOM 3983 HN ALA I 288 −19.077 −22.554 −11.279 0.00 0.00
I
ATOM 3984 CA ALA I 288 −17.436 −21.360 −10.879 0.00 0.00
I
ATOM 3985 HA ALA I 288 −16.442 −21.781 −10.905 0.00 0.00
I
ATOM 3986 CB ALA I 288 −17.728 −20.843 −12.369 0.00 0.00
I
ATOM 3987 HB1 ALA I 288 −16.882 −20.197 −12.689 0.00 0.00
I
ATOM 3988 HB2 ALA I 288 −17.873 −21.652 −13.117 0.00 0.00
I
ATOM 3989 HB3 ALA I 288 −18.580 −20.131 −12.414 0.00 0.00
I
ATOM 3990 C ALA I 288 −17.368 −20.120 −10.000 0.00 0.00
I
ATOM 3991 O ALA I 288 −16.352 −19.601 −9.743 0.00 0.00
I
ATOM 3992 N SER I 289 −18.538 −19.585 −9.499 0.00 0.00
I
ATOM 3993 HN SER I 289 −19.431 −20.028 −9.537 0.00 0.00
I
ATOM 3994 CA SER I 289 −18.643 −18.410 −8.642 0.00 0.00
I
ATOM 3995 HA SER I 289 −18.165 −17.637 −9.225 0.00 0.00
I

```
ATOM   3996  CB  SER I 289     -20.055 -17.932  -8.340  0.00  0.00
I
ATOM   3997  HB1 SER I 289     -20.685 -18.604  -7.719  0.00  0.00
I
ATOM   3998  HB2 SER I 289     -20.018 -17.018  -7.709  0.00  0.00
I
ATOM   3999  OG  SER I 289     -20.724 -17.719  -9.572  0.00  0.00
I
ATOM   4000  HG1 SER I 289     -20.889 -18.580  -9.962  0.00  0.00
I
ATOM   4001  C   SER I 289     -17.911 -18.648  -7.310  0.00  0.00
I
ATOM   4002  O   SER I 289     -17.158 -17.821  -6.780  0.00  0.00
I
ATOM   4003  N   TYR I 290     -18.053 -19.861  -6.715  0.00  0.00
I
ATOM   4004  HN  TYR I 290     -18.739 -20.565  -6.880  0.00  0.00
I
ATOM   4005  CA  TYR I 290     -17.270 -20.282  -5.523  0.00  0.00
I
ATOM   4006  HA  TYR I 290     -17.315 -19.486  -4.795  0.00  0.00
I
ATOM   4007  CB  TYR I 290     -17.959 -21.529  -4.867  0.00  0.00
I
ATOM   4008  HB1 TYR I 290     -19.037 -21.315  -4.705  0.00  0.00
I
ATOM   4009  HB2 TYR I 290     -17.968 -22.402  -5.554  0.00  0.00
I
ATOM   4010  CG  TYR I 290     -17.347 -21.958  -3.644  0.00  0.00
I
ATOM   4011  CD1 TYR I 290     -16.511 -23.133  -3.547  0.00  0.00
I
ATOM   4012  HD1 TYR I 290     -16.290 -23.573  -4.508  0.00  0.00
I
ATOM   4013  CE1 TYR I 290     -16.075 -23.640  -2.313  0.00  0.00
I
ATOM   4014  HE1 TYR I 290     -15.567 -24.593  -2.335  0.00  0.00
I
ATOM   4015  CZ  TYR I 290     -16.403 -22.929  -1.164  0.00  0.00
I
ATOM   4016  OH  TYR I 290     -15.923 -23.416   0.077  0.00  0.00
I
ATOM   4017  HH  TYR I 290     -15.236 -24.049  -0.140  0.00  0.00
I
ATOM   4018  CD2 TYR I 290     -17.452 -21.248  -2.443  0.00  0.00
I
ATOM   4019  HD2 TYR I 290     -18.084 -20.374  -2.503  0.00  0.00
I
ATOM   4020  CE2 TYR I 290     -17.078 -21.729  -1.197  0.00  0.00
I
ATOM   4021  HE2 TYR I 290     -17.225 -21.154  -0.295  0.00  0.00
I
ATOM   4022  C   TYR I 290     -15.841 -20.462  -5.931  0.00  0.00
I
```

ATOM 4023 O TYR I 290 -14.960 -20.045 -5.170 0.00 0.00
I
ATOM 4024 N PHE I 291 -15.604 -21.017 -7.109 0.00 0.00
I
ATOM 4025 HN PHE I 291 -16.273 -21.562 -7.609 0.00 0.00
I
ATOM 4026 CA PHE I 291 -14.207 -21.266 -7.516 0.00 0.00
I
ATOM 4027 HA PHE I 291 -13.679 -21.718 -6.689 0.00 0.00
I
ATOM 4028 CB PHE I 291 -14.269 -22.267 -8.753 0.00 0.00
I
ATOM 4029 HB1 PHE I 291 -15.034 -23.042 -8.533 0.00 0.00
I
ATOM 4030 HB2 PHE I 291 -14.619 -21.801 -9.699 0.00 0.00
I
ATOM 4031 CG PHE I 291 -12.976 -22.931 -9.067 0.00 0.00
I
ATOM 4032 CD1 PHE I 291 -12.798 -24.304 -8.801 0.00 0.00
I
ATOM 4033 HD1 PHE I 291 -13.625 -24.843 -8.364 0.00 0.00
I
ATOM 4034 CE1 PHE I 291 -11.565 -24.899 -9.005 0.00 0.00
I
ATOM 4035 HE1 PHE I 291 -11.497 -25.943 -8.739 0.00 0.00
I
ATOM 4036 CZ PHE I 291 -10.558 -24.126 -9.538 0.00 0.00
I
ATOM 4037 HZ PHE I 291 -9.613 -24.590 -9.780 0.00 0.00
I
ATOM 4038 CD2 PHE I 291 -11.929 -22.165 -9.555 0.00 0.00
I
ATOM 4039 HD2 PHE I 291 -12.058 -21.094 -9.610 0.00 0.00
I
ATOM 4040 CE2 PHE I 291 -10.741 -22.799 -9.862 0.00 0.00
I
ATOM 4041 HE2 PHE I 291 -9.946 -22.181 -10.254 0.00 0.00
I
ATOM 4042 C PHE I 291 -13.358 -19.932 -7.686 0.00 0.00
I
ATOM 4043 O PHE I 291 -12.245 -19.758 -7.275 0.00 0.00
I
ATOM 4044 N GLN I 292 -14.001 -18.950 -8.342 0.00 0.00
I
ATOM 4045 HN GLN I 292 -14.917 -19.050 -8.723 0.00 0.00
I
ATOM 4046 CA GLN I 292 -13.357 -17.683 -8.602 0.00 0.00
I
ATOM 4047 HA GLN I 292 -12.288 -17.723 -8.755 0.00 0.00
I
ATOM 4048 CB GLN I 292 -14.002 -17.020 -9.857 0.00 0.00
I
ATOM 4049 HB1 GLN I 292 -15.000 -17.506 -9.915 0.00 0.00
I

```
ATOM   4050  HB2 GLN I 292     -14.063 -15.929  -9.661  0.00  0.00
I
ATOM   4051  CG  GLN I 292     -13.132 -17.188 -11.139  0.00  0.00
I
ATOM   4052  HG1 GLN I 292     -13.701 -16.739 -11.981  0.00  0.00
I
ATOM   4053  HG2 GLN I 292     -12.128 -16.717 -11.065  0.00  0.00
I
ATOM   4054  CD  GLN I 292     -12.879 -18.642 -11.473  0.00  0.00
I
ATOM   4055  OE1 GLN I 292     -11.777 -19.166 -11.612  0.00  0.00
I
ATOM   4056  NE2 GLN I 292     -13.974 -19.440 -11.501  0.00  0.00
I
ATOM   4057 HE21 GLN I 292     -13.763 -20.305 -11.955  0.00  0.00
I
ATOM   4058 HE22 GLN I 292     -14.896 -19.152 -11.241  0.00  0.00
I
ATOM   4059  C   GLN I 292     -13.415 -16.762  -7.352  0.00  0.00
I
ATOM   4060  O   GLN I 292     -12.619 -15.798  -7.259  0.00  0.00
I
ATOM   4061  N   ALA I 293     -14.396 -16.990  -6.445  0.00  0.00
I
ATOM   4062  HN  ALA I 293     -15.143 -17.618  -6.647  0.00  0.00
I
ATOM   4063  CA  ALA I 293     -14.334 -16.265  -5.190  0.00  0.00
I
ATOM   4064  HA  ALA I 293     -14.152 -15.209  -5.324  0.00  0.00
I
ATOM   4065  CB  ALA I 293     -15.732 -16.385  -4.537  0.00  0.00
I
ATOM   4066  HB1 ALA I 293     -15.807 -15.618  -3.736  0.00  0.00
I
ATOM   4067  HB2 ALA I 293     -16.450 -16.130  -5.347  0.00  0.00
I
ATOM   4068  HB3 ALA I 293     -16.062 -17.279  -3.967  0.00  0.00
I
ATOM   4069  C   ALA I 293     -13.249 -16.713  -4.155  0.00  0.00
I
ATOM   4070  O   ALA I 293     -12.622 -15.935  -3.428  0.00  0.00
I
ATOM   4071  N   VAL I 294     -12.961 -18.101  -4.128  0.00  0.00
I
ATOM   4072  HN  VAL I 294     -13.430 -18.749  -4.723  0.00  0.00
I
ATOM   4073  CA  VAL I 294     -11.953 -18.615  -3.274  0.00  0.00
I
ATOM   4074  HA  VAL I 294     -11.878 -17.849  -2.517  0.00  0.00
I
ATOM   4075  CB  VAL I 294     -12.244 -19.873  -2.383  0.00  0.00
I
ATOM   4076  HB  VAL I 294     -11.616 -19.875  -1.466  0.00  0.00
I
```

ATOM 4077 CG1 VAL I 294 -13.706 -19.878 -1.890 0.00 0.00
I
ATOM 4078 HG11 VAL I 294 -13.985 -18.886 -1.476 0.00 0.00
I
ATOM 4079 HG12 VAL I 294 -14.404 -20.163 -2.707 0.00 0.00
I
ATOM 4080 HG13 VAL I 294 -13.800 -20.546 -1.007 0.00 0.00
I
ATOM 4081 CG2 VAL I 294 -12.014 -21.135 -3.155 0.00 0.00
I
ATOM 4082 HG21 VAL I 294 -12.320 -22.039 -2.586 0.00 0.00
I
ATOM 4083 HG22 VAL I 294 -12.756 -21.076 -3.980 0.00 0.00
I
ATOM 4084 HG23 VAL I 294 -11.000 -21.300 -3.578 0.00 0.00
I
ATOM 4085 C VAL I 294 -10.491 -18.588 -3.832 0.00 0.00
I
ATOM 4086 O VAL I 294 -9.599 -18.400 -3.041 0.00 0.00
I
ATOM 4087 N THR I 295 -10.304 -18.756 -5.090 0.00 0.00
I
ATOM 4088 HN THR I 295 -11.044 -18.807 -5.756 0.00 0.00
I
ATOM 4089 CA THR I 295 -9.006 -18.870 -5.745 0.00 0.00
I
ATOM 4090 HA THR I 295 -8.419 -19.592 -5.196 0.00 0.00
I
ATOM 4091 CB THR I 295 -9.086 -19.259 -7.162 0.00 0.00
I
ATOM 4092 HB THR I 295 -9.721 -20.170 -7.208 0.00 0.00
I
ATOM 4093 OG1 THR I 295 -7.859 -19.825 -7.644 0.00 0.00
I
ATOM 4094 HG1 THR I 295 -8.129 -20.226 -8.474 0.00 0.00
I
ATOM 4095 CG2 THR I 295 -9.443 -18.055 -8.148 0.00 0.00
I
ATOM 4096 HG21 THR I 295 -9.699 -18.290 -9.203 0.00 0.00
I
ATOM 4097 HG22 THR I 295 -10.414 -17.679 -7.761 0.00 0.00
I
ATOM 4098 HG23 THR I 295 -8.735 -17.198 -8.152 0.00 0.00
I
ATOM 4099 C THR I 295 -8.058 -17.652 -5.632 0.00 0.00
I
ATOM 4100 O THR I 295 -6.832 -17.911 -5.802 0.00 0.00
I
ATOM 4101 N PRO I 296 -8.409 -16.334 -5.547 0.00 0.00
I
ATOM 4102 CD PRO I 296 -9.788 -15.825 -5.206 0.00 0.00
I
ATOM 4103 HD1 PRO I 296 -10.621 -16.324 -5.746 0.00 0.00
I

```
ATOM   4104  HD2 PRO I 296      -9.932 -15.943  -4.111  0.00  0.00
I
ATOM   4105  CA  PRO I 296      -7.399 -15.311  -5.472  0.00  0.00
I
ATOM   4106  HA  PRO I 296      -6.662 -15.522  -6.233  0.00  0.00
I
ATOM   4107  CB  PRO I 296      -8.227 -14.047  -5.667  0.00  0.00
I
ATOM   4108  HB1 PRO I 296      -8.105 -13.806  -6.745  0.00  0.00
I
ATOM   4109  HB2 PRO I 296      -7.832 -13.126  -5.186  0.00  0.00
I
ATOM   4110  CG  PRO I 296      -9.731 -14.330  -5.499  0.00  0.00
I
ATOM   4111  HG1 PRO I 296     -10.289 -13.799  -4.698  0.00  0.00
I
ATOM   4112  HG2 PRO I 296     -10.237 -14.009  -6.434  0.00  0.00
I
ATOM   4113  C   PRO I 296      -6.720 -15.276  -4.088  0.00  0.00
I
ATOM   4114  O   PRO I 296      -5.734 -14.530  -3.956  0.00  0.00
I
ATOM   4115  N   ARG I 297      -7.282 -15.966  -3.005  0.00  0.00
I
ATOM   4116  HN  ARG I 297      -8.191 -16.368  -2.924  0.00  0.00
I
ATOM   4117  CA  ARG I 297      -6.568 -16.065  -1.836  0.00  0.00
I
ATOM   4118  HA  ARG I 297      -5.926 -15.199  -1.774  0.00  0.00
I
ATOM   4119  CB  ARG I 297      -7.513 -16.424  -0.533  0.00  0.00
I
ATOM   4120  HB1 ARG I 297      -8.048 -17.338  -0.868  0.00  0.00
I
ATOM   4121  HB2 ARG I 297      -6.867 -16.687   0.332  0.00  0.00
I
ATOM   4122  CG  ARG I 297      -8.376 -15.174  -0.292  0.00  0.00
I
ATOM   4123  HG1 ARG I 297      -7.820 -14.220  -0.169  0.00  0.00
I
ATOM   4124  HG2 ARG I 297      -9.011 -15.005  -1.187  0.00  0.00
I
ATOM   4125  CD  ARG I 297      -9.340 -15.351   0.853  0.00  0.00
I
ATOM   4126  HD1 ARG I 297     -10.052 -14.501   0.929  0.00  0.00
I
ATOM   4127  HD2 ARG I 297      -9.954 -16.276   0.808  0.00  0.00
I
ATOM   4128  NE  ARG I 297      -8.580 -15.418   2.149  0.00  0.00
I
ATOM   4129  HE  ARG I 297      -7.709 -14.927   2.166  0.00  0.00
I
ATOM   4130  CZ  ARG I 297      -8.692 -16.305   3.109  0.00  0.00
I
```

ATOM 4131 NH1 ARG I 297 -9.595 -17.245 3.137 0.00 0.00
I
ATOM 4132 HH11 ARG I 297 -9.311 -17.908 3.829 0.00 0.00
I
ATOM 4133 HH12 ARG I 297 -10.310 -17.290 2.440 0.00 0.00
I
ATOM 4134 NH2 ARG I 297 -7.950 -16.252 4.202 0.00 0.00
I
ATOM 4135 HH21 ARG I 297 -8.091 -16.827 5.008 0.00 0.00
I
ATOM 4136 HH22 ARG I 297 -7.226 -15.566 4.271 0.00 0.00
I
ATOM 4137 C ARG I 297 -5.544 -17.194 -2.085 0.00 0.00
I
ATOM 4138 O ARG I 297 -5.927 -18.342 -1.794 0.00 0.00
I
ATOM 4139 N THR I 298 -4.314 -16.938 -2.651 0.00 0.00
I
ATOM 4140 HN THR I 298 -4.188 -16.137 -3.230 0.00 0.00
I
ATOM 4141 CA THR I 298 -3.030 -17.798 -2.576 0.00 0.00
I
ATOM 4142 HA THR I 298 -2.153 -17.241 -2.870 0.00 0.00
I
ATOM 4143 CB THR I 298 -2.689 -18.254 -1.218 0.00 0.00
I
ATOM 4144 HB THR I 298 -3.516 -18.786 -0.700 0.00 0.00
I
ATOM 4145 OG1 THR I 298 -2.405 -17.084 -0.461 0.00 0.00
I
ATOM 4146 HG1 THR I 298 -3.161 -16.501 -0.353 0.00 0.00
I
ATOM 4147 CG2 THR I 298 -1.382 -19.082 -1.278 0.00 0.00
I
ATOM 4148 HG21 THR I 298 -1.016 -19.319 -0.256 0.00 0.00
I
ATOM 4149 HG22 THR I 298 -1.592 -20.041 -1.799 0.00 0.00
I
ATOM 4150 HG23 THR I 298 -0.554 -18.597 -1.838 0.00 0.00
I
ATOM 4151 C THR I 298 -3.043 -18.891 -3.618 0.00 0.00
I
ATOM 4152 O THR I 298 -2.068 -19.077 -4.323 0.00 0.00
I
ATOM 4153 N ALA I 299 -4.183 -19.577 -3.790 0.00 0.00
I
ATOM 4154 HN ALA I 299 -4.952 -19.325 -3.208 0.00 0.00
I
ATOM 4155 CA ALA I 299 -4.417 -20.787 -4.617 0.00 0.00
I
ATOM 4156 HA ALA I 299 -3.914 -21.664 -4.239 0.00 0.00
I
ATOM 4157 CB ALA I 299 -5.958 -21.125 -4.567 0.00 0.00
I

ATOM 4158 HB1 ALA I 299 −6.620 −20.267 −4.812 0.00 0.00
I
ATOM 4159 HB2 ALA I 299 −6.200 −21.800 −5.416 0.00 0.00
I
ATOM 4160 HB3 ALA I 299 −6.239 −21.551 −3.580 0.00 0.00
I
ATOM 4161 C ALA I 299 −4.012 −20.709 −6.089 0.00 0.00
I
ATOM 4162 O ALA I 299 −3.294 −21.573 −6.476 0.00 0.00
I
ATOM 4163 N GLY I 300 −4.480 −19.692 −6.796 0.00 0.00
I
ATOM 4164 HN GLY I 300 −5.160 −19.079 −6.402 0.00 0.00
I
ATOM 4165 CA GLY I 300 −4.028 −19.442 −8.191 0.00 0.00
I
ATOM 4166 HA1 GLY I 300 −2.959 −19.558 −8.094 0.00 0.00
I
ATOM 4167 HA2 GLY I 300 −4.388 −18.448 −8.410 0.00 0.00
I
ATOM 4168 C GLY I 300 −4.480 −20.286 −9.429 0.00 0.00
I
ATOM 4169 O GLY I 300 −3.840 −20.122 −10.487 0.00 0.00
I
ATOM 4170 N PHE I 301 −5.527 −21.099 −9.353 0.00 0.00
I
ATOM 4171 HN PHE I 301 −6.016 −21.208 −8.490 0.00 0.00
I
ATOM 4172 CA PHE I 301 −6.148 −21.833 −10.499 0.00 0.00
I
ATOM 4173 HA PHE I 301 −5.413 −21.908 −11.286 0.00 0.00
I
ATOM 4174 CB PHE I 301 −6.523 −23.299 −10.235 0.00 0.00
I
ATOM 4175 HB1 PHE I 301 −7.140 −23.123 −9.328 0.00 0.00
I
ATOM 4176 HB2 PHE I 301 −7.150 −23.714 −11.052 0.00 0.00
I
ATOM 4177 CG PHE I 301 −5.387 −24.107 −9.993 0.00 0.00
I
ATOM 4178 CD1 PHE I 301 −4.701 −24.111 −8.777 0.00 0.00
I
ATOM 4179 HD1 PHE I 301 −4.926 −23.360 −8.034 0.00 0.00
I
ATOM 4180 CE1 PHE I 301 −3.677 −24.996 −8.505 0.00 0.00
I
ATOM 4181 HE1 PHE I 301 −3.108 −24.890 −7.594 0.00 0.00
I
ATOM 4182 CZ PHE I 301 −3.329 −25.916 −9.538 0.00 0.00
I
ATOM 4183 HZ PHE I 301 −2.512 −26.608 −9.402 0.00 0.00
I
ATOM 4184 CD2 PHE I 301 −5.003 −24.992 −11.034 0.00 0.00
I

```
ATOM   4185  HD2 PHE I 301      -5.562 -25.018 -11.958  0.00  0.00
I
ATOM   4186  CE2 PHE I 301      -3.946 -25.888 -10.792  0.00  0.00
I
ATOM   4187  HE2 PHE I 301      -3.617 -26.600 -11.534  0.00  0.00
I
ATOM   4188  C   PHE I 301      -7.284 -21.024 -11.026  0.00  0.00
I
ATOM   4189  O   PHE I 301      -7.875 -20.283 -10.209  0.00  0.00
I
ATOM   4190  N   ASN I 302      -7.652 -21.117 -12.311  0.00  0.00
I
ATOM   4191  HN  ASN I 302      -7.327 -21.787 -12.975  0.00  0.00
I
ATOM   4192  CA  ASN I 302      -8.763 -20.331 -12.859  0.00  0.00
I
ATOM   4193  HA  ASN I 302      -9.455 -20.136 -12.053  0.00  0.00
I
ATOM   4194  CB  ASN I 302      -8.246 -18.968 -13.479  0.00  0.00
I
ATOM   4195  HB1 ASN I 302      -8.974 -18.356 -14.053  0.00  0.00
I
ATOM   4196  HB2 ASN I 302      -7.931 -18.370 -12.598  0.00  0.00
I
ATOM   4197  CG  ASN I 302      -6.892 -19.186 -14.301  0.00  0.00
I
ATOM   4198  OD1 ASN I 302      -5.876 -18.597 -13.890  0.00  0.00
I
ATOM   4199  ND2 ASN I 302      -7.042 -19.676 -15.548  0.00  0.00
I
ATOM   4200 HD21 ASN I 302      -6.300 -19.437 -16.174  0.00  0.00
I
ATOM   4201 HD22 ASN I 302      -7.879 -20.191 -15.734  0.00  0.00
I
ATOM   4202  C   ASN I 302      -9.596 -21.065 -13.837  0.00  0.00
I
ATOM   4203  O   ASN I 302      -9.112 -21.747 -14.723  0.00  0.00
I
ATOM   4204  N   SER I 303     -10.891 -20.974 -13.668  0.00  0.00
I
ATOM   4205  HN  SER I 303     -11.366 -20.601 -12.875  0.00  0.00
I
ATOM   4206  CA  SER I 303     -11.836 -21.691 -14.497  0.00  0.00
I
ATOM   4207  HA  SER I 303     -11.311 -22.351 -15.172  0.00  0.00
I
ATOM   4208  CB  SER I 303     -12.718 -22.617 -13.669  0.00  0.00
I
ATOM   4209  HB1 SER I 303     -13.507 -23.179 -14.213  0.00  0.00
I
ATOM   4210  HB2 SER I 303     -12.066 -23.462 -13.358  0.00  0.00
I
ATOM   4211  OG  SER I 303     -13.314 -21.990 -12.587  0.00  0.00
I
```

ATOM 4212 HG1 SER I 303 −14.010 −22.603 −12.342 0.00 0.00
I
ATOM 4213 C SER I 303 −12.638 −20.630 −15.321 0.00 0.00
I
ATOM 4214 O SER I 303 −13.518 −20.970 −16.165 0.00 0.00
I
ATOM 4215 N LEU I 304 −12.200 −19.350 −15.108 0.00 0.00
I
ATOM 4216 HN LEU I 304 −11.565 −19.244 −14.347 0.00 0.00
I
ATOM 4217 CA LEU I 304 −12.749 −18.165 −15.778 0.00 0.00
I
ATOM 4218 HA LEU I 304 −13.237 −18.490 −16.685 0.00 0.00
I
ATOM 4219 CB LEU I 304 −13.762 −17.381 −14.984 0.00 0.00
I
ATOM 4220 HB1 LEU I 304 −13.296 −17.056 −14.029 0.00 0.00
I
ATOM 4221 HB2 LEU I 304 −14.055 −16.431 −15.481 0.00 0.00
I
ATOM 4222 CG LEU I 304 −15.130 −18.238 −14.608 0.00 0.00
I
ATOM 4223 HG LEU I 304 −14.822 −19.210 −14.166 0.00 0.00
I
ATOM 4224 CD1 LEU I 304 −15.943 −17.489 −13.565 0.00 0.00
I
ATOM 4225 HD11 LEU I 304 −15.449 −17.708 −12.594 0.00 0.00
I
ATOM 4226 HD12 LEU I 304 −15.945 −16.394 −13.751 0.00 0.00
I
ATOM 4227 HD13 LEU I 304 −16.990 −17.859 −13.590 0.00 0.00
I
ATOM 4228 CD2 LEU I 304 −15.937 −18.489 −15.903 0.00 0.00
I
ATOM 4229 HD21 LEU I 304 −15.846 −19.493 −16.372 0.00 0.00
I
ATOM 4230 HD22 LEU I 304 −17.018 −18.391 −15.664 0.00 0.00
I
ATOM 4231 HD23 LEU I 304 −15.695 −17.742 −16.689 0.00 0.00
I
ATOM 4232 C LEU I 304 −11.604 −17.266 −16.193 0.00 0.00
I
ATOM 4233 O LEU I 304 −10.552 −17.284 −15.522 0.00 0.00
I
ATOM 4234 N ASP I 305 −11.753 −16.511 −17.317 0.00 0.00
I
ATOM 4235 HN ASP I 305 −12.559 −16.534 −17.904 0.00 0.00
I
ATOM 4236 CA ASP I 305 −10.781 −15.580 −17.762 0.00 0.00
I
ATOM 4237 HA ASP I 305 −9.808 −16.000 −17.554 0.00 0.00
I
ATOM 4238 CB ASP I 305 −10.832 −15.531 −19.312 0.00 0.00
I

```
ATOM   4239  HB1 ASP I 305     -10.689 -16.523 -19.792  0.00  0.00
I
ATOM   4240  HB2 ASP I 305     -11.804 -15.111 -19.650  0.00  0.00
I
ATOM   4241  CG  ASP I 305      -9.884 -14.509 -19.932  0.00  0.00
I
ATOM   4242  OD1 ASP I 305     -10.330 -13.705 -20.755  0.00  0.00
I
ATOM   4243  OD2 ASP I 305      -8.682 -14.550 -19.688  0.00  0.00
I
ATOM   4244  C   ASP I 305     -10.883 -14.172 -17.103  0.00  0.00
I
ATOM   4245  O   ASP I 305     -11.910 -13.495 -17.377  0.00  0.00
I
ATOM   4246  N   PHE I 306      -9.807 -13.744 -16.413  0.00  0.00
I
ATOM   4247  HN  PHE I 306      -8.986 -14.284 -16.248  0.00  0.00
I
ATOM   4248  CA  PHE I 306      -9.814 -12.425 -15.768  0.00  0.00
I
ATOM   4249  HA  PHE I 306     -10.702 -12.507 -15.158  0.00  0.00
I
ATOM   4250  CB  PHE I 306      -8.609 -12.269 -14.920  0.00  0.00
I
ATOM   4251  HB1 PHE I 306      -7.757 -12.365 -15.627  0.00  0.00
I
ATOM   4252  HB2 PHE I 306      -8.601 -11.265 -14.444  0.00  0.00
I
ATOM   4253  CG  PHE I 306      -8.595 -13.273 -13.809  0.00  0.00
I
ATOM   4254  CD1 PHE I 306      -9.360 -13.048 -12.667  0.00  0.00
I
ATOM   4255  HD1 PHE I 306      -9.852 -12.091 -12.575  0.00  0.00
I
ATOM   4256  CE1 PHE I 306      -9.458 -13.984 -11.627  0.00  0.00
I
ATOM   4257  HE1 PHE I 306     -10.083 -13.878 -10.752  0.00  0.00
I
ATOM   4258  CZ  PHE I 306      -8.862 -15.245 -11.756  0.00  0.00
I
ATOM   4259  HZ  PHE I 306      -8.948 -16.032 -11.021  0.00  0.00
I
ATOM   4260  CD2 PHE I 306      -7.714 -14.423 -13.764  0.00  0.00
I
ATOM   4261  HD2 PHE I 306      -7.037 -14.690 -14.562  0.00  0.00
I
ATOM   4262  CE2 PHE I 306      -7.885 -15.365 -12.746  0.00  0.00
I
ATOM   4263  HE2 PHE I 306      -7.477 -16.365 -12.768  0.00  0.00
I
ATOM   4264  C   PHE I 306      -9.917 -11.253 -16.629  0.00  0.00
I
ATOM   4265  O   PHE I 306     -10.389 -10.281 -16.165  0.00  0.00
I
```

ATOM   4266 N   GLY I 307   -9.403 -11.319 -17.940 0.00 0.00
I
ATOM   4267 HN GLY I 307   -8.829 -12.097 -18.183 0.00 0.00
I
ATOM   4268 CA GLY I 307   -9.460 -10.120 -18.810 0.00 0.00
I
ATOM   4269 HA1 GLY I 307   -8.740 -10.242 -19.606 0.00 0.00
I
ATOM   4270 HA2 GLY I 307   -9.280 -9.246 -18.202 0.00 0.00
I
ATOM   4271 C   GLY I 307   -10.820 -9.922 -19.442 0.00 0.00
I
ATOM   4272 O   GLY I 307   -11.035 -9.003 -20.227 0.00 0.00
I
ATOM   4273 N   SER I 308   -11.774 -10.877 -19.109 0.00 0.00
I
ATOM   4274 HN SER I 308   -11.475 -11.624 -18.520 0.00 0.00
I
ATOM   4275 CA SER I 308   -13.223 -10.853 -19.496 0.00 0.00
I
ATOM   4276 HA SER I 308   -13.295 -10.089 -20.257 0.00 0.00
I
ATOM   4277 CB SER I 308   -13.695 -12.204 -19.953 0.00 0.00
I
ATOM   4278 HB1 SER I 308   -13.674 -13.080 -19.271 0.00 0.00
I
ATOM   4279 HB2 SER I 308   -14.770 -12.207 -20.234 0.00 0.00
I
ATOM   4280 OG SER I 308   -12.940 -12.587 -21.137 0.00 0.00
I
ATOM   4281 HG1 SER I 308   -12.134 -12.965 -20.779 0.00 0.00
I
ATOM   4282 C   SER I 308   -14.033 -10.404 -18.273 0.00 0.00
I
ATOM   4283 O   SER I 308   -15.093 -9.880 -18.410 0.00 0.00
I
ATOM   4284 N   MET I 309   -13.498 -10.557 -17.025 0.00 0.00
I
ATOM   4285 HN MET I 309   -12.672 -11.108 -16.945 0.00 0.00
I
ATOM   4286 CA MET I 309   -14.036 -9.797 -15.979 0.00 0.00
I
ATOM   4287 HA MET I 309   -15.110 -9.912 -16.003 0.00 0.00
I
ATOM   4288 CB MET I 309   -13.432 -10.269 -14.595 0.00 0.00
I
ATOM   4289 HB1 MET I 309   -12.334 -10.133 -14.694 0.00 0.00
I
ATOM   4290 HB2 MET I 309   -13.826 -9.654 -13.757 0.00 0.00
I
ATOM   4291 CG MET I 309   -13.610 -11.785 -14.328 0.00 0.00
I
ATOM   4292 HG1 MET I 309   -14.702 -11.919 -14.485 0.00 0.00
I

ATOM 4293 HG2 MET I 309 −13.032 −12.267 −15.145 0.00 0.00
I
ATOM 4294 SD MET I 309 −13.133 −12.336 −12.761 0.00 0.00
I
ATOM 4295 CE MET I 309 −13.778 −13.997 −12.975 0.00 0.00
I
ATOM 4296 HE1 MET I 309 −13.334 −14.435 −13.895 0.00 0.00
I
ATOM 4297 HE2 MET I 309 −13.667 −14.609 −12.054 0.00 0.00
I
ATOM 4298 HE3 MET I 309 −14.854 −14.087 −13.234 0.00 0.00
I
ATOM 4299 C MET I 309 −13.792 −8.293 −16.167 0.00 0.00
I
ATOM 4300 O MET I 309 −12.817 −7.886 −16.828 0.00 0.00
I
ATOM 4301 N ARG I 310 −14.679 −7.458 −15.632 0.00 0.00
I
ATOM 4302 HN ARG I 310 −15.435 −7.748 −15.051 0.00 0.00
I
ATOM 4303 CA ARG I 310 −14.593 −5.993 −15.787 0.00 0.00
I
ATOM 4304 HA ARG I 310 −14.073 −5.651 −16.669 0.00 0.00
I
ATOM 4305 CB ARG I 310 −16.066 −5.458 −15.837 0.00 0.00
I
ATOM 4306 HB1 ARG I 310 −16.792 −5.840 −15.087 0.00 0.00
I
ATOM 4307 HB2 ARG I 310 −16.057 −4.347 −15.819 0.00 0.00
I
ATOM 4308 CG ARG I 310 −16.829 −5.864 −17.098 0.00 0.00
I
ATOM 4309 HG1 ARG I 310 −16.302 −5.465 −17.991 0.00 0.00
I
ATOM 4310 HG2 ARG I 310 −16.922 −6.963 −17.237 0.00 0.00
I
ATOM 4311 CD ARG I 310 −18.275 −5.367 −17.169 0.00 0.00
I
ATOM 4312 HD1 ARG I 310 −18.390 −4.291 −16.919 0.00 0.00
I
ATOM 4313 HD2 ARG I 310 −18.662 −5.559 −18.193 0.00 0.00
I
ATOM 4314 NE ARG I 310 −19.072 −6.114 −16.135 0.00 0.00
I
ATOM 4315 HE ARG I 310 −18.616 −6.713 −15.477 0.00 0.00
I
ATOM 4316 CZ ARG I 310 −20.386 −5.946 −16.023 0.00 0.00
I
ATOM 4317 NH1 ARG I 310 −21.238 −5.163 −16.745 0.00 0.00
I
ATOM 4318 HH11 ARG I 310 −22.197 −5.072 −16.475 0.00 0.00
I
ATOM 4319 HH12 ARG I 310 −20.902 −4.649 −17.535 0.00 0.00
I

ATOM 4320 NH2 ARG I 310 -21.014 -6.624 -15.025 0.00 0.00
I
ATOM 4321 HH21 ARG I 310 -21.958 -6.417 -14.771 0.00 0.00
I
ATOM 4322 HH22 ARG I 310 -20.408 -7.145 -14.422 0.00 0.00
I
ATOM 4323 C ARG I 310 -13.878 -5.367 -14.651 0.00 0.00
I
ATOM 4324 O ARG I 310 -13.604 -6.092 -13.684 0.00 0.00
I
ATOM 4325 N GLU I 311 -13.475 -4.128 -14.671 0.00 0.00
I
ATOM 4326 HN GLU I 311 -13.814 -3.483 -15.351 0.00 0.00
I
ATOM 4327 CA GLU I 311 -12.363 -3.573 -13.865 0.00 0.00
I
ATOM 4328 HA GLU I 311 -11.442 -4.126 -13.976 0.00 0.00
I
ATOM 4329 CB GLU I 311 -12.061 -2.129 -14.313 0.00 0.00
I
ATOM 4330 HB1 GLU I 311 -12.849 -1.381 -14.080 0.00 0.00
I
ATOM 4331 HB2 GLU I 311 -11.175 -1.739 -13.767 0.00 0.00
I
ATOM 4332 CG GLU I 311 -11.596 -2.055 -15.796 0.00 0.00
I
ATOM 4333 HG1 GLU I 311 -10.918 -1.176 -15.841 0.00 0.00
I
ATOM 4334 HG2 GLU I 311 -10.913 -2.851 -16.163 0.00 0.00
I
ATOM 4335 CD GLU I 311 -12.696 -1.907 -16.766 0.00 0.00
I
ATOM 4336 OE1 GLU I 311 -12.326 -1.858 -17.970 0.00 0.00
I
ATOM 4337 OE2 GLU I 311 -13.871 -1.827 -16.415 0.00 0.00
I
ATOM 4338 C GLU I 311 -12.790 -3.698 -12.398 0.00 0.00
I
ATOM 4339 O GLU I 311 -11.911 -3.902 -11.555 0.00 0.00
I
ATOM 4340 N GLY I 312 -14.058 -3.384 -12.046 0.00 0.00
I
ATOM 4341 HN GLY I 312 -14.696 -3.161 -12.779 0.00 0.00
I
ATOM 4342 CA GLY I 312 -14.514 -3.340 -10.645 0.00 0.00
I
ATOM 4343 HA1 GLY I 312 -15.569 -3.108 -10.652 0.00 0.00
I
ATOM 4344 HA2 GLY I 312 -13.893 -2.594 -10.171 0.00 0.00
I
ATOM 4345 C GLY I 312 -14.357 -4.639 -9.942 0.00 0.00
I
ATOM 4346 O GLY I 312 -14.176 -4.880 -8.757 0.00 0.00
I

```
ATOM   4347  N   THR I 313     -14.449  -5.666 -10.770  0.00  0.00
I
ATOM   4348  HN  THR I 313     -14.594  -5.433 -11.728  0.00  0.00
I
ATOM   4349  CA  THR I 313     -14.351  -7.086 -10.446  0.00  0.00
I
ATOM   4350  HA  THR I 313     -15.043  -7.329  -9.654  0.00  0.00
I
ATOM   4351  CB  THR I 313     -14.816  -8.011 -11.573  0.00  0.00
I
ATOM   4352  HB  THR I 313     -14.229  -8.073 -12.515  0.00  0.00
I
ATOM   4353  OG1 THR I 313     -16.083  -7.624 -12.026  0.00  0.00
I
ATOM   4354  HG1 THR I 313     -16.254  -8.050 -12.870  0.00  0.00
I
ATOM   4355  CG2 THR I 313     -15.099  -9.416 -11.069  0.00  0.00
I
ATOM   4356 HG21 THR I 313     -14.138  -9.873 -10.748  0.00  0.00
I
ATOM   4357 HG22 THR I 313     -15.728  -9.446 -10.154  0.00  0.00
I
ATOM   4358 HG23 THR I 313     -15.620 -10.033 -11.832  0.00  0.00
I
ATOM   4359  C   THR I 313     -12.897  -7.459 -10.053  0.00  0.00
I
ATOM   4360  O   THR I 313     -12.614  -8.169  -9.129  0.00  0.00
I
ATOM   4361  N   ILE I 314     -11.953  -6.858 -10.793  0.00  0.00
I
ATOM   4362  HN  ILE I 314     -12.283  -6.171 -11.435  0.00  0.00
I
ATOM   4363  CA  ILE I 314     -10.561  -6.987 -10.559  0.00  0.00
I
ATOM   4364  HA  ILE I 314     -10.413  -8.043 -10.386  0.00  0.00
I
ATOM   4365  CB  ILE I 314      -9.845  -6.574 -11.843  0.00  0.00
I
ATOM   4366  HB  ILE I 314     -10.230  -5.554 -12.058  0.00  0.00
I
ATOM   4367  CG2 ILE I 314      -8.313  -6.596 -11.593  0.00  0.00
I
ATOM   4368 HG21 ILE I 314      -7.787  -6.525 -12.569  0.00  0.00
I
ATOM   4369 HG22 ILE I 314      -8.079  -5.750 -10.912  0.00  0.00
I
ATOM   4370 HG23 ILE I 314      -7.977  -7.602 -11.261  0.00  0.00
I
ATOM   4371  CG1 ILE I 314     -10.243  -7.547 -12.992  0.00  0.00
I
ATOM   4372 HG11 ILE I 314     -11.310  -7.383 -13.251  0.00  0.00
I
ATOM   4373 HG12 ILE I 314      -9.730  -7.089 -13.865  0.00  0.00
I
```

ATOM 4374 CD ILE I 314 -9.923 -9.023 -12.906 0.00 0.00
I
ATOM 4375 HD1 ILE I 314 -10.378 -9.459 -11.992 0.00 0.00
I
ATOM 4376 HD2 ILE I 314 -10.417 -9.553 -13.748 0.00 0.00
I
ATOM 4377 HD3 ILE I 314 -8.832 -9.231 -12.888 0.00 0.00
I
ATOM 4378 C ILE I 314 -10.092 -6.224 -9.323 0.00 0.00
I
ATOM 4379 O ILE I 314 -9.319 -6.718 -8.520 0.00 0.00
I
ATOM 4380 N VAL I 315 -10.696 -5.034 -9.075 0.00 0.00
I
ATOM 4381 HN VAL I 315 -11.324 -4.709 -9.778 0.00 0.00
I
ATOM 4382 CA VAL I 315 -10.529 -4.155 -7.866 0.00 0.00
I
ATOM 4383 HA VAL I 315 -9.471 -4.040 -7.682 0.00 0.00
I
ATOM 4384 CB VAL I 315 -11.184 -2.799 -8.010 0.00 0.00
I
ATOM 4385 HB VAL I 315 -12.181 -2.816 -8.500 0.00 0.00
I
ATOM 4386 CG1 VAL I 315 -11.474 -2.071 -6.659 0.00 0.00
I
ATOM 4387 HG11 VAL I 315 -11.986 -1.106 -6.859 0.00 0.00
I
ATOM 4388 HG12 VAL I 315 -12.171 -2.606 -5.978 0.00 0.00
I
ATOM 4389 HG13 VAL I 315 -10.490 -1.848 -6.194 0.00 0.00
I
ATOM 4390 CG2 VAL I 315 -10.252 -2.021 -8.900 0.00 0.00
I
ATOM 4391 HG21 VAL I 315 -9.277 -1.841 -8.399 0.00 0.00
I
ATOM 4392 HG22 VAL I 315 -10.092 -2.545 -9.867 0.00 0.00
I
ATOM 4393 HG23 VAL I 315 -10.802 -1.092 -9.165 0.00 0.00
I
ATOM 4394 C VAL I 315 -11.021 -4.946 -6.694 0.00 0.00
I
ATOM 4395 O VAL I 315 -10.338 -4.907 -5.652 0.00 0.00
I
ATOM 4396 N PHE I 316 -12.157 -5.709 -6.719 0.00 0.00
I
ATOM 4397 HN PHE I 316 -12.716 -5.658 -7.542 0.00 0.00
I
ATOM 4398 CA PHE I 316 -12.538 -6.604 -5.662 0.00 0.00
I
ATOM 4399 HA PHE I 316 -12.647 -5.975 -4.791 0.00 0.00
I
ATOM 4400 CB PHE I 316 -13.972 -7.149 -6.016 0.00 0.00
I

ATOM 4401 HB1 PHE I 316 -14.651 -6.284 -6.169 0.00 0.00
I
ATOM 4402 HB2 PHE I 316 -13.853 -7.539 -7.049 0.00 0.00
I
ATOM 4403 CG PHE I 316 -14.622 -8.133 -5.087 0.00 0.00
I
ATOM 4404 CD1 PHE I 316 -14.557 -9.480 -5.389 0.00 0.00
I
ATOM 4405 HD1 PHE I 316 -13.926 -9.832 -6.192 0.00 0.00
I
ATOM 4406 CE1 PHE I 316 -15.228 -10.358 -4.519 0.00 0.00
I
ATOM 4407 HE1 PHE I 316 -15.232 -11.425 -4.684 0.00 0.00
I
ATOM 4408 CZ PHE I 316 -15.812 -9.888 -3.346 0.00 0.00
I
ATOM 4409 HZ PHE I 316 -16.215 -10.583 -2.625 0.00 0.00
I
ATOM 4410 CD2 PHE I 316 -15.095 -7.627 -3.851 0.00 0.00
I
ATOM 4411 HD2 PHE I 316 -15.032 -6.586 -3.569 0.00 0.00
I
ATOM 4412 CE2 PHE I 316 -15.683 -8.591 -2.979 0.00 0.00
I
ATOM 4413 HE2 PHE I 316 -16.149 -8.348 -2.035 0.00 0.00
I
ATOM 4414 C PHE I 316 -11.453 -7.641 -5.400 0.00 0.00
I
ATOM 4415 O PHE I 316 -11.010 -7.968 -4.301 0.00 0.00
I
ATOM 4416 N THR I 317 -11.047 -8.367 -6.462 0.00 0.00
I
ATOM 4417 HN THR I 317 -11.577 -8.174 -7.285 0.00 0.00
I
ATOM 4418 CA THR I 317 -9.972 -9.450 -6.497 0.00 0.00
I
ATOM 4419 HA THR I 317 -10.248 -10.295 -5.883 0.00 0.00
I
ATOM 4420 CB THR I 317 -9.747 -9.981 -7.871 0.00 0.00
I
ATOM 4421 HB THR I 317 -9.455 -9.203 -8.609 0.00 0.00
I
ATOM 4422 OG1 THR I 317 -10.902 -10.558 -8.495 0.00 0.00
I
ATOM 4423 HG1 THR I 317 -11.568 -9.883 -8.345 0.00 0.00
I
ATOM 4424 CG2 THR I 317 -8.752 -11.127 -7.897 0.00 0.00
I
ATOM 4425 HG21 THR I 317 -9.132 -12.075 -7.459 0.00 0.00
I
ATOM 4426 HG22 THR I 317 -8.510 -11.371 -8.954 0.00 0.00
I
ATOM 4427 HG23 THR I 317 -7.815 -10.960 -7.324 0.00 0.00
I

ATOM 4428 C THR I 317 -8.710 -8.924 -6.000 0.00 0.00
I
ATOM 4429 O THR I 317 -8.030 -9.621 -5.280 0.00 0.00
I
ATOM 4430 N LEU I 318 -8.277 -7.636 -6.239 0.00 0.00
I
ATOM 4431 HN LEU I 318 -8.670 -6.947 -6.844 0.00 0.00
I
ATOM 4432 CA LEU I 318 -7.021 -7.067 -5.712 0.00 0.00
I
ATOM 4433 HA LEU I 318 -6.292 -7.800 -6.023 0.00 0.00
I
ATOM 4434 CB LEU I 318 -6.747 -5.580 -6.311 0.00 0.00
I
ATOM 4435 HB1 LEU I 318 -7.631 -4.995 -5.981 0.00 0.00
I
ATOM 4436 HB2 LEU I 318 -5.846 -5.213 -5.774 0.00 0.00
I
ATOM 4437 CG LEU I 318 -6.361 -5.432 -7.793 0.00 0.00
I
ATOM 4438 HG LEU I 318 -7.117 -5.745 -8.544 0.00 0.00
I
ATOM 4439 CD1 LEU I 318 -6.071 -3.904 -7.982 0.00 0.00
I
ATOM 4440 HD11 LEU I 318 -7.032 -3.366 -7.835 0.00 0.00
I
ATOM 4441 HD12 LEU I 318 -5.404 -3.645 -7.132 0.00 0.00
I
ATOM 4442 HD13 LEU I 318 -5.567 -3.515 -8.892 0.00 0.00
I
ATOM 4443 CD2 LEU I 318 -5.134 -6.287 -8.139 0.00 0.00
I
ATOM 4444 HD21 LEU I 318 -4.832 -6.022 -9.175 0.00 0.00
I
ATOM 4445 HD22 LEU I 318 -4.245 -6.089 -7.503 0.00 0.00
I
ATOM 4446 HD23 LEU I 318 -5.296 -7.377 -8.003 0.00 0.00
I
ATOM 4447 C LEU I 318 -6.988 -7.106 -4.071 0.00 0.00
I
ATOM 4448 O LEU I 318 -6.047 -7.588 -3.489 0.00 0.00
I
ATOM 4449 N LEU I 319 -8.111 -6.767 -3.435 0.00 0.00
I
ATOM 4450 HN LEU I 319 -8.884 -6.651 -4.054 0.00 0.00
I
ATOM 4451 CA LEU I 319 -8.317 -6.932 -1.974 0.00 0.00
I
ATOM 4452 HA LEU I 319 -7.561 -6.299 -1.533 0.00 0.00
I
ATOM 4453 CB LEU I 319 -9.754 -6.578 -1.536 0.00 0.00
I
ATOM 4454 HB1 LEU I 319 -10.344 -7.477 -1.816 0.00 0.00
I

ATOM   4455 HB2 LEU I 319   -9.864 -6.349 -0.455 0.00 0.00
I
ATOM   4456 CG LEU I 319   -10.306 -5.336 -2.312 0.00 0.00
I
ATOM   4457 HG LEU I 319   -10.099 -5.495 -3.392 0.00 0.00
I
ATOM   4458 CD1 LEU I 319   -11.868 -5.167 -1.997 0.00 0.00
I
ATOM   4459 HD11 LEU I 319   -12.078 -4.121 -2.307 0.00 0.00
I
ATOM   4460 HD12 LEU I 319   -12.422 -6.010 -2.463 0.00 0.00
I
ATOM   4461 HD13 LEU I 319   -11.968 -5.300 -0.899 0.00 0.00
I
ATOM   4462 CD2 LEU I 319   -9.464 -4.099 -1.944 0.00 0.00
I
ATOM   4463 HD21 LEU I 319   -9.854 -3.219 -2.499 0.00 0.00
I
ATOM   4464 HD22 LEU I 319   -9.413 -3.889 -0.855 0.00 0.00
I
ATOM   4465 HD23 LEU I 319   -8.403 -4.205 -2.258 0.00 0.00
I
ATOM   4466 C   LEU I 319   -8.077 -8.376 -1.417 0.00 0.00
I
ATOM   4467 O   LEU I 319   -7.437 -8.643 -0.395 0.00 0.00
I
ATOM   4468 N   LEU I 320   -8.690 -9.302 -2.147 0.00 0.00
I
ATOM   4469 HN LEU I 320   -9.286 -9.058 -2.909 0.00 0.00
I
ATOM   4470 CA LEU I 320   -8.643 -10.765 -1.835 0.00 0.00
I
ATOM   4471 HA LEU I 320   -8.981 -10.793 -0.809 0.00 0.00
I
ATOM   4472 CB LEU I 320   -9.776 -11.592 -2.565 0.00 0.00
I
ATOM   4473 HB1 LEU I 320   -9.471 -11.627 -3.633 0.00 0.00
I
ATOM   4474 HB2 LEU I 320   -9.862 -12.606 -2.119 0.00 0.00
I
ATOM   4475 CG LEU I 320   -11.192 -10.996 -2.526 0.00 0.00
I
ATOM   4476 HG LEU I 320   -11.196 -9.973 -2.959 0.00 0.00
I
ATOM   4477 CD1 LEU I 320   -12.277 -11.801 -3.315 0.00 0.00
I
ATOM   4478 HD11 LEU I 320   -12.280 -11.617 -4.410 0.00 0.00
I
ATOM   4479 HD12 LEU I 320   -12.005 -12.858 -3.103 0.00 0.00
I
ATOM   4480 HD13 LEU I 320   -13.287 -11.658 -2.875 0.00 0.00
I
ATOM   4481 CD2 LEU I 320   -11.807 -10.766 -1.165 0.00 0.00
I

ATOM 4482 HD21 LEU I 320 -11.329 -9.971 -0.553 0.00 0.00
I
ATOM 4483 HD22 LEU I 320 -12.866 -10.439 -1.095 0.00 0.00
I
ATOM 4484 HD23 LEU I 320 -11.604 -11.667 -0.548 0.00 0.00
I
ATOM 4485 C LEU I 320 -7.237 -11.399 -1.833 0.00 0.00
I
ATOM 4486 O LEU I 320 -6.993 -12.129 -0.903 0.00 0.00
I
ATOM 4487 N MET I 321 -6.303 -11.094 -2.795 0.00 0.00
I
ATOM 4488 HN MET I 321 -6.657 -10.757 -3.664 0.00 0.00
I
ATOM 4489 CA MET I 321 -4.893 -11.499 -2.871 0.00 0.00
I
ATOM 4490 HA MET I 321 -4.874 -12.563 -2.686 0.00 0.00
I
ATOM 4491 CB MET I 321 -4.104 -10.905 -4.066 0.00 0.00
I
ATOM 4492 HB1 MET I 321 -4.020 -9.805 -3.931 0.00 0.00
I
ATOM 4493 HB2 MET I 321 -3.072 -11.304 -4.166 0.00 0.00
I
ATOM 4494 CG MET I 321 -4.746 -11.080 -5.437 0.00 0.00
I
ATOM 4495 HG1 MET I 321 -4.676 -12.122 -5.816 0.00 0.00
I
ATOM 4496 HG2 MET I 321 -5.814 -10.787 -5.353 0.00 0.00
I
ATOM 4497 SD MET I 321 -3.874 -10.020 -6.692 0.00 0.00
I
ATOM 4498 CE MET I 321 -4.589 -10.572 -8.205 0.00 0.00
I
ATOM 4499 HE1 MET I 321 -5.680 -10.368 -8.238 0.00 0.00
I
ATOM 4500 HE2 MET I 321 -4.000 -10.028 -8.975 0.00 0.00
I
ATOM 4501 HE3 MET I 321 -4.366 -11.629 -8.462 0.00 0.00
I
ATOM 4502 C MET I 321 -4.207 -10.908 -1.577 0.00 0.00
I
ATOM 4503 O MET I 321 -3.340 -11.545 -0.934 0.00 0.00
I
ATOM 4504 N PHE I 322 -4.653 -9.753 -1.128 0.00 0.00
I
ATOM 4505 HN PHE I 322 -5.376 -9.210 -1.548 0.00 0.00
I
ATOM 4506 CA PHE I 322 -4.036 -9.181 0.028 0.00 0.00
I
ATOM 4507 HA PHE I 322 -2.972 -9.306 0.158 0.00 0.00
I
ATOM 4508 CB PHE I 322 -4.440 -7.648 -0.145 0.00 0.00
I

ATOM 4509 HB1 PHE I 322 -4.481 -7.447 -1.237 0.00 0.00
I
ATOM 4510 HB2 PHE I 322 -5.421 -7.422 0.325 0.00 0.00
I
ATOM 4511 CG PHE I 322 -3.515 -6.718 0.548 0.00 0.00
I
ATOM 4512 CD1 PHE I 322 -4.054 -5.839 1.460 0.00 0.00
I
ATOM 4513 HD1 PHE I 322 -5.084 -5.921 1.773 0.00 0.00
I
ATOM 4514 CE1 PHE I 322 -3.275 -4.775 1.932 0.00 0.00
I
ATOM 4515 HE1 PHE I 322 -3.688 -4.048 2.616 0.00 0.00
I
ATOM 4516 CZ PHE I 322 -1.990 -4.467 1.361 0.00 0.00
I
ATOM 4517 HZ PHE I 322 -1.415 -3.570 1.536 0.00 0.00
I
ATOM 4518 CD2 PHE I 322 -2.241 -6.512 0.050 0.00 0.00
I
ATOM 4519 HD2 PHE I 322 -1.851 -7.235 -0.651 0.00 0.00
I
ATOM 4520 CE2 PHE I 322 -1.482 -5.353 0.392 0.00 0.00
I
ATOM 4521 HE2 PHE I 322 -0.548 -5.042 -0.053 0.00 0.00
I
ATOM 4522 C PHE I 322 -4.723 -9.632 1.273 0.00 0.00
I
ATOM 4523 O PHE I 322 -4.483 -9.175 2.385 0.00 0.00
I
ATOM 4524 N ILE I 323 -5.637 -10.587 1.270 0.00 0.00
I
ATOM 4525 HN ILE I 323 -5.907 -10.976 0.392 0.00 0.00
I
ATOM 4526 CA ILE I 323 -6.119 -11.182 2.524 0.00 0.00
I
ATOM 4527 HA ILE I 323 -5.893 -10.538 3.361 0.00 0.00
I
ATOM 4528 CB ILE I 323 -7.627 -11.592 2.551 0.00 0.00
I
ATOM 4529 HB ILE I 323 -7.791 -12.208 1.642 0.00 0.00
I
ATOM 4530 CG2 ILE I 323 -7.993 -12.164 3.951 0.00 0.00
I
ATOM 4531 HG21 ILE I 323 -7.292 -12.920 4.365 0.00 0.00
I
ATOM 4532 HG22 ILE I 323 -7.886 -11.353 4.703 0.00 0.00
I
ATOM 4533 HG23 ILE I 323 -8.948 -12.725 3.870 0.00 0.00
I
ATOM 4534 CG1 ILE I 323 -8.564 -10.407 2.364 0.00 0.00
I
ATOM 4535 HG11 ILE I 323 -8.692 -9.779 3.272 0.00 0.00
I

ATOM 4536 HG12 ILE I 323 -8.279 -9.724 1.535 0.00 0.00
I
ATOM 4537 CD ILE I 323 -10.027 -10.666 1.999 0.00 0.00
I
ATOM 4538 HD1 ILE I 323 -10.539 -11.119 2.874 0.00 0.00
I
ATOM 4539 HD2 ILE I 323 -10.553 -9.716 1.762 0.00 0.00
I
ATOM 4540 HD3 ILE I 323 -10.111 -11.415 1.183 0.00 0.00
I
ATOM 4541 C ILE I 323 -5.328 -12.524 2.740 0.00 0.00
I
ATOM 4542 O ILE I 323 -5.578 -13.498 1.997 0.00 0.00
I
ATOM 4543 N GLY I 324 -4.429 -12.628 3.787 0.00 0.00
I
ATOM 4544 HN GLY I 324 -4.485 -11.887 4.452 0.00 0.00
I
ATOM 4545 CA GLY I 324 -3.633 -13.792 4.147 0.00 0.00
I
ATOM 4546 HA1 GLY I 324 -2.788 -13.386 4.684 0.00 0.00
I
ATOM 4547 HA2 GLY I 324 -3.383 -14.345 3.255 0.00 0.00
I
ATOM 4548 C GLY I 324 -4.466 -14.783 4.924 0.00 0.00
I
ATOM 4549 O GLY I 324 -5.702 -14.695 4.898 0.00 0.00
I
ATOM 4550 N ALA I 325 -3.889 -15.764 5.604 0.00 0.00
I
ATOM 4551 HN ALA I 325 -2.907 -15.930 5.554 0.00 0.00
I
ATOM 4552 CA ALA I 325 -4.646 -16.687 6.525 0.00 0.00
I
ATOM 4553 HA ALA I 325 -5.689 -16.475 6.706 0.00 0.00
I
ATOM 4554 CB ALA I 325 -4.406 -18.191 6.103 0.00 0.00
I
ATOM 4555 HB1 ALA I 325 -3.314 -18.374 6.006 0.00 0.00
I
ATOM 4556 HB2 ALA I 325 -4.833 -18.803 6.925 0.00 0.00
I
ATOM 4557 HB3 ALA I 325 -4.931 -18.311 5.131 0.00 0.00
I
ATOM 4558 C ALA I 325 -4.243 -16.574 7.927 0.00 0.00
I
ATOM 4559 O ALA I 325 -4.885 -15.856 8.705 0.00 0.00
I
ATOM 4560 N GLY I 326 -3.092 -17.172 8.311 0.00 0.00
I
ATOM 4561 HN GLY I 326 -2.549 -17.692 7.656 0.00 0.00
I
ATOM 4562 CA GLY I 326 -2.601 -17.037 9.711 0.00 0.00
I

ATOM 4563 HA1 GLY I 326 -3.085 -17.773 10.337 0.00 0.00
I
ATOM 4564 HA2 GLY I 326 -2.766 -16.035 10.077 0.00 0.00
I
ATOM 4565 C GLY I 326 -1.174 -17.441 9.802 0.00 0.00
I
ATOM 4566 O GLY I 326 -0.568 -17.792 8.750 0.00 0.00
I
ATOM 4567 N SER I 327 -0.557 -17.301 10.962 0.00 0.00
I
ATOM 4568 HN SER I 327 -1.044 -17.043 11.792 0.00 0.00
I
ATOM 4569 CA SER I 327 0.909 -17.556 11.135 0.00 0.00
I
ATOM 4570 HA SER I 327 1.325 -16.961 10.336 0.00 0.00
I
ATOM 4571 CB SER I 327 1.479 -16.918 12.400 0.00 0.00
I
ATOM 4572 HB1 SER I 327 1.114 -17.429 13.317 0.00 0.00
I
ATOM 4573 HB2 SER I 327 2.587 -16.877 12.325 0.00 0.00
I
ATOM 4574 OG SER I 327 1.126 -15.542 12.348 0.00 0.00
I
ATOM 4575 HG1 SER I 327 0.789 -15.251 13.198 0.00 0.00
I
ATOM 4576 C SER I 327 1.440 -18.949 11.123 0.00 0.00
I
ATOM 4577 O SER I 327 2.578 -19.241 10.785 0.00 0.00
I
ATOM 4578 N ALA I 328 0.433 -19.848 11.331 0.00 0.00
I
ATOM 4579 HN ALA I 328 -0.455 -19.499 11.618 0.00 0.00
I
ATOM 4580 CA ALA I 328 0.499 -21.300 11.388 0.00 0.00
I
ATOM 4581 HA ALA I 328 1.477 -21.659 11.671 0.00 0.00
I
ATOM 4582 CB ALA I 328 -0.372 -21.835 12.456 0.00 0.00
I
ATOM 4583 HB1 ALA I 328 -0.285 -22.935 12.585 0.00 0.00
I
ATOM 4584 HB2 ALA I 328 -0.115 -21.237 13.356 0.00 0.00
I
ATOM 4585 HB3 ALA I 328 -1.437 -21.639 12.205 0.00 0.00
I
ATOM 4586 C ALA I 328 0.200 -21.967 10.040 0.00 0.00
I
ATOM 4587 O ALA I 328 0.128 -23.195 9.915 0.00 0.00
I
ATOM 4588 N SER I 329 -0.002 -21.108 8.968 0.00 0.00
I
ATOM 4589 HN SER I 329 0.148 -20.139 9.149 0.00 0.00
I

ATOM 4590 CA SER I 329 -0.262 -21.543 7.641 0.00 0.00
I
ATOM 4591 HA SER I 329 0.038 -22.579 7.576 0.00 0.00
I
ATOM 4592 CB SER I 329 -1.758 -21.565 7.256 0.00 0.00
I
ATOM 4593 HB1 SER I 329 -1.801 -21.894 6.195 0.00 0.00
I
ATOM 4594 HB2 SER I 329 -2.280 -22.345 7.850 0.00 0.00
I
ATOM 4595 OG SER I 329 -2.494 -20.322 7.507 0.00 0.00
I
ATOM 4596 HG1 SER I 329 -3.273 -20.491 6.972 0.00 0.00
I
ATOM 4597 C SER I 329 0.601 -20.813 6.564 0.00 0.00
I
ATOM 4598 O SER I 329 1.144 -19.792 6.870 0.00 0.00
I
ATOM 4599 N THR I 330 0.659 -21.413 5.351 0.00 0.00
I
ATOM 4600 HN THR I 330 0.339 -22.331 5.128 0.00 0.00
I
ATOM 4601 CA THR I 330 1.553 -20.963 4.223 0.00 0.00
I
ATOM 4602 HA THR I 330 2.401 -20.452 4.655 0.00 0.00
I
ATOM 4603 CB THR I 330 2.161 -22.116 3.383 0.00 0.00
I
ATOM 4604 HB THR I 330 3.061 -21.763 2.835 0.00 0.00
I
ATOM 4605 OG1 THR I 330 1.301 -22.713 2.419 0.00 0.00
I
ATOM 4606 HG1 THR I 330 1.808 -23.063 1.683 0.00 0.00
I
ATOM 4607 CG2 THR I 330 2.674 -23.276 4.187 0.00 0.00
I
ATOM 4608 HG21 THR I 330 1.803 -23.594 4.799 0.00 0.00
I
ATOM 4609 HG22 THR I 330 3.149 -24.099 3.611 0.00 0.00
I
ATOM 4610 HG23 THR I 330 3.464 -22.907 4.877 0.00 0.00
I
ATOM 4611 C THR I 330 0.899 -19.907 3.263 0.00 0.00
I
ATOM 4612 O THR I 330 1.436 -19.636 2.167 0.00 0.00
I
ATOM 4613 N ALA I 331 -0.252 -19.266 3.556 0.00 0.00
I
ATOM 4614 HN ALA I 331 -0.637 -19.509 4.443 0.00 0.00
I
ATOM 4615 CA ALA I 331 -0.713 -18.108 2.810 0.00 0.00
I
ATOM 4616 HA ALA I 331 -0.508 -18.222 1.756 0.00 0.00
I

```
ATOM   4617  CB  ALA I 331      -2.179 -17.976   3.148  0.00  0.00
I
ATOM   4618  HB1 ALA I 331      -2.654 -18.903   2.762  0.00  0.00
I
ATOM   4619  HB2 ALA I 331      -2.342 -17.853   4.240  0.00  0.00
I
ATOM   4620  HB3 ALA I 331      -2.643 -17.114   2.622  0.00  0.00
I
ATOM   4621  C   ALA I 331      -0.034 -16.755   3.120  0.00  0.00
I
ATOM   4622  O   ALA I 331       0.592 -16.582   4.131  0.00  0.00
I
ATOM   4623  N   SER I 332      -0.214 -15.824   2.183  0.00  0.00
I
ATOM   4624  HN  SER I 332      -0.665 -15.967   1.306  0.00  0.00
I
ATOM   4625  CA  SER I 332       0.356 -14.447   2.468  0.00  0.00
I
ATOM   4626  HA  SER I 332       0.185 -14.222   3.510  0.00  0.00
I
ATOM   4627  CB  SER I 332       1.843 -14.261   2.088  0.00  0.00
I
ATOM   4628  HB1 SER I 332       2.431 -15.158   2.381  0.00  0.00
I
ATOM   4629  HB2 SER I 332       1.925 -14.116   0.990  0.00  0.00
I
ATOM   4630  OG  SER I 332       2.408 -13.182   2.746  0.00  0.00
I
ATOM   4631  HG1 SER I 332       3.318 -13.101   2.454  0.00  0.00
I
ATOM   4632  C   SER I 332      -0.587 -13.434   1.904  0.00  0.00
I
ATOM   4633  O   SER I 332      -1.677 -13.834   1.453  0.00  0.00
I
ATOM   4634  N   GLY I 333      -0.183 -12.112   2.093  0.00  0.00
I
ATOM   4635  HN  GLY I 333       0.740 -11.838   2.352  0.00  0.00
I
ATOM   4636  CA  GLY I 333      -1.196 -11.099   2.221  0.00  0.00
I
ATOM   4637  HA1 GLY I 333      -2.081 -11.583   1.836  0.00  0.00
I
ATOM   4638  HA2 GLY I 333      -0.787 -10.236   1.717  0.00  0.00
I
ATOM   4639  C   GLY I 333      -1.396 -10.795   3.663  0.00  0.00
I
ATOM   4640  O   GLY I 333      -0.824 -11.469   4.551  0.00  0.00
I
ATOM   4641  N   ILE I 334      -2.167  -9.752   4.137  0.00  0.00
I
ATOM   4642  HN  ILE I 334      -2.572  -9.106   3.494  0.00  0.00
I
ATOM   4643  CA  ILE I 334      -2.309  -9.603   5.542  0.00  0.00
I
```

ATOM	4644	HA	ILE	I	334	−1.340	−9.692	6.011	0.00	0.00	I
ATOM	4645	CB	ILE	I	334	−2.843	−8.231	5.912	0.00	0.00	I
ATOM	4646	HB	ILE	I	334	−3.873	−8.075	5.526	0.00	0.00	I
ATOM	4647	CG2	ILE	I	334	−2.888	−8.047	7.459	0.00	0.00	I
ATOM	4648	HG21	ILE	I	334	−1.802	−8.061	7.695	0.00	0.00	I
ATOM	4649	HG22	ILE	I	334	−3.332	−7.078	7.774	0.00	0.00	I
ATOM	4650	HG23	ILE	I	334	−3.572	−8.732	8.004	0.00	0.00	I
ATOM	4651	CG1	ILE	I	334	−1.940	−7.196	5.221	0.00	0.00	I
ATOM	4652	HG11	ILE	I	334	−0.905	−7.403	5.567	0.00	0.00	I
ATOM	4653	HG12	ILE	I	334	−1.970	−7.345	4.120	0.00	0.00	I
ATOM	4654	CD	ILE	I	334	−2.320	−5.684	5.420	0.00	0.00	I
ATOM	4655	HD1	ILE	I	334	−3.328	−5.427	5.032	0.00	0.00	I
ATOM	4656	HD2	ILE	I	334	−2.189	−5.420	6.491	0.00	0.00	I
ATOM	4657	HD3	ILE	I	334	−1.718	−5.037	4.746	0.00	0.00	I
ATOM	4658	C	ILE	I	334	−3.261	−10.594	6.161	0.00	0.00	I
ATOM	4659	O	ILE	I	334	−4.412	−10.661	5.753	0.00	0.00	I
ATOM	4660	N	LYS	I	335	−2.773	−11.365	7.169	0.00	0.00	I
ATOM	4661	HN	LYS	I	335	−1.817	−11.242	7.424	0.00	0.00	I
ATOM	4662	CA	LYS	I	335	−3.556	−12.406	7.788	0.00	0.00	I
ATOM	4663	HA	LYS	I	335	−3.771	−13.097	6.986	0.00	0.00	I
ATOM	4664	CB	LYS	I	335	−2.569	−13.052	8.880	0.00	0.00	I
ATOM	4665	HB1	LYS	I	335	−2.429	−12.227	9.610	0.00	0.00	I
ATOM	4666	HB2	LYS	I	335	−3.152	−13.843	9.398	0.00	0.00	I
ATOM	4667	CG	LYS	I	335	−1.275	−13.611	8.295	0.00	0.00	I
ATOM	4668	HG1	LYS	I	335	−1.444	−14.599	7.817	0.00	0.00	I
ATOM	4669	HG2	LYS	I	335	−0.823	−12.978	7.502	0.00	0.00	I
ATOM	4670	CD	LYS	I	335	−0.282	−14.020	9.378	0.00	0.00	I

ATOM   4671 HD1 LYS I 335   0.019 -13.200 10.064 0.00 0.00
I
ATOM   4672 HD2 LYS I 335   -0.783 -14.751 10.048 0.00 0.00
I
ATOM   4673 CE LYS I 335   0.927 -14.642   8.808 0.00 0.00
I
ATOM   4674 HE1 LYS I 335   1.727 -15.006   9.488 0.00 0.00
I
ATOM   4675 HE2 LYS I 335   0.460 -15.443   8.196 0.00 0.00
I
ATOM   4676 NZ LYS I 335   1.702 -13.686   8.029 0.00 0.00
I
ATOM   4677 HZ1 LYS I 335   1.117 -13.220   7.307 0.00 0.00
I
ATOM   4678 HZ2 LYS I 335   2.003 -12.933   8.680 0.00 0.00
I
ATOM   4679 HZ3 LYS I 335   2.453 -14.241   7.571 0.00 0.00
I
ATOM   4680 C   LYS I 335   -4.857 -11.999   8.491 0.00 0.00
I
ATOM   4681 O   LYS I 335   -5.046 -10.835   8.874 0.00 0.00
I
ATOM   4682 N   LEU I 336   -5.752 -12.953   8.611 0.00 0.00
I
ATOM   4683 HN LEU I 336   -5.508 -13.891   8.377 0.00 0.00
I
ATOM   4684 CA LEU I 336   -7.122 -12.847   9.066 0.00 0.00
I
ATOM   4685 HA LEU I 336   -7.605 -12.130   8.419 0.00 0.00
I
ATOM   4686 CB LEU I 336   -7.903 -14.150   8.814 0.00 0.00
I
ATOM   4687 HB1 LEU I 336   -7.675 -14.511   7.788 0.00 0.00
I
ATOM   4688 HB2 LEU I 336   -7.535 -14.879   9.567 0.00 0.00
I
ATOM   4689 CG LEU I 336   -9.482 -14.199   9.013 0.00 0.00
I
ATOM   4690 HG LEU I 336   -9.829 -13.187   8.715 0.00 0.00
I
ATOM   4691 CD1 LEU I 336   -10.094 -15.215   8.097 0.00 0.00
I
ATOM   4692 HD11 LEU I 336   -9.692 -16.245   8.205 0.00 0.00
I
ATOM   4693 HD12 LEU I 336   -11.197 -15.158   8.215 0.00 0.00
I
ATOM   4694 HD13 LEU I 336   -9.996 -15.034   7.005 0.00 0.00
I
ATOM   4695 CD2 LEU I 336   -9.833 -14.483 10.430 0.00 0.00
I
ATOM   4696 HD21 LEU I 336   -9.286 -13.770 11.083 0.00 0.00
I
ATOM   4697 HD22 LEU I 336   -10.906 -14.222 10.547 0.00 0.00
I

ATOM 4698 HD23 LEU I 336 -9.604 -15.528 10.729 0.00 0.00
I
ATOM 4699 C LEU I 336 -7.238 -12.331 10.510 0.00 0.00
I
ATOM 4700 O LEU I 336 -7.973 -11.374 10.845 0.00 0.00
I
ATOM 4701 N THR I 337 -6.416 -12.891 11.366 0.00 0.00
I
ATOM 4702 HN THR I 337 -5.806 -13.622 11.072 0.00 0.00
I
ATOM 4703 CA THR I 337 -6.070 -12.507 12.699 0.00 0.00
I
ATOM 4704 HA THR I 337 -7.024 -12.429 13.198 0.00 0.00
I
ATOM 4705 CB THR I 337 -5.128 -13.504 13.318 0.00 0.00
I
ATOM 4706 HB THR I 337 -4.656 -13.113 14.244 0.00 0.00
I
ATOM 4707 OG1 THR I 337 -4.148 -13.795 12.336 0.00 0.00
I
ATOM 4708 HG1 THR I 337 -3.518 -14.413 12.713 0.00 0.00
I
ATOM 4709 CG2 THR I 337 -6.065 -14.720 13.651 0.00 0.00
I
ATOM 4710 HG21 THR I 337 -6.960 -14.297 14.156 0.00 0.00
I
ATOM 4711 HG22 THR I 337 -6.506 -15.204 12.753 0.00 0.00
I
ATOM 4712 HG23 THR I 337 -5.552 -15.504 14.249 0.00 0.00
I
ATOM 4713 C THR I 337 -5.443 -11.178 12.868 0.00 0.00
I
ATOM 4714 O THR I 337 -5.873 -10.512 13.785 0.00 0.00
I
ATOM 4715 N THR I 338 -4.451 -10.768 12.005 0.00 0.00
I
ATOM 4716 HN THR I 338 -4.088 -11.366 11.294 0.00 0.00
I
ATOM 4717 CA THR I 338 -3.784 -9.421 12.027 0.00 0.00
I
ATOM 4718 HA THR I 338 -3.528 -9.200 13.053 0.00 0.00
I
ATOM 4719 CB THR I 338 -2.461 -9.400 11.207 0.00 0.00
I
ATOM 4720 HB THR I 338 -2.622 -9.447 10.108 0.00 0.00
I
ATOM 4721 OG1 THR I 338 -1.787 -10.587 11.534 0.00 0.00
I
ATOM 4722 HG1 THR I 338 -0.942 -10.465 11.095 0.00 0.00
I
ATOM 4723 CG2 THR I 338 -1.536 -8.315 11.656 0.00 0.00
I
ATOM 4724 HG21 THR I 338 -1.896 -7.298 11.391 0.00 0.00
I

ATOM 4725 HG22 THR I 338 -1.239 -8.289 12.726 0.00 0.00
I
ATOM 4726 HG23 THR I 338 -0.601 -8.409 11.063 0.00 0.00
I
ATOM 4727 C THR I 338 -4.670 -8.341 11.523 0.00 0.00
I
ATOM 4728 O THR I 338 -4.706 -7.279 12.123 0.00 0.00
I
ATOM 4729 N PHE I 339 -5.532 -8.551 10.512 0.00 0.00
I
ATOM 4730 HN PHE I 339 -5.503 -9.477 10.144 0.00 0.00
I
ATOM 4731 CA PHE I 339 -6.497 -7.636 9.921 0.00 0.00
I
ATOM 4732 HA PHE I 339 -5.906 -6.750 9.740 0.00 0.00
I
ATOM 4733 CB PHE I 339 -7.026 -8.223 8.575 0.00 0.00
I
ATOM 4734 HB1 PHE I 339 -6.218 -8.809 8.087 0.00 0.00
I
ATOM 4735 HB2 PHE I 339 -7.853 -8.965 8.603 0.00 0.00
I
ATOM 4736 CG PHE I 339 -7.456 -7.147 7.616 0.00 0.00
I
ATOM 4737 CD1 PHE I 339 -8.810 -6.766 7.479 0.00 0.00
I
ATOM 4738 HD1 PHE I 339 -9.630 -7.145 8.070 0.00 0.00
I
ATOM 4739 CE1 PHE I 339 -9.153 -5.621 6.752 0.00 0.00
I
ATOM 4740 HE1 PHE I 339 -10.146 -5.198 6.778 0.00 0.00
I
ATOM 4741 CZ PHE I 339 -8.286 -5.091 5.800 0.00 0.00
I
ATOM 4742 HZ PHE I 339 -8.557 -4.233 5.202 0.00 0.00
I
ATOM 4743 CD2 PHE I 339 -6.514 -6.554 6.726 0.00 0.00
I
ATOM 4744 HD2 PHE I 339 -5.454 -6.760 6.720 0.00 0.00
I
ATOM 4745 CE2 PHE I 339 -6.947 -5.609 5.793 0.00 0.00
I
ATOM 4746 HE2 PHE I 339 -6.232 -5.024 5.233 0.00 0.00
I
ATOM 4747 C PHE I 339 -7.605 -7.271 10.888 0.00 0.00
I
ATOM 4748 O PHE I 339 -7.871 -6.071 11.109 0.00 0.00
I
ATOM 4749 N ILE I 340 -8.168 -8.246 11.620 0.00 0.00
I
ATOM 4750 HN ILE I 340 -7.993 -9.223 11.532 0.00 0.00
I
ATOM 4751 CA ILE I 340 -9.189 -7.915 12.622 0.00 0.00
I

```
ATOM   4752  HA  ILE I 340      -9.842  -7.199  12.146  0.00  0.00
I
ATOM   4753  CB  ILE I 340      -9.988  -9.200  12.923  0.00  0.00
I
ATOM   4754  HB  ILE I 340     -10.161  -9.796  12.001  0.00  0.00
I
ATOM   4755  CG2 ILE I 340      -9.231 -10.066  13.991  0.00  0.00
I
ATOM   4756 HG21 ILE I 340      -9.466  -9.526  14.933  0.00  0.00
I
ATOM   4757 HG22 ILE I 340      -9.699 -11.071  13.919  0.00  0.00
I
ATOM   4758 HG23 ILE I 340      -8.174 -10.310  13.751  0.00  0.00
I
ATOM   4759  CG1 ILE I 340     -11.524  -8.974  13.361  0.00  0.00
I
ATOM   4760 HG11 ILE I 340     -11.919  -9.974  13.639  0.00  0.00
I
ATOM   4761 HG12 ILE I 340     -11.580  -8.423  14.323  0.00  0.00
I
ATOM   4762  CD  ILE I 340     -12.468  -8.376  12.284  0.00  0.00
I
ATOM   4763  HD1 ILE I 340     -12.321  -8.958  11.350  0.00  0.00
I
ATOM   4764  HD2 ILE I 340     -13.556  -8.380  12.510  0.00  0.00
I
ATOM   4765  HD3 ILE I 340     -12.355  -7.293  12.063  0.00  0.00
I
ATOM   4766  C   ILE I 340      -8.710  -7.239  13.893  0.00  0.00
I
ATOM   4767  O   ILE I 340      -9.434  -6.506  14.551  0.00  0.00
I
ATOM   4768  N   VAL I 341      -7.412  -7.372  14.221  0.00  0.00
I
ATOM   4769  HN  VAL I 341      -6.956  -8.145  13.786  0.00  0.00
I
ATOM   4770  CA  VAL I 341      -6.743  -6.586  15.251  0.00  0.00
I
ATOM   4771  HA  VAL I 341      -7.269  -6.746  16.180  0.00  0.00
I
ATOM   4772  CB  VAL I 341      -5.340  -7.128  15.529  0.00  0.00
I
ATOM   4773  HB  VAL I 341      -4.660  -7.213  14.654  0.00  0.00
I
ATOM   4774  CG1 VAL I 341      -4.630  -6.188  16.511  0.00  0.00
I
ATOM   4775 HG11 VAL I 341      -5.226  -6.016  17.433  0.00  0.00
I
ATOM   4776 HG12 VAL I 341      -3.609  -6.590  16.685  0.00  0.00
I
ATOM   4777 HG13 VAL I 341      -4.564  -5.172  16.067  0.00  0.00
I
ATOM   4778  CG2 VAL I 341      -5.440  -8.527  16.125  0.00  0.00
I
```

```
ATOM   4779 HG21 VAL I 341      -4.697  -9.241  15.710  0.00  0.00
I
ATOM   4780 HG22 VAL I 341      -5.177  -8.475  17.203  0.00  0.00
I
ATOM   4781 HG23 VAL I 341      -6.381  -9.068  15.886  0.00  0.00
I
ATOM   4782  C   VAL I 341      -6.896  -5.119  14.928  0.00  0.00
I
ATOM   4783  O   VAL I 341      -7.150  -4.411  15.853  0.00  0.00
I
ATOM   4784  N   ILE I 342      -6.758  -4.762  13.629  0.00  0.00
I
ATOM   4785  HN  ILE I 342      -6.469  -5.407  12.926  0.00  0.00
I
ATOM   4786  CA  ILE I 342      -6.825  -3.370  13.153  0.00  0.00
I
ATOM   4787  HA  ILE I 342      -6.256  -2.766  13.844  0.00  0.00
I
ATOM   4788  CB  ILE I 342      -6.279  -3.263  11.695  0.00  0.00
I
ATOM   4789  HB  ILE I 342      -6.918  -3.807  10.967  0.00  0.00
I
ATOM   4790  CG2 ILE I 342      -6.258  -1.804  11.248  0.00  0.00
I
ATOM   4791 HG21 ILE I 342      -7.280  -1.372  11.213  0.00  0.00
I
ATOM   4792 HG22 ILE I 342      -5.696  -1.113  11.911  0.00  0.00
I
ATOM   4793 HG23 ILE I 342      -5.847  -1.653  10.227  0.00  0.00
I
ATOM   4794  CG1 ILE I 342      -4.825  -3.902  11.634  0.00  0.00
I
ATOM   4795 HG11 ILE I 342      -4.221  -3.346  12.383  0.00  0.00
I
ATOM   4796 HG12 ILE I 342      -4.954  -4.953  11.971  0.00  0.00
I
ATOM   4797  CD  ILE I 342      -4.274  -3.749  10.250  0.00  0.00
I
ATOM   4798  HD1 ILE I 342      -4.956  -4.185   9.490  0.00  0.00
I
ATOM   4799  HD2 ILE I 342      -3.965  -2.719   9.973  0.00  0.00
I
ATOM   4800  HD3 ILE I 342      -3.376  -4.396  10.149  0.00  0.00
I
ATOM   4801  C   ILE I 342      -8.244  -2.884  13.138  0.00  0.00
I
ATOM   4802  O   ILE I 342      -8.440  -1.880  13.820  0.00  0.00
I
ATOM   4803  N   LEU I 343      -9.228  -3.587  12.480  0.00  0.00
I
ATOM   4804  HN  LEU I 343      -8.997  -4.363  11.898  0.00  0.00
I
ATOM   4805  CA  LEU I 343     -10.656  -3.209  12.448  0.00  0.00
I
```

ATOM	4806	HA	LEU	I	343	−10.693	−2.219	12.018	0.00	0.00	I
ATOM	4807	CB	LEU	I	343	−11.586	−4.258	11.650	0.00	0.00	I
ATOM	4808	HB1	LEU	I	343	−11.621	−5.184	12.263	0.00	0.00	I
ATOM	4809	HB2	LEU	I	343	−12.580	−3.764	11.613	0.00	0.00	I
ATOM	4810	CG	LEU	I	343	−11.195	−4.618	10.214	0.00	0.00	I
ATOM	4811	HG	LEU	I	343	−10.256	−5.204	10.117	0.00	0.00	I
ATOM	4812	CD1	LEU	I	343	−12.274	−5.588	9.558	0.00	0.00	I
ATOM	4813	HD11	LEU	I	343	−13.287	−5.155	9.698	0.00	0.00	I
ATOM	4814	HD12	LEU	I	343	−11.959	−5.693	8.497	0.00	0.00	I
ATOM	4815	HD13	LEU	I	343	−12.341	−6.597	10.017	0.00	0.00	I
ATOM	4816	CD2	LEU	I	343	−10.889	−3.364	9.349	0.00	0.00	I
ATOM	4817	HD21	LEU	I	343	−10.750	−3.588	8.270	0.00	0.00	I
ATOM	4818	HD22	LEU	I	343	−11.757	−2.681	9.472	0.00	0.00	I
ATOM	4819	HD23	LEU	I	343	−9.950	−2.874	9.684	0.00	0.00	I
ATOM	4820	C	LEU	I	343	−11.245	−3.062	13.755	0.00	0.00	I
ATOM	4821	O	LEU	I	343	−11.921	−2.023	14.013	0.00	0.00	I
ATOM	4822	N	THR	I	344	−10.955	−3.903	14.784	0.00	0.00	I
ATOM	4823	HN	THR	I	344	−10.366	−4.655	14.498	0.00	0.00	I
ATOM	4824	CA	THR	I	344	−11.525	−3.762	16.169	0.00	0.00	I
ATOM	4825	HA	THR	I	344	−12.595	−3.746	16.022	0.00	0.00	I
ATOM	4826	CB	THR	I	344	−11.269	−4.936	17.086	0.00	0.00	I
ATOM	4827	HB	THR	I	344	−11.540	−4.853	18.160	0.00	0.00	I
ATOM	4828	OG1	THR	I	344	−9.881	−5.256	16.972	0.00	0.00	I
ATOM	4829	HG1	THR	I	344	−9.850	−5.840	16.211	0.00	0.00	I
ATOM	4830	CG2	THR	I	344	−12.077	−6.125	16.531	0.00	0.00	I
ATOM	4831	HG21	THR	I	344	−11.843	−6.393	15.478	0.00	0.00	I
ATOM	4832	HG22	THR	I	344	−11.864	−7.007	17.172	0.00	0.00	I

ATOM   4833 HG23 THR I 344   -13.154 -5.873 16.637 0.00 0.00
I
ATOM   4834 C   THR I 344   -10.996 -2.460 16.825 0.00 0.00
I
ATOM   4835 O   THR I 344   -11.683 -1.731 17.524 0.00 0.00
I
ATOM   4836 N   SER I 345   -9.666 -2.305 16.648 0.00 0.00
I
ATOM   4837 HN SER I 345   -9.175 -3.099 16.299 0.00 0.00
I
ATOM   4838 CA SER I 345   -8.966 -1.163 17.197 0.00 0.00
I
ATOM   4839 HA SER I 345   -9.163 -1.058 18.253 0.00 0.00
I
ATOM   4840 CB SER I 345   -7.433 -1.150 17.064 0.00 0.00
I
ATOM   4841 HB1 SER I 345   -7.000 -1.102 16.042 0.00 0.00
I
ATOM   4842 HB2 SER I 345   -7.036 -0.237 17.558 0.00 0.00
I
ATOM   4843 OG SER I 345   -6.820 -2.297 17.678 0.00 0.00
I
ATOM   4844 HG1 SER I 345   -7.051 -3.036 17.111 0.00 0.00
I
ATOM   4845 C   SER I 345   -9.331   0.205 16.592 0.00 0.00
I
ATOM   4846 O   SER I 345   -9.277   1.168 17.359 0.00 0.00
I
ATOM   4847 N   VAL I 346   -9.658   0.236 15.261 0.00 0.00
I
ATOM   4848 HN VAL I 346   -9.449 -0.481 14.601 0.00 0.00
I
ATOM   4849 CA VAL I 346   -10.304   1.370 14.611 0.00 0.00
I
ATOM   4850 HA VAL I 346   -9.608   2.194 14.662 0.00 0.00
I
ATOM   4851 CB VAL I 346   -10.643   1.120 13.107 0.00 0.00
I
ATOM   4852 HB VAL I 346   -11.200   0.162 13.029 0.00 0.00
I
ATOM   4853 CG1 VAL I 346   -11.517   2.239 12.541 0.00 0.00
I
ATOM   4854 HG11 VAL I 346   -11.947   1.999 11.545 0.00 0.00
I
ATOM   4855 HG12 VAL I 346   -12.440   2.336 13.152 0.00 0.00
I
ATOM   4856 HG13 VAL I 346   -10.830   3.102 12.406 0.00 0.00
I
ATOM   4857 CG2 VAL I 346   -9.410   0.927 12.298 0.00 0.00
I
ATOM   4858 HG21 VAL I 346   -9.566   0.563 11.260 0.00 0.00
I
ATOM   4859 HG22 VAL I 346   -8.777   1.833 12.182 0.00 0.00
I

```
ATOM   4860 HG23 VAL I 346      -8.844   0.138  12.838  0.00  0.00
I
ATOM   4861  C   VAL I 346     -11.557   1.767  15.353  0.00  0.00
I
ATOM   4862  O   VAL I 346     -11.692   2.900  15.853  0.00  0.00
I
ATOM   4863  N   ILE I 347     -12.634   0.895  15.387  0.00  0.00
I
ATOM   4864  HN  ILE I 347     -12.598  -0.018  14.989  0.00  0.00
I
ATOM   4865  CA  ILE I 347     -13.825   1.276  16.146  0.00  0.00
I
ATOM   4866  HA  ILE I 347     -14.110   2.265  15.821  0.00  0.00
I
ATOM   4867  CB  ILE I 347     -14.984   0.300  15.892  0.00  0.00
I
ATOM   4868  HB  ILE I 347     -14.676  -0.687  16.297  0.00  0.00
I
ATOM   4869  CG2 ILE I 347     -16.273   0.683  16.675  0.00  0.00
I
ATOM   4870 HG21 ILE I 347     -16.420   1.780  16.766  0.00  0.00
I
ATOM   4871 HG22 ILE I 347     -17.211   0.156  16.401  0.00  0.00
I
ATOM   4872 HG23 ILE I 347     -15.914   0.404  17.689  0.00  0.00
I
ATOM   4873  CG1 ILE I 347     -15.297   0.049  14.390  0.00  0.00
I
ATOM   4874 HG11 ILE I 347     -14.383  -0.316  13.873  0.00  0.00
I
ATOM   4875 HG12 ILE I 347     -16.212  -0.561  14.231  0.00  0.00
I
ATOM   4876  CD  ILE I 347     -15.688   1.363  13.679  0.00  0.00
I
ATOM   4877  HD1 ILE I 347     -15.988   1.128  12.635  0.00  0.00
I
ATOM   4878  HD2 ILE I 347     -16.600   1.882  14.043  0.00  0.00
I
ATOM   4879  HD3 ILE I 347     -14.926   2.171  13.660  0.00  0.00
I
ATOM   4880  C   ILE I 347     -13.602   1.416  17.703  0.00  0.00
I
ATOM   4881  O   ILE I 347     -14.139   2.362  18.281  0.00  0.00
I
ATOM   4882  N   ALA I 348     -12.781   0.633  18.377  0.00  0.00
I
ATOM   4883  HN  ALA I 348     -12.437  -0.221  17.996  0.00  0.00
I
ATOM   4884  CA  ALA I 348     -12.295   0.927  19.772  0.00  0.00
I
ATOM   4885  HA  ALA I 348     -13.102   0.810  20.480  0.00  0.00
I
ATOM   4886  CB  ALA I 348     -11.269  -0.216  20.211  0.00  0.00
I
```

ATOM 4887 HB1 ALA I 348 -11.220 -0.155 21.319 0.00 0.00
I
ATOM 4888 HB2 ALA I 348 -11.653 -1.215 19.915 0.00 0.00
I
ATOM 4889 HB3 ALA I 348 -10.256 -0.096 19.771 0.00 0.00
I
ATOM 4890 C ALA I 348 -11.755 2.327 20.043 0.00 0.00
I
ATOM 4891 O ALA I 348 -12.178 2.946 20.995 0.00 0.00
I
ATOM 4892 N TYR I 349 -10.872 2.785 19.145 0.00 0.00
I
ATOM 4893 HN TYR I 349 -10.464 2.138 18.506 0.00 0.00
I
ATOM 4894 CA TYR I 349 -10.406 4.224 19.208 0.00 0.00
I
ATOM 4895 HA TYR I 349 -9.988 4.339 20.198 0.00 0.00
I
ATOM 4896 CB TYR I 349 -9.258 4.483 18.255 0.00 0.00
I
ATOM 4897 HB1 TYR I 349 -8.426 3.777 18.463 0.00 0.00
I
ATOM 4898 HB2 TYR I 349 -9.587 4.449 17.194 0.00 0.00
I
ATOM 4899 CG TYR I 349 -8.680 5.750 18.593 0.00 0.00
I
ATOM 4900 CD1 TYR I 349 -7.972 5.975 19.720 0.00 0.00
I
ATOM 4901 HD1 TYR I 349 -7.605 5.204 20.381 0.00 0.00
I
ATOM 4902 CE1 TYR I 349 -7.577 7.276 20.127 0.00 0.00
I
ATOM 4903 HE1 TYR I 349 -7.015 7.397 21.041 0.00 0.00
I
ATOM 4904 CZ TYR I 349 -7.950 8.367 19.362 0.00 0.00
I
ATOM 4905 OH TYR I 349 -7.528 9.636 19.707 0.00 0.00
I
ATOM 4906 HH TYR I 349 -7.375 9.552 20.651 0.00 0.00
I
ATOM 4907 CD2 TYR I 349 -8.902 6.893 17.776 0.00 0.00
I
ATOM 4908 HD2 TYR I 349 -9.412 6.728 16.839 0.00 0.00
I
ATOM 4909 CE2 TYR I 349 -8.618 8.175 18.161 0.00 0.00
I
ATOM 4910 HE2 TYR I 349 -9.034 8.968 17.558 0.00 0.00
I
ATOM 4911 C TYR I 349 -11.514 5.268 19.099 0.00 0.00
I
ATOM 4912 O TYR I 349 -11.598 6.142 19.982 0.00 0.00
I
ATOM 4913 N LEU I 350 -12.457 5.077 18.138 0.00 0.00
I

ATOM	4914	HN	LEU	I	350	−12.439	4.209	17.649	0.00	0.00	I
ATOM	4915	CA	LEU	I	350	−13.549	5.953	17.855	0.00	0.00	I
ATOM	4916	HA	LEU	I	350	−13.153	6.958	17.874	0.00	0.00	I
ATOM	4917	CB	LEU	I	350	−14.307	5.717	16.568	0.00	0.00	I
ATOM	4918	HB1	LEU	I	350	−14.896	4.775	16.585	0.00	0.00	I
ATOM	4919	HB2	LEU	I	350	−14.975	6.604	16.540	0.00	0.00	I
ATOM	4920	CG	LEU	I	350	−13.429	5.776	15.252	0.00	0.00	I
ATOM	4921	HG	LEU	I	350	−12.474	5.268	15.504	0.00	0.00	I
ATOM	4922	CD1	LEU	I	350	−14.069	5.107	14.059	0.00	0.00	I
ATOM	4923	HD11	LEU	I	350	−14.974	5.601	13.647	0.00	0.00	I
ATOM	4924	HD12	LEU	I	350	−13.324	5.181	13.238	0.00	0.00	I
ATOM	4925	HD13	LEU	I	350	−14.249	4.023	14.228	0.00	0.00	I
ATOM	4926	CD2	LEU	I	350	−13.070	7.242	14.957	0.00	0.00	I
ATOM	4927	HD21	LEU	I	350	−13.999	7.803	14.718	0.00	0.00	I
ATOM	4928	HD22	LEU	I	350	−12.565	7.687	15.841	0.00	0.00	I
ATOM	4929	HD23	LEU	I	350	−12.390	7.128	14.085	0.00	0.00	I
ATOM	4930	C	LEU	I	350	−14.510	6.078	18.984	0.00	0.00	I
ATOM	4931	O	LEU	I	350	−14.933	7.190	19.458	0.00	0.00	I
ATOM	4932	N	ARG	I	351	−14.773	4.879	19.634	0.00	0.00	I
ATOM	4933	HN	ARG	I	351	−14.380	4.043	19.261	0.00	0.00	I
ATOM	4934	CA	ARG	I	351	−15.657	4.725	20.803	0.00	0.00	I
ATOM	4935	HA	ARG	I	351	−16.578	5.213	20.518	0.00	0.00	I
ATOM	4936	CB	ARG	I	351	−15.876	3.167	21.049	0.00	0.00	I
ATOM	4937	HB1	ARG	I	351	−14.953	2.557	21.144	0.00	0.00	I
ATOM	4938	HB2	ARG	I	351	−16.354	3.130	22.051	0.00	0.00	I
ATOM	4939	CG	ARG	I	351	−16.775	2.554	19.977	0.00	0.00	I
ATOM	4940	HG1	ARG	I	351	−17.766	3.055	19.964	0.00	0.00	I

ATOM 4941 HG2 ARG I 351 −16.387 2.724 18.950 0.00 0.00
I
ATOM 4942 CD ARG I 351 −17.188 1.089 20.098 0.00 0.00
I
ATOM 4943 HD1 ARG I 351 −17.908 0.858 19.283 0.00 0.00
I
ATOM 4944 HD2 ARG I 351 −16.211 0.568 20.005 0.00 0.00
I
ATOM 4945 NE ARG I 351 −17.845 0.751 21.407 0.00 0.00
I
ATOM 4946 HE ARG I 351 −18.066 1.491 22.042 0.00 0.00
I
ATOM 4947 CZ ARG I 351 −18.132 −0.449 21.882 0.00 0.00
I
ATOM 4948 NH1 ARG I 351 −17.773 −1.565 21.252 0.00 0.00
I
ATOM 4949 HH11 ARG I 351 −17.942 −2.476 21.626 0.00 0.00
I
ATOM 4950 HH12 ARG I 351 −17.173 −1.378 20.473 0.00 0.00
I
ATOM 4951 NH2 ARG I 351 −18.704 −0.551 23.035 0.00 0.00
I
ATOM 4952 HH21 ARG I 351 −18.647 −1.391 23.575 0.00 0.00
I
ATOM 4953 HH22 ARG I 351 −18.996 0.302 23.466 0.00 0.00
I
ATOM 4954 C ARG I 351 −15.053 5.296 22.075 0.00 0.00
I
ATOM 4955 O ARG I 351 −15.637 5.919 22.946 0.00 0.00
I
ATOM 4956 N GLY I 352 −13.777 5.110 22.167 0.00 0.00
I
ATOM 4957 HN GLY I 352 −13.404 4.568 21.417 0.00 0.00
I
ATOM 4958 CA GLY I 352 −12.892 5.493 23.269 0.00 0.00
I
ATOM 4959 HA1 GLY I 352 −13.344 6.279 23.854 0.00 0.00
I
ATOM 4960 HA2 GLY I 352 −11.938 5.726 22.820 0.00 0.00
I
ATOM 4961 C GLY I 352 −12.508 4.439 24.291 0.00 0.00
I
ATOM 4962 O GLY I 352 −12.393 4.785 25.464 0.00 0.00
I
ATOM 4963 N LYS I 353 −12.402 3.128 23.964 0.00 0.00
I
ATOM 4964 HN LYS I 353 −12.706 2.955 23.031 0.00 0.00
I
ATOM 4965 CA LYS I 353 −11.842 2.039 24.813 0.00 0.00
I
ATOM 4966 HA LYS I 353 −12.456 1.981 25.699 0.00 0.00
I
ATOM 4967 CB LYS I 353 −11.902 0.655 24.119 0.00 0.00
I

ATOM	4968	HB1	LYS	I	353	−11.412	0.793	23.131	0.00	0.00	I
ATOM	4969	HB2	LYS	I	353	−11.415	−0.139	24.724	0.00	0.00	I
ATOM	4970	CG	LYS	I	353	−13.360	0.280	23.970	0.00	0.00	I
ATOM	4971	HG1	LYS	I	353	−13.913	0.164	24.926	0.00	0.00	I
ATOM	4972	HG2	LYS	I	353	−13.818	1.016	23.275	0.00	0.00	I
ATOM	4973	CD	LYS	I	353	−13.516	−1.059	23.249	0.00	0.00	I
ATOM	4974	HD1	LYS	I	353	−14.536	−1.029	22.811	0.00	0.00	I
ATOM	4975	HD2	LYS	I	353	−12.747	−1.092	22.448	0.00	0.00	I
ATOM	4976	CE	LYS	I	353	−13.434	−2.328	24.189	0.00	0.00	I
ATOM	4977	HE1	LYS	I	353	−13.067	−3.182	23.580	0.00	0.00	I
ATOM	4978	HE2	LYS	I	353	−12.609	−2.140	24.908	0.00	0.00	I
ATOM	4979	NZ	LYS	I	353	−14.684	−2.600	24.849	0.00	0.00	I
ATOM	4980	HZ1	LYS	I	353	−14.574	−3.044	25.783	0.00	0.00	I
ATOM	4981	HZ2	LYS	I	353	−15.194	−1.714	25.037	0.00	0.00	I
ATOM	4982	HZ3	LYS	I	353	−15.275	−3.204	24.242	0.00	0.00	I
ATOM	4983	C	LYS	I	353	−10.396	2.290	25.229	0.00	0.00	I
ATOM	4984	O	LYS	I	353	−9.597	2.845	24.492	0.00	0.00	I
ATOM	4985	N	LYS	I	354	−10.064	1.805	26.496	0.00	0.00	I
ATOM	4986	HN	LYS	I	354	−10.631	1.315	27.153	0.00	0.00	I
ATOM	4987	CA	LYS	I	354	−8.655	1.591	26.833	0.00	0.00	I
ATOM	4988	HA	LYS	I	354	−8.067	2.329	26.307	0.00	0.00	I
ATOM	4989	CB	LYS	I	354	−8.491	1.682	28.349	0.00	0.00	I
ATOM	4990	HB1	LYS	I	354	−9.024	2.614	28.632	0.00	0.00	I
ATOM	4991	HB2	LYS	I	354	−8.935	0.835	28.913	0.00	0.00	I
ATOM	4992	CG	LYS	I	354	−7.087	1.849	28.901	0.00	0.00	I
ATOM	4993	HG1	LYS	I	354	−6.600	0.850	28.912	0.00	0.00	I
ATOM	4994	HG2	LYS	I	354	−6.567	2.570	28.234	0.00	0.00	I

ATOM 4995 CD LYS I 354 -6.998 2.551 30.227 0.00 0.00
I
ATOM 4996 HD1 LYS I 354 -7.608 3.479 30.211 0.00 0.00
I
ATOM 4997 HD2 LYS I 354 -7.419 1.907 31.028 0.00 0.00
I
ATOM 4998 CE LYS I 354 -5.529 2.782 30.636 0.00 0.00
I
ATOM 4999 HE1 LYS I 354 -4.940 1.846 30.531 0.00 0.00
I
ATOM 5000 HE2 LYS I 354 -5.170 3.574 29.944 0.00 0.00
I
ATOM 5001 NZ LYS I 354 -5.359 3.139 32.036 0.00 0.00
I
ATOM 5002 HZ1 LYS I 354 -5.957 2.453 32.540 0.00 0.00
I
ATOM 5003 HZ2 LYS I 354 -4.384 3.134 32.395 0.00 0.00
I
ATOM 5004 HZ3 LYS I 354 -5.692 4.113 32.184 0.00 0.00
I
ATOM 5005 C LYS I 354 -8.065 0.194 26.291 0.00 0.00
I
ATOM 5006 O LYS I 354 -6.957 0.051 25.770 0.00 0.00
I
ATOM 5007 N GLU I 355 -8.856 -0.822 26.382 0.00 0.00
I
ATOM 5008 HN GLU I 355 -9.701 -0.775 26.909 0.00 0.00
I
ATOM 5009 CA GLU I 355 -8.538 -2.151 25.883 0.00 0.00
I
ATOM 5010 HA GLU I 355 -7.476 -2.317 25.780 0.00 0.00
I
ATOM 5011 CB GLU I 355 -8.978 -3.364 26.734 0.00 0.00
I
ATOM 5012 HB1 GLU I 355 -10.078 -3.440 26.602 0.00 0.00
I
ATOM 5013 HB2 GLU I 355 -8.597 -4.344 26.375 0.00 0.00
I
ATOM 5014 CG GLU I 355 -8.564 -2.982 28.233 0.00 0.00
I
ATOM 5015 HG1 GLU I 355 -7.471 -2.794 28.167 0.00 0.00
I
ATOM 5016 HG2 GLU I 355 -9.139 -2.155 28.701 0.00 0.00
I
ATOM 5017 CD GLU I 355 -8.584 -4.216 29.139 0.00 0.00
I
ATOM 5018 OE1 GLU I 355 -9.498 -5.078 29.034 0.00 0.00
I
ATOM 5019 OE2 GLU I 355 -7.630 -4.372 29.940 0.00 0.00
I
ATOM 5020 C GLU I 355 -9.081 -2.426 24.493 0.00 0.00
I
ATOM 5021 O GLU I 355 -10.293 -2.289 24.295 0.00 0.00
I

ATOM 5022 N THR I 356 -8.321 -2.827 23.468 0.00 0.00
I
ATOM 5023 HN THR I 356 -7.326 -2.766 23.505 0.00 0.00
I
ATOM 5024 CA THR I 356 -8.959 -3.387 22.242 0.00 0.00
I
ATOM 5025 HA THR I 356 -9.812 -2.737 22.110 0.00 0.00
I
ATOM 5026 CB THR I 356 -7.995 -3.350 21.042 0.00 0.00
I
ATOM 5027 HB THR I 356 -7.015 -3.842 21.219 0.00 0.00
I
ATOM 5028 OG1 THR I 356 -7.820 -1.943 20.770 0.00 0.00
I
ATOM 5029 HG1 THR I 356 -7.194 -1.994 20.043 0.00 0.00
I
ATOM 5030 CG2 THR I 356 -8.605 -3.990 19.815 0.00 0.00
I
ATOM 5031 HG21 THR I 356 -7.793 -4.146 19.073 0.00 0.00
I
ATOM 5032 HG22 THR I 356 -9.098 -4.984 19.873 0.00 0.00
I
ATOM 5033 HG23 THR I 356 -9.431 -3.341 19.456 0.00 0.00
I
ATOM 5034 C THR I 356 -9.405 -4.835 22.446 0.00 0.00
I
ATOM 5035 O THR I 356 -8.622 -5.574 23.045 0.00 0.00
I
ATOM 5036 N VAL I 357 -10.644 -5.139 22.081 0.00 0.00
I
ATOM 5037 HN VAL I 357 -11.217 -4.509 21.564 0.00 0.00
I
ATOM 5038 CA VAL I 357 -11.238 -6.419 22.295 0.00 0.00
I
ATOM 5039 HA VAL I 357 -10.539 -6.947 22.926 0.00 0.00
I
ATOM 5040 CB VAL I 357 -12.514 -6.198 23.187 0.00 0.00
I
ATOM 5041 HB VAL I 357 -13.100 -5.289 22.932 0.00 0.00
I
ATOM 5042 CG1 VAL I 357 -13.497 -7.368 23.212 0.00 0.00
I
ATOM 5043 HG11 VAL I 357 -12.922 -8.252 23.562 0.00 0.00
I
ATOM 5044 HG12 VAL I 357 -14.372 -7.222 23.881 0.00 0.00
I
ATOM 5045 HG13 VAL I 357 -13.921 -7.604 22.212 0.00 0.00
I
ATOM 5046 CG2 VAL I 357 -11.911 -5.932 24.549 0.00 0.00
I
ATOM 5047 HG21 VAL I 357 -12.721 -5.727 25.281 0.00 0.00
I
ATOM 5048 HG22 VAL I 357 -11.344 -6.840 24.848 0.00 0.00
I

```
ATOM   5049 HG23 VAL I 357     -11.238  -5.054  24.650  0.00  0.00
I
ATOM   5050  C   VAL I 357     -11.636  -6.981  20.993  0.00  0.00
I
ATOM   5051  O   VAL I 357     -12.264  -6.231  20.197  0.00  0.00
I
ATOM   5052  N   ILE I 358     -11.392  -8.251  20.716  0.00  0.00
I
ATOM   5053  HN  ILE I 358     -10.830  -8.709  21.400  0.00  0.00
I
ATOM   5054  CA  ILE I 358     -12.002  -9.186  19.826  0.00  0.00
I
ATOM   5055  HA  ILE I 358     -12.776  -8.771  19.197  0.00  0.00
I
ATOM   5056  CB  ILE I 358     -10.903  -9.788  18.977  0.00  0.00
I
ATOM   5057  HB  ILE I 358     -10.160 -10.296  19.630  0.00  0.00
I
ATOM   5058  CG2 ILE I 358     -11.612 -10.925  18.083  0.00  0.00
I
ATOM   5059 HG21 ILE I 358     -12.618 -10.639  17.707  0.00  0.00
I
ATOM   5060 HG22 ILE I 358     -10.950 -11.105  17.210  0.00  0.00
I
ATOM   5061 HG23 ILE I 358     -11.713 -11.948  18.503  0.00  0.00
I
ATOM   5062  CG1 ILE I 358     -10.131  -8.783  18.030  0.00  0.00
I
ATOM   5063 HG11 ILE I 358     -10.618  -8.642  17.042  0.00  0.00
I
ATOM   5064 HG12 ILE I 358      -9.977  -7.824  18.569  0.00  0.00
I
ATOM   5065  CD  ILE I 358      -8.756  -9.316  17.600  0.00  0.00
I
ATOM   5066  HD1 ILE I 358      -8.781 -10.422  17.705  0.00  0.00
I
ATOM   5067  HD2 ILE I 358      -8.395  -9.043  16.586  0.00  0.00
I
ATOM   5068  HD3 ILE I 358      -7.913  -8.986  18.244  0.00  0.00
I
ATOM   5069  C   ILE I 358     -12.491 -10.363  20.753  0.00  0.00
I
ATOM   5070  O   ILE I 358     -11.755 -10.738  21.634  0.00  0.00
I
ATOM   5071  N   PHE I 359     -13.788 -10.810  20.545  0.00  0.00
I
ATOM   5072  HN  PHE I 359     -14.281 -10.416  19.772  0.00  0.00
I
ATOM   5073  CA  PHE I 359     -14.437 -11.996  20.993  0.00  0.00
I
ATOM   5074  HA  PHE I 359     -15.499 -11.832  20.881  0.00  0.00
I
ATOM   5075  CB  PHE I 359     -13.961 -13.292  20.334  0.00  0.00
I
```

ATOM 5076 HB1 PHE I 359 −12.851 −13.337 20.345 0.00 0.00
I
ATOM 5077 HB2 PHE I 359 −14.332 −14.205 20.847 0.00 0.00
I
ATOM 5078 CG PHE I 359 −14.468 −13.607 18.902 0.00 0.00
I
ATOM 5079 CD1 PHE I 359 −13.661 −14.344 18.084 0.00 0.00
I
ATOM 5080 HD1 PHE I 359 −12.684 −14.562 18.489 0.00 0.00
I
ATOM 5081 CE1 PHE I 359 −14.090 −14.616 16.755 0.00 0.00
I
ATOM 5082 HE1 PHE I 359 −13.442 −15.174 16.095 0.00 0.00
I
ATOM 5083 CZ PHE I 359 −15.323 −14.224 16.289 0.00 0.00
I
ATOM 5084 HZ PHE I 359 −15.592 −14.499 15.280 0.00 0.00
I
ATOM 5085 CD2 PHE I 359 −15.735 −13.209 18.472 0.00 0.00
I
ATOM 5086 HD2 PHE I 359 −16.455 −12.766 19.144 0.00 0.00
I
ATOM 5087 CE2 PHE I 359 −16.137 −13.530 17.190 0.00 0.00
I
ATOM 5088 HE2 PHE I 359 −17.091 −13.260 16.764 0.00 0.00
I
ATOM 5089 C PHE I 359 −14.349 −12.124 22.438 0.00 0.00
I
ATOM 5090 O PHE I 359 −13.952 −13.150 23.064 0.00 0.00
I
ATOM 5091 N ARG I 360 −14.748 −11.025 23.107 0.00 0.00
I
ATOM 5092 HN ARG I 360 −14.933 −10.258 22.497 0.00 0.00
I
ATOM 5093 CA ARG I 360 −14.996 −10.877 24.572 0.00 0.00
I
ATOM 5094 HA ARG I 360 −15.292 −9.880 24.863 0.00 0.00
I
ATOM 5095 CB ARG I 360 −16.098 −11.758 25.080 0.00 0.00
I
ATOM 5096 HB1 ARG I 360 −15.654 −12.775 25.139 0.00 0.00
I
ATOM 5097 HB2 ARG I 360 −16.228 −11.541 26.162 0.00 0.00
I
ATOM 5098 CG ARG I 360 −17.416 −11.618 24.321 0.00 0.00
I
ATOM 5099 HG1 ARG I 360 −17.653 −10.532 24.305 0.00 0.00
I
ATOM 5100 HG2 ARG I 360 −17.275 −11.819 23.238 0.00 0.00
I
ATOM 5101 CD ARG I 360 −18.607 −12.412 24.849 0.00 0.00
I
ATOM 5102 HD1 ARG I 360 −18.674 −12.292 25.951 0.00 0.00
I

ATOM 5103 HD2 ARG I 360 −19.494 −11.982 24.337 0.00 0.00
I
ATOM 5104 NE ARG I 360 −18.483 −13.821 24.403 0.00 0.00
I
ATOM 5105 HE ARG I 360 −17.692 −14.060 23.840 0.00 0.00
I
ATOM 5106 CZ ARG I 360 −19.105 −14.844 25.049 0.00 0.00
I
ATOM 5107 NH1 ARG I 360 −20.243 −14.683 25.712 0.00 0.00
I
ATOM 5108 HH11 ARG I 360 −20.668 −15.573 25.872 0.00 0.00
I
ATOM 5109 HH12 ARG I 360 −20.780 −13.889 25.427 0.00 0.00
I
ATOM 5110 NH2 ARG I 360 −18.600 −16.068 24.881 0.00 0.00
I
ATOM 5111 HH21 ARG I 360 −19.137 −16.782 25.330 0.00 0.00
I
ATOM 5112 HH22 ARG I 360 −17.616 −16.070 24.708 0.00 0.00
I
ATOM 5113 C ARG I 360 −13.657 −11.115 25.375 0.00 0.00
I
ATOM 5114 O ARG I 360 −13.698 −11.431 26.555 0.00 0.00
I
ATOM 5115 N ARG I 361 −12.485 −10.902 24.776 0.00 0.00
I
ATOM 5116 HN ARG I 361 −12.405 −10.751 23.793 0.00 0.00
I
ATOM 5117 CA ARG I 361 −11.258 −11.010 25.475 0.00 0.00
I
ATOM 5118 HA ARG I 361 −11.339 −11.061 26.550 0.00 0.00
I
ATOM 5119 CB ARG I 361 −10.655 −12.467 25.120 0.00 0.00
I
ATOM 5120 HB1 ARG I 361 −10.759 −12.648 24.029 0.00 0.00
I
ATOM 5121 HB2 ARG I 361 −9.555 −12.445 25.273 0.00 0.00
I
ATOM 5122 CG ARG I 361 −11.332 −13.689 25.792 0.00 0.00
I
ATOM 5123 HG1 ARG I 361 −11.355 −13.603 26.899 0.00 0.00
I
ATOM 5124 HG2 ARG I 361 −12.410 −13.623 25.532 0.00 0.00
I
ATOM 5125 CD ARG I 361 −10.688 −15.005 25.361 0.00 0.00
I
ATOM 5126 HD1 ARG I 361 −11.298 −15.800 25.840 0.00 0.00
I
ATOM 5127 HD2 ARG I 361 −10.660 −15.260 24.280 0.00 0.00
I
ATOM 5128 NE ARG I 361 −9.313 −14.971 26.007 0.00 0.00
I
ATOM 5129 HE ARG I 361 −8.938 −14.165 26.465 0.00 0.00
I

```
ATOM   5130  CZ  ARG I 361      -8.348 -15.896  25.739  0.00  0.00
I
ATOM   5131  NH1 ARG I 361      -8.703 -17.016  25.110  0.00  0.00
I
ATOM   5132 HH11 ARG I 361      -8.006 -17.717  24.966  0.00  0.00
I
ATOM   5133 HH12 ARG I 361      -9.646 -17.140  24.800  0.00  0.00
I
ATOM   5134  NH2 ARG I 361      -7.239 -15.891  26.481  0.00  0.00
I
ATOM   5135 HH21 ARG I 361      -6.640 -16.687  26.566  0.00  0.00
I
ATOM   5136 HH22 ARG I 361      -7.084 -15.111  27.087  0.00  0.00
I
ATOM   5137  C   ARG I 361     -10.361  -9.864  24.953  0.00  0.00
I
ATOM   5138  O   ARG I 361     -10.639  -9.320  23.830  0.00  0.00
I
ATOM   5139  N   SER I 362      -9.279  -9.454  25.738  0.00  0.00
I
ATOM   5140  HN  SER I 362      -9.060  -9.817  26.641  0.00  0.00
I
ATOM   5141  CA  SER I 362      -8.554  -8.165  25.443  0.00  0.00
I
ATOM   5142  HA  SER I 362      -9.042  -7.553  24.698  0.00  0.00
I
ATOM   5143  CB  SER I 362      -8.459  -7.277  26.692  0.00  0.00
I
ATOM   5144  HB1 SER I 362      -7.916  -7.710  27.560  0.00  0.00
I
ATOM   5145  HB2 SER I 362      -7.993  -6.327  26.353  0.00  0.00
I
ATOM   5146  OG  SER I 362      -9.817  -6.948  27.219  0.00  0.00
I
ATOM   5147  HG1 SER I 362      -9.746  -6.286  27.911  0.00  0.00
I
ATOM   5148  C   SER I 362      -7.084  -8.314  25.030  0.00  0.00
I
ATOM   5149  O   SER I 362      -6.326  -9.085  25.653  0.00  0.00
I
ATOM   5150  N   ILE I 363      -6.711  -7.551  23.996  0.00  0.00
I
ATOM   5151  HN  ILE I 363      -7.278  -6.822  23.619  0.00  0.00
I
ATOM   5152  CA  ILE I 363      -5.296  -7.596  23.537  0.00  0.00
I
ATOM   5153  HA  ILE I 363      -4.780  -8.435  23.982  0.00  0.00
I
ATOM   5154  CB  ILE I 363      -5.352  -7.814  21.976  0.00  0.00
I
ATOM   5155  HB  ILE I 363      -4.295  -7.794  21.636  0.00  0.00
I
ATOM   5156  CG2 ILE I 363      -5.998  -9.157  21.541  0.00  0.00
I
```

ATOM   5157 HG21 ILE I 363   -5.846 -9.262 20.445 0.00 0.00
I
ATOM   5158 HG22 ILE I 363   -5.507 -10.005 22.065 0.00 0.00
I
ATOM   5159 HG23 ILE I 363   -7.072 -9.379 21.718 0.00 0.00
I
ATOM   5160 CG1 ILE I 363   -6.066 -6.736 21.207 0.00 0.00
I
ATOM   5161 HG11 ILE I 363   -7.162 -6.912 21.247 0.00 0.00
I
ATOM   5162 HG12 ILE I 363   -6.037 -5.747 21.712 0.00 0.00
I
ATOM   5163 CD ILE I 363   -5.718 -6.742 19.753 0.00 0.00
I
ATOM   5164 HD1 ILE I 363   -4.622 -6.579 19.669 0.00 0.00
I
ATOM   5165 HD2 ILE I 363   -5.998 -7.650 19.178 0.00 0.00
I
ATOM   5166 HD3 ILE I 363   -6.209 -5.875 19.263 0.00 0.00
I
ATOM   5167 C   ILE I 363   -4.542 -6.352 23.947 0.00 0.00
I
ATOM   5168 O   ILE I 363   -5.057 -5.189 23.927 0.00 0.00
I
ATOM   5169 N   LYS I 364   -3.228 -6.532 24.146 0.00 0.00
I
ATOM   5170 HN LYS I 364   -2.811 -7.437 24.181 0.00 0.00
I
ATOM   5171 CA LYS I 364   -2.346 -5.463 24.516 0.00 0.00
I
ATOM   5172 HA LYS I 364   -3.046 -4.688 24.790 0.00 0.00
I
ATOM   5173 CB LYS I 364   -1.403 -5.890 25.735 0.00 0.00
I
ATOM   5174 HB1 LYS I 364   -0.775 -6.699 25.305 0.00 0.00
I
ATOM   5175 HB2 LYS I 364   -0.872 -4.987 26.104 0.00 0.00
I
ATOM   5176 CG LYS I 364   -2.164 -6.328 26.945 0.00 0.00
I
ATOM   5177 HG1 LYS I 364   -2.761 -5.537 27.446 0.00 0.00
I
ATOM   5178 HG2 LYS I 364   -2.817 -7.188 26.680 0.00 0.00
I
ATOM   5179 CD LYS I 364   -1.312 -6.700 28.191 0.00 0.00
I
ATOM   5180 HD1 LYS I 364   -0.797 -7.672 28.037 0.00 0.00
I
ATOM   5181 HD2 LYS I 364   -0.584 -5.877 28.354 0.00 0.00
I
ATOM   5182 CE LYS I 364   -2.174 -6.741 29.423 0.00 0.00
I
ATOM   5183 HE1 LYS I 364   -2.321 -5.727 29.852 0.00 0.00
I

ATOM 5184 HE2 LYS I 364 -3.168 -7.222 29.303 0.00 0.00
I
ATOM 5185 NZ LYS I 364 -1.508 -7.519 30.537 0.00 0.00
I
ATOM 5186 HZ1 LYS I 364 -0.705 -7.016 30.966 0.00 0.00
I
ATOM 5187 HZ2 LYS I 364 -2.174 -7.944 31.214 0.00 0.00
I
ATOM 5188 HZ3 LYS I 364 -1.028 -8.287 30.025 0.00 0.00
I
ATOM 5189 C LYS I 364 -1.668 -4.843 23.223 0.00 0.00
I
ATOM 5190 O LYS I 364 -1.723 -5.429 22.121 0.00 0.00
I
ATOM 5191 N TYR I 365 -1.024 -3.688 23.411 0.00 0.00
I
ATOM 5192 HN TYR I 365 -1.158 -3.233 24.288 0.00 0.00
I
ATOM 5193 CA TYR I 365 -0.009 -3.081 22.505 0.00 0.00
I
ATOM 5194 HA TYR I 365 -0.474 -2.858 21.556 0.00 0.00
I
ATOM 5195 CB TYR I 365 0.572 -1.744 23.024 0.00 0.00
I
ATOM 5196 HB1 TYR I 365 -0.379 -1.271 23.351 0.00 0.00
I
ATOM 5197 HB2 TYR I 365 1.276 -1.946 23.860 0.00 0.00
I
ATOM 5198 CG TYR I 365 1.069 -0.786 21.979 0.00 0.00
I
ATOM 5199 CD1 TYR I 365 0.387 -0.390 20.815 0.00 0.00
I
ATOM 5200 HD1 TYR I 365 -0.675 -0.571 20.746 0.00 0.00
I
ATOM 5201 CE1 TYR I 365 1.077 0.394 19.802 0.00 0.00
I
ATOM 5202 HE1 TYR I 365 0.622 0.809 18.914 0.00 0.00
I
ATOM 5203 CZ TYR I 365 2.340 0.889 20.035 0.00 0.00
I
ATOM 5204 OH TYR I 365 2.991 1.782 19.088 0.00 0.00
I
ATOM 5205 HH TYR I 365 3.779 2.259 19.361 0.00 0.00
I
ATOM 5206 CD2 TYR I 365 2.398 -0.316 22.125 0.00 0.00
I
ATOM 5207 HD2 TYR I 365 3.024 -0.655 22.937 0.00 0.00
I
ATOM 5208 CE2 TYR I 365 3.013 0.475 21.159 0.00 0.00
I
ATOM 5209 HE2 TYR I 365 4.010 0.846 21.347 0.00 0.00
I
ATOM 5210 C TYR I 365 1.222 -3.906 21.954 0.00 0.00
I

ATOM 5211 O TYR I 365 1.504 -3.625 20.807 0.00 0.00
I
ATOM 5212 N PRO I 366 1.935 -4.872 22.563 0.00 0.00
I
ATOM 5213 CD PRO I 366 1.930 -5.269 23.995 0.00 0.00
I
ATOM 5214 HD1 PRO I 366 1.889 -4.355 24.625 0.00 0.00
I
ATOM 5215 HD2 PRO I 366 1.052 -5.912 24.218 0.00 0.00
I
ATOM 5216 CA PRO I 366 2.807 -5.759 21.850 0.00 0.00
I
ATOM 5217 HA PRO I 366 3.624 -5.117 21.558 0.00 0.00
I
ATOM 5218 CB PRO I 366 3.377 -6.701 22.830 0.00 0.00
I
ATOM 5219 HB1 PRO I 366 4.396 -7.042 22.550 0.00 0.00
I
ATOM 5220 HB2 PRO I 366 2.670 -7.554 22.912 0.00 0.00
I
ATOM 5221 CG PRO I 366 3.307 -5.991 24.129 0.00 0.00
I
ATOM 5222 HG1 PRO I 366 3.313 -6.728 24.961 0.00 0.00
I
ATOM 5223 HG2 PRO I 366 4.156 -5.284 24.246 0.00 0.00
I
ATOM 5224 C PRO I 366 2.232 -6.484 20.682 0.00 0.00
I
ATOM 5225 O PRO I 366 2.933 -6.833 19.696 0.00 0.00
I
ATOM 5226 N ILE I 367 0.894 -6.794 20.730 0.00 0.00
I
ATOM 5227 HN ILE I 367 0.382 -6.565 21.555 0.00 0.00
I
ATOM 5228 CA ILE I 367 0.107 -7.359 19.660 0.00 0.00
I
ATOM 5229 HA ILE I 367 0.658 -8.127 19.136 0.00 0.00
I
ATOM 5230 CB ILE I 367 -1.182 -7.968 20.139 0.00 0.00
I
ATOM 5231 HB ILE I 367 -1.751 -7.116 20.570 0.00 0.00
I
ATOM 5232 CG2 ILE I 367 -2.018 -8.457 18.990 0.00 0.00
I
ATOM 5233 HG21 ILE I 367 -2.428 -7.589 18.431 0.00 0.00
I
ATOM 5234 HG22 ILE I 367 -1.423 -9.174 18.384 0.00 0.00
I
ATOM 5235 HG23 ILE I 367 -2.886 -9.067 19.319 0.00 0.00
I
ATOM 5236 CG1 ILE I 367 -0.884 -9.093 21.138 0.00 0.00
I
ATOM 5237 HG11 ILE I 367 -0.372 -9.830 20.483 0.00 0.00
I

ATOM 5238 HG12 ILE I 367 -0.216 -8.724 21.946 0.00 0.00
I
ATOM 5239 CD ILE I 367 -2.110 -9.725 21.674 0.00 0.00
I
ATOM 5240 HD1 ILE I 367 -1.823 -10.391 22.516 0.00 0.00
I
ATOM 5241 HD2 ILE I 367 -2.904 -9.124 22.168 0.00 0.00
I
ATOM 5242 HD3 ILE I 367 -2.576 -10.431 20.953 0.00 0.00
I
ATOM 5243 C ILE I 367 -0.093 -6.365 18.452 0.00 0.00
I
ATOM 5244 O ILE I 367 0.129 -6.684 17.297 0.00 0.00
I
ATOM 5245 N ILE I 368 -0.587 -5.164 18.810 0.00 0.00
I
ATOM 5246 HN ILE I 368 -0.652 -4.943 19.780 0.00 0.00
I
ATOM 5247 CA ILE I 368 -0.913 -4.032 17.975 0.00 0.00
I
ATOM 5248 HA ILE I 368 -1.525 -4.470 17.200 0.00 0.00
I
ATOM 5249 CB ILE I 368 -1.596 -2.875 18.825 0.00 0.00
I
ATOM 5250 HB ILE I 368 -0.957 -2.430 19.617 0.00 0.00
I
ATOM 5251 CG2 ILE I 368 -2.083 -1.738 17.820 0.00 0.00
I
ATOM 5252 HG21 ILE I 368 -2.516 -2.293 16.961 0.00 0.00
I
ATOM 5253 HG22 ILE I 368 -2.756 -0.984 18.282 0.00 0.00
I
ATOM 5254 HG23 ILE I 368 -1.235 -1.218 17.326 0.00 0.00
I
ATOM 5255 CG1 ILE I 368 -2.830 -3.457 19.558 0.00 0.00
I
ATOM 5256 HG11 ILE I 368 -3.569 -3.770 18.789 0.00 0.00
I
ATOM 5257 HG12 ILE I 368 -2.594 -4.416 20.067 0.00 0.00
I
ATOM 5258 CD ILE I 368 -3.481 -2.508 20.576 0.00 0.00
I
ATOM 5259 HD1 ILE I 368 -2.799 -2.340 21.437 0.00 0.00
I
ATOM 5260 HD2 ILE I 368 -3.850 -1.569 20.109 0.00 0.00
I
ATOM 5261 HD3 ILE I 368 -4.325 -3.080 21.016 0.00 0.00
I
ATOM 5262 C ILE I 368 0.289 -3.417 17.289 0.00 0.00
I
ATOM 5263 O ILE I 368 0.205 -3.153 16.073 0.00 0.00
I
ATOM 5264 N ILE I 369 1.470 -3.243 17.948 0.00 0.00
I

ATOM	5265	HN	ILE	I	369	1.517	-3.573	18.888	0.00	0.00	I
ATOM	5266	CA	ILE	I	369	2.727	-2.888	17.288	0.00	0.00	I
ATOM	5267	HA	ILE	I	369	2.539	-1.947	16.791	0.00	0.00	I
ATOM	5268	CB	ILE	I	369	3.817	-2.538	18.240	0.00	0.00	I
ATOM	5269	HB	ILE	I	369	3.388	-1.796	18.946	0.00	0.00	I
ATOM	5270	CG2	ILE	I	369	4.219	-3.851	18.966	0.00	0.00	I
ATOM	5271	HG21	ILE	I	369	4.805	-3.515	19.848	0.00	0.00	I
ATOM	5272	HG22	ILE	I	369	3.294	-4.363	19.308	0.00	0.00	I
ATOM	5273	HG23	ILE	I	369	4.821	-4.472	18.268	0.00	0.00	I
ATOM	5274	CG1	ILE	I	369	5.111	-1.851	17.600	0.00	0.00	I
ATOM	5275	HG11	ILE	I	369	5.860	-1.737	18.412	0.00	0.00	I
ATOM	5276	HG12	ILE	I	369	5.742	-2.419	16.883	0.00	0.00	I
ATOM	5277	CD	ILE	I	369	4.922	-0.492	16.981	0.00	0.00	I
ATOM	5278	HD1	ILE	I	369	4.451	-0.679	15.993	0.00	0.00	I
ATOM	5279	HD2	ILE	I	369	4.167	0.091	17.551	0.00	0.00	I
ATOM	5280	HD3	ILE	I	369	5.838	0.132	16.901	0.00	0.00	I
ATOM	5281	C	ILE	I	369	3.131	-3.791	16.162	0.00	0.00	I
ATOM	5282	O	ILE	I	369	3.605	-3.327	15.171	0.00	0.00	I
ATOM	5283	N	LYS	I	370	2.950	-5.137	16.245	0.00	0.00	I
ATOM	5284	HN	LYS	I	370	2.546	-5.498	17.082	0.00	0.00	I
ATOM	5285	CA	LYS	I	370	3.117	-6.113	15.150	0.00	0.00	I
ATOM	5286	HA	LYS	I	370	4.132	-5.941	14.825	0.00	0.00	I
ATOM	5287	CB	LYS	I	370	2.992	-7.547	15.704	0.00	0.00	I
ATOM	5288	HB1	LYS	I	370	2.037	-7.689	16.255	0.00	0.00	I
ATOM	5289	HB2	LYS	I	370	3.023	-8.274	14.865	0.00	0.00	I
ATOM	5290	CG	LYS	I	370	4.272	-7.808	16.488	0.00	0.00	I
ATOM	5291	HG1	LYS	I	370	5.176	-7.494	15.924	0.00	0.00	I

ATOM	5292	HG2	LYS	I	370	4.276	-7.334	17.492	0.00	0.00	I
ATOM	5293	CD	LYS	I	370	4.417	-9.303	16.750	0.00	0.00	I
ATOM	5294	HD1	LYS	I	370	4.168	-9.815	15.796	0.00	0.00	I
ATOM	5295	HD2	LYS	I	370	5.475	-9.608	16.896	0.00	0.00	I
ATOM	5296	CE	LYS	I	370	3.444	-9.909	17.789	0.00	0.00	I
ATOM	5297	HE1	LYS	I	370	2.413	-9.549	17.582	0.00	0.00	I
ATOM	5298	HE2	LYS	I	370	3.503	-11.016	17.865	0.00	0.00	I
ATOM	5299	NZ	LYS	I	370	3.815	-9.423	19.136	0.00	0.00	I
ATOM	5300	HZ1	LYS	I	370	3.546	-8.425	19.254	0.00	0.00	I
ATOM	5301	HZ2	LYS	I	370	3.230	-9.968	19.802	0.00	0.00	I
ATOM	5302	HZ3	LYS	I	370	4.789	-9.689	19.386	0.00	0.00	I
ATOM	5303	C	LYS	I	370	2.220	-5.773	13.993	0.00	0.00	I
ATOM	5304	O	LYS	I	370	2.584	-5.617	12.796	0.00	0.00	I
ATOM	5305	N	ALA	I	371	0.884	-5.652	14.236	0.00	0.00	I
ATOM	5306	HN	ALA	I	371	0.468	-5.845	15.122	0.00	0.00	I
ATOM	5307	CA	ALA	I	371	-0.185	-5.338	13.320	0.00	0.00	I
ATOM	5308	HA	ALA	I	371	-0.260	-6.002	12.471	0.00	0.00	I
ATOM	5309	CB	ALA	I	371	-1.517	-5.313	14.025	0.00	0.00	I
ATOM	5310	HB1	ALA	I	371	-1.738	-6.319	14.443	0.00	0.00	I
ATOM	5311	HB2	ALA	I	371	-1.550	-4.611	14.885	0.00	0.00	I
ATOM	5312	HB3	ALA	I	371	-2.347	-4.952	13.381	0.00	0.00	I
ATOM	5313	C	ALA	I	371	0.147	-3.952	12.586	0.00	0.00	I
ATOM	5314	O	ALA	I	371	0.131	-3.928	11.352	0.00	0.00	I
ATOM	5315	N	LEU	I	372	0.591	-2.895	13.334	0.00	0.00	I
ATOM	5316	HN	LEU	I	372	0.743	-3.055	14.307	0.00	0.00	I
ATOM	5317	CA	LEU	I	372	0.992	-1.606	12.902	0.00	0.00	I
ATOM	5318	HA	LEU	I	372	0.231	-1.069	12.355	0.00	0.00	I

ATOM 5319 CB LEU I 372 1.360 -0.801 14.187 0.00 0.00
I
ATOM 5320 HB1 LEU I 372 0.547 -0.761 14.943 0.00 0.00
I
ATOM 5321 HB2 LEU I 372 2.267 -1.226 14.667 0.00 0.00
I
ATOM 5322 CG LEU I 372 1.792 0.627 13.899 0.00 0.00
I
ATOM 5323 HG LEU I 372 2.847 0.643 13.553 0.00 0.00
I
ATOM 5324 CD1 LEU I 372 1.016 1.459 12.914 0.00 0.00
I
ATOM 5325 HD11 LEU I 372 1.208 1.105 11.879 0.00 0.00
I
ATOM 5326 HD12 LEU I 372 -0.067 1.439 13.163 0.00 0.00
I
ATOM 5327 HD13 LEU I 372 1.201 2.555 12.892 0.00 0.00
I
ATOM 5328 CD2 LEU I 372 1.907 1.461 15.217 0.00 0.00
I
ATOM 5329 HD21 LEU I 372 0.858 1.604 15.554 0.00 0.00
I
ATOM 5330 HD22 LEU I 372 2.331 0.817 16.017 0.00 0.00
I
ATOM 5331 HD23 LEU I 372 2.481 2.410 15.142 0.00 0.00
I
ATOM 5332 C LEU I 372 2.242 -1.686 12.017 0.00 0.00
I
ATOM 5333 O LEU I 372 2.289 -1.180 10.872 0.00 0.00
I
ATOM 5334 N ALA I 373 3.234 -2.464 12.313 0.00 0.00
I
ATOM 5335 HN ALA I 373 3.394 -2.849 13.219 0.00 0.00
I
ATOM 5336 CA ALA I 373 4.367 -2.679 11.432 0.00 0.00
I
ATOM 5337 HA ALA I 373 4.739 -1.699 11.170 0.00 0.00
I
ATOM 5338 CB ALA I 373 5.335 -3.534 12.080 0.00 0.00
I
ATOM 5339 HB1 ALA I 373 4.940 -4.561 12.232 0.00 0.00
I
ATOM 5340 HB2 ALA I 373 6.273 -3.610 11.489 0.00 0.00
I
ATOM 5341 HB3 ALA I 373 5.630 -3.217 13.103 0.00 0.00
I
ATOM 5342 C ALA I 373 3.972 -3.322 10.150 0.00 0.00
I
ATOM 5343 O ALA I 373 4.354 -2.946 8.968 0.00 0.00
I
ATOM 5344 N VAL I 374 3.168 -4.369 10.175 0.00 0.00
I
ATOM 5345 HN VAL I 374 2.818 -4.676 11.056 0.00 0.00
I

ATOM 5346 CA VAL I 374 2.750 -5.229 9.028 0.00 0.00
I
ATOM 5347 HA VAL I 374 3.643 -5.671 8.612 0.00 0.00
I
ATOM 5348 CB VAL I 374 1.738 -6.412 9.526 0.00 0.00
I
ATOM 5349 HB VAL I 374 1.182 -6.138 10.448 0.00 0.00
I
ATOM 5350 CG1 VAL I 374 0.740 -6.864 8.404 0.00 0.00
I
ATOM 5351 HG11 VAL I 374 -0.058 -6.091 8.419 0.00 0.00
I
ATOM 5352 HG12 VAL I 374 1.256 -6.889 7.420 0.00 0.00
I
ATOM 5353 HG13 VAL I 374 0.268 -7.862 8.532 0.00 0.00
I
ATOM 5354 CG2 VAL I 374 2.698 -7.551 10.046 0.00 0.00
I
ATOM 5355 HG21 VAL I 374 3.319 -7.923 9.204 0.00 0.00
I
ATOM 5356 HG22 VAL I 374 3.351 -7.265 10.899 0.00 0.00
I
ATOM 5357 HG23 VAL I 374 1.982 -8.320 10.409 0.00 0.00
I
ATOM 5358 C VAL I 374 1.937 -4.269 8.121 0.00 0.00
I
ATOM 5359 O VAL I 374 2.253 -4.121 6.973 0.00 0.00
I
ATOM 5360 N SER I 375 0.960 -3.441 8.655 0.00 0.00
I
ATOM 5361 HN SER I 375 0.856 -3.549 9.640 0.00 0.00
I
ATOM 5362 CA SER I 375 0.027 -2.547 7.962 0.00 0.00
I
ATOM 5363 HA SER I 375 -0.543 -3.137 7.259 0.00 0.00
I
ATOM 5364 CB SER I 375 -1.085 -1.949 8.886 0.00 0.00
I
ATOM 5365 HB1 SER I 375 -1.415 -2.788 9.536 0.00 0.00
I
ATOM 5366 HB2 SER I 375 -0.722 -1.125 9.536 0.00 0.00
I
ATOM 5367 OG SER I 375 -2.186 -1.431 8.115 0.00 0.00
I
ATOM 5368 HG1 SER I 375 -2.642 -2.152 7.675 0.00 0.00
I
ATOM 5369 C SER I 375 0.819 -1.533 7.273 0.00 0.00
I
ATOM 5370 O SER I 375 0.679 -1.256 6.111 0.00 0.00
I
ATOM 5371 N VAL I 376 1.821 -0.966 8.034 0.00 0.00
I
ATOM 5372 HN VAL I 376 1.964 -1.204 8.991 0.00 0.00
I

ATOM 5373 CA VAL I 376 2.623 0.121 7.499 0.00 0.00
I
ATOM 5374 HA VAL I 376 1.952 0.796 6.988 0.00 0.00
I
ATOM 5375 CB VAL I 376 3.454 0.894 8.494 0.00 0.00
I
ATOM 5376 HB VAL I 376 4.078 0.194 9.089 0.00 0.00
I
ATOM 5377 CG1 VAL I 376 4.289 2.097 7.893 0.00 0.00
I
ATOM 5378 HG11 VAL I 376 5.049 1.678 7.200 0.00 0.00
I
ATOM 5379 HG12 VAL I 376 3.602 2.830 7.419 0.00 0.00
I
ATOM 5380 HG13 VAL I 376 4.875 2.476 8.758 0.00 0.00
I
ATOM 5381 CG2 VAL I 376 2.562 1.606 9.447 0.00 0.00
I
ATOM 5382 HG21 VAL I 376 3.267 1.938 10.239 0.00 0.00
I
ATOM 5383 HG22 VAL I 376 2.057 2.520 9.067 0.00 0.00
I
ATOM 5384 HG23 VAL I 376 1.831 0.946 9.959 0.00 0.00
I
ATOM 5385 C VAL I 376 3.486 -0.310 6.340 0.00 0.00
I
ATOM 5386 O VAL I 376 3.511 0.213 5.217 0.00 0.00
I
ATOM 5387 N THR I 377 4.170 -1.403 6.570 0.00 0.00
I
ATOM 5388 HN THR I 377 4.031 -1.860 7.445 0.00 0.00
I
ATOM 5389 CA THR I 377 5.232 -1.900 5.686 0.00 0.00
I
ATOM 5390 HA THR I 377 5.846 -1.029 5.510 0.00 0.00
I
ATOM 5391 CB THR I 377 6.046 -2.996 6.401 0.00 0.00
I
ATOM 5392 HB THR I 377 5.388 -3.799 6.795 0.00 0.00
I
ATOM 5393 OG1 THR I 377 6.730 -2.448 7.553 0.00 0.00
I
ATOM 5394 HG1 THR I 377 6.138 -2.684 8.271 0.00 0.00
I
ATOM 5395 CG2 THR I 377 7.065 -3.663 5.547 0.00 0.00
I
ATOM 5396 HG21 THR I 377 7.619 -4.418 6.144 0.00 0.00
I
ATOM 5397 HG22 THR I 377 6.641 -4.128 4.631 0.00 0.00
I
ATOM 5398 HG23 THR I 377 7.841 -2.923 5.255 0.00 0.00
I
ATOM 5399 C THR I 377 4.638 -2.417 4.331 0.00 0.00
I

```
ATOM   5400  O   THR I 377       4.995  -2.067   3.223  0.00  0.00
I
ATOM   5401  N   SER I 378       3.452  -3.090   4.437  0.00  0.00
I
ATOM   5402  HN  SER I 378       3.087  -3.238   5.353  0.00  0.00
I
ATOM   5403  CA  SER I 378       2.661  -3.705   3.354  0.00  0.00
I
ATOM   5404  HA  SER I 378       3.357  -4.435   2.968  0.00  0.00
I
ATOM   5405  CB  SER I 378       1.292  -4.411   3.792  0.00  0.00
I
ATOM   5406  HB1 SER I 378       0.586  -3.747   4.335  0.00  0.00
I
ATOM   5407  HB2 SER I 378       0.735  -4.727   2.885  0.00  0.00
I
ATOM   5408  OG  SER I 378       1.614  -5.501   4.638  0.00  0.00
I
ATOM   5409  HG1 SER I 378       1.620  -5.141   5.527  0.00  0.00
I
ATOM   5410  C   SER I 378       2.353  -2.665   2.270  0.00  0.00
I
ATOM   5411  O   SER I 378       2.273  -3.015   1.043  0.00  0.00
I
ATOM   5412  N   LEU I 379       2.081  -1.385   2.612  0.00  0.00
I
ATOM   5413  HN  LEU I 379       2.077  -1.105   3.569  0.00  0.00
I
ATOM   5414  CA  LEU I 379       1.826  -0.237   1.774  0.00  0.00
I
ATOM   5415  HA  LEU I 379       0.924  -0.364   1.194  0.00  0.00
I
ATOM   5416  CB  LEU I 379       1.497   0.988   2.670  0.00  0.00
I
ATOM   5417  HB1 LEU I 379       0.506   0.792   3.131  0.00  0.00
I
ATOM   5418  HB2 LEU I 379       2.275   1.057   3.461  0.00  0.00
I
ATOM   5419  CG  LEU I 379       1.388   2.377   1.995  0.00  0.00
I
ATOM   5420  HG  LEU I 379       2.195   2.621   1.271  0.00  0.00
I
ATOM   5421  CD1 LEU I 379       0.102   2.590   1.151  0.00  0.00
I
ATOM   5422 HD11 LEU I 379       0.247   2.096   0.166  0.00  0.00
I
ATOM   5423 HD12 LEU I 379      -0.779   2.208   1.709  0.00  0.00
I
ATOM   5424 HD13 LEU I 379       0.021   3.673   0.919  0.00  0.00
I
ATOM   5425  CD2 LEU I 379       1.549   3.523   3.000  0.00  0.00
I
ATOM   5426 HD21 LEU I 379       1.248   4.427   2.429  0.00  0.00
I
```

ATOM 5427 HD22 LEU I 379 0.954 3.379 3.927 0.00 0.00
I
ATOM 5428 HD23 LEU I 379 2.638 3.535 3.222 0.00 0.00
I
ATOM 5429 C LEU I 379 3.030 0.124 0.963 0.00 0.00
I
ATOM 5430 O LEU I 379 2.941 0.455 -0.198 0.00 0.00
I
ATOM 5431 N PHE I 380 4.173 0.202 1.679 0.00 0.00
I
ATOM 5432 HN PHE I 380 4.139 0.028 2.660 0.00 0.00
I
ATOM 5433 CA PHE I 380 5.332 0.696 0.996 0.00 0.00
I
ATOM 5434 HA PHE I 380 5.025 1.503 0.347 0.00 0.00
I
ATOM 5435 CB PHE I 380 6.527 1.102 1.924 0.00 0.00
I
ATOM 5436 HB1 PHE I 380 6.975 0.181 2.355 0.00 0.00
I
ATOM 5437 HB2 PHE I 380 7.311 1.641 1.352 0.00 0.00
I
ATOM 5438 CG PHE I 380 6.156 2.040 3.036 0.00 0.00
I
ATOM 5439 CD1 PHE I 380 6.775 1.962 4.243 0.00 0.00
I
ATOM 5440 HD1 PHE I 380 7.507 1.191 4.433 0.00 0.00
I
ATOM 5441 CE1 PHE I 380 6.520 2.868 5.270 0.00 0.00
I
ATOM 5442 HE1 PHE I 380 7.062 2.791 6.201 0.00 0.00
I
ATOM 5443 CZ PHE I 380 5.600 3.926 5.050 0.00 0.00
I
ATOM 5444 HZ PHE I 380 5.532 4.777 5.712 0.00 0.00
I
ATOM 5445 CD2 PHE I 380 5.330 3.153 2.765 0.00 0.00
I
ATOM 5446 HD2 PHE I 380 4.787 3.276 1.840 0.00 0.00
I
ATOM 5447 CE2 PHE I 380 5.084 4.135 3.750 0.00 0.00
I
ATOM 5448 HE2 PHE I 380 4.381 4.899 3.452 0.00 0.00
I
ATOM 5449 C PHE I 380 5.938 -0.326 -0.005 0.00 0.00
I
ATOM 5450 O PHE I 380 6.591 0.079 -0.969 0.00 0.00
I
ATOM 5451 N ILE I 381 5.656 -1.674 0.229 0.00 0.00
I
ATOM 5452 HN ILE I 381 5.052 -1.960 0.969 0.00 0.00
I
ATOM 5453 CA ILE I 381 6.050 -2.756 -0.645 0.00 0.00
I

```
ATOM   5454  HA  ILE I 381       7.120  -2.772  -0.792  0.00  0.00
I
ATOM   5455  CB  ILE I 381       5.871  -4.185   0.020  0.00  0.00
I
ATOM   5456  HB  ILE I 381       4.819  -4.207   0.378  0.00  0.00
I
ATOM   5457  CG2 ILE I 381       6.188  -5.387  -0.938  0.00  0.00
I
ATOM   5458 HG21 ILE I 381       5.662  -6.170  -0.351  0.00  0.00
I
ATOM   5459 HG22 ILE I 381       5.775  -5.350  -1.968  0.00  0.00
I
ATOM   5460 HG23 ILE I 381       7.259  -5.678  -0.990  0.00  0.00
I
ATOM   5461  CG1 ILE I 381       6.931  -4.275   1.266  0.00  0.00
I
ATOM   5462 HG11 ILE I 381       7.920  -4.566   0.852  0.00  0.00
I
ATOM   5463 HG12 ILE I 381       7.087  -3.344   1.852  0.00  0.00
I
ATOM   5464  CD  ILE I 381       6.466  -5.390   2.259  0.00  0.00
I
ATOM   5465  HD1 ILE I 381       7.051  -5.337   3.202  0.00  0.00
I
ATOM   5466  HD2 ILE I 381       5.418  -5.283   2.612  0.00  0.00
I
ATOM   5467  HD3 ILE I 381       6.638  -6.375   1.775  0.00  0.00
I
ATOM   5468  C   ILE I 381       5.369  -2.800  -2.015  0.00  0.00
I
ATOM   5469  O   ILE I 381       5.930  -2.969  -3.105  0.00  0.00
I
ATOM   5470  N   VAL I 382       4.072  -2.582  -1.986  0.00  0.00
I
ATOM   5471  HN  VAL I 382       3.533  -2.564  -1.147  0.00  0.00
I
ATOM   5472  CA  VAL I 382       3.285  -2.434  -3.200  0.00  0.00
I
ATOM   5473  HA  VAL I 382       3.585  -3.150  -3.950  0.00  0.00
I
ATOM   5474  CB  VAL I 382       1.784  -2.720  -2.911  0.00  0.00
I
ATOM   5475  HB  VAL I 382       1.232  -2.543  -3.859  0.00  0.00
I
ATOM   5476  CG1 VAL I 382       1.579  -4.179  -2.589  0.00  0.00
I
ATOM   5477 HG11 VAL I 382       1.908  -4.427  -1.557  0.00  0.00
I
ATOM   5478 HG12 VAL I 382       0.532  -4.533  -2.707  0.00  0.00
I
ATOM   5479 HG13 VAL I 382       2.158  -4.759  -3.339  0.00  0.00
I
ATOM   5480  CG2 VAL I 382       1.090  -1.828  -1.794  0.00  0.00
I
```

ATOM   5481 HG21 VAL I 382   0.030 -2.131 -1.662 0.00 0.00
I
ATOM   5482 HG22 VAL I 382   1.643 -1.867 -0.831 0.00 0.00
I
ATOM   5483 HG23 VAL I 382   1.028 -0.750 -2.054 0.00 0.00
I
ATOM   5484 C   VAL I 382   3.476 -1.161 -3.874 0.00 0.00
I
ATOM   5485 O   VAL I 382   3.498 -1.176 -5.142 0.00 0.00
I
ATOM   5486 N   PHE I 383   3.812 -0.015 -3.177 0.00 0.00
I
ATOM   5487 HN PHE I 383   3.942 -0.020 -2.189 0.00 0.00
I
ATOM   5488 CA PHE I 383   3.898   1.321 -3.818 0.00 0.00
I
ATOM   5489 HA PHE I 383   3.124   1.341 -4.572 0.00 0.00
I
ATOM   5490 CB PHE I 383   3.632   2.451 -2.786 0.00 0.00
I
ATOM   5491 HB1 PHE I 383   2.921   2.041 -2.036 0.00 0.00
I
ATOM   5492 HB2 PHE I 383   4.532   2.886 -2.302 0.00 0.00
I
ATOM   5493 CG PHE I 383   2.894   3.571 -3.467 0.00 0.00
I
ATOM   5494 CD1 PHE I 383   3.520   4.545 -4.291 0.00 0.00
I
ATOM   5495 HD1 PHE I 383   4.576   4.403 -4.470 0.00 0.00
I
ATOM   5496 CE1 PHE I 383   2.801   5.556 -4.868 0.00 0.00
I
ATOM   5497 HE1 PHE I 383   3.300   6.187 -5.590 0.00 0.00
I
ATOM   5498 CZ PHE I 383   1.427   5.659 -4.664 0.00 0.00
I
ATOM   5499 HZ PHE I 383   0.794   6.342 -5.212 0.00 0.00
I
ATOM   5500 CD2 PHE I 383   1.498   3.716 -3.274 0.00 0.00
I
ATOM   5501 HD2 PHE I 383   1.039   2.997 -2.611 0.00 0.00
I
ATOM   5502 CE2 PHE I 383   0.747   4.687 -3.851 0.00 0.00
I
ATOM   5503 HE2 PHE I 383   -0.286   4.767 -3.548 0.00 0.00
I
ATOM   5504 C   PHE I 383   5.253   1.513 -4.414 0.00 0.00
I
ATOM   5505 O   PHE I 383   5.437   2.146 -5.461 0.00 0.00
I
ATOM   5506 N   LEU I 384   6.355   0.973 -3.854 0.00 0.00
I
ATOM   5507 HN LEU I 384   6.302   0.598 -2.932 0.00 0.00
I

```
ATOM   5508  CA  LEU I 384       7.655   0.803  -4.527  0.00  0.00
I
ATOM   5509  HA  LEU I 384       7.889   1.735  -5.020  0.00  0.00
I
ATOM   5510  CB  LEU I 384       8.780   0.485  -3.478  0.00  0.00
I
ATOM   5511  HB1 LEU I 384       8.607  -0.481  -2.958  0.00  0.00
I
ATOM   5512  HB2 LEU I 384       9.738   0.350  -4.024  0.00  0.00
I
ATOM   5513  CG  LEU I 384       8.970   1.657  -2.490  0.00  0.00
I
ATOM   5514  HG  LEU I 384       8.061   1.880  -1.892  0.00  0.00
I
ATOM   5515  CD1 LEU I 384      10.042   1.321  -1.454  0.00  0.00
I
ATOM   5516 HD11 LEU I 384       9.652   0.389  -0.991  0.00  0.00
I
ATOM   5517 HD12 LEU I 384      11.018   1.031  -1.897  0.00  0.00
I
ATOM   5518 HD13 LEU I 384      10.163   1.994  -0.578  0.00  0.00
I
ATOM   5519  CD2 LEU I 384       9.382   2.927  -3.220  0.00  0.00
I
ATOM   5520 HD21 LEU I 384       9.582   3.809  -2.575  0.00  0.00
I
ATOM   5521 HD22 LEU I 384      10.325   2.769  -3.785  0.00  0.00
I
ATOM   5522 HD23 LEU I 384       8.477   3.272  -3.765  0.00  0.00
I
ATOM   5523  C   LEU I 384       7.648  -0.329  -5.620  0.00  0.00
I
ATOM   5524  O   LEU I 384       8.103  -0.120  -6.736  0.00  0.00
I
ATOM   5525  N   GLY I 385       6.987  -1.555  -5.422  0.00  0.00
I
ATOM   5526  HN  GLY I 385       6.724  -1.826  -4.500  0.00  0.00
I
ATOM   5527  CA  GLY I 385       6.957  -2.485  -6.527  0.00  0.00
I
ATOM   5528  HA1 GLY I 385       6.647  -3.425  -6.095  0.00  0.00
I
ATOM   5529  HA2 GLY I 385       7.933  -2.566  -6.983  0.00  0.00
I
ATOM   5530  C   GLY I 385       6.067  -2.164  -7.702  0.00  0.00
I
ATOM   5531  O   GLY I 385       6.455  -2.681  -8.810  0.00  0.00
I
ATOM   5532  N   ILE I 386       5.020  -1.268  -7.570  0.00  0.00
I
ATOM   5533  HN  ILE I 386       4.644  -1.141  -6.655  0.00  0.00
I
ATOM   5534  CA  ILE I 386       4.299  -0.844  -8.819  0.00  0.00
I
```

```
ATOM   5535  HA  ILE I 386       4.323  -1.667  -9.518  0.00  0.00
I
ATOM   5536  CB  ILE I 386       2.903  -0.310  -8.662  0.00  0.00
I
ATOM   5537  HB  ILE I 386       2.602  -0.137  -9.718  0.00  0.00
I
ATOM   5538  CG2 ILE I 386       2.033  -1.498  -8.243  0.00  0.00
I
ATOM   5539 HG21 ILE I 386       2.201  -2.380  -8.897  0.00  0.00
I
ATOM   5540 HG22 ILE I 386       2.163  -1.714  -7.161  0.00  0.00
I
ATOM   5541 HG23 ILE I 386       0.942  -1.301  -8.303  0.00  0.00
I
ATOM   5542  CG1 ILE I 386       2.748   0.886  -7.772  0.00  0.00
I
ATOM   5543 HG11 ILE I 386       2.726   0.590  -6.701  0.00  0.00
I
ATOM   5544 HG12 ILE I 386       3.588   1.596  -7.930  0.00  0.00
I
ATOM   5545  CD  ILE I 386       1.387   1.619  -7.875  0.00  0.00
I
ATOM   5546  HD1 ILE I 386       1.185   2.348  -7.062  0.00  0.00
I
ATOM   5547  HD2 ILE I 386       1.418   2.176  -8.836  0.00  0.00
I
ATOM   5548  HD3 ILE I 386       0.451   1.033  -7.998  0.00  0.00
I
ATOM   5549  C   ILE I 386       5.113   0.230  -9.566  0.00  0.00
I
ATOM   5550  O   ILE I 386       5.255   0.175 -10.758  0.00  0.00
I
ATOM   5551  N   PHE I 387       5.749   1.169  -8.891  0.00  0.00
I
ATOM   5552  HN  PHE I 387       5.490   1.273  -7.934  0.00  0.00
I
ATOM   5553  CA  PHE I 387       6.825   2.040  -9.401  0.00  0.00
I
ATOM   5554  HA  PHE I 387       6.397   2.641 -10.190  0.00  0.00
I
ATOM   5555  CB  PHE I 387       7.160   3.012  -8.263  0.00  0.00
I
ATOM   5556  HB1 PHE I 387       6.234   3.466  -7.849  0.00  0.00
I
ATOM   5557  HB2 PHE I 387       7.562   2.578  -7.323  0.00  0.00
I
ATOM   5558  CG  PHE I 387       8.169   4.065  -8.682  0.00  0.00
I
ATOM   5559  CD1 PHE I 387       9.532   3.795  -8.485  0.00  0.00
I
ATOM   5560  HD1 PHE I 387       9.872   2.826  -8.151  0.00  0.00
I
ATOM   5561  CE1 PHE I 387      10.492   4.658  -9.066  0.00  0.00
I
```

ATOM 5562 HE1 PHE I 387 11.501 4.285 −9.164 0.00 0.00
I
ATOM 5563 CZ PHE I 387 10.084 5.830 −9.771 0.00 0.00
I
ATOM 5564 HZ PHE I 387 10.799 6.467 −10.272 0.00 0.00
I
ATOM 5565 CD2 PHE I 387 7.745 5.259 −9.375 0.00 0.00
I
ATOM 5566 HD2 PHE I 387 6.701 5.529 −9.433 0.00 0.00
I
ATOM 5567 CE2 PHE I 387 8.714 6.159 −9.884 0.00 0.00
I
ATOM 5568 HE2 PHE I 387 8.415 7.018 −10.467 0.00 0.00
I
ATOM 5569 C PHE I 387 7.989 1.404 −10.139 0.00 0.00
I
ATOM 5570 O PHE I 387 8.336 1.609 −11.266 0.00 0.00
I
ATOM 5571 N ALA I 388 8.589 0.398 −9.452 0.00 0.00
I
ATOM 5572 HN ALA I 388 8.338 0.219 −8.503 0.00 0.00
I
ATOM 5573 CA ALA I 388 9.691 −0.411 −10.071 0.00 0.00
I
ATOM 5574 HA ALA I 388 10.459 0.280 −10.385 0.00 0.00
I
ATOM 5575 CB ALA I 388 10.264 −1.392 −9.016 0.00 0.00
I
ATOM 5576 HB1 ALA I 388 9.602 −2.264 −8.824 0.00 0.00
I
ATOM 5577 HB2 ALA I 388 11.063 −1.887 −9.609 0.00 0.00
I
ATOM 5578 HB3 ALA I 388 10.560 −0.844 −8.096 0.00 0.00
I
ATOM 5579 C ALA I 388 9.354 −1.149 −11.295 0.00 0.00
I
ATOM 5580 O ALA I 388 10.109 −1.037 −12.268 0.00 0.00
I
ATOM 5581 N LEU I 389 8.201 −1.855 −11.412 0.00 0.00
I
ATOM 5582 HN LEU I 389 7.670 −2.055 −10.592 0.00 0.00
I
ATOM 5583 CA LEU I 389 7.731 −2.447 −12.678 0.00 0.00
I
ATOM 5584 HA LEU I 389 8.533 −2.905 −13.238 0.00 0.00
I
ATOM 5585 CB LEU I 389 6.566 −3.485 −12.275 0.00 0.00
I
ATOM 5586 HB1 LEU I 389 5.726 −3.010 −11.725 0.00 0.00
I
ATOM 5587 HB2 LEU I 389 6.121 −3.802 −13.243 0.00 0.00
I
ATOM 5588 CG LEU I 389 7.008 −4.642 −11.347 0.00 0.00
I

ATOM 5589 HG LEU I 389 7.689 -4.283 -10.545 0.00 0.00
I
ATOM 5590 CD1 LEU I 389 6.021 -5.381 -10.562 0.00 0.00
I
ATOM 5591 HD11 LEU I 389 5.292 -5.969 -11.159 0.00 0.00
I
ATOM 5592 HD12 LEU I 389 6.517 -6.023 -9.804 0.00 0.00
I
ATOM 5593 HD13 LEU I 389 5.383 -4.606 -10.086 0.00 0.00
I
ATOM 5594 CD2 LEU I 389 7.842 -5.686 -12.158 0.00 0.00
I
ATOM 5595 HD21 LEU I 389 7.329 -6.041 -13.077 0.00 0.00
I
ATOM 5596 HD22 LEU I 389 8.700 -5.085 -12.528 0.00 0.00
I
ATOM 5597 HD23 LEU I 389 8.272 -6.512 -11.552 0.00 0.00
I
ATOM 5598 C LEU I 389 7.179 -1.415 -13.636 0.00 0.00
I
ATOM 5599 O LEU I 389 7.602 -1.532 -14.761 0.00 0.00
I
ATOM 5600 N THR I 390 6.403 -0.317 -13.242 0.00 0.00
I
ATOM 5601 HN THR I 390 6.090 -0.087 -12.323 0.00 0.00
I
ATOM 5602 CA THR I 390 6.125 0.783 -14.242 0.00 0.00
I
ATOM 5603 HA THR I 390 5.697 0.359 -15.138 0.00 0.00
I
ATOM 5604 CB THR I 390 5.126 1.886 -13.834 0.00 0.00
I
ATOM 5605 HB THR I 390 5.101 2.688 -14.602 0.00 0.00
I
ATOM 5606 OG1 THR I 390 5.522 2.460 -12.601 0.00 0.00
I
ATOM 5607 HG1 THR I 390 5.214 1.847 -11.929 0.00 0.00
I
ATOM 5608 CG2 THR I 390 3.711 1.358 -13.610 0.00 0.00
I
ATOM 5609 HG21 THR I 390 3.624 0.734 -12.694 0.00 0.00
I
ATOM 5610 HG22 THR I 390 3.028 2.191 -13.341 0.00 0.00
I
ATOM 5611 HG23 THR I 390 3.337 0.823 -14.509 0.00 0.00
I
ATOM 5612 C THR I 390 7.242 1.525 -14.895 0.00 0.00
I
ATOM 5613 O THR I 390 7.273 1.763 -16.118 0.00 0.00
I
ATOM 5614 N ILE I 391 8.299 1.803 -14.162 0.00 0.00
I
ATOM 5615 HN ILE I 391 8.319 1.842 -13.166 0.00 0.00
I

ATOM 5616 CA ILE I 391 9.539 2.302 −14.806 0.00 0.00
I
ATOM 5617 HA ILE I 391 9.198 3.076 −15.478 0.00 0.00
I
ATOM 5618 CB ILE I 391 10.470 3.006 −13.881 0.00 0.00
I
ATOM 5619 HB ILE I 391 11.259 3.511 −14.478 0.00 0.00
I
ATOM 5620 CG2 ILE I 391 9.863 4.190 −13.137 0.00 0.00
I
ATOM 5621 HG21 ILE I 391 8.860 3.925 −12.740 0.00 0.00
I
ATOM 5622 HG22 ILE I 391 10.604 4.569 −12.401 0.00 0.00
I
ATOM 5623 HG23 ILE I 391 9.616 4.967 −13.891 0.00 0.00
I
ATOM 5624 CG1 ILE I 391 11.165 2.110 −12.881 0.00 0.00
I
ATOM 5625 HG11 ILE I 391 10.540 1.943 −11.977 0.00 0.00
I
ATOM 5626 HG12 ILE I 391 11.344 1.117 −13.346 0.00 0.00
I
ATOM 5627 CD ILE I 391 12.584 2.532 −12.305 0.00 0.00
I
ATOM 5628 HD1 ILE I 391 12.521 3.554 −11.873 0.00 0.00
I
ATOM 5629 HD2 ILE I 391 12.798 1.829 −11.471 0.00 0.00
I
ATOM 5630 HD3 ILE I 391 13.245 2.599 −13.195 0.00 0.00
I
ATOM 5631 C ILE I 391 10.177 1.317 −15.705 0.00 0.00
I
ATOM 5632 O ILE I 391 10.558 1.656 −16.827 0.00 0.00
I
ATOM 5633 N THR I 392 10.233 0.019 −15.288 0.00 0.00
I
ATOM 5634 HN THR I 392 9.860 −0.269 −14.409 0.00 0.00
I
ATOM 5635 CA THR I 392 10.914 −1.067 −16.076 0.00 0.00
I
ATOM 5636 HA THR I 392 11.885 −0.728 −16.406 0.00 0.00
I
ATOM 5637 CB THR I 392 11.244 −2.263 −15.218 0.00 0.00
I
ATOM 5638 HB THR I 392 10.341 −2.858 −14.961 0.00 0.00
I
ATOM 5639 OG1 THR I 392 11.927 −1.854 −14.063 0.00 0.00
I
ATOM 5640 HG1 THR I 392 11.329 −1.468 −13.418 0.00 0.00
I
ATOM 5641 CG2 THR I 392 12.284 −3.058 −15.955 0.00 0.00
I
ATOM 5642 HG21 THR I 392 12.800 −3.854 −15.378 0.00 0.00
I

```
ATOM   5643 HG22 THR I 392      11.874  -3.509 -16.884  0.00  0.00
I
ATOM   5644 HG23 THR I 392      13.006  -2.334 -16.390  0.00  0.00
I
ATOM   5645  C   THR I 392      10.238  -1.493 -17.337  0.00  0.00
I
ATOM   5646  O   THR I 392      10.777  -1.533 -18.416  0.00  0.00
I
ATOM   5647  N   GLU I 393       8.914  -1.872 -17.157  0.00  0.00
I
ATOM   5648  HN  GLU I 393       8.459  -1.831 -16.270  0.00  0.00
I
ATOM   5649  CA  GLU I 393       8.090  -2.616 -18.090  0.00  0.00
I
ATOM   5650  HA  GLU I 393       8.700  -2.865 -18.946  0.00  0.00
I
ATOM   5651  CB  GLU I 393       7.529  -3.872 -17.388  0.00  0.00
I
ATOM   5652  HB1 GLU I 393       6.918  -3.557 -16.515  0.00  0.00
I
ATOM   5653  HB2 GLU I 393       6.976  -4.339 -18.231  0.00  0.00
I
ATOM   5654  CG  GLU I 393       8.544  -4.961 -16.945  0.00  0.00
I
ATOM   5655  HG1 GLU I 393       9.390  -4.546 -16.355  0.00  0.00
I
ATOM   5656  HG2 GLU I 393       7.942  -5.703 -16.378  0.00  0.00
I
ATOM   5657  CD  GLU I 393       9.226  -5.660 -18.139  0.00  0.00
I
ATOM   5658  OE1 GLU I 393      10.485  -5.655 -18.252  0.00  0.00
I
ATOM   5659  OE2 GLU I 393       8.473  -6.117 -19.044  0.00  0.00
I
ATOM   5660  C   GLU I 393       6.865  -1.762 -18.613  0.00  0.00
I
ATOM   5661  O   GLU I 393       6.221  -1.068 -17.833  0.00  0.00
I
ATOM   5662  N   GLN I 394       6.433  -1.897 -19.877  0.00  0.00
I
ATOM   5663  HN  GLN I 394       7.026  -2.377 -20.520  0.00  0.00
I
ATOM   5664  CA  GLN I 394       5.363  -1.287 -20.614  0.00  0.00
I
ATOM   5665  HA  GLN I 394       5.009  -0.483 -19.987  0.00  0.00
I
ATOM   5666  CB  GLN I 394       5.714  -0.721 -22.016  0.00  0.00
I
ATOM   5667  HB1 GLN I 394       6.641  -0.117 -21.915  0.00  0.00
I
ATOM   5668  HB2 GLN I 394       5.949  -1.526 -22.744  0.00  0.00
I
ATOM   5669  CG  GLN I 394       4.600   0.138 -22.659  0.00  0.00
I
```

```
ATOM   5670  HG1 GLN I 394       3.710  -0.513 -22.791  0.00  0.00
I
ATOM   5671  HG2 GLN I 394       4.285   0.941 -21.959  0.00  0.00
I
ATOM   5672  CD  GLN I 394       4.965   0.734 -24.061  0.00  0.00
I
ATOM   5673  OE1 GLN I 394       4.770   1.933 -24.112  0.00  0.00
I
ATOM   5674  NE2 GLN I 394       5.244  -0.104 -25.026  0.00  0.00
I
ATOM   5675 HE21 GLN I 394       5.311   0.275 -25.949  0.00  0.00
I
ATOM   5676 HE22 GLN I 394       5.189  -1.089 -24.864  0.00  0.00
I
ATOM   5677  C   GLN I 394       4.259  -2.274 -20.636  0.00  0.00
I
ATOM   5678  O   GLN I 394       4.104  -3.153 -21.577  0.00  0.00
I
ATOM   5679  N   ALA I 395       3.327  -2.220 -19.674  0.00  0.00
I
ATOM   5680  HN  ALA I 395       3.664  -1.768 -18.852  0.00  0.00
I
ATOM   5681  CA  ALA I 395       2.108  -2.970 -19.664  0.00  0.00
I
ATOM   5682  HA  ALA I 395       1.840  -3.419 -20.609  0.00  0.00
I
ATOM   5683  CB  ALA I 395       2.266  -4.112 -18.672  0.00  0.00
I
ATOM   5684  HB1 ALA I 395       3.036  -4.780 -19.115  0.00  0.00
I
ATOM   5685  HB2 ALA I 395       2.640  -3.709 -17.707  0.00  0.00
I
ATOM   5686  HB3 ALA I 395       1.293  -4.605 -18.460  0.00  0.00
I
ATOM   5687  C   ALA I 395       1.084  -1.979 -19.145  0.00  0.00
I
ATOM   5688  O   ALA I 395       1.460  -1.094 -18.409  0.00  0.00
I
ATOM   5689  N   PRO I 396      -0.191  -2.033 -19.535  0.00  0.00
I
ATOM   5690  CD  PRO I 396      -0.621  -2.908 -20.570  0.00  0.00
I
ATOM   5691  HD1 PRO I 396      -0.079  -2.674 -21.511  0.00  0.00
I
ATOM   5692  HD2 PRO I 396      -0.557  -3.970 -20.247  0.00  0.00
I
ATOM   5693  CA  PRO I 396      -1.231  -1.324 -18.929  0.00  0.00
I
ATOM   5694  HA  PRO I 396      -1.042  -0.283 -19.141  0.00  0.00
I
ATOM   5695  CB  PRO I 396      -2.548  -1.970 -19.515  0.00  0.00
I
ATOM   5696  HB1 PRO I 396      -3.399  -1.255 -19.509  0.00  0.00
I
```

```
ATOM   5697  HB2 PRO I 396      -2.971  -2.802 -18.913  0.00  0.00
I
ATOM   5698  CG  PRO I 396      -2.158  -2.555 -20.843  0.00  0.00
I
ATOM   5699  HG1 PRO I 396      -2.251  -1.761 -21.614  0.00  0.00
I
ATOM   5700  HG2 PRO I 396      -2.795  -3.455 -20.984  0.00  0.00
I
ATOM   5701  C   PRO I 396      -1.228  -1.344 -17.432  0.00  0.00
I
ATOM   5702  O   PRO I 396      -0.880  -2.367 -16.826  0.00  0.00
I
ATOM   5703  N   PHE I 397      -1.749  -0.269 -16.805  0.00  0.00
I
ATOM   5704  HN  PHE I 397      -2.140   0.532 -17.251  0.00  0.00
I
ATOM   5705  CA  PHE I 397      -1.669  -0.121 -15.340  0.00  0.00
I
ATOM   5706  HA  PHE I 397      -0.628  -0.037 -15.065  0.00  0.00
I
ATOM   5707  CB  PHE I 397      -2.200   1.284 -14.876  0.00  0.00
I
ATOM   5708  HB1 PHE I 397      -1.751   2.101 -15.480  0.00  0.00
I
ATOM   5709  HB2 PHE I 397      -3.289   1.395 -15.067  0.00  0.00
I
ATOM   5710  CG  PHE I 397      -1.726   1.609 -13.481  0.00  0.00
I
ATOM   5711  CD1 PHE I 397      -0.437   1.648 -13.151  0.00  0.00
I
ATOM   5712  HD1 PHE I 397       0.391   1.504 -13.828  0.00  0.00
I
ATOM   5713  CE1 PHE I 397       0.043   2.159 -11.900  0.00  0.00
I
ATOM   5714  HE1 PHE I 397       1.033   2.369 -11.524  0.00  0.00
I
ATOM   5715  CZ  PHE I 397      -0.932   2.461 -10.937  0.00  0.00
I
ATOM   5716  HZ  PHE I 397      -0.711   2.678  -9.902  0.00  0.00
I
ATOM   5717  CD2 PHE I 397      -2.675   1.973 -12.565  0.00  0.00
I
ATOM   5718  HD2 PHE I 397      -3.717   2.058 -12.835  0.00  0.00
I
ATOM   5719  CE2 PHE I 397      -2.281   2.281 -11.274  0.00  0.00
I
ATOM   5720  HE2 PHE I 397      -3.089   2.500 -10.592  0.00  0.00
I
ATOM   5721  C   PHE I 397      -2.216  -1.253 -14.482  0.00  0.00
I
ATOM   5722  O   PHE I 397      -1.583  -1.856 -13.647  0.00  0.00
I
ATOM   5723  N   LEU I 398      -3.446  -1.577 -14.789  0.00  0.00
I
```

ATOM 5724 HN LEU I 398 -3.855 -1.167 -15.600 0.00 0.00
I
ATOM 5725 CA LEU I 398 -4.093 -2.758 -14.264 0.00 0.00
I
ATOM 5726 HA LEU I 398 -4.401 -2.446 -13.277 0.00 0.00
I
ATOM 5727 CB LEU I 398 -5.385 -3.084 -14.961 0.00 0.00
I
ATOM 5728 HB1 LEU I 398 -5.991 -2.156 -14.884 0.00 0.00
I
ATOM 5729 HB2 LEU I 398 -5.245 -3.391 -16.020 0.00 0.00
I
ATOM 5730 CG LEU I 398 -6.321 -4.038 -14.186 0.00 0.00
I
ATOM 5731 HG LEU I 398 -5.711 -4.893 -13.824 0.00 0.00
I
ATOM 5732 CD1 LEU I 398 -6.778 -3.402 -12.910 0.00 0.00
I
ATOM 5733 HD11 LEU I 398 -7.457 -2.532 -13.034 0.00 0.00
I
ATOM 5734 HD12 LEU I 398 -7.379 -4.139 -12.336 0.00 0.00
I
ATOM 5735 HD13 LEU I 398 -5.954 -2.981 -12.294 0.00 0.00
I
ATOM 5736 CD2 LEU I 398 -7.437 -4.462 -14.999 0.00 0.00
I
ATOM 5737 HD21 LEU I 398 -7.959 -5.331 -14.544 0.00 0.00
I
ATOM 5738 HD22 LEU I 398 -8.129 -3.631 -15.251 0.00 0.00
I
ATOM 5739 HD23 LEU I 398 -7.117 -4.931 -15.954 0.00 0.00
I
ATOM 5740 C LEU I 398 -3.298 -4.051 -14.032 0.00 0.00
I
ATOM 5741 O LEU I 398 -3.185 -4.584 -12.921 0.00 0.00
I
ATOM 5742 N GLN I 399 -2.586 -4.480 -15.056 0.00 0.00
I
ATOM 5743 HN GLN I 399 -2.703 -3.969 -15.904 0.00 0.00
I
ATOM 5744 CA GLN I 399 -1.740 -5.639 -15.107 0.00 0.00
I
ATOM 5745 HA GLN I 399 -2.390 -6.485 -14.942 0.00 0.00
I
ATOM 5746 CB GLN I 399 -1.155 -6.044 -16.578 0.00 0.00
I
ATOM 5747 HB1 GLN I 399 -0.382 -5.297 -16.858 0.00 0.00
I
ATOM 5748 HB2 GLN I 399 -0.813 -7.101 -16.584 0.00 0.00
I
ATOM 5749 CG GLN I 399 -2.195 -5.857 -17.704 0.00 0.00
I
ATOM 5750 HG1 GLN I 399 -3.162 -6.353 -17.472 0.00 0.00
I

ATOM 5751 HG2 GLN I 399 −2.267 −4.748 −17.703 0.00 0.00
I
ATOM 5752 CD GLN I 399 −1.500 −6.336 −18.994 0.00 0.00
I
ATOM 5753 OE1 GLN I 399 −0.305 −6.146 −19.113 0.00 0.00
I
ATOM 5754 NE2 GLN I 399 −2.238 −7.059 −19.820 0.00 0.00
I
ATOM 5755 HE21 GLN I 399 −1.885 −7.393 −20.695 0.00 0.00
I
ATOM 5756 HE22 GLN I 399 −3.119 −7.331 −19.434 0.00 0.00
I
ATOM 5757 C GLN I 399 −0.605 −5.519 −14.087 0.00 0.00
I
ATOM 5758 O GLN I 399 −0.212 −6.438 −13.438 0.00 0.00
I
ATOM 5759 N ILE I 400 0.084 −4.414 −14.017 0.00 0.00
I
ATOM 5760 HN ILE I 400 −0.209 −3.652 −14.590 0.00 0.00
I
ATOM 5761 CA ILE I 400 1.184 −4.140 −13.064 0.00 0.00
I
ATOM 5762 HA ILE I 400 1.936 −4.913 −13.118 0.00 0.00
I
ATOM 5763 CB ILE I 400 2.000 −2.940 −13.491 0.00 0.00
I
ATOM 5764 HB ILE I 400 1.412 −2.021 −13.703 0.00 0.00
I
ATOM 5765 CG2 ILE I 400 3.085 −2.613 −12.421 0.00 0.00
I
ATOM 5766 HG21 ILE I 400 3.595 −3.563 −12.152 0.00 0.00
I
ATOM 5767 HG22 ILE I 400 3.867 −1.897 −12.753 0.00 0.00
I
ATOM 5768 HG23 ILE I 400 2.666 −2.149 −11.502 0.00 0.00
I
ATOM 5769 CG1 ILE I 400 2.730 −3.291 −14.808 0.00 0.00
I
ATOM 5770 HG11 ILE I 400 3.509 −4.032 −14.530 0.00 0.00
I
ATOM 5771 HG12 ILE I 400 1.910 −3.609 −15.488 0.00 0.00
I
ATOM 5772 CD ILE I 400 3.435 −2.083 −15.492 0.00 0.00
I
ATOM 5773 HD1 ILE I 400 4.284 −2.300 −16.175 0.00 0.00
I
ATOM 5774 HD2 ILE I 400 2.732 −1.538 −16.157 0.00 0.00
I
ATOM 5775 HD3 ILE I 400 4.002 −1.436 −14.789 0.00 0.00
I
ATOM 5776 C ILE I 400 0.679 −4.151 −11.621 0.00 0.00
I
ATOM 5777 O ILE I 400 1.227 −4.852 −10.769 0.00 0.00
I

```
ATOM   5778  N   VAL I 401      -0.468  -3.471 -11.340  0.00  0.00
I
ATOM   5779  HN  VAL I 401      -0.976  -2.999 -12.056  0.00  0.00
I
ATOM   5780  CA  VAL I 401      -1.121  -3.613 -10.055  0.00  0.00
I
ATOM   5781  HA  VAL I 401      -0.465  -3.363  -9.234  0.00  0.00
I
ATOM   5782  CB  VAL I 401      -2.170  -2.509  -9.976  0.00  0.00
I
ATOM   5783  HB  VAL I 401      -2.921  -2.616 -10.788  0.00  0.00
I
ATOM   5784  CG1 VAL I 401      -2.899  -2.586  -8.667  0.00  0.00
I
ATOM   5785 HG11 VAL I 401      -3.720  -1.861  -8.487  0.00  0.00
I
ATOM   5786 HG12 VAL I 401      -3.376  -3.589  -8.636  0.00  0.00
I
ATOM   5787 HG13 VAL I 401      -2.126  -2.552  -7.870  0.00  0.00
I
ATOM   5788  CG2 VAL I 401      -1.556  -1.127 -10.188  0.00  0.00
I
ATOM   5789 HG21 VAL I 401      -2.268  -0.323  -9.904  0.00  0.00
I
ATOM   5790 HG22 VAL I 401      -0.781  -0.914  -9.422  0.00  0.00
I
ATOM   5791 HG23 VAL I 401      -1.332  -0.752 -11.210  0.00  0.00
I
ATOM   5792  C   VAL I 401      -1.640  -5.070  -9.757  0.00  0.00
I
ATOM   5793  O   VAL I 401      -1.311  -5.536  -8.670  0.00  0.00
I
ATOM   5794  N   PHE I 402      -2.190  -5.874 -10.706  0.00  0.00
I
ATOM   5795  HN  PHE I 402      -2.322  -5.458 -11.602  0.00  0.00
I
ATOM   5796  CA  PHE I 402      -2.513  -7.286 -10.488  0.00  0.00
I
ATOM   5797  HA  PHE I 402      -3.129  -7.373  -9.605  0.00  0.00
I
ATOM   5798  CB  PHE I 402      -3.380  -7.695 -11.749  0.00  0.00
I
ATOM   5799  HB1 PHE I 402      -4.057  -6.878 -12.079  0.00  0.00
I
ATOM   5800  HB2 PHE I 402      -2.720  -7.935 -12.609  0.00  0.00
I
ATOM   5801  CG  PHE I 402      -4.241  -8.934 -11.642  0.00  0.00
I
ATOM   5802  CD1 PHE I 402      -5.558  -8.770 -11.020  0.00  0.00
I
ATOM   5803  HD1 PHE I 402      -5.791  -7.764 -10.701  0.00  0.00
I
ATOM   5804  CE1 PHE I 402      -6.433  -9.873 -10.932  0.00  0.00
I
```

ATOM 5805 HE1 PHE I 402 -7.348 -9.804 -10.362 0.00 0.00
I
ATOM 5806 CZ PHE I 402 -6.083 -11.097 -11.496 0.00 0.00
I
ATOM 5807 HZ PHE I 402 -6.656 -12.011 -11.539 0.00 0.00
I
ATOM 5808 CD2 PHE I 402 -3.896 -10.141 -12.179 0.00 0.00
I
ATOM 5809 HD2 PHE I 402 -2.955 -10.301 -12.685 0.00 0.00
I
ATOM 5810 CE2 PHE I 402 -4.843 -11.198 -12.072 0.00 0.00
I
ATOM 5811 HE2 PHE I 402 -4.733 -12.034 -12.747 0.00 0.00
I
ATOM 5812 C PHE I 402 -1.266 -8.219 -10.380 0.00 0.00
I
ATOM 5813 O PHE I 402 -1.254 -9.169 -9.598 0.00 0.00
I
ATOM 5814 N GLU I 403 -0.174 -7.902 -11.012 0.00 0.00
I
ATOM 5815 HN GLU I 403 -0.082 -7.171 -11.684 0.00 0.00
I
ATOM 5816 CA GLU I 403 0.969 -8.814 -10.828 0.00 0.00
I
ATOM 5817 HA GLU I 403 0.653 -9.826 -10.620 0.00 0.00
I
ATOM 5818 CB GLU I 403 1.598 -9.179 -12.191 0.00 0.00
I
ATOM 5819 HB1 GLU I 403 1.711 -8.346 -12.918 0.00 0.00
I
ATOM 5820 HB2 GLU I 403 2.586 -9.675 -12.081 0.00 0.00
I
ATOM 5821 CG GLU I 403 0.622 -10.195 -12.846 0.00 0.00
I
ATOM 5822 HG1 GLU I 403 0.871 -11.189 -12.416 0.00 0.00
I
ATOM 5823 HG2 GLU I 403 -0.454 -9.993 -12.657 0.00 0.00
I
ATOM 5824 CD GLU I 403 0.682 -10.454 -14.338 0.00 0.00
I
ATOM 5825 OE1 GLU I 403 -0.166 -9.906 -15.087 0.00 0.00
I
ATOM 5826 OE2 GLU I 403 1.380 -11.394 -14.869 0.00 0.00
I
ATOM 5827 C GLU I 403 2.008 -8.502 -9.709 0.00 0.00
I
ATOM 5828 O GLU I 403 2.658 -9.372 -9.194 0.00 0.00
I
ATOM 5829 N THR I 404 1.981 -7.274 -9.189 0.00 0.00
I
ATOM 5830 HN THR I 404 1.449 -6.598 -9.694 0.00 0.00
I
ATOM 5831 CA THR I 404 2.579 -6.785 -7.961 0.00 0.00
I

ATOM 5832 HA THR I 404 3.606 -7.118 -7.940 0.00 0.00
I
ATOM 5833 CB THR I 404 2.484 -5.289 -7.836 0.00 0.00
I
ATOM 5834 HB THR I 404 1.477 -4.820 -7.866 0.00 0.00
I
ATOM 5835 OG1 THR I 404 3.180 -4.737 -8.904 0.00 0.00
I
ATOM 5836 HG1 THR I 404 2.517 -4.792 -9.596 0.00 0.00
I
ATOM 5837 CG2 THR I 404 3.269 -4.734 -6.655 0.00 0.00
I
ATOM 5838 HG21 THR I 404 3.557 -3.675 -6.832 0.00 0.00
I
ATOM 5839 HG22 THR I 404 2.793 -4.925 -5.670 0.00 0.00
I
ATOM 5840 HG23 THR I 404 4.186 -5.358 -6.601 0.00 0.00
I
ATOM 5841 C THR I 404 1.788 -7.369 -6.787 0.00 0.00
I
ATOM 5842 O THR I 404 2.400 -7.752 -5.810 0.00 0.00
I
ATOM 5843 N PHE I 405 0.414 -7.391 -6.815 0.00 0.00
I
ATOM 5844 HN PHE I 405 -0.085 -7.014 -7.591 0.00 0.00
I
ATOM 5845 CA PHE I 405 -0.359 -7.961 -5.618 0.00 0.00
I
ATOM 5846 HA PHE I 405 0.208 -7.671 -4.746 0.00 0.00
I
ATOM 5847 CB PHE I 405 -1.885 -7.339 -5.525 0.00 0.00
I
ATOM 5848 HB1 PHE I 405 -2.249 -7.107 -6.549 0.00 0.00
I
ATOM 5849 HB2 PHE I 405 -2.511 -8.021 -4.911 0.00 0.00
I
ATOM 5850 CG PHE I 405 -1.897 -5.982 -4.904 0.00 0.00
I
ATOM 5851 CD1 PHE I 405 -1.527 -4.807 -5.546 0.00 0.00
I
ATOM 5852 HD1 PHE I 405 -1.076 -4.866 -6.526 0.00 0.00
I
ATOM 5853 CE1 PHE I 405 -1.703 -3.528 -4.884 0.00 0.00
I
ATOM 5854 HE1 PHE I 405 -1.370 -2.642 -5.404 0.00 0.00
I
ATOM 5855 CZ PHE I 405 -2.097 -3.450 -3.544 0.00 0.00
I
ATOM 5856 HZ PHE I 405 -2.109 -2.569 -2.919 0.00 0.00
I
ATOM 5857 CD2 PHE I 405 -2.268 -5.888 -3.560 0.00 0.00
I
ATOM 5858 HD2 PHE I 405 -2.373 -6.774 -2.950 0.00 0.00
I

ATOM 5859 CE2 PHE I 405 -2.416 -4.620 -2.901 0.00 0.00
I
ATOM 5860 HE2 PHE I 405 -2.759 -4.531 -1.881 0.00 0.00
I
ATOM 5861 C PHE I 405 -0.472 -9.419 -5.627 0.00 0.00
I
ATOM 5862 O PHE I 405 -0.543 -10.057 -4.544 0.00 0.00
I
ATOM 5863 N SER I 406 -0.324 -10.084 -6.789 0.00 0.00
I
ATOM 5864 HN SER I 406 -0.379 -9.475 -7.577 0.00 0.00
I
ATOM 5865 CA SER I 406 -0.172 -11.525 -7.082 0.00 0.00
I
ATOM 5866 HA SER I 406 -0.899 -11.987 -6.432 0.00 0.00
I
ATOM 5867 CB SER I 406 -0.376 -11.893 -8.583 0.00 0.00
I
ATOM 5868 HB1 SER I 406 -1.318 -11.493 -9.015 0.00 0.00
I
ATOM 5869 HB2 SER I 406 0.551 -11.543 -9.084 0.00 0.00
I
ATOM 5870 OG SER I 406 -0.498 -13.319 -8.733 0.00 0.00
I
ATOM 5871 HG1 SER I 406 -0.702 -13.407 -9.667 0.00 0.00
I
ATOM 5872 C SER I 406 1.190 -12.008 -6.582 0.00 0.00
I
ATOM 5873 O SER I 406 1.360 -12.969 -5.861 0.00 0.00
I
ATOM 5874 N ALA I 407 2.259 -11.208 -6.860 0.00 0.00
I
ATOM 5875 HN ALA I 407 2.161 -10.448 -7.497 0.00 0.00
I
ATOM 5876 CA ALA I 407 3.559 -11.481 -6.289 0.00 0.00
I
ATOM 5877 HA ALA I 407 3.728 -12.523 -6.520 0.00 0.00
I
ATOM 5878 CB ALA I 407 4.662 -10.763 -6.992 0.00 0.00
I
ATOM 5879 HB1 ALA I 407 5.618 -11.030 -6.494 0.00 0.00
I
ATOM 5880 HB2 ALA I 407 4.806 -11.052 -8.055 0.00 0.00
I
ATOM 5881 HB3 ALA I 407 4.581 -9.656 -6.933 0.00 0.00
I
ATOM 5882 C ALA I 407 3.545 -11.276 -4.801 0.00 0.00
I
ATOM 5883 O ALA I 407 3.983 -12.142 -4.038 0.00 0.00
I
ATOM 5884 N PHE I 408 2.871 -10.227 -4.258 0.00 0.00
I
ATOM 5885 HN PHE I 408 2.529 -9.461 -4.797 0.00 0.00
I

ATOM 5886 CA PHE I 408 2.852 -9.894 -2.820 0.00 0.00
I
ATOM 5887 HA PHE I 408 3.858 -9.853 -2.429 0.00 0.00
I
ATOM 5888 CB PHE I 408 2.415 -8.434 -2.380 0.00 0.00
I
ATOM 5889 HB1 PHE I 408 3.064 -7.672 -2.862 0.00 0.00
I
ATOM 5890 HB2 PHE I 408 1.378 -8.292 -2.753 0.00 0.00
I
ATOM 5891 CG PHE I 408 2.451 -8.198 -0.891 0.00 0.00
I
ATOM 5892 CD1 PHE I 408 3.637 -7.931 -0.323 0.00 0.00
I
ATOM 5893 HD1 PHE I 408 4.549 -7.929 -0.901 0.00 0.00
I
ATOM 5894 CE1 PHE I 408 3.692 -7.807 1.068 0.00 0.00
I
ATOM 5895 HE1 PHE I 408 4.670 -7.830 1.525 0.00 0.00
I
ATOM 5896 CZ PHE I 408 2.535 -7.842 1.824 0.00 0.00
I
ATOM 5897 HZ PHE I 408 2.705 -7.692 2.879 0.00 0.00
I
ATOM 5898 CD2 PHE I 408 1.216 -8.364 -0.071 0.00 0.00
I
ATOM 5899 HD2 PHE I 408 0.295 -8.523 -0.613 0.00 0.00
I
ATOM 5900 CE2 PHE I 408 1.267 -8.057 1.292 0.00 0.00
I
ATOM 5901 HE2 PHE I 408 0.373 -8.046 1.898 0.00 0.00
I
ATOM 5902 C PHE I 408 2.067 -10.976 -2.072 0.00 0.00
I
ATOM 5903 O PHE I 408 2.583 -11.489 -1.047 0.00 0.00
I
ATOM 5904 N GLY I 409 0.868 -11.335 -2.472 0.00 0.00
I
ATOM 5905 HN GLY I 409 0.487 -10.845 -3.252 0.00 0.00
I
ATOM 5906 CA GLY I 409 0.122 -12.407 -1.905 0.00 0.00
I
ATOM 5907 HA1 GLY I 409 -0.901 -12.374 -2.248 0.00 0.00
I
ATOM 5908 HA2 GLY I 409 0.356 -12.301 -0.856 0.00 0.00
I
ATOM 5909 C GLY I 409 0.606 -13.790 -2.105 0.00 0.00
I
ATOM 5910 O GLY I 409 0.083 -14.742 -1.472 0.00 0.00
I
ATOM 5911 N THR I 410 1.601 -14.059 -2.988 0.00 0.00
I
ATOM 5912 HN THR I 410 2.012 -13.242 -3.382 0.00 0.00
I

```
ATOM   5913  CA  THR I 410       2.027 -15.393  -3.518  0.00  0.00
I
ATOM   5914  HA  THR I 410       2.676 -15.130  -4.340  0.00  0.00
I
ATOM   5915  CB  THR I 410       2.895 -16.228  -2.517  0.00  0.00
I
ATOM   5916  HB  THR I 410       3.290 -17.065  -3.132  0.00  0.00
I
ATOM   5917  OG1 THR I 410       2.161 -16.548  -1.301  0.00  0.00
I
ATOM   5918  HG1 THR I 410       1.353 -16.037  -1.381  0.00  0.00
I
ATOM   5919  CG2 THR I 410       4.054 -15.326  -2.101  0.00  0.00
I
ATOM   5920 HG21 THR I 410       4.498 -14.648  -2.862  0.00  0.00
I
ATOM   5921 HG22 THR I 410       3.837 -14.699  -1.210  0.00  0.00
I
ATOM   5922 HG23 THR I 410       4.880 -16.000  -1.790  0.00  0.00
I
ATOM   5923  C   THR I 410       0.898 -16.154  -4.317  0.00  0.00
I
ATOM   5924  O   THR I 410       0.717 -17.372  -4.120  0.00  0.00
I
ATOM   5925  N   VAL I 411       0.239 -15.487  -5.257  0.00  0.00
I
ATOM   5926  HN  VAL I 411       0.416 -14.507  -5.284  0.00  0.00
I
ATOM   5927  CA  VAL I 411      -0.982 -16.134  -5.896  0.00  0.00
I
ATOM   5928  HA  VAL I 411      -1.541 -16.795  -5.249  0.00  0.00
I
ATOM   5929  CB  VAL I 411      -2.054 -15.143  -6.137  0.00  0.00
I
ATOM   5930  HB  VAL I 411      -1.799 -14.557  -7.046  0.00  0.00
I
ATOM   5931  CG1 VAL I 411      -3.384 -15.970  -6.343  0.00  0.00
I
ATOM   5932 HG11 VAL I 411      -4.201 -15.229  -6.476  0.00  0.00
I
ATOM   5933 HG12 VAL I 411      -3.369 -16.712  -7.170  0.00  0.00
I
ATOM   5934 HG13 VAL I 411      -3.707 -16.453  -5.396  0.00  0.00
I
ATOM   5935  CG2 VAL I 411      -2.380 -14.270  -4.894  0.00  0.00
I
ATOM   5936 HG21 VAL I 411      -3.139 -13.509  -5.173  0.00  0.00
I
ATOM   5937 HG22 VAL I 411      -2.655 -14.938  -4.049  0.00  0.00
I
ATOM   5938 HG23 VAL I 411      -1.566 -13.604  -4.535  0.00  0.00
I
ATOM   5939  C   VAL I 411      -0.726 -16.903  -7.129  0.00  0.00
I
```

ATOM   5940 O   VAL I 411   -1.090 -18.088 -7.208 0.00 0.00
I
ATOM   5941 N   GLY I 412   -0.108 -16.301 -8.170 0.00 0.00
I
ATOM   5942 HN GLY I 412   0.193 -15.361 -8.030 0.00 0.00
I
ATOM   5943 CA GLY I 412   0.141 -16.887 -9.435 0.00 0.00
I
ATOM   5944 HA1 GLY I 412   0.074 -17.963 -9.376 0.00 0.00
I
ATOM   5945 HA2 GLY I 412   1.059 -16.484 -9.837 0.00 0.00
I
ATOM   5946 C   GLY I 412   -0.863 -16.497 -10.458 0.00 0.00
I
ATOM   5947 O   GLY I 412   -0.705 -17.057 -11.549 0.00 0.00
I
ATOM   5948 N   LEU I 413   -1.827 -15.652 -10.101 0.00 0.00
I
ATOM   5949 HN LEU I 413   -1.848 -15.106 -9.266 0.00 0.00
I
ATOM   5950 CA LEU I 413   -2.785 -15.106 -10.986 0.00 0.00
I
ATOM   5951 HA LEU I 413   -3.096 -15.907 -11.641 0.00 0.00
I
ATOM   5952 CB LEU I 413   -4.039 -14.607 -10.279 0.00 0.00
I
ATOM   5953 HB1 LEU I 413   -3.712 -13.752 -9.649 0.00 0.00
I
ATOM   5954 HB2 LEU I 413   -4.660 -14.154 -11.080 0.00 0.00
I
ATOM   5955 CG LEU I 413   -4.834 -15.740 -9.540 0.00 0.00
I
ATOM   5956 HG LEU I 413   -4.235 -16.188 -8.719 0.00 0.00
I
ATOM   5957 CD1 LEU I 413   -6.164 -15.192 -9.008 0.00 0.00
I
ATOM   5958 HD11 LEU I 413   -6.588 -14.476 -9.744 0.00 0.00
I
ATOM   5959 HD12 LEU I 413   -6.886 -16.019 -8.834 0.00 0.00
I
ATOM   5960 HD13 LEU I 413   -5.921 -14.784 -8.004 0.00 0.00
I
ATOM   5961 CD2 LEU I 413   -4.950 -16.937 -10.494 0.00 0.00
I
ATOM   5962 HD21 LEU I 413   -5.732 -17.652 -10.161 0.00 0.00
I
ATOM   5963 HD22 LEU I 413   -5.222 -16.491 -11.475 0.00 0.00
I
ATOM   5964 HD23 LEU I 413   -4.045 -17.575 -10.583 0.00 0.00
I
ATOM   5965 C   LEU I 413   -2.153 -14.000 -11.767 0.00 0.00
I
ATOM   5966 O   LEU I 413   -1.276 -13.318 -11.262 0.00 0.00
I

```
ATOM   5967  N   THR I 414       -2.531 -13.820 -13.035  0.00  0.00
I
ATOM   5968  HN  THR I 414       -3.207 -14.390 -13.497  0.00  0.00
I
ATOM   5969  CA  THR I 414       -1.776 -13.106 -13.963  0.00  0.00
I
ATOM   5970  HA  THR I 414       -1.114 -12.401 -13.483  0.00  0.00
I
ATOM   5971  CB  THR I 414       -0.717 -14.081 -14.669  0.00  0.00
I
ATOM   5972  HB  THR I 414        0.005 -14.478 -13.924  0.00  0.00
I
ATOM   5973  OG1 THR I 414        0.018 -13.457 -15.725  0.00  0.00
I
ATOM   5974  HG1 THR I 414        0.581 -12.757 -15.386  0.00  0.00
I
ATOM   5975  CG2 THR I 414       -1.413 -15.368 -15.213  0.00  0.00
I
ATOM   5976 HG21 THR I 414       -0.694 -16.145 -15.549  0.00  0.00
I
ATOM   5977 HG22 THR I 414       -2.070 -15.817 -14.437  0.00  0.00
I
ATOM   5978 HG23 THR I 414       -1.975 -15.021 -16.107  0.00  0.00
I
ATOM   5979  C   THR I 414       -2.720 -12.590 -14.945  0.00  0.00
I
ATOM   5980  O   THR I 414       -3.772 -13.178 -15.237  0.00  0.00
I
ATOM   5981  N   MET I 415       -2.363 -11.457 -15.530  0.00  0.00
I
ATOM   5982  HN  MET I 415       -1.636 -10.821 -15.284  0.00  0.00
I
ATOM   5983  CA  MET I 415       -3.173 -10.772 -16.496  0.00  0.00
I
ATOM   5984  HA  MET I 415       -4.061 -11.326 -16.760  0.00  0.00
I
ATOM   5985  CB  MET I 415       -3.661  -9.409 -15.910  0.00  0.00
I
ATOM   5986  HB1 MET I 415       -3.966  -9.665 -14.873  0.00  0.00
I
ATOM   5987  HB2 MET I 415       -2.813  -8.691 -15.891  0.00  0.00
I
ATOM   5988  CG  MET I 415       -4.742  -8.899 -16.802  0.00  0.00
I
ATOM   5989  HG1 MET I 415       -4.420  -8.732 -17.852  0.00  0.00
I
ATOM   5990  HG2 MET I 415       -5.446  -9.757 -16.842  0.00  0.00
I
ATOM   5991  SD  MET I 415       -5.515  -7.416 -16.141  0.00  0.00
I
ATOM   5992  CE  MET I 415       -6.380  -8.323 -14.670  0.00  0.00
I
ATOM   5993  HE1 MET I 415       -6.905  -7.668 -13.942  0.00  0.00
I
```

ATOM 5994 HE2 MET I 415 -7.050 -9.157 -14.970 0.00 0.00
I
ATOM 5995 HE3 MET I 415 -5.737 -9.037 -14.111 0.00 0.00
I
ATOM 5996 C MET I 415 -2.356 -10.547 -17.836 0.00 0.00
I
ATOM 5997 O MET I 415 -2.898 -10.532 -18.910 0.00 0.00
I
ATOM 5998 N GLY I 416 -1.061 -10.265 -17.646 0.00 0.00
I
ATOM 5999 HN GLY I 416 -0.636 -10.367 -16.750 0.00 0.00
I
ATOM 6000 CA GLY I 416 -0.229 -9.927 -18.858 0.00 0.00
I
ATOM 6001 HA1 GLY I 416 -0.653 -9.118 -19.435 0.00 0.00
I
ATOM 6002 HA2 GLY I 416 -0.229 -10.791 -19.507 0.00 0.00
I
ATOM 6003 C GLY I 416 1.142 -9.595 -18.588 0.00 0.00
I
ATOM 6004 O GLY I 416 1.979 -9.717 -19.480 0.00 0.00
I
ATOM 6005 N LEU I 417 1.550 -9.163 -17.391 0.00 0.00
I
ATOM 6006 HN LEU I 417 0.935 -9.217 -16.608 0.00 0.00
I
ATOM 6007 CA LEU I 417 2.892 -8.626 -17.119 0.00 0.00
I
ATOM 6008 HA LEU I 417 2.968 -7.798 -17.808 0.00 0.00
I
ATOM 6009 CB LEU I 417 2.838 -8.077 -15.664 0.00 0.00
I
ATOM 6010 HB1 LEU I 417 1.880 -7.549 -15.471 0.00 0.00
I
ATOM 6011 HB2 LEU I 417 2.900 -9.008 -15.061 0.00 0.00
I
ATOM 6012 CG LEU I 417 4.099 -7.212 -15.376 0.00 0.00
I
ATOM 6013 HG LEU I 417 4.914 -7.937 -15.587 0.00 0.00
I
ATOM 6014 CD1 LEU I 417 4.228 -6.072 -16.354 0.00 0.00
I
ATOM 6015 HD11 LEU I 417 5.059 -5.435 -15.985 0.00 0.00
I
ATOM 6016 HD12 LEU I 417 4.505 -6.435 -17.367 0.00 0.00
I
ATOM 6017 HD13 LEU I 417 3.281 -5.511 -16.503 0.00 0.00
I
ATOM 6018 CD2 LEU I 417 4.096 -6.943 -13.856 0.00 0.00
I
ATOM 6019 HD21 LEU I 417 3.139 -6.496 -13.510 0.00 0.00
I
ATOM 6020 HD22 LEU I 417 4.157 -7.930 -13.351 0.00 0.00
I

```
ATOM   6021 HD23 LEU I 417       4.952  -6.302 -13.552  0.00  0.00
I
ATOM   6022  C   LEU I 417       4.060  -9.586 -17.207  0.00  0.00
I
ATOM   6023  O   LEU I 417       5.154  -9.287 -17.717  0.00  0.00
I
ATOM   6024  N   THR I 418       3.867 -10.783 -16.609  0.00  0.00
I
ATOM   6025  HN  THR I 418       2.988 -10.892 -16.151  0.00  0.00
I
ATOM   6026  CA  THR I 418       4.935 -11.831 -16.433  0.00  0.00
I
ATOM   6027  HA  THR I 418       5.839 -11.332 -16.115  0.00  0.00
I
ATOM   6028  CB  THR I 418       4.500 -12.808 -15.323  0.00  0.00
I
ATOM   6029  HB  THR I 418       3.761 -13.552 -15.689  0.00  0.00
I
ATOM   6030  OG1 THR I 418       4.040 -12.030 -14.172  0.00  0.00
I
ATOM   6031  HG1 THR I 418       3.204 -11.612 -14.388  0.00  0.00
I
ATOM   6032  CG2 THR I 418       5.734 -13.593 -14.763  0.00  0.00
I
ATOM   6033 HG21 THR I 418       5.431 -14.143 -13.847  0.00  0.00
I
ATOM   6034 HG22 THR I 418       6.210 -14.277 -15.498  0.00  0.00
I
ATOM   6035 HG23 THR I 418       6.536 -12.899 -14.432  0.00  0.00
I
ATOM   6036  C   THR I 418       5.303 -12.595 -17.723  0.00  0.00
I
ATOM   6037  O   THR I 418       6.558 -12.840 -17.793  0.00  0.00
I
ATOM   6038  N   PRO I 419       4.515 -13.130 -18.696  0.00  0.00
I
ATOM   6039  CD  PRO I 419       3.103 -13.377 -18.505  0.00  0.00
I
ATOM   6040  HD1 PRO I 419       2.940 -13.904 -17.540  0.00  0.00
I
ATOM   6041  HD2 PRO I 419       2.553 -12.412 -18.497  0.00  0.00
I
ATOM   6042  CA  PRO I 419       4.995 -13.724 -19.955  0.00  0.00
I
ATOM   6043  HA  PRO I 419       5.544 -14.598 -19.637  0.00  0.00
I
ATOM   6044  CB  PRO I 419       3.698 -13.977 -20.816  0.00  0.00
I
ATOM   6045  HB1 PRO I 419       3.969 -14.849 -21.449  0.00  0.00
I
ATOM   6046  HB2 PRO I 419       3.387 -13.133 -21.468  0.00  0.00
I
ATOM   6047  CG  PRO I 419       2.644 -14.340 -19.632  0.00  0.00
I
```

ATOM   6048 HG1 PRO I 419   1.590 -14.049 -19.827 0.00 0.00
I
ATOM   6049 HG2 PRO I 419   2.824 -15.379 -19.282 0.00 0.00
I
ATOM   6050 C   PRO I 419   6.073 -13.122 -20.774 0.00 0.00
I
ATOM   6051 O   PRO I 419   6.651 -13.865 -21.601 0.00 0.00
I
ATOM   6052 N   GLU I 420   6.266 -11.770 -20.760 0.00 0.00
I
ATOM   6053 HN GLU I 420   5.794 -11.262 -20.044 0.00 0.00
I
ATOM   6054 CA GLU I 420   7.039 -11.081 -21.654 0.00 0.00
I
ATOM   6055 HA GLU I 420   7.614 -11.716 -22.313 0.00 0.00
I
ATOM   6056 CB GLU I 420   5.936 -10.454 -22.716 0.00 0.00
I
ATOM   6057 HB1 GLU I 420   6.571 -10.059 -23.538 0.00 0.00
I
ATOM   6058 HB2 GLU I 420   5.306 -11.262 -23.147 0.00 0.00
I
ATOM   6059 CG GLU I 420   4.964 -9.523 -22.049 0.00 0.00
I
ATOM   6060 HG1 GLU I 420   4.277 -10.136 -21.427 0.00 0.00
I
ATOM   6061 HG2 GLU I 420   5.430 -8.824 -21.322 0.00 0.00
I
ATOM   6062 CD GLU I 420   4.004 -8.876 -23.038 0.00 0.00
I
ATOM   6063 OE1 GLU I 420   4.162 -7.699 -23.352 0.00 0.00
I
ATOM   6064 OE2 GLU I 420   3.059 -9.617 -23.531 0.00 0.00
I
ATOM   6065 C   GLU I 420   8.103 -10.049 -21.036 0.00 0.00
I
ATOM   6066 O   GLU I 420   8.735 -9.324 -21.805 0.00 0.00
I
ATOM   6067 N   LEU I 421   8.330 -10.175 -19.724 0.00 0.00
I
ATOM   6068 HN LEU I 421   7.860 -10.805 -19.111 0.00 0.00
I
ATOM   6069 CA LEU I 421   9.315 -9.359 -18.936 0.00 0.00
I
ATOM   6070 HA LEU I 421   8.995 -8.327 -18.926 0.00 0.00
I
ATOM   6071 CB LEU I 421   9.526 -9.979 -17.476 0.00 0.00
I
ATOM   6072 HB1 LEU I 421   9.910 -11.011 -17.623 0.00 0.00
I
ATOM   6073 HB2 LEU I 421   10.229 -9.259 -17.004 0.00 0.00
I
ATOM   6074 CG LEU I 421   8.314 -10.036 -16.549 0.00 0.00
I

ATOM 6075 HG LEU I 421 7.430 −10.460 −17.070 0.00 0.00
I
ATOM 6076 CD1 LEU I 421 8.586 −10.923 −15.310 0.00 0.00
I
ATOM 6077 HD11 LEU I 421 8.911 −11.960 −15.539 0.00 0.00
I
ATOM 6078 HD12 LEU I 421 9.208 −10.329 −14.606 0.00 0.00
I
ATOM 6079 HD13 LEU I 421 7.604 −11.020 −14.799 0.00 0.00
I
ATOM 6080 CD2 LEU I 421 7.943 −8.623 −16.077 0.00 0.00
I
ATOM 6081 HD21 LEU I 421 7.350 −8.028 −16.805 0.00 0.00
I
ATOM 6082 HD22 LEU I 421 7.380 −8.717 −15.125 0.00 0.00
I
ATOM 6083 HD23 LEU I 421 8.850 −8.023 −15.848 0.00 0.00
I
ATOM 6084 C LEU I 421 10.856 −9.261 −19.377 0.00 0.00
I
ATOM 6085 O LEU I 421 11.488 −10.079 −20.068 0.00 0.00
I
ATOM 6086 N THR I 422 11.539 −8.166 −19.005 0.00 0.00
I
ATOM 6087 HN THR I 422 10.977 −7.404 −18.690 0.00 0.00
I
ATOM 6088 CA THR I 422 12.968 −7.967 −19.190 0.00 0.00
I
ATOM 6089 HA THR I 422 13.364 −8.606 −19.966 0.00 0.00
I
ATOM 6090 CB THR I 422 13.438 −6.591 −19.320 0.00 0.00
I
ATOM 6091 HB THR I 422 14.548 −6.615 −19.359 0.00 0.00
I
ATOM 6092 OG1 THR I 422 13.031 −5.827 −18.137 0.00 0.00
I
ATOM 6093 HG1 THR I 422 12.098 −5.620 −18.229 0.00 0.00
I
ATOM 6094 CG2 THR I 422 12.936 −6.013 −20.630 0.00 0.00
I
ATOM 6095 HG21 THR I 422 11.850 −5.827 −20.484 0.00 0.00
I
ATOM 6096 HG22 THR I 422 13.423 −5.040 −20.853 0.00 0.00
I
ATOM 6097 HG23 THR I 422 13.049 −6.661 −21.526 0.00 0.00
I
ATOM 6098 C THR I 422 13.768 −8.582 −18.038 0.00 0.00
I
ATOM 6099 O THR I 422 13.162 −9.023 −17.063 0.00 0.00
I
ATOM 6100 N THR I 423 15.118 −8.687 −18.085 0.00 0.00
I
ATOM 6101 HN THR I 423 15.589 −8.590 −18.959 0.00 0.00
I

```
ATOM   6102  CA  THR I 423      15.896  -9.230 -16.989  0.00  0.00
I
ATOM   6103  HA  THR I 423      15.683 -10.266 -16.772  0.00  0.00
I
ATOM   6104  CB  THR I 423      17.463  -9.208 -17.347  0.00  0.00
I
ATOM   6105  HB  THR I 423      17.766  -8.177 -17.631  0.00  0.00
I
ATOM   6106  OG1 THR I 423      17.819 -10.039 -18.419  0.00  0.00
I
ATOM   6107  HG1 THR I 423      18.768 -10.152 -18.502  0.00  0.00
I
ATOM   6108  CG2 THR I 423      18.425  -9.650 -16.206  0.00  0.00
I
ATOM   6109 HG21 THR I 423      18.174 -10.648 -15.787  0.00  0.00
I
ATOM   6110 HG22 THR I 423      19.421  -9.748 -16.688  0.00  0.00
I
ATOM   6111 HG23 THR I 423      18.526  -8.949 -15.351  0.00  0.00
I
ATOM   6112  C   THR I 423      15.695  -8.466 -15.757  0.00  0.00
I
ATOM   6113  O   THR I 423      15.585  -9.127 -14.705  0.00  0.00
I
ATOM   6114  N   ALA I 424      15.780  -7.133 -15.770  0.00  0.00
I
ATOM   6115  HN  ALA I 424      16.041  -6.658 -16.606  0.00  0.00
I
ATOM   6116  CA  ALA I 424      15.503  -6.363 -14.647  0.00  0.00
I
ATOM   6117  HA  ALA I 424      16.030  -6.779 -13.801  0.00  0.00
I
ATOM   6118  CB  ALA I 424      15.751  -4.875 -15.001  0.00  0.00
I
ATOM   6119  HB1 ALA I 424      15.135  -4.361 -15.770  0.00  0.00
I
ATOM   6120  HB2 ALA I 424      15.923  -4.302 -14.065  0.00  0.00
I
ATOM   6121  HB3 ALA I 424      16.744  -4.834 -15.498  0.00  0.00
I
ATOM   6122  C   ALA I 424      14.101  -6.602 -14.040  0.00  0.00
I
ATOM   6123  O   ALA I 424      13.903  -7.040 -12.903  0.00  0.00
I
ATOM   6124  N   GLY I 425      13.082  -6.572 -14.968  0.00  0.00
I
ATOM   6125  HN  GLY I 425      13.304  -6.406 -15.926  0.00  0.00
I
ATOM   6126  CA  GLY I 425      11.728  -6.884 -14.614  0.00  0.00
I
ATOM   6127  HA1 GLY I 425      11.086  -6.706 -15.465  0.00  0.00
I
ATOM   6128  HA2 GLY I 425      11.491  -6.132 -13.876  0.00  0.00
I
```

ATOM 6129 C GLY I 425 11.465 -8.225 -13.904 0.00 0.00
I
ATOM 6130 O GLY I 425 10.507 -8.258 -13.113 0.00 0.00
I
ATOM 6131 N LYS I 426 12.234 -9.264 -14.266 0.00 0.00
I
ATOM 6132 HN LYS I 426 12.984 -9.057 -14.890 0.00 0.00
I
ATOM 6133 CA LYS I 426 12.223 -10.569 -13.620 0.00 0.00
I
ATOM 6134 HA LYS I 426 11.224 -10.977 -13.653 0.00 0.00
I
ATOM 6135 CB LYS I 426 13.053 -11.489 -14.468 0.00 0.00
I
ATOM 6136 HB1 LYS I 426 14.052 -11.065 -14.709 0.00 0.00
I
ATOM 6137 HB2 LYS I 426 13.088 -12.461 -13.932 0.00 0.00
I
ATOM 6138 CG LYS I 426 12.321 -11.886 -15.721 0.00 0.00
I
ATOM 6139 HG1 LYS I 426 11.354 -12.378 -15.484 0.00 0.00
I
ATOM 6140 HG2 LYS I 426 12.071 -10.951 -16.265 0.00 0.00
I
ATOM 6141 CD LYS I 426 13.308 -12.698 -16.647 0.00 0.00
I
ATOM 6142 HD1 LYS I 426 14.286 -12.181 -16.750 0.00 0.00
I
ATOM 6143 HD2 LYS I 426 13.436 -13.738 -16.277 0.00 0.00
I
ATOM 6144 CE LYS I 426 12.669 -12.768 -18.015 0.00 0.00
I
ATOM 6145 HE1 LYS I 426 11.737 -13.361 -17.901 0.00 0.00
I
ATOM 6146 HE2 LYS I 426 12.304 -11.781 -18.370 0.00 0.00
I
ATOM 6147 NZ LYS I 426 13.580 -13.278 -19.060 0.00 0.00
I
ATOM 6148 HZ1 LYS I 426 13.225 -13.267 -20.038 0.00 0.00
I
ATOM 6149 HZ2 LYS I 426 14.511 -12.830 -19.174 0.00 0.00
I
ATOM 6150 HZ3 LYS I 426 13.689 -14.302 -18.914 0.00 0.00
I
ATOM 6151 C LYS I 426 12.668 -10.610 -12.172 0.00 0.00
I
ATOM 6152 O LYS I 426 12.089 -11.263 -11.336 0.00 0.00
I
ATOM 6153 N CYS I 427 13.797 -9.920 -11.986 0.00 0.00
I
ATOM 6154 HN CYS I 427 14.428 -9.675 -12.719 0.00 0.00
I
ATOM 6155 CA CYS I 427 14.421 -9.801 -10.741 0.00 0.00
I

```
ATOM   6156  HA  CYS I 427      14.604 -10.808 -10.395  0.00  0.00
I
ATOM   6157  CB  CYS I 427      15.687  -8.886 -10.758  0.00  0.00
I
ATOM   6158  HB1 CYS I 427      15.443  -7.850 -11.076  0.00  0.00
I
ATOM   6159  HB2 CYS I 427      15.979  -8.872  -9.687  0.00  0.00
I
ATOM   6160  SG  CYS I 427      17.083  -9.498 -11.728  0.00  0.00
I
ATOM   6161  HG1 CYS I 427      16.563  -9.537 -12.946  0.00  0.00
I
ATOM   6162  C   CYS I 427      13.490  -9.062  -9.794  0.00  0.00
I
ATOM   6163  O   CYS I 427      13.256  -9.423  -8.692  0.00  0.00
I
ATOM   6164  N   ILE I 428      12.838  -7.983 -10.285  0.00  0.00
I
ATOM   6165  HN  ILE I 428      12.983  -7.638 -11.209  0.00  0.00
I
ATOM   6166  CA  ILE I 428      12.019  -7.191  -9.424  0.00  0.00
I
ATOM   6167  HA  ILE I 428      12.545  -6.996  -8.501  0.00  0.00
I
ATOM   6168  CB  ILE I 428      11.743  -5.833 -10.017  0.00  0.00
I
ATOM   6169  HB  ILE I 428      11.569  -6.117 -11.077  0.00  0.00
I
ATOM   6170  CG2 ILE I 428      10.633  -5.020  -9.425  0.00  0.00
I
ATOM   6171 HG21 ILE I 428      10.877  -4.824  -8.359  0.00  0.00
I
ATOM   6172 HG22 ILE I 428      10.494  -4.073  -9.991  0.00  0.00
I
ATOM   6173 HG23 ILE I 428       9.659  -5.554  -9.450  0.00  0.00
I
ATOM   6174  CG1 ILE I 428      13.099  -5.012 -10.158  0.00  0.00
I
ATOM   6175 HG11 ILE I 428      13.419  -4.530  -9.209  0.00  0.00
I
ATOM   6176 HG12 ILE I 428      13.933  -5.726 -10.327  0.00  0.00
I
ATOM   6177  CD  ILE I 428      13.137  -3.985 -11.256  0.00  0.00
I
ATOM   6178  HD1 ILE I 428      14.108  -3.626 -11.658  0.00  0.00
I
ATOM   6179  HD2 ILE I 428      12.639  -4.488 -12.112  0.00  0.00
I
ATOM   6180  HD3 ILE I 428      12.575  -3.066 -10.983  0.00  0.00
I
ATOM   6181  C   ILE I 428      10.738  -7.849  -9.013  0.00  0.00
I
ATOM   6182  O   ILE I 428      10.469  -7.762  -7.811  0.00  0.00
I
```

ATOM   6183 N   ILE I 429   10.017 -8.649 -9.881 0.00 0.00
I
ATOM   6184 HN ILE I 429   10.180 -8.787 -10.855 0.00 0.00
I
ATOM   6185 CA ILE I 429   8.969 -9.568 -9.390 0.00 0.00
I
ATOM   6186 HA ILE I 429   8.348 -8.870 -8.847 0.00 0.00
I
ATOM   6187 CB ILE I 429   8.184 -10.335 -10.488 0.00 0.00
I
ATOM   6188 HB ILE I 429   9.003 -10.772 -11.100 0.00 0.00
I
ATOM   6189 CG2 ILE I 429   7.220 -11.377 -9.902 0.00 0.00
I
ATOM   6190 HG21 ILE I 429   6.742 -10.992 -8.975 0.00 0.00
I
ATOM   6191 HG22 ILE I 429   6.453 -11.639 -10.661 0.00 0.00
I
ATOM   6192 HG23 ILE I 429   7.704 -12.325 -9.585 0.00 0.00
I
ATOM   6193 CG1 ILE I 429   7.441 -9.398 -11.408 0.00 0.00
I
ATOM   6194 HG11 ILE I 429   8.101 -8.546 -11.679 0.00 0.00
I
ATOM   6195 HG12 ILE I 429   7.226 -9.888 -12.382 0.00 0.00
I
ATOM   6196 CD ILE I 429   6.145 -8.838 -10.849 0.00 0.00
I
ATOM   6197 HD1 ILE I 429   5.396 -9.659 -10.864 0.00 0.00
I
ATOM   6198 HD2 ILE I 429   6.201 -8.486 -9.796 0.00 0.00
I
ATOM   6199 HD3 ILE I 429   5.991 -7.960 -11.512 0.00 0.00
I
ATOM   6200 C   ILE I 429   9.479 -10.510 -8.331 0.00 0.00
I
ATOM   6201 O   ILE I 429   8.796 -10.661 -7.336 0.00 0.00
I
ATOM   6202 N   ILE I 430   10.625 -11.156 -8.535 0.00 0.00
I
ATOM   6203 HN ILE I 430   11.268 -11.076 -9.293 0.00 0.00
I
ATOM   6204 CA ILE I 430   11.140 -12.102 -7.521 0.00 0.00
I
ATOM   6205 HA ILE I 430   10.379 -12.867 -7.471 0.00 0.00
I
ATOM   6206 CB ILE I 430   12.540 -12.811 -7.792 0.00 0.00
I
ATOM   6207 HB ILE I 430   13.185 -11.940 -8.037 0.00 0.00
I
ATOM   6208 CG2 ILE I 430   13.004 -13.683 -6.671 0.00 0.00
I
ATOM   6209 HG21 ILE I 430   12.215 -14.348 -6.259 0.00 0.00
I

ATOM 6210 HG22 ILE I 430 13.920 -14.173 -7.065 0.00 0.00
I
ATOM 6211 HG23 ILE I 430 13.438 -12.940 -5.968 0.00 0.00
I
ATOM 6212 CG1 ILE I 430 12.496 -13.747 -9.072 0.00 0.00
I
ATOM 6213 HG11 ILE I 430 12.067 -14.741 -8.822 0.00 0.00
I
ATOM 6214 HG12 ILE I 430 11.966 -13.190 -9.874 0.00 0.00
I
ATOM 6215 CD ILE I 430 13.887 -14.015 -9.747 0.00 0.00
I
ATOM 6216 HD1 ILE I 430 14.416 -13.043 -9.839 0.00 0.00
I
ATOM 6217 HD2 ILE I 430 14.523 -14.712 -9.160 0.00 0.00
I
ATOM 6218 HD3 ILE I 430 13.689 -14.431 -10.758 0.00 0.00
I
ATOM 6219 C ILE I 430 11.259 -11.533 -6.104 0.00 0.00
I
ATOM 6220 O ILE I 430 10.906 -12.152 -5.090 0.00 0.00
I
ATOM 6221 N VAL I 431 11.764 -10.220 -6.043 0.00 0.00
I
ATOM 6222 HN VAL I 431 12.163 -9.744 -6.822 0.00 0.00
I
ATOM 6223 CA VAL I 431 11.958 -9.502 -4.785 0.00 0.00
I
ATOM 6224 HA VAL I 431 12.513 -10.143 -4.116 0.00 0.00
I
ATOM 6225 CB VAL I 431 12.683 -8.228 -5.104 0.00 0.00
I
ATOM 6226 HB VAL I 431 12.248 -7.710 -5.985 0.00 0.00
I
ATOM 6227 CG1 VAL I 431 12.763 -7.228 -4.022 0.00 0.00
I
ATOM 6228 HG11 VAL I 431 12.984 -7.624 -3.008 0.00 0.00
I
ATOM 6229 HG12 VAL I 431 13.597 -6.560 -4.327 0.00 0.00
I
ATOM 6230 HG13 VAL I 431 11.784 -6.710 -3.940 0.00 0.00
I
ATOM 6231 CG2 VAL I 431 14.130 -8.682 -5.410 0.00 0.00
I
ATOM 6232 HG21 VAL I 431 14.544 -9.241 -4.544 0.00 0.00
I
ATOM 6233 HG22 VAL I 431 14.101 -9.266 -6.355 0.00 0.00
I
ATOM 6234 HG23 VAL I 431 14.861 -7.849 -5.491 0.00 0.00
I
ATOM 6235 C VAL I 431 10.631 -9.359 -4.058 0.00 0.00
I
ATOM 6236 O VAL I 431 10.560 -9.622 -2.834 0.00 0.00
I

ATOM 6237 N ILE I 432 9.568 -8.915 -4.782 0.00 0.00
I
ATOM 6238 HN ILE I 432 9.654 -8.524 -5.695 0.00 0.00
I
ATOM 6239 CA ILE I 432 8.183 -8.855 -4.243 0.00 0.00
I
ATOM 6240 HA ILE I 432 8.178 -8.142 -3.432 0.00 0.00
I
ATOM 6241 CB ILE I 432 7.266 -8.251 -5.207 0.00 0.00
I
ATOM 6242 HB ILE I 432 7.276 -8.839 -6.149 0.00 0.00
I
ATOM 6243 CG2 ILE I 432 5.868 -7.952 -4.690 0.00 0.00
I
ATOM 6244 HG21 ILE I 432 5.321 -8.900 -4.497 0.00 0.00
I
ATOM 6245 HG22 ILE I 432 6.087 -7.567 -3.671 0.00 0.00
I
ATOM 6246 HG23 ILE I 432 5.307 -7.161 -5.232 0.00 0.00
I
ATOM 6247 CG1 ILE I 432 7.788 -6.880 -5.780 0.00 0.00
I
ATOM 6248 HG11 ILE I 432 7.587 -6.000 -5.133 0.00 0.00
I
ATOM 6249 HG12 ILE I 432 8.893 -6.929 -5.889 0.00 0.00
I
ATOM 6250 CD ILE I 432 7.311 -6.598 -7.274 0.00 0.00
I
ATOM 6251 HD1 ILE I 432 7.428 -7.484 -7.934 0.00 0.00
I
ATOM 6252 HD2 ILE I 432 6.293 -6.152 -7.281 0.00 0.00
I
ATOM 6253 HD3 ILE I 432 7.922 -5.846 -7.817 0.00 0.00
I
ATOM 6254 C ILE I 432 7.671 -10.206 -3.771 0.00 0.00
I
ATOM 6255 O ILE I 432 7.143 -10.260 -2.646 0.00 0.00
I
ATOM 6256 N MET I 433 7.844 -11.278 -4.515 0.00 0.00
I
ATOM 6257 HN MET I 433 8.243 -11.244 -5.428 0.00 0.00
I
ATOM 6258 CA MET I 433 7.493 -12.676 -4.135 0.00 0.00
I
ATOM 6259 HA MET I 433 6.464 -12.746 -3.815 0.00 0.00
I
ATOM 6260 CB MET I 433 7.889 -13.633 -5.320 0.00 0.00
I
ATOM 6261 HB1 MET I 433 8.979 -13.542 -5.519 0.00 0.00
I
ATOM 6262 HB2 MET I 433 7.866 -14.678 -4.942 0.00 0.00
I
ATOM 6263 CG MET I 433 7.204 -13.529 -6.644 0.00 0.00
I

ATOM 6264 HG1 MET I 433 6.180 -13.209 -6.356 0.00 0.00
I
ATOM 6265 HG2 MET I 433 7.585 -12.647 -7.202 0.00 0.00
I
ATOM 6266 SD MET I 433 7.194 -14.927 -7.851 0.00 0.00
I
ATOM 6267 CE MET I 433 8.804 -14.801 -8.675 0.00 0.00
I
ATOM 6268 HE1 MET I 433 9.630 -14.997 -7.959 0.00 0.00
I
ATOM 6269 HE2 MET I 433 8.866 -15.442 -9.581 0.00 0.00
I
ATOM 6270 HE3 MET I 433 8.962 -13.738 -8.959 0.00 0.00
I
ATOM 6271 C MET I 433 8.343 -13.151 -2.880 0.00 0.00
I
ATOM 6272 O MET I 433 7.850 -13.942 -2.105 0.00 0.00
I
ATOM 6273 N PHE I 434 9.647 -12.788 -2.786 0.00 0.00
I
ATOM 6274 HN PHE I 434 10.014 -12.173 -3.479 0.00 0.00
I
ATOM 6275 CA PHE I 434 10.482 -13.149 -1.671 0.00 0.00
I
ATOM 6276 HA PHE I 434 10.543 -14.226 -1.618 0.00 0.00
I
ATOM 6277 CB PHE I 434 11.996 -12.664 -1.854 0.00 0.00
I
ATOM 6278 HB1 PHE I 434 12.264 -12.982 -2.884 0.00 0.00
I
ATOM 6279 HB2 PHE I 434 12.117 -11.564 -1.758 0.00 0.00
I
ATOM 6280 CG PHE I 434 12.918 -13.383 -0.897 0.00 0.00
I
ATOM 6281 CD1 PHE I 434 13.610 -14.467 -1.327 0.00 0.00
I
ATOM 6282 HD1 PHE I 434 13.399 -14.878 -2.303 0.00 0.00
I
ATOM 6283 CE1 PHE I 434 14.541 -15.176 -0.390 0.00 0.00
I
ATOM 6284 HE1 PHE I 434 15.189 -15.933 -0.807 0.00 0.00
I
ATOM 6285 CZ PHE I 434 14.704 -14.667 0.874 0.00 0.00
I
ATOM 6286 HZ PHE I 434 15.502 -15.118 1.446 0.00 0.00
I
ATOM 6287 CD2 PHE I 434 12.985 -12.982 0.448 0.00 0.00
I
ATOM 6288 HD2 PHE I 434 12.424 -12.142 0.829 0.00 0.00
I
ATOM 6289 CE2 PHE I 434 13.922 -13.614 1.302 0.00 0.00
I
ATOM 6290 HE2 PHE I 434 13.975 -13.371 2.352 0.00 0.00
I

```
ATOM   6291  C   PHE I 434       9.981 -12.508  -0.381  0.00  0.00
I
ATOM   6292  O   PHE I 434       9.634 -13.175   0.579  0.00  0.00
I
ATOM   6293  N   ILE I 435       9.707 -11.222  -0.439  0.00  0.00
I
ATOM   6294  HN  ILE I 435       9.984 -10.798  -1.298  0.00  0.00
I
ATOM   6295  CA  ILE I 435       9.185 -10.420   0.657  0.00  0.00
I
ATOM   6296  HA  ILE I 435       9.760 -10.718   1.521  0.00  0.00
I
ATOM   6297  CB  ILE I 435       9.421  -8.905   0.480  0.00  0.00
I
ATOM   6298  HB  ILE I 435       9.013  -8.597  -0.506  0.00  0.00
I
ATOM   6299  CG2 ILE I 435       8.691  -8.075   1.587  0.00  0.00
I
ATOM   6300 HG21 ILE I 435       7.636  -8.416   1.662  0.00  0.00
I
ATOM   6301 HG22 ILE I 435       9.162  -8.287   2.571  0.00  0.00
I
ATOM   6302 HG23 ILE I 435       8.586  -6.998   1.335  0.00  0.00
I
ATOM   6303  CG1 ILE I 435      10.861  -8.454   0.466  0.00  0.00
I
ATOM   6304 HG11 ILE I 435      11.173  -8.690   1.505  0.00  0.00
I
ATOM   6305 HG12 ILE I 435      11.412  -9.132  -0.221  0.00  0.00
I
ATOM   6306  CD  ILE I 435      11.062  -7.014   0.135  0.00  0.00
I
ATOM   6307  HD1 ILE I 435      10.910  -6.864  -0.955  0.00  0.00
I
ATOM   6308  HD2 ILE I 435      10.470  -6.327   0.778  0.00  0.00
I
ATOM   6309  HD3 ILE I 435      12.138  -6.781   0.281  0.00  0.00
I
ATOM   6310  C   ILE I 435       7.675 -10.811   0.962  0.00  0.00
I
ATOM   6311  O   ILE I 435       7.223 -11.003   2.156  0.00  0.00
I
ATOM   6312  N   GLY I 436       6.886 -11.287  -0.130  0.00  0.00
I
ATOM   6313  HN  GLY I 436       7.198 -11.031  -1.042  0.00  0.00
I
ATOM   6314  CA  GLY I 436       5.614 -11.995   0.062  0.00  0.00
I
ATOM   6315  HA1 GLY I 436       5.324 -12.273  -0.941  0.00  0.00
I
ATOM   6316  HA2 GLY I 436       4.793 -11.407   0.444  0.00  0.00
I
ATOM   6317  C   GLY I 436       5.603 -13.279   0.849  0.00  0.00
I
```

ATOM 6318 O GLY I 436 4.876 -13.395 1.833 0.00 0.00
I
ATOM 6319 N ARG I 437 6.482 -14.231 0.464 0.00 0.00
I
ATOM 6320 HN ARG I 437 6.929 -13.996 -0.396 0.00 0.00
I
ATOM 6321 CA ARG I 437 6.548 -15.488 1.154 0.00 0.00
I
ATOM 6322 HA ARG I 437 5.601 -16.009 1.149 0.00 0.00
I
ATOM 6323 CB ARG I 437 7.553 -16.385 0.346 0.00 0.00
I
ATOM 6324 HB1 ARG I 437 7.134 -16.332 -0.682 0.00 0.00
I
ATOM 6325 HB2 ARG I 437 8.581 -15.985 0.478 0.00 0.00
I
ATOM 6326 CG ARG I 437 7.666 -17.823 0.761 0.00 0.00
I
ATOM 6327 HG1 ARG I 437 8.507 -18.274 0.193 0.00 0.00
I
ATOM 6328 HG2 ARG I 437 7.955 -17.923 1.829 0.00 0.00
I
ATOM 6329 CD ARG I 437 6.278 -18.458 0.642 0.00 0.00
I
ATOM 6330 HD1 ARG I 437 5.623 -18.039 1.436 0.00 0.00
I
ATOM 6331 HD2 ARG I 437 5.666 -18.311 -0.273 0.00 0.00
I
ATOM 6332 NE ARG I 437 6.540 -19.894 0.939 0.00 0.00
I
ATOM 6333 HE ARG I 437 7.496 -20.121 1.122 0.00 0.00
I
ATOM 6334 CZ ARG I 437 5.703 -20.902 0.635 0.00 0.00
I
ATOM 6335 NH1 ARG I 437 4.390 -20.709 0.328 0.00 0.00
I
ATOM 6336 HH11 ARG I 437 3.750 -21.403 -0.002 0.00 0.00
I
ATOM 6337 HH12 ARG I 437 4.023 -19.786 0.213 0.00 0.00
I
ATOM 6338 NH2 ARG I 437 6.183 -22.179 0.621 0.00 0.00
I
ATOM 6339 HH21 ARG I 437 5.499 -22.896 0.483 0.00 0.00
I
ATOM 6340 HH22 ARG I 437 7.093 -22.425 0.954 0.00 0.00
I
ATOM 6341 C ARG I 437 7.011 -15.407 2.618 0.00 0.00
I
ATOM 6342 O ARG I 437 6.264 -15.871 3.442 0.00 0.00
I
ATOM 6343 N ILE I 438 8.041 -14.611 2.910 0.00 0.00
I
ATOM 6344 HN ILE I 438 8.608 -14.184 2.209 0.00 0.00
I

ATOM 6345 CA ILE I 438 8.415 -14.277 4.314 0.00 0.00
I
ATOM 6346 HA ILE I 438 8.477 -15.171 4.917 0.00 0.00
I
ATOM 6347 CB ILE I 438 9.829 -13.605 4.521 0.00 0.00
I
ATOM 6348 HB ILE I 438 10.003 -13.394 5.598 0.00 0.00
I
ATOM 6349 CG2 ILE I 438 10.855 -14.605 3.975 0.00 0.00
I
ATOM 6350 HG21 ILE I 438 10.497 -15.612 4.278 0.00 0.00
I
ATOM 6351 HG22 ILE I 438 10.888 -14.581 2.865 0.00 0.00
I
ATOM 6352 HG23 ILE I 438 11.917 -14.510 4.288 0.00 0.00
I
ATOM 6353 CG1 ILE I 438 9.966 -12.213 3.961 0.00 0.00
I
ATOM 6354 HG11 ILE I 438 9.392 -12.159 3.011 0.00 0.00
I
ATOM 6355 HG12 ILE I 438 9.356 -11.716 4.746 0.00 0.00
I
ATOM 6356 CD ILE I 438 11.297 -11.565 3.799 0.00 0.00
I
ATOM 6357 HD1 ILE I 438 11.898 -11.797 4.705 0.00 0.00
I
ATOM 6358 HD2 ILE I 438 11.643 -12.154 2.923 0.00 0.00
I
ATOM 6359 HD3 ILE I 438 11.282 -10.490 3.521 0.00 0.00
I
ATOM 6360 C ILE I 438 7.357 -13.526 5.220 0.00 0.00
I
ATOM 6361 O ILE I 438 7.164 -13.705 6.421 0.00 0.00
I
ATOM 6362 N GLY I 439 6.594 -12.677 4.516 0.00 0.00
I
ATOM 6363 HN GLY I 439 6.658 -12.466 3.544 0.00 0.00
I
ATOM 6364 CA GLY I 439 5.785 -11.674 5.191 0.00 0.00
I
ATOM 6365 HA1 GLY I 439 5.277 -12.085 6.051 0.00 0.00
I
ATOM 6366 HA2 GLY I 439 5.170 -11.305 4.382 0.00 0.00
I
ATOM 6367 C GLY I 439 6.529 -10.447 5.683 0.00 0.00
I
ATOM 6368 O GLY I 439 7.754 -10.423 5.854 0.00 0.00
I
ATOM 6369 N PRO I 440 5.717 -9.382 6.056 0.00 0.00
I
ATOM 6370 CD PRO I 440 4.269 -9.300 5.758 0.00 0.00
I
ATOM 6371 HD1 PRO I 440 4.164 -9.339 4.653 0.00 0.00
I

```
ATOM   6372  HD2 PRO I 440       3.719 -10.231   6.014  0.00  0.00
I
ATOM   6373  CA  PRO I 440       6.314  -8.058   6.157  0.00  0.00
I
ATOM   6374  HA  PRO I 440       7.123  -7.998   5.443  0.00  0.00
I
ATOM   6375  CB  PRO I 440       5.085  -7.079   5.868  0.00  0.00
I
ATOM   6376  HB1 PRO I 440       4.884  -6.919   4.787  0.00  0.00
I
ATOM   6377  HB2 PRO I 440       5.208  -6.123   6.420  0.00  0.00
I
ATOM   6378  CG  PRO I 440       3.862  -7.950   6.270  0.00  0.00
I
ATOM   6379  HG1 PRO I 440       2.933  -7.484   5.875  0.00  0.00
I
ATOM   6380  HG2 PRO I 440       3.780  -8.026   7.375  0.00  0.00
I
ATOM   6381  C   PRO I 440       6.912  -7.900   7.589  0.00  0.00
I
ATOM   6382  O   PRO I 440       7.759  -6.989   7.764  0.00  0.00
I
ATOM   6383  N   LEU I 441       6.476  -8.698   8.599  0.00  0.00
I
ATOM   6384  HN  LEU I 441       5.693  -9.311   8.527  0.00  0.00
I
ATOM   6385  CA  LEU I 441       7.024  -8.482   9.961  0.00  0.00
I
ATOM   6386  HA  LEU I 441       7.095  -7.432  10.201  0.00  0.00
I
ATOM   6387  CB  LEU I 441       6.093  -9.182  11.009  0.00  0.00
I
ATOM   6388  HB1 LEU I 441       5.040  -8.973  10.720  0.00  0.00
I
ATOM   6389  HB2 LEU I 441       6.385 -10.254  10.999  0.00  0.00
I
ATOM   6390  CG  LEU I 441       6.274  -8.817  12.490  0.00  0.00
I
ATOM   6391  HG  LEU I 441       7.328  -9.039  12.766  0.00  0.00
I
ATOM   6392  CD1 LEU I 441       5.984  -7.358  12.751  0.00  0.00
I
ATOM   6393 HD11 LEU I 441       4.896  -7.214  12.582  0.00  0.00
I
ATOM   6394 HD12 LEU I 441       6.303  -7.118  13.788  0.00  0.00
I
ATOM   6395 HD13 LEU I 441       6.483  -6.759  11.958  0.00  0.00
I
ATOM   6396  CD2 LEU I 441       5.518  -9.832  13.443  0.00  0.00
I
ATOM   6397 HD21 LEU I 441       4.433  -9.672  13.265  0.00  0.00
I
ATOM   6398 HD22 LEU I 441       5.687 -10.846  13.022  0.00  0.00
I
```

ATOM 6399 HD23 LEU I 441 5.818 -9.771 14.511 0.00 0.00
I
ATOM 6400 C LEU I 441 8.406 -9.095 10.019 0.00 0.00
I
ATOM 6401 O LEU I 441 9.292 -8.384 10.448 0.00 0.00
I
ATOM 6402 N THR I 442 8.578 -10.332 9.439 0.00 0.00
I
ATOM 6403 HN THR I 442 7.751 -10.688 9.010 0.00 0.00
I
ATOM 6404 CA THR I 442 9.790 -11.090 9.197 0.00 0.00
I
ATOM 6405 HA THR I 442 10.254 -11.304 10.149 0.00 0.00
I
ATOM 6406 CB THR I 442 9.598 -12.356 8.415 0.00 0.00
I
ATOM 6407 HB THR I 442 9.847 -12.353 7.332 0.00 0.00
I
ATOM 6408 OG1 THR I 442 8.308 -12.990 8.750 0.00 0.00
I
ATOM 6409 HG1 THR I 442 8.018 -13.359 7.912 0.00 0.00
I
ATOM 6410 CG2 THR I 442 10.558 -13.404 9.067 0.00 0.00
I
ATOM 6411 HG21 THR I 442 10.263 -14.361 8.587 0.00 0.00
I
ATOM 6412 HG22 THR I 442 11.607 -13.181 8.779 0.00 0.00
I
ATOM 6413 HG23 THR I 442 10.421 -13.340 10.168 0.00 0.00
I
ATOM 6414 C THR I 442 10.915 -10.355 8.430 0.00 0.00
I
ATOM 6415 O THR I 442 12.001 -10.288 8.974 0.00 0.00
I
ATOM 6416 N PHE I 443 10.503 -9.590 7.353 0.00 0.00
I
ATOM 6417 HN PHE I 443 9.566 -9.812 7.095 0.00 0.00
I
ATOM 6418 CA PHE I 443 11.219 -8.577 6.695 0.00 0.00
I
ATOM 6419 HA PHE I 443 12.075 -8.981 6.174 0.00 0.00
I
ATOM 6420 CB PHE I 443 10.234 -8.119 5.447 0.00 0.00
I
ATOM 6421 HB1 PHE I 443 9.971 -8.924 4.728 0.00 0.00
I
ATOM 6422 HB2 PHE I 443 9.307 -7.802 5.971 0.00 0.00
I
ATOM 6423 CG PHE I 443 10.690 -6.933 4.543 0.00 0.00
I
ATOM 6424 CD1 PHE I 443 9.980 -5.725 4.427 0.00 0.00
I
ATOM 6425 HD1 PHE I 443 9.072 -5.640 5.006 0.00 0.00
I

ATOM 6426 CE1 PHE I 443 10.434 -4.702 3.629 0.00 0.00
I
ATOM 6427 HE1 PHE I 443 9.761 -3.858 3.602 0.00 0.00
I
ATOM 6428 CZ PHE I 443 11.664 -4.835 3.028 0.00 0.00
I
ATOM 6429 HZ PHE I 443 12.061 -4.010 2.456 0.00 0.00
I
ATOM 6430 CD2 PHE I 443 11.895 -7.072 3.794 0.00 0.00
I
ATOM 6431 HD2 PHE I 443 12.511 -7.958 3.831 0.00 0.00
I
ATOM 6432 CE2 PHE I 443 12.426 -5.999 3.120 0.00 0.00
I
ATOM 6433 HE2 PHE I 443 13.416 -6.084 2.695 0.00 0.00
I
ATOM 6434 C PHE I 443 11.775 -7.500 7.580 0.00 0.00
I
ATOM 6435 O PHE I 443 13.056 -7.388 7.629 0.00 0.00
I
ATOM 6436 N VAL I 444 11.037 -6.725 8.419 0.00 0.00
I
ATOM 6437 HN VAL I 444 10.054 -6.855 8.522 0.00 0.00
I
ATOM 6438 CA VAL I 444 11.582 -5.744 9.368 0.00 0.00
I
ATOM 6439 HA VAL I 444 12.080 -4.955 8.823 0.00 0.00
I
ATOM 6440 CB VAL I 444 10.420 -4.967 10.029 0.00 0.00
I
ATOM 6441 HB VAL I 444 9.696 -5.643 10.534 0.00 0.00
I
ATOM 6442 CG1 VAL I 444 10.983 -4.047 11.053 0.00 0.00
I
ATOM 6443 HG11 VAL I 444 11.211 -4.639 11.966 0.00 0.00
I
ATOM 6444 HG12 VAL I 444 11.982 -3.708 10.706 0.00 0.00
I
ATOM 6445 HG13 VAL I 444 10.284 -3.256 11.399 0.00 0.00
I
ATOM 6446 CG2 VAL I 444 9.812 -4.231 8.907 0.00 0.00
I
ATOM 6447 HG21 VAL I 444 8.990 -3.638 9.363 0.00 0.00
I
ATOM 6448 HG22 VAL I 444 10.550 -3.538 8.448 0.00 0.00
I
ATOM 6449 HG23 VAL I 444 9.396 -4.950 8.170 0.00 0.00
I
ATOM 6450 C VAL I 444 12.489 -6.317 10.361 0.00 0.00
I
ATOM 6451 O VAL I 444 13.551 -5.807 10.721 0.00 0.00
I
ATOM 6452 N PHE I 445 12.089 -7.575 10.810 0.00 0.00
I

ATOM 6453 HN PHE I 445 11.167 -7.920 10.654 0.00 0.00
I
ATOM 6454 CA PHE I 445 12.755 -8.181 11.898 0.00 0.00
I
ATOM 6455 HA PHE I 445 13.032 -7.403 12.594 0.00 0.00
I
ATOM 6456 CB PHE I 445 11.742 -9.120 12.675 0.00 0.00
I
ATOM 6457 HB1 PHE I 445 10.782 -8.563 12.733 0.00 0.00
I
ATOM 6458 HB2 PHE I 445 11.591 -10.108 12.189 0.00 0.00
I
ATOM 6459 CG PHE I 445 12.083 -9.423 14.089 0.00 0.00
I
ATOM 6460 CD1 PHE I 445 12.260 -10.768 14.538 0.00 0.00
I
ATOM 6461 HD1 PHE I 445 12.375 -11.545 13.797 0.00 0.00
I
ATOM 6462 CE1 PHE I 445 12.409 -11.069 15.889 0.00 0.00
I
ATOM 6463 HE1 PHE I 445 12.706 -12.079 16.130 0.00 0.00
I
ATOM 6464 CZ PHE I 445 12.373 -10.043 16.847 0.00 0.00
I
ATOM 6465 HZ PHE I 445 12.646 -10.362 17.843 0.00 0.00
I
ATOM 6466 CD2 PHE I 445 12.038 -8.392 15.099 0.00 0.00
I
ATOM 6467 HD2 PHE I 445 11.889 -7.377 14.762 0.00 0.00
I
ATOM 6468 CE2 PHE I 445 12.100 -8.701 16.450 0.00 0.00
I
ATOM 6469 HE2 PHE I 445 11.936 -7.981 17.238 0.00 0.00
I
ATOM 6470 C PHE I 445 14.016 -8.878 11.588 0.00 0.00
I
ATOM 6471 O PHE I 445 14.867 -9.172 12.427 0.00 0.00
I
ATOM 6472 N SER I 446 14.206 -9.157 10.289 0.00 0.00
I
ATOM 6473 HN SER I 446 13.447 -8.947 9.677 0.00 0.00
I
ATOM 6474 CA SER I 446 15.488 -9.505 9.738 0.00 0.00
I
ATOM 6475 HA SER I 446 15.913 -10.321 10.303 0.00 0.00
I
ATOM 6476 CB SER I 446 15.496 -9.744 8.122 0.00 0.00
I
ATOM 6477 HB1 SER I 446 15.194 -8.805 7.612 0.00 0.00
I
ATOM 6478 HB2 SER I 446 16.502 -10.064 7.778 0.00 0.00
I
ATOM 6479 OG SER I 446 14.554 -10.784 7.886 0.00 0.00
I

```
ATOM   6480  HG1 SER I 446      13.668 -10.415   7.916  0.00  0.00
I
ATOM   6481  C   SER I 446      16.523  -8.420   9.768  0.00  0.00
I
ATOM   6482  O   SER I 446      17.762  -8.697   9.695  0.00  0.00
I
ATOM   6483  N   PHE I 447      16.136  -7.158  10.035  0.00  0.00
I
ATOM   6484  HN  PHE I 447      15.175  -6.901  10.089  0.00  0.00
I
ATOM   6485  CA  PHE I 447      17.056  -6.099  10.282  0.00  0.00
I
ATOM   6486  HA  PHE I 447      18.041  -6.416   9.973  0.00  0.00
I
ATOM   6487  CB  PHE I 447      16.659  -4.802   9.453  0.00  0.00
I
ATOM   6488  HB1 PHE I 447      15.597  -4.559   9.669  0.00  0.00
I
ATOM   6489  HB2 PHE I 447      17.375  -3.974   9.643  0.00  0.00
I
ATOM   6490  CG  PHE I 447      16.768  -5.230   7.922  0.00  0.00
I
ATOM   6491  CD1 PHE I 447      15.681  -5.353   7.096  0.00  0.00
I
ATOM   6492  HD1 PHE I 447      14.677  -5.337   7.494  0.00  0.00
I
ATOM   6493  CE1 PHE I 447      15.870  -5.564   5.698  0.00  0.00
I
ATOM   6494  HE1 PHE I 447      14.968  -5.653   5.110  0.00  0.00
I
ATOM   6495  CZ  PHE I 447      17.161  -5.729   5.107  0.00  0.00
I
ATOM   6496  HZ  PHE I 447      17.331  -5.897   4.054  0.00  0.00
I
ATOM   6497  CD2 PHE I 447      18.074  -5.347   7.329  0.00  0.00
I
ATOM   6498  HD2 PHE I 447      18.975  -5.242   7.915  0.00  0.00
I
ATOM   6499  CE2 PHE I 447      18.277  -5.579   5.959  0.00  0.00
I
ATOM   6500  HE2 PHE I 447      19.223  -5.952   5.594  0.00  0.00
I
ATOM   6501  C   PHE I 447      17.098  -5.648  11.739  0.00  0.00
I
ATOM   6502  O   PHE I 447      17.685  -4.597  12.031  0.00  0.00
I
ATOM   6503  N   ALA I 448      16.386  -6.337  12.605  0.00  0.00
I
ATOM   6504  HN  ALA I 448      16.008  -7.243  12.432  0.00  0.00
I
ATOM   6505  CA  ALA I 448      16.146  -5.780  13.999  0.00  0.00
I
ATOM   6506  HA  ALA I 448      15.914  -4.739  13.830  0.00  0.00
I
```

ATOM 6507 CB ALA I 448 14.879 -6.323 14.651 0.00 0.00
I
ATOM 6508 HB1 ALA I 448 13.955 -6.017 14.116 0.00 0.00
I
ATOM 6509 HB2 ALA I 448 14.884 -7.421 14.821 0.00 0.00
I
ATOM 6510 HB3 ALA I 448 14.738 -5.936 15.683 0.00 0.00
I
ATOM 6511 C ALA I 448 17.283 -5.820 14.998 0.00 0.00
I
ATOM 6512 O ALA I 448 18.249 -6.612 14.945 0.00 0.00
I
ATOM 6513 N LYS I 449 17.225 -4.881 15.969 0.00 0.00
I
ATOM 6514 HN LYS I 449 16.518 -4.208 15.768 0.00 0.00
I
ATOM 6515 CA LYS I 449 18.031 -4.801 17.132 0.00 0.00
I
ATOM 6516 HA LYS I 449 18.842 -5.511 17.083 0.00 0.00
I
ATOM 6517 CB LYS I 449 18.477 -3.369 17.371 0.00 0.00
I
ATOM 6518 HB1 LYS I 449 17.577 -2.723 17.444 0.00 0.00
I
ATOM 6519 HB2 LYS I 449 18.878 -3.392 18.407 0.00 0.00
I
ATOM 6520 CG LYS I 449 19.544 -2.846 16.382 0.00 0.00
I
ATOM 6521 HG1 LYS I 449 20.506 -3.298 16.705 0.00 0.00
I
ATOM 6522 HG2 LYS I 449 19.316 -3.010 15.307 0.00 0.00
I
ATOM 6523 CD LYS I 449 19.625 -1.340 16.486 0.00 0.00
I
ATOM 6524 HD1 LYS I 449 18.817 -0.742 16.014 0.00 0.00
I
ATOM 6525 HD2 LYS I 449 19.740 -1.006 17.539 0.00 0.00
I
ATOM 6526 CE LYS I 449 20.812 -0.744 15.766 0.00 0.00
I
ATOM 6527 HE1 LYS I 449 21.041 0.300 16.069 0.00 0.00
I
ATOM 6528 HE2 LYS I 449 21.741 -1.288 16.041 0.00 0.00
I
ATOM 6529 NZ LYS I 449 20.615 -0.723 14.313 0.00 0.00
I
ATOM 6530 HZ1 LYS I 449 21.523 -0.558 13.834 0.00 0.00
I
ATOM 6531 HZ2 LYS I 449 20.331 -1.657 13.954 0.00 0.00
I
ATOM 6532 HZ3 LYS I 449 19.870 -0.041 14.064 0.00 0.00
I
ATOM 6533 C LYS I 449 17.254 -5.216 18.406 0.00 0.00
I

ATOM 6534 O LYS I 449 15.999 -4.999 18.515 0.00 0.00
I
ATOM 6535 N THR I 450 17.970 -5.815 19.387 0.00 0.00
I
ATOM 6536 HN THR I 450 18.921 -6.063 19.220 0.00 0.00
I
ATOM 6537 CA THR I 450 17.519 -6.355 20.631 0.00 0.00
I
ATOM 6538 HA THR I 450 16.441 -6.323 20.581 0.00 0.00
I
ATOM 6539 CB THR I 450 18.017 -7.749 20.947 0.00 0.00
I
ATOM 6540 HB THR I 450 19.052 -7.721 21.352 0.00 0.00
I
ATOM 6541 OG1 THR I 450 18.034 -8.577 19.775 0.00 0.00
I
ATOM 6542 HG1 THR I 450 18.485 -9.393 20.002 0.00 0.00
I
ATOM 6543 CG2 THR I 450 17.037 -8.386 21.936 0.00 0.00
I
ATOM 6544 HG21 THR I 450 16.043 -8.206 21.474 0.00 0.00
I
ATOM 6545 HG22 THR I 450 17.234 -9.470 22.073 0.00 0.00
I
ATOM 6546 HG23 THR I 450 17.163 -7.986 22.965 0.00 0.00
I
ATOM 6547 C THR I 450 17.850 -5.504 21.752 0.00 0.00
I
ATOM 6548 O THR I 450 19.016 -5.028 21.897 0.00 0.00
I
ATOM 6549 N GLU I 451 16.846 -5.187 22.558 0.00 0.00
I
ATOM 6550 HN GLU I 451 16.000 -5.698 22.426 0.00 0.00
I
ATOM 6551 CA GLU I 451 16.878 -4.065 23.464 0.00 0.00
I
ATOM 6552 HA GLU I 451 17.828 -3.573 23.315 0.00 0.00
I
ATOM 6553 CB GLU I 451 15.792 -3.086 23.096 0.00 0.00
I
ATOM 6554 HB1 GLU I 451 15.939 -2.933 22.005 0.00 0.00
I
ATOM 6555 HB2 GLU I 451 14.793 -3.553 23.230 0.00 0.00
I
ATOM 6556 CG GLU I 451 15.774 -1.729 23.804 0.00 0.00
I
ATOM 6557 HG1 GLU I 451 14.960 -1.172 23.292 0.00 0.00
I
ATOM 6558 HG2 GLU I 451 15.440 -1.873 24.854 0.00 0.00
I
ATOM 6559 CD GLU I 451 17.091 -0.939 23.844 0.00 0.00
I
ATOM 6560 OE1 GLU I 451 17.166 0.030 23.126 0.00 0.00
I

ATOM 6561 OE2 GLU I 451 18.041 -1.308 24.580 0.00 0.00
I
ATOM 6562 C GLU I 451 16.859 -4.528 24.904 0.00 0.00
I
ATOM 6563 O GLU I 451 16.090 -5.432 25.282 0.00 0.00
I
ATOM 6564 N GLN I 452 17.583 -3.844 25.834 0.00 0.00
I
ATOM 6565 HN GLN I 452 18.016 -3.011 25.498 0.00 0.00
I
ATOM 6566 CA GLN I 452 17.586 -4.099 27.283 0.00 0.00
I
ATOM 6567 HA GLN I 452 16.842 -4.806 27.621 0.00 0.00
I
ATOM 6568 CB GLN I 452 19.012 -4.586 27.738 0.00 0.00
I
ATOM 6569 HB1 GLN I 452 19.361 -5.499 27.210 0.00 0.00
I
ATOM 6570 HB2 GLN I 452 19.776 -3.849 27.409 0.00 0.00
I
ATOM 6571 CG GLN I 452 19.136 -4.772 29.246 0.00 0.00
I
ATOM 6572 HG1 GLN I 452 19.015 -3.808 29.784 0.00 0.00
I
ATOM 6573 HG2 GLN I 452 18.338 -5.454 29.611 0.00 0.00
I
ATOM 6574 CD GLN I 452 20.503 -5.325 29.774 0.00 0.00
I
ATOM 6575 OE1 GLN I 452 21.450 -5.557 28.985 0.00 0.00
I
ATOM 6576 NE2 GLN I 452 20.495 -5.574 31.072 0.00 0.00
I
ATOM 6577 HE21 GLN I 452 21.203 -6.138 31.495 0.00 0.00
I
ATOM 6578 HE22 GLN I 452 19.770 -5.143 31.610 0.00 0.00
I
ATOM 6579 C GLN I 452 17.327 -2.814 27.986 0.00 0.00
I
ATOM 6580 O GLN I 452 16.851 -2.801 29.122 0.00 0.00
I
ATOM 6581 N SER I 453 17.540 -1.642 27.323 0.00 0.00
I
ATOM 6582 HN SER I 453 17.953 -1.575 26.418 0.00 0.00
I
ATOM 6583 CA SER I 453 17.388 -0.368 27.969 0.00 0.00
I
ATOM 6584 HA SER I 453 17.897 -0.540 28.905 0.00 0.00
I
ATOM 6585 CB SER I 453 18.114 0.754 27.246 0.00 0.00
I
ATOM 6586 HB1 SER I 453 19.104 0.369 26.920 0.00 0.00
I
ATOM 6587 HB2 SER I 453 17.554 1.048 26.332 0.00 0.00
I

ATOM	6588	OG	SER	I	453	18.336	1.824	28.165	0.00	0.00
I										
ATOM	6589	HG1	SER	I	453	18.766	1.544	28.976	0.00	0.00
I										
ATOM	6590	C	SER	I	453	15.983	0.027	28.233	0.00	0.00
I										
ATOM	6591	O	SER	I	453	15.126	-0.115	27.333	0.00	0.00
I										
ATOM	6592	N	ASN	I	454	15.676	0.573	29.433	0.00	0.00
I										
ATOM	6593	HN	ASN	I	454	16.485	0.665	30.008	0.00	0.00
I										
ATOM	6594	CA	ASN	I	454	14.410	1.122	29.944	0.00	0.00
I										
ATOM	6595	HA	ASN	I	454	13.638	0.707	29.312	0.00	0.00
I										
ATOM	6596	CB	ASN	I	454	14.128	0.840	31.420	0.00	0.00
I										
ATOM	6597	HB1	ASN	I	454	14.990	0.991	32.104	0.00	0.00
I										
ATOM	6598	HB2	ASN	I	454	13.240	1.424	31.742	0.00	0.00
I										
ATOM	6599	CG	ASN	I	454	13.869	-0.614	31.535	0.00	0.00
I										
ATOM	6600	OD1	ASN	I	454	14.532	-1.244	32.403	0.00	0.00
I										
ATOM	6601	ND2	ASN	I	454	13.016	-1.195	30.665	0.00	0.00
I										
ATOM	6602	HD21	ASN	I	454	12.802	-2.170	30.727	0.00	0.00
I										
ATOM	6603	HD22	ASN	I	454	12.461	-0.598	30.086	0.00	0.00
I										
ATOM	6604	C	ASN	I	454	14.392	2.627	29.766	0.00	0.00
I										
ATOM	6605	O	ASN	I	454	13.356	3.243	30.082	0.00	0.00
I										
ATOM	6606	N	ILE	I	455	15.349	3.327	29.204	0.00	0.00
I										
ATOM	6607	HN	ILE	I	455	16.241	2.929	29.005	0.00	0.00
I										
ATOM	6608	CA	ILE	I	455	15.320	4.772	28.995	0.00	0.00
I										
ATOM	6609	HA	ILE	I	455	15.056	5.152	29.972	0.00	0.00
I										
ATOM	6610	CB	ILE	I	455	16.665	5.420	28.703	0.00	0.00
I										
ATOM	6611	HB	ILE	I	455	16.622	6.529	28.750	0.00	0.00
I										
ATOM	6612	CG2	ILE	I	455	17.655	4.915	29.769	0.00	0.00
I										
ATOM	6613	HG21	ILE	I	455	18.009	3.863	29.731	0.00	0.00
I										
ATOM	6614	HG22	ILE	I	455	18.544	5.577	29.689	0.00	0.00
I										

ATOM   6615 HG23 ILE I 455   17.313   5.083 30.813 0.00 0.00
I
ATOM   6616 CG1 ILE I 455   17.153   5.069 27.247 0.00 0.00
I
ATOM   6617 HG11 ILE I 455   17.555   4.057 27.469 0.00 0.00
I
ATOM   6618 HG12 ILE I 455   16.287   5.018 26.553 0.00 0.00
I
ATOM   6619 CD ILE I 455   18.156   6.047 26.585 0.00 0.00
I
ATOM   6620 HD1 ILE I 455   17.658   7.010 26.343 0.00 0.00
I
ATOM   6621 HD2 ILE I 455   18.995   6.271 27.278 0.00 0.00
I
ATOM   6622 HD3 ILE I 455   18.484   5.566 25.639 0.00 0.00
I
ATOM   6623 C   ILE I 455   14.174   5.210 28.112 0.00 0.00
I
ATOM   6624 O   ILE I 455   13.681   4.525 27.200 0.00 0.00
I
ATOM   6625 N   ARG I 456   13.652   6.391 28.427 0.00 0.00
I
ATOM   6626 HN ARG I 456   13.974   6.845 29.255 0.00 0.00
I
ATOM   6627 CA ARG I 456   12.607   7.007 27.709 0.00 0.00
I
ATOM   6628 HA ARG I 456   12.818   6.864 26.659 0.00 0.00
I
ATOM   6629 CB ARG I 456   11.197   6.518 28.109 0.00 0.00
I
ATOM   6630 HB1 ARG I 456   10.465   6.995 27.423 0.00 0.00
I
ATOM   6631 HB2 ARG I 456   11.128   5.419 27.962 0.00 0.00
I
ATOM   6632 CG ARG I 456   10.777   6.884 29.616 0.00 0.00
I
ATOM   6633 HG1 ARG I 456   11.553   6.514 30.320 0.00 0.00
I
ATOM   6634 HG2 ARG I 456   10.747   7.993 29.674 0.00 0.00
I
ATOM   6635 CD ARG I 456   9.394   6.424 30.082 0.00 0.00
I
ATOM   6636 HD1 ARG I 456   9.329   5.378 30.448 0.00 0.00
I
ATOM   6637 HD2 ARG I 456   9.171   6.966 31.026 0.00 0.00
I
ATOM   6638 NE ARG I 456   8.368   6.786 29.072 0.00 0.00
I
ATOM   6639 HE ARG I 456   8.501   6.277 28.222 0.00 0.00
I
ATOM   6640 CZ ARG I 456   7.733   7.968 28.965 0.00 0.00
I
ATOM   6641 NH1 ARG I 456   7.080   8.195 27.783 0.00 0.00
I

```
ATOM   6642 HH11 ARG I 456       6.324   8.842  27.887  0.00  0.00
I
ATOM   6643 HH12 ARG I 456       7.056   7.412  27.162  0.00  0.00
I
ATOM   6644  NH2 ARG I 456       7.509   8.747  29.976  0.00  0.00
I
ATOM   6645 HH21 ARG I 456       6.762   9.411  29.987  0.00  0.00
I
ATOM   6646 HH22 ARG I 456       7.747   8.303  30.840  0.00  0.00
I
ATOM   6647  C   ARG I 456      12.605   8.542  27.906  0.00  0.00
I
ATOM   6648  O   ARG I 456      12.929   9.063  28.955  0.00  0.00
I
ATOM   6649  N   TYR I 457      12.283   9.271  26.872  0.00  0.00
I
ATOM   6650  HN  TYR I 457      12.083   8.829  26.001  0.00  0.00
I
ATOM   6651  CA  TYR I 457      12.134  10.719  26.955  0.00  0.00
I
ATOM   6652  HA  TYR I 457      12.938  11.080  27.579  0.00  0.00
I
ATOM   6653  CB  TYR I 457      12.327  11.271  25.459  0.00  0.00
I
ATOM   6654  HB1 TYR I 457      11.911  10.505  24.771  0.00  0.00
I
ATOM   6655  HB2 TYR I 457      11.812  12.251  25.369  0.00  0.00
I
ATOM   6656  CG  TYR I 457      13.804  11.373  25.254  0.00  0.00
I
ATOM   6657  CD1 TYR I 457      14.585  12.457  25.660  0.00  0.00
I
ATOM   6658  HD1 TYR I 457      14.168  13.187  26.339  0.00  0.00
I
ATOM   6659  CE1 TYR I 457      15.969  12.511  25.393  0.00  0.00
I
ATOM   6660  HE1 TYR I 457      16.549  13.332  25.786  0.00  0.00
I
ATOM   6661  CZ  TYR I 457      16.603  11.556  24.640  0.00  0.00
I
ATOM   6662  OH  TYR I 457      17.857  11.801  24.129  0.00  0.00
I
ATOM   6663  HH  TYR I 457      17.984  12.747  24.232  0.00  0.00
I
ATOM   6664  CD2 TYR I 457      14.407  10.327  24.517  0.00  0.00
I
ATOM   6665  HD2 TYR I 457      13.809   9.473  24.236  0.00  0.00
I
ATOM   6666  CE2 TYR I 457      15.800  10.482  24.165  0.00  0.00
I
ATOM   6667  HE2 TYR I 457      16.265   9.612  23.727  0.00  0.00
I
ATOM   6668  C   TYR I 457      10.765  11.018  27.597  0.00  0.00
I
```

ATOM 6669 O TYR I 457 9.824 10.225 27.575 0.00 0.00
I
ATOM 6670 N PRO I 458 10.500 12.302 28.007 0.00 0.00
I
ATOM 6671 CD PRO I 458 11.508 13.250 28.384 0.00 0.00
I
ATOM 6672 HD1 PRO I 458 12.061 12.847 29.259 0.00 0.00
I
ATOM 6673 HD2 PRO I 458 12.291 13.374 27.606 0.00 0.00
I
ATOM 6674 CA PRO I 458 9.206 12.744 28.562 0.00 0.00
I
ATOM 6675 HA PRO I 458 8.938 12.036 29.333 0.00 0.00
I
ATOM 6676 CB PRO I 458 9.425 14.113 29.149 0.00 0.00
I
ATOM 6677 HB1 PRO I 458 9.596 14.062 30.245 0.00 0.00
I
ATOM 6678 HB2 PRO I 458 8.556 14.799 29.047 0.00 0.00
I
ATOM 6679 CG PRO I 458 10.773 14.569 28.580 0.00 0.00
I
ATOM 6680 HG1 PRO I 458 11.374 15.327 29.127 0.00 0.00
I
ATOM 6681 HG2 PRO I 458 10.418 14.960 27.603 0.00 0.00
I
ATOM 6682 C PRO I 458 8.141 12.684 27.479 0.00 0.00
I
ATOM 6683 O PRO I 458 8.351 12.894 26.271 0.00 0.00
I
ATOM 6684 N ASP I 459 6.927 12.245 27.866 0.00 0.00
I
ATOM 6685 HN ASP I 459 6.676 11.905 28.769 0.00 0.00
I
ATOM 6686 CA ASP I 459 5.690 12.244 27.027 0.00 0.00
I
ATOM 6687 HA ASP I 459 5.835 11.586 26.183 0.00 0.00
I
ATOM 6688 CB ASP I 459 4.533 11.840 27.938 0.00 0.00
I
ATOM 6689 HB1 ASP I 459 4.386 12.540 28.788 0.00 0.00
I
ATOM 6690 HB2 ASP I 459 3.549 11.605 27.478 0.00 0.00
I
ATOM 6691 CG ASP I 459 4.926 10.522 28.573 0.00 0.00
I
ATOM 6692 OD1 ASP I 459 5.469 10.611 29.722 0.00 0.00
I
ATOM 6693 OD2 ASP I 459 4.713 9.468 27.928 0.00 0.00
I
ATOM 6694 C ASP I 459 5.269 13.536 26.380 0.00 0.00
I
ATOM 6695 O ASP I 459 5.293 14.604 26.960 0.00 0.00
I

ATOM 6696 N GLY I 460 4.774 13.396 25.102 0.00 0.00
I
ATOM 6697 HN GLY I 460 4.991 12.526 24.666 0.00 0.00
I
ATOM 6698 CA GLY I 460 4.425 14.614 24.379 0.00 0.00
I
ATOM 6699 HA1 GLY I 460 5.313 14.996 23.898 0.00 0.00
I
ATOM 6700 HA2 GLY I 460 3.871 15.245 25.058 0.00 0.00
I
ATOM 6701 C GLY I 460 3.537 14.286 23.243 0.00 0.00
I
ATOM 6702 O GLY I 460 3.029 13.169 23.210 0.00 0.00
I
ATOM 6703 N GLU I 461 3.141 15.275 22.397 0.00 0.00
I
ATOM 6704 HN GLU I 461 3.623 16.146 22.355 0.00 0.00
I
ATOM 6705 CA GLU I 461 1.893 15.106 21.604 0.00 0.00
I
ATOM 6706 HA GLU I 461 1.687 14.067 21.395 0.00 0.00
I
ATOM 6707 CB GLU I 461 0.783 15.755 22.447 0.00 0.00
I
ATOM 6708 HB1 GLU I 461 0.822 15.304 23.461 0.00 0.00
I
ATOM 6709 HB2 GLU I 461 0.952 16.834 22.647 0.00 0.00
I
ATOM 6710 CG GLU I 461 -0.740 15.490 21.976 0.00 0.00
I
ATOM 6711 HG1 GLU I 461 -0.864 16.031 21.014 0.00 0.00
I
ATOM 6712 HG2 GLU I 461 -1.064 14.429 21.908 0.00 0.00
I
ATOM 6713 CD GLU I 461 -1.793 16.230 22.879 0.00 0.00
I
ATOM 6714 OE1 GLU I 461 -1.782 16.191 24.131 0.00 0.00
I
ATOM 6715 OE2 GLU I 461 -2.766 16.751 22.291 0.00 0.00
I
ATOM 6716 C GLU I 461 2.066 16.005 20.359 0.00 0.00
I
ATOM 6717 O GLU I 461 2.643 17.102 20.374 0.00 0.00
I
ATOM 6718 N VAL I 462 1.511 15.436 19.227 0.00 0.00
I
ATOM 6719 HN VAL I 462 1.000 14.580 19.203 0.00 0.00
I
ATOM 6720 CA VAL I 462 1.364 16.164 17.971 0.00 0.00
I
ATOM 6721 HA VAL I 462 1.595 17.199 18.176 0.00 0.00
I
ATOM 6722 CB VAL I 462 2.279 15.735 16.768 0.00 0.00
I

ATOM 6723 HB VAL I 462 2.064 16.380 15.890 0.00 0.00
I
ATOM 6724 CG1 VAL I 462 3.791 15.968 17.178 0.00 0.00
I
ATOM 6725 HG11 VAL I 462 3.889 17.072 17.265 0.00 0.00
I
ATOM 6726 HG12 VAL I 462 3.963 15.509 18.175 0.00 0.00
I
ATOM 6727 HG13 VAL I 462 4.535 15.564 16.460 0.00 0.00
I
ATOM 6728 CG2 VAL I 462 1.920 14.333 16.277 0.00 0.00
I
ATOM 6729 HG21 VAL I 462 0.955 14.467 15.744 0.00 0.00
I
ATOM 6730 HG22 VAL I 462 2.696 13.872 15.629 0.00 0.00
I
ATOM 6731 HG23 VAL I 462 1.762 13.589 17.087 0.00 0.00
I
ATOM 6732 C VAL I 462 −0.044 15.999 17.499 0.00 0.00
I
ATOM 6733 O VAL I 462 −0.728 15.188 18.155 0.00 0.00
I
ATOM 6734 N PHE I 463 −0.469 16.625 16.431 0.00 0.00
I
ATOM 6735 HN PHE I 463 0.058 17.349 15.993 0.00 0.00
I
ATOM 6736 CA PHE I 463 −1.821 16.477 15.874 0.00 0.00
I
ATOM 6737 HA PHE I 463 −2.554 16.234 16.629 0.00 0.00
I
ATOM 6738 CB PHE I 463 −2.318 17.800 15.212 0.00 0.00
I
ATOM 6739 HB1 PHE I 463 −2.165 18.681 15.871 0.00 0.00
I
ATOM 6740 HB2 PHE I 463 −1.818 17.984 14.237 0.00 0.00
I
ATOM 6741 CG PHE I 463 −3.854 17.741 15.078 0.00 0.00
I
ATOM 6742 CD1 PHE I 463 −4.377 17.049 13.942 0.00 0.00
I
ATOM 6743 HD1 PHE I 463 −3.706 16.683 13.180 0.00 0.00
I
ATOM 6744 CE1 PHE I 463 −5.765 17.056 13.729 0.00 0.00
I
ATOM 6745 HE1 PHE I 463 −6.084 16.613 12.797 0.00 0.00
I
ATOM 6746 CZ PHE I 463 −6.629 17.558 14.684 0.00 0.00
I
ATOM 6747 HZ PHE I 463 −7.680 17.467 14.456 0.00 0.00
I
ATOM 6748 CD2 PHE I 463 −4.657 18.131 16.121 0.00 0.00
I
ATOM 6749 HD2 PHE I 463 −4.201 18.322 17.081 0.00 0.00
I

ATOM 6750 CE2 PHE I 463 -6.069 18.059 15.903 0.00 0.00
I
ATOM 6751 HE2 PHE I 463 -6.721 18.479 16.655 0.00 0.00
I
ATOM 6752 C PHE I 463 -1.857 15.369 14.800 0.00 0.00
I
ATOM 6753 O PHE I 463 -0.913 15.420 13.980 0.00 0.00
I
ATOM 6754 N THR I 464 -2.792 14.410 14.703 0.00 0.00
I
ATOM 6755 HN THR I 464 -3.475 14.247 15.411 0.00 0.00
I
ATOM 6756 CA THR I 464 -2.853 13.356 13.702 0.00 0.00
I
ATOM 6757 HA THR I 464 -2.359 13.662 12.791 0.00 0.00
I
ATOM 6758 CB THR I 464 -2.161 11.971 13.993 0.00 0.00
I
ATOM 6759 HB THR I 464 -2.345 11.320 13.112 0.00 0.00
I
ATOM 6760 OG1 THR I 464 -2.642 11.465 15.250 0.00 0.00
I
ATOM 6761 HG1 THR I 464 -2.298 10.576 15.362 0.00 0.00
I
ATOM 6762 CG2 THR I 464 -0.658 11.995 14.218 0.00 0.00
I
ATOM 6763 HG21 THR I 464 -0.118 12.523 13.403 0.00 0.00
I
ATOM 6764 HG22 THR I 464 -0.369 12.555 15.133 0.00 0.00
I
ATOM 6765 HG23 THR I 464 -0.225 10.974 14.274 0.00 0.00
I
ATOM 6766 C THR I 464 -4.299 13.192 13.464 0.00 0.00
I
ATOM 6767 O THR I 464 -5.110 13.491 14.320 0.00 0.00
I
ATOM 6768 N GLY I 465 -4.755 12.860 12.273 0.00 0.00
I
ATOM 6769 HN GLY I 465 -4.152 12.552 11.542 0.00 0.00
I
ATOM 6770 CA GLY I 465 -6.169 12.762 12.019 0.00 0.00
I
ATOM 6771 HA1 GLY I 465 -6.652 13.636 12.432 0.00 0.00
I
ATOM 6772 HA2 GLY I 465 -6.599 11.807 12.283 0.00 0.00
I
ATOM 6773 C GLY I 465 -6.425 12.975 10.587 0.00 0.00
I
ATOM 6774 OT1 GLY I 465 -5.480 13.214 9.843 0.00 0.00
I
ATOM 6775 OT2 GLY I 465 -7.615 12.990 10.163 0.00 0.00
I
ATOM 6776 N THR J 35 -24.417 21.398 17.993 0.00 0.00
J

ATOM 6777 HT1 THR J 35 −24.455 20.399 17.707 0.00 0.00
J
ATOM 6778 HT2 THR J 35 −24.879 22.095 17.375 0.00 0.00
J
ATOM 6779 HT3 THR J 35 −24.829 21.576 18.932 0.00 0.00
J
ATOM 6780 CA THR J 35 −22.957 21.748 18.132 0.00 0.00
J
ATOM 6781 HA THR J 35 −22.895 22.337 19.035 0.00 0.00
J
ATOM 6782 CB THR J 35 −22.216 20.499 18.526 0.00 0.00
J
ATOM 6783 HB THR J 35 −21.186 20.762 18.848 0.00 0.00
J
ATOM 6784 OG1 THR J 35 −22.056 19.580 17.459 0.00 0.00
J
ATOM 6785 HG1 THR J 35 −21.315 19.048 17.758 0.00 0.00
J
ATOM 6786 CG2 THR J 35 −22.786 19.761 19.741 0.00 0.00
J
ATOM 6787 HG21 THR J 35 −22.976 20.419 20.615 0.00 0.00
J
ATOM 6788 HG22 THR J 35 −23.748 19.226 19.586 0.00 0.00
J
ATOM 6789 HG23 THR J 35 −22.088 18.954 20.051 0.00 0.00
J
ATOM 6790 C THR J 35 −22.308 22.460 16.900 0.00 0.00
J
ATOM 6791 O THR J 35 −22.758 22.203 15.790 0.00 0.00
J
ATOM 6792 N PRO J 36 −21.172 23.127 17.006 0.00 0.00
J
ATOM 6793 CD PRO J 36 −20.888 23.756 18.323 0.00 0.00
J
ATOM 6794 HD1 PRO J 36 −21.773 23.997 18.949 0.00 0.00
J
ATOM 6795 HD2 PRO J 36 −20.083 23.265 18.911 0.00 0.00
J
ATOM 6796 CA PRO J 36 −20.490 23.879 15.883 0.00 0.00
J
ATOM 6797 HA PRO J 36 −21.214 24.497 15.372 0.00 0.00
J
ATOM 6798 CB PRO J 36 −19.408 24.764 16.598 0.00 0.00
J
ATOM 6799 HB1 PRO J 36 −19.139 25.689 16.045 0.00 0.00
J
ATOM 6800 HB2 PRO J 36 −18.434 24.254 16.755 0.00 0.00
J
ATOM 6801 CG PRO J 36 −20.182 25.077 17.928 0.00 0.00
J
ATOM 6802 HG1 PRO J 36 −20.936 25.859 17.694 0.00 0.00
J
ATOM 6803 HG2 PRO J 36 −19.450 25.507 18.644 0.00 0.00
J

ATOM	6804	C	PRO	J	36	−19.876	23.013	14.762	0.00	0.00	J
ATOM	6805	O	PRO	J	36	−19.853	23.568	13.658	0.00	0.00	J
ATOM	6806	N	PRO	J	37	−19.380	21.701	14.940	0.00	0.00	J
ATOM	6807	CD	PRO	J	37	−18.920	21.173	16.233	0.00	0.00	J
ATOM	6808	HD1	PRO	J	37	−18.507	22.006	16.841	0.00	0.00	J
ATOM	6809	HD2	PRO	J	37	−19.862	20.752	16.645	0.00	0.00	J
ATOM	6810	CA	PRO	J	37	−19.121	20.786	13.850	0.00	0.00	J
ATOM	6811	HA	PRO	J	37	−18.420	21.324	13.229	0.00	0.00	J
ATOM	6812	CB	PRO	J	37	−18.617	19.517	14.535	0.00	0.00	J
ATOM	6813	HB1	PRO	J	37	−18.012	18.961	13.787	0.00	0.00	J
ATOM	6814	HB2	PRO	J	37	−19.529	18.884	14.581	0.00	0.00	J
ATOM	6815	CG	PRO	J	37	−18.013	19.973	15.825	0.00	0.00	J
ATOM	6816	HG1	PRO	J	37	−17.051	20.421	15.494	0.00	0.00	J
ATOM	6817	HG2	PRO	J	37	−17.896	19.125	16.533	0.00	0.00	J
ATOM	6818	C	PRO	J	37	−20.363	20.646	12.955	0.00	0.00	J
ATOM	6819	O	PRO	J	37	−20.150	20.357	11.788	0.00	0.00	J
ATOM	6820	N	GLN	J	38	−21.604	20.730	13.455	0.00	0.00	J
ATOM	6821	HN	GLN	J	38	−21.883	20.891	14.398	0.00	0.00	J
ATOM	6822	CA	GLN	J	38	−22.807	20.631	12.644	0.00	0.00	J
ATOM	6823	HA	GLN	J	38	−22.650	19.828	11.940	0.00	0.00	J
ATOM	6824	CB	GLN	J	38	−24.022	20.329	13.532	0.00	0.00	J
ATOM	6825	HB1	GLN	J	38	−24.300	21.100	14.281	0.00	0.00	J
ATOM	6826	HB2	GLN	J	38	−25.029	20.230	13.073	0.00	0.00	J
ATOM	6827	CG	GLN	J	38	−23.837	19.016	14.328	0.00	0.00	J
ATOM	6828	HG1	GLN	J	38	−23.707	18.197	13.589	0.00	0.00	J
ATOM	6829	HG2	GLN	J	38	−22.885	19.087	14.898	0.00	0.00	J
ATOM	6830	CD	GLN	J	38	−24.969	18.675	15.288	0.00	0.00	J

ATOM 6831 OE1 GLN J 38 -25.181 19.451 16.249 0.00 0.00
J
ATOM 6832 NE2 GLN J 38 -25.498 17.381 15.181 0.00 0.00
J
ATOM 6833 HE21 GLN J 38 -26.084 16.967 15.879 0.00 0.00
J
ATOM 6834 HE22 GLN J 38 -25.103 16.822 14.452 0.00 0.00
J
ATOM 6835 C GLN J 38 -23.145 21.879 11.876 0.00 0.00
J
ATOM 6836 O GLN J 38 -23.586 21.784 10.725 0.00 0.00
J
ATOM 6837 N VAL J 39 -22.790 23.050 12.402 0.00 0.00
J
ATOM 6838 HN VAL J 39 -22.404 23.133 13.317 0.00 0.00
J
ATOM 6839 CA VAL J 39 -22.902 24.281 11.721 0.00 0.00
J
ATOM 6840 HA VAL J 39 -23.855 24.432 11.235 0.00 0.00
J
ATOM 6841 CB VAL J 39 -22.455 25.483 12.608 0.00 0.00
J
ATOM 6842 HB VAL J 39 -21.508 25.157 13.089 0.00 0.00
J
ATOM 6843 CG1 VAL J 39 -22.277 26.867 11.888 0.00 0.00
J
ATOM 6844 HG11 VAL J 39 -21.311 26.844 11.341 0.00 0.00
J
ATOM 6845 HG12 VAL J 39 -23.099 27.066 11.168 0.00 0.00
J
ATOM 6846 HG13 VAL J 39 -22.257 27.673 12.652 0.00 0.00
J
ATOM 6847 CG2 VAL J 39 -23.468 25.730 13.662 0.00 0.00
J
ATOM 6848 HG21 VAL J 39 -23.723 24.795 14.205 0.00 0.00
J
ATOM 6849 HG22 VAL J 39 -23.159 26.519 14.381 0.00 0.00
J
ATOM 6850 HG23 VAL J 39 -24.442 26.006 13.204 0.00 0.00
J
ATOM 6851 C VAL J 39 -21.916 24.290 10.509 0.00 0.00
J
ATOM 6852 O VAL J 39 -22.198 24.705 9.385 0.00 0.00
J
ATOM 6853 N LEU J 40 -20.693 23.764 10.701 0.00 0.00
J
ATOM 6854 HN LEU J 40 -20.287 23.679 11.608 0.00 0.00
J
ATOM 6855 CA LEU J 40 -19.809 23.605 9.584 0.00 0.00
J
ATOM 6856 HA LEU J 40 -19.639 24.510 9.020 0.00 0.00
J
ATOM 6857 CB LEU J 40 -18.424 23.156 10.215 0.00 0.00
J

ATOM 6858 HB1 LEU J 40 −18.199 23.848 11.054 0.00 0.00
J
ATOM 6859 HB2 LEU J 40 −18.563 22.156 10.680 0.00 0.00
J
ATOM 6860 CG LEU J 40 −17.228 23.014 9.289 0.00 0.00
J
ATOM 6861 HG LEU J 40 −17.535 22.545 8.330 0.00 0.00
J
ATOM 6862 CD1 LEU J 40 −16.580 24.397 8.953 0.00 0.00
J
ATOM 6863 HD11 LEU J 40 −16.521 24.977 9.899 0.00 0.00
J
ATOM 6864 HD12 LEU J 40 −15.545 24.261 8.574 0.00 0.00
J
ATOM 6865 HD13 LEU J 40 −17.230 25.092 8.379 0.00 0.00
J
ATOM 6866 CD2 LEU J 40 −16.338 21.996 10.031 0.00 0.00
J
ATOM 6867 HD21 LEU J 40 −15.870 22.451 10.931 0.00 0.00
J
ATOM 6868 HD22 LEU J 40 −17.049 21.188 10.305 0.00 0.00
J
ATOM 6869 HD23 LEU J 40 −15.483 21.656 9.408 0.00 0.00
J
ATOM 6870 C LEU J 40 −20.429 22.680 8.504 0.00 0.00
J
ATOM 6871 O LEU J 40 −20.420 23.161 7.351 0.00 0.00
J
ATOM 6872 N ALA J 41 −21.038 21.510 8.806 0.00 0.00
J
ATOM 6873 HN ALA J 41 −21.027 21.120 9.723 0.00 0.00
J
ATOM 6874 CA ALA J 41 −21.590 20.594 7.839 0.00 0.00
J
ATOM 6875 HA ALA J 41 −20.756 20.369 7.191 0.00 0.00
J
ATOM 6876 CB ALA J 41 −22.008 19.304 8.498 0.00 0.00
J
ATOM 6877 HB1 ALA J 41 −22.871 19.388 9.191 0.00 0.00
J
ATOM 6878 HB2 ALA J 41 −22.283 18.495 7.787 0.00 0.00
J
ATOM 6879 HB3 ALA J 41 −21.237 18.818 9.133 0.00 0.00
J
ATOM 6880 C ALA J 41 −22.728 21.094 6.959 0.00 0.00
J
ATOM 6881 O ALA J 41 −22.803 20.801 5.799 0.00 0.00
J
ATOM 6882 N ILE J 42 −23.657 21.758 7.590 0.00 0.00
J
ATOM 6883 HN ILE J 42 −23.475 21.909 8.558 0.00 0.00
J
ATOM 6884 CA ILE J 42 −24.850 22.365 7.026 0.00 0.00
J

ATOM 6885 HA ILE J 42 −25.297 21.686 6.315 0.00 0.00
J
ATOM 6886 CB ILE J 42 −25.940 22.571 7.986 0.00 0.00
J
ATOM 6887 HB ILE J 42 −25.980 21.716 8.695 0.00 0.00
J
ATOM 6888 CG2 ILE J 42 −25.720 23.782 8.891 0.00 0.00
J
ATOM 6889 HG21 ILE J 42 −24.665 23.831 9.235 0.00 0.00
J
ATOM 6890 HG22 ILE J 42 −26.083 24.669 8.329 0.00 0.00
J
ATOM 6891 HG23 ILE J 42 −26.411 23.736 9.760 0.00 0.00
J
ATOM 6892 CG1 ILE J 42 −27.328 22.571 7.268 0.00 0.00
J
ATOM 6893 HG11 ILE J 42 −27.470 23.474 6.637 0.00 0.00
J
ATOM 6894 HG12 ILE J 42 −27.359 21.605 6.720 0.00 0.00
J
ATOM 6895 CD ILE J 42 −28.543 22.465 8.217 0.00 0.00
J
ATOM 6896 HD1 ILE J 42 −29.421 22.639 7.558 0.00 0.00
J
ATOM 6897 HD2 ILE J 42 −28.550 21.412 8.570 0.00 0.00
J
ATOM 6898 HD3 ILE J 42 −28.588 23.168 9.076 0.00 0.00
J
ATOM 6899 C ILE J 42 −24.472 23.519 6.177 0.00 0.00
J
ATOM 6900 O ILE J 42 −24.944 23.663 5.076 0.00 0.00
J
ATOM 6901 N GLY J 43 −23.508 24.372 6.662 0.00 0.00
J
ATOM 6902 HN GLY J 43 −22.937 24.128 7.442 0.00 0.00
J
ATOM 6903 CA GLY J 43 −22.974 25.560 6.036 0.00 0.00
J
ATOM 6904 HA1 GLY J 43 −22.262 25.965 6.740 0.00 0.00
J
ATOM 6905 HA2 GLY J 43 −23.809 26.218 5.845 0.00 0.00
J
ATOM 6906 C GLY J 43 −22.235 25.219 4.785 0.00 0.00
J
ATOM 6907 O GLY J 43 −22.433 25.781 3.718 0.00 0.00
J
ATOM 6908 N PHE J 44 −21.379 24.222 4.886 0.00 0.00
J
ATOM 6909 HN PHE J 44 −21.294 23.723 5.745 0.00 0.00
J
ATOM 6910 CA PHE J 44 −20.679 23.687 3.764 0.00 0.00
J
ATOM 6911 HA PHE J 44 −20.219 24.571 3.347 0.00 0.00
J

ATOM	6912	CB	PHE	J	44	−19.738	22.497	4.177	0.00	0.00	J
ATOM	6913	HB1	PHE	J	44	−19.377	22.620	5.220	0.00	0.00	J
ATOM	6914	HB2	PHE	J	44	−20.205	21.490	4.229	0.00	0.00	J
ATOM	6915	CG	PHE	J	44	−18.532	22.317	3.225	0.00	0.00	J
ATOM	6916	CD1	PHE	J	44	−17.352	22.995	3.567	0.00	0.00	J
ATOM	6917	HD1	PHE	J	44	−17.248	23.691	4.385	0.00	0.00	J
ATOM	6918	CE1	PHE	J	44	−16.263	22.888	2.728	0.00	0.00	J
ATOM	6919	HE1	PHE	J	44	−15.353	23.437	2.924	0.00	0.00	J
ATOM	6920	CZ	PHE	J	44	−16.315	21.995	1.619	0.00	0.00	J
ATOM	6921	HZ	PHE	J	44	−15.387	21.864	1.083	0.00	0.00	J
ATOM	6922	CD2	PHE	J	44	−18.617	21.534	2.023	0.00	0.00	J
ATOM	6923	HD2	PHE	J	44	−19.546	21.077	1.713	0.00	0.00	J
ATOM	6924	CE2	PHE	J	44	−17.475	21.389	1.251	0.00	0.00	J
ATOM	6925	HE2	PHE	J	44	−17.489	20.694	0.424	0.00	0.00	J
ATOM	6926	C	PHE	J	44	−21.578	23.141	2.611	0.00	0.00	J
ATOM	6927	O	PHE	J	44	−21.487	23.539	1.444	0.00	0.00	J
ATOM	6928	N	PHE	J	45	−22.673	22.330	2.972	0.00	0.00	J
ATOM	6929	HN	PHE	J	45	−22.760	22.153	3.950	0.00	0.00	J
ATOM	6930	CA	PHE	J	45	−23.703	21.873	2.059	0.00	0.00	J
ATOM	6931	HA	PHE	J	45	−23.310	21.407	1.168	0.00	0.00	J
ATOM	6932	CB	PHE	J	45	−24.547	20.834	2.811	0.00	0.00	J
ATOM	6933	HB1	PHE	J	45	−24.016	19.932	3.183	0.00	0.00	J
ATOM	6934	HB2	PHE	J	45	−24.927	21.312	3.739	0.00	0.00	J
ATOM	6935	CG	PHE	J	45	−25.632	20.263	1.933	0.00	0.00	J
ATOM	6936	CD1	PHE	J	45	−25.323	19.581	0.719	0.00	0.00	J
ATOM	6937	HD1	PHE	J	45	−24.316	19.318	0.427	0.00	0.00	J
ATOM	6938	CE1	PHE	J	45	−26.400	19.170	−0.098	0.00	0.00	J

ATOM	6939	HE1	PHE	J	45	−26.298	18.588	−1.003	0.00	0.00	J
ATOM	6940	CZ	PHE	J	45	−27.709	19.472	0.258	0.00	0.00	J
ATOM	6941	HZ	PHE	J	45	−28.487	19.145	−0.415	0.00	0.00	J
ATOM	6942	CD2	PHE	J	45	−27.037	20.429	2.310	0.00	0.00	J
ATOM	6943	HD2	PHE	J	45	−27.219	21.047	3.176	0.00	0.00	J
ATOM	6944	CE2	PHE	J	45	−28.017	20.032	1.407	0.00	0.00	J
ATOM	6945	HE2	PHE	J	45	−29.064	20.144	1.648	0.00	0.00	J
ATOM	6946	C	PHE	J	45	−24.495	23.093	1.494	0.00	0.00	J
ATOM	6947	O	PHE	J	45	−24.573	23.280	0.258	0.00	0.00	J
ATOM	6948	N	LEU	J	46	−24.962	24.083	2.376	0.00	0.00	J
ATOM	6949	HN	LEU	J	46	−24.696	23.982	3.331	0.00	0.00	J
ATOM	6950	CA	LEU	J	46	−25.711	25.314	1.912	0.00	0.00	J
ATOM	6951	HA	LEU	J	46	−26.609	24.998	1.401	0.00	0.00	J
ATOM	6952	CB	LEU	J	46	−26.063	26.234	3.080	0.00	0.00	J
ATOM	6953	HB1	LEU	J	46	−25.135	26.480	3.640	0.00	0.00	J
ATOM	6954	HB2	LEU	J	46	−26.341	27.255	2.742	0.00	0.00	J
ATOM	6955	CG	LEU	J	46	−27.105	25.785	4.070	0.00	0.00	J
ATOM	6956	HG	LEU	J	46	−26.930	24.688	4.063	0.00	0.00	J
ATOM	6957	CD1	LEU	J	46	−26.907	26.338	5.518	0.00	0.00	J
ATOM	6958	HD11	LEU	J	46	−25.876	26.480	5.908	0.00	0.00	J
ATOM	6959	HD12	LEU	J	46	−27.338	27.360	5.471	0.00	0.00	J
ATOM	6960	HD13	LEU	J	46	−27.539	25.682	6.154	0.00	0.00	J
ATOM	6961	CD2	LEU	J	46	−28.483	26.289	3.562	0.00	0.00	J
ATOM	6962	HD21	LEU	J	46	−29.313	25.979	4.232	0.00	0.00	J
ATOM	6963	HD22	LEU	J	46	−28.483	27.399	3.515	0.00	0.00	J
ATOM	6964	HD23	LEU	J	46	−28.728	26.029	2.510	0.00	0.00	J
ATOM	6965	C	LEU	J	46	−24.853	26.080	0.924	0.00	0.00	J

ATOM 6966 O LEU J 46 -25.378 26.322 -0.144 0.00 0.00
J
ATOM 6967 N THR J 47 -23.545 26.356 1.192 0.00 0.00
J
ATOM 6968 HN THR J 47 -23.182 26.058 2.072 0.00 0.00
J
ATOM 6969 CA THR J 47 -22.627 27.012 0.183 0.00 0.00
J
ATOM 6970 HA THR J 47 -23.027 27.999 0.002 0.00 0.00
J
ATOM 6971 CB THR J 47 -21.204 27.157 0.755 0.00 0.00
J
ATOM 6972 HB THR J 47 -20.789 26.157 1.005 0.00 0.00
J
ATOM 6973 OG1 THR J 47 -21.247 27.845 1.994 0.00 0.00
J
ATOM 6974 HG1 THR J 47 -21.414 27.178 2.665 0.00 0.00
J
ATOM 6975 CG2 THR J 47 -20.364 27.909 -0.238 0.00 0.00
J
ATOM 6976 HG21 THR J 47 -20.238 27.239 -1.115 0.00 0.00
J
ATOM 6977 HG22 THR J 47 -20.858 28.855 -0.546 0.00 0.00
J
ATOM 6978 HG23 THR J 47 -19.367 28.162 0.182 0.00 0.00
J
ATOM 6979 C THR J 47 -22.569 26.298 -1.162 0.00 0.00
J
ATOM 6980 O THR J 47 -22.608 26.802 -2.274 0.00 0.00
J
ATOM 6981 N ILE J 48 -22.512 24.898 -1.130 0.00 0.00
J
ATOM 6982 HN ILE J 48 -22.420 24.281 -0.351 0.00 0.00
J
ATOM 6983 CA ILE J 48 -22.505 24.194 -2.350 0.00 0.00
J
ATOM 6984 HA ILE J 48 -21.761 24.584 -3.029 0.00 0.00
J
ATOM 6985 CB ILE J 48 -22.114 22.718 -2.243 0.00 0.00
J
ATOM 6986 HB ILE J 48 -22.720 22.223 -1.454 0.00 0.00
J
ATOM 6987 CG2 ILE J 48 -22.365 21.984 -3.585 0.00 0.00
J
ATOM 6988 HG21 ILE J 48 -23.457 22.002 -3.785 0.00 0.00
J
ATOM 6989 HG22 ILE J 48 -21.925 22.491 -4.471 0.00 0.00
J
ATOM 6990 HG23 ILE J 48 -22.030 20.925 -3.545 0.00 0.00
J
ATOM 6991 CG1 ILE J 48 -20.541 22.614 -1.868 0.00 0.00
J
ATOM 6992 HG11 ILE J 48 -20.043 23.027 -2.771 0.00 0.00
J

ATOM 6993 HG12 ILE J 48 −20.264 23.270 −1.015 0.00 0.00
J
ATOM 6994 CD ILE J 48 −20.094 21.216 −1.350 0.00 0.00
J
ATOM 6995 HD1 ILE J 48 −20.649 20.927 −0.432 0.00 0.00
J
ATOM 6996 HD2 ILE J 48 −20.279 20.511 −2.189 0.00 0.00
J
ATOM 6997 HD3 ILE J 48 −18.990 21.094 −1.336 0.00 0.00
J
ATOM 6998 C ILE J 48 −23.895 24.373 −3.121 0.00 0.00
J
ATOM 6999 O ILE J 48 −23.936 24.656 −4.320 0.00 0.00
J
ATOM 7000 N ILE J 49 −25.069 24.098 −2.524 0.00 0.00
J
ATOM 7001 HN ILE J 49 −24.984 23.882 −1.555 0.00 0.00
J
ATOM 7002 CA ILE J 49 −26.298 24.053 −3.211 0.00 0.00
J
ATOM 7003 HA ILE J 49 −26.133 23.649 −4.199 0.00 0.00
J
ATOM 7004 CB ILE J 49 −27.311 23.209 −2.463 0.00 0.00
J
ATOM 7005 HB ILE J 49 −28.282 23.305 −2.994 0.00 0.00
J
ATOM 7006 CG2 ILE J 49 −26.875 21.744 −2.496 0.00 0.00
J
ATOM 7007 HG21 ILE J 49 −27.711 21.177 −2.032 0.00 0.00
J
ATOM 7008 HG22 ILE J 49 −26.688 21.533 −3.571 0.00 0.00
J
ATOM 7009 HG23 ILE J 49 −26.007 21.531 −1.837 0.00 0.00
J
ATOM 7010 CG1 ILE J 49 −27.566 23.657 −1.010 0.00 0.00
J
ATOM 7011 HG11 ILE J 49 −26.944 23.080 −0.294 0.00 0.00
J
ATOM 7012 HG12 ILE J 49 −27.293 24.732 −0.940 0.00 0.00
J
ATOM 7013 CD ILE J 49 −29.099 23.464 −0.629 0.00 0.00
J
ATOM 7014 HD1 ILE J 49 −29.672 24.251 −1.163 0.00 0.00
J
ATOM 7015 HD2 ILE J 49 −29.468 22.501 −1.041 0.00 0.00
J
ATOM 7016 HD3 ILE J 49 −29.354 23.381 0.450 0.00 0.00
J
ATOM 7017 C ILE J 49 −26.863 25.435 −3.632 0.00 0.00
J
ATOM 7018 O ILE J 49 −27.559 25.576 −4.627 0.00 0.00
J
ATOM 7019 N ILE J 50 −26.597 26.475 −2.790 0.00 0.00
J

ATOM 7020 HN ILE J 50 −26.133 26.323 −1.921 0.00 0.00
J
ATOM 7021 CA ILE J 50 −26.880 27.884 −3.087 0.00 0.00
J
ATOM 7022 HA ILE J 50 −27.807 27.825 −3.639 0.00 0.00
J
ATOM 7023 CB ILE J 50 −26.695 28.524 −1.798 0.00 0.00
J
ATOM 7024 HB ILE J 50 −25.755 28.216 −1.294 0.00 0.00
J
ATOM 7025 CG2 ILE J 50 −26.634 30.060 −1.940 0.00 0.00
J
ATOM 7026 HG21 ILE J 50 −27.480 30.605 −2.411 0.00 0.00
J
ATOM 7027 HG22 ILE J 50 −26.705 30.394 −0.882 0.00 0.00
J
ATOM 7028 HG23 ILE J 50 −25.642 30.408 −2.301 0.00 0.00
J
ATOM 7029 CG1 ILE J 50 −27.883 28.136 −0.848 0.00 0.00
J
ATOM 7030 HG11 ILE J 50 −27.834 27.040 −0.671 0.00 0.00
J
ATOM 7031 HG12 ILE J 50 −27.727 28.572 0.161 0.00 0.00
J
ATOM 7032 CD ILE J 50 −29.216 28.502 −1.528 0.00 0.00
J
ATOM 7033 HD1 ILE J 50 −30.010 28.339 −0.769 0.00 0.00
J
ATOM 7034 HD2 ILE J 50 −29.322 29.564 −1.839 0.00 0.00
J
ATOM 7035 HD3 ILE J 50 −29.491 27.848 −2.383 0.00 0.00
J
ATOM 7036 C ILE J 50 −25.994 28.446 −4.139 0.00 0.00
J
ATOM 7037 O ILE J 50 −26.379 29.274 −4.920 0.00 0.00
J
ATOM 7038 N GLY J 51 −24.743 28.026 −4.092 0.00 0.00
J
ATOM 7039 HN GLY J 51 −24.523 27.299 −3.446 0.00 0.00
J
ATOM 7040 CA GLY J 51 −23.705 28.410 −5.056 0.00 0.00
J
ATOM 7041 HA1 GLY J 51 −22.817 27.904 −4.708 0.00 0.00
J
ATOM 7042 HA2 GLY J 51 −23.577 29.481 −5.115 0.00 0.00
J
ATOM 7043 C GLY J 51 −23.983 27.807 −6.440 0.00 0.00
J
ATOM 7044 O GLY J 51 −23.809 28.490 −7.462 0.00 0.00
J
ATOM 7045 N ALA J 52 −24.503 26.562 −6.497 0.00 0.00
J
ATOM 7046 HN ALA J 52 −24.634 25.953 −5.719 0.00 0.00
J

ATOM 7047 CA ALA J 52 −24.948 25.810 −7.698 0.00 0.00
J
ATOM 7048 HA ALA J 52 −24.068 25.772 −8.323 0.00 0.00
J
ATOM 7049 CB ALA J 52 −25.463 24.394 −7.377 0.00 0.00
J
ATOM 7050 HB1 ALA J 52 −26.321 24.670 −6.727 0.00 0.00
J
ATOM 7051 HB2 ALA J 52 −25.884 23.830 −8.237 0.00 0.00
J
ATOM 7052 HB3 ALA J 52 −24.643 23.801 −6.921 0.00 0.00
J
ATOM 7053 C ALA J 52 −25.977 26.543 −8.517 0.00 0.00
J
ATOM 7054 O ALA J 52 −25.938 26.516 −9.739 0.00 0.00
J
ATOM 7055 N VAL J 53 −26.882 27.321 −7.963 0.00 0.00
J
ATOM 7056 HN VAL J 53 −26.902 27.350 −6.966 0.00 0.00
J
ATOM 7057 CA VAL J 53 −27.849 28.037 −8.752 0.00 0.00
J
ATOM 7058 HA VAL J 53 −28.068 27.592 −9.712 0.00 0.00
J
ATOM 7059 CB VAL J 53 −29.177 28.071 −7.997 0.00 0.00
J
ATOM 7060 HB VAL J 53 −29.876 28.718 −8.568 0.00 0.00
J
ATOM 7061 CG1 VAL J 53 −29.930 26.674 −8.126 0.00 0.00
J
ATOM 7062 HG11 VAL J 53 −30.142 26.449 −9.193 0.00 0.00
J
ATOM 7063 HG12 VAL J 53 −29.290 25.882 −7.680 0.00 0.00
J
ATOM 7064 HG13 VAL J 53 −30.893 26.783 −7.583 0.00 0.00
J
ATOM 7065 CG2 VAL J 53 −29.110 28.423 −6.493 0.00 0.00
J
ATOM 7066 HG21 VAL J 53 −30.123 28.497 −6.043 0.00 0.00
J
ATOM 7067 HG22 VAL J 53 −28.599 27.644 −5.889 0.00 0.00
J
ATOM 7068 HG23 VAL J 53 −28.532 29.322 −6.192 0.00 0.00
J
ATOM 7069 C VAL J 53 −27.419 29.488 −9.163 0.00 0.00
J
ATOM 7070 O VAL J 53 −27.785 29.936 −10.256 0.00 0.00
J
ATOM 7071 N LEU J 54 −26.537 30.137 −8.349 0.00 0.00
J
ATOM 7072 HN LEU J 54 −26.204 29.687 −7.524 0.00 0.00
J
ATOM 7073 CA LEU J 54 −25.925 31.392 −8.604 0.00 0.00
J

ATOM 7074 HA LEU J 54 -26.740 32.051 -8.868 0.00 0.00
J
ATOM 7075 CB LEU J 54 -25.151 32.014 -7.495 0.00 0.00
J
ATOM 7076 HB1 LEU J 54 -24.354 31.287 -7.229 0.00 0.00
J
ATOM 7077 HB2 LEU J 54 -24.664 32.986 -7.724 0.00 0.00
J
ATOM 7078 CG LEU J 54 -25.904 32.333 -6.286 0.00 0.00
J
ATOM 7079 HG LEU J 54 -26.374 31.504 -5.715 0.00 0.00
J
ATOM 7080 CD1 LEU J 54 -24.967 32.990 -5.334 0.00 0.00
J
ATOM 7081 HD11 LEU J 54 -24.115 32.311 -5.118 0.00 0.00
J
ATOM 7082 HD12 LEU J 54 -24.618 33.975 -5.712 0.00 0.00
J
ATOM 7083 HD13 LEU J 54 -25.553 33.214 -4.417 0.00 0.00
J
ATOM 7084 CD2 LEU J 54 -27.146 33.208 -6.669 0.00 0.00
J
ATOM 7085 HD21 LEU J 54 -28.035 32.571 -6.864 0.00 0.00
J
ATOM 7086 HD22 LEU J 54 -27.491 33.872 -5.848 0.00 0.00
J
ATOM 7087 HD23 LEU J 54 -26.900 33.816 -7.566 0.00 0.00
J
ATOM 7088 C LEU J 54 -24.992 31.361 -9.912 0.00 0.00
J
ATOM 7089 O LEU J 54 -25.084 32.254 -10.732 0.00 0.00
J
ATOM 7090 N LEU J 55 -24.149 30.328 -9.985 0.00 0.00
J
ATOM 7091 HN LEU J 55 -23.951 29.830 -9.144 0.00 0.00
J
ATOM 7092 CA LEU J 55 -23.407 30.002 -11.136 0.00 0.00
J
ATOM 7093 HA LEU J 55 -22.642 30.739 -11.332 0.00 0.00
J
ATOM 7094 CB LEU J 55 -22.581 28.694 -10.866 0.00 0.00
J
ATOM 7095 HB1 LEU J 55 -23.353 27.929 -10.636 0.00 0.00
J
ATOM 7096 HB2 LEU J 55 -22.133 28.364 -11.827 0.00 0.00
J
ATOM 7097 CG LEU J 55 -21.523 28.790 -9.786 0.00 0.00
J
ATOM 7098 HG LEU J 55 -21.792 29.434 -8.922 0.00 0.00
J
ATOM 7099 CD1 LEU J 55 -21.224 27.391 -9.269 0.00 0.00
J
ATOM 7100 HD11 LEU J 55 -20.574 27.691 -8.419 0.00 0.00
J

```
ATOM   7101 HD12 LEU J  55      -22.061  26.811  -8.825  0.00  0.00
J
ATOM   7102 HD13 LEU J  55      -20.774  26.886 -10.151  0.00  0.00
J
ATOM   7103  CD2 LEU J  55      -20.185  29.410 -10.211  0.00  0.00
J
ATOM   7104 HD21 LEU J  55      -19.578  29.603  -9.301  0.00  0.00
J
ATOM   7105 HD22 LEU J  55      -19.637  28.727 -10.895  0.00  0.00
J
ATOM   7106 HD23 LEU J  55      -20.301  30.336 -10.815  0.00  0.00
J
ATOM   7107  C   LEU J  55      -24.234  29.759 -12.406  0.00  0.00
J
ATOM   7108  O   LEU J  55      -23.963  30.299 -13.447  0.00  0.00
J
ATOM   7109  N   MET J  56      -25.228  28.887 -12.239  0.00  0.00
J
ATOM   7110  HN  MET J  56      -25.532  28.447 -11.397  0.00  0.00
J
ATOM   7111  CA  MET J  56      -26.017  28.443 -13.376  0.00  0.00
J
ATOM   7112  HA  MET J  56      -25.433  28.160 -14.239  0.00  0.00
J
ATOM   7113  CB  MET J  56      -26.862  27.222 -12.925  0.00  0.00
J
ATOM   7114  HB1 MET J  56      -26.186  26.425 -12.547  0.00  0.00
J
ATOM   7115  HB2 MET J  56      -27.415  27.544 -12.018  0.00  0.00
J
ATOM   7116  CG  MET J  56      -27.752  26.661 -14.006  0.00  0.00
J
ATOM   7117  HG1 MET J  56      -28.576  27.366 -14.247  0.00  0.00
J
ATOM   7118  HG2 MET J  56      -27.148  26.600 -14.937  0.00  0.00
J
ATOM   7119  SD  MET J  56      -28.542  25.090 -13.618  0.00  0.00
J
ATOM   7120  CE  MET J  56      -29.018  25.511 -11.892  0.00  0.00
J
ATOM   7121  HE1 MET J  56      -29.250  26.581 -11.701  0.00  0.00
J
ATOM   7122  HE2 MET J  56      -30.036  25.109 -11.704  0.00  0.00
J
ATOM   7123  HE3 MET J  56      -28.313  25.270 -11.068  0.00  0.00
J
ATOM   7124  C   MET J  56      -26.863  29.578 -13.895  0.00  0.00
J
ATOM   7125  O   MET J  56      -27.121  29.579 -15.114  0.00  0.00
J
ATOM   7126  N   LEU J  57      -27.333  30.580 -13.064  0.00  0.00
J
ATOM   7127  HN  LEU J  57      -27.212  30.385 -12.094  0.00  0.00
J
```

ATOM 7128 CA LEU J 57 -27.989 31.868 -13.515 0.00 0.00
J
ATOM 7129 HA LEU J 57 -29.013 31.554 -13.655 0.00 0.00
J
ATOM 7130 CB LEU J 57 -27.813 32.908 -12.354 0.00 0.00
J
ATOM 7131 HB1 LEU J 57 -28.044 32.308 -11.448 0.00 0.00
J
ATOM 7132 HB2 LEU J 57 -26.730 33.151 -12.289 0.00 0.00
J
ATOM 7133 CG LEU J 57 -28.570 34.251 -12.445 0.00 0.00
J
ATOM 7134 HG LEU J 57 -28.470 34.709 -13.452 0.00 0.00
J
ATOM 7135 CD1 LEU J 57 -30.033 33.999 -12.111 0.00 0.00
J
ATOM 7136 HD11 LEU J 57 -30.042 33.411 -11.168 0.00 0.00
J
ATOM 7137 HD12 LEU J 57 -30.649 34.923 -12.083 0.00 0.00
J
ATOM 7138 HD13 LEU J 57 -30.529 33.293 -12.811 0.00 0.00
J
ATOM 7139 CD2 LEU J 57 -27.974 35.337 -11.571 0.00 0.00
J
ATOM 7140 HD21 LEU J 57 -28.594 36.257 -11.517 0.00 0.00
J
ATOM 7141 HD22 LEU J 57 -27.938 34.954 -10.529 0.00 0.00
J
ATOM 7142 HD23 LEU J 57 -26.906 35.589 -11.744 0.00 0.00
J
ATOM 7143 C LEU J 57 -27.611 32.491 -14.850 0.00 0.00
J
ATOM 7144 O LEU J 57 -26.439 32.454 -15.181 0.00 0.00
J
ATOM 7145 N PRO J 58 -28.455 32.944 -15.809 0.00 0.00
J
ATOM 7146 CD PRO J 58 -29.918 33.021 -15.719 0.00 0.00
J
ATOM 7147 HD1 PRO J 58 -30.279 32.213 -15.048 0.00 0.00
J
ATOM 7148 HD2 PRO J 58 -30.125 34.028 -15.298 0.00 0.00
J
ATOM 7149 CA PRO J 58 -28.078 33.290 -17.193 0.00 0.00
J
ATOM 7150 HA PRO J 58 -27.410 32.540 -17.589 0.00 0.00
J
ATOM 7151 CB PRO J 58 -29.386 33.469 -18.007 0.00 0.00
J
ATOM 7152 HB1 PRO J 58 -29.310 32.955 -18.989 0.00 0.00
J
ATOM 7153 HB2 PRO J 58 -29.735 34.506 -18.199 0.00 0.00
J
ATOM 7154 CG PRO J 58 -30.463 32.880 -17.166 0.00 0.00
J

ATOM 7155 HG1 PRO J 58 -30.487 31.793 -17.394 0.00 0.00
J
ATOM 7156 HG2 PRO J 58 -31.481 33.313 -17.270 0.00 0.00
J
ATOM 7157 C PRO J 58 -27.297 34.552 -17.276 0.00 0.00
J
ATOM 7158 O PRO J 58 -26.743 34.765 -18.387 0.00 0.00
J
ATOM 7159 N ILE J 59 -27.153 35.407 -16.177 0.00 0.00
J
ATOM 7160 HN ILE J 59 -27.452 35.171 -15.256 0.00 0.00
J
ATOM 7161 CA ILE J 59 -26.257 36.598 -16.223 0.00 0.00
J
ATOM 7162 HA ILE J 59 -26.146 36.842 -17.269 0.00 0.00
J
ATOM 7163 CB ILE J 59 -26.900 37.925 -15.591 0.00 0.00
J
ATOM 7164 HB ILE J 59 -26.162 38.753 -15.523 0.00 0.00
J
ATOM 7165 CG2 ILE J 59 -28.001 38.459 -16.527 0.00 0.00
J
ATOM 7166 HG21 ILE J 59 -27.733 38.753 -17.564 0.00 0.00
J
ATOM 7167 HG22 ILE J 59 -28.792 37.688 -16.653 0.00 0.00
J
ATOM 7168 HG23 ILE J 59 -28.572 39.327 -16.134 0.00 0.00
J
ATOM 7169 CG1 ILE J 59 -27.456 37.704 -14.154 0.00 0.00
J
ATOM 7170 HG11 ILE J 59 -28.543 37.475 -14.170 0.00 0.00
J
ATOM 7171 HG12 ILE J 59 -26.933 36.837 -13.697 0.00 0.00
J
ATOM 7172 CD ILE J 59 -27.246 38.949 -13.331 0.00 0.00
J
ATOM 7173 HD1 ILE J 59 -26.192 39.296 -13.395 0.00 0.00
J
ATOM 7174 HD2 ILE J 59 -28.011 39.707 -13.604 0.00 0.00
J
ATOM 7175 HD3 ILE J 59 -27.513 38.721 -12.277 0.00 0.00
J
ATOM 7176 C ILE J 59 -24.839 36.366 -15.644 0.00 0.00
J
ATOM 7177 O ILE J 59 -23.979 37.265 -15.737 0.00 0.00
J
ATOM 7178 N SER J 60 -24.559 35.189 -15.051 0.00 0.00
J
ATOM 7179 HN SER J 60 -25.454 34.763 -14.939 0.00 0.00
J
ATOM 7180 CA SER J 60 -23.368 34.563 -14.426 0.00 0.00
J
ATOM 7181 HA SER J 60 -22.656 35.296 -14.078 0.00 0.00
J

ATOM 7182 CB SER J 60 -23.779 33.574 -13.238 0.00 0.00
J
ATOM 7183 HB1 SER J 60 -24.261 32.675 -13.679 0.00 0.00
J
ATOM 7184 HB2 SER J 60 -22.874 33.216 -12.702 0.00 0.00
J
ATOM 7185 OG SER J 60 -24.732 34.311 -12.461 0.00 0.00
J
ATOM 7186 HG1 SER J 60 -24.726 33.798 -11.649 0.00 0.00
J
ATOM 7187 C SER J 60 -22.677 33.807 -15.537 0.00 0.00
J
ATOM 7188 O SER J 60 -21.505 33.568 -15.465 0.00 0.00
J
ATOM 7189 N THR J 61 -23.351 33.576 -16.637 0.00 0.00
J
ATOM 7190 HN THR J 61 -24.311 33.823 -16.743 0.00 0.00
J
ATOM 7191 CA THR J 61 -22.846 32.650 -17.680 0.00 0.00
J
ATOM 7192 HA THR J 61 -21.778 32.558 -17.546 0.00 0.00
J
ATOM 7193 CB THR J 61 -23.354 31.307 -17.511 0.00 0.00
J
ATOM 7194 HB THR J 61 -23.257 30.752 -18.468 0.00 0.00
J
ATOM 7195 OG1 THR J 61 -24.704 31.307 -17.183 0.00 0.00
J
ATOM 7196 HG1 THR J 61 -24.916 31.628 -16.304 0.00 0.00
J
ATOM 7197 CG2 THR J 61 -22.633 30.558 -16.398 0.00 0.00
J
ATOM 7198 HG21 THR J 61 -21.524 30.514 -16.443 0.00 0.00
J
ATOM 7199 HG22 THR J 61 -22.827 30.916 -15.365 0.00 0.00
J
ATOM 7200 HG23 THR J 61 -22.934 29.492 -16.480 0.00 0.00
J
ATOM 7201 C THR J 61 -22.966 33.030 -19.195 0.00 0.00
J
ATOM 7202 O THR J 61 -23.891 33.774 -19.567 0.00 0.00
J
ATOM 7203 N THR J 62 -22.115 32.580 -20.136 0.00 0.00
J
ATOM 7204 HN THR J 62 -21.381 31.972 -19.845 0.00 0.00
J
ATOM 7205 CA THR J 62 -22.086 32.946 -21.556 0.00 0.00
J
ATOM 7206 HA THR J 62 -22.357 33.987 -21.649 0.00 0.00
J
ATOM 7207 CB THR J 62 -20.737 32.863 -22.085 0.00 0.00
J
ATOM 7208 HB THR J 62 -20.787 33.139 -23.160 0.00 0.00
J

ATOM 7209 OG1 THR J 62 −20.243 31.520 −21.788 0.00 0.00
J
ATOM 7210 HG1 THR J 62 −19.653 31.236 −22.490 0.00 0.00
J
ATOM 7211 CG2 THR J 62 −19.848 33.901 −21.308 0.00 0.00
J
ATOM 7212 HG21 THR J 62 −20.026 33.698 −20.231 0.00 0.00
J
ATOM 7213 HG22 THR J 62 −18.752 33.862 −21.487 0.00 0.00
J
ATOM 7214 HG23 THR J 62 −20.174 34.916 −21.620 0.00 0.00
J
ATOM 7215 C THR J 62 −23.033 32.161 −22.478 0.00 0.00
J
ATOM 7216 O THR J 62 −23.326 32.609 −23.596 0.00 0.00
J
ATOM 7217 N LYS J 63 −23.470 30.953 −22.010 0.00 0.00
J
ATOM 7218 HN LYS J 63 −23.308 30.782 −21.041 0.00 0.00
J
ATOM 7219 CA LYS J 63 −24.379 30.060 −22.729 0.00 0.00
J
ATOM 7220 HA LYS J 63 −25.177 30.644 −23.162 0.00 0.00
J
ATOM 7221 CB LYS J 63 −23.645 29.281 −23.848 0.00 0.00
J
ATOM 7222 HB1 LYS J 63 −24.342 28.588 −24.365 0.00 0.00
J
ATOM 7223 HB2 LYS J 63 −23.400 30.114 −24.541 0.00 0.00
J
ATOM 7224 CG LYS J 63 −22.489 28.433 −23.339 0.00 0.00
J
ATOM 7225 HG1 LYS J 63 −21.829 29.146 −22.800 0.00 0.00
J
ATOM 7226 HG2 LYS J 63 −22.921 27.618 −22.719 0.00 0.00
J
ATOM 7227 CD LYS J 63 −21.839 27.692 −24.525 0.00 0.00
J
ATOM 7228 HD1 LYS J 63 −22.580 26.985 −24.957 0.00 0.00
J
ATOM 7229 HD2 LYS J 63 −21.496 28.462 −25.249 0.00 0.00
J
ATOM 7230 CE LYS J 63 −20.558 26.918 −24.045 0.00 0.00
J
ATOM 7231 HE1 LYS J 63 −19.701 27.571 −23.773 0.00 0.00
J
ATOM 7232 HE2 LYS J 63 −20.740 26.360 −23.102 0.00 0.00
J
ATOM 7233 NZ LYS J 63 −20.231 26.129 −25.299 0.00 0.00
J
ATOM 7234 HZ1 LYS J 63 −19.702 25.236 −25.222 0.00 0.00
J
ATOM 7235 HZ2 LYS J 63 −21.099 25.841 −25.793 0.00 0.00
J

ATOM 7236 HZ3 LYS J 63 −19.622 26.762 −25.856 0.00 0.00
J
ATOM 7237 C LYS J 63 −24.924 29.190 −21.654 0.00 0.00
J
ATOM 7238 O LYS J 63 −24.437 29.395 −20.515 0.00 0.00
J
ATOM 7239 N PRO J 64 −25.974 28.306 −21.882 0.00 0.00
J
ATOM 7240 CD PRO J 64 −26.823 28.360 −23.091 0.00 0.00
J
ATOM 7241 HD1 PRO J 64 −27.179 29.369 −23.390 0.00 0.00
J
ATOM 7242 HD2 PRO J 64 −26.442 28.011 −24.075 0.00 0.00
J
ATOM 7243 CA PRO J 64 −26.584 27.450 −20.868 0.00 0.00
J
ATOM 7244 HA PRO J 64 −27.075 28.054 −20.119 0.00 0.00
J
ATOM 7245 CB PRO J 64 −27.533 26.542 −21.586 0.00 0.00
J
ATOM 7246 HB1 PRO J 64 −28.397 26.169 −20.994 0.00 0.00
J
ATOM 7247 HB2 PRO J 64 −27.008 25.706 −22.093 0.00 0.00
J
ATOM 7248 CG PRO J 64 −28.015 27.510 −22.644 0.00 0.00
J
ATOM 7249 HG1 PRO J 64 −28.783 28.249 −22.330 0.00 0.00
J
ATOM 7250 HG2 PRO J 64 −28.574 26.997 −23.456 0.00 0.00
J
ATOM 7251 C PRO J 64 −25.519 26.553 −20.061 0.00 0.00
J
ATOM 7252 O PRO J 64 −24.590 25.947 −20.681 0.00 0.00
J
ATOM 7253 N LEU J 65 −25.602 26.399 −18.738 0.00 0.00
J
ATOM 7254 HN LEU J 65 −26.286 26.931 −18.244 0.00 0.00
J
ATOM 7255 CA LEU J 65 −24.803 25.531 −17.928 0.00 0.00
J
ATOM 7256 HA LEU J 65 −24.197 24.953 −18.610 0.00 0.00
J
ATOM 7257 CB LEU J 65 −23.948 26.434 −16.977 0.00 0.00
J
ATOM 7258 HB1 LEU J 65 −23.307 26.995 −17.689 0.00 0.00
J
ATOM 7259 HB2 LEU J 65 −24.644 27.179 −16.534 0.00 0.00
J
ATOM 7260 CG LEU J 65 −23.039 25.639 −15.999 0.00 0.00
J
ATOM 7261 HG LEU J 65 −23.682 24.863 −15.532 0.00 0.00
J
ATOM 7262 CD1 LEU J 65 −21.894 24.779 −16.722 0.00 0.00
J

```
ATOM   7263 HD11 LEU J  65      -21.293  25.461 -17.361  0.00  0.00
J
ATOM   7264 HD12 LEU J  65      -21.167  24.384 -15.980  0.00  0.00
J
ATOM   7265 HD13 LEU J  65      -22.168  23.909 -17.356  0.00  0.00
J
ATOM   7266  CD2 LEU J  65      -22.461  26.497 -14.820  0.00  0.00
J
ATOM   7267 HD21 LEU J  65      -21.891  27.356 -15.235  0.00  0.00
J
ATOM   7268 HD22 LEU J  65      -23.227  26.803 -14.075  0.00  0.00
J
ATOM   7269 HD23 LEU J  65      -21.775  25.899 -14.183  0.00  0.00
J
ATOM   7270  C   LEU J  65      -25.667  24.523 -17.229  0.00  0.00
J
ATOM   7271  O   LEU J  65      -26.615  24.843 -16.506  0.00  0.00
J
ATOM   7272  N   SER J  66      -25.301  23.247 -17.309  0.00  0.00
J
ATOM   7273  HN  SER J  66      -24.507  23.006 -17.862  0.00  0.00
J
ATOM   7274  CA  SER J  66      -25.961  22.201 -16.587  0.00  0.00
J
ATOM   7275  HA  SER J  66      -26.999  22.232 -16.884  0.00  0.00
J
ATOM   7276  CB  SER J  66      -25.472  20.848 -17.190  0.00  0.00
J
ATOM   7277  HB1 SER J  66      -25.765  20.751 -18.257  0.00  0.00
J
ATOM   7278  HB2 SER J  66      -24.368  20.847 -17.064  0.00  0.00
J
ATOM   7279  OG  SER J  66      -25.938  19.732 -16.441  0.00  0.00
J
ATOM   7280  HG1 SER J  66      -26.364  19.191 -17.109  0.00  0.00
J
ATOM   7281  C   SER J  66      -26.002  22.288 -15.034  0.00  0.00
J
ATOM   7282  O   SER J  66      -25.096  22.772 -14.402  0.00  0.00
J
ATOM   7283  N   TRP J  67      -27.097  21.824 -14.444  0.00  0.00
J
ATOM   7284  HN  TRP J  67      -27.866  21.407 -14.921  0.00  0.00
J
ATOM   7285  CA  TRP J  67      -27.223  21.638 -12.980  0.00  0.00
J
ATOM   7286  HA  TRP J  67      -27.026  22.657 -12.682  0.00  0.00
J
ATOM   7287  CB  TRP J  67      -28.563  21.104 -12.543  0.00  0.00
J
ATOM   7288  HB1 TRP J  67      -29.409  21.553 -13.106  0.00  0.00
J
ATOM   7289  HB2 TRP J  67      -28.742  20.008 -12.532  0.00  0.00
J
```

ATOM 7290 CG TRP J 67 -29.013 21.766 -11.160 0.00 0.00
J
ATOM 7291 CD1 TRP J 67 -28.288 22.519 -10.300 0.00 0.00
J
ATOM 7292 HD1 TRP J 67 -27.239 22.688 -10.494 0.00 0.00
J
ATOM 7293 NE1 TRP J 67 -29.098 23.104 -9.356 0.00 0.00
J
ATOM 7294 HE1 TRP J 67 -28.743 23.542 -8.559 0.00 0.00
J
ATOM 7295 CE2 TRP J 67 -30.324 22.460 -9.411 0.00 0.00
J
ATOM 7296 CD2 TRP J 67 -30.288 21.593 -10.497 0.00 0.00
J
ATOM 7297 CE3 TRP J 67 -31.347 20.719 -10.822 0.00 0.00
J
ATOM 7298 HE3 TRP J 67 -31.305 19.919 -11.547 0.00 0.00
J
ATOM 7299 CZ3 TRP J 67 -32.566 20.890 -10.060 0.00 0.00
J
ATOM 7300 HZ3 TRP J 67 -33.433 20.282 -10.272 0.00 0.00
J
ATOM 7301 CZ2 TRP J 67 -31.494 22.565 -8.685 0.00 0.00
J
ATOM 7302 HZ2 TRP J 67 -31.711 23.305 -7.929 0.00 0.00
J
ATOM 7303 CH2 TRP J 67 -32.653 21.854 -9.077 0.00 0.00
J
ATOM 7304 HH2 TRP J 67 -33.599 22.012 -8.580 0.00 0.00
J
ATOM 7305 C TRP J 67 -26.160 20.740 -12.410 0.00 0.00
J
ATOM 7306 O TRP J 67 -25.505 21.076 -11.433 0.00 0.00
J
ATOM 7307 N ILE J 68 -25.754 19.603 -13.029 0.00 0.00
J
ATOM 7308 HN ILE J 68 -26.081 19.269 -13.910 0.00 0.00
J
ATOM 7309 CA ILE J 68 -24.589 18.813 -12.403 0.00 0.00
J
ATOM 7310 HA ILE J 68 -24.733 18.813 -11.332 0.00 0.00
J
ATOM 7311 CB ILE J 68 -24.699 17.370 -12.994 0.00 0.00
J
ATOM 7312 HB ILE J 68 -25.772 17.108 -12.881 0.00 0.00
J
ATOM 7313 CG2 ILE J 68 -24.442 17.338 -14.472 0.00 0.00
J
ATOM 7314 HG21 ILE J 68 -24.361 16.265 -14.747 0.00 0.00
J
ATOM 7315 HG22 ILE J 68 -25.284 17.751 -15.067 0.00 0.00
J
ATOM 7316 HG23 ILE J 68 -23.543 17.928 -14.752 0.00 0.00
J

ATOM	7317	CG1	ILE	J	68	−23.852	16.342	−12.272	0.00	0.00	J
ATOM	7318	HG11	ILE	J	68	−23.964	15.424	−12.887	0.00	0.00	J
ATOM	7319	HG12	ILE	J	68	−22.799	16.689	−12.197	0.00	0.00	J
ATOM	7320	CD	ILE	J	68	−24.300	16.161	−10.814	0.00	0.00	J
ATOM	7321	HD1	ILE	J	68	−25.334	15.785	−10.663	0.00	0.00	J
ATOM	7322	HD2	ILE	J	68	−23.662	15.418	−10.290	0.00	0.00	J
ATOM	7323	HD3	ILE	J	68	−24.212	17.163	−10.342	0.00	0.00	J
ATOM	7324	C	ILE	J	68	−23.242	19.475	−12.600	0.00	0.00	J
ATOM	7325	O	ILE	J	68	−22.354	19.410	−11.722	0.00	0.00	J
ATOM	7326	N	ASP	J	69	−22.997	20.188	−13.686	0.00	0.00	J
ATOM	7327	HN	ASP	J	69	−23.710	20.132	−14.380	0.00	0.00	J
ATOM	7328	CA	ASP	J	69	−21.810	20.992	−13.908	0.00	0.00	J
ATOM	7329	HA	ASP	J	69	−20.969	20.366	−13.650	0.00	0.00	J
ATOM	7330	CB	ASP	J	69	−21.696	21.404	−15.357	0.00	0.00	J
ATOM	7331	HB1	ASP	J	69	−22.615	21.963	−15.634	0.00	0.00	J
ATOM	7332	HB2	ASP	J	69	−20.831	22.043	−15.636	0.00	0.00	J
ATOM	7333	CG	ASP	J	69	−21.557	20.114	−16.240	0.00	0.00	J
ATOM	7334	OD1	ASP	J	69	−21.933	20.175	−17.463	0.00	0.00	J
ATOM	7335	OD2	ASP	J	69	−21.127	19.066	−15.761	0.00	0.00	J
ATOM	7336	C	ASP	J	69	−21.613	22.181	−12.958	0.00	0.00	J
ATOM	7337	O	ASP	J	69	−20.570	22.266	−12.340	0.00	0.00	J
ATOM	7338	N	ALA	J	70	−22.770	22.962	−12.746	0.00	0.00	J
ATOM	7339	HN	ALA	J	70	−23.564	22.767	−13.316	0.00	0.00	J
ATOM	7340	CA	ALA	J	70	−22.996	24.007	−11.843	0.00	0.00	J
ATOM	7341	HA	ALA	J	70	−22.213	24.711	−12.083	0.00	0.00	J
ATOM	7342	CB	ALA	J	70	−24.361	24.648	−11.973	0.00	0.00	J
ATOM	7343	HB1	ALA	J	70	−24.492	24.906	−13.046	0.00	0.00	J

ATOM 7344 HB2 ALA J 70 −25.115 23.873 −11.720 0.00 0.00
J
ATOM 7345 HB3 ALA J 70 −24.465 25.621 −11.446 0.00 0.00
J
ATOM 7346 C ALA J 70 −22.665 23.580 −10.430 0.00 0.00
J
ATOM 7347 O ALA J 70 −21.864 24.184 −9.757 0.00 0.00
J
ATOM 7348 N LEU J 71 −23.214 22.429 −9.981 0.00 0.00
J
ATOM 7349 HN LEU J 71 −23.853 21.954 −10.582 0.00 0.00
J
ATOM 7350 CA LEU J 71 −23.021 21.737 −8.675 0.00 0.00
J
ATOM 7351 HA LEU J 71 −23.312 22.479 −7.946 0.00 0.00
J
ATOM 7352 CB LEU J 71 −23.976 20.527 −8.525 0.00 0.00
J
ATOM 7353 HB1 LEU J 71 −24.998 20.893 −8.760 0.00 0.00
J
ATOM 7354 HB2 LEU J 71 −23.701 19.757 −9.277 0.00 0.00
J
ATOM 7355 CG LEU J 71 −24.077 19.995 −7.080 0.00 0.00
J
ATOM 7356 HG LEU J 71 −23.045 19.810 −6.713 0.00 0.00
J
ATOM 7357 CD1 LEU J 71 −24.865 20.933 −6.211 0.00 0.00
J
ATOM 7358 HD11 LEU J 71 −24.349 21.911 −6.104 0.00 0.00
J
ATOM 7359 HD12 LEU J 71 −25.793 20.953 −6.821 0.00 0.00
J
ATOM 7360 HD13 LEU J 71 −25.054 20.521 −5.196 0.00 0.00
J
ATOM 7361 CD2 LEU J 71 −24.700 18.567 −7.088 0.00 0.00
J
ATOM 7362 HD21 LEU J 71 −23.950 17.848 −7.482 0.00 0.00
J
ATOM 7363 HD22 LEU J 71 −25.021 18.289 −6.062 0.00 0.00
J
ATOM 7364 HD23 LEU J 71 −25.650 18.528 −7.662 0.00 0.00
J
ATOM 7365 C LEU J 71 −21.614 21.387 −8.466 0.00 0.00
J
ATOM 7366 O LEU J 71 −21.089 21.595 −7.404 0.00 0.00
J
ATOM 7367 N PHE J 72 −21.055 20.603 −9.420 0.00 0.00
J
ATOM 7368 HN PHE J 72 −21.661 20.228 −10.116 0.00 0.00
J
ATOM 7369 CA PHE J 72 −19.727 20.071 −9.406 0.00 0.00
J
ATOM 7370 HA PHE J 72 −19.603 19.414 −8.558 0.00 0.00
J

ATOM 7371 CB PHE J 72 -19.456 19.128 -10.597 0.00 0.00
J
ATOM 7372 HB1 PHE J 72 -20.169 18.290 -10.442 0.00 0.00
J
ATOM 7373 HB2 PHE J 72 -19.659 19.694 -11.531 0.00 0.00
J
ATOM 7374 CG PHE J 72 -17.998 18.629 -10.604 0.00 0.00
J
ATOM 7375 CD1 PHE J 72 -16.995 19.508 -11.067 0.00 0.00
J
ATOM 7376 HD1 PHE J 72 -17.201 20.536 -11.323 0.00 0.00
J
ATOM 7377 CE1 PHE J 72 -15.696 19.122 -11.114 0.00 0.00
J
ATOM 7378 HE1 PHE J 72 -14.999 19.918 -11.328 0.00 0.00
J
ATOM 7379 CZ PHE J 72 -15.315 17.762 -10.958 0.00 0.00
J
ATOM 7380 HZ PHE J 72 -14.266 17.541 -11.097 0.00 0.00
J
ATOM 7381 CD2 PHE J 72 -17.644 17.271 -10.303 0.00 0.00
J
ATOM 7382 HD2 PHE J 72 -18.351 16.536 -9.949 0.00 0.00
J
ATOM 7383 CE2 PHE J 72 -16.316 16.874 -10.521 0.00 0.00
J
ATOM 7384 HE2 PHE J 72 -15.981 15.852 -10.415 0.00 0.00
J
ATOM 7385 C PHE J 72 -18.720 21.232 -9.201 0.00 0.00
J
ATOM 7386 O PHE J 72 -17.776 21.105 -8.405 0.00 0.00
J
ATOM 7387 N THR J 73 -18.956 22.320 -9.966 0.00 0.00
J
ATOM 7388 HN THR J 73 -19.720 22.309 -10.607 0.00 0.00
J
ATOM 7389 CA THR J 73 -18.241 23.568 -9.864 0.00 0.00
J
ATOM 7390 HA THR J 73 -17.212 23.279 -10.018 0.00 0.00
J
ATOM 7391 CB THR J 73 -18.631 24.640 -10.910 0.00 0.00
J
ATOM 7392 HB THR J 73 -19.650 25.060 -10.766 0.00 0.00
J
ATOM 7393 OG1 THR J 73 -18.541 24.036 -12.211 0.00 0.00
J
ATOM 7394 HG1 THR J 73 -19.260 23.415 -12.347 0.00 0.00
J
ATOM 7395 CG2 THR J 73 -17.697 25.868 -10.814 0.00 0.00
J
ATOM 7396 HG21 THR J 73 -16.629 25.617 -10.642 0.00 0.00
J
ATOM 7397 HG22 THR J 73 -17.675 26.484 -11.738 0.00 0.00
J

ATOM 7398 HG23 THR J 73 −17.893 26.467 −9.899 0.00 0.00
J
ATOM 7399 C THR J 73 −18.263 24.268 −8.531 0.00 0.00
J
ATOM 7400 O THR J 73 −17.229 24.726 −7.994 0.00 0.00
J
ATOM 7401 N ALA J 74 −19.462 24.385 −7.895 0.00 0.00
J
ATOM 7402 HN ALA J 74 −20.325 24.116 −8.316 0.00 0.00
J
ATOM 7403 CA ALA J 74 −19.502 24.873 −6.458 0.00 0.00
J
ATOM 7404 HA ALA J 74 −18.917 25.781 −6.484 0.00 0.00
J
ATOM 7405 CB ALA J 74 −20.951 25.309 −6.082 0.00 0.00
J
ATOM 7406 HB1 ALA J 74 −21.414 24.419 −5.606 0.00 0.00
J
ATOM 7407 HB2 ALA J 74 −20.997 26.281 −5.546 0.00 0.00
J
ATOM 7408 HB3 ALA J 74 −21.548 25.463 −7.006 0.00 0.00
J
ATOM 7409 C ALA J 74 −18.731 23.941 −5.451 0.00 0.00
J
ATOM 7410 O ALA J 74 −17.907 24.411 −4.689 0.00 0.00
J
ATOM 7411 N ALA J 75 −18.927 22.565 −5.565 0.00 0.00
J
ATOM 7412 HN ALA J 75 −19.706 22.358 −6.152 0.00 0.00
J
ATOM 7413 CA ALA J 75 −18.226 21.521 −4.787 0.00 0.00
J
ATOM 7414 HA ALA J 75 −18.542 21.702 −3.770 0.00 0.00
J
ATOM 7415 CB ALA J 75 −18.799 20.111 −5.159 0.00 0.00
J
ATOM 7416 HB1 ALA J 75 −18.276 19.349 −4.543 0.00 0.00
J
ATOM 7417 HB2 ALA J 75 −19.860 20.089 −4.827 0.00 0.00
J
ATOM 7418 HB3 ALA J 75 −18.616 19.875 −6.229 0.00 0.00
J
ATOM 7419 C ALA J 75 −16.771 21.621 −4.950 0.00 0.00
J
ATOM 7420 O ALA J 75 −16.101 21.512 −3.919 0.00 0.00
J
ATOM 7421 N SER J 76 −16.275 21.779 −6.176 0.00 0.00
J
ATOM 7422 HN SER J 76 −16.861 21.940 −6.966 0.00 0.00
J
ATOM 7423 CA SER J 76 −14.848 22.022 −6.434 0.00 0.00
J
ATOM 7424 HA SER J 76 −14.267 21.191 −6.061 0.00 0.00
J

ATOM 7425 CB SER J 76 −14.441 22.138 −7.961 0.00 0.00
J
ATOM 7426 HB1 SER J 76 −14.868 21.232 −8.441 0.00 0.00
J
ATOM 7427 HB2 SER J 76 −14.796 23.061 −8.469 0.00 0.00
J
ATOM 7428 OG SER J 76 −12.995 22.025 −8.031 0.00 0.00
J
ATOM 7429 HG1 SER J 76 −12.846 21.831 −8.959 0.00 0.00
J
ATOM 7430 C SER J 76 −14.320 23.319 −5.761 0.00 0.00
J
ATOM 7431 O SER J 76 −13.293 23.368 −5.132 0.00 0.00
J
ATOM 7432 N ALA J 77 −14.959 24.497 −6.000 0.00 0.00
J
ATOM 7433 HN ALA J 77 −15.645 24.549 −6.721 0.00 0.00
J
ATOM 7434 CA ALA J 77 −14.542 25.751 −5.367 0.00 0.00
J
ATOM 7435 HA ALA J 77 −13.519 25.889 −5.684 0.00 0.00
J
ATOM 7436 CB ALA J 77 −15.332 26.971 −5.943 0.00 0.00
J
ATOM 7437 HB1 ALA J 77 −16.425 26.779 −5.886 0.00 0.00
J
ATOM 7438 HB2 ALA J 77 −14.983 27.921 −5.484 0.00 0.00
J
ATOM 7439 HB3 ALA J 77 −15.114 27.013 −7.032 0.00 0.00
J
ATOM 7440 C ALA J 77 −14.634 25.780 −3.871 0.00 0.00
J
ATOM 7441 O ALA J 77 −13.705 26.260 −3.261 0.00 0.00
J
ATOM 7442 N THR J 78 −15.673 25.288 −3.243 0.00 0.00
J
ATOM 7443 HN THR J 78 −16.460 24.941 −3.748 0.00 0.00
J
ATOM 7444 CA THR J 78 −15.822 25.294 −1.808 0.00 0.00
J
ATOM 7445 HA THR J 78 −15.622 26.288 −1.434 0.00 0.00
J
ATOM 7446 CB THR J 78 −17.255 24.908 −1.423 0.00 0.00
J
ATOM 7447 HB THR J 78 −17.404 23.817 −1.573 0.00 0.00
J
ATOM 7448 OG1 THR J 78 −18.152 25.675 −2.242 0.00 0.00
J
ATOM 7449 HG1 THR J 78 −18.130 25.373 −3.153 0.00 0.00
J
ATOM 7450 CG2 THR J 78 −17.582 25.203 0.007 0.00 0.00
J
ATOM 7451 HG21 THR J 78 −18.585 24.808 0.277 0.00 0.00
J

ATOM 7452 HG22 THR J 78 −16.840 24.656 0.628 0.00 0.00
J
ATOM 7453 HG23 THR J 78 −17.664 26.287 0.235 0.00 0.00
J
ATOM 7454 C THR J 78 −14.863 24.303 −1.076 0.00 0.00
J
ATOM 7455 O THR J 78 −14.571 24.458 0.089 0.00 0.00
J
ATOM 7456 N THR J 79 −14.412 23.298 −1.851 0.00 0.00
J
ATOM 7457 HN THR J 79 −14.463 23.355 −2.845 0.00 0.00
J
ATOM 7458 CA THR J 79 −13.362 22.458 −1.303 0.00 0.00
J
ATOM 7459 HA THR J 79 −13.516 22.513 −0.236 0.00 0.00
J
ATOM 7460 CB THR J 79 −13.408 20.993 −1.748 0.00 0.00
J
ATOM 7461 HB THR J 79 −12.485 20.408 −1.548 0.00 0.00
J
ATOM 7462 OG1 THR J 79 −13.462 20.898 −3.144 0.00 0.00
J
ATOM 7463 HG1 THR J 79 −14.403 20.981 −3.317 0.00 0.00
J
ATOM 7464 CG2 THR J 79 −14.639 20.358 −1.176 0.00 0.00
J
ATOM 7465 HG21 THR J 79 −14.730 19.296 −1.493 0.00 0.00
J
ATOM 7466 HG22 THR J 79 −14.543 20.443 −0.073 0.00 0.00
J
ATOM 7467 HG23 THR J 79 −15.520 21.007 −1.370 0.00 0.00
J
ATOM 7468 C THR J 79 −11.951 22.948 −1.674 0.00 0.00
J
ATOM 7469 O THR J 79 −11.037 22.277 −1.323 0.00 0.00
J
ATOM 7470 N VAL J 80 −11.798 24.062 −2.413 0.00 0.00
J
ATOM 7471 HN VAL J 80 −12.646 24.581 −2.489 0.00 0.00
J
ATOM 7472 CA VAL J 80 −10.620 24.534 −3.106 0.00 0.00
J
ATOM 7473 HA VAL J 80 −11.209 25.048 −3.852 0.00 0.00
J
ATOM 7474 CB VAL J 80 −9.733 25.550 −2.252 0.00 0.00
J
ATOM 7475 HB VAL J 80 −9.039 25.978 −3.007 0.00 0.00
J
ATOM 7476 CG1 VAL J 80 −10.642 26.718 −1.793 0.00 0.00
J
ATOM 7477 HG11 VAL J 80 −11.117 27.187 −2.682 0.00 0.00
J
ATOM 7478 HG12 VAL J 80 −11.474 26.448 −1.108 0.00 0.00
J

ATOM 7479 HG13 VAL J 80 -9.983 27.464 -1.299 0.00 0.00
J
ATOM 7480 CG2 VAL J 80 -9.089 24.892 -1.000 0.00 0.00
J
ATOM 7481 HG21 VAL J 80 -8.552 23.926 -1.113 0.00 0.00
J
ATOM 7482 HG22 VAL J 80 -8.343 25.615 -0.607 0.00 0.00
J
ATOM 7483 HG23 VAL J 80 -9.753 24.703 -0.130 0.00 0.00
J
ATOM 7484 C VAL J 80 -9.857 23.510 -3.896 0.00 0.00
J
ATOM 7485 O VAL J 80 -8.608 23.483 -3.837 0.00 0.00
J
ATOM 7486 N THR J 81 -10.514 22.558 -4.638 0.00 0.00
J
ATOM 7487 HN THR J 81 -11.510 22.604 -4.648 0.00 0.00
J
ATOM 7488 CA THR J 81 -9.875 21.389 -5.247 0.00 0.00
J
ATOM 7489 HA THR J 81 -9.104 21.001 -4.598 0.00 0.00
J
ATOM 7490 CB THR J 81 -10.760 20.177 -5.459 0.00 0.00
J
ATOM 7491 HB THR J 81 -11.629 20.350 -6.128 0.00 0.00
J
ATOM 7492 OG1 THR J 81 -11.158 19.673 -4.199 0.00 0.00
J
ATOM 7493 HG1 THR J 81 -11.815 20.225 -3.768 0.00 0.00
J
ATOM 7494 CG2 THR J 81 -10.035 18.978 -6.140 0.00 0.00
J
ATOM 7495 HG21 THR J 81 -9.284 19.346 -6.871 0.00 0.00
J
ATOM 7496 HG22 THR J 81 -9.612 18.258 -5.407 0.00 0.00
J
ATOM 7497 HG23 THR J 81 -10.653 18.299 -6.766 0.00 0.00
J
ATOM 7498 C THR J 81 -9.196 21.739 -6.551 0.00 0.00
J
ATOM 7499 O THR J 81 -8.078 21.354 -6.718 0.00 0.00
J
ATOM 7500 N GLY J 82 -9.883 22.363 -7.538 0.00 0.00
J
ATOM 7501 HN GLY J 82 -10.774 22.582 -7.148 0.00 0.00
J
ATOM 7502 CA GLY J 82 -9.293 22.885 -8.732 0.00 0.00
J
ATOM 7503 HA1 GLY J 82 -8.231 22.800 -8.550 0.00 0.00
J
ATOM 7504 HA2 GLY J 82 -9.676 23.849 -9.030 0.00 0.00
J
ATOM 7505 C GLY J 82 -9.585 22.005 -9.959 0.00 0.00
J

ATOM 7506 O GLY J 82 -9.287 22.289 -11.106 0.00 0.00
J
ATOM 7507 N LEU J 83 -10.325 20.906 -9.696 0.00 0.00
J
ATOM 7508 HN LEU J 83 -10.453 20.774 -8.716 0.00 0.00
J
ATOM 7509 CA LEU J 83 -10.965 20.102 -10.654 0.00 0.00
J
ATOM 7510 HA LEU J 83 -10.227 19.855 -11.403 0.00 0.00
J
ATOM 7511 CB LEU J 83 -11.663 18.868 -10.041 0.00 0.00
J
ATOM 7512 HB1 LEU J 83 -12.203 19.321 -9.182 0.00 0.00
J
ATOM 7513 HB2 LEU J 83 -12.306 18.358 -10.790 0.00 0.00
J
ATOM 7514 CG LEU J 83 -10.610 17.811 -9.620 0.00 0.00
J
ATOM 7515 HG LEU J 83 -9.912 18.268 -8.886 0.00 0.00
J
ATOM 7516 CD1 LEU J 83 -11.383 16.650 -8.887 0.00 0.00
J
ATOM 7517 HD11 LEU J 83 -12.191 16.256 -9.540 0.00 0.00
J
ATOM 7518 HD12 LEU J 83 -10.603 15.866 -8.781 0.00 0.00
J
ATOM 7519 HD13 LEU J 83 -11.835 16.932 -7.912 0.00 0.00
J
ATOM 7520 CD2 LEU J 83 -9.740 17.245 -10.814 0.00 0.00
J
ATOM 7521 HD21 LEU J 83 -9.248 18.100 -11.325 0.00 0.00
J
ATOM 7522 HD22 LEU J 83 -8.981 16.655 -10.256 0.00 0.00
J
ATOM 7523 HD23 LEU J 83 -10.482 16.687 -11.425 0.00 0.00
J
ATOM 7524 C LEU J 83 -12.076 20.940 -11.384 0.00 0.00
J
ATOM 7525 O LEU J 83 -12.985 21.571 -10.749 0.00 0.00
J
ATOM 7526 N ALA J 84 -12.154 20.771 -12.765 0.00 0.00
J
ATOM 7527 HN ALA J 84 -11.551 20.111 -13.206 0.00 0.00
J
ATOM 7528 CA ALA J 84 -12.877 21.711 -13.550 0.00 0.00
J
ATOM 7529 HA ALA J 84 -13.716 22.112 -13.001 0.00 0.00
J
ATOM 7530 CB ALA J 84 -11.927 22.854 -14.013 0.00 0.00
J
ATOM 7531 HB1 ALA J 84 -12.423 23.758 -14.426 0.00 0.00
J
ATOM 7532 HB2 ALA J 84 -11.454 23.194 -13.067 0.00 0.00
J

ATOM 7533 HB3 ALA J 84 −11.159 22.538 −14.751 0.00 0.00
J
ATOM 7534 C ALA J 84 −13.557 20.961 −14.677 0.00 0.00
J
ATOM 7535 O ALA J 84 −12.942 20.268 −15.491 0.00 0.00
J
ATOM 7536 N VAL J 85 −14.952 21.018 −14.768 0.00 0.00
J
ATOM 7537 HN VAL J 85 −15.381 21.641 −14.119 0.00 0.00
J
ATOM 7538 CA VAL J 85 −15.735 20.411 −15.814 0.00 0.00
J
ATOM 7539 HA VAL J 85 −15.074 19.759 −16.366 0.00 0.00
J
ATOM 7540 CB VAL J 85 −16.860 19.571 −15.227 0.00 0.00
J
ATOM 7541 HB VAL J 85 −17.453 19.180 −16.081 0.00 0.00
J
ATOM 7542 CG1 VAL J 85 −16.234 18.450 −14.465 0.00 0.00
J
ATOM 7543 HG11 VAL J 85 −15.645 18.910 −13.643 0.00 0.00
J
ATOM 7544 HG12 VAL J 85 −17.082 17.844 −14.078 0.00 0.00
J
ATOM 7545 HG13 VAL J 85 −15.559 17.823 −15.086 0.00 0.00
J
ATOM 7546 CG2 VAL J 85 −17.904 20.339 −14.402 0.00 0.00
J
ATOM 7547 HG21 VAL J 85 −18.773 19.693 −14.152 0.00 0.00
J
ATOM 7548 HG22 VAL J 85 −17.460 20.772 −13.480 0.00 0.00
J
ATOM 7549 HG23 VAL J 85 −18.280 21.207 −14.984 0.00 0.00
J
ATOM 7550 C VAL J 85 −16.266 21.378 −16.852 0.00 0.00
J
ATOM 7551 O VAL J 85 −17.068 20.965 −17.779 0.00 0.00
J
ATOM 7552 N VAL J 86 −15.797 22.652 −16.754 0.00 0.00
J
ATOM 7553 HN VAL J 86 −15.119 22.790 −16.036 0.00 0.00
J
ATOM 7554 CA VAL J 86 −16.159 23.660 −17.647 0.00 0.00
J
ATOM 7555 HA VAL J 86 −16.197 23.212 −18.629 0.00 0.00
J
ATOM 7556 CB VAL J 86 −17.464 24.544 −17.380 0.00 0.00
J
ATOM 7557 HB VAL J 86 −17.364 25.485 −17.962 0.00 0.00
J
ATOM 7558 CG1 VAL J 86 −18.730 23.844 −18.015 0.00 0.00
J
ATOM 7559 HG11 VAL J 86 −19.182 23.060 −17.370 0.00 0.00
J

ATOM 7560 HG12 VAL J 86 −19.588 24.538 −18.136 0.00 0.00
J
ATOM 7561 HG13 VAL J 86 −18.481 23.416 −19.010 0.00 0.00
J
ATOM 7562 CG2 VAL J 86 −17.767 24.651 −15.855 0.00 0.00
J
ATOM 7563 HG21 VAL J 86 −18.541 25.430 −15.687 0.00 0.00
J
ATOM 7564 HG22 VAL J 86 −18.255 23.778 −15.371 0.00 0.00
J
ATOM 7565 HG23 VAL J 86 −16.856 24.943 −15.290 0.00 0.00
J
ATOM 7566 C VAL J 86 −15.014 24.611 −17.595 0.00 0.00
J
ATOM 7567 O VAL J 86 −14.321 24.761 −16.607 0.00 0.00
J
ATOM 7568 N ASP J 87 −14.837 25.422 −18.657 0.00 0.00
J
ATOM 7569 HN ASP J 87 −15.316 25.380 −19.531 0.00 0.00
J
ATOM 7570 CA ASP J 87 −13.794 26.455 −18.609 0.00 0.00
J
ATOM 7571 HA ASP J 87 −12.900 25.980 −18.232 0.00 0.00
J
ATOM 7572 CB ASP J 87 −13.577 26.952 −20.099 0.00 0.00
J
ATOM 7573 HB1 ASP J 87 −13.589 26.020 −20.705 0.00 0.00
J
ATOM 7574 HB2 ASP J 87 −14.451 27.540 −20.450 0.00 0.00
J
ATOM 7575 CG ASP J 87 −12.283 27.706 −20.181 0.00 0.00
J
ATOM 7576 OD1 ASP J 87 −11.182 27.100 −20.227 0.00 0.00
J
ATOM 7577 OD2 ASP J 87 −12.309 28.969 −20.137 0.00 0.00
J
ATOM 7578 C ASP J 87 −14.096 27.548 −17.526 0.00 0.00
J
ATOM 7579 O ASP J 87 −15.011 28.322 −17.597 0.00 0.00
J
ATOM 7580 N THR J 88 −13.180 27.695 −16.560 0.00 0.00
J
ATOM 7581 HN THR J 88 −12.343 27.154 −16.577 0.00 0.00
J
ATOM 7582 CA THR J 88 −13.195 28.669 −15.481 0.00 0.00
J
ATOM 7583 HA THR J 88 −14.070 28.469 −14.880 0.00 0.00
J
ATOM 7584 CB THR J 88 −11.931 28.526 −14.703 0.00 0.00
J
ATOM 7585 HB THR J 88 −11.001 28.950 −15.139 0.00 0.00
J
ATOM 7586 OG1 THR J 88 −11.702 27.124 −14.293 0.00 0.00
J

ATOM 7587 HG1 THR J 88 −10.750 27.099 −14.172 0.00 0.00
J
ATOM 7588 CG2 THR J 88 −12.132 29.371 −13.367 0.00 0.00
J
ATOM 7589 HG21 THR J 88 −13.142 29.221 −12.928 0.00 0.00
J
ATOM 7590 HG22 THR J 88 −11.315 29.102 −12.664 0.00 0.00
J
ATOM 7591 HG23 THR J 88 −12.156 30.481 −13.424 0.00 0.00
J
ATOM 7592 C THR J 88 −13.281 30.122 −15.968 0.00 0.00
J
ATOM 7593 O THR J 88 −14.101 30.911 −15.565 0.00 0.00
J
ATOM 7594 N GLY J 89 −12.424 30.487 −16.939 0.00 0.00
J
ATOM 7595 HN GLY J 89 −11.854 29.839 −17.438 0.00 0.00
J
ATOM 7596 CA GLY J 89 −12.509 31.770 −17.630 0.00 0.00
J
ATOM 7597 HA1 GLY J 89 −11.523 31.882 −18.057 0.00 0.00
J
ATOM 7598 HA2 GLY J 89 −12.715 32.463 −16.829 0.00 0.00
J
ATOM 7599 C GLY J 89 −13.653 31.913 −18.637 0.00 0.00
J
ATOM 7600 O GLY J 89 −14.560 32.744 −18.503 0.00 0.00
J
ATOM 7601 N THR J 90 −13.778 31.124 −19.690 0.00 0.00
J
ATOM 7602 HN THR J 90 −13.159 30.371 −19.899 0.00 0.00
J
ATOM 7603 CA THR J 90 −14.762 31.312 −20.846 0.00 0.00
J
ATOM 7604 HA THR J 90 −14.614 32.297 −21.264 0.00 0.00
J
ATOM 7605 CB THR J 90 −14.419 30.370 −22.028 0.00 0.00
J
ATOM 7606 HB THR J 90 −14.677 29.378 −21.598 0.00 0.00
J
ATOM 7607 OG1 THR J 90 −13.022 30.369 −22.312 0.00 0.00
J
ATOM 7608 HG1 THR J 90 −12.636 29.876 −21.584 0.00 0.00
J
ATOM 7609 CG2 THR J 90 −15.061 30.737 −23.334 0.00 0.00
J
ATOM 7610 HG21 THR J 90 −14.624 31.731 −23.570 0.00 0.00
J
ATOM 7611 HG22 THR J 90 −14.835 30.024 −24.155 0.00 0.00
J
ATOM 7612 HG23 THR J 90 −16.138 31.006 −23.278 0.00 0.00
J
ATOM 7613 C THR J 90 −16.305 31.168 −20.615 0.00 0.00
J

ATOM 7614 O THR J 90 -17.204 31.796 -21.142 0.00 0.00
J
ATOM 7615 N GLN J 91 -16.669 30.294 -19.638 0.00 0.00
J
ATOM 7616 HN GLN J 91 -15.993 29.783 -19.113 0.00 0.00
J
ATOM 7617 CA GLN J 91 -18.146 30.106 -19.373 0.00 0.00
J
ATOM 7618 HA GLN J 91 -18.655 30.067 -20.324 0.00 0.00
J
ATOM 7619 CB GLN J 91 -18.345 28.611 -18.861 0.00 0.00
J
ATOM 7620 HB1 GLN J 91 -17.831 27.821 -19.448 0.00 0.00
J
ATOM 7621 HB2 GLN J 91 -17.713 28.664 -17.949 0.00 0.00
J
ATOM 7622 CG GLN J 91 -19.742 28.184 -18.302 0.00 0.00
J
ATOM 7623 HG1 GLN J 91 -19.787 27.327 -17.597 0.00 0.00
J
ATOM 7624 HG2 GLN J 91 -20.255 29.008 -17.761 0.00 0.00
J
ATOM 7625 CD GLN J 91 -20.652 27.892 -19.425 0.00 0.00
J
ATOM 7626 OE1 GLN J 91 -20.519 26.801 -19.947 0.00 0.00
J
ATOM 7627 NE2 GLN J 91 -21.439 28.848 -19.917 0.00 0.00
J
ATOM 7628 HE21 GLN J 91 -21.964 28.640 -20.742 0.00 0.00
J
ATOM 7629 HE22 GLN J 91 -21.468 29.777 -19.549 0.00 0.00
J
ATOM 7630 C GLN J 91 -18.895 31.233 -18.625 0.00 0.00
J
ATOM 7631 O GLN J 91 -20.093 31.304 -18.500 0.00 0.00
J
ATOM 7632 N PHE J 92 -18.088 32.020 -17.978 0.00 0.00
J
ATOM 7633 HN PHE J 92 -17.138 31.818 -18.204 0.00 0.00
J
ATOM 7634 CA PHE J 92 -18.552 32.872 -16.939 0.00 0.00
J
ATOM 7635 HA PHE J 92 -19.628 32.795 -16.887 0.00 0.00
J
ATOM 7636 CB PHE J 92 -17.901 32.415 -15.635 0.00 0.00
J
ATOM 7637 HB1 PHE J 92 -16.800 32.421 -15.788 0.00 0.00
J
ATOM 7638 HB2 PHE J 92 -18.128 33.142 -14.825 0.00 0.00
J
ATOM 7639 CG PHE J 92 -18.255 30.992 -15.136 0.00 0.00
J
ATOM 7640 CD1 PHE J 92 -19.409 30.770 -14.279 0.00 0.00
J

ATOM 7641 HD1 PHE J 92 −20.043 31.592 −13.981 0.00 0.00
J
ATOM 7642 CE1 PHE J 92 −19.729 29.473 −13.896 0.00 0.00
J
ATOM 7643 HE1 PHE J 92 −20.581 29.281 −13.260 0.00 0.00
J
ATOM 7644 CZ PHE J 92 −18.877 28.424 −14.127 0.00 0.00
J
ATOM 7645 HZ PHE J 92 −19.234 27.504 −13.689 0.00 0.00
J
ATOM 7646 CD2 PHE J 92 −17.503 29.841 −15.518 0.00 0.00
J
ATOM 7647 HD2 PHE J 92 −16.726 30.176 −16.189 0.00 0.00
J
ATOM 7648 CE2 PHE J 92 −17.779 28.539 −14.971 0.00 0.00
J
ATOM 7649 HE2 PHE J 92 −17.225 27.703 −15.372 0.00 0.00
J
ATOM 7650 C PHE J 92 −18.275 34.347 −17.144 0.00 0.00
J
ATOM 7651 O PHE J 92 −17.242 34.713 −17.637 0.00 0.00
J
ATOM 7652 N THR J 93 −19.265 35.177 −16.847 0.00 0.00
J
ATOM 7653 HN THR J 93 −20.074 34.819 −16.387 0.00 0.00
J
ATOM 7654 CA THR J 93 −19.227 36.683 −17.059 0.00 0.00
J
ATOM 7655 HA THR J 93 −18.860 37.003 −18.023 0.00 0.00
J
ATOM 7656 CB THR J 93 −20.603 37.218 −16.982 0.00 0.00
J
ATOM 7657 HB THR J 93 −20.682 38.326 −16.979 0.00 0.00
J
ATOM 7658 OG1 THR J 93 −21.325 36.743 −15.823 0.00 0.00
J
ATOM 7659 HG1 THR J 93 −22.157 37.216 −15.895 0.00 0.00
J
ATOM 7660 CG2 THR J 93 −21.374 36.542 −18.142 0.00 0.00
J
ATOM 7661 HG21 THR J 93 −21.589 35.458 −18.025 0.00 0.00
J
ATOM 7662 HG22 THR J 93 −22.408 36.941 −18.217 0.00 0.00
J
ATOM 7663 HG23 THR J 93 −20.864 36.534 −19.128 0.00 0.00
J
ATOM 7664 C THR J 93 −18.438 37.369 −15.995 0.00 0.00
J
ATOM 7665 O THR J 93 −17.917 36.726 −15.094 0.00 0.00
J
ATOM 7666 N VAL J 94 −18.213 38.681 −15.991 0.00 0.00
J
ATOM 7667 HN VAL J 94 −18.688 39.202 −16.695 0.00 0.00
J

ATOM 7668 CA VAL J 94 −17.330 39.276 −14.960 0.00 0.00
J
ATOM 7669 HA VAL J 94 −16.412 38.708 −14.953 0.00 0.00
J
ATOM 7670 CB VAL J 94 −16.916 40.686 −15.240 0.00 0.00
J
ATOM 7671 HB VAL J 94 −17.789 41.371 −15.188 0.00 0.00
J
ATOM 7672 CG1 VAL J 94 −15.939 41.160 −14.102 0.00 0.00
J
ATOM 7673 HG11 VAL J 94 −14.989 40.616 −13.911 0.00 0.00
J
ATOM 7674 HG12 VAL J 94 −15.549 42.096 −14.555 0.00 0.00
J
ATOM 7675 HG13 VAL J 94 −16.505 41.372 −13.170 0.00 0.00
J
ATOM 7676 CG2 VAL J 94 −16.321 40.961 −16.623 0.00 0.00
J
ATOM 7677 HG21 VAL J 94 −15.416 40.363 −16.861 0.00 0.00
J
ATOM 7678 HG22 VAL J 94 −16.924 40.792 −17.541 0.00 0.00
J
ATOM 7679 HG23 VAL J 94 −16.083 42.042 −16.719 0.00 0.00
J
ATOM 7680 C VAL J 94 −17.970 39.082 −13.515 0.00 0.00
J
ATOM 7681 O VAL J 94 −17.332 38.863 −12.513 0.00 0.00
J
ATOM 7682 N PHE J 95 −19.335 39.190 −13.490 0.00 0.00
J
ATOM 7683 HN PHE J 95 −19.897 39.494 −14.255 0.00 0.00
J
ATOM 7684 CA PHE J 95 −20.132 38.721 −12.367 0.00 0.00
J
ATOM 7685 HA PHE J 95 −19.878 39.389 −11.557 0.00 0.00
J
ATOM 7686 CB PHE J 95 −21.610 38.986 −12.784 0.00 0.00
J
ATOM 7687 HB1 PHE J 95 −21.626 40.081 −12.968 0.00 0.00
J
ATOM 7688 HB2 PHE J 95 −21.968 38.548 −13.740 0.00 0.00
J
ATOM 7689 CG PHE J 95 −22.563 38.733 −11.601 0.00 0.00
J
ATOM 7690 CD1 PHE J 95 −22.253 39.186 −10.319 0.00 0.00
J
ATOM 7691 HD1 PHE J 95 −21.301 39.638 −10.079 0.00 0.00
J
ATOM 7692 CE1 PHE J 95 −23.192 39.033 −9.249 0.00 0.00
J
ATOM 7693 HE1 PHE J 95 −23.012 39.318 −8.223 0.00 0.00
J
ATOM 7694 CZ PHE J 95 −24.466 38.472 −9.533 0.00 0.00
J

ATOM 7695 HZ PHE J 95 -25.154 38.221 -8.739 0.00 0.00
J
ATOM 7696 CD2 PHE J 95 -23.776 38.043 -11.810 0.00 0.00
J
ATOM 7697 HD2 PHE J 95 -24.019 37.676 -12.796 0.00 0.00
J
ATOM 7698 CE2 PHE J 95 -24.725 37.850 -10.801 0.00 0.00
J
ATOM 7699 HE2 PHE J 95 -25.692 37.392 -10.945 0.00 0.00
J
ATOM 7700 C PHE J 95 -19.985 37.198 -12.036 0.00 0.00
J
ATOM 7701 O PHE J 95 -19.796 36.776 -10.892 0.00 0.00
J
ATOM 7702 N GLY J 96 -20.018 36.210 -13.024 0.00 0.00
J
ATOM 7703 HN GLY J 96 -20.386 36.475 -13.912 0.00 0.00
J
ATOM 7704 CA GLY J 96 -19.851 34.760 -12.897 0.00 0.00
J
ATOM 7705 HA1 GLY J 96 -19.883 34.351 -13.896 0.00 0.00
J
ATOM 7706 HA2 GLY J 96 -20.669 34.321 -12.346 0.00 0.00
J
ATOM 7707 C GLY J 96 -18.491 34.405 -12.333 0.00 0.00
J
ATOM 7708 O GLY J 96 -18.417 33.596 -11.382 0.00 0.00
J
ATOM 7709 N GLN J 97 -17.399 34.991 -12.897 0.00 0.00
J
ATOM 7710 HN GLN J 97 -17.553 35.558 -13.702 0.00 0.00
J
ATOM 7711 CA GLN J 97 -16.055 34.820 -12.488 0.00 0.00
J
ATOM 7712 HA GLN J 97 -15.760 33.784 -12.556 0.00 0.00
J
ATOM 7713 CB GLN J 97 -14.952 35.481 -13.423 0.00 0.00
J
ATOM 7714 HB1 GLN J 97 -15.201 36.556 -13.549 0.00 0.00
J
ATOM 7715 HB2 GLN J 97 -13.913 35.378 -13.042 0.00 0.00
J
ATOM 7716 CG GLN J 97 -15.107 34.817 -14.873 0.00 0.00
J
ATOM 7717 HG1 GLN J 97 -14.838 33.746 -14.757 0.00 0.00
J
ATOM 7718 HG2 GLN J 97 -16.196 34.802 -15.094 0.00 0.00
J
ATOM 7719 CD GLN J 97 -14.241 35.487 -15.919 0.00 0.00
J
ATOM 7720 OE1 GLN J 97 -13.330 36.216 -15.577 0.00 0.00
J
ATOM 7721 NE2 GLN J 97 -14.411 35.219 -17.221 0.00 0.00
J

ATOM 7722 HE21 GLN J 97 −13.568 35.132 −17.751 0.00 0.00
J
ATOM 7723 HE22 GLN J 97 −15.189 34.662 −17.512 0.00 0.00
J
ATOM 7724 C GLN J 97 −15.908 35.302 −11.103 0.00 0.00
J
ATOM 7725 O GLN J 97 −15.287 34.666 −10.244 0.00 0.00
J
ATOM 7726 N THR J 98 −16.495 36.471 −10.748 0.00 0.00
J
ATOM 7727 HN THR J 98 −17.008 37.024 −11.399 0.00 0.00
J
ATOM 7728 CA THR J 98 −16.706 36.986 −9.308 0.00 0.00
J
ATOM 7729 HA THR J 98 −15.736 37.106 −8.849 0.00 0.00
J
ATOM 7730 CB THR J 98 −17.379 38.347 −9.172 0.00 0.00
J
ATOM 7731 HB THR J 98 −18.359 38.353 −9.696 0.00 0.00
J
ATOM 7732 OG1 THR J 98 −16.604 39.345 −9.838 0.00 0.00
J
ATOM 7733 HG1 THR J 98 −16.928 39.277 −10.739 0.00 0.00
J
ATOM 7734 CG2 THR J 98 −17.533 38.818 −7.708 0.00 0.00
J
ATOM 7735 HG21 THR J 98 −16.578 38.521 −7.223 0.00 0.00
J
ATOM 7736 HG22 THR J 98 −17.729 39.911 −7.711 0.00 0.00
J
ATOM 7737 HG23 THR J 98 −18.375 38.248 −7.263 0.00 0.00
J
ATOM 7738 C THR J 98 −17.480 36.006 −8.344 0.00 0.00
J
ATOM 7739 O THR J 98 −17.165 35.835 −7.192 0.00 0.00
J
ATOM 7740 N VAL J 99 −18.545 35.296 −8.806 0.00 0.00
J
ATOM 7741 HN VAL J 99 −18.857 35.449 −9.740 0.00 0.00
J
ATOM 7742 CA VAL J 99 −19.219 34.204 −8.056 0.00 0.00
J
ATOM 7743 HA VAL J 99 −19.592 34.657 −7.150 0.00 0.00
J
ATOM 7744 CB VAL J 99 −20.384 33.620 −8.804 0.00 0.00
J
ATOM 7745 HB VAL J 99 −20.057 33.298 −9.816 0.00 0.00
J
ATOM 7746 CG1 VAL J 99 −21.017 32.409 −8.026 0.00 0.00
J
ATOM 7747 HG11 VAL J 99 −21.219 32.772 −6.996 0.00 0.00
J
ATOM 7748 HG12 VAL J 99 −21.957 31.978 −8.431 0.00 0.00
J

ATOM 7749 HG13 VAL J 99 -20.355 31.517 -8.007 0.00 0.00
J
ATOM 7750 CG2 VAL J 99 -21.401 34.708 -8.785 0.00 0.00
J
ATOM 7751 HG21 VAL J 99 -21.074 35.583 -9.388 0.00 0.00
J
ATOM 7752 HG22 VAL J 99 -22.443 34.440 -9.063 0.00 0.00
J
ATOM 7753 HG23 VAL J 99 -21.608 35.109 -7.769 0.00 0.00
J
ATOM 7754 C VAL J 99 -18.286 33.067 -7.738 0.00 0.00
J
ATOM 7755 O VAL J 99 -18.240 32.514 -6.666 0.00 0.00
J
ATOM 7756 N ILE J 100 -17.484 32.609 -8.738 0.00 0.00
J
ATOM 7757 HN ILE J 100 -17.643 32.811 -9.701 0.00 0.00
J
ATOM 7758 CA ILE J 100 -16.390 31.693 -8.428 0.00 0.00
J
ATOM 7759 HA ILE J 100 -16.854 30.862 -7.918 0.00 0.00
J
ATOM 7760 CB ILE J 100 -15.683 31.257 -9.662 0.00 0.00
J
ATOM 7761 HB ILE J 100 -15.290 32.117 -10.244 0.00 0.00
J
ATOM 7762 CG2 ILE J 100 -14.495 30.321 -9.260 0.00 0.00
J
ATOM 7763 HG21 ILE J 100 -14.758 29.631 -8.429 0.00 0.00
J
ATOM 7764 HG22 ILE J 100 -14.225 29.717 -10.152 0.00 0.00
J
ATOM 7765 HG23 ILE J 100 -13.600 30.890 -8.930 0.00 0.00
J
ATOM 7766 CG1 ILE J 100 -16.740 30.399 -10.481 0.00 0.00
J
ATOM 7767 HG11 ILE J 100 -17.037 29.549 -9.830 0.00 0.00
J
ATOM 7768 HG12 ILE J 100 -17.703 30.930 -10.640 0.00 0.00
J
ATOM 7769 CD ILE J 100 -16.207 30.027 -11.881 0.00 0.00
J
ATOM 7770 HD1 ILE J 100 -15.491 30.795 -12.244 0.00 0.00
J
ATOM 7771 HD2 ILE J 100 -15.653 29.070 -11.775 0.00 0.00
J
ATOM 7772 HD3 ILE J 100 -17.061 29.777 -12.545 0.00 0.00
J
ATOM 7773 C ILE J 100 -15.385 32.225 -7.343 0.00 0.00
J
ATOM 7774 O ILE J 100 -15.101 31.423 -6.409 0.00 0.00
J
ATOM 7775 N MET J 101 -14.931 33.497 -7.301 0.00 0.00
J

ATOM	7776	HN	MET	J	101	−15.169	34.113	−8.048	0.00	0.00
J										
ATOM	7777	CA	MET	J	101	−14.099	34.047	−6.178	0.00	0.00
J										
ATOM	7778	HA	MET	J	101	−13.254	33.377	−6.237	0.00	0.00
J										
ATOM	7779	CB	MET	J	101	−13.660	35.414	−6.587	0.00	0.00
J										
ATOM	7780	HB1	MET	J	101	−13.111	35.205	−7.530	0.00	0.00
J										
ATOM	7781	HB2	MET	J	101	−14.573	36.031	−6.730	0.00	0.00
J										
ATOM	7782	CG	MET	J	101	−12.633	36.161	−5.658	0.00	0.00
J										
ATOM	7783	HG1	MET	J	101	−12.973	36.037	−4.608	0.00	0.00
J										
ATOM	7784	HG2	MET	J	101	−11.607	35.753	−5.778	0.00	0.00
J										
ATOM	7785	SD	MET	J	101	−12.731	37.999	−5.898	0.00	0.00
J										
ATOM	7786	CE	MET	J	101	−11.770	38.132	−7.460	0.00	0.00
J										
ATOM	7787	HE1	MET	J	101	−11.788	39.133	−7.941	0.00	0.00
J										
ATOM	7788	HE2	MET	J	101	−10.757	37.759	−7.198	0.00	0.00
J										
ATOM	7789	HE3	MET	J	101	−12.128	37.352	−8.165	0.00	0.00
J										
ATOM	7790	C	MET	J	101	−14.910	34.030	−4.894	0.00	0.00
J										
ATOM	7791	O	MET	J	101	−14.287	33.692	−3.859	0.00	0.00
J										
ATOM	7792	N	GLY	J	102	−16.176	34.310	−4.812	0.00	0.00
J										
ATOM	7793	HN	GLY	J	102	−16.452	34.788	−5.643	0.00	0.00
J										
ATOM	7794	CA	GLY	J	102	−17.002	34.276	−3.643	0.00	0.00
J										
ATOM	7795	HA1	GLY	J	102	−18.030	34.553	−3.825	0.00	0.00
J										
ATOM	7796	HA2	GLY	J	102	−16.398	34.908	−3.008	0.00	0.00
J										
ATOM	7797	C	GLY	J	102	−16.872	32.919	−3.003	0.00	0.00
J										
ATOM	7798	O	GLY	J	102	−16.498	32.838	−1.842	0.00	0.00
J										
ATOM	7799	N	LEU	J	103	−17.139	31.846	−3.816	0.00	0.00
J										
ATOM	7800	HN	LEU	J	103	−17.574	32.014	−4.697	0.00	0.00
J										
ATOM	7801	CA	LEU	J	103	−17.048	30.419	−3.475	0.00	0.00
J										
ATOM	7802	HA	LEU	J	103	−17.788	30.265	−2.704	0.00	0.00
J										

ATOM 7803 CB LEU J 103 -17.340 29.462 -4.629 0.00 0.00
J
ATOM 7804 HB1 LEU J 103 -16.632 29.829 -5.403 0.00 0.00
J
ATOM 7805 HB2 LEU J 103 -17.061 28.406 -4.427 0.00 0.00
J
ATOM 7806 CG LEU J 103 -18.805 29.584 -5.008 0.00 0.00
J
ATOM 7807 HG LEU J 103 -19.056 30.663 -5.082 0.00 0.00
J
ATOM 7808 CD1 LEU J 103 -18.985 28.938 -6.414 0.00 0.00
J
ATOM 7809 HD11 LEU J 103 -20.064 28.830 -6.656 0.00 0.00
J
ATOM 7810 HD12 LEU J 103 -18.514 29.521 -7.234 0.00 0.00
J
ATOM 7811 HD13 LEU J 103 -18.352 28.026 -6.455 0.00 0.00
J
ATOM 7812 CD2 LEU J 103 -19.674 28.866 -4.012 0.00 0.00
J
ATOM 7813 HD21 LEU J 103 -20.645 28.660 -4.511 0.00 0.00
J
ATOM 7814 HD22 LEU J 103 -19.202 27.891 -3.766 0.00 0.00
J
ATOM 7815 HD23 LEU J 103 -19.743 29.268 -2.979 0.00 0.00
J
ATOM 7816 C LEU J 103 -15.728 29.978 -2.875 0.00 0.00
J
ATOM 7817 O LEU J 103 -15.725 29.333 -1.844 0.00 0.00
J
ATOM 7818 N ILE J 104 -14.617 30.391 -3.480 0.00 0.00
J
ATOM 7819 HN ILE J 104 -14.684 30.907 -4.331 0.00 0.00
J
ATOM 7820 CA ILE J 104 -13.228 30.244 -2.920 0.00 0.00
J
ATOM 7821 HA ILE J 104 -13.254 29.209 -2.611 0.00 0.00
J
ATOM 7822 CB ILE J 104 -12.196 30.488 -4.067 0.00 0.00
J
ATOM 7823 HB ILE J 104 -12.445 31.510 -4.423 0.00 0.00
J
ATOM 7824 CG2 ILE J 104 -10.731 30.402 -3.623 0.00 0.00
J
ATOM 7825 HG21 ILE J 104 -10.526 29.443 -3.102 0.00 0.00
J
ATOM 7826 HG22 ILE J 104 -10.025 30.426 -4.480 0.00 0.00
J
ATOM 7827 HG23 ILE J 104 -10.513 31.179 -2.859 0.00 0.00
J
ATOM 7828 CG1 ILE J 104 -12.393 29.495 -5.290 0.00 0.00
J
ATOM 7829 HG11 ILE J 104 -11.980 28.517 -4.963 0.00 0.00
J

```
ATOM   7830 HG12 ILE J 104     -13.486  29.325  -5.396  0.00  0.00
J
ATOM   7831  CD  ILE J 104     -11.808  29.937  -6.595  0.00  0.00
J
ATOM   7832  HD1 ILE J 104     -11.771  29.057  -7.272  0.00  0.00
J
ATOM   7833  HD2 ILE J 104     -12.364  30.843  -6.917  0.00  0.00
J
ATOM   7834  HD3 ILE J 104     -10.737  30.220  -6.507  0.00  0.00
J
ATOM   7835  C   ILE J 104     -13.039  30.997  -1.645  0.00  0.00
J
ATOM   7836  O   ILE J 104     -12.306  30.507  -0.789  0.00  0.00
J
ATOM   7837  N   GLN J 105     -13.578  32.247  -1.558  0.00  0.00
J
ATOM   7838  HN  GLN J 105     -14.058  32.649  -2.334  0.00  0.00
J
ATOM   7839  CA  GLN J 105     -13.294  33.067  -0.413  0.00  0.00
J
ATOM   7840  HA  GLN J 105     -12.227  33.145  -0.266  0.00  0.00
J
ATOM   7841  CB  GLN J 105     -13.648  34.489  -0.663  0.00  0.00
J
ATOM   7842  HB1 GLN J 105     -13.267  34.723  -1.680  0.00  0.00
J
ATOM   7843  HB2 GLN J 105     -14.745  34.546  -0.824  0.00  0.00
J
ATOM   7844  CG  GLN J 105     -13.021  35.545   0.352  0.00  0.00
J
ATOM   7845  HG1 GLN J 105     -13.579  36.503   0.275  0.00  0.00
J
ATOM   7846  HG2 GLN J 105     -13.065  35.226   1.415  0.00  0.00
J
ATOM   7847  CD  GLN J 105     -11.597  35.926   0.000  0.00  0.00
J
ATOM   7848  OE1 GLN J 105     -11.333  36.810  -0.790  0.00  0.00
J
ATOM   7849  NE2 GLN J 105     -10.586  35.240   0.508  0.00  0.00
J
ATOM   7850 HE21 GLN J 105      -9.650  35.582   0.430  0.00  0.00
J
ATOM   7851 HE22 GLN J 105     -10.673  34.407   1.054  0.00  0.00
J
ATOM   7852  C   GLN J 105     -14.046  32.471   0.805  0.00  0.00
J
ATOM   7853  O   GLN J 105     -13.477  32.333   1.911  0.00  0.00
J
ATOM   7854  N   ILE J 106     -15.357  32.094   0.677  0.00  0.00
J
ATOM   7855  HN  ILE J 106     -15.769  32.391  -0.181  0.00  0.00
J
ATOM   7856  CA  ILE J 106     -16.197  31.432   1.668  0.00  0.00
J
```

ATOM	7857	HA	ILE	J	106	−16.038	31.897	2.630	0.00	0.00	J
ATOM	7858	CB	ILE	J	106	−17.701	31.386	1.239	0.00	0.00	J
ATOM	7859	HB	ILE	J	106	−17.724	31.060	0.177	0.00	0.00	J
ATOM	7860	CG2	ILE	J	106	−18.621	30.429	1.948	0.00	0.00	J
ATOM	7861	HG21	ILE	J	106	−18.609	30.609	3.044	0.00	0.00	J
ATOM	7862	HG22	ILE	J	106	−19.656	30.505	1.550	0.00	0.00	J
ATOM	7863	HG23	ILE	J	106	−18.169	29.445	1.703	0.00	0.00	J
ATOM	7864	CG1	ILE	J	106	−18.266	32.816	1.271	0.00	0.00	J
ATOM	7865	HG11	ILE	J	106	−17.738	33.517	0.590	0.00	0.00	J
ATOM	7866	HG12	ILE	J	106	−19.275	32.862	0.808	0.00	0.00	J
ATOM	7867	CD	ILE	J	106	−18.465	33.523	2.634	0.00	0.00	J
ATOM	7868	HD1	ILE	J	106	−19.010	34.490	2.590	0.00	0.00	J
ATOM	7869	HD2	ILE	J	106	−19.070	32.890	3.318	0.00	0.00	J
ATOM	7870	HD3	ILE	J	106	−17.503	33.693	3.164	0.00	0.00	J
ATOM	7871	C	ILE	J	106	−15.638	30.077	2.001	0.00	0.00	J
ATOM	7872	O	ILE	J	106	−15.383	29.685	3.177	0.00	0.00	J
ATOM	7873	N	GLY	J	107	−15.319	29.266	0.944	0.00	0.00	J
ATOM	7874	HN	GLY	J	107	−15.470	29.571	0.006	0.00	0.00	J
ATOM	7875	CA	GLY	J	107	−14.788	27.949	1.168	0.00	0.00	J
ATOM	7876	HA1	GLY	J	107	−14.848	27.413	0.233	0.00	0.00	J
ATOM	7877	HA2	GLY	J	107	−15.426	27.560	1.948	0.00	0.00	J
ATOM	7878	C	GLY	J	107	−13.345	27.818	1.629	0.00	0.00	J
ATOM	7879	O	GLY	J	107	−12.860	26.727	1.967	0.00	0.00	J
ATOM	7880	N	GLY	J	108	−12.616	28.924	1.595	0.00	0.00	J
ATOM	7881	HN	GLY	J	108	−12.931	29.814	1.273	0.00	0.00	J
ATOM	7882	CA	GLY	J	108	−11.270	28.979	2.116	0.00	0.00	J
ATOM	7883	HA1	GLY	J	108	−10.766	29.687	1.475	0.00	0.00	J

ATOM	7884	HA2	GLY	J	108	−10.880	27.972	2.123	0.00	0.00	J
ATOM	7885	C	GLY	J	108	−11.271	29.572	3.525	0.00	0.00	J
ATOM	7886	O	GLY	J	108	−11.316	28.913	4.598	0.00	0.00	J
ATOM	7887	N	LEU	J	109	−11.281	30.942	3.577	0.00	0.00	J
ATOM	7888	HN	LEU	J	109	−11.254	31.484	2.740	0.00	0.00	J
ATOM	7889	CA	LEU	J	109	−11.131	31.785	4.755	0.00	0.00	J
ATOM	7890	HA	LEU	J	109	−10.232	31.576	5.316	0.00	0.00	J
ATOM	7891	CB	LEU	J	109	−11.260	33.298	4.481	0.00	0.00	J
ATOM	7892	HB1	LEU	J	109	−10.458	33.534	3.748	0.00	0.00	J
ATOM	7893	HB2	LEU	J	109	−12.170	33.472	3.868	0.00	0.00	J
ATOM	7894	CG	LEU	J	109	−11.198	34.368	5.608	0.00	0.00	J
ATOM	7895	HG	LEU	J	109	−12.110	34.222	6.225	0.00	0.00	J
ATOM	7896	CD1	LEU	J	109	−11.400	35.838	5.069	0.00	0.00	J
ATOM	7897	HD11	LEU	J	109	−10.558	36.103	4.395	0.00	0.00	J
ATOM	7898	HD12	LEU	J	109	−12.391	36.068	4.622	0.00	0.00	J
ATOM	7899	HD13	LEU	J	109	−11.246	36.434	5.993	0.00	0.00	J
ATOM	7900	CD2	LEU	J	109	−9.913	34.247	6.419	0.00	0.00	J
ATOM	7901	HD21	LEU	J	109	−9.006	34.604	5.887	0.00	0.00	J
ATOM	7902	HD22	LEU	J	109	−10.053	34.746	7.402	0.00	0.00	J
ATOM	7903	HD23	LEU	J	109	−9.737	33.154	6.504	0.00	0.00	J
ATOM	7904	C	LEU	J	109	−12.232	31.525	5.701	0.00	0.00	J
ATOM	7905	O	LEU	J	109	−11.977	31.409	6.916	0.00	0.00	J
ATOM	7906	N	GLY	J	110	−13.478	31.414	5.257	0.00	0.00	J
ATOM	7907	HN	GLY	J	110	−13.814	31.587	4.334	0.00	0.00	J
ATOM	7908	CA	GLY	J	110	−14.559	30.990	6.113	0.00	0.00	J
ATOM	7909	HA1	GLY	J	110	−15.523	31.027	5.627	0.00	0.00	J
ATOM	7910	HA2	GLY	J	110	−14.508	31.613	6.993	0.00	0.00	J

ATOM 7911 C GLY J 110 -14.505 29.578 6.651 0.00 0.00
J
ATOM 7912 O GLY J 110 -14.798 29.423 7.847 0.00 0.00
J
ATOM 7913 N PHE J 111 -14.164 28.536 5.857 0.00 0.00
J
ATOM 7914 HN PHE J 111 -13.954 28.625 4.887 0.00 0.00
J
ATOM 7915 CA PHE J 111 -13.846 27.240 6.426 0.00 0.00
J
ATOM 7916 HA PHE J 111 -14.616 27.007 7.146 0.00 0.00
J
ATOM 7917 CB PHE J 111 -13.733 26.308 5.168 0.00 0.00
J
ATOM 7918 HB1 PHE J 111 -14.604 26.443 4.492 0.00 0.00
J
ATOM 7919 HB2 PHE J 111 -12.787 26.397 4.591 0.00 0.00
J
ATOM 7920 CG PHE J 111 -13.811 24.903 5.691 0.00 0.00
J
ATOM 7921 CD1 PHE J 111 -15.069 24.284 5.702 0.00 0.00
J
ATOM 7922 HD1 PHE J 111 -15.929 24.824 5.335 0.00 0.00
J
ATOM 7923 CE1 PHE J 111 -15.232 22.987 6.175 0.00 0.00
J
ATOM 7924 HE1 PHE J 111 -16.215 22.545 6.116 0.00 0.00
J
ATOM 7925 CZ PHE J 111 -14.085 22.241 6.603 0.00 0.00
J
ATOM 7926 HZ PHE J 111 -14.098 21.203 6.899 0.00 0.00
J
ATOM 7927 CD2 PHE J 111 -12.752 24.216 6.206 0.00 0.00
J
ATOM 7928 HD2 PHE J 111 -11.791 24.659 6.422 0.00 0.00
J
ATOM 7929 CE2 PHE J 111 -12.844 22.878 6.659 0.00 0.00
J
ATOM 7930 HE2 PHE J 111 -12.000 22.266 6.941 0.00 0.00
J
ATOM 7931 C PHE J 111 -12.648 27.112 7.331 0.00 0.00
J
ATOM 7932 O PHE J 111 -12.716 26.652 8.478 0.00 0.00
J
ATOM 7933 N MET J 112 -11.482 27.571 6.880 0.00 0.00
J
ATOM 7934 HN MET J 112 -11.364 28.097 6.042 0.00 0.00
J
ATOM 7935 CA MET J 112 -10.268 27.229 7.607 0.00 0.00
J
ATOM 7936 HA MET J 112 -10.149 26.159 7.689 0.00 0.00
J
ATOM 7937 CB MET J 112 -9.037 27.858 6.884 0.00 0.00
J

ATOM 7938 HB1 MET J 112 -9.133 28.955 6.739 0.00 0.00
J
ATOM 7939 HB2 MET J 112 -8.191 27.671 7.580 0.00 0.00
J
ATOM 7940 CG MET J 112 -8.690 27.233 5.533 0.00 0.00
J
ATOM 7941 HG1 MET J 112 -9.479 27.495 4.797 0.00 0.00
J
ATOM 7942 HG2 MET J 112 -7.815 27.759 5.096 0.00 0.00
J
ATOM 7943 SD MET J 112 -8.431 25.384 5.593 0.00 0.00
J
ATOM 7944 CE MET J 112 -9.916 24.892 4.625 0.00 0.00
J
ATOM 7945 HE1 MET J 112 -10.139 23.811 4.751 0.00 0.00
J
ATOM 7946 HE2 MET J 112 -10.766 25.448 5.075 0.00 0.00
J
ATOM 7947 HE3 MET J 112 -9.698 25.236 3.591 0.00 0.00
J
ATOM 7948 C MET J 112 -10.170 27.976 8.977 0.00 0.00
J
ATOM 7949 O MET J 112 -9.493 27.517 9.858 0.00 0.00
J
ATOM 7950 N THR J 113 -10.758 29.186 9.064 0.00 0.00
J
ATOM 7951 HN THR J 113 -11.295 29.560 8.312 0.00 0.00
J
ATOM 7952 CA THR J 113 -10.604 30.001 10.290 0.00 0.00
J
ATOM 7953 HA THR J 113 -9.564 29.830 10.524 0.00 0.00
J
ATOM 7954 CB THR J 113 -10.811 31.532 10.165 0.00 0.00
J
ATOM 7955 HB THR J 113 -10.671 31.936 11.190 0.00 0.00
J
ATOM 7956 OG1 THR J 113 -12.162 31.803 9.798 0.00 0.00
J
ATOM 7957 HG1 THR J 113 -12.108 31.856 8.841 0.00 0.00
J
ATOM 7958 CG2 THR J 113 -9.754 32.185 9.253 0.00 0.00
J
ATOM 7959 HG21 THR J 113 -9.849 31.626 8.298 0.00 0.00
J
ATOM 7960 HG22 THR J 113 -9.961 33.234 8.952 0.00 0.00
J
ATOM 7961 HG23 THR J 113 -8.723 32.059 9.648 0.00 0.00
J
ATOM 7962 C THR J 113 -11.538 29.438 11.403 0.00 0.00
J
ATOM 7963 O THR J 113 -11.261 29.375 12.614 0.00 0.00
J
ATOM 7964 N PHE J 114 -12.685 28.935 10.976 0.00 0.00
J

ATOM 7965 HN PHE J 114 -12.959 29.023 10.022 0.00 0.00
J
ATOM 7966 CA PHE J 114 -13.577 28.218 11.792 0.00 0.00
J
ATOM 7967 HA PHE J 114 -13.831 28.903 12.587 0.00 0.00
J
ATOM 7968 CB PHE J 114 -14.879 27.923 10.917 0.00 0.00
J
ATOM 7969 HB1 PHE J 114 -15.120 28.836 10.332 0.00 0.00
J
ATOM 7970 HB2 PHE J 114 -14.737 27.110 10.174 0.00 0.00
J
ATOM 7971 CG PHE J 114 -16.094 27.608 11.788 0.00 0.00
J
ATOM 7972 CD1 PHE J 114 -16.200 27.826 13.192 0.00 0.00
J
ATOM 7973 HD1 PHE J 114 -15.304 28.126 13.717 0.00 0.00
J
ATOM 7974 CE1 PHE J 114 -17.243 27.467 13.963 0.00 0.00
J
ATOM 7975 HE1 PHE J 114 -17.173 27.627 15.029 0.00 0.00
J
ATOM 7976 CZ PHE J 114 -18.364 26.835 13.368 0.00 0.00
J
ATOM 7977 HZ PHE J 114 -19.160 26.528 14.030 0.00 0.00
J
ATOM 7978 CD2 PHE J 114 -17.238 26.971 11.186 0.00 0.00
J
ATOM 7979 HD2 PHE J 114 -17.333 26.765 10.130 0.00 0.00
J
ATOM 7980 CE2 PHE J 114 -18.372 26.631 11.945 0.00 0.00
J
ATOM 7981 HE2 PHE J 114 -19.175 26.115 11.441 0.00 0.00
J
ATOM 7982 C PHE J 114 -12.978 26.820 12.174 0.00 0.00
J
ATOM 7983 O PHE J 114 -13.044 26.411 13.334 0.00 0.00
J
ATOM 7984 N ALA J 115 -12.394 26.019 11.280 0.00 0.00
J
ATOM 7985 HN ALA J 115 -12.394 26.200 10.300 0.00 0.00
J
ATOM 7986 CA ALA J 115 -11.702 24.800 11.533 0.00 0.00
J
ATOM 7987 HA ALA J 115 -12.482 24.241 12.029 0.00 0.00
J
ATOM 7988 CB ALA J 115 -11.117 24.156 10.237 0.00 0.00
J
ATOM 7989 HB1 ALA J 115 -11.961 23.949 9.544 0.00 0.00
J
ATOM 7990 HB2 ALA J 115 -10.314 24.760 9.763 0.00 0.00
J
ATOM 7991 HB3 ALA J 115 -10.675 23.167 10.482 0.00 0.00
J

ATOM 7992 C ALA J 115 -10.544 24.905 12.522 0.00 0.00
J
ATOM 7993 O ALA J 115 -10.341 24.072 13.412 0.00 0.00
J
ATOM 7994 N VAL J 116 -9.786 25.966 12.436 0.00 0.00
J
ATOM 7995 HN VAL J 116 -10.063 26.588 11.707 0.00 0.00
J
ATOM 7996 CA VAL J 116 -8.704 26.275 13.332 0.00 0.00
J
ATOM 7997 HA VAL J 116 -7.945 25.507 13.304 0.00 0.00
J
ATOM 7998 CB VAL J 116 -8.017 27.465 12.853 0.00 0.00
J
ATOM 7999 HB VAL J 116 -8.690 28.177 12.330 0.00 0.00
J
ATOM 8000 CG1 VAL J 116 -7.244 28.167 14.004 0.00 0.00
J
ATOM 8001 HG11 VAL J 116 -6.459 28.769 13.499 0.00 0.00
J
ATOM 8002 HG12 VAL J 116 -7.838 28.921 14.563 0.00 0.00
J
ATOM 8003 HG13 VAL J 116 -6.744 27.416 14.653 0.00 0.00
J
ATOM 8004 CG2 VAL J 116 -6.902 27.078 11.774 0.00 0.00
J
ATOM 8005 HG21 VAL J 116 -6.215 26.325 12.216 0.00 0.00
J
ATOM 8006 HG22 VAL J 116 -7.416 26.643 10.890 0.00 0.00
J
ATOM 8007 HG23 VAL J 116 -6.310 27.991 11.550 0.00 0.00
J
ATOM 8008 C VAL J 116 -9.215 26.517 14.717 0.00 0.00
J
ATOM 8009 O VAL J 116 -8.681 26.063 15.720 0.00 0.00
J
ATOM 8010 N LEU J 117 -10.350 27.330 14.841 0.00 0.00
J
ATOM 8011 HN LEU J 117 -10.751 27.735 14.023 0.00 0.00
J
ATOM 8012 CA LEU J 117 -10.848 27.663 16.129 0.00 0.00
J
ATOM 8013 HA LEU J 117 -10.182 28.245 16.748 0.00 0.00
J
ATOM 8014 CB LEU J 117 -12.044 28.550 15.784 0.00 0.00
J
ATOM 8015 HB1 LEU J 117 -11.649 29.444 15.256 0.00 0.00
J
ATOM 8016 HB2 LEU J 117 -12.655 28.118 14.962 0.00 0.00
J
ATOM 8017 CG LEU J 117 -12.956 29.155 16.871 0.00 0.00
J
ATOM 8018 HG LEU J 117 -12.403 29.154 17.835 0.00 0.00
J

ATOM 8019 CD1 LEU J 117 −14.279 28.392 16.984 0.00 0.00
J
ATOM 8020 HD11 LEU J 117 −14.074 27.387 17.411 0.00 0.00
J
ATOM 8021 HD12 LEU J 117 −14.946 28.904 17.710 0.00 0.00
J
ATOM 8022 HD13 LEU J 117 −14.705 28.437 15.959 0.00 0.00
J
ATOM 8023 CD2 LEU J 117 −13.230 30.673 16.570 0.00 0.00
J
ATOM 8024 HD21 LEU J 117 −13.636 30.782 15.541 0.00 0.00
J
ATOM 8025 HD22 LEU J 117 −14.047 31.126 17.171 0.00 0.00
J
ATOM 8026 HD23 LEU J 117 −12.284 31.253 16.626 0.00 0.00
J
ATOM 8027 C LEU J 117 −11.375 26.516 16.843 0.00 0.00
J
ATOM 8028 O LEU J 117 −11.133 26.411 18.043 0.00 0.00
J
ATOM 8029 N ILE J 118 −11.965 25.528 16.101 0.00 0.00
J
ATOM 8030 HN ILE J 118 −12.141 25.660 15.129 0.00 0.00
J
ATOM 8031 CA ILE J 118 −12.542 24.303 16.617 0.00 0.00
J
ATOM 8032 HA ILE J 118 −13.128 24.522 17.497 0.00 0.00
J
ATOM 8033 CB ILE J 118 −13.576 23.620 15.692 0.00 0.00
J
ATOM 8034 HB ILE J 118 −13.165 23.463 14.671 0.00 0.00
J
ATOM 8035 CG2 ILE J 118 −13.970 22.224 16.359 0.00 0.00
J
ATOM 8036 HG21 ILE J 118 −14.828 21.742 15.845 0.00 0.00
J
ATOM 8037 HG22 ILE J 118 −13.089 21.548 16.314 0.00 0.00
J
ATOM 8038 HG23 ILE J 118 −14.281 22.499 17.389 0.00 0.00
J
ATOM 8039 CG1 ILE J 118 −14.809 24.573 15.435 0.00 0.00
J
ATOM 8040 HG11 ILE J 118 −15.137 24.925 16.436 0.00 0.00
J
ATOM 8041 HG12 ILE J 118 −14.542 25.517 14.914 0.00 0.00
J
ATOM 8042 CD ILE J 118 −16.035 23.925 14.709 0.00 0.00
J
ATOM 8043 HD1 ILE J 118 −15.819 23.779 13.630 0.00 0.00
J
ATOM 8044 HD2 ILE J 118 −16.536 23.132 15.305 0.00 0.00
J
ATOM 8045 HD3 ILE J 118 −16.825 24.705 14.678 0.00 0.00
J

ATOM 8046 C ILE J 118 -11.441 23.399 17.139 0.00 0.00
J
ATOM 8047 O ILE J 118 -11.487 22.907 18.272 0.00 0.00
J
ATOM 8048 N VAL J 119 -10.332 23.301 16.413 0.00 0.00
J
ATOM 8049 HN VAL J 119 -10.301 23.710 15.504 0.00 0.00
J
ATOM 8050 CA VAL J 119 -9.144 22.569 16.762 0.00 0.00
J
ATOM 8051 HA VAL J 119 -9.478 21.573 17.013 0.00 0.00
J
ATOM 8052 CB VAL J 119 -8.264 22.481 15.497 0.00 0.00
J
ATOM 8053 HB VAL J 119 -8.827 23.141 14.803 0.00 0.00
J
ATOM 8054 CG1 VAL J 119 -6.753 22.825 15.576 0.00 0.00
J
ATOM 8055 HG11 VAL J 119 -6.268 22.657 14.591 0.00 0.00
J
ATOM 8056 HG12 VAL J 119 -6.669 23.874 15.932 0.00 0.00
J
ATOM 8057 HG13 VAL J 119 -6.197 22.200 16.308 0.00 0.00
J
ATOM 8058 CG2 VAL J 119 -8.433 21.192 14.746 0.00 0.00
J
ATOM 8059 HG21 VAL J 119 -7.959 20.386 15.347 0.00 0.00
J
ATOM 8060 HG22 VAL J 119 -9.497 20.952 14.533 0.00 0.00
J
ATOM 8061 HG23 VAL J 119 -7.908 21.208 13.767 0.00 0.00
J
ATOM 8062 C VAL J 119 -8.422 23.135 18.039 0.00 0.00
J
ATOM 8063 O VAL J 119 -7.975 22.343 18.870 0.00 0.00
J
ATOM 8064 N MET J 120 -8.348 24.466 18.176 0.00 0.00
J
ATOM 8065 HN MET J 120 -8.649 25.070 17.442 0.00 0.00
J
ATOM 8066 CA MET J 120 -7.691 25.109 19.328 0.00 0.00
J
ATOM 8067 HA MET J 120 -6.787 24.528 19.446 0.00 0.00
J
ATOM 8068 CB MET J 120 -7.304 26.509 19.028 0.00 0.00
J
ATOM 8069 HB1 MET J 120 -8.183 26.973 18.532 0.00 0.00
J
ATOM 8070 HB2 MET J 120 -6.867 27.006 19.921 0.00 0.00
J
ATOM 8071 CG MET J 120 -6.166 26.657 17.965 0.00 0.00
J
ATOM 8072 HG1 MET J 120 -6.396 26.147 17.006 0.00 0.00
J

ATOM 8073 HG2 MET J 120 −6.031 27.743 17.775 0.00 0.00
J
ATOM 8074 SD MET J 120 −4.661 25.949 18.768 0.00 0.00
J
ATOM 8075 CE MET J 120 −4.066 27.518 19.668 0.00 0.00
J
ATOM 8076 HE1 MET J 120 −3.416 27.434 20.565 0.00 0.00
J
ATOM 8077 HE2 MET J 120 −3.489 28.236 19.046 0.00 0.00
J
ATOM 8078 HE3 MET J 120 −4.900 28.098 20.118 0.00 0.00
J
ATOM 8079 C MET J 120 −8.505 25.059 20.685 0.00 0.00
J
ATOM 8080 O MET J 120 −7.937 24.605 21.665 0.00 0.00
J
ATOM 8081 N ILE J 121 −9.783 25.417 20.711 0.00 0.00
J
ATOM 8082 HN ILE J 121 −10.233 25.760 19.890 0.00 0.00
J
ATOM 8083 CA ILE J 121 −10.703 25.200 21.795 0.00 0.00
J
ATOM 8084 HA ILE J 121 −10.383 25.812 22.625 0.00 0.00
J
ATOM 8085 CB ILE J 121 −12.143 25.766 21.363 0.00 0.00
J
ATOM 8086 HB ILE J 121 −12.361 25.476 20.312 0.00 0.00
J
ATOM 8087 CG2 ILE J 121 −13.204 25.319 22.422 0.00 0.00
J
ATOM 8088 HG21 ILE J 121 −12.836 25.641 23.419 0.00 0.00
J
ATOM 8089 HG22 ILE J 121 −14.203 25.780 22.267 0.00 0.00
J
ATOM 8090 HG23 ILE J 121 −13.308 24.217 22.521 0.00 0.00
J
ATOM 8091 CG1 ILE J 121 −12.180 27.302 21.216 0.00 0.00
J
ATOM 8092 HG11 ILE J 121 −11.920 27.816 22.166 0.00 0.00
J
ATOM 8093 HG12 ILE J 121 −11.311 27.496 20.551 0.00 0.00
J
ATOM 8094 CD ILE J 121 −13.495 27.822 20.643 0.00 0.00
J
ATOM 8095 HD1 ILE J 121 −13.809 27.263 19.736 0.00 0.00
J
ATOM 8096 HD2 ILE J 121 −14.220 27.522 21.430 0.00 0.00
J
ATOM 8097 HD3 ILE J 121 −13.453 28.920 20.477 0.00 0.00
J
ATOM 8098 C ILE J 121 −10.845 23.725 22.226 0.00 0.00
J
ATOM 8099 O ILE J 121 −10.665 23.471 23.402 0.00 0.00
J

ATOM 8100 N LEU J 122 −10.885 22.713 21.306 0.00 0.00
J
ATOM 8101 HN LEU J 122 −11.086 22.951 20.359 0.00 0.00
J
ATOM 8102 CA LEU J 122 −10.869 21.309 21.539 0.00 0.00
J
ATOM 8103 HA LEU J 122 −11.665 21.069 22.227 0.00 0.00
J
ATOM 8104 CB LEU J 122 −11.081 20.569 20.222 0.00 0.00
J
ATOM 8105 HB1 LEU J 122 −12.036 20.942 19.794 0.00 0.00
J
ATOM 8106 HB2 LEU J 122 −10.349 20.831 19.429 0.00 0.00
J
ATOM 8107 CG LEU J 122 −11.121 18.991 20.243 0.00 0.00
J
ATOM 8108 HG LEU J 122 −10.071 18.689 20.441 0.00 0.00
J
ATOM 8109 CD1 LEU J 122 −12.001 18.335 21.267 0.00 0.00
J
ATOM 8110 HD11 LEU J 122 −11.808 17.245 21.357 0.00 0.00
J
ATOM 8111 HD12 LEU J 122 −11.663 18.727 22.249 0.00 0.00
J
ATOM 8112 HD13 LEU J 122 −13.069 18.631 21.341 0.00 0.00
J
ATOM 8113 CD2 LEU J 122 −11.505 18.490 18.850 0.00 0.00
J
ATOM 8114 HD21 LEU J 122 −12.507 18.863 18.546 0.00 0.00
J
ATOM 8115 HD22 LEU J 122 −10.690 18.839 18.181 0.00 0.00
J
ATOM 8116 HD23 LEU J 122 −11.520 17.379 18.867 0.00 0.00
J
ATOM 8117 C LEU J 122 −9.529 20.950 22.158 0.00 0.00
J
ATOM 8118 O LEU J 122 −9.521 20.357 23.231 0.00 0.00
J
ATOM 8119 N GLY J 123 −8.390 21.482 21.559 0.00 0.00
J
ATOM 8120 HN GLY J 123 −8.292 21.968 20.694 0.00 0.00
J
ATOM 8121 CA GLY J 123 −7.098 21.161 22.225 0.00 0.00
J
ATOM 8122 HA1 GLY J 123 −6.361 21.595 21.566 0.00 0.00
J
ATOM 8123 HA2 GLY J 123 −7.077 20.085 22.311 0.00 0.00
J
ATOM 8124 C GLY J 123 −6.806 21.716 23.617 0.00 0.00
J
ATOM 8125 O GLY J 123 −6.238 21.079 24.473 0.00 0.00
J
ATOM 8126 N LYS J 124 −7.347 22.867 23.937 0.00 0.00
J

ATOM 8127 HN LYS J 124 -7.806 23.503 23.322 0.00 0.00
J
ATOM 8128 CA LYS J 124 -7.406 23.356 25.291 0.00 0.00
J
ATOM 8129 HA LYS J 124 -6.458 23.168 25.772 0.00 0.00
J
ATOM 8130 CB LYS J 124 -7.782 24.864 25.365 0.00 0.00
J
ATOM 8131 HB1 LYS J 124 -8.830 25.050 25.048 0.00 0.00
J
ATOM 8132 HB2 LYS J 124 -7.744 25.187 26.427 0.00 0.00
J
ATOM 8133 CG LYS J 124 -6.866 25.848 24.600 0.00 0.00
J
ATOM 8134 HG1 LYS J 124 -6.832 25.755 23.493 0.00 0.00
J
ATOM 8135 HG2 LYS J 124 -7.226 26.889 24.740 0.00 0.00
J
ATOM 8136 CD LYS J 124 -5.418 25.619 25.133 0.00 0.00
J
ATOM 8137 HD1 LYS J 124 -5.286 25.415 26.217 0.00 0.00
J
ATOM 8138 HD2 LYS J 124 -5.060 24.693 24.635 0.00 0.00
J
ATOM 8139 CE LYS J 124 -4.319 26.780 24.867 0.00 0.00
J
ATOM 8140 HE1 LYS J 124 -4.040 26.892 23.797 0.00 0.00
J
ATOM 8141 HE2 LYS J 124 -4.693 27.797 25.113 0.00 0.00
J
ATOM 8142 NZ LYS J 124 -2.953 26.564 25.483 0.00 0.00
J
ATOM 8143 HZ1 LYS J 124 -2.520 25.671 25.172 0.00 0.00
J
ATOM 8144 HZ2 LYS J 124 -2.308 27.366 25.333 0.00 0.00
J
ATOM 8145 HZ3 LYS J 124 -3.077 26.536 26.515 0.00 0.00
J
ATOM 8146 C LYS J 124 -8.400 22.544 26.220 0.00 0.00
J
ATOM 8147 O LYS J 124 -8.166 22.083 27.323 0.00 0.00
J
ATOM 8148 N LYS J 125 -9.603 22.257 25.607 0.00 0.00
J
ATOM 8149 HN LYS J 125 -9.842 22.669 24.731 0.00 0.00
J
ATOM 8150 CA LYS J 125 -10.675 21.545 26.375 0.00 0.00
J
ATOM 8151 HA LYS J 125 -10.755 22.142 27.272 0.00 0.00
J
ATOM 8152 CB LYS J 125 -12.011 21.699 25.517 0.00 0.00
J
ATOM 8153 HB1 LYS J 125 -12.081 22.747 25.157 0.00 0.00
J

ATOM 8154 HB2 LYS J 125 -11.841 20.993 24.676 0.00 0.00
J
ATOM 8155 CG LYS J 125 -13.297 21.207 26.206 0.00 0.00
J
ATOM 8156 HG1 LYS J 125 -14.112 21.149 25.453 0.00 0.00
J
ATOM 8157 HG2 LYS J 125 -13.107 20.159 26.522 0.00 0.00
J
ATOM 8158 CD LYS J 125 -13.708 21.974 27.476 0.00 0.00
J
ATOM 8159 HD1 LYS J 125 -12.921 21.919 28.258 0.00 0.00
J
ATOM 8160 HD2 LYS J 125 -13.885 23.034 27.191 0.00 0.00
J
ATOM 8161 CE LYS J 125 -14.901 21.363 28.148 0.00 0.00
J
ATOM 8162 HE1 LYS J 125 -15.728 21.302 27.408 0.00 0.00
J
ATOM 8163 HE2 LYS J 125 -14.729 20.347 28.563 0.00 0.00
J
ATOM 8164 NZ LYS J 125 -15.399 22.335 29.190 0.00 0.00
J
ATOM 8165 HZ1 LYS J 125 -15.750 21.909 30.071 0.00 0.00
J
ATOM 8166 HZ2 LYS J 125 -14.581 22.910 29.476 0.00 0.00
J
ATOM 8167 HZ3 LYS J 125 -16.010 23.017 28.697 0.00 0.00
J
ATOM 8168 C LYS J 125 -10.304 20.182 26.730 0.00 0.00
J
ATOM 8169 O LYS J 125 -10.562 19.802 27.874 0.00 0.00
J
ATOM 8170 N ILE J 126 -9.738 19.340 25.836 0.00 0.00
J
ATOM 8171 HN ILE J 126 -9.775 19.653 24.890 0.00 0.00
J
ATOM 8172 CA ILE J 126 -9.369 17.985 26.256 0.00 0.00
J
ATOM 8173 HA ILE J 126 -10.110 17.612 26.947 0.00 0.00
J
ATOM 8174 CB ILE J 126 -9.292 17.024 25.051 0.00 0.00
J
ATOM 8175 HB ILE J 126 -10.216 17.336 24.518 0.00 0.00
J
ATOM 8176 CG2 ILE J 126 -8.046 17.336 24.185 0.00 0.00
J
ATOM 8177 HG21 ILE J 126 -8.033 16.771 23.229 0.00 0.00
J
ATOM 8178 HG22 ILE J 126 -8.109 18.427 23.983 0.00 0.00
J
ATOM 8179 HG23 ILE J 126 -7.108 17.148 24.750 0.00 0.00
J
ATOM 8180 CG1 ILE J 126 -9.512 15.466 25.365 0.00 0.00
J

ATOM 8181 HG11 ILE J 126 -9.768 14.983 24.398 0.00 0.00
J
ATOM 8182 HG12 ILE J 126 -8.585 14.953 25.700 0.00 0.00
J
ATOM 8183 CD ILE J 126 -10.675 15.082 26.283 0.00 0.00
J
ATOM 8184 HD1 ILE J 126 -11.686 15.426 25.974 0.00 0.00
J
ATOM 8185 HD2 ILE J 126 -10.968 14.017 26.166 0.00 0.00
J
ATOM 8186 HD3 ILE J 126 -10.464 15.334 27.344 0.00 0.00
J
ATOM 8187 C ILE J 126 -8.065 18.034 27.047 0.00 0.00
J
ATOM 8188 O ILE J 126 -7.850 17.119 27.881 0.00 0.00
J
ATOM 8189 N GLY J 127 -7.286 19.069 26.907 0.00 0.00
J
ATOM 8190 HN GLY J 127 -7.488 19.645 26.119 0.00 0.00
J
ATOM 8191 CA GLY J 127 -6.060 19.237 27.666 0.00 0.00
J
ATOM 8192 HA1 GLY J 127 -5.599 20.183 27.422 0.00 0.00
J
ATOM 8193 HA2 GLY J 127 -5.513 18.309 27.593 0.00 0.00
J
ATOM 8194 C GLY J 127 -6.330 19.465 29.126 0.00 0.00
J
ATOM 8195 O GLY J 127 -5.627 18.947 29.945 0.00 0.00
J
ATOM 8196 N LEU J 128 -7.407 20.247 29.492 0.00 0.00
J
ATOM 8197 HN LEU J 128 -7.887 20.859 28.869 0.00 0.00
J
ATOM 8198 CA LEU J 128 -7.952 20.236 30.798 0.00 0.00
J
ATOM 8199 HA LEU J 128 -7.174 20.572 31.468 0.00 0.00
J
ATOM 8200 CB LEU J 128 -9.084 21.317 30.720 0.00 0.00
J
ATOM 8201 HB1 LEU J 128 -8.630 22.319 30.560 0.00 0.00
J
ATOM 8202 HB2 LEU J 128 -9.691 21.277 29.790 0.00 0.00
J
ATOM 8203 CG LEU J 128 -9.876 21.454 31.991 0.00 0.00
J
ATOM 8204 HG LEU J 128 -10.385 20.509 32.278 0.00 0.00
J
ATOM 8205 CD1 LEU J 128 -8.945 21.607 33.244 0.00 0.00
J
ATOM 8206 HD11 LEU J 128 -9.517 21.924 34.142 0.00 0.00
J
ATOM 8207 HD12 LEU J 128 -8.476 20.629 33.484 0.00 0.00
J

ATOM 8208 HD13 LEU J 128 -8.154 22.339 32.973 0.00 0.00
J
ATOM 8209 CD2 LEU J 128 -10.756 22.669 31.869 0.00 0.00
J
ATOM 8210 HD21 LEU J 128 -10.126 23.557 31.652 0.00 0.00
J
ATOM 8211 HD22 LEU J 128 -11.318 22.447 30.937 0.00 0.00
J
ATOM 8212 HD23 LEU J 128 -11.407 22.765 32.764 0.00 0.00
J
ATOM 8213 C LEU J 128 -8.483 18.909 31.331 0.00 0.00
J
ATOM 8214 O LEU J 128 -8.114 18.523 32.457 0.00 0.00
J
ATOM 8215 N LYS J 129 -9.335 18.161 30.521 0.00 0.00
J
ATOM 8216 HN LYS J 129 -9.636 18.691 29.732 0.00 0.00
J
ATOM 8217 CA LYS J 129 -10.011 16.966 30.984 0.00 0.00
J
ATOM 8218 HA LYS J 129 -10.467 17.189 31.937 0.00 0.00
J
ATOM 8219 CB LYS J 129 -11.051 16.423 29.969 0.00 0.00
J
ATOM 8220 HB1 LYS J 129 -10.547 16.000 29.074 0.00 0.00
J
ATOM 8221 HB2 LYS J 129 -11.593 15.635 30.534 0.00 0.00
J
ATOM 8222 CG LYS J 129 -12.182 17.426 29.606 0.00 0.00
J
ATOM 8223 HG1 LYS J 129 -11.852 18.321 29.035 0.00 0.00
J
ATOM 8224 HG2 LYS J 129 -13.002 16.950 29.027 0.00 0.00
J
ATOM 8225 CD LYS J 129 -12.931 17.964 30.833 0.00 0.00
J
ATOM 8226 HD1 LYS J 129 -12.218 18.472 31.517 0.00 0.00
J
ATOM 8227 HD2 LYS J 129 -13.664 18.675 30.396 0.00 0.00
J
ATOM 8228 CE LYS J 129 -13.848 16.930 31.475 0.00 0.00
J
ATOM 8229 HE1 LYS J 129 -14.664 16.657 30.772 0.00 0.00
J
ATOM 8230 HE2 LYS J 129 -13.319 16.077 31.952 0.00 0.00
J
ATOM 8231 NZ LYS J 129 -14.525 17.719 32.557 0.00 0.00
J
ATOM 8232 HZ1 LYS J 129 -15.381 18.122 32.125 0.00 0.00
J
ATOM 8233 HZ2 LYS J 129 -14.867 17.129 33.343 0.00 0.00
J
ATOM 8234 HZ3 LYS J 129 -13.918 18.473 32.936 0.00 0.00
J

ATOM	8235	C	LYS	J	129	-9.122	15.804	31.245	0.00	0.00	J
ATOM	8236	O	LYS	J	129	-9.330	15.119	32.180	0.00	0.00	J
ATOM	8237	N	GLU	J	130	-8.096	15.672	30.413	0.00	0.00	J
ATOM	8238	HN	GLU	J	130	-8.050	16.243	29.597	0.00	0.00	J
ATOM	8239	CA	GLU	J	130	-7.058	14.670	30.614	0.00	0.00	J
ATOM	8240	HA	GLU	J	130	-7.607	13.764	30.824	0.00	0.00	J
ATOM	8241	CB	GLU	J	130	-6.337	14.560	29.302	0.00	0.00	J
ATOM	8242	HB1	GLU	J	130	-7.070	14.195	28.551	0.00	0.00	J
ATOM	8243	HB2	GLU	J	130	-6.052	15.581	28.968	0.00	0.00	J
ATOM	8244	CG	GLU	J	130	-5.147	13.608	29.317	0.00	0.00	J
ATOM	8245	HG1	GLU	J	130	-4.389	14.027	30.013	0.00	0.00	J
ATOM	8246	HG2	GLU	J	130	-5.313	12.575	29.689	0.00	0.00	J
ATOM	8247	CD	GLU	J	130	-4.455	13.442	27.944	0.00	0.00	J
ATOM	8248	OE1	GLU	J	130	-5.154	13.057	27.006	0.00	0.00	J
ATOM	8249	OE2	GLU	J	130	-3.207	13.629	27.836	0.00	0.00	J
ATOM	8250	C	GLU	J	130	-6.141	14.900	31.771	0.00	0.00	J
ATOM	8251	O	GLU	J	130	-5.795	13.970	32.531	0.00	0.00	J
ATOM	8252	N	ARG	J	131	-5.931	16.245	31.990	0.00	0.00	J
ATOM	8253	HN	ARG	J	131	-6.211	16.923	31.314	0.00	0.00	J
ATOM	8254	CA	ARG	J	131	-5.143	16.709	33.131	0.00	0.00	J
ATOM	8255	HA	ARG	J	131	-4.259	16.097	33.238	0.00	0.00	J
ATOM	8256	CB	ARG	J	131	-4.851	18.254	32.993	0.00	0.00	J
ATOM	8257	HB1	ARG	J	131	-4.118	18.481	32.189	0.00	0.00	J
ATOM	8258	HB2	ARG	J	131	-5.814	18.728	32.707	0.00	0.00	J
ATOM	8259	CG	ARG	J	131	-4.297	18.930	34.206	0.00	0.00	J
ATOM	8260	HG1	ARG	J	131	-5.087	18.989	34.986	0.00	0.00	J
ATOM	8261	HG2	ARG	J	131	-3.430	18.320	34.538	0.00	0.00	J

ATOM 8262 CD ARG J 131 -3.810 20.403 34.106 0.00 0.00
J
ATOM 8263 HD1 ARG J 131 -4.625 20.994 33.635 0.00 0.00
J
ATOM 8264 HD2 ARG J 131 -3.515 20.896 35.056 0.00 0.00
J
ATOM 8265 NE ARG J 131 -2.582 20.390 33.160 0.00 0.00
J
ATOM 8266 HE ARG J 131 -2.125 19.534 32.916 0.00 0.00
J
ATOM 8267 CZ ARG J 131 -2.056 21.458 32.564 0.00 0.00
J
ATOM 8268 NH1 ARG J 131 -1.009 21.319 31.782 0.00 0.00
J
ATOM 8269 HH11 ARG J 131 -0.715 22.117 31.256 0.00 0.00
J
ATOM 8270 HH12 ARG J 131 -0.688 20.383 31.636 0.00 0.00
J
ATOM 8271 NH2 ARG J 131 -2.564 22.741 32.798 0.00 0.00
J
ATOM 8272 HH21 ARG J 131 -2.191 23.469 32.222 0.00 0.00
J
ATOM 8273 HH22 ARG J 131 -3.149 22.923 33.588 0.00 0.00
J
ATOM 8274 C ARG J 131 -5.773 16.490 34.451 0.00 0.00
J
ATOM 8275 O ARG J 131 -5.127 16.049 35.358 0.00 0.00
J
ATOM 8276 N MET J 132 -7.096 16.757 34.573 0.00 0.00
J
ATOM 8277 HN MET J 132 -7.467 17.278 33.808 0.00 0.00
J
ATOM 8278 CA MET J 132 -7.818 16.303 35.738 0.00 0.00
J
ATOM 8279 HA MET J 132 -7.316 16.802 36.554 0.00 0.00
J
ATOM 8280 CB MET J 132 -9.341 16.778 35.768 0.00 0.00
J
ATOM 8281 HB1 MET J 132 -9.779 16.546 34.774 0.00 0.00
J
ATOM 8282 HB2 MET J 132 -9.886 16.315 36.618 0.00 0.00
J
ATOM 8283 CG MET J 132 -9.440 18.311 35.939 0.00 0.00
J
ATOM 8284 HG1 MET J 132 -8.873 18.728 35.079 0.00 0.00
J
ATOM 8285 HG2 MET J 132 -10.506 18.607 35.830 0.00 0.00
J
ATOM 8286 SD MET J 132 -8.787 18.875 37.552 0.00 0.00
J
ATOM 8287 CE MET J 132 -8.910 20.630 36.999 0.00 0.00
J
ATOM 8288 HE1 MET J 132 -9.863 21.159 37.217 0.00 0.00
J

```
ATOM   8289  HE2 MET J 132      -8.084  21.249  37.410  0.00  0.00
J
ATOM   8290  HE3 MET J 132      -8.743  20.710  35.904  0.00  0.00
J
ATOM   8291  C   MET J 132      -7.942  14.827  35.939  0.00  0.00
J
ATOM   8292  O   MET J 132      -7.911  14.340  37.073  0.00  0.00
J
ATOM   8293  N   LEU J 133      -8.132  14.017  34.883  0.00  0.00
J
ATOM   8294  HN  LEU J 133      -8.339  14.400  33.986  0.00  0.00
J
ATOM   8295  CA  LEU J 133      -8.332  12.585  34.982  0.00  0.00
J
ATOM   8296  HA  LEU J 133      -9.083  12.316  35.710  0.00  0.00
J
ATOM   8297  CB  LEU J 133      -8.632  12.140  33.567  0.00  0.00
J
ATOM   8298  HB1 LEU J 133      -9.487  12.745  33.197  0.00  0.00
J
ATOM   8299  HB2 LEU J 133      -7.907  12.407  32.769  0.00  0.00
J
ATOM   8300  CG  LEU J 133      -9.113  10.725  33.337  0.00  0.00
J
ATOM   8301  HG  LEU J 133      -9.561  10.383  34.294  0.00  0.00
J
ATOM   8302  CD1 LEU J 133     -10.165  10.675  32.200  0.00  0.00
J
ATOM   8303 HD11 LEU J 133      -9.643  11.001  31.275  0.00  0.00
J
ATOM   8304 HD12 LEU J 133     -10.568   9.651  32.048  0.00  0.00
J
ATOM   8305 HD13 LEU J 133     -10.991  11.407  32.320  0.00  0.00
J
ATOM   8306  CD2 LEU J 133      -7.904   9.714  33.042  0.00  0.00
J
ATOM   8307 HD21 LEU J 133      -7.115   9.733  33.823  0.00  0.00
J
ATOM   8308 HD22 LEU J 133      -8.326   8.690  32.956  0.00  0.00
J
ATOM   8309 HD23 LEU J 133      -7.482   9.919  32.035  0.00  0.00
J
ATOM   8310  C   LEU J 133      -7.116  11.908  35.456  0.00  0.00
J
ATOM   8311  O   LEU J 133      -7.214  11.003  36.270  0.00  0.00
J
ATOM   8312  N   VAL J 134      -5.896  12.309  35.005  0.00  0.00
J
ATOM   8313  HN  VAL J 134      -5.895  12.943  34.236  0.00  0.00
J
ATOM   8314  CA  VAL J 134      -4.646  11.660  35.403  0.00  0.00
J
ATOM   8315  HA  VAL J 134      -4.941  10.669  35.715  0.00  0.00
J
```

ATOM 8316 CB VAL J 134 −3.582 11.556 34.347 0.00 0.00
J
ATOM 8317 HB VAL J 134 −2.815 10.835 34.703 0.00 0.00
J
ATOM 8318 CG1 VAL J 134 −4.197 10.881 33.107 0.00 0.00
J
ATOM 8319 HG11 VAL J 134 −4.763 9.984 33.440 0.00 0.00
J
ATOM 8320 HG12 VAL J 134 −5.076 11.433 32.709 0.00 0.00
J
ATOM 8321 HG13 VAL J 134 −3.511 10.801 32.237 0.00 0.00
J
ATOM 8322 CG2 VAL J 134 −2.841 12.902 34.144 0.00 0.00
J
ATOM 8323 HG21 VAL J 134 −2.154 12.988 33.275 0.00 0.00
J
ATOM 8324 HG22 VAL J 134 −3.620 13.665 33.929 0.00 0.00
J
ATOM 8325 HG23 VAL J 134 −2.358 13.271 35.073 0.00 0.00
J
ATOM 8326 C VAL J 134 −4.035 12.308 36.644 0.00 0.00
J
ATOM 8327 O VAL J 134 −3.073 11.890 37.359 0.00 0.00
J
ATOM 8328 N GLN J 135 −4.625 13.478 36.945 0.00 0.00
J
ATOM 8329 HN GLN J 135 −5.441 13.620 36.390 0.00 0.00
J
ATOM 8330 CA GLN J 135 −4.364 14.375 38.044 0.00 0.00
J
ATOM 8331 HA GLN J 135 −5.086 15.157 37.858 0.00 0.00
J
ATOM 8332 CB GLN J 135 −4.663 13.714 39.390 0.00 0.00
J
ATOM 8333 HB1 GLN J 135 −4.266 12.678 39.336 0.00 0.00
J
ATOM 8334 HB2 GLN J 135 −4.066 14.077 40.253 0.00 0.00
J
ATOM 8335 CG GLN J 135 −6.174 13.501 39.719 0.00 0.00
J
ATOM 8336 HG1 GLN J 135 −6.737 14.457 39.661 0.00 0.00
J
ATOM 8337 HG2 GLN J 135 −6.668 12.845 38.970 0.00 0.00
J
ATOM 8338 CD GLN J 135 −6.275 12.886 41.085 0.00 0.00
J
ATOM 8339 OE1 GLN J 135 −5.282 12.441 41.696 0.00 0.00
J
ATOM 8340 NE2 GLN J 135 −7.516 12.881 41.516 0.00 0.00
J
ATOM 8341 HE21 GLN J 135 −7.763 12.586 42.439 0.00 0.00
J
ATOM 8342 HE22 GLN J 135 −8.323 12.994 40.937 0.00 0.00
J

ATOM	8343	C	GLN	J	135	-3.081	15.113	38.011	0.00	0.00	J
ATOM	8344	O	GLN	J	135	-2.486	15.228	39.040	0.00	0.00	J
ATOM	8345	N	GLU	J	136	-2.642	15.535	36.819	0.00	0.00	J
ATOM	8346	HN	GLU	J	136	-3.248	15.445	36.032	0.00	0.00	J
ATOM	8347	CA	GLU	J	136	-1.270	16.162	36.647	0.00	0.00	J
ATOM	8348	HA	GLU	J	136	-0.453	15.568	37.027	0.00	0.00	J
ATOM	8349	CB	GLU	J	136	-1.052	16.395	35.113	0.00	0.00	J
ATOM	8350	HB1	GLU	J	136	-0.928	15.386	34.664	0.00	0.00	J
ATOM	8351	HB2	GLU	J	136	-2.023	16.725	34.684	0.00	0.00	J
ATOM	8352	CG	GLU	J	136	0.071	17.384	34.617	0.00	0.00	J
ATOM	8353	HG1	GLU	J	136	0.148	18.395	35.072	0.00	0.00	J
ATOM	8354	HG2	GLU	J	136	1.059	16.878	34.659	0.00	0.00	J
ATOM	8355	CD	GLU	J	136	0.038	17.643	33.107	0.00	0.00	J
ATOM	8356	OE1	GLU	J	136	-1.059	18.088	32.645	0.00	0.00	J
ATOM	8357	OE2	GLU	J	136	1.111	17.437	32.402	0.00	0.00	J
ATOM	8358	C	GLU	J	136	-1.085	17.498	37.324	0.00	0.00	J
ATOM	8359	O	GLU	J	136	-0.010	17.829	37.880	0.00	0.00	J
ATOM	8360	N	ALA	J	137	-2.136	18.327	37.374	0.00	0.00	J
ATOM	8361	HN	ALA	J	137	-2.978	18.111	36.886	0.00	0.00	J
ATOM	8362	CA	ALA	J	137	-2.152	19.575	38.078	0.00	0.00	J
ATOM	8363	HA	ALA	J	137	-1.812	19.257	39.052	0.00	0.00	J
ATOM	8364	CB	ALA	J	137	-1.353	20.733	37.442	0.00	0.00	J
ATOM	8365	HB1	ALA	J	137	-1.716	21.006	36.428	0.00	0.00	J
ATOM	8366	HB2	ALA	J	137	-1.295	21.648	38.070	0.00	0.00	J
ATOM	8367	HB3	ALA	J	137	-0.315	20.375	37.272	0.00	0.00	J
ATOM	8368	C	ALA	J	137	-3.631	20.025	38.232	0.00	0.00	J
ATOM	8369	O	ALA	J	137	-4.504	19.597	37.454	0.00	0.00	J

ATOM 8370 N LEU J 138 -3.972 20.980 39.143 0.00 0.00
J
ATOM 8371 HN LEU J 138 -3.256 21.267 39.775 0.00 0.00
J
ATOM 8372 CA LEU J 138 -5.227 21.585 39.247 0.00 0.00
J
ATOM 8373 HA LEU J 138 -6.049 20.962 38.929 0.00 0.00
J
ATOM 8374 CB LEU J 138 -5.547 21.979 40.683 0.00 0.00
J
ATOM 8375 HB1 LEU J 138 -4.657 22.511 41.082 0.00 0.00
J
ATOM 8376 HB2 LEU J 138 -6.469 22.598 40.706 0.00 0.00
J
ATOM 8377 CG LEU J 138 -5.752 20.780 41.625 0.00 0.00
J
ATOM 8378 HG LEU J 138 -4.948 20.022 41.510 0.00 0.00
J
ATOM 8379 CD1 LEU J 138 -5.611 21.321 43.023 0.00 0.00
J
ATOM 8380 HD11 LEU J 138 -4.564 21.647 43.201 0.00 0.00
J
ATOM 8381 HD12 LEU J 138 -6.306 22.166 43.218 0.00 0.00
J
ATOM 8382 HD13 LEU J 138 -5.794 20.536 43.788 0.00 0.00
J
ATOM 8383 CD2 LEU J 138 -7.153 20.124 41.478 0.00 0.00
J
ATOM 8384 HD21 LEU J 138 -7.360 19.281 42.172 0.00 0.00
J
ATOM 8385 HD22 LEU J 138 -7.898 20.911 41.720 0.00 0.00
J
ATOM 8386 HD23 LEU J 138 -7.278 19.763 40.435 0.00 0.00
J
ATOM 8387 C LEU J 138 -5.290 22.802 38.338 0.00 0.00
J
ATOM 8388 O LEU J 138 -6.336 23.319 38.016 0.00 0.00
J
ATOM 8389 N ASN J 139 -4.037 23.308 37.857 0.00 0.00
J
ATOM 8390 HN ASN J 139 -3.241 22.940 38.332 0.00 0.00
J
ATOM 8391 CA ASN J 139 -3.919 24.489 37.011 0.00 0.00
J
ATOM 8392 HA ASN J 139 -4.436 25.322 37.462 0.00 0.00
J
ATOM 8393 CB ASN J 139 -2.387 24.672 36.979 0.00 0.00
J
ATOM 8394 HB1 ASN J 139 -1.943 23.746 36.556 0.00 0.00
J
ATOM 8395 HB2 ASN J 139 -2.070 25.370 36.174 0.00 0.00
J
ATOM 8396 CG ASN J 139 -1.744 24.940 38.319 0.00 0.00
J

ATOM 8397 OD1 ASN J 139 -0.926 24.093 38.682 0.00 0.00
J
ATOM 8398 ND2 ASN J 139 -2.113 26.064 38.969 0.00 0.00
J
ATOM 8399 HD21 ASN J 139 -1.479 26.434 39.648 0.00 0.00
J
ATOM 8400 HD22 ASN J 139 -2.553 26.774 38.419 0.00 0.00
J
ATOM 8401 C ASN J 139 -4.479 24.327 35.556 0.00 0.00
J
ATOM 8402 O ASN J 139 -3.968 23.590 34.741 0.00 0.00
J
ATOM 8403 N GLN J 140 -5.542 25.031 35.211 0.00 0.00
J
ATOM 8404 HN GLN J 140 -6.034 25.634 35.834 0.00 0.00
J
ATOM 8405 CA GLN J 140 -6.239 24.858 33.964 0.00 0.00
J
ATOM 8406 HA GLN J 140 -6.440 23.802 33.858 0.00 0.00
J
ATOM 8407 CB GLN J 140 -7.601 25.523 34.005 0.00 0.00
J
ATOM 8408 HB1 GLN J 140 -7.475 26.608 34.205 0.00 0.00
J
ATOM 8409 HB2 GLN J 140 -8.209 25.428 33.080 0.00 0.00
J
ATOM 8410 CG GLN J 140 -8.481 25.048 35.236 0.00 0.00
J
ATOM 8411 HG1 GLN J 140 -8.574 23.946 35.131 0.00 0.00
J
ATOM 8412 HG2 GLN J 140 -7.971 25.295 36.192 0.00 0.00
J
ATOM 8413 CD GLN J 140 -9.986 25.478 35.224 0.00 0.00
J
ATOM 8414 OE1 GLN J 140 -10.755 24.784 34.579 0.00 0.00
J
ATOM 8415 NE2 GLN J 140 -10.282 26.624 35.764 0.00 0.00
J
ATOM 8416 HE21 GLN J 140 -11.207 26.989 35.654 0.00 0.00
J
ATOM 8417 HE22 GLN J 140 -9.608 27.190 36.239 0.00 0.00
J
ATOM 8418 C GLN J 140 -5.491 25.495 32.753 0.00 0.00
J
ATOM 8419 O GLN J 140 -4.976 26.567 32.866 0.00 0.00
J
ATOM 8420 N PRO J 141 -5.476 24.883 31.550 0.00 0.00
J
ATOM 8421 CD PRO J 141 -5.553 23.428 31.182 0.00 0.00
J
ATOM 8422 HD1 PRO J 141 -5.244 22.735 31.994 0.00 0.00
J
ATOM 8423 HD2 PRO J 141 -6.623 23.242 30.951 0.00 0.00
J

ATOM 8424 CA PRO J 141 -5.090 25.653 30.355 0.00 0.00
J
ATOM 8425 HA PRO J 141 -4.116 26.021 30.642 0.00 0.00
J
ATOM 8426 CB PRO J 141 -5.068 24.655 29.215 0.00 0.00
J
ATOM 8427 HB1 PRO J 141 -4.207 24.798 28.528 0.00 0.00
J
ATOM 8428 HB2 PRO J 141 -6.067 24.542 28.743 0.00 0.00
J
ATOM 8429 CG PRO J 141 -4.677 23.370 29.978 0.00 0.00
J
ATOM 8430 HG1 PRO J 141 -3.596 23.447 30.221 0.00 0.00
J
ATOM 8431 HG2 PRO J 141 -4.879 22.471 29.358 0.00 0.00
J
ATOM 8432 C PRO J 141 -6.072 26.814 29.988 0.00 0.00
J
ATOM 8433 O PRO J 141 -7.198 26.831 30.557 0.00 0.00
J
ATOM 8434 N THR J 142 -5.721 27.734 29.125 0.00 0.00
J
ATOM 8435 HN THR J 142 -4.850 27.695 28.642 0.00 0.00
J
ATOM 8436 CA THR J 142 -6.575 28.901 28.723 0.00 0.00
J
ATOM 8437 HA THR J 142 -7.326 29.084 29.477 0.00 0.00
J
ATOM 8438 CB THR J 142 -5.786 30.273 28.664 0.00 0.00
J
ATOM 8439 HB THR J 142 -5.336 30.442 29.666 0.00 0.00
J
ATOM 8440 OG1 THR J 142 -6.713 31.288 28.421 0.00 0.00
J
ATOM 8441 HG1 THR J 142 -6.211 32.106 28.450 0.00 0.00
J
ATOM 8442 CG2 THR J 142 -4.705 30.398 27.662 0.00 0.00
J
ATOM 8443 HG21 THR J 142 -4.438 31.470 27.545 0.00 0.00
J
ATOM 8444 HG22 THR J 142 -3.805 29.813 27.947 0.00 0.00
J
ATOM 8445 HG23 THR J 142 -5.049 30.029 26.672 0.00 0.00
J
ATOM 8446 C THR J 142 -7.378 28.625 27.477 0.00 0.00
J
ATOM 8447 O THR J 142 -6.930 28.872 26.322 0.00 0.00
J
ATOM 8448 N ILE J 143 -8.596 28.115 27.687 0.00 0.00
J
ATOM 8449 HN ILE J 143 -8.935 27.819 28.576 0.00 0.00
J
ATOM 8450 CA ILE J 143 -9.526 27.733 26.596 0.00 0.00
J

ATOM 8451 HA ILE J 143 -9.087 27.079 25.857 0.00 0.00
J
ATOM 8452 CB ILE J 143 -10.760 27.092 27.206 0.00 0.00
J
ATOM 8453 HB ILE J 143 -11.402 27.754 27.826 0.00 0.00
J
ATOM 8454 CG2 ILE J 143 -11.800 26.625 26.071 0.00 0.00
J
ATOM 8455 HG21 ILE J 143 -12.180 27.441 25.418 0.00 0.00
J
ATOM 8456 HG22 ILE J 143 -11.257 25.820 25.531 0.00 0.00
J
ATOM 8457 HG23 ILE J 143 -12.693 26.220 26.593 0.00 0.00
J
ATOM 8458 CG1 ILE J 143 -10.453 25.727 28.018 0.00 0.00
J
ATOM 8459 HG11 ILE J 143 -9.895 24.955 27.445 0.00 0.00
J
ATOM 8460 HG12 ILE J 143 -9.702 25.960 28.803 0.00 0.00
J
ATOM 8461 CD ILE J 143 -11.696 25.125 28.728 0.00 0.00
J
ATOM 8462 HD1 ILE J 143 -11.339 24.330 29.418 0.00 0.00
J
ATOM 8463 HD2 ILE J 143 -12.282 25.768 29.421 0.00 0.00
J
ATOM 8464 HD3 ILE J 143 -12.412 24.661 28.017 0.00 0.00
J
ATOM 8465 C ILE J 143 -9.941 28.987 25.816 0.00 0.00
J
ATOM 8466 O ILE J 143 -10.054 29.046 24.598 0.00 0.00
J
ATOM 8467 N GLY J 144 -10.157 30.043 26.656 0.00 0.00
J
ATOM 8468 HN GLY J 144 -10.163 29.904 27.643 0.00 0.00
J
ATOM 8469 CA GLY J 144 -10.616 31.363 26.241 0.00 0.00
J
ATOM 8470 HA1 GLY J 144 -10.848 31.872 27.164 0.00 0.00
J
ATOM 8471 HA2 GLY J 144 -11.370 31.187 25.488 0.00 0.00
J
ATOM 8472 C GLY J 144 -9.508 32.129 25.551 0.00 0.00
J
ATOM 8473 O GLY J 144 -9.789 33.131 24.861 0.00 0.00
J
ATOM 8474 N GLY J 145 -8.253 31.661 25.538 0.00 0.00
J
ATOM 8475 HN GLY J 145 -8.094 30.923 26.188 0.00 0.00
J
ATOM 8476 CA GLY J 145 -7.170 32.359 24.838 0.00 0.00
J
ATOM 8477 HA1 GLY J 145 -6.314 31.757 25.107 0.00 0.00
J

ATOM 8478 HA2 GLY J 145 -7.221 33.432 24.949 0.00 0.00
J
ATOM 8479 C GLY J 145 -7.352 32.158 23.337 0.00 0.00
J
ATOM 8480 O GLY J 145 -6.709 32.734 22.476 0.00 0.00
J
ATOM 8481 N VAL J 146 -8.103 31.125 22.947 0.00 0.00
J
ATOM 8482 HN VAL J 146 -8.447 30.514 23.655 0.00 0.00
J
ATOM 8483 CA VAL J 146 -8.383 30.807 21.548 0.00 0.00
J
ATOM 8484 HA VAL J 146 -7.448 30.663 21.027 0.00 0.00
J
ATOM 8485 CB VAL J 146 -9.133 29.635 21.487 0.00 0.00
J
ATOM 8486 HB VAL J 146 -10.046 29.790 22.100 0.00 0.00
J
ATOM 8487 CG1 VAL J 146 -9.364 29.387 19.949 0.00 0.00
J
ATOM 8488 HG11 VAL J 146 -8.542 29.706 19.273 0.00 0.00
J
ATOM 8489 HG12 VAL J 146 -9.709 28.342 19.796 0.00 0.00
J
ATOM 8490 HG13 VAL J 146 -10.186 30.039 19.583 0.00 0.00
J
ATOM 8491 CG2 VAL J 146 -8.263 28.486 22.022 0.00 0.00
J
ATOM 8492 HG21 VAL J 146 -7.186 28.603 21.776 0.00 0.00
J
ATOM 8493 HG22 VAL J 146 -8.260 28.534 23.132 0.00 0.00
J
ATOM 8494 HG23 VAL J 146 -8.637 27.498 21.680 0.00 0.00
J
ATOM 8495 C VAL J 146 -9.095 32.082 20.845 0.00 0.00
J
ATOM 8496 O VAL J 146 -8.782 32.460 19.735 0.00 0.00
J
ATOM 8497 N ILE J 147 -10.051 32.752 21.620 0.00 0.00
J
ATOM 8498 HN ILE J 147 -10.030 32.700 22.615 0.00 0.00
J
ATOM 8499 CA ILE J 147 -11.171 33.543 21.018 0.00 0.00
J
ATOM 8500 HA ILE J 147 -11.731 32.862 20.393 0.00 0.00
J
ATOM 8501 CB ILE J 147 -12.249 34.008 22.014 0.00 0.00
J
ATOM 8502 HB ILE J 147 -11.958 34.767 22.771 0.00 0.00
J
ATOM 8503 CG2 ILE J 147 -13.484 34.604 21.361 0.00 0.00
J
ATOM 8504 HG21 ILE J 147 -13.335 35.605 20.902 0.00 0.00
J

```
ATOM   8505 HG22 ILE J 147     -13.807  33.857  20.605  0.00  0.00
J
ATOM   8506 HG23 ILE J 147     -14.259  34.713  22.150  0.00  0.00
J
ATOM   8507  CG1 ILE J 147     -12.593  32.692  22.907  0.00  0.00
J
ATOM   8508 HG11 ILE J 147     -11.697  32.356  23.470  0.00  0.00
J
ATOM   8509 HG12 ILE J 147     -13.274  33.153  23.654  0.00  0.00
J
ATOM   8510  CD  ILE J 147     -13.326  31.533  22.275  0.00  0.00
J
ATOM   8511  HD1 ILE J 147     -14.370  31.815  22.019  0.00  0.00
J
ATOM   8512  HD2 ILE J 147     -12.962  31.230  21.270  0.00  0.00
J
ATOM   8513  HD3 ILE J 147     -13.318  30.661  22.963  0.00  0.00
J
ATOM   8514  C   ILE J 147     -10.674  34.762  20.210  0.00  0.00
J
ATOM   8515  O   ILE J 147     -11.092  34.996  19.067  0.00  0.00
J
ATOM   8516  N   GLY J 148      -9.760  35.551  20.795  0.00  0.00
J
ATOM   8517  HN  GLY J 148      -9.448  35.288  21.704  0.00  0.00
J
ATOM   8518  CA  GLY J 148      -9.023  36.686  20.240  0.00  0.00
J
ATOM   8519  HA1 GLY J 148      -8.361  36.969  21.045  0.00  0.00
J
ATOM   8520  HA2 GLY J 148      -9.753  37.419  19.930  0.00  0.00
J
ATOM   8521  C   GLY J 148      -8.047  36.249  19.152  0.00  0.00
J
ATOM   8522  O   GLY J 148      -7.739  37.035  18.280  0.00  0.00
J
ATOM   8523  N   LEU J 149      -7.437  35.044  19.152  0.00  0.00
J
ATOM   8524  HN  LEU J 149      -7.814  34.438  19.848  0.00  0.00
J
ATOM   8525  CA  LEU J 149      -6.429  34.572  18.283  0.00  0.00
J
ATOM   8526  HA  LEU J 149      -5.627  35.292  18.346  0.00  0.00
J
ATOM   8527  CB  LEU J 149      -5.968  33.277  18.904  0.00  0.00
J
ATOM   8528  HB1 LEU J 149      -5.812  33.229  20.003  0.00  0.00
J
ATOM   8529  HB2 LEU J 149      -6.786  32.538  18.761  0.00  0.00
J
ATOM   8530  CG  LEU J 149      -4.603  32.724  18.268  0.00  0.00
J
ATOM   8531  HG  LEU J 149      -4.702  32.874  17.172  0.00  0.00
J
```

ATOM 8532 CD1 LEU J 149 -3.394 33.380 18.899 0.00 0.00
J
ATOM 8533 HD11 LEU J 149 -3.645 33.421 19.981 0.00 0.00
J
ATOM 8534 HD12 LEU J 149 -2.530 32.703 18.726 0.00 0.00
J
ATOM 8535 HD13 LEU J 149 -3.260 34.418 18.526 0.00 0.00
J
ATOM 8536 CD2 LEU J 149 -4.538 31.206 18.406 0.00 0.00
J
ATOM 8537 HD21 LEU J 149 -3.665 30.757 17.887 0.00 0.00
J
ATOM 8538 HD22 LEU J 149 -4.484 30.981 19.493 0.00 0.00
J
ATOM 8539 HD23 LEU J 149 -5.393 30.720 17.889 0.00 0.00
J
ATOM 8540 C LEU J 149 -6.894 34.383 16.919 0.00 0.00
J
ATOM 8541 O LEU J 149 -6.268 34.879 15.926 0.00 0.00
J
ATOM 8542 N VAL J 150 -8.063 33.791 16.859 0.00 0.00
J
ATOM 8543 HN VAL J 150 -8.494 33.443 17.688 0.00 0.00
J
ATOM 8544 CA VAL J 150 -8.729 33.522 15.627 0.00 0.00
J
ATOM 8545 HA VAL J 150 -8.173 32.956 14.894 0.00 0.00
J
ATOM 8546 CB VAL J 150 -9.995 32.641 15.877 0.00 0.00
J
ATOM 8547 HB VAL J 150 -10.733 33.128 16.550 0.00 0.00
J
ATOM 8548 CG1 VAL J 150 -10.650 32.336 14.481 0.00 0.00
J
ATOM 8549 HG11 VAL J 150 -10.831 33.345 14.052 0.00 0.00
J
ATOM 8550 HG12 VAL J 150 -9.973 31.840 13.754 0.00 0.00
J
ATOM 8551 HG13 VAL J 150 -11.615 31.791 14.559 0.00 0.00
J
ATOM 8552 CG2 VAL J 150 -9.368 31.349 16.423 0.00 0.00
J
ATOM 8553 HG21 VAL J 150 -8.876 31.674 17.365 0.00 0.00
J
ATOM 8554 HG22 VAL J 150 -10.178 30.612 16.606 0.00 0.00
J
ATOM 8555 HG23 VAL J 150 -8.707 30.966 15.617 0.00 0.00
J
ATOM 8556 C VAL J 150 -9.168 34.820 14.954 0.00 0.00
J
ATOM 8557 O VAL J 150 -9.016 34.933 13.716 0.00 0.00
J
ATOM 8558 N LYS J 151 -9.658 35.872 15.654 0.00 0.00
J

ATOM 8559 HN LYS J 151 -9.732 35.856 16.648 0.00 0.00
J
ATOM 8560 CA LYS J 151 -9.903 37.171 15.117 0.00 0.00
J
ATOM 8561 HA LYS J 151 -10.571 37.030 14.279 0.00 0.00
J
ATOM 8562 CB LYS J 151 -10.539 38.067 16.222 0.00 0.00
J
ATOM 8563 HB1 LYS J 151 -9.773 38.348 16.976 0.00 0.00
J
ATOM 8564 HB2 LYS J 151 -10.875 38.950 15.637 0.00 0.00
J
ATOM 8565 CG LYS J 151 -11.829 37.412 16.784 0.00 0.00
J
ATOM 8566 HG1 LYS J 151 -12.667 37.434 16.054 0.00 0.00
J
ATOM 8567 HG2 LYS J 151 -11.621 36.354 17.052 0.00 0.00
J
ATOM 8568 CD LYS J 151 -12.441 38.240 17.978 0.00 0.00
J
ATOM 8569 HD1 LYS J 151 -11.674 38.381 18.769 0.00 0.00
J
ATOM 8570 HD2 LYS J 151 -12.758 39.216 17.554 0.00 0.00
J
ATOM 8571 CE LYS J 151 -13.608 37.581 18.631 0.00 0.00
J
ATOM 8572 HE1 LYS J 151 -14.306 37.268 17.825 0.00 0.00
J
ATOM 8573 HE2 LYS J 151 -13.338 36.700 19.252 0.00 0.00
J
ATOM 8574 NZ LYS J 151 -14.325 38.568 19.433 0.00 0.00
J
ATOM 8575 HZ1 LYS J 151 -15.314 38.273 19.556 0.00 0.00
J
ATOM 8576 HZ2 LYS J 151 -14.085 38.459 20.439 0.00 0.00
J
ATOM 8577 HZ3 LYS J 151 -14.209 39.546 19.099 0.00 0.00
J
ATOM 8578 C LYS J 151 -8.694 37.822 14.513 0.00 0.00
J
ATOM 8579 O LYS J 151 -8.793 38.539 13.511 0.00 0.00
J
ATOM 8580 N VAL J 152 -7.435 37.593 15.054 0.00 0.00
J
ATOM 8581 HN VAL J 152 -7.313 37.016 15.858 0.00 0.00
J
ATOM 8582 CA VAL J 152 -6.186 38.058 14.468 0.00 0.00
J
ATOM 8583 HA VAL J 152 -6.369 38.924 13.849 0.00 0.00
J
ATOM 8584 CB VAL J 152 -5.082 38.509 15.406 0.00 0.00
J
ATOM 8585 HB VAL J 152 -4.270 38.923 14.771 0.00 0.00
J

ATOM 8586 CG1 VAL J 152 -5.690 39.601 16.307 0.00 0.00
J
ATOM 8587 HG11 VAL J 152 -4.947 40.110 16.957 0.00 0.00
J
ATOM 8588 HG12 VAL J 152 -6.053 40.394 15.618 0.00 0.00
J
ATOM 8589 HG13 VAL J 152 -6.579 39.296 16.899 0.00 0.00
J
ATOM 8590 CG2 VAL J 152 -4.513 37.325 16.253 0.00 0.00
J
ATOM 8591 HG21 VAL J 152 -4.190 36.479 15.609 0.00 0.00
J
ATOM 8592 HG22 VAL J 152 -3.705 37.664 16.937 0.00 0.00
J
ATOM 8593 HG23 VAL J 152 -5.262 36.886 16.946 0.00 0.00
J
ATOM 8594 C VAL J 152 -5.535 37.154 13.418 0.00 0.00
J
ATOM 8595 O VAL J 152 -4.996 37.735 12.498 0.00 0.00
J
ATOM 8596 N LEU J 153 -5.686 35.821 13.398 0.00 0.00
J
ATOM 8597 HN LEU J 153 -5.906 35.401 14.275 0.00 0.00
J
ATOM 8598 CA LEU J 153 -5.429 34.849 12.294 0.00 0.00
J
ATOM 8599 HA LEU J 153 -4.386 34.923 12.025 0.00 0.00
J
ATOM 8600 CB LEU J 153 -5.728 33.392 12.757 0.00 0.00
J
ATOM 8601 HB1 LEU J 153 -6.725 33.367 13.246 0.00 0.00
J
ATOM 8602 HB2 LEU J 153 -5.744 32.751 11.850 0.00 0.00
J
ATOM 8603 CG LEU J 153 -4.672 32.845 13.703 0.00 0.00
J
ATOM 8604 HG LEU J 153 -4.579 33.763 14.322 0.00 0.00
J
ATOM 8605 CD1 LEU J 153 -5.340 31.663 14.548 0.00 0.00
J
ATOM 8606 HD11 LEU J 153 -4.735 31.494 15.464 0.00 0.00
J
ATOM 8607 HD12 LEU J 153 -6.272 32.091 14.975 0.00 0.00
J
ATOM 8608 HD13 LEU J 153 -5.579 30.707 14.034 0.00 0.00
J
ATOM 8609 CD2 LEU J 153 -3.402 32.382 13.038 0.00 0.00
J
ATOM 8610 HD21 LEU J 153 -2.906 33.203 12.478 0.00 0.00
J
ATOM 8611 HD22 LEU J 153 -2.671 31.886 13.711 0.00 0.00
J
ATOM 8612 HD23 LEU J 153 -3.627 31.658 12.225 0.00 0.00
J

```
ATOM   8613  C   LEU J 153      -6.293  35.124  11.032  0.00  0.00
J
ATOM   8614  O   LEU J 153      -5.978  34.828   9.890  0.00  0.00
J
ATOM   8615  N   PHE J 154      -7.465  35.731  11.244  0.00  0.00
J
ATOM   8616  HN  PHE J 154      -7.920  35.851  12.123  0.00  0.00
J
ATOM   8617  CA  PHE J 154      -8.373  36.194  10.167  0.00  0.00
J
ATOM   8618  HA  PHE J 154      -8.423  35.500   9.341  0.00  0.00
J
ATOM   8619  CB  PHE J 154      -9.766  36.395  10.750  0.00  0.00
J
ATOM   8620  HB1 PHE J 154     -10.028  35.465  11.299  0.00  0.00
J
ATOM   8621  HB2 PHE J 154      -9.717  37.266  11.438  0.00  0.00
J
ATOM   8622  CG  PHE J 154     -10.839  36.703   9.708  0.00  0.00
J
ATOM   8623  CD1 PHE J 154     -10.814  37.893   9.061  0.00  0.00
J
ATOM   8624  HD1 PHE J 154     -10.065  38.565   9.454  0.00  0.00
J
ATOM   8625  CE1 PHE J 154     -11.871  38.338   8.177  0.00  0.00
J
ATOM   8626  HE1 PHE J 154     -11.975  39.370   7.877  0.00  0.00
J
ATOM   8627  CZ  PHE J 154     -12.862  37.458   7.868  0.00  0.00
J
ATOM   8628  HZ  PHE J 154     -13.598  37.743   7.131  0.00  0.00
J
ATOM   8629  CD2 PHE J 154     -11.945  35.847   9.406  0.00  0.00
J
ATOM   8630  HD2 PHE J 154     -11.978  34.854   9.828  0.00  0.00
J
ATOM   8631  CE2 PHE J 154     -12.901  36.176   8.506  0.00  0.00
J
ATOM   8632  HE2 PHE J 154     -13.724  35.523   8.256  0.00  0.00
J
ATOM   8633  C   PHE J 154      -7.756  37.406   9.545  0.00  0.00
J
ATOM   8634  O   PHE J 154      -7.570  37.526   8.335  0.00  0.00
J
ATOM   8635  N   LEU J 155      -7.233  38.344  10.377  0.00  0.00
J
ATOM   8636  HN  LEU J 155      -7.298  38.215  11.363  0.00  0.00
J
ATOM   8637  CA  LEU J 155      -6.641  39.624   9.871  0.00  0.00
J
ATOM   8638  HA  LEU J 155      -7.376  40.028   9.190  0.00  0.00
J
ATOM   8639  CB  LEU J 155      -6.416  40.489  11.091  0.00  0.00
J
```

ATOM 8640 HB1 LEU J 155 -7.290 40.647 11.760 0.00 0.00
J
ATOM 8641 HB2 LEU J 155 -5.742 39.938 11.781 0.00 0.00
J
ATOM 8642 CG LEU J 155 -5.666 41.892 10.863 0.00 0.00
J
ATOM 8643 HG LEU J 155 -4.685 41.604 10.429 0.00 0.00
J
ATOM 8644 CD1 LEU J 155 -6.464 42.741 9.871 0.00 0.00
J
ATOM 8645 HD11 LEU J 155 -6.145 43.805 9.844 0.00 0.00
J
ATOM 8646 HD12 LEU J 155 -6.364 42.232 8.889 0.00 0.00
J
ATOM 8647 HD13 LEU J 155 -7.542 42.707 10.136 0.00 0.00
J
ATOM 8648 CD2 LEU J 155 -5.520 42.640 12.158 0.00 0.00
J
ATOM 8649 HD21 LEU J 155 -4.982 43.576 11.895 0.00 0.00
J
ATOM 8650 HD22 LEU J 155 -6.502 42.953 12.573 0.00 0.00
J
ATOM 8651 HD23 LEU J 155 -5.001 42.019 12.919 0.00 0.00
J
ATOM 8652 C LEU J 155 -5.362 39.310 9.091 0.00 0.00
J
ATOM 8653 O LEU J 155 -5.017 39.896 8.133 0.00 0.00
J
ATOM 8654 N PHE J 156 -4.512 38.341 9.618 0.00 0.00
J
ATOM 8655 HN PHE J 156 -4.711 37.975 10.524 0.00 0.00
J
ATOM 8656 CA PHE J 156 -3.344 37.813 8.969 0.00 0.00
J
ATOM 8657 HA PHE J 156 -2.626 38.615 8.880 0.00 0.00
J
ATOM 8658 CB PHE J 156 -2.736 36.731 9.963 0.00 0.00
J
ATOM 8659 HB1 PHE J 156 -2.778 37.126 11.001 0.00 0.00
J
ATOM 8660 HB2 PHE J 156 -3.399 35.841 10.009 0.00 0.00
J
ATOM 8661 CG PHE J 156 -1.229 36.420 9.741 0.00 0.00
J
ATOM 8662 CD1 PHE J 156 -0.273 37.428 9.941 0.00 0.00
J
ATOM 8663 HD1 PHE J 156 -0.573 38.444 10.149 0.00 0.00
J
ATOM 8664 CE1 PHE J 156 1.138 37.234 9.886 0.00 0.00
J
ATOM 8665 HE1 PHE J 156 1.704 38.138 10.057 0.00 0.00
J
ATOM 8666 CZ PHE J 156 1.542 35.957 9.693 0.00 0.00
J

ATOM	8667	HZ	PHE	J	156	2.574	35.653	9.790	0.00	0.00
J										
ATOM	8668	CD2	PHE	J	156	−0.783	35.133	9.500	0.00	0.00
J										
ATOM	8669	HD2	PHE	J	156	−1.412	34.268	9.349	0.00	0.00
J										
ATOM	8670	CE2	PHE	J	156	0.598	34.937	9.453	0.00	0.00
J										
ATOM	8671	HE2	PHE	J	156	0.995	33.946	9.291	0.00	0.00
J										
ATOM	8672	C	PHE	J	156	−3.547	37.125	7.613	0.00	0.00
J										
ATOM	8673	O	PHE	J	156	−2.904	37.302	6.582	0.00	0.00
J										
ATOM	8674	N	SER	J	157	−4.590	36.307	7.549	0.00	0.00
J										
ATOM	8675	HN	SER	J	157	−5.104	36.123	8.383	0.00	0.00
J										
ATOM	8676	CA	SER	J	157	−5.105	35.632	6.272	0.00	0.00
J										
ATOM	8677	HA	SER	J	157	−4.298	35.050	5.852	0.00	0.00
J										
ATOM	8678	CB	SER	J	157	−6.234	34.673	6.633	0.00	0.00
J										
ATOM	8679	HB1	SER	J	157	−5.884	34.030	7.469	0.00	0.00
J										
ATOM	8680	HB2	SER	J	157	−7.187	35.079	7.032	0.00	0.00
J										
ATOM	8681	OG	SER	J	157	−6.562	33.739	5.568	0.00	0.00
J										
ATOM	8682	HG1	SER	J	157	−7.033	34.252	4.908	0.00	0.00
J										
ATOM	8683	C	SER	J	157	−5.589	36.657	5.243	0.00	0.00
J										
ATOM	8684	O	SER	J	157	−5.220	36.554	4.077	0.00	0.00
J										
ATOM	8685	N	ILE	J	158	−6.419	37.665	5.625	0.00	0.00
J										
ATOM	8686	HN	ILE	J	158	−6.720	37.910	6.543	0.00	0.00
J										
ATOM	8687	CA	ILE	J	158	−6.867	38.654	4.620	0.00	0.00
J										
ATOM	8688	HA	ILE	J	158	−7.263	38.072	3.800	0.00	0.00
J										
ATOM	8689	CB	ILE	J	158	−8.024	39.576	5.091	0.00	0.00
J										
ATOM	8690	HB	ILE	J	158	−8.292	40.227	4.232	0.00	0.00
J										
ATOM	8691	CG2	ILE	J	158	−9.221	38.640	5.507	0.00	0.00
J										
ATOM	8692	HG21	ILE	J	158	−9.086	38.080	6.457	0.00	0.00
J										
ATOM	8693	HG22	ILE	J	158	−10.192	39.131	5.730	0.00	0.00
J										

ATOM 8694 HG23 ILE J 158 -9.379 37.835 4.757 0.00 0.00
J
ATOM 8695 CG1 ILE J 158 -7.647 40.521 6.224 0.00 0.00
J
ATOM 8696 HG11 ILE J 158 -7.548 39.940 7.166 0.00 0.00
J
ATOM 8697 HG12 ILE J 158 -6.681 41.056 6.105 0.00 0.00
J
ATOM 8698 CD ILE J 158 -8.613 41.703 6.399 0.00 0.00
J
ATOM 8699 HD1 ILE J 158 -8.329 42.125 7.386 0.00 0.00
J
ATOM 8700 HD2 ILE J 158 -8.481 42.538 5.678 0.00 0.00
J
ATOM 8701 HD3 ILE J 158 -9.639 41.303 6.548 0.00 0.00
J
ATOM 8702 C ILE J 158 -5.630 39.438 4.153 0.00 0.00
J
ATOM 8703 O ILE J 158 -5.479 39.794 2.946 0.00 0.00
J
ATOM 8704 N SER J 159 -4.752 39.799 5.124 0.00 0.00
J
ATOM 8705 HN SER J 159 -4.828 39.480 6.065 0.00 0.00
J
ATOM 8706 CA SER J 159 -3.547 40.543 4.676 0.00 0.00
J
ATOM 8707 HA SER J 159 -4.066 41.279 4.080 0.00 0.00
J
ATOM 8708 CB SER J 159 -2.712 41.134 5.780 0.00 0.00
J
ATOM 8709 HB1 SER J 159 -2.213 40.327 6.358 0.00 0.00
J
ATOM 8710 HB2 SER J 159 -1.857 41.745 5.419 0.00 0.00
J
ATOM 8711 OG SER J 159 -3.560 41.872 6.702 0.00 0.00
J
ATOM 8712 HG1 SER J 159 -4.044 41.219 7.213 0.00 0.00
J
ATOM 8713 C SER J 159 -2.634 39.783 3.725 0.00 0.00
J
ATOM 8714 O SER J 159 -2.136 40.297 2.744 0.00 0.00
J
ATOM 8715 N ILE J 160 -2.303 38.454 4.015 0.00 0.00
J
ATOM 8716 HN ILE J 160 -2.740 38.031 4.804 0.00 0.00
J
ATOM 8717 CA ILE J 160 -1.444 37.653 3.162 0.00 0.00
J
ATOM 8718 HA ILE J 160 -0.547 38.225 2.970 0.00 0.00
J
ATOM 8719 CB ILE J 160 -0.999 36.307 3.815 0.00 0.00
J
ATOM 8720 HB ILE J 160 -1.928 35.743 4.045 0.00 0.00
J

ATOM 8721 CG2 ILE J 160 -0.102 35.641 2.791 0.00 0.00
J
ATOM 8722 HG21 ILE J 160 -0.728 35.257 1.958 0.00 0.00
J
ATOM 8723 HG22 ILE J 160 0.751 36.291 2.502 0.00 0.00
J
ATOM 8724 HG23 ILE J 160 0.324 34.714 3.231 0.00 0.00
J
ATOM 8725 CG1 ILE J 160 -0.244 36.488 5.117 0.00 0.00
J
ATOM 8726 HG11 ILE J 160 0.716 37.010 4.916 0.00 0.00
J
ATOM 8727 HG12 ILE J 160 -0.806 37.004 5.924 0.00 0.00
J
ATOM 8728 CD ILE J 160 -0.071 35.121 5.815 0.00 0.00
J
ATOM 8729 HD1 ILE J 160 -1.041 34.858 6.288 0.00 0.00
J
ATOM 8730 HD2 ILE J 160 0.317 34.311 5.160 0.00 0.00
J
ATOM 8731 HD3 ILE J 160 0.729 35.288 6.568 0.00 0.00
J
ATOM 8732 C ILE J 160 -2.068 37.547 1.738 0.00 0.00
J
ATOM 8733 O ILE J 160 -1.383 37.863 0.750 0.00 0.00
J
ATOM 8734 N GLU J 161 -3.431 37.388 1.578 0.00 0.00
J
ATOM 8735 HN GLU J 161 -4.020 37.135 2.342 0.00 0.00
J
ATOM 8736 CA GLU J 161 -4.019 37.335 0.213 0.00 0.00
J
ATOM 8737 HA GLU J 161 -3.453 36.690 -0.445 0.00 0.00
J
ATOM 8738 CB GLU J 161 -5.479 36.835 0.173 0.00 0.00
J
ATOM 8739 HB1 GLU J 161 -6.145 37.464 0.801 0.00 0.00
J
ATOM 8740 HB2 GLU J 161 -5.869 36.888 -0.866 0.00 0.00
J
ATOM 8741 CG GLU J 161 -5.613 35.427 0.558 0.00 0.00
J
ATOM 8742 HG1 GLU J 161 -5.143 34.806 -0.234 0.00 0.00
J
ATOM 8743 HG2 GLU J 161 -5.107 35.225 1.527 0.00 0.00
J
ATOM 8744 CD GLU J 161 -7.015 35.017 0.765 0.00 0.00
J
ATOM 8745 OE1 GLU J 161 -7.312 33.837 1.124 0.00 0.00
J
ATOM 8746 OE2 GLU J 161 -7.912 35.942 0.581 0.00 0.00
J
ATOM 8747 C GLU J 161 -3.996 38.621 -0.573 0.00 0.00
J

```
ATOM   8748  O   GLU J 161      -3.792  38.629  -1.785  0.00  0.00
J
ATOM   8749  N   LEU J 162      -4.263  39.798   0.020  0.00  0.00
J
ATOM   8750  HN  LEU J 162      -4.538  39.904   0.973  0.00  0.00
J
ATOM   8751  CA  LEU J 162      -3.972  41.056  -0.574  0.00  0.00
J
ATOM   8752  HA  LEU J 162      -4.322  41.093  -1.595  0.00  0.00
J
ATOM   8753  CB  LEU J 162      -4.774  42.113   0.229  0.00  0.00
J
ATOM   8754  HB1 LEU J 162      -4.449  42.002   1.286  0.00  0.00
J
ATOM   8755  HB2 LEU J 162      -4.489  43.125  -0.132  0.00  0.00
J
ATOM   8756  CG  LEU J 162      -6.299  41.907   0.277  0.00  0.00
J
ATOM   8757  HG  LEU J 162      -6.581  41.003   0.858  0.00  0.00
J
ATOM   8758  CD1 LEU J 162      -6.879  43.081   1.132  0.00  0.00
J
ATOM   8759 HD11 LEU J 162      -7.944  42.868   1.370  0.00  0.00
J
ATOM   8760 HD12 LEU J 162      -6.502  43.085   2.177  0.00  0.00
J
ATOM   8761 HD13 LEU J 162      -6.616  44.029   0.617  0.00  0.00
J
ATOM   8762  CD2 LEU J 162      -7.101  41.857  -1.047  0.00  0.00
J
ATOM   8763 HD21 LEU J 162      -6.791  42.697  -1.705  0.00  0.00
J
ATOM   8764 HD22 LEU J 162      -7.007  40.833  -1.469  0.00  0.00
J
ATOM   8765 HD23 LEU J 162      -8.205  41.943  -0.956  0.00  0.00
J
ATOM   8766  C   LEU J 162      -2.518  41.514  -0.876  0.00  0.00
J
ATOM   8767  O   LEU J 162      -2.352  42.110  -1.928  0.00  0.00
J
ATOM   8768  N   ILE J 163      -1.475  41.296   0.029  0.00  0.00
J
ATOM   8769  HN  ILE J 163      -1.716  40.925   0.922  0.00  0.00
J
ATOM   8770  CA  ILE J 163      -0.027  41.572  -0.253  0.00  0.00
J
ATOM   8771  HA  ILE J 163      -0.047  42.632  -0.461  0.00  0.00
J
ATOM   8772  CB  ILE J 163       0.933  41.470   0.944  0.00  0.00
J
ATOM   8773  HB  ILE J 163       0.258  41.894   1.718  0.00  0.00
J
ATOM   8774  CG2 ILE J 163       1.225  39.972   1.229  0.00  0.00
J
```

ATOM 8775 HG21 ILE J 163 1.475 39.820 2.301 0.00 0.00
J
ATOM 8776 HG22 ILE J 163 0.332 39.353 0.996 0.00 0.00
J
ATOM 8777 HG23 ILE J 163 2.029 39.650 0.533 0.00 0.00
J
ATOM 8778 CG1 ILE J 163 2.155 42.404 0.825 0.00 0.00
J
ATOM 8779 HG11 ILE J 163 2.797 41.894 0.075 0.00 0.00
J
ATOM 8780 HG12 ILE J 163 1.886 43.447 0.550 0.00 0.00
J
ATOM 8781 CD ILE J 163 2.936 42.533 2.145 0.00 0.00
J
ATOM 8782 HD1 ILE J 163 3.666 43.351 1.966 0.00 0.00
J
ATOM 8783 HD2 ILE J 163 2.294 42.845 2.996 0.00 0.00
J
ATOM 8784 HD3 ILE J 163 3.508 41.618 2.411 0.00 0.00
J
ATOM 8785 C ILE J 163 0.444 40.879 -1.487 0.00 0.00
J
ATOM 8786 O ILE J 163 1.208 41.495 -2.288 0.00 0.00
J
ATOM 8787 N ALA J 164 -0.082 39.681 -1.727 0.00 0.00
J
ATOM 8788 HN ALA J 164 -0.574 39.090 -1.093 0.00 0.00
J
ATOM 8789 CA ALA J 164 0.218 38.814 -2.869 0.00 0.00
J
ATOM 8790 HA ALA J 164 1.294 38.772 -2.942 0.00 0.00
J
ATOM 8791 CB ALA J 164 -0.234 37.338 -2.535 0.00 0.00
J
ATOM 8792 HB1 ALA J 164 0.368 37.137 -1.623 0.00 0.00
J
ATOM 8793 HB2 ALA J 164 -1.318 37.168 -2.364 0.00 0.00
J
ATOM 8794 HB3 ALA J 164 0.126 36.725 -3.389 0.00 0.00
J
ATOM 8795 C ALA J 164 -0.241 39.417 -4.110 0.00 0.00
J
ATOM 8796 O ALA J 164 0.412 39.331 -5.144 0.00 0.00
J
ATOM 8797 N ALA J 165 -1.415 40.151 -4.124 0.00 0.00
J
ATOM 8798 HN ALA J 165 -1.816 40.426 -3.254 0.00 0.00
J
ATOM 8799 CA ALA J 165 -1.830 40.867 -5.333 0.00 0.00
J
ATOM 8800 HA ALA J 165 -1.748 40.163 -6.149 0.00 0.00
J
ATOM 8801 CB ALA J 165 -3.232 41.349 -5.188 0.00 0.00
J

ATOM 8802 HB1 ALA J 165 -3.898 40.611 -4.691 0.00 0.00
J
ATOM 8803 HB2 ALA J 165 -3.311 42.270 -4.570 0.00 0.00
J
ATOM 8804 HB3 ALA J 165 -3.687 41.697 -6.140 0.00 0.00
J
ATOM 8805 C ALA J 165 -0.850 42.079 -5.730 0.00 0.00
J
ATOM 8806 O ALA J 165 -0.582 42.219 -6.910 0.00 0.00
J
ATOM 8807 N LEU J 166 -0.352 42.810 -4.752 0.00 0.00
J
ATOM 8808 HN LEU J 166 -0.629 42.626 -3.813 0.00 0.00
J
ATOM 8809 CA LEU J 166 0.490 43.973 -4.909 0.00 0.00
J
ATOM 8810 HA LEU J 166 0.152 44.567 -5.745 0.00 0.00
J
ATOM 8811 CB LEU J 166 0.502 45.005 -3.677 0.00 0.00
J
ATOM 8812 HB1 LEU J 166 1.081 44.530 -2.857 0.00 0.00
J
ATOM 8813 HB2 LEU J 166 0.992 45.989 -3.834 0.00 0.00
J
ATOM 8814 CG LEU J 166 -0.975 45.268 -3.217 0.00 0.00
J
ATOM 8815 HG LEU J 166 -1.550 44.325 -3.098 0.00 0.00
J
ATOM 8816 CD1 LEU J 166 -0.868 46.064 -1.902 0.00 0.00
J
ATOM 8817 HD11 LEU J 166 -1.697 46.784 -1.734 0.00 0.00
J
ATOM 8818 HD12 LEU J 166 -0.825 45.349 -1.052 0.00 0.00
J
ATOM 8819 HD13 LEU J 166 0.151 46.507 -1.945 0.00 0.00
J
ATOM 8820 CD2 LEU J 166 -1.805 46.045 -4.238 0.00 0.00
J
ATOM 8821 HD21 LEU J 166 -2.758 46.313 -3.734 0.00 0.00
J
ATOM 8822 HD22 LEU J 166 -1.290 47.003 -4.461 0.00 0.00
J
ATOM 8823 HD23 LEU J 166 -1.844 45.524 -5.219 0.00 0.00
J
ATOM 8824 C LEU J 166 1.874 43.461 -5.351 0.00 0.00
J
ATOM 8825 O LEU J 166 2.474 44.092 -6.195 0.00 0.00
J
ATOM 8826 N ILE J 167 2.362 42.330 -4.822 0.00 0.00
J
ATOM 8827 HN ILE J 167 1.982 41.923 -3.995 0.00 0.00
J
ATOM 8828 CA ILE J 167 3.521 41.591 -5.359 0.00 0.00
J

ATOM 8829 HA ILE J 167 4.290 42.343 -5.260 0.00 0.00
J
ATOM 8830 CB ILE J 167 3.883 40.326 -4.551 0.00 0.00
J
ATOM 8831 HB ILE J 167 2.966 39.758 -4.284 0.00 0.00
J
ATOM 8832 CG2 ILE J 167 4.852 39.369 -5.296 0.00 0.00
J
ATOM 8833 HG21 ILE J 167 4.934 38.428 -4.711 0.00 0.00
J
ATOM 8834 HG22 ILE J 167 4.515 38.988 -6.284 0.00 0.00
J
ATOM 8835 HG23 ILE J 167 5.866 39.818 -5.376 0.00 0.00
J
ATOM 8836 CG1 ILE J 167 4.583 40.618 -3.187 0.00 0.00
J
ATOM 8837 HG11 ILE J 167 5.514 41.202 -3.346 0.00 0.00
J
ATOM 8838 HG12 ILE J 167 4.027 41.264 -2.474 0.00 0.00
J
ATOM 8839 CD ILE J 167 5.002 39.420 -2.350 0.00 0.00
J
ATOM 8840 HD1 ILE J 167 4.106 38.978 -1.864 0.00 0.00
J
ATOM 8841 HD2 ILE J 167 5.686 38.632 -2.731 0.00 0.00
J
ATOM 8842 HD3 ILE J 167 5.605 39.838 -1.515 0.00 0.00
J
ATOM 8843 C ILE J 167 3.274 41.203 -6.861 0.00 0.00
J
ATOM 8844 O ILE J 167 4.100 41.579 -7.695 0.00 0.00
J
ATOM 8845 N LEU J 168 2.180 40.513 -7.258 0.00 0.00
J
ATOM 8846 HN LEU J 168 1.469 40.226 -6.620 0.00 0.00
J
ATOM 8847 CA LEU J 168 1.948 40.131 -8.660 0.00 0.00
J
ATOM 8848 HA LEU J 168 2.894 39.776 -9.040 0.00 0.00
J
ATOM 8849 CB LEU J 168 0.887 38.997 -8.788 0.00 0.00
J
ATOM 8850 HB1 LEU J 168 0.052 39.201 -8.084 0.00 0.00
J
ATOM 8851 HB2 LEU J 168 0.399 39.118 -9.779 0.00 0.00
J
ATOM 8852 CG LEU J 168 1.390 37.582 -8.330 0.00 0.00
J
ATOM 8853 HG LEU J 168 1.478 37.590 -7.222 0.00 0.00
J
ATOM 8854 CD1 LEU J 168 0.320 36.538 -8.553 0.00 0.00
J
ATOM 8855 HD11 LEU J 168 -0.045 36.583 -9.601 0.00 0.00
J

```
ATOM   8856 HD12 LEU J 168       0.693  35.523  -8.294  0.00  0.00
J
ATOM   8857 HD13 LEU J 168      -0.539  36.775  -7.888  0.00  0.00
J
ATOM   8858  CD2 LEU J 168       2.726  37.132  -8.931  0.00  0.00
J
ATOM   8859 HD21 LEU J 168       2.747  36.029  -9.061  0.00  0.00
J
ATOM   8860 HD22 LEU J 168       2.716  37.433 -10.000  0.00  0.00
J
ATOM   8861 HD23 LEU J 168       3.621  37.668  -8.546  0.00  0.00
J
ATOM   8862  C   LEU J 168       1.530  41.301  -9.589  0.00  0.00
J
ATOM   8863  O   LEU J 168       1.687  41.218 -10.781  0.00  0.00
J
ATOM   8864  N   SER J 169       1.090  42.438  -9.031  0.00  0.00
J
ATOM   8865  HN  SER J 169       0.953  42.540  -8.049  0.00  0.00
J
ATOM   8866  CA  SER J 169       0.604  43.613  -9.799  0.00  0.00
J
ATOM   8867  HA  SER J 169      -0.195  43.215 -10.407  0.00  0.00
J
ATOM   8868  CB  SER J 169      -0.004  44.740  -8.995  0.00  0.00
J
ATOM   8869  HB1 SER J 169       0.796  45.215  -8.389  0.00  0.00
J
ATOM   8870  HB2 SER J 169      -0.425  45.516  -9.669  0.00  0.00
J
ATOM   8871  OG  SER J 169      -1.145  44.327  -8.281  0.00  0.00
J
ATOM   8872  HG1 SER J 169      -1.020  43.391  -8.105  0.00  0.00
J
ATOM   8873  C   SER J 169       1.689  44.184 -10.702  0.00  0.00
J
ATOM   8874  O   SER J 169       1.436  44.503 -11.856  0.00  0.00
J
ATOM   8875  N   ILE J 170       2.952  44.403 -10.274  0.00  0.00
J
ATOM   8876  HN  ILE J 170       3.214  44.213  -9.331  0.00  0.00
J
ATOM   8877  CA  ILE J 170       3.953  44.990 -11.187  0.00  0.00
J
ATOM   8878  HA  ILE J 170       3.426  45.862 -11.546  0.00  0.00
J
ATOM   8879  CB  ILE J 170       5.146  45.430 -10.403  0.00  0.00
J
ATOM   8880  HB  ILE J 170       4.755  46.069  -9.582  0.00  0.00
J
ATOM   8881  CG2 ILE J 170       5.981  44.229  -9.795  0.00  0.00
J
ATOM   8882 HG21 ILE J 170       6.469  43.601 -10.571  0.00  0.00
J
```

ATOM 8883 HG22 ILE J 170 6.837 44.616 -9.202 0.00 0.00
J
ATOM 8884 HG23 ILE J 170 5.343 43.631 -9.110 0.00 0.00
J
ATOM 8885 CG1 ILE J 170 6.124 46.301 -11.232 0.00 0.00
J
ATOM 8886 HG11 ILE J 170 6.868 46.829 -10.597 0.00 0.00
J
ATOM 8887 HG12 ILE J 170 6.583 45.625 -11.984 0.00 0.00
J
ATOM 8888 CD ILE J 170 5.474 47.522 -11.994 0.00 0.00
J
ATOM 8889 HD1 ILE J 170 4.816 47.961 -11.214 0.00 0.00
J
ATOM 8890 HD2 ILE J 170 6.301 48.252 -12.131 0.00 0.00
J
ATOM 8891 HD3 ILE J 170 5.005 47.223 -12.956 0.00 0.00
J
ATOM 8892 C ILE J 170 4.365 44.047 -12.325 0.00 0.00
J
ATOM 8893 O ILE J 170 4.825 44.435 -13.453 0.00 0.00
J
ATOM 8894 N ARG J 171 4.111 42.760 -12.148 0.00 0.00
J
ATOM 8895 HN ARG J 171 3.665 42.444 -11.314 0.00 0.00
J
ATOM 8896 CA ARG J 171 4.306 41.782 -13.165 0.00 0.00
J
ATOM 8897 HA ARG J 171 5.161 41.985 -13.793 0.00 0.00
J
ATOM 8898 CB ARG J 171 4.496 40.330 -12.489 0.00 0.00
J
ATOM 8899 HB1 ARG J 171 3.631 39.980 -11.887 0.00 0.00
J
ATOM 8900 HB2 ARG J 171 4.482 39.606 -13.332 0.00 0.00
J
ATOM 8901 CG ARG J 171 5.830 40.168 -11.689 0.00 0.00
J
ATOM 8902 HG1 ARG J 171 5.656 40.795 -10.788 0.00 0.00
J
ATOM 8903 HG2 ARG J 171 6.041 39.106 -11.439 0.00 0.00
J
ATOM 8904 CD ARG J 171 7.064 40.710 -12.376 0.00 0.00
J
ATOM 8905 HD1 ARG J 171 6.676 41.645 -12.834 0.00 0.00
J
ATOM 8906 HD2 ARG J 171 7.898 40.964 -11.688 0.00 0.00
J
ATOM 8907 NE ARG J 171 7.600 39.789 -13.432 0.00 0.00
J
ATOM 8908 HE ARG J 171 7.370 39.946 -14.392 0.00 0.00
J
ATOM 8909 CZ ARG J 171 8.666 38.973 -13.310 0.00 0.00
J

ATOM 8910 NH1 ARG J 171 9.370 38.937 −12.162 0.00 0.00
J
ATOM 8911 HH11 ARG J 171 10.126 38.284 −12.135 0.00 0.00
J
ATOM 8912 HH12 ARG J 171 9.003 39.203 −11.271 0.00 0.00
J
ATOM 8913 NH2 ARG J 171 9.118 38.213 −14.299 0.00 0.00
J
ATOM 8914 HH21 ARG J 171 9.884 37.597 −14.117 0.00 0.00
J
ATOM 8915 HH22 ARG J 171 8.614 38.093 −15.154 0.00 0.00
J
ATOM 8916 C ARG J 171 3.137 41.743 −14.128 0.00 0.00
J
ATOM 8917 O ARG J 171 3.387 41.598 −15.293 0.00 0.00
J
ATOM 8918 N LEU J 172 1.890 41.927 −13.774 0.00 0.00
J
ATOM 8919 HN LEU J 172 1.676 42.073 −12.811 0.00 0.00
J
ATOM 8920 CA LEU J 172 0.732 41.737 −14.597 0.00 0.00
J
ATOM 8921 HA LEU J 172 0.946 41.019 −15.375 0.00 0.00
J
ATOM 8922 CB LEU J 172 −0.489 41.057 −13.897 0.00 0.00
J
ATOM 8923 HB1 LEU J 172 −0.667 41.522 −12.904 0.00 0.00
J
ATOM 8924 HB2 LEU J 172 −1.337 41.139 −14.609 0.00 0.00
J
ATOM 8925 CG LEU J 172 −0.417 39.469 −13.796 0.00 0.00
J
ATOM 8926 HG LEU J 172 0.121 39.053 −14.675 0.00 0.00
J
ATOM 8927 CD1 LEU J 172 0.442 39.008 −12.562 0.00 0.00
J
ATOM 8928 HD11 LEU J 172 0.493 37.902 −12.652 0.00 0.00
J
ATOM 8929 HD12 LEU J 172 1.441 39.490 −12.618 0.00 0.00
J
ATOM 8930 HD13 LEU J 172 −0.017 39.403 −11.631 0.00 0.00
J
ATOM 8931 CD2 LEU J 172 −1.857 38.904 −13.784 0.00 0.00
J
ATOM 8932 HD21 LEU J 172 −2.386 39.151 −14.729 0.00 0.00
J
ATOM 8933 HD22 LEU J 172 −1.794 37.797 −13.701 0.00 0.00
J
ATOM 8934 HD23 LEU J 172 −2.391 39.292 −12.891 0.00 0.00
J
ATOM 8935 C LEU J 172 0.210 42.971 −15.249 0.00 0.00
J
ATOM 8936 O LEU J 172 −0.141 43.023 −16.415 0.00 0.00
J

```
ATOM   8937  N   VAL J 173       0.107  44.111 -14.526  0.00  0.00
J
ATOM   8938  HN  VAL J 173       0.392  44.116 -13.571  0.00  0.00
J
ATOM   8939  CA  VAL J 173      -0.495  45.392 -14.904  0.00  0.00
J
ATOM   8940  HA  VAL J 173      -1.446  45.108 -15.329  0.00  0.00
J
ATOM   8941  CB  VAL J 173      -0.729  46.262 -13.731  0.00  0.00
J
ATOM   8942  HB  VAL J 173       0.209  46.404 -13.154  0.00  0.00
J
ATOM   8943  CG1 VAL J 173      -1.310  47.599 -14.215  0.00  0.00
J
ATOM   8944 HG11 VAL J 173      -2.339  47.358 -14.559  0.00  0.00
J
ATOM   8945 HG12 VAL J 173      -1.456  48.235 -13.316  0.00  0.00
J
ATOM   8946 HG13 VAL J 173      -0.738  48.084 -15.034  0.00  0.00
J
ATOM   8947  CG2 VAL J 173      -1.715  45.508 -12.874  0.00  0.00
J
ATOM   8948 HG21 VAL J 173      -1.478  44.443 -12.666  0.00  0.00
J
ATOM   8949 HG22 VAL J 173      -1.965  46.016 -11.918  0.00  0.00
J
ATOM   8950 HG23 VAL J 173      -2.669  45.438 -13.439  0.00  0.00
J
ATOM   8951  C   VAL J 173       0.266  45.989 -16.044  0.00  0.00
J
ATOM   8952  O   VAL J 173      -0.313  46.375 -17.047  0.00  0.00
J
ATOM   8953  N   PRO J 174       1.572  46.083 -16.130  0.00  0.00
J
ATOM   8954  CD  PRO J 174       2.535  46.160 -14.989  0.00  0.00
J
ATOM   8955  HD1 PRO J 174       2.013  46.596 -14.110  0.00  0.00
J
ATOM   8956  HD2 PRO J 174       2.828  45.143 -14.652  0.00  0.00
J
ATOM   8957  CA  PRO J 174       2.205  46.634 -17.395  0.00  0.00
J
ATOM   8958  HA  PRO J 174       1.748  47.578 -17.652  0.00  0.00
J
ATOM   8959  CB  PRO J 174       3.723  46.868 -16.964  0.00  0.00
J
ATOM   8960  HB1 PRO J 174       4.258  47.665 -17.523  0.00  0.00
J
ATOM   8961  HB2 PRO J 174       4.256  45.896 -17.038  0.00  0.00
J
ATOM   8962  CG  PRO J 174       3.658  47.098 -15.439  0.00  0.00
J
ATOM   8963  HG1 PRO J 174       3.417  48.135 -15.120  0.00  0.00
J
```

ATOM 8964 HG2 PRO J 174 4.586 46.816 −14.896 0.00 0.00
J
ATOM 8965 C PRO J 174 2.120 45.733 −18.596 0.00 0.00
J
ATOM 8966 O PRO J 174 2.113 46.145 −19.795 0.00 0.00
J
ATOM 8967 N GLN J 175 1.939 44.469 −18.308 0.00 0.00
J
ATOM 8968 HN GLN J 175 1.774 44.180 −17.368 0.00 0.00
J
ATOM 8969 CA GLN J 175 1.915 43.531 −19.432 0.00 0.00
J
ATOM 8970 HA GLN J 175 2.624 43.875 −20.171 0.00 0.00
J
ATOM 8971 CB GLN J 175 2.497 42.111 −19.122 0.00 0.00
J
ATOM 8972 HB1 GLN J 175 3.506 42.156 −18.659 0.00 0.00
J
ATOM 8973 HB2 GLN J 175 1.714 41.638 −18.492 0.00 0.00
J
ATOM 8974 CG GLN J 175 2.812 41.155 −20.374 0.00 0.00
J
ATOM 8975 HG1 GLN J 175 2.703 40.083 −20.103 0.00 0.00
J
ATOM 8976 HG2 GLN J 175 2.019 41.289 −21.140 0.00 0.00
J
ATOM 8977 CD GLN J 175 4.045 41.625 −21.079 0.00 0.00
J
ATOM 8978 OE1 GLN J 175 3.997 42.283 −22.140 0.00 0.00
J
ATOM 8979 NE2 GLN J 175 5.176 41.126 −20.572 0.00 0.00
J
ATOM 8980 HE21 GLN J 175 6.061 41.246 −21.023 0.00 0.00
J
ATOM 8981 HE22 GLN J 175 5.085 40.909 −19.600 0.00 0.00
J
ATOM 8982 C GLN J 175 0.591 43.424 −20.133 0.00 0.00
J
ATOM 8983 O GLN J 175 0.554 43.432 −21.390 0.00 0.00
J
ATOM 8984 N TYR J 176 −0.501 43.354 −19.343 0.00 0.00
J
ATOM 8985 HN TYR J 176 −0.506 43.413 −18.348 0.00 0.00
J
ATOM 8986 CA TYR J 176 −1.763 43.045 −19.826 0.00 0.00
J
ATOM 8987 HA TYR J 176 −1.812 42.453 −20.728 0.00 0.00
J
ATOM 8988 CB TYR J 176 −2.496 42.042 −18.774 0.00 0.00
J
ATOM 8989 HB1 TYR J 176 −2.508 42.367 −17.712 0.00 0.00
J
ATOM 8990 HB2 TYR J 176 −3.572 42.107 −19.041 0.00 0.00
J

ATOM 8991 CG TYR J 176 −1.998 40.621 −18.816 0.00 0.00
J
ATOM 8992 CD1 TYR J 176 −2.009 40.002 −20.083 0.00 0.00
J
ATOM 8993 HD1 TYR J 176 −2.582 40.435 −20.890 0.00 0.00
J
ATOM 8994 CE1 TYR J 176 −1.606 38.634 −20.280 0.00 0.00
J
ATOM 8995 HE1 TYR J 176 −1.611 38.237 −21.284 0.00 0.00
J
ATOM 8996 CZ TYR J 176 −0.964 37.978 −19.191 0.00 0.00
J
ATOM 8997 OH TYR J 176 −0.437 36.723 −19.456 0.00 0.00
J
ATOM 8998 HH TYR J 176 −0.888 36.359 −20.221 0.00 0.00
J
ATOM 8999 CD2 TYR J 176 −1.381 39.842 −17.777 0.00 0.00
J
ATOM 9000 HD2 TYR J 176 −1.277 40.362 −16.836 0.00 0.00
J
ATOM 9001 CE2 TYR J 176 −0.822 38.578 −17.960 0.00 0.00
J
ATOM 9002 HE2 TYR J 176 −0.390 38.086 −17.101 0.00 0.00
J
ATOM 9003 C TYR J 176 −2.650 44.319 −19.981 0.00 0.00
J
ATOM 9004 O TYR J 176 −3.690 44.335 −20.615 0.00 0.00
J
ATOM 9005 N GLY J 177 −2.244 45.447 −19.301 0.00 0.00
J
ATOM 9006 HN GLY J 177 −1.314 45.517 −18.948 0.00 0.00
J
ATOM 9007 CA GLY J 177 −3.148 46.567 −19.045 0.00 0.00
J
ATOM 9008 HA1 GLY J 177 −3.885 46.512 −19.833 0.00 0.00
J
ATOM 9009 HA2 GLY J 177 −2.581 47.486 −19.058 0.00 0.00
J
ATOM 9010 C GLY J 177 −3.741 46.459 −17.724 0.00 0.00
J
ATOM 9011 O GLY J 177 −3.879 45.389 −17.107 0.00 0.00
J
ATOM 9012 N TRP J 178 −4.315 47.577 −17.163 0.00 0.00
J
ATOM 9013 HN TRP J 178 −4.438 48.302 −17.836 0.00 0.00
J
ATOM 9014 CA TRP J 178 −4.944 47.684 −15.867 0.00 0.00
J
ATOM 9015 HA TRP J 178 −4.201 47.297 −15.186 0.00 0.00
J
ATOM 9016 CB TRP J 178 −5.280 49.136 −15.586 0.00 0.00
J
ATOM 9017 HB1 TRP J 178 −5.706 49.588 −16.507 0.00 0.00
J

ATOM 9018 HB2 TRP J 178 −5.997 49.156 −14.738 0.00 0.00
J
ATOM 9019 CG TRP J 178 −4.170 50.052 −15.039 0.00 0.00
J
ATOM 9020 CD1 TRP J 178 −3.397 50.926 −15.712 0.00 0.00
J
ATOM 9021 HD1 TRP J 178 −3.677 51.171 −16.726 0.00 0.00
J
ATOM 9022 NE1 TRP J 178 −2.556 51.590 −14.875 0.00 0.00
J
ATOM 9023 HE1 TRP J 178 −1.884 52.271 −15.070 0.00 0.00
J
ATOM 9024 CE2 TRP J 178 −2.631 50.902 −13.610 0.00 0.00
J
ATOM 9025 CD2 TRP J 178 −3.664 49.994 −13.773 0.00 0.00
J
ATOM 9026 CE3 TRP J 178 −4.063 49.217 −12.627 0.00 0.00
J
ATOM 9027 HE3 TRP J 178 −4.807 48.439 −12.715 0.00 0.00
J
ATOM 9028 CZ3 TRP J 178 −3.236 49.339 −11.503 0.00 0.00
J
ATOM 9029 HZ3 TRP J 178 −3.484 48.778 −10.615 0.00 0.00
J
ATOM 9030 CZ2 TRP J 178 −1.876 51.130 −12.400 0.00 0.00
J
ATOM 9031 HZ2 TRP J 178 −1.165 51.937 −12.298 0.00 0.00
J
ATOM 9032 CH2 TRP J 178 −2.166 50.273 −11.384 0.00 0.00
J
ATOM 9033 HH2 TRP J 178 −1.629 50.297 −10.447 0.00 0.00
J
ATOM 9034 C TRP J 178 −6.145 46.873 −15.515 0.00 0.00
J
ATOM 9035 O TRP J 178 −6.098 46.294 −14.415 0.00 0.00
J
ATOM 9036 N SER J 179 −7.197 46.768 −16.329 0.00 0.00
J
ATOM 9037 HN SER J 179 −7.147 47.290 −17.177 0.00 0.00
J
ATOM 9038 CA SER J 179 −8.318 45.913 −15.987 0.00 0.00
J
ATOM 9039 HA SER J 179 −8.657 46.213 −15.006 0.00 0.00
J
ATOM 9040 CB SER J 179 −9.545 46.281 −16.888 0.00 0.00
J
ATOM 9041 HB1 SER J 179 −10.422 45.630 −16.683 0.00 0.00
J
ATOM 9042 HB2 SER J 179 −9.845 47.320 −16.631 0.00 0.00
J
ATOM 9043 OG SER J 179 −9.210 46.143 −18.253 0.00 0.00
J
ATOM 9044 HG1 SER J 179 −9.569 46.930 −18.668 0.00 0.00
J

ATOM 9045 C SER J 179 −8.104 44.406 −15.909 0.00 0.00
J
ATOM 9046 O SER J 179 −8.416 43.800 −14.876 0.00 0.00
J
ATOM 9047 N SER J 180 −7.529 43.800 −16.979 0.00 0.00
J
ATOM 9048 HN SER J 180 −7.339 44.215 −17.866 0.00 0.00
J
ATOM 9049 CA SER J 180 −7.164 42.404 −16.879 0.00 0.00
J
ATOM 9050 HA SER J 180 −8.073 41.912 −16.566 0.00 0.00
J
ATOM 9051 CB SER J 180 −6.954 41.654 −18.236 0.00 0.00
J
ATOM 9052 HB1 SER J 180 −6.239 42.303 −18.784 0.00 0.00
J
ATOM 9053 HB2 SER J 180 −6.633 40.620 −17.984 0.00 0.00
J
ATOM 9054 OG SER J 180 −8.168 41.615 −18.947 0.00 0.00
J
ATOM 9055 HG1 SER J 180 −8.133 40.777 −19.413 0.00 0.00
J
ATOM 9056 C SER J 180 −6.055 42.139 −15.935 0.00 0.00
J
ATOM 9057 O SER J 180 −6.162 41.172 −15.136 0.00 0.00
J
ATOM 9058 N GLY J 181 −4.950 42.903 −15.922 0.00 0.00
J
ATOM 9059 HN GLY J 181 −4.878 43.688 −16.532 0.00 0.00
J
ATOM 9060 CA GLY J 181 −3.812 42.636 −15.091 0.00 0.00
J
ATOM 9061 HA1 GLY J 181 −2.932 43.218 −15.319 0.00 0.00
J
ATOM 9062 HA2 GLY J 181 −3.653 41.571 −15.176 0.00 0.00
J
ATOM 9063 C GLY J 181 −4.109 42.776 −13.558 0.00 0.00
J
ATOM 9064 O GLY J 181 −3.559 42.015 −12.758 0.00 0.00
J
ATOM 9065 N LEU J 182 −4.887 43.756 −13.020 0.00 0.00
J
ATOM 9066 HN LEU J 182 −5.453 44.349 −13.587 0.00 0.00
J
ATOM 9067 CA LEU J 182 −5.150 43.836 −11.610 0.00 0.00
J
ATOM 9068 HA LEU J 182 −4.260 43.532 −11.078 0.00 0.00
J
ATOM 9069 CB LEU J 182 −5.442 45.321 −11.191 0.00 0.00
J
ATOM 9070 HB1 LEU J 182 −4.540 45.807 −11.621 0.00 0.00
J
ATOM 9071 HB2 LEU J 182 −6.348 45.686 −11.720 0.00 0.00
J

ATOM 9072 CG LEU J 182 −5.687 45.636 −9.677 0.00 0.00
J
ATOM 9073 HG LEU J 182 −6.480 45.047 −9.168 0.00 0.00
J
ATOM 9074 CD1 LEU J 182 −4.371 45.362 −8.932 0.00 0.00
J
ATOM 9075 HD11 LEU J 182 −4.186 44.266 −8.930 0.00 0.00
J
ATOM 9076 HD12 LEU J 182 −3.574 46.040 −9.304 0.00 0.00
J
ATOM 9077 HD13 LEU J 182 −4.429 45.647 −7.859 0.00 0.00
J
ATOM 9078 CD2 LEU J 182 −6.251 47.052 −9.595 0.00 0.00
J
ATOM 9079 HD21 LEU J 182 −6.516 47.324 −8.551 0.00 0.00
J
ATOM 9080 HD22 LEU J 182 −5.532 47.742 −10.088 0.00 0.00
J
ATOM 9081 HD23 LEU J 182 −7.189 47.101 −10.188 0.00 0.00
J
ATOM 9082 C LEU J 182 −6.222 42.909 −11.090 0.00 0.00
J
ATOM 9083 O LEU J 182 −6.069 42.387 −9.996 0.00 0.00
J
ATOM 9084 N PHE J 183 −7.257 42.596 −11.912 0.00 0.00
J
ATOM 9085 HN PHE J 183 −7.262 42.894 −12.864 0.00 0.00
J
ATOM 9086 CA PHE J 183 −8.190 41.508 −11.557 0.00 0.00
J
ATOM 9087 HA PHE J 183 −8.619 41.728 −10.590 0.00 0.00
J
ATOM 9088 CB PHE J 183 −9.286 41.526 −12.603 0.00 0.00
J
ATOM 9089 HB1 PHE J 183 −9.452 42.584 −12.898 0.00 0.00
J
ATOM 9090 HB2 PHE J 183 −9.060 40.946 −13.524 0.00 0.00
J
ATOM 9091 CG PHE J 183 −10.558 40.949 −12.180 0.00 0.00
J
ATOM 9092 CD1 PHE J 183 −11.744 41.713 −11.891 0.00 0.00
J
ATOM 9093 HD1 PHE J 183 −11.665 42.790 −11.910 0.00 0.00
J
ATOM 9094 CE1 PHE J 183 −12.925 41.103 −11.393 0.00 0.00
J
ATOM 9095 HE1 PHE J 183 −13.736 41.744 −11.080 0.00 0.00
J
ATOM 9096 CZ PHE J 183 −12.992 39.677 −11.468 0.00 0.00
J
ATOM 9097 HZ PHE J 183 −13.891 39.183 −11.131 0.00 0.00
J
ATOM 9098 CD2 PHE J 183 −10.723 39.536 −12.201 0.00 0.00
J

ATOM 9099 HD2 PHE J 183 -9.884 38.920 -12.489 0.00 0.00
J
ATOM 9100 CE2 PHE J 183 -11.928 38.883 -11.823 0.00 0.00
J
ATOM 9101 HE2 PHE J 183 -12.057 37.813 -11.892 0.00 0.00
J
ATOM 9102 C PHE J 183 -7.564 40.128 -11.503 0.00 0.00
J
ATOM 9103 O PHE J 183 -7.726 39.386 -10.476 0.00 0.00
J
ATOM 9104 N ALA J 184 -6.664 39.814 -12.461 0.00 0.00
J
ATOM 9105 HN ALA J 184 -6.512 40.450 -13.213 0.00 0.00
J
ATOM 9106 CA ALA J 184 -5.973 38.600 -12.549 0.00 0.00
J
ATOM 9107 HA ALA J 184 -6.752 37.855 -12.477 0.00 0.00
J
ATOM 9108 CB ALA J 184 -5.295 38.516 -13.921 0.00 0.00
J
ATOM 9109 HB1 ALA J 184 -4.757 39.461 -14.149 0.00 0.00
J
ATOM 9110 HB2 ALA J 184 -4.634 37.624 -13.943 0.00 0.00
J
ATOM 9111 HB3 ALA J 184 -6.038 38.566 -14.745 0.00 0.00
J
ATOM 9112 C ALA J 184 -5.012 38.455 -11.381 0.00 0.00
J
ATOM 9113 O ALA J 184 -4.807 37.394 -10.813 0.00 0.00
J
ATOM 9114 N SER J 185 -4.359 39.563 -10.968 0.00 0.00
J
ATOM 9115 HN SER J 185 -4.326 40.378 -11.542 0.00 0.00
J
ATOM 9116 CA SER J 185 -3.432 39.625 -9.834 0.00 0.00
J
ATOM 9117 HA SER J 185 -2.659 38.883 -9.963 0.00 0.00
J
ATOM 9118 CB SER J 185 -2.841 41.004 -9.631 0.00 0.00
J
ATOM 9119 HB1 SER J 185 -3.571 41.810 -9.401 0.00 0.00
J
ATOM 9120 HB2 SER J 185 -2.167 40.996 -8.749 0.00 0.00
J
ATOM 9121 OG SER J 185 -2.044 41.411 -10.740 0.00 0.00
J
ATOM 9122 HG1 SER J 185 -2.559 41.752 -11.475 0.00 0.00
J
ATOM 9123 C SER J 185 -4.157 39.293 -8.488 0.00 0.00
J
ATOM 9124 O SER J 185 -3.611 38.527 -7.705 0.00 0.00
J
ATOM 9125 N LEU J 186 -5.341 39.881 -8.299 0.00 0.00
J

ATOM 9126 HN LEU J 186 -5.752 40.511 -8.953 0.00 0.00
J
ATOM 9127 CA LEU J 186 -6.155 39.687 -7.075 0.00 0.00
J
ATOM 9128 HA LEU J 186 -5.591 40.066 -6.236 0.00 0.00
J
ATOM 9129 CB LEU J 186 -7.473 40.515 -7.295 0.00 0.00
J
ATOM 9130 HB1 LEU J 186 -7.202 41.438 -7.851 0.00 0.00
J
ATOM 9131 HB2 LEU J 186 -8.177 40.023 -8.000 0.00 0.00
J
ATOM 9132 CG LEU J 186 -8.415 40.741 -6.073 0.00 0.00
J
ATOM 9133 HG LEU J 186 -8.617 39.772 -5.569 0.00 0.00
J
ATOM 9134 CD1 LEU J 186 -7.811 41.691 -5.046 0.00 0.00
J
ATOM 9135 HD11 LEU J 186 -7.723 42.741 -5.399 0.00 0.00
J
ATOM 9136 HD12 LEU J 186 -8.344 41.573 -4.079 0.00 0.00
J
ATOM 9137 HD13 LEU J 186 -6.762 41.342 -4.929 0.00 0.00
J
ATOM 9138 CD2 LEU J 186 -9.719 41.331 -6.577 0.00 0.00
J
ATOM 9139 HD21 LEU J 186 -10.285 41.683 -5.688 0.00 0.00
J
ATOM 9140 HD22 LEU J 186 -9.491 42.270 -7.125 0.00 0.00
J
ATOM 9141 HD23 LEU J 186 -10.365 40.650 -7.172 0.00 0.00
J
ATOM 9142 C LEU J 186 -6.589 38.203 -6.996 0.00 0.00
J
ATOM 9143 O LEU J 186 -6.503 37.533 -5.967 0.00 0.00
J
ATOM 9144 N PHE J 187 -6.981 37.679 -8.119 0.00 0.00
J
ATOM 9145 HN PHE J 187 -7.097 38.290 -8.898 0.00 0.00
J
ATOM 9146 CA PHE J 187 -7.419 36.293 -8.328 0.00 0.00
J
ATOM 9147 HA PHE J 187 -8.128 36.158 -7.524 0.00 0.00
J
ATOM 9148 CB PHE J 187 -8.076 36.193 -9.758 0.00 0.00
J
ATOM 9149 HB1 PHE J 187 -8.678 37.094 -10.002 0.00 0.00
J
ATOM 9150 HB2 PHE J 187 -7.234 36.133 -10.481 0.00 0.00
J
ATOM 9151 CG PHE J 187 -9.042 35.111 -9.933 0.00 0.00
J
ATOM 9152 CD1 PHE J 187 -10.435 35.351 -10.137 0.00 0.00
J

ATOM 9153 HD1 PHE J 187 −10.884 36.326 −10.015 0.00 0.00
J
ATOM 9154 CE1 PHE J 187 −11.294 34.389 −10.635 0.00 0.00
J
ATOM 9155 HE1 PHE J 187 −12.352 34.568 −10.759 0.00 0.00
J
ATOM 9156 CZ PHE J 187 −10.834 33.083 −10.757 0.00 0.00
J
ATOM 9157 HZ PHE J 187 −11.466 32.217 −10.887 0.00 0.00
J
ATOM 9158 CD2 PHE J 187 −8.543 33.831 −10.284 0.00 0.00
J
ATOM 9159 HD2 PHE J 187 −7.466 33.750 −10.274 0.00 0.00
J
ATOM 9160 CE2 PHE J 187 −9.433 32.841 −10.556 0.00 0.00
J
ATOM 9161 HE2 PHE J 187 −9.026 31.841 −10.529 0.00 0.00
J
ATOM 9162 C PHE J 187 −6.292 35.256 −8.168 0.00 0.00
J
ATOM 9163 O PHE J 187 −6.461 34.260 −7.388 0.00 0.00
J
ATOM 9164 N HSE J 188 −5.090 35.521 −8.720 0.00 0.00
J
ATOM 9165 HN HSE J 188 −5.044 36.339 −9.289 0.00 0.00
J
ATOM 9166 CA HSE J 188 −3.917 34.594 −8.700 0.00 0.00
J
ATOM 9167 HA HSE J 188 −4.195 33.600 −9.019 0.00 0.00
J
ATOM 9168 CB HSE J 188 −2.747 35.041 −9.607 0.00 0.00
J
ATOM 9169 HB1 HSE J 188 −2.521 36.110 −9.405 0.00 0.00
J
ATOM 9170 HB2 HSE J 188 −1.874 34.428 −9.297 0.00 0.00
J
ATOM 9171 ND1 HSE J 188 −2.930 33.477 −11.523 0.00 0.00
J
ATOM 9172 CG HSE J 188 −2.918 34.797 −11.089 0.00 0.00
J
ATOM 9173 CE1 HSE J 188 −2.611 33.531 −12.801 0.00 0.00
J
ATOM 9174 HE1 HSE J 188 −2.503 32.661 −13.449 0.00 0.00
J
ATOM 9175 NE2 HSE J 188 −2.328 34.845 −13.163 0.00 0.00
J
ATOM 9176 HE2 HSE J 188 −1.824 35.173 −13.962 0.00 0.00
J
ATOM 9177 CD2 HSE J 188 −2.613 35.630 −12.067 0.00 0.00
J
ATOM 9178 HD2 HSE J 188 −2.110 36.586 −11.994 0.00 0.00
J
ATOM 9179 C HSE J 188 −3.301 34.423 −7.308 0.00 0.00
J

ATOM 9180 O HSE J 188 −2.953 33.274 −6.968 0.00 0.00
J
ATOM 9181 N ALA J 189 −3.251 35.531 −6.557 0.00 0.00
J
ATOM 9182 HN ALA J 189 −3.579 36.346 −7.028 0.00 0.00
J
ATOM 9183 CA ALA J 189 −2.893 35.606 −5.203 0.00 0.00
J
ATOM 9184 HA ALA J 189 −1.881 35.241 −5.100 0.00 0.00
J
ATOM 9185 CB ALA J 189 −2.830 37.164 −4.895 0.00 0.00
J
ATOM 9186 HB1 ALA J 189 −2.040 37.632 −5.520 0.00 0.00
J
ATOM 9187 HB2 ALA J 189 −3.851 37.583 −5.023 0.00 0.00
J
ATOM 9188 HB3 ALA J 189 −2.693 37.354 −3.809 0.00 0.00
J
ATOM 9189 C ALA J 189 −3.817 34.828 −4.253 0.00 0.00
J
ATOM 9190 O ALA J 189 −3.324 34.105 −3.410 0.00 0.00
J
ATOM 9191 N ILE J 190 −5.138 34.998 −4.402 0.00 0.00
J
ATOM 9192 HN ILE J 190 −5.407 35.608 −5.143 0.00 0.00
J
ATOM 9193 CA ILE J 190 −6.153 34.272 −3.685 0.00 0.00
J
ATOM 9194 HA ILE J 190 −5.899 34.296 −2.635 0.00 0.00
J
ATOM 9195 CB ILE J 190 −7.570 34.800 −3.797 0.00 0.00
J
ATOM 9196 HB ILE J 190 −7.758 34.939 −4.883 0.00 0.00
J
ATOM 9197 CG2 ILE J 190 −8.583 33.809 −3.200 0.00 0.00
J
ATOM 9198 HG21 ILE J 190 −9.571 34.217 −3.507 0.00 0.00
J
ATOM 9199 HG22 ILE J 190 −8.647 32.754 −3.542 0.00 0.00
J
ATOM 9200 HG23 ILE J 190 −8.517 33.914 −2.096 0.00 0.00
J
ATOM 9201 CG1 ILE J 190 −7.778 36.192 −3.093 0.00 0.00
J
ATOM 9202 HG11 ILE J 190 −7.711 36.174 −1.984 0.00 0.00
J
ATOM 9203 HG12 ILE J 190 −6.931 36.809 −3.462 0.00 0.00
J
ATOM 9204 CD ILE J 190 −9.115 36.872 −3.449 0.00 0.00
J
ATOM 9205 HD1 ILE J 190 −9.109 37.862 −2.944 0.00 0.00
J
ATOM 9206 HD2 ILE J 190 −9.277 36.977 −4.543 0.00 0.00
J

ATOM 9207 HD3 ILE J 190 -9.939 36.278 -2.999 0.00 0.00
J
ATOM 9208 C ILE J 190 -6.065 32.737 -3.937 0.00 0.00
J
ATOM 9209 O ILE J 190 -5.903 31.906 -3.059 0.00 0.00
J
ATOM 9210 N SER J 191 -6.089 32.314 -5.240 0.00 0.00
J
ATOM 9211 HN SER J 191 -6.250 32.966 -5.977 0.00 0.00
J
ATOM 9212 CA SER J 191 -5.917 30.882 -5.691 0.00 0.00
J
ATOM 9213 HA SER J 191 -6.667 30.169 -5.381 0.00 0.00
J
ATOM 9214 CB SER J 191 -6.099 30.873 -7.261 0.00 0.00
J
ATOM 9215 HB1 SER J 191 -7.162 31.036 -7.542 0.00 0.00
J
ATOM 9216 HB2 SER J 191 -5.465 31.628 -7.773 0.00 0.00
J
ATOM 9217 OG SER J 191 -5.978 29.617 -7.875 0.00 0.00
J
ATOM 9218 HG1 SER J 191 -5.809 29.710 -8.816 0.00 0.00
J
ATOM 9219 C SER J 191 -4.586 30.336 -5.216 0.00 0.00
J
ATOM 9220 O SER J 191 -4.544 29.221 -4.728 0.00 0.00
J
ATOM 9221 N ALA J 192 -3.489 31.038 -5.279 0.00 0.00
J
ATOM 9222 HN ALA J 192 -3.458 31.947 -5.686 0.00 0.00
J
ATOM 9223 CA ALA J 192 -2.160 30.552 -4.735 0.00 0.00
J
ATOM 9224 HA ALA J 192 -1.921 29.683 -5.331 0.00 0.00
J
ATOM 9225 CB ALA J 192 -1.067 31.620 -5.043 0.00 0.00
J
ATOM 9226 HB1 ALA J 192 -0.949 32.023 -6.071 0.00 0.00
J
ATOM 9227 HB2 ALA J 192 -1.383 32.455 -4.381 0.00 0.00
J
ATOM 9228 HB3 ALA J 192 -0.048 31.337 -4.704 0.00 0.00
J
ATOM 9229 C ALA J 192 -2.127 30.218 -3.290 0.00 0.00
J
ATOM 9230 O ALA J 192 -1.643 29.169 -2.833 0.00 0.00
J
ATOM 9231 N PHE J 193 -2.639 31.165 -2.505 0.00 0.00
J
ATOM 9232 HN PHE J 193 -3.004 32.017 -2.870 0.00 0.00
J
ATOM 9233 CA PHE J 193 -2.621 31.049 -1.021 0.00 0.00
J

ATOM 9234 HA PHE J 193 -1.632 30.820 -0.653 0.00 0.00
J
ATOM 9235 CB PHE J 193 -2.992 32.471 -0.359 0.00 0.00
J
ATOM 9236 HB1 PHE J 193 -2.314 33.228 -0.809 0.00 0.00
J
ATOM 9237 HB2 PHE J 193 -4.048 32.763 -0.541 0.00 0.00
J
ATOM 9238 CG PHE J 193 -2.738 32.513 1.124 0.00 0.00
J
ATOM 9239 CD1 PHE J 193 -1.479 32.459 1.676 0.00 0.00
J
ATOM 9240 HD1 PHE J 193 -0.621 32.418 1.022 0.00 0.00
J
ATOM 9241 CE1 PHE J 193 -1.230 32.365 3.055 0.00 0.00
J
ATOM 9242 HE1 PHE J 193 -0.234 32.328 3.470 0.00 0.00
J
ATOM 9243 CZ PHE J 193 -2.366 32.258 3.888 0.00 0.00
J
ATOM 9244 HZ PHE J 193 -2.226 32.123 4.951 0.00 0.00
J
ATOM 9245 CD2 PHE J 193 -3.804 32.490 1.981 0.00 0.00
J
ATOM 9246 HD2 PHE J 193 -4.803 32.637 1.598 0.00 0.00
J
ATOM 9247 CE2 PHE J 193 -3.666 32.174 3.326 0.00 0.00
J
ATOM 9248 HE2 PHE J 193 -4.488 32.056 4.016 0.00 0.00
J
ATOM 9249 C PHE J 193 -3.548 29.970 -0.547 0.00 0.00
J
ATOM 9250 O PHE J 193 -3.249 29.308 0.445 0.00 0.00
J
ATOM 9251 N ASN J 194 -4.736 29.739 -1.157 0.00 0.00
J
ATOM 9252 HN ASN J 194 -4.943 30.199 -2.017 0.00 0.00
J
ATOM 9253 CA ASN J 194 -5.725 28.649 -0.764 0.00 0.00
J
ATOM 9254 HA ASN J 194 -5.688 28.535 0.310 0.00 0.00
J
ATOM 9255 CB ASN J 194 -7.082 29.079 -1.142 0.00 0.00
J
ATOM 9256 HB1 ASN J 194 -7.111 29.393 -2.207 0.00 0.00
J
ATOM 9257 HB2 ASN J 194 -7.821 28.276 -0.933 0.00 0.00
J
ATOM 9258 CG ASN J 194 -7.482 30.267 -0.300 0.00 0.00
J
ATOM 9259 OD1 ASN J 194 -8.276 30.014 0.622 0.00 0.00
J
ATOM 9260 ND2 ASN J 194 -7.038 31.537 -0.589 0.00 0.00
J

ATOM 9261 HD21 ASN J 194 -7.296 32.300 0.004 0.00 0.00
J
ATOM 9262 HD22 ASN J 194 -6.839 31.712 -1.553 0.00 0.00
J
ATOM 9263 C ASN J 194 -5.352 27.259 -1.328 0.00 0.00
J
ATOM 9264 O ASN J 194 -5.781 26.207 -0.889 0.00 0.00
J
ATOM 9265 N ASN J 195 -4.432 27.307 -2.292 0.00 0.00
J
ATOM 9266 HN ASN J 195 -4.097 28.191 -2.609 0.00 0.00
J
ATOM 9267 CA ASN J 195 -3.904 26.273 -3.120 0.00 0.00
J
ATOM 9268 HA ASN J 195 -3.168 26.699 -3.787 0.00 0.00
J
ATOM 9269 CB ASN J 195 -3.286 25.082 -2.357 0.00 0.00
J
ATOM 9270 HB1 ASN J 195 -4.021 24.815 -1.568 0.00 0.00
J
ATOM 9271 HB2 ASN J 195 -3.018 24.185 -2.954 0.00 0.00
J
ATOM 9272 CG ASN J 195 -1.956 25.354 -1.686 0.00 0.00
J
ATOM 9273 OD1 ASN J 195 -1.069 24.574 -1.868 0.00 0.00
J
ATOM 9274 ND2 ASN J 195 -1.779 26.475 -0.893 0.00 0.00
J
ATOM 9275 HD21 ASN J 195 -0.890 26.459 -0.435 0.00 0.00
J
ATOM 9276 HD22 ASN J 195 -2.442 27.224 -0.906 0.00 0.00
J
ATOM 9277 C ASN J 195 -5.057 25.718 -4.013 0.00 0.00
J
ATOM 9278 O ASN J 195 -5.256 24.523 -4.115 0.00 0.00
J
ATOM 9279 N ALA J 196 -5.919 26.588 -4.604 0.00 0.00
J
ATOM 9280 HN ALA J 196 -5.804 27.556 -4.394 0.00 0.00
J
ATOM 9281 CA ALA J 196 -7.111 26.259 -5.393 0.00 0.00
J
ATOM 9282 HA ALA J 196 -7.665 25.460 -4.923 0.00 0.00
J
ATOM 9283 CB ALA J 196 -8.019 27.483 -5.513 0.00 0.00
J
ATOM 9284 HB1 ALA J 196 -8.263 27.929 -4.526 0.00 0.00
J
ATOM 9285 HB2 ALA J 196 -7.446 28.259 -6.064 0.00 0.00
J
ATOM 9286 HB3 ALA J 196 -8.981 27.339 -6.050 0.00 0.00
J
ATOM 9287 C ALA J 196 -6.847 25.728 -6.825 0.00 0.00
J

ATOM 9288 O ALA J 196 −7.502 24.810 −7.221 0.00 0.00
J
ATOM 9289 N GLY J 197 −5.879 26.276 −7.579 0.00 0.00
J
ATOM 9290 HN GLY J 197 −5.496 27.102 −7.174 0.00 0.00
J
ATOM 9291 CA GLY J 197 −5.454 25.767 −8.933 0.00 0.00
J
ATOM 9292 HA1 GLY J 197 −5.620 24.709 −9.070 0.00 0.00
J
ATOM 9293 HA2 GLY J 197 −4.392 25.902 −9.076 0.00 0.00
J
ATOM 9294 C GLY J 197 −6.117 26.406 −10.125 0.00 0.00
J
ATOM 9295 O GLY J 197 −6.005 26.042 −11.309 0.00 0.00
J
ATOM 9296 N PHE J 198 −6.879 27.475 −9.850 0.00 0.00
J
ATOM 9297 HN PHE J 198 −6.817 27.863 −8.934 0.00 0.00
J
ATOM 9298 CA PHE J 198 −7.609 28.140 −10.825 0.00 0.00
J
ATOM 9299 HA PHE J 198 −7.752 27.540 −11.712 0.00 0.00
J
ATOM 9300 CB PHE J 198 −8.923 28.674 −10.263 0.00 0.00
J
ATOM 9301 HB1 PHE J 198 −8.829 29.314 −9.361 0.00 0.00
J
ATOM 9302 HB2 PHE J 198 −9.408 29.305 −11.038 0.00 0.00
J
ATOM 9303 CG PHE J 198 −9.877 27.527 −9.921 0.00 0.00
J
ATOM 9304 CD1 PHE J 198 −10.075 27.114 −8.628 0.00 0.00
J
ATOM 9305 HD1 PHE J 198 −9.540 27.728 −7.919 0.00 0.00
J
ATOM 9306 CE1 PHE J 198 −11.032 26.186 −8.246 0.00 0.00
J
ATOM 9307 HE1 PHE J 198 −11.171 25.809 −7.244 0.00 0.00
J
ATOM 9308 CZ PHE J 198 −11.840 25.582 −9.230 0.00 0.00
J
ATOM 9309 HZ PHE J 198 −12.579 24.860 −8.917 0.00 0.00
J
ATOM 9310 CD2 PHE J 198 −10.653 26.898 −10.886 0.00 0.00
J
ATOM 9311 HD2 PHE J 198 −10.496 27.204 −11.910 0.00 0.00
J
ATOM 9312 CE2 PHE J 198 −11.681 25.973 −10.528 0.00 0.00
J
ATOM 9313 HE2 PHE J 198 −12.185 25.573 −11.395 0.00 0.00
J
ATOM 9314 C PHE J 198 −6.762 29.356 −11.295 0.00 0.00
J

ATOM 9315 O PHE J 198 -5.900 30.035 -10.604 0.00 0.00
J
ATOM 9316 N SER J 199 -7.010 29.705 -12.610 0.00 0.00
J
ATOM 9317 HN SER J 199 -7.702 29.222 -13.141 0.00 0.00
J
ATOM 9318 CA SER J 199 -6.400 30.854 -13.257 0.00 0.00
J
ATOM 9319 HA SER J 199 -6.519 31.606 -12.491 0.00 0.00
J
ATOM 9320 CB SER J 199 -4.977 30.494 -13.729 0.00 0.00
J
ATOM 9321 HB1 SER J 199 -4.325 30.360 -12.840 0.00 0.00
J
ATOM 9322 HB2 SER J 199 -5.110 29.546 -14.293 0.00 0.00
J
ATOM 9323 OG SER J 199 -4.298 31.500 -14.431 0.00 0.00
J
ATOM 9324 HG1 SER J 199 -3.513 31.077 -14.786 0.00 0.00
J
ATOM 9325 C SER J 199 -7.250 31.151 -14.355 0.00 0.00
J
ATOM 9326 O SER J 199 -7.960 30.271 -14.888 0.00 0.00
J
ATOM 9327 N LEU J 200 -7.211 32.428 -14.842 0.00 0.00
J
ATOM 9328 HN LEU J 200 -6.771 33.113 -14.267 0.00 0.00
J
ATOM 9329 CA LEU J 200 -8.035 32.897 -15.952 0.00 0.00
J
ATOM 9330 HA LEU J 200 -8.932 32.301 -16.044 0.00 0.00
J
ATOM 9331 CB LEU J 200 -8.542 34.343 -15.807 0.00 0.00
J
ATOM 9332 HB1 LEU J 200 -7.709 35.075 -15.747 0.00 0.00
J
ATOM 9333 HB2 LEU J 200 -9.226 34.721 -16.597 0.00 0.00
J
ATOM 9334 CG LEU J 200 -9.429 34.613 -14.556 0.00 0.00
J
ATOM 9335 HG LEU J 200 -8.726 34.402 -13.722 0.00 0.00
J
ATOM 9336 CD1 LEU J 200 -9.835 36.087 -14.563 0.00 0.00
J
ATOM 9337 HD11 LEU J 200 -10.735 36.114 -15.214 0.00 0.00
J
ATOM 9338 HD12 LEU J 200 -10.053 36.258 -13.487 0.00 0.00
J
ATOM 9339 HD13 LEU J 200 -9.023 36.768 -14.896 0.00 0.00
J
ATOM 9340 CD2 LEU J 200 -10.621 33.721 -14.270 0.00 0.00
J
ATOM 9341 HD21 LEU J 200 -11.098 34.195 -13.385 0.00 0.00
J

ATOM 9342 HD22 LEU J 200 −11.243 33.796 −15.188 0.00 0.00
J
ATOM 9343 HD23 LEU J 200 −10.380 32.666 −14.018 0.00 0.00
J
ATOM 9344 C LEU J 200 −7.323 32.950 −17.319 0.00 0.00
J
ATOM 9345 O LEU J 200 −7.829 33.571 −18.249 0.00 0.00
J
ATOM 9346 N TRP J 201 −6.117 32.343 −17.441 0.00 0.00
J
ATOM 9347 HN TRP J 201 −5.674 31.951 −16.639 0.00 0.00
J
ATOM 9348 CA TRP J 201 −5.494 31.994 −18.700 0.00 0.00
J
ATOM 9349 HA TRP J 201 −6.302 31.997 −19.417 0.00 0.00
J
ATOM 9350 CB TRP J 201 −4.352 32.955 −19.084 0.00 0.00
J
ATOM 9351 HB1 TRP J 201 −3.588 32.906 −18.280 0.00 0.00
J
ATOM 9352 HB2 TRP J 201 −3.964 32.555 −20.045 0.00 0.00
J
ATOM 9353 CG TRP J 201 −4.643 34.387 −19.428 0.00 0.00
J
ATOM 9354 CD1 TRP J 201 −5.214 34.972 −20.569 0.00 0.00
J
ATOM 9355 HD1 TRP J 201 −5.520 34.450 −21.464 0.00 0.00
J
ATOM 9356 NE1 TRP J 201 −5.180 36.327 −20.515 0.00 0.00
J
ATOM 9357 HE1 TRP J 201 −5.330 36.995 −21.210 0.00 0.00
J
ATOM 9358 CE2 TRP J 201 −4.816 36.645 −19.263 0.00 0.00
J
ATOM 9359 CD2 TRP J 201 −4.406 35.493 −18.547 0.00 0.00
J
ATOM 9360 CE3 TRP J 201 −3.925 35.539 −17.284 0.00 0.00
J
ATOM 9361 HE3 TRP J 201 −3.705 34.698 −16.642 0.00 0.00
J
ATOM 9362 CZ3 TRP J 201 −3.800 36.804 −16.638 0.00 0.00
J
ATOM 9363 HZ3 TRP J 201 −3.238 36.967 −15.730 0.00 0.00
J
ATOM 9364 CZ2 TRP J 201 −4.763 37.813 −18.587 0.00 0.00
J
ATOM 9365 HZ2 TRP J 201 −4.882 38.670 −19.234 0.00 0.00
J
ATOM 9366 CH2 TRP J 201 −4.228 37.929 −17.344 0.00 0.00
J
ATOM 9367 HH2 TRP J 201 −4.146 38.867 −16.814 0.00 0.00
J
ATOM 9368 C TRP J 201 −4.921 30.608 −18.604 0.00 0.00
J

ATOM 9369 O TRP J 201 −4.577 30.068 −17.545 0.00 0.00
J
ATOM 9370 N PRO J 202 −4.804 29.826 −19.720 0.00 0.00
J
ATOM 9371 CD PRO J 202 −5.519 30.191 −20.945 0.00 0.00
J
ATOM 9372 HD1 PRO J 202 −6.599 30.345 −20.738 0.00 0.00
J
ATOM 9373 HD2 PRO J 202 −5.012 30.959 −21.568 0.00 0.00
J
ATOM 9374 CA PRO J 202 −4.760 28.411 −19.584 0.00 0.00
J
ATOM 9375 HA PRO J 202 −5.211 28.005 −18.691 0.00 0.00
J
ATOM 9376 CB PRO J 202 −5.516 27.852 −20.793 0.00 0.00
J
ATOM 9377 HB1 PRO J 202 −6.586 27.791 −20.501 0.00 0.00
J
ATOM 9378 HB2 PRO J 202 −5.124 26.872 −21.139 0.00 0.00
J
ATOM 9379 CG PRO J 202 −5.419 28.895 −21.831 0.00 0.00
J
ATOM 9380 HG1 PRO J 202 −6.157 28.753 −22.649 0.00 0.00
J
ATOM 9381 HG2 PRO J 202 −4.422 28.958 −22.318 0.00 0.00
J
ATOM 9382 C PRO J 202 −3.313 27.951 −19.551 0.00 0.00
J
ATOM 9383 O PRO J 202 −3.104 26.754 −19.509 0.00 0.00
J
ATOM 9384 N ASP J 203 −2.315 28.835 −19.537 0.00 0.00
J
ATOM 9385 HN ASP J 203 −2.480 29.816 −19.611 0.00 0.00
J
ATOM 9386 CA ASP J 203 −0.987 28.441 −19.222 0.00 0.00
J
ATOM 9387 HA ASP J 203 −0.693 27.602 −19.836 0.00 0.00
J
ATOM 9388 CB ASP J 203 0.074 29.513 −19.555 0.00 0.00
J
ATOM 9389 HB1 ASP J 203 1.128 29.168 −19.491 0.00 0.00
J
ATOM 9390 HB2 ASP J 203 −0.189 29.852 −20.580 0.00 0.00
J
ATOM 9391 CG ASP J 203 −0.006 30.688 −18.611 0.00 0.00
J
ATOM 9392 OD1 ASP J 203 −0.952 31.509 −18.649 0.00 0.00
J
ATOM 9393 OD2 ASP J 203 0.945 30.859 −17.767 0.00 0.00
J
ATOM 9394 C ASP J 203 −0.718 27.951 −17.812 0.00 0.00
J
ATOM 9395 O ASP J 203 0.210 27.233 −17.576 0.00 0.00
J

```
ATOM   9396  N    ASN J 204      -1.467  28.352 -16.784  0.00  0.00
J
ATOM   9397  HN   ASN J 204      -2.268  28.916 -16.973  0.00  0.00
J
ATOM   9398  CA   ASN J 204      -1.408  28.005 -15.381  0.00  0.00
J
ATOM   9399  HA   ASN J 204      -2.105  28.664 -14.885  0.00  0.00
J
ATOM   9400  CB   ASN J 204      -1.789  26.533 -15.211  0.00  0.00
J
ATOM   9401  HB1  ASN J 204      -1.149  25.810 -15.761  0.00  0.00
J
ATOM   9402  HB2  ASN J 204      -1.686  26.210 -14.153  0.00  0.00
J
ATOM   9403  CG   ASN J 204      -3.226  26.274 -15.571  0.00  0.00
J
ATOM   9404  OD1  ASN J 204      -4.133  26.969 -15.031  0.00  0.00
J
ATOM   9405  ND2  ASN J 204      -3.515  25.457 -16.613  0.00  0.00
J
ATOM   9406 HD21  ASN J 204      -4.480  25.250 -16.771  0.00  0.00
J
ATOM   9407 HD22  ASN J 204      -2.823  24.979 -17.156  0.00  0.00
J
ATOM   9408  C    ASN J 204      -0.157  28.312 -14.693  0.00  0.00
J
ATOM   9409  O    ASN J 204       0.392  27.418 -14.073  0.00  0.00
J
ATOM   9410  N    LEU J 205       0.351  29.570 -14.860  0.00  0.00
J
ATOM   9411  HN   LEU J 205      -0.142  30.221 -15.431  0.00  0.00
J
ATOM   9412  CA   LEU J 205       1.504  30.146 -14.226  0.00  0.00
J
ATOM   9413  HA   LEU J 205       1.555  31.187 -14.508  0.00  0.00
J
ATOM   9414  CB   LEU J 205       1.553  30.047 -12.655  0.00  0.00
J
ATOM   9415  HB1  LEU J 205       1.812  29.025 -12.303  0.00  0.00
J
ATOM   9416  HB2  LEU J 205       2.421  30.689 -12.392  0.00  0.00
J
ATOM   9417  CG   LEU J 205       0.298  30.475 -11.919  0.00  0.00
J
ATOM   9418  HG   LEU J 205      -0.552  29.808 -12.179  0.00  0.00
J
ATOM   9419  CD1  LEU J 205       0.565  30.363 -10.451  0.00  0.00
J
ATOM   9420 HD11  LEU J 205       0.776  29.311 -10.163  0.00  0.00
J
ATOM   9421 HD12  LEU J 205       1.287  31.041  -9.949  0.00  0.00
J
ATOM   9422 HD13  LEU J 205      -0.433  30.624 -10.038  0.00  0.00
J
```

ATOM 9423 CD2 LEU J 205 -0.069 31.916 -12.437 0.00 0.00
J
ATOM 9424 HD21 LEU J 205 -0.400 31.783 -13.489 0.00 0.00
J
ATOM 9425 HD22 LEU J 205 -0.874 32.231 -11.738 0.00 0.00
J
ATOM 9426 HD23 LEU J 205 0.722 32.689 -12.337 0.00 0.00
J
ATOM 9427 C LEU J 205 2.820 29.724 -14.761 0.00 0.00
J
ATOM 9428 O LEU J 205 3.890 29.851 -14.150 0.00 0.00
J
ATOM 9429 N MET J 206 2.791 29.103 -15.952 0.00 0.00
J
ATOM 9430 HN MET J 206 1.864 28.983 -16.299 0.00 0.00
J
ATOM 9431 CA MET J 206 3.957 28.589 -16.666 0.00 0.00
J
ATOM 9432 HA MET J 206 4.572 27.975 -16.025 0.00 0.00
J
ATOM 9433 CB MET J 206 3.445 27.806 -17.861 0.00 0.00
J
ATOM 9434 HB1 MET J 206 2.720 27.045 -17.500 0.00 0.00
J
ATOM 9435 HB2 MET J 206 2.905 28.433 -18.603 0.00 0.00
J
ATOM 9436 CG MET J 206 4.536 27.009 -18.639 0.00 0.00
J
ATOM 9437 HG1 MET J 206 4.068 26.524 -19.522 0.00 0.00
J
ATOM 9438 HG2 MET J 206 5.272 27.707 -19.092 0.00 0.00
J
ATOM 9439 SD MET J 206 5.400 25.695 -17.596 0.00 0.00
J
ATOM 9440 CE MET J 206 6.717 25.359 -18.783 0.00 0.00
J
ATOM 9441 HE1 MET J 206 6.296 24.976 -19.737 0.00 0.00
J
ATOM 9442 HE2 MET J 206 7.149 26.330 -19.108 0.00 0.00
J
ATOM 9443 HE3 MET J 206 7.515 24.640 -18.497 0.00 0.00
J
ATOM 9444 C MET J 206 4.825 29.718 -17.131 0.00 0.00
J
ATOM 9445 O MET J 206 6.015 29.569 -17.272 0.00 0.00
J
ATOM 9446 N SER J 207 4.209 30.875 -17.320 0.00 0.00
J
ATOM 9447 HN SER J 207 3.230 30.883 -17.133 0.00 0.00
J
ATOM 9448 CA SER J 207 4.942 32.118 -17.700 0.00 0.00
J
ATOM 9449 HA SER J 207 5.388 31.907 -18.661 0.00 0.00
J

ATOM 9450 CB SER J 207 3.959 33.284 -17.912 0.00 0.00
J
ATOM 9451 HB1 SER J 207 3.121 32.858 -18.503 0.00 0.00
J
ATOM 9452 HB2 SER J 207 3.549 33.648 -16.946 0.00 0.00
J
ATOM 9453 OG SER J 207 4.502 34.419 -18.610 0.00 0.00
J
ATOM 9454 HG1 SER J 207 3.807 35.082 -18.620 0.00 0.00
J
ATOM 9455 C SER J 207 6.122 32.589 -16.767 0.00 0.00
J
ATOM 9456 O SER J 207 7.096 33.234 -17.138 0.00 0.00
J
ATOM 9457 N TYR J 208 5.936 32.270 -15.532 0.00 0.00
J
ATOM 9458 HN TYR J 208 5.155 31.710 -15.267 0.00 0.00
J
ATOM 9459 CA TYR J 208 6.636 32.823 -14.405 0.00 0.00
J
ATOM 9460 HA TYR J 208 7.387 33.506 -14.775 0.00 0.00
J
ATOM 9461 CB TYR J 208 5.685 33.451 -13.286 0.00 0.00
J
ATOM 9462 HB1 TYR J 208 5.093 32.673 -12.758 0.00 0.00
J
ATOM 9463 HB2 TYR J 208 6.416 33.997 -12.653 0.00 0.00
J
ATOM 9464 CG TYR J 208 4.571 34.294 -13.972 0.00 0.00
J
ATOM 9465 CD1 TYR J 208 3.156 34.276 -13.649 0.00 0.00
J
ATOM 9466 HD1 TYR J 208 2.841 33.634 -12.841 0.00 0.00
J
ATOM 9467 CE1 TYR J 208 2.206 35.146 -14.289 0.00 0.00
J
ATOM 9468 HE1 TYR J 208 1.188 35.221 -13.937 0.00 0.00
J
ATOM 9469 CZ TYR J 208 2.566 36.003 -15.268 0.00 0.00
J
ATOM 9470 OH TYR J 208 1.677 36.865 -15.880 0.00 0.00
J
ATOM 9471 HH TYR J 208 2.246 37.631 -15.990 0.00 0.00
J
ATOM 9472 CD2 TYR J 208 4.953 35.279 -14.961 0.00 0.00
J
ATOM 9473 HD2 TYR J 208 5.970 35.412 -15.300 0.00 0.00
J
ATOM 9474 CE2 TYR J 208 3.958 36.100 -15.552 0.00 0.00
J
ATOM 9475 HE2 TYR J 208 4.313 36.852 -16.241 0.00 0.00
J
ATOM 9476 C TYR J 208 7.444 31.744 -13.649 0.00 0.00
J

```
ATOM   9477  O   TYR J 208       7.955  31.973 -12.536  0.00  0.00
J
ATOM   9478  N   VAL J 209       7.552  30.567 -14.364  0.00  0.00
J
ATOM   9479  HN  VAL J 209       6.963  30.466 -15.162  0.00  0.00
J
ATOM   9480  CA  VAL J 209       8.090  29.359 -13.799  0.00  0.00
J
ATOM   9481  HA  VAL J 209       7.604  29.151 -12.857  0.00  0.00
J
ATOM   9482  CB  VAL J 209       7.725  28.140 -14.683  0.00  0.00
J
ATOM   9483  HB  VAL J 209       6.685  28.249 -15.058  0.00  0.00
J
ATOM   9484  CG1 VAL J 209       8.689  28.041 -15.957  0.00  0.00
J
ATOM   9485 HG11 VAL J 209       8.466  27.192 -16.638  0.00  0.00
J
ATOM   9486 HG12 VAL J 209       8.500  29.031 -16.425  0.00  0.00
J
ATOM   9487 HG13 VAL J 209       9.749  27.958 -15.633  0.00  0.00
J
ATOM   9488  CG2 VAL J 209       7.661  26.829 -13.845  0.00  0.00
J
ATOM   9489 HG21 VAL J 209       7.377  25.921 -14.420  0.00  0.00
J
ATOM   9490 HG22 VAL J 209       8.635  26.688 -13.329  0.00  0.00
J
ATOM   9491 HG23 VAL J 209       6.868  26.987 -13.084  0.00  0.00
J
ATOM   9492  C   VAL J 209       9.522  29.375 -13.407  0.00  0.00
J
ATOM   9493  O   VAL J 209       9.888  28.818 -12.422  0.00  0.00
J
ATOM   9494  N   GLY J 210      10.369  30.119 -14.255  0.00  0.00
J
ATOM   9495  HN  GLY J 210      10.007  30.438 -15.128  0.00  0.00
J
ATOM   9496  CA  GLY J 210      11.809  30.223 -14.080  0.00  0.00
J
ATOM   9497  HA1 GLY J 210      12.205  29.907 -15.034  0.00  0.00
J
ATOM   9498  HA2 GLY J 210      12.065  29.611 -13.228  0.00  0.00
J
ATOM   9499  C   GLY J 210      12.320  31.627 -13.717  0.00  0.00
J
ATOM   9500  O   GLY J 210      13.458  31.968 -13.899  0.00  0.00
J
ATOM   9501  N   ASP J 211      11.391  32.406 -13.103  0.00  0.00
J
ATOM   9502  HN  ASP J 211      10.475  32.124 -12.831  0.00  0.00
J
ATOM   9503  CA  ASP J 211      11.783  33.731 -12.619  0.00  0.00
J
```

ATOM 9504 HA ASP J 211 12.675 34.063 −13.130 0.00 0.00
J
ATOM 9505 CB ASP J 211 10.647 34.613 −13.117 0.00 0.00
J
ATOM 9506 HB1 ASP J 211 10.557 34.457 −14.213 0.00 0.00
J
ATOM 9507 HB2 ASP J 211 9.711 34.266 −12.629 0.00 0.00
J
ATOM 9508 CG ASP J 211 10.948 36.046 −12.930 0.00 0.00
J
ATOM 9509 OD1 ASP J 211 11.241 36.687 −13.972 0.00 0.00
J
ATOM 9510 OD2 ASP J 211 10.863 36.610 −11.778 0.00 0.00
J
ATOM 9511 C ASP J 211 11.904 33.620 −11.099 0.00 0.00
J
ATOM 9512 O ASP J 211 10.977 33.309 −10.345 0.00 0.00
J
ATOM 9513 N PRO J 212 13.133 33.863 −10.499 0.00 0.00
J
ATOM 9514 CD PRO J 212 14.381 34.286 −11.216 0.00 0.00
J
ATOM 9515 HD1 PRO J 212 14.801 33.353 −11.648 0.00 0.00
J
ATOM 9516 HD2 PRO J 212 14.268 35.077 −11.988 0.00 0.00
J
ATOM 9517 CA PRO J 212 13.447 33.626 −9.104 0.00 0.00
J
ATOM 9518 HA PRO J 212 13.389 32.551 −9.017 0.00 0.00
J
ATOM 9519 CB PRO J 212 14.923 34.037 −8.941 0.00 0.00
J
ATOM 9520 HB1 PRO J 212 15.509 33.096 −8.862 0.00 0.00
J
ATOM 9521 HB2 PRO J 212 15.097 34.537 −7.964 0.00 0.00
J
ATOM 9522 CG PRO J 212 15.280 34.912 −10.091 0.00 0.00
J
ATOM 9523 HG1 PRO J 212 16.365 34.923 −10.330 0.00 0.00
J
ATOM 9524 HG2 PRO J 212 14.877 35.946 −10.032 0.00 0.00
J
ATOM 9525 C PRO J 212 12.555 34.297 −8.080 0.00 0.00
J
ATOM 9526 O PRO J 212 12.113 33.577 −7.189 0.00 0.00
J
ATOM 9527 N THR J 213 12.249 35.617 −8.207 0.00 0.00
J
ATOM 9528 HN THR J 213 12.566 36.201 −8.951 0.00 0.00
J
ATOM 9529 CA THR J 213 11.454 36.295 −7.151 0.00 0.00
J
ATOM 9530 HA THR J 213 11.940 35.950 −6.250 0.00 0.00
J

ATOM 9531 CB THR J 213 11.503 37.826 -7.069 0.00 0.00
J
ATOM 9532 HB THR J 213 10.979 38.207 -6.167 0.00 0.00
J
ATOM 9533 OG1 THR J 213 10.916 38.523 -8.185 0.00 0.00
J
ATOM 9534 HG1 THR J 213 11.526 38.310 -8.896 0.00 0.00
J
ATOM 9535 CG2 THR J 213 12.912 38.350 -6.846 0.00 0.00
J
ATOM 9536 HG21 THR J 213 12.820 39.458 -6.833 0.00 0.00
J
ATOM 9537 HG22 THR J 213 13.303 37.975 -5.876 0.00 0.00
J
ATOM 9538 HG23 THR J 213 13.551 38.088 -7.716 0.00 0.00
J
ATOM 9539 C THR J 213 10.067 35.820 -7.104 0.00 0.00
J
ATOM 9540 O THR J 213 9.507 35.569 -6.045 0.00 0.00
J
ATOM 9541 N VAL J 214 9.417 35.684 -8.295 0.00 0.00
J
ATOM 9542 HN VAL J 214 9.817 36.058 -9.128 0.00 0.00
J
ATOM 9543 CA VAL J 214 8.053 35.101 -8.421 0.00 0.00
J
ATOM 9544 HA VAL J 214 7.447 35.793 -7.855 0.00 0.00
J
ATOM 9545 CB VAL J 214 7.432 35.219 -9.846 0.00 0.00
J
ATOM 9546 HB VAL J 214 7.915 34.513 -10.555 0.00 0.00
J
ATOM 9547 CG1 VAL J 214 5.923 35.080 -9.784 0.00 0.00
J
ATOM 9548 HG11 VAL J 214 5.556 34.032 -9.756 0.00 0.00
J
ATOM 9549 HG12 VAL J 214 5.464 35.578 -8.903 0.00 0.00
J
ATOM 9550 HG13 VAL J 214 5.472 35.556 -10.681 0.00 0.00
J
ATOM 9551 CG2 VAL J 214 7.772 36.694 -10.242 0.00 0.00
J
ATOM 9552 HG21 VAL J 214 7.465 36.887 -11.293 0.00 0.00
J
ATOM 9553 HG22 VAL J 214 7.154 37.340 -9.582 0.00 0.00
J
ATOM 9554 HG23 VAL J 214 8.868 36.864 -10.187 0.00 0.00
J
ATOM 9555 C VAL J 214 7.945 33.622 -7.951 0.00 0.00
J
ATOM 9556 O VAL J 214 7.066 33.239 -7.143 0.00 0.00
J
ATOM 9557 N ASN J 215 8.903 32.782 -8.435 0.00 0.00
J

ATOM 9558 HN ASN J 215 9.476 33.047 -9.206 0.00 0.00
J
ATOM 9559 CA ASN J 215 8.915 31.394 -7.930 0.00 0.00
J
ATOM 9560 HA ASN J 215 7.977 30.934 -8.204 0.00 0.00
J
ATOM 9561 CB ASN J 215 10.049 30.618 -8.670 0.00 0.00
J
ATOM 9562 HB1 ASN J 215 9.806 30.669 -9.753 0.00 0.00
J
ATOM 9563 HB2 ASN J 215 11.034 31.099 -8.490 0.00 0.00
J
ATOM 9564 CG ASN J 215 10.071 29.122 -8.377 0.00 0.00
J
ATOM 9565 OD1 ASN J 215 10.265 28.607 -7.294 0.00 0.00
J
ATOM 9566 ND2 ASN J 215 9.798 28.285 -9.437 0.00 0.00
J
ATOM 9567 HD21 ASN J 215 10.007 27.326 -9.247 0.00 0.00
J
ATOM 9568 HD22 ASN J 215 9.751 28.568 -10.395 0.00 0.00
J
ATOM 9569 C ASN J 215 9.108 31.275 -6.388 0.00 0.00
J
ATOM 9570 O ASN J 215 8.368 30.544 -5.787 0.00 0.00
J
ATOM 9571 N LEU J 216 9.928 32.008 -5.734 0.00 0.00
J
ATOM 9572 HN LEU J 216 10.474 32.688 -6.217 0.00 0.00
J
ATOM 9573 CA LEU J 216 9.912 32.090 -4.251 0.00 0.00
J
ATOM 9574 HA LEU J 216 9.988 31.064 -3.923 0.00 0.00
J
ATOM 9575 CB LEU J 216 11.306 32.705 -3.978 0.00 0.00
J
ATOM 9576 HB1 LEU J 216 12.075 32.158 -4.563 0.00 0.00
J
ATOM 9577 HB2 LEU J 216 11.300 33.765 -4.311 0.00 0.00
J
ATOM 9578 CG LEU J 216 11.569 32.769 -2.442 0.00 0.00
J
ATOM 9579 HG LEU J 216 10.699 33.133 -1.856 0.00 0.00
J
ATOM 9580 CD1 LEU J 216 11.983 31.442 -1.768 0.00 0.00
J
ATOM 9581 HD11 LEU J 216 12.952 31.067 -2.162 0.00 0.00
J
ATOM 9582 HD12 LEU J 216 12.101 31.580 -0.672 0.00 0.00
J
ATOM 9583 HD13 LEU J 216 11.147 30.744 -1.988 0.00 0.00
J
ATOM 9584 CD2 LEU J 216 12.614 33.885 -2.147 0.00 0.00
J

ATOM 9585 HD21 LEU J 216 12.375 34.836 -2.670 0.00 0.00
J
ATOM 9586 HD22 LEU J 216 12.613 34.135 -1.065 0.00 0.00
J
ATOM 9587 HD23 LEU J 216 13.580 33.573 -2.599 0.00 0.00
J
ATOM 9588 C LEU J 216 8.678 32.644 -3.580 0.00 0.00
J
ATOM 9589 O LEU J 216 8.178 32.187 -2.600 0.00 0.00
J
ATOM 9590 N VAL J 217 8.094 33.726 -4.093 0.00 0.00
J
ATOM 9591 HN VAL J 217 8.316 34.223 -4.928 0.00 0.00
J
ATOM 9592 CA VAL J 217 6.875 34.216 -3.398 0.00 0.00
J
ATOM 9593 HA VAL J 217 6.990 34.309 -2.328 0.00 0.00
J
ATOM 9594 CB VAL J 217 6.492 35.650 -3.745 0.00 0.00
J
ATOM 9595 HB VAL J 217 5.551 35.941 -3.231 0.00 0.00
J
ATOM 9596 CG1 VAL J 217 7.526 36.704 -3.291 0.00 0.00
J
ATOM 9597 HG11 VAL J 217 7.319 37.619 -3.886 0.00 0.00
J
ATOM 9598 HG12 VAL J 217 7.547 37.057 -2.237 0.00 0.00
J
ATOM 9599 HG13 VAL J 217 8.523 36.389 -3.667 0.00 0.00
J
ATOM 9600 CG2 VAL J 217 6.228 35.779 -5.259 0.00 0.00
J
ATOM 9601 HG21 VAL J 217 5.280 35.255 -5.509 0.00 0.00
J
ATOM 9602 HG22 VAL J 217 6.313 36.826 -5.620 0.00 0.00
J
ATOM 9603 HG23 VAL J 217 6.945 35.118 -5.791 0.00 0.00
J
ATOM 9604 C VAL J 217 5.760 33.287 -3.521 0.00 0.00
J
ATOM 9605 O VAL J 217 5.076 33.043 -2.502 0.00 0.00
J
ATOM 9606 N ILE J 218 5.510 32.719 -4.721 0.00 0.00
J
ATOM 9607 HN ILE J 218 6.085 32.948 -5.502 0.00 0.00
J
ATOM 9608 CA ILE J 218 4.408 31.806 -4.915 0.00 0.00
J
ATOM 9609 HA ILE J 218 3.518 32.216 -4.461 0.00 0.00
J
ATOM 9610 CB ILE J 218 4.120 31.562 -6.410 0.00 0.00
J
ATOM 9611 HB ILE J 218 4.954 31.126 -7.001 0.00 0.00
J

ATOM 9612 CG2 ILE J 218 2.877 30.571 -6.581 0.00 0.00
J
ATOM 9613 HG21 ILE J 218 1.938 30.943 -6.118 0.00 0.00
J
ATOM 9614 HG22 ILE J 218 2.786 30.582 -7.688 0.00 0.00
J
ATOM 9615 HG23 ILE J 218 3.233 29.564 -6.275 0.00 0.00
J
ATOM 9616 CG1 ILE J 218 3.823 32.860 -7.159 0.00 0.00
J
ATOM 9617 HG11 ILE J 218 4.746 33.464 -7.288 0.00 0.00
J
ATOM 9618 HG12 ILE J 218 3.626 32.631 -8.228 0.00 0.00
J
ATOM 9619 CD ILE J 218 2.588 33.603 -6.546 0.00 0.00
J
ATOM 9620 HD1 ILE J 218 2.746 34.030 -5.533 0.00 0.00
J
ATOM 9621 HD2 ILE J 218 2.279 34.497 -7.129 0.00 0.00
J
ATOM 9622 HD3 ILE J 218 1.656 32.999 -6.558 0.00 0.00
J
ATOM 9623 C ILE J 218 4.491 30.541 -4.169 0.00 0.00
J
ATOM 9624 O ILE J 218 3.521 30.059 -3.615 0.00 0.00
J
ATOM 9625 N THR J 219 5.652 29.915 -4.098 0.00 0.00
J
ATOM 9626 HN THR J 219 6.382 30.311 -4.649 0.00 0.00
J
ATOM 9627 CA THR J 219 5.848 28.722 -3.338 0.00 0.00
J
ATOM 9628 HA THR J 219 5.020 28.065 -3.558 0.00 0.00
J
ATOM 9629 CB THR J 219 7.122 27.994 -3.587 0.00 0.00
J
ATOM 9630 HB THR J 219 7.094 27.103 -2.924 0.00 0.00
J
ATOM 9631 OG1 THR J 219 8.266 28.771 -3.343 0.00 0.00
J
ATOM 9632 HG1 THR J 219 8.524 29.092 -4.210 0.00 0.00
J
ATOM 9633 CG2 THR J 219 7.163 27.630 -5.031 0.00 0.00
J
ATOM 9634 HG21 THR J 219 7.938 26.857 -5.218 0.00 0.00
J
ATOM 9635 HG22 THR J 219 6.192 27.114 -5.187 0.00 0.00
J
ATOM 9636 HG23 THR J 219 7.294 28.542 -5.653 0.00 0.00
J
ATOM 9637 C THR J 219 5.727 28.926 -1.899 0.00 0.00
J
ATOM 9638 O THR J 219 5.244 28.013 -1.171 0.00 0.00
J

ATOM 9639 N PHE J 220 6.232 30.084 -1.422 0.00 0.00
J
ATOM 9640 HN PHE J 220 6.574 30.728 -2.102 0.00 0.00
J
ATOM 9641 CA PHE J 220 6.047 30.488 0.017 0.00 0.00
J
ATOM 9642 HA PHE J 220 6.565 29.733 0.590 0.00 0.00
J
ATOM 9643 CB PHE J 220 6.678 31.851 0.325 0.00 0.00
J
ATOM 9644 HB1 PHE J 220 7.694 31.935 -0.118 0.00 0.00
J
ATOM 9645 HB2 PHE J 220 6.128 32.705 -0.124 0.00 0.00
J
ATOM 9646 CG PHE J 220 6.876 32.089 1.713 0.00 0.00
J
ATOM 9647 CD1 PHE J 220 5.747 32.676 2.425 0.00 0.00
J
ATOM 9648 HD1 PHE J 220 4.795 32.794 1.929 0.00 0.00
J
ATOM 9649 CE1 PHE J 220 5.894 32.838 3.822 0.00 0.00
J
ATOM 9650 HE1 PHE J 220 5.067 33.231 4.396 0.00 0.00
J
ATOM 9651 CZ PHE J 220 7.017 32.442 4.516 0.00 0.00
J
ATOM 9652 HZ PHE J 220 7.017 32.625 5.581 0.00 0.00
J
ATOM 9653 CD2 PHE J 220 7.977 31.673 2.433 0.00 0.00
J
ATOM 9654 HD2 PHE J 220 8.783 31.088 2.015 0.00 0.00
J
ATOM 9655 CE2 PHE J 220 8.049 31.923 3.836 0.00 0.00
J
ATOM 9656 HE2 PHE J 220 8.940 31.515 4.290 0.00 0.00
J
ATOM 9657 C PHE J 220 4.537 30.498 0.336 0.00 0.00
J
ATOM 9658 O PHE J 220 4.083 30.052 1.372 0.00 0.00
J
ATOM 9659 N LEU J 221 3.672 31.072 -0.556 0.00 0.00
J
ATOM 9660 HN LEU J 221 3.880 31.421 -1.466 0.00 0.00
J
ATOM 9661 CA LEU J 221 2.197 30.992 -0.437 0.00 0.00
J
ATOM 9662 HA LEU J 221 1.895 31.390 0.520 0.00 0.00
J
ATOM 9663 CB LEU J 221 1.552 31.895 -1.481 0.00 0.00
J
ATOM 9664 HB1 LEU J 221 1.798 31.638 -2.533 0.00 0.00
J
ATOM 9665 HB2 LEU J 221 0.447 31.792 -1.527 0.00 0.00
J

ATOM   9666 CG LEU J 221   1.845 33.383 -1.338 0.00 0.00
J
ATOM   9667 HG LEU J 221   2.932 33.497 -1.142 0.00 0.00
J
ATOM   9668 CD1 LEU J 221   1.342 34.030 -2.634 0.00 0.00
J
ATOM   9669 HD11 LEU J 221   1.558 35.117 -2.713 0.00 0.00
J
ATOM   9670 HD12 LEU J 221   1.789 33.537 -3.524 0.00 0.00
J
ATOM   9671 HD13 LEU J 221   0.240 33.934 -2.733 0.00 0.00
J
ATOM   9672 CD2 LEU J 221   1.066 34.092 -0.208 0.00 0.00
J
ATOM   9673 HD21 LEU J 221   -0.004 33.829 -0.349 0.00 0.00
J
ATOM   9674 HD22 LEU J 221   1.552 33.608   0.666 0.00 0.00
J
ATOM   9675 HD23 LEU J 221   1.231 35.180 -0.051 0.00 0.00
J
ATOM   9676 C   LEU J 221   1.614 29.588 -0.555 0.00 0.00
J
ATOM   9677 O   LEU J 221   0.740 29.248   0.208 0.00 0.00
J
ATOM   9678 N   PHE J 222   2.025 28.709 -1.450 0.00 0.00
J
ATOM   9679 HN PHE J 222   2.679 28.986 -2.150 0.00 0.00
J
ATOM   9680 CA PHE J 222   1.564 27.316 -1.566 0.00 0.00
J
ATOM   9681 HA PHE J 222   0.549 27.244 -1.930 0.00 0.00
J
ATOM   9682 CB PHE J 222   2.324 26.657 -2.671 0.00 0.00
J
ATOM   9683 HB1 PHE J 222   3.384 26.914 -2.464 0.00 0.00
J
ATOM   9684 HB2 PHE J 222   2.255 25.548 -2.656 0.00 0.00
J
ATOM   9685 CG PHE J 222   2.025 26.994 -4.170 0.00 0.00
J
ATOM   9686 CD1 PHE J 222   2.838 26.329 -5.148 0.00 0.00
J
ATOM   9687 HD1 PHE J 222   3.534 25.551 -4.873 0.00 0.00
J
ATOM   9688 CE1 PHE J 222   2.595 26.585 -6.465 0.00 0.00
J
ATOM   9689 HE1 PHE J 222   3.182 26.076 -7.215 0.00 0.00
J
ATOM   9690 CZ PHE J 222   1.568 27.447 -6.848 0.00 0.00
J
ATOM   9691 HZ PHE J 222   1.401 27.704 -7.883 0.00 0.00
J
ATOM   9692 CD2 PHE J 222   1.016 27.877 -4.504 0.00 0.00
J

ATOM 9693 HD2 PHE J 222 0.515 28.401 -3.704 0.00 0.00
J
ATOM 9694 CE2 PHE J 222 0.844 28.101 -5.880 0.00 0.00
J
ATOM 9695 HE2 PHE J 222 0.121 28.848 -6.172 0.00 0.00
J
ATOM 9696 C PHE J 222 1.821 26.541 -0.340 0.00 0.00
J
ATOM 9697 O PHE J 222 0.904 25.995 0.375 0.00 0.00
J
ATOM 9698 N ILE J 223 3.077 26.664 0.166 0.00 0.00
J
ATOM 9699 HN ILE J 223 3.628 27.247 -0.426 0.00 0.00
J
ATOM 9700 CA ILE J 223 3.548 25.997 1.384 0.00 0.00
J
ATOM 9701 HA ILE J 223 3.291 24.963 1.211 0.00 0.00
J
ATOM 9702 CB ILE J 223 5.024 26.188 1.510 0.00 0.00
J
ATOM 9703 HB ILE J 223 5.151 27.289 1.431 0.00 0.00
J
ATOM 9704 CG2 ILE J 223 5.316 25.732 3.011 0.00 0.00
J
ATOM 9705 HG21 ILE J 223 4.983 24.672 3.016 0.00 0.00
J
ATOM 9706 HG22 ILE J 223 6.390 25.596 3.262 0.00 0.00
J
ATOM 9707 HG23 ILE J 223 4.843 26.227 3.887 0.00 0.00
J
ATOM 9708 CG1 ILE J 223 5.772 25.400 0.400 0.00 0.00
J
ATOM 9709 HG11 ILE J 223 5.607 24.302 0.416 0.00 0.00
J
ATOM 9710 HG12 ILE J 223 5.327 25.786 -0.543 0.00 0.00
J
ATOM 9711 CD ILE J 223 7.274 25.748 0.387 0.00 0.00
J
ATOM 9712 HD1 ILE J 223 7.714 25.159 -0.445 0.00 0.00
J
ATOM 9713 HD2 ILE J 223 7.335 26.847 0.235 0.00 0.00
J
ATOM 9714 HD3 ILE J 223 7.710 25.491 1.376 0.00 0.00
J
ATOM 9715 C ILE J 223 2.797 26.569 2.560 0.00 0.00
J
ATOM 9716 O ILE J 223 2.252 25.769 3.294 0.00 0.00
J
ATOM 9717 N THR J 224 2.615 27.925 2.708 0.00 0.00
J
ATOM 9718 HN THR J 224 3.092 28.645 2.210 0.00 0.00
J
ATOM 9719 CA THR J 224 2.010 28.506 3.921 0.00 0.00
J

ATOM 9720 HA THR J 224 2.596 28.040 4.699 0.00 0.00
J
ATOM 9721 CB THR J 224 2.026 30.068 3.936 0.00 0.00
J
ATOM 9722 HB THR J 224 1.609 30.356 2.947 0.00 0.00
J
ATOM 9723 OG1 THR J 224 3.329 30.582 3.999 0.00 0.00
J
ATOM 9724 HG1 THR J 224 3.676 30.608 3.104 0.00 0.00
J
ATOM 9725 CG2 THR J 224 1.290 30.704 5.129 0.00 0.00
J
ATOM 9726 HG21 THR J 224 1.563 31.779 5.072 0.00 0.00
J
ATOM 9727 HG22 THR J 224 0.199 30.598 4.952 0.00 0.00
J
ATOM 9728 HG23 THR J 224 1.586 30.190 6.069 0.00 0.00
J
ATOM 9729 C THR J 224 0.538 28.081 4.058 0.00 0.00
J
ATOM 9730 O THR J 224 0.136 27.790 5.180 0.00 0.00
J
ATOM 9731 N GLY J 225 -0.238 28.125 2.985 0.00 0.00
J
ATOM 9732 HN GLY J 225 0.008 28.557 2.121 0.00 0.00
J
ATOM 9733 CA GLY J 225 -1.583 27.588 2.992 0.00 0.00
J
ATOM 9734 HA1 GLY J 225 -1.946 27.534 1.977 0.00 0.00
J
ATOM 9735 HA2 GLY J 225 -2.251 28.136 3.640 0.00 0.00
J
ATOM 9736 C GLY J 225 -1.565 26.131 3.451 0.00 0.00
J
ATOM 9737 O GLY J 225 -2.174 25.704 4.427 0.00 0.00
J
ATOM 9738 N GLY J 226 -0.633 25.320 2.835 0.00 0.00
J
ATOM 9739 HN GLY J 226 0.060 25.668 2.208 0.00 0.00
J
ATOM 9740 CA GLY J 226 -0.519 23.879 3.203 0.00 0.00
J
ATOM 9741 HA1 GLY J 226 0.313 23.442 2.671 0.00 0.00
J
ATOM 9742 HA2 GLY J 226 -1.435 23.350 2.985 0.00 0.00
J
ATOM 9743 C GLY J 226 -0.253 23.543 4.635 0.00 0.00
J
ATOM 9744 O GLY J 226 -0.888 22.642 5.174 0.00 0.00
J
ATOM 9745 N ILE J 227 0.743 24.211 5.257 0.00 0.00
J
ATOM 9746 HN ILE J 227 1.331 24.862 4.783 0.00 0.00
J

ATOM 9747 CA ILE J 227 1.145 23.985 6.616 0.00 0.00
J
ATOM 9748 HA ILE J 227 0.990 22.957 6.910 0.00 0.00
J
ATOM 9749 CB ILE J 227 2.563 24.461 6.871 0.00 0.00
J
ATOM 9750 HB ILE J 227 2.847 24.104 7.884 0.00 0.00
J
ATOM 9751 CG2 ILE J 227 3.486 23.651 5.984 0.00 0.00
J
ATOM 9752 HG21 ILE J 227 3.380 23.887 4.904 0.00 0.00
J
ATOM 9753 HG22 ILE J 227 4.540 23.800 6.301 0.00 0.00
J
ATOM 9754 HG23 ILE J 227 3.282 22.572 6.158 0.00 0.00
J
ATOM 9755 CG1 ILE J 227 2.788 26.022 6.770 0.00 0.00
J
ATOM 9756 HG11 ILE J 227 2.628 26.292 5.704 0.00 0.00
J
ATOM 9757 HG12 ILE J 227 1.979 26.489 7.372 0.00 0.00
J
ATOM 9758 CD ILE J 227 4.123 26.513 7.248 0.00 0.00
J
ATOM 9759 HD1 ILE J 227 4.967 26.185 6.603 0.00 0.00
J
ATOM 9760 HD2 ILE J 227 4.027 27.620 7.242 0.00 0.00
J
ATOM 9761 HD3 ILE J 227 4.254 26.160 8.293 0.00 0.00
J
ATOM 9762 C ILE J 227 0.081 24.659 7.513 0.00 0.00
J
ATOM 9763 O ILE J 227 −0.039 24.257 8.665 0.00 0.00
J
ATOM 9764 N GLY J 228 −0.630 25.691 6.928 0.00 0.00
J
ATOM 9765 HN GLY J 228 −0.571 26.035 5.994 0.00 0.00
J
ATOM 9766 CA GLY J 228 −1.491 26.604 7.718 0.00 0.00
J
ATOM 9767 HA1 GLY J 228 −1.824 26.037 8.575 0.00 0.00
J
ATOM 9768 HA2 GLY J 228 −2.285 26.863 7.034 0.00 0.00
J
ATOM 9769 C GLY J 228 −0.807 27.900 8.210 0.00 0.00
J
ATOM 9770 O GLY J 228 0.266 27.943 8.676 0.00 0.00
J
ATOM 9771 N PHE J 229 −1.539 28.990 8.058 0.00 0.00
J
ATOM 9772 HN PHE J 229 −2.430 29.057 7.617 0.00 0.00
J
ATOM 9773 CA PHE J 229 −1.077 30.322 8.491 0.00 0.00
J

ATOM 9774 HA PHE J 229 -0.044 30.502 8.231 0.00 0.00
J
ATOM 9775 CB PHE J 229 -1.832 31.426 7.656 0.00 0.00
J
ATOM 9776 HB1 PHE J 229 -1.567 32.486 7.854 0.00 0.00
J
ATOM 9777 HB2 PHE J 229 -1.521 31.237 6.606 0.00 0.00
J
ATOM 9778 CG PHE J 229 -3.311 31.259 7.689 0.00 0.00
J
ATOM 9779 CD1 PHE J 229 -3.857 30.490 6.628 0.00 0.00
J
ATOM 9780 HD1 PHE J 229 -3.201 30.117 5.855 0.00 0.00
J
ATOM 9781 CE1 PHE J 229 -5.241 30.118 6.571 0.00 0.00
J
ATOM 9782 HE1 PHE J 229 -5.591 29.555 5.718 0.00 0.00
J
ATOM 9783 CZ PHE J 229 -6.116 30.805 7.394 0.00 0.00
J
ATOM 9784 HZ PHE J 229 -7.163 30.565 7.280 0.00 0.00
J
ATOM 9785 CD2 PHE J 229 -4.239 31.799 8.612 0.00 0.00
J
ATOM 9786 HD2 PHE J 229 -3.950 32.540 9.343 0.00 0.00
J
ATOM 9787 CE2 PHE J 229 -5.613 31.573 8.501 0.00 0.00
J
ATOM 9788 HE2 PHE J 229 -6.342 31.987 9.182 0.00 0.00
J
ATOM 9789 C PHE J 229 -1.004 30.571 9.969 0.00 0.00
J
ATOM 9790 O PHE J 229 -0.212 31.394 10.443 0.00 0.00
J
ATOM 9791 N THR J 230 -1.670 29.634 10.750 0.00 0.00
J
ATOM 9792 HN THR J 230 -2.074 28.810 10.358 0.00 0.00
J
ATOM 9793 CA THR J 230 -1.375 29.397 12.213 0.00 0.00
J
ATOM 9794 HA THR J 230 -1.763 30.262 12.731 0.00 0.00
J
ATOM 9795 CB THR J 230 -2.148 28.102 12.566 0.00 0.00
J
ATOM 9796 HB THR J 230 -1.687 27.620 13.454 0.00 0.00
J
ATOM 9797 OG1 THR J 230 -2.040 27.152 11.488 0.00 0.00
J
ATOM 9798 HG1 THR J 230 -2.179 26.335 11.973 0.00 0.00
J
ATOM 9799 CG2 THR J 230 -3.614 28.420 12.843 0.00 0.00
J
ATOM 9800 HG21 THR J 230 -4.079 27.419 12.971 0.00 0.00
J

ATOM 9801 HG22 THR J 230 -3.821 29.066 13.722 0.00 0.00
J
ATOM 9802 HG23 THR J 230 -3.950 28.976 11.942 0.00 0.00
J
ATOM 9803 C THR J 230 0.064 29.099 12.566 0.00 0.00
J
ATOM 9804 O THR J 230 0.612 29.658 13.500 0.00 0.00
J
ATOM 9805 N VAL J 231 0.714 28.157 11.828 0.00 0.00
J
ATOM 9806 HN VAL J 231 0.341 27.658 11.049 0.00 0.00
J
ATOM 9807 CA VAL J 231 2.133 27.623 11.998 0.00 0.00
J
ATOM 9808 HA VAL J 231 2.250 27.366 13.040 0.00 0.00
J
ATOM 9809 CB VAL J 231 2.481 26.483 11.045 0.00 0.00
J
ATOM 9810 HB VAL J 231 2.302 26.756 9.983 0.00 0.00
J
ATOM 9811 CG1 VAL J 231 3.874 25.949 11.340 0.00 0.00
J
ATOM 9812 HG11 VAL J 231 4.079 24.998 10.802 0.00 0.00
J
ATOM 9813 HG12 VAL J 231 4.602 26.638 10.860 0.00 0.00
J
ATOM 9814 HG13 VAL J 231 4.031 25.831 12.433 0.00 0.00
J
ATOM 9815 CG2 VAL J 231 1.423 25.375 11.315 0.00 0.00
J
ATOM 9816 HG21 VAL J 231 1.860 24.419 10.954 0.00 0.00
J
ATOM 9817 HG22 VAL J 231 1.205 25.318 12.403 0.00 0.00
J
ATOM 9818 HG23 VAL J 231 0.547 25.632 10.682 0.00 0.00
J
ATOM 9819 C VAL J 231 3.087 28.757 11.799 0.00 0.00
J
ATOM 9820 O VAL J 231 3.883 29.108 12.613 0.00 0.00
J
ATOM 9821 N LEU J 232 2.872 29.520 10.714 0.00 0.00
J
ATOM 9822 HN LEU J 232 2.094 29.318 10.123 0.00 0.00
J
ATOM 9823 CA LEU J 232 3.576 30.804 10.360 0.00 0.00
J
ATOM 9824 HA LEU J 232 4.622 30.557 10.252 0.00 0.00
J
ATOM 9825 CB LEU J 232 3.059 31.250 8.970 0.00 0.00
J
ATOM 9826 HB1 LEU J 232 3.024 30.455 8.195 0.00 0.00
J
ATOM 9827 HB2 LEU J 232 2.010 31.550 9.178 0.00 0.00
J

```
ATOM   9828  CG  LEU J 232       3.659  32.591   8.420  0.00  0.00
J
ATOM   9829  HG  LEU J 232       3.556  33.306   9.264  0.00  0.00
J
ATOM   9830  CD1 LEU J 232       5.142  32.428   8.269  0.00  0.00
J
ATOM   9831 HD11 LEU J 232       5.491  32.924   7.338  0.00  0.00
J
ATOM   9832 HD12 LEU J 232       5.657  32.943   9.108  0.00  0.00
J
ATOM   9833 HD13 LEU J 232       5.471  31.371   8.170  0.00  0.00
J
ATOM   9834  CD2 LEU J 232       2.836  33.076   7.188  0.00  0.00
J
ATOM   9835 HD21 LEU J 232       2.913  34.184   7.145  0.00  0.00
J
ATOM   9836 HD22 LEU J 232       3.296  32.676   6.259  0.00  0.00
J
ATOM   9837 HD23 LEU J 232       1.738  32.934   7.279  0.00  0.00
J
ATOM   9838  C   LEU J 232       3.439  31.899  11.355  0.00  0.00
J
ATOM   9839  O   LEU J 232       4.337  32.598  11.754  0.00  0.00
J
ATOM   9840  N   PHE J 233       2.237  31.981  11.934  0.00  0.00
J
ATOM   9841  HN  PHE J 233       1.434  31.468  11.640  0.00  0.00
J
ATOM   9842  CA  PHE J 233       1.990  32.850  13.040  0.00  0.00
J
ATOM   9843  HA  PHE J 233       2.284  33.808  12.637  0.00  0.00
J
ATOM   9844  CB  PHE J 233       0.441  32.930  13.175  0.00  0.00
J
ATOM   9845  HB1 PHE J 233      -0.059  33.209  12.223  0.00  0.00
J
ATOM   9846  HB2 PHE J 233       0.040  31.937  13.469  0.00  0.00
J
ATOM   9847  CG  PHE J 233      -0.004  33.880  14.332  0.00  0.00
J
ATOM   9848  CD1 PHE J 233      -0.312  33.321  15.570  0.00  0.00
J
ATOM   9849  HD1 PHE J 233      -0.210  32.249  15.643  0.00  0.00
J
ATOM   9850  CE1 PHE J 233      -0.713  34.171  16.613  0.00  0.00
J
ATOM   9851  HE1 PHE J 233      -1.060  33.873  17.591  0.00  0.00
J
ATOM   9852  CZ  PHE J 233      -0.865  35.583  16.389  0.00  0.00
J
ATOM   9853  HZ  PHE J 233      -1.256  36.226  17.164  0.00  0.00
J
ATOM   9854  CD2 PHE J 233      -0.190  35.209  14.079  0.00  0.00
J
```

ATOM 9855 HD2 PHE J 233 -0.083 35.565 13.064 0.00 0.00
J
ATOM 9856 CE2 PHE J 233 -0.549 36.061 15.107 0.00 0.00
J
ATOM 9857 HE2 PHE J 233 -0.761 37.103 14.922 0.00 0.00
J
ATOM 9858 C PHE J 233 2.653 32.459 14.385 0.00 0.00
J
ATOM 9859 O PHE J 233 3.282 33.293 15.077 0.00 0.00
J
ATOM 9860 N ASP J 234 2.778 31.101 14.725 0.00 0.00
J
ATOM 9861 HN ASP J 234 2.371 30.451 14.088 0.00 0.00
J
ATOM 9862 CA ASP J 234 3.516 30.557 15.856 0.00 0.00
J
ATOM 9863 HA ASP J 234 3.289 31.128 16.744 0.00 0.00
J
ATOM 9864 CB ASP J 234 3.153 29.051 15.965 0.00 0.00
J
ATOM 9865 HB1 ASP J 234 2.128 28.780 15.632 0.00 0.00
J
ATOM 9866 HB2 ASP J 234 3.859 28.572 15.254 0.00 0.00
J
ATOM 9867 CG ASP J 234 3.403 28.504 17.378 0.00 0.00
J
ATOM 9868 OD1 ASP J 234 4.003 27.396 17.532 0.00 0.00
J
ATOM 9869 OD2 ASP J 234 3.030 29.127 18.406 0.00 0.00
J
ATOM 9870 C ASP J 234 5.009 30.790 15.691 0.00 0.00
J
ATOM 9871 O ASP J 234 5.776 31.128 16.615 0.00 0.00
J
ATOM 9872 N VAL J 235 5.635 30.602 14.450 0.00 0.00
J
ATOM 9873 HN VAL J 235 5.064 30.223 13.726 0.00 0.00
J
ATOM 9874 CA VAL J 235 7.035 30.799 14.272 0.00 0.00
J
ATOM 9875 HA VAL J 235 7.553 30.490 15.168 0.00 0.00
J
ATOM 9876 CB VAL J 235 7.562 29.940 13.176 0.00 0.00
J
ATOM 9877 HB VAL J 235 7.335 28.901 13.498 0.00 0.00
J
ATOM 9878 CG1 VAL J 235 6.968 30.340 11.816 0.00 0.00
J
ATOM 9879 HG11 VAL J 235 7.112 29.481 11.126 0.00 0.00
J
ATOM 9880 HG12 VAL J 235 5.880 30.567 11.810 0.00 0.00
J
ATOM 9881 HG13 VAL J 235 7.491 31.273 11.515 0.00 0.00
J

```
ATOM   9882  CG2 VAL J 235       9.128  30.078  13.148  0.00  0.00
J
ATOM   9883 HG21 VAL J 235       9.631  29.347  12.479  0.00  0.00
J
ATOM   9884 HG22 VAL J 235       9.334  31.091  12.741  0.00  0.00
J
ATOM   9885 HG23 VAL J 235       9.546  30.122  14.176  0.00  0.00
J
ATOM   9886  C   VAL J 235       7.318  32.304  14.127  0.00  0.00
J
ATOM   9887  O   VAL J 235       8.490  32.716  14.166  0.00  0.00
J
ATOM   9888  N   MET J 236       6.357  33.265  13.903  0.00  0.00
J
ATOM   9889  HN  MET J 236       5.532  32.933  13.452  0.00  0.00
J
ATOM   9890  CA  MET J 236       6.527  34.642  14.009  0.00  0.00
J
ATOM   9891  HA  MET J 236       7.543  34.789  13.673  0.00  0.00
J
ATOM   9892  CB  MET J 236       5.611  35.408  13.021  0.00  0.00
J
ATOM   9893  HB1 MET J 236       5.829  34.980  12.019  0.00  0.00
J
ATOM   9894  HB2 MET J 236       4.563  35.132  13.267  0.00  0.00
J
ATOM   9895  CG  MET J 236       5.921  36.975  13.065  0.00  0.00
J
ATOM   9896  HG1 MET J 236       5.583  37.517  13.974  0.00  0.00
J
ATOM   9897  HG2 MET J 236       7.025  37.057  13.159  0.00  0.00
J
ATOM   9898  SD  MET J 236       5.541  37.974  11.626  0.00  0.00
J
ATOM   9899  CE  MET J 236       6.288  39.472  12.290  0.00  0.00
J
ATOM   9900  HE1 MET J 236       5.906  39.656  13.316  0.00  0.00
J
ATOM   9901  HE2 MET J 236       7.370  39.271  12.438  0.00  0.00
J
ATOM   9902  HE3 MET J 236       6.106  40.294  11.565  0.00  0.00
J
ATOM   9903  C   MET J 236       6.636  35.268  15.424  0.00  0.00
J
ATOM   9904  O   MET J 236       7.433  36.140  15.769  0.00  0.00
J
ATOM   9905  N   LYS J 237       5.662  34.867  16.268  0.00  0.00
J
ATOM   9906  HN  LYS J 237       4.839  34.525  15.822  0.00  0.00
J
ATOM   9907  CA  LYS J 237       5.507  35.146  17.649  0.00  0.00
J
ATOM   9908  HA  LYS J 237       5.506  36.226  17.684  0.00  0.00
J
```

ATOM 9909 CB LYS J 237 4.284 34.491 18.100 0.00 0.00
J
ATOM 9910 HB1 LYS J 237 3.543 34.435 17.274 0.00 0.00
J
ATOM 9911 HB2 LYS J 237 4.429 33.443 18.440 0.00 0.00
J
ATOM 9912 CG LYS J 237 3.507 35.176 19.244 0.00 0.00
J
ATOM 9913 HG1 LYS J 237 4.079 35.344 20.181 0.00 0.00
J
ATOM 9914 HG2 LYS J 237 3.001 36.100 18.893 0.00 0.00
J
ATOM 9915 CD LYS J 237 2.349 34.326 19.727 0.00 0.00
J
ATOM 9916 HD1 LYS J 237 1.619 34.011 18.951 0.00 0.00
J
ATOM 9917 HD2 LYS J 237 2.654 33.360 20.185 0.00 0.00
J
ATOM 9918 CE LYS J 237 1.301 34.990 20.644 0.00 0.00
J
ATOM 9919 HE1 LYS J 237 0.851 35.855 20.111 0.00 0.00
J
ATOM 9920 HE2 LYS J 237 0.557 34.330 21.139 0.00 0.00
J
ATOM 9921 NZ LYS J 237 2.009 35.703 21.773 0.00 0.00
J
ATOM 9922 HZ1 LYS J 237 2.792 35.065 22.020 0.00 0.00
J
ATOM 9923 HZ2 LYS J 237 2.395 36.591 21.393 0.00 0.00
J
ATOM 9924 HZ3 LYS J 237 1.451 35.995 22.601 0.00 0.00
J
ATOM 9925 C LYS J 237 6.633 34.643 18.592 0.00 0.00
J
ATOM 9926 O LYS J 237 7.149 35.353 19.442 0.00 0.00
J
ATOM 9927 N ASN J 238 7.192 33.363 18.385 0.00 0.00
J
ATOM 9928 HN ASN J 238 6.963 32.789 17.602 0.00 0.00
J
ATOM 9929 CA ASN J 238 8.162 32.654 19.288 0.00 0.00
J
ATOM 9930 HA ASN J 238 8.412 33.342 20.081 0.00 0.00
J
ATOM 9931 CB ASN J 238 7.498 31.367 19.690 0.00 0.00
J
ATOM 9932 HB1 ASN J 238 7.238 30.732 18.816 0.00 0.00
J
ATOM 9933 HB2 ASN J 238 8.184 30.799 20.353 0.00 0.00
J
ATOM 9934 CG ASN J 238 6.194 31.656 20.483 0.00 0.00
J
ATOM 9935 OD1 ASN J 238 6.139 32.330 21.511 0.00 0.00
J

```
ATOM   9936  ND2 ASN J 238       5.036  31.038  20.101  0.00  0.00
J
ATOM   9937 HD21 ASN J 238       4.338  31.119  20.812  0.00  0.00
J
ATOM   9938 HD22 ASN J 238       5.081  30.187  19.578  0.00  0.00
J
ATOM   9939  C   ASN J 238       9.507  32.415  18.590  0.00  0.00
J
ATOM   9940  O   ASN J 238      10.277  31.526  18.876  0.00  0.00
J
ATOM   9941  N   ARG J 239       9.789  33.271  17.622  0.00  0.00
J
ATOM   9942  HN  ARG J 239       9.113  33.992  17.494  0.00  0.00
J
ATOM   9943  CA  ARG J 239      10.953  33.264  16.731  0.00  0.00
J
ATOM   9944  HA  ARG J 239      10.948  32.389  16.099  0.00  0.00
J
ATOM   9945  CB  ARG J 239      10.769  34.477  15.726  0.00  0.00
J
ATOM   9946  HB1 ARG J 239      11.599  34.451  14.989  0.00  0.00
J
ATOM   9947  HB2 ARG J 239       9.891  34.253  15.083  0.00  0.00
J
ATOM   9948  CG  ARG J 239      10.723  35.845  16.423  0.00  0.00
J
ATOM   9949  HG1 ARG J 239       9.781  35.863  17.012  0.00  0.00
J
ATOM   9950  HG2 ARG J 239      11.630  35.882  17.065  0.00  0.00
J
ATOM   9951  CD  ARG J 239      10.628  37.040  15.400  0.00  0.00
J
ATOM   9952  HD1 ARG J 239      11.454  36.959  14.662  0.00  0.00
J
ATOM   9953  HD2 ARG J 239       9.708  36.992  14.779  0.00  0.00
J
ATOM   9954  NE  ARG J 239      10.784  38.373  16.066  0.00  0.00
J
ATOM   9955  HE  ARG J 239      11.689  38.770  16.216  0.00  0.00
J
ATOM   9956  CZ  ARG J 239       9.757  39.192  16.515  0.00  0.00
J
ATOM   9957  NH1 ARG J 239       8.540  38.784  16.672  0.00  0.00
J
ATOM   9958 HH11 ARG J 239       7.877  39.291  17.224  0.00  0.00
J
ATOM   9959 HH12 ARG J 239       8.259  37.861  16.408  0.00  0.00
J
ATOM   9960  NH2 ARG J 239      10.088  40.358  17.044  0.00  0.00
J
ATOM   9961 HH21 ARG J 239       9.348  40.989  17.279  0.00  0.00
J
ATOM   9962 HH22 ARG J 239      10.846  40.870  16.641  0.00  0.00
J
```

ATOM 9963 C ARG J 239 12.364 33.329 17.307 0.00 0.00
J
ATOM 9964 O ARG J 239 13.359 33.176 16.564 0.00 0.00
J
ATOM 9965 N ARG J 240 12.485 33.585 18.616 0.00 0.00
J
ATOM 9966 HN ARG J 240 11.677 33.702 19.187 0.00 0.00
J
ATOM 9967 CA ARG J 240 13.847 33.624 19.268 0.00 0.00
J
ATOM 9968 HA ARG J 240 14.575 33.584 18.472 0.00 0.00
J
ATOM 9969 CB ARG J 240 13.994 34.902 20.114 0.00 0.00
J
ATOM 9970 HB1 ARG J 240 13.075 34.949 20.737 0.00 0.00
J
ATOM 9971 HB2 ARG J 240 14.884 34.811 20.772 0.00 0.00
J
ATOM 9972 CG ARG J 240 13.969 36.148 19.252 0.00 0.00
J
ATOM 9973 HG1 ARG J 240 14.905 36.118 18.653 0.00 0.00
J
ATOM 9974 HG2 ARG J 240 13.108 36.176 18.550 0.00 0.00
J
ATOM 9975 CD ARG J 240 13.799 37.455 20.045 0.00 0.00
J
ATOM 9976 HD1 ARG J 240 12.750 37.475 20.411 0.00 0.00
J
ATOM 9977 HD2 ARG J 240 14.574 37.593 20.828 0.00 0.00
J
ATOM 9978 NE ARG J 240 13.909 38.611 19.136 0.00 0.00
J
ATOM 9979 HE ARG J 240 14.493 38.460 18.338 0.00 0.00
J
ATOM 9980 CZ ARG J 240 13.398 39.824 19.463 0.00 0.00
J
ATOM 9981 NH1 ARG J 240 13.797 40.874 18.716 0.00 0.00
J
ATOM 9982 HH11 ARG J 240 13.568 41.800 19.016 0.00 0.00
J
ATOM 9983 HH12 ARG J 240 14.677 40.758 18.256 0.00 0.00
J
ATOM 9984 NH2 ARG J 240 12.578 40.015 20.463 0.00 0.00
J
ATOM 9985 HH21 ARG J 240 12.253 40.948 20.618 0.00 0.00
J
ATOM 9986 HH22 ARG J 240 12.496 39.339 21.195 0.00 0.00
J
ATOM 9987 C ARG J 240 13.948 32.347 20.161 0.00 0.00
J
ATOM 9988 O ARG J 240 15.004 32.110 20.763 0.00 0.00
J
ATOM 9989 N PHE J 241 12.912 31.457 20.198 0.00 0.00
J

ATOM 9990 HN PHE J 241 12.050 31.735 19.780 0.00 0.00
J
ATOM 9991 CA PHE J 241 12.994 30.096 20.778 0.00 0.00
J
ATOM 9992 HA PHE J 241 11.988 29.709 20.727 0.00 0.00
J
ATOM 9993 CB PHE J 241 14.008 29.123 20.007 0.00 0.00
J
ATOM 9994 HB1 PHE J 241 14.963 29.662 19.831 0.00 0.00
J
ATOM 9995 HB2 PHE J 241 14.235 28.221 20.613 0.00 0.00
J
ATOM 9996 CG PHE J 241 13.451 28.821 18.640 0.00 0.00
J
ATOM 9997 CD1 PHE J 241 13.350 29.773 17.582 0.00 0.00
J
ATOM 9998 HD1 PHE J 241 13.849 30.728 17.649 0.00 0.00
J
ATOM 9999 CE1 PHE J 241 12.817 29.432 16.346 0.00 0.00
J
ATOM 10000 HE1 PHE J 241 12.680 30.158 15.559 0.00 0.00
J
ATOM 10001 CZ PHE J 241 12.100 28.239 16.212 0.00 0.00
J
ATOM 10002 HZ PHE J 241 11.668 27.952 15.264 0.00 0.00
J
ATOM 10003 CD2 PHE J 241 12.881 27.546 18.390 0.00 0.00
J
ATOM 10004 HD2 PHE J 241 13.178 26.708 19.004 0.00 0.00
J
ATOM 10005 CE2 PHE J 241 12.281 27.201 17.150 0.00 0.00
J
ATOM 10006 HE2 PHE J 241 11.839 26.222 17.048 0.00 0.00
J
ATOM 10007 C PHE J 241 13.200 30.145 22.274 0.00 0.00
J
ATOM 10008 O PHE J 241 13.975 29.291 22.730 0.00 0.00
J
ATOM 10009 N LYS J 242 12.599 31.035 23.042 0.00 0.00
J
ATOM 10010 HN LYS J 242 12.251 31.927 22.764 0.00 0.00
J
ATOM 10011 CA LYS J 242 12.583 30.804 24.498 0.00 0.00
J
ATOM 10012 HA LYS J 242 13.630 30.778 24.764 0.00 0.00
J
ATOM 10013 CB LYS J 242 11.846 31.971 25.190 0.00 0.00
J
ATOM 10014 HB1 LYS J 242 12.404 32.885 24.896 0.00 0.00
J
ATOM 10015 HB2 LYS J 242 10.838 32.062 24.732 0.00 0.00
J
ATOM 10016 CG LYS J 242 11.622 31.911 26.710 0.00 0.00
J

ATOM 10017 HG1 LYS J 242   10.983 31.060 27.029 0.00 0.00
J
ATOM 10018 HG2 LYS J 242   12.572 31.626 27.212 0.00 0.00
J
ATOM 10019 CD LYS J 242   11.128 33.255 27.201 0.00 0.00
J
ATOM 10020 HD1 LYS J 242   11.655 34.055 26.638 0.00 0.00
J
ATOM 10021 HD2 LYS J 242   10.065 33.328 26.889 0.00 0.00
J
ATOM 10022 CE LYS J 242   11.258 33.465 28.710 0.00 0.00
J
ATOM 10023 HE1 LYS J 242   10.781 32.576 29.174 0.00 0.00
J
ATOM 10024 HE2 LYS J 242   12.297 33.563 29.091 0.00 0.00
J
ATOM 10025 NZ LYS J 242   10.632 34.732 29.225 0.00 0.00
J
ATOM 10026 HZ1 LYS J 242   10.742 35.550 28.591 0.00 0.00
J
ATOM 10027 HZ2 LYS J 242   9.628 34.594 29.457 0.00 0.00
J
ATOM 10028 HZ3 LYS J 242   11.135 35.054 30.076 0.00 0.00
J
ATOM 10029 C   LYS J 242   12.027 29.398 25.024 0.00 0.00
J
ATOM 10030 O   LYS J 242   12.494 28.880 26.044 0.00 0.00
J
ATOM 10031 N   THR J 243   11.039 28.824 24.347 0.00 0.00
J
ATOM 10032 HN THR J 243   10.609 29.359 23.624 0.00 0.00
J
ATOM 10033 CA THR J 243   10.624 27.435 24.450 0.00 0.00
J
ATOM 10034 HA THR J 243   11.439 26.726 24.422 0.00 0.00
J
ATOM 10035 CB THR J 243   9.765 26.978 25.622 0.00 0.00
J
ATOM 10036 HB THR J 243   10.374 27.075 26.546 0.00 0.00
J
ATOM 10037 OG1 THR J 243   9.610 25.542 25.634 0.00 0.00
J
ATOM 10038 HG1 THR J 243   9.053 25.199 26.337 0.00 0.00
J
ATOM 10039 CG2 THR J 243   8.440 27.685 25.742 0.00 0.00
J
ATOM 10040 HG21 THR J 243   7.719 27.453 24.930 0.00 0.00
J
ATOM 10041 HG22 THR J 243   7.828 27.474 26.645 0.00 0.00
J
ATOM 10042 HG23 THR J 243   8.512 28.784 25.596 0.00 0.00
J
ATOM 10043 C   THR J 243   9.827 27.390 23.164 0.00 0.00
J

ATOM 10044 O   THR J 243   9.494 28.411 22.606 0.00 0.00
J
ATOM 10045 N   PHE J 244   9.542 26.200 22.488 0.00 0.00
J
ATOM 10046 HN PHE J 244   9.845 25.399 22.999 0.00 0.00
J
ATOM 10047 CA PHE J 244   8.874 26.178 21.234 0.00 0.00
J
ATOM 10048 HA PHE J 244   8.081 26.911 21.247 0.00 0.00
J
ATOM 10049 CB PHE J 244   9.942 26.427 20.130 0.00 0.00
J
ATOM 10050 HB1 PHE J 244   10.539 27.316 20.425 0.00 0.00
J
ATOM 10051 HB2 PHE J 244   10.631 25.578 19.932 0.00 0.00
J
ATOM 10052 CG PHE J 244   9.295 26.739 18.795 0.00 0.00
J
ATOM 10053 CD1 PHE J 244   9.088 28.056 18.380 0.00 0.00
J
ATOM 10054 HD1 PHE J 244   9.276 28.863 19.072 0.00 0.00
J
ATOM 10055 CE1 PHE J 244   8.722 28.348 17.040 0.00 0.00
J
ATOM 10056 HE1 PHE J 244   8.579 29.404 16.868 0.00 0.00
J
ATOM 10057 CZ PHE J 244   8.675 27.359 16.101 0.00 0.00
J
ATOM 10058 HZ PHE J 244   8.390 27.698 15.116 0.00 0.00
J
ATOM 10059 CD2 PHE J 244   9.027 25.724 17.866 0.00 0.00
J
ATOM 10060 HD2 PHE J 244   9.327 24.731 18.166 0.00 0.00
J
ATOM 10061 CE2 PHE J 244   8.749 26.025 16.508 0.00 0.00
J
ATOM 10062 HE2 PHE J 244   8.947 25.217 15.819 0.00 0.00
J
ATOM 10063 C   PHE J 244   8.131 24.799 21.002 0.00 0.00
J
ATOM 10064 O   PHE J 244   8.713 23.724 21.357 0.00 0.00
J
ATOM 10065 N   SER J 245   7.021 24.803 20.328 0.00 0.00
J
ATOM 10066 HN SER J 245   6.588 25.613 19.940 0.00 0.00
J
ATOM 10067 CA SER J 245   6.133 23.645 20.085 0.00 0.00
J
ATOM 10068 HA SER J 245   5.808 23.301 21.056 0.00 0.00
J
ATOM 10069 CB SER J 245   4.859 24.107 19.361 0.00 0.00
J
ATOM 10070 HB1 SER J 245   4.444 24.969 19.925 0.00 0.00
J

ATOM 10071 HB2 SER J 245   5.121 24.445 18.335 0.00 0.00
J
ATOM 10072 OG SER J 245   3.741 23.200 19.305 0.00 0.00
J
ATOM 10073 HG1 SER J 245   3.026 23.578 18.787 0.00 0.00
J
ATOM 10074 C   SER J 245   6.696 22.498 19.305 0.00 0.00
J
ATOM 10075 O   SER J 245   7.411 22.684 18.318 0.00 0.00
J
ATOM 10076 N   LEU J 246   6.279 21.295 19.667 0.00 0.00
J
ATOM 10077 HN LEU J 246   5.698 21.211 20.473 0.00 0.00
J
ATOM 10078 CA LEU J 246   6.734 20.004 19.046 0.00 0.00
J
ATOM 10079 HA LEU J 246   7.799 20.125 18.918 0.00 0.00
J
ATOM 10080 CB LEU J 246   6.314 18.748 19.921 0.00 0.00
J
ATOM 10081 HB1 LEU J 246   6.437 18.990 20.998 0.00 0.00
J
ATOM 10082 HB2 LEU J 246   5.272 18.429 19.706 0.00 0.00
J
ATOM 10083 CG LEU J 246   7.254 17.527 19.707 0.00 0.00
J
ATOM 10084 HG LEU J 246   7.532 17.407 18.638 0.00 0.00
J
ATOM 10085 CD1 LEU J 246   8.596 17.506 20.450 0.00 0.00
J
ATOM 10086 HD11 LEU J 246   8.521 17.925 21.476 0.00 0.00
J
ATOM 10087 HD12 LEU J 246   9.262 16.618 20.424 0.00 0.00
J
ATOM 10088 HD13 LEU J 246   9.134 18.334 19.941 0.00 0.00
J
ATOM 10089 CD2 LEU J 246   6.512 16.315 20.256 0.00 0.00
J
ATOM 10090 HD21 LEU J 246   5.707 15.773 19.714 0.00 0.00
J
ATOM 10091 HD22 LEU J 246   7.182 15.450 20.447 0.00 0.00
J
ATOM 10092 HD23 LEU J 246   6.088 16.470 21.272 0.00 0.00
J
ATOM 10093 C   LEU J 246   6.111 19.796 17.677 0.00 0.00
J
ATOM 10094 O   LEU J 246   6.780 19.421 16.697 0.00 0.00
J
ATOM 10095 N   HSE J 247   4.801 20.139 17.579 0.00 0.00
J
ATOM 10096 HN HSE J 247   4.313 20.346 18.423 0.00 0.00
J
ATOM 10097 CA HSE J 247   4.131 20.077 16.339 0.00 0.00
J

ATOM 10098 HA HSE J 247   4.305 19.074 15.976 0.00 0.00
J
ATOM 10099 CB HSE J 247   2.610 20.364 16.573 0.00 0.00
J
ATOM 10100 HB1 HSE J 247   2.181 19.699 17.352 0.00 0.00
J
ATOM 10101 HB2 HSE J 247   2.494 21.415 16.916 0.00 0.00
J
ATOM 10102 ND1 HSE J 247   0.947 19.017 15.202 0.00 0.00
J
ATOM 10103 CG HSE J 247   1.663 20.214 15.376 0.00 0.00
J
ATOM 10104 CE1 HSE J 247   0.305 19.209 14.083 0.00 0.00
J
ATOM 10105 HE1 HSE J 247   -0.346 18.448 13.653 0.00 0.00
J
ATOM 10106 NE2 HSE J 247   0.495 20.466 13.572 0.00 0.00
J
ATOM 10107 HE2 HSE J 247   0.097 20.943 12.788 0.00 0.00
J
ATOM 10108 CD2 HSE J 247   1.375 21.090 14.382 0.00 0.00
J
ATOM 10109 HD2 HSE J 247   1.855 22.031 14.145 0.00 0.00
J
ATOM 10110 C   HSE J 247   4.711 21.146 15.338 0.00 0.00
J
ATOM 10111 O   HSE J 247   5.024 20.746 14.250 0.00 0.00
J
ATOM 10112 N   THR J 248   4.884 22.435 15.712 0.00 0.00
J
ATOM 10113 HN THR J 248   4.701 22.705 16.654 0.00 0.00
J
ATOM 10114 CA THR J 248   5.427 23.574 14.892 0.00 0.00
J
ATOM 10115 HA THR J 248   4.759 23.722 14.057 0.00 0.00
J
ATOM 10116 CB THR J 248   5.519 24.907 15.659 0.00 0.00
J
ATOM 10117 HB THR J 248   6.232 24.767 16.500 0.00 0.00
J
ATOM 10118 OG1 THR J 248   4.178 25.070 16.118 0.00 0.00
J
ATOM 10119 HG1 THR J 248   4.050 25.896 16.591 0.00 0.00
J
ATOM 10120 CG2 THR J 248   5.844 26.118 14.812 0.00 0.00
J
ATOM 10121 HG21 THR J 248   5.946 26.996 15.486 0.00 0.00
J
ATOM 10122 HG22 THR J 248   6.810 25.844 14.335 0.00 0.00
J
ATOM 10123 HG23 THR J 248   5.024 26.363 14.104 0.00 0.00
J
ATOM 10124 C   THR J 248   6.858 23.197 14.399 0.00 0.00
J

ATOM 10125 O THR J 248 7.232 23.452 13.239 0.00 0.00
J
ATOM 10126 N LYS J 249 7.657 22.557 15.286 0.00 0.00
J
ATOM 10127 HN LYS J 249 7.383 22.447 16.239 0.00 0.00
J
ATOM 10128 CA LYS J 249 8.975 22.129 14.883 0.00 0.00
J
ATOM 10129 HA LYS J 249 9.428 22.968 14.376 0.00 0.00
J
ATOM 10130 CB LYS J 249 9.731 21.708 16.170 0.00 0.00
J
ATOM 10131 HB1 LYS J 249 9.915 22.662 16.709 0.00 0.00
J
ATOM 10132 HB2 LYS J 249 9.105 20.975 16.722 0.00 0.00
J
ATOM 10133 CG LYS J 249 11.104 21.008 15.985 0.00 0.00
J
ATOM 10134 HG1 LYS J 249 11.059 20.094 15.354 0.00 0.00
J
ATOM 10135 HG2 LYS J 249 11.878 21.605 15.456 0.00 0.00
J
ATOM 10136 CD LYS J 249 11.781 20.691 17.397 0.00 0.00
J
ATOM 10137 HD1 LYS J 249 11.769 21.654 17.952 0.00 0.00
J
ATOM 10138 HD2 LYS J 249 11.024 20.065 17.915 0.00 0.00
J
ATOM 10139 CE LYS J 249 13.170 20.003 17.443 0.00 0.00
J
ATOM 10140 HE1 LYS J 249 13.578 19.921 18.473 0.00 0.00
J
ATOM 10141 HE2 LYS J 249 13.083 19.051 16.878 0.00 0.00
J
ATOM 10142 NZ LYS J 249 14.100 20.841 16.759 0.00 0.00
J
ATOM 10143 HZ1 LYS J 249 14.173 21.696 17.347 0.00 0.00
J
ATOM 10144 HZ2 LYS J 249 15.023 20.364 16.812 0.00 0.00
J
ATOM 10145 HZ3 LYS J 249 13.966 21.183 15.786 0.00 0.00
J
ATOM 10146 C LYS J 249 8.982 20.941 13.963 0.00 0.00
J
ATOM 10147 O LYS J 249 9.720 20.979 12.925 0.00 0.00
J
ATOM 10148 N LEU J 250 8.147 19.870 14.200 0.00 0.00
J
ATOM 10149 HN LEU J 250 7.519 20.017 14.961 0.00 0.00
J
ATOM 10150 CA LEU J 250 8.002 18.717 13.336 0.00 0.00
J
ATOM 10151 HA LEU J 250 8.964 18.244 13.206 0.00 0.00
J

ATOM 10152 CB LEU J 250   7.028 17.731 13.912 0.00 0.00
J
ATOM 10153 HB1 LEU J 250   7.308 17.511 14.965 0.00 0.00
J
ATOM 10154 HB2 LEU J 250   6.019 18.154 14.105 0.00 0.00
J
ATOM 10155 CG LEU J 250   6.798 16.312 13.190 0.00 0.00
J
ATOM 10156 HG LEU J 250   6.416 16.520 12.168 0.00 0.00
J
ATOM 10157 CD1 LEU J 250   8.059 15.643 12.804 0.00 0.00
J
ATOM 10158 HD11 LEU J 250   8.567 16.109 11.933 0.00 0.00
J
ATOM 10159 HD12 LEU J 250   8.745 15.537 13.672 0.00 0.00
J
ATOM 10160 HD13 LEU J 250   7.895 14.619 12.406 0.00 0.00
J
ATOM 10161 CD2 LEU J 250   5.855 15.461 14.032 0.00 0.00
J
ATOM 10162 HD21 LEU J 250   4.847 15.871 14.255 0.00 0.00
J
ATOM 10163 HD22 LEU J 250   5.583 14.492 13.561 0.00 0.00
J
ATOM 10164 HD23 LEU J 250   6.418 15.171 14.945 0.00 0.00
J
ATOM 10165 C   LEU J 250   7.413 19.089 11.989 0.00 0.00
J
ATOM 10166 O   LEU J 250   7.834 18.524 11.013 0.00 0.00
J
ATOM 10167 N   MET J 251   6.370 19.925 11.938 0.00 0.00
J
ATOM 10168 HN MET J 251   6.069 20.442 12.735 0.00 0.00
J
ATOM 10169 CA MET J 251   5.736 20.387 10.744 0.00 0.00
J
ATOM 10170 HA MET J 251   5.248 19.529 10.305 0.00 0.00
J
ATOM 10171 CB MET J 251   4.608 21.470 11.124 0.00 0.00
J
ATOM 10172 HB1 MET J 251   3.897 21.049 11.867 0.00 0.00
J
ATOM 10173 HB2 MET J 251   5.166 22.226 11.717 0.00 0.00
J
ATOM 10174 CG MET J 251   3.788 22.089   9.963 0.00 0.00
J
ATOM 10175 HG1 MET J 251   2.867 22.605 10.310 0.00 0.00
J
ATOM 10176 HG2 MET J 251   4.486 22.685   9.337 0.00 0.00
J
ATOM 10177 SD MET J 251   2.940 20.826   9.011 0.00 0.00
J
ATOM 10178 CE MET J 251   1.971 19.926 10.197 0.00 0.00
J

ATOM 10179 HE1 MET J 251   1.352 20.635 10.787 0.00 0.00
J
ATOM 10180 HE2 MET J 251   1.338 19.127   9.755 0.00 0.00
J
ATOM 10181 HE3 MET J 251   2.706 19.386 10.832 0.00 0.00
J
ATOM 10182 C   MET J 251   6.631 21.047   9.774 0.00 0.00
J
ATOM 10183 O   MET J 251   6.723 20.710   8.596 0.00 0.00
J
ATOM 10184 N   LEU J 252   7.414 22.002 10.312 0.00 0.00
J
ATOM 10185 HN LEU J 252   7.377 22.114 11.302 0.00 0.00
J
ATOM 10186 CA LEU J 252   8.525 22.658   9.596 0.00 0.00
J
ATOM 10187 HA LEU J 252   8.091 22.937   8.648 0.00 0.00
J
ATOM 10188 CB LEU J 252   9.070 23.908 10.369 0.00 0.00
J
ATOM 10189 HB1 LEU J 252   9.206 23.722 11.456 0.00 0.00
J
ATOM 10190 HB2 LEU J 252   10.095 24.165 10.024 0.00 0.00
J
ATOM 10191 CG LEU J 252   8.096 25.178 10.203 0.00 0.00
J
ATOM 10192 HG LEU J 252   7.087 24.912 10.587 0.00 0.00
J
ATOM 10193 CD1 LEU J 252   8.632 26.311 11.046 0.00 0.00
J
ATOM 10194 HD11 LEU J 252   9.587 26.750 10.688 0.00 0.00
J
ATOM 10195 HD12 LEU J 252   7.959 27.186 11.174 0.00 0.00
J
ATOM 10196 HD13 LEU J 252   9.018 25.980 12.034 0.00 0.00
J
ATOM 10197 CD2 LEU J 252   7.807 25.742   8.782 0.00 0.00
J
ATOM 10198 HD21 LEU J 252   7.226 26.685   8.876 0.00 0.00
J
ATOM 10199 HD22 LEU J 252   8.711 26.044   8.212 0.00 0.00
J
ATOM 10200 HD23 LEU J 252   7.082 25.070   8.274 0.00 0.00
J
ATOM 10201 C   LEU J 252   9.612 21.709   9.214 0.00 0.00
J
ATOM 10202 O   LEU J 252   10.069 21.705   8.127 0.00 0.00
J
ATOM 10203 N   THR J 253   10.028 20.893 10.140 0.00 0.00
J
ATOM 10204 HN THR J 253   9.672 20.846 11.070 0.00 0.00
J
ATOM 10205 CA THR J 253   11.192 20.033   9.882 0.00 0.00
J

```
ATOM  10206  HA  THR J 253      11.991  20.642   9.486  0.00  0.00
J
ATOM  10207  CB  THR J 253      11.876  19.471  11.076  0.00  0.00
J
ATOM  10208  HB  THR J 253      12.671  18.765  10.751  0.00  0.00
J
ATOM  10209  OG1 THR J 253      11.080  18.747  11.995  0.00  0.00
J
ATOM  10210  HG1 THR J 253      10.519  19.343  12.496  0.00  0.00
J
ATOM  10211  CG2 THR J 253      12.616  20.657  11.799  0.00  0.00
J
ATOM  10212 HG21 THR J 253      11.981  21.566  11.866  0.00  0.00
J
ATOM  10213 HG22 THR J 253      12.855  20.289  12.820  0.00  0.00
J
ATOM  10214 HG23 THR J 253      13.544  20.924  11.250  0.00  0.00
J
ATOM  10215  C   THR J 253      10.886  18.903   9.016  0.00  0.00
J
ATOM  10216  O   THR J 253      11.744  18.550   8.218  0.00  0.00
J
ATOM  10217  N   GLY J 254       9.577  18.347   8.892  0.00  0.00
J
ATOM  10218  HN  GLY J 254       8.910  18.566   9.599  0.00  0.00
J
ATOM  10219  CA  GLY J 254       9.170  17.537   7.814  0.00  0.00
J
ATOM  10220  HA1 GLY J 254       8.118  17.371   7.996  0.00  0.00
J
ATOM  10221  HA2 GLY J 254       9.702  16.598   7.776  0.00  0.00
J
ATOM  10222  C   GLY J 254       9.044  18.125   6.429  0.00  0.00
J
ATOM  10223  O   GLY J 254       9.296  17.450   5.422  0.00  0.00
J
ATOM  10224  N   THR J 255       8.594  19.409   6.364  0.00  0.00
J
ATOM  10225  HN  THR J 255       8.459  19.914   7.213  0.00  0.00
J
ATOM  10226  CA  THR J 255       8.269  20.201   5.093  0.00  0.00
J
ATOM  10227  HA  THR J 255       7.733  19.422   4.572  0.00  0.00
J
ATOM  10228  CB  THR J 255       7.364  21.468   5.274  0.00  0.00
J
ATOM  10229  HB  THR J 255       7.834  22.106   6.052  0.00  0.00
J
ATOM  10230  OG1 THR J 255       6.121  21.039   5.800  0.00  0.00
J
ATOM  10231  HG1 THR J 255       6.326  20.890   6.726  0.00  0.00
J
ATOM  10232  CG2 THR J 255       6.913  22.139   3.902  0.00  0.00
J
```

ATOM 10233 HG21 THR J 255   6.648 21.374   3.140 0.00 0.00
J
ATOM 10234 HG22 THR J 255   5.991 22.753   3.988 0.00 0.00
J
ATOM 10235 HG23 THR J 255   7.723 22.712   3.403 0.00 0.00
J
ATOM 10236 C   THR J 255   9.405 20.483   4.200 0.00 0.00
J
ATOM 10237 O   THR J 255   9.328 20.423   2.991 0.00 0.00
J
ATOM 10238 N   LEU J 256   10.508 20.878   4.806 0.00 0.00
J
ATOM 10239 HN LEU J 256   10.528 21.034   5.791 0.00 0.00
J
ATOM 10240 CA LEU J 256   11.747 21.237   4.146 0.00 0.00
J
ATOM 10241 HA LEU J 256   11.502 21.854   3.294 0.00 0.00
J
ATOM 10242 CB LEU J 256   12.639 22.100   5.045 0.00 0.00
J
ATOM 10243 HB1 LEU J 256   13.099 21.508   5.865 0.00 0.00
J
ATOM 10244 HB2 LEU J 256   13.453 22.522   4.418 0.00 0.00
J
ATOM 10245 CG LEU J 256   11.906 23.278   5.593 0.00 0.00
J
ATOM 10246 HG LEU J 256   10.873 23.055   5.938 0.00 0.00
J
ATOM 10247 CD1 LEU J 256   12.604 23.879   6.840 0.00 0.00
J
ATOM 10248 HD11 LEU J 256   12.268 24.920   7.038 0.00 0.00
J
ATOM 10249 HD12 LEU J 256   12.556 23.292   7.782 0.00 0.00
J
ATOM 10250 HD13 LEU J 256   13.680 23.867   6.567 0.00 0.00
J
ATOM 10251 CD2 LEU J 256   11.607 24.490   4.547 0.00 0.00
J
ATOM 10252 HD21 LEU J 256   10.849 24.106   3.831 0.00 0.00
J
ATOM 10253 HD22 LEU J 256   11.232 25.347   5.146 0.00 0.00
J
ATOM 10254 HD23 LEU J 256   12.522 24.831   4.018 0.00 0.00
J
ATOM 10255 C   LEU J 256   12.445 20.009   3.633 0.00 0.00
J
ATOM 10256 O   LEU J 256   12.726 20.011   2.437 0.00 0.00
J
ATOM 10257 N   MET J 257   12.640 18.899   4.403 0.00 0.00
J
ATOM 10258 HN MET J 257   12.258 18.952   5.322 0.00 0.00
J
ATOM 10259 CA MET J 257   13.115 17.705   3.831 0.00 0.00
J

```
ATOM  10260  HA  MET J 257      13.916  18.032   3.185  0.00  0.00
J
ATOM  10261  CB  MET J 257      13.455  16.748   4.994  0.00  0.00
J
ATOM  10262  HB1 MET J 257      12.459  16.377   5.316  0.00  0.00
J
ATOM  10263  HB2 MET J 257      14.126  15.952   4.607  0.00  0.00
J
ATOM  10264  CG  MET J 257      14.063  17.313   6.267  0.00  0.00
J
ATOM  10265  HG1 MET J 257      13.294  17.967   6.730  0.00  0.00
J
ATOM  10266  HG2 MET J 257      14.429  16.514   6.948  0.00  0.00
J
ATOM  10267  SD  MET J 257      15.537  18.300   6.087  0.00  0.00
J
ATOM  10268  CE  MET J 257      16.024  18.136   7.788  0.00  0.00
J
ATOM  10269  HE1 MET J 257      15.171  18.518   8.387  0.00  0.00
J
ATOM  10270  HE2 MET J 257      16.070  17.070   8.097  0.00  0.00
J
ATOM  10271  HE3 MET J 257      16.939  18.713   8.042  0.00  0.00
J
ATOM  10272  C   MET J 257      12.178  17.044   2.829  0.00  0.00
J
ATOM  10273  O   MET J 257      12.710  16.803   1.762  0.00  0.00
J
ATOM  10274  N   LEU J 258      10.886  16.806   3.111  0.00  0.00
J
ATOM  10275  HN  LEU J 258      10.367  17.201   3.865  0.00  0.00
J
ATOM  10276  CA  LEU J 258       9.987  16.325   2.045  0.00  0.00
J
ATOM  10277  HA  LEU J 258      10.246  15.317   1.758  0.00  0.00
J
ATOM  10278  CB  LEU J 258       8.526  16.071   2.564  0.00  0.00
J
ATOM  10279  HB1 LEU J 258       8.717  15.491   3.492  0.00  0.00
J
ATOM  10280  HB2 LEU J 258       7.995  17.042   2.662  0.00  0.00
J
ATOM  10281  CG  LEU J 258       7.617  15.163   1.813  0.00  0.00
J
ATOM  10282  HG  LEU J 258       8.181  14.239   1.563  0.00  0.00
J
ATOM  10283  CD1 LEU J 258       6.449  14.806   2.694  0.00  0.00
J
ATOM  10284 HD11 LEU J 258       5.826  15.705   2.889  0.00  0.00
J
ATOM  10285 HD12 LEU J 258       5.853  13.940   2.336  0.00  0.00
J
ATOM  10286 HD13 LEU J 258       6.793  14.529   3.714  0.00  0.00
J
```

ATOM 10287 CD2 LEU J 258   6.981 15.774   0.531 0.00 0.00
J
ATOM 10288 HD21 LEU J 258   6.298 14.991   0.137 0.00 0.00
J
ATOM 10289 HD22 LEU J 258   6.452 16.750   0.493 0.00 0.00
J
ATOM 10290 HD23 LEU J 258   7.736 15.725 -0.282 0.00 0.00
J
ATOM 10291 C   LEU J 258   9.953 17.099   0.691 0.00 0.00
J
ATOM 10292 O   LEU J 258   10.156 16.460 -0.365 0.00 0.00
J
ATOM 10293 N   ASN J 259   9.879 18.404   0.706 0.00 0.00
J
ATOM 10294 HN ASN J 259   9.633 18.779   1.596 0.00 0.00
J
ATOM 10295 CA ASN J 259   9.945 19.221 -0.507 0.00 0.00
J
ATOM 10296 HA ASN J 259   9.271 18.878 -1.278 0.00 0.00
J
ATOM 10297 CB ASN J 259   9.711 20.714 -0.198 0.00 0.00
J
ATOM 10298 HB1 ASN J 259   10.289 21.081   0.677 0.00 0.00
J
ATOM 10299 HB2 ASN J 259   10.054 21.362 -1.032 0.00 0.00
J
ATOM 10300 CG ASN J 259   8.255 21.062   0.066 0.00 0.00
J
ATOM 10301 OD1 ASN J 259   7.298 20.287 -0.026 0.00 0.00
J
ATOM 10302 ND2 ASN J 259   8.079 22.394   0.441 0.00 0.00
J
ATOM 10303 HD21 ASN J 259   7.190 22.646   0.823 0.00 0.00
J
ATOM 10304 HD22 ASN J 259   8.839 23.037   0.343 0.00 0.00
J
ATOM 10305 C   ASN J 259   11.201 19.126 -1.301 0.00 0.00
J
ATOM 10306 O   ASN J 259   11.205 19.194 -2.538 0.00 0.00
J
ATOM 10307 N   ALA J 260   12.379 19.031 -0.625 0.00 0.00
J
ATOM 10308 HN ALA J 260   12.373 19.037   0.372 0.00 0.00
J
ATOM 10309 CA ALA J 260   13.664 18.884 -1.323 0.00 0.00
J
ATOM 10310 HA ALA J 260   13.709 19.572 -2.154 0.00 0.00
J
ATOM 10311 CB ALA J 260   14.730 19.317 -0.325 0.00 0.00
J
ATOM 10312 HB1 ALA J 260   14.701 18.687   0.589 0.00 0.00
J
ATOM 10313 HB2 ALA J 260   15.727 19.228 -0.807 0.00 0.00
J

ATOM 10314 HB3 ALA J 260   14.538 20.374 -0.042 0.00 0.00
J
ATOM 10315 C   ALA J 260   14.021 17.447 -1.829 0.00 0.00
J
ATOM 10316 O   ALA J 260   14.655 17.266 -2.849 0.00 0.00
J
ATOM 10317 N   ILE J 261   13.586 16.444 -1.161 0.00 0.00
J
ATOM 10318 HN ILE J 261   13.168 16.517 -0.259 0.00 0.00
J
ATOM 10319 CA ILE J 261   13.715 15.063 -1.497 0.00 0.00
J
ATOM 10320 HA ILE J 261   14.747 14.828 -1.711 0.00 0.00
J
ATOM 10321 CB ILE J 261   13.409 14.162 -0.251 0.00 0.00
J
ATOM 10322 HB ILE J 261   12.457 14.598   0.122 0.00 0.00
J
ATOM 10323 CG2 ILE J 261   13.430 12.668 -0.738 0.00 0.00
J
ATOM 10324 HG21 ILE J 261   13.263 11.957   0.099 0.00 0.00
J
ATOM 10325 HG22 ILE J 261   12.696 12.509 -1.556 0.00 0.00
J
ATOM 10326 HG23 ILE J 261   14.439 12.331 -1.057 0.00 0.00
J
ATOM 10327 CG1 ILE J 261   14.534 14.395   0.940 0.00 0.00
J
ATOM 10328 HG11 ILE J 261   15.450 13.865   0.602 0.00 0.00
J
ATOM 10329 HG12 ILE J 261   14.597 15.484   1.152 0.00 0.00
J
ATOM 10330 CD ILE J 261   14.132 13.821   2.250 0.00 0.00
J
ATOM 10331 HD1 ILE J 261   13.351 14.504   2.648 0.00 0.00
J
ATOM 10332 HD2 ILE J 261   13.639 12.839   2.085 0.00 0.00
J
ATOM 10333 HD3 ILE J 261   14.983 13.780   2.962 0.00 0.00
J
ATOM 10334 C   ILE J 261   12.931 14.727 -2.765 0.00 0.00
J
ATOM 10335 O   ILE J 261   13.338 14.049 -3.661 0.00 0.00
J
ATOM 10336 N   ALA J 262   11.773 15.335 -2.829 0.00 0.00
J
ATOM 10337 HN ALA J 262   11.472 15.816 -2.009 0.00 0.00
J
ATOM 10338 CA ALA J 262   10.963 15.249 -4.032 0.00 0.00
J
ATOM 10339 HA ALA J 262   10.764 14.225 -4.312 0.00 0.00
J
ATOM 10340 CB ALA J 262   9.524 15.875 -3.683 0.00 0.00
J

ATOM 10341 HB1 ALA J 262 9.111 15.295 -2.831 0.00 0.00
J
ATOM 10342 HB2 ALA J 262 9.536 16.966 -3.471 0.00 0.00
J
ATOM 10343 HB3 ALA J 262 8.807 15.618 -4.492 0.00 0.00
J
ATOM 10344 C ALA J 262 11.524 15.964 -5.235 0.00 0.00
J
ATOM 10345 O ALA J 262 11.573 15.422 -6.336 0.00 0.00
J
ATOM 10346 N MET J 263 12.046 17.228 -5.132 0.00 0.00
J
ATOM 10347 HN MET J 263 12.003 17.709 -4.260 0.00 0.00
J
ATOM 10348 CA MET J 263 12.755 17.883 -6.138 0.00 0.00
J
ATOM 10349 HA MET J 263 12.106 18.000 -6.993 0.00 0.00
J
ATOM 10350 CB MET J 263 13.193 19.277 -5.726 0.00 0.00
J
ATOM 10351 HB1 MET J 263 12.368 19.968 -5.452 0.00 0.00
J
ATOM 10352 HB2 MET J 263 13.977 19.143 -4.950 0.00 0.00
J
ATOM 10353 CG MET J 263 13.743 20.039 -6.961 0.00 0.00
J
ATOM 10354 HG1 MET J 263 14.424 19.454 -7.615 0.00 0.00
J
ATOM 10355 HG2 MET J 263 12.894 20.341 -7.612 0.00 0.00
J
ATOM 10356 SD MET J 263 14.653 21.425 -6.429 0.00 0.00
J
ATOM 10357 CE MET J 263 15.473 21.839 -7.993 0.00 0.00
J
ATOM 10358 HE1 MET J 263 16.033 20.940 -8.329 0.00 0.00
J
ATOM 10359 HE2 MET J 263 14.797 22.183 -8.805 0.00 0.00
J
ATOM 10360 HE3 MET J 263 16.348 22.490 -7.782 0.00 0.00
J
ATOM 10361 C MET J 263 13.958 17.059 -6.588 0.00 0.00
J
ATOM 10362 O MET J 263 14.089 16.907 -7.769 0.00 0.00
J
ATOM 10363 N LEU J 264 14.876 16.569 -5.750 0.00 0.00
J
ATOM 10364 HN LEU J 264 14.888 16.700 -4.762 0.00 0.00
J
ATOM 10365 CA LEU J 264 16.017 15.853 -6.263 0.00 0.00
J
ATOM 10366 HA LEU J 264 16.346 16.522 -7.045 0.00 0.00
J
ATOM 10367 CB LEU J 264 17.043 15.825 -5.144 0.00 0.00
J

ATOM 10368 HB1 LEU J 264   17.279 16.869 -4.843 0.00 0.00
J
ATOM 10369 HB2 LEU J 264   16.565 15.269 -4.309 0.00 0.00
J
ATOM 10370 CG LEU J 264   18.421 15.119 -5.508 0.00 0.00
J
ATOM 10371 HG LEU J 264   18.293 14.040 -5.739 0.00 0.00
J
ATOM 10372 CD1 LEU J 264   19.026 15.755 -6.767 0.00 0.00
J
ATOM 10373 HD11 LEU J 264   20.063 15.361 -6.819 0.00 0.00
J
ATOM 10374 HD12 LEU J 264   18.422 15.631 -7.692 0.00 0.00
J
ATOM 10375 HD13 LEU J 264   18.930 16.848 -6.594 0.00 0.00
J
ATOM 10376 CD2 LEU J 264   19.408 15.223 -4.266 0.00 0.00
J
ATOM 10377 HD21 LEU J 264   20.242 14.489 -4.297 0.00 0.00
J
ATOM 10378 HD22 LEU J 264   19.772 16.270 -4.330 0.00 0.00
J
ATOM 10379 HD23 LEU J 264   18.809 15.071 -3.343 0.00 0.00
J
ATOM 10380 C   LEU J 264   15.671 14.569 -6.892 0.00 0.00
J
ATOM 10381 O   LEU J 264   16.148 14.159 -7.948 0.00 0.00
J
ATOM 10382 N   THR J 265   14.643 13.919 -6.354 0.00 0.00
J
ATOM 10383 HN THR J 265   14.406 14.183 -5.422 0.00 0.00
J
ATOM 10384 CA THR J 265   13.903 12.803 -7.080 0.00 0.00
J
ATOM 10385 HA THR J 265   14.672 12.046 -7.085 0.00 0.00
J
ATOM 10386 CB THR J 265   12.690 12.171 -6.308 0.00 0.00
J
ATOM 10387 HB THR J 265   11.888 12.937 -6.367 0.00 0.00
J
ATOM 10388 OG1 THR J 265   12.934 11.760 -4.997 0.00 0.00
J
ATOM 10389 HG1 THR J 265   13.133 12.561 -4.507 0.00 0.00
J
ATOM 10390 CG2 THR J 265   12.176 10.880 -6.925 0.00 0.00
J
ATOM 10391 HG21 THR J 265   11.757 11.069 -7.937 0.00 0.00
J
ATOM 10392 HG22 THR J 265   12.978 10.139 -7.128 0.00 0.00
J
ATOM 10393 HG23 THR J 265   11.296 10.455 -6.396 0.00 0.00
J
ATOM 10394 C   THR J 265   13.447 13.133 -8.488 0.00 0.00
J

ATOM 10395 O THR J 265 13.888 12.444 -9.397 0.00 0.00
J
ATOM 10396 N VAL J 266 12.536 14.138 -8.697 0.00 0.00
J
ATOM 10397 HN VAL J 266 12.437 14.784 -7.944 0.00 0.00
J
ATOM 10398 CA VAL J 266 12.000 14.587 -9.974 0.00 0.00
J
ATOM 10399 HA VAL J 266 11.576 13.695 -10.412 0.00 0.00
J
ATOM 10400 CB VAL J 266 10.835 15.520 -9.819 0.00 0.00
J
ATOM 10401 HB VAL J 266 10.154 15.325 -8.963 0.00 0.00
J
ATOM 10402 CG1 VAL J 266 11.229 16.995 -9.677 0.00 0.00
J
ATOM 10403 HG11 VAL J 266 11.402 17.469 -10.667 0.00 0.00
J
ATOM 10404 HG12 VAL J 266 10.467 17.635 -9.182 0.00 0.00
J
ATOM 10405 HG13 VAL J 266 12.117 17.057 -9.012 0.00 0.00
J
ATOM 10406 CG2 VAL J 266 9.995 15.263 -11.143 0.00 0.00
J
ATOM 10407 HG21 VAL J 266 9.332 16.154 -11.156 0.00 0.00
J
ATOM 10408 HG22 VAL J 266 10.617 15.223 -12.063 0.00 0.00
J
ATOM 10409 HG23 VAL J 266 9.346 14.362 -11.151 0.00 0.00
J
ATOM 10410 C VAL J 266 13.152 15.078 -10.861 0.00 0.00
J
ATOM 10411 O VAL J 266 13.035 14.857 -12.040 0.00 0.00
J
ATOM 10412 N PHE J 267 14.177 15.753 -10.386 0.00 0.00
J
ATOM 10413 HN PHE J 267 14.194 15.949 -9.409 0.00 0.00
J
ATOM 10414 CA PHE J 267 15.269 16.217 -11.237 0.00 0.00
J
ATOM 10415 HA PHE J 267 14.755 16.744 -12.027 0.00 0.00
J
ATOM 10416 CB PHE J 267 16.194 17.175 -10.509 0.00 0.00
J
ATOM 10417 HB1 PHE J 267 15.564 18.028 -10.176 0.00 0.00
J
ATOM 10418 HB2 PHE J 267 16.496 16.651 -9.578 0.00 0.00
J
ATOM 10419 CG PHE J 267 17.306 17.916 -11.216 0.00 0.00
J
ATOM 10420 CD1 PHE J 267 18.625 17.665 -10.901 0.00 0.00
J
ATOM 10421 HD1 PHE J 267 18.975 16.899 -10.225 0.00 0.00
J

ATOM 10422 CE1 PHE J 267   19.704 18.401 -11.497 0.00 0.00
J
ATOM 10423 HE1 PHE J 267   20.681 18.193 -11.087 0.00 0.00
J
ATOM 10424 CZ PHE J 267   19.413 19.328 -12.502 0.00 0.00
J
ATOM 10425 HZ PHE J 267   20.196 19.784 -13.090 0.00 0.00
J
ATOM 10426 CD2 PHE J 267   17.001 18.963 -12.170 0.00 0.00
J
ATOM 10427 HD2 PHE J 267   16.045 19.434 -12.347 0.00 0.00
J
ATOM 10428 CE2 PHE J 267   18.045 19.624 -12.801 0.00 0.00
J
ATOM 10429 HE2 PHE J 267   17.923 20.402 -13.541 0.00 0.00
J
ATOM 10430 C   PHE J 267   16.053 15.125 -11.915 0.00 0.00
J
ATOM 10431 O   PHE J 267   16.418 15.184 -13.121 0.00 0.00
J
ATOM 10432 N   ILE J 268   16.419 14.055 -11.181 0.00 0.00
J
ATOM 10433 HN ILE J 268   16.438 14.223 -10.198 0.00 0.00
J
ATOM 10434 CA ILE J 268   17.068 12.832 -11.642 0.00 0.00
J
ATOM 10435 HA ILE J 268   17.873 13.098 -12.311 0.00 0.00
J
ATOM 10436 CB ILE J 268   17.806 12.146 -10.482 0.00 0.00
J
ATOM 10437 HB ILE J 268   16.988 11.963 -9.753 0.00 0.00
J
ATOM 10438 CG2 ILE J 268   18.392 10.718 -10.937 0.00 0.00
J
ATOM 10439 HG21 ILE J 268   17.575   9.991 -11.128 0.00 0.00
J
ATOM 10440 HG22 ILE J 268   18.930 10.838 -11.902 0.00 0.00
J
ATOM 10441 HG23 ILE J 268   19.074 10.354 -10.139 0.00 0.00
J
ATOM 10442 CG1 ILE J 268   18.900 13.029 -9.990 0.00 0.00
J
ATOM 10443 HG11 ILE J 268   19.767 13.011 -10.684 0.00 0.00
J
ATOM 10444 HG12 ILE J 268   18.509 14.067 -9.928 0.00 0.00
J
ATOM 10445 CD ILE J 268   19.418 12.605 -8.628 0.00 0.00
J
ATOM 10446 HD1 ILE J 268   19.789 11.561 -8.712 0.00 0.00
J
ATOM 10447 HD2 ILE J 268   20.201 13.301 -8.257 0.00 0.00
J
ATOM 10448 HD3 ILE J 268   18.641 12.804 -7.860 0.00 0.00
J

```
ATOM  10449  C   ILE J 268      16.076  11.904 -12.386  0.00  0.00
J
ATOM  10450  O   ILE J 268      16.428  11.332 -13.411  0.00  0.00
J
ATOM  10451  N   LEU J 269      14.850  11.819 -11.913  0.00  0.00
J
ATOM  10452  HN  LEU J 269      14.513  12.298 -11.106  0.00  0.00
J
ATOM  10453  CA  LEU J 269      13.796  11.021 -12.528  0.00  0.00
J
ATOM  10454  HA  LEU J 269      14.058   9.974 -12.576  0.00  0.00
J
ATOM  10455  CB  LEU J 269      12.558  10.978 -11.637  0.00  0.00
J
ATOM  10456  HB1 LEU J 269      12.882  10.605 -10.642  0.00  0.00
J
ATOM  10457  HB2 LEU J 269      12.310  12.054 -11.516  0.00  0.00
J
ATOM  10458  CG  LEU J 269      11.363  10.088 -12.102  0.00  0.00
J
ATOM  10459  HG  LEU J 269      11.042  10.585 -13.042  0.00  0.00
J
ATOM  10460  CD1 LEU J 269      11.817   8.641 -12.286  0.00  0.00
J
ATOM  10461 HD11 LEU J 269      12.494   8.557 -13.163  0.00  0.00
J
ATOM  10462 HD12 LEU J 269      12.368   8.240 -11.408  0.00  0.00
J
ATOM  10463 HD13 LEU J 269      10.928   7.980 -12.365  0.00  0.00
J
ATOM  10464  CD2 LEU J 269      10.198  10.086 -11.115  0.00  0.00
J
ATOM  10465 HD21 LEU J 269       9.410   9.393 -11.480  0.00  0.00
J
ATOM  10466 HD22 LEU J 269      10.620   9.580 -10.220  0.00  0.00
J
ATOM  10467 HD23 LEU J 269       9.794  11.049 -10.734  0.00  0.00
J
ATOM  10468  C   LEU J 269      13.509  11.393 -14.053  0.00  0.00
J
ATOM  10469  O   LEU J 269      13.459  10.519 -14.892  0.00  0.00
J
ATOM  10470  N   GLU J 270      13.427  12.767 -14.288  0.00  0.00
J
ATOM  10471  HN  GLU J 270      13.568  13.375 -13.511  0.00  0.00
J
ATOM  10472  CA  GLU J 270      13.112  13.408 -15.591  0.00  0.00
J
ATOM  10473  HA  GLU J 270      12.585  12.702 -16.216  0.00  0.00
J
ATOM  10474  CB  GLU J 270      12.148  14.572 -15.440  0.00  0.00
J
ATOM  10475  HB1 GLU J 270      12.364  15.257 -14.592  0.00  0.00
J
```

```
ATOM  10476  HB2 GLU J 270      12.012  15.089 -16.414  0.00  0.00
J
ATOM  10477  CG  GLU J 270      10.661  14.151 -14.976  0.00  0.00
J
ATOM  10478  HG1 GLU J 270      10.817  13.711 -13.968  0.00  0.00
J
ATOM  10479  HG2 GLU J 270      10.271  15.185 -14.863  0.00  0.00
J
ATOM  10480  CD  GLU J 270       9.828  13.301 -15.832  0.00  0.00
J
ATOM  10481  OE1 GLU J 270       9.519  12.132 -15.455  0.00  0.00
J
ATOM  10482  OE2 GLU J 270       9.514  13.710 -16.983  0.00  0.00
J
ATOM  10483  C   GLU J 270      14.361  13.798 -16.357  0.00  0.00
J
ATOM  10484  O   GLU J 270      14.303  14.492 -17.360  0.00  0.00
J
ATOM  10485  N   TYR J 271      15.605  13.342 -15.963  0.00  0.00
J
ATOM  10486  HN  TYR J 271      15.723  12.839 -15.110  0.00  0.00
J
ATOM  10487  CA  TYR J 271      16.828  13.893 -16.511  0.00  0.00
J
ATOM  10488  HA  TYR J 271      16.844  14.966 -16.392  0.00  0.00
J
ATOM  10489  CB  TYR J 271      18.045  13.274 -15.685  0.00  0.00
J
ATOM  10490  HB1 TYR J 271      17.827  13.170 -14.600  0.00  0.00
J
ATOM  10491  HB2 TYR J 271      18.320  12.324 -16.192  0.00  0.00
J
ATOM  10492  CG  TYR J 271      19.287  14.099 -15.741  0.00  0.00
J
ATOM  10493  CD1 TYR J 271      20.135  14.044 -16.840  0.00  0.00
J
ATOM  10494  HD1 TYR J 271      19.959  13.406 -17.694  0.00  0.00
J
ATOM  10495  CE1 TYR J 271      21.152  14.997 -17.022  0.00  0.00
J
ATOM  10496  HE1 TYR J 271      21.801  14.865 -17.876  0.00  0.00
J
ATOM  10497  CZ  TYR J 271      21.370  15.986 -16.023  0.00  0.00
J
ATOM  10498  OH  TYR J 271      22.368  16.953 -16.252  0.00  0.00
J
ATOM  10499  HH  TYR J 271      22.978  16.547 -16.872  0.00  0.00
J
ATOM  10500  CD2 TYR J 271      19.488  15.092 -14.753  0.00  0.00
J
ATOM  10501  HD2 TYR J 271      18.782  15.177 -13.940  0.00  0.00
J
ATOM  10502  CE2 TYR J 271      20.441  16.061 -14.950  0.00  0.00
J
```

```
ATOM  10503  HE2 TYR J 271      20.463  16.946 -14.330  0.00  0.00
J
ATOM  10504  C   TYR J 271      17.010  13.559 -18.008  0.00  0.00
J
ATOM  10505  O   TYR J 271      17.678  14.288 -18.717  0.00  0.00
J
ATOM  10506  N   SER J 272      16.621  12.304 -18.355  0.00  0.00
J
ATOM  10507  HN  SER J 272      16.265  11.654 -17.688  0.00  0.00
J
ATOM  10508  CA  SER J 272      16.690  11.958 -19.782  0.00  0.00
J
ATOM  10509  HA  SER J 272      17.215  12.689 -20.379  0.00  0.00
J
ATOM  10510  CB  SER J 272      17.342  10.563 -19.885  0.00  0.00
J
ATOM  10511  HB1 SER J 272      17.595  10.335 -20.943  0.00  0.00
J
ATOM  10512  HB2 SER J 272      18.316  10.720 -19.375  0.00  0.00
J
ATOM  10513  OG  SER J 272      16.720   9.499 -19.198  0.00  0.00
J
ATOM  10514  HG1 SER J 272      17.262   8.715 -19.316  0.00  0.00
J
ATOM  10515  C   SER J 272      15.345  11.875 -20.435  0.00  0.00
J
ATOM  10516  O   SER J 272      15.185  11.093 -21.405  0.00  0.00
J
ATOM  10517  N   ASN J 273      14.355  12.616 -19.870  0.00  0.00
J
ATOM  10518  HN  ASN J 273      14.472  13.031 -18.971  0.00  0.00
J
ATOM  10519  CA  ASN J 273      13.093  12.839 -20.626  0.00  0.00
J
ATOM  10520  HA  ASN J 273      12.989  12.109 -21.415  0.00  0.00
J
ATOM  10521  CB  ASN J 273      11.917  12.587 -19.628  0.00  0.00
J
ATOM  10522  HB1 ASN J 273      11.829  11.580 -19.167  0.00  0.00
J
ATOM  10523  HB2 ASN J 273      11.936  13.302 -18.777  0.00  0.00
J
ATOM  10524  CG  ASN J 273      10.697  12.747 -20.469  0.00  0.00
J
ATOM  10525  OD1 ASN J 273      10.564  12.351 -21.656  0.00  0.00
J
ATOM  10526  ND2 ASN J 273       9.654  13.320 -19.843  0.00  0.00
J
ATOM  10527 HD21 ASN J 273       8.745  13.211 -20.247  0.00  0.00
J
ATOM  10528 HD22 ASN J 273       9.875  13.818 -19.005  0.00  0.00
J
ATOM  10529  C   ASN J 273      13.124  14.277 -21.072  0.00  0.00
J
```

ATOM 10530 O ASN J 273 13.360 15.108 -20.235 0.00 0.00
J
ATOM 10531 N PRO J 274 13.071 14.504 -22.387 0.00 0.00
J
ATOM 10532 CD PRO J 274 13.424 13.498 -23.349 0.00 0.00
J
ATOM 10533 HD1 PRO J 274 14.484 13.191 -23.226 0.00 0.00
J
ATOM 10534 HD2 PRO J 274 12.739 12.663 -23.610 0.00 0.00
J
ATOM 10535 CA PRO J 274 13.254 15.905 -22.883 0.00 0.00
J
ATOM 10536 HA PRO J 274 14.016 16.357 -22.266 0.00 0.00
J
ATOM 10537 CB PRO J 274 13.752 15.650 -24.300 0.00 0.00
J
ATOM 10538 HB1 PRO J 274 14.851 15.813 -24.315 0.00 0.00
J
ATOM 10539 HB2 PRO J 274 13.280 16.405 -24.966 0.00 0.00
J
ATOM 10540 CG PRO J 274 13.355 14.251 -24.660 0.00 0.00
J
ATOM 10541 HG1 PRO J 274 14.113 13.809 -25.342 0.00 0.00
J
ATOM 10542 HG2 PRO J 274 12.346 14.238 -25.124 0.00 0.00
J
ATOM 10543 C PRO J 274 12.048 16.793 -22.691 0.00 0.00
J
ATOM 10544 O PRO J 274 12.267 18.017 -22.467 0.00 0.00
J
ATOM 10545 N GLY J 275 10.843 16.152 -22.746 0.00 0.00
J
ATOM 10546 HN GLY J 275 10.906 15.157 -22.773 0.00 0.00
J
ATOM 10547 CA GLY J 275 9.520 16.803 -22.810 0.00 0.00
J
ATOM 10548 HA1 GLY J 275 8.655 16.169 -22.940 0.00 0.00
J
ATOM 10549 HA2 GLY J 275 9.681 17.572 -23.550 0.00 0.00
J
ATOM 10550 C GLY J 275 9.234 17.548 -21.497 0.00 0.00
J
ATOM 10551 O GLY J 275 8.320 18.415 -21.403 0.00 0.00
J
ATOM 10552 N THR J 276 9.937 17.153 -20.416 0.00 0.00
J
ATOM 10553 HN THR J 276 10.422 16.286 -20.500 0.00 0.00
J
ATOM 10554 CA THR J 276 9.911 18.024 -19.241 0.00 0.00
J
ATOM 10555 HA THR J 276 8.993 18.584 -19.135 0.00 0.00
J
ATOM 10556 CB THR J 276 9.929 17.143 -17.980 0.00 0.00
J

ATOM 10557 HB THR J 276 10.753 16.409 -18.109 0.00 0.00
J
ATOM 10558 OG1 THR J 276 8.851 16.252 -17.944 0.00 0.00
J
ATOM 10559 HG1 THR J 276 9.076 15.471 -17.433 0.00 0.00
J
ATOM 10560 CG2 THR J 276 10.125 17.926 -16.645 0.00 0.00
J
ATOM 10561 HG21 THR J 276 9.277 18.643 -16.616 0.00 0.00
J
ATOM 10562 HG22 THR J 276 10.118 17.135 -15.864 0.00 0.00
J
ATOM 10563 HG23 THR J 276 11.130 18.392 -16.569 0.00 0.00
J
ATOM 10564 C THR J 276 11.069 18.880 -19.230 0.00 0.00
J
ATOM 10565 O THR J 276 12.216 18.460 -19.036 0.00 0.00
J
ATOM 10566 N LEU J 277 10.857 20.166 -19.503 0.00 0.00
J
ATOM 10567 HN LEU J 277 9.896 20.339 -19.705 0.00 0.00
J
ATOM 10568 CA LEU J 277 11.689 21.273 -19.289 0.00 0.00
J
ATOM 10569 HA LEU J 277 11.155 22.095 -19.742 0.00 0.00
J
ATOM 10570 CB LEU J 277 11.885 21.452 -17.731 0.00 0.00
J
ATOM 10571 HB1 LEU J 277 12.398 20.503 -17.466 0.00 0.00
J
ATOM 10572 HB2 LEU J 277 12.628 22.223 -17.437 0.00 0.00
J
ATOM 10573 CG LEU J 277 10.666 21.760 -16.914 0.00 0.00
J
ATOM 10574 HG LEU J 277 9.912 20.995 -17.198 0.00 0.00
J
ATOM 10575 CD1 LEU J 277 10.897 21.595 -15.400 0.00 0.00
J
ATOM 10576 HD11 LEU J 277 11.567 22.332 -14.907 0.00 0.00
J
ATOM 10577 HD12 LEU J 277 9.905 21.481 -14.913 0.00 0.00
J
ATOM 10578 HD13 LEU J 277 11.458 20.652 -15.230 0.00 0.00
J
ATOM 10579 CD2 LEU J 277 10.137 23.190 -17.362 0.00 0.00
J
ATOM 10580 HD21 LEU J 277 9.358 23.487 -16.627 0.00 0.00
J
ATOM 10581 HD22 LEU J 277 11.003 23.882 -17.287 0.00 0.00
J
ATOM 10582 HD23 LEU J 277 9.599 23.257 -18.331 0.00 0.00
J
ATOM 10583 C LEU J 277 13.072 21.257 -19.972 0.00 0.00
J

ATOM 10584 O   LEU J 277   14.089 21.780 -19.489 0.00 0.00
J
ATOM 10585 N   GLY J 278   13.209 20.670 -21.247 0.00 0.00
J
ATOM 10586 HN GLY J 278   12.293 20.427 -21.555 0.00 0.00
J
ATOM 10587 CA GLY J 278   14.340 20.622 -22.180 0.00 0.00
J
ATOM 10588 HA1 GLY J 278   14.102 19.930 -22.974 0.00 0.00
J
ATOM 10589 HA2 GLY J 278   15.271 20.411 -21.676 0.00 0.00
J
ATOM 10590 C   GLY J 278   14.624 21.991 -22.898 0.00 0.00
J
ATOM 10591 O   GLY J 278   15.652 22.123 -23.583 0.00 0.00
J
ATOM 10592 N   HSE J 279   13.796 23.051 -22.634 0.00 0.00
J
ATOM 10593 HN HSE J 279   13.100 23.020 -21.921 0.00 0.00
J
ATOM 10594 CA HSE J 279   13.982 24.336 -23.304 0.00 0.00
J
ATOM 10595 HA HSE J 279   14.649 24.224 -24.146 0.00 0.00
J
ATOM 10596 CB HSE J 279   12.598 24.822 -23.861 0.00 0.00
J
ATOM 10597 HB1 HSE J 279   12.577 25.799 -24.391 0.00 0.00
J
ATOM 10598 HB2 HSE J 279   12.418 23.955 -24.532 0.00 0.00
J
ATOM 10599 ND1 HSE J 279   10.896 23.976 -22.105 0.00 0.00
J
ATOM 10600 CG HSE J 279   11.511 25.039 -22.818 0.00 0.00
J
ATOM 10601 CE1 HSE J 279   9.930 24.493 -21.358 0.00 0.00
J
ATOM 10602 HE1 HSE J 279   9.103 23.995 -20.850 0.00 0.00
J
ATOM 10603 NE2 HSE J 279   9.881 25.826 -21.607 0.00 0.00
J
ATOM 10604 HE2 HSE J 279   9.346 26.552 -21.176 0.00 0.00
J
ATOM 10605 CD2 HSE J 279   10.855 26.176 -22.523 0.00 0.00
J
ATOM 10606 HD2 HSE J 279   11.102 27.186 -22.824 0.00 0.00
J
ATOM 10607 C   HSE J 279   14.576 25.439 -22.397 0.00 0.00
J
ATOM 10608 O   HSE J 279   14.686 26.611 -22.753 0.00 0.00
J
ATOM 10609 N   LEU J 280   15.024 25.018 -21.236 0.00 0.00
J
ATOM 10610 HN LEU J 280   14.996 24.059 -20.968 0.00 0.00
J

ATOM 10611 CA LEU J 280   15.456 25.898 -20.149 0.00 0.00
J
ATOM 10612 HA LEU J 280   15.390 26.918 -20.497 0.00 0.00
J
ATOM 10613 CB LEU J 280   14.665 25.841 -18.856 0.00 0.00
J
ATOM 10614 HB1 LEU J 280   14.570 24.781 -18.538 0.00 0.00
J
ATOM 10615 HB2 LEU J 280   15.230 26.334 -18.036 0.00 0.00
J
ATOM 10616 CG LEU J 280   13.211 26.417 -18.964 0.00 0.00
J
ATOM 10617 HG LEU J 280   12.581 25.735 -19.573 0.00 0.00
J
ATOM 10618 CD1 LEU J 280   12.524 26.438 -17.528 0.00 0.00
J
ATOM 10619 HD11 LEU J 280   12.759 25.524 -16.942 0.00 0.00
J
ATOM 10620 HD12 LEU J 280   13.028 27.232 -16.937 0.00 0.00
J
ATOM 10621 HD13 LEU J 280   11.487 26.838 -17.517 0.00 0.00
J
ATOM 10622 CD2 LEU J 280   13.067 27.789 -19.706 0.00 0.00
J
ATOM 10623 HD21 LEU J 280   13.210 27.684 -20.802 0.00 0.00
J
ATOM 10624 HD22 LEU J 280   12.079 28.281 -19.581 0.00 0.00
J
ATOM 10625 HD23 LEU J 280   13.824 28.497 -19.305 0.00 0.00
J
ATOM 10626 C   LEU J 280   16.904 25.529 -19.849 0.00 0.00
J
ATOM 10627 O   LEU J 280   17.228 24.411 -19.538 0.00 0.00
J
ATOM 10628 N   HSE J 281   17.794 26.554 -19.842 0.00 0.00
J
ATOM 10629 HN HSE J 281   17.521 27.486 -20.067 0.00 0.00
J
ATOM 10630 CA HSE J 281   19.052 26.522 -19.191 0.00 0.00
J
ATOM 10631 HA HSE J 281   19.573 25.648 -19.552 0.00 0.00
J
ATOM 10632 CB HSE J 281   19.916 27.667 -19.673 0.00 0.00
J
ATOM 10633 HB1 HSE J 281   19.419 28.660 -19.615 0.00 0.00
J
ATOM 10634 HB2 HSE J 281   20.895 27.669 -19.148 0.00 0.00
J
ATOM 10635 ND1 HSE J 281   20.389 28.521 -22.012 0.00 0.00
J
ATOM 10636 CG HSE J 281   20.236 27.485 -21.175 0.00 0.00
J
ATOM 10637 CE1 HSE J 281   20.963 28.070 -23.146 0.00 0.00
J

ATOM 10638 HE1 HSE J 281   21.233 28.807 -23.902 0.00 0.00
J
ATOM 10639 NE2 HSE J 281   21.128 26.730 -23.080 0.00 0.00
J
ATOM 10640 HE2 HSE J 281   21.458 26.253 -23.895 0.00 0.00
J
ATOM 10641 CD2 HSE J 281   20.688 26.323 -21.827 0.00 0.00
J
ATOM 10642 HD2 HSE J 281   20.782 25.339 -21.386 0.00 0.00
J
ATOM 10643 C   HSE J 281   19.057 26.293 -17.636 0.00 0.00
J
ATOM 10644 O   HSE J 281   18.006 26.505 -17.056 0.00 0.00
J
ATOM 10645 N   ILE J 282   20.159 25.799 -17.071 0.00 0.00
J
ATOM 10646 HN ILE J 282   21.027 25.794 -17.563 0.00 0.00
J
ATOM 10647 CA ILE J 282   20.295 25.252 -15.729 0.00 0.00
J
ATOM 10648 HA ILE J 282   19.777 24.305 -15.745 0.00 0.00
J
ATOM 10649 CB ILE J 282   21.792 24.921 -15.473 0.00 0.00
J
ATOM 10650 HB ILE J 282   22.179 24.415 -16.383 0.00 0.00
J
ATOM 10651 CG2 ILE J 282   22.648 26.169 -15.320 0.00 0.00
J
ATOM 10652 HG21 ILE J 282   22.514 26.933 -16.116 0.00 0.00
J
ATOM 10653 HG22 ILE J 282   22.421 26.788 -14.426 0.00 0.00
J
ATOM 10654 HG23 ILE J 282   23.727 25.914 -15.390 0.00 0.00
J
ATOM 10655 CG1 ILE J 282   22.072 23.905 -14.292 0.00 0.00
J
ATOM 10656 HG11 ILE J 282   23.155 23.843 -14.051 0.00 0.00
J
ATOM 10657 HG12 ILE J 282   21.590 24.288 -13.367 0.00 0.00
J
ATOM 10658 CD ILE J 282   21.413 22.561 -14.470 0.00 0.00
J
ATOM 10659 HD1 ILE J 282   21.445 21.947 -13.544 0.00 0.00
J
ATOM 10660 HD2 ILE J 282   20.345 22.659 -14.758 0.00 0.00
J
ATOM 10661 HD3 ILE J 282   21.962 22.015 -15.267 0.00 0.00
J
ATOM 10662 C   ILE J 282   19.567 25.988 -14.580 0.00 0.00
J
ATOM 10663 O   ILE J 282   18.842 25.354 -13.772 0.00 0.00
J
ATOM 10664 N   VAL J 283   19.691 27.373 -14.503 0.00 0.00
J

ATOM 10665 HN VAL J 283   20.305 27.784 -15.172 0.00 0.00
J
ATOM 10666 CA VAL J 283   18.936 28.141 -13.347 0.00 0.00
J
ATOM 10667 HA VAL J 283   19.170 27.722 -12.379 0.00 0.00
J
ATOM 10668 CB VAL J 283   19.453 29.557 -13.402 0.00 0.00
J
ATOM 10669 HB VAL J 283   19.339 30.118 -14.354 0.00 0.00
J
ATOM 10670 CG1 VAL J 283   18.796 30.368 -12.321 0.00 0.00
J
ATOM 10671 HG11 VAL J 283   18.919 29.836 -11.353 0.00 0.00
J
ATOM 10672 HG12 VAL J 283   19.261 31.376 -12.287 0.00 0.00
J
ATOM 10673 HG13 VAL J 283   17.747 30.728 -12.386 0.00 0.00
J
ATOM 10674 CG2 VAL J 283   20.974 29.463 -13.041 0.00 0.00
J
ATOM 10675 HG21 VAL J 283   21.623 29.183 -13.898 0.00 0.00
J
ATOM 10676 HG22 VAL J 283   21.498 30.372 -12.676 0.00 0.00
J
ATOM 10677 HG23 VAL J 283   21.024 28.699 -12.236 0.00 0.00
J
ATOM 10678 C   VAL J 283   17.476 28.098 -13.476 0.00 0.00
J
ATOM 10679 O   VAL J 283   16.767 27.732 -12.541 0.00 0.00
J
ATOM 10680 N   ASP J 284   16.858 28.491 -14.640 0.00 0.00
J
ATOM 10681 HN ASP J 284   17.386 29.111 -15.215 0.00 0.00
J
ATOM 10682 CA ASP J 284   15.499 28.435 -15.055 0.00 0.00
J
ATOM 10683 HA ASP J 284   14.883 29.122 -14.494 0.00 0.00
J
ATOM 10684 CB ASP J 284   15.420 28.923 -16.540 0.00 0.00
J
ATOM 10685 HB1 ASP J 284   16.141 28.442 -17.235 0.00 0.00
J
ATOM 10686 HB2 ASP J 284   14.437 28.653 -16.982 0.00 0.00
J
ATOM 10687 CG ASP J 284   15.600 30.392 -16.640 0.00 0.00
J
ATOM 10688 OD1 ASP J 284   16.632 30.909 -16.151 0.00 0.00
J
ATOM 10689 OD2 ASP J 284   14.832 31.109 -17.301 0.00 0.00
J
ATOM 10690 C   ASP J 284   15.027 27.002 -14.898 0.00 0.00
J
ATOM 10691 O   ASP J 284   13.925 26.787 -14.378 0.00 0.00
J

ATOM 10692 N LYS J 285 15.764 25.977 −15.309 0.00 0.00
J
ATOM 10693 HN LYS J 285 16.588 26.209 −15.819 0.00 0.00
J
ATOM 10694 CA LYS J 285 15.354 24.586 −15.223 0.00 0.00
J
ATOM 10695 HA LYS J 285 14.363 24.509 −15.644 0.00 0.00
J
ATOM 10696 CB LYS J 285 16.200 23.697 −16.134 0.00 0.00
J
ATOM 10697 HB1 LYS J 285 16.028 24.145 −17.136 0.00 0.00
J
ATOM 10698 HB2 LYS J 285 17.270 23.836 −15.871 0.00 0.00
J
ATOM 10699 CG LYS J 285 15.757 22.188 −16.103 0.00 0.00
J
ATOM 10700 HG1 LYS J 285 15.935 21.717 −15.112 0.00 0.00
J
ATOM 10701 HG2 LYS J 285 14.678 22.185 −16.368 0.00 0.00
J
ATOM 10702 CD LYS J 285 16.346 21.370 −17.253 0.00 0.00
J
ATOM 10703 HD1 LYS J 285 16.244 21.947 −18.197 0.00 0.00
J
ATOM 10704 HD2 LYS J 285 17.408 21.167 −16.995 0.00 0.00
J
ATOM 10705 CE LYS J 285 15.656 20.032 −17.365 0.00 0.00
J
ATOM 10706 HE1 LYS J 285 15.682 19.496 −16.393 0.00 0.00
J
ATOM 10707 HE2 LYS J 285 14.585 20.199 −17.609 0.00 0.00
J
ATOM 10708 NZ LYS J 285 16.300 19.269 −18.415 0.00 0.00
J
ATOM 10709 HZ1 LYS J 285 17.210 18.832 −18.165 0.00 0.00
J
ATOM 10710 HZ2 LYS J 285 15.742 18.465 −18.768 0.00 0.00
J
ATOM 10711 HZ3 LYS J 285 16.510 19.830 −19.266 0.00 0.00
J
ATOM 10712 C LYS J 285 15.188 24.189 −13.758 0.00 0.00
J
ATOM 10713 O LYS J 285 14.217 23.577 −13.345 0.00 0.00
J
ATOM 10714 N LEU J 286 16.169 24.487 −12.935 0.00 0.00
J
ATOM 10715 HN LEU J 286 16.963 24.948 −13.323 0.00 0.00
J
ATOM 10716 CA LEU J 286 16.255 24.201 −11.470 0.00 0.00
J
ATOM 10717 HA LEU J 286 16.131 23.159 −11.217 0.00 0.00
J
ATOM 10718 CB LEU J 286 17.587 24.633 −10.923 0.00 0.00
J

ATOM 10719 HB1 LEU J 286   17.893 25.559 -11.455 0.00 0.00
J
ATOM 10720 HB2 LEU J 286   17.507 24.827 -9.832 0.00 0.00
J
ATOM 10721 CG LEU J 286   18.787 23.645 -11.058 0.00 0.00
J
ATOM 10722 HG LEU J 286   18.927 23.461 -12.144 0.00 0.00
J
ATOM 10723 CD1 LEU J 286   19.987 24.393 -10.451 0.00 0.00
J
ATOM 10724 HD11 LEU J 286   20.235 25.248 -11.116 0.00 0.00
J
ATOM 10725 HD12 LEU J 286   19.798 24.822 -9.444 0.00 0.00
J
ATOM 10726 HD13 LEU J 286   20.881 23.735 -10.443 0.00 0.00
J
ATOM 10727 CD2 LEU J 286   18.656 22.262 -10.361 0.00 0.00
J
ATOM 10728 HD21 LEU J 286   18.655 22.400 -9.258 0.00 0.00
J
ATOM 10729 HD22 LEU J 286   17.694 21.780 -10.641 0.00 0.00
J
ATOM 10730 HD23 LEU J 286   19.514 21.617 -10.649 0.00 0.00
J
ATOM 10731 C   LEU J 286   15.143 24.964 -10.711 0.00 0.00
J
ATOM 10732 O   LEU J 286   14.437 24.332 -9.947 0.00 0.00
J
ATOM 10733 N   TRP J 287   14.859 26.247 -10.974 0.00 0.00
J
ATOM 10734 HN TRP J 287   15.318 26.778 -11.682 0.00 0.00
J
ATOM 10735 CA TRP J 287   13.624 26.803 -10.409 0.00 0.00
J
ATOM 10736 HA TRP J 287   13.650 26.778 -9.330 0.00 0.00
J
ATOM 10737 CB TRP J 287   13.550 28.309 -10.792 0.00 0.00
J
ATOM 10738 HB1 TRP J 287   13.724 28.361 -11.888 0.00 0.00
J
ATOM 10739 HB2 TRP J 287   12.531 28.713 -10.612 0.00 0.00
J
ATOM 10740 CG TRP J 287   14.342 29.258 -9.893 0.00 0.00
J
ATOM 10741 CD1 TRP J 287   15.359 30.000 -10.260 0.00 0.00
J
ATOM 10742 HD1 TRP J 287   15.732 29.945 -11.272 0.00 0.00
J
ATOM 10743 NE1 TRP J 287   16.022 30.541 -9.196 0.00 0.00
J
ATOM 10744 HE1 TRP J 287   16.757 31.175 -9.298 0.00 0.00
J
ATOM 10745 CE2 TRP J 287   15.217 30.358 -8.088 0.00 0.00
J

ATOM 10746 CD2 TRP J 287 14.219 29.430 -8.461 0.00 0.00
J
ATOM 10747 CE3 TRP J 287 13.396 28.937 -7.554 0.00 0.00
J
ATOM 10748 HE3 TRP J 287 12.653 28.190 -7.787 0.00 0.00
J
ATOM 10749 CZ3 TRP J 287 13.532 29.260 -6.160 0.00 0.00
J
ATOM 10750 HZ3 TRP J 287 12.675 29.014 -5.550 0.00 0.00
J
ATOM 10751 CZ2 TRP J 287 15.303 30.820 -6.729 0.00 0.00
J
ATOM 10752 HZ2 TRP J 287 15.970 31.614 -6.427 0.00 0.00
J
ATOM 10753 CH2 TRP J 287 14.431 30.298 -5.756 0.00 0.00
J
ATOM 10754 HH2 TRP J 287 14.454 30.657 -4.738 0.00 0.00
J
ATOM 10755 C TRP J 287 12.289 26.106 -10.911 0.00 0.00
J
ATOM 10756 O TRP J 287 11.415 25.778 -10.129 0.00 0.00
J
ATOM 10757 N ALA J 288 12.067 25.867 -12.189 0.00 0.00
J
ATOM 10758 HN ALA J 288 12.791 26.132 -12.822 0.00 0.00
J
ATOM 10759 CA ALA J 288 10.937 25.087 -12.687 0.00 0.00
J
ATOM 10760 HA ALA J 288 10.048 25.622 -12.387 0.00 0.00
J
ATOM 10761 CB ALA J 288 11.184 25.009 -14.218 0.00 0.00
J
ATOM 10762 HB1 ALA J 288 10.289 24.543 -14.682 0.00 0.00
J
ATOM 10763 HB2 ALA J 288 11.514 25.945 -14.718 0.00 0.00
J
ATOM 10764 HB3 ALA J 288 12.082 24.376 -14.383 0.00 0.00
J
ATOM 10765 C ALA J 288 10.835 23.622 -12.129 0.00 0.00
J
ATOM 10766 O ALA J 288 9.737 23.078 -11.941 0.00 0.00
J
ATOM 10767 N SER J 289 11.926 22.975 -11.784 0.00 0.00
J
ATOM 10768 HN SER J 289 12.798 23.432 -11.940 0.00 0.00
J
ATOM 10769 CA SER J 289 11.918 21.637 -11.307 0.00 0.00
J
ATOM 10770 HA SER J 289 11.147 21.092 -11.830 0.00 0.00
J
ATOM 10771 CB SER J 289 13.323 20.863 -11.434 0.00 0.00
J
ATOM 10772 HB1 SER J 289 14.054 21.339 -10.745 0.00 0.00
J

ATOM 10773 HB2 SER J 289   13.235 19.838 -11.014 0.00 0.00
J
ATOM 10774 OG SER J 289   13.842 20.887 -12.710 0.00 0.00
J
ATOM 10775 HG1 SER J 289   13.832 21.818 -12.947 0.00 0.00
J
ATOM 10776 C   SER J 289   11.613 21.603 -9.856 0.00 0.00
J
ATOM 10777 O   SER J 289   11.073 20.565 -9.389 0.00 0.00
J
ATOM 10778 N   TYR J 290   11.745 22.712 -9.140 0.00 0.00
J
ATOM 10779 HN TYR J 290   12.250 23.506 -9.471 0.00 0.00
J
ATOM 10780 CA TYR J 290   11.145 22.869 -7.833 0.00 0.00
J
ATOM 10781 HA TYR J 290   11.298 21.982 -7.237 0.00 0.00
J
ATOM 10782 CB TYR J 290   11.951 23.978 -7.010 0.00 0.00
J
ATOM 10783 HB1 TYR J 290   13.012 23.650 -7.020 0.00 0.00
J
ATOM 10784 HB2 TYR J 290   11.802 24.998 -7.425 0.00 0.00
J
ATOM 10785 CG TYR J 290   11.554 24.104 -5.581 0.00 0.00
J
ATOM 10786 CD1 TYR J 290   10.937 25.353 -5.192 0.00 0.00
J
ATOM 10787 HD1 TYR J 290   10.839 26.095 -5.971 0.00 0.00
J
ATOM 10788 CE1 TYR J 290   10.356 25.461 -3.937 0.00 0.00
J
ATOM 10789 HE1 TYR J 290   9.861 26.365 -3.617 0.00 0.00
J
ATOM 10790 CZ TYR J 290   10.493 24.400 -2.989 0.00 0.00
J
ATOM 10791 OH TYR J 290   10.005 24.580 -1.703 0.00 0.00
J
ATOM 10792 HH TYR J 290   9.811 25.505 -1.532 0.00 0.00
J
ATOM 10793 CD2 TYR J 290   11.676 23.035 -4.656 0.00 0.00
J
ATOM 10794 HD2 TYR J 290   12.056 22.049 -4.881 0.00 0.00
J
ATOM 10795 CE2 TYR J 290   11.174 23.215 -3.306 0.00 0.00
J
ATOM 10796 HE2 TYR J 290   11.228 22.378 -2.625 0.00 0.00
J
ATOM 10797 C   TYR J 290   9.688 23.052 -7.879 0.00 0.00
J
ATOM 10798 O   TYR J 290   9.026 22.497 -7.083 0.00 0.00
J
ATOM 10799 N   PHE J 291   9.232 23.882 -8.882 0.00 0.00
J

```
ATOM  10800  HN  PHE J 291       9.911  24.216  -9.531  0.00  0.00
J
ATOM  10801  CA  PHE J 291       7.856  24.222  -9.152  0.00  0.00
J
ATOM  10802  HA  PHE J 291       7.533  24.554  -8.176  0.00  0.00
J
ATOM  10803  CB  PHE J 291       7.780  25.354 -10.282  0.00  0.00
J
ATOM  10804  HB1 PHE J 291       8.706  25.958 -10.166  0.00  0.00
J
ATOM  10805  HB2 PHE J 291       7.724  24.928 -11.306  0.00  0.00
J
ATOM  10806  CG  PHE J 291       6.506  26.225  -9.985  0.00  0.00
J
ATOM  10807  CD1 PHE J 291       6.488  27.113  -8.970  0.00  0.00
J
ATOM  10808  HD1 PHE J 291       7.329  27.324  -8.326  0.00  0.00
J
ATOM  10809  CE1 PHE J 291       5.267  27.797  -8.686  0.00  0.00
J
ATOM  10810  HE1 PHE J 291       5.275  28.521  -7.885  0.00  0.00
J
ATOM  10811  CZ  PHE J 291       4.156  27.623  -9.439  0.00  0.00
J
ATOM  10812  HZ  PHE J 291       3.228  28.163  -9.321  0.00  0.00
J
ATOM  10813  CD2 PHE J 291       5.344  25.919 -10.664  0.00  0.00
J
ATOM  10814  HD2 PHE J 291       5.295  25.105 -11.372  0.00  0.00
J
ATOM  10815  CE2 PHE J 291       4.136  26.606 -10.408  0.00  0.00
J
ATOM  10816  HE2 PHE J 291       3.307  26.565 -11.099  0.00  0.00
J
ATOM  10817  C   PHE J 291       7.049  22.976  -9.541  0.00  0.00
J
ATOM  10818  O   PHE J 291       5.912  22.873  -9.082  0.00  0.00
J
ATOM  10819  N   GLN J 292       7.685  22.003 -10.223  0.00  0.00
J
ATOM  10820  HN  GLN J 292       8.627  22.048 -10.549  0.00  0.00
J
ATOM  10821  CA  GLN J 292       7.064  20.757 -10.639  0.00  0.00
J
ATOM  10822  HA  GLN J 292       6.080  20.998 -11.013  0.00  0.00
J
ATOM  10823  CB  GLN J 292       7.932  19.997 -11.635  0.00  0.00
J
ATOM  10824  HB1 GLN J 292       8.200  20.599 -12.529  0.00  0.00
J
ATOM  10825  HB2 GLN J 292       8.899  19.669 -11.198  0.00  0.00
J
ATOM  10826  CG  GLN J 292       7.354  18.580 -12.109  0.00  0.00
J
```

ATOM 10827 HG1 GLN J 292   7.060 17.923 -11.262 0.00 0.00
J
ATOM 10828 HG2 GLN J 292   6.492 18.904 -12.730 0.00 0.00
J
ATOM 10829 CD GLN J 292   8.249 17.830 -13.051 0.00 0.00
J
ATOM 10830 OE1 GLN J 292   9.495 17.982 -13.085 0.00 0.00
J
ATOM 10831 NE2 GLN J 292   7.687 16.792 -13.759 0.00 0.00
J
ATOM 10832 HE21 GLN J 292   8.241 16.201 -14.346 0.00 0.00
J
ATOM 10833 HE22 GLN J 292   6.702 16.670 -13.633 0.00 0.00
J
ATOM 10834 C   GLN J 292   6.882 19.879 -9.459 0.00 0.00
J
ATOM 10835 O   GLN J 292   5.921 19.183 -9.347 0.00 0.00
J
ATOM 10836 N   ALA J 293   7.830 19.858 -8.533 0.00 0.00
J
ATOM 10837 HN ALA J 293   8.602 20.483 -8.622 0.00 0.00
J
ATOM 10838 CA ALA J 293   7.809 19.102 -7.319 0.00 0.00
J
ATOM 10839 HA ALA J 293   7.557 18.073 -7.525 0.00 0.00
J
ATOM 10840 CB ALA J 293   9.203 19.121 -6.669 0.00 0.00
J
ATOM 10841 HB1 ALA J 293   9.609 20.122 -6.411 0.00 0.00
J
ATOM 10842 HB2 ALA J 293   9.202 18.548 -5.717 0.00 0.00
J
ATOM 10843 HB3 ALA J 293   9.943 18.692 -7.378 0.00 0.00
J
ATOM 10844 C   ALA J 293   6.792 19.500 -6.378 0.00 0.00
J
ATOM 10845 O   ALA J 293   6.163 18.700 -5.695 0.00 0.00
J
ATOM 10846 N   VAL J 294   6.529 20.820 -6.230 0.00 0.00
J
ATOM 10847 HN VAL J 294   7.167 21.505 -6.573 0.00 0.00
J
ATOM 10848 CA VAL J 294   5.616 21.334 -5.218 0.00 0.00
J
ATOM 10849 HA VAL J 294   5.629 20.693 -4.349 0.00 0.00
J
ATOM 10850 CB VAL J 294   6.011 22.699 -4.619 0.00 0.00
J
ATOM 10851 HB VAL J 294   5.468 22.879 -3.667 0.00 0.00
J
ATOM 10852 CG1 VAL J 294   7.452 22.562 -4.243 0.00 0.00
J
ATOM 10853 HG11 VAL J 294   7.799 23.332 -3.521 0.00 0.00
J

ATOM 10854 HG12 VAL J 294   7.551 21.537 -3.825 0.00 0.00
J
ATOM 10855 HG13 VAL J 294   8.108 22.579 -5.140 0.00 0.00
J
ATOM 10856 CG2 VAL J 294   5.818 23.812 -5.643 0.00 0.00
J
ATOM 10857 HG21 VAL J 294   6.159 23.552 -6.668 0.00 0.00
J
ATOM 10858 HG22 VAL J 294   4.799 24.248 -5.716 0.00 0.00
J
ATOM 10859 HG23 VAL J 294   6.449 24.715 -5.501 0.00 0.00
J
ATOM 10860 C   VAL J 294   4.159 21.447 -5.682 0.00 0.00
J
ATOM 10861 O   VAL J 294   3.202 21.535 -4.886 0.00 0.00
J
ATOM 10862 N   THR J 295   3.927 21.377 -7.016 0.00 0.00
J
ATOM 10863 HN THR J 295   4.710 21.158 -7.594 0.00 0.00
J
ATOM 10864 CA THR J 295   2.668 21.226 -7.728 0.00 0.00
J
ATOM 10865 HA THR J 295   2.054 22.019 -7.326 0.00 0.00
J
ATOM 10866 CB THR J 295   2.902 21.457 -9.136 0.00 0.00
J
ATOM 10867 HB THR J 295   3.888 20.998 -9.363 0.00 0.00
J
ATOM 10868 OG1 THR J 295   3.243 22.824 -9.261 0.00 0.00
J
ATOM 10869 HG1 THR J 295   4.191 22.801 -9.109 0.00 0.00
J
ATOM 10870 CG2 THR J 295   1.784 21.186 -10.087 0.00 0.00
J
ATOM 10871 HG21 THR J 295   2.121 21.281 -11.141 0.00 0.00
J
ATOM 10872 HG22 THR J 295   1.413 20.142 -9.999 0.00 0.00
J
ATOM 10873 HG23 THR J 295   0.855 21.769 -9.914 0.00 0.00
J
ATOM 10874 C   THR J 295   1.720 20.122 -7.463 0.00 0.00
J
ATOM 10875 O   THR J 295   0.552 20.421 -7.231 0.00 0.00
J
ATOM 10876 N   PRO J 296   2.119 18.858 -7.283 0.00 0.00
J
ATOM 10877 CD PRO J 296   3.320 18.208 -7.864 0.00 0.00
J
ATOM 10878 HD1 PRO J 296   3.251 18.258 -8.972 0.00 0.00
J
ATOM 10879 HD2 PRO J 296   4.211 18.786 -7.538 0.00 0.00
J
ATOM 10880 CA PRO J 296   1.107 17.875 -6.987 0.00 0.00
J

ATOM 10881 HA   PRO J 296   0.266 18.159 -7.602 0.00 0.00
J
ATOM 10882 CB   PRO J 296   1.757 16.564 -7.403 0.00 0.00
J
ATOM 10883 HB1 PRO J 296   1.351 16.240 -8.385 0.00 0.00
J
ATOM 10884 HB2 PRO J 296   1.475 15.712 -6.747 0.00 0.00
J
ATOM 10885 CG   PRO J 296   3.242 16.775 -7.458 0.00 0.00
J
ATOM 10886 HG1 PRO J 296   3.537 16.771 -6.387 0.00 0.00
J
ATOM 10887 HG2 PRO J 296   3.799 16.058 -8.097 0.00 0.00
J
ATOM 10888 C   PRO J 296   0.792 17.827 -5.480 0.00 0.00
J
ATOM 10889 O   PRO J 296   -0.180 17.181 -5.022 0.00 0.00
J
ATOM 10890 N   ARG J 297   1.608 18.461 -4.642 0.00 0.00
J
ATOM 10891 HN   ARG J 297   2.450 18.836 -5.021 0.00 0.00
J
ATOM 10892 CA   ARG J 297   1.422 18.721 -3.230 0.00 0.00
J
ATOM 10893 HA   ARG J 297   1.001 17.859 -2.734 0.00 0.00
J
ATOM 10894 CB   ARG J 297   2.701 19.088 -2.477 0.00 0.00
J
ATOM 10895 HB1 ARG J 297   3.141 19.967 -2.996 0.00 0.00
J
ATOM 10896 HB2 ARG J 297   2.395 19.344 -1.440 0.00 0.00
J
ATOM 10897 CG   ARG J 297   3.865 18.010 -2.544 0.00 0.00
J
ATOM 10898 HG1 ARG J 297   3.330 17.052 -2.721 0.00 0.00
J
ATOM 10899 HG2 ARG J 297   4.471 18.159 -3.463 0.00 0.00
J
ATOM 10900 CD   ARG J 297   4.833 17.978 -1.346 0.00 0.00
J
ATOM 10901 HD1 ARG J 297   5.503 17.095 -1.421 0.00 0.00
J
ATOM 10902 HD2 ARG J 297   5.395 18.902 -1.092 0.00 0.00
J
ATOM 10903 NE   ARG J 297   4.007 17.887 -0.159 0.00 0.00
J
ATOM 10904 HE   ARG J 297   3.359 17.132 -0.057 0.00 0.00
J
ATOM 10905 CZ   ARG J 297   4.012 18.629   0.943 0.00 0.00
J
ATOM 10906 NH1 ARG J 297   4.713 19.782   1.048 0.00 0.00
J
ATOM 10907 HH11 ARG J 297   4.742 20.334   1.881 0.00 0.00
J

```
ATOM  10908 HH12 ARG J 297       5.410  20.010   0.368  0.00  0.00
J
ATOM  10909  NH2 ARG J 297       3.370  18.199   2.035  0.00  0.00
J
ATOM  10910 HH21 ARG J 297       3.431  18.766   2.857  0.00  0.00
J
ATOM  10911 HH22 ARG J 297       2.599  17.563   1.996  0.00  0.00
J
ATOM  10912  C   ARG J 297       0.425  19.863  -2.980  0.00  0.00
J
ATOM  10913  O   ARG J 297       0.693  20.901  -2.415  0.00  0.00
J
ATOM  10914  N   THR J 298      -0.830  19.559  -3.340  0.00  0.00
J
ATOM  10915  HN  THR J 298      -0.916  18.684  -3.809  0.00  0.00
J
ATOM  10916  CA  THR J 298      -2.096  20.265  -3.061  0.00  0.00
J
ATOM  10917  HA  THR J 298      -2.812  19.481  -3.257  0.00  0.00
J
ATOM  10918  CB  THR J 298      -2.263  20.795  -1.631  0.00  0.00
J
ATOM  10919  HB  THR J 298      -1.542  21.603  -1.385  0.00  0.00
J
ATOM  10920  OG1 THR J 298      -2.056  19.772  -0.628  0.00  0.00
J
ATOM  10921  HG1 THR J 298      -1.503  19.160  -1.119  0.00  0.00
J
ATOM  10922  CG2 THR J 298      -3.727  21.138  -1.506  0.00  0.00
J
ATOM  10923 HG21 THR J 298      -4.043  21.277  -0.450  0.00  0.00
J
ATOM  10924 HG22 THR J 298      -4.052  22.058  -2.036  0.00  0.00
J
ATOM  10925 HG23 THR J 298      -4.400  20.304  -1.797  0.00  0.00
J
ATOM  10926  C   THR J 298      -2.430  21.402  -4.138  0.00  0.00
J
ATOM  10927  O   THR J 298      -3.549  21.760  -4.565  0.00  0.00
J
ATOM  10928  N   ALA J 299      -1.367  22.090  -4.536  0.00  0.00
J
ATOM  10929  HN  ALA J 299      -0.477  21.733  -4.264  0.00  0.00
J
ATOM  10930  CA  ALA J 299      -1.383  23.340  -5.237  0.00  0.00
J
ATOM  10931  HA  ALA J 299      -1.995  23.996  -4.636  0.00  0.00
J
ATOM  10932  CB  ALA J 299       0.033  23.829  -5.485  0.00  0.00
J
ATOM  10933  HB1 ALA J 299       0.366  23.971  -4.435  0.00  0.00
J
ATOM  10934  HB2 ALA J 299       0.598  23.082  -6.083  0.00  0.00
J
```

```
ATOM  10935  HB3 ALA J 299       0.228  24.787  -6.013  0.00  0.00
J
ATOM  10936  C   ALA J 299      -2.037  23.264  -6.534  0.00  0.00
J
ATOM  10937  O   ALA J 299      -2.947  24.052  -6.798  0.00  0.00
J
ATOM  10938  N   GLY J 300      -1.762  22.238  -7.376  0.00  0.00
J
ATOM  10939  HN  GLY J 300      -0.993  21.646  -7.147  0.00  0.00
J
ATOM  10940  CA  GLY J 300      -2.631  21.735  -8.420  0.00  0.00
J
ATOM  10941  HA1 GLY J 300      -3.689  21.790  -8.212  0.00  0.00
J
ATOM  10942  HA2 GLY J 300      -2.360  20.699  -8.562  0.00  0.00
J
ATOM  10943  C   GLY J 300      -2.580  22.382  -9.790  0.00  0.00
J
ATOM  10944  O   GLY J 300      -3.243  21.928 -10.719  0.00  0.00
J
ATOM  10945  N   PHE J 301      -1.794  23.453  -9.944  0.00  0.00
J
ATOM  10946  HN  PHE J 301      -1.425  23.805  -9.088  0.00  0.00
J
ATOM  10947  CA  PHE J 301      -1.634  24.112 -11.247  0.00  0.00
J
ATOM  10948  HA  PHE J 301      -2.603  24.441 -11.593  0.00  0.00
J
ATOM  10949  CB  PHE J 301      -0.725  25.291 -11.133  0.00  0.00
J
ATOM  10950  HB1 PHE J 301       0.244  24.982 -10.685  0.00  0.00
J
ATOM  10951  HB2 PHE J 301      -0.521  25.809 -12.095  0.00  0.00
J
ATOM  10952  CG  PHE J 301      -1.412  26.391 -10.381  0.00  0.00
J
ATOM  10953  CD1 PHE J 301      -1.117  26.660  -9.015  0.00  0.00
J
ATOM  10954  HD1 PHE J 301      -0.299  26.087  -8.605  0.00  0.00
J
ATOM  10955  CE1 PHE J 301      -1.674  27.727  -8.392  0.00  0.00
J
ATOM  10956  HE1 PHE J 301      -1.507  28.013  -7.364  0.00  0.00
J
ATOM  10957  CZ  PHE J 301      -2.559  28.595  -9.025  0.00  0.00
J
ATOM  10958  HZ  PHE J 301      -3.079  29.286  -8.378  0.00  0.00
J
ATOM  10959  CD2 PHE J 301      -2.400  27.191 -11.036  0.00  0.00
J
ATOM  10960  HD2 PHE J 301      -2.790  27.034 -12.031  0.00  0.00
J
ATOM  10961  CE2 PHE J 301      -2.886  28.321 -10.348  0.00  0.00
J
```

ATOM 10962 HE2 PHE J 301   -3.619 28.961 -10.816 0.00 0.00
J
ATOM 10963 C   PHE J 301   -1.077 23.165 -12.388 0.00 0.00
J
ATOM 10964 O   PHE J 301   0.012 22.685 -12.315 0.00 0.00
J
ATOM 10965 N   ASN J 302   -1.773 22.934 -13.460 0.00 0.00
J
ATOM 10966 HN ASN J 302   -2.652 23.399 -13.522 0.00 0.00
J
ATOM 10967 CA ASN J 302   -1.425 21.998 -14.489 0.00 0.00
J
ATOM 10968 HA ASN J 302   -0.902 21.185 -14.008 0.00 0.00
J
ATOM 10969 CB ASN J 302   -2.707 21.513 -15.172 0.00 0.00
J
ATOM 10970 HB1 ASN J 302   -3.313 22.408 -15.428 0.00 0.00
J
ATOM 10971 HB2 ASN J 302   -2.542 21.025 -16.156 0.00 0.00
J
ATOM 10972 CG ASN J 302   -3.612 20.570 -14.347 0.00 0.00
J
ATOM 10973 OD1 ASN J 302   -3.502 19.381 -14.397 0.00 0.00
J
ATOM 10974 ND2 ASN J 302   -4.680 21.133 -13.766 0.00 0.00
J
ATOM 10975 HD21 ASN J 302   -5.242 20.687 -13.070 0.00 0.00
J
ATOM 10976 HD22 ASN J 302   -4.766 22.128 -13.720 0.00 0.00
J
ATOM 10977 C   ASN J 302   -0.525 22.629 -15.527 0.00 0.00
J
ATOM 10978 O   ASN J 302   -0.739 22.660 -16.732 0.00 0.00
J
ATOM 10979 N   SER J 303   0.632 23.136 -15.055 0.00 0.00
J
ATOM 10980 HN SER J 303   0.893 23.204 -14.095 0.00 0.00
J
ATOM 10981 CA SER J 303   1.401 23.989 -15.917 0.00 0.00
J
ATOM 10982 HA SER J 303   0.718 24.681 -16.387 0.00 0.00
J
ATOM 10983 CB SER J 303   2.347 24.890 -15.079 0.00 0.00
J
ATOM 10984 HB1 SER J 303   3.307 24.392 -14.821 0.00 0.00
J
ATOM 10985 HB2 SER J 303   2.461 25.795 -15.712 0.00 0.00
J
ATOM 10986 OG SER J 303   1.807 25.173 -13.756 0.00 0.00
J
ATOM 10987 HG1 SER J 303   1.233 25.934 -13.866 0.00 0.00
J
ATOM 10988 C   SER J 303   2.241 23.323 -16.930 0.00 0.00
J

ATOM 10989 O SER J 303 2.220 23.682 −18.099 0.00 0.00
J
ATOM 10990 N LEU J 304 2.923 22.283 −16.533 0.00 0.00
J
ATOM 10991 HN LEU J 304 2.919 22.028 −15.569 0.00 0.00
J
ATOM 10992 CA LEU J 304 3.547 21.381 −17.492 0.00 0.00
J
ATOM 10993 HA LEU J 304 4.038 21.959 −18.261 0.00 0.00
J
ATOM 10994 CB LEU J 304 4.496 20.367 −16.758 0.00 0.00
J
ATOM 10995 HB1 LEU J 304 3.943 19.797 −15.980 0.00 0.00
J
ATOM 10996 HB2 LEU J 304 4.935 19.676 −17.509 0.00 0.00
J
ATOM 10997 CG LEU J 304 5.728 20.887 −15.953 0.00 0.00
J
ATOM 10998 HG LEU J 304 5.351 21.311 −14.998 0.00 0.00
J
ATOM 10999 CD1 LEU J 304 6.822 19.819 −15.700 0.00 0.00
J
ATOM 11000 HD11 LEU J 304 7.639 20.169 −15.034 0.00 0.00
J
ATOM 11001 HD12 LEU J 304 6.319 18.953 −15.219 0.00 0.00
J
ATOM 11002 HD13 LEU J 304 7.411 19.420 −16.552 0.00 0.00
J
ATOM 11003 CD2 LEU J 304 6.424 22.192 −16.606 0.00 0.00
J
ATOM 11004 HD21 LEU J 304 5.804 23.064 −16.308 0.00 0.00
J
ATOM 11005 HD22 LEU J 304 7.358 22.418 −16.050 0.00 0.00
J
ATOM 11006 HD23 LEU J 304 6.625 22.192 −17.699 0.00 0.00
J
ATOM 11007 C LEU J 304 2.459 20.531 −18.210 0.00 0.00
J
ATOM 11008 O LEU J 304 2.639 20.175 −19.329 0.00 0.00
J
ATOM 11009 N ASP J 305 1.377 20.117 −17.467 0.00 0.00
J
ATOM 11010 HN ASP J 305 1.276 20.456 −16.534 0.00 0.00
J
ATOM 11011 CA ASP J 305 0.440 19.208 −17.943 0.00 0.00
J
ATOM 11012 HA ASP J 305 −0.360 19.340 −17.229 0.00 0.00
J
ATOM 11013 CB ASP J 305 −0.333 19.651 −19.240 0.00 0.00
J
ATOM 11014 HB1 ASP J 305 −0.777 20.656 −19.073 0.00 0.00
J
ATOM 11015 HB2 ASP J 305 0.307 19.611 −20.148 0.00 0.00
J

ATOM 11016 CG ASP J 305 -1.391 18.548 -19.444 0.00 0.00
J
ATOM 11017 OD1 ASP J 305 -2.477 18.587 -18.757 0.00 0.00
J
ATOM 11018 OD2 ASP J 305 -1.184 17.587 -20.194 0.00 0.00
J
ATOM 11019 C ASP J 305 0.978 17.789 -17.780 0.00 0.00
J
ATOM 11020 O ASP J 305 1.943 17.320 -18.372 0.00 0.00
J
ATOM 11021 N PHE J 306 0.278 16.944 -16.970 0.00 0.00
J
ATOM 11022 HN PHE J 306 -0.684 17.070 -16.741 0.00 0.00
J
ATOM 11023 CA PHE J 306 0.742 15.619 -16.565 0.00 0.00
J
ATOM 11024 HA PHE J 306 1.801 15.494 -16.738 0.00 0.00
J
ATOM 11025 CB PHE J 306 0.428 15.359 -15.027 0.00 0.00
J
ATOM 11026 HB1 PHE J 306 -0.641 15.640 -14.921 0.00 0.00
J
ATOM 11027 HB2 PHE J 306 0.722 14.314 -14.792 0.00 0.00
J
ATOM 11028 CG PHE J 306 1.165 16.307 -14.118 0.00 0.00
J
ATOM 11029 CD1 PHE J 306 0.694 17.633 -13.866 0.00 0.00
J
ATOM 11030 HD1 PHE J 306 -0.092 18.090 -14.448 0.00 0.00
J
ATOM 11031 CE1 PHE J 306 1.299 18.496 -12.921 0.00 0.00
J
ATOM 11032 HE1 PHE J 306 0.755 19.265 -12.393 0.00 0.00
J
ATOM 11033 CZ PHE J 306 2.424 18.005 -12.231 0.00 0.00
J
ATOM 11034 HZ PHE J 306 2.770 18.612 -11.407 0.00 0.00
J
ATOM 11035 CD2 PHE J 306 2.282 15.929 -13.406 0.00 0.00
J
ATOM 11036 HD2 PHE J 306 2.649 14.943 -13.649 0.00 0.00
J
ATOM 11037 CE2 PHE J 306 2.878 16.741 -12.459 0.00 0.00
J
ATOM 11038 HE2 PHE J 306 3.712 16.250 -11.980 0.00 0.00
J
ATOM 11039 C PHE J 306 0.128 14.599 -17.487 0.00 0.00
J
ATOM 11040 O PHE J 306 0.316 13.387 -17.333 0.00 0.00
J
ATOM 11041 N GLY J 307 -0.596 15.046 -18.604 0.00 0.00
J
ATOM 11042 HN GLY J 307 -0.755 16.000 -18.846 0.00 0.00
J

ATOM 11043 CA GLY J 307 -1.261 14.137 -19.478 0.00 0.00
J
ATOM 11044 HA1 GLY J 307 -1.975 14.571 -20.163 0.00 0.00
J
ATOM 11045 HA2 GLY J 307 -1.711 13.362 -18.875 0.00 0.00
J
ATOM 11046 C GLY J 307 -0.244 13.279 -20.271 0.00 0.00
J
ATOM 11047 O GLY J 307 -0.385 12.068 -20.414 0.00 0.00
J
ATOM 11048 N SER J 308 0.854 13.910 -20.752 0.00 0.00
J
ATOM 11049 HN SER J 308 1.009 14.874 -20.550 0.00 0.00
J
ATOM 11050 CA SER J 308 1.906 13.371 -21.605 0.00 0.00
J
ATOM 11051 HA SER J 308 1.421 12.600 -22.185 0.00 0.00
J
ATOM 11052 CB SER J 308 2.534 14.439 -22.565 0.00 0.00
J
ATOM 11053 HB1 SER J 308 3.413 13.933 -23.017 0.00 0.00
J
ATOM 11054 HB2 SER J 308 1.859 14.786 -23.377 0.00 0.00
J
ATOM 11055 OG SER J 308 3.054 15.628 -21.954 0.00 0.00
J
ATOM 11056 HG1 SER J 308 2.463 16.385 -21.959 0.00 0.00
J
ATOM 11057 C SER J 308 3.056 12.683 -20.873 0.00 0.00
J
ATOM 11058 O SER J 308 3.925 12.077 -21.529 0.00 0.00
J
ATOM 11059 N MET J 309 3.071 12.703 -19.520 0.00 0.00
J
ATOM 11060 HN MET J 309 2.379 13.246 -19.051 0.00 0.00
J
ATOM 11061 CA MET J 309 4.184 12.081 -18.829 0.00 0.00
J
ATOM 11062 HA MET J 309 5.107 12.144 -19.384 0.00 0.00
J
ATOM 11063 CB MET J 309 4.254 12.661 -17.399 0.00 0.00
J
ATOM 11064 HB1 MET J 309 3.242 12.749 -16.950 0.00 0.00
J
ATOM 11065 HB2 MET J 309 4.886 12.090 -16.685 0.00 0.00
J
ATOM 11066 CG MET J 309 4.743 14.133 -17.327 0.00 0.00
J
ATOM 11067 HG1 MET J 309 5.628 14.114 -17.999 0.00 0.00
J
ATOM 11068 HG2 MET J 309 3.905 14.673 -17.818 0.00 0.00
J
ATOM 11069 SD MET J 309 5.079 14.798 -15.721 0.00 0.00
J

ATOM 11070 CE MET J 309   4.648 16.573 -16.135 0.00 0.00
J
ATOM 11071 HE1 MET J 309   5.214 17.110 -16.926 0.00 0.00
J
ATOM 11072 HE2 MET J 309   3.569 16.537 -16.395 0.00 0.00
J
ATOM 11073 HE3 MET J 309   4.630 17.230 -15.239 0.00 0.00
J
ATOM 11074 C   MET J 309   4.159 10.593 -18.669 0.00 0.00
J
ATOM 11075 O   MET J 309   3.174   9.938 -19.040 0.00 0.00
J
ATOM 11076 N   ARG J 310   5.274   9.936 -18.179 0.00 0.00
J
ATOM 11077 HN ARG J 310   6.034 10.504 -17.871 0.00 0.00
J
ATOM 11078 CA ARG J 310   5.487   8.504 -18.108 0.00 0.00
J
ATOM 11079 HA ARG J 310   4.990   8.097 -18.976 0.00 0.00
J
ATOM 11080 CB ARG J 310   7.029   8.272 -17.934 0.00 0.00
J
ATOM 11081 HB1 ARG J 310   7.437   8.867 -17.089 0.00 0.00
J
ATOM 11082 HB2 ARG J 310   7.229   7.197 -17.737 0.00 0.00
J
ATOM 11083 CG ARG J 310   7.795   8.605 -19.242 0.00 0.00
J
ATOM 11084 HG1 ARG J 310   7.327   7.893 -19.955 0.00 0.00
J
ATOM 11085 HG2 ARG J 310   7.472   9.593 -19.635 0.00 0.00
J
ATOM 11086 CD ARG J 310   9.313   8.614 -19.267 0.00 0.00
J
ATOM 11087 HD1 ARG J 310   9.651   7.559 -19.182 0.00 0.00
J
ATOM 11088 HD2 ARG J 310   9.681   9.027 -20.230 0.00 0.00
J
ATOM 11089 NE ARG J 310   9.725   9.601 -18.179 0.00 0.00
J
ATOM 11090 HE ARG J 310   9.074 10.223 -17.743 0.00 0.00
J
ATOM 11091 CZ ARG J 310   10.944   9.578 -17.589 0.00 0.00
J
ATOM 11092 NH1 ARG J 310   11.907   8.874 -18.045 0.00 0.00
J
ATOM 11093 HH11 ARG J 310   12.812   9.057 -17.663 0.00 0.00
J
ATOM 11094 HH12 ARG J 310   11.801   8.567 -18.991 0.00 0.00
J
ATOM 11095 NH2 ARG J 310   11.167 10.421 -16.587 0.00 0.00
J
ATOM 11096 HH21 ARG J 310   11.962 10.172 -16.034 0.00 0.00
J

ATOM 11097 HH22 ARG J 310   10.530 11.098 -16.219 0.00 0.00
J
ATOM 11098 C   ARG J 310   4.721   7.888 -16.948 0.00 0.00
J
ATOM 11099 O   ARG J 310   4.701   8.396 -15.808 0.00 0.00
J
ATOM 11100 N   GLU J 311   4.161   6.690 -17.094 0.00 0.00
J
ATOM 11101 HN GLU J 311   4.427   6.193 -17.917 0.00 0.00
J
ATOM 11102 CA GLU J 311   3.423   6.084 -16.042 0.00 0.00
J
ATOM 11103 HA GLU J 311   2.598   6.769 -15.913 0.00 0.00
J
ATOM 11104 CB GLU J 311   2.801   4.743 -16.506 0.00 0.00
J
ATOM 11105 HB1 GLU J 311   3.586   3.977 -16.328 0.00 0.00
J
ATOM 11106 HB2 GLU J 311   1.899   4.407 -15.951 0.00 0.00
J
ATOM 11107 CG GLU J 311   2.382   4.576 -18.001 0.00 0.00
J
ATOM 11108 HG1 GLU J 311   1.760   3.659 -17.925 0.00 0.00
J
ATOM 11109 HG2 GLU J 311   1.760   5.467 -18.232 0.00 0.00
J
ATOM 11110 CD GLU J 311   3.480   4.423 -19.033 0.00 0.00
J
ATOM 11111 OE1 GLU J 311   4.697   4.426 -18.667 0.00 0.00
J
ATOM 11112 OE2 GLU J 311   3.303   4.448 -20.245 0.00 0.00
J
ATOM 11113 C   GLU J 311   4.134   5.926 -14.669 0.00 0.00
J
ATOM 11114 O   GLU J 311   3.483   6.063 -13.640 0.00 0.00
J
ATOM 11115 N   GLY J 312   5.464   5.622 -14.663 0.00 0.00
J
ATOM 11116 HN GLY J 312   5.892   5.259 -15.488 0.00 0.00
J
ATOM 11117 CA GLY J 312   6.348   5.659 -13.541 0.00 0.00
J
ATOM 11118 HA1 GLY J 312   7.352   5.505 -13.907 0.00 0.00
J
ATOM 11119 HA2 GLY J 312   6.100   4.842 -12.879 0.00 0.00
J
ATOM 11120 C   GLY J 312   6.336   6.932 -12.825 0.00 0.00
J
ATOM 11121 O   GLY J 312   6.127   6.981 -11.585 0.00 0.00
J
ATOM 11122 N   THR J 313   6.475   8.062 -13.537 0.00 0.00
J
ATOM 11123 HN THR J 313   6.707   8.062 -14.506 0.00 0.00
J

```
ATOM  11124  CA  THR J 313       6.452   9.414 -12.983  0.00  0.00
J
ATOM  11125  HA  THR J 313       7.166   9.461 -12.175  0.00  0.00
J
ATOM  11126  CB  THR J 313       6.808  10.510 -13.969  0.00  0.00
J
ATOM  11127  HB  THR J 313       6.096  10.520 -14.821  0.00  0.00
J
ATOM  11128  OG1 THR J 313       8.136  10.170 -14.320  0.00  0.00
J
ATOM  11129  HG1 THR J 313       8.634  10.977 -14.470  0.00  0.00
J
ATOM  11130  CG2 THR J 313       6.859  11.902 -13.247  0.00  0.00
J
ATOM  11131 HG21 THR J 313       7.683  11.906 -12.501  0.00  0.00
J
ATOM  11132 HG22 THR J 313       7.194  12.636 -14.010  0.00  0.00
J
ATOM  11133 HG23 THR J 313       5.849  12.171 -12.872  0.00  0.00
J
ATOM  11134  C   THR J 313       5.099   9.660 -12.476  0.00  0.00
J
ATOM  11135  O   THR J 313       5.020  10.286 -11.457  0.00  0.00
J
ATOM  11136  N   ILE J 314       4.002   9.289 -13.164  0.00  0.00
J
ATOM  11137  HN  ILE J 314       3.872   8.764 -14.002  0.00  0.00
J
ATOM  11138  CA  ILE J 314       2.670   9.519 -12.727  0.00  0.00
J
ATOM  11139  HA  ILE J 314       2.503  10.552 -12.460  0.00  0.00
J
ATOM  11140  CB  ILE J 314       1.621   9.100 -13.799  0.00  0.00
J
ATOM  11141  HB  ILE J 314       1.963   8.123 -14.202  0.00  0.00
J
ATOM  11142  CG2 ILE J 314       0.183   9.040 -13.244  0.00  0.00
J
ATOM  11143 HG21 ILE J 314      -0.097   9.951 -12.673  0.00  0.00
J
ATOM  11144 HG22 ILE J 314      -0.496   9.000 -14.122  0.00  0.00
J
ATOM  11145 HG23 ILE J 314       0.013   8.032 -12.810  0.00  0.00
J
ATOM  11146  CG1 ILE J 314       1.760  10.188 -14.951  0.00  0.00
J
ATOM  11147 HG11 ILE J 314       1.350  11.186 -14.689  0.00  0.00
J
ATOM  11148 HG12 ILE J 314       2.850  10.314 -15.130  0.00  0.00
J
ATOM  11149  CD  ILE J 314       1.102   9.785 -16.221  0.00  0.00
J
ATOM  11150  HD1 ILE J 314       1.114   8.703 -16.469  0.00  0.00
J
```

ATOM 11151 HD2 ILE J 314   0.014 10.012 -16.201 0.00 0.00
J
ATOM 11152 HD3 ILE J 314   1.499 10.376 -17.074 0.00 0.00
J
ATOM 11153 C   ILE J 314   2.433   8.780 -11.408 0.00 0.00
J
ATOM 11154 O   ILE J 314   1.765   9.316 -10.525 0.00 0.00
J
ATOM 11155 N   VAL J 315   3.033   7.552 -11.215 0.00 0.00
J
ATOM 11156 HN VAL J 315   3.434   7.018 -11.956 0.00 0.00
J
ATOM 11157 CA VAL J 315   3.039   6.793 -9.968 0.00 0.00
J
ATOM 11158 HA VAL J 315   2.000   6.798 -9.672 0.00 0.00
J
ATOM 11159 CB VAL J 315   3.511   5.315 -10.096 0.00 0.00
J
ATOM 11160 HB VAL J 315   4.398   5.357 -10.764 0.00 0.00
J
ATOM 11161 CG1 VAL J 315   3.824   4.611 -8.750 0.00 0.00
J
ATOM 11162 HG11 VAL J 315   4.244   3.597 -8.921 0.00 0.00
J
ATOM 11163 HG12 VAL J 315   4.656   5.059 -8.167 0.00 0.00
J
ATOM 11164 HG13 VAL J 315   2.882   4.387 -8.205 0.00 0.00
J
ATOM 11165 CG2 VAL J 315   2.376   4.528 -10.779 0.00 0.00
J
ATOM 11166 HG21 VAL J 315   1.503   4.618 -10.099 0.00 0.00
J
ATOM 11167 HG22 VAL J 315   2.209   5.134 -11.695 0.00 0.00
J
ATOM 11168 HG23 VAL J 315   2.646   3.490 -11.071 0.00 0.00
J
ATOM 11169 C   VAL J 315   3.787   7.536 -8.839 0.00 0.00
J
ATOM 11170 O   VAL J 315   3.281   7.756 -7.752 0.00 0.00
J
ATOM 11171 N   PHE J 316   4.952   8.207 -9.098 0.00 0.00
J
ATOM 11172 HN PHE J 316   5.356   8.051 -9.996 0.00 0.00
J
ATOM 11173 CA PHE J 316   5.629   9.146 -8.202 0.00 0.00
J
ATOM 11174 HA PHE J 316   5.736   8.587 -7.284 0.00 0.00
J
ATOM 11175 CB PHE J 316   7.019   9.477 -8.709 0.00 0.00
J
ATOM 11176 HB1 PHE J 316   7.654   8.581 -8.538 0.00 0.00
J
ATOM 11177 HB2 PHE J 316   7.005   9.701 -9.797 0.00 0.00
J

ATOM 11178 CG PHE J 316 7.587 10.620 -7.959 0.00 0.00
J
ATOM 11179 CD1 PHE J 316 7.953 10.473 -6.558 0.00 0.00
J
ATOM 11180 HD1 PHE J 316 7.723 9.517 -6.111 0.00 0.00
J
ATOM 11181 CE1 PHE J 316 8.565 11.529 -5.889 0.00 0.00
J
ATOM 11182 HE1 PHE J 316 8.819 11.372 -4.851 0.00 0.00
J
ATOM 11183 CZ PHE J 316 8.732 12.777 -6.448 0.00 0.00
J
ATOM 11184 HZ PHE J 316 9.092 13.673 -5.964 0.00 0.00
J
ATOM 11185 CD2 PHE J 316 7.758 11.872 -8.485 0.00 0.00
J
ATOM 11186 HD2 PHE J 316 7.544 12.009 -9.534 0.00 0.00
J
ATOM 11187 CE2 PHE J 316 8.294 12.975 -7.763 0.00 0.00
J
ATOM 11188 HE2 PHE J 316 8.342 13.911 -8.299 0.00 0.00
J
ATOM 11189 C PHE J 316 4.675 10.347 -7.889 0.00 0.00
J
ATOM 11190 O PHE J 316 4.469 10.649 -6.764 0.00 0.00
J
ATOM 11191 N THR J 317 4.025 10.952 -8.859 0.00 0.00
J
ATOM 11192 HN THR J 317 4.190 10.561 -9.761 0.00 0.00
J
ATOM 11193 CA THR J 317 3.063 12.063 -8.788 0.00 0.00
J
ATOM 11194 HA THR J 317 3.521 12.858 -8.218 0.00 0.00
J
ATOM 11195 CB THR J 317 2.559 12.562 -10.148 0.00 0.00
J
ATOM 11196 HB THR J 317 1.894 11.837 -10.664 0.00 0.00
J
ATOM 11197 OG1 THR J 317 3.656 12.805 -10.972 0.00 0.00
J
ATOM 11198 HG1 THR J 317 4.244 12.052 -10.873 0.00 0.00
J
ATOM 11199 CG2 THR J 317 1.729 13.906 -9.984 0.00 0.00
J
ATOM 11200 HG21 THR J 317 1.384 14.274 -10.974 0.00 0.00
J
ATOM 11201 HG22 THR J 317 0.768 13.866 -9.429 0.00 0.00
J
ATOM 11202 HG23 THR J 317 2.330 14.713 -9.513 0.00 0.00
J
ATOM 11203 C THR J 317 1.834 11.689 -7.995 0.00 0.00
J
ATOM 11204 O THR J 317 1.367 12.537 -7.224 0.00 0.00
J

```
ATOM  11205  N   LEU J 318       1.329  10.436  -8.082  0.00  0.00
J
ATOM  11206  HN  LEU J 318       1.737   9.734  -8.660  0.00  0.00
J
ATOM  11207  CA  LEU J 318       0.103   9.960  -7.338  0.00  0.00
J
ATOM  11208  HA  LEU J 318      -0.682  10.646  -7.618  0.00  0.00
J
ATOM  11209  CB  LEU J 318      -0.141   8.459  -7.866  0.00  0.00
J
ATOM  11210  HB1 LEU J 318      -0.328   8.464  -8.961  0.00  0.00
J
ATOM  11211  HB2 LEU J 318       0.783   7.857  -7.736  0.00  0.00
J
ATOM  11212  CG  LEU J 318      -1.390   7.810  -7.161  0.00  0.00
J
ATOM  11213  HG  LEU J 318      -1.340   7.844  -6.052  0.00  0.00
J
ATOM  11214  CD1 LEU J 318      -2.525   8.658  -7.523  0.00  0.00
J
ATOM  11215 HD11 LEU J 318      -3.470   8.075  -7.546  0.00  0.00
J
ATOM  11216 HD12 LEU J 318      -2.680   9.404  -6.715  0.00  0.00
J
ATOM  11217 HD13 LEU J 318      -2.438   9.093  -8.542  0.00  0.00
J
ATOM  11218  CD2 LEU J 318      -1.648   6.336  -7.601  0.00  0.00
J
ATOM  11219 HD21 LEU J 318      -2.022   6.203  -8.639  0.00  0.00
J
ATOM  11220 HD22 LEU J 318      -0.658   5.835  -7.640  0.00  0.00
J
ATOM  11221 HD23 LEU J 318      -2.302   5.688  -6.979  0.00  0.00
J
ATOM  11222  C   LEU J 318       0.296  10.004  -5.889  0.00  0.00
J
ATOM  11223  O   LEU J 318      -0.644  10.364  -5.115  0.00  0.00
J
ATOM  11224  N   LEU J 319       1.488   9.657  -5.338  0.00  0.00
J
ATOM  11225  HN  LEU J 319       2.230   9.505  -5.988  0.00  0.00
J
ATOM  11226  CA  LEU J 319       1.853   9.664  -3.954  0.00  0.00
J
ATOM  11227  HA  LEU J 319       1.129   9.186  -3.311  0.00  0.00
J
ATOM  11228  CB  LEU J 319       3.223   8.980  -3.766  0.00  0.00
J
ATOM  11229  HB1 LEU J 319       3.247   7.977  -4.244  0.00  0.00
J
ATOM  11230  HB2 LEU J 319       3.974   9.522  -4.380  0.00  0.00
J
ATOM  11231  CG  LEU J 319       3.746   8.575  -2.344  0.00  0.00
J
```

ATOM 11232 HG LEU J 319   3.779   9.557 -1.826 0.00 0.00
J
ATOM 11233 CD1 LEU J 319   2.776   7.700 -1.575 0.00 0.00
J
ATOM 11234 HD11 LEU J 319   2.361   6.871 -2.187 0.00 0.00
J
ATOM 11235 HD12 LEU J 319   3.256   7.286 -0.662 0.00 0.00
J
ATOM 11236 HD13 LEU J 319   1.804   8.216 -1.426 0.00 0.00
J
ATOM 11237 CD2 LEU J 319   5.151   8.001 -2.324 0.00 0.00
J
ATOM 11238 HD21 LEU J 319   5.889   8.762 -2.657 0.00 0.00
J
ATOM 11239 HD22 LEU J 319   5.296   7.688 -1.268 0.00 0.00
J
ATOM 11240 HD23 LEU J 319   5.234   7.123 -2.999 0.00 0.00
J
ATOM 11241 C   LEU J 319   1.927 11.030 -3.361 0.00 0.00
J
ATOM 11242 O   LEU J 319   1.466 11.346 -2.226 0.00 0.00
J
ATOM 11243 N   LEU J 320   2.504 12.016 -4.095 0.00 0.00
J
ATOM 11244 HN LEU J 320   2.998 11.877 -4.949 0.00 0.00
J
ATOM 11245 CA LEU J 320   2.466 13.390 -3.706 0.00 0.00
J
ATOM 11246 HA LEU J 320   2.860 13.412 -2.700 0.00 0.00
J
ATOM 11247 CB LEU J 320   3.408 14.201 -4.579 0.00 0.00
J
ATOM 11248 HB1 LEU J 320   3.065 14.034 -5.622 0.00 0.00
J
ATOM 11249 HB2 LEU J 320   3.264 15.294 -4.435 0.00 0.00
J
ATOM 11250 CG LEU J 320   4.888 13.810 -4.535 0.00 0.00
J
ATOM 11251 HG LEU J 320   4.997 12.795 -4.973 0.00 0.00
J
ATOM 11252 CD1 LEU J 320   5.753 14.736 -5.440 0.00 0.00
J
ATOM 11253 HD11 LEU J 320   6.857 14.620 -5.475 0.00 0.00
J
ATOM 11254 HD12 LEU J 320   5.349 14.640 -6.471 0.00 0.00
J
ATOM 11255 HD13 LEU J 320   5.691 15.767 -5.032 0.00 0.00
J
ATOM 11256 CD2 LEU J 320   5.580 13.685 -3.162 0.00 0.00
J
ATOM 11257 HD21 LEU J 320   5.099 12.827 -2.647 0.00 0.00
J
ATOM 11258 HD22 LEU J 320   6.669 13.477 -3.240 0.00 0.00
J

```
ATOM  11259 HD23 LEU J 320       5.381  14.621  -2.597  0.00  0.00
J
ATOM  11260  C   LEU J 320       1.159  13.994  -3.568  0.00  0.00
J
ATOM  11261  O   LEU J 320       0.972  14.839  -2.750  0.00  0.00
J
ATOM  11262  N   MET J 321       0.084  13.579  -4.346  0.00  0.00
J
ATOM  11263  HN  MET J 321       0.140  13.008  -5.161  0.00  0.00
J
ATOM  11264  CA  MET J 321      -1.252  14.047  -3.980  0.00  0.00
J
ATOM  11265  HA  MET J 321      -1.175  15.121  -3.890  0.00  0.00
J
ATOM  11266  CB  MET J 321      -2.196  13.722  -5.116  0.00  0.00
J
ATOM  11267  HB1 MET J 321      -2.273  12.629  -5.303  0.00  0.00
J
ATOM  11268  HB2 MET J 321      -3.228  14.027  -4.842  0.00  0.00
J
ATOM  11269  CG  MET J 321      -1.775  14.405  -6.430  0.00  0.00
J
ATOM  11270  HG1 MET J 321      -1.325  15.406  -6.260  0.00  0.00
J
ATOM  11271  HG2 MET J 321      -0.941  13.796  -6.841  0.00  0.00
J
ATOM  11272  SD  MET J 321      -3.106  14.509  -7.676  0.00  0.00
J
ATOM  11273  CE  MET J 321      -2.837  12.831  -8.162  0.00  0.00
J
ATOM  11274  HE1 MET J 321      -3.130  12.120  -7.360  0.00  0.00
J
ATOM  11275  HE2 MET J 321      -3.532  12.539  -8.979  0.00  0.00
J
ATOM  11276  HE3 MET J 321      -1.775  12.583  -8.376  0.00  0.00
J
ATOM  11277  C   MET J 321      -1.837  13.628  -2.625  0.00  0.00
J
ATOM  11278  O   MET J 321      -2.648  14.373  -2.084  0.00  0.00
J
ATOM  11279  N   PHE J 322      -1.376  12.413  -2.076  0.00  0.00
J
ATOM  11280  HN  PHE J 322      -0.639  11.905  -2.516  0.00  0.00
J
ATOM  11281  CA  PHE J 322      -1.956  11.777  -0.964  0.00  0.00
J
ATOM  11282  HA  PHE J 322      -2.983  12.104  -0.897  0.00  0.00
J
ATOM  11283  CB  PHE J 322      -2.116  10.263  -1.164  0.00  0.00
J
ATOM  11284  HB1 PHE J 322      -2.071   9.921  -2.220  0.00  0.00
J
ATOM  11285  HB2 PHE J 322      -1.287   9.699  -0.687  0.00  0.00
J
```

```
ATOM  11286  CG  PHE J 322      -3.437   9.764  -0.592  0.00  0.00
J
ATOM  11287  CD1 PHE J 322      -4.640  10.175  -1.132  0.00  0.00
J
ATOM  11288  HD1 PHE J 322      -4.660  10.848  -1.976  0.00  0.00
J
ATOM  11289  CE1 PHE J 322      -5.856   9.714  -0.715  0.00  0.00
J
ATOM  11290  HE1 PHE J 322      -6.758  10.134  -1.136  0.00  0.00
J
ATOM  11291  CZ  PHE J 322      -5.939   8.917   0.392  0.00  0.00
J
ATOM  11292  HZ  PHE J 322      -6.885   8.633   0.830  0.00  0.00
J
ATOM  11293  CD2 PHE J 322      -3.505   8.771   0.452  0.00  0.00
J
ATOM  11294  HD2 PHE J 322      -2.594   8.435   0.925  0.00  0.00
J
ATOM  11295  CE2 PHE J 322      -4.729   8.429   1.014  0.00  0.00
J
ATOM  11296  HE2 PHE J 322      -4.661   7.840   1.917  0.00  0.00
J
ATOM  11297  C   PHE J 322      -1.265  12.052   0.333  0.00  0.00
J
ATOM  11298  O   PHE J 322      -1.772  11.584   1.317  0.00  0.00
J
ATOM  11299  N   ILE J 323      -0.202  12.873   0.298  0.00  0.00
J
ATOM  11300  HN  ILE J 323       0.183  13.276  -0.528  0.00  0.00
J
ATOM  11301  CA  ILE J 323       0.383  13.276   1.607  0.00  0.00
J
ATOM  11302  HA  ILE J 323      -0.139  12.845   2.448  0.00  0.00
J
ATOM  11303  CB  ILE J 323       1.870  12.954   1.666  0.00  0.00
J
ATOM  11304  HB  ILE J 323       2.371  13.200   0.706  0.00  0.00
J
ATOM  11305  CG2 ILE J 323       2.654  13.724   2.860  0.00  0.00
J
ATOM  11306 HG21 ILE J 323       2.732  14.824   2.727  0.00  0.00
J
ATOM  11307 HG22 ILE J 323       2.218  13.610   3.875  0.00  0.00
J
ATOM  11308 HG23 ILE J 323       3.689  13.326   2.932  0.00  0.00
J
ATOM  11309  CG1 ILE J 323       2.105  11.470   1.741  0.00  0.00
J
ATOM  11310 HG11 ILE J 323       1.826  10.938   0.807  0.00  0.00
J
ATOM  11311 HG12 ILE J 323       3.179  11.214   1.870  0.00  0.00
J
ATOM  11312  CD  ILE J 323       1.270  10.813   2.875  0.00  0.00
J
```

ATOM 11313 HD1 ILE J 323   1.416 11.372   3.825 0.00 0.00
J
ATOM 11314 HD2 ILE J 323   0.173 10.962   2.781 0.00 0.00
J
ATOM 11315 HD3 ILE J 323   1.521   9.749   3.070 0.00 0.00
J
ATOM 11316 C   ILE J 323   0.018 14.749   1.749 0.00 0.00
J
ATOM 11317 O   ILE J 323   0.518 15.596   1.114 0.00 0.00
J
ATOM 11318 N   GLY J 324   -0.788 15.021   2.748 0.00 0.00
J
ATOM 11319 HN GLY J 324   -1.173 14.254   3.254 0.00 0.00
J
ATOM 11320 CA GLY J 324   -1.097 16.405   3.168 0.00 0.00
J
ATOM 11321 HA1 GLY J 324   -1.966 16.316   3.803 0.00 0.00
J
ATOM 11322 HA2 GLY J 324   -1.162 16.987   2.260 0.00 0.00
J
ATOM 11323 C   GLY J 324   -0.010 17.038   4.037 0.00 0.00
J
ATOM 11324 O   GLY J 324   1.205 16.834   4.004 0.00 0.00
J
ATOM 11325 N   ALA J 325   -0.520 17.883   4.987 0.00 0.00
J
ATOM 11326 HN ALA J 325   -1.490 17.923   5.215 0.00 0.00
J
ATOM 11327 CA ALA J 325   0.303 18.689   5.937 0.00 0.00
J
ATOM 11328 HA ALA J 325   1.281 18.293   6.169 0.00 0.00
J
ATOM 11329 CB ALA J 325   0.571 20.010   5.254 0.00 0.00
J
ATOM 11330 HB1 ALA J 325   1.179 19.883   4.332 0.00 0.00
J
ATOM 11331 HB2 ALA J 325   -0.386 20.502   4.979 0.00 0.00
J
ATOM 11332 HB3 ALA J 325   1.170 20.734   5.848 0.00 0.00
J
ATOM 11333 C   ALA J 325   -0.283 18.843   7.338 0.00 0.00
J
ATOM 11334 O   ALA J 325   -0.424 17.885   8.061 0.00 0.00
J
ATOM 11335 N   GLY J 326   -0.670 20.145   7.680 0.00 0.00
J
ATOM 11336 HN GLY J 326   -0.691 20.879   7.005 0.00 0.00
J
ATOM 11337 CA GLY J 326   -1.395 20.449   8.892 0.00 0.00
J
ATOM 11338 HA1 GLY J 326   -1.286 21.510   9.063 0.00 0.00
J
ATOM 11339 HA2 GLY J 326   -0.876 19.913   9.673 0.00 0.00
J

ATOM 11340 C   GLY J 326   -2.840 19.977   9.158 0.00 0.00
J
ATOM 11341 O   GLY J 326   -3.599 19.471   8.328 0.00 0.00
J
ATOM 11342 N   SER J 327   -3.205 20.185 10.415 0.00 0.00
J
ATOM 11343 HN SER J 327   -2.519 20.607 11.003 0.00 0.00
J
ATOM 11344 CA SER J 327   -4.474 19.811 11.055 0.00 0.00
J
ATOM 11345 HA SER J 327   -4.543 18.734 11.021 0.00 0.00
J
ATOM 11346 CB SER J 327   -4.427 20.149 12.535 0.00 0.00
J
ATOM 11347 HB1 SER J 327   -5.332 19.808 13.083 0.00 0.00
J
ATOM 11348 HB2 SER J 327   -3.630 19.509 12.971 0.00 0.00
J
ATOM 11349 OG SER J 327   -4.374 21.540 12.780 0.00 0.00
J
ATOM 11350 HG1 SER J 327   -4.471 21.645 13.730 0.00 0.00
J
ATOM 11351 C   SER J 327   -5.770 20.510 10.407 0.00 0.00
J
ATOM 11352 O   SER J 327   -6.863 19.902 10.213 0.00 0.00
J
ATOM 11353 N   ALA J 328   -5.654 21.782   9.982 0.00 0.00
J
ATOM 11354 HN ALA J 328   -4.743 22.186   9.974 0.00 0.00
J
ATOM 11355 CA ALA J 328   -6.716 22.634   9.584 0.00 0.00
J
ATOM 11356 HA ALA J 328   -7.579 22.065   9.271 0.00 0.00
J
ATOM 11357 CB ALA J 328   -7.091 23.517 10.823 0.00 0.00
J
ATOM 11358 HB1 ALA J 328   -7.632 22.775 11.449 0.00 0.00
J
ATOM 11359 HB2 ALA J 328   -6.158 23.809 11.351 0.00 0.00
J
ATOM 11360 HB3 ALA J 328   -7.765 24.351 10.532 0.00 0.00
J
ATOM 11361 C   ALA J 328   -6.281 23.478   8.493 0.00 0.00
J
ATOM 11362 O   ALA J 328   -6.809 24.632   8.330 0.00 0.00
J
ATOM 11363 N   SER J 329   -5.388 22.988   7.617 0.00 0.00
J
ATOM 11364 HN SER J 329   -4.811 22.191   7.776 0.00 0.00
J
ATOM 11365 CA SER J 329   -5.158 23.560   6.399 0.00 0.00
J
ATOM 11366 HA SER J 329   -5.416 24.609   6.388 0.00 0.00
J

ATOM 11367 CB SER J 329   -3.713 23.475   6.185 0.00 0.00
J
ATOM 11368 HB1 SER J 329   -3.478 23.920   5.195 0.00 0.00
J
ATOM 11369 HB2 SER J 329   -3.138 24.156   6.848 0.00 0.00
J
ATOM 11370 OG SER J 329   -3.261 22.109   6.212 0.00 0.00
J
ATOM 11371 HG1 SER J 329   -2.416 22.170   5.761 0.00 0.00
J
ATOM 11372 C   SER J 329   -5.905 22.937   5.183 0.00 0.00
J
ATOM 11373 O   SER J 329   -6.752 22.029   5.294 0.00 0.00
J
ATOM 11374 N   THR J 330   -5.741 23.582   3.984 0.00 0.00
J
ATOM 11375 HN THR J 330   -4.941 24.170   3.894 0.00 0.00
J
ATOM 11376 CA THR J 330   -6.472 23.191   2.761 0.00 0.00
J
ATOM 11377 HA THR J 330   -7.497 22.932   2.983 0.00 0.00
J
ATOM 11378 CB THR J 330   -6.377 24.304   1.648 0.00 0.00
J
ATOM 11379 HB THR J 330   -6.951 23.962   0.761 0.00 0.00
J
ATOM 11380 OG1 THR J 330   -5.046 24.630   1.307 0.00 0.00
J
ATOM 11381 HG1 THR J 330   -5.174 25.217   0.558 0.00 0.00
J
ATOM 11382 CG2 THR J 330   -6.923 25.674   2.191 0.00 0.00
J
ATOM 11383 HG21 THR J 330   -8.017 25.517   2.076 0.00 0.00
J
ATOM 11384 HG22 THR J 330   -6.716 25.969   3.242 0.00 0.00
J
ATOM 11385 HG23 THR J 330   -6.633 26.467   1.470 0.00 0.00
J
ATOM 11386 C   THR J 330   -5.885 21.879   2.258 0.00 0.00
J
ATOM 11387 O   THR J 330   -6.505 21.076   1.624 0.00 0.00
J
ATOM 11388 N   ALA J 331   -4.591 21.596   2.615 0.00 0.00
J
ATOM 11389 HN ALA J 331   -4.048 22.334   3.006 0.00 0.00
J
ATOM 11390 CA ALA J 331   -3.937 20.347   2.250 0.00 0.00
J
ATOM 11391 HA ALA J 331   -4.120 20.251   1.190 0.00 0.00
J
ATOM 11392 CB ALA J 331   -2.414 20.323   2.483 0.00 0.00
J
ATOM 11393 HB1 ALA J 331   -2.199 20.579   3.543 0.00 0.00
J

ATOM 11394 HB2 ALA J 331 -1.865 19.379 2.275 0.00 0.00
J
ATOM 11395 HB3 ALA J 331 -2.176 21.160 1.793 0.00 0.00
J
ATOM 11396 C ALA J 331 -4.613 19.193 2.927 0.00 0.00
J
ATOM 11397 O ALA J 331 -5.095 19.247 4.060 0.00 0.00
J
ATOM 11398 N SER J 332 -4.689 18.073 2.162 0.00 0.00
J
ATOM 11399 HN SER J 332 -4.275 18.153 1.258 0.00 0.00
J
ATOM 11400 CA SER J 332 -5.325 16.873 2.636 0.00 0.00
J
ATOM 11401 HA SER J 332 -5.087 16.776 3.685 0.00 0.00
J
ATOM 11402 CB SER J 332 -6.838 16.876 2.428 0.00 0.00
J
ATOM 11403 HB1 SER J 332 -7.092 17.816 2.962 0.00 0.00
J
ATOM 11404 HB2 SER J 332 -7.056 17.004 1.347 0.00 0.00
J
ATOM 11405 OG SER J 332 -7.450 15.769 3.162 0.00 0.00
J
ATOM 11406 HG1 SER J 332 -8.402 15.764 3.040 0.00 0.00
J
ATOM 11407 C SER J 332 -4.667 15.617 2.041 0.00 0.00
J
ATOM 11408 O SER J 332 -3.675 15.728 1.335 0.00 0.00
J
ATOM 11409 N GLY J 333 -5.139 14.443 2.517 0.00 0.00
J
ATOM 11410 HN GLY J 333 -5.979 14.382 3.051 0.00 0.00
J
ATOM 11411 CA GLY J 333 -4.415 13.166 2.401 0.00 0.00
J
ATOM 11412 HA1 GLY J 333 -3.421 13.356 2.023 0.00 0.00
J
ATOM 11413 HA2 GLY J 333 -4.985 12.545 1.725 0.00 0.00
J
ATOM 11414 C GLY J 333 -4.324 12.343 3.632 0.00 0.00
J
ATOM 11415 O GLY J 333 -5.114 12.609 4.545 0.00 0.00
J
ATOM 11416 N ILE J 334 -3.246 11.657 3.753 0.00 0.00
J
ATOM 11417 HN ILE J 334 -2.731 11.685 2.899 0.00 0.00
J
ATOM 11418 CA ILE J 334 -2.638 11.255 4.989 0.00 0.00
J
ATOM 11419 HA ILE J 334 -3.323 11.144 5.816 0.00 0.00
J
ATOM 11420 CB ILE J 334 -1.904 9.859 4.818 0.00 0.00
J

ATOM 11421 HB ILE J 334   −1.217   9.851   3.946 0.00 0.00
J
ATOM 11422 CG2 ILE J 334   −1.061   9.481   6.031 0.00 0.00
J
ATOM 11423 HG21 ILE J 334   −0.258 10.240   6.149 0.00 0.00
J
ATOM 11424 HG22 ILE J 334   −1.604   9.394   6.996 0.00 0.00
J
ATOM 11425 HG23 ILE J 334   −0.517   8.513   5.999 0.00 0.00
J
ATOM 11426 CG1 ILE J 334   −2.978   8.723   4.486 0.00 0.00
J
ATOM 11427 HG11 ILE J 334   −3.592   8.768   5.411 0.00 0.00
J
ATOM 11428 HG12 ILE J 334   −3.529   8.985   3.557 0.00 0.00
J
ATOM 11429 CD ILE J 334   −2.383   7.331   4.126 0.00 0.00
J
ATOM 11430 HD1 ILE J 334   −1.669   7.055   4.931 0.00 0.00
J
ATOM 11431 HD2 ILE J 334   −2.998   6.445   3.861 0.00 0.00
J
ATOM 11432 HD3 ILE J 334   −1.749   7.469   3.224 0.00 0.00
J
ATOM 11433 C   ILE J 334   −1.727 12.352   5.438 0.00 0.00
J
ATOM 11434 O   ILE J 334   −0.734 12.606   4.776 0.00 0.00
J
ATOM 11435 N   LYS J 335   −2.063 13.083   6.554 0.00 0.00
J
ATOM 11436 HN LYS J 335   −2.743 12.777   7.216 0.00 0.00
J
ATOM 11437 CA LYS J 335   −1.256 14.215   7.035 0.00 0.00
J
ATOM 11438 HA LYS J 335   −1.303 14.960   6.254 0.00 0.00
J
ATOM 11439 CB LYS J 335   −1.979 14.802   8.317 0.00 0.00
J
ATOM 11440 HB1 LYS J 335   −2.118 14.112   9.176 0.00 0.00
J
ATOM 11441 HB2 LYS J 335   −1.387 15.665   8.691 0.00 0.00
J
ATOM 11442 CG LYS J 335   −3.367 15.242   7.839 0.00 0.00
J
ATOM 11443 HG1 LYS J 335   −3.496 15.752   6.860 0.00 0.00
J
ATOM 11444 HG2 LYS J 335   −3.880 14.257   7.847 0.00 0.00
J
ATOM 11445 CD LYS J 335   −4.012 16.072   8.894 0.00 0.00
J
ATOM 11446 HD1 LYS J 335   −4.052 15.520   9.858 0.00 0.00
J
ATOM 11447 HD2 LYS J 335   −3.516 17.021   9.190 0.00 0.00
J

ATOM 11448 CE LYS J 335 −5.432 16.477 8.518 0.00 0.00
J
ATOM 11449 HE1 LYS J 335 −5.879 17.016 9.381 0.00 0.00
J
ATOM 11450 HE2 LYS J 335 −5.626 17.086 7.609 0.00 0.00
J
ATOM 11451 NZ LYS J 335 −6.346 15.339 8.356 0.00 0.00
J
ATOM 11452 HZ1 LYS J 335 −6.319 15.167 7.331 0.00 0.00
J
ATOM 11453 HZ2 LYS J 335 −6.065 14.485 8.879 0.00 0.00
J
ATOM 11454 HZ3 LYS J 335 −7.266 15.695 8.686 0.00 0.00
J
ATOM 11455 C LYS J 335 0.213 13.926 7.290 0.00 0.00
J
ATOM 11456 O LYS J 335 0.637 12.815 7.635 0.00 0.00
J
ATOM 11457 N LEU J 336 1.110 14.878 7.053 0.00 0.00
J
ATOM 11458 HN LEU J 336 0.761 15.631 6.501 0.00 0.00
J
ATOM 11459 CA LEU J 336 2.571 14.764 7.186 0.00 0.00
J
ATOM 11460 HA LEU J 336 3.090 14.096 6.514 0.00 0.00
J
ATOM 11461 CB LEU J 336 3.194 16.151 7.043 0.00 0.00
J
ATOM 11462 HB1 LEU J 336 2.885 16.606 6.078 0.00 0.00
J
ATOM 11463 HB2 LEU J 336 2.774 16.810 7.833 0.00 0.00
J
ATOM 11464 CG LEU J 336 4.683 16.262 7.074 0.00 0.00
J
ATOM 11465 HG LEU J 336 5.077 15.880 8.040 0.00 0.00
J
ATOM 11466 CD1 LEU J 336 5.319 15.488 5.881 0.00 0.00
J
ATOM 11467 HD11 LEU J 336 6.426 15.545 5.799 0.00 0.00
J
ATOM 11468 HD12 LEU J 336 5.023 14.421 5.976 0.00 0.00
J
ATOM 11469 HD13 LEU J 336 4.818 15.818 4.946 0.00 0.00
J
ATOM 11470 CD2 LEU J 336 5.059 17.753 6.900 0.00 0.00
J
ATOM 11471 HD21 LEU J 336 4.444 18.332 6.177 0.00 0.00
J
ATOM 11472 HD22 LEU J 336 4.880 18.213 7.895 0.00 0.00
J
ATOM 11473 HD23 LEU J 336 6.113 17.918 6.592 0.00 0.00
J
ATOM 11474 C LEU J 336 2.970 14.297 8.542 0.00 0.00
J

```
ATOM  11475  O   LEU J 336       3.899  13.522   8.762  0.00  0.00
J
ATOM  11476  N   THR J 337       2.309  14.939   9.586  0.00  0.00
J
ATOM  11477  HN  THR J 337       1.502  15.435   9.276  0.00  0.00
J
ATOM  11478  CA  THR J 337       2.461  14.535  10.929  0.00  0.00
J
ATOM  11479  HA  THR J 337       3.506  14.754  11.093  0.00  0.00
J
ATOM  11480  CB  THR J 337       1.657  15.280  11.997  0.00  0.00
J
ATOM  11481  HB  THR J 337       1.730  14.740  12.964  0.00  0.00
J
ATOM  11482  OG1 THR J 337       0.368  15.499  11.619  0.00  0.00
J
ATOM  11483  HG1 THR J 337      -0.194  15.269  12.363  0.00  0.00
J
ATOM  11484  CG2 THR J 337       2.287  16.639  12.072  0.00  0.00
J
ATOM  11485 HG21 THR J 337       2.298  17.211  11.120  0.00  0.00
J
ATOM  11486 HG22 THR J 337       1.751  17.225  12.849  0.00  0.00
J
ATOM  11487 HG23 THR J 337       3.358  16.606  12.367  0.00  0.00
J
ATOM  11488  C   THR J 337       2.159  13.080  11.167  0.00  0.00
J
ATOM  11489  O   THR J 337       3.010  12.397  11.698  0.00  0.00
J
ATOM  11490  N   THR J 338       1.048  12.535  10.693  0.00  0.00
J
ATOM  11491  HN  THR J 338       0.380  13.114  10.230  0.00  0.00
J
ATOM  11492  CA  THR J 338       0.585  11.176  10.657  0.00  0.00
J
ATOM  11493  HA  THR J 338       0.520  10.793  11.664  0.00  0.00
J
ATOM  11494  CB  THR J 338      -0.825  10.953  10.044  0.00  0.00
J
ATOM  11495  HB  THR J 338      -0.806  11.350   9.007  0.00  0.00
J
ATOM  11496  OG1 THR J 338      -1.802  11.749  10.729  0.00  0.00
J
ATOM  11497  HG1 THR J 338      -2.424  11.915  10.018  0.00  0.00
J
ATOM  11498  CG2 THR J 338      -1.142   9.439  10.243  0.00  0.00
J
ATOM  11499 HG21 THR J 338      -2.114   9.243   9.742  0.00  0.00
J
ATOM  11500 HG22 THR J 338      -0.381   8.731   9.850  0.00  0.00
J
ATOM  11501 HG23 THR J 338      -1.353   9.244  11.316  0.00  0.00
J
```

ATOM 11502 C   THR J 338   1.564 10.339   9.947 0.00 0.00
J
ATOM 11503 O   THR J 338   1.986   9.321 10.526 0.00 0.00
J
ATOM 11504 N   PHE J 339   1.995 10.683   8.700 0.00 0.00
J
ATOM 11505 HN PHE J 339   1.643 11.518   8.285 0.00 0.00
J
ATOM 11506 CA PHE J 339   2.887   9.920   7.825 0.00 0.00
J
ATOM 11507 HA PHE J 339   2.460   8.933   7.720 0.00 0.00
J
ATOM 11508 CB PHE J 339   3.014 10.730   6.469 0.00 0.00
J
ATOM 11509 HB1 PHE J 339   1.984 10.952   6.115 0.00 0.00
J
ATOM 11510 HB2 PHE J 339   3.623 11.652   6.586 0.00 0.00
J
ATOM 11511 CG PHE J 339   3.723   9.918   5.366 0.00 0.00
J
ATOM 11512 CD1 PHE J 339   3.410   8.590   5.184 0.00 0.00
J
ATOM 11513 HD1 PHE J 339   2.547   8.186   5.693 0.00 0.00
J
ATOM 11514 CE1 PHE J 339   4.072   7.774   4.224 0.00 0.00
J
ATOM 11515 HE1 PHE J 339   3.735   6.790   3.933 0.00 0.00
J
ATOM 11516 CZ PHE J 339   5.083   8.335   3.444 0.00 0.00
J
ATOM 11517 HZ PHE J 339   5.724   7.741   2.810 0.00 0.00
J
ATOM 11518 CD2 PHE J 339   4.771 10.383   4.547 0.00 0.00
J
ATOM 11519 HD2 PHE J 339   5.130 11.392   4.689 0.00 0.00
J
ATOM 11520 CE2 PHE J 339   5.335   9.668   3.480 0.00 0.00
J
ATOM 11521 HE2 PHE J 339   6.160 10.059   2.902 0.00 0.00
J
ATOM 11522 C   PHE J 339   4.269   9.663   8.431 0.00 0.00
J
ATOM 11523 O   PHE J 339   4.812   8.557   8.411 0.00 0.00
J
ATOM 11524 N   ILE J 340   4.952 10.632   9.056 0.00 0.00
J
ATOM 11525 HN ILE J 340   4.548 11.543   9.037 0.00 0.00
J
ATOM 11526 CA ILE J 340   6.104 10.332   9.874 0.00 0.00
J
ATOM 11527 HA ILE J 340   6.870   9.832   9.301 0.00 0.00
J
ATOM 11528 CB ILE J 340   6.740 11.724 10.257 0.00 0.00
J

ATOM 11529 HB ILE J 340   6.084 12.332 10.916 0.00 0.00
J
ATOM 11530 CG2 ILE J 340   8.087 11.534 11.089 0.00 0.00
J
ATOM 11531 HG21 ILE J 340   7.859 10.942 12.000 0.00 0.00
J
ATOM 11532 HG22 ILE J 340   8.763 10.949 10.429 0.00 0.00
J
ATOM 11533 HG23 ILE J 340   8.527 12.518 11.357 0.00 0.00
J
ATOM 11534 CG1 ILE J 340   7.164 12.571   9.013 0.00 0.00
J
ATOM 11535 HG11 ILE J 340   8.101 12.165   8.575 0.00 0.00
J
ATOM 11536 HG12 ILE J 340   6.393 12.491   8.217 0.00 0.00
J
ATOM 11537 CD ILE J 340   7.160 14.061   9.323 0.00 0.00
J
ATOM 11538 HD1 ILE J 340   6.189 14.437   9.712 0.00 0.00
J
ATOM 11539 HD2 ILE J 340   7.975 14.314 10.034 0.00 0.00
J
ATOM 11540 HD3 ILE J 340   7.439 14.637   8.415 0.00 0.00
J
ATOM 11541 C   ILE J 340   5.907   9.482 11.177 0.00 0.00
J
ATOM 11542 O   ILE J 340   6.771   8.697 11.428 0.00 0.00
J
ATOM 11543 N   VAL J 341   4.772   9.681 11.905 0.00 0.00
J
ATOM 11544 HN VAL J 341   4.016 10.274 11.638 0.00 0.00
J
ATOM 11545 CA VAL J 341   4.530   9.063 13.164 0.00 0.00
J
ATOM 11546 HA VAL J 341   5.399   9.267 13.771 0.00 0.00
J
ATOM 11547 CB VAL J 341   3.301   9.726 13.868 0.00 0.00
J
ATOM 11548 HB VAL J 341   2.652 10.086 13.041 0.00 0.00
J
ATOM 11549 CG1 VAL J 341   2.440   8.851 14.756 0.00 0.00
J
ATOM 11550 HG11 VAL J 341   2.974   8.471 15.653 0.00 0.00
J
ATOM 11551 HG12 VAL J 341   1.554   9.413 15.118 0.00 0.00
J
ATOM 11552 HG13 VAL J 341   2.212   8.004 14.073 0.00 0.00
J
ATOM 11553 CG2 VAL J 341   3.811 11.016 14.559 0.00 0.00
J
ATOM 11554 HG21 VAL J 341   4.525 11.623 13.962 0.00 0.00
J
ATOM 11555 HG22 VAL J 341   2.991 11.742 14.741 0.00 0.00
J

ATOM 11556 HG23 VAL J 341   4.289 10.716 15.516 0.00 0.00
J
ATOM 11557 C   VAL J 341   4.357   7.518 13.019 0.00 0.00
J
ATOM 11558 O   VAL J 341   4.769   6.639 13.810 0.00 0.00
J
ATOM 11559 N   ILE J 342   3.690   7.078 11.874 0.00 0.00
J
ATOM 11560 HN ILE J 342   3.183   7.776 11.374 0.00 0.00
J
ATOM 11561 CA ILE J 342   3.622   5.655 11.508 0.00 0.00
J
ATOM 11562 HA ILE J 342   3.480   5.133 12.443 0.00 0.00
J
ATOM 11563 CB ILE J 342   2.531   5.218 10.577 0.00 0.00
J
ATOM 11564 HB ILE J 342   2.803   4.161 10.373 0.00 0.00
J
ATOM 11565 CG2 ILE J 342   1.119   5.165 11.322 0.00 0.00
J
ATOM 11566 HG21 ILE J 342   1.093   4.671 12.317 0.00 0.00
J
ATOM 11567 HG22 ILE J 342   0.803   6.228 11.399 0.00 0.00
J
ATOM 11568 HG23 ILE J 342   0.408   4.605 10.678 0.00 0.00
J
ATOM 11569 CG1 ILE J 342   2.285   6.077   9.337 0.00 0.00
J
ATOM 11570 HG11 ILE J 342   2.006   7.117   9.609 0.00 0.00
J
ATOM 11571 HG12 ILE J 342   3.326   6.104   8.950 0.00 0.00
J
ATOM 11572 CD ILE J 342   1.365   5.351   8.310 0.00 0.00
J
ATOM 11573 HD1 ILE J 342   0.409   5.018   8.769 0.00 0.00
J
ATOM 11574 HD2 ILE J 342   1.213   6.058   7.467 0.00 0.00
J
ATOM 11575 HD3 ILE J 342   1.954   4.493   7.921 0.00 0.00
J
ATOM 11576 C   ILE J 342   5.038   5.207 11.036 0.00 0.00
J
ATOM 11577 O   ILE J 342   5.499   4.146 11.468 0.00 0.00
J
ATOM 11578 N   LEU J 343   5.830   6.091 10.364 0.00 0.00
J
ATOM 11579 HN LEU J 343   5.627   7.023 10.074 0.00 0.00
J
ATOM 11580 CA LEU J 343   7.219   5.643 10.035 0.00 0.00
J
ATOM 11581 HA LEU J 343   7.228   4.754   9.421 0.00 0.00
J
ATOM 11582 CB LEU J 343   7.900   6.607   9.046 0.00 0.00
J

```
ATOM  11583  HB1 LEU J 343       7.246   6.750   8.159  0.00  0.00
J
ATOM  11584  HB2 LEU J 343       7.957   7.620   9.499  0.00  0.00
J
ATOM  11585  CG  LEU J 343       9.290   6.160   8.711  0.00  0.00
J
ATOM  11586  HG  LEU J 343       9.840   6.101   9.674  0.00  0.00
J
ATOM  11587  CD1 LEU J 343       9.401   4.886   7.868  0.00  0.00
J
ATOM  11588 HD11 LEU J 343       9.126   3.944   8.391  0.00  0.00
J
ATOM  11589 HD12 LEU J 343       8.664   4.880   7.036  0.00  0.00
J
ATOM  11590 HD13 LEU J 343      10.416   4.714   7.451  0.00  0.00
J
ATOM  11591  CD2 LEU J 343       9.943   7.321   7.963  0.00  0.00
J
ATOM  11592 HD21 LEU J 343      10.118   8.112   8.724  0.00  0.00
J
ATOM  11593 HD22 LEU J 343      10.921   7.054   7.508  0.00  0.00
J
ATOM  11594 HD23 LEU J 343       9.255   7.709   7.182  0.00  0.00
J
ATOM  11595  C   LEU J 343       8.084   5.453  11.244  0.00  0.00
J
ATOM  11596  O   LEU J 343       8.760   4.441  11.539  0.00  0.00
J
ATOM  11597  N   THR J 344       8.033   6.436  12.218  0.00  0.00
J
ATOM  11598  HN  THR J 344       7.451   7.223  12.026  0.00  0.00
J
ATOM  11599  CA  THR J 344       8.918   6.431  13.391  0.00  0.00
J
ATOM  11600  HA  THR J 344       9.921   6.100  13.164  0.00  0.00
J
ATOM  11601  CB  THR J 344       9.004   7.743  14.142  0.00  0.00
J
ATOM  11602  HB  THR J 344       9.728   7.660  14.981  0.00  0.00
J
ATOM  11603  OG1 THR J 344       7.736   8.255  14.556  0.00  0.00
J
ATOM  11604  HG1 THR J 344       7.984   8.848  15.269  0.00  0.00
J
ATOM  11605  CG2 THR J 344       9.656   8.736  13.215  0.00  0.00
J
ATOM  11606 HG21 THR J 344       8.966   9.003  12.387  0.00  0.00
J
ATOM  11607 HG22 THR J 344       9.930   9.680  13.733  0.00  0.00
J
ATOM  11608 HG23 THR J 344      10.560   8.272  12.766  0.00  0.00
J
ATOM  11609  C   THR J 344       8.492   5.391  14.452  0.00  0.00
J
```

ATOM 11610 O   THR J 344   9.336   4.946 15.212 0.00 0.00
J
ATOM 11611 N   SER J 345   7.179   4.916 14.427 0.00 0.00
J
ATOM 11612 HN SER J 345   6.621   5.356 13.727 0.00 0.00
J
ATOM 11613 CA SER J 345   6.765   3.809 15.180 0.00 0.00
J
ATOM 11614 HA SER J 345   7.153   3.733 16.185 0.00 0.00
J
ATOM 11615 CB SER J 345   5.210   3.757 15.285 0.00 0.00
J
ATOM 11616 HB1 SER J 345   4.791   3.919 14.269 0.00 0.00
J
ATOM 11617 HB2 SER J 345   4.925   2.746 15.647 0.00 0.00
J
ATOM 11618 OG SER J 345   4.638   4.868 16.086 0.00 0.00
J
ATOM 11619 HG1 SER J 345   4.525   5.603 15.479 0.00 0.00
J
ATOM 11620 C   SER J 345   7.261   2.448 14.689 0.00 0.00
J
ATOM 11621 O   SER J 345   7.473   1.505 15.495 0.00 0.00
J
ATOM 11622 N   VAL J 346   7.424   2.303 13.302 0.00 0.00
J
ATOM 11623 HN VAL J 346   7.280   3.029 12.634 0.00 0.00
J
ATOM 11624 CA VAL J 346   7.859   1.034 12.769 0.00 0.00
J
ATOM 11625 HA VAL J 346   7.488   0.255 13.419 0.00 0.00
J
ATOM 11626 CB VAL J 346   7.407   0.623 11.396 0.00 0.00
J
ATOM 11627 HB VAL J 346   7.758 -0.419 11.237 0.00 0.00
J
ATOM 11628 CG1 VAL J 346   5.830   0.724 11.442 0.00 0.00
J
ATOM 11629 HG11 VAL J 346   5.571   1.802 11.368 0.00 0.00
J
ATOM 11630 HG12 VAL J 346   5.656   0.139 10.514 0.00 0.00
J
ATOM 11631 HG13 VAL J 346   5.419   0.141 12.294 0.00 0.00
J
ATOM 11632 CG2 VAL J 346   7.982   1.568 10.348 0.00 0.00
J
ATOM 11633 HG21 VAL J 346   9.090   1.547 10.424 0.00 0.00
J
ATOM 11634 HG22 VAL J 346   7.783   1.181   9.325 0.00 0.00
J
ATOM 11635 HG23 VAL J 346   7.631   2.621 10.400 0.00 0.00
J
ATOM 11636 C   VAL J 346   9.391   0.905 12.960 0.00 0.00
J

ATOM 11637 O   VAL J 346   9.868 -0.218 13.233 0.00 0.00
J
ATOM 11638 N   ILE J 347   10.127   2.031 12.840 0.00 0.00
J
ATOM 11639 HN ILE J 347   9.688   2.871 12.529 0.00 0.00
J
ATOM 11640 CA ILE J 347   11.498   2.239 13.284 0.00 0.00
J
ATOM 11641 HA ILE J 347   12.159   1.552 12.776 0.00 0.00
J
ATOM 11642 CB ILE J 347   12.076   3.624 12.930 0.00 0.00
J
ATOM 11643 HB ILE J 347   11.294   4.330 13.283 0.00 0.00
J
ATOM 11644 CG2 ILE J 347   13.430   3.879 13.685 0.00 0.00
J
ATOM 11645 HG21 ILE J 347   13.954   4.740 13.217 0.00 0.00
J
ATOM 11646 HG22 ILE J 347   13.366   4.021 14.785 0.00 0.00
J
ATOM 11647 HG23 ILE J 347   14.100   3.003 13.549 0.00 0.00
J
ATOM 11648 CG1 ILE J 347   12.175   3.663 11.422 0.00 0.00
J
ATOM 11649 HG11 ILE J 347   12.870   2.838 11.156 0.00 0.00
J
ATOM 11650 HG12 ILE J 347   11.185   3.507 10.944 0.00 0.00
J
ATOM 11651 CD ILE J 347   12.646   4.917 10.826 0.00 0.00
J
ATOM 11652 HD1 ILE J 347   13.664   5.136 11.213 0.00 0.00
J
ATOM 11653 HD2 ILE J 347   12.971   4.721   9.782 0.00 0.00
J
ATOM 11654 HD3 ILE J 347   11.980   5.806 10.832 0.00 0.00
J
ATOM 11655 C   ILE J 347   11.736   1.959 14.741 0.00 0.00
J
ATOM 11656 O   ILE J 347   12.754   1.332 15.144 0.00 0.00
J
ATOM 11657 N   ALA J 348   10.798   2.322 15.662 0.00 0.00
J
ATOM 11658 HN ALA J 348   9.990   2.834 15.381 0.00 0.00
J
ATOM 11659 CA ALA J 348   10.930   2.104 17.084 0.00 0.00
J
ATOM 11660 HA ALA J 348   11.837   2.630 17.345 0.00 0.00
J
ATOM 11661 CB ALA J 348   9.694   2.658 17.716 0.00 0.00
J
ATOM 11662 HB1 ALA J 348   8.841   1.972 17.524 0.00 0.00
J
ATOM 11663 HB2 ALA J 348   9.718   2.717 18.825 0.00 0.00
J

```
ATOM  11664  HB3 ALA J 348       9.403   3.674  17.375  0.00  0.00
J
ATOM  11665  C   ALA J 348      11.007   0.612  17.305  0.00  0.00
J
ATOM  11666  O   ALA J 348      11.950   0.147  17.885  0.00  0.00
J
ATOM  11667  N   TYR J 349      10.059  -0.097  16.660  0.00  0.00
J
ATOM  11668  HN  TYR J 349       9.375   0.412  16.144  0.00  0.00
J
ATOM  11669  CA  TYR J 349       9.997  -1.565  16.601  0.00  0.00
J
ATOM  11670  HA  TYR J 349       9.876  -1.887  17.625  0.00  0.00
J
ATOM  11671  CB  TYR J 349       8.672  -2.050  15.754  0.00  0.00
J
ATOM  11672  HB1 TYR J 349       7.845  -1.606  16.348  0.00  0.00
J
ATOM  11673  HB2 TYR J 349       8.790  -1.682  14.713  0.00  0.00
J
ATOM  11674  CG  TYR J 349       8.628  -3.586  15.759  0.00  0.00
J
ATOM  11675  CD1 TYR J 349       8.408  -4.177  16.984  0.00  0.00
J
ATOM  11676  HD1 TYR J 349       8.120  -3.600  17.851  0.00  0.00
J
ATOM  11677  CE1 TYR J 349       8.408  -5.573  17.152  0.00  0.00
J
ATOM  11678  HE1 TYR J 349       7.982  -5.997  18.049  0.00  0.00
J
ATOM  11679  CZ  TYR J 349       8.569  -6.366  16.001  0.00  0.00
J
ATOM  11680  OH  TYR J 349       8.421  -7.785  16.143  0.00  0.00
J
ATOM  11681  HH  TYR J 349       8.336  -7.996  17.076  0.00  0.00
J
ATOM  11682  CD2 TYR J 349       8.877  -4.411  14.644  0.00  0.00
J
ATOM  11683  HD2 TYR J 349       9.205  -4.054  13.679  0.00  0.00
J
ATOM  11684  CE2 TYR J 349       8.977  -5.779  14.774  0.00  0.00
J
ATOM  11685  HE2 TYR J 349       9.224  -6.439  13.955  0.00  0.00
J
ATOM  11686  C   TYR J 349      11.253  -2.234  16.053  0.00  0.00
J
ATOM  11687  O   TYR J 349      11.773  -3.156  16.663  0.00  0.00
J
ATOM  11688  N   LEU J 350      11.806  -1.757  14.912  0.00  0.00
J
ATOM  11689  HN  LEU J 350      11.448  -0.973  14.411  0.00  0.00
J
ATOM  11690  CA  LEU J 350      13.116  -2.280  14.431  0.00  0.00
J
```

ATOM 11691 HA LEU J 350   13.012 -3.339 14.250 0.00 0.00
J
ATOM 11692 CB LEU J 350   13.444 -1.669 13.031 0.00 0.00
J
ATOM 11693 HB1 LEU J 350   12.477 -1.644 12.484 0.00 0.00
J
ATOM 11694 HB2 LEU J 350   13.649 -0.579 13.091 0.00 0.00
J
ATOM 11695 CG LEU J 350   14.600 -2.294 12.327 0.00 0.00
J
ATOM 11696 HG LEU J 350   14.706 -3.379 12.541 0.00 0.00
J
ATOM 11697 CD1 LEU J 350   14.344 -1.993 10.797 0.00 0.00
J
ATOM 11698 HD11 LEU J 350   13.322 -2.339 10.533 0.00 0.00
J
ATOM 11699 HD12 LEU J 350   14.473 -0.897 10.670 0.00 0.00
J
ATOM 11700 HD13 LEU J 350   15.064 -2.536 10.148 0.00 0.00
J
ATOM 11701 CD2 LEU J 350   16.009 -1.705 12.605 0.00 0.00
J
ATOM 11702 HD21 LEU J 350   16.272 -1.927 13.661 0.00 0.00
J
ATOM 11703 HD22 LEU J 350   16.695 -2.130 11.843 0.00 0.00
J
ATOM 11704 HD23 LEU J 350   15.782 -0.635 12.407 0.00 0.00
J
ATOM 11705 C   LEU J 350   14.261 -1.984 15.485 0.00 0.00
J
ATOM 11706 O   LEU J 350   15.102 -2.906 15.801 0.00 0.00
J
ATOM 11707 N   ARG J 351   14.297 -0.905 16.262 0.00 0.00
J
ATOM 11708 HN ARG J 351   13.569 -0.225 16.222 0.00 0.00
J
ATOM 11709 CA ARG J 351   15.164 -0.827 17.364 0.00 0.00
J
ATOM 11710 HA ARG J 351   16.135 -1.209 17.087 0.00 0.00
J
ATOM 11711 CB ARG J 351   15.352   0.673 17.807 0.00 0.00
J
ATOM 11712 HB1 ARG J 351   14.281   0.960 17.884 0.00 0.00
J
ATOM 11713 HB2 ARG J 351   15.864   0.677 18.792 0.00 0.00
J
ATOM 11714 CG ARG J 351   16.155   1.522 16.815 0.00 0.00
J
ATOM 11715 HG1 ARG J 351   17.058   0.945 16.521 0.00 0.00
J
ATOM 11716 HG2 ARG J 351   15.703   1.520 15.800 0.00 0.00
J
ATOM 11717 CD ARG J 351   16.396   2.954 17.386 0.00 0.00
J

ATOM 11718 HD1 ARG J 351   15.520   3.367 17.929 0.00 0.00
J
ATOM 11719 HD2 ARG J 351   17.223   2.872 18.123 0.00 0.00
J
ATOM 11720 NE ARG J 351   16.784   3.791 16.242 0.00 0.00
J
ATOM 11721 HE ARG J 351   17.756   3.804 16.007 0.00 0.00
J
ATOM 11722 CZ ARG J 351   16.247   4.974 15.922 0.00 0.00
J
ATOM 11723 NH1 ARG J 351   16.707   5.876 15.033 0.00 0.00
J
ATOM 11724 HH11 ARG J 351   16.171   6.720 15.031 0.00 0.00
J
ATOM 11725 HH12 ARG J 351   17.674   5.878 14.781 0.00 0.00
J
ATOM 11726 NH2 ARG J 351   15.096   5.383 16.485 0.00 0.00
J
ATOM 11727 HH21 ARG J 351   14.794   6.332 16.575 0.00 0.00
J
ATOM 11728 HH22 ARG J 351   14.622   4.687 17.025 0.00 0.00
J
ATOM 11729 C   ARG J 351   14.854 -1.587 18.706 0.00 0.00
J
ATOM 11730 O   ARG J 351   15.708 -1.747 19.599 0.00 0.00
J
ATOM 11731 N   GLY J 352   13.652 -2.125 18.831 0.00 0.00
J
ATOM 11732 HN GLY J 352   12.939 -2.081 18.135 0.00 0.00
J
ATOM 11733 CA GLY J 352   13.287 -2.965 20.042 0.00 0.00
J
ATOM 11734 HA1 GLY J 352   14.188 -3.403 20.445 0.00 0.00
J
ATOM 11735 HA2 GLY J 352   12.563 -3.724 19.782 0.00 0.00
J
ATOM 11736 C   GLY J 352   12.594 -2.084 21.110 0.00 0.00
J
ATOM 11737 O   GLY J 352   12.324 -2.429 22.260 0.00 0.00
J
ATOM 11738 N   LYS J 353   12.156 -0.888 20.701 0.00 0.00
J
ATOM 11739 HN LYS J 353   12.296 -0.580 19.763 0.00 0.00
J
ATOM 11740 CA LYS J 353   11.238   0.006 21.395 0.00 0.00
J
ATOM 11741 HA LYS J 353   11.532 -0.113 22.427 0.00 0.00
J
ATOM 11742 CB LYS J 353   11.434   1.468 20.876 0.00 0.00
J
ATOM 11743 HB1 LYS J 353   11.027   1.414 19.843 0.00 0.00
J
ATOM 11744 HB2 LYS J 353   10.736   1.997 21.559 0.00 0.00
J

ATOM 11745 CG LYS J 353 12.860 1.996 20.734 0.00 0.00
J
ATOM 11746 HG1 LYS J 353 13.345 1.346 19.974 0.00 0.00
J
ATOM 11747 HG2 LYS J 353 12.689 3.001 20.292 0.00 0.00
J
ATOM 11748 CD LYS J 353 13.706 1.969 22.035 0.00 0.00
J
ATOM 11749 HD1 LYS J 353 13.049 2.440 22.797 0.00 0.00
J
ATOM 11750 HD2 LYS J 353 13.642 0.895 22.313 0.00 0.00
J
ATOM 11751 CE LYS J 353 15.098 2.571 21.785 0.00 0.00
J
ATOM 11752 HE1 LYS J 353 15.603 2.033 20.955 0.00 0.00
J
ATOM 11753 HE2 LYS J 353 14.985 3.622 21.441 0.00 0.00
J
ATOM 11754 NZ LYS J 353 15.899 2.578 22.997 0.00 0.00
J
ATOM 11755 HZ1 LYS J 353 15.238 2.511 23.796 0.00 0.00
J
ATOM 11756 HZ2 LYS J 353 16.527 1.749 23.014 0.00 0.00
J
ATOM 11757 HZ3 LYS J 353 16.474 3.437 23.109 0.00 0.00
J
ATOM 11758 C LYS J 353 9.790 -0.451 21.458 0.00 0.00
J
ATOM 11759 O LYS J 353 9.212 -0.960 20.492 0.00 0.00
J
ATOM 11760 N LYS J 354 9.176 -0.133 22.656 0.00 0.00
J
ATOM 11761 HN LYS J 354 9.772 0.230 23.369 0.00 0.00
J
ATOM 11762 CA LYS J 354 7.701 -0.244 22.909 0.00 0.00
J
ATOM 11763 HA LYS J 354 7.203 -0.691 22.062 0.00 0.00
J
ATOM 11764 CB LYS J 354 7.503 -1.027 24.232 0.00 0.00
J
ATOM 11765 HB1 LYS J 354 8.149 -1.923 24.115 0.00 0.00
J
ATOM 11766 HB2 LYS J 354 8.006 -0.528 25.087 0.00 0.00
J
ATOM 11767 CG LYS J 354 6.102 -1.401 24.691 0.00 0.00
J
ATOM 11768 HG1 LYS J 354 5.592 -0.540 25.174 0.00 0.00
J
ATOM 11769 HG2 LYS J 354 5.497 -1.584 23.778 0.00 0.00
J
ATOM 11770 CD LYS J 354 5.968 -2.648 25.586 0.00 0.00
J
ATOM 11771 HD1 LYS J 354 4.895 -2.819 25.819 0.00 0.00
J

ATOM 11772 HD2 LYS J 354 6.501 -3.515 25.140 0.00 0.00
J
ATOM 11773 CE LYS J 354 6.672 -2.518 26.999 0.00 0.00
J
ATOM 11774 HE1 LYS J 354 6.571 -3.470 27.562 0.00 0.00
J
ATOM 11775 HE2 LYS J 354 7.772 -2.378 26.925 0.00 0.00
J
ATOM 11776 NZ LYS J 354 6.134 -1.469 27.881 0.00 0.00
J
ATOM 11777 HZ1 LYS J 354 6.445 -0.479 27.804 0.00 0.00
J
ATOM 11778 HZ2 LYS J 354 5.095 -1.506 27.873 0.00 0.00
J
ATOM 11779 HZ3 LYS J 354 6.379 -1.691 28.867 0.00 0.00
J
ATOM 11780 C LYS J 354 7.082 1.135 23.016 0.00 0.00
J
ATOM 11781 O LYS J 354 5.928 1.316 23.279 0.00 0.00
J
ATOM 11782 N GLU J 355 7.894 2.179 22.743 0.00 0.00
J
ATOM 11783 HN GLU J 355 8.868 2.119 22.535 0.00 0.00
J
ATOM 11784 CA GLU J 355 7.425 3.543 22.635 0.00 0.00
J
ATOM 11785 HA GLU J 355 6.347 3.577 22.591 0.00 0.00
J
ATOM 11786 CB GLU J 355 8.000 4.355 23.780 0.00 0.00
J
ATOM 11787 HB1 GLU J 355 9.065 4.114 23.985 0.00 0.00
J
ATOM 11788 HB2 GLU J 355 7.921 5.451 23.613 0.00 0.00
J
ATOM 11789 CG GLU J 355 7.084 4.223 24.994 0.00 0.00
J
ATOM 11790 HG1 GLU J 355 6.028 4.332 24.664 0.00 0.00
J
ATOM 11791 HG2 GLU J 355 7.237 3.272 25.548 0.00 0.00
J
ATOM 11792 CD GLU J 355 7.251 5.342 26.007 0.00 0.00
J
ATOM 11793 OE1 GLU J 355 6.279 6.060 26.362 0.00 0.00
J
ATOM 11794 OE2 GLU J 355 8.391 5.500 26.532 0.00 0.00
J
ATOM 11795 C GLU J 355 8.032 4.202 21.364 0.00 0.00
J
ATOM 11796 O GLU J 355 9.168 3.862 21.022 0.00 0.00
J
ATOM 11797 N THR J 356 7.417 5.300 20.944 0.00 0.00
J
ATOM 11798 HN THR J 356 6.644 5.754 21.380 0.00 0.00
J

```
ATOM  11799  CA  THR J 356       8.018   6.102  19.801  0.00  0.00
J
ATOM  11800  HA  THR J 356       8.874   5.599  19.377  0.00  0.00
J
ATOM  11801  CB  THR J 356       6.937   6.251  18.700  0.00  0.00
J
ATOM  11802  HB  THR J 356       5.975   6.684  19.048  0.00  0.00
J
ATOM  11803  OG1 THR J 356       6.726   4.897  18.275  0.00  0.00
J
ATOM  11804  HG1 THR J 356       6.269   5.011  17.439  0.00  0.00
J
ATOM  11805  CG2 THR J 356       7.385   7.081  17.486  0.00  0.00
J
ATOM  11806 HG21 THR J 356       8.138   6.556  16.861  0.00  0.00
J
ATOM  11807 HG22 THR J 356       6.476   7.112  16.848  0.00  0.00
J
ATOM  11808 HG23 THR J 356       7.624   8.104  17.850  0.00  0.00
J
ATOM  11809  C   THR J 356       8.492   7.453  20.317  0.00  0.00
J
ATOM  11810  O   THR J 356       7.687   8.326  20.812  0.00  0.00
J
ATOM  11811  N   VAL J 357       9.809   7.803  20.213  0.00  0.00
J
ATOM  11812  HN  VAL J 357      10.517   7.103  20.256  0.00  0.00
J
ATOM  11813  CA  VAL J 357      10.342   9.182  20.518  0.00  0.00
J
ATOM  11814  HA  VAL J 357       9.592   9.637  21.149  0.00  0.00
J
ATOM  11815  CB  VAL J 357      11.715   9.056  21.157  0.00  0.00
J
ATOM  11816  HB  VAL J 357      12.344   8.508  20.423  0.00  0.00
J
ATOM  11817  CG1 VAL J 357      12.267  10.467  21.305  0.00  0.00
J
ATOM  11818 HG11 VAL J 357      11.591  11.192  21.807  0.00  0.00
J
ATOM  11819 HG12 VAL J 357      13.255  10.611  21.792  0.00  0.00
J
ATOM  11820 HG13 VAL J 357      12.525  10.844  20.292  0.00  0.00
J
ATOM  11821  CG2 VAL J 357      11.639   8.363  22.442  0.00  0.00
J
ATOM  11822 HG21 VAL J 357      11.096   7.394  22.427  0.00  0.00
J
ATOM  11823 HG22 VAL J 357      12.678   8.244  22.817  0.00  0.00
J
ATOM  11824 HG23 VAL J 357      11.194   9.017  23.221  0.00  0.00
J
ATOM  11825  C   VAL J 357      10.394  10.053  19.261  0.00  0.00
J
```

ATOM 11826 O   VAL J 357   10.891   9.676 18.202 0.00 0.00
J
ATOM 11827 N   ILE J 358   9.943 11.380 19.258 0.00 0.00
J
ATOM 11828 HN ILE J 358   9.506 11.679 20.103 0.00 0.00
J
ATOM 11829 CA ILE J 358   10.137 12.338 18.233 0.00 0.00
J
ATOM 11830 HA ILE J 358   10.930 11.917 17.633 0.00 0.00
J
ATOM 11831 CB ILE J 358   8.913 12.837 17.494 0.00 0.00
J
ATOM 11832 HB ILE J 358   8.243 13.378 18.196 0.00 0.00
J
ATOM 11833 CG2 ILE J 358   9.278 13.966 16.498 0.00 0.00
J
ATOM 11834 HG21 ILE J 358   9.353 14.884 17.120 0.00 0.00
J
ATOM 11835 HG22 ILE J 358   10.220 13.785 15.938 0.00 0.00
J
ATOM 11836 HG23 ILE J 358   8.429 14.093 15.792 0.00 0.00
J
ATOM 11837 CG1 ILE J 358   8.275 11.590 16.723 0.00 0.00
J
ATOM 11838 HG11 ILE J 358   9.106 11.112 16.162 0.00 0.00
J
ATOM 11839 HG12 ILE J 358   7.942 10.885 17.514 0.00 0.00
J
ATOM 11840 CD ILE J 358   7.186 11.886 15.752 0.00 0.00
J
ATOM 11841 HD1 ILE J 358   6.729 10.976 15.307 0.00 0.00
J
ATOM 11842 HD2 ILE J 358   6.411 12.599 16.104 0.00 0.00
J
ATOM 11843 HD3 ILE J 358   7.768 12.291 14.897 0.00 0.00
J
ATOM 11844 C   ILE J 358   10.752 13.603 18.843 0.00 0.00
J
ATOM 11845 O   ILE J 358   10.120 14.317 19.584 0.00 0.00
J
ATOM 11846 N   PHE J 359   12.088 13.785 18.570 0.00 0.00
J
ATOM 11847 HN PHE J 359   12.494 13.197 17.874 0.00 0.00
J
ATOM 11848 CA PHE J 359   12.863 14.936 19.055 0.00 0.00
J
ATOM 11849 HA PHE J 359   13.883 14.660 18.833 0.00 0.00
J
ATOM 11850 CB PHE J 359   12.622 16.298 18.254 0.00 0.00
J
ATOM 11851 HB1 PHE J 359   11.631 16.694 18.563 0.00 0.00
J
ATOM 11852 HB2 PHE J 359   13.309 17.125 18.534 0.00 0.00
J

ATOM 11853 CG PHE J 359 12.778 16.307 16.760 0.00 0.00
J
ATOM 11854 CD1 PHE J 359 11.631 16.381 15.907 0.00 0.00
J
ATOM 11855 HD1 PHE J 359 10.720 16.601 16.443 0.00 0.00
J
ATOM 11856 CE1 PHE J 359 11.724 16.495 14.552 0.00 0.00
J
ATOM 11857 HE1 PHE J 359 10.890 16.791 13.932 0.00 0.00
J
ATOM 11858 CZ PHE J 359 13.024 16.353 14.003 0.00 0.00
J
ATOM 11859 HZ PHE J 359 13.119 16.263 12.931 0.00 0.00
J
ATOM 11860 CD2 PHE J 359 14.092 16.208 16.182 0.00 0.00
J
ATOM 11861 HD2 PHE J 359 14.982 16.135 16.790 0.00 0.00
J
ATOM 11862 CE2 PHE J 359 14.169 16.132 14.777 0.00 0.00
J
ATOM 11863 HE2 PHE J 359 15.185 16.051 14.422 0.00 0.00
J
ATOM 11864 C PHE J 359 12.846 15.148 20.545 0.00 0.00
J
ATOM 11865 O PHE J 359 12.565 16.234 21.031 0.00 0.00
J
ATOM 11866 N ARG J 360 13.271 14.109 21.289 0.00 0.00
J
ATOM 11867 HN ARG J 360 13.686 13.340 20.809 0.00 0.00
J
ATOM 11868 CA ARG J 360 13.444 13.913 22.702 0.00 0.00
J
ATOM 11869 HA ARG J 360 13.745 12.901 22.929 0.00 0.00
J
ATOM 11870 CB ARG J 360 14.544 14.835 23.251 0.00 0.00
J
ATOM 11871 HB1 ARG J 360 14.096 15.815 22.982 0.00 0.00
J
ATOM 11872 HB2 ARG J 360 14.639 14.655 24.343 0.00 0.00
J
ATOM 11873 CG ARG J 360 15.871 14.829 22.425 0.00 0.00
J
ATOM 11874 HG1 ARG J 360 16.539 14.021 22.793 0.00 0.00
J
ATOM 11875 HG2 ARG J 360 15.563 14.535 21.399 0.00 0.00
J
ATOM 11876 CD ARG J 360 16.474 16.178 22.471 0.00 0.00
J
ATOM 11877 HD1 ARG J 360 15.672 16.944 22.399 0.00 0.00
J
ATOM 11878 HD2 ARG J 360 16.954 16.257 23.470 0.00 0.00
J
ATOM 11879 NE ARG J 360 17.469 16.275 21.341 0.00 0.00
J

```
ATOM  11880  HE  ARG J 360      17.332  15.724  20.518  0.00  0.00
J
ATOM  11881  CZ  ARG J 360      18.373  17.209  21.204  0.00  0.00
J
ATOM  11882  NH1 ARG J 360      18.334  18.285  21.965  0.00  0.00
J
ATOM  11883 HH11 ARG J 360      19.007  18.996  21.759  0.00  0.00
J
ATOM  11884 HH12 ARG J 360      17.489  18.532  22.439  0.00  0.00
J
ATOM  11885  NH2 ARG J 360      19.314  17.169  20.250  0.00  0.00
J
ATOM  11886 HH21 ARG J 360      19.962  17.930  20.290  0.00  0.00
J
ATOM  11887 HH22 ARG J 360      19.281  16.318  19.725  0.00  0.00
J
ATOM  11888  C   ARG J 360      12.120  14.144  23.419  0.00  0.00
J
ATOM  11889  O   ARG J 360      12.109  14.484  24.634  0.00  0.00
J
ATOM  11890  N   ARG J 361      10.969  13.951  22.782  0.00  0.00
J
ATOM  11891  HN  ARG J 361      10.930  13.819  21.795  0.00  0.00
J
ATOM  11892  CA  ARG J 361       9.741  13.634  23.402  0.00  0.00
J
ATOM  11893  HA  ARG J 361       9.946  13.355  24.425  0.00  0.00
J
ATOM  11894  CB  ARG J 361       8.823  14.890  23.215  0.00  0.00
J
ATOM  11895  HB1 ARG J 361       8.615  14.991  22.128  0.00  0.00
J
ATOM  11896  HB2 ARG J 361       7.893  14.562  23.727  0.00  0.00
J
ATOM  11897  CG  ARG J 361       9.253  16.223  23.817  0.00  0.00
J
ATOM  11898  HG1 ARG J 361      10.307  16.382  23.504  0.00  0.00
J
ATOM  11899  HG2 ARG J 361       8.649  17.050  23.386  0.00  0.00
J
ATOM  11900  CD  ARG J 361       9.186  16.338  25.351  0.00  0.00
J
ATOM  11901  HD1 ARG J 361       8.126  16.372  25.682  0.00  0.00
J
ATOM  11902  HD2 ARG J 361       9.708  15.510  25.875  0.00  0.00
J
ATOM  11903  NE  ARG J 361       9.862  17.555  25.896  0.00  0.00
J
ATOM  11904  HE  ARG J 361       9.280  18.206  26.384  0.00  0.00
J
ATOM  11905  CZ  ARG J 361      11.168  17.787  26.012  0.00  0.00
J
ATOM  11906  NH1 ARG J 361      11.522  18.940  26.582  0.00  0.00
J
```

ATOM 11907 HH11 ARG J 361   12.463 19.272 26.521 0.00 0.00
J
ATOM 11908 HH12 ARG J 361   10.845 19.667 26.694 0.00 0.00
J
ATOM 11909 NH2 ARG J 361   12.117 16.958 25.613 0.00 0.00
J
ATOM 11910 HH21 ARG J 361   13.070 17.211 25.777 0.00 0.00
J
ATOM 11911 HH22 ARG J 361   11.848 16.010 25.439 0.00 0.00
J
ATOM 11912 C   ARG J 361   9.040 12.445 22.797 0.00 0.00
J
ATOM 11913 O   ARG J 361   8.978 12.193 21.603 0.00 0.00
J
ATOM 11914 N   SER J 362   8.524 11.606 23.695 0.00 0.00
J
ATOM 11915 HN SER J 362   8.464 11.845 24.661 0.00 0.00
J
ATOM 11916 CA SER J 362   7.746 10.381 23.442 0.00 0.00
J
ATOM 11917 HA SER J 362   8.379   9.887 22.719 0.00 0.00
J
ATOM 11918 CB SER J 362   7.502   9.529 24.666 0.00 0.00
J
ATOM 11919 HB1 SER J 362   6.927 10.145 25.389 0.00 0.00
J
ATOM 11920 HB2 SER J 362   6.935   8.658 24.271 0.00 0.00
J
ATOM 11921 OG SER J 362   8.718   9.079 25.191 0.00 0.00
J
ATOM 11922 HG1 SER J 362   8.647   9.035 26.147 0.00 0.00
J
ATOM 11923 C   SER J 362   6.381 10.691 22.846 0.00 0.00
J
ATOM 11924 O   SER J 362   5.838 11.719 23.201 0.00 0.00
J
ATOM 11925 N   ILE J 363   5.740   9.804 22.024 0.00 0.00
J
ATOM 11926 HN ILE J 363   6.147   8.980 21.636 0.00 0.00
J
ATOM 11927 CA ILE J 363   4.369 10.198 21.577 0.00 0.00
J
ATOM 11928 HA ILE J 363   4.145 11.243 21.732 0.00 0.00
J
ATOM 11929 CB ILE J 363   4.267 10.064 20.090 0.00 0.00
J
ATOM 11930 HB ILE J 363   4.668   9.046 19.896 0.00 0.00
J
ATOM 11931 CG2 ILE J 363   2.881 10.084 19.417 0.00 0.00
J
ATOM 11932 HG21 ILE J 363   2.886 10.200 18.312 0.00 0.00
J
ATOM 11933 HG22 ILE J 363   2.515   9.039 19.501 0.00 0.00
J

```
ATOM  11934 HG23 ILE J 363       2.254  10.796  19.995  0.00  0.00
J
ATOM  11935  CG1 ILE J 363       5.281  11.062  19.356  0.00  0.00
J
ATOM  11936 HG11 ILE J 363       6.277  10.942  19.833  0.00  0.00
J
ATOM  11937 HG12 ILE J 363       5.286  10.668  18.317  0.00  0.00
J
ATOM  11938  CD  ILE J 363       4.919  12.572  19.293  0.00  0.00
J
ATOM  11939  HD1 ILE J 363       5.382  13.074  20.169  0.00  0.00
J
ATOM  11940  HD2 ILE J 363       5.336  13.074  18.393  0.00  0.00
J
ATOM  11941  HD3 ILE J 363       3.812  12.621  19.370  0.00  0.00
J
ATOM  11942  C   ILE J 363       3.353   9.400  22.360  0.00  0.00
J
ATOM  11943  O   ILE J 363       3.450   8.188  22.471  0.00  0.00
J
ATOM  11944  N   LYS J 364       2.510  10.175  23.025  0.00  0.00
J
ATOM  11945  HN  LYS J 364       2.535  11.160  22.878  0.00  0.00
J
ATOM  11946  CA  LYS J 364       1.453   9.506  23.820  0.00  0.00
J
ATOM  11947  HA  LYS J 364       1.881   9.062  24.707  0.00  0.00
J
ATOM  11948  CB  LYS J 364       0.600  10.592  24.567  0.00  0.00
J
ATOM  11949  HB1 LYS J 364      -0.137  10.180  25.288  0.00  0.00
J
ATOM  11950  HB2 LYS J 364       1.322  11.148  25.202  0.00  0.00
J
ATOM  11951  CG  LYS J 364      -0.082  11.643  23.746  0.00  0.00
J
ATOM  11952  HG1 LYS J 364       0.687  12.275  23.252  0.00  0.00
J
ATOM  11953  HG2 LYS J 364      -0.608  11.063  22.959  0.00  0.00
J
ATOM  11954  CD  LYS J 364      -1.043  12.482  24.570  0.00  0.00
J
ATOM  11955  HD1 LYS J 364      -1.575  13.069  23.791  0.00  0.00
J
ATOM  11956  HD2 LYS J 364      -1.800  11.882  25.119  0.00  0.00
J
ATOM  11957  CE  LYS J 364      -0.300  13.296  25.635  0.00  0.00
J
ATOM  11958  HE1 LYS J 364       0.110  12.600  26.397  0.00  0.00
J
ATOM  11959  HE2 LYS J 364       0.626  13.782  25.257  0.00  0.00
J
ATOM  11960  NZ  LYS J 364      -1.121  14.371  26.226  0.00  0.00
J
```

ATOM 11961 HZ1 LYS J 364   -0.575 15.049 26.795 0.00 0.00
J
ATOM 11962 HZ2 LYS J 364   -1.547 14.865 25.415 0.00 0.00
J
ATOM 11963 HZ3 LYS J 364   -1.896 14.094 26.862 0.00 0.00
J
ATOM 11964 C   LYS J 364   0.541   8.669 23.046 0.00 0.00
J
ATOM 11965 O   LYS J 364   0.343   8.832 21.818 0.00 0.00
J
ATOM 11966 N   TYR J 365   -0.059   7.625 23.764 0.00 0.00
J
ATOM 11967 HN TYR J 365   0.174   7.571 24.732 0.00 0.00
J
ATOM 11968 CA TYR J 365   -0.779   6.571 23.171 0.00 0.00
J
ATOM 11969 HA TYR J 365   -0.036   6.172 22.497 0.00 0.00
J
ATOM 11970 CB TYR J 365   -1.265   5.562 24.294 0.00 0.00
J
ATOM 11971 HB1 TYR J 365   -0.493   5.382 25.072 0.00 0.00
J
ATOM 11972 HB2 TYR J 365   -2.079   6.011 24.902 0.00 0.00
J
ATOM 11973 CG TYR J 365   -1.642   4.222 23.730 0.00 0.00
J
ATOM 11974 CD1 TYR J 365   -0.665   3.331 23.366 0.00 0.00
J
ATOM 11975 HD1 TYR J 365   0.358   3.652 23.242 0.00 0.00
J
ATOM 11976 CE1 TYR J 365   -0.964   2.054 23.043 0.00 0.00
J
ATOM 11977 HE1 TYR J 365   -0.167   1.402 22.715 0.00 0.00
J
ATOM 11978 CZ TYR J 365   -2.316   1.594 23.087 0.00 0.00
J
ATOM 11979 OH TYR J 365   -2.514   0.209 22.819 0.00 0.00
J
ATOM 11980 HH TYR J 365   -3.446 -0.009 22.742 0.00 0.00
J
ATOM 11981 CD2 TYR J 365   -2.985   3.781 23.840 0.00 0.00
J
ATOM 11982 HD2 TYR J 365   -3.723   4.441 24.271 0.00 0.00
J
ATOM 11983 CE2 TYR J 365   -3.239   2.463 23.568 0.00 0.00
J
ATOM 11984 HE2 TYR J 365   -4.303   2.283 23.550 0.00 0.00
J
ATOM 11985 C   TYR J 365   -1.948   6.881 22.187 0.00 0.00
J
ATOM 11986 O   TYR J 365   -1.877   6.250 21.101 0.00 0.00
J
ATOM 11987 N   PRO J 366   -3.015   7.636 22.421 0.00 0.00
J

ATOM 11988 CD PRO J 366   -3.382   8.136 23.694 0.00 0.00
J
ATOM 11989 HD1 PRO J 366   -3.730   7.245 24.259 0.00 0.00
J
ATOM 11990 HD2 PRO J 366   -2.506   8.532 24.250 0.00 0.00
J
ATOM 11991 CA PRO J 366   -4.044   8.043 21.410 0.00 0.00
J
ATOM 11992 HA PRO J 366   -4.661   7.161 21.316 0.00 0.00
J
ATOM 11993 CB PRO J 366   -4.720   9.297 22.000 0.00 0.00
J
ATOM 11994 HB1 PRO J 366   -5.723   9.571 21.609 0.00 0.00
J
ATOM 11995 HB2 PRO J 366   -3.988 10.130 21.934 0.00 0.00
J
ATOM 11996 CG PRO J 366   -4.666   8.889 23.491 0.00 0.00
J
ATOM 11997 HG1 PRO J 366   -4.780   9.777 24.149 0.00 0.00
J
ATOM 11998 HG2 PRO J 366   -5.564   8.281 23.732 0.00 0.00
J
ATOM 11999 C   PRO J 366   -3.584   8.301 20.012 0.00 0.00
J
ATOM 12000 O   PRO J 366   -4.083   7.686 19.093 0.00 0.00
J
ATOM 12001 N   ILE J 367   -2.653   9.209 19.895 0.00 0.00
J
ATOM 12002 HN ILE J 367   -2.405   9.659 20.749 0.00 0.00
J
ATOM 12003 CA ILE J 367   -2.031   9.717 18.655 0.00 0.00
J
ATOM 12004 HA ILE J 367   -2.767 10.235 18.057 0.00 0.00
J
ATOM 12005 CB ILE J 367   -0.822 10.625 18.956 0.00 0.00
J
ATOM 12006 HB ILE J 367   -0.119 10.045 19.592 0.00 0.00
J
ATOM 12007 CG2 ILE J 367   -0.135 10.964 17.597 0.00 0.00
J
ATOM 12008 HG21 ILE J 367   0.371 10.082 17.149 0.00 0.00
J
ATOM 12009 HG22 ILE J 367   -0.795 11.447 16.845 0.00 0.00
J
ATOM 12010 HG23 ILE J 367   0.703 11.652 17.836 0.00 0.00
J
ATOM 12011 CG1 ILE J 367   -1.028 11.815 19.888 0.00 0.00
J
ATOM 12012 HG11 ILE J 367   -1.171 11.450 20.928 0.00 0.00
J
ATOM 12013 HG12 ILE J 367   -0.104 12.429 19.816 0.00 0.00
J
ATOM 12014 CD ILE J 367   -2.283 12.583 19.577 0.00 0.00
J

ATOM 12015 HD1 ILE J 367   -2.390 13.018 18.561 0.00 0.00
J
ATOM 12016 HD2 ILE J 367   -3.111 11.844 19.619 0.00 0.00
J
ATOM 12017 HD3 ILE J 367   -2.460 13.376 20.335 0.00 0.00
J
ATOM 12018 C   ILE J 367   -1.544   8.482 17.834 0.00 0.00
J
ATOM 12019 O   ILE J 367   -1.619   8.448 16.601 0.00 0.00
J
ATOM 12020 N   ILE J 368   -0.999   7.453 18.525 0.00 0.00
J
ATOM 12021 HN ILE J 368   -0.934   7.450 19.519 0.00 0.00
J
ATOM 12022 CA ILE J 368   -0.360   6.341 17.787 0.00 0.00
J
ATOM 12023 HA ILE J 368   0.150   6.757 16.931 0.00 0.00
J
ATOM 12024 CB ILE J 368   0.528   5.340 18.606 0.00 0.00
J
ATOM 12025 HB ILE J 368   -0.068   4.692 19.283 0.00 0.00
J
ATOM 12026 CG2 ILE J 368   1.382   4.469 17.547 0.00 0.00
J
ATOM 12027 HG21 ILE J 368   0.716   3.867 16.892 0.00 0.00
J
ATOM 12028 HG22 ILE J 368   1.904   5.140 16.831 0.00 0.00
J
ATOM 12029 HG23 ILE J 368   2.191   3.825 17.953 0.00 0.00
J
ATOM 12030 CG1 ILE J 368   1.422   6.185 19.575 0.00 0.00
J
ATOM 12031 HG11 ILE J 368   2.159   6.730 18.947 0.00 0.00
J
ATOM 12032 HG12 ILE J 368   0.712   6.914 20.019 0.00 0.00
J
ATOM 12033 CD ILE J 368   2.310   5.347 20.527 0.00 0.00
J
ATOM 12034 HD1 ILE J 368   3.318   5.333 20.061 0.00 0.00
J
ATOM 12035 HD2 ILE J 368   2.354   5.914 21.481 0.00 0.00
J
ATOM 12036 HD3 ILE J 368   1.859   4.353 20.733 0.00 0.00
J
ATOM 12037 C   ILE J 368   -1.472   5.511 17.104 0.00 0.00
J
ATOM 12038 O   ILE J 368   -1.445   5.195 15.961 0.00 0.00
J
ATOM 12039 N   ILE J 369   -2.559   5.114 17.887 0.00 0.00
J
ATOM 12040 HN ILE J 369   -2.591   5.458 18.822 0.00 0.00
J
ATOM 12041 CA ILE J 369   -3.520   4.160 17.464 0.00 0.00
J

ATOM 12042 HA ILE J 369   -2.993   3.306 17.066 0.00 0.00
J
ATOM 12043 CB ILE J 369   -4.383   3.598 18.672 0.00 0.00
J
ATOM 12044 HB ILE J 369   -4.999   4.416 19.104 0.00 0.00
J
ATOM 12045 CG2 ILE J 369   -5.177   2.443 17.971 0.00 0.00
J
ATOM 12046 HG21 ILE J 369   -5.821   2.058 18.790 0.00 0.00
J
ATOM 12047 HG22 ILE J 369   -5.850   2.778 17.153 0.00 0.00
J
ATOM 12048 HG23 ILE J 369   -4.583   1.571 17.623 0.00 0.00
J
ATOM 12049 CG1 ILE J 369   -3.440   3.144 19.808 0.00 0.00
J
ATOM 12050 HG11 ILE J 369   -2.810   4.002 20.127 0.00 0.00
J
ATOM 12051 HG12 ILE J 369   -4.059   2.899 20.697 0.00 0.00
J
ATOM 12052 CD ILE J 369   -2.557   1.958 19.328 0.00 0.00
J
ATOM 12053 HD1 ILE J 369   -1.683   2.268 18.716 0.00 0.00
J
ATOM 12054 HD2 ILE J 369   -2.104   1.699 20.309 0.00 0.00
J
ATOM 12055 HD3 ILE J 369   -3.173   1.076 19.052 0.00 0.00
J
ATOM 12056 C   ILE J 369   -4.408   4.832 16.321 0.00 0.00
J
ATOM 12057 O   ILE J 369   -4.947   4.137 15.448 0.00 0.00
J
ATOM 12058 N   LYS J 370   -4.549   6.220 16.471 0.00 0.00
J
ATOM 12059 HN LYS J 370   -4.103   6.651 17.252 0.00 0.00
J
ATOM 12060 CA LYS J 370   -5.254   7.145 15.649 0.00 0.00
J
ATOM 12061 HA LYS J 370   -6.228   6.728 15.439 0.00 0.00
J
ATOM 12062 CB LYS J 370   -5.430   8.455 16.401 0.00 0.00
J
ATOM 12063 HB1 LYS J 370   -5.788   8.326 17.445 0.00 0.00
J
ATOM 12064 HB2 LYS J 370   -4.410   8.884 16.499 0.00 0.00
J
ATOM 12065 CG LYS J 370   -6.319   9.459 15.580 0.00 0.00
J
ATOM 12066 HG1 LYS J 370   -5.873   9.588 14.571 0.00 0.00
J
ATOM 12067 HG2 LYS J 370   -7.327   9.008 15.466 0.00 0.00
J
ATOM 12068 CD LYS J 370   -6.729 10.722 16.361 0.00 0.00
J

ATOM 12069 HD1 LYS J 370   -7.175 11.361 15.569 0.00 0.00
J
ATOM 12070 HD2 LYS J 370   -7.595 10.517 17.027 0.00 0.00
J
ATOM 12071 CE LYS J 370   -5.638 11.457 17.162 0.00 0.00
J
ATOM 12072 HE1 LYS J 370   -5.105 10.825 17.905 0.00 0.00
J
ATOM 12073 HE2 LYS J 370   -4.754 11.852 16.618 0.00 0.00
J
ATOM 12074 NZ LYS J 370   -6.280 12.568 17.871 0.00 0.00
J
ATOM 12075 HZ1 LYS J 370   -7.320 12.546 17.844 0.00 0.00
J
ATOM 12076 HZ2 LYS J 370   -5.973 12.545 18.865 0.00 0.00
J
ATOM 12077 HZ3 LYS J 370   -5.961 13.521 17.606 0.00 0.00
J
ATOM 12078 C   LYS J 370   -4.565   7.308 14.350 0.00 0.00
J
ATOM 12079 O   LYS J 370   -5.162   7.375 13.289 0.00 0.00
J
ATOM 12080 N   ALA J 371   -3.152   7.356 14.344 0.00 0.00
J
ATOM 12081 HN ALA J 371   -2.619   7.380 15.186 0.00 0.00
J
ATOM 12082 CA ALA J 371   -2.409   7.480 13.091 0.00 0.00
J
ATOM 12083 HA ALA J 371   -2.728   8.357 12.547 0.00 0.00
J
ATOM 12084 CB ALA J 371   -0.895   7.440 13.367 0.00 0.00
J
ATOM 12085 HB1 ALA J 371   -0.365   7.435 12.390 0.00 0.00
J
ATOM 12086 HB2 ALA J 371   -0.733   8.401 13.900 0.00 0.00
J
ATOM 12087 HB3 ALA J 371   -0.499   6.619 14.002 0.00 0.00
J
ATOM 12088 C   ALA J 371   -2.607   6.282 12.210 0.00 0.00
J
ATOM 12089 O   ALA J 371   -2.862   6.332 10.969 0.00 0.00
J
ATOM 12090 N   LEU J 372   -2.674   5.059 12.800 0.00 0.00
J
ATOM 12091 HN LEU J 372   -2.346   4.997 13.739 0.00 0.00
J
ATOM 12092 CA LEU J 372   -3.063   3.854 12.116 0.00 0.00
J
ATOM 12093 HA LEU J 372   -2.434   3.781 11.241 0.00 0.00
J
ATOM 12094 CB LEU J 372   -2.778   2.572 12.957 0.00 0.00
J
ATOM 12095 HB1 LEU J 372   -1.757   2.639 13.391 0.00 0.00
J

ATOM 12096 HB2 LEU J 372   -3.475   2.673 13.816 0.00 0.00
J
ATOM 12097 CG LEU J 372   -3.093   1.209 12.256 0.00 0.00
J
ATOM 12098 HG LEU J 372   -4.164   1.131 11.970 0.00 0.00
J
ATOM 12099 CD1 LEU J 372   -2.215   1.026 10.961 0.00 0.00
J
ATOM 12100 HD11 LEU J 372   -2.783   0.207 10.469 0.00 0.00
J
ATOM 12101 HD12 LEU J 372   -2.272   1.910 10.290 0.00 0.00
J
ATOM 12102 HD13 LEU J 372   -1.133   0.821 11.105 0.00 0.00
J
ATOM 12103 CD2 LEU J 372   -2.865   0.002 13.188 0.00 0.00
J
ATOM 12104 HD21 LEU J 372   -3.714   0.009 13.905 0.00 0.00
J
ATOM 12105 HD22 LEU J 372   -2.951 -0.893 12.534 0.00 0.00
J
ATOM 12106 HD23 LEU J 372   -1.831   0.143 13.567 0.00 0.00
J
ATOM 12107 C   LEU J 372   -4.477   3.960 11.600 0.00 0.00
J
ATOM 12108 O   LEU J 372   -4.589   3.783 10.400 0.00 0.00
J
ATOM 12109 N   ALA J 373   -5.491   4.268 12.458 0.00 0.00
J
ATOM 12110 HN ALA J 373   -5.310   4.575 13.389 0.00 0.00
J
ATOM 12111 CA ALA J 373   -6.930   4.399 12.058 0.00 0.00
J
ATOM 12112 HA ALA J 373   -7.324   3.448 11.733 0.00 0.00
J
ATOM 12113 CB ALA J 373   -7.752   4.547 13.291 0.00 0.00
J
ATOM 12114 HB1 ALA J 373   -7.633   5.558 13.735 0.00 0.00
J
ATOM 12115 HB2 ALA J 373   -8.835   4.337 13.160 0.00 0.00
J
ATOM 12116 HB3 ALA J 373   -7.413   3.847 14.085 0.00 0.00
J
ATOM 12117 C   ALA J 373   -7.303   5.452 10.990 0.00 0.00
J
ATOM 12118 O   ALA J 373   -8.184   5.242 10.127 0.00 0.00
J
ATOM 12119 N   VAL J 374   -6.754   6.604 10.906 0.00 0.00
J
ATOM 12120 HN VAL J 374   -6.027   6.804 11.558 0.00 0.00
J
ATOM 12121 CA VAL J 374   -6.987   7.601   9.843 0.00 0.00
J
ATOM 12122 HA VAL J 374   -8.052   7.587   9.665 0.00 0.00
J

ATOM 12123 CB VAL J 374   -6.654   9.000 10.204 0.00 0.00
J
ATOM 12124 HB VAL J 374   -6.856   9.573   9.274 0.00 0.00
J
ATOM 12125 CG1 VAL J 374   -7.579   9.529 11.267 0.00 0.00
J
ATOM 12126 HG11 VAL J 374   -7.658 10.634 11.184 0.00 0.00
J
ATOM 12127 HG12 VAL J 374   -8.632   9.181 11.331 0.00 0.00
J
ATOM 12128 HG13 VAL J 374   -7.092   9.340 12.248 0.00 0.00
J
ATOM 12129 CG2 VAL J 374   -5.224   9.295 10.559 0.00 0.00
J
ATOM 12130 HG21 VAL J 374   -4.576   9.173   9.665 0.00 0.00
J
ATOM 12131 HG22 VAL J 374   -5.199 10.365 10.856 0.00 0.00
J
ATOM 12132 HG23 VAL J 374   -4.936   8.613 11.388 0.00 0.00
J
ATOM 12133 C   VAL J 374   -6.346   7.084   8.542 0.00 0.00
J
ATOM 12134 O   VAL J 374   -6.880   7.114   7.429 0.00 0.00
J
ATOM 12135 N   SER J 375   -5.132   6.457   8.601 0.00 0.00
J
ATOM 12136 HN SER J 375   -4.619   6.526   9.453 0.00 0.00
J
ATOM 12137 CA SER J 375   -4.417   5.936   7.472 0.00 0.00
J
ATOM 12138 HA SER J 375   -4.384   6.753   6.766 0.00 0.00
J
ATOM 12139 CB SER J 375   -2.997   5.402   7.829 0.00 0.00
J
ATOM 12140 HB1 SER J 375   -3.156   4.493   8.448 0.00 0.00
J
ATOM 12141 HB2 SER J 375   -2.571   5.057   6.863 0.00 0.00
J
ATOM 12142 OG SER J 375   -2.190   6.455   8.418 0.00 0.00
J
ATOM 12143 HG1 SER J 375   -2.304   6.432   9.371 0.00 0.00
J
ATOM 12144 C   SER J 375   -5.122   4.860   6.831 0.00 0.00
J
ATOM 12145 O   SER J 375   -5.211   4.978   5.633 0.00 0.00
J
ATOM 12146 N   VAL J 376   -5.613   3.838   7.484 0.00 0.00
J
ATOM 12147 HN VAL J 376   -5.357   3.563   8.408 0.00 0.00
J
ATOM 12148 CA VAL J 376   -6.382   2.739   6.862 0.00 0.00
J
ATOM 12149 HA VAL J 376   -5.837   2.397   5.995 0.00 0.00
J

ATOM 12150 CB VAL J 376 -6.462 1.515 7.750 0.00 0.00
J
ATOM 12151 HB VAL J 376 -6.977 0.722 7.168 0.00 0.00
J
ATOM 12152 CG1 VAL J 376 -5.028 0.963 8.055 0.00 0.00
J
ATOM 12153 HG11 VAL J 376 -4.672 1.411 9.007 0.00 0.00
J
ATOM 12154 HG12 VAL J 376 -5.118 -0.132 8.218 0.00 0.00
J
ATOM 12155 HG13 VAL J 376 -4.459 1.069 7.107 0.00 0.00
J
ATOM 12156 CG2 VAL J 376 -7.241 1.804 8.998 0.00 0.00
J
ATOM 12157 HG21 VAL J 376 -6.761 2.657 9.523 0.00 0.00
J
ATOM 12158 HG22 VAL J 376 -8.321 1.989 8.814 0.00 0.00
J
ATOM 12159 HG23 VAL J 376 -7.169 1.039 9.801 0.00 0.00
J
ATOM 12160 C VAL J 376 -7.765 3.233 6.292 0.00 0.00
J
ATOM 12161 O VAL J 376 -8.226 2.821 5.245 0.00 0.00
J
ATOM 12162 N THR J 377 -8.391 4.177 7.023 0.00 0.00
J
ATOM 12163 HN THR J 377 -7.915 4.378 7.876 0.00 0.00
J
ATOM 12164 CA THR J 377 -9.724 4.732 6.676 0.00 0.00
J
ATOM 12165 HA THR J 377 -10.463 3.956 6.536 0.00 0.00
J
ATOM 12166 CB THR J 377 -10.329 5.660 7.708 0.00 0.00
J
ATOM 12167 HB THR J 377 -9.592 6.461 7.930 0.00 0.00
J
ATOM 12168 OG1 THR J 377 -10.462 4.936 8.905 0.00 0.00
J
ATOM 12169 HG1 THR J 377 -9.660 5.079 9.413 0.00 0.00
J
ATOM 12170 CG2 THR J 377 -11.695 6.165 7.335 0.00 0.00
J
ATOM 12171 HG21 THR J 377 -12.460 5.368 7.455 0.00 0.00
J
ATOM 12172 HG22 THR J 377 -12.046 7.024 7.946 0.00 0.00
J
ATOM 12173 HG23 THR J 377 -11.722 6.548 6.293 0.00 0.00
J
ATOM 12174 C THR J 377 -9.581 5.485 5.350 0.00 0.00
J
ATOM 12175 O THR J 377 -10.328 5.293 4.413 0.00 0.00
J
ATOM 12176 N SER J 378 -8.539 6.308 5.180 0.00 0.00
J

ATOM 12177 HN SER J 378 -7.958 6.648 5.915 0.00 0.00
J
ATOM 12178 CA SER J 378 -8.318 7.086 3.914 0.00 0.00
J
ATOM 12179 HA SER J 378 -9.159 7.730 3.698 0.00 0.00
J
ATOM 12180 CB SER J 378 -7.014 7.858 4.063 0.00 0.00
J
ATOM 12181 HB1 SER J 378 -6.149 7.180 4.218 0.00 0.00
J
ATOM 12182 HB2 SER J 378 -6.801 8.557 3.226 0.00 0.00
J
ATOM 12183 OG SER J 378 -7.034 8.695 5.182 0.00 0.00
J
ATOM 12184 HG1 SER J 378 -7.019 8.205 6.008 0.00 0.00
J
ATOM 12185 C SER J 378 -7.978 6.173 2.774 0.00 0.00
J
ATOM 12186 O SER J 378 -8.444 6.392 1.667 0.00 0.00
J
ATOM 12187 N LEU J 379 -7.184 5.118 2.926 0.00 0.00
J
ATOM 12188 HN LEU J 379 -6.776 5.066 3.834 0.00 0.00
J
ATOM 12189 CA LEU J 379 -7.008 4.122 1.904 0.00 0.00
J
ATOM 12190 HA LEU J 379 -6.696 4.556 0.966 0.00 0.00
J
ATOM 12191 CB LEU J 379 -5.930 3.048 2.335 0.00 0.00
J
ATOM 12192 HB1 LEU J 379 -6.328 2.481 3.204 0.00 0.00
J
ATOM 12193 HB2 LEU J 379 -5.850 2.295 1.522 0.00 0.00
J
ATOM 12194 CG LEU J 379 -4.559 3.657 2.668 0.00 0.00
J
ATOM 12195 HG LEU J 379 -4.733 4.427 3.450 0.00 0.00
J
ATOM 12196 CD1 LEU J 379 -3.708 2.478 3.157 0.00 0.00
J
ATOM 12197 HD11 LEU J 379 -4.180 1.712 3.809 0.00 0.00
J
ATOM 12198 HD12 LEU J 379 -3.234 1.947 2.303 0.00 0.00
J
ATOM 12199 HD13 LEU J 379 -2.921 2.933 3.795 0.00 0.00
J
ATOM 12200 CD2 LEU J 379 -3.937 4.490 1.599 0.00 0.00
J
ATOM 12201 HD21 LEU J 379 -4.623 5.266 1.197 0.00 0.00
J
ATOM 12202 HD22 LEU J 379 -3.110 5.124 1.985 0.00 0.00
J
ATOM 12203 HD23 LEU J 379 -3.544 3.826 0.800 0.00 0.00
J

ATOM 12204 C   LEU J 379   -8.304   3.422   1.469 0.00 0.00
J
ATOM 12205 O   LEU J 379   -8.687   3.230   0.337 0.00 0.00
J
ATOM 12206 N   PHE J 380   -9.126   3.062   2.489 0.00 0.00
J
ATOM 12207 HN PHE J 380   -8.874   3.179   3.447 0.00 0.00
J
ATOM 12208 CA PHE J 380   -10.356   2.289   2.163 0.00 0.00
J
ATOM 12209 HA PHE J 380   -10.051   1.526   1.462 0.00 0.00
J
ATOM 12210 CB PHE J 380   -11.021   1.805   3.539 0.00 0.00
J
ATOM 12211 HB1 PHE J 380   -10.253   1.167   4.027 0.00 0.00
J
ATOM 12212 HB2 PHE J 380   -11.422   2.722   4.020 0.00 0.00
J
ATOM 12213 CG PHE J 380   -12.193   0.849   3.231 0.00 0.00
J
ATOM 12214 CD1 PHE J 380   -12.159 -0.094   2.218 0.00 0.00
J
ATOM 12215 HD1 PHE J 380   -11.364 -0.213   1.497 0.00 0.00
J
ATOM 12216 CE1 PHE J 380   -13.291 -0.829   1.956 0.00 0.00
J
ATOM 12217 HE1 PHE J 380   -13.296 -1.447   1.070 0.00 0.00
J
ATOM 12218 CZ PHE J 380   -14.392 -0.785   2.843 0.00 0.00
J
ATOM 12219 HZ PHE J 380   -15.190 -1.512   2.799 0.00 0.00
J
ATOM 12220 CD2 PHE J 380   -13.233   0.845   4.168 0.00 0.00
J
ATOM 12221 HD2 PHE J 380   -13.085   1.517   5.001 0.00 0.00
J
ATOM 12222 CE2 PHE J 380   -14.357   0.082   3.963 0.00 0.00
J
ATOM 12223 HE2 PHE J 380   -15.234   0.065   4.593 0.00 0.00
J
ATOM 12224 C   PHE J 380   -11.382   3.180   1.484 0.00 0.00
J
ATOM 12225 O   PHE J 380   -12.015   2.787   0.487 0.00 0.00
J
ATOM 12226 N   ILE J 381   -11.654   4.403   1.998 0.00 0.00
J
ATOM 12227 HN ILE J 381   -11.101   4.614   2.801 0.00 0.00
J
ATOM 12228 CA ILE J 381   -12.600   5.421   1.505 0.00 0.00
J
ATOM 12229 HA ILE J 381   -13.584   4.981   1.578 0.00 0.00
J
ATOM 12230 CB ILE J 381   -12.742   6.734   2.276 0.00 0.00
J

ATOM 12231 HB ILE J 381 -11.670 7.016 2.343 0.00 0.00
J
ATOM 12232 CG2 ILE J 381 -13.431 7.879 1.508 0.00 0.00
J
ATOM 12233 HG21 ILE J 381 -12.905 8.142 0.565 0.00 0.00
J
ATOM 12234 HG22 ILE J 381 -14.474 7.605 1.241 0.00 0.00
J
ATOM 12235 HG23 ILE J 381 -13.497 8.705 2.248 0.00 0.00
J
ATOM 12236 CG1 ILE J 381 -13.327 6.526 3.626 0.00 0.00
J
ATOM 12237 HG11 ILE J 381 -12.910 5.788 4.344 0.00 0.00
J
ATOM 12238 HG12 ILE J 381 -13.155 7.468 4.191 0.00 0.00
J
ATOM 12239 CD ILE J 381 -14.809 6.385 3.518 0.00 0.00
J
ATOM 12240 HD1 ILE J 381 -15.449 7.205 3.127 0.00 0.00
J
ATOM 12241 HD2 ILE J 381 -15.028 5.532 2.841 0.00 0.00
J
ATOM 12242 HD3 ILE J 381 -15.191 5.986 4.482 0.00 0.00
J
ATOM 12243 C ILE J 381 -12.329 5.745 0.029 0.00 0.00
J
ATOM 12244 O ILE J 381 -13.258 5.833 -0.816 0.00 0.00
J
ATOM 12245 N VAL J 382 -11.028 5.896 -0.365 0.00 0.00
J
ATOM 12246 HN VAL J 382 -10.236 5.920 0.239 0.00 0.00
J
ATOM 12247 CA VAL J 382 -10.712 6.091 -1.804 0.00 0.00
J
ATOM 12248 HA VAL J 382 -11.377 6.753 -2.338 0.00 0.00
J
ATOM 12249 CB VAL J 382 -9.353 6.715 -2.137 0.00 0.00
J
ATOM 12250 HB VAL J 382 -9.305 6.887 -3.234 0.00 0.00
J
ATOM 12251 CG1 VAL J 382 -9.490 8.121 -1.455 0.00 0.00
J
ATOM 12252 HG11 VAL J 382 -9.686 8.024 -0.366 0.00 0.00
J
ATOM 12253 HG12 VAL J 382 -8.584 8.696 -1.743 0.00 0.00
J
ATOM 12254 HG13 VAL J 382 -10.337 8.671 -1.920 0.00 0.00
J
ATOM 12255 CG2 VAL J 382 -8.106 5.909 -1.697 0.00 0.00
J
ATOM 12256 HG21 VAL J 382 -7.184 6.505 -1.871 0.00 0.00
J
ATOM 12257 HG22 VAL J 382 -8.148 5.532 -0.653 0.00 0.00
J

ATOM 12258 HG23 VAL J 382 -8.008 4.984 -2.306 0.00 0.00
J
ATOM 12259 C VAL J 382 -10.933 4.812 -2.597 0.00 0.00
J
ATOM 12260 O VAL J 382 -11.246 4.856 -3.839 0.00 0.00
J
ATOM 12261 N PHE J 383 -10.748 3.645 -1.994 0.00 0.00
J
ATOM 12262 HN PHE J 383 -10.465 3.625 -1.038 0.00 0.00
J
ATOM 12263 CA PHE J 383 -11.038 2.340 -2.696 0.00 0.00
J
ATOM 12264 HA PHE J 383 -10.497 2.329 -3.631 0.00 0.00
J
ATOM 12265 CB PHE J 383 -10.580 1.158 -1.907 0.00 0.00
J
ATOM 12266 HB1 PHE J 383 -10.511 1.414 -0.829 0.00 0.00
J
ATOM 12267 HB2 PHE J 383 -11.316 0.337 -1.771 0.00 0.00
J
ATOM 12268 CG PHE J 383 -9.261 0.682 -2.357 0.00 0.00
J
ATOM 12269 CD1 PHE J 383 -9.143 -0.551 -2.866 0.00 0.00
J
ATOM 12270 HD1 PHE J 383 -9.940 -1.277 -2.928 0.00 0.00
J
ATOM 12271 CE1 PHE J 383 -7.925 -1.006 -3.290 0.00 0.00
J
ATOM 12272 HE1 PHE J 383 -7.807 -2.063 -3.478 0.00 0.00
J
ATOM 12273 CZ PHE J 383 -6.755 -0.197 -3.135 0.00 0.00
J
ATOM 12274 HZ PHE J 383 -5.826 -0.650 -3.445 0.00 0.00
J
ATOM 12275 CD2 PHE J 383 -8.181 1.616 -2.388 0.00 0.00
J
ATOM 12276 HD2 PHE J 383 -8.321 2.641 -2.079 0.00 0.00
J
ATOM 12277 CE2 PHE J 383 -6.905 1.176 -2.811 0.00 0.00
J
ATOM 12278 HE2 PHE J 383 -6.034 1.809 -2.734 0.00 0.00
J
ATOM 12279 C PHE J 383 -12.529 2.219 -2.947 0.00 0.00
J
ATOM 12280 O PHE J 383 -12.823 1.725 -4.040 0.00 0.00
J
ATOM 12281 N LEU J 384 -13.465 2.535 -2.056 0.00 0.00
J
ATOM 12282 HN LEU J 384 -13.154 2.740 -1.131 0.00 0.00
J
ATOM 12283 CA LEU J 384 -14.902 2.676 -2.449 0.00 0.00
J
ATOM 12284 HA LEU J 384 -15.077 1.704 -2.886 0.00 0.00
J

ATOM	12285	CB	LEU	J	384	-15.795	2.697	-1.186	0.00	0.00	J
ATOM	12286	HB1	LEU	J	384	-15.579	3.659	-0.675	0.00	0.00	J
ATOM	12287	HB2	LEU	J	384	-16.879	2.794	-1.412	0.00	0.00	J
ATOM	12288	CG	LEU	J	384	-15.636	1.581	-0.211	0.00	0.00	J
ATOM	12289	HG	LEU	J	384	-14.557	1.491	0.038	0.00	0.00	J
ATOM	12290	CD1	LEU	J	384	-16.466	1.802	1.072	0.00	0.00	J
ATOM	12291	HD11	LEU	J	384	-16.172	2.770	1.531	0.00	0.00	J
ATOM	12292	HD12	LEU	J	384	-17.472	1.956	0.626	0.00	0.00	J
ATOM	12293	HD13	LEU	J	384	-16.402	0.990	1.827	0.00	0.00	J
ATOM	12294	CD2	LEU	J	384	-15.994	0.191	-0.810	0.00	0.00	J
ATOM	12295	HD21	LEU	J	384	-15.474	-0.050	-1.762	0.00	0.00	J
ATOM	12296	HD22	LEU	J	384	-15.722	-0.540	-0.019	0.00	0.00	J
ATOM	12297	HD23	LEU	J	384	-17.086	0.141	-1.009	0.00	0.00	J
ATOM	12298	C	LEU	J	384	-15.283	3.642	-3.524	0.00	0.00	J
ATOM	12299	O	LEU	J	384	-16.135	3.393	-4.324	0.00	0.00	J
ATOM	12300	N	GLY	J	385	-14.659	4.805	-3.630	0.00	0.00	J
ATOM	12301	HN	GLY	J	385	-14.108	5.113	-2.858	0.00	0.00	J
ATOM	12302	CA	GLY	J	385	-14.765	5.614	-4.824	0.00	0.00	J
ATOM	12303	HA1	GLY	J	385	-14.027	6.371	-4.601	0.00	0.00	J
ATOM	12304	HA2	GLY	J	385	-15.772	5.983	-4.945	0.00	0.00	J
ATOM	12305	C	GLY	J	385	-14.231	5.032	-6.161	0.00	0.00	J
ATOM	12306	O	GLY	J	385	-14.915	5.274	-7.148	0.00	0.00	J
ATOM	12307	N	ILE	J	386	-13.050	4.357	-6.106	0.00	0.00	J
ATOM	12308	HN	ILE	J	386	-12.657	4.334	-5.190	0.00	0.00	J
ATOM	12309	CA	ILE	J	386	-12.449	3.780	-7.290	0.00	0.00	J
ATOM	12310	HA	ILE	J	386	-12.435	4.511	-8.086	0.00	0.00	J
ATOM	12311	CB	ILE	J	386	-10.985	3.290	-6.971	0.00	0.00	J

ATOM 12312 HB ILE J 386 -10.825 2.705 -6.040 0.00 0.00
J
ATOM 12313 CG2 ILE J 386 -10.434 2.423 -8.139 0.00 0.00
J
ATOM 12314 HG21 ILE J 386 -10.508 2.934 -9.123 0.00 0.00
J
ATOM 12315 HG22 ILE J 386 -9.381 2.131 -7.938 0.00 0.00
J
ATOM 12316 HG23 ILE J 386 -10.916 1.426 -8.227 0.00 0.00
J
ATOM 12317 CG1 ILE J 386 -10.136 4.526 -6.905 0.00 0.00
J
ATOM 12318 HG11 ILE J 386 -9.855 4.967 -7.885 0.00 0.00
J
ATOM 12319 HG12 ILE J 386 -10.641 5.347 -6.352 0.00 0.00
J
ATOM 12320 CD ILE J 386 -8.852 4.275 -6.166 0.00 0.00
J
ATOM 12321 HD1 ILE J 386 -8.380 3.358 -6.580 0.00 0.00
J
ATOM 12322 HD2 ILE J 386 -8.123 5.112 -6.118 0.00 0.00
J
ATOM 12323 HD3 ILE J 386 -9.082 4.059 -5.101 0.00 0.00
J
ATOM 12324 C ILE J 386 -13.231 2.598 -7.840 0.00 0.00
J
ATOM 12325 O ILE J 386 -13.425 2.341 -9.037 0.00 0.00
J
ATOM 12326 N PHE J 387 -13.761 1.838 -6.875 0.00 0.00
J
ATOM 12327 HN PHE J 387 -13.466 1.976 -5.933 0.00 0.00
J
ATOM 12328 CA PHE J 387 -14.771 0.879 -7.023 0.00 0.00
J
ATOM 12329 HA PHE J 387 -14.426 0.113 -7.701 0.00 0.00
J
ATOM 12330 CB PHE J 387 -15.063 0.197 -5.623 0.00 0.00
J
ATOM 12331 HB1 PHE J 387 -14.139 -0.201 -5.152 0.00 0.00
J
ATOM 12332 HB2 PHE J 387 -15.648 0.981 -5.096 0.00 0.00
J
ATOM 12333 CG PHE J 387 -15.906 -1.013 -5.863 0.00 0.00
J
ATOM 12334 CD1 PHE J 387 -17.105 -0.968 -5.183 0.00 0.00
J
ATOM 12335 HD1 PHE J 387 -17.464 -0.125 -4.611 0.00 0.00
J
ATOM 12336 CE1 PHE J 387 -18.070 -1.987 -5.361 0.00 0.00
J
ATOM 12337 HE1 PHE J 387 -19.010 -2.064 -4.837 0.00 0.00
J
ATOM 12338 CZ PHE J 387 -17.749 -3.006 -6.198 0.00 0.00
J

ATOM 12339 HZ PHE J 387   -18.554 -3.600 -6.606 0.00 0.00
J
ATOM 12340 CD2 PHE J 387   -15.536 -2.119 -6.655 0.00 0.00
J
ATOM 12341 HD2 PHE J 387   -14.556 -2.163 -7.107 0.00 0.00
J
ATOM 12342 CE2 PHE J 387   -16.517 -3.119 -6.902 0.00 0.00
J
ATOM 12343 HE2 PHE J 387   -16.251 -3.902 -7.596 0.00 0.00
J
ATOM 12344 C   PHE J 387   -15.989   1.394 -7.676 0.00 0.00
J
ATOM 12345 O   PHE J 387   -16.281   0.979 -8.794 0.00 0.00
J
ATOM 12346 N   ALA J 388   -16.617   2.446 -7.059 0.00 0.00
J
ATOM 12347 HN ALA J 388   -16.443   2.718 -6.116 0.00 0.00
J
ATOM 12348 CA ALA J 388   -17.680   3.048 -7.778 0.00 0.00
J
ATOM 12349 HA ALA J 388   -18.449   2.316 -7.977 0.00 0.00
J
ATOM 12350 CB ALA J 388   -18.427   4.052 -6.850 0.00 0.00
J
ATOM 12351 HB1 ALA J 388   -18.441   3.731 -5.786 0.00 0.00
J
ATOM 12352 HB2 ALA J 388   -17.876   5.015 -6.885 0.00 0.00
J
ATOM 12353 HB3 ALA J 388   -19.510   4.239 -7.011 0.00 0.00
J
ATOM 12354 C   ALA J 388   -17.454   3.703 -9.137 0.00 0.00
J
ATOM 12355 O   ALA J 388   -18.276   3.562 -10.057 0.00 0.00
J
ATOM 12356 N   LEU J 389   -16.426   4.498 -9.372 0.00 0.00
J
ATOM 12357 HN LEU J 389   -15.910   4.806 -8.576 0.00 0.00
J
ATOM 12358 CA LEU J 389   -15.999   5.036 -10.673 0.00 0.00
J
ATOM 12359 HA LEU J 389   -16.793   5.692 -10.998 0.00 0.00
J
ATOM 12360 CB LEU J 389   -14.740   5.839 -10.544 0.00 0.00
J
ATOM 12361 HB1 LEU J 389   -14.075   5.238 -9.887 0.00 0.00
J
ATOM 12362 HB2 LEU J 389   -14.141   6.091 -11.445 0.00 0.00
J
ATOM 12363 CG LEU J 389   -14.842   7.229 -9.885 0.00 0.00
J
ATOM 12364 HG LEU J 389   -15.525   7.105 -9.017 0.00 0.00
J
ATOM 12365 CD1 LEU J 389   -13.406   7.751 -9.586 0.00 0.00
J

```
ATOM  12366 HD11 LEU J 389     -13.472   8.719  -9.045  0.00  0.00
J
ATOM  12367 HD12 LEU J 389     -12.826   6.968  -9.053  0.00  0.00
J
ATOM  12368 HD13 LEU J 389     -12.963   7.952 -10.585  0.00  0.00
J
ATOM  12369  CD2 LEU J 389     -15.547   8.252 -10.773  0.00  0.00
J
ATOM  12370 HD21 LEU J 389     -16.553   7.884 -11.066  0.00  0.00
J
ATOM  12371 HD22 LEU J 389     -15.615   9.205 -10.207  0.00  0.00
J
ATOM  12372 HD23 LEU J 389     -15.064   8.504 -11.742  0.00  0.00
J
ATOM  12373  C   LEU J 389     -15.785   3.998 -11.780  0.00  0.00
J
ATOM  12374  O   LEU J 389     -16.317   4.165 -12.913  0.00  0.00
J
ATOM  12375  N   THR J 390     -15.169   2.840 -11.445  0.00  0.00
J
ATOM  12376  HN  THR J 390     -14.873   2.760 -10.496  0.00  0.00
J
ATOM  12377  CA  THR J 390     -14.994   1.662 -12.404  0.00  0.00
J
ATOM  12378  HA  THR J 390     -14.734   2.187 -13.312  0.00  0.00
J
ATOM  12379  CB  THR J 390     -13.880   0.753 -12.069  0.00  0.00
J
ATOM  12380  HB  THR J 390     -13.873  -0.049 -12.838  0.00  0.00
J
ATOM  12381  OG1 THR J 390     -14.016   0.149 -10.788  0.00  0.00
J
ATOM  12382  HG1 THR J 390     -13.769   0.821 -10.148  0.00  0.00
J
ATOM  12383  CG2 THR J 390     -12.537   1.407 -12.120  0.00  0.00
J
ATOM  12384 HG21 THR J 390     -12.361   2.064 -11.242  0.00  0.00
J
ATOM  12385 HG22 THR J 390     -11.757   0.622 -12.214  0.00  0.00
J
ATOM  12386 HG23 THR J 390     -12.266   2.026 -13.002  0.00  0.00
J
ATOM  12387  C   THR J 390     -16.305   0.969 -12.610  0.00  0.00
J
ATOM  12388  O   THR J 390     -16.534   0.464 -13.690  0.00  0.00
J
ATOM  12389  N   ILE J 391     -17.212   0.815 -11.619  0.00  0.00
J
ATOM  12390  HN  ILE J 391     -17.128   1.139 -10.679  0.00  0.00
J
ATOM  12391  CA  ILE J 391     -18.563   0.353 -12.113  0.00  0.00
J
ATOM  12392  HA  ILE J 391     -18.371  -0.535 -12.697  0.00  0.00
J
```

```
ATOM  12393  CB  ILE J 391     -19.347   0.043 -10.890  0.00  0.00
J
ATOM  12394  HB  ILE J 391     -19.510   1.013 -10.373  0.00  0.00
J
ATOM  12395  CG2 ILE J 391     -20.796  -0.569 -11.211  0.00  0.00
J
ATOM  12396 HG21 ILE J 391     -20.698  -1.328 -12.017  0.00  0.00
J
ATOM  12397 HG22 ILE J 391     -21.416  -0.969 -10.381  0.00  0.00
J
ATOM  12398 HG23 ILE J 391     -21.385   0.115 -11.859  0.00  0.00
J
ATOM  12399  CG1 ILE J 391     -18.650  -0.897  -9.912  0.00  0.00
J
ATOM  12400 HG11 ILE J 391     -17.653  -0.463  -9.684  0.00  0.00
J
ATOM  12401 HG12 ILE J 391     -19.119  -0.971  -8.908  0.00  0.00
J
ATOM  12402  CD  ILE J 391     -18.348  -2.333 -10.495  0.00  0.00
J
ATOM  12403  HD1 ILE J 391     -19.345  -2.750 -10.750  0.00  0.00
J
ATOM  12404  HD2 ILE J 391     -17.795  -2.159 -11.443  0.00  0.00
J
ATOM  12405  HD3 ILE J 391     -17.879  -2.945  -9.695  0.00  0.00
J
ATOM  12406  C   ILE J 391     -19.357   1.340 -13.055  0.00  0.00
J
ATOM  12407  O   ILE J 391     -20.025   0.958 -14.046  0.00  0.00
J
ATOM  12408  N   THR J 392     -19.183   2.654 -12.846  0.00  0.00
J
ATOM  12409  HN  THR J 392     -18.533   2.969 -12.159  0.00  0.00
J
ATOM  12410  CA  THR J 392     -19.910   3.640 -13.679  0.00  0.00
J
ATOM  12411  HA  THR J 392     -20.937   3.319 -13.779  0.00  0.00
J
ATOM  12412  CB  THR J 392     -19.902   4.950 -13.025  0.00  0.00
J
ATOM  12413  HB  THR J 392     -18.897   5.389 -12.850  0.00  0.00
J
ATOM  12414  OG1 THR J 392     -20.410   4.918 -11.728  0.00  0.00
J
ATOM  12415  HG1 THR J 392     -19.971   4.177 -11.304  0.00  0.00
J
ATOM  12416  CG2 THR J 392     -20.706   5.994 -13.851  0.00  0.00
J
ATOM  12417 HG21 THR J 392     -21.764   5.690 -14.005  0.00  0.00
J
ATOM  12418 HG22 THR J 392     -20.666   6.923 -13.244  0.00  0.00
J
ATOM  12419 HG23 THR J 392     -20.326   6.236 -14.866  0.00  0.00
J
```

ATOM 12420 C THR J 392 -19.220 3.861 -15.063 0.00 0.00
J
ATOM 12421 O THR J 392 -19.924 3.943 -16.105 0.00 0.00
J
ATOM 12422 N GLU J 393 -17.871 3.924 -15.103 0.00 0.00
J
ATOM 12423 HN GLU J 393 -17.378 4.028 -14.242 0.00 0.00
J
ATOM 12424 CA GLU J 393 -17.266 4.535 -16.305 0.00 0.00
J
ATOM 12425 HA GLU J 393 -18.014 4.797 -17.038 0.00 0.00
J
ATOM 12426 CB GLU J 393 -16.484 5.835 -16.021 0.00 0.00
J
ATOM 12427 HB1 GLU J 393 -15.694 5.492 -15.320 0.00 0.00
J
ATOM 12428 HB2 GLU J 393 -16.043 6.051 -17.017 0.00 0.00
J
ATOM 12429 CG GLU J 393 -17.384 7.018 -15.509 0.00 0.00
J
ATOM 12430 HG1 GLU J 393 -18.189 6.740 -14.797 0.00 0.00
J
ATOM 12431 HG2 GLU J 393 -16.770 7.718 -14.903 0.00 0.00
J
ATOM 12432 CD GLU J 393 -18.062 8.033 -16.464 0.00 0.00
J
ATOM 12433 OE1 GLU J 393 -17.311 8.497 -17.355 0.00 0.00
J
ATOM 12434 OE2 GLU J 393 -19.299 8.188 -16.435 0.00 0.00
J
ATOM 12435 C GLU J 393 -16.268 3.567 -16.886 0.00 0.00
J
ATOM 12436 O GLU J 393 -15.682 2.694 -16.273 0.00 0.00
J
ATOM 12437 N GLN J 394 -16.003 3.700 -18.189 0.00 0.00
J
ATOM 12438 HN GLN J 394 -16.328 4.526 -18.642 0.00 0.00
J
ATOM 12439 CA GLN J 394 -15.368 2.815 -19.168 0.00 0.00
J
ATOM 12440 HA GLN J 394 -15.488 1.825 -18.754 0.00 0.00
J
ATOM 12441 CB GLN J 394 -16.182 2.858 -20.508 0.00 0.00
J
ATOM 12442 HB1 GLN J 394 -16.063 3.804 -21.078 0.00 0.00
J
ATOM 12443 HB2 GLN J 394 -15.880 1.943 -21.060 0.00 0.00
J
ATOM 12444 CG GLN J 394 -17.666 2.474 -20.229 0.00 0.00
J
ATOM 12445 HG1 GLN J 394 -17.706 1.590 -19.557 0.00 0.00
J
ATOM 12446 HG2 GLN J 394 -18.215 3.313 -19.752 0.00 0.00
J

ATOM 12447 CD   GLN J 394   -18.332   2.194 -21.560 0.00 0.00
J
ATOM 12448 OE1 GLN J 394   -18.846   1.108 -21.775 0.00 0.00
J
ATOM 12449 NE2 GLN J 394   -18.322   3.183 -22.444 0.00 0.00
J
ATOM 12450 HE21 GLN J 394   -18.739   2.915 -23.312 0.00 0.00
J
ATOM 12451 HE22 GLN J 394   -18.159   4.117 -22.127 0.00 0.00
J
ATOM 12452 C   GLN J 394   -13.867   3.048 -19.430 0.00 0.00
J
ATOM 12453 O   GLN J 394   -13.299   2.565 -20.403 0.00 0.00
J
ATOM 12454 N   ALA J 395   -13.279   3.948 -18.749 0.00 0.00
J
ATOM 12455 HN ALA J 395   -13.772   4.398 -18.008 0.00 0.00
J
ATOM 12456 CA ALA J 395   -12.051   4.570 -19.108 0.00 0.00
J
ATOM 12457 HA ALA J 395   -11.891   4.505 -20.174 0.00 0.00
J
ATOM 12458 CB ALA J 395   -12.157   6.067 -18.680 0.00 0.00
J
ATOM 12459 HB1 ALA J 395   -12.587   6.123 -17.657 0.00 0.00
J
ATOM 12460 HB2 ALA J 395   -11.129   6.487 -18.650 0.00 0.00
J
ATOM 12461 HB3 ALA J 395   -12.760   6.535 -19.487 0.00 0.00
J
ATOM 12462 C   ALA J 395   -10.902   3.911 -18.411 0.00 0.00
J
ATOM 12463 O   ALA J 395   -11.131   3.219 -17.429 0.00 0.00
J
ATOM 12464 N   PRO J 396   -9.720   4.046 -19.035 0.00 0.00
J
ATOM 12465 CD PRO J 396   -9.489   4.496 -20.463 0.00 0.00
J
ATOM 12466 HD1 PRO J 396   -10.096   3.886 -21.165 0.00 0.00
J
ATOM 12467 HD2 PRO J 396   -10.002   5.476 -20.571 0.00 0.00
J
ATOM 12468 CA PRO J 396   -8.527   3.595 -18.512 0.00 0.00
J
ATOM 12469 HA PRO J 396   -8.447   2.533 -18.687 0.00 0.00
J
ATOM 12470 CB PRO J 396   -7.442   4.250 -19.391 0.00 0.00
J
ATOM 12471 HB1 PRO J 396   -6.476   3.704 -19.433 0.00 0.00
J
ATOM 12472 HB2 PRO J 396   -7.278   5.254 -18.945 0.00 0.00
J
ATOM 12473 CG PRO J 396   -8.044   4.425 -20.832 0.00 0.00
J

ATOM 12474 HG1 PRO J 396   -7.877   3.516 -21.449 0.00 0.00
J
ATOM 12475 HG2 PRO J 396   -7.647   5.315 -21.365 0.00 0.00
J
ATOM 12476 C   PRO J 396   -8.277   3.816 -16.990 0.00 0.00
J
ATOM 12477 O   PRO J 396   -8.564   4.881 -16.483 0.00 0.00
J
ATOM 12478 N   PHE J 397   -7.723   2.802 -16.357 0.00 0.00
J
ATOM 12479 HN PHE J 397   -7.357   2.006 -16.833 0.00 0.00
J
ATOM 12480 CA PHE J 397   -7.525   2.632 -14.943 0.00 0.00
J
ATOM 12481 HA PHE J 397   -8.525   2.627 -14.534 0.00 0.00
J
ATOM 12482 CB PHE J 397   -6.942   1.263 -14.701 0.00 0.00
J
ATOM 12483 HB1 PHE J 397   -7.559   0.512 -15.238 0.00 0.00
J
ATOM 12484 HB2 PHE J 397   -5.928   1.147 -15.139 0.00 0.00
J
ATOM 12485 CG PHE J 397   -6.886   0.863 -13.291 0.00 0.00
J
ATOM 12486 CD1 PHE J 397   -8.020   0.766 -12.440 0.00 0.00
J
ATOM 12487 HD1 PHE J 397   -8.970   1.199 -12.712 0.00 0.00
J
ATOM 12488 CE1 PHE J 397   -8.002   0.136 -11.218 0.00 0.00
J
ATOM 12489 HE1 PHE J 397   -8.911   0.148 -10.635 0.00 0.00
J
ATOM 12490 CZ PHE J 397   -6.790 -0.376 -10.759 0.00 0.00
J
ATOM 12491 HZ PHE J 397   -6.674 -0.778 -9.763 0.00 0.00
J
ATOM 12492 CD2 PHE J 397   -5.699   0.337 -12.823 0.00 0.00
J
ATOM 12493 HD2 PHE J 397   -4.791   0.357 -13.408 0.00 0.00
J
ATOM 12494 CE2 PHE J 397   -5.632 -0.273 -11.580 0.00 0.00
J
ATOM 12495 HE2 PHE J 397   -4.644 -0.522 -11.222 0.00 0.00
J
ATOM 12496 C   PHE J 397   -6.732   3.717 -14.237 0.00 0.00
J
ATOM 12497 O   PHE J 397   -7.176   4.168 -13.200 0.00 0.00
J
ATOM 12498 N   LEU J 398   -5.573   4.178 -14.843 0.00 0.00
J
ATOM 12499 HN LEU J 398   -5.366   3.870 -15.768 0.00 0.00
J
ATOM 12500 CA LEU J 398   -4.579   5.108 -14.334 0.00 0.00
J

ATOM 12501 HA LEU J 398 -4.468 4.749 -13.321 0.00 0.00
J
ATOM 12502 CB LEU J 398 -3.270 5.205 -15.194 0.00 0.00
J
ATOM 12503 HB1 LEU J 398 -2.980 4.199 -15.566 0.00 0.00
J
ATOM 12504 HB2 LEU J 398 -3.696 5.697 -16.095 0.00 0.00
J
ATOM 12505 CG LEU J 398 -2.025 6.031 -14.773 0.00 0.00
J
ATOM 12506 HG LEU J 398 -2.332 6.958 -14.243 0.00 0.00
J
ATOM 12507 CD1 LEU J 398 -1.159 5.106 -13.804 0.00 0.00
J
ATOM 12508 HD11 LEU J 398 -1.849 4.439 -13.245 0.00 0.00
J
ATOM 12509 HD12 LEU J 398 -0.543 4.405 -14.406 0.00 0.00
J
ATOM 12510 HD13 LEU J 398 -0.635 5.685 -13.014 0.00 0.00
J
ATOM 12511 CD2 LEU J 398 -1.191 6.356 -16.022 0.00 0.00
J
ATOM 12512 HD21 LEU J 398 -0.918 5.415 -16.546 0.00 0.00
J
ATOM 12513 HD22 LEU J 398 -1.675 7.159 -16.618 0.00 0.00
J
ATOM 12514 HD23 LEU J 398 -0.251 6.890 -15.765 0.00 0.00
J
ATOM 12515 C LEU J 398 -5.216 6.510 -14.127 0.00 0.00
J
ATOM 12516 O LEU J 398 -5.127 7.061 -13.032 0.00 0.00
J
ATOM 12517 N GLN J 399 -6.054 6.991 -15.064 0.00 0.00
J
ATOM 12518 HN GLN J 399 -6.282 6.460 -15.876 0.00 0.00
J
ATOM 12519 CA GLN J 399 -6.841 8.193 -14.864 0.00 0.00
J
ATOM 12520 HA GLN J 399 -6.162 9.024 -14.743 0.00 0.00
J
ATOM 12521 CB GLN J 399 -7.575 8.477 -16.187 0.00 0.00
J
ATOM 12522 HB1 GLN J 399 -8.044 7.501 -16.437 0.00 0.00
J
ATOM 12523 HB2 GLN J 399 -8.431 9.185 -16.164 0.00 0.00
J
ATOM 12524 CG GLN J 399 -6.610 8.880 -17.293 0.00 0.00
J
ATOM 12525 HG1 GLN J 399 -5.802 9.625 -17.128 0.00 0.00
J
ATOM 12526 HG2 GLN J 399 -6.076 7.921 -17.467 0.00 0.00
J
ATOM 12527 CD GLN J 399 -7.408 9.074 -18.571 0.00 0.00
J

```
ATOM  12528  OE1 GLN J 399      -7.472   8.245 -19.480  0.00  0.00
J
ATOM  12529  NE2 GLN J 399      -7.976  10.293 -18.830  0.00  0.00
J
ATOM  12530 HE21 GLN J 399      -8.211  10.485 -19.783  0.00  0.00
J
ATOM  12531 HE22 GLN J 399      -7.819  11.068 -18.217  0.00  0.00
J
ATOM  12532  C   GLN J 399      -7.916   8.160 -13.747  0.00  0.00
J
ATOM  12533  O   GLN J 399      -7.985   9.175 -13.029  0.00  0.00
J
ATOM  12534  N   ILE J 400      -8.647   7.060 -13.551  0.00  0.00
J
ATOM  12535  HN  ILE J 400      -8.540   6.287 -14.172  0.00  0.00
J
ATOM  12536  CA  ILE J 400      -9.577   6.753 -12.371  0.00  0.00
J
ATOM  12537  HA  ILE J 400     -10.207   7.626 -12.283  0.00  0.00
J
ATOM  12538  CB  ILE J 400     -10.443   5.484 -12.572  0.00  0.00
J
ATOM  12539  HB  ILE J 400      -9.916   4.607 -13.006  0.00  0.00
J
ATOM  12540  CG2 ILE J 400     -11.152   5.113 -11.230  0.00  0.00
J
ATOM  12541 HG21 ILE J 400     -10.526   4.652 -10.436  0.00  0.00
J
ATOM  12542 HG22 ILE J 400     -11.727   5.972 -10.823  0.00  0.00
J
ATOM  12543 HG23 ILE J 400     -11.875   4.304 -11.470  0.00  0.00
J
ATOM  12544  CG1 ILE J 400     -11.534   5.915 -13.657  0.00  0.00
J
ATOM  12545 HG11 ILE J 400     -12.371   6.523 -13.253  0.00  0.00
J
ATOM  12546 HG12 ILE J 400     -10.966   6.505 -14.407  0.00  0.00
J
ATOM  12547  CD  ILE J 400     -12.130   4.684 -14.377  0.00  0.00
J
ATOM  12548  HD1 ILE J 400     -12.597   5.132 -15.280  0.00  0.00
J
ATOM  12549  HD2 ILE J 400     -11.312   4.009 -14.709  0.00  0.00
J
ATOM  12550  HD3 ILE J 400     -12.909   4.145 -13.797  0.00  0.00
J
ATOM  12551  C   ILE J 400      -8.840   6.650 -10.949  0.00  0.00
J
ATOM  12552  O   ILE J 400      -9.209   7.260  -9.956  0.00  0.00
J
ATOM  12553  N   VAL J 401      -7.730   5.893 -10.920  0.00  0.00
J
ATOM  12554  HN  VAL J 401      -7.551   5.308 -11.708  0.00  0.00
J
```

ATOM 12555 CA VAL J 401   -6.915   5.935 -9.756 0.00 0.00
J
ATOM 12556 HA VAL J 401   -7.526   5.661 -8.909 0.00 0.00
J
ATOM 12557 CB VAL J 401   -5.931   4.771 -9.923 0.00 0.00
J
ATOM 12558 HB VAL J 401   -5.399   5.057 -10.855 0.00 0.00
J
ATOM 12559 CG1 VAL J 401   -4.948   4.667 -8.721 0.00 0.00
J
ATOM 12560 HG11 VAL J 401   -4.285   3.793 -8.896 0.00 0.00
J
ATOM 12561 HG12 VAL J 401   -4.353   5.570 -8.468 0.00 0.00
J
ATOM 12562 HG13 VAL J 401   -5.385   4.366 -7.745 0.00 0.00
J
ATOM 12563 CG2 VAL J 401   -6.642   3.441 -10.088 0.00 0.00
J
ATOM 12564 HG21 VAL J 401   -5.853   2.726 -10.406 0.00 0.00
J
ATOM 12565 HG22 VAL J 401   -7.245   3.240 -9.178 0.00 0.00
J
ATOM 12566 HG23 VAL J 401   -7.274   3.500 -11.000 0.00 0.00
J
ATOM 12567 C   VAL J 401   -6.226   7.309 -9.396 0.00 0.00
J
ATOM 12568 O   VAL J 401   -6.301   7.693 -8.234 0.00 0.00
J
ATOM 12569 N   PHE J 402   -5.635   8.063 -10.422 0.00 0.00
J
ATOM 12570 HN PHE J 402   -5.481   7.668 -11.325 0.00 0.00
J
ATOM 12571 CA PHE J 402   -5.070   9.393 -10.312 0.00 0.00
J
ATOM 12572 HA PHE J 402   -4.401   9.336 -9.467 0.00 0.00
J
ATOM 12573 CB PHE J 402   -4.154   9.643 -11.564 0.00 0.00
J
ATOM 12574 HB1 PHE J 402   -3.398   8.839 -11.432 0.00 0.00
J
ATOM 12575 HB2 PHE J 402   -4.524   9.349 -12.569 0.00 0.00
J
ATOM 12576 CG PHE J 402   -3.516 11.019 -11.621 0.00 0.00
J
ATOM 12577 CD1 PHE J 402   -2.205 11.163 -11.015 0.00 0.00
J
ATOM 12578 HD1 PHE J 402   -1.689 10.367 -10.498 0.00 0.00
J
ATOM 12579 CE1 PHE J 402   -1.564 12.393 -11.222 0.00 0.00
J
ATOM 12580 HE1 PHE J 402   -0.617 12.576 -10.736 0.00 0.00
J
ATOM 12581 CZ PHE J 402   -2.152 13.468 -11.961 0.00 0.00
J

ATOM 12582 HZ PHE J 402   -1.708 14.444 -12.086 0.00 0.00
J
ATOM 12583 CD2 PHE J 402   -4.103 12.089 -12.453 0.00 0.00
J
ATOM 12584 HD2 PHE J 402   -4.959 11.892 -13.082 0.00 0.00
J
ATOM 12585 CE2 PHE J 402   -3.324 13.229 -12.622 0.00 0.00
J
ATOM 12586 HE2 PHE J 402   -3.731 14.028 -13.224 0.00 0.00
J
ATOM 12587 C   PHE J 402   -5.985 10.575 -9.959 0.00 0.00
J
ATOM 12588 O   PHE J 402   -5.744 11.349 -9.037 0.00 0.00
J
ATOM 12589 N   GLU J 403   -7.184 10.641 -10.552 0.00 0.00
J
ATOM 12590 HN GLU J 403   -7.363   9.981 -11.278 0.00 0.00
J
ATOM 12591 CA GLU J 403   -8.193 11.648 -10.314 0.00 0.00
J
ATOM 12592 HA GLU J 403   -7.765 12.609 -10.067 0.00 0.00
J
ATOM 12593 CB GLU J 403   -8.923 12.080 -11.599 0.00 0.00
J
ATOM 12594 HB1 GLU J 403   -9.382 11.215 -12.123 0.00 0.00
J
ATOM 12595 HB2 GLU J 403   -9.761 12.733 -11.272 0.00 0.00
J
ATOM 12596 CG GLU J 403   -7.934 12.777 -12.555 0.00 0.00
J
ATOM 12597 HG1 GLU J 403   -7.504 13.724 -12.164 0.00 0.00
J
ATOM 12598 HG2 GLU J 403   -7.129 12.070 -12.847 0.00 0.00
J
ATOM 12599 CD GLU J 403   -8.575 12.963 -13.955 0.00 0.00
J
ATOM 12600 OE1 GLU J 403   -8.055 12.439 -14.987 0.00 0.00
J
ATOM 12601 OE2 GLU J 403   -9.596 13.738 -14.024 0.00 0.00
J
ATOM 12602 C   GLU J 403   -9.004 11.435 -9.039 0.00 0.00
J
ATOM 12603 O   GLU J 403   -9.486 12.345 -8.417 0.00 0.00
J
ATOM 12604 N   THR J 404   -9.105 10.191 -8.540 0.00 0.00
J
ATOM 12605 HN THR J 404   -8.599   9.462 -8.995 0.00 0.00
J
ATOM 12606 CA THR J 404   -9.654   9.934 -7.200 0.00 0.00
J
ATOM 12607 HA THR J 404   -10.668 10.290 -7.101 0.00 0.00
J
ATOM 12608 CB THR J 404   -9.883   8.482 -6.845 0.00 0.00
J

```
ATOM  12609  HB  THR J 404      -8.941   7.895  -6.812  0.00  0.00
J
ATOM  12610  OG1 THR J 404     -10.705   8.036  -7.850  0.00  0.00
J
ATOM  12611  HG1 THR J 404     -10.133   7.749  -8.566  0.00  0.00
J
ATOM  12612  CG2 THR J 404     -10.670   8.183  -5.531  0.00  0.00
J
ATOM  12613 HG21 THR J 404     -11.687   8.597  -5.702  0.00  0.00
J
ATOM  12614 HG22 THR J 404     -10.838   7.113  -5.282  0.00  0.00
J
ATOM  12615 HG23 THR J 404     -10.293   8.764  -4.662  0.00  0.00
J
ATOM  12616  C   THR J 404      -8.780  10.437  -6.040  0.00  0.00
J
ATOM  12617  O   THR J 404      -9.189  11.196  -5.168  0.00  0.00
J
ATOM  12618  N   PHE J 405      -7.468  10.143  -6.140  0.00  0.00
J
ATOM  12619  HN  PHE J 405      -7.263   9.682  -7.000  0.00  0.00
J
ATOM  12620  CA  PHE J 405      -6.455  10.731  -5.257  0.00  0.00
J
ATOM  12621  HA  PHE J 405      -6.642  10.490  -4.221  0.00  0.00
J
ATOM  12622  CB  PHE J 405      -5.142   9.978  -5.462  0.00  0.00
J
ATOM  12623  HB1 PHE J 405      -4.959   9.896  -6.555  0.00  0.00
J
ATOM  12624  HB2 PHE J 405      -4.257  10.583  -5.169  0.00  0.00
J
ATOM  12625  CG  PHE J 405      -4.894   8.573  -4.928  0.00  0.00
J
ATOM  12626  CD1 PHE J 405      -3.614   8.206  -4.523  0.00  0.00
J
ATOM  12627  HD1 PHE J 405      -2.821   8.939  -4.559  0.00  0.00
J
ATOM  12628  CE1 PHE J 405      -3.285   6.965  -3.938  0.00  0.00
J
ATOM  12629  HE1 PHE J 405      -2.276   6.849  -3.571  0.00  0.00
J
ATOM  12630  CZ  PHE J 405      -4.234   5.963  -4.019  0.00  0.00
J
ATOM  12631  HZ  PHE J 405      -3.972   5.003  -3.599  0.00  0.00
J
ATOM  12632  CD2 PHE J 405      -5.888   7.517  -4.955  0.00  0.00
J
ATOM  12633  HD2 PHE J 405      -6.882   7.769  -5.294  0.00  0.00
J
ATOM  12634  CE2 PHE J 405      -5.564   6.203  -4.527  0.00  0.00
J
ATOM  12635  HE2 PHE J 405      -6.216   5.345  -4.604  0.00  0.00
J
```

ATOM 12636 C PHE J 405 -6.356 12.270 -5.346 0.00 0.00
J
ATOM 12637 O PHE J 405 -6.160 13.014 -4.366 0.00 0.00
J
ATOM 12638 N SER J 406 -6.420 12.775 -6.604 0.00 0.00
J
ATOM 12639 HN SER J 406 -6.393 12.190 -7.411 0.00 0.00
J
ATOM 12640 CA SER J 406 -6.511 14.296 -6.842 0.00 0.00
J
ATOM 12641 HA SER J 406 -5.581 14.699 -6.470 0.00 0.00
J
ATOM 12642 CB SER J 406 -6.733 14.574 -8.402 0.00 0.00
J
ATOM 12643 HB1 SER J 406 -6.063 14.022 -9.095 0.00 0.00
J
ATOM 12644 HB2 SER J 406 -7.777 14.330 -8.692 0.00 0.00
J
ATOM 12645 OG SER J 406 -6.410 15.882 -8.799 0.00 0.00
J
ATOM 12646 HG1 SER J 406 -6.334 15.849 -9.755 0.00 0.00
J
ATOM 12647 C SER J 406 -7.687 14.964 -6.184 0.00 0.00
J
ATOM 12648 O SER J 406 -7.426 15.997 -5.598 0.00 0.00
J
ATOM 12649 N ALA J 407 -8.888 14.316 -6.222 0.00 0.00
J
ATOM 12650 HN ALA J 407 -8.968 13.493 -6.779 0.00 0.00
J
ATOM 12651 CA ALA J 407 -10.095 14.746 -5.529 0.00 0.00
J
ATOM 12652 HA ALA J 407 -10.209 15.770 -5.851 0.00 0.00
J
ATOM 12653 CB ALA J 407 -11.226 13.785 -5.976 0.00 0.00
J
ATOM 12654 HB1 ALA J 407 -12.265 14.056 -5.690 0.00 0.00
J
ATOM 12655 HB2 ALA J 407 -11.237 13.736 -7.085 0.00 0.00
J
ATOM 12656 HB3 ALA J 407 -10.936 12.775 -5.614 0.00 0.00
J
ATOM 12657 C ALA J 407 -9.923 14.737 -4.018 0.00 0.00
J
ATOM 12658 O ALA J 407 -10.270 15.713 -3.345 0.00 0.00
J
ATOM 12659 N PHE J 408 -9.473 13.576 -3.431 0.00 0.00
J
ATOM 12660 HN PHE J 408 -9.115 12.850 -4.012 0.00 0.00
J
ATOM 12661 CA PHE J 408 -9.296 13.447 -2.009 0.00 0.00
J
ATOM 12662 HA PHE J 408 -10.292 13.503 -1.597 0.00 0.00
J

ATOM 12663 CB PHE J 408 -8.869 12.017 -1.749 0.00 0.00
J
ATOM 12664 HB1 PHE J 408 -9.426 11.299 -2.388 0.00 0.00
J
ATOM 12665 HB2 PHE J 408 -7.791 11.852 -1.963 0.00 0.00
J
ATOM 12666 CG PHE J 408 -9.204 11.638 -0.293 0.00 0.00
J
ATOM 12667 CD1 PHE J 408 -10.530 11.386 0.142 0.00 0.00
J
ATOM 12668 HD1 PHE J 408 -11.424 11.633 -0.412 0.00 0.00
J
ATOM 12669 CE1 PHE J 408 -10.681 10.700 1.418 0.00 0.00
J
ATOM 12670 HE1 PHE J 408 -11.603 10.304 1.815 0.00 0.00
J
ATOM 12671 CZ PHE J 408 -9.614 10.566 2.235 0.00 0.00
J
ATOM 12672 HZ PHE J 408 -9.638 10.209 3.254 0.00 0.00
J
ATOM 12673 CD2 PHE J 408 -8.132 11.559 0.633 0.00 0.00
J
ATOM 12674 HD2 PHE J 408 -7.132 11.818 0.319 0.00 0.00
J
ATOM 12675 CE2 PHE J 408 -8.337 11.022 1.854 0.00 0.00
J
ATOM 12676 HE2 PHE J 408 -7.574 10.841 2.596 0.00 0.00
J
ATOM 12677 C PHE J 408 -8.319 14.390 -1.356 0.00 0.00
J
ATOM 12678 O PHE J 408 -8.521 14.936 -0.308 0.00 0.00
J
ATOM 12679 N GLY J 409 -7.132 14.644 -1.979 0.00 0.00
J
ATOM 12680 HN GLY J 409 -6.942 14.124 -2.808 0.00 0.00
J
ATOM 12681 CA GLY J 409 -6.084 15.520 -1.357 0.00 0.00
J
ATOM 12682 HA1 GLY J 409 -5.102 15.419 -1.797 0.00 0.00
J
ATOM 12683 HA2 GLY J 409 -6.127 15.328 -0.295 0.00 0.00
J
ATOM 12684 C GLY J 409 -6.442 16.961 -1.533 0.00 0.00
J
ATOM 12685 O GLY J 409 -5.800 17.887 -1.005 0.00 0.00
J
ATOM 12686 N THR J 410 -7.557 17.172 -2.271 0.00 0.00
J
ATOM 12687 HN THR J 410 -8.187 16.405 -2.367 0.00 0.00
J
ATOM 12688 CA THR J 410 -7.930 18.477 -2.796 0.00 0.00
J
ATOM 12689 HA THR J 410 -8.722 18.225 -3.485 0.00 0.00
J

ATOM 12690 CB THR J 410 -8.477 19.572 -1.843 0.00 0.00
J
ATOM 12691 HB THR J 410 -8.743 20.486 -2.415 0.00 0.00
J
ATOM 12692 OG1 THR J 410 -7.572 19.933 -0.850 0.00 0.00
J
ATOM 12693 HG1 THR J 410 -6.875 19.279 -0.759 0.00 0.00
J
ATOM 12694 CG2 THR J 410 -9.733 19.025 -1.106 0.00 0.00
J
ATOM 12695 HG21 THR J 410 -10.279 18.311 -1.758 0.00 0.00
J
ATOM 12696 HG22 THR J 410 -9.460 18.472 -0.182 0.00 0.00
J
ATOM 12697 HG23 THR J 410 -10.436 19.830 -0.801 0.00 0.00
J
ATOM 12698 C THR J 410 -6.823 19.128 -3.654 0.00 0.00
J
ATOM 12699 O THR J 410 -6.683 20.331 -3.714 0.00 0.00
J
ATOM 12700 N VAL J 411 -6.254 18.317 -4.519 0.00 0.00
J
ATOM 12701 HN VAL J 411 -6.609 17.388 -4.592 0.00 0.00
J
ATOM 12702 CA VAL J 411 -5.213 18.720 -5.511 0.00 0.00
J
ATOM 12703 HA VAL J 411 -4.645 19.495 -5.018 0.00 0.00
J
ATOM 12704 CB VAL J 411 -4.173 17.659 -5.902 0.00 0.00
J
ATOM 12705 HB VAL J 411 -4.566 16.849 -6.553 0.00 0.00
J
ATOM 12706 CG1 VAL J 411 -2.922 18.323 -6.570 0.00 0.00
J
ATOM 12707 HG11 VAL J 411 -2.434 18.968 -5.808 0.00 0.00
J
ATOM 12708 HG12 VAL J 411 -2.265 17.457 -6.796 0.00 0.00
J
ATOM 12709 HG13 VAL J 411 -3.048 18.954 -7.475 0.00 0.00
J
ATOM 12710 CG2 VAL J 411 -3.767 16.932 -4.673 0.00 0.00
J
ATOM 12711 HG21 VAL J 411 -3.116 16.089 -4.988 0.00 0.00
J
ATOM 12712 HG22 VAL J 411 -3.174 17.562 -3.976 0.00 0.00
J
ATOM 12713 HG23 VAL J 411 -4.500 16.392 -4.035 0.00 0.00
J
ATOM 12714 C VAL J 411 -5.694 19.345 -6.762 0.00 0.00
J
ATOM 12715 O VAL J 411 -5.255 20.458 -7.039 0.00 0.00
J
ATOM 12716 N GLY J 412 -6.578 18.769 -7.550 0.00 0.00
J

ATOM 12717 HN GLY J 412   -6.929 17.913 -7.179 0.00 0.00
J
ATOM 12718 CA GLY J 412   -7.042 19.408 -8.796 0.00 0.00
J
ATOM 12719 HA1 GLY J 412   -7.118 20.485 -8.783 0.00 0.00
J
ATOM 12720 HA2 GLY J 412   -8.030 18.999 -8.942 0.00 0.00
J
ATOM 12721 C   GLY J 412   -6.226 19.127 -10.097 0.00 0.00
J
ATOM 12722 O   GLY J 412   -6.566 19.701 -11.144 0.00 0.00
J
ATOM 12723 N   LEU J 413   -5.262 18.222 -10.017 0.00 0.00
J
ATOM 12724 HN LEU J 413   -5.113 17.784 -9.133 0.00 0.00
J
ATOM 12725 CA LEU J 413   -4.669 17.603 -11.247 0.00 0.00
J
ATOM 12726 HA LEU J 413   -4.473 18.458 -11.877 0.00 0.00
J
ATOM 12727 CB LEU J 413   -3.332 16.857 -10.972 0.00 0.00
J
ATOM 12728 HB1 LEU J 413   -3.512 15.897 -10.442 0.00 0.00
J
ATOM 12729 HB2 LEU J 413   -2.814 16.652 -11.933 0.00 0.00
J
ATOM 12730 CG LEU J 413   -2.253 17.759 -10.267 0.00 0.00
J
ATOM 12731 HG LEU J 413   -2.699 18.024 -9.285 0.00 0.00
J
ATOM 12732 CD1 LEU J 413   -0.922 17.000 -9.992 0.00 0.00
J
ATOM 12733 HD11 LEU J 413   -1.071 16.075 -9.395 0.00 0.00
J
ATOM 12734 HD12 LEU J 413   -0.491 16.648 -10.953 0.00 0.00
J
ATOM 12735 HD13 LEU J 413   -0.112 17.610 -9.539 0.00 0.00
J
ATOM 12736 CD2 LEU J 413   -1.964 18.930 -11.183 0.00 0.00
J
ATOM 12737 HD21 LEU J 413   -1.210 19.601 -10.718 0.00 0.00
J
ATOM 12738 HD22 LEU J 413   -1.474 18.589 -12.119 0.00 0.00
J
ATOM 12739 HD23 LEU J 413   -2.827 19.583 -11.436 0.00 0.00
J
ATOM 12740 C   LEU J 413   -5.557 16.714 -12.048 0.00 0.00
J
ATOM 12741 O   LEU J 413   -6.330 15.900 -11.536 0.00 0.00
J
ATOM 12742 N   THR J 414   -5.410 16.741 -13.359 0.00 0.00
J
ATOM 12743 HN THR J 414   -4.776 17.362 -13.814 0.00 0.00
J

ATOM 12744 CA THR J 414   -6.236 15.855 -14.271 0.00 0.00
J
ATOM 12745 HA THR J 414   -6.397 14.890 -13.816 0.00 0.00
J
ATOM 12746 CB THR J 414   -7.636 16.442 -14.568 0.00 0.00
J
ATOM 12747 HB THR J 414   -8.283 16.451 -13.665 0.00 0.00
J
ATOM 12748 OG1 THR J 414   -8.455 15.772 -15.546 0.00 0.00
J
ATOM 12749 HG1 THR J 414   -8.944 15.104 -15.061 0.00 0.00
J
ATOM 12750 CG2 THR J 414   -7.452 17.808 -15.191 0.00 0.00
J
ATOM 12751 HG21 THR J 414   -6.932 17.692 -16.166 0.00 0.00
J
ATOM 12752 HG22 THR J 414   -8.368 18.250 -15.638 0.00 0.00
J
ATOM 12753 HG23 THR J 414   -6.764 18.430 -14.580 0.00 0.00
J
ATOM 12754 C   THR J 414   -5.576 15.435 -15.564 0.00 0.00
J
ATOM 12755 O   THR J 414   -4.697 16.151 -16.087 0.00 0.00
J
ATOM 12756 N   MET J 415   -5.966 14.273 -16.179 0.00 0.00
J
ATOM 12757 HN MET J 415   -6.538 13.676 -15.622 0.00 0.00
J
ATOM 12758 CA MET J 415   -5.489 13.788 -17.414 0.00 0.00
J
ATOM 12759 HA MET J 415   -4.650 14.369 -17.767 0.00 0.00
J
ATOM 12760 CB MET J 415   -5.000 12.378 -17.212 0.00 0.00
J
ATOM 12761 HB1 MET J 415   -5.675 11.695 -16.654 0.00 0.00
J
ATOM 12762 HB2 MET J 415   -4.816 11.842 -18.168 0.00 0.00
J
ATOM 12763 CG MET J 415   -3.663 12.291 -16.419 0.00 0.00
J
ATOM 12764 HG1 MET J 415   -2.835 12.858 -16.897 0.00 0.00
J
ATOM 12765 HG2 MET J 415   -3.825 12.821 -15.457 0.00 0.00
J
ATOM 12766 SD MET J 415   -3.054 10.667 -15.813 0.00 0.00
J
ATOM 12767 CE MET J 415   -2.384 10.367 -17.524 0.00 0.00
J
ATOM 12768 HE1 MET J 415   -3.178 10.318 -18.300 0.00 0.00
J
ATOM 12769 HE2 MET J 415   -1.571 11.024 -17.898 0.00 0.00
J
ATOM 12770 HE3 MET J 415   -1.952   9.344 -17.486 0.00 0.00
J

```
ATOM  12771  C   MET J 415      -6.632  13.710 -18.345  0.00  0.00
J
ATOM  12772  O   MET J 415      -6.448  13.236 -19.470  0.00  0.00
J
ATOM  12773  N   GLY J 416      -7.897  14.035 -17.899  0.00  0.00
J
ATOM  12774  HN  GLY J 416      -8.046  14.461 -17.010  0.00  0.00
J
ATOM  12775  CA  GLY J 416      -9.038  13.930 -18.767  0.00  0.00
J
ATOM  12776  HA1 GLY J 416      -8.731  13.500 -19.709  0.00  0.00
J
ATOM  12777  HA2 GLY J 416      -9.427  14.931 -18.879  0.00  0.00
J
ATOM  12778  C   GLY J 416     -10.140  12.992 -18.290  0.00  0.00
J
ATOM  12779  O   GLY J 416     -11.056  12.669 -19.087  0.00  0.00
J
ATOM  12780  N   LEU J 417     -10.133  12.385 -17.056  0.00  0.00
J
ATOM  12781  HN  LEU J 417      -9.360  12.444 -16.429  0.00  0.00
J
ATOM  12782  CA  LEU J 417     -11.368  11.633 -16.604  0.00  0.00
J
ATOM  12783  HA  LEU J 417     -11.783  11.126 -17.462  0.00  0.00
J
ATOM  12784  CB  LEU J 417     -11.017  10.664 -15.512  0.00  0.00
J
ATOM  12785  HB1 LEU J 417     -10.281   9.902 -15.847  0.00  0.00
J
ATOM  12786  HB2 LEU J 417     -10.458  11.224 -14.732  0.00  0.00
J
ATOM  12787  CG  LEU J 417     -12.170   9.937 -14.786  0.00  0.00
J
ATOM  12788  HG  LEU J 417     -12.932  10.724 -14.603  0.00  0.00
J
ATOM  12789  CD1 LEU J 417     -12.659   8.815 -15.594  0.00  0.00
J
ATOM  12790 HD11 LEU J 417     -12.933   9.297 -16.557  0.00  0.00
J
ATOM  12791 HD12 LEU J 417     -11.880   8.047 -15.786  0.00  0.00
J
ATOM  12792 HD13 LEU J 417     -13.461   8.306 -15.017  0.00  0.00
J
ATOM  12793  CD2 LEU J 417     -11.740   9.506 -13.340  0.00  0.00
J
ATOM  12794 HD21 LEU J 417     -11.760  10.411 -12.695  0.00  0.00
J
ATOM  12795 HD22 LEU J 417     -12.554   8.895 -12.896  0.00  0.00
J
ATOM  12796 HD23 LEU J 417     -10.711   9.086 -13.327  0.00  0.00
J
ATOM  12797  C   LEU J 417     -12.463  12.543 -16.130  0.00  0.00
J
```

ATOM 12798 O LEU J 417 -13.605 12.435 -16.619 0.00 0.00
J
ATOM 12799 N THR J 418 -12.076 13.478 -15.237 0.00 0.00
J
ATOM 12800 HN THR J 418 -11.158 13.547 -14.855 0.00 0.00
J
ATOM 12801 CA THR J 418 -13.016 14.402 -14.711 0.00 0.00
J
ATOM 12802 HA THR J 418 -13.653 13.763 -14.117 0.00 0.00
J
ATOM 12803 CB THR J 418 -12.562 15.247 -13.520 0.00 0.00
J
ATOM 12804 HB THR J 418 -13.294 16.014 -13.191 0.00 0.00
J
ATOM 12805 OG1 THR J 418 -11.333 15.792 -13.782 0.00 0.00
J
ATOM 12806 HG1 THR J 418 -10.657 15.147 -14.001 0.00 0.00
J
ATOM 12807 CG2 THR J 418 -12.460 14.373 -12.256 0.00 0.00
J
ATOM 12808 HG21 THR J 418 -11.897 13.434 -12.448 0.00 0.00
J
ATOM 12809 HG22 THR J 418 -11.932 15.031 -11.534 0.00 0.00
J
ATOM 12810 HG23 THR J 418 -13.502 14.150 -11.939 0.00 0.00
J
ATOM 12811 C THR J 418 -13.986 15.175 -15.644 0.00 0.00
J
ATOM 12812 O THR J 418 -15.189 15.275 -15.384 0.00 0.00
J
ATOM 12813 N PRO J 419 -13.706 15.799 -16.787 0.00 0.00
J
ATOM 12814 CD PRO J 419 -12.386 16.329 -17.168 0.00 0.00
J
ATOM 12815 HD1 PRO J 419 -11.653 16.415 -16.338 0.00 0.00
J
ATOM 12816 HD2 PRO J 419 -11.928 15.687 -17.950 0.00 0.00
J
ATOM 12817 CA PRO J 419 -14.750 16.461 -17.577 0.00 0.00
J
ATOM 12818 HA PRO J 419 -15.504 16.900 -16.941 0.00 0.00
J
ATOM 12819 CB PRO J 419 -13.980 17.504 -18.463 0.00 0.00
J
ATOM 12820 HB1 PRO J 419 -14.509 18.466 -18.637 0.00 0.00
J
ATOM 12821 HB2 PRO J 419 -13.727 17.108 -19.469 0.00 0.00
J
ATOM 12822 CG PRO J 419 -12.691 17.736 -17.681 0.00 0.00
J
ATOM 12823 HG1 PRO J 419 -11.802 18.104 -18.236 0.00 0.00
J
ATOM 12824 HG2 PRO J 419 -12.826 18.234 -16.697 0.00 0.00
J

ATOM 12825 C PRO J 419 −15.475 15.476 −18.430 0.00 0.00
J
ATOM 12826 O PRO J 419 −16.536 15.850 −18.827 0.00 0.00
J
ATOM 12827 N GLU J 420 −15.032 14.225 −18.708 0.00 0.00
J
ATOM 12828 HN GLU J 420 −14.192 13.800 −18.379 0.00 0.00
J
ATOM 12829 CA GLU J 420 −15.773 13.360 −19.658 0.00 0.00
J
ATOM 12830 HA GLU J 420 −16.373 13.980 −20.307 0.00 0.00
J
ATOM 12831 CB GLU J 420 −14.850 12.663 −20.637 0.00 0.00
J
ATOM 12832 HB1 GLU J 420 −14.254 11.886 −20.113 0.00 0.00
J
ATOM 12833 HB2 GLU J 420 −15.503 11.995 −21.239 0.00 0.00
J
ATOM 12834 CG GLU J 420 −13.977 13.559 −21.600 0.00 0.00
J
ATOM 12835 HG1 GLU J 420 −14.607 14.379 −22.006 0.00 0.00
J
ATOM 12836 HG2 GLU J 420 −13.182 13.984 −20.951 0.00 0.00
J
ATOM 12837 CD GLU J 420 −13.433 12.819 −22.805 0.00 0.00
J
ATOM 12838 OE1 GLU J 420 −14.188 11.925 −23.284 0.00 0.00
J
ATOM 12839 OE2 GLU J 420 −12.285 13.175 −23.188 0.00 0.00
J
ATOM 12840 C GLU J 420 −16.676 12.303 −18.917 0.00 0.00
J
ATOM 12841 O GLU J 420 −17.123 11.389 −19.550 0.00 0.00
J
ATOM 12842 N LEU J 421 −17.006 12.597 −17.598 0.00 0.00
J
ATOM 12843 HN LEU J 421 −16.552 13.363 −17.149 0.00 0.00
J
ATOM 12844 CA LEU J 421 −17.878 11.778 −16.783 0.00 0.00
J
ATOM 12845 HA LEU J 421 −17.646 10.729 −16.888 0.00 0.00
J
ATOM 12846 CB LEU J 421 −17.684 12.113 −15.290 0.00 0.00
J
ATOM 12847 HB1 LEU J 421 −17.808 13.206 −15.136 0.00 0.00
J
ATOM 12848 HB2 LEU J 421 −18.416 11.573 −14.652 0.00 0.00
J
ATOM 12849 CG LEU J 421 −16.272 11.818 −14.762 0.00 0.00
J
ATOM 12850 HG LEU J 421 −15.549 12.156 −15.535 0.00 0.00
J
ATOM 12851 CD1 LEU J 421 −16.136 12.683 −13.541 0.00 0.00
J

ATOM 12852 HD11 LEU J 421   −16.864 12.438 −12.738 0.00 0.00
J
ATOM 12853 HD12 LEU J 421   −15.094 12.622 −13.160 0.00 0.00
J
ATOM 12854 HD13 LEU J 421   −16.172 13.754 −13.836 0.00 0.00
J
ATOM 12855 CD2 LEU J 421   −16.048 10.332 −14.361 0.00 0.00
J
ATOM 12856 HD21 LEU J 421   −16.517   9.626 −15.079 0.00 0.00
J
ATOM 12857 HD22 LEU J 421   −14.976 10.066 −14.236 0.00 0.00
J
ATOM 12858 HD23 LEU J 421   −16.575 10.103 −13.410 0.00 0.00
J
ATOM 12859 C   LEU J 421   −19.342 11.996 −17.168 0.00 0.00
J
ATOM 12860 O   LEU J 421   −19.709 13.006 −17.652 0.00 0.00
J
ATOM 12861 N   THR J 422   −20.190 10.988 −16.871 0.00 0.00
J
ATOM 12862 HN THR J 422   −19.771 10.103 −16.683 0.00 0.00
J
ATOM 12863 CA THR J 422   −21.666 11.088 −16.883 0.00 0.00
J
ATOM 12864 HA THR J 422   −22.025 11.923 −17.467 0.00 0.00
J
ATOM 12865 CB THR J 422   −22.310   9.780 −17.225 0.00 0.00
J
ATOM 12866 HB THR J 422   −23.417   9.830 −17.135 0.00 0.00
J
ATOM 12867 OG1 THR J 422   −21.882   8.671 −16.379 0.00 0.00
J
ATOM 12868 HG1 THR J 422   −20.929   8.554 −16.403 0.00 0.00
J
ATOM 12869 CG2 THR J 422   −22.028   9.311 −18.690 0.00 0.00
J
ATOM 12870 HG21 THR J 422   −22.670   8.417 −18.839 0.00 0.00
J
ATOM 12871 HG22 THR J 422   −22.200 10.124 −19.427 0.00 0.00
J
ATOM 12872 HG23 THR J 422   −20.947   9.067 −18.768 0.00 0.00
J
ATOM 12873 C   THR J 422   −22.057 11.410 −15.436 0.00 0.00
J
ATOM 12874 O   THR J 422   −21.210 11.465 −14.551 0.00 0.00
J
ATOM 12875 N   THR J 423   −23.366 11.622 −15.248 0.00 0.00
J
ATOM 12876 HN THR J 423   −24.130 11.500 −15.876 0.00 0.00
J
ATOM 12877 CA THR J 423   −23.782 12.199 −13.994 0.00 0.00
J
ATOM 12878 HA THR J 423   −23.298 13.164 −13.998 0.00 0.00
J

ATOM 12879 CB THR J 423   -25.284 12.431 -14.066 0.00 0.00
J
ATOM 12880 HB THR J 423   -25.804 11.480 -14.313 0.00 0.00
J
ATOM 12881 OG1 THR J 423   -25.700 13.291 -15.162 0.00 0.00
J
ATOM 12882 HG1 THR J 423   -26.183 12.699 -15.742 0.00 0.00
J
ATOM 12883 CG2 THR J 423   -25.844 12.951 -12.670 0.00 0.00
J
ATOM 12884 HG21 THR J 423   -25.185 13.695 -12.174 0.00 0.00
J
ATOM 12885 HG22 THR J 423   -26.739 13.603 -12.759 0.00 0.00
J
ATOM 12886 HG23 THR J 423   -25.982 12.077 -11.997 0.00 0.00
J
ATOM 12887 C   THR J 423   -23.484 11.569 -12.690 0.00 0.00
J
ATOM 12888 O   THR J 423   -23.038 12.189 -11.738 0.00 0.00
J
ATOM 12889 N   ALA J 424   -23.520 10.156 -12.710 0.00 0.00
J
ATOM 12890 HN ALA J 424   -24.034   9.787 -13.479 0.00 0.00
J
ATOM 12891 CA ALA J 424   -23.086   9.288 -11.635 0.00 0.00
J
ATOM 12892 HA ALA J 424   -23.706   9.469 -10.769 0.00 0.00
J
ATOM 12893 CB ALA J 424   -23.324   7.829 -11.898 0.00 0.00
J
ATOM 12894 HB1 ALA J 424   -22.947   7.282 -11.007 0.00 0.00
J
ATOM 12895 HB2 ALA J 424   -24.416   7.630 -11.919 0.00 0.00
J
ATOM 12896 HB3 ALA J 424   -22.753   7.433 -12.765 0.00 0.00
J
ATOM 12897 C   ALA J 424   -21.603   9.475 -11.262 0.00 0.00
J
ATOM 12898 O   ALA J 424   -21.278   9.663 -10.124 0.00 0.00
J
ATOM 12899 N   GLY J 425   -20.729   9.486 -12.305 0.00 0.00
J
ATOM 12900 HN GLY J 425   -21.016   9.278 -13.236 0.00 0.00
J
ATOM 12901 CA GLY J 425   -19.279   9.712 -12.231 0.00 0.00
J
ATOM 12902 HA1 GLY J 425   -19.010   9.845 -13.269 0.00 0.00
J
ATOM 12903 HA2 GLY J 425   -18.734   9.021 -11.605 0.00 0.00
J
ATOM 12904 C   GLY J 425   -18.965 11.046 -11.503 0.00 0.00
J
ATOM 12905 O   GLY J 425   -18.172 11.021 -10.517 0.00 0.00
J

ATOM 12906 N LYS J 426 -19.639 12.158 -11.839 0.00 0.00
J
ATOM 12907 HN LYS J 426 -20.188 12.241 -12.667 0.00 0.00
J
ATOM 12908 CA LYS J 426 -19.359 13.449 -11.183 0.00 0.00
J
ATOM 12909 HA LYS J 426 -18.282 13.499 -11.232 0.00 0.00
J
ATOM 12910 CB LYS J 426 -20.004 14.596 -11.979 0.00 0.00
J
ATOM 12911 HB1 LYS J 426 -21.045 14.300 -12.226 0.00 0.00
J
ATOM 12912 HB2 LYS J 426 -20.013 15.441 -11.257 0.00 0.00
J
ATOM 12913 CG LYS J 426 -19.213 14.880 -13.296 0.00 0.00
J
ATOM 12914 HG1 LYS J 426 -18.235 15.408 -13.262 0.00 0.00
J
ATOM 12915 HG2 LYS J 426 -19.020 13.858 -13.686 0.00 0.00
J
ATOM 12916 CD LYS J 426 -20.151 15.670 -14.210 0.00 0.00
J
ATOM 12917 HD1 LYS J 426 -21.092 15.086 -14.304 0.00 0.00
J
ATOM 12918 HD2 LYS J 426 -20.415 16.630 -13.719 0.00 0.00
J
ATOM 12919 CE LYS J 426 -19.573 15.918 -15.669 0.00 0.00
J
ATOM 12920 HE1 LYS J 426 -18.685 16.584 -15.629 0.00 0.00
J
ATOM 12921 HE2 LYS J 426 -19.247 14.923 -16.042 0.00 0.00
J
ATOM 12922 NZ LYS J 426 -20.573 16.621 -16.486 0.00 0.00
J
ATOM 12923 HZ1 LYS J 426 -20.808 17.531 -16.039 0.00 0.00
J
ATOM 12924 HZ2 LYS J 426 -20.111 16.907 -17.373 0.00 0.00
J
ATOM 12925 HZ3 LYS J 426 -21.422 16.043 -16.649 0.00 0.00
J
ATOM 12926 C LYS J 426 -19.805 13.419 -9.700 0.00 0.00
J
ATOM 12927 O LYS J 426 -19.114 13.978 -8.844 0.00 0.00
J
ATOM 12928 N CYS J 427 -20.963 12.792 -9.404 0.00 0.00
J
ATOM 12929 HN CYS J 427 -21.546 12.349 -10.080 0.00 0.00
J
ATOM 12930 CA CYS J 427 -21.372 12.661 -8.058 0.00 0.00
J
ATOM 12931 HA CYS J 427 -21.464 13.594 -7.523 0.00 0.00
J
ATOM 12932 CB CYS J 427 -22.776 12.010 -8.093 0.00 0.00
J

ATOM 12933 HB1 CYS J 427   -22.677 11.047 -8.639 0.00 0.00
J
ATOM 12934 HB2 CYS J 427   -23.161 11.927 -7.055 0.00 0.00
J
ATOM 12935 SG CYS J 427   -24.025 12.984 -8.920 0.00 0.00
J
ATOM 12936 HG1 CYS J 427   -23.530 12.562 -10.075 0.00 0.00
J
ATOM 12937 C   CYS J 427   -20.544 11.863 -7.124 0.00 0.00
J
ATOM 12938 O   CYS J 427   -20.323 12.296 -5.955 0.00 0.00
J
ATOM 12939 N   ILE J 428   -20.011 10.734 -7.630 0.00 0.00
J
ATOM 12940 HN ILE J 428   -20.405 10.329 -8.451 0.00 0.00
J
ATOM 12941 CA ILE J 428   -18.930 10.065 -6.902 0.00 0.00
J
ATOM 12942 HA ILE J 428   -19.257   9.734 -5.927 0.00 0.00
J
ATOM 12943 CB ILE J 428   -18.417   8.789 -7.587 0.00 0.00
J
ATOM 12944 HB ILE J 428   -17.904   9.210 -8.478 0.00 0.00
J
ATOM 12945 CG2 ILE J 428   -17.471   7.978 -6.688 0.00 0.00
J
ATOM 12946 HG21 ILE J 428   -18.018   7.833 -5.731 0.00 0.00
J
ATOM 12947 HG22 ILE J 428   -17.224   6.982 -7.115 0.00 0.00
J
ATOM 12948 HG23 ILE J 428   -16.566   8.605 -6.543 0.00 0.00
J
ATOM 12949 CG1 ILE J 428   -19.587   7.794 -7.834 0.00 0.00
J
ATOM 12950 HG11 ILE J 428   -19.815   7.181 -6.935 0.00 0.00
J
ATOM 12951 HG12 ILE J 428   -20.524   8.353 -8.046 0.00 0.00
J
ATOM 12952 CD ILE J 428   -19.337   6.931 -9.070 0.00 0.00
J
ATOM 12953 HD1 ILE J 428   -18.268   6.658 -9.199 0.00 0.00
J
ATOM 12954 HD2 ILE J 428   -19.955   6.011 -9.140 0.00 0.00
J
ATOM 12955 HD3 ILE J 428   -19.580   7.570 -9.946 0.00 0.00
J
ATOM 12956 C   ILE J 428   -17.684 10.928 -6.556 0.00 0.00
J
ATOM 12957 O   ILE J 428   -17.213 10.936 -5.408 0.00 0.00
J
ATOM 12958 N   ILE J 429   -17.277 11.764 -7.525 0.00 0.00
J
ATOM 12959 HN ILE J 429   -17.856 11.836 -8.334 0.00 0.00
J

```
ATOM  12960  CA  ILE J 429     -16.093  12.663  -7.419  0.00  0.00
J
ATOM  12961  HA  ILE J 429     -15.306  12.034  -7.030  0.00  0.00
J
ATOM  12962  CB  ILE J 429     -15.633  13.329  -8.680  0.00  0.00
J
ATOM  12963  HB  ILE J 429     -16.535  13.895  -8.997  0.00  0.00
J
ATOM  12964  CG2 ILE J 429     -14.521  14.400  -8.438  0.00  0.00
J
ATOM  12965 HG21 ILE J 429     -14.177  14.798  -9.417  0.00  0.00
J
ATOM  12966 HG22 ILE J 429     -14.878  15.302  -7.897  0.00  0.00
J
ATOM  12967 HG23 ILE J 429     -13.656  13.962  -7.895  0.00  0.00
J
ATOM  12968  CG1 ILE J 429     -15.195  12.299  -9.724  0.00  0.00
J
ATOM  12969 HG11 ILE J 429     -15.930  11.466  -9.689  0.00  0.00
J
ATOM  12970 HG12 ILE J 429     -15.185  12.762 -10.734  0.00  0.00
J
ATOM  12971  CD  ILE J 429     -13.798  11.651  -9.584  0.00  0.00
J
ATOM  12972  HD1 ILE J 429     -13.593  10.999 -10.460  0.00  0.00
J
ATOM  12973  HD2 ILE J 429     -13.034  12.453  -9.497  0.00  0.00
J
ATOM  12974  HD3 ILE J 429     -13.812  11.032  -8.662  0.00  0.00
J
ATOM  12975  C   ILE J 429     -16.312  13.688  -6.253  0.00  0.00
J
ATOM  12976  O   ILE J 429     -15.366  14.043  -5.559  0.00  0.00
J
ATOM  12977  N   ILE J 430     -17.551  14.257  -6.179  0.00  0.00
J
ATOM  12978  HN  ILE J 430     -18.199  13.966  -6.878  0.00  0.00
J
ATOM  12979  CA  ILE J 430     -17.942  15.273  -5.254  0.00  0.00
J
ATOM  12980  HA  ILE J 430     -17.363  16.183  -5.314  0.00  0.00
J
ATOM  12981  CB  ILE J 430     -19.325  15.795  -5.582  0.00  0.00
J
ATOM  12982  HB  ILE J 430     -19.989  14.916  -5.729  0.00  0.00
J
ATOM  12983  CG2 ILE J 430     -19.871  16.567  -4.251  0.00  0.00
J
ATOM  12984 HG21 ILE J 430     -20.234  15.759  -3.580  0.00  0.00
J
ATOM  12985 HG22 ILE J 430     -19.088  17.151  -3.721  0.00  0.00
J
ATOM  12986 HG23 ILE J 430     -20.685  17.236  -4.602  0.00  0.00
J
```

ATOM 12987 CG1 ILE J 430   -19.356 16.617 -6.899 0.00 0.00
J
ATOM 12988 HG11 ILE J 430   -18.817 17.546 -6.617 0.00 0.00
J
ATOM 12989 HG12 ILE J 430   -18.751 16.093 -7.670 0.00 0.00
J
ATOM 12990 CD ILE J 430   -20.819 16.884 -7.351 0.00 0.00
J
ATOM 12991 HD1 ILE J 430   -21.303 15.942 -7.686 0.00 0.00
J
ATOM 12992 HD2 ILE J 430   -21.432 17.334 -6.542 0.00 0.00
J
ATOM 12993 HD3 ILE J 430   -20.866 17.678 -8.126 0.00 0.00
J
ATOM 12994 C   ILE J 430   -17.903 14.668 -3.785 0.00 0.00
J
ATOM 12995 O   ILE J 430   -17.360 15.313 -2.886 0.00 0.00
J
ATOM 12996 N   VAL J 431   -18.418 13.447 -3.553 0.00 0.00
J
ATOM 12997 HN VAL J 431   -18.729 12.816 -4.258 0.00 0.00
J
ATOM 12998 CA VAL J 431   -18.288 12.773 -2.313 0.00 0.00
J
ATOM 12999 HA VAL J 431   -18.683 13.389 -1.519 0.00 0.00
J
ATOM 13000 CB VAL J 431   -19.027 11.492 -2.460 0.00 0.00
J
ATOM 13001 HB VAL J 431   -18.735 10.874 -3.336 0.00 0.00
J
ATOM 13002 CG1 VAL J 431   -18.921 10.587 -1.198 0.00 0.00
J
ATOM 13003 HG11 VAL J 431   -18.023   9.933 -1.186 0.00 0.00
J
ATOM 13004 HG12 VAL J 431   -18.911 11.141 -0.235 0.00 0.00
J
ATOM 13005 HG13 VAL J 431   -19.787   9.891 -1.189 0.00 0.00
J
ATOM 13006 CG2 VAL J 431   -20.545 11.785 -2.485 0.00 0.00
J
ATOM 13007 HG21 VAL J 431   -20.893 12.347 -3.377 0.00 0.00
J
ATOM 13008 HG22 VAL J 431   -21.020 10.784 -2.564 0.00 0.00
J
ATOM 13009 HG23 VAL J 431   -21.006 12.154 -1.543 0.00 0.00
J
ATOM 13010 C   VAL J 431   -16.820 12.596 -1.873 0.00 0.00
J
ATOM 13011 O   VAL J 431   -16.418 12.943 -0.824 0.00 0.00
J
ATOM 13012 N   ILE J 432   -15.900 12.112 -2.763 0.00 0.00
J
ATOM 13013 HN ILE J 432   -16.099 11.852 -3.704 0.00 0.00
J

ATOM 13014 CA ILE J 432   -14.500 11.973 -2.413 0.00 0.00
J
ATOM 13015 HA ILE J 432   -14.324 11.343 -1.554 0.00 0.00
J
ATOM 13016 CB ILE J 432   -13.731 11.214 -3.457 0.00 0.00
J
ATOM 13017 HB ILE J 432   -13.895 11.745 -4.419 0.00 0.00
J
ATOM 13018 CG2 ILE J 432   -12.238 11.093 -3.295 0.00 0.00
J
ATOM 13019 HG21 ILE J 432   -11.880 10.682 -2.326 0.00 0.00
J
ATOM 13020 HG22 ILE J 432   -11.840 10.531 -4.167 0.00 0.00
J
ATOM 13021 HG23 ILE J 432   -11.721 12.062 -3.467 0.00 0.00
J
ATOM 13022 CG1 ILE J 432   -14.194   9.699 -3.628 0.00 0.00
J
ATOM 13023 HG11 ILE J 432   -15.273   9.674 -3.894 0.00 0.00
J
ATOM 13024 HG12 ILE J 432   -13.623   9.229 -4.457 0.00 0.00
J
ATOM 13025 CD ILE J 432   -14.005   8.846 -2.393 0.00 0.00
J
ATOM 13026 HD1 ILE J 432   -14.848   8.931 -1.675 0.00 0.00
J
ATOM 13027 HD2 ILE J 432   -14.028   7.795 -2.753 0.00 0.00
J
ATOM 13028 HD3 ILE J 432   -13.065   9.019 -1.827 0.00 0.00
J
ATOM 13029 C   ILE J 432   -13.724 13.338 -2.119 0.00 0.00
J
ATOM 13030 O   ILE J 432   -13.001 13.506 -1.098 0.00 0.00
J
ATOM 13031 N   MET J 433   -13.896 14.424 -2.913 0.00 0.00
J
ATOM 13032 HN MET J 433   -14.119 14.234 -3.866 0.00 0.00
J
ATOM 13033 CA MET J 433   -13.395 15.773 -2.545 0.00 0.00
J
ATOM 13034 HA MET J 433   -12.328 15.847 -2.392 0.00 0.00
J
ATOM 13035 CB MET J 433   -14.013 16.817 -3.590 0.00 0.00
J
ATOM 13036 HB1 MET J 433   -15.080 16.540 -3.730 0.00 0.00
J
ATOM 13037 HB2 MET J 433   -13.923 17.868 -3.241 0.00 0.00
J
ATOM 13038 CG MET J 433   -13.281 16.712 -5.012 0.00 0.00
J
ATOM 13039 HG1 MET J 433   -12.186 16.744 -4.826 0.00 0.00
J
ATOM 13040 HG2 MET J 433   -13.548 15.735 -5.469 0.00 0.00
J

ATOM 13041 SD MET J 433 -13.726 18.187 -6.039 0.00 0.00
J
ATOM 13042 CE MET J 433 -15.453 17.874 -6.548 0.00 0.00
J
ATOM 13043 HE1 MET J 433 -15.775 16.845 -6.283 0.00 0.00
J
ATOM 13044 HE2 MET J 433 -16.170 18.622 -6.145 0.00 0.00
J
ATOM 13045 HE3 MET J 433 -15.614 18.091 -7.625 0.00 0.00
J
ATOM 13046 C MET J 433 -13.989 16.375 -1.235 0.00 0.00
J
ATOM 13047 O MET J 433 -13.265 16.982 -0.465 0.00 0.00
J
ATOM 13048 N PHE J 434 -15.312 16.152 -0.945 0.00 0.00
J
ATOM 13049 HN PHE J 434 -15.984 15.615 -1.449 0.00 0.00
J
ATOM 13050 CA PHE J 434 -15.989 16.531 0.266 0.00 0.00
J
ATOM 13051 HA PHE J 434 -15.947 17.600 0.415 0.00 0.00
J
ATOM 13052 CB PHE J 434 -17.552 16.227 0.194 0.00 0.00
J
ATOM 13053 HB1 PHE J 434 -18.075 16.690 -0.670 0.00 0.00
J
ATOM 13054 HB2 PHE J 434 -17.609 15.122 0.092 0.00 0.00
J
ATOM 13055 CG PHE J 434 -18.424 16.687 1.319 0.00 0.00
J
ATOM 13056 CD1 PHE J 434 -19.234 15.683 1.819 0.00 0.00
J
ATOM 13057 HD1 PHE J 434 -19.215 14.731 1.309 0.00 0.00
J
ATOM 13058 CE1 PHE J 434 -20.181 15.948 2.880 0.00 0.00
J
ATOM 13059 HE1 PHE J 434 -20.873 15.207 3.251 0.00 0.00
J
ATOM 13060 CZ PHE J 434 -20.314 17.251 3.318 0.00 0.00
J
ATOM 13061 HZ PHE J 434 -21.082 17.463 4.047 0.00 0.00
J
ATOM 13062 CD2 PHE J 434 -18.586 17.968 1.839 0.00 0.00
J
ATOM 13063 HD2 PHE J 434 -17.990 18.731 1.362 0.00 0.00
J
ATOM 13064 CE2 PHE J 434 -19.611 18.315 2.694 0.00 0.00
J
ATOM 13065 HE2 PHE J 434 -19.724 19.324 3.063 0.00 0.00
J
ATOM 13066 C PHE J 434 -15.395 15.771 1.503 0.00 0.00
J
ATOM 13067 O PHE J 434 -15.033 16.467 2.434 0.00 0.00
J

ATOM 13068 N ILE J 435 -15.124 14.457 1.428 0.00 0.00
J
ATOM 13069 HN ILE J 435 -15.340 13.833 0.681 0.00 0.00
J
ATOM 13070 CA ILE J 435 -14.610 13.707 2.579 0.00 0.00
J
ATOM 13071 HA ILE J 435 -15.164 14.103 3.417 0.00 0.00
J
ATOM 13072 CB ILE J 435 -14.755 12.212 2.423 0.00 0.00
J
ATOM 13073 HB ILE J 435 -14.196 11.945 1.500 0.00 0.00
J
ATOM 13074 CG2 ILE J 435 -13.973 11.433 3.563 0.00 0.00
J
ATOM 13075 HG21 ILE J 435 -12.865 11.423 3.482 0.00 0.00
J
ATOM 13076 HG22 ILE J 435 -14.504 11.860 4.440 0.00 0.00
J
ATOM 13077 HG23 ILE J 435 -14.269 10.362 3.569 0.00 0.00
J
ATOM 13078 CG1 ILE J 435 -16.227 11.680 2.428 0.00 0.00
J
ATOM 13079 HG11 ILE J 435 -16.641 11.725 3.458 0.00 0.00
J
ATOM 13080 HG12 ILE J 435 -16.746 12.337 1.697 0.00 0.00
J
ATOM 13081 CD ILE J 435 -16.417 10.317 1.835 0.00 0.00
J
ATOM 13082 HD1 ILE J 435 -17.492 10.063 1.718 0.00 0.00
J
ATOM 13083 HD2 ILE J 435 -15.797 10.277 0.914 0.00 0.00
J
ATOM 13084 HD3 ILE J 435 -16.081 9.459 2.457 0.00 0.00
J
ATOM 13085 C ILE J 435 -13.110 14.228 2.779 0.00 0.00
J
ATOM 13086 O ILE J 435 -12.765 14.540 3.923 0.00 0.00
J
ATOM 13087 N GLY J 436 -12.328 14.418 1.655 0.00 0.00
J
ATOM 13088 HN GLY J 436 -12.654 14.160 0.748 0.00 0.00
J
ATOM 13089 CA GLY J 436 -11.061 15.242 1.497 0.00 0.00
J
ATOM 13090 HA1 GLY J 436 -11.035 15.586 0.474 0.00 0.00
J
ATOM 13091 HA2 GLY J 436 -10.317 14.525 1.811 0.00 0.00
J
ATOM 13092 C GLY J 436 -11.032 16.414 2.322 0.00 0.00
J
ATOM 13093 O GLY J 436 -10.018 16.618 2.887 0.00 0.00
J
ATOM 13094 N ARG J 437 -12.161 17.184 2.611 0.00 0.00
J

ATOM 13095 HN ARG J 437 -12.996 16.864 2.172 0.00 0.00
J
ATOM 13096 CA ARG J 437 -12.046 18.455 3.316 0.00 0.00
J
ATOM 13097 HA ARG J 437 -11.033 18.823 3.247 0.00 0.00
J
ATOM 13098 CB ARG J 437 -13.023 19.432 2.568 0.00 0.00
J
ATOM 13099 HB1 ARG J 437 -12.694 19.361 1.509 0.00 0.00
J
ATOM 13100 HB2 ARG J 437 -14.029 18.960 2.587 0.00 0.00
J
ATOM 13101 CG ARG J 437 -12.963 20.839 3.081 0.00 0.00
J
ATOM 13102 HG1 ARG J 437 -13.547 21.557 2.467 0.00 0.00
J
ATOM 13103 HG2 ARG J 437 -13.495 20.844 4.056 0.00 0.00
J
ATOM 13104 CD ARG J 437 -11.567 21.509 3.358 0.00 0.00
J
ATOM 13105 HD1 ARG J 437 -11.666 22.417 3.990 0.00 0.00
J
ATOM 13106 HD2 ARG J 437 -10.888 20.793 3.871 0.00 0.00
J
ATOM 13107 NE ARG J 437 -10.985 21.802 2.019 0.00 0.00
J
ATOM 13108 HE ARG J 437 -11.421 22.558 1.531 0.00 0.00
J
ATOM 13109 CZ ARG J 437 -9.765 21.379 1.596 0.00 0.00
J
ATOM 13110 NH1 ARG J 437 -8.988 20.545 2.301 0.00 0.00
J
ATOM 13111 HH11 ARG J 437 -8.035 20.632 2.013 0.00 0.00
J
ATOM 13112 HH12 ARG J 437 -9.267 20.317 3.234 0.00 0.00
J
ATOM 13113 NH2 ARG J 437 -9.231 21.877 0.498 0.00 0.00
J
ATOM 13114 HH21 ARG J 437 -8.499 21.302 0.131 0.00 0.00
J
ATOM 13115 HH22 ARG J 437 -9.898 22.154 -0.193 0.00 0.00
J
ATOM 13116 C ARG J 437 -12.502 18.347 4.736 0.00 0.00
J
ATOM 13117 O ARG J 437 -11.972 18.876 5.718 0.00 0.00
J
ATOM 13118 N ILE J 438 -13.672 17.740 4.905 0.00 0.00
J
ATOM 13119 HN ILE J 438 -13.967 17.243 4.092 0.00 0.00
J
ATOM 13120 CA ILE J 438 -14.233 17.623 6.238 0.00 0.00
J
ATOM 13121 HA ILE J 438 -14.264 18.584 6.730 0.00 0.00
J

ATOM 13122 CB ILE J 438 -15.685 17.265 6.194 0.00 0.00
J
ATOM 13123 HB ILE J 438 -15.653 16.174 5.987 0.00 0.00
J
ATOM 13124 CG2 ILE J 438 -16.318 17.521 7.596 0.00 0.00
J
ATOM 13125 HG21 ILE J 438 -17.413 17.334 7.609 0.00 0.00
J
ATOM 13126 HG22 ILE J 438 -15.848 16.805 8.305 0.00 0.00
J
ATOM 13127 HG23 ILE J 438 -16.077 18.552 7.933 0.00 0.00
J
ATOM 13128 CG1 ILE J 438 -16.436 18.020 5.079 0.00 0.00
J
ATOM 13129 HG11 ILE J 438 -16.043 17.829 4.058 0.00 0.00
J
ATOM 13130 HG12 ILE J 438 -17.473 17.632 4.988 0.00 0.00
J
ATOM 13131 CD ILE J 438 -16.468 19.534 5.302 0.00 0.00
J
ATOM 13132 HD1 ILE J 438 -16.943 19.885 6.243 0.00 0.00
J
ATOM 13133 HD2 ILE J 438 -15.406 19.853 5.226 0.00 0.00
J
ATOM 13134 HD3 ILE J 438 -16.986 20.055 4.469 0.00 0.00
J
ATOM 13135 C ILE J 438 -13.510 16.668 7.055 0.00 0.00
J
ATOM 13136 O ILE J 438 -13.183 16.944 8.203 0.00 0.00
J
ATOM 13137 N GLY J 439 -13.043 15.591 6.548 0.00 0.00
J
ATOM 13138 HN GLY J 439 -13.220 15.360 5.594 0.00 0.00
J
ATOM 13139 CA GLY J 439 -12.234 14.673 7.254 0.00 0.00
J
ATOM 13140 HA1 GLY J 439 -11.589 15.157 7.971 0.00 0.00
J
ATOM 13141 HA2 GLY J 439 -11.690 14.248 6.423 0.00 0.00
J
ATOM 13142 C GLY J 439 -13.053 13.577 7.963 0.00 0.00
J
ATOM 13143 O GLY J 439 -13.901 13.864 8.791 0.00 0.00
J
ATOM 13144 N PRO J 440 -12.766 12.266 7.733 0.00 0.00
J
ATOM 13145 CD PRO J 440 -11.913 11.779 6.674 0.00 0.00
J
ATOM 13146 HD1 PRO J 440 -12.276 12.164 5.697 0.00 0.00
J
ATOM 13147 HD2 PRO J 440 -10.856 11.887 6.997 0.00 0.00
J
ATOM 13148 CA PRO J 440 -13.356 11.175 8.481 0.00 0.00
J

ATOM 13149 HA PRO J 440   -14.333 11.028   8.044 0.00 0.00
J
ATOM 13150 CB PRO J 440   -12.471   9.987   8.210 0.00 0.00
J
ATOM 13151 HB1 PRO J 440   -13.003   9.030   8.396 0.00 0.00
J
ATOM 13152 HB2 PRO J 440   -11.548   9.933   8.825 0.00 0.00
J
ATOM 13153 CG PRO J 440   -12.197 10.280   6.721 0.00 0.00
J
ATOM 13154 HG1 PRO J 440   -13.166 10.109   6.204 0.00 0.00
J
ATOM 13155 HG2 PRO J 440   -11.374   9.727   6.222 0.00 0.00
J
ATOM 13156 C   PRO J 440   -13.671 11.387 10.020 0.00 0.00
J
ATOM 13157 O   PRO J 440   -14.787 11.180 10.443 0.00 0.00
J
ATOM 13158 N   LEU J 441   -12.738 11.829 10.789 0.00 0.00
J
ATOM 13159 HN LEU J 441   -11.804 12.022 10.497 0.00 0.00
J
ATOM 13160 CA LEU J 441   -13.016 11.977 12.199 0.00 0.00
J
ATOM 13161 HA LEU J 441   -13.440 11.091 12.648 0.00 0.00
J
ATOM 13162 CB LEU J 441   -11.772 12.402 12.872 0.00 0.00
J
ATOM 13163 HB1 LEU J 441   -11.299 13.248 12.328 0.00 0.00
J
ATOM 13164 HB2 LEU J 441   -11.989 12.644 13.934 0.00 0.00
J
ATOM 13165 CG LEU J 441   -10.745 11.234 12.910 0.00 0.00
J
ATOM 13166 HG LEU J 441   -10.470 10.974 11.865 0.00 0.00
J
ATOM 13167 CD1 LEU J 441   -9.380 11.793 13.464 0.00 0.00
J
ATOM 13168 HD11 LEU J 441   -8.528 11.081 13.504 0.00 0.00
J
ATOM 13169 HD12 LEU J 441   -8.996 12.647 12.866 0.00 0.00
J
ATOM 13170 HD13 LEU J 441   -9.630 12.056 14.514 0.00 0.00
J
ATOM 13171 CD2 LEU J 441   -11.240 10.013 13.755 0.00 0.00
J
ATOM 13172 HD21 LEU J 441   -10.417   9.324 14.039 0.00 0.00
J
ATOM 13173 HD22 LEU J 441   -11.809 10.247 14.680 0.00 0.00
J
ATOM 13174 HD23 LEU J 441   -11.840   9.424 13.028 0.00 0.00
J
ATOM 13175 C   LEU J 441   -14.110 12.973 12.518 0.00 0.00
J

ATOM 13176 O LEU J 441 -14.864 12.808 13.439 0.00 0.00
J
ATOM 13177 N THR J 442 -14.204 14.129 11.769 0.00 0.00
J
ATOM 13178 HN THR J 442 -13.602 14.238 10.981 0.00 0.00
J
ATOM 13179 CA THR J 442 -15.190 15.232 11.824 0.00 0.00
J
ATOM 13180 HA THR J 442 -15.231 15.524 12.864 0.00 0.00
J
ATOM 13181 CB THR J 442 -14.777 16.373 10.998 0.00 0.00
J
ATOM 13182 HB THR J 442 -14.909 16.176 9.913 0.00 0.00
J
ATOM 13183 OG1 THR J 442 -13.430 16.690 11.169 0.00 0.00
J
ATOM 13184 HG1 THR J 442 -13.213 17.419 10.584 0.00 0.00
J
ATOM 13185 CG2 THR J 442 -15.604 17.615 11.413 0.00 0.00
J
ATOM 13186 HG21 THR J 442 -16.647 17.482 11.052 0.00 0.00
J
ATOM 13187 HG22 THR J 442 -15.561 17.837 12.500 0.00 0.00
J
ATOM 13188 HG23 THR J 442 -15.239 18.486 10.828 0.00 0.00
J
ATOM 13189 C THR J 442 -16.621 14.784 11.638 0.00 0.00
J
ATOM 13190 O THR J 442 -17.589 15.311 12.226 0.00 0.00
J
ATOM 13191 N PHE J 443 -16.844 13.722 10.883 0.00 0.00
J
ATOM 13192 HN PHE J 443 -16.042 13.275 10.494 0.00 0.00
J
ATOM 13193 CA PHE J 443 -18.168 13.156 10.701 0.00 0.00
J
ATOM 13194 HA PHE J 443 -18.860 13.956 10.482 0.00 0.00
J
ATOM 13195 CB PHE J 443 -18.438 12.170 9.528 0.00 0.00
J
ATOM 13196 HB1 PHE J 443 -17.829 11.242 9.574 0.00 0.00
J
ATOM 13197 HB2 PHE J 443 -19.519 11.916 9.495 0.00 0.00
J
ATOM 13198 CG PHE J 443 -18.126 12.916 8.135 0.00 0.00
J
ATOM 13199 CD1 PHE J 443 -19.136 13.679 7.450 0.00 0.00
J
ATOM 13200 HD1 PHE J 443 -20.163 13.771 7.770 0.00 0.00
J
ATOM 13201 CE1 PHE J 443 -18.867 14.161 6.147 0.00 0.00
J
ATOM 13202 HE1 PHE J 443 -19.499 14.748 5.498 0.00 0.00
J

```
ATOM  13203  CZ  PHE J 443     -17.644  13.974   5.581  0.00  0.00
J
ATOM  13204  HZ  PHE J 443     -17.427  14.376   4.603  0.00  0.00
J
ATOM  13205  CD2 PHE J 443     -16.835  12.889   7.545  0.00  0.00
J
ATOM  13206  HD2 PHE J 443     -16.147  12.240   8.066  0.00  0.00
J
ATOM  13207  CE2 PHE J 443     -16.609  13.353   6.272  0.00  0.00
J
ATOM  13208  HE2 PHE J 443     -15.659  13.181   5.789  0.00  0.00
J
ATOM  13209  C   PHE J 443     -18.536  12.435  11.915  0.00  0.00
J
ATOM  13210  O   PHE J 443     -19.666  12.641  12.425  0.00  0.00
J
ATOM  13211  N   VAL J 444     -17.635  11.592  12.496  0.00  0.00
J
ATOM  13212  HN  VAL J 444     -16.758  11.540  12.025  0.00  0.00
J
ATOM  13213  CA  VAL J 444     -17.898  10.828  13.732  0.00  0.00
J
ATOM  13214  HA  VAL J 444     -18.801  10.283  13.501  0.00  0.00
J
ATOM  13215  CB  VAL J 444     -16.864   9.772  14.115  0.00  0.00
J
ATOM  13216  HB  VAL J 444     -15.972  10.352  14.437  0.00  0.00
J
ATOM  13217  CG1 VAL J 444     -17.401   8.956  15.263  0.00  0.00
J
ATOM  13218 HG11 VAL J 444     -17.474   9.484  16.238  0.00  0.00
J
ATOM  13219 HG12 VAL J 444     -18.449   8.690  15.007  0.00  0.00
J
ATOM  13220 HG13 VAL J 444     -16.636   8.171  15.444  0.00  0.00
J
ATOM  13221  CG2 VAL J 444     -16.482   8.983  12.865  0.00  0.00
J
ATOM  13222 HG21 VAL J 444     -17.372   8.431  12.494  0.00  0.00
J
ATOM  13223 HG22 VAL J 444     -16.033   9.532  12.010  0.00  0.00
J
ATOM  13224 HG23 VAL J 444     -15.732   8.174  13.001  0.00  0.00
J
ATOM  13225  C   VAL J 444     -18.169  11.825  14.855  0.00  0.00
J
ATOM  13226  O   VAL J 444     -18.987  11.569  15.694  0.00  0.00
J
ATOM  13227  N   PHE J 445     -17.417  12.906  14.913  0.00  0.00
J
ATOM  13228  HN  PHE J 445     -16.596  12.873  14.348  0.00  0.00
J
ATOM  13229  CA  PHE J 445     -17.505  13.931  15.891  0.00  0.00
J
```

ATOM	13230	HA	PHE	J	445	−17.508	13.357	16.805	0.00	0.00	J
ATOM	13231	CB	PHE	J	445	−16.278	14.799	15.611	0.00	0.00	J
ATOM	13232	HB1	PHE	J	445	−15.373	14.154	15.586	0.00	0.00	J
ATOM	13233	HB2	PHE	J	445	−16.285	15.359	14.651	0.00	0.00	J
ATOM	13234	CG	PHE	J	445	−15.967	15.778	16.712	0.00	0.00	J
ATOM	13235	CD1	PHE	J	445	−15.906	17.127	16.509	0.00	0.00	J
ATOM	13236	HD1	PHE	J	445	−15.951	17.474	15.487	0.00	0.00	J
ATOM	13237	CE1	PHE	J	445	−15.365	18.017	17.464	0.00	0.00	J
ATOM	13238	HE1	PHE	J	445	−15.219	19.060	17.222	0.00	0.00	J
ATOM	13239	CZ	PHE	J	445	−14.884	17.549	18.720	0.00	0.00	J
ATOM	13240	HZ	PHE	J	445	−14.483	18.208	19.476	0.00	0.00	J
ATOM	13241	CD2	PHE	J	445	−15.698	15.333	18.076	0.00	0.00	J
ATOM	13242	HD2	PHE	J	445	−15.829	14.279	18.271	0.00	0.00	J
ATOM	13243	CE2	PHE	J	445	−15.112	16.175	19.015	0.00	0.00	J
ATOM	13244	HE2	PHE	J	445	−14.683	15.734	19.902	0.00	0.00	J
ATOM	13245	C	PHE	J	445	−18.779	14.708	15.924	0.00	0.00	J
ATOM	13246	O	PHE	J	445	−19.266	14.971	17.010	0.00	0.00	J
ATOM	13247	N	SER	J	446	−19.373	15.045	14.751	0.00	0.00	J
ATOM	13248	HN	SER	J	446	−19.001	14.943	13.832	0.00	0.00	J
ATOM	13249	CA	SER	J	446	−20.567	15.859	14.523	0.00	0.00	J
ATOM	13250	HA	SER	J	446	−20.486	16.793	15.059	0.00	0.00	J
ATOM	13251	CB	SER	J	446	−20.868	16.146	12.965	0.00	0.00	J
ATOM	13252	HB1	SER	J	446	−21.020	15.191	12.417	0.00	0.00	J
ATOM	13253	HB2	SER	J	446	−21.826	16.663	12.744	0.00	0.00	J
ATOM	13254	OG	SER	J	446	−19.877	16.935	12.356	0.00	0.00	J
ATOM	13255	HG1	SER	J	446	−19.074	16.432	12.511	0.00	0.00	J
ATOM	13256	C	SER	J	446	−21.773	15.152	15.051	0.00	0.00	J

ATOM 13257 O SER J 446 -22.758 15.871 15.229 0.00 0.00
J
ATOM 13258 N PHE J 447 -21.904 13.786 15.179 0.00 0.00
J
ATOM 13259 HN PHE J 447 -21.198 13.241 14.732 0.00 0.00
J
ATOM 13260 CA PHE J 447 -23.104 13.087 15.541 0.00 0.00
J
ATOM 13261 HA PHE J 447 -23.929 13.755 15.740 0.00 0.00
J
ATOM 13262 CB PHE J 447 -23.552 12.254 14.326 0.00 0.00
J
ATOM 13263 HB1 PHE J 447 -22.759 11.519 14.071 0.00 0.00
J
ATOM 13264 HB2 PHE J 447 -24.559 11.877 14.606 0.00 0.00
J
ATOM 13265 CG PHE J 447 -23.874 13.095 12.987 0.00 0.00
J
ATOM 13266 CD1 PHE J 447 -22.902 13.153 11.936 0.00 0.00
J
ATOM 13267 HD1 PHE J 447 -21.899 12.804 12.137 0.00 0.00
J
ATOM 13268 CE1 PHE J 447 -23.059 13.970 10.791 0.00 0.00
J
ATOM 13269 HE1 PHE J 447 -22.361 14.015 9.968 0.00 0.00
J
ATOM 13270 CZ PHE J 447 -24.403 14.511 10.605 0.00 0.00
J
ATOM 13271 HZ PHE J 447 -24.672 14.869 9.622 0.00 0.00
J
ATOM 13272 CD2 PHE J 447 -25.119 13.743 12.764 0.00 0.00
J
ATOM 13273 HD2 PHE J 447 -25.864 13.704 13.544 0.00 0.00
J
ATOM 13274 CE2 PHE J 447 -25.414 14.395 11.604 0.00 0.00
J
ATOM 13275 HE2 PHE J 447 -26.374 14.862 11.442 0.00 0.00
J
ATOM 13276 C PHE J 447 -22.893 12.191 16.778 0.00 0.00
J
ATOM 13277 O PHE J 447 -23.779 11.511 17.259 0.00 0.00
J
ATOM 13278 N ALA J 448 -21.637 12.209 17.265 0.00 0.00
J
ATOM 13279 HN ALA J 448 -20.905 12.717 16.817 0.00 0.00
J
ATOM 13280 CA ALA J 448 -21.080 11.465 18.328 0.00 0.00
J
ATOM 13281 HA ALA J 448 -20.940 10.448 17.990 0.00 0.00
J
ATOM 13282 CB ALA J 448 -19.757 12.161 18.751 0.00 0.00
J
ATOM 13283 HB1 ALA J 448 -19.236 11.385 19.351 0.00 0.00
J

ATOM 13284 HB2 ALA J 448 −19.221 12.301 17.788 0.00 0.00
J
ATOM 13285 HB3 ALA J 448 −19.832 13.043 19.423 0.00 0.00
J
ATOM 13286 C ALA J 448 −21.953 11.193 19.593 0.00 0.00
J
ATOM 13287 O ALA J 448 −22.466 12.093 20.209 0.00 0.00
J
ATOM 13288 N LYS J 449 −22.219 9.965 19.937 0.00 0.00
J
ATOM 13289 HN LYS J 449 −21.825 9.267 19.344 0.00 0.00
J
ATOM 13290 CA LYS J 449 −23.314 9.496 20.777 0.00 0.00
J
ATOM 13291 HA LYS J 449 −23.799 10.361 21.204 0.00 0.00
J
ATOM 13292 CB LYS J 449 −24.301 8.577 19.876 0.00 0.00
J
ATOM 13293 HB1 LYS J 449 −24.809 9.352 19.264 0.00 0.00
J
ATOM 13294 HB2 LYS J 449 −23.736 7.907 19.193 0.00 0.00
J
ATOM 13295 CG LYS J 449 −25.462 7.831 20.586 0.00 0.00
J
ATOM 13296 HG1 LYS J 449 −25.080 6.939 21.127 0.00 0.00
J
ATOM 13297 HG2 LYS J 449 −25.818 8.501 21.397 0.00 0.00
J
ATOM 13298 CD LYS J 449 −26.666 7.568 19.665 0.00 0.00
J
ATOM 13299 HD1 LYS J 449 −26.923 8.479 19.083 0.00 0.00
J
ATOM 13300 HD2 LYS J 449 −26.455 6.737 18.958 0.00 0.00
J
ATOM 13301 CE LYS J 449 −27.870 7.184 20.564 0.00 0.00
J
ATOM 13302 HE1 LYS J 449 −27.493 6.335 21.172 0.00 0.00
J
ATOM 13303 HE2 LYS J 449 −28.047 8.121 21.136 0.00 0.00
J
ATOM 13304 NZ LYS J 449 −28.972 6.905 19.798 0.00 0.00
J
ATOM 13305 HZ1 LYS J 449 −28.682 6.608 18.844 0.00 0.00
J
ATOM 13306 HZ2 LYS J 449 −29.642 6.226 20.213 0.00 0.00
J
ATOM 13307 HZ3 LYS J 449 −29.564 7.758 19.734 0.00 0.00
J
ATOM 13308 C LYS J 449 −22.809 8.670 22.008 0.00 0.00
J
ATOM 13309 O LYS J 449 −22.165 7.617 21.946 0.00 0.00
J
ATOM 13310 N THR J 450 −23.099 9.198 23.272 0.00 0.00
J

ATOM 13311 HN THR J 450 -23.415 10.111 23.518 0.00 0.00
J
ATOM 13312 CA THR J 450 -22.591 8.584 24.493 0.00 0.00
J
ATOM 13313 HA THR J 450 -21.523 8.545 24.332 0.00 0.00
J
ATOM 13314 CB THR J 450 -22.641 9.599 25.685 0.00 0.00
J
ATOM 13315 HB THR J 450 -23.720 9.628 25.947 0.00 0.00
J
ATOM 13316 OG1 THR J 450 -22.238 10.904 25.219 0.00 0.00
J
ATOM 13317 HG1 THR J 450 -21.380 10.761 24.812 0.00 0.00
J
ATOM 13318 CG2 THR J 450 -21.844 9.171 26.969 0.00 0.00
J
ATOM 13319 HG21 THR J 450 -21.905 9.943 27.766 0.00 0.00
J
ATOM 13320 HG22 THR J 450 -22.345 8.231 27.286 0.00 0.00
J
ATOM 13321 HG23 THR J 450 -20.777 9.006 26.711 0.00 0.00
J
ATOM 13322 C THR J 450 -23.013 7.137 24.767 0.00 0.00
J
ATOM 13323 O THR J 450 -24.191 6.838 24.622 0.00 0.00
J
ATOM 13324 N GLU J 451 -22.144 6.200 25.176 0.00 0.00
J
ATOM 13325 HN GLU J 451 -21.224 6.469 25.452 0.00 0.00
J
ATOM 13326 CA GLU J 451 -22.584 4.807 25.440 0.00 0.00
J
ATOM 13327 HA GLU J 451 -23.651 4.703 25.308 0.00 0.00
J
ATOM 13328 CB GLU J 451 -21.787 3.784 24.507 0.00 0.00
J
ATOM 13329 HB1 GLU J 451 -22.367 2.837 24.536 0.00 0.00
J
ATOM 13330 HB2 GLU J 451 -21.835 4.142 23.457 0.00 0.00
J
ATOM 13331 CG GLU J 451 -20.242 3.565 24.822 0.00 0.00
J
ATOM 13332 HG1 GLU J 451 -19.635 4.463 24.580 0.00 0.00
J
ATOM 13333 HG2 GLU J 451 -20.136 3.251 25.883 0.00 0.00
J
ATOM 13334 CD GLU J 451 -19.757 2.323 24.235 0.00 0.00
J
ATOM 13335 OE1 GLU J 451 -18.776 2.394 23.459 0.00 0.00
J
ATOM 13336 OE2 GLU J 451 -20.133 1.177 24.600 0.00 0.00
J
ATOM 13337 C GLU J 451 -22.330 4.538 26.927 0.00 0.00
J

ATOM 13338 O   GLU J 451   -21.909   5.478 27.678 0.00 0.00
J
ATOM 13339 N   GLN J 452   -22.711   3.407 27.454 0.00 0.00
J
ATOM 13340 HN GLN J 452   -23.164   2.779 26.826 0.00 0.00
J
ATOM 13341 CA GLN J 452   -22.519   2.782 28.783 0.00 0.00
J
ATOM 13342 HA GLN J 452   -23.036   3.392 29.510 0.00 0.00
J
ATOM 13343 CB GLN J 452   -23.170   1.345 28.895 0.00 0.00
J
ATOM 13344 HB1 GLN J 452   -23.057   0.984 29.939 0.00 0.00
J
ATOM 13345 HB2 GLN J 452   -24.267   1.360 28.715 0.00 0.00
J
ATOM 13346 CG GLN J 452   -22.472   0.233 28.068 0.00 0.00
J
ATOM 13347 HG1 GLN J 452   -22.173   0.595 27.061 0.00 0.00
J
ATOM 13348 HG2 GLN J 452   -21.595 -0.149 28.634 0.00 0.00
J
ATOM 13349 CD GLN J 452   -23.349 -0.961 27.786 0.00 0.00
J
ATOM 13350 OE1 GLN J 452   -24.540 -0.700 27.667 0.00 0.00
J
ATOM 13351 NE2 GLN J 452   -22.945 -2.223 27.578 0.00 0.00
J
ATOM 13352 HE21 GLN J 452   -23.615 -2.872 27.217 0.00 0.00
J
ATOM 13353 HE22 GLN J 452   -22.065 -2.598 27.868 0.00 0.00
J
ATOM 13354 C   GLN J 452   -21.038   2.774 29.257 0.00 0.00
J
ATOM 13355 O   GLN J 452   -20.158   2.560 28.490 0.00 0.00
J
ATOM 13356 N   SER J 453   -20.796   2.876 30.626 0.00 0.00
J
ATOM 13357 HN SER J 453   -21.528   3.080 31.272 0.00 0.00
J
ATOM 13358 CA SER J 453   -19.495   2.902 31.178 0.00 0.00
J
ATOM 13359 HA SER J 453   -18.971   3.762 30.788 0.00 0.00
J
ATOM 13360 CB SER J 453   -19.571   3.013 32.722 0.00 0.00
J
ATOM 13361 HB1 SER J 453   -20.351   2.348 33.152 0.00 0.00
J
ATOM 13362 HB2 SER J 453   -18.579   3.001 33.222 0.00 0.00
J
ATOM 13363 OG SER J 453   -20.062   4.273 33.107 0.00 0.00
J
ATOM 13364 HG1 SER J 453   -19.403   4.543 33.752 0.00 0.00
J

ATOM 13365 C SER J 453 −18.550 1.732 30.939 0.00 0.00
J
ATOM 13366 O SER J 453 −18.962 0.576 31.023 0.00 0.00
J
ATOM 13367 N ASN J 454 −17.253 1.982 30.696 0.00 0.00
J
ATOM 13368 HN ASN J 454 −16.884 2.907 30.747 0.00 0.00
J
ATOM 13369 CA ASN J 454 −16.239 1.027 30.314 0.00 0.00
J
ATOM 13370 HA ASN J 454 −16.581 0.555 29.405 0.00 0.00
J
ATOM 13371 CB ASN J 454 −14.887 1.665 29.891 0.00 0.00
J
ATOM 13372 HB1 ASN J 454 −14.535 2.327 30.710 0.00 0.00
J
ATOM 13373 HB2 ASN J 454 −14.066 0.941 29.703 0.00 0.00
J
ATOM 13374 CG ASN J 454 −15.091 2.496 28.631 0.00 0.00
J
ATOM 13375 OD1 ASN J 454 −15.339 3.684 28.712 0.00 0.00
J
ATOM 13376 ND2 ASN J 454 −14.991 1.845 27.469 0.00 0.00
J
ATOM 13377 HD21 ASN J 454 −15.206 2.380 26.652 0.00 0.00
J
ATOM 13378 HD22 ASN J 454 −14.810 0.862 27.452 0.00 0.00
J
ATOM 13379 C ASN J 454 −15.863 −0.109 31.299 0.00 0.00
J
ATOM 13380 O ASN J 454 −15.564 0.081 32.508 0.00 0.00
J
ATOM 13381 N ILE J 455 −15.858 −1.333 30.836 0.00 0.00
J
ATOM 13382 HN ILE J 455 −16.280 −1.464 29.942 0.00 0.00
J
ATOM 13383 CA ILE J 455 −15.437 −2.496 31.539 0.00 0.00
J
ATOM 13384 HA ILE J 455 −15.068 −2.089 32.469 0.00 0.00
J
ATOM 13385 CB ILE J 455 −16.640 −3.444 31.807 0.00 0.00
J
ATOM 13386 HB ILE J 455 −16.429 −4.365 32.392 0.00 0.00
J
ATOM 13387 CG2 ILE J 455 −17.671 −2.733 32.793 0.00 0.00
J
ATOM 13388 HG21 ILE J 455 −18.129 −1.858 32.285 0.00 0.00
J
ATOM 13389 HG22 ILE J 455 −18.592 −3.303 33.042 0.00 0.00
J
ATOM 13390 HG23 ILE J 455 −17.175 −2.272 33.674 0.00 0.00
J
ATOM 13391 CG1 ILE J 455 −17.251 −3.924 30.431 0.00 0.00
J

```
ATOM  13392 HG11 ILE J 455     -17.361  -3.152  29.640  0.00  0.00
J
ATOM  13393 HG12 ILE J 455     -16.450  -4.614  30.089  0.00  0.00
J
ATOM  13394  CD  ILE J 455     -18.549  -4.748  30.589  0.00  0.00
J
ATOM  13395  HD1 ILE J 455     -18.817  -5.267  29.644  0.00  0.00
J
ATOM  13396  HD2 ILE J 455     -18.557  -5.470  31.433  0.00  0.00
J
ATOM  13397  HD3 ILE J 455     -19.354  -4.009  30.787  0.00  0.00
J
ATOM  13398  C   ILE J 455     -14.347  -3.250  30.879  0.00  0.00
J
ATOM  13399  O   ILE J 455     -13.962  -2.985  29.730  0.00  0.00
J
ATOM  13400  N   ARG J 456     -13.911  -4.362  31.484  0.00  0.00
J
ATOM  13401  HN  ARG J 456     -14.508  -4.632  32.236  0.00  0.00
J
ATOM  13402  CA  ARG J 456     -12.820  -5.150  31.053  0.00  0.00
J
ATOM  13403  HA  ARG J 456     -12.595  -4.808  30.054  0.00  0.00
J
ATOM  13404  CB  ARG J 456     -11.623  -4.950  31.973  0.00  0.00
J
ATOM  13405  HB1 ARG J 456     -11.971  -5.284  32.974  0.00  0.00
J
ATOM  13406  HB2 ARG J 456     -10.750  -5.525  31.599  0.00  0.00
J
ATOM  13407  CG  ARG J 456     -11.047  -3.539  32.273  0.00  0.00
J
ATOM  13408  HG1 ARG J 456     -10.789  -3.091  31.289  0.00  0.00
J
ATOM  13409  HG2 ARG J 456     -11.828  -2.903  32.743  0.00  0.00
J
ATOM  13410  CD  ARG J 456      -9.892  -3.534  33.271  0.00  0.00
J
ATOM  13411  HD1 ARG J 456      -9.651  -2.527  33.673  0.00  0.00
J
ATOM  13412  HD2 ARG J 456     -10.271  -3.996  34.207  0.00  0.00
J
ATOM  13413  NE  ARG J 456      -8.798  -4.284  32.650  0.00  0.00
J
ATOM  13414  HE  ARG J 456      -8.784  -4.286  31.650  0.00  0.00
J
ATOM  13415  CZ  ARG J 456      -7.807  -4.838  33.303  0.00  0.00
J
ATOM  13416  NH1 ARG J 456      -7.727  -4.887  34.599  0.00  0.00
J
ATOM  13417 HH11 ARG J 456      -6.980  -5.398  35.024  0.00  0.00
J
ATOM  13418 HH12 ARG J 456      -8.539  -4.536  35.065  0.00  0.00
J
```

ATOM 13419 NH2 ARG J 456 -6.848 -5.464 32.634 0.00 0.00
J
ATOM 13420 HH21 ARG J 456 -6.231 -6.058 33.150 0.00 0.00
J
ATOM 13421 HH22 ARG J 456 -7.123 -5.560 31.677 0.00 0.00
J
ATOM 13422 C ARG J 456 -13.143 -6.646 31.056 0.00 0.00
J
ATOM 13423 O ARG J 456 -14.094 -7.111 31.732 0.00 0.00
J
ATOM 13424 N TYR J 457 -12.319 -7.456 30.285 0.00 0.00
J
ATOM 13425 HN TYR J 457 -11.705 -6.968 29.668 0.00 0.00
J
ATOM 13426 CA TYR J 457 -12.375 -8.910 30.132 0.00 0.00
J
ATOM 13427 HA TYR J 457 -12.924 -9.321 30.967 0.00 0.00
J
ATOM 13428 CB TYR J 457 -13.007 -9.347 28.776 0.00 0.00
J
ATOM 13429 HB1 TYR J 457 -12.314 -9.129 27.935 0.00 0.00
J
ATOM 13430 HB2 TYR J 457 -13.241 -10.433 28.769 0.00 0.00
J
ATOM 13431 CG TYR J 457 -14.285 -8.653 28.392 0.00 0.00
J
ATOM 13432 CD1 TYR J 457 -15.415 -8.715 29.219 0.00 0.00
J
ATOM 13433 HD1 TYR J 457 -15.349 -9.332 30.103 0.00 0.00
J
ATOM 13434 CE1 TYR J 457 -16.666 -8.079 28.874 0.00 0.00
J
ATOM 13435 HE1 TYR J 457 -17.485 -8.248 29.557 0.00 0.00
J
ATOM 13436 CZ TYR J 457 -16.636 -7.222 27.696 0.00 0.00
J
ATOM 13437 OH TYR J 457 -17.784 -6.469 27.359 0.00 0.00
J
ATOM 13438 HH TYR J 457 -18.489 -6.897 27.849 0.00 0.00
J
ATOM 13439 CD2 TYR J 457 -14.335 -7.846 27.191 0.00 0.00
J
ATOM 13440 HD2 TYR J 457 -13.453 -7.823 26.568 0.00 0.00
J
ATOM 13441 CE2 TYR J 457 -15.523 -7.150 26.899 0.00 0.00
J
ATOM 13442 HE2 TYR J 457 -15.675 -6.513 26.040 0.00 0.00
J
ATOM 13443 C TYR J 457 -10.967 -9.579 30.211 0.00 0.00
J
ATOM 13444 O TYR J 457 -10.027 -8.889 29.868 0.00 0.00
J
ATOM 13445 N PRO J 458 -10.876 -10.921 30.418 0.00 0.00
J

ATOM 13446 CD PRO J 458   -12.039 -11.753 30.934 0.00 0.00
J
ATOM 13447 HD1 PRO J 458   -12.660 -11.350 31.762 0.00 0.00
J
ATOM 13448 HD2 PRO J 458   -12.740 -11.866 30.080 0.00 0.00
J
ATOM 13449 CA PRO J 458   -9.698 -11.742 30.337 0.00 0.00
J
ATOM 13450 HA PRO J 458   -9.180 -11.567 31.268 0.00 0.00
J
ATOM 13451 CB PRO J 458   -10.171 -13.209 30.429 0.00 0.00
J
ATOM 13452 HB1 PRO J 458   -9.385 -13.885 30.830 0.00 0.00
J
ATOM 13453 HB2 PRO J 458   -10.586 -13.589 29.471 0.00 0.00
J
ATOM 13454 CG PRO J 458   -11.331 -13.027 31.387 0.00 0.00
J
ATOM 13455 HG1 PRO J 458   -10.841 -12.897 32.376 0.00 0.00
J
ATOM 13456 HG2 PRO J 458   -12.014 -13.898 31.480 0.00 0.00
J
ATOM 13457 C   PRO J 458   -8.740 -11.467 29.169 0.00 0.00
J
ATOM 13458 O   PRO J 458   -9.177 -11.134 28.109 0.00 0.00
J
ATOM 13459 N   ASP J 459   -7.403 -11.636 29.413 0.00 0.00
J
ATOM 13460 HN ASP J 459   -7.066 -11.883 30.318 0.00 0.00
J
ATOM 13461 CA ASP J 459   -6.326 -11.479 28.446 0.00 0.00
J
ATOM 13462 HA ASP J 459   -6.361 -10.436 28.168 0.00 0.00
J
ATOM 13463 CB ASP J 459   -5.011 -11.795 29.273 0.00 0.00
J
ATOM 13464 HB1 ASP J 459   -5.212 -12.603 30.008 0.00 0.00
J
ATOM 13465 HB2 ASP J 459   -4.028 -11.830 28.757 0.00 0.00
J
ATOM 13466 CG ASP J 459   -4.896 -10.603 30.262 0.00 0.00
J
ATOM 13467 OD1 ASP J 459   -5.032 -10.817 31.484 0.00 0.00
J
ATOM 13468 OD2 ASP J 459   -4.581 -9.395 29.893 0.00 0.00
J
ATOM 13469 C   ASP J 459   -6.445 -12.291 27.186 0.00 0.00
J
ATOM 13470 O   ASP J 459   -7.264 -13.203 27.194 0.00 0.00
J
ATOM 13471 N   GLY J 460   -5.794 -11.933 26.014 0.00 0.00
J
ATOM 13472 HN GLY J 460   -5.254 -11.095 26.033 0.00 0.00
J

ATOM 13473 CA GLY J 460   -6.083 -12.636 24.728 0.00 0.00
J
ATOM 13474 HA1 GLY J 460   -6.764 -12.023 24.157 0.00 0.00
J
ATOM 13475 HA2 GLY J 460   -6.339 -13.644 25.020 0.00 0.00
J
ATOM 13476 C   GLY J 460   -4.798 -12.677 23.959 0.00 0.00
J
ATOM 13477 O   GLY J 460   -3.826 -11.950 24.045 0.00 0.00
J
ATOM 13478 N   GLU J 461   -4.644 -13.679 23.027 0.00 0.00
J
ATOM 13479 HN GLU J 461   -5.454 -14.219 22.812 0.00 0.00
J
ATOM 13480 CA GLU J 461   -3.500 -13.878 22.156 0.00 0.00
J
ATOM 13481 HA GLU J 461   -3.032 -12.934 21.921 0.00 0.00
J
ATOM 13482 CB GLU J 461   -2.285 -14.547 22.849 0.00 0.00
J
ATOM 13483 HB1 GLU J 461   -2.038 -14.065 23.819 0.00 0.00
J
ATOM 13484 HB2 GLU J 461   -2.602 -15.581 23.103 0.00 0.00
J
ATOM 13485 CG GLU J 461   -1.101 -14.576 21.966 0.00 0.00
J
ATOM 13486 HG1 GLU J 461   -0.274 -15.046 22.540 0.00 0.00
J
ATOM 13487 HG2 GLU J 461   -1.344 -15.295 21.154 0.00 0.00
J
ATOM 13488 CD GLU J 461   -0.680 -13.187 21.467 0.00 0.00
J
ATOM 13489 OE1 GLU J 461   -0.011 -12.405 22.225 0.00 0.00
J
ATOM 13490 OE2 GLU J 461   -0.947 -12.852 20.273 0.00 0.00
J
ATOM 13491 C   GLU J 461   -3.946 -14.482 20.863 0.00 0.00
J
ATOM 13492 O   GLU J 461   -4.886 -15.268 20.852 0.00 0.00
J
ATOM 13493 N   VAL J 462   -3.406 -13.979 19.786 0.00 0.00
J
ATOM 13494 HN VAL J 462   -2.564 -13.447 19.837 0.00 0.00
J
ATOM 13495 CA VAL J 462   -3.972 -14.238 18.487 0.00 0.00
J
ATOM 13496 HA VAL J 462   -4.535 -15.160 18.472 0.00 0.00
J
ATOM 13497 CB VAL J 462   -4.914 -13.076 17.986 0.00 0.00
J
ATOM 13498 HB VAL J 462   -5.087 -13.238 16.900 0.00 0.00
J
ATOM 13499 CG1 VAL J 462   -6.292 -13.203 18.636 0.00 0.00
J

```
ATOM  13500 HG11 VAL J 462      -7.036 -12.453  18.292  0.00  0.00
J
ATOM  13501 HG12 VAL J 462      -6.683 -14.232  18.482  0.00  0.00
J
ATOM  13502 HG13 VAL J 462      -6.135 -13.153  19.734  0.00  0.00
J
ATOM  13503  CG2 VAL J 462      -4.289 -11.821  18.429  0.00  0.00
J
ATOM  13504 HG21 VAL J 462      -4.864 -10.934  18.085  0.00  0.00
J
ATOM  13505 HG22 VAL J 462      -4.160 -11.659  19.520  0.00  0.00
J
ATOM  13506 HG23 VAL J 462      -3.279 -11.756  17.970  0.00  0.00
J
ATOM  13507  C   VAL J 462      -2.859 -14.505  17.443  0.00  0.00
J
ATOM  13508  O   VAL J 462      -3.093 -14.611  16.192  0.00  0.00
J
ATOM  13509  N   PHE J 463      -1.634 -14.524  17.943  0.00  0.00
J
ATOM  13510  HN  PHE J 463      -1.525 -14.372  18.923  0.00  0.00
J
ATOM  13511  CA  PHE J 463      -0.429 -14.689  17.077  0.00  0.00
J
ATOM  13512  HA  PHE J 463       0.456 -14.385  17.617  0.00  0.00
J
ATOM  13513  CB  PHE J 463      -0.193 -16.163  16.845  0.00  0.00
J
ATOM  13514  HB1 PHE J 463      -1.081 -16.629  16.367  0.00  0.00
J
ATOM  13515  HB2 PHE J 463       0.631 -16.269  16.108  0.00  0.00
J
ATOM  13516  CG  PHE J 463       0.221 -17.064  18.025  0.00  0.00
J
ATOM  13517  CD1 PHE J 463       0.676 -16.516  19.262  0.00  0.00
J
ATOM  13518  HD1 PHE J 463       0.839 -15.456  19.393  0.00  0.00
J
ATOM  13519  CE1 PHE J 463       1.021 -17.322  20.337  0.00  0.00
J
ATOM  13520  HE1 PHE J 463       1.337 -16.964  21.306  0.00  0.00
J
ATOM  13521  CZ  PHE J 463       0.833 -18.704  20.269  0.00  0.00
J
ATOM  13522  HZ  PHE J 463       1.144 -19.394  21.039  0.00  0.00
J
ATOM  13523  CD2 PHE J 463       0.027 -18.440  17.961  0.00  0.00
J
ATOM  13524  HD2 PHE J 463      -0.487 -18.844  17.101  0.00  0.00
J
ATOM  13525  CE2 PHE J 463       0.261 -19.260  19.063  0.00  0.00
J
ATOM  13526  HE2 PHE J 463       0.055 -20.320  19.059  0.00  0.00
J
```

ATOM 13527 C PHE J 463 -0.272 -13.851 15.833 0.00 0.00
J
ATOM 13528 O PHE J 463 -0.117 -14.408 14.726 0.00 0.00
J
ATOM 13529 N THR J 464 -0.202 -12.492 15.852 0.00 0.00
J
ATOM 13530 HN THR J 464 -0.142 -12.051 16.745 0.00 0.00
J
ATOM 13531 CA THR J 464 -0.049 -11.761 14.688 0.00 0.00
J
ATOM 13532 HA THR J 464 -0.800 -12.083 13.982 0.00 0.00
J
ATOM 13533 CB THR J 464 -0.599 -10.358 14.826 0.00 0.00
J
ATOM 13534 HB THR J 464 -0.325 -9.902 13.851 0.00 0.00
J
ATOM 13535 OG1 THR J 464 0.152 -9.721 15.855 0.00 0.00
J
ATOM 13536 HG1 THR J 464 -0.010 -10.326 16.582 0.00 0.00
J
ATOM 13537 CG2 THR J 464 -2.168 -10.375 15.091 0.00 0.00
J
ATOM 13538 HG21 THR J 464 -2.298 -10.836 16.094 0.00 0.00
J
ATOM 13539 HG22 THR J 464 -2.532 -9.328 15.157 0.00 0.00
J
ATOM 13540 HG23 THR J 464 -2.620 -11.007 14.296 0.00 0.00
J
ATOM 13541 C THR J 464 1.284 -11.836 13.882 0.00 0.00
J
ATOM 13542 O THR J 464 2.332 -12.093 14.459 0.00 0.00
J
ATOM 13543 N GLY J 465 1.261 -11.665 12.610 0.00 0.00
J
ATOM 13544 HN GLY J 465 0.409 -11.533 12.108 0.00 0.00
J
ATOM 13545 CA GLY J 465 2.416 -11.266 11.798 0.00 0.00
J
ATOM 13546 HA1 GLY J 465 3.156 -12.048 11.719 0.00 0.00
J
ATOM 13547 HA2 GLY J 465 2.804 -10.350 12.218 0.00 0.00
J
ATOM 13548 C GLY J 465 1.861 -11.135 10.407 0.00 0.00
J
ATOM 13549 OT1 GLY J 465 0.673 -10.676 10.285 0.00 0.00
J
ATOM 13550 OT2 GLY J 465 2.489 -11.455 9.427 0.00 0.00
J
ATOM 13551 N GLN A 8 -35.080 11.419 80.384 0.00 0.00
A
ATOM 13552 HT1 GLN A 8 -35.249 10.417 80.163 0.00 0.00
A
ATOM 13553 HT2 GLN A 8 -35.565 11.697 81.261 0.00 0.00
A

ATOM 13554 HT3 GLN A 8 −34.067 11.466 80.619 0.00 0.00
A
ATOM 13555 CA GLN A 8 −35.327 12.316 79.174 0.00 0.00
A
ATOM 13556 HA GLN A 8 −36.288 12.058 78.754 0.00 0.00
A
ATOM 13557 CB GLN A 8 −35.291 13.725 79.702 0.00 0.00
A
ATOM 13558 HB1 GLN A 8 −35.829 13.773 80.672 0.00 0.00
A
ATOM 13559 HB2 GLN A 8 −34.278 14.087 79.979 0.00 0.00
A
ATOM 13560 CG GLN A 8 −36.039 14.682 78.725 0.00 0.00
A
ATOM 13561 HG1 GLN A 8 −35.587 14.707 77.710 0.00 0.00
A
ATOM 13562 HG2 GLN A 8 −37.087 14.328 78.625 0.00 0.00
A
ATOM 13563 CD GLN A 8 −35.980 16.077 79.312 0.00 0.00
A
ATOM 13564 OE1 GLN A 8 −34.895 16.488 79.794 0.00 0.00
A
ATOM 13565 NE2 GLN A 8 −37.121 16.804 79.314 0.00 0.00
A
ATOM 13566 HE21 GLN A 8 −37.169 17.780 79.527 0.00 0.00
A
ATOM 13567 HE22 GLN A 8 −37.915 16.241 79.085 0.00 0.00
A
ATOM 13568 C GLN A 8 −34.440 11.961 77.989 0.00 0.00
A
ATOM 13569 O GLN A 8 −34.663 10.974 77.320 0.00 0.00
A
ATOM 13570 N PHE A 9 −33.338 12.709 77.680 0.00 0.00
A
ATOM 13571 HN PHE A 9 −33.022 13.516 78.173 0.00 0.00
A
ATOM 13572 CA PHE A 9 −32.581 12.473 76.490 0.00 0.00
A
ATOM 13573 HA PHE A 9 −33.148 11.972 75.720 0.00 0.00
A
ATOM 13574 CB PHE A 9 −32.006 13.826 75.945 0.00 0.00
A
ATOM 13575 HB1 PHE A 9 −31.375 14.314 76.718 0.00 0.00
A
ATOM 13576 HB2 PHE A 9 −31.307 13.694 75.091 0.00 0.00
A
ATOM 13577 CG PHE A 9 −33.125 14.638 75.524 0.00 0.00
A
ATOM 13578 CD1 PHE A 9 −34.126 14.232 74.631 0.00 0.00
A
ATOM 13579 HD1 PHE A 9 −33.941 13.228 74.279 0.00 0.00
A
ATOM 13580 CE1 PHE A 9 −35.240 14.977 74.258 0.00 0.00
A

ATOM	13581	HE1	PHE	A	9	−36.011	14.614	73.594	0.00	0.00	A
ATOM	13582	CZ	PHE	A	9	−35.380	16.277	74.906	0.00	0.00	A
ATOM	13583	HZ	PHE	A	9	−36.152	16.933	74.531	0.00	0.00	A
ATOM	13584	CD2	PHE	A	9	−33.313	15.916	76.146	0.00	0.00	A
ATOM	13585	HD2	PHE	A	9	−32.627	16.210	76.926	0.00	0.00	A
ATOM	13586	CE2	PHE	A	9	−34.392	16.812	75.752	0.00	0.00	A
ATOM	13587	HE2	PHE	A	9	−34.464	17.777	76.231	0.00	0.00	A
ATOM	13588	C	PHE	A	9	−31.406	11.564	76.701	0.00	0.00	A
ATOM	13589	O	PHE	A	9	−30.919	11.587	77.824	0.00	0.00	A
ATOM	13590	N	ALA	A	10	−30.936	10.764	75.713	0.00	0.00	A
ATOM	13591	HN	ALA	A	10	−31.442	10.628	74.865	0.00	0.00	A
ATOM	13592	CA	ALA	A	10	−29.766	9.994	75.935	0.00	0.00	A
ATOM	13593	HA	ALA	A	10	−29.227	10.158	76.856	0.00	0.00	A
ATOM	13594	CB	ALA	A	10	−30.041	8.508	75.623	0.00	0.00	A
ATOM	13595	HB1	ALA	A	10	−29.122	7.965	75.316	0.00	0.00	A
ATOM	13596	HB2	ALA	A	10	−30.385	7.963	76.528	0.00	0.00	A
ATOM	13597	HB3	ALA	A	10	−30.832	8.493	74.843	0.00	0.00	A
ATOM	13598	C	ALA	A	10	−28.818	10.480	74.853	0.00	0.00	A
ATOM	13599	O	ALA	A	10	−29.221	10.532	73.670	0.00	0.00	A
ATOM	13600	N	VAL	A	11	−27.557	10.703	75.152	0.00	0.00	A
ATOM	13601	HN	VAL	A	11	−27.185	10.479	76.049	0.00	0.00	A
ATOM	13602	CA	VAL	A	11	−26.516	10.889	74.158	0.00	0.00	A
ATOM	13603	HA	VAL	A	11	−26.919	10.872	73.156	0.00	0.00	A
ATOM	13604	CB	VAL	A	11	−25.883	12.238	74.261	0.00	0.00	A
ATOM	13605	HB	VAL	A	11	−25.566	12.524	75.287	0.00	0.00	A
ATOM	13606	CG1	VAL	A	11	−24.648	12.368	73.410	0.00	0.00	A
ATOM	13607	HG11	VAL	A	11	−23.772	11.858	73.863	0.00	0.00	A

```
ATOM  13608 HG12 VAL A  11      −24.731  11.958  72.380  0.00  0.00
A
ATOM  13609 HG13 VAL A  11      −24.333  13.420  73.237  0.00  0.00
A
ATOM  13610  CG2 VAL A  11      −26.808  13.354  73.667  0.00  0.00
A
ATOM  13611 HG21 VAL A  11      −27.814  13.470  74.125  0.00  0.00
A
ATOM  13612 HG22 VAL A  11      −26.305  14.343  73.616  0.00  0.00
A
ATOM  13613 HG23 VAL A  11      −27.038  13.036  72.628  0.00  0.00
A
ATOM  13614  C   VAL A  11      −25.512   9.724  74.237  0.00  0.00
A
ATOM  13615  O   VAL A  11      −24.771   9.470  75.213  0.00  0.00
A
ATOM  13616  N   ILE A  12      −25.385   8.932  73.113  0.00  0.00
A
ATOM  13617  HN  ILE A  12      −26.055   8.974  72.376  0.00  0.00
A
ATOM  13618  CA  ILE A  12      −24.440   7.817  73.100  0.00  0.00
A
ATOM  13619  HA  ILE A  12      −24.038   7.477  74.043  0.00  0.00
A
ATOM  13620  CB  ILE A  12      −24.882   6.496  72.381  0.00  0.00
A
ATOM  13621  HB  ILE A  12      −25.141   6.763  71.334  0.00  0.00
A
ATOM  13622  CG2 ILE A  12      −23.791   5.398  72.458  0.00  0.00
A
ATOM  13623 HG21 ILE A  12      −22.803   5.860  72.246  0.00  0.00
A
ATOM  13624 HG22 ILE A  12      −23.789   4.905  73.453  0.00  0.00
A
ATOM  13625 HG23 ILE A  12      −24.012   4.583  71.736  0.00  0.00
A
ATOM  13626  CG1 ILE A  12      −26.211   6.007  73.089  0.00  0.00
A
ATOM  13627 HG11 ILE A  12      −25.874   5.846  74.135  0.00  0.00
A
ATOM  13628 HG12 ILE A  12      −26.898   6.879  73.138  0.00  0.00
A
ATOM  13629  CD  ILE A  12      −26.842   4.753  72.425  0.00  0.00
A
ATOM  13630  HD1 ILE A  12      −26.181   3.908  72.137  0.00  0.00
A
ATOM  13631  HD2 ILE A  12      −27.541   4.213  73.099  0.00  0.00
A
ATOM  13632  HD3 ILE A  12      −27.465   5.038  71.550  0.00  0.00
A
ATOM  13633  C   ILE A  12      −23.207   8.301  72.219  0.00  0.00
A
ATOM  13634  O   ILE A  12      −23.244   8.656  71.060  0.00  0.00
A
```

ATOM 13635 N   GLY A 13   -22.026   8.258 72.782 0.00 0.00
A
ATOM 13636 HN GLY A 13   -21.977   7.854 73.692 0.00 0.00
A
ATOM 13637 CA GLY A 13   -20.712   8.791 72.354 0.00 0.00
A
ATOM 13638 HA1 GLY A 13   -20.754   8.781 71.275 0.00 0.00
A
ATOM 13639 HA2 GLY A 13   -19.956   8.127 72.749 0.00 0.00
A
ATOM 13640 C   GLY A 13   -20.557 10.254 72.773 0.00 0.00
A
ATOM 13641 O   GLY A 13   -21.355 11.135 72.439 0.00 0.00
A
ATOM 13642 N   LEU A 14   -19.470 10.613 73.524 0.00 0.00
A
ATOM 13643 HN LEU A 14   -18.772   9.924 73.703 0.00 0.00
A
ATOM 13644 CA LEU A 14   -19.322 12.015 74.129 0.00 0.00
A
ATOM 13645 HA LEU A 14   -20.245 12.573 74.069 0.00 0.00
A
ATOM 13646 CB LEU A 14   -19.068 11.943 75.668 0.00 0.00
A
ATOM 13647 HB1 LEU A 14   -18.069 11.465 75.754 0.00 0.00
A
ATOM 13648 HB2 LEU A 14   -18.951 13.016 75.931 0.00 0.00
A
ATOM 13649 CG LEU A 14   -20.248 11.242 76.413 0.00 0.00
A
ATOM 13650 HG LEU A 14   -20.504 10.328 75.837 0.00 0.00
A
ATOM 13651 CD1 LEU A 14   -19.837 10.860 77.811 0.00 0.00
A
ATOM 13652 HD11 LEU A 14   -19.405 11.687 78.414 0.00 0.00
A
ATOM 13653 HD12 LEU A 14   -20.610 10.253 78.329 0.00 0.00
A
ATOM 13654 HD13 LEU A 14   -19.118 10.070 77.507 0.00 0.00
A
ATOM 13655 CD2 LEU A 14   -21.513 12.036 76.424 0.00 0.00
A
ATOM 13656 HD21 LEU A 14   -22.400 11.384 76.571 0.00 0.00
A
ATOM 13657 HD22 LEU A 14   -21.382 12.732 77.279 0.00 0.00
A
ATOM 13658 HD23 LEU A 14   -21.730 12.657 75.528 0.00 0.00
A
ATOM 13659 C   LEU A 14   -18.198 12.779 73.415 0.00 0.00
A
ATOM 13660 O   LEU A 14   -17.450 13.607 73.959 0.00 0.00
A
ATOM 13661 N   GLY A 15   -17.927 12.385 72.122 0.00 0.00
A

ATOM 13662 HN GLY A 15 −18.499 11.709 71.664 0.00 0.00
A
ATOM 13663 CA GLY A 15 −17.009 13.171 71.225 0.00 0.00
A
ATOM 13664 HA1 GLY A 15 −17.102 12.673 70.271 0.00 0.00
A
ATOM 13665 HA2 GLY A 15 −16.041 13.221 71.700 0.00 0.00
A
ATOM 13666 C GLY A 15 −17.329 14.620 70.936 0.00 0.00
A
ATOM 13667 O GLY A 15 −18.259 15.142 71.504 0.00 0.00
A
ATOM 13668 N ARG A 16 −16.572 15.320 70.068 0.00 0.00
A
ATOM 13669 HN ARG A 16 −15.860 14.884 69.524 0.00 0.00
A
ATOM 13670 CA ARG A 16 −16.836 16.721 69.950 0.00 0.00
A
ATOM 13671 HA ARG A 16 −16.771 17.246 70.892 0.00 0.00
A
ATOM 13672 CB ARG A 16 −15.822 17.256 68.931 0.00 0.00
A
ATOM 13673 HB1 ARG A 16 −14.833 17.209 69.434 0.00 0.00
A
ATOM 13674 HB2 ARG A 16 −15.813 16.661 67.993 0.00 0.00
A
ATOM 13675 CG ARG A 16 −16.088 18.705 68.524 0.00 0.00
A
ATOM 13676 HG1 ARG A 16 −17.037 18.751 67.949 0.00 0.00
A
ATOM 13677 HG2 ARG A 16 −16.134 19.378 69.407 0.00 0.00
A
ATOM 13678 CD ARG A 16 −14.963 19.279 67.685 0.00 0.00
A
ATOM 13679 HD1 ARG A 16 −15.066 20.339 67.367 0.00 0.00
A
ATOM 13680 HD2 ARG A 16 −14.071 19.039 68.302 0.00 0.00
A
ATOM 13681 NE ARG A 16 −14.973 18.449 66.422 0.00 0.00
A
ATOM 13682 HE ARG A 16 −15.660 17.768 66.171 0.00 0.00
A
ATOM 13683 CZ ARG A 16 −14.262 18.796 65.346 0.00 0.00
A
ATOM 13684 NH1 ARG A 16 −13.386 19.796 65.331 0.00 0.00
A
ATOM 13685 HH11 ARG A 16 −12.772 19.897 64.548 0.00 0.00
A
ATOM 13686 HH12 ARG A 16 −13.026 20.113 66.209 0.00 0.00
A
ATOM 13687 NH2 ARG A 16 −14.592 18.275 64.216 0.00 0.00
A
ATOM 13688 HH21 ARG A 16 −13.926 18.214 63.473 0.00 0.00
A

ATOM	13689	HH22	ARG	A	16	−15.288	17.577	64.386	0.00	0.00	A
ATOM	13690	C	ARG	A	16	−18.289	17.093	69.533	0.00	0.00	A
ATOM	13691	O	ARG	A	16	−18.880	18.039	69.985	0.00	0.00	A
ATOM	13692	N	PHE	A	17	−18.843	16.252	68.635	0.00	0.00	A
ATOM	13693	HN	PHE	A	17	−18.309	15.509	68.241	0.00	0.00	A
ATOM	13694	CA	PHE	A	17	−20.291	16.408	68.344	0.00	0.00	A
ATOM	13695	HA	PHE	A	17	−20.428	17.429	68.021	0.00	0.00	A
ATOM	13696	CB	PHE	A	17	−20.658	15.394	67.303	0.00	0.00	A
ATOM	13697	HB1	PHE	A	17	−20.087	15.495	66.356	0.00	0.00	A
ATOM	13698	HB2	PHE	A	17	−20.517	14.385	67.747	0.00	0.00	A
ATOM	13699	CG	PHE	A	17	−22.105	15.452	66.840	0.00	0.00	A
ATOM	13700	CD1	PHE	A	17	−22.689	16.693	66.463	0.00	0.00	A
ATOM	13701	HD1	PHE	A	17	−21.990	17.516	66.488	0.00	0.00	A
ATOM	13702	CE1	PHE	A	17	−23.987	16.826	65.987	0.00	0.00	A
ATOM	13703	HE1	PHE	A	17	−24.445	17.771	65.734	0.00	0.00	A
ATOM	13704	CZ	PHE	A	17	−24.727	15.611	65.868	0.00	0.00	A
ATOM	13705	HZ	PHE	A	17	−25.731	15.518	65.481	0.00	0.00	A
ATOM	13706	CD2	PHE	A	17	−22.814	14.327	66.707	0.00	0.00	A
ATOM	13707	HD2	PHE	A	17	−22.374	13.353	66.867	0.00	0.00	A
ATOM	13708	CE2	PHE	A	17	−24.142	14.407	66.238	0.00	0.00	A
ATOM	13709	HE2	PHE	A	17	−24.704	13.491	66.133	0.00	0.00	A
ATOM	13710	C	PHE	A	17	−21.205	16.107	69.585	0.00	0.00	A
ATOM	13711	O	PHE	A	17	−22.041	16.853	70.067	0.00	0.00	A
ATOM	13712	N	GLY	A	18	−20.974	14.934	70.154	0.00	0.00	A
ATOM	13713	HN	GLY	A	18	−20.127	14.485	69.880	0.00	0.00	A
ATOM	13714	CA	GLY	A	18	−21.864	14.520	71.349	0.00	0.00	A
ATOM	13715	HA1	GLY	A	18	−21.638	13.464	71.368	0.00	0.00	A

ATOM 13716 HA2 GLY A 18 −22.929 14.560 71.170 0.00 0.00
A
ATOM 13717 C GLY A 18 −21.709 15.189 72.667 0.00 0.00
A
ATOM 13718 O GLY A 18 −22.657 15.407 73.433 0.00 0.00
A
ATOM 13719 N GLY A 19 −20.473 15.644 73.036 0.00 0.00
A
ATOM 13720 HN GLY A 19 −19.667 15.556 72.455 0.00 0.00
A
ATOM 13721 CA GLY A 19 −20.258 16.353 74.286 0.00 0.00
A
ATOM 13722 HA1 GLY A 19 −19.189 16.329 74.433 0.00 0.00
A
ATOM 13723 HA2 GLY A 19 −20.820 15.951 75.116 0.00 0.00
A
ATOM 13724 C GLY A 19 −20.668 17.791 74.142 0.00 0.00
A
ATOM 13725 O GLY A 19 −21.059 18.432 75.089 0.00 0.00
A
ATOM 13726 N SER A 20 −20.627 18.349 72.912 0.00 0.00
A
ATOM 13727 HN SER A 20 −20.175 17.897 72.146 0.00 0.00
A
ATOM 13728 CA SER A 20 −21.273 19.652 72.599 0.00 0.00
A
ATOM 13729 HA SER A 20 −20.835 20.388 73.258 0.00 0.00
A
ATOM 13730 CB SER A 20 −20.892 20.125 71.118 0.00 0.00
A
ATOM 13731 HB1 SER A 20 −19.788 20.151 71.003 0.00 0.00
A
ATOM 13732 HB2 SER A 20 −21.316 19.520 70.288 0.00 0.00
A
ATOM 13733 OG SER A 20 −21.291 21.499 70.829 0.00 0.00
A
ATOM 13734 HG1 SER A 20 −21.490 21.399 69.896 0.00 0.00
A
ATOM 13735 C SER A 20 −22.771 19.643 72.675 0.00 0.00
A
ATOM 13736 O SER A 20 −23.360 20.576 73.199 0.00 0.00
A
ATOM 13737 N ILE A 21 −23.437 18.517 72.208 0.00 0.00
A
ATOM 13738 HN ILE A 21 −22.951 17.847 71.652 0.00 0.00
A
ATOM 13739 CA ILE A 21 −24.900 18.292 72.400 0.00 0.00
A
ATOM 13740 HA ILE A 21 −25.447 19.162 72.069 0.00 0.00
A
ATOM 13741 CB ILE A 21 −25.323 17.061 71.720 0.00 0.00
A
ATOM 13742 HB ILE A 21 −24.573 16.288 71.991 0.00 0.00
A

ATOM 13743 CG2 ILE A 21 −26.843 16.639 71.940 0.00 0.00
A
ATOM 13744 HG21 ILE A 21 −27.060 15.685 71.414 0.00 0.00
A
ATOM 13745 HG22 ILE A 21 −27.082 16.557 73.022 0.00 0.00
A
ATOM 13746 HG23 ILE A 21 −27.514 17.413 71.509 0.00 0.00
A
ATOM 13747 CG1 ILE A 21 −25.162 17.117 70.126 0.00 0.00
A
ATOM 13748 HG11 ILE A 21 −25.982 17.719 69.679 0.00 0.00
A
ATOM 13749 HG12 ILE A 21 −24.154 17.544 69.937 0.00 0.00
A
ATOM 13750 CD ILE A 21 −25.203 15.821 69.437 0.00 0.00
A
ATOM 13751 HD1 ILE A 21 −24.677 15.865 68.459 0.00 0.00
A
ATOM 13752 HD2 ILE A 21 −24.768 15.010 70.059 0.00 0.00
A
ATOM 13753 HD3 ILE A 21 −26.232 15.435 69.274 0.00 0.00
A
ATOM 13754 C ILE A 21 −25.316 18.173 73.882 0.00 0.00
A
ATOM 13755 O ILE A 21 −26.169 18.939 74.331 0.00 0.00
A
ATOM 13756 N CYS A 22 −24.611 17.305 74.681 0.00 0.00
A
ATOM 13757 HN CYS A 22 −24.127 16.569 74.215 0.00 0.00
A
ATOM 13758 CA CYS A 22 −24.650 17.080 76.121 0.00 0.00
A
ATOM 13759 HA CYS A 22 −25.602 16.612 76.322 0.00 0.00
A
ATOM 13760 CB CYS A 22 −23.447 16.223 76.503 0.00 0.00
A
ATOM 13761 HB1 CYS A 22 −23.567 15.315 75.875 0.00 0.00
A
ATOM 13762 HB2 CYS A 22 −22.510 16.726 76.182 0.00 0.00
A
ATOM 13763 SG CYS A 22 −23.248 15.659 78.259 0.00 0.00
A
ATOM 13764 HG1 CYS A 22 −22.652 16.745 78.730 0.00 0.00
A
ATOM 13765 C CYS A 22 −24.534 18.344 76.917 0.00 0.00
A
ATOM 13766 O CYS A 22 −25.353 18.551 77.834 0.00 0.00
A
ATOM 13767 N LYS A 23 −23.550 19.169 76.597 0.00 0.00
A
ATOM 13768 HN LYS A 23 −22.747 18.857 76.095 0.00 0.00
A
ATOM 13769 CA LYS A 23 −23.344 20.419 77.360 0.00 0.00
A

ATOM 13770 HA LYS A 23 -23.438 20.127 78.395 0.00 0.00
A
ATOM 13771 CB LYS A 23 -21.997 21.026 76.973 0.00 0.00
A
ATOM 13772 HB1 LYS A 23 -21.241 20.232 77.151 0.00 0.00
A
ATOM 13773 HB2 LYS A 23 -21.879 21.175 75.878 0.00 0.00
A
ATOM 13774 CG LYS A 23 -21.595 22.349 77.784 0.00 0.00
A
ATOM 13775 HG1 LYS A 23 -22.195 23.232 77.477 0.00 0.00
A
ATOM 13776 HG2 LYS A 23 -22.018 22.156 78.793 0.00 0.00
A
ATOM 13777 CD LYS A 23 -20.116 22.830 77.777 0.00 0.00
A
ATOM 13778 HD1 LYS A 23 -19.917 23.376 78.724 0.00 0.00
A
ATOM 13779 HD2 LYS A 23 -19.361 22.032 77.946 0.00 0.00
A
ATOM 13780 CE LYS A 23 -19.680 23.542 76.566 0.00 0.00
A
ATOM 13781 HE1 LYS A 23 -18.594 23.776 76.575 0.00 0.00
A
ATOM 13782 HE2 LYS A 23 -19.880 23.102 75.566 0.00 0.00
A
ATOM 13783 NZ LYS A 23 -20.290 24.887 76.511 0.00 0.00
A
ATOM 13784 HZ1 LYS A 23 -20.495 25.241 77.467 0.00 0.00
A
ATOM 13785 HZ2 LYS A 23 -19.722 25.621 76.040 0.00 0.00
A
ATOM 13786 HZ3 LYS A 23 -21.138 24.925 75.910 0.00 0.00
A
ATOM 13787 C LYS A 23 -24.396 21.490 77.133 0.00 0.00
A
ATOM 13788 O LYS A 23 -24.845 22.068 78.087 0.00 0.00
A
ATOM 13789 N GLU A 24 -24.765 21.644 75.870 0.00 0.00
A
ATOM 13790 HN GLU A 24 -24.490 21.033 75.131 0.00 0.00
A
ATOM 13791 CA GLU A 24 -25.793 22.625 75.448 0.00 0.00
A
ATOM 13792 HA GLU A 24 -25.634 23.539 76.001 0.00 0.00
A
ATOM 13793 CB GLU A 24 -25.726 22.936 73.897 0.00 0.00
A
ATOM 13794 HB1 GLU A 24 -24.748 23.359 73.584 0.00 0.00
A
ATOM 13795 HB2 GLU A 24 -25.914 22.009 73.314 0.00 0.00
A
ATOM 13796 CG GLU A 24 -26.814 24.108 73.497 0.00 0.00
A

ATOM 13797 HG1 GLU A 24 −26.833 24.039 72.389 0.00 0.00
A
ATOM 13798 HG2 GLU A 24 −27.846 23.840 73.812 0.00 0.00
A
ATOM 13799 CD GLU A 24 −26.446 25.536 73.786 0.00 0.00
A
ATOM 13800 OE1 GLU A 24 −27.018 26.431 73.123 0.00 0.00
A
ATOM 13801 OE2 GLU A 24 −25.454 25.805 74.551 0.00 0.00
A
ATOM 13802 C GLU A 24 −27.234 22.293 75.896 0.00 0.00
A
ATOM 13803 O GLU A 24 −27.975 23.130 76.367 0.00 0.00
A
ATOM 13804 N LEU A 25 −27.441 21.009 75.984 0.00 0.00
A
ATOM 13805 HN LEU A 25 −26.922 20.347 75.450 0.00 0.00
A
ATOM 13806 CA LEU A 25 −28.610 20.392 76.689 0.00 0.00
A
ATOM 13807 HA LEU A 25 −29.492 20.855 76.271 0.00 0.00
A
ATOM 13808 CB LEU A 25 −28.767 18.904 76.480 0.00 0.00
A
ATOM 13809 HB1 LEU A 25 −27.751 18.486 76.649 0.00 0.00
A
ATOM 13810 HB2 LEU A 25 −29.463 18.396 77.182 0.00 0.00
A
ATOM 13811 CG LEU A 25 −29.283 18.571 75.084 0.00 0.00
A
ATOM 13812 HG LEU A 25 −28.666 19.203 74.410 0.00 0.00
A
ATOM 13813 CD1 LEU A 25 −29.022 17.063 74.890 0.00 0.00
A
ATOM 13814 HD11 LEU A 25 −29.570 16.535 75.700 0.00 0.00
A
ATOM 13815 HD12 LEU A 25 −29.191 16.761 73.834 0.00 0.00
A
ATOM 13816 HD13 LEU A 25 −27.923 16.972 75.028 0.00 0.00
A
ATOM 13817 CD2 LEU A 25 −30.777 18.781 74.926 0.00 0.00
A
ATOM 13818 HD21 LEU A 25 −31.096 18.287 73.984 0.00 0.00
A
ATOM 13819 HD22 LEU A 25 −31.320 18.386 75.811 0.00 0.00
A
ATOM 13820 HD23 LEU A 25 −31.005 19.868 74.888 0.00 0.00
A
ATOM 13821 C LEU A 25 −28.842 20.656 78.128 0.00 0.00
A
ATOM 13822 O LEU A 25 −29.972 21.061 78.504 0.00 0.00
A
ATOM 13823 N HSE A 26 −27.774 20.479 78.940 0.00 0.00
A

ATOM	13824	HN	HSE	A	26	-26.922	20.178	78.520	0.00	0.00	A
ATOM	13825	CA	HSE	A	26	-27.827	20.807	80.321	0.00	0.00	A
ATOM	13826	HA	HSE	A	26	-28.623	20.311	80.857	0.00	0.00	A
ATOM	13827	CB	HSE	A	26	-26.381	20.431	80.857	0.00	0.00	A
ATOM	13828	HB1	HSE	A	26	-26.178	19.422	80.437	0.00	0.00	A
ATOM	13829	HB2	HSE	A	26	-25.547	21.091	80.536	0.00	0.00	A
ATOM	13830	ND1	HSE	A	26	-27.273	19.677	82.998	0.00	0.00	A
ATOM	13831	CG	HSE	A	26	-26.310	20.341	82.350	0.00	0.00	A
ATOM	13832	CE1	HSE	A	26	-26.997	19.778	84.244	0.00	0.00	A
ATOM	13833	HE1	HSE	A	26	-27.469	19.217	85.051	0.00	0.00	A
ATOM	13834	NE2	HSE	A	26	-25.897	20.539	84.391	0.00	0.00	A
ATOM	13835	HE2	HSE	A	26	-25.550	20.912	85.251	0.00	0.00	A
ATOM	13836	CD2	HSE	A	26	-25.445	20.853	83.183	0.00	0.00	A
ATOM	13837	HD2	HSE	A	26	-24.719	21.608	82.907	0.00	0.00	A
ATOM	13838	C	HSE	A	26	-28.059	22.238	80.610	0.00	0.00	A
ATOM	13839	O	HSE	A	26	-28.907	22.634	81.428	0.00	0.00	A
ATOM	13840	N	ARG	A	27	-27.406	23.098	79.830	0.00	0.00	A
ATOM	13841	HN	ARG	A	27	-26.679	22.660	79.309	0.00	0.00	A
ATOM	13842	CA	ARG	A	27	-27.608	24.588	79.857	0.00	0.00	A
ATOM	13843	HA	ARG	A	27	-27.407	24.892	80.874	0.00	0.00	A
ATOM	13844	CB	ARG	A	27	-26.610	25.170	78.876	0.00	0.00	A
ATOM	13845	HB1	ARG	A	27	-25.751	24.486	79.046	0.00	0.00	A
ATOM	13846	HB2	ARG	A	27	-26.804	25.013	77.793	0.00	0.00	A
ATOM	13847	CG	ARG	A	27	-26.261	26.714	78.956	0.00	0.00	A
ATOM	13848	HG1	ARG	A	27	-27.258	27.187	78.827	0.00	0.00	A
ATOM	13849	HG2	ARG	A	27	-25.992	26.816	80.029	0.00	0.00	A
ATOM	13850	CD	ARG	A	27	-25.219	27.334	77.951	0.00	0.00	A

ATOM 13851 HD1 ARG A 27 −25.150 28.386 78.303 0.00 0.00
A
ATOM 13852 HD2 ARG A 27 −24.221 26.846 77.961 0.00 0.00
A
ATOM 13853 NE ARG A 27 −25.959 27.329 76.614 0.00 0.00
A
ATOM 13854 HE ARG A 27 −25.722 26.711 75.864 0.00 0.00
A
ATOM 13855 CZ ARG A 27 −27.124 27.981 76.416 0.00 0.00
A
ATOM 13856 NH1 ARG A 27 −27.608 29.010 77.099 0.00 0.00
A
ATOM 13857 HH11 ARG A 27 −28.511 29.311 76.791 0.00 0.00
A
ATOM 13858 HH12 ARG A 27 −27.013 29.481 77.750 0.00 0.00
A
ATOM 13859 NH2 ARG A 27 −27.871 27.550 75.420 0.00 0.00
A
ATOM 13860 HH21 ARG A 27 −28.523 28.221 75.067 0.00 0.00
A
ATOM 13861 HH22 ARG A 27 −27.402 26.912 74.810 0.00 0.00
A
ATOM 13862 C ARG A 27 −28.981 25.154 79.645 0.00 0.00
A
ATOM 13863 O ARG A 27 −29.463 26.075 80.287 0.00 0.00
A
ATOM 13864 N MET A 28 −29.675 24.428 78.729 0.00 0.00
A
ATOM 13865 HN MET A 28 −29.249 23.698 78.200 0.00 0.00
A
ATOM 13866 CA MET A 28 −31.056 24.590 78.384 0.00 0.00
A
ATOM 13867 HA MET A 28 −31.220 25.654 78.475 0.00 0.00
A
ATOM 13868 CB MET A 28 −31.386 24.219 76.923 0.00 0.00
A
ATOM 13869 HB1 MET A 28 −31.030 23.175 76.790 0.00 0.00
A
ATOM 13870 HB2 MET A 28 −32.452 24.235 76.610 0.00 0.00
A
ATOM 13871 CG MET A 28 −30.594 25.103 75.859 0.00 0.00
A
ATOM 13872 HG1 MET A 28 −30.908 26.151 76.053 0.00 0.00
A
ATOM 13873 HG2 MET A 28 −29.491 25.060 75.976 0.00 0.00
A
ATOM 13874 SD MET A 28 −31.018 24.629 74.118 0.00 0.00
A
ATOM 13875 CE MET A 28 −32.576 25.632 74.208 0.00 0.00
A
ATOM 13876 HE1 MET A 28 −33.075 25.599 75.200 0.00 0.00
A
ATOM 13877 HE2 MET A 28 −32.353 26.682 73.920 0.00 0.00
A

ATOM 13878 HE3 MET A 28 −33.347 25.115 73.597 0.00 0.00
A
ATOM 13879 C MET A 28 −31.994 23.795 79.340 0.00 0.00
A
ATOM 13880 O MET A 28 −33.216 23.684 79.074 0.00 0.00
A
ATOM 13881 N GLY A 29 −31.477 23.276 80.466 0.00 0.00
A
ATOM 13882 HN GLY A 29 −30.484 23.271 80.556 0.00 0.00
A
ATOM 13883 CA GLY A 29 −32.308 22.726 81.584 0.00 0.00
A
ATOM 13884 HA1 GLY A 29 −33.185 23.353 81.650 0.00 0.00
A
ATOM 13885 HA2 GLY A 29 −31.586 22.742 82.388 0.00 0.00
A
ATOM 13886 C GLY A 29 −32.701 21.302 81.460 0.00 0.00
A
ATOM 13887 O GLY A 29 −33.464 20.764 82.288 0.00 0.00
A
ATOM 13888 N HSE A 30 −32.194 20.624 80.421 0.00 0.00
A
ATOM 13889 HN HSE A 30 −31.636 21.142 79.777 0.00 0.00
A
ATOM 13890 CA HSE A 30 −32.635 19.294 80.077 0.00 0.00
A
ATOM 13891 HA HSE A 30 −33.619 19.153 80.498 0.00 0.00
A
ATOM 13892 CB HSE A 30 −32.778 19.033 78.554 0.00 0.00
A
ATOM 13893 HB1 HSE A 30 −31.830 18.775 78.035 0.00 0.00
A
ATOM 13894 HB2 HSE A 30 −33.403 18.120 78.449 0.00 0.00
A
ATOM 13895 ND1 HSE A 30 −34.704 20.286 78.047 0.00 0.00
A
ATOM 13896 CG HSE A 30 −33.336 20.166 77.899 0.00 0.00
A
ATOM 13897 CE1 HSE A 30 −34.987 21.378 77.375 0.00 0.00
A
ATOM 13898 HE1 HSE A 30 −35.956 21.846 77.198 0.00 0.00
A
ATOM 13899 NE2 HSE A 30 −33.863 21.847 76.731 0.00 0.00
A
ATOM 13900 HE2 HSE A 30 −33.860 22.698 76.205 0.00 0.00
A
ATOM 13901 CD2 HSE A 30 −32.834 21.071 77.043 0.00 0.00
A
ATOM 13902 HD2 HSE A 30 −31.849 21.240 76.627 0.00 0.00
A
ATOM 13903 C HSE A 30 −31.809 18.182 80.797 0.00 0.00
A
ATOM 13904 O HSE A 30 −30.832 18.451 81.551 0.00 0.00
A

ATOM 13905 N GLU A 31 −32.319 16.939 80.691 0.00 0.00
A
ATOM 13906 HN GLU A 31 −33.236 16.830 80.316 0.00 0.00
A
ATOM 13907 CA GLU A 31 −31.776 15.810 81.447 0.00 0.00
A
ATOM 13908 HA GLU A 31 −31.027 16.266 82.077 0.00 0.00
A
ATOM 13909 CB GLU A 31 −32.827 15.195 82.336 0.00 0.00
A
ATOM 13910 HB1 GLU A 31 −33.804 15.035 81.832 0.00 0.00
A
ATOM 13911 HB2 GLU A 31 −32.446 14.224 82.718 0.00 0.00
A
ATOM 13912 CG GLU A 31 −33.318 16.175 83.408 0.00 0.00
A
ATOM 13913 HG1 GLU A 31 −32.506 16.588 84.043 0.00 0.00
A
ATOM 13914 HG2 GLU A 31 −33.703 17.117 82.964 0.00 0.00
A
ATOM 13915 CD GLU A 31 −34.436 15.479 84.255 0.00 0.00
A
ATOM 13916 OE1 GLU A 31 −35.520 15.164 83.689 0.00 0.00
A
ATOM 13917 OE2 GLU A 31 −34.234 15.144 85.490 0.00 0.00
A
ATOM 13918 C GLU A 31 −31.053 14.856 80.538 0.00 0.00
A
ATOM 13919 O GLU A 31 −31.689 14.215 79.687 0.00 0.00
A
ATOM 13920 N VAL A 32 −29.727 14.813 80.659 0.00 0.00
A
ATOM 13921 HN VAL A 32 −29.307 15.562 81.166 0.00 0.00
A
ATOM 13922 CA VAL A 32 −28.917 14.032 79.793 0.00 0.00
A
ATOM 13923 HA VAL A 32 −29.405 13.885 78.841 0.00 0.00
A
ATOM 13924 CB VAL A 32 −27.727 14.769 79.226 0.00 0.00
A
ATOM 13925 HB VAL A 32 −27.014 14.954 80.058 0.00 0.00
A
ATOM 13926 CG1 VAL A 32 −27.055 13.968 78.047 0.00 0.00
A
ATOM 13927 HG11 VAL A 32 −26.981 12.942 78.468 0.00 0.00
A
ATOM 13928 HG12 VAL A 32 −27.736 13.804 77.184 0.00 0.00
A
ATOM 13929 HG13 VAL A 32 −26.043 14.248 77.687 0.00 0.00
A
ATOM 13930 CG2 VAL A 32 −28.069 16.159 78.598 0.00 0.00
A
ATOM 13931 HG21 VAL A 32 −28.600 16.840 79.298 0.00 0.00
A

ATOM 13932 HG22 VAL A 32   -27.206 16.700 78.154 0.00 0.00
A
ATOM 13933 HG23 VAL A 32   -28.712 16.026 77.702 0.00 0.00
A
ATOM 13934 C   VAL A 32   -28.518 12.652 80.392 0.00 0.00
A
ATOM 13935 O   VAL A 32   -28.163 12.577 81.641 0.00 0.00
A
ATOM 13936 N   LEU A 33   -28.648 11.491 79.742 0.00 0.00
A
ATOM 13937 HN LEU A 33   -29.084 11.438 78.847 0.00 0.00
A
ATOM 13938 CA LEU A 33   -27.868 10.293 80.007 0.00 0.00
A
ATOM 13939 HA LEU A 33   -27.495 10.394 81.016 0.00 0.00
A
ATOM 13940 CB LEU A 33   -28.747   9.007 79.743 0.00 0.00
A
ATOM 13941 HB1 LEU A 33   -29.580   8.966 80.476 0.00 0.00
A
ATOM 13942 HB2 LEU A 33   -29.113   9.106 78.699 0.00 0.00
A
ATOM 13943 CG LEU A 33   -27.991   7.657 79.845 0.00 0.00
A
ATOM 13944 HG LEU A 33   -27.353   7.679 78.935 0.00 0.00
A
ATOM 13945 CD1 LEU A 33   -27.177   7.444 81.034 0.00 0.00
A
ATOM 13946 HD11 LEU A 33   -26.784   6.406 81.078 0.00 0.00
A
ATOM 13947 HD12 LEU A 33   -26.360   8.195 81.076 0.00 0.00
A
ATOM 13948 HD13 LEU A 33   -27.725   7.491 81.999 0.00 0.00
A
ATOM 13949 CD2 LEU A 33   -28.964   6.440 79.624 0.00 0.00
A
ATOM 13950 HD21 LEU A 33   -29.716   6.458 80.441 0.00 0.00
A
ATOM 13951 HD22 LEU A 33   -29.564   6.521 78.692 0.00 0.00
A
ATOM 13952 HD23 LEU A 33   -28.404   5.484 79.704 0.00 0.00
A
ATOM 13953 C   LEU A 33   -26.626 10.342 79.067 0.00 0.00
A
ATOM 13954 O   LEU A 33   -26.829 10.554 77.916 0.00 0.00
A
ATOM 13955 N   ALA A 34   -25.401 10.158 79.619 0.00 0.00
A
ATOM 13956 HN ALA A 34   -25.271 10.249 80.603 0.00 0.00
A
ATOM 13957 CA ALA A 34   -24.197 10.286 78.883 0.00 0.00
A
ATOM 13958 HA ALA A 34   -24.346 10.609 77.863 0.00 0.00
A

ATOM 13959 CB ALA A 34 -23.262 11.248 79.630 0.00 0.00
A
ATOM 13960 HB1 ALA A 34 -23.650 12.288 79.583 0.00 0.00
A
ATOM 13961 HB2 ALA A 34 -23.142 11.015 80.710 0.00 0.00
A
ATOM 13962 HB3 ALA A 34 -22.266 11.256 79.137 0.00 0.00
A
ATOM 13963 C ALA A 34 -23.423 8.964 78.820 0.00 0.00
A
ATOM 13964 O ALA A 34 -23.021 8.447 79.808 0.00 0.00
A
ATOM 13965 N VAL A 35 -23.261 8.358 77.552 0.00 0.00
A
ATOM 13966 HN VAL A 35 -23.668 8.684 76.703 0.00 0.00
A
ATOM 13967 CA VAL A 35 -22.863 6.996 77.392 0.00 0.00
A
ATOM 13968 HA VAL A 35 -22.499 6.678 78.358 0.00 0.00
A
ATOM 13969 CB VAL A 35 -23.979 5.987 76.811 0.00 0.00
A
ATOM 13970 HB VAL A 35 -24.143 6.238 75.741 0.00 0.00
A
ATOM 13971 CG1 VAL A 35 -23.633 4.490 76.894 0.00 0.00
A
ATOM 13972 HG11 VAL A 35 -24.485 3.923 76.462 0.00 0.00
A
ATOM 13973 HG12 VAL A 35 -22.831 4.257 76.162 0.00 0.00
A
ATOM 13974 HG13 VAL A 35 -23.304 4.264 77.930 0.00 0.00
A
ATOM 13975 CG2 VAL A 35 -25.189 6.388 77.672 0.00 0.00
A
ATOM 13976 HG21 VAL A 35 -25.006 6.254 78.760 0.00 0.00
A
ATOM 13977 HG22 VAL A 35 -25.493 7.446 77.522 0.00 0.00
A
ATOM 13978 HG23 VAL A 35 -26.031 5.733 77.362 0.00 0.00
A
ATOM 13979 C VAL A 35 -21.639 6.866 76.482 0.00 0.00
A
ATOM 13980 O VAL A 35 -21.780 7.138 75.310 0.00 0.00
A
ATOM 13981 N ASP A 36 -20.461 6.297 76.929 0.00 0.00
A
ATOM 13982 HN ASP A 36 -20.351 5.997 77.874 0.00 0.00
A
ATOM 13983 CA ASP A 36 -19.317 6.190 76.087 0.00 0.00
A
ATOM 13984 HA ASP A 36 -19.565 5.834 75.098 0.00 0.00
A
ATOM 13985 CB ASP A 36 -18.511 7.533 76.053 0.00 0.00
A

ATOM 13986 HB1 ASP A 36 −19.339 8.185 75.700 0.00 0.00
A
ATOM 13987 HB2 ASP A 36 −18.209 8.014 77.007 0.00 0.00
A
ATOM 13988 CG ASP A 36 −17.424 7.661 74.963 0.00 0.00
A
ATOM 13989 OD1 ASP A 36 −17.664 8.535 74.080 0.00 0.00
A
ATOM 13990 OD2 ASP A 36 −16.376 6.969 74.987 0.00 0.00
A
ATOM 13991 C ASP A 36 −18.485 5.128 76.819 0.00 0.00
A
ATOM 13992 O ASP A 36 −18.792 4.771 77.966 0.00 0.00
A
ATOM 13993 N ILE A 37 −17.384 4.726 76.243 0.00 0.00
A
ATOM 13994 HN ILE A 37 −17.041 5.212 75.444 0.00 0.00
A
ATOM 13995 CA ILE A 37 −16.428 3.765 76.829 0.00 0.00
A
ATOM 13996 HA ILE A 37 −16.907 3.214 77.625 0.00 0.00
A
ATOM 13997 CB ILE A 37 −15.942 2.722 75.765 0.00 0.00
A
ATOM 13998 HB ILE A 37 −15.054 2.190 76.168 0.00 0.00
A
ATOM 13999 CG2 ILE A 37 −17.112 1.725 75.649 0.00 0.00
A
ATOM 14000 HG21 ILE A 37 −18.017 2.194 75.207 0.00 0.00
A
ATOM 14001 HG22 ILE A 37 −16.701 0.864 75.080 0.00 0.00
A
ATOM 14002 HG23 ILE A 37 −17.384 1.321 76.647 0.00 0.00
A
ATOM 14003 CG1 ILE A 37 −15.525 3.306 74.427 0.00 0.00
A
ATOM 14004 HG11 ILE A 37 −16.429 3.886 74.139 0.00 0.00
A
ATOM 14005 HG12 ILE A 37 −14.642 3.976 74.504 0.00 0.00
A
ATOM 14006 CD ILE A 37 −15.389 2.283 73.243 0.00 0.00
A
ATOM 14007 HD1 ILE A 37 −14.760 1.420 73.552 0.00 0.00
A
ATOM 14008 HD2 ILE A 37 −16.319 1.696 73.088 0.00 0.00
A
ATOM 14009 HD3 ILE A 37 −14.916 2.709 72.333 0.00 0.00
A
ATOM 14010 C ILE A 37 −15.188 4.531 77.287 0.00 0.00
A
ATOM 14011 O ILE A 37 −14.441 3.934 78.052 0.00 0.00
A
ATOM 14012 N ASN A 38 −14.937 5.748 76.839 0.00 0.00
A

ATOM	14013	HN	ASN	A	38	−15.431	6.175	76.085	0.00	0.00	A
ATOM	14014	CA	ASN	A	38	−13.812	6.480	77.323	0.00	0.00	A
ATOM	14015	HA	ASN	A	38	−12.960	5.821	77.243	0.00	0.00	A
ATOM	14016	CB	ASN	A	38	−13.409	7.570	76.294	0.00	0.00	A
ATOM	14017	HB1	ASN	A	38	−13.547	7.258	75.237	0.00	0.00	A
ATOM	14018	HB2	ASN	A	38	−14.009	8.467	76.558	0.00	0.00	A
ATOM	14019	CG	ASN	A	38	−11.851	7.937	76.459	0.00	0.00	A
ATOM	14020	OD1	ASN	A	38	−11.503	8.533	77.469	0.00	0.00	A
ATOM	14021	ND2	ASN	A	38	−10.943	7.709	75.416	0.00	0.00	A
ATOM	14022	HD21	ASN	A	38	−11.263	7.186	74.625	0.00	0.00	A
ATOM	14023	HD22	ASN	A	38	−9.998	8.029	75.477	0.00	0.00	A
ATOM	14024	C	ASN	A	38	−14.045	7.125	78.675	0.00	0.00	A
ATOM	14025	O	ASN	A	38	−14.848	8.022	78.958	0.00	0.00	A
ATOM	14026	N	GLU	A	39	−13.291	6.562	79.626	0.00	0.00	A
ATOM	14027	HN	GLU	A	39	−12.585	5.893	79.408	0.00	0.00	A
ATOM	14028	CA	GLU	A	39	−13.580	6.734	81.081	0.00	0.00	A
ATOM	14029	HA	GLU	A	39	−14.565	6.347	81.295	0.00	0.00	A
ATOM	14030	CB	GLU	A	39	−12.709	5.887	81.920	0.00	0.00	A
ATOM	14031	HB1	GLU	A	39	−11.613	6.065	81.877	0.00	0.00	A
ATOM	14032	HB2	GLU	A	39	−13.016	6.100	82.966	0.00	0.00	A
ATOM	14033	CG	GLU	A	39	−12.929	4.403	81.614	0.00	0.00	A
ATOM	14034	HG1	GLU	A	39	−13.996	4.098	81.669	0.00	0.00	A
ATOM	14035	HG2	GLU	A	39	−12.635	4.203	80.561	0.00	0.00	A
ATOM	14036	CD	GLU	A	39	−12.038	3.409	82.275	0.00	0.00	A
ATOM	14037	OE1	GLU	A	39	−10.894	3.756	82.691	0.00	0.00	A
ATOM	14038	OE2	GLU	A	39	−12.392	2.236	82.424	0.00	0.00	A
ATOM	14039	C	GLU	A	39	−13.409	8.188	81.496	0.00	0.00	A

ATOM 14040 O GLU A 39 −14.107 8.759 82.356 0.00 0.00
A
ATOM 14041 N GLU A 40 −12.404 8.943 80.947 0.00 0.00
A
ATOM 14042 HN GLU A 40 −11.752 8.473 80.357 0.00 0.00
A
ATOM 14043 CA GLU A 40 −12.152 10.405 81.157 0.00 0.00
A
ATOM 14044 HA GLU A 40 −12.155 10.476 82.235 0.00 0.00
A
ATOM 14045 CB GLU A 40 −10.787 10.792 80.454 0.00 0.00
A
ATOM 14046 HB1 GLU A 40 −10.039 10.031 80.764 0.00 0.00
A
ATOM 14047 HB2 GLU A 40 −10.996 10.598 79.380 0.00 0.00
A
ATOM 14048 CG GLU A 40 −10.298 12.281 80.446 0.00 0.00
A
ATOM 14049 HG1 GLU A 40 −11.129 12.886 80.024 0.00 0.00
A
ATOM 14050 HG2 GLU A 40 −10.123 12.605 81.494 0.00 0.00
A
ATOM 14051 CD GLU A 40 −9.010 12.468 79.739 0.00 0.00
A
ATOM 14052 OE1 GLU A 40 −8.809 13.493 79.013 0.00 0.00
A
ATOM 14053 OE2 GLU A 40 −8.120 11.554 79.856 0.00 0.00
A
ATOM 14054 C GLU A 40 −13.299 11.224 80.579 0.00 0.00
A
ATOM 14055 O GLU A 40 −13.573 12.322 81.063 0.00 0.00
A
ATOM 14056 N LYS A 41 −13.979 10.742 79.504 0.00 0.00
A
ATOM 14057 HN LYS A 41 −13.854 9.816 79.157 0.00 0.00
A
ATOM 14058 CA LYS A 41 −15.133 11.504 79.001 0.00 0.00
A
ATOM 14059 HA LYS A 41 −14.852 12.540 78.878 0.00 0.00
A
ATOM 14060 CB LYS A 41 −15.513 10.995 77.544 0.00 0.00
A
ATOM 14061 HB1 LYS A 41 −15.272 9.920 77.402 0.00 0.00
A
ATOM 14062 HB2 LYS A 41 −16.584 11.223 77.357 0.00 0.00
A
ATOM 14063 CG LYS A 41 −14.534 11.599 76.498 0.00 0.00
A
ATOM 14064 HG1 LYS A 41 −14.633 12.700 76.603 0.00 0.00
A
ATOM 14065 HG2 LYS A 41 −13.516 11.223 76.737 0.00 0.00
A
ATOM 14066 CD LYS A 41 −14.865 11.182 75.095 0.00 0.00
A

ATOM 14067 HD1 LYS A 41 −14.612 10.100 75.070 0.00 0.00
A
ATOM 14068 HD2 LYS A 41 −15.946 11.382 74.934 0.00 0.00
A
ATOM 14069 CE LYS A 41 −13.925 11.818 74.128 0.00 0.00
A
ATOM 14070 HE1 LYS A 41 −14.157 12.903 74.069 0.00 0.00
A
ATOM 14071 HE2 LYS A 41 −12.869 11.723 74.459 0.00 0.00
A
ATOM 14072 NZ LYS A 41 −14.037 11.191 72.779 0.00 0.00
A
ATOM 14073 HZ1 LYS A 41 −13.699 11.922 72.121 0.00 0.00
A
ATOM 14074 HZ2 LYS A 41 −13.453 10.355 72.575 0.00 0.00
A
ATOM 14075 HZ3 LYS A 41 −15.049 11.008 72.624 0.00 0.00
A
ATOM 14076 C LYS A 41 −16.328 11.400 79.927 0.00 0.00
A
ATOM 14077 O LYS A 41 −17.033 12.373 80.058 0.00 0.00
A
ATOM 14078 N VAL A 42 −16.504 10.193 80.460 0.00 0.00
A
ATOM 14079 HN VAL A 42 −15.912 9.417 80.255 0.00 0.00
A
ATOM 14080 CA VAL A 42 −17.479 9.975 81.466 0.00 0.00
A
ATOM 14081 HA VAL A 42 −18.464 10.175 81.069 0.00 0.00
A
ATOM 14082 CB VAL A 42 −17.533 8.479 81.764 0.00 0.00
A
ATOM 14083 HB VAL A 42 −16.558 8.140 82.176 0.00 0.00
A
ATOM 14084 CG1 VAL A 42 −18.465 8.131 82.926 0.00 0.00
A
ATOM 14085 HG11 VAL A 42 −17.956 8.396 83.877 0.00 0.00
A
ATOM 14086 HG12 VAL A 42 −19.443 8.659 82.924 0.00 0.00
A
ATOM 14087 HG13 VAL A 42 −18.694 7.044 82.904 0.00 0.00
A
ATOM 14088 CG2 VAL A 42 −18.052 7.661 80.578 0.00 0.00
A
ATOM 14089 HG21 VAL A 42 −17.421 7.853 79.684 0.00 0.00
A
ATOM 14090 HG22 VAL A 42 −18.089 6.561 80.730 0.00 0.00
A
ATOM 14091 HG23 VAL A 42 −19.090 7.975 80.336 0.00 0.00
A
ATOM 14092 C VAL A 42 −17.270 10.844 82.714 0.00 0.00
A
ATOM 14093 O VAL A 42 −18.171 11.440 83.228 0.00 0.00
A

ATOM	14094	N	ASN	A	43	−16.005	10.944	83.112	0.00	0.00	A
ATOM	14095	HN	ASN	A	43	−15.306	10.337	82.741	0.00	0.00	A
ATOM	14096	CA	ASN	A	43	−15.611	12.006	84.002	0.00	0.00	A
ATOM	14097	HA	ASN	A	43	−16.157	11.910	84.929	0.00	0.00	A
ATOM	14098	CB	ASN	A	43	−14.111	11.979	84.355	0.00	0.00	A
ATOM	14099	HB1	ASN	A	43	−13.443	11.961	83.467	0.00	0.00	A
ATOM	14100	HB2	ASN	A	43	−13.846	12.930	84.866	0.00	0.00	A
ATOM	14101	CG	ASN	A	43	−13.663	10.767	85.231	0.00	0.00	A
ATOM	14102	OD1	ASN	A	43	−12.970	9.833	84.803	0.00	0.00	A
ATOM	14103	ND2	ASN	A	43	−14.251	10.776	86.473	0.00	0.00	A
ATOM	14104	HD21	ASN	A	43	−13.825	10.283	87.232	0.00	0.00	A
ATOM	14105	HD22	ASN	A	43	−14.897	11.455	86.821	0.00	0.00	A
ATOM	14106	C	ASN	A	43	−15.945	13.419	83.584	0.00	0.00	A
ATOM	14107	O	ASN	A	43	−16.559	14.164	84.304	0.00	0.00	A
ATOM	14108	N	ALA	A	44	−15.512	13.827	82.363	0.00	0.00	A
ATOM	14109	HN	ALA	A	44	−14.998	13.146	81.848	0.00	0.00	A
ATOM	14110	CA	ALA	A	44	−15.722	15.112	81.680	0.00	0.00	A
ATOM	14111	HA	ALA	A	44	−15.142	15.802	82.274	0.00	0.00	A
ATOM	14112	CB	ALA	A	44	−15.301	15.126	80.173	0.00	0.00	A
ATOM	14113	HB1	ALA	A	44	−15.245	16.122	79.685	0.00	0.00	A
ATOM	14114	HB2	ALA	A	44	−14.306	14.640	80.090	0.00	0.00	A
ATOM	14115	HB3	ALA	A	44	−16.060	14.467	79.699	0.00	0.00	A
ATOM	14116	C	ALA	A	44	−17.160	15.618	81.695	0.00	0.00	A
ATOM	14117	O	ALA	A	44	−17.415	16.797	82.012	0.00	0.00	A
ATOM	14118	N	TYR	A	45	−18.048	14.698	81.318	0.00	0.00	A
ATOM	14119	HN	TYR	A	45	−17.766	13.777	81.058	0.00	0.00	A
ATOM	14120	CA	TYR	A	45	−19.455	15.004	81.164	0.00	0.00	A

ATOM 14121 HA TYR A 45 −19.560 16.076 81.088 0.00 0.00
A
ATOM 14122 CB TYR A 45 −19.866 14.456 79.768 0.00 0.00
A
ATOM 14123 HB1 TYR A 45 −19.499 13.407 79.776 0.00 0.00
A
ATOM 14124 HB2 TYR A 45 −20.965 14.355 79.634 0.00 0.00
A
ATOM 14125 CG TYR A 45 −19.287 15.310 78.641 0.00 0.00
A
ATOM 14126 CD1 TYR A 45 −19.760 16.602 78.222 0.00 0.00
A
ATOM 14127 HD1 TYR A 45 −20.740 16.909 78.556 0.00 0.00
A
ATOM 14128 CE1 TYR A 45 −18.997 17.422 77.369 0.00 0.00
A
ATOM 14129 HE1 TYR A 45 −19.395 18.407 77.176 0.00 0.00
A
ATOM 14130 CZ TYR A 45 −17.813 16.965 76.858 0.00 0.00
A
ATOM 14131 OH TYR A 45 −17.181 17.744 75.816 0.00 0.00
A
ATOM 14132 HH TYR A 45 −17.490 18.650 75.884 0.00 0.00
A
ATOM 14133 CD2 TYR A 45 −18.082 14.926 78.087 0.00 0.00
A
ATOM 14134 HD2 TYR A 45 −17.890 13.874 78.234 0.00 0.00
A
ATOM 14135 CE2 TYR A 45 −17.345 15.629 77.130 0.00 0.00
A
ATOM 14136 HE2 TYR A 45 −16.680 15.158 76.421 0.00 0.00
A
ATOM 14137 C TYR A 45 −20.402 14.585 82.315 0.00 0.00
A
ATOM 14138 O TYR A 45 −21.590 14.768 82.269 0.00 0.00
A
ATOM 14139 N ALA A 46 −19.866 14.125 83.446 0.00 0.00
A
ATOM 14140 HN ALA A 46 −18.878 14.012 83.373 0.00 0.00
A
ATOM 14141 CA ALA A 46 −20.418 13.959 84.762 0.00 0.00
A
ATOM 14142 HA ALA A 46 −21.185 13.199 84.740 0.00 0.00
A
ATOM 14143 CB ALA A 46 −19.245 13.693 85.673 0.00 0.00
A
ATOM 14144 HB1 ALA A 46 −19.683 13.421 86.657 0.00 0.00
A
ATOM 14145 HB2 ALA A 46 −18.568 12.962 85.182 0.00 0.00
A
ATOM 14146 HB3 ALA A 46 −18.544 14.537 85.851 0.00 0.00
A
ATOM 14147 C ALA A 46 −21.161 15.259 85.116 0.00 0.00
A

ATOM	14148	O	ALA	A	46	−22.289	15.244	85.543	0.00	0.00	A
ATOM	14149	N	SER	A	47	−20.433	16.411	84.911	0.00	0.00	A
ATOM	14150	HN	SER	A	47	−19.452	16.287	84.784	0.00	0.00	A
ATOM	14151	CA	SER	A	47	−20.840	17.751	85.364	0.00	0.00	A
ATOM	14152	HA	SER	A	47	−21.248	17.610	86.354	0.00	0.00	A
ATOM	14153	CB	SER	A	47	−19.693	18.838	85.250	0.00	0.00	A
ATOM	14154	HB1	SER	A	47	−19.341	18.890	84.198	0.00	0.00	A
ATOM	14155	HB2	SER	A	47	−20.064	19.805	85.651	0.00	0.00	A
ATOM	14156	OG	SER	A	47	−18.668	18.462	86.149	0.00	0.00	A
ATOM	14157	HG1	SER	A	47	−17.938	18.046	85.686	0.00	0.00	A
ATOM	14158	C	SER	A	47	−21.873	18.330	84.407	0.00	0.00	A
ATOM	14159	O	SER	A	47	−22.356	19.455	84.589	0.00	0.00	A
ATOM	14160	N	TYR	A	48	−22.109	17.698	83.276	0.00	0.00	A
ATOM	14161	HN	TYR	A	48	−21.683	16.808	83.138	0.00	0.00	A
ATOM	14162	CA	TYR	A	48	−23.029	18.090	82.195	0.00	0.00	A
ATOM	14163	HA	TYR	A	48	−23.558	19.007	82.408	0.00	0.00	A
ATOM	14164	CB	TYR	A	48	−22.211	18.300	80.862	0.00	0.00	A
ATOM	14165	HB1	TYR	A	48	−21.613	17.373	80.731	0.00	0.00	A
ATOM	14166	HB2	TYR	A	48	−22.848	18.508	79.975	0.00	0.00	A
ATOM	14167	CG	TYR	A	48	−21.347	19.552	81.021	0.00	0.00	A
ATOM	14168	CD1	TYR	A	48	−19.983	19.509	80.714	0.00	0.00	A
ATOM	14169	HD1	TYR	A	48	−19.487	18.549	80.715	0.00	0.00	A
ATOM	14170	CE1	TYR	A	48	−19.115	20.630	80.857	0.00	0.00	A
ATOM	14171	HE1	TYR	A	48	−18.044	20.531	80.760	0.00	0.00	A
ATOM	14172	CZ	TYR	A	48	−19.681	21.874	81.202	0.00	0.00	A
ATOM	14173	OH	TYR	A	48	−18.974	23.136	81.322	0.00	0.00	A
ATOM	14174	HH	TYR	A	48	−18.150	23.114	80.829	0.00	0.00	A

ATOM 14175 CD2 TYR A 48 −21.873 20.764 81.409 0.00 0.00
A
ATOM 14176 HD2 TYR A 48 −22.914 20.845 81.682 0.00 0.00
A
ATOM 14177 CE2 TYR A 48 −21.093 21.931 81.366 0.00 0.00
A
ATOM 14178 HE2 TYR A 48 −21.536 22.835 81.758 0.00 0.00
A
ATOM 14179 C TYR A 48 −24.227 17.281 81.944 0.00 0.00
A
ATOM 14180 O TYR A 48 −25.058 17.524 81.120 0.00 0.00
A
ATOM 14181 N ALA A 49 −24.407 16.245 82.770 0.00 0.00
A
ATOM 14182 HN ALA A 49 −23.697 15.873 83.364 0.00 0.00
A
ATOM 14183 CA ALA A 49 −25.606 15.360 82.524 0.00 0.00
A
ATOM 14184 HA ALA A 49 −26.345 15.913 81.963 0.00 0.00
A
ATOM 14185 CB ALA A 49 −25.054 14.135 81.733 0.00 0.00
A
ATOM 14186 HB1 ALA A 49 −25.897 13.439 81.534 0.00 0.00
A
ATOM 14187 HB2 ALA A 49 −24.604 14.512 80.790 0.00 0.00
A
ATOM 14188 HB3 ALA A 49 −24.404 13.572 82.436 0.00 0.00
A
ATOM 14189 C ALA A 49 −26.138 14.993 83.851 0.00 0.00
A
ATOM 14190 O ALA A 49 −25.494 15.144 84.906 0.00 0.00
A
ATOM 14191 N THR A 50 −27.381 14.516 83.907 0.00 0.00
A
ATOM 14192 HN THR A 50 −27.983 14.503 83.113 0.00 0.00
A
ATOM 14193 CA THR A 50 −27.973 14.037 85.135 0.00 0.00
A
ATOM 14194 HA THR A 50 −27.782 14.656 86.000 0.00 0.00
A
ATOM 14195 CB THR A 50 −29.491 13.967 85.078 0.00 0.00
A
ATOM 14196 HB THR A 50 −29.859 13.483 86.008 0.00 0.00
A
ATOM 14197 OG1 THR A 50 −30.030 13.351 83.861 0.00 0.00
A
ATOM 14198 HG1 THR A 50 −30.944 13.175 84.097 0.00 0.00
A
ATOM 14199 CG2 THR A 50 −29.973 15.464 85.093 0.00 0.00
A
ATOM 14200 HG21 THR A 50 −31.073 15.624 85.096 0.00 0.00
A
ATOM 14201 HG22 THR A 50 −29.579 15.967 86.002 0.00 0.00
A

ATOM 14202 HG23 THR A 50 −29.605 16.003 84.194 0.00 0.00
A
ATOM 14203 C THR A 50 −27.456 12.651 85.494 0.00 0.00
A
ATOM 14204 O THR A 50 −27.260 12.301 86.687 0.00 0.00
A
ATOM 14205 N HSE A 51 −27.177 11.869 84.521 0.00 0.00
A
ATOM 14206 HN HSE A 51 −27.348 12.180 83.590 0.00 0.00
A
ATOM 14207 CA HSE A 51 −26.537 10.552 84.724 0.00 0.00
A
ATOM 14208 HA HSE A 51 −25.995 10.624 85.656 0.00 0.00
A
ATOM 14209 CB HSE A 51 −27.465 9.319 84.985 0.00 0.00
A
ATOM 14210 HB1 HSE A 51 −28.275 9.625 85.681 0.00 0.00
A
ATOM 14211 HB2 HSE A 51 −28.001 9.030 84.056 0.00 0.00
A
ATOM 14212 ND1 HSE A 51 −25.894 8.397 86.662 0.00 0.00
A
ATOM 14213 CG HSE A 51 −26.769 8.120 85.601 0.00 0.00
A
ATOM 14214 CE1 HSE A 51 −25.186 7.234 86.702 0.00 0.00
A
ATOM 14215 HE1 HSE A 51 −24.368 6.987 87.379 0.00 0.00
A
ATOM 14216 NE2 HSE A 51 −25.528 6.350 85.732 0.00 0.00
A
ATOM 14217 HE2 HSE A 51 −25.074 5.484 85.521 0.00 0.00
A
ATOM 14218 CD2 HSE A 51 −26.598 6.872 85.097 0.00 0.00
A
ATOM 14219 HD2 HSE A 51 −27.097 6.520 84.203 0.00 0.00
A
ATOM 14220 C HSE A 51 −25.386 10.245 83.751 0.00 0.00
A
ATOM 14221 O HSE A 51 −25.581 10.514 82.502 0.00 0.00
A
ATOM 14222 N ALA A 52 −24.159 9.888 84.273 0.00 0.00
A
ATOM 14223 HN ALA A 52 −24.040 9.865 85.262 0.00 0.00
A
ATOM 14224 CA ALA A 52 −23.044 9.617 83.379 0.00 0.00
A
ATOM 14225 HA ALA A 52 −23.383 9.575 82.355 0.00 0.00
A
ATOM 14226 CB ALA A 52 −21.960 10.664 83.288 0.00 0.00
A
ATOM 14227 HB1 ALA A 52 −22.271 11.705 83.053 0.00 0.00
A
ATOM 14228 HB2 ALA A 52 −21.343 10.712 84.211 0.00 0.00
A

ATOM	14229	HB3	ALA	A	52	−21.174	10.443	82.536	0.00	0.00	A
ATOM	14230	C	ALA	A	52	−22.473	8.165	83.577	0.00	0.00	A
ATOM	14231	O	ALA	A	52	−22.261	7.701	84.659	0.00	0.00	A
ATOM	14232	N	VAL	A	53	−22.218	7.423	82.581	0.00	0.00	A
ATOM	14233	HN	VAL	A	53	−22.292	7.767	81.648	0.00	0.00	A
ATOM	14234	CA	VAL	A	53	−21.997	6.007	82.665	0.00	0.00	A
ATOM	14235	HA	VAL	A	53	−21.552	5.883	83.641	0.00	0.00	A
ATOM	14236	CB	VAL	A	53	−23.331	5.141	82.505	0.00	0.00	A
ATOM	14237	HB	VAL	A	53	−24.103	5.453	83.240	0.00	0.00	A
ATOM	14238	CG1	VAL	A	53	−24.101	5.555	81.191	0.00	0.00	A
ATOM	14239	HG11	VAL	A	53	−24.551	6.568	81.119	0.00	0.00	A
ATOM	14240	HG12	VAL	A	53	−23.387	5.500	80.342	0.00	0.00	A
ATOM	14241	HG13	VAL	A	53	−24.875	4.764	81.091	0.00	0.00	A
ATOM	14242	CG2	VAL	A	53	−23.045	3.678	82.643	0.00	0.00	A
ATOM	14243	HG21	VAL	A	53	−22.430	3.375	81.768	0.00	0.00	A
ATOM	14244	HG22	VAL	A	53	−22.411	3.318	83.481	0.00	0.00	A
ATOM	14245	HG23	VAL	A	53	−23.978	3.074	82.637	0.00	0.00	A
ATOM	14246	C	VAL	A	53	−20.989	5.583	81.602	0.00	0.00	A
ATOM	14247	O	VAL	A	53	−21.032	5.869	80.434	0.00	0.00	A
ATOM	14248	N	ILE	A	54	−20.123	4.767	82.085	0.00	0.00	A
ATOM	14249	HN	ILE	A	54	−20.140	4.581	83.064	0.00	0.00	A
ATOM	14250	CA	ILE	A	54	−19.171	4.010	81.325	0.00	0.00	A
ATOM	14251	HA	ILE	A	54	−18.973	4.698	80.516	0.00	0.00	A
ATOM	14252	CB	ILE	A	54	−17.846	3.631	81.992	0.00	0.00	A
ATOM	14253	HB	ILE	A	54	−18.114	2.789	82.665	0.00	0.00	A
ATOM	14254	CG2	ILE	A	54	−16.756	3.133	80.928	0.00	0.00	A
ATOM	14255	HG21	ILE	A	54	−17.139	2.243	80.384	0.00	0.00	A

ATOM 14256 HG22 ILE A 54 −16.482 4.035 80.339 0.00 0.00
A
ATOM 14257 HG23 ILE A 54 −15.890 2.832 81.555 0.00 0.00
A
ATOM 14258 CG1 ILE A 54 −17.228 4.753 82.899 0.00 0.00
A
ATOM 14259 HG11 ILE A 54 −16.841 5.558 82.239 0.00 0.00
A
ATOM 14260 HG12 ILE A 54 −17.997 5.307 83.478 0.00 0.00
A
ATOM 14261 CD ILE A 54 −16.168 4.370 83.926 0.00 0.00
A
ATOM 14262 HD1 ILE A 54 −15.251 4.109 83.356 0.00 0.00
A
ATOM 14263 HD2 ILE A 54 −15.892 5.135 84.683 0.00 0.00
A
ATOM 14264 HD3 ILE A 54 −16.425 3.386 84.373 0.00 0.00
A
ATOM 14265 C ILE A 54 −19.815 2.767 80.860 0.00 0.00
A
ATOM 14266 O ILE A 54 −19.992 1.826 81.616 0.00 0.00
A
ATOM 14267 N ALA A 55 −20.251 2.717 79.609 0.00 0.00
A
ATOM 14268 HN ALA A 55 −20.009 3.448 78.976 0.00 0.00
A
ATOM 14269 CA ALA A 55 −20.959 1.622 79.040 0.00 0.00
A
ATOM 14270 HA ALA A 55 −20.553 0.686 79.393 0.00 0.00
A
ATOM 14271 CB ALA A 55 −22.397 1.472 79.444 0.00 0.00
A
ATOM 14272 HB1 ALA A 55 −22.821 2.497 79.500 0.00 0.00
A
ATOM 14273 HB2 ALA A 55 −22.975 0.760 78.817 0.00 0.00
A
ATOM 14274 HB3 ALA A 55 −22.316 1.203 80.519 0.00 0.00
A
ATOM 14275 C ALA A 55 −20.922 1.466 77.560 0.00 0.00
A
ATOM 14276 O ALA A 55 −20.793 2.399 76.744 0.00 0.00
A
ATOM 14277 N ASN A 56 −20.869 0.220 77.047 0.00 0.00
A
ATOM 14278 HN ASN A 56 −20.919 −0.582 77.638 0.00 0.00
A
ATOM 14279 CA ASN A 56 −20.727 −0.059 75.642 0.00 0.00
A
ATOM 14280 HA ASN A 56 −20.228 0.744 75.119 0.00 0.00
A
ATOM 14281 CB ASN A 56 −20.067 −1.354 75.291 0.00 0.00
A
ATOM 14282 HB1 ASN A 56 −20.569 −2.165 75.861 0.00 0.00
A

ATOM 14283 HB2 ASN A 56 −20.264 −1.470 74.204 0.00 0.00
A
ATOM 14284 CG ASN A 56 −18.587 −1.452 75.538 0.00 0.00
A
ATOM 14285 OD1 ASN A 56 −17.773 −1.503 74.635 0.00 0.00
A
ATOM 14286 ND2 ASN A 56 −18.188 −1.337 76.838 0.00 0.00
A
ATOM 14287 HD21 ASN A 56 −17.232 −1.115 77.031 0.00 0.00
A
ATOM 14288 HD22 ASN A 56 −18.856 −1.405 77.578 0.00 0.00
A
ATOM 14289 C ASN A 56 −22.136 −0.178 75.011 0.00 0.00
A
ATOM 14290 O ASN A 56 −23.058 −0.850 75.514 0.00 0.00
A
ATOM 14291 N ALA A 57 −22.293 0.529 73.848 0.00 0.00
A
ATOM 14292 HN ALA A 57 −21.544 1.071 73.477 0.00 0.00
A
ATOM 14293 CA ALA A 57 −23.613 0.736 73.341 0.00 0.00
A
ATOM 14294 HA ALA A 57 −24.366 0.529 74.086 0.00 0.00
A
ATOM 14295 CB ALA A 57 −23.772 2.113 72.823 0.00 0.00
A
ATOM 14296 HB1 ALA A 57 −23.869 2.831 73.665 0.00 0.00
A
ATOM 14297 HB2 ALA A 57 −22.910 2.353 72.164 0.00 0.00
A
ATOM 14298 HB3 ALA A 57 −24.675 2.374 72.229 0.00 0.00
A
ATOM 14299 C ALA A 57 −23.777 −0.293 72.181 0.00 0.00
A
ATOM 14300 O ALA A 57 −24.855 −0.401 71.656 0.00 0.00
A
ATOM 14301 N THR A 58 −22.734 −1.130 71.878 0.00 0.00
A
ATOM 14302 HN THR A 58 −21.855 −0.787 72.199 0.00 0.00
A
ATOM 14303 CA THR A 58 −22.831 −1.957 70.758 0.00 0.00
A
ATOM 14304 HA THR A 58 −23.763 −1.790 70.240 0.00 0.00
A
ATOM 14305 CB THR A 58 −21.556 −1.802 69.882 0.00 0.00
A
ATOM 14306 HB THR A 58 −21.528 −2.559 69.069 0.00 0.00
A
ATOM 14307 OG1 THR A 58 −20.350 −2.028 70.705 0.00 0.00
A
ATOM 14308 HG1 THR A 58 −19.720 −2.211 70.004 0.00 0.00
A
ATOM 14309 CG2 THR A 58 −21.506 −0.391 69.178 0.00 0.00
A

ATOM 14310 HG21 THR A 58 -22.365 -0.521 68.486 0.00 0.00
A
ATOM 14311 HG22 THR A 58 -21.610 0.445 69.902 0.00 0.00
A
ATOM 14312 HG23 THR A 58 -20.560 -0.264 68.610 0.00 0.00
A
ATOM 14313 C THR A 58 -22.940 -3.365 71.301 0.00 0.00
A
ATOM 14314 O THR A 58 -23.195 -4.356 70.539 0.00 0.00
A
ATOM 14315 N GLU A 59 -22.816 -3.515 72.708 0.00 0.00
A
ATOM 14316 HN GLU A 59 -22.780 -2.701 73.282 0.00 0.00
A
ATOM 14317 CA GLU A 59 -22.954 -4.803 73.412 0.00 0.00
A
ATOM 14318 HA GLU A 59 -22.616 -5.590 72.753 0.00 0.00
A
ATOM 14319 CB GLU A 59 -22.088 -4.820 74.740 0.00 0.00
A
ATOM 14320 HB1 GLU A 59 -21.100 -4.500 74.347 0.00 0.00
A
ATOM 14321 HB2 GLU A 59 -22.481 -3.978 75.348 0.00 0.00
A
ATOM 14322 CG GLU A 59 -21.942 -6.189 75.480 0.00 0.00
A
ATOM 14323 HG1 GLU A 59 -21.007 -6.142 76.078 0.00 0.00
A
ATOM 14324 HG2 GLU A 59 -22.824 -6.401 76.121 0.00 0.00
A
ATOM 14325 CD GLU A 59 -21.827 -7.286 74.428 0.00 0.00
A
ATOM 14326 OE1 GLU A 59 -22.758 -8.152 74.371 0.00 0.00
A
ATOM 14327 OE2 GLU A 59 -20.823 -7.303 73.661 0.00 0.00
A
ATOM 14328 C GLU A 59 -24.417 -5.022 73.781 0.00 0.00
A
ATOM 14329 O GLU A 59 -24.992 -4.323 74.633 0.00 0.00
A
ATOM 14330 N GLU A 60 -24.992 -6.101 73.238 0.00 0.00
A
ATOM 14331 HN GLU A 60 -24.533 -6.612 72.516 0.00 0.00
A
ATOM 14332 CA GLU A 60 -26.382 -6.505 73.646 0.00 0.00
A
ATOM 14333 HA GLU A 60 -27.056 -5.716 73.346 0.00 0.00
A
ATOM 14334 CB GLU A 60 -26.870 -7.634 72.670 0.00 0.00
A
ATOM 14335 HB1 GLU A 60 -27.923 -7.784 72.991 0.00 0.00
A
ATOM 14336 HB2 GLU A 60 -26.801 -7.337 71.602 0.00 0.00
A

ATOM 14337 CG GLU A 60 -26.061 -8.944 72.829 0.00 0.00
A
ATOM 14338 HG1 GLU A 60 -25.035 -8.709 72.472 0.00 0.00
A
ATOM 14339 HG2 GLU A 60 -26.003 -9.191 73.910 0.00 0.00
A
ATOM 14340 CD GLU A 60 -26.622 -10.033 71.970 0.00 0.00
A
ATOM 14341 OE1 GLU A 60 -26.951 -11.096 72.538 0.00 0.00
A
ATOM 14342 OE2 GLU A 60 -26.559 -9.851 70.726 0.00 0.00
A
ATOM 14343 C GLU A 60 -26.615 -6.797 75.139 0.00 0.00
A
ATOM 14344 O GLU A 60 -27.710 -6.483 75.662 0.00 0.00
A
ATOM 14345 N ASN A 61 -25.545 -7.459 75.819 0.00 0.00
A
ATOM 14346 HN ASN A 61 -24.749 -7.790 75.320 0.00 0.00
A
ATOM 14347 CA ASN A 61 -25.590 -7.646 77.273 0.00 0.00
A
ATOM 14348 HA ASN A 61 -26.359 -8.302 77.653 0.00 0.00
A
ATOM 14349 CB ASN A 61 -24.334 -8.468 77.682 0.00 0.00
A
ATOM 14350 HB1 ASN A 61 -23.434 -7.851 77.473 0.00 0.00
A
ATOM 14351 HB2 ASN A 61 -24.317 -8.675 78.773 0.00 0.00
A
ATOM 14352 CG ASN A 61 -24.254 -9.694 76.905 0.00 0.00
A
ATOM 14353 OD1 ASN A 61 -25.245 -10.380 76.566 0.00 0.00
A
ATOM 14354 ND2 ASN A 61 -23.057 -10.161 76.623 0.00 0.00
A
ATOM 14355 HD21 ASN A 61 -23.050 -10.953 76.013 0.00 0.00
A
ATOM 14356 HD22 ASN A 61 -22.211 -9.782 76.996 0.00 0.00
A
ATOM 14357 C ASN A 61 -25.528 -6.329 78.103 0.00 0.00
A
ATOM 14358 O ASN A 61 -26.381 -6.061 79.013 0.00 0.00
A
ATOM 14359 N GLU A 62 -24.592 -5.444 77.825 0.00 0.00
A
ATOM 14360 HN GLU A 62 -23.886 -5.704 77.170 0.00 0.00
A
ATOM 14361 CA GLU A 62 -24.276 -4.203 78.476 0.00 0.00
A
ATOM 14362 HA GLU A 62 -24.406 -4.386 79.532 0.00 0.00
A
ATOM 14363 CB GLU A 62 -22.799 -3.749 78.346 0.00 0.00
A

ATOM 14364 HB1 GLU A 62   -22.138 -4.510 78.814 0.00 0.00
A
ATOM 14365 HB2 GLU A 62   -22.464 -3.692 77.288 0.00 0.00
A
ATOM 14366 CG GLU A 62   -22.359 -2.443 79.181 0.00 0.00
A
ATOM 14367 HG1 GLU A 62   -22.480 -1.492 78.619 0.00 0.00
A
ATOM 14368 HG2 GLU A 62   -22.848 -2.555 80.172 0.00 0.00
A
ATOM 14369 CD GLU A 62   -20.846 -2.452 79.517 0.00 0.00
A
ATOM 14370 OE1 GLU A 62   -20.096 -1.965 78.686 0.00 0.00
A
ATOM 14371 OE2 GLU A 62   -20.552 -2.752 80.695 0.00 0.00
A
ATOM 14372 C   GLU A 62   -25.303 -3.030 78.251 0.00 0.00
A
ATOM 14373 O   GLU A 62   -25.578 -2.232 79.099 0.00 0.00
A
ATOM 14374 N   LEU A 63   -25.890 -3.000 77.021 0.00 0.00
A
ATOM 14375 HN LEU A 63   -25.574 -3.629 76.315 0.00 0.00
A
ATOM 14376 CA LEU A 63   -27.042 -2.114 76.674 0.00 0.00
A
ATOM 14377 HA LEU A 63   -26.732 -1.117 76.950 0.00 0.00
A
ATOM 14378 CB LEU A 63   -27.359 -2.169 75.147 0.00 0.00
A
ATOM 14379 HB1 LEU A 63   -26.367 -2.053 74.660 0.00 0.00
A
ATOM 14380 HB2 LEU A 63   -27.683 -3.205 74.909 0.00 0.00
A
ATOM 14381 CG LEU A 63   -28.349 -1.127 74.629 0.00 0.00
A
ATOM 14382 HG LEU A 63   -29.289 -1.160 75.221 0.00 0.00
A
ATOM 14383 CD1 LEU A 63   -27.782   0.250 74.612 0.00 0.00
A
ATOM 14384 HD11 LEU A 63   -28.419   1.023 74.130 0.00 0.00
A
ATOM 14385 HD12 LEU A 63   -27.600   0.454 75.689 0.00 0.00
A
ATOM 14386 HD13 LEU A 63   -26.834   0.239 74.033 0.00 0.00
A
ATOM 14387 CD2 LEU A 63   -28.730 -1.408 73.209 0.00 0.00
A
ATOM 14388 HD21 LEU A 63   -29.392 -0.598 72.835 0.00 0.00
A
ATOM 14389 HD22 LEU A 63   -27.839 -1.251 72.566 0.00 0.00
A
ATOM 14390 HD23 LEU A 63   -29.212 -2.399 73.068 0.00 0.00
A

ATOM 14391 C LEU A 63 −28.239 −2.540 77.452 0.00 0.00
A
ATOM 14392 O LEU A 63 −29.023 −1.658 77.761 0.00 0.00
A
ATOM 14393 N LEU A 64 −28.573 −3.842 77.596 0.00 0.00
A
ATOM 14394 HN LEU A 64 −27.988 −4.521 77.161 0.00 0.00
A
ATOM 14395 CA LEU A 64 −29.573 −4.319 78.518 0.00 0.00
A
ATOM 14396 HA LEU A 64 −30.499 −3.814 78.289 0.00 0.00
A
ATOM 14397 CB LEU A 64 −29.706 −5.841 78.375 0.00 0.00
A
ATOM 14398 HB1 LEU A 64 −29.753 −6.082 77.291 0.00 0.00
A
ATOM 14399 HB2 LEU A 64 −28.849 −6.351 78.864 0.00 0.00
A
ATOM 14400 CG LEU A 64 −30.943 −6.398 79.006 0.00 0.00
A
ATOM 14401 HG LEU A 64 −31.096 −5.935 80.004 0.00 0.00
A
ATOM 14402 CD1 LEU A 64 −32.071 −6.119 78.101 0.00 0.00
A
ATOM 14403 HD11 LEU A 64 −32.992 −6.599 78.496 0.00 0.00
A
ATOM 14404 HD12 LEU A 64 −32.356 −5.058 77.938 0.00 0.00
A
ATOM 14405 HD13 LEU A 64 −31.894 −6.479 77.065 0.00 0.00
A
ATOM 14406 CD2 LEU A 64 −30.851 −7.932 79.172 0.00 0.00
A
ATOM 14407 HD21 LEU A 64 −31.752 −8.264 79.730 0.00 0.00
A
ATOM 14408 HD22 LEU A 64 −30.728 −8.375 78.161 0.00 0.00
A
ATOM 14409 HD23 LEU A 64 −29.979 −8.299 79.755 0.00 0.00
A
ATOM 14410 C LEU A 64 −29.161 −4.013 79.987 0.00 0.00
A
ATOM 14411 O LEU A 64 −30.050 −3.576 80.759 0.00 0.00
A
ATOM 14412 N SER A 65 −27.829 −4.164 80.387 0.00 0.00
A
ATOM 14413 HN SER A 65 −27.131 −4.534 79.779 0.00 0.00
A
ATOM 14414 CA SER A 65 −27.331 −3.946 81.817 0.00 0.00
A
ATOM 14415 HA SER A 65 −28.040 −4.540 82.375 0.00 0.00
A
ATOM 14416 CB SER A 65 −25.861 −4.467 82.054 0.00 0.00
A
ATOM 14417 HB1 SER A 65 −25.825 −5.491 81.626 0.00 0.00
A

ATOM 14418 HB2 SER A 65 −25.114 −3.779 81.604 0.00 0.00
A
ATOM 14419 OG SER A 65 −25.566 −4.695 83.415 0.00 0.00
A
ATOM 14420 HG1 SER A 65 −24.670 −5.040 83.426 0.00 0.00
A
ATOM 14421 C SER A 65 −27.513 −2.567 82.354 0.00 0.00
A
ATOM 14422 O SER A 65 −27.933 −2.286 83.492 0.00 0.00
A
ATOM 14423 N LEU A 66 −27.230 −1.532 81.449 0.00 0.00
A
ATOM 14424 HN LEU A 66 −26.725 −1.785 80.627 0.00 0.00
A
ATOM 14425 CA LEU A 66 −27.587 −0.140 81.759 0.00 0.00
A
ATOM 14426 HA LEU A 66 −27.196 0.172 82.716 0.00 0.00
A
ATOM 14427 CB LEU A 66 −26.901 0.673 80.621 0.00 0.00
A
ATOM 14428 HB1 LEU A 66 −25.883 0.829 81.036 0.00 0.00
A
ATOM 14429 HB2 LEU A 66 −26.827 0.162 79.637 0.00 0.00
A
ATOM 14430 CG LEU A 66 −27.442 2.115 80.346 0.00 0.00
A
ATOM 14431 HG LEU A 66 −28.524 1.946 80.163 0.00 0.00
A
ATOM 14432 CD1 LEU A 66 −27.234 3.075 81.575 0.00 0.00
A
ATOM 14433 HD11 LEU A 66 −27.869 3.986 81.550 0.00 0.00
A
ATOM 14434 HD12 LEU A 66 −27.481 2.544 82.519 0.00 0.00
A
ATOM 14435 HD13 LEU A 66 −26.227 3.518 81.732 0.00 0.00
A
ATOM 14436 CD2 LEU A 66 −26.727 2.777 79.204 0.00 0.00
A
ATOM 14437 HD21 LEU A 66 −26.642 2.115 78.316 0.00 0.00
A
ATOM 14438 HD22 LEU A 66 −26.994 3.850 79.093 0.00 0.00
A
ATOM 14439 HD23 LEU A 66 −25.700 2.917 79.605 0.00 0.00
A
ATOM 14440 C LEU A 66 −29.066 0.205 81.768 0.00 0.00
A
ATOM 14441 O LEU A 66 −29.684 0.880 82.592 0.00 0.00
A
ATOM 14442 N GLY A 67 −29.707 −0.413 80.776 0.00 0.00
A
ATOM 14443 HN GLY A 67 −29.261 −1.080 80.185 0.00 0.00
A
ATOM 14444 CA GLY A 67 −31.196 −0.389 80.573 0.00 0.00
A

ATOM 14445 HA1 GLY A 67 -31.710 -0.384 81.523 0.00 0.00
A
ATOM 14446 HA2 GLY A 67 -31.451 -1.214 79.924 0.00 0.00
A
ATOM 14447 C GLY A 67 -31.646 0.816 79.904 0.00 0.00
A
ATOM 14448 O GLY A 67 -31.789 1.878 80.507 0.00 0.00
A
ATOM 14449 N ILE A 68 -31.739 0.819 78.593 0.00 0.00
A
ATOM 14450 HN ILE A 68 -31.515 -0.063 78.187 0.00 0.00
A
ATOM 14451 CA ILE A 68 -32.030 1.916 77.743 0.00 0.00
A
ATOM 14452 HA ILE A 68 -32.014 2.781 78.389 0.00 0.00
A
ATOM 14453 CB ILE A 68 -30.919 2.080 76.743 0.00 0.00
A
ATOM 14454 HB ILE A 68 -29.986 1.836 77.296 0.00 0.00
A
ATOM 14455 CG2 ILE A 68 -31.054 1.009 75.611 0.00 0.00
A
ATOM 14456 HG21 ILE A 68 -30.941 -0.001 76.061 0.00 0.00
A
ATOM 14457 HG22 ILE A 68 -32.091 1.024 75.214 0.00 0.00
A
ATOM 14458 HG23 ILE A 68 -30.347 1.189 74.773 0.00 0.00
A
ATOM 14459 CG1 ILE A 68 -30.750 3.400 75.980 0.00 0.00
A
ATOM 14460 HG11 ILE A 68 -29.792 3.407 75.417 0.00 0.00
A
ATOM 14461 HG12 ILE A 68 -31.633 3.429 75.307 0.00 0.00
A
ATOM 14462 CD ILE A 68 -30.718 4.564 76.916 0.00 0.00
A
ATOM 14463 HD1 ILE A 68 -30.107 4.379 77.825 0.00 0.00
A
ATOM 14464 HD2 ILE A 68 -30.503 5.550 76.451 0.00 0.00
A
ATOM 14465 HD3 ILE A 68 -31.802 4.613 77.157 0.00 0.00
A
ATOM 14466 C ILE A 68 -33.466 1.877 77.160 0.00 0.00
A
ATOM 14467 O ILE A 68 -33.963 2.785 76.480 0.00 0.00
A
ATOM 14468 N ARG A 69 -34.127 0.704 77.280 0.00 0.00
A
ATOM 14469 HN ARG A 69 -33.646 -0.058 77.707 0.00 0.00
A
ATOM 14470 CA ARG A 69 -35.321 0.315 76.462 0.00 0.00
A
ATOM 14471 HA ARG A 69 -35.108 0.199 75.409 0.00 0.00
A

ATOM 14472 CB ARG A 69 -35.801 -1.184 76.796 0.00 0.00
A
ATOM 14473 HB1 ARG A 69 -34.881 -1.799 76.887 0.00 0.00
A
ATOM 14474 HB2 ARG A 69 -36.242 -1.128 77.814 0.00 0.00
A
ATOM 14475 CG ARG A 69 -36.806 -1.688 75.818 0.00 0.00
A
ATOM 14476 HG1 ARG A 69 -37.758 -1.128 75.936 0.00 0.00
A
ATOM 14477 HG2 ARG A 69 -36.488 -1.703 74.754 0.00 0.00
A
ATOM 14478 CD ARG A 69 -37.263 -3.051 76.249 0.00 0.00
A
ATOM 14479 HD1 ARG A 69 -36.323 -3.617 76.426 0.00 0.00
A
ATOM 14480 HD2 ARG A 69 -37.813 -2.976 77.211 0.00 0.00
A
ATOM 14481 NE ARG A 69 -38.122 -3.516 75.012 0.00 0.00
A
ATOM 14482 HE ARG A 69 -38.880 -2.937 74.712 0.00 0.00
A
ATOM 14483 CZ ARG A 69 -37.816 -4.530 74.248 0.00 0.00
A
ATOM 14484 NH1 ARG A 69 -36.788 -5.364 74.527 0.00 0.00
A
ATOM 14485 HH11 ARG A 69 -36.697 -6.236 74.046 0.00 0.00
A
ATOM 14486 HH12 ARG A 69 -36.141 -5.318 75.288 0.00 0.00
A
ATOM 14487 NH2 ARG A 69 -38.565 -4.719 73.167 0.00 0.00
A
ATOM 14488 HH21 ARG A 69 -38.160 -5.400 72.557 0.00 0.00
A
ATOM 14489 HH22 ARG A 69 -38.936 -3.879 72.772 0.00 0.00
A
ATOM 14490 C ARG A 69 -36.455 1.235 76.463 0.00 0.00
A
ATOM 14491 O ARG A 69 -37.149 1.457 75.452 0.00 0.00
A
ATOM 14492 N ASN A 70 -36.676 2.002 77.558 0.00 0.00
A
ATOM 14493 HN ASN A 70 -36.134 1.879 78.386 0.00 0.00
A
ATOM 14494 CA ASN A 70 -37.827 2.809 77.814 0.00 0.00
A
ATOM 14495 HA ASN A 70 -38.715 2.407 77.349 0.00 0.00
A
ATOM 14496 CB ASN A 70 -38.080 2.913 79.357 0.00 0.00
A
ATOM 14497 HB1 ASN A 70 -37.140 2.959 79.947 0.00 0.00
A
ATOM 14498 HB2 ASN A 70 -38.610 3.882 79.482 0.00 0.00
A

ATOM	14499	CG	ASN	A	70	−39.003	1.787	79.777	0.00	0.00	A
ATOM	14500	OD1	ASN	A	70	−38.587	0.708	79.973	0.00	0.00	A
ATOM	14501	ND2	ASN	A	70	−40.255	2.018	80.078	0.00	0.00	A
ATOM	14502	HD21	ASN	A	70	−40.582	1.354	80.751	0.00	0.00	A
ATOM	14503	HD22	ASN	A	70	−40.758	2.862	79.892	0.00	0.00	A
ATOM	14504	C	ASN	A	70	−37.725	4.239	77.235	0.00	0.00	A
ATOM	14505	O	ASN	A	70	−38.698	4.971	77.178	0.00	0.00	A
ATOM	14506	N	PHE	A	71	−36.514	4.628	76.722	0.00	0.00	A
ATOM	14507	HN	PHE	A	71	−35.813	3.918	76.705	0.00	0.00	A
ATOM	14508	CA	PHE	A	71	−36.177	5.973	76.157	0.00	0.00	A
ATOM	14509	HA	PHE	A	71	−36.342	6.722	76.918	0.00	0.00	A
ATOM	14510	CB	PHE	A	71	−34.660	6.152	75.898	0.00	0.00	A
ATOM	14511	HB1	PHE	A	71	−34.205	5.179	75.614	0.00	0.00	A
ATOM	14512	HB2	PHE	A	71	−34.604	6.988	75.168	0.00	0.00	A
ATOM	14513	CG	PHE	A	71	−33.908	6.632	77.133	0.00	0.00	A
ATOM	14514	CD1	PHE	A	71	−33.665	5.860	78.267	0.00	0.00	A
ATOM	14515	HD1	PHE	A	71	−34.254	4.962	78.385	0.00	0.00	A
ATOM	14516	CE1	PHE	A	71	−32.782	6.269	79.268	0.00	0.00	A
ATOM	14517	HE1	PHE	A	71	−32.721	5.594	80.108	0.00	0.00	A
ATOM	14518	CZ	PHE	A	71	−32.256	7.524	79.250	0.00	0.00	A
ATOM	14519	HZ	PHE	A	71	−31.558	7.857	80.005	0.00	0.00	A
ATOM	14520	CD2	PHE	A	71	−33.286	7.879	77.107	0.00	0.00	A
ATOM	14521	HD2	PHE	A	71	−33.385	8.580	76.291	0.00	0.00	A
ATOM	14522	CE2	PHE	A	71	−32.555	8.367	78.172	0.00	0.00	A
ATOM	14523	HE2	PHE	A	71	−32.100	9.340	78.064	0.00	0.00	A
ATOM	14524	C	PHE	A	71	−37.049	6.249	74.936	0.00	0.00	A
ATOM	14525	O	PHE	A	71	−37.277	5.355	74.174	0.00	0.00	A

ATOM	14526	N	GLU	A	72	−37.524	7.522	74.765	0.00	0.00	A
ATOM	14527	HN	GLU	A	72	−37.401	8.202	75.484	0.00	0.00	A
ATOM	14528	CA	GLU	A	72	−38.349	7.998	73.674	0.00	0.00	A
ATOM	14529	HA	GLU	A	72	−38.947	7.245	73.183	0.00	0.00	A
ATOM	14530	CB	GLU	A	72	−39.310	9.086	74.168	0.00	0.00	A
ATOM	14531	HB1	GLU	A	72	−38.833	9.814	74.858	0.00	0.00	A
ATOM	14532	HB2	GLU	A	72	−39.522	9.637	73.227	0.00	0.00	A
ATOM	14533	CG	GLU	A	72	−40.564	8.533	74.842	0.00	0.00	A
ATOM	14534	HG1	GLU	A	72	−41.040	7.787	74.170	0.00	0.00	A
ATOM	14535	HG2	GLU	A	72	−40.375	8.041	75.820	0.00	0.00	A
ATOM	14536	CD	GLU	A	72	−41.584	9.634	75.179	0.00	0.00	A
ATOM	14537	OE1	GLU	A	72	−42.486	9.988	74.430	0.00	0.00	A
ATOM	14538	OE2	GLU	A	72	−41.388	10.294	76.215	0.00	0.00	A
ATOM	14539	C	GLU	A	72	−37.374	8.547	72.579	0.00	0.00	A
ATOM	14540	O	GLU	A	72	−37.501	8.281	71.392	0.00	0.00	A
ATOM	14541	N	TYR	A	73	−36.258	9.279	72.969	0.00	0.00	A
ATOM	14542	HN	TYR	A	73	−36.088	9.565	73.909	0.00	0.00	A
ATOM	14543	CA	TYR	A	73	−35.296	9.905	72.097	0.00	0.00	A
ATOM	14544	HA	TYR	A	73	−35.510	9.536	71.105	0.00	0.00	A
ATOM	14545	CB	TYR	A	73	−35.264	11.445	72.177	0.00	0.00	A
ATOM	14546	HB1	TYR	A	73	−35.018	11.853	73.181	0.00	0.00	A
ATOM	14547	HB2	TYR	A	73	−34.371	11.872	71.672	0.00	0.00	A
ATOM	14548	CG	TYR	A	73	−36.497	12.049	71.592	0.00	0.00	A
ATOM	14549	CD1	TYR	A	73	−37.632	12.277	72.396	0.00	0.00	A
ATOM	14550	HD1	TYR	A	73	−37.636	11.874	73.397	0.00	0.00	A
ATOM	14551	CE1	TYR	A	73	−38.784	12.898	71.926	0.00	0.00	A
ATOM	14552	HE1	TYR	A	73	−39.716	12.905	72.470	0.00	0.00	A

ATOM 14553 CZ TYR A 73 −38.785 13.379 70.576 0.00 0.00
A
ATOM 14554 OH TYR A 73 −39.927 13.793 69.973 0.00 0.00
A
ATOM 14555 HH TYR A 73 −40.640 13.414 70.491 0.00 0.00
A
ATOM 14556 CD2 TYR A 73 −36.430 12.714 70.339 0.00 0.00
A
ATOM 14557 HD2 TYR A 73 −35.549 12.599 69.726 0.00 0.00
A
ATOM 14558 CE2 TYR A 73 −37.569 13.350 69.793 0.00 0.00
A
ATOM 14559 HE2 TYR A 73 −37.585 13.776 68.801 0.00 0.00
A
ATOM 14560 C TYR A 73 −33.895 9.444 72.487 0.00 0.00
A
ATOM 14561 O TYR A 73 −33.421 9.520 73.630 0.00 0.00
A
ATOM 14562 N VAL A 74 −33.113 8.908 71.522 0.00 0.00
A
ATOM 14563 HN VAL A 74 −33.444 8.923 70.582 0.00 0.00
A
ATOM 14564 CA VAL A 74 −31.762 8.462 71.838 0.00 0.00
A
ATOM 14565 HA VAL A 74 −31.430 9.019 72.701 0.00 0.00
A
ATOM 14566 CB VAL A 74 −31.714 6.905 71.980 0.00 0.00
A
ATOM 14567 HB VAL A 74 −32.138 6.582 71.005 0.00 0.00
A
ATOM 14568 CG1 VAL A 74 −30.297 6.407 72.262 0.00 0.00
A
ATOM 14569 HG11 VAL A 74 −30.304 5.327 72.522 0.00 0.00
A
ATOM 14570 HG12 VAL A 74 −29.608 6.647 71.423 0.00 0.00
A
ATOM 14571 HG13 VAL A 74 −29.883 7.024 73.088 0.00 0.00
A
ATOM 14572 CG2 VAL A 74 −32.672 6.488 73.155 0.00 0.00
A
ATOM 14573 HG21 VAL A 74 −33.744 6.732 72.996 0.00 0.00
A
ATOM 14574 HG22 VAL A 74 −32.557 5.414 73.414 0.00 0.00
A
ATOM 14575 HG23 VAL A 74 −32.229 6.899 74.087 0.00 0.00
A
ATOM 14576 C VAL A 74 −30.993 8.981 70.692 0.00 0.00
A
ATOM 14577 O VAL A 74 −31.387 8.975 69.551 0.00 0.00
A
ATOM 14578 N ILE A 75 −29.815 9.614 70.956 0.00 0.00
A
ATOM 14579 HN ILE A 75 −29.506 9.737 71.896 0.00 0.00
A

ATOM 14580 CA ILE A 75 -28.987 10.170 69.954 0.00 0.00
A
ATOM 14581 HA ILE A 75 -29.413 10.072 68.966 0.00 0.00
A
ATOM 14582 CB ILE A 75 -28.651 11.623 70.267 0.00 0.00
A
ATOM 14583 HB ILE A 75 -27.868 11.722 71.049 0.00 0.00
A
ATOM 14584 CG2 ILE A 75 -27.916 12.251 69.048 0.00 0.00
A
ATOM 14585 HG21 ILE A 75 -26.960 11.804 68.701 0.00 0.00
A
ATOM 14586 HG22 ILE A 75 -28.479 12.179 68.093 0.00 0.00
A
ATOM 14587 HG23 ILE A 75 -27.677 13.315 69.262 0.00 0.00
A
ATOM 14588 CG1 ILE A 75 -29.890 12.488 70.692 0.00 0.00
A
ATOM 14589 HG11 ILE A 75 -30.342 12.048 71.607 0.00 0.00
A
ATOM 14590 HG12 ILE A 75 -29.476 13.479 70.973 0.00 0.00
A
ATOM 14591 CD ILE A 75 -30.962 12.550 69.614 0.00 0.00
A
ATOM 14592 HD1 ILE A 75 -31.825 13.128 70.011 0.00 0.00
A
ATOM 14593 HD2 ILE A 75 -30.581 13.080 68.715 0.00 0.00
A
ATOM 14594 HD3 ILE A 75 -31.179 11.536 69.215 0.00 0.00
A
ATOM 14595 C ILE A 75 -27.674 9.448 69.824 0.00 0.00
A
ATOM 14596 O ILE A 75 -26.863 9.428 70.807 0.00 0.00
A
ATOM 14597 N VAL A 76 -27.302 8.879 68.658 0.00 0.00
A
ATOM 14598 HN VAL A 76 -27.935 9.117 67.925 0.00 0.00
A
ATOM 14599 CA VAL A 76 -26.016 8.196 68.404 0.00 0.00
A
ATOM 14600 HA VAL A 76 -25.566 7.979 69.361 0.00 0.00
A
ATOM 14601 CB VAL A 76 -26.235 6.921 67.574 0.00 0.00
A
ATOM 14602 HB VAL A 76 -26.469 7.284 66.550 0.00 0.00
A
ATOM 14603 CG1 VAL A 76 -24.911 6.116 67.600 0.00 0.00
A
ATOM 14604 HG11 VAL A 76 -24.437 6.143 68.605 0.00 0.00
A
ATOM 14605 HG12 VAL A 76 -25.091 5.046 67.361 0.00 0.00
A
ATOM 14606 HG13 VAL A 76 -24.309 6.599 66.802 0.00 0.00
A

ATOM 14607 CG2 VAL A 76 −27.378 6.200 68.229 0.00 0.00
A
ATOM 14608 HG21 VAL A 76 −27.422 6.249 69.338 0.00 0.00
A
ATOM 14609 HG22 VAL A 76 −28.304 6.663 67.826 0.00 0.00
A
ATOM 14610 HG23 VAL A 76 −27.264 5.131 67.951 0.00 0.00
A
ATOM 14611 C VAL A 76 −25.062 9.075 67.732 0.00 0.00
A
ATOM 14612 O VAL A 76 −25.153 9.415 66.572 0.00 0.00
A
ATOM 14613 N ALA A 77 −24.040 9.476 68.593 0.00 0.00
A
ATOM 14614 HN ALA A 77 −23.948 9.062 69.495 0.00 0.00
A
ATOM 14615 CA ALA A 77 −23.064 10.449 68.233 0.00 0.00
A
ATOM 14616 HA ALA A 77 −23.429 10.944 67.345 0.00 0.00
A
ATOM 14617 CB ALA A 77 −22.679 11.318 69.459 0.00 0.00
A
ATOM 14618 HB1 ALA A 77 −22.164 10.751 70.263 0.00 0.00
A
ATOM 14619 HB2 ALA A 77 −21.998 12.149 69.175 0.00 0.00
A
ATOM 14620 HB3 ALA A 77 −23.482 11.738 70.101 0.00 0.00
A
ATOM 14621 C ALA A 77 −21.821 9.748 67.779 0.00 0.00
A
ATOM 14622 O ALA A 77 −20.837 10.373 67.298 0.00 0.00
A
ATOM 14623 N ILE A 78 −21.884 8.378 67.811 0.00 0.00
A
ATOM 14624 HN ILE A 78 −22.760 8.008 68.112 0.00 0.00
A
ATOM 14625 CA ILE A 78 −20.890 7.496 67.165 0.00 0.00
A
ATOM 14626 HA ILE A 78 −19.891 7.761 67.477 0.00 0.00
A
ATOM 14627 CB ILE A 78 −21.038 6.071 67.597 0.00 0.00
A
ATOM 14628 HB ILE A 78 −21.923 5.627 67.093 0.00 0.00
A
ATOM 14629 CG2 ILE A 78 −19.803 5.224 67.135 0.00 0.00
A
ATOM 14630 HG21 ILE A 78 −19.760 4.196 67.556 0.00 0.00
A
ATOM 14631 HG22 ILE A 78 −19.735 5.236 66.026 0.00 0.00
A
ATOM 14632 HG23 ILE A 78 −18.880 5.661 67.573 0.00 0.00
A
ATOM 14633 CG1 ILE A 78 −21.341 5.908 69.145 0.00 0.00
A

ATOM 14634 HG11 ILE A 78 −20.547 6.519 69.627 0.00 0.00
A
ATOM 14635 HG12 ILE A 78 −22.268 6.471 69.387 0.00 0.00
A
ATOM 14636 CD ILE A 78 −21.462 4.493 69.650 0.00 0.00
A
ATOM 14637 HD1 ILE A 78 −22.377 4.006 69.252 0.00 0.00
A
ATOM 14638 HD2 ILE A 78 −20.591 3.818 69.509 0.00 0.00
A
ATOM 14639 HD3 ILE A 78 −21.588 4.534 70.754 0.00 0.00
A
ATOM 14640 C ILE A 78 −21.122 7.516 65.637 0.00 0.00
A
ATOM 14641 O ILE A 78 −22.216 7.278 65.104 0.00 0.00
A
ATOM 14642 N GLY A 79 −20.050 7.764 64.846 0.00 0.00
A
ATOM 14643 HN GLY A 79 −19.217 8.036 65.323 0.00 0.00
A
ATOM 14644 CA GLY A 79 −20.307 7.925 63.411 0.00 0.00
A
ATOM 14645 HA1 GLY A 79 −20.850 8.856 63.340 0.00 0.00
A
ATOM 14646 HA2 GLY A 79 −20.871 7.051 63.120 0.00 0.00
A
ATOM 14647 C GLY A 79 −19.133 8.020 62.529 0.00 0.00
A
ATOM 14648 O GLY A 79 −19.213 7.819 61.313 0.00 0.00
A
ATOM 14649 N ALA A 80 −17.899 8.190 63.114 0.00 0.00
A
ATOM 14650 HN ALA A 80 −17.997 8.526 64.048 0.00 0.00
A
ATOM 14651 CA ALA A 80 −16.626 7.989 62.401 0.00 0.00
A
ATOM 14652 HA ALA A 80 −16.792 8.519 61.475 0.00 0.00
A
ATOM 14653 CB ALA A 80 −15.472 8.514 63.177 0.00 0.00
A
ATOM 14654 HB1 ALA A 80 −14.470 8.307 62.744 0.00 0.00
A
ATOM 14655 HB2 ALA A 80 −15.676 9.573 63.442 0.00 0.00
A
ATOM 14656 HB3 ALA A 80 −15.508 7.958 64.138 0.00 0.00
A
ATOM 14657 C ALA A 80 −16.481 6.650 61.913 0.00 0.00
A
ATOM 14658 O ALA A 80 −16.142 6.416 60.705 0.00 0.00
A
ATOM 14659 N ASN A 81 −16.863 5.630 62.797 0.00 0.00
A
ATOM 14660 HN ASN A 81 −17.206 5.853 63.707 0.00 0.00
A

ATOM 14661 CA ASN A 81 −16.944 4.234 62.581 0.00 0.00
A
ATOM 14662 HA ASN A 81 −16.281 3.962 61.773 0.00 0.00
A
ATOM 14663 CB ASN A 81 −16.619 3.385 63.748 0.00 0.00
A
ATOM 14664 HB1 ASN A 81 −15.675 3.777 64.182 0.00 0.00
A
ATOM 14665 HB2 ASN A 81 −17.483 3.450 64.443 0.00 0.00
A
ATOM 14666 CG ASN A 81 −16.271 1.914 63.497 0.00 0.00
A
ATOM 14667 OD1 ASN A 81 −17.010 1.007 63.878 0.00 0.00
A
ATOM 14668 ND2 ASN A 81 −15.115 1.750 62.748 0.00 0.00
A
ATOM 14669 HD21 ASN A 81 −14.407 2.409 63.002 0.00 0.00
A
ATOM 14670 HD22 ASN A 81 −14.894 0.916 62.242 0.00 0.00
A
ATOM 14671 C ASN A 81 −18.316 3.911 61.972 0.00 0.00
A
ATOM 14672 O ASN A 81 −19.333 4.011 62.665 0.00 0.00
A
ATOM 14673 N ILE A 82 −18.411 3.418 60.777 0.00 0.00
A
ATOM 14674 HN ILE A 82 −17.579 3.317 60.238 0.00 0.00
A
ATOM 14675 CA ILE A 82 −19.592 2.694 60.263 0.00 0.00
A
ATOM 14676 HA ILE A 82 −20.331 3.481 60.271 0.00 0.00
A
ATOM 14677 CB ILE A 82 −19.325 2.115 58.848 0.00 0.00
A
ATOM 14678 HB ILE A 82 −18.671 1.217 58.818 0.00 0.00
A
ATOM 14679 CG2 ILE A 82 −20.666 1.476 58.316 0.00 0.00
A
ATOM 14680 HG21 ILE A 82 −21.527 2.096 58.645 0.00 0.00
A
ATOM 14681 HG22 ILE A 82 −20.638 1.216 57.236 0.00 0.00
A
ATOM 14682 HG23 ILE A 82 −20.866 0.472 58.749 0.00 0.00
A
ATOM 14683 CG1 ILE A 82 −18.649 3.229 57.971 0.00 0.00
A
ATOM 14684 HG11 ILE A 82 −19.343 4.093 58.046 0.00 0.00
A
ATOM 14685 HG12 ILE A 82 −17.663 3.524 58.390 0.00 0.00
A
ATOM 14686 CD ILE A 82 −18.545 2.864 56.408 0.00 0.00
A
ATOM 14687 HD1 ILE A 82 −18.124 1.864 56.167 0.00 0.00
A

ATOM 14688 HD2 ILE A 82 −19.550 3.050 55.972 0.00 0.00
A
ATOM 14689 HD3 ILE A 82 −17.770 3.527 55.968 0.00 0.00
A
ATOM 14690 C ILE A 82 −20.187 1.540 61.147 0.00 0.00
A
ATOM 14691 O ILE A 82 −21.355 1.494 61.513 0.00 0.00
A
ATOM 14692 N GLN A 83 −19.342 0.566 61.583 0.00 0.00
A
ATOM 14693 HN GLN A 83 −18.364 0.637 61.402 0.00 0.00
A
ATOM 14694 CA GLN A 83 −19.752 −0.597 62.388 0.00 0.00
A
ATOM 14695 HA GLN A 83 −20.431 −1.157 61.763 0.00 0.00
A
ATOM 14696 CB GLN A 83 −18.610 −1.593 62.505 0.00 0.00
A
ATOM 14697 HB1 GLN A 83 −18.175 −1.719 61.490 0.00 0.00
A
ATOM 14698 HB2 GLN A 83 −17.840 −1.178 63.190 0.00 0.00
A
ATOM 14699 CG GLN A 83 −19.040 −2.916 63.201 0.00 0.00
A
ATOM 14700 HG1 GLN A 83 −19.139 −2.805 64.302 0.00 0.00
A
ATOM 14701 HG2 GLN A 83 −19.934 −3.285 62.654 0.00 0.00
A
ATOM 14702 CD GLN A 83 −18.027 −4.066 63.111 0.00 0.00
A
ATOM 14703 OE1 GLN A 83 −18.147 −4.945 63.962 0.00 0.00
A
ATOM 14704 NE2 GLN A 83 −17.069 −4.032 62.171 0.00 0.00
A
ATOM 14705 HE21 GLN A 83 −16.598 −4.912 62.108 0.00 0.00
A
ATOM 14706 HE22 GLN A 83 −16.954 −3.237 61.577 0.00 0.00
A
ATOM 14707 C GLN A 83 −20.382 −0.251 63.710 0.00 0.00
A
ATOM 14708 O GLN A 83 −21.586 −0.547 63.907 0.00 0.00
A
ATOM 14709 N ALA A 84 −19.664 0.467 64.681 0.00 0.00
A
ATOM 14710 HN ALA A 84 −18.793 0.890 64.441 0.00 0.00
A
ATOM 14711 CA ALA A 84 −20.215 0.847 66.016 0.00 0.00
A
ATOM 14712 HA ALA A 84 −20.347 −0.041 66.615 0.00 0.00
A
ATOM 14713 CB ALA A 84 −19.112 1.615 66.786 0.00 0.00
A
ATOM 14714 HB1 ALA A 84 −18.204 0.979 66.847 0.00 0.00
A

```
ATOM  14715  HB2 ALA A  84     -18.836   2.549  66.250  0.00  0.00
A
ATOM  14716  HB3 ALA A  84     -19.434   1.938  67.799  0.00  0.00
A
ATOM  14717  C   ALA A  84     -21.545   1.581  65.938  0.00  0.00
A
ATOM  14718  O   ALA A  84     -22.424   1.324  66.754  0.00  0.00
A
ATOM  14719  N   SER A  85     -21.682   2.551  64.938  0.00  0.00
A
ATOM  14720  HN  SER A  85     -20.933   2.600  64.281  0.00  0.00
A
ATOM  14721  CA  SER A  85     -22.879   3.361  64.667  0.00  0.00
A
ATOM  14722  HA  SER A  85     -23.210   3.789  65.602  0.00  0.00
A
ATOM  14723  CB  SER A  85     -22.499   4.319  63.597  0.00  0.00
A
ATOM  14724  HB1 SER A  85     -21.626   4.926  63.922  0.00  0.00
A
ATOM  14725  HB2 SER A  85     -22.267   3.772  62.659  0.00  0.00
A
ATOM  14726  OG  SER A  85     -23.528   5.304  63.364  0.00  0.00
A
ATOM  14727  HG1 SER A  85     -23.375   6.051  63.948  0.00  0.00
A
ATOM  14728  C   SER A  85     -24.032   2.396  64.167  0.00  0.00
A
ATOM  14729  O   SER A  85     -25.104   2.368  64.808  0.00  0.00
A
ATOM  14730  N   THR A  86     -23.860   1.626  63.081  0.00  0.00
A
ATOM  14731  HN  THR A  86     -23.002   1.651  62.574  0.00  0.00
A
ATOM  14732  CA  THR A  86     -24.921   0.820  62.497  0.00  0.00
A
ATOM  14733  HA  THR A  86     -25.844   1.356  62.331  0.00  0.00
A
ATOM  14734  CB  THR A  86     -24.592   0.263  61.128  0.00  0.00
A
ATOM  14735  HB  THR A  86     -25.330  -0.511  60.827  0.00  0.00
A
ATOM  14736  OG1 THR A  86     -23.343  -0.344  61.070  0.00  0.00
A
ATOM  14737  HG1 THR A  86     -22.654   0.324  61.105  0.00  0.00
A
ATOM  14738  CG2 THR A  86     -24.694   1.334  60.040  0.00  0.00
A
ATOM  14739 HG21 THR A  86     -23.962   2.134  60.279  0.00  0.00
A
ATOM  14740 HG22 THR A  86     -24.465   0.984  59.011  0.00  0.00
A
ATOM  14741 HG23 THR A  86     -25.695   1.812  60.097  0.00  0.00
A
```

ATOM 14742 C THR A 86 -25.341 -0.246 63.435 0.00 0.00
A
ATOM 14743 O THR A 86 -26.529 -0.536 63.643 0.00 0.00
A
ATOM 14744 N LEU A 87 -24.398 -0.865 64.150 0.00 0.00
A
ATOM 14745 HN LEU A 87 -23.452 -0.638 63.929 0.00 0.00
A
ATOM 14746 CA LEU A 87 -24.627 -1.850 65.179 0.00 0.00
A
ATOM 14747 HA LEU A 87 -25.400 -2.565 64.938 0.00 0.00
A
ATOM 14748 CB LEU A 87 -23.362 -2.639 65.405 0.00 0.00
A
ATOM 14749 HB1 LEU A 87 -23.096 -2.989 64.385 0.00 0.00
A
ATOM 14750 HB2 LEU A 87 -22.571 -1.943 65.758 0.00 0.00
A
ATOM 14751 CG LEU A 87 -23.418 -3.894 66.353 0.00 0.00
A
ATOM 14752 HG LEU A 87 -23.838 -3.531 67.315 0.00 0.00
A
ATOM 14753 CD1 LEU A 87 -24.195 -5.141 65.852 0.00 0.00
A
ATOM 14754 HD11 LEU A 87 -24.608 -5.749 66.685 0.00 0.00
A
ATOM 14755 HD12 LEU A 87 -25.061 -4.898 65.200 0.00 0.00
A
ATOM 14756 HD13 LEU A 87 -23.592 -5.886 65.289 0.00 0.00
A
ATOM 14757 CD2 LEU A 87 -22.077 -4.385 66.754 0.00 0.00
A
ATOM 14758 HD21 LEU A 87 -21.374 -3.595 67.095 0.00 0.00
A
ATOM 14759 HD22 LEU A 87 -22.059 -5.075 67.624 0.00 0.00
A
ATOM 14760 HD23 LEU A 87 -21.521 -4.846 65.910 0.00 0.00
A
ATOM 14761 C LEU A 87 -25.209 -1.355 66.448 0.00 0.00
A
ATOM 14762 O LEU A 87 -26.147 -1.954 66.988 0.00 0.00
A
ATOM 14763 N THR A 88 -24.893 -0.155 66.886 0.00 0.00
A
ATOM 14764 HN THR A 88 -24.145 0.369 66.488 0.00 0.00
A
ATOM 14765 CA THR A 88 -25.738 0.645 67.865 0.00 0.00
A
ATOM 14766 HA THR A 88 -25.827 0.049 68.761 0.00 0.00
A
ATOM 14767 CB THR A 88 -25.126 1.935 68.526 0.00 0.00
A
ATOM 14768 HB THR A 88 -24.903 2.744 67.798 0.00 0.00
A

ATOM 14769 OG1 THR A 88 -23.889 1.666 68.986 0.00 0.00
A
ATOM 14770 HG1 THR A 88 -23.282 1.801 68.255 0.00 0.00
A
ATOM 14771 CG2 THR A 88 -26.017 2.521 69.737 0.00 0.00
A
ATOM 14772 HG21 THR A 88 -26.869 2.990 69.201 0.00 0.00
A
ATOM 14773 HG22 THR A 88 -26.390 1.646 70.311 0.00 0.00
A
ATOM 14774 HG23 THR A 88 -25.572 3.378 70.286 0.00 0.00
A
ATOM 14775 C THR A 88 -27.146 0.928 67.337 0.00 0.00
A
ATOM 14776 O THR A 88 -28.161 0.761 68.023 0.00 0.00
A
ATOM 14777 N THR A 89 -27.333 1.379 66.084 0.00 0.00
A
ATOM 14778 HN THR A 89 -26.503 1.673 65.616 0.00 0.00
A
ATOM 14779 CA THR A 89 -28.673 1.678 65.486 0.00 0.00
A
ATOM 14780 HA THR A 89 -29.093 2.357 66.213 0.00 0.00
A
ATOM 14781 CB THR A 89 -28.638 2.297 64.119 0.00 0.00
A
ATOM 14782 HB THR A 89 -28.494 1.510 63.349 0.00 0.00
A
ATOM 14783 OG1 THR A 89 -27.623 3.341 64.008 0.00 0.00
A
ATOM 14784 HG1 THR A 89 -26.819 2.918 64.315 0.00 0.00
A
ATOM 14785 CG2 THR A 89 -30.005 2.856 63.800 0.00 0.00
A
ATOM 14786 HG21 THR A 89 -30.328 3.485 64.657 0.00 0.00
A
ATOM 14787 HG22 THR A 89 -29.973 3.633 63.006 0.00 0.00
A
ATOM 14788 HG23 THR A 89 -30.716 2.040 63.550 0.00 0.00
A
ATOM 14789 C THR A 89 -29.607 0.472 65.393 0.00 0.00
A
ATOM 14790 O THR A 89 -30.825 0.589 65.531 0.00 0.00
A
ATOM 14791 N LEU A 90 -28.992 -0.681 65.065 0.00 0.00
A
ATOM 14792 HN LEU A 90 -28.012 -0.511 64.990 0.00 0.00
A
ATOM 14793 CA LEU A 90 -29.612 -1.991 64.924 0.00 0.00
A
ATOM 14794 HA LEU A 90 -30.476 -1.840 64.294 0.00 0.00
A
ATOM 14795 CB LEU A 90 -28.596 -2.930 64.257 0.00 0.00
A

```
ATOM  14796  HB1 LEU A  90     -28.156  -2.285  63.466  0.00  0.00
A
ATOM  14797  HB2 LEU A  90     -27.798  -3.302  64.935  0.00  0.00
A
ATOM  14798  CG  LEU A  90     -29.095  -4.097  63.423  0.00  0.00
A
ATOM  14799  HG  LEU A  90     -29.476  -4.959  64.010  0.00  0.00
A
ATOM  14800  CD1 LEU A  90     -30.182  -3.577  62.465  0.00  0.00
A
ATOM  14801 HD11 LEU A  90     -29.831  -2.704  61.874  0.00  0.00
A
ATOM  14802 HD12 LEU A  90     -30.354  -4.221  61.577  0.00  0.00
A
ATOM  14803 HD13 LEU A  90     -31.158  -3.245  62.881  0.00  0.00
A
ATOM  14804  CD2 LEU A  90     -28.056  -4.623  62.474  0.00  0.00
A
ATOM  14805 HD21 LEU A  90     -28.517  -5.382  61.807  0.00  0.00
A
ATOM  14806 HD22 LEU A  90     -27.690  -3.719  61.940  0.00  0.00
A
ATOM  14807 HD23 LEU A  90     -27.216  -5.029  63.077  0.00  0.00
A
ATOM  14808  C   LEU A  90     -30.139  -2.534  66.285  0.00  0.00
A
ATOM  14809  O   LEU A  90     -31.294  -2.996  66.381  0.00  0.00
A
ATOM  14810  N   LEU A  91     -29.346  -2.302  67.363  0.00  0.00
A
ATOM  14811  HN  LEU A  91     -28.399  -1.993  67.320  0.00  0.00
A
ATOM  14812  CA  LEU A  91     -29.798  -2.531  68.683  0.00  0.00
A
ATOM  14813  HA  LEU A  91     -30.175  -3.542  68.630  0.00  0.00
A
ATOM  14814  CB  LEU A  91     -28.621  -2.587  69.798  0.00  0.00
A
ATOM  14815  HB1 LEU A  91     -28.065  -1.625  69.775  0.00  0.00
A
ATOM  14816  HB2 LEU A  91     -29.076  -2.620  70.811  0.00  0.00
A
ATOM  14817  CG  LEU A  91     -27.666  -3.756  69.607  0.00  0.00
A
ATOM  14818  HG  LEU A  91     -27.187  -3.663  68.609  0.00  0.00
A
ATOM  14819  CD1 LEU A  91     -26.557  -3.600  70.648  0.00  0.00
A
ATOM  14820 HD11 LEU A  91     -26.996  -3.828  71.642  0.00  0.00
A
ATOM  14821 HD12 LEU A  91     -25.766  -4.375  70.558  0.00  0.00
A
ATOM  14822 HD13 LEU A  91     -26.025  -2.624  70.636  0.00  0.00
A
```

ATOM 14823 CD2 LEU A 91 −28.292 −5.160 69.708 0.00 0.00
A
ATOM 14824 HD21 LEU A 91 −28.724 −5.302 70.721 0.00 0.00
A
ATOM 14825 HD22 LEU A 91 −29.173 −5.281 69.043 0.00 0.00
A
ATOM 14826 HD23 LEU A 91 −27.516 −5.923 69.484 0.00 0.00
A
ATOM 14827 C LEU A 91 −31.007 −1.690 69.152 0.00 0.00
A
ATOM 14828 O LEU A 91 −31.922 −2.177 69.760 0.00 0.00
A
ATOM 14829 N LEU A 92 −30.981 −0.366 68.848 0.00 0.00
A
ATOM 14830 HN LEU A 92 −30.308 0.085 68.268 0.00 0.00
A
ATOM 14831 CA LEU A 92 −32.072 0.499 69.206 0.00 0.00
A
ATOM 14832 HA LEU A 92 −32.411 0.299 70.212 0.00 0.00
A
ATOM 14833 CB LEU A 92 −31.652 1.999 69.064 0.00 0.00
A
ATOM 14834 HB1 LEU A 92 −31.463 2.204 67.988 0.00 0.00
A
ATOM 14835 HB2 LEU A 92 −32.455 2.716 69.338 0.00 0.00
A
ATOM 14836 CG LEU A 92 −30.451 2.377 69.884 0.00 0.00
A
ATOM 14837 HG LEU A 92 −29.540 1.866 69.506 0.00 0.00
A
ATOM 14838 CD1 LEU A 92 −30.161 3.873 69.652 0.00 0.00
A
ATOM 14839 HD11 LEU A 92 −29.184 4.182 70.082 0.00 0.00
A
ATOM 14840 HD12 LEU A 92 −30.008 4.053 68.567 0.00 0.00
A
ATOM 14841 HD13 LEU A 92 −30.994 4.546 69.949 0.00 0.00
A
ATOM 14842 CD2 LEU A 92 −30.604 2.238 71.434 0.00 0.00
A
ATOM 14843 HD21 LEU A 92 −29.563 2.356 71.805 0.00 0.00
A
ATOM 14844 HD22 LEU A 92 −31.208 3.128 71.714 0.00 0.00
A
ATOM 14845 HD23 LEU A 92 −31.032 1.251 71.711 0.00 0.00
A
ATOM 14846 C LEU A 92 −33.360 0.292 68.366 0.00 0.00
A
ATOM 14847 O LEU A 92 −34.440 0.388 68.896 0.00 0.00
A
ATOM 14848 N LYS A 93 −33.243 −0.096 67.038 0.00 0.00
A
ATOM 14849 HN LYS A 93 −32.343 −0.122 66.611 0.00 0.00
A

ATOM 14850 CA LYS A 93 -34.409 -0.471 66.293 0.00 0.00
A
ATOM 14851 HA LYS A 93 -35.162 0.283 66.470 0.00 0.00
A
ATOM 14852 CB LYS A 93 -34.058 -0.449 64.752 0.00 0.00
A
ATOM 14853 HB1 LYS A 93 -33.481 0.485 64.579 0.00 0.00
A
ATOM 14854 HB2 LYS A 93 -33.383 -1.253 64.388 0.00 0.00
A
ATOM 14855 CG LYS A 93 -35.234 -0.270 63.704 0.00 0.00
A
ATOM 14856 HG1 LYS A 93 -35.030 -0.477 62.632 0.00 0.00
A
ATOM 14857 HG2 LYS A 93 -36.026 -1.026 63.895 0.00 0.00
A
ATOM 14858 CD LYS A 93 -35.830 1.124 63.872 0.00 0.00
A
ATOM 14859 HD1 LYS A 93 -35.804 1.351 64.959 0.00 0.00
A
ATOM 14860 HD2 LYS A 93 -35.096 1.805 63.390 0.00 0.00
A
ATOM 14861 CE LYS A 93 -37.203 1.377 63.200 0.00 0.00
A
ATOM 14862 HE1 LYS A 93 -37.169 1.385 62.090 0.00 0.00
A
ATOM 14863 HE2 LYS A 93 -37.876 0.564 63.547 0.00 0.00
A
ATOM 14864 NZ LYS A 93 -37.806 2.634 63.672 0.00 0.00
A
ATOM 14865 HZ1 LYS A 93 -37.296 3.497 63.396 0.00 0.00
A
ATOM 14866 HZ2 LYS A 93 -38.818 2.774 63.477 0.00 0.00
A
ATOM 14867 HZ3 LYS A 93 -37.649 2.557 64.697 0.00 0.00
A
ATOM 14868 C LYS A 93 -34.963 -1.874 66.663 0.00 0.00
A
ATOM 14869 O LYS A 93 -36.134 -2.226 66.429 0.00 0.00
A
ATOM 14870 N GLU A 94 -34.138 -2.796 67.220 0.00 0.00
A
ATOM 14871 HN GLU A 94 -33.150 -2.708 67.320 0.00 0.00
A
ATOM 14872 CA GLU A 94 -34.550 -4.127 67.739 0.00 0.00
A
ATOM 14873 HA GLU A 94 -35.142 -4.570 66.952 0.00 0.00
A
ATOM 14874 CB GLU A 94 -33.389 -5.062 67.943 0.00 0.00
A
ATOM 14875 HB1 GLU A 94 -32.650 -4.936 67.123 0.00 0.00
A
ATOM 14876 HB2 GLU A 94 -32.939 -4.879 68.942 0.00 0.00
A

ATOM 14877 CG GLU A 94 −33.797 −6.569 67.742 0.00 0.00
A
ATOM 14878 HG1 GLU A 94 −34.536 −6.960 68.474 0.00 0.00
A
ATOM 14879 HG2 GLU A 94 −34.219 −6.708 66.724 0.00 0.00
A
ATOM 14880 CD GLU A 94 −32.598 −7.507 67.913 0.00 0.00
A
ATOM 14881 OE1 GLU A 94 −32.780 −8.571 68.573 0.00 0.00
A
ATOM 14882 OE2 GLU A 94 −31.577 −7.343 67.235 0.00 0.00
A
ATOM 14883 C GLU A 94 −35.341 −3.928 69.014 0.00 0.00
A
ATOM 14884 O GLU A 94 −36.297 −4.620 69.246 0.00 0.00
A
ATOM 14885 N LEU A 95 −34.895 −2.973 69.900 0.00 0.00
A
ATOM 14886 HN LEU A 95 −34.028 −2.488 69.819 0.00 0.00
A
ATOM 14887 CA LEU A 95 −35.595 −2.496 71.107 0.00 0.00
A
ATOM 14888 HA LEU A 95 −35.739 −3.407 71.668 0.00 0.00
A
ATOM 14889 CB LEU A 95 −34.768 −1.481 71.922 0.00 0.00
A
ATOM 14890 HB1 LEU A 95 −34.391 −0.649 71.290 0.00 0.00
A
ATOM 14891 HB2 LEU A 95 −35.463 −1.073 72.687 0.00 0.00
A
ATOM 14892 CG LEU A 95 −33.525 −2.071 72.665 0.00 0.00
A
ATOM 14893 HG LEU A 95 −32.773 −2.443 71.937 0.00 0.00
A
ATOM 14894 CD1 LEU A 95 −32.750 −0.928 73.407 0.00 0.00
A
ATOM 14895 HD11 LEU A 95 −32.194 −0.241 72.733 0.00 0.00
A
ATOM 14896 HD12 LEU A 95 −33.386 −0.274 74.042 0.00 0.00
A
ATOM 14897 HD13 LEU A 95 −32.005 −1.408 74.076 0.00 0.00
A
ATOM 14898 CD2 LEU A 95 −33.843 −3.241 73.597 0.00 0.00
A
ATOM 14899 HD21 LEU A 95 −34.121 −4.184 73.080 0.00 0.00
A
ATOM 14900 HD22 LEU A 95 −33.060 −3.337 74.379 0.00 0.00
A
ATOM 14901 HD23 LEU A 95 −34.792 −2.959 74.101 0.00 0.00
A
ATOM 14902 C LEU A 95 −36.930 −1.849 70.946 0.00 0.00
A
ATOM 14903 O LEU A 95 −37.777 −2.020 71.845 0.00 0.00
A

ATOM 14904 N   ASP A 96   −37.098 −1.102 69.878 0.00 0.00
A
ATOM 14905 HN   ASP A 96   −36.279 −1.137 69.311 0.00 0.00
A
ATOM 14906 CA   ASP A 96   −38.211 −0.325 69.408 0.00 0.00
A
ATOM 14907 HA   ASP A 96   −38.035 −0.093 68.368 0.00 0.00
A
ATOM 14908 CB   ASP A 96   −39.483 −1.206 69.476 0.00 0.00
A
ATOM 14909 HB1 ASP A 96   −39.120 −2.215 69.184 0.00 0.00
A
ATOM 14910 HB2 ASP A 96   −39.920 −1.184 70.498 0.00 0.00
A
ATOM 14911 CG   ASP A 96   −40.511 −0.737 68.474 0.00 0.00
A
ATOM 14912 OD1 ASP A 96   −40.143   0.030 67.529 0.00 0.00
A
ATOM 14913 OD2 ASP A 96   −41.675 −1.088 68.656 0.00 0.00
A
ATOM 14914 C   ASP A 96   −38.325   1.065 70.007 0.00 0.00
A
ATOM 14915 O   ASP A 96   −39.440   1.460 70.431 0.00 0.00
A
ATOM 14916 N   ILE A 97   −37.173   1.773 70.162 0.00 0.00
A
ATOM 14917 HN   ILE A 97   −36.286   1.483 69.812 0.00 0.00
A
ATOM 14918 CA   ILE A 97   −37.111   3.135 70.652 0.00 0.00
A
ATOM 14919 HA   ILE A 97   −37.467   3.234 71.667 0.00 0.00
A
ATOM 14920 CB   ILE A 97   −35.650   3.654 70.636 0.00 0.00
A
ATOM 14921 HB   ILE A 97   −35.347   3.318 69.621 0.00 0.00
A
ATOM 14922 CG2 ILE A 97   −35.416   5.173 70.845 0.00 0.00
A
ATOM 14923 HG21 ILE A 97   −34.320   5.287 70.989 0.00 0.00
A
ATOM 14924 HG22 ILE A 97   −35.593   5.670 69.868 0.00 0.00
A
ATOM 14925 HG23 ILE A 97   −36.066   5.527 71.674 0.00 0.00
A
ATOM 14926 CG1 ILE A 97   −34.769   2.854 71.688 0.00 0.00
A
ATOM 14927 HG11 ILE A 97   −35.061   1.782 71.684 0.00 0.00
A
ATOM 14928 HG12 ILE A 97   −33.689   2.892 71.428 0.00 0.00
A
ATOM 14929 CD   ILE A 97   −34.933   3.302 73.129 0.00 0.00
A
ATOM 14930 HD1 ILE A 97   −34.556   2.475 73.769 0.00 0.00
A

ATOM 14931 HD2 ILE A 97 −34.392 4.234 73.398 0.00 0.00
A
ATOM 14932 HD3 ILE A 97 −36.015 3.360 73.374 0.00 0.00
A
ATOM 14933 C ILE A 97 −38.022 3.975 69.776 0.00 0.00
A
ATOM 14934 O ILE A 97 −37.979 3.768 68.557 0.00 0.00
A
ATOM 14935 N PRO A 98 −38.773 4.920 70.266 0.00 0.00
A
ATOM 14936 CD PRO A 98 −39.398 4.790 71.528 0.00 0.00
A
ATOM 14937 HD1 PRO A 98 −39.552 3.729 71.817 0.00 0.00
A
ATOM 14938 HD2 PRO A 98 −38.555 5.107 72.179 0.00 0.00
A
ATOM 14939 CA PRO A 98 −39.399 5.871 69.370 0.00 0.00
A
ATOM 14940 HA PRO A 98 −39.971 5.159 68.795 0.00 0.00
A
ATOM 14941 CB PRO A 98 −40.303 6.665 70.310 0.00 0.00
A
ATOM 14942 HB1 PRO A 98 −41.181 7.133 69.816 0.00 0.00
A
ATOM 14943 HB2 PRO A 98 −39.761 7.530 70.748 0.00 0.00
A
ATOM 14944 CG PRO A 98 −40.621 5.665 71.447 0.00 0.00
A
ATOM 14945 HG1 PRO A 98 −40.857 6.129 72.428 0.00 0.00
A
ATOM 14946 HG2 PRO A 98 −41.485 5.086 71.056 0.00 0.00
A
ATOM 14947 C PRO A 98 −38.565 6.748 68.404 0.00 0.00
A
ATOM 14948 O PRO A 98 −38.763 6.726 67.224 0.00 0.00
A
ATOM 14949 N ASN A 99 −37.556 7.468 68.790 0.00 0.00
A
ATOM 14950 HN ASN A 99 −37.295 7.465 69.752 0.00 0.00
A
ATOM 14951 CA ASN A 99 −36.770 8.381 67.939 0.00 0.00
A
ATOM 14952 HA ASN A 99 −37.025 8.343 66.890 0.00 0.00
A
ATOM 14953 CB ASN A 99 −36.914 9.793 68.470 0.00 0.00
A
ATOM 14954 HB1 ASN A 99 −36.465 9.831 69.485 0.00 0.00
A
ATOM 14955 HB2 ASN A 99 −36.314 10.469 67.824 0.00 0.00
A
ATOM 14956 CG ASN A 99 −38.301 10.176 68.454 0.00 0.00
A
ATOM 14957 OD1 ASN A 99 −38.819 10.475 67.396 0.00 0.00
A

ATOM 14958 ND2 ASN A 99   -38.909 10.215 69.638 0.00 0.00
A
ATOM 14959 HD21 ASN A 99   -39.713 10.807 69.689 0.00 0.00
A
ATOM 14960 HD22 ASN A 99   -38.382   9.865 70.413 0.00 0.00
A
ATOM 14961 C   ASN A 99   -35.279   8.083 68.013 0.00 0.00
A
ATOM 14962 O   ASN A 99   -34.616   8.280 69.052 0.00 0.00
A
ATOM 14963 N   ILE A 100   -34.750   7.655 66.927 0.00 0.00
A
ATOM 14964 HN ILE A 100   -35.436   7.421 66.243 0.00 0.00
A
ATOM 14965 CA ILE A 100   -33.336   7.353 66.870 0.00 0.00
A
ATOM 14966 HA ILE A 100   -32.919   7.586 67.839 0.00 0.00
A
ATOM 14967 CB ILE A 100   -33.146   5.860 66.411 0.00 0.00
A
ATOM 14968 HB ILE A 100   -33.515   5.690 65.377 0.00 0.00
A
ATOM 14969 CG2 ILE A 100   -31.682   5.561 66.460 0.00 0.00
A
ATOM 14970 HG21 ILE A 100   -31.142   6.203 65.732 0.00 0.00
A
ATOM 14971 HG22 ILE A 100   -31.110   5.777 67.387 0.00 0.00
A
ATOM 14972 HG23 ILE A 100   -31.410   4.496 66.295 0.00 0.00
A
ATOM 14973 CG1 ILE A 100   -33.976   4.879 67.345 0.00 0.00
A
ATOM 14974 HG11 ILE A 100   -33.590   4.930 68.386 0.00 0.00
A
ATOM 14975 HG12 ILE A 100   -35.052   5.142 67.435 0.00 0.00
A
ATOM 14976 CD ILE A 100   -33.974   3.529 66.698 0.00 0.00
A
ATOM 14977 HD1 ILE A 100   -32.965   3.069 66.647 0.00 0.00
A
ATOM 14978 HD2 ILE A 100   -34.482   2.764 67.323 0.00 0.00
A
ATOM 14979 HD3 ILE A 100   -34.321   3.602 65.645 0.00 0.00
A
ATOM 14980 C   ILE A 100   -32.665   8.190 65.818 0.00 0.00
A
ATOM 14981 O   ILE A 100   -32.958   8.047 64.645 0.00 0.00
A
ATOM 14982 N   TRP A 101   -31.738   9.104 66.215 0.00 0.00
A
ATOM 14983 HN TRP A 101   -31.404   9.138 67.154 0.00 0.00
A
ATOM 14984 CA TRP A 101   -31.078 10.013 65.378 0.00 0.00
A

ATOM 14985 HA TRP A 101 −31.357 9.789 64.359 0.00 0.00
A
ATOM 14986 CB TRP A 101 −31.068 11.556 65.792 0.00 0.00
A
ATOM 14987 HB1 TRP A 101 −30.384 11.718 66.653 0.00 0.00
A
ATOM 14988 HB2 TRP A 101 −30.555 12.138 64.996 0.00 0.00
A
ATOM 14989 CG TRP A 101 −32.508 12.249 66.078 0.00 0.00
A
ATOM 14990 CD1 TRP A 101 −33.755 11.664 66.085 0.00 0.00
A
ATOM 14991 HD1 TRP A 101 −33.974 10.664 65.738 0.00 0.00
A
ATOM 14992 NE1 TRP A 101 −34.771 12.600 66.316 0.00 0.00
A
ATOM 14993 HE1 TRP A 101 −35.727 12.572 66.125 0.00 0.00
A
ATOM 14994 CE2 TRP A 101 −34.117 13.795 66.537 0.00 0.00
A
ATOM 14995 CD2 TRP A 101 −32.725 13.580 66.473 0.00 0.00
A
ATOM 14996 CE3 TRP A 101 −31.807 14.623 66.608 0.00 0.00
A
ATOM 14997 HE3 TRP A 101 −30.752 14.405 66.541 0.00 0.00
A
ATOM 14998 CZ3 TRP A 101 −32.268 15.931 66.754 0.00 0.00
A
ATOM 14999 HZ3 TRP A 101 −31.515 16.706 66.727 0.00 0.00
A
ATOM 15000 CZ2 TRP A 101 −34.554 15.108 66.666 0.00 0.00
A
ATOM 15001 HZ2 TRP A 101 −35.584 15.428 66.599 0.00 0.00
A
ATOM 15002 CH2 TRP A 101 −33.626 16.152 66.934 0.00 0.00
A
ATOM 15003 HH2 TRP A 101 −34.018 17.158 66.906 0.00 0.00
A
ATOM 15004 C TRP A 101 −29.632 9.570 65.437 0.00 0.00
A
ATOM 15005 O TRP A 101 −29.137 9.238 66.492 0.00 0.00
A
ATOM 15006 N VAL A 102 −28.882 9.490 64.336 0.00 0.00
A
ATOM 15007 HN VAL A 102 −29.370 9.842 63.541 0.00 0.00
A
ATOM 15008 CA VAL A 102 −27.562 8.851 64.092 0.00 0.00
A
ATOM 15009 HA VAL A 102 −27.050 8.692 65.029 0.00 0.00
A
ATOM 15010 CB VAL A 102 −27.694 7.522 63.375 0.00 0.00
A
ATOM 15011 HB VAL A 102 −27.796 7.603 62.272 0.00 0.00
A

ATOM	15012	CG1	VAL	A	102	−26.483	6.569	63.612	0.00	0.00	A
ATOM	15013	HG11	VAL	A	102	−26.309	6.343	64.686	0.00	0.00	A
ATOM	15014	HG12	VAL	A	102	−26.675	5.657	63.008	0.00	0.00	A
ATOM	15015	HG13	VAL	A	102	−25.570	7.100	63.269	0.00	0.00	A
ATOM	15016	CG2	VAL	A	102	−28.860	6.758	63.923	0.00	0.00	A
ATOM	15017	HG21	VAL	A	102	−29.884	7.091	63.649	0.00	0.00	A
ATOM	15018	HG22	VAL	A	102	−28.831	5.717	63.534	0.00	0.00	A
ATOM	15019	HG23	VAL	A	102	−28.833	6.674	65.030	0.00	0.00	A
ATOM	15020	C	VAL	A	102	−26.627	9.753	63.192	0.00	0.00	A
ATOM	15021	O	VAL	A	102	−27.008	10.237	62.102	0.00	0.00	A
ATOM	15022	N	LYS	A	103	−25.359	10.006	63.644	0.00	0.00	A
ATOM	15023	HN	LYS	A	103	−25.086	9.656	64.537	0.00	0.00	A
ATOM	15024	CA	LYS	A	103	−24.285	10.592	62.887	0.00	0.00	A
ATOM	15025	HA	LYS	A	103	−24.593	11.537	62.463	0.00	0.00	A
ATOM	15026	CB	LYS	A	103	−23.021	10.856	63.708	0.00	0.00	A
ATOM	15027	HB1	LYS	A	103	−23.282	11.205	64.731	0.00	0.00	A
ATOM	15028	HB2	LYS	A	103	−22.559	9.895	64.019	0.00	0.00	A
ATOM	15029	CG	LYS	A	103	−21.796	11.628	63.092	0.00	0.00	A
ATOM	15030	HG1	LYS	A	103	−21.243	11.022	62.343	0.00	0.00	A
ATOM	15031	HG2	LYS	A	103	−22.119	12.584	62.628	0.00	0.00	A
ATOM	15032	CD	LYS	A	103	−20.671	11.906	64.198	0.00	0.00	A
ATOM	15033	HD1	LYS	A	103	−21.258	12.564	64.874	0.00	0.00	A
ATOM	15034	HD2	LYS	A	103	−20.525	10.991	64.811	0.00	0.00	A
ATOM	15035	CE	LYS	A	103	−19.376	12.474	63.664	0.00	0.00	A
ATOM	15036	HE1	LYS	A	103	−18.717	11.785	63.093	0.00	0.00	A
ATOM	15037	HE2	LYS	A	103	−19.291	13.428	63.101	0.00	0.00	A
ATOM	15038	NZ	LYS	A	103	−18.528	12.773	64.868	0.00	0.00	A

ATOM	15039	HZ1	LYS	A	103	−17.557	12.480	64.639	0.00	0.00	A
ATOM	15040	HZ2	LYS	A	103	−18.381	13.785	65.059	0.00	0.00	A
ATOM	15041	HZ3	LYS	A	103	−18.679	12.224	65.738	0.00	0.00	A
ATOM	15042	C	LYS	A	103	−23.766	9.749	61.746	0.00	0.00	A
ATOM	15043	O	LYS	A	103	−23.385	8.661	62.001	0.00	0.00	A
ATOM	15044	N	ALA	A	104	−23.595	10.298	60.523	0.00	0.00	A
ATOM	15045	HN	ALA	A	104	−23.917	11.217	60.311	0.00	0.00	A
ATOM	15046	CA	ALA	A	104	−22.906	9.600	59.396	0.00	0.00	A
ATOM	15047	HA	ALA	A	104	−22.264	8.938	59.957	0.00	0.00	A
ATOM	15048	CB	ALA	A	104	−23.858	8.928	58.399	0.00	0.00	A
ATOM	15049	HB1	ALA	A	104	−24.647	9.663	58.135	0.00	0.00	A
ATOM	15050	HB2	ALA	A	104	−23.217	8.570	57.565	0.00	0.00	A
ATOM	15051	HB3	ALA	A	104	−24.242	8.052	58.964	0.00	0.00	A
ATOM	15052	C	ALA	A	104	−21.986	10.549	58.680	0.00	0.00	A
ATOM	15053	O	ALA	A	104	−22.173	11.763	58.620	0.00	0.00	A
ATOM	15054	N	GLN	A	105	−20.926	9.930	58.273	0.00	0.00	A
ATOM	15055	HN	GLN	A	105	−20.959	8.943	58.405	0.00	0.00	A
ATOM	15056	CA	GLN	A	105	−19.790	10.521	57.543	0.00	0.00	A
ATOM	15057	HA	GLN	A	105	−19.755	11.598	57.616	0.00	0.00	A
ATOM	15058	CB	GLN	A	105	−18.534	10.086	58.336	0.00	0.00	A
ATOM	15059	HB1	GLN	A	105	−18.394	8.988	58.436	0.00	0.00	A
ATOM	15060	HB2	GLN	A	105	−17.593	10.357	57.812	0.00	0.00	A
ATOM	15061	CG	GLN	A	105	−18.445	10.620	59.831	0.00	0.00	A
ATOM	15062	HG1	GLN	A	105	−19.401	10.292	60.291	0.00	0.00	A
ATOM	15063	HG2	GLN	A	105	−17.566	10.100	60.268	0.00	0.00	A
ATOM	15064	CD	GLN	A	105	−18.348	12.181	60.046	0.00	0.00	A
ATOM	15065	OE1	GLN	A	105	−17.316	12.616	60.613	0.00	0.00	A

ATOM 15066 NE2 GLN A 105 −19.374 12.927 59.799 0.00 0.00
A
ATOM 15067 HE21 GLN A 105 −19.344 13.882 60.096 0.00 0.00
A
ATOM 15068 HE22 GLN A 105 −20.205 12.548 59.392 0.00 0.00
A
ATOM 15069 C GLN A 105 −19.590 10.124 56.036 0.00 0.00
A
ATOM 15070 O GLN A 105 −18.750 10.723 55.358 0.00 0.00
A
ATOM 15071 N ASN A 106 −20.357 9.166 55.586 0.00 0.00
A
ATOM 15072 HN ASN A 106 −21.025 8.668 56.133 0.00 0.00
A
ATOM 15073 CA ASN A 106 −20.286 8.654 54.184 0.00 0.00
A
ATOM 15074 HA ASN A 106 −20.120 9.592 53.675 0.00 0.00
A
ATOM 15075 CB ASN A 106 −19.114 7.692 53.931 0.00 0.00
A
ATOM 15076 HB1 ASN A 106 −19.110 7.294 52.894 0.00 0.00
A
ATOM 15077 HB2 ASN A 106 −18.213 8.330 54.055 0.00 0.00
A
ATOM 15078 CG ASN A 106 −19.093 6.621 55.025 0.00 0.00
A
ATOM 15079 OD1 ASN A 106 −19.963 5.730 54.956 0.00 0.00
A
ATOM 15080 ND2 ASN A 106 −18.035 6.619 55.826 0.00 0.00
A
ATOM 15081 HD21 ASN A 106 −17.856 5.843 56.430 0.00 0.00
A
ATOM 15082 HD22 ASN A 106 −17.282 7.273 55.754 0.00 0.00
A
ATOM 15083 C ASN A 106 −21.600 8.034 53.637 0.00 0.00
A
ATOM 15084 O ASN A 106 −22.594 7.849 54.369 0.00 0.00
A
ATOM 15085 N TYR A 107 −21.593 7.793 52.303 0.00 0.00
A
ATOM 15086 HN TYR A 107 −20.816 8.144 51.786 0.00 0.00
A
ATOM 15087 CA TYR A 107 −22.774 7.377 51.565 0.00 0.00
A
ATOM 15088 HA TYR A 107 −23.573 8.083 51.736 0.00 0.00
A
ATOM 15089 CB TYR A 107 −22.263 7.384 50.126 0.00 0.00
A
ATOM 15090 HB1 TYR A 107 −21.868 8.397 49.897 0.00 0.00
A
ATOM 15091 HB2 TYR A 107 −21.478 6.600 50.055 0.00 0.00
A
ATOM 15092 CG TYR A 107 −23.354 6.950 49.201 0.00 0.00
A

ATOM	15093	CD1	TYR	A	107	−24.355	7.884	48.870	0.00	0.00	A
ATOM	15094	HD1	TYR	A	107	−24.395	8.886	49.271	0.00	0.00	A
ATOM	15095	CE1	TYR	A	107	−25.238	7.580	47.780	0.00	0.00	A
ATOM	15096	HE1	TYR	A	107	−26.015	8.282	47.516	0.00	0.00	A
ATOM	15097	CZ	TYR	A	107	−25.250	6.300	47.235	0.00	0.00	A
ATOM	15098	OH	TYR	A	107	−26.371	5.951	46.503	0.00	0.00	A
ATOM	15099	HH	TYR	A	107	−26.276	5.084	46.103	0.00	0.00	A
ATOM	15100	CD2	TYR	A	107	−23.329	5.720	48.549	0.00	0.00	A
ATOM	15101	HD2	TYR	A	107	−22.552	5.015	48.806	0.00	0.00	A
ATOM	15102	CE2	TYR	A	107	−24.324	5.338	47.604	0.00	0.00	A
ATOM	15103	HE2	TYR	A	107	−24.272	4.380	47.108	0.00	0.00	A
ATOM	15104	C	TYR	A	107	−23.335	5.976	51.972	0.00	0.00	A
ATOM	15105	O	TYR	A	107	−24.472	5.819	52.325	0.00	0.00	A
ATOM	15106	N	TYR	A	108	−22.488	5.013	52.063	0.00	0.00	A
ATOM	15107	HN	TYR	A	108	−21.550	5.186	51.774	0.00	0.00	A
ATOM	15108	CA	TYR	A	108	−22.820	3.648	52.493	0.00	0.00	A
ATOM	15109	HA	TYR	A	108	−23.585	3.183	51.889	0.00	0.00	A
ATOM	15110	CB	TYR	A	108	−21.471	2.794	52.406	0.00	0.00	A
ATOM	15111	HB1	TYR	A	108	−21.278	2.767	51.313	0.00	0.00	A
ATOM	15112	HB2	TYR	A	108	−20.584	3.392	52.706	0.00	0.00	A
ATOM	15113	CG	TYR	A	108	−21.436	1.426	53.052	0.00	0.00	A
ATOM	15114	CD1	TYR	A	108	−22.508	0.556	52.961	0.00	0.00	A
ATOM	15115	HD1	TYR	A	108	−23.425	0.790	52.440	0.00	0.00	A
ATOM	15116	CE1	TYR	A	108	−22.464	−0.667	53.707	0.00	0.00	A
ATOM	15117	HE1	TYR	A	108	−23.309	−1.340	53.724	0.00	0.00	A
ATOM	15118	CZ	TYR	A	108	−21.380	−0.906	54.533	0.00	0.00	A
ATOM	15119	OH	TYR	A	108	−21.410	−1.986	55.465	0.00	0.00	A

ATOM 15120 HH   TYR A 108   -20.759 -1.788 56.143 0.00 0.00
A
ATOM 15121 CD2 TYR A 108   -20.253   1.009 53.636 0.00 0.00
A
ATOM 15122 HD2 TYR A 108   -19.457   1.726 53.498 0.00 0.00
A
ATOM 15123 CE2 TYR A 108   -20.206 -0.128 54.453 0.00 0.00
A
ATOM 15124 HE2 TYR A 108   -19.331 -0.319 55.056 0.00 0.00
A
ATOM 15125 C   TYR A 108   -23.301   3.537 53.922 0.00 0.00
A
ATOM 15126 O   TYR A 108   -24.321   2.949 54.168 0.00 0.00
A
ATOM 15127 N   HSE A 109   -22.809   4.359 54.869 0.00 0.00
A
ATOM 15128 HN HSE A 109   -21.905   4.769 54.774 0.00 0.00
A
ATOM 15129 CA HSE A 109   -23.410   4.527 56.171 0.00 0.00
A
ATOM 15130 HA HSE A 109   -23.538   3.588 56.689 0.00 0.00
A
ATOM 15131 CB HSE A 109   -22.408   5.594 56.887 0.00 0.00
A
ATOM 15132 HB1 HSE A 109   -21.411   5.145 56.692 0.00 0.00
A
ATOM 15133 HB2 HSE A 109   -22.542   6.613 56.464 0.00 0.00
A
ATOM 15134 ND1 HSE A 109   -21.526   6.655 59.002 0.00 0.00
A
ATOM 15135 CG HSE A 109   -22.487   5.806 58.350 0.00 0.00
A
ATOM 15136 CE1 HSE A 109   -21.876   6.561 60.260 0.00 0.00
A
ATOM 15137 HE1 HSE A 109   -21.652   7.322 61.008 0.00 0.00
A
ATOM 15138 NE2 HSE A 109   -22.869   5.682 60.540 0.00 0.00
A
ATOM 15139 HE2 HSE A 109   -23.323   5.546 61.420 0.00 0.00
A
ATOM 15140 CD2 HSE A 109   -23.252   5.212 59.297 0.00 0.00
A
ATOM 15141 HD2 HSE A 109   -24.054   4.506 59.120 0.00 0.00
A
ATOM 15142 C   HSE A 109   -24.749   5.212 56.111 0.00 0.00
A
ATOM 15143 O   HSE A 109   -25.740   4.887 56.790 0.00 0.00
A
ATOM 15144 N   HSE A 110   -24.917   6.311 55.275 0.00 0.00
A
ATOM 15145 HN HSE A 110   -24.109   6.675 54.816 0.00 0.00
A
ATOM 15146 CA HSE A 110   -26.229   6.943 55.095 0.00 0.00
A

ATOM	15147	HA	HSE	A	110	−26.506	7.252	56.092	0.00	0.00	A
ATOM	15148	CB	HSE	A	110	−26.041	8.135	54.104	0.00	0.00	A
ATOM	15149	HB1	HSE	A	110	−25.421	9.002	54.419	0.00	0.00	A
ATOM	15150	HB2	HSE	A	110	−25.740	7.686	53.134	0.00	0.00	A
ATOM	15151	ND1	HSE	A	110	−27.540	9.569	52.731	0.00	0.00	A
ATOM	15152	CG	HSE	A	110	−27.276	8.798	53.833	0.00	0.00	A
ATOM	15153	CE1	HSE	A	110	−28.779	10.027	52.883	0.00	0.00	A
ATOM	15154	HE1	HSE	A	110	−29.321	10.591	52.123	0.00	0.00	A
ATOM	15155	NE2	HSE	A	110	−29.322	9.622	54.066	0.00	0.00	A
ATOM	15156	HE2	HSE	A	110	−30.302	9.732	54.231	0.00	0.00	A
ATOM	15157	CD2	HSE	A	110	−28.387	8.808	54.669	0.00	0.00	A
ATOM	15158	HD2	HSE	A	110	−28.582	8.475	55.681	0.00	0.00	A
ATOM	15159	C	HSE	A	110	−27.322	5.972	54.520	0.00	0.00	A
ATOM	15160	O	HSE	A	110	−28.397	5.851	55.017	0.00	0.00	A
ATOM	15161	N	LYS	A	111	−26.862	5.133	53.531	0.00	0.00	A
ATOM	15162	HN	LYS	A	111	−25.918	5.225	53.223	0.00	0.00	A
ATOM	15163	CA	LYS	A	111	−27.685	4.189	52.820	0.00	0.00	A
ATOM	15164	HA	LYS	A	111	−28.539	4.719	52.422	0.00	0.00	A
ATOM	15165	CB	LYS	A	111	−26.879	3.645	51.571	0.00	0.00	A
ATOM	15166	HB1	LYS	A	111	−26.243	4.490	51.231	0.00	0.00	A
ATOM	15167	HB2	LYS	A	111	−26.192	2.850	51.930	0.00	0.00	A
ATOM	15168	CG	LYS	A	111	−27.721	3.149	50.378	0.00	0.00	A
ATOM	15169	HG1	LYS	A	111	−26.920	2.492	49.978	0.00	0.00	A
ATOM	15170	HG2	LYS	A	111	−28.477	2.472	50.831	0.00	0.00	A
ATOM	15171	CD	LYS	A	111	−28.178	4.204	49.295	0.00	0.00	A
ATOM	15172	HD1	LYS	A	111	−28.932	4.830	49.819	0.00	0.00	A
ATOM	15173	HD2	LYS	A	111	−27.248	4.793	49.147	0.00	0.00	A

```
ATOM  15174  CE  LYS A 111     -28.767   3.741  47.996  0.00  0.00
A
ATOM  15175  HE1 LYS A 111     -28.673   4.552  47.243  0.00  0.00
A
ATOM  15176  HE2 LYS A 111     -28.254   2.839  47.598  0.00  0.00
A
ATOM  15177  NZ  LYS A 111     -30.248   3.605  48.082  0.00  0.00
A
ATOM  15178  HZ1 LYS A 111     -30.676   3.052  47.312  0.00  0.00
A
ATOM  15179  HZ2 LYS A 111     -30.649   3.233  48.966  0.00  0.00
A
ATOM  15180  HZ3 LYS A 111     -30.541   4.602  48.037  0.00  0.00
A
ATOM  15181  C   LYS A 111     -28.255   3.110  53.718  0.00  0.00
A
ATOM  15182  O   LYS A 111     -29.447   2.770  53.663  0.00  0.00
A
ATOM  15183  N   VAL A 112     -27.368   2.464  54.527  0.00  0.00
A
ATOM  15184  HN  VAL A 112     -26.426   2.560  54.214  0.00  0.00
A
ATOM  15185  CA  VAL A 112     -27.792   1.451  55.434  0.00  0.00
A
ATOM  15186  HA  VAL A 112     -28.392   0.664  55.002  0.00  0.00
A
ATOM  15187  CB  VAL A 112     -26.576   0.804  56.172  0.00  0.00
A
ATOM  15188  HB  VAL A 112     -25.892   1.593  56.553  0.00  0.00
A
ATOM  15189  CG1 VAL A 112     -26.952  -0.097  57.387  0.00  0.00
A
ATOM  15190 HG11 VAL A 112     -27.668  -0.864  57.022  0.00  0.00
A
ATOM  15191 HG12 VAL A 112     -25.994  -0.545  57.728  0.00  0.00
A
ATOM  15192 HG13 VAL A 112     -27.267   0.470  58.289  0.00  0.00
A
ATOM  15193  CG2 VAL A 112     -25.903  -0.068  55.113  0.00  0.00
A
ATOM  15194 HG21 VAL A 112     -26.714  -0.720  54.724  0.00  0.00
A
ATOM  15195 HG22 VAL A 112     -25.360   0.428  54.279  0.00  0.00
A
ATOM  15196 HG23 VAL A 112     -25.176  -0.683  55.686  0.00  0.00
A
ATOM  15197  C   VAL A 112     -28.670   2.005  56.511  0.00  0.00
A
ATOM  15198  O   VAL A 112     -29.674   1.446  56.884  0.00  0.00
A
ATOM  15199  N   LEU A 113     -28.417   3.230  57.071  0.00  0.00
A
ATOM  15200  HN  LEU A 113     -27.623   3.683  56.671  0.00  0.00
A
```

ATOM	15201	CA	LEU	A	113	−29.218	3.865	58.126	0.00	0.00	A
ATOM	15202	HA	LEU	A	113	−29.551	3.120	58.834	0.00	0.00	A
ATOM	15203	CB	LEU	A	113	−28.654	5.124	58.803	0.00	0.00	A
ATOM	15204	HB1	LEU	A	113	−28.499	5.998	58.135	0.00	0.00	A
ATOM	15205	HB2	LEU	A	113	−29.535	5.308	59.455	0.00	0.00	A
ATOM	15206	CG	LEU	A	113	−27.452	4.751	59.667	0.00	0.00	A
ATOM	15207	HG	LEU	A	113	−26.850	4.131	58.969	0.00	0.00	A
ATOM	15208	CD1	LEU	A	113	−26.659	5.967	59.986	0.00	0.00	A
ATOM	15209	HD11	LEU	A	113	−27.129	6.692	60.684	0.00	0.00	A
ATOM	15210	HD12	LEU	A	113	−25.661	5.644	60.354	0.00	0.00	A
ATOM	15211	HD13	LEU	A	113	−26.438	6.551	59.067	0.00	0.00	A
ATOM	15212	CD2	LEU	A	113	−27.733	3.955	60.957	0.00	0.00	A
ATOM	15213	HD21	LEU	A	113	−28.473	4.408	61.651	0.00	0.00	A
ATOM	15214	HD22	LEU	A	113	−28.103	2.941	60.697	0.00	0.00	A
ATOM	15215	HD23	LEU	A	113	−26.773	3.844	61.506	0.00	0.00	A
ATOM	15216	C	LEU	A	113	−30.606	4.196	57.644	0.00	0.00	A
ATOM	15217	O	LEU	A	113	−31.630	3.968	58.287	0.00	0.00	A
ATOM	15218	N	GLU	A	114	−30.681	4.814	56.347	0.00	0.00	A
ATOM	15219	HN	GLU	A	114	−29.883	5.218	55.907	0.00	0.00	A
ATOM	15220	CA	GLU	A	114	−31.915	5.012	55.578	0.00	0.00	A
ATOM	15221	HA	GLU	A	114	−32.559	5.726	56.068	0.00	0.00	A
ATOM	15222	CB	GLU	A	114	−31.536	5.547	54.170	0.00	0.00	A
ATOM	15223	HB1	GLU	A	114	−30.929	6.473	54.250	0.00	0.00	A
ATOM	15224	HB2	GLU	A	114	−30.760	4.947	53.648	0.00	0.00	A
ATOM	15225	CG	GLU	A	114	−32.714	5.951	53.157	0.00	0.00	A
ATOM	15226	HG1	GLU	A	114	−33.458	5.126	53.182	0.00	0.00	A
ATOM	15227	HG2	GLU	A	114	−33.299	6.851	53.441	0.00	0.00	A

ATOM 15228 CD GLU A 114 −32.228 6.244 51.770 0.00 0.00
A
ATOM 15229 OE1 GLU A 114 −31.681 7.382 51.523 0.00 0.00
A
ATOM 15230 OE2 GLU A 114 −32.560 5.410 50.842 0.00 0.00
A
ATOM 15231 C GLU A 114 −32.664 3.735 55.381 0.00 0.00
A
ATOM 15232 O GLU A 114 −33.843 3.701 55.724 0.00 0.00
A
ATOM 15233 N LYS A 115 −32.004 2.640 54.871 0.00 0.00
A
ATOM 15234 HN LYS A 115 −31.050 2.706 54.589 0.00 0.00
A
ATOM 15235 CA LYS A 115 −32.685 1.355 54.703 0.00 0.00
A
ATOM 15236 HA LYS A 115 −33.501 1.543 54.020 0.00 0.00
A
ATOM 15237 CB LYS A 115 −31.715 0.348 54.087 0.00 0.00
A
ATOM 15238 HB1 LYS A 115 −31.278 0.746 53.146 0.00 0.00
A
ATOM 15239 HB2 LYS A 115 −30.791 0.160 54.674 0.00 0.00
A
ATOM 15240 CG LYS A 115 −32.292 −1.036 53.704 0.00 0.00
A
ATOM 15241 HG1 LYS A 115 −31.414 −1.682 53.486 0.00 0.00
A
ATOM 15242 HG2 LYS A 115 −32.834 −1.491 54.560 0.00 0.00
A
ATOM 15243 CD LYS A 115 −33.246 −1.053 52.574 0.00 0.00
A
ATOM 15244 HD1 LYS A 115 −34.116 −0.440 52.892 0.00 0.00
A
ATOM 15245 HD2 LYS A 115 −32.779 −0.638 51.655 0.00 0.00
A
ATOM 15246 CE LYS A 115 −33.765 −2.475 52.427 0.00 0.00
A
ATOM 15247 HE1 LYS A 115 −33.097 −3.232 51.963 0.00 0.00
A
ATOM 15248 HE2 LYS A 115 −33.998 −2.677 53.494 0.00 0.00
A
ATOM 15249 NZ LYS A 115 −34.973 −2.647 51.610 0.00 0.00
A
ATOM 15250 HZ1 LYS A 115 −35.174 −3.666 51.659 0.00 0.00
A
ATOM 15251 HZ2 LYS A 115 −35.788 −2.223 52.099 0.00 0.00
A
ATOM 15252 HZ3 LYS A 115 −34.994 −2.231 50.657 0.00 0.00
A
ATOM 15253 C LYS A 115 −33.328 0.783 56.000 0.00 0.00
A
ATOM 15254 O LYS A 115 −34.521 0.428 56.086 0.00 0.00
A

ATOM	15255	N	ILE	A	116	−32.612	0.731	57.152	0.00	0.00	A
ATOM	15256	HN	ILE	A	116	−31.652	0.972	57.271	0.00	0.00	A
ATOM	15257	CA	ILE	A	116	−33.257	0.421	58.398	0.00	0.00	A
ATOM	15258	HA	ILE	A	116	−33.815	−0.488	58.226	0.00	0.00	A
ATOM	15259	CB	ILE	A	116	−32.255	0.079	59.519	0.00	0.00	A
ATOM	15260	HB	ILE	A	116	−32.773	−0.042	60.494	0.00	0.00	A
ATOM	15261	CG2	ILE	A	116	−31.422	−1.169	59.033	0.00	0.00	A
ATOM	15262	HG21	ILE	A	116	−30.947	−1.701	59.886	0.00	0.00	A
ATOM	15263	HG22	ILE	A	116	−32.094	−1.857	58.477	0.00	0.00	A
ATOM	15264	HG23	ILE	A	116	−30.593	−0.903	58.343	0.00	0.00	A
ATOM	15265	CG1	ILE	A	116	−31.158	1.186	59.902	0.00	0.00	A
ATOM	15266	HG11	ILE	A	116	−30.571	1.488	59.008	0.00	0.00	A
ATOM	15267	HG12	ILE	A	116	−31.669	2.024	60.422	0.00	0.00	A
ATOM	15268	CD	ILE	A	116	−30.096	0.713	60.955	0.00	0.00	A
ATOM	15269	HD1	ILE	A	116	−30.659	0.266	61.803	0.00	0.00	A
ATOM	15270	HD2	ILE	A	116	−29.401	−0.095	60.639	0.00	0.00	A
ATOM	15271	HD3	ILE	A	116	−29.450	1.534	61.331	0.00	0.00	A
ATOM	15272	C	ILE	A	116	−34.162	1.439	59.068	0.00	0.00	A
ATOM	15273	O	ILE	A	116	−34.987	1.125	59.929	0.00	0.00	A
ATOM	15274	N	GLY	A	117	−34.081	2.688	58.588	0.00	0.00	A
ATOM	15275	HN	GLY	A	117	−33.434	2.901	57.859	0.00	0.00	A
ATOM	15276	CA	GLY	A	117	−35.273	3.561	58.733	0.00	0.00	A
ATOM	15277	HA1	GLY	A	117	−36.165	2.977	58.908	0.00	0.00	A
ATOM	15278	HA2	GLY	A	117	−35.297	3.972	57.734	0.00	0.00	A
ATOM	15279	C	GLY	A	117	−35.179	4.665	59.792	0.00	0.00	A
ATOM	15280	O	GLY	A	117	−36.152	5.305	60.215	0.00	0.00	A
ATOM	15281	N	ALA	A	118	−33.928	4.945	60.212	0.00	0.00	A

ATOM 15282 HN ALA A 118 −33.184 4.369 59.883 0.00 0.00
A
ATOM 15283 CA ALA A 118 −33.540 5.822 61.307 0.00 0.00
A
ATOM 15284 HA ALA A 118 −33.969 5.473 62.235 0.00 0.00
A
ATOM 15285 CB ALA A 118 −32.055 5.856 61.537 0.00 0.00
A
ATOM 15286 HB1 ALA A 118 −31.773 6.444 62.436 0.00 0.00
A
ATOM 15287 HB2 ALA A 118 −31.730 4.866 61.923 0.00 0.00
A
ATOM 15288 HB3 ALA A 118 −31.545 6.147 60.594 0.00 0.00
A
ATOM 15289 C ALA A 118 −34.132 7.254 61.182 0.00 0.00
A
ATOM 15290 O ALA A 118 −34.325 7.796 60.057 0.00 0.00
A
ATOM 15291 N ASP A 119 −34.627 7.859 62.278 0.00 0.00
A
ATOM 15292 HN ASP A 119 −34.350 7.461 63.149 0.00 0.00
A
ATOM 15293 CA ASP A 119 −35.517 9.022 62.295 0.00 0.00
A
ATOM 15294 HA ASP A 119 −36.313 8.795 61.603 0.00 0.00
A
ATOM 15295 CB ASP A 119 −36.080 9.104 63.676 0.00 0.00
A
ATOM 15296 HB1 ASP A 119 −35.175 9.123 64.319 0.00 0.00
A
ATOM 15297 HB2 ASP A 119 −36.682 10.019 63.864 0.00 0.00
A
ATOM 15298 CG ASP A 119 −36.914 7.851 64.014 0.00 0.00
A
ATOM 15299 OD1 ASP A 119 −38.037 7.644 63.429 0.00 0.00
A
ATOM 15300 OD2 ASP A 119 −36.457 6.986 64.796 0.00 0.00
A
ATOM 15301 C ASP A 119 −34.903 10.346 61.813 0.00 0.00
A
ATOM 15302 O ASP A 119 −35.515 11.152 61.112 0.00 0.00
A
ATOM 15303 N ARG A 120 −33.654 10.623 62.242 0.00 0.00
A
ATOM 15304 HN ARG A 120 −33.156 9.997 62.837 0.00 0.00
A
ATOM 15305 CA ARG A 120 −32.890 11.649 61.631 0.00 0.00
A
ATOM 15306 HA ARG A 120 −33.313 11.864 60.661 0.00 0.00
A
ATOM 15307 CB ARG A 120 −33.034 12.887 62.474 0.00 0.00
A
ATOM 15308 HB1 ARG A 120 −34.016 13.399 62.383 0.00 0.00
A

ATOM 15309 HB2 ARG A 120   -32.797 12.673 63.537 0.00 0.00
A
ATOM 15310 CG ARG A 120   -32.207 14.024 61.926 0.00 0.00
A
ATOM 15311 HG1 ARG A 120   -31.146 13.811 62.178 0.00 0.00
A
ATOM 15312 HG2 ARG A 120   -32.280 14.076 60.818 0.00 0.00
A
ATOM 15313 CD ARG A 120   -32.431 15.395 62.509 0.00 0.00
A
ATOM 15314 HD1 ARG A 120   -33.505 15.671 62.437 0.00 0.00
A
ATOM 15315 HD2 ARG A 120   -32.289 15.419 63.610 0.00 0.00
A
ATOM 15316 NE ARG A 120   -31.478 16.337 61.800 0.00 0.00
A
ATOM 15317 HE ARG A 120   -30.865 15.964 61.104 0.00 0.00
A
ATOM 15318 CZ ARG A 120   -31.504 17.652 61.921 0.00 0.00
A
ATOM 15319 NH1 ARG A 120   -32.460 18.281 62.579 0.00 0.00
A
ATOM 15320 HH11 ARG A 120   -32.253 19.216 62.866 0.00 0.00
A
ATOM 15321 HH12 ARG A 120   -33.189 17.852 63.112 0.00 0.00
A
ATOM 15322 NH2 ARG A 120   -30.649 18.408 61.248 0.00 0.00
A
ATOM 15323 HH21 ARG A 120   -30.759 19.392 61.104 0.00 0.00
A
ATOM 15324 HH22 ARG A 120   -29.872 17.940 60.827 0.00 0.00
A
ATOM 15325 C   ARG A 120   -31.428 11.245 61.423 0.00 0.00
A
ATOM 15326 O   ARG A 120   -30.798 10.629 62.314 0.00 0.00
A
ATOM 15327 N   ILE A 121   -30.908 11.440 60.193 0.00 0.00
A
ATOM 15328 HN ILE A 121   -31.405 11.918 59.472 0.00 0.00
A
ATOM 15329 CA ILE A 121   -29.642 10.911 59.896 0.00 0.00
A
ATOM 15330 HA ILE A 121   -29.228 10.533 60.819 0.00 0.00
A
ATOM 15331 CB ILE A 121   -29.473   9.821 58.843 0.00 0.00
A
ATOM 15332 HB ILE A 121   -29.543 10.231 57.813 0.00 0.00
A
ATOM 15333 CG2 ILE A 121   -28.145   9.111 59.129 0.00 0.00
A
ATOM 15334 HG21 ILE A 121   -28.123   8.136 58.597 0.00 0.00
A
ATOM 15335 HG22 ILE A 121   -27.299   9.764 58.824 0.00 0.00
A

ATOM 15336 HG23 ILE A 121   -28.024   8.937 60.220 0.00 0.00
A
ATOM 15337 CG1 ILE A 121   -30.687   8.830 58.977 0.00 0.00
A
ATOM 15338 HG11 ILE A 121   -30.431   8.161 59.826 0.00 0.00
A
ATOM 15339 HG12 ILE A 121   -31.635   9.328 59.273 0.00 0.00
A
ATOM 15340 CD ILE A 121   -30.933   7.992 57.715 0.00 0.00
A
ATOM 15341 HD1 ILE A 121   -30.043   7.661 57.139 0.00 0.00
A
ATOM 15342 HD2 ILE A 121   -31.560   7.090 57.881 0.00 0.00
A
ATOM 15343 HD3 ILE A 121   -31.333   8.654 56.918 0.00 0.00
A
ATOM 15344 C   ILE A 121   -28.812 12.171 59.565 0.00 0.00
A
ATOM 15345 O   ILE A 121   -29.187 13.014 58.748 0.00 0.00
A
ATOM 15346 N   ILE A 122   -27.744 12.479 60.328 0.00 0.00
A
ATOM 15347 HN ILE A 122   -27.487 11.854 61.061 0.00 0.00
A
ATOM 15348 CA ILE A 122   -27.108 13.788 60.334 0.00 0.00
A
ATOM 15349 HA ILE A 122   -27.743 14.523 59.862 0.00 0.00
A
ATOM 15350 CB ILE A 122   -26.995 14.191 61.813 0.00 0.00
A
ATOM 15351 HB ILE A 122   -26.217 13.538 62.263 0.00 0.00
A
ATOM 15352 CG2 ILE A 122   -26.513 15.623 61.907 0.00 0.00
A
ATOM 15353 HG21 ILE A 122   -26.264 15.990 62.926 0.00 0.00
A
ATOM 15354 HG22 ILE A 122   -25.657 15.758 61.211 0.00 0.00
A
ATOM 15355 HG23 ILE A 122   -27.346 16.237 61.504 0.00 0.00
A
ATOM 15356 CG1 ILE A 122   -28.298 13.999 62.661 0.00 0.00
A
ATOM 15357 HG11 ILE A 122   -29.079 14.668 62.242 0.00 0.00
A
ATOM 15358 HG12 ILE A 122   -28.754 12.997 62.512 0.00 0.00
A
ATOM 15359 CD ILE A 122   -28.118 14.060 64.158 0.00 0.00
A
ATOM 15360 HD1 ILE A 122   -27.686 15.018 64.520 0.00 0.00
A
ATOM 15361 HD2 ILE A 122   -29.119 13.984 64.635 0.00 0.00
A
ATOM 15362 HD3 ILE A 122   -27.546 13.181 64.523 0.00 0.00
A

```
ATOM  15363  C   ILE A 122     -25.710  13.715  59.754  0.00  0.00
A
ATOM  15364  O   ILE A 122     -25.027  12.736  60.124  0.00  0.00
A
ATOM  15365  N   HSE A 123     -25.259  14.705  58.866  0.00  0.00
A
ATOM  15366  HN  HSE A 123     -25.825  15.456  58.535  0.00  0.00
A
ATOM  15367  CA  HSE A 123     -23.863  14.896  58.576  0.00  0.00
A
ATOM  15368  HA  HSE A 123     -23.201  14.149  58.986  0.00  0.00
A
ATOM  15369  CB  HSE A 123     -23.525  14.797  57.083  0.00  0.00
A
ATOM  15370  HB1 HSE A 123     -23.669  13.746  56.753  0.00  0.00
A
ATOM  15371  HB2 HSE A 123     -24.109  15.459  56.408  0.00  0.00
A
ATOM  15372  ND1 HSE A 123     -21.650  16.233  56.549  0.00  0.00
A
ATOM  15373  CG  HSE A 123     -22.115  14.981  56.707  0.00  0.00
A
ATOM  15374  CE1 HSE A 123     -20.336  16.039  56.256  0.00  0.00
A
ATOM  15375  HE1 HSE A 123     -19.675  16.778  55.803  0.00  0.00
A
ATOM  15376  NE2 HSE A 123     -19.990  14.704  56.283  0.00  0.00
A
ATOM  15377  HE2 HSE A 123     -19.058  14.364  56.407  0.00  0.00
A
ATOM  15378  CD2 HSE A 123     -21.138  14.060  56.484  0.00  0.00
A
ATOM  15379  HD2 HSE A 123     -21.266  12.998  56.655  0.00  0.00
A
ATOM  15380  C   HSE A 123     -23.522  16.244  59.155  0.00  0.00
A
ATOM  15381  O   HSE A 123     -24.103  17.197  58.710  0.00  0.00
A
ATOM  15382  N   PRO A 124     -22.648  16.405  60.114  0.00  0.00
A
ATOM  15383  CD  PRO A 124     -22.039  15.297  60.872  0.00  0.00
A
ATOM  15384  HD1 PRO A 124     -22.766  14.466  61.002  0.00  0.00
A
ATOM  15385  HD2 PRO A 124     -21.244  14.921  60.194  0.00  0.00
A
ATOM  15386  CA  PRO A 124     -22.418  17.657  60.819  0.00  0.00
A
ATOM  15387  HA  PRO A 124     -23.339  17.813  61.360  0.00  0.00
A
ATOM  15388  CB  PRO A 124     -21.217  17.293  61.555  0.00  0.00
A
ATOM  15389  HB1 PRO A 124     -21.024  17.949  62.431  0.00  0.00
A
```

ATOM 15390 HB2 PRO A 124 -20.355 17.334 60.856 0.00 0.00
A
ATOM 15391 CG PRO A 124 -21.504 15.882 62.162 0.00 0.00
A
ATOM 15392 HG1 PRO A 124 -20.683 15.244 62.554 0.00 0.00
A
ATOM 15393 HG2 PRO A 124 -22.279 16.029 62.945 0.00 0.00
A
ATOM 15394 C PRO A 124 -22.112 18.788 59.913 0.00 0.00
A
ATOM 15395 O PRO A 124 -22.559 19.873 60.252 0.00 0.00
A
ATOM 15396 N GLU A 125 -21.412 18.641 58.785 0.00 0.00
A
ATOM 15397 HN GLU A 125 -21.140 17.762 58.400 0.00 0.00
A
ATOM 15398 CA GLU A 125 -21.127 19.792 57.909 0.00 0.00
A
ATOM 15399 HA GLU A 125 -21.080 20.689 58.509 0.00 0.00
A
ATOM 15400 CB GLU A 125 -19.864 19.668 57.130 0.00 0.00
A
ATOM 15401 HB1 GLU A 125 -19.914 18.745 56.513 0.00 0.00
A
ATOM 15402 HB2 GLU A 125 -19.768 20.494 56.394 0.00 0.00
A
ATOM 15403 CG GLU A 125 -18.598 19.673 58.005 0.00 0.00
A
ATOM 15404 HG1 GLU A 125 -18.392 20.585 58.604 0.00 0.00
A
ATOM 15405 HG2 GLU A 125 -18.630 18.798 58.690 0.00 0.00
A
ATOM 15406 CD GLU A 125 -17.414 19.426 57.103 0.00 0.00
A
ATOM 15407 OE1 GLU A 125 -17.568 19.588 55.886 0.00 0.00
A
ATOM 15408 OE2 GLU A 125 -16.314 19.086 57.626 0.00 0.00
A
ATOM 15409 C GLU A 125 -22.263 20.084 56.948 0.00 0.00
A
ATOM 15410 O GLU A 125 -22.648 21.266 56.791 0.00 0.00
A
ATOM 15411 N LYS A 126 -22.953 19.123 56.409 0.00 0.00
A
ATOM 15412 HN LYS A 126 -22.717 18.167 56.565 0.00 0.00
A
ATOM 15413 CA LYS A 126 -24.128 19.294 55.566 0.00 0.00
A
ATOM 15414 HA LYS A 126 -23.820 19.966 54.779 0.00 0.00
A
ATOM 15415 CB LYS A 126 -24.480 17.941 54.853 0.00 0.00
A
ATOM 15416 HB1 LYS A 126 -23.562 17.433 54.487 0.00 0.00
A

ATOM 15417 HB2 LYS A 126 −24.949 17.262 55.596 0.00 0.00
A
ATOM 15418 CG LYS A 126 −25.465 18.143 53.656 0.00 0.00
A
ATOM 15419 HG1 LYS A 126 −26.483 18.363 54.041 0.00 0.00
A
ATOM 15420 HG2 LYS A 126 −25.151 18.942 52.950 0.00 0.00
A
ATOM 15421 CD LYS A 126 −25.585 16.844 52.790 0.00 0.00
A
ATOM 15422 HD1 LYS A 126 −26.191 16.971 51.868 0.00 0.00
A
ATOM 15423 HD2 LYS A 126 −24.594 16.585 52.360 0.00 0.00
A
ATOM 15424 CE LYS A 126 −26.201 15.667 53.614 0.00 0.00
A
ATOM 15425 HE1 LYS A 126 −26.275 14.800 52.922 0.00 0.00
A
ATOM 15426 HE2 LYS A 126 −25.566 15.574 54.521 0.00 0.00
A
ATOM 15427 NZ LYS A 126 −27.643 15.944 53.932 0.00 0.00
A
ATOM 15428 HZ1 LYS A 126 −28.242 15.112 54.107 0.00 0.00
A
ATOM 15429 HZ2 LYS A 126 −27.768 16.671 54.665 0.00 0.00
A
ATOM 15430 HZ3 LYS A 126 −28.081 16.251 53.040 0.00 0.00
A
ATOM 15431 C LYS A 126 −25.410 19.840 56.288 0.00 0.00
A
ATOM 15432 O LYS A 126 −26.104 20.711 55.831 0.00 0.00
A
ATOM 15433 N ASP A 127 −25.666 19.315 57.481 0.00 0.00
A
ATOM 15434 HN ASP A 127 −25.126 18.635 57.971 0.00 0.00
A
ATOM 15435 CA ASP A 127 −26.768 19.814 58.282 0.00 0.00
A
ATOM 15436 HA ASP A 127 −27.689 19.871 57.720 0.00 0.00
A
ATOM 15437 CB ASP A 127 −26.950 19.002 59.541 0.00 0.00
A
ATOM 15438 HB1 ASP A 127 −26.004 18.772 60.076 0.00 0.00
A
ATOM 15439 HB2 ASP A 127 −27.620 19.521 60.258 0.00 0.00
A
ATOM 15440 CG ASP A 127 −27.635 17.681 59.200 0.00 0.00
A
ATOM 15441 OD1 ASP A 127 −28.719 17.465 59.827 0.00 0.00
A
ATOM 15442 OD2 ASP A 127 −27.202 16.795 58.387 0.00 0.00
A
ATOM 15443 C ASP A 127 −26.575 21.310 58.632 0.00 0.00
A

ATOM 15444 O ASP A 127 −27.499 22.074 58.366 0.00 0.00
A
ATOM 15445 N MET A 128 −25.317 21.657 58.950 0.00 0.00
A
ATOM 15446 HN MET A 128 −24.629 20.942 58.857 0.00 0.00
A
ATOM 15447 CA MET A 128 −25.001 22.993 59.466 0.00 0.00
A
ATOM 15448 HA MET A 128 −25.781 23.371 60.110 0.00 0.00
A
ATOM 15449 CB MET A 128 −23.573 23.026 60.168 0.00 0.00
A
ATOM 15450 HB1 MET A 128 −23.441 22.113 60.787 0.00 0.00
A
ATOM 15451 HB2 MET A 128 −22.860 22.849 59.334 0.00 0.00
A
ATOM 15452 CG MET A 128 −23.127 24.305 60.903 0.00 0.00
A
ATOM 15453 HG1 MET A 128 −22.274 23.897 61.487 0.00 0.00
A
ATOM 15454 HG2 MET A 128 −22.782 25.103 60.211 0.00 0.00
A
ATOM 15455 SD MET A 128 −24.378 24.842 62.135 0.00 0.00
A
ATOM 15456 CE MET A 128 −23.724 26.472 61.870 0.00 0.00
A
ATOM 15457 HE1 MET A 128 −24.268 27.238 62.462 0.00 0.00
A
ATOM 15458 HE2 MET A 128 −22.635 26.616 62.039 0.00 0.00
A
ATOM 15459 HE3 MET A 128 −23.945 26.782 60.826 0.00 0.00
A
ATOM 15460 C MET A 128 −24.964 24.120 58.324 0.00 0.00
A
ATOM 15461 O MET A 128 −25.149 25.296 58.609 0.00 0.00
A
ATOM 15462 N GLY A 129 −24.819 23.737 57.015 0.00 0.00
A
ATOM 15463 HN GLY A 129 −24.493 22.803 56.893 0.00 0.00
A
ATOM 15464 CA GLY A 129 −24.790 24.673 55.897 0.00 0.00
A
ATOM 15465 HA1 GLY A 129 −24.830 24.082 54.994 0.00 0.00
A
ATOM 15466 HA2 GLY A 129 −23.919 25.308 55.963 0.00 0.00
A
ATOM 15467 C GLY A 129 −26.001 25.535 55.824 0.00 0.00
A
ATOM 15468 O GLY A 129 −25.848 26.783 55.590 0.00 0.00
A
ATOM 15469 N VAL A 130 −27.201 25.002 56.108 0.00 0.00
A
ATOM 15470 HN VAL A 130 −27.152 24.041 56.366 0.00 0.00
A

ATOM 15471 CA VAL A 130 -28.517 25.627 56.099 0.00 0.00
A
ATOM 15472 HA VAL A 130 -28.650 26.138 55.157 0.00 0.00
A
ATOM 15473 CB VAL A 130 -29.572 24.578 56.397 0.00 0.00
A
ATOM 15474 HB VAL A 130 -29.236 24.066 57.324 0.00 0.00
A
ATOM 15475 CG1 VAL A 130 -30.943 25.118 56.566 0.00 0.00
A
ATOM 15476 HG11 VAL A 130 -31.760 24.376 56.696 0.00 0.00
A
ATOM 15477 HG12 VAL A 130 -31.145 25.997 57.216 0.00 0.00
A
ATOM 15478 HG13 VAL A 130 -31.103 25.488 55.531 0.00 0.00
A
ATOM 15479 CG2 VAL A 130 -29.696 23.529 55.308 0.00 0.00
A
ATOM 15480 HG21 VAL A 130 -30.207 23.975 54.427 0.00 0.00
A
ATOM 15481 HG22 VAL A 130 -28.746 23.241 54.809 0.00 0.00
A
ATOM 15482 HG23 VAL A 130 -30.207 22.635 55.725 0.00 0.00
A
ATOM 15483 C VAL A 130 -28.595 26.801 57.053 0.00 0.00
A
ATOM 15484 O VAL A 130 -29.075 27.828 56.784 0.00 0.00
A
ATOM 15485 N LYS A 131 -27.998 26.557 58.204 0.00 0.00
A
ATOM 15486 HN LYS A 131 -27.295 25.863 58.338 0.00 0.00
A
ATOM 15487 CA LYS A 131 -27.877 27.535 59.265 0.00 0.00
A
ATOM 15488 HA LYS A 131 -28.883 27.881 59.448 0.00 0.00
A
ATOM 15489 CB LYS A 131 -27.431 26.691 60.584 0.00 0.00
A
ATOM 15490 HB1 LYS A 131 -28.213 25.996 60.960 0.00 0.00
A
ATOM 15491 HB2 LYS A 131 -26.502 26.119 60.376 0.00 0.00
A
ATOM 15492 CG LYS A 131 -27.072 27.627 61.801 0.00 0.00
A
ATOM 15493 HG1 LYS A 131 -26.615 26.981 62.581 0.00 0.00
A
ATOM 15494 HG2 LYS A 131 -26.309 28.369 61.481 0.00 0.00
A
ATOM 15495 CD LYS A 131 -28.326 28.396 62.216 0.00 0.00
A
ATOM 15496 HD1 LYS A 131 -28.780 29.044 61.436 0.00 0.00
A
ATOM 15497 HD2 LYS A 131 -28.984 27.520 62.404 0.00 0.00
A

ATOM 15498 CE LYS A 131   -28.152 29.258 63.520 0.00 0.00
A
ATOM 15499 HE1 LYS A 131   -27.963 28.564 64.367 0.00 0.00
A
ATOM 15500 HE2 LYS A 131   -27.433 30.103 63.575 0.00 0.00
A
ATOM 15501 NZ LYS A 131   -29.447 29.848 63.806 0.00 0.00
A
ATOM 15502 HZ1 LYS A 131   -29.514 30.325 64.727 0.00 0.00
A
ATOM 15503 HZ2 LYS A 131   -29.710 30.476 63.019 0.00 0.00
A
ATOM 15504 HZ3 LYS A 131   -30.136 29.070 63.760 0.00 0.00
A
ATOM 15505 C   LYS A 131   -26.840 28.599 58.973 0.00 0.00
A
ATOM 15506 O   LYS A 131   -27.032 29.772 59.314 0.00 0.00
A
ATOM 15507 N   ILE A 132   -25.728 28.333 58.232 0.00 0.00
A
ATOM 15508 HN ILE A 132   -25.674 27.424 57.826 0.00 0.00
A
ATOM 15509 CA ILE A 132   -24.841 29.414 57.679 0.00 0.00
A
ATOM 15510 HA ILE A 132   -24.545 30.162 58.400 0.00 0.00
A
ATOM 15511 CB ILE A 132   -23.499 28.853 57.181 0.00 0.00
A
ATOM 15512 HB ILE A 132   -23.676 28.039 56.446 0.00 0.00
A
ATOM 15513 CG2 ILE A 132   -22.610 29.976 56.538 0.00 0.00
A
ATOM 15514 HG21 ILE A 132   -21.561 29.610 56.518 0.00 0.00
A
ATOM 15515 HG22 ILE A 132   -22.834 30.320 55.506 0.00 0.00
A
ATOM 15516 HG23 ILE A 132   -22.539 30.939 57.086 0.00 0.00
A
ATOM 15517 CG1 ILE A 132   -22.727 28.242 58.418 0.00 0.00
A
ATOM 15518 HG11 ILE A 132   -23.384 27.399 58.719 0.00 0.00
A
ATOM 15519 HG12 ILE A 132   -21.755 27.786 58.130 0.00 0.00
A
ATOM 15520 CD ILE A 132   -22.409 29.337 59.456 0.00 0.00
A
ATOM 15521 HD1 ILE A 132   -21.689 28.835 60.138 0.00 0.00
A
ATOM 15522 HD2 ILE A 132   -21.786 30.174 59.075 0.00 0.00
A
ATOM 15523 HD3 ILE A 132   -23.275 29.555 60.118 0.00 0.00
A
ATOM 15524 C   ILE A 132   -25.444 30.332 56.695 0.00 0.00
A

ATOM 15525 O ILE A 132 -25.256 31.561 56.646 0.00 0.00
A
ATOM 15526 N ALA A 133 -26.267 29.705 55.795 0.00 0.00
A
ATOM 15527 HN ALA A 133 -26.213 28.717 55.666 0.00 0.00
A
ATOM 15528 CA ALA A 133 -27.067 30.494 54.839 0.00 0.00
A
ATOM 15529 HA ALA A 133 -26.353 31.102 54.304 0.00 0.00
A
ATOM 15530 CB ALA A 133 -27.868 29.439 53.926 0.00 0.00
A
ATOM 15531 HB1 ALA A 133 -27.204 28.843 53.264 0.00 0.00
A
ATOM 15532 HB2 ALA A 133 -28.531 28.797 54.546 0.00 0.00
A
ATOM 15533 HB3 ALA A 133 -28.484 29.970 53.170 0.00 0.00
A
ATOM 15534 C ALA A 133 -28.026 31.415 55.497 0.00 0.00
A
ATOM 15535 O ALA A 133 -28.123 32.562 55.126 0.00 0.00
A
ATOM 15536 N GLN A 134 -28.658 30.949 56.502 0.00 0.00
A
ATOM 15537 HN GLN A 134 -28.524 29.987 56.726 0.00 0.00
A
ATOM 15538 CA GLN A 134 -29.638 31.720 57.357 0.00 0.00
A
ATOM 15539 HA GLN A 134 -30.389 32.121 56.691 0.00 0.00
A
ATOM 15540 CB GLN A 134 -30.422 30.762 58.293 0.00 0.00
A
ATOM 15541 HB1 GLN A 134 -30.539 29.934 57.562 0.00 0.00
A
ATOM 15542 HB2 GLN A 134 -29.712 30.426 59.079 0.00 0.00
A
ATOM 15543 CG GLN A 134 -31.728 31.302 58.869 0.00 0.00
A
ATOM 15544 HG1 GLN A 134 -32.191 30.476 59.450 0.00 0.00
A
ATOM 15545 HG2 GLN A 134 -31.618 32.289 59.367 0.00 0.00
A
ATOM 15546 CD GLN A 134 -32.813 31.577 57.803 0.00 0.00
A
ATOM 15547 OE1 GLN A 134 -33.271 30.758 56.999 0.00 0.00
A
ATOM 15548 NE2 GLN A 134 -33.161 32.864 57.761 0.00 0.00
A
ATOM 15549 HE21 GLN A 134 -33.604 33.247 56.950 0.00 0.00
A
ATOM 15550 HE22 GLN A 134 -32.932 33.368 58.594 0.00 0.00
A
ATOM 15551 C GLN A 134 -29.018 32.811 58.137 0.00 0.00
A

ATOM 15552 O GLN A 134 -29.510 33.931 58.180 0.00 0.00
A
ATOM 15553 N SER A 135 -27.908 32.474 58.809 0.00 0.00
A
ATOM 15554 HN SER A 135 -27.513 31.565 58.911 0.00 0.00
A
ATOM 15555 CA SER A 135 -27.177 33.479 59.607 0.00 0.00
A
ATOM 15556 HA SER A 135 -27.909 33.949 60.248 0.00 0.00
A
ATOM 15557 CB SER A 135 -26.084 32.869 60.494 0.00 0.00
A
ATOM 15558 HB1 SER A 135 -25.463 32.194 59.867 0.00 0.00
A
ATOM 15559 HB2 SER A 135 -25.521 33.674 61.013 0.00 0.00
A
ATOM 15560 OG SER A 135 -26.648 32.032 61.500 0.00 0.00
A
ATOM 15561 HG1 SER A 135 -26.003 31.439 61.890 0.00 0.00
A
ATOM 15562 C SER A 135 -26.444 34.557 58.800 0.00 0.00
A
ATOM 15563 O SER A 135 -25.772 35.440 59.277 0.00 0.00
A
ATOM 15564 N LEU A 136 -26.403 34.390 57.483 0.00 0.00
A
ATOM 15565 HN LEU A 136 -26.765 33.558 57.069 0.00 0.00
A
ATOM 15566 CA LEU A 136 -25.759 35.313 56.450 0.00 0.00
A
ATOM 15567 HA LEU A 136 -25.106 35.997 56.972 0.00 0.00
A
ATOM 15568 CB LEU A 136 -24.957 34.513 55.430 0.00 0.00
A
ATOM 15569 HB1 LEU A 136 -24.296 33.780 55.940 0.00 0.00
A
ATOM 15570 HB2 LEU A 136 -25.695 33.935 54.834 0.00 0.00
A
ATOM 15571 CG LEU A 136 -24.046 35.181 54.380 0.00 0.00
A
ATOM 15572 HG LEU A 136 -24.614 36.005 53.899 0.00 0.00
A
ATOM 15573 CD1 LEU A 136 -22.835 35.698 55.178 0.00 0.00
A
ATOM 15574 HD11 LEU A 136 -22.118 36.229 54.516 0.00 0.00
A
ATOM 15575 HD12 LEU A 136 -23.176 36.442 55.929 0.00 0.00
A
ATOM 15576 HD13 LEU A 136 -22.432 34.788 55.673 0.00 0.00
A
ATOM 15577 CD2 LEU A 136 -23.726 34.277 53.257 0.00 0.00
A
ATOM 15578 HD21 LEU A 136 -24.620 34.001 52.658 0.00 0.00
A

ATOM 15579 HD22 LEU A 136 -22.952 34.730 52.602 0.00 0.00
A
ATOM 15580 HD23 LEU A 136 -23.274 33.302 53.539 0.00 0.00
A
ATOM 15581 C LEU A 136 -26.770 36.205 55.690 0.00 0.00
A
ATOM 15582 O LEU A 136 -26.473 37.197 55.010 0.00 0.00
A
ATOM 15583 N SER A 137 -28.076 35.874 55.821 0.00 0.00
A
ATOM 15584 HN SER A 137 -28.261 35.078 56.392 0.00 0.00
A
ATOM 15585 CA SER A 137 -29.248 36.531 55.219 0.00 0.00
A
ATOM 15586 HA SER A 137 -29.166 36.505 54.142 0.00 0.00
A
ATOM 15587 CB SER A 137 -30.622 35.829 55.696 0.00 0.00
A
ATOM 15588 HB1 SER A 137 -30.471 34.735 55.570 0.00 0.00
A
ATOM 15589 HB2 SER A 137 -30.746 35.982 56.789 0.00 0.00
A
ATOM 15590 OG SER A 137 -31.717 36.258 54.867 0.00 0.00
A
ATOM 15591 HG1 SER A 137 -32.499 35.956 55.334 0.00 0.00
A
ATOM 15592 C SER A 137 -29.311 37.963 55.611 0.00 0.00
A
ATOM 15593 O SER A 137 -29.492 38.851 54.810 0.00 0.00
A
ATOM 15594 N ASP A 138 -29.073 38.202 56.843 0.00 0.00
A
ATOM 15595 HN ASP A 138 -28.909 37.366 57.360 0.00 0.00
A
ATOM 15596 CA ASP A 138 -29.051 39.523 57.521 0.00 0.00
A
ATOM 15597 HA ASP A 138 -29.107 40.363 56.844 0.00 0.00
A
ATOM 15598 CB ASP A 138 -30.280 39.447 58.532 0.00 0.00
A
ATOM 15599 HB1 ASP A 138 -31.069 38.765 58.150 0.00 0.00
A
ATOM 15600 HB2 ASP A 138 -30.071 38.994 59.525 0.00 0.00
A
ATOM 15601 CG ASP A 138 -30.965 40.743 58.782 0.00 0.00
A
ATOM 15602 OD1 ASP A 138 -30.403 41.586 59.503 0.00 0.00
A
ATOM 15603 OD2 ASP A 138 -32.064 40.962 58.145 0.00 0.00
A
ATOM 15604 C ASP A 138 -27.874 39.902 58.433 0.00 0.00
A
ATOM 15605 O ASP A 138 -27.290 39.064 59.091 0.00 0.00
A

ATOM 15606 N GLU A 139 -27.540 41.140 58.637 0.00 0.00
A
ATOM 15607 HN GLU A 139 -28.039 41.802 58.085 0.00 0.00
A
ATOM 15608 CA GLU A 139 -26.384 41.559 59.432 0.00 0.00
A
ATOM 15609 HA GLU A 139 -25.814 40.654 59.578 0.00 0.00
A
ATOM 15610 CB GLU A 139 -25.537 42.546 58.600 0.00 0.00
A
ATOM 15611 HB1 GLU A 139 -26.260 43.281 58.187 0.00 0.00
A
ATOM 15612 HB2 GLU A 139 -24.793 43.043 59.260 0.00 0.00
A
ATOM 15613 CG GLU A 139 -24.741 41.795 57.482 0.00 0.00
A
ATOM 15614 HG1 GLU A 139 -23.868 41.191 57.808 0.00 0.00
A
ATOM 15615 HG2 GLU A 139 -25.447 41.159 56.906 0.00 0.00
A
ATOM 15616 CD GLU A 139 -24.273 42.870 56.457 0.00 0.00
A
ATOM 15617 OE1 GLU A 139 -24.701 44.043 56.639 0.00 0.00
A
ATOM 15618 OE2 GLU A 139 -23.469 42.630 55.527 0.00 0.00
A
ATOM 15619 C GLU A 139 -26.846 42.169 60.766 0.00 0.00
A
ATOM 15620 O GLU A 139 -26.039 42.716 61.481 0.00 0.00
A
ATOM 15621 N ASN A 140 -28.124 42.291 61.046 0.00 0.00
A
ATOM 15622 HN ASN A 140 -28.668 42.001 60.262 0.00 0.00
A
ATOM 15623 CA ASN A 140 -28.684 42.972 62.200 0.00 0.00
A
ATOM 15624 HA ASN A 140 -27.843 43.216 62.833 0.00 0.00
A
ATOM 15625 CB ASN A 140 -29.544 44.146 61.737 0.00 0.00
A
ATOM 15626 HB1 ASN A 140 -30.266 43.866 60.940 0.00 0.00
A
ATOM 15627 HB2 ASN A 140 -30.210 44.592 62.506 0.00 0.00
A
ATOM 15628 CG ASN A 140 -28.602 45.194 61.199 0.00 0.00
A
ATOM 15629 OD1 ASN A 140 -28.282 45.317 59.975 0.00 0.00
A
ATOM 15630 ND2 ASN A 140 -28.131 46.025 62.126 0.00 0.00
A
ATOM 15631 HD21 ASN A 140 -27.193 46.309 61.926 0.00 0.00
A
ATOM 15632 HD22 ASN A 140 -28.322 45.939 63.104 0.00 0.00
A

ATOM 15633 C ASN A 140 −29.607 42.088 63.044 0.00 0.00
A
ATOM 15634 O ASN A 140 −29.681 42.256 64.261 0.00 0.00
A
ATOM 15635 N VAL A 141 −30.433 41.229 62.416 0.00 0.00
A
ATOM 15636 HN VAL A 141 −30.277 41.096 61.441 0.00 0.00
A
ATOM 15637 CA VAL A 141 −31.556 40.616 63.032 0.00 0.00
A
ATOM 15638 HA VAL A 141 −31.920 41.185 63.874 0.00 0.00
A
ATOM 15639 CB VAL A 141 −32.641 40.500 61.917 0.00 0.00
A
ATOM 15640 HB VAL A 141 −32.364 39.785 61.113 0.00 0.00
A
ATOM 15641 CG1 VAL A 141 −33.940 40.007 62.616 0.00 0.00
A
ATOM 15642 HG11 VAL A 141 −34.279 40.707 63.409 0.00 0.00
A
ATOM 15643 HG12 VAL A 141 −34.833 39.887 61.966 0.00 0.00
A
ATOM 15644 HG13 VAL A 141 −33.707 39.061 63.150 0.00 0.00
A
ATOM 15645 CG2 VAL A 141 −32.986 41.860 61.349 0.00 0.00
A
ATOM 15646 HG21 VAL A 141 −33.752 41.742 60.553 0.00 0.00
A
ATOM 15647 HG22 VAL A 141 −33.328 42.581 62.122 0.00 0.00
A
ATOM 15648 HG23 VAL A 141 −32.200 42.387 60.767 0.00 0.00
A
ATOM 15649 C VAL A 141 −31.102 39.256 63.449 0.00 0.00
A
ATOM 15650 O VAL A 141 −31.102 38.285 62.651 0.00 0.00
A
ATOM 15651 N LEU A 142 −30.586 39.156 64.710 0.00 0.00
A
ATOM 15652 HN LEU A 142 −30.704 39.851 65.415 0.00 0.00
A
ATOM 15653 CA LEU A 142 −29.999 37.990 65.173 0.00 0.00
A
ATOM 15654 HA LEU A 142 −29.314 37.647 64.413 0.00 0.00
A
ATOM 15655 CB LEU A 142 −29.200 38.309 66.426 0.00 0.00
A
ATOM 15656 HB1 LEU A 142 −29.840 38.727 67.232 0.00 0.00
A
ATOM 15657 HB2 LEU A 142 −28.697 37.445 66.911 0.00 0.00
A
ATOM 15658 CG LEU A 142 −28.005 39.251 66.157 0.00 0.00
A
ATOM 15659 HG LEU A 142 −28.388 40.252 65.865 0.00 0.00
A

ATOM 15660 CD1 LEU A 142   -27.265 39.509 67.489 0.00 0.00
A
ATOM 15661 HD11 LEU A 142   -26.934 38.539 67.916 0.00 0.00
A
ATOM 15662 HD12 LEU A 142   -26.311 40.038 67.278 0.00 0.00
A
ATOM 15663 HD13 LEU A 142   -27.763 40.173 68.227 0.00 0.00
A
ATOM 15664 CD2 LEU A 142   -27.094 38.840 65.017 0.00 0.00
A
ATOM 15665 HD21 LEU A 142   -26.199 39.471 64.826 0.00 0.00
A
ATOM 15666 HD22 LEU A 142   -26.710 37.799 64.973 0.00 0.00
A
ATOM 15667 HD23 LEU A 142   -27.613 38.856 64.035 0.00 0.00
A
ATOM 15668 C   LEU A 142   -31.024 36.833 65.415 0.00 0.00
A
ATOM 15669 O   LEU A 142   -30.713 35.744 64.967 0.00 0.00
A
ATOM 15670 N   ASN A 143   -32.205 37.074 65.970 0.00 0.00
A
ATOM 15671 HN ASN A 143   -32.476 37.969 66.314 0.00 0.00
A
ATOM 15672 CA ASN A 143   -33.201 36.066 66.191 0.00 0.00
A
ATOM 15673 HA ASN A 143   -33.112 35.303 65.432 0.00 0.00
A
ATOM 15674 CB ASN A 143   -33.193 35.340 67.545 0.00 0.00
A
ATOM 15675 HB1 ASN A 143   -33.052 35.944 68.467 0.00 0.00
A
ATOM 15676 HB2 ASN A 143   -34.128 34.766 67.720 0.00 0.00
A
ATOM 15677 CG ASN A 143   -31.958 34.280 67.535 0.00 0.00
A
ATOM 15678 OD1 ASN A 143   -31.935 33.204 66.986 0.00 0.00
A
ATOM 15679 ND2 ASN A 143   -30.874 34.710 68.195 0.00 0.00
A
ATOM 15680 HD21 ASN A 143   -30.022 34.192 68.122 0.00 0.00
A
ATOM 15681 HD22 ASN A 143   -30.904 35.481 68.830 0.00 0.00
A
ATOM 15682 C   ASN A 143   -34.608 36.632 66.064 0.00 0.00
A
ATOM 15683 O   ASN A 143   -34.801 37.859 66.113 0.00 0.00
A
ATOM 15684 N   TYR A 144   -35.503 35.709 65.700 0.00 0.00
A
ATOM 15685 HN TYR A 144   -35.401 34.724 65.820 0.00 0.00
A
ATOM 15686 CA TYR A 144   -36.759 35.969 65.049 0.00 0.00
A

ATOM 15687 HA TYR A 144 −37.023 37.010 65.167 0.00 0.00
A
ATOM 15688 CB TYR A 144 −36.667 35.736 63.494 0.00 0.00
A
ATOM 15689 HB1 TYR A 144 −35.857 36.458 63.253 0.00 0.00
A
ATOM 15690 HB2 TYR A 144 −36.330 34.699 63.279 0.00 0.00
A
ATOM 15691 CG TYR A 144 −37.914 36.065 62.768 0.00 0.00
A
ATOM 15692 CD1 TYR A 144 −38.390 35.129 61.875 0.00 0.00
A
ATOM 15693 HD1 TYR A 144 −38.013 34.117 61.900 0.00 0.00
A
ATOM 15694 CE1 TYR A 144 −39.499 35.463 61.075 0.00 0.00
A
ATOM 15695 HE1 TYR A 144 −39.889 34.842 60.282 0.00 0.00
A
ATOM 15696 CZ TYR A 144 −40.053 36.763 61.138 0.00 0.00
A
ATOM 15697 OH TYR A 144 −41.076 37.087 60.242 0.00 0.00
A
ATOM 15698 HH TYR A 144 −41.455 36.282 59.882 0.00 0.00
A
ATOM 15699 CD2 TYR A 144 −38.533 37.357 62.847 0.00 0.00
A
ATOM 15700 HD2 TYR A 144 −38.197 38.193 63.443 0.00 0.00
A
ATOM 15701 CE2 TYR A 144 −39.586 37.721 62.021 0.00 0.00
A
ATOM 15702 HE2 TYR A 144 −39.965 38.730 62.093 0.00 0.00
A
ATOM 15703 C TYR A 144 −37.896 35.101 65.601 0.00 0.00
A
ATOM 15704 O TYR A 144 −37.657 33.893 65.708 0.00 0.00
A
ATOM 15705 N ILE A 145 −39.096 35.682 65.859 0.00 0.00
A
ATOM 15706 HN ILE A 145 −39.150 36.674 65.780 0.00 0.00
A
ATOM 15707 CA ILE A 145 −40.274 34.889 66.064 0.00 0.00
A
ATOM 15708 HA ILE A 145 −40.088 33.837 65.904 0.00 0.00
A
ATOM 15709 CB ILE A 145 −40.922 34.922 67.520 0.00 0.00
A
ATOM 15710 HB ILE A 145 −41.136 35.982 67.774 0.00 0.00
A
ATOM 15711 CG2 ILE A 145 −42.307 34.194 67.492 0.00 0.00
A
ATOM 15712 HG21 ILE A 145 −42.864 34.737 66.699 0.00 0.00
A
ATOM 15713 HG22 ILE A 145 −42.193 33.116 67.248 0.00 0.00
A

ATOM 15714 HG23 ILE A 145 -42.835 34.417 68.443 0.00 0.00
A
ATOM 15715 CG1 ILE A 145 -39.838 34.226 68.525 0.00 0.00
A
ATOM 15716 HG11 ILE A 145 -39.568 33.222 68.133 0.00 0.00
A
ATOM 15717 HG12 ILE A 145 -38.965 34.912 68.574 0.00 0.00
A
ATOM 15718 CD ILE A 145 -40.218 34.061 69.981 0.00 0.00
A
ATOM 15719 HD1 ILE A 145 -40.833 33.151 70.148 0.00 0.00
A
ATOM 15720 HD2 ILE A 145 -39.371 33.912 70.685 0.00 0.00
A
ATOM 15721 HD3 ILE A 145 -40.665 35.016 70.331 0.00 0.00
A
ATOM 15722 C ILE A 145 -41.306 35.521 65.019 0.00 0.00
A
ATOM 15723 O ILE A 145 -41.390 36.720 65.003 0.00 0.00
A
ATOM 15724 N ASP A 146 -41.927 34.772 64.176 0.00 0.00
A
ATOM 15725 HN ASP A 146 -41.836 33.789 64.319 0.00 0.00
A
ATOM 15726 CA ASP A 146 -42.803 35.214 63.141 0.00 0.00
A
ATOM 15727 HA ASP A 146 -42.264 36.034 62.691 0.00 0.00
A
ATOM 15728 CB ASP A 146 -43.019 33.925 62.221 0.00 0.00
A
ATOM 15729 HB1 ASP A 146 -41.976 33.573 62.069 0.00 0.00
A
ATOM 15730 HB2 ASP A 146 -43.651 33.186 62.757 0.00 0.00
A
ATOM 15731 CG ASP A 146 -43.549 34.272 60.897 0.00 0.00
A
ATOM 15732 OD1 ASP A 146 -42.823 34.715 59.946 0.00 0.00
A
ATOM 15733 OD2 ASP A 146 -44.781 34.134 60.705 0.00 0.00
A
ATOM 15734 C ASP A 146 -44.211 35.653 63.684 0.00 0.00
A
ATOM 15735 O ASP A 146 -45.044 34.811 63.946 0.00 0.00
A
ATOM 15736 N LEU A 147 -44.424 36.991 63.795 0.00 0.00
A
ATOM 15737 HN LEU A 147 -43.665 37.581 63.530 0.00 0.00
A
ATOM 15738 CA LEU A 147 -45.451 37.550 64.605 0.00 0.00
A
ATOM 15739 HA LEU A 147 -46.377 37.061 64.339 0.00 0.00
A
ATOM 15740 CB LEU A 147 -45.255 37.402 66.062 0.00 0.00
A

ATOM 15741 HB1 LEU A 147 −45.024 36.316 66.047 0.00 0.00
A
ATOM 15742 HB2 LEU A 147 −44.359 38.008 66.314 0.00 0.00
A
ATOM 15743 CG LEU A 147 −46.361 37.612 67.043 0.00 0.00
A
ATOM 15744 HG LEU A 147 −46.663 38.673 66.909 0.00 0.00
A
ATOM 15745 CD1 LEU A 147 −47.528 36.663 66.812 0.00 0.00
A
ATOM 15746 HD11 LEU A 147 −48.077 36.864 65.867 0.00 0.00
A
ATOM 15747 HD12 LEU A 147 −47.181 35.608 66.796 0.00 0.00
A
ATOM 15748 HD13 LEU A 147 −48.196 36.824 67.686 0.00 0.00
A
ATOM 15749 CD2 LEU A 147 −45.772 37.420 68.454 0.00 0.00
A
ATOM 15750 HD21 LEU A 147 −46.610 37.536 69.174 0.00 0.00
A
ATOM 15751 HD22 LEU A 147 −45.210 36.467 68.561 0.00 0.00
A
ATOM 15752 HD23 LEU A 147 −45.052 38.257 68.583 0.00 0.00
A
ATOM 15753 C LEU A 147 −45.622 39.114 64.466 0.00 0.00
A
ATOM 15754 O LEU A 147 −46.729 39.593 64.132 0.00 0.00
A
ATOM 15755 N SER A 152 −44.572 39.983 64.668 0.00 0.00
A
ATOM 15756 HN SER A 152 −44.798 40.949 64.765 0.00 0.00
A
ATOM 15757 CA SER A 152 −43.160 39.649 64.757 0.00 0.00
A
ATOM 15758 HA SER A 152 −43.066 38.596 64.978 0.00 0.00
A
ATOM 15759 CB SER A 152 −42.337 39.953 63.553 0.00 0.00
A
ATOM 15760 HB1 SER A 152 −42.324 41.060 63.465 0.00 0.00
A
ATOM 15761 HB2 SER A 152 −41.285 39.632 63.706 0.00 0.00
A
ATOM 15762 OG SER A 152 −42.878 39.213 62.383 0.00 0.00
A
ATOM 15763 HG1 SER A 152 −42.398 39.386 61.570 0.00 0.00
A
ATOM 15764 C SER A 152 −42.373 40.280 65.905 0.00 0.00
A
ATOM 15765 O SER A 152 −42.489 41.429 66.317 0.00 0.00
A
ATOM 15766 N ILE A 153 −41.414 39.456 66.398 0.00 0.00
A
ATOM 15767 HN ILE A 153 −41.273 38.561 65.982 0.00 0.00
A

ATOM 15768 CA ILE A 153 −40.467 39.794 67.460 0.00 0.00
A
ATOM 15769 HA ILE A 153 −40.580 40.785 67.874 0.00 0.00
A
ATOM 15770 CB ILE A 153 −40.542 38.916 68.685 0.00 0.00
A
ATOM 15771 HB ILE A 153 −40.272 37.889 68.356 0.00 0.00
A
ATOM 15772 CG2 ILE A 153 −39.612 39.649 69.689 0.00 0.00
A
ATOM 15773 HG21 ILE A 153 −38.530 39.617 69.439 0.00 0.00
A
ATOM 15774 HG22 ILE A 153 −39.918 40.705 69.849 0.00 0.00
A
ATOM 15775 HG23 ILE A 153 −39.761 39.093 70.639 0.00 0.00
A
ATOM 15776 CG1 ILE A 153 −41.969 38.797 69.198 0.00 0.00
A
ATOM 15777 HG11 ILE A 153 −42.450 39.724 69.577 0.00 0.00
A
ATOM 15778 HG12 ILE A 153 −42.640 38.456 68.381 0.00 0.00
A
ATOM 15779 CD ILE A 153 −42.170 37.811 70.394 0.00 0.00
A
ATOM 15780 HD1 ILE A 153 −41.706 38.244 71.306 0.00 0.00
A
ATOM 15781 HD2 ILE A 153 −43.253 37.659 70.588 0.00 0.00
A
ATOM 15782 HD3 ILE A 153 −41.808 36.795 70.128 0.00 0.00
A
ATOM 15783 C ILE A 153 −39.033 39.664 66.812 0.00 0.00
A
ATOM 15784 O ILE A 153 −38.776 38.713 66.054 0.00 0.00
A
ATOM 15785 N VAL A 154 −38.087 40.651 67.034 0.00 0.00
A
ATOM 15786 HN VAL A 154 −38.387 41.501 67.460 0.00 0.00
A
ATOM 15787 CA VAL A 154 −36.688 40.464 66.632 0.00 0.00
A
ATOM 15788 HA VAL A 154 −36.501 39.472 66.247 0.00 0.00
A
ATOM 15789 CB VAL A 154 −36.435 41.401 65.427 0.00 0.00
A
ATOM 15790 HB VAL A 154 −35.355 41.447 65.169 0.00 0.00
A
ATOM 15791 CG1 VAL A 154 −37.266 40.863 64.205 0.00 0.00
A
ATOM 15792 HG11 VAL A 154 −38.361 40.958 64.368 0.00 0.00
A
ATOM 15793 HG12 VAL A 154 −36.932 41.326 63.252 0.00 0.00
A
ATOM 15794 HG13 VAL A 154 −37.120 39.766 64.111 0.00 0.00
A

ATOM 15795 CG2 VAL A 154   -36.740 42.931 65.679 0.00 0.00
A
ATOM 15796 HG21 VAL A 154   -36.634 43.486 64.723 0.00 0.00
A
ATOM 15797 HG22 VAL A 154   -37.812 43.043 65.948 0.00 0.00
A
ATOM 15798 HG23 VAL A 154   -36.256 43.434 66.544 0.00 0.00
A
ATOM 15799 C   VAL A 154   -35.712 40.759 67.806 0.00 0.00
A
ATOM 15800 O   VAL A 154   -35.985 41.696 68.560 0.00 0.00
A
ATOM 15801 N   GLU A 155   -34.646 39.988 67.898 0.00 0.00
A
ATOM 15802 HN GLU A 155   -34.583 39.309 67.171 0.00 0.00
A
ATOM 15803 CA GLU A 155   -33.507 40.314 68.670 0.00 0.00
A
ATOM 15804 HA GLU A 155   -33.797 40.938 69.503 0.00 0.00
A
ATOM 15805 CB GLU A 155   -33.096 38.889 69.252 0.00 0.00
A
ATOM 15806 HB1 GLU A 155   -34.002 38.394 69.663 0.00 0.00
A
ATOM 15807 HB2 GLU A 155   -32.811 38.316 68.344 0.00 0.00
A
ATOM 15808 CG GLU A 155   -31.972 38.836 70.296 0.00 0.00
A
ATOM 15809 HG1 GLU A 155   -31.157 39.556 70.069 0.00 0.00
A
ATOM 15810 HG2 GLU A 155   -32.439 39.252 71.214 0.00 0.00
A
ATOM 15811 CD GLU A 155   -31.259 37.513 70.371 0.00 0.00
A
ATOM 15812 OE1 GLU A 155   -30.498 37.100 69.470 0.00 0.00
A
ATOM 15813 OE2 GLU A 155   -31.470 36.919 71.430 0.00 0.00
A
ATOM 15814 C   GLU A 155   -32.470 41.018 67.789 0.00 0.00
A
ATOM 15815 O   GLU A 155   -32.107 40.467 66.747 0.00 0.00
A
ATOM 15816 N   LEU A 156   -32.180 42.314 67.933 0.00 0.00
A
ATOM 15817 HN LEU A 156   -32.518 42.801 68.735 0.00 0.00
A
ATOM 15818 CA LEU A 156   -31.597 43.181 66.960 0.00 0.00
A
ATOM 15819 HA LEU A 156   -31.470 42.688 66.007 0.00 0.00
A
ATOM 15820 CB LEU A 156   -32.433 44.398 66.772 0.00 0.00
A
ATOM 15821 HB1 LEU A 156   -33.517 44.163 66.708 0.00 0.00
A

ATOM 15822 HB2 LEU A 156   -32.347 44.962 67.725 0.00 0.00
A
ATOM 15823 CG LEU A 156   -32.158 45.328 65.540 0.00 0.00
A
ATOM 15824 HG LEU A 156   -31.116 45.707 65.621 0.00 0.00
A
ATOM 15825 CD1 LEU A 156   -32.339 44.527 64.215 0.00 0.00
A
ATOM 15826 HD11 LEU A 156   -31.474 43.836 64.118 0.00 0.00
A
ATOM 15827 HD12 LEU A 156   -33.315 44.009 64.105 0.00 0.00
A
ATOM 15828 HD13 LEU A 156   -32.306 45.208 63.337 0.00 0.00
A
ATOM 15829 CD2 LEU A 156   -33.060 46.517 65.635 0.00 0.00
A
ATOM 15830 HD21 LEU A 156   -33.128 46.978 66.643 0.00 0.00
A
ATOM 15831 HD22 LEU A 156   -32.822 47.277 64.860 0.00 0.00
A
ATOM 15832 HD23 LEU A 156   -34.113 46.213 65.449 0.00 0.00
A
ATOM 15833 C   LEU A 156   -30.176 43.606 67.486 0.00 0.00
A
ATOM 15834 O   LEU A 156   -30.028 44.186 68.546 0.00 0.00
A
ATOM 15835 N   LEU A 157   -29.083 43.341 66.746 0.00 0.00
A
ATOM 15836 HN LEU A 157   -29.268 42.804 65.926 0.00 0.00
A
ATOM 15837 CA LEU A 157   -27.764 43.952 66.888 0.00 0.00
A
ATOM 15838 HA LEU A 157   -27.548 44.083 67.938 0.00 0.00
A
ATOM 15839 CB LEU A 157   -26.583 42.952 66.509 0.00 0.00
A
ATOM 15840 HB1 LEU A 157   -26.681 42.156 67.278 0.00 0.00
A
ATOM 15841 HB2 LEU A 157   -26.749 42.597 65.469 0.00 0.00
A
ATOM 15842 CG LEU A 157   -25.112 43.439 66.714 0.00 0.00
A
ATOM 15843 HG LEU A 157   -24.870 44.216 65.957 0.00 0.00
A
ATOM 15844 CD1 LEU A 157   -24.762 44.124 68.038 0.00 0.00
A
ATOM 15845 HD11 LEU A 157   -25.009 43.400 68.844 0.00 0.00
A
ATOM 15846 HD12 LEU A 157   -23.677 44.366 68.037 0.00 0.00
A
ATOM 15847 HD13 LEU A 157   -25.357 45.059 68.113 0.00 0.00
A
ATOM 15848 CD2 LEU A 157   -24.153 42.269 66.488 0.00 0.00
A

ATOM 15849 HD21 LEU A 157 -24.394 41.803 65.509 0.00 0.00
A
ATOM 15850 HD22 LEU A 157 -23.095 42.604 66.555 0.00 0.00
A
ATOM 15851 HD23 LEU A 157 -24.218 41.582 67.359 0.00 0.00
A
ATOM 15852 C LEU A 157 -27.692 45.263 66.066 0.00 0.00
A
ATOM 15853 O LEU A 157 -28.100 45.272 64.900 0.00 0.00
A
ATOM 15854 N LEU A 162 -27.168 46.393 66.639 0.00 0.00
A
ATOM 15855 HN LEU A 162 -26.902 46.349 67.599 0.00 0.00
A
ATOM 15856 CA LEU A 162 -26.976 47.688 65.966 0.00 0.00
A
ATOM 15857 HA LEU A 162 -27.241 47.648 64.920 0.00 0.00
A
ATOM 15858 CB LEU A 162 -27.732 48.892 66.662 0.00 0.00
A
ATOM 15859 HB1 LEU A 162 -27.521 48.879 67.753 0.00 0.00
A
ATOM 15860 HB2 LEU A 162 -27.360 49.894 66.356 0.00 0.00
A
ATOM 15861 CG LEU A 162 -29.296 48.866 66.509 0.00 0.00
A
ATOM 15862 HG LEU A 162 -29.660 47.935 66.995 0.00 0.00
A
ATOM 15863 CD1 LEU A 162 -29.713 50.050 67.383 0.00 0.00
A
ATOM 15864 HD11 LEU A 162 -30.817 50.127 67.479 0.00 0.00
A
ATOM 15865 HD12 LEU A 162 -29.468 49.928 68.460 0.00 0.00
A
ATOM 15866 HD13 LEU A 162 -29.346 51.011 66.965 0.00 0.00
A
ATOM 15867 CD2 LEU A 162 -29.857 48.996 65.095 0.00 0.00
A
ATOM 15868 HD21 LEU A 162 -29.528 49.869 64.491 0.00 0.00
A
ATOM 15869 HD22 LEU A 162 -29.546 48.111 64.501 0.00 0.00
A
ATOM 15870 HD23 LEU A 162 -30.967 48.982 65.127 0.00 0.00
A
ATOM 15871 C LEU A 162 -25.526 48.138 66.170 0.00 0.00
A
ATOM 15872 O LEU A 162 -24.734 47.551 66.943 0.00 0.00
A
ATOM 15873 N ASP A 163 -25.032 49.077 65.376 0.00 0.00
A
ATOM 15874 HN ASP A 163 -25.575 49.391 64.601 0.00 0.00
A
ATOM 15875 CA ASP A 163 -23.711 49.597 65.447 0.00 0.00
A

ATOM 15876 HA ASP A 163 -23.413 49.539 66.484 0.00 0.00
A
ATOM 15877 CB ASP A 163 -22.829 48.711 64.513 0.00 0.00
A
ATOM 15878 HB1 ASP A 163 -23.069 47.628 64.573 0.00 0.00
A
ATOM 15879 HB2 ASP A 163 -22.994 49.074 63.476 0.00 0.00
A
ATOM 15880 CG ASP A 163 -21.448 48.905 65.005 0.00 0.00
A
ATOM 15881 OD1 ASP A 163 -20.917 48.123 65.799 0.00 0.00
A
ATOM 15882 OD2 ASP A 163 -20.754 49.827 64.460 0.00 0.00
A
ATOM 15883 C ASP A 163 -23.673 51.028 65.016 0.00 0.00
A
ATOM 15884 O ASP A 163 -24.170 51.344 63.967 0.00 0.00
A
ATOM 15885 N SER A 164 -22.946 51.922 65.792 0.00 0.00
A
ATOM 15886 HN SER A 164 -22.654 51.674 66.713 0.00 0.00
A
ATOM 15887 CA SER A 164 -22.382 53.179 65.295 0.00 0.00
A
ATOM 15888 HA SER A 164 -21.785 53.474 66.145 0.00 0.00
A
ATOM 15889 CB SER A 164 -21.345 53.096 64.214 0.00 0.00
A
ATOM 15890 HB1 SER A 164 -21.763 52.737 63.249 0.00 0.00
A
ATOM 15891 HB2 SER A 164 -20.991 54.135 64.045 0.00 0.00
A
ATOM 15892 OG SER A 164 -20.245 52.308 64.628 0.00 0.00
A
ATOM 15893 HG1 SER A 164 -20.507 51.392 64.511 0.00 0.00
A
ATOM 15894 C SER A 164 -23.499 54.233 65.091 0.00 0.00
A
ATOM 15895 O SER A 164 -23.458 55.241 64.358 0.00 0.00
A
ATOM 15896 N LYS A 165 -24.613 54.027 65.853 0.00 0.00
A
ATOM 15897 HN LYS A 165 -24.704 53.272 66.497 0.00 0.00
A
ATOM 15898 CA LYS A 165 -25.753 54.968 65.992 0.00 0.00
A
ATOM 15899 HA LYS A 165 -25.210 55.897 65.904 0.00 0.00
A
ATOM 15900 CB LYS A 165 -26.797 54.614 64.878 0.00 0.00
A
ATOM 15901 HB1 LYS A 165 -27.499 55.458 65.045 0.00 0.00
A
ATOM 15902 HB2 LYS A 165 -26.267 54.797 63.919 0.00 0.00
A

ATOM 15903 CG LYS A 165 -27.479 53.257 64.979 0.00 0.00
A
ATOM 15904 HG1 LYS A 165 -26.797 52.381 64.936 0.00 0.00
A
ATOM 15905 HG2 LYS A 165 -27.967 53.215 65.976 0.00 0.00
A
ATOM 15906 CD LYS A 165 -28.536 53.074 63.899 0.00 0.00
A
ATOM 15907 HD1 LYS A 165 -28.957 52.059 64.067 0.00 0.00
A
ATOM 15908 HD2 LYS A 165 -29.292 53.885 63.955 0.00 0.00
A
ATOM 15909 CE LYS A 165 -27.874 53.197 62.492 0.00 0.00
A
ATOM 15910 HE1 LYS A 165 -27.371 54.173 62.318 0.00 0.00
A
ATOM 15911 HE2 LYS A 165 -27.153 52.386 62.256 0.00 0.00
A
ATOM 15912 NZ LYS A 165 -28.884 53.100 61.436 0.00 0.00
A
ATOM 15913 HZ1 LYS A 165 -28.512 53.470 60.538 0.00 0.00
A
ATOM 15914 HZ2 LYS A 165 -29.087 52.098 61.246 0.00 0.00
A
ATOM 15915 HZ3 LYS A 165 -29.723 53.672 61.664 0.00 0.00
A
ATOM 15916 C LYS A 165 -26.353 54.817 67.372 0.00 0.00
A
ATOM 15917 O LYS A 165 -26.030 53.788 67.996 0.00 0.00
A
ATOM 15918 N SER A 166 -27.240 55.787 67.881 0.00 0.00
A
ATOM 15919 HN SER A 166 -27.563 56.479 67.240 0.00 0.00
A
ATOM 15920 CA SER A 166 -28.027 55.571 69.141 0.00 0.00
A
ATOM 15921 HA SER A 166 -27.453 54.889 69.750 0.00 0.00
A
ATOM 15922 CB SER A 166 -28.104 56.862 70.028 0.00 0.00
A
ATOM 15923 HB1 SER A 166 -28.624 56.747 71.003 0.00 0.00
A
ATOM 15924 HB2 SER A 166 -27.066 57.160 70.290 0.00 0.00
A
ATOM 15925 OG SER A 166 -28.761 57.926 69.273 0.00 0.00
A
ATOM 15926 HG1 SER A 166 -28.311 57.991 68.428 0.00 0.00
A
ATOM 15927 C SER A 166 -29.389 55.101 68.863 0.00 0.00
A
ATOM 15928 O SER A 166 -29.840 55.033 67.704 0.00 0.00
A
ATOM 15929 N ILE A 167 -30.192 54.707 69.876 0.00 0.00
A

ATOM 15930 HN ILE A 167 -29.829 54.702 70.805 0.00 0.00
A
ATOM 15931 CA ILE A 167 -31.615 54.339 69.733 0.00 0.00
A
ATOM 15932 HA ILE A 167 -31.674 53.501 69.054 0.00 0.00
A
ATOM 15933 CB ILE A 167 -32.284 53.831 71.051 0.00 0.00
A
ATOM 15934 HB ILE A 167 -31.740 52.959 71.472 0.00 0.00
A
ATOM 15935 CG2 ILE A 167 -32.185 54.854 72.165 0.00 0.00
A
ATOM 15936 HG21 ILE A 167 -32.930 55.673 72.075 0.00 0.00
A
ATOM 15937 HG22 ILE A 167 -32.400 54.433 73.171 0.00 0.00
A
ATOM 15938 HG23 ILE A 167 -31.186 55.335 72.084 0.00 0.00
A
ATOM 15939 CG1 ILE A 167 -33.746 53.281 70.928 0.00 0.00
A
ATOM 15940 HG11 ILE A 167 -34.190 53.050 71.921 0.00 0.00
A
ATOM 15941 HG12 ILE A 167 -34.451 53.998 70.456 0.00 0.00
A
ATOM 15942 CD ILE A 167 -33.886 52.001 70.066 0.00 0.00
A
ATOM 15943 HD1 ILE A 167 -34.818 51.462 70.338 0.00 0.00
A
ATOM 15944 HD2 ILE A 167 -33.947 52.106 68.962 0.00 0.00
A
ATOM 15945 HD3 ILE A 167 -33.156 51.250 70.438 0.00 0.00
A
ATOM 15946 C ILE A 167 -32.393 55.475 69.167 0.00 0.00
A
ATOM 15947 O ILE A 167 -33.369 55.284 68.476 0.00 0.00
A
ATOM 15948 N ILE A 168 -31.962 56.732 69.364 0.00 0.00
A
ATOM 15949 HN ILE A 168 -31.168 57.058 69.871 0.00 0.00
A
ATOM 15950 CA ILE A 168 -32.636 57.935 68.838 0.00 0.00
A
ATOM 15951 HA ILE A 168 -33.676 57.971 69.125 0.00 0.00
A
ATOM 15952 CB ILE A 168 -32.191 59.206 69.546 0.00 0.00
A
ATOM 15953 HB ILE A 168 -31.191 59.535 69.193 0.00 0.00
A
ATOM 15954 CG2 ILE A 168 -33.172 60.413 69.334 0.00 0.00
A
ATOM 15955 HG21 ILE A 168 -33.309 60.588 68.245 0.00 0.00
A
ATOM 15956 HG22 ILE A 168 -34.212 60.214 69.670 0.00 0.00
A

ATOM 15957 HG23 ILE A 168 −32.723 61.288 69.851 0.00 0.00
A
ATOM 15958 CG1 ILE A 168 −32.125 58.923 71.062 0.00 0.00
A
ATOM 15959 HG11 ILE A 168 −31.401 58.138 71.368 0.00 0.00
A
ATOM 15960 HG12 ILE A 168 −31.804 59.896 71.492 0.00 0.00
A
ATOM 15961 CD ILE A 168 −33.426 58.527 71.663 0.00 0.00
A
ATOM 15962 HD1 ILE A 168 −34.144 59.373 71.616 0.00 0.00
A
ATOM 15963 HD2 ILE A 168 −33.929 57.647 71.208 0.00 0.00
A
ATOM 15964 HD3 ILE A 168 −33.292 58.397 72.758 0.00 0.00
A
ATOM 15965 C ILE A 168 −32.493 57.938 67.322 0.00 0.00
A
ATOM 15966 O ILE A 168 −33.475 58.181 66.622 0.00 0.00
A
ATOM 15967 N ASP A 169 −31.305 57.559 66.837 0.00 0.00
A
ATOM 15968 HN ASP A 169 −30.543 57.377 67.452 0.00 0.00
A
ATOM 15969 CA ASP A 169 −30.987 57.498 65.393 0.00 0.00
A
ATOM 15970 HA ASP A 169 −31.397 58.402 64.968 0.00 0.00
A
ATOM 15971 CB ASP A 169 −29.496 57.391 65.064 0.00 0.00
A
ATOM 15972 HB1 ASP A 169 −29.201 56.404 65.482 0.00 0.00
A
ATOM 15973 HB2 ASP A 169 −29.209 57.407 63.991 0.00 0.00
A
ATOM 15974 CG ASP A 169 −28.672 58.453 65.776 0.00 0.00
A
ATOM 15975 OD1 ASP A 169 −28.987 59.667 65.635 0.00 0.00
A
ATOM 15976 OD2 ASP A 169 −27.739 58.145 66.554 0.00 0.00
A
ATOM 15977 C ASP A 169 −31.700 56.397 64.688 0.00 0.00
A
ATOM 15978 O ASP A 169 −32.141 56.594 63.507 0.00 0.00
A
ATOM 15979 N LEU A 170 −31.895 55.215 65.349 0.00 0.00
A
ATOM 15980 HN LEU A 170 −31.508 55.126 66.263 0.00 0.00
A
ATOM 15981 CA LEU A 170 −32.719 54.135 64.848 0.00 0.00
A
ATOM 15982 HA LEU A 170 −32.505 54.006 63.798 0.00 0.00
A
ATOM 15983 CB LEU A 170 −32.357 52.850 65.713 0.00 0.00
A

ATOM 15984 HB1 LEU A 170 −31.319 52.510 65.506 0.00 0.00
A
ATOM 15985 HB2 LEU A 170 −32.585 53.116 66.767 0.00 0.00
A
ATOM 15986 CG LEU A 170 −33.206 51.610 65.255 0.00 0.00
A
ATOM 15987 HG LEU A 170 −34.270 51.931 65.268 0.00 0.00
A
ATOM 15988 CD1 LEU A 170 −32.953 51.107 63.857 0.00 0.00
A
ATOM 15989 HD11 LEU A 170 −33.641 50.234 63.843 0.00 0.00
A
ATOM 15990 HD12 LEU A 170 −33.331 51.794 63.070 0.00 0.00
A
ATOM 15991 HD13 LEU A 170 −31.906 50.769 63.705 0.00 0.00
A
ATOM 15992 CD2 LEU A 170 −33.218 50.467 66.268 0.00 0.00
A
ATOM 15993 HD21 LEU A 170 −34.097 49.805 66.120 0.00 0.00
A
ATOM 15994 HD22 LEU A 170 −32.292 49.853 66.284 0.00 0.00
A
ATOM 15995 HD23 LEU A 170 −33.325 50.999 67.238 0.00 0.00
A
ATOM 15996 C LEU A 170 −34.231 54.423 64.673 0.00 0.00
A
ATOM 15997 O LEU A 170 −34.757 54.231 63.601 0.00 0.00
A
ATOM 15998 N ASN A 171 −34.907 55.059 65.676 0.00 0.00
A
ATOM 15999 HN ASN A 171 −34.617 55.342 66.587 0.00 0.00
A
ATOM 16000 CA ASN A 171 −36.184 55.702 65.448 0.00 0.00
A
ATOM 16001 HA ASN A 171 −36.387 56.250 66.356 0.00 0.00
A
ATOM 16002 CB ASN A 171 −36.307 56.643 64.240 0.00 0.00
A
ATOM 16003 HB1 ASN A 171 −35.484 57.384 64.327 0.00 0.00
A
ATOM 16004 HB2 ASN A 171 −36.165 56.114 63.274 0.00 0.00
A
ATOM 16005 CG ASN A 171 −37.665 57.384 64.303 0.00 0.00
A
ATOM 16006 OD1 ASN A 171 −38.350 57.618 65.330 0.00 0.00
A
ATOM 16007 ND2 ASN A 171 −37.985 57.996 63.101 0.00 0.00
A
ATOM 16008 HD21 ASN A 171 −38.942 58.245 62.953 0.00 0.00
A
ATOM 16009 HD22 ASN A 171 −37.408 57.972 62.285 0.00 0.00
A
ATOM 16010 C ASN A 171 −37.259 54.565 65.462 0.00 0.00
A

ATOM 16011 O ASN A 171 -37.928 54.312 64.498 0.00 0.00
A
ATOM 16012 N VAL A 172 -37.500 53.927 66.597 0.00 0.00
A
ATOM 16013 HN VAL A 172 -36.984 54.286 67.370 0.00 0.00
A
ATOM 16014 CA VAL A 172 -38.473 52.823 66.889 0.00 0.00
A
ATOM 16015 HA VAL A 172 -38.607 52.244 65.987 0.00 0.00
A
ATOM 16016 CB VAL A 172 -38.016 51.853 67.956 0.00 0.00
A
ATOM 16017 HB VAL A 172 -38.110 52.265 68.983 0.00 0.00
A
ATOM 16018 CG1 VAL A 172 -38.868 50.601 67.845 0.00 0.00
A
ATOM 16019 HG11 VAL A 172 -38.705 50.205 66.820 0.00 0.00
A
ATOM 16020 HG12 VAL A 172 -38.478 49.848 68.563 0.00 0.00
A
ATOM 16021 HG13 VAL A 172 -39.908 50.811 68.174 0.00 0.00
A
ATOM 16022 CG2 VAL A 172 -36.573 51.633 67.548 0.00 0.00
A
ATOM 16023 HG21 VAL A 172 -36.504 51.395 66.465 0.00 0.00
A
ATOM 16024 HG22 VAL A 172 -35.916 52.500 67.773 0.00 0.00
A
ATOM 16025 HG23 VAL A 172 -36.052 50.818 68.094 0.00 0.00
A
ATOM 16026 C VAL A 172 -39.748 53.418 67.377 0.00 0.00
A
ATOM 16027 O VAL A 172 -39.747 54.153 68.381 0.00 0.00
A
ATOM 16028 N ARG A 173 -40.875 53.046 66.728 0.00 0.00
A
ATOM 16029 HN ARG A 173 -40.792 52.285 66.090 0.00 0.00
A
ATOM 16030 CA ARG A 173 -42.114 53.768 67.096 0.00 0.00
A
ATOM 16031 HA ARG A 173 -42.198 53.706 68.171 0.00 0.00
A
ATOM 16032 CB ARG A 173 -42.136 55.239 66.594 0.00 0.00
A
ATOM 16033 HB1 ARG A 173 -43.059 55.691 67.014 0.00 0.00
A
ATOM 16034 HB2 ARG A 173 -41.280 55.824 66.996 0.00 0.00
A
ATOM 16035 CG ARG A 173 -42.069 55.398 65.014 0.00 0.00
A
ATOM 16036 HG1 ARG A 173 -42.877 54.749 64.615 0.00 0.00
A
ATOM 16037 HG2 ARG A 173 -42.414 56.429 64.785 0.00 0.00
A

ATOM 16038 CD ARG A 173 −40.701 55.160 64.323 0.00 0.00
A
ATOM 16039 HD1 ARG A 173 −40.123 56.031 64.698 0.00 0.00
A
ATOM 16040 HD2 ARG A 173 −40.225 54.177 64.529 0.00 0.00
A
ATOM 16041 NE ARG A 173 −40.941 55.297 62.921 0.00 0.00
A
ATOM 16042 HE ARG A 173 −41.840 55.640 62.648 0.00 0.00
A
ATOM 16043 CZ ARG A 173 −40.152 54.896 61.922 0.00 0.00
A
ATOM 16044 NH1 ARG A 173 −39.002 54.328 62.048 0.00 0.00
A
ATOM 16045 HH11 ARG A 173 −38.555 53.964 61.232 0.00 0.00
A
ATOM 16046 HH12 ARG A 173 −38.593 54.256 62.958 0.00 0.00
A
ATOM 16047 NH2 ARG A 173 −40.474 55.263 60.634 0.00 0.00
A
ATOM 16048 HH21 ARG A 173 −39.853 54.983 59.902 0.00 0.00
A
ATOM 16049 HH22 ARG A 173 −41.266 55.847 60.453 0.00 0.00
A
ATOM 16050 C ARG A 173 −43.285 53.067 66.642 0.00 0.00
A
ATOM 16051 O ARG A 173 −43.227 52.360 65.600 0.00 0.00
A
ATOM 16052 N ALA A 174 −44.361 53.273 67.367 0.00 0.00
A
ATOM 16053 HN ALA A 174 −44.242 53.823 68.190 0.00 0.00
A
ATOM 16054 CA ALA A 174 −45.672 52.655 67.148 0.00 0.00
A
ATOM 16055 HA ALA A 174 −45.497 51.771 67.743 0.00 0.00
A
ATOM 16056 CB ALA A 174 −46.627 53.419 68.012 0.00 0.00
A
ATOM 16057 HB1 ALA A 174 −47.610 52.924 68.158 0.00 0.00
A
ATOM 16058 HB2 ALA A 174 −46.186 53.630 69.010 0.00 0.00
A
ATOM 16059 HB3 ALA A 174 −46.882 54.353 67.467 0.00 0.00
A
ATOM 16060 C ALA A 174 −46.160 52.261 65.797 0.00 0.00
A
ATOM 16061 O ALA A 174 −45.998 52.942 64.783 0.00 0.00
A
ATOM 16062 N GLY A 177 −46.607 50.985 65.536 0.00 0.00
A
ATOM 16063 HN GLY A 177 −46.898 50.768 64.607 0.00 0.00
A
ATOM 16064 CA GLY A 177 −46.995 49.977 66.508 0.00 0.00
A

ATOM 16065 HA1 GLY A 177   -47.695 49.266 66.095 0.00 0.00
A
ATOM 16066 HA2 GLY A 177   -47.373 50.573 67.326 0.00 0.00
A
ATOM 16067 C   GLY A 177   -45.884 49.130 67.122 0.00 0.00
A
ATOM 16068 O   GLY A 177   -46.028 48.347 68.055 0.00 0.00
A
ATOM 16069 N   CYS A 178   -44.647 49.338 66.570 0.00 0.00
A
ATOM 16070 HN CYS A 178   -44.634 50.030 65.851 0.00 0.00
A
ATOM 16071 CA CYS A 178   -43.404 48.616 67.030 0.00 0.00
A
ATOM 16072 HA CYS A 178   -43.569 47.549 67.037 0.00 0.00
A
ATOM 16073 CB CYS A 178   -42.115 49.009 66.298 0.00 0.00
A
ATOM 16074 HB1 CYS A 178   -42.030 50.107 66.147 0.00 0.00
A
ATOM 16075 HB2 CYS A 178   -41.165 48.839 66.849 0.00 0.00
A
ATOM 16076 SG CYS A 178   -41.979 48.134 64.672 0.00 0.00
A
ATOM 16077 HG1 CYS A 178   -42.902 48.826 64.020 0.00 0.00
A
ATOM 16078 C   CYS A 178   -43.111 49.158 68.439 0.00 0.00
A
ATOM 16079 O   CYS A 178   -43.496 50.302 68.702 0.00 0.00
A
ATOM 16080 N   THR A 179   -42.593 48.311 69.360 0.00 0.00
A
ATOM 16081 HN THR A 179   -42.449 47.360 69.097 0.00 0.00
A
ATOM 16082 CA THR A 179   -42.166 48.841 70.691 0.00 0.00
A
ATOM 16083 HA THR A 179   -41.729 49.822 70.577 0.00 0.00
A
ATOM 16084 CB THR A 179   -43.365 48.945 71.648 0.00 0.00
A
ATOM 16085 HB THR A 179   -44.139 49.595 71.186 0.00 0.00
A
ATOM 16086 OG1 THR A 179   -43.001 49.685 72.762 0.00 0.00
A
ATOM 16087 HG1 THR A 179   -43.737 49.825 73.363 0.00 0.00
A
ATOM 16088 CG2 THR A 179   -43.978 47.599 72.127 0.00 0.00
A
ATOM 16089 HG21 THR A 179   -44.763 47.810 72.885 0.00 0.00
A
ATOM 16090 HG22 THR A 179   -44.440 47.049 71.279 0.00 0.00
A
ATOM 16091 HG23 THR A 179   -43.229 46.903 72.561 0.00 0.00
A

ATOM 16092 C THR A 179 −41.180 47.888 71.204 0.00 0.00
A
ATOM 16093 O THR A 179 −40.822 46.898 70.606 0.00 0.00
A
ATOM 16094 N ILE A 180 −40.489 48.297 72.323 0.00 0.00
A
ATOM 16095 HN ILE A 180 −40.780 49.126 72.796 0.00 0.00
A
ATOM 16096 CA ILE A 180 −39.230 47.757 72.839 0.00 0.00
A
ATOM 16097 HA ILE A 180 −38.987 47.084 72.030 0.00 0.00
A
ATOM 16098 CB ILE A 180 −38.169 48.741 73.099 0.00 0.00
A
ATOM 16099 HB ILE A 180 −38.348 49.271 74.059 0.00 0.00
A
ATOM 16100 CG2 ILE A 180 −36.832 47.876 73.116 0.00 0.00
A
ATOM 16101 HG21 ILE A 180 −36.428 47.518 72.145 0.00 0.00
A
ATOM 16102 HG22 ILE A 180 −36.031 48.366 73.710 0.00 0.00
A
ATOM 16103 HG23 ILE A 180 −37.071 46.898 73.586 0.00 0.00
A
ATOM 16104 CG1 ILE A 180 −38.114 49.874 72.010 0.00 0.00
A
ATOM 16105 HG11 ILE A 180 −38.161 49.439 70.989 0.00 0.00
A
ATOM 16106 HG12 ILE A 180 −39.082 50.373 72.234 0.00 0.00
A
ATOM 16107 CD ILE A 180 −36.966 50.816 72.173 0.00 0.00
A
ATOM 16108 HD1 ILE A 180 −36.877 51.249 73.193 0.00 0.00
A
ATOM 16109 HD2 ILE A 180 −35.954 50.373 72.059 0.00 0.00
A
ATOM 16110 HD3 ILE A 180 −37.065 51.633 71.427 0.00 0.00
A
ATOM 16111 C ILE A 180 −39.476 46.936 74.084 0.00 0.00
A
ATOM 16112 O ILE A 180 −39.972 47.399 75.130 0.00 0.00
A
ATOM 16113 N LEU A 181 −39.216 45.567 74.033 0.00 0.00
A
ATOM 16114 HN LEU A 181 −38.852 45.115 73.222 0.00 0.00
A
ATOM 16115 CA LEU A 181 −39.422 44.755 75.220 0.00 0.00
A
ATOM 16116 HA LEU A 181 −40.171 45.164 75.882 0.00 0.00
A
ATOM 16117 CB LEU A 181 −39.688 43.354 74.624 0.00 0.00
A
ATOM 16118 HB1 LEU A 181 −38.808 43.056 74.014 0.00 0.00
A

ATOM 16119 HB2 LEU A 181 -39.757 42.693 75.514 0.00 0.00
A
ATOM 16120 CG LEU A 181 -40.905 43.156 73.688 0.00 0.00
A
ATOM 16121 HG LEU A 181 -40.846 43.765 72.760 0.00 0.00
A
ATOM 16122 CD1 LEU A 181 -40.907 41.714 73.304 0.00 0.00
A
ATOM 16123 HD11 LEU A 181 -40.924 41.095 74.227 0.00 0.00
A
ATOM 16124 HD12 LEU A 181 -41.759 41.458 72.638 0.00 0.00
A
ATOM 16125 HD13 LEU A 181 -39.984 41.442 72.749 0.00 0.00
A
ATOM 16126 CD2 LEU A 181 -42.202 43.660 74.340 0.00 0.00
A
ATOM 16127 HD21 LEU A 181 -42.347 43.170 75.326 0.00 0.00
A
ATOM 16128 HD22 LEU A 181 -42.214 44.769 74.417 0.00 0.00
A
ATOM 16129 HD23 LEU A 181 -43.007 43.363 73.635 0.00 0.00
A
ATOM 16130 C LEU A 181 -38.240 44.747 76.219 0.00 0.00
A
ATOM 16131 O LEU A 181 -38.406 44.783 77.434 0.00 0.00
A
ATOM 16132 N ALA A 182 -37.018 44.564 75.694 0.00 0.00
A
ATOM 16133 HN ALA A 182 -36.881 44.498 74.709 0.00 0.00
A
ATOM 16134 CA ALA A 182 -35.882 44.290 76.565 0.00 0.00
A
ATOM 16135 HA ALA A 182 -35.890 44.841 77.494 0.00 0.00
A
ATOM 16136 CB ALA A 182 -35.914 42.768 76.968 0.00 0.00
A
ATOM 16137 HB1 ALA A 182 -35.107 42.631 77.720 0.00 0.00
A
ATOM 16138 HB2 ALA A 182 -36.900 42.514 77.413 0.00 0.00
A
ATOM 16139 HB3 ALA A 182 -35.773 42.035 76.145 0.00 0.00
A
ATOM 16140 C ALA A 182 -34.613 44.695 75.817 0.00 0.00
A
ATOM 16141 O ALA A 182 -34.684 44.779 74.578 0.00 0.00
A
ATOM 16142 N ILE A 183 -33.455 44.849 76.445 0.00 0.00
A
ATOM 16143 HN ILE A 183 -33.459 44.757 77.438 0.00 0.00
A
ATOM 16144 CA ILE A 183 -32.117 44.849 75.899 0.00 0.00
A
ATOM 16145 HA ILE A 183 -32.167 44.489 74.882 0.00 0.00
A

ATOM 16146 CB ILE A 183 −31.542 46.273 75.774 0.00 0.00
A
ATOM 16147 HB ILE A 183 −32.299 47.024 75.466 0.00 0.00
A
ATOM 16148 CG2 ILE A 183 −31.227 46.884 77.219 0.00 0.00
A
ATOM 16149 HG21 ILE A 183 −30.481 46.303 77.803 0.00 0.00
A
ATOM 16150 HG22 ILE A 183 −30.780 47.892 77.081 0.00 0.00
A
ATOM 16151 HG23 ILE A 183 −32.172 47.099 77.763 0.00 0.00
A
ATOM 16152 CG1 ILE A 183 −30.250 46.373 74.920 0.00 0.00
A
ATOM 16153 HG11 ILE A 183 −29.492 45.718 75.399 0.00 0.00
A
ATOM 16154 HG12 ILE A 183 −30.427 46.027 73.879 0.00 0.00
A
ATOM 16155 CD ILE A 183 −29.778 47.832 74.651 0.00 0.00
A
ATOM 16156 HD1 ILE A 183 −28.865 47.783 74.020 0.00 0.00
A
ATOM 16157 HD2 ILE A 183 −30.631 48.314 74.127 0.00 0.00
A
ATOM 16158 HD3 ILE A 183 −29.445 48.365 75.568 0.00 0.00
A
ATOM 16159 C ILE A 183 −31.226 43.896 76.716 0.00 0.00
A
ATOM 16160 O ILE A 183 −31.334 43.798 77.969 0.00 0.00
A
ATOM 16161 N LYS A 184 −30.459 43.107 75.992 0.00 0.00
A
ATOM 16162 HN LYS A 184 −30.369 43.243 75.008 0.00 0.00
A
ATOM 16163 CA LYS A 184 −29.629 42.052 76.512 0.00 0.00
A
ATOM 16164 HA LYS A 184 −30.157 41.638 77.358 0.00 0.00
A
ATOM 16165 CB LYS A 184 −29.529 40.915 75.520 0.00 0.00
A
ATOM 16166 HB1 LYS A 184 −29.285 41.306 74.508 0.00 0.00
A
ATOM 16167 HB2 LYS A 184 −28.668 40.255 75.757 0.00 0.00
A
ATOM 16168 CG LYS A 184 −30.872 40.205 75.322 0.00 0.00
A
ATOM 16169 HG1 LYS A 184 −31.195 39.754 76.285 0.00 0.00
A
ATOM 16170 HG2 LYS A 184 −31.629 40.992 75.118 0.00 0.00
A
ATOM 16171 CD LYS A 184 −30.822 39.037 74.257 0.00 0.00
A
ATOM 16172 HD1 LYS A 184 −31.847 38.667 74.041 0.00 0.00
A

ATOM 16173 HD2 LYS A 184 −30.574 39.419 73.243 0.00 0.00
A
ATOM 16174 CE LYS A 184 −29.820 38.035 74.655 0.00 0.00
A
ATOM 16175 HE1 LYS A 184 −28.770 38.391 74.574 0.00 0.00
A
ATOM 16176 HE2 LYS A 184 −29.987 37.814 75.731 0.00 0.00
A
ATOM 16177 NZ LYS A 184 −30.032 36.828 73.863 0.00 0.00
A
ATOM 16178 HZ1 LYS A 184 −29.150 36.285 73.766 0.00 0.00
A
ATOM 16179 HZ2 LYS A 184 −30.685 36.249 74.428 0.00 0.00
A
ATOM 16180 HZ3 LYS A 184 −30.610 37.092 73.040 0.00 0.00
A
ATOM 16181 C LYS A 184 −28.247 42.482 77.029 0.00 0.00
A
ATOM 16182 O LYS A 184 −27.683 43.472 76.612 0.00 0.00
A
ATOM 16183 N HSE A 185 −27.763 41.727 78.036 0.00 0.00
A
ATOM 16184 HN HSE A 185 −28.294 41.056 78.548 0.00 0.00
A
ATOM 16185 CA HSE A 185 −26.389 41.782 78.442 0.00 0.00
A
ATOM 16186 HA HSE A 185 −25.799 42.055 77.580 0.00 0.00
A
ATOM 16187 CB HSE A 185 −26.128 42.777 79.654 0.00 0.00
A
ATOM 16188 HB1 HSE A 185 −26.739 43.690 79.484 0.00 0.00
A
ATOM 16189 HB2 HSE A 185 −26.510 42.296 80.579 0.00 0.00
A
ATOM 16190 ND1 HSE A 185 −23.889 43.643 79.085 0.00 0.00
A
ATOM 16191 CG HSE A 185 −24.714 42.956 80.035 0.00 0.00
A
ATOM 16192 CE1 HSE A 185 −22.673 43.693 79.714 0.00 0.00
A
ATOM 16193 HE1 HSE A 185 −21.804 44.274 79.404 0.00 0.00
A
ATOM 16194 NE2 HSE A 185 −22.679 43.161 80.952 0.00 0.00
A
ATOM 16195 HE2 HSE A 185 −21.882 43.136 81.555 0.00 0.00
A
ATOM 16196 CD2 HSE A 185 −23.957 42.725 81.141 0.00 0.00
A
ATOM 16197 HD2 HSE A 185 −24.213 42.340 82.120 0.00 0.00
A
ATOM 16198 C HSE A 185 −25.883 40.474 78.968 0.00 0.00
A
ATOM 16199 O HSE A 185 −26.650 39.627 79.472 0.00 0.00
A

ATOM 16200 N HSE A 186 -24.554 40.250 78.981 0.00 0.00
A
ATOM 16201 HN HSE A 186 -23.895 40.865 78.555 0.00 0.00
A
ATOM 16202 CA HSE A 186 -23.991 39.114 79.605 0.00 0.00
A
ATOM 16203 HA HSE A 186 -24.517 38.238 79.255 0.00 0.00
A
ATOM 16204 CB HSE A 186 -22.460 39.073 79.308 0.00 0.00
A
ATOM 16205 HB1 HSE A 186 -22.372 39.138 78.203 0.00 0.00
A
ATOM 16206 HB2 HSE A 186 -21.769 39.811 79.770 0.00 0.00
A
ATOM 16207 ND1 HSE A 186 -22.566 36.516 79.269 0.00 0.00
A
ATOM 16208 CG HSE A 186 -21.891 37.707 79.603 0.00 0.00
A
ATOM 16209 CE1 HSE A 186 -21.717 35.500 79.497 0.00 0.00
A
ATOM 16210 HE1 HSE A 186 -21.891 34.424 79.473 0.00 0.00
A
ATOM 16211 NE2 HSE A 186 -20.557 36.016 80.006 0.00 0.00
A
ATOM 16212 HE2 HSE A 186 -19.752 35.489 80.277 0.00 0.00
A
ATOM 16213 CD2 HSE A 186 -20.667 37.410 80.100 0.00 0.00
A
ATOM 16214 HD2 HSE A 186 -19.917 38.095 80.474 0.00 0.00
A
ATOM 16215 C HSE A 186 -24.064 39.053 81.139 0.00 0.00
A
ATOM 16216 O HSE A 186 -23.503 39.802 81.941 0.00 0.00
A
ATOM 16217 N GLY A 187 -24.951 38.196 81.637 0.00 0.00
A
ATOM 16218 HN GLY A 187 -25.411 37.558 81.024 0.00 0.00
A
ATOM 16219 CA GLY A 187 -25.400 38.167 82.997 0.00 0.00
A
ATOM 16220 HA1 GLY A 187 -24.496 38.118 83.586 0.00 0.00
A
ATOM 16221 HA2 GLY A 187 -25.946 37.240 83.093 0.00 0.00
A
ATOM 16222 C GLY A 187 -26.365 39.232 83.414 0.00 0.00
A
ATOM 16223 O GLY A 187 -26.523 39.496 84.580 0.00 0.00
A
ATOM 16224 N ASP A 188 -26.960 39.992 82.526 0.00 0.00
A
ATOM 16225 HN ASP A 188 -26.964 39.683 81.579 0.00 0.00
A
ATOM 16226 CA ASP A 188 -27.910 41.005 82.957 0.00 0.00
A

ATOM 16227 HA ASP A 188 −28.483 40.801 83.850 0.00 0.00
A
ATOM 16228 CB ASP A 188 −27.278 42.377 83.202 0.00 0.00
A
ATOM 16229 HB1 ASP A 188 −26.184 42.185 83.234 0.00 0.00
A
ATOM 16230 HB2 ASP A 188 −27.538 43.077 82.380 0.00 0.00
A
ATOM 16231 CG ASP A 188 −27.712 43.010 84.567 0.00 0.00
A
ATOM 16232 OD1 ASP A 188 −26.795 43.424 85.328 0.00 0.00
A
ATOM 16233 OD2 ASP A 188 −28.908 42.872 84.913 0.00 0.00
A
ATOM 16234 C ASP A 188 −29.035 41.271 81.939 0.00 0.00
A
ATOM 16235 O ASP A 188 −28.937 40.790 80.758 0.00 0.00
A
ATOM 16236 N ILE A 189 −30.035 42.042 82.419 0.00 0.00
A
ATOM 16237 HN ILE A 189 −30.051 42.324 83.375 0.00 0.00
A
ATOM 16238 CA ILE A 189 −31.134 42.544 81.603 0.00 0.00
A
ATOM 16239 HA ILE A 189 −30.802 42.613 80.577 0.00 0.00
A
ATOM 16240 CB ILE A 189 −32.301 41.508 81.562 0.00 0.00
A
ATOM 16241 HB ILE A 189 −31.749 40.560 81.384 0.00 0.00
A
ATOM 16242 CG2 ILE A 189 −32.920 41.427 82.939 0.00 0.00
A
ATOM 16243 HG21 ILE A 189 −33.509 40.492 83.053 0.00 0.00
A
ATOM 16244 HG22 ILE A 189 −32.178 41.443 83.765 0.00 0.00
A
ATOM 16245 HG23 ILE A 189 −33.589 42.314 82.922 0.00 0.00
A
ATOM 16246 CG1 ILE A 189 −33.332 41.676 80.369 0.00 0.00
A
ATOM 16247 HG11 ILE A 189 −34.218 41.095 80.702 0.00 0.00
A
ATOM 16248 HG12 ILE A 189 −33.579 42.738 80.155 0.00 0.00
A
ATOM 16249 CD ILE A 189 −32.779 40.980 79.121 0.00 0.00
A
ATOM 16250 HD1 ILE A 189 −33.471 41.156 78.270 0.00 0.00
A
ATOM 16251 HD2 ILE A 189 −31.969 41.662 78.787 0.00 0.00
A
ATOM 16252 HD3 ILE A 189 −32.550 39.894 79.167 0.00 0.00
A
ATOM 16253 C ILE A 189 −31.547 43.886 82.077 0.00 0.00
A

ATOM 16254 O ILE A 189 −31.406 44.186 83.277 0.00 0.00
A
ATOM 16255 N CYS A 190 −32.105 44.623 81.084 0.00 0.00
A
ATOM 16256 HN CYS A 190 −32.322 44.297 80.168 0.00 0.00
A
ATOM 16257 CA CYS A 190 −32.668 45.931 81.317 0.00 0.00
A
ATOM 16258 HA CYS A 190 −33.069 45.901 82.319 0.00 0.00
A
ATOM 16259 CB CYS A 190 −31.643 47.133 81.064 0.00 0.00
A
ATOM 16260 HB1 CYS A 190 −30.722 46.894 81.638 0.00 0.00
A
ATOM 16261 HB2 CYS A 190 −31.381 47.147 79.984 0.00 0.00
A
ATOM 16262 SG CYS A 190 −32.316 48.720 81.659 0.00 0.00
A
ATOM 16263 HG1 CYS A 190 −31.298 49.566 81.708 0.00 0.00
A
ATOM 16264 C CYS A 190 −33.813 45.944 80.433 0.00 0.00
A
ATOM 16265 O CYS A 190 −33.791 45.482 79.343 0.00 0.00
A
ATOM 16266 N LEU A 191 −34.941 46.585 80.858 0.00 0.00
A
ATOM 16267 HN LEU A 191 −35.075 46.935 81.782 0.00 0.00
A
ATOM 16268 CA LEU A 191 −36.107 46.550 79.985 0.00 0.00
A
ATOM 16269 HA LEU A 191 −36.058 45.614 79.449 0.00 0.00
A
ATOM 16270 CB LEU A 191 −37.383 46.620 80.839 0.00 0.00
A
ATOM 16271 HB1 LEU A 191 −37.062 47.282 81.671 0.00 0.00
A
ATOM 16272 HB2 LEU A 191 −38.088 47.246 80.251 0.00 0.00
A
ATOM 16273 CG LEU A 191 −38.037 45.224 81.233 0.00 0.00
A
ATOM 16274 HG LEU A 191 −38.404 44.767 80.290 0.00 0.00
A
ATOM 16275 CD1 LEU A 191 −37.232 44.227 82.035 0.00 0.00
A
ATOM 16276 HD11 LEU A 191 −37.714 43.226 82.057 0.00 0.00
A
ATOM 16277 HD12 LEU A 191 −36.236 44.130 81.553 0.00 0.00
A
ATOM 16278 HD13 LEU A 191 −36.982 44.553 83.068 0.00 0.00
A
ATOM 16279 CD2 LEU A 191 −39.241 45.485 82.038 0.00 0.00
A
ATOM 16280 HD21 LEU A 191 −39.016 46.185 82.870 0.00 0.00
A

ATOM 16281 HD22 LEU A 191 -40.010 45.933 81.373 0.00 0.00
A
ATOM 16282 HD23 LEU A 191 -39.616 44.540 82.487 0.00 0.00
A
ATOM 16283 C LEU A 191 -36.131 47.697 78.971 0.00 0.00
A
ATOM 16284 O LEU A 191 -36.868 47.665 77.986 0.00 0.00
A
ATOM 16285 N SER A 192 -35.351 48.766 79.193 0.00 0.00
A
ATOM 16286 HN SER A 192 -34.580 48.725 79.824 0.00 0.00
A
ATOM 16287 CA SER A 192 -35.442 50.072 78.531 0.00 0.00
A
ATOM 16288 HA SER A 192 -36.045 49.973 77.640 0.00 0.00
A
ATOM 16289 CB SER A 192 -36.066 51.184 79.447 0.00 0.00
A
ATOM 16290 HB1 SER A 192 -36.963 50.736 79.927 0.00 0.00
A
ATOM 16291 HB2 SER A 192 -35.386 51.710 80.150 0.00 0.00
A
ATOM 16292 OG SER A 192 -36.647 52.222 78.601 0.00 0.00
A
ATOM 16293 HG1 SER A 192 -37.244 52.759 79.126 0.00 0.00
A
ATOM 16294 C SER A 192 -34.080 50.524 78.003 0.00 0.00
A
ATOM 16295 O SER A 192 -33.136 50.799 78.784 0.00 0.00
A
ATOM 16296 N PRO A 193 -33.817 50.499 76.646 0.00 0.00
A
ATOM 16297 CD PRO A 193 -34.807 50.199 75.620 0.00 0.00
A
ATOM 16298 HD1 PRO A 193 -35.002 49.106 75.585 0.00 0.00
A
ATOM 16299 HD2 PRO A 193 -35.651 50.885 75.848 0.00 0.00
A
ATOM 16300 CA PRO A 193 -32.487 50.904 76.110 0.00 0.00
A
ATOM 16301 HA PRO A 193 -31.736 50.283 76.576 0.00 0.00
A
ATOM 16302 CB PRO A 193 -32.599 50.729 74.602 0.00 0.00
A
ATOM 16303 HB1 PRO A 193 -32.169 49.743 74.325 0.00 0.00
A
ATOM 16304 HB2 PRO A 193 -32.021 51.517 74.073 0.00 0.00
A
ATOM 16305 CG PRO A 193 -34.094 50.598 74.328 0.00 0.00
A
ATOM 16306 HG1 PRO A 193 -34.313 49.985 73.428 0.00 0.00
A
ATOM 16307 HG2 PRO A 193 -34.433 51.648 74.195 0.00 0.00
A

ATOM 16308 C PRO A 193 -32.094 52.314 76.578 0.00 0.00
A
ATOM 16309 O PRO A 193 -32.886 53.201 76.349 0.00 0.00
A
ATOM 16310 N ALA A 194 -30.929 52.603 77.172 0.00 0.00
A
ATOM 16311 HN ALA A 194 -30.362 51.849 77.495 0.00 0.00
A
ATOM 16312 CA ALA A 194 -30.610 53.999 77.515 0.00 0.00
A
ATOM 16313 HA ALA A 194 -31.516 54.415 77.930 0.00 0.00
A
ATOM 16314 CB ALA A 194 -29.469 54.077 78.517 0.00 0.00
A
ATOM 16315 HB1 ALA A 194 -29.606 54.987 79.139 0.00 0.00
A
ATOM 16316 HB2 ALA A 194 -29.588 53.162 79.136 0.00 0.00
A
ATOM 16317 HB3 ALA A 194 -28.479 54.000 78.018 0.00 0.00
A
ATOM 16318 C ALA A 194 -30.292 54.832 76.157 0.00 0.00
A
ATOM 16319 O ALA A 194 -29.848 54.275 75.152 0.00 0.00
A
ATOM 16320 N PRO A 195 -30.640 56.141 76.123 0.00 0.00
A
ATOM 16321 CD PRO A 195 -31.205 56.840 77.260 0.00 0.00
A
ATOM 16322 HD1 PRO A 195 -31.891 56.160 77.809 0.00 0.00
A
ATOM 16323 HD2 PRO A 195 -30.282 57.035 77.847 0.00 0.00
A
ATOM 16324 CA PRO A 195 -30.799 56.907 74.910 0.00 0.00
A
ATOM 16325 HA PRO A 195 -31.179 56.307 74.097 0.00 0.00
A
ATOM 16326 CB PRO A 195 -31.987 57.880 75.169 0.00 0.00
A
ATOM 16327 HB1 PRO A 195 -32.932 57.334 74.961 0.00 0.00
A
ATOM 16328 HB2 PRO A 195 -31.806 58.847 74.653 0.00 0.00
A
ATOM 16329 CG PRO A 195 -31.975 58.011 76.736 0.00 0.00
A
ATOM 16330 HG1 PRO A 195 -31.379 58.893 77.053 0.00 0.00
A
ATOM 16331 HG2 PRO A 195 -32.966 58.055 77.236 0.00 0.00
A
ATOM 16332 C PRO A 195 -29.629 57.682 74.318 0.00 0.00
A
ATOM 16333 O PRO A 195 -29.741 58.126 73.224 0.00 0.00
A
ATOM 16334 N GLU A 196 -28.551 57.872 75.126 0.00 0.00
A

ATOM 16335 HN GLU A 196 −28.462 57.379 75.988 0.00 0.00
A
ATOM 16336 CA GLU A 196 −27.425 58.753 74.834 0.00 0.00
A
ATOM 16337 HA GLU A 196 −27.587 59.506 74.078 0.00 0.00
A
ATOM 16338 CB GLU A 196 −27.163 59.532 76.130 0.00 0.00
A
ATOM 16339 HB1 GLU A 196 −26.723 58.994 76.996 0.00 0.00
A
ATOM 16340 HB2 GLU A 196 −26.265 60.148 75.910 0.00 0.00
A
ATOM 16341 CG GLU A 196 −28.300 60.406 76.493 0.00 0.00
A
ATOM 16342 HG1 GLU A 196 −28.822 60.832 75.609 0.00 0.00
A
ATOM 16343 HG2 GLU A 196 −29.124 59.851 76.990 0.00 0.00
A
ATOM 16344 CD GLU A 196 −27.792 61.455 77.398 0.00 0.00
A
ATOM 16345 OE1 GLU A 196 −27.483 61.219 78.567 0.00 0.00
A
ATOM 16346 OE2 GLU A 196 −27.804 62.655 76.943 0.00 0.00
A
ATOM 16347 C GLU A 196 −26.263 57.831 74.522 0.00 0.00
A
ATOM 16348 O GLU A 196 −25.222 58.328 74.090 0.00 0.00
A
ATOM 16349 N ASP A 197 −26.442 56.470 74.589 0.00 0.00
A
ATOM 16350 HN ASP A 197 −27.324 56.146 74.922 0.00 0.00
A
ATOM 16351 CA ASP A 197 −25.384 55.500 74.330 0.00 0.00
A
ATOM 16352 HA ASP A 197 −24.445 55.949 74.620 0.00 0.00
A
ATOM 16353 CB ASP A 197 −25.733 54.217 75.129 0.00 0.00
A
ATOM 16354 HB1 ASP A 197 −26.757 53.935 74.803 0.00 0.00
A
ATOM 16355 HB2 ASP A 197 −25.131 53.370 74.735 0.00 0.00
A
ATOM 16356 CG ASP A 197 −25.517 54.344 76.605 0.00 0.00
A
ATOM 16357 OD1 ASP A 197 −25.765 53.337 77.282 0.00 0.00
A
ATOM 16358 OD2 ASP A 197 −25.072 55.351 77.152 0.00 0.00
A
ATOM 16359 C ASP A 197 −25.397 55.309 72.878 0.00 0.00
A
ATOM 16360 O ASP A 197 −26.427 54.991 72.265 0.00 0.00
A
ATOM 16361 N ILE A 198 −24.326 55.538 72.184 0.00 0.00
A

ATOM 16362 HN ILE A 198 -23.557 56.060 72.545 0.00 0.00
A
ATOM 16363 CA ILE A 198 -24.133 55.027 70.860 0.00 0.00
A
ATOM 16364 HA ILE A 198 -25.026 55.207 70.278 0.00 0.00
A
ATOM 16365 CB ILE A 198 -22.904 55.580 70.163 0.00 0.00
A
ATOM 16366 HB ILE A 198 -21.958 55.347 70.697 0.00 0.00
A
ATOM 16367 CG2 ILE A 198 -22.787 54.943 68.710 0.00 0.00
A
ATOM 16368 HG21 ILE A 198 -22.532 53.864 68.636 0.00 0.00
A
ATOM 16369 HG22 ILE A 198 -23.688 55.228 68.126 0.00 0.00
A
ATOM 16370 HG23 ILE A 198 -21.902 55.360 68.183 0.00 0.00
A
ATOM 16371 CG1 ILE A 198 -23.046 57.130 70.102 0.00 0.00
A
ATOM 16372 HG11 ILE A 198 -23.867 57.344 69.385 0.00 0.00
A
ATOM 16373 HG12 ILE A 198 -23.302 57.440 71.137 0.00 0.00
A
ATOM 16374 CD ILE A 198 -21.943 57.917 69.455 0.00 0.00
A
ATOM 16375 HD1 ILE A 198 -21.635 57.414 68.514 0.00 0.00
A
ATOM 16376 HD2 ILE A 198 -22.410 58.853 69.081 0.00 0.00
A
ATOM 16377 HD3 ILE A 198 -21.114 58.094 70.172 0.00 0.00
A
ATOM 16378 C ILE A 198 -23.965 53.505 70.969 0.00 0.00
A
ATOM 16379 O ILE A 198 -23.087 53.037 71.736 0.00 0.00
A
ATOM 16380 N ILE A 199 -24.821 52.778 70.278 0.00 0.00
A
ATOM 16381 HN ILE A 199 -25.413 53.125 69.555 0.00 0.00
A
ATOM 16382 CA ILE A 199 -24.915 51.349 70.437 0.00 0.00
A
ATOM 16383 HA ILE A 199 -24.682 50.987 71.428 0.00 0.00
A
ATOM 16384 CB ILE A 199 -26.351 50.856 70.155 0.00 0.00
A
ATOM 16385 HB ILE A 199 -26.753 51.121 69.153 0.00 0.00
A
ATOM 16386 CG2 ILE A 199 -26.388 49.256 70.194 0.00 0.00
A
ATOM 16387 HG21 ILE A 199 -26.305 48.907 71.246 0.00 0.00
A
ATOM 16388 HG22 ILE A 199 -27.330 48.825 69.794 0.00 0.00
A

ATOM 16389 HG23 ILE A 199   −25.652 48.778 69.514 0.00 0.00
A
ATOM 16390 CG1 ILE A 199   −27.279 51.514 71.189 0.00 0.00
A
ATOM 16391 HG11 ILE A 199   −26.852 51.402 72.209 0.00 0.00
A
ATOM 16392 HG12 ILE A 199   −27.324 52.600 70.961 0.00 0.00
A
ATOM 16393 CD ILE A 199   −28.744 50.976 71.306 0.00 0.00
A
ATOM 16394 HD1 ILE A 199   −28.617 49.879 71.423 0.00 0.00
A
ATOM 16395 HD2 ILE A 199   −29.115 51.431 72.249 0.00 0.00
A
ATOM 16396 HD3 ILE A 199   −29.316 51.227 70.387 0.00 0.00
A
ATOM 16397 C   ILE A 199   −23.946 50.752 69.493 0.00 0.00
A
ATOM 16398 O   ILE A 199   −23.726 51.226 68.379 0.00 0.00
A
ATOM 16399 N   ARG A 200   −23.118 49.863 69.984 0.00 0.00
A
ATOM 16400 HN ARG A 200   −23.302 49.173 70.680 0.00 0.00
A
ATOM 16401 CA ARG A 200   −21.738 49.786 69.522 0.00 0.00
A
ATOM 16402 HA ARG A 200   −21.735 49.453 68.494 0.00 0.00
A
ATOM 16403 CB ARG A 200   −20.862 51.028 69.574 0.00 0.00
A
ATOM 16404 HB1 ARG A 200   −21.372 51.877 69.070 0.00 0.00
A
ATOM 16405 HB2 ARG A 200   −20.931 51.278 70.655 0.00 0.00
A
ATOM 16406 CG ARG A 200   −19.362 50.857 69.176 0.00 0.00
A
ATOM 16407 HG1 ARG A 200   −18.840 51.764 69.550 0.00 0.00
A
ATOM 16408 HG2 ARG A 200   −18.931 49.985 69.712 0.00 0.00
A
ATOM 16409 CD ARG A 200   −19.115 50.730 67.629 0.00 0.00
A
ATOM 16410 HD1 ARG A 200   −19.884 51.271 67.038 0.00 0.00
A
ATOM 16411 HD2 ARG A 200   −18.086 51.095 67.421 0.00 0.00
A
ATOM 16412 NE ARG A 200   −19.120 49.278 67.322 0.00 0.00
A
ATOM 16413 HE ARG A 200   −19.784 48.885 66.685 0.00 0.00
A
ATOM 16414 CZ ARG A 200   −18.173 48.405 67.804 0.00 0.00
A
ATOM 16415 NH1 ARG A 200   −17.097 48.774 68.522 0.00 0.00
A

ATOM 16416 HH11 ARG A 200   −16.566 48.064 68.985 0.00 0.00
A
ATOM 16417 HH12 ARG A 200   −16.953 49.730 68.779 0.00 0.00
A
ATOM 16418 NH2 ARG A 200   −18.231 47.123 67.463 0.00 0.00
A
ATOM 16419 HH21 ARG A 200   −17.864 46.367 68.004 0.00 0.00
A
ATOM 16420 HH22 ARG A 200   −18.930 46.790 66.830 0.00 0.00
A
ATOM 16421 C   ARG A 200   −21.000 48.717 70.353 0.00 0.00
A
ATOM 16422 O   ARG A 200   −20.422 48.970 71.438 0.00 0.00
A
ATOM 16423 N   GLU A 201   −21.055 47.476 69.743 0.00 0.00
A
ATOM 16424 HN GLU A 201   −21.657 47.494 68.949 0.00 0.00
A
ATOM 16425 CA GLU A 201   −20.522 46.197 70.241 0.00 0.00
A
ATOM 16426 HA GLU A 201   −20.356 45.581 69.370 0.00 0.00
A
ATOM 16427 CB GLU A 201   −19.097 46.348 70.777 0.00 0.00
A
ATOM 16428 HB1 GLU A 201   −18.529 47.120 70.215 0.00 0.00
A
ATOM 16429 HB2 GLU A 201   −19.135 46.772 71.804 0.00 0.00
A
ATOM 16430 CG GLU A 201   −18.355 44.955 70.919 0.00 0.00
A
ATOM 16431 HG1 GLU A 201   −17.509 45.225 71.587 0.00 0.00
A
ATOM 16432 HG2 GLU A 201   −19.086 44.325 71.469 0.00 0.00
A
ATOM 16433 CD GLU A 201   −17.905 44.229 69.648 0.00 0.00
A
ATOM 16434 OE1 GLU A 201   −18.207 43.010 69.488 0.00 0.00
A
ATOM 16435 OE2 GLU A 201   −17.292 44.801 68.768 0.00 0.00
A
ATOM 16436 C   GLU A 201   −21.457 45.595 71.264 0.00 0.00
A
ATOM 16437 O   GLU A 201   −21.730 46.148 72.359 0.00 0.00
A
ATOM 16438 N   GLN A 202   −22.033 44.432 70.990 0.00 0.00
A
ATOM 16439 HN GLN A 202   −21.680 43.781 70.322 0.00 0.00
A
ATOM 16440 CA GLN A 202   −23.138 43.859 71.771 0.00 0.00
A
ATOM 16441 HA GLN A 202   −23.461 42.976 71.241 0.00 0.00
A
ATOM 16442 CB GLN A 202   −22.440 43.180 72.981 0.00 0.00
A

ATOM 16443 HB1 GLN A 202 -22.071 43.954 73.688 0.00 0.00
A
ATOM 16444 HB2 GLN A 202 -23.262 42.698 73.551 0.00 0.00
A
ATOM 16445 CG GLN A 202 -21.259 42.183 72.692 0.00 0.00
A
ATOM 16446 HG1 GLN A 202 -21.632 41.379 72.023 0.00 0.00
A
ATOM 16447 HG2 GLN A 202 -20.396 42.707 72.228 0.00 0.00
A
ATOM 16448 CD GLN A 202 -20.794 41.568 73.993 0.00 0.00
A
ATOM 16449 OE1 GLN A 202 -20.209 42.297 74.868 0.00 0.00
A
ATOM 16450 NE2 GLN A 202 -20.993 40.275 74.076 0.00 0.00
A
ATOM 16451 HE21 GLN A 202 -20.796 39.812 74.940 0.00 0.00
A
ATOM 16452 HE22 GLN A 202 -21.277 39.787 73.250 0.00 0.00
A
ATOM 16453 C GLN A 202 -24.303 44.704 72.169 0.00 0.00
A
ATOM 16454 O GLN A 202 -24.755 45.522 71.315 0.00 0.00
A
ATOM 16455 N ASP A 203 -24.889 44.633 73.401 0.00 0.00
A
ATOM 16456 HN ASP A 203 -24.621 44.101 74.200 0.00 0.00
A
ATOM 16457 CA ASP A 203 -26.105 45.322 73.851 0.00 0.00
A
ATOM 16458 HA ASP A 203 -26.380 44.899 74.806 0.00 0.00
A
ATOM 16459 CB ASP A 203 -25.810 46.828 74.243 0.00 0.00
A
ATOM 16460 HB1 ASP A 203 -25.742 47.309 73.244 0.00 0.00
A
ATOM 16461 HB2 ASP A 203 -26.641 47.274 74.830 0.00 0.00
A
ATOM 16462 CG ASP A 203 -24.523 47.095 75.031 0.00 0.00
A
ATOM 16463 OD1 ASP A 203 -23.796 48.048 74.831 0.00 0.00
A
ATOM 16464 OD2 ASP A 203 -24.389 46.308 76.082 0.00 0.00
A
ATOM 16465 C ASP A 203 -27.323 45.169 72.864 0.00 0.00
A
ATOM 16466 O ASP A 203 -27.857 46.153 72.362 0.00 0.00
A
ATOM 16467 N CYS A 204 -27.752 44.004 72.579 0.00 0.00
A
ATOM 16468 HN CYS A 204 -27.395 43.193 73.037 0.00 0.00
A
ATOM 16469 CA CYS A 204 -28.780 43.636 71.646 0.00 0.00
A

ATOM 16470 HA CYS A 204 −28.667 44.255 70.768 0.00 0.00
A
ATOM 16471 CB CYS A 204 −28.791 42.096 71.299 0.00 0.00
A
ATOM 16472 HB1 CYS A 204 −29.261 41.558 72.150 0.00 0.00
A
ATOM 16473 HB2 CYS A 204 −29.520 42.023 70.463 0.00 0.00
A
ATOM 16474 SG CYS A 204 −27.241 41.495 70.731 0.00 0.00
A
ATOM 16475 HG1 CYS A 204 −26.708 41.234 71.915 0.00 0.00
A
ATOM 16476 C CYS A 204 −30.113 43.945 72.248 0.00 0.00
A
ATOM 16477 O CYS A 204 −30.560 43.402 73.300 0.00 0.00
A
ATOM 16478 N LEU A 205 −30.917 44.675 71.482 0.00 0.00
A
ATOM 16479 HN LEU A 205 −30.505 44.978 70.627 0.00 0.00
A
ATOM 16480 CA LEU A 205 −32.229 45.155 71.686 0.00 0.00
A
ATOM 16481 HA LEU A 205 −32.467 45.019 72.731 0.00 0.00
A
ATOM 16482 CB LEU A 205 −32.541 46.649 71.448 0.00 0.00
A
ATOM 16483 HB1 LEU A 205 −33.553 46.824 71.873 0.00 0.00
A
ATOM 16484 HB2 LEU A 205 −31.902 47.284 72.098 0.00 0.00
A
ATOM 16485 CG LEU A 205 −32.423 47.182 69.999 0.00 0.00
A
ATOM 16486 HG LEU A 205 −32.940 46.533 69.261 0.00 0.00
A
ATOM 16487 CD1 LEU A 205 −33.071 48.567 70.037 0.00 0.00
A
ATOM 16488 HD11 LEU A 205 −32.404 49.237 70.620 0.00 0.00
A
ATOM 16489 HD12 LEU A 205 −33.301 48.919 69.009 0.00 0.00
A
ATOM 16490 HD13 LEU A 205 −34.072 48.390 70.486 0.00 0.00
A
ATOM 16491 CD2 LEU A 205 −30.994 47.269 69.375 0.00 0.00
A
ATOM 16492 HD21 LEU A 205 −31.173 47.550 68.315 0.00 0.00
A
ATOM 16493 HD22 LEU A 205 −30.472 48.078 69.929 0.00 0.00
A
ATOM 16494 HD23 LEU A 205 −30.480 46.287 69.461 0.00 0.00
A
ATOM 16495 C LEU A 205 −33.286 44.256 71.092 0.00 0.00
A
ATOM 16496 O LEU A 205 −33.194 43.680 70.013 0.00 0.00
A

ATOM 16497 N VAL A 206 −34.406 44.129 71.831 0.00 0.00
A
ATOM 16498 HN VAL A 206 −34.583 44.574 72.705 0.00 0.00
A
ATOM 16499 CA VAL A 206 −35.494 43.306 71.479 0.00 0.00
A
ATOM 16500 HA VAL A 206 −35.176 42.719 70.630 0.00 0.00
A
ATOM 16501 CB VAL A 206 −35.845 42.384 72.615 0.00 0.00
A
ATOM 16502 HB VAL A 206 −36.209 42.879 73.541 0.00 0.00
A
ATOM 16503 CG1 VAL A 206 −36.759 41.300 72.093 0.00 0.00
A
ATOM 16504 HG11 VAL A 206 −36.937 40.423 72.752 0.00 0.00
A
ATOM 16505 HG12 VAL A 206 −37.733 41.813 71.947 0.00 0.00
A
ATOM 16506 HG13 VAL A 206 −36.427 40.884 71.118 0.00 0.00
A
ATOM 16507 CG2 VAL A 206 −34.579 41.673 73.057 0.00 0.00
A
ATOM 16508 HG21 VAL A 206 −34.742 40.800 73.724 0.00 0.00
A
ATOM 16509 HG22 VAL A 206 −34.158 41.225 72.131 0.00 0.00
A
ATOM 16510 HG23 VAL A 206 −33.844 42.406 73.452 0.00 0.00
A
ATOM 16511 C VAL A 206 −36.736 44.074 71.114 0.00 0.00
A
ATOM 16512 O VAL A 206 −37.334 44.720 72.012 0.00 0.00
A
ATOM 16513 N ILE A 207 −37.159 44.044 69.851 0.00 0.00
A
ATOM 16514 HN ILE A 207 −36.610 43.536 69.192 0.00 0.00
A
ATOM 16515 CA ILE A 207 −38.221 44.837 69.349 0.00 0.00
A
ATOM 16516 HA ILE A 207 −38.621 45.485 70.115 0.00 0.00
A
ATOM 16517 CB ILE A 207 −37.835 45.742 68.166 0.00 0.00
A
ATOM 16518 HB ILE A 207 −37.413 45.068 67.389 0.00 0.00
A
ATOM 16519 CG2 ILE A 207 −39.075 46.370 67.499 0.00 0.00
A
ATOM 16520 HG21 ILE A 207 −38.704 47.138 66.787 0.00 0.00
A
ATOM 16521 HG22 ILE A 207 −39.781 45.690 66.976 0.00 0.00
A
ATOM 16522 HG23 ILE A 207 −39.704 46.881 68.259 0.00 0.00
A
ATOM 16523 CG1 ILE A 207 −36.882 46.795 68.588 0.00 0.00
A

ATOM 16524 HG11 ILE A 207   -37.444 47.707 68.881 0.00 0.00
A
ATOM 16525 HG12 ILE A 207   -36.159 46.406 69.336 0.00 0.00
A
ATOM 16526 CD ILE A 207   -35.925 47.104 67.468 0.00 0.00
A
ATOM 16527 HD1 ILE A 207   -35.098 47.762 67.811 0.00 0.00
A
ATOM 16528 HD2 ILE A 207   -35.541 46.161 67.023 0.00 0.00
A
ATOM 16529 HD3 ILE A 207   -36.370 47.694 66.638 0.00 0.00
A
ATOM 16530 C   ILE A 207   -39.323 43.955 68.915 0.00 0.00
A
ATOM 16531 O   ILE A 207   -39.141 42.852 68.392 0.00 0.00
A
ATOM 16532 N   MET A 208   -40.555 44.307 69.189 0.00 0.00
A
ATOM 16533 HN MET A 208   -40.683 45.188 69.639 0.00 0.00
A
ATOM 16534 CA MET A 208   -41.809 43.648 68.827 0.00 0.00
A
ATOM 16535 HA MET A 208   -41.649 42.789 68.192 0.00 0.00
A
ATOM 16536 CB MET A 208   -42.675 43.301 70.163 0.00 0.00
A
ATOM 16537 HB1 MET A 208   -42.056 42.577 70.735 0.00 0.00
A
ATOM 16538 HB2 MET A 208   -42.760 44.190 70.824 0.00 0.00
A
ATOM 16539 CG MET A 208   -44.116 42.770 69.934 0.00 0.00
A
ATOM 16540 HG1 MET A 208   -44.903 43.499 69.644 0.00 0.00
A
ATOM 16541 HG2 MET A 208   -44.070 41.920 69.220 0.00 0.00
A
ATOM 16542 SD MET A 208   -44.755 42.104 71.494 0.00 0.00
A
ATOM 16543 CE MET A 208   -46.322 41.400 70.899 0.00 0.00
A
ATOM 16544 HE1 MET A 208   -46.199 40.383 70.469 0.00 0.00
A
ATOM 16545 HE2 MET A 208   -47.050 41.288 71.731 0.00 0.00
A
ATOM 16546 HE3 MET A 208   -46.788 42.109 70.182 0.00 0.00
A
ATOM 16547 C   MET A 208   -42.672 44.567 68.091 0.00 0.00
A
ATOM 16548 O   MET A 208   -42.763 45.741 68.421 0.00 0.00
A
ATOM 16549 N   GLY A 209   -43.243 44.027 67.109 0.00 0.00
A
ATOM 16550 HN GLY A 209   -43.102 43.066 66.885 0.00 0.00
A

ATOM 16551 CA GLY A 209 −44.318 44.663 66.375 0.00 0.00
A
ATOM 16552 HA1 GLY A 209 −43.998 45.322 65.582 0.00 0.00
A
ATOM 16553 HA2 GLY A 209 −45.010 45.185 67.020 0.00 0.00
A
ATOM 16554 C GLY A 209 −45.250 43.609 65.783 0.00 0.00
A
ATOM 16555 O GLY A 209 −45.168 42.403 65.986 0.00 0.00
A
ATOM 16556 N HSE A 210 −46.229 44.031 64.895 0.00 0.00
A
ATOM 16557 HN HSE A 210 −46.406 44.987 64.678 0.00 0.00
A
ATOM 16558 CA HSE A 210 −46.929 43.051 64.044 0.00 0.00
A
ATOM 16559 HA HSE A 210 −47.094 42.132 64.585 0.00 0.00
A
ATOM 16560 CB HSE A 210 −48.289 43.519 63.529 0.00 0.00
A
ATOM 16561 HB1 HSE A 210 −48.714 43.516 64.555 0.00 0.00
A
ATOM 16562 HB2 HSE A 210 −48.236 44.576 63.192 0.00 0.00
A
ATOM 16563 ND1 HSE A 210 −49.029 42.543 61.365 0.00 0.00
A
ATOM 16564 CG HSE A 210 −49.067 42.546 62.762 0.00 0.00
A
ATOM 16565 CE1 HSE A 210 −49.947 41.654 61.023 0.00 0.00
A
ATOM 16566 HE1 HSE A 210 −50.244 41.279 60.043 0.00 0.00
A
ATOM 16567 NE2 HSE A 210 −50.512 41.145 62.171 0.00 0.00
A
ATOM 16568 HE2 HSE A 210 −51.396 40.697 62.301 0.00 0.00
A
ATOM 16569 CD2 HSE A 210 −49.933 41.651 63.303 0.00 0.00
A
ATOM 16570 HD2 HSE A 210 −50.100 41.449 64.354 0.00 0.00
A
ATOM 16571 C HSE A 210 −46.064 42.688 62.911 0.00 0.00
A
ATOM 16572 O HSE A 210 −45.123 43.425 62.593 0.00 0.00
A
ATOM 16573 N LYS A 211 −46.250 41.530 62.299 0.00 0.00
A
ATOM 16574 HN LYS A 211 −46.943 40.941 62.708 0.00 0.00
A
ATOM 16575 CA LYS A 211 −45.513 41.013 61.197 0.00 0.00
A
ATOM 16576 HA LYS A 211 −44.489 40.777 61.447 0.00 0.00
A
ATOM 16577 CB LYS A 211 −46.177 39.691 60.727 0.00 0.00
A

ATOM 16578 HB1 LYS A 211 -46.241 38.965 61.565 0.00 0.00
A
ATOM 16579 HB2 LYS A 211 -47.231 39.863 60.421 0.00 0.00
A
ATOM 16580 CG LYS A 211 -45.482 39.074 59.460 0.00 0.00
A
ATOM 16581 HG1 LYS A 211 -45.808 39.503 58.489 0.00 0.00
A
ATOM 16582 HG2 LYS A 211 -44.434 39.387 59.656 0.00 0.00
A
ATOM 16583 CD LYS A 211 -45.562 37.498 59.440 0.00 0.00
A
ATOM 16584 HD1 LYS A 211 -45.183 36.985 60.349 0.00 0.00
A
ATOM 16585 HD2 LYS A 211 -46.634 37.217 59.514 0.00 0.00
A
ATOM 16586 CE LYS A 211 -44.773 36.782 58.299 0.00 0.00
A
ATOM 16587 HE1 LYS A 211 -45.120 37.154 57.311 0.00 0.00
A
ATOM 16588 HE2 LYS A 211 -43.707 37.068 58.425 0.00 0.00
A
ATOM 16589 NZ LYS A 211 -45.083 35.311 58.312 0.00 0.00
A
ATOM 16590 HZ1 LYS A 211 -46.053 35.048 58.046 0.00 0.00
A
ATOM 16591 HZ2 LYS A 211 -44.408 34.880 57.648 0.00 0.00
A
ATOM 16592 HZ3 LYS A 211 -44.845 34.948 59.257 0.00 0.00
A
ATOM 16593 C LYS A 211 -45.322 41.960 60.028 0.00 0.00
A
ATOM 16594 O LYS A 211 -44.216 42.141 59.519 0.00 0.00
A
ATOM 16595 N LYS A 212 -46.386 42.767 59.663 0.00 0.00
A
ATOM 16596 HN LYS A 212 -47.300 42.589 60.019 0.00 0.00
A
ATOM 16597 CA LYS A 212 -46.211 43.702 58.503 0.00 0.00
A
ATOM 16598 HA LYS A 212 -45.596 43.245 57.741 0.00 0.00
A
ATOM 16599 CB LYS A 212 -47.617 44.112 58.048 0.00 0.00
A
ATOM 16600 HB1 LYS A 212 -48.203 44.635 58.834 0.00 0.00
A
ATOM 16601 HB2 LYS A 212 -47.479 44.855 57.233 0.00 0.00
A
ATOM 16602 CG LYS A 212 -48.577 42.925 57.586 0.00 0.00
A
ATOM 16603 HG1 LYS A 212 -47.932 42.315 56.919 0.00 0.00
A
ATOM 16604 HG2 LYS A 212 -48.826 42.475 58.571 0.00 0.00
A

ATOM 16605 CD LYS A 212 −49.896 43.429 56.854 0.00 0.00
A
ATOM 16606 HD1 LYS A 212 −50.038 44.333 57.485 0.00 0.00
A
ATOM 16607 HD2 LYS A 212 −49.741 43.786 55.814 0.00 0.00
A
ATOM 16608 CE LYS A 212 −51.149 42.574 57.063 0.00 0.00
A
ATOM 16609 HE1 LYS A 212 −51.462 42.516 58.127 0.00 0.00
A
ATOM 16610 HE2 LYS A 212 −52.044 42.979 56.543 0.00 0.00
A
ATOM 16611 NZ LYS A 212 −50.998 41.167 56.718 0.00 0.00
A
ATOM 16612 HZ1 LYS A 212 −50.907 41.029 55.691 0.00 0.00
A
ATOM 16613 HZ2 LYS A 212 −50.248 40.663 57.233 0.00 0.00
A
ATOM 16614 HZ3 LYS A 212 −51.815 40.610 57.041 0.00 0.00
A
ATOM 16615 C LYS A 212 −45.432 44.998 58.816 0.00 0.00
A
ATOM 16616 O LYS A 212 −44.702 45.639 58.007 0.00 0.00
A
ATOM 16617 N ASP A 213 −45.550 45.457 60.096 0.00 0.00
A
ATOM 16618 HN ASP A 213 −46.171 44.927 60.667 0.00 0.00
A
ATOM 16619 CA ASP A 213 −44.877 46.587 60.677 0.00 0.00
A
ATOM 16620 HA ASP A 213 −45.081 47.403 60.000 0.00 0.00
A
ATOM 16621 CB ASP A 213 −45.492 46.902 62.060 0.00 0.00
A
ATOM 16622 HB1 ASP A 213 −45.592 46.053 62.769 0.00 0.00
A
ATOM 16623 HB2 ASP A 213 −44.865 47.702 62.508 0.00 0.00
A
ATOM 16624 CG ASP A 213 −46.829 47.537 62.026 0.00 0.00
A
ATOM 16625 OD1 ASP A 213 −47.760 47.330 62.844 0.00 0.00
A
ATOM 16626 OD2 ASP A 213 −47.043 48.425 61.137 0.00 0.00
A
ATOM 16627 C ASP A 213 −43.416 46.375 60.829 0.00 0.00
A
ATOM 16628 O ASP A 213 −42.628 47.281 60.616 0.00 0.00
A
ATOM 16629 N ILE A 214 −43.026 45.042 61.132 0.00 0.00
A
ATOM 16630 HN ILE A 214 −43.610 44.273 61.383 0.00 0.00
A
ATOM 16631 CA ILE A 214 −41.644 44.727 61.148 0.00 0.00
A

ATOM 16632 HA ILE A 214 −41.119 45.565 61.582 0.00 0.00
A
ATOM 16633 CB ILE A 214 −41.372 43.556 62.104 0.00 0.00
A
ATOM 16634 HB ILE A 214 −42.234 42.856 62.083 0.00 0.00
A
ATOM 16635 CG2 ILE A 214 −40.210 42.764 61.688 0.00 0.00
A
ATOM 16636 HG21 ILE A 214 −40.314 42.319 60.675 0.00 0.00
A
ATOM 16637 HG22 ILE A 214 −39.181 43.179 61.649 0.00 0.00
A
ATOM 16638 HG23 ILE A 214 −40.047 41.890 62.355 0.00 0.00
A
ATOM 16639 CG1 ILE A 214 −41.217 44.029 63.572 0.00 0.00
A
ATOM 16640 HG11 ILE A 214 −42.212 44.452 63.829 0.00 0.00
A
ATOM 16641 HG12 ILE A 214 −41.082 43.142 64.227 0.00 0.00
A
ATOM 16642 CD ILE A 214 −40.031 44.992 63.890 0.00 0.00
A
ATOM 16643 HD1 ILE A 214 −40.204 45.882 63.248 0.00 0.00
A
ATOM 16644 HD2 ILE A 214 −40.230 45.359 64.920 0.00 0.00
A
ATOM 16645 HD3 ILE A 214 −39.009 44.617 63.668 0.00 0.00
A
ATOM 16646 C ILE A 214 −41.075 44.700 59.802 0.00 0.00
A
ATOM 16647 O ILE A 214 −40.032 45.297 59.502 0.00 0.00
A
ATOM 16648 N LYS A 215 −41.868 44.197 58.860 0.00 0.00
A
ATOM 16649 HN LYS A 215 −42.752 43.818 59.122 0.00 0.00
A
ATOM 16650 CA LYS A 215 −41.458 44.324 57.433 0.00 0.00
A
ATOM 16651 HA LYS A 215 −40.548 43.742 57.438 0.00 0.00
A
ATOM 16652 CB LYS A 215 −42.345 43.486 56.527 0.00 0.00
A
ATOM 16653 HB1 LYS A 215 −42.537 42.458 56.903 0.00 0.00
A
ATOM 16654 HB2 LYS A 215 −43.340 43.981 56.523 0.00 0.00
A
ATOM 16655 CG LYS A 215 −41.888 43.480 55.065 0.00 0.00
A
ATOM 16656 HG1 LYS A 215 −42.083 44.468 54.594 0.00 0.00
A
ATOM 16657 HG2 LYS A 215 −40.811 43.257 54.909 0.00 0.00
A
ATOM 16658 CD LYS A 215 −42.643 42.576 54.192 0.00 0.00
A

ATOM	16659	HD1	LYS	A	215	−42.759	41.567	54.642	0.00	0.00	A
ATOM	16660	HD2	LYS	A	215	−43.669	42.924	53.946	0.00	0.00	A
ATOM	16661	CE	LYS	A	215	−41.994	42.490	52.787	0.00	0.00	A
ATOM	16662	HE1	LYS	A	215	−42.591	41.881	52.074	0.00	0.00	A
ATOM	16663	HE2	LYS	A	215	−41.882	43.506	52.351	0.00	0.00	A
ATOM	16664	NZ	LYS	A	215	−40.707	41.785	52.978	0.00	0.00	A
ATOM	16665	HZ1	LYS	A	215	−40.672	41.075	53.737	0.00	0.00	A
ATOM	16666	HZ2	LYS	A	215	−40.471	41.204	52.148	0.00	0.00	A
ATOM	16667	HZ3	LYS	A	215	−39.861	42.388	53.031	0.00	0.00	A
ATOM	16668	C	LYS	A	215	−41.223	45.805	56.955	0.00	0.00	A
ATOM	16669	O	LYS	A	215	−40.181	46.093	56.345	0.00	0.00	A
ATOM	16670	N	ARG	A	216	−42.195	46.664	57.263	0.00	0.00	A
ATOM	16671	HN	ARG	A	216	−43.076	46.434	57.669	0.00	0.00	A
ATOM	16672	CA	ARG	A	216	−42.169	48.031	56.873	0.00	0.00	A
ATOM	16673	HA	ARG	A	216	−42.254	48.147	55.803	0.00	0.00	A
ATOM	16674	CB	ARG	A	216	−43.465	48.750	57.377	0.00	0.00	A
ATOM	16675	HB1	ARG	A	216	−44.274	48.363	56.722	0.00	0.00	A
ATOM	16676	HB2	ARG	A	216	−43.758	48.396	58.389	0.00	0.00	A
ATOM	16677	CG	ARG	A	216	−43.465	50.335	57.367	0.00	0.00	A
ATOM	16678	HG1	ARG	A	216	−42.778	50.745	58.137	0.00	0.00	A
ATOM	16679	HG2	ARG	A	216	−43.149	50.802	56.410	0.00	0.00	A
ATOM	16680	CD	ARG	A	216	−44.901	50.948	57.526	0.00	0.00	A
ATOM	16681	HD1	ARG	A	216	−44.839	52.056	57.466	0.00	0.00	A
ATOM	16682	HD2	ARG	A	216	−45.627	50.513	56.806	0.00	0.00	A
ATOM	16683	NE	ARG	A	216	−45.225	50.577	58.856	0.00	0.00	A
ATOM	16684	HE	ARG	A	216	−45.726	49.721	58.984	0.00	0.00	A
ATOM	16685	CZ	ARG	A	216	−44.956	51.232	59.995	0.00	0.00	A

ATOM 16686 NH1 ARG A 216 −44.376 52.448 60.075 0.00 0.00
A
ATOM 16687 HH11 ARG A 216 −44.512 53.022 60.883 0.00 0.00
A
ATOM 16688 HH12 ARG A 216 −44.329 53.071 59.294 0.00 0.00
A
ATOM 16689 NH2 ARG A 216 −45.445 50.752 61.111 0.00 0.00
A
ATOM 16690 HH21 ARG A 216 −45.309 51.232 61.977 0.00 0.00
A
ATOM 16691 HH22 ARG A 216 −45.833 49.831 61.140 0.00 0.00
A
ATOM 16692 C ARG A 216 −40.912 48.722 57.309 0.00 0.00
A
ATOM 16693 O ARG A 216 −40.243 49.379 56.519 0.00 0.00
A
ATOM 16694 N PHE A 217 −40.534 48.548 58.565 0.00 0.00
A
ATOM 16695 HN PHE A 217 −41.187 47.985 59.065 0.00 0.00
A
ATOM 16696 CA PHE A 217 −39.375 48.977 59.385 0.00 0.00
A
ATOM 16697 HA PHE A 217 −39.278 50.040 59.217 0.00 0.00
A
ATOM 16698 CB PHE A 217 −39.673 48.740 60.875 0.00 0.00
A
ATOM 16699 HB1 PHE A 217 −40.735 49.011 61.056 0.00 0.00
A
ATOM 16700 HB2 PHE A 217 −39.641 47.657 61.121 0.00 0.00
A
ATOM 16701 CG PHE A 217 −38.654 49.423 61.835 0.00 0.00
A
ATOM 16702 CD1 PHE A 217 −38.155 50.764 61.609 0.00 0.00
A
ATOM 16703 HD1 PHE A 217 −38.371 51.227 60.657 0.00 0.00
A
ATOM 16704 CE1 PHE A 217 −37.241 51.272 62.552 0.00 0.00
A
ATOM 16705 HE1 PHE A 217 −36.905 52.287 62.404 0.00 0.00
A
ATOM 16706 CZ PHE A 217 −36.889 50.564 63.720 0.00 0.00
A
ATOM 16707 HZ PHE A 217 −36.314 51.123 64.445 0.00 0.00
A
ATOM 16708 CD2 PHE A 217 −38.329 48.761 63.073 0.00 0.00
A
ATOM 16709 HD2 PHE A 217 −38.644 47.743 63.253 0.00 0.00
A
ATOM 16710 CE2 PHE A 217 −37.452 49.331 63.928 0.00 0.00
A
ATOM 16711 HE2 PHE A 217 −37.193 48.874 64.872 0.00 0.00
A
ATOM 16712 C PHE A 217 −38.165 48.255 58.840 0.00 0.00
A

ATOM 16713 O PHE A 217 -37.077 48.818 58.770 0.00 0.00
A
ATOM 16714 N GLU A 218 -38.272 47.019 58.312 0.00 0.00
A
ATOM 16715 HN GLU A 218 -39.014 46.365 58.439 0.00 0.00
A
ATOM 16716 CA GLU A 218 -37.139 46.355 57.650 0.00 0.00
A
ATOM 16717 HA GLU A 218 -36.238 46.468 58.233 0.00 0.00
A
ATOM 16718 CB GLU A 218 -37.324 44.801 57.437 0.00 0.00
A
ATOM 16719 HB1 GLU A 218 -37.005 44.367 58.409 0.00 0.00
A
ATOM 16720 HB2 GLU A 218 -38.358 44.594 57.088 0.00 0.00
A
ATOM 16721 CG GLU A 218 -36.344 44.101 56.439 0.00 0.00
A
ATOM 16722 HG1 GLU A 218 -36.520 44.440 55.396 0.00 0.00
A
ATOM 16723 HG2 GLU A 218 -35.319 44.257 56.839 0.00 0.00
A
ATOM 16724 CD GLU A 218 -36.621 42.588 56.478 0.00 0.00
A
ATOM 16725 OE1 GLU A 218 -35.779 41.789 56.955 0.00 0.00
A
ATOM 16726 OE2 GLU A 218 -37.642 42.184 55.853 0.00 0.00
A
ATOM 16727 C GLU A 218 -36.820 46.907 56.210 0.00 0.00
A
ATOM 16728 O GLU A 218 -35.664 47.210 55.909 0.00 0.00
A
ATOM 16729 N ASN A 219 -37.864 47.290 55.420 0.00 0.00
A
ATOM 16730 HN ASN A 219 -38.753 46.897 55.644 0.00 0.00
A
ATOM 16731 CA ASN A 219 -37.664 48.121 54.216 0.00 0.00
A
ATOM 16732 HA ASN A 219 -36.946 47.619 53.585 0.00 0.00
A
ATOM 16733 CB ASN A 219 -39.106 48.253 53.566 0.00 0.00
A
ATOM 16734 HB1 ASN A 219 -39.957 48.511 54.231 0.00 0.00
A
ATOM 16735 HB2 ASN A 219 -39.041 49.073 52.818 0.00 0.00
A
ATOM 16736 CG ASN A 219 -39.440 46.851 52.997 0.00 0.00
A
ATOM 16737 OD1 ASN A 219 -38.593 46.073 52.538 0.00 0.00
A
ATOM 16738 ND2 ASN A 219 -40.748 46.515 52.992 0.00 0.00
A
ATOM 16739 HD21 ASN A 219 -41.022 45.632 52.610 0.00 0.00
A

ATOM 16740 HD22 ASN A 219 −41.425 47.164 53.337 0.00 0.00
A
ATOM 16741 C ASN A 219 −37.009 49.513 54.486 0.00 0.00
A
ATOM 16742 O ASN A 219 −36.255 50.010 53.654 0.00 0.00
A
ATOM 16743 N GLU A 220 −37.346 50.129 55.626 0.00 0.00
A
ATOM 16744 HN GLU A 220 −37.998 49.699 56.247 0.00 0.00
A
ATOM 16745 CA GLU A 220 −36.767 51.400 56.059 0.00 0.00
A
ATOM 16746 HA GLU A 220 −36.760 52.101 55.238 0.00 0.00
A
ATOM 16747 CB GLU A 220 −37.462 51.839 57.365 0.00 0.00
A
ATOM 16748 HB1 GLU A 220 −38.542 51.862 57.103 0.00 0.00
A
ATOM 16749 HB2 GLU A 220 −37.344 51.098 58.184 0.00 0.00
A
ATOM 16750 CG GLU A 220 −36.975 53.186 57.789 0.00 0.00
A
ATOM 16751 HG1 GLU A 220 −35.903 53.225 58.080 0.00 0.00
A
ATOM 16752 HG2 GLU A 220 −37.070 53.959 56.997 0.00 0.00
A
ATOM 16753 CD GLU A 220 −37.793 53.642 59.004 0.00 0.00
A
ATOM 16754 OE1 GLU A 220 −39.012 53.880 58.832 0.00 0.00
A
ATOM 16755 OE2 GLU A 220 −37.271 53.777 60.154 0.00 0.00
A
ATOM 16756 C GLU A 220 −35.214 51.168 56.315 0.00 0.00
A
ATOM 16757 O GLU A 220 −34.353 51.881 55.785 0.00 0.00
A
ATOM 16758 N GLY A 221 −34.870 50.135 57.048 0.00 0.00
A
ATOM 16759 HN GLY A 221 −35.568 49.642 57.560 0.00 0.00
A
ATOM 16760 CA GLY A 221 −33.523 49.744 57.356 0.00 0.00
A
ATOM 16761 HA1 GLY A 221 −32.776 50.350 56.865 0.00 0.00
A
ATOM 16762 HA2 GLY A 221 −33.493 48.751 56.933 0.00 0.00
A
ATOM 16763 C GLY A 221 −33.215 49.754 58.837 0.00 0.00
A
ATOM 16764 O GLY A 221 −32.512 50.649 59.353 0.00 0.00
A
ATOM 16765 N MET A 222 −33.747 48.827 59.524 0.00 0.00
A
ATOM 16766 HN MET A 222 −34.351 48.111 59.182 0.00 0.00
A

ATOM 16767 CA MET A 222 −33.768 48.925 60.945 0.00 0.00
A
ATOM 16768 HA MET A 222 −33.961 49.947 61.234 0.00 0.00
A
ATOM 16769 CB MET A 222 −34.924 48.054 61.432 0.00 0.00
A
ATOM 16770 HB1 MET A 222 −34.965 48.059 62.543 0.00 0.00
A
ATOM 16771 HB2 MET A 222 −35.819 48.604 61.072 0.00 0.00
A
ATOM 16772 CG MET A 222 −34.851 46.593 60.968 0.00 0.00
A
ATOM 16773 HG1 MET A 222 −35.383 46.512 59.996 0.00 0.00
A
ATOM 16774 HG2 MET A 222 −33.817 46.364 60.633 0.00 0.00
A
ATOM 16775 SD MET A 222 −35.400 45.310 62.199 0.00 0.00
A
ATOM 16776 CE MET A 222 −35.897 43.904 61.198 0.00 0.00
A
ATOM 16777 HE1 MET A 222 −36.777 44.239 60.608 0.00 0.00
A
ATOM 16778 HE2 MET A 222 −35.072 43.532 60.554 0.00 0.00
A
ATOM 16779 HE3 MET A 222 −36.319 43.067 61.795 0.00 0.00
A
ATOM 16780 C MET A 222 −32.404 48.495 61.558 0.00 0.00
A
ATOM 16781 O MET A 222 −31.330 48.579 60.970 0.00 0.00
A
ATOM 16782 N GLN B 8 −23.060 39.935 45.577 0.00 0.00
B
ATOM 16783 HT1 GLN B 8 −23.905 40.455 45.267 0.00 0.00
B
ATOM 16784 HT2 GLN B 8 −23.277 39.016 45.142 0.00 0.00
B
ATOM 16785 HT3 GLN B 8 −22.204 40.424 45.246 0.00 0.00
B
ATOM 16786 CA GLN B 8 −23.090 39.921 47.051 0.00 0.00
B
ATOM 16787 HA GLN B 8 −22.534 40.799 47.344 0.00 0.00
B
ATOM 16788 CB GLN B 8 −24.530 40.144 47.592 0.00 0.00
B
ATOM 16789 HB1 GLN B 8 −24.984 40.942 46.967 0.00 0.00
B
ATOM 16790 HB2 GLN B 8 −25.254 39.374 47.248 0.00 0.00
B
ATOM 16791 CG GLN B 8 −24.707 40.470 49.107 0.00 0.00
B
ATOM 16792 HG1 GLN B 8 −25.761 40.447 49.458 0.00 0.00
B
ATOM 16793 HG2 GLN B 8 −24.058 39.796 49.707 0.00 0.00
B

ATOM 16794 CD GLN B 8 −24.369 41.923 49.389 0.00 0.00
B
ATOM 16795 OE1 GLN B 8 −23.740 42.704 48.657 0.00 0.00
B
ATOM 16796 NE2 GLN B 8 −24.759 42.325 50.614 0.00 0.00
B
ATOM 16797 HE21 GLN B 8 −24.268 43.083 51.044 0.00 0.00
B
ATOM 16798 HE22 GLN B 8 −25.661 42.052 50.948 0.00 0.00
B
ATOM 16799 C GLN B 8 −22.296 38.815 47.784 0.00 0.00
B
ATOM 16800 O GLN B 8 −21.383 39.103 48.561 0.00 0.00
B
ATOM 16801 N PHE B 9 −22.648 37.542 47.585 0.00 0.00
B
ATOM 16802 HN PHE B 9 −23.282 37.247 46.874 0.00 0.00
B
ATOM 16803 CA PHE B 9 −22.112 36.454 48.375 0.00 0.00
B
ATOM 16804 HA PHE B 9 −21.425 36.826 49.120 0.00 0.00
B
ATOM 16805 CB PHE B 9 −23.271 35.659 49.100 0.00 0.00
B
ATOM 16806 HB1 PHE B 9 −23.969 35.300 48.313 0.00 0.00
B
ATOM 16807 HB2 PHE B 9 −22.836 34.840 49.711 0.00 0.00
B
ATOM 16808 CG PHE B 9 −24.153 36.469 50.000 0.00 0.00
B
ATOM 16809 CD1 PHE B 9 −23.616 37.528 50.836 0.00 0.00
B
ATOM 16810 HD1 PHE B 9 −22.573 37.806 50.795 0.00 0.00
B
ATOM 16811 CE1 PHE B 9 −24.439 38.179 51.764 0.00 0.00
B
ATOM 16812 HE1 PHE B 9 −23.985 38.927 52.397 0.00 0.00
B
ATOM 16813 CZ PHE B 9 −25.751 37.893 51.856 0.00 0.00
B
ATOM 16814 HZ PHE B 9 −26.330 38.522 52.516 0.00 0.00
B
ATOM 16815 CD2 PHE B 9 −25.520 36.127 50.298 0.00 0.00
B
ATOM 16816 HD2 PHE B 9 −25.982 35.306 49.771 0.00 0.00
B
ATOM 16817 CE2 PHE B 9 −26.350 36.895 51.100 0.00 0.00
B
ATOM 16818 HE2 PHE B 9 −27.400 36.680 51.232 0.00 0.00
B
ATOM 16819 C PHE B 9 −21.357 35.459 47.406 0.00 0.00
B
ATOM 16820 O PHE B 9 −21.978 35.034 46.434 0.00 0.00
B

ATOM 16821 N ALA B 10 −20.110 35.107 47.653 0.00 0.00
B
ATOM 16822 HN ALA B 10 −19.681 35.724 48.309 0.00 0.00
B
ATOM 16823 CA ALA B 10 −19.323 34.022 47.052 0.00 0.00
B
ATOM 16824 HA ALA B 10 −20.029 33.630 46.335 0.00 0.00
B
ATOM 16825 CB ALA B 10 −18.023 34.556 46.403 0.00 0.00
B
ATOM 16826 HB1 ALA B 10 −18.255 35.079 45.450 0.00 0.00
B
ATOM 16827 HB2 ALA B 10 −17.598 35.342 47.064 0.00 0.00
B
ATOM 16828 HB3 ALA B 10 −17.372 33.710 46.095 0.00 0.00
B
ATOM 16829 C ALA B 10 −18.971 32.903 48.030 0.00 0.00
B
ATOM 16830 O ALA B 10 −18.668 33.011 49.196 0.00 0.00
B
ATOM 16831 N VAL B 11 −19.049 31.698 47.444 0.00 0.00
B
ATOM 16832 HN VAL B 11 −19.257 31.573 46.477 0.00 0.00
B
ATOM 16833 CA VAL B 11 −18.960 30.429 48.168 0.00 0.00
B
ATOM 16834 HA VAL B 11 −18.536 30.526 49.157 0.00 0.00
B
ATOM 16835 CB VAL B 11 −20.261 29.688 48.248 0.00 0.00
B
ATOM 16836 HB VAL B 11 −20.484 29.381 47.204 0.00 0.00
B
ATOM 16837 CG1 VAL B 11 −20.051 28.450 49.151 0.00 0.00
B
ATOM 16838 HG11 VAL B 11 −19.142 27.928 48.781 0.00 0.00
B
ATOM 16839 HG12 VAL B 11 −19.842 28.703 50.212 0.00 0.00
B
ATOM 16840 HG13 VAL B 11 −20.843 27.672 49.100 0.00 0.00
B
ATOM 16841 CG2 VAL B 11 −21.378 30.647 48.805 0.00 0.00
B
ATOM 16842 HG21 VAL B 11 −22.300 30.076 49.047 0.00 0.00
B
ATOM 16843 HG22 VAL B 11 −20.979 30.977 49.788 0.00 0.00
B
ATOM 16844 HG23 VAL B 11 −21.593 31.437 48.053 0.00 0.00
B
ATOM 16845 C VAL B 11 −17.961 29.549 47.412 0.00 0.00
B
ATOM 16846 O VAL B 11 −18.189 29.269 46.248 0.00 0.00
B
ATOM 16847 N ILE B 12 −16.920 29.015 48.014 0.00 0.00
B

ATOM 16848 HN ILE B 12 −16.841 29.133 49.000 0.00 0.00
B
ATOM 16849 CA ILE B 12 −15.708 28.463 47.327 0.00 0.00
B
ATOM 16850 HA ILE B 12 −15.926 28.255 46.290 0.00 0.00
B
ATOM 16851 CB ILE B 12 −14.437 29.294 47.396 0.00 0.00
B
ATOM 16852 HB ILE B 12 −14.073 29.543 48.416 0.00 0.00
B
ATOM 16853 CG2 ILE B 12 −13.331 28.618 46.617 0.00 0.00
B
ATOM 16854 HG21 ILE B 12 −12.361 29.157 46.573 0.00 0.00
B
ATOM 16855 HG22 ILE B 12 −13.125 27.572 46.930 0.00 0.00
B
ATOM 16856 HG23 ILE B 12 −13.638 28.534 45.552 0.00 0.00
B
ATOM 16857 CG1 ILE B 12 −14.746 30.692 46.810 0.00 0.00
B
ATOM 16858 HG11 ILE B 12 −15.318 30.612 45.861 0.00 0.00
B
ATOM 16859 HG12 ILE B 12 −15.402 31.231 47.526 0.00 0.00
B
ATOM 16860 CD ILE B 12 −13.553 31.690 46.583 0.00 0.00
B
ATOM 16861 HD1 ILE B 12 −13.894 32.534 45.946 0.00 0.00
B
ATOM 16862 HD2 ILE B 12 −13.270 32.151 47.553 0.00 0.00
B
ATOM 16863 HD3 ILE B 12 −12.649 31.290 46.076 0.00 0.00
B
ATOM 16864 C ILE B 12 −15.406 27.095 47.955 0.00 0.00
B
ATOM 16865 O ILE B 12 −14.961 26.898 49.097 0.00 0.00
B
ATOM 16866 N GLY B 13 −15.680 26.058 47.161 0.00 0.00
B
ATOM 16867 HN GLY B 13 −16.108 26.365 46.314 0.00 0.00
B
ATOM 16868 CA GLY B 13 −15.520 24.678 47.505 0.00 0.00
B
ATOM 16869 HA1 GLY B 13 −14.948 24.609 48.419 0.00 0.00
B
ATOM 16870 HA2 GLY B 13 −14.972 24.215 46.698 0.00 0.00
B
ATOM 16871 C GLY B 13 −16.822 24.019 47.741 0.00 0.00
B
ATOM 16872 O GLY B 13 −17.421 23.976 48.801 0.00 0.00
B
ATOM 16873 N LEU B 14 −17.447 23.529 46.647 0.00 0.00
B
ATOM 16874 HN LEU B 14 −16.926 23.487 45.798 0.00 0.00
B

ATOM 16875 CA LEU B 14 −18.859 23.199 46.525 0.00 0.00
B
ATOM 16876 HA LEU B 14 −19.443 23.809 47.198 0.00 0.00
B
ATOM 16877 CB LEU B 14 −19.456 23.480 45.102 0.00 0.00
B
ATOM 16878 HB1 LEU B 14 −19.098 22.814 44.288 0.00 0.00
B
ATOM 16879 HB2 LEU B 14 −20.552 23.315 45.180 0.00 0.00
B
ATOM 16880 CG LEU B 14 −19.374 24.945 44.571 0.00 0.00
B
ATOM 16881 HG LEU B 14 −18.291 25.179 44.488 0.00 0.00
B
ATOM 16882 CD1 LEU B 14 −19.847 24.909 43.174 0.00 0.00
B
ATOM 16883 HD11 LEU B 14 −19.538 25.796 42.580 0.00 0.00
B
ATOM 16884 HD12 LEU B 14 −19.455 24.011 42.650 0.00 0.00
B
ATOM 16885 HD13 LEU B 14 −20.946 24.785 43.072 0.00 0.00
B
ATOM 16886 CD2 LEU B 14 −20.206 25.827 45.460 0.00 0.00
B
ATOM 16887 HD21 LEU B 14 −21.236 25.410 45.453 0.00 0.00
B
ATOM 16888 HD22 LEU B 14 −19.801 26.091 46.461 0.00 0.00
B
ATOM 16889 HD23 LEU B 14 −20.149 26.877 45.102 0.00 0.00
B
ATOM 16890 C LEU B 14 −19.225 21.784 46.917 0.00 0.00
B
ATOM 16891 O LEU B 14 −20.184 21.209 46.465 0.00 0.00
B
ATOM 16892 N GLY B 15 −18.387 21.318 47.827 0.00 0.00
B
ATOM 16893 HN GLY B 15 −17.637 21.826 48.243 0.00 0.00
B
ATOM 16894 CA GLY B 15 −18.640 20.130 48.633 0.00 0.00
B
ATOM 16895 HA1 GLY B 15 −17.782 19.872 49.236 0.00 0.00
B
ATOM 16896 HA2 GLY B 15 −18.784 19.267 47.999 0.00 0.00
B
ATOM 16897 C GLY B 15 −19.942 20.122 49.518 0.00 0.00
B
ATOM 16898 O GLY B 15 −20.887 20.866 49.180 0.00 0.00
B
ATOM 16899 N ARG B 16 −20.082 19.305 50.567 0.00 0.00
B
ATOM 16900 HN ARG B 16 −19.346 18.656 50.743 0.00 0.00
B
ATOM 16901 CA ARG B 16 −21.296 19.216 51.410 0.00 0.00
B

ATOM 16902 HA ARG B 16 -22.132 19.052 50.747 0.00 0.00
B
ATOM 16903 CB ARG B 16 -21.065 18.005 52.350 0.00 0.00
B
ATOM 16904 HB1 ARG B 16 -20.009 18.009 52.695 0.00 0.00
B
ATOM 16905 HB2 ARG B 16 -21.655 18.022 53.291 0.00 0.00
B
ATOM 16906 CG ARG B 16 -21.293 16.707 51.574 0.00 0.00
B
ATOM 16907 HG1 ARG B 16 -22.360 16.517 51.332 0.00 0.00
B
ATOM 16908 HG2 ARG B 16 -20.823 16.632 50.570 0.00 0.00
B
ATOM 16909 CD ARG B 16 -20.743 15.402 52.217 0.00 0.00
B
ATOM 16910 HD1 ARG B 16 -21.141 15.349 53.253 0.00 0.00
B
ATOM 16911 HD2 ARG B 16 -21.110 14.556 51.597 0.00 0.00
B
ATOM 16912 NE ARG B 16 -19.265 15.293 52.146 0.00 0.00
B
ATOM 16913 HE ARG B 16 -18.718 15.996 51.690 0.00 0.00
B
ATOM 16914 CZ ARG B 16 -18.599 14.282 52.686 0.00 0.00
B
ATOM 16915 NH1 ARG B 16 -19.234 13.236 53.234 0.00 0.00
B
ATOM 16916 HH11 ARG B 16 -18.715 12.502 53.672 0.00 0.00
B
ATOM 16917 HH12 ARG B 16 -20.213 13.280 53.435 0.00 0.00
B
ATOM 16918 NH2 ARG B 16 -17.292 14.070 52.386 0.00 0.00
B
ATOM 16919 HH21 ARG B 16 -16.812 13.599 53.126 0.00 0.00
B
ATOM 16920 HH22 ARG B 16 -16.904 14.776 51.795 0.00 0.00
B
ATOM 16921 C ARG B 16 -21.843 20.448 52.101 0.00 0.00
B
ATOM 16922 O ARG B 16 -23.030 20.765 51.944 0.00 0.00
B
ATOM 16923 N PHE B 17 -21.014 21.236 52.743 0.00 0.00
B
ATOM 16924 HN PHE B 17 -20.057 20.958 52.716 0.00 0.00
B
ATOM 16925 CA PHE B 17 -21.306 22.565 53.305 0.00 0.00
B
ATOM 16926 HA PHE B 17 -22.168 22.468 53.948 0.00 0.00
B
ATOM 16927 CB PHE B 17 -20.190 22.827 54.322 0.00 0.00
B
ATOM 16928 HB1 PHE B 17 -20.243 22.037 55.102 0.00 0.00
B

ATOM 16929 HB2 PHE B 17 −19.240 22.645 53.776 0.00 0.00
B
ATOM 16930 CG PHE B 17 −20.198 24.185 54.960 0.00 0.00
B
ATOM 16931 CD1 PHE B 17 −20.746 24.377 56.214 0.00 0.00
B
ATOM 16932 HD1 PHE B 17 −21.455 23.625 56.527 0.00 0.00
B
ATOM 16933 CE1 PHE B 17 −20.413 25.488 56.985 0.00 0.00
B
ATOM 16934 HE1 PHE B 17 −20.976 25.741 57.871 0.00 0.00
B
ATOM 16935 CZ PHE B 17 −19.573 26.456 56.449 0.00 0.00
B
ATOM 16936 HZ PHE B 17 −19.362 27.266 57.131 0.00 0.00
B
ATOM 16937 CD2 PHE B 17 −19.289 25.159 54.447 0.00 0.00
B
ATOM 16938 HD2 PHE B 17 −18.794 24.758 53.574 0.00 0.00
B
ATOM 16939 CE2 PHE B 17 −18.966 26.263 55.146 0.00 0.00
B
ATOM 16940 HE2 PHE B 17 −18.371 27.053 54.713 0.00 0.00
B
ATOM 16941 C PHE B 17 −21.587 23.681 52.294 0.00 0.00
B
ATOM 16942 O PHE B 17 −22.626 24.317 52.336 0.00 0.00
B
ATOM 16943 N GLY B 18 −20.705 23.836 51.300 0.00 0.00
B
ATOM 16944 HN GLY B 18 −19.904 23.243 51.338 0.00 0.00
B
ATOM 16945 CA GLY B 18 −20.776 24.849 50.254 0.00 0.00
B
ATOM 16946 HA1 GLY B 18 −19.847 24.830 49.703 0.00 0.00
B
ATOM 16947 HA2 GLY B 18 −20.892 25.816 50.721 0.00 0.00
B
ATOM 16948 C GLY B 18 −21.914 24.706 49.316 0.00 0.00
B
ATOM 16949 O GLY B 18 −22.554 25.663 48.968 0.00 0.00
B
ATOM 16950 N GLY B 19 −22.197 23.457 48.788 0.00 0.00
B
ATOM 16951 HN GLY B 19 −21.616 22.679 49.012 0.00 0.00
B
ATOM 16952 CA GLY B 19 −23.452 23.262 47.989 0.00 0.00
B
ATOM 16953 HA1 GLY B 19 −23.346 22.274 47.567 0.00 0.00
B
ATOM 16954 HA2 GLY B 19 −23.571 24.013 47.222 0.00 0.00
B
ATOM 16955 C GLY B 19 −24.749 23.314 48.829 0.00 0.00
B

ATOM 16956 O GLY B 19 −25.819 23.415 48.228 0.00 0.00
B
ATOM 16957 N SER B 20 −24.719 23.258 50.143 0.00 0.00
B
ATOM 16958 HN SER B 20 −23.816 23.192 50.562 0.00 0.00
B
ATOM 16959 CA SER B 20 −25.837 23.384 51.058 0.00 0.00
B
ATOM 16960 HA SER B 20 −26.725 22.912 50.665 0.00 0.00
B
ATOM 16961 CB SER B 20 −25.653 22.519 52.338 0.00 0.00
B
ATOM 16962 HB1 SER B 20 −25.724 21.447 52.056 0.00 0.00
B
ATOM 16963 HB2 SER B 20 −24.717 22.791 52.870 0.00 0.00
B
ATOM 16964 OG SER B 20 −26.691 22.827 53.346 0.00 0.00
B
ATOM 16965 HG1 SER B 20 −26.785 22.103 53.969 0.00 0.00
B
ATOM 16966 C SER B 20 −26.155 24.847 51.337 0.00 0.00
B
ATOM 16967 O SER B 20 −27.327 25.260 51.338 0.00 0.00
B
ATOM 16968 N ILE B 21 −25.107 25.690 51.526 0.00 0.00
B
ATOM 16969 HN ILE B 21 −24.205 25.292 51.673 0.00 0.00
B
ATOM 16970 CA ILE B 21 −25.244 27.112 51.574 0.00 0.00
B
ATOM 16971 HA ILE B 21 −26.024 27.374 52.273 0.00 0.00
B
ATOM 16972 CB ILE B 21 −23.911 27.755 51.834 0.00 0.00
B
ATOM 16973 HB ILE B 21 −23.158 27.446 51.078 0.00 0.00
B
ATOM 16974 CG2 ILE B 21 −23.980 29.311 51.829 0.00 0.00
B
ATOM 16975 HG21 ILE B 21 −22.967 29.763 51.890 0.00 0.00
B
ATOM 16976 HG22 ILE B 21 −24.570 29.659 50.954 0.00 0.00
B
ATOM 16977 HG23 ILE B 21 −24.590 29.701 52.672 0.00 0.00
B
ATOM 16978 CG1 ILE B 21 −23.368 27.266 53.221 0.00 0.00
B
ATOM 16979 HG11 ILE B 21 −24.122 27.747 53.880 0.00 0.00
B
ATOM 16980 HG12 ILE B 21 −23.480 26.179 53.426 0.00 0.00
B
ATOM 16981 CD ILE B 21 −21.976 27.855 53.496 0.00 0.00
B
ATOM 16982 HD1 ILE B 21 −22.132 28.944 53.656 0.00 0.00
B

ATOM 16983 HD2 ILE B 21 −21.634 27.393 54.447 0.00 0.00
B
ATOM 16984 HD3 ILE B 21 −21.220 27.681 52.701 0.00 0.00
B
ATOM 16985 C ILE B 21 −25.791 27.685 50.323 0.00 0.00
B
ATOM 16986 O ILE B 21 −26.698 28.496 50.285 0.00 0.00
B
ATOM 16987 N CYS B 22 −25.260 27.359 49.127 0.00 0.00
B
ATOM 16988 HN CYS B 22 −24.418 26.835 49.228 0.00 0.00
B
ATOM 16989 CA CYS B 22 −25.654 27.884 47.815 0.00 0.00
B
ATOM 16990 HA CYS B 22 −25.588 28.962 47.827 0.00 0.00
B
ATOM 16991 CB CYS B 22 −24.932 27.231 46.676 0.00 0.00
B
ATOM 16992 HB1 CYS B 22 −25.039 26.133 46.807 0.00 0.00
B
ATOM 16993 HB2 CYS B 22 −25.326 27.548 45.687 0.00 0.00
B
ATOM 16994 SG CYS B 22 −23.124 27.538 46.620 0.00 0.00
B
ATOM 16995 HG1 CYS B 22 −22.692 26.832 47.655 0.00 0.00
B
ATOM 16996 C CYS B 22 −27.095 27.590 47.490 0.00 0.00
B
ATOM 16997 O CYS B 22 −27.905 28.423 47.211 0.00 0.00
B
ATOM 16998 N LYS B 23 −27.513 26.308 47.717 0.00 0.00
B
ATOM 16999 HN LYS B 23 −26.834 25.593 47.866 0.00 0.00
B
ATOM 17000 CA LYS B 23 −28.872 25.939 47.362 0.00 0.00
B
ATOM 17001 HA LYS B 23 −29.015 26.204 46.325 0.00 0.00
B
ATOM 17002 CB LYS B 23 −29.009 24.385 47.534 0.00 0.00
B
ATOM 17003 HB1 LYS B 23 −28.175 23.973 46.927 0.00 0.00
B
ATOM 17004 HB2 LYS B 23 −29.103 24.068 48.595 0.00 0.00
B
ATOM 17005 CG LYS B 23 −30.328 23.855 46.966 0.00 0.00
B
ATOM 17006 HG1 LYS B 23 −31.171 24.100 47.646 0.00 0.00
B
ATOM 17007 HG2 LYS B 23 −30.525 24.452 46.050 0.00 0.00
B
ATOM 17008 CD LYS B 23 −30.263 22.375 46.764 0.00 0.00
B
ATOM 17009 HD1 LYS B 23 −29.567 22.127 45.934 0.00 0.00
B

ATOM 17010 HD2 LYS B 23 −29.774 22.019 47.696 0.00 0.00
B
ATOM 17011 CE LYS B 23 −31.639 21.682 46.595 0.00 0.00
B
ATOM 17012 HE1 LYS B 23 −32.342 21.774 47.450 0.00 0.00
B
ATOM 17013 HE2 LYS B 23 −32.210 22.067 45.723 0.00 0.00
B
ATOM 17014 NZ LYS B 23 −31.518 20.269 46.346 0.00 0.00
B
ATOM 17015 HZ1 LYS B 23 −30.790 19.894 46.988 0.00 0.00
B
ATOM 17016 HZ2 LYS B 23 −32.430 19.769 46.355 0.00 0.00
B
ATOM 17017 HZ3 LYS B 23 −31.123 20.114 45.396 0.00 0.00
B
ATOM 17018 C LYS B 23 −29.859 26.686 48.312 0.00 0.00
B
ATOM 17019 O LYS B 23 −30.905 27.206 47.853 0.00 0.00
B
ATOM 17020 N GLU B 24 −29.549 26.802 49.579 0.00 0.00
B
ATOM 17021 HN GLU B 24 −28.689 26.372 49.843 0.00 0.00
B
ATOM 17022 CA GLU B 24 −30.409 27.429 50.596 0.00 0.00
B
ATOM 17023 HA GLU B 24 −31.410 27.062 50.422 0.00 0.00
B
ATOM 17024 CB GLU B 24 −30.094 26.993 51.987 0.00 0.00
B
ATOM 17025 HB1 GLU B 24 −30.348 25.911 52.015 0.00 0.00
B
ATOM 17026 HB2 GLU B 24 −29.038 27.255 52.212 0.00 0.00
B
ATOM 17027 CG GLU B 24 −30.965 27.488 53.217 0.00 0.00
B
ATOM 17028 HG1 GLU B 24 −30.468 27.287 54.190 0.00 0.00
B
ATOM 17029 HG2 GLU B 24 −30.983 28.597 53.164 0.00 0.00
B
ATOM 17030 CD GLU B 24 −32.391 26.915 53.170 0.00 0.00
B
ATOM 17031 OE1 GLU B 24 −33.178 27.439 54.005 0.00 0.00
B
ATOM 17032 OE2 GLU B 24 −32.780 26.007 52.410 0.00 0.00
B
ATOM 17033 C GLU B 24 −30.498 28.959 50.428 0.00 0.00
B
ATOM 17034 O GLU B 24 −31.527 29.607 50.532 0.00 0.00
B
ATOM 17035 N LEU B 25 −29.277 29.630 50.227 0.00 0.00
B
ATOM 17036 HN LEU B 25 −28.459 29.069 50.120 0.00 0.00
B

```
ATOM  17037  CA  LEU B  25      -29.203  30.987  49.854  0.00  0.00
B
ATOM  17038  HA  LEU B  25      -29.691  31.518  50.657  0.00  0.00
B
ATOM  17039  CB  LEU B  25      -27.765  31.480  49.726  0.00  0.00
B
ATOM  17040  HB1 LEU B  25      -27.187  30.782  49.083  0.00  0.00
B
ATOM  17041  HB2 LEU B  25      -27.768  32.500  49.285  0.00  0.00
B
ATOM  17042  CG  LEU B  25      -26.905  31.765  51.002  0.00  0.00
B
ATOM  17043  HG  LEU B  25      -26.767  30.788  51.512  0.00  0.00
B
ATOM  17044  CD1 LEU B  25      -25.519  32.250  50.446  0.00  0.00
B
ATOM  17045 HD11 LEU B  25      -25.126  31.412  49.830  0.00  0.00
B
ATOM  17046 HD12 LEU B  25      -25.601  33.251  49.970  0.00  0.00
B
ATOM  17047 HD13 LEU B  25      -24.957  32.255  51.404  0.00  0.00
B
ATOM  17048  CD2 LEU B  25      -27.614  32.713  51.940  0.00  0.00
B
ATOM  17049 HD21 LEU B  25      -28.534  32.267  52.374  0.00  0.00
B
ATOM  17050 HD22 LEU B  25      -26.876  32.969  52.731  0.00  0.00
B
ATOM  17051 HD23 LEU B  25      -27.749  33.637  51.339  0.00  0.00
B
ATOM  17052  C   LEU B  25      -29.966  31.358  48.552  0.00  0.00
B
ATOM  17053  O   LEU B  25      -30.798  32.302  48.494  0.00  0.00
B
ATOM  17054  N   HSE B  26      -29.900  30.531  47.510  0.00  0.00
B
ATOM  17055  HN  HSE B  26      -29.158  29.874  47.401  0.00  0.00
B
ATOM  17056  CA  HSE B  26      -30.859  30.548  46.400  0.00  0.00
B
ATOM  17057  HA  HSE B  26      -30.719  31.527  45.967  0.00  0.00
B
ATOM  17058  CB  HSE B  26      -30.475  29.509  45.339  0.00  0.00
B
ATOM  17059  HB1 HSE B  26      -29.376  29.464  45.183  0.00  0.00
B
ATOM  17060  HB2 HSE B  26      -30.648  28.509  45.791  0.00  0.00
B
ATOM  17061  ND1 HSE B  26      -30.917  30.548  43.150  0.00  0.00
B
ATOM  17062  CG  HSE B  26      -31.241  29.593  44.058  0.00  0.00
B
ATOM  17063  CE1 HSE B  26      -31.786  30.341  42.142  0.00  0.00
B
```

```
ATOM  17064  HE1 HSE B  26     -31.791  30.953  41.240  0.00  0.00
B
ATOM  17065  NE2 HSE B  26     -32.484  29.207  42.277  0.00  0.00
B
ATOM  17066  HE2 HSE B  26     -33.214  28.854  41.692  0.00  0.00
B
ATOM  17067  CD2 HSE B  26     -32.103  28.692  43.494  0.00  0.00
B
ATOM  17068  HD2 HSE B  26     -32.632  27.853  43.929  0.00  0.00
B
ATOM  17069  C   HSE B  26     -32.374  30.282  46.820  0.00  0.00
B
ATOM  17070  O   HSE B  26     -33.275  30.967  46.272  0.00  0.00
B
ATOM  17071  N   ARG B  27     -32.579  29.440  47.839  0.00  0.00
B
ATOM  17072  HN  ARG B  27     -31.856  29.116  48.445  0.00  0.00
B
ATOM  17073  CA  ARG B  27     -33.986  29.258  48.341  0.00  0.00
B
ATOM  17074  HA  ARG B  27     -34.589  29.006  47.481  0.00  0.00
B
ATOM  17075  CB  ARG B  27     -34.133  28.072  49.302  0.00  0.00
B
ATOM  17076  HB1 ARG B  27     -33.637  27.244  48.752  0.00  0.00
B
ATOM  17077  HB2 ARG B  27     -33.471  28.203  50.184  0.00  0.00
B
ATOM  17078  CG  ARG B  27     -35.579  27.640  49.562  0.00  0.00
B
ATOM  17079  HG1 ARG B  27     -36.303  28.428  49.859  0.00  0.00
B
ATOM  17080  HG2 ARG B  27     -35.902  27.330  48.545  0.00  0.00
B
ATOM  17081  CD  ARG B  27     -35.584  26.429  50.503  0.00  0.00
B
ATOM  17082  HD1 ARG B  27     -36.583  25.944  50.491  0.00  0.00
B
ATOM  17083  HD2 ARG B  27     -34.856  25.619  50.284  0.00  0.00
B
ATOM  17084  NE  ARG B  27     -35.325  26.909  51.873  0.00  0.00
B
ATOM  17085  HE  ARG B  27     -34.364  26.815  52.131  0.00  0.00
B
ATOM  17086  CZ  ARG B  27     -36.098  27.711  52.618  0.00  0.00
B
ATOM  17087  NH1 ARG B  27     -37.406  27.789  52.512  0.00  0.00
B
ATOM  17088 HH11 ARG B  27     -37.837  28.438  53.139  0.00  0.00
B
ATOM  17089 HH12 ARG B  27     -37.815  26.984  52.081  0.00  0.00
B
ATOM  17090  NH2 ARG B  27     -35.535  28.550  53.421  0.00  0.00
B
```

ATOM 17091 HH21 ARG B 27 −36.157 29.132 53.945 0.00 0.00
B
ATOM 17092 HH22 ARG B 27 −34.576 28.394 53.656 0.00 0.00
B
ATOM 17093 C ARG B 27 −34.584 30.413 49.080 0.00 0.00
B
ATOM 17094 O ARG B 27 −35.781 30.675 48.921 0.00 0.00
B
ATOM 17095 N MET B 28 −33.713 31.208 49.709 0.00 0.00
B
ATOM 17096 HN MET B 28 −32.770 30.919 49.852 0.00 0.00
B
ATOM 17097 CA MET B 28 −34.142 32.512 50.199 0.00 0.00
B
ATOM 17098 HA MET B 28 −35.203 32.369 50.342 0.00 0.00
B
ATOM 17099 CB MET B 28 −33.405 32.945 51.554 0.00 0.00
B
ATOM 17100 HB1 MET B 28 −32.337 32.993 51.253 0.00 0.00
B
ATOM 17101 HB2 MET B 28 −33.772 33.952 51.846 0.00 0.00
B
ATOM 17102 CG MET B 28 −33.712 31.975 52.776 0.00 0.00
B
ATOM 17103 HG1 MET B 28 −34.785 31.699 52.856 0.00 0.00
B
ATOM 17104 HG2 MET B 28 −33.099 31.055 52.666 0.00 0.00
B
ATOM 17105 SD MET B 28 −33.093 32.730 54.323 0.00 0.00
B
ATOM 17106 CE MET B 28 −31.313 32.372 54.036 0.00 0.00
B
ATOM 17107 HE1 MET B 28 −30.925 31.511 54.620 0.00 0.00
B
ATOM 17108 HE2 MET B 28 −31.209 31.972 53.004 0.00 0.00
B
ATOM 17109 HE3 MET B 28 −30.788 33.345 54.142 0.00 0.00
B
ATOM 17110 C MET B 28 −33.868 33.653 49.235 0.00 0.00
B
ATOM 17111 O MET B 28 −33.966 34.880 49.578 0.00 0.00
B
ATOM 17112 N GLY B 29 −33.605 33.333 47.982 0.00 0.00
B
ATOM 17113 HN GLY B 29 −33.400 32.417 47.645 0.00 0.00
B
ATOM 17114 CA GLY B 29 −33.607 34.301 46.873 0.00 0.00
B
ATOM 17115 HA1 GLY B 29 −34.481 34.927 46.974 0.00 0.00
B
ATOM 17116 HA2 GLY B 29 −33.600 33.761 45.938 0.00 0.00
B
ATOM 17117 C GLY B 29 −32.431 35.254 46.738 0.00 0.00
B

ATOM 17118 O GLY B 29 −32.456 36.187 45.933 0.00 0.00
B
ATOM 17119 N HSE B 30 −31.381 35.003 47.559 0.00 0.00
B
ATOM 17120 HN HSE B 30 −31.382 34.228 48.186 0.00 0.00
B
ATOM 17121 CA HSE B 30 −30.198 35.856 47.549 0.00 0.00
B
ATOM 17122 HA HSE B 30 −30.498 36.891 47.483 0.00 0.00
B
ATOM 17123 CB HSE B 30 −29.512 35.682 48.958 0.00 0.00
B
ATOM 17124 HB1 HSE B 30 −28.971 34.716 49.052 0.00 0.00
B
ATOM 17125 HB2 HSE B 30 −28.730 36.464 49.074 0.00 0.00
B
ATOM 17126 ND1 HSE B 30 −30.933 37.182 50.276 0.00 0.00
B
ATOM 17127 CG HSE B 30 −30.271 36.000 50.181 0.00 0.00
B
ATOM 17128 CE1 HSE B 30 −31.487 37.144 51.430 0.00 0.00
B
ATOM 17129 HE1 HSE B 30 −32.115 37.967 51.770 0.00 0.00
B
ATOM 17130 NE2 HSE B 30 −31.300 35.979 52.045 0.00 0.00
B
ATOM 17131 HE2 HSE B 30 −31.454 35.701 52.994 0.00 0.00
B
ATOM 17132 CD2 HSE B 30 −30.512 35.257 51.285 0.00 0.00
B
ATOM 17133 HD2 HSE B 30 −30.143 34.298 51.629 0.00 0.00
B
ATOM 17134 C HSE B 30 −29.256 35.666 46.410 0.00 0.00
B
ATOM 17135 O HSE B 30 −29.145 34.584 45.797 0.00 0.00
B
ATOM 17136 N GLU B 31 −28.548 36.727 46.052 0.00 0.00
B
ATOM 17137 HN GLU B 31 −28.733 37.611 46.473 0.00 0.00
B
ATOM 17138 CA GLU B 31 −27.487 36.757 45.049 0.00 0.00
B
ATOM 17139 HA GLU B 31 −27.921 36.426 44.117 0.00 0.00
B
ATOM 17140 CB GLU B 31 −27.094 38.188 44.896 0.00 0.00
B
ATOM 17141 HB1 GLU B 31 −28.076 38.653 44.667 0.00 0.00
B
ATOM 17142 HB2 GLU B 31 −26.704 38.679 45.813 0.00 0.00
B
ATOM 17143 CG GLU B 31 −26.214 38.494 43.683 0.00 0.00
B
ATOM 17144 HG1 GLU B 31 −25.251 37.957 43.820 0.00 0.00
B

ATOM 17145 HG2 GLU B 31 −26.634 38.232 42.689 0.00 0.00
B
ATOM 17146 CD GLU B 31 −25.896 39.985 43.648 0.00 0.00
B
ATOM 17147 OE1 GLU B 31 −26.176 40.746 42.661 0.00 0.00
B
ATOM 17148 OE2 GLU B 31 −25.389 40.535 44.673 0.00 0.00
B
ATOM 17149 C GLU B 31 −26.257 35.924 45.529 0.00 0.00
B
ATOM 17150 O GLU B 31 −25.904 36.072 46.722 0.00 0.00
B
ATOM 17151 N VAL B 32 −25.704 35.108 44.614 0.00 0.00
B
ATOM 17152 HN VAL B 32 −26.078 35.103 43.690 0.00 0.00
B
ATOM 17153 CA VAL B 32 −24.869 34.066 45.103 0.00 0.00
B
ATOM 17154 HA VAL B 32 −24.211 34.560 45.802 0.00 0.00
B
ATOM 17155 CB VAL B 32 −25.665 33.005 45.908 0.00 0.00
B
ATOM 17156 HB VAL B 32 −26.427 33.492 46.554 0.00 0.00
B
ATOM 17157 CG1 VAL B 32 −26.479 32.066 45.032 0.00 0.00
B
ATOM 17158 HG11 VAL B 32 −27.026 31.275 45.587 0.00 0.00
B
ATOM 17159 HG12 VAL B 32 −27.110 32.664 44.340 0.00 0.00
B
ATOM 17160 HG13 VAL B 32 −25.827 31.467 44.361 0.00 0.00
B
ATOM 17161 CG2 VAL B 32 −24.713 32.159 46.844 0.00 0.00
B
ATOM 17162 HG21 VAL B 32 −24.195 31.593 46.040 0.00 0.00
B
ATOM 17163 HG22 VAL B 32 −23.981 32.786 47.397 0.00 0.00
B
ATOM 17164 HG23 VAL B 32 −25.254 31.437 47.491 0.00 0.00
B
ATOM 17165 C VAL B 32 −23.975 33.592 44.015 0.00 0.00
B
ATOM 17166 O VAL B 32 −24.252 33.632 42.835 0.00 0.00
B
ATOM 17167 N LEU B 33 −22.822 33.060 44.484 0.00 0.00
B
ATOM 17168 HN LEU B 33 −22.797 32.938 45.473 0.00 0.00
B
ATOM 17169 CA LEU B 33 −21.722 32.643 43.585 0.00 0.00
B
ATOM 17170 HA LEU B 33 −22.108 32.435 42.598 0.00 0.00
B
ATOM 17171 CB LEU B 33 −20.608 33.635 43.335 0.00 0.00
B

ATOM 17172 HB1 LEU B 33 −20.990 34.644 43.067 0.00 0.00
B
ATOM 17173 HB2 LEU B 33 −19.918 33.713 44.202 0.00 0.00
B
ATOM 17174 CG LEU B 33 −19.600 33.250 42.266 0.00 0.00
B
ATOM 17175 HG LEU B 33 −19.277 32.187 42.303 0.00 0.00
B
ATOM 17176 CD1 LEU B 33 −20.083 33.361 40.798 0.00 0.00
B
ATOM 17177 HD11 LEU B 33 −19.287 33.259 40.029 0.00 0.00
B
ATOM 17178 HD12 LEU B 33 −20.877 32.603 40.630 0.00 0.00
B
ATOM 17179 HD13 LEU B 33 −20.557 34.332 40.540 0.00 0.00
B
ATOM 17180 CD2 LEU B 33 −18.224 34.052 42.429 0.00 0.00
B
ATOM 17181 HD21 LEU B 33 −17.540 33.643 41.655 0.00 0.00
B
ATOM 17182 HD22 LEU B 33 −18.391 35.099 42.096 0.00 0.00
B
ATOM 17183 HD23 LEU B 33 −17.844 33.899 43.461 0.00 0.00
B
ATOM 17184 C LEU B 33 −21.139 31.333 44.089 0.00 0.00
B
ATOM 17185 O LEU B 33 −20.736 31.204 45.271 0.00 0.00
B
ATOM 17186 N ALA B 34 −21.035 30.400 43.100 0.00 0.00
B
ATOM 17187 HN ALA B 34 −21.282 30.590 42.153 0.00 0.00
B
ATOM 17188 CA ALA B 34 −20.469 29.062 43.323 0.00 0.00
B
ATOM 17189 HA ALA B 34 −20.150 29.049 44.355 0.00 0.00
B
ATOM 17190 CB ALA B 34 −21.471 28.088 42.941 0.00 0.00
B
ATOM 17191 HB1 ALA B 34 −21.204 27.024 43.121 0.00 0.00
B
ATOM 17192 HB2 ALA B 34 −22.429 28.128 43.503 0.00 0.00
B
ATOM 17193 HB3 ALA B 34 −21.627 28.085 41.841 0.00 0.00
B
ATOM 17194 C ALA B 34 −19.191 28.793 42.549 0.00 0.00
B
ATOM 17195 O ALA B 34 −19.127 28.935 41.319 0.00 0.00
B
ATOM 17196 N VAL B 35 −18.112 28.449 43.202 0.00 0.00
B
ATOM 17197 HN VAL B 35 −18.189 28.338 44.189 0.00 0.00
B
ATOM 17198 CA VAL B 35 −16.771 28.408 42.596 0.00 0.00
B

ATOM 17199 HA VAL B 35 −16.860 28.424 41.519 0.00 0.00
B
ATOM 17200 CB VAL B 35 −15.896 29.451 43.181 0.00 0.00
B
ATOM 17201 HB VAL B 35 −15.507 29.257 44.203 0.00 0.00
B
ATOM 17202 CG1 VAL B 35 −14.705 29.551 42.227 0.00 0.00
B
ATOM 17203 HG11 VAL B 35 −14.085 30.321 42.734 0.00 0.00
B
ATOM 17204 HG12 VAL B 35 −14.045 28.658 42.217 0.00 0.00
B
ATOM 17205 HG13 VAL B 35 −14.915 29.948 41.211 0.00 0.00
B
ATOM 17206 CG2 VAL B 35 −16.585 30.829 43.164 0.00 0.00
B
ATOM 17207 HG21 VAL B 35 −15.964 31.657 43.570 0.00 0.00
B
ATOM 17208 HG22 VAL B 35 −16.793 31.138 42.117 0.00 0.00
B
ATOM 17209 HG23 VAL B 35 −17.539 30.857 43.733 0.00 0.00
B
ATOM 17210 C VAL B 35 −16.223 27.036 43.064 0.00 0.00
B
ATOM 17211 O VAL B 35 −16.233 26.682 44.214 0.00 0.00
B
ATOM 17212 N ASP B 36 −15.740 26.250 42.098 0.00 0.00
B
ATOM 17213 HN ASP B 36 −15.885 26.584 41.170 0.00 0.00
B
ATOM 17214 CA ASP B 36 −15.175 24.982 42.339 0.00 0.00
B
ATOM 17215 HA ASP B 36 −14.607 25.026 43.257 0.00 0.00
B
ATOM 17216 CB ASP B 36 −16.191 23.858 42.455 0.00 0.00
B
ATOM 17217 HB1 ASP B 36 −17.093 24.218 42.995 0.00 0.00
B
ATOM 17218 HB2 ASP B 36 −16.489 23.609 41.414 0.00 0.00
B
ATOM 17219 CG ASP B 36 −15.659 22.756 43.348 0.00 0.00
B
ATOM 17220 OD1 ASP B 36 −16.074 22.608 44.542 0.00 0.00
B
ATOM 17221 OD2 ASP B 36 −14.804 21.953 42.858 0.00 0.00
B
ATOM 17222 C ASP B 36 −14.188 24.693 41.161 0.00 0.00
B
ATOM 17223 O ASP B 36 −14.162 25.389 40.155 0.00 0.00
B
ATOM 17224 N ILE B 37 −13.266 23.744 41.314 0.00 0.00
B
ATOM 17225 HN ILE B 37 −13.305 23.176 42.133 0.00 0.00
B

ATOM 17226 CA ILE B 37 -12.379 23.310 40.261 0.00 0.00
B
ATOM 17227 HA ILE B 37 -12.236 24.158 39.608 0.00 0.00
B
ATOM 17228 CB ILE B 37 -10.968 22.898 40.813 0.00 0.00
B
ATOM 17229 HB ILE B 37 -10.597 23.740 41.437 0.00 0.00
B
ATOM 17230 CG2 ILE B 37 -11.126 21.624 41.764 0.00 0.00
B
ATOM 17231 HG21 ILE B 37 -11.720 21.909 42.659 0.00 0.00
B
ATOM 17232 HG22 ILE B 37 -11.493 20.694 41.279 0.00 0.00
B
ATOM 17233 HG23 ILE B 37 -10.128 21.426 42.210 0.00 0.00
B
ATOM 17234 CG1 ILE B 37 -9.812 22.735 39.680 0.00 0.00
B
ATOM 17235 HG11 ILE B 37 -8.912 22.403 40.240 0.00 0.00
B
ATOM 17236 HG12 ILE B 37 -10.225 21.931 39.034 0.00 0.00
B
ATOM 17237 CD ILE B 37 -9.604 23.990 38.867 0.00 0.00
B
ATOM 17238 HD1 ILE B 37 -9.252 24.803 39.538 0.00 0.00
B
ATOM 17239 HD2 ILE B 37 -8.804 23.820 38.115 0.00 0.00
B
ATOM 17240 HD3 ILE B 37 -10.453 24.353 38.250 0.00 0.00
B
ATOM 17241 C ILE B 37 -13.019 22.173 39.465 0.00 0.00
B
ATOM 17242 O ILE B 37 -12.700 21.876 38.322 0.00 0.00
B
ATOM 17243 N ASN B 38 -14.056 21.551 40.093 0.00 0.00
B
ATOM 17244 HN ASN B 38 -14.375 21.848 40.990 0.00 0.00
B
ATOM 17245 CA ASN B 38 -14.795 20.471 39.495 0.00 0.00
B
ATOM 17246 HA ASN B 38 -14.196 19.981 38.741 0.00 0.00
B
ATOM 17247 CB ASN B 38 -15.254 19.496 40.629 0.00 0.00
B
ATOM 17248 HB1 ASN B 38 -14.358 19.284 41.251 0.00 0.00
B
ATOM 17249 HB2 ASN B 38 -15.904 19.986 41.385 0.00 0.00
B
ATOM 17250 CG ASN B 38 -15.800 18.159 40.132 0.00 0.00
B
ATOM 17251 OD1 ASN B 38 -16.024 17.927 38.953 0.00 0.00
B
ATOM 17252 ND2 ASN B 38 -15.991 17.209 41.119 0.00 0.00
B

ATOM 17253 HD21 ASN B 38 −16.369 16.301 40.936 0.00 0.00
B
ATOM 17254 HD22 ASN B 38 −15.710 17.426 42.054 0.00 0.00
B
ATOM 17255 C ASN B 38 −15.982 20.950 38.670 0.00 0.00
B
ATOM 17256 O ASN B 38 −16.979 21.558 39.185 0.00 0.00
B
ATOM 17257 N GLU B 39 −15.992 20.580 37.324 0.00 0.00
B
ATOM 17258 HN GLU B 39 −15.171 20.060 37.098 0.00 0.00
B
ATOM 17259 CA GLU B 39 −16.938 20.888 36.290 0.00 0.00
B
ATOM 17260 HA GLU B 39 −16.962 21.957 36.142 0.00 0.00
B
ATOM 17261 CB GLU B 39 −16.416 20.264 34.950 0.00 0.00
B
ATOM 17262 HB1 GLU B 39 −15.399 20.614 34.671 0.00 0.00
B
ATOM 17263 HB2 GLU B 39 −16.347 19.161 35.070 0.00 0.00
B
ATOM 17264 CG GLU B 39 −17.408 20.506 33.726 0.00 0.00
B
ATOM 17265 HG1 GLU B 39 −18.352 19.921 33.757 0.00 0.00
B
ATOM 17266 HG2 GLU B 39 −17.767 21.556 33.684 0.00 0.00
B
ATOM 17267 CD GLU B 39 −16.749 20.254 32.356 0.00 0.00
B
ATOM 17268 OE1 GLU B 39 −16.724 19.074 31.958 0.00 0.00
B
ATOM 17269 OE2 GLU B 39 −16.456 21.244 31.645 0.00 0.00
B
ATOM 17270 C GLU B 39 −18.326 20.483 36.494 0.00 0.00
B
ATOM 17271 O GLU B 39 −19.277 21.217 36.341 0.00 0.00
B
ATOM 17272 N GLU B 40 −18.536 19.205 37.016 0.00 0.00
B
ATOM 17273 HN GLU B 40 −17.813 18.547 37.212 0.00 0.00
B
ATOM 17274 CA GLU B 40 −19.867 18.636 37.218 0.00 0.00
B
ATOM 17275 HA GLU B 40 −20.424 18.821 36.311 0.00 0.00
B
ATOM 17276 CB GLU B 40 −19.816 17.123 37.620 0.00 0.00
B
ATOM 17277 HB1 GLU B 40 −19.552 16.865 38.668 0.00 0.00
B
ATOM 17278 HB2 GLU B 40 −20.856 16.746 37.523 0.00 0.00
B
ATOM 17279 CG GLU B 40 −18.841 16.297 36.666 0.00 0.00
B

ATOM 17280 HG1 GLU B 40 −17.889 16.869 36.678 0.00 0.00 B
ATOM 17281 HG2 GLU B 40 −18.684 15.336 37.200 0.00 0.00 B
ATOM 17282 CD GLU B 40 −19.351 16.013 35.193 0.00 0.00 B
ATOM 17283 OE1 GLU B 40 −18.535 15.907 34.207 0.00 0.00 B
ATOM 17284 OE2 GLU B 40 −20.574 15.827 35.047 0.00 0.00 B
ATOM 17285 C GLU B 40 −20.665 19.372 38.281 0.00 0.00 B
ATOM 17286 O GLU B 40 −21.877 19.562 38.118 0.00 0.00 B
ATOM 17287 N LYS B 41 −19.971 19.779 39.406 0.00 0.00 B
ATOM 17288 HN LYS B 41 −19.025 19.477 39.316 0.00 0.00 B
ATOM 17289 CA LYS B 41 −20.556 20.410 40.583 0.00 0.00 B
ATOM 17290 HA LYS B 41 −21.426 19.860 40.909 0.00 0.00 B
ATOM 17291 CB LYS B 41 −19.536 20.603 41.775 0.00 0.00 B
ATOM 17292 HB1 LYS B 41 −18.579 20.956 41.335 0.00 0.00 B
ATOM 17293 HB2 LYS B 41 −19.918 21.398 42.450 0.00 0.00 B
ATOM 17294 CG LYS B 41 −19.534 19.369 42.626 0.00 0.00 B
ATOM 17295 HG1 LYS B 41 −20.347 19.433 43.380 0.00 0.00 B
ATOM 17296 HG2 LYS B 41 −19.832 18.492 42.012 0.00 0.00 B
ATOM 17297 CD LYS B 41 −18.226 18.922 43.261 0.00 0.00 B
ATOM 17298 HD1 LYS B 41 −17.778 18.301 42.456 0.00 0.00 B
ATOM 17299 HD2 LYS B 41 −17.554 19.806 43.307 0.00 0.00 B
ATOM 17300 CE LYS B 41 −18.362 18.311 44.639 0.00 0.00 B
ATOM 17301 HE1 LYS B 41 −18.719 19.077 45.361 0.00 0.00 B
ATOM 17302 HE2 LYS B 41 −19.045 17.435 44.651 0.00 0.00 B
ATOM 17303 NZ LYS B 41 −17.080 17.834 45.218 0.00 0.00 B
ATOM 17304 HZ1 LYS B 41 −16.879 16.870 44.883 0.00 0.00 B
ATOM 17305 HZ2 LYS B 41 −16.287 18.468 44.992 0.00 0.00 B
ATOM 17306 HZ3 LYS B 41 −17.232 17.724 46.241 0.00 0.00 B

ATOM 17307 C LYS B 41 −20.988 21.839 40.223 0.00 0.00
B
ATOM 17308 O LYS B 41 −22.008 22.275 40.595 0.00 0.00
B
ATOM 17309 N VAL B 42 −20.123 22.527 39.390 0.00 0.00
B
ATOM 17310 HN VAL B 42 −19.305 22.020 39.130 0.00 0.00
B
ATOM 17311 CA VAL B 42 −20.396 23.826 38.818 0.00 0.00
B
ATOM 17312 HA VAL B 42 −20.724 24.462 39.628 0.00 0.00
B
ATOM 17313 CB VAL B 42 −19.096 24.449 38.218 0.00 0.00
B
ATOM 17314 HB VAL B 42 −18.660 23.639 37.594 0.00 0.00
B
ATOM 17315 CG1 VAL B 42 −19.405 25.609 37.237 0.00 0.00
B
ATOM 17316 HG11 VAL B 42 −18.468 26.029 36.813 0.00 0.00
B
ATOM 17317 HG12 VAL B 42 −20.106 25.410 36.398 0.00 0.00
B
ATOM 17318 HG13 VAL B 42 −19.895 26.411 37.829 0.00 0.00
B
ATOM 17319 CG2 VAL B 42 −18.259 24.753 39.458 0.00 0.00
B
ATOM 17320 HG21 VAL B 42 −18.790 25.509 40.076 0.00 0.00
B
ATOM 17321 HG22 VAL B 42 −17.913 23.836 39.982 0.00 0.00
B
ATOM 17322 HG23 VAL B 42 −17.268 25.175 39.188 0.00 0.00
B
ATOM 17323 C VAL B 42 −21.540 23.808 37.882 0.00 0.00
B
ATOM 17324 O VAL B 42 −22.490 24.543 38.127 0.00 0.00
B
ATOM 17325 N ASN B 43 −21.593 22.880 36.857 0.00 0.00
B
ATOM 17326 HN ASN B 43 −20.764 22.356 36.676 0.00 0.00
B
ATOM 17327 CA ASN B 43 −22.720 22.713 36.081 0.00 0.00
B
ATOM 17328 HA ASN B 43 −22.979 23.678 35.668 0.00 0.00
B
ATOM 17329 CB ASN B 43 −22.245 21.689 34.923 0.00 0.00
B
ATOM 17330 HB1 ASN B 43 −21.387 22.059 34.323 0.00 0.00
B
ATOM 17331 HB2 ASN B 43 −22.012 20.701 35.375 0.00 0.00
B
ATOM 17332 CG ASN B 43 −23.261 21.397 33.812 0.00 0.00
B
ATOM 17333 OD1 ASN B 43 −23.101 21.943 32.710 0.00 0.00
B

ATOM 17334 ND2 ASN B 43 −24.289 20.581 34.091 0.00 0.00
B
ATOM 17335 HD21 ASN B 43 −24.257 20.001 34.904 0.00 0.00
B
ATOM 17336 HD22 ASN B 43 −25.020 20.394 33.435 0.00 0.00
B
ATOM 17337 C ASN B 43 −23.955 22.247 36.754 0.00 0.00
B
ATOM 17338 O ASN B 43 −25.062 22.617 36.362 0.00 0.00
B
ATOM 17339 N ALA B 44 −23.809 21.405 37.829 0.00 0.00
B
ATOM 17340 HN ALA B 44 −22.911 21.017 38.017 0.00 0.00
B
ATOM 17341 CA ALA B 44 −24.945 21.123 38.739 0.00 0.00
B
ATOM 17342 HA ALA B 44 −25.661 20.603 38.120 0.00 0.00
B
ATOM 17343 CB ALA B 44 −24.557 20.078 39.804 0.00 0.00
B
ATOM 17344 HB1 ALA B 44 −25.395 19.791 40.475 0.00 0.00
B
ATOM 17345 HB2 ALA B 44 −24.280 19.127 39.301 0.00 0.00
B
ATOM 17346 HB3 ALA B 44 −23.600 20.310 40.319 0.00 0.00
B
ATOM 17347 C ALA B 44 −25.560 22.304 39.540 0.00 0.00
B
ATOM 17348 O ALA B 44 −26.789 22.392 39.645 0.00 0.00
B
ATOM 17349 N TYR B 45 −24.752 23.216 40.149 0.00 0.00
B
ATOM 17350 HN TYR B 45 −23.806 22.903 40.114 0.00 0.00
B
ATOM 17351 CA TYR B 45 −25.193 24.284 41.060 0.00 0.00
B
ATOM 17352 HA TYR B 45 −26.149 24.078 41.517 0.00 0.00
B
ATOM 17353 CB TYR B 45 −24.165 24.544 42.181 0.00 0.00
B
ATOM 17354 HB1 TYR B 45 −23.141 24.684 41.774 0.00 0.00
B
ATOM 17355 HB2 TYR B 45 −24.518 25.502 42.618 0.00 0.00
B
ATOM 17356 CG TYR B 45 −24.167 23.457 43.223 0.00 0.00
B
ATOM 17357 CD1 TYR B 45 −22.959 22.835 43.607 0.00 0.00
B
ATOM 17358 HD1 TYR B 45 −21.968 23.031 43.223 0.00 0.00
B
ATOM 17359 CE1 TYR B 45 −22.968 21.731 44.456 0.00 0.00
B
ATOM 17360 HE1 TYR B 45 −22.013 21.294 44.708 0.00 0.00
B

ATOM 17361 CZ TYR B 45 −24.209 21.237 44.868 0.00 0.00
B
ATOM 17362 OH TYR B 45 −24.206 20.119 45.639 0.00 0.00
B
ATOM 17363 HH TYR B 45 −23.320 19.827 45.865 0.00 0.00
B
ATOM 17364 CD2 TYR B 45 −25.326 23.101 43.817 0.00 0.00
B
ATOM 17365 HD2 TYR B 45 −26.251 23.632 43.645 0.00 0.00
B
ATOM 17366 CE2 TYR B 45 −25.427 21.923 44.548 0.00 0.00
B
ATOM 17367 HE2 TYR B 45 −26.416 21.660 44.892 0.00 0.00
B
ATOM 17368 C TYR B 45 −25.395 25.532 40.303 0.00 0.00
B
ATOM 17369 O TYR B 45 −25.741 26.566 40.890 0.00 0.00
B
ATOM 17370 N ALA B 46 −25.174 25.524 38.930 0.00 0.00
B
ATOM 17371 HN ALA B 46 −24.786 24.739 38.453 0.00 0.00
B
ATOM 17372 CA ALA B 46 −25.293 26.697 38.132 0.00 0.00
B
ATOM 17373 HA ALA B 46 −24.703 27.423 38.673 0.00 0.00
B
ATOM 17374 CB ALA B 46 −24.684 26.384 36.714 0.00 0.00
B
ATOM 17375 HB1 ALA B 46 −25.281 25.634 36.153 0.00 0.00
B
ATOM 17376 HB2 ALA B 46 −24.599 27.335 36.145 0.00 0.00
B
ATOM 17377 HB3 ALA B 46 −23.692 25.907 36.870 0.00 0.00
B
ATOM 17378 C ALA B 46 −26.785 27.186 37.995 0.00 0.00
B
ATOM 17379 O ALA B 46 −26.976 28.314 37.527 0.00 0.00
B
ATOM 17380 N SER B 47 −27.811 26.414 38.337 0.00 0.00
B
ATOM 17381 HN SER B 47 −27.821 25.443 38.564 0.00 0.00
B
ATOM 17382 CA SER B 47 −29.197 26.866 38.381 0.00 0.00
B
ATOM 17383 HA SER B 47 −29.349 27.655 37.659 0.00 0.00
B
ATOM 17384 CB SER B 47 −30.098 25.689 38.010 0.00 0.00
B
ATOM 17385 HB1 SER B 47 −31.127 25.966 38.325 0.00 0.00
B
ATOM 17386 HB2 SER B 47 −30.284 25.349 36.969 0.00 0.00
B
ATOM 17387 OG SER B 47 −29.710 24.551 38.706 0.00 0.00
B

ATOM 17388 HG1 SER B 47 −30.392 23.885 38.598 0.00 0.00
B
ATOM 17389 C SER B 47 −29.606 27.296 39.689 0.00 0.00
B
ATOM 17390 O SER B 47 −30.678 27.962 39.845 0.00 0.00
B
ATOM 17391 N TYR B 48 −28.870 26.946 40.670 0.00 0.00
B
ATOM 17392 HN TYR B 48 −28.079 26.378 40.453 0.00 0.00
B
ATOM 17393 CA TYR B 48 −29.175 27.178 42.065 0.00 0.00
B
ATOM 17394 HA TYR B 48 −30.185 27.524 42.229 0.00 0.00
B
ATOM 17395 CB TYR B 48 −28.906 25.878 42.830 0.00 0.00
B
ATOM 17396 HB1 TYR B 48 −27.915 25.418 42.626 0.00 0.00
B
ATOM 17397 HB2 TYR B 48 −29.036 26.126 43.905 0.00 0.00
B
ATOM 17398 CG TYR B 48 −30.006 24.881 42.605 0.00 0.00
B
ATOM 17399 CD1 TYR B 48 −31.275 24.983 43.246 0.00 0.00
B
ATOM 17400 HD1 TYR B 48 −31.406 25.858 43.866 0.00 0.00
B
ATOM 17401 CE1 TYR B 48 −32.288 24.140 42.904 0.00 0.00
B
ATOM 17402 HE1 TYR B 48 −33.269 24.275 43.335 0.00 0.00
B
ATOM 17403 CZ TYR B 48 −32.042 23.004 42.159 0.00 0.00
B
ATOM 17404 OH TYR B 48 −33.013 22.078 41.933 0.00 0.00
B
ATOM 17405 HH TYR B 48 −32.705 21.358 41.378 0.00 0.00
B
ATOM 17406 CD2 TYR B 48 −29.780 23.801 41.777 0.00 0.00
B
ATOM 17407 HD2 TYR B 48 −28.797 23.812 41.331 0.00 0.00
B
ATOM 17408 CE2 TYR B 48 −30.747 22.824 41.644 0.00 0.00
B
ATOM 17409 HE2 TYR B 48 −30.532 21.991 40.991 0.00 0.00
B
ATOM 17410 C TYR B 48 −28.191 28.217 42.558 0.00 0.00
B
ATOM 17411 O TYR B 48 −28.023 28.232 43.798 0.00 0.00
B
ATOM 17412 N ALA B 49 −27.574 28.981 41.666 0.00 0.00
B
ATOM 17413 HN ALA B 49 −27.697 28.807 40.692 0.00 0.00
B
ATOM 17414 CA ALA B 49 −26.900 30.145 42.026 0.00 0.00
B

ATOM 17415 HA ALA B 49 −27.306 30.478 42.970 0.00 0.00
B
ATOM 17416 CB ALA B 49 −25.413 29.959 41.985 0.00 0.00
B
ATOM 17417 HB1 ALA B 49 −24.812 30.889 42.077 0.00 0.00
B
ATOM 17418 HB2 ALA B 49 −24.987 29.239 42.716 0.00 0.00
B
ATOM 17419 HB3 ALA B 49 −25.111 29.523 41.009 0.00 0.00
B
ATOM 17420 C ALA B 49 −27.287 31.247 40.950 0.00 0.00
B
ATOM 17421 O ALA B 49 −28.071 30.958 40.002 0.00 0.00
B
ATOM 17422 N THR B 50 −26.732 32.420 41.109 0.00 0.00
B
ATOM 17423 HN THR B 50 −26.076 32.619 41.833 0.00 0.00
B
ATOM 17424 CA THR B 50 −26.974 33.497 40.135 0.00 0.00
B
ATOM 17425 HA THR B 50 −27.855 33.332 39.533 0.00 0.00
B
ATOM 17426 CB THR B 50 −26.741 34.882 40.769 0.00 0.00
B
ATOM 17427 HB THR B 50 −25.708 35.240 40.577 0.00 0.00
B
ATOM 17428 OG1 THR B 50 −26.989 34.800 42.131 0.00 0.00
B
ATOM 17429 HG1 THR B 50 −27.884 34.470 42.238 0.00 0.00
B
ATOM 17430 CG2 THR B 50 −27.683 35.746 39.984 0.00 0.00
B
ATOM 17431 HG21 THR B 50 −28.735 35.430 40.151 0.00 0.00
B
ATOM 17432 HG22 THR B 50 −27.557 36.824 40.223 0.00 0.00
B
ATOM 17433 HG23 THR B 50 −27.430 35.505 38.929 0.00 0.00
B
ATOM 17434 C THR B 50 −25.870 33.331 39.100 0.00 0.00
B
ATOM 17435 O THR B 50 −26.042 33.469 37.901 0.00 0.00
B
ATOM 17436 N HSE B 51 −24.687 32.843 39.541 0.00 0.00
B
ATOM 17437 HN HSE B 51 −24.487 32.531 40.467 0.00 0.00
B
ATOM 17438 CA HSE B 51 −23.669 32.537 38.560 0.00 0.00
B
ATOM 17439 HA HSE B 51 −23.987 31.984 37.689 0.00 0.00
B
ATOM 17440 CB HSE B 51 −22.789 33.853 38.081 0.00 0.00
B
ATOM 17441 HB1 HSE B 51 −23.419 34.577 37.520 0.00 0.00
B

ATOM 17442 HB2 HSE B 51 −22.342 34.320 38.985 0.00 0.00
B
ATOM 17443 ND1 HSE B 51 −22.032 32.792 36.013 0.00 0.00
B
ATOM 17444 CG HSE B 51 −21.662 33.501 37.160 0.00 0.00
B
ATOM 17445 CE1 HSE B 51 −20.938 32.718 35.302 0.00 0.00
B
ATOM 17446 HE1 HSE B 51 −20.824 32.289 34.307 0.00 0.00
B
ATOM 17447 NE2 HSE B 51 −19.936 33.449 35.942 0.00 0.00
B
ATOM 17448 HE2 HSE B 51 −18.998 33.630 35.646 0.00 0.00
B
ATOM 17449 CD2 HSE B 51 −20.372 33.929 37.172 0.00 0.00
B
ATOM 17450 HD2 HSE B 51 −19.797 34.548 37.850 0.00 0.00
B
ATOM 17451 C HSE B 51 −22.735 31.380 39.180 0.00 0.00
B
ATOM 17452 O HSE B 51 −22.843 31.117 40.372 0.00 0.00
B
ATOM 17453 N ALA B 52 −22.057 30.598 38.245 0.00 0.00
B
ATOM 17454 HN ALA B 52 −22.192 30.793 37.276 0.00 0.00
B
ATOM 17455 CA ALA B 52 −21.146 29.520 38.663 0.00 0.00
B
ATOM 17456 HA ALA B 52 −20.712 29.599 39.648 0.00 0.00
B
ATOM 17457 CB ALA B 52 −21.948 28.215 38.713 0.00 0.00
B
ATOM 17458 HB1 ALA B 52 −22.768 28.518 39.399 0.00 0.00
B
ATOM 17459 HB2 ALA B 52 −22.340 27.742 37.787 0.00 0.00
B
ATOM 17460 HB3 ALA B 52 −21.308 27.463 39.222 0.00 0.00
B
ATOM 17461 C ALA B 52 −20.091 29.437 37.621 0.00 0.00
B
ATOM 17462 O ALA B 52 −20.395 29.644 36.415 0.00 0.00
B
ATOM 17463 N VAL B 53 −18.844 29.192 38.095 0.00 0.00
B
ATOM 17464 HN VAL B 53 −18.690 28.992 39.060 0.00 0.00
B
ATOM 17465 CA VAL B 53 −17.662 29.269 37.222 0.00 0.00
B
ATOM 17466 HA VAL B 53 −17.943 28.997 36.216 0.00 0.00
B
ATOM 17467 CB VAL B 53 −17.216 30.709 37.106 0.00 0.00
B
ATOM 17468 HB VAL B 53 −17.911 31.269 36.445 0.00 0.00
B

ATOM 17469 CG1 VAL B 53 -17.222 31.444 38.473 0.00 0.00
B
ATOM 17470 HG11 VAL B 53 -17.027 32.521 38.283 0.00 0.00
B
ATOM 17471 HG12 VAL B 53 -18.220 31.449 38.961 0.00 0.00
B
ATOM 17472 HG13 VAL B 53 -16.444 31.101 39.188 0.00 0.00
B
ATOM 17473 CG2 VAL B 53 -15.806 30.876 36.448 0.00 0.00
B
ATOM 17474 HG21 VAL B 53 -15.499 31.929 36.269 0.00 0.00
B
ATOM 17475 HG22 VAL B 53 -14.990 30.471 37.084 0.00 0.00
B
ATOM 17476 HG23 VAL B 53 -15.687 30.244 35.543 0.00 0.00
B
ATOM 17477 C VAL B 53 -16.602 28.343 37.718 0.00 0.00
B
ATOM 17478 O VAL B 53 -16.311 28.271 38.859 0.00 0.00
B
ATOM 17479 N ILE B 54 -15.958 27.559 36.768 0.00 0.00
B
ATOM 17480 HN ILE B 54 -16.113 27.673 35.790 0.00 0.00
B
ATOM 17481 CA ILE B 54 -14.947 26.552 37.069 0.00 0.00
B
ATOM 17482 HA ILE B 54 -15.218 26.013 37.965 0.00 0.00
B
ATOM 17483 CB ILE B 54 -14.854 25.440 35.978 0.00 0.00
B
ATOM 17484 HB ILE B 54 -14.401 25.853 35.051 0.00 0.00
B
ATOM 17485 CG2 ILE B 54 -13.945 24.267 36.465 0.00 0.00
B
ATOM 17486 HG21 ILE B 54 -13.079 24.647 37.049 0.00 0.00
B
ATOM 17487 HG22 ILE B 54 -14.536 23.676 37.197 0.00 0.00
B
ATOM 17488 HG23 ILE B 54 -13.669 23.602 35.620 0.00 0.00
B
ATOM 17489 CG1 ILE B 54 -16.246 24.941 35.440 0.00 0.00
B
ATOM 17490 HG11 ILE B 54 -16.798 24.366 36.213 0.00 0.00
B
ATOM 17491 HG12 ILE B 54 -16.878 25.802 35.133 0.00 0.00
B
ATOM 17492 CD ILE B 54 -16.003 24.253 34.098 0.00 0.00
B
ATOM 17493 HD1 ILE B 54 -15.812 25.028 33.325 0.00 0.00
B
ATOM 17494 HD2 ILE B 54 -15.334 23.366 34.114 0.00 0.00
B
ATOM 17495 HD3 ILE B 54 -16.998 23.834 33.836 0.00 0.00
B

ATOM	17496	C	ILE	B	54	−13.595	27.258	37.245	0.00	0.00
B										
ATOM	17497	O	ILE	B	54	−13.130	27.889	36.290	0.00	0.00
B										
ATOM	17498	N	ALA	B	55	−13.063	27.289	38.497	0.00	0.00
B										
ATOM	17499	HN	ALA	B	55	−13.638	26.977	39.250	0.00	0.00
B										
ATOM	17500	CA	ALA	B	55	−11.849	28.102	38.761	0.00	0.00
B										
ATOM	17501	HA	ALA	B	55	−11.301	28.087	37.831	0.00	0.00
B										
ATOM	17502	CB	ALA	B	55	−12.135	29.593	39.110	0.00	0.00
B										
ATOM	17503	HB1	ALA	B	55	−11.191	30.144	39.310	0.00	0.00
B										
ATOM	17504	HB2	ALA	B	55	−12.626	30.112	38.260	0.00	0.00
B										
ATOM	17505	HB3	ALA	B	55	−12.824	29.616	39.981	0.00	0.00
B										
ATOM	17506	C	ALA	B	55	−10.920	27.419	39.731	0.00	0.00
B										
ATOM	17507	O	ALA	B	55	−11.337	26.788	40.689	0.00	0.00
B										
ATOM	17508	N	ASN	B	56	−9.611	27.393	39.526	0.00	0.00
B										
ATOM	17509	HN	ASN	B	56	−9.307	27.683	38.622	0.00	0.00
B										
ATOM	17510	CA	ASN	B	56	−8.632	27.173	40.535	0.00	0.00
B										
ATOM	17511	HA	ASN	B	56	−8.850	26.286	41.111	0.00	0.00
B										
ATOM	17512	CB	ASN	B	56	−7.244	26.931	39.911	0.00	0.00
B										
ATOM	17513	HB1	ASN	B	56	−7.348	26.057	39.233	0.00	0.00
B										
ATOM	17514	HB2	ASN	B	56	−6.944	27.871	39.400	0.00	0.00
B										
ATOM	17515	CG	ASN	B	56	−6.280	26.366	40.938	0.00	0.00
B										
ATOM	17516	OD1	ASN	B	56	−6.652	26.242	42.117	0.00	0.00
B										
ATOM	17517	ND2	ASN	B	56	−5.070	25.900	40.579	0.00	0.00
B										
ATOM	17518	HD21	ASN	B	56	−4.417	25.782	41.328	0.00	0.00
B										
ATOM	17519	HD22	ASN	B	56	−4.858	25.766	39.611	0.00	0.00
B										
ATOM	17520	C	ASN	B	56	−8.602	28.401	41.464	0.00	0.00
B										
ATOM	17521	O	ASN	B	56	−8.301	29.504	41.017	0.00	0.00
B										
ATOM	17522	N	ALA	B	57	−8.917	28.193	42.770	0.00	0.00
B										

ATOM 17523 HN ALA B 57 -9.296 27.305 43.019 0.00 0.00
B
ATOM 17524 CA ALA B 57 -9.047 29.161 43.816 0.00 0.00
B
ATOM 17525 HA ALA B 57 -9.393 30.132 43.493 0.00 0.00
B
ATOM 17526 CB ALA B 57 -9.905 28.674 45.009 0.00 0.00
B
ATOM 17527 HB1 ALA B 57 -9.626 29.201 45.946 0.00 0.00
B
ATOM 17528 HB2 ALA B 57 -10.980 28.847 44.792 0.00 0.00
B
ATOM 17529 HB3 ALA B 57 -9.588 27.644 45.280 0.00 0.00
B
ATOM 17530 C ALA B 57 -7.644 29.492 44.435 0.00 0.00
B
ATOM 17531 O ALA B 57 -7.518 30.356 45.301 0.00 0.00
B
ATOM 17532 N THR B 58 -6.547 28.776 44.022 0.00 0.00
B
ATOM 17533 HN THR B 58 -6.624 28.148 43.251 0.00 0.00
B
ATOM 17534 CA THR B 58 -5.246 28.894 44.534 0.00 0.00
B
ATOM 17535 HA THR B 58 -5.254 29.510 45.421 0.00 0.00
B
ATOM 17536 CB THR B 58 -4.619 27.624 45.124 0.00 0.00
B
ATOM 17537 HB THR B 58 -3.703 27.988 45.637 0.00 0.00
B
ATOM 17538 OG1 THR B 58 -4.446 26.688 44.075 0.00 0.00
B
ATOM 17539 HG1 THR B 58 -3.574 26.838 43.702 0.00 0.00
B
ATOM 17540 CG2 THR B 58 -5.632 27.201 46.162 0.00 0.00
B
ATOM 17541 HG21 THR B 58 -6.526 26.721 45.709 0.00 0.00
B
ATOM 17542 HG22 THR B 58 -5.104 26.572 46.910 0.00 0.00
B
ATOM 17543 HG23 THR B 58 -5.994 28.085 46.728 0.00 0.00
B
ATOM 17544 C THR B 58 -4.323 29.764 43.637 0.00 0.00
B
ATOM 17545 O THR B 58 -3.142 29.873 43.871 0.00 0.00
B
ATOM 17546 N GLU B 59 -4.837 30.497 42.627 0.00 0.00
B
ATOM 17547 HN GLU B 59 -5.823 30.580 42.503 0.00 0.00
B
ATOM 17548 CA GLU B 59 -3.997 31.399 41.780 0.00 0.00
B
ATOM 17549 HA GLU B 59 -2.970 31.400 42.114 0.00 0.00
B

ATOM	17550	CB	GLU	B	59	-4.108	30.846	40.333	0.00	0.00	B
ATOM	17551	HB1	GLU	B	59	-3.672	29.831	40.450	0.00	0.00	B
ATOM	17552	HB2	GLU	B	59	-5.206	30.707	40.237	0.00	0.00	B
ATOM	17553	CG	GLU	B	59	-3.358	31.811	39.289	0.00	0.00	B
ATOM	17554	HG1	GLU	B	59	-3.668	32.878	39.256	0.00	0.00	B
ATOM	17555	HG2	GLU	B	59	-2.286	31.756	39.576	0.00	0.00	B
ATOM	17556	CD	GLU	B	59	-3.509	31.406	37.812	0.00	0.00	B
ATOM	17557	OE1	GLU	B	59	-2.546	30.772	37.326	0.00	0.00	B
ATOM	17558	OE2	GLU	B	59	-4.529	31.719	37.147	0.00	0.00	B
ATOM	17559	C	GLU	B	59	-4.639	32.818	41.756	0.00	0.00	B
ATOM	17560	O	GLU	B	59	-5.843	33.043	41.740	0.00	0.00	B
ATOM	17561	N	GLU	B	60	-3.810	33.888	41.779	0.00	0.00	B
ATOM	17562	HN	GLU	B	60	-2.843	33.797	41.555	0.00	0.00	B
ATOM	17563	CA	GLU	B	60	-4.315	35.233	41.839	0.00	0.00	B
ATOM	17564	HA	GLU	B	60	-5.095	35.133	42.580	0.00	0.00	B
ATOM	17565	CB	GLU	B	60	-3.165	36.184	42.357	0.00	0.00	B
ATOM	17566	HB1	GLU	B	60	-3.623	37.188	42.482	0.00	0.00	B
ATOM	17567	HB2	GLU	B	60	-2.899	35.796	43.363	0.00	0.00	B
ATOM	17568	CG	GLU	B	60	-1.839	36.387	41.636	0.00	0.00	B
ATOM	17569	HG1	GLU	B	60	-1.997	36.620	40.561	0.00	0.00	B
ATOM	17570	HG2	GLU	B	60	-1.224	37.173	42.125	0.00	0.00	B
ATOM	17571	CD	GLU	B	60	-0.856	35.227	41.665	0.00	0.00	B
ATOM	17572	OE1	GLU	B	60	-1.121	34.277	40.910	0.00	0.00	B
ATOM	17573	OE2	GLU	B	60	0.182	35.300	42.330	0.00	0.00	B
ATOM	17574	C	GLU	B	60	-5.053	35.779	40.585	0.00	0.00	B
ATOM	17575	O	GLU	B	60	-6.168	36.401	40.550	0.00	0.00	B
ATOM	17576	N	ASN	B	61	-4.454	35.469	39.398	0.00	0.00	B

ATOM 17577 HN ASN B 61 -3.573 35.007 39.469 0.00 0.00
B
ATOM 17578 CA ASN B 61 -4.951 35.778 38.099 0.00 0.00
B
ATOM 17579 HA ASN B 61 -5.129 36.841 38.172 0.00 0.00
B
ATOM 17580 CB ASN B 61 -3.826 35.605 37.036 0.00 0.00
B
ATOM 17581 HB1 ASN B 61 -3.303 34.640 37.207 0.00 0.00
B
ATOM 17582 HB2 ASN B 61 -4.325 35.553 36.045 0.00 0.00
B
ATOM 17583 CG ASN B 61 -2.769 36.793 37.106 0.00 0.00
B
ATOM 17584 OD1 ASN B 61 -2.136 37.038 38.149 0.00 0.00
B
ATOM 17585 ND2 ASN B 61 -2.543 37.504 35.938 0.00 0.00
B
ATOM 17586 HD21 ASN B 61 -1.716 38.014 35.704 0.00 0.00
B
ATOM 17587 HD22 ASN B 61 -3.137 37.366 35.146 0.00 0.00
B
ATOM 17588 C ASN B 61 -6.261 35.141 37.787 0.00 0.00
B
ATOM 17589 O ASN B 61 -7.174 35.724 37.170 0.00 0.00
B
ATOM 17590 N GLU B 62 -6.447 33.857 38.186 0.00 0.00
B
ATOM 17591 HN GLU B 62 -5.681 33.434 38.664 0.00 0.00
B
ATOM 17592 CA GLU B 62 -7.734 33.136 38.155 0.00 0.00
B
ATOM 17593 HA GLU B 62 -8.083 33.219 37.136 0.00 0.00
B
ATOM 17594 CB GLU B 62 -7.651 31.591 38.588 0.00 0.00
B
ATOM 17595 HB1 GLU B 62 -6.739 31.068 38.230 0.00 0.00
B
ATOM 17596 HB2 GLU B 62 -7.583 31.665 39.694 0.00 0.00
B
ATOM 17597 CG GLU B 62 -8.818 30.736 38.092 0.00 0.00
B
ATOM 17598 HG1 GLU B 62 -9.050 30.031 38.918 0.00 0.00
B
ATOM 17599 HG2 GLU B 62 -9.774 31.293 37.983 0.00 0.00
B
ATOM 17600 CD GLU B 62 -8.557 30.029 36.744 0.00 0.00
B
ATOM 17601 OE1 GLU B 62 -7.985 30.677 35.793 0.00 0.00
B
ATOM 17602 OE2 GLU B 62 -8.783 28.826 36.509 0.00 0.00
B
ATOM 17603 C GLU B 62 -8.819 33.933 38.960 0.00 0.00
B

ATOM 17604 O GLU B 62 -9.884 34.165 38.431 0.00 0.00
B
ATOM 17605 N LEU B 63 -8.560 34.432 40.198 0.00 0.00
B
ATOM 17606 HN LEU B 63 -7.732 34.214 40.708 0.00 0.00
B
ATOM 17607 CA LEU B 63 -9.497 35.246 40.996 0.00 0.00
B
ATOM 17608 HA LEU B 63 -10.432 34.712 41.078 0.00 0.00
B
ATOM 17609 CB LEU B 63 -8.982 35.386 42.456 0.00 0.00
B
ATOM 17610 HB1 LEU B 63 -7.985 35.874 42.516 0.00 0.00
B
ATOM 17611 HB2 LEU B 63 -9.708 36.000 43.031 0.00 0.00
B
ATOM 17612 CG LEU B 63 -8.946 34.002 43.112 0.00 0.00
B
ATOM 17613 HG LEU B 63 -8.401 33.278 42.470 0.00 0.00
B
ATOM 17614 CD1 LEU B 63 -8.060 34.187 44.329 0.00 0.00
B
ATOM 17615 HD11 LEU B 63 -7.007 34.496 44.154 0.00 0.00
B
ATOM 17616 HD12 LEU B 63 -8.533 34.864 45.072 0.00 0.00
B
ATOM 17617 HD13 LEU B 63 -7.915 33.258 44.921 0.00 0.00
B
ATOM 17618 CD2 LEU B 63 -10.275 33.387 43.527 0.00 0.00
B
ATOM 17619 HD21 LEU B 63 -9.967 32.431 44.002 0.00 0.00
B
ATOM 17620 HD22 LEU B 63 -10.770 34.048 44.270 0.00 0.00
B
ATOM 17621 HD23 LEU B 63 -10.883 33.221 42.612 0.00 0.00
B
ATOM 17622 C LEU B 63 -9.742 36.580 40.300 0.00 0.00
B
ATOM 17623 O LEU B 63 -10.843 37.158 40.203 0.00 0.00
B
ATOM 17624 N LEU B 64 -8.714 37.222 39.760 0.00 0.00
B
ATOM 17625 HN LEU B 64 -7.866 36.702 39.829 0.00 0.00
B
ATOM 17626 CA LEU B 64 -8.739 38.485 39.033 0.00 0.00
B
ATOM 17627 HA LEU B 64 -9.320 39.157 39.648 0.00 0.00
B
ATOM 17628 CB LEU B 64 -7.318 39.044 38.847 0.00 0.00
B
ATOM 17629 HB1 LEU B 64 -6.803 38.105 38.553 0.00 0.00
B
ATOM 17630 HB2 LEU B 64 -7.362 39.875 38.110 0.00 0.00
B

ATOM 17631 CG LEU B 64 -6.698 39.572 40.169 0.00 0.00
B
ATOM 17632 HG LEU B 64 -6.830 38.856 41.008 0.00 0.00
B
ATOM 17633 CD1 LEU B 64 -5.149 39.705 40.068 0.00 0.00
B
ATOM 17634 HD11 LEU B 64 -4.767 40.369 40.872 0.00 0.00
B
ATOM 17635 HD12 LEU B 64 -4.650 38.712 40.066 0.00 0.00
B
ATOM 17636 HD13 LEU B 64 -4.835 40.310 39.190 0.00 0.00
B
ATOM 17637 CD2 LEU B 64 -7.312 40.922 40.618 0.00 0.00
B
ATOM 17638 HD21 LEU B 64 -8.270 40.780 41.162 0.00 0.00
B
ATOM 17639 HD22 LEU B 64 -6.560 41.534 41.161 0.00 0.00
B
ATOM 17640 HD23 LEU B 64 -7.435 41.638 39.777 0.00 0.00
B
ATOM 17641 C LEU B 64 -9.500 38.465 37.730 0.00 0.00
B
ATOM 17642 O LEU B 64 -10.352 39.380 37.539 0.00 0.00
B
ATOM 17643 N SER B 65 -9.277 37.476 36.927 0.00 0.00
B
ATOM 17644 HN SER B 65 -8.427 36.995 37.128 0.00 0.00
B
ATOM 17645 CA SER B 65 -10.205 37.227 35.786 0.00 0.00
B
ATOM 17646 HA SER B 65 -10.304 38.130 35.202 0.00 0.00
B
ATOM 17647 CB SER B 65 -9.598 36.260 34.755 0.00 0.00
B
ATOM 17648 HB1 SER B 65 -9.458 35.284 35.266 0.00 0.00
B
ATOM 17649 HB2 SER B 65 -10.383 36.106 33.984 0.00 0.00
B
ATOM 17650 OG SER B 65 -8.498 36.855 34.083 0.00 0.00
B
ATOM 17651 HG1 SER B 65 -7.921 36.157 33.764 0.00 0.00
B
ATOM 17652 C SER B 65 -11.616 36.682 35.993 0.00 0.00
B
ATOM 17653 O SER B 65 -12.454 36.850 35.091 0.00 0.00
B
ATOM 17654 N LEU B 66 -11.854 36.094 37.186 0.00 0.00
B
ATOM 17655 HN LEU B 66 -11.137 35.914 37.856 0.00 0.00
B
ATOM 17656 CA LEU B 66 -13.178 35.912 37.747 0.00 0.00
B
ATOM 17657 HA LEU B 66 -13.751 35.338 37.034 0.00 0.00
B

ATOM 17658 CB LEU B 66 -13.008 35.187 39.128 0.00 0.00
B
ATOM 17659 HB1 LEU B 66 -12.181 34.455 39.003 0.00 0.00
B
ATOM 17660 HB2 LEU B 66 -12.679 35.749 40.028 0.00 0.00
B
ATOM 17661 CG LEU B 66 -14.243 34.339 39.578 0.00 0.00
B
ATOM 17662 HG LEU B 66 -14.596 33.668 38.766 0.00 0.00
B
ATOM 17663 CD1 LEU B 66 -13.844 33.380 40.671 0.00 0.00
B
ATOM 17664 HD11 LEU B 66 -13.558 33.911 41.604 0.00 0.00
B
ATOM 17665 HD12 LEU B 66 -14.642 32.655 40.936 0.00 0.00
B
ATOM 17666 HD13 LEU B 66 -12.943 32.812 40.355 0.00 0.00
B
ATOM 17667 CD2 LEU B 66 -15.596 35.048 40.032 0.00 0.00
B
ATOM 17668 HD21 LEU B 66 -16.215 35.328 39.152 0.00 0.00
B
ATOM 17669 HD22 LEU B 66 -16.140 34.288 40.633 0.00 0.00
B
ATOM 17670 HD23 LEU B 66 -15.295 35.920 40.652 0.00 0.00
B
ATOM 17671 C LEU B 66 -14.063 37.166 37.937 0.00 0.00
B
ATOM 17672 O LEU B 66 -15.284 37.199 37.628 0.00 0.00
B
ATOM 17673 N GLY B 67 -13.509 38.225 38.560 0.00 0.00
B
ATOM 17674 HN GLY B 67 -12.547 38.152 38.814 0.00 0.00
B
ATOM 17675 CA GLY B 67 -14.267 39.345 39.063 0.00 0.00
B
ATOM 17676 HA1 GLY B 67 -15.310 39.240 38.801 0.00 0.00
B
ATOM 17677 HA2 GLY B 67 -13.913 40.244 38.581 0.00 0.00
B
ATOM 17678 C GLY B 67 -14.254 39.391 40.546 0.00 0.00
B
ATOM 17679 O GLY B 67 -15.146 39.991 41.130 0.00 0.00
B
ATOM 17680 N ILE B 68 -13.252 38.808 41.311 0.00 0.00
B
ATOM 17681 HN ILE B 68 -12.573 38.261 40.828 0.00 0.00
B
ATOM 17682 CA ILE B 68 -13.332 38.841 42.751 0.00 0.00
B
ATOM 17683 HA ILE B 68 -14.226 38.284 42.989 0.00 0.00
B
ATOM 17684 CB ILE B 68 -12.224 37.863 43.216 0.00 0.00
B

ATOM 17685 HB ILE B 68 −12.156 37.036 42.477 0.00 0.00
B
ATOM 17686 CG2 ILE B 68 −10.828 38.591 43.136 0.00 0.00
B
ATOM 17687 HG21 ILE B 68 −10.735 39.320 43.969 0.00 0.00
B
ATOM 17688 HG22 ILE B 68 −10.007 37.939 43.504 0.00 0.00
B
ATOM 17689 HG23 ILE B 68 −10.570 39.062 42.163 0.00 0.00
B
ATOM 17690 CG1 ILE B 68 −12.617 37.207 44.611 0.00 0.00
B
ATOM 17691 HG11 ILE B 68 −11.710 36.724 45.033 0.00 0.00
B
ATOM 17692 HG12 ILE B 68 −12.787 37.990 45.381 0.00 0.00
B
ATOM 17693 CD ILE B 68 −13.859 36.373 44.548 0.00 0.00
B
ATOM 17694 HD1 ILE B 68 −14.817 36.882 44.310 0.00 0.00
B
ATOM 17695 HD2 ILE B 68 −13.670 35.534 43.844 0.00 0.00
B
ATOM 17696 HD3 ILE B 68 −14.089 35.961 45.553 0.00 0.00
B
ATOM 17697 C ILE B 68 −13.409 40.093 43.468 0.00 0.00
B
ATOM 17698 O ILE B 68 −14.015 40.179 44.508 0.00 0.00
B
ATOM 17699 N ARG B 69 −12.892 41.250 43.024 0.00 0.00
B
ATOM 17700 HN ARG B 69 −12.320 41.233 42.208 0.00 0.00
B
ATOM 17701 CA ARG B 69 −12.868 42.497 43.774 0.00 0.00
B
ATOM 17702 HA ARG B 69 −12.876 42.190 44.810 0.00 0.00
B
ATOM 17703 CB ARG B 69 −11.655 43.412 43.444 0.00 0.00
B
ATOM 17704 HB1 ARG B 69 −11.548 43.845 42.427 0.00 0.00
B
ATOM 17705 HB2 ARG B 69 −11.700 44.203 44.223 0.00 0.00
B
ATOM 17706 CG ARG B 69 −10.348 42.655 43.716 0.00 0.00
B
ATOM 17707 HG1 ARG B 69 −10.258 42.379 44.789 0.00 0.00
B
ATOM 17708 HG2 ARG B 69 −10.046 41.803 43.070 0.00 0.00
B
ATOM 17709 CD ARG B 69 −9.095 43.535 43.733 0.00 0.00
B
ATOM 17710 HD1 ARG B 69 −9.159 44.411 44.414 0.00 0.00
B
ATOM 17711 HD2 ARG B 69 −8.218 42.971 44.117 0.00 0.00
B

ATOM 17712 NE ARG B 69 -8.778 43.904 42.392 0.00 0.00
B
ATOM 17713 HE ARG B 69 -9.419 43.753 41.640 0.00 0.00
B
ATOM 17714 CZ ARG B 69 -7.682 44.495 41.984 0.00 0.00
B
ATOM 17715 NH1 ARG B 69 -6.711 44.868 42.830 0.00 0.00
B
ATOM 17716 HH11 ARG B 69 -6.110 45.566 42.440 0.00 0.00
B
ATOM 17717 HH12 ARG B 69 -6.845 44.906 43.821 0.00 0.00
B
ATOM 17718 NH2 ARG B 69 -7.550 44.806 40.718 0.00 0.00
B
ATOM 17719 HH21 ARG B 69 -6.683 45.156 40.365 0.00 0.00
B
ATOM 17720 HH22 ARG B 69 -8.285 44.377 40.193 0.00 0.00
B
ATOM 17721 C ARG B 69 -14.133 43.301 43.609 0.00 0.00
B
ATOM 17722 O ARG B 69 -14.085 44.543 43.601 0.00 0.00
B
ATOM 17723 N ASN B 70 -15.223 42.625 43.412 0.00 0.00
B
ATOM 17724 HN ASN B 70 -15.083 41.644 43.311 0.00 0.00
B
ATOM 17725 CA ASN B 70 -16.512 43.201 43.421 0.00 0.00
B
ATOM 17726 HA ASN B 70 -16.463 44.261 43.620 0.00 0.00
B
ATOM 17727 CB ASN B 70 -17.086 42.985 41.920 0.00 0.00
B
ATOM 17728 HB1 ASN B 70 -17.154 41.880 41.833 0.00 0.00
B
ATOM 17729 HB2 ASN B 70 -18.058 43.508 41.794 0.00 0.00
B
ATOM 17730 CG ASN B 70 -16.209 43.635 40.897 0.00 0.00
B
ATOM 17731 OD1 ASN B 70 -16.079 44.857 40.773 0.00 0.00
B
ATOM 17732 ND2 ASN B 70 -15.683 42.761 40.038 0.00 0.00
B
ATOM 17733 HD21 ASN B 70 -15.087 43.224 39.383 0.00 0.00
B
ATOM 17734 HD22 ASN B 70 -15.816 41.770 40.017 0.00 0.00
B
ATOM 17735 C ASN B 70 -17.404 42.523 44.412 0.00 0.00
B
ATOM 17736 O ASN B 70 -18.526 42.900 44.670 0.00 0.00
B
ATOM 17737 N PHE B 71 -16.786 41.518 45.122 0.00 0.00
B
ATOM 17738 HN PHE B 71 -15.908 41.197 44.776 0.00 0.00
B

ATOM 17739 CA PHE B 71 −17.500 40.802 46.152 0.00 0.00
B
ATOM 17740 HA PHE B 71 −18.554 40.967 45.990 0.00 0.00
B
ATOM 17741 CB PHE B 71 −17.222 39.277 46.000 0.00 0.00
B
ATOM 17742 HB1 PHE B 71 −16.137 39.117 45.828 0.00 0.00
B
ATOM 17743 HB2 PHE B 71 −17.406 38.589 46.853 0.00 0.00
B
ATOM 17744 CG PHE B 71 −18.020 38.613 44.956 0.00 0.00
B
ATOM 17745 CD1 PHE B 71 −19.261 38.093 45.165 0.00 0.00
B
ATOM 17746 HD1 PHE B 71 −19.704 38.197 46.144 0.00 0.00
B
ATOM 17747 CE1 PHE B 71 −20.027 37.556 44.121 0.00 0.00
B
ATOM 17748 HE1 PHE B 71 −21.018 37.161 44.290 0.00 0.00
B
ATOM 17749 CZ PHE B 71 −19.453 37.449 42.835 0.00 0.00
B
ATOM 17750 HZ PHE B 71 −20.001 36.906 42.079 0.00 0.00
B
ATOM 17751 CD2 PHE B 71 −17.490 38.506 43.641 0.00 0.00
B
ATOM 17752 HD2 PHE B 71 −16.527 38.981 43.517 0.00 0.00
B
ATOM 17753 CE2 PHE B 71 −18.189 37.950 42.560 0.00 0.00
B
ATOM 17754 HE2 PHE B 71 −17.660 37.713 41.649 0.00 0.00
B
ATOM 17755 C PHE B 71 −17.182 41.273 47.527 0.00 0.00
B
ATOM 17756 O PHE B 71 −16.052 41.645 47.760 0.00 0.00
B
ATOM 17757 N GLU B 72 −18.162 41.070 48.469 0.00 0.00
B
ATOM 17758 HN GLU B 72 −18.969 40.550 48.198 0.00 0.00
B
ATOM 17759 CA GLU B 72 −18.011 41.589 49.825 0.00 0.00
B
ATOM 17760 HA GLU B 72 −17.081 42.137 49.867 0.00 0.00
B
ATOM 17761 CB GLU B 72 −19.187 42.498 50.162 0.00 0.00
B
ATOM 17762 HB1 GLU B 72 −19.276 43.211 49.315 0.00 0.00
B
ATOM 17763 HB2 GLU B 72 −20.110 41.883 50.113 0.00 0.00
B
ATOM 17764 CG GLU B 72 −18.968 43.399 51.397 0.00 0.00
B
ATOM 17765 HG1 GLU B 72 −19.033 42.881 52.377 0.00 0.00
B

ATOM 17766 HG2 GLU B 72 −17.976 43.894 51.323 0.00 0.00
B
ATOM 17767 CD GLU B 72 −20.020 44.480 51.517 0.00 0.00
B
ATOM 17768 OE1 GLU B 72 −20.943 44.554 52.336 0.00 0.00
B
ATOM 17769 OE2 GLU B 72 −19.974 45.265 50.499 0.00 0.00
B
ATOM 17770 C GLU B 72 −17.828 40.387 50.782 0.00 0.00
B
ATOM 17771 O GLU B 72 −16.851 40.408 51.538 0.00 0.00
B
ATOM 17772 N TYR B 73 −18.670 39.272 50.766 0.00 0.00
B
ATOM 17773 HN TYR B 73 −19.384 39.236 50.070 0.00 0.00
B
ATOM 17774 CA TYR B 73 −18.419 38.111 51.589 0.00 0.00
B
ATOM 17775 HA TYR B 73 −17.718 38.382 52.365 0.00 0.00
B
ATOM 17776 CB TYR B 73 −19.639 37.521 52.279 0.00 0.00
B
ATOM 17777 HB1 TYR B 73 −20.398 37.319 51.493 0.00 0.00
B
ATOM 17778 HB2 TYR B 73 −19.405 36.582 52.824 0.00 0.00
B
ATOM 17779 CG TYR B 73 −20.190 38.505 53.309 0.00 0.00
B
ATOM 17780 CD1 TYR B 73 −21.131 39.469 53.006 0.00 0.00
B
ATOM 17781 HD1 TYR B 73 −21.449 39.647 51.990 0.00 0.00
B
ATOM 17782 CE1 TYR B 73 −21.515 40.304 54.015 0.00 0.00
B
ATOM 17783 HE1 TYR B 73 −22.067 41.221 53.869 0.00 0.00
B
ATOM 17784 CZ TYR B 73 −21.055 40.136 55.386 0.00 0.00
B
ATOM 17785 OH TYR B 73 −21.442 41.023 56.344 0.00 0.00
B
ATOM 17786 HH TYR B 73 −22.131 41.532 55.910 0.00 0.00
B
ATOM 17787 CD2 TYR B 73 −19.701 38.381 54.631 0.00 0.00
B
ATOM 17788 HD2 TYR B 73 −18.990 37.589 54.814 0.00 0.00
B
ATOM 17789 CE2 TYR B 73 −20.154 39.142 55.673 0.00 0.00
B
ATOM 17790 HE2 TYR B 73 −19.790 38.937 56.669 0.00 0.00
B
ATOM 17791 C TYR B 73 −17.944 37.050 50.699 0.00 0.00
B
ATOM 17792 O TYR B 73 −18.426 36.805 49.585 0.00 0.00
B

ATOM 17793 N VAL B 74 −16.910 36.336 51.158 0.00 0.00
B
ATOM 17794 HN VAL B 74 −16.367 36.661 51.928 0.00 0.00
B
ATOM 17795 CA VAL B 74 −16.340 35.167 50.522 0.00 0.00
B
ATOM 17796 HA VAL B 74 −16.999 34.869 49.720 0.00 0.00
B
ATOM 17797 CB VAL B 74 −14.963 35.474 49.831 0.00 0.00
B
ATOM 17798 HB VAL B 74 −14.305 36.073 50.497 0.00 0.00
B
ATOM 17799 CG1 VAL B 74 −14.231 34.149 49.484 0.00 0.00
B
ATOM 17800 HG11 VAL B 74 −13.568 34.345 48.615 0.00 0.00
B
ATOM 17801 HG12 VAL B 74 −13.492 33.757 50.216 0.00 0.00
B
ATOM 17802 HG13 VAL B 74 −14.887 33.270 49.307 0.00 0.00
B
ATOM 17803 CG2 VAL B 74 −15.292 36.406 48.719 0.00 0.00
B
ATOM 17804 HG21 VAL B 74 −16.068 35.965 48.057 0.00 0.00
B
ATOM 17805 HG22 VAL B 74 −15.737 37.328 49.148 0.00 0.00
B
ATOM 17806 HG23 VAL B 74 −14.341 36.593 48.175 0.00 0.00
B
ATOM 17807 C VAL B 74 −16.290 34.131 51.593 0.00 0.00
B
ATOM 17808 O VAL B 74 −15.638 34.354 52.601 0.00 0.00
B
ATOM 17809 N ILE B 75 −16.936 33.021 51.384 0.00 0.00
B
ATOM 17810 HN ILE B 75 −17.456 32.834 50.555 0.00 0.00
B
ATOM 17811 CA ILE B 75 −16.927 31.944 52.361 0.00 0.00
B
ATOM 17812 HA ILE B 75 −16.343 32.263 53.212 0.00 0.00
B
ATOM 17813 CB ILE B 75 −18.339 31.509 52.693 0.00 0.00
B
ATOM 17814 HB ILE B 75 −18.793 30.868 51.908 0.00 0.00
B
ATOM 17815 CG2 ILE B 75 −18.419 30.585 53.989 0.00 0.00
B
ATOM 17816 HG21 ILE B 75 −17.554 30.735 54.670 0.00 0.00
B
ATOM 17817 HG22 ILE B 75 −19.372 30.855 54.491 0.00 0.00
B
ATOM 17818 HG23 ILE B 75 −18.542 29.530 53.664 0.00 0.00
B
ATOM 17819 CG1 ILE B 75 −19.380 32.701 52.890 0.00 0.00
B

ATOM 17820 HG11 ILE B 75 -19.246 33.247 53.847 0.00 0.00
B
ATOM 17821 HG12 ILE B 75 -19.212 33.510 52.147 0.00 0.00
B
ATOM 17822 CD ILE B 75 -20.828 32.372 52.681 0.00 0.00
B
ATOM 17823 HD1 ILE B 75 -20.780 31.553 51.932 0.00 0.00
B
ATOM 17824 HD2 ILE B 75 -21.349 32.038 53.604 0.00 0.00
B
ATOM 17825 HD3 ILE B 75 -21.287 33.295 52.266 0.00 0.00
B
ATOM 17826 C ILE B 75 -16.059 30.836 51.678 0.00 0.00
B
ATOM 17827 O ILE B 75 -16.428 30.157 50.742 0.00 0.00
B
ATOM 17828 N VAL B 76 -14.891 30.512 52.293 0.00 0.00
B
ATOM 17829 HN VAL B 76 -14.549 30.937 53.127 0.00 0.00
B
ATOM 17830 CA VAL B 76 -14.111 29.408 51.862 0.00 0.00
B
ATOM 17831 HA VAL B 76 -14.283 29.237 50.809 0.00 0.00
B
ATOM 17832 CB VAL B 76 -12.595 29.656 51.889 0.00 0.00
B
ATOM 17833 HB VAL B 76 -12.303 30.005 52.903 0.00 0.00
B
ATOM 17834 CG1 VAL B 76 -11.711 28.376 51.629 0.00 0.00
B
ATOM 17835 HG11 VAL B 76 -11.936 28.005 50.606 0.00 0.00
B
ATOM 17836 HG12 VAL B 76 -10.629 28.611 51.716 0.00 0.00
B
ATOM 17837 HG13 VAL B 76 -11.902 27.530 52.323 0.00 0.00
B
ATOM 17838 CG2 VAL B 76 -12.336 30.701 50.779 0.00 0.00
B
ATOM 17839 HG21 VAL B 76 -12.813 30.483 49.800 0.00 0.00
B
ATOM 17840 HG22 VAL B 76 -12.674 31.684 51.170 0.00 0.00
B
ATOM 17841 HG23 VAL B 76 -11.254 30.753 50.533 0.00 0.00
B
ATOM 17842 C VAL B 76 -14.547 28.142 52.594 0.00 0.00
B
ATOM 17843 O VAL B 76 -14.451 27.991 53.831 0.00 0.00
B
ATOM 17844 N ALA B 77 -15.127 27.226 51.796 0.00 0.00
B
ATOM 17845 HN ALA B 77 -15.276 27.345 50.817 0.00 0.00
B
ATOM 17846 CA ALA B 77 -15.770 26.023 52.267 0.00 0.00
B

ATOM	17847	HA	ALA	B	77	−16.013	26.028	53.319	0.00	0.00	B
ATOM	17848	CB	ALA	B	77	−16.988	25.873	51.316	0.00	0.00	B
ATOM	17849	HB1	ALA	B	77	−17.533	26.840	51.269	0.00	0.00	B
ATOM	17850	HB2	ALA	B	77	−16.625	25.727	50.276	0.00	0.00	B
ATOM	17851	HB3	ALA	B	77	−17.667	25.049	51.624	0.00	0.00	B
ATOM	17852	C	ALA	B	77	−14.814	24.788	52.167	0.00	0.00	B
ATOM	17853	O	ALA	B	77	−14.969	23.788	52.779	0.00	0.00	B
ATOM	17854	N	ILE	B	78	−13.738	24.899	51.366	0.00	0.00	B
ATOM	17855	HN	ILE	B	78	−13.579	25.615	50.689	0.00	0.00	B
ATOM	17856	CA	ILE	B	78	−12.661	23.970	51.438	0.00	0.00	B
ATOM	17857	HA	ILE	B	78	−13.184	23.036	51.301	0.00	0.00	B
ATOM	17858	CB	ILE	B	78	−11.554	24.283	50.427	0.00	0.00	B
ATOM	17859	HB	ILE	B	78	−10.827	25.029	50.815	0.00	0.00	B
ATOM	17860	CG2	ILE	B	78	−10.742	23.001	50.087	0.00	0.00	B
ATOM	17861	HG21	ILE	B	78	−10.415	22.498	51.021	0.00	0.00	B
ATOM	17862	HG22	ILE	B	78	−11.473	22.288	49.650	0.00	0.00	B
ATOM	17863	HG23	ILE	B	78	−9.834	23.112	49.457	0.00	0.00	B
ATOM	17864	CG1	ILE	B	78	−12.060	25.073	49.188	0.00	0.00	B
ATOM	17865	HG11	ILE	B	78	−12.891	24.558	48.662	0.00	0.00	B
ATOM	17866	HG12	ILE	B	78	−12.499	26.030	49.543	0.00	0.00	B
ATOM	17867	CD	ILE	B	78	−10.969	25.305	48.165	0.00	0.00	B
ATOM	17868	HD1	ILE	B	78	−11.466	25.804	47.306	0.00	0.00	B
ATOM	17869	HD2	ILE	B	78	−10.243	25.997	48.643	0.00	0.00	B
ATOM	17870	HD3	ILE	B	78	−10.450	24.353	47.923	0.00	0.00	B
ATOM	17871	C	ILE	B	78	−12.008	23.733	52.873	0.00	0.00	B
ATOM	17872	O	ILE	B	78	−11.914	24.628	53.708	0.00	0.00	B
ATOM	17873	N	GLY	B	79	−11.678	22.461	53.178	0.00	0.00	B

ATOM 17874 HN GLY B 79 -11.745 21.728 52.506 0.00 0.00
B
ATOM 17875 CA GLY B 79 -11.061 22.178 54.456 0.00 0.00
B
ATOM 17876 HA1 GLY B 79 -11.766 22.445 55.229 0.00 0.00
B
ATOM 17877 HA2 GLY B 79 -10.109 22.688 54.421 0.00 0.00
B
ATOM 17878 C GLY B 79 -10.563 20.800 54.751 0.00 0.00
B
ATOM 17879 O GLY B 79 -9.782 20.651 55.672 0.00 0.00
B
ATOM 17880 N ALA B 80 -10.995 19.722 53.988 0.00 0.00
B
ATOM 17881 HN ALA B 80 -11.501 19.890 53.146 0.00 0.00
B
ATOM 17882 CA ALA B 80 -10.515 18.353 54.238 0.00 0.00
B
ATOM 17883 HA ALA B 80 -10.416 18.263 55.309 0.00 0.00
B
ATOM 17884 CB ALA B 80 -11.378 17.218 53.769 0.00 0.00
B
ATOM 17885 HB1 ALA B 80 -11.115 16.252 54.252 0.00 0.00
B
ATOM 17886 HB2 ALA B 80 -12.455 17.344 54.009 0.00 0.00
B
ATOM 17887 HB3 ALA B 80 -11.229 17.204 52.668 0.00 0.00
B
ATOM 17888 C ALA B 80 -9.075 18.250 53.686 0.00 0.00
B
ATOM 17889 O ALA B 80 -8.116 17.705 54.297 0.00 0.00
B
ATOM 17890 N ASN B 81 -8.837 19.063 52.691 0.00 0.00
B
ATOM 17891 HN ASN B 81 -9.640 19.513 52.310 0.00 0.00
B
ATOM 17892 CA ASN B 81 -7.530 19.387 52.092 0.00 0.00
B
ATOM 17893 HA ASN B 81 -6.776 18.677 52.398 0.00 0.00
B
ATOM 17894 CB ASN B 81 -7.756 19.683 50.548 0.00 0.00
B
ATOM 17895 HB1 ASN B 81 -8.196 18.809 50.021 0.00 0.00
B
ATOM 17896 HB2 ASN B 81 -8.482 20.519 50.452 0.00 0.00
B
ATOM 17897 CG ASN B 81 -6.484 20.048 49.902 0.00 0.00
B
ATOM 17898 OD1 ASN B 81 -5.857 21.114 50.133 0.00 0.00
B
ATOM 17899 ND2 ASN B 81 -5.961 19.104 49.116 0.00 0.00
B
ATOM 17900 HD21 ASN B 81 -4.973 18.952 49.165 0.00 0.00
B

ATOM 17901 HD22 ASN B 81 −6.486 18.270 48.946 0.00 0.00
B
ATOM 17902 C ASN B 81 −7.133 20.689 52.885 0.00 0.00
B
ATOM 17903 O ASN B 81 −7.753 21.757 52.861 0.00 0.00
B
ATOM 17904 N ILE B 82 −6.025 20.667 53.587 0.00 0.00
B
ATOM 17905 HN ILE B 82 −5.474 19.838 53.639 0.00 0.00
B
ATOM 17906 CA ILE B 82 −5.581 21.879 54.275 0.00 0.00
B
ATOM 17907 HA ILE B 82 −6.427 22.400 54.698 0.00 0.00
B
ATOM 17908 CB ILE B 82 −4.557 21.589 55.346 0.00 0.00
B
ATOM 17909 HB ILE B 82 −3.589 21.238 54.930 0.00 0.00
B
ATOM 17910 CG2 ILE B 82 −4.151 22.884 56.107 0.00 0.00
B
ATOM 17911 HG21 ILE B 82 −3.428 23.538 55.574 0.00 0.00
B
ATOM 17912 HG22 ILE B 82 −5.004 23.568 56.306 0.00 0.00
B
ATOM 17913 HG23 ILE B 82 −3.743 22.543 57.082 0.00 0.00
B
ATOM 17914 CG1 ILE B 82 −5.023 20.551 56.347 0.00 0.00
B
ATOM 17915 HG11 ILE B 82 −5.177 19.572 55.845 0.00 0.00
B
ATOM 17916 HG12 ILE B 82 −4.239 20.450 57.128 0.00 0.00
B
ATOM 17917 CD ILE B 82 −6.416 20.872 56.890 0.00 0.00
B
ATOM 17918 HD1 ILE B 82 −6.854 20.098 57.555 0.00 0.00
B
ATOM 17919 HD2 ILE B 82 −6.411 21.723 57.605 0.00 0.00
B
ATOM 17920 HD3 ILE B 82 −7.160 21.050 56.085 0.00 0.00
B
ATOM 17921 C ILE B 82 −4.961 22.941 53.428 0.00 0.00
B
ATOM 17922 O ILE B 82 −5.392 24.080 53.437 0.00 0.00
B
ATOM 17923 N GLN B 83 −4.080 22.469 52.448 0.00 0.00
B
ATOM 17924 HN GLN B 83 −3.774 21.521 52.447 0.00 0.00
B
ATOM 17925 CA GLN B 83 −3.383 23.342 51.547 0.00 0.00
B
ATOM 17926 HA GLN B 83 −2.826 24.067 52.123 0.00 0.00
B
ATOM 17927 CB GLN B 83 −2.432 22.554 50.586 0.00 0.00
B

ATOM 17928 HB1 GLN B 83 −1.752 21.885 51.155 0.00 0.00
B
ATOM 17929 HB2 GLN B 83 −3.103 21.986 49.907 0.00 0.00
B
ATOM 17930 CG GLN B 83 −1.609 23.503 49.599 0.00 0.00
B
ATOM 17931 HG1 GLN B 83 −2.287 24.168 49.022 0.00 0.00
B
ATOM 17932 HG2 GLN B 83 −0.969 24.225 50.150 0.00 0.00
B
ATOM 17933 CD GLN B 83 −0.841 22.783 48.481 0.00 0.00
B
ATOM 17934 OE1 GLN B 83 −1.260 22.638 47.340 0.00 0.00
B
ATOM 17935 NE2 GLN B 83 0.208 22.145 49.016 0.00 0.00
B
ATOM 17936 HE21 GLN B 83 0.752 21.596 48.382 0.00 0.00
B
ATOM 17937 HE22 GLN B 83 0.517 22.359 49.943 0.00 0.00
B
ATOM 17938 C GLN B 83 −4.229 24.186 50.683 0.00 0.00
B
ATOM 17939 O GLN B 83 −3.942 25.383 50.568 0.00 0.00
B
ATOM 17940 N ALA B 84 −5.335 23.737 50.136 0.00 0.00
B
ATOM 17941 HN ALA B 84 −5.519 22.773 50.309 0.00 0.00
B
ATOM 17942 CA ALA B 84 −6.202 24.584 49.347 0.00 0.00
B
ATOM 17943 HA ALA B 84 −5.595 25.028 48.572 0.00 0.00
B
ATOM 17944 CB ALA B 84 −7.260 23.708 48.634 0.00 0.00
B
ATOM 17945 HB1 ALA B 84 −7.642 24.187 47.707 0.00 0.00
B
ATOM 17946 HB2 ALA B 84 −6.666 22.822 48.325 0.00 0.00
B
ATOM 17947 HB3 ALA B 84 −8.035 23.483 49.397 0.00 0.00
B
ATOM 17948 C ALA B 84 −6.926 25.710 50.046 0.00 0.00
B
ATOM 17949 O ALA B 84 −6.980 26.867 49.699 0.00 0.00
B
ATOM 17950 N SER B 85 −7.519 25.287 51.246 0.00 0.00
B
ATOM 17951 HN SER B 85 −7.389 24.339 51.528 0.00 0.00
B
ATOM 17952 CA SER B 85 −8.180 26.229 52.134 0.00 0.00
B
ATOM 17953 HA SER B 85 −9.012 26.736 51.668 0.00 0.00
B
ATOM 17954 CB SER B 85 −8.766 25.496 53.302 0.00 0.00
B

ATOM 17955 HB1 SER B 85 -9.437 24.731 52.855 0.00 0.00
B
ATOM 17956 HB2 SER B 85 -7.969 24.982 53.882 0.00 0.00
B
ATOM 17957 OG SER B 85 -9.530 26.323 54.198 0.00 0.00
B
ATOM 17958 HG1 SER B 85 -10.315 25.805 54.390 0.00 0.00
B
ATOM 17959 C SER B 85 -7.275 27.372 52.695 0.00 0.00
B
ATOM 17960 O SER B 85 -7.552 28.557 52.634 0.00 0.00
B
ATOM 17961 N THR B 86 -6.111 27.018 53.190 0.00 0.00
B
ATOM 17962 HN THR B 86 -6.081 26.022 53.193 0.00 0.00
B
ATOM 17963 CA THR B 86 -4.976 28.012 53.601 0.00 0.00
B
ATOM 17964 HA THR B 86 -5.444 28.569 54.400 0.00 0.00
B
ATOM 17965 CB THR B 86 -3.868 27.271 54.288 0.00 0.00
B
ATOM 17966 HB THR B 86 -3.127 28.076 54.481 0.00 0.00
B
ATOM 17967 OG1 THR B 86 -3.359 26.160 53.541 0.00 0.00
B
ATOM 17968 HG1 THR B 86 -4.075 25.556 53.330 0.00 0.00
B
ATOM 17969 CG2 THR B 86 -4.274 26.621 55.639 0.00 0.00
B
ATOM 17970 HG21 THR B 86 -3.370 26.248 56.167 0.00 0.00
B
ATOM 17971 HG22 THR B 86 -4.884 27.290 56.283 0.00 0.00
B
ATOM 17972 HG23 THR B 86 -4.941 25.774 55.370 0.00 0.00
B
ATOM 17973 C THR B 86 -4.512 28.936 52.555 0.00 0.00
B
ATOM 17974 O THR B 86 -4.472 30.130 52.734 0.00 0.00
B
ATOM 17975 N LEU B 87 -4.184 28.379 51.369 0.00 0.00
B
ATOM 17976 HN LEU B 87 -4.116 27.388 51.283 0.00 0.00
B
ATOM 17977 CA LEU B 87 -3.891 29.154 50.153 0.00 0.00
B
ATOM 17978 HA LEU B 87 -3.210 29.958 50.389 0.00 0.00
B
ATOM 17979 CB LEU B 87 -3.250 28.357 48.940 0.00 0.00
B
ATOM 17980 HB1 LEU B 87 -3.771 27.387 48.790 0.00 0.00
B
ATOM 17981 HB2 LEU B 87 -3.263 29.026 48.053 0.00 0.00
B

ATOM 17982 CG LEU B 87 -1.794 27.897 49.115 0.00 0.00
B
ATOM 17983 HG LEU B 87 -1.700 27.192 49.968 0.00 0.00
B
ATOM 17984 CD1 LEU B 87 -1.308 26.950 47.949 0.00 0.00
B
ATOM 17985 HD11 LEU B 87 -2.152 26.243 47.799 0.00 0.00
B
ATOM 17986 HD12 LEU B 87 -1.209 27.592 47.048 0.00 0.00
B
ATOM 17987 HD13 LEU B 87 -0.445 26.339 48.289 0.00 0.00
B
ATOM 17988 CD2 LEU B 87 -0.852 29.063 49.218 0.00 0.00
B
ATOM 17989 HD21 LEU B 87 -0.962 29.650 50.154 0.00 0.00
B
ATOM 17990 HD22 LEU B 87 0.165 28.621 49.141 0.00 0.00
B
ATOM 17991 HD23 LEU B 87 -0.885 29.749 48.345 0.00 0.00
B
ATOM 17992 C LEU B 87 -5.020 30.022 49.600 0.00 0.00
B
ATOM 17993 O LEU B 87 -4.728 31.159 49.186 0.00 0.00
B
ATOM 17994 N THR B 88 -6.288 29.508 49.562 0.00 0.00
B
ATOM 17995 HN THR B 88 -6.489 28.565 49.815 0.00 0.00
B
ATOM 17996 CA THR B 88 -7.387 30.336 49.191 0.00 0.00
B
ATOM 17997 HA THR B 88 -7.182 30.821 48.248 0.00 0.00
B
ATOM 17998 CB THR B 88 -8.741 29.556 49.073 0.00 0.00
B
ATOM 17999 HB THR B 88 -9.126 29.299 50.083 0.00 0.00
B
ATOM 18000 OG1 THR B 88 -8.607 28.328 48.329 0.00 0.00
B
ATOM 18001 HG1 THR B 88 -8.163 27.646 48.837 0.00 0.00
B
ATOM 18002 CG2 THR B 88 -9.777 30.364 48.304 0.00 0.00
B
ATOM 18003 HG21 THR B 88 -9.887 31.383 48.733 0.00 0.00
B
ATOM 18004 HG22 THR B 88 -9.293 30.582 47.328 0.00 0.00
B
ATOM 18005 HG23 THR B 88 -10.759 29.855 48.200 0.00 0.00
B
ATOM 18006 C THR B 88 -7.623 31.482 50.123 0.00 0.00
B
ATOM 18007 O THR B 88 -7.977 32.544 49.629 0.00 0.00
B
ATOM 18008 N THR B 89 -7.511 31.327 51.454 0.00 0.00
B

ATOM 18009 HN THR B 89 −7.290 30.437 51.843 0.00 0.00
B
ATOM 18010 CA THR B 89 −7.582 32.364 52.461 0.00 0.00
B
ATOM 18011 HA THR B 89 −8.559 32.784 52.274 0.00 0.00
B
ATOM 18012 CB THR B 89 −7.444 31.885 53.847 0.00 0.00
B
ATOM 18013 HB THR B 89 −6.458 31.383 53.947 0.00 0.00
B
ATOM 18014 OG1 THR B 89 −8.476 30.897 54.100 0.00 0.00
B
ATOM 18015 HG1 THR B 89 −8.156 30.084 53.704 0.00 0.00
B
ATOM 18016 CG2 THR B 89 −7.657 33.011 54.908 0.00 0.00
B
ATOM 18017 HG21 THR B 89 −8.516 33.665 54.644 0.00 0.00
B
ATOM 18018 HG22 THR B 89 −7.782 32.532 55.902 0.00 0.00
B
ATOM 18019 HG23 THR B 89 −6.766 33.675 54.915 0.00 0.00
B
ATOM 18020 C THR B 89 −6.501 33.466 52.289 0.00 0.00
B
ATOM 18021 O THR B 89 −6.806 34.688 52.176 0.00 0.00
B
ATOM 18022 N LEU B 90 −5.229 33.104 52.204 0.00 0.00
B
ATOM 18023 HN LEU B 90 −5.024 32.136 52.325 0.00 0.00
B
ATOM 18024 CA LEU B 90 −4.188 34.026 52.079 0.00 0.00
B
ATOM 18025 HA LEU B 90 −4.339 34.688 52.918 0.00 0.00
B
ATOM 18026 CB LEU B 90 −2.801 33.389 52.477 0.00 0.00
B
ATOM 18027 HB1 LEU B 90 −2.003 34.144 52.314 0.00 0.00
B
ATOM 18028 HB2 LEU B 90 −2.799 33.029 53.527 0.00 0.00
B
ATOM 18029 CG LEU B 90 −2.326 32.248 51.575 0.00 0.00
B
ATOM 18030 HG LEU B 90 −3.088 31.618 51.069 0.00 0.00
B
ATOM 18031 CD1 LEU B 90 −1.468 32.704 50.347 0.00 0.00
B
ATOM 18032 HD11 LEU B 90 −0.545 33.179 50.745 0.00 0.00
B
ATOM 18033 HD12 LEU B 90 −0.989 31.864 49.799 0.00 0.00
B
ATOM 18034 HD13 LEU B 90 −2.028 33.400 49.687 0.00 0.00
B
ATOM 18035 CD2 LEU B 90 −1.519 31.218 52.291 0.00 0.00
B

ATOM 18036 HD21 LEU B 90   -0.422 31.386 52.341 0.00 0.00
B
ATOM 18037 HD22 LEU B 90   -1.841 31.189 53.354 0.00 0.00
B
ATOM 18038 HD23 LEU B 90   -1.689 30.212 51.850 0.00 0.00
B
ATOM 18039 C   LEU B 90   -4.174 34.953 50.890 0.00 0.00
B
ATOM 18040 O   LEU B 90   -3.956 36.185 51.011 0.00 0.00
B
ATOM 18041 N   LEU B 91   -4.561 34.338 49.730 0.00 0.00
B
ATOM 18042 HN LEU B 91   -4.505 33.350 49.612 0.00 0.00
B
ATOM 18043 CA LEU B 91   -4.696 35.146 48.527 0.00 0.00
B
ATOM 18044 HA LEU B 91   -3.760 35.648 48.326 0.00 0.00
B
ATOM 18045 CB LEU B 91   -5.049 34.205 47.320 0.00 0.00
B
ATOM 18046 HB1 LEU B 91   -6.020 33.728 47.570 0.00 0.00
B
ATOM 18047 HB2 LEU B 91   -5.252 34.821 46.418 0.00 0.00
B
ATOM 18048 CG LEU B 91   -3.874 33.248 46.855 0.00 0.00
B
ATOM 18049 HG LEU B 91   -3.369 32.794 47.735 0.00 0.00
B
ATOM 18050 CD1 LEU B 91   -4.339 32.117 46.004 0.00 0.00
B
ATOM 18051 HD11 LEU B 91   -3.497 31.507 45.613 0.00 0.00
B
ATOM 18052 HD12 LEU B 91   -5.026 31.453 46.572 0.00 0.00
B
ATOM 18053 HD13 LEU B 91   -5.044 32.529 45.250 0.00 0.00
B
ATOM 18054 CD2 LEU B 91   -2.710 34.058 46.217 0.00 0.00
B
ATOM 18055 HD21 LEU B 91   -2.757 35.078 46.655 0.00 0.00
B
ATOM 18056 HD22 LEU B 91   -1.761 33.481 46.227 0.00 0.00
B
ATOM 18057 HD23 LEU B 91   -2.814 34.167 45.116 0.00 0.00
B
ATOM 18058 C   LEU B 91   -5.784 36.235 48.522 0.00 0.00
B
ATOM 18059 O   LEU B 91   -5.618 37.340 48.061 0.00 0.00
B
ATOM 18060 N   LEU B 92   -6.992 35.882 48.993 0.00 0.00
B
ATOM 18061 HN LEU B 92   -7.224 34.924 49.140 0.00 0.00
B
ATOM 18062 CA LEU B 92   -8.014 36.871 49.205 0.00 0.00
B

ATOM 18063 HA LEU B 92 -8.314 37.265 48.245 0.00 0.00
B
ATOM 18064 CB LEU B 92 -9.306 36.193 49.758 0.00 0.00
B
ATOM 18065 HB1 LEU B 92 -8.935 35.624 50.637 0.00 0.00
B
ATOM 18066 HB2 LEU B 92 -10.077 36.840 50.228 0.00 0.00
B
ATOM 18067 CG LEU B 92 -9.995 35.103 48.879 0.00 0.00
B
ATOM 18068 HG LEU B 92 -9.225 34.340 48.639 0.00 0.00
B
ATOM 18069 CD1 LEU B 92 -10.877 34.374 49.892 0.00 0.00
B
ATOM 18070 HD11 LEU B 92 -11.676 35.023 50.311 0.00 0.00
B
ATOM 18071 HD12 LEU B 92 -11.468 33.681 49.256 0.00 0.00
B
ATOM 18072 HD13 LEU B 92 -10.190 33.841 50.583 0.00 0.00
B
ATOM 18073 CD2 LEU B 92 -10.686 35.621 47.662 0.00 0.00
B
ATOM 18074 HD21 LEU B 92 -10.021 36.101 46.912 0.00 0.00
B
ATOM 18075 HD22 LEU B 92 -11.214 34.848 47.063 0.00 0.00
B
ATOM 18076 HD23 LEU B 92 -11.481 36.358 47.908 0.00 0.00
B
ATOM 18077 C LEU B 92 -7.715 38.068 50.018 0.00 0.00
B
ATOM 18078 O LEU B 92 -8.044 39.226 49.595 0.00 0.00
B
ATOM 18079 N LYS B 93 -6.972 37.828 51.158 0.00 0.00
B
ATOM 18080 HN LYS B 93 -6.841 36.882 51.443 0.00 0.00
B
ATOM 18081 CA LYS B 93 -6.466 38.920 52.042 0.00 0.00
B
ATOM 18082 HA LYS B 93 -7.358 39.497 52.241 0.00 0.00
B
ATOM 18083 CB LYS B 93 -5.708 38.442 53.334 0.00 0.00
B
ATOM 18084 HB1 LYS B 93 -6.161 37.491 53.685 0.00 0.00
B
ATOM 18085 HB2 LYS B 93 -4.638 38.230 53.125 0.00 0.00
B
ATOM 18086 CG LYS B 93 -5.833 39.422 54.482 0.00 0.00
B
ATOM 18087 HG1 LYS B 93 -5.172 39.136 55.328 0.00 0.00
B
ATOM 18088 HG2 LYS B 93 -5.421 40.389 54.123 0.00 0.00
B
ATOM 18089 CD LYS B 93 -7.272 39.718 54.979 0.00 0.00
B

ATOM 18090 HD1 LYS B 93 -7.907 40.211 54.213 0.00 0.00
B
ATOM 18091 HD2 LYS B 93 -7.714 38.711 55.136 0.00 0.00
B
ATOM 18092 CE LYS B 93 -7.351 40.337 56.377 0.00 0.00
B
ATOM 18093 HE1 LYS B 93 -6.796 39.612 57.010 0.00 0.00
B
ATOM 18094 HE2 LYS B 93 -6.975 41.380 56.451 0.00 0.00
B
ATOM 18095 NZ LYS B 93 -8.764 40.451 56.866 0.00 0.00
B
ATOM 18096 HZ1 LYS B 93 -8.920 39.728 57.597 0.00 0.00
B
ATOM 18097 HZ2 LYS B 93 -8.908 41.430 57.185 0.00 0.00
B
ATOM 18098 HZ3 LYS B 93 -9.442 40.238 56.107 0.00 0.00
B
ATOM 18099 C LYS B 93 -5.437 39.763 51.323 0.00 0.00
B
ATOM 18100 O LYS B 93 -5.421 40.983 51.426 0.00 0.00
B
ATOM 18101 N GLU B 94 -4.679 39.104 50.439 0.00 0.00
B
ATOM 18102 HN GLU B 94 -4.865 38.132 50.323 0.00 0.00
B
ATOM 18103 CA GLU B 94 -3.706 39.802 49.638 0.00 0.00
B
ATOM 18104 HA GLU B 94 -3.105 40.430 50.279 0.00 0.00
B
ATOM 18105 CB GLU B 94 -2.753 38.842 48.942 0.00 0.00
B
ATOM 18106 HB1 GLU B 94 -2.500 38.139 49.765 0.00 0.00
B
ATOM 18107 HB2 GLU B 94 -3.192 38.308 48.072 0.00 0.00
B
ATOM 18108 CG GLU B 94 -1.499 39.603 48.452 0.00 0.00
B
ATOM 18109 HG1 GLU B 94 -1.816 40.219 47.584 0.00 0.00
B
ATOM 18110 HG2 GLU B 94 -0.995 40.213 49.232 0.00 0.00
B
ATOM 18111 CD GLU B 94 -0.404 38.602 47.991 0.00 0.00
B
ATOM 18112 OE1 GLU B 94 0.000 38.735 46.847 0.00 0.00
B
ATOM 18113 OE2 GLU B 94 0.053 37.732 48.767 0.00 0.00
B
ATOM 18114 C GLU B 94 -4.276 40.742 48.545 0.00 0.00
B
ATOM 18115 O GLU B 94 -3.733 41.811 48.271 0.00 0.00
B
ATOM 18116 N LEU B 95 -5.400 40.403 48.002 0.00 0.00
B

ATOM 18117 HN LEU B 95 −5.718 39.508 48.304 0.00 0.00
B
ATOM 18118 CA LEU B 95 −6.218 41.052 47.009 0.00 0.00
B
ATOM 18119 HA LEU B 95 −5.590 41.745 46.468 0.00 0.00
B
ATOM 18120 CB LEU B 95 −6.816 39.990 46.129 0.00 0.00
B
ATOM 18121 HB1 LEU B 95 −7.447 39.240 46.651 0.00 0.00
B
ATOM 18122 HB2 LEU B 95 −7.528 40.522 45.462 0.00 0.00
B
ATOM 18123 CG LEU B 95 −5.830 39.288 45.184 0.00 0.00
B
ATOM 18124 HG LEU B 95 −4.897 38.913 45.657 0.00 0.00
B
ATOM 18125 CD1 LEU B 95 −6.481 37.944 44.607 0.00 0.00
B
ATOM 18126 HD11 LEU B 95 −5.828 37.448 43.857 0.00 0.00
B
ATOM 18127 HD12 LEU B 95 −6.664 37.198 45.410 0.00 0.00
B
ATOM 18128 HD13 LEU B 95 −7.484 38.130 44.168 0.00 0.00
B
ATOM 18129 CD2 LEU B 95 −5.387 40.182 43.982 0.00 0.00
B
ATOM 18130 HD21 LEU B 95 −4.742 41.027 44.306 0.00 0.00
B
ATOM 18131 HD22 LEU B 95 −4.681 39.538 43.416 0.00 0.00
B
ATOM 18132 HD23 LEU B 95 −6.330 40.424 43.447 0.00 0.00
B
ATOM 18133 C LEU B 95 −7.354 41.868 47.579 0.00 0.00
B
ATOM 18134 O LEU B 95 −8.300 42.362 46.895 0.00 0.00
B
ATOM 18135 N ASP B 96 −7.375 42.149 48.886 0.00 0.00
B
ATOM 18136 HN ASP B 96 −6.649 41.781 49.462 0.00 0.00
B
ATOM 18137 CA ASP B 96 −8.191 43.175 49.538 0.00 0.00
B
ATOM 18138 HA ASP B 96 −7.951 43.324 50.580 0.00 0.00
B
ATOM 18139 CB ASP B 96 −8.043 44.630 49.013 0.00 0.00
B
ATOM 18140 HB1 ASP B 96 −8.452 44.786 47.992 0.00 0.00
B
ATOM 18141 HB2 ASP B 96 −8.568 45.341 49.686 0.00 0.00
B
ATOM 18142 CG ASP B 96 −6.639 45.031 49.074 0.00 0.00
B
ATOM 18143 OD1 ASP B 96 −6.116 45.015 50.172 0.00 0.00
B

ATOM 18144 OD2 ASP B 96 −6.159 45.518 48.008 0.00 0.00
B
ATOM 18145 C ASP B 96 −9.656 42.845 49.461 0.00 0.00
B
ATOM 18146 O ASP B 96 −10.553 43.696 49.535 0.00 0.00
B
ATOM 18147 N ILE B 97 −10.001 41.516 49.566 0.00 0.00
B
ATOM 18148 HN ILE B 97 −9.316 40.792 49.543 0.00 0.00
B
ATOM 18149 CA ILE B 97 −11.353 41.064 49.677 0.00 0.00
B
ATOM 18150 HA ILE B 97 −11.895 41.589 48.905 0.00 0.00
B
ATOM 18151 CB ILE B 97 −11.480 39.612 49.191 0.00 0.00
B
ATOM 18152 HB ILE B 97 −10.760 39.037 49.811 0.00 0.00
B
ATOM 18153 CG2 ILE B 97 −12.845 38.870 49.364 0.00 0.00
B
ATOM 18154 HG21 ILE B 97 −12.680 37.839 48.986 0.00 0.00
B
ATOM 18155 HG22 ILE B 97 −13.324 38.754 50.359 0.00 0.00
B
ATOM 18156 HG23 ILE B 97 −13.570 39.500 48.805 0.00 0.00
B
ATOM 18157 CG1 ILE B 97 −11.036 39.508 47.729 0.00 0.00
B
ATOM 18158 HG11 ILE B 97 −9.938 39.382 47.616 0.00 0.00
B
ATOM 18159 HG12 ILE B 97 −11.392 38.527 47.346 0.00 0.00
B
ATOM 18160 CD ILE B 97 −11.591 40.554 46.710 0.00 0.00
B
ATOM 18161 HD1 ILE B 97 −11.424 40.155 45.687 0.00 0.00
B
ATOM 18162 HD2 ILE B 97 −12.678 40.770 46.798 0.00 0.00
B
ATOM 18163 HD3 ILE B 97 −10.980 41.475 46.816 0.00 0.00
B
ATOM 18164 C ILE B 97 −11.931 41.326 51.099 0.00 0.00
B
ATOM 18165 O ILE B 97 −11.458 40.658 52.015 0.00 0.00
B
ATOM 18166 N PRO B 98 −13.048 42.110 51.285 0.00 0.00
B
ATOM 18167 CD PRO B 98 −13.671 42.916 50.298 0.00 0.00
B
ATOM 18168 HD1 PRO B 98 −12.948 43.335 49.567 0.00 0.00
B
ATOM 18169 HD2 PRO B 98 −14.212 42.232 49.609 0.00 0.00
B
ATOM 18170 CA PRO B 98 −13.393 42.643 52.591 0.00 0.00
B

ATOM 18171 HA PRO B 98 −12.641 43.342 52.926 0.00 0.00
B
ATOM 18172 CB PRO B 98 −14.706 43.424 52.475 0.00 0.00
B
ATOM 18173 HB1 PRO B 98 −14.816 44.238 53.222 0.00 0.00
B
ATOM 18174 HB2 PRO B 98 −15.553 42.707 52.536 0.00 0.00
B
ATOM 18175 CG PRO B 98 −14.587 43.937 51.020 0.00 0.00
B
ATOM 18176 HG1 PRO B 98 −15.534 43.977 50.440 0.00 0.00
B
ATOM 18177 HG2 PRO B 98 −14.109 44.935 50.922 0.00 0.00
B
ATOM 18178 C PRO B 98 −13.516 41.513 53.679 0.00 0.00
B
ATOM 18179 O PRO B 98 −13.019 41.645 54.752 0.00 0.00
B
ATOM 18180 N ASN B 99 −14.405 40.488 53.473 0.00 0.00
B
ATOM 18181 HN ASN B 99 −14.866 40.320 52.606 0.00 0.00
B
ATOM 18182 CA ASN B 99 −14.807 39.457 54.502 0.00 0.00
B
ATOM 18183 HA ASN B 99 −14.125 39.533 55.337 0.00 0.00
B
ATOM 18184 CB ASN B 99 −16.300 39.520 54.836 0.00 0.00
B
ATOM 18185 HB1 ASN B 99 −16.947 39.489 53.933 0.00 0.00
B
ATOM 18186 HB2 ASN B 99 −16.587 38.670 55.491 0.00 0.00
B
ATOM 18187 CG ASN B 99 −16.546 40.753 55.684 0.00 0.00
B
ATOM 18188 OD1 ASN B 99 −16.157 40.697 56.855 0.00 0.00
B
ATOM 18189 ND2 ASN B 99 −17.054 41.884 55.120 0.00 0.00
B
ATOM 18190 HD21 ASN B 99 −17.309 42.644 55.717 0.00 0.00
B
ATOM 18191 HD22 ASN B 99 −17.190 41.906 54.130 0.00 0.00
B
ATOM 18192 C ASN B 99 −14.536 38.035 53.944 0.00 0.00
B
ATOM 18193 O ASN B 99 −15.142 37.500 52.985 0.00 0.00
B
ATOM 18194 N ILE B 100 −13.643 37.291 54.616 0.00 0.00
B
ATOM 18195 HN ILE B 100 −13.067 37.627 55.357 0.00 0.00
B
ATOM 18196 CA ILE B 100 −13.211 35.932 54.272 0.00 0.00
B
ATOM 18197 HA ILE B 100 −13.675 35.605 53.353 0.00 0.00
B

ATOM 18198 CB ILE B 100   −11.730 35.742 54.186 0.00 0.00
B
ATOM 18199 HB ILE B 100   −11.310 35.786 55.213 0.00 0.00
B
ATOM 18200 CG2 ILE B 100   −11.415 34.504 53.441 0.00 0.00
B
ATOM 18201 HG21 ILE B 100   −10.305 34.461 53.408 0.00 0.00
B
ATOM 18202 HG22 ILE B 100   −11.774 33.577 53.937 0.00 0.00
B
ATOM 18203 HG23 ILE B 100   −11.784 34.465 52.393 0.00 0.00
B
ATOM 18204 CG1 ILE B 100   −11.141 36.865 53.307 0.00 0.00
B
ATOM 18205 HG11 ILE B 100   −11.275 36.676 52.221 0.00 0.00
B
ATOM 18206 HG12 ILE B 100   −11.507 37.893 53.516 0.00 0.00
B
ATOM 18207 CD ILE B 100   −9.598 37.009 53.529 0.00 0.00
B
ATOM 18208 HD1 ILE B 100   −9.219 37.770 52.813 0.00 0.00
B
ATOM 18209 HD2 ILE B 100   −9.303 37.378 54.534 0.00 0.00
B
ATOM 18210 HD3 ILE B 100   −8.978 36.097 53.396 0.00 0.00
B
ATOM 18211 C   ILE B 100   −13.693 35.117 55.406 0.00 0.00
B
ATOM 18212 O   ILE B 100   −13.116 35.243 56.493 0.00 0.00
B
ATOM 18213 N   TRP B 101   −14.707 34.296 55.223 0.00 0.00
B
ATOM 18214 HN TRP B 101   −15.143 34.211 54.331 0.00 0.00
B
ATOM 18215 CA TRP B 101   −15.111 33.291 56.256 0.00 0.00
B
ATOM 18216 HA TRP B 101   −14.542 33.517 57.146 0.00 0.00
B
ATOM 18217 CB TRP B 101   −16.631 33.260 56.440 0.00 0.00
B
ATOM 18218 HB1 TRP B 101   −17.083 33.041 55.449 0.00 0.00
B
ATOM 18219 HB2 TRP B 101   −16.844 32.421 57.137 0.00 0.00
B
ATOM 18220 CG TRP B 101   −17.361 34.529 56.989 0.00 0.00
B
ATOM 18221 CD1 TRP B 101   −16.930 35.838 57.120 0.00 0.00
B
ATOM 18222 HD1 TRP B 101   −15.992 36.211 56.737 0.00 0.00
B
ATOM 18223 NE1 TRP B 101   −17.937 36.597 57.744 0.00 0.00
B
ATOM 18224 HE1 TRP B 101   −17.817 37.523 58.028 0.00 0.00
B

ATOM 18225 CE2 TRP B 101 −19.085 35.815 57.843 0.00 0.00
B
ATOM 18226 CD2 TRP B 101 −18.768 34.536 57.365 0.00 0.00
B
ATOM 18227 CE3 TRP B 101 −19.759 33.585 57.245 0.00 0.00
B
ATOM 18228 HE3 TRP B 101 −19.638 32.553 56.949 0.00 0.00
B
ATOM 18229 CZ3 TRP B 101 −21.070 33.895 57.820 0.00 0.00
B
ATOM 18230 HZ3 TRP B 101 −21.938 33.261 57.920 0.00 0.00
B
ATOM 18231 CZ2 TRP B 101 −20.345 36.174 58.276 0.00 0.00
B
ATOM 18232 HZ2 TRP B 101 −20.472 37.186 58.632 0.00 0.00
B
ATOM 18233 CH2 TRP B 101 −21.333 35.199 58.298 0.00 0.00
B
ATOM 18234 HH2 TRP B 101 −22.253 35.520 58.764 0.00 0.00
B
ATOM 18235 C TRP B 101 −14.593 31.926 55.847 0.00 0.00
B
ATOM 18236 O TRP B 101 −14.635 31.567 54.669 0.00 0.00
B
ATOM 18237 N VAL B 102 −14.038 31.170 56.780 0.00 0.00
B
ATOM 18238 HN VAL B 102 −14.173 31.353 57.751 0.00 0.00
B
ATOM 18239 CA VAL B 102 −13.398 29.935 56.418 0.00 0.00
B
ATOM 18240 HA VAL B 102 −13.719 29.632 55.433 0.00 0.00
B
ATOM 18241 CB VAL B 102 −11.846 30.058 56.412 0.00 0.00
B
ATOM 18242 HB VAL B 102 −11.521 30.096 57.473 0.00 0.00
B
ATOM 18243 CG1 VAL B 102 −11.225 28.848 55.750 0.00 0.00
B
ATOM 18244 HG11 VAL B 102 −11.332 27.829 56.181 0.00 0.00
B
ATOM 18245 HG12 VAL B 102 −11.485 28.784 54.672 0.00 0.00
B
ATOM 18246 HG13 VAL B 102 −10.117 28.898 55.698 0.00 0.00
B
ATOM 18247 CG2 VAL B 102 −11.344 31.419 55.772 0.00 0.00
B
ATOM 18248 HG21 VAL B 102 −10.234 31.438 55.805 0.00 0.00
B
ATOM 18249 HG22 VAL B 102 −11.808 31.667 54.794 0.00 0.00
B
ATOM 18250 HG23 VAL B 102 −11.650 32.273 56.415 0.00 0.00
B
ATOM 18251 C VAL B 102 −13.664 28.776 57.367 0.00 0.00
B

ATOM	18252	O	VAL	B	102	−13.748	29.017	58.605	0.00	0.00
B										
ATOM	18253	N	LYS	B	103	−13.999	27.545	56.819	0.00	0.00
B										
ATOM	18254	HN	LYS	B	103	−13.868	27.398	55.842	0.00	0.00
B										
ATOM	18255	CA	LYS	B	103	−14.289	26.397	57.642	0.00	0.00
B										
ATOM	18256	HA	LYS	B	103	−15.028	26.658	58.385	0.00	0.00
B										
ATOM	18257	CB	LYS	B	103	−14.857	25.262	56.725	0.00	0.00
B										
ATOM	18258	HB1	LYS	B	103	−15.545	25.828	56.062	0.00	0.00
B										
ATOM	18259	HB2	LYS	B	103	−14.060	24.745	56.148	0.00	0.00
B										
ATOM	18260	CG	LYS	B	103	−15.672	24.172	57.464	0.00	0.00
B										
ATOM	18261	HG1	LYS	B	103	−15.028	23.598	58.163	0.00	0.00
B										
ATOM	18262	HG2	LYS	B	103	−16.573	24.551	57.992	0.00	0.00
B										
ATOM	18263	CD	LYS	B	103	−16.207	23.227	56.396	0.00	0.00
B										
ATOM	18264	HD1	LYS	B	103	−16.843	22.491	56.935	0.00	0.00
B										
ATOM	18265	HD2	LYS	B	103	−16.914	23.689	55.675	0.00	0.00
B										
ATOM	18266	CE	LYS	B	103	−15.130	22.303	55.784	0.00	0.00
B										
ATOM	18267	HE1	LYS	B	103	−14.298	22.741	55.192	0.00	0.00
B										
ATOM	18268	HE2	LYS	B	103	−14.625	21.826	56.652	0.00	0.00
B										
ATOM	18269	NZ	LYS	B	103	−15.622	21.237	54.932	0.00	0.00
B										
ATOM	18270	HZ1	LYS	B	103	−14.925	20.470	54.845	0.00	0.00
B										
ATOM	18271	HZ2	LYS	B	103	−16.502	20.764	55.221	0.00	0.00
B										
ATOM	18272	HZ3	LYS	B	103	−15.842	21.668	54.011	0.00	0.00
B										
ATOM	18273	C	LYS	B	103	−12.990	25.901	58.296	0.00	0.00
B										
ATOM	18274	O	LYS	B	103	−11.923	25.895	57.716	0.00	0.00
B										
ATOM	18275	N	ALA	B	104	−13.044	25.487	59.526	0.00	0.00
B										
ATOM	18276	HN	ALA	B	104	−13.879	25.579	60.063	0.00	0.00
B										
ATOM	18277	CA	ALA	B	104	−11.933	24.894	60.255	0.00	0.00
B										
ATOM	18278	HA	ALA	B	104	−11.091	24.725	59.601	0.00	0.00
B										

ATOM 18279 CB ALA B 104 −11.522 25.850 61.381 0.00 0.00
B
ATOM 18280 HB1 ALA B 104 −10.714 25.440 62.024 0.00 0.00
B
ATOM 18281 HB2 ALA B 104 −11.236 26.823 60.929 0.00 0.00
B
ATOM 18282 HB3 ALA B 104 −12.329 26.062 62.115 0.00 0.00
B
ATOM 18283 C ALA B 104 −12.101 23.498 60.789 0.00 0.00
B
ATOM 18284 O ALA B 104 −13.150 23.196 61.387 0.00 0.00
B
ATOM 18285 N GLN B 105 −11.112 22.605 60.569 0.00 0.00
B
ATOM 18286 HN GLN B 105 −10.297 22.817 60.036 0.00 0.00
B
ATOM 18287 CA GLN B 105 −11.080 21.246 61.170 0.00 0.00
B
ATOM 18288 HA GLN B 105 −12.054 20.781 61.136 0.00 0.00
B
ATOM 18289 CB GLN B 105 −10.127 20.404 60.259 0.00 0.00
B
ATOM 18290 HB1 GLN B 105 −9.116 20.750 60.564 0.00 0.00
B
ATOM 18291 HB2 GLN B 105 −10.222 19.328 60.518 0.00 0.00
B
ATOM 18292 CG GLN B 105 −10.393 20.533 58.743 0.00 0.00
B
ATOM 18293 HG1 GLN B 105 −10.248 21.598 58.461 0.00 0.00
B
ATOM 18294 HG2 GLN B 105 −9.698 19.942 58.109 0.00 0.00
B
ATOM 18295 CD GLN B 105 −11.812 20.204 58.388 0.00 0.00
B
ATOM 18296 OE1 GLN B 105 −12.691 20.982 57.995 0.00 0.00
B
ATOM 18297 NE2 GLN B 105 −12.002 18.862 58.392 0.00 0.00
B
ATOM 18298 HE21 GLN B 105 −12.828 18.439 58.018 0.00 0.00
B
ATOM 18299 HE22 GLN B 105 −11.325 18.266 58.823 0.00 0.00
B
ATOM 18300 C GLN B 105 −10.653 21.088 62.580 0.00 0.00
B
ATOM 18301 O GLN B 105 −11.303 20.344 63.286 0.00 0.00
B
ATOM 18302 N ASN B 106 −9.536 21.722 62.965 0.00 0.00
B
ATOM 18303 HN ASN B 106 −9.010 22.134 62.224 0.00 0.00
B
ATOM 18304 CA ASN B 106 −8.901 21.489 64.260 0.00 0.00
B
ATOM 18305 HA ASN B 106 −9.708 21.252 64.937 0.00 0.00
B

ATOM 18306 CB ASN B 106 -7.820 20.365 64.132 0.00 0.00
B
ATOM 18307 HB1 ASN B 106 -7.163 20.370 65.028 0.00 0.00
B
ATOM 18308 HB2 ASN B 106 -8.404 19.428 64.014 0.00 0.00
B
ATOM 18309 CG ASN B 106 -7.051 20.595 62.854 0.00 0.00
B
ATOM 18310 OD1 ASN B 106 -6.407 21.595 62.649 0.00 0.00
B
ATOM 18311 ND2 ASN B 106 -7.055 19.641 61.935 0.00 0.00
B
ATOM 18312 HD21 ASN B 106 -6.682 19.845 61.030 0.00 0.00
B
ATOM 18313 HD22 ASN B 106 -7.578 18.810 62.121 0.00 0.00
B
ATOM 18314 C ASN B 106 -8.336 22.845 64.695 0.00 0.00
B
ATOM 18315 O ASN B 106 -8.330 23.787 63.963 0.00 0.00
B
ATOM 18316 N TYR B 107 -7.789 22.922 65.890 0.00 0.00
B
ATOM 18317 HN TYR B 107 -7.732 22.099 66.451 0.00 0.00
B
ATOM 18318 CA TYR B 107 -7.167 24.110 66.385 0.00 0.00
B
ATOM 18319 HA TYR B 107 -7.938 24.855 66.251 0.00 0.00
B
ATOM 18320 CB TYR B 107 -6.708 23.903 67.850 0.00 0.00
B
ATOM 18321 HB1 TYR B 107 -7.568 23.521 68.442 0.00 0.00
B
ATOM 18322 HB2 TYR B 107 -5.964 23.092 67.999 0.00 0.00
B
ATOM 18323 CG TYR B 107 -6.030 25.155 68.427 0.00 0.00
B
ATOM 18324 CD1 TYR B 107 -6.662 26.473 68.390 0.00 0.00
B
ATOM 18325 HD1 TYR B 107 -7.641 26.529 67.936 0.00 0.00
B
ATOM 18326 CE1 TYR B 107 -6.107 27.590 69.135 0.00 0.00
B
ATOM 18327 HE1 TYR B 107 -6.688 28.495 69.232 0.00 0.00
B
ATOM 18328 CZ TYR B 107 -4.817 27.457 69.728 0.00 0.00
B
ATOM 18329 OH TYR B 107 -4.112 28.455 70.291 0.00 0.00
B
ATOM 18330 HH TYR B 107 -4.554 29.287 70.105 0.00 0.00
B
ATOM 18331 CD2 TYR B 107 -4.869 25.108 69.165 0.00 0.00
B
ATOM 18332 HD2 TYR B 107 -4.270 24.212 69.097 0.00 0.00
B

ATOM 18333 CE2 TYR B 107 -4.253 26.183 69.771 0.00 0.00
B
ATOM 18334 HE2 TYR B 107 -3.235 26.056 70.106 0.00 0.00
B
ATOM 18335 C TYR B 107 -6.002 24.572 65.474 0.00 0.00
B
ATOM 18336 O TYR B 107 -5.902 25.766 65.226 0.00 0.00
B
ATOM 18337 N TYR B 108 -5.166 23.731 64.880 0.00 0.00
B
ATOM 18338 HN TYR B 108 -5.229 22.754 65.066 0.00 0.00
B
ATOM 18339 CA TYR B 108 -4.092 24.109 64.027 0.00 0.00
B
ATOM 18340 HA TYR B 108 -3.405 24.693 64.622 0.00 0.00
B
ATOM 18341 CB TYR B 108 -3.478 22.674 63.593 0.00 0.00
B
ATOM 18342 HB1 TYR B 108 -3.051 22.180 64.492 0.00 0.00
B
ATOM 18343 HB2 TYR B 108 -4.264 21.963 63.261 0.00 0.00
B
ATOM 18344 CG TYR B 108 -2.459 22.716 62.365 0.00 0.00
B
ATOM 18345 CD1 TYR B 108 -1.236 23.325 62.519 0.00 0.00
B
ATOM 18346 HD1 TYR B 108 -0.905 23.692 63.479 0.00 0.00
B
ATOM 18347 CE1 TYR B 108 -0.482 23.580 61.390 0.00 0.00
B
ATOM 18348 HE1 TYR B 108 0.473 24.078 61.469 0.00 0.00
B
ATOM 18349 CZ TYR B 108 -0.926 23.200 60.116 0.00 0.00
B
ATOM 18350 OH TYR B 108 -0.107 23.399 59.024 0.00 0.00
B
ATOM 18351 HH TYR B 108 0.692 23.895 59.216 0.00 0.00
B
ATOM 18352 CD2 TYR B 108 -2.824 22.135 61.185 0.00 0.00
B
ATOM 18353 HD2 TYR B 108 -3.815 21.709 61.136 0.00 0.00
B
ATOM 18354 CE2 TYR B 108 -2.091 22.407 60.013 0.00 0.00
B
ATOM 18355 HE2 TYR B 108 -2.491 22.143 59.045 0.00 0.00
B
ATOM 18356 C TYR B 108 -4.431 24.944 62.878 0.00 0.00
B
ATOM 18357 O TYR B 108 -3.979 26.052 62.633 0.00 0.00
B
ATOM 18358 N HSE B 109 -5.439 24.496 62.160 0.00 0.00
B
ATOM 18359 HN HSE B 109 -5.837 23.615 62.404 0.00 0.00
B

ATOM 18360 CA HSE B 109 -6.075 25.101 61.012 0.00 0.00
B
ATOM 18361 HA HSE B 109 -5.256 25.174 60.311 0.00 0.00
B
ATOM 18362 CB HSE B 109 -7.106 24.128 60.396 0.00 0.00
B
ATOM 18363 HB1 HSE B 109 -6.644 23.118 60.386 0.00 0.00
B
ATOM 18364 HB2 HSE B 109 -7.999 23.982 61.040 0.00 0.00
B
ATOM 18365 ND1 HSE B 109 -8.593 23.573 58.609 0.00 0.00
B
ATOM 18366 CG HSE B 109 -7.622 24.417 58.950 0.00 0.00
B
ATOM 18367 CE1 HSE B 109 -9.150 24.040 57.503 0.00 0.00
B
ATOM 18368 HE1 HSE B 109 -10.111 23.705 57.112 0.00 0.00
B
ATOM 18369 NE2 HSE B 109 -8.498 25.186 57.111 0.00 0.00
B
ATOM 18370 HE2 HSE B 109 -8.630 25.760 56.303 0.00 0.00
B
ATOM 18371 CD2 HSE B 109 -7.558 25.468 58.037 0.00 0.00
B
ATOM 18372 HD2 HSE B 109 -6.847 26.282 58.092 0.00 0.00
B
ATOM 18373 C HSE B 109 -6.785 26.405 61.338 0.00 0.00
B
ATOM 18374 O HSE B 109 -6.642 27.437 60.675 0.00 0.00
B
ATOM 18375 N HSE B 110 -7.473 26.464 62.516 0.00 0.00
B
ATOM 18376 HN HSE B 110 -7.649 25.611 63.001 0.00 0.00
B
ATOM 18377 CA HSE B 110 -8.026 27.695 63.107 0.00 0.00
B
ATOM 18378 HA HSE B 110 -8.677 28.131 62.364 0.00 0.00
B
ATOM 18379 CB HSE B 110 -8.877 27.370 64.346 0.00 0.00
B
ATOM 18380 HB1 HSE B 110 -9.579 26.582 63.998 0.00 0.00
B
ATOM 18381 HB2 HSE B 110 -8.204 26.858 65.067 0.00 0.00
B
ATOM 18382 ND1 HSE B 110 -10.263 28.170 66.233 0.00 0.00
B
ATOM 18383 CG HSE B 110 -9.610 28.457 65.063 0.00 0.00
B
ATOM 18384 CE1 HSE B 110 -10.647 29.304 66.731 0.00 0.00
B
ATOM 18385 HE1 HSE B 110 -11.300 29.454 67.591 0.00 0.00
B
ATOM 18386 NE2 HSE B 110 -10.354 30.360 65.927 0.00 0.00
B

ATOM 18387 HE2 HSE B 110 −10.631 31.318 66.001 0.00 0.00
B
ATOM 18388 CD2 HSE B 110 −9.692 29.833 64.869 0.00 0.00
B
ATOM 18389 HD2 HSE B 110 −9.311 30.433 64.052 0.00 0.00
B
ATOM 18390 C HSE B 110 −6.893 28.752 63.499 0.00 0.00
B
ATOM 18391 O HSE B 110 −6.943 29.959 63.139 0.00 0.00
B
ATOM 18392 N LYS B 111 −5.834 28.312 64.082 0.00 0.00
B
ATOM 18393 HN LYS B 111 −5.945 27.418 64.510 0.00 0.00
B
ATOM 18394 CA LYS B 111 −4.761 29.162 64.562 0.00 0.00
B
ATOM 18395 HA LYS B 111 −5.276 29.981 65.041 0.00 0.00
B
ATOM 18396 CB LYS B 111 −3.960 28.269 65.595 0.00 0.00
B
ATOM 18397 HB1 LYS B 111 −4.554 27.899 66.458 0.00 0.00
B
ATOM 18398 HB2 LYS B 111 −3.693 27.355 65.023 0.00 0.00
B
ATOM 18399 CG LYS B 111 −2.724 28.865 66.301 0.00 0.00
B
ATOM 18400 HG1 LYS B 111 −2.150 28.100 66.866 0.00 0.00
B
ATOM 18401 HG2 LYS B 111 −2.085 29.376 65.550 0.00 0.00
B
ATOM 18402 CD LYS B 111 −3.095 29.894 67.391 0.00 0.00
B
ATOM 18403 HD1 LYS B 111 −3.994 29.688 68.009 0.00 0.00
B
ATOM 18404 HD2 LYS B 111 −2.229 29.989 68.080 0.00 0.00
B
ATOM 18405 CE LYS B 111 −3.261 31.324 66.834 0.00 0.00
B
ATOM 18406 HE1 LYS B 111 −2.258 31.688 66.524 0.00 0.00
B
ATOM 18407 HE2 LYS B 111 −3.952 31.354 65.964 0.00 0.00
B
ATOM 18408 NZ LYS B 111 −3.748 32.194 67.949 0.00 0.00
B
ATOM 18409 HZ1 LYS B 111 −4.244 32.937 67.416 0.00 0.00
B
ATOM 18410 HZ2 LYS B 111 −4.402 31.833 68.674 0.00 0.00
B
ATOM 18411 HZ3 LYS B 111 −2.909 32.547 68.452 0.00 0.00
B
ATOM 18412 C LYS B 111 −3.927 29.746 63.422 0.00 0.00
B
ATOM 18413 O LYS B 111 −3.496 30.911 63.442 0.00 0.00
B

ATOM 18414 N VAL B 112 -3.723 29.018 62.274 0.00 0.00
B
ATOM 18415 HN VAL B 112 -3.894 28.037 62.246 0.00 0.00
B
ATOM 18416 CA VAL B 112 -3.108 29.512 61.070 0.00 0.00
B
ATOM 18417 HA VAL B 112 -2.191 30.059 61.231 0.00 0.00
B
ATOM 18418 CB VAL B 112 -2.876 28.334 60.132 0.00 0.00
B
ATOM 18419 HB VAL B 112 -3.804 27.723 60.146 0.00 0.00
B
ATOM 18420 CG1 VAL B 112 -2.640 28.739 58.651 0.00 0.00
B
ATOM 18421 HG11 VAL B 112 -3.605 28.629 58.111 0.00 0.00
B
ATOM 18422 HG12 VAL B 112 -2.283 29.791 58.659 0.00 0.00
B
ATOM 18423 HG13 VAL B 112 -1.935 28.079 58.102 0.00 0.00
B
ATOM 18424 CG2 VAL B 112 -1.700 27.487 60.612 0.00 0.00
B
ATOM 18425 HG21 VAL B 112 -1.950 27.120 61.630 0.00 0.00
B
ATOM 18426 HG22 VAL B 112 -1.326 26.583 60.086 0.00 0.00
B
ATOM 18427 HG23 VAL B 112 -0.834 28.167 60.757 0.00 0.00
B
ATOM 18428 C VAL B 112 -4.062 30.426 60.382 0.00 0.00
B
ATOM 18429 O VAL B 112 -3.723 31.536 60.044 0.00 0.00
B
ATOM 18430 N LEU B 113 -5.334 29.973 60.243 0.00 0.00
B
ATOM 18431 HN LEU B 113 -5.540 29.070 60.611 0.00 0.00
B
ATOM 18432 CA LEU B 113 -6.399 30.829 59.591 0.00 0.00
B
ATOM 18433 HA LEU B 113 -6.020 31.099 58.617 0.00 0.00
B
ATOM 18434 CB LEU B 113 -7.887 30.193 59.473 0.00 0.00
B
ATOM 18435 HB1 LEU B 113 -8.197 29.827 60.475 0.00 0.00
B
ATOM 18436 HB2 LEU B 113 -8.470 31.034 59.039 0.00 0.00
B
ATOM 18437 CG LEU B 113 -7.977 29.006 58.536 0.00 0.00
B
ATOM 18438 HG LEU B 113 -7.049 28.404 58.645 0.00 0.00
B
ATOM 18439 CD1 LEU B 113 -9.297 28.258 58.789 0.00 0.00
B
ATOM 18440 HD11 LEU B 113 -9.480 27.523 57.977 0.00 0.00
B

ATOM 18441 HD12 LEU B 113   -9.208 27.654 59.717 0.00 0.00
B
ATOM 18442 HD13 LEU B 113   -10.260 28.810 58.742 0.00 0.00
B
ATOM 18443 CD2 LEU B 113   -7.869 29.431 57.069 0.00 0.00
B
ATOM 18444 HD21 LEU B 113   -8.745 30.059 56.802 0.00 0.00
B
ATOM 18445 HD22 LEU B 113   -6.906 29.961 56.906 0.00 0.00
B
ATOM 18446 HD23 LEU B 113   -7.854 28.555 56.385 0.00 0.00
B
ATOM 18447 C   LEU B 113   -6.580 32.158 60.285 0.00 0.00
B
ATOM 18448 O   LEU B 113   -6.755 33.182 59.574 0.00 0.00
B
ATOM 18449 N   GLU B 114   -6.520 32.220 61.610 0.00 0.00
B
ATOM 18450 HN GLU B 114   -6.486 31.391 62.163 0.00 0.00
B
ATOM 18451 CA GLU B 114   -6.461 33.460 62.323 0.00 0.00
B
ATOM 18452 HA GLU B 114   -7.397 33.946 62.087 0.00 0.00
B
ATOM 18453 CB GLU B 114   -6.344 33.238 63.801 0.00 0.00
B
ATOM 18454 HB1 GLU B 114   -6.939 32.356 64.121 0.00 0.00
B
ATOM 18455 HB2 GLU B 114   -5.292 32.925 63.970 0.00 0.00
B
ATOM 18456 CG GLU B 114   -6.700 34.379 64.770 0.00 0.00
B
ATOM 18457 HG1 GLU B 114   -6.300 35.361 64.439 0.00 0.00
B
ATOM 18458 HG2 GLU B 114   -7.796 34.556 64.816 0.00 0.00
B
ATOM 18459 CD GLU B 114   -6.182 34.088 66.118 0.00 0.00
B
ATOM 18460 OE1 GLU B 114   -4.936 34.055 66.356 0.00 0.00
B
ATOM 18461 OE2 GLU B 114   -7.005 33.692 67.068 0.00 0.00
B
ATOM 18462 C   GLU B 114   -5.426 34.416 61.890 0.00 0.00
B
ATOM 18463 O   GLU B 114   -5.639 35.534 61.418 0.00 0.00
B
ATOM 18464 N   LYS B 115   -4.092 33.978 61.826 0.00 0.00
B
ATOM 18465 HN LYS B 115   -3.865 33.017 61.963 0.00 0.00
B
ATOM 18466 CA LYS B 115   -2.873 34.775 61.445 0.00 0.00
B
ATOM 18467 HA LYS B 115   -2.779 35.721 61.958 0.00 0.00
B

ATOM 18468 CB LYS B 115 -1.642 33.977 61.794 0.00 0.00
B
ATOM 18469 HB1 LYS B 115 -1.750 33.772 62.881 0.00 0.00
B
ATOM 18470 HB2 LYS B 115 -1.595 33.026 61.221 0.00 0.00
B
ATOM 18471 CG LYS B 115 -0.318 34.688 61.662 0.00 0.00
B
ATOM 18472 HG1 LYS B 115 0.484 33.974 61.377 0.00 0.00
B
ATOM 18473 HG2 LYS B 115 -0.291 35.478 60.882 0.00 0.00
B
ATOM 18474 CD LYS B 115 0.112 35.237 63.021 0.00 0.00
B
ATOM 18475 HD1 LYS B 115 -0.702 35.871 63.433 0.00 0.00
B
ATOM 18476 HD2 LYS B 115 0.154 34.451 63.805 0.00 0.00
B
ATOM 18477 CE LYS B 115 1.420 36.015 63.128 0.00 0.00
B
ATOM 18478 HE1 LYS B 115 1.875 35.878 64.133 0.00 0.00
B
ATOM 18479 HE2 LYS B 115 2.068 35.609 62.322 0.00 0.00
B
ATOM 18480 NZ LYS B 115 1.230 37.433 62.964 0.00 0.00
B
ATOM 18481 HZ1 LYS B 115 1.556 37.864 62.076 0.00 0.00
B
ATOM 18482 HZ2 LYS B 115 0.237 37.632 63.200 0.00 0.00
B
ATOM 18483 HZ3 LYS B 115 1.722 37.953 63.719 0.00 0.00
B
ATOM 18484 C LYS B 115 -2.845 35.254 60.018 0.00 0.00
B
ATOM 18485 O LYS B 115 -2.544 36.399 59.753 0.00 0.00
B
ATOM 18486 N ILE B 116 -3.286 34.388 59.041 0.00 0.00
B
ATOM 18487 HN ILE B 116 -3.596 33.479 59.308 0.00 0.00
B
ATOM 18488 CA ILE B 116 -3.188 34.575 57.579 0.00 0.00
B
ATOM 18489 HA ILE B 116 -2.343 35.241 57.480 0.00 0.00
B
ATOM 18490 CB ILE B 116 -2.924 33.285 56.741 0.00 0.00
B
ATOM 18491 HB ILE B 116 -2.677 33.627 55.713 0.00 0.00
B
ATOM 18492 CG2 ILE B 116 -1.698 32.637 57.310 0.00 0.00
B
ATOM 18493 HG21 ILE B 116 -1.784 32.544 58.414 0.00 0.00
B
ATOM 18494 HG22 ILE B 116 -1.593 31.603 56.917 0.00 0.00
B

ATOM 18495 HG23 ILE B 116 -0.808 33.288 57.182 0.00 0.00
B
ATOM 18496 CG1 ILE B 116 -4.183 32.389 56.680 0.00 0.00
B
ATOM 18497 HG11 ILE B 116 -4.228 31.974 57.709 0.00 0.00
B
ATOM 18498 HG12 ILE B 116 -4.999 33.098 56.422 0.00 0.00
B
ATOM 18499 CD ILE B 116 -4.131 31.243 55.691 0.00 0.00
B
ATOM 18500 HD1 ILE B 116 -3.894 31.611 54.670 0.00 0.00
B
ATOM 18501 HD2 ILE B 116 -3.296 30.553 55.940 0.00 0.00
B
ATOM 18502 HD3 ILE B 116 -5.083 30.671 55.732 0.00 0.00
B
ATOM 18503 C ILE B 116 -4.409 35.371 57.023 0.00 0.00
B
ATOM 18504 O ILE B 116 -4.630 35.723 55.868 0.00 0.00
B
ATOM 18505 N GLY B 117 -5.289 35.694 57.988 0.00 0.00
B
ATOM 18506 HN GLY B 117 -5.145 35.429 58.939 0.00 0.00
B
ATOM 18507 CA GLY B 117 -6.317 36.773 57.934 0.00 0.00
B
ATOM 18508 HA1 GLY B 117 -6.126 37.396 57.073 0.00 0.00
B
ATOM 18509 HA2 GLY B 117 -6.157 37.406 58.794 0.00 0.00
B
ATOM 18510 C GLY B 117 -7.746 36.440 57.680 0.00 0.00
B
ATOM 18511 O GLY B 117 -8.352 37.189 56.985 0.00 0.00
B
ATOM 18512 N ALA B 118 -8.272 35.365 58.291 0.00 0.00
B
ATOM 18513 HN ALA B 118 -7.763 34.613 58.702 0.00 0.00
B
ATOM 18514 CA ALA B 118 -9.699 35.151 58.390 0.00 0.00
B
ATOM 18515 HA ALA B 118 -10.194 35.135 57.430 0.00 0.00
B
ATOM 18516 CB ALA B 118 -10.034 33.688 59.000 0.00 0.00
B
ATOM 18517 HB1 ALA B 118 -9.553 33.546 59.991 0.00 0.00
B
ATOM 18518 HB2 ALA B 118 -11.135 33.602 59.118 0.00 0.00
B
ATOM 18519 HB3 ALA B 118 -9.705 32.914 58.274 0.00 0.00
B
ATOM 18520 C ALA B 118 -10.483 36.192 59.228 0.00 0.00
B
ATOM 18521 O ALA B 118 -10.048 36.679 60.310 0.00 0.00
B

ATOM 18522 N ASP B 119 -11.613 36.574 58.660 0.00 0.00
B
ATOM 18523 HN ASP B 119 -11.804 36.313 57.716 0.00 0.00
B
ATOM 18524 CA ASP B 119 -12.678 37.399 59.314 0.00 0.00
B
ATOM 18525 HA ASP B 119 -12.121 37.929 60.072 0.00 0.00
B
ATOM 18526 CB ASP B 119 -13.321 38.349 58.289 0.00 0.00
B
ATOM 18527 HB1 ASP B 119 -13.823 37.746 57.502 0.00 0.00
B
ATOM 18528 HB2 ASP B 119 -14.063 39.022 58.769 0.00 0.00
B
ATOM 18529 CG ASP B 119 -12.248 39.170 57.630 0.00 0.00
B
ATOM 18530 OD1 ASP B 119 -11.964 39.022 56.424 0.00 0.00
B
ATOM 18531 OD2 ASP B 119 -11.659 39.905 58.445 0.00 0.00
B
ATOM 18532 C ASP B 119 -13.650 36.632 60.145 0.00 0.00
B
ATOM 18533 O ASP B 119 -14.029 37.108 61.236 0.00 0.00
B
ATOM 18534 N ARG B 120 -14.065 35.469 59.723 0.00 0.00
B
ATOM 18535 HN ARG B 120 -13.866 34.969 58.884 0.00 0.00
B
ATOM 18536 CA ARG B 120 -14.854 34.588 60.638 0.00 0.00
B
ATOM 18537 HA ARG B 120 -14.523 34.758 61.652 0.00 0.00
B
ATOM 18538 CB ARG B 120 -16.376 34.772 60.383 0.00 0.00
B
ATOM 18539 HB1 ARG B 120 -16.629 35.811 60.685 0.00 0.00
B
ATOM 18540 HB2 ARG B 120 -16.511 34.699 59.283 0.00 0.00
B
ATOM 18541 CG ARG B 120 -17.336 33.772 61.079 0.00 0.00
B
ATOM 18542 HG1 ARG B 120 -17.079 32.699 60.947 0.00 0.00
B
ATOM 18543 HG2 ARG B 120 -17.191 33.906 62.172 0.00 0.00
B
ATOM 18544 CD ARG B 120 -18.865 34.027 60.780 0.00 0.00
B
ATOM 18545 HD1 ARG B 120 -19.035 35.079 61.093 0.00 0.00
B
ATOM 18546 HD2 ARG B 120 -19.164 33.752 59.746 0.00 0.00
B
ATOM 18547 NE ARG B 120 -19.563 33.172 61.665 0.00 0.00
B
ATOM 18548 HE ARG B 120 -19.016 32.516 62.184 0.00 0.00
B

ATOM 18549 CZ ARG B 120 -20.889 33.285 61.922 0.00 0.00
B
ATOM 18550 NH1 ARG B 120 -21.625 34.218 61.488 0.00 0.00
B
ATOM 18551 HH11 ARG B 120 -22.578 34.268 61.788 0.00 0.00
B
ATOM 18552 HH12 ARG B 120 -21.259 35.084 61.148 0.00 0.00
B
ATOM 18553 NH2 ARG B 120 -21.289 32.290 62.717 0.00 0.00
B
ATOM 18554 HH21 ARG B 120 -22.019 32.476 63.374 0.00 0.00
B
ATOM 18555 HH22 ARG B 120 -20.534 31.736 63.067 0.00 0.00
B
ATOM 18556 C ARG B 120 -14.411 33.091 60.349 0.00 0.00
B
ATOM 18557 O ARG B 120 -14.460 32.563 59.259 0.00 0.00
B
ATOM 18558 N ILE B 121 -13.966 32.410 61.436 0.00 0.00
B
ATOM 18559 HN ILE B 121 -14.028 32.712 62.384 0.00 0.00
B
ATOM 18560 CA ILE B 121 -13.596 31.033 61.336 0.00 0.00
B
ATOM 18561 HA ILE B 121 -13.426 30.857 60.284 0.00 0.00
B
ATOM 18562 CB ILE B 121 -12.453 30.548 62.218 0.00 0.00
B
ATOM 18563 HB ILE B 121 -12.638 30.912 63.251 0.00 0.00
B
ATOM 18564 CG2 ILE B 121 -12.210 29.072 62.148 0.00 0.00
B
ATOM 18565 HG21 ILE B 121 -13.006 28.338 62.399 0.00 0.00
B
ATOM 18566 HG22 ILE B 121 -12.063 28.827 61.074 0.00 0.00
B
ATOM 18567 HG23 ILE B 121 -11.314 28.775 62.733 0.00 0.00
B
ATOM 18568 CG1 ILE B 121 -11.201 31.237 61.592 0.00 0.00
B
ATOM 18569 HG11 ILE B 121 -10.355 30.564 61.845 0.00 0.00
B
ATOM 18570 HG12 ILE B 121 -11.104 31.229 60.486 0.00 0.00
B
ATOM 18571 CD ILE B 121 -10.839 32.640 62.199 0.00 0.00
B
ATOM 18572 HD1 ILE B 121 -9.746 32.722 62.383 0.00 0.00
B
ATOM 18573 HD2 ILE B 121 -11.297 33.465 61.612 0.00 0.00
B
ATOM 18574 HD3 ILE B 121 -11.241 32.642 63.234 0.00 0.00
B
ATOM 18575 C ILE B 121 -14.939 30.259 61.764 0.00 0.00
B

ATOM 18576 O ILE B 121 −15.504 30.436 62.840 0.00 0.00
B
ATOM 18577 N ILE B 122 −15.412 29.259 60.939 0.00 0.00
B
ATOM 18578 HN ILE B 122 −15.004 29.197 60.032 0.00 0.00
B
ATOM 18579 CA ILE B 122 −16.562 28.381 61.014 0.00 0.00
B
ATOM 18580 HA ILE B 122 −17.271 28.776 61.726 0.00 0.00
B
ATOM 18581 CB ILE B 122 −17.396 28.281 59.766 0.00 0.00
B
ATOM 18582 HB ILE B 122 −16.786 27.745 59.007 0.00 0.00
B
ATOM 18583 CG2 ILE B 122 −18.602 27.417 60.056 0.00 0.00
B
ATOM 18584 HG21 ILE B 122 −19.257 28.050 60.692 0.00 0.00
B
ATOM 18585 HG22 ILE B 122 −19.123 27.233 59.093 0.00 0.00
B
ATOM 18586 HG23 ILE B 122 −18.307 26.484 60.584 0.00 0.00
B
ATOM 18587 CG1 ILE B 122 −17.843 29.668 59.149 0.00 0.00
B
ATOM 18588 HG11 ILE B 122 −18.379 30.262 59.920 0.00 0.00
B
ATOM 18589 HG12 ILE B 122 −17.008 30.295 58.769 0.00 0.00
B
ATOM 18590 CD ILE B 122 −18.794 29.660 57.893 0.00 0.00
B
ATOM 18591 HD1 ILE B 122 −19.688 29.063 58.174 0.00 0.00
B
ATOM 18592 HD2 ILE B 122 −19.023 30.706 57.598 0.00 0.00
B
ATOM 18593 HD3 ILE B 122 −18.376 29.047 57.065 0.00 0.00
B
ATOM 18594 C ILE B 122 −15.977 27.114 61.442 0.00 0.00
B
ATOM 18595 O ILE B 122 −15.231 26.469 60.718 0.00 0.00
B
ATOM 18596 N HSE B 123 −16.362 26.677 62.672 0.00 0.00
B
ATOM 18597 HN HSE B 123 −17.090 27.162 63.151 0.00 0.00
B
ATOM 18598 CA HSE B 123 −16.187 25.278 63.174 0.00 0.00
B
ATOM 18599 HA HSE B 123 −15.379 24.822 62.621 0.00 0.00
B
ATOM 18600 CB HSE B 123 −15.631 25.363 64.640 0.00 0.00
B
ATOM 18601 HB1 HSE B 123 −16.172 26.161 65.193 0.00 0.00
B
ATOM 18602 HB2 HSE B 123 −15.682 24.376 65.147 0.00 0.00
B

ATOM 18603 ND1 HSE B 123 −13.407 24.459 64.622 0.00 0.00
B
ATOM 18604 CG HSE B 123 −14.168 25.624 64.656 0.00 0.00
B
ATOM 18605 CE1 HSE B 123 −12.201 24.832 64.961 0.00 0.00
B
ATOM 18606 HE1 HSE B 123 −11.273 24.262 65.015 0.00 0.00
B
ATOM 18607 NE2 HSE B 123 −12.166 26.144 65.245 0.00 0.00
B
ATOM 18608 HE2 HSE B 123 −11.391 26.659 65.610 0.00 0.00
B
ATOM 18609 CD2 HSE B 123 −13.459 26.688 65.071 0.00 0.00
B
ATOM 18610 HD2 HSE B 123 −13.818 27.687 65.287 0.00 0.00
B
ATOM 18611 C HSE B 123 −17.527 24.569 63.151 0.00 0.00
B
ATOM 18612 O HSE B 123 −18.464 24.922 63.845 0.00 0.00
B
ATOM 18613 N PRO B 124 −17.790 23.664 62.183 0.00 0.00
B
ATOM 18614 CD PRO B 124 −16.975 23.386 61.040 0.00 0.00
B
ATOM 18615 HD1 PRO B 124 −16.361 24.277 60.786 0.00 0.00
B
ATOM 18616 HD2 PRO B 124 −16.255 22.577 61.287 0.00 0.00
B
ATOM 18617 CA PRO B 124 −19.147 23.166 61.888 0.00 0.00
B
ATOM 18618 HA PRO B 124 −19.796 24.029 61.903 0.00 0.00
B
ATOM 18619 CB PRO B 124 −19.076 22.476 60.493 0.00 0.00
B
ATOM 18620 HB1 PRO B 124 −20.009 22.745 59.953 0.00 0.00
B
ATOM 18621 HB2 PRO B 124 −19.082 21.365 60.491 0.00 0.00
B
ATOM 18622 CG PRO B 124 −17.896 23.183 59.835 0.00 0.00
B
ATOM 18623 HG1 PRO B 124 −18.283 24.189 59.565 0.00 0.00
B
ATOM 18624 HG2 PRO B 124 −17.275 22.730 59.032 0.00 0.00
B
ATOM 18625 C PRO B 124 −19.603 22.104 62.814 0.00 0.00
B
ATOM 18626 O PRO B 124 −20.776 22.186 63.235 0.00 0.00
B
ATOM 18627 N GLU B 125 −18.836 21.127 63.211 0.00 0.00
B
ATOM 18628 HN GLU B 125 −17.960 20.982 62.758 0.00 0.00
B
ATOM 18629 CA GLU B 125 −19.243 19.983 64.051 0.00 0.00
B

ATOM 18630 HA GLU B 125 -20.080 19.565 63.511 0.00 0.00
B
ATOM 18631 CB GLU B 125 -18.091 18.894 64.331 0.00 0.00
B
ATOM 18632 HB1 GLU B 125 -17.682 18.523 63.367 0.00 0.00
B
ATOM 18633 HB2 GLU B 125 -17.228 19.299 64.900 0.00 0.00
B
ATOM 18634 CG GLU B 125 -18.620 17.675 65.199 0.00 0.00
B
ATOM 18635 HG1 GLU B 125 -18.545 18.031 66.249 0.00 0.00
B
ATOM 18636 HG2 GLU B 125 -19.697 17.495 64.994 0.00 0.00
B
ATOM 18637 CD GLU B 125 -17.733 16.481 64.989 0.00 0.00
B
ATOM 18638 OE1 GLU B 125 -18.210 15.408 64.555 0.00 0.00
B
ATOM 18639 OE2 GLU B 125 -16.495 16.656 65.170 0.00 0.00
B
ATOM 18640 C GLU B 125 -19.735 20.458 65.362 0.00 0.00
B
ATOM 18641 O GLU B 125 -20.781 20.085 65.904 0.00 0.00
B
ATOM 18642 N LYS B 126 -19.004 21.420 65.909 0.00 0.00
B
ATOM 18643 HN LYS B 126 -18.241 21.807 65.397 0.00 0.00
B
ATOM 18644 CA LYS B 126 -19.292 22.058 67.206 0.00 0.00
B
ATOM 18645 HA LYS B 126 -19.397 21.293 67.961 0.00 0.00
B
ATOM 18646 CB LYS B 126 -18.160 23.007 67.597 0.00 0.00
B
ATOM 18647 HB1 LYS B 126 -17.154 22.546 67.498 0.00 0.00
B
ATOM 18648 HB2 LYS B 126 -18.011 23.896 66.948 0.00 0.00
B
ATOM 18649 CG LYS B 126 -18.243 23.300 69.053 0.00 0.00
B
ATOM 18650 HG1 LYS B 126 -18.931 24.151 69.243 0.00 0.00
B
ATOM 18651 HG2 LYS B 126 -18.506 22.352 69.569 0.00 0.00
B
ATOM 18652 CD LYS B 126 -16.935 24.008 69.626 0.00 0.00
B
ATOM 18653 HD1 LYS B 126 -16.974 24.215 70.717 0.00 0.00
B
ATOM 18654 HD2 LYS B 126 -16.093 23.305 69.448 0.00 0.00
B
ATOM 18655 CE LYS B 126 -16.427 25.270 68.849 0.00 0.00
B
ATOM 18656 HE1 LYS B 126 -15.547 25.661 69.403 0.00 0.00
B

ATOM 18657 HE2 LYS B 126 −16.247 24.842 67.840 0.00 0.00
B
ATOM 18658 NZ LYS B 126 −17.477 26.314 68.715 0.00 0.00
B
ATOM 18659 HZ1 LYS B 126 −17.084 27.207 68.355 0.00 0.00
B
ATOM 18660 HZ2 LYS B 126 −18.168 26.051 67.985 0.00 0.00
B
ATOM 18661 HZ3 LYS B 126 −17.968 26.490 69.615 0.00 0.00
B
ATOM 18662 C LYS B 126 −20.600 22.850 67.256 0.00 0.00
B
ATOM 18663 O LYS B 126 −21.474 22.533 68.123 0.00 0.00
B
ATOM 18664 N ASP B 127 −20.855 23.793 66.247 0.00 0.00
B
ATOM 18665 HN ASP B 127 −20.247 23.916 65.467 0.00 0.00
B
ATOM 18666 CA ASP B 127 −22.119 24.541 66.227 0.00 0.00
B
ATOM 18667 HA ASP B 127 −22.359 24.852 67.233 0.00 0.00
B
ATOM 18668 CB ASP B 127 −21.854 25.691 65.233 0.00 0.00
B
ATOM 18669 HB1 ASP B 127 −20.885 25.539 64.712 0.00 0.00
B
ATOM 18670 HB2 ASP B 127 −22.594 25.727 64.404 0.00 0.00
B
ATOM 18671 CG ASP B 127 −21.925 27.047 65.856 0.00 0.00
B
ATOM 18672 OD1 ASP B 127 −23.000 27.688 65.750 0.00 0.00
B
ATOM 18673 OD2 ASP B 127 −20.863 27.578 66.292 0.00 0.00
B
ATOM 18674 C ASP B 127 −23.285 23.718 65.824 0.00 0.00
B
ATOM 18675 O ASP B 127 −24.420 23.950 66.311 0.00 0.00
B
ATOM 18676 N MET B 128 −23.095 22.671 64.956 0.00 0.00
B
ATOM 18677 HN MET B 128 −22.172 22.538 64.605 0.00 0.00
B
ATOM 18678 CA MET B 128 −24.196 21.724 64.734 0.00 0.00
B
ATOM 18679 HA MET B 128 −25.021 22.251 64.277 0.00 0.00
B
ATOM 18680 CB MET B 128 −23.873 20.507 63.755 0.00 0.00
B
ATOM 18681 HB1 MET B 128 −23.384 20.940 62.857 0.00 0.00
B
ATOM 18682 HB2 MET B 128 −23.150 19.862 64.300 0.00 0.00
B
ATOM 18683 CG MET B 128 −25.073 19.656 63.244 0.00 0.00
B

ATOM 18684 HG1 MET B 128 −24.698 18.915 62.507 0.00 0.00
B
ATOM 18685 HG2 MET B 128 −25.483 19.079 64.101 0.00 0.00
B
ATOM 18686 SD MET B 128 −26.283 20.615 62.468 0.00 0.00
B
ATOM 18687 CE MET B 128 −27.659 19.575 63.120 0.00 0.00
B
ATOM 18688 HE1 MET B 128 −28.548 19.693 62.464 0.00 0.00
B
ATOM 18689 HE2 MET B 128 −27.317 18.530 62.955 0.00 0.00
B
ATOM 18690 HE3 MET B 128 −27.961 19.704 64.181 0.00 0.00
B
ATOM 18691 C MET B 128 −24.782 21.037 65.990 0.00 0.00
B
ATOM 18692 O MET B 128 −25.931 20.812 66.207 0.00 0.00
B
ATOM 18693 N GLY B 129 −23.892 20.569 66.942 0.00 0.00
B
ATOM 18694 HN GLY B 129 −22.928 20.571 66.685 0.00 0.00
B
ATOM 18695 CA GLY B 129 −24.330 20.122 68.293 0.00 0.00
B
ATOM 18696 HA1 GLY B 129 −23.474 20.041 68.947 0.00 0.00
B
ATOM 18697 HA2 GLY B 129 −24.744 19.126 68.241 0.00 0.00
B
ATOM 18698 C GLY B 129 −25.272 21.019 69.036 0.00 0.00
B
ATOM 18699 O GLY B 129 −26.274 20.666 69.635 0.00 0.00
B
ATOM 18700 N VAL B 130 −24.932 22.337 69.070 0.00 0.00
B
ATOM 18701 HN VAL B 130 −24.180 22.717 68.537 0.00 0.00
B
ATOM 18702 CA VAL B 130 −25.739 23.393 69.648 0.00 0.00
B
ATOM 18703 HA VAL B 130 −25.854 23.206 70.706 0.00 0.00
B
ATOM 18704 CB VAL B 130 −25.038 24.810 69.546 0.00 0.00
B
ATOM 18705 HB VAL B 130 −24.720 24.924 68.487 0.00 0.00
B
ATOM 18706 CG1 VAL B 130 −26.031 25.953 70.036 0.00 0.00
B
ATOM 18707 HG11 VAL B 130 −26.692 25.757 70.907 0.00 0.00
B
ATOM 18708 HG12 VAL B 130 −25.583 26.896 70.416 0.00 0.00
B
ATOM 18709 HG13 VAL B 130 −26.717 26.203 69.199 0.00 0.00
B
ATOM 18710 CG2 VAL B 130 −23.855 24.787 70.541 0.00 0.00
B

ATOM 18711 HG21 VAL B 130 −23.190 23.993 70.140 0.00 0.00
B
ATOM 18712 HG22 VAL B 130 −23.407 25.802 70.472 0.00 0.00
B
ATOM 18713 HG23 VAL B 130 −24.248 24.512 71.543 0.00 0.00
B
ATOM 18714 C VAL B 130 −27.125 23.419 68.992 0.00 0.00
B
ATOM 18715 O VAL B 130 −28.179 23.403 69.639 0.00 0.00
B
ATOM 18716 N LYS B 131 −27.015 23.452 67.679 0.00 0.00
B
ATOM 18717 HN LYS B 131 −26.093 23.539 67.310 0.00 0.00
B
ATOM 18718 CA LYS B 131 −28.178 23.252 66.847 0.00 0.00
B
ATOM 18719 HA LYS B 131 −28.778 24.147 66.919 0.00 0.00
B
ATOM 18720 CB LYS B 131 −27.716 23.405 65.362 0.00 0.00
B
ATOM 18721 HB1 LYS B 131 −26.703 23.862 65.341 0.00 0.00
B
ATOM 18722 HB2 LYS B 131 −27.607 22.371 64.970 0.00 0.00
B
ATOM 18723 CG LYS B 131 −28.716 24.153 64.459 0.00 0.00
B
ATOM 18724 HG1 LYS B 131 −28.907 25.135 64.942 0.00 0.00
B
ATOM 18725 HG2 LYS B 131 −28.176 24.445 63.533 0.00 0.00
B
ATOM 18726 CD LYS B 131 −30.102 23.502 64.174 0.00 0.00
B
ATOM 18727 HD1 LYS B 131 −29.900 22.585 63.580 0.00 0.00
B
ATOM 18728 HD2 LYS B 131 −30.678 23.104 65.036 0.00 0.00
B
ATOM 18729 CE LYS B 131 −30.974 24.307 63.207 0.00 0.00
B
ATOM 18730 HE1 LYS B 131 −30.318 24.713 62.407 0.00 0.00
B
ATOM 18731 HE2 LYS B 131 −31.752 23.676 62.726 0.00 0.00
B
ATOM 18732 NZ LYS B 131 −31.718 25.431 63.864 0.00 0.00
B
ATOM 18733 HZ1 LYS B 131 −31.171 26.263 64.166 0.00 0.00
B
ATOM 18734 HZ2 LYS B 131 −32.542 25.635 63.262 0.00 0.00
B
ATOM 18735 HZ3 LYS B 131 −32.150 25.104 64.752 0.00 0.00
B
ATOM 18736 C LYS B 131 −29.158 22.082 67.175 0.00 0.00
B
ATOM 18737 O LYS B 131 −30.347 22.304 67.462 0.00 0.00
B

ATOM 18738 N   ILE B 132   -28.597 20.850 67.229 0.00 0.00
B
ATOM 18739 HN ILE B 132   -27.620 20.678 67.127 0.00 0.00
B
ATOM 18740 CA ILE B 132   -29.371 19.620 67.634 0.00 0.00
B
ATOM 18741 HA ILE B 132   -30.105 19.479 66.854 0.00 0.00
B
ATOM 18742 CB ILE B 132   -28.502 18.379 67.706 0.00 0.00
B
ATOM 18743 HB ILE B 132   -27.686 18.686 68.394 0.00 0.00
B
ATOM 18744 CG2 ILE B 132   -29.064 17.197 68.567 0.00 0.00
B
ATOM 18745 HG21 ILE B 132   -28.332 16.374 68.425 0.00 0.00
B
ATOM 18746 HG22 ILE B 132   -29.177 17.443 69.645 0.00 0.00
B
ATOM 18747 HG23 ILE B 132   -30.041 16.935 68.108 0.00 0.00
B
ATOM 18748 CG1 ILE B 132   -27.993 17.868 66.271 0.00 0.00
B
ATOM 18749 HG11 ILE B 132   -27.445 18.711 65.797 0.00 0.00
B
ATOM 18750 HG12 ILE B 132   -27.148 17.157 66.391 0.00 0.00
B
ATOM 18751 CD ILE B 132   -29.122 17.349 65.381 0.00 0.00
B
ATOM 18752 HD1 ILE B 132   -29.708 16.580 65.929 0.00 0.00
B
ATOM 18753 HD2 ILE B 132   -29.924 18.086 65.163 0.00 0.00
B
ATOM 18754 HD3 ILE B 132   -28.728 16.842 64.475 0.00 0.00
B
ATOM 18755 C   ILE B 132   -30.007 19.790 69.050 0.00 0.00
B
ATOM 18756 O   ILE B 132   -31.176 19.432 69.288 0.00 0.00
B
ATOM 18757 N   ALA B 133   -29.305 20.368 70.073 0.00 0.00
B
ATOM 18758 HN ALA B 133   -28.316 20.476 70.019 0.00 0.00
B
ATOM 18759 CA ALA B 133   -29.806 20.587 71.394 0.00 0.00
B
ATOM 18760 HA ALA B 133   -30.271 19.668 71.720 0.00 0.00
B
ATOM 18761 CB ALA B 133   -28.598 20.978 72.266 0.00 0.00
B
ATOM 18762 HB1 ALA B 133   -28.878 20.970 73.341 0.00 0.00
B
ATOM 18763 HB2 ALA B 133   -27.733 20.299 72.103 0.00 0.00
B
ATOM 18764 HB3 ALA B 133   -28.281 22.037 72.155 0.00 0.00
B

ATOM	18765	C	ALA	B	133	−30.894	21.521	71.469	0.00	0.00	B
ATOM	18766	O	ALA	B	133	−31.910	21.375	72.085	0.00	0.00	B
ATOM	18767	N	GLN	B	134	−30.786	22.557	70.598	0.00	0.00	B
ATOM	18768	HN	GLN	B	134	−29.929	22.584	70.089	0.00	0.00	B
ATOM	18769	CA	GLN	B	134	−31.789	23.645	70.452	0.00	0.00	B
ATOM	18770	HA	GLN	B	134	−32.152	24.010	71.401	0.00	0.00	B
ATOM	18771	CB	GLN	B	134	−31.073	24.867	69.667	0.00	0.00	B
ATOM	18772	HB1	GLN	B	134	−30.286	25.119	70.410	0.00	0.00	B
ATOM	18773	HB2	GLN	B	134	−30.587	24.377	68.796	0.00	0.00	B
ATOM	18774	CG	GLN	B	134	−31.968	26.067	69.359	0.00	0.00	B
ATOM	18775	HG1	GLN	B	134	−31.415	26.763	68.694	0.00	0.00	B
ATOM	18776	HG2	GLN	B	134	−32.964	25.798	68.946	0.00	0.00	B
ATOM	18777	CD	GLN	B	134	−32.339	26.677	70.692	0.00	0.00	B
ATOM	18778	OE1	GLN	B	134	−33.486	26.669	71.090	0.00	0.00	B
ATOM	18779	NE2	GLN	B	134	−31.380	27.463	71.222	0.00	0.00	B
ATOM	18780	HE21	GLN	B	134	−31.512	27.745	72.172	0.00	0.00	B
ATOM	18781	HE22	GLN	B	134	−30.506	27.507	70.738	0.00	0.00	B
ATOM	18782	C	GLN	B	134	−32.982	23.178	69.639	0.00	0.00	B
ATOM	18783	O	GLN	B	134	−34.045	23.807	69.688	0.00	0.00	B
ATOM	18784	N	SER	B	135	−32.787	22.087	68.926	0.00	0.00	B
ATOM	18785	HN	SER	B	135	−31.820	21.858	68.841	0.00	0.00	B
ATOM	18786	CA	SER	B	135	−33.816	21.167	68.263	0.00	0.00	B
ATOM	18787	HA	SER	B	135	−34.590	21.821	67.892	0.00	0.00	B
ATOM	18788	CB	SER	B	135	−33.069	20.495	67.070	0.00	0.00	B
ATOM	18789	HB1	SER	B	135	−32.388	21.177	66.517	0.00	0.00	B
ATOM	18790	HB2	SER	B	135	−32.620	19.593	67.538	0.00	0.00	B
ATOM	18791	OG	SER	B	135	−34.033	20.059	66.095	0.00	0.00	B

ATOM 18792 HG1 SER B 135 -34.228 20.739 65.446 0.00 0.00
B
ATOM 18793 C SER B 135 -34.604 20.152 69.117 0.00 0.00
B
ATOM 18794 O SER B 135 -35.774 20.149 68.973 0.00 0.00
B
ATOM 18795 N LEU B 136 -33.887 19.521 70.061 0.00 0.00
B
ATOM 18796 HN LEU B 136 -32.924 19.771 70.123 0.00 0.00
B
ATOM 18797 CA LEU B 136 -34.449 18.532 71.016 0.00 0.00
B
ATOM 18798 HA LEU B 136 -35.134 17.872 70.505 0.00 0.00
B
ATOM 18799 CB LEU B 136 -33.529 17.597 71.782 0.00 0.00
B
ATOM 18800 HB1 LEU B 136 -32.827 18.129 72.460 0.00 0.00
B
ATOM 18801 HB2 LEU B 136 -34.082 16.883 72.428 0.00 0.00
B
ATOM 18802 CG LEU B 136 -32.666 16.631 70.867 0.00 0.00
B
ATOM 18803 HG LEU B 136 -32.411 17.230 69.967 0.00 0.00
B
ATOM 18804 CD1 LEU B 136 -31.376 16.220 71.541 0.00 0.00
B
ATOM 18805 HD11 LEU B 136 -30.903 15.499 70.840 0.00 0.00
B
ATOM 18806 HD12 LEU B 136 -30.757 17.140 71.600 0.00 0.00
B
ATOM 18807 HD13 LEU B 136 -31.498 15.726 72.529 0.00 0.00
B
ATOM 18808 CD2 LEU B 136 -33.554 15.376 70.555 0.00 0.00
B
ATOM 18809 HD21 LEU B 136 -33.649 14.548 71.291 0.00 0.00
B
ATOM 18810 HD22 LEU B 136 -34.617 15.601 70.325 0.00 0.00
B
ATOM 18811 HD23 LEU B 136 -33.206 14.973 69.580 0.00 0.00
B
ATOM 18812 C LEU B 136 -35.299 19.241 72.047 0.00 0.00
B
ATOM 18813 O LEU B 136 -36.490 18.993 72.106 0.00 0.00
B
ATOM 18814 N SER B 137 -34.721 20.270 72.652 0.00 0.00
B
ATOM 18815 HN SER B 137 -33.779 20.478 72.400 0.00 0.00
B
ATOM 18816 CA SER B 137 -35.362 21.301 73.451 0.00 0.00
B
ATOM 18817 HA SER B 137 -34.648 21.675 74.170 0.00 0.00
B
ATOM 18818 CB SER B 137 -35.605 22.593 72.602 0.00 0.00
B

ATOM 18819 HB1 SER B 137 −34.838 22.552 71.798 0.00 0.00
B
ATOM 18820 HB2 SER B 137 −36.593 22.508 72.102 0.00 0.00
B
ATOM 18821 OG SER B 137 −35.552 23.760 73.356 0.00 0.00
B
ATOM 18822 HG1 SER B 137 −35.618 24.529 72.786 0.00 0.00
B
ATOM 18823 C SER B 137 −36.549 20.991 74.367 0.00 0.00
B
ATOM 18824 O SER B 137 −36.520 20.000 75.080 0.00 0.00
B
ATOM 18825 N ASP B 138 −37.517 21.850 74.571 0.00 0.00
B
ATOM 18826 HN ASP B 138 −37.527 22.684 74.025 0.00 0.00
B
ATOM 18827 CA ASP B 138 −38.727 21.638 75.348 0.00 0.00
B
ATOM 18828 HA ASP B 138 −38.565 20.784 75.989 0.00 0.00
B
ATOM 18829 CB ASP B 138 −38.980 22.857 76.201 0.00 0.00
B
ATOM 18830 HB1 ASP B 138 −39.018 23.787 75.595 0.00 0.00
B
ATOM 18831 HB2 ASP B 138 −39.891 22.654 76.805 0.00 0.00
B
ATOM 18832 CG ASP B 138 −37.967 23.164 77.282 0.00 0.00
B
ATOM 18833 OD1 ASP B 138 −37.504 24.337 77.221 0.00 0.00
B
ATOM 18834 OD2 ASP B 138 −37.759 22.367 78.190 0.00 0.00
B
ATOM 18835 C ASP B 138 −39.872 21.333 74.400 0.00 0.00
B
ATOM 18836 O ASP B 138 −39.878 21.915 73.343 0.00 0.00
B
ATOM 18837 N GLU B 139 −40.753 20.374 74.676 0.00 0.00
B
ATOM 18838 HN GLU B 139 −40.712 19.988 75.595 0.00 0.00
B
ATOM 18839 CA GLU B 139 −41.529 19.529 73.809 0.00 0.00
B
ATOM 18840 HA GLU B 139 −40.925 18.907 73.165 0.00 0.00
B
ATOM 18841 CB GLU B 139 −42.425 18.654 74.721 0.00 0.00
B
ATOM 18842 HB1 GLU B 139 −43.115 19.295 75.310 0.00 0.00
B
ATOM 18843 HB2 GLU B 139 −43.113 18.044 74.097 0.00 0.00
B
ATOM 18844 CG GLU B 139 −41.591 17.562 75.530 0.00 0.00
B
ATOM 18845 HG1 GLU B 139 −41.231 16.677 74.963 0.00 0.00
B

ATOM 18846 HG2 GLU B 139 -40.707 18.102 75.931 0.00 0.00
B
ATOM 18847 CD GLU B 139 -42.393 16.945 76.682 0.00 0.00
B
ATOM 18848 OE1 GLU B 139 -43.613 17.196 76.696 0.00 0.00
B
ATOM 18849 OE2 GLU B 139 -41.843 16.252 77.593 0.00 0.00
B
ATOM 18850 C GLU B 139 -42.437 20.245 72.811 0.00 0.00
B
ATOM 18851 O GLU B 139 -42.423 19.836 71.637 0.00 0.00
B
ATOM 18852 N ASN B 140 -43.222 21.215 73.228 0.00 0.00
B
ATOM 18853 HN ASN B 140 -43.068 21.617 74.127 0.00 0.00
B
ATOM 18854 CA ASN B 140 -44.246 21.914 72.494 0.00 0.00
B
ATOM 18855 HA ASN B 140 -44.584 21.400 71.606 0.00 0.00
B
ATOM 18856 CB ASN B 140 -45.512 22.210 73.318 0.00 0.00
B
ATOM 18857 HB1 ASN B 140 -45.370 22.916 74.163 0.00 0.00
B
ATOM 18858 HB2 ASN B 140 -46.235 22.711 72.639 0.00 0.00
B
ATOM 18859 CG ASN B 140 -46.113 20.920 73.766 0.00 0.00
B
ATOM 18860 OD1 ASN B 140 -46.818 20.191 73.060 0.00 0.00
B
ATOM 18861 ND2 ASN B 140 -45.816 20.438 74.980 0.00 0.00
B
ATOM 18862 HD21 ASN B 140 -45.934 19.455 75.118 0.00 0.00
B
ATOM 18863 HD22 ASN B 140 -45.218 20.890 75.642 0.00 0.00
B
ATOM 18864 C ASN B 140 -43.771 23.378 72.096 0.00 0.00
B
ATOM 18865 O ASN B 140 -44.561 24.203 71.565 0.00 0.00
B
ATOM 18866 N VAL B 141 -42.462 23.707 72.279 0.00 0.00
B
ATOM 18867 HN VAL B 141 -41.796 23.157 72.777 0.00 0.00
B
ATOM 18868 CA VAL B 141 -41.842 24.924 71.916 0.00 0.00
B
ATOM 18869 HA VAL B 141 -42.545 25.740 71.999 0.00 0.00
B
ATOM 18870 CB VAL B 141 -40.636 25.267 72.860 0.00 0.00
B
ATOM 18871 HB VAL B 141 -40.032 24.337 72.800 0.00 0.00
B
ATOM 18872 CG1 VAL B 141 -39.811 26.450 72.296 0.00 0.00
B

ATOM 18873 HG11 VAL B 141   −38.880 26.630 72.875 0.00 0.00
B
ATOM 18874 HG12 VAL B 141   −39.428 26.224 71.278 0.00 0.00
B
ATOM 18875 HG13 VAL B 141   −40.450 27.358 72.290 0.00 0.00
B
ATOM 18876 CG2 VAL B 141   −41.137 25.395 74.340 0.00 0.00
B
ATOM 18877 HG21 VAL B 141   −41.617 24.428 74.600 0.00 0.00
B
ATOM 18878 HG22 VAL B 141   −40.252 25.416 75.012 0.00 0.00
B
ATOM 18879 HG23 VAL B 141   −41.821 26.271 74.336 0.00 0.00
B
ATOM 18880 C   VAL B 141   −41.459 24.826 70.407 0.00 0.00
B
ATOM 18881 O   VAL B 141   −40.657 24.051 69.931 0.00 0.00
B
ATOM 18882 N   LEU B 142   −42.094 25.669 69.583 0.00 0.00
B
ATOM 18883 HN LEU B 142   −42.713 26.348 69.971 0.00 0.00
B
ATOM 18884 CA LEU B 142   −41.720 25.740 68.222 0.00 0.00
B
ATOM 18885 HA LEU B 142   −41.483 24.786 67.776 0.00 0.00
B
ATOM 18886 CB LEU B 142   −42.849 26.264 67.250 0.00 0.00
B
ATOM 18887 HB1 LEU B 142   −43.036 27.340 67.451 0.00 0.00
B
ATOM 18888 HB2 LEU B 142   −42.589 26.339 66.172 0.00 0.00
B
ATOM 18889 CG LEU B 142   −44.156 25.477 67.445 0.00 0.00
B
ATOM 18890 HG LEU B 142   −44.530 25.396 68.488 0.00 0.00
B
ATOM 18891 CD1 LEU B 142   −45.299 26.382 66.871 0.00 0.00
B
ATOM 18892 HD11 LEU B 142   −46.223 25.767 66.923 0.00 0.00
B
ATOM 18893 HD12 LEU B 142   −45.348 27.307 67.485 0.00 0.00
B
ATOM 18894 HD13 LEU B 142   −45.289 26.786 65.836 0.00 0.00
B
ATOM 18895 CD2 LEU B 142   −44.131 24.032 66.834 0.00 0.00
B
ATOM 18896 HD21 LEU B 142   −43.239 23.492 67.215 0.00 0.00
B
ATOM 18897 HD22 LEU B 142   −45.067 23.537 67.169 0.00 0.00
B
ATOM 18898 HD23 LEU B 142   −44.045 23.980 65.727 0.00 0.00
B
ATOM 18899 C   LEU B 142   −40.524 26.679 67.980 0.00 0.00
B

ATOM 18900 O LEU B 142 −39.486 26.357 67.397 0.00 0.00
B
ATOM 18901 N ASN B 143 −40.618 27.980 68.430 0.00 0.00
B
ATOM 18902 HN ASN B 143 −41.478 28.345 68.779 0.00 0.00
B
ATOM 18903 CA ASN B 143 −39.656 29.040 68.233 0.00 0.00
B
ATOM 18904 HA ASN B 143 −38.695 28.681 67.894 0.00 0.00
B
ATOM 18905 CB ASN B 143 −40.235 30.099 67.190 0.00 0.00
B
ATOM 18906 HB1 ASN B 143 −41.252 30.447 67.470 0.00 0.00
B
ATOM 18907 HB2 ASN B 143 −39.638 31.030 67.077 0.00 0.00
B
ATOM 18908 CG ASN B 143 −40.270 29.451 65.758 0.00 0.00
B
ATOM 18909 OD1 ASN B 143 −39.226 29.324 65.111 0.00 0.00
B
ATOM 18910 ND2 ASN B 143 −41.398 28.877 65.378 0.00 0.00
B
ATOM 18911 HD21 ASN B 143 −41.279 28.229 64.625 0.00 0.00
B
ATOM 18912 HD22 ASN B 143 −42.254 28.935 65.892 0.00 0.00
B
ATOM 18913 C ASN B 143 −39.481 29.727 69.514 0.00 0.00
B
ATOM 18914 O ASN B 143 −40.326 29.580 70.421 0.00 0.00
B
ATOM 18915 N TYR B 144 −38.324 30.391 69.676 0.00 0.00
B
ATOM 18916 HN TYR B 144 −37.698 30.527 68.911 0.00 0.00
B
ATOM 18917 CA TYR B 144 −37.834 30.761 70.964 0.00 0.00
B
ATOM 18918 HA TYR B 144 −38.646 30.958 71.648 0.00 0.00
B
ATOM 18919 CB TYR B 144 −36.918 29.504 71.401 0.00 0.00
B
ATOM 18920 HB1 TYR B 144 −37.613 28.649 71.537 0.00 0.00
B
ATOM 18921 HB2 TYR B 144 −36.249 29.280 70.542 0.00 0.00
B
ATOM 18922 CG TYR B 144 −36.142 29.635 72.701 0.00 0.00
B
ATOM 18923 CD1 TYR B 144 −36.985 30.163 73.817 0.00 0.00
B
ATOM 18924 HD1 TYR B 144 −38.045 30.337 73.701 0.00 0.00
B
ATOM 18925 CE1 TYR B 144 −36.316 30.217 75.084 0.00 0.00
B
ATOM 18926 HE1 TYR B 144 −36.881 30.653 75.894 0.00 0.00
B

ATOM 18927 CZ TYR B 144   −34.927 30.011 75.174 0.00 0.00
B
ATOM 18928 OH TYR B 144   −34.476 30.106 76.454 0.00 0.00
B
ATOM 18929 HH TYR B 144   −33.524 29.996 76.512 0.00 0.00
B
ATOM 18930 CD2 TYR B 144   −34.737 29.548 72.823 0.00 0.00
B
ATOM 18931 HD2 TYR B 144   −34.017 29.391 72.033 0.00 0.00
B
ATOM 18932 CE2 TYR B 144   −34.153 29.583 74.096 0.00 0.00
B
ATOM 18933 HE2 TYR B 144   −33.085 29.469 74.209 0.00 0.00
B
ATOM 18934 C   TYR B 144   −36.889 31.977 70.937 0.00 0.00
B
ATOM 18935 O   TYR B 144   −36.130 32.220 70.026 0.00 0.00
B
ATOM 18936 N   ILE B 145   −36.863 32.871 71.916 0.00 0.00
B
ATOM 18937 HN ILE B 145   −37.593 32.892 72.594 0.00 0.00
B
ATOM 18938 CA ILE B 145   −35.822 33.936 71.993 0.00 0.00
B
ATOM 18939 HA ILE B 145   −34.919 33.741 71.434 0.00 0.00
B
ATOM 18940 CB ILE B 145   −36.343 35.331 71.719 0.00 0.00
B
ATOM 18941 HB ILE B 145   −37.254 35.501 72.332 0.00 0.00
B
ATOM 18942 CG2 ILE B 145   −35.194 36.375 72.176 0.00 0.00
B
ATOM 18943 HG21 ILE B 145   −35.583 37.353 71.821 0.00 0.00
B
ATOM 18944 HG22 ILE B 145   −34.897 36.323 73.245 0.00 0.00
B
ATOM 18945 HG23 ILE B 145   −34.312 36.124 71.548 0.00 0.00
B
ATOM 18946 CG1 ILE B 145   −36.837 35.469 70.288 0.00 0.00
B
ATOM 18947 HG11 ILE B 145   −35.944 35.590 69.638 0.00 0.00
B
ATOM 18948 HG12 ILE B 145   −37.416 34.567 69.997 0.00 0.00
B
ATOM 18949 CD ILE B 145   −37.672 36.769 70.024 0.00 0.00
B
ATOM 18950 HD1 ILE B 145   −38.178 36.714 69.036 0.00 0.00
B
ATOM 18951 HD2 ILE B 145   −38.419 36.964 70.823 0.00 0.00
B
ATOM 18952 HD3 ILE B 145   −36.983 37.636 70.108 0.00 0.00
B
ATOM 18953 C   ILE B 145   −35.319 33.807 73.460 0.00 0.00
B

ATOM	18954	O	ILE	B	145	−36.118	33.850	74.424	0.00	0.00	B
ATOM	18955	N	ASP	B	146	−33.989	33.691	73.650	0.00	0.00	B
ATOM	18956	HN	ASP	B	146	−33.327	33.460	72.942	0.00	0.00	B
ATOM	18957	CA	ASP	B	146	−33.381	33.619	74.970	0.00	0.00	B
ATOM	18958	HA	ASP	B	146	−33.991	33.107	75.700	0.00	0.00	B
ATOM	18959	CB	ASP	B	146	−32.047	32.744	74.773	0.00	0.00	B
ATOM	18960	HB1	ASP	B	146	−32.335	31.809	74.246	0.00	0.00	B
ATOM	18961	HB2	ASP	B	146	−31.309	33.379	74.237	0.00	0.00	B
ATOM	18962	CG	ASP	B	146	−31.357	32.325	76.100	0.00	0.00	B
ATOM	18963	OD1	ASP	B	146	−30.122	32.140	76.020	0.00	0.00	B
ATOM	18964	OD2	ASP	B	146	−32.051	32.122	77.137	0.00	0.00	B
ATOM	18965	C	ASP	B	146	−32.985	34.981	75.517	0.00	0.00	B
ATOM	18966	O	ASP	B	146	−31.905	35.526	75.182	0.00	0.00	B
ATOM	18967	N	LEU	B	147	−33.798	35.645	76.404	0.00	0.00	B
ATOM	18968	HN	LEU	B	147	−34.670	35.361	76.794	0.00	0.00	B
ATOM	18969	CA	LEU	B	147	−33.469	37.042	76.728	0.00	0.00	B
ATOM	18970	HA	LEU	B	147	−33.061	37.550	75.868	0.00	0.00	B
ATOM	18971	CB	LEU	B	147	−34.732	37.775	77.397	0.00	0.00	B
ATOM	18972	HB1	LEU	B	147	−34.993	37.315	78.375	0.00	0.00	B
ATOM	18973	HB2	LEU	B	147	−34.404	38.774	77.757	0.00	0.00	B
ATOM	18974	CG	LEU	B	147	−36.006	37.858	76.499	0.00	0.00	B
ATOM	18975	HG	LEU	B	147	−36.433	36.833	76.469	0.00	0.00	B
ATOM	18976	CD1	LEU	B	147	−37.098	38.716	77.273	0.00	0.00	B
ATOM	18977	HD11	LEU	B	147	−36.724	39.754	77.397	0.00	0.00	B
ATOM	18978	HD12	LEU	B	147	−38.036	38.728	76.677	0.00	0.00	B
ATOM	18979	HD13	LEU	B	147	−37.251	38.178	78.233	0.00	0.00	B
ATOM	18980	CD2	LEU	B	147	−35.793	38.553	75.197	0.00	0.00	B

ATOM 18981 HD21 LEU B 147 −35.444 39.572 75.470 0.00 0.00
B
ATOM 18982 HD22 LEU B 147 −35.061 38.011 74.562 0.00 0.00
B
ATOM 18983 HD23 LEU B 147 −36.709 38.757 74.602 0.00 0.00
B
ATOM 18984 C LEU B 147 −32.284 37.150 77.801 0.00 0.00
B
ATOM 18985 O LEU B 147 −31.438 38.054 77.705 0.00 0.00
B
ATOM 18986 N SER B 148 −32.293 36.195 78.797 0.00 0.00
B
ATOM 18987 HN SER B 148 −32.985 35.494 78.955 0.00 0.00
B
ATOM 18988 CA SER B 148 −31.185 36.056 79.763 0.00 0.00
B
ATOM 18989 HA SER B 148 −30.288 36.187 79.176 0.00 0.00
B
ATOM 18990 CB SER B 148 −31.319 36.997 80.972 0.00 0.00
B
ATOM 18991 HB1 SER B 148 −30.378 36.975 81.564 0.00 0.00
B
ATOM 18992 HB2 SER B 148 −31.437 38.056 80.657 0.00 0.00
B
ATOM 18993 OG SER B 148 −32.441 36.636 81.848 0.00 0.00
B
ATOM 18994 HG1 SER B 148 −32.468 37.241 82.592 0.00 0.00
B
ATOM 18995 C SER B 148 −31.174 34.577 80.189 0.00 0.00
B
ATOM 18996 O SER B 148 −31.998 33.805 79.602 0.00 0.00
B
ATOM 18997 N ASP B 149 −30.465 34.105 81.243 0.00 0.00
B
ATOM 18998 HN ASP B 149 −29.773 34.643 81.719 0.00 0.00
B
ATOM 18999 CA ASP B 149 −30.574 32.792 81.844 0.00 0.00
B
ATOM 19000 HA ASP B 149 −30.814 32.040 81.106 0.00 0.00
B
ATOM 19001 CB ASP B 149 −29.262 32.410 82.548 0.00 0.00
B
ATOM 19002 HB1 ASP B 149 −28.981 33.117 83.359 0.00 0.00
B
ATOM 19003 HB2 ASP B 149 −29.307 31.413 83.035 0.00 0.00
B
ATOM 19004 CG ASP B 149 −28.011 32.475 81.636 0.00 0.00
B
ATOM 19005 OD1 ASP B 149 −28.055 32.052 80.424 0.00 0.00
B
ATOM 19006 OD2 ASP B 149 −26.968 33.067 82.081 0.00 0.00
B
ATOM 19007 C ASP B 149 −31.780 32.684 82.834 0.00 0.00
B

ATOM	19008	O	ASP	B	149	−32.119	31.577	83.319	0.00	0.00	B
ATOM	19009	N	GLU	B	150	−32.433	33.884	83.112	0.00	0.00	B
ATOM	19010	HN	GLU	B	150	−32.118	34.670	82.586	0.00	0.00	B
ATOM	19011	CA	GLU	B	150	−33.645	34.074	83.899	0.00	0.00	B
ATOM	19012	HA	GLU	B	150	−33.747	33.283	84.627	0.00	0.00	B
ATOM	19013	CB	GLU	B	150	−33.560	35.361	84.654	0.00	0.00	B
ATOM	19014	HB1	GLU	B	150	−33.552	36.254	83.993	0.00	0.00	B
ATOM	19015	HB2	GLU	B	150	−34.459	35.452	85.300	0.00	0.00	B
ATOM	19016	CG	GLU	B	150	−32.381	35.420	85.659	0.00	0.00	B
ATOM	19017	HG1	GLU	B	150	−31.521	35.015	85.084	0.00	0.00	B
ATOM	19018	HG2	GLU	B	150	−32.213	36.464	85.998	0.00	0.00	B
ATOM	19019	CD	GLU	B	150	−32.562	34.478	86.858	0.00	0.00	B
ATOM	19020	OE1	GLU	B	150	−31.859	33.423	86.953	0.00	0.00	B
ATOM	19021	OE2	GLU	B	150	−33.514	34.652	87.620	0.00	0.00	B
ATOM	19022	C	GLU	B	150	−34.933	34.136	83.028	0.00	0.00	B
ATOM	19023	O	GLU	B	150	−35.932	33.456	83.397	0.00	0.00	B
ATOM	19024	N	TYR	B	151	−35.000	34.907	81.909	0.00	0.00	B
ATOM	19025	HN	TYR	B	151	−34.210	35.374	81.521	0.00	0.00	B
ATOM	19026	CA	TYR	B	151	−36.148	35.229	81.169	0.00	0.00	B
ATOM	19027	HA	TYR	B	151	−37.065	34.782	81.523	0.00	0.00	B
ATOM	19028	CB	TYR	B	151	−36.401	36.772	80.934	0.00	0.00	B
ATOM	19029	HB1	TYR	B	151	−35.518	37.179	80.396	0.00	0.00	B
ATOM	19030	HB2	TYR	B	151	−37.266	36.908	80.250	0.00	0.00	B
ATOM	19031	CG	TYR	B	151	−36.725	37.475	82.233	0.00	0.00	B
ATOM	19032	CD1	TYR	B	151	−38.053	37.529	82.776	0.00	0.00	B
ATOM	19033	HD1	TYR	B	151	−38.887	37.129	82.220	0.00	0.00	B
ATOM	19034	CE1	TYR	B	151	−38.248	38.086	84.048	0.00	0.00	B

ATOM 19035 HE1 TYR B 151 −39.224 38.070 84.511 0.00 0.00
B
ATOM 19036 CZ TYR B 151 −37.087 38.503 84.776 0.00 0.00
B
ATOM 19037 OH TYR B 151 −37.295 38.927 86.106 0.00 0.00
B
ATOM 19038 HH TYR B 151 −36.423 39.024 86.494 0.00 0.00
B
ATOM 19039 CD2 TYR B 151 −35.691 37.982 82.945 0.00 0.00
B
ATOM 19040 HD2 TYR B 151 −34.664 37.957 82.610 0.00 0.00
B
ATOM 19041 CE2 TYR B 151 −35.871 38.572 84.179 0.00 0.00
B
ATOM 19042 HE2 TYR B 151 −35.039 38.930 84.768 0.00 0.00
B
ATOM 19043 C TYR B 151 −36.089 34.663 79.738 0.00 0.00
B
ATOM 19044 O TYR B 151 −35.054 34.297 79.283 0.00 0.00
B
ATOM 19045 N SER B 152 −37.307 34.442 79.162 0.00 0.00
B
ATOM 19046 HN SER B 152 −38.140 34.689 79.651 0.00 0.00
B
ATOM 19047 CA SER B 152 −37.513 33.758 77.882 0.00 0.00
B
ATOM 19048 HA SER B 152 −36.655 33.910 77.245 0.00 0.00
B
ATOM 19049 CB SER B 152 −37.785 32.229 78.011 0.00 0.00
B
ATOM 19050 HB1 SER B 152 −38.451 32.082 78.887 0.00 0.00
B
ATOM 19051 HB2 SER B 152 −38.247 31.769 77.111 0.00 0.00
B
ATOM 19052 OG SER B 152 −36.629 31.549 78.419 0.00 0.00
B
ATOM 19053 HG1 SER B 152 −36.777 30.640 78.147 0.00 0.00
B
ATOM 19054 C SER B 152 −38.688 34.327 77.166 0.00 0.00
B
ATOM 19055 O SER B 152 −39.581 34.829 77.822 0.00 0.00
B
ATOM 19056 N ILE B 153 −38.654 34.341 75.846 0.00 0.00
B
ATOM 19057 HN ILE B 153 −37.862 33.978 75.362 0.00 0.00
B
ATOM 19058 CA ILE B 153 −39.920 34.498 75.066 0.00 0.00
B
ATOM 19059 HA ILE B 153 −40.771 34.688 75.703 0.00 0.00
B
ATOM 19060 CB ILE B 153 −39.877 35.550 73.926 0.00 0.00
B
ATOM 19061 HB ILE B 153 −39.227 35.322 73.054 0.00 0.00
B

ATOM 19062 CG2 ILE B 153 −41.335 35.693 73.310 0.00 0.00
B
ATOM 19063 HG21 ILE B 153 −41.624 34.755 72.790 0.00 0.00
B
ATOM 19064 HG22 ILE B 153 −41.994 35.713 74.205 0.00 0.00
B
ATOM 19065 HG23 ILE B 153 −41.488 36.661 72.787 0.00 0.00
B
ATOM 19066 CG1 ILE B 153 −39.320 36.862 74.503 0.00 0.00
B
ATOM 19067 HG11 ILE B 153 −40.059 37.283 75.218 0.00 0.00
B
ATOM 19068 HG12 ILE B 153 −38.413 36.783 75.140 0.00 0.00
B
ATOM 19069 CD ILE B 153 −39.036 37.950 73.493 0.00 0.00
B
ATOM 19070 HD1 ILE B 153 −37.987 37.780 73.170 0.00 0.00
B
ATOM 19071 HD2 ILE B 153 −39.778 37.810 72.678 0.00 0.00
B
ATOM 19072 HD3 ILE B 153 −39.098 38.955 73.962 0.00 0.00
B
ATOM 19073 C ILE B 153 −40.207 33.185 74.358 0.00 0.00
B
ATOM 19074 O ILE B 153 −39.292 32.712 73.703 0.00 0.00
B
ATOM 19075 N VAL B 154 −41.414 32.574 74.429 0.00 0.00
B
ATOM 19076 HN VAL B 154 −42.225 32.948 74.872 0.00 0.00
B
ATOM 19077 CA VAL B 154 −41.706 31.241 73.924 0.00 0.00
B
ATOM 19078 HA VAL B 154 −40.820 30.854 73.442 0.00 0.00
B
ATOM 19079 CB VAL B 154 −41.884 30.393 75.152 0.00 0.00
B
ATOM 19080 HB VAL B 154 −42.645 30.895 75.786 0.00 0.00
B
ATOM 19081 CG1 VAL B 154 −42.327 28.994 74.758 0.00 0.00
B
ATOM 19082 HG11 VAL B 154 −42.426 28.339 75.650 0.00 0.00
B
ATOM 19083 HG12 VAL B 154 −43.381 29.008 74.409 0.00 0.00
B
ATOM 19084 HG13 VAL B 154 −41.605 28.540 74.047 0.00 0.00
B
ATOM 19085 CG2 VAL B 154 −40.627 30.198 75.950 0.00 0.00
B
ATOM 19086 HG21 VAL B 154 −39.972 29.530 75.350 0.00 0.00
B
ATOM 19087 HG22 VAL B 154 −40.012 31.073 76.248 0.00 0.00
B
ATOM 19088 HG23 VAL B 154 −40.975 29.688 76.874 0.00 0.00
B

ATOM 19089 C VAL B 154 −42.829 31.406 72.947 0.00 0.00
B
ATOM 19090 O VAL B 154 −43.842 32.000 73.296 0.00 0.00
B
ATOM 19091 N GLU B 155 −42.847 30.757 71.733 0.00 0.00
B
ATOM 19092 HN GLU B 155 −41.996 30.396 71.359 0.00 0.00
B
ATOM 19093 CA GLU B 155 −43.986 30.469 70.944 0.00 0.00
B
ATOM 19094 HA GLU B 155 −44.809 31.135 71.160 0.00 0.00
B
ATOM 19095 CB GLU B 155 −43.655 30.548 69.453 0.00 0.00
B
ATOM 19096 HB1 GLU B 155 −43.352 31.604 69.287 0.00 0.00
B
ATOM 19097 HB2 GLU B 155 −42.894 29.768 69.238 0.00 0.00
B
ATOM 19098 CG GLU B 155 −44.901 30.173 68.558 0.00 0.00
B
ATOM 19099 HG1 GLU B 155 −45.137 29.088 68.576 0.00 0.00
B
ATOM 19100 HG2 GLU B 155 −45.738 30.764 68.986 0.00 0.00
B
ATOM 19101 CD GLU B 155 −44.593 30.517 67.073 0.00 0.00
B
ATOM 19102 OE1 GLU B 155 −45.419 31.285 66.538 0.00 0.00
B
ATOM 19103 OE2 GLU B 155 −43.668 30.019 66.456 0.00 0.00
B
ATOM 19104 C GLU B 155 −44.397 29.057 71.262 0.00 0.00
B
ATOM 19105 O GLU B 155 −43.818 28.090 70.816 0.00 0.00
B
ATOM 19106 N LEU B 156 −45.440 28.894 72.094 0.00 0.00
B
ATOM 19107 HN LEU B 156 −45.989 29.689 72.340 0.00 0.00
B
ATOM 19108 CA LEU B 156 −45.714 27.609 72.625 0.00 0.00
B
ATOM 19109 HA LEU B 156 −44.862 26.951 72.537 0.00 0.00
B
ATOM 19110 CB LEU B 156 −46.159 27.789 74.128 0.00 0.00
B
ATOM 19111 HB1 LEU B 156 −45.582 28.452 74.808 0.00 0.00
B
ATOM 19112 HB2 LEU B 156 −47.143 28.300 74.194 0.00 0.00
B
ATOM 19113 CG LEU B 156 −46.281 26.479 74.878 0.00 0.00
B
ATOM 19114 HG LEU B 156 −47.080 25.851 74.429 0.00 0.00
B
ATOM 19115 CD1 LEU B 156 −44.966 25.651 74.900 0.00 0.00
B

ATOM 19116 HD11 LEU B 156 −45.059 24.608 75.271 0.00 0.00
B
ATOM 19117 HD12 LEU B 156 −44.766 25.441 73.827 0.00 0.00
B
ATOM 19118 HD13 LEU B 156 −44.039 26.127 75.284 0.00 0.00
B
ATOM 19119 CD2 LEU B 156 −46.679 26.758 76.338 0.00 0.00
B
ATOM 19120 HD21 LEU B 156 −47.668 27.261 76.280 0.00 0.00
B
ATOM 19121 HD22 LEU B 156 −46.892 25.841 76.928 0.00 0.00
B
ATOM 19122 HD23 LEU B 156 −45.948 27.417 76.854 0.00 0.00
B
ATOM 19123 C LEU B 156 −46.877 27.028 71.948 0.00 0.00
B
ATOM 19124 O LEU B 156 −47.946 27.662 71.894 0.00 0.00
B
ATOM 19125 N LEU B 157 −46.793 25.792 71.401 0.00 0.00
B
ATOM 19126 HN LEU B 157 −45.950 25.278 71.538 0.00 0.00
B
ATOM 19127 CA LEU B 157 −47.905 25.109 70.791 0.00 0.00
B
ATOM 19128 HA LEU B 157 −48.438 25.862 70.230 0.00 0.00
B
ATOM 19129 CB LEU B 157 −47.397 23.967 69.918 0.00 0.00
B
ATOM 19130 HB1 LEU B 157 −46.541 24.290 69.288 0.00 0.00
B
ATOM 19131 HB2 LEU B 157 −47.041 23.159 70.592 0.00 0.00
B
ATOM 19132 CG LEU B 157 −48.473 23.296 69.069 0.00 0.00
B
ATOM 19133 HG LEU B 157 −49.404 23.210 69.669 0.00 0.00
B
ATOM 19134 CD1 LEU B 157 −48.817 24.229 67.933 0.00 0.00
B
ATOM 19135 HD11 LEU B 157 −49.540 25.008 68.258 0.00 0.00
B
ATOM 19136 HD12 LEU B 157 −47.880 24.733 67.615 0.00 0.00
B
ATOM 19137 HD13 LEU B 157 −49.388 23.765 67.100 0.00 0.00
B
ATOM 19138 CD2 LEU B 157 −48.041 21.933 68.476 0.00 0.00
B
ATOM 19139 HD21 LEU B 157 −47.133 22.035 67.844 0.00 0.00
B
ATOM 19140 HD22 LEU B 157 −47.873 21.178 69.273 0.00 0.00
B
ATOM 19141 HD23 LEU B 157 −48.818 21.512 67.803 0.00 0.00
B
ATOM 19142 C LEU B 157 −48.860 24.532 71.858 0.00 0.00
B

ATOM 19143 O LEU B 157 −48.407 23.831 72.768 0.00 0.00
B
ATOM 19144 N ALA B 158 −50.119 24.891 71.801 0.00 0.00
B
ATOM 19145 HN ALA B 158 −50.413 25.434 71.018 0.00 0.00
B
ATOM 19146 CA ALA B 158 −51.144 24.391 72.713 0.00 0.00
B
ATOM 19147 HA ALA B 158 −50.817 24.690 73.698 0.00 0.00
B
ATOM 19148 CB ALA B 158 −52.486 25.068 72.430 0.00 0.00
B
ATOM 19149 HB1 ALA B 158 −53.222 24.921 73.249 0.00 0.00
B
ATOM 19150 HB2 ALA B 158 −52.327 26.161 72.307 0.00 0.00
B
ATOM 19151 HB3 ALA B 158 −52.948 24.748 71.472 0.00 0.00
B
ATOM 19152 C ALA B 158 −51.470 22.923 72.778 0.00 0.00
B
ATOM 19153 O ALA B 158 −51.635 22.184 71.806 0.00 0.00
B
ATOM 19154 N THR B 159 −51.517 22.429 73.972 0.00 0.00
B
ATOM 19155 HN THR B 159 −51.318 22.978 74.781 0.00 0.00
B
ATOM 19156 CA THR B 159 −51.684 20.972 74.299 0.00 0.00
B
ATOM 19157 HA THR B 159 −51.261 20.357 73.519 0.00 0.00
B
ATOM 19158 CB THR B 159 −50.732 20.463 75.460 0.00 0.00
B
ATOM 19159 HB THR B 159 −50.780 19.354 75.501 0.00 0.00
B
ATOM 19160 OG1 THR B 159 −51.246 21.033 76.677 0.00 0.00
B
ATOM 19161 HG1 THR B 159 −50.722 20.707 77.412 0.00 0.00
B
ATOM 19162 CG2 THR B 159 −49.261 20.933 75.283 0.00 0.00
B
ATOM 19163 HG21 THR B 159 −49.165 22.001 75.574 0.00 0.00
B
ATOM 19164 HG22 THR B 159 −48.564 20.296 75.868 0.00 0.00
B
ATOM 19165 HG23 THR B 159 −48.982 20.901 74.209 0.00 0.00
B
ATOM 19166 C THR B 159 −53.178 20.609 74.446 0.00 0.00
B
ATOM 19167 O THR B 159 −54.015 21.426 74.745 0.00 0.00
B
ATOM 19168 N ARG B 160 −53.543 19.307 74.379 0.00 0.00
B
ATOM 19169 HN ARG B 160 −52.896 18.581 74.157 0.00 0.00
B

ATOM 19170 CA ARG B 160 −54.923 19.030 74.655 0.00 0.00
B
ATOM 19171 HA ARG B 160 −55.483 19.769 74.101 0.00 0.00
B
ATOM 19172 CB ARG B 160 −55.487 17.584 74.208 0.00 0.00
B
ATOM 19173 HB1 ARG B 160 −55.234 17.508 73.129 0.00 0.00
B
ATOM 19174 HB2 ARG B 160 −54.896 16.749 74.640 0.00 0.00
B
ATOM 19175 CG ARG B 160 −57.091 17.368 74.303 0.00 0.00
B
ATOM 19176 HG1 ARG B 160 −57.417 17.405 75.364 0.00 0.00
B
ATOM 19177 HG2 ARG B 160 −57.594 18.123 73.662 0.00 0.00
B
ATOM 19178 CD ARG B 160 −57.684 16.013 73.889 0.00 0.00
B
ATOM 19179 HD1 ARG B 160 −57.322 15.179 74.528 0.00 0.00
B
ATOM 19180 HD2 ARG B 160 −58.790 16.058 73.992 0.00 0.00
B
ATOM 19181 NE ARG B 160 −57.386 15.980 72.442 0.00 0.00
B
ATOM 19182 HE ARG B 160 −57.056 16.795 71.965 0.00 0.00
B
ATOM 19183 CZ ARG B 160 −56.971 14.832 71.855 0.00 0.00
B
ATOM 19184 NH1 ARG B 160 −57.331 13.598 72.323 0.00 0.00
B
ATOM 19185 HH11 ARG B 160 −57.154 12.861 71.670 0.00 0.00
B
ATOM 19186 HH12 ARG B 160 −57.977 13.483 73.078 0.00 0.00
B
ATOM 19187 NH2 ARG B 160 −56.346 14.881 70.681 0.00 0.00
B
ATOM 19188 HH21 ARG B 160 −56.070 14.052 70.194 0.00 0.00
B
ATOM 19189 HH22 ARG B 160 −56.014 15.790 70.427 0.00 0.00
B
ATOM 19190 C ARG B 160 −55.283 19.274 76.143 0.00 0.00
B
ATOM 19191 O ARG B 160 −56.385 19.514 76.516 0.00 0.00
B
ATOM 19192 N LYS B 161 −54.255 19.244 77.041 0.00 0.00
B
ATOM 19193 HN LYS B 161 −53.356 18.981 76.700 0.00 0.00
B
ATOM 19194 CA LYS B 161 −54.326 19.730 78.385 0.00 0.00
B
ATOM 19195 HA LYS B 161 −55.184 19.312 78.892 0.00 0.00
B
ATOM 19196 CB LYS B 161 −53.079 19.079 79.046 0.00 0.00
B

ATOM 19197 HB1 LYS B 161 -52.123 19.514 78.683 0.00 0.00
B
ATOM 19198 HB2 LYS B 161 -52.945 19.305 80.125 0.00 0.00
B
ATOM 19199 CG LYS B 161 -52.983 17.543 78.948 0.00 0.00
B
ATOM 19200 HG1 LYS B 161 -53.870 17.149 79.488 0.00 0.00
B
ATOM 19201 HG2 LYS B 161 -52.900 17.107 77.929 0.00 0.00
B
ATOM 19202 CD LYS B 161 -51.717 17.156 79.775 0.00 0.00
B
ATOM 19203 HD1 LYS B 161 -51.260 16.230 79.366 0.00 0.00
B
ATOM 19204 HD2 LYS B 161 -51.026 17.970 79.469 0.00 0.00
B
ATOM 19205 CE LYS B 161 -51.830 17.199 81.223 0.00 0.00
B
ATOM 19206 HE1 LYS B 161 -52.292 18.108 81.664 0.00 0.00
B
ATOM 19207 HE2 LYS B 161 -52.496 16.321 81.364 0.00 0.00
B
ATOM 19208 NZ LYS B 161 -50.481 17.038 81.948 0.00 0.00
B
ATOM 19209 HZ1 LYS B 161 -49.819 16.368 81.507 0.00 0.00
B
ATOM 19210 HZ2 LYS B 161 -50.081 17.992 82.053 0.00 0.00
B
ATOM 19211 HZ3 LYS B 161 -50.791 16.700 82.881 0.00 0.00
B
ATOM 19212 C LYS B 161 -54.460 21.255 78.564 0.00 0.00
B
ATOM 19213 O LYS B 161 -55.136 21.769 79.430 0.00 0.00
B
ATOM 19214 N LEU B 162 -53.908 22.048 77.628 0.00 0.00
B
ATOM 19215 HN LEU B 162 -53.243 21.646 77.003 0.00 0.00
B
ATOM 19216 CA LEU B 162 -54.110 23.510 77.661 0.00 0.00
B
ATOM 19217 HA LEU B 162 -54.167 23.881 78.674 0.00 0.00
B
ATOM 19218 CB LEU B 162 -52.893 24.111 76.976 0.00 0.00
B
ATOM 19219 HB1 LEU B 162 -51.935 23.569 77.129 0.00 0.00
B
ATOM 19220 HB2 LEU B 162 -53.133 24.028 75.895 0.00 0.00
B
ATOM 19221 CG LEU B 162 -52.637 25.552 77.239 0.00 0.00
B
ATOM 19222 HG LEU B 162 -53.596 26.091 77.084 0.00 0.00
B
ATOM 19223 CD1 LEU B 162 -52.074 25.869 78.680 0.00 0.00
B

ATOM 19224 HD11 LEU B 162 −52.489 25.086 79.351 0.00 0.00
B
ATOM 19225 HD12 LEU B 162 −50.963 25.857 78.708 0.00 0.00
B
ATOM 19226 HD13 LEU B 162 −52.337 26.890 79.029 0.00 0.00
B
ATOM 19227 CD2 LEU B 162 −51.746 26.144 76.143 0.00 0.00
B
ATOM 19228 HD21 LEU B 162 −50.768 25.621 76.091 0.00 0.00
B
ATOM 19229 HD22 LEU B 162 −52.215 26.153 75.135 0.00 0.00
B
ATOM 19230 HD23 LEU B 162 −51.418 27.179 76.378 0.00 0.00
B
ATOM 19231 C LEU B 162 −55.456 23.927 77.040 0.00 0.00
B
ATOM 19232 O LEU B 162 −56.089 24.947 77.413 0.00 0.00
B
ATOM 19233 N ASP B 163 −55.959 23.093 76.138 0.00 0.00
B
ATOM 19234 HN ASP B 163 −55.525 22.199 76.064 0.00 0.00
B
ATOM 19235 CA ASP B 163 −57.110 23.369 75.229 0.00 0.00
B
ATOM 19236 HA ASP B 163 −56.825 24.142 74.531 0.00 0.00
B
ATOM 19237 CB ASP B 163 −57.244 22.026 74.372 0.00 0.00
B
ATOM 19238 HB1 ASP B 163 −56.299 22.024 73.789 0.00 0.00
B
ATOM 19239 HB2 ASP B 163 −57.181 21.190 75.102 0.00 0.00
B
ATOM 19240 CG ASP B 163 −58.507 21.900 73.613 0.00 0.00
B
ATOM 19241 OD1 ASP B 163 −59.521 21.447 74.175 0.00 0.00
B
ATOM 19242 OD2 ASP B 163 −58.502 22.183 72.436 0.00 0.00
B
ATOM 19243 C ASP B 163 −58.461 23.766 75.914 0.00 0.00
B
ATOM 19244 O ASP B 163 −58.832 23.127 76.935 0.00 0.00
B
ATOM 19245 N SER B 164 −59.257 24.626 75.158 0.00 0.00
B
ATOM 19246 HN SER B 164 −58.876 25.027 74.328 0.00 0.00
B
ATOM 19247 CA SER B 164 −60.672 24.888 75.498 0.00 0.00
B
ATOM 19248 HA SER B 164 −60.982 25.671 74.821 0.00 0.00
B
ATOM 19249 CB SER B 164 −61.623 23.737 75.356 0.00 0.00
B
ATOM 19250 HB1 SER B 164 −61.533 23.096 76.259 0.00 0.00
B

ATOM 19251 HB2 SER B 164 −62.644 24.163 75.256 0.00 0.00
B
ATOM 19252 OG SER B 164 −61.471 23.025 74.143 0.00 0.00
B
ATOM 19253 HG1 SER B 164 −60.668 22.503 74.202 0.00 0.00
B
ATOM 19254 C SER B 164 −60.859 25.426 76.890 0.00 0.00
B
ATOM 19255 O SER B 164 −61.764 25.114 77.661 0.00 0.00
B
ATOM 19256 N LYS B 165 −59.853 26.265 77.296 0.00 0.00
B
ATOM 19257 HN LYS B 165 −59.062 26.319 76.691 0.00 0.00
B
ATOM 19258 CA LYS B 165 −59.912 27.168 78.442 0.00 0.00
B
ATOM 19259 HA LYS B 165 −60.945 27.222 78.752 0.00 0.00
B
ATOM 19260 CB LYS B 165 −58.977 26.604 79.528 0.00 0.00
B
ATOM 19261 HB1 LYS B 165 −58.018 26.494 78.976 0.00 0.00
B
ATOM 19262 HB2 LYS B 165 −58.953 27.280 80.409 0.00 0.00
B
ATOM 19263 CG LYS B 165 −59.336 25.176 79.970 0.00 0.00
B
ATOM 19264 HG1 LYS B 165 −60.236 25.246 80.617 0.00 0.00
B
ATOM 19265 HG2 LYS B 165 −59.605 24.539 79.100 0.00 0.00
B
ATOM 19266 CD LYS B 165 −58.161 24.566 80.742 0.00 0.00
B
ATOM 19267 HD1 LYS B 165 −57.228 24.702 80.154 0.00 0.00
B
ATOM 19268 HD2 LYS B 165 −58.059 25.126 81.696 0.00 0.00
B
ATOM 19269 CE LYS B 165 −58.418 23.037 80.898 0.00 0.00
B
ATOM 19270 HE1 LYS B 165 −59.183 22.992 81.703 0.00 0.00
B
ATOM 19271 HE2 LYS B 165 −58.849 22.536 80.004 0.00 0.00
B
ATOM 19272 NZ LYS B 165 −57.205 22.385 81.232 0.00 0.00
B
ATOM 19273 HZ1 LYS B 165 −56.745 22.043 80.364 0.00 0.00
B
ATOM 19274 HZ2 LYS B 165 −56.484 22.960 81.712 0.00 0.00
B
ATOM 19275 HZ3 LYS B 165 −57.280 21.522 81.807 0.00 0.00
B
ATOM 19276 C LYS B 165 −59.570 28.618 77.937 0.00 0.00
B
ATOM 19277 O LYS B 165 −58.601 28.909 77.253 0.00 0.00
B

ATOM 19278 N SER B 166 −60.385 29.593 78.348 0.00 0.00
B
ATOM 19279 HN SER B 166 −61.187 29.442 78.921 0.00 0.00
B
ATOM 19280 CA SER B 166 −60.084 31.050 78.364 0.00 0.00
B
ATOM 19281 HA SER B 166 −59.856 31.250 77.328 0.00 0.00
B
ATOM 19282 CB SER B 166 −61.327 31.917 78.677 0.00 0.00
B
ATOM 19283 HB1 SER B 166 −61.081 32.980 78.467 0.00 0.00
B
ATOM 19284 HB2 SER B 166 −62.185 31.561 78.068 0.00 0.00
B
ATOM 19285 OG SER B 166 −61.841 31.864 79.988 0.00 0.00
B
ATOM 19286 HG1 SER B 166 −61.888 30.934 80.219 0.00 0.00
B
ATOM 19287 C SER B 166 −58.885 31.380 79.279 0.00 0.00
B
ATOM 19288 O SER B 166 −58.401 30.650 80.147 0.00 0.00
B
ATOM 19289 N ILE B 167 −58.249 32.537 79.010 0.00 0.00
B
ATOM 19290 HN ILE B 167 −58.419 33.105 78.208 0.00 0.00
B
ATOM 19291 CA ILE B 167 −57.062 32.972 79.786 0.00 0.00
B
ATOM 19292 HA ILE B 167 −56.340 32.169 79.761 0.00 0.00
B
ATOM 19293 CB ILE B 167 −56.419 34.133 79.100 0.00 0.00
B
ATOM 19294 HB ILE B 167 −57.096 34.918 78.702 0.00 0.00
B
ATOM 19295 CG2 ILE B 167 −55.418 34.838 80.034 0.00 0.00
B
ATOM 19296 HG21 ILE B 167 −54.618 34.164 80.407 0.00 0.00
B
ATOM 19297 HG22 ILE B 167 −54.964 35.665 79.448 0.00 0.00
B
ATOM 19298 HG23 ILE B 167 −56.015 35.295 80.852 0.00 0.00
B
ATOM 19299 CG1 ILE B 167 −55.704 33.573 77.839 0.00 0.00
B
ATOM 19300 HG11 ILE B 167 −54.875 32.885 78.112 0.00 0.00
B
ATOM 19301 HG12 ILE B 167 −56.459 33.094 77.180 0.00 0.00
B
ATOM 19302 CD ILE B 167 −55.097 34.718 76.885 0.00 0.00
B
ATOM 19303 HD1 ILE B 167 −54.681 34.402 75.905 0.00 0.00
B
ATOM 19304 HD2 ILE B 167 −55.866 35.507 76.742 0.00 0.00
B

ATOM 19305 HD3 ILE B 167 −54.341 35.308 77.448 0.00 0.00
B
ATOM 19306 C ILE B 167 −57.360 33.134 81.288 0.00 0.00
B
ATOM 19307 O ILE B 167 −56.507 32.832 82.130 0.00 0.00
B
ATOM 19308 N ILE B 168 −58.554 33.521 81.705 0.00 0.00
B
ATOM 19309 HN ILE B 168 −59.220 33.873 81.052 0.00 0.00
B
ATOM 19310 CA ILE B 168 −58.927 33.652 83.123 0.00 0.00
B
ATOM 19311 HA ILE B 168 −58.081 33.971 83.713 0.00 0.00
B
ATOM 19312 CB ILE B 168 −59.836 34.897 83.230 0.00 0.00
B
ATOM 19313 HB ILE B 168 −59.506 35.609 82.443 0.00 0.00
B
ATOM 19314 CG2 ILE B 168 −61.335 34.493 83.046 0.00 0.00
B
ATOM 19315 HG21 ILE B 168 −61.960 35.411 83.021 0.00 0.00
B
ATOM 19316 HG22 ILE B 168 −61.467 33.989 82.065 0.00 0.00
B
ATOM 19317 HG23 ILE B 168 −61.717 33.924 83.921 0.00 0.00
B
ATOM 19318 CG1 ILE B 168 −59.627 35.483 84.658 0.00 0.00
B
ATOM 19319 HG11 ILE B 168 −60.080 34.746 85.355 0.00 0.00
B
ATOM 19320 HG12 ILE B 168 −58.541 35.644 84.830 0.00 0.00
B
ATOM 19321 CD ILE B 168 −60.307 36.829 84.867 0.00 0.00
B
ATOM 19322 HD1 ILE B 168 −59.805 37.361 85.703 0.00 0.00
B
ATOM 19323 HD2 ILE B 168 −60.424 37.520 84.005 0.00 0.00
B
ATOM 19324 HD3 ILE B 168 −61.350 36.618 85.186 0.00 0.00
B
ATOM 19325 C ILE B 168 −59.503 32.348 83.707 0.00 0.00
B
ATOM 19326 O ILE B 168 −59.720 32.193 84.874 0.00 0.00
B
ATOM 19327 N ASP B 169 −59.773 31.311 82.810 0.00 0.00
B
ATOM 19328 HN ASP B 169 −59.716 31.447 81.824 0.00 0.00
B
ATOM 19329 CA ASP B 169 −59.841 29.991 83.286 0.00 0.00
B
ATOM 19330 HA ASP B 169 −60.380 29.927 84.220 0.00 0.00
B
ATOM 19331 CB ASP B 169 −60.483 28.908 82.363 0.00 0.00
B

ATOM 19332 HB1 ASP B 169 -59.817 28.908 81.474 0.00 0.00
B
ATOM 19333 HB2 ASP B 169 -60.407 27.873 82.760 0.00 0.00
B
ATOM 19334 CG ASP B 169 -61.893 29.221 81.926 0.00 0.00
B
ATOM 19335 OD1 ASP B 169 -62.210 29.391 80.743 0.00 0.00
B
ATOM 19336 OD2 ASP B 169 -62.690 29.218 82.876 0.00 0.00
B
ATOM 19337 C ASP B 169 -58.524 29.324 83.719 0.00 0.00
B
ATOM 19338 O ASP B 169 -58.404 28.738 84.811 0.00 0.00
B
ATOM 19339 N LEU B 170 -57.495 29.538 82.840 0.00 0.00
B
ATOM 19340 HN LEU B 170 -57.509 30.011 81.962 0.00 0.00
B
ATOM 19341 CA LEU B 170 -56.149 29.074 83.131 0.00 0.00
B
ATOM 19342 HA LEU B 170 -56.299 28.055 83.455 0.00 0.00
B
ATOM 19343 CB LEU B 170 -55.215 29.118 81.809 0.00 0.00
B
ATOM 19344 HB1 LEU B 170 -55.093 30.186 81.528 0.00 0.00
B
ATOM 19345 HB2 LEU B 170 -54.257 28.698 82.182 0.00 0.00
B
ATOM 19346 CG LEU B 170 -55.753 28.384 80.540 0.00 0.00
B
ATOM 19347 HG LEU B 170 -56.852 28.541 80.527 0.00 0.00
B
ATOM 19348 CD1 LEU B 170 -55.030 28.835 79.246 0.00 0.00
B
ATOM 19349 HD11 LEU B 170 -53.969 28.816 79.575 0.00 0.00
B
ATOM 19350 HD12 LEU B 170 -55.122 28.162 78.368 0.00 0.00
B
ATOM 19351 HD13 LEU B 170 -55.188 29.880 78.904 0.00 0.00
B
ATOM 19352 CD2 LEU B 170 -55.592 26.875 80.839 0.00 0.00
B
ATOM 19353 HD21 LEU B 170 -56.196 26.583 81.725 0.00 0.00
B
ATOM 19354 HD22 LEU B 170 -55.704 26.186 79.975 0.00 0.00
B
ATOM 19355 HD23 LEU B 170 -54.524 26.768 81.125 0.00 0.00
B
ATOM 19356 C LEU B 170 -55.527 29.939 84.233 0.00 0.00
B
ATOM 19357 O LEU B 170 -54.719 29.485 85.043 0.00 0.00
B
ATOM 19358 N ASN B 171 -55.904 31.276 84.307 0.00 0.00
B

ATOM 19359 HN ASN B 171 -56.561 31.725 83.707 0.00 0.00
B
ATOM 19360 CA ASN B 171 -55.427 32.175 85.351 0.00 0.00
B
ATOM 19361 HA ASN B 171 -55.820 33.136 85.055 0.00 0.00
B
ATOM 19362 CB ASN B 171 -55.959 31.729 86.730 0.00 0.00
B
ATOM 19363 HB1 ASN B 171 -56.937 31.210 86.636 0.00 0.00
B
ATOM 19364 HB2 ASN B 171 -55.305 30.896 87.065 0.00 0.00
B
ATOM 19365 CG ASN B 171 -55.997 32.968 87.532 0.00 0.00
B
ATOM 19366 OD1 ASN B 171 -56.339 34.083 87.036 0.00 0.00
B
ATOM 19367 ND2 ASN B 171 -55.660 32.942 88.821 0.00 0.00
B
ATOM 19368 HD21 ASN B 171 -55.947 33.777 89.291 0.00 0.00
B
ATOM 19369 HD22 ASN B 171 -55.241 32.142 89.250 0.00 0.00
B
ATOM 19370 C ASN B 171 -53.891 32.409 85.280 0.00 0.00
B
ATOM 19371 O ASN B 171 -53.262 32.515 86.346 0.00 0.00
B
ATOM 19372 N VAL B 172 -53.278 32.516 84.029 0.00 0.00
B
ATOM 19373 HN VAL B 172 -53.819 32.426 83.197 0.00 0.00
B
ATOM 19374 CA VAL B 172 -51.910 32.561 83.832 0.00 0.00
B
ATOM 19375 HA VAL B 172 -51.564 31.576 84.110 0.00 0.00
B
ATOM 19376 CB VAL B 172 -51.619 32.628 82.267 0.00 0.00
B
ATOM 19377 HB VAL B 172 -51.840 33.662 81.925 0.00 0.00
B
ATOM 19378 CG1 VAL B 172 -50.172 32.290 81.986 0.00 0.00
B
ATOM 19379 HG11 VAL B 172 -49.562 32.997 82.588 0.00 0.00
B
ATOM 19380 HG12 VAL B 172 -49.923 31.271 82.350 0.00 0.00
B
ATOM 19381 HG13 VAL B 172 -50.006 32.411 80.894 0.00 0.00
B
ATOM 19382 CG2 VAL B 172 -52.461 31.574 81.453 0.00 0.00
B
ATOM 19383 HG21 VAL B 172 -53.535 31.832 81.566 0.00 0.00
B
ATOM 19384 HG22 VAL B 172 -52.164 31.669 80.386 0.00 0.00
B
ATOM 19385 HG23 VAL B 172 -52.417 30.534 81.840 0.00 0.00
B

ATOM 19386 C VAL B 172 -51.177 33.733 84.546 0.00 0.00
B
ATOM 19387 O VAL B 172 -50.253 33.526 85.273 0.00 0.00
B
ATOM 19388 N ARG B 173 -51.659 35.002 84.427 0.00 0.00
B
ATOM 19389 HN ARG B 173 -52.420 35.359 83.891 0.00 0.00
B
ATOM 19390 CA ARG B 173 -50.873 36.183 84.881 0.00 0.00
B
ATOM 19391 HA ARG B 173 -49.805 36.099 84.742 0.00 0.00
B
ATOM 19392 CB ARG B 173 -51.419 37.483 84.109 0.00 0.00
B
ATOM 19393 HB1 ARG B 173 -51.423 37.231 83.027 0.00 0.00
B
ATOM 19394 HB2 ARG B 173 -52.516 37.558 84.267 0.00 0.00
B
ATOM 19395 CG ARG B 173 -50.801 38.902 84.243 0.00 0.00
B
ATOM 19396 HG1 ARG B 173 -51.349 39.574 83.549 0.00 0.00
B
ATOM 19397 HG2 ARG B 173 -51.105 39.105 85.292 0.00 0.00
B
ATOM 19398 CD ARG B 173 -49.312 38.996 84.251 0.00 0.00
B
ATOM 19399 HD1 ARG B 173 -48.702 38.351 84.919 0.00 0.00
B
ATOM 19400 HD2 ARG B 173 -48.870 38.769 83.257 0.00 0.00
B
ATOM 19401 NE ARG B 173 -49.016 40.458 84.490 0.00 0.00
B
ATOM 19402 HE ARG B 173 -49.781 41.061 84.262 0.00 0.00
B
ATOM 19403 CZ ARG B 173 -47.775 40.971 84.687 0.00 0.00
B
ATOM 19404 NH1 ARG B 173 -47.712 42.267 84.711 0.00 0.00
B
ATOM 19405 HH11 ARG B 173 -46.812 42.702 84.748 0.00 0.00
B
ATOM 19406 HH12 ARG B 173 -48.520 42.775 85.009 0.00 0.00
B
ATOM 19407 NH2 ARG B 173 -46.683 40.237 84.829 0.00 0.00
B
ATOM 19408 HH21 ARG B 173 -45.817 40.663 85.089 0.00 0.00
B
ATOM 19409 HH22 ARG B 173 -46.849 39.255 84.919 0.00 0.00
B
ATOM 19410 C ARG B 173 -50.898 36.330 86.407 0.00 0.00
B
ATOM 19411 O ARG B 173 -49.889 36.455 87.060 0.00 0.00
B
ATOM 19412 N ALA B 174 -52.070 36.176 87.071 0.00 0.00
B

ATOM 19413 HN ALA B 174 -52.875 35.822 86.602 0.00 0.00
B
ATOM 19414 CA ALA B 174 -52.185 36.138 88.557 0.00 0.00
B
ATOM 19415 HA ALA B 174 -51.835 37.049 89.019 0.00 0.00
B
ATOM 19416 CB ALA B 174 -53.664 35.867 88.904 0.00 0.00
B
ATOM 19417 HB1 ALA B 174 -54.250 36.810 88.956 0.00 0.00
B
ATOM 19418 HB2 ALA B 174 -54.065 35.100 88.208 0.00 0.00
B
ATOM 19419 HB3 ALA B 174 -53.814 35.451 89.923 0.00 0.00
B
ATOM 19420 C ALA B 174 -51.390 34.949 89.239 0.00 0.00
B
ATOM 19421 O ALA B 174 -50.740 35.113 90.285 0.00 0.00
B
ATOM 19422 N LYS B 175 -51.429 33.722 88.622 0.00 0.00
B
ATOM 19423 HN LYS B 175 -52.050 33.595 87.852 0.00 0.00
B
ATOM 19424 CA LYS B 175 -50.698 32.514 89.204 0.00 0.00
B
ATOM 19425 HA LYS B 175 -50.687 32.591 90.281 0.00 0.00
B
ATOM 19426 CB LYS B 175 -51.500 31.224 88.875 0.00 0.00
B
ATOM 19427 HB1 LYS B 175 -52.570 31.347 89.150 0.00 0.00
B
ATOM 19428 HB2 LYS B 175 -51.462 30.994 87.789 0.00 0.00
B
ATOM 19429 CG LYS B 175 -51.032 29.977 89.645 0.00 0.00
B
ATOM 19430 HG1 LYS B 175 -50.022 29.716 89.262 0.00 0.00
B
ATOM 19431 HG2 LYS B 175 -50.789 30.146 90.716 0.00 0.00
B
ATOM 19432 CD LYS B 175 -52.062 28.839 89.404 0.00 0.00
B
ATOM 19433 HD1 LYS B 175 -53.044 29.279 89.678 0.00 0.00
B
ATOM 19434 HD2 LYS B 175 -51.986 28.774 88.298 0.00 0.00
B
ATOM 19435 CE LYS B 175 -51.849 27.568 90.245 0.00 0.00
B
ATOM 19436 HE1 LYS B 175 -50.931 27.047 89.898 0.00 0.00
B
ATOM 19437 HE2 LYS B 175 -51.989 27.790 91.325 0.00 0.00
B
ATOM 19438 NZ LYS B 175 -52.932 26.555 90.127 0.00 0.00
B
ATOM 19439 HZ1 LYS B 175 -52.827 25.712 90.728 0.00 0.00
B

ATOM 19440 HZ2 LYS B 175 −53.897 26.934 90.203 0.00 0.00
B
ATOM 19441 HZ3 LYS B 175 −52.727 26.172 89.183 0.00 0.00
B
ATOM 19442 C LYS B 175 −49.174 32.280 88.920 0.00 0.00
B
ATOM 19443 O LYS B 175 −48.417 31.697 89.737 0.00 0.00
B
ATOM 19444 N TYR B 176 −48.656 32.862 87.791 0.00 0.00
B
ATOM 19445 HN TYR B 176 −49.234 33.301 87.107 0.00 0.00
B
ATOM 19446 CA TYR B 176 −47.232 32.568 87.360 0.00 0.00
B
ATOM 19447 HA TYR B 176 −46.682 32.128 88.178 0.00 0.00
B
ATOM 19448 CB TYR B 176 −47.254 31.895 85.996 0.00 0.00
B
ATOM 19449 HB1 TYR B 176 −47.667 32.544 85.195 0.00 0.00
B
ATOM 19450 HB2 TYR B 176 −46.299 31.415 85.691 0.00 0.00
B
ATOM 19451 CG TYR B 176 −48.061 30.602 86.036 0.00 0.00
B
ATOM 19452 CD1 TYR B 176 −47.683 29.592 86.841 0.00 0.00
B
ATOM 19453 HD1 TYR B 176 −46.783 29.588 87.438 0.00 0.00
B
ATOM 19454 CE1 TYR B 176 −48.520 28.429 87.006 0.00 0.00
B
ATOM 19455 HE1 TYR B 176 −48.148 27.610 87.603 0.00 0.00
B
ATOM 19456 CZ TYR B 176 −49.647 28.308 86.288 0.00 0.00
B
ATOM 19457 OH TYR B 176 −50.391 27.139 86.424 0.00 0.00
B
ATOM 19458 HH TYR B 176 −51.298 27.406 86.256 0.00 0.00
B
ATOM 19459 CD2 TYR B 176 −49.296 30.465 85.262 0.00 0.00
B
ATOM 19460 HD2 TYR B 176 −49.634 31.214 84.561 0.00 0.00
B
ATOM 19461 CE2 TYR B 176 −50.093 29.357 85.433 0.00 0.00
B
ATOM 19462 HE2 TYR B 176 −51.097 29.305 85.040 0.00 0.00
B
ATOM 19463 C TYR B 176 −46.458 33.858 87.262 0.00 0.00
B
ATOM 19464 O TYR B 176 −45.260 33.864 87.394 0.00 0.00
B
ATOM 19465 N GLY B 177 −47.119 35.051 87.113 0.00 0.00
B
ATOM 19466 HN GLY B 177 −48.102 35.157 87.246 0.00 0.00
B

ATOM 19467 CA GLY B 177 −46.539 36.307 86.803 0.00 0.00
B
ATOM 19468 HA1 GLY B 177 −45.611 36.445 87.338 0.00 0.00
B
ATOM 19469 HA2 GLY B 177 −47.373 36.935 87.081 0.00 0.00
B
ATOM 19470 C GLY B 177 −46.197 36.460 85.370 0.00 0.00
B
ATOM 19471 O GLY B 177 −45.922 37.617 84.931 0.00 0.00
B
ATOM 19472 N CYS B 178 −46.286 35.386 84.611 0.00 0.00
B
ATOM 19473 HN CYS B 178 −46.417 34.516 85.079 0.00 0.00
B
ATOM 19474 CA CYS B 178 −46.074 35.380 83.183 0.00 0.00
B
ATOM 19475 HA CYS B 178 −45.093 35.769 82.951 0.00 0.00
B
ATOM 19476 CB CYS B 178 −45.948 34.015 82.483 0.00 0.00
B
ATOM 19477 HB1 CYS B 178 −46.928 33.502 82.377 0.00 0.00
B
ATOM 19478 HB2 CYS B 178 −45.612 34.235 81.447 0.00 0.00
B
ATOM 19479 SG CYS B 178 −44.886 32.761 83.384 0.00 0.00
B
ATOM 19480 HG1 CYS B 178 −45.182 31.719 82.621 0.00 0.00
B
ATOM 19481 C CYS B 178 −47.088 36.210 82.311 0.00 0.00
B
ATOM 19482 O CYS B 178 −48.258 36.298 82.574 0.00 0.00
B
ATOM 19483 N THR B 179 −46.603 36.744 81.150 0.00 0.00
B
ATOM 19484 HN THR B 179 −45.670 36.588 80.834 0.00 0.00
B
ATOM 19485 CA THR B 179 −47.422 37.610 80.339 0.00 0.00
B
ATOM 19486 HA THR B 179 −48.425 37.605 80.740 0.00 0.00
B
ATOM 19487 CB THR B 179 −46.696 38.996 80.188 0.00 0.00
B
ATOM 19488 HB THR B 179 −45.713 39.076 79.675 0.00 0.00
B
ATOM 19489 OG1 THR B 179 −46.512 39.602 81.453 0.00 0.00
B
ATOM 19490 HG1 THR B 179 −45.866 40.297 81.309 0.00 0.00
B
ATOM 19491 CG2 THR B 179 −47.594 39.927 79.435 0.00 0.00
B
ATOM 19492 HG21 THR B 179 −47.113 40.926 79.504 0.00 0.00
B
ATOM 19493 HG22 THR B 179 −47.860 39.568 78.418 0.00 0.00
B

ATOM 19494 HG23 THR B 179 −48.578 40.050 79.937 0.00 0.00
B
ATOM 19495 C THR B 179 −47.509 37.097 78.935 0.00 0.00
B
ATOM 19496 O THR B 179 −46.477 36.772 78.301 0.00 0.00
B
ATOM 19497 N ILE B 180 −48.729 37.095 78.414 0.00 0.00
B
ATOM 19498 HN ILE B 180 −49.529 37.409 78.920 0.00 0.00
B
ATOM 19499 CA ILE B 180 −49.033 36.442 77.175 0.00 0.00
B
ATOM 19500 HA ILE B 180 −48.308 35.674 76.948 0.00 0.00
B
ATOM 19501 CB ILE B 180 −50.398 35.879 77.096 0.00 0.00
B
ATOM 19502 HB ILE B 180 −51.215 36.568 77.398 0.00 0.00
B
ATOM 19503 CG2 ILE B 180 −50.800 35.396 75.644 0.00 0.00
B
ATOM 19504 HG21 ILE B 180 −50.047 34.637 75.340 0.00 0.00
B
ATOM 19505 HG22 ILE B 180 −51.816 34.951 75.580 0.00 0.00
B
ATOM 19506 HG23 ILE B 180 −50.879 36.167 74.848 0.00 0.00
B
ATOM 19507 CG1 ILE B 180 −50.373 34.650 78.055 0.00 0.00
B
ATOM 19508 HG11 ILE B 180 −49.710 33.919 77.544 0.00 0.00
B
ATOM 19509 HG12 ILE B 180 −49.915 35.039 78.990 0.00 0.00
B
ATOM 19510 CD ILE B 180 −51.753 34.161 78.494 0.00 0.00
B
ATOM 19511 HD1 ILE B 180 −52.255 33.566 77.701 0.00 0.00
B
ATOM 19512 HD2 ILE B 180 −51.563 33.483 79.353 0.00 0.00
B
ATOM 19513 HD3 ILE B 180 −52.329 35.069 78.770 0.00 0.00
B
ATOM 19514 C ILE B 180 −48.969 37.487 76.112 0.00 0.00
B
ATOM 19515 O ILE B 180 −49.669 38.480 76.199 0.00 0.00
B
ATOM 19516 N LEU B 181 −48.041 37.320 75.163 0.00 0.00
B
ATOM 19517 HN LEU B 181 −47.465 36.506 75.167 0.00 0.00
B
ATOM 19518 CA LEU B 181 −47.866 38.309 74.123 0.00 0.00
B
ATOM 19519 HA LEU B 181 −48.044 39.317 74.467 0.00 0.00
B
ATOM 19520 CB LEU B 181 −46.490 38.166 73.455 0.00 0.00
B

ATOM 19521 HB1 LEU B 181 -46.434 37.108 73.119 0.00 0.00
B
ATOM 19522 HB2 LEU B 181 -46.496 38.932 72.651 0.00 0.00
B
ATOM 19523 CG LEU B 181 -45.179 38.513 74.322 0.00 0.00
B
ATOM 19524 HG LEU B 181 -45.048 37.806 75.169 0.00 0.00
B
ATOM 19525 CD1 LEU B 181 -43.887 38.447 73.487 0.00 0.00
B
ATOM 19526 HD11 LEU B 181 -43.077 38.756 74.182 0.00 0.00
B
ATOM 19527 HD12 LEU B 181 -43.760 37.510 72.905 0.00 0.00
B
ATOM 19528 HD13 LEU B 181 -44.068 39.232 72.721 0.00 0.00
B
ATOM 19529 CD2 LEU B 181 -45.256 39.902 74.950 0.00 0.00
B
ATOM 19530 HD21 LEU B 181 -44.255 40.229 75.304 0.00 0.00
B
ATOM 19531 HD22 LEU B 181 -45.575 40.623 74.167 0.00 0.00
B
ATOM 19532 HD23 LEU B 181 -46.052 39.938 75.724 0.00 0.00
B
ATOM 19533 C LEU B 181 -48.888 38.107 73.003 0.00 0.00
B
ATOM 19534 O LEU B 181 -49.398 39.070 72.561 0.00 0.00
B
ATOM 19535 N ALA B 182 -49.179 36.821 72.581 0.00 0.00
B
ATOM 19536 HN ALA B 182 -48.834 36.009 73.046 0.00 0.00
B
ATOM 19537 CA ALA B 182 -50.132 36.597 71.461 0.00 0.00
B
ATOM 19538 HA ALA B 182 -50.968 37.271 71.575 0.00 0.00
B
ATOM 19539 CB ALA B 182 -49.466 36.966 70.164 0.00 0.00
B
ATOM 19540 HB1 ALA B 182 -49.112 38.013 70.278 0.00 0.00
B
ATOM 19541 HB2 ALA B 182 -48.684 36.216 69.915 0.00 0.00
B
ATOM 19542 HB3 ALA B 182 -50.186 36.962 69.318 0.00 0.00
B
ATOM 19543 C ALA B 182 -50.834 35.267 71.378 0.00 0.00
B
ATOM 19544 O ALA B 182 -50.516 34.250 71.959 0.00 0.00
B
ATOM 19545 N ILE B 183 -51.842 35.254 70.515 0.00 0.00
B
ATOM 19546 HN ILE B 183 -51.961 36.058 69.938 0.00 0.00
B
ATOM 19547 CA ILE B 183 -52.486 33.982 70.070 0.00 0.00
B

ATOM 19548 HA ILE B 183 -51.970 33.055 70.268 0.00 0.00
B
ATOM 19549 CB ILE B 183 -53.964 33.745 70.523 0.00 0.00
B
ATOM 19550 HB ILE B 183 -54.536 34.657 70.247 0.00 0.00
B
ATOM 19551 CG2 ILE B 183 -54.562 32.504 69.813 0.00 0.00
B
ATOM 19552 HG21 ILE B 183 -54.024 31.570 70.080 0.00 0.00
B
ATOM 19553 HG22 ILE B 183 -55.605 32.447 70.193 0.00 0.00
B
ATOM 19554 HG23 ILE B 183 -54.511 32.550 68.704 0.00 0.00
B
ATOM 19555 CG1 ILE B 183 -54.064 33.625 72.112 0.00 0.00
B
ATOM 19556 HG11 ILE B 183 -53.435 32.768 72.434 0.00 0.00
B
ATOM 19557 HG12 ILE B 183 -53.693 34.538 72.625 0.00 0.00
B
ATOM 19558 CD ILE B 183 -55.519 33.299 72.582 0.00 0.00
B
ATOM 19559 HD1 ILE B 183 -55.935 32.399 72.081 0.00 0.00
B
ATOM 19560 HD2 ILE B 183 -55.466 33.015 73.654 0.00 0.00
B
ATOM 19561 HD3 ILE B 183 -56.059 34.255 72.410 0.00 0.00
B
ATOM 19562 C ILE B 183 -52.421 34.035 68.556 0.00 0.00
B
ATOM 19563 O ILE B 183 -52.796 34.985 67.878 0.00 0.00
B
ATOM 19564 N LYS B 184 -51.973 32.913 67.969 0.00 0.00
B
ATOM 19565 HN LYS B 184 -51.710 32.124 68.519 0.00 0.00
B
ATOM 19566 CA LYS B 184 -51.964 32.642 66.560 0.00 0.00
B
ATOM 19567 HA LYS B 184 -52.299 33.559 66.100 0.00 0.00
B
ATOM 19568 CB LYS B 184 -50.621 32.303 65.984 0.00 0.00
B
ATOM 19569 HB1 LYS B 184 -50.217 31.582 66.727 0.00 0.00
B
ATOM 19570 HB2 LYS B 184 -50.688 31.711 65.046 0.00 0.00
B
ATOM 19571 CG LYS B 184 -49.706 33.550 65.972 0.00 0.00
B
ATOM 19572 HG1 LYS B 184 -50.050 34.197 65.136 0.00 0.00
B
ATOM 19573 HG2 LYS B 184 -49.803 34.095 66.935 0.00 0.00
B
ATOM 19574 CD LYS B 184 -48.184 33.072 65.887 0.00 0.00
B

ATOM 19575 HD1 LYS B 184 −47.466 33.889 66.116 0.00 0.00
B
ATOM 19576 HD2 LYS B 184 −48.007 32.252 66.616 0.00 0.00
B
ATOM 19577 CE LYS B 184 −47.795 32.531 64.508 0.00 0.00
B
ATOM 19578 HE1 LYS B 184 −48.370 31.591 64.365 0.00 0.00
B
ATOM 19579 HE2 LYS B 184 −48.090 33.256 63.720 0.00 0.00
B
ATOM 19580 NZ LYS B 184 −46.324 32.363 64.389 0.00 0.00
B
ATOM 19581 HZ1 LYS B 184 −46.227 31.829 63.502 0.00 0.00
B
ATOM 19582 HZ2 LYS B 184 −45.766 33.227 64.237 0.00 0.00
B
ATOM 19583 HZ3 LYS B 184 −45.821 31.896 65.170 0.00 0.00
B
ATOM 19584 C LYS B 184 −52.908 31.533 66.243 0.00 0.00
B
ATOM 19585 O LYS B 184 −52.653 30.397 66.596 0.00 0.00
B
ATOM 19586 N HSE B 185 −53.883 31.782 65.438 0.00 0.00
B
ATOM 19587 HN HSE B 185 −54.020 32.656 64.978 0.00 0.00
B
ATOM 19588 CA HSE B 185 −54.960 30.839 65.240 0.00 0.00
B
ATOM 19589 HA HSE B 185 −54.790 29.900 65.746 0.00 0.00
B
ATOM 19590 CB HSE B 185 −56.359 31.312 65.792 0.00 0.00
B
ATOM 19591 HB1 HSE B 185 −57.090 30.476 65.782 0.00 0.00
B
ATOM 19592 HB2 HSE B 185 −56.278 31.804 66.785 0.00 0.00
B
ATOM 19593 ND1 HSE B 185 −58.123 32.722 64.550 0.00 0.00
B
ATOM 19594 CG HSE B 185 −56.920 32.609 65.204 0.00 0.00
B
ATOM 19595 CE1 HSE B 185 −58.235 34.025 64.293 0.00 0.00
B
ATOM 19596 HE1 HSE B 185 −59.180 34.398 63.897 0.00 0.00
B
ATOM 19597 NE2 HSE B 185 −57.165 34.717 64.680 0.00 0.00
B
ATOM 19598 HE2 HSE B 185 −57.095 35.711 64.766 0.00 0.00
B
ATOM 19599 CD2 HSE B 185 −56.342 33.842 65.269 0.00 0.00
B
ATOM 19600 HD2 HSE B 185 −55.400 34.084 65.747 0.00 0.00
B
ATOM 19601 C HSE B 185 −55.117 30.574 63.729 0.00 0.00
B

ATOM 19602 O HSE B 185 -55.459 31.443 62.943 0.00 0.00
B
ATOM 19603 N HSE B 186 -54.857 29.354 63.310 0.00 0.00
B
ATOM 19604 HN HSE B 186 -54.555 28.636 63.932 0.00 0.00
B
ATOM 19605 CA HSE B 186 -54.833 28.892 61.849 0.00 0.00
B
ATOM 19606 HA HSE B 186 -54.348 27.929 61.907 0.00 0.00
B
ATOM 19607 CB HSE B 186 -56.300 28.645 61.326 0.00 0.00
B
ATOM 19608 HB1 HSE B 186 -56.861 28.110 62.122 0.00 0.00
B
ATOM 19609 HB2 HSE B 186 -56.829 29.608 61.158 0.00 0.00
B
ATOM 19610 ND1 HSE B 186 -56.746 26.494 60.326 0.00 0.00
B
ATOM 19611 CG HSE B 186 -56.492 27.782 60.083 0.00 0.00
B
ATOM 19612 CE1 HSE B 186 -56.938 25.995 59.087 0.00 0.00
B
ATOM 19613 HE1 HSE B 186 -57.233 24.991 58.780 0.00 0.00
B
ATOM 19614 NE2 HSE B 186 -56.834 26.986 58.091 0.00 0.00
B
ATOM 19615 HE2 HSE B 186 -56.610 26.751 57.145 0.00 0.00
B
ATOM 19616 CD2 HSE B 186 -56.620 28.148 58.770 0.00 0.00
B
ATOM 19617 HD2 HSE B 186 -56.652 29.121 58.296 0.00 0.00
B
ATOM 19618 C HSE B 186 -54.016 29.748 60.931 0.00 0.00
B
ATOM 19619 O HSE B 186 -54.463 30.096 59.828 0.00 0.00
B
ATOM 19620 N GLY B 187 -52.780 30.105 61.329 0.00 0.00
B
ATOM 19621 HN GLY B 187 -52.311 29.789 62.150 0.00 0.00
B
ATOM 19622 CA GLY B 187 -51.989 31.003 60.506 0.00 0.00
B
ATOM 19623 HA1 GLY B 187 -52.150 30.663 59.494 0.00 0.00
B
ATOM 19624 HA2 GLY B 187 -50.951 30.999 60.802 0.00 0.00
B
ATOM 19625 C GLY B 187 -52.305 32.452 60.542 0.00 0.00
B
ATOM 19626 O GLY B 187 -51.583 33.195 59.939 0.00 0.00
B
ATOM 19627 N ASP B 188 -53.386 32.907 61.176 0.00 0.00
B
ATOM 19628 HN ASP B 188 -54.022 32.231 61.540 0.00 0.00
B

ATOM 19629 CA ASP B 188   -53.737 34.262 61.430 0.00 0.00
B
ATOM 19630 HA ASP B 188   -53.188 34.833 60.696 0.00 0.00
B
ATOM 19631 CB ASP B 188   -55.258 34.719 61.284 0.00 0.00
B
ATOM 19632 HB1 ASP B 188   -55.917 33.890 61.621 0.00 0.00
B
ATOM 19633 HB2 ASP B 188   -55.486 35.639 61.865 0.00 0.00
B
ATOM 19634 CG ASP B 188   -55.579 35.008 59.904 0.00 0.00
B
ATOM 19635 OD1 ASP B 188   -56.637 35.726 59.683 0.00 0.00
B
ATOM 19636 OD2 ASP B 188   -54.931 34.527 58.946 0.00 0.00
B
ATOM 19637 C   ASP B 188   -53.320 34.782 62.936 0.00 0.00
B
ATOM 19638 O   ASP B 188   -52.926 33.987 63.789 0.00 0.00
B
ATOM 19639 N   ILE B 189   -53.487 36.074 63.317 0.00 0.00
B
ATOM 19640 HN ILE B 189   -53.721 36.691 62.569 0.00 0.00
B
ATOM 19641 CA ILE B 189   -52.729 36.680 64.436 0.00 0.00
B
ATOM 19642 HA ILE B 189   -52.436 35.917 65.142 0.00 0.00
B
ATOM 19643 CB ILE B 189   -51.490 37.565 64.033 0.00 0.00
B
ATOM 19644 HB ILE B 189   -51.883 38.318 63.317 0.00 0.00
B
ATOM 19645 CG2 ILE B 189   -50.808 38.270 65.275 0.00 0.00
B
ATOM 19646 HG21 ILE B 189   -51.562 38.881 65.817 0.00 0.00
B
ATOM 19647 HG22 ILE B 189   -50.542 37.458 65.984 0.00 0.00
B
ATOM 19648 HG23 ILE B 189   -49.965 38.935 64.988 0.00 0.00
B
ATOM 19649 CG1 ILE B 189   -50.494 36.775 63.231 0.00 0.00
B
ATOM 19650 HG11 ILE B 189   -49.785 36.239 63.897 0.00 0.00
B
ATOM 19651 HG12 ILE B 189   -50.944 36.041 62.529 0.00 0.00
B
ATOM 19652 CD ILE B 189   -49.637 37.708 62.373 0.00 0.00
B
ATOM 19653 HD1 ILE B 189   -49.014 38.278 63.095 0.00 0.00
B
ATOM 19654 HD2 ILE B 189   -48.956 37.038 61.806 0.00 0.00
B
ATOM 19655 HD3 ILE B 189   -50.216 38.387 61.711 0.00 0.00
B

ATOM 19656 C ILE B 189 −53.663 37.543 65.250 0.00 0.00
B
ATOM 19657 O ILE B 189 −54.318 38.407 64.736 0.00 0.00
B
ATOM 19658 N CYS B 190 −53.661 37.311 66.580 0.00 0.00
B
ATOM 19659 HN CYS B 190 −53.019 36.705 67.044 0.00 0.00
B
ATOM 19660 CA CYS B 190 −54.359 38.224 67.481 0.00 0.00
B
ATOM 19661 HA CYS B 190 −54.916 39.035 67.036 0.00 0.00
B
ATOM 19662 CB CYS B 190 −55.492 37.400 68.229 0.00 0.00
B
ATOM 19663 HB1 CYS B 190 −55.171 36.412 68.623 0.00 0.00
B
ATOM 19664 HB2 CYS B 190 −55.919 37.972 69.081 0.00 0.00
B
ATOM 19665 SG CYS B 190 −56.905 37.061 67.148 0.00 0.00
B
ATOM 19666 HG1 CYS B 190 −57.427 38.265 67.324 0.00 0.00
B
ATOM 19667 C CYS B 190 −53.348 38.675 68.554 0.00 0.00
B
ATOM 19668 O CYS B 190 −52.836 37.888 69.365 0.00 0.00
B
ATOM 19669 N LEU B 191 −53.011 39.956 68.677 0.00 0.00
B
ATOM 19670 HN LEU B 191 −53.299 40.654 68.026 0.00 0.00
B
ATOM 19671 CA LEU B 191 −52.027 40.370 69.641 0.00 0.00
B
ATOM 19672 HA LEU B 191 −51.373 39.532 69.834 0.00 0.00
B
ATOM 19673 CB LEU B 191 −51.124 41.495 68.916 0.00 0.00
B
ATOM 19674 HB1 LEU B 191 −51.785 42.225 68.400 0.00 0.00
B
ATOM 19675 HB2 LEU B 191 −50.405 41.930 69.642 0.00 0.00
B
ATOM 19676 CG LEU B 191 −50.170 41.062 67.799 0.00 0.00
B
ATOM 19677 HG LEU B 191 −50.686 40.616 66.922 0.00 0.00
B
ATOM 19678 CD1 LEU B 191 −49.373 42.237 67.256 0.00 0.00
B
ATOM 19679 HD11 LEU B 191 −48.738 41.930 66.398 0.00 0.00
B
ATOM 19680 HD12 LEU B 191 −50.129 43.009 66.996 0.00 0.00
B
ATOM 19681 HD13 LEU B 191 −48.628 42.652 67.968 0.00 0.00
B
ATOM 19682 CD2 LEU B 191 −49.205 40.014 68.353 0.00 0.00
B

ATOM 19683 HD21 LEU B 191 −48.355 39.864 67.653 0.00 0.00
B
ATOM 19684 HD22 LEU B 191 −48.832 40.301 69.360 0.00 0.00
B
ATOM 19685 HD23 LEU B 191 −49.664 39.007 68.258 0.00 0.00
B
ATOM 19686 C LEU B 191 −52.718 40.916 70.855 0.00 0.00
B
ATOM 19687 O LEU B 191 −53.805 41.409 70.770 0.00 0.00
B
ATOM 19688 N SER B 192 −52.089 40.912 72.065 0.00 0.00
B
ATOM 19689 HN SER B 192 −51.153 40.569 72.050 0.00 0.00
B
ATOM 19690 CA SER B 192 −52.631 41.434 73.302 0.00 0.00
B
ATOM 19691 HA SER B 192 −51.899 41.098 74.021 0.00 0.00
B
ATOM 19692 CB SER B 192 −52.550 42.993 73.503 0.00 0.00
B
ATOM 19693 HB1 SER B 192 −51.643 43.361 72.977 0.00 0.00
B
ATOM 19694 HB2 SER B 192 −53.342 43.575 72.985 0.00 0.00
B
ATOM 19695 OG SER B 192 −52.375 43.312 74.873 0.00 0.00
B
ATOM 19696 HG1 SER B 192 −52.781 44.176 74.968 0.00 0.00
B
ATOM 19697 C SER B 192 −53.942 40.912 73.802 0.00 0.00
B
ATOM 19698 O SER B 192 −54.847 41.678 73.966 0.00 0.00
B
ATOM 19699 N PRO B 193 −54.104 39.606 73.926 0.00 0.00
B
ATOM 19700 CD PRO B 193 −53.044 38.590 73.941 0.00 0.00
B
ATOM 19701 HD1 PRO B 193 −52.650 38.519 72.905 0.00 0.00
B
ATOM 19702 HD2 PRO B 193 −52.290 38.726 74.745 0.00 0.00
B
ATOM 19703 CA PRO B 193 −55.498 39.088 73.911 0.00 0.00
B
ATOM 19704 HA PRO B 193 −56.002 39.684 73.164 0.00 0.00
B
ATOM 19705 CB PRO B 193 −55.251 37.629 73.619 0.00 0.00
B
ATOM 19706 HB1 PRO B 193 −55.297 37.382 72.537 0.00 0.00
B
ATOM 19707 HB2 PRO B 193 −56.071 36.969 73.977 0.00 0.00
B
ATOM 19708 CG PRO B 193 −53.925 37.359 74.243 0.00 0.00
B
ATOM 19709 HG1 PRO B 193 −53.459 36.455 73.794 0.00 0.00
B

ATOM 19710 HG2 PRO B 193 -54.003 37.246 75.345 0.00 0.00
B
ATOM 19711 C PRO B 193 -56.122 39.226 75.280 0.00 0.00
B
ATOM 19712 O PRO B 193 -55.397 39.491 76.218 0.00 0.00
B
ATOM 19713 N ALA B 194 -57.425 39.018 75.368 0.00 0.00
B
ATOM 19714 HN ALA B 194 -57.971 38.601 74.646 0.00 0.00
B
ATOM 19715 CA ALA B 194 -58.149 39.270 76.616 0.00 0.00
B
ATOM 19716 HA ALA B 194 -57.890 40.228 77.043 0.00 0.00
B
ATOM 19717 CB ALA B 194 -59.673 39.409 76.221 0.00 0.00
B
ATOM 19718 HB1 ALA B 194 -59.821 40.248 75.509 0.00 0.00
B
ATOM 19719 HB2 ALA B 194 -60.093 38.486 75.765 0.00 0.00
B
ATOM 19720 HB3 ALA B 194 -60.317 39.529 77.118 0.00 0.00
B
ATOM 19721 C ALA B 194 -57.958 38.100 77.615 0.00 0.00
B
ATOM 19722 O ALA B 194 -57.600 37.016 77.200 0.00 0.00
B
ATOM 19723 N PRO B 195 -58.298 38.222 78.899 0.00 0.00
B
ATOM 19724 CD PRO B 195 -58.240 39.499 79.587 0.00 0.00
B
ATOM 19725 HD1 PRO B 195 -57.284 40.002 79.330 0.00 0.00
B
ATOM 19726 HD2 PRO B 195 -59.176 40.070 79.407 0.00 0.00
B
ATOM 19727 CA PRO B 195 -58.708 37.099 79.759 0.00 0.00
B
ATOM 19728 HA PRO B 195 -57.877 36.446 79.980 0.00 0.00
B
ATOM 19729 CB PRO B 195 -58.993 37.871 81.115 0.00 0.00
B
ATOM 19730 HB1 PRO B 195 -58.654 37.302 82.007 0.00 0.00
B
ATOM 19731 HB2 PRO B 195 -60.065 37.878 81.409 0.00 0.00
B
ATOM 19732 CG PRO B 195 -58.258 39.192 81.037 0.00 0.00
B
ATOM 19733 HG1 PRO B 195 -58.805 39.952 81.635 0.00 0.00
B
ATOM 19734 HG2 PRO B 195 -57.239 39.014 81.444 0.00 0.00
B
ATOM 19735 C PRO B 195 -59.778 36.220 79.273 0.00 0.00
B
ATOM 19736 O PRO B 195 -59.797 35.018 79.526 0.00 0.00
B

ATOM	19737	N	GLU	B	196	−60.770	36.680	78.451	0.00	0.00	B
ATOM	19738	HN	GLU	B	196	−60.841	37.674	78.482	0.00	0.00	B
ATOM	19739	CA	GLU	B	196	−61.726	35.800	77.882	0.00	0.00	B
ATOM	19740	HA	GLU	B	196	−61.883	34.992	78.582	0.00	0.00	B
ATOM	19741	CB	GLU	B	196	−63.025	36.616	77.875	0.00	0.00	B
ATOM	19742	HB1	GLU	B	196	−63.901	35.944	77.748	0.00	0.00	B
ATOM	19743	HB2	GLU	B	196	−63.095	36.947	78.933	0.00	0.00	B
ATOM	19744	CG	GLU	B	196	−62.992	37.882	76.964	0.00	0.00	B
ATOM	19745	HG1	GLU	B	196	−62.213	38.560	77.373	0.00	0.00	B
ATOM	19746	HG2	GLU	B	196	−62.692	37.586	75.936	0.00	0.00	B
ATOM	19747	CD	GLU	B	196	−64.354	38.507	76.774	0.00	0.00	B
ATOM	19748	OE1	GLU	B	196	−65.126	38.167	75.856	0.00	0.00	B
ATOM	19749	OE2	GLU	B	196	−64.614	39.485	77.500	0.00	0.00	B
ATOM	19750	C	GLU	B	196	−61.449	35.177	76.559	0.00	0.00	B
ATOM	19751	O	GLU	B	196	−62.166	34.318	75.981	0.00	0.00	B
ATOM	19752	N	ASP	B	197	−60.190	35.399	76.017	0.00	0.00	B
ATOM	19753	HN	ASP	B	197	−59.527	35.972	76.492	0.00	0.00	B
ATOM	19754	CA	ASP	B	197	−59.851	34.774	74.785	0.00	0.00	B
ATOM	19755	HA	ASP	B	197	−60.697	34.730	74.115	0.00	0.00	B
ATOM	19756	CB	ASP	B	197	−58.845	35.496	73.820	0.00	0.00	B
ATOM	19757	HB1	ASP	B	197	−58.057	35.847	74.520	0.00	0.00	B
ATOM	19758	HB2	ASP	B	197	−58.625	34.841	72.949	0.00	0.00	B
ATOM	19759	CG	ASP	B	197	−59.495	36.744	73.157	0.00	0.00	B
ATOM	19760	OD1	ASP	B	197	−60.627	36.628	72.593	0.00	0.00	B
ATOM	19761	OD2	ASP	B	197	−58.910	37.862	73.215	0.00	0.00	B
ATOM	19762	C	ASP	B	197	−59.361	33.396	74.967	0.00	0.00	B
ATOM	19763	O	ASP	B	197	−58.561	33.083	75.858	0.00	0.00	B

ATOM 19764 N ILE B 198 −59.927 32.450 74.166 0.00 0.00
B
ATOM 19765 HN ILE B 198 −60.690 32.719 73.583 0.00 0.00
B
ATOM 19766 CA ILE B 198 −59.788 30.993 74.379 0.00 0.00
B
ATOM 19767 HA ILE B 198 −59.475 30.759 75.386 0.00 0.00
B
ATOM 19768 CB ILE B 198 −61.150 30.253 74.126 0.00 0.00
B
ATOM 19769 HB ILE B 198 −61.432 30.350 73.056 0.00 0.00
B
ATOM 19770 CG2 ILE B 198 −60.936 28.772 74.311 0.00 0.00
B
ATOM 19771 HG21 ILE B 198 −61.796 28.171 73.947 0.00 0.00
B
ATOM 19772 HG22 ILE B 198 −60.081 28.367 73.729 0.00 0.00
B
ATOM 19773 HG23 ILE B 198 −60.826 28.535 75.391 0.00 0.00
B
ATOM 19774 CG1 ILE B 198 −62.258 30.844 75.068 0.00 0.00
B
ATOM 19775 HG11 ILE B 198 −61.910 30.590 76.092 0.00 0.00
B
ATOM 19776 HG12 ILE B 198 −62.493 31.912 74.873 0.00 0.00
B
ATOM 19777 CD ILE B 198 −63.614 30.201 74.928 0.00 0.00
B
ATOM 19778 HD1 ILE B 198 −63.873 30.304 73.852 0.00 0.00
B
ATOM 19779 HD2 ILE B 198 −63.598 29.146 75.275 0.00 0.00
B
ATOM 19780 HD3 ILE B 198 −64.400 30.779 75.458 0.00 0.00
B
ATOM 19781 C ILE B 198 −58.635 30.426 73.601 0.00 0.00
B
ATOM 19782 O ILE B 198 −58.406 30.776 72.430 0.00 0.00
B
ATOM 19783 N ILE B 199 −57.732 29.567 74.162 0.00 0.00
B
ATOM 19784 HN ILE B 199 −57.885 29.213 75.081 0.00 0.00
B
ATOM 19785 CA ILE B 199 −56.728 28.779 73.439 0.00 0.00
B
ATOM 19786 HA ILE B 199 −56.655 29.228 72.460 0.00 0.00
B
ATOM 19787 CB ILE B 199 −55.434 28.776 74.152 0.00 0.00
B
ATOM 19788 HB ILE B 199 −55.246 29.838 74.418 0.00 0.00
B
ATOM 19789 CG2 ILE B 199 −55.399 27.869 75.391 0.00 0.00
B
ATOM 19790 HG21 ILE B 199 −54.366 27.875 75.801 0.00 0.00
B

ATOM 19791 HG22 ILE B 199 −56.094 28.279 76.155 0.00 0.00
B
ATOM 19792 HG23 ILE B 199 −55.638 26.817 75.126 0.00 0.00
B
ATOM 19793 CG1 ILE B 199 −54.211 28.446 73.342 0.00 0.00
B
ATOM 19794 HG11 ILE B 199 −53.420 28.468 74.122 0.00 0.00
B
ATOM 19795 HG12 ILE B 199 −54.266 27.390 73.001 0.00 0.00
B
ATOM 19796 CD ILE B 199 −53.856 29.567 72.310 0.00 0.00
B
ATOM 19797 HD1 ILE B 199 −54.618 29.623 71.503 0.00 0.00
B
ATOM 19798 HD2 ILE B 199 −53.904 30.584 72.754 0.00 0.00
B
ATOM 19799 HD3 ILE B 199 −52.872 29.319 71.859 0.00 0.00
B
ATOM 19800 C ILE B 199 −57.201 27.321 73.187 0.00 0.00
B
ATOM 19801 O ILE B 199 −57.810 26.574 73.981 0.00 0.00
B
ATOM 19802 N ARG B 200 −56.884 26.730 72.002 0.00 0.00
B
ATOM 19803 HN ARG B 200 −56.578 27.373 71.304 0.00 0.00
B
ATOM 19804 CA ARG B 200 −57.310 25.397 71.600 0.00 0.00
B
ATOM 19805 HA ARG B 200 −57.719 24.892 72.463 0.00 0.00
B
ATOM 19806 CB ARG B 200 −58.345 25.377 70.411 0.00 0.00
B
ATOM 19807 HB1 ARG B 200 −57.759 25.766 69.551 0.00 0.00
B
ATOM 19808 HB2 ARG B 200 −58.650 24.403 69.972 0.00 0.00
B
ATOM 19809 CG ARG B 200 −59.451 26.395 70.629 0.00 0.00
B
ATOM 19810 HG1 ARG B 200 −60.194 25.958 71.330 0.00 0.00
B
ATOM 19811 HG2 ARG B 200 −58.972 27.279 71.101 0.00 0.00
B
ATOM 19812 CD ARG B 200 −60.173 26.844 69.345 0.00 0.00
B
ATOM 19813 HD1 ARG B 200 −60.776 26.105 68.775 0.00 0.00
B
ATOM 19814 HD2 ARG B 200 −60.925 27.604 69.646 0.00 0.00
B
ATOM 19815 NE ARG B 200 −59.168 27.516 68.548 0.00 0.00
B
ATOM 19816 HE ARG B 200 −58.232 27.568 68.897 0.00 0.00
B
ATOM 19817 CZ ARG B 200 −59.178 27.618 67.195 0.00 0.00
B

ATOM 19818 NH1 ARG B 200 −60.265 27.472 66.439 0.00 0.00
B
ATOM 19819 HH11 ARG B 200 −60.198 27.690 65.465 0.00 0.00
B
ATOM 19820 HH12 ARG B 200 −61.171 27.302 66.827 0.00 0.00
B
ATOM 19821 NH2 ARG B 200 −58.039 27.775 66.520 0.00 0.00
B
ATOM 19822 HH21 ARG B 200 −58.104 27.524 65.554 0.00 0.00
B
ATOM 19823 HH22 ARG B 200 −57.175 27.631 67.004 0.00 0.00
B
ATOM 19824 C ARG B 200 −56.086 24.509 71.192 0.00 0.00
B
ATOM 19825 O ARG B 200 −55.006 25.071 70.845 0.00 0.00
B
ATOM 19826 N GLU B 201 −56.283 23.220 71.123 0.00 0.00
B
ATOM 19827 HN GLU B 201 −57.067 22.780 71.555 0.00 0.00
B
ATOM 19828 CA GLU B 201 −55.302 22.165 70.681 0.00 0.00
B
ATOM 19829 HA GLU B 201 −54.466 22.190 71.365 0.00 0.00
B
ATOM 19830 CB GLU B 201 −55.990 20.829 70.600 0.00 0.00
B
ATOM 19831 HB1 GLU B 201 −56.303 20.638 71.648 0.00 0.00
B
ATOM 19832 HB2 GLU B 201 −56.855 20.906 69.907 0.00 0.00
B
ATOM 19833 CG GLU B 201 −55.058 19.707 70.148 0.00 0.00
B
ATOM 19834 HG1 GLU B 201 −54.782 19.794 69.075 0.00 0.00
B
ATOM 19835 HG2 GLU B 201 −54.164 19.832 70.796 0.00 0.00
B
ATOM 19836 CD GLU B 201 −55.654 18.342 70.388 0.00 0.00
B
ATOM 19837 OE1 GLU B 201 −56.761 18.269 71.017 0.00 0.00
B
ATOM 19838 OE2 GLU B 201 −54.991 17.326 70.038 0.00 0.00
B
ATOM 19839 C GLU B 201 −54.700 22.524 69.314 0.00 0.00
B
ATOM 19840 O GLU B 201 −55.342 22.611 68.279 0.00 0.00
B
ATOM 19841 N GLN B 202 −53.348 22.807 69.336 0.00 0.00
B
ATOM 19842 HN GLN B 202 −52.790 22.626 70.143 0.00 0.00
B
ATOM 19843 CA GLN B 202 −52.508 23.199 68.166 0.00 0.00
B
ATOM 19844 HA GLN B 202 −51.498 23.134 68.545 0.00 0.00
B

ATOM 19845 CB GLN B 202 -52.563 22.128 67.033 0.00 0.00
B
ATOM 19846 HB1 GLN B 202 -53.590 22.066 66.615 0.00 0.00
B
ATOM 19847 HB2 GLN B 202 -51.982 22.630 66.230 0.00 0.00
B
ATOM 19848 CG GLN B 202 -52.002 20.791 67.522 0.00 0.00
B
ATOM 19849 HG1 GLN B 202 -51.056 20.951 68.083 0.00 0.00
B
ATOM 19850 HG2 GLN B 202 -52.736 20.355 68.232 0.00 0.00
B
ATOM 19851 CD GLN B 202 -51.606 19.923 66.336 0.00 0.00
B
ATOM 19852 OE1 GLN B 202 -50.618 20.142 65.628 0.00 0.00
B
ATOM 19853 NE2 GLN B 202 -52.445 18.951 65.974 0.00 0.00
B
ATOM 19854 HE21 GLN B 202 -52.123 18.479 65.153 0.00 0.00
B
ATOM 19855 HE22 GLN B 202 -53.085 18.585 66.649 0.00 0.00
B
ATOM 19856 C GLN B 202 -52.788 24.639 67.718 0.00 0.00
B
ATOM 19857 O GLN B 202 -52.280 25.125 66.695 0.00 0.00
B
ATOM 19858 N ASP B 203 -53.424 25.523 68.534 0.00 0.00
B
ATOM 19859 HN ASP B 203 -53.856 25.081 69.316 0.00 0.00
B
ATOM 19860 CA ASP B 203 -53.212 26.937 68.574 0.00 0.00
B
ATOM 19861 HA ASP B 203 -53.229 27.333 67.570 0.00 0.00
B
ATOM 19862 CB ASP B 203 -54.262 27.720 69.386 0.00 0.00
B
ATOM 19863 HB1 ASP B 203 -54.289 27.467 70.467 0.00 0.00
B
ATOM 19864 HB2 ASP B 203 -53.970 28.792 69.378 0.00 0.00
B
ATOM 19865 CG ASP B 203 -55.670 27.592 68.744 0.00 0.00
B
ATOM 19866 OD1 ASP B 203 -55.716 27.050 67.613 0.00 0.00
B
ATOM 19867 OD2 ASP B 203 -56.686 28.023 69.355 0.00 0.00
B
ATOM 19868 C ASP B 203 -51.852 27.201 69.143 0.00 0.00
B
ATOM 19869 O ASP B 203 -51.318 26.418 69.908 0.00 0.00
B
ATOM 19870 N CYS B 204 -51.209 28.352 68.691 0.00 0.00
B
ATOM 19871 HN CYS B 204 -51.565 28.860 67.910 0.00 0.00
B

ATOM 19872 CA CYS B 204 −49.900 28.712 69.207 0.00 0.00
B
ATOM 19873 HA CYS B 204 −49.467 27.968 69.859 0.00 0.00
B
ATOM 19874 CB CYS B 204 −48.866 29.281 68.144 0.00 0.00
B
ATOM 19875 HB1 CYS B 204 −49.312 30.037 67.464 0.00 0.00
B
ATOM 19876 HB2 CYS B 204 −47.932 29.708 68.570 0.00 0.00
B
ATOM 19877 SG CYS B 204 −48.284 27.906 67.142 0.00 0.00
B
ATOM 19878 HG1 CYS B 204 −49.491 27.467 66.813 0.00 0.00
B
ATOM 19879 C CYS B 204 −50.229 29.907 70.159 0.00 0.00
B
ATOM 19880 O CYS B 204 −50.907 30.832 69.770 0.00 0.00
B
ATOM 19881 N LEU B 205 −49.657 29.866 71.438 0.00 0.00
B
ATOM 19882 HN LEU B 205 −49.087 29.115 71.763 0.00 0.00
B
ATOM 19883 CA LEU B 205 −49.845 30.826 72.485 0.00 0.00
B
ATOM 19884 HA LEU B 205 −50.395 31.674 72.104 0.00 0.00
B
ATOM 19885 CB LEU B 205 −50.358 30.203 73.836 0.00 0.00
B
ATOM 19886 HB1 LEU B 205 −51.191 29.575 73.454 0.00 0.00
B
ATOM 19887 HB2 LEU B 205 −49.583 29.522 74.247 0.00 0.00
B
ATOM 19888 CG LEU B 205 −50.842 31.140 74.967 0.00 0.00
B
ATOM 19889 HG LEU B 205 −50.989 32.162 74.555 0.00 0.00
B
ATOM 19890 CD1 LEU B 205 −52.026 30.597 75.700 0.00 0.00
B
ATOM 19891 HD11 LEU B 205 −52.397 31.338 76.441 0.00 0.00
B
ATOM 19892 HD12 LEU B 205 −52.800 30.391 74.931 0.00 0.00
B
ATOM 19893 HD13 LEU B 205 −51.802 29.646 76.228 0.00 0.00
B
ATOM 19894 CD2 LEU B 205 −49.689 31.437 75.963 0.00 0.00
B
ATOM 19895 HD21 LEU B 205 −50.079 32.044 76.808 0.00 0.00
B
ATOM 19896 HD22 LEU B 205 −49.314 30.475 76.373 0.00 0.00
B
ATOM 19897 HD23 LEU B 205 −48.907 32.080 75.505 0.00 0.00
B
ATOM 19898 C LEU B 205 −48.445 31.416 72.788 0.00 0.00
B

ATOM 19899 O   LEU B 205   -47.586 30.857 73.406 0.00 0.00
B
ATOM 19900 N   VAL B 206   -48.236 32.688 72.366 0.00 0.00
B
ATOM 19901 HN VAL B 206   -48.915 33.165 71.814 0.00 0.00
B
ATOM 19902 CA VAL B 206   -46.927 33.339 72.591 0.00 0.00
B
ATOM 19903 HA VAL B 206   -46.130 32.624 72.450 0.00 0.00
B
ATOM 19904 CB VAL B 206   -46.644 34.396 71.501 0.00 0.00
B
ATOM 19905 HB VAL B 206   -47.327 35.268 71.582 0.00 0.00
B
ATOM 19906 CG1 VAL B 206   -45.198 34.871 71.733 0.00 0.00
B
ATOM 19907 HG11 VAL B 206   -44.783 35.268 70.782 0.00 0.00
B
ATOM 19908 HG12 VAL B 206   -44.959 35.491 72.623 0.00 0.00
B
ATOM 19909 HG13 VAL B 206   -44.519 33.998 71.832 0.00 0.00
B
ATOM 19910 CG2 VAL B 206   -46.889 33.749 70.101 0.00 0.00
B
ATOM 19911 HG21 VAL B 206   -46.666 34.527 69.339 0.00 0.00
B
ATOM 19912 HG22 VAL B 206   -46.188 32.887 70.085 0.00 0.00
B
ATOM 19913 HG23 VAL B 206   -47.933 33.381 69.999 0.00 0.00
B
ATOM 19914 C   VAL B 206   -46.785 34.020 73.927 0.00 0.00
B
ATOM 19915 O   VAL B 206   -47.630 34.758 74.455 0.00 0.00
B
ATOM 19916 N   ILE B 207   -45.759 33.716 74.724 0.00 0.00
B
ATOM 19917 HN ILE B 207   -45.043 33.144 74.332 0.00 0.00
B
ATOM 19918 CA ILE B 207   -45.659 34.083 76.087 0.00 0.00
B
ATOM 19919 HA ILE B 207   -46.268 34.955 76.273 0.00 0.00
B
ATOM 19920 CB ILE B 207   -46.177 32.911 76.904 0.00 0.00
B
ATOM 19921 HB ILE B 207   -47.194 32.684 76.519 0.00 0.00
B
ATOM 19922 CG2 ILE B 207   -45.348 31.608 76.517 0.00 0.00
B
ATOM 19923 HG21 ILE B 207   -44.298 31.664 76.878 0.00 0.00
B
ATOM 19924 HG22 ILE B 207   -45.844 30.681 76.877 0.00 0.00
B
ATOM 19925 HG23 ILE B 207   -45.304 31.496 75.413 0.00 0.00
B

ATOM 19926 CG1 ILE B 207 -46.235 33.148 78.401 0.00 0.00
B
ATOM 19927 HG11 ILE B 207 -45.189 33.043 78.760 0.00 0.00
B
ATOM 19928 HG12 ILE B 207 -46.646 34.156 78.623 0.00 0.00
B
ATOM 19929 CD ILE B 207 -47.000 32.089 79.142 0.00 0.00
B
ATOM 19930 HD1 ILE B 207 -46.949 32.282 80.235 0.00 0.00
B
ATOM 19931 HD2 ILE B 207 -48.042 31.916 78.797 0.00 0.00
B
ATOM 19932 HD3 ILE B 207 -46.660 31.056 78.918 0.00 0.00
B
ATOM 19933 C ILE B 207 -44.277 34.441 76.425 0.00 0.00
B
ATOM 19934 O ILE B 207 -43.407 33.813 75.838 0.00 0.00
B
ATOM 19935 N MET B 208 -44.179 35.552 77.276 0.00 0.00
B
ATOM 19936 HN MET B 208 -45.040 35.941 77.594 0.00 0.00
B
ATOM 19937 CA MET B 208 -42.928 36.142 77.813 0.00 0.00
B
ATOM 19938 HA MET B 208 -42.086 35.629 77.372 0.00 0.00
B
ATOM 19939 CB MET B 208 -42.762 37.629 77.340 0.00 0.00
B
ATOM 19940 HB1 MET B 208 -42.744 37.619 76.229 0.00 0.00
B
ATOM 19941 HB2 MET B 208 -43.627 38.290 77.561 0.00 0.00
B
ATOM 19942 CG MET B 208 -41.550 38.451 77.829 0.00 0.00
B
ATOM 19943 HG1 MET B 208 -41.469 38.466 78.937 0.00 0.00
B
ATOM 19944 HG2 MET B 208 -40.630 37.966 77.439 0.00 0.00
B
ATOM 19945 SD MET B 208 -41.496 40.195 77.332 0.00 0.00
B
ATOM 19946 CE MET B 208 -42.767 41.007 78.465 0.00 0.00
B
ATOM 19947 HE1 MET B 208 -42.604 42.082 78.693 0.00 0.00
B
ATOM 19948 HE2 MET B 208 -43.789 40.958 78.031 0.00 0.00
B
ATOM 19949 HE3 MET B 208 -42.750 40.423 79.410 0.00 0.00
B
ATOM 19950 C MET B 208 -42.896 36.123 79.279 0.00 0.00
B
ATOM 19951 O MET B 208 -43.907 36.515 79.896 0.00 0.00
B
ATOM 19952 N GLY B 209 -41.804 35.772 79.942 0.00 0.00
B

ATOM 19953 HN GLY B 209 −40.974 35.625 79.410 0.00 0.00
B
ATOM 19954 CA GLY B 209 −41.671 35.757 81.341 0.00 0.00
B
ATOM 19955 HA1 GLY B 209 −42.512 35.211 81.742 0.00 0.00
B
ATOM 19956 HA2 GLY B 209 −41.632 36.766 81.722 0.00 0.00
B
ATOM 19957 C GLY B 209 −40.478 34.953 81.851 0.00 0.00
B
ATOM 19958 O GLY B 209 −39.512 34.702 81.180 0.00 0.00
B
ATOM 19959 N HSE B 210 −40.553 34.481 83.103 0.00 0.00
B
ATOM 19960 HN HSE B 210 −41.406 34.646 83.591 0.00 0.00
B
ATOM 19961 CA HSE B 210 −39.405 33.914 83.729 0.00 0.00
B
ATOM 19962 HA HSE B 210 −38.588 34.492 83.324 0.00 0.00
B
ATOM 19963 CB HSE B 210 −39.449 34.105 85.177 0.00 0.00
B
ATOM 19964 HB1 HSE B 210 −39.579 35.208 85.190 0.00 0.00
B
ATOM 19965 HB2 HSE B 210 −40.250 33.553 85.713 0.00 0.00
B
ATOM 19966 ND1 HSE B 210 −37.857 32.570 86.171 0.00 0.00
B
ATOM 19967 CG HSE B 210 −38.182 33.895 85.893 0.00 0.00
B
ATOM 19968 CE1 HSE B 210 −36.638 32.624 86.691 0.00 0.00
B
ATOM 19969 HE1 HSE B 210 −36.025 31.779 87.005 0.00 0.00
B
ATOM 19970 NE2 HSE B 210 −36.134 33.868 86.667 0.00 0.00
B
ATOM 19971 HE2 HSE B 210 −35.205 34.071 86.977 0.00 0.00
B
ATOM 19972 CD2 HSE B 210 −37.173 34.705 86.223 0.00 0.00
B
ATOM 19973 HD2 HSE B 210 −37.110 35.785 86.171 0.00 0.00
B
ATOM 19974 C HSE B 210 −39.342 32.395 83.337 0.00 0.00
B
ATOM 19975 O HSE B 210 −40.366 31.740 83.338 0.00 0.00
B
ATOM 19976 N LYS B 211 −38.140 31.821 83.167 0.00 0.00
B
ATOM 19977 HN LYS B 211 −37.261 32.272 83.038 0.00 0.00
B
ATOM 19978 CA LYS B 211 −37.938 30.488 82.658 0.00 0.00
B
ATOM 19979 HA LYS B 211 −38.227 30.403 81.621 0.00 0.00
B

ATOM 19980 CB LYS B 211 −36.360 30.211 82.692 0.00 0.00
B
ATOM 19981 HB1 LYS B 211 −35.851 30.896 83.403 0.00 0.00
B
ATOM 19982 HB2 LYS B 211 −36.161 29.148 82.950 0.00 0.00
B
ATOM 19983 CG LYS B 211 −35.750 30.487 81.228 0.00 0.00
B
ATOM 19984 HG1 LYS B 211 −36.070 29.606 80.632 0.00 0.00
B
ATOM 19985 HG2 LYS B 211 −36.292 31.378 80.845 0.00 0.00
B
ATOM 19986 CD LYS B 211 −34.277 30.471 81.266 0.00 0.00
B
ATOM 19987 HD1 LYS B 211 −33.962 31.346 81.874 0.00 0.00
B
ATOM 19988 HD2 LYS B 211 −33.958 29.587 81.859 0.00 0.00
B
ATOM 19989 CE LYS B 211 −33.660 30.402 79.845 0.00 0.00
B
ATOM 19990 HE1 LYS B 211 −32.566 30.267 79.983 0.00 0.00
B
ATOM 19991 HE2 LYS B 211 −34.119 29.527 79.337 0.00 0.00
B
ATOM 19992 NZ LYS B 211 −33.923 31.586 78.948 0.00 0.00
B
ATOM 19993 HZ1 LYS B 211 −33.372 31.663 78.069 0.00 0.00
B
ATOM 19994 HZ2 LYS B 211 −34.927 31.530 78.682 0.00 0.00
B
ATOM 19995 HZ3 LYS B 211 −33.627 32.463 79.423 0.00 0.00
B
ATOM 19996 C LYS B 211 −38.563 29.460 83.663 0.00 0.00
B
ATOM 19997 O LYS B 211 −39.172 28.501 83.174 0.00 0.00
B
ATOM 19998 N LYS B 212 −38.367 29.581 84.968 0.00 0.00
B
ATOM 19999 HN LYS B 212 −37.963 30.416 85.333 0.00 0.00
B
ATOM 20000 CA LYS B 212 −38.888 28.743 85.984 0.00 0.00
B
ATOM 20001 HA LYS B 212 −38.636 27.735 85.692 0.00 0.00
B
ATOM 20002 CB LYS B 212 −38.242 29.126 87.299 0.00 0.00
B
ATOM 20003 HB1 LYS B 212 −38.390 30.193 87.571 0.00 0.00
B
ATOM 20004 HB2 LYS B 212 −38.554 28.417 88.096 0.00 0.00
B
ATOM 20005 CG LYS B 212 −36.692 28.797 87.220 0.00 0.00
B
ATOM 20006 HG1 LYS B 212 −36.544 27.710 87.040 0.00 0.00
B

ATOM 20007 HG2 LYS B 212 −36.238 29.332 86.359 0.00 0.00
B
ATOM 20008 CD LYS B 212 −35.985 28.963 88.622 0.00 0.00
B
ATOM 20009 HD1 LYS B 212 −36.067 30.049 88.841 0.00 0.00
B
ATOM 20010 HD2 LYS B 212 −36.637 28.365 89.294 0.00 0.00
B
ATOM 20011 CE LYS B 212 −34.612 28.419 88.649 0.00 0.00
B
ATOM 20012 HE1 LYS B 212 −34.310 28.471 89.717 0.00 0.00
B
ATOM 20013 HE2 LYS B 212 −34.561 27.350 88.354 0.00 0.00
B
ATOM 20014 NZ LYS B 212 −33.709 29.310 87.965 0.00 0.00
B
ATOM 20015 HZ1 LYS B 212 −33.711 29.131 86.941 0.00 0.00
B
ATOM 20016 HZ2 LYS B 212 −34.038 30.294 88.033 0.00 0.00
B
ATOM 20017 HZ3 LYS B 212 −32.718 29.219 88.266 0.00 0.00
B
ATOM 20018 C LYS B 212 −40.523 28.853 86.013 0.00 0.00
B
ATOM 20019 O LYS B 212 −41.167 27.778 86.160 0.00 0.00
B
ATOM 20020 N ASP B 213 −41.106 30.077 85.885 0.00 0.00
B
ATOM 20021 HN ASP B 213 −40.509 30.859 86.044 0.00 0.00
B
ATOM 20022 CA ASP B 213 −42.570 30.313 85.812 0.00 0.00
B
ATOM 20023 HA ASP B 213 −43.029 29.590 86.471 0.00 0.00
B
ATOM 20024 CB ASP B 213 −43.069 31.702 86.226 0.00 0.00
B
ATOM 20025 HB1 ASP B 213 −42.724 32.479 85.511 0.00 0.00
B
ATOM 20026 HB2 ASP B 213 −44.167 31.870 86.264 0.00 0.00
B
ATOM 20027 CG ASP B 213 −42.492 31.952 87.688 0.00 0.00
B
ATOM 20028 OD1 ASP B 213 −42.871 31.188 88.594 0.00 0.00
B
ATOM 20029 OD2 ASP B 213 −41.723 32.850 87.897 0.00 0.00
B
ATOM 20030 C ASP B 213 −43.145 29.834 84.439 0.00 0.00
B
ATOM 20031 O ASP B 213 −44.226 29.271 84.301 0.00 0.00
B
ATOM 20032 N ILE B 214 −42.410 29.932 83.388 0.00 0.00
B
ATOM 20033 HN ILE B 214 −41.559 30.452 83.372 0.00 0.00
B

ATOM 20034 CA ILE B 214 −42.821 29.353 82.129 0.00 0.00
B
ATOM 20035 HA ILE B 214 −43.825 29.613 81.827 0.00 0.00
B
ATOM 20036 CB ILE B 214 −41.956 29.895 80.988 0.00 0.00
B
ATOM 20037 HB ILE B 214 −40.902 29.791 81.324 0.00 0.00
B
ATOM 20038 CG2 ILE B 214 −42.222 29.162 79.627 0.00 0.00
B
ATOM 20039 HG21 ILE B 214 −43.210 29.474 79.226 0.00 0.00
B
ATOM 20040 HG22 ILE B 214 −41.408 29.268 78.879 0.00 0.00
B
ATOM 20041 HG23 ILE B 214 −42.263 28.062 79.781 0.00 0.00
B
ATOM 20042 CG1 ILE B 214 −42.091 31.490 80.815 0.00 0.00
B
ATOM 20043 HG11 ILE B 214 −43.063 31.655 80.302 0.00 0.00
B
ATOM 20044 HG12 ILE B 214 −42.088 32.040 81.781 0.00 0.00
B
ATOM 20045 CD ILE B 214 −40.943 32.020 79.944 0.00 0.00
B
ATOM 20046 HD1 ILE B 214 −41.103 33.118 79.885 0.00 0.00
B
ATOM 20047 HD2 ILE B 214 −39.991 31.782 80.463 0.00 0.00
B
ATOM 20048 HD3 ILE B 214 −41.094 31.554 78.947 0.00 0.00
B
ATOM 20049 C ILE B 214 −42.882 27.803 82.102 0.00 0.00
B
ATOM 20050 O ILE B 214 −43.854 27.190 81.634 0.00 0.00
B
ATOM 20051 N LYS B 215 −41.824 27.209 82.714 0.00 0.00
B
ATOM 20052 HN LYS B 215 −41.012 27.731 82.963 0.00 0.00
B
ATOM 20053 CA LYS B 215 −41.836 25.807 83.026 0.00 0.00
B
ATOM 20054 HA LYS B 215 −42.011 25.226 82.132 0.00 0.00
B
ATOM 20055 CB LYS B 215 −40.423 25.388 83.424 0.00 0.00
B
ATOM 20056 HB1 LYS B 215 −39.713 25.780 82.664 0.00 0.00
B
ATOM 20057 HB2 LYS B 215 −40.253 25.937 84.375 0.00 0.00
B
ATOM 20058 CG LYS B 215 −40.065 23.883 83.567 0.00 0.00
B
ATOM 20059 HG1 LYS B 215 −38.987 23.860 83.834 0.00 0.00
B
ATOM 20060 HG2 LYS B 215 −40.490 23.334 84.435 0.00 0.00
B

ATOM 20061 CD LYS B 215 −40.279 23.031 82.302 0.00 0.00
B
ATOM 20062 HD1 LYS B 215 −40.311 21.931 82.457 0.00 0.00
B
ATOM 20063 HD2 LYS B 215 −41.258 23.333 81.872 0.00 0.00
B
ATOM 20064 CE LYS B 215 −39.247 23.242 81.206 0.00 0.00
B
ATOM 20065 HE1 LYS B 215 −39.095 24.271 80.817 0.00 0.00
B
ATOM 20066 HE2 LYS B 215 −38.249 22.911 81.565 0.00 0.00
B
ATOM 20067 NZ LYS B 215 −39.648 22.344 80.061 0.00 0.00
B
ATOM 20068 HZ1 LYS B 215 −40.621 22.639 79.846 0.00 0.00
B
ATOM 20069 HZ2 LYS B 215 −38.981 22.572 79.295 0.00 0.00
B
ATOM 20070 HZ3 LYS B 215 −39.538 21.369 80.405 0.00 0.00
B
ATOM 20071 C LYS B 215 −42.918 25.379 84.001 0.00 0.00
B
ATOM 20072 O LYS B 215 −43.560 24.279 83.802 0.00 0.00
B
ATOM 20073 N ARG B 216 −43.225 26.145 85.054 0.00 0.00
B
ATOM 20074 HN ARG B 216 −42.662 26.908 85.363 0.00 0.00
B
ATOM 20075 CA ARG B 216 −44.379 25.764 85.896 0.00 0.00
B
ATOM 20076 HA ARG B 216 −44.284 24.782 86.335 0.00 0.00
B
ATOM 20077 CB ARG B 216 −44.474 26.803 87.088 0.00 0.00
B
ATOM 20078 HB1 ARG B 216 −43.486 26.814 87.597 0.00 0.00
B
ATOM 20079 HB2 ARG B 216 −44.819 27.726 86.575 0.00 0.00
B
ATOM 20080 CG ARG B 216 −45.616 26.381 88.073 0.00 0.00
B
ATOM 20081 HG1 ARG B 216 −46.537 26.291 87.459 0.00 0.00
B
ATOM 20082 HG2 ARG B 216 −45.318 25.399 88.501 0.00 0.00
B
ATOM 20083 CD ARG B 216 −45.716 27.382 89.214 0.00 0.00
B
ATOM 20084 HD1 ARG B 216 −44.788 27.440 89.822 0.00 0.00
B
ATOM 20085 HD2 ARG B 216 −45.917 28.436 88.927 0.00 0.00
B
ATOM 20086 NE ARG B 216 −46.760 26.824 90.084 0.00 0.00
B
ATOM 20087 HE ARG B 216 −46.920 25.838 90.139 0.00 0.00
B

ATOM 20088 CZ ARG B 216 −47.368 27.584 91.037 0.00 0.00
B
ATOM 20089 NH1 ARG B 216 −47.151 28.846 91.218 0.00 0.00
B
ATOM 20090 HH11 ARG B 216 −47.673 29.292 91.945 0.00 0.00
B
ATOM 20091 HH12 ARG B 216 −46.376 29.292 90.771 0.00 0.00
B
ATOM 20092 NH2 ARG B 216 −48.344 26.987 91.773 0.00 0.00
B
ATOM 20093 HH21 ARG B 216 −48.602 27.345 92.671 0.00 0.00
B
ATOM 20094 HH22 ARG B 216 −48.381 25.988 91.738 0.00 0.00
B
ATOM 20095 C ARG B 216 −45.668 25.749 85.060 0.00 0.00
B
ATOM 20096 O ARG B 216 −46.419 24.833 85.256 0.00 0.00
B
ATOM 20097 N PHE B 217 −45.846 26.702 84.103 0.00 0.00
B
ATOM 20098 HN PHE B 217 −45.183 27.446 84.077 0.00 0.00
B
ATOM 20099 CA PHE B 217 −46.997 26.681 83.066 0.00 0.00
B
ATOM 20100 HA PHE B 217 −47.920 26.796 83.615 0.00 0.00
B
ATOM 20101 CB PHE B 217 −46.962 28.015 82.214 0.00 0.00
B
ATOM 20102 HB1 PHE B 217 −46.882 28.900 82.880 0.00 0.00
B
ATOM 20103 HB2 PHE B 217 −46.017 27.999 81.631 0.00 0.00
B
ATOM 20104 CG PHE B 217 −48.171 28.194 81.284 0.00 0.00
B
ATOM 20105 CD1 PHE B 217 −47.944 28.546 79.939 0.00 0.00
B
ATOM 20106 HD1 PHE B 217 −46.939 28.654 79.560 0.00 0.00
B
ATOM 20107 CE1 PHE B 217 −49.056 28.830 79.089 0.00 0.00
B
ATOM 20108 HE1 PHE B 217 −48.943 29.041 78.036 0.00 0.00
B
ATOM 20109 CZ PHE B 217 −50.324 28.865 79.642 0.00 0.00
B
ATOM 20110 HZ PHE B 217 −51.138 29.181 79.006 0.00 0.00
B
ATOM 20111 CD2 PHE B 217 −49.487 28.063 81.739 0.00 0.00
B
ATOM 20112 HD2 PHE B 217 −49.655 27.770 82.765 0.00 0.00
B
ATOM 20113 CE2 PHE B 217 −50.546 28.512 81.007 0.00 0.00
B
ATOM 20114 HE2 PHE B 217 −51.512 28.419 81.479 0.00 0.00
B

ATOM 20115 C PHE B 217 -47.068 25.421 82.317 0.00 0.00
B
ATOM 20116 O PHE B 217 -48.093 24.987 81.914 0.00 0.00
B
ATOM 20117 N GLU B 218 -45.895 24.829 81.970 0.00 0.00
B
ATOM 20118 HN GLU B 218 -44.987 25.078 82.297 0.00 0.00
B
ATOM 20119 CA GLU B 218 -45.866 23.708 81.067 0.00 0.00
B
ATOM 20120 HA GLU B 218 -46.430 23.904 80.167 0.00 0.00
B
ATOM 20121 CB GLU B 218 -44.449 23.527 80.600 0.00 0.00
B
ATOM 20122 HB1 GLU B 218 -44.204 24.470 80.065 0.00 0.00
B
ATOM 20123 HB2 GLU B 218 -43.787 23.520 81.492 0.00 0.00
B
ATOM 20124 CG GLU B 218 -44.222 22.286 79.721 0.00 0.00
B
ATOM 20125 HG1 GLU B 218 -44.445 21.440 80.406 0.00 0.00
B
ATOM 20126 HG2 GLU B 218 -44.821 22.287 78.785 0.00 0.00
B
ATOM 20127 CD GLU B 218 -42.739 22.123 79.444 0.00 0.00
B
ATOM 20128 OE1 GLU B 218 -42.214 21.033 79.764 0.00 0.00
B
ATOM 20129 OE2 GLU B 218 -42.119 23.081 78.920 0.00 0.00
B
ATOM 20130 C GLU B 218 -46.321 22.407 81.787 0.00 0.00
B
ATOM 20131 O GLU B 218 -46.940 21.480 81.287 0.00 0.00
B
ATOM 20132 N ASN B 219 -46.038 22.378 83.033 0.00 0.00
B
ATOM 20133 HN ASN B 219 -45.490 23.167 83.300 0.00 0.00
B
ATOM 20134 CA ASN B 219 -46.250 21.168 83.812 0.00 0.00
B
ATOM 20135 HA ASN B 219 -46.423 20.268 83.242 0.00 0.00
B
ATOM 20136 CB ASN B 219 -45.077 20.971 84.769 0.00 0.00
B
ATOM 20137 HB1 ASN B 219 -44.828 21.815 85.447 0.00 0.00
B
ATOM 20138 HB2 ASN B 219 -45.387 20.156 85.458 0.00 0.00
B
ATOM 20139 CG ASN B 219 -43.945 20.381 83.938 0.00 0.00
B
ATOM 20140 OD1 ASN B 219 -44.151 19.674 82.994 0.00 0.00
B
ATOM 20141 ND2 ASN B 219 -42.704 20.635 84.401 0.00 0.00
B

ATOM 20142 HD21 ASN B 219 −41.861 20.441 83.900 0.00 0.00
B
ATOM 20143 HD22 ASN B 219 −42.520 21.153 85.236 0.00 0.00
B
ATOM 20144 C ASN B 219 −47.348 21.297 84.821 0.00 0.00
B
ATOM 20145 O ASN B 219 −48.007 20.256 85.072 0.00 0.00
B
ATOM 20146 N GLU B 220 −47.703 22.434 85.480 0.00 0.00
B
ATOM 20147 HN GLU B 220 −47.165 23.258 85.319 0.00 0.00
B
ATOM 20148 CA GLU B 220 −48.771 22.474 86.428 0.00 0.00
B
ATOM 20149 HA GLU B 220 −48.946 21.443 86.700 0.00 0.00
B
ATOM 20150 CB GLU B 220 −48.351 23.367 87.627 0.00 0.00
B
ATOM 20151 HB1 GLU B 220 −48.137 24.410 87.309 0.00 0.00
B
ATOM 20152 HB2 GLU B 220 −49.230 23.464 88.300 0.00 0.00
B
ATOM 20153 CG GLU B 220 −47.130 22.738 88.414 0.00 0.00
B
ATOM 20154 HG1 GLU B 220 −47.355 21.665 88.596 0.00 0.00
B
ATOM 20155 HG2 GLU B 220 −46.261 22.909 87.743 0.00 0.00
B
ATOM 20156 CD GLU B 220 −46.840 23.387 89.748 0.00 0.00
B
ATOM 20157 OE1 GLU B 220 −45.721 23.097 90.199 0.00 0.00
B
ATOM 20158 OE2 GLU B 220 −47.642 24.226 90.251 0.00 0.00
B
ATOM 20159 C GLU B 220 −49.969 23.210 85.806 0.00 0.00
B
ATOM 20160 O GLU B 220 −51.090 23.220 86.316 0.00 0.00
B
ATOM 20161 N GLY B 221 −49.719 23.983 84.721 0.00 0.00
B
ATOM 20162 HN GLY B 221 −48.808 24.132 84.345 0.00 0.00
B
ATOM 20163 CA GLY B 221 −50.776 24.873 84.343 0.00 0.00
B
ATOM 20164 HA1 GLY B 221 −50.421 25.842 84.026 0.00 0.00
B
ATOM 20165 HA2 GLY B 221 −51.396 25.117 85.194 0.00 0.00
B
ATOM 20166 C GLY B 221 −51.696 24.285 83.233 0.00 0.00
B
ATOM 20167 O GLY B 221 −52.780 24.771 82.813 0.00 0.00
B
ATOM 20168 N MET B 222 −51.244 23.129 82.718 0.00 0.00
B

ATOM 20169 HN MET B 222 -50.430 22.694 83.094 0.00 0.00
B
ATOM 20170 CA MET B 222 -51.897 22.414 81.628 0.00 0.00
B
ATOM 20171 HA MET B 222 -52.500 23.095 81.046 0.00 0.00
B
ATOM 20172 CB MET B 222 -50.933 21.873 80.555 0.00 0.00
B
ATOM 20173 HB1 MET B 222 -50.232 21.103 80.941 0.00 0.00
B
ATOM 20174 HB2 MET B 222 -51.456 21.344 79.730 0.00 0.00
B
ATOM 20175 CG MET B 222 -50.005 22.962 80.033 0.00 0.00
B
ATOM 20176 HG1 MET B 222 -50.610 23.824 79.681 0.00 0.00
B
ATOM 20177 HG2 MET B 222 -49.367 23.421 80.819 0.00 0.00
B
ATOM 20178 SD MET B 222 -49.000 22.455 78.663 0.00 0.00
B
ATOM 20179 CE MET B 222 -48.783 24.211 77.969 0.00 0.00
B
ATOM 20180 HE1 MET B 222 -48.371 24.854 78.776 0.00 0.00
B
ATOM 20181 HE2 MET B 222 -48.142 24.269 77.063 0.00 0.00
B
ATOM 20182 HE3 MET B 222 -49.832 24.489 77.731 0.00 0.00
B
ATOM 20183 C MET B 222 -52.735 21.259 82.120 0.00 0.00
B
ATOM 20184 O MET B 222 -53.511 21.453 83.066 0.00 0.00
B
ATOM 20185 N GLN C 8 28.173 20.148 26.753 0.00 0.00
C
ATOM 20186 HT1 GLN C 8 28.703 20.870 27.283 0.00 0.00
C
ATOM 20187 HT2 GLN C 8 27.989 20.774 25.943 0.00 0.00
C
ATOM 20188 HT3 GLN C 8 28.736 19.291 26.585 0.00 0.00
C
ATOM 20189 CA GLN C 8 26.886 19.725 27.475 0.00 0.00
C
ATOM 20190 HA GLN C 8 26.345 20.660 27.487 0.00 0.00
C
ATOM 20191 CB GLN C 8 26.171 18.495 26.713 0.00 0.00
C
ATOM 20192 HB1 GLN C 8 26.770 17.573 26.869 0.00 0.00
C
ATOM 20193 HB2 GLN C 8 25.227 18.292 27.262 0.00 0.00
C
ATOM 20194 CG GLN C 8 25.885 18.834 25.230 0.00 0.00
C
ATOM 20195 HG1 GLN C 8 25.325 19.778 25.057 0.00 0.00
C

ATOM 20196 HG2 GLN C 8 26.822 18.790 24.635 0.00 0.00
C
ATOM 20197 CD GLN C 8 25.080 17.690 24.604 0.00 0.00
C
ATOM 20198 OE1 GLN C 8 25.211 16.580 25.208 0.00 0.00
C
ATOM 20199 NE2 GLN C 8 24.167 17.863 23.648 0.00 0.00
C
ATOM 20200 HE21 GLN C 8 23.642 17.071 23.335 0.00 0.00
C
ATOM 20201 HE22 GLN C 8 23.879 18.800 23.452 0.00 0.00
C
ATOM 20202 C GLN C 8 27.201 19.429 28.892 0.00 0.00
C
ATOM 20203 O GLN C 8 28.174 18.759 29.230 0.00 0.00
C
ATOM 20204 N PHE C 9 26.321 19.746 29.889 0.00 0.00
C
ATOM 20205 HN PHE C 9 25.479 20.248 29.705 0.00 0.00
C
ATOM 20206 CA PHE C 9 26.550 19.512 31.352 0.00 0.00
C
ATOM 20207 HA PHE C 9 27.591 19.266 31.500 0.00 0.00
C
ATOM 20208 CB PHE C 9 26.244 20.726 32.207 0.00 0.00
C
ATOM 20209 HB1 PHE C 9 25.287 21.226 31.943 0.00 0.00
C
ATOM 20210 HB2 PHE C 9 26.157 20.457 33.281 0.00 0.00
C
ATOM 20211 CG PHE C 9 27.300 21.789 32.116 0.00 0.00
C
ATOM 20212 CD1 PHE C 9 28.389 21.778 33.021 0.00 0.00
C
ATOM 20213 HD1 PHE C 9 28.546 21.040 33.794 0.00 0.00
C
ATOM 20214 CE1 PHE C 9 29.407 22.776 32.981 0.00 0.00
C
ATOM 20215 HE1 PHE C 9 30.258 22.630 33.631 0.00 0.00
C
ATOM 20216 CZ PHE C 9 29.314 23.800 32.037 0.00 0.00
C
ATOM 20217 HZ PHE C 9 30.055 24.585 32.059 0.00 0.00
C
ATOM 20218 CD2 PHE C 9 27.248 22.789 31.133 0.00 0.00
C
ATOM 20219 HD2 PHE C 9 26.392 22.893 30.483 0.00 0.00
C
ATOM 20220 CE2 PHE C 9 28.276 23.783 31.094 0.00 0.00
C
ATOM 20221 HE2 PHE C 9 28.224 24.628 30.422 0.00 0.00
C
ATOM 20222 C PHE C 9 25.603 18.448 31.813 0.00 0.00
C

```
ATOM  20223  O   PHE C   9       24.591  18.316  31.143  0.00  0.00
C
ATOM  20224  N   ALA C  10       25.833  17.665  32.830  0.00  0.00
C
ATOM  20225  HN  ALA C  10       26.560  17.753  33.507  0.00  0.00
C
ATOM  20226  CA  ALA C  10       24.784  16.665  33.158  0.00  0.00
C
ATOM  20227  HA  ALA C  10       23.968  16.522  32.467  0.00  0.00
C
ATOM  20228  CB  ALA C  10       25.426  15.325  32.798  0.00  0.00
C
ATOM  20229  HB1 ALA C  10       24.900  14.473  33.280  0.00  0.00
C
ATOM  20230  HB2 ALA C  10       25.343  15.119  31.709  0.00  0.00
C
ATOM  20231  HB3 ALA C  10       26.512  15.397  33.021  0.00  0.00
C
ATOM  20232  C   ALA C  10       24.457  16.689  34.649  0.00  0.00
C
ATOM  20233  O   ALA C  10       25.406  16.673  35.522  0.00  0.00
C
ATOM  20234  N   VAL C  11       23.162  16.740  34.981  0.00  0.00
C
ATOM  20235  HN  VAL C  11       22.432  16.774  34.303  0.00  0.00
C
ATOM  20236  CA  VAL C  11       22.600  16.732  36.296  0.00  0.00
C
ATOM  20237  HA  VAL C  11       23.354  16.618  37.061  0.00  0.00
C
ATOM  20238  CB  VAL C  11       21.695  17.871  36.610  0.00  0.00
C
ATOM  20239  HB  VAL C  11       20.809  17.961  35.945  0.00  0.00
C
ATOM  20240  CG1 VAL C  11       21.152  17.923  38.065  0.00  0.00
C
ATOM  20241 HG11 VAL C  11       20.522  18.822  38.232  0.00  0.00
C
ATOM  20242 HG12 VAL C  11       20.535  17.056  38.384  0.00  0.00
C
ATOM  20243 HG13 VAL C  11       22.051  18.086  38.697  0.00  0.00
C
ATOM  20244  CG2 VAL C  11       22.456  19.198  36.235  0.00  0.00
C
ATOM  20245 HG21 VAL C  11       23.507  19.232  36.594  0.00  0.00
C
ATOM  20246 HG22 VAL C  11       22.429  19.410  35.145  0.00  0.00
C
ATOM  20247 HG23 VAL C  11       21.949  20.075  36.693  0.00  0.00
C
ATOM  20248  C   VAL C  11       21.687  15.511  36.446  0.00  0.00
C
ATOM  20249  O   VAL C  11       20.664  15.361  35.807  0.00  0.00
C
```

ATOM 20250 N   ILE C 12   22.087 14.567 37.320 0.00 0.00
C
ATOM 20251 HN ILE C 12   22.946 14.699 37.809 0.00 0.00
C
ATOM 20252 CA ILE C 12   21.362 13.313 37.652 0.00 0.00
C
ATOM 20253 HA ILE C 12   20.536 13.177 36.971 0.00 0.00
C
ATOM 20254 CB ILE C 12   22.327 12.062 37.562 0.00 0.00
C
ATOM 20255 HB ILE C 12   23.105 12.242 38.335 0.00 0.00
C
ATOM 20256 CG2 ILE C 12   21.621 10.644 37.784 0.00 0.00
C
ATOM 20257 HG21 ILE C 12   20.818 10.580 37.019 0.00 0.00
C
ATOM 20258 HG22 ILE C 12   22.425   9.881 37.715 0.00 0.00
C
ATOM 20259 HG23 ILE C 12   21.151 10.595 38.789 0.00 0.00
C
ATOM 20260 CG1 ILE C 12   23.060 12.054 36.168 0.00 0.00
C
ATOM 20261 HG11 ILE C 12   22.365 12.096 35.302 0.00 0.00
C
ATOM 20262 HG12 ILE C 12   23.725 12.944 36.173 0.00 0.00
C
ATOM 20263 CD ILE C 12   24.044 10.863 35.898 0.00 0.00
C
ATOM 20264 HD1 ILE C 12   24.425 10.421 36.844 0.00 0.00
C
ATOM 20265 HD2 ILE C 12   23.412 10.151 35.326 0.00 0.00
C
ATOM 20266 HD3 ILE C 12   24.796 11.216 35.160 0.00 0.00
C
ATOM 20267 C   ILE C 12   20.774 13.490 38.942 0.00 0.00
C
ATOM 20268 O   ILE C 12   21.489 13.599 39.935 0.00 0.00
C
ATOM 20269 N   GLY C 13   19.405 13.381 39.064 0.00 0.00
C
ATOM 20270 HN GLY C 13   18.758 13.159 38.338 0.00 0.00
C
ATOM 20271 CA GLY C 13   18.729 13.803 40.279 0.00 0.00
C
ATOM 20272 HA1 GLY C 13   19.377 13.982 41.125 0.00 0.00
C
ATOM 20273 HA2 GLY C 13   17.897 13.137 40.454 0.00 0.00
C
ATOM 20274 C   GLY C 13   18.020 15.176 40.057 0.00 0.00
C
ATOM 20275 O   GLY C 13   18.754 16.142 39.922 0.00 0.00
C
ATOM 20276 N   LEU C 14   16.707 15.234 40.099 0.00 0.00
C

ATOM 20277 HN LEU C 14 16.173 14.392 40.128 0.00 0.00
C
ATOM 20278 CA LEU C 14 16.051 16.555 39.748 0.00 0.00
C
ATOM 20279 HA LEU C 14 16.821 17.312 39.751 0.00 0.00
C
ATOM 20280 CB LEU C 14 15.326 16.631 38.434 0.00 0.00
C
ATOM 20281 HB1 LEU C 14 14.465 15.929 38.441 0.00 0.00
C
ATOM 20282 HB2 LEU C 14 14.983 17.671 38.245 0.00 0.00
C
ATOM 20283 CG LEU C 14 16.160 16.092 37.232 0.00 0.00
C
ATOM 20284 HG LEU C 14 16.725 15.206 37.592 0.00 0.00
C
ATOM 20285 CD1 LEU C 14 15.429 15.692 36.004 0.00 0.00
C
ATOM 20286 HD11 LEU C 14 14.729 14.865 36.250 0.00 0.00
C
ATOM 20287 HD12 LEU C 14 14.869 16.522 35.522 0.00 0.00
C
ATOM 20288 HD13 LEU C 14 16.173 15.263 35.298 0.00 0.00
C
ATOM 20289 CD2 LEU C 14 17.276 17.034 36.874 0.00 0.00
C
ATOM 20290 HD21 LEU C 14 16.930 17.717 36.069 0.00 0.00
C
ATOM 20291 HD22 LEU C 14 17.658 17.650 37.717 0.00 0.00
C
ATOM 20292 HD23 LEU C 14 18.108 16.416 36.473 0.00 0.00
C
ATOM 20293 C LEU C 14 15.154 16.978 40.921 0.00 0.00
C
ATOM 20294 O LEU C 14 14.179 17.649 40.763 0.00 0.00
C
ATOM 20295 N GLY C 15 15.733 16.818 42.149 0.00 0.00
C
ATOM 20296 HN GLY C 15 16.578 16.290 42.141 0.00 0.00
C
ATOM 20297 CA GLY C 15 15.242 17.370 43.400 0.00 0.00
C
ATOM 20298 HA1 GLY C 15 15.804 16.803 44.128 0.00 0.00
C
ATOM 20299 HA2 GLY C 15 14.202 17.088 43.478 0.00 0.00
C
ATOM 20300 C GLY C 15 15.456 18.790 43.670 0.00 0.00
C
ATOM 20301 O GLY C 15 15.993 19.502 42.839 0.00 0.00
C
ATOM 20302 N ARG C 16 15.210 19.248 44.921 0.00 0.00
C
ATOM 20303 HN ARG C 16 14.877 18.625 45.625 0.00 0.00
C

ATOM 20304 CA ARG C 16 15.327 20.662 45.464 0.00 0.00
C
ATOM 20305 HA ARG C 16 14.543 21.265 45.030 0.00 0.00
C
ATOM 20306 CB ARG C 16 15.109 20.685 47.034 0.00 0.00
C
ATOM 20307 HB1 ARG C 16 15.923 20.144 47.562 0.00 0.00
C
ATOM 20308 HB2 ARG C 16 15.048 21.759 47.311 0.00 0.00
C
ATOM 20309 CG ARG C 16 13.802 20.051 47.497 0.00 0.00
C
ATOM 20310 HG1 ARG C 16 12.952 20.500 46.939 0.00 0.00
C
ATOM 20311 HG2 ARG C 16 13.924 19.001 47.158 0.00 0.00
C
ATOM 20312 CD ARG C 16 13.578 20.188 49.090 0.00 0.00
C
ATOM 20313 HD1 ARG C 16 13.810 21.244 49.347 0.00 0.00
C
ATOM 20314 HD2 ARG C 16 12.540 19.871 49.328 0.00 0.00
C
ATOM 20315 NE ARG C 16 14.545 19.214 49.784 0.00 0.00
C
ATOM 20316 HE ARG C 16 15.430 19.627 49.997 0.00 0.00
C
ATOM 20317 CZ ARG C 16 14.497 17.913 49.985 0.00 0.00
C
ATOM 20318 NH1 ARG C 16 13.388 17.274 49.564 0.00 0.00
C
ATOM 20319 HH11 ARG C 16 13.774 16.367 49.394 0.00 0.00
C
ATOM 20320 HH12 ARG C 16 12.660 17.680 49.012 0.00 0.00
C
ATOM 20321 NH2 ARG C 16 15.517 17.247 50.420 0.00 0.00
C
ATOM 20322 HH21 ARG C 16 15.394 16.257 50.482 0.00 0.00
C
ATOM 20323 HH22 ARG C 16 16.404 17.704 50.478 0.00 0.00
C
ATOM 20324 C ARG C 16 16.711 21.209 45.131 0.00 0.00
C
ATOM 20325 O ARG C 16 16.838 22.316 44.584 0.00 0.00
C
ATOM 20326 N PHE C 17 17.758 20.375 45.502 0.00 0.00
C
ATOM 20327 HN PHE C 17 17.448 19.478 45.807 0.00 0.00
C
ATOM 20328 CA PHE C 17 19.074 20.718 45.069 0.00 0.00
C
ATOM 20329 HA PHE C 17 19.468 21.685 45.345 0.00 0.00
C
ATOM 20330 CB PHE C 17 20.029 19.711 45.796 0.00 0.00
C

ATOM 20331 HB1 PHE C 17 19.856 19.839 46.886 0.00 0.00
C
ATOM 20332 HB2 PHE C 17 19.726 18.736 45.358 0.00 0.00
C
ATOM 20333 CG PHE C 17 21.432 19.793 45.399 0.00 0.00
C
ATOM 20334 CD1 PHE C 17 22.256 20.705 46.140 0.00 0.00
C
ATOM 20335 HD1 PHE C 17 21.763 21.432 46.768 0.00 0.00
C
ATOM 20336 CE1 PHE C 17 23.611 20.824 46.026 0.00 0.00
C
ATOM 20337 HE1 PHE C 17 24.157 21.547 46.613 0.00 0.00
C
ATOM 20338 CZ PHE C 17 24.242 19.896 45.163 0.00 0.00
C
ATOM 20339 HZ PHE C 17 25.312 19.781 45.256 0.00 0.00
C
ATOM 20340 CD2 PHE C 17 22.129 18.920 44.516 0.00 0.00
C
ATOM 20341 HD2 PHE C 17 21.547 18.234 43.919 0.00 0.00
C
ATOM 20342 CE2 PHE C 17 23.530 19.008 44.402 0.00 0.00
C
ATOM 20343 HE2 PHE C 17 24.047 18.421 43.656 0.00 0.00
C
ATOM 20344 C PHE C 17 19.341 20.680 43.601 0.00 0.00
C
ATOM 20345 O PHE C 17 19.838 21.696 43.203 0.00 0.00
C
ATOM 20346 N GLY C 18 19.059 19.607 42.901 0.00 0.00
C
ATOM 20347 HN GLY C 18 18.402 18.989 43.327 0.00 0.00
C
ATOM 20348 CA GLY C 18 19.319 19.340 41.483 0.00 0.00
C
ATOM 20349 HA1 GLY C 18 19.210 18.276 41.339 0.00 0.00
C
ATOM 20350 HA2 GLY C 18 20.343 19.646 41.328 0.00 0.00
C
ATOM 20351 C GLY C 18 18.528 20.107 40.457 0.00 0.00
C
ATOM 20352 O GLY C 18 18.972 20.323 39.358 0.00 0.00
C
ATOM 20353 N GLY C 19 17.326 20.664 40.739 0.00 0.00
C
ATOM 20354 HN GLY C 19 16.915 20.560 41.641 0.00 0.00
C
ATOM 20355 CA GLY C 19 16.593 21.359 39.757 0.00 0.00
C
ATOM 20356 HA1 GLY C 19 15.553 21.440 40.037 0.00 0.00
C
ATOM 20357 HA2 GLY C 19 16.572 20.848 38.806 0.00 0.00
C

ATOM 20358 C   GLY C 19   16.985 22.761 39.612 0.00 0.00
C
ATOM 20359 O   GLY C 19   16.836 23.375 38.493 0.00 0.00
C
ATOM 20360 N   SER C 20   17.575 23.298 40.758 0.00 0.00
C
ATOM 20361 HN SER C 20   17.699 22.770 41.594 0.00 0.00
C
ATOM 20362 CA SER C 20   18.234 24.531 40.922 0.00 0.00
C
ATOM 20363 HA SER C 20   17.592 25.334 40.592 0.00 0.00
C
ATOM 20364 CB SER C 20   18.563 24.936 42.362 0.00 0.00
C
ATOM 20365 HB1 SER C 20   17.695 24.989 43.053 0.00 0.00
C
ATOM 20366 HB2 SER C 20   19.149 24.151 42.886 0.00 0.00
C
ATOM 20367 OG SER C 20   19.218 26.237 42.498 0.00 0.00
C
ATOM 20368 HG1 SER C 20   19.240 26.502 43.420 0.00 0.00
C
ATOM 20369 C   SER C 20   19.438 24.635 40.082 0.00 0.00
C
ATOM 20370 O   SER C 20   19.648 25.663 39.353 0.00 0.00
C
ATOM 20371 N   ILE C 21   20.210 23.536 40.045 0.00 0.00
C
ATOM 20372 HN ILE C 21   19.891 22.701 40.488 0.00 0.00
C
ATOM 20373 CA ILE C 21   21.382 23.532 39.160 0.00 0.00
C
ATOM 20374 HA ILE C 21   21.905 24.450 39.385 0.00 0.00
C
ATOM 20375 CB ILE C 21   22.188 22.302 39.419 0.00 0.00
C
ATOM 20376 HB ILE C 21   21.543 21.399 39.466 0.00 0.00
C
ATOM 20377 CG2 ILE C 21   23.298 22.010 38.303 0.00 0.00
C
ATOM 20378 HG21 ILE C 21   23.788 21.051 38.575 0.00 0.00
C
ATOM 20379 HG22 ILE C 21   22.790 21.834 37.331 0.00 0.00
C
ATOM 20380 HG23 ILE C 21   24.085 22.787 38.193 0.00 0.00
C
ATOM 20381 CG1 ILE C 21   22.765 22.434 40.855 0.00 0.00
C
ATOM 20382 HG11 ILE C 21   23.604 23.162 40.824 0.00 0.00
C
ATOM 20383 HG12 ILE C 21   22.024 22.782 41.607 0.00 0.00
C
ATOM 20384 CD ILE C 21   23.398 21.160 41.317 0.00 0.00
C

ATOM	20385	HD1	ILE	C	21	22.698	20.297	41.320	0.00	0.00	C
ATOM	20386	HD2	ILE	C	21	24.233	20.944	40.617	0.00	0.00	C
ATOM	20387	HD3	ILE	C	21	23.877	21.153	42.320	0.00	0.00	C
ATOM	20388	C	ILE	C	21	20.949	23.517	37.719	0.00	0.00	C
ATOM	20389	O	ILE	C	21	21.513	24.294	36.940	0.00	0.00	C
ATOM	20390	N	CYS	C	22	19.830	22.770	37.281	0.00	0.00	C
ATOM	20391	HN	CYS	C	22	19.350	22.222	37.962	0.00	0.00	C
ATOM	20392	CA	CYS	C	22	19.364	22.763	35.885	0.00	0.00	C
ATOM	20393	HA	CYS	C	22	20.243	22.494	35.319	0.00	0.00	C
ATOM	20394	CB	CYS	C	22	18.314	21.717	35.523	0.00	0.00	C
ATOM	20395	HB1	CYS	C	22	17.439	21.980	36.155	0.00	0.00	C
ATOM	20396	HB2	CYS	C	22	18.032	21.836	34.455	0.00	0.00	C
ATOM	20397	SG	CYS	C	22	18.975	20.134	35.916	0.00	0.00	C
ATOM	20398	HG1	CYS	C	22	18.777	20.210	37.223	0.00	0.00	C
ATOM	20399	C	CYS	C	22	18.950	24.124	35.421	0.00	0.00	C
ATOM	20400	O	CYS	C	22	19.511	24.609	34.448	0.00	0.00	C
ATOM	20401	N	LYS	C	23	18.014	24.736	36.227	0.00	0.00	C
ATOM	20402	HN	LYS	C	23	17.595	24.369	37.053	0.00	0.00	C
ATOM	20403	CA	LYS	C	23	17.459	26.039	35.931	0.00	0.00	C
ATOM	20404	HA	LYS	C	23	16.995	25.951	34.960	0.00	0.00	C
ATOM	20405	CB	LYS	C	23	16.369	26.310	37.028	0.00	0.00	C
ATOM	20406	HB1	LYS	C	23	15.508	25.627	36.869	0.00	0.00	C
ATOM	20407	HB2	LYS	C	23	16.808	26.181	38.041	0.00	0.00	C
ATOM	20408	CG	LYS	C	23	15.885	27.763	37.048	0.00	0.00	C
ATOM	20409	HG1	LYS	C	23	16.654	28.489	37.390	0.00	0.00	C
ATOM	20410	HG2	LYS	C	23	15.707	28.086	36.000	0.00	0.00	C
ATOM	20411	CD	LYS	C	23	14.580	27.879	37.858	0.00	0.00	C

ATOM 20412 HD1 LYS C 23 13.845 27.302 37.258 0.00 0.00
C
ATOM 20413 HD2 LYS C 23 14.711 27.451 38.875 0.00 0.00
C
ATOM 20414 CE LYS C 23 14.013 29.272 38.078 0.00 0.00
C
ATOM 20415 HE1 LYS C 23 13.865 29.913 37.182 0.00 0.00
C
ATOM 20416 HE2 LYS C 23 13.024 29.302 38.584 0.00 0.00
C
ATOM 20417 NZ LYS C 23 14.988 29.996 38.978 0.00 0.00
C
ATOM 20418 HZ1 LYS C 23 14.497 30.794 39.429 0.00 0.00
C
ATOM 20419 HZ2 LYS C 23 15.232 29.395 39.791 0.00 0.00
C
ATOM 20420 HZ3 LYS C 23 15.859 30.309 38.503 0.00 0.00
C
ATOM 20421 C LYS C 23 18.473 27.141 35.788 0.00 0.00
C
ATOM 20422 O LYS C 23 18.368 27.924 34.831 0.00 0.00
C
ATOM 20423 N GLU C 24 19.453 27.179 36.734 0.00 0.00
C
ATOM 20424 HN GLU C 24 19.581 26.416 37.362 0.00 0.00
C
ATOM 20425 CA GLU C 24 20.468 28.207 36.700 0.00 0.00
C
ATOM 20426 HA GLU C 24 20.054 29.192 36.540 0.00 0.00
C
ATOM 20427 CB GLU C 24 21.193 28.209 38.044 0.00 0.00
C
ATOM 20428 HB1 GLU C 24 20.454 28.256 38.872 0.00 0.00
C
ATOM 20429 HB2 GLU C 24 21.902 27.356 38.097 0.00 0.00
C
ATOM 20430 CG GLU C 24 22.140 29.388 38.315 0.00 0.00
C
ATOM 20431 HG1 GLU C 24 22.654 29.237 39.288 0.00 0.00
C
ATOM 20432 HG2 GLU C 24 22.977 29.375 37.586 0.00 0.00
C
ATOM 20433 CD GLU C 24 21.493 30.729 38.275 0.00 0.00
C
ATOM 20434 OE1 GLU C 24 22.277 31.711 38.546 0.00 0.00
C
ATOM 20435 OE2 GLU C 24 20.270 30.834 38.081 0.00 0.00
C
ATOM 20436 C GLU C 24 21.624 28.101 35.622 0.00 0.00
C
ATOM 20437 O GLU C 24 22.165 29.084 35.029 0.00 0.00
C
ATOM 20438 N LEU C 25 22.077 26.884 35.297 0.00 0.00
C

ATOM 20439 HN LEU C 25 21.777 26.124 35.868 0.00 0.00
C
ATOM 20440 CA LEU C 25 22.887 26.541 34.114 0.00 0.00
C
ATOM 20441 HA LEU C 25 23.798 27.121 34.102 0.00 0.00
C
ATOM 20442 CB LEU C 25 23.210 25.057 34.093 0.00 0.00
C
ATOM 20443 HB1 LEU C 25 22.266 24.471 34.091 0.00 0.00
C
ATOM 20444 HB2 LEU C 25 23.757 24.805 33.159 0.00 0.00
C
ATOM 20445 CG LEU C 25 24.070 24.439 35.265 0.00 0.00
C
ATOM 20446 HG LEU C 25 23.670 24.659 36.278 0.00 0.00
C
ATOM 20447 CD1 LEU C 25 24.131 22.887 35.025 0.00 0.00
C
ATOM 20448 HD11 LEU C 25 24.654 22.711 34.061 0.00 0.00
C
ATOM 20449 HD12 LEU C 25 24.890 22.402 35.675 0.00 0.00
C
ATOM 20450 HD13 LEU C 25 23.162 22.342 35.028 0.00 0.00
C
ATOM 20451 CD2 LEU C 25 25.516 24.935 35.235 0.00 0.00
C
ATOM 20452 HD21 LEU C 25 25.975 24.399 34.377 0.00 0.00
C
ATOM 20453 HD22 LEU C 25 25.633 26.019 35.019 0.00 0.00
C
ATOM 20454 HD23 LEU C 25 26.043 24.665 36.175 0.00 0.00
C
ATOM 20455 C LEU C 25 22.115 26.797 32.873 0.00 0.00
C
ATOM 20456 O LEU C 25 22.604 27.366 31.904 0.00 0.00
C
ATOM 20457 N HSE C 26 20.858 26.420 32.830 0.00 0.00
C
ATOM 20458 HN HSE C 26 20.597 25.784 33.552 0.00 0.00
C
ATOM 20459 CA HSE C 26 20.078 26.812 31.645 0.00 0.00
C
ATOM 20460 HA HSE C 26 20.609 26.463 30.772 0.00 0.00
C
ATOM 20461 CB HSE C 26 18.711 26.111 31.621 0.00 0.00
C
ATOM 20462 HB1 HSE C 26 18.862 25.012 31.559 0.00 0.00
C
ATOM 20463 HB2 HSE C 26 18.025 26.391 32.450 0.00 0.00
C
ATOM 20464 ND1 HSE C 26 17.902 25.812 29.317 0.00 0.00
C
ATOM 20465 CG HSE C 26 17.834 26.479 30.529 0.00 0.00
C

ATOM 20466 CE1 HSE C 26 16.962 26.336 28.537 0.00 0.00
C
ATOM 20467 HE1 HSE C 26 16.818 26.083 27.487 0.00 0.00
C
ATOM 20468 NE2 HSE C 26 16.261 27.328 29.248 0.00 0.00
C
ATOM 20469 HE2 HSE C 26 15.456 27.880 29.029 0.00 0.00
C
ATOM 20470 CD2 HSE C 26 16.790 27.370 30.522 0.00 0.00
C
ATOM 20471 HD2 HSE C 26 16.429 28.039 31.294 0.00 0.00
C
ATOM 20472 C HSE C 26 20.033 28.360 31.404 0.00 0.00
C
ATOM 20473 O HSE C 26 20.203 28.842 30.263 0.00 0.00
C
ATOM 20474 N ARG C 27 19.756 29.061 32.502 0.00 0.00
C
ATOM 20475 HN ARG C 27 19.639 28.682 33.416 0.00 0.00
C
ATOM 20476 CA ARG C 27 19.777 30.483 32.551 0.00 0.00
C
ATOM 20477 HA ARG C 27 19.049 30.894 31.866 0.00 0.00
C
ATOM 20478 CB ARG C 27 19.395 31.108 33.936 0.00 0.00
C
ATOM 20479 HB1 ARG C 27 18.451 30.641 34.290 0.00 0.00
C
ATOM 20480 HB2 ARG C 27 20.249 30.974 34.633 0.00 0.00
C
ATOM 20481 CG ARG C 27 19.002 32.606 33.925 0.00 0.00
C
ATOM 20482 HG1 ARG C 27 19.935 33.147 33.660 0.00 0.00
C
ATOM 20483 HG2 ARG C 27 18.171 32.903 33.250 0.00 0.00
C
ATOM 20484 CD ARG C 27 18.498 33.093 35.311 0.00 0.00
C
ATOM 20485 HD1 ARG C 27 18.149 34.134 35.140 0.00 0.00
C
ATOM 20486 HD2 ARG C 27 17.593 32.519 35.603 0.00 0.00
C
ATOM 20487 NE ARG C 27 19.638 32.981 36.230 0.00 0.00
C
ATOM 20488 HE ARG C 27 19.889 32.159 36.741 0.00 0.00
C
ATOM 20489 CZ ARG C 27 20.557 33.994 36.326 0.00 0.00
C
ATOM 20490 NH1 ARG C 27 20.433 35.084 35.529 0.00 0.00
C
ATOM 20491 HH11 ARG C 27 21.146 35.775 35.647 0.00 0.00
C
ATOM 20492 HH12 ARG C 27 19.762 35.229 34.802 0.00 0.00
C

ATOM	20493	NH2	ARG	C	27	21.598	33.882	37.121	0.00	0.00	C
ATOM	20494	HH21	ARG	C	27	22.293	34.596	37.047	0.00	0.00	C
ATOM	20495	HH22	ARG	C	27	21.697	33.033	37.640	0.00	0.00	C
ATOM	20496	C	ARG	C	27	21.139	31.034	32.171	0.00	0.00	C
ATOM	20497	O	ARG	C	27	21.249	32.114	31.562	0.00	0.00	C
ATOM	20498	N	MET	C	28	22.261	30.338	32.456	0.00	0.00	C
ATOM	20499	HN	MET	C	28	22.226	29.630	33.156	0.00	0.00	C
ATOM	20500	CA	MET	C	28	23.560	30.754	32.069	0.00	0.00	C
ATOM	20501	HA	MET	C	28	23.656	31.829	32.102	0.00	0.00	C
ATOM	20502	CB	MET	C	28	24.621	30.154	33.014	0.00	0.00	C
ATOM	20503	HB1	MET	C	28	24.423	29.078	33.208	0.00	0.00	C
ATOM	20504	HB2	MET	C	28	25.608	30.204	32.507	0.00	0.00	C
ATOM	20505	CG	MET	C	28	24.835	31.018	34.281	0.00	0.00	C
ATOM	20506	HG1	MET	C	28	25.034	32.044	33.905	0.00	0.00	C
ATOM	20507	HG2	MET	C	28	23.929	30.994	34.923	0.00	0.00	C
ATOM	20508	SD	MET	C	28	26.318	30.540	35.200	0.00	0.00	C
ATOM	20509	CE	MET	C	28	25.647	29.337	36.320	0.00	0.00	C
ATOM	20510	HE1	MET	C	28	26.409	28.975	37.043	0.00	0.00	C
ATOM	20511	HE2	MET	C	28	24.892	29.891	36.916	0.00	0.00	C
ATOM	20512	HE3	MET	C	28	25.164	28.426	35.907	0.00	0.00	C
ATOM	20513	C	MET	C	28	23.899	30.212	30.640	0.00	0.00	C
ATOM	20514	O	MET	C	28	25.076	30.336	30.213	0.00	0.00	C
ATOM	20515	N	GLY	C	29	22.866	29.749	29.920	0.00	0.00	C
ATOM	20516	HN	GLY	C	29	21.954	29.692	30.319	0.00	0.00	C
ATOM	20517	CA	GLY	C	29	22.926	29.529	28.377	0.00	0.00	C
ATOM	20518	HA1	GLY	C	29	23.528	30.332	27.977	0.00	0.00	C
ATOM	20519	HA2	GLY	C	29	21.891	29.414	28.091	0.00	0.00	C

ATOM 20520 C GLY C 29 23.522 28.291 27.880 0.00 0.00
C
ATOM 20521 O GLY C 29 23.807 27.996 26.691 0.00 0.00
C
ATOM 20522 N HSE C 30 23.814 27.412 28.875 0.00 0.00
C
ATOM 20523 HN HSE C 30 23.400 27.518 29.775 0.00 0.00
C
ATOM 20524 CA HSE C 30 24.663 26.253 28.750 0.00 0.00
C
ATOM 20525 HA HSE C 30 25.311 26.301 27.888 0.00 0.00
C
ATOM 20526 CB HSE C 30 25.412 25.857 30.035 0.00 0.00
C
ATOM 20527 HB1 HSE C 30 24.650 26.019 30.827 0.00 0.00
C
ATOM 20528 HB2 HSE C 30 25.785 24.810 30.049 0.00 0.00
C
ATOM 20529 ND1 HSE C 30 27.548 26.870 29.276 0.00 0.00
C
ATOM 20530 CG HSE C 30 26.673 26.643 30.346 0.00 0.00
C
ATOM 20531 CE1 HSE C 30 28.635 27.495 29.893 0.00 0.00
C
ATOM 20532 HE1 HSE C 30 29.452 27.963 29.343 0.00 0.00
C
ATOM 20533 NE2 HSE C 30 28.373 27.723 31.194 0.00 0.00
C
ATOM 20534 HE2 HSE C 30 28.925 28.349 31.745 0.00 0.00
C
ATOM 20535 CD2 HSE C 30 27.185 27.131 31.518 0.00 0.00
C
ATOM 20536 HD2 HSE C 30 26.694 27.264 32.474 0.00 0.00
C
ATOM 20537 C HSE C 30 23.737 25.083 28.562 0.00 0.00
C
ATOM 20538 O HSE C 30 22.597 25.083 29.023 0.00 0.00
C
ATOM 20539 N GLU C 31 24.210 24.025 27.859 0.00 0.00
C
ATOM 20540 HN GLU C 31 25.158 24.038 27.549 0.00 0.00
C
ATOM 20541 CA GLU C 31 23.354 22.920 27.342 0.00 0.00
C
ATOM 20542 HA GLU C 31 22.348 23.244 27.119 0.00 0.00
C
ATOM 20543 CB GLU C 31 23.866 22.301 25.931 0.00 0.00
C
ATOM 20544 HB1 GLU C 31 24.959 22.180 26.090 0.00 0.00
C
ATOM 20545 HB2 GLU C 31 23.251 21.393 25.749 0.00 0.00
C
ATOM 20546 CG GLU C 31 23.599 23.322 24.843 0.00 0.00
C

ATOM 20547 HG1 GLU C 31   22.572 23.411 24.428 0.00 0.00
C
ATOM 20548 HG2 GLU C 31   23.886 24.351 25.150 0.00 0.00
C
ATOM 20549 CD GLU C 31   24.454 22.949 23.643 0.00 0.00
C
ATOM 20550 OE1 GLU C 31   23.992 22.451 22.601 0.00 0.00
C
ATOM 20551 OE2 GLU C 31   25.677 23.319 23.712 0.00 0.00
C
ATOM 20552 C   GLU C 31   23.327 21.885 28.480 0.00 0.00
C
ATOM 20553 O   GLU C 31   24.401 21.351 28.783 0.00 0.00
C
ATOM 20554 N   VAL C 32   22.177 21.565 29.170 0.00 0.00
C
ATOM 20555 HN VAL C 32   21.312 21.944 28.850 0.00 0.00
C
ATOM 20556 CA VAL C 32   21.972 20.662 30.341 0.00 0.00
C
ATOM 20557 HA VAL C 32   22.944 20.347 30.690 0.00 0.00
C
ATOM 20558 CB VAL C 32   21.244 21.396 31.498 0.00 0.00
C
ATOM 20559 HB VAL C 32   20.162 21.524 31.279 0.00 0.00
C
ATOM 20560 CG1 VAL C 32   21.251 20.522 32.781 0.00 0.00
C
ATOM 20561 HG11 VAL C 32   20.705 21.080 33.572 0.00 0.00
C
ATOM 20562 HG12 VAL C 32   20.856 19.502 32.585 0.00 0.00
C
ATOM 20563 HG13 VAL C 32   22.313 20.538 33.106 0.00 0.00
C
ATOM 20564 CG2 VAL C 32   21.915 22.766 31.855 0.00 0.00
C
ATOM 20565 HG21 VAL C 32   21.320 23.159 32.707 0.00 0.00
C
ATOM 20566 HG22 VAL C 32   23.005 22.630 32.019 0.00 0.00
C
ATOM 20567 HG23 VAL C 32   21.828 23.542 31.065 0.00 0.00
C
ATOM 20568 C   VAL C 32   21.274 19.367 29.977 0.00 0.00
C
ATOM 20569 O   VAL C 32   20.340 19.350 29.183 0.00 0.00
C
ATOM 20570 N   LEU C 33   21.831 18.233 30.399 0.00 0.00
C
ATOM 20571 HN LEU C 33   22.691 18.312 30.898 0.00 0.00
C
ATOM 20572 CA LEU C 33   21.269 16.908 30.291 0.00 0.00
C
ATOM 20573 HA LEU C 33   20.411 16.882 29.636 0.00 0.00
C

ATOM 20574 CB LEU C 33   22.308 15.783 29.887 0.00 0.00
C
ATOM 20575 HB1 LEU C 33   22.541 15.883 28.806 0.00 0.00
C
ATOM 20576 HB2 LEU C 33   23.226 15.936 30.495 0.00 0.00
C
ATOM 20577 CG LEU C 33   21.849 14.302 30.065 0.00 0.00
C
ATOM 20578 HG LEU C 33   21.557 14.088 31.116 0.00 0.00
C
ATOM 20579 CD1 LEU C 33   20.640 13.981 29.214 0.00 0.00
C
ATOM 20580 HD11 LEU C 33   19.915 14.814 29.335 0.00 0.00
C
ATOM 20581 HD12 LEU C 33   20.908 13.759 28.159 0.00 0.00
C
ATOM 20582 HD13 LEU C 33   20.140 13.076 29.620 0.00 0.00
C
ATOM 20583 CD2 LEU C 33   23.006 13.292 29.749 0.00 0.00
C
ATOM 20584 HD21 LEU C 33   22.550 12.314 29.483 0.00 0.00
C
ATOM 20585 HD22 LEU C 33   23.405 13.619 28.765 0.00 0.00
C
ATOM 20586 HD23 LEU C 33   23.876 13.308 30.440 0.00 0.00
C
ATOM 20587 C   LEU C 33   20.762 16.465 31.634 0.00 0.00
C
ATOM 20588 O   LEU C 33   21.493 16.526 32.623 0.00 0.00
C
ATOM 20589 N   ALA C 34   19.454 16.143 31.721 0.00 0.00
C
ATOM 20590 HN ALA C 34   18.756 16.221 31.013 0.00 0.00
C
ATOM 20591 CA ALA C 34   18.767 16.143 33.048 0.00 0.00
C
ATOM 20592 HA ALA C 34   19.519 16.190 33.822 0.00 0.00
C
ATOM 20593 CB ALA C 34   17.731 17.269 33.054 0.00 0.00
C
ATOM 20594 HB1 ALA C 34   17.414 17.533 34.086 0.00 0.00
C
ATOM 20595 HB2 ALA C 34   18.079 18.216 32.588 0.00 0.00
C
ATOM 20596 HB3 ALA C 34   16.795 16.976 32.534 0.00 0.00
C
ATOM 20597 C   ALA C 34   18.086 14.816 33.167 0.00 0.00
C
ATOM 20598 O   ALA C 34   17.188 14.341 32.463 0.00 0.00
C
ATOM 20599 N   VAL C 35   18.509 14.125 34.219 0.00 0.00
C
ATOM 20600 HN VAL C 35   19.161 14.529 34.856 0.00 0.00
C

ATOM 20601 CA VAL C 35 18.298 12.686 34.384 0.00 0.00
C
ATOM 20602 HA VAL C 35 17.783 12.281 33.526 0.00 0.00
C
ATOM 20603 CB VAL C 35 19.600 11.836 34.256 0.00 0.00
C
ATOM 20604 HB VAL C 35 20.325 12.123 35.047 0.00 0.00
C
ATOM 20605 CG1 VAL C 35 19.241 10.324 34.454 0.00 0.00
C
ATOM 20606 HG11 VAL C 35 20.005 9.595 34.111 0.00 0.00
C
ATOM 20607 HG12 VAL C 35 18.903 10.204 35.506 0.00 0.00
C
ATOM 20608 HG13 VAL C 35 18.424 9.983 33.783 0.00 0.00
C
ATOM 20609 CG2 VAL C 35 20.318 12.099 32.898 0.00 0.00
C
ATOM 20610 HG21 VAL C 35 20.636 13.158 32.792 0.00 0.00
C
ATOM 20611 HG22 VAL C 35 21.222 11.458 32.981 0.00 0.00
C
ATOM 20612 HG23 VAL C 35 19.733 11.784 32.008 0.00 0.00
C
ATOM 20613 C VAL C 35 17.601 12.471 35.726 0.00 0.00
C
ATOM 20614 O VAL C 35 18.100 12.921 36.782 0.00 0.00
C
ATOM 20615 N ASP C 36 16.436 11.728 35.704 0.00 0.00
C
ATOM 20616 HN ASP C 36 16.105 11.235 34.903 0.00 0.00
C
ATOM 20617 CA ASP C 36 15.847 11.332 36.998 0.00 0.00
C
ATOM 20618 HA ASP C 36 16.641 10.860 37.557 0.00 0.00
C
ATOM 20619 CB ASP C 36 15.061 12.466 37.771 0.00 0.00
C
ATOM 20620 HB1 ASP C 36 15.747 13.339 37.731 0.00 0.00
C
ATOM 20621 HB2 ASP C 36 14.108 12.726 37.263 0.00 0.00
C
ATOM 20622 CG ASP C 36 14.706 12.197 39.178 0.00 0.00
C
ATOM 20623 OD1 ASP C 36 15.171 12.965 40.077 0.00 0.00
C
ATOM 20624 OD2 ASP C 36 13.797 11.329 39.450 0.00 0.00
C
ATOM 20625 C ASP C 36 14.871 10.221 36.539 0.00 0.00
C
ATOM 20626 O ASP C 36 14.802 9.839 35.379 0.00 0.00
C
ATOM 20627 N ILE C 37 14.289 9.493 37.554 0.00 0.00
C

ATOM 20628 HN ILE C 37   14.432   9.805 38.490 0.00 0.00
C
ATOM 20629 CA ILE C 37   13.405   8.307 37.419 0.00 0.00
C
ATOM 20630 HA ILE C 37   13.412   7.995 36.385 0.00 0.00
C
ATOM 20631 CB ILE C 37   13.978   7.099 38.169 0.00 0.00
C
ATOM 20632 HB ILE C 37   15.065   6.942 37.998 0.00 0.00
C
ATOM 20633 CG2 ILE C 37   13.814   7.273 39.672 0.00 0.00
C
ATOM 20634 HG21 ILE C 37   14.446   6.613 40.305 0.00 0.00
C
ATOM 20635 HG22 ILE C 37   14.126   8.258 40.081 0.00 0.00
C
ATOM 20636 HG23 ILE C 37   12.789   7.082 40.056 0.00 0.00
C
ATOM 20637 CG1 ILE C 37   13.379   5.742 37.678 0.00 0.00
C
ATOM 20638 HG11 ILE C 37   13.701   4.935 38.371 0.00 0.00
C
ATOM 20639 HG12 ILE C 37   12.269   5.714 37.718 0.00 0.00
C
ATOM 20640 CD ILE C 37   13.809   5.347 36.228 0.00 0.00
C
ATOM 20641 HD1 ILE C 37   13.029   4.783 35.674 0.00 0.00
C
ATOM 20642 HD2 ILE C 37   14.089   6.281 35.696 0.00 0.00
C
ATOM 20643 HD3 ILE C 37   14.723   4.716 36.239 0.00 0.00
C
ATOM 20644 C   ILE C 37   12.024   8.720 37.708 0.00 0.00
C
ATOM 20645 O   ILE C 37   11.102   8.126 37.213 0.00 0.00
C
ATOM 20646 N   ASN C 38   11.763   9.886 38.375 0.00 0.00
C
ATOM 20647 HN ASN C 38   12.467 10.329 38.923 0.00 0.00
C
ATOM 20648 CA ASN C 38   10.460 10.553 38.585 0.00 0.00
C
ATOM 20649 HA ASN C 38   9.890   9.658 38.787 0.00 0.00
C
ATOM 20650 CB ASN C 38   10.546 11.536 39.768 0.00 0.00
C
ATOM 20651 HB1 ASN C 38   11.510 12.077 39.651 0.00 0.00
C
ATOM 20652 HB2 ASN C 38   9.742 12.302 39.725 0.00 0.00
C
ATOM 20653 CG ASN C 38   10.403 10.804 41.027 0.00 0.00
C
ATOM 20654 OD1 ASN C 38   9.371 10.928 41.617 0.00 0.00
C

ATOM 20655 ND2 ASN C 38 11.418 10.176 41.608 0.00 0.00
C
ATOM 20656 HD21 ASN C 38 11.309 9.616 42.429 0.00 0.00
C
ATOM 20657 HD22 ASN C 38 12.326 10.244 41.194 0.00 0.00
C
ATOM 20658 C ASN C 38 9.897 11.293 37.336 0.00 0.00
C
ATOM 20659 O ASN C 38 10.285 12.412 37.058 0.00 0.00
C
ATOM 20660 N GLU C 39 8.952 10.671 36.514 0.00 0.00
C
ATOM 20661 HN GLU C 39 8.574 9.811 36.849 0.00 0.00
C
ATOM 20662 CA GLU C 39 8.461 11.263 35.256 0.00 0.00
C
ATOM 20663 HA GLU C 39 9.311 11.288 34.590 0.00 0.00
C
ATOM 20664 CB GLU C 39 7.409 10.289 34.571 0.00 0.00
C
ATOM 20665 HB1 GLU C 39 6.475 10.552 35.113 0.00 0.00
C
ATOM 20666 HB2 GLU C 39 7.352 10.465 33.476 0.00 0.00
C
ATOM 20667 CG GLU C 39 7.680 8.787 34.770 0.00 0.00
C
ATOM 20668 HG1 GLU C 39 8.692 8.476 34.431 0.00 0.00
C
ATOM 20669 HG2 GLU C 39 7.532 8.490 35.831 0.00 0.00
C
ATOM 20670 CD GLU C 39 6.781 7.946 33.916 0.00 0.00
C
ATOM 20671 OE1 GLU C 39 6.037 7.217 34.525 0.00 0.00
C
ATOM 20672 OE2 GLU C 39 6.927 7.873 32.623 0.00 0.00
C
ATOM 20673 C GLU C 39 7.879 12.629 35.389 0.00 0.00
C
ATOM 20674 O GLU C 39 8.085 13.550 34.556 0.00 0.00
C
ATOM 20675 N GLU C 40 7.159 12.909 36.472 0.00 0.00
C
ATOM 20676 HN GLU C 40 6.887 12.134 37.038 0.00 0.00
C
ATOM 20677 CA GLU C 40 6.658 14.210 36.850 0.00 0.00
C
ATOM 20678 HA GLU C 40 6.090 14.584 36.011 0.00 0.00
C
ATOM 20679 CB GLU C 40 5.756 14.318 38.132 0.00 0.00
C
ATOM 20680 HB1 GLU C 40 5.387 15.354 38.291 0.00 0.00
C
ATOM 20681 HB2 GLU C 40 4.813 13.777 37.900 0.00 0.00
C

ATOM 20682 CG GLU C 40 6.370 13.819 39.522 0.00 0.00
C
ATOM 20683 HG1 GLU C 40 6.327 12.730 39.309 0.00 0.00
C
ATOM 20684 HG2 GLU C 40 7.458 14.031 39.600 0.00 0.00
C
ATOM 20685 CD GLU C 40 5.469 14.138 40.700 0.00 0.00
C
ATOM 20686 OE1 GLU C 40 5.453 13.319 41.622 0.00 0.00
C
ATOM 20687 OE2 GLU C 40 4.843 15.226 40.735 0.00 0.00
C
ATOM 20688 C GLU C 40 7.853 15.264 37.040 0.00 0.00
C
ATOM 20689 O GLU C 40 7.848 16.408 36.634 0.00 0.00
C
ATOM 20690 N LYS C 41 8.969 14.807 37.703 0.00 0.00
C
ATOM 20691 HN LYS C 41 9.030 13.826 37.865 0.00 0.00
C
ATOM 20692 CA LYS C 41 10.063 15.813 37.953 0.00 0.00
C
ATOM 20693 HA LYS C 41 9.661 16.780 38.215 0.00 0.00
C
ATOM 20694 CB LYS C 41 11.088 15.409 39.002 0.00 0.00
C
ATOM 20695 HB1 LYS C 41 11.540 14.434 38.721 0.00 0.00
C
ATOM 20696 HB2 LYS C 41 11.880 16.188 38.982 0.00 0.00
C
ATOM 20697 CG LYS C 41 10.616 15.351 40.405 0.00 0.00
C
ATOM 20698 HG1 LYS C 41 10.246 16.343 40.744 0.00 0.00
C
ATOM 20699 HG2 LYS C 41 9.755 14.650 40.400 0.00 0.00
C
ATOM 20700 CD LYS C 41 11.670 14.870 41.407 0.00 0.00
C
ATOM 20701 HD1 LYS C 41 12.159 13.948 41.025 0.00 0.00
C
ATOM 20702 HD2 LYS C 41 12.484 15.618 41.518 0.00 0.00
C
ATOM 20703 CE LYS C 41 11.096 14.705 42.794 0.00 0.00
C
ATOM 20704 HE1 LYS C 41 10.421 15.532 43.101 0.00 0.00
C
ATOM 20705 HE2 LYS C 41 10.457 13.799 42.868 0.00 0.00
C
ATOM 20706 NZ LYS C 41 12.207 14.489 43.745 0.00 0.00
C
ATOM 20707 HZ1 LYS C 41 12.564 15.400 44.098 0.00 0.00
C
ATOM 20708 HZ2 LYS C 41 11.821 13.821 44.443 0.00 0.00
C

ATOM 20709 HZ3 LYS C 41   12.976 13.922 43.335 0.00 0.00
C
ATOM 20710 C   LYS C 41   10.710 15.970 36.584 0.00 0.00
C
ATOM 20711 O   LYS C 41   11.027 17.104 36.190 0.00 0.00
C
ATOM 20712 N   VAL C 42   10.870 14.876 35.722 0.00 0.00
C
ATOM 20713 HN VAL C 42   10.525 14.018 36.094 0.00 0.00
C
ATOM 20714 CA VAL C 42   11.427 15.079 34.458 0.00 0.00
C
ATOM 20715 HA VAL C 42   12.356 15.604 34.621 0.00 0.00
C
ATOM 20716 CB VAL C 42   11.817 13.775 33.837 0.00 0.00
C
ATOM 20717 HB VAL C 42   10.880 13.218 33.625 0.00 0.00
C
ATOM 20718 CG1 VAL C 42   12.500 13.861 32.460 0.00 0.00
C
ATOM 20719 HG11 VAL C 42   13.317 14.610 32.379 0.00 0.00
C
ATOM 20720 HG12 VAL C 42   12.820 12.839 32.167 0.00 0.00
C
ATOM 20721 HG13 VAL C 42   11.768 14.134 31.670 0.00 0.00
C
ATOM 20722 CG2 VAL C 42   12.711 12.921 34.767 0.00 0.00
C
ATOM 20723 HG21 VAL C 42   13.712 13.393 34.858 0.00 0.00
C
ATOM 20724 HG22 VAL C 42   12.268 12.851 35.783 0.00 0.00
C
ATOM 20725 HG23 VAL C 42   13.029 11.915 34.417 0.00 0.00
C
ATOM 20726 C   VAL C 42   10.637 16.025 33.495 0.00 0.00
C
ATOM 20727 O   VAL C 42   11.230 16.828 32.758 0.00 0.00
C
ATOM 20728 N   ASN C 43   9.261 15.979 33.485 0.00 0.00
C
ATOM 20729 HN ASN C 43   8.822 15.257 34.014 0.00 0.00
C
ATOM 20730 CA ASN C 43   8.451 17.013 32.835 0.00 0.00
C
ATOM 20731 HA ASN C 43   8.764 17.249 31.828 0.00 0.00
C
ATOM 20732 CB ASN C 43   6.951 16.581 32.754 0.00 0.00
C
ATOM 20733 HB1 ASN C 43   6.687 16.183 33.757 0.00 0.00
C
ATOM 20734 HB2 ASN C 43   6.248 17.413 32.538 0.00 0.00
C
ATOM 20735 CG ASN C 43   6.738 15.488 31.709 0.00 0.00
C

ATOM 20736 OD1 ASN C 43   6.396 15.688 30.568 0.00 0.00
C
ATOM 20737 ND2 ASN C 43   6.891 14.211 32.149 0.00 0.00
C
ATOM 20738 HD21 ASN C 43   6.437 13.484 31.633 0.00 0.00
C
ATOM 20739 HD22 ASN C 43   7.178 14.044 33.092 0.00 0.00
C
ATOM 20740 C   ASN C 43   8.611 18.460 33.387 0.00 0.00
C
ATOM 20741 O   ASN C 43   8.729 19.410 32.601 0.00 0.00
C
ATOM 20742 N   ALA C 44   8.892 18.655 34.645 0.00 0.00
C
ATOM 20743 HN ALA C 44   8.847 17.995 35.391 0.00 0.00
C
ATOM 20744 CA ALA C 44   9.029 20.000 35.048 0.00 0.00
C
ATOM 20745 HA ALA C 44   8.280 20.556 34.503 0.00 0.00
C
ATOM 20746 CB ALA C 44   8.691 20.190 36.486 0.00 0.00
C
ATOM 20747 HB1 ALA C 44   8.603 21.263 36.760 0.00 0.00
C
ATOM 20748 HB2 ALA C 44   7.673 19.785 36.668 0.00 0.00
C
ATOM 20749 HB3 ALA C 44   9.416 19.730 37.191 0.00 0.00
C
ATOM 20750 C   ALA C 44   10.443 20.484 34.817 0.00 0.00
C
ATOM 20751 O   ALA C 44   10.734 21.699 34.993 0.00 0.00
C
ATOM 20752 N   TYR C 45   11.364 19.684 34.359 0.00 0.00
C
ATOM 20753 HN TYR C 45   11.309 18.688 34.359 0.00 0.00
C
ATOM 20754 CA TYR C 45   12.634 20.230 33.861 0.00 0.00
C
ATOM 20755 HA TYR C 45   12.494 21.298 33.789 0.00 0.00
C
ATOM 20756 CB TYR C 45   13.851 19.894 34.754 0.00 0.00
C
ATOM 20757 HB1 TYR C 45   13.930 18.789 34.843 0.00 0.00
C
ATOM 20758 HB2 TYR C 45   14.826 20.170 34.298 0.00 0.00
C
ATOM 20759 CG TYR C 45   13.701 20.497 36.086 0.00 0.00
C
ATOM 20760 CD1 TYR C 45   13.865 21.868 36.325 0.00 0.00
C
ATOM 20761 HD1 TYR C 45   14.200 22.502 35.517 0.00 0.00
C
ATOM 20762 CE1 TYR C 45   13.568 22.452 37.537 0.00 0.00
C

ATOM 20763 HE1 TYR C 45 13.850 23.454 37.824 0.00 0.00
C
ATOM 20764 CZ TYR C 45 13.102 21.670 38.610 0.00 0.00
C
ATOM 20765 OH TYR C 45 12.766 22.082 39.862 0.00 0.00
C
ATOM 20766 HH TYR C 45 12.855 23.028 39.992 0.00 0.00
C
ATOM 20767 CD2 TYR C 45 13.163 19.750 37.174 0.00 0.00
C
ATOM 20768 HD2 TYR C 45 12.966 18.691 37.090 0.00 0.00
C
ATOM 20769 CE2 TYR C 45 12.794 20.319 38.391 0.00 0.00
C
ATOM 20770 HE2 TYR C 45 12.360 19.668 39.135 0.00 0.00
C
ATOM 20771 C TYR C 45 12.947 19.980 32.468 0.00 0.00
C
ATOM 20772 O TYR C 45 13.961 20.370 31.927 0.00 0.00
C
ATOM 20773 N ALA C 46 11.947 19.467 31.703 0.00 0.00
C
ATOM 20774 HN ALA C 46 11.074 19.317 32.160 0.00 0.00
C
ATOM 20775 CA ALA C 46 12.071 19.165 30.278 0.00 0.00
C
ATOM 20776 HA ALA C 46 12.843 18.427 30.119 0.00 0.00
C
ATOM 20777 CB ALA C 46 10.666 18.739 29.832 0.00 0.00
C
ATOM 20778 HB1 ALA C 46 10.739 18.264 28.830 0.00 0.00
C
ATOM 20779 HB2 ALA C 46 10.376 17.900 30.499 0.00 0.00
C
ATOM 20780 HB3 ALA C 46 9.849 19.492 29.822 0.00 0.00
C
ATOM 20781 C ALA C 46 12.429 20.370 29.431 0.00 0.00
C
ATOM 20782 O ALA C 46 13.099 20.288 28.396 0.00 0.00
C
ATOM 20783 N SER C 47 11.978 21.648 29.841 0.00 0.00
C
ATOM 20784 HN SER C 47 11.434 21.740 30.671 0.00 0.00
C
ATOM 20785 CA SER C 47 12.155 22.913 29.183 0.00 0.00
C
ATOM 20786 HA SER C 47 12.341 22.801 28.125 0.00 0.00
C
ATOM 20787 CB SER C 47 10.852 23.796 29.305 0.00 0.00
C
ATOM 20788 HB1 SER C 47 10.569 23.880 30.376 0.00 0.00
C
ATOM 20789 HB2 SER C 47 10.948 24.811 28.864 0.00 0.00
C

ATOM 20790 OG SER C 47   9.826 23.200 28.615 0.00 0.00
C
ATOM 20791 HG1 SER C 47   9.162 23.886 28.514 0.00 0.00
C
ATOM 20792 C   SER C 47   13.235 23.754 29.895 0.00 0.00
C
ATOM 20793 O   SER C 47   13.321 24.983 29.707 0.00 0.00
C
ATOM 20794 N   TYR C 48   14.162 23.170 30.743 0.00 0.00
C
ATOM 20795 HN TYR C 48   14.118 22.220 31.042 0.00 0.00
C
ATOM 20796 CA TYR C 48   15.329 23.780 31.422 0.00 0.00
C
ATOM 20797 HA TYR C 48   15.477 24.746 30.962 0.00 0.00
C
ATOM 20798 CB TYR C 48   15.121 23.915 32.957 0.00 0.00
C
ATOM 20799 HB1 TYR C 48   14.769 22.955 33.391 0.00 0.00
C
ATOM 20800 HB2 TYR C 48   16.064 24.176 33.485 0.00 0.00
C
ATOM 20801 CG TYR C 48   14.171 25.019 33.273 0.00 0.00
C
ATOM 20802 CD1 TYR C 48   14.463 26.389 33.358 0.00 0.00
C
ATOM 20803 HD1 TYR C 48   15.460 26.686 33.066 0.00 0.00
C
ATOM 20804 CE1 TYR C 48   13.519 27.351 33.811 0.00 0.00
C
ATOM 20805 HE1 TYR C 48   13.781 28.398 33.803 0.00 0.00
C
ATOM 20806 CZ TYR C 48   12.260 26.918 34.173 0.00 0.00
C
ATOM 20807 OH TYR C 48   11.339 27.845 34.546 0.00 0.00
C
ATOM 20808 HH TYR C 48   11.776 28.679 34.734 0.00 0.00
C
ATOM 20809 CD2 TYR C 48   12.916 24.616 33.819 0.00 0.00
C
ATOM 20810 HD2 TYR C 48   12.726 23.575 34.035 0.00 0.00
C
ATOM 20811 CE2 TYR C 48   11.954 25.625 34.107 0.00 0.00
C
ATOM 20812 HE2 TYR C 48   10.953 25.330 34.382 0.00 0.00
C
ATOM 20813 C   TYR C 48   16.481 22.858 31.139 0.00 0.00
C
ATOM 20814 O   TYR C 48   17.464 22.860 31.902 0.00 0.00
C
ATOM 20815 N   ALA C 49   16.301 22.072 30.083 0.00 0.00
C
ATOM 20816 HN ALA C 49   15.502 22.049 29.486 0.00 0.00
C

ATOM	20817	CA	ALA	C	49	17.396	21.313	29.557	0.00	0.00	C
ATOM	20818	HA	ALA	C	49	18.338	21.840	29.559	0.00	0.00	C
ATOM	20819	CB	ALA	C	49	17.591	20.000	30.388	0.00	0.00	C
ATOM	20820	HB1	ALA	C	49	18.099	20.293	31.332	0.00	0.00	C
ATOM	20821	HB2	ALA	C	49	16.649	19.451	30.600	0.00	0.00	C
ATOM	20822	HB3	ALA	C	49	18.239	19.239	29.902	0.00	0.00	C
ATOM	20823	C	ALA	C	49	17.211	21.019	28.062	0.00	0.00	C
ATOM	20824	O	ALA	C	49	16.076	21.266	27.516	0.00	0.00	C
ATOM	20825	N	THR	C	50	18.292	20.571	27.394	0.00	0.00	C
ATOM	20826	HN	THR	C	50	19.120	20.354	27.906	0.00	0.00	C
ATOM	20827	CA	THR	C	50	18.320	20.288	25.975	0.00	0.00	C
ATOM	20828	HA	THR	C	50	17.394	20.746	25.659	0.00	0.00	C
ATOM	20829	CB	THR	C	50	19.568	20.650	25.230	0.00	0.00	C
ATOM	20830	HB	THR	C	50	19.571	20.276	24.184	0.00	0.00	C
ATOM	20831	OG1	THR	C	50	20.649	20.160	25.931	0.00	0.00	C
ATOM	20832	HG1	THR	C	50	21.273	19.944	25.234	0.00	0.00	C
ATOM	20833	CG2	THR	C	50	19.660	22.162	25.323	0.00	0.00	C
ATOM	20834	HG21	THR	C	50	18.740	22.590	24.872	0.00	0.00	C
ATOM	20835	HG22	THR	C	50	19.734	22.429	26.399	0.00	0.00	C
ATOM	20836	HG23	THR	C	50	20.586	22.452	24.782	0.00	0.00	C
ATOM	20837	C	THR	C	50	18.034	18.788	25.929	0.00	0.00	C
ATOM	20838	O	THR	C	50	17.592	18.294	24.875	0.00	0.00	C
ATOM	20839	N	HSE	C	51	18.333	18.014	27.021	0.00	0.00	C
ATOM	20840	HN	HSE	C	51	18.783	18.546	27.734	0.00	0.00	C
ATOM	20841	CA	HSE	C	51	17.977	16.615	27.163	0.00	0.00	C
ATOM	20842	HA	HSE	C	51	17.241	16.429	26.395	0.00	0.00	C
ATOM	20843	CB	HSE	C	51	19.108	15.585	26.812	0.00	0.00	C

ATOM 20844 HB1 HSE C 51   20.027 15.948 27.320 0.00 0.00
C
ATOM 20845 HB2 HSE C 51   18.786 14.544 27.031 0.00 0.00
C
ATOM 20846 ND1 HSE C 51   18.911 14.649 24.502 0.00 0.00
C
ATOM 20847 CG HSE C 51   19.478 15.587 25.365 0.00 0.00
C
ATOM 20848 CE1 HSE C 51   19.561 14.777 23.330 0.00 0.00
C
ATOM 20849 HE1 HSE C 51   19.164 14.195 22.499 0.00 0.00
C
ATOM 20850 NE2 HSE C 51   20.539 15.687 23.450 0.00 0.00
C
ATOM 20851 HE2 HSE C 51   21.304 15.815 22.818 0.00 0.00
C
ATOM 20852 CD2 HSE C 51   20.518 16.145 24.709 0.00 0.00
C
ATOM 20853 HD2 HSE C 51   21.337 16.766 25.050 0.00 0.00
C
ATOM 20854 C   HSE C 51   17.340 16.349 28.487 0.00 0.00
C
ATOM 20855 O   HSE C 51   17.979 16.558 29.502 0.00 0.00
C
ATOM 20856 N   ALA C 52   16.077 15.917 28.592 0.00 0.00
C
ATOM 20857 HN ALA C 52   15.549 15.727 27.768 0.00 0.00
C
ATOM 20858 CA ALA C 52   15.498 15.492 29.839 0.00 0.00
C
ATOM 20859 HA ALA C 52   16.216 15.610 30.637 0.00 0.00
C
ATOM 20860 CB ALA C 52   14.292 16.515 29.998 0.00 0.00
C
ATOM 20861 HB1 ALA C 52   14.601 17.563 30.201 0.00 0.00
C
ATOM 20862 HB2 ALA C 52   13.598 16.416 29.136 0.00 0.00
C
ATOM 20863 HB3 ALA C 52   13.746 16.199 30.912 0.00 0.00
C
ATOM 20864 C   ALA C 52   15.150 14.035 29.659 0.00 0.00
C
ATOM 20865 O   ALA C 52   14.451 13.689 28.732 0.00 0.00
C
ATOM 20866 N   VAL C 53   15.672 13.200 30.586 0.00 0.00
C
ATOM 20867 HN VAL C 53   16.338 13.600 31.210 0.00 0.00
C
ATOM 20868 CA VAL C 53   15.635 11.789 30.450 0.00 0.00
C
ATOM 20869 HA VAL C 53   15.081 11.588 29.545 0.00 0.00
C
ATOM 20870 CB VAL C 53   17.034 11.183 30.128 0.00 0.00
C

```
ATOM  20871  HB  VAL C  53      17.782  11.360  30.931  0.00  0.00
C
ATOM  20872  CG1 VAL C  53      16.871   9.680  29.846  0.00  0.00
C
ATOM  20873 HG11 VAL C  53      17.797   9.287  29.374  0.00  0.00
C
ATOM  20874 HG12 VAL C  53      16.610   9.142  30.783  0.00  0.00
C
ATOM  20875 HG13 VAL C  53      16.036   9.407  29.167  0.00  0.00
C
ATOM  20876  CG2 VAL C  53      17.505  11.928  28.869  0.00  0.00
C
ATOM  20877 HG21 VAL C  53      16.694  11.790  28.123  0.00  0.00
C
ATOM  20878 HG22 VAL C  53      17.638  13.013  29.064  0.00  0.00
C
ATOM  20879 HG23 VAL C  53      18.449  11.494  28.475  0.00  0.00
C
ATOM  20880  C   VAL C  53      15.072  11.143  31.656  0.00  0.00
C
ATOM  20881  O   VAL C  53      15.396  11.383  32.825  0.00  0.00
C
ATOM  20882  N   ILE C  54      14.124  10.197  31.418  0.00  0.00
C
ATOM  20883  HN  ILE C  54      13.762  10.055  30.500  0.00  0.00
C
ATOM  20884  CA  ILE C  54      13.631   9.298  32.463  0.00  0.00
C
ATOM  20885  HA  ILE C  54      13.625   9.751  33.443  0.00  0.00
C
ATOM  20886  CB  ILE C  54      12.123   8.850  32.339  0.00  0.00
C
ATOM  20887  HB  ILE C  54      11.933   8.383  31.349  0.00  0.00
C
ATOM  20888  CG2 ILE C  54      11.732   7.979  33.519  0.00  0.00
C
ATOM  20889 HG21 ILE C  54      12.271   7.020  33.673  0.00  0.00
C
ATOM  20890 HG22 ILE C  54      11.797   8.511  34.493  0.00  0.00
C
ATOM  20891 HG23 ILE C  54      10.666   7.669  33.566  0.00  0.00
C
ATOM  20892  CG1 ILE C  54      11.314  10.170  32.279  0.00  0.00
C
ATOM  20893 HG11 ILE C  54      11.267  10.598  33.304  0.00  0.00
C
ATOM  20894 HG12 ILE C  54      11.832  10.903  31.624  0.00  0.00
C
ATOM  20895  CD  ILE C  54       9.928  10.011  31.562  0.00  0.00
C
ATOM  20896  HD1 ILE C  54       9.372   9.227  32.119  0.00  0.00
C
ATOM  20897  HD2 ILE C  54       9.348  10.939  31.754  0.00  0.00
C
```

ATOM 20898 HD3 ILE C 54 10.014 9.907 30.459 0.00 0.00
C
ATOM 20899 C ILE C 54 14.356 7.950 32.386 0.00 0.00
C
ATOM 20900 O ILE C 54 14.220 7.169 31.486 0.00 0.00
C
ATOM 20901 N ALA C 55 15.214 7.798 33.454 0.00 0.00
C
ATOM 20902 HN ALA C 55 15.338 8.500 34.151 0.00 0.00
C
ATOM 20903 CA ALA C 55 16.131 6.709 33.384 0.00 0.00
C
ATOM 20904 HA ALA C 55 15.718 5.850 32.877 0.00 0.00
C
ATOM 20905 CB ALA C 55 17.379 7.095 32.499 0.00 0.00
C
ATOM 20906 HB1 ALA C 55 17.130 7.261 31.429 0.00 0.00
C
ATOM 20907 HB2 ALA C 55 17.971 7.973 32.836 0.00 0.00
C
ATOM 20908 HB3 ALA C 55 18.088 6.269 32.722 0.00 0.00
C
ATOM 20909 C ALA C 55 16.731 6.336 34.772 0.00 0.00
C
ATOM 20910 O ALA C 55 16.745 7.124 35.706 0.00 0.00
C
ATOM 20911 N ASN C 56 17.120 5.060 34.996 0.00 0.00
C
ATOM 20912 HN ASN C 56 17.012 4.360 34.295 0.00 0.00
C
ATOM 20913 CA ASN C 56 17.671 4.581 36.232 0.00 0.00
C
ATOM 20914 HA ASN C 56 17.296 5.197 37.036 0.00 0.00
C
ATOM 20915 CB ASN C 56 17.224 3.092 36.325 0.00 0.00
C
ATOM 20916 HB1 ASN C 56 16.137 2.862 36.304 0.00 0.00
C
ATOM 20917 HB2 ASN C 56 17.642 2.399 35.564 0.00 0.00
C
ATOM 20918 CG ASN C 56 17.655 2.622 37.801 0.00 0.00
C
ATOM 20919 OD1 ASN C 56 17.026 2.969 38.794 0.00 0.00
C
ATOM 20920 ND2 ASN C 56 18.530 1.653 37.844 0.00 0.00
C
ATOM 20921 HD21 ASN C 56 18.692 1.213 38.726 0.00 0.00
C
ATOM 20922 HD22 ASN C 56 19.084 1.594 37.013 0.00 0.00
C
ATOM 20923 C ASN C 56 19.131 4.782 36.177 0.00 0.00
C
ATOM 20924 O ASN C 56 19.893 4.281 35.350 0.00 0.00
C

ATOM 20925 N ALA C 57 19.573 5.579 37.152 0.00 0.00
C
ATOM 20926 HN ALA C 57 18.965 5.968 37.839 0.00 0.00
C
ATOM 20927 CA ALA C 57 20.973 5.816 37.228 0.00 0.00
C
ATOM 20928 HA ALA C 57 21.289 6.220 36.277 0.00 0.00
C
ATOM 20929 CB ALA C 57 21.105 6.951 38.235 0.00 0.00
C
ATOM 20930 HB1 ALA C 57 22.162 7.266 38.372 0.00 0.00
C
ATOM 20931 HB2 ALA C 57 20.568 7.829 37.816 0.00 0.00
C
ATOM 20932 HB3 ALA C 57 20.721 6.634 39.227 0.00 0.00
C
ATOM 20933 C ALA C 57 21.863 4.609 37.602 0.00 0.00
C
ATOM 20934 O ALA C 57 22.947 4.487 37.117 0.00 0.00
C
ATOM 20935 N THR C 58 21.388 3.723 38.478 0.00 0.00
C
ATOM 20936 HN THR C 58 20.491 3.874 38.888 0.00 0.00
C
ATOM 20937 CA THR C 58 22.196 2.782 39.198 0.00 0.00
C
ATOM 20938 HA THR C 58 23.072 3.338 39.496 0.00 0.00
C
ATOM 20939 CB THR C 58 21.468 2.215 40.457 0.00 0.00
C
ATOM 20940 HB THR C 58 21.992 1.377 40.964 0.00 0.00
C
ATOM 20941 OG1 THR C 58 20.055 1.850 40.267 0.00 0.00
C
ATOM 20942 HG1 THR C 58 19.747 1.371 41.040 0.00 0.00
C
ATOM 20943 CG2 THR C 58 21.285 3.411 41.387 0.00 0.00
C
ATOM 20944 HG21 THR C 58 22.268 3.866 41.634 0.00 0.00
C
ATOM 20945 HG22 THR C 58 20.717 4.189 40.833 0.00 0.00
C
ATOM 20946 HG23 THR C 58 20.827 3.058 42.336 0.00 0.00
C
ATOM 20947 C THR C 58 22.462 1.547 38.274 0.00 0.00
C
ATOM 20948 O THR C 58 22.472 0.446 38.659 0.00 0.00
C
ATOM 20949 N GLU C 59 22.714 1.796 36.989 0.00 0.00
C
ATOM 20950 HN GLU C 59 22.625 2.745 36.696 0.00 0.00
C
ATOM 20951 CA GLU C 59 22.915 0.828 36.001 0.00 0.00
C

ATOM 20952 HA GLU C 59 23.553 0.085 36.455 0.00 0.00
C
ATOM 20953 CB GLU C 59 21.673 0.294 35.359 0.00 0.00
C
ATOM 20954 HB1 GLU C 59 21.087 -0.092 36.220 0.00 0.00
C
ATOM 20955 HB2 GLU C 59 21.097 1.107 34.867 0.00 0.00
C
ATOM 20956 CG GLU C 59 21.773 -0.737 34.203 0.00 0.00
C
ATOM 20957 HG1 GLU C 59 20.787 -1.141 33.889 0.00 0.00
C
ATOM 20958 HG2 GLU C 59 22.217 -0.321 33.273 0.00 0.00
C
ATOM 20959 CD GLU C 59 22.505 -1.966 34.562 0.00 0.00
C
ATOM 20960 OE1 GLU C 59 21.945 -2.732 35.467 0.00 0.00
C
ATOM 20961 OE2 GLU C 59 23.672 -2.177 34.129 0.00 0.00
C
ATOM 20962 C GLU C 59 23.910 1.345 34.909 0.00 0.00
C
ATOM 20963 O GLU C 59 23.645 2.346 34.183 0.00 0.00
C
ATOM 20964 N GLU C 60 25.026 0.525 34.655 0.00 0.00
C
ATOM 20965 HN GLU C 60 25.224 -0.281 35.208 0.00 0.00
C
ATOM 20966 CA GLU C 60 25.927 0.927 33.570 0.00 0.00
C
ATOM 20967 HA GLU C 60 26.433 1.840 33.845 0.00 0.00
C
ATOM 20968 CB GLU C 60 26.908 -0.281 33.475 0.00 0.00
C
ATOM 20969 HB1 GLU C 60 27.466 -0.381 34.430 0.00 0.00
C
ATOM 20970 HB2 GLU C 60 26.256 -1.156 33.267 0.00 0.00
C
ATOM 20971 CG GLU C 60 28.101 -0.127 32.372 0.00 0.00
C
ATOM 20972 HG1 GLU C 60 27.840 0.293 31.377 0.00 0.00
C
ATOM 20973 HG2 GLU C 60 28.874 0.541 32.810 0.00 0.00
C
ATOM 20974 CD GLU C 60 28.603 -1.550 32.194 0.00 0.00
C
ATOM 20975 OE1 GLU C 60 29.092 -2.106 33.259 0.00 0.00
C
ATOM 20976 OE2 GLU C 60 28.507 -2.170 31.138 0.00 0.00
C
ATOM 20977 C GLU C 60 25.243 0.977 32.208 0.00 0.00
C
ATOM 20978 O GLU C 60 25.363 1.935 31.440 0.00 0.00
C

ATOM 20979 N ASN C 61 24.497 -0.071 31.868 0.00 0.00
C
ATOM 20980 HN ASN C 61 24.336 -0.745 32.586 0.00 0.00
C
ATOM 20981 CA ASN C 61 23.819 -0.274 30.590 0.00 0.00
C
ATOM 20982 HA ASN C 61 24.586 -0.191 29.833 0.00 0.00
C
ATOM 20983 CB ASN C 61 23.059 -1.582 30.565 0.00 0.00
C
ATOM 20984 HB1 ASN C 61 23.801 -2.394 30.719 0.00 0.00
C
ATOM 20985 HB2 ASN C 61 22.227 -1.572 31.301 0.00 0.00
C
ATOM 20986 CG ASN C 61 22.602 -1.810 29.070 0.00 0.00
C
ATOM 20987 OD1 ASN C 61 23.534 -1.857 28.263 0.00 0.00
C
ATOM 20988 ND2 ASN C 61 21.344 -1.974 28.727 0.00 0.00
C
ATOM 20989 HD21 ASN C 61 21.140 -1.763 27.771 0.00 0.00
C
ATOM 20990 HD22 ASN C 61 20.535 -1.934 29.313 0.00 0.00
C
ATOM 20991 C ASN C 61 22.855 0.933 30.301 0.00 0.00
C
ATOM 20992 O ASN C 61 22.952 1.433 29.160 0.00 0.00
C
ATOM 20993 N GLU C 62 22.057 1.586 31.231 0.00 0.00
C
ATOM 20994 HN GLU C 62 22.207 1.218 32.145 0.00 0.00
C
ATOM 20995 CA GLU C 62 21.279 2.746 31.080 0.00 0.00
C
ATOM 20996 HA GLU C 62 20.647 2.610 30.215 0.00 0.00
C
ATOM 20997 CB GLU C 62 20.375 2.976 32.343 0.00 0.00
C
ATOM 20998 HB1 GLU C 62 20.979 3.207 33.246 0.00 0.00
C
ATOM 20999 HB2 GLU C 62 19.795 3.916 32.217 0.00 0.00
C
ATOM 21000 CG GLU C 62 19.447 1.809 32.683 0.00 0.00
C
ATOM 21001 HG1 GLU C 62 20.039 0.907 32.949 0.00 0.00
C
ATOM 21002 HG2 GLU C 62 19.008 2.072 33.669 0.00 0.00
C
ATOM 21003 CD GLU C 62 18.396 1.564 31.612 0.00 0.00
C
ATOM 21004 OE1 GLU C 62 17.241 1.809 31.905 0.00 0.00
C
ATOM 21005 OE2 GLU C 62 18.728 1.024 30.515 0.00 0.00
C

ATOM 21006 C   GLU C 62   22.083   3.972 30.806 0.00 0.00
C
ATOM 21007 O   GLU C 62   21.765   4.805 29.957 0.00 0.00
C
ATOM 21008 N   LEU C 63   23.184   4.066 31.490 0.00 0.00
C
ATOM 21009 HN LEU C 63   23.387   3.452 32.249 0.00 0.00
C
ATOM 21010 CA LEU C 63   24.054   5.187 31.352 0.00 0.00
C
ATOM 21011 HA LEU C 63   23.432   6.068 31.422 0.00 0.00
C
ATOM 21012 CB LEU C 63   25.135   5.329 32.533 0.00 0.00
C
ATOM 21013 HB1 LEU C 63   25.668   4.356 32.590 0.00 0.00
C
ATOM 21014 HB2 LEU C 63   25.950   6.009 32.204 0.00 0.00
C
ATOM 21015 CG LEU C 63   24.670   5.500 33.944 0.00 0.00
C
ATOM 21016 HG LEU C 63   23.769   4.870 34.108 0.00 0.00
C
ATOM 21017 CD1 LEU C 63   25.675   5.266 35.071 0.00 0.00
C
ATOM 21018 HD11 LEU C 63   26.369   6.119 35.234 0.00 0.00
C
ATOM 21019 HD12 LEU C 63   25.094   5.073 35.999 0.00 0.00
C
ATOM 21020 HD13 LEU C 63   26.219   4.310 34.916 0.00 0.00
C
ATOM 21021 CD2 LEU C 63   24.198   6.950 34.175 0.00 0.00
C
ATOM 21022 HD21 LEU C 63   25.087   7.612 34.243 0.00 0.00
C
ATOM 21023 HD22 LEU C 63   23.347   7.174 33.496 0.00 0.00
C
ATOM 21024 HD23 LEU C 63   23.756   7.076 35.186 0.00 0.00
C
ATOM 21025 C   LEU C 63   24.667   5.348 29.959 0.00 0.00
C
ATOM 21026 O   LEU C 63   24.663   6.448 29.361 0.00 0.00
C
ATOM 21027 N   LEU C 64   25.193   4.202 29.433 0.00 0.00
C
ATOM 21028 HN LEU C 64   25.165   3.554 30.191 0.00 0.00
C
ATOM 21029 CA LEU C 64   25.697   4.010 28.106 0.00 0.00
C
ATOM 21030 HA LEU C 64   26.392   4.791 27.836 0.00 0.00
C
ATOM 21031 CB LEU C 64   26.277   2.506 27.869 0.00 0.00
C
ATOM 21032 HB1 LEU C 64   25.446   1.843 28.192 0.00 0.00
C

ATOM 21033 HB2 LEU C 64 26.470 2.394 26.781 0.00 0.00
C
ATOM 21034 CG LEU C 64 27.571 2.204 28.634 0.00 0.00
C
ATOM 21035 HG LEU C 64 27.390 2.520 29.684 0.00 0.00
C
ATOM 21036 CD1 LEU C 64 27.956 0.721 28.689 0.00 0.00
C
ATOM 21037 HD11 LEU C 64 28.764 0.544 29.431 0.00 0.00
C
ATOM 21038 HD12 LEU C 64 27.127 0.055 29.010 0.00 0.00
C
ATOM 21039 HD13 LEU C 64 28.350 0.435 27.690 0.00 0.00
C
ATOM 21040 CD2 LEU C 64 28.626 3.114 28.044 0.00 0.00
C
ATOM 21041 HD21 LEU C 64 28.471 4.160 28.384 0.00 0.00
C
ATOM 21042 HD22 LEU C 64 29.562 2.885 28.597 0.00 0.00
C
ATOM 21043 HD23 LEU C 64 28.832 2.976 26.961 0.00 0.00
C
ATOM 21044 C LEU C 64 24.683 4.146 27.010 0.00 0.00
C
ATOM 21045 O LEU C 64 24.883 4.647 25.915 0.00 0.00
C
ATOM 21046 N SER C 65 23.475 3.865 27.331 0.00 0.00
C
ATOM 21047 HN SER C 65 23.376 3.680 28.305 0.00 0.00
C
ATOM 21048 CA SER C 65 22.241 4.168 26.562 0.00 0.00
C
ATOM 21049 HA SER C 65 22.394 3.796 25.559 0.00 0.00
C
ATOM 21050 CB SER C 65 21.010 3.478 27.234 0.00 0.00
C
ATOM 21051 HB1 SER C 65 21.344 2.685 27.937 0.00 0.00
C
ATOM 21052 HB2 SER C 65 20.286 4.147 27.747 0.00 0.00
C
ATOM 21053 OG SER C 65 20.342 2.814 26.142 0.00 0.00
C
ATOM 21054 HG1 SER C 65 19.481 2.501 26.429 0.00 0.00
C
ATOM 21055 C SER C 65 21.855 5.581 26.450 0.00 0.00
C
ATOM 21056 O SER C 65 21.628 6.069 25.386 0.00 0.00
C
ATOM 21057 N LEU C 66 21.911 6.444 27.521 0.00 0.00
C
ATOM 21058 HN LEU C 66 22.040 6.032 28.420 0.00 0.00
C
ATOM 21059 CA LEU C 66 21.784 7.949 27.356 0.00 0.00
C

ATOM 21060 HA LEU C 66   21.146   8.064 26.492 0.00 0.00
C
ATOM 21061 CB LEU C 66   21.037   8.639 28.484 0.00 0.00
C
ATOM 21062 HB1 LEU C 66   20.944   9.683 28.116 0.00 0.00
C
ATOM 21063 HB2 LEU C 66   20.013   8.248 28.307 0.00 0.00
C
ATOM 21064 CG LEU C 66   21.525   8.446 29.962 0.00 0.00
C
ATOM 21065 HG LEU C 66   21.806   7.384 30.127 0.00 0.00
C
ATOM 21066 CD1 LEU C 66   22.869   9.213 30.377 0.00 0.00
C
ATOM 21067 HD11 LEU C 66   22.748 10.305 30.212 0.00 0.00
C
ATOM 21068 HD12 LEU C 66   23.073   8.923 31.430 0.00 0.00
C
ATOM 21069 HD13 LEU C 66   23.739   9.038 29.708 0.00 0.00
C
ATOM 21070 CD2 LEU C 66   20.492   8.897 30.977 0.00 0.00
C
ATOM 21071 HD21 LEU C 66   20.971   8.579 31.928 0.00 0.00
C
ATOM 21072 HD22 LEU C 66   20.379 10.000 30.919 0.00 0.00
C
ATOM 21073 HD23 LEU C 66   19.539   8.338 30.861 0.00 0.00
C
ATOM 21074 C   LEU C 66   23.090   8.557 26.849 0.00 0.00
C
ATOM 21075 O   LEU C 66   23.118   9.673 26.366 0.00 0.00
C
ATOM 21076 N   GLY C 67   24.225   7.898 26.907 0.00 0.00
C
ATOM 21077 HN GLY C 67   24.297   6.993 27.319 0.00 0.00
C
ATOM 21078 CA GLY C 67   25.432   8.343 26.258 0.00 0.00
C
ATOM 21079 HA1 GLY C 67   25.275   9.006 25.419 0.00 0.00
C
ATOM 21080 HA2 GLY C 67   26.051   7.476 26.083 0.00 0.00
C
ATOM 21081 C   GLY C 67   26.193   9.274 27.183 0.00 0.00
C
ATOM 21082 O   GLY C 67   26.712 10.286 26.773 0.00 0.00
C
ATOM 21083 N   ILE C 68   26.356   8.855 28.453 0.00 0.00
C
ATOM 21084 HN ILE C 68   26.010   7.966 28.745 0.00 0.00
C
ATOM 21085 CA ILE C 68   26.994   9.636 29.484 0.00 0.00
C
ATOM 21086 HA ILE C 68   26.552 10.619 29.413 0.00 0.00
C

```
ATOM  21087  CB  ILE C  68      26.679   9.017  30.910  0.00  0.00
C
ATOM  21088  HB  ILE C  68      25.620   8.686  30.853  0.00  0.00
C
ATOM  21089  CG2 ILE C  68      27.566   7.849  31.162  0.00  0.00
C
ATOM  21090 HG21 ILE C  68      27.326   7.071  30.407  0.00  0.00
C
ATOM  21091 HG22 ILE C  68      28.613   8.217  31.106  0.00  0.00
C
ATOM  21092 HG23 ILE C  68      27.547   7.448  32.198  0.00  0.00
C
ATOM  21093  CG1 ILE C  68      26.775  10.088  32.040  0.00  0.00
C
ATOM  21094 HG11 ILE C  68      26.800   9.549  33.011  0.00  0.00
C
ATOM  21095 HG12 ILE C  68      27.706  10.687  31.952  0.00  0.00
C
ATOM  21096  CD  ILE C  68      25.649  11.093  31.994  0.00  0.00
C
ATOM  21097  HD1 ILE C  68      25.509  11.559  30.996  0.00  0.00
C
ATOM  21098  HD2 ILE C  68      24.691  10.610  32.284  0.00  0.00
C
ATOM  21099  HD3 ILE C  68      25.831  11.920  32.713  0.00  0.00
C
ATOM  21100  C   ILE C  68      28.530   9.891  29.337  0.00  0.00
C
ATOM  21101  O   ILE C  68      29.029  10.865  29.849  0.00  0.00
C
ATOM  21102  N   ARG C  69      29.336   9.078  28.635  0.00  0.00
C
ATOM  21103  HN  ARG C  69      28.940   8.287  28.175  0.00  0.00
C
ATOM  21104  CA  ARG C  69      30.806   9.156  28.633  0.00  0.00
C
ATOM  21105  HA  ARG C  69      31.106   9.354  29.651  0.00  0.00
C
ATOM  21106  CB  ARG C  69      31.492   7.882  27.952  0.00  0.00
C
ATOM  21107  HB1 ARG C  69      31.183   6.967  28.502  0.00  0.00
C
ATOM  21108  HB2 ARG C  69      31.160   7.827  26.893  0.00  0.00
C
ATOM  21109  CG  ARG C  69      32.985   7.828  27.892  0.00  0.00
C
ATOM  21110  HG1 ARG C  69      33.434   8.751  27.466  0.00  0.00
C
ATOM  21111  HG2 ARG C  69      33.372   7.660  28.919  0.00  0.00
C
ATOM  21112  CD  ARG C  69      33.485   6.785  26.948  0.00  0.00
C
ATOM  21113  HD1 ARG C  69      33.147   5.772  27.257  0.00  0.00
C
```

ATOM 21114 HD2 ARG C 69 33.020 6.964 25.955 0.00 0.00
C
ATOM 21115 NE ARG C 69 34.919 6.704 26.915 0.00 0.00
C
ATOM 21116 HE ARG C 69 35.258 6.710 27.856 0.00 0.00
C
ATOM 21117 CZ ARG C 69 35.691 6.776 25.833 0.00 0.00
C
ATOM 21118 NH1 ARG C 69 35.216 7.223 24.682 0.00 0.00
C
ATOM 21119 HH11 ARG C 69 35.809 7.163 23.879 0.00 0.00
C
ATOM 21120 HH12 ARG C 69 34.442 7.851 24.599 0.00 0.00
C
ATOM 21121 NH2 ARG C 69 36.929 6.339 25.890 0.00 0.00
C
ATOM 21122 HH21 ARG C 69 37.484 6.496 25.074 0.00 0.00
C
ATOM 21123 HH22 ARG C 69 37.455 6.608 26.697 0.00 0.00
C
ATOM 21124 C ARG C 69 31.317 10.411 27.946 0.00 0.00
C
ATOM 21125 O ARG C 69 32.452 10.878 28.239 0.00 0.00
C
ATOM 21126 N ASN C 70 30.587 11.168 27.066 0.00 0.00
C
ATOM 21127 HN ASN C 70 29.659 10.919 26.801 0.00 0.00
C
ATOM 21128 CA ASN C 70 31.202 12.328 26.401 0.00 0.00
C
ATOM 21129 HA ASN C 70 32.244 12.121 26.211 0.00 0.00
C
ATOM 21130 CB ASN C 70 30.442 12.836 25.129 0.00 0.00
C
ATOM 21131 HB1 ASN C 70 29.343 12.981 25.193 0.00 0.00
C
ATOM 21132 HB2 ASN C 70 30.966 13.757 24.795 0.00 0.00
C
ATOM 21133 CG ASN C 70 30.699 11.812 24.061 0.00 0.00
C
ATOM 21134 OD1 ASN C 70 31.846 11.503 23.708 0.00 0.00
C
ATOM 21135 ND2 ASN C 70 29.622 11.386 23.336 0.00 0.00
C
ATOM 21136 HD21 ASN C 70 29.784 10.773 22.563 0.00 0.00
C
ATOM 21137 HD22 ASN C 70 28.724 11.521 23.755 0.00 0.00
C
ATOM 21138 C ASN C 70 31.143 13.571 27.313 0.00 0.00
C
ATOM 21139 O ASN C 70 31.710 14.594 27.127 0.00 0.00
C
ATOM 21140 N PHE C 71 30.314 13.462 28.407 0.00 0.00
C

ATOM 21141 HN PHE C 71 29.981 12.576 28.721 0.00 0.00
C
ATOM 21142 CA PHE C 71 30.192 14.509 29.378 0.00 0.00
C
ATOM 21143 HA PHE C 71 30.023 15.436 28.850 0.00 0.00
C
ATOM 21144 CB PHE C 71 28.935 14.425 30.264 0.00 0.00
C
ATOM 21145 HB1 PHE C 71 28.855 13.371 30.605 0.00 0.00
C
ATOM 21146 HB2 PHE C 71 28.989 14.994 31.217 0.00 0.00
C
ATOM 21147 CG PHE C 71 27.719 14.639 29.436 0.00 0.00
C
ATOM 21148 CD1 PHE C 71 27.330 13.751 28.429 0.00 0.00
C
ATOM 21149 HD1 PHE C 71 27.919 12.884 28.172 0.00 0.00
C
ATOM 21150 CE1 PHE C 71 26.050 13.952 27.782 0.00 0.00
C
ATOM 21151 HE1 PHE C 71 25.617 13.253 27.081 0.00 0.00
C
ATOM 21152 CZ PHE C 71 25.354 15.104 28.077 0.00 0.00
C
ATOM 21153 HZ PHE C 71 24.400 15.323 27.619 0.00 0.00
C
ATOM 21154 CD2 PHE C 71 27.008 15.782 29.611 0.00 0.00
C
ATOM 21155 HD2 PHE C 71 27.383 16.535 30.289 0.00 0.00
C
ATOM 21156 CE2 PHE C 71 25.866 16.026 28.946 0.00 0.00
C
ATOM 21157 HE2 PHE C 71 25.205 16.860 29.129 0.00 0.00
C
ATOM 21158 C PHE C 71 31.447 14.630 30.191 0.00 0.00
C
ATOM 21159 O PHE C 71 32.069 13.603 30.583 0.00 0.00
C
ATOM 21160 N GLU C 72 31.924 15.868 30.472 0.00 0.00
C
ATOM 21161 HN GLU C 72 31.478 16.677 30.098 0.00 0.00
C
ATOM 21162 CA GLU C 72 33.129 16.048 31.178 0.00 0.00
C
ATOM 21163 HA GLU C 72 33.741 15.159 31.148 0.00 0.00
C
ATOM 21164 CB GLU C 72 33.970 17.292 30.638 0.00 0.00
C
ATOM 21165 HB1 GLU C 72 34.005 17.248 29.529 0.00 0.00
C
ATOM 21166 HB2 GLU C 72 33.336 18.141 30.971 0.00 0.00
C
ATOM 21167 CG GLU C 72 35.354 17.473 31.257 0.00 0.00
C

ATOM 21168 HG1 GLU C 72   35.104 18.006 32.199 0.00 0.00
C
ATOM 21169 HG2 GLU C 72   35.887 16.562 31.604 0.00 0.00
C
ATOM 21170 CD GLU C 72   36.278 18.369 30.432 0.00 0.00
C
ATOM 21171 OE1 GLU C 72   35.951 18.407 29.149 0.00 0.00
C
ATOM 21172 OE2 GLU C 72   37.216 19.074 30.835 0.00 0.00
C
ATOM 21173 C   GLU C 72   32.861 16.290 32.657 0.00 0.00
C
ATOM 21174 O   GLU C 72   33.654 15.889 33.505 0.00 0.00
C
ATOM 21175 N   TYR C 73   31.608 16.772 32.991 0.00 0.00
C
ATOM 21176 HN TYR C 73   30.970 17.019 32.266 0.00 0.00
C
ATOM 21177 CA TYR C 73   31.189 16.901 34.337 0.00 0.00
C
ATOM 21178 HA TYR C 73   31.829 16.353 35.012 0.00 0.00
C
ATOM 21179 CB TYR C 73   31.069 18.374 34.754 0.00 0.00
C
ATOM 21180 HB1 TYR C 73   30.479 18.939 34.000 0.00 0.00
C
ATOM 21181 HB2 TYR C 73   30.572 18.496 35.740 0.00 0.00
C
ATOM 21182 CG TYR C 73   32.470 19.017 34.830 0.00 0.00
C
ATOM 21183 CD1 TYR C 73   33.197 19.531 33.729 0.00 0.00
C
ATOM 21184 HD1 TYR C 73   32.841 19.527 32.710 0.00 0.00
C
ATOM 21185 CE1 TYR C 73   34.484 20.098 33.894 0.00 0.00
C
ATOM 21186 HE1 TYR C 73   35.146 20.398 33.095 0.00 0.00
C
ATOM 21187 CZ TYR C 73   34.938 20.246 35.182 0.00 0.00
C
ATOM 21188 OH TYR C 73   36.191 20.819 35.419 0.00 0.00
C
ATOM 21189 HH TYR C 73   36.622 20.917 34.566 0.00 0.00
C
ATOM 21190 CD2 TYR C 73   32.985 19.240 36.101 0.00 0.00
C
ATOM 21191 HD2 TYR C 73   32.398 18.884 36.934 0.00 0.00
C
ATOM 21192 CE2 TYR C 73   34.217 19.854 36.294 0.00 0.00
C
ATOM 21193 HE2 TYR C 73   34.667 19.977 37.268 0.00 0.00
C
ATOM 21194 C   TYR C 73   29.837 16.265 34.529 0.00 0.00
C

ATOM 21195 O TYR C 73 28.918 16.663 33.825 0.00 0.00
C
ATOM 21196 N VAL C 74 29.737 15.342 35.513 0.00 0.00
C
ATOM 21197 HN VAL C 74 30.499 14.982 36.047 0.00 0.00
C
ATOM 21198 CA VAL C 74 28.440 14.900 36.010 0.00 0.00
C
ATOM 21199 HA VAL C 74 27.667 15.363 35.415 0.00 0.00
C
ATOM 21200 CB VAL C 74 28.281 13.375 35.832 0.00 0.00
C
ATOM 21201 HB VAL C 74 29.002 12.721 36.367 0.00 0.00
C
ATOM 21202 CG1 VAL C 74 26.803 13.033 36.221 0.00 0.00
C
ATOM 21203 HG11 VAL C 74 26.456 12.036 35.877 0.00 0.00
C
ATOM 21204 HG12 VAL C 74 26.674 13.254 37.302 0.00 0.00
C
ATOM 21205 HG13 VAL C 74 26.192 13.790 35.684 0.00 0.00
C
ATOM 21206 CG2 VAL C 74 28.653 13.140 34.316 0.00 0.00
C
ATOM 21207 HG21 VAL C 74 28.015 12.300 33.967 0.00 0.00
C
ATOM 21208 HG22 VAL C 74 28.239 14.013 33.768 0.00 0.00
C
ATOM 21209 HG23 VAL C 74 29.753 13.089 34.168 0.00 0.00
C
ATOM 21210 C VAL C 74 28.233 15.198 37.483 0.00 0.00
C
ATOM 21211 O VAL C 74 29.140 14.992 38.324 0.00 0.00
C
ATOM 21212 N ILE C 75 27.046 15.704 37.811 0.00 0.00
C
ATOM 21213 HN ILE C 75 26.385 16.000 37.126 0.00 0.00
C
ATOM 21214 CA ILE C 75 26.648 15.953 39.196 0.00 0.00
C
ATOM 21215 HA ILE C 75 27.435 15.689 39.886 0.00 0.00
C
ATOM 21216 CB ILE C 75 26.268 17.426 39.494 0.00 0.00
C
ATOM 21217 HB ILE C 75 27.146 17.966 39.079 0.00 0.00
C
ATOM 21218 CG2 ILE C 75 25.011 17.792 38.649 0.00 0.00
C
ATOM 21219 HG21 ILE C 75 24.072 17.312 38.999 0.00 0.00
C
ATOM 21220 HG22 ILE C 75 24.689 18.838 38.842 0.00 0.00
C
ATOM 21221 HG23 ILE C 75 25.170 17.631 37.561 0.00 0.00
C

```
ATOM  21222  CG1 ILE C  75      26.244  17.576  41.040  0.00  0.00
C
ATOM  21223 HG11 ILE C  75      25.317  17.251  41.558  0.00  0.00
C
ATOM  21224 HG12 ILE C  75      27.035  16.986  41.550  0.00  0.00
C
ATOM  21225  CD  ILE C  75      26.529  19.093  41.399  0.00  0.00
C
ATOM  21226  HD1 ILE C  75      26.028  19.858  40.768  0.00  0.00
C
ATOM  21227  HD2 ILE C  75      26.351  19.239  42.486  0.00  0.00
C
ATOM  21228  HD3 ILE C  75      27.610  19.311  41.266  0.00  0.00
C
ATOM  21229  C   ILE C  75      25.607  14.914  39.529  0.00  0.00
C
ATOM  21230  O   ILE C  75      24.462  14.889  38.994  0.00  0.00
C
ATOM  21231  N   VAL C  76      25.892  14.081  40.579  0.00  0.00
C
ATOM  21232  HN  VAL C  76      26.779  14.216  41.015  0.00  0.00
C
ATOM  21233  CA  VAL C  76      25.066  13.203  41.302  0.00  0.00
C
ATOM  21234  HA  VAL C  76      24.362  12.908  40.537  0.00  0.00
C
ATOM  21235  CB  VAL C  76      25.814  12.100  42.010  0.00  0.00
C
ATOM  21236  HB  VAL C  76      26.523  12.445  42.793  0.00  0.00
C
ATOM  21237  CG1 VAL C  76      24.746  11.094  42.654  0.00  0.00
C
ATOM  21238 HG11 VAL C  76      24.010  10.787  41.881  0.00  0.00
C
ATOM  21239 HG12 VAL C  76      25.173  10.169  43.098  0.00  0.00
C
ATOM  21240 HG13 VAL C  76      24.126  11.594  43.429  0.00  0.00
C
ATOM  21241  CG2 VAL C  76      26.674  11.362  40.963  0.00  0.00
C
ATOM  21242 HG21 VAL C  76      27.457  11.985  40.481  0.00  0.00
C
ATOM  21243 HG22 VAL C  76      27.057  10.670  41.743  0.00  0.00
C
ATOM  21244 HG23 VAL C  76      26.129  10.925  40.099  0.00  0.00
C
ATOM  21245  C   VAL C  76      24.229  13.999  42.301  0.00  0.00
C
ATOM  21246  O   VAL C  76      24.665  14.150  43.437  0.00  0.00
C
ATOM  21247  N   ALA C  77      22.979  14.496  41.968  0.00  0.00
C
ATOM  21248  HN  ALA C  77      22.654  14.300  41.046  0.00  0.00
C
```

ATOM 21249 CA ALA C 77   22.099 15.281 42.805 0.00 0.00
C
ATOM 21250 HA ALA C 77   22.759 15.882 43.413 0.00 0.00
C
ATOM 21251 CB ALA C 77   21.196 16.135 41.872 0.00 0.00
C
ATOM 21252 HB1 ALA C 77   20.605 16.822 42.516 0.00 0.00
C
ATOM 21253 HB2 ALA C 77   21.816 16.821 41.257 0.00 0.00
C
ATOM 21254 HB3 ALA C 77   20.643 15.451 41.193 0.00 0.00
C
ATOM 21255 C   ALA C 77   21.269 14.352 43.560 0.00 0.00
C
ATOM 21256 O   ALA C 77   20.610 14.734 44.536 0.00 0.00
C
ATOM 21257 N   ILE C 78   21.316 13.088 43.292 0.00 0.00
C
ATOM 21258 HN ILE C 78   21.883 12.587 42.642 0.00 0.00
C
ATOM 21259 CA ILE C 78   20.883 12.020 44.268 0.00 0.00
C
ATOM 21260 HA ILE C 78   19.816 12.157 44.366 0.00 0.00
C
ATOM 21261 CB ILE C 78   21.079 10.669 43.548 0.00 0.00
C
ATOM 21262 HB ILE C 78   22.140 10.342 43.503 0.00 0.00
C
ATOM 21263 CG2 ILE C 78   20.441   9.586 44.333 0.00 0.00
C
ATOM 21264 HG21 ILE C 78   21.140   9.320 45.154 0.00 0.00
C
ATOM 21265 HG22 ILE C 78   19.513 10.014 44.769 0.00 0.00
C
ATOM 21266 HG23 ILE C 78   20.297   8.611 43.820 0.00 0.00
C
ATOM 21267 CG1 ILE C 78   20.682 10.560 42.030 0.00 0.00
C
ATOM 21268 HG11 ILE C 78   19.645 10.879 41.791 0.00 0.00
C
ATOM 21269 HG12 ILE C 78   21.351 11.336 41.601 0.00 0.00
C
ATOM 21270 CD ILE C 78   21.097   9.291 41.325 0.00 0.00
C
ATOM 21271 HD1 ILE C 78   21.433   8.608 42.134 0.00 0.00
C
ATOM 21272 HD2 ILE C 78   20.201   8.742 40.965 0.00 0.00
C
ATOM 21273 HD3 ILE C 78   21.778   9.507 40.474 0.00 0.00
C
ATOM 21274 C   ILE C 78   21.642 11.946 45.574 0.00 0.00
C
ATOM 21275 O   ILE C 78   22.868 11.850 45.625 0.00 0.00
C

ATOM 21276 N GLY C 79 20.893 12.091 46.689 0.00 0.00
C
ATOM 21277 HN GLY C 79 19.903 12.140 46.586 0.00 0.00
C
ATOM 21278 CA GLY C 79 21.517 12.178 48.035 0.00 0.00
C
ATOM 21279 HA1 GLY C 79 21.419 13.177 48.432 0.00 0.00
C
ATOM 21280 HA2 GLY C 79 22.526 11.877 47.797 0.00 0.00
C
ATOM 21281 C GLY C 79 20.862 11.196 49.018 0.00 0.00
C
ATOM 21282 O GLY C 79 21.571 10.330 49.488 0.00 0.00
C
ATOM 21283 N ALA C 80 19.601 11.546 49.380 0.00 0.00
C
ATOM 21284 HN ALA C 80 19.088 12.280 48.941 0.00 0.00
C
ATOM 21285 CA ALA C 80 18.886 10.956 50.406 0.00 0.00
C
ATOM 21286 HA ALA C 80 19.450 10.947 51.327 0.00 0.00
C
ATOM 21287 CB ALA C 80 17.512 11.562 50.531 0.00 0.00
C
ATOM 21288 HB1 ALA C 80 16.847 11.265 49.692 0.00 0.00
C
ATOM 21289 HB2 ALA C 80 17.050 11.198 51.473 0.00 0.00
C
ATOM 21290 HB3 ALA C 80 17.693 12.655 50.440 0.00 0.00
C
ATOM 21291 C ALA C 80 18.766 9.428 50.155 0.00 0.00
C
ATOM 21292 O ALA C 80 18.773 8.644 51.109 0.00 0.00
C
ATOM 21293 N ASN C 81 18.662 8.972 48.906 0.00 0.00
C
ATOM 21294 HN ASN C 81 18.583 9.628 48.159 0.00 0.00
C
ATOM 21295 CA ASN C 81 18.842 7.553 48.702 0.00 0.00
C
ATOM 21296 HA ASN C 81 18.594 6.945 49.560 0.00 0.00
C
ATOM 21297 CB ASN C 81 18.124 7.144 47.356 0.00 0.00
C
ATOM 21298 HB1 ASN C 81 17.113 7.594 47.453 0.00 0.00
C
ATOM 21299 HB2 ASN C 81 18.687 7.594 46.510 0.00 0.00
C
ATOM 21300 CG ASN C 81 18.017 5.673 47.148 0.00 0.00
C
ATOM 21301 OD1 ASN C 81 18.815 4.939 47.754 0.00 0.00
C
ATOM 21302 ND2 ASN C 81 17.079 5.124 46.310 0.00 0.00
C

ATOM	21303	HD21	ASN	C	81	17.030	4.126	46.272	0.00	0.00	C
ATOM	21304	HD22	ASN	C	81	16.445	5.721	45.817	0.00	0.00	C
ATOM	21305	C	ASN	C	81	20.399	7.285	48.552	0.00	0.00	C
ATOM	21306	O	ASN	C	81	21.041	7.350	47.507	0.00	0.00	C
ATOM	21307	N	ILE	C	82	21.005	6.938	49.717	0.00	0.00	C
ATOM	21308	HN	ILE	C	82	20.503	7.075	50.567	0.00	0.00	C
ATOM	21309	CA	ILE	C	82	22.434	6.551	49.954	0.00	0.00	C
ATOM	21310	HA	ILE	C	82	23.024	7.407	49.661	0.00	0.00	C
ATOM	21311	CB	ILE	C	82	22.681	6.127	51.368	0.00	0.00	C
ATOM	21312	HB	ILE	C	82	22.243	5.133	51.602	0.00	0.00	C
ATOM	21313	CG2	ILE	C	82	24.237	6.081	51.558	0.00	0.00	C
ATOM	21314	HG21	ILE	C	82	24.503	5.852	52.612	0.00	0.00	C
ATOM	21315	HG22	ILE	C	82	24.568	5.194	50.977	0.00	0.00	C
ATOM	21316	HG23	ILE	C	82	24.731	6.918	51.021	0.00	0.00	C
ATOM	21317	CG1	ILE	C	82	22.047	6.998	52.539	0.00	0.00	C
ATOM	21318	HG11	ILE	C	82	20.945	6.968	52.407	0.00	0.00	C
ATOM	21319	HG12	ILE	C	82	22.413	6.532	53.479	0.00	0.00	C
ATOM	21320	CD	ILE	C	82	22.486	8.430	52.413	0.00	0.00	C
ATOM	21321	HD1	ILE	C	82	23.585	8.439	52.250	0.00	0.00	C
ATOM	21322	HD2	ILE	C	82	22.068	9.023	51.573	0.00	0.00	C
ATOM	21323	HD3	ILE	C	82	22.165	9.092	53.246	0.00	0.00	C
ATOM	21324	C	ILE	C	82	22.890	5.483	48.970	0.00	0.00	C
ATOM	21325	O	ILE	C	82	23.968	5.562	48.402	0.00	0.00	C
ATOM	21326	N	GLN	C	83	22.141	4.366	48.813	0.00	0.00	C
ATOM	21327	HN	GLN	C	83	21.258	4.228	49.256	0.00	0.00	C
ATOM	21328	CA	GLN	C	83	22.508	3.201	47.907	0.00	0.00	C
ATOM	21329	HA	GLN	C	83	23.507	2.957	48.238	0.00	0.00	C

ATOM 21330 CB GLN C 83   21.529   2.142 48.006 0.00 0.00
C
ATOM 21331 HB1 GLN C 83   21.402   1.749 49.037 0.00 0.00
C
ATOM 21332 HB2 GLN C 83   20.522   2.526 47.736 0.00 0.00
C
ATOM 21333 CG GLN C 83   21.817   0.828 47.117 0.00 0.00
C
ATOM 21334 HG1 GLN C 83   21.929   1.042 46.033 0.00 0.00
C
ATOM 21335 HG2 GLN C 83   22.676   0.340 47.624 0.00 0.00
C
ATOM 21336 CD GLN C 83   20.611 -0.108 47.297 0.00 0.00
C
ATOM 21337 OE1 GLN C 83   19.450   0.340 47.240 0.00 0.00
C
ATOM 21338 NE2 GLN C 83   20.966 -1.426 47.482 0.00 0.00
C
ATOM 21339 HE21 GLN C 83   20.280 -2.150 47.550 0.00 0.00
C
ATOM 21340 HE22 GLN C 83   21.905 -1.684 47.256 0.00 0.00
C
ATOM 21341 C   GLN C 83   22.670   3.713 46.454 0.00 0.00
C
ATOM 21342 O   GLN C 83   23.691   3.439 45.814 0.00 0.00
C
ATOM 21343 N   ALA C 84   21.642   4.477 45.901 0.00 0.00
C
ATOM 21344 HN ALA C 84   20.859   4.749 46.456 0.00 0.00
C
ATOM 21345 CA ALA C 84   21.700   5.013 44.599 0.00 0.00
C
ATOM 21346 HA ALA C 84   21.902   4.136 44.002 0.00 0.00
C
ATOM 21347 CB ALA C 84   20.426   5.758 44.163 0.00 0.00
C
ATOM 21348 HB1 ALA C 84   20.561   6.232 43.168 0.00 0.00
C
ATOM 21349 HB2 ALA C 84   19.599   5.045 43.957 0.00 0.00
C
ATOM 21350 HB3 ALA C 84   20.053   6.387 45.000 0.00 0.00
C
ATOM 21351 C   ALA C 84   22.783   5.923 44.398 0.00 0.00
C
ATOM 21352 O   ALA C 84   23.491   5.846 43.414 0.00 0.00
C
ATOM 21353 N   SER C 85   23.080   6.853 45.380 0.00 0.00
C
ATOM 21354 HN SER C 85   22.598   6.897 46.251 0.00 0.00
C
ATOM 21355 CA SER C 85   24.222   7.786 45.288 0.00 0.00
C
ATOM 21356 HA SER C 85   24.067   8.300 44.351 0.00 0.00
C

ATOM 21357 CB SER C 85   24.097   8.760 46.458 0.00 0.00
C
ATOM 21358 HB1 SER C 85   23.112   9.237 46.268 0.00 0.00
C
ATOM 21359 HB2 SER C 85   24.189   8.233 47.432 0.00 0.00
C
ATOM 21360 OG SER C 85   25.117   9.736 46.553 0.00 0.00
C
ATOM 21361 HG1 SER C 85   24.722 10.570 46.821 0.00 0.00
C
ATOM 21362 C   SER C 85   25.552   6.992 45.150 0.00 0.00
C
ATOM 21363 O   SER C 85   26.302   7.221 44.186 0.00 0.00
C
ATOM 21364 N   THR C 86   25.860   6.008 46.049 0.00 0.00
C
ATOM 21365 HN THR C 86   25.250   5.731 46.788 0.00 0.00
C
ATOM 21366 CA THR C 86   26.981   5.106 45.979 0.00 0.00
C
ATOM 21367 HA THR C 86   27.869   5.699 45.815 0.00 0.00
C
ATOM 21368 CB THR C 86   27.230   4.272 47.218 0.00 0.00
C
ATOM 21369 HB THR C 86   27.931   3.443 46.981 0.00 0.00
C
ATOM 21370 OG1 THR C 86   26.003   3.702 47.642 0.00 0.00
C
ATOM 21371 HG1 THR C 86   25.419   4.372 48.004 0.00 0.00
C
ATOM 21372 CG2 THR C 86   27.767   5.184 48.378 0.00 0.00
C
ATOM 21373 HG21 THR C 86   27.296   6.176 48.208 0.00 0.00
C
ATOM 21374 HG22 THR C 86   27.434   4.817 49.372 0.00 0.00
C
ATOM 21375 HG23 THR C 86   28.872   5.266 48.302 0.00 0.00
C
ATOM 21376 C   THR C 86   27.024   4.185 44.798 0.00 0.00
C
ATOM 21377 O   THR C 86   28.008   4.189 44.079 0.00 0.00
C
ATOM 21378 N   LEU C 87   25.951   3.415 44.469 0.00 0.00
C
ATOM 21379 HN LEU C 87   25.116   3.589 44.984 0.00 0.00
C
ATOM 21380 CA LEU C 87   25.799   2.556 43.309 0.00 0.00
C
ATOM 21381 HA LEU C 87   26.630   1.867 43.287 0.00 0.00
C
ATOM 21382 CB LEU C 87   24.514   1.748 43.376 0.00 0.00
C
ATOM 21383 HB1 LEU C 87   24.488   1.300 44.393 0.00 0.00
C

ATOM 21384 HB2 LEU C 87   23.577   2.342 43.429 0.00 0.00
C
ATOM 21385 CG LEU C 87   24.340   0.627 42.341 0.00 0.00
C
ATOM 21386 HG LEU C 87   24.099   1.146 41.389 0.00 0.00
C
ATOM 21387 CD1 LEU C 87   25.561 -0.296 42.138 0.00 0.00
C
ATOM 21388 HD11 LEU C 87   25.355 -1.339 41.815 0.00 0.00
C
ATOM 21389 HD12 LEU C 87   26.167   0.107 41.299 0.00 0.00
C
ATOM 21390 HD13 LEU C 87   26.030 -0.423 43.137 0.00 0.00
C
ATOM 21391 CD2 LEU C 87   23.104 -0.199 42.766 0.00 0.00
C
ATOM 21392 HD21 LEU C 87   23.407 -0.855 43.610 0.00 0.00
C
ATOM 21393 HD22 LEU C 87   22.187   0.393 42.973 0.00 0.00
C
ATOM 21394 HD23 LEU C 87   22.897 -0.864 41.900 0.00 0.00
C
ATOM 21395 C   LEU C 87   25.948   3.311 41.949 0.00 0.00
C
ATOM 21396 O   LEU C 87   26.627   2.913 40.988 0.00 0.00
C
ATOM 21397 N   THR C 88   25.281   4.532 41.719 0.00 0.00
C
ATOM 21398 HN THR C 88   24.752   4.834 42.509 0.00 0.00
C
ATOM 21399 CA THR C 88   25.536   5.416 40.623 0.00 0.00
C
ATOM 21400 HA THR C 88   25.349   4.949 39.667 0.00 0.00
C
ATOM 21401 CB THR C 88   24.751   6.758 40.612 0.00 0.00
C
ATOM 21402 HB THR C 88   25.125   7.440 41.406 0.00 0.00
C
ATOM 21403 OG1 THR C 88   23.369   6.426 40.831 0.00 0.00
C
ATOM 21404 HG1 THR C 88   23.237   6.081 41.717 0.00 0.00
C
ATOM 21405 CG2 THR C 88   24.850   7.518 39.226 0.00 0.00
C
ATOM 21406 HG21 THR C 88   24.563   8.584 39.348 0.00 0.00
C
ATOM 21407 HG22 THR C 88   25.909   7.589 38.898 0.00 0.00
C
ATOM 21408 HG23 THR C 88   24.200   7.061 38.450 0.00 0.00
C
ATOM 21409 C   THR C 88   26.993   5.869 40.501 0.00 0.00
C
ATOM 21410 O   THR C 88   27.571   5.744 39.436 0.00 0.00
C

ATOM 21411 N THR C 89 27.627 6.357 41.632 0.00 0.00
C
ATOM 21412 HN THR C 89 27.235 6.351 42.548 0.00 0.00
C
ATOM 21413 CA THR C 89 28.996 6.846 41.573 0.00 0.00
C
ATOM 21414 HA THR C 89 29.138 7.601 40.814 0.00 0.00
C
ATOM 21415 CB THR C 89 29.308 7.644 42.792 0.00 0.00
C
ATOM 21416 HB THR C 89 29.252 6.917 43.630 0.00 0.00
C
ATOM 21417 OG1 THR C 89 28.267 8.699 42.909 0.00 0.00
C
ATOM 21418 HG1 THR C 89 27.607 8.281 43.465 0.00 0.00
C
ATOM 21419 CG2 THR C 89 30.633 8.289 42.757 0.00 0.00
C
ATOM 21420 HG21 THR C 89 30.745 9.058 43.551 0.00 0.00
C
ATOM 21421 HG22 THR C 89 31.426 7.532 42.943 0.00 0.00
C
ATOM 21422 HG23 THR C 89 30.705 8.945 41.864 0.00 0.00
C
ATOM 21423 C THR C 89 30.077 5.823 41.340 0.00 0.00
C
ATOM 21424 O THR C 89 31.080 5.954 40.648 0.00 0.00
C
ATOM 21425 N LEU C 90 29.764 4.628 41.914 0.00 0.00
C
ATOM 21426 HN LEU C 90 29.096 4.525 42.647 0.00 0.00
C
ATOM 21427 CA LEU C 90 30.519 3.464 41.595 0.00 0.00
C
ATOM 21428 HA LEU C 90 31.508 3.709 41.954 0.00 0.00
C
ATOM 21429 CB LEU C 90 29.998 2.245 42.298 0.00 0.00
C
ATOM 21430 HB1 LEU C 90 28.909 2.423 42.421 0.00 0.00
C
ATOM 21431 HB2 LEU C 90 30.168 1.278 41.778 0.00 0.00
C
ATOM 21432 CG LEU C 90 30.589 2.261 43.741 0.00 0.00
C
ATOM 21433 HG LEU C 90 30.501 3.297 44.131 0.00 0.00
C
ATOM 21434 CD1 LEU C 90 29.819 1.400 44.773 0.00 0.00
C
ATOM 21435 HD11 LEU C 90 30.029 0.318 44.636 0.00 0.00
C
ATOM 21436 HD12 LEU C 90 29.880 1.740 45.829 0.00 0.00
C
ATOM 21437 HD13 LEU C 90 28.729 1.514 44.590 0.00 0.00
C

ATOM 21438 CD2 LEU C 90 32.013 1.849 43.816 0.00 0.00
C
ATOM 21439 HD21 LEU C 90 32.069 0.883 43.270 0.00 0.00
C
ATOM 21440 HD22 LEU C 90 32.699 2.574 43.328 0.00 0.00
C
ATOM 21441 HD23 LEU C 90 32.210 1.598 44.880 0.00 0.00
C
ATOM 21442 C LEU C 90 30.624 3.025 40.116 0.00 0.00
C
ATOM 21443 O LEU C 90 31.681 2.764 39.602 0.00 0.00
C
ATOM 21444 N LEU C 91 29.448 3.104 39.372 0.00 0.00
C
ATOM 21445 HN LEU C 91 28.663 3.354 39.934 0.00 0.00
C
ATOM 21446 CA LEU C 91 29.494 2.832 37.971 0.00 0.00
C
ATOM 21447 HA LEU C 91 30.228 2.054 37.821 0.00 0.00
C
ATOM 21448 CB LEU C 91 28.048 2.411 37.737 0.00 0.00
C
ATOM 21449 HB1 LEU C 91 27.513 3.349 37.997 0.00 0.00
C
ATOM 21450 HB2 LEU C 91 27.910 2.251 36.646 0.00 0.00
C
ATOM 21451 CG LEU C 91 27.477 1.238 38.501 0.00 0.00
C
ATOM 21452 HG LEU C 91 27.440 1.389 39.601 0.00 0.00
C
ATOM 21453 CD1 LEU C 91 26.023 0.950 38.049 0.00 0.00
C
ATOM 21454 HD11 LEU C 91 26.048 0.828 36.945 0.00 0.00
C
ATOM 21455 HD12 LEU C 91 25.596 0.006 38.449 0.00 0.00
C
ATOM 21456 HD13 LEU C 91 25.378 1.783 38.404 0.00 0.00
C
ATOM 21457 CD2 LEU C 91 28.333 0.009 38.249 0.00 0.00
C
ATOM 21458 HD21 LEU C 91 27.928 -0.999 38.481 0.00 0.00
C
ATOM 21459 HD22 LEU C 91 28.725 -0.063 37.212 0.00 0.00
C
ATOM 21460 HD23 LEU C 91 29.177 0.156 38.957 0.00 0.00
C
ATOM 21461 C LEU C 91 29.932 4.037 37.146 0.00 0.00
C
ATOM 21462 O LEU C 91 30.760 3.796 36.328 0.00 0.00
C
ATOM 21463 N LEU C 92 29.696 5.347 37.538 0.00 0.00
C
ATOM 21464 HN LEU C 92 29.025 5.552 38.246 0.00 0.00
C

ATOM 21465 CA LEU C 92   30.291   6.535 36.904 0.00 0.00
C
ATOM 21466 HA LEU C 92   29.986   6.452 35.871 0.00 0.00
C
ATOM 21467 CB LEU C 92   29.794   7.874 37.474 0.00 0.00
C
ATOM 21468 HB1 LEU C 92   30.084   8.003 38.539 0.00 0.00
C
ATOM 21469 HB2 LEU C 92   30.325   8.658 36.892 0.00 0.00
C
ATOM 21470 CG LEU C 92   28.317   8.130 37.285 0.00 0.00
C
ATOM 21471 HG LEU C 92   27.762   7.202 37.538 0.00 0.00
C
ATOM 21472 CD1 LEU C 92   27.826   9.291 38.034 0.00 0.00
C
ATOM 21473 HD11 LEU C 92   26.802   9.453 37.634 0.00 0.00
C
ATOM 21474 HD12 LEU C 92   27.751   9.122 39.129 0.00 0.00
C
ATOM 21475 HD13 LEU C 92   28.526 10.151 37.962 0.00 0.00
C
ATOM 21476 CD2 LEU C 92   28.110   8.496 35.837 0.00 0.00
C
ATOM 21477 HD21 LEU C 92   28.563   9.434 35.452 0.00 0.00
C
ATOM 21478 HD22 LEU C 92   28.398   7.652 35.174 0.00 0.00
C
ATOM 21479 HD23 LEU C 92   27.032   8.599 35.586 0.00 0.00
C
ATOM 21480 C   LEU C 92   31.798   6.550 36.830 0.00 0.00
C
ATOM 21481 O   LEU C 92   32.488   6.776 35.793 0.00 0.00
C
ATOM 21482 N   LYS C 93   32.401   6.243 37.974 0.00 0.00
C
ATOM 21483 HN LYS C 93   31.932   6.082 38.838 0.00 0.00
C
ATOM 21484 CA LYS C 93   33.845   6.155 37.974 0.00 0.00
C
ATOM 21485 HA LYS C 93   34.184   7.049 37.472 0.00 0.00
C
ATOM 21486 CB LYS C 93   34.274   5.981 39.467 0.00 0.00
C
ATOM 21487 HB1 LYS C 93   33.901   6.877 40.008 0.00 0.00
C
ATOM 21488 HB2 LYS C 93   33.631   5.207 39.937 0.00 0.00
C
ATOM 21489 CG LYS C 93   35.680   5.716 39.730 0.00 0.00
C
ATOM 21490 HG1 LYS C 93   35.784   5.767 40.835 0.00 0.00
C
ATOM 21491 HG2 LYS C 93   36.051   4.728 39.382 0.00 0.00
C

ATOM 21492 CD LYS C 93 36.765 6.702 39.174 0.00 0.00
C
ATOM 21493 HD1 LYS C 93 37.779 6.454 39.554 0.00 0.00
C
ATOM 21494 HD2 LYS C 93 36.798 6.715 38.064 0.00 0.00
C
ATOM 21495 CE LYS C 93 36.610 8.209 39.669 0.00 0.00
C
ATOM 21496 HE1 LYS C 93 35.783 8.667 39.085 0.00 0.00
C
ATOM 21497 HE2 LYS C 93 36.554 8.136 40.777 0.00 0.00
C
ATOM 21498 NZ LYS C 93 37.786 8.951 39.301 0.00 0.00
C
ATOM 21499 HZ1 LYS C 93 37.657 9.947 39.571 0.00 0.00
C
ATOM 21500 HZ2 LYS C 93 38.587 8.612 39.872 0.00 0.00
C
ATOM 21501 HZ3 LYS C 93 37.931 8.966 38.272 0.00 0.00
C
ATOM 21502 C LYS C 93 34.452 4.931 37.219 0.00 0.00
C
ATOM 21503 O LYS C 93 35.539 4.913 36.719 0.00 0.00
C
ATOM 21504 N GLU C 94 33.667 3.804 37.130 0.00 0.00
C
ATOM 21505 HN GLU C 94 32.838 3.850 37.681 0.00 0.00
C
ATOM 21506 CA GLU C 94 33.850 2.636 36.214 0.00 0.00
C
ATOM 21507 HA GLU C 94 34.856 2.296 36.415 0.00 0.00
C
ATOM 21508 CB GLU C 94 32.936 1.437 36.521 0.00 0.00
C
ATOM 21509 HB1 GLU C 94 32.997 1.171 37.598 0.00 0.00
C
ATOM 21510 HB2 GLU C 94 31.877 1.537 36.200 0.00 0.00
C
ATOM 21511 CG GLU C 94 33.400 0.024 35.803 0.00 0.00
C
ATOM 21512 HG1 GLU C 94 33.032 -0.045 34.757 0.00 0.00
C
ATOM 21513 HG2 GLU C 94 34.497 -0.123 35.905 0.00 0.00
C
ATOM 21514 CD GLU C 94 32.726 -1.113 36.526 0.00 0.00
C
ATOM 21515 OE1 GLU C 94 32.022 -1.903 35.920 0.00 0.00
C
ATOM 21516 OE2 GLU C 94 32.957 -1.267 37.758 0.00 0.00
C
ATOM 21517 C GLU C 94 33.819 2.991 34.731 0.00 0.00
C
ATOM 21518 O GLU C 94 34.721 2.604 33.996 0.00 0.00
C

ATOM 21519 N   LEU C 95   32.874   3.877 34.328 0.00 0.00
C
ATOM 21520 HN LEU C 95   32.131   4.105 34.953 0.00 0.00
C
ATOM 21521 CA LEU C 95   32.829   4.467 32.962 0.00 0.00
C
ATOM 21522 HA LEU C 95   32.994   3.702 32.218 0.00 0.00
C
ATOM 21523 CB LEU C 95   31.487   5.124 32.741 0.00 0.00
C
ATOM 21524 HB1 LEU C 95   31.320   5.825 33.586 0.00 0.00
C
ATOM 21525 HB2 LEU C 95   31.410   5.662 31.772 0.00 0.00
C
ATOM 21526 CG LEU C 95   30.256   4.164 32.662 0.00 0.00
C
ATOM 21527 HG LEU C 95   30.346   3.431 33.492 0.00 0.00
C
ATOM 21528 CD1 LEU C 95   28.874   4.910 32.509 0.00 0.00
C
ATOM 21529 HD11 LEU C 95   28.068   4.176 32.294 0.00 0.00
C
ATOM 21530 HD12 LEU C 95   28.686   5.449 33.463 0.00 0.00
C
ATOM 21531 HD13 LEU C 95   28.965   5.672 31.706 0.00 0.00
C
ATOM 21532 CD2 LEU C 95   30.525   3.317 31.377 0.00 0.00
C
ATOM 21533 HD21 LEU C 95   30.750   3.979 30.514 0.00 0.00
C
ATOM 21534 HD22 LEU C 95   31.456   2.722 31.487 0.00 0.00
C
ATOM 21535 HD23 LEU C 95   29.606   2.713 31.221 0.00 0.00
C
ATOM 21536 C   LEU C 95   33.934   5.491 32.716 0.00 0.00
C
ATOM 21537 O   LEU C 95   34.127   5.907 31.557 0.00 0.00
C
ATOM 21538 N   ASP C 96   34.679   5.865 33.788 0.00 0.00
C
ATOM 21539 HN ASP C 96   34.526   5.398 34.655 0.00 0.00
C
ATOM 21540 CA ASP C 96   35.857   6.769 33.828 0.00 0.00
C
ATOM 21541 HA ASP C 96   36.174   6.834 34.858 0.00 0.00
C
ATOM 21542 CB ASP C 96   37.181   6.242 33.141 0.00 0.00
C
ATOM 21543 HB1 ASP C 96   37.365   5.249 33.605 0.00 0.00
C
ATOM 21544 HB2 ASP C 96   37.063   6.233 32.037 0.00 0.00
C
ATOM 21545 CG ASP C 96   38.424   7.025 33.387 0.00 0.00
C

ATOM 21546 OD1 ASP C 96   38.533   7.609 34.524 0.00 0.00
C
ATOM 21547 OD2 ASP C 96   39.372   7.170 32.537 0.00 0.00
C
ATOM 21548 C   ASP C 96   35.525   8.224 33.430 0.00 0.00
C
ATOM 21549 O   ASP C 96   36.236   8.885 32.704 0.00 0.00
C
ATOM 21550 N   ILE C 97   34.345   8.688 33.984 0.00 0.00
C
ATOM 21551 HN ILE C 97   33.738   8.056 34.461 0.00 0.00
C
ATOM 21552 CA ILE C 97   33.839 10.046 33.977 0.00 0.00
C
ATOM 21553 HA ILE C 97   33.771 10.224 32.914 0.00 0.00
C
ATOM 21554 CB ILE C 97   32.508 10.309 34.689 0.00 0.00
C
ATOM 21555 HB ILE C 97   32.599   9.904 35.720 0.00 0.00
C
ATOM 21556 CG2 ILE C 97   32.123 11.813 34.913 0.00 0.00
C
ATOM 21557 HG21 ILE C 97   32.890 12.406 35.455 0.00 0.00
C
ATOM 21558 HG22 ILE C 97   31.947 12.214 33.891 0.00 0.00
C
ATOM 21559 HG23 ILE C 97   31.162 11.829 35.471 0.00 0.00
C
ATOM 21560 CG1 ILE C 97   31.371   9.553 33.940 0.00 0.00
C
ATOM 21561 HG11 ILE C 97   31.423   8.473 34.196 0.00 0.00
C
ATOM 21562 HG12 ILE C 97   30.442   9.888 34.451 0.00 0.00
C
ATOM 21563 CD ILE C 97   31.212   9.725 32.446 0.00 0.00
C
ATOM 21564 HD1 ILE C 97   31.952   9.135 31.865 0.00 0.00
C
ATOM 21565 HD2 ILE C 97   30.252   9.196 32.262 0.00 0.00
C
ATOM 21566 HD3 ILE C 97   31.150 10.771 32.074 0.00 0.00
C
ATOM 21567 C   ILE C 97   34.875 10.990 34.559 0.00 0.00
C
ATOM 21568 O   ILE C 97   35.330 10.717 35.673 0.00 0.00
C
ATOM 21569 N   PRO C 98   35.328 12.115 33.957 0.00 0.00
C
ATOM 21570 CD PRO C 98   34.908 12.697 32.710 0.00 0.00
C
ATOM 21571 HD1 PRO C 98   34.206 12.058 32.132 0.00 0.00
C
ATOM 21572 HD2 PRO C 98   34.351 13.635 32.921 0.00 0.00
C

ATOM 21573 CA PRO C 98   36.571 12.671 34.451 0.00 0.00
C
ATOM 21574 HA PRO C 98   37.324 11.956 34.748 0.00 0.00
C
ATOM 21575 CB PRO C 98   37.094 13.441 33.196 0.00 0.00
C
ATOM 21576 HB1 PRO C 98   38.141 13.237 32.883 0.00 0.00
C
ATOM 21577 HB2 PRO C 98   36.904 14.524 33.351 0.00 0.00
C
ATOM 21578 CG PRO C 98   36.225 12.954 32.006 0.00 0.00
C
ATOM 21579 HG1 PRO C 98   36.226 13.713 31.195 0.00 0.00
C
ATOM 21580 HG2 PRO C 98   36.668 12.042 31.553 0.00 0.00
C
ATOM 21581 C   PRO C 98   36.274 13.470 35.736 0.00 0.00
C
ATOM 21582 O   PRO C 98   37.096 13.329 36.633 0.00 0.00
C
ATOM 21583 N   ASN C 99   35.184 14.237 35.824 0.00 0.00
C
ATOM 21584 HN ASN C 99   34.600 14.399 35.032 0.00 0.00
C
ATOM 21585 CA ASN C 99   34.725 14.858 37.050 0.00 0.00
C
ATOM 21586 HA ASN C 99   35.362 14.587 37.878 0.00 0.00
C
ATOM 21587 CB ASN C 99   34.718 16.402 36.894 0.00 0.00
C
ATOM 21588 HB1 ASN C 99   34.052 16.705 36.059 0.00 0.00
C
ATOM 21589 HB2 ASN C 99   34.395 16.750 37.899 0.00 0.00
C
ATOM 21590 CG ASN C 99   36.117 16.891 36.530 0.00 0.00
C
ATOM 21591 OD1 ASN C 99   37.003 17.025 37.399 0.00 0.00
C
ATOM 21592 ND2 ASN C 99   36.398 17.264 35.267 0.00 0.00
C
ATOM 21593 HD21 ASN C 99   37.321 17.517 34.976 0.00 0.00
C
ATOM 21594 HD22 ASN C 99   35.636 17.158 34.628 0.00 0.00
C
ATOM 21595 C   ASN C 99   33.382 14.318 37.545 0.00 0.00
C
ATOM 21596 O   ASN C 99   32.328 14.453 36.845 0.00 0.00
C
ATOM 21597 N   ILE C 100   33.191 13.812 38.757 0.00 0.00
C
ATOM 21598 HN ILE C 100   33.981 13.632 39.338 0.00 0.00
C
ATOM 21599 CA ILE C 100   31.928 13.492 39.361 0.00 0.00
C

ATOM 21600 HA ILE C 100   31.015 13.639 38.803 0.00 0.00
C
ATOM 21601 CB ILE C 100   31.715 11.991 39.716 0.00 0.00
C
ATOM 21602 HB ILE C 100   32.467 11.777 40.506 0.00 0.00
C
ATOM 21603 CG2 ILE C 100   30.242 11.828 40.153 0.00 0.00
C
ATOM 21604 HG21 ILE C 100   30.047 12.329 41.125 0.00 0.00
C
ATOM 21605 HG22 ILE C 100   29.575 12.345 39.431 0.00 0.00
C
ATOM 21606 HG23 ILE C 100   30.002 10.745 40.220 0.00 0.00
C
ATOM 21607 CG1 ILE C 100   32.081 10.949 38.701 0.00 0.00
C
ATOM 21608 HG11 ILE C 100   31.295 10.923 37.917 0.00 0.00
C
ATOM 21609 HG12 ILE C 100   32.992 11.290 38.164 0.00 0.00
C
ATOM 21610 CD ILE C 100   32.262   9.516 39.246 0.00 0.00
C
ATOM 21611 HD1 ILE C 100   32.967   9.380 40.093 0.00 0.00
C
ATOM 21612 HD2 ILE C 100   31.269   9.087 39.498 0.00 0.00
C
ATOM 21613 HD3 ILE C 100   32.586   8.786 38.474 0.00 0.00
C
ATOM 21614 C   ILE C 100   31.792 14.351 40.650 0.00 0.00
C
ATOM 21615 O   ILE C 100   32.645 14.443 41.517 0.00 0.00
C
ATOM 21616 N   TRP C 101   30.680 15.021 40.778 0.00 0.00
C
ATOM 21617 HN TRP C 101   29.864 14.946 40.210 0.00 0.00
C
ATOM 21618 CA TRP C 101   30.381 15.880 41.964 0.00 0.00
C
ATOM 21619 HA TRP C 101   31.211 15.869 42.655 0.00 0.00
C
ATOM 21620 CB TRP C 101   30.200 17.380 41.566 0.00 0.00
C
ATOM 21621 HB1 TRP C 101   29.384 17.362 40.813 0.00 0.00
C
ATOM 21622 HB2 TRP C 101   29.868 18.007 42.421 0.00 0.00
C
ATOM 21623 CG TRP C 101   31.422 18.060 41.067 0.00 0.00
C
ATOM 21624 CD1 TRP C 101   32.565 17.643 40.427 0.00 0.00
C
ATOM 21625 HD1 TRP C 101   32.692 16.633 40.065 0.00 0.00
C
ATOM 21626 NE1 TRP C 101   33.239 18.717 39.895 0.00 0.00
C

```
ATOM  21627  HE1 TRP C 101      34.099  18.790  39.440  0.00  0.00
C
ATOM  21628  CE2 TRP C 101      32.635  19.871  40.326  0.00  0.00
C
ATOM  21629  CD2 TRP C 101      31.470  19.532  41.004  0.00  0.00
C
ATOM  21630  CE3 TRP C 101      30.575  20.541  41.454  0.00  0.00
C
ATOM  21631  HE3 TRP C 101      29.687  20.415  42.056  0.00  0.00
C
ATOM  21632  CZ3 TRP C 101      30.992  21.902  41.281  0.00  0.00
C
ATOM  21633  HZ3 TRP C 101      30.267  22.692  41.412  0.00  0.00
C
ATOM  21634  CZ2 TRP C 101      32.913  21.171  40.027  0.00  0.00
C
ATOM  21635  HZ2 TRP C 101      33.873  21.371  39.575  0.00  0.00
C
ATOM  21636  CH2 TRP C 101      32.121  22.186  40.572  0.00  0.00
C
ATOM  21637  HH2 TRP C 101      32.360  23.239  40.580  0.00  0.00
C
ATOM  21638  C   TRP C 101      29.162  15.164  42.603  0.00  0.00
C
ATOM  21639  O   TRP C 101      28.165  14.848  41.960  0.00  0.00
C
ATOM  21640  N   VAL C 102      29.210  14.785  43.923  0.00  0.00
C
ATOM  21641  HN  VAL C 102      29.913  15.214  44.485  0.00  0.00
C
ATOM  21642  CA  VAL C 102      28.240  13.947  44.658  0.00  0.00
C
ATOM  21643  HA  VAL C 102      27.523  13.670  43.899  0.00  0.00
C
ATOM  21644  CB  VAL C 102      28.866  12.736  45.259  0.00  0.00
C
ATOM  21645  HB  VAL C 102      29.610  13.047  46.023  0.00  0.00
C
ATOM  21646  CG1 VAL C 102      27.837  11.774  45.788  0.00  0.00
C
ATOM  21647 HG11 VAL C 102      27.129  11.423  45.007  0.00  0.00
C
ATOM  21648 HG12 VAL C 102      28.393  10.932  46.252  0.00  0.00
C
ATOM  21649 HG13 VAL C 102      27.364  12.222  46.688  0.00  0.00
C
ATOM  21650  CG2 VAL C 102      29.770  11.951  44.199  0.00  0.00
C
ATOM  21651 HG21 VAL C 102      30.542  11.348  44.725  0.00  0.00
C
ATOM  21652 HG22 VAL C 102      29.120  11.220  43.673  0.00  0.00
C
ATOM  21653 HG23 VAL C 102      30.225  12.627  43.444  0.00  0.00
C
```

ATOM 21654 C VAL C 102 27.513 14.619 45.782 0.00 0.00
C
ATOM 21655 O VAL C 102 28.145 15.204 46.660 0.00 0.00
C
ATOM 21656 N LYS C 103 26.177 14.509 45.722 0.00 0.00
C
ATOM 21657 HN LYS C 103 25.677 14.034 45.002 0.00 0.00
C
ATOM 21658 CA LYS C 103 25.359 14.834 46.918 0.00 0.00
C
ATOM 21659 HA LYS C 103 25.810 15.677 47.420 0.00 0.00
C
ATOM 21660 CB LYS C 103 23.933 15.321 46.533 0.00 0.00
C
ATOM 21661 HB1 LYS C 103 23.928 16.188 45.839 0.00 0.00
C
ATOM 21662 HB2 LYS C 103 23.440 14.430 46.090 0.00 0.00
C
ATOM 21663 CG LYS C 103 23.084 15.829 47.675 0.00 0.00
C
ATOM 21664 HG1 LYS C 103 23.103 15.045 48.462 0.00 0.00
C
ATOM 21665 HG2 LYS C 103 23.707 16.658 48.072 0.00 0.00
C
ATOM 21666 CD LYS C 103 21.644 16.182 47.198 0.00 0.00
C
ATOM 21667 HD1 LYS C 103 21.778 17.023 46.485 0.00 0.00
C
ATOM 21668 HD2 LYS C 103 21.366 15.307 46.572 0.00 0.00
C
ATOM 21669 CE LYS C 103 20.522 16.492 48.180 0.00 0.00
C
ATOM 21670 HE1 LYS C 103 20.795 17.428 48.712 0.00 0.00
C
ATOM 21671 HE2 LYS C 103 19.586 16.816 47.676 0.00 0.00
C
ATOM 21672 NZ LYS C 103 20.148 15.447 49.119 0.00 0.00
C
ATOM 21673 HZ1 LYS C 103 20.851 15.220 49.852 0.00 0.00
C
ATOM 21674 HZ2 LYS C 103 19.405 15.737 49.787 0.00 0.00
C
ATOM 21675 HZ3 LYS C 103 19.889 14.545 48.669 0.00 0.00
C
ATOM 21676 C LYS C 103 25.350 13.827 48.023 0.00 0.00
C
ATOM 21677 O LYS C 103 25.188 12.607 47.809 0.00 0.00
C
ATOM 21678 N ALA C 104 25.559 14.216 49.273 0.00 0.00
C
ATOM 21679 HN ALA C 104 25.842 15.168 49.356 0.00 0.00
C
ATOM 21680 CA ALA C 104 25.899 13.307 50.365 0.00 0.00
C

ATOM 21681 HA ALA C 104   25.642 12.289 50.112 0.00 0.00
C
ATOM 21682 CB ALA C 104   27.386 13.339 50.658 0.00 0.00
C
ATOM 21683 HB1 ALA C 104   27.516 12.792 51.616 0.00 0.00
C
ATOM 21684 HB2 ALA C 104   27.983 12.817 49.879 0.00 0.00
C
ATOM 21685 HB3 ALA C 104   27.879 14.323 50.811 0.00 0.00
C
ATOM 21686 C   ALA C 104   25.111 13.639 51.602 0.00 0.00
C
ATOM 21687 O   ALA C 104   24.888 14.830 51.869 0.00 0.00
C
ATOM 21688 N   GLN C 105   24.733 12.700 52.450 0.00 0.00
C
ATOM 21689 HN GLN C 105   25.062 11.795 52.192 0.00 0.00
C
ATOM 21690 CA GLN C 105   23.991 12.988 53.672 0.00 0.00
C
ATOM 21691 HA GLN C 105   23.549 13.973 53.665 0.00 0.00
C
ATOM 21692 CB GLN C 105   22.698 12.102 53.567 0.00 0.00
C
ATOM 21693 HB1 GLN C 105   23.222 11.128 53.677 0.00 0.00
C
ATOM 21694 HB2 GLN C 105   21.995 12.366 54.385 0.00 0.00
C
ATOM 21695 CG GLN C 105   21.975 12.147 52.208 0.00 0.00
C
ATOM 21696 HG1 GLN C 105   22.730 11.939 51.420 0.00 0.00
C
ATOM 21697 HG2 GLN C 105   21.118 11.439 52.191 0.00 0.00
C
ATOM 21698 CD GLN C 105   21.215 13.452 51.896 0.00 0.00
C
ATOM 21699 OE1 GLN C 105   21.703 14.163 51.033 0.00 0.00
C
ATOM 21700 NE2 GLN C 105   20.082 13.744 52.586 0.00 0.00
C
ATOM 21701 HE21 GLN C 105   19.307 14.066 52.043 0.00 0.00
C
ATOM 21702 HE22 GLN C 105   19.956 13.209 53.422 0.00 0.00
C
ATOM 21703 C   GLN C 105   24.736 12.609 54.978 0.00 0.00
C
ATOM 21704 O   GLN C 105   24.419 13.112 56.031 0.00 0.00
C
ATOM 21705 N   ASN C 106   25.702 11.670 54.896 0.00 0.00
C
ATOM 21706 HN ASN C 106   26.048 11.282 54.045 0.00 0.00
C
ATOM 21707 CA ASN C 106   26.204 11.258 56.194 0.00 0.00
C

ATOM 21708 HA ASN C 106   26.233 12.100 56.869 0.00 0.00
C
ATOM 21709 CB ASN C 106   25.238 10.242 56.850 0.00 0.00
C
ATOM 21710 HB1 ASN C 106   25.432   9.952 57.905 0.00 0.00
C
ATOM 21711 HB2 ASN C 106   24.224 10.683 56.737 0.00 0.00
C
ATOM 21712 CG ASN C 106   25.178   8.896 56.086 0.00 0.00
C
ATOM 21713 OD1 ASN C 106   26.130   8.098 56.190 0.00 0.00
C
ATOM 21714 ND2 ASN C 106   24.035   8.587 55.452 0.00 0.00
C
ATOM 21715 HD21 ASN C 106   23.853   7.659 55.127 0.00 0.00
C
ATOM 21716 HD22 ASN C 106   23.335   9.299 55.390 0.00 0.00
C
ATOM 21717 C   ASN C 106   27.718 10.790 56.219 0.00 0.00
C
ATOM 21718 O   ASN C 106   28.308 10.747 55.158 0.00 0.00
C
ATOM 21719 N   TYR C 107   28.259 10.425 57.338 0.00 0.00
C
ATOM 21720 HN TYR C 107   27.727 10.292 58.171 0.00 0.00
C
ATOM 21721 CA TYR C 107   29.635 10.050 57.522 0.00 0.00
C
ATOM 21722 HA TYR C 107   30.293 10.776 57.068 0.00 0.00
C
ATOM 21723 CB TYR C 107   30.118   9.815 59.018 0.00 0.00
C
ATOM 21724 HB1 TYR C 107   29.478   9.007 59.432 0.00 0.00
C
ATOM 21725 HB2 TYR C 107   31.163   9.446 59.095 0.00 0.00
C
ATOM 21726 CG TYR C 107   29.919 11.088 59.787 0.00 0.00
C
ATOM 21727 CD1 TYR C 107   28.881 11.291 60.695 0.00 0.00
C
ATOM 21728 HD1 TYR C 107   28.105 10.564 60.889 0.00 0.00
C
ATOM 21729 CE1 TYR C 107   29.014 12.464 61.529 0.00 0.00
C
ATOM 21730 HE1 TYR C 107   28.202 12.638 62.220 0.00 0.00
C
ATOM 21731 CZ TYR C 107   30.062 13.415 61.383 0.00 0.00
C
ATOM 21732 OH TYR C 107   30.124 14.577 62.176 0.00 0.00
C
ATOM 21733 HH TYR C 107   29.209 14.810 62.352 0.00 0.00
C
ATOM 21734 CD2 TYR C 107   30.916 12.070 59.621 0.00 0.00
C

ATOM 21735 HD2 TYR C 107 31.703 11.890 58.904 0.00 0.00
C
ATOM 21736 CE2 TYR C 107 31.013 13.210 60.430 0.00 0.00
C
ATOM 21737 HE2 TYR C 107 31.848 13.856 60.203 0.00 0.00
C
ATOM 21738 C TYR C 107 30.133 8.831 56.735 0.00 0.00
C
ATOM 21739 O TYR C 107 31.134 9.014 55.981 0.00 0.00
C
ATOM 21740 N TYR C 108 29.309 7.746 56.822 0.00 0.00
C
ATOM 21741 HN TYR C 108 28.484 7.795 57.380 0.00 0.00
C
ATOM 21742 CA TYR C 108 29.650 6.512 56.171 0.00 0.00
C
ATOM 21743 HA TYR C 108 30.634 6.126 56.391 0.00 0.00
C
ATOM 21744 CB TYR C 108 28.626 5.371 56.704 0.00 0.00
C
ATOM 21745 HB1 TYR C 108 29.006 4.995 57.678 0.00 0.00
C
ATOM 21746 HB2 TYR C 108 27.621 5.840 56.763 0.00 0.00
C
ATOM 21747 CG TYR C 108 28.428 4.170 55.780 0.00 0.00
C
ATOM 21748 CD1 TYR C 108 29.237 3.088 55.805 0.00 0.00
C
ATOM 21749 HD1 TYR C 108 30.050 3.098 56.516 0.00 0.00
C
ATOM 21750 CE1 TYR C 108 29.145 2.047 54.941 0.00 0.00
C
ATOM 21751 HE1 TYR C 108 29.740 1.161 55.106 0.00 0.00
C
ATOM 21752 CZ TYR C 108 28.246 2.103 53.872 0.00 0.00
C
ATOM 21753 OH TYR C 108 28.049 1.090 52.908 0.00 0.00
C
ATOM 21754 HH TYR C 108 28.781 0.472 52.956 0.00 0.00
C
ATOM 21755 CD2 TYR C 108 27.498 4.243 54.684 0.00 0.00
C
ATOM 21756 HD2 TYR C 108 26.833 5.094 54.696 0.00 0.00
C
ATOM 21757 CE2 TYR C 108 27.410 3.199 53.787 0.00 0.00
C
ATOM 21758 HE2 TYR C 108 26.807 3.282 52.895 0.00 0.00
C
ATOM 21759 C TYR C 108 29.570 6.785 54.698 0.00 0.00
C
ATOM 21760 O TYR C 108 30.472 6.457 53.984 0.00 0.00
C
ATOM 21761 N HSE C 109 28.486 7.483 54.257 0.00 0.00
C

ATOM 21762 HN HSE C 109   27.833   7.884 54.896 0.00 0.00
C
ATOM 21763 CA HSE C 109   28.322   7.942 52.922 0.00 0.00
C
ATOM 21764 HA HSE C 109   28.263   7.033 52.342 0.00 0.00
C
ATOM 21765 CB HSE C 109   27.018   8.739 52.838 0.00 0.00
C
ATOM 21766 HB1 HSE C 109   26.187   8.115 53.232 0.00 0.00
C
ATOM 21767 HB2 HSE C 109   27.012   9.634 53.495 0.00 0.00
C
ATOM 21768 ND1 HSE C 109   25.401   9.959 51.486 0.00 0.00
C
ATOM 21769 CG HSE C 109   26.573   9.192 51.513 0.00 0.00
C
ATOM 21770 CE1 HSE C 109   25.046   9.993 50.181 0.00 0.00
C
ATOM 21771 HE1 HSE C 109   24.292 10.686 49.807 0.00 0.00
C
ATOM 21772 NE2 HSE C 109   25.939   9.296 49.432 0.00 0.00
C
ATOM 21773 HE2 HSE C 109   26.004   9.252 48.435 0.00 0.00
C
ATOM 21774 CD2 HSE C 109   26.827   8.739 50.288 0.00 0.00
C
ATOM 21775 HD2 HSE C 109   27.731   8.185 50.069 0.00 0.00
C
ATOM 21776 C   HSE C 109   29.456   8.682 52.341 0.00 0.00
C
ATOM 21777 O   HSE C 109   30.019   8.443 51.276 0.00 0.00
C
ATOM 21778 N   HSE C 110   30.022   9.667 53.163 0.00 0.00
C
ATOM 21779 HN HSE C 110   29.593   9.902 54.032 0.00 0.00
C
ATOM 21780 CA HSE C 110   31.221 10.550 52.748 0.00 0.00
C
ATOM 21781 HA HSE C 110   30.868 11.068 51.869 0.00 0.00
C
ATOM 21782 CB HSE C 110   31.532 11.651 53.809 0.00 0.00
C
ATOM 21783 HB1 HSE C 110   30.595 12.157 54.129 0.00 0.00
C
ATOM 21784 HB2 HSE C 110   32.000 11.163 54.690 0.00 0.00
C
ATOM 21785 ND1 HSE C 110   31.992 14.073 53.332 0.00 0.00
C
ATOM 21786 CG HSE C 110   32.452 12.770 53.349 0.00 0.00
C
ATOM 21787 CE1 HSE C 110   33.068 14.799 52.988 0.00 0.00
C
ATOM 21788 HE1 HSE C 110   33.142 15.886 53.021 0.00 0.00
C

ATOM 21789 NE2 HSE C 110   34.202 13.987 52.764 0.00 0.00
C
ATOM 21790 HE2 HSE C 110   35.152 14.252 52.597 0.00 0.00
C
ATOM 21791 CD2 HSE C 110   33.800 12.678 53.043 0.00 0.00
C
ATOM 21792 HD2 HSE C 110   34.516 11.871 52.950 0.00 0.00
C
ATOM 21793 C   HSE C 110   32.523   9.839 52.522 0.00 0.00
C
ATOM 21794 O   HSE C 110   33.216 10.036 51.590 0.00 0.00
C
ATOM 21795 N   LYS C 111   32.752   8.828 53.444 0.00 0.00
C
ATOM 21796 HN LYS C 111   32.041   8.515 54.070 0.00 0.00
C
ATOM 21797 CA LYS C 111   33.960   7.996 53.607 0.00 0.00
C
ATOM 21798 HA LYS C 111   34.913   8.502 53.565 0.00 0.00
C
ATOM 21799 CB LYS C 111   33.958   7.210 54.978 0.00 0.00
C
ATOM 21800 HB1 LYS C 111   32.949   6.784 55.168 0.00 0.00
C
ATOM 21801 HB2 LYS C 111   34.601   6.312 55.092 0.00 0.00
C
ATOM 21802 CG LYS C 111   34.261   8.121 56.153 0.00 0.00
C
ATOM 21803 HG1 LYS C 111   35.359   8.279 56.084 0.00 0.00
C
ATOM 21804 HG2 LYS C 111   33.579   8.976 55.956 0.00 0.00
C
ATOM 21805 CD LYS C 111   33.833   7.660 57.633 0.00 0.00
C
ATOM 21806 HD1 LYS C 111   32.733   7.504 57.641 0.00 0.00
C
ATOM 21807 HD2 LYS C 111   34.397   6.743 57.908 0.00 0.00
C
ATOM 21808 CE LYS C 111   34.255   8.653 58.719 0.00 0.00
C
ATOM 21809 HE1 LYS C 111   33.815   9.673 58.729 0.00 0.00
C
ATOM 21810 HE2 LYS C 111   34.052   8.311 59.757 0.00 0.00
C
ATOM 21811 NZ LYS C 111   35.703   8.915 58.705 0.00 0.00
C
ATOM 21812 HZ1 LYS C 111   36.160   8.010 58.471 0.00 0.00
C
ATOM 21813 HZ2 LYS C 111   35.944   9.597 57.957 0.00 0.00
C
ATOM 21814 HZ3 LYS C 111   36.096   9.263 59.603 0.00 0.00
C
ATOM 21815 C   LYS C 111   33.995   7.033 52.408 0.00 0.00
C

ATOM 21816 O   LYS C 111   34.917   6.962 51.590 0.00 0.00
C
ATOM 21817 N   VAL C 112   32.880   6.344 52.162 0.00 0.00
C
ATOM 21818 HN VAL C 112   32.118   6.415 52.801 0.00 0.00
C
ATOM 21819 CA VAL C 112   32.755   5.498 50.993 0.00 0.00
C
ATOM 21820 HA VAL C 112   33.590   4.838 51.180 0.00 0.00
C
ATOM 21821 CB VAL C 112   31.456   4.817 51.006 0.00 0.00
C
ATOM 21822 HB VAL C 112   30.583   5.502 51.062 0.00 0.00
C
ATOM 21823 CG1 VAL C 112   31.158   3.955 49.741 0.00 0.00
C
ATOM 21824 HG11 VAL C 112   30.235   3.348 49.854 0.00 0.00
C
ATOM 21825 HG12 VAL C 112   30.914   4.632 48.894 0.00 0.00
C
ATOM 21826 HG13 VAL C 112   32.023   3.333 49.424 0.00 0.00
C
ATOM 21827 CG2 VAL C 112   31.345   3.837 52.224 0.00 0.00
C
ATOM 21828 HG21 VAL C 112   32.198   3.126 52.247 0.00 0.00
C
ATOM 21829 HG22 VAL C 112   31.105   4.356 53.177 0.00 0.00
C
ATOM 21830 HG23 VAL C 112   30.501   3.150 51.999 0.00 0.00
C
ATOM 21831 C   VAL C 112   32.995   6.082 49.544 0.00 0.00
C
ATOM 21832 O   VAL C 112   33.711   5.527 48.725 0.00 0.00
C
ATOM 21833 N   LEU C 113   32.498   7.375 49.362 0.00 0.00
C
ATOM 21834 HN LEU C 113   32.108   7.737 50.205 0.00 0.00
C
ATOM 21835 CA LEU C 113   32.641   8.254 48.176 0.00 0.00
C
ATOM 21836 HA LEU C 113   32.455   7.635 47.310 0.00 0.00
C
ATOM 21837 CB LEU C 113   31.639   9.473 48.235 0.00 0.00
C
ATOM 21838 HB1 LEU C 113   31.780 10.041 49.179 0.00 0.00
C
ATOM 21839 HB2 LEU C 113   31.820 10.194 47.409 0.00 0.00
C
ATOM 21840 CG LEU C 113   30.173   9.007 48.082 0.00 0.00
C
ATOM 21841 HG LEU C 113   30.058   8.226 48.863 0.00 0.00
C
ATOM 21842 CD1 LEU C 113   29.135 10.028 48.484 0.00 0.00
C

ATOM 21843 HD11 LEU C 113   29.211 10.141 49.586 0.00 0.00
C
ATOM 21844 HD12 LEU C 113   29.372 10.968 47.942 0.00 0.00
C
ATOM 21845 HD13 LEU C 113   28.104   9.791 48.146 0.00 0.00
C
ATOM 21846 CD2 LEU C 113   29.855   8.301 46.758 0.00 0.00
C
ATOM 21847 HD21 LEU C 113   28.799   7.956 46.751 0.00 0.00
C
ATOM 21848 HD22 LEU C 113   29.988   9.073 45.970 0.00 0.00
C
ATOM 21849 HD23 LEU C 113   30.601   7.483 46.668 0.00 0.00
C
ATOM 21850 C   LEU C 113   34.038   8.784 48.016 0.00 0.00
C
ATOM 21851 O   LEU C 113   34.560   8.814 46.930 0.00 0.00
C
ATOM 21852 N   GLU C 114   34.663   9.322 49.043 0.00 0.00
C
ATOM 21853 HN GLU C 114   34.159   9.103 49.875 0.00 0.00
C
ATOM 21854 CA GLU C 114   36.068   9.756 49.095 0.00 0.00
C
ATOM 21855 HA GLU C 114   36.099 10.626 48.455 0.00 0.00
C
ATOM 21856 CB GLU C 114   36.302 10.276 50.471 0.00 0.00
C
ATOM 21857 HB1 GLU C 114   35.444 10.923 50.753 0.00 0.00
C
ATOM 21858 HB2 GLU C 114   36.258   9.353 51.088 0.00 0.00
C
ATOM 21859 CG GLU C 114   37.678 10.891 50.717 0.00 0.00
C
ATOM 21860 HG1 GLU C 114   38.481 10.268 50.267 0.00 0.00
C
ATOM 21861 HG2 GLU C 114   37.657 11.898 50.248 0.00 0.00
C
ATOM 21862 CD GLU C 114   37.944 11.071 52.252 0.00 0.00
C
ATOM 21863 OE1 GLU C 114   38.729 10.340 52.771 0.00 0.00
C
ATOM 21864 OE2 GLU C 114   37.331 11.948 52.817 0.00 0.00
C
ATOM 21865 C   GLU C 114   37.103   8.669 48.681 0.00 0.00
C
ATOM 21866 O   GLU C 114   37.972   8.918 47.831 0.00 0.00
C
ATOM 21867 N   LYS C 115   36.953   7.449 49.207 0.00 0.00
C
ATOM 21868 HN LYS C 115   36.341   7.223 49.960 0.00 0.00
C
ATOM 21869 CA LYS C 115   37.930   6.384 48.955 0.00 0.00
C

ATOM 21870 HA LYS C 115   38.898   6.848 48.834 0.00 0.00
C
ATOM 21871 CB LYS C 115   37.852   5.239 50.041 0.00 0.00
C
ATOM 21872 HB1 LYS C 115   36.774   5.010 50.180 0.00 0.00
C
ATOM 21873 HB2 LYS C 115   38.457   4.388 49.662 0.00 0.00
C
ATOM 21874 CG LYS C 115   38.215   5.817 51.478 0.00 0.00
C
ATOM 21875 HG1 LYS C 115   39.245   6.206 51.329 0.00 0.00
C
ATOM 21876 HG2 LYS C 115   37.625   6.699 51.809 0.00 0.00
C
ATOM 21877 CD LYS C 115   38.206   4.679 52.569 0.00 0.00
C
ATOM 21878 HD1 LYS C 115   37.157   4.369 52.762 0.00 0.00
C
ATOM 21879 HD2 LYS C 115   38.780   3.811 52.180 0.00 0.00
C
ATOM 21880 CE LYS C 115   38.812   4.954 53.919 0.00 0.00
C
ATOM 21881 HE1 LYS C 115   38.317   5.757 54.507 0.00 0.00
C
ATOM 21882 HE2 LYS C 115   38.839   4.011 54.506 0.00 0.00
C
ATOM 21883 NZ LYS C 115   40.221   5.436 53.843 0.00 0.00
C
ATOM 21884 HZ1 LYS C 115   40.314   6.457 53.667 0.00 0.00
C
ATOM 21885 HZ2 LYS C 115   40.765   5.254 54.710 0.00 0.00
C
ATOM 21886 HZ3 LYS C 115   40.728   5.000 53.046 0.00 0.00
C
ATOM 21887 C   LYS C 115   37.719   5.716 47.610 0.00 0.00
C
ATOM 21888 O   LYS C 115   38.610   5.060 47.086 0.00 0.00
C
ATOM 21889 N   ILE C 116   36.570   5.945 46.929 0.00 0.00
C
ATOM 21890 HN ILE C 116   35.761   6.373 47.325 0.00 0.00
C
ATOM 21891 CA ILE C 116   36.301   5.316 45.589 0.00 0.00
C
ATOM 21892 HA ILE C 116   37.002   4.516 45.403 0.00 0.00
C
ATOM 21893 CB ILE C 116   34.935   4.738 45.364 0.00 0.00
C
ATOM 21894 HB ILE C 116   34.992   4.327 44.333 0.00 0.00
C
ATOM 21895 CG2 ILE C 116   34.781   3.506 46.237 0.00 0.00
C
ATOM 21896 HG21 ILE C 116   35.576   2.734 46.152 0.00 0.00
C

ATOM 21897 HG22 ILE C 116   34.757   3.759 47.318 0.00 0.00
C
ATOM 21898 HG23 ILE C 116   33.881   2.873 46.083 0.00 0.00
C
ATOM 21899 CG1 ILE C 116   33.781   5.871 45.457 0.00 0.00
C
ATOM 21900 HG11 ILE C 116   33.862   6.410 46.425 0.00 0.00
C
ATOM 21901 HG12 ILE C 116   33.931   6.610 44.642 0.00 0.00
C
ATOM 21902 CD ILE C 116   32.358   5.347 45.319 0.00 0.00
C
ATOM 21903 HD1 ILE C 116   32.248   5.181 44.226 0.00 0.00
C
ATOM 21904 HD2 ILE C 116   32.263   4.357 45.814 0.00 0.00
C
ATOM 21905 HD3 ILE C 116   31.584   6.070 45.654 0.00 0.00
C
ATOM 21906 C   ILE C 116   36.650   6.418 44.574 0.00 0.00
C
ATOM 21907 O   ILE C 116   36.787   6.163 43.353 0.00 0.00
C
ATOM 21908 N   GLY C 117   36.728   7.654 45.035 0.00 0.00
C
ATOM 21909 HN GLY C 117   36.632   7.636 46.027 0.00 0.00
C
ATOM 21910 CA GLY C 117   37.473   8.793 44.432 0.00 0.00
C
ATOM 21911 HA1 GLY C 117   38.154   8.470 43.658 0.00 0.00
C
ATOM 21912 HA2 GLY C 117   37.905   9.272 45.298 0.00 0.00
C
ATOM 21913 C   GLY C 117   36.658   9.836 43.802 0.00 0.00
C
ATOM 21914 O   GLY C 117   36.871 10.299 42.683 0.00 0.00
C
ATOM 21915 N   ALA C 118   35.557 10.307 44.501 0.00 0.00
C
ATOM 21916 HN ALA C 118   35.351 10.037 45.438 0.00 0.00
C
ATOM 21917 CA ALA C 118   34.781 11.424 44.050 0.00 0.00
C
ATOM 21918 HA ALA C 118   34.586 11.353 42.990 0.00 0.00
C
ATOM 21919 CB ALA C 118   33.459 11.408 44.892 0.00 0.00
C
ATOM 21920 HB1 ALA C 118   33.663 11.749 45.929 0.00 0.00
C
ATOM 21921 HB2 ALA C 118   32.805 12.168 44.414 0.00 0.00
C
ATOM 21922 HB3 ALA C 118   33.080 10.366 44.825 0.00 0.00
C
ATOM 21923 C   ALA C 118   35.513 12.759 44.298 0.00 0.00
C

ATOM 21924 O ALA C 118 36.144 12.946 45.347 0.00 0.00
C
ATOM 21925 N ASP C 119 35.462 13.634 43.215 0.00 0.00
C
ATOM 21926 HN ASP C 119 35.111 13.344 42.328 0.00 0.00
C
ATOM 21927 CA ASP C 119 36.222 14.879 43.218 0.00 0.00
C
ATOM 21928 HA ASP C 119 37.186 14.523 43.551 0.00 0.00
C
ATOM 21929 CB ASP C 119 36.387 15.414 41.734 0.00 0.00
C
ATOM 21930 HB1 ASP C 119 35.458 15.880 41.343 0.00 0.00
C
ATOM 21931 HB2 ASP C 119 37.223 16.143 41.676 0.00 0.00
C
ATOM 21932 CG ASP C 119 36.835 14.260 40.878 0.00 0.00
C
ATOM 21933 OD1 ASP C 119 38.116 14.066 40.909 0.00 0.00
C
ATOM 21934 OD2 ASP C 119 36.016 13.530 40.267 0.00 0.00
C
ATOM 21935 C ASP C 119 35.695 15.924 44.165 0.00 0.00
C
ATOM 21936 O ASP C 119 36.546 16.403 44.921 0.00 0.00
C
ATOM 21937 N ARG C 120 34.367 16.140 44.212 0.00 0.00
C
ATOM 21938 HN ARG C 120 33.819 15.494 43.685 0.00 0.00
C
ATOM 21939 CA ARG C 120 33.715 17.105 45.146 0.00 0.00
C
ATOM 21940 HA ARG C 120 34.490 17.405 45.836 0.00 0.00
C
ATOM 21941 CB ARG C 120 33.198 18.404 44.345 0.00 0.00
C
ATOM 21942 HB1 ARG C 120 34.044 18.734 43.704 0.00 0.00
C
ATOM 21943 HB2 ARG C 120 32.371 18.057 43.689 0.00 0.00
C
ATOM 21944 CG ARG C 120 32.794 19.547 45.229 0.00 0.00
C
ATOM 21945 HG1 ARG C 120 31.980 19.217 45.910 0.00 0.00
C
ATOM 21946 HG2 ARG C 120 33.642 19.765 45.913 0.00 0.00
C
ATOM 21947 CD ARG C 120 32.504 20.790 44.514 0.00 0.00
C
ATOM 21948 HD1 ARG C 120 33.372 21.166 43.930 0.00 0.00
C
ATOM 21949 HD2 ARG C 120 31.610 20.663 43.867 0.00 0.00
C
ATOM 21950 NE ARG C 120 32.101 21.685 45.668 0.00 0.00
C

ATOM 21951 HE ARG C 120   31.731 21.202 46.462 0.00 0.00
C
ATOM 21952 CZ ARG C 120   32.130 23.025 45.658 0.00 0.00
C
ATOM 21953 NH1 ARG C 120   32.801 23.782 44.744 0.00 0.00
C
ATOM 21954 HH11 ARG C 120   32.926 24.757 44.927 0.00 0.00
C
ATOM 21955 HH12 ARG C 120   33.549 23.312 44.275 0.00 0.00
C
ATOM 21956 NH2 ARG C 120   31.557 23.687 46.663 0.00 0.00
C
ATOM 21957 HH21 ARG C 120   31.936 24.606 46.777 0.00 0.00
C
ATOM 21958 HH22 ARG C 120   31.241 23.120 47.423 0.00 0.00
C
ATOM 21959 C   ARG C 120   32.575 16.419 45.828 0.00 0.00
C
ATOM 21960 O   ARG C 120   31.640 15.929 45.149 0.00 0.00
C
ATOM 21961 N   ILE C 121   32.580 16.457 47.171 0.00 0.00
C
ATOM 21962 HN ILE C 121   33.260 16.943 47.714 0.00 0.00
C
ATOM 21963 CA ILE C 121   31.470 15.828 47.893 0.00 0.00
C
ATOM 21964 HA ILE C 121   30.824 15.390 47.146 0.00 0.00
C
ATOM 21965 CB ILE C 121   31.911 14.760 48.893 0.00 0.00
C
ATOM 21966 HB ILE C 121   32.641 15.125 49.648 0.00 0.00
C
ATOM 21967 CG2 ILE C 121   30.759 13.974 49.530 0.00 0.00
C
ATOM 21968 HG21 ILE C 121   30.129 13.556 48.716 0.00 0.00
C
ATOM 21969 HG22 ILE C 121   31.141 13.151 50.170 0.00 0.00
C
ATOM 21970 HG23 ILE C 121   30.121 14.567 50.219 0.00 0.00
C
ATOM 21971 CG1 ILE C 121   32.969 13.836 48.253 0.00 0.00
C
ATOM 21972 HG11 ILE C 121   32.418 13.275 47.469 0.00 0.00
C
ATOM 21973 HG12 ILE C 121   33.808 14.298 47.690 0.00 0.00
C
ATOM 21974 CD ILE C 121   33.709 13.006 49.181 0.00 0.00
C
ATOM 21975 HD1 ILE C 121   33.087 12.287 49.754 0.00 0.00
C
ATOM 21976 HD2 ILE C 121   34.330 12.438 48.456 0.00 0.00
C
ATOM 21977 HD3 ILE C 121   34.480 13.518 49.796 0.00 0.00
C

ATOM 21978 C   ILE C 121   30.668 16.899 48.591 0.00 0.00
C
ATOM 21979 O   ILE C 121   31.246 17.720 49.351 0.00 0.00
C
ATOM 21980 N   ILE C 122   29.340 17.143 48.270 0.00 0.00
C
ATOM 21981 HN ILE C 122   28.932 16.435 47.699 0.00 0.00
C
ATOM 21982 CA ILE C 122   28.450 18.146 48.758 0.00 0.00
C
ATOM 21983 HA ILE C 122   29.071 18.846 49.295 0.00 0.00
C
ATOM 21984 CB ILE C 122   27.634 18.943 47.689 0.00 0.00
C
ATOM 21985 HB ILE C 122   26.914 18.229 47.236 0.00 0.00
C
ATOM 21986 CG2 ILE C 122   26.855 20.157 48.427 0.00 0.00
C
ATOM 21987 HG21 ILE C 122   27.490 21.026 48.706 0.00 0.00
C
ATOM 21988 HG22 ILE C 122   26.067 20.417 47.688 0.00 0.00
C
ATOM 21989 HG23 ILE C 122   26.248 19.928 49.328 0.00 0.00
C
ATOM 21990 CG1 ILE C 122   28.551 19.561 46.637 0.00 0.00
C
ATOM 21991 HG11 ILE C 122   28.020 20.359 46.075 0.00 0.00
C
ATOM 21992 HG12 ILE C 122   29.409 20.098 47.097 0.00 0.00
C
ATOM 21993 CD ILE C 122   28.993 18.702 45.429 0.00 0.00
C
ATOM 21994 HD1 ILE C 122   28.078 18.153 45.119 0.00 0.00
C
ATOM 21995 HD2 ILE C 122   29.287 19.384 44.602 0.00 0.00
C
ATOM 21996 HD3 ILE C 122   29.773 17.944 45.655 0.00 0.00
C
ATOM 21997 C   ILE C 122   27.389 17.526 49.725 0.00 0.00
C
ATOM 21998 O   ILE C 122   26.481 16.792 49.365 0.00 0.00
C
ATOM 21999 N   HSE C 123   27.457 17.913 50.892 0.00 0.00
C
ATOM 22000 HN HSE C 123   28.229 18.492 51.141 0.00 0.00
C
ATOM 22001 CA HSE C 123   26.425 17.792 51.938 0.00 0.00
C
ATOM 22002 HA HSE C 123   25.764 16.956 51.763 0.00 0.00
C
ATOM 22003 CB HSE C 123   27.050 17.494 53.319 0.00 0.00
C
ATOM 22004 HB1 HSE C 123   27.845 18.231 53.564 0.00 0.00
C

ATOM 22005 HB2 HSE C 123 26.260 17.587 54.095 0.00 0.00
C
ATOM 22006 ND1 HSE C 123 27.306 15.014 54.080 0.00 0.00
C
ATOM 22007 CG HSE C 123 27.780 16.165 53.381 0.00 0.00
C
ATOM 22008 CE1 HSE C 123 28.358 14.174 53.995 0.00 0.00
C
ATOM 22009 HE1 HSE C 123 28.434 13.152 54.364 0.00 0.00
C
ATOM 22010 NE2 HSE C 123 29.337 14.724 53.255 0.00 0.00
C
ATOM 22011 HE2 HSE C 123 30.250 14.341 53.113 0.00 0.00
C
ATOM 22012 CD2 HSE C 123 28.996 15.926 52.813 0.00 0.00
C
ATOM 22013 HD2 HSE C 123 29.559 16.485 52.076 0.00 0.00
C
ATOM 22014 C HSE C 123 25.809 19.151 51.989 0.00 0.00
C
ATOM 22015 O HSE C 123 26.400 19.983 52.660 0.00 0.00
C
ATOM 22016 N PRO C 124 24.680 19.442 51.350 0.00 0.00
C
ATOM 22017 CD PRO C 124 23.976 18.614 50.395 0.00 0.00
C
ATOM 22018 HD1 PRO C 124 24.562 18.523 49.455 0.00 0.00
C
ATOM 22019 HD2 PRO C 124 23.686 17.628 50.816 0.00 0.00
C
ATOM 22020 CA PRO C 124 24.307 20.807 51.193 0.00 0.00
C
ATOM 22021 HA PRO C 124 25.109 21.465 50.892 0.00 0.00
C
ATOM 22022 CB PRO C 124 23.154 20.876 50.254 0.00 0.00
C
ATOM 22023 HB1 PRO C 124 23.624 21.167 49.290 0.00 0.00
C
ATOM 22024 HB2 PRO C 124 22.374 21.640 50.457 0.00 0.00
C
ATOM 22025 CG PRO C 124 22.699 19.415 50.037 0.00 0.00
C
ATOM 22026 HG1 PRO C 124 21.889 19.289 50.787 0.00 0.00
C
ATOM 22027 HG2 PRO C 124 22.283 19.280 49.016 0.00 0.00
C
ATOM 22028 C PRO C 124 23.856 21.445 52.477 0.00 0.00
C
ATOM 22029 O PRO C 124 23.938 22.672 52.620 0.00 0.00
C
ATOM 22030 N GLU C 125 23.333 20.743 53.509 0.00 0.00
C
ATOM 22031 HN GLU C 125 23.280 19.748 53.468 0.00 0.00
C

```
ATOM  22032  CA  GLU C 125      22.785  21.430  54.634  0.00  0.00
C
ATOM  22033  HA  GLU C 125      22.350  22.354  54.284  0.00  0.00
C
ATOM  22034  CB  GLU C 125      21.573  20.725  55.302  0.00  0.00
C
ATOM  22035  HB1 GLU C 125      21.943  19.686  55.433  0.00  0.00
C
ATOM  22036  HB2 GLU C 125      21.264  21.180  56.267  0.00  0.00
C
ATOM  22037  CG  GLU C 125      20.369  20.803  54.365  0.00  0.00
C
ATOM  22038  HG1 GLU C 125      19.809  21.756  54.480  0.00  0.00
C
ATOM  22039  HG2 GLU C 125      20.656  20.705  53.296  0.00  0.00
C
ATOM  22040  CD  GLU C 125      19.539  19.567  54.685  0.00  0.00
C
ATOM  22041  OE1 GLU C 125      19.651  18.505  53.999  0.00  0.00
C
ATOM  22042  OE2 GLU C 125      18.673  19.558  55.600  0.00  0.00
C
ATOM  22043  C   GLU C 125      23.841  21.544  55.718  0.00  0.00
C
ATOM  22044  O   GLU C 125      23.828  22.467  56.504  0.00  0.00
C
ATOM  22045  N   LYS C 126      24.755  20.628  55.720  0.00  0.00
C
ATOM  22046  HN  LYS C 126      24.609  19.826  55.147  0.00  0.00
C
ATOM  22047  CA  LYS C 126      25.889  20.707  56.676  0.00  0.00
C
ATOM  22048  HA  LYS C 126      25.553  20.907  57.683  0.00  0.00
C
ATOM  22049  CB  LYS C 126      26.711  19.348  56.592  0.00  0.00
C
ATOM  22050  HB1 LYS C 126      25.982  18.512  56.672  0.00  0.00
C
ATOM  22051  HB2 LYS C 126      27.155  19.248  55.579  0.00  0.00
C
ATOM  22052  CG  LYS C 126      27.841  19.149  57.595  0.00  0.00
C
ATOM  22053  HG1 LYS C 126      28.746  19.606  57.139  0.00  0.00
C
ATOM  22054  HG2 LYS C 126      27.474  19.572  58.554  0.00  0.00
C
ATOM  22055  CD  LYS C 126      28.042  17.581  57.719  0.00  0.00
C
ATOM  22056  HD1 LYS C 126      27.049  17.087  57.780  0.00  0.00
C
ATOM  22057  HD2 LYS C 126      28.252  17.342  56.655  0.00  0.00
C
ATOM  22058  CE  LYS C 126      29.018  17.252  58.878  0.00  0.00
C
```

ATOM 22059 HE1 LYS C 126 28.624 17.815 59.752 0.00 0.00
C
ATOM 22060 HE2 LYS C 126 28.872 16.166 59.056 0.00 0.00
C
ATOM 22061 NZ LYS C 126 30.389 17.588 58.583 0.00 0.00
C
ATOM 22062 HZ1 LYS C 126 30.524 18.617 58.509 0.00 0.00
C
ATOM 22063 HZ2 LYS C 126 30.972 17.188 59.345 0.00 0.00
C
ATOM 22064 HZ3 LYS C 126 30.773 17.094 57.753 0.00 0.00
C
ATOM 22065 C LYS C 126 26.778 21.889 56.411 0.00 0.00
C
ATOM 22066 O LYS C 126 27.232 22.589 57.333 0.00 0.00
C
ATOM 22067 N ASP C 127 26.999 22.130 55.061 0.00 0.00
C
ATOM 22068 HN ASP C 127 26.885 21.315 54.497 0.00 0.00
C
ATOM 22069 CA ASP C 127 27.656 23.286 54.567 0.00 0.00
C
ATOM 22070 HA ASP C 127 28.561 23.496 55.118 0.00 0.00
C
ATOM 22071 CB ASP C 127 27.976 23.101 53.065 0.00 0.00
C
ATOM 22072 HB1 ASP C 127 27.767 22.039 52.812 0.00 0.00
C
ATOM 22073 HB2 ASP C 127 27.301 23.722 52.439 0.00 0.00
C
ATOM 22074 CG ASP C 127 29.425 23.307 52.669 0.00 0.00
C
ATOM 22075 OD1 ASP C 127 29.971 24.391 52.629 0.00 0.00
C
ATOM 22076 OD2 ASP C 127 30.080 22.245 52.376 0.00 0.00
C
ATOM 22077 C ASP C 127 26.963 24.566 54.904 0.00 0.00
C
ATOM 22078 O ASP C 127 27.542 25.582 55.227 0.00 0.00
C
ATOM 22079 N MET C 128 25.664 24.674 54.749 0.00 0.00
C
ATOM 22080 HN MET C 128 25.148 23.871 54.459 0.00 0.00
C
ATOM 22081 CA MET C 128 24.837 25.874 54.953 0.00 0.00
C
ATOM 22082 HA MET C 128 25.391 26.703 54.538 0.00 0.00
C
ATOM 22083 CB MET C 128 23.491 25.719 54.312 0.00 0.00
C
ATOM 22084 HB1 MET C 128 23.782 25.434 53.279 0.00 0.00
C
ATOM 22085 HB2 MET C 128 22.896 24.849 54.665 0.00 0.00
C

ATOM 22086 CG MET C 128   22.634 26.955 54.108 0.00 0.00
C
ATOM 22087 HG1 MET C 128   21.618 26.619 53.810 0.00 0.00
C
ATOM 22088 HG2 MET C 128   22.550 27.596 55.011 0.00 0.00
C
ATOM 22089 SD MET C 128   23.323 27.892 52.820 0.00 0.00
C
ATOM 22090 CE MET C 128   22.382 29.393 53.206 0.00 0.00
C
ATOM 22091 HE1 MET C 128   22.433 30.058 52.317 0.00 0.00
C
ATOM 22092 HE2 MET C 128   21.348 29.038 53.403 0.00 0.00
C
ATOM 22093 HE3 MET C 128   22.973 29.763 54.070 0.00 0.00
C
ATOM 22094 C   MET C 128   24.625 26.296 56.410 0.00 0.00
C
ATOM 22095 O   MET C 128   24.459 27.416 56.774 0.00 0.00
C
ATOM 22096 N   GLY C 129   24.698 25.264 57.232 0.00 0.00
C
ATOM 22097 HN GLY C 129   24.984 24.385 56.858 0.00 0.00
C
ATOM 22098 CA GLY C 129   24.651 25.424 58.729 0.00 0.00
C
ATOM 22099 HA1 GLY C 129   24.437 24.470 59.189 0.00 0.00
C
ATOM 22100 HA2 GLY C 129   23.904 26.163 58.976 0.00 0.00
C
ATOM 22101 C   GLY C 129   25.900 26.023 59.312 0.00 0.00
C
ATOM 22102 O   GLY C 129   25.749 26.969 60.134 0.00 0.00
C
ATOM 22103 N   VAL C 130   27.122 25.659 58.831 0.00 0.00
C
ATOM 22104 HN VAL C 130   27.347 24.828 58.328 0.00 0.00
C
ATOM 22105 CA VAL C 130   28.314 26.376 59.246 0.00 0.00
C
ATOM 22106 HA VAL C 130   28.165 26.464 60.312 0.00 0.00
C
ATOM 22107 CB VAL C 130   29.667 25.766 58.952 0.00 0.00
C
ATOM 22108 HB VAL C 130   30.410 26.427 59.446 0.00 0.00
C
ATOM 22109 CG1 VAL C 130   29.762 24.451 59.774 0.00 0.00
C
ATOM 22110 HG11 VAL C 130   30.834 24.157 59.755 0.00 0.00
C
ATOM 22111 HG12 VAL C 130   29.551 24.587 60.856 0.00 0.00
C
ATOM 22112 HG13 VAL C 130   29.158 23.610 59.372 0.00 0.00
C

ATOM 22113 CG2 VAL C 130 30.025 25.512 57.441 0.00 0.00
C
ATOM 22114 HG21 VAL C 130 29.321 24.729 57.087 0.00 0.00
C
ATOM 22115 HG22 VAL C 130 29.844 26.446 56.867 0.00 0.00
C
ATOM 22116 HG23 VAL C 130 31.038 25.060 57.378 0.00 0.00
C
ATOM 22117 C VAL C 130 28.262 27.772 58.817 0.00 0.00
C
ATOM 22118 O VAL C 130 28.547 28.741 59.536 0.00 0.00
C
ATOM 22119 N LYS C 131 27.804 28.022 57.586 0.00 0.00
C
ATOM 22120 HN LYS C 131 27.458 27.299 56.994 0.00 0.00
C
ATOM 22121 CA LYS C 131 27.542 29.421 57.047 0.00 0.00
C
ATOM 22122 HA LYS C 131 28.428 30.036 57.080 0.00 0.00
C
ATOM 22123 CB LYS C 131 27.230 29.315 55.523 0.00 0.00
C
ATOM 22124 HB1 LYS C 131 26.404 28.574 55.470 0.00 0.00
C
ATOM 22125 HB2 LYS C 131 26.834 30.317 55.252 0.00 0.00
C
ATOM 22126 CG LYS C 131 28.442 28.953 54.696 0.00 0.00
C
ATOM 22127 HG1 LYS C 131 29.187 29.777 54.705 0.00 0.00
C
ATOM 22128 HG2 LYS C 131 28.967 28.107 55.189 0.00 0.00
C
ATOM 22129 CD LYS C 131 28.092 28.280 53.340 0.00 0.00
C
ATOM 22130 HD1 LYS C 131 27.417 27.399 53.376 0.00 0.00
C
ATOM 22131 HD2 LYS C 131 27.448 29.078 52.911 0.00 0.00
C
ATOM 22132 CE LYS C 131 29.261 27.919 52.459 0.00 0.00
C
ATOM 22133 HE1 LYS C 131 28.883 27.542 51.485 0.00 0.00
C
ATOM 22134 HE2 LYS C 131 29.808 28.870 52.281 0.00 0.00
C
ATOM 22135 NZ LYS C 131 30.187 26.941 53.081 0.00 0.00
C
ATOM 22136 HZ1 LYS C 131 30.502 27.300 54.005 0.00 0.00
C
ATOM 22137 HZ2 LYS C 131 29.969 25.925 53.123 0.00 0.00
C
ATOM 22138 HZ3 LYS C 131 31.010 27.070 52.459 0.00 0.00
C
ATOM 22139 C LYS C 131 26.516 30.283 57.809 0.00 0.00
C

ATOM 22140 O   LYS C 131   26.754 31.492 57.937 0.00 0.00
C
ATOM 22141 N   ILE C 132   25.358 29.780 58.229 0.00 0.00
C
ATOM 22142 HN ILE C 132   25.138 28.817 58.092 0.00 0.00
C
ATOM 22143 CA ILE C 132   24.371 30.491 59.047 0.00 0.00
C
ATOM 22144 HA ILE C 132   24.258 31.474 58.613 0.00 0.00
C
ATOM 22145 CB ILE C 132   23.015 29.749 58.893 0.00 0.00
C
ATOM 22146 HB ILE C 132   23.180 28.663 59.059 0.00 0.00
C
ATOM 22147 CG2 ILE C 132   21.956 30.170 59.958 0.00 0.00
C
ATOM 22148 HG21 ILE C 132   21.972 31.278 60.033 0.00 0.00
C
ATOM 22149 HG22 ILE C 132   20.995 29.705 59.651 0.00 0.00
C
ATOM 22150 HG23 ILE C 132   22.293 29.794 60.948 0.00 0.00
C
ATOM 22151 CG1 ILE C 132   22.350 29.989 57.544 0.00 0.00
C
ATOM 22152 HG11 ILE C 132   22.829 29.449 56.699 0.00 0.00
C
ATOM 22153 HG12 ILE C 132   21.337 29.555 57.679 0.00 0.00
C
ATOM 22154 CD ILE C 132   22.067 31.464 57.077 0.00 0.00
C
ATOM 22155 HD1 ILE C 132   21.764 32.247 57.805 0.00 0.00
C
ATOM 22156 HD2 ILE C 132   22.911 31.883 56.489 0.00 0.00
C
ATOM 22157 HD3 ILE C 132   21.317 31.318 56.270 0.00 0.00
C
ATOM 22158 C   ILE C 132   24.887 30.634 60.479 0.00 0.00
C
ATOM 22159 O   ILE C 132   24.508 31.653 61.148 0.00 0.00
C
ATOM 22160 N   ALA C 133   25.792 29.742 61.070 0.00 0.00
C
ATOM 22161 HN ALA C 133   25.900 28.794 60.778 0.00 0.00
C
ATOM 22162 CA ALA C 133   26.526 30.252 62.246 0.00 0.00
C
ATOM 22163 HA ALA C 133   25.828 30.494 63.034 0.00 0.00
C
ATOM 22164 CB ALA C 133   27.441 29.100 62.730 0.00 0.00
C
ATOM 22165 HB1 ALA C 133   28.274 28.863 62.035 0.00 0.00
C
ATOM 22166 HB2 ALA C 133   27.980 29.251 63.689 0.00 0.00
C

ATOM 22167 HB3 ALA C 133 26.955 28.108 62.857 0.00 0.00
C
ATOM 22168 C ALA C 133 27.410 31.492 62.029 0.00 0.00
C
ATOM 22169 O ALA C 133 27.343 32.513 62.730 0.00 0.00
C
ATOM 22170 N GLN C 134 28.184 31.435 60.991 0.00 0.00
C
ATOM 22171 HN GLN C 134 28.365 30.584 60.503 0.00 0.00
C
ATOM 22172 CA GLN C 134 29.182 32.462 60.650 0.00 0.00
C
ATOM 22173 HA GLN C 134 29.797 32.656 61.515 0.00 0.00
C
ATOM 22174 CB GLN C 134 30.070 31.993 59.508 0.00 0.00
C
ATOM 22175 HB1 GLN C 134 30.494 31.050 59.916 0.00 0.00
C
ATOM 22176 HB2 GLN C 134 29.529 31.655 58.599 0.00 0.00
C
ATOM 22177 CG GLN C 134 31.217 33.053 59.185 0.00 0.00
C
ATOM 22178 HG1 GLN C 134 30.659 33.952 58.846 0.00 0.00
C
ATOM 22179 HG2 GLN C 134 31.852 33.362 60.043 0.00 0.00
C
ATOM 22180 CD GLN C 134 32.215 32.468 58.235 0.00 0.00
C
ATOM 22181 OE1 GLN C 134 31.838 31.995 57.139 0.00 0.00
C
ATOM 22182 NE2 GLN C 134 33.515 32.284 58.649 0.00 0.00
C
ATOM 22183 HE21 GLN C 134 33.996 31.531 58.200 0.00 0.00
C
ATOM 22184 HE22 GLN C 134 33.713 32.656 59.556 0.00 0.00
C
ATOM 22185 C GLN C 134 28.396 33.766 60.235 0.00 0.00
C
ATOM 22186 O GLN C 134 28.807 34.849 60.510 0.00 0.00
C
ATOM 22187 N SER C 135 27.264 33.640 59.590 0.00 0.00
C
ATOM 22188 HN SER C 135 27.075 32.755 59.171 0.00 0.00
C
ATOM 22189 CA SER C 135 26.372 34.849 59.400 0.00 0.00
C
ATOM 22190 HA SER C 135 27.014 35.571 58.919 0.00 0.00
C
ATOM 22191 CB SER C 135 25.018 34.655 58.542 0.00 0.00
C
ATOM 22192 HB1 SER C 135 24.349 34.068 59.207 0.00 0.00
C
ATOM 22193 HB2 SER C 135 24.510 35.577 58.186 0.00 0.00
C

ATOM 22194 OG   SER C 135   25.350 33.828 57.435 0.00 0.00
C
ATOM 22195 HG1 SER C 135   25.606 33.003 57.854 0.00 0.00
C
ATOM 22196 C   SER C 135   25.897 35.419 60.640 0.00 0.00
C
ATOM 22197 O   SER C 135   25.716 36.631 60.624 0.00 0.00
C
ATOM 22198 N   LEU C 136   25.578 34.640 61.702 0.00 0.00
C
ATOM 22199 HN LEU C 136   25.828 33.675 61.678 0.00 0.00
C
ATOM 22200 CA LEU C 136   25.068 35.186 62.989 0.00 0.00
C
ATOM 22201 HA LEU C 136   24.230 35.850 62.840 0.00 0.00
C
ATOM 22202 CB LEU C 136   24.629 34.021 63.874 0.00 0.00
C
ATOM 22203 HB1 LEU C 136   25.442 33.271 63.981 0.00 0.00
C
ATOM 22204 HB2 LEU C 136   24.351 34.375 64.890 0.00 0.00
C
ATOM 22205 CG LEU C 136   23.272 33.403 63.529 0.00 0.00
C
ATOM 22206 HG LEU C 136   23.319 32.935 62.523 0.00 0.00
C
ATOM 22207 CD1 LEU C 136   22.865 32.275 64.515 0.00 0.00
C
ATOM 22208 HD11 LEU C 136   23.526 31.397 64.354 0.00 0.00
C
ATOM 22209 HD12 LEU C 136   22.860 32.524 65.598 0.00 0.00
C
ATOM 22210 HD13 LEU C 136   21.833 31.986 64.222 0.00 0.00
C
ATOM 22211 CD2 LEU C 136   22.129 34.432 63.673 0.00 0.00
C
ATOM 22212 HD21 LEU C 136   22.328 35.078 64.554 0.00 0.00
C
ATOM 22213 HD22 LEU C 136   22.214 35.155 62.833 0.00 0.00
C
ATOM 22214 HD23 LEU C 136   21.136 33.971 63.862 0.00 0.00
C
ATOM 22215 C   LEU C 136   26.197 35.916 63.689 0.00 0.00
C
ATOM 22216 O   LEU C 136   25.977 36.880 64.363 0.00 0.00
C
ATOM 22217 N   SER C 137   27.444 35.442 63.502 0.00 0.00
C
ATOM 22218 HN SER C 137   27.601 34.530 63.130 0.00 0.00
C
ATOM 22219 CA SER C 137   28.628 36.066 64.037 0.00 0.00
C
ATOM 22220 HA SER C 137   28.429 36.119 65.097 0.00 0.00
C

ATOM 22221 CB SER C 137 29.892 35.088 63.838 0.00 0.00
C
ATOM 22222 HB1 SER C 137 29.392 34.105 63.967 0.00 0.00
C
ATOM 22223 HB2 SER C 137 30.208 35.125 62.773 0.00 0.00
C
ATOM 22224 OG SER C 137 30.962 35.473 64.803 0.00 0.00
C
ATOM 22225 HG1 SER C 137 31.452 36.198 64.409 0.00 0.00
C
ATOM 22226 C SER C 137 28.935 37.428 63.328 0.00 0.00
C
ATOM 22227 O SER C 137 29.048 38.468 63.950 0.00 0.00
C
ATOM 22228 N ASP C 138 29.042 37.427 61.972 0.00 0.00
C
ATOM 22229 HN ASP C 138 29.001 36.527 61.546 0.00 0.00
C
ATOM 22230 CA ASP C 138 29.422 38.506 61.064 0.00 0.00
C
ATOM 22231 HA ASP C 138 29.676 39.395 61.622 0.00 0.00
C
ATOM 22232 CB ASP C 138 30.566 37.983 60.160 0.00 0.00
C
ATOM 22233 HB1 ASP C 138 30.207 37.008 59.766 0.00 0.00
C
ATOM 22234 HB2 ASP C 138 30.746 38.652 59.292 0.00 0.00
C
ATOM 22235 CG ASP C 138 31.786 37.712 60.961 0.00 0.00
C
ATOM 22236 OD1 ASP C 138 32.195 38.705 61.560 0.00 0.00
C
ATOM 22237 OD2 ASP C 138 32.289 36.598 60.985 0.00 0.00
C
ATOM 22238 C ASP C 138 28.179 38.835 60.182 0.00 0.00
C
ATOM 22239 O ASP C 138 28.069 38.399 59.057 0.00 0.00
C
ATOM 22240 N GLU C 139 27.378 39.707 60.793 0.00 0.00
C
ATOM 22241 HN GLU C 139 27.606 40.045 61.702 0.00 0.00
C
ATOM 22242 CA GLU C 139 26.154 40.094 60.182 0.00 0.00
C
ATOM 22243 HA GLU C 139 25.614 39.183 59.972 0.00 0.00
C
ATOM 22244 CB GLU C 139 25.391 40.870 61.342 0.00 0.00
C
ATOM 22245 HB1 GLU C 139 26.122 41.632 61.688 0.00 0.00
C
ATOM 22246 HB2 GLU C 139 24.526 41.420 60.914 0.00 0.00
C
ATOM 22247 CG GLU C 139 24.957 39.904 62.475 0.00 0.00
C

ATOM 22248 HG1 GLU C 139   24.222 39.229 61.987 0.00 0.00
C
ATOM 22249 HG2 GLU C 139   25.871 39.441 62.902 0.00 0.00
C
ATOM 22250 CD GLU C 139   24.370 40.555 63.711 0.00 0.00
C
ATOM 22251 OE1 GLU C 139   24.407 40.090 64.870 0.00 0.00
C
ATOM 22252 OE2 GLU C 139   23.618 41.528 63.439 0.00 0.00
C
ATOM 22253 C   GLU C 139   26.277 40.981 58.832 0.00 0.00
C
ATOM 22254 O   GLU C 139   25.510 40.785 57.841 0.00 0.00
C
ATOM 22255 N   ASN C 140   27.302 41.856 58.756 0.00 0.00
C
ATOM 22256 HN ASN C 140   27.915 41.809 59.541 0.00 0.00
C
ATOM 22257 CA ASN C 140   27.682 42.743 57.657 0.00 0.00
C
ATOM 22258 HA ASN C 140   26.703 43.112 57.388 0.00 0.00
C
ATOM 22259 CB ASN C 140   28.718 43.795 58.177 0.00 0.00
C
ATOM 22260 HB1 ASN C 140   29.686 43.323 58.451 0.00 0.00
C
ATOM 22261 HB2 ASN C 140   28.887 44.460 57.303 0.00 0.00
C
ATOM 22262 CG ASN C 140   28.094 44.430 59.420 0.00 0.00
C
ATOM 22263 OD1 ASN C 140   28.585 44.088 60.496 0.00 0.00
C
ATOM 22264 ND2 ASN C 140   27.058 45.261 59.253 0.00 0.00
C
ATOM 22265 HD21 ASN C 140   26.647 45.674 60.066 0.00 0.00
C
ATOM 22266 HD22 ASN C 140   26.594 45.440 58.385 0.00 0.00
C
ATOM 22267 C   ASN C 140   28.228 42.095 56.428 0.00 0.00
C
ATOM 22268 O   ASN C 140   28.065 42.624 55.305 0.00 0.00
C
ATOM 22269 N   VAL C 141   28.954 40.998 56.593 0.00 0.00
C
ATOM 22270 HN VAL C 141   29.123 40.535 57.460 0.00 0.00
C
ATOM 22271 CA VAL C 141   29.853 40.571 55.514 0.00 0.00
C
ATOM 22272 HA VAL C 141   30.067 41.435 54.902 0.00 0.00
C
ATOM 22273 CB VAL C 141   31.174 39.962 56.094 0.00 0.00
C
ATOM 22274 HB VAL C 141   30.932 39.006 56.607 0.00 0.00
C

ATOM 22275 CG1 VAL C 141 32.053 39.587 54.896 0.00 0.00
C
ATOM 22276 HG11 VAL C 141 32.650 40.473 54.594 0.00 0.00
C
ATOM 22277 HG12 VAL C 141 32.742 38.800 55.270 0.00 0.00
C
ATOM 22278 HG13 VAL C 141 31.514 39.154 54.027 0.00 0.00
C
ATOM 22279 CG2 VAL C 141 31.902 40.877 57.038 0.00 0.00
C
ATOM 22280 HG21 VAL C 141 31.151 41.130 57.816 0.00 0.00
C
ATOM 22281 HG22 VAL C 141 32.701 40.411 57.655 0.00 0.00
C
ATOM 22282 HG23 VAL C 141 32.349 41.788 56.587 0.00 0.00
C
ATOM 22283 C VAL C 141 29.099 39.398 54.816 0.00 0.00
C
ATOM 22284 O VAL C 141 28.957 38.298 55.369 0.00 0.00
C
ATOM 22285 N LEU C 142 28.736 39.596 53.570 0.00 0.00
C
ATOM 22286 HN LEU C 142 28.781 40.464 53.080 0.00 0.00
C
ATOM 22287 CA LEU C 142 28.025 38.630 52.788 0.00 0.00
C
ATOM 22288 HA LEU C 142 27.385 38.073 53.456 0.00 0.00
C
ATOM 22289 CB LEU C 142 26.764 39.264 51.923 0.00 0.00
C
ATOM 22290 HB1 LEU C 142 27.122 39.493 50.896 0.00 0.00
C
ATOM 22291 HB2 LEU C 142 26.039 38.424 51.859 0.00 0.00
C
ATOM 22292 CG LEU C 142 25.860 40.441 52.447 0.00 0.00
C
ATOM 22293 HG LEU C 142 26.456 41.356 52.655 0.00 0.00
C
ATOM 22294 CD1 LEU C 142 24.710 40.645 51.473 0.00 0.00
C
ATOM 22295 HD11 LEU C 142 25.065 41.100 50.524 0.00 0.00
C
ATOM 22296 HD12 LEU C 142 24.164 39.720 51.189 0.00 0.00
C
ATOM 22297 HD13 LEU C 142 23.983 41.309 51.988 0.00 0.00
C
ATOM 22298 CD2 LEU C 142 25.467 40.152 53.919 0.00 0.00
C
ATOM 22299 HD21 LEU C 142 26.322 40.136 54.629 0.00 0.00
C
ATOM 22300 HD22 LEU C 142 24.792 40.985 54.209 0.00 0.00
C
ATOM 22301 HD23 LEU C 142 24.828 39.244 53.959 0.00 0.00
C

```
ATOM  22302  C   LEU C 142      28.796  37.791  51.765  0.00  0.00
C
ATOM  22303  O   LEU C 142      28.398  36.624  51.442  0.00  0.00
C
ATOM  22304  N   ASN C 143      29.910  38.342  51.276  0.00  0.00
C
ATOM  22305  HN  ASN C 143      30.127  39.286  51.515  0.00  0.00
C
ATOM  22306  CA  ASN C 143      30.883  37.468  50.698  0.00  0.00
C
ATOM  22307  HA  ASN C 143      30.818  36.446  51.042  0.00  0.00
C
ATOM  22308  CB  ASN C 143      30.954  37.673  49.193  0.00  0.00
C
ATOM  22309  HB1 ASN C 143      30.458  38.617  48.879  0.00  0.00
C
ATOM  22310  HB2 ASN C 143      31.972  37.608  48.753  0.00  0.00
C
ATOM  22311  CG  ASN C 143      30.104  36.561  48.486  0.00  0.00
C
ATOM  22312  OD1 ASN C 143      30.449  35.388  48.437  0.00  0.00
C
ATOM  22313  ND2 ASN C 143      28.980  37.002  47.875  0.00  0.00
C
ATOM  22314 HD21 ASN C 143      28.408  36.266  47.515  0.00  0.00
C
ATOM  22315 HD22 ASN C 143      28.691  37.959  47.908  0.00  0.00
C
ATOM  22316  C   ASN C 143      32.261  37.867  51.245  0.00  0.00
C
ATOM  22317  O   ASN C 143      32.427  39.002  51.590  0.00  0.00
C
ATOM  22318  N   TYR C 144      33.233  36.921  51.385  0.00  0.00
C
ATOM  22319  HN  TYR C 144      32.958  35.994  51.144  0.00  0.00
C
ATOM  22320  CA  TYR C 144      34.505  37.257  51.919  0.00  0.00
C
ATOM  22321  HA  TYR C 144      34.619  38.331  51.915  0.00  0.00
C
ATOM  22322  CB  TYR C 144      34.640  36.878  53.404  0.00  0.00
C
ATOM  22323  HB1 TYR C 144      34.033  37.516  54.081  0.00  0.00
C
ATOM  22324  HB2 TYR C 144      34.250  35.850  53.559  0.00  0.00
C
ATOM  22325  CG  TYR C 144      36.001  36.793  54.060  0.00  0.00
C
ATOM  22326  CD1 TYR C 144      36.997  37.691  53.705  0.00  0.00
C
ATOM  22327  HD1 TYR C 144      36.877  38.540  53.047  0.00  0.00
C
ATOM  22328  CE1 TYR C 144      38.315  37.491  54.169  0.00  0.00
C
```

ATOM 22329 HE1 TYR C 144 39.116 38.161 53.894 0.00 0.00
C
ATOM 22330 CZ TYR C 144 38.562 36.501 55.048 0.00 0.00
C
ATOM 22331 OH TYR C 144 39.910 36.224 55.464 0.00 0.00
C
ATOM 22332 HH TYR C 144 39.810 35.541 56.131 0.00 0.00
C
ATOM 22333 CD2 TYR C 144 36.269 35.897 55.110 0.00 0.00
C
ATOM 22334 HD2 TYR C 144 35.541 35.232 55.551 0.00 0.00
C
ATOM 22335 CE2 TYR C 144 37.530 35.625 55.504 0.00 0.00
C
ATOM 22336 HE2 TYR C 144 37.642 34.913 56.308 0.00 0.00
C
ATOM 22337 C TYR C 144 35.455 36.316 51.179 0.00 0.00
C
ATOM 22338 O TYR C 144 35.151 35.103 51.082 0.00 0.00
C
ATOM 22339 N ILE C 145 36.596 36.826 50.611 0.00 0.00
C
ATOM 22340 HN ILE C 145 36.812 37.798 50.647 0.00 0.00
C
ATOM 22341 CA ILE C 145 37.660 35.988 50.156 0.00 0.00
C
ATOM 22342 HA ILE C 145 37.566 34.966 50.491 0.00 0.00
C
ATOM 22343 CB ILE C 145 37.775 35.867 48.618 0.00 0.00
C
ATOM 22344 HB ILE C 145 38.656 35.194 48.545 0.00 0.00
C
ATOM 22345 CG2 ILE C 145 36.531 35.100 48.137 0.00 0.00
C
ATOM 22346 HG21 ILE C 145 35.556 35.555 48.412 0.00 0.00
C
ATOM 22347 HG22 ILE C 145 36.565 35.059 47.027 0.00 0.00
C
ATOM 22348 HG23 ILE C 145 36.493 34.081 48.578 0.00 0.00
C
ATOM 22349 CG1 ILE C 145 38.167 37.057 47.811 0.00 0.00
C
ATOM 22350 HG11 ILE C 145 37.292 37.742 47.838 0.00 0.00
C
ATOM 22351 HG12 ILE C 145 38.982 37.689 48.224 0.00 0.00
C
ATOM 22352 CD ILE C 145 38.680 36.808 46.322 0.00 0.00
C
ATOM 22353 HD1 ILE C 145 38.066 36.037 45.809 0.00 0.00
C
ATOM 22354 HD2 ILE C 145 38.585 37.782 45.797 0.00 0.00
C
ATOM 22355 HD3 ILE C 145 39.709 36.390 46.286 0.00 0.00
C

ATOM 22356 C ILE C 145 38.955 36.543 50.781 0.00 0.00
C
ATOM 22357 O ILE C 145 39.090 37.690 51.210 0.00 0.00
C
ATOM 22358 N ASP C 146 39.954 35.639 50.867 0.00 0.00
C
ATOM 22359 HN ASP C 146 39.780 34.685 50.636 0.00 0.00
C
ATOM 22360 CA ASP C 146 41.216 35.926 51.508 0.00 0.00
C
ATOM 22361 HA ASP C 146 41.124 36.818 52.110 0.00 0.00
C
ATOM 22362 CB ASP C 146 41.540 34.733 52.420 0.00 0.00
C
ATOM 22363 HB1 ASP C 146 40.679 34.539 53.095 0.00 0.00
C
ATOM 22364 HB2 ASP C 146 41.686 33.809 51.820 0.00 0.00
C
ATOM 22365 CG ASP C 146 42.735 35.120 53.316 0.00 0.00
C
ATOM 22366 OD1 ASP C 146 42.839 36.277 53.775 0.00 0.00
C
ATOM 22367 OD2 ASP C 146 43.693 34.235 53.541 0.00 0.00
C
ATOM 22368 C ASP C 146 42.284 36.083 50.473 0.00 0.00
C
ATOM 22369 O ASP C 146 42.115 35.537 49.335 0.00 0.00
C
ATOM 22370 N LEU C 147 43.302 36.967 50.744 0.00 0.00
C
ATOM 22371 HN LEU C 147 43.279 37.561 51.544 0.00 0.00
C
ATOM 22372 CA LEU C 147 44.448 37.250 49.847 0.00 0.00
C
ATOM 22373 HA LEU C 147 44.523 36.543 49.034 0.00 0.00
C
ATOM 22374 CB LEU C 147 44.445 38.578 49.107 0.00 0.00
C
ATOM 22375 HB1 LEU C 147 44.572 39.452 49.781 0.00 0.00
C
ATOM 22376 HB2 LEU C 147 45.331 38.628 48.439 0.00 0.00
C
ATOM 22377 CG LEU C 147 43.194 38.851 48.308 0.00 0.00
C
ATOM 22378 HG LEU C 147 42.831 37.859 47.965 0.00 0.00
C
ATOM 22379 CD1 LEU C 147 42.004 39.368 49.117 0.00 0.00
C
ATOM 22380 HD11 LEU C 147 41.720 38.626 49.893 0.00 0.00
C
ATOM 22381 HD12 LEU C 147 42.199 40.300 49.689 0.00 0.00
C
ATOM 22382 HD13 LEU C 147 41.098 39.512 48.490 0.00 0.00
C

ATOM 22383 CD2 LEU C 147   43.437 39.745 47.115 0.00 0.00
C
ATOM 22384 HD21 LEU C 147   43.523 40.777 47.516 0.00 0.00
C
ATOM 22385 HD22 LEU C 147   44.362 39.435 46.582 0.00 0.00
C
ATOM 22386 HD23 LEU C 147   42.550 39.624 46.457 0.00 0.00
C
ATOM 22387 C   LEU C 147   45.672 37.133 50.673 0.00 0.00
C
ATOM 22388 O   LEU C 147   46.344 38.123 51.123 0.00 0.00
C
ATOM 22389 N   SER C 148   46.041 35.844 50.906 0.00 0.00
C
ATOM 22390 HN SER C 148   45.392 35.137 50.638 0.00 0.00
C
ATOM 22391 CA SER C 148   47.275 35.410 51.550 0.00 0.00
C
ATOM 22392 HA SER C 148   47.124 34.352 51.709 0.00 0.00
C
ATOM 22393 CB SER C 148   48.475 35.761 50.618 0.00 0.00
C
ATOM 22394 HB1 SER C 148   48.454 36.839 50.352 0.00 0.00
C
ATOM 22395 HB2 SER C 148   49.446 35.606 51.134 0.00 0.00
C
ATOM 22396 OG SER C 148   48.492 34.971 49.386 0.00 0.00
C
ATOM 22397 HG1 SER C 148   49.055 35.385 48.728 0.00 0.00
C
ATOM 22398 C   SER C 148   47.399 35.957 52.967 0.00 0.00
C
ATOM 22399 O   SER C 148   48.464 36.455 53.230 0.00 0.00
C
ATOM 22400 N   ASP C 149   46.310 36.060 53.739 0.00 0.00
C
ATOM 22401 HN ASP C 149   45.441 35.818 53.315 0.00 0.00
C
ATOM 22402 CA ASP C 149   46.312 36.582 55.141 0.00 0.00
C
ATOM 22403 HA ASP C 149   45.372 36.551 55.672 0.00 0.00
C
ATOM 22404 CB ASP C 149   47.243 35.632 56.084 0.00 0.00
C
ATOM 22405 HB1 ASP C 149   48.238 35.411 55.641 0.00 0.00
C
ATOM 22406 HB2 ASP C 149   47.535 36.246 56.962 0.00 0.00
C
ATOM 22407 CG ASP C 149   46.568 34.407 56.521 0.00 0.00
C
ATOM 22408 OD1 ASP C 149   45.562 34.550 57.187 0.00 0.00
C
ATOM 22409 OD2 ASP C 149   47.017 33.254 56.359 0.00 0.00
C

ATOM 22410 C   ASP C 149   46.577 38.052 55.343 0.00 0.00
C
ATOM 22411 O   ASP C 149   46.033 38.636 56.342 0.00 0.00
C
ATOM 22412 N   GLU C 150   47.320 38.709 54.455 0.00 0.00
C
ATOM 22413 HN GLU C 150   47.914 38.284 53.776 0.00 0.00
C
ATOM 22414 CA GLU C 150   47.496 40.176 54.419 0.00 0.00
C
ATOM 22415 HA GLU C 150   47.570 40.468 55.456 0.00 0.00
C
ATOM 22416 CB GLU C 150   48.665 40.587 53.532 0.00 0.00
C
ATOM 22417 HB1 GLU C 150   48.542 39.983 52.608 0.00 0.00
C
ATOM 22418 HB2 GLU C 150   48.617 41.693 53.444 0.00 0.00
C
ATOM 22419 CG GLU C 150   50.002 40.339 54.234 0.00 0.00
C
ATOM 22420 HG1 GLU C 150   50.212 39.265 54.429 0.00 0.00
C
ATOM 22421 HG2 GLU C 150   50.887 40.599 53.615 0.00 0.00
C
ATOM 22422 CD GLU C 150   50.058 41.155 55.474 0.00 0.00
C
ATOM 22423 OE1 GLU C 150   50.512 42.264 55.352 0.00 0.00
C
ATOM 22424 OE2 GLU C 150   49.668 40.797 56.619 0.00 0.00
C
ATOM 22425 C   GLU C 150   46.206 40.891 53.990 0.00 0.00
C
ATOM 22426 O   GLU C 150   45.720 41.811 54.619 0.00 0.00
C
ATOM 22427 N   TYR C 151   45.522 40.551 52.836 0.00 0.00
C
ATOM 22428 HN TYR C 151   45.905 39.768 52.353 0.00 0.00
C
ATOM 22429 CA TYR C 151   44.397 41.394 52.405 0.00 0.00
C
ATOM 22430 HA TYR C 151   44.140 42.152 53.130 0.00 0.00
C
ATOM 22431 CB TYR C 151   44.643 42.061 50.949 0.00 0.00
C
ATOM 22432 HB1 TYR C 151   45.044 41.323 50.222 0.00 0.00
C
ATOM 22433 HB2 TYR C 151   43.718 42.500 50.518 0.00 0.00
C
ATOM 22434 CG TYR C 151   45.549 43.275 50.996 0.00 0.00
C
ATOM 22435 CD1 TYR C 151   45.021 44.504 50.545 0.00 0.00
C
ATOM 22436 HD1 TYR C 151   44.038 44.471 50.098 0.00 0.00
C

ATOM 22437 CE1 TYR C 151 45.906 45.597 50.437 0.00 0.00
C
ATOM 22438 HE1 TYR C 151 45.495 46.496 50.003 0.00 0.00
C
ATOM 22439 CZ TYR C 151 47.196 45.616 50.969 0.00 0.00
C
ATOM 22440 OH TYR C 151 47.971 46.773 50.974 0.00 0.00
C
ATOM 22441 HH TYR C 151 48.665 46.535 51.592 0.00 0.00
C
ATOM 22442 CD2 TYR C 151 46.879 43.272 51.462 0.00 0.00
C
ATOM 22443 HD2 TYR C 151 47.398 42.388 51.803 0.00 0.00
C
ATOM 22444 CE2 TYR C 151 47.641 44.442 51.455 0.00 0.00
C
ATOM 22445 HE2 TYR C 151 48.696 44.299 51.635 0.00 0.00
C
ATOM 22446 C TYR C 151 43.168 40.598 52.367 0.00 0.00
C
ATOM 22447 O TYR C 151 43.164 39.352 52.521 0.00 0.00
C
ATOM 22448 N SER C 152 41.996 41.312 52.350 0.00 0.00
C
ATOM 22449 HN SER C 152 42.145 42.283 52.179 0.00 0.00
C
ATOM 22450 CA SER C 152 40.670 40.789 52.357 0.00 0.00
C
ATOM 22451 HA SER C 152 40.725 39.756 52.046 0.00 0.00
C
ATOM 22452 CB SER C 152 39.995 41.002 53.828 0.00 0.00
C
ATOM 22453 HB1 SER C 152 40.239 42.075 53.984 0.00 0.00
C
ATOM 22454 HB2 SER C 152 38.915 40.774 53.709 0.00 0.00
C
ATOM 22455 OG SER C 152 40.352 40.018 54.869 0.00 0.00
C
ATOM 22456 HG1 SER C 152 39.697 39.875 55.557 0.00 0.00
C
ATOM 22457 C SER C 152 39.892 41.552 51.358 0.00 0.00
C
ATOM 22458 O SER C 152 39.999 42.760 51.224 0.00 0.00
C
ATOM 22459 N ILE C 153 39.102 40.840 50.546 0.00 0.00
C
ATOM 22460 HN ILE C 153 39.027 39.856 50.690 0.00 0.00
C
ATOM 22461 CA ILE C 153 38.149 41.558 49.673 0.00 0.00
C
ATOM 22462 HA ILE C 153 38.148 42.638 49.676 0.00 0.00
C
ATOM 22463 CB ILE C 153 38.179 41.129 48.229 0.00 0.00
C

ATOM 22464 HB ILE C 153   38.136 40.020 48.172 0.00 0.00
C
ATOM 22465 CG2 ILE C 153   36.969 41.697 47.426 0.00 0.00
C
ATOM 22466 HG21 ILE C 153   37.026 42.805 47.367 0.00 0.00
C
ATOM 22467 HG22 ILE C 153   37.012 41.488 46.336 0.00 0.00
C
ATOM 22468 HG23 ILE C 153   36.019 41.281 47.827 0.00 0.00
C
ATOM 22469 CG1 ILE C 153   39.467 41.432 47.560 0.00 0.00
C
ATOM 22470 HG11 ILE C 153   39.400 42.483 47.206 0.00 0.00
C
ATOM 22471 HG12 ILE C 153   40.256 41.412 48.342 0.00 0.00
C
ATOM 22472 CD ILE C 153   39.762 40.494 46.370 0.00 0.00
C
ATOM 22473 HD1 ILE C 153   38.978 40.499 45.584 0.00 0.00
C
ATOM 22474 HD2 ILE C 153   40.707 40.855 45.911 0.00 0.00
C
ATOM 22475 HD3 ILE C 153   39.878 39.439 46.700 0.00 0.00
C
ATOM 22476 C   ILE C 153   36.845 41.118 50.229 0.00 0.00
C
ATOM 22477 O   ILE C 153   36.634 39.971 50.556 0.00 0.00
C
ATOM 22478 N   VAL C 154   35.882 42.032 50.487 0.00 0.00
C
ATOM 22479 HN VAL C 154   36.066 42.977 50.226 0.00 0.00
C
ATOM 22480 CA VAL C 154   34.680 41.758 51.343 0.00 0.00
C
ATOM 22481 HA VAL C 154   34.424 40.709 51.303 0.00 0.00
C
ATOM 22482 CB VAL C 154   34.769 42.261 52.771 0.00 0.00
C
ATOM 22483 HB VAL C 154   33.770 42.322 53.253 0.00 0.00
C
ATOM 22484 CG1 VAL C 154   35.564 41.167 53.543 0.00 0.00
C
ATOM 22485 HG11 VAL C 154   35.710 41.484 54.598 0.00 0.00
C
ATOM 22486 HG12 VAL C 154   35.069 40.173 53.541 0.00 0.00
C
ATOM 22487 HG13 VAL C 154   36.547 41.024 53.045 0.00 0.00
C
ATOM 22488 CG2 VAL C 154   35.522 43.549 52.852 0.00 0.00
C
ATOM 22489 HG21 VAL C 154   36.564 43.480 52.474 0.00 0.00
C
ATOM 22490 HG22 VAL C 154   35.047 44.276 52.160 0.00 0.00
C

ATOM 22491 HG23 VAL C 154 35.458 43.984 53.873 0.00 0.00
C
ATOM 22492 C VAL C 154 33.545 42.563 50.671 0.00 0.00
C
ATOM 22493 O VAL C 154 33.752 43.633 50.084 0.00 0.00
C
ATOM 22494 N GLU C 155 32.295 42.059 50.892 0.00 0.00
C
ATOM 22495 HN GLU C 155 32.179 41.163 51.313 0.00 0.00
C
ATOM 22496 CA GLU C 155 31.022 42.598 50.477 0.00 0.00
C
ATOM 22497 HA GLU C 155 31.137 43.402 49.765 0.00 0.00
C
ATOM 22498 CB GLU C 155 30.228 41.563 49.589 0.00 0.00
C
ATOM 22499 HB1 GLU C 155 30.733 41.445 48.606 0.00 0.00
C
ATOM 22500 HB2 GLU C 155 30.272 40.560 50.065 0.00 0.00
C
ATOM 22501 CG GLU C 155 28.687 41.871 49.255 0.00 0.00
C
ATOM 22502 HG1 GLU C 155 28.048 41.779 50.159 0.00 0.00
C
ATOM 22503 HG2 GLU C 155 28.502 42.896 48.868 0.00 0.00
C
ATOM 22504 CD GLU C 155 28.323 40.862 48.245 0.00 0.00
C
ATOM 22505 OE1 GLU C 155 28.135 41.142 47.099 0.00 0.00
C
ATOM 22506 OE2 GLU C 155 28.131 39.642 48.605 0.00 0.00
C
ATOM 22507 C GLU C 155 30.195 42.972 51.643 0.00 0.00
C
ATOM 22508 O GLU C 155 29.797 42.100 52.412 0.00 0.00
C
ATOM 22509 N LEU C 156 29.975 44.263 51.864 0.00 0.00
C
ATOM 22510 HN LEU C 156 30.302 44.941 51.210 0.00 0.00
C
ATOM 22511 CA LEU C 156 29.519 44.772 53.129 0.00 0.00
C
ATOM 22512 HA LEU C 156 29.475 43.977 53.858 0.00 0.00
C
ATOM 22513 CB LEU C 156 30.452 45.916 53.649 0.00 0.00
C
ATOM 22514 HB1 LEU C 156 30.603 46.630 52.811 0.00 0.00
C
ATOM 22515 HB2 LEU C 156 30.021 46.493 54.495 0.00 0.00
C
ATOM 22516 CG LEU C 156 31.903 45.408 53.974 0.00 0.00
C
ATOM 22517 HG LEU C 156 32.374 45.095 53.018 0.00 0.00
C

ATOM 22518 CD1 LEU C 156   32.640 46.688 54.449 0.00 0.00
C
ATOM 22519 HD11 LEU C 156   33.745 46.586 54.402 0.00 0.00
C
ATOM 22520 HD12 LEU C 156   32.500 47.464 53.666 0.00 0.00
C
ATOM 22521 HD13 LEU C 156   32.344 47.003 55.473 0.00 0.00
C
ATOM 22522 CD2 LEU C 156   32.071 44.342 55.020 0.00 0.00
C
ATOM 22523 HD21 LEU C 156   31.822 43.308 54.701 0.00 0.00
C
ATOM 22524 HD22 LEU C 156   33.169 44.259 55.162 0.00 0.00
C
ATOM 22525 HD23 LEU C 156   31.434 44.592 55.896 0.00 0.00
C
ATOM 22526 C   LEU C 156   28.181 45.434 53.036 0.00 0.00
C
ATOM 22527 O   LEU C 156   28.072 46.407 52.198 0.00 0.00
C
ATOM 22528 N   LEU C 157   27.209 45.078 53.878 0.00 0.00
C
ATOM 22529 HN LEU C 157   27.447 44.433 54.600 0.00 0.00
C
ATOM 22530 CA LEU C 157   25.789 45.581 53.911 0.00 0.00
C
ATOM 22531 HA LEU C 157   25.653 46.368 53.184 0.00 0.00
C
ATOM 22532 CB LEU C 157   24.731 44.472 53.559 0.00 0.00
C
ATOM 22533 HB1 LEU C 157   24.886 44.076 52.532 0.00 0.00
C
ATOM 22534 HB2 LEU C 157   24.900 43.647 54.284 0.00 0.00
C
ATOM 22535 CG LEU C 157   23.212 44.889 53.592 0.00 0.00
C
ATOM 22536 HG LEU C 157   23.131 45.992 53.703 0.00 0.00
C
ATOM 22537 CD1 LEU C 157   22.489 44.652 52.218 0.00 0.00
C
ATOM 22538 HD11 LEU C 157   22.396 43.557 52.053 0.00 0.00
C
ATOM 22539 HD12 LEU C 157   21.453 45.052 52.252 0.00 0.00
C
ATOM 22540 HD13 LEU C 157   23.024 45.024 51.319 0.00 0.00
C
ATOM 22541 CD2 LEU C 157   22.541 44.235 54.812 0.00 0.00
C
ATOM 22542 HD21 LEU C 157   21.462 44.067 54.608 0.00 0.00
C
ATOM 22543 HD22 LEU C 157   22.951 43.221 55.008 0.00 0.00
C
ATOM 22544 HD23 LEU C 157   22.605 44.880 55.714 0.00 0.00
C

ATOM 22545 C LEU C 157 25.452 46.086 55.372 0.00 0.00
C
ATOM 22546 O LEU C 157 25.903 45.504 56.384 0.00 0.00
C
ATOM 22547 N LYS C 161 24.676 47.197 55.459 0.00 0.00
C
ATOM 22548 HN LYS C 161 24.510 47.697 54.613 0.00 0.00
C
ATOM 22549 CA LYS C 161 24.240 47.995 56.626 0.00 0.00
C
ATOM 22550 HA LYS C 161 23.468 48.593 56.164 0.00 0.00
C
ATOM 22551 CB LYS C 161 23.466 47.067 57.571 0.00 0.00
C
ATOM 22552 HB1 LYS C 161 22.751 46.486 56.951 0.00 0.00
C
ATOM 22553 HB2 LYS C 161 24.140 46.413 58.165 0.00 0.00
C
ATOM 22554 CG LYS C 161 22.740 47.868 58.652 0.00 0.00
C
ATOM 22555 HG1 LYS C 161 23.339 48.420 59.408 0.00 0.00
C
ATOM 22556 HG2 LYS C 161 22.160 48.655 58.126 0.00 0.00
C
ATOM 22557 CD LYS C 161 21.812 46.925 59.467 0.00 0.00
C
ATOM 22558 HD1 LYS C 161 21.217 46.417 58.678 0.00 0.00
C
ATOM 22559 HD2 LYS C 161 22.348 46.118 60.011 0.00 0.00
C
ATOM 22560 CE LYS C 161 20.639 47.532 60.254 0.00 0.00
C
ATOM 22561 HE1 LYS C 161 20.189 48.421 59.762 0.00 0.00
C
ATOM 22562 HE2 LYS C 161 19.900 46.706 60.328 0.00 0.00
C
ATOM 22563 NZ LYS C 161 21.015 47.920 61.618 0.00 0.00
C
ATOM 22564 HZ1 LYS C 161 21.251 47.152 62.279 0.00 0.00
C
ATOM 22565 HZ2 LYS C 161 21.897 48.470 61.585 0.00 0.00
C
ATOM 22566 HZ3 LYS C 161 20.378 48.569 62.122 0.00 0.00
C
ATOM 22567 C LYS C 161 25.319 48.931 57.017 0.00 0.00
C
ATOM 22568 O LYS C 161 25.516 49.239 58.204 0.00 0.00
C
ATOM 22569 N LEU C 162 25.996 49.501 56.050 0.00 0.00
C
ATOM 22570 HN LEU C 162 25.828 49.266 55.096 0.00 0.00
C
ATOM 22571 CA LEU C 162 26.840 50.729 56.235 0.00 0.00
C

ATOM 22572 HA LEU C 162   27.438 50.621 57.127 0.00 0.00
C
ATOM 22573 CB LEU C 162   27.775 50.867 54.940 0.00 0.00
C
ATOM 22574 HB1 LEU C 162   27.201 51.016 54.000 0.00 0.00
C
ATOM 22575 HB2 LEU C 162   28.350 51.785 55.188 0.00 0.00
C
ATOM 22576 CG LEU C 162   28.781 49.716 54.859 0.00 0.00
C
ATOM 22577 HG LEU C 162   28.113 48.834 54.765 0.00 0.00
C
ATOM 22578 CD1 LEU C 162   29.721 49.782 53.676 0.00 0.00
C
ATOM 22579 HD11 LEU C 162   30.334 50.707 53.735 0.00 0.00
C
ATOM 22580 HD12 LEU C 162   30.409 48.910 53.690 0.00 0.00
C
ATOM 22581 HD13 LEU C 162   29.086 49.893 52.771 0.00 0.00
C
ATOM 22582 CD2 LEU C 162   29.643 49.435 56.102 0.00 0.00
C
ATOM 22583 HD21 LEU C 162   30.223 48.492 56.017 0.00 0.00
C
ATOM 22584 HD22 LEU C 162   30.377 50.262 56.213 0.00 0.00
C
ATOM 22585 HD23 LEU C 162   29.061 49.315 57.041 0.00 0.00
C
ATOM 22586 C   LEU C 162   25.999 51.919 56.506 0.00 0.00
C
ATOM 22587 O   LEU C 162   24.782 51.909 56.254 0.00 0.00
C
ATOM 22588 N   ASP C 163   26.556 52.991 57.092 0.00 0.00
C
ATOM 22589 HN ASP C 163   27.534 52.982 57.284 0.00 0.00
C
ATOM 22590 CA ASP C 163   25.876 54.246 57.400 0.00 0.00
C
ATOM 22591 HA ASP C 163   25.085 54.058 58.111 0.00 0.00
C
ATOM 22592 CB ASP C 163   26.996 55.115 57.985 0.00 0.00
C
ATOM 22593 HB1 ASP C 163   27.691 55.349 57.151 0.00 0.00
C
ATOM 22594 HB2 ASP C 163   26.453 55.991 58.398 0.00 0.00
C
ATOM 22595 CG ASP C 163   27.706 54.397 59.128 0.00 0.00
C
ATOM 22596 OD1 ASP C 163   28.800 53.938 58.946 0.00 0.00
C
ATOM 22597 OD2 ASP C 163   27.095 54.365 60.210 0.00 0.00
C
ATOM 22598 C   ASP C 163   25.245 54.902 56.164 0.00 0.00
C

ATOM 22599 O   ASP C 163   25.788 54.768 55.044 0.00 0.00
C
ATOM 22600 N   SER C 164   24.057 55.543 56.266 0.00 0.00
C
ATOM 22601 HN SER C 164   23.549 55.483 57.122 0.00 0.00
C
ATOM 22602 CA SER C 164   23.307 56.125 55.217 0.00 0.00
C
ATOM 22603 HA SER C 164   23.550 55.581 54.316 0.00 0.00
C
ATOM 22604 CB SER C 164   21.757 56.024 55.379 0.00 0.00
C
ATOM 22605 HB1 SER C 164   21.412 56.561 56.289 0.00 0.00
C
ATOM 22606 HB2 SER C 164   21.144 56.415 54.539 0.00 0.00
C
ATOM 22607 OG SER C 164   21.366 54.656 55.442 0.00 0.00
C
ATOM 22608 HG1 SER C 164   21.124 54.456 56.349 0.00 0.00
C
ATOM 22609 C   SER C 164   23.784 57.505 54.885 0.00 0.00
C
ATOM 22610 O   SER C 164   23.273 58.602 55.186 0.00 0.00
C
ATOM 22611 N   LYS C 165   24.864 57.666 54.081 0.00 0.00
C
ATOM 22612 HN LYS C 165   25.288 56.914 53.582 0.00 0.00
C
ATOM 22613 CA LYS C 165   25.511 58.985 54.045 0.00 0.00
C
ATOM 22614 HA LYS C 165   24.695 59.639 53.777 0.00 0.00
C
ATOM 22615 CB LYS C 165   26.377 59.206 55.388 0.00 0.00
C
ATOM 22616 HB1 LYS C 165   26.982 60.100 55.124 0.00 0.00
C
ATOM 22617 HB2 LYS C 165   25.677 59.461 56.212 0.00 0.00
C
ATOM 22618 CG LYS C 165   27.316 57.995 55.654 0.00 0.00
C
ATOM 22619 HG1 LYS C 165   26.600 57.176 55.879 0.00 0.00
C
ATOM 22620 HG2 LYS C 165   27.864 57.826 54.703 0.00 0.00
C
ATOM 22621 CD LYS C 165   28.327 58.401 56.741 0.00 0.00
C
ATOM 22622 HD1 LYS C 165   29.124 57.637 56.868 0.00 0.00
C
ATOM 22623 HD2 LYS C 165   28.769 59.400 56.541 0.00 0.00
C
ATOM 22624 CE LYS C 165   27.738 58.678 58.208 0.00 0.00
C
ATOM 22625 HE1 LYS C 165   26.999 59.505 58.142 0.00 0.00
C

ATOM 22626 HE2 LYS C 165   27.305 57.743 58.622 0.00 0.00
C
ATOM 22627 NZ LYS C 165   28.906 59.204 58.949 0.00 0.00
C
ATOM 22628 HZ1 LYS C 165   28.820 59.430 59.961 0.00 0.00
C
ATOM 22629 HZ2 LYS C 165   29.724 58.569 58.856 0.00 0.00
C
ATOM 22630 HZ3 LYS C 165   29.108 60.117 58.493 0.00 0.00
C
ATOM 22631 C   LYS C 165   26.511 58.882 52.830 0.00 0.00
C
ATOM 22632 O   LYS C 165   26.763 57.829 52.204 0.00 0.00
C
ATOM 22633 N   SER C 166   27.126 60.001 52.451 0.00 0.00
C
ATOM 22634 HN SER C 166   26.984 60.759 53.082 0.00 0.00
C
ATOM 22635 CA SER C 166   28.134 60.190 51.426 0.00 0.00
C
ATOM 22636 HA SER C 166   27.893 59.610 50.547 0.00 0.00
C
ATOM 22637 CB SER C 166   28.489 61.602 50.931 0.00 0.00
C
ATOM 22638 HB1 SER C 166   29.385 61.474 50.286 0.00 0.00
C
ATOM 22639 HB2 SER C 166   27.556 61.930 50.424 0.00 0.00
C
ATOM 22640 OG SER C 166   28.909 62.365 52.061 0.00 0.00
C
ATOM 22641 HG1 SER C 166   28.858 63.282 51.782 0.00 0.00
C
ATOM 22642 C   SER C 166   29.475 59.498 51.883 0.00 0.00
C
ATOM 22643 O   SER C 166   29.747 59.361 53.083 0.00 0.00
C
ATOM 22644 N   ILE C 167   30.211 58.974 50.886 0.00 0.00
C
ATOM 22645 HN ILE C 167   29.919 59.198 49.959 0.00 0.00
C
ATOM 22646 CA ILE C 167   31.296 58.019 51.124 0.00 0.00
C
ATOM 22647 HA ILE C 167   31.023 57.319 51.899 0.00 0.00
C
ATOM 22648 CB ILE C 167   31.679 57.377 49.776 0.00 0.00
C
ATOM 22649 HB ILE C 167   31.593 58.117 48.951 0.00 0.00
C
ATOM 22650 CG2 ILE C 167   33.076 56.761 49.727 0.00 0.00
C
ATOM 22651 HG21 ILE C 167   33.291 56.441 48.684 0.00 0.00
C
ATOM 22652 HG22 ILE C 167   33.946 57.388 50.016 0.00 0.00
C

```
ATOM  22653 HG23 ILE C 167      33.025  55.909  50.438  0.00  0.00
C
ATOM  22654  CG1 ILE C 167      30.640  56.399  49.320  0.00  0.00
C
ATOM  22655 HG11 ILE C 167      30.766  55.566  50.044  0.00  0.00
C
ATOM  22656 HG12 ILE C 167      29.574  56.679  49.459  0.00  0.00
C
ATOM  22657  CD  ILE C 167      30.810  55.867  47.860  0.00  0.00
C
ATOM  22658  HD1 ILE C 167      31.786  55.349  47.744  0.00  0.00
C
ATOM  22659  HD2 ILE C 167      29.966  55.165  47.685  0.00  0.00
C
ATOM  22660  HD3 ILE C 167      30.713  56.686  47.116  0.00  0.00
C
ATOM  22661  C   ILE C 167      32.560  58.702  51.787  0.00  0.00
C
ATOM  22662  O   ILE C 167      33.382  58.121  52.472  0.00  0.00
C
ATOM  22663  N   ILE C 168      32.675  60.034  51.609  0.00  0.00
C
ATOM  22664  HN  ILE C 168      31.980  60.504  51.070  0.00  0.00
C
ATOM  22665  CA  ILE C 168      33.696  60.968  52.199  0.00  0.00
C
ATOM  22666  HA  ILE C 168      34.625  60.424  52.290  0.00  0.00
C
ATOM  22667  CB  ILE C 168      33.874  62.225  51.291  0.00  0.00
C
ATOM  22668  HB  ILE C 168      34.034  61.842  50.260  0.00  0.00
C
ATOM  22669  CG2 ILE C 168      32.665  63.056  51.155  0.00  0.00
C
ATOM  22670 HG21 ILE C 168      31.786  62.538  50.716  0.00  0.00
C
ATOM  22671 HG22 ILE C 168      32.392  63.528  52.123  0.00  0.00
C
ATOM  22672 HG23 ILE C 168      32.903  63.913  50.490  0.00  0.00
C
ATOM  22673  CG1 ILE C 168      35.079  63.167  51.548  0.00  0.00
C
ATOM  22674 HG11 ILE C 168      35.133  63.907  50.721  0.00  0.00
C
ATOM  22675 HG12 ILE C 168      35.025  63.766  52.482  0.00  0.00
C
ATOM  22676  CD  ILE C 168      36.409  62.401  51.379  0.00  0.00
C
ATOM  22677  HD1 ILE C 168      37.188  63.171  51.190  0.00  0.00
C
ATOM  22678  HD2 ILE C 168      36.710  61.847  52.293  0.00  0.00
C
ATOM  22679  HD3 ILE C 168      36.450  61.710  50.510  0.00  0.00
C
```

ATOM 22680 C ILE C 168 33.207 61.415 53.607 0.00 0.00
C
ATOM 22681 O ILE C 168 34.019 61.889 54.382 0.00 0.00
C
ATOM 22682 N ASP C 169 31.915 61.207 53.979 0.00 0.00
C
ATOM 22683 HN ASP C 169 31.194 60.983 53.328 0.00 0.00
C
ATOM 22684 CA ASP C 169 31.382 61.305 55.378 0.00 0.00
C
ATOM 22685 HA ASP C 169 31.940 62.078 55.886 0.00 0.00
C
ATOM 22686 CB ASP C 169 29.919 61.727 55.303 0.00 0.00
C
ATOM 22687 HB1 ASP C 169 29.763 62.601 54.636 0.00 0.00
C
ATOM 22688 HB2 ASP C 169 29.374 60.820 54.963 0.00 0.00
C
ATOM 22689 CG ASP C 169 29.370 62.228 56.562 0.00 0.00
C
ATOM 22690 OD1 ASP C 169 29.323 61.470 57.621 0.00 0.00
C
ATOM 22691 OD2 ASP C 169 28.908 63.410 56.640 0.00 0.00
C
ATOM 22692 C ASP C 169 31.613 59.990 56.125 0.00 0.00
C
ATOM 22693 O ASP C 169 31.808 59.858 57.374 0.00 0.00
C
ATOM 22694 N LEU C 170 31.599 58.909 55.387 0.00 0.00
C
ATOM 22695 HN LEU C 170 31.335 59.138 54.453 0.00 0.00
C
ATOM 22696 CA LEU C 170 32.111 57.616 55.794 0.00 0.00
C
ATOM 22697 HA LEU C 170 31.642 57.464 56.755 0.00 0.00
C
ATOM 22698 CB LEU C 170 31.645 56.434 54.823 0.00 0.00
C
ATOM 22699 HB1 LEU C 170 30.657 56.627 54.354 0.00 0.00
C
ATOM 22700 HB2 LEU C 170 32.374 56.375 53.987 0.00 0.00
C
ATOM 22701 CG LEU C 170 31.638 55.070 55.476 0.00 0.00
C
ATOM 22702 HG LEU C 170 32.447 54.998 56.235 0.00 0.00
C
ATOM 22703 CD1 LEU C 170 30.214 54.907 56.100 0.00 0.00
C
ATOM 22704 HD11 LEU C 170 30.026 53.865 56.437 0.00 0.00
C
ATOM 22705 HD12 LEU C 170 29.983 55.561 56.968 0.00 0.00
C
ATOM 22706 HD13 LEU C 170 29.474 55.161 55.311 0.00 0.00
C

```
ATOM  22707  CD2 LEU C 170      31.879  54.002  54.432  0.00  0.00
C
ATOM  22708 HD21 LEU C 170      32.953  54.092  54.163  0.00  0.00
C
ATOM  22709 HD22 LEU C 170      31.524  53.058  54.898  0.00  0.00
C
ATOM  22710 HD23 LEU C 170      31.255  54.208  53.536  0.00  0.00
C
ATOM  22711  C   LEU C 170      33.677  57.707  55.929  0.00  0.00
C
ATOM  22712  O   LEU C 170      34.364  58.478  55.339  0.00  0.00
C
ATOM  22713  N   ASN C 171      34.223  56.771  56.780  0.00  0.00
C
ATOM  22714  HN  ASN C 171      33.602  56.062  57.104  0.00  0.00
C
ATOM  22715  CA  ASN C 171      35.684  56.761  57.159  0.00  0.00
C
ATOM  22716  HA  ASN C 171      36.108  57.751  57.082  0.00  0.00
C
ATOM  22717  CB  ASN C 171      35.710  56.171  58.671  0.00  0.00
C
ATOM  22718  HB1 ASN C 171      35.328  55.128  58.621  0.00  0.00
C
ATOM  22719  HB2 ASN C 171      36.769  56.165  59.006  0.00  0.00
C
ATOM  22720  CG  ASN C 171      34.858  57.020  59.677  0.00  0.00
C
ATOM  22721  OD1 ASN C 171      35.220  58.208  59.807  0.00  0.00
C
ATOM  22722  ND2 ASN C 171      33.911  56.464  60.429  0.00  0.00
C
ATOM  22723 HD21 ASN C 171      33.159  57.063  60.706  0.00  0.00
C
ATOM  22724 HD22 ASN C 171      33.973  55.470  60.511  0.00  0.00
C
ATOM  22725  C   ASN C 171      36.618  55.790  56.359  0.00  0.00
C
ATOM  22726  O   ASN C 171      37.732  55.442  56.759  0.00  0.00
C
ATOM  22727  N   VAL C 172      36.153  55.307  55.210  0.00  0.00
C
ATOM  22728  HN  VAL C 172      35.282  55.674  54.892  0.00  0.00
C
ATOM  22729  CA  VAL C 172      36.544  54.173  54.472  0.00  0.00
C
ATOM  22730  HA  VAL C 172      36.722  53.430  55.234  0.00  0.00
C
ATOM  22731  CB  VAL C 172      35.532  53.565  53.528  0.00  0.00
C
ATOM  22732  HB  VAL C 172      34.633  53.139  54.021  0.00  0.00
C
ATOM  22733  CG1 VAL C 172      34.990  54.570  52.518  0.00  0.00
C
```

ATOM 22734 HG11 VAL C 172   34.496 55.462 52.958 0.00 0.00
C
ATOM 22735 HG12 VAL C 172   35.765 54.901 51.794 0.00 0.00
C
ATOM 22736 HG13 VAL C 172   34.205 54.094 51.893 0.00 0.00
C
ATOM 22737 CG2 VAL C 172   36.055 52.225 52.864 0.00 0.00
C
ATOM 22738 HG21 VAL C 172   36.349 51.369 53.510 0.00 0.00
C
ATOM 22739 HG22 VAL C 172   35.225 51.787 52.269 0.00 0.00
C
ATOM 22740 HG23 VAL C 172   36.935 52.424 52.215 0.00 0.00
C
ATOM 22741 C   VAL C 172   37.873 54.332 53.779 0.00 0.00
C
ATOM 22742 O   VAL C 172   38.774 53.493 53.929 0.00 0.00
C
ATOM 22743 N   ARG C 173   37.997 55.443 53.112 0.00 0.00
C
ATOM 22744 HN ARG C 173   37.189 56.019 53.017 0.00 0.00
C
ATOM 22745 CA ARG C 173   39.286 55.941 52.671 0.00 0.00
C
ATOM 22746 HA ARG C 173   39.837 55.132 52.215 0.00 0.00
C
ATOM 22747 CB ARG C 173   39.054 56.956 51.508 0.00 0.00
C
ATOM 22748 HB1 ARG C 173   38.540 56.421 50.682 0.00 0.00
C
ATOM 22749 HB2 ARG C 173   38.331 57.754 51.780 0.00 0.00
C
ATOM 22750 CG ARG C 173   40.312 57.606 50.942 0.00 0.00
C
ATOM 22751 HG1 ARG C 173   40.685 58.233 51.780 0.00 0.00
C
ATOM 22752 HG2 ARG C 173   41.062 56.847 50.633 0.00 0.00
C
ATOM 22753 CD ARG C 173   40.141 58.462 49.623 0.00 0.00
C
ATOM 22754 HD1 ARG C 173   39.386 59.276 49.673 0.00 0.00
C
ATOM 22755 HD2 ARG C 173   41.147 58.848 49.349 0.00 0.00
C
ATOM 22756 NE ARG C 173   39.714 57.465 48.585 0.00 0.00
C
ATOM 22757 HE ARG C 173   40.016 56.524 48.737 0.00 0.00
C
ATOM 22758 CZ ARG C 173   39.379 57.662 47.301 0.00 0.00
C
ATOM 22759 NH1 ARG C 173   39.320 58.853 46.755 0.00 0.00
C
ATOM 22760 HH11 ARG C 173   39.396 58.984 45.766 0.00 0.00
C

ATOM 22761 HH12 ARG C 173   39.629 59.647 47.278 0.00 0.00
C
ATOM 22762 NH2 ARG C 173   39.189 56.578 46.537 0.00 0.00
C
ATOM 22763 HH21 ARG C 173   38.808 56.711 45.622 0.00 0.00
C
ATOM 22764 HH22 ARG C 173   39.182 55.661 46.935 0.00 0.00
C
ATOM 22765 C   ARG C 173   40.070 56.684 53.763 0.00 0.00
C
ATOM 22766 O   ARG C 173   41.270 56.446 53.851 0.00 0.00
C
ATOM 22767 N   ALA C 174   39.492 57.606 54.460 0.00 0.00
C
ATOM 22768 HN ALA C 174   38.513 57.748 54.334 0.00 0.00
C
ATOM 22769 CA ALA C 174   40.220 58.438 55.443 0.00 0.00
C
ATOM 22770 HA ALA C 174   41.040 58.896 54.910 0.00 0.00
C
ATOM 22771 CB ALA C 174   39.284 59.588 55.894 0.00 0.00
C
ATOM 22772 HB1 ALA C 174   39.997 60.417 56.093 0.00 0.00
C
ATOM 22773 HB2 ALA C 174   38.633 59.898 55.049 0.00 0.00
C
ATOM 22774 HB3 ALA C 174   38.656 59.287 56.759 0.00 0.00
C
ATOM 22775 C   ALA C 174   40.900 57.768 56.664 0.00 0.00
C
ATOM 22776 O   ALA C 174   42.092 58.048 56.874 0.00 0.00
C
ATOM 22777 N   LYS C 175   40.150 56.952 57.407 0.00 0.00
C
ATOM 22778 HN LYS C 175   39.235 56.713 57.091 0.00 0.00
C
ATOM 22779 CA LYS C 175   40.817 56.237 58.384 0.00 0.00
C
ATOM 22780 HA LYS C 175   41.674 56.753 58.791 0.00 0.00
C
ATOM 22781 CB LYS C 175   39.762 55.936 59.492 0.00 0.00
C
ATOM 22782 HB1 LYS C 175   38.900 55.392 59.050 0.00 0.00
C
ATOM 22783 HB2 LYS C 175   40.289 55.390 60.303 0.00 0.00
C
ATOM 22784 CG LYS C 175   39.286 57.263 60.135 0.00 0.00
C
ATOM 22785 HG1 LYS C 175   40.040 58.003 60.478 0.00 0.00
C
ATOM 22786 HG2 LYS C 175   38.653 57.848 59.434 0.00 0.00
C
ATOM 22787 CD LYS C 175   38.351 56.949 61.400 0.00 0.00
C

ATOM 22788 HD1 LYS C 175 37.604 57.770 61.448 0.00 0.00
C
ATOM 22789 HD2 LYS C 175 37.798 55.993 61.274 0.00 0.00
C
ATOM 22790 CE LYS C 175 39.180 56.809 62.688 0.00 0.00
C
ATOM 22791 HE1 LYS C 175 39.897 55.969 62.567 0.00 0.00
C
ATOM 22792 HE2 LYS C 175 39.806 57.705 62.887 0.00 0.00
C
ATOM 22793 NZ LYS C 175 38.403 56.539 63.860 0.00 0.00
C
ATOM 22794 HZ1 LYS C 175 38.798 57.034 64.685 0.00 0.00
C
ATOM 22795 HZ2 LYS C 175 37.462 56.964 63.733 0.00 0.00
C
ATOM 22796 HZ3 LYS C 175 38.274 55.524 64.046 0.00 0.00
C
ATOM 22797 C LYS C 175 41.287 54.808 57.896 0.00 0.00
C
ATOM 22798 O LYS C 175 42.337 54.377 58.316 0.00 0.00
C
ATOM 22799 N TYR C 176 40.471 54.146 57.048 0.00 0.00
C
ATOM 22800 HN TYR C 176 39.616 54.569 56.755 0.00 0.00
C
ATOM 22801 CA TYR C 176 40.826 52.698 56.836 0.00 0.00
C
ATOM 22802 HA TYR C 176 41.374 52.360 57.703 0.00 0.00
C
ATOM 22803 CB TYR C 176 39.564 51.884 56.853 0.00 0.00
C
ATOM 22804 HB1 TYR C 176 38.984 52.222 55.968 0.00 0.00
C
ATOM 22805 HB2 TYR C 176 39.768 50.792 56.830 0.00 0.00
C
ATOM 22806 CG TYR C 176 38.857 52.121 58.169 0.00 0.00
C
ATOM 22807 CD1 TYR C 176 37.573 52.716 58.184 0.00 0.00
C
ATOM 22808 HD1 TYR C 176 37.121 52.984 57.241 0.00 0.00
C
ATOM 22809 CE1 TYR C 176 36.983 53.039 59.388 0.00 0.00
C
ATOM 22810 HE1 TYR C 176 36.054 53.590 59.384 0.00 0.00
C
ATOM 22811 CZ TYR C 176 37.556 52.764 60.543 0.00 0.00
C
ATOM 22812 OH TYR C 176 36.806 53.053 61.701 0.00 0.00
C
ATOM 22813 HH TYR C 176 37.432 53.135 62.424 0.00 0.00
C
ATOM 22814 CD2 TYR C 176 39.443 51.833 59.419 0.00 0.00
C

ATOM 22815 HD2 TYR C 176 40.390 51.314 59.413 0.00 0.00
C
ATOM 22816 CE2 TYR C 176 38.881 52.185 60.674 0.00 0.00
C
ATOM 22817 HE2 TYR C 176 39.414 52.047 61.603 0.00 0.00
C
ATOM 22818 C TYR C 176 41.604 52.363 55.571 0.00 0.00
C
ATOM 22819 O TYR C 176 42.284 51.301 55.456 0.00 0.00
C
ATOM 22820 N GLY C 177 41.654 53.338 54.622 0.00 0.00
C
ATOM 22821 HN GLY C 177 41.263 54.228 54.844 0.00 0.00
C
ATOM 22822 CA GLY C 177 42.512 53.283 53.457 0.00 0.00
C
ATOM 22823 HA1 GLY C 177 43.551 53.184 53.730 0.00 0.00
C
ATOM 22824 HA2 GLY C 177 42.391 54.260 53.012 0.00 0.00
C
ATOM 22825 C GLY C 177 42.123 52.250 52.486 0.00 0.00
C
ATOM 22826 O GLY C 177 42.995 51.867 51.724 0.00 0.00
C
ATOM 22827 N CYS C 178 40.829 51.798 52.413 0.00 0.00
C
ATOM 22828 HN CYS C 178 40.061 52.272 52.837 0.00 0.00
C
ATOM 22829 CA CYS C 178 40.474 50.615 51.573 0.00 0.00
C
ATOM 22830 HA CYS C 178 41.309 49.960 51.368 0.00 0.00
C
ATOM 22831 CB CYS C 178 39.231 49.761 52.107 0.00 0.00
C
ATOM 22832 HB1 CYS C 178 38.371 50.462 52.154 0.00 0.00
C
ATOM 22833 HB2 CYS C 178 39.055 48.878 51.456 0.00 0.00
C
ATOM 22834 SG CYS C 178 39.502 49.221 53.821 0.00 0.00
C
ATOM 22835 HG1 CYS C 178 39.698 50.463 54.240 0.00 0.00
C
ATOM 22836 C CYS C 178 40.116 51.128 50.146 0.00 0.00
C
ATOM 22837 O CYS C 178 39.579 52.186 49.976 0.00 0.00
C
ATOM 22838 N THR C 179 40.373 50.240 49.140 0.00 0.00
C
ATOM 22839 HN THR C 179 40.743 49.346 49.382 0.00 0.00
C
ATOM 22840 CA THR C 179 40.027 50.390 47.750 0.00 0.00
C
ATOM 22841 HA THR C 179 40.120 51.421 47.443 0.00 0.00
C

ATOM 22842 CB THR C 179 40.870 49.393 47.022 0.00 0.00
C
ATOM 22843 HB THR C 179 40.937 48.406 47.527 0.00 0.00
C
ATOM 22844 OG1 THR C 179 42.197 49.924 46.987 0.00 0.00
C
ATOM 22845 HG1 THR C 179 42.705 49.549 47.710 0.00 0.00
C
ATOM 22846 CG2 THR C 179 40.539 49.257 45.520 0.00 0.00
C
ATOM 22847 HG21 THR C 179 40.703 50.228 45.007 0.00 0.00
C
ATOM 22848 HG22 THR C 179 41.173 48.545 44.950 0.00 0.00
C
ATOM 22849 HG23 THR C 179 39.487 48.909 45.437 0.00 0.00
C
ATOM 22850 C THR C 179 38.608 49.959 47.537 0.00 0.00
C
ATOM 22851 O THR C 179 38.254 48.799 47.722 0.00 0.00
C
ATOM 22852 N ILE C 180 37.790 50.912 47.040 0.00 0.00
C
ATOM 22853 HN ILE C 180 38.006 51.815 46.676 0.00 0.00
C
ATOM 22854 CA ILE C 180 36.402 50.678 46.897 0.00 0.00
C
ATOM 22855 HA ILE C 180 35.996 49.981 47.616 0.00 0.00
C
ATOM 22856 CB ILE C 180 35.613 51.995 47.072 0.00 0.00
C
ATOM 22857 HB ILE C 180 35.829 52.658 46.207 0.00 0.00
C
ATOM 22858 CG2 ILE C 180 34.072 51.707 47.070 0.00 0.00
C
ATOM 22859 HG21 ILE C 180 33.827 51.009 47.899 0.00 0.00
C
ATOM 22860 HG22 ILE C 180 33.545 52.677 47.192 0.00 0.00
C
ATOM 22861 HG23 ILE C 180 33.782 51.306 46.075 0.00 0.00
C
ATOM 22862 CG1 ILE C 180 35.890 52.766 48.337 0.00 0.00
C
ATOM 22863 HG11 ILE C 180 34.905 53.075 48.748 0.00 0.00
C
ATOM 22864 HG12 ILE C 180 36.413 52.024 48.976 0.00 0.00
C
ATOM 22865 CD ILE C 180 36.708 54.131 48.065 0.00 0.00
C
ATOM 22866 HD1 ILE C 180 36.904 54.745 48.970 0.00 0.00
C
ATOM 22867 HD2 ILE C 180 37.774 53.853 47.921 0.00 0.00
C
ATOM 22868 HD3 ILE C 180 36.308 54.839 47.308 0.00 0.00
C

ATOM 22869 C ILE C 180 36.029 50.101 45.569 0.00 0.00
C
ATOM 22870 O ILE C 180 35.979 50.837 44.571 0.00 0.00
C
ATOM 22871 N LEU C 181 35.753 48.814 45.399 0.00 0.00
C
ATOM 22872 HN LEU C 181 35.520 48.219 46.164 0.00 0.00
C
ATOM 22873 CA LEU C 181 35.706 48.113 44.150 0.00 0.00
C
ATOM 22874 HA LEU C 181 36.552 48.498 43.599 0.00 0.00
C
ATOM 22875 CB LEU C 181 35.829 46.543 44.455 0.00 0.00
C
ATOM 22876 HB1 LEU C 181 34.954 46.228 45.064 0.00 0.00
C
ATOM 22877 HB2 LEU C 181 35.700 46.066 43.460 0.00 0.00
C
ATOM 22878 CG LEU C 181 37.200 46.091 45.017 0.00 0.00
C
ATOM 22879 HG LEU C 181 37.310 46.600 45.998 0.00 0.00
C
ATOM 22880 CD1 LEU C 181 37.001 44.609 45.304 0.00 0.00
C
ATOM 22881 HD11 LEU C 181 36.008 44.524 45.794 0.00 0.00
C
ATOM 22882 HD12 LEU C 181 36.978 44.000 44.375 0.00 0.00
C
ATOM 22883 HD13 LEU C 181 37.826 44.138 45.879 0.00 0.00
C
ATOM 22884 CD2 LEU C 181 38.416 46.346 44.168 0.00 0.00
C
ATOM 22885 HD21 LEU C 181 38.613 47.392 43.849 0.00 0.00
C
ATOM 22886 HD22 LEU C 181 39.283 46.027 44.785 0.00 0.00
C
ATOM 22887 HD23 LEU C 181 38.358 45.748 43.233 0.00 0.00
C
ATOM 22888 C LEU C 181 34.427 48.393 43.384 0.00 0.00
C
ATOM 22889 O LEU C 181 34.380 48.566 42.183 0.00 0.00
C
ATOM 22890 N ALA C 182 33.277 48.443 44.093 0.00 0.00
C
ATOM 22891 HN ALA C 182 33.287 48.268 45.075 0.00 0.00
C
ATOM 22892 CA ALA C 182 31.939 48.708 43.483 0.00 0.00
C
ATOM 22893 HA ALA C 182 31.990 49.553 42.813 0.00 0.00
C
ATOM 22894 CB ALA C 182 31.508 47.563 42.623 0.00 0.00
C
ATOM 22895 HB1 ALA C 182 30.447 47.615 42.297 0.00 0.00
C

ATOM 22896 HB2 ALA C 182   32.140 47.574 41.709 0.00 0.00
C
ATOM 22897 HB3 ALA C 182   31.758 46.571 43.056 0.00 0.00
C
ATOM 22898 C   ALA C 182   30.943 49.122 44.471 0.00 0.00
C
ATOM 22899 O   ALA C 182   30.966 48.716 45.672 0.00 0.00
C
ATOM 22900 N   ILE C 183   29.960 49.851 43.973 0.00 0.00
C
ATOM 22901 HN ILE C 183   29.954 49.988 42.985 0.00 0.00
C
ATOM 22902 CA ILE C 183   28.782 50.224 44.698 0.00 0.00
C
ATOM 22903 HA ILE C 183   28.665 49.788 45.679 0.00 0.00
C
ATOM 22904 CB ILE C 183   28.613 51.691 44.953 0.00 0.00
C
ATOM 22905 HB ILE C 183   29.376 51.984 45.706 0.00 0.00
C
ATOM 22906 CG2 ILE C 183   28.914 52.553 43.742 0.00 0.00
C
ATOM 22907 HG21 ILE C 183   29.835 52.280 43.184 0.00 0.00
C
ATOM 22908 HG22 ILE C 183   28.057 52.510 43.036 0.00 0.00
C
ATOM 22909 HG23 ILE C 183   28.982 53.638 43.975 0.00 0.00
C
ATOM 22910 CG1 ILE C 183   27.291 52.004 45.654 0.00 0.00
C
ATOM 22911 HG11 ILE C 183   26.436 51.695 45.015 0.00 0.00
C
ATOM 22912 HG12 ILE C 183   27.286 51.326 46.534 0.00 0.00
C
ATOM 22913 CD ILE C 183   27.119 53.417 46.080 0.00 0.00
C
ATOM 22914 HD1 ILE C 183   27.964 53.743 46.723 0.00 0.00
C
ATOM 22915 HD2 ILE C 183   27.077 54.132 45.231 0.00 0.00
C
ATOM 22916 HD3 ILE C 183   26.147 53.473 46.616 0.00 0.00
C
ATOM 22917 C   ILE C 183   27.595 49.716 43.933 0.00 0.00
C
ATOM 22918 O   ILE C 183   27.340 50.026 42.777 0.00 0.00
C
ATOM 22919 N   LYS C 184   26.742 48.931 44.615 0.00 0.00
C
ATOM 22920 HN LYS C 184   26.791 48.687 45.581 0.00 0.00
C
ATOM 22921 CA LYS C 184   25.560 48.218 44.005 0.00 0.00
C
ATOM 22922 HA LYS C 184   25.724 48.220 42.937 0.00 0.00
C

ATOM 22923 CB LYS C 184 25.289 46.701 44.367 0.00 0.00
C
ATOM 22924 HB1 LYS C 184 24.785 46.671 45.357 0.00 0.00
C
ATOM 22925 HB2 LYS C 184 24.567 46.261 43.646 0.00 0.00
C
ATOM 22926 CG LYS C 184 26.561 45.755 44.503 0.00 0.00
C
ATOM 22927 HG1 LYS C 184 27.073 45.885 43.525 0.00 0.00
C
ATOM 22928 HG2 LYS C 184 27.102 46.218 45.356 0.00 0.00
C
ATOM 22929 CD LYS C 184 26.102 44.312 44.638 0.00 0.00
C
ATOM 22930 HD1 LYS C 184 25.270 44.284 45.375 0.00 0.00
C
ATOM 22931 HD2 LYS C 184 25.829 43.937 43.629 0.00 0.00
C
ATOM 22932 CE LYS C 184 27.117 43.286 45.173 0.00 0.00
C
ATOM 22933 HE1 LYS C 184 28.130 43.294 44.719 0.00 0.00
C
ATOM 22934 HE2 LYS C 184 27.207 43.659 46.216 0.00 0.00
C
ATOM 22935 NZ LYS C 184 26.490 41.964 45.260 0.00 0.00
C
ATOM 22936 HZ1 LYS C 184 27.111 41.407 45.881 0.00 0.00
C
ATOM 22937 HZ2 LYS C 184 25.527 41.953 45.653 0.00 0.00
C
ATOM 22938 HZ3 LYS C 184 26.371 41.427 44.378 0.00 0.00
C
ATOM 22939 C LYS C 184 24.266 48.954 44.137 0.00 0.00
C
ATOM 22940 O LYS C 184 24.017 49.570 45.156 0.00 0.00
C
ATOM 22941 N HSE C 185 23.405 48.866 43.076 0.00 0.00
C
ATOM 22942 HN HSE C 185 23.775 48.636 42.179 0.00 0.00
C
ATOM 22943 CA HSE C 185 22.090 49.488 42.985 0.00 0.00
C
ATOM 22944 HA HSE C 185 21.748 49.732 43.980 0.00 0.00
C
ATOM 22945 CB HSE C 185 22.088 50.712 42.043 0.00 0.00
C
ATOM 22946 HB1 HSE C 185 22.213 50.284 41.025 0.00 0.00
C
ATOM 22947 HB2 HSE C 185 21.121 51.243 42.177 0.00 0.00
C
ATOM 22948 ND1 HSE C 185 24.223 51.842 41.083 0.00 0.00
C
ATOM 22949 CG HSE C 185 23.345 51.579 42.183 0.00 0.00
C

ATOM 22950 CE1 HSE C 185 25.120 52.678 41.523 0.00 0.00
C
ATOM 22951 HE1 HSE C 185 26.007 52.927 40.942 0.00 0.00
C
ATOM 22952 NE2 HSE C 185 24.953 52.910 42.833 0.00 0.00
C
ATOM 22953 HE2 HSE C 185 25.582 53.430 43.411 0.00 0.00
C
ATOM 22954 CD2 HSE C 185 23.766 52.350 43.269 0.00 0.00
C
ATOM 22955 HD2 HSE C 185 23.396 52.489 44.277 0.00 0.00
C
ATOM 22956 C HSE C 185 21.116 48.469 42.547 0.00 0.00
C
ATOM 22957 O HSE C 185 20.421 48.587 41.534 0.00 0.00
C
ATOM 22958 N HSE C 186 21.151 47.331 43.282 0.00 0.00
C
ATOM 22959 HN HSE C 186 21.742 47.282 44.084 0.00 0.00
C
ATOM 22960 CA HSE C 186 20.257 46.117 43.187 0.00 0.00
C
ATOM 22961 HA HSE C 186 20.645 45.294 43.770 0.00 0.00
C
ATOM 22962 CB HSE C 186 18.944 46.344 43.946 0.00 0.00
C
ATOM 22963 HB1 HSE C 186 18.484 47.229 43.455 0.00 0.00
C
ATOM 22964 HB2 HSE C 186 18.209 45.524 43.802 0.00 0.00
C
ATOM 22965 ND1 HSE C 186 19.100 45.888 46.355 0.00 0.00
C
ATOM 22966 CG HSE C 186 19.020 46.809 45.413 0.00 0.00
C
ATOM 22967 CE1 HSE C 186 19.221 46.553 47.546 0.00 0.00
C
ATOM 22968 HE1 HSE C 186 19.650 46.109 48.445 0.00 0.00
C
ATOM 22969 NE2 HSE C 186 19.103 47.878 47.342 0.00 0.00
C
ATOM 22970 HE2 HSE C 186 19.243 48.567 48.053 0.00 0.00
C
ATOM 22971 CD2 HSE C 186 18.957 48.084 45.980 0.00 0.00
C
ATOM 22972 HD2 HSE C 186 18.735 49.037 45.515 0.00 0.00
C
ATOM 22973 C HSE C 186 19.976 45.676 41.809 0.00 0.00
C
ATOM 22974 O HSE C 186 18.843 45.676 41.412 0.00 0.00
C
ATOM 22975 N GLY C 187 21.001 45.264 41.007 0.00 0.00
C
ATOM 22976 HN GLY C 187 21.951 45.314 41.305 0.00 0.00
C

ATOM 22977 CA GLY C 187   20.664 44.900 39.586 0.00 0.00
C
ATOM 22978 HA1 GLY C 187   19.596 44.839 39.436 0.00 0.00
C
ATOM 22979 HA2 GLY C 187   21.106 43.943 39.350 0.00 0.00
C
ATOM 22980 C   GLY C 187   21.270 45.888 38.658 0.00 0.00
C
ATOM 22981 O   GLY C 187   21.553 45.592 37.475 0.00 0.00
C
ATOM 22982 N   ASP C 188   21.656 47.119 39.135 0.00 0.00
C
ATOM 22983 HN ASP C 188   21.299 47.374 40.030 0.00 0.00
C
ATOM 22984 CA ASP C 188   22.463 48.072 38.364 0.00 0.00
C
ATOM 22985 HA ASP C 188   22.809 47.628 37.442 0.00 0.00
C
ATOM 22986 CB ASP C 188   21.830 49.496 38.084 0.00 0.00
C
ATOM 22987 HB1 ASP C 188   21.836 49.870 39.130 0.00 0.00
C
ATOM 22988 HB2 ASP C 188   22.536 50.126 37.501 0.00 0.00
C
ATOM 22989 CG ASP C 188   20.489 49.520 37.475 0.00 0.00
C
ATOM 22990 OD1 ASP C 188   20.388 49.714 36.208 0.00 0.00
C
ATOM 22991 OD2 ASP C 188   19.456 49.400 38.187 0.00 0.00
C
ATOM 22992 C   ASP C 188   23.755 48.166 39.066 0.00 0.00
C
ATOM 22993 O   ASP C 188   23.811 47.836 40.250 0.00 0.00
C
ATOM 22994 N   ILE C 189   24.863 48.674 38.390 0.00 0.00
C
ATOM 22995 HN ILE C 189   24.907 49.055 37.470 0.00 0.00
C
ATOM 22996 CA ILE C 189   26.169 48.587 38.974 0.00 0.00
C
ATOM 22997 HA ILE C 189   26.139 48.673 40.050 0.00 0.00
C
ATOM 22998 CB ILE C 189   26.753 47.246 38.621 0.00 0.00
C
ATOM 22999 HB ILE C 189   26.072 46.508 39.097 0.00 0.00
C
ATOM 23000 CG2 ILE C 189   26.752 47.002 37.072 0.00 0.00
C
ATOM 23001 HG21 ILE C 189   27.290 46.086 36.749 0.00 0.00
C
ATOM 23002 HG22 ILE C 189   25.720 46.895 36.674 0.00 0.00
C
ATOM 23003 HG23 ILE C 189   27.214 47.930 36.670 0.00 0.00
C

ATOM 23004 CG1 ILE C 189   28.125 46.962 39.264 0.00 0.00
C
ATOM 23005 HG11 ILE C 189   28.561 46.049 38.804 0.00 0.00
C
ATOM 23006 HG12 ILE C 189   28.943 47.687 39.070 0.00 0.00
C
ATOM 23007 CD ILE C 189   28.038 46.756 40.754 0.00 0.00
C
ATOM 23008 HD1 ILE C 189   27.438 45.862 41.030 0.00 0.00
C
ATOM 23009 HD2 ILE C 189   29.059 46.574 41.152 0.00 0.00
C
ATOM 23010 HD3 ILE C 189   27.668 47.635 41.324 0.00 0.00
C
ATOM 23011 C   ILE C 189   27.080 49.847 38.612 0.00 0.00
C
ATOM 23012 O   ILE C 189   26.892 50.456 37.597 0.00 0.00
C
ATOM 23013 N   CYS C 190   28.035 50.215 39.547 0.00 0.00
C
ATOM 23014 HN CYS C 190   28.105 49.734 40.418 0.00 0.00
C
ATOM 23015 CA CYS C 190   29.078 51.240 39.217 0.00 0.00
C
ATOM 23016 HA CYS C 190   29.123 51.469 38.162 0.00 0.00
C
ATOM 23017 CB CYS C 190   28.590 52.590 39.873 0.00 0.00
C
ATOM 23018 HB1 CYS C 190   27.711 52.954 39.300 0.00 0.00
C
ATOM 23019 HB2 CYS C 190   28.350 52.403 40.942 0.00 0.00
C
ATOM 23020 SG CYS C 190   29.804 54.017 39.800 0.00 0.00
C
ATOM 23021 HG1 CYS C 190   28.972 54.878 40.368 0.00 0.00
C
ATOM 23022 C   CYS C 190   30.367 50.765 39.847 0.00 0.00
C
ATOM 23023 O   CYS C 190   30.367 50.346 41.016 0.00 0.00
C
ATOM 23024 N   LEU C 191   31.492 50.707 39.138 0.00 0.00
C
ATOM 23025 HN LEU C 191   31.687 50.965 38.195 0.00 0.00
C
ATOM 23026 CA LEU C 191   32.755 50.222 39.653 0.00 0.00
C
ATOM 23027 HA LEU C 191   32.589 49.510 40.448 0.00 0.00
C
ATOM 23028 CB LEU C 191   33.439 49.427 38.516 0.00 0.00
C
ATOM 23029 HB1 LEU C 191   33.698 50.175 37.737 0.00 0.00
C
ATOM 23030 HB2 LEU C 191   34.323 48.932 38.970 0.00 0.00
C

ATOM 23031 CG LEU C 191   32.559 48.350 37.809 0.00 0.00
C
ATOM 23032 HG LEU C 191   31.721 48.888 37.316 0.00 0.00
C
ATOM 23033 CD1 LEU C 191   33.438 47.547 36.839 0.00 0.00
C
ATOM 23034 HD11 LEU C 191   34.140 48.151 36.225 0.00 0.00
C
ATOM 23035 HD12 LEU C 191   34.054 46.851 37.447 0.00 0.00
C
ATOM 23036 HD13 LEU C 191   32.812 47.020 36.088 0.00 0.00
C
ATOM 23037 CD2 LEU C 191   31.916 47.330 38.732 0.00 0.00
C
ATOM 23038 HD21 LEU C 191   31.404 46.615 38.053 0.00 0.00
C
ATOM 23039 HD22 LEU C 191   32.651 46.775 39.354 0.00 0.00
C
ATOM 23040 HD23 LEU C 191   31.165 47.793 39.408 0.00 0.00
C
ATOM 23041 C   LEU C 191   33.623 51.451 39.888 0.00 0.00
C
ATOM 23042 O   LEU C 191   33.589 52.455 39.118 0.00 0.00
C
ATOM 23043 N   SER C 192   34.348 51.475 41.013 0.00 0.00
C
ATOM 23044 HN SER C 192   34.406 50.733 41.676 0.00 0.00
C
ATOM 23045 CA SER C 192   35.174 52.569 41.500 0.00 0.00
C
ATOM 23046 HA SER C 192   35.390 52.376 42.541 0.00 0.00
C
ATOM 23047 CB SER C 192   36.491 52.608 40.626 0.00 0.00
C
ATOM 23048 HB1 SER C 192   36.205 52.908 39.596 0.00 0.00
C
ATOM 23049 HB2 SER C 192   37.165 53.450 40.891 0.00 0.00
C
ATOM 23050 OG SER C 192   37.190 51.339 40.487 0.00 0.00
C
ATOM 23051 HG1 SER C 192   37.228 51.142 39.548 0.00 0.00
C
ATOM 23052 C   SER C 192   34.547 53.952 41.503 0.00 0.00
C
ATOM 23053 O   SER C 192   34.904 54.834 40.728 0.00 0.00
C
ATOM 23054 N   PRO C 193   33.524 54.184 42.332 0.00 0.00
C
ATOM 23055 CD PRO C 193   33.345 53.331 43.504 0.00 0.00
C
ATOM 23056 HD1 PRO C 193   32.917 52.334 43.265 0.00 0.00
C
ATOM 23057 HD2 PRO C 193   34.321 53.220 44.024 0.00 0.00
C

ATOM 23058 CA PRO C 193 32.774 55.416 42.393 0.00 0.00
C
ATOM 23059 HA PRO C 193 32.276 55.676 41.470 0.00 0.00
C
ATOM 23060 CB PRO C 193 31.637 55.083 43.351 0.00 0.00
C
ATOM 23061 HB1 PRO C 193 30.792 54.661 42.766 0.00 0.00
C
ATOM 23062 HB2 PRO C 193 31.186 55.966 43.853 0.00 0.00
C
ATOM 23063 CG PRO C 193 32.267 54.055 44.344 0.00 0.00
C
ATOM 23064 HG1 PRO C 193 31.539 53.396 44.863 0.00 0.00
C
ATOM 23065 HG2 PRO C 193 32.717 54.652 45.166 0.00 0.00
C
ATOM 23066 C PRO C 193 33.535 56.582 42.987 0.00 0.00
C
ATOM 23067 O PRO C 193 34.613 56.412 43.596 0.00 0.00
C
ATOM 23068 N ALA C 194 33.007 57.817 42.769 0.00 0.00
C
ATOM 23069 HN ALA C 194 32.197 57.911 42.195 0.00 0.00
C
ATOM 23070 CA ALA C 194 33.539 59.042 43.328 0.00 0.00
C
ATOM 23071 HA ALA C 194 34.619 59.049 43.332 0.00 0.00
C
ATOM 23072 CB ALA C 194 33.065 60.323 42.613 0.00 0.00
C
ATOM 23073 HB1 ALA C 194 33.321 60.175 41.542 0.00 0.00
C
ATOM 23074 HB2 ALA C 194 31.966 60.489 42.612 0.00 0.00
C
ATOM 23075 HB3 ALA C 194 33.584 61.197 43.061 0.00 0.00
C
ATOM 23076 C ALA C 194 33.042 59.120 44.788 0.00 0.00
C
ATOM 23077 O ALA C 194 31.892 58.883 45.040 0.00 0.00
C
ATOM 23078 N PRO C 195 33.818 59.535 45.838 0.00 0.00
C
ATOM 23079 CD PRO C 195 35.320 59.503 45.720 0.00 0.00
C
ATOM 23080 HD1 PRO C 195 35.594 58.594 45.142 0.00 0.00
C
ATOM 23081 HD2 PRO C 195 35.620 60.429 45.185 0.00 0.00
C
ATOM 23082 CA PRO C 195 33.438 59.565 47.207 0.00 0.00
C
ATOM 23083 HA PRO C 195 33.175 58.542 47.435 0.00 0.00
C
ATOM 23084 CB PRO C 195 34.621 59.905 48.007 0.00 0.00
C

ATOM 23085 HB1 PRO C 195 34.536 59.437 49.012 0.00 0.00
C
ATOM 23086 HB2 PRO C 195 34.752 61.006 48.075 0.00 0.00
C
ATOM 23087 CG PRO C 195 35.810 59.389 47.199 0.00 0.00
C
ATOM 23088 HG1 PRO C 195 36.773 59.923 47.347 0.00 0.00
C
ATOM 23089 HG2 PRO C 195 35.903 58.289 47.325 0.00 0.00
C
ATOM 23090 C PRO C 195 32.361 60.545 47.436 0.00 0.00
C
ATOM 23091 O PRO C 195 31.905 60.562 48.570 0.00 0.00
C
ATOM 23092 N GLU C 196 32.011 61.454 46.466 0.00 0.00
C
ATOM 23093 HN GLU C 196 32.372 61.527 45.539 0.00 0.00
C
ATOM 23094 CA GLU C 196 30.853 62.282 46.556 0.00 0.00
C
ATOM 23095 HA GLU C 196 30.902 62.950 47.403 0.00 0.00
C
ATOM 23096 CB GLU C 196 30.710 63.100 45.217 0.00 0.00
C
ATOM 23097 HB1 GLU C 196 30.655 62.437 44.327 0.00 0.00
C
ATOM 23098 HB2 GLU C 196 29.717 63.589 45.313 0.00 0.00
C
ATOM 23099 CG GLU C 196 31.732 64.263 44.986 0.00 0.00
C
ATOM 23100 HG1 GLU C 196 31.808 64.930 45.872 0.00 0.00
C
ATOM 23101 HG2 GLU C 196 32.769 63.964 44.724 0.00 0.00
C
ATOM 23102 CD GLU C 196 31.210 65.064 43.801 0.00 0.00
C
ATOM 23103 OE1 GLU C 196 29.962 65.337 43.760 0.00 0.00
C
ATOM 23104 OE2 GLU C 196 31.988 65.397 42.868 0.00 0.00
C
ATOM 23105 C GLU C 196 29.532 61.414 46.746 0.00 0.00
C
ATOM 23106 O GLU C 196 28.570 61.704 47.404 0.00 0.00
C
ATOM 23107 N ASP C 197 29.408 60.303 46.068 0.00 0.00
C
ATOM 23108 HN ASP C 197 30.161 59.908 45.549 0.00 0.00
C
ATOM 23109 CA ASP C 197 28.258 59.472 46.051 0.00 0.00
C
ATOM 23110 HA ASP C 197 27.401 60.079 45.799 0.00 0.00
C
ATOM 23111 CB ASP C 197 28.376 58.513 44.813 0.00 0.00
C

ATOM 23112 HB1 ASP C 197 29.238 57.816 44.883 0.00 0.00
C
ATOM 23113 HB2 ASP C 197 27.543 57.782 44.886 0.00 0.00
C
ATOM 23114 CG ASP C 197 28.199 59.235 43.482 0.00 0.00
C
ATOM 23115 OD1 ASP C 197 27.065 59.746 43.252 0.00 0.00
C
ATOM 23116 OD2 ASP C 197 29.177 59.275 42.713 0.00 0.00
C
ATOM 23117 C ASP C 197 27.927 58.738 47.344 0.00 0.00
C
ATOM 23118 O ASP C 197 28.695 58.715 48.318 0.00 0.00
C
ATOM 23119 N ILE C 198 26.701 58.273 47.422 0.00 0.00
C
ATOM 23120 HN ILE C 198 26.166 58.351 46.584 0.00 0.00
C
ATOM 23121 CA ILE C 198 25.963 57.907 48.640 0.00 0.00
C
ATOM 23122 HA ILE C 198 26.477 58.244 49.528 0.00 0.00
C
ATOM 23123 CB ILE C 198 24.697 58.736 48.550 0.00 0.00
C
ATOM 23124 HB ILE C 198 24.252 58.679 47.534 0.00 0.00
C
ATOM 23125 CG2 ILE C 198 23.695 58.241 49.644 0.00 0.00
C
ATOM 23126 HG21 ILE C 198 23.308 57.233 49.382 0.00 0.00
C
ATOM 23127 HG22 ILE C 198 24.127 58.147 50.663 0.00 0.00
C
ATOM 23128 HG23 ILE C 198 22.817 58.910 49.770 0.00 0.00
C
ATOM 23129 CG1 ILE C 198 25.109 60.203 48.829 0.00 0.00
C
ATOM 23130 HG11 ILE C 198 25.205 60.288 49.933 0.00 0.00
C
ATOM 23131 HG12 ILE C 198 26.058 60.521 48.348 0.00 0.00
C
ATOM 23132 CD ILE C 198 23.929 61.236 48.407 0.00 0.00
C
ATOM 23133 HD1 ILE C 198 23.585 60.959 47.387 0.00 0.00
C
ATOM 23134 HD2 ILE C 198 23.131 61.152 49.175 0.00 0.00
C
ATOM 23135 HD3 ILE C 198 24.227 62.306 48.410 0.00 0.00
C
ATOM 23136 C ILE C 198 25.709 56.440 48.794 0.00 0.00
C
ATOM 23137 O ILE C 198 25.371 55.738 47.869 0.00 0.00
C
ATOM 23138 N ILE C 199 25.894 55.839 50.006 0.00 0.00
C

```
ATOM  23139  HN  ILE C 199      26.198  56.403  50.770  0.00  0.00
C
ATOM  23140  CA  ILE C 199      25.618  54.431  50.307  0.00  0.00
C
ATOM  23141  HA  ILE C 199      25.179  53.873  49.492  0.00  0.00
C
ATOM  23142  CB  ILE C 199      26.863  53.718  50.833  0.00  0.00
C
ATOM  23143  HB  ILE C 199      27.622  53.739  50.021  0.00  0.00
C
ATOM  23144  CG2 ILE C 199      27.429  54.401  52.063  0.00  0.00
C
ATOM  23145 HG21 ILE C 199      27.604  55.492  51.952  0.00  0.00
C
ATOM  23146 HG22 ILE C 199      26.870  54.225  53.006  0.00  0.00
C
ATOM  23147 HG23 ILE C 199      28.464  54.049  52.266  0.00  0.00
C
ATOM  23148  CG1 ILE C 199      26.646  52.176  50.995  0.00  0.00
C
ATOM  23149 HG11 ILE C 199      27.552  51.863  51.557  0.00  0.00
C
ATOM  23150 HG12 ILE C 199      25.834  52.022  51.737  0.00  0.00
C
ATOM  23151  CD  ILE C 199      26.387  51.516  49.622  0.00  0.00
C
ATOM  23152  HD1 ILE C 199      26.396  50.427  49.842  0.00  0.00
C
ATOM  23153  HD2 ILE C 199      25.323  51.713  49.368  0.00  0.00
C
ATOM  23154  HD3 ILE C 199      27.055  51.807  48.784  0.00  0.00
C
ATOM  23155  C   ILE C 199      24.463  54.404  51.224  0.00  0.00
C
ATOM  23156  O   ILE C 199      24.019  55.471  51.698  0.00  0.00
C
ATOM  23157  N   ARG C 200      23.728  53.260  51.447  0.00  0.00
C
ATOM  23158  HN  ARG C 200      23.951  52.369  51.059  0.00  0.00
C
ATOM  23159  CA  ARG C 200      22.426  53.014  52.125  0.00  0.00
C
ATOM  23160  HA  ARG C 200      22.311  53.795  52.863  0.00  0.00
C
ATOM  23161  CB  ARG C 200      21.198  52.891  51.159  0.00  0.00
C
ATOM  23162  HB1 ARG C 200      21.101  51.886  50.694  0.00  0.00
C
ATOM  23163  HB2 ARG C 200      20.312  53.147  51.779  0.00  0.00
C
ATOM  23164  CG  ARG C 200      21.125  54.086  50.159  0.00  0.00
C
ATOM  23165  HG1 ARG C 200      21.184  55.044  50.718  0.00  0.00
C
```

ATOM 23166 HG2 ARG C 200   21.902 54.089 49.365 0.00 0.00
C
ATOM 23167 CD ARG C 200   19.747 54.176 49.384 0.00 0.00
C
ATOM 23168 HD1 ARG C 200   18.919 54.038 50.112 0.00 0.00
C
ATOM 23169 HD2 ARG C 200   19.761 55.180 48.908 0.00 0.00
C
ATOM 23170 NE ARG C 200   19.763 53.065 48.344 0.00 0.00
C
ATOM 23171 HE ARG C 200   20.492 52.383 48.395 0.00 0.00
C
ATOM 23172 CZ ARG C 200   19.136 53.052 47.192 0.00 0.00
C
ATOM 23173 NH1 ARG C 200   18.150 53.986 46.933 0.00 0.00
C
ATOM 23174 HH11 ARG C 200   17.554 53.796 46.154 0.00 0.00
C
ATOM 23175 HH12 ARG C 200   17.761 54.580 47.638 0.00 0.00
C
ATOM 23176 NH2 ARG C 200   19.412 52.169 46.234 0.00 0.00
C
ATOM 23177 HH21 ARG C 200   18.887 52.310 45.395 0.00 0.00
C
ATOM 23178 HH22 ARG C 200   20.087 51.431 46.256 0.00 0.00
C
ATOM 23179 C   ARG C 200   22.355 51.750 52.876 0.00 0.00
C
ATOM 23180 O   ARG C 200   23.095 50.799 52.589 0.00 0.00
C
ATOM 23181 N   GLU C 201   21.557 51.825 53.965 0.00 0.00
C
ATOM 23182 HN GLU C 201   20.986 52.637 54.064 0.00 0.00
C
ATOM 23183 CA GLU C 201   21.395 50.799 54.981 0.00 0.00
C
ATOM 23184 HA GLU C 201   22.352 50.659 55.461 0.00 0.00
C
ATOM 23185 CB GLU C 201   20.352 51.347 56.053 0.00 0.00
C
ATOM 23186 HB1 GLU C 201   20.614 52.392 56.323 0.00 0.00
C
ATOM 23187 HB2 GLU C 201   19.369 51.281 55.540 0.00 0.00
C
ATOM 23188 CG GLU C 201   20.426 50.555 57.380 0.00 0.00
C
ATOM 23189 HG1 GLU C 201   20.046 49.553 57.086 0.00 0.00
C
ATOM 23190 HG2 GLU C 201   21.388 50.579 57.935 0.00 0.00
C
ATOM 23191 CD GLU C 201   19.370 51.167 58.274 0.00 0.00
C
ATOM 23192 OE1 GLU C 201   19.766 51.943 59.174 0.00 0.00
C

ATOM 23193 OE2 GLU C 201   18.114 50.894 58.079 0.00 0.00
C
ATOM 23194 C   GLU C 201   21.161 49.409 54.358 0.00 0.00
C
ATOM 23195 O   GLU C 201   21.517 48.447 55.040 0.00 0.00
C
ATOM 23196 N   GLN C 202   20.470 49.253 53.203 0.00 0.00
C
ATOM 23197 HN GLN C 202   20.045 50.008 52.710 0.00 0.00
C
ATOM 23198 CA GLN C 202   20.233 47.992 52.575 0.00 0.00
C
ATOM 23199 HA GLN C 202   20.662 47.207 53.180 0.00 0.00
C
ATOM 23200 CB GLN C 202   18.758 47.618 52.510 0.00 0.00
C
ATOM 23201 HB1 GLN C 202   18.183 48.438 52.028 0.00 0.00
C
ATOM 23202 HB2 GLN C 202   18.566 46.716 51.889 0.00 0.00
C
ATOM 23203 CG GLN C 202   18.195 47.357 53.888 0.00 0.00
C
ATOM 23204 HG1 GLN C 202   18.256 48.192 54.618 0.00 0.00
C
ATOM 23205 HG2 GLN C 202   17.094 47.263 53.782 0.00 0.00
C
ATOM 23206 CD GLN C 202   18.704 46.122 54.524 0.00 0.00
C
ATOM 23207 OE1 GLN C 202   18.192 44.995 54.313 0.00 0.00
C
ATOM 23208 NE2 GLN C 202   19.735 46.218 55.413 0.00 0.00
C
ATOM 23209 HE21 GLN C 202   19.889 45.392 55.955 0.00 0.00
C
ATOM 23210 HE22 GLN C 202   20.391 46.972 55.385 0.00 0.00
C
ATOM 23211 C   GLN C 202   20.878 47.746 51.225 0.00 0.00
C
ATOM 23212 O   GLN C 202   20.412 46.887 50.511 0.00 0.00
C
ATOM 23213 N   ASP C 203   21.887 48.552 50.778 0.00 0.00
C
ATOM 23214 HN ASP C 203   22.291 49.254 51.360 0.00 0.00
C
ATOM 23215 CA ASP C 203   22.622 48.424 49.525 0.00 0.00
C
ATOM 23216 HA ASP C 203   22.228 47.628 48.912 0.00 0.00
C
ATOM 23217 CB ASP C 203   22.736 49.807 48.861 0.00 0.00
C
ATOM 23218 HB1 ASP C 203   23.163 50.641 49.457 0.00 0.00
C
ATOM 23219 HB2 ASP C 203   23.486 49.637 48.059 0.00 0.00
C

ATOM 23220 CG ASP C 203 21.425 50.149 48.176 0.00 0.00
C
ATOM 23221 OD1 ASP C 203 21.394 50.323 46.857 0.00 0.00
C
ATOM 23222 OD2 ASP C 203 20.396 50.311 48.822 0.00 0.00
C
ATOM 23223 C ASP C 203 23.923 47.881 49.855 0.00 0.00
C
ATOM 23224 O ASP C 203 24.647 48.394 50.753 0.00 0.00
C
ATOM 23225 N CYS C 204 24.471 46.800 49.152 0.00 0.00
C
ATOM 23226 HN CYS C 204 23.861 46.285 48.555 0.00 0.00
C
ATOM 23227 CA CYS C 204 25.790 46.332 49.382 0.00 0.00
C
ATOM 23228 HA CYS C 204 26.029 46.381 50.434 0.00 0.00
C
ATOM 23229 CB CYS C 204 25.972 44.892 48.654 0.00 0.00
C
ATOM 23230 HB1 CYS C 204 26.007 45.027 47.551 0.00 0.00
C
ATOM 23231 HB2 CYS C 204 26.996 44.486 48.797 0.00 0.00
C
ATOM 23232 SG CYS C 204 24.703 43.608 48.999 0.00 0.00
C
ATOM 23233 HG1 CYS C 204 23.686 44.335 48.558 0.00 0.00
C
ATOM 23234 C CYS C 204 26.889 47.220 48.759 0.00 0.00
C
ATOM 23235 O CYS C 204 26.807 47.628 47.628 0.00 0.00
C
ATOM 23236 N LEU C 205 28.054 47.353 49.407 0.00 0.00
C
ATOM 23237 HN LEU C 205 28.108 47.086 50.366 0.00 0.00
C
ATOM 23238 CA LEU C 205 29.219 47.894 48.876 0.00 0.00
C
ATOM 23239 HA LEU C 205 29.145 48.251 47.860 0.00 0.00
C
ATOM 23240 CB LEU C 205 29.596 49.101 49.825 0.00 0.00
C
ATOM 23241 HB1 LEU C 205 28.752 49.814 49.942 0.00 0.00
C
ATOM 23242 HB2 LEU C 205 29.715 48.678 50.845 0.00 0.00
C
ATOM 23243 CG LEU C 205 30.768 50.036 49.371 0.00 0.00
C
ATOM 23244 HG LEU C 205 31.592 49.475 48.880 0.00 0.00
C
ATOM 23245 CD1 LEU C 205 30.278 51.066 48.383 0.00 0.00
C
ATOM 23246 HD11 LEU C 205 31.067 51.833 48.229 0.00 0.00
C

```
ATOM  23247 HD12 LEU C 205      29.817  50.699  47.441  0.00  0.00
C
ATOM  23248 HD13 LEU C 205      29.400  51.612  48.791  0.00  0.00
C
ATOM  23249  CD2 LEU C 205      31.311  50.797  50.588  0.00  0.00
C
ATOM  23250 HD21 LEU C 205      32.250  51.340  50.346  0.00  0.00
C
ATOM  23251 HD22 LEU C 205      30.470  51.444  50.917  0.00  0.00
C
ATOM  23252 HD23 LEU C 205      31.572  50.027  51.346  0.00  0.00
C
ATOM  23253  C   LEU C 205      30.406  46.824  48.951  0.00  0.00
C
ATOM  23254  O   LEU C 205      30.661  46.142  49.975  0.00  0.00
C
ATOM  23255  N   VAL C 206      31.268  46.701  47.922  0.00  0.00
C
ATOM  23256  HN  VAL C 206      31.263  47.427  47.239  0.00  0.00
C
ATOM  23257  CA  VAL C 206      32.253  45.668  47.893  0.00  0.00
C
ATOM  23258  HA  VAL C 206      32.234  44.983  48.728  0.00  0.00
C
ATOM  23259  CB  VAL C 206      32.083  44.794  46.659  0.00  0.00
C
ATOM  23260  HB  VAL C 206      32.333  45.402  45.764  0.00  0.00
C
ATOM  23261  CG1 VAL C 206      32.996  43.592  46.628  0.00  0.00
C
ATOM  23262 HG11 VAL C 206      33.625  43.448  47.532  0.00  0.00
C
ATOM  23263 HG12 VAL C 206      32.410  42.654  46.524  0.00  0.00
C
ATOM  23264 HG13 VAL C 206      33.757  43.650  45.821  0.00  0.00
C
ATOM  23265  CG2 VAL C 206      30.667  44.390  46.437  0.00  0.00
C
ATOM  23266 HG21 VAL C 206      30.022  45.273  46.237  0.00  0.00
C
ATOM  23267 HG22 VAL C 206      30.440  43.723  45.578  0.00  0.00
C
ATOM  23268 HG23 VAL C 206      30.192  43.777  47.232  0.00  0.00
C
ATOM  23269  C   VAL C 206      33.564  46.355  47.839  0.00  0.00
C
ATOM  23270  O   VAL C 206      33.866  47.074  46.915  0.00  0.00
C
ATOM  23271  N   ILE C 207      34.430  46.113  48.853  0.00  0.00
C
ATOM  23272  HN  ILE C 207      34.180  45.544  49.633  0.00  0.00
C
ATOM  23273  CA  ILE C 207      35.706  46.790  49.110  0.00  0.00
C
```

ATOM 23274 HA ILE C 207   36.005 47.305 48.209 0.00 0.00
C
ATOM 23275 CB ILE C 207   35.599 47.819 50.315 0.00 0.00
C
ATOM 23276 HB ILE C 207   36.498 48.467 50.380 0.00 0.00
C
ATOM 23277 CG2 ILE C 207   34.402 48.783 50.042 0.00 0.00
C
ATOM 23278 HG21 ILE C 207   33.402 48.308 50.136 0.00 0.00
C
ATOM 23279 HG22 ILE C 207   34.506 49.613 50.773 0.00 0.00
C
ATOM 23280 HG23 ILE C 207   34.618 49.267 49.066 0.00 0.00
C
ATOM 23281 CG1 ILE C 207   35.220 47.218 51.674 0.00 0.00
C
ATOM 23282 HG11 ILE C 207   34.535 47.888 52.236 0.00 0.00
C
ATOM 23283 HG12 ILE C 207   34.531 46.407 51.355 0.00 0.00
C
ATOM 23284 CD ILE C 207   36.402 46.876 52.575 0.00 0.00
C
ATOM 23285 HD1 ILE C 207   36.038 46.580 53.582 0.00 0.00
C
ATOM 23286 HD2 ILE C 207   37.106 46.120 52.166 0.00 0.00
C
ATOM 23287 HD3 ILE C 207   37.058 47.765 52.693 0.00 0.00
C
ATOM 23288 C   ILE C 207   36.890 45.864 49.279 0.00 0.00
C
ATOM 23289 O   ILE C 207   36.808 44.680 49.508 0.00 0.00
C
ATOM 23290 N   MET C 208   38.086 46.400 49.137 0.00 0.00
C
ATOM 23291 HN MET C 208   38.161 47.308 48.732 0.00 0.00
C
ATOM 23292 CA MET C 208   39.343 45.714 49.357 0.00 0.00
C
ATOM 23293 HA MET C 208   39.192 44.782 49.881 0.00 0.00
C
ATOM 23294 CB MET C 208   40.101 45.494 48.000 0.00 0.00
C
ATOM 23295 HB1 MET C 208   39.497 44.830 47.346 0.00 0.00
C
ATOM 23296 HB2 MET C 208   40.041 46.493 47.516 0.00 0.00
C
ATOM 23297 CG MET C 208   41.560 44.939 47.969 0.00 0.00
C
ATOM 23298 HG1 MET C 208   42.332 45.498 48.539 0.00 0.00
C
ATOM 23299 HG2 MET C 208   41.435 43.927 48.412 0.00 0.00
C
ATOM 23300 SD MET C 208   42.097 44.788 46.214 0.00 0.00
C

ATOM 23301 CE MET C 208 43.706 43.981 46.546 0.00 0.00
C
ATOM 23302 HE1 MET C 208 44.408 43.770 45.711 0.00 0.00
C
ATOM 23303 HE2 MET C 208 44.229 44.527 47.360 0.00 0.00
C
ATOM 23304 HE3 MET C 208 43.547 42.959 46.951 0.00 0.00
C
ATOM 23305 C MET C 208 40.282 46.404 50.372 0.00 0.00
C
ATOM 23306 O MET C 208 40.918 47.471 50.186 0.00 0.00
C
ATOM 23307 N GLY C 209 40.518 45.856 51.551 0.00 0.00
C
ATOM 23308 HN GLY C 209 40.204 44.912 51.617 0.00 0.00
C
ATOM 23309 CA GLY C 209 41.219 46.315 52.694 0.00 0.00
C
ATOM 23310 HA1 GLY C 209 40.473 46.315 53.475 0.00 0.00
C
ATOM 23311 HA2 GLY C 209 41.712 47.262 52.538 0.00 0.00
C
ATOM 23312 C GLY C 209 42.227 45.365 53.274 0.00 0.00
C
ATOM 23313 O GLY C 209 42.083 44.131 53.037 0.00 0.00
C
ATOM 23314 N HSE C 210 43.260 45.873 53.965 0.00 0.00
C
ATOM 23315 HN HSE C 210 43.413 46.858 53.994 0.00 0.00
C
ATOM 23316 CA HSE C 210 44.173 45.037 54.762 0.00 0.00
C
ATOM 23317 HA HSE C 210 44.457 44.177 54.173 0.00 0.00
C
ATOM 23318 CB HSE C 210 45.492 45.779 55.137 0.00 0.00
C
ATOM 23319 HB1 HSE C 210 45.882 46.206 54.188 0.00 0.00
C
ATOM 23320 HB2 HSE C 210 45.228 46.612 55.823 0.00 0.00
C
ATOM 23321 ND1 HSE C 210 46.577 44.730 57.179 0.00 0.00
C
ATOM 23322 CG HSE C 210 46.564 44.949 55.837 0.00 0.00
C
ATOM 23323 CE1 HSE C 210 47.502 43.860 57.433 0.00 0.00
C
ATOM 23324 HE1 HSE C 210 47.787 43.406 58.381 0.00 0.00
C
ATOM 23325 NE2 HSE C 210 48.162 43.545 56.323 0.00 0.00
C
ATOM 23326 HE2 HSE C 210 48.660 42.680 56.383 0.00 0.00
C
ATOM 23327 CD2 HSE C 210 47.569 44.180 55.288 0.00 0.00
C

ATOM 23328 HD2 HSE C 210 48.029 43.964 54.332 0.00 0.00
C
ATOM 23329 C HSE C 210 43.514 44.494 56.015 0.00 0.00
C
ATOM 23330 O HSE C 210 42.575 45.083 56.546 0.00 0.00
C
ATOM 23331 N LYS C 211 43.895 43.260 56.479 0.00 0.00
C
ATOM 23332 HN LYS C 211 44.612 42.783 55.976 0.00 0.00
C
ATOM 23333 CA LYS C 211 43.162 42.550 57.536 0.00 0.00
C
ATOM 23334 HA LYS C 211 42.148 42.340 57.229 0.00 0.00
C
ATOM 23335 CB LYS C 211 43.956 41.282 57.846 0.00 0.00
C
ATOM 23336 HB1 LYS C 211 44.269 40.850 56.872 0.00 0.00
C
ATOM 23337 HB2 LYS C 211 44.928 41.435 58.362 0.00 0.00
C
ATOM 23338 CG LYS C 211 43.141 40.140 58.455 0.00 0.00
C
ATOM 23339 HG1 LYS C 211 43.731 39.336 58.943 0.00 0.00
C
ATOM 23340 HG2 LYS C 211 42.559 40.560 59.303 0.00 0.00
C
ATOM 23341 CD LYS C 211 42.183 39.433 57.384 0.00 0.00
C
ATOM 23342 HD1 LYS C 211 41.513 38.871 58.069 0.00 0.00
C
ATOM 23343 HD2 LYS C 211 41.578 40.244 56.926 0.00 0.00
C
ATOM 23344 CE LYS C 211 42.965 38.598 56.344 0.00 0.00
C
ATOM 23345 HE1 LYS C 211 43.815 39.159 55.900 0.00 0.00
C
ATOM 23346 HE2 LYS C 211 43.520 37.797 56.878 0.00 0.00
C
ATOM 23347 NZ LYS C 211 42.021 38.069 55.431 0.00 0.00
C
ATOM 23348 HZ1 LYS C 211 41.330 37.411 55.844 0.00 0.00
C
ATOM 23349 HZ2 LYS C 211 41.492 38.835 54.967 0.00 0.00
C
ATOM 23350 HZ3 LYS C 211 42.546 37.583 54.677 0.00 0.00
C
ATOM 23351 C LYS C 211 43.191 43.336 58.831 0.00 0.00
C
ATOM 23352 O LYS C 211 42.126 43.459 59.419 0.00 0.00
C
ATOM 23353 N LYS C 212 44.353 44.050 59.236 0.00 0.00
C
ATOM 23354 HN LYS C 212 45.085 44.034 58.561 0.00 0.00
C

ATOM 23355 CA LYS C 212 44.498 44.996 60.431 0.00 0.00
C
ATOM 23356 HA LYS C 212 44.093 44.484 61.292 0.00 0.00
C
ATOM 23357 CB LYS C 212 45.963 45.538 60.630 0.00 0.00
C
ATOM 23358 HB1 LYS C 212 46.455 45.846 59.683 0.00 0.00
C
ATOM 23359 HB2 LYS C 212 46.038 46.331 61.405 0.00 0.00
C
ATOM 23360 CG LYS C 212 46.891 44.434 61.275 0.00 0.00
C
ATOM 23361 HG1 LYS C 212 46.660 43.491 60.736 0.00 0.00
C
ATOM 23362 HG2 LYS C 212 47.920 44.763 61.016 0.00 0.00
C
ATOM 23363 CD LYS C 212 46.616 44.437 62.807 0.00 0.00
C
ATOM 23364 HD1 LYS C 212 46.805 45.391 63.344 0.00 0.00
C
ATOM 23365 HD2 LYS C 212 45.530 44.254 62.951 0.00 0.00
C
ATOM 23366 CE LYS C 212 47.315 43.296 63.596 0.00 0.00
C
ATOM 23367 HE1 LYS C 212 46.762 42.377 63.304 0.00 0.00
C
ATOM 23368 HE2 LYS C 212 48.417 43.201 63.490 0.00 0.00
C
ATOM 23369 NZ LYS C 212 47.151 43.515 64.964 0.00 0.00
C
ATOM 23370 HZ1 LYS C 212 47.715 44.362 65.179 0.00 0.00
C
ATOM 23371 HZ2 LYS C 212 46.161 43.753 65.174 0.00 0.00
C
ATOM 23372 HZ3 LYS C 212 47.436 42.719 65.569 0.00 0.00
C
ATOM 23373 C LYS C 212 43.523 46.196 60.286 0.00 0.00
C
ATOM 23374 O LYS C 212 42.803 46.550 61.206 0.00 0.00
C
ATOM 23375 N ASP C 213 43.353 46.770 59.100 0.00 0.00
C
ATOM 23376 HN ASP C 213 43.718 46.340 58.278 0.00 0.00
C
ATOM 23377 CA ASP C 213 42.433 47.867 58.892 0.00 0.00
C
ATOM 23378 HA ASP C 213 42.524 48.598 59.681 0.00 0.00
C
ATOM 23379 CB ASP C 213 43.001 48.503 57.605 0.00 0.00
C
ATOM 23380 HB1 ASP C 213 43.207 47.814 56.758 0.00 0.00
C
ATOM 23381 HB2 ASP C 213 42.300 49.290 57.254 0.00 0.00
C

ATOM 23382 CG ASP C 213 44.249 49.306 57.940 0.00 0.00
C
ATOM 23383 OD1 ASP C 213 45.065 49.294 57.010 0.00 0.00
C
ATOM 23384 OD2 ASP C 213 44.450 49.798 59.069 0.00 0.00
C
ATOM 23385 C ASP C 213 41.063 47.484 58.884 0.00 0.00
C
ATOM 23386 O ASP C 213 40.248 48.079 59.595 0.00 0.00
C
ATOM 23387 N ILE C 214 40.779 46.258 58.190 0.00 0.00
C
ATOM 23388 HN ILE C 214 41.496 45.872 57.614 0.00 0.00
C
ATOM 23389 CA ILE C 214 39.434 45.663 58.237 0.00 0.00
C
ATOM 23390 HA ILE C 214 38.744 46.427 57.911 0.00 0.00
C
ATOM 23391 CB ILE C 214 39.508 44.417 57.244 0.00 0.00
C
ATOM 23392 HB ILE C 214 40.523 44.028 57.473 0.00 0.00
C
ATOM 23393 CG2 ILE C 214 38.394 43.440 57.474 0.00 0.00
C
ATOM 23394 HG21 ILE C 214 38.476 42.913 58.449 0.00 0.00
C
ATOM 23395 HG22 ILE C 214 37.432 43.995 57.468 0.00 0.00
C
ATOM 23396 HG23 ILE C 214 38.348 42.723 56.627 0.00 0.00
C
ATOM 23397 CG1 ILE C 214 39.452 44.854 55.775 0.00 0.00
C
ATOM 23398 HG11 ILE C 214 40.309 45.549 55.640 0.00 0.00
C
ATOM 23399 HG12 ILE C 214 39.657 43.952 55.159 0.00 0.00
C
ATOM 23400 CD ILE C 214 38.180 45.480 55.334 0.00 0.00
C
ATOM 23401 HD1 ILE C 214 37.376 44.722 55.213 0.00 0.00
C
ATOM 23402 HD2 ILE C 214 37.929 46.255 56.089 0.00 0.00
C
ATOM 23403 HD3 ILE C 214 38.222 46.032 54.371 0.00 0.00
C
ATOM 23404 C ILE C 214 39.076 45.313 59.672 0.00 0.00
C
ATOM 23405 O ILE C 214 37.973 45.647 60.096 0.00 0.00
C
ATOM 23406 N LYS C 215 40.051 44.900 60.499 0.00 0.00
C
ATOM 23407 HN LYS C 215 40.936 44.785 60.054 0.00 0.00
C
ATOM 23408 CA LYS C 215 39.852 44.721 61.875 0.00 0.00
C

ATOM	23409	HA	LYS	C	215	38.952	44.133	61.978	0.00	0.00	C
ATOM	23410	CB	LYS	C	215	41.068	44.072	62.566	0.00	0.00	C
ATOM	23411	HB1	LYS	C	215	41.369	43.185	61.968	0.00	0.00	C
ATOM	23412	HB2	LYS	C	215	41.951	44.744	62.633	0.00	0.00	C
ATOM	23413	CG	LYS	C	215	40.965	43.582	64.029	0.00	0.00	C
ATOM	23414	HG1	LYS	C	215	41.958	43.131	64.242	0.00	0.00	C
ATOM	23415	HG2	LYS	C	215	40.869	44.526	64.607	0.00	0.00	C
ATOM	23416	CD	LYS	C	215	39.806	42.685	64.394	0.00	0.00	C
ATOM	23417	HD1	LYS	C	215	40.009	42.291	65.413	0.00	0.00	C
ATOM	23418	HD2	LYS	C	215	38.797	43.146	64.330	0.00	0.00	C
ATOM	23419	CE	LYS	C	215	39.779	41.318	63.574	0.00	0.00	C
ATOM	23420	HE1	LYS	C	215	39.652	41.476	62.482	0.00	0.00	C
ATOM	23421	HE2	LYS	C	215	40.750	40.793	63.699	0.00	0.00	C
ATOM	23422	NZ	LYS	C	215	38.644	40.424	63.996	0.00	0.00	C
ATOM	23423	HZ1	LYS	C	215	38.426	39.710	63.272	0.00	0.00	C
ATOM	23424	HZ2	LYS	C	215	38.817	40.013	64.936	0.00	0.00	C
ATOM	23425	HZ3	LYS	C	215	37.749	40.926	64.163	0.00	0.00	C
ATOM	23426	C	LYS	C	215	39.536	45.976	62.721	0.00	0.00	C
ATOM	23427	O	LYS	C	215	38.626	45.908	63.572	0.00	0.00	C
ATOM	23428	N	ARG	C	216	40.169	47.160	62.461	0.00	0.00	C
ATOM	23429	HN	ARG	C	216	40.778	47.014	61.684	0.00	0.00	C
ATOM	23430	CA	ARG	C	216	39.800	48.446	63.038	0.00	0.00	C
ATOM	23431	HA	ARG	C	216	39.736	48.330	64.110	0.00	0.00	C
ATOM	23432	CB	ARG	C	216	40.877	49.540	62.594	0.00	0.00	C
ATOM	23433	HB1	ARG	C	216	40.811	49.551	61.485	0.00	0.00	C
ATOM	23434	HB2	ARG	C	216	40.435	50.497	62.945	0.00	0.00	C
ATOM	23435	CG	ARG	C	216	42.322	49.332	63.147	0.00	0.00	C

ATOM 23436 HG1 ARG C 216   42.305 48.948 64.189 0.00 0.00
C
ATOM 23437 HG2 ARG C 216   42.699 48.511 62.500 0.00 0.00
C
ATOM 23438 CD ARG C 216   43.110 50.536 63.024 0.00 0.00
C
ATOM 23439 HD1 ARG C 216   42.616 51.420 63.481 0.00 0.00
C
ATOM 23440 HD2 ARG C 216   44.112 50.408 63.487 0.00 0.00
C
ATOM 23441 NE ARG C 216   43.236 50.778 61.512 0.00 0.00
C
ATOM 23442 HE ARG C 216   43.636 50.104 60.890 0.00 0.00
C
ATOM 23443 CZ ARG C 216   43.075 51.959 60.952 0.00 0.00
C
ATOM 23444 NH1 ARG C 216   42.894 52.990 61.724 0.00 0.00
C
ATOM 23445 HH11 ARG C 216   42.563 53.818 61.271 0.00 0.00
C
ATOM 23446 HH12 ARG C 216   42.731 52.771 62.686 0.00 0.00
C
ATOM 23447 NH2 ARG C 216   43.360 52.105 59.679 0.00 0.00
C
ATOM 23448 HH21 ARG C 216   43.561 52.983 59.244 0.00 0.00
C
ATOM 23449 HH22 ARG C 216   43.697 51.239 59.311 0.00 0.00
C
ATOM 23450 C   ARG C 216   38.493 48.969 62.515 0.00 0.00
C
ATOM 23451 O   ARG C 216   37.647 49.568 63.180 0.00 0.00
C
ATOM 23452 N   PHE C 217   38.121 48.671 61.239 0.00 0.00
C
ATOM 23453 HN PHE C 217   38.702 48.178 60.597 0.00 0.00
C
ATOM 23454 CA PHE C 217   36.890 49.098 60.647 0.00 0.00
C
ATOM 23455 HA PHE C 217   36.667 50.091 61.010 0.00 0.00
C
ATOM 23456 CB PHE C 217   37.055 48.948 59.191 0.00 0.00
C
ATOM 23457 HB1 PHE C 217   37.866 49.652 58.906 0.00 0.00
C
ATOM 23458 HB2 PHE C 217   37.357 47.943 58.826 0.00 0.00
C
ATOM 23459 CG PHE C 217   35.969 49.375 58.216 0.00 0.00
C
ATOM 23460 CD1 PHE C 217   34.878 50.228 58.516 0.00 0.00
C
ATOM 23461 HD1 PHE C 217   34.652 50.546 59.523 0.00 0.00
C
ATOM 23462 CE1 PHE C 217   33.903 50.432 57.536 0.00 0.00
C

ATOM 23463 HE1 PHE C 217 33.121 51.166 57.668 0.00 0.00
C
ATOM 23464 CZ PHE C 217 34.044 50.024 56.223 0.00 0.00
C
ATOM 23465 HZ PHE C 217 33.342 50.331 55.462 0.00 0.00
C
ATOM 23466 CD2 PHE C 217 36.256 49.089 56.817 0.00 0.00
C
ATOM 23467 HD2 PHE C 217 37.044 48.368 56.662 0.00 0.00
C
ATOM 23468 CE2 PHE C 217 35.207 49.327 55.890 0.00 0.00
C
ATOM 23469 HE2 PHE C 217 35.194 48.867 54.913 0.00 0.00
C
ATOM 23470 C PHE C 217 35.781 48.226 61.284 0.00 0.00
C
ATOM 23471 O PHE C 217 34.698 48.712 61.395 0.00 0.00
C
ATOM 23472 N GLU C 218 36.076 46.958 61.759 0.00 0.00
C
ATOM 23473 HN GLU C 218 36.990 46.655 61.499 0.00 0.00
C
ATOM 23474 CA GLU C 218 35.212 45.967 62.414 0.00 0.00
C
ATOM 23475 HA GLU C 218 34.229 45.821 61.992 0.00 0.00
C
ATOM 23476 CB GLU C 218 35.788 44.478 62.228 0.00 0.00
C
ATOM 23477 HB1 GLU C 218 35.665 44.239 61.149 0.00 0.00
C
ATOM 23478 HB2 GLU C 218 36.869 44.396 62.468 0.00 0.00
C
ATOM 23479 CG GLU C 218 34.862 43.358 62.884 0.00 0.00
C
ATOM 23480 HG1 GLU C 218 34.837 43.748 63.924 0.00 0.00
C
ATOM 23481 HG2 GLU C 218 33.897 43.177 62.364 0.00 0.00
C
ATOM 23482 CD GLU C 218 35.619 42.011 62.763 0.00 0.00
C
ATOM 23483 OE1 GLU C 218 34.914 40.986 62.737 0.00 0.00
C
ATOM 23484 OE2 GLU C 218 36.851 41.922 62.652 0.00 0.00
C
ATOM 23485 C GLU C 218 35.071 46.366 63.813 0.00 0.00
C
ATOM 23486 O GLU C 218 33.951 46.490 64.317 0.00 0.00
C
ATOM 23487 N ASN C 219 36.175 46.512 64.564 0.00 0.00
C
ATOM 23488 HN ASN C 219 37.013 46.470 64.025 0.00 0.00
C
ATOM 23489 CA ASN C 219 36.131 47.085 65.882 0.00 0.00
C

ATOM 23490 HA ASN C 219   35.488 46.433 66.455 0.00 0.00
C
ATOM 23491 CB ASN C 219   37.563 47.108 66.502 0.00 0.00
C
ATOM 23492 HB1 ASN C 219   38.266 47.670 65.850 0.00 0.00
C
ATOM 23493 HB2 ASN C 219   37.475 47.515 67.532 0.00 0.00
C
ATOM 23494 CG ASN C 219   38.104 45.693 66.747 0.00 0.00
C
ATOM 23495 OD1 ASN C 219   37.350 44.710 66.801 0.00 0.00
C
ATOM 23496 ND2 ASN C 219   39.474 45.648 66.829 0.00 0.00
C
ATOM 23497 HD21 ASN C 219   39.799 44.748 67.119 0.00 0.00
C
ATOM 23498 HD22 ASN C 219   39.950 46.527 66.794 0.00 0.00
C
ATOM 23499 C   ASN C 219   35.564 48.520 66.110 0.00 0.00
C
ATOM 23500 O   ASN C 219   34.864 48.643 67.062 0.00 0.00
C
ATOM 23501 N   GLU C 220   35.776 49.600 65.248 0.00 0.00
C
ATOM 23502 HN GLU C 220   36.371 49.544 64.449 0.00 0.00
C
ATOM 23503 CA GLU C 220   35.026 50.871 65.344 0.00 0.00
C
ATOM 23504 HA GLU C 220   34.837 51.264 66.332 0.00 0.00
C
ATOM 23505 CB GLU C 220   35.831 52.019 64.685 0.00 0.00
C
ATOM 23506 HB1 GLU C 220   35.796 51.893 63.581 0.00 0.00
C
ATOM 23507 HB2 GLU C 220   35.192 52.925 64.753 0.00 0.00
C
ATOM 23508 CG GLU C 220   37.239 52.136 65.171 0.00 0.00
C
ATOM 23509 HG1 GLU C 220   37.354 51.964 66.263 0.00 0.00
C
ATOM 23510 HG2 GLU C 220   37.940 51.538 64.551 0.00 0.00
C
ATOM 23511 CD GLU C 220   37.757 53.511 64.945 0.00 0.00
C
ATOM 23512 OE1 GLU C 220   37.771 54.369 65.794 0.00 0.00
C
ATOM 23513 OE2 GLU C 220   38.074 53.850 63.751 0.00 0.00
C
ATOM 23514 C   GLU C 220   33.605 50.727 64.852 0.00 0.00
C
ATOM 23515 O   GLU C 220   32.670 51.048 65.573 0.00 0.00
C
ATOM 23516 N   GLY C 221   33.287 50.093 63.641 0.00 0.00
C

ATOM 23517 HN GLY C 221 34.068 49.684 63.174 0.00 0.00
C
ATOM 23518 CA GLY C 221 31.963 50.011 63.141 0.00 0.00
C
ATOM 23519 HA1 GLY C 221 31.393 50.782 63.638 0.00 0.00
C
ATOM 23520 HA2 GLY C 221 31.577 49.064 63.488 0.00 0.00
C
ATOM 23521 C GLY C 221 31.852 50.053 61.632 0.00 0.00
C
ATOM 23522 O GLY C 221 32.027 51.049 61.029 0.00 0.00
C
ATOM 23523 N MET C 222 31.513 48.888 61.024 0.00 0.00
C
ATOM 23524 HN MET C 222 31.431 48.038 61.539 0.00 0.00
C
ATOM 23525 CA MET C 222 30.935 48.808 59.718 0.00 0.00
C
ATOM 23526 HA MET C 222 31.338 49.599 59.103 0.00 0.00
C
ATOM 23527 CB MET C 222 31.150 47.379 59.232 0.00 0.00
C
ATOM 23528 HB1 MET C 222 30.764 46.596 59.919 0.00 0.00
C
ATOM 23529 HB2 MET C 222 30.556 47.292 58.297 0.00 0.00
C
ATOM 23530 CG MET C 222 32.651 47.160 58.781 0.00 0.00
C
ATOM 23531 HG1 MET C 222 32.918 47.902 57.998 0.00 0.00
C
ATOM 23532 HG2 MET C 222 33.375 47.251 59.619 0.00 0.00
C
ATOM 23533 SD MET C 222 33.093 45.548 58.211 0.00 0.00
C
ATOM 23534 CE MET C 222 34.883 45.780 58.072 0.00 0.00
C
ATOM 23535 HE1 MET C 222 35.345 44.893 57.586 0.00 0.00
C
ATOM 23536 HE2 MET C 222 34.967 46.689 57.440 0.00 0.00
C
ATOM 23537 HE3 MET C 222 35.350 45.986 59.059 0.00 0.00
C
ATOM 23538 C MET C 222 29.518 49.218 59.709 0.00 0.00
C
ATOM 23539 O MET C 222 29.197 50.368 59.386 0.00 0.00
C
ATOM 23540 N GLN D 8 22.549 35.272 72.988 0.00 0.00
D
ATOM 23541 HT1 GLN D 8 22.902 35.560 72.053 0.00 0.00
D
ATOM 23542 HT2 GLN D 8 23.255 35.665 73.642 0.00 0.00
D
ATOM 23543 HT3 GLN D 8 21.642 35.751 73.163 0.00 0.00
D

ATOM 23544 CA GLN D 8 22.474 33.769 73.089 0.00 0.00
D
ATOM 23545 HA GLN D 8 23.489 33.401 73.089 0.00 0.00
D
ATOM 23546 CB GLN D 8 21.791 33.193 74.401 0.00 0.00
D
ATOM 23547 HB1 GLN D 8 20.732 33.511 74.301 0.00 0.00
D
ATOM 23548 HB2 GLN D 8 22.056 32.118 74.501 0.00 0.00
D
ATOM 23549 CG GLN D 8 22.233 33.858 75.705 0.00 0.00
D
ATOM 23550 HG1 GLN D 8 23.317 33.696 75.883 0.00 0.00
D
ATOM 23551 HG2 GLN D 8 21.960 34.921 75.535 0.00 0.00
D
ATOM 23552 CD GLN D 8 21.525 33.225 76.885 0.00 0.00
D
ATOM 23553 OE1 GLN D 8 22.165 32.624 77.735 0.00 0.00
D
ATOM 23554 NE2 GLN D 8 20.188 33.457 76.969 0.00 0.00
D
ATOM 23555 HE21 GLN D 8 19.750 33.219 77.836 0.00 0.00
D
ATOM 23556 HE22 GLN D 8 19.629 33.874 76.253 0.00 0.00
D
ATOM 23557 C GLN D 8 21.755 33.288 71.824 0.00 0.00
D
ATOM 23558 O GLN D 8 20.894 34.025 71.363 0.00 0.00
D
ATOM 23559 N PHE D 9 21.829 32.056 71.368 0.00 0.00
D
ATOM 23560 HN PHE D 9 22.484 31.388 71.712 0.00 0.00
D
ATOM 23561 CA PHE D 9 21.126 31.613 70.160 0.00 0.00
D
ATOM 23562 HA PHE D 9 20.338 32.310 69.917 0.00 0.00
D
ATOM 23563 CB PHE D 9 22.121 31.378 68.994 0.00 0.00
D
ATOM 23564 HB1 PHE D 9 22.864 30.721 69.493 0.00 0.00
D
ATOM 23565 HB2 PHE D 9 21.628 30.833 68.161 0.00 0.00
D
ATOM 23566 CG PHE D 9 22.828 32.599 68.493 0.00 0.00
D
ATOM 23567 CD1 PHE D 9 22.014 33.625 68.029 0.00 0.00
D
ATOM 23568 HD1 PHE D 9 20.943 33.508 68.107 0.00 0.00
D
ATOM 23569 CE1 PHE D 9 22.625 34.838 67.648 0.00 0.00
D
ATOM 23570 HE1 PHE D 9 22.083 35.722 67.347 0.00 0.00
D

ATOM 23571 CZ PHE D   9   24.008 34.954 67.726 0.00 0.00
D
ATOM 23572 HZ PHE D   9   24.493 35.905 67.568 0.00 0.00
D
ATOM 23573 CD2 PHE D   9   24.214 32.720 68.596 0.00 0.00
D
ATOM 23574 HD2 PHE D   9   24.735 31.887 69.043 0.00 0.00
D
ATOM 23575 CE2 PHE D   9   24.797 33.913 68.219 0.00 0.00
D
ATOM 23576 HE2 PHE D   9   25.865 34.035 68.330 0.00 0.00
D
ATOM 23577 C   PHE D   9   20.460 30.317 70.532 0.00 0.00
D
ATOM 23578 O   PHE D   9   21.008 29.569 71.293 0.00 0.00
D
ATOM 23579 N   ALA D 10   19.264 30.047 69.996 0.00 0.00
D
ATOM 23580 HN ALA D 10   18.691 30.757 69.593 0.00 0.00
D
ATOM 23581 CA ALA D 10   18.575 28.785 70.255 0.00 0.00
D
ATOM 23582 HA ALA D 10   19.173 28.129 70.869 0.00 0.00
D
ATOM 23583 CB ALA D 10   17.267 28.950 71.037 0.00 0.00
D
ATOM 23584 HB1 ALA D 10   17.526 29.193 72.090 0.00 0.00
D
ATOM 23585 HB2 ALA D 10   16.598 29.700 70.565 0.00 0.00
D
ATOM 23586 HB3 ALA D 10   16.809 27.943 70.934 0.00 0.00
D
ATOM 23587 C   ALA D 10   18.304 28.117 68.954 0.00 0.00
D
ATOM 23588 O   ALA D 10   17.934 28.727 67.930 0.00 0.00
D
ATOM 23589 N   VAL D 11   18.645 26.811 68.944 0.00 0.00
D
ATOM 23590 HN VAL D 11   18.960 26.443 69.814 0.00 0.00
D
ATOM 23591 CA VAL D 11   18.625 25.969 67.727 0.00 0.00
D
ATOM 23592 HA VAL D 11   18.172 26.592 66.971 0.00 0.00
D
ATOM 23593 CB VAL D 11   19.954 25.452 67.225 0.00 0.00
D
ATOM 23594 HB VAL D 11   20.331 24.750 67.999 0.00 0.00
D
ATOM 23595 CG1 VAL D 11   19.815 24.739 65.848 0.00 0.00
D
ATOM 23596 HG11 VAL D 11   20.795 24.357 65.491 0.00 0.00
D
ATOM 23597 HG12 VAL D 11   19.227 23.813 66.020 0.00 0.00
D

ATOM 23598 HG13 VAL D 11   19.413 25.357 65.016 0.00 0.00
D
ATOM 23599 CG2 VAL D 11   20.899 26.699 67.211 0.00 0.00
D
ATOM 23600 HG21 VAL D 11   21.926 26.352 66.966 0.00 0.00
D
ATOM 23601 HG22 VAL D 11   20.499 27.410 66.458 0.00 0.00
D
ATOM 23602 HG23 VAL D 11   21.021 27.168 68.211 0.00 0.00
D
ATOM 23603 C   VAL D 11   17.656 24.819 67.882 0.00 0.00
D
ATOM 23604 O   VAL D 11   17.829 24.114 68.858 0.00 0.00
D
ATOM 23605 N   ILE D 12   16.558 24.712 67.086 0.00 0.00
D
ATOM 23606 HN ILE D 12   16.484 25.345 66.319 0.00 0.00
D
ATOM 23607 CA ILE D 12   15.476 23.775 67.265 0.00 0.00
D
ATOM 23608 HA ILE D 12   15.801 23.031 67.977 0.00 0.00
D
ATOM 23609 CB ILE D 12   14.125 24.373 67.698 0.00 0.00
D
ATOM 23610 HB ILE D 12   13.626 24.802 66.802 0.00 0.00
D
ATOM 23611 CG2 ILE D 12   13.164 23.268 68.175 0.00 0.00
D
ATOM 23612 HG21 ILE D 12   13.521 22.893 69.158 0.00 0.00
D
ATOM 23613 HG22 ILE D 12   12.099 23.561 68.295 0.00 0.00
D
ATOM 23614 HG23 ILE D 12   13.048 22.415 67.473 0.00 0.00
D
ATOM 23615 CG1 ILE D 12   14.223 25.446 68.801 0.00 0.00
D
ATOM 23616 HG11 ILE D 12   14.737 25.031 69.694 0.00 0.00
D
ATOM 23617 HG12 ILE D 12   14.873 26.242 68.378 0.00 0.00
D
ATOM 23618 CD ILE D 12   12.858 26.166 69.238 0.00 0.00
D
ATOM 23619 HD1 ILE D 12   11.998 25.467 69.319 0.00 0.00
D
ATOM 23620 HD2 ILE D 12   13.032 26.596 70.247 0.00 0.00
D
ATOM 23621 HD3 ILE D 12   12.671 27.000 68.528 0.00 0.00
D
ATOM 23622 C   ILE D 12   15.437 22.981 66.045 0.00 0.00
D
ATOM 23623 O   ILE D 12   15.104 23.497 64.972 0.00 0.00
D
ATOM 23624 N   GLY D 13   15.809 21.689 66.163 0.00 0.00
D

ATOM 23625 HN GLY D 13 16.097 21.171 66.964 0.00 0.00
D
ATOM 23626 CA GLY D 13 15.965 20.851 64.982 0.00 0.00
D
ATOM 23627 HA1 GLY D 13 15.610 21.280 64.056 0.00 0.00
D
ATOM 23628 HA2 GLY D 13 15.428 19.922 65.108 0.00 0.00
D
ATOM 23629 C GLY D 13 17.378 20.543 64.829 0.00 0.00
D
ATOM 23630 O GLY D 13 17.998 21.093 63.970 0.00 0.00
D
ATOM 23631 N LEU D 14 17.967 19.501 65.514 0.00 0.00
D
ATOM 23632 HN LEU D 14 17.409 18.818 65.980 0.00 0.00
D
ATOM 23633 CA LEU D 14 19.360 19.245 65.410 0.00 0.00
D
ATOM 23634 HA LEU D 14 19.888 20.137 65.108 0.00 0.00
D
ATOM 23635 CB LEU D 14 19.800 18.543 66.787 0.00 0.00
D
ATOM 23636 HB1 LEU D 14 19.016 17.786 67.003 0.00 0.00
D
ATOM 23637 HB2 LEU D 14 20.782 18.051 66.620 0.00 0.00
D
ATOM 23638 CG LEU D 14 19.684 19.427 68.043 0.00 0.00
D
ATOM 23639 HG LEU D 14 18.606 19.607 68.244 0.00 0.00
D
ATOM 23640 CD1 LEU D 14 20.272 18.756 69.256 0.00 0.00
D
ATOM 23641 HD11 LEU D 14 19.847 19.303 70.125 0.00 0.00
D
ATOM 23642 HD12 LEU D 14 20.073 17.668 69.361 0.00 0.00
D
ATOM 23643 HD13 LEU D 14 21.383 18.773 69.275 0.00 0.00
D
ATOM 23644 CD2 LEU D 14 20.258 20.816 67.768 0.00 0.00
D
ATOM 23645 HD21 LEU D 14 20.094 21.563 68.575 0.00 0.00
D
ATOM 23646 HD22 LEU D 14 21.334 20.707 67.515 0.00 0.00
D
ATOM 23647 HD23 LEU D 14 19.730 21.359 66.955 0.00 0.00
D
ATOM 23648 C LEU D 14 19.746 18.346 64.256 0.00 0.00
D
ATOM 23649 O LEU D 14 20.487 17.432 64.403 0.00 0.00
D
ATOM 23650 N GLY D 15 19.262 18.685 63.045 0.00 0.00
D
ATOM 23651 HN GLY D 15 18.657 19.477 63.054 0.00 0.00
D

ATOM 23652 CA GLY D 15 19.470 18.018 61.761 0.00 0.00
D
ATOM 23653 HA1 GLY D 15 18.643 18.271 61.114 0.00 0.00
D
ATOM 23654 HA2 GLY D 15 19.549 16.951 61.906 0.00 0.00
D
ATOM 23655 C GLY D 15 20.756 18.312 61.124 0.00 0.00
D
ATOM 23656 O GLY D 15 21.723 18.740 61.818 0.00 0.00
D
ATOM 23657 N ARG D 16 20.838 17.988 59.792 0.00 0.00
D
ATOM 23658 HN ARG D 16 20.045 17.585 59.342 0.00 0.00
D
ATOM 23659 CA ARG D 16 22.054 18.211 59.009 0.00 0.00
D
ATOM 23660 HA ARG D 16 22.886 17.649 59.408 0.00 0.00
D
ATOM 23661 CB ARG D 16 21.801 17.567 57.604 0.00 0.00
D
ATOM 23662 HB1 ARG D 16 20.973 17.987 56.993 0.00 0.00
D
ATOM 23663 HB2 ARG D 16 22.767 17.823 57.118 0.00 0.00
D
ATOM 23664 CG ARG D 16 21.749 16.058 57.718 0.00 0.00
D
ATOM 23665 HG1 ARG D 16 22.665 15.673 58.216 0.00 0.00
D
ATOM 23666 HG2 ARG D 16 20.863 15.798 58.336 0.00 0.00
D
ATOM 23667 CD ARG D 16 21.488 15.307 56.373 0.00 0.00
D
ATOM 23668 HD1 ARG D 16 22.454 15.350 55.825 0.00 0.00
D
ATOM 23669 HD2 ARG D 16 21.249 14.229 56.497 0.00 0.00
D
ATOM 23670 NE ARG D 16 20.425 16.009 55.618 0.00 0.00
D
ATOM 23671 HE ARG D 16 20.612 16.868 55.141 0.00 0.00
D
ATOM 23672 CZ ARG D 16 19.097 15.866 55.881 0.00 0.00
D
ATOM 23673 NH1 ARG D 16 18.568 14.975 56.680 0.00 0.00
D
ATOM 23674 HH11 ARG D 16 17.651 15.146 57.039 0.00 0.00
D
ATOM 23675 HH12 ARG D 16 19.057 14.156 56.980 0.00 0.00
D
ATOM 23676 NH2 ARG D 16 18.329 16.878 55.564 0.00 0.00
D
ATOM 23677 HH21 ARG D 16 17.394 17.010 55.895 0.00 0.00
D
ATOM 23678 HH22 ARG D 16 18.752 17.661 55.108 0.00 0.00
D

ATOM	23679	C	ARG	D	16	22.322	19.666	58.981	0.00	0.00	D
ATOM	23680	O	ARG	D	16	23.454	20.168	59.143	0.00	0.00	D
ATOM	23681	N	PHE	D	17	21.344	20.560	58.815	0.00	0.00	D
ATOM	23682	HN	PHE	D	17	20.399	20.269	58.683	0.00	0.00	D
ATOM	23683	CA	PHE	D	17	21.541	21.975	58.889	0.00	0.00	D
ATOM	23684	HA	PHE	D	17	22.370	22.243	58.250	0.00	0.00	D
ATOM	23685	CB	PHE	D	17	20.179	22.574	58.382	0.00	0.00	D
ATOM	23686	HB1	PHE	D	17	19.819	22.251	57.382	0.00	0.00	D
ATOM	23687	HB2	PHE	D	17	19.384	22.286	59.102	0.00	0.00	D
ATOM	23688	CG	PHE	D	17	20.139	24.063	58.239	0.00	0.00	D
ATOM	23689	CD1	PHE	D	17	21.026	24.740	57.393	0.00	0.00	D
ATOM	23690	HD1	PHE	D	17	21.803	24.262	56.815	0.00	0.00	D
ATOM	23691	CE1	PHE	D	17	20.890	26.171	57.267	0.00	0.00	D
ATOM	23692	HE1	PHE	D	17	21.589	26.804	56.741	0.00	0.00	D
ATOM	23693	CZ	PHE	D	17	19.740	26.797	57.773	0.00	0.00	D
ATOM	23694	HZ	PHE	D	17	19.626	27.871	57.765	0.00	0.00	D
ATOM	23695	CD2	PHE	D	17	18.954	24.745	58.714	0.00	0.00	D
ATOM	23696	HD2	PHE	D	17	18.252	24.140	59.270	0.00	0.00	D
ATOM	23697	CE2	PHE	D	17	18.887	26.108	58.616	0.00	0.00	D
ATOM	23698	HE2	PHE	D	17	18.134	26.661	59.158	0.00	0.00	D
ATOM	23699	C	PHE	D	17	21.814	22.371	60.407	0.00	0.00	D
ATOM	23700	O	PHE	D	17	22.843	22.891	60.821	0.00	0.00	D
ATOM	23701	N	GLY	D	18	20.885	22.006	61.347	0.00	0.00	D
ATOM	23702	HN	GLY	D	18	20.004	21.618	61.088	0.00	0.00	D
ATOM	23703	CA	GLY	D	18	20.770	22.580	62.716	0.00	0.00	D
ATOM	23704	HA1	GLY	D	18	19.790	22.358	63.113	0.00	0.00	D
ATOM	23705	HA2	GLY	D	18	20.856	23.650	62.593	0.00	0.00	D

ATOM 23706 C GLY D 18 21.825 22.064 63.718 0.00 0.00
D
ATOM 23707 O GLY D 18 22.303 22.818 64.580 0.00 0.00
D
ATOM 23708 N GLY D 19 22.201 20.804 63.599 0.00 0.00
D
ATOM 23709 HN GLY D 19 21.819 20.130 62.972 0.00 0.00
D
ATOM 23710 CA GLY D 19 23.221 20.318 64.585 0.00 0.00
D
ATOM 23711 HA1 GLY D 19 23.233 19.246 64.457 0.00 0.00
D
ATOM 23712 HA2 GLY D 19 22.931 20.647 65.571 0.00 0.00
D
ATOM 23713 C GLY D 19 24.510 20.802 64.307 0.00 0.00
D
ATOM 23714 O GLY D 19 25.335 21.000 65.215 0.00 0.00
D
ATOM 23715 N SER D 20 24.764 21.090 63.004 0.00 0.00
D
ATOM 23716 HN SER D 20 24.129 20.578 62.431 0.00 0.00
D
ATOM 23717 CA SER D 20 25.965 21.662 62.419 0.00 0.00
D
ATOM 23718 HA SER D 20 26.835 21.300 62.947 0.00 0.00
D
ATOM 23719 CB SER D 20 26.135 21.558 60.891 0.00 0.00
D
ATOM 23720 HB1 SER D 20 25.440 22.260 60.383 0.00 0.00
D
ATOM 23721 HB2 SER D 20 27.201 21.694 60.609 0.00 0.00
D
ATOM 23722 OG SER D 20 25.860 20.216 60.461 0.00 0.00
D
ATOM 23723 HG1 SER D 20 25.096 20.301 59.886 0.00 0.00
D
ATOM 23724 C SER D 20 26.041 23.176 62.743 0.00 0.00
D
ATOM 23725 O SER D 20 27.129 23.696 62.930 0.00 0.00
D
ATOM 23726 N ILE D 21 24.870 23.926 62.720 0.00 0.00
D
ATOM 23727 HN ILE D 21 23.982 23.528 62.502 0.00 0.00
D
ATOM 23728 CA ILE D 21 24.857 25.122 63.353 0.00 0.00
D
ATOM 23729 HA ILE D 21 25.508 25.838 62.874 0.00 0.00
D
ATOM 23730 CB ILE D 21 23.489 25.912 63.376 0.00 0.00
D
ATOM 23731 HB ILE D 21 22.825 25.215 63.930 0.00 0.00
D
ATOM 23732 CG2 ILE D 21 23.551 27.258 64.129 0.00 0.00
D

ATOM 23733 HG21 ILE D 21   22.591 27.816 64.096 0.00 0.00
D
ATOM 23734 HG22 ILE D 21   23.900 27.041 65.161 0.00 0.00
D
ATOM 23735 HG23 ILE D 21   24.276 27.945 63.643 0.00 0.00
D
ATOM 23736 CG1 ILE D 21   23.009 26.092 61.891 0.00 0.00
D
ATOM 23737 HG11 ILE D 21   23.807 26.625 61.332 0.00 0.00
D
ATOM 23738 HG12 ILE D 21   22.895 25.068 61.476 0.00 0.00
D
ATOM 23739 CD ILE D 21   21.800 26.975 61.696 0.00 0.00
D
ATOM 23740 HD1 ILE D 21   20.995 26.457 62.260 0.00 0.00
D
ATOM 23741 HD2 ILE D 21   21.990 27.959 62.174 0.00 0.00
D
ATOM 23742 HD3 ILE D 21   21.531 27.035 60.619 0.00 0.00
D
ATOM 23743 C   ILE D 21   25.342 25.111 64.833 0.00 0.00
D
ATOM 23744 O   ILE D 21   26.320 25.855 65.129 0.00 0.00
D
ATOM 23745 N   CYS D 22   24.715 24.354 65.704 0.00 0.00
D
ATOM 23746 HN CYS D 22   23.924 23.853 65.361 0.00 0.00
D
ATOM 23747 CA CYS D 22   25.046 24.148 67.147 0.00 0.00
D
ATOM 23748 HA CYS D 22   24.816 25.125 67.546 0.00 0.00
D
ATOM 23749 CB CYS D 22   24.251 22.916 67.754 0.00 0.00
D
ATOM 23750 HB1 CYS D 22   24.294 22.061 67.046 0.00 0.00
D
ATOM 23751 HB2 CYS D 22   24.617 22.630 68.763 0.00 0.00
D
ATOM 23752 SG CYS D 22   22.620 23.305 67.956 0.00 0.00
D
ATOM 23753 HG1 CYS D 22   22.089 22.264 67.332 0.00 0.00
D
ATOM 23754 C   CYS D 22   26.493 23.848 67.409 0.00 0.00
D
ATOM 23755 O   CYS D 22   27.055 24.670 68.165 0.00 0.00
D
ATOM 23756 N   LYS D 23   27.035 22.887 66.635 0.00 0.00
D
ATOM 23757 HN LYS D 23   26.385 22.366 66.087 0.00 0.00
D
ATOM 23758 CA LYS D 23   28.408 22.476 66.620 0.00 0.00
D
ATOM 23759 HA LYS D 23   28.528 22.363 67.688 0.00 0.00
D

ATOM 23760 CB LYS D 23 28.395 21.040 65.825 0.00 0.00
D
ATOM 23761 HB1 LYS D 23 27.556 20.437 66.233 0.00 0.00
D
ATOM 23762 HB2 LYS D 23 28.206 21.100 64.732 0.00 0.00
D
ATOM 23763 CG LYS D 23 29.653 20.206 66.001 0.00 0.00
D
ATOM 23764 HG1 LYS D 23 30.515 20.726 65.533 0.00 0.00
D
ATOM 23765 HG2 LYS D 23 29.992 20.093 67.053 0.00 0.00
D
ATOM 23766 CD LYS D 23 29.662 18.795 65.368 0.00 0.00
D
ATOM 23767 HD1 LYS D 23 28.971 18.113 65.908 0.00 0.00
D
ATOM 23768 HD2 LYS D 23 29.111 18.942 64.415 0.00 0.00
D
ATOM 23769 CE LYS D 23 31.043 18.070 65.322 0.00 0.00
D
ATOM 23770 HE1 LYS D 23 31.710 18.833 64.866 0.00 0.00
D
ATOM 23771 HE2 LYS D 23 31.503 17.893 66.317 0.00 0.00
D
ATOM 23772 NZ LYS D 23 30.931 16.867 64.541 0.00 0.00
D
ATOM 23773 HZ1 LYS D 23 31.751 16.227 64.530 0.00 0.00
D
ATOM 23774 HZ2 LYS D 23 30.139 16.327 64.942 0.00 0.00
D
ATOM 23775 HZ3 LYS D 23 30.669 16.988 63.542 0.00 0.00
D
ATOM 23776 C LYS D 23 29.465 23.493 66.157 0.00 0.00
D
ATOM 23777 O LYS D 23 30.498 23.504 66.792 0.00 0.00
D
ATOM 23778 N GLU D 24 29.211 24.321 65.117 0.00 0.00
D
ATOM 23779 HN GLU D 24 28.459 24.215 64.471 0.00 0.00
D
ATOM 23780 CA GLU D 24 30.161 25.314 64.702 0.00 0.00
D
ATOM 23781 HA GLU D 24 31.151 24.881 64.704 0.00 0.00
D
ATOM 23782 CB GLU D 24 29.766 25.786 63.380 0.00 0.00
D
ATOM 23783 HB1 GLU D 24 29.811 25.008 62.587 0.00 0.00
D
ATOM 23784 HB2 GLU D 24 28.706 26.119 63.388 0.00 0.00
D
ATOM 23785 CG GLU D 24 30.685 26.883 62.808 0.00 0.00
D
ATOM 23786 HG1 GLU D 24 30.258 27.047 61.796 0.00 0.00
D

ATOM 23787 HG2 GLU D 24   30.548 27.808 63.408 0.00 0.00
D
ATOM 23788 CD GLU D 24   32.105 26.390 62.557 0.00 0.00
D
ATOM 23789 OE1 GLU D 24   32.292 25.170 62.391 0.00 0.00
D
ATOM 23790 OE2 GLU D 24   33.055 27.216 62.700 0.00 0.00
D
ATOM 23791 C   GLU D 24   30.107 26.582 65.616 0.00 0.00
D
ATOM 23792 O   GLU D 24   31.084 27.039 66.244 0.00 0.00
D
ATOM 23793 N   LEU D 25   28.902 27.076 65.800 0.00 0.00
D
ATOM 23794 HN LEU D 25   28.162 26.536 65.407 0.00 0.00
D
ATOM 23795 CA LEU D 25   28.634 28.304 66.583 0.00 0.00
D
ATOM 23796 HA LEU D 25   29.059 29.135 66.040 0.00 0.00
D
ATOM 23797 CB LEU D 25   27.115 28.418 66.634 0.00 0.00
D
ATOM 23798 HB1 LEU D 25   26.745 28.157 65.619 0.00 0.00
D
ATOM 23799 HB2 LEU D 25   26.601 27.690 67.298 0.00 0.00
D
ATOM 23800 CG LEU D 25   26.448 29.830 67.028 0.00 0.00
D
ATOM 23801 HG LEU D 25   26.828 30.070 68.044 0.00 0.00
D
ATOM 23802 CD1 LEU D 25   26.778 31.004 66.002 0.00 0.00
D
ATOM 23803 HD11 LEU D 25   26.551 32.022 66.384 0.00 0.00
D
ATOM 23804 HD12 LEU D 25   27.882 31.035 65.881 0.00 0.00
D
ATOM 23805 HD13 LEU D 25   26.137 30.810 65.115 0.00 0.00
D
ATOM 23806 CD2 LEU D 25   24.923 29.639 67.033 0.00 0.00
D
ATOM 23807 HD21 LEU D 25   24.412 30.580 67.328 0.00 0.00
D
ATOM 23808 HD22 LEU D 25   24.464 29.276 66.089 0.00 0.00
D
ATOM 23809 HD23 LEU D 25   24.767 28.860 67.810 0.00 0.00
D
ATOM 23810 C   LEU D 25   29.095 28.411 68.031 0.00 0.00
D
ATOM 23811 O   LEU D 25   29.607 29.335 68.600 0.00 0.00
D
ATOM 23812 N   HSE D 26   28.961 27.204 68.705 0.00 0.00
D
ATOM 23813 HN HSE D 26   28.626 26.487 68.099 0.00 0.00
D

ATOM 23814 CA HSE D 26 29.483 26.884 70.020 0.00 0.00
D
ATOM 23815 HA HSE D 26 29.136 27.521 70.821 0.00 0.00
D
ATOM 23816 CB HSE D 26 29.024 25.491 70.561 0.00 0.00
D
ATOM 23817 HB1 HSE D 26 27.918 25.383 70.592 0.00 0.00
D
ATOM 23818 HB2 HSE D 26 29.318 24.637 69.913 0.00 0.00
D
ATOM 23819 ND1 HSE D 26 28.941 25.720 73.017 0.00 0.00
D
ATOM 23820 CG HSE D 26 29.546 25.076 71.914 0.00 0.00
D
ATOM 23821 CE1 HSE D 26 29.454 24.965 74.022 0.00 0.00
D
ATOM 23822 HE1 HSE D 26 29.147 25.158 75.050 0.00 0.00
D
ATOM 23823 NE2 HSE D 26 30.225 23.957 73.559 0.00 0.00
D
ATOM 23824 HE2 HSE D 26 30.637 23.218 74.092 0.00 0.00
D
ATOM 23825 CD2 HSE D 26 30.316 24.045 72.244 0.00 0.00
D
ATOM 23826 HD2 HSE D 26 30.958 23.445 71.611 0.00 0.00
D
ATOM 23827 C HSE D 26 30.986 26.837 70.178 0.00 0.00
D
ATOM 23828 O HSE D 26 31.645 27.194 71.122 0.00 0.00
D
ATOM 23829 N ARG D 27 31.647 26.444 69.111 0.00 0.00
D
ATOM 23830 HN ARG D 27 31.189 26.393 68.227 0.00 0.00
D
ATOM 23831 CA ARG D 27 33.049 26.479 69.034 0.00 0.00
D
ATOM 23832 HA ARG D 27 33.544 26.136 69.930 0.00 0.00
D
ATOM 23833 CB ARG D 27 33.601 25.536 67.919 0.00 0.00
D
ATOM 23834 HB1 ARG D 27 32.921 24.657 67.925 0.00 0.00
D
ATOM 23835 HB2 ARG D 27 33.463 26.101 66.973 0.00 0.00
D
ATOM 23836 CG ARG D 27 35.017 25.203 68.148 0.00 0.00
D
ATOM 23837 HG1 ARG D 27 35.670 26.101 68.189 0.00 0.00
D
ATOM 23838 HG2 ARG D 27 35.187 24.580 69.051 0.00 0.00
D
ATOM 23839 CD ARG D 27 35.701 24.403 67.012 0.00 0.00
D
ATOM 23840 HD1 ARG D 27 36.617 23.998 67.492 0.00 0.00
D

ATOM 23841 HD2 ARG D 27 34.981 23.612 66.711 0.00 0.00
D
ATOM 23842 NE ARG D 27 35.887 25.345 65.900 0.00 0.00
D
ATOM 23843 HE ARG D 27 36.528 26.098 66.043 0.00 0.00
D
ATOM 23844 CZ ARG D 27 35.137 25.389 64.735 0.00 0.00
D
ATOM 23845 NH1 ARG D 27 34.179 24.486 64.500 0.00 0.00
D
ATOM 23846 HH11 ARG D 27 33.657 24.543 63.649 0.00 0.00
D
ATOM 23847 HH12 ARG D 27 34.012 23.781 65.189 0.00 0.00
D
ATOM 23848 NH2 ARG D 27 35.260 26.465 63.990 0.00 0.00
D
ATOM 23849 HH21 ARG D 27 34.433 26.699 63.478 0.00 0.00
D
ATOM 23850 HH22 ARG D 27 35.780 27.221 64.388 0.00 0.00
D
ATOM 23851 C ARG D 27 33.509 27.845 68.753 0.00 0.00
D
ATOM 23852 O ARG D 27 34.702 28.186 69.013 0.00 0.00
D
ATOM 23853 N MET D 28 32.653 28.698 68.230 0.00 0.00
D
ATOM 23854 HN MET D 28 31.871 28.334 67.729 0.00 0.00
D
ATOM 23855 CA MET D 28 32.900 30.144 68.053 0.00 0.00
D
ATOM 23856 HA MET D 28 33.967 30.157 67.888 0.00 0.00
D
ATOM 23857 CB MET D 28 32.033 30.800 66.932 0.00 0.00
D
ATOM 23858 HB1 MET D 28 30.976 30.537 67.149 0.00 0.00
D
ATOM 23859 HB2 MET D 28 32.089 31.896 67.104 0.00 0.00
D
ATOM 23860 CG MET D 28 32.482 30.486 65.501 0.00 0.00
D
ATOM 23861 HG1 MET D 28 33.569 30.713 65.496 0.00 0.00
D
ATOM 23862 HG2 MET D 28 32.383 29.408 65.251 0.00 0.00
D
ATOM 23863 SD MET D 28 31.760 31.398 64.056 0.00 0.00
D
ATOM 23864 CE MET D 28 32.779 30.622 62.789 0.00 0.00
D
ATOM 23865 HE1 MET D 28 33.140 29.646 63.178 0.00 0.00
D
ATOM 23866 HE2 MET D 28 32.194 30.411 61.867 0.00 0.00
D
ATOM 23867 HE3 MET D 28 33.730 31.188 62.698 0.00 0.00
D

ATOM	23868	C	MET	D	28	32.732	30.862	69.367	0.00	0.00	D
ATOM	23869	O	MET	D	28	32.940	32.069	69.452	0.00	0.00	D
ATOM	23870	N	GLY	D	29	32.347	30.187	70.409	0.00	0.00	D
ATOM	23871	HN	GLY	D	29	32.218	29.204	70.308	0.00	0.00	D
ATOM	23872	CA	GLY	D	29	32.113	30.675	71.769	0.00	0.00	D
ATOM	23873	HA1	GLY	D	29	32.829	31.433	72.050	0.00	0.00	D
ATOM	23874	HA2	GLY	D	29	32.133	29.810	72.415	0.00	0.00	D
ATOM	23875	C	GLY	D	29	30.897	31.401	71.971	0.00	0.00	D
ATOM	23876	O	GLY	D	29	30.789	32.048	72.992	0.00	0.00	D
ATOM	23877	N	HSE	D	30	29.919	31.322	71.072	0.00	0.00	D
ATOM	23878	HN	HSE	D	30	30.049	30.686	70.316	0.00	0.00	D
ATOM	23879	CA	HSE	D	30	28.618	31.925	71.323	0.00	0.00	D
ATOM	23880	HA	HSE	D	30	28.690	32.872	71.839	0.00	0.00	D
ATOM	23881	CB	HSE	D	30	27.840	32.094	70.008	0.00	0.00	D
ATOM	23882	HB1	HSE	D	30	27.598	31.103	69.569	0.00	0.00	D
ATOM	23883	HB2	HSE	D	30	26.840	32.544	70.187	0.00	0.00	D
ATOM	23884	ND1	HSE	D	30	28.702	34.333	69.127	0.00	0.00	D
ATOM	23885	CG	HSE	D	30	28.623	32.933	69.048	0.00	0.00	D
ATOM	23886	CE1	HSE	D	30	29.502	34.704	68.110	0.00	0.00	D
ATOM	23887	HE1	HSE	D	30	29.760	35.734	67.863	0.00	0.00	D
ATOM	23888	NE2	HSE	D	30	29.823	33.627	67.351	0.00	0.00	D
ATOM	23889	HE2	HSE	D	30	30.449	33.677	66.573	0.00	0.00	D
ATOM	23890	CD2	HSE	D	30	29.279	32.529	67.940	0.00	0.00	D
ATOM	23891	HD2	HSE	D	30	29.330	31.486	67.652	0.00	0.00	D
ATOM	23892	C	HSE	D	30	27.865	31.049	72.245	0.00	0.00	D
ATOM	23893	O	HSE	D	30	28.101	29.864	72.491	0.00	0.00	D
ATOM	23894	N	GLU	D	31	26.788	31.567	72.797	0.00	0.00	D

ATOM 23895 HN GLU D 31 26.592 32.541 72.717 0.00 0.00
D
ATOM 23896 CA GLU D 31 26.020 30.750 73.749 0.00 0.00
D
ATOM 23897 HA GLU D 31 26.646 29.924 74.052 0.00 0.00
D
ATOM 23898 CB GLU D 31 25.648 31.478 75.108 0.00 0.00
D
ATOM 23899 HB1 GLU D 31 25.201 32.404 74.686 0.00 0.00
D
ATOM 23900 HB2 GLU D 31 24.903 30.868 75.663 0.00 0.00
D
ATOM 23901 CG GLU D 31 26.829 32.045 75.951 0.00 0.00
D
ATOM 23902 HG1 GLU D 31 27.545 31.333 76.413 0.00 0.00
D
ATOM 23903 HG2 GLU D 31 27.466 32.732 75.353 0.00 0.00
D
ATOM 23904 CD GLU D 31 26.204 32.684 77.161 0.00 0.00
D
ATOM 23905 OE1 GLU D 31 25.354 32.077 77.856 0.00 0.00
D
ATOM 23906 OE2 GLU D 31 26.731 33.776 77.472 0.00 0.00
D
ATOM 23907 C GLU D 31 24.812 30.152 73.064 0.00 0.00
D
ATOM 23908 O GLU D 31 23.858 30.823 72.590 0.00 0.00
D
ATOM 23909 N VAL D 32 24.845 28.858 72.903 0.00 0.00
D
ATOM 23910 HN VAL D 32 25.617 28.371 73.304 0.00 0.00
D
ATOM 23911 CA VAL D 32 23.868 28.121 72.150 0.00 0.00
D
ATOM 23912 HA VAL D 32 23.054 28.780 71.884 0.00 0.00
D
ATOM 23913 CB VAL D 32 24.424 27.608 70.797 0.00 0.00
D
ATOM 23914 HB VAL D 32 24.728 28.547 70.289 0.00 0.00
D
ATOM 23915 CG1 VAL D 32 25.713 26.715 70.925 0.00 0.00
D
ATOM 23916 HG11 VAL D 32 26.555 27.065 71.560 0.00 0.00
D
ATOM 23917 HG12 VAL D 32 25.523 25.745 71.433 0.00 0.00
D
ATOM 23918 HG13 VAL D 32 26.175 26.604 69.921 0.00 0.00
D
ATOM 23919 CG2 VAL D 32 23.383 27.043 69.887 0.00 0.00
D
ATOM 23920 HG21 VAL D 32 23.043 26.042 70.225 0.00 0.00
D
ATOM 23921 HG22 VAL D 32 22.542 27.768 69.836 0.00 0.00
D

```
ATOM  23922 HG23 VAL D  32      23.738  26.963  68.837  0.00  0.00
D
ATOM  23923  C   VAL D  32      23.052  27.046  72.834  0.00  0.00
D
ATOM  23924  O   VAL D  32      23.624  26.135  73.400  0.00  0.00
D
ATOM  23925  N   LEU D  33      21.685  27.131  72.913  0.00  0.00
D
ATOM  23926  HN  LEU D  33      21.233  27.905  72.475  0.00  0.00
D
ATOM  23927  CA  LEU D  33      20.770  26.163  73.430  0.00  0.00
D
ATOM  23928  HA  LEU D  33      21.293  25.534  74.136  0.00  0.00
D
ATOM  23929  CB  LEU D  33      19.497  26.738  74.051  0.00  0.00
D
ATOM  23930  HB1 LEU D  33      19.803  27.383  74.902  0.00  0.00
D
ATOM  23931  HB2 LEU D  33      18.814  27.209  73.312  0.00  0.00
D
ATOM  23932  CG  LEU D  33      18.532  25.705  74.623  0.00  0.00
D
ATOM  23933  HG  LEU D  33      18.202  25.022  73.811  0.00  0.00
D
ATOM  23934  CD1 LEU D  33      19.005  24.778  75.684  0.00  0.00
D
ATOM  23935 HD11 LEU D  33      19.925  24.212  75.422  0.00  0.00
D
ATOM  23936 HD12 LEU D  33      19.269  25.450  76.529  0.00  0.00
D
ATOM  23937 HD13 LEU D  33      18.293  24.095  76.194  0.00  0.00
D
ATOM  23938  CD2 LEU D  33      17.286  26.374  75.173  0.00  0.00
D
ATOM  23939 HD21 LEU D  33      17.589  27.001  76.038  0.00  0.00
D
ATOM  23940 HD22 LEU D  33      16.863  26.990  74.350  0.00  0.00
D
ATOM  23941 HD23 LEU D  33      16.580  25.532  75.334  0.00  0.00
D
ATOM  23942  C   LEU D  33      20.368  25.199  72.372  0.00  0.00
D
ATOM  23943  O   LEU D  33      19.873  25.674  71.368  0.00  0.00
D
ATOM  23944  N   ALA D  34      20.615  23.879  72.584  0.00  0.00
D
ATOM  23945  HN  ALA D  34      20.975  23.633  73.480  0.00  0.00
D
ATOM  23946  CA  ALA D  34      20.144  22.959  71.612  0.00  0.00
D
ATOM  23947  HA  ALA D  34      19.760  23.475  70.745  0.00  0.00
D
ATOM  23948  CB  ALA D  34      21.282  22.111  71.053  0.00  0.00
D
```

ATOM 23949 HB1 ALA D 34 22.124 22.754 70.717 0.00 0.00
D
ATOM 23950 HB2 ALA D 34 21.631 21.456 71.880 0.00 0.00
D
ATOM 23951 HB3 ALA D 34 21.000 21.454 70.203 0.00 0.00
D
ATOM 23952 C ALA D 34 18.923 22.118 71.968 0.00 0.00
D
ATOM 23953 O ALA D 34 18.901 21.387 72.883 0.00 0.00
D
ATOM 23954 N VAL D 35 17.876 22.219 71.141 0.00 0.00
D
ATOM 23955 HN VAL D 35 17.909 22.905 70.417 0.00 0.00
D
ATOM 23956 CA VAL D 35 16.627 21.477 71.244 0.00 0.00
D
ATOM 23957 HA VAL D 35 16.615 20.894 72.153 0.00 0.00
D
ATOM 23958 CB VAL D 35 15.399 22.395 71.379 0.00 0.00
D
ATOM 23959 HB VAL D 35 15.196 22.718 70.336 0.00 0.00
D
ATOM 23960 CG1 VAL D 35 14.277 21.581 71.959 0.00 0.00
D
ATOM 23961 HG11 VAL D 35 14.637 21.100 72.894 0.00 0.00
D
ATOM 23962 HG12 VAL D 35 13.452 22.231 72.321 0.00 0.00
D
ATOM 23963 HG13 VAL D 35 13.869 20.809 71.273 0.00 0.00
D
ATOM 23964 CG2 VAL D 35 15.708 23.674 72.199 0.00 0.00
D
ATOM 23965 HG21 VAL D 35 15.932 23.313 73.225 0.00 0.00
D
ATOM 23966 HG22 VAL D 35 16.554 24.259 71.778 0.00 0.00
D
ATOM 23967 HG23 VAL D 35 14.803 24.315 72.134 0.00 0.00
D
ATOM 23968 C VAL D 35 16.394 20.419 70.099 0.00 0.00
D
ATOM 23969 O VAL D 35 16.427 20.696 68.900 0.00 0.00
D
ATOM 23970 N ASP D 36 15.982 19.182 70.466 0.00 0.00
D
ATOM 23971 HN ASP D 36 15.982 18.964 71.439 0.00 0.00
D
ATOM 23972 CA ASP D 36 15.431 18.216 69.597 0.00 0.00
D
ATOM 23973 HA ASP D 36 14.894 18.815 68.876 0.00 0.00
D
ATOM 23974 CB ASP D 36 16.497 17.419 68.792 0.00 0.00
D
ATOM 23975 HB1 ASP D 36 17.444 17.991 68.700 0.00 0.00
D

ATOM 23976 HB2 ASP D 36   16.885 16.556 69.374 0.00 0.00
D
ATOM 23977 CG ASP D 36   16.157 16.730 67.485 0.00 0.00
D
ATOM 23978 OD1 ASP D 36   16.718 17.051 66.381 0.00 0.00
D
ATOM 23979 OD2 ASP D 36   15.261 15.860 67.495 0.00 0.00
D
ATOM 23980 C   ASP D 36   14.535 17.323 70.386 0.00 0.00
D
ATOM 23981 O   ASP D 36   14.559 17.263 71.615 0.00 0.00
D
ATOM 23982 N   ILE D 37   13.673 16.514 69.718 0.00 0.00
D
ATOM 23983 HN ILE D 37   13.745 16.401 68.730 0.00 0.00
D
ATOM 23984 CA ILE D 37   12.725 15.651 70.317 0.00 0.00
D
ATOM 23985 HA ILE D 37   12.805 15.731 71.391 0.00 0.00
D
ATOM 23986 CB ILE D 37   11.372 16.043 69.759 0.00 0.00
D
ATOM 23987 HB ILE D 37   11.346 17.124 70.017 0.00 0.00
D
ATOM 23988 CG2 ILE D 37   11.281 15.789 68.262 0.00 0.00
D
ATOM 23989 HG21 ILE D 37   10.284 16.123 67.902 0.00 0.00
D
ATOM 23990 HG22 ILE D 37   11.964 16.402 67.635 0.00 0.00
D
ATOM 23991 HG23 ILE D 37   11.302 14.715 67.979 0.00 0.00
D
ATOM 23992 CG1 ILE D 37   10.179 15.409 70.529 0.00 0.00
D
ATOM 23993 HG11 ILE D 37   9.253 15.947 70.231 0.00 0.00
D
ATOM 23994 HG12 ILE D 37   10.155 14.352 70.189 0.00 0.00
D
ATOM 23995 CD ILE D 37   10.270 15.537 72.084 0.00 0.00
D
ATOM 23996 HD1 ILE D 37   11.054 14.827 72.424 0.00 0.00
D
ATOM 23997 HD2 ILE D 37   10.569 16.570 72.362 0.00 0.00
D
ATOM 23998 HD3 ILE D 37   9.316 15.266 72.584 0.00 0.00
D
ATOM 23999 C   ILE D 37   13.186 14.202 70.182 0.00 0.00
D
ATOM 24000 O   ILE D 37   12.576 13.245 70.599 0.00 0.00
D
ATOM 24001 N   ASN D 38   14.363 14.009 69.519 0.00 0.00
D
ATOM 24002 HN ASN D 38   14.873 14.789 69.165 0.00 0.00
D

ATOM 24003 CA ASN D 38   14.978 12.719 69.286 0.00 0.00
D
ATOM 24004 HA ASN D 38   14.249 11.970 69.562 0.00 0.00
D
ATOM 24005 CB ASN D 38   15.228 12.686 67.761 0.00 0.00
D
ATOM 24006 HB1 ASN D 38   14.238 12.794 67.269 0.00 0.00
D
ATOM 24007 HB2 ASN D 38   15.929 13.465 67.392 0.00 0.00
D
ATOM 24008 CG ASN D 38   15.842 11.364 67.349 0.00 0.00
D
ATOM 24009 OD1 ASN D 38   17.073 11.214 67.586 0.00 0.00
D
ATOM 24010 ND2 ASN D 38   15.061 10.483 66.692 0.00 0.00
D
ATOM 24011 HD21 ASN D 38   15.587   9.792 66.196 0.00 0.00
D
ATOM 24012 HD22 ASN D 38   14.080 10.571 66.521 0.00 0.00
D
ATOM 24013 C   ASN D 38   16.207 12.486 70.150 0.00 0.00
D
ATOM 24014 O   ASN D 38   17.053 13.375 70.260 0.00 0.00
D
ATOM 24015 N   GLU D 39   16.275 11.371 70.889 0.00 0.00
D
ATOM 24016 HN GLU D 39   15.697 10.569 70.762 0.00 0.00
D
ATOM 24017 CA GLU D 39   17.183 11.080 71.997 0.00 0.00
D
ATOM 24018 HA GLU D 39   17.046 11.890 72.698 0.00 0.00
D
ATOM 24019 CB GLU D 39   16.711   9.743 72.640 0.00 0.00
D
ATOM 24020 HB1 GLU D 39   15.616   9.788 72.820 0.00 0.00
D
ATOM 24021 HB2 GLU D 39   16.826   8.843 71.998 0.00 0.00
D
ATOM 24022 CG GLU D 39   17.310   9.552 74.088 0.00 0.00
D
ATOM 24023 HG1 GLU D 39   18.399   9.334 74.106 0.00 0.00
D
ATOM 24024 HG2 GLU D 39   17.269 10.540 74.594 0.00 0.00
D
ATOM 24025 CD GLU D 39   16.587   8.481 74.900 0.00 0.00
D
ATOM 24026 OE1 GLU D 39   17.136   7.365 75.141 0.00 0.00
D
ATOM 24027 OE2 GLU D 39   15.436   8.702 75.313 0.00 0.00
D
ATOM 24028 C   GLU D 39   18.603 11.028 71.588 0.00 0.00
D
ATOM 24029 O   GLU D 39   19.474 11.618 72.204 0.00 0.00
D

ATOM 24030 N   GLU D 40   18.930 10.439 70.411 0.00 0.00
D
ATOM 24031 HN GLU D 40   18.168   9.971 69.971 0.00 0.00
D
ATOM 24032 CA GLU D 40   20.258 10.448 69.770 0.00 0.00
D
ATOM 24033 HA GLU D 40   21.079 10.188 70.421 0.00 0.00
D
ATOM 24034 CB GLU D 40   20.305   9.529 68.553 0.00 0.00
D
ATOM 24035 HB1 GLU D 40   19.900   8.532 68.832 0.00 0.00
D
ATOM 24036 HB2 GLU D 40   19.637   9.962 67.778 0.00 0.00
D
ATOM 24037 CG GLU D 40   21.701   9.295 67.844 0.00 0.00
D
ATOM 24038 HG1 GLU D 40   22.167 10.278 67.616 0.00 0.00
D
ATOM 24039 HG2 GLU D 40   22.259   8.705 68.602 0.00 0.00
D
ATOM 24040 CD GLU D 40   21.572   8.517 66.563 0.00 0.00
D
ATOM 24041 OE1 GLU D 40   21.572   7.242 66.605 0.00 0.00
D
ATOM 24042 OE2 GLU D 40   21.523   9.184 65.486 0.00 0.00
D
ATOM 24043 C   GLU D 40   20.690 11.884 69.285 0.00 0.00
D
ATOM 24044 O   GLU D 40   21.788 12.198 69.603 0.00 0.00
D
ATOM 24045 N   LYS D 41   19.840 12.713 68.751 0.00 0.00
D
ATOM 24046 HN LYS D 41   18.873 12.519 68.601 0.00 0.00
D
ATOM 24047 CA LYS D 41   20.347 14.014 68.335 0.00 0.00
D
ATOM 24048 HA LYS D 41   21.253 13.955 67.749 0.00 0.00
D
ATOM 24049 CB LYS D 41   19.405 14.796 67.413 0.00 0.00
D
ATOM 24050 HB1 LYS D 41   18.417 14.822 67.921 0.00 0.00
D
ATOM 24051 HB2 LYS D 41   19.631 15.884 67.432 0.00 0.00
D
ATOM 24052 CG LYS D 41   19.067 14.221 66.036 0.00 0.00
D
ATOM 24053 HG1 LYS D 41   19.018 13.124 66.202 0.00 0.00
D
ATOM 24054 HG2 LYS D 41   18.120 14.593 65.589 0.00 0.00
D
ATOM 24055 CD LYS D 41   20.237 14.339 65.054 0.00 0.00
D
ATOM 24056 HD1 LYS D 41   20.717 15.316 65.276 0.00 0.00
D

ATOM 24057 HD2 LYS D 41 21.058 13.627 65.282 0.00 0.00
D
ATOM 24058 CE LYS D 41 19.832 14.411 63.619 0.00 0.00
D
ATOM 24059 HE1 LYS D 41 19.006 15.138 63.471 0.00 0.00
D
ATOM 24060 HE2 LYS D 41 20.737 14.518 62.983 0.00 0.00
D
ATOM 24061 NZ LYS D 41 19.217 13.117 63.188 0.00 0.00
D
ATOM 24062 HZ1 LYS D 41 19.762 12.317 63.570 0.00 0.00
D
ATOM 24063 HZ2 LYS D 41 18.243 12.990 63.531 0.00 0.00
D
ATOM 24064 HZ3 LYS D 41 19.262 13.025 62.153 0.00 0.00
D
ATOM 24065 C LYS D 41 20.736 14.974 69.528 0.00 0.00
D
ATOM 24066 O LYS D 41 21.762 15.639 69.536 0.00 0.00
D
ATOM 24067 N VAL D 42 19.879 15.055 70.605 0.00 0.00
D
ATOM 24068 HN VAL D 42 19.030 14.532 70.580 0.00 0.00
D
ATOM 24069 CA VAL D 42 20.206 15.834 71.843 0.00 0.00
D
ATOM 24070 HA VAL D 42 20.258 16.888 71.613 0.00 0.00
D
ATOM 24071 CB VAL D 42 19.096 15.721 72.870 0.00 0.00
D
ATOM 24072 HB VAL D 42 18.844 14.658 73.067 0.00 0.00
D
ATOM 24073 CG1 VAL D 42 19.507 16.260 74.231 0.00 0.00
D
ATOM 24074 HG11 VAL D 42 19.914 17.288 74.119 0.00 0.00
D
ATOM 24075 HG12 VAL D 42 18.615 16.313 74.891 0.00 0.00
D
ATOM 24076 HG13 VAL D 42 20.216 15.616 74.793 0.00 0.00
D
ATOM 24077 CG2 VAL D 42 17.937 16.511 72.305 0.00 0.00
D
ATOM 24078 HG21 VAL D 42 17.037 16.475 72.955 0.00 0.00
D
ATOM 24079 HG22 VAL D 42 18.355 17.538 72.241 0.00 0.00
D
ATOM 24080 HG23 VAL D 42 17.566 16.261 71.288 0.00 0.00
D
ATOM 24081 C VAL D 42 21.554 15.423 72.502 0.00 0.00
D
ATOM 24082 O VAL D 42 22.372 16.265 72.847 0.00 0.00
D
ATOM 24083 N ASN D 43 21.712 14.135 72.657 0.00 0.00
D

ATOM 24084 HN ASN D 43 21.066 13.413 72.422 0.00 0.00
D
ATOM 24085 CA ASN D 43 22.906 13.523 73.245 0.00 0.00
D
ATOM 24086 HA ASN D 43 23.159 14.093 74.126 0.00 0.00
D
ATOM 24087 CB ASN D 43 22.757 12.045 73.800 0.00 0.00
D
ATOM 24088 HB1 ASN D 43 22.442 11.382 72.966 0.00 0.00
D
ATOM 24089 HB2 ASN D 43 23.740 11.721 74.204 0.00 0.00
D
ATOM 24090 CG ASN D 43 21.753 12.014 74.978 0.00 0.00
D
ATOM 24091 OD1 ASN D 43 22.135 12.463 76.066 0.00 0.00
D
ATOM 24092 ND2 ASN D 43 20.445 11.617 74.754 0.00 0.00
D
ATOM 24093 HD21 ASN D 43 19.921 11.588 75.605 0.00 0.00
D
ATOM 24094 HD22 ASN D 43 20.128 11.479 73.816 0.00 0.00
D
ATOM 24095 C ASN D 43 24.081 13.645 72.328 0.00 0.00
D
ATOM 24096 O ASN D 43 25.146 13.875 72.776 0.00 0.00
D
ATOM 24097 N ALA D 44 23.833 13.464 71.087 0.00 0.00
D
ATOM 24098 HN ALA D 44 22.955 13.277 70.653 0.00 0.00
D
ATOM 24099 CA ALA D 44 24.902 13.584 70.144 0.00 0.00
D
ATOM 24100 HA ALA D 44 25.654 12.897 70.504 0.00 0.00
D
ATOM 24101 CB ALA D 44 24.621 12.992 68.746 0.00 0.00
D
ATOM 24102 HB1 ALA D 44 24.144 11.994 68.853 0.00 0.00
D
ATOM 24103 HB2 ALA D 44 23.869 13.672 68.292 0.00 0.00
D
ATOM 24104 HB3 ALA D 44 25.552 12.898 68.146 0.00 0.00
D
ATOM 24105 C ALA D 44 25.594 14.955 69.935 0.00 0.00
D
ATOM 24106 O ALA D 44 26.800 15.071 69.727 0.00 0.00
D
ATOM 24107 N TYR D 45 24.784 16.071 70.087 0.00 0.00
D
ATOM 24108 HN TYR D 45 23.794 15.964 70.042 0.00 0.00
D
ATOM 24109 CA TYR D 45 25.274 17.452 69.987 0.00 0.00
D
ATOM 24110 HA TYR D 45 26.260 17.472 69.547 0.00 0.00
D

ATOM 24111 CB TYR D 45 24.375 18.171 69.044 0.00 0.00
D
ATOM 24112 HB1 TYR D 45 23.337 17.971 69.383 0.00 0.00
D
ATOM 24113 HB2 TYR D 45 24.518 19.272 69.000 0.00 0.00
D
ATOM 24114 CG TYR D 45 24.618 17.788 67.584 0.00 0.00
D
ATOM 24115 CD1 TYR D 45 23.620 17.149 66.862 0.00 0.00
D
ATOM 24116 HD1 TYR D 45 22.773 16.835 67.454 0.00 0.00
D
ATOM 24117 CE1 TYR D 45 23.654 16.977 65.490 0.00 0.00
D
ATOM 24118 HE1 TYR D 45 22.816 16.625 64.906 0.00 0.00
D
ATOM 24119 CZ TYR D 45 24.883 17.347 64.867 0.00 0.00
D
ATOM 24120 OH TYR D 45 25.031 17.243 63.422 0.00 0.00
D
ATOM 24121 HH TYR D 45 24.194 16.834 63.190 0.00 0.00
D
ATOM 24122 CD2 TYR D 45 25.817 18.051 66.953 0.00 0.00
D
ATOM 24123 HD2 TYR D 45 26.609 18.417 67.590 0.00 0.00
D
ATOM 24124 CE2 TYR D 45 25.993 17.775 65.601 0.00 0.00
D
ATOM 24125 HE2 TYR D 45 26.920 17.854 65.053 0.00 0.00
D
ATOM 24126 C TYR D 45 25.450 18.022 71.338 0.00 0.00
D
ATOM 24127 O TYR D 45 25.850 19.173 71.394 0.00 0.00
D
ATOM 24128 N ALA D 46 25.174 17.267 72.406 0.00 0.00
D
ATOM 24129 HN ALA D 46 24.816 16.337 72.363 0.00 0.00
D
ATOM 24130 CA ALA D 46 25.456 17.663 73.774 0.00 0.00
D
ATOM 24131 HA ALA D 46 24.752 18.462 73.953 0.00 0.00
D
ATOM 24132 CB ALA D 46 25.040 16.606 74.750 0.00 0.00
D
ATOM 24133 HB1 ALA D 46 23.985 16.309 74.566 0.00 0.00
D
ATOM 24134 HB2 ALA D 46 25.689 15.711 74.645 0.00 0.00
D
ATOM 24135 HB3 ALA D 46 25.087 16.925 75.813 0.00 0.00
D
ATOM 24136 C ALA D 46 26.878 18.061 74.218 0.00 0.00
D
ATOM 24137 O ALA D 46 27.096 19.129 74.693 0.00 0.00
D

ATOM 24138 N SER D 47 27.859 17.265 73.904 0.00 0.00
D
ATOM 24139 HN SER D 47 27.705 16.363 73.508 0.00 0.00
D
ATOM 24140 CA SER D 47 29.285 17.633 74.070 0.00 0.00
D
ATOM 24141 HA SER D 47 29.366 18.061 75.059 0.00 0.00
D
ATOM 24142 CB SER D 47 30.323 16.468 73.946 0.00 0.00
D
ATOM 24143 HB1 SER D 47 30.614 16.195 72.909 0.00 0.00
D
ATOM 24144 HB2 SER D 47 31.236 16.878 74.429 0.00 0.00
D
ATOM 24145 OG SER D 47 29.891 15.275 74.639 0.00 0.00
D
ATOM 24146 HG1 SER D 47 29.278 14.816 74.060 0.00 0.00
D
ATOM 24147 C SER D 47 29.739 18.671 73.054 0.00 0.00
D
ATOM 24148 O SER D 47 30.922 18.993 73.099 0.00 0.00
D
ATOM 24149 N TYR D 48 28.840 19.254 72.195 0.00 0.00
D
ATOM 24150 HN TYR D 48 27.963 18.779 72.171 0.00 0.00
D
ATOM 24151 CA TYR D 48 29.094 20.274 71.204 0.00 0.00
D
ATOM 24152 HA TYR D 48 30.117 20.567 71.388 0.00 0.00
D
ATOM 24153 CB TYR D 48 28.821 19.620 69.799 0.00 0.00
D
ATOM 24154 HB1 TYR D 48 27.794 19.223 69.648 0.00 0.00
D
ATOM 24155 HB2 TYR D 48 28.967 20.382 69.004 0.00 0.00
D
ATOM 24156 CG TYR D 48 29.775 18.474 69.517 0.00 0.00
D
ATOM 24157 CD1 TYR D 48 29.264 17.280 68.977 0.00 0.00
D
ATOM 24158 HD1 TYR D 48 28.194 17.129 68.963 0.00 0.00
D
ATOM 24159 CE1 TYR D 48 30.163 16.291 68.581 0.00 0.00
D
ATOM 24160 HE1 TYR D 48 29.816 15.411 68.060 0.00 0.00
D
ATOM 24161 CZ TYR D 48 31.520 16.548 68.628 0.00 0.00
D
ATOM 24162 OH TYR D 48 32.365 15.570 68.151 0.00 0.00
D
ATOM 24163 HH TYR D 48 33.136 16.114 67.973 0.00 0.00
D
ATOM 24164 CD2 TYR D 48 31.110 18.658 69.669 0.00 0.00
D

ATOM 24165 HD2 TYR D 48 31.440 19.606 70.068 0.00 0.00
D
ATOM 24166 CE2 TYR D 48 32.027 17.691 69.197 0.00 0.00
D
ATOM 24167 HE2 TYR D 48 33.100 17.812 69.211 0.00 0.00
D
ATOM 24168 C TYR D 48 28.272 21.500 71.325 0.00 0.00
D
ATOM 24169 O TYR D 48 28.163 22.279 70.364 0.00 0.00
D
ATOM 24170 N ALA D 49 27.698 21.688 72.504 0.00 0.00
D
ATOM 24171 HN ALA D 49 27.586 20.949 73.162 0.00 0.00
D
ATOM 24172 CA ALA D 49 26.724 22.763 72.655 0.00 0.00
D
ATOM 24173 HA ALA D 49 27.085 23.660 72.173 0.00 0.00
D
ATOM 24174 CB ALA D 49 25.346 22.250 72.084 0.00 0.00
D
ATOM 24175 HB1 ALA D 49 24.560 22.994 72.334 0.00 0.00
D
ATOM 24176 HB2 ALA D 49 25.600 21.943 71.046 0.00 0.00
D
ATOM 24177 HB3 ALA D 49 25.006 21.410 72.726 0.00 0.00
D
ATOM 24178 C ALA D 49 26.716 23.116 74.062 0.00 0.00
D
ATOM 24179 O ALA D 49 27.287 22.492 74.934 0.00 0.00
D
ATOM 24180 N THR D 50 26.168 24.274 74.363 0.00 0.00
D
ATOM 24181 HN THR D 50 25.556 24.673 73.684 0.00 0.00
D
ATOM 24182 CA THR D 50 26.171 24.845 75.726 0.00 0.00
D
ATOM 24183 HA THR D 50 27.133 24.645 76.175 0.00 0.00
D
ATOM 24184 CB THR D 50 25.932 26.300 75.745 0.00 0.00
D
ATOM 24185 HB THR D 50 24.846 26.528 75.791 0.00 0.00
D
ATOM 24186 OG1 THR D 50 26.471 26.928 74.545 0.00 0.00
D
ATOM 24187 HG1 THR D 50 26.993 26.268 74.081 0.00 0.00
D
ATOM 24188 CG2 THR D 50 26.605 26.984 76.948 0.00 0.00
D
ATOM 24189 HG21 THR D 50 26.318 26.371 77.829 0.00 0.00
D
ATOM 24190 HG22 THR D 50 27.695 26.967 76.738 0.00 0.00
D
ATOM 24191 HG23 THR D 50 26.254 28.031 77.070 0.00 0.00
D

ATOM	24192	C	THR	D	50	25.222	24.136	76.681	0.00	0.00	D
ATOM	24193	O	THR	D	50	25.504	23.716	77.809	0.00	0.00	D
ATOM	24194	N	HSE	D	51	23.939	23.887	76.217	0.00	0.00	D
ATOM	24195	HN	HSE	D	51	23.657	24.064	75.277	0.00	0.00	D
ATOM	24196	CA	HSE	D	51	23.129	22.925	76.810	0.00	0.00	D
ATOM	24197	HA	HSE	D	51	23.722	22.137	77.251	0.00	0.00	D
ATOM	24198	CB	HSE	D	51	22.285	23.609	77.929	0.00	0.00	D
ATOM	24199	HB1	HSE	D	51	22.972	24.060	78.675	0.00	0.00	D
ATOM	24200	HB2	HSE	D	51	21.672	24.345	77.366	0.00	0.00	D
ATOM	24201	ND1	HSE	D	51	22.021	21.913	79.800	0.00	0.00	D
ATOM	24202	CG	HSE	D	51	21.456	22.661	78.793	0.00	0.00	D
ATOM	24203	CE1	HSE	D	51	21.031	21.367	80.441	0.00	0.00	D
ATOM	24204	HE1	HSE	D	51	21.185	20.703	81.292	0.00	0.00	D
ATOM	24205	NE2	HSE	D	51	19.823	21.831	79.889	0.00	0.00	D
ATOM	24206	HE2	HSE	D	51	18.908	21.549	80.178	0.00	0.00	D
ATOM	24207	CD2	HSE	D	51	20.133	22.649	78.886	0.00	0.00	D
ATOM	24208	HD2	HSE	D	51	19.416	23.119	78.224	0.00	0.00	D
ATOM	24209	C	HSE	D	51	22.255	22.264	75.749	0.00	0.00	D
ATOM	24210	O	HSE	D	51	22.136	22.704	74.648	0.00	0.00	D
ATOM	24211	N	ALA	D	52	21.674	21.060	76.070	0.00	0.00	D
ATOM	24212	HN	ALA	D	52	21.892	20.746	76.991	0.00	0.00	D
ATOM	24213	CA	ALA	D	52	21.045	20.102	75.120	0.00	0.00	D
ATOM	24214	HA	ALA	D	52	20.760	20.616	74.214	0.00	0.00	D
ATOM	24215	CB	ALA	D	52	22.141	19.114	74.854	0.00	0.00	D
ATOM	24216	HB1	ALA	D	52	22.752	18.877	75.751	0.00	0.00	D
ATOM	24217	HB2	ALA	D	52	21.727	18.126	74.559	0.00	0.00	D
ATOM	24218	HB3	ALA	D	52	22.887	19.596	74.187	0.00	0.00	D

ATOM 24219 C   ALA D 52   19.870 19.494 75.838 0.00 0.00
D
ATOM 24220 O   ALA D 52   20.009 18.916 76.920 0.00 0.00
D
ATOM 24221 N   VAL D 53   18.668 19.623 75.282 0.00 0.00
D
ATOM 24222 HN VAL D 53   18.590 20.077 74.398 0.00 0.00
D
ATOM 24223 CA VAL D 53   17.401 19.324 75.956 0.00 0.00
D
ATOM 24224 HA VAL D 53   17.659 18.729 76.820 0.00 0.00
D
ATOM 24225 CB VAL D 53   16.756 20.662 76.478 0.00 0.00
D
ATOM 24226 HB VAL D 53   17.525 21.151 77.113 0.00 0.00
D
ATOM 24227 CG1 VAL D 53   16.367 21.688 75.426 0.00 0.00
D
ATOM 24228 HG11 VAL D 53   15.994 22.606 75.930 0.00 0.00
D
ATOM 24229 HG12 VAL D 53   17.283 21.787 74.805 0.00 0.00
D
ATOM 24230 HG13 VAL D 53   15.536 21.251 74.833 0.00 0.00
D
ATOM 24231 CG2 VAL D 53   15.590 20.348 77.415 0.00 0.00
D
ATOM 24232 HG21 VAL D 53   15.188 21.226 77.965 0.00 0.00
D
ATOM 24233 HG22 VAL D 53   14.730 19.967 76.824 0.00 0.00
D
ATOM 24234 HG23 VAL D 53   15.899 19.563 78.137 0.00 0.00
D
ATOM 24235 C   VAL D 53   16.491 18.618 75.052 0.00 0.00
D
ATOM 24236 O   VAL D 53   16.185 19.046 73.922 0.00 0.00
D
ATOM 24237 N   ILE D 54   16.053 17.414 75.494 0.00 0.00
D
ATOM 24238 HN ILE D 54   16.576 16.956 76.209 0.00 0.00
D
ATOM 24239 CA ILE D 54   14.931 16.794 74.898 0.00 0.00
D
ATOM 24240 HA ILE D 54   15.119 16.703 73.839 0.00 0.00
D
ATOM 24241 CB ILE D 54   14.806 15.347 75.353 0.00 0.00
D
ATOM 24242 HB ILE D 54   14.721 15.451 76.456 0.00 0.00
D
ATOM 24243 CG2 ILE D 54   13.533 14.703 74.695 0.00 0.00
D
ATOM 24244 HG21 ILE D 54   12.574 15.185 74.982 0.00 0.00
D
ATOM 24245 HG22 ILE D 54   13.667 14.664 73.593 0.00 0.00
D

ATOM 24246 HG23 ILE D 54   13.330 13.685 75.090 0.00 0.00
D
ATOM 24247 CG1 ILE D 54   16.049 14.530 74.936 0.00 0.00
D
ATOM 24248 HG11 ILE D 54   16.335 14.699 73.875 0.00 0.00
D
ATOM 24249 HG12 ILE D 54   16.946 14.854 75.506 0.00 0.00
D
ATOM 24250 CD ILE D 54   15.947 12.982 75.091 0.00 0.00
D
ATOM 24251 HD1 ILE D 54   16.937 12.582 75.399 0.00 0.00
D
ATOM 24252 HD2 ILE D 54   15.280 12.812 75.963 0.00 0.00
D
ATOM 24253 HD3 ILE D 54   15.563 12.389 74.233 0.00 0.00
D
ATOM 24254 C   ILE D 54   13.557 17.531 75.051 0.00 0.00
D
ATOM 24255 O   ILE D 54   12.857 17.560 76.082 0.00 0.00
D
ATOM 24256 N   ALA D 55   13.152 18.305 74.034 0.00 0.00
D
ATOM 24257 HN ALA D 55   13.569 18.207 73.134 0.00 0.00
D
ATOM 24258 CA ALA D 55   12.008 19.173 74.150 0.00 0.00
D
ATOM 24259 HA ALA D 55   11.253 18.742 74.791 0.00 0.00
D
ATOM 24260 CB ALA D 55   12.392 20.518 74.809 0.00 0.00
D
ATOM 24261 HB1 ALA D 55   13.067 21.252 74.319 0.00 0.00
D
ATOM 24262 HB2 ALA D 55   11.376 20.963 74.872 0.00 0.00
D
ATOM 24263 HB3 ALA D 55   12.764 20.283 75.829 0.00 0.00
D
ATOM 24264 C   ALA D 55   11.338 19.217 72.830 0.00 0.00
D
ATOM 24265 O   ALA D 55   11.949 19.300 71.756 0.00 0.00
D
ATOM 24266 N   ASN D 56   9.992 19.162 72.952 0.00 0.00
D
ATOM 24267 HN ASN D 56   9.544 19.036 73.833 0.00 0.00
D
ATOM 24268 CA ASN D 56   9.130 19.187 71.830 0.00 0.00
D
ATOM 24269 HA ASN D 56   9.632 18.800 70.956 0.00 0.00
D
ATOM 24270 CB ASN D 56   7.824 18.438 72.054 0.00 0.00
D
ATOM 24271 HB1 ASN D 56   8.188 17.399 72.202 0.00 0.00
D
ATOM 24272 HB2 ASN D 56   7.261 18.678 72.981 0.00 0.00
D

ATOM 24273 CG ASN D 56 6.938 18.182 70.766 0.00 0.00
D
ATOM 24274 OD1 ASN D 56 6.709 19.142 70.056 0.00 0.00
D
ATOM 24275 ND2 ASN D 56 6.368 16.994 70.456 0.00 0.00
D
ATOM 24276 HD21 ASN D 56 5.711 17.074 69.706 0.00 0.00
D
ATOM 24277 HD22 ASN D 56 6.377 16.215 71.083 0.00 0.00
D
ATOM 24278 C ASN D 56 8.764 20.638 71.532 0.00 0.00
D
ATOM 24279 O ASN D 56 8.226 21.293 72.333 0.00 0.00
D
ATOM 24280 N ALA D 57 9.015 21.052 70.298 0.00 0.00
D
ATOM 24281 HN ALA D 57 9.575 20.448 69.736 0.00 0.00
D
ATOM 24282 CA ALA D 57 8.755 22.359 69.730 0.00 0.00
D
ATOM 24283 HA ALA D 57 9.259 22.944 70.485 0.00 0.00
D
ATOM 24284 CB ALA D 57 9.580 22.739 68.439 0.00 0.00
D
ATOM 24285 HB1 ALA D 57 9.946 23.786 68.365 0.00 0.00
D
ATOM 24286 HB2 ALA D 57 10.514 22.149 68.558 0.00 0.00
D
ATOM 24287 HB3 ALA D 57 9.028 22.503 67.504 0.00 0.00
D
ATOM 24288 C ALA D 57 7.289 22.836 69.669 0.00 0.00
D
ATOM 24289 O ALA D 57 6.959 24.017 69.874 0.00 0.00
D
ATOM 24290 N THR D 58 6.414 21.816 69.588 0.00 0.00
D
ATOM 24291 HN THR D 58 6.752 20.880 69.651 0.00 0.00
D
ATOM 24292 CA THR D 58 4.996 22.048 69.461 0.00 0.00
D
ATOM 24293 HA THR D 58 4.910 22.998 68.955 0.00 0.00
D
ATOM 24294 CB THR D 58 4.222 21.223 68.471 0.00 0.00
D
ATOM 24295 HB THR D 58 3.191 21.601 68.302 0.00 0.00
D
ATOM 24296 OG1 THR D 58 4.179 19.893 68.962 0.00 0.00
D
ATOM 24297 HG1 THR D 58 5.011 19.657 69.377 0.00 0.00
D
ATOM 24298 CG2 THR D 58 4.983 21.167 67.159 0.00 0.00
D
ATOM 24299 HG21 THR D 58 5.016 22.184 66.713 0.00 0.00
D

ATOM 24300 HG22 THR D 58   5.989 20.722 67.313 0.00 0.00
D
ATOM 24301 HG23 THR D 58   4.355 20.596 66.442 0.00 0.00
D
ATOM 24302 C   THR D 58   4.238 21.989 70.770 0.00 0.00
D
ATOM 24303 O   THR D 58   3.047 22.223 70.755 0.00 0.00
D
ATOM 24304 N   GLU D 59   4.942 21.861 71.891 0.00 0.00
D
ATOM 24305 HN GLU D 59   5.909 21.626 71.823 0.00 0.00
D
ATOM 24306 CA GLU D 59   4.359 21.969 73.265 0.00 0.00
D
ATOM 24307 HA GLU D 59   3.308 21.749 73.149 0.00 0.00
D
ATOM 24308 CB GLU D 59   4.900 20.920 74.218 0.00 0.00
D
ATOM 24309 HB1 GLU D 59   4.896 19.937 73.700 0.00 0.00
D
ATOM 24310 HB2 GLU D 59   5.961 21.157 74.445 0.00 0.00
D
ATOM 24311 CG GLU D 59   4.062 20.681 75.524 0.00 0.00
D
ATOM 24312 HG1 GLU D 59   4.558 20.074 76.311 0.00 0.00
D
ATOM 24313 HG2 GLU D 59   3.745 21.617 76.033 0.00 0.00
D
ATOM 24314 CD GLU D 59   2.846 19.795 75.221 0.00 0.00
D
ATOM 24315 OE1 GLU D 59   3.008 18.564 75.092 0.00 0.00
D
ATOM 24316 OE2 GLU D 59   1.711 20.360 75.192 0.00 0.00
D
ATOM 24317 C   GLU D 59   4.568 23.282 73.880 0.00 0.00
D
ATOM 24318 O   GLU D 59   5.725 23.597 74.099 0.00 0.00
D
ATOM 24319 N   GLU D 60   3.482 23.981 74.256 0.00 0.00
D
ATOM 24320 HN GLU D 60   2.549 23.634 74.210 0.00 0.00
D
ATOM 24321 CA GLU D 60   3.504 25.346 74.736 0.00 0.00
D
ATOM 24322 HA GLU D 60   4.096 25.892 74.016 0.00 0.00
D
ATOM 24323 CB GLU D 60   2.029 25.871 74.753 0.00 0.00
D
ATOM 24324 HB1 GLU D 60   1.625 25.650 73.743 0.00 0.00
D
ATOM 24325 HB2 GLU D 60   1.384 25.262 75.422 0.00 0.00
D
ATOM 24326 CG GLU D 60   1.790 27.358 75.112 0.00 0.00
D

ATOM 24327 HG1 GLU D 60   2.255 27.648 76.078 0.00 0.00
D
ATOM 24328 HG2 GLU D 60   2.370 28.055 74.470 0.00 0.00
D
ATOM 24329 CD GLU D 60   0.374 27.760 75.350 0.00 0.00
D
ATOM 24330 OE1 GLU D 60   -0.241 27.246 76.301 0.00 0.00
D
ATOM 24331 OE2 GLU D 60   -0.076 28.654 74.524 0.00 0.00
D
ATOM 24332 C   GLU D 60   4.002 25.508 76.165 0.00 0.00
D
ATOM 24333 O   GLU D 60   4.914 26.266 76.435 0.00 0.00
D
ATOM 24334 N   ASN D 61   3.600 24.607 77.079 0.00 0.00
D
ATOM 24335 HN ASN D 61   2.817 24.033 76.854 0.00 0.00
D
ATOM 24336 CA ASN D 61   4.022 24.415 78.436 0.00 0.00
D
ATOM 24337 HA ASN D 61   4.011 25.391 78.898 0.00 0.00
D
ATOM 24338 CB ASN D 61   3.072 23.344 79.192 0.00 0.00
D
ATOM 24339 HB1 ASN D 61   2.957 22.513 78.464 0.00 0.00
D
ATOM 24340 HB2 ASN D 61   3.590 23.046 80.129 0.00 0.00
D
ATOM 24341 CG ASN D 61   1.741 23.918 79.493 0.00 0.00
D
ATOM 24342 OD1 ASN D 61   1.591 25.096 79.826 0.00 0.00
D
ATOM 24343 ND2 ASN D 61   0.715 23.055 79.323 0.00 0.00
D
ATOM 24344 HD21 ASN D 61   -0.176 23.509 79.337 0.00 0.00
D
ATOM 24345 HD22 ASN D 61   0.855 22.187 78.848 0.00 0.00
D
ATOM 24346 C   ASN D 61   5.432 24.067 78.510 0.00 0.00
D
ATOM 24347 O   ASN D 61   6.113 24.291 79.524 0.00 0.00
D
ATOM 24348 N   GLU D 62   6.063 23.420 77.445 0.00 0.00
D
ATOM 24349 HN GLU D 62   5.547 23.472 76.593 0.00 0.00
D
ATOM 24350 CA GLU D 62   7.397 23.020 77.509 0.00 0.00
D
ATOM 24351 HA GLU D 62   7.713 22.635 78.467 0.00 0.00
D
ATOM 24352 CB GLU D 62   7.682 21.938 76.547 0.00 0.00
D
ATOM 24353 HB1 GLU D 62   6.976 21.109 76.767 0.00 0.00
D

```
ATOM  24354  HB2 GLU D  62       7.613  22.283  75.494  0.00  0.00
D
ATOM  24355  CG  GLU D  62       9.072  21.194  76.734  0.00  0.00
D
ATOM  24356  HG1 GLU D  62       9.219  20.552  75.840  0.00  0.00
D
ATOM  24357  HG2 GLU D  62       9.904  21.928  76.793  0.00  0.00
D
ATOM  24358  CD  GLU D  62       9.168  20.254  77.905  0.00  0.00
D
ATOM  24359  OE1 GLU D  62       9.129  19.027  77.746  0.00  0.00
D
ATOM  24360  OE2 GLU D  62       9.424  20.726  79.094  0.00  0.00
D
ATOM  24361  C   GLU D  62       8.356  24.253  77.203  0.00  0.00
D
ATOM  24362  O   GLU D  62       9.421  24.537  77.812  0.00  0.00
D
ATOM  24363  N   LEU D  63       8.058  25.081  76.163  0.00  0.00
D
ATOM  24364  HN  LEU D  63       7.194  24.941  75.685  0.00  0.00
D
ATOM  24365  CA  LEU D  63       8.766  26.310  75.794  0.00  0.00
D
ATOM  24366  HA  LEU D  63       9.832  26.133  75.780  0.00  0.00
D
ATOM  24367  CB  LEU D  63       8.384  26.609  74.398  0.00  0.00
D
ATOM  24368  HB1 LEU D  63       7.276  26.679  74.369  0.00  0.00
D
ATOM  24369  HB2 LEU D  63       8.785  27.618  74.164  0.00  0.00
D
ATOM  24370  CG  LEU D  63       8.733  25.623  73.272  0.00  0.00
D
ATOM  24371  HG  LEU D  63       8.167  24.683  73.451  0.00  0.00
D
ATOM  24372  CD1 LEU D  63       8.332  26.361  72.001  0.00  0.00
D
ATOM  24373 HD11 LEU D  63       8.531  25.778  71.075  0.00  0.00
D
ATOM  24374 HD12 LEU D  63       7.262  26.657  71.981  0.00  0.00
D
ATOM  24375 HD13 LEU D  63       8.953  27.282  71.994  0.00  0.00
D
ATOM  24376  CD2 LEU D  63      10.224  25.280  73.169  0.00  0.00
D
ATOM  24377 HD21 LEU D  63      10.301  24.474  72.408  0.00  0.00
D
ATOM  24378 HD22 LEU D  63      10.744  26.208  72.848  0.00  0.00
D
ATOM  24379 HD23 LEU D  63      10.577  24.849  74.130  0.00  0.00
D
ATOM  24380  C   LEU D  63       8.489  27.389  76.885  0.00  0.00
D
```

ATOM 24381 O   LEU D 64   9.384 28.156 77.221 0.00 0.00
D
ATOM 24382 N   LEU D 64   7.337 27.492 77.530 0.00 0.00
D
ATOM 24383 HN LEU D 64   6.454 27.090 77.304 0.00 0.00
D
ATOM 24384 CA LEU D 64   7.261 28.092 78.877 0.00 0.00
D
ATOM 24385 HA LEU D 64   7.451 29.140 78.699 0.00 0.00
D
ATOM 24386 CB LEU D 64   5.805 28.212 79.403 0.00 0.00
D
ATOM 24387 HB1 LEU D 64   5.404 27.192 79.219 0.00 0.00
D
ATOM 24388 HB2 LEU D 64   5.783 28.378 80.502 0.00 0.00
D
ATOM 24389 CG LEU D 64   5.066 29.327 78.602 0.00 0.00
D
ATOM 24390 HG LEU D 64   5.368 29.225 77.537 0.00 0.00
D
ATOM 24391 CD1 LEU D 64   3.613 28.933 78.661 0.00 0.00
D
ATOM 24392 HD11 LEU D 64   3.484 28.264 77.784 0.00 0.00
D
ATOM 24393 HD12 LEU D 64   3.356 28.439 79.623 0.00 0.00
D
ATOM 24394 HD13 LEU D 64   2.963 29.833 78.620 0.00 0.00
D
ATOM 24395 CD2 LEU D 64   5.257 30.752 79.049 0.00 0.00
D
ATOM 24396 HD21 LEU D 64   4.907 31.396 78.215 0.00 0.00
D
ATOM 24397 HD22 LEU D 64   4.762 31.023 80.006 0.00 0.00
D
ATOM 24398 HD23 LEU D 64   6.332 31.001 79.177 0.00 0.00
D
ATOM 24399 C   LEU D 64   8.097 27.670 80.064 0.00 0.00
D
ATOM 24400 O   LEU D 64   8.663 28.527 80.711 0.00 0.00
D
ATOM 24401 N   SER D 65   8.265 26.365 80.320 0.00 0.00
D
ATOM 24402 HN SER D 65   7.762 25.626 79.877 0.00 0.00
D
ATOM 24403 CA SER D 65   9.302 25.958 81.246 0.00 0.00
D
ATOM 24404 HA SER D 65   9.073 26.486 82.160 0.00 0.00
D
ATOM 24405 CB SER D 65   9.301 24.403 81.540 0.00 0.00
D
ATOM 24406 HB1 SER D 65   9.503 23.793 80.633 0.00 0.00
D
ATOM 24407 HB2 SER D 65   10.063 24.212 82.326 0.00 0.00
D

ATOM 24408 OG SER D 65 8.020 23.947 81.963 0.00 0.00
D
ATOM 24409 HG1 SER D 65 8.067 22.990 81.907 0.00 0.00
D
ATOM 24410 C SER D 65 10.716 26.340 80.797 0.00 0.00
D
ATOM 24411 O SER D 65 11.506 26.898 81.565 0.00 0.00
D
ATOM 24412 N LEU D 66 10.997 26.035 79.486 0.00 0.00
D
ATOM 24413 HN LEU D 66 10.313 25.566 78.932 0.00 0.00
D
ATOM 24414 CA LEU D 66 12.327 26.226 78.862 0.00 0.00
D
ATOM 24415 HA LEU D 66 13.016 25.601 79.410 0.00 0.00
D
ATOM 24416 CB LEU D 66 12.322 25.738 77.400 0.00 0.00
D
ATOM 24417 HB1 LEU D 66 11.850 24.733 77.363 0.00 0.00
D
ATOM 24418 HB2 LEU D 66 11.810 26.418 76.686 0.00 0.00
D
ATOM 24419 CG LEU D 66 13.702 25.572 76.698 0.00 0.00
D
ATOM 24420 HG LEU D 66 14.081 26.616 76.690 0.00 0.00
D
ATOM 24421 CD1 LEU D 66 14.690 24.660 77.509 0.00 0.00
D
ATOM 24422 HD11 LEU D 66 15.528 24.365 76.841 0.00 0.00
D
ATOM 24423 HD12 LEU D 66 14.978 25.225 78.421 0.00 0.00
D
ATOM 24424 HD13 LEU D 66 14.147 23.751 77.847 0.00 0.00
D
ATOM 24425 CD2 LEU D 66 13.567 25.209 75.188 0.00 0.00
D
ATOM 24426 HD21 LEU D 66 13.320 24.126 75.184 0.00 0.00
D
ATOM 24427 HD22 LEU D 66 12.751 25.769 74.683 0.00 0.00
D
ATOM 24428 HD23 LEU D 66 14.483 25.413 74.592 0.00 0.00
D
ATOM 24429 C LEU D 66 12.875 27.604 79.004 0.00 0.00
D
ATOM 24430 O LEU D 66 14.020 27.765 79.270 0.00 0.00
D
ATOM 24431 N GLY D 67 12.069 28.621 78.856 0.00 0.00
D
ATOM 24432 HN GLY D 67 11.186 28.334 78.495 0.00 0.00
D
ATOM 24433 CA GLY D 67 12.436 30.051 78.792 0.00 0.00
D
ATOM 24434 HA1 GLY D 67 12.939 30.241 79.729 0.00 0.00
D

ATOM 24435 HA2 GLY D 67   11.462 30.516 78.771 0.00 0.00
D
ATOM 24436 C   GLY D 67   13.240 30.379 77.535 0.00 0.00
D
ATOM 24437 O   GLY D 67   14.449 30.614 77.555 0.00 0.00
D
ATOM 24438 N   ILE D 68   12.473 30.329 76.406 0.00 0.00
D
ATOM 24439 HN ILE D 68   11.493 30.147 76.360 0.00 0.00
D
ATOM 24440 CA ILE D 68   13.075 30.316 75.075 0.00 0.00
D
ATOM 24441 HA ILE D 68   14.124 30.065 75.124 0.00 0.00
D
ATOM 24442 CB ILE D 68   12.521 29.204 74.249 0.00 0.00
D
ATOM 24443 HB ILE D 68   12.633 28.236 74.783 0.00 0.00
D
ATOM 24444 CG2 ILE D 68   11.139 29.469 73.856 0.00 0.00
D
ATOM 24445 HG21 ILE D 68   11.188 30.281 73.099 0.00 0.00
D
ATOM 24446 HG22 ILE D 68   10.648 28.554 73.460 0.00 0.00
D
ATOM 24447 HG23 ILE D 68   10.386 29.718 74.634 0.00 0.00
D
ATOM 24448 CG1 ILE D 68   13.359 28.870 73.018 0.00 0.00
D
ATOM 24449 HG11 ILE D 68   12.871 28.000 72.529 0.00 0.00
D
ATOM 24450 HG12 ILE D 68   13.262 29.602 72.188 0.00 0.00
D
ATOM 24451 CD ILE D 68   14.815 28.507 73.366 0.00 0.00
D
ATOM 24452 HD1 ILE D 68   14.791 27.895 74.293 0.00 0.00
D
ATOM 24453 HD2 ILE D 68   15.292 27.860 72.599 0.00 0.00
D
ATOM 24454 HD3 ILE D 68   15.503 29.373 73.470 0.00 0.00
D
ATOM 24455 C   ILE D 68   12.884 31.721 74.428 0.00 0.00
D
ATOM 24456 O   ILE D 68   13.536 32.107 73.501 0.00 0.00
D
ATOM 24457 N   ARG D 69   12.101 32.552 75.161 0.00 0.00
D
ATOM 24458 HN ARG D 69   11.621 32.150 75.938 0.00 0.00
D
ATOM 24459 CA ARG D 69   11.851 33.920 74.748 0.00 0.00
D
ATOM 24460 HA ARG D 69   11.812 33.965 73.670 0.00 0.00
D
ATOM 24461 CB ARG D 69   10.574 34.465 75.440 0.00 0.00
D

ATOM 24462 HB1 ARG D 69   9.719 33.817 75.149 0.00 0.00
D
ATOM 24463 HB2 ARG D 69   10.749 34.407 76.536 0.00 0.00
D
ATOM 24464 CG ARG D 69   10.184 35.969 75.121 0.00 0.00
D
ATOM 24465 HG1 ARG D 69   11.102 36.593 75.088 0.00 0.00
D
ATOM 24466 HG2 ARG D 69   9.875 36.029 74.055 0.00 0.00
D
ATOM 24467 CD ARG D 69   9.042 36.695 76.002 0.00 0.00
D
ATOM 24468 HD1 ARG D 69   9.530 36.846 76.988 0.00 0.00
D
ATOM 24469 HD2 ARG D 69   8.744 37.547 75.354 0.00 0.00
D
ATOM 24470 NE ARG D 69   7.745 35.863 76.238 0.00 0.00
D
ATOM 24471 HE ARG D 69   7.277 35.454 75.454 0.00 0.00
D
ATOM 24472 CZ ARG D 69   7.188 35.564 77.432 0.00 0.00
D
ATOM 24473 NH1 ARG D 69   7.776 35.865 78.553 0.00 0.00
D
ATOM 24474 HH11 ARG D 69   7.226 35.700 79.372 0.00 0.00
D
ATOM 24475 HH12 ARG D 69   8.767 35.993 78.585 0.00 0.00
D
ATOM 24476 NH2 ARG D 69   5.959 35.057 77.521 0.00 0.00
D
ATOM 24477 HH21 ARG D 69   5.566 35.084 78.440 0.00 0.00
D
ATOM 24478 HH22 ARG D 69   5.375 35.182 76.719 0.00 0.00
D
ATOM 24479 C   ARG D 69   13.035 34.772 75.113 0.00 0.00
D
ATOM 24480 O   ARG D 69   13.215 35.914 74.702 0.00 0.00
D
ATOM 24481 N   ASN D 70   13.948 34.239 75.870 0.00 0.00
D
ATOM 24482 HN ASN D 70   13.933 33.276 76.125 0.00 0.00
D
ATOM 24483 CA ASN D 70   15.039 34.924 76.492 0.00 0.00
D
ATOM 24484 HA ASN D 70   14.874 35.991 76.492 0.00 0.00
D
ATOM 24485 CB ASN D 70   15.225 34.610 77.989 0.00 0.00
D
ATOM 24486 HB1 ASN D 70   15.291 33.538 78.271 0.00 0.00
D
ATOM 24487 HB2 ASN D 70   16.167 35.103 78.312 0.00 0.00
D
ATOM 24488 CG ASN D 70   14.176 35.323 78.844 0.00 0.00
D

ATOM 24489 OD1 ASN D 70   13.230 35.945 78.383 0.00 0.00
D
ATOM 24490 ND2 ASN D 70   14.310 35.278 80.162 0.00 0.00
D
ATOM 24491 HD21 ASN D 70   13.579 35.802 80.597 0.00 0.00
D
ATOM 24492 HD22 ASN D 70   15.066 34.754 80.555 0.00 0.00
D
ATOM 24493 C   ASN D 70   16.429 34.632 75.693 0.00 0.00
D
ATOM 24494 O   ASN D 70   17.541 34.722 76.245 0.00 0.00
D
ATOM 24495 N   PHE D 71   16.214 34.276 74.426 0.00 0.00
D
ATOM 24496 HN PHE D 71   15.309 34.248 74.009 0.00 0.00
D
ATOM 24497 CA PHE D 71   17.187 34.123 73.428 0.00 0.00
D
ATOM 24498 HA PHE D 71   18.109 34.383 73.928 0.00 0.00
D
ATOM 24499 CB PHE D 71   17.183 32.670 72.896 0.00 0.00
D
ATOM 24500 HB1 PHE D 71   16.128 32.399 72.679 0.00 0.00
D
ATOM 24501 HB2 PHE D 71   17.756 32.542 71.953 0.00 0.00
D
ATOM 24502 CG PHE D 71   17.720 31.639 73.864 0.00 0.00
D
ATOM 24503 CD1 PHE D 71   19.087 31.289 73.793 0.00 0.00
D
ATOM 24504 HD1 PHE D 71   19.701 31.639 72.977 0.00 0.00
D
ATOM 24505 CE1 PHE D 71   19.643 30.308 74.706 0.00 0.00
D
ATOM 24506 HE1 PHE D 71   20.710 30.166 74.612 0.00 0.00
D
ATOM 24507 CZ PHE D 71   18.885 29.889 75.723 0.00 0.00
D
ATOM 24508 HZ PHE D 71   19.323 29.261 76.485 0.00 0.00
D
ATOM 24509 CD2 PHE D 71   16.910 31.061 74.850 0.00 0.00
D
ATOM 24510 HD2 PHE D 71   15.848 31.238 74.935 0.00 0.00
D
ATOM 24511 CE2 PHE D 71   17.501 30.195 75.822 0.00 0.00
D
ATOM 24512 HE2 PHE D 71   16.949 29.786 76.656 0.00 0.00
D
ATOM 24513 C   PHE D 71   16.895 35.125 72.339 0.00 0.00
D
ATOM 24514 O   PHE D 71   15.796 35.392 71.970 0.00 0.00
D
ATOM 24515 N   GLU D 72   17.867 35.874 71.832 0.00 0.00
D

ATOM 24516 HN GLU D 72 18.724 35.887 72.342 0.00 0.00
D
ATOM 24517 CA GLU D 72 17.831 36.928 70.828 0.00 0.00
D
ATOM 24518 HA GLU D 72 17.104 37.638 71.192 0.00 0.00
D
ATOM 24519 CB GLU D 72 19.257 37.507 70.734 0.00 0.00
D
ATOM 24520 HB1 GLU D 72 20.095 36.778 70.709 0.00 0.00
D
ATOM 24521 HB2 GLU D 72 19.273 38.207 69.871 0.00 0.00
D
ATOM 24522 CG GLU D 72 19.586 38.381 71.977 0.00 0.00
D
ATOM 24523 HG1 GLU D 72 20.497 38.939 71.672 0.00 0.00
D
ATOM 24524 HG2 GLU D 72 18.702 39.037 72.126 0.00 0.00
D
ATOM 24525 CD GLU D 72 19.946 37.677 73.319 0.00 0.00
D
ATOM 24526 OE1 GLU D 72 20.278 38.480 74.209 0.00 0.00
D
ATOM 24527 OE2 GLU D 72 20.103 36.458 73.453 0.00 0.00
D
ATOM 24528 C GLU D 72 17.297 36.503 69.482 0.00 0.00
D
ATOM 24529 O GLU D 72 16.405 37.060 68.844 0.00 0.00
D
ATOM 24530 N TYR D 73 17.834 35.347 68.955 0.00 0.00
D
ATOM 24531 HN TYR D 73 18.365 34.874 69.653 0.00 0.00
D
ATOM 24532 CA TYR D 73 17.584 34.718 67.650 0.00 0.00
D
ATOM 24533 HA TYR D 73 16.643 35.124 67.309 0.00 0.00
D
ATOM 24534 CB TYR D 73 18.676 34.815 66.491 0.00 0.00
D
ATOM 24535 HB1 TYR D 73 19.635 34.336 66.781 0.00 0.00
D
ATOM 24536 HB2 TYR D 73 18.226 34.190 65.690 0.00 0.00
D
ATOM 24537 CG TYR D 73 18.944 36.219 66.045 0.00 0.00
D
ATOM 24538 CD1 TYR D 73 19.349 37.225 66.877 0.00 0.00
D
ATOM 24539 HD1 TYR D 73 19.460 37.026 67.932 0.00 0.00
D
ATOM 24540 CE1 TYR D 73 19.494 38.535 66.469 0.00 0.00
D
ATOM 24541 HE1 TYR D 73 19.877 39.363 67.048 0.00 0.00
D
ATOM 24542 CZ TYR D 73 19.154 38.836 65.127 0.00 0.00
D

ATOM 24543 OH TYR D 73   19.210 40.171 64.700 0.00 0.00
D
ATOM 24544 HH TYR D 73   19.888 40.570 65.251 0.00 0.00
D
ATOM 24545 CD2 TYR D 73   18.612 36.559 64.768 0.00 0.00
D
ATOM 24546 HD2 TYR D 73   18.139 35.800 64.163 0.00 0.00
D
ATOM 24547 CE2 TYR D 73   18.834 37.798 64.267 0.00 0.00
D
ATOM 24548 HE2 TYR D 73   18.538 38.083 63.269 0.00 0.00
D
ATOM 24549 C   TYR D 73   17.322 33.223 67.965 0.00 0.00
D
ATOM 24550 O   TYR D 73   17.974 32.624 68.815 0.00 0.00
D
ATOM 24551 N   VAL D 74   16.243 32.682 67.372 0.00 0.00
D
ATOM 24552 HN VAL D 74   15.669 33.245 66.782 0.00 0.00
D
ATOM 24553 CA VAL D 74   15.795 31.330 67.567 0.00 0.00
D
ATOM 24554 HA VAL D 74   16.554 30.712 68.025 0.00 0.00
D
ATOM 24555 CB VAL D 74   14.421 31.117 68.307 0.00 0.00
D
ATOM 24556 HB VAL D 74   13.522 31.506 67.783 0.00 0.00
D
ATOM 24557 CG1 VAL D 74   14.312 29.626 68.479 0.00 0.00
D
ATOM 24558 HG11 VAL D 74   13.545 29.455 69.264 0.00 0.00
D
ATOM 24559 HG12 VAL D 74   14.102 29.134 67.506 0.00 0.00
D
ATOM 24560 HG13 VAL D 74   15.241 29.180 68.893 0.00 0.00
D
ATOM 24561 CG2 VAL D 74   14.474 31.984 69.570 0.00 0.00
D
ATOM 24562 HG21 VAL D 74   15.184 31.582 70.324 0.00 0.00
D
ATOM 24563 HG22 VAL D 74   14.657 33.057 69.349 0.00 0.00
D
ATOM 24564 HG23 VAL D 74   13.483 32.043 70.069 0.00 0.00
D
ATOM 24565 C   VAL D 74   15.784 30.789 66.196 0.00 0.00
D
ATOM 24566 O   VAL D 74   14.973 31.267 65.426 0.00 0.00
D
ATOM 24567 N   ILE D 75   16.664 29.834 65.782 0.00 0.00
D
ATOM 24568 HN ILE D 75   17.208 29.412 66.504 0.00 0.00
D
ATOM 24569 CA ILE D 75   16.743 29.250 64.449 0.00 0.00
D

ATOM 24570 HA ILE D 75   16.233 29.884 63.739 0.00 0.00
D
ATOM 24571 CB ILE D 75   18.205 28.962 63.989 0.00 0.00
D
ATOM 24572 HB ILE D 75   18.658 28.193 64.651 0.00 0.00
D
ATOM 24573 CG2 ILE D 75   18.186 28.429 62.536 0.00 0.00
D
ATOM 24574 HG21 ILE D 75   19.222 28.436 62.136 0.00 0.00
D
ATOM 24575 HG22 ILE D 75   17.808 27.385 62.510 0.00 0.00
D
ATOM 24576 HG23 ILE D 75   17.466 29.012 61.923 0.00 0.00
D
ATOM 24577 CG1 ILE D 75   19.146 30.176 64.264 0.00 0.00
D
ATOM 24578 HG11 ILE D 75   19.254 30.556 65.302 0.00 0.00
D
ATOM 24579 HG12 ILE D 75   20.136 29.861 63.871 0.00 0.00
D
ATOM 24580 CD ILE D 75   18.729 31.458 63.446 0.00 0.00
D
ATOM 24581 HD1 ILE D 75   18.612 31.221 62.367 0.00 0.00
D
ATOM 24582 HD2 ILE D 75   17.862 31.939 63.947 0.00 0.00
D
ATOM 24583 HD3 ILE D 75   19.560 32.194 63.474 0.00 0.00
D
ATOM 24584 C   ILE D 75   16.013 27.948 64.486 0.00 0.00
D
ATOM 24585 O   ILE D 75   16.306 27.067 65.287 0.00 0.00
D
ATOM 24586 N   VAL D 76   15.068 27.764 63.558 0.00 0.00
D
ATOM 24587 HN VAL D 76   14.980 28.550 62.951 0.00 0.00
D
ATOM 24588 CA VAL D 76   14.193 26.653 63.450 0.00 0.00
D
ATOM 24589 HA VAL D 76   14.391 26.087 64.349 0.00 0.00
D
ATOM 24590 CB VAL D 76   12.715 27.114 63.618 0.00 0.00
D
ATOM 24591 HB VAL D 76   12.591 27.859 62.802 0.00 0.00
D
ATOM 24592 CG1 VAL D 76   11.715 25.912 63.503 0.00 0.00
D
ATOM 24593 HG11 VAL D 76   10.788 26.406 63.866 0.00 0.00
D
ATOM 24594 HG12 VAL D 76   11.534 25.645 62.440 0.00 0.00
D
ATOM 24595 HG13 VAL D 76   11.973 25.030 64.127 0.00 0.00
D
ATOM 24596 CG2 VAL D 76   12.595 27.918 64.984 0.00 0.00
D

ATOM 24597 HG21 VAL D 76   11.640 28.480 65.061 0.00 0.00
D
ATOM 24598 HG22 VAL D 76   12.734 27.153 65.778 0.00 0.00
D
ATOM 24599 HG23 VAL D 76   13.345 28.737 64.943 0.00 0.00
D
ATOM 24600 C   VAL D 76   14.599 25.813 62.217 0.00 0.00
D
ATOM 24601 O   VAL D 76   14.238 26.168 61.100 0.00 0.00
D
ATOM 24602 N   ALA D 77   15.331 24.748 62.407 0.00 0.00
D
ATOM 24603 HN ALA D 77   15.608 24.431 63.311 0.00 0.00
D
ATOM 24604 CA ALA D 77   15.898 24.012 61.337 0.00 0.00
D
ATOM 24605 HA ALA D 77   16.064 24.708 60.528 0.00 0.00
D
ATOM 24606 CB ALA D 77   17.267 23.487 61.827 0.00 0.00
D
ATOM 24607 HB1 ALA D 77   17.072 22.880 62.737 0.00 0.00
D
ATOM 24608 HB2 ALA D 77   17.712 22.846 61.037 0.00 0.00
D
ATOM 24609 HB3 ALA D 77   17.919 24.330 62.143 0.00 0.00
D
ATOM 24610 C   ALA D 77   14.980 22.893 61.045 0.00 0.00
D
ATOM 24611 O   ALA D 77   15.227 22.027 60.246 0.00 0.00
D
ATOM 24612 N   ILE D 78   13.823 22.811 61.717 0.00 0.00
D
ATOM 24613 HN ILE D 78   13.692 23.529 62.397 0.00 0.00
D
ATOM 24614 CA ILE D 78   12.708 21.891 61.490 0.00 0.00
D
ATOM 24615 HA ILE D 78   13.048 20.875 61.353 0.00 0.00
D
ATOM 24616 CB ILE D 78   11.542 21.925 62.453 0.00 0.00
D
ATOM 24617 HB ILE D 78   10.976 22.881 62.482 0.00 0.00
D
ATOM 24618 CG2 ILE D 78   10.504 20.785 62.007 0.00 0.00
D
ATOM 24619 HG21 ILE D 78   11.224 20.006 61.677 0.00 0.00
D
ATOM 24620 HG22 ILE D 78   9.892 20.308 62.802 0.00 0.00
D
ATOM 24621 HG23 ILE D 78   9.880 21.257 61.217 0.00 0.00
D
ATOM 24622 CG1 ILE D 78   12.197 21.591 63.849 0.00 0.00
D
ATOM 24623 HG11 ILE D 78   12.588 20.554 63.783 0.00 0.00
D

ATOM 24624 HG12 ILE D 78   13.111 22.205 63.994 0.00 0.00
D
ATOM 24625 CD ILE D 78   11.287 21.644 65.080 0.00 0.00
D
ATOM 24626 HD1 ILE D 78   11.666 21.334 66.078 0.00 0.00
D
ATOM 24627 HD2 ILE D 78   10.870 22.673 65.107 0.00 0.00
D
ATOM 24628 HD3 ILE D 78   10.357 21.047 64.969 0.00 0.00
D
ATOM 24629 C   ILE D 78   12.061 22.293 60.166 0.00 0.00
D
ATOM 24630 O   ILE D 78   11.368 23.337 60.219 0.00 0.00
D
ATOM 24631 N   GLY D 79   12.291 21.532 59.099 0.00 0.00
D
ATOM 24632 HN GLY D 79   12.823 20.691 59.169 0.00 0.00
D
ATOM 24633 CA GLY D 79   11.552 21.808 57.836 0.00 0.00
D
ATOM 24634 HA1 GLY D 79   12.219 21.935 56.996 0.00 0.00
D
ATOM 24635 HA2 GLY D 79   10.859 22.629 57.946 0.00 0.00
D
ATOM 24636 C   GLY D 79   10.710 20.734 57.324 0.00 0.00
D
ATOM 24637 O   GLY D 79   9.609 20.954 56.717 0.00 0.00
D
ATOM 24638 N   ALA D 80   11.062 19.514 57.642 0.00 0.00
D
ATOM 24639 HN ALA D 80   11.953 19.383 58.070 0.00 0.00
D
ATOM 24640 CA ALA D 80   10.167 18.381 57.364 0.00 0.00
D
ATOM 24641 HA ALA D 80   9.796 18.499 56.356 0.00 0.00
D
ATOM 24642 CB ALA D 80   10.815 17.050 57.590 0.00 0.00
D
ATOM 24643 HB1 ALA D 80   10.061 16.306 57.258 0.00 0.00
D
ATOM 24644 HB2 ALA D 80   11.748 17.108 56.989 0.00 0.00
D
ATOM 24645 HB3 ALA D 80   11.042 16.967 58.674 0.00 0.00
D
ATOM 24646 C   ALA D 80   8.989 18.380 58.345 0.00 0.00
D
ATOM 24647 O   ALA D 80   9.186 18.575 59.570 0.00 0.00
D
ATOM 24648 N   ASN D 81   7.804 18.100 57.732 0.00 0.00
D
ATOM 24649 HN ASN D 81   7.765 18.119 56.736 0.00 0.00
D
ATOM 24650 CA ASN D 81   6.494 17.996 58.357 0.00 0.00
D

ATOM 24651 HA ASN D 81 5.986 17.340 57.666 0.00 0.00
D
ATOM 24652 CB ASN D 81 6.502 17.316 59.730 0.00 0.00
D
ATOM 24653 HB1 ASN D 81 7.159 16.423 59.667 0.00 0.00
D
ATOM 24654 HB2 ASN D 81 6.966 18.004 60.468 0.00 0.00
D
ATOM 24655 CG ASN D 81 5.120 16.947 60.118 0.00 0.00
D
ATOM 24656 OD1 ASN D 81 4.439 17.634 60.948 0.00 0.00
D
ATOM 24657 ND2 ASN D 81 4.495 15.999 59.495 0.00 0.00
D
ATOM 24658 HD21 ASN D 81 3.501 15.942 59.582 0.00 0.00
D
ATOM 24659 HD22 ASN D 81 4.953 15.568 58.717 0.00 0.00
D
ATOM 24660 C ASN D 81 5.891 19.347 58.335 0.00 0.00
D
ATOM 24661 O ASN D 81 6.259 20.209 59.163 0.00 0.00
D
ATOM 24662 N ILE D 82 4.875 19.639 57.468 0.00 0.00
D
ATOM 24663 HN ILE D 82 4.575 18.904 56.865 0.00 0.00
D
ATOM 24664 CA ILE D 82 4.122 20.848 57.382 0.00 0.00
D
ATOM 24665 HA ILE D 82 4.915 21.528 57.106 0.00 0.00
D
ATOM 24666 CB ILE D 82 3.216 20.814 56.266 0.00 0.00
D
ATOM 24667 HB ILE D 82 3.571 20.229 55.390 0.00 0.00
D
ATOM 24668 CG2 ILE D 82 1.928 20.100 56.465 0.00 0.00
D
ATOM 24669 HG21 ILE D 82 1.379 20.144 55.501 0.00 0.00
D
ATOM 24670 HG22 ILE D 82 2.124 19.101 56.911 0.00 0.00
D
ATOM 24671 HG23 ILE D 82 1.272 20.618 57.197 0.00 0.00
D
ATOM 24672 CG1 ILE D 82 2.832 22.170 55.901 0.00 0.00
D
ATOM 24673 HG11 ILE D 82 1.886 22.138 55.320 0.00 0.00
D
ATOM 24674 HG12 ILE D 82 2.424 22.744 56.760 0.00 0.00
D
ATOM 24675 CD ILE D 82 3.832 23.088 55.144 0.00 0.00
D
ATOM 24676 HD1 ILE D 82 4.661 23.553 55.719 0.00 0.00
D
ATOM 24677 HD2 ILE D 82 4.399 22.344 54.544 0.00 0.00
D

ATOM 24678 HD3 ILE D 82   3.316 23.845 54.515 0.00 0.00
D
ATOM 24679 C   ILE D 82   3.580 21.307 58.720 0.00 0.00
D
ATOM 24680 O   ILE D 82   3.825 22.457 59.150 0.00 0.00
D
ATOM 24681 N   GLN D 83   2.988 20.377 59.484 0.00 0.00
D
ATOM 24682 HN GLN D 83   2.787 19.445 59.192 0.00 0.00
D
ATOM 24683 CA GLN D 83   2.389 20.726 60.764 0.00 0.00
D
ATOM 24684 HA GLN D 83   1.762 21.599 60.656 0.00 0.00
D
ATOM 24685 CB GLN D 83   1.503 19.540 61.227 0.00 0.00
D
ATOM 24686 HB1 GLN D 83   2.097 18.615 61.388 0.00 0.00
D
ATOM 24687 HB2 GLN D 83   1.024 19.758 62.206 0.00 0.00
D
ATOM 24688 CG GLN D 83   0.370 19.085 60.324 0.00 0.00
D
ATOM 24689 HG1 GLN D 83   -0.362 18.516 60.936 0.00 0.00
D
ATOM 24690 HG2 GLN D 83   -0.204 19.956 59.941 0.00 0.00
D
ATOM 24691 CD GLN D 83   0.722 18.181 59.162 0.00 0.00
D
ATOM 24692 OE1 GLN D 83   1.908 17.940 58.844 0.00 0.00
D
ATOM 24693 NE2 GLN D 83   -0.315 17.578 58.530 0.00 0.00
D
ATOM 24694 HE21 GLN D 83   -0.044 17.005 57.757 0.00 0.00
D
ATOM 24695 HE22 GLN D 83   -1.234 17.908 58.748 0.00 0.00
D
ATOM 24696 C   GLN D 83   3.359 21.080 61.860 0.00 0.00
D
ATOM 24697 O   GLN D 83   3.360 22.151 62.453 0.00 0.00
D
ATOM 24698 N   ALA D 84   4.361 20.232 62.218 0.00 0.00
D
ATOM 24699 HN ALA D 84   4.427 19.360 61.740 0.00 0.00
D
ATOM 24700 CA ALA D 84   5.502 20.584 63.128 0.00 0.00
D
ATOM 24701 HA ALA D 84   5.106 20.573 64.132 0.00 0.00
D
ATOM 24702 CB ALA D 84   6.611 19.527 63.167 0.00 0.00
D
ATOM 24703 HB1 ALA D 84   6.173 18.546 63.451 0.00 0.00
D
ATOM 24704 HB2 ALA D 84   7.169 19.320 62.229 0.00 0.00
D

ATOM 24705 HB3 ALA D 84   7.282 19.789 64.013 0.00 0.00
D
ATOM 24706 C   ALA D 84   6.164 21.839 62.773 0.00 0.00
D
ATOM 24707 O   ALA D 84   6.296 22.724 63.666 0.00 0.00
D
ATOM 24708 N   SER D 85   6.631 21.999 61.523 0.00 0.00
D
ATOM 24709 HN SER D 85   6.363 21.380 60.788 0.00 0.00
D
ATOM 24710 CA SER D 85   7.389 23.197 61.101 0.00 0.00
D
ATOM 24711 HA SER D 85   8.258 23.410 61.706 0.00 0.00
D
ATOM 24712 CB SER D 85   7.962 22.924 59.695 0.00 0.00
D
ATOM 24713 HB1 SER D 85   8.432 21.918 59.731 0.00 0.00
D
ATOM 24714 HB2 SER D 85   7.134 22.839 58.959 0.00 0.00
D
ATOM 24715 OG SER D 85   8.940 23.926 59.322 0.00 0.00
D
ATOM 24716 HG1 SER D 85   9.803 23.627 59.617 0.00 0.00
D
ATOM 24717 C   SER D 85   6.520 24.526 61.118 0.00 0.00
D
ATOM 24718 O   SER D 85   6.809 25.551 61.830 0.00 0.00
D
ATOM 24719 N   THR D 86   5.327 24.512 60.393 0.00 0.00
D
ATOM 24720 HN THR D 86   4.971 23.736 59.879 0.00 0.00
D
ATOM 24721 CA THR D 86   4.493 25.734 60.558 0.00 0.00
D
ATOM 24722 HA THR D 86   5.037 26.659 60.442 0.00 0.00
D
ATOM 24723 CB THR D 86   3.343 25.816 59.580 0.00 0.00
D
ATOM 24724 HB THR D 86   2.835 26.776 59.814 0.00 0.00
D
ATOM 24725 OG1 THR D 86   2.549 24.659 59.716 0.00 0.00
D
ATOM 24726 HG1 THR D 86   3.105 23.926 59.439 0.00 0.00
D
ATOM 24727 CG2 THR D 86   3.997 25.850 58.146 0.00 0.00
D
ATOM 24728 HG21 THR D 86   4.957 26.409 58.135 0.00 0.00
D
ATOM 24729 HG22 THR D 86   4.246 24.821 57.810 0.00 0.00
D
ATOM 24730 HG23 THR D 86   3.153 26.224 57.529 0.00 0.00
D
ATOM 24731 C   THR D 86   3.913 25.921 62.007 0.00 0.00
D

ATOM 24732 O THR D 86 3.947 27.081 62.441 0.00 0.00
D
ATOM 24733 N LEU D 87 3.409 24.893 62.710 0.00 0.00
D
ATOM 24734 HN LEU D 87 3.369 23.950 62.390 0.00 0.00
D
ATOM 24735 CA LEU D 87 2.870 25.149 64.125 0.00 0.00
D
ATOM 24736 HA LEU D 87 2.076 25.876 64.044 0.00 0.00
D
ATOM 24737 CB LEU D 87 2.223 23.824 64.820 0.00 0.00
D
ATOM 24738 HB1 LEU D 87 1.559 23.422 64.026 0.00 0.00
D
ATOM 24739 HB2 LEU D 87 3.083 23.121 64.857 0.00 0.00
D
ATOM 24740 CG LEU D 87 1.533 24.075 66.092 0.00 0.00
D
ATOM 24741 HG LEU D 87 2.320 24.358 66.824 0.00 0.00
D
ATOM 24742 CD1 LEU D 87 0.479 25.208 66.095 0.00 0.00
D
ATOM 24743 HD11 LEU D 87 0.294 25.390 67.175 0.00 0.00
D
ATOM 24744 HD12 LEU D 87 0.848 26.198 65.750 0.00 0.00
D
ATOM 24745 HD13 LEU D 87 -0.478 24.937 65.600 0.00 0.00
D
ATOM 24746 CD2 LEU D 87 0.857 22.764 66.589 0.00 0.00
D
ATOM 24747 HD21 LEU D 87 1.478 21.847 66.501 0.00 0.00
D
ATOM 24748 HD22 LEU D 87 0.369 22.793 67.587 0.00 0.00
D
ATOM 24749 HD23 LEU D 87 0.071 22.587 65.824 0.00 0.00
D
ATOM 24750 C LEU D 87 3.878 25.591 65.150 0.00 0.00
D
ATOM 24751 O LEU D 87 3.670 26.549 65.901 0.00 0.00
D
ATOM 24752 N THR D 88 5.080 24.952 65.072 0.00 0.00
D
ATOM 24753 HN THR D 88 5.145 24.046 64.661 0.00 0.00
D
ATOM 24754 CA THR D 88 6.248 25.460 65.840 0.00 0.00
D
ATOM 24755 HA THR D 88 6.087 25.391 66.905 0.00 0.00
D
ATOM 24756 CB THR D 88 7.525 24.642 65.471 0.00 0.00
D
ATOM 24757 HB THR D 88 7.552 24.819 64.375 0.00 0.00
D
ATOM 24758 OG1 THR D 88 7.319 23.328 65.959 0.00 0.00
D

ATOM 24759 HG1 THR D 88   6.612 23.035 65.380 0.00 0.00
D
ATOM 24760 CG2 THR D 88   8.827 25.188 66.276 0.00 0.00
D
ATOM 24761 HG21 THR D 88   9.616 24.457 66.000 0.00 0.00
D
ATOM 24762 HG22 THR D 88   9.227 26.180 65.976 0.00 0.00
D
ATOM 24763 HG23 THR D 88   8.612 25.212 67.366 0.00 0.00
D
ATOM 24764 C   THR D 88   6.601 26.923 65.532 0.00 0.00
D
ATOM 24765 O   THR D 88   6.748 27.668 66.485 0.00 0.00
D
ATOM 24766 N   THR D 89   6.733 27.394 64.229 0.00 0.00
D
ATOM 24767 HN THR D 89   6.569 26.687 63.546 0.00 0.00
D
ATOM 24768 CA THR D 89   6.999 28.775 63.868 0.00 0.00
D
ATOM 24769 HA THR D 89   7.890 29.096 64.389 0.00 0.00
D
ATOM 24770 CB THR D 89   7.010 28.956 62.331 0.00 0.00
D
ATOM 24771 HB THR D 89   6.014 28.737 61.890 0.00 0.00
D
ATOM 24772 OG1 THR D 89   7.870 27.946 61.756 0.00 0.00
D
ATOM 24773 HG1 THR D 89   7.409 27.105 61.727 0.00 0.00
D
ATOM 24774 CG2 THR D 89   7.466 30.333 61.771 0.00 0.00
D
ATOM 24775 HG21 THR D 89   8.526 30.541 62.030 0.00 0.00
D
ATOM 24776 HG22 THR D 89   7.284 30.461 60.682 0.00 0.00
D
ATOM 24777 HG23 THR D 89   6.825 31.158 62.150 0.00 0.00
D
ATOM 24778 C   THR D 89   5.928 29.746 64.308 0.00 0.00
D
ATOM 24779 O   THR D 89   6.188 30.857 64.774 0.00 0.00
D
ATOM 24780 N   LEU D 90   4.659 29.327 64.266 0.00 0.00
D
ATOM 24781 HN LEU D 90   4.427 28.414 63.938 0.00 0.00
D
ATOM 24782 CA LEU D 90   3.626 30.108 64.869 0.00 0.00
D
ATOM 24783 HA LEU D 90   3.719 31.134 64.546 0.00 0.00
D
ATOM 24784 CB LEU D 90   2.378 29.601 64.175 0.00 0.00
D
ATOM 24785 HB1 LEU D 90   2.693 29.477 63.117 0.00 0.00
D

ATOM 24786 HB2 LEU D 90   2.250 28.526 64.425 0.00 0.00
D
ATOM 24787 CG LEU D 90   1.082 30.275 64.376 0.00 0.00
D
ATOM 24788 HG LEU D 90   0.713 30.342 65.421 0.00 0.00
D
ATOM 24789 CD1 LEU D 90   0.899 31.665 63.693 0.00 0.00
D
ATOM 24790 HD11 LEU D 90   -0.118 32.068 63.888 0.00 0.00
D
ATOM 24791 HD12 LEU D 90   1.569 32.413 64.167 0.00 0.00
D
ATOM 24792 HD13 LEU D 90   1.081 31.616 62.598 0.00 0.00
D
ATOM 24793 CD2 LEU D 90   0.103 29.315 63.693 0.00 0.00
D
ATOM 24794 HD21 LEU D 90   0.196 29.450 62.594 0.00 0.00
D
ATOM 24795 HD22 LEU D 90   0.182 28.235 63.942 0.00 0.00
D
ATOM 24796 HD23 LEU D 90   -0.965 29.562 63.876 0.00 0.00
D
ATOM 24797 C   LEU D 90   3.526 30.109 66.387 0.00 0.00
D
ATOM 24798 O   LEU D 90   3.353 31.191 66.947 0.00 0.00
D
ATOM 24799 N   LEU D 91   3.816 28.938 67.025 0.00 0.00
D
ATOM 24800 HN LEU D 91   3.770 28.062 66.552 0.00 0.00
D
ATOM 24801 CA LEU D 91   3.866 28.811 68.479 0.00 0.00
D
ATOM 24802 HA LEU D 91   2.946 29.161 68.924 0.00 0.00
D
ATOM 24803 CB LEU D 91   4.118 27.385 69.028 0.00 0.00
D
ATOM 24804 HB1 LEU D 91   3.476 26.638 68.514 0.00 0.00
D
ATOM 24805 HB2 LEU D 91   5.176 27.112 68.824 0.00 0.00
D
ATOM 24806 CG LEU D 91   3.955 27.074 70.523 0.00 0.00
D
ATOM 24807 HG LEU D 91   4.862 27.444 71.046 0.00 0.00
D
ATOM 24808 CD1 LEU D 91   2.699 27.654 71.136 0.00 0.00
D
ATOM 24809 HD11 LEU D 91   2.566 28.739 70.939 0.00 0.00
D
ATOM 24810 HD12 LEU D 91   1.763 27.228 70.714 0.00 0.00
D
ATOM 24811 HD13 LEU D 91   2.618 27.459 72.226 0.00 0.00
D
ATOM 24812 CD2 LEU D 91   4.114 25.562 70.822 0.00 0.00
D

ATOM 24813 HD21 LEU D 91   5.165 25.282 70.596 0.00 0.00
D
ATOM 24814 HD22 LEU D 91   3.923 25.238 71.867 0.00 0.00
D
ATOM 24815 HD23 LEU D 91   3.436 25.065 70.095 0.00 0.00
D
ATOM 24816 C   LEU D 91   4.915 29.769 69.097 0.00 0.00
D
ATOM 24817 O   LEU D 91   4.582 30.530 70.039 0.00 0.00
D
ATOM 24818 N   LEU D 92   6.145 29.859 68.444 0.00 0.00
D
ATOM 24819 HN LEU D 92   6.335 29.195 67.725 0.00 0.00
D
ATOM 24820 CA LEU D 92   7.131 30.717 68.926 0.00 0.00
D
ATOM 24821 HA LEU D 92   7.161 30.562 69.994 0.00 0.00
D
ATOM 24822 CB LEU D 92   8.487 30.424 68.229 0.00 0.00
D
ATOM 24823 HB1 LEU D 92   8.289 30.149 67.171 0.00 0.00
D
ATOM 24824 HB2 LEU D 92   9.229 31.223 68.445 0.00 0.00
D
ATOM 24825 CG LEU D 92   9.140 29.121 68.838 0.00 0.00
D
ATOM 24826 HG LEU D 92   8.364 28.329 68.898 0.00 0.00
D
ATOM 24827 CD1 LEU D 92   10.187 28.646 67.806 0.00 0.00
D
ATOM 24828 HD11 LEU D 92   11.052 29.343 67.817 0.00 0.00
D
ATOM 24829 HD12 LEU D 92   10.596 27.706 68.235 0.00 0.00
D
ATOM 24830 HD13 LEU D 92   9.815 28.416 66.785 0.00 0.00
D
ATOM 24831 CD2 LEU D 92   9.911 29.488 70.120 0.00 0.00
D
ATOM 24832 HD21 LEU D 92   10.848 29.915 69.703 0.00 0.00
D
ATOM 24833 HD22 LEU D 92   9.264 30.173 70.708 0.00 0.00
D
ATOM 24834 HD23 LEU D 92   10.036 28.521 70.653 0.00 0.00
D
ATOM 24835 C   LEU D 92   6.783 32.204 68.669 0.00 0.00
D
ATOM 24836 O   LEU D 92   7.192 33.040 69.495 0.00 0.00
D
ATOM 24837 N   LYS D 93   6.066 32.470 67.562 0.00 0.00
D
ATOM 24838 HN LYS D 93   5.798 31.646 67.069 0.00 0.00
D
ATOM 24839 CA LYS D 93   5.403 33.799 67.320 0.00 0.00
D

ATOM 24840 HA LYS D 93 6.232 34.474 67.471 0.00 0.00
D
ATOM 24841 CB LYS D 93 5.104 33.935 65.835 0.00 0.00
D
ATOM 24842 HB1 LYS D 93 5.971 33.594 65.230 0.00 0.00
D
ATOM 24843 HB2 LYS D 93 4.247 33.280 65.572 0.00 0.00
D
ATOM 24844 CG LYS D 93 4.847 35.324 65.233 0.00 0.00
D
ATOM 24845 HG1 LYS D 93 4.402 35.064 64.248 0.00 0.00
D
ATOM 24846 HG2 LYS D 93 4.108 35.897 65.833 0.00 0.00
D
ATOM 24847 CD LYS D 93 6.015 36.235 64.999 0.00 0.00
D
ATOM 24848 HD1 LYS D 93 6.517 36.452 65.966 0.00 0.00
D
ATOM 24849 HD2 LYS D 93 6.650 35.619 64.326 0.00 0.00
D
ATOM 24850 CE LYS D 93 5.861 37.605 64.282 0.00 0.00
D
ATOM 24851 HE1 LYS D 93 5.277 37.486 63.345 0.00 0.00
D
ATOM 24852 HE2 LYS D 93 5.193 38.223 64.919 0.00 0.00
D
ATOM 24853 NZ LYS D 93 7.125 38.246 63.982 0.00 0.00
D
ATOM 24854 HZ1 LYS D 93 6.948 38.885 63.181 0.00 0.00
D
ATOM 24855 HZ2 LYS D 93 7.440 38.820 64.790 0.00 0.00
D
ATOM 24856 HZ3 LYS D 93 7.866 37.612 63.623 0.00 0.00
D
ATOM 24857 C LYS D 93 4.351 34.309 68.302 0.00 0.00
D
ATOM 24858 O LYS D 93 4.390 35.469 68.653 0.00 0.00
D
ATOM 24859 N GLU D 94 3.477 33.445 68.865 0.00 0.00
D
ATOM 24860 HN GLU D 94 3.499 32.498 68.554 0.00 0.00
D
ATOM 24861 CA GLU D 94 2.533 33.680 69.930 0.00 0.00
D
ATOM 24862 HA GLU D 94 1.856 34.492 69.710 0.00 0.00
D
ATOM 24863 CB GLU D 94 1.532 32.497 70.107 0.00 0.00
D
ATOM 24864 HB1 GLU D 94 2.219 31.659 70.353 0.00 0.00
D
ATOM 24865 HB2 GLU D 94 0.890 32.645 71.002 0.00 0.00
D
ATOM 24866 CG GLU D 94 0.638 31.998 68.990 0.00 0.00
D

ATOM 24867 HG1 GLU D 94 1.246 31.852 68.072 0.00 0.00
D
ATOM 24868 HG2 GLU D 94 0.170 31.002 69.144 0.00 0.00
D
ATOM 24869 CD GLU D 94 -0.548 32.912 68.777 0.00 0.00
D
ATOM 24870 OE1 GLU D 94 -1.582 32.633 69.434 0.00 0.00
D
ATOM 24871 OE2 GLU D 94 -0.478 34.018 68.138 0.00 0.00
D
ATOM 24872 C GLU D 94 3.161 34.009 71.299 0.00 0.00
D
ATOM 24873 O GLU D 94 2.588 34.574 72.168 0.00 0.00
D
ATOM 24874 N LEU D 95 4.390 33.520 71.463 0.00 0.00
D
ATOM 24875 HN LEU D 95 4.719 32.969 70.700 0.00 0.00
D
ATOM 24876 CA LEU D 95 5.263 33.587 72.576 0.00 0.00
D
ATOM 24877 HA LEU D 95 4.689 33.717 73.481 0.00 0.00
D
ATOM 24878 CB LEU D 95 6.103 32.291 72.672 0.00 0.00
D
ATOM 24879 HB1 LEU D 95 6.640 32.063 71.727 0.00 0.00
D
ATOM 24880 HB2 LEU D 95 6.970 32.322 73.367 0.00 0.00
D
ATOM 24881 CG LEU D 95 5.274 31.127 73.112 0.00 0.00
D
ATOM 24882 HG LEU D 95 4.438 30.915 72.412 0.00 0.00
D
ATOM 24883 CD1 LEU D 95 6.195 29.859 73.160 0.00 0.00
D
ATOM 24884 HD11 LEU D 95 6.552 29.457 72.188 0.00 0.00
D
ATOM 24885 HD12 LEU D 95 7.084 30.077 73.790 0.00 0.00
D
ATOM 24886 HD13 LEU D 95 5.676 28.918 73.440 0.00 0.00
D
ATOM 24887 CD2 LEU D 95 4.664 31.195 74.554 0.00 0.00
D
ATOM 24888 HD21 LEU D 95 4.159 32.163 74.758 0.00 0.00
D
ATOM 24889 HD22 LEU D 95 4.073 30.294 74.823 0.00 0.00
D
ATOM 24890 HD23 LEU D 95 5.445 31.218 75.344 0.00 0.00
D
ATOM 24891 C LEU D 95 6.199 34.684 72.565 0.00 0.00
D
ATOM 24892 O LEU D 95 7.043 34.911 73.502 0.00 0.00
D
ATOM 24893 N ASP D 96 6.061 35.597 71.603 0.00 0.00
D

```
ATOM  24894  HN  ASP D  96       5.369  35.513  70.891  0.00  0.00
D
ATOM  24895  CA  ASP D  96       6.819  36.865  71.633  0.00  0.00
D
ATOM  24896  HA  ASP D  96       6.428  37.371  70.762  0.00  0.00
D
ATOM  24897  CB  ASP D  96       6.618  37.763  72.934  0.00  0.00
D
ATOM  24898  HB1 ASP D  96       7.039  37.209  73.800  0.00  0.00
D
ATOM  24899  HB2 ASP D  96       7.111  38.746  72.775  0.00  0.00
D
ATOM  24900  CG  ASP D  96       5.110  38.007  73.014  0.00  0.00
D
ATOM  24901  OD1 ASP D  96       4.463  37.719  74.056  0.00  0.00
D
ATOM  24902  OD2 ASP D  96       4.607  38.573  72.057  0.00  0.00
D
ATOM  24903  C   ASP D  96       8.354  36.760  71.384  0.00  0.00
D
ATOM  24904  O   ASP D  96       9.199  37.562  71.862  0.00  0.00
D
ATOM  24905  N   ILE D  97       8.811  35.667  70.664  0.00  0.00
D
ATOM  24906  HN  ILE D  97       8.221  34.963  70.273  0.00  0.00
D
ATOM  24907  CA  ILE D  97      10.243  35.445  70.400  0.00  0.00
D
ATOM  24908  HA  ILE D  97      10.727  35.450  71.366  0.00  0.00
D
ATOM  24909  CB  ILE D  97      10.557  34.047  69.888  0.00  0.00
D
ATOM  24910  HB  ILE D  97       9.743  33.641  69.250  0.00  0.00
D
ATOM  24911  CG2 ILE D  97      11.930  33.949  69.128  0.00  0.00
D
ATOM  24912 HG21 ILE D  97      12.162  32.892  68.876  0.00  0.00
D
ATOM  24913 HG22 ILE D  97      11.915  34.533  68.183  0.00  0.00
D
ATOM  24914 HG23 ILE D  97      12.808  34.345  69.680  0.00  0.00
D
ATOM  24915  CG1 ILE D  97      10.671  33.051  71.087  0.00  0.00
D
ATOM  24916 HG11 ILE D  97      10.920  32.091  70.586  0.00  0.00
D
ATOM  24917 HG12 ILE D  97      11.586  33.194  71.701  0.00  0.00
D
ATOM  24918  CD  ILE D  97       9.432  32.874  72.026  0.00  0.00
D
ATOM  24919  HD1 ILE D  97       8.884  33.802  72.296  0.00  0.00
D
ATOM  24920  HD2 ILE D  97       8.700  32.261  71.458  0.00  0.00
D
```

ATOM 24921 HD3 ILE D 97 9.689 32.313 72.951 0.00 0.00
D
ATOM 24922 C ILE D 97 10.911 36.677 69.621 0.00 0.00
D
ATOM 24923 O ILE D 97 10.407 36.979 68.552 0.00 0.00
D
ATOM 24924 N PRO D 98 12.046 37.293 70.009 0.00 0.00
D
ATOM 24925 CD PRO D 98 12.613 37.122 71.381 0.00 0.00
D
ATOM 24926 HD1 PRO D 98 11.808 37.270 72.132 0.00 0.00
D
ATOM 24927 HD2 PRO D 98 13.032 36.120 71.613 0.00 0.00
D
ATOM 24928 CA PRO D 98 12.598 38.469 69.328 0.00 0.00
D
ATOM 24929 HA PRO D 98 11.947 39.325 69.429 0.00 0.00
D
ATOM 24930 CB PRO D 98 13.874 38.703 70.145 0.00 0.00
D
ATOM 24931 HB1 PRO D 98 13.867 39.811 70.236 0.00 0.00
D
ATOM 24932 HB2 PRO D 98 14.823 38.416 69.643 0.00 0.00
D
ATOM 24933 CG PRO D 98 13.676 38.176 71.549 0.00 0.00
D
ATOM 24934 HG1 PRO D 98 14.600 37.755 72.000 0.00 0.00
D
ATOM 24935 HG2 PRO D 98 13.314 38.964 72.244 0.00 0.00
D
ATOM 24936 C PRO D 98 12.887 38.309 67.853 0.00 0.00
D
ATOM 24937 O PRO D 98 12.453 39.179 67.114 0.00 0.00
D
ATOM 24938 N ASN D 99 13.474 37.189 67.376 0.00 0.00
D
ATOM 24939 HN ASN D 99 13.822 36.629 68.124 0.00 0.00
D
ATOM 24940 CA ASN D 99 13.668 36.962 65.984 0.00 0.00
D
ATOM 24941 HA ASN D 99 12.892 37.448 65.411 0.00 0.00
D
ATOM 24942 CB ASN D 99 15.040 37.528 65.514 0.00 0.00
D
ATOM 24943 HB1 ASN D 99 15.877 36.946 65.955 0.00 0.00
D
ATOM 24944 HB2 ASN D 99 14.923 37.552 64.410 0.00 0.00
D
ATOM 24945 CG ASN D 99 15.370 38.998 65.857 0.00 0.00
D
ATOM 24946 OD1 ASN D 99 14.986 39.970 65.158 0.00 0.00
D
ATOM 24947 ND2 ASN D 99 15.989 39.187 67.086 0.00 0.00
D

ATOM 24948 HD21 ASN D 99   16.290 40.119 67.287 0.00 0.00
D
ATOM 24949 HD22 ASN D 99   16.082 38.372 67.658 0.00 0.00
D
ATOM 24950 C   ASN D 99   13.638 35.465 65.572 0.00 0.00
D
ATOM 24951 O   ASN D 99   14.343 34.603 66.128 0.00 0.00
D
ATOM 24952 N   ILE D 100   12.856 35.037 64.529 0.00 0.00
D
ATOM 24953 HN ILE D 100   12.263 35.626 63.985 0.00 0.00
D
ATOM 24954 CA ILE D 100   12.889 33.588 64.194 0.00 0.00
D
ATOM 24955 HA ILE D 100   13.621 33.072 64.796 0.00 0.00
D
ATOM 24956 CB ILE D 100   11.472 33.024 64.300 0.00 0.00
D
ATOM 24957 HB ILE D 100   10.854 33.554 63.545 0.00 0.00
D
ATOM 24958 CG2 ILE D 100   11.333 31.503 64.115 0.00 0.00
D
ATOM 24959 HG21 ILE D 100   10.289 31.124 64.078 0.00 0.00
D
ATOM 24960 HG22 ILE D 100   11.711 31.257 63.099 0.00 0.00
D
ATOM 24961 HG23 ILE D 100   11.827 30.960 64.948 0.00 0.00
D
ATOM 24962 CG1 ILE D 100   10.843 33.356 65.663 0.00 0.00
D
ATOM 24963 HG11 ILE D 100   11.360 32.562 66.245 0.00 0.00
D
ATOM 24964 HG12 ILE D 100   11.085 34.351 66.092 0.00 0.00
D
ATOM 24965 CD ILE D 100   9.236 33.210 65.777 0.00 0.00
D
ATOM 24966 HD1 ILE D 100   8.778 34.058 65.224 0.00 0.00
D
ATOM 24967 HD2 ILE D 100   8.844 32.274 65.325 0.00 0.00
D
ATOM 24968 HD3 ILE D 100   8.959 33.339 66.845 0.00 0.00
D
ATOM 24969 C   ILE D 100   13.354 33.461 62.720 0.00 0.00
D
ATOM 24970 O   ILE D 100   12.766 34.107 61.842 0.00 0.00
D
ATOM 24971 N   TRP D 101   14.251 32.555 62.436 0.00 0.00
D
ATOM 24972 HN TRP D 101   14.624 31.945 63.131 0.00 0.00
D
ATOM 24973 CA TRP D 101   14.630 32.105 61.109 0.00 0.00
D
ATOM 24974 HA TRP D 101   14.112 32.673 60.351 0.00 0.00
D

ATOM 24975 CB TRP D 101 16.144 32.395 61.014 0.00 0.00
D
ATOM 24976 HB1 TRP D 101 16.680 31.730 61.725 0.00 0.00
D
ATOM 24977 HB2 TRP D 101 16.512 32.077 60.016 0.00 0.00
D
ATOM 24978 CG TRP D 101 16.696 33.814 61.119 0.00 0.00
D
ATOM 24979 CD1 TRP D 101 16.067 35.001 61.397 0.00 0.00
D
ATOM 24980 HD1 TRP D 101 15.087 35.178 61.814 0.00 0.00
D
ATOM 24981 NE1 TRP D 101 17.095 36.016 61.257 0.00 0.00
D
ATOM 24982 HE1 TRP D 101 16.882 36.936 61.503 0.00 0.00
D
ATOM 24983 CE2 TRP D 101 18.371 35.484 60.882 0.00 0.00
D
ATOM 24984 CD2 TRP D 101 18.134 34.076 60.814 0.00 0.00
D
ATOM 24985 CE3 TRP D 101 19.166 33.290 60.399 0.00 0.00
D
ATOM 24986 HE3 TRP D 101 19.013 32.223 60.344 0.00 0.00
D
ATOM 24987 CZ3 TRP D 101 20.446 33.837 60.043 0.00 0.00
D
ATOM 24988 HZ3 TRP D 101 21.229 33.213 59.638 0.00 0.00
D
ATOM 24989 CZ2 TRP D 101 19.616 36.024 60.690 0.00 0.00
D
ATOM 24990 HZ2 TRP D 101 19.818 37.024 61.043 0.00 0.00
D
ATOM 24991 CH2 TRP D 101 20.679 35.261 60.299 0.00 0.00
D
ATOM 24992 HH2 TRP D 101 21.652 35.729 60.304 0.00 0.00
D
ATOM 24993 C TRP D 101 14.301 30.663 60.918 0.00 0.00
D
ATOM 24994 O TRP D 101 14.437 29.928 61.869 0.00 0.00
D
ATOM 24995 N VAL D 102 13.813 30.191 59.721 0.00 0.00
D
ATOM 24996 HN VAL D 102 13.828 30.723 58.878 0.00 0.00
D
ATOM 24997 CA VAL D 102 13.154 28.871 59.635 0.00 0.00
D
ATOM 24998 HA VAL D 102 13.484 28.236 60.443 0.00 0.00
D
ATOM 24999 CB VAL D 102 11.617 28.980 59.531 0.00 0.00
D
ATOM 25000 HB VAL D 102 11.324 29.101 58.466 0.00 0.00
D
ATOM 25001 CG1 VAL D 102 10.900 27.697 59.983 0.00 0.00
D

ATOM 25002 HG11 VAL D 102   9.915 27.727 59.470 0.00 0.00
D
ATOM 25003 HG12 VAL D 102   11.446 26.802 59.616 0.00 0.00
D
ATOM 25004 HG13 VAL D 102   10.859 27.659 61.093 0.00 0.00
D
ATOM 25005 CG2 VAL D 102   11.023 30.177 60.367 0.00 0.00
D
ATOM 25006 HG21 VAL D 102   11.411 31.094 59.875 0.00 0.00
D
ATOM 25007 HG22 VAL D 102   9.915 30.234 60.431 0.00 0.00
D
ATOM 25008 HG23 VAL D 102   11.424 29.996 61.387 0.00 0.00
D
ATOM 25009 C   VAL D 102   13.639 28.209 58.413 0.00 0.00
D
ATOM 25010 O   VAL D 102   13.759 28.817 57.355 0.00 0.00
D
ATOM 25011 N   LYS D 103   13.990 26.908 58.434 0.00 0.00
D
ATOM 25012 HN LYS D 103   13.848 26.529 59.345 0.00 0.00
D
ATOM 25013 CA LYS D 103   14.231 26.029 57.226 0.00 0.00
D
ATOM 25014 HA LYS D 103   14.879 26.633 56.609 0.00 0.00
D
ATOM 25015 CB LYS D 103   14.962 24.823 57.721 0.00 0.00
D
ATOM 25016 HB1 LYS D 103   15.805 25.146 58.369 0.00 0.00
D
ATOM 25017 HB2 LYS D 103   14.298 24.238 58.393 0.00 0.00
D
ATOM 25018 CG LYS D 103   15.434 23.940 56.517 0.00 0.00
D
ATOM 25019 HG1 LYS D 103   14.639 23.764 55.761 0.00 0.00
D
ATOM 25020 HG2 LYS D 103   16.338 24.430 56.097 0.00 0.00
D
ATOM 25021 CD LYS D 103   15.780 22.499 57.072 0.00 0.00
D
ATOM 25022 HD1 LYS D 103   16.599 22.583 57.818 0.00 0.00
D
ATOM 25023 HD2 LYS D 103   14.925 22.123 57.674 0.00 0.00
D
ATOM 25024 CE LYS D 103   16.102 21.437 55.999 0.00 0.00
D
ATOM 25025 HE1 LYS D 103   15.329 21.454 55.201 0.00 0.00
D
ATOM 25026 HE2 LYS D 103   17.105 21.767 55.654 0.00 0.00
D
ATOM 25027 NZ LYS D 103   16.102 19.973 56.404 0.00 0.00
D
ATOM 25028 HZ1 LYS D 103   15.128 19.614 56.335 0.00 0.00
D

ATOM 25029 HZ2 LYS D 103 16.703 19.389 55.788 0.00 0.00
D
ATOM 25030 HZ3 LYS D 103 16.230 19.666 57.389 0.00 0.00
D
ATOM 25031 C LYS D 103 12.950 25.683 56.413 0.00 0.00
D
ATOM 25032 O LYS D 103 11.982 25.219 56.973 0.00 0.00
D
ATOM 25033 N ALA D 104 12.999 26.031 55.124 0.00 0.00
D
ATOM 25034 HN ALA D 104 13.779 26.433 54.650 0.00 0.00
D
ATOM 25035 CA ALA D 104 11.842 25.857 54.282 0.00 0.00
D
ATOM 25036 HA ALA D 104 11.056 25.454 54.904 0.00 0.00
D
ATOM 25037 CB ALA D 104 11.328 27.202 53.865 0.00 0.00
D
ATOM 25038 HB1 ALA D 104 11.322 27.857 54.762 0.00 0.00
D
ATOM 25039 HB2 ALA D 104 12.029 27.745 53.196 0.00 0.00
D
ATOM 25040 HB3 ALA D 104 10.273 27.180 53.517 0.00 0.00
D
ATOM 25041 C ALA D 104 12.056 24.892 53.066 0.00 0.00
D
ATOM 25042 O ALA D 104 13.172 24.852 52.574 0.00 0.00
D
ATOM 25043 N GLN D 105 11.100 24.054 52.674 0.00 0.00
D
ATOM 25044 HN GLN D 105 10.239 24.005 53.173 0.00 0.00
D
ATOM 25045 CA GLN D 105 11.350 22.997 51.783 0.00 0.00
D
ATOM 25046 HA GLN D 105 12.340 22.892 51.365 0.00 0.00
D
ATOM 25047 CB GLN D 105 11.184 21.584 52.540 0.00 0.00
D
ATOM 25048 HB1 GLN D 105 10.103 21.571 52.796 0.00 0.00
D
ATOM 25049 HB2 GLN D 105 11.395 20.782 51.802 0.00 0.00
D
ATOM 25050 CG GLN D 105 12.009 21.284 53.780 0.00 0.00
D
ATOM 25051 HG1 GLN D 105 12.067 22.157 54.465 0.00 0.00
D
ATOM 25052 HG2 GLN D 105 11.538 20.444 54.334 0.00 0.00
D
ATOM 25053 CD GLN D 105 13.453 21.024 53.313 0.00 0.00
D
ATOM 25054 OE1 GLN D 105 14.328 21.921 53.357 0.00 0.00
D
ATOM 25055 NE2 GLN D 105 13.654 19.859 52.694 0.00 0.00
D

ATOM 25056 HE21 GLN D 105   14.565 19.559 52.412 0.00 0.00
D
ATOM 25057 HE22 GLN D 105   12.849 19.357 52.379 0.00 0.00
D
ATOM 25058 C   GLN D 105   10.371 22.866 50.588 0.00 0.00
D
ATOM 25059 O   GLN D 105   10.401 21.911 49.784 0.00 0.00
D
ATOM 25060 N   ASN D 106   9.492 23.820 50.439 0.00 0.00
D
ATOM 25061 HN ASN D 106   9.365 24.456 51.196 0.00 0.00
D
ATOM 25062 CA ASN D 106   8.548 23.754 49.368 0.00 0.00
D
ATOM 25063 HA ASN D 106   9.096 23.638 48.445 0.00 0.00
D
ATOM 25064 CB ASN D 106   7.573 22.540 49.355 0.00 0.00
D
ATOM 25065 HB1 ASN D 106   6.755 22.626 48.608 0.00 0.00
D
ATOM 25066 HB2 ASN D 106   8.215 21.660 49.140 0.00 0.00
D
ATOM 25067 CG ASN D 106   6.839 22.338 50.635 0.00 0.00
D
ATOM 25068 OD1 ASN D 106   5.855 23.039 50.816 0.00 0.00
D
ATOM 25069 ND2 ASN D 106   7.388 21.447 51.467 0.00 0.00
D
ATOM 25070 HD21 ASN D 106   6.937 21.218 52.329 0.00 0.00
D
ATOM 25071 HD22 ASN D 106   8.029 20.825 51.018 0.00 0.00
D
ATOM 25072 C   ASN D 106   7.867 25.077 49.172 0.00 0.00
D
ATOM 25073 O   ASN D 106   7.803 25.879 50.097 0.00 0.00
D
ATOM 25074 N   TYR D 107   7.246 25.335 47.979 0.00 0.00
D
ATOM 25075 HN TYR D 107   7.427 24.667 47.261 0.00 0.00
D
ATOM 25076 CA TYR D 107   6.345 26.453 47.578 0.00 0.00
D
ATOM 25077 HA TYR D 107   7.044 27.262 47.420 0.00 0.00
D
ATOM 25078 CB TYR D 107   5.590 26.206 46.234 0.00 0.00
D
ATOM 25079 HB1 TYR D 107   4.919 25.332 46.374 0.00 0.00
D
ATOM 25080 HB2 TYR D 107   4.864 27.004 45.968 0.00 0.00
D
ATOM 25081 CG TYR D 107   6.489 25.913 45.049 0.00 0.00
D
ATOM 25082 CD1 TYR D 107   7.663 26.622 44.802 0.00 0.00
D

ATOM 25083 HD1 TYR D 107 8.068 27.424 45.400 0.00 0.00
D
ATOM 25084 CE1 TYR D 107 8.414 26.205 43.643 0.00 0.00
D
ATOM 25085 HE1 TYR D 107 9.300 26.764 43.380 0.00 0.00
D
ATOM 25086 CZ TYR D 107 7.902 25.234 42.775 0.00 0.00
D
ATOM 25087 OH TYR D 107 8.671 24.702 41.693 0.00 0.00
D
ATOM 25088 HH TYR D 107 8.402 23.804 41.485 0.00 0.00
D
ATOM 25089 CD2 TYR D 107 6.016 24.904 44.156 0.00 0.00
D
ATOM 25090 HD2 TYR D 107 5.128 24.326 44.367 0.00 0.00
D
ATOM 25091 CE2 TYR D 107 6.721 24.550 43.040 0.00 0.00
D
ATOM 25092 HE2 TYR D 107 6.393 23.771 42.368 0.00 0.00
D
ATOM 25093 C TYR D 107 5.286 26.790 48.624 0.00 0.00
D
ATOM 25094 O TYR D 107 5.119 27.916 49.062 0.00 0.00
D
ATOM 25095 N TYR D 108 4.531 25.778 49.026 0.00 0.00
D
ATOM 25096 HN TYR D 108 4.830 24.881 48.711 0.00 0.00
D
ATOM 25097 CA TYR D 108 3.563 25.908 50.104 0.00 0.00
D
ATOM 25098 HA TYR D 108 2.887 26.736 49.948 0.00 0.00
D
ATOM 25099 CB TYR D 108 2.702 24.588 50.252 0.00 0.00
D
ATOM 25100 HB1 TYR D 108 2.171 24.500 49.279 0.00 0.00
D
ATOM 25101 HB2 TYR D 108 3.375 23.705 50.226 0.00 0.00
D
ATOM 25102 CG TYR D 108 1.656 24.680 51.348 0.00 0.00
D
ATOM 25103 CD1 TYR D 108 0.778 25.728 51.397 0.00 0.00
D
ATOM 25104 HD1 TYR D 108 0.649 26.528 50.683 0.00 0.00
D
ATOM 25105 CE1 TYR D 108 −0.185 25.764 52.431 0.00 0.00
D
ATOM 25106 HE1 TYR D 108 −0.877 26.590 52.493 0.00 0.00
D
ATOM 25107 CZ TYR D 108 −0.276 24.647 53.301 0.00 0.00
D
ATOM 25108 OH TYR D 108 −1.332 24.652 54.308 0.00 0.00
D
ATOM 25109 HH TYR D 108 −2.006 25.286 54.053 0.00 0.00
D

ATOM 25110 CD2 TYR D 108 1.527 23.528 52.237 0.00 0.00
D
ATOM 25111 HD2 TYR D 108 2.302 22.778 52.192 0.00 0.00
D
ATOM 25112 CE2 TYR D 108 0.591 23.540 53.226 0.00 0.00
D
ATOM 25113 HE2 TYR D 108 0.523 22.783 53.993 0.00 0.00
D
ATOM 25114 C TYR D 108 4.103 26.236 51.435 0.00 0.00
D
ATOM 25115 O TYR D 108 3.632 27.164 52.120 0.00 0.00
D
ATOM 25116 N HSE D 109 5.129 25.482 51.928 0.00 0.00
D
ATOM 25117 HN HSE D 109 5.538 24.733 51.413 0.00 0.00
D
ATOM 25118 CA HSE D 109 5.648 25.678 53.243 0.00 0.00
D
ATOM 25119 HA HSE D 109 4.848 25.507 53.948 0.00 0.00
D
ATOM 25120 CB HSE D 109 6.748 24.619 53.291 0.00 0.00
D
ATOM 25121 HB1 HSE D 109 6.238 23.633 53.272 0.00 0.00
D
ATOM 25122 HB2 HSE D 109 7.411 24.592 52.400 0.00 0.00
D
ATOM 25123 ND1 HSE D 109 8.821 23.944 54.578 0.00 0.00
D
ATOM 25124 CG HSE D 109 7.624 24.625 54.595 0.00 0.00
D
ATOM 25125 CE1 HSE D 109 9.258 24.000 55.825 0.00 0.00
D
ATOM 25126 HE1 HSE D 109 10.145 23.573 56.293 0.00 0.00
D
ATOM 25127 NE2 HSE D 109 8.356 24.667 56.618 0.00 0.00
D
ATOM 25128 HE2 HSE D 109 8.301 24.715 57.616 0.00 0.00
D
ATOM 25129 CD2 HSE D 109 7.381 25.159 55.774 0.00 0.00
D
ATOM 25130 HD2 HSE D 109 6.594 25.865 56.008 0.00 0.00
D
ATOM 25131 C HSE D 109 6.295 27.005 53.448 0.00 0.00
D
ATOM 25132 O HSE D 109 6.051 27.668 54.403 0.00 0.00
D
ATOM 25133 N HSE D 110 7.014 27.492 52.362 0.00 0.00
D
ATOM 25134 HN HSE D 110 7.122 26.824 51.630 0.00 0.00
D
ATOM 25135 CA HSE D 110 7.633 28.836 52.216 0.00 0.00
D
ATOM 25136 HA HSE D 110 8.289 28.998 53.058 0.00 0.00
D

ATOM 25137 CB HSE D 110   8.383 28.997 50.915 0.00 0.00
D
ATOM 25138 HB1 HSE D 110   9.115 28.193 50.689 0.00 0.00
D
ATOM 25139 HB2 HSE D 110   7.642 28.851 50.100 0.00 0.00
D
ATOM 25140 ND1 HSE D 110   9.765 30.499 49.475 0.00 0.00
D
ATOM 25141 CG HSE D 110   9.177 30.226 50.703 0.00 0.00
D
ATOM 25142 CE1 HSE D 110   10.288 31.673 49.654 0.00 0.00
D
ATOM 25143 HE1 HSE D 110   10.541 32.230 48.752 0.00 0.00
D
ATOM 25144 NE2 HSE D 110   9.969 32.239 50.816 0.00 0.00
D
ATOM 25145 HE2 HSE D 110   10.090 33.154 51.201 0.00 0.00
D
ATOM 25146 CD2 HSE D 110   9.336 31.253 51.533 0.00 0.00
D
ATOM 25147 HD2 HSE D 110   8.993 31.399 52.550 0.00 0.00
D
ATOM 25148 C   HSE D 110   6.571 29.884 52.218 0.00 0.00
D
ATOM 25149 O   HSE D 110   6.585 30.900 52.979 0.00 0.00
D
ATOM 25150 N   LYS D 111   5.455 29.650 51.514 0.00 0.00
D
ATOM 25151 HN LYS D 111   5.333 28.842 50.942 0.00 0.00
D
ATOM 25152 CA LYS D 111   4.260 30.493 51.526 0.00 0.00
D
ATOM 25153 HA LYS D 111   4.543 31.474 51.172 0.00 0.00
D
ATOM 25154 CB LYS D 111   3.273 30.006 50.537 0.00 0.00
D
ATOM 25155 HB1 LYS D 111   3.737 29.880 49.535 0.00 0.00
D
ATOM 25156 HB2 LYS D 111   2.859 28.999 50.757 0.00 0.00
D
ATOM 25157 CG LYS D 111   2.081 30.987 50.390 0.00 0.00
D
ATOM 25158 HG1 LYS D 111   1.458 30.575 49.568 0.00 0.00
D
ATOM 25159 HG2 LYS D 111   1.455 30.801 51.289 0.00 0.00
D
ATOM 25160 CD LYS D 111   2.452 32.422 50.208 0.00 0.00
D
ATOM 25161 HD1 LYS D 111   1.549 33.068 50.173 0.00 0.00
D
ATOM 25162 HD2 LYS D 111   3.232 32.804 50.901 0.00 0.00
D
ATOM 25163 CE LYS D 111   2.948 32.649 48.769 0.00 0.00
D

ATOM 25164 HE1 LYS D 111   3.786 31.927 48.669 0.00 0.00
D
ATOM 25165 HE2 LYS D 111   2.096 32.426 48.093 0.00 0.00
D
ATOM 25166 NZ LYS D 111   3.552 33.973 48.498 0.00 0.00
D
ATOM 25167 HZ1 LYS D 111   4.058 34.046 47.593 0.00 0.00
D
ATOM 25168 HZ2 LYS D 111   2.864 34.752 48.459 0.00 0.00
D
ATOM 25169 HZ3 LYS D 111   4.256 34.129 49.248 0.00 0.00
D
ATOM 25170 C   LYS D 111   3.588 30.714 52.816 0.00 0.00
D
ATOM 25171 O   LYS D 111   3.174 31.812 53.187 0.00 0.00
D
ATOM 25172 N   VAL D 112   3.426 29.609 53.640 0.00 0.00
D
ATOM 25173 HN VAL D 112   3.753 28.714 53.348 0.00 0.00
D
ATOM 25174 CA VAL D 112   2.800 29.830 54.992 0.00 0.00
D
ATOM 25175 HA VAL D 112   1.949 30.481 54.860 0.00 0.00
D
ATOM 25176 CB VAL D 112   2.216 28.512 55.505 0.00 0.00
D
ATOM 25177 HB VAL D 112   2.984 27.713 55.427 0.00 0.00
D
ATOM 25178 CG1 VAL D 112   1.617 28.575 56.904 0.00 0.00
D
ATOM 25179 HG11 VAL D 112   0.873 27.757 57.008 0.00 0.00
D
ATOM 25180 HG12 VAL D 112   2.329 28.403 57.739 0.00 0.00
D
ATOM 25181 HG13 VAL D 112   1.036 29.517 56.999 0.00 0.00
D
ATOM 25182 CG2 VAL D 112   1.100 28.030 54.571 0.00 0.00
D
ATOM 25183 HG21 VAL D 112   1.422 27.998 53.508 0.00 0.00
D
ATOM 25184 HG22 VAL D 112   0.888 26.977 54.855 0.00 0.00
D
ATOM 25185 HG23 VAL D 112   0.192 28.661 54.673 0.00 0.00
D
ATOM 25186 C   VAL D 112   3.737 30.569 55.965 0.00 0.00
D
ATOM 25187 O   VAL D 112   3.235 31.352 56.745 0.00 0.00
D
ATOM 25188 N   LEU D 113   5.091 30.338 55.874 0.00 0.00
D
ATOM 25189 HN LEU D 113   5.367 29.715 55.146 0.00 0.00
D
ATOM 25190 CA LEU D 113   5.996 30.991 56.717 0.00 0.00
D

```
ATOM  25191  HA  LEU D 113       5.828  30.865  57.776  0.00  0.00
D
ATOM  25192  CB  LEU D 113       7.396  30.461  56.548  0.00  0.00
D
ATOM  25193  HB1 LEU D 113       7.707  30.339  55.489  0.00  0.00
D
ATOM  25194  HB2 LEU D 113       8.122  31.021  57.175  0.00  0.00
D
ATOM  25195  CG  LEU D 113       7.569  28.991  56.997  0.00  0.00
D
ATOM  25196  HG  LEU D 113       6.773  28.376  56.524  0.00  0.00
D
ATOM  25197  CD1 LEU D 113       8.910  28.309  56.688  0.00  0.00
D
ATOM  25198 HD11 LEU D 113       8.928  27.307  57.168  0.00  0.00
D
ATOM  25199 HD12 LEU D 113       8.957  28.044  55.611  0.00  0.00
D
ATOM  25200 HD13 LEU D 113       9.805  28.872  57.031  0.00  0.00
D
ATOM  25201  CD2 LEU D 113       7.271  28.772  58.522  0.00  0.00
D
ATOM  25202 HD21 LEU D 113       6.195  28.853  58.788  0.00  0.00
D
ATOM  25203 HD22 LEU D 113       7.619  27.770  58.853  0.00  0.00
D
ATOM  25204 HD23 LEU D 113       7.897  29.483  59.102  0.00  0.00
D
ATOM  25205  C   LEU D 113       6.103  32.497  56.507  0.00  0.00
D
ATOM  25206  O   LEU D 113       6.244  33.321  57.392  0.00  0.00
D
ATOM  25207  N   GLU D 114       5.930  32.875  55.238  0.00  0.00
D
ATOM  25208  HN  GLU D 114       5.905  32.120  54.587  0.00  0.00
D
ATOM  25209  CA  GLU D 114       5.792  34.221  54.800  0.00  0.00
D
ATOM  25210  HA  GLU D 114       6.563  34.819  55.262  0.00  0.00
D
ATOM  25211  CB  GLU D 114       5.860  34.247  53.250  0.00  0.00
D
ATOM  25212  HB1 GLU D 114       6.879  33.999  52.883  0.00  0.00
D
ATOM  25213  HB2 GLU D 114       5.246  33.432  52.808  0.00  0.00
D
ATOM  25214  CG  GLU D 114       5.568  35.568  52.544  0.00  0.00
D
ATOM  25215  HG1 GLU D 114       4.574  35.942  52.871  0.00  0.00
D
ATOM  25216  HG2 GLU D 114       6.409  36.282  52.679  0.00  0.00
D
ATOM  25217  CD  GLU D 114       5.463  35.593  50.931  0.00  0.00
D
```

ATOM 25218 OE1 GLU D 114 5.354 36.737 50.402 0.00 0.00
D
ATOM 25219 OE2 GLU D 114 5.445 34.514 50.281 0.00 0.00
D
ATOM 25220 C GLU D 114 4.450 34.784 55.325 0.00 0.00
D
ATOM 25221 O GLU D 114 4.531 35.945 55.745 0.00 0.00
D
ATOM 25222 N LYS D 115 3.322 34.088 55.356 0.00 0.00
D
ATOM 25223 HN LYS D 115 3.259 33.367 54.670 0.00 0.00
D
ATOM 25224 CA LYS D 115 2.124 34.690 55.731 0.00 0.00
D
ATOM 25225 HA LYS D 115 2.171 35.720 55.410 0.00 0.00
D
ATOM 25226 CB LYS D 115 0.981 34.020 54.903 0.00 0.00
D
ATOM 25227 HB1 LYS D 115 1.074 32.932 55.107 0.00 0.00
D
ATOM 25228 HB2 LYS D 115 -0.039 34.268 55.265 0.00 0.00
D
ATOM 25229 CG LYS D 115 0.976 34.288 53.387 0.00 0.00
D
ATOM 25230 HG1 LYS D 115 1.977 34.204 52.911 0.00 0.00
D
ATOM 25231 HG2 LYS D 115 0.311 33.489 52.996 0.00 0.00
D
ATOM 25232 CD LYS D 115 0.341 35.685 53.035 0.00 0.00
D
ATOM 25233 HD1 LYS D 115 -0.727 35.679 53.341 0.00 0.00
D
ATOM 25234 HD2 LYS D 115 0.949 36.505 53.476 0.00 0.00
D
ATOM 25235 CE LYS D 115 0.233 36.133 51.549 0.00 0.00
D
ATOM 25236 HE1 LYS D 115 1.060 35.744 50.917 0.00 0.00
D
ATOM 25237 HE2 LYS D 115 -0.686 35.648 51.157 0.00 0.00
D
ATOM 25238 NZ LYS D 115 0.120 37.566 51.301 0.00 0.00
D
ATOM 25239 HZ1 LYS D 115 0.997 38.015 51.633 0.00 0.00
D
ATOM 25240 HZ2 LYS D 115 0.072 37.726 50.275 0.00 0.00
D
ATOM 25241 HZ3 LYS D 115 -0.591 38.155 51.781 0.00 0.00
D
ATOM 25242 C LYS D 115 1.858 34.771 57.264 0.00 0.00
D
ATOM 25243 O LYS D 115 1.098 35.573 57.751 0.00 0.00
D
ATOM 25244 N ILE D 116 2.606 34.038 58.029 0.00 0.00
D

ATOM 25245 HN ILE D 116   3.221 33.413 57.553 0.00 0.00
D
ATOM 25246 CA ILE D 116   2.586 33.957 59.486 0.00 0.00
D
ATOM 25247 HA ILE D 116   1.653 34.411 59.789 0.00 0.00
D
ATOM 25248 CB ILE D 116   2.438 32.532 60.175 0.00 0.00
D
ATOM 25249 HB ILE D 116   2.347 32.796 61.250 0.00 0.00
D
ATOM 25250 CG2 ILE D 116   1.111 31.817 59.736 0.00 0.00
D
ATOM 25251 HG21 ILE D 116   0.238 32.499 59.826 0.00 0.00
D
ATOM 25252 HG22 ILE D 116   1.215 31.698 58.636 0.00 0.00
D
ATOM 25253 HG23 ILE D 116   0.984 30.834 60.237 0.00 0.00
D
ATOM 25254 CG1 ILE D 116   3.634 31.719 60.065 0.00 0.00
D
ATOM 25255 HG11 ILE D 116   4.298 31.922 59.197 0.00 0.00
D
ATOM 25256 HG12 ILE D 116   4.313 32.025 60.889 0.00 0.00
D
ATOM 25257 CD ILE D 116   3.462 30.276 60.274 0.00 0.00
D
ATOM 25258 HD1 ILE D 116   4.450 29.826 60.508 0.00 0.00
D
ATOM 25259 HD2 ILE D 116   2.744 30.066 61.095 0.00 0.00
D
ATOM 25260 HD3 ILE D 116   3.154 29.772 59.333 0.00 0.00
D
ATOM 25261 C   ILE D 116   3.681 34.844 60.037 0.00 0.00
D
ATOM 25262 O   ILE D 116   3.955 34.817 61.223 0.00 0.00
D
ATOM 25263 N   GLY D 117   4.383 35.619 59.112 0.00 0.00
D
ATOM 25264 HN GLY D 117   4.123 35.607 58.150 0.00 0.00
D
ATOM 25265 CA GLY D 117   5.504 36.494 59.470 0.00 0.00
D
ATOM 25266 HA1 GLY D 117   5.113 37.306 60.064 0.00 0.00
D
ATOM 25267 HA2 GLY D 117   5.937 36.832 58.540 0.00 0.00
D
ATOM 25268 C   GLY D 117   6.667 35.874 60.252 0.00 0.00
D
ATOM 25269 O   GLY D 117   7.112 36.492 61.172 0.00 0.00
D
ATOM 25270 N   ALA D 118   7.238 34.772 59.815 0.00 0.00
D
ATOM 25271 HN ALA D 118   6.987 34.286 58.981 0.00 0.00
D

ATOM 25272 CA ALA D 118 8.578 34.476 60.227 0.00 0.00
D
ATOM 25273 HA ALA D 118 8.769 34.599 61.283 0.00 0.00
D
ATOM 25274 CB ALA D 118 8.967 33.101 59.746 0.00 0.00
D
ATOM 25275 HB1 ALA D 118 8.286 32.320 60.146 0.00 0.00
D
ATOM 25276 HB2 ALA D 118 8.916 33.060 58.636 0.00 0.00
D
ATOM 25277 HB3 ALA D 118 10.032 32.881 59.972 0.00 0.00
D
ATOM 25278 C ALA D 118 9.606 35.464 59.552 0.00 0.00
D
ATOM 25279 O ALA D 118 9.370 35.880 58.443 0.00 0.00
D
ATOM 25280 N ASP D 119 10.702 35.837 60.258 0.00 0.00
D
ATOM 25281 HN ASP D 119 11.152 35.331 60.990 0.00 0.00
D
ATOM 25282 CA ASP D 119 11.516 36.939 59.858 0.00 0.00
D
ATOM 25283 HA ASP D 119 10.834 37.756 59.678 0.00 0.00
D
ATOM 25284 CB ASP D 119 12.631 37.477 60.940 0.00 0.00
D
ATOM 25285 HB1 ASP D 119 13.325 36.626 61.109 0.00 0.00
D
ATOM 25286 HB2 ASP D 119 13.117 38.380 60.510 0.00 0.00
D
ATOM 25287 CG ASP D 119 11.963 37.910 62.242 0.00 0.00
D
ATOM 25288 OD1 ASP D 119 11.697 37.047 63.132 0.00 0.00
D
ATOM 25289 OD2 ASP D 119 11.666 39.115 62.428 0.00 0.00
D
ATOM 25290 C ASP D 119 12.430 36.626 58.632 0.00 0.00
D
ATOM 25291 O ASP D 119 12.617 37.427 57.740 0.00 0.00
D
ATOM 25292 N ARG D 120 13.046 35.430 58.576 0.00 0.00
D
ATOM 25293 HN ARG D 120 12.954 34.879 59.402 0.00 0.00
D
ATOM 25294 CA ARG D 120 13.973 34.968 57.564 0.00 0.00
D
ATOM 25295 HA ARG D 120 13.872 35.611 56.702 0.00 0.00
D
ATOM 25296 CB ARG D 120 15.493 34.984 58.112 0.00 0.00
D
ATOM 25297 HB1 ARG D 120 15.515 35.971 58.623 0.00 0.00
D
ATOM 25298 HB2 ARG D 120 15.670 34.174 58.852 0.00 0.00
D

ATOM 25299 CG ARG D 120   16.554 34.838 57.055 0.00 0.00
D
ATOM 25300 HG1 ARG D 120   16.629 33.854 56.544 0.00 0.00
D
ATOM 25301 HG2 ARG D 120   16.214 35.560 56.282 0.00 0.00
D
ATOM 25302 CD ARG D 120   17.937 35.225 57.396 0.00 0.00
D
ATOM 25303 HD1 ARG D 120   18.022 36.069 58.112 0.00 0.00
D
ATOM 25304 HD2 ARG D 120   18.511 34.330 57.718 0.00 0.00
D
ATOM 25305 NE ARG D 120   18.515 35.682 56.104 0.00 0.00
D
ATOM 25306 HE ARG D 120   18.031 35.463 55.256 0.00 0.00
D
ATOM 25307 CZ ARG D 120   19.713 36.252 55.963 0.00 0.00
D
ATOM 25308 NH1 ARG D 120   20.366 36.832 56.999 0.00 0.00
D
ATOM 25309 HH11 ARG D 120   21.106 37.405 56.647 0.00 0.00
D
ATOM 25310 HH12 ARG D 120   19.793 37.046 57.791 0.00 0.00
D
ATOM 25311 NH2 ARG D 120   20.238 36.423 54.769 0.00 0.00
D
ATOM 25312 HH21 ARG D 120   21.214 36.625 54.850 0.00 0.00
D
ATOM 25313 HH22 ARG D 120   19.708 36.068 54.000 0.00 0.00
D
ATOM 25314 C   ARG D 120   13.610 33.471 57.157 0.00 0.00
D
ATOM 25315 O   ARG D 120   13.382 32.591 57.963 0.00 0.00
D
ATOM 25316 N   ILE D 121   13.689 33.150 55.878 0.00 0.00
D
ATOM 25317 HN ILE D 121   13.927 33.903 55.269 0.00 0.00
D
ATOM 25318 CA ILE D 121   13.394 31.871 55.343 0.00 0.00
D
ATOM 25319 HA ILE D 121   12.960 31.268 56.126 0.00 0.00
D
ATOM 25320 CB ILE D 121   12.327 32.020 54.197 0.00 0.00
D
ATOM 25321 HB ILE D 121   12.797 32.708 53.462 0.00 0.00
D
ATOM 25322 CG2 ILE D 121   12.052 30.651 53.640 0.00 0.00
D
ATOM 25323 HG21 ILE D 121   12.989 30.169 53.289 0.00 0.00
D
ATOM 25324 HG22 ILE D 121   11.579 30.019 54.423 0.00 0.00
D
ATOM 25325 HG23 ILE D 121   11.264 30.671 52.857 0.00 0.00
D

ATOM 25326 CG1 ILE D 121   11.049 32.713 54.574 0.00 0.00
D
ATOM 25327 HG11 ILE D 121   11.352 33.777 54.673 0.00 0.00
D
ATOM 25328 HG12 ILE D 121   10.279 32.632 53.778 0.00 0.00
D
ATOM 25329 CD ILE D 121   10.430 32.242 55.888 0.00 0.00
D
ATOM 25330 HD1 ILE D 121   9.906 33.111 56.340 0.00 0.00
D
ATOM 25331 HD2 ILE D 121   9.815 31.336 55.701 0.00 0.00
D
ATOM 25332 HD3 ILE D 121   11.185 31.918 56.636 0.00 0.00
D
ATOM 25333 C   ILE D 121   14.750 31.332 54.844 0.00 0.00
D
ATOM 25334 O   ILE D 121   15.417 32.000 54.105 0.00 0.00
D
ATOM 25335 N   ILE D 122   15.246 30.201 55.297 0.00 0.00
D
ATOM 25336 HN ILE D 122   14.855 29.655 56.034 0.00 0.00
D
ATOM 25337 CA ILE D 122   16.467 29.556 54.701 0.00 0.00
D
ATOM 25338 HA ILE D 122   16.944 30.190 53.969 0.00 0.00
D
ATOM 25339 CB ILE D 122   17.605 29.145 55.622 0.00 0.00
D
ATOM 25340 HB ILE D 122   17.282 28.179 56.067 0.00 0.00
D
ATOM 25341 CG2 ILE D 122   18.908 28.843 54.871 0.00 0.00
D
ATOM 25342 HG21 ILE D 122   18.731 28.062 54.101 0.00 0.00
D
ATOM 25343 HG22 ILE D 122   19.496 29.695 54.467 0.00 0.00
D
ATOM 25344 HG23 ILE D 122   19.540 28.432 55.688 0.00 0.00
D
ATOM 25345 CG1 ILE D 122   17.681 30.066 56.866 0.00 0.00
D
ATOM 25346 HG11 ILE D 122   16.656 30.106 57.293 0.00 0.00
D
ATOM 25347 HG12 ILE D 122   18.298 29.463 57.565 0.00 0.00
D
ATOM 25348 CD ILE D 122   18.247 31.459 56.499 0.00 0.00
D
ATOM 25349 HD1 ILE D 122   18.332 32.166 57.352 0.00 0.00
D
ATOM 25350 HD2 ILE D 122   19.224 31.489 55.970 0.00 0.00
D
ATOM 25351 HD3 ILE D 122   17.521 31.894 55.780 0.00 0.00
D
ATOM 25352 C   ILE D 122   16.016 28.399 53.759 0.00 0.00
D

ATOM 25353 O ILE D 122 15.279 27.532 54.173 0.00 0.00
D
ATOM 25354 N HSE D 123 16.550 28.369 52.486 0.00 0.00
D
ATOM 25355 HN HSE D 123 17.161 29.091 52.168 0.00 0.00
D
ATOM 25356 CA HSE D 123 16.324 27.215 51.636 0.00 0.00
D
ATOM 25357 HA HSE D 123 15.618 26.633 52.209 0.00 0.00
D
ATOM 25358 CB HSE D 123 15.682 27.550 50.218 0.00 0.00
D
ATOM 25359 HB1 HSE D 123 16.208 28.355 49.661 0.00 0.00
D
ATOM 25360 HB2 HSE D 123 15.702 26.641 49.579 0.00 0.00
D
ATOM 25361 ND1 HSE D 123 13.242 27.407 50.034 0.00 0.00
D
ATOM 25362 CG HSE D 123 14.311 28.135 50.384 0.00 0.00
D
ATOM 25363 CE1 HSE D 123 12.220 28.206 50.107 0.00 0.00
D
ATOM 25364 HE1 HSE D 123 11.180 27.917 49.961 0.00 0.00
D
ATOM 25365 NE2 HSE D 123 12.604 29.458 50.311 0.00 0.00
D
ATOM 25366 HE2 HSE D 123 12.016 30.266 50.265 0.00 0.00
D
ATOM 25367 CD2 HSE D 123 13.928 29.436 50.549 0.00 0.00
D
ATOM 25368 HD2 HSE D 123 14.557 30.267 50.844 0.00 0.00
D
ATOM 25369 C HSE D 123 17.642 26.426 51.433 0.00 0.00
D
ATOM 25370 O HSE D 123 18.070 26.562 50.300 0.00 0.00
D
ATOM 25371 N PRO D 124 18.360 25.758 52.366 0.00 0.00
D
ATOM 25372 CD PRO D 124 17.897 25.415 53.661 0.00 0.00
D
ATOM 25373 HD1 PRO D 124 17.526 26.316 54.195 0.00 0.00
D
ATOM 25374 HD2 PRO D 124 17.068 24.679 53.579 0.00 0.00
D
ATOM 25375 CA PRO D 124 19.666 25.128 52.055 0.00 0.00
D
ATOM 25376 HA PRO D 124 20.418 25.892 52.181 0.00 0.00
D
ATOM 25377 CB PRO D 124 19.904 24.188 53.210 0.00 0.00
D
ATOM 25378 HB1 PRO D 124 20.969 23.917 53.376 0.00 0.00
D
ATOM 25379 HB2 PRO D 124 19.483 23.208 52.901 0.00 0.00
D

ATOM 25380 CG PRO D 124 19.163 24.761 54.384 0.00 0.00
D
ATOM 25381 HG1 PRO D 124 18.797 24.119 55.213 0.00 0.00
D
ATOM 25382 HG2 PRO D 124 19.749 25.578 54.858 0.00 0.00
D
ATOM 25383 C PRO D 124 19.960 24.582 50.664 0.00 0.00
D
ATOM 25384 O PRO D 124 20.689 25.180 49.875 0.00 0.00
D
ATOM 25385 N GLU D 125 19.332 23.401 50.258 0.00 0.00
D
ATOM 25386 HN GLU D 125 18.620 22.976 50.811 0.00 0.00
D
ATOM 25387 CA GLU D 125 19.583 22.682 49.040 0.00 0.00
D
ATOM 25388 HA GLU D 125 20.624 22.413 49.137 0.00 0.00
D
ATOM 25389 CB GLU D 125 18.653 21.439 49.077 0.00 0.00
D
ATOM 25390 HB1 GLU D 125 17.566 21.649 49.177 0.00 0.00
D
ATOM 25391 HB2 GLU D 125 18.772 20.969 48.078 0.00 0.00
D
ATOM 25392 CG GLU D 125 19.176 20.364 50.043 0.00 0.00
D
ATOM 25393 HG1 GLU D 125 19.932 19.722 49.543 0.00 0.00
D
ATOM 25394 HG2 GLU D 125 19.696 20.746 50.948 0.00 0.00
D
ATOM 25395 CD GLU D 125 18.011 19.435 50.295 0.00 0.00
D
ATOM 25396 OE1 GLU D 125 17.014 19.941 50.942 0.00 0.00
D
ATOM 25397 OE2 GLU D 125 18.030 18.201 49.998 0.00 0.00
D
ATOM 25398 C GLU D 125 19.320 23.481 47.791 0.00 0.00
D
ATOM 25399 O GLU D 125 20.170 23.532 46.904 0.00 0.00
D
ATOM 25400 N LYS D 126 18.241 24.226 47.673 0.00 0.00
D
ATOM 25401 HN LYS D 126 17.570 24.248 48.410 0.00 0.00
D
ATOM 25402 CA LYS D 126 18.022 25.146 46.562 0.00 0.00
D
ATOM 25403 HA LYS D 126 18.128 24.454 45.739 0.00 0.00
D
ATOM 25404 CB LYS D 126 16.546 25.542 46.667 0.00 0.00
D
ATOM 25405 HB1 LYS D 126 15.885 24.651 46.725 0.00 0.00
D
ATOM 25406 HB2 LYS D 126 16.284 26.150 47.560 0.00 0.00
D

ATOM 25407 CG LYS D 126 16.045 26.424 45.447 0.00 0.00
D
ATOM 25408 HG1 LYS D 126 16.585 27.390 45.355 0.00 0.00
D
ATOM 25409 HG2 LYS D 126 16.209 25.940 44.461 0.00 0.00
D
ATOM 25410 CD LYS D 126 14.545 26.594 45.727 0.00 0.00
D
ATOM 25411 HD1 LYS D 126 14.102 25.651 46.112 0.00 0.00
D
ATOM 25412 HD2 LYS D 126 14.396 27.297 46.574 0.00 0.00
D
ATOM 25413 CE LYS D 126 13.850 27.111 44.441 0.00 0.00
D
ATOM 25414 HE1 LYS D 126 14.130 26.384 43.649 0.00 0.00
D
ATOM 25415 HE2 LYS D 126 12.744 27.194 44.514 0.00 0.00
D
ATOM 25416 NZ LYS D 126 14.383 28.412 44.092 0.00 0.00
D
ATOM 25417 HZ1 LYS D 126 14.140 29.051 44.876 0.00 0.00
D
ATOM 25418 HZ2 LYS D 126 15.400 28.410 43.873 0.00 0.00
D
ATOM 25419 HZ3 LYS D 126 13.848 28.608 43.222 0.00 0.00
D
ATOM 25420 C LYS D 126 18.919 26.346 46.403 0.00 0.00
D
ATOM 25421 O LYS D 126 19.417 26.545 45.276 0.00 0.00
D
ATOM 25422 N ASP D 127 19.234 27.150 47.481 0.00 0.00
D
ATOM 25423 HN ASP D 127 18.711 26.951 48.306 0.00 0.00
D
ATOM 25424 CA ASP D 127 20.300 28.159 47.509 0.00 0.00
D
ATOM 25425 HA ASP D 127 20.147 28.844 46.688 0.00 0.00
D
ATOM 25426 CB ASP D 127 20.312 28.967 48.804 0.00 0.00
D
ATOM 25427 HB1 ASP D 127 19.830 28.433 49.651 0.00 0.00
D
ATOM 25428 HB2 ASP D 127 21.276 29.331 49.220 0.00 0.00
D
ATOM 25429 CG ASP D 127 19.498 30.194 48.641 0.00 0.00
D
ATOM 25430 OD1 ASP D 127 18.366 30.264 49.180 0.00 0.00
D
ATOM 25431 OD2 ASP D 127 20.047 31.170 48.114 0.00 0.00
D
ATOM 25432 C ASP D 127 21.713 27.588 47.164 0.00 0.00
D
ATOM 25433 O ASP D 127 22.460 28.215 46.405 0.00 0.00
D

ATOM 25434 N   MET D 128   22.056 26.409 47.705 0.00 0.00
D
ATOM 25435 HN MET D 128   21.423 25.942 48.318 0.00 0.00
D
ATOM 25436 CA MET D 128   23.372 25.831 47.435 0.00 0.00
D
ATOM 25437 HA MET D 128   24.080 26.598 47.715 0.00 0.00
D
ATOM 25438 CB MET D 128   23.625 24.621 48.365 0.00 0.00
D
ATOM 25439 HB1 MET D 128   23.066 24.817 49.305 0.00 0.00
D
ATOM 25440 HB2 MET D 128   23.021 23.739 48.062 0.00 0.00
D
ATOM 25441 CG MET D 128   25.093 24.220 48.525 0.00 0.00
D
ATOM 25442 HG1 MET D 128   25.171 23.516 49.380 0.00 0.00
D
ATOM 25443 HG2 MET D 128   25.455 23.570 47.700 0.00 0.00
D
ATOM 25444 SD MET D 128   26.285 25.514 48.774 0.00 0.00
D
ATOM 25445 CE MET D 128   26.068 25.825 50.577 0.00 0.00
D
ATOM 25446 HE1 MET D 128   24.970 25.818 50.751 0.00 0.00
D
ATOM 25447 HE2 MET D 128   26.615 25.033 51.131 0.00 0.00
D
ATOM 25448 HE3 MET D 128   26.501 26.796 50.897 0.00 0.00
D
ATOM 25449 C   MET D 128   23.504 25.475 45.980 0.00 0.00
D
ATOM 25450 O   MET D 128   24.613 25.616 45.419 0.00 0.00
D
ATOM 25451 N   GLY D 129   22.447 25.005 45.275 0.00 0.00
D
ATOM 25452 HN GLY D 129   21.663 24.772 45.844 0.00 0.00
D
ATOM 25453 CA GLY D 129   22.423 24.623 43.899 0.00 0.00
D
ATOM 25454 HA1 GLY D 129   21.384 24.414 43.688 0.00 0.00
D
ATOM 25455 HA2 GLY D 129   23.037 23.739 43.812 0.00 0.00
D
ATOM 25456 C   GLY D 129   22.871 25.768 43.053 0.00 0.00
D
ATOM 25457 O   GLY D 129   23.541 25.549 42.029 0.00 0.00
D
ATOM 25458 N   VAL D 130   22.628 27.017 43.471 0.00 0.00
D
ATOM 25459 HN VAL D 130   22.225 27.200 44.365 0.00 0.00
D
ATOM 25460 CA VAL D 130   23.009 28.152 42.663 0.00 0.00
D

ATOM 25461 HA VAL D 130 22.738 28.061 41.622 0.00 0.00
D
ATOM 25462 CB VAL D 130 22.316 29.425 43.154 0.00 0.00
D
ATOM 25463 HB VAL D 130 22.687 29.512 44.197 0.00 0.00
D
ATOM 25464 CG1 VAL D 130 22.598 30.571 42.125 0.00 0.00
D
ATOM 25465 HG11 VAL D 130 22.268 30.201 41.131 0.00 0.00
D
ATOM 25466 HG12 VAL D 130 22.007 31.474 42.389 0.00 0.00
D
ATOM 25467 HG13 VAL D 130 23.677 30.816 42.024 0.00 0.00
D
ATOM 25468 CG2 VAL D 130 20.807 29.242 43.256 0.00 0.00
D
ATOM 25469 HG21 VAL D 130 20.390 28.609 42.444 0.00 0.00
D
ATOM 25470 HG22 VAL D 130 20.531 28.706 44.189 0.00 0.00
D
ATOM 25471 HG23 VAL D 130 20.378 30.266 43.292 0.00 0.00
D
ATOM 25472 C VAL D 130 24.523 28.334 42.757 0.00 0.00
D
ATOM 25473 O VAL D 130 25.164 28.507 41.734 0.00 0.00
D
ATOM 25474 N LYS D 131 25.111 28.383 43.968 0.00 0.00
D
ATOM 25475 HN LYS D 131 24.521 28.341 44.771 0.00 0.00
D
ATOM 25476 CA LYS D 131 26.501 28.493 44.260 0.00 0.00
D
ATOM 25477 HA LYS D 131 26.850 29.340 43.690 0.00 0.00
D
ATOM 25478 CB LYS D 131 26.657 28.453 45.791 0.00 0.00
D
ATOM 25479 HB1 LYS D 131 26.513 27.412 46.152 0.00 0.00
D
ATOM 25480 HB2 LYS D 131 27.666 28.827 46.068 0.00 0.00
D
ATOM 25481 CG LYS D 131 25.637 29.329 46.522 0.00 0.00
D
ATOM 25482 HG1 LYS D 131 25.821 30.334 46.086 0.00 0.00
D
ATOM 25483 HG2 LYS D 131 24.557 29.145 46.338 0.00 0.00
D
ATOM 25484 CD LYS D 131 25.700 29.366 48.061 0.00 0.00
D
ATOM 25485 HD1 LYS D 131 25.489 28.333 48.411 0.00 0.00
D
ATOM 25486 HD2 LYS D 131 26.704 29.529 48.508 0.00 0.00
D
ATOM 25487 CE LYS D 131 24.643 30.248 48.671 0.00 0.00
D

ATOM 25488 HE1 LYS D 131   23.658 30.021 48.211 0.00 0.00
D
ATOM 25489 HE2 LYS D 131   24.582 30.112 49.772 0.00 0.00
D
ATOM 25490 NZ LYS D 131   24.865 31.658 48.249 0.00 0.00
D
ATOM 25491 HZ1 LYS D 131   25.696 31.693 47.625 0.00 0.00
D
ATOM 25492 HZ2 LYS D 131   23.969 32.009 47.853 0.00 0.00
D
ATOM 25493 HZ3 LYS D 131   25.041 32.287 49.058 0.00 0.00
D
ATOM 25494 C   LYS D 131   27.218 27.345 43.577 0.00 0.00
D
ATOM 25495 O   LYS D 131   28.246 27.626 42.975 0.00 0.00
D
ATOM 25496 N   ILE D 132   26.662 26.082 43.594 0.00 0.00
D
ATOM 25497 HN ILE D 132   25.891 25.981 44.218 0.00 0.00
D
ATOM 25498 CA ILE D 132   27.266 24.891 42.972 0.00 0.00
D
ATOM 25499 HA ILE D 132   28.324 24.971 43.173 0.00 0.00
D
ATOM 25500 CB ILE D 132   26.641 23.496 43.395 0.00 0.00
D
ATOM 25501 HB ILE D 132   25.530 23.480 43.424 0.00 0.00
D
ATOM 25502 CG2 ILE D 132   27.222 22.387 42.496 0.00 0.00
D
ATOM 25503 HG21 ILE D 132   26.981 21.430 43.006 0.00 0.00
D
ATOM 25504 HG22 ILE D 132   26.628 22.345 41.558 0.00 0.00
D
ATOM 25505 HG23 ILE D 132   28.302 22.533 42.280 0.00 0.00
D
ATOM 25506 CG1 ILE D 132   27.053 23.243 44.875 0.00 0.00
D
ATOM 25507 HG11 ILE D 132   26.549 24.026 45.481 0.00 0.00
D
ATOM 25508 HG12 ILE D 132   26.577 22.268 45.115 0.00 0.00
D
ATOM 25509 CD ILE D 132   28.543 23.128 45.241 0.00 0.00
D
ATOM 25510 HD1 ILE D 132   28.523 22.627 46.233 0.00 0.00
D
ATOM 25511 HD2 ILE D 132   29.183 22.626 44.484 0.00 0.00
D
ATOM 25512 HD3 ILE D 132   28.984 24.123 45.462 0.00 0.00
D
ATOM 25513 C   ILE D 132   27.171 25.066 41.469 0.00 0.00
D
ATOM 25514 O   ILE D 132   28.163 24.797 40.792 0.00 0.00
D

ATOM 25515 N ALA D 133 26.077 25.527 40.830 0.00 0.00
D
ATOM 25516 HN ALA D 133 25.295 25.790 41.389 0.00 0.00
D
ATOM 25517 CA ALA D 133 26.107 25.722 39.335 0.00 0.00
D
ATOM 25518 HA ALA D 133 26.360 24.819 38.799 0.00 0.00
D
ATOM 25519 CB ALA D 133 24.698 26.170 38.883 0.00 0.00
D
ATOM 25520 HB1 ALA D 133 24.055 25.287 39.090 0.00 0.00
D
ATOM 25521 HB2 ALA D 133 24.299 26.969 39.544 0.00 0.00
D
ATOM 25522 HB3 ALA D 133 24.745 26.457 37.811 0.00 0.00
D
ATOM 25523 C ALA D 133 27.143 26.740 38.966 0.00 0.00
D
ATOM 25524 O ALA D 133 27.731 26.538 37.880 0.00 0.00
D
ATOM 25525 N GLN D 134 27.281 27.907 39.728 0.00 0.00
D
ATOM 25526 HN GLN D 134 26.817 28.069 40.595 0.00 0.00
D
ATOM 25527 CA GLN D 134 28.325 28.903 39.422 0.00 0.00
D
ATOM 25528 HA GLN D 134 28.251 29.198 38.386 0.00 0.00
D
ATOM 25529 CB GLN D 134 28.139 30.146 40.382 0.00 0.00
D
ATOM 25530 HB1 GLN D 134 28.155 29.742 41.416 0.00 0.00
D
ATOM 25531 HB2 GLN D 134 29.031 30.805 40.322 0.00 0.00
D
ATOM 25532 CG GLN D 134 26.835 30.946 40.058 0.00 0.00
D
ATOM 25533 HG1 GLN D 134 26.818 31.281 38.999 0.00 0.00
D
ATOM 25534 HG2 GLN D 134 25.912 30.347 40.217 0.00 0.00
D
ATOM 25535 CD GLN D 134 26.761 32.219 40.766 0.00 0.00
D
ATOM 25536 OE1 GLN D 134 25.784 32.314 41.487 0.00 0.00
D
ATOM 25537 NE2 GLN D 134 27.632 33.225 40.547 0.00 0.00
D
ATOM 25538 HE21 GLN D 134 27.460 34.107 40.986 0.00 0.00
D
ATOM 25539 HE22 GLN D 134 28.128 33.226 39.679 0.00 0.00
D
ATOM 25540 C GLN D 134 29.688 28.342 39.675 0.00 0.00
D
ATOM 25541 O GLN D 134 30.655 28.652 39.063 0.00 0.00
D

ATOM 25542 N   SER D 135   29.853 27.470 40.691 0.00 0.00
D
ATOM 25543 HN SER D 135   29.232 27.260 41.443 0.00 0.00
D
ATOM 25544 CA SER D 135   31.122 26.644 40.718 0.00 0.00
D
ATOM 25545 HA SER D 135   31.995 27.276 40.665 0.00 0.00
D
ATOM 25546 CB SER D 135   31.150 25.662 41.942 0.00 0.00
D
ATOM 25547 HB1 SER D 135   30.202 25.084 41.983 0.00 0.00
D
ATOM 25548 HB2 SER D 135   32.038 24.997 41.883 0.00 0.00
D
ATOM 25549 OG SER D 135   31.181 26.415 43.164 0.00 0.00
D
ATOM 25550 HG1 SER D 135   32.091 26.331 43.459 0.00 0.00
D
ATOM 25551 C   SER D 135   31.392 25.714 39.532 0.00 0.00
D
ATOM 25552 O   SER D 135   32.459 25.695 38.978 0.00 0.00
D
ATOM 25553 N   LEU D 136   30.342 24.941 39.100 0.00 0.00
D
ATOM 25554 HN LEU D 136   29.476 25.044 39.584 0.00 0.00
D
ATOM 25555 CA LEU D 136   30.421 24.092 37.891 0.00 0.00
D
ATOM 25556 HA LEU D 136   31.248 23.397 37.894 0.00 0.00
D
ATOM 25557 CB LEU D 136   29.103 23.318 37.803 0.00 0.00
D
ATOM 25558 HB1 LEU D 136   28.824 22.979 38.824 0.00 0.00
D
ATOM 25559 HB2 LEU D 136   28.338 24.047 37.461 0.00 0.00
D
ATOM 25560 CG LEU D 136   29.170 22.006 36.951 0.00 0.00
D
ATOM 25561 HG LEU D 136   29.528 22.199 35.917 0.00 0.00
D
ATOM 25562 CD1 LEU D 136   29.982 20.970 37.725 0.00 0.00
D
ATOM 25563 HD11 LEU D 136   31.051 21.247 37.846 0.00 0.00
D
ATOM 25564 HD12 LEU D 136   29.633 20.771 38.761 0.00 0.00
D
ATOM 25565 HD13 LEU D 136   29.803 19.923 37.401 0.00 0.00
D
ATOM 25566 CD2 LEU D 136   27.689 21.495 36.871 0.00 0.00
D
ATOM 25567 HD21 LEU D 136   27.052 22.122 36.211 0.00 0.00
D
ATOM 25568 HD22 LEU D 136   27.589 20.483 36.425 0.00 0.00
D

ATOM 25569 HD23 LEU D 136 27.276 21.624 37.895 0.00 0.00
D
ATOM 25570 C LEU D 136 30.693 24.805 36.585 0.00 0.00
D
ATOM 25571 O LEU D 136 31.315 24.273 35.673 0.00 0.00
D
ATOM 25572 N SER D 137 30.190 26.084 36.439 0.00 0.00
D
ATOM 25573 HN SER D 137 29.496 26.404 37.079 0.00 0.00
D
ATOM 25574 CA SER D 137 30.206 26.805 35.159 0.00 0.00
D
ATOM 25575 HA SER D 137 29.717 26.163 34.442 0.00 0.00
D
ATOM 25576 CB SER D 137 29.353 28.159 35.192 0.00 0.00
D
ATOM 25577 HB1 SER D 137 28.338 27.918 35.574 0.00 0.00
D
ATOM 25578 HB2 SER D 137 29.855 28.791 35.956 0.00 0.00
D
ATOM 25579 OG SER D 137 29.099 28.745 33.892 0.00 0.00
D
ATOM 25580 HG1 SER D 137 28.666 29.589 34.039 0.00 0.00
D
ATOM 25581 C SER D 137 31.623 27.108 34.708 0.00 0.00
D
ATOM 25582 O SER D 137 32.001 27.152 33.539 0.00 0.00
D
ATOM 25583 N ASP D 138 32.550 27.351 35.720 0.00 0.00
D
ATOM 25584 HN ASP D 138 32.257 27.310 36.672 0.00 0.00
D
ATOM 25585 CA ASP D 138 33.931 27.796 35.409 0.00 0.00
D
ATOM 25586 HA ASP D 138 34.108 27.702 34.347 0.00 0.00
D
ATOM 25587 CB ASP D 138 34.163 29.239 35.833 0.00 0.00
D
ATOM 25588 HB1 ASP D 138 33.633 29.386 36.799 0.00 0.00
D
ATOM 25589 HB2 ASP D 138 35.239 29.413 36.050 0.00 0.00
D
ATOM 25590 CG ASP D 138 33.746 30.139 34.756 0.00 0.00
D
ATOM 25591 OD1 ASP D 138 32.778 31.012 34.926 0.00 0.00
D
ATOM 25592 OD2 ASP D 138 34.371 30.206 33.675 0.00 0.00
D
ATOM 25593 C ASP D 138 35.004 26.938 36.060 0.00 0.00
D
ATOM 25594 O ASP D 138 35.086 26.605 37.264 0.00 0.00
D
ATOM 25595 N GLU D 139 35.831 26.378 35.198 0.00 0.00
D

ATOM 25596 HN GLU D 139 35.696 26.427 34.211 0.00 0.00
D
ATOM 25597 CA GLU D 139 36.649 25.248 35.634 0.00 0.00
D
ATOM 25598 HA GLU D 139 36.289 24.842 36.568 0.00 0.00
D
ATOM 25599 CB GLU D 139 36.695 24.222 34.412 0.00 0.00
D
ATOM 25600 HB1 GLU D 139 37.442 23.449 34.691 0.00 0.00
D
ATOM 25601 HB2 GLU D 139 35.700 23.738 34.506 0.00 0.00
D
ATOM 25602 CG GLU D 139 36.872 24.859 33.014 0.00 0.00
D
ATOM 25603 HG1 GLU D 139 35.964 25.433 32.730 0.00 0.00
D
ATOM 25604 HG2 GLU D 139 37.736 25.500 32.735 0.00 0.00
D
ATOM 25605 CD GLU D 139 36.789 23.730 32.092 0.00 0.00
D
ATOM 25606 OE1 GLU D 139 35.834 23.651 31.254 0.00 0.00
D
ATOM 25607 OE2 GLU D 139 37.853 23.045 31.946 0.00 0.00
D
ATOM 25608 C GLU D 139 38.045 25.691 35.997 0.00 0.00
D
ATOM 25609 O GLU D 139 38.748 24.832 36.539 0.00 0.00
D
ATOM 25610 N ASN D 140 38.439 26.993 35.909 0.00 0.00
D
ATOM 25611 HN ASN D 140 37.778 27.562 35.427 0.00 0.00
D
ATOM 25612 CA ASN D 140 39.647 27.645 36.332 0.00 0.00
D
ATOM 25613 HA ASN D 140 40.344 26.894 36.674 0.00 0.00
D
ATOM 25614 CB ASN D 140 40.257 28.451 35.132 0.00 0.00
D
ATOM 25615 HB1 ASN D 140 39.521 29.000 34.507 0.00 0.00
D
ATOM 25616 HB2 ASN D 140 41.025 29.185 35.456 0.00 0.00
D
ATOM 25617 CG ASN D 140 40.948 27.448 34.157 0.00 0.00
D
ATOM 25618 OD1 ASN D 140 41.657 26.542 34.607 0.00 0.00
D
ATOM 25619 ND2 ASN D 140 40.732 27.639 32.862 0.00 0.00
D
ATOM 25620 HD21 ASN D 140 41.022 26.986 32.162 0.00 0.00
D
ATOM 25621 HD22 ASN D 140 40.156 28.380 32.518 0.00 0.00
D
ATOM 25622 C ASN D 140 39.421 28.695 37.407 0.00 0.00
D

ATOM 25623 O   ASN D 140   40.398 29.288 37.906 0.00 0.00
D
ATOM 25624 N   VAL D 141   38.191 28.962 37.737 0.00 0.00
D
ATOM 25625 HN VAL D 141   37.478 28.384 37.346 0.00 0.00
D
ATOM 25626 CA VAL D 141   37.909 30.001 38.726 0.00 0.00
D
ATOM 25627 HA VAL D 141   38.669 30.757 38.858 0.00 0.00
D
ATOM 25628 CB VAL D 141   36.590 30.774 38.416 0.00 0.00
D
ATOM 25629 HB VAL D 141   35.863 29.963 38.196 0.00 0.00
D
ATOM 25630 CG1 VAL D 141   35.965 31.532 39.676 0.00 0.00
D
ATOM 25631 HG11 VAL D 141   35.686 30.945 40.577 0.00 0.00
D
ATOM 25632 HG12 VAL D 141   36.709 32.313 39.944 0.00 0.00
D
ATOM 25633 HG13 VAL D 141   35.102 32.087 39.251 0.00 0.00
D
ATOM 25634 CG2 VAL D 141   36.659 31.686 37.209 0.00 0.00
D
ATOM 25635 HG21 VAL D 141   37.301 32.592 37.249 0.00 0.00
D
ATOM 25636 HG22 VAL D 141   37.022 31.037 36.384 0.00 0.00
D
ATOM 25637 HG23 VAL D 141   35.683 32.132 36.922 0.00 0.00
D
ATOM 25638 C   VAL D 141   37.659 29.311 40.017 0.00 0.00
D
ATOM 25639 O   VAL D 141   36.729 28.504 40.204 0.00 0.00
D
ATOM 25640 N   LEU D 142   38.527 29.565 41.027 0.00 0.00
D
ATOM 25641 HN LEU D 142   39.269 30.190 40.795 0.00 0.00
D
ATOM 25642 CA LEU D 142   38.323 29.017 42.351 0.00 0.00
D
ATOM 25643 HA LEU D 142   38.056 27.972 42.299 0.00 0.00
D
ATOM 25644 CB LEU D 142   39.581 29.313 43.156 0.00 0.00
D
ATOM 25645 HB1 LEU D 142   39.773 30.398 43.297 0.00 0.00
D
ATOM 25646 HB2 LEU D 142   39.405 28.947 44.190 0.00 0.00
D
ATOM 25647 CG LEU D 142   40.745 28.529 42.557 0.00 0.00
D
ATOM 25648 HG LEU D 142   41.044 28.902 41.554 0.00 0.00
D
ATOM 25649 CD1 LEU D 142   41.934 28.506 43.484 0.00 0.00
D

ATOM 25650 HD11 LEU D 142   42.776 27.861 43.153 0.00 0.00
D
ATOM 25651 HD12 LEU D 142   42.118 29.571 43.742 0.00 0.00
D
ATOM 25652 HD13 LEU D 142   41.694 27.986 44.436 0.00 0.00
D
ATOM 25653 CD2 LEU D 142   40.455 27.027 42.173 0.00 0.00
D
ATOM 25654 HD21 LEU D 142   40.316 26.443 43.108 0.00 0.00
D
ATOM 25655 HD22 LEU D 142   39.621 26.914 41.448 0.00 0.00
D
ATOM 25656 HD23 LEU D 142   41.368 26.508 41.809 0.00 0.00
D
ATOM 25657 C   LEU D 142   37.162 29.571 43.090 0.00 0.00
D
ATOM 25658 O   LEU D 142   36.314 28.857 43.634 0.00 0.00
D
ATOM 25659 N   ASN D 143   37.049 30.913 43.070 0.00 0.00
D
ATOM 25660 HN ASN D 143   37.668 31.528 42.587 0.00 0.00
D
ATOM 25661 CA ASN D 143   36.094 31.573 43.998 0.00 0.00
D
ATOM 25662 HA ASN D 143   35.303 30.875 44.227 0.00 0.00
D
ATOM 25663 CB ASN D 143   36.905 32.134 45.208 0.00 0.00
D
ATOM 25664 HB1 ASN D 143   37.982 32.160 44.936 0.00 0.00
D
ATOM 25665 HB2 ASN D 143   36.653 33.162 45.546 0.00 0.00
D
ATOM 25666 CG ASN D 143   36.679 31.251 46.454 0.00 0.00
D
ATOM 25667 OD1 ASN D 143   37.595 30.675 47.049 0.00 0.00
D
ATOM 25668 ND2 ASN D 143   35.403 31.020 46.856 0.00 0.00
D
ATOM 25669 HD21 ASN D 143   35.241 30.361 47.591 0.00 0.00
D
ATOM 25670 HD22 ASN D 143   34.768 31.753 46.611 0.00 0.00
D
ATOM 25671 C   ASN D 143   35.514 32.736 43.160 0.00 0.00
D
ATOM 25672 O   ASN D 143   36.140 33.292 42.266 0.00 0.00
D
ATOM 25673 N   TYR D 144   34.285 33.163 43.469 0.00 0.00
D
ATOM 25674 HN TYR D 144   33.699 32.765 44.170 0.00 0.00
D
ATOM 25675 CA TYR D 144   33.570 34.160 42.719 0.00 0.00
D
ATOM 25676 HA TYR D 144   34.239 34.862 42.243 0.00 0.00
D

ATOM 25677 CB TYR D 144   32.621 33.502 41.680 0.00 0.00
D
ATOM 25678 HB1 TYR D 144   31.974 34.238 41.155 0.00 0.00
D
ATOM 25679 HB2 TYR D 144   33.224 32.969 40.914 0.00 0.00
D
ATOM 25680 CG TYR D 144   31.720 32.447 42.203 0.00 0.00
D
ATOM 25681 CD1 TYR D 144   30.362 32.689 42.563 0.00 0.00
D
ATOM 25682 HD1 TYR D 144   29.919 33.634 42.285 0.00 0.00
D
ATOM 25683 CE1 TYR D 144   29.687 31.659 43.254 0.00 0.00
D
ATOM 25684 HE1 TYR D 144   28.669 31.950 43.470 0.00 0.00
D
ATOM 25685 CZ TYR D 144   30.269 30.410 43.440 0.00 0.00
D
ATOM 25686 OH TYR D 144   29.752 29.573 44.438 0.00 0.00
D
ATOM 25687 HH TYR D 144   30.075 28.684 44.275 0.00 0.00
D
ATOM 25688 CD2 TYR D 144   32.246 31.081 42.409 0.00 0.00
D
ATOM 25689 HD2 TYR D 144   33.249 30.891 42.055 0.00 0.00
D
ATOM 25690 CE2 TYR D 144   31.538 30.110 43.055 0.00 0.00
D
ATOM 25691 HE2 TYR D 144   31.910 29.123 43.290 0.00 0.00
D
ATOM 25692 C   TYR D 144   32.768 35.039 43.577 0.00 0.00
D
ATOM 25693 O   TYR D 144   32.287 34.582 44.608 0.00 0.00
D
ATOM 25694 N   ILE D 145   32.532 36.324 43.214 0.00 0.00
D
ATOM 25695 HN ILE D 145   33.087 36.524 42.410 0.00 0.00
D
ATOM 25696 CA ILE D 145   31.762 37.322 43.933 0.00 0.00
D
ATOM 25697 HA ILE D 145   30.955 36.869 44.490 0.00 0.00
D
ATOM 25698 CB ILE D 145   32.588 38.270 44.818 0.00 0.00
D
ATOM 25699 HB ILE D 145   33.396 38.693 44.183 0.00 0.00
D
ATOM 25700 CG2 ILE D 145   31.734 39.395 45.401 0.00 0.00
D
ATOM 25701 HG21 ILE D 145   31.286 40.101 44.670 0.00 0.00
D
ATOM 25702 HG22 ILE D 145   31.025 38.977 46.146 0.00 0.00
D
ATOM 25703 HG23 ILE D 145   32.392 39.981 46.078 0.00 0.00
D

ATOM 25704 CG1 ILE D 145 33.277 37.507 46.007 0.00 0.00
D
ATOM 25705 HG11 ILE D 145 32.492 37.347 46.776 0.00 0.00
D
ATOM 25706 HG12 ILE D 145 33.668 36.495 45.767 0.00 0.00
D
ATOM 25707 CD ILE D 145 34.530 38.299 46.426 0.00 0.00
D
ATOM 25708 HD1 ILE D 145 35.023 37.731 47.244 0.00 0.00
D
ATOM 25709 HD2 ILE D 145 35.265 38.103 45.616 0.00 0.00
D
ATOM 25710 HD3 ILE D 145 34.449 39.326 46.840 0.00 0.00
D
ATOM 25711 C ILE D 145 31.078 38.123 42.919 0.00 0.00
D
ATOM 25712 O ILE D 145 31.692 38.967 42.238 0.00 0.00
D
ATOM 25713 N ASP D 146 29.711 37.985 42.772 0.00 0.00
D
ATOM 25714 HN ASP D 146 29.156 37.385 43.342 0.00 0.00
D
ATOM 25715 CA ASP D 146 28.919 38.656 41.789 0.00 0.00
D
ATOM 25716 HA ASP D 146 29.359 38.575 40.806 0.00 0.00
D
ATOM 25717 CB ASP D 146 27.461 38.048 41.724 0.00 0.00
D
ATOM 25718 HB1 ASP D 146 26.971 38.132 42.718 0.00 0.00
D
ATOM 25719 HB2 ASP D 146 26.854 38.559 40.946 0.00 0.00
D
ATOM 25720 CG ASP D 146 27.524 36.558 41.291 0.00 0.00
D
ATOM 25721 OD1 ASP D 146 27.934 36.350 40.099 0.00 0.00
D
ATOM 25722 OD2 ASP D 146 26.943 35.680 41.949 0.00 0.00
D
ATOM 25723 C ASP D 146 28.862 40.177 42.185 0.00 0.00
D
ATOM 25724 O ASP D 146 28.572 40.529 43.321 0.00 0.00
D
ATOM 25725 N LEU D 147 29.049 41.002 41.141 0.00 0.00
D
ATOM 25726 HN LEU D 147 29.195 40.609 40.236 0.00 0.00
D
ATOM 25727 CA LEU D 147 28.789 42.379 41.312 0.00 0.00
D
ATOM 25728 HA LEU D 147 28.693 42.647 42.354 0.00 0.00
D
ATOM 25729 CB LEU D 147 29.956 43.183 40.679 0.00 0.00
D
ATOM 25730 HB1 LEU D 147 29.923 42.968 39.589 0.00 0.00
D

ATOM 25731 HB2 LEU D 147 29.632 44.211 40.946 0.00 0.00
D
ATOM 25732 CG LEU D 147 31.346 42.868 41.381 0.00 0.00
D
ATOM 25733 HG LEU D 147 31.458 41.763 41.337 0.00 0.00
D
ATOM 25734 CD1 LEU D 147 32.433 43.564 40.670 0.00 0.00
D
ATOM 25735 HD11 LEU D 147 32.704 43.197 39.657 0.00 0.00
D
ATOM 25736 HD12 LEU D 147 32.112 44.623 40.575 0.00 0.00
D
ATOM 25737 HD13 LEU D 147 33.406 43.563 41.206 0.00 0.00
D
ATOM 25738 CD2 LEU D 147 31.383 43.101 42.935 0.00 0.00
D
ATOM 25739 HD21 LEU D 147 30.586 42.563 43.492 0.00 0.00
D
ATOM 25740 HD22 LEU D 147 32.332 42.697 43.348 0.00 0.00
D
ATOM 25741 HD23 LEU D 147 31.289 44.186 43.159 0.00 0.00
D
ATOM 25742 C LEU D 147 27.478 42.664 40.582 0.00 0.00
D
ATOM 25743 O LEU D 147 26.704 43.477 40.959 0.00 0.00
D
ATOM 25744 N SER D 148 27.213 41.782 39.607 0.00 0.00
D
ATOM 25745 HN SER D 148 27.917 41.159 39.275 0.00 0.00
D
ATOM 25746 CA SER D 148 25.965 41.808 38.993 0.00 0.00
D
ATOM 25747 HA SER D 148 25.186 41.849 39.740 0.00 0.00
D
ATOM 25748 CB SER D 148 25.669 42.990 38.116 0.00 0.00
D
ATOM 25749 HB1 SER D 148 24.571 42.939 37.952 0.00 0.00
D
ATOM 25750 HB2 SER D 148 25.904 43.901 38.707 0.00 0.00
D
ATOM 25751 OG SER D 148 26.312 42.973 36.827 0.00 0.00
D
ATOM 25752 HG1 SER D 148 25.768 43.481 36.221 0.00 0.00
D
ATOM 25753 C SER D 148 25.737 40.494 38.327 0.00 0.00
D
ATOM 25754 O SER D 148 26.649 39.670 38.387 0.00 0.00
D
ATOM 25755 N ASP D 149 24.574 40.216 37.668 0.00 0.00
D
ATOM 25756 HN ASP D 149 23.746 40.763 37.764 0.00 0.00
D
ATOM 25757 CA ASP D 149 24.414 39.264 36.653 0.00 0.00
D

ATOM 25758 HA ASP D 149   24.540 38.258 37.026 0.00 0.00
D
ATOM 25759 CB ASP D 149   22.986 39.444 36.013 0.00 0.00
D
ATOM 25760 HB1 ASP D 149   22.090 39.634 36.642 0.00 0.00
D
ATOM 25761 HB2 ASP D 149   22.990 40.223 35.221 0.00 0.00
D
ATOM 25762 CG ASP D 149   22.513 38.141 35.223 0.00 0.00
D
ATOM 25763 OD1 ASP D 149   22.121 37.156 35.927 0.00 0.00
D
ATOM 25764 OD2 ASP D 149   22.674 38.159 33.924 0.00 0.00
D
ATOM 25765 C   ASP D 149   25.465 39.294 35.546 0.00 0.00
D
ATOM 25766 O   ASP D 149   25.879 38.285 34.971 0.00 0.00
D
ATOM 25767 N   GLU D 150   25.942 40.500 35.144 0.00 0.00
D
ATOM 25768 HN GLU D 150   25.507 41.319 35.511 0.00 0.00
D
ATOM 25769 CA GLU D 150   26.956 40.649 34.091 0.00 0.00
D
ATOM 25770 HA GLU D 150   26.843 39.838 33.387 0.00 0.00
D
ATOM 25771 CB GLU D 150   26.645 41.899 33.120 0.00 0.00
D
ATOM 25772 HB1 GLU D 150   26.812 42.761 33.800 0.00 0.00
D
ATOM 25773 HB2 GLU D 150   27.495 41.996 32.411 0.00 0.00
D
ATOM 25774 CG GLU D 150   25.277 41.976 32.502 0.00 0.00
D
ATOM 25775 HG1 GLU D 150   24.770 41.047 32.164 0.00 0.00
D
ATOM 25776 HG2 GLU D 150   24.574 42.187 33.336 0.00 0.00
D
ATOM 25777 CD GLU D 150   25.254 43.181 31.576 0.00 0.00
D
ATOM 25778 OE1 GLU D 150   25.705 43.050 30.401 0.00 0.00
D
ATOM 25779 OE2 GLU D 150   24.948 44.354 32.027 0.00 0.00
D
ATOM 25780 C   GLU D 150   28.409 40.780 34.538 0.00 0.00
D
ATOM 25781 O   GLU D 150   29.275 40.280 33.830 0.00 0.00
D
ATOM 25782 N   TYR D 151   28.625 41.483 35.669 0.00 0.00
D
ATOM 25783 HN TYR D 151   27.846 41.686 36.257 0.00 0.00
D
ATOM 25784 CA TYR D 151   29.994 41.839 36.203 0.00 0.00
D

ATOM 25785 HA TYR D 151 30.797 41.560 35.536 0.00 0.00
D
ATOM 25786 CB TYR D 151 29.953 43.333 36.506 0.00 0.00
D
ATOM 25787 HB1 TYR D 151 29.130 43.867 37.027 0.00 0.00
D
ATOM 25788 HB2 TYR D 151 30.820 43.671 37.114 0.00 0.00
D
ATOM 25789 CG TYR D 151 30.060 44.082 35.207 0.00 0.00
D
ATOM 25790 CD1 TYR D 151 31.347 44.392 34.780 0.00 0.00
D
ATOM 25791 HD1 TYR D 151 32.138 43.988 35.395 0.00 0.00
D
ATOM 25792 CE1 TYR D 151 31.556 45.141 33.622 0.00 0.00
D
ATOM 25793 HE1 TYR D 151 32.592 45.214 33.328 0.00 0.00
D
ATOM 25794 CZ TYR D 151 30.523 45.752 32.960 0.00 0.00
D
ATOM 25795 OH TYR D 151 30.611 46.395 31.788 0.00 0.00
D
ATOM 25796 HH TYR D 151 31.541 46.321 31.562 0.00 0.00
D
ATOM 25797 CD2 TYR D 151 29.020 44.710 34.594 0.00 0.00
D
ATOM 25798 HD2 TYR D 151 28.013 44.627 34.975 0.00 0.00
D
ATOM 25799 CE2 TYR D 151 29.254 45.512 33.491 0.00 0.00
D
ATOM 25800 HE2 TYR D 151 28.348 45.935 33.082 0.00 0.00
D
ATOM 25801 C TYR D 151 30.311 41.035 37.541 0.00 0.00
D
ATOM 25802 O TYR D 151 29.477 40.768 38.393 0.00 0.00
D
ATOM 25803 N SER D 152 31.593 40.652 37.762 0.00 0.00
D
ATOM 25804 HN SER D 152 32.184 40.907 37.001 0.00 0.00
D
ATOM 25805 CA SER D 152 32.015 39.811 38.850 0.00 0.00
D
ATOM 25806 HA SER D 152 31.695 40.355 39.727 0.00 0.00
D
ATOM 25807 CB SER D 152 31.437 38.405 38.990 0.00 0.00
D
ATOM 25808 HB1 SER D 152 31.742 37.920 39.942 0.00 0.00
D
ATOM 25809 HB2 SER D 152 30.370 38.410 38.680 0.00 0.00
D
ATOM 25810 OG SER D 152 32.025 37.567 37.958 0.00 0.00
D
ATOM 25811 HG1 SER D 152 32.379 36.773 38.365 0.00 0.00
D

ATOM 25812 C SER D 152 33.517 39.845 39.199 0.00 0.00
D
ATOM 25813 O SER D 152 34.252 40.257 38.358 0.00 0.00
D
ATOM 25814 N ILE D 153 33.882 39.475 40.454 0.00 0.00
D
ATOM 25815 HN ILE D 153 33.232 39.156 41.139 0.00 0.00
D
ATOM 25816 CA ILE D 153 35.253 39.163 40.817 0.00 0.00
D
ATOM 25817 HA ILE D 153 35.980 39.663 40.195 0.00 0.00
D
ATOM 25818 CB ILE D 153 35.538 39.648 42.273 0.00 0.00
D
ATOM 25819 HB ILE D 153 34.705 39.178 42.839 0.00 0.00
D
ATOM 25820 CG2 ILE D 153 36.942 39.135 42.751 0.00 0.00
D
ATOM 25821 HG21 ILE D 153 37.607 39.184 41.862 0.00 0.00
D
ATOM 25822 HG22 ILE D 153 37.309 39.487 43.738 0.00 0.00
D
ATOM 25823 HG23 ILE D 153 36.687 38.067 42.923 0.00 0.00
D
ATOM 25824 CG1 ILE D 153 35.458 41.172 42.310 0.00 0.00
D
ATOM 25825 HG11 ILE D 153 36.451 41.614 42.076 0.00 0.00
D
ATOM 25826 HG12 ILE D 153 34.669 41.481 41.591 0.00 0.00
D
ATOM 25827 CD ILE D 153 35.019 41.635 43.660 0.00 0.00
D
ATOM 25828 HD1 ILE D 153 35.035 42.746 43.629 0.00 0.00
D
ATOM 25829 HD2 ILE D 153 33.998 41.303 43.945 0.00 0.00
D
ATOM 25830 HD3 ILE D 153 35.833 41.255 44.314 0.00 0.00
D
ATOM 25831 C ILE D 153 35.309 37.636 40.641 0.00 0.00
D
ATOM 25832 O ILE D 153 34.355 36.937 40.965 0.00 0.00
D
ATOM 25833 N VAL D 154 36.458 37.230 40.065 0.00 0.00
D
ATOM 25834 HN VAL D 154 37.172 37.924 40.103 0.00 0.00
D
ATOM 25835 CA VAL D 154 36.853 35.896 39.924 0.00 0.00
D
ATOM 25836 HA VAL D 154 36.345 35.185 40.559 0.00 0.00
D
ATOM 25837 CB VAL D 154 36.992 35.347 38.456 0.00 0.00
D
ATOM 25838 HB VAL D 154 37.417 34.320 38.460 0.00 0.00
D

ATOM 25839 CG1 VAL D 154   35.667 35.142 37.780 0.00 0.00
D
ATOM 25840 HG11 VAL D 154   34.946 34.668 38.480 0.00 0.00
D
ATOM 25841 HG12 VAL D 154   35.131 36.066 37.472 0.00 0.00
D
ATOM 25842 HG13 VAL D 154   35.704 34.551 36.840 0.00 0.00
D
ATOM 25843 CG2 VAL D 154   37.825 36.192 37.542 0.00 0.00
D
ATOM 25844 HG21 VAL D 154   37.321 37.178 37.447 0.00 0.00
D
ATOM 25845 HG22 VAL D 154   38.804 36.423 38.014 0.00 0.00
D
ATOM 25846 HG23 VAL D 154   38.004 35.692 36.567 0.00 0.00
D
ATOM 25847 C   VAL D 154   38.239 35.741 40.638 0.00 0.00
D
ATOM 25848 O   VAL D 154   39.022 36.667 40.756 0.00 0.00
D
ATOM 25849 N   GLU D 155   38.613 34.539 41.154 0.00 0.00
D
ATOM 25850 HN GLU D 155   38.034 33.728 41.207 0.00 0.00
D
ATOM 25851 CA GLU D 155   40.001 34.335 41.581 0.00 0.00
D
ATOM 25852 HA GLU D 155   40.625 35.196 41.394 0.00 0.00
D
ATOM 25853 CB GLU D 155   40.159 33.862 43.003 0.00 0.00
D
ATOM 25854 HB1 GLU D 155   39.880 34.686 43.694 0.00 0.00
D
ATOM 25855 HB2 GLU D 155   39.394 33.064 43.109 0.00 0.00
D
ATOM 25856 CG GLU D 155   41.517 33.222 43.317 0.00 0.00
D
ATOM 25857 HG1 GLU D 155   41.572 32.273 42.743 0.00 0.00
D
ATOM 25858 HG2 GLU D 155   42.350 33.904 43.042 0.00 0.00
D
ATOM 25859 CD GLU D 155   41.818 32.888 44.803 0.00 0.00
D
ATOM 25860 OE1 GLU D 155   41.531 31.731 45.105 0.00 0.00
D
ATOM 25861 OE2 GLU D 155   42.231 33.723 45.600 0.00 0.00
D
ATOM 25862 C   GLU D 155   40.454 33.158 40.644 0.00 0.00
D
ATOM 25863 O   GLU D 155   39.733 32.156 40.547 0.00 0.00
D
ATOM 25864 N   LEU D 156   41.470 33.368 39.727 0.00 0.00
D
ATOM 25865 HN LEU D 156   41.978 34.221 39.821 0.00 0.00
D

ATOM 25866 CA LEU D 156 41.683 32.451 38.622 0.00 0.00
D
ATOM 25867 HA LEU D 156 40.892 31.721 38.532 0.00 0.00
D
ATOM 25868 CB LEU D 156 41.812 33.286 37.277 0.00 0.00
D
ATOM 25869 HB1 LEU D 156 40.895 33.910 37.344 0.00 0.00
D
ATOM 25870 HB2 LEU D 156 42.654 34.010 37.241 0.00 0.00
D
ATOM 25871 CG LEU D 156 41.730 32.448 35.987 0.00 0.00
D
ATOM 25872 HG LEU D 156 42.325 31.531 36.184 0.00 0.00
D
ATOM 25873 CD1 LEU D 156 40.252 32.079 35.873 0.00 0.00
D
ATOM 25874 HD11 LEU D 156 39.976 31.597 36.835 0.00 0.00
D
ATOM 25875 HD12 LEU D 156 39.536 32.929 35.889 0.00 0.00
D
ATOM 25876 HD13 LEU D 156 40.010 31.483 34.968 0.00 0.00
D
ATOM 25877 CD2 LEU D 156 42.336 33.206 34.752 0.00 0.00
D
ATOM 25878 HD21 LEU D 156 43.418 33.353 34.955 0.00 0.00
D
ATOM 25879 HD22 LEU D 156 42.244 32.749 33.744 0.00 0.00
D
ATOM 25880 HD23 LEU D 156 42.061 34.279 34.661 0.00 0.00
D
ATOM 25881 C LEU D 156 42.945 31.620 38.842 0.00 0.00
D
ATOM 25882 O LEU D 156 43.905 32.173 39.337 0.00 0.00
D
ATOM 25883 N LEU D 157 42.940 30.282 38.709 0.00 0.00
D
ATOM 25884 HN LEU D 157 42.112 29.822 38.398 0.00 0.00
D
ATOM 25885 CA LEU D 157 44.127 29.472 38.965 0.00 0.00
D
ATOM 25886 HA LEU D 157 44.795 29.902 39.696 0.00 0.00
D
ATOM 25887 CB LEU D 157 43.551 28.114 39.544 0.00 0.00
D
ATOM 25888 HB1 LEU D 157 42.876 28.328 40.401 0.00 0.00
D
ATOM 25889 HB2 LEU D 157 42.900 27.681 38.754 0.00 0.00
D
ATOM 25890 CG LEU D 157 44.658 27.213 40.054 0.00 0.00
D
ATOM 25891 HG LEU D 157 45.400 27.116 39.233 0.00 0.00
D
ATOM 25892 CD1 LEU D 157 45.354 27.768 41.276 0.00 0.00
D

ATOM 25893 HD11 LEU D 157   46.061 26.995 41.648 0.00 0.00
D
ATOM 25894 HD12 LEU D 157   45.919 28.713 41.131 0.00 0.00
D
ATOM 25895 HD13 LEU D 157   44.583 27.947 42.055 0.00 0.00
D
ATOM 25896 CD2 LEU D 157   44.178 25.785 40.431 0.00 0.00
D
ATOM 25897 HD21 LEU D 157   45.078 25.144 40.540 0.00 0.00
D
ATOM 25898 HD22 LEU D 157   43.571 25.712 41.359 0.00 0.00
D
ATOM 25899 HD23 LEU D 157   43.642 25.328 39.572 0.00 0.00
D
ATOM 25900 C   LEU D 157   44.851 29.373 37.667 0.00 0.00
D
ATOM 25901 O   LEU D 157   44.112 29.143 36.664 0.00 0.00
D
ATOM 25902 N   ALA D 158   46.273 29.472 37.638 0.00 0.00
D
ATOM 25903 HN ALA D 158   46.762 29.745 38.462 0.00 0.00
D
ATOM 25904 CA ALA D 158   47.036 29.390 36.457 0.00 0.00
D
ATOM 25905 HA ALA D 158   46.474 29.610 35.561 0.00 0.00
D
ATOM 25906 CB ALA D 158   48.098 30.558 36.568 0.00 0.00
D
ATOM 25907 HB1 ALA D 158   47.555 31.522 36.466 0.00 0.00
D
ATOM 25908 HB2 ALA D 158   48.641 30.442 37.530 0.00 0.00
D
ATOM 25909 HB3 ALA D 158   48.868 30.441 35.776 0.00 0.00
D
ATOM 25910 C   ALA D 158   47.669 28.020 36.108 0.00 0.00
D
ATOM 25911 O   ALA D 158   47.580 27.040 36.828 0.00 0.00
D
ATOM 25912 N   THR D 159   48.209 28.013 34.939 0.00 0.00
D
ATOM 25913 HN THR D 159   48.324 28.873 34.447 0.00 0.00
D
ATOM 25914 CA THR D 159   48.369 26.870 34.066 0.00 0.00
D
ATOM 25915 HA THR D 159   48.184 25.966 34.629 0.00 0.00
D
ATOM 25916 CB THR D 159   47.394 26.726 32.939 0.00 0.00
D
ATOM 25917 HB THR D 159   47.470 25.771 32.376 0.00 0.00
D
ATOM 25918 OG1 THR D 159   47.508 27.820 32.132 0.00 0.00
D
ATOM 25919 HG1 THR D 159   46.927 27.679 31.382 0.00 0.00
D

ATOM 25920 CG2 THR D 159   45.986 26.945 33.625 0.00 0.00
D
ATOM 25921 HG21 THR D 159   45.810 27.932 34.103 0.00 0.00
D
ATOM 25922 HG22 THR D 159   45.124 26.666 32.983 0.00 0.00
D
ATOM 25923 HG23 THR D 159   46.069 26.216 34.460 0.00 0.00
D
ATOM 25924 C   THR D 159   49.749 26.856 33.414 0.00 0.00
D
ATOM 25925 O   THR D 159   50.508 27.771 33.631 0.00 0.00
D
ATOM 25926 N   ARG D 160   50.102 25.868 32.547 0.00 0.00
D
ATOM 25927 HN ARG D 160   49.548 25.044 32.457 0.00 0.00
D
ATOM 25928 CA ARG D 160   51.389 25.788 32.027 0.00 0.00
D
ATOM 25929 HA ARG D 160   52.093 25.875 32.842 0.00 0.00
D
ATOM 25930 CB ARG D 160   51.647 24.381 31.282 0.00 0.00
D
ATOM 25931 HB1 ARG D 160   51.110 23.573 31.824 0.00 0.00
D
ATOM 25932 HB2 ARG D 160   51.194 24.424 30.268 0.00 0.00
D
ATOM 25933 CG ARG D 160   53.175 24.165 30.973 0.00 0.00
D
ATOM 25934 HG1 ARG D 160   53.313 23.210 30.422 0.00 0.00
D
ATOM 25935 HG2 ARG D 160   53.464 24.958 30.250 0.00 0.00
D
ATOM 25936 CD ARG D 160   54.184 24.097 32.083 0.00 0.00
D
ATOM 25937 HD1 ARG D 160   55.194 23.736 31.792 0.00 0.00
D
ATOM 25938 HD2 ARG D 160   54.243 25.134 32.478 0.00 0.00
D
ATOM 25939 NE ARG D 160   53.657 23.197 33.147 0.00 0.00
D
ATOM 25940 HE ARG D 160   53.243 23.472 34.014 0.00 0.00
D
ATOM 25941 CZ ARG D 160   53.701 21.897 33.112 0.00 0.00
D
ATOM 25942 NH1 ARG D 160   54.379 21.216 32.175 0.00 0.00
D
ATOM 25943 HH11 ARG D 160   54.468 20.221 32.220 0.00 0.00
D
ATOM 25944 HH12 ARG D 160   55.041 21.774 31.676 0.00 0.00
D
ATOM 25945 NH2 ARG D 160   53.041 21.208 34.084 0.00 0.00
D
ATOM 25946 HH21 ARG D 160   52.909 20.222 33.985 0.00 0.00
D

ATOM 25947 HH22 ARG D 160   52.477 21.763 34.696 0.00 0.00
D
ATOM 25948 C   ARG D 160   51.745 26.911 31.075 0.00 0.00
D
ATOM 25949 O   ARG D 160   52.883 27.433 31.071 0.00 0.00
D
ATOM 25950 N   LYS D 161   50.737 27.399 30.335 0.00 0.00
D
ATOM 25951 HN LYS D 161   49.907 26.846 30.301 0.00 0.00
D
ATOM 25952 CA LYS D 161   50.831 28.549 29.453 0.00 0.00
D
ATOM 25953 HA LYS D 161   51.617 28.379 28.732 0.00 0.00
D
ATOM 25954 CB LYS D 161   49.504 28.714 28.637 0.00 0.00
D
ATOM 25955 HB1 LYS D 161   49.195 27.745 28.190 0.00 0.00
D
ATOM 25956 HB2 LYS D 161   48.675 28.934 29.343 0.00 0.00
D
ATOM 25957 CG LYS D 161   49.564 29.787 27.466 0.00 0.00
D
ATOM 25958 HG1 LYS D 161   48.527 30.134 27.270 0.00 0.00
D
ATOM 25959 HG2 LYS D 161   50.129 30.694 27.769 0.00 0.00
D
ATOM 25960 CD LYS D 161   50.157 29.185 26.165 0.00 0.00
D
ATOM 25961 HD1 LYS D 161   51.172 28.825 26.435 0.00 0.00
D
ATOM 25962 HD2 LYS D 161   49.570 28.272 25.930 0.00 0.00
D
ATOM 25963 CE LYS D 161   50.227 30.277 24.951 0.00 0.00
D
ATOM 25964 HE1 LYS D 161   50.784 29.868 24.081 0.00 0.00
D
ATOM 25965 HE2 LYS D 161   49.212 30.434 24.528 0.00 0.00
D
ATOM 25966 NZ LYS D 161   50.768 31.569 25.382 0.00 0.00
D
ATOM 25967 HZ1 LYS D 161   51.416 31.330 26.160 0.00 0.00
D
ATOM 25968 HZ2 LYS D 161   51.232 32.078 24.602 0.00 0.00
D
ATOM 25969 HZ3 LYS D 161   49.996 32.170 25.733 0.00 0.00
D
ATOM 25970 C   LYS D 161   51.082 29.907 30.163 0.00 0.00
D
ATOM 25971 O   LYS D 161   51.766 30.787 29.641 0.00 0.00
D
ATOM 25972 N   LEU D 162   50.596 30.122 31.460 0.00 0.00
D
ATOM 25973 HN LEU D 162   49.855 29.531 31.769 0.00 0.00
D

ATOM 25974 CA LEU D 162   50.916 31.231 32.239 0.00 0.00
D
ATOM 25975 HA LEU D 162   51.059 32.141 31.676 0.00 0.00
D
ATOM 25976 CB LEU D 162   49.688 31.553 33.129 0.00 0.00
D
ATOM 25977 HB1 LEU D 162   49.563 30.608 33.700 0.00 0.00
D
ATOM 25978 HB2 LEU D 162   49.983 32.357 33.837 0.00 0.00
D
ATOM 25979 CG LEU D 162   48.449 31.977 32.421 0.00 0.00
D
ATOM 25980 HG LEU D 162   48.299 31.287 31.563 0.00 0.00
D
ATOM 25981 CD1 LEU D 162   47.201 31.684 33.389 0.00 0.00
D
ATOM 25982 HD11 LEU D 162   47.182 32.235 34.353 0.00 0.00
D
ATOM 25983 HD12 LEU D 162   46.326 32.034 32.801 0.00 0.00
D
ATOM 25984 HD13 LEU D 162   47.051 30.620 33.671 0.00 0.00
D
ATOM 25985 CD2 LEU D 162   48.565 33.510 31.959 0.00 0.00
D
ATOM 25986 HD21 LEU D 162   47.562 33.837 31.609 0.00 0.00
D
ATOM 25987 HD22 LEU D 162   48.794 34.194 32.804 0.00 0.00
D
ATOM 25988 HD23 LEU D 162   49.264 33.681 31.113 0.00 0.00
D
ATOM 25989 C   LEU D 162   52.129 31.138 33.163 0.00 0.00
D
ATOM 25990 O   LEU D 162   52.444 32.070 33.902 0.00 0.00
D
ATOM 25991 N   ASP D 163   52.875 29.974 33.191 0.00 0.00
D
ATOM 25992 HN ASP D 163   52.649 29.212 32.589 0.00 0.00
D
ATOM 25993 CA ASP D 163   54.135 29.753 33.904 0.00 0.00
D
ATOM 25994 HA ASP D 163   53.918 30.135 34.890 0.00 0.00
D
ATOM 25995 CB ASP D 163   54.450 28.205 33.797 0.00 0.00
D
ATOM 25996 HB1 ASP D 163   53.517 27.609 33.890 0.00 0.00
D
ATOM 25997 HB2 ASP D 163   54.988 27.970 32.854 0.00 0.00
D
ATOM 25998 CG ASP D 163   55.457 27.895 34.841 0.00 0.00
D
ATOM 25999 OD1 ASP D 163   56.344 27.078 34.631 0.00 0.00
D
ATOM 26000 OD2 ASP D 163   55.368 28.420 35.997 0.00 0.00
D

ATOM 26001 C ASP D 163 55.320 30.609 33.466 0.00 0.00
D
ATOM 26002 O ASP D 163 55.756 30.411 32.363 0.00 0.00
D
ATOM 26003 N SER D 164 55.884 31.494 34.334 0.00 0.00
D
ATOM 26004 HN SER D 164 55.369 31.528 35.188 0.00 0.00
D
ATOM 26005 CA SER D 164 57.082 32.336 34.059 0.00 0.00
D
ATOM 26006 HA SER D 164 57.251 32.889 34.971 0.00 0.00
D
ATOM 26007 CB SER D 164 58.434 31.654 33.710 0.00 0.00
D
ATOM 26008 HB1 SER D 164 58.409 31.046 32.780 0.00 0.00
D
ATOM 26009 HB2 SER D 164 59.193 32.406 33.406 0.00 0.00
D
ATOM 26010 OG SER D 164 58.914 30.862 34.821 0.00 0.00
D
ATOM 26011 HG1 SER D 164 59.834 30.602 34.732 0.00 0.00
D
ATOM 26012 C SER D 164 56.756 33.481 33.131 0.00 0.00
D
ATOM 26013 O SER D 164 57.516 33.966 32.294 0.00 0.00
D
ATOM 26014 N LYS D 165 55.501 33.916 33.279 0.00 0.00
D
ATOM 26015 HN LYS D 165 54.887 33.596 33.996 0.00 0.00
D
ATOM 26016 CA LYS D 165 54.958 34.963 32.391 0.00 0.00
D
ATOM 26017 HA LYS D 165 55.576 35.033 31.508 0.00 0.00
D
ATOM 26018 CB LYS D 165 53.599 34.508 31.830 0.00 0.00
D
ATOM 26019 HB1 LYS D 165 52.988 34.196 32.704 0.00 0.00
D
ATOM 26020 HB2 LYS D 165 52.981 35.264 31.301 0.00 0.00
D
ATOM 26021 CG LYS D 165 53.643 33.377 30.772 0.00 0.00
D
ATOM 26022 HG1 LYS D 165 54.492 32.671 30.897 0.00 0.00
D
ATOM 26023 HG2 LYS D 165 52.695 32.811 30.893 0.00 0.00
D
ATOM 26024 CD LYS D 165 53.617 33.903 29.322 0.00 0.00
D
ATOM 26025 HD1 LYS D 165 53.237 32.976 28.840 0.00 0.00
D
ATOM 26026 HD2 LYS D 165 52.863 34.711 29.207 0.00 0.00
D
ATOM 26027 CE LYS D 165 55.032 34.221 28.805 0.00 0.00
D

ATOM 26028 HE1 LYS D 165   55.598 34.941 29.435 0.00 0.00
D
ATOM 26029 HE2 LYS D 165   55.672 33.315 28.747 0.00 0.00
D
ATOM 26030 NZ LYS D 165   54.996 34.761 27.341 0.00 0.00
D
ATOM 26031 HZ1 LYS D 165   54.389 35.606 27.351 0.00 0.00
D
ATOM 26032 HZ2 LYS D 165   55.874 35.113 26.911 0.00 0.00
D
ATOM 26033 HZ3 LYS D 165   54.454 34.082 26.769 0.00 0.00
D
ATOM 26034 C   LYS D 165   54.867 36.292 33.097 0.00 0.00
D
ATOM 26035 O   LYS D 165   54.683 36.314 34.318 0.00 0.00
D
ATOM 26036 N   SER D 166   55.162 37.470 32.447 0.00 0.00
D
ATOM 26037 HN SER D 166   55.265 37.493 31.455 0.00 0.00
D
ATOM 26038 CA SER D 166   55.049 38.764 33.160 0.00 0.00
D
ATOM 26039 HA SER D 166   55.354 38.599 34.183 0.00 0.00
D
ATOM 26040 CB SER D 166   56.191 39.708 32.596 0.00 0.00
D
ATOM 26041 HB1 SER D 166   57.163 39.216 32.815 0.00 0.00
D
ATOM 26042 HB2 SER D 166   56.105 39.747 31.489 0.00 0.00
D
ATOM 26043 OG SER D 166   56.126 41.011 33.118 0.00 0.00
D
ATOM 26044 HG1 SER D 166   56.960 41.448 32.931 0.00 0.00
D
ATOM 26045 C   SER D 166   53.716 39.376 32.974 0.00 0.00
D
ATOM 26046 O   SER D 166   53.291 39.456 31.827 0.00 0.00
D
ATOM 26047 N   ILE D 167   53.043 39.770 34.065 0.00 0.00
D
ATOM 26048 HN ILE D 167   53.471 39.650 34.957 0.00 0.00
D
ATOM 26049 CA ILE D 167   51.688 40.320 33.959 0.00 0.00
D
ATOM 26050 HA ILE D 167   51.093 39.531 33.524 0.00 0.00
D
ATOM 26051 CB ILE D 167   51.020 40.625 35.317 0.00 0.00
D
ATOM 26052 HB ILE D 167   51.865 41.081 35.877 0.00 0.00
D
ATOM 26053 CG2 ILE D 167   49.838 41.612 35.335 0.00 0.00
D
ATOM 26054 HG21 ILE D 167   50.269 42.595 35.046 0.00 0.00
D

ATOM 26055 HG22 ILE D 167   49.022 41.411 34.609 0.00 0.00
D
ATOM 26056 HG23 ILE D 167   49.383 41.779 36.335 0.00 0.00
D
ATOM 26057 CG1 ILE D 167   50.591 39.251 35.984 0.00 0.00
D
ATOM 26058 HG11 ILE D 167   51.419 38.532 36.165 0.00 0.00
D
ATOM 26059 HG12 ILE D 167   50.218 39.590 36.973 0.00 0.00
D
ATOM 26060 CD ILE D 167   49.484 38.499 35.299 0.00 0.00
D
ATOM 26061 HD1 ILE D 167   49.158 37.519 35.711 0.00 0.00
D
ATOM 26062 HD2 ILE D 167   48.643 39.178 35.042 0.00 0.00
D
ATOM 26063 HD3 ILE D 167   49.960 38.166 34.353 0.00 0.00
D
ATOM 26064 C   ILE D 167   51.465 41.472 33.041 0.00 0.00
D
ATOM 26065 O   ILE D 167   50.481 41.529 32.330 0.00 0.00
D
ATOM 26066 N   ILE D 168   52.546 42.343 33.016 0.00 0.00
D
ATOM 26067 HN ILE D 168   53.345 42.186 33.591 0.00 0.00
D
ATOM 26068 CA ILE D 168   52.510 43.552 32.204 0.00 0.00
D
ATOM 26069 HA ILE D 168   51.490 43.892 32.311 0.00 0.00
D
ATOM 26070 CB ILE D 168   53.352 44.680 32.793 0.00 0.00
D
ATOM 26071 HB ILE D 168   53.248 45.625 32.219 0.00 0.00
D
ATOM 26072 CG2 ILE D 168   52.785 45.080 34.131 0.00 0.00
D
ATOM 26073 HG21 ILE D 168   51.761 45.496 34.016 0.00 0.00
D
ATOM 26074 HG22 ILE D 168   52.749 44.211 34.822 0.00 0.00
D
ATOM 26075 HG23 ILE D 168   53.464 45.882 34.493 0.00 0.00
D
ATOM 26076 CG1 ILE D 168   54.884 44.432 33.053 0.00 0.00
D
ATOM 26077 HG11 ILE D 168   55.347 45.203 33.706 0.00 0.00
D
ATOM 26078 HG12 ILE D 168   54.992 43.442 33.546 0.00 0.00
D
ATOM 26079 CD ILE D 168   55.736 44.416 31.668 0.00 0.00
D
ATOM 26080 HD1 ILE D 168   55.802 45.430 31.219 0.00 0.00
D
ATOM 26081 HD2 ILE D 168   56.693 44.041 32.090 0.00 0.00
D

ATOM 26082 HD3 ILE D 168   55.329 43.758 30.871 0.00 0.00
D
ATOM 26083 C   ILE D 168   52.788 43.219 30.740 0.00 0.00
D
ATOM 26084 O   ILE D 168   52.385 43.933 29.835 0.00 0.00
D
ATOM 26085 N   ASP D 169   53.296 42.055 30.392 0.00 0.00
D
ATOM 26086 HN ASP D 169   53.579 41.425 31.112 0.00 0.00
D
ATOM 26087 CA ASP D 169   53.538 41.604 29.037 0.00 0.00
D
ATOM 26088 HA ASP D 169   53.801 42.394 28.349 0.00 0.00
D
ATOM 26089 CB ASP D 169   54.786 40.559 29.209 0.00 0.00
D
ATOM 26090 HB1 ASP D 169   55.554 40.870 29.948 0.00 0.00
D
ATOM 26091 HB2 ASP D 169   54.425 39.633 29.706 0.00 0.00
D
ATOM 26092 CG ASP D 169   55.483 40.415 27.880 0.00 0.00
D
ATOM 26093 OD1 ASP D 169   55.907 41.432 27.294 0.00 0.00
D
ATOM 26094 OD2 ASP D 169   55.704 39.248 27.508 0.00 0.00
D
ATOM 26095 C   ASP D 169   52.351 40.840 28.499 0.00 0.00
D
ATOM 26096 O   ASP D 169   52.374 40.334 27.398 0.00 0.00
D
ATOM 26097 N   LEU D 170   51.250 40.835 29.181 0.00 0.00
D
ATOM 26098 HN LEU D 170   51.298 40.976 30.166 0.00 0.00
D
ATOM 26099 CA LEU D 170   50.080 40.120 28.759 0.00 0.00
D
ATOM 26100 HA LEU D 170   50.279 39.464 27.925 0.00 0.00
D
ATOM 26101 CB LEU D 170   49.362 39.144 29.812 0.00 0.00
D
ATOM 26102 HB1 LEU D 170   48.730 39.723 30.519 0.00 0.00
D
ATOM 26103 HB2 LEU D 170   48.625 38.562 29.217 0.00 0.00
D
ATOM 26104 CG LEU D 170   50.329 38.164 30.603 0.00 0.00
D
ATOM 26105 HG LEU D 170   51.164 38.724 31.076 0.00 0.00
D
ATOM 26106 CD1 LEU D 170   49.459 37.268 31.498 0.00 0.00
D
ATOM 26107 HD11 LEU D 170   48.686 36.721 30.916 0.00 0.00
D
ATOM 26108 HD12 LEU D 170   49.989 36.411 31.966 0.00 0.00
D

ATOM 26109 HD13 LEU D 170   49.036 37.817 32.366 0.00 0.00
D
ATOM 26110 CD2 LEU D 170   51.030 37.247 29.579 0.00 0.00
D
ATOM 26111 HD21 LEU D 170   51.732 36.625 30.174 0.00 0.00
D
ATOM 26112 HD22 LEU D 170   50.311 36.663 28.965 0.00 0.00
D
ATOM 26113 HD23 LEU D 170   51.642 37.834 28.862 0.00 0.00
D
ATOM 26114 C   LEU D 170   49.055 41.150 28.151 0.00 0.00
D
ATOM 26115 O   LEU D 170   48.627 42.064 28.809 0.00 0.00
D
ATOM 26116 N   ASN D 171   48.600 40.968 26.911 0.00 0.00
D
ATOM 26117 HN ASN D 171   48.923 40.200 26.364 0.00 0.00
D
ATOM 26118 CA ASN D 171   47.864 42.073 26.319 0.00 0.00
D
ATOM 26119 HA ASN D 171   48.175 42.980 26.817 0.00 0.00
D
ATOM 26120 CB ASN D 171   48.171 42.108 24.791 0.00 0.00
D
ATOM 26121 HB1 ASN D 171   48.035 41.060 24.447 0.00 0.00
D
ATOM 26122 HB2 ASN D 171   47.607 42.773 24.102 0.00 0.00
D
ATOM 26123 CG ASN D 171   49.681 42.471 24.623 0.00 0.00
D
ATOM 26124 OD1 ASN D 171   50.443 41.549 24.233 0.00 0.00
D
ATOM 26125 ND2 ASN D 171   50.134 43.725 24.839 0.00 0.00
D
ATOM 26126 HD21 ASN D 171   51.105 43.929 24.715 0.00 0.00
D
ATOM 26127 HD22 ASN D 171   49.471 44.362 25.232 0.00 0.00
D
ATOM 26128 C   ASN D 171   46.351 41.937 26.588 0.00 0.00
D
ATOM 26129 O   ASN D 171   45.593 42.837 26.309 0.00 0.00
D
ATOM 26130 N   VAL D 172   45.887 40.833 27.246 0.00 0.00
D
ATOM 26131 HN VAL D 172   46.616 40.193 27.479 0.00 0.00
D
ATOM 26132 CA VAL D 172   44.475 40.636 27.604 0.00 0.00
D
ATOM 26133 HA VAL D 172   43.956 40.726 26.661 0.00 0.00
D
ATOM 26134 CB VAL D 172   44.302 39.233 28.090 0.00 0.00
D
ATOM 26135 HB VAL D 172   43.201 39.161 28.218 0.00 0.00
D

ATOM 26136 CG1 VAL D 172   44.579 38.266 26.896 0.00 0.00
D
ATOM 26137 HG11 VAL D 172   43.736 38.326 26.175 0.00 0.00
D
ATOM 26138 HG12 VAL D 172   45.534 38.372 26.338 0.00 0.00
D
ATOM 26139 HG13 VAL D 172   44.700 37.279 27.390 0.00 0.00
D
ATOM 26140 CG2 VAL D 172   45.094 39.001 29.415 0.00 0.00
D
ATOM 26141 HG21 VAL D 172   44.615 39.708 30.126 0.00 0.00
D
ATOM 26142 HG22 VAL D 172   44.966 37.977 29.827 0.00 0.00
D
ATOM 26143 HG23 VAL D 172   46.177 39.247 29.441 0.00 0.00
D
ATOM 26144 C   VAL D 172   43.787 41.584 28.571 0.00 0.00
D
ATOM 26145 O   VAL D 172   42.557 41.735 28.477 0.00 0.00
D
ATOM 26146 N   ARG D 173   44.501 42.283 29.499 0.00 0.00
D
ATOM 26147 HN ARG D 173   45.458 42.030 29.618 0.00 0.00
D
ATOM 26148 CA ARG D 173   43.897 43.356 30.266 0.00 0.00
D
ATOM 26149 HA ARG D 173   42.971 43.035 30.719 0.00 0.00
D
ATOM 26150 CB ARG D 173   44.837 43.726 31.425 0.00 0.00
D
ATOM 26151 HB1 ARG D 173   44.842 42.771 31.994 0.00 0.00
D
ATOM 26152 HB2 ARG D 173   45.886 43.820 31.071 0.00 0.00
D
ATOM 26153 CG ARG D 173   44.330 44.949 32.220 0.00 0.00
D
ATOM 26154 HG1 ARG D 173   44.520 45.930 31.734 0.00 0.00
D
ATOM 26155 HG2 ARG D 173   43.230 44.791 32.218 0.00 0.00
D
ATOM 26156 CD ARG D 173   44.727 45.146 33.678 0.00 0.00
D
ATOM 26157 HD1 ARG D 173   44.257 46.061 34.097 0.00 0.00
D
ATOM 26158 HD2 ARG D 173   44.396 44.302 34.321 0.00 0.00
D
ATOM 26159 NE ARG D 173   46.202 45.401 33.448 0.00 0.00
D
ATOM 26160 HE ARG D 173   46.521 45.798 32.587 0.00 0.00
D
ATOM 26161 CZ ARG D 173   47.097 45.560 34.392 0.00 0.00
D
ATOM 26162 NH1 ARG D 173   46.828 45.157 35.687 0.00 0.00
D

ATOM 26163 HH11 ARG D 173   47.483 45.388 36.407 0.00 0.00
D
ATOM 26164 HH12 ARG D 173   45.873 45.211 35.979 0.00 0.00
D
ATOM 26165 NH2 ARG D 173   48.319 45.772 34.049 0.00 0.00
D
ATOM 26166 HH21 ARG D 173   49.044 45.582 34.711 0.00 0.00
D
ATOM 26167 HH22 ARG D 173   48.483 46.061 33.106 0.00 0.00
D
ATOM 26168 C   ARG D 173   43.377 44.584 29.564 0.00 0.00
D
ATOM 26169 O   ARG D 173   42.277 45.013 29.835 0.00 0.00
D
ATOM 26170 N   ALA D 174   44.120 45.116 28.639 0.00 0.00
D
ATOM 26171 HN ALA D 174   45.029 44.707 28.619 0.00 0.00
D
ATOM 26172 CA ALA D 174   43.731 46.207 27.719 0.00 0.00
D
ATOM 26173 HA ALA D 174   43.273 47.019 28.265 0.00 0.00
D
ATOM 26174 CB ALA D 174   45.029 46.647 26.878 0.00 0.00
D
ATOM 26175 HB1 ALA D 174   45.964 46.485 27.455 0.00 0.00
D
ATOM 26176 HB2 ALA D 174   45.264 45.911 26.080 0.00 0.00
D
ATOM 26177 HB3 ALA D 174   45.012 47.693 26.502 0.00 0.00
D
ATOM 26178 C   ALA D 174   42.599 45.843 26.769 0.00 0.00
D
ATOM 26179 O   ALA D 174   41.692 46.633 26.606 0.00 0.00
D
ATOM 26180 N   LYS D 175   42.699 44.652 26.137 0.00 0.00
D
ATOM 26181 HN LYS D 175   43.529 44.112 26.252 0.00 0.00
D
ATOM 26182 CA LYS D 175   41.918 44.244 25.058 0.00 0.00
D
ATOM 26183 HA LYS D 175   41.847 45.026 24.316 0.00 0.00
D
ATOM 26184 CB LYS D 175   42.463 42.920 24.463 0.00 0.00
D
ATOM 26185 HB1 LYS D 175   43.559 43.021 24.312 0.00 0.00
D
ATOM 26186 HB2 LYS D 175   42.432 41.991 25.072 0.00 0.00
D
ATOM 26187 CG LYS D 175   41.925 42.591 23.093 0.00 0.00
D
ATOM 26188 HG1 LYS D 175   40.881 42.252 23.267 0.00 0.00
D
ATOM 26189 HG2 LYS D 175   41.943 43.475 22.420 0.00 0.00
D

ATOM 26190 CD LYS D 175 42.634 41.401 22.536 0.00 0.00
D
ATOM 26191 HD1 LYS D 175 42.594 40.529 23.223 0.00 0.00
D
ATOM 26192 HD2 LYS D 175 42.069 41.148 21.614 0.00 0.00
D
ATOM 26193 CE LYS D 175 44.024 41.618 22.123 0.00 0.00
D
ATOM 26194 HE1 LYS D 175 44.092 42.502 21.454 0.00 0.00
D
ATOM 26195 HE2 LYS D 175 44.718 41.790 22.973 0.00 0.00
D
ATOM 26196 NZ LYS D 175 44.477 40.542 21.232 0.00 0.00
D
ATOM 26197 HZ1 LYS D 175 44.251 40.746 20.238 0.00 0.00
D
ATOM 26198 HZ2 LYS D 175 45.517 40.523 21.239 0.00 0.00
D
ATOM 26199 HZ3 LYS D 175 44.080 39.631 21.539 0.00 0.00
D
ATOM 26200 C LYS D 175 40.491 43.920 25.496 0.00 0.00
D
ATOM 26201 O LYS D 175 39.538 44.369 24.784 0.00 0.00
D
ATOM 26202 N TYR D 176 40.331 43.337 26.683 0.00 0.00
D
ATOM 26203 HN TYR D 176 41.127 43.030 27.198 0.00 0.00
D
ATOM 26204 CA TYR D 176 38.977 42.954 27.102 0.00 0.00
D
ATOM 26205 HA TYR D 176 38.228 43.178 26.357 0.00 0.00
D
ATOM 26206 CB TYR D 176 39.120 41.382 27.460 0.00 0.00
D
ATOM 26207 HB1 TYR D 176 39.953 41.269 28.187 0.00 0.00
D
ATOM 26208 HB2 TYR D 176 38.187 41.033 27.952 0.00 0.00
D
ATOM 26209 CG TYR D 176 39.505 40.629 26.190 0.00 0.00
D
ATOM 26210 CD1 TYR D 176 38.659 40.672 25.081 0.00 0.00
D
ATOM 26211 HD1 TYR D 176 37.778 41.292 25.150 0.00 0.00
D
ATOM 26212 CE1 TYR D 176 38.947 39.818 24.056 0.00 0.00
D
ATOM 26213 HE1 TYR D 176 38.227 39.739 23.256 0.00 0.00
D
ATOM 26214 CZ TYR D 176 40.142 39.074 23.997 0.00 0.00
D
ATOM 26215 OH TYR D 176 40.440 38.313 22.895 0.00 0.00
D
ATOM 26216 HH TYR D 176 41.246 37.810 23.032 0.00 0.00
D

ATOM 26217 CD2 TYR D 176 40.628 39.791 26.247 0.00 0.00
D
ATOM 26218 HD2 TYR D 176 41.348 39.752 27.051 0.00 0.00
D
ATOM 26219 CE2 TYR D 176 40.956 39.068 25.136 0.00 0.00
D
ATOM 26220 HE2 TYR D 176 41.797 38.391 25.108 0.00 0.00
D
ATOM 26221 C TYR D 176 38.565 43.677 28.337 0.00 0.00
D
ATOM 26222 O TYR D 176 37.483 43.481 28.947 0.00 0.00
D
ATOM 26223 N GLY D 177 39.353 44.717 28.819 0.00 0.00
D
ATOM 26224 HN GLY D 177 40.186 44.871 28.292 0.00 0.00
D
ATOM 26225 CA GLY D 177 38.982 45.688 29.846 0.00 0.00
D
ATOM 26226 HA1 GLY D 177 38.040 46.148 29.587 0.00 0.00
D
ATOM 26227 HA2 GLY D 177 39.736 46.461 29.862 0.00 0.00
D
ATOM 26228 C GLY D 177 38.986 45.254 31.298 0.00 0.00
D
ATOM 26229 O GLY D 177 38.683 46.080 32.171 0.00 0.00
D
ATOM 26230 N CYS D 178 39.316 43.981 31.571 0.00 0.00
D
ATOM 26231 HN CYS D 178 39.694 43.571 30.744 0.00 0.00
D
ATOM 26232 CA CYS D 178 39.269 43.371 32.886 0.00 0.00
D
ATOM 26233 HA CYS D 178 38.371 43.765 33.339 0.00 0.00
D
ATOM 26234 CB CYS D 178 39.323 41.771 32.851 0.00 0.00
D
ATOM 26235 HB1 CYS D 178 40.372 41.414 32.777 0.00 0.00
D
ATOM 26236 HB2 CYS D 178 38.919 41.367 33.804 0.00 0.00
D
ATOM 26237 SG CYS D 178 38.194 41.199 31.445 0.00 0.00
D
ATOM 26238 HG1 CYS D 178 37.486 42.311 31.311 0.00 0.00
D
ATOM 26239 C CYS D 178 40.442 43.776 33.770 0.00 0.00
D
ATOM 26240 O CYS D 178 41.564 43.523 33.400 0.00 0.00
D
ATOM 26241 N THR D 179 40.215 44.549 34.854 0.00 0.00
D
ATOM 26242 HN THR D 179 39.246 44.569 35.089 0.00 0.00
D
ATOM 26243 CA THR D 179 41.281 44.895 35.839 0.00 0.00
D

ATOM 26244 HA THR D 179 41.995 45.352 35.170 0.00 0.00
D
ATOM 26245 CB THR D 179 40.841 45.959 36.819 0.00 0.00
D
ATOM 26246 HB THR D 179 40.235 46.728 36.294 0.00 0.00
D
ATOM 26247 OG1 THR D 179 41.882 46.625 37.379 0.00 0.00
D
ATOM 26248 HG1 THR D 179 41.622 47.537 37.525 0.00 0.00
D
ATOM 26249 CG2 THR D 179 39.996 45.391 37.881 0.00 0.00
D
ATOM 26250 HG21 THR D 179 40.567 44.648 38.478 0.00 0.00
D
ATOM 26251 HG22 THR D 179 39.708 46.152 38.636 0.00 0.00
D
ATOM 26252 HG23 THR D 179 39.048 44.891 37.587 0.00 0.00
D
ATOM 26253 C THR D 179 41.908 43.688 36.560 0.00 0.00
D
ATOM 26254 O THR D 179 41.330 42.622 36.611 0.00 0.00
D
ATOM 26255 N ILE D 180 43.110 43.844 37.102 0.00 0.00
D
ATOM 26256 HN ILE D 180 43.454 44.773 37.216 0.00 0.00
D
ATOM 26257 CA ILE D 180 43.764 42.794 37.927 0.00 0.00
D
ATOM 26258 HA ILE D 180 43.065 41.979 38.047 0.00 0.00
D
ATOM 26259 CB ILE D 180 45.185 42.409 37.440 0.00 0.00
D
ATOM 26260 HB ILE D 180 45.731 43.373 37.359 0.00 0.00
D
ATOM 26261 CG2 ILE D 180 45.863 41.545 38.542 0.00 0.00
D
ATOM 26262 HG21 ILE D 180 45.161 40.719 38.785 0.00 0.00
D
ATOM 26263 HG22 ILE D 180 46.840 41.233 38.116 0.00 0.00
D
ATOM 26264 HG23 ILE D 180 46.110 42.078 39.485 0.00 0.00
D
ATOM 26265 CG1 ILE D 180 45.091 41.624 36.135 0.00 0.00
D
ATOM 26266 HG11 ILE D 180 44.714 40.581 36.209 0.00 0.00
D
ATOM 26267 HG12 ILE D 180 44.517 42.167 35.354 0.00 0.00
D
ATOM 26268 CD ILE D 180 46.470 41.426 35.599 0.00 0.00
D
ATOM 26269 HD1 ILE D 180 47.040 42.355 35.813 0.00 0.00
D
ATOM 26270 HD2 ILE D 180 46.993 40.622 36.160 0.00 0.00
D

ATOM 26271 HD3 ILE D 180 46.593 41.408 34.494 0.00 0.00
D
ATOM 26272 C ILE D 180 43.980 43.335 39.320 0.00 0.00
D
ATOM 26273 O ILE D 180 44.689 44.289 39.522 0.00 0.00
D
ATOM 26274 N LEU D 181 43.293 42.820 40.351 0.00 0.00
D
ATOM 26275 HN LEU D 181 42.749 41.998 40.204 0.00 0.00
D
ATOM 26276 CA LEU D 181 43.289 43.374 41.632 0.00 0.00
D
ATOM 26277 HA LEU D 181 43.278 44.452 41.556 0.00 0.00
D
ATOM 26278 CB LEU D 181 42.045 43.038 42.508 0.00 0.00
D
ATOM 26279 HB1 LEU D 181 41.911 41.946 42.662 0.00 0.00
D
ATOM 26280 HB2 LEU D 181 42.078 43.513 43.511 0.00 0.00
D
ATOM 26281 CG LEU D 181 40.751 43.561 41.919 0.00 0.00
D
ATOM 26282 HG LEU D 181 40.656 43.209 40.870 0.00 0.00
D
ATOM 26283 CD1 LEU D 181 39.516 43.113 42.729 0.00 0.00
D
ATOM 26284 HD11 LEU D 181 39.741 43.167 43.816 0.00 0.00
D
ATOM 26285 HD12 LEU D 181 38.659 43.774 42.474 0.00 0.00
D
ATOM 26286 HD13 LEU D 181 39.245 42.076 42.438 0.00 0.00
D
ATOM 26287 CD2 LEU D 181 40.785 45.090 41.805 0.00 0.00
D
ATOM 26288 HD21 LEU D 181 39.787 45.373 41.408 0.00 0.00
D
ATOM 26289 HD22 LEU D 181 40.942 45.634 42.761 0.00 0.00
D
ATOM 26290 HD23 LEU D 181 41.494 45.478 41.043 0.00 0.00
D
ATOM 26291 C LEU D 181 44.560 42.973 42.425 0.00 0.00
D
ATOM 26292 O LEU D 181 45.125 43.737 43.161 0.00 0.00
D
ATOM 26293 N ALA D 182 44.997 41.744 42.331 0.00 0.00
D
ATOM 26294 HN ALA D 182 44.411 41.034 41.947 0.00 0.00
D
ATOM 26295 CA ALA D 182 46.131 41.171 42.978 0.00 0.00
D
ATOM 26296 HA ALA D 182 46.938 41.885 42.906 0.00 0.00
D
ATOM 26297 CB ALA D 182 45.880 40.669 44.454 0.00 0.00
D

ATOM 26298 HB1 ALA D 182 44.968 40.036 44.502 0.00 0.00
D
ATOM 26299 HB2 ALA D 182 46.798 40.177 44.841 0.00 0.00
D
ATOM 26300 HB3 ALA D 182 45.900 41.512 45.176 0.00 0.00
D
ATOM 26301 C ALA D 182 46.634 39.856 42.313 0.00 0.00
D
ATOM 26302 O ALA D 182 45.989 39.092 41.561 0.00 0.00
D
ATOM 26303 N ILE D 183 47.940 39.485 42.653 0.00 0.00
D
ATOM 26304 HN ILE D 183 48.533 39.933 43.317 0.00 0.00
D
ATOM 26305 CA ILE D 183 48.486 38.201 42.295 0.00 0.00
D
ATOM 26306 HA ILE D 183 47.749 37.629 41.749 0.00 0.00
D
ATOM 26307 CB ILE D 183 49.740 38.164 41.458 0.00 0.00
D
ATOM 26308 HB ILE D 183 50.464 38.556 42.204 0.00 0.00
D
ATOM 26309 CG2 ILE D 183 49.983 36.690 41.039 0.00 0.00
D
ATOM 26310 HG21 ILE D 183 50.108 36.042 41.933 0.00 0.00
D
ATOM 26311 HG22 ILE D 183 49.152 36.357 40.380 0.00 0.00
D
ATOM 26312 HG23 ILE D 183 50.938 36.558 40.486 0.00 0.00
D
ATOM 26313 CG1 ILE D 183 49.733 39.272 40.365 0.00 0.00
D
ATOM 26314 HG11 ILE D 183 49.049 38.867 39.588 0.00 0.00
D
ATOM 26315 HG12 ILE D 183 49.371 40.260 40.721 0.00 0.00
D
ATOM 26316 CD ILE D 183 50.995 39.407 39.597 0.00 0.00
D
ATOM 26317 HD1 ILE D 183 51.191 38.519 38.959 0.00 0.00
D
ATOM 26318 HD2 ILE D 183 50.919 40.217 38.841 0.00 0.00
D
ATOM 26319 HD3 ILE D 183 51.850 39.642 40.267 0.00 0.00
D
ATOM 26320 C ILE D 183 48.714 37.577 43.697 0.00 0.00
D
ATOM 26321 O ILE D 183 49.006 38.243 44.660 0.00 0.00
D
ATOM 26322 N LYS D 184 48.646 36.275 43.828 0.00 0.00
D
ATOM 26323 HN LYS D 184 48.494 35.632 43.081 0.00 0.00
D
ATOM 26324 CA LYS D 184 48.633 35.676 45.076 0.00 0.00
D

ATOM 26325 HA LYS D 184 49.103 36.223 45.880 0.00 0.00
D
ATOM 26326 CB LYS D 184 47.143 35.369 45.337 0.00 0.00
D
ATOM 26327 HB1 LYS D 184 46.757 36.282 45.838 0.00 0.00
D
ATOM 26328 HB2 LYS D 184 46.593 35.133 44.401 0.00 0.00
D
ATOM 26329 CG LYS D 184 46.783 34.279 46.347 0.00 0.00
D
ATOM 26330 HG1 LYS D 184 46.872 33.285 45.859 0.00 0.00
D
ATOM 26331 HG2 LYS D 184 47.563 34.294 47.138 0.00 0.00
D
ATOM 26332 CD LYS D 184 45.472 34.430 47.142 0.00 0.00
D
ATOM 26333 HD1 LYS D 184 45.607 35.203 47.929 0.00 0.00
D
ATOM 26334 HD2 LYS D 184 44.774 34.910 46.422 0.00 0.00
D
ATOM 26335 CE LYS D 184 44.758 33.146 47.508 0.00 0.00
D
ATOM 26336 HE1 LYS D 184 44.880 32.410 46.685 0.00 0.00
D
ATOM 26337 HE2 LYS D 184 45.392 32.653 48.276 0.00 0.00
D
ATOM 26338 NZ LYS D 184 43.380 33.180 47.930 0.00 0.00
D
ATOM 26339 HZ1 LYS D 184 42.707 33.423 47.175 0.00 0.00
D
ATOM 26340 HZ2 LYS D 184 43.179 32.220 48.276 0.00 0.00
D
ATOM 26341 HZ3 LYS D 184 43.155 33.813 48.723 0.00 0.00
D
ATOM 26342 C LYS D 184 49.296 34.347 44.777 0.00 0.00
D
ATOM 26343 O LYS D 184 49.011 33.657 43.799 0.00 0.00
D
ATOM 26344 N HSE D 185 50.302 33.994 45.541 0.00 0.00
D
ATOM 26345 HN HSE D 185 50.536 34.473 46.383 0.00 0.00
D
ATOM 26346 CA HSE D 185 51.191 32.949 45.278 0.00 0.00
D
ATOM 26347 HA HSE D 185 50.695 32.127 44.784 0.00 0.00
D
ATOM 26348 CB HSE D 185 52.392 33.322 44.359 0.00 0.00
D
ATOM 26349 HB1 HSE D 185 52.961 32.392 44.146 0.00 0.00
D
ATOM 26350 HB2 HSE D 185 51.895 33.895 43.547 0.00 0.00
D
ATOM 26351 ND1 HSE D 185 54.565 33.936 45.060 0.00 0.00
D

ATOM 26352 CG HSE D 185 53.240 34.300 45.026 0.00 0.00
D
ATOM 26353 CE1 HSE D 185 55.138 34.794 45.792 0.00 0.00
D
ATOM 26354 HE1 HSE D 185 56.205 34.848 46.006 0.00 0.00
D
ATOM 26355 NE2 HSE D 185 54.284 35.707 46.234 0.00 0.00
D
ATOM 26356 HE2 HSE D 185 54.473 36.199 47.083 0.00 0.00
D
ATOM 26357 CD2 HSE D 185 53.047 35.457 45.697 0.00 0.00
D
ATOM 26358 HD2 HSE D 185 52.174 36.090 45.798 0.00 0.00
D
ATOM 26359 C HSE D 185 51.617 32.355 46.623 0.00 0.00
D
ATOM 26360 O HSE D 185 52.168 33.060 47.438 0.00 0.00
D
ATOM 26361 N HSE D 186 51.245 31.122 46.866 0.00 0.00
D
ATOM 26362 HN HSE D 186 50.788 30.609 46.143 0.00 0.00
D
ATOM 26363 CA HSE D 186 51.615 30.318 48.021 0.00 0.00
D
ATOM 26364 HA HSE D 186 50.918 29.494 48.070 0.00 0.00
D
ATOM 26365 CB HSE D 186 52.953 29.524 47.737 0.00 0.00
D
ATOM 26366 HB1 HSE D 186 53.737 30.201 47.336 0.00 0.00
D
ATOM 26367 HB2 HSE D 186 53.299 29.080 48.695 0.00 0.00
D
ATOM 26368 ND1 HSE D 186 53.219 28.301 45.676 0.00 0.00
D
ATOM 26369 CG HSE D 186 52.645 28.390 46.910 0.00 0.00
D
ATOM 26370 CE1 HSE D 186 52.883 27.131 45.223 0.00 0.00
D
ATOM 26371 HE1 HSE D 186 53.079 26.671 44.255 0.00 0.00
D
ATOM 26372 NE2 HSE D 186 52.124 26.440 46.106 0.00 0.00
D
ATOM 26373 HE2 HSE D 186 51.449 25.721 45.941 0.00 0.00
D
ATOM 26374 CD2 HSE D 186 51.991 27.240 47.168 0.00 0.00
D
ATOM 26375 HD2 HSE D 186 51.366 26.801 47.936 0.00 0.00
D
ATOM 26376 C HSE D 186 51.603 30.922 49.413 0.00 0.00
D
ATOM 26377 O HSE D 186 52.562 30.722 50.204 0.00 0.00
D
ATOM 26378 N GLY D 187 50.589 31.701 49.747 0.00 0.00
D

ATOM 26379 HN GLY D 187   49.834 31.899 49.127 0.00 0.00
D
ATOM 26380 CA GLY D 187   50.610 32.311 51.085 0.00 0.00
D
ATOM 26381 HA1 GLY D 187   51.031 31.651 51.829 0.00 0.00
D
ATOM 26382 HA2 GLY D 187   49.601 32.592 51.347 0.00 0.00
D
ATOM 26383 C   GLY D 187   51.262 33.633 51.163 0.00 0.00
D
ATOM 26384 O   GLY D 187   51.451 34.225 52.221 0.00 0.00
D
ATOM 26385 N   ASP D 188   51.686 34.125 50.019 0.00 0.00
D
ATOM 26386 HN ASP D 188   51.711 33.516 49.230 0.00 0.00
D
ATOM 26387 CA ASP D 188   52.222 35.443 49.875 0.00 0.00
D
ATOM 26388 HA ASP D 188   51.938 36.055 50.718 0.00 0.00
D
ATOM 26389 CB ASP D 188   53.766 35.374 49.681 0.00 0.00
D
ATOM 26390 HB1 ASP D 188   54.293 34.840 50.501 0.00 0.00
D
ATOM 26391 HB2 ASP D 188   53.996 34.851 48.728 0.00 0.00
D
ATOM 26392 CG ASP D 188   54.280 36.804 49.704 0.00 0.00
D
ATOM 26393 OD1 ASP D 188   54.330 37.503 50.777 0.00 0.00
D
ATOM 26394 OD2 ASP D 188   54.722 37.188 48.595 0.00 0.00
D
ATOM 26395 C   ASP D 188   51.469 36.137 48.739 0.00 0.00
D
ATOM 26396 O   ASP D 188   50.662 35.622 47.982 0.00 0.00
D
ATOM 26397 N   ILE D 189   51.601 37.500 48.745 0.00 0.00
D
ATOM 26398 HN ILE D 189   52.344 37.936 49.246 0.00 0.00
D
ATOM 26399 CA ILE D 189   50.760 38.436 47.900 0.00 0.00
D
ATOM 26400 HA ILE D 189   50.268 37.881 47.114 0.00 0.00
D
ATOM 26401 CB ILE D 189   49.739 39.329 48.708 0.00 0.00
D
ATOM 26402 HB ILE D 189   49.009 38.645 49.191 0.00 0.00
D
ATOM 26403 CG2 ILE D 189   50.518 40.252 49.771 0.00 0.00
D
ATOM 26404 HG21 ILE D 189   51.120 39.775 50.573 0.00 0.00
D
ATOM 26405 HG22 ILE D 189   51.100 40.959 49.142 0.00 0.00
D

ATOM 26406 HG23 ILE D 189   49.890 41.030 50.257 0.00 0.00
D
ATOM 26407 CG1 ILE D 189   48.823 40.227 47.799 0.00 0.00
D
ATOM 26408 HG11 ILE D 189   49.421 41.012 47.289 0.00 0.00
D
ATOM 26409 HG12 ILE D 189   48.426 39.524 47.036 0.00 0.00
D
ATOM 26410 CD ILE D 189   47.684 40.816 48.579 0.00 0.00
D
ATOM 26411 HD1 ILE D 189   47.946 41.765 49.094 0.00 0.00
D
ATOM 26412 HD2 ILE D 189   46.953 41.224 47.848 0.00 0.00
D
ATOM 26413 HD3 ILE D 189   47.168 40.077 49.228 0.00 0.00
D
ATOM 26414 C   ILE D 189   51.725 39.356 47.168 0.00 0.00
D
ATOM 26415 O   ILE D 189   52.699 39.874 47.690 0.00 0.00
D
ATOM 26416 N   CYS D 190   51.410 39.622 45.914 0.00 0.00
D
ATOM 26417 HN CYS D 190   50.650 39.169 45.454 0.00 0.00
D
ATOM 26418 CA CYS D 190   52.028 40.672 45.061 0.00 0.00
D
ATOM 26419 HA CYS D 190   52.790 41.229 45.584 0.00 0.00
D
ATOM 26420 CB CYS D 190   52.628 39.822 43.908 0.00 0.00
D
ATOM 26421 HB1 CYS D 190   53.458 39.231 44.350 0.00 0.00
D
ATOM 26422 HB2 CYS D 190   51.838 39.120 43.564 0.00 0.00
D
ATOM 26423 SG CYS D 190   53.205 40.836 42.550 0.00 0.00
D
ATOM 26424 HG1 CYS D 190   53.427 39.797 41.759 0.00 0.00
D
ATOM 26425 C   CYS D 190   51.046 41.626 44.570 0.00 0.00
D
ATOM 26426 O   CYS D 190   50.067 41.204 43.991 0.00 0.00
D
ATOM 26427 N   LEU D 191   51.229 42.910 44.818 0.00 0.00
D
ATOM 26428 HN LEU D 191   52.035 43.210 45.323 0.00 0.00
D
ATOM 26429 CA LEU D 191   50.396 44.013 44.237 0.00 0.00
D
ATOM 26430 HA LEU D 191   49.510 43.521 43.863 0.00 0.00
D
ATOM 26431 CB LEU D 191   49.934 45.005 45.313 0.00 0.00
D
ATOM 26432 HB1 LEU D 191   50.813 45.570 45.692 0.00 0.00
D

ATOM 26433 HB2 LEU D 191 49.249 45.687 44.764 0.00 0.00
D
ATOM 26434 CG LEU D 191 49.186 44.437 46.539 0.00 0.00
D
ATOM 26435 HG LEU D 191 49.882 43.665 46.932 0.00 0.00
D
ATOM 26436 CD1 LEU D 191 49.031 45.487 47.689 0.00 0.00
D
ATOM 26437 HD11 LEU D 191 48.026 45.955 47.613 0.00 0.00
D
ATOM 26438 HD12 LEU D 191 48.992 44.887 48.624 0.00 0.00
D
ATOM 26439 HD13 LEU D 191 49.835 46.252 47.638 0.00 0.00
D
ATOM 26440 CD2 LEU D 191 47.858 43.779 46.077 0.00 0.00
D
ATOM 26441 HD21 LEU D 191 47.240 43.662 46.993 0.00 0.00
D
ATOM 26442 HD22 LEU D 191 47.390 44.435 45.312 0.00 0.00
D
ATOM 26443 HD23 LEU D 191 47.976 42.812 45.543 0.00 0.00
D
ATOM 26444 C LEU D 191 51.211 44.786 43.111 0.00 0.00
D
ATOM 26445 O LEU D 191 50.750 45.666 42.441 0.00 0.00
D
ATOM 26446 N SER D 192 52.496 44.490 42.895 0.00 0.00
D
ATOM 26447 HN SER D 192 52.912 43.786 43.466 0.00 0.00
D
ATOM 26448 CA SER D 192 53.407 45.246 42.036 0.00 0.00
D
ATOM 26449 HA SER D 192 53.077 46.272 42.115 0.00 0.00
D
ATOM 26450 CB SER D 192 54.896 45.133 42.421 0.00 0.00
D
ATOM 26451 HB1 SER D 192 55.482 45.652 41.633 0.00 0.00
D
ATOM 26452 HB2 SER D 192 54.959 45.726 43.359 0.00 0.00
D
ATOM 26453 OG SER D 192 55.393 43.804 42.686 0.00 0.00
D
ATOM 26454 HG1 SER D 192 56.004 43.653 41.961 0.00 0.00
D
ATOM 26455 C SER D 192 53.348 44.764 40.575 0.00 0.00
D
ATOM 26456 O SER D 192 54.053 45.373 39.758 0.00 0.00
D
ATOM 26457 N GLU D 196 52.612 43.702 40.310 0.00 0.00
D
ATOM 26458 HN GLU D 196 52.242 43.254 41.120 0.00 0.00
D
ATOM 26459 CA GLU D 196 52.324 43.035 39.005 0.00 0.00
D

ATOM 26460 HA GLU D 196 51.700 42.205 39.303 0.00 0.00
D
ATOM 26461 CB GLU D 196 51.552 43.959 38.060 0.00 0.00
D
ATOM 26462 HB1 GLU D 196 51.322 43.381 37.140 0.00 0.00
D
ATOM 26463 HB2 GLU D 196 52.313 44.714 37.767 0.00 0.00
D
ATOM 26464 CG GLU D 196 50.300 44.486 38.574 0.00 0.00
D
ATOM 26465 HG1 GLU D 196 49.776 43.588 38.964 0.00 0.00
D
ATOM 26466 HG2 GLU D 196 50.435 45.226 39.392 0.00 0.00
D
ATOM 26467 CD GLU D 196 49.399 45.145 37.565 0.00 0.00
D
ATOM 26468 OE1 GLU D 196 48.218 45.486 37.928 0.00 0.00
D
ATOM 26469 OE2 GLU D 196 49.819 45.343 36.434 0.00 0.00
D
ATOM 26470 C GLU D 196 53.526 42.425 38.308 0.00 0.00
D
ATOM 26471 O GLU D 196 53.691 42.541 37.091 0.00 0.00
D
ATOM 26472 N ASP D 197 54.349 41.641 38.984 0.00 0.00
D
ATOM 26473 HN ASP D 197 54.212 41.636 39.972 0.00 0.00
D
ATOM 26474 CA ASP D 197 55.480 41.071 38.401 0.00 0.00
D
ATOM 26475 HA ASP D 197 55.835 41.828 37.717 0.00 0.00
D
ATOM 26476 CB ASP D 197 56.700 40.940 39.411 0.00 0.00
D
ATOM 26477 HB1 ASP D 197 56.525 40.190 40.212 0.00 0.00
D
ATOM 26478 HB2 ASP D 197 57.535 40.530 38.803 0.00 0.00
D
ATOM 26479 CG ASP D 197 57.278 42.160 39.919 0.00 0.00
D
ATOM 26480 OD1 ASP D 197 57.046 42.546 41.086 0.00 0.00
D
ATOM 26481 OD2 ASP D 197 58.063 42.808 39.116 0.00 0.00
D
ATOM 26482 C ASP D 197 55.100 39.826 37.683 0.00 0.00
D
ATOM 26483 O ASP D 197 54.094 39.853 36.951 0.00 0.00
D
ATOM 26484 N ILE D 198 55.843 38.757 37.969 0.00 0.00
D
ATOM 26485 HN ILE D 198 56.401 38.693 38.792 0.00 0.00
D
ATOM 26486 CA ILE D 198 55.833 37.512 37.177 0.00 0.00
D

ATOM 26487 HA ILE D 198 55.471 37.730 36.183 0.00 0.00
D
ATOM 26488 CB ILE D 198 57.276 36.840 37.019 0.00 0.00
D
ATOM 26489 HB ILE D 198 57.457 36.565 38.080 0.00 0.00
D
ATOM 26490 CG2 ILE D 198 57.312 35.701 36.087 0.00 0.00
D
ATOM 26491 HG21 ILE D 198 56.834 36.023 35.137 0.00 0.00
D
ATOM 26492 HG22 ILE D 198 58.373 35.538 35.801 0.00 0.00
D
ATOM 26493 HG23 ILE D 198 56.850 34.776 36.494 0.00 0.00
D
ATOM 26494 CG1 ILE D 198 58.340 37.912 36.787 0.00 0.00
D
ATOM 26495 HG11 ILE D 198 58.493 38.526 37.701 0.00 0.00
D
ATOM 26496 HG12 ILE D 198 59.301 37.419 36.527 0.00 0.00
D
ATOM 26497 CD ILE D 198 58.054 38.829 35.596 0.00 0.00
D
ATOM 26498 HD1 ILE D 198 57.131 39.446 35.648 0.00 0.00
D
ATOM 26499 HD2 ILE D 198 58.844 39.577 35.371 0.00 0.00
D
ATOM 26500 HD3 ILE D 198 57.981 38.185 34.694 0.00 0.00
D
ATOM 26501 C ILE D 198 54.894 36.536 37.839 0.00 0.00
D
ATOM 26502 O ILE D 198 54.698 36.571 39.045 0.00 0.00
D
ATOM 26503 N ILE D 199 54.347 35.567 37.047 0.00 0.00
D
ATOM 26504 HN ILE D 199 54.392 35.615 36.053 0.00 0.00
D
ATOM 26505 CA ILE D 199 53.373 34.665 37.652 0.00 0.00
D
ATOM 26506 HA ILE D 199 53.500 34.686 38.725 0.00 0.00
D
ATOM 26507 CB ILE D 199 51.926 34.973 37.229 0.00 0.00
D
ATOM 26508 HB ILE D 199 51.808 36.032 37.542 0.00 0.00
D
ATOM 26509 CG2 ILE D 199 51.779 34.963 35.690 0.00 0.00
D
ATOM 26510 HG21 ILE D 199 52.529 35.664 35.265 0.00 0.00
D
ATOM 26511 HG22 ILE D 199 52.027 33.966 35.267 0.00 0.00
D
ATOM 26512 HG23 ILE D 199 50.797 35.223 35.241 0.00 0.00
D
ATOM 26513 CG1 ILE D 199 50.963 34.033 37.941 0.00 0.00
D

ATOM 26514 HG11 ILE D 199 51.101 33.021 37.502 0.00 0.00
D
ATOM 26515 HG12 ILE D 199 51.183 34.079 39.029 0.00 0.00
D
ATOM 26516 CD ILE D 199 49.499 34.513 37.812 0.00 0.00
D
ATOM 26517 HD1 ILE D 199 49.298 35.481 38.320 0.00 0.00
D
ATOM 26518 HD2 ILE D 199 49.389 34.600 36.710 0.00 0.00
D
ATOM 26519 HD3 ILE D 199 48.877 33.687 38.218 0.00 0.00
D
ATOM 26520 C ILE D 199 53.909 33.220 37.322 0.00 0.00
D
ATOM 26521 O ILE D 199 54.687 33.037 36.410 0.00 0.00
D
ATOM 26522 N ARG D 200 53.466 32.180 38.128 0.00 0.00
D
ATOM 26523 HN ARG D 200 52.797 32.414 38.829 0.00 0.00
D
ATOM 26524 CA ARG D 200 53.846 30.836 37.928 0.00 0.00
D
ATOM 26525 HA ARG D 200 54.611 30.748 37.171 0.00 0.00
D
ATOM 26526 CB ARG D 200 54.454 30.207 39.253 0.00 0.00
D
ATOM 26527 HB1 ARG D 200 53.759 30.344 40.109 0.00 0.00
D
ATOM 26528 HB2 ARG D 200 54.677 29.150 38.994 0.00 0.00
D
ATOM 26529 CG ARG D 200 55.826 30.832 39.607 0.00 0.00
D
ATOM 26530 HG1 ARG D 200 56.659 30.412 39.004 0.00 0.00
D
ATOM 26531 HG2 ARG D 200 55.691 31.912 39.827 0.00 0.00
D
ATOM 26532 CD ARG D 200 56.380 30.323 40.925 0.00 0.00
D
ATOM 26533 HD1 ARG D 200 56.583 29.231 40.931 0.00 0.00
D
ATOM 26534 HD2 ARG D 200 57.327 30.842 41.186 0.00 0.00
D
ATOM 26535 NE ARG D 200 55.429 30.593 41.955 0.00 0.00
D
ATOM 26536 HE ARG D 200 54.527 30.979 41.761 0.00 0.00
D
ATOM 26537 CZ ARG D 200 55.460 30.054 43.196 0.00 0.00
D
ATOM 26538 NH1 ARG D 200 56.483 29.325 43.653 0.00 0.00
D
ATOM 26539 HH11 ARG D 200 56.379 28.960 44.578 0.00 0.00
D
ATOM 26540 HH12 ARG D 200 57.296 29.266 43.072 0.00 0.00
D

ATOM 26541 NH2 ARG D 200   54.421 30.233 43.973 0.00 0.00
D
ATOM 26542 HH21 ARG D 200   54.227 29.693 44.792 0.00 0.00
D
ATOM 26543 HH22 ARG D 200   53.589 30.512 43.494 0.00 0.00
D
ATOM 26544 C   ARG D 200   52.680 29.929 37.616 0.00 0.00
D
ATOM 26545 O   ARG D 200   51.524 30.363 37.723 0.00 0.00
D
ATOM 26546 N   GLU D 201   52.863 28.741 37.125 0.00 0.00
D
ATOM 26547 HN GLU D 201   53.762 28.377 36.896 0.00 0.00
D
ATOM 26548 CA GLU D 201   51.779 27.727 37.187 0.00 0.00
D
ATOM 26549 HA GLU D 201   50.968 28.080 36.566 0.00 0.00
D
ATOM 26550 CB GLU D 201   52.301 26.512 36.391 0.00 0.00
D
ATOM 26551 HB1 GLU D 201   52.430 26.637 35.295 0.00 0.00
D
ATOM 26552 HB2 GLU D 201   53.341 26.375 36.759 0.00 0.00
D
ATOM 26553 CG GLU D 201   51.463 25.180 36.459 0.00 0.00
D
ATOM 26554 HG1 GLU D 201   51.259 24.952 37.527 0.00 0.00
D
ATOM 26555 HG2 GLU D 201   50.512 25.337 35.907 0.00 0.00
D
ATOM 26556 CD GLU D 201   52.212 23.973 35.791 0.00 0.00
D
ATOM 26557 OE1 GLU D 201   53.434 23.878 35.694 0.00 0.00
D
ATOM 26558 OE2 GLU D 201   51.388 23.075 35.537 0.00 0.00
D
ATOM 26559 C   GLU D 201   51.226 27.372 38.582 0.00 0.00
D
ATOM 26560 O   GLU D 201   51.976 27.042 39.475 0.00 0.00
D
ATOM 26561 N   GLN D 202   49.866 27.374 38.779 0.00 0.00
D
ATOM 26562 HN GLN D 202   49.338 27.392 37.934 0.00 0.00
D
ATOM 26563 CA GLN D 202   49.068 27.118 39.926 0.00 0.00
D
ATOM 26564 HA GLN D 202   48.027 26.939 39.699 0.00 0.00
D
ATOM 26565 CB GLN D 202   49.565 25.834 40.601 0.00 0.00
D
ATOM 26566 HB1 GLN D 202   50.109 25.183 39.884 0.00 0.00
D
ATOM 26567 HB2 GLN D 202   50.211 26.058 41.477 0.00 0.00
D

ATOM 26568 CG GLN D 202 48.371 25.031 41.188 0.00 0.00
D
ATOM 26569 HG1 GLN D 202 47.698 25.708 41.756 0.00 0.00
D
ATOM 26570 HG2 GLN D 202 47.815 24.648 40.305 0.00 0.00
D
ATOM 26571 CD GLN D 202 48.714 23.897 42.064 0.00 0.00
D
ATOM 26572 OE1 GLN D 202 49.616 23.997 42.932 0.00 0.00
D
ATOM 26573 NE2 GLN D 202 48.011 22.714 41.890 0.00 0.00
D
ATOM 26574 HE21 GLN D 202 48.570 21.954 42.223 0.00 0.00
D
ATOM 26575 HE22 GLN D 202 47.337 22.728 41.152 0.00 0.00
D
ATOM 26576 C GLN D 202 49.074 28.220 40.928 0.00 0.00
D
ATOM 26577 O GLN D 202 48.684 28.029 42.159 0.00 0.00
D
ATOM 26578 N ASP D 203 49.498 29.386 40.505 0.00 0.00
D
ATOM 26579 HN ASP D 203 49.915 29.510 39.608 0.00 0.00
D
ATOM 26580 CA ASP D 203 49.403 30.633 41.228 0.00 0.00
D
ATOM 26581 HA ASP D 203 49.274 30.556 42.297 0.00 0.00
D
ATOM 26582 CB ASP D 203 50.663 31.467 40.866 0.00 0.00
D
ATOM 26583 HB1 ASP D 203 50.810 31.511 39.766 0.00 0.00
D
ATOM 26584 HB2 ASP D 203 50.528 32.555 41.044 0.00 0.00
D
ATOM 26585 CG ASP D 203 51.965 31.176 41.653 0.00 0.00
D
ATOM 26586 OD1 ASP D 203 52.874 31.967 41.496 0.00 0.00
D
ATOM 26587 OD2 ASP D 203 52.111 30.231 42.453 0.00 0.00
D
ATOM 26588 C ASP D 203 48.025 31.127 40.870 0.00 0.00
D
ATOM 26589 O ASP D 203 47.485 30.602 39.946 0.00 0.00
D
ATOM 26590 N CYS D 204 47.481 32.036 41.683 0.00 0.00
D
ATOM 26591 HN CYS D 204 47.996 32.341 42.481 0.00 0.00
D
ATOM 26592 CA CYS D 204 46.201 32.616 41.435 0.00 0.00
D
ATOM 26593 HA CYS D 204 45.702 32.030 40.678 0.00 0.00
D
ATOM 26594 CB CYS D 204 45.231 32.504 42.656 0.00 0.00
D

ATOM 26595 HB1 CYS D 204 45.677 33.149 43.443 0.00 0.00
D
ATOM 26596 HB2 CYS D 204 44.238 32.920 42.384 0.00 0.00
D
ATOM 26597 SG CYS D 204 45.049 30.892 43.335 0.00 0.00
D
ATOM 26598 HG1 CYS D 204 44.391 31.214 44.439 0.00 0.00
D
ATOM 26599 C CYS D 204 46.377 34.112 41.095 0.00 0.00
D
ATOM 26600 O CYS D 204 47.180 34.828 41.687 0.00 0.00
D
ATOM 26601 N LEU D 205 45.535 34.600 40.127 0.00 0.00
D
ATOM 26602 HN LEU D 205 44.837 34.018 39.717 0.00 0.00
D
ATOM 26603 CA LEU D 205 45.440 36.008 39.645 0.00 0.00
D
ATOM 26604 HA LEU D 205 46.094 36.637 40.231 0.00 0.00
D
ATOM 26605 CB LEU D 205 45.624 36.130 38.101 0.00 0.00
D
ATOM 26606 HB1 LEU D 205 46.581 35.586 37.954 0.00 0.00
D
ATOM 26607 HB2 LEU D 205 44.776 35.587 37.630 0.00 0.00
D
ATOM 26608 CG LEU D 205 45.670 37.576 37.567 0.00 0.00
D
ATOM 26609 HG LEU D 205 44.798 38.126 37.983 0.00 0.00
D
ATOM 26610 CD1 LEU D 205 46.926 38.217 38.050 0.00 0.00
D
ATOM 26611 HD11 LEU D 205 47.026 39.231 37.608 0.00 0.00
D
ATOM 26612 HD12 LEU D 205 47.054 38.241 39.154 0.00 0.00
D
ATOM 26613 HD13 LEU D 205 47.777 37.586 37.716 0.00 0.00
D
ATOM 26614 CD2 LEU D 205 45.677 37.672 35.984 0.00 0.00
D
ATOM 26615 HD21 LEU D 205 44.679 37.492 35.529 0.00 0.00
D
ATOM 26616 HD22 LEU D 205 45.941 38.670 35.572 0.00 0.00
D
ATOM 26617 HD23 LEU D 205 46.363 36.863 35.651 0.00 0.00
D
ATOM 26618 C LEU D 205 44.052 36.485 39.976 0.00 0.00
D
ATOM 26619 O LEU D 205 43.088 35.827 39.804 0.00 0.00
D
ATOM 26620 N VAL D 206 43.845 37.672 40.640 0.00 0.00
D
ATOM 26621 HN VAL D 206 44.640 38.198 40.931 0.00 0.00
D

ATOM 26622 CA VAL D 206 42.583 38.068 41.188 0.00 0.00
D
ATOM 26623 HA VAL D 206 41.836 37.288 41.196 0.00 0.00
D
ATOM 26624 CB VAL D 206 42.524 38.459 42.646 0.00 0.00
D
ATOM 26625 HB VAL D 206 42.973 39.464 42.797 0.00 0.00
D
ATOM 26626 CG1 VAL D 206 41.050 38.415 43.165 0.00 0.00
D
ATOM 26627 HG11 VAL D 206 40.480 39.261 42.726 0.00 0.00
D
ATOM 26628 HG12 VAL D 206 40.589 37.512 42.710 0.00 0.00
D
ATOM 26629 HG13 VAL D 206 40.933 38.457 44.269 0.00 0.00
D
ATOM 26630 CG2 VAL D 206 43.298 37.398 43.520 0.00 0.00
D
ATOM 26631 HG21 VAL D 206 42.700 36.463 43.582 0.00 0.00
D
ATOM 26632 HG22 VAL D 206 44.315 37.175 43.133 0.00 0.00
D
ATOM 26633 HG23 VAL D 206 43.436 37.808 44.543 0.00 0.00
D
ATOM 26634 C VAL D 206 42.000 39.147 40.205 0.00 0.00
D
ATOM 26635 O VAL D 206 42.525 40.263 40.151 0.00 0.00
D
ATOM 26636 N ILE D 207 40.920 38.816 39.361 0.00 0.00
D
ATOM 26637 HN ILE D 207 40.437 37.965 39.550 0.00 0.00
D
ATOM 26638 CA ILE D 207 40.495 39.578 38.077 0.00 0.00
D
ATOM 26639 HA ILE D 207 41.130 40.429 37.882 0.00 0.00
D
ATOM 26640 CB ILE D 207 40.485 38.634 36.792 0.00 0.00
D
ATOM 26641 HB ILE D 207 39.610 37.966 36.645 0.00 0.00
D
ATOM 26642 CG2 ILE D 207 40.363 39.618 35.573 0.00 0.00
D
ATOM 26643 HG21 ILE D 207 40.364 38.925 34.705 0.00 0.00
D
ATOM 26644 HG22 ILE D 207 39.438 40.232 35.559 0.00 0.00
D
ATOM 26645 HG23 ILE D 207 41.218 40.324 35.644 0.00 0.00
D
ATOM 26646 CG1 ILE D 207 41.725 37.862 36.763 0.00 0.00
D
ATOM 26647 HG11 ILE D 207 42.674 38.440 36.752 0.00 0.00
D
ATOM 26648 HG12 ILE D 207 41.644 37.259 37.692 0.00 0.00
D

ATOM 26649 CD ILE D 207 41.784 36.764 35.646 0.00 0.00
D
ATOM 26650 HD1 ILE D 207 42.804 36.328 35.699 0.00 0.00
D
ATOM 26651 HD2 ILE D 207 41.021 35.986 35.859 0.00 0.00
D
ATOM 26652 HD3 ILE D 207 41.578 37.270 34.678 0.00 0.00
D
ATOM 26653 C ILE D 207 39.139 40.001 38.366 0.00 0.00
D
ATOM 26654 O ILE D 207 38.337 39.233 39.012 0.00 0.00
D
ATOM 26655 N MET D 208 38.752 41.164 37.902 0.00 0.00
D
ATOM 26656 HN MET D 208 39.350 41.702 37.314 0.00 0.00
D
ATOM 26657 CA MET D 208 37.388 41.597 37.955 0.00 0.00
D
ATOM 26658 HA MET D 208 36.667 40.820 38.163 0.00 0.00
D
ATOM 26659 CB MET D 208 37.321 42.575 39.188 0.00 0.00
D
ATOM 26660 HB1 MET D 208 37.468 41.979 40.115 0.00 0.00
D
ATOM 26661 HB2 MET D 208 38.191 43.257 39.083 0.00 0.00
D
ATOM 26662 CG MET D 208 36.125 43.583 39.333 0.00 0.00
D
ATOM 26663 HG1 MET D 208 36.011 44.125 38.369 0.00 0.00
D
ATOM 26664 HG2 MET D 208 35.140 43.102 39.513 0.00 0.00
D
ATOM 26665 SD MET D 208 36.450 44.843 40.586 0.00 0.00
D
ATOM 26666 CE MET D 208 35.087 46.061 40.249 0.00 0.00
D
ATOM 26667 HE1 MET D 208 35.454 46.977 39.738 0.00 0.00
D
ATOM 26668 HE2 MET D 208 34.416 45.450 39.606 0.00 0.00
D
ATOM 26669 HE3 MET D 208 34.456 46.371 41.109 0.00 0.00
D
ATOM 26670 C MET D 208 36.952 42.290 36.739 0.00 0.00
D
ATOM 26671 O MET D 208 37.691 43.154 36.307 0.00 0.00
D
ATOM 26672 N GLY D 209 35.726 42.010 36.212 0.00 0.00
D
ATOM 26673 HN GLY D 209 35.047 41.503 36.737 0.00 0.00
D
ATOM 26674 CA GLY D 209 35.300 42.540 34.912 0.00 0.00
D
ATOM 26675 HA1 GLY D 209 35.903 42.089 34.137 0.00 0.00
D

ATOM 26676 HA2 GLY D 209 35.271 43.619 34.892 0.00 0.00
D
ATOM 26677 C GLY D 209 33.916 41.998 34.593 0.00 0.00
D
ATOM 26678 O GLY D 209 33.225 41.605 35.523 0.00 0.00
D
ATOM 26679 N HSE D 210 33.587 41.953 33.274 0.00 0.00
D
ATOM 26680 HN HSE D 210 34.307 42.310 32.684 0.00 0.00
D
ATOM 26681 CA HSE D 210 32.374 41.387 32.747 0.00 0.00
D
ATOM 26682 HA HSE D 210 31.481 41.491 33.345 0.00 0.00
D
ATOM 26683 CB HSE D 210 31.929 42.081 31.396 0.00 0.00
D
ATOM 26684 HB1 HSE D 210 31.939 43.150 31.697 0.00 0.00
D
ATOM 26685 HB2 HSE D 210 32.701 41.881 30.622 0.00 0.00
D
ATOM 26686 ND1 HSE D 210 30.418 40.407 30.135 0.00 0.00
D
ATOM 26687 CG HSE D 210 30.590 41.660 30.908 0.00 0.00
D
ATOM 26688 CE1 HSE D 210 29.218 40.399 29.654 0.00 0.00
D
ATOM 26689 HE1 HSE D 210 28.877 39.623 28.967 0.00 0.00
D
ATOM 26690 NE2 HSE D 210 28.523 41.538 30.033 0.00 0.00
D
ATOM 26691 HE2 HSE D 210 27.600 41.845 29.801 0.00 0.00
D
ATOM 26692 CD2 HSE D 210 29.340 42.319 30.852 0.00 0.00
D
ATOM 26693 HD2 HSE D 210 29.013 43.225 31.347 0.00 0.00
D
ATOM 26694 C HSE D 210 32.663 39.913 32.534 0.00 0.00
D
ATOM 26695 O HSE D 210 33.735 39.555 32.127 0.00 0.00
D
ATOM 26696 N LYS D 211 31.681 39.020 32.785 0.00 0.00
D
ATOM 26697 HN LYS D 211 30.763 39.216 33.120 0.00 0.00
D
ATOM 26698 CA LYS D 211 31.867 37.538 32.676 0.00 0.00
D
ATOM 26699 HA LYS D 211 32.739 37.283 33.260 0.00 0.00
D
ATOM 26700 CB LYS D 211 30.570 36.892 33.225 0.00 0.00
D
ATOM 26701 HB1 LYS D 211 29.771 37.299 32.569 0.00 0.00
D
ATOM 26702 HB2 LYS D 211 30.618 35.782 33.214 0.00 0.00
D

ATOM 26703 CG LYS D 211 30.228 37.231 34.738 0.00 0.00
D
ATOM 26704 HG1 LYS D 211 30.944 36.686 35.389 0.00 0.00
D
ATOM 26705 HG2 LYS D 211 30.413 38.241 35.162 0.00 0.00
D
ATOM 26706 CD LYS D 211 28.780 36.785 35.071 0.00 0.00
D
ATOM 26707 HD1 LYS D 211 28.139 37.488 34.496 0.00 0.00
D
ATOM 26708 HD2 LYS D 211 28.484 35.744 34.820 0.00 0.00
D
ATOM 26709 CE LYS D 211 28.256 37.010 36.539 0.00 0.00
D
ATOM 26710 HE1 LYS D 211 28.592 38.023 36.847 0.00 0.00
D
ATOM 26711 HE2 LYS D 211 27.167 36.860 36.698 0.00 0.00
D
ATOM 26712 NZ LYS D 211 28.891 36.028 37.401 0.00 0.00
D
ATOM 26713 HZ1 LYS D 211 28.748 36.197 38.418 0.00 0.00
D
ATOM 26714 HZ2 LYS D 211 28.431 35.141 37.115 0.00 0.00
D
ATOM 26715 HZ3 LYS D 211 29.907 35.914 37.214 0.00 0.00
D
ATOM 26716 C LYS D 211 32.298 37.103 31.294 0.00 0.00
D
ATOM 26717 O LYS D 211 33.098 36.197 31.148 0.00 0.00
D
ATOM 26718 N LYS D 212 31.733 37.672 30.156 0.00 0.00
D
ATOM 26719 HN LYS D 212 30.977 38.321 30.179 0.00 0.00
D
ATOM 26720 CA LYS D 212 32.084 37.171 28.858 0.00 0.00
D
ATOM 26721 HA LYS D 212 32.227 36.104 28.952 0.00 0.00
D
ATOM 26722 CB LYS D 212 30.995 37.471 27.786 0.00 0.00
D
ATOM 26723 HB1 LYS D 212 30.944 38.575 27.893 0.00 0.00
D
ATOM 26724 HB2 LYS D 212 31.456 37.307 26.789 0.00 0.00
D
ATOM 26725 CG LYS D 212 29.777 36.579 28.002 0.00 0.00
D
ATOM 26726 HG1 LYS D 212 30.015 35.533 27.712 0.00 0.00
D
ATOM 26727 HG2 LYS D 212 29.464 36.658 29.066 0.00 0.00
D
ATOM 26728 CD LYS D 212 28.658 37.152 27.223 0.00 0.00
D
ATOM 26729 HD1 LYS D 212 28.517 38.244 27.370 0.00 0.00
D

ATOM 26730 HD2 LYS D 212 28.877 37.177 26.135 0.00 0.00
D
ATOM 26731 CE LYS D 212 27.346 36.311 27.464 0.00 0.00
D
ATOM 26732 HE1 LYS D 212 26.547 37.030 27.183 0.00 0.00
D
ATOM 26733 HE2 LYS D 212 27.207 35.452 26.772 0.00 0.00
D
ATOM 26734 NZ LYS D 212 26.942 35.839 28.806 0.00 0.00
D
ATOM 26735 HZ1 LYS D 212 27.043 36.592 29.516 0.00 0.00
D
ATOM 26736 HZ2 LYS D 212 25.987 35.437 28.719 0.00 0.00
D
ATOM 26737 HZ3 LYS D 212 27.528 35.121 29.277 0.00 0.00
D
ATOM 26738 C LYS D 212 33.423 37.661 28.316 0.00 0.00
D
ATOM 26739 O LYS D 212 34.140 37.094 27.473 0.00 0.00
D
ATOM 26740 N ASP D 213 33.806 38.832 28.932 0.00 0.00
D
ATOM 26741 HN ASP D 213 33.235 39.274 29.620 0.00 0.00
D
ATOM 26742 CA ASP D 213 35.113 39.459 28.723 0.00 0.00
D
ATOM 26743 HA ASP D 213 35.401 39.199 27.715 0.00 0.00
D
ATOM 26744 CB ASP D 213 34.964 40.929 28.867 0.00 0.00
D
ATOM 26745 HB1 ASP D 213 34.605 41.179 29.888 0.00 0.00
D
ATOM 26746 HB2 ASP D 213 35.895 41.518 28.719 0.00 0.00
D
ATOM 26747 CG ASP D 213 34.051 41.604 27.847 0.00 0.00
D
ATOM 26748 OD1 ASP D 213 34.147 41.300 26.634 0.00 0.00
D
ATOM 26749 OD2 ASP D 213 33.361 42.587 28.205 0.00 0.00
D
ATOM 26750 C ASP D 213 36.127 38.785 29.591 0.00 0.00
D
ATOM 26751 O ASP D 213 37.301 38.581 29.194 0.00 0.00
D
ATOM 26752 N ILE D 214 35.840 38.344 30.869 0.00 0.00
D
ATOM 26753 HN ILE D 214 34.938 38.455 31.279 0.00 0.00
D
ATOM 26754 CA ILE D 214 36.656 37.449 31.689 0.00 0.00
D
ATOM 26755 HA ILE D 214 37.574 37.918 32.011 0.00 0.00
D
ATOM 26756 CB ILE D 214 35.991 37.216 33.055 0.00 0.00
D

ATOM 26757 HB ILE D 214   34.938 36.924 32.858 0.00 0.00
D
ATOM 26758 CG2 ILE D 214   36.709 36.102 33.834 0.00 0.00
D
ATOM 26759 HG21 ILE D 214   37.761 36.435 33.962 0.00 0.00
D
ATOM 26760 HG22 ILE D 214   36.287 36.023 34.859 0.00 0.00
D
ATOM 26761 HG23 ILE D 214   36.713 35.110 33.334 0.00 0.00
D
ATOM 26762 CG1 ILE D 214   36.109 38.383 33.967 0.00 0.00
D
ATOM 26763 HG11 ILE D 214   37.186 38.406 34.241 0.00 0.00
D
ATOM 26764 HG12 ILE D 214   35.891 39.273 33.340 0.00 0.00
D
ATOM 26765 CD ILE D 214   35.180 38.525 35.221 0.00 0.00
D
ATOM 26766 HD1 ILE D 214   35.387 39.526 35.657 0.00 0.00
D
ATOM 26767 HD2 ILE D 214   34.112 38.494 34.919 0.00 0.00
D
ATOM 26768 HD3 ILE D 214   35.366 37.729 35.973 0.00 0.00
D
ATOM 26769 C   ILE D 214   36.962 36.114 30.985 0.00 0.00
D
ATOM 26770 O   ILE D 214   38.123 35.610 30.940 0.00 0.00
D
ATOM 26771 N   LYS D 215   35.953 35.501 30.381 0.00 0.00
D
ATOM 26772 HN LYS D 215   35.007 35.779 30.533 0.00 0.00
D
ATOM 26773 CA LYS D 215   36.080 34.268 29.571 0.00 0.00
D
ATOM 26774 HA LYS D 215   36.706 33.596 30.139 0.00 0.00
D
ATOM 26775 CB LYS D 215   34.680 33.644 29.290 0.00 0.00
D
ATOM 26776 HB1 LYS D 215   34.088 33.705 30.229 0.00 0.00
D
ATOM 26777 HB2 LYS D 215   34.146 34.212 28.498 0.00 0.00
D
ATOM 26778 CG LYS D 215   34.722 32.074 29.025 0.00 0.00
D
ATOM 26779 HG1 LYS D 215   33.707 31.624 28.995 0.00 0.00
D
ATOM 26780 HG2 LYS D 215   35.251 31.778 28.094 0.00 0.00
D
ATOM 26781 CD LYS D 215   35.355 31.300 30.183 0.00 0.00
D
ATOM 26782 HD1 LYS D 215   36.459 31.369 30.081 0.00 0.00
D
ATOM 26783 HD2 LYS D 215   34.850 31.770 31.054 0.00 0.00
D

ATOM 26784 CE LYS D 215 34.913 29.866 30.228 0.00 0.00
D
ATOM 26785 HE1 LYS D 215 33.827 29.696 30.389 0.00 0.00
D
ATOM 26786 HE2 LYS D 215 35.219 29.304 29.320 0.00 0.00
D
ATOM 26787 NZ LYS D 215 35.574 29.234 31.365 0.00 0.00
D
ATOM 26788 HZ1 LYS D 215 35.042 29.397 32.244 0.00 0.00
D
ATOM 26789 HZ2 LYS D 215 35.695 28.226 31.138 0.00 0.00
D
ATOM 26790 HZ3 LYS D 215 36.566 29.536 31.444 0.00 0.00
D
ATOM 26791 C LYS D 215 36.876 34.452 28.340 0.00 0.00
D
ATOM 26792 O LYS D 215 37.701 33.616 28.052 0.00 0.00
D
ATOM 26793 N ARG D 216 36.815 35.579 27.635 0.00 0.00
D
ATOM 26794 HN ARG D 216 36.042 36.189 27.792 0.00 0.00
D
ATOM 26795 CA ARG D 216 37.793 35.960 26.552 0.00 0.00
D
ATOM 26796 HA ARG D 216 37.817 35.181 25.804 0.00 0.00
D
ATOM 26797 CB ARG D 216 37.132 37.204 25.916 0.00 0.00
D
ATOM 26798 HB1 ARG D 216 36.752 37.788 26.782 0.00 0.00
D
ATOM 26799 HB2 ARG D 216 37.822 37.828 25.310 0.00 0.00
D
ATOM 26800 CG ARG D 216 35.999 36.902 24.875 0.00 0.00
D
ATOM 26801 HG1 ARG D 216 36.277 36.089 24.172 0.00 0.00
D
ATOM 26802 HG2 ARG D 216 35.108 36.524 25.421 0.00 0.00
D
ATOM 26803 CD ARG D 216 35.450 38.100 24.112 0.00 0.00
D
ATOM 26804 HD1 ARG D 216 36.266 38.725 23.692 0.00 0.00
D
ATOM 26805 HD2 ARG D 216 34.885 37.799 23.204 0.00 0.00
D
ATOM 26806 NE ARG D 216 34.477 38.931 24.957 0.00 0.00
D
ATOM 26807 HE ARG D 216 34.783 39.559 25.672 0.00 0.00
D
ATOM 26808 CZ ARG D 216 33.147 38.896 24.755 0.00 0.00
D
ATOM 26809 NH1 ARG D 216 32.639 38.038 23.856 0.00 0.00
D
ATOM 26810 HH11 ARG D 216 31.651 37.889 23.852 0.00 0.00
D

ATOM 26811 HH12 ARG D 216   33.182 37.230 23.628 0.00 0.00
D
ATOM 26812 NH2 ARG D 216   32.439 39.758 25.450 0.00 0.00
D
ATOM 26813 HH21 ARG D 216   31.477 39.929 25.239 0.00 0.00
D
ATOM 26814 HH22 ARG D 216   32.984 40.419 25.965 0.00 0.00
D
ATOM 26815 C   ARG D 216   39.199 36.241 26.946 0.00 0.00
D
ATOM 26816 O   ARG D 216   40.136 35.843 26.286 0.00 0.00
D
ATOM 26817 N   PHE D 217   39.382 36.853 28.168 0.00 0.00
D
ATOM 26818 HN PHE D 217   38.610 37.337 28.572 0.00 0.00
D
ATOM 26819 CA PHE D 217   40.639 37.130 28.836 0.00 0.00
D
ATOM 26820 HA PHE D 217   41.313 37.589 28.128 0.00 0.00
D
ATOM 26821 CB PHE D 217   40.250 38.129 30.014 0.00 0.00
D
ATOM 26822 HB1 PHE D 217   39.977 39.084 29.516 0.00 0.00
D
ATOM 26823 HB2 PHE D 217   39.364 37.912 30.647 0.00 0.00
D
ATOM 26824 CG PHE D 217   41.332 38.535 30.968 0.00 0.00
D
ATOM 26825 CD1 PHE D 217   41.999 37.572 31.747 0.00 0.00
D
ATOM 26826 HD1 PHE D 217   41.722 36.536 31.618 0.00 0.00
D
ATOM 26827 CE1 PHE D 217   43.069 37.908 32.537 0.00 0.00
D
ATOM 26828 HE1 PHE D 217   43.448 37.211 33.270 0.00 0.00
D
ATOM 26829 CZ PHE D 217   43.437 39.232 32.676 0.00 0.00
D
ATOM 26830 HZ PHE D 217   44.076 39.487 33.509 0.00 0.00
D
ATOM 26831 CD2 PHE D 217   41.753 39.883 31.071 0.00 0.00
D
ATOM 26832 HD2 PHE D 217   41.129 40.622 30.590 0.00 0.00
D
ATOM 26833 CE2 PHE D 217   42.716 40.214 32.051 0.00 0.00
D
ATOM 26834 HE2 PHE D 217   42.889 41.258 32.263 0.00 0.00
D
ATOM 26835 C   PHE D 217   41.278 35.777 29.195 0.00 0.00
D
ATOM 26836 O   PHE D 217   42.483 35.544 28.960 0.00 0.00
D
ATOM 26837 N   GLU D 218   40.431 34.894 29.739 0.00 0.00
D

ATOM 26838 HN GLU D 218 39.536 35.298 29.912 0.00 0.00
D
ATOM 26839 CA GLU D 218 40.903 33.523 30.015 0.00 0.00
D
ATOM 26840 HA GLU D 218 41.815 33.579 30.591 0.00 0.00
D
ATOM 26841 CB GLU D 218 39.759 32.799 30.811 0.00 0.00
D
ATOM 26842 HB1 GLU D 218 39.527 33.342 31.753 0.00 0.00
D
ATOM 26843 HB2 GLU D 218 38.913 32.849 30.093 0.00 0.00
D
ATOM 26844 CG GLU D 218 39.943 31.289 31.190 0.00 0.00
D
ATOM 26845 HG1 GLU D 218 40.314 30.733 30.302 0.00 0.00
D
ATOM 26846 HG2 GLU D 218 40.764 31.172 31.929 0.00 0.00
D
ATOM 26847 CD GLU D 218 38.630 30.561 31.576 0.00 0.00
D
ATOM 26848 OE1 GLU D 218 37.956 31.056 32.563 0.00 0.00
D
ATOM 26849 OE2 GLU D 218 38.257 29.578 30.943 0.00 0.00
D
ATOM 26850 C GLU D 218 41.323 32.705 28.799 0.00 0.00
D
ATOM 26851 O GLU D 218 42.321 32.013 28.782 0.00 0.00
D
ATOM 26852 N ASN D 219 40.507 32.757 27.740 0.00 0.00
D
ATOM 26853 HN ASN D 219 39.654 33.262 27.635 0.00 0.00
D
ATOM 26854 CA ASN D 219 40.840 32.016 26.459 0.00 0.00
D
ATOM 26855 HA ASN D 219 40.886 31.007 26.840 0.00 0.00
D
ATOM 26856 CB ASN D 219 39.721 32.367 25.382 0.00 0.00
D
ATOM 26857 HB1 ASN D 219 38.723 32.191 25.836 0.00 0.00
D
ATOM 26858 HB2 ASN D 219 39.760 33.402 24.980 0.00 0.00
D
ATOM 26859 CG ASN D 219 39.746 31.512 24.111 0.00 0.00
D
ATOM 26860 OD1 ASN D 219 39.650 32.082 22.994 0.00 0.00
D
ATOM 26861 ND2 ASN D 219 39.903 30.218 24.338 0.00 0.00
D
ATOM 26862 HD21 ASN D 219 39.807 29.644 23.525 0.00 0.00
D
ATOM 26863 HD22 ASN D 219 39.654 29.892 25.250 0.00 0.00
D
ATOM 26864 C ASN D 219 42.195 32.322 25.855 0.00 0.00
D

```
ATOM  26865  O   ASN D 219      43.103  31.528  25.660  0.00  0.00
D
ATOM  26866  N   GLU D 220      42.447  33.614  25.678  0.00  0.00
D
ATOM  26867  HN  GLU D 220      41.735  34.235  25.995  0.00  0.00
D
ATOM  26868  CA  GLU D 220      43.651  34.196  25.205  0.00  0.00
D
ATOM  26869  HA  GLU D 220      43.806  33.546  24.356  0.00  0.00
D
ATOM  26870  CB  GLU D 220      43.377  35.608  24.588  0.00  0.00
D
ATOM  26871  HB1 GLU D 220      42.508  35.475  23.910  0.00  0.00
D
ATOM  26872  HB2 GLU D 220      42.855  36.244  25.334  0.00  0.00
D
ATOM  26873  CG  GLU D 220      44.458  36.293  23.769  0.00  0.00
D
ATOM  26874  HG1 GLU D 220      45.198  36.863  24.372  0.00  0.00
D
ATOM  26875  HG2 GLU D 220      45.048  35.513  23.243  0.00  0.00
D
ATOM  26876  CD  GLU D 220      43.932  37.317  22.857  0.00  0.00
D
ATOM  26877  OE1 GLU D 220      44.427  38.510  22.918  0.00  0.00
D
ATOM  26878  OE2 GLU D 220      42.958  37.034  22.178  0.00  0.00
D
ATOM  26879  C   GLU D 220      44.779  34.197  26.180  0.00  0.00
D
ATOM  26880  O   GLU D 220      45.933  33.991  25.864  0.00  0.00
D
ATOM  26881  N   GLY D 221      44.559  34.505  27.467  0.00  0.00
D
ATOM  26882  HN  GLY D 221      43.629  34.749  27.731  0.00  0.00
D
ATOM  26883  CA  GLY D 221      45.552  34.291  28.553  0.00  0.00
D
ATOM  26884  HA1 GLY D 221      45.144  34.654  29.484  0.00  0.00
D
ATOM  26885  HA2 GLY D 221      46.364  34.976  28.357  0.00  0.00
D
ATOM  26886  C   GLY D 221      46.100  32.860  28.634  0.00  0.00
D
ATOM  26887  O   GLY D 221      47.289  32.524  28.908  0.00  0.00
D
ATOM  26888  N   MET D 222      45.275  31.823  28.436  0.00  0.00
D
ATOM  26889  HN  MET D 222      44.284  31.932  28.468  0.00  0.00
D
ATOM  26890  CA  MET D 222      45.653  30.442  28.167  0.00  0.00
D
ATOM  26891  HA  MET D 222      46.575  30.192  28.671  0.00  0.00
D
```

ATOM 26892 CB MET D 222   44.560 29.504 28.805 0.00 0.00
D
ATOM 26893 HB1 MET D 222   43.542 29.689 28.400 0.00 0.00
D
ATOM 26894 HB2 MET D 222   44.787 28.429 28.640 0.00 0.00
D
ATOM 26895 CG MET D 222   44.439 29.820 30.252 0.00 0.00
D
ATOM 26896 HG1 MET D 222   45.493 29.808 30.602 0.00 0.00
D
ATOM 26897 HG2 MET D 222   44.061 30.848 30.438 0.00 0.00
D
ATOM 26898 SD MET D 222   43.620 28.568 31.233 0.00 0.00
D
ATOM 26899 CE MET D 222   43.907 29.454 32.776 0.00 0.00
D
ATOM 26900 HE1 MET D 222   45.005 29.612 32.832 0.00 0.00
D
ATOM 26901 HE2 MET D 222   43.327 30.400 32.828 0.00 0.00
D
ATOM 26902 HE3 MET D 222   43.610 28.859 33.666 0.00 0.00
D
ATOM 26903 C   MET D 222   45.772 30.168 26.704 0.00 0.00
D
ATOM 26904 O   MET D 222   46.651 30.682 26.001 0.00 0.00
D
ATOM 26905 N   GLN E   8   48.827 −16.545 63.021 0.00 0.00
E
ATOM 26906 HT1 GLN E   8   49.495 −17.111 63.582 0.00 0.00
E
ATOM 26907 HT2 GLN E   8   49.207 −15.579 62.959 0.00 0.00
E
ATOM 26908 HT3 GLN E   8   48.915 −17.022 62.100 0.00 0.00
E
ATOM 26909 CA GLN E   8   47.413 −16.391 63.496 0.00 0.00
E
ATOM 26910 HA GLN E   8   46.992 −17.367 63.687 0.00 0.00
E
ATOM 26911 CB GLN E   8   47.364 −15.724 64.811 0.00 0.00
E
ATOM 26912 HB1 GLN E   8   48.222 −16.180 65.348 0.00 0.00
E
ATOM 26913 HB2 GLN E   8   47.701 −14.672 64.693 0.00 0.00
E
ATOM 26914 CG GLN E   8   46.053 −15.879 65.630 0.00 0.00
E
ATOM 26915 HG1 GLN E   8   45.233 −15.303 65.151 0.00 0.00
E
ATOM 26916 HG2 GLN E   8   45.733 −16.941 65.695 0.00 0.00
E
ATOM 26917 CD GLN E   8   46.220 −15.369 67.021 0.00 0.00
E
ATOM 26918 OE1 GLN E   8   45.604 −14.349 67.400 0.00 0.00
E

ATOM 26919 NE2 GLN E   8   47.113 -15.938 67.839 0.00 0.00
E
ATOM 26920 HE21 GLN E   8   47.727 -16.661 67.522 0.00 0.00
E
ATOM 26921 HE22 GLN E   8   47.104 -15.716 68.814 0.00 0.00
E
ATOM 26922 C   GLN E   8   46.335 -15.797 62.602 0.00 0.00
E
ATOM 26923 O   GLN E   8   46.462 -14.701 62.094 0.00 0.00
E
ATOM 26924 N   PHE E   9   45.182 -16.422 62.374 0.00 0.00
E
ATOM 26925 HN PHE E   9   44.941 -17.296 62.787 0.00 0.00
E
ATOM 26926 CA PHE E   9   44.149 -15.910 61.543 0.00 0.00
E
ATOM 26927 HA PHE E   9   44.435 -15.114 60.872 0.00 0.00
E
ATOM 26928 CB PHE E   9   43.411 -17.058 60.734 0.00 0.00
E
ATOM 26929 HB1 PHE E   9   42.994 -17.778 61.470 0.00 0.00
E
ATOM 26930 HB2 PHE E   9   42.551 -16.727 60.113 0.00 0.00
E
ATOM 26931 CG PHE E   9   44.406 -17.748 59.824 0.00 0.00
E
ATOM 26932 CD1 PHE E   9   44.689 -17.276 58.542 0.00 0.00
E
ATOM 26933 HD1 PHE E   9   44.156 -16.457 58.082 0.00 0.00
E
ATOM 26934 CE1 PHE E   9   45.504 -17.960 57.616 0.00 0.00
E
ATOM 26935 HE1 PHE E   9   45.609 -17.549 56.623 0.00 0.00
E
ATOM 26936 CZ PHE E   9   46.108 -19.110 58.055 0.00 0.00
E
ATOM 26937 HZ PHE E   9   46.714 -19.683 57.368 0.00 0.00
E
ATOM 26938 CD2 PHE E   9   44.910 -19.016 60.158 0.00 0.00
E
ATOM 26939 HD2 PHE E   9   44.548 -19.359 61.116 0.00 0.00
E
ATOM 26940 CE2 PHE E   9   45.814 -19.652 59.330 0.00 0.00
E
ATOM 26941 HE2 PHE E   9   46.392 -20.511 59.637 0.00 0.00
E
ATOM 26942 C   PHE E   9   43.140 -15.201 62.445 0.00 0.00
E
ATOM 26943 O   PHE E   9   43.022 -15.433 63.680 0.00 0.00
E
ATOM 26944 N   ALA E 10   42.335 -14.409 61.815 0.00 0.00
E
ATOM 26945 HN ALA E 10   42.326 -14.271 60.828 0.00 0.00
E

ATOM 26946 CA ALA E 10 41.213 -13.760 62.415 0.00 0.00
E
ATOM 26947 HA ALA E 10 40.955 -14.255 63.339 0.00 0.00
E
ATOM 26948 CB ALA E 10 41.319 -12.274 62.844 0.00 0.00
E
ATOM 26949 HB1 ALA E 10 42.294 -12.142 63.359 0.00 0.00
E
ATOM 26950 HB2 ALA E 10 41.487 -11.644 61.945 0.00 0.00
E
ATOM 26951 HB3 ALA E 10 40.505 -11.924 63.514 0.00 0.00
E
ATOM 26952 C ALA E 10 39.981 -13.893 61.625 0.00 0.00
E
ATOM 26953 O ALA E 10 40.012 -13.652 60.444 0.00 0.00
E
ATOM 26954 N VAL E 11 38.952 -14.249 62.297 0.00 0.00
E
ATOM 26955 HN VAL E 11 38.915 -14.283 63.293 0.00 0.00
E
ATOM 26956 CA VAL E 11 37.714 -14.390 61.605 0.00 0.00
E
ATOM 26957 HA VAL E 11 37.728 -13.843 60.673 0.00 0.00
E
ATOM 26958 CB VAL E 11 37.102 -15.786 61.341 0.00 0.00
E
ATOM 26959 HB VAL E 11 36.959 -16.182 62.369 0.00 0.00
E
ATOM 26960 CG1 VAL E 11 35.718 -15.823 60.692 0.00 0.00
E
ATOM 26961 HG11 VAL E 11 34.938 -15.218 61.203 0.00 0.00
E
ATOM 26962 HG12 VAL E 11 35.768 -15.472 59.639 0.00 0.00
E
ATOM 26963 HG13 VAL E 11 35.306 -16.851 60.779 0.00 0.00
E
ATOM 26964 CG2 VAL E 11 38.155 -16.611 60.501 0.00 0.00
E
ATOM 26965 HG21 VAL E 11 38.522 -15.987 59.659 0.00 0.00
E
ATOM 26966 HG22 VAL E 11 38.979 -16.887 61.193 0.00 0.00
E
ATOM 26967 HG23 VAL E 11 37.725 -17.559 60.113 0.00 0.00
E
ATOM 26968 C VAL E 11 36.678 -13.651 62.334 0.00 0.00
E
ATOM 26969 O VAL E 11 36.560 -13.795 63.546 0.00 0.00
E
ATOM 26970 N ILE E 12 35.979 -12.674 61.689 0.00 0.00
E
ATOM 26971 HN ILE E 12 36.272 -12.456 60.761 0.00 0.00
E
ATOM 26972 CA ILE E 12 35.101 -11.756 62.361 0.00 0.00
E

ATOM 26973 HA ILE E 12 35.136 -11.905 63.430 0.00 0.00
E
ATOM 26974 CB ILE E 12 35.523 -10.339 62.108 0.00 0.00
E
ATOM 26975 HB ILE E 12 35.735 -10.206 61.025 0.00 0.00
E
ATOM 26976 CG2 ILE E 12 34.472 -9.317 62.593 0.00 0.00
E
ATOM 26977 HG21 ILE E 12 33.537 -9.511 62.025 0.00 0.00
E
ATOM 26978 HG22 ILE E 12 34.280 -9.176 63.678 0.00 0.00
E
ATOM 26979 HG23 ILE E 12 34.635 -8.266 62.274 0.00 0.00
E
ATOM 26980 CG1 ILE E 12 36.809 -10.107 62.901 0.00 0.00
E
ATOM 26981 HG11 ILE E 12 36.457 -9.872 63.928 0.00 0.00
E
ATOM 26982 HG12 ILE E 12 37.422 -11.033 62.861 0.00 0.00
E
ATOM 26983 CD ILE E 12 37.572 -8.919 62.492 0.00 0.00
E
ATOM 26984 HD1 ILE E 12 36.925 -8.020 62.408 0.00 0.00
E
ATOM 26985 HD2 ILE E 12 38.383 -8.700 63.219 0.00 0.00
E
ATOM 26986 HD3 ILE E 12 38.004 -9.076 61.480 0.00 0.00
E
ATOM 26987 C ILE E 12 33.800 -12.102 61.854 0.00 0.00
E
ATOM 26988 O ILE E 12 33.475 -11.927 60.649 0.00 0.00
E
ATOM 26989 N GLY E 13 32.928 -12.551 62.771 0.00 0.00
E
ATOM 26990 HN GLY E 13 33.310 -12.806 63.656 0.00 0.00
E
ATOM 26991 CA GLY E 13 31.618 -13.014 62.395 0.00 0.00
E
ATOM 26992 HA1 GLY E 13 31.370 -12.753 61.377 0.00 0.00
E
ATOM 26993 HA2 GLY E 13 30.901 -12.719 63.147 0.00 0.00
E
ATOM 26994 C GLY E 13 31.548 -14.551 62.526 0.00 0.00
E
ATOM 26995 O GLY E 13 32.247 -15.316 61.808 0.00 0.00
E
ATOM 26996 N LEU E 14 30.661 -15.146 63.303 0.00 0.00
E
ATOM 26997 HN LEU E 14 30.024 -14.593 63.835 0.00 0.00
E
ATOM 26998 CA LEU E 14 30.597 -16.619 63.554 0.00 0.00
E
ATOM 26999 HA LEU E 14 31.425 -17.073 63.029 0.00 0.00
E

ATOM 27000 CB LEU E 14 30.899 −16.905 65.086 0.00 0.00
E
ATOM 27001 HB1 LEU E 14 30.040 −16.472 65.642 0.00 0.00
E
ATOM 27002 HB2 LEU E 14 31.004 −17.963 65.406 0.00 0.00
E
ATOM 27003 CG LEU E 14 32.188 −16.205 65.551 0.00 0.00
E
ATOM 27004 HG LEU E 14 32.263 −15.154 65.202 0.00 0.00
E
ATOM 27005 CD1 LEU E 14 32.376 −16.279 67.098 0.00 0.00
E
ATOM 27006 HD11 LEU E 14 32.812 −17.253 67.409 0.00 0.00
E
ATOM 27007 HD12 LEU E 14 32.963 −15.421 67.490 0.00 0.00
E
ATOM 27008 HD13 LEU E 14 31.384 −16.016 67.523 0.00 0.00
E
ATOM 27009 CD2 LEU E 14 33.410 −16.921 64.930 0.00 0.00
E
ATOM 27010 HD21 LEU E 14 33.306 −17.172 63.852 0.00 0.00
E
ATOM 27011 HD22 LEU E 14 34.369 −16.374 65.054 0.00 0.00
E
ATOM 27012 HD23 LEU E 14 33.612 −17.900 65.414 0.00 0.00
E
ATOM 27013 C LEU E 14 29.339 −17.292 63.059 0.00 0.00
E
ATOM 27014 O LEU E 14 28.923 −18.324 63.567 0.00 0.00
E
ATOM 27015 N GLY E 15 28.781 −16.809 61.925 0.00 0.00
E
ATOM 27016 HN GLY E 15 29.146 −15.990 61.490 0.00 0.00
E
ATOM 27017 CA GLY E 15 27.795 −17.534 61.179 0.00 0.00
E
ATOM 27018 HA1 GLY E 15 27.450 −16.889 60.384 0.00 0.00
E
ATOM 27019 HA2 GLY E 15 26.918 −17.750 61.772 0.00 0.00
E
ATOM 27020 C GLY E 15 28.183 −18.834 60.605 0.00 0.00
E
ATOM 27021 O GLY E 15 29.175 −19.444 60.881 0.00 0.00
E
ATOM 27022 N ARG E 16 27.374 −19.273 59.676 0.00 0.00
E
ATOM 27023 HN ARG E 16 26.639 −18.669 59.376 0.00 0.00
E
ATOM 27024 CA ARG E 16 27.407 −20.542 58.891 0.00 0.00
E
ATOM 27025 HA ARG E 16 27.342 −21.430 59.502 0.00 0.00
E
ATOM 27026 CB ARG E 16 26.283 −20.474 57.826 0.00 0.00
E

ATOM 27027 HB1 ARG E 16 26.195 -19.608 57.136 0.00 0.00
E
ATOM 27028 HB2 ARG E 16 26.328 -21.321 57.109 0.00 0.00
E
ATOM 27029 CG ARG E 16 24.841 -20.506 58.486 0.00 0.00
E
ATOM 27030 HG1 ARG E 16 24.948 -21.510 58.949 0.00 0.00
E
ATOM 27031 HG2 ARG E 16 24.736 -19.674 59.215 0.00 0.00
E
ATOM 27032 CD ARG E 16 23.653 -20.559 57.509 0.00 0.00
E
ATOM 27033 HD1 ARG E 16 23.764 -21.407 56.800 0.00 0.00
E
ATOM 27034 HD2 ARG E 16 22.687 -20.609 58.057 0.00 0.00
E
ATOM 27035 NE ARG E 16 23.632 -19.469 56.528 0.00 0.00
E
ATOM 27036 HE ARG E 16 24.253 -19.546 55.748 0.00 0.00
E
ATOM 27037 CZ ARG E 16 23.431 -18.254 56.878 0.00 0.00
E
ATOM 27038 NH1 ARG E 16 22.716 -17.929 57.979 0.00 0.00
E
ATOM 27039 HH11 ARG E 16 22.722 -16.982 58.298 0.00 0.00
E
ATOM 27040 HH12 ARG E 16 22.499 -18.663 58.623 0.00 0.00
E
ATOM 27041 NH2 ARG E 16 24.014 -17.249 56.156 0.00 0.00
E
ATOM 27042 HH21 ARG E 16 24.101 -16.396 56.671 0.00 0.00
E
ATOM 27043 HH22 ARG E 16 24.749 -17.512 55.531 0.00 0.00
E
ATOM 27044 C ARG E 16 28.738 -20.646 58.235 0.00 0.00
E
ATOM 27045 O ARG E 16 29.403 -21.654 58.359 0.00 0.00
E
ATOM 27046 N PHE E 17 29.195 -19.556 57.601 0.00 0.00
E
ATOM 27047 HN PHE E 17 28.537 -18.811 57.667 0.00 0.00
E
ATOM 27048 CA PHE E 17 30.494 -19.361 57.106 0.00 0.00
E
ATOM 27049 HA PHE E 17 30.699 -20.143 56.390 0.00 0.00
E
ATOM 27050 CB PHE E 17 30.519 -18.154 56.158 0.00 0.00
E
ATOM 27051 HB1 PHE E 17 29.708 -18.164 55.400 0.00 0.00
E
ATOM 27052 HB2 PHE E 17 30.435 -17.210 56.738 0.00 0.00
E
ATOM 27053 CG PHE E 17 31.753 -17.908 55.264 0.00 0.00
E

ATOM 27054 CD1 PHE E 17 32.197 −18.893 54.348 0.00 0.00
E
ATOM 27055 HD1 PHE E 17 31.658 −19.809 54.159 0.00 0.00
E
ATOM 27056 CE1 PHE E 17 33.180 −18.454 53.413 0.00 0.00
E
ATOM 27057 HE1 PHE E 17 33.455 −19.137 52.623 0.00 0.00
E
ATOM 27058 CZ PHE E 17 33.746 −17.195 53.411 0.00 0.00
E
ATOM 27059 HZ PHE E 17 34.471 −16.950 52.649 0.00 0.00
E
ATOM 27060 CD2 PHE E 17 32.445 −16.712 55.323 0.00 0.00
E
ATOM 27061 HD2 PHE E 17 32.129 −15.985 56.057 0.00 0.00
E
ATOM 27062 CE2 PHE E 17 33.449 −16.350 54.422 0.00 0.00
E
ATOM 27063 HE2 PHE E 17 33.890 −15.365 54.379 0.00 0.00
E
ATOM 27064 C PHE E 17 31.636 −19.239 58.090 0.00 0.00
E
ATOM 27065 O PHE E 17 32.535 −20.022 58.026 0.00 0.00
E
ATOM 27066 N GLY E 18 31.839 −18.155 58.923 0.00 0.00
E
ATOM 27067 HN GLY E 18 31.056 −17.541 58.983 0.00 0.00
E
ATOM 27068 CA GLY E 18 33.013 −17.879 59.772 0.00 0.00
E
ATOM 27069 HA1 GLY E 18 32.997 −16.837 60.058 0.00 0.00
E
ATOM 27070 HA2 GLY E 18 33.808 −17.909 59.042 0.00 0.00
E
ATOM 27071 C GLY E 18 33.206 −18.875 60.950 0.00 0.00
E
ATOM 27072 O GLY E 18 34.335 −19.173 61.385 0.00 0.00
E
ATOM 27073 N GLY E 19 32.089 −19.419 61.467 0.00 0.00
E
ATOM 27074 HN GLY E 19 31.224 −18.997 61.206 0.00 0.00
E
ATOM 27075 CA GLY E 19 32.098 −20.590 62.298 0.00 0.00
E
ATOM 27076 HA1 GLY E 19 31.071 −20.830 62.532 0.00 0.00
E
ATOM 27077 HA2 GLY E 19 32.565 −20.343 63.240 0.00 0.00
E
ATOM 27078 C GLY E 19 32.659 −21.787 61.687 0.00 0.00
E
ATOM 27079 O GLY E 19 33.428 −22.551 62.286 0.00 0.00
E
ATOM 27080 N SER E 20 32.248 −22.053 60.448 0.00 0.00
E

ATOM 27081 HN SER E 20 31.644 -21.409 59.984 0.00 0.00
E
ATOM 27082 CA SER E 20 32.880 -23.175 59.769 0.00 0.00
E
ATOM 27083 HA SER E 20 32.784 -24.078 60.354 0.00 0.00
E
ATOM 27084 CB SER E 20 32.205 -23.558 58.420 0.00 0.00
E
ATOM 27085 HB1 SER E 20 31.146 -23.871 58.540 0.00 0.00
E
ATOM 27086 HB2 SER E 20 32.260 -22.683 57.738 0.00 0.00
E
ATOM 27087 OG SER E 20 32.864 -24.656 57.748 0.00 0.00
E
ATOM 27088 HG1 SER E 20 32.276 -24.922 57.037 0.00 0.00
E
ATOM 27089 C SER E 20 34.356 -22.876 59.568 0.00 0.00
E
ATOM 27090 O SER E 20 35.201 -23.774 59.746 0.00 0.00
E
ATOM 27091 N ILE E 21 34.764 -21.601 59.283 0.00 0.00
E
ATOM 27092 HN ILE E 21 34.078 -20.890 59.149 0.00 0.00
E
ATOM 27093 CA ILE E 21 36.207 -21.281 59.023 0.00 0.00
E
ATOM 27094 HA ILE E 21 36.550 -21.995 58.288 0.00 0.00
E
ATOM 27095 CB ILE E 21 36.517 -19.907 58.473 0.00 0.00
E
ATOM 27096 HB ILE E 21 36.176 -19.136 59.197 0.00 0.00
E
ATOM 27097 CG2 ILE E 21 38.009 -19.733 58.143 0.00 0.00
E
ATOM 27098 HG21 ILE E 21 38.659 -19.851 59.036 0.00 0.00
E
ATOM 27099 HG22 ILE E 21 38.356 -20.502 57.420 0.00 0.00
E
ATOM 27100 HG23 ILE E 21 38.277 -18.726 57.756 0.00 0.00
E
ATOM 27101 CG1 ILE E 21 35.644 -19.713 57.187 0.00 0.00
E
ATOM 27102 HG11 ILE E 21 36.240 -20.207 56.390 0.00 0.00
E
ATOM 27103 HG12 ILE E 21 34.659 -20.225 57.224 0.00 0.00
E
ATOM 27104 CD ILE E 21 35.476 -18.253 56.772 0.00 0.00
E
ATOM 27105 HD1 ILE E 21 35.445 -17.636 57.695 0.00 0.00
E
ATOM 27106 HD2 ILE E 21 36.356 -17.896 56.195 0.00 0.00
E
ATOM 27107 HD3 ILE E 21 34.499 -18.045 56.286 0.00 0.00
E

ATOM	27108	C	ILE	E	21	36.945	-21.533	60.282	0.00	0.00	E
ATOM	27109	O	ILE	E	21	37.971	-22.201	60.286	0.00	0.00	E
ATOM	27110	N	CYS	E	22	36.519	-20.934	61.357	0.00	0.00	E
ATOM	27111	HN	CYS	E	22	35.624	-20.495	61.366	0.00	0.00	E
ATOM	27112	CA	CYS	E	22	37.211	-21.115	62.656	0.00	0.00	E
ATOM	27113	HA	CYS	E	22	38.139	-20.563	62.657	0.00	0.00	E
ATOM	27114	CB	CYS	E	22	36.384	-20.555	63.825	0.00	0.00	E
ATOM	27115	HB1	CYS	E	22	35.379	-20.951	63.564	0.00	0.00	E
ATOM	27116	HB2	CYS	E	22	36.574	-20.919	64.858	0.00	0.00	E
ATOM	27117	SG	CYS	E	22	36.376	-18.772	63.900	0.00	0.00	E
ATOM	27118	HG1	CYS	E	22	35.567	-18.494	62.888	0.00	0.00	E
ATOM	27119	C	CYS	E	22	37.495	-22.581	63.054	0.00	0.00	E
ATOM	27120	O	CYS	E	22	38.565	-22.840	63.586	0.00	0.00	E
ATOM	27121	N	LYS	E	23	36.547	-23.581	62.905	0.00	0.00	E
ATOM	27122	HN	LYS	E	23	35.634	-23.467	62.520	0.00	0.00	E
ATOM	27123	CA	LYS	E	23	36.785	-24.846	63.548	0.00	0.00	E
ATOM	27124	HA	LYS	E	23	37.364	-24.714	64.450	0.00	0.00	E
ATOM	27125	CB	LYS	E	23	35.465	-25.579	63.927	0.00	0.00	E
ATOM	27126	HB1	LYS	E	23	35.918	-26.367	64.566	0.00	0.00	E
ATOM	27127	HB2	LYS	E	23	34.948	-24.819	64.550	0.00	0.00	E
ATOM	27128	CG	LYS	E	23	34.591	-26.032	62.724	0.00	0.00	E
ATOM	27129	HG1	LYS	E	23	34.585	-25.273	61.913	0.00	0.00	E
ATOM	27130	HG2	LYS	E	23	34.917	-26.999	62.285	0.00	0.00	E
ATOM	27131	CD	LYS	E	23	33.248	-26.153	63.209	0.00	0.00	E
ATOM	27132	HD1	LYS	E	23	33.258	-26.912	64.020	0.00	0.00	E
ATOM	27133	HD2	LYS	E	23	32.825	-25.168	63.503	0.00	0.00	E
ATOM	27134	CE	LYS	E	23	32.208	-26.719	62.199	0.00	0.00	E

ATOM 27135 HE1 LYS E 23 32.274 -26.037 61.324 0.00 0.00
E
ATOM 27136 HE2 LYS E 23 32.533 -27.764 62.010 0.00 0.00
E
ATOM 27137 NZ LYS E 23 30.820 -26.685 62.712 0.00 0.00
E
ATOM 27138 HZ1 LYS E 23 30.343 -25.812 62.409 0.00 0.00
E
ATOM 27139 HZ2 LYS E 23 30.203 -27.474 62.432 0.00 0.00
E
ATOM 27140 HZ3 LYS E 23 30.807 -26.666 63.752 0.00 0.00
E
ATOM 27141 C LYS E 23 37.675 -25.696 62.647 0.00 0.00
E
ATOM 27142 O LYS E 23 38.455 -26.493 63.166 0.00 0.00
E
ATOM 27143 N GLU E 24 37.680 -25.487 61.257 0.00 0.00
E
ATOM 27144 HN GLU E 24 37.270 -24.657 60.886 0.00 0.00
E
ATOM 27145 CA GLU E 24 38.522 -26.177 60.379 0.00 0.00
E
ATOM 27146 HA GLU E 24 38.398 -27.219 60.632 0.00 0.00
E
ATOM 27147 CB GLU E 24 38.100 -26.005 58.862 0.00 0.00
E
ATOM 27148 HB1 GLU E 24 37.054 -26.377 58.814 0.00 0.00
E
ATOM 27149 HB2 GLU E 24 38.095 -24.937 58.555 0.00 0.00
E
ATOM 27150 CG GLU E 24 38.899 -26.743 57.797 0.00 0.00
E
ATOM 27151 HG1 GLU E 24 38.726 -26.427 56.746 0.00 0.00
E
ATOM 27152 HG2 GLU E 24 39.995 -26.685 57.969 0.00 0.00
E
ATOM 27153 CD GLU E 24 38.540 -28.242 57.838 0.00 0.00
E
ATOM 27154 OE1 GLU E 24 37.796 -28.744 58.703 0.00 0.00
E
ATOM 27155 OE2 GLU E 24 39.022 -28.949 56.896 0.00 0.00
E
ATOM 27156 C GLU E 24 39.960 -25.752 60.543 0.00 0.00
E
ATOM 27157 O GLU E 24 40.881 -26.534 60.497 0.00 0.00
E
ATOM 27158 N LEU E 25 40.222 -24.483 60.805 0.00 0.00
E
ATOM 27159 HN LEU E 25 39.507 -23.787 60.811 0.00 0.00
E
ATOM 27160 CA LEU E 25 41.618 -23.992 61.093 0.00 0.00
E
ATOM 27161 HA LEU E 25 42.366 -24.328 60.389 0.00 0.00
E

ATOM	27162	CB	LEU	E	25	41.661	-22.368	61.170	0.00	0.00	E
ATOM	27163	HB1	LEU	E	25	40.850	-22.104	61.881	0.00	0.00	E
ATOM	27164	HB2	LEU	E	25	42.547	-22.025	61.746	0.00	0.00	E
ATOM	27165	CG	LEU	E	25	41.353	-21.641	59.858	0.00	0.00	E
ATOM	27166	HG	LEU	E	25	40.365	-21.947	59.453	0.00	0.00	E
ATOM	27167	CD1	LEU	E	25	41.188	-20.109	60.109	0.00	0.00	E
ATOM	27168	HD11	LEU	E	25	40.630	-19.573	59.312	0.00	0.00	E
ATOM	27169	HD12	LEU	E	25	40.608	-20.001	61.051	0.00	0.00	E
ATOM	27170	HD13	LEU	E	25	42.112	-19.572	60.412	0.00	0.00	E
ATOM	27171	CD2	LEU	E	25	42.297	-22.019	58.738	0.00	0.00	E
ATOM	27172	HD21	LEU	E	25	41.994	-21.514	57.796	0.00	0.00	E
ATOM	27173	HD22	LEU	E	25	43.350	-21.812	59.025	0.00	0.00	E
ATOM	27174	HD23	LEU	E	25	42.166	-23.117	58.623	0.00	0.00	E
ATOM	27175	C	LEU	E	25	41.982	-24.422	62.409	0.00	0.00	E
ATOM	27176	O	LEU	E	25	43.143	-24.931	62.579	0.00	0.00	E
ATOM	27177	N	HSE	E	26	41.137	-24.398	63.493	0.00	0.00	E
ATOM	27178	HN	HSE	E	26	40.193	-24.090	63.403	0.00	0.00	E
ATOM	27179	CA	HSE	E	26	41.525	-25.015	64.709	0.00	0.00	E
ATOM	27180	HA	HSE	E	26	42.501	-24.652	64.994	0.00	0.00	E
ATOM	27181	CB	HSE	E	26	40.432	-24.719	65.784	0.00	0.00	E
ATOM	27182	HB1	HSE	E	26	40.170	-23.644	65.687	0.00	0.00	E
ATOM	27183	HB2	HSE	E	26	39.483	-25.247	65.551	0.00	0.00	E
ATOM	27184	ND1	HSE	E	26	41.838	-24.432	67.840	0.00	0.00	E
ATOM	27185	CG	HSE	E	26	40.822	-25.101	67.138	0.00	0.00	E
ATOM	27186	CE1	HSE	E	26	41.780	-24.963	69.041	0.00	0.00	E
ATOM	27187	HE1	HSE	E	26	42.433	-24.646	69.855	0.00	0.00	E
ATOM	27188	NE2	HSE	E	26	40.802	-25.920	69.148	0.00	0.00	E

ATOM 27189 HE2 HSE E 26   40.571 −26.502 69.928 0.00 0.00
E
ATOM 27190 CD2 HSE E 26   40.219 −26.038 67.922 0.00 0.00
E
ATOM 27191 HD2 HSE E 26   39.362 −26.674 67.739 0.00 0.00
E
ATOM 27192 C   HSE E 26   41.801 −26.499 64.568 0.00 0.00
E
ATOM 27193 O   HSE E 26   42.796 −26.970 65.092 0.00 0.00
E
ATOM 27194 N   ARG E 27   41.035 −27.201 63.767 0.00 0.00
E
ATOM 27195 HN ARG E 27   40.242 −26.813 63.302 0.00 0.00
E
ATOM 27196 CA ARG E 27   41.223 −28.611 63.494 0.00 0.00
E
ATOM 27197 HA ARG E 27   41.242 −29.173 64.416 0.00 0.00
E
ATOM 27198 CB ARG E 27   39.953 −29.062 62.722 0.00 0.00
E
ATOM 27199 HB1 ARG E 27   38.982 −28.865 63.224 0.00 0.00
E
ATOM 27200 HB2 ARG E 27   39.993 −28.434 61.807 0.00 0.00
E
ATOM 27201 CG ARG E 27   39.985 −30.552 62.352 0.00 0.00
E
ATOM 27202 HG1 ARG E 27   40.972 −30.988 62.087 0.00 0.00
E
ATOM 27203 HG2 ARG E 27   39.667 −31.156 63.229 0.00 0.00
E
ATOM 27204 CD ARG E 27   39.081 −30.892 61.148 0.00 0.00
E
ATOM 27205 HD1 ARG E 27   38.888 −31.985 61.107 0.00 0.00
E
ATOM 27206 HD2 ARG E 27   38.104 −30.408 61.361 0.00 0.00
E
ATOM 27207 NE ARG E 27   39.579 −30.314 59.879 0.00 0.00
E
ATOM 27208 HE ARG E 27   39.017 −29.689 59.337 0.00 0.00
E
ATOM 27209 CZ ARG E 27   40.616 −30.815 59.158 0.00 0.00
E
ATOM 27210 NH1 ARG E 27   41.269 −31.887 59.575 0.00 0.00
E
ATOM 27211 HH11 ARG E 27   42.242 −31.834 59.351 0.00 0.00
E
ATOM 27212 HH12 ARG E 27   41.171 −32.151 60.534 0.00 0.00
E
ATOM 27213 NH2 ARG E 27   40.900 −30.394 57.934 0.00 0.00
E
ATOM 27214 HH21 ARG E 27   41.435 −30.969 57.315 0.00 0.00
E
ATOM 27215 HH22 ARG E 27   40.208 −29.752 57.605 0.00 0.00
E

ATOM 27216 C ARG E 27 42.429 −29.019 62.711 0.00 0.00
E
ATOM 27217 O ARG E 27 43.158 −29.921 63.078 0.00 0.00
E
ATOM 27218 N MET E 28 42.684 −28.204 61.678 0.00 0.00
E
ATOM 27219 HN MET E 28 42.033 −27.470 61.502 0.00 0.00
E
ATOM 27220 CA MET E 28 43.952 −28.345 61.010 0.00 0.00
E
ATOM 27221 HA MET E 28 44.070 −29.313 60.546 0.00 0.00
E
ATOM 27222 CB MET E 28 44.081 −27.325 59.781 0.00 0.00
E
ATOM 27223 HB1 MET E 28 43.835 −26.311 60.161 0.00 0.00
E
ATOM 27224 HB2 MET E 28 45.096 −27.328 59.330 0.00 0.00
E
ATOM 27225 CG MET E 28 43.270 −27.755 58.482 0.00 0.00
E
ATOM 27226 HG1 MET E 28 43.870 −28.564 58.014 0.00 0.00
E
ATOM 27227 HG2 MET E 28 42.317 −28.266 58.739 0.00 0.00
E
ATOM 27228 SD MET E 28 43.040 −26.394 57.296 0.00 0.00
E
ATOM 27229 CE MET E 28 42.685 −27.490 55.930 0.00 0.00
E
ATOM 27230 HE1 MET E 28 42.215 −26.955 55.077 0.00 0.00
E
ATOM 27231 HE2 MET E 28 43.624 −28.020 55.661 0.00 0.00
E
ATOM 27232 HE3 MET E 28 42.098 −28.329 56.360 0.00 0.00
E
ATOM 27233 C MET E 28 45.223 −28.046 61.857 0.00 0.00
E
ATOM 27234 O MET E 28 46.298 −28.589 61.596 0.00 0.00
E
ATOM 27235 N GLY E 29 45.159 −27.308 62.885 0.00 0.00
E
ATOM 27236 HN GLY E 29 44.287 −27.085 63.314 0.00 0.00
E
ATOM 27237 CA GLY E 29 46.186 −26.986 63.821 0.00 0.00
E
ATOM 27238 HA1 GLY E 29 47.056 −27.567 63.552 0.00 0.00
E
ATOM 27239 HA2 GLY E 29 45.919 −27.352 64.801 0.00 0.00
E
ATOM 27240 C GLY E 29 46.581 −25.562 63.863 0.00 0.00
E
ATOM 27241 O GLY E 29 47.507 −25.230 64.601 0.00 0.00
E
ATOM 27242 N HSE E 30 45.919 −24.634 63.159 0.00 0.00
E

ATOM 27243 HN HSE E 30 45.061 -24.801 62.680 0.00 0.00
E
ATOM 27244 CA HSE E 30 46.356 -23.258 63.151 0.00 0.00
E
ATOM 27245 HA HSE E 30 47.431 -23.206 63.070 0.00 0.00
E
ATOM 27246 CB HSE E 30 45.678 -22.566 61.978 0.00 0.00
E
ATOM 27247 HB1 HSE E 30 44.577 -22.712 61.988 0.00 0.00
E
ATOM 27248 HB2 HSE E 30 45.943 -21.489 61.910 0.00 0.00
E
ATOM 27249 ND1 HSE E 30 47.505 -22.920 60.368 0.00 0.00
E
ATOM 27250 CG HSE E 30 46.190 -23.025 60.656 0.00 0.00
E
ATOM 27251 CE1 HSE E 30 47.625 -23.371 59.156 0.00 0.00
E
ATOM 27252 HE1 HSE E 30 48.539 -23.251 58.576 0.00 0.00
E
ATOM 27253 NE2 HSE E 30 46.491 -23.914 58.671 0.00 0.00
E
ATOM 27254 HE2 HSE E 30 46.465 -24.588 57.933 0.00 0.00
E
ATOM 27255 CD2 HSE E 30 45.518 -23.663 59.641 0.00 0.00
E
ATOM 27256 HD2 HSE E 30 44.502 -24.023 59.541 0.00 0.00
E
ATOM 27257 C HSE E 30 45.787 -22.540 64.315 0.00 0.00
E
ATOM 27258 O HSE E 30 44.775 -22.885 64.923 0.00 0.00
E
ATOM 27259 N GLU E 31 46.406 -21.362 64.675 0.00 0.00
E
ATOM 27260 HN GLU E 31 47.232 -21.095 64.184 0.00 0.00
E
ATOM 27261 CA GLU E 31 45.824 -20.472 65.643 0.00 0.00
E
ATOM 27262 HA GLU E 31 45.322 -21.048 66.407 0.00 0.00
E
ATOM 27263 CB GLU E 31 46.974 -19.563 66.217 0.00 0.00
E
ATOM 27264 HB1 GLU E 31 47.487 -19.114 65.340 0.00 0.00
E
ATOM 27265 HB2 GLU E 31 46.449 -18.746 66.756 0.00 0.00
E
ATOM 27266 CG GLU E 31 47.950 -20.260 67.191 0.00 0.00
E
ATOM 27267 HG1 GLU E 31 47.402 -20.795 67.995 0.00 0.00
E
ATOM 27268 HG2 GLU E 31 48.429 -21.099 66.643 0.00 0.00
E
ATOM 27269 CD GLU E 31 48.926 -19.244 67.790 0.00 0.00
E

ATOM 27270 OE1 GLU E 31   49.586 -19.535 68.842 0.00 0.00
E
ATOM 27271 OE2 GLU E 31   49.250 -18.190 67.202 0.00 0.00
E
ATOM 27272 C   GLU E 31   44.816 -19.659 64.961 0.00 0.00
E
ATOM 27273 O   GLU E 31   44.868 -19.361 63.793 0.00 0.00
E
ATOM 27274 N   VAL E 32   43.712 -19.269 65.662 0.00 0.00
E
ATOM 27275 HN VAL E 32   43.702 -19.482 66.636 0.00 0.00
E
ATOM 27276 CA VAL E 32   42.593 -18.513 65.142 0.00 0.00
E
ATOM 27277 HA VAL E 32   42.978 -17.811 64.417 0.00 0.00
E
ATOM 27278 CB VAL E 32   41.565 -19.359 64.378 0.00 0.00
E
ATOM 27279 HB VAL E 32   42.140 -19.726 63.500 0.00 0.00
E
ATOM 27280 CG1 VAL E 32   41.144 -20.669 65.164 0.00 0.00
E
ATOM 27281 HG11 VAL E 32   40.783 -20.346 66.163 0.00 0.00
E
ATOM 27282 HG12 VAL E 32   40.377 -21.202 64.562 0.00 0.00
E
ATOM 27283 HG13 VAL E 32   42.016 -21.350 65.268 0.00 0.00
E
ATOM 27284 CG2 VAL E 32   40.261 -18.621 63.990 0.00 0.00
E
ATOM 27285 HG21 VAL E 32   40.402 -17.527 63.861 0.00 0.00
E
ATOM 27286 HG22 VAL E 32   39.795 -19.203 63.166 0.00 0.00
E
ATOM 27287 HG23 VAL E 32   39.557 -18.840 64.821 0.00 0.00
E
ATOM 27288 C   VAL E 32   41.813 -17.626 66.165 0.00 0.00
E
ATOM 27289 O   VAL E 32   41.332 -18.003 67.270 0.00 0.00
E
ATOM 27290 N   LEU E 33   41.743 -16.347 65.901 0.00 0.00
E
ATOM 27291 HN LEU E 33   42.192 -15.917 65.121 0.00 0.00
E
ATOM 27292 CA LEU E 33   40.873 -15.437 66.540 0.00 0.00
E
ATOM 27293 HA LEU E 33   40.886 -15.635 67.601 0.00 0.00
E
ATOM 27294 CB LEU E 33   41.524 -14.050 66.443 0.00 0.00
E
ATOM 27295 HB1 LEU E 33   42.505 -14.153 66.954 0.00 0.00
E
ATOM 27296 HB2 LEU E 33   41.810 -13.778 65.405 0.00 0.00
E

ATOM 27297 CG LEU E 33 40.716 -12.875 66.946 0.00 0.00
E
ATOM 27298 HG LEU E 33 39.777 -12.758 66.366 0.00 0.00
E
ATOM 27299 CD1 LEU E 33 40.181 -13.018 68.387 0.00 0.00
E
ATOM 27300 HD11 LEU E 33 39.834 -12.023 68.739 0.00 0.00
E
ATOM 27301 HD12 LEU E 33 39.314 -13.709 68.445 0.00 0.00
E
ATOM 27302 HD13 LEU E 33 41.007 -13.348 69.053 0.00 0.00
E
ATOM 27303 CD2 LEU E 33 41.375 -11.406 66.742 0.00 0.00
E
ATOM 27304 HD21 LEU E 33 42.025 -11.398 65.841 0.00 0.00
E
ATOM 27305 HD22 LEU E 33 40.591 -10.624 66.657 0.00 0.00
E
ATOM 27306 HD23 LEU E 33 42.108 -11.221 67.557 0.00 0.00
E
ATOM 27307 C LEU E 33 39.444 -15.484 66.039 0.00 0.00
E
ATOM 27308 O LEU E 33 39.121 -15.302 64.838 0.00 0.00
E
ATOM 27309 N ALA E 34 38.484 -15.866 66.851 0.00 0.00
E
ATOM 27310 HN ALA E 34 38.675 -16.144 67.790 0.00 0.00
E
ATOM 27311 CA ALA E 34 37.051 -15.930 66.504 0.00 0.00
E
ATOM 27312 HA ALA E 34 36.896 -15.958 65.435 0.00 0.00
E
ATOM 27313 CB ALA E 34 36.510 -17.240 67.077 0.00 0.00
E
ATOM 27314 HB1 ALA E 34 35.446 -17.336 66.772 0.00 0.00
E
ATOM 27315 HB2 ALA E 34 36.996 -18.167 66.704 0.00 0.00
E
ATOM 27316 HB3 ALA E 34 36.436 -17.385 68.176 0.00 0.00
E
ATOM 27317 C ALA E 34 36.348 -14.719 67.158 0.00 0.00
E
ATOM 27318 O ALA E 34 36.097 -14.667 68.396 0.00 0.00
E
ATOM 27319 N VAL E 35 35.895 -13.737 66.350 0.00 0.00
E
ATOM 27320 HN VAL E 35 36.034 -13.826 65.367 0.00 0.00
E
ATOM 27321 CA VAL E 35 35.226 -12.523 66.818 0.00 0.00
E
ATOM 27322 HA VAL E 35 35.236 -12.560 67.897 0.00 0.00
E
ATOM 27323 CB VAL E 35 35.808 -11.233 66.351 0.00 0.00
E

ATOM 27324 HB VAL E 35   35.885 -11.231 65.242 0.00 0.00
E
ATOM 27325 CG1 VAL E 35   34.979 -9.978 66.671 0.00 0.00
E
ATOM 27326 HG11 VAL E 35   35.566 -9.073 66.401 0.00 0.00
E
ATOM 27327 HG12 VAL E 35   33.974 -9.931 66.200 0.00 0.00
E
ATOM 27328 HG13 VAL E 35   34.930 -10.052 67.778 0.00 0.00
E
ATOM 27329 CG2 VAL E 35   37.234 -11.105 66.850 0.00 0.00
E
ATOM 27330 HG21 VAL E 35   37.754 -11.951 66.351 0.00 0.00
E
ATOM 27331 HG22 VAL E 35   37.760 -10.143 66.669 0.00 0.00
E
ATOM 27332 HG23 VAL E 35   37.170 -11.213 67.954 0.00 0.00
E
ATOM 27333 C   VAL E 35   33.852 -12.569 66.366 0.00 0.00
E
ATOM 27334 O   VAL E 35   33.616 -12.967 65.230 0.00 0.00
E
ATOM 27335 N   ASP E 36   32.885 -12.214 67.184 0.00 0.00
E
ATOM 27336 HN ASP E 36   33.101 -11.988 68.130 0.00 0.00
E
ATOM 27337 CA ASP E 36   31.489 -11.926 66.881 0.00 0.00
E
ATOM 27338 HA ASP E 36   31.361 -11.487 65.902 0.00 0.00
E
ATOM 27339 CB ASP E 36   30.497 -13.148 66.874 0.00 0.00
E
ATOM 27340 HB1 ASP E 36   30.949 -14.067 66.442 0.00 0.00
E
ATOM 27341 HB2 ASP E 36   30.199 -13.597 67.845 0.00 0.00
E
ATOM 27342 CG ASP E 36   29.238 -12.818 66.111 0.00 0.00
E
ATOM 27343 OD1 ASP E 36   28.941 -13.524 65.115 0.00 0.00
E
ATOM 27344 OD2 ASP E 36   28.448 -11.910 66.498 0.00 0.00
E
ATOM 27345 C   ASP E 36   31.143 -10.838 67.866 0.00 0.00
E
ATOM 27346 O   ASP E 36   31.879 -10.582 68.852 0.00 0.00
E
ATOM 27347 N   ILE E 37   30.075 -10.136 67.733 0.00 0.00
E
ATOM 27348 HN ILE E 37   29.653 -10.193 66.832 0.00 0.00
E
ATOM 27349 CA ILE E 37   29.448 -9.229 68.717 0.00 0.00
E
ATOM 27350 HA ILE E 37   30.215 -8.866 69.386 0.00 0.00
E

ATOM 27351 CB ILE E 37   28.924 -7.926 68.089 0.00 0.00
E
ATOM 27352 HB ILE E 37   29.763 -7.470 67.521 0.00 0.00
E
ATOM 27353 CG2 ILE E 37   27.795 -8.373 67.144 0.00 0.00
E
ATOM 27354 HG21 ILE E 37   28.187 -9.148 66.451 0.00 0.00
E
ATOM 27355 HG22 ILE E 37   26.910 -8.792 67.669 0.00 0.00
E
ATOM 27356 HG23 ILE E 37   27.343 -7.483 66.656 0.00 0.00
E
ATOM 27357 CG1 ILE E 37   28.536 -6.826 69.126 0.00 0.00
E
ATOM 27358 HG11 ILE E 37   27.624 -7.057 69.717 0.00 0.00
E
ATOM 27359 HG12 ILE E 37   29.307 -6.715 69.919 0.00 0.00
E
ATOM 27360 CD ILE E 37   28.249 -5.437 68.481 0.00 0.00
E
ATOM 27361 HD1 ILE E 37   27.600 -5.473 67.580 0.00 0.00
E
ATOM 27362 HD2 ILE E 37   27.801 -4.776 69.254 0.00 0.00
E
ATOM 27363 HD3 ILE E 37   29.231 -5.017 68.176 0.00 0.00
E
ATOM 27364 C   ILE E 37   28.321 -9.932 69.505 0.00 0.00
E
ATOM 27365 O   ILE E 37   28.004 -9.530 70.629 0.00 0.00
E
ATOM 27366 N   ASN E 38   27.850 -11.076 69.030 0.00 0.00
E
ATOM 27367 HN ASN E 38   28.080 -11.304 68.087 0.00 0.00
E
ATOM 27368 CA ASN E 38   26.938 -11.945 69.672 0.00 0.00
E
ATOM 27369 HA ASN E 38   26.173 -11.336 70.131 0.00 0.00
E
ATOM 27370 CB ASN E 38   26.254 -12.772 68.551 0.00 0.00
E
ATOM 27371 HB1 ASN E 38   26.020 -12.087 67.709 0.00 0.00
E
ATOM 27372 HB2 ASN E 38   27.080 -13.359 68.095 0.00 0.00
E
ATOM 27373 CG ASN E 38   25.185 -13.665 68.972 0.00 0.00
E
ATOM 27374 OD1 ASN E 38   25.408 -14.836 69.317 0.00 0.00
E
ATOM 27375 ND2 ASN E 38   23.940 -13.203 69.144 0.00 0.00
E
ATOM 27376 HD21 ASN E 38   23.206 -13.879 69.205 0.00 0.00
E
ATOM 27377 HD22 ASN E 38   23.689 -12.327 68.732 0.00 0.00
E

ATOM 27378 C ASN E 38 27.533 -12.882 70.785 0.00 0.00
E
ATOM 27379 O ASN E 38 28.460 -13.765 70.526 0.00 0.00
E
ATOM 27380 N GLU E 39 26.845 -12.833 72.002 0.00 0.00
E
ATOM 27381 HN GLU E 39 26.121 -12.161 72.139 0.00 0.00
E
ATOM 27382 CA GLU E 39 27.326 -13.557 73.140 0.00 0.00
E
ATOM 27383 HA GLU E 39 28.347 -13.230 73.274 0.00 0.00
E
ATOM 27384 CB GLU E 39 26.519 -13.297 74.458 0.00 0.00
E
ATOM 27385 HB1 GLU E 39 25.434 -13.519 74.372 0.00 0.00
E
ATOM 27386 HB2 GLU E 39 26.762 -13.945 75.327 0.00 0.00
E
ATOM 27387 CG GLU E 39 26.600 -11.817 74.835 0.00 0.00
E
ATOM 27388 HG1 GLU E 39 27.668 -11.636 75.084 0.00 0.00
E
ATOM 27389 HG2 GLU E 39 26.352 -11.042 74.080 0.00 0.00
E
ATOM 27390 CD GLU E 39 25.737 -11.374 76.052 0.00 0.00
E
ATOM 27391 OE1 GLU E 39 26.041 -11.674 77.220 0.00 0.00
E
ATOM 27392 OE2 GLU E 39 24.783 -10.560 75.801 0.00 0.00
E
ATOM 27393 C GLU E 39 27.279 -15.088 72.983 0.00 0.00
E
ATOM 27394 O GLU E 39 28.194 -15.869 73.293 0.00 0.00
E
ATOM 27395 N GLU E 40 26.118 -15.577 72.495 0.00 0.00
E
ATOM 27396 HN GLU E 40 25.387 -14.958 72.221 0.00 0.00
E
ATOM 27397 CA GLU E 40 25.856 -17.016 72.319 0.00 0.00
E
ATOM 27398 HA GLU E 40 25.901 -17.525 73.270 0.00 0.00
E
ATOM 27399 CB GLU E 40 24.512 -17.281 71.730 0.00 0.00
E
ATOM 27400 HB1 GLU E 40 23.793 -16.753 72.392 0.00 0.00
E
ATOM 27401 HB2 GLU E 40 24.407 -16.746 70.762 0.00 0.00
E
ATOM 27402 CG GLU E 40 24.169 -18.768 71.437 0.00 0.00
E
ATOM 27403 HG1 GLU E 40 24.698 -19.075 70.509 0.00 0.00
E
ATOM 27404 HG2 GLU E 40 24.486 -19.537 72.173 0.00 0.00
E

ATOM 27405 CD GLU E 40 22.687 -19.040 71.239 0.00 0.00
E
ATOM 27406 OE1 GLU E 40 22.180 -18.412 70.214 0.00 0.00
E
ATOM 27407 OE2 GLU E 40 21.959 -19.747 71.977 0.00 0.00
E
ATOM 27408 C GLU E 40 26.891 -17.631 71.441 0.00 0.00
E
ATOM 27409 O GLU E 40 27.560 -18.607 71.776 0.00 0.00
E
ATOM 27410 N LYS E 41 27.194 -17.079 70.284 0.00 0.00
E
ATOM 27411 HN LYS E 41 26.671 -16.268 70.034 0.00 0.00
E
ATOM 27412 CA LYS E 41 28.244 -17.485 69.370 0.00 0.00
E
ATOM 27413 HA LYS E 41 28.161 -18.544 69.173 0.00 0.00
E
ATOM 27414 CB LYS E 41 28.183 -16.757 67.925 0.00 0.00
E
ATOM 27415 HB1 LYS E 41 28.037 -15.659 68.014 0.00 0.00
E
ATOM 27416 HB2 LYS E 41 29.087 -17.086 67.369 0.00 0.00
E
ATOM 27417 CG LYS E 41 26.931 -17.296 67.189 0.00 0.00
E
ATOM 27418 HG1 LYS E 41 26.999 -18.405 67.161 0.00 0.00
E
ATOM 27419 HG2 LYS E 41 26.072 -16.993 67.825 0.00 0.00
E
ATOM 27420 CD LYS E 41 26.654 -16.552 65.798 0.00 0.00
E
ATOM 27421 HD1 LYS E 41 26.622 -15.448 65.915 0.00 0.00
E
ATOM 27422 HD2 LYS E 41 27.475 -16.854 65.113 0.00 0.00
E
ATOM 27423 CE LYS E 41 25.294 -16.836 65.146 0.00 0.00
E
ATOM 27424 HE1 LYS E 41 25.457 -16.431 64.125 0.00 0.00
E
ATOM 27425 HE2 LYS E 41 25.169 -17.936 65.062 0.00 0.00
E
ATOM 27426 NZ LYS E 41 24.147 -16.150 65.697 0.00 0.00
E
ATOM 27427 HZ1 LYS E 41 24.235 -15.127 65.535 0.00 0.00
E
ATOM 27428 HZ2 LYS E 41 23.254 -16.475 65.272 0.00 0.00
E
ATOM 27429 HZ3 LYS E 41 23.931 -16.355 66.693 0.00 0.00
E
ATOM 27430 C LYS E 41 29.694 -17.376 69.897 0.00 0.00
E
ATOM 27431 O LYS E 41 30.481 -18.294 69.699 0.00 0.00
E

ATOM 27432 N VAL E 42 30.069 -16.301 70.592 0.00 0.00
E
ATOM 27433 HN VAL E 42 29.402 -15.571 70.714 0.00 0.00
E
ATOM 27434 CA VAL E 42 31.384 -16.207 71.103 0.00 0.00
E
ATOM 27435 HA VAL E 42 32.033 -16.476 70.284 0.00 0.00
E
ATOM 27436 CB VAL E 42 31.676 -14.808 71.574 0.00 0.00
E
ATOM 27437 HB VAL E 42 30.779 -14.476 72.139 0.00 0.00
E
ATOM 27438 CG1 VAL E 42 32.946 -14.655 72.413 0.00 0.00
E
ATOM 27439 HG11 VAL E 42 33.143 -13.563 72.474 0.00 0.00
E
ATOM 27440 HG12 VAL E 42 32.933 -14.940 73.487 0.00 0.00
E
ATOM 27441 HG13 VAL E 42 33.830 -15.084 71.894 0.00 0.00
E
ATOM 27442 CG2 VAL E 42 32.107 -13.882 70.389 0.00 0.00
E
ATOM 27443 HG21 VAL E 42 33.190 -13.905 70.144 0.00 0.00
E
ATOM 27444 HG22 VAL E 42 31.614 -14.190 69.442 0.00 0.00
E
ATOM 27445 HG23 VAL E 42 31.711 -12.850 70.508 0.00 0.00
E
ATOM 27446 C VAL E 42 31.678 -17.362 72.169 0.00 0.00
E
ATOM 27447 O VAL E 42 32.687 -17.940 72.296 0.00 0.00
E
ATOM 27448 N ASN E 43 30.611 -17.670 72.965 0.00 0.00
E
ATOM 27449 HN ASN E 43 29.773 -17.138 72.869 0.00 0.00
E
ATOM 27450 CA ASN E 43 30.538 -18.721 73.910 0.00 0.00
E
ATOM 27451 HA ASN E 43 31.433 -18.638 74.509 0.00 0.00
E
ATOM 27452 CB ASN E 43 29.257 -18.675 74.795 0.00 0.00
E
ATOM 27453 HB1 ASN E 43 29.117 -17.634 75.156 0.00 0.00
E
ATOM 27454 HB2 ASN E 43 28.344 -18.918 74.210 0.00 0.00
E
ATOM 27455 CG ASN E 43 29.566 -19.519 76.095 0.00 0.00
E
ATOM 27456 OD1 ASN E 43 30.384 -19.150 76.901 0.00 0.00
E
ATOM 27457 ND2 ASN E 43 29.024 -20.721 76.114 0.00 0.00
E
ATOM 27458 HD21 ASN E 43 28.985 -21.258 76.956 0.00 0.00
E

ATOM 27459 HD22 ASN E 43 28.673 -20.969 75.211 0.00 0.00
E
ATOM 27460 C ASN E 43 30.536 -20.123 73.205 0.00 0.00
E
ATOM 27461 O ASN E 43 31.336 -21.024 73.593 0.00 0.00
E
ATOM 27462 N ALA E 44 29.675 -20.369 72.201 0.00 0.00
E
ATOM 27463 HN ALA E 44 29.066 -19.639 71.903 0.00 0.00
E
ATOM 27464 CA ALA E 44 29.549 -21.573 71.370 0.00 0.00
E
ATOM 27465 HA ALA E 44 29.233 -22.359 72.040 0.00 0.00
E
ATOM 27466 CB ALA E 44 28.635 -21.117 70.205 0.00 0.00
E
ATOM 27467 HB1 ALA E 44 28.539 -21.879 69.402 0.00 0.00
E
ATOM 27468 HB2 ALA E 44 27.687 -20.692 70.600 0.00 0.00
E
ATOM 27469 HB3 ALA E 44 29.060 -20.235 69.680 0.00 0.00
E
ATOM 27470 C ALA E 44 30.807 -22.083 70.708 0.00 0.00
E
ATOM 27471 O ALA E 44 31.037 -23.300 70.587 0.00 0.00
E
ATOM 27472 N TYR E 45 31.657 -21.117 70.254 0.00 0.00
E
ATOM 27473 HN TYR E 45 31.322 -20.179 70.201 0.00 0.00
E
ATOM 27474 CA TYR E 45 32.872 -21.495 69.567 0.00 0.00
E
ATOM 27475 HA TYR E 45 32.722 -22.488 69.171 0.00 0.00
E
ATOM 27476 CB TYR E 45 33.046 -20.598 68.313 0.00 0.00
E
ATOM 27477 HB1 TYR E 45 32.686 -19.572 68.542 0.00 0.00
E
ATOM 27478 HB2 TYR E 45 34.101 -20.578 67.967 0.00 0.00
E
ATOM 27479 CG TYR E 45 32.212 -21.201 67.236 0.00 0.00
E
ATOM 27480 CD1 TYR E 45 30.970 -20.683 66.962 0.00 0.00
E
ATOM 27481 HD1 TYR E 45 30.673 -19.751 67.421 0.00 0.00
E
ATOM 27482 CE1 TYR E 45 30.191 -21.305 66.004 0.00 0.00
E
ATOM 27483 HE1 TYR E 45 29.186 -20.982 65.779 0.00 0.00
E
ATOM 27484 CZ TYR E 45 30.655 -22.400 65.334 0.00 0.00
E
ATOM 27485 OH TYR E 45 29.775 -22.913 64.364 0.00 0.00
E

ATOM	27486	HH	TYR	E	45	29.181	−22.182	64.180	0.00	0.00	E
ATOM	27487	CD2	TYR	E	45	32.761	−22.252	66.511	0.00	0.00	E
ATOM	27488	HD2	TYR	E	45	33.708	−22.648	66.846	0.00	0.00	E
ATOM	27489	CE2	TYR	E	45	31.961	−22.864	65.579	0.00	0.00	E
ATOM	27490	HE2	TYR	E	45	32.326	−23.693	64.991	0.00	0.00	E
ATOM	27491	C	TYR	E	45	34.175	−21.476	70.332	0.00	0.00	E
ATOM	27492	O	TYR	E	45	35.293	−21.667	69.816	0.00	0.00	E
ATOM	27493	N	ALA	E	46	34.086	−21.281	71.714	0.00	0.00	E
ATOM	27494	HN	ALA	E	46	33.206	−21.281	72.182	0.00	0.00	E
ATOM	27495	CA	ALA	E	46	35.184	−21.016	72.553	0.00	0.00	E
ATOM	27496	HA	ALA	E	46	35.750	−20.244	72.053	0.00	0.00	E
ATOM	27497	CB	ALA	E	46	34.825	−20.511	73.970	0.00	0.00	E
ATOM	27498	HB1	ALA	E	46	34.477	−19.456	73.960	0.00	0.00	E
ATOM	27499	HB2	ALA	E	46	34.104	−21.094	74.582	0.00	0.00	E
ATOM	27500	HB3	ALA	E	46	35.779	−20.389	74.525	0.00	0.00	E
ATOM	27501	C	ALA	E	46	36.055	−22.306	72.789	0.00	0.00	E
ATOM	27502	O	ALA	E	46	37.215	−22.161	73.167	0.00	0.00	E
ATOM	27503	N	SER	E	47	35.623	−23.539	72.548	0.00	0.00	E
ATOM	27504	HN	SER	E	47	34.648	−23.695	72.410	0.00	0.00	E
ATOM	27505	CA	SER	E	47	36.609	−24.643	72.590	0.00	0.00	E
ATOM	27506	HA	SER	E	47	37.531	−24.383	73.089	0.00	0.00	E
ATOM	27507	CB	SER	E	47	36.014	−25.852	73.407	0.00	0.00	E
ATOM	27508	HB1	SER	E	47	35.241	−26.264	72.725	0.00	0.00	E
ATOM	27509	HB2	SER	E	47	36.727	−26.675	73.628	0.00	0.00	E
ATOM	27510	OG	SER	E	47	35.525	−25.471	74.702	0.00	0.00	E
ATOM	27511	HG1	SER	E	47	36.030	−25.999	75.325	0.00	0.00	E
ATOM	27512	C	SER	E	47	36.926	−25.209	71.229	0.00	0.00	E

ATOM 27513 O   SER E 47   37.482 -26.294 71.081 0.00 0.00
E
ATOM 27514 N   TYR E 48   36.526 -24.370 70.191 0.00 0.00
E
ATOM 27515 HN TYR E 48   36.085 -23.478 70.257 0.00 0.00
E
ATOM 27516 CA TYR E 48   36.768 -24.679 68.765 0.00 0.00
E
ATOM 27517 HA TYR E 48   37.344 -25.589 68.683 0.00 0.00
E
ATOM 27518 CB TYR E 48   35.415 -24.926 68.025 0.00 0.00
E
ATOM 27519 HB1 TYR E 48   34.751 -24.044 68.154 0.00 0.00
E
ATOM 27520 HB2 TYR E 48   35.482 -25.121 66.933 0.00 0.00
E
ATOM 27521 CG TYR E 48   34.797 -26.088 68.631 0.00 0.00
E
ATOM 27522 CD1 TYR E 48   35.117 -27.350 68.215 0.00 0.00
E
ATOM 27523 HD1 TYR E 48   35.993 -27.508 67.603 0.00 0.00
E
ATOM 27524 CE1 TYR E 48   34.344 -28.507 68.590 0.00 0.00
E
ATOM 27525 HE1 TYR E 48   34.696 -29.428 68.151 0.00 0.00
E
ATOM 27526 CZ TYR E 48   33.308 -28.371 69.473 0.00 0.00
E
ATOM 27527 OH TYR E 48   32.508 -29.503 69.770 0.00 0.00
E
ATOM 27528 HH TYR E 48   32.626 -30.190 69.110 0.00 0.00
E
ATOM 27529 CD2 TYR E 48   33.745 -25.986 69.602 0.00 0.00
E
ATOM 27530 HD2 TYR E 48   33.405 -24.995 69.863 0.00 0.00
E
ATOM 27531 CE2 TYR E 48   32.977 -27.120 70.036 0.00 0.00
E
ATOM 27532 HE2 TYR E 48   32.170 -26.989 70.742 0.00 0.00
E
ATOM 27533 C   TYR E 48   37.547 -23.569 68.043 0.00 0.00
E
ATOM 27534 O   TYR E 48   37.603 -23.521 66.809 0.00 0.00
E
ATOM 27535 N   ALA E 49   38.368 -22.807 68.849 0.00 0.00
E
ATOM 27536 HN ALA E 49   38.466 -22.955 69.830 0.00 0.00
E
ATOM 27537 CA ALA E 49   39.149 -21.766 68.388 0.00 0.00
E
ATOM 27538 HA ALA E 49   39.580 -22.187 67.492 0.00 0.00
E
ATOM 27539 CB ALA E 49   38.401 -20.459 68.010 0.00 0.00
E

```
ATOM  27540  HB1 ALA E  49      37.916 -19.958  68.875  0.00  0.00
E
ATOM  27541  HB2 ALA E  49      39.189 -19.753  67.671  0.00  0.00
E
ATOM  27542  HB3 ALA E  49      37.526 -20.607  67.341  0.00  0.00
E
ATOM  27543  C   ALA E  49      40.253 -21.441  69.325  0.00  0.00
E
ATOM  27544  O   ALA E  49      40.005 -21.647  70.522  0.00  0.00
E
ATOM  27545  N   THR E  50      41.491 -20.957  68.973  0.00  0.00
E
ATOM  27546  HN  THR E  50      41.848 -20.708  68.075  0.00  0.00
E
ATOM  27547  CA  THR E  50      42.483 -20.481  69.968  0.00  0.00
E
ATOM  27548  HA  THR E  50      42.464 -21.241  70.735  0.00  0.00
E
ATOM  27549  CB  THR E  50      43.881 -20.263  69.553  0.00  0.00
E
ATOM  27550  HB  THR E  50      44.568 -20.072  70.405  0.00  0.00
E
ATOM  27551  OG1 THR E  50      44.053 -19.212  68.626  0.00  0.00
E
ATOM  27552  HG1 THR E  50      44.379 -18.466  69.135  0.00  0.00
E
ATOM  27553  CG2 THR E  50      44.414 -21.592  68.921  0.00  0.00
E
ATOM  27554 HG21 THR E  50      44.123 -22.533  69.434  0.00  0.00
E
ATOM  27555 HG22 THR E  50      43.970 -21.730  67.912  0.00  0.00
E
ATOM  27556 HG23 THR E  50      45.521 -21.524  68.872  0.00  0.00
E
ATOM  27557  C   THR E  50      42.095 -19.296  70.860  0.00  0.00
E
ATOM  27558  O   THR E  50      42.309 -19.399  72.071  0.00  0.00
E
ATOM  27559  N   HSE E  51      41.311 -18.346  70.346  0.00  0.00
E
ATOM  27560  HN  HSE E  51      41.187 -18.253  69.362  0.00  0.00
E
ATOM  27561  CA  HSE E  51      40.872 -17.201  71.101  0.00  0.00
E
ATOM  27562  HA  HSE E  51      40.781 -17.475  72.141  0.00  0.00
E
ATOM  27563  CB  HSE E  51      41.900 -16.048  71.031  0.00  0.00
E
ATOM  27564  HB1 HSE E  51      42.929 -16.461  71.106  0.00  0.00
E
ATOM  27565  HB2 HSE E  51      41.911 -15.710  69.973  0.00  0.00
E
ATOM  27566  ND1 HSE E  51      42.393 -15.224  73.306  0.00  0.00
E
```

ATOM 27567 CG HSE E 51 41.874 -14.991 72.041 0.00 0.00
E
ATOM 27568 CE1 HSE E 51 42.331 -14.084 73.966 0.00 0.00
E
ATOM 27569 HE1 HSE E 51 42.767 -13.841 74.935 0.00 0.00
E
ATOM 27570 NE2 HSE E 51 41.770 -13.133 73.128 0.00 0.00
E
ATOM 27571 HE2 HSE E 51 41.616 -12.173 73.363 0.00 0.00
E
ATOM 27572 CD2 HSE E 51 41.482 -13.717 71.912 0.00 0.00
E
ATOM 27573 HD2 HSE E 51 41.070 -13.138 71.095 0.00 0.00
E
ATOM 27574 C HSE E 51 39.599 -16.716 70.604 0.00 0.00
E
ATOM 27575 O HSE E 51 39.373 -16.577 69.419 0.00 0.00
E
ATOM 27576 N ALA E 52 38.666 -16.437 71.516 0.00 0.00
E
ATOM 27577 HN ALA E 52 38.717 -16.801 72.442 0.00 0.00
E
ATOM 27578 CA ALA E 52 37.373 -15.857 71.266 0.00 0.00
E
ATOM 27579 HA ALA E 52 37.358 -15.358 70.309 0.00 0.00
E
ATOM 27580 CB ALA E 52 36.206 -16.872 71.465 0.00 0.00
E
ATOM 27581 HB1 ALA E 52 36.133 -17.532 72.356 0.00 0.00
E
ATOM 27582 HB2 ALA E 52 35.270 -16.300 71.283 0.00 0.00
E
ATOM 27583 HB3 ALA E 52 36.301 -17.515 70.564 0.00 0.00
E
ATOM 27584 C ALA E 52 37.122 -14.734 72.289 0.00 0.00
E
ATOM 27585 O ALA E 52 37.467 -14.833 73.508 0.00 0.00
E
ATOM 27586 N VAL E 53 36.628 -13.600 71.751 0.00 0.00
E
ATOM 27587 HN VAL E 53 36.404 -13.543 70.781 0.00 0.00
E
ATOM 27588 CA VAL E 53 36.439 -12.361 72.401 0.00 0.00
E
ATOM 27589 HA VAL E 53 36.093 -12.605 73.394 0.00 0.00
E
ATOM 27590 CB VAL E 53 37.758 -11.617 72.426 0.00 0.00
E
ATOM 27591 HB VAL E 53 38.490 -12.348 72.830 0.00 0.00
E
ATOM 27592 CG1 VAL E 53 38.285 -11.182 70.976 0.00 0.00
E
ATOM 27593 HG11 VAL E 53 38.007 -11.901 70.176 0.00 0.00
E

ATOM 27594 HG12 VAL E 53 37.815 -10.245 70.607 0.00 0.00
E
ATOM 27595 HG13 VAL E 53 39.386 -11.034 70.983 0.00 0.00
E
ATOM 27596 CG2 VAL E 53 37.845 -10.440 73.356 0.00 0.00
E
ATOM 27597 HG21 VAL E 53 38.920 -10.175 73.448 0.00 0.00
E
ATOM 27598 HG22 VAL E 53 37.279 -9.640 72.833 0.00 0.00
E
ATOM 27599 HG23 VAL E 53 37.426 -10.801 74.320 0.00 0.00
E
ATOM 27600 C VAL E 53 35.231 -11.648 71.760 0.00 0.00
E
ATOM 27601 O VAL E 53 34.959 -11.915 70.653 0.00 0.00
E
ATOM 27602 N ILE E 54 34.562 -10.685 72.428 0.00 0.00
E
ATOM 27603 HN ILE E 54 34.898 -10.479 73.344 0.00 0.00
E
ATOM 27604 CA ILE E 54 33.482 -10.041 71.839 0.00 0.00
E
ATOM 27605 HA ILE E 54 33.026 -10.588 71.027 0.00 0.00
E
ATOM 27606 CB ILE E 54 32.349 -10.039 72.883 0.00 0.00
E
ATOM 27607 HB ILE E 54 32.750 -9.410 73.707 0.00 0.00
E
ATOM 27608 CG2 ILE E 54 31.101 -9.243 72.387 0.00 0.00
E
ATOM 27609 HG21 ILE E 54 31.356 -8.233 72.004 0.00 0.00
E
ATOM 27610 HG22 ILE E 54 30.550 -9.831 71.622 0.00 0.00
E
ATOM 27611 HG23 ILE E 54 30.404 -9.161 73.249 0.00 0.00
E
ATOM 27612 CG1 ILE E 54 31.868 -11.408 73.526 0.00 0.00
E
ATOM 27613 HG11 ILE E 54 31.446 -12.041 72.716 0.00 0.00
E
ATOM 27614 HG12 ILE E 54 32.676 -12.008 73.996 0.00 0.00
E
ATOM 27615 CD ILE E 54 30.869 -11.319 74.712 0.00 0.00
E
ATOM 27616 HD1 ILE E 54 29.996 -10.731 74.357 0.00 0.00
E
ATOM 27617 HD2 ILE E 54 30.531 -12.297 75.117 0.00 0.00
E
ATOM 27618 HD3 ILE E 54 31.391 -10.761 75.518 0.00 0.00
E
ATOM 27619 C ILE E 54 33.909 -8.626 71.448 0.00 0.00
E
ATOM 27620 O ILE E 54 34.381 -7.834 72.302 0.00 0.00
E

ATOM	27621	N	ALA	E	55	33.796	-8.339	70.164	0.00	0.00	E
ATOM	27622	HN	ALA	E	55	33.538	-9.029	69.492	0.00	0.00	E
ATOM	27623	CA	ALA	E	55	34.202	-6.986	69.761	0.00	0.00	E
ATOM	27624	HA	ALA	E	55	33.871	-6.285	70.514	0.00	0.00	E
ATOM	27625	CB	ALA	E	55	35.730	-7.029	69.520	0.00	0.00	E
ATOM	27626	HB1	ALA	E	55	36.034	-7.765	68.745	0.00	0.00	E
ATOM	27627	HB2	ALA	E	55	36.073	-5.983	69.367	0.00	0.00	E
ATOM	27628	HB3	ALA	E	55	36.350	-7.286	70.405	0.00	0.00	E
ATOM	27629	C	ALA	E	55	33.533	-6.487	68.475	0.00	0.00	E
ATOM	27630	O	ALA	E	55	33.324	-7.246	67.519	0.00	0.00	E
ATOM	27631	N	ASN	E	56	33.171	-5.201	68.497	0.00	0.00	E
ATOM	27632	HN	ASN	E	56	33.255	-4.735	69.374	0.00	0.00	E
ATOM	27633	CA	ASN	E	56	32.723	-4.432	67.350	0.00	0.00	E
ATOM	27634	HA	ASN	E	56	32.014	-5.056	66.827	0.00	0.00	E
ATOM	27635	CB	ASN	E	56	31.976	-3.120	67.836	0.00	0.00	E
ATOM	27636	HB1	ASN	E	56	31.312	-3.566	68.608	0.00	0.00	E
ATOM	27637	HB2	ASN	E	56	32.571	-2.243	68.168	0.00	0.00	E
ATOM	27638	CG	ASN	E	56	31.015	-2.708	66.742	0.00	0.00	E
ATOM	27639	OD1	ASN	E	56	30.990	-3.251	65.628	0.00	0.00	E
ATOM	27640	ND2	ASN	E	56	30.142	-1.743	67.032	0.00	0.00	E
ATOM	27641	HD21	ASN	E	56	29.446	-1.504	66.356	0.00	0.00	E
ATOM	27642	HD22	ASN	E	56	30.146	-1.210	67.878	0.00	0.00	E
ATOM	27643	C	ASN	E	56	33.807	-4.060	66.364	0.00	0.00	E
ATOM	27644	O	ASN	E	56	34.714	-3.190	66.597	0.00	0.00	E
ATOM	27645	N	ALA	E	57	33.788	-4.614	65.111	0.00	0.00	E
ATOM	27646	HN	ALA	E	57	33.109	-5.341	65.041	0.00	0.00	E
ATOM	27647	CA	ALA	E	57	34.702	-4.462	64.013	0.00	0.00	E

ATOM 27648 HA ALA E 57 35.665 -4.134 64.374 0.00 0.00
E
ATOM 27649 CB ALA E 57 34.745 -5.729 63.241 0.00 0.00
E
ATOM 27650 HB1 ALA E 57 35.665 -5.669 62.621 0.00 0.00
E
ATOM 27651 HB2 ALA E 57 34.901 -6.512 64.014 0.00 0.00
E
ATOM 27652 HB3 ALA E 57 33.909 -5.923 62.536 0.00 0.00
E
ATOM 27653 C ALA E 57 34.501 -3.221 63.193 0.00 0.00
E
ATOM 27654 O ALA E 57 35.265 -3.021 62.197 0.00 0.00
E
ATOM 27655 N THR E 58 33.503 -2.399 63.695 0.00 0.00
E
ATOM 27656 HN THR E 58 32.958 -2.624 64.499 0.00 0.00
E
ATOM 27657 CA THR E 58 33.173 -1.201 62.932 0.00 0.00
E
ATOM 27658 HA THR E 58 33.662 -1.255 61.971 0.00 0.00
E
ATOM 27659 CB THR E 58 31.667 -0.884 62.834 0.00 0.00
E
ATOM 27660 HB THR E 58 31.545 0.120 62.375 0.00 0.00
E
ATOM 27661 OG1 THR E 58 31.034 -0.815 64.087 0.00 0.00
E
ATOM 27662 HG1 THR E 58 31.020 -1.738 64.352 0.00 0.00
E
ATOM 27663 CG2 THR E 58 31.035 -1.991 62.040 0.00 0.00
E
ATOM 27664 HG21 THR E 58 31.655 -2.261 61.158 0.00 0.00
E
ATOM 27665 HG22 THR E 58 31.133 -2.855 62.732 0.00 0.00
E
ATOM 27666 HG23 THR E 58 30.047 -1.710 61.619 0.00 0.00
E
ATOM 27667 C THR E 58 33.789 0.029 63.577 0.00 0.00
E
ATOM 27668 O THR E 58 33.835 1.142 63.003 0.00 0.00
E
ATOM 27669 N GLU E 59 34.480 -0.263 64.711 0.00 0.00
E
ATOM 27670 HN GLU E 59 34.731 -1.208 64.907 0.00 0.00
E
ATOM 27671 CA GLU E 59 34.949 0.746 65.720 0.00 0.00
E
ATOM 27672 HA GLU E 59 34.823 1.775 65.415 0.00 0.00
E
ATOM 27673 CB GLU E 59 34.254 0.404 67.047 0.00 0.00
E
ATOM 27674 HB1 GLU E 59 33.170 0.280 66.837 0.00 0.00
E

ATOM 27675 HB2 GLU E 59 34.578 −0.583 67.442 0.00 0.00
E
ATOM 27676 CG GLU E 59 34.381 1.462 68.154 0.00 0.00
E
ATOM 27677 HG1 GLU E 59 33.939 1.005 69.065 0.00 0.00
E
ATOM 27678 HG2 GLU E 59 35.484 1.546 68.256 0.00 0.00
E
ATOM 27679 CD GLU E 59 33.680 2.794 67.999 0.00 0.00
E
ATOM 27680 OE1 GLU E 59 32.672 3.057 68.790 0.00 0.00
E
ATOM 27681 OE2 GLU E 59 34.145 3.643 67.192 0.00 0.00
E
ATOM 27682 C GLU E 59 36.481 0.632 66.006 0.00 0.00
E
ATOM 27683 O GLU E 59 37.052 −0.383 66.526 0.00 0.00
E
ATOM 27684 N GLU E 60 37.167 1.694 65.661 0.00 0.00
E
ATOM 27685 HN GLU E 60 36.693 2.410 65.155 0.00 0.00
E
ATOM 27686 CA GLU E 60 38.607 1.841 65.707 0.00 0.00
E
ATOM 27687 HA GLU E 60 39.019 1.201 64.941 0.00 0.00
E
ATOM 27688 CB GLU E 60 39.040 3.284 65.391 0.00 0.00
E
ATOM 27689 HB1 GLU E 60 38.802 3.598 64.353 0.00 0.00
E
ATOM 27690 HB2 GLU E 60 38.461 3.936 66.079 0.00 0.00
E
ATOM 27691 CG GLU E 60 40.562 3.599 65.490 0.00 0.00
E
ATOM 27692 HG1 GLU E 60 40.996 3.138 66.402 0.00 0.00
E
ATOM 27693 HG2 GLU E 60 41.153 3.206 64.635 0.00 0.00
E
ATOM 27694 CD GLU E 60 40.849 5.107 65.471 0.00 0.00
E
ATOM 27695 OE1 GLU E 60 41.309 5.641 66.526 0.00 0.00
E
ATOM 27696 OE2 GLU E 60 40.731 5.681 64.334 0.00 0.00
E
ATOM 27697 C GLU E 60 39.354 1.402 66.975 0.00 0.00
E
ATOM 27698 O GLU E 60 40.258 0.575 66.920 0.00 0.00
E
ATOM 27699 N ASN E 61 39.013 1.914 68.127 0.00 0.00
E
ATOM 27700 HN ASN E 61 38.245 2.542 68.028 0.00 0.00
E
ATOM 27701 CA ASN E 61 39.607 1.626 69.412 0.00 0.00
E

ATOM 27702 HA ASN E 61 40.649 1.840 69.223 0.00 0.00
E
ATOM 27703 CB ASN E 61 38.954 2.720 70.369 0.00 0.00
E
ATOM 27704 HB1 ASN E 61 39.156 3.724 69.938 0.00 0.00
E
ATOM 27705 HB2 ASN E 61 37.874 2.529 70.546 0.00 0.00
E
ATOM 27706 CG ASN E 61 39.690 2.547 71.690 0.00 0.00
E
ATOM 27707 OD1 ASN E 61 39.309 1.746 72.577 0.00 0.00
E
ATOM 27708 ND2 ASN E 61 40.874 3.239 71.823 0.00 0.00
E
ATOM 27709 HD21 ASN E 61 41.333 3.278 72.711 0.00 0.00
E
ATOM 27710 HD22 ASN E 61 41.118 3.819 71.046 0.00 0.00
E
ATOM 27711 C ASN E 61 39.459 0.138 69.862 0.00 0.00
E
ATOM 27712 O ASN E 61 40.505 -0.302 70.365 0.00 0.00
E
ATOM 27713 N GLU E 62 38.333 -0.482 69.494 0.00 0.00
E
ATOM 27714 HN GLU E 62 37.670 -0.031 68.901 0.00 0.00
E
ATOM 27715 CA GLU E 62 38.048 -1.949 69.698 0.00 0.00
E
ATOM 27716 HA GLU E 62 38.159 -2.238 70.732 0.00 0.00
E
ATOM 27717 CB GLU E 62 36.547 -2.218 69.383 0.00 0.00
E
ATOM 27718 HB1 GLU E 62 36.269 -2.110 68.313 0.00 0.00
E
ATOM 27719 HB2 GLU E 62 36.449 -3.317 69.520 0.00 0.00
E
ATOM 27720 CG GLU E 62 35.622 -1.449 70.321 0.00 0.00
E
ATOM 27721 HG1 GLU E 62 35.841 -0.360 70.320 0.00 0.00
E
ATOM 27722 HG2 GLU E 62 34.561 -1.663 70.071 0.00 0.00
E
ATOM 27723 CD GLU E 62 35.905 -1.882 71.776 0.00 0.00
E
ATOM 27724 OE1 GLU E 62 36.404 -1.057 72.593 0.00 0.00
E
ATOM 27725 OE2 GLU E 62 35.684 -3.086 72.119 0.00 0.00
E
ATOM 27726 C GLU E 62 38.966 -2.791 68.860 0.00 0.00
E
ATOM 27727 O GLU E 62 39.674 -3.694 69.396 0.00 0.00
E
ATOM 27728 N LEU E 63 39.134 -2.412 67.626 0.00 0.00
E

ATOM 27729 HN LEU E 63   38.457 -1.780 67.258 0.00 0.00
E
ATOM 27730 CA LEU E 63   40.181 -2.799 66.724 0.00 0.00
E
ATOM 27731 HA LEU E 63   40.103 -3.876 66.757 0.00 0.00
E
ATOM 27732 CB LEU E 63   40.018 -2.279 65.246 0.00 0.00
E
ATOM 27733 HB1 LEU E 63   40.161 -1.177 65.267 0.00 0.00
E
ATOM 27734 HB2 LEU E 63   40.925 -2.755 64.816 0.00 0.00
E
ATOM 27735 CG LEU E 63   38.647 -2.638 64.593 0.00 0.00
E
ATOM 27736 HG LEU E 63   37.916 -2.589 65.428 0.00 0.00
E
ATOM 27737 CD1 LEU E 63   38.187 -1.588 63.546 0.00 0.00
E
ATOM 27738 HD11 LEU E 63   37.099 -1.640 63.332 0.00 0.00
E
ATOM 27739 HD12 LEU E 63   38.499 -0.567 63.854 0.00 0.00
E
ATOM 27740 HD13 LEU E 63   38.752 -1.779 62.608 0.00 0.00
E
ATOM 27741 CD2 LEU E 63   38.818 -4.044 64.043 0.00 0.00
E
ATOM 27742 HD21 LEU E 63   39.189 -4.896 64.654 0.00 0.00
E
ATOM 27743 HD22 LEU E 63   37.854 -4.358 63.589 0.00 0.00
E
ATOM 27744 HD23 LEU E 63   39.554 -4.032 63.211 0.00 0.00
E
ATOM 27745 C   LEU E 63   41.612 -2.702 67.214 0.00 0.00
E
ATOM 27746 O   LEU E 63   42.410 -3.684 67.211 0.00 0.00
E
ATOM 27747 N   LEU E 64   42.056 -1.460 67.715 0.00 0.00
E
ATOM 27748 HN LEU E 64   41.427 -0.688 67.666 0.00 0.00
E
ATOM 27749 CA LEU E 64   43.376 -1.277 68.307 0.00 0.00
E
ATOM 27750 HA LEU E 64   44.129 -1.670 67.640 0.00 0.00
E
ATOM 27751 CB LEU E 64   43.645   0.185 68.596 0.00 0.00
E
ATOM 27752 HB1 LEU E 64   42.982   0.460 69.444 0.00 0.00
E
ATOM 27753 HB2 LEU E 64   44.712   0.211 68.905 0.00 0.00
E
ATOM 27754 CG LEU E 64   43.505   1.265 67.504 0.00 0.00
E
ATOM 27755 HG LEU E 64   42.430   1.152 67.248 0.00 0.00
E

ATOM 27756 CD1 LEU E 64   43.752   2.643 68.100 0.00 0.00
E
ATOM 27757 HD11 LEU E 64   44.681   2.771 68.696 0.00 0.00
E
ATOM 27758 HD12 LEU E 64   43.763   3.427 67.313 0.00 0.00
E
ATOM 27759 HD13 LEU E 64   42.888   2.755 68.790 0.00 0.00
E
ATOM 27760 CD2 LEU E 64   44.300   1.016 66.244 0.00 0.00
E
ATOM 27761 HD21 LEU E 64   45.254   0.505 66.499 0.00 0.00
E
ATOM 27762 HD22 LEU E 64   43.878   0.285 65.521 0.00 0.00
E
ATOM 27763 HD23 LEU E 64   44.570   1.971 65.745 0.00 0.00
E
ATOM 27764 C   LEU E 64   43.496 -2.085 69.612 0.00 0.00
E
ATOM 27765 O   LEU E 64   44.519 -2.695 69.818 0.00 0.00
E
ATOM 27766 N   SER E 65   42.470 -2.149 70.532 0.00 0.00
E
ATOM 27767 HN SER E 65   41.681 -1.552 70.411 0.00 0.00
E
ATOM 27768 CA SER E 65   42.490 -2.863 71.758 0.00 0.00
E
ATOM 27769 HA SER E 65   43.233 -2.383 72.378 0.00 0.00
E
ATOM 27770 CB SER E 65   41.155 -2.638 72.514 0.00 0.00
E
ATOM 27771 HB1 SER E 65   40.934 -1.549 72.514 0.00 0.00
E
ATOM 27772 HB2 SER E 65   40.370 -3.224 71.989 0.00 0.00
E
ATOM 27773 OG SER E 65   41.253 -2.984 73.837 0.00 0.00
E
ATOM 27774 HG1 SER E 65   40.425 -2.698 74.230 0.00 0.00
E
ATOM 27775 C   SER E 65   42.751 -4.382 71.608 0.00 0.00
E
ATOM 27776 O   SER E 65   43.629 -4.981 72.251 0.00 0.00
E
ATOM 27777 N   LEU E 66   42.108 -5.009 70.547 0.00 0.00
E
ATOM 27778 HN LEU E 66   41.402 -4.503 70.058 0.00 0.00
E
ATOM 27779 CA LEU E 66   42.334 -6.329 70.010 0.00 0.00
E
ATOM 27780 HA LEU E 66   42.117 -6.877 70.916 0.00 0.00
E
ATOM 27781 CB LEU E 66   41.269 -6.547 68.891 0.00 0.00
E
ATOM 27782 HB1 LEU E 66   40.311 -6.195 69.329 0.00 0.00
E

ATOM 27783 HB2 LEU E 66 41.597 -5.921 68.034 0.00 0.00
E
ATOM 27784 CG LEU E 66 41.043 -7.880 68.236 0.00 0.00
E
ATOM 27785 HG LEU E 66 41.918 -8.076 67.580 0.00 0.00
E
ATOM 27786 CD1 LEU E 66 40.922 -9.072 69.209 0.00 0.00
E
ATOM 27787 HD11 LEU E 66 40.454 -9.971 68.754 0.00 0.00
E
ATOM 27788 HD12 LEU E 66 41.978 -9.296 69.474 0.00 0.00
E
ATOM 27789 HD13 LEU E 66 40.410 -8.930 70.184 0.00 0.00
E
ATOM 27790 CD2 LEU E 66 39.819 -7.800 67.201 0.00 0.00
E
ATOM 27791 HD21 LEU E 66 39.938 -8.621 66.462 0.00 0.00
E
ATOM 27792 HD22 LEU E 66 38.804 -7.824 67.652 0.00 0.00
E
ATOM 27793 HD23 LEU E 66 39.869 -6.870 66.595 0.00 0.00
E
ATOM 27794 C LEU E 66 43.787 -6.610 69.445 0.00 0.00
E
ATOM 27795 O LEU E 66 44.325 -7.705 69.461 0.00 0.00
E
ATOM 27796 N GLY E 67 44.380 -5.535 68.882 0.00 0.00
E
ATOM 27797 HN GLY E 67 43.989 -4.618 68.864 0.00 0.00
E
ATOM 27798 CA GLY E 67 45.726 -5.610 68.372 0.00 0.00
E
ATOM 27799 HA1 GLY E 67 46.249 -6.482 68.736 0.00 0.00
E
ATOM 27800 HA2 GLY E 67 46.153 -4.624 68.484 0.00 0.00
E
ATOM 27801 C GLY E 67 45.838 -6.041 66.913 0.00 0.00
E
ATOM 27802 O GLY E 67 46.702 -6.891 66.604 0.00 0.00
E
ATOM 27803 N ILE E 68 44.958 -5.452 66.038 0.00 0.00
E
ATOM 27804 HN ILE E 68 44.449 -4.653 66.351 0.00 0.00
E
ATOM 27805 CA ILE E 68 44.752 -5.810 64.631 0.00 0.00
E
ATOM 27806 HA ILE E 68 44.489 -6.856 64.568 0.00 0.00
E
ATOM 27807 CB ILE E 68 43.539 -5.021 64.196 0.00 0.00
E
ATOM 27808 HB ILE E 68 42.844 -5.059 65.063 0.00 0.00
E
ATOM 27809 CG2 ILE E 68 43.897 -3.530 64.029 0.00 0.00
E

ATOM 27810 HG21 ILE E 68   42.919 -3.018 63.902 0.00 0.00
E
ATOM 27811 HG22 ILE E 68   44.377 -3.179 64.967 0.00 0.00
E
ATOM 27812 HG23 ILE E 68   44.468 -3.398 63.086 0.00 0.00
E
ATOM 27813 CG1 ILE E 68   42.816 -5.456 62.943 0.00 0.00
E
ATOM 27814 HG11 ILE E 68   42.000 -4.770 62.630 0.00 0.00
E
ATOM 27815 HG12 ILE E 68   43.491 -5.420 62.061 0.00 0.00
E
ATOM 27816 CD ILE E 68   42.233 -6.923 62.936 0.00 0.00
E
ATOM 27817 HD1 ILE E 68   43.023 -7.705 62.928 0.00 0.00
E
ATOM 27818 HD2 ILE E 68   41.695 -7.084 63.894 0.00 0.00
E
ATOM 27819 HD3 ILE E 68   41.509 -7.311 62.189 0.00 0.00
E
ATOM 27820 C   ILE E 68   46.016 -5.635 63.745 0.00 0.00
E
ATOM 27821 O   ILE E 68   46.217 -6.493 62.840 0.00 0.00
E
ATOM 27822 N   ARG E 69   46.943 -4.730 64.081 0.00 0.00
E
ATOM 27823 HN ARG E 69   46.884 -4.143 64.884 0.00 0.00
E
ATOM 27824 CA ARG E 69   48.105 -4.422 63.193 0.00 0.00
E
ATOM 27825 HA ARG E 69   47.677 -4.441 62.202 0.00 0.00
E
ATOM 27826 CB ARG E 69   48.551 -2.995 63.572 0.00 0.00
E
ATOM 27827 HB1 ARG E 69   47.750 -2.280 63.286 0.00 0.00
E
ATOM 27828 HB2 ARG E 69   48.760 -2.915 64.660 0.00 0.00
E
ATOM 27829 CG ARG E 69   49.826 -2.459 62.918 0.00 0.00
E
ATOM 27830 HG1 ARG E 69   50.207 -1.567 63.460 0.00 0.00
E
ATOM 27831 HG2 ARG E 69   50.517 -3.318 63.058 0.00 0.00
E
ATOM 27832 CD ARG E 69   49.816 -2.168 61.420 0.00 0.00
E
ATOM 27833 HD1 ARG E 69   49.720 -3.140 60.891 0.00 0.00
E
ATOM 27834 HD2 ARG E 69   48.956 -1.467 61.364 0.00 0.00
E
ATOM 27835 NE ARG E 69   50.995 -1.378 61.069 0.00 0.00
E
ATOM 27836 HE ARG E 69   51.549 -0.976 61.799 0.00 0.00
E

ATOM 27837 CZ ARG E 69 51.046 -0.613 60.006 0.00 0.00
E
ATOM 27838 NH1 ARG E 69 50.243 -0.656 58.943 0.00 0.00
E
ATOM 27839 HH11 ARG E 69 50.558 -0.178 58.123 0.00 0.00
E
ATOM 27840 HH12 ARG E 69 49.653 -1.463 58.935 0.00 0.00
E
ATOM 27841 NH2 ARG E 69 52.005 0.353 59.985 0.00 0.00
E
ATOM 27842 HH21 ARG E 69 52.110 0.914 59.164 0.00 0.00
E
ATOM 27843 HH22 ARG E 69 52.374 0.552 60.893 0.00 0.00
E
ATOM 27844 C ARG E 69 49.155 -5.514 63.170 0.00 0.00
E
ATOM 27845 O ARG E 69 50.150 -5.594 62.406 0.00 0.00
E
ATOM 27846 N ASN E 70 48.987 -6.480 64.046 0.00 0.00
E
ATOM 27847 HN ASN E 70 48.188 -6.472 64.642 0.00 0.00
E
ATOM 27848 CA ASN E 70 49.813 -7.638 64.173 0.00 0.00
E
ATOM 27849 HA ASN E 70 50.748 -7.427 63.673 0.00 0.00
E
ATOM 27850 CB ASN E 70 50.088 -8.009 65.672 0.00 0.00
E
ATOM 27851 HB1 ASN E 70 49.198 -8.447 66.172 0.00 0.00
E
ATOM 27852 HB2 ASN E 70 50.993 -8.652 65.725 0.00 0.00
E
ATOM 27853 CG ASN E 70 50.488 -6.646 66.322 0.00 0.00
E
ATOM 27854 OD1 ASN E 70 51.622 -6.280 66.174 0.00 0.00
E
ATOM 27855 ND2 ASN E 70 49.681 -6.055 67.234 0.00 0.00
E
ATOM 27856 HD21 ASN E 70 50.145 -5.512 67.934 0.00 0.00
E
ATOM 27857 HD22 ASN E 70 48.741 -6.393 67.272 0.00 0.00
E
ATOM 27858 C ASN E 70 49.210 -8.875 63.518 0.00 0.00
E
ATOM 27859 O ASN E 70 49.785 -9.969 63.500 0.00 0.00
E
ATOM 27860 N PHE E 71 48.138 -8.757 62.902 0.00 0.00
E
ATOM 27861 HN PHE E 71 47.582 -7.929 62.906 0.00 0.00
E
ATOM 27862 CA PHE E 71 47.585 -9.825 62.062 0.00 0.00
E
ATOM 27863 HA PHE E 71 48.012 -10.787 62.303 0.00 0.00
E

ATOM 27864 CB PHE E 71   46.041 -9.986 62.294 0.00 0.00
E
ATOM 27865 HB1 PHE E 71   45.530 -9.005 62.186 0.00 0.00
E
ATOM 27866 HB2 PHE E 71   45.767 -10.685 61.475 0.00 0.00
E
ATOM 27867 CG PHE E 71   45.644 -10.650 63.639 0.00 0.00
E
ATOM 27868 CD1 PHE E 71   45.279 -11.983 63.787 0.00 0.00
E
ATOM 27869 HD1 PHE E 71   45.325 -12.690 62.972 0.00 0.00
E
ATOM 27870 CE1 PHE E 71   44.722 -12.415 65.019 0.00 0.00
E
ATOM 27871 HE1 PHE E 71   44.246 -13.377 65.145 0.00 0.00
E
ATOM 27872 CZ PHE E 71   44.573 -11.557 66.146 0.00 0.00
E
ATOM 27873 HZ PHE E 71   44.235 -11.943 67.096 0.00 0.00
E
ATOM 27874 CD2 PHE E 71   45.433 -9.752 64.786 0.00 0.00
E
ATOM 27875 HD2 PHE E 71   45.743 -8.725 64.666 0.00 0.00
E
ATOM 27876 CE2 PHE E 71   44.967 -10.266 65.989 0.00 0.00
E
ATOM 27877 HE2 PHE E 71   45.028 -9.554 66.799 0.00 0.00
E
ATOM 27878 C   PHE E 71   47.916 -9.552 60.577 0.00 0.00
E
ATOM 27879 O   PHE E 71   47.992 -8.377 60.144 0.00 0.00
E
ATOM 27880 N   GLU E 72   48.099 -10.600 59.685 0.00 0.00
E
ATOM 27881 HN GLU E 72   48.117 -11.571 59.911 0.00 0.00
E
ATOM 27882 CA GLU E 72   48.235 -10.413 58.309 0.00 0.00
E
ATOM 27883 HA GLU E 72   48.423 -9.363 58.136 0.00 0.00
E
ATOM 27884 CB GLU E 72   49.496 -11.159 57.790 0.00 0.00
E
ATOM 27885 HB1 GLU E 72   50.343 -10.634 58.281 0.00 0.00
E
ATOM 27886 HB2 GLU E 72   49.363 -12.176 58.216 0.00 0.00
E
ATOM 27887 CG GLU E 72   49.787 -11.121 56.246 0.00 0.00
E
ATOM 27888 HG1 GLU E 72   49.318 -12.020 55.794 0.00 0.00
E
ATOM 27889 HG2 GLU E 72   49.449 -10.084 56.034 0.00 0.00
E
ATOM 27890 CD GLU E 72   51.197 -11.326 55.947 0.00 0.00
E

ATOM 27891 OE1 GLU E 72 51.938 -10.496 55.377 0.00 0.00
E
ATOM 27892 OE2 GLU E 72 51.656 -12.453 56.170 0.00 0.00
E
ATOM 27893 C GLU E 72 46.906 -10.768 57.561 0.00 0.00
E
ATOM 27894 O GLU E 72 46.622 -10.258 56.479 0.00 0.00
E
ATOM 27895 N TYR E 73 46.068 -11.640 58.220 0.00 0.00
E
ATOM 27896 HN TYR E 73 46.419 -12.022 59.071 0.00 0.00
E
ATOM 27897 CA TYR E 73 44.904 -12.127 57.524 0.00 0.00
E
ATOM 27898 HA TYR E 73 44.621 -11.487 56.701 0.00 0.00
E
ATOM 27899 CB TYR E 73 44.790 -13.641 57.177 0.00 0.00
E
ATOM 27900 HB1 TYR E 73 45.249 -14.314 57.933 0.00 0.00
E
ATOM 27901 HB2 TYR E 73 43.718 -13.874 57.001 0.00 0.00
E
ATOM 27902 CG TYR E 73 45.499 -13.984 55.872 0.00 0.00
E
ATOM 27903 CD1 TYR E 73 46.909 -14.185 55.825 0.00 0.00
E
ATOM 27904 HD1 TYR E 73 47.461 -13.842 56.688 0.00 0.00
E
ATOM 27905 CE1 TYR E 73 47.443 -14.747 54.691 0.00 0.00
E
ATOM 27906 HE1 TYR E 73 48.503 -14.945 54.764 0.00 0.00
E
ATOM 27907 CZ TYR E 73 46.713 -15.105 53.579 0.00 0.00
E
ATOM 27908 OH TYR E 73 47.184 -15.875 52.588 0.00 0.00
E
ATOM 27909 HH TYR E 73 48.132 -16.027 52.576 0.00 0.00
E
ATOM 27910 CD2 TYR E 73 44.775 -14.151 54.670 0.00 0.00
E
ATOM 27911 HD2 TYR E 73 43.702 -14.029 54.703 0.00 0.00
E
ATOM 27912 CE2 TYR E 73 45.325 -14.674 53.539 0.00 0.00
E
ATOM 27913 HE2 TYR E 73 44.758 -14.942 52.660 0.00 0.00
E
ATOM 27914 C TYR E 73 43.732 -11.864 58.469 0.00 0.00
E
ATOM 27915 O TYR E 73 43.666 -12.244 59.597 0.00 0.00
E
ATOM 27916 N VAL E 74 42.773 -11.183 57.958 0.00 0.00
E
ATOM 27917 HN VAL E 74 42.930 -10.831 57.038 0.00 0.00
E

ATOM 27918 CA VAL E 74 41.460 -10.924 58.571 0.00 0.00
E
ATOM 27919 HA VAL E 74 41.361 -11.554 59.443 0.00 0.00
E
ATOM 27920 CB VAL E 74 41.206 -9.481 58.839 0.00 0.00
E
ATOM 27921 HB VAL E 74 41.109 -8.840 57.937 0.00 0.00
E
ATOM 27922 CG1 VAL E 74 39.916 -9.321 59.540 0.00 0.00
E
ATOM 27923 HG11 VAL E 74 39.939 -9.884 60.498 0.00 0.00
E
ATOM 27924 HG12 VAL E 74 39.850 -8.264 59.876 0.00 0.00
E
ATOM 27925 HG13 VAL E 74 39.013 -9.551 58.935 0.00 0.00
E
ATOM 27926 CG2 VAL E 74 42.310 -9.014 59.777 0.00 0.00
E
ATOM 27927 HG21 VAL E 74 43.321 -9.088 59.321 0.00 0.00
E
ATOM 27928 HG22 VAL E 74 42.108 -7.964 60.079 0.00 0.00
E
ATOM 27929 HG23 VAL E 74 42.415 -9.684 60.656 0.00 0.00
E
ATOM 27930 C VAL E 74 40.400 -11.335 57.650 0.00 0.00
E
ATOM 27931 O VAL E 74 40.304 -10.746 56.545 0.00 0.00
E
ATOM 27932 N ILE E 75 39.584 -12.364 58.072 0.00 0.00
E
ATOM 27933 HN ILE E 75 39.589 -12.816 58.961 0.00 0.00
E
ATOM 27934 CA ILE E 75 38.436 -12.811 57.346 0.00 0.00
E
ATOM 27935 HA ILE E 75 38.595 -12.459 56.338 0.00 0.00
E
ATOM 27936 CB ILE E 75 38.244 -14.350 57.247 0.00 0.00
E
ATOM 27937 HB ILE E 75 37.681 -14.708 58.135 0.00 0.00
E
ATOM 27938 CG2 ILE E 75 37.270 -14.521 56.065 0.00 0.00
E
ATOM 27939 HG21 ILE E 75 36.990 -15.586 55.920 0.00 0.00
E
ATOM 27940 HG22 ILE E 75 36.312 -13.987 56.242 0.00 0.00
E
ATOM 27941 HG23 ILE E 75 37.683 -14.155 55.100 0.00 0.00
E
ATOM 27942 CG1 ILE E 75 39.553 -15.144 57.210 0.00 0.00
E
ATOM 27943 HG11 ILE E 75 40.216 -14.865 58.057 0.00 0.00
E
ATOM 27944 HG12 ILE E 75 39.383 -16.242 57.192 0.00 0.00
E

ATOM 27945 CD ILE E 75 40.451 −14.742 56.009 0.00 0.00
E
ATOM 27946 HD1 ILE E 75 41.365 −15.343 56.201 0.00 0.00
E
ATOM 27947 HD2 ILE E 75 39.999 −15.086 55.054 0.00 0.00
E
ATOM 27948 HD3 ILE E 75 40.813 −13.692 56.027 0.00 0.00
E
ATOM 27949 C ILE E 75 37.283 −12.085 57.910 0.00 0.00
E
ATOM 27950 O ILE E 75 36.922 −12.186 59.118 0.00 0.00
E
ATOM 27951 N VAL E 76 36.555 −11.354 57.096 0.00 0.00
E
ATOM 27952 HN VAL E 76 36.856 −11.177 56.162 0.00 0.00
E
ATOM 27953 CA VAL E 76 35.184 −10.776 57.422 0.00 0.00
E
ATOM 27954 HA VAL E 76 35.118 −10.653 58.493 0.00 0.00
E
ATOM 27955 CB VAL E 76 34.892 −9.299 56.928 0.00 0.00
E
ATOM 27956 HB VAL E 76 35.143 −9.329 55.846 0.00 0.00
E
ATOM 27957 CG1 VAL E 76 33.362 −8.887 57.076 0.00 0.00
E
ATOM 27958 HG11 VAL E 76 33.001 −9.129 58.099 0.00 0.00
E
ATOM 27959 HG12 VAL E 76 33.358 −7.791 56.894 0.00 0.00
E
ATOM 27960 HG13 VAL E 76 32.709 −9.424 56.354 0.00 0.00
E
ATOM 27961 CG2 VAL E 76 35.794 −8.368 57.678 0.00 0.00
E
ATOM 27962 HG21 VAL E 76 35.386 −8.358 58.711 0.00 0.00
E
ATOM 27963 HG22 VAL E 76 36.870 −8.565 57.487 0.00 0.00
E
ATOM 27964 HG23 VAL E 76 35.730 −7.319 57.317 0.00 0.00
E
ATOM 27965 C VAL E 76 34.061 −11.658 56.942 0.00 0.00
E
ATOM 27966 O VAL E 76 33.817 −11.791 55.782 0.00 0.00
E
ATOM 27967 N ALA E 77 33.261 −12.330 57.872 0.00 0.00
E
ATOM 27968 HN ALA E 77 33.390 −12.069 58.825 0.00 0.00
E
ATOM 27969 CA ALA E 77 32.270 −13.337 57.505 0.00 0.00
E
ATOM 27970 HA ALA E 77 32.493 −13.744 56.530 0.00 0.00
E
ATOM 27971 CB ALA E 77 32.511 −14.579 58.370 0.00 0.00
E

ATOM 27972 HB1 ALA E 77 33.585 -14.811 58.209 0.00 0.00
E
ATOM 27973 HB2 ALA E 77 32.440 -14.220 59.419 0.00 0.00
E
ATOM 27974 HB3 ALA E 77 31.952 -15.510 58.137 0.00 0.00
E
ATOM 27975 C ALA E 77 30.876 -12.826 57.624 0.00 0.00
E
ATOM 27976 O ALA E 77 29.922 -13.543 57.551 0.00 0.00
E
ATOM 27977 N ILE E 78 30.817 -11.500 57.776 0.00 0.00
E
ATOM 27978 HN ILE E 78 31.731 -11.104 57.728 0.00 0.00
E
ATOM 27979 CA ILE E 78 29.659 -10.677 57.846 0.00 0.00
E
ATOM 27980 HA ILE E 78 28.975 -11.353 58.337 0.00 0.00
E
ATOM 27981 CB ILE E 78 29.900 -9.356 58.564 0.00 0.00
E
ATOM 27982 HB ILE E 78 30.566 -8.647 58.027 0.00 0.00
E
ATOM 27983 CG2 ILE E 78 28.517 -8.637 58.718 0.00 0.00
E
ATOM 27984 HG21 ILE E 78 27.881 -8.551 57.811 0.00 0.00
E
ATOM 27985 HG22 ILE E 78 27.997 -9.026 59.619 0.00 0.00
E
ATOM 27986 HG23 ILE E 78 28.729 -7.596 59.044 0.00 0.00
E
ATOM 27987 CG1 ILE E 78 30.645 -9.537 59.945 0.00 0.00
E
ATOM 27988 HG11 ILE E 78 29.991 -10.050 60.682 0.00 0.00
E
ATOM 27989 HG12 ILE E 78 31.558 -10.142 59.762 0.00 0.00
E
ATOM 27990 CD ILE E 78 31.120 -8.171 60.582 0.00 0.00
E
ATOM 27991 HD1 ILE E 78 31.914 -7.816 59.891 0.00 0.00
E
ATOM 27992 HD2 ILE E 78 30.278 -7.447 60.624 0.00 0.00
E
ATOM 27993 HD3 ILE E 78 31.426 -8.412 61.622 0.00 0.00
E
ATOM 27994 C ILE E 78 29.171 -10.371 56.442 0.00 0.00
E
ATOM 27995 O ILE E 78 29.861 -9.701 55.681 0.00 0.00
E
ATOM 27996 N GLY E 79 27.948 -10.768 56.020 0.00 0.00
E
ATOM 27997 HN GLY E 79 27.335 -11.283 56.613 0.00 0.00
E
ATOM 27998 CA GLY E 79 27.326 -10.246 54.767 0.00 0.00
E

ATOM 27999 HA1 GLY E 79 27.464 -10.938 53.950 0.00 0.00
E
ATOM 28000 HA2 GLY E 79 27.858 -9.367 54.435 0.00 0.00
E
ATOM 28001 C GLY E 79 25.887 -9.901 54.938 0.00 0.00
E
ATOM 28002 O GLY E 79 25.236 -9.485 53.984 0.00 0.00
E
ATOM 28003 N ALA E 80 25.315 -10.121 56.119 0.00 0.00
E
ATOM 28004 HN ALA E 80 25.746 -10.675 56.827 0.00 0.00
E
ATOM 28005 CA ALA E 80 23.876 -9.999 56.361 0.00 0.00
E
ATOM 28006 HA ALA E 80 23.420 -10.599 55.588 0.00 0.00
E
ATOM 28007 CB ALA E 80 23.571 -10.422 57.758 0.00 0.00
E
ATOM 28008 HB1 ALA E 80 23.996 -11.418 58.006 0.00 0.00
E
ATOM 28009 HB2 ALA E 80 23.843 -9.770 58.616 0.00 0.00
E
ATOM 28010 HB3 ALA E 80 22.490 -10.518 57.997 0.00 0.00
E
ATOM 28011 C ALA E 80 23.277 -8.599 56.182 0.00 0.00
E
ATOM 28012 O ALA E 80 22.225 -8.458 55.553 0.00 0.00
E
ATOM 28013 N ASN E 81 23.987 -7.618 56.669 0.00 0.00
E
ATOM 28014 HN ASN E 81 24.828 -7.818 57.165 0.00 0.00
E
ATOM 28015 CA ASN E 81 23.763 -6.234 56.387 0.00 0.00
E
ATOM 28016 HA ASN E 81 22.978 -6.182 55.647 0.00 0.00
E
ATOM 28017 CB ASN E 81 23.492 -5.614 57.729 0.00 0.00
E
ATOM 28018 HB1 ASN E 81 22.783 -6.274 58.273 0.00 0.00
E
ATOM 28019 HB2 ASN E 81 24.424 -5.558 58.330 0.00 0.00
E
ATOM 28020 CG ASN E 81 22.951 -4.212 57.578 0.00 0.00
E
ATOM 28021 OD1 ASN E 81 23.060 -3.495 56.599 0.00 0.00
E
ATOM 28022 ND2 ASN E 81 22.289 -3.718 58.733 0.00 0.00
E
ATOM 28023 HD21 ASN E 81 21.961 -2.788 58.894 0.00 0.00
E
ATOM 28024 HD22 ASN E 81 21.842 -4.372 59.344 0.00 0.00
E
ATOM 28025 C ASN E 81 25.019 -5.676 55.716 0.00 0.00
E

ATOM 28026 O ASN E 81 26.178 −5.957 56.079 0.00 0.00
E
ATOM 28027 N ILE E 82 24.721 −4.970 54.643 0.00 0.00
E
ATOM 28028 HN ILE E 82 23.780 −4.938 54.317 0.00 0.00
E
ATOM 28029 CA ILE E 82 25.742 −4.352 53.767 0.00 0.00
E
ATOM 28030 HA ILE E 82 26.482 −5.132 53.664 0.00 0.00
E
ATOM 28031 CB ILE E 82 25.208 −4.044 52.380 0.00 0.00
E
ATOM 28032 HB ILE E 82 24.511 −3.184 52.473 0.00 0.00
E
ATOM 28033 CG2 ILE E 82 26.286 −3.661 51.450 0.00 0.00
E
ATOM 28034 HG21 ILE E 82 26.904 −2.777 51.717 0.00 0.00
E
ATOM 28035 HG22 ILE E 82 27.112 −4.387 51.286 0.00 0.00
E
ATOM 28036 HG23 ILE E 82 25.868 −3.586 50.423 0.00 0.00
E
ATOM 28037 CG1 ILE E 82 24.416 −5.250 51.900 0.00 0.00
E
ATOM 28038 HG11 ILE E 82 24.976 −6.209 51.866 0.00 0.00
E
ATOM 28039 HG12 ILE E 82 23.539 −5.399 52.566 0.00 0.00
E
ATOM 28040 CD ILE E 82 23.854 −5.084 50.454 0.00 0.00
E
ATOM 28041 HD1 ILE E 82 24.639 −4.912 49.688 0.00 0.00
E
ATOM 28042 HD2 ILE E 82 23.337 −5.985 50.059 0.00 0.00
E
ATOM 28043 HD3 ILE E 82 23.229 −4.168 50.386 0.00 0.00
E
ATOM 28044 C ILE E 82 26.504 −3.249 54.495 0.00 0.00
E
ATOM 28045 O ILE E 82 27.741 −3.174 54.441 0.00 0.00
E
ATOM 28046 N GLN E 83 25.764 −2.387 55.201 0.00 0.00
E
ATOM 28047 HN GLN E 83 24.769 −2.441 55.172 0.00 0.00
E
ATOM 28048 CA GLN E 83 26.274 −1.210 55.945 0.00 0.00
E
ATOM 28049 HA GLN E 83 26.894 −0.686 55.233 0.00 0.00
E
ATOM 28050 CB GLN E 83 25.023 −0.306 56.389 0.00 0.00
E
ATOM 28051 HB1 GLN E 83 24.364 −0.059 55.529 0.00 0.00
E
ATOM 28052 HB2 GLN E 83 24.362 −0.916 57.042 0.00 0.00
E

ATOM 28053 CG   GLN E 83   25.427   1.036 56.960 0.00 0.00
E
ATOM 28054 HG1 GLN E 83   26.150   0.996 57.802 0.00 0.00
E
ATOM 28055 HG2 GLN E 83   25.814   1.856 56.318 0.00 0.00
E
ATOM 28056 CD GLN E 83   24.214   1.590 57.621 0.00 0.00
E
ATOM 28057 OE1 GLN E 83   24.017   1.401 58.806 0.00 0.00
E
ATOM 28058 NE2 GLN E 83   23.425   2.368 56.896 0.00 0.00
E
ATOM 28059 HE21 GLN E 83   22.599   2.745 57.314 0.00 0.00
E
ATOM 28060 HE22 GLN E 83   23.474   2.378 55.897 0.00 0.00
E
ATOM 28061 C   GLN E 83   27.247 -1.577 57.064 0.00 0.00
E
ATOM 28062 O   GLN E 83   28.419 -1.094 57.178 0.00 0.00
E
ATOM 28063 N   ALA E 84   26.858 -2.650 57.774 0.00 0.00
E
ATOM 28064 HN ALA E 84   25.916 -2.945 57.628 0.00 0.00
E
ATOM 28065 CA ALA E 84   27.746 -3.332 58.714 0.00 0.00
E
ATOM 28066 HA ALA E 84   28.052 -2.622 59.468 0.00 0.00
E
ATOM 28067 CB ALA E 84   26.939 -4.409 59.443 0.00 0.00
E
ATOM 28068 HB1 ALA E 84   26.673 -5.272 58.796 0.00 0.00
E
ATOM 28069 HB2 ALA E 84   27.415 -4.877 60.331 0.00 0.00
E
ATOM 28070 HB3 ALA E 84   26.066 -3.865 59.863 0.00 0.00
E
ATOM 28071 C   ALA E 84   29.030 -4.024 58.124 0.00 0.00
E
ATOM 28072 O   ALA E 84   30.145 -3.739 58.522 0.00 0.00
E
ATOM 28073 N   SER E 85   28.897 -4.832 57.014 0.00 0.00
E
ATOM 28074 HN SER E 85   27.996 -4.951 56.604 0.00 0.00
E
ATOM 28075 CA SER E 85   29.968 -5.527 56.343 0.00 0.00
E
ATOM 28076 HA SER E 85   30.485 -6.076 57.116 0.00 0.00
E
ATOM 28077 CB SER E 85   29.437 -6.457 55.130 0.00 0.00
E
ATOM 28078 HB1 SER E 85   28.637 -7.099 55.557 0.00 0.00
E
ATOM 28079 HB2 SER E 85   29.001 -5.767 54.378 0.00 0.00
E

```
ATOM  28080  OG  SER E  85      30.449  -7.317  54.618  0.00  0.00
E
ATOM  28081  HG1 SER E  85      30.337  -8.131  55.115  0.00  0.00
E
ATOM  28082  C   SER E  85      30.988  -4.587  55.719  0.00  0.00
E
ATOM  28083  O   SER E  85      32.184  -4.723  55.975  0.00  0.00
E
ATOM  28084  N   THR E  86      30.505  -3.568  54.960  0.00  0.00
E
ATOM  28085  HN  THR E  86      29.522  -3.444  54.850  0.00  0.00
E
ATOM  28086  CA  THR E  86      31.392  -2.719  54.274  0.00  0.00
E
ATOM  28087  HA  THR E  86      32.146  -3.296  53.760  0.00  0.00
E
ATOM  28088  CB  THR E  86      30.802  -2.053  53.021  0.00  0.00
E
ATOM  28089  HB  THR E  86      31.526  -1.397  52.493  0.00  0.00
E
ATOM  28090  OG1 THR E  86      29.658  -1.319  53.234  0.00  0.00
E
ATOM  28091  HG1 THR E  86      28.970  -1.788  53.713  0.00  0.00
E
ATOM  28092  CG2 THR E  86      30.279  -3.216  52.079  0.00  0.00
E
ATOM  28093 HG21 THR E  86      29.557  -3.949  52.497  0.00  0.00
E
ATOM  28094 HG22 THR E  86      29.739  -2.807  51.199  0.00  0.00
E
ATOM  28095 HG23 THR E  86      31.225  -3.673  51.718  0.00  0.00
E
ATOM  28096  C   THR E  86      32.061  -1.579  55.201  0.00  0.00
E
ATOM  28097  O   THR E  86      33.217  -1.296  54.995  0.00  0.00
E
ATOM  28098  N   LEU E  87      31.374  -1.045  56.207  0.00  0.00
E
ATOM  28099  HN  LEU E  87      30.386  -1.153  56.283  0.00  0.00
E
ATOM  28100  CA  LEU E  87      32.045  -0.237  57.228  0.00  0.00
E
ATOM  28101  HA  LEU E  87      32.659   0.580  56.879  0.00  0.00
E
ATOM  28102  CB  LEU E  87      31.011   0.411  58.206  0.00  0.00
E
ATOM  28103  HB1 LEU E  87      30.521   1.259  57.682  0.00  0.00
E
ATOM  28104  HB2 LEU E  87      30.292  -0.366  58.541  0.00  0.00
E
ATOM  28105  CG  LEU E  87      31.629   1.374  59.329  0.00  0.00
E
ATOM  28106  HG  LEU E  87      32.315   0.783  59.973  0.00  0.00
E
```

ATOM 28107 CD1 LEU E 87 32.330 2.612 58.659 0.00 0.00
E
ATOM 28108 HD11 LEU E 87 32.439 3.388 59.446 0.00 0.00
E
ATOM 28109 HD12 LEU E 87 33.308 2.337 58.208 0.00 0.00
E
ATOM 28110 HD13 LEU E 87 31.657 3.009 57.870 0.00 0.00
E
ATOM 28111 CD2 LEU E 87 30.511 1.842 60.232 0.00 0.00
E
ATOM 28112 HD21 LEU E 87 29.833 2.347 59.511 0.00 0.00
E
ATOM 28113 HD22 LEU E 87 30.008 0.978 60.716 0.00 0.00
E
ATOM 28114 HD23 LEU E 87 30.871 2.541 61.017 0.00 0.00
E
ATOM 28115 C LEU E 87 33.073 −1.044 58.040 0.00 0.00
E
ATOM 28116 O LEU E 87 34.139 −0.538 58.419 0.00 0.00
E
ATOM 28117 N THR E 88 32.861 −2.337 58.217 0.00 0.00
E
ATOM 28118 HN THR E 88 32.029 −2.806 57.933 0.00 0.00
E
ATOM 28119 CA THR E 88 33.892 −3.191 58.813 0.00 0.00
E
ATOM 28120 HA THR E 88 34.104 −2.694 59.748 0.00 0.00
E
ATOM 28121 CB THR E 88 33.445 −4.655 59.201 0.00 0.00
E
ATOM 28122 HB THR E 88 33.137 −5.140 58.251 0.00 0.00
E
ATOM 28123 OG1 THR E 88 32.412 −4.469 60.175 0.00 0.00
E
ATOM 28124 HG1 THR E 88 31.870 −3.769 59.803 0.00 0.00
E
ATOM 28125 CG2 THR E 88 34.480 −5.473 59.836 0.00 0.00
E
ATOM 28126 HG21 THR E 88 35.183 −4.877 60.456 0.00 0.00
E
ATOM 28127 HG22 THR E 88 34.059 −6.408 60.263 0.00 0.00
E
ATOM 28128 HG23 THR E 88 35.052 −5.931 59.000 0.00 0.00
E
ATOM 28129 C THR E 88 35.159 −3.337 57.914 0.00 0.00
E
ATOM 28130 O THR E 88 36.238 −3.161 58.454 0.00 0.00
E
ATOM 28131 N THR E 89 34.973 −3.606 56.589 0.00 0.00
E
ATOM 28132 HN THR E 89 34.023 −3.856 56.421 0.00 0.00
E
ATOM 28133 CA THR E 89 36.007 −3.599 55.510 0.00 0.00
E

ATOM 28134 HA THR E 89 36.785 -4.295 55.785 0.00 0.00
E
ATOM 28135 CB THR E 89 35.460 -4.044 54.097 0.00 0.00
E
ATOM 28136 HB THR E 89 34.645 -3.344 53.811 0.00 0.00
E
ATOM 28137 OG1 THR E 89 34.961 -5.356 54.053 0.00 0.00
E
ATOM 28138 HG1 THR E 89 34.186 -5.409 54.618 0.00 0.00
E
ATOM 28139 CG2 THR E 89 36.636 -4.104 53.089 0.00 0.00
E
ATOM 28140 HG21 THR E 89 37.012 -3.067 52.951 0.00 0.00
E
ATOM 28141 HG22 THR E 89 37.510 -4.727 53.376 0.00 0.00
E
ATOM 28142 HG23 THR E 89 36.264 -4.521 52.129 0.00 0.00
E
ATOM 28143 C THR E 89 36.753 -2.321 55.330 0.00 0.00
E
ATOM 28144 O THR E 89 37.961 -2.290 55.183 0.00 0.00
E
ATOM 28145 N LEU E 90 36.066 -1.221 55.384 0.00 0.00
E
ATOM 28146 HN LEU E 90 35.078 -1.281 55.268 0.00 0.00
E
ATOM 28147 CA LEU E 90 36.608 0.106 55.333 0.00 0.00
E
ATOM 28148 HA LEU E 90 37.171 0.232 54.420 0.00 0.00
E
ATOM 28149 CB LEU E 90 35.498 1.199 55.185 0.00 0.00
E
ATOM 28150 HB1 LEU E 90 34.649 0.884 54.540 0.00 0.00
E
ATOM 28151 HB2 LEU E 90 35.105 1.323 56.216 0.00 0.00
E
ATOM 28152 CG LEU E 90 35.838 2.629 54.809 0.00 0.00
E
ATOM 28153 HG LEU E 90 36.670 2.562 54.075 0.00 0.00
E
ATOM 28154 CD1 LEU E 90 34.673 3.416 54.013 0.00 0.00
E
ATOM 28155 HD11 LEU E 90 33.750 3.626 54.596 0.00 0.00
E
ATOM 28156 HD12 LEU E 90 35.014 4.322 53.468 0.00 0.00
E
ATOM 28157 HD13 LEU E 90 34.138 2.863 53.211 0.00 0.00
E
ATOM 28158 CD2 LEU E 90 36.229 3.501 56.035 0.00 0.00
E
ATOM 28159 HD21 LEU E 90 37.054 2.973 56.559 0.00 0.00
E
ATOM 28160 HD22 LEU E 90 36.456 4.529 55.678 0.00 0.00
E

ATOM 28161 HD23 LEU E 90 35.310 3.614 56.651 0.00 0.00
E
ATOM 28162 C LEU E 90 37.507 0.392 56.440 0.00 0.00
E
ATOM 28163 O LEU E 90 38.550 0.945 56.303 0.00 0.00
E
ATOM 28164 N LEU E 91 37.148 0.063 57.747 0.00 0.00
E
ATOM 28165 HN LEU E 91 36.302 -0.459 57.823 0.00 0.00
E
ATOM 28166 CA LEU E 91 37.965 0.207 58.950 0.00 0.00
E
ATOM 28167 HA LEU E 91 38.295 1.235 58.896 0.00 0.00
E
ATOM 28168 CB LEU E 91 37.351 -0.083 60.370 0.00 0.00
E
ATOM 28169 HB1 LEU E 91 36.346 -0.492 60.130 0.00 0.00
E
ATOM 28170 HB2 LEU E 91 37.971 -0.799 60.951 0.00 0.00
E
ATOM 28171 CG LEU E 91 37.055 1.218 61.146 0.00 0.00
E
ATOM 28172 HG LEU E 91 36.394 0.940 61.995 0.00 0.00
E
ATOM 28173 CD1 LEU E 91 38.353 1.800 61.839 0.00 0.00
E
ATOM 28174 HD11 LEU E 91 38.047 2.646 62.491 0.00 0.00
E
ATOM 28175 HD12 LEU E 91 38.943 1.084 62.450 0.00 0.00
E
ATOM 28176 HD13 LEU E 91 39.075 2.098 61.049 0.00 0.00
E
ATOM 28177 CD2 LEU E 91 36.327 2.295 60.283 0.00 0.00
E
ATOM 28178 HD21 LEU E 91 37.031 2.961 59.741 0.00 0.00
E
ATOM 28179 HD22 LEU E 91 35.635 1.819 59.556 0.00 0.00
E
ATOM 28180 HD23 LEU E 91 35.694 2.978 60.889 0.00 0.00
E
ATOM 28181 C LEU E 91 39.228 -0.652 58.748 0.00 0.00
E
ATOM 28182 O LEU E 91 40.383 -0.250 58.944 0.00 0.00
E
ATOM 28183 N LEU E 92 39.073 -1.886 58.353 0.00 0.00
E
ATOM 28184 HN LEU E 92 38.182 -2.323 58.256 0.00 0.00
E
ATOM 28185 CA LEU E 92 40.188 -2.788 58.163 0.00 0.00
E
ATOM 28186 HA LEU E 92 40.682 -2.777 59.123 0.00 0.00
E
ATOM 28187 CB LEU E 92 39.751 -4.268 57.841 0.00 0.00
E

ATOM	28188	HB1	LEU	E	92	39.141	-4.195	56.916	0.00	0.00	E
ATOM	28189	HB2	LEU	E	92	40.663	-4.892	57.726	0.00	0.00	E
ATOM	28190	CG	LEU	E	92	38.931	-4.990	58.968	0.00	0.00	E
ATOM	28191	HG	LEU	E	92	38.142	-4.320	59.373	0.00	0.00	E
ATOM	28192	CD1	LEU	E	92	38.187	-6.166	58.334	0.00	0.00	E
ATOM	28193	HD11	LEU	E	92	37.478	-6.597	59.074	0.00	0.00	E
ATOM	28194	HD12	LEU	E	92	37.556	-5.821	57.487	0.00	0.00	E
ATOM	28195	HD13	LEU	E	92	38.856	-6.994	58.017	0.00	0.00	E
ATOM	28196	CD2	LEU	E	92	39.804	-5.320	60.186	0.00	0.00	E
ATOM	28197	HD21	LEU	E	92	39.167	-5.941	60.852	0.00	0.00	E
ATOM	28198	HD22	LEU	E	92	40.638	-5.930	59.778	0.00	0.00	E
ATOM	28199	HD23	LEU	E	92	40.162	-4.407	60.708	0.00	0.00	E
ATOM	28200	C	LEU	E	92	41.132	-2.261	57.044	0.00	0.00	E
ATOM	28201	O	LEU	E	92	42.376	-2.279	57.050	0.00	0.00	E
ATOM	28202	N	LYS	E	93	40.494	-1.755	55.995	0.00	0.00	E
ATOM	28203	HN	LYS	E	93	39.498	-1.739	55.959	0.00	0.00	E
ATOM	28204	CA	LYS	E	93	41.305	-1.186	54.892	0.00	0.00	E
ATOM	28205	HA	LYS	E	93	42.087	-1.892	54.652	0.00	0.00	E
ATOM	28206	CB	LYS	E	93	40.305	-0.979	53.683	0.00	0.00	E
ATOM	28207	HB1	LYS	E	93	39.905	-1.991	53.460	0.00	0.00	E
ATOM	28208	HB2	LYS	E	93	39.487	-0.332	54.067	0.00	0.00	E
ATOM	28209	CG	LYS	E	93	41.042	-0.416	52.485	0.00	0.00	E
ATOM	28210	HG1	LYS	E	93	40.375	-0.112	51.651	0.00	0.00	E
ATOM	28211	HG2	LYS	E	93	41.416	0.580	52.804	0.00	0.00	E
ATOM	28212	CD	LYS	E	93	42.263	-1.071	51.978	0.00	0.00	E
ATOM	28213	HD1	LYS	E	93	42.708	-0.377	51.234	0.00	0.00	E
ATOM	28214	HD2	LYS	E	93	43.009	-1.268	52.776	0.00	0.00	E

ATOM 28215 CE LYS E 93 42.221 -2.384 51.178 0.00 0.00
E
ATOM 28216 HE1 LYS E 93 41.769 -3.195 51.788 0.00 0.00
E
ATOM 28217 HE2 LYS E 93 41.818 -2.326 50.144 0.00 0.00
E
ATOM 28218 NZ LYS E 93 43.575 -2.846 50.904 0.00 0.00
E
ATOM 28219 HZ1 LYS E 93 43.942 -2.554 49.976 0.00 0.00
E
ATOM 28220 HZ2 LYS E 93 44.217 -2.564 51.671 0.00 0.00
E
ATOM 28221 HZ3 LYS E 93 43.494 -3.883 50.941 0.00 0.00
E
ATOM 28222 C LYS E 93 42.067 0.071 55.335 0.00 0.00
E
ATOM 28223 O LYS E 93 43.213 0.230 54.975 0.00 0.00
E
ATOM 28224 N GLU E 94 41.349 1.018 55.993 0.00 0.00
E
ATOM 28225 HN GLU E 94 40.358 0.989 56.103 0.00 0.00
E
ATOM 28226 CA GLU E 94 41.960 2.164 56.647 0.00 0.00
E
ATOM 28227 HA GLU E 94 42.263 2.818 55.843 0.00 0.00
E
ATOM 28228 CB GLU E 94 40.868 2.804 57.424 0.00 0.00
E
ATOM 28229 HB1 GLU E 94 40.043 2.997 56.706 0.00 0.00
E
ATOM 28230 HB2 GLU E 94 40.390 2.068 58.105 0.00 0.00
E
ATOM 28231 CG GLU E 94 41.188 4.071 58.228 0.00 0.00
E
ATOM 28232 HG1 GLU E 94 40.251 4.267 58.793 0.00 0.00
E
ATOM 28233 HG2 GLU E 94 41.922 3.885 59.040 0.00 0.00
E
ATOM 28234 CD GLU E 94 41.586 5.363 57.464 0.00 0.00
E
ATOM 28235 OE1 GLU E 94 41.781 5.350 56.218 0.00 0.00
E
ATOM 28236 OE2 GLU E 94 41.716 6.417 58.125 0.00 0.00
E
ATOM 28237 C GLU E 94 43.035 1.909 57.649 0.00 0.00
E
ATOM 28238 O GLU E 94 44.049 2.625 57.845 0.00 0.00
E
ATOM 28239 N LEU E 95 42.919 0.736 58.364 0.00 0.00
E
ATOM 28240 HN LEU E 95 42.069 0.223 58.456 0.00 0.00
E
ATOM 28241 CA LEU E 95 43.981 0.133 59.211 0.00 0.00
E

ATOM 28242 HA LEU E 95 44.465 0.920 59.771 0.00 0.00
E
ATOM 28243 CB LEU E 95 43.320 −0.949 60.160 0.00 0.00
E
ATOM 28244 HB1 LEU E 95 42.801 −1.699 59.526 0.00 0.00
E
ATOM 28245 HB2 LEU E 95 44.112 −1.623 60.550 0.00 0.00
E
ATOM 28246 CG LEU E 95 42.596 −0.506 61.385 0.00 0.00
E
ATOM 28247 HG LEU E 95 41.825 0.245 61.110 0.00 0.00
E
ATOM 28248 CD1 LEU E 95 41.725 −1.770 61.750 0.00 0.00
E
ATOM 28249 HD11 LEU E 95 42.428 −2.617 61.603 0.00 0.00
E
ATOM 28250 HD12 LEU E 95 41.377 −1.827 62.804 0.00 0.00
E
ATOM 28251 HD13 LEU E 95 40.822 −1.779 61.102 0.00 0.00
E
ATOM 28252 CD2 LEU E 95 43.525 −0.110 62.572 0.00 0.00
E
ATOM 28253 HD21 LEU E 95 44.398 −0.797 62.549 0.00 0.00
E
ATOM 28254 HD22 LEU E 95 43.878 0.931 62.407 0.00 0.00
E
ATOM 28255 HD23 LEU E 95 42.944 −0.235 63.510 0.00 0.00
E
ATOM 28256 C LEU E 95 45.171 −0.532 58.430 0.00 0.00
E
ATOM 28257 O LEU E 95 46.205 −0.904 59.028 0.00 0.00
E
ATOM 28258 N ASP E 96 45.040 −0.655 57.098 0.00 0.00
E
ATOM 28259 HN ASP E 96 44.278 −0.221 56.626 0.00 0.00
E
ATOM 28260 CA ASP E 96 45.943 −1.155 56.111 0.00 0.00
E
ATOM 28261 HA ASP E 96 45.420 −1.131 55.166 0.00 0.00
E
ATOM 28262 CB ASP E 96 47.264 −0.233 56.126 0.00 0.00
E
ATOM 28263 HB1 ASP E 96 46.928 0.820 56.236 0.00 0.00
E
ATOM 28264 HB2 ASP E 96 47.911 −0.531 56.979 0.00 0.00
E
ATOM 28265 CG ASP E 96 48.052 −0.492 54.921 0.00 0.00
E
ATOM 28266 OD1 ASP E 96 47.575 −0.356 53.745 0.00 0.00
E
ATOM 28267 OD2 ASP E 96 49.229 −0.943 55.150 0.00 0.00
E
ATOM 28268 C ASP E 96 46.238 −2.694 56.296 0.00 0.00
E

ATOM 28269 O ASP E 96 47.440 -3.086 56.241 0.00 0.00 E
ATOM 28270 N ILE E 97 45.193 -3.551 56.655 0.00 0.00 E
ATOM 28271 HN ILE E 97 44.264 -3.225 56.808 0.00 0.00 E
ATOM 28272 CA ILE E 97 45.494 -4.984 56.896 0.00 0.00 E
ATOM 28273 HA ILE E 97 46.327 -4.932 57.581 0.00 0.00 E
ATOM 28274 CB ILE E 97 44.398 -5.741 57.669 0.00 0.00 E
ATOM 28275 HB ILE E 97 43.537 -5.896 56.983 0.00 0.00 E
ATOM 28276 CG2 ILE E 97 44.936 -7.097 58.133 0.00 0.00 E
ATOM 28277 HG21 ILE E 97 46.013 -6.949 58.361 0.00 0.00 E
ATOM 28278 HG22 ILE E 97 44.311 -7.359 59.013 0.00 0.00 E
ATOM 28279 HG23 ILE E 97 44.848 -7.888 57.357 0.00 0.00 E
ATOM 28280 CG1 ILE E 97 43.910 -4.927 58.925 0.00 0.00 E
ATOM 28281 HG11 ILE E 97 43.400 -3.982 58.641 0.00 0.00 E
ATOM 28282 HG12 ILE E 97 43.236 -5.634 59.454 0.00 0.00 E
ATOM 28283 CD ILE E 97 45.072 -4.611 59.928 0.00 0.00 E
ATOM 28284 HD1 ILE E 97 44.524 -4.349 60.858 0.00 0.00 E
ATOM 28285 HD2 ILE E 97 45.718 -5.465 60.225 0.00 0.00 E
ATOM 28286 HD3 ILE E 97 45.807 -3.825 59.649 0.00 0.00 E
ATOM 28287 C ILE E 97 45.908 -5.644 55.590 0.00 0.00 E
ATOM 28288 O ILE E 97 45.106 -5.644 54.653 0.00 0.00 E
ATOM 28289 N PRO E 98 47.114 -6.236 55.469 0.00 0.00 E
ATOM 28290 CD PRO E 98 48.055 -6.228 56.605 0.00 0.00 E
ATOM 28291 HD1 PRO E 98 48.433 -5.222 56.886 0.00 0.00 E
ATOM 28292 HD2 PRO E 98 47.721 -6.705 57.551 0.00 0.00 E
ATOM 28293 CA PRO E 98 47.713 -6.808 54.323 0.00 0.00 E
ATOM 28294 HA PRO E 98 48.047 -5.970 53.730 0.00 0.00 E
ATOM 28295 CB PRO E 98 48.919 -7.590 54.777 0.00 0.00 E

```
ATOM  28296  HB1 PRO E  98      49.805  -7.470  54.117  0.00  0.00
E
ATOM  28297  HB2 PRO E  98      48.658  -8.669  54.804  0.00  0.00
E
ATOM  28298  CG  PRO E  98      49.342  -6.951  56.116  0.00  0.00
E
ATOM  28299  HG1 PRO E  98      49.904  -7.695  56.721  0.00  0.00
E
ATOM  28300  HG2 PRO E  98      49.971  -6.040  56.025  0.00  0.00
E
ATOM  28301  C   PRO E  98      46.793  -7.654  53.507  0.00  0.00
E
ATOM  28302  O   PRO E  98      46.674  -7.288  52.353  0.00  0.00
E
ATOM  28303  N   ASN E  99      46.042  -8.636  54.039  0.00  0.00
E
ATOM  28304  HN  ASN E  99      46.136  -8.850  55.008  0.00  0.00
E
ATOM  28305  CA  ASN E  99      45.105  -9.427  53.310  0.00  0.00
E
ATOM  28306  HA  ASN E  99      44.903  -8.988  52.344  0.00  0.00
E
ATOM  28307  CB  ASN E  99      45.432 -10.934  53.249  0.00  0.00
E
ATOM  28308  HB1 ASN E  99      45.385 -11.499  54.205  0.00  0.00
E
ATOM  28309  HB2 ASN E  99      44.690 -11.492  52.641  0.00  0.00
E
ATOM  28310  CG  ASN E  99      46.791 -11.268  52.647  0.00  0.00
E
ATOM  28311  OD1 ASN E  99      46.975 -11.355  51.464  0.00  0.00
E
ATOM  28312  ND2 ASN E  99      47.750 -11.420  53.517  0.00  0.00
E
ATOM  28313 HD21 ASN E  99      48.540 -11.917  53.157  0.00  0.00
E
ATOM  28314 HD22 ASN E  99      47.616 -11.400  54.507  0.00  0.00
E
ATOM  28315  C   ASN E  99      43.707  -9.422  54.061  0.00  0.00
E
ATOM  28316  O   ASN E  99      43.535  -9.903  55.156  0.00  0.00
E
ATOM  28317  N   ILE E 100      42.731  -8.815  53.311  0.00  0.00
E
ATOM  28318  HN  ILE E 100      42.983  -8.384  52.447  0.00  0.00
E
ATOM  28319  CA  ILE E 100      41.307  -8.599  53.758  0.00  0.00
E
ATOM  28320  HA  ILE E 100      41.134  -8.973  54.756  0.00  0.00
E
ATOM  28321  CB  ILE E 100      40.838  -7.110  53.686  0.00  0.00
E
ATOM  28322  HB  ILE E 100      40.999  -6.657  52.685  0.00  0.00
E
```

ATOM 28323 CG2 ILE E 100   39.293 -7.027 53.850 0.00 0.00
E
ATOM 28324 HG21 ILE E 100   38.767 -7.663 53.106 0.00 0.00
E
ATOM 28325 HG22 ILE E 100   39.020 -7.306 54.890 0.00 0.00
E
ATOM 28326 HG23 ILE E 100   38.941 -5.991 53.658 0.00 0.00
E
ATOM 28327 CG1 ILE E 100   41.751 -6.374 54.685 0.00 0.00
E
ATOM 28328 HG11 ILE E 100   41.375 -6.651 55.693 0.00 0.00
E
ATOM 28329 HG12 ILE E 100   42.820 -6.650 54.564 0.00 0.00
E
ATOM 28330 CD ILE E 100   41.592 -4.820 54.540 0.00 0.00
E
ATOM 28331 HD1 ILE E 100   40.529 -4.553 54.721 0.00 0.00
E
ATOM 28332 HD2 ILE E 100   42.224 -4.203 55.213 0.00 0.00
E
ATOM 28333 HD3 ILE E 100   41.842 -4.532 53.496 0.00 0.00
E
ATOM 28334 C   ILE E 100   40.462 -9.457 52.839 0.00 0.00
E
ATOM 28335 O   ILE E 100   40.433 -9.265 51.615 0.00 0.00
E
ATOM 28336 N   TRP E 101   39.757 -10.419 53.375 0.00 0.00
E
ATOM 28337 HN TRP E 101   39.852 -10.616 54.348 0.00 0.00
E
ATOM 28338 CA TRP E 101   38.773 -11.321 52.634 0.00 0.00
E
ATOM 28339 HA TRP E 101   38.737 -10.982 51.609 0.00 0.00
E
ATOM 28340 CB TRP E 101   39.217 -12.867 52.687 0.00 0.00
E
ATOM 28341 HB1 TRP E 101   39.196 -13.191 53.750 0.00 0.00
E
ATOM 28342 HB2 TRP E 101   38.365 -13.433 52.252 0.00 0.00
E
ATOM 28343 CG TRP E 101   40.601 -13.162 51.995 0.00 0.00
E
ATOM 28344 CD1 TRP E 101   41.727 -12.388 51.616 0.00 0.00
E
ATOM 28345 HD1 TRP E 101   41.859 -11.327 51.459 0.00 0.00
E
ATOM 28346 NE1 TRP E 101   42.695 -13.233 51.220 0.00 0.00
E
ATOM 28347 HE1 TRP E 101   43.576 -12.928 50.932 0.00 0.00
E
ATOM 28348 CE2 TRP E 101   42.278 -14.507 51.256 0.00 0.00
E
ATOM 28349 CD2 TRP E 101   40.978 -14.503 51.784 0.00 0.00
E

```
ATOM  28350  CE3 TRP E 101      40.254 -15.700  51.940  0.00  0.00
E
ATOM  28351  HE3 TRP E 101      39.206 -15.733  52.200  0.00  0.00
E
ATOM  28352  CZ3 TRP E 101      40.857 -16.873  51.445  0.00  0.00
E
ATOM  28353  HZ3 TRP E 101      40.378 -17.840  51.498  0.00  0.00
E
ATOM  28354  CZ2 TRP E 101      42.941 -15.684  50.978  0.00  0.00
E
ATOM  28355  HZ2 TRP E 101      43.890 -15.700  50.463  0.00  0.00
E
ATOM  28356  CH2 TRP E 101      42.201 -16.877  51.041  0.00  0.00
E
ATOM  28357  HH2 TRP E 101      42.584 -17.832  50.713  0.00  0.00
E
ATOM  28358  C   TRP E 101      37.413 -11.142  53.184  0.00  0.00
E
ATOM  28359  O   TRP E 101      37.213 -11.195  54.364  0.00  0.00
E
ATOM  28360  N   VAL E 102      36.473 -10.782  52.336  0.00  0.00
E
ATOM  28361  HN  VAL E 102      36.718 -10.681  51.375  0.00  0.00
E
ATOM  28362  CA  VAL E 102      35.099 -10.401  52.688  0.00  0.00
E
ATOM  28363  HA  VAL E 102      35.028 -10.619  53.744  0.00  0.00
E
ATOM  28364  CB  VAL E 102      34.880  -8.912  52.483  0.00  0.00
E
ATOM  28365  HB  VAL E 102      34.589  -8.703  51.431  0.00  0.00
E
ATOM  28366  CG1 VAL E 102      33.755  -8.343  53.304  0.00  0.00
E
ATOM  28367 HG11 VAL E 102      33.858  -7.241  53.211  0.00  0.00
E
ATOM  28368 HG12 VAL E 102      32.733  -8.700  53.052  0.00  0.00
E
ATOM  28369 HG13 VAL E 102      33.893  -8.759  54.325  0.00  0.00
E
ATOM  28370  CG2 VAL E 102      36.193  -8.091  52.829  0.00  0.00
E
ATOM  28371 HG21 VAL E 102      36.427  -8.193  53.911  0.00  0.00
E
ATOM  28372 HG22 VAL E 102      37.079  -8.462  52.272  0.00  0.00
E
ATOM  28373 HG23 VAL E 102      35.952  -7.055  52.507  0.00  0.00
E
ATOM  28374  C   VAL E 102      34.002 -11.182  51.946  0.00  0.00
E
ATOM  28375  O   VAL E 102      34.023 -11.257  50.726  0.00  0.00
E
ATOM  28376  N   LYS E 103      33.116 -11.747  52.740  0.00  0.00
E
```

ATOM 28377 HN LYS E 103 33.388 −11.693 53.698 0.00 0.00
E
ATOM 28378 CA LYS E 103 31.782 −12.203 52.302 0.00 0.00
E
ATOM 28379 HA LYS E 103 31.979 −13.003 51.604 0.00 0.00
E
ATOM 28380 CB LYS E 103 30.952 −12.888 53.441 0.00 0.00
E
ATOM 28381 HB1 LYS E 103 31.786 −13.425 53.941 0.00 0.00
E
ATOM 28382 HB2 LYS E 103 30.599 −12.223 54.259 0.00 0.00
E
ATOM 28383 CG LYS E 103 29.921 −13.896 52.975 0.00 0.00
E
ATOM 28384 HG1 LYS E 103 29.046 −13.329 52.592 0.00 0.00
E
ATOM 28385 HG2 LYS E 103 30.366 −14.352 52.065 0.00 0.00
E
ATOM 28386 CD LYS E 103 29.452 −14.841 54.146 0.00 0.00
E
ATOM 28387 HD1 LYS E 103 29.129 −15.821 53.734 0.00 0.00
E
ATOM 28388 HD2 LYS E 103 30.276 −15.031 54.866 0.00 0.00
E
ATOM 28389 CE LYS E 103 28.154 −14.473 54.888 0.00 0.00
E
ATOM 28390 HE1 LYS E 103 28.055 −15.102 55.799 0.00 0.00
E
ATOM 28391 HE2 LYS E 103 28.131 −13.391 55.137 0.00 0.00
E
ATOM 28392 NZ LYS E 103 26.946 −14.709 54.098 0.00 0.00
E
ATOM 28393 HZ1 LYS E 103 27.101 −14.435 53.107 0.00 0.00
E
ATOM 28394 HZ2 LYS E 103 26.619 −15.689 54.216 0.00 0.00
E
ATOM 28395 HZ3 LYS E 103 26.157 −14.109 54.412 0.00 0.00
E
ATOM 28396 C LYS E 103 30.983 −11.183 51.571 0.00 0.00
E
ATOM 28397 O LYS E 103 30.860 −10.028 52.036 0.00 0.00
E
ATOM 28398 N ALA E 104 30.376 −11.592 50.408 0.00 0.00
E
ATOM 28399 HN ALA E 104 30.420 −12.527 50.063 0.00 0.00
E
ATOM 28400 CA ALA E 104 29.520 −10.708 49.509 0.00 0.00
E
ATOM 28401 HA ALA E 104 29.342 −9.739 49.951 0.00 0.00
E
ATOM 28402 CB ALA E 104 30.306 −10.577 48.208 0.00 0.00
E
ATOM 28403 HB1 ALA E 104 30.624 −11.561 47.803 0.00 0.00
E

```
ATOM  28404  HB2 ALA E 104      29.680 -10.016  47.481  0.00  0.00
E
ATOM  28405  HB3 ALA E 104      31.155  -9.861  48.238  0.00  0.00
E
ATOM  28406  C   ALA E 104      28.144 -11.327  49.223  0.00  0.00
E
ATOM  28407  O   ALA E 104      28.012 -12.585  49.177  0.00  0.00
E
ATOM  28408  N   GLN E 105      27.045 -10.550  49.220  0.00  0.00
E
ATOM  28409  HN  GLN E 105      27.178  -9.587  49.444  0.00  0.00
E
ATOM  28410  CA  GLN E 105      25.725 -11.078  49.018  0.00  0.00
E
ATOM  28411  HA  GLN E 105      25.762 -12.153  48.923  0.00  0.00
E
ATOM  28412  CB  GLN E 105      24.773 -10.804  50.245  0.00  0.00
E
ATOM  28413  HB1 GLN E 105      23.847 -11.347  49.961  0.00  0.00
E
ATOM  28414  HB2 GLN E 105      25.232 -11.361  51.090  0.00  0.00
E
ATOM  28415  CG  GLN E 105      24.549  -9.371  50.658  0.00  0.00
E
ATOM  28416  HG1 GLN E 105      25.222  -8.950  51.435  0.00  0.00
E
ATOM  28417  HG2 GLN E 105      24.527  -8.675  49.793  0.00  0.00
E
ATOM  28418  CD  GLN E 105      23.078  -9.303  51.212  0.00  0.00
E
ATOM  28419  OE1 GLN E 105      22.133  -8.957  50.517  0.00  0.00
E
ATOM  28420  NE2 GLN E 105      22.895  -9.594  52.544  0.00  0.00
E
ATOM  28421 HE21 GLN E 105      21.970  -9.827  52.845  0.00  0.00
E
ATOM  28422 HE22 GLN E 105      23.654  -9.852  53.141  0.00  0.00
E
ATOM  28423  C   GLN E 105      25.069 -10.543  47.779  0.00  0.00
E
ATOM  28424  O   GLN E 105      24.133 -11.238  47.393  0.00  0.00
E
ATOM  28425  N   ASN E 106      25.541  -9.488  47.138  0.00  0.00
E
ATOM  28426  HN  ASN E 106      26.415  -9.062  47.359  0.00  0.00
E
ATOM  28427  CA  ASN E 106      24.973  -9.045  45.903  0.00  0.00
E
ATOM  28428  HA  ASN E 106      24.821  -9.958  45.347  0.00  0.00
E
ATOM  28429  CB  ASN E 106      23.566  -8.386  46.099  0.00  0.00
E
ATOM  28430  HB1 ASN E 106      23.167  -8.191  45.080  0.00  0.00
E
```

ATOM 28431 HB2 ASN E 106 22.910 -9.093 46.649 0.00 0.00
E
ATOM 28432 CG ASN E 106 23.571 -7.021 46.856 0.00 0.00
E
ATOM 28433 OD1 ASN E 106 24.591 -6.359 47.123 0.00 0.00
E
ATOM 28434 ND2 ASN E 106 22.369 -6.555 47.204 0.00 0.00
E
ATOM 28435 HD21 ASN E 106 22.357 -5.596 47.489 0.00 0.00
E
ATOM 28436 HD22 ASN E 106 21.503 -7.048 47.117 0.00 0.00
E
ATOM 28437 C ASN E 106 25.938 -8.169 45.091 0.00 0.00
E
ATOM 28438 O ASN E 106 27.096 -8.002 45.456 0.00 0.00
E
ATOM 28439 N TYR E 107 25.563 -7.741 43.878 0.00 0.00
E
ATOM 28440 HN TYR E 107 24.655 -7.981 43.546 0.00 0.00
E
ATOM 28441 CA TYR E 107 26.281 -6.871 43.013 0.00 0.00
E
ATOM 28442 HA TYR E 107 27.248 -7.324 42.850 0.00 0.00
E
ATOM 28443 CB TYR E 107 25.557 -6.703 41.616 0.00 0.00
E
ATOM 28444 HB1 TYR E 107 25.113 -7.643 41.222 0.00 0.00
E
ATOM 28445 HB2 TYR E 107 24.669 -6.069 41.823 0.00 0.00
E
ATOM 28446 CG TYR E 107 26.338 -6.139 40.465 0.00 0.00
E
ATOM 28447 CD1 TYR E 107 27.359 -6.902 39.941 0.00 0.00
E
ATOM 28448 HD1 TYR E 107 27.523 -7.859 40.414 0.00 0.00
E
ATOM 28449 CE1 TYR E 107 28.095 -6.469 38.849 0.00 0.00
E
ATOM 28450 HE1 TYR E 107 28.990 -6.964 38.501 0.00 0.00
E
ATOM 28451 CZ TYR E 107 27.894 -5.190 38.351 0.00 0.00
E
ATOM 28452 OH TYR E 107 28.417 -4.783 37.109 0.00 0.00
E
ATOM 28453 HH TYR E 107 28.328 -3.830 37.039 0.00 0.00
E
ATOM 28454 CD2 TYR E 107 26.136 -4.880 39.926 0.00 0.00
E
ATOM 28455 HD2 TYR E 107 25.319 -4.254 40.256 0.00 0.00
E
ATOM 28456 CE2 TYR E 107 26.906 -4.372 38.891 0.00 0.00
E
ATOM 28457 HE2 TYR E 107 26.752 -3.435 38.377 0.00 0.00
E

ATOM 28458 C TYR E 107 26.745 -5.460 43.650 0.00 0.00
E
ATOM 28459 O TYR E 107 27.891 -5.059 43.642 0.00 0.00
E
ATOM 28460 N TYR E 108 25.801 -4.690 44.275 0.00 0.00
E
ATOM 28461 HN TYR E 108 24.871 -5.050 44.263 0.00 0.00
E
ATOM 28462 CA TYR E 108 25.939 -3.397 44.932 0.00 0.00
E
ATOM 28463 HA TYR E 108 26.292 -2.669 44.217 0.00 0.00
E
ATOM 28464 CB TYR E 108 24.603 -3.022 45.545 0.00 0.00
E
ATOM 28465 HB1 TYR E 108 23.969 -2.791 44.661 0.00 0.00
E
ATOM 28466 HB2 TYR E 108 24.176 -3.858 46.140 0.00 0.00
E
ATOM 28467 CG TYR E 108 24.833 -1.767 46.384 0.00 0.00
E
ATOM 28468 CD1 TYR E 108 25.241 -0.521 45.917 0.00 0.00
E
ATOM 28469 HD1 TYR E 108 25.451 -0.392 44.866 0.00 0.00
E
ATOM 28470 CE1 TYR E 108 25.463 0.494 46.883 0.00 0.00
E
ATOM 28471 HE1 TYR E 108 25.651 1.527 46.631 0.00 0.00
E
ATOM 28472 CZ TYR E 108 25.513 0.191 48.274 0.00 0.00
E
ATOM 28473 OH TYR E 108 25.795 1.145 49.219 0.00 0.00
E
ATOM 28474 HH TYR E 108 26.168 1.947 48.845 0.00 0.00
E
ATOM 28475 CD2 TYR E 108 24.890 -2.069 47.757 0.00 0.00
E
ATOM 28476 HD2 TYR E 108 24.649 -3.014 48.221 0.00 0.00
E
ATOM 28477 CE2 TYR E 108 25.273 -1.077 48.718 0.00 0.00
E
ATOM 28478 HE2 TYR E 108 25.279 -1.333 49.767 0.00 0.00
E
ATOM 28479 C TYR E 108 27.049 -3.566 46.028 0.00 0.00
E
ATOM 28480 O TYR E 108 27.957 -2.812 46.092 0.00 0.00
E
ATOM 28481 N HSE E 109 26.981 -4.545 46.895 0.00 0.00
E
ATOM 28482 HN HSE E 109 26.222 -5.193 46.889 0.00 0.00
E
ATOM 28483 CA HSE E 109 27.939 -4.903 47.918 0.00 0.00
E
ATOM 28484 HA HSE E 109 28.045 -4.119 48.654 0.00 0.00
E

ATOM 28485 CB HSE E 109 27.338 -6.173 48.653 0.00 0.00
E
ATOM 28486 HB1 HSE E 109 26.283 -5.949 48.919 0.00 0.00
E
ATOM 28487 HB2 HSE E 109 27.240 -6.927 47.843 0.00 0.00
E
ATOM 28488 ND1 HSE E 109 27.514 -7.868 50.442 0.00 0.00
E
ATOM 28489 CG HSE E 109 28.084 -6.756 49.800 0.00 0.00
E
ATOM 28490 CE1 HSE E 109 28.286 -8.116 51.498 0.00 0.00
E
ATOM 28491 HE1 HSE E 109 28.040 -8.783 52.325 0.00 0.00
E
ATOM 28492 NE2 HSE E 109 29.308 -7.195 51.560 0.00 0.00
E
ATOM 28493 HE2 HSE E 109 29.967 -7.067 52.301 0.00 0.00
E
ATOM 28494 CD2 HSE E 109 29.112 -6.321 50.527 0.00 0.00
E
ATOM 28495 HD2 HSE E 109 29.777 -5.495 50.304 0.00 0.00
E
ATOM 28496 C HSE E 109 29.354 -5.248 47.368 0.00 0.00
E
ATOM 28497 O HSE E 109 30.363 -4.678 47.733 0.00 0.00
E
ATOM 28498 N HSE E 110 29.392 -6.125 46.353 0.00 0.00
E
ATOM 28499 HN HSE E 110 28.616 -6.692 46.088 0.00 0.00
E
ATOM 28500 CA HSE E 110 30.597 -6.547 45.619 0.00 0.00
E
ATOM 28501 HA HSE E 110 31.391 -6.811 46.302 0.00 0.00
E
ATOM 28502 CB HSE E 110 30.190 -7.607 44.488 0.00 0.00
E
ATOM 28503 HB1 HSE E 110 30.106 -8.608 44.962 0.00 0.00
E
ATOM 28504 HB2 HSE E 110 29.186 -7.357 44.083 0.00 0.00
E
ATOM 28505 ND1 HSE E 110 31.858 -8.779 43.006 0.00 0.00
E
ATOM 28506 CG HSE E 110 31.118 -7.614 43.285 0.00 0.00
E
ATOM 28507 CE1 HSE E 110 32.654 -8.452 42.058 0.00 0.00
E
ATOM 28508 HE1 HSE E 110 33.317 -9.058 41.440 0.00 0.00
E
ATOM 28509 NE2 HSE E 110 32.412 -7.174 41.617 0.00 0.00
E
ATOM 28510 HE2 HSE E 110 33.077 -6.658 41.076 0.00 0.00
E
ATOM 28511 CD2 HSE E 110 31.384 -6.675 42.378 0.00 0.00
E

ATOM 28512 HD2 HSE E 110 31.114 -5.638 42.224 0.00 0.00
E
ATOM 28513 C HSE E 110 31.201 -5.270 45.008 0.00 0.00
E
ATOM 28514 O HSE E 110 32.392 -5.079 45.124 0.00 0.00
E
ATOM 28515 N LYS E 111 30.407 -4.311 44.382 0.00 0.00
E
ATOM 28516 HN LYS E 111 29.431 -4.509 44.341 0.00 0.00
E
ATOM 28517 CA LYS E 111 30.919 -3.051 43.823 0.00 0.00
E
ATOM 28518 HA LYS E 111 31.811 -3.430 43.348 0.00 0.00
E
ATOM 28519 CB LYS E 111 30.008 -2.399 42.787 0.00 0.00
E
ATOM 28520 HB1 LYS E 111 28.985 -2.308 43.212 0.00 0.00
E
ATOM 28521 HB2 LYS E 111 30.385 -1.419 42.422 0.00 0.00
E
ATOM 28522 CG LYS E 111 29.904 -3.205 41.453 0.00 0.00
E
ATOM 28523 HG1 LYS E 111 29.678 -4.280 41.625 0.00 0.00
E
ATOM 28524 HG2 LYS E 111 29.099 -2.839 40.781 0.00 0.00
E
ATOM 28525 CD LYS E 111 31.169 -3.091 40.498 0.00 0.00
E
ATOM 28526 HD1 LYS E 111 31.499 -2.032 40.436 0.00 0.00
E
ATOM 28527 HD2 LYS E 111 31.888 -3.610 41.167 0.00 0.00
E
ATOM 28528 CE LYS E 111 30.980 -3.706 39.150 0.00 0.00
E
ATOM 28529 HE1 LYS E 111 30.673 -4.773 39.189 0.00 0.00
E
ATOM 28530 HE2 LYS E 111 30.205 -3.162 38.568 0.00 0.00
E
ATOM 28531 NZ LYS E 111 32.240 -3.746 38.455 0.00 0.00
E
ATOM 28532 HZ1 LYS E 111 32.556 -2.783 38.218 0.00 0.00
E
ATOM 28533 HZ2 LYS E 111 32.995 -4.118 39.065 0.00 0.00
E
ATOM 28534 HZ3 LYS E 111 32.258 -4.237 37.538 0.00 0.00
E
ATOM 28535 C LYS E 111 31.500 -2.067 44.902 0.00 0.00
E
ATOM 28536 O LYS E 111 32.484 -1.392 44.642 0.00 0.00
E
ATOM 28537 N VAL E 112 31.031 -2.074 46.213 0.00 0.00
E
ATOM 28538 HN VAL E 112 30.204 -2.585 46.437 0.00 0.00
E

```
ATOM  28539  CA  VAL E 112      31.733  -1.451  47.234  0.00  0.00
E
ATOM  28540  HA  VAL E 112      31.730  -0.437  46.861  0.00  0.00
E
ATOM  28541  CB  VAL E 112      30.983  -1.276  48.502  0.00  0.00
E
ATOM  28542  HB  VAL E 112      30.887  -2.181  49.139  0.00  0.00
E
ATOM  28543  CG1 VAL E 112      31.723  -0.271  49.383  0.00  0.00
E
ATOM  28544 HG11 VAL E 112      31.085  -0.004  50.253  0.00  0.00
E
ATOM  28545 HG12 VAL E 112      32.612  -0.760  49.836  0.00  0.00
E
ATOM  28546 HG13 VAL E 112      32.055   0.524  48.682  0.00  0.00
E
ATOM  28547  CG2 VAL E 112      29.601  -0.677  48.268  0.00  0.00
E
ATOM  28548 HG21 VAL E 112      28.898  -1.397  47.797  0.00  0.00
E
ATOM  28549 HG22 VAL E 112      29.088  -0.353  49.198  0.00  0.00
E
ATOM  28550 HG23 VAL E 112      29.690   0.195  47.585  0.00  0.00
E
ATOM  28551  C   VAL E 112      33.119  -2.086  47.480  0.00  0.00
E
ATOM  28552  O   VAL E 112      34.163  -1.454  47.430  0.00  0.00
E
ATOM  28553  N   LEU E 113      33.192  -3.463  47.732  0.00  0.00
E
ATOM  28554  HN  LEU E 113      32.342  -3.962  47.581  0.00  0.00
E
ATOM  28555  CA  LEU E 113      34.461  -4.125  48.128  0.00  0.00
E
ATOM  28556  HA  LEU E 113      34.868  -3.582  48.968  0.00  0.00
E
ATOM  28557  CB  LEU E 113      34.015  -5.553  48.507  0.00  0.00
E
ATOM  28558  HB1 LEU E 113      33.457  -5.995  47.654  0.00  0.00
E
ATOM  28559  HB2 LEU E 113      34.859  -6.207  48.814  0.00  0.00
E
ATOM  28560  CG  LEU E 113      33.057  -5.728  49.677  0.00  0.00
E
ATOM  28561  HG  LEU E 113      32.338  -4.881  49.666  0.00  0.00
E
ATOM  28562  CD1 LEU E 113      32.274  -7.074  49.690  0.00  0.00
E
ATOM  28563 HD11 LEU E 113      31.587  -7.130  50.561  0.00  0.00
E
ATOM  28564 HD12 LEU E 113      31.608  -7.095  48.801  0.00  0.00
E
ATOM  28565 HD13 LEU E 113      32.899  -7.992  49.711  0.00  0.00
E
```

```
ATOM  28566  CD2 LEU E 113      33.687  -5.484  51.056  0.00  0.00
E
ATOM  28567 HD21 LEU E 113      34.544  -6.139  51.319  0.00  0.00
E
ATOM  28568 HD22 LEU E 113      34.003  -4.420  51.007  0.00  0.00
E
ATOM  28569 HD23 LEU E 113      33.038  -5.644  51.943  0.00  0.00
E
ATOM  28570  C   LEU E 113      35.523  -4.195  47.049  0.00  0.00
E
ATOM  28571  O   LEU E 113      36.734  -4.099  47.226  0.00  0.00
E
ATOM  28572  N   GLU E 114      35.076  -4.246  45.765  0.00  0.00
E
ATOM  28573  HN  GLU E 114      34.136  -4.507  45.561  0.00  0.00
E
ATOM  28574  CA  GLU E 114      35.888  -4.061  44.562  0.00  0.00
E
ATOM  28575  HA  GLU E 114      36.637  -4.839  44.582  0.00  0.00
E
ATOM  28576  CB  GLU E 114      35.023  -4.301  43.278  0.00  0.00
E
ATOM  28577  HB1 GLU E 114      34.656  -5.349  43.299  0.00  0.00
E
ATOM  28578  HB2 GLU E 114      34.147  -3.640  43.450  0.00  0.00
E
ATOM  28579  CG  GLU E 114      35.614  -4.060  41.898  0.00  0.00
E
ATOM  28580  HG1 GLU E 114      36.159  -3.093  41.940  0.00  0.00
E
ATOM  28581  HG2 GLU E 114      36.386  -4.853  41.803  0.00  0.00
E
ATOM  28582  CD  GLU E 114      34.621  -4.028  40.752  0.00  0.00
E
ATOM  28583  OE1 GLU E 114      34.116  -5.122  40.386  0.00  0.00
E
ATOM  28584  OE2 GLU E 114      34.337  -2.962  40.164  0.00  0.00
E
ATOM  28585  C   GLU E 114      36.780  -2.825  44.462  0.00  0.00
E
ATOM  28586  O   GLU E 114      37.867  -2.865  43.927  0.00  0.00
E
ATOM  28587  N   LYS E 115      36.283  -1.677  44.856  0.00  0.00
E
ATOM  28588  HN  LYS E 115      35.327  -1.738  45.132  0.00  0.00
E
ATOM  28589  CA  LYS E 115      36.906  -0.418  44.525  0.00  0.00
E
ATOM  28590  HA  LYS E 115      37.584  -0.536  43.692  0.00  0.00
E
ATOM  28591  CB  LYS E 115      35.932   0.608  43.913  0.00  0.00
E
ATOM  28592  HB1 LYS E 115      35.034   0.798  44.540  0.00  0.00
E
```

ATOM 28593 HB2 LYS E 115   36.472   1.549 43.673 0.00 0.00
E
ATOM 28594 CG LYS E 115   35.360   0.008 42.517 0.00 0.00
E
ATOM 28595 HG1 LYS E 115   36.070 -0.600 41.917 0.00 0.00
E
ATOM 28596 HG2 LYS E 115   34.591 -0.754 42.766 0.00 0.00
E
ATOM 28597 CD LYS E 115   34.966   1.086 41.430 0.00 0.00
E
ATOM 28598 HD1 LYS E 115   34.396   1.875 41.965 0.00 0.00
E
ATOM 28599 HD2 LYS E 115   35.979   1.478 41.196 0.00 0.00
E
ATOM 28600 CE LYS E 115   34.075   0.701 40.233 0.00 0.00
E
ATOM 28601 HE1 LYS E 115   33.070   0.326 40.521 0.00 0.00
E
ATOM 28602 HE2 LYS E 115   34.007   1.610 39.598 0.00 0.00
E
ATOM 28603 NZ LYS E 115   34.741 -0.388 39.535 0.00 0.00
E
ATOM 28604 HZ1 LYS E 115   35.769 -0.236 39.485 0.00 0.00
E
ATOM 28605 HZ2 LYS E 115   34.688 -1.211 40.169 0.00 0.00
E
ATOM 28606 HZ3 LYS E 115   34.303 -0.755 38.666 0.00 0.00
E
ATOM 28607 C   LYS E 115   37.642   0.221 45.664 0.00 0.00
E
ATOM 28608 O   LYS E 115   38.326   1.240 45.408 0.00 0.00
E
ATOM 28609 N   ILE E 116   37.570 -0.398 46.869 0.00 0.00
E
ATOM 28610 HN ILE E 116   36.814 -1.044 46.938 0.00 0.00
E
ATOM 28611 CA ILE E 116   38.396   0.020 48.039 0.00 0.00
E
ATOM 28612 HA ILE E 116   38.774   0.996 47.772 0.00 0.00
E
ATOM 28613 CB ILE E 116   37.621   0.262 49.316 0.00 0.00
E
ATOM 28614 HB ILE E 116   38.408   0.375 50.092 0.00 0.00
E
ATOM 28615 CG2 ILE E 116   36.939   1.586 49.195 0.00 0.00
E
ATOM 28616 HG21 ILE E 116   36.074   1.881 49.827 0.00 0.00
E
ATOM 28617 HG22 ILE E 116   37.721   2.355 49.367 0.00 0.00
E
ATOM 28618 HG23 ILE E 116   36.570   1.661 48.150 0.00 0.00
E
ATOM 28619 CG1 ILE E 116   36.651 -0.889 49.682 0.00 0.00
E

ATOM 28620 HG11 ILE E 116 35.820 -0.880 48.945 0.00 0.00
E
ATOM 28621 HG12 ILE E 116 37.183 -1.858 49.784 0.00 0.00
E
ATOM 28622 CD ILE E 116 36.068 -0.668 51.135 0.00 0.00
E
ATOM 28623 HD1 ILE E 116 35.587 0.332 51.199 0.00 0.00
E
ATOM 28624 HD2 ILE E 116 35.418 -1.527 51.405 0.00 0.00
E
ATOM 28625 HD3 ILE E 116 36.894 -0.613 51.876 0.00 0.00
E
ATOM 28626 C ILE E 116 39.652 -0.802 48.224 0.00 0.00
E
ATOM 28627 O ILE E 116 40.532 -0.535 49.034 0.00 0.00
E
ATOM 28628 N GLY E 117 39.792 -1.717 47.306 0.00 0.00
E
ATOM 28629 HN GLY E 117 39.152 -1.854 46.553 0.00 0.00
E
ATOM 28630 CA GLY E 117 40.998 -2.447 47.231 0.00 0.00
E
ATOM 28631 HA1 GLY E 117 41.874 -1.960 47.634 0.00 0.00
E
ATOM 28632 HA2 GLY E 117 41.147 -2.602 46.172 0.00 0.00
E
ATOM 28633 C GLY E 117 41.107 -3.731 48.067 0.00 0.00
E
ATOM 28634 O GLY E 117 42.140 -4.199 48.598 0.00 0.00
E
ATOM 28635 N ALA E 118 39.943 -4.408 48.341 0.00 0.00
E
ATOM 28636 HN ALA E 118 39.095 -4.191 47.863 0.00 0.00
E
ATOM 28637 CA ALA E 118 39.882 -5.602 49.151 0.00 0.00
E
ATOM 28638 HA ALA E 118 40.540 -5.421 49.988 0.00 0.00
E
ATOM 28639 CB ALA E 118 38.542 -5.881 49.784 0.00 0.00
E
ATOM 28640 HB1 ALA E 118 38.083 -6.812 49.389 0.00 0.00
E
ATOM 28641 HB2 ALA E 118 38.613 -5.786 50.889 0.00 0.00
E
ATOM 28642 HB3 ALA E 118 37.947 -5.021 49.411 0.00 0.00
E
ATOM 28643 C ALA E 118 40.382 -6.807 48.394 0.00 0.00
E
ATOM 28644 O ALA E 118 40.085 -6.893 47.176 0.00 0.00
E
ATOM 28645 N ASP E 119 41.158 -7.718 48.990 0.00 0.00
E
ATOM 28646 HN ASP E 119 41.309 -7.764 49.974 0.00 0.00
E

ATOM 28647 CA ASP E 119 41.882 -8.734 48.317 0.00 0.00
E
ATOM 28648 HA ASP E 119 42.477 -8.155 47.626 0.00 0.00
E
ATOM 28649 CB ASP E 119 42.795 -9.373 49.344 0.00 0.00
E
ATOM 28650 HB1 ASP E 119 42.299 -9.862 50.210 0.00 0.00
E
ATOM 28651 HB2 ASP E 119 43.332 -10.196 48.824 0.00 0.00
E
ATOM 28652 CG ASP E 119 43.921 -8.427 49.886 0.00 0.00
E
ATOM 28653 OD1 ASP E 119 43.676 -7.785 50.899 0.00 0.00
E
ATOM 28654 OD2 ASP E 119 44.977 -8.397 49.262 0.00 0.00
E
ATOM 28655 C ASP E 119 41.106 -9.829 47.720 0.00 0.00
E
ATOM 28656 O ASP E 119 41.419 -10.303 46.667 0.00 0.00
E
ATOM 28657 N ARG E 120 40.053 -10.125 48.331 0.00 0.00
E
ATOM 28658 HN ARG E 120 39.852 -9.754 49.234 0.00 0.00
E
ATOM 28659 CA ARG E 120 39.238 -11.188 47.872 0.00 0.00
E
ATOM 28660 HA ARG E 120 39.247 -11.262 46.794 0.00 0.00
E
ATOM 28661 CB ARG E 120 39.974 -12.542 48.257 0.00 0.00
E
ATOM 28662 HB1 ARG E 120 41.028 -12.413 47.928 0.00 0.00
E
ATOM 28663 HB2 ARG E 120 39.736 -12.854 49.296 0.00 0.00
E
ATOM 28664 CG ARG E 120 39.428 -13.707 47.382 0.00 0.00
E
ATOM 28665 HG1 ARG E 120 38.330 -13.715 47.551 0.00 0.00
E
ATOM 28666 HG2 ARG E 120 39.629 -13.542 46.302 0.00 0.00
E
ATOM 28667 CD ARG E 120 39.976 -15.025 47.794 0.00 0.00
E
ATOM 28668 HD1 ARG E 120 41.040 -15.033 47.476 0.00 0.00
E
ATOM 28669 HD2 ARG E 120 39.833 -15.101 48.894 0.00 0.00
E
ATOM 28670 NE ARG E 120 39.101 -16.102 47.141 0.00 0.00
E
ATOM 28671 HE ARG E 120 38.166 -15.801 46.951 0.00 0.00
E
ATOM 28672 CZ ARG E 120 39.560 -17.375 46.984 0.00 0.00
E
ATOM 28673 NH1 ARG E 120 40.871 -17.616 46.981 0.00 0.00
E

ATOM 28674 HH11 ARG E 120 41.131 −18.555 46.755 0.00 0.00
E
ATOM 28675 HH12 ARG E 120 41.587 −16.921 46.910 0.00 0.00
E
ATOM 28676 NH2 ARG E 120 38.746 −18.418 47.002 0.00 0.00
E
ATOM 28677 HH21 ARG E 120 39.078 −19.291 46.645 0.00 0.00
E
ATOM 28678 HH22 ARG E 120 37.765 −18.258 46.898 0.00 0.00
E
ATOM 28679 C ARG E 120 37.822 −11.186 48.254 0.00 0.00
E
ATOM 28680 O ARG E 120 37.558 −10.772 49.375 0.00 0.00
E
ATOM 28681 N ILE E 121 36.913 −11.489 47.292 0.00 0.00
E
ATOM 28682 HN ILE E 121 37.109 −11.632 46.325 0.00 0.00
E
ATOM 28683 CA ILE E 121 35.470 −11.185 47.518 0.00 0.00
E
ATOM 28684 HA ILE E 121 35.298 −10.744 48.489 0.00 0.00
E
ATOM 28685 CB ILE E 121 34.949 −10.225 46.477 0.00 0.00
E
ATOM 28686 HB ILE E 121 34.933 −10.645 45.448 0.00 0.00
E
ATOM 28687 CG2 ILE E 121 33.457 −10.008 46.754 0.00 0.00
E
ATOM 28688 HG21 ILE E 121 32.802 −10.894 46.612 0.00 0.00
E
ATOM 28689 HG22 ILE E 121 33.230 −9.661 47.784 0.00 0.00
E
ATOM 28690 HG23 ILE E 121 33.180 −9.108 46.165 0.00 0.00
E
ATOM 28691 CG1 ILE E 121 35.661 −8.847 46.543 0.00 0.00
E
ATOM 28692 HG11 ILE E 121 35.529 −8.529 47.600 0.00 0.00
E
ATOM 28693 HG12 ILE E 121 36.743 −8.973 46.323 0.00 0.00
E
ATOM 28694 CD ILE E 121 35.248 −7.701 45.514 0.00 0.00
E
ATOM 28695 HD1 ILE E 121 35.219 −8.111 44.482 0.00 0.00
E
ATOM 28696 HD2 ILE E 121 34.208 −7.387 45.746 0.00 0.00
E
ATOM 28697 HD3 ILE E 121 36.032 −6.915 45.454 0.00 0.00
E
ATOM 28698 C ILE E 121 34.685 −12.492 47.389 0.00 0.00
E
ATOM 28699 O ILE E 121 34.607 −13.054 46.322 0.00 0.00
E
ATOM 28700 N ILE E 122 34.148 −13.060 48.532 0.00 0.00
E

ATOM 28701 HN ILE E 122 34.153 -12.543 49.385 0.00 0.00
E
ATOM 28702 CA ILE E 122 33.591 -14.432 48.646 0.00 0.00
E
ATOM 28703 HA ILE E 122 34.124 -15.048 47.937 0.00 0.00
E
ATOM 28704 CB ILE E 122 33.788 -15.169 50.035 0.00 0.00
E
ATOM 28705 HB ILE E 122 33.301 -14.535 50.806 0.00 0.00
E
ATOM 28706 CG2 ILE E 122 33.202 -16.637 50.016 0.00 0.00
E
ATOM 28707 HG21 ILE E 122 34.032 -17.302 49.694 0.00 0.00
E
ATOM 28708 HG22 ILE E 122 32.721 -16.921 50.977 0.00 0.00
E
ATOM 28709 HG23 ILE E 122 32.429 -16.716 49.222 0.00 0.00
E
ATOM 28710 CG1 ILE E 122 35.275 -15.133 50.445 0.00 0.00
E
ATOM 28711 HG11 ILE E 122 35.670 -16.158 50.612 0.00 0.00
E
ATOM 28712 HG12 ILE E 122 35.902 -14.761 49.607 0.00 0.00
E
ATOM 28713 CD ILE E 122 35.632 -14.304 51.659 0.00 0.00
E
ATOM 28714 HD1 ILE E 122 35.259 -14.898 52.520 0.00 0.00
E
ATOM 28715 HD2 ILE E 122 36.733 -14.241 51.794 0.00 0.00
E
ATOM 28716 HD3 ILE E 122 35.147 -13.307 51.587 0.00 0.00
E
ATOM 28717 C ILE E 122 32.121 -14.457 48.482 0.00 0.00
E
ATOM 28718 O ILE E 122 31.266 -14.069 49.344 0.00 0.00
E
ATOM 28719 N HSE E 123 31.685 -15.102 47.370 0.00 0.00
E
ATOM 28720 HN HSE E 123 32.342 -15.544 46.763 0.00 0.00
E
ATOM 28721 CA HSE E 123 30.247 -15.181 46.992 0.00 0.00
E
ATOM 28722 HA HSE E 123 29.703 -14.428 47.544 0.00 0.00
E
ATOM 28723 CB HSE E 123 30.023 -14.791 45.585 0.00 0.00
E
ATOM 28724 HB1 HSE E 123 30.646 -15.389 44.886 0.00 0.00
E
ATOM 28725 HB2 HSE E 123 28.958 -14.943 45.310 0.00 0.00
E
ATOM 28726 ND1 HSE E 123 29.066 -12.586 45.252 0.00 0.00
E
ATOM 28727 CG HSE E 123 30.215 -13.363 45.134 0.00 0.00
E

ATOM 28728 CE1 HSE E 123 29.412 -11.369 44.778 0.00 0.00
E
ATOM 28729 HE1 HSE E 123 28.753 -10.524 44.579 0.00 0.00
E
ATOM 28730 NE2 HSE E 123 30.731 -11.364 44.322 0.00 0.00
E
ATOM 28731 HE2 HSE E 123 31.173 -10.685 43.737 0.00 0.00
E
ATOM 28732 CD2 HSE E 123 31.219 -12.611 44.535 0.00 0.00
E
ATOM 28733 HD2 HSE E 123 32.172 -12.965 44.160 0.00 0.00
E
ATOM 28734 C HSE E 123 29.670 -16.603 47.297 0.00 0.00
E
ATOM 28735 O HSE E 123 29.721 -17.394 46.396 0.00 0.00
E
ATOM 28736 N PRO E 124 29.059 -17.013 48.365 0.00 0.00
E
ATOM 28737 CD PRO E 124 28.566 -16.141 49.457 0.00 0.00
E
ATOM 28738 HD1 PRO E 124 29.375 -15.494 49.860 0.00 0.00
E
ATOM 28739 HD2 PRO E 124 27.724 -15.543 49.048 0.00 0.00
E
ATOM 28740 CA PRO E 124 29.213 -18.417 48.736 0.00 0.00
E
ATOM 28741 HA PRO E 124 30.220 -18.725 48.498 0.00 0.00
E
ATOM 28742 CB PRO E 124 29.101 -18.367 50.242 0.00 0.00
E
ATOM 28743 HB1 PRO E 124 30.143 -18.257 50.612 0.00 0.00
E
ATOM 28744 HB2 PRO E 124 28.624 -19.237 50.742 0.00 0.00
E
ATOM 28745 CG PRO E 124 28.152 -17.082 50.533 0.00 0.00
E
ATOM 28746 HG1 PRO E 124 27.119 -17.375 50.248 0.00 0.00
E
ATOM 28747 HG2 PRO E 124 28.270 -16.821 51.606 0.00 0.00
E
ATOM 28748 C PRO E 124 28.229 -19.399 48.100 0.00 0.00
E
ATOM 28749 O PRO E 124 28.572 -20.580 48.090 0.00 0.00
E
ATOM 28750 N GLU E 125 27.055 -18.939 47.667 0.00 0.00
E
ATOM 28751 HN GLU E 125 26.864 -17.964 47.740 0.00 0.00
E
ATOM 28752 CA GLU E 125 26.085 -19.822 46.988 0.00 0.00
E
ATOM 28753 HA GLU E 125 26.214 -20.825 47.369 0.00 0.00
E
ATOM 28754 CB GLU E 125 24.593 -19.580 47.210 0.00 0.00
E

ATOM 28755 HB1 GLU E 125 24.086 -18.862 46.530 0.00 0.00
E
ATOM 28756 HB2 GLU E 125 24.206 -20.603 47.015 0.00 0.00
E
ATOM 28757 CG GLU E 125 24.081 -19.267 48.625 0.00 0.00
E
ATOM 28758 HG1 GLU E 125 24.074 -20.199 49.229 0.00 0.00
E
ATOM 28759 HG2 GLU E 125 24.702 -18.486 49.114 0.00 0.00
E
ATOM 28760 CD GLU E 125 22.691 -18.686 48.581 0.00 0.00
E
ATOM 28761 OE1 GLU E 125 21.746 -19.366 49.078 0.00 0.00
E
ATOM 28762 OE2 GLU E 125 22.480 -17.585 47.934 0.00 0.00
E
ATOM 28763 C GLU E 125 26.282 -19.768 45.507 0.00 0.00
E
ATOM 28764 O GLU E 125 25.921 -20.719 44.814 0.00 0.00
E
ATOM 28765 N LYS E 126 26.717 -18.670 44.867 0.00 0.00
E
ATOM 28766 HN LYS E 126 26.710 -17.800 45.354 0.00 0.00
E
ATOM 28767 CA LYS E 126 27.121 -18.593 43.453 0.00 0.00
E
ATOM 28768 HA LYS E 126 26.238 -18.879 42.901 0.00 0.00
E
ATOM 28769 CB LYS E 126 27.578 -17.132 43.290 0.00 0.00
E
ATOM 28770 HB1 LYS E 126 26.733 -16.428 43.450 0.00 0.00
E
ATOM 28771 HB2 LYS E 126 28.428 -17.015 43.996 0.00 0.00
E
ATOM 28772 CG LYS E 126 28.084 -16.697 41.884 0.00 0.00
E
ATOM 28773 HG1 LYS E 126 28.732 -17.480 41.436 0.00 0.00
E
ATOM 28774 HG2 LYS E 126 27.241 -16.579 41.171 0.00 0.00
E
ATOM 28775 CD LYS E 126 28.832 -15.269 41.879 0.00 0.00
E
ATOM 28776 HD1 LYS E 126 28.217 -14.422 42.251 0.00 0.00
E
ATOM 28777 HD2 LYS E 126 29.760 -15.460 42.459 0.00 0.00
E
ATOM 28778 CE LYS E 126 29.390 -15.069 40.469 0.00 0.00
E
ATOM 28779 HE1 LYS E 126 29.937 -14.104 40.403 0.00 0.00
E
ATOM 28780 HE2 LYS E 126 30.074 -15.921 40.271 0.00 0.00
E
ATOM 28781 NZ LYS E 126 28.251 -15.015 39.532 0.00 0.00
E

ATOM 28782 HZ1 LYS E 126 27.501 −15.706 39.736 0.00 0.00
E
ATOM 28783 HZ2 LYS E 126 27.837 −14.073 39.380 0.00 0.00
E
ATOM 28784 HZ3 LYS E 126 28.658 −15.227 38.599 0.00 0.00
E
ATOM 28785 C LYS E 126 28.322 −19.446 43.176 0.00 0.00
E
ATOM 28786 O LYS E 126 28.303 −20.138 42.130 0.00 0.00
E
ATOM 28787 N ASP E 127 29.398 −19.441 44.009 0.00 0.00
E
ATOM 28788 HN ASP E 127 29.351 −18.861 44.819 0.00 0.00
E
ATOM 28789 CA ASP E 127 30.666 −20.202 43.889 0.00 0.00
E
ATOM 28790 HA ASP E 127 31.016 −20.030 42.882 0.00 0.00
E
ATOM 28791 CB ASP E 127 31.690 −19.813 44.961 0.00 0.00
E
ATOM 28792 HB1 ASP E 127 31.658 −18.705 45.038 0.00 0.00
E
ATOM 28793 HB2 ASP E 127 31.352 −20.316 45.892 0.00 0.00
E
ATOM 28794 CG ASP E 127 33.173 −20.109 44.707 0.00 0.00
E
ATOM 28795 OD1 ASP E 127 33.446 −21.258 44.269 0.00 0.00
E
ATOM 28796 OD2 ASP E 127 33.995 −19.157 44.855 0.00 0.00
E
ATOM 28797 C ASP E 127 30.367 −21.703 43.988 0.00 0.00
E
ATOM 28798 O ASP E 127 30.753 −22.542 43.164 0.00 0.00
E
ATOM 28799 N MET E 128 29.437 −22.048 44.995 0.00 0.00
E
ATOM 28800 HN MET E 128 28.940 −21.372 45.533 0.00 0.00
E
ATOM 28801 CA MET E 128 28.944 −23.368 45.216 0.00 0.00
E
ATOM 28802 HA MET E 128 29.830 −23.976 45.323 0.00 0.00
E
ATOM 28803 CB MET E 128 28.109 −23.374 46.508 0.00 0.00
E
ATOM 28804 HB1 MET E 128 28.649 −22.883 47.346 0.00 0.00
E
ATOM 28805 HB2 MET E 128 27.160 −22.849 46.266 0.00 0.00
E
ATOM 28806 CG MET E 128 27.689 −24.711 47.098 0.00 0.00
E
ATOM 28807 HG1 MET E 128 26.958 −24.678 47.934 0.00 0.00
E
ATOM 28808 HG2 MET E 128 27.143 −25.265 46.305 0.00 0.00
E

ATOM 28809 SD MET E 128 29.183 -25.701 47.518 0.00 0.00
E
ATOM 28810 CE MET E 128 28.389 -27.345 47.747 0.00 0.00
E
ATOM 28811 HE1 MET E 128 27.673 -27.589 46.933 0.00 0.00
E
ATOM 28812 HE2 MET E 128 29.215 -28.085 47.811 0.00 0.00
E
ATOM 28813 HE3 MET E 128 27.946 -27.320 48.766 0.00 0.00
E
ATOM 28814 C MET E 128 28.102 -23.986 44.053 0.00 0.00
E
ATOM 28815 O MET E 128 28.171 -25.123 43.692 0.00 0.00
E
ATOM 28816 N GLY E 129 27.322 -23.100 43.330 0.00 0.00
E
ATOM 28817 HN GLY E 129 27.323 -22.149 43.628 0.00 0.00
E
ATOM 28818 CA GLY E 129 26.405 -23.498 42.230 0.00 0.00
E
ATOM 28819 HA1 GLY E 129 25.818 -22.639 41.938 0.00 0.00
E
ATOM 28820 HA2 GLY E 129 25.833 -24.335 42.604 0.00 0.00
E
ATOM 28821 C GLY E 129 27.176 -24.067 41.039 0.00 0.00
E
ATOM 28822 O GLY E 129 26.831 -25.047 40.292 0.00 0.00
E
ATOM 28823 N VAL E 130 28.314 -23.468 40.823 0.00 0.00
E
ATOM 28824 HN VAL E 130 28.619 -22.723 41.410 0.00 0.00
E
ATOM 28825 CA VAL E 130 29.393 -23.937 39.977 0.00 0.00
E
ATOM 28826 HA VAL E 130 29.039 -24.094 38.969 0.00 0.00
E
ATOM 28827 CB VAL E 130 30.519 -22.949 39.880 0.00 0.00
E
ATOM 28828 HB VAL E 130 31.047 -22.728 40.832 0.00 0.00
E
ATOM 28829 CG1 VAL E 130 31.659 -23.234 38.873 0.00 0.00
E
ATOM 28830 HG11 VAL E 130 32.193 -24.190 39.065 0.00 0.00
E
ATOM 28831 HG12 VAL E 130 31.242 -23.382 37.855 0.00 0.00
E
ATOM 28832 HG13 VAL E 130 32.443 -22.449 38.940 0.00 0.00
E
ATOM 28833 CG2 VAL E 130 29.880 -21.605 39.391 0.00 0.00
E
ATOM 28834 HG21 VAL E 130 29.342 -20.992 40.145 0.00 0.00
E
ATOM 28835 HG22 VAL E 130 30.721 -20.942 39.096 0.00 0.00
E

ATOM 28836 HG23 VAL E 130 29.217 −21.819 38.526 0.00 0.00
E
ATOM 28837 C VAL E 130 30.067 −25.220 40.368 0.00 0.00
E
ATOM 28838 O VAL E 130 30.298 −26.120 39.524 0.00 0.00
E
ATOM 28839 N LYS E 131 30.431 −25.445 41.603 0.00 0.00
E
ATOM 28840 HN LYS E 131 30.443 −24.640 42.191 0.00 0.00
E
ATOM 28841 CA LYS E 131 30.848 −26.726 42.129 0.00 0.00
E
ATOM 28842 HA LYS E 131 31.745 −27.000 41.593 0.00 0.00
E
ATOM 28843 CB LYS E 131 31.423 −26.611 43.547 0.00 0.00
E
ATOM 28844 HB1 LYS E 131 32.352 −26.003 43.506 0.00 0.00
E
ATOM 28845 HB2 LYS E 131 30.695 −26.197 44.278 0.00 0.00
E
ATOM 28846 CG LYS E 131 31.987 −27.881 44.204 0.00 0.00
E
ATOM 28847 HG1 LYS E 131 32.175 −27.611 45.266 0.00 0.00
E
ATOM 28848 HG2 LYS E 131 31.125 −28.576 44.294 0.00 0.00
E
ATOM 28849 CD LYS E 131 33.126 −28.582 43.489 0.00 0.00
E
ATOM 28850 HD1 LYS E 131 32.831 −28.767 42.434 0.00 0.00
E
ATOM 28851 HD2 LYS E 131 34.049 −27.976 43.369 0.00 0.00
E
ATOM 28852 CE LYS E 131 33.603 −29.903 44.175 0.00 0.00
E
ATOM 28853 HE1 LYS E 131 34.042 −29.669 45.169 0.00 0.00
E
ATOM 28854 HE2 LYS E 131 32.797 −30.668 44.192 0.00 0.00
E
ATOM 28855 NZ LYS E 131 34.671 −30.442 43.396 0.00 0.00
E
ATOM 28856 HZ1 LYS E 131 35.048 −31.207 43.991 0.00 0.00
E
ATOM 28857 HZ2 LYS E 131 34.344 −30.886 42.515 0.00 0.00
E
ATOM 28858 HZ3 LYS E 131 35.526 −29.863 43.268 0.00 0.00
E
ATOM 28859 C LYS E 131 29.735 −27.773 42.006 0.00 0.00
E
ATOM 28860 O LYS E 131 30.034 −28.974 41.720 0.00 0.00
E
ATOM 28861 N ILE E 132 28.482 −27.465 42.311 0.00 0.00
E
ATOM 28862 HN ILE E 132 28.323 −26.547 42.666 0.00 0.00
E

ATOM 28863 CA ILE E 132 27.434 -28.418 42.214 0.00 0.00
E
ATOM 28864 HA ILE E 132 27.706 -29.284 42.799 0.00 0.00
E
ATOM 28865 CB ILE E 132 26.137 -27.983 42.846 0.00 0.00
E
ATOM 28866 HB ILE E 132 25.935 -26.937 42.528 0.00 0.00
E
ATOM 28867 CG2 ILE E 132 24.924 -28.779 42.288 0.00 0.00
E
ATOM 28868 HG21 ILE E 132 24.801 -28.637 41.193 0.00 0.00
E
ATOM 28869 HG22 ILE E 132 25.193 -29.856 42.315 0.00 0.00
E
ATOM 28870 HG23 ILE E 132 23.936 -28.569 42.751 0.00 0.00
E
ATOM 28871 CG1 ILE E 132 26.218 -28.007 44.391 0.00 0.00
E
ATOM 28872 HG11 ILE E 132 26.907 -27.214 44.752 0.00 0.00
E
ATOM 28873 HG12 ILE E 132 25.263 -27.620 44.805 0.00 0.00
E
ATOM 28874 CD ILE E 132 26.689 -29.298 45.082 0.00 0.00
E
ATOM 28875 HD1 ILE E 132 27.769 -29.523 44.946 0.00 0.00
E
ATOM 28876 HD2 ILE E 132 26.562 -29.129 46.173 0.00 0.00
E
ATOM 28877 HD3 ILE E 132 26.102 -30.204 44.822 0.00 0.00
E
ATOM 28878 C ILE E 132 27.310 -28.872 40.725 0.00 0.00
E
ATOM 28879 O ILE E 132 27.334 -30.072 40.466 0.00 0.00
E
ATOM 28880 N ALA E 133 27.235 -27.931 39.745 0.00 0.00
E
ATOM 28881 HN ALA E 133 27.316 -27.008 40.112 0.00 0.00
E
ATOM 28882 CA ALA E 133 27.252 -28.063 38.286 0.00 0.00
E
ATOM 28883 HA ALA E 133 26.373 -28.600 37.960 0.00 0.00
E
ATOM 28884 CB ALA E 133 27.285 -26.695 37.642 0.00 0.00
E
ATOM 28885 HB1 ALA E 133 27.008 -26.730 36.566 0.00 0.00
E
ATOM 28886 HB2 ALA E 133 26.474 -26.098 38.111 0.00 0.00
E
ATOM 28887 HB3 ALA E 133 28.264 -26.187 37.779 0.00 0.00
E
ATOM 28888 C ALA E 133 28.326 -28.979 37.763 0.00 0.00
E
ATOM 28889 O ALA E 133 28.161 -29.842 36.862 0.00 0.00
E

ATOM 28890 N GLN E 134 29.575 −28.835 38.334 0.00 0.00
E
ATOM 28891 HN GLN E 134 29.649 −28.065 38.964 0.00 0.00
E
ATOM 28892 CA GLN E 134 30.632 −29.743 38.041 0.00 0.00
E
ATOM 28893 HA GLN E 134 30.722 −29.685 36.966 0.00 0.00
E
ATOM 28894 CB GLN E 134 31.866 −29.159 38.605 0.00 0.00
E
ATOM 28895 HB1 GLN E 134 32.013 −28.062 38.502 0.00 0.00
E
ATOM 28896 HB2 GLN E 134 31.864 −29.470 39.671 0.00 0.00
E
ATOM 28897 CG GLN E 134 33.139 −29.841 38.075 0.00 0.00
E
ATOM 28898 HG1 GLN E 134 33.143 −30.894 38.429 0.00 0.00
E
ATOM 28899 HG2 GLN E 134 33.142 −29.878 36.965 0.00 0.00
E
ATOM 28900 CD GLN E 134 34.416 −29.270 38.625 0.00 0.00
E
ATOM 28901 OE1 GLN E 134 35.029 −28.498 37.835 0.00 0.00
E
ATOM 28902 NE2 GLN E 134 34.841 −29.618 39.847 0.00 0.00
E
ATOM 28903 HE21 GLN E 134 35.597 −29.069 40.203 0.00 0.00
E
ATOM 28904 HE22 GLN E 134 34.310 −30.286 40.369 0.00 0.00
E
ATOM 28905 C GLN E 134 30.401 −31.173 38.431 0.00 0.00
E
ATOM 28906 O GLN E 134 30.547 −32.164 37.731 0.00 0.00
E
ATOM 28907 N SER E 135 30.099 −31.352 39.716 0.00 0.00
E
ATOM 28908 HN SER E 135 30.037 −30.542 40.293 0.00 0.00
E
ATOM 28909 CA SER E 135 29.850 −32.665 40.213 0.00 0.00
E
ATOM 28910 HA SER E 135 30.677 −33.295 39.923 0.00 0.00
E
ATOM 28911 CB SER E 135 29.612 −32.736 41.827 0.00 0.00
E
ATOM 28912 HB1 SER E 135 28.794 −32.071 42.179 0.00 0.00
E
ATOM 28913 HB2 SER E 135 29.278 −33.780 42.004 0.00 0.00
E
ATOM 28914 OG SER E 135 30.847 −32.578 42.516 0.00 0.00
E
ATOM 28915 HG1 SER E 135 30.680 −32.858 43.419 0.00 0.00
E
ATOM 28916 C SER E 135 28.612 −33.395 39.708 0.00 0.00
E

ATOM 28917 O SER E 135 28.499 -34.647 39.696 0.00 0.00
E
ATOM 28918 N LEU E 136 27.637 -32.572 39.289 0.00 0.00
E
ATOM 28919 HN LEU E 136 27.601 -31.608 39.540 0.00 0.00
E
ATOM 28920 CA LEU E 136 26.523 -33.112 38.433 0.00 0.00
E
ATOM 28921 HA LEU E 136 25.998 -33.867 39.001 0.00 0.00
E
ATOM 28922 CB LEU E 136 25.553 -32.002 38.060 0.00 0.00
E
ATOM 28923 HB1 LEU E 136 26.079 -31.080 37.733 0.00 0.00
E
ATOM 28924 HB2 LEU E 136 25.099 -32.367 37.114 0.00 0.00
E
ATOM 28925 CG LEU E 136 24.418 -31.638 39.042 0.00 0.00
E
ATOM 28926 HG LEU E 136 24.873 -31.169 39.940 0.00 0.00
E
ATOM 28927 CD1 LEU E 136 23.458 -30.577 38.512 0.00 0.00
E
ATOM 28928 HD11 LEU E 136 22.975 -30.988 37.600 0.00 0.00
E
ATOM 28929 HD12 LEU E 136 22.679 -30.372 39.278 0.00 0.00
E
ATOM 28930 HD13 LEU E 136 23.959 -29.599 38.350 0.00 0.00
E
ATOM 28931 CD2 LEU E 136 23.570 -32.859 39.372 0.00 0.00
E
ATOM 28932 HD21 LEU E 136 24.105 -33.611 39.991 0.00 0.00
E
ATOM 28933 HD22 LEU E 136 22.572 -32.663 39.819 0.00 0.00
E
ATOM 28934 HD23 LEU E 136 23.397 -33.373 38.402 0.00 0.00
E
ATOM 28935 C LEU E 136 27.045 -33.757 37.160 0.00 0.00
E
ATOM 28936 O LEU E 136 26.775 -34.939 36.903 0.00 0.00
E
ATOM 28937 N SER E 137 27.896 -33.042 36.349 0.00 0.00
E
ATOM 28938 HN SER E 137 28.090 -32.101 36.613 0.00 0.00
E
ATOM 28939 CA SER E 137 28.569 -33.388 35.099 0.00 0.00
E
ATOM 28940 HA SER E 137 27.816 -33.576 34.348 0.00 0.00
E
ATOM 28941 CB SER E 137 29.420 -32.170 34.572 0.00 0.00
E
ATOM 28942 HB1 SER E 137 28.661 -31.362 34.499 0.00 0.00
E
ATOM 28943 HB2 SER E 137 30.247 -31.962 35.284 0.00 0.00
E

ATOM 28944 OG SER E 137   30.060 -32.418 33.374 0.00 0.00
E
ATOM 28945 HG1 SER E 137   30.805 -31.818 33.301 0.00 0.00
E
ATOM 28946 C   SER E 137   29.549 -34.582 35.306 0.00 0.00
E
ATOM 28947 O   SER E 137   29.491 -35.583 34.568 0.00 0.00
E
ATOM 28948 N   ASP E 138   30.385 -34.566 36.388 0.00 0.00
E
ATOM 28949 HN ASP E 138   30.361 -33.803 37.029 0.00 0.00
E
ATOM 28950 CA ASP E 138   31.345 -35.573 36.637 0.00 0.00
E
ATOM 28951 HA ASP E 138   31.144 -36.386 35.955 0.00 0.00
E
ATOM 28952 CB ASP E 138   32.790 -35.072 36.372 0.00 0.00
E
ATOM 28953 HB1 ASP E 138   32.807 -34.005 36.682 0.00 0.00
E
ATOM 28954 HB2 ASP E 138   33.570 -35.707 36.844 0.00 0.00
E
ATOM 28955 CG ASP E 138   33.101 -35.036 34.910 0.00 0.00
E
ATOM 28956 OD1 ASP E 138   33.097 -36.148 34.308 0.00 0.00
E
ATOM 28957 OD2 ASP E 138   33.357 -33.953 34.259 0.00 0.00
E
ATOM 28958 C   ASP E 138   31.268 -36.073 38.117 0.00 0.00
E
ATOM 28959 O   ASP E 138   31.659 -35.443 39.067 0.00 0.00
E
ATOM 28960 N   GLU E 139   30.956 -37.348 38.265 0.00 0.00
E
ATOM 28961 HN GLU E 139   30.870 -37.937 37.465 0.00 0.00
E
ATOM 28962 CA GLU E 139   30.857 -37.891 39.609 0.00 0.00
E
ATOM 28963 HA GLU E 139   30.964 -37.177 40.413 0.00 0.00
E
ATOM 28964 CB GLU E 139   29.393 -38.359 39.793 0.00 0.00
E
ATOM 28965 HB1 GLU E 139   29.330 -38.725 40.840 0.00 0.00
E
ATOM 28966 HB2 GLU E 139   28.785 -37.439 39.654 0.00 0.00
E
ATOM 28967 CG GLU E 139   28.916 -39.498 38.804 0.00 0.00
E
ATOM 28968 HG1 GLU E 139   29.060 -39.259 37.728 0.00 0.00
E
ATOM 28969 HG2 GLU E 139   29.568 -40.332 39.139 0.00 0.00
E
ATOM 28970 CD GLU E 139   27.508 -39.917 39.103 0.00 0.00
E

ATOM 28971 OE1 GLU E 139 26.649 −40.027 38.199 0.00 0.00
E
ATOM 28972 OE2 GLU E 139 27.193 −40.300 40.306 0.00 0.00
E
ATOM 28973 C GLU E 139 31.878 −39.001 39.812 0.00 0.00
E
ATOM 28974 O GLU E 139 31.921 −39.530 40.931 0.00 0.00
E
ATOM 28975 N ASN E 140 32.788 −39.370 38.892 0.00 0.00
E
ATOM 28976 HN ASN E 140 32.728 −38.932 37.998 0.00 0.00
E
ATOM 28977 CA ASN E 140 33.876 −40.227 39.201 0.00 0.00
E
ATOM 28978 HA ASN E 140 33.744 −40.659 40.182 0.00 0.00
E
ATOM 28979 CB ASN E 140 33.793 −41.559 38.315 0.00 0.00
E
ATOM 28980 HB1 ASN E 140 34.040 −41.333 37.256 0.00 0.00
E
ATOM 28981 HB2 ASN E 140 34.525 −42.297 38.709 0.00 0.00
E
ATOM 28982 CG ASN E 140 32.451 −42.281 38.423 0.00 0.00
E
ATOM 28983 OD1 ASN E 140 31.979 −42.697 39.507 0.00 0.00
E
ATOM 28984 ND2 ASN E 140 31.724 −42.454 37.272 0.00 0.00
E
ATOM 28985 HD21 ASN E 140 30.824 −42.876 37.379 0.00 0.00
E
ATOM 28986 HD22 ASN E 140 32.068 −42.014 36.443 0.00 0.00
E
ATOM 28987 C ASN E 140 35.200 −39.528 39.138 0.00 0.00
E
ATOM 28988 O ASN E 140 36.073 −40.103 39.796 0.00 0.00
E
ATOM 28989 N VAL E 141 35.364 −38.379 38.493 0.00 0.00
E
ATOM 28990 HN VAL E 141 34.636 −37.979 37.941 0.00 0.00
E
ATOM 28991 CA VAL E 141 36.521 −37.549 38.570 0.00 0.00
E
ATOM 28992 HA VAL E 141 37.359 −38.219 38.440 0.00 0.00
E
ATOM 28993 CB VAL E 141 36.625 −36.535 37.461 0.00 0.00
E
ATOM 28994 HB VAL E 141 35.755 −35.853 37.568 0.00 0.00
E
ATOM 28995 CG1 VAL E 141 37.910 −35.685 37.788 0.00 0.00
E
ATOM 28996 HG11 VAL E 141 37.732 −35.057 38.687 0.00 0.00
E
ATOM 28997 HG12 VAL E 141 38.762 −36.330 38.090 0.00 0.00
E

ATOM 28998 HG13 VAL E 141   38.275 -35.091 36.923 0.00 0.00
E
ATOM 28999 CG2 VAL E 141   36.826 -37.319 36.156 0.00 0.00
E
ATOM 29000 HG21 VAL E 141   37.807 -37.832 36.254 0.00 0.00
E
ATOM 29001 HG22 VAL E 141   35.896 -37.907 36.004 0.00 0.00
E
ATOM 29002 HG23 VAL E 141   37.004 -36.800 35.190 0.00 0.00
E
ATOM 29003 C   VAL E 141   36.568 -36.883 39.905 0.00 0.00
E
ATOM 29004 O   VAL E 141   35.760 -35.992 40.048 0.00 0.00
E
ATOM 29005 N   LEU E 142   37.433 -37.192 40.838 0.00 0.00
E
ATOM 29006 HN LEU E 142   38.186 -37.825 40.681 0.00 0.00
E
ATOM 29007 CA LEU E 142   37.389 -36.519 42.098 0.00 0.00
E
ATOM 29008 HA LEU E 142   36.366 -36.206 42.250 0.00 0.00
E
ATOM 29009 CB LEU E 142   37.659 -37.532 43.187 0.00 0.00
E
ATOM 29010 HB1 LEU E 142   38.742 -37.696 43.003 0.00 0.00
E
ATOM 29011 HB2 LEU E 142   37.439 -37.273 44.244 0.00 0.00
E
ATOM 29012 CG LEU E 142   37.022 -38.978 43.126 0.00 0.00
E
ATOM 29013 HG LEU E 142   37.285 -39.490 42.176 0.00 0.00
E
ATOM 29014 CD1 LEU E 142   37.535 -39.862 44.267 0.00 0.00
E
ATOM 29015 HD11 LEU E 142   38.645 -39.847 44.221 0.00 0.00
E
ATOM 29016 HD12 LEU E 142   37.326 -39.346 45.229 0.00 0.00
E
ATOM 29017 HD13 LEU E 142   37.202 -40.922 44.273 0.00 0.00
E
ATOM 29018 CD2 LEU E 142   35.482 -38.851 43.320 0.00 0.00
E
ATOM 29019 HD21 LEU E 142   35.290 -38.476 44.348 0.00 0.00
E
ATOM 29020 HD22 LEU E 142   34.981 -38.238 42.540 0.00 0.00
E
ATOM 29021 HD23 LEU E 142   35.081 -39.886 43.343 0.00 0.00
E
ATOM 29022 C   LEU E 142   38.202 -35.219 42.227 0.00 0.00
E
ATOM 29023 O   LEU E 142   37.798 -34.324 42.931 0.00 0.00
E
ATOM 29024 N   ASN E 143   39.331 -35.135 41.523 0.00 0.00
E

```
ATOM  29025  HN  ASN E 143      39.511 -35.806  40.808  0.00  0.00
E
ATOM  29026  CA  ASN E 143      40.156 -33.997  41.380  0.00  0.00
E
ATOM  29027  HA  ASN E 143      39.591 -33.078  41.440  0.00  0.00
E
ATOM  29028  CB  ASN E 143      41.379 -33.961  42.355  0.00  0.00
E
ATOM  29029  HB1 ASN E 143      41.996 -34.883  42.297  0.00  0.00
E
ATOM  29030  HB2 ASN E 143      42.142 -33.183  42.139  0.00  0.00
E
ATOM  29031  CG  ASN E 143      40.977 -33.594  43.781  0.00  0.00
E
ATOM  29032  OD1 ASN E 143      40.752 -32.405  44.091  0.00  0.00
E
ATOM  29033  ND2 ASN E 143      40.924 -34.590  44.703  0.00  0.00
E
ATOM  29034 HD21 ASN E 143      40.590 -34.364  45.618  0.00  0.00
E
ATOM  29035 HD22 ASN E 143      41.270 -35.504  44.493  0.00  0.00
E
ATOM  29036  C   ASN E 143      40.609 -34.040  39.921  0.00  0.00
E
ATOM  29037  O   ASN E 143      40.778 -35.060  39.290  0.00  0.00
E
ATOM  29038  N   TYR E 144      40.928 -32.896  39.363  0.00  0.00
E
ATOM  29039  HN  TYR E 144      40.791 -32.048  39.870  0.00  0.00
E
ATOM  29040  CA  TYR E 144      41.641 -32.797  38.120  0.00  0.00
E
ATOM  29041  HA  TYR E 144      42.245 -33.691  38.065  0.00  0.00
E
ATOM  29042  CB  TYR E 144      40.689 -32.914  36.855  0.00  0.00
E
ATOM  29043  HB1 TYR E 144      41.246 -33.171  35.929  0.00  0.00
E
ATOM  29044  HB2 TYR E 144      39.881 -33.663  37.002  0.00  0.00
E
ATOM  29045  CG  TYR E 144      39.795 -31.667  36.748  0.00  0.00
E
ATOM  29046  CD1 TYR E 144      38.545 -31.760  37.346  0.00  0.00
E
ATOM  29047  HD1 TYR E 144      38.359 -32.486  38.123  0.00  0.00
E
ATOM  29048  CE1 TYR E 144      37.696 -30.620  37.159  0.00  0.00
E
ATOM  29049  HE1 TYR E 144      36.766 -30.666  37.706  0.00  0.00
E
ATOM  29050  CZ  TYR E 144      37.996 -29.526  36.334  0.00  0.00
E
ATOM  29051  OH  TYR E 144      37.100 -28.498  36.185  0.00  0.00
E
```

ATOM 29052 HH TYR E 144 36.257 -28.683 36.607 0.00 0.00
E
ATOM 29053 CD2 TYR E 144 40.106 -30.685 35.803 0.00 0.00
E
ATOM 29054 HD2 TYR E 144 40.959 -30.775 35.147 0.00 0.00
E
ATOM 29055 CE2 TYR E 144 39.204 -29.657 35.502 0.00 0.00
E
ATOM 29056 HE2 TYR E 144 39.585 -28.863 34.877 0.00 0.00
E
ATOM 29057 C TYR E 144 42.616 -31.692 37.998 0.00 0.00
E
ATOM 29058 O TYR E 144 42.709 -30.748 38.813 0.00 0.00
E
ATOM 29059 N ILE E 145 43.518 -31.780 37.006 0.00 0.00
E
ATOM 29060 HN ILE E 145 43.610 -32.639 36.508 0.00 0.00
E
ATOM 29061 CA ILE E 145 44.268 -30.663 36.469 0.00 0.00
E
ATOM 29062 HA ILE E 145 43.922 -29.748 36.927 0.00 0.00
E
ATOM 29063 CB ILE E 145 45.784 -30.782 36.776 0.00 0.00
E
ATOM 29064 HB ILE E 145 46.262 -31.651 36.276 0.00 0.00
E
ATOM 29065 CG2 ILE E 145 46.488 -29.543 36.110 0.00 0.00
E
ATOM 29066 HG21 ILE E 145 47.563 -29.616 36.378 0.00 0.00
E
ATOM 29067 HG22 ILE E 145 46.448 -29.501 35.000 0.00 0.00
E
ATOM 29068 HG23 ILE E 145 46.103 -28.632 36.617 0.00 0.00
E
ATOM 29069 CG1 ILE E 145 45.966 -30.817 38.391 0.00 0.00
E
ATOM 29070 HG11 ILE E 145 45.514 -29.888 38.798 0.00 0.00
E
ATOM 29071 HG12 ILE E 145 45.435 -31.729 38.738 0.00 0.00
E
ATOM 29072 CD ILE E 145 47.412 -30.990 38.735 0.00 0.00
E
ATOM 29073 HD1 ILE E 145 47.421 -31.213 39.824 0.00 0.00
E
ATOM 29074 HD2 ILE E 145 47.949 -31.854 38.289 0.00 0.00
E
ATOM 29075 HD3 ILE E 145 47.962 -30.039 38.568 0.00 0.00
E
ATOM 29076 C ILE E 145 43.983 -30.585 35.032 0.00 0.00
E
ATOM 29077 O ILE E 145 44.079 -31.535 34.282 0.00 0.00
E
ATOM 29078 N ASP E 146 43.440 -29.491 34.538 0.00 0.00
E

ATOM 29079 HN ASP E 146 43.228 -28.734 35.152 0.00 0.00
E
ATOM 29080 CA ASP E 146 43.126 -29.430 33.091 0.00 0.00
E
ATOM 29081 HA ASP E 146 42.624 -30.357 32.858 0.00 0.00
E
ATOM 29082 CB ASP E 146 42.068 -28.333 32.839 0.00 0.00
E
ATOM 29083 HB1 ASP E 146 41.172 -28.493 33.475 0.00 0.00
E
ATOM 29084 HB2 ASP E 146 42.559 -27.430 33.260 0.00 0.00
E
ATOM 29085 CG ASP E 146 41.573 -28.217 31.430 0.00 0.00
E
ATOM 29086 OD1 ASP E 146 40.410 -28.632 31.141 0.00 0.00
E
ATOM 29087 OD2 ASP E 146 42.281 -27.639 30.590 0.00 0.00
E
ATOM 29088 C ASP E 146 44.398 -29.402 32.262 0.00 0.00
E
ATOM 29089 O ASP E 146 45.290 -28.572 32.472 0.00 0.00
E
ATOM 29090 N LEU E 147 44.495 -30.286 31.178 0.00 0.00
E
ATOM 29091 HN LEU E 147 43.724 -30.880 30.961 0.00 0.00
E
ATOM 29092 CA LEU E 147 45.715 -30.571 30.490 0.00 0.00
E
ATOM 29093 HA LEU E 147 45.925 -29.771 29.795 0.00 0.00
E
ATOM 29094 CB LEU E 147 46.960 -30.761 31.429 0.00 0.00
E
ATOM 29095 HB1 LEU E 147 47.205 -29.799 31.928 0.00 0.00
E
ATOM 29096 HB2 LEU E 147 46.936 -31.618 32.135 0.00 0.00
E
ATOM 29097 CG LEU E 147 48.400 -31.168 30.853 0.00 0.00
E
ATOM 29098 HG LEU E 147 48.226 -32.141 30.345 0.00 0.00
E
ATOM 29099 CD1 LEU E 147 48.816 -30.105 29.791 0.00 0.00
E
ATOM 29100 HD11 LEU E 147 49.825 -30.291 29.367 0.00 0.00
E
ATOM 29101 HD12 LEU E 147 48.090 -29.941 28.966 0.00 0.00
E
ATOM 29102 HD13 LEU E 147 48.650 -29.094 30.221 0.00 0.00
E
ATOM 29103 CD2 LEU E 147 49.456 -31.339 31.938 0.00 0.00
E
ATOM 29104 HD21 LEU E 147 50.440 -31.620 31.506 0.00 0.00
E
ATOM 29105 HD22 LEU E 147 49.641 -30.484 32.623 0.00 0.00
E

ATOM 29106 HD23 LEU E 147 49.101 -32.237 32.488 0.00 0.00
E
ATOM 29107 C LEU E 147 45.515 -31.897 29.626 0.00 0.00
E
ATOM 29108 O LEU E 147 46.045 -31.973 28.532 0.00 0.00
E
ATOM 29109 N SER E 152 44.825 -32.973 30.117 0.00 0.00
E
ATOM 29110 HN SER E 152 44.946 -33.780 29.543 0.00 0.00
E
ATOM 29111 CA SER E 152 44.136 -33.153 31.350 0.00 0.00
E
ATOM 29112 HA SER E 152 44.329 -32.359 32.056 0.00 0.00
E
ATOM 29113 CB SER E 152 42.600 -33.303 31.225 0.00 0.00
E
ATOM 29114 HB1 SER E 152 42.388 -34.224 30.641 0.00 0.00
E
ATOM 29115 HB2 SER E 152 42.184 -33.164 32.245 0.00 0.00
E
ATOM 29116 OG SER E 152 42.163 -32.225 30.401 0.00 0.00
E
ATOM 29117 HG1 SER E 152 42.096 -32.482 29.478 0.00 0.00
E
ATOM 29118 C SER E 152 44.722 -34.282 32.163 0.00 0.00
E
ATOM 29119 O SER E 152 45.055 -35.298 31.611 0.00 0.00
E
ATOM 29120 N ILE E 153 44.792 -34.187 33.501 0.00 0.00
E
ATOM 29121 HN ILE E 153 44.493 -33.371 33.989 0.00 0.00
E
ATOM 29122 CA ILE E 153 45.145 -35.287 34.451 0.00 0.00
E
ATOM 29123 HA ILE E 153 45.143 -36.265 33.992 0.00 0.00
E
ATOM 29124 CB ILE E 153 46.390 -35.006 35.269 0.00 0.00
E
ATOM 29125 HB ILE E 153 46.157 -34.142 35.927 0.00 0.00
E
ATOM 29126 CG2 ILE E 153 46.786 -36.247 36.052 0.00 0.00
E
ATOM 29127 HG21 ILE E 153 46.949 -37.064 35.318 0.00 0.00
E
ATOM 29128 HG22 ILE E 153 47.666 -36.141 36.723 0.00 0.00
E
ATOM 29129 HG23 ILE E 153 45.906 -36.519 36.674 0.00 0.00
E
ATOM 29130 CG1 ILE E 153 47.602 -34.727 34.495 0.00 0.00
E
ATOM 29131 HG11 ILE E 153 48.125 -35.587 34.026 0.00 0.00
E
ATOM 29132 HG12 ILE E 153 47.288 -33.994 33.721 0.00 0.00
E

ATOM 29133 CD ILE E 153 48.645 -34.029 35.325 0.00 0.00
E
ATOM 29134 HD1 ILE E 153 48.260 -33.207 35.965 0.00 0.00
E
ATOM 29135 HD2 ILE E 153 49.143 -34.774 35.982 0.00 0.00
E
ATOM 29136 HD3 ILE E 153 49.419 -33.550 34.690 0.00 0.00
E
ATOM 29137 C ILE E 153 44.032 -35.325 35.477 0.00 0.00
E
ATOM 29138 O ILE E 153 43.551 -34.279 35.958 0.00 0.00
E
ATOM 29139 N VAL E 154 43.484 -36.517 35.763 0.00 0.00
E
ATOM 29140 HN VAL E 154 43.830 -37.369 35.378 0.00 0.00
E
ATOM 29141 CA VAL E 154 42.440 -36.802 36.696 0.00 0.00
E
ATOM 29142 HA VAL E 154 42.148 -35.883 37.182 0.00 0.00
E
ATOM 29143 CB VAL E 154 41.187 -37.349 36.007 0.00 0.00
E
ATOM 29144 HB VAL E 154 40.437 -37.580 36.793 0.00 0.00
E
ATOM 29145 CG1 VAL E 154 40.533 -36.265 35.072 0.00 0.00
E
ATOM 29146 HG11 VAL E 154 39.722 -36.761 34.497 0.00 0.00
E
ATOM 29147 HG12 VAL E 154 40.083 -35.341 35.494 0.00 0.00
E
ATOM 29148 HG13 VAL E 154 41.254 -35.863 34.328 0.00 0.00
E
ATOM 29149 CG2 VAL E 154 41.569 -38.531 35.245 0.00 0.00
E
ATOM 29150 HG21 VAL E 154 42.067 -39.308 35.863 0.00 0.00
E
ATOM 29151 HG22 VAL E 154 40.580 -38.959 34.971 0.00 0.00
E
ATOM 29152 HG23 VAL E 154 42.236 -38.400 34.367 0.00 0.00
E
ATOM 29153 C VAL E 154 42.836 -37.686 37.814 0.00 0.00
E
ATOM 29154 O VAL E 154 43.668 -38.594 37.647 0.00 0.00
E
ATOM 29155 N GLU E 155 42.162 -37.556 38.933 0.00 0.00
E
ATOM 29156 HN GLU E 155 41.709 -36.670 39.005 0.00 0.00
E
ATOM 29157 CA GLU E 155 41.967 -38.625 39.926 0.00 0.00
E
ATOM 29158 HA GLU E 155 42.743 -39.372 39.850 0.00 0.00
E
ATOM 29159 CB GLU E 155 42.028 -38.025 41.361 0.00 0.00
E

ATOM 29160 HB1 GLU E 155   43.082 -37.710 41.519 0.00 0.00
E
ATOM 29161 HB2 GLU E 155   41.368 -37.134 41.430 0.00 0.00
E
ATOM 29162 CG GLU E 155   41.758 -39.062 42.421 0.00 0.00
E
ATOM 29163 HG1 GLU E 155   40.686 -39.325 42.297 0.00 0.00
E
ATOM 29164 HG2 GLU E 155   42.423 -39.938 42.264 0.00 0.00
E
ATOM 29165 CD GLU E 155   41.873 -38.470 43.829 0.00 0.00
E
ATOM 29166 OE1 GLU E 155   42.473 -39.119 44.711 0.00 0.00
E
ATOM 29167 OE2 GLU E 155   41.173 -37.407 44.058 0.00 0.00
E
ATOM 29168 C   GLU E 155   40.651 -39.255 39.477 0.00 0.00
E
ATOM 29169 O   GLU E 155   39.650 -38.586 39.474 0.00 0.00
E
ATOM 29170 N   LEU E 156   40.647 -40.521 39.133 0.00 0.00
E
ATOM 29171 HN LEU E 156   41.400 -41.116 39.405 0.00 0.00
E
ATOM 29172 CA LEU E 156   39.422 -41.165 38.762 0.00 0.00
E
ATOM 29173 HA LEU E 156   38.534 -40.561 38.875 0.00 0.00
E
ATOM 29174 CB LEU E 156   39.490 -41.602 37.360 0.00 0.00
E
ATOM 29175 HB1 LEU E 156   39.815 -40.746 36.731 0.00 0.00
E
ATOM 29176 HB2 LEU E 156   40.201 -42.440 37.196 0.00 0.00
E
ATOM 29177 CG LEU E 156   38.076 -42.086 36.760 0.00 0.00
E
ATOM 29178 HG LEU E 156   37.486 -42.608 37.543 0.00 0.00
E
ATOM 29179 CD1 LEU E 156   37.266 -40.891 36.377 0.00 0.00
E
ATOM 29180 HD11 LEU E 156   37.753 -40.430 35.492 0.00 0.00
E
ATOM 29181 HD12 LEU E 156   36.246 -41.170 36.035 0.00 0.00
E
ATOM 29182 HD13 LEU E 156   37.109 -40.127 37.168 0.00 0.00
E
ATOM 29183 CD2 LEU E 156   38.280 -42.973 35.525 0.00 0.00
E
ATOM 29184 HD21 LEU E 156   38.609 -43.985 35.843 0.00 0.00
E
ATOM 29185 HD22 LEU E 156   37.284 -43.081 35.044 0.00 0.00
E
ATOM 29186 HD23 LEU E 156   39.068 -42.651 34.811 0.00 0.00
E

ATOM 29187 C LEU E 156 39.053 -42.394 39.609 0.00 0.00
E
ATOM 29188 O LEU E 156 39.825 -43.326 39.779 0.00 0.00
E
ATOM 29189 N LEU E 157 37.837 -42.443 40.157 0.00 0.00
E
ATOM 29190 HN LEU E 157 37.273 -41.622 40.105 0.00 0.00
E
ATOM 29191 CA LEU E 157 37.355 -43.584 40.993 0.00 0.00
E
ATOM 29192 HA LEU E 157 38.183 -44.223 41.265 0.00 0.00
E
ATOM 29193 CB LEU E 157 36.599 -43.060 42.250 0.00 0.00
E
ATOM 29194 HB1 LEU E 157 37.247 -42.233 42.609 0.00 0.00
E
ATOM 29195 HB2 LEU E 157 35.635 -42.601 41.941 0.00 0.00
E
ATOM 29196 CG LEU E 157 36.376 -44.183 43.289 0.00 0.00
E
ATOM 29197 HG LEU E 157 35.795 -44.952 42.737 0.00 0.00
E
ATOM 29198 CD1 LEU E 157 37.668 -44.851 43.863 0.00 0.00
E
ATOM 29199 HD11 LEU E 157 38.246 -43.970 44.215 0.00 0.00
E
ATOM 29200 HD12 LEU E 157 37.581 -45.584 44.693 0.00 0.00
E
ATOM 29201 HD13 LEU E 157 38.129 -45.432 43.036 0.00 0.00
E
ATOM 29202 CD2 LEU E 157 35.535 -43.656 44.470 0.00 0.00
E
ATOM 29203 HD21 LEU E 157 36.210 -43.028 45.090 0.00 0.00
E
ATOM 29204 HD22 LEU E 157 34.557 -43.271 44.109 0.00 0.00
E
ATOM 29205 HD23 LEU E 157 35.242 -44.474 45.162 0.00 0.00
E
ATOM 29206 C LEU E 157 36.515 -44.643 40.121 0.00 0.00
E
ATOM 29207 O LEU E 157 35.564 -44.247 39.506 0.00 0.00
E
ATOM 29208 N LEU E 162 36.921 -45.943 39.904 0.00 0.00
E
ATOM 29209 HN LEU E 162 37.771 -46.334 40.248 0.00 0.00
E
ATOM 29210 CA LEU E 162 36.421 -46.688 38.858 0.00 0.00
E
ATOM 29211 HA LEU E 162 35.432 -46.352 38.587 0.00 0.00
E
ATOM 29212 CB LEU E 162 37.301 -46.610 37.610 0.00 0.00
E
ATOM 29213 HB1 LEU E 162 37.864 -45.655 37.540 0.00 0.00
E

ATOM 29214 HB2 LEU E 162   38.184 -47.283 37.663 0.00 0.00
E
ATOM 29215 CG LEU E 162   36.431 -46.735 36.300 0.00 0.00
E
ATOM 29216 HG LEU E 162   35.945 -47.734 36.311 0.00 0.00
E
ATOM 29217 CD1 LEU E 162   35.312 -45.695 36.184 0.00 0.00
E
ATOM 29218 HD11 LEU E 162   34.615 -45.800 37.043 0.00 0.00
E
ATOM 29219 HD12 LEU E 162   35.642 -44.634 36.156 0.00 0.00
E
ATOM 29220 HD13 LEU E 162   34.665 -45.867 35.297 0.00 0.00
E
ATOM 29221 CD2 LEU E 162   37.319 -46.720 35.037 0.00 0.00
E
ATOM 29222 HD21 LEU E 162   36.750 -46.910 34.102 0.00 0.00
E
ATOM 29223 HD22 LEU E 162   37.744 -45.694 35.033 0.00 0.00
E
ATOM 29224 HD23 LEU E 162   38.197 -47.388 35.171 0.00 0.00
E
ATOM 29225 C   LEU E 162   36.224 -48.170 39.217 0.00 0.00
E
ATOM 29226 O   LEU E 162   37.025 -48.794 39.914 0.00 0.00
E
ATOM 29227 N   ASP E 163   35.146 -48.756 38.832 0.00 0.00
E
ATOM 29228 HN ASP E 163   34.404 -48.255 38.394 0.00 0.00
E
ATOM 29229 CA ASP E 163   34.876 -50.226 38.855 0.00 0.00
E
ATOM 29230 HA ASP E 163   34.901 -50.498 39.900 0.00 0.00
E
ATOM 29231 CB ASP E 163   33.472 -50.528 38.382 0.00 0.00
E
ATOM 29232 HB1 ASP E 163   33.341 -50.217 37.324 0.00 0.00
E
ATOM 29233 HB2 ASP E 163   33.319 -51.629 38.369 0.00 0.00
E
ATOM 29234 CG ASP E 163   32.370 -49.980 39.268 0.00 0.00
E
ATOM 29235 OD1 ASP E 163   31.666 -49.063 38.810 0.00 0.00
E
ATOM 29236 OD2 ASP E 163   32.101 -50.527 40.350 0.00 0.00
E
ATOM 29237 C   ASP E 163   35.887 -51.053 38.058 0.00 0.00
E
ATOM 29238 O   ASP E 163   36.386 -50.742 36.994 0.00 0.00
E
ATOM 29239 N   SER E 164   36.221 -52.236 38.601 0.00 0.00
E
ATOM 29240 HN SER E 164   35.939 -52.678 39.450 0.00 0.00
E

ATOM 29241 CA SER E 164 37.078 -53.131 37.803 0.00 0.00
E
ATOM 29242 HA SER E 164 37.943 -52.631 37.393 0.00 0.00
E
ATOM 29243 CB SER E 164 37.593 -54.388 38.512 0.00 0.00
E
ATOM 29244 HB1 SER E 164 38.319 -54.131 39.312 0.00 0.00
E
ATOM 29245 HB2 SER E 164 36.739 -54.980 38.903 0.00 0.00
E
ATOM 29246 OG SER E 164 38.413 -55.252 37.634 0.00 0.00
E
ATOM 29247 HG1 SER E 164 39.250 -55.168 38.097 0.00 0.00
E
ATOM 29248 C SER E 164 36.236 -53.722 36.596 0.00 0.00
E
ATOM 29249 O SER E 164 35.047 -53.991 36.582 0.00 0.00
E
ATOM 29250 N LYS E 165 36.989 -53.951 35.461 0.00 0.00
E
ATOM 29251 HN LYS E 165 37.977 -53.813 35.485 0.00 0.00
E
ATOM 29252 CA LYS E 165 36.435 -54.542 34.274 0.00 0.00
E
ATOM 29253 HA LYS E 165 35.760 -55.313 34.614 0.00 0.00
E
ATOM 29254 CB LYS E 165 35.785 -53.592 33.349 0.00 0.00
E
ATOM 29255 HB1 LYS E 165 35.088 -53.189 34.115 0.00 0.00
E
ATOM 29256 HB2 LYS E 165 36.500 -52.834 32.965 0.00 0.00
E
ATOM 29257 CG LYS E 165 35.104 -54.200 32.117 0.00 0.00
E
ATOM 29258 HG1 LYS E 165 35.860 -54.667 31.450 0.00 0.00
E
ATOM 29259 HG2 LYS E 165 34.473 -55.014 32.534 0.00 0.00
E
ATOM 29260 CD LYS E 165 34.454 -53.118 31.324 0.00 0.00
E
ATOM 29261 HD1 LYS E 165 33.768 -52.555 31.992 0.00 0.00
E
ATOM 29262 HD2 LYS E 165 35.247 -52.487 30.867 0.00 0.00
E
ATOM 29263 CE LYS E 165 33.553 -53.681 30.174 0.00 0.00
E
ATOM 29264 HE1 LYS E 165 34.009 -53.583 29.166 0.00 0.00
E
ATOM 29265 HE2 LYS E 165 33.208 -54.663 30.564 0.00 0.00
E
ATOM 29266 NZ LYS E 165 32.275 -52.879 29.970 0.00 0.00
E
ATOM 29267 HZ1 LYS E 165 31.566 -53.460 29.479 0.00 0.00
E

ATOM 29268 HZ2 LYS E 165 31.906 -52.514 30.871 0.00 0.00
E
ATOM 29269 HZ3 LYS E 165 32.503 -52.222 29.197 0.00 0.00
E
ATOM 29270 C LYS E 165 37.569 -55.294 33.627 0.00 0.00
E
ATOM 29271 O LYS E 165 38.775 -54.951 33.820 0.00 0.00
E
ATOM 29272 N SER E 166 37.315 -56.411 32.912 0.00 0.00
E
ATOM 29273 HN SER E 166 36.429 -56.853 33.030 0.00 0.00
E
ATOM 29274 CA SER E 166 38.298 -57.228 32.238 0.00 0.00
E
ATOM 29275 HA SER E 166 38.925 -57.695 32.983 0.00 0.00
E
ATOM 29276 CB SER E 166 37.580 -58.364 31.453 0.00 0.00
E
ATOM 29277 HB1 SER E 166 37.038 -57.835 30.640 0.00 0.00
E
ATOM 29278 HB2 SER E 166 38.371 -58.908 30.894 0.00 0.00
E
ATOM 29279 OG SER E 166 36.740 -59.230 32.232 0.00 0.00
E
ATOM 29280 HG1 SER E 166 36.238 -59.728 31.583 0.00 0.00
E
ATOM 29281 C SER E 166 39.092 -56.465 31.221 0.00 0.00
E
ATOM 29282 O SER E 166 38.641 -55.510 30.605 0.00 0.00
E
ATOM 29283 N ILE E 167 40.371 -56.864 31.049 0.00 0.00
E
ATOM 29284 HN ILE E 167 40.659 -57.736 31.437 0.00 0.00
E
ATOM 29285 CA ILE E 167 41.343 -56.007 30.513 0.00 0.00
E
ATOM 29286 HA ILE E 167 41.261 -55.067 31.038 0.00 0.00
E
ATOM 29287 CB ILE E 167 42.721 -56.543 30.765 0.00 0.00
E
ATOM 29288 HB ILE E 167 42.634 -56.920 31.806 0.00 0.00
E
ATOM 29289 CG2 ILE E 167 43.129 -57.658 29.793 0.00 0.00
E
ATOM 29290 HG21 ILE E 167 42.377 -58.469 29.690 0.00 0.00
E
ATOM 29291 HG22 ILE E 167 43.364 -57.332 28.758 0.00 0.00
E
ATOM 29292 HG23 ILE E 167 44.092 -58.113 30.109 0.00 0.00
E
ATOM 29293 CG1 ILE E 167 43.775 -55.401 30.756 0.00 0.00
E
ATOM 29294 HG11 ILE E 167 44.807 -55.779 30.592 0.00 0.00
E

```
ATOM  29295 HG12 ILE E 167      43.601 -54.653  29.954  0.00  0.00
E
ATOM  29296  CD  ILE E 167      43.850 -54.639  32.120  0.00  0.00
E
ATOM  29297  HD1 ILE E 167      44.157 -55.327  32.938  0.00  0.00
E
ATOM  29298  HD2 ILE E 167      44.602 -53.826  32.204  0.00  0.00
E
ATOM  29299  HD3 ILE E 167      42.809 -54.270  32.240  0.00  0.00
E
ATOM  29300  C   ILE E 167      41.093 -55.599  29.077  0.00  0.00
E
ATOM  29301  O   ILE E 167      41.336 -54.407  28.805  0.00  0.00
E
ATOM  29302  N   ILE E 168      40.623 -56.454  28.138  0.00  0.00
E
ATOM  29303  HN  ILE E 168      40.436 -57.420  28.296  0.00  0.00
E
ATOM  29304  CA  ILE E 168      40.603 -56.081  26.769  0.00  0.00
E
ATOM  29305  HA  ILE E 168      41.411 -55.421  26.492  0.00  0.00
E
ATOM  29306  CB  ILE E 168      40.768 -57.296  25.945  0.00  0.00
E
ATOM  29307  HB  ILE E 168      39.740 -57.689  25.789  0.00  0.00
E
ATOM  29308  CG2 ILE E 168      41.295 -56.864  24.554  0.00  0.00
E
ATOM  29309 HG21 ILE E 168      40.772 -55.949  24.201  0.00  0.00
E
ATOM  29310 HG22 ILE E 168      42.381 -56.706  24.380  0.00  0.00
E
ATOM  29311 HG23 ILE E 168      40.937 -57.665  23.874  0.00  0.00
E
ATOM  29312  CG1 ILE E 168      41.582 -58.403  26.624  0.00  0.00
E
ATOM  29313 HG11 ILE E 168      42.610 -58.066  26.879  0.00  0.00
E
ATOM  29314 HG12 ILE E 168      41.137 -58.745  27.582  0.00  0.00
E
ATOM  29315  CD  ILE E 168      41.862 -59.636  25.749  0.00  0.00
E
ATOM  29316  HD1 ILE E 168      42.402 -59.309  24.834  0.00  0.00
E
ATOM  29317  HD2 ILE E 168      42.398 -60.454  26.276  0.00  0.00
E
ATOM  29318  HD3 ILE E 168      40.922 -60.112  25.398  0.00  0.00
E
ATOM  29319  C   ILE E 168      39.308 -55.226  26.578  0.00  0.00
E
ATOM  29320  O   ILE E 168      39.196 -54.329  25.764  0.00  0.00
E
ATOM  29321  N   ASP E 169      38.309 -55.396  27.461  0.00  0.00
E
```

ATOM 29322 HN ASP E 169   38.497 -56.089 28.153 0.00 0.00
E
ATOM 29323 CA ASP E 169   37.023 -54.766 27.547 0.00 0.00
E
ATOM 29324 HA ASP E 169   36.518 -54.746 26.593 0.00 0.00
E
ATOM 29325 CB ASP E 169   36.117 -55.688 28.449 0.00 0.00
E
ATOM 29326 HB1 ASP E 169   36.545 -55.981 29.432 0.00 0.00
E
ATOM 29327 HB2 ASP E 169   35.207 -55.125 28.744 0.00 0.00
E
ATOM 29328 CG ASP E 169   35.727 -56.978 27.752 0.00 0.00
E
ATOM 29329 OD1 ASP E 169   36.039 -58.055 28.275 0.00 0.00
E
ATOM 29330 OD2 ASP E 169   35.010 -56.983 26.726 0.00 0.00
E
ATOM 29331 C   ASP E 169   37.221 -53.402 28.142 0.00 0.00
E
ATOM 29332 O   ASP E 169   36.533 -52.503 27.639 0.00 0.00
E
ATOM 29333 N   LEU E 170   38.094 -53.265 29.094 0.00 0.00
E
ATOM 29334 HN LEU E 170   38.417 -54.042 29.629 0.00 0.00
E
ATOM 29335 CA LEU E 170   38.423 -51.998 29.680 0.00 0.00
E
ATOM 29336 HA LEU E 170   37.509 -51.533 30.019 0.00 0.00
E
ATOM 29337 CB LEU E 170   39.428 -52.182 30.842 0.00 0.00
E
ATOM 29338 HB1 LEU E 170   38.902 -52.952 31.447 0.00 0.00
E
ATOM 29339 HB2 LEU E 170   40.343 -52.762 30.597 0.00 0.00
E
ATOM 29340 CG LEU E 170   39.764 -50.979 31.829 0.00 0.00
E
ATOM 29341 HG LEU E 170   40.529 -50.325 31.359 0.00 0.00
E
ATOM 29342 CD1 LEU E 170   38.469 -50.249 32.166 0.00 0.00
E
ATOM 29343 HD11 LEU E 170   37.674 -50.841 32.669 0.00 0.00
E
ATOM 29344 HD12 LEU E 170   38.724 -49.299 32.682 0.00 0.00
E
ATOM 29345 HD13 LEU E 170   38.099 -49.778 31.230 0.00 0.00
E
ATOM 29346 CD2 LEU E 170   40.248 -51.606 33.152 0.00 0.00
E
ATOM 29347 HD21 LEU E 170   41.008 -52.415 33.111 0.00 0.00
E
ATOM 29348 HD22 LEU E 170   40.645 -50.758 33.749 0.00 0.00
E

ATOM 29349 HD23 LEU E 170 39.324 -52.027 33.604 0.00 0.00
E
ATOM 29350 C LEU E 170 39.118 -51.066 28.790 0.00 0.00
E
ATOM 29351 O LEU E 170 38.924 -49.861 28.803 0.00 0.00
E
ATOM 29352 N ASN E 171 39.955 -51.637 27.969 0.00 0.00
E
ATOM 29353 HN ASN E 171 39.958 -52.633 27.924 0.00 0.00
E
ATOM 29354 CA ASN E 171 40.676 -51.053 26.872 0.00 0.00
E
ATOM 29355 HA ASN E 171 41.289 -51.881 26.548 0.00 0.00
E
ATOM 29356 CB ASN E 171 39.842 -50.587 25.645 0.00 0.00
E
ATOM 29357 HB1 ASN E 171 39.092 -51.403 25.565 0.00 0.00
E
ATOM 29358 HB2 ASN E 171 39.271 -49.660 25.868 0.00 0.00
E
ATOM 29359 CG ASN E 171 40.573 -50.341 24.351 0.00 0.00
E
ATOM 29360 OD1 ASN E 171 41.592 -50.976 24.067 0.00 0.00
E
ATOM 29361 ND2 ASN E 171 39.983 -49.538 23.400 0.00 0.00
E
ATOM 29362 HD21 ASN E 171 40.549 -49.126 22.686 0.00 0.00
E
ATOM 29363 HD22 ASN E 171 39.046 -49.264 23.619 0.00 0.00
E
ATOM 29364 C ASN E 171 41.765 -50.109 27.337 0.00 0.00
E
ATOM 29365 O ASN E 171 41.465 -48.957 27.475 0.00 0.00
E
ATOM 29366 N VAL E 172 43.007 -50.625 27.730 0.00 0.00
E
ATOM 29367 HN VAL E 172 43.084 -51.601 27.545 0.00 0.00
E
ATOM 29368 CA VAL E 172 44.249 -49.954 28.122 0.00 0.00
E
ATOM 29369 HA VAL E 172 44.083 -48.905 28.320 0.00 0.00
E
ATOM 29370 CB VAL E 172 44.863 -50.340 29.389 0.00 0.00
E
ATOM 29371 HB VAL E 172 45.590 -49.550 29.674 0.00 0.00
E
ATOM 29372 CG1 VAL E 172 43.774 -50.304 30.412 0.00 0.00
E
ATOM 29373 HG11 VAL E 172 43.346 -49.284 30.509 0.00 0.00
E
ATOM 29374 HG12 VAL E 172 42.942 -51.017 30.227 0.00 0.00
E
ATOM 29375 HG13 VAL E 172 44.133 -50.601 31.421 0.00 0.00
E

```
ATOM  29376  CG2 VAL E 172      45.527 -51.775  29.365  0.00  0.00
E
ATOM  29377 HG21 VAL E 172      46.166 -51.782  28.456  0.00  0.00
E
ATOM  29378 HG22 VAL E 172      46.190 -51.935  30.242  0.00  0.00
E
ATOM  29379 HG23 VAL E 172      44.792 -52.600  29.247  0.00  0.00
E
ATOM  29380  C   VAL E 172      45.259 -49.841  26.992  0.00  0.00
E
ATOM  29381  O   VAL E 172      46.304 -49.219  27.161  0.00  0.00
E
ATOM  29382  N   ARG E 173      44.884 -50.407  25.794  0.00  0.00
E
ATOM  29383  HN  ARG E 173      44.112 -51.038  25.805  0.00  0.00
E
ATOM  29384  CA  ARG E 173      45.641 -50.197  24.592  0.00  0.00
E
ATOM  29385  HA  ARG E 173      46.627 -50.576  24.813  0.00  0.00
E
ATOM  29386  CB  ARG E 173      45.177 -51.004  23.295  0.00  0.00
E
ATOM  29387  HB1 ARG E 173      44.082 -50.820  23.280  0.00  0.00
E
ATOM  29388  HB2 ARG E 173      45.681 -50.698  22.353  0.00  0.00
E
ATOM  29389  CG  ARG E 173      45.309 -52.542  23.503  0.00  0.00
E
ATOM  29390  HG1 ARG E 173      46.400 -52.749  23.510  0.00  0.00
E
ATOM  29391  HG2 ARG E 173      45.038 -52.827  24.543  0.00  0.00
E
ATOM  29392  CD  ARG E 173      44.442 -53.363  22.469  0.00  0.00
E
ATOM  29393  HD1 ARG E 173      44.711 -52.903  21.494  0.00  0.00
E
ATOM  29394  HD2 ARG E 173      44.792 -54.406  22.626  0.00  0.00
E
ATOM  29395  NE  ARG E 173      42.974 -53.250  22.867  0.00  0.00
E
ATOM  29396  HE  ARG E 173      42.744 -52.393  23.328  0.00  0.00
E
ATOM  29397  CZ  ARG E 173      41.957 -53.826  22.264  0.00  0.00
E
ATOM  29398  NH1 ARG E 173      42.056 -54.828  21.482  0.00  0.00
E
ATOM  29399 HH11 ARG E 173      41.233 -54.997  20.941  0.00  0.00
E
ATOM  29400 HH12 ARG E 173      42.913 -55.278  21.229  0.00  0.00
E
ATOM  29401  NH2 ARG E 173      40.722 -53.365  22.596  0.00  0.00
E
ATOM  29402 HH21 ARG E 173      39.905 -53.798  22.216  0.00  0.00
E
```

ATOM 29403 HH22 ARG E 173 40.721 −52.629 23.272 0.00 0.00
E
ATOM 29404 C ARG E 173 45.662 −48.782 24.220 0.00 0.00
E
ATOM 29405 O ARG E 173 46.695 −48.156 23.861 0.00 0.00
E
ATOM 29406 N ALA E 174 44.473 −48.116 24.282 0.00 0.00
E
ATOM 29407 HN ALA E 174 43.687 −48.485 24.773 0.00 0.00
E
ATOM 29408 CA ALA E 174 44.389 −46.676 23.927 0.00 0.00
E
ATOM 29409 HA ALA E 174 45.237 −46.099 24.267 0.00 0.00
E
ATOM 29410 CB ALA E 174 44.072 −46.430 22.391 0.00 0.00
E
ATOM 29411 HB1 ALA E 174 44.866 −46.777 21.695 0.00 0.00
E
ATOM 29412 HB2 ALA E 174 43.130 −46.922 22.065 0.00 0.00
E
ATOM 29413 HB3 ALA E 174 43.848 −45.367 22.157 0.00 0.00
E
ATOM 29414 C ALA E 174 43.178 −46.175 24.718 0.00 0.00
E
ATOM 29415 O ALA E 174 42.349 −47.059 24.995 0.00 0.00
E
ATOM 29416 N GLY E 177 42.979 −44.917 25.033 0.00 0.00
E
ATOM 29417 HN GLY E 177 42.258 −44.869 25.721 0.00 0.00
E
ATOM 29418 CA GLY E 177 43.705 −43.789 24.551 0.00 0.00
E
ATOM 29419 HA1 GLY E 177 43.146 −43.163 23.871 0.00 0.00
E
ATOM 29420 HA2 GLY E 177 44.637 −44.152 24.145 0.00 0.00
E
ATOM 29421 C GLY E 177 44.176 −42.829 25.749 0.00 0.00
E
ATOM 29422 O GLY E 177 44.809 −41.783 25.495 0.00 0.00
E
ATOM 29423 N CYS E 178 43.897 −43.142 27.044 0.00 0.00
E
ATOM 29424 HN CYS E 178 43.312 −43.919 27.264 0.00 0.00
E
ATOM 29425 CA CYS E 178 44.361 −42.343 28.174 0.00 0.00
E
ATOM 29426 HA CYS E 178 45.040 −41.570 27.846 0.00 0.00
E
ATOM 29427 CB CYS E 178 43.302 −41.638 29.035 0.00 0.00
E
ATOM 29428 HB1 CYS E 178 43.770 −41.087 29.879 0.00 0.00
E
ATOM 29429 HB2 CYS E 178 42.739 −40.942 28.377 0.00 0.00
E

ATOM 29430 SG CYS E 178   42.050 -42.894 29.718 0.00 0.00
E
ATOM 29431 HG1 CYS E 178   42.961 -43.801 30.042 0.00 0.00
E
ATOM 29432 C   CYS E 178   45.293 -43.178 29.022 0.00 0.00
E
ATOM 29433 O   CYS E 178   45.246 -44.359 29.226 0.00 0.00
E
ATOM 29434 N   THR E 179   46.226 -42.544 29.624 0.00 0.00
E
ATOM 29435 HN THR E 179   46.205 -41.552 29.728 0.00 0.00
E
ATOM 29436 CA THR E 179   47.412 -43.217 30.208 0.00 0.00
E
ATOM 29437 HA THR E 179   47.490 -44.264 29.955 0.00 0.00
E
ATOM 29438 CB THR E 179   48.760 -42.706 29.827 0.00 0.00
E
ATOM 29439 HB THR E 179   48.882 -41.711 30.307 0.00 0.00
E
ATOM 29440 OG1 THR E 179   48.911 -42.656 28.471 0.00 0.00
E
ATOM 29441 HG1 THR E 179   48.466 -41.856 28.181 0.00 0.00
E
ATOM 29442 CG2 THR E 179   49.915 -43.578 30.410 0.00 0.00
E
ATOM 29443 HG21 THR E 179   50.915 -43.274 30.033 0.00 0.00
E
ATOM 29444 HG22 THR E 179   49.854 -43.464 31.514 0.00 0.00
E
ATOM 29445 HG23 THR E 179   49.574 -44.628 30.287 0.00 0.00
E
ATOM 29446 C   THR E 179   47.193 -43.228 31.703 0.00 0.00
E
ATOM 29447 O   THR E 179   47.388 -42.135 32.311 0.00 0.00
E
ATOM 29448 N   ILE E 180   47.030 -44.345 32.323 0.00 0.00
E
ATOM 29449 HN ILE E 180   46.764 -45.124 31.759 0.00 0.00
E
ATOM 29450 CA ILE E 180   47.023 -44.452 33.814 0.00 0.00
E
ATOM 29451 HA ILE E 180   46.465 -43.560 34.053 0.00 0.00
E
ATOM 29452 CB ILE E 180   46.360 -45.784 34.272 0.00 0.00
E
ATOM 29453 HB ILE E 180   47.083 -46.573 33.975 0.00 0.00
E
ATOM 29454 CG2 ILE E 180   46.404 -46.017 35.831 0.00 0.00
E
ATOM 29455 HG21 ILE E 180   46.846 -47.032 35.930 0.00 0.00
E
ATOM 29456 HG22 ILE E 180   47.133 -45.299 36.264 0.00 0.00
E

```
ATOM  29457 HG23 ILE E 180      45.373 -46.031  36.244  0.00  0.00
E
ATOM  29458  CG1 ILE E 180      45.006 -46.107  33.672  0.00  0.00
E
ATOM  29459 HG11 ILE E 180      44.266 -45.826  34.451  0.00  0.00
E
ATOM  29460 HG12 ILE E 180      44.749 -45.448  32.816  0.00  0.00
E
ATOM  29461  CD  ILE E 180      44.720 -47.555  33.375  0.00  0.00
E
ATOM  29462  HD1 ILE E 180      43.632 -47.751  33.485  0.00  0.00
E
ATOM  29463  HD2 ILE E 180      45.041 -47.677  32.319  0.00  0.00
E
ATOM  29464  HD3 ILE E 180      45.324 -48.131  34.108  0.00  0.00
E
ATOM  29465  C   ILE E 180      48.447 -44.343  34.377  0.00  0.00
E
ATOM  29466  O   ILE E 180      49.323 -45.194  34.170  0.00  0.00
E
ATOM  29467  N   LEU E 181      48.665 -43.309  35.236  0.00  0.00
E
ATOM  29468  HN  LEU E 181      47.858 -42.836  35.582  0.00  0.00
E
ATOM  29469  CA  LEU E 181      49.957 -42.946  35.741  0.00  0.00
E
ATOM  29470  HA  LEU E 181      50.755 -43.091  35.027  0.00  0.00
E
ATOM  29471  CB  LEU E 181      50.202 -41.558  36.231  0.00  0.00
E
ATOM  29472  HB1 LEU E 181      49.564 -41.431  37.132  0.00  0.00
E
ATOM  29473  HB2 LEU E 181      51.230 -41.492  36.648  0.00  0.00
E
ATOM  29474  CG  LEU E 181      49.815 -40.456  35.189  0.00  0.00
E
ATOM  29475  HG  LEU E 181      48.824 -40.534  34.694  0.00  0.00
E
ATOM  29476  CD1 LEU E 181      49.920 -38.967  35.728  0.00  0.00
E
ATOM  29477 HD11 LEU E 181      50.947 -38.728  36.077  0.00  0.00
E
ATOM  29478 HD12 LEU E 181      49.667 -38.180  34.986  0.00  0.00
E
ATOM  29479 HD13 LEU E 181      49.296 -38.846  36.639  0.00  0.00
E
ATOM  29480  CD2 LEU E 181      50.788 -40.494  33.954  0.00  0.00
E
ATOM  29481 HD21 LEU E 181      50.457 -39.785  33.165  0.00  0.00
E
ATOM  29482 HD22 LEU E 181      51.830 -40.404  34.330  0.00  0.00
E
ATOM  29483 HD23 LEU E 181      50.744 -41.518  33.525  0.00  0.00
E
```

ATOM 29484 C LEU E 181 50.349 -43.812 36.993 0.00 0.00
E
ATOM 29485 O LEU E 181 51.534 -43.961 37.297 0.00 0.00
E
ATOM 29486 N ALA E 182 49.332 -44.256 37.817 0.00 0.00
E
ATOM 29487 HN ALA E 182 48.405 -43.942 37.631 0.00 0.00
E
ATOM 29488 CA ALA E 182 49.529 -45.132 38.965 0.00 0.00
E
ATOM 29489 HA ALA E 182 49.957 -46.052 38.596 0.00 0.00
E
ATOM 29490 CB ALA E 182 50.274 -44.342 40.049 0.00 0.00
E
ATOM 29491 HB1 ALA E 182 51.273 -43.934 39.784 0.00 0.00
E
ATOM 29492 HB2 ALA E 182 49.602 -43.481 40.253 0.00 0.00
E
ATOM 29493 HB3 ALA E 182 50.338 -44.991 40.948 0.00 0.00
E
ATOM 29494 C ALA E 182 48.171 -45.478 39.457 0.00 0.00
E
ATOM 29495 O ALA E 182 47.263 -44.662 39.482 0.00 0.00
E
ATOM 29496 N ILE E 183 48.072 -46.730 39.948 0.00 0.00
E
ATOM 29497 HN ILE E 183 48.885 -47.306 39.986 0.00 0.00
E
ATOM 29498 CA ILE E 183 46.899 -47.155 40.728 0.00 0.00
E
ATOM 29499 HA ILE E 183 46.012 -46.553 40.596 0.00 0.00
E
ATOM 29500 CB ILE E 183 46.605 -48.655 40.538 0.00 0.00
E
ATOM 29501 HB ILE E 183 47.385 -49.266 41.041 0.00 0.00
E
ATOM 29502 CG2 ILE E 183 45.318 -48.948 41.359 0.00 0.00
E
ATOM 29503 HG21 ILE E 183 45.182 -50.051 41.357 0.00 0.00
E
ATOM 29504 HG22 ILE E 183 45.384 -48.628 42.420 0.00 0.00
E
ATOM 29505 HG23 ILE E 183 44.509 -48.398 40.832 0.00 0.00
E
ATOM 29506 CG1 ILE E 183 46.364 -49.115 39.033 0.00 0.00
E
ATOM 29507 HG11 ILE E 183 45.714 -50.013 39.107 0.00 0.00
E
ATOM 29508 HG12 ILE E 183 45.802 -48.318 38.501 0.00 0.00
E
ATOM 29509 CD ILE E 183 47.641 -49.534 38.206 0.00 0.00
E
ATOM 29510 HD1 ILE E 183 48.406 -48.733 38.289 0.00 0.00
E

ATOM 29511 HD2 ILE E 183 48.266 -50.306 38.703 0.00 0.00
E
ATOM 29512 HD3 ILE E 183 47.440 -49.574 37.114 0.00 0.00
E
ATOM 29513 C ILE E 183 47.253 -46.866 42.184 0.00 0.00
E
ATOM 29514 O ILE E 183 48.163 -47.524 42.650 0.00 0.00
E
ATOM 29515 N LYS E 184 46.471 -46.043 42.874 0.00 0.00
E
ATOM 29516 HN LYS E 184 45.683 -45.652 42.406 0.00 0.00
E
ATOM 29517 CA LYS E 184 46.701 -45.745 44.268 0.00 0.00
E
ATOM 29518 HA LYS E 184 47.737 -45.441 44.267 0.00 0.00
E
ATOM 29519 CB LYS E 184 45.882 -44.442 44.643 0.00 0.00
E
ATOM 29520 HB1 LYS E 184 44.805 -44.707 44.592 0.00 0.00
E
ATOM 29521 HB2 LYS E 184 46.149 -44.185 45.690 0.00 0.00
E
ATOM 29522 CG LYS E 184 46.156 -43.320 43.679 0.00 0.00
E
ATOM 29523 HG1 LYS E 184 47.235 -43.324 43.414 0.00 0.00
E
ATOM 29524 HG2 LYS E 184 45.551 -43.288 42.748 0.00 0.00
E
ATOM 29525 CD LYS E 184 46.217 -41.933 44.345 0.00 0.00
E
ATOM 29526 HD1 LYS E 184 46.788 -42.031 45.294 0.00 0.00
E
ATOM 29527 HD2 LYS E 184 46.836 -41.247 43.730 0.00 0.00
E
ATOM 29528 CE LYS E 184 44.790 -41.406 44.593 0.00 0.00
E
ATOM 29529 HE1 LYS E 184 44.237 -41.437 43.630 0.00 0.00
E
ATOM 29530 HE2 LYS E 184 44.287 -42.026 45.365 0.00 0.00
E
ATOM 29531 NZ LYS E 184 44.884 -40.008 45.033 0.00 0.00
E
ATOM 29532 HZ1 LYS E 184 45.806 -39.809 44.595 0.00 0.00
E
ATOM 29533 HZ2 LYS E 184 44.083 -39.380 44.819 0.00 0.00
E
ATOM 29534 HZ3 LYS E 184 45.027 -39.930 46.060 0.00 0.00
E
ATOM 29535 C LYS E 184 46.547 -46.967 45.216 0.00 0.00
E
ATOM 29536 O LYS E 184 45.793 -47.963 44.961 0.00 0.00
E
ATOM 29537 N HSE E 185 47.238 -46.964 46.441 0.00 0.00
E

ATOM 29538 HN HSE E 185 47.965 -46.299 46.591 0.00 0.00
E
ATOM 29539 CA HSE E 185 47.185 -48.036 47.372 0.00 0.00
E
ATOM 29540 HA HSE E 185 46.146 -48.079 47.664 0.00 0.00
E
ATOM 29541 CB HSE E 185 47.954 -49.372 46.904 0.00 0.00
E
ATOM 29542 HB1 HSE E 185 47.489 -49.833 46.006 0.00 0.00
E
ATOM 29543 HB2 HSE E 185 48.994 -49.050 46.685 0.00 0.00
E
ATOM 29544 ND1 HSE E 185 46.902 -50.714 48.574 0.00 0.00
E
ATOM 29545 CG HSE E 185 48.064 -50.379 48.004 0.00 0.00
E
ATOM 29546 CE1 HSE E 185 47.132 -51.828 49.316 0.00 0.00
E
ATOM 29547 HE1 HSE E 185 46.415 -52.395 49.910 0.00 0.00
E
ATOM 29548 NE2 HSE E 185 48.439 -52.194 49.269 0.00 0.00
E
ATOM 29549 HE2 HSE E 185 48.811 -53.096 49.488 0.00 0.00
E
ATOM 29550 CD2 HSE E 185 49.023 -51.295 48.363 0.00 0.00
E
ATOM 29551 HD2 HSE E 185 49.996 -51.502 47.934 0.00 0.00
E
ATOM 29552 C HSE E 185 47.903 -47.602 48.662 0.00 0.00
E
ATOM 29553 O HSE E 185 48.767 -46.704 48.594 0.00 0.00
E
ATOM 29554 N HSE E 186 47.562 -48.115 49.890 0.00 0.00
E
ATOM 29555 HN HSE E 186 47.010 -48.944 49.939 0.00 0.00
E
ATOM 29556 CA HSE E 186 48.252 -47.722 51.084 0.00 0.00
E
ATOM 29557 HA HSE E 186 48.216 -46.643 51.048 0.00 0.00
E
ATOM 29558 CB HSE E 186 47.531 -48.188 52.384 0.00 0.00
E
ATOM 29559 HB1 HSE E 186 48.128 -47.968 53.295 0.00 0.00
E
ATOM 29560 HB2 HSE E 186 46.504 -47.765 52.405 0.00 0.00
E
ATOM 29561 ND1 HSE E 186 46.147 -50.150 51.718 0.00 0.00
E
ATOM 29562 CG HSE E 186 47.177 -49.653 52.433 0.00 0.00
E
ATOM 29563 CE1 HSE E 186 46.158 -51.494 52.080 0.00 0.00
E
ATOM 29564 HE1 HSE E 186 45.406 -52.204 51.738 0.00 0.00
E

ATOM 29565 NE2 HSE E 186 47.191 -51.799 52.913 0.00 0.00
E
ATOM 29566 HE2 HSE E 186 47.345 -52.639 53.433 0.00 0.00
E
ATOM 29567 CD2 HSE E 186 47.840 -50.632 53.132 0.00 0.00
E
ATOM 29568 HD2 HSE E 186 48.645 -50.569 53.854 0.00 0.00
E
ATOM 29569 C HSE E 186 49.639 -48.102 51.095 0.00 0.00
E
ATOM 29570 O HSE E 186 50.373 -47.385 51.749 0.00 0.00
E
ATOM 29571 N GLY E 187 50.051 -49.255 50.530 0.00 0.00
E
ATOM 29572 HN GLY E 187 49.310 -49.711 50.043 0.00 0.00
E
ATOM 29573 CA GLY E 187 51.432 -49.647 50.248 0.00 0.00
E
ATOM 29574 HA1 GLY E 187 51.405 -50.722 50.141 0.00 0.00
E
ATOM 29575 HA2 GLY E 187 52.003 -49.276 51.087 0.00 0.00
E
ATOM 29576 C GLY E 187 51.951 -49.059 49.035 0.00 0.00
E
ATOM 29577 O GLY E 187 51.421 -48.092 48.441 0.00 0.00
E
ATOM 29578 N ASP E 188 52.976 -49.673 48.473 0.00 0.00
E
ATOM 29579 HN ASP E 188 53.485 -50.400 48.928 0.00 0.00
E
ATOM 29580 CA ASP E 188 53.641 -49.158 47.347 0.00 0.00
E
ATOM 29581 HA ASP E 188 53.472 -48.094 47.423 0.00 0.00
E
ATOM 29582 CB ASP E 188 55.097 -49.595 47.158 0.00 0.00
E
ATOM 29583 HB1 ASP E 188 55.130 -50.706 47.180 0.00 0.00
E
ATOM 29584 HB2 ASP E 188 55.384 -49.224 46.152 0.00 0.00
E
ATOM 29585 CG ASP E 188 56.008 -48.983 48.079 0.00 0.00
E
ATOM 29586 OD1 ASP E 188 55.927 -47.769 48.338 0.00 0.00
E
ATOM 29587 OD2 ASP E 188 56.958 -49.703 48.576 0.00 0.00
E
ATOM 29588 C ASP E 188 52.863 -49.403 46.030 0.00 0.00
E
ATOM 29589 O ASP E 188 52.165 -50.395 45.909 0.00 0.00
E
ATOM 29590 N ILE E 189 52.856 -48.447 45.128 0.00 0.00
E
ATOM 29591 HN ILE E 189 53.321 -47.593 45.347 0.00 0.00
E

ATOM	29592	CA	ILE	E	189	52.013	-48.412	43.985	0.00	0.00 E
ATOM	29593	HA	ILE	E	189	51.142	-49.008	44.215	0.00	0.00 E
ATOM	29594	CB	ILE	E	189	51.458	-47.058	43.990	0.00	0.00 E
ATOM	29595	HB	ILE	E	189	50.915	-46.801	43.055	0.00	0.00 E
ATOM	29596	CG2	ILE	E	189	50.223	-46.976	45.031	0.00	0.00 E
ATOM	29597	HG21	ILE	E	189	49.438	-47.659	44.641	0.00	0.00 E
ATOM	29598	HG22	ILE	E	189	50.450	-47.132	46.107	0.00	0.00 E
ATOM	29599	HG23	ILE	E	189	49.755	-45.973	44.941	0.00	0.00 E
ATOM	29600	CG1	ILE	E	189	52.635	-45.972	44.104	0.00	0.00 E
ATOM	29601	HG11	ILE	E	189	53.101	-45.970	45.112	0.00	0.00 E
ATOM	29602	HG12	ILE	E	189	53.387	-46.212	43.322	0.00	0.00 E
ATOM	29603	CD	ILE	E	189	52.084	-44.600	43.875	0.00	0.00 E
ATOM	29604	HD1	ILE	E	189	52.900	-43.847	43.858	0.00	0.00 E
ATOM	29605	HD2	ILE	E	189	51.395	-44.548	43.005	0.00	0.00 E
ATOM	29606	HD3	ILE	E	189	51.345	-44.375	44.673	0.00	0.00 E
ATOM	29607	C	ILE	E	189	52.598	-48.815	42.621	0.00	0.00 E
ATOM	29608	O	ILE	E	189	53.684	-48.395	42.258	0.00	0.00 E
ATOM	29609	N	CYS	E	190	51.860	-49.617	41.837	0.00	0.00 E
ATOM	29610	HN	CYS	E	190	50.949	-49.900	42.125	0.00	0.00 E
ATOM	29611	CA	CYS	E	190	52.102	-49.891	40.483	0.00	0.00 E
ATOM	29612	HA	CYS	E	190	53.177	-49.950	40.389	0.00	0.00 E
ATOM	29613	CB	CYS	E	190	51.408	-51.289	40.019	0.00	0.00 E
ATOM	29614	HB1	CYS	E	190	51.478	-52.024	40.849	0.00	0.00 E
ATOM	29615	HB2	CYS	E	190	50.319	-51.135	39.863	0.00	0.00 E
ATOM	29616	SG	CYS	E	190	52.326	-52.110	38.762	0.00	0.00 E
ATOM	29617	HG1	CYS	E	190	51.619	-53.230	38.721	0.00	0.00 E
ATOM	29618	C	CYS	E	190	51.765	-48.689	39.594	0.00	0.00 E

ATOM 29619 O CYS E 190 50.685 -48.074 39.621 0.00 0.00
E
ATOM 29620 N LEU E 191 52.682 -48.450 38.706 0.00 0.00
E
ATOM 29621 HN LEU E 191 53.549 -48.941 38.664 0.00 0.00
E
ATOM 29622 CA LEU E 191 52.679 -47.306 37.886 0.00 0.00
E
ATOM 29623 HA LEU E 191 52.047 -46.525 38.281 0.00 0.00
E
ATOM 29624 CB LEU E 191 54.169 -46.781 37.886 0.00 0.00
E
ATOM 29625 HB1 LEU E 191 54.856 -47.511 37.408 0.00 0.00
E
ATOM 29626 HB2 LEU E 191 54.296 -45.953 37.156 0.00 0.00
E
ATOM 29627 CG LEU E 191 54.811 -46.229 39.246 0.00 0.00
E
ATOM 29628 HG LEU E 191 55.164 -47.118 39.811 0.00 0.00
E
ATOM 29629 CD1 LEU E 191 56.076 -45.449 38.841 0.00 0.00
E
ATOM 29630 HD11 LEU E 191 56.518 -44.807 39.632 0.00 0.00
E
ATOM 29631 HD12 LEU E 191 56.862 -46.176 38.544 0.00 0.00
E
ATOM 29632 HD13 LEU E 191 55.839 -44.709 38.047 0.00 0.00
E
ATOM 29633 CD2 LEU E 191 53.838 -45.245 39.988 0.00 0.00
E
ATOM 29634 HD21 LEU E 191 54.362 -44.620 40.742 0.00 0.00
E
ATOM 29635 HD22 LEU E 191 53.271 -44.632 39.255 0.00 0.00
E
ATOM 29636 HD23 LEU E 191 53.065 -45.790 40.572 0.00 0.00
E
ATOM 29637 C LEU E 191 52.211 -47.616 36.476 0.00 0.00
E
ATOM 29638 O LEU E 191 52.255 -46.814 35.558 0.00 0.00
E
ATOM 29639 N SER E 192 51.699 -48.843 36.177 0.00 0.00
E
ATOM 29640 HN SER E 192 51.656 -49.587 36.839 0.00 0.00
E
ATOM 29641 CA SER E 192 51.090 -49.135 34.857 0.00 0.00
E
ATOM 29642 HA SER E 192 50.545 -48.278 34.489 0.00 0.00
E
ATOM 29643 CB SER E 192 52.218 -49.507 33.825 0.00 0.00
E
ATOM 29644 HB1 SER E 192 53.099 -48.830 33.831 0.00 0.00
E
ATOM 29645 HB2 SER E 192 52.589 -50.525 34.074 0.00 0.00
E

ATOM 29646 OG SER E 192 51.923 −49.494 32.422 0.00 0.00
E
ATOM 29647 HG1 SER E 192 52.614 −50.101 32.148 0.00 0.00
E
ATOM 29648 C SER E 192 50.210 −50.309 35.038 0.00 0.00
E
ATOM 29649 O SER E 192 50.592 −51.258 35.739 0.00 0.00
E
ATOM 29650 N PRO E 193 48.967 −50.321 34.505 0.00 0.00
E
ATOM 29651 CD PRO E 193 48.416 −49.354 33.494 0.00 0.00
E
ATOM 29652 HD1 PRO E 193 47.907 −48.564 34.086 0.00 0.00
E
ATOM 29653 HD2 PRO E 193 49.150 −48.970 32.753 0.00 0.00
E
ATOM 29654 CA PRO E 193 48.187 −51.574 34.399 0.00 0.00
E
ATOM 29655 HA PRO E 193 48.128 −51.923 35.419 0.00 0.00
E
ATOM 29656 CB PRO E 193 46.822 −51.111 33.804 0.00 0.00
E
ATOM 29657 HB1 PRO E 193 46.243 −50.683 34.650 0.00 0.00
E
ATOM 29658 HB2 PRO E 193 46.296 −51.974 33.342 0.00 0.00
E
ATOM 29659 CG PRO E 193 47.261 −50.110 32.735 0.00 0.00
E
ATOM 29660 HG1 PRO E 193 46.434 −49.428 32.444 0.00 0.00
E
ATOM 29661 HG2 PRO E 193 47.644 −50.688 31.867 0.00 0.00
E
ATOM 29662 C PRO E 193 48.776 −52.682 33.600 0.00 0.00
E
ATOM 29663 O PRO E 193 49.696 −52.427 32.784 0.00 0.00
E
ATOM 29664 N ALA E 194 48.227 −53.893 33.695 0.00 0.00
E
ATOM 29665 HN ALA E 194 47.460 −53.920 34.332 0.00 0.00
E
ATOM 29666 CA ALA E 194 48.794 −55.160 33.234 0.00 0.00
E
ATOM 29667 HA ALA E 194 48.710 −55.208 32.158 0.00 0.00
E
ATOM 29668 CB ALA E 194 50.286 −55.387 33.649 0.00 0.00
E
ATOM 29669 HB1 ALA E 194 50.290 −55.562 34.746 0.00 0.00
E
ATOM 29670 HB2 ALA E 194 50.732 −56.258 33.122 0.00 0.00
E
ATOM 29671 HB3 ALA E 194 50.975 −54.552 33.396 0.00 0.00
E
ATOM 29672 C ALA E 194 47.812 −56.165 33.853 0.00 0.00
E

ATOM 29673 O ALA E 194 47.326 −55.939 34.927 0.00 0.00
E
ATOM 29674 N PRO E 195 47.456 −57.330 33.300 0.00 0.00
E
ATOM 29675 CD PRO E 195 47.931 −57.745 31.934 0.00 0.00
E
ATOM 29676 HD1 PRO E 195 47.968 −56.953 31.156 0.00 0.00
E
ATOM 29677 HD2 PRO E 195 48.941 −58.183 32.080 0.00 0.00
E
ATOM 29678 CA PRO E 195 46.475 −58.300 33.766 0.00 0.00
E
ATOM 29679 HA PRO E 195 45.515 −57.848 33.565 0.00 0.00
E
ATOM 29680 CB PRO E 195 46.694 −59.551 32.848 0.00 0.00
E
ATOM 29681 HB1 PRO E 195 45.824 −60.242 32.863 0.00 0.00
E
ATOM 29682 HB2 PRO E 195 47.621 −60.061 33.185 0.00 0.00
E
ATOM 29683 CG PRO E 195 46.971 −58.934 31.506 0.00 0.00
E
ATOM 29684 HG1 PRO E 195 47.464 −59.554 30.727 0.00 0.00
E
ATOM 29685 HG2 PRO E 195 46.083 −58.436 31.059 0.00 0.00
E
ATOM 29686 C PRO E 195 46.414 −58.697 35.219 0.00 0.00
E
ATOM 29687 O PRO E 195 45.297 −58.921 35.773 0.00 0.00
E
ATOM 29688 N GLU E 196 47.546 −58.788 35.920 0.00 0.00
E
ATOM 29689 HN GLU E 196 48.440 −58.625 35.510 0.00 0.00
E
ATOM 29690 CA GLU E 196 47.625 −59.110 37.389 0.00 0.00
E
ATOM 29691 HA GLU E 196 46.647 −59.457 37.691 0.00 0.00
E
ATOM 29692 CB GLU E 196 48.683 −60.209 37.514 0.00 0.00
E
ATOM 29693 HB1 GLU E 196 49.668 −59.748 37.286 0.00 0.00
E
ATOM 29694 HB2 GLU E 196 48.695 −60.423 38.604 0.00 0.00
E
ATOM 29695 CG GLU E 196 48.569 −61.401 36.585 0.00 0.00
E
ATOM 29696 HG1 GLU E 196 47.603 −61.950 36.586 0.00 0.00
E
ATOM 29697 HG2 GLU E 196 48.726 −61.080 35.532 0.00 0.00
E
ATOM 29698 CD GLU E 196 49.724 −62.351 36.722 0.00 0.00
E
ATOM 29699 OE1 GLU E 196 49.684 −63.197 37.649 0.00 0.00
E

```
ATOM  29700  OE2 GLU E 196      50.769 -62.296  36.018  0.00  0.00
E
ATOM  29701  C   GLU E 196      48.087 -57.941  38.290  0.00  0.00
E
ATOM  29702  O   GLU E 196      48.327 -58.103  39.515  0.00  0.00
E
ATOM  29703  N   ASP E 197      48.027 -56.700  37.686  0.00  0.00
E
ATOM  29704  HN  ASP E 197      47.837 -56.652  36.708  0.00  0.00
E
ATOM  29705  CA  ASP E 197      48.319 -55.418  38.292  0.00  0.00
E
ATOM  29706  HA  ASP E 197      48.290 -55.352  39.369  0.00  0.00
E
ATOM  29707  CB  ASP E 197      49.638 -54.759  37.712  0.00  0.00
E
ATOM  29708  HB1 ASP E 197      49.433 -54.582  36.634  0.00  0.00
E
ATOM  29709  HB2 ASP E 197      49.775 -53.715  38.066  0.00  0.00
E
ATOM  29710  CG  ASP E 197      50.848 -55.726  37.819  0.00  0.00
E
ATOM  29711  OD1 ASP E 197      51.436 -55.779  38.929  0.00  0.00
E
ATOM  29712  OD2 ASP E 197      51.237 -56.465  36.863  0.00  0.00
E
ATOM  29713  C   ASP E 197      47.283 -54.339  37.920  0.00  0.00
E
ATOM  29714  O   ASP E 197      47.464 -53.211  38.361  0.00  0.00
E
ATOM  29715  N   ILE E 198      46.165 -54.737  37.332  0.00  0.00
E
ATOM  29716  HN  ILE E 198      46.073 -55.639  36.918  0.00  0.00
E
ATOM  29717  CA  ILE E 198      44.973 -53.889  37.198  0.00  0.00
E
ATOM  29718  HA  ILE E 198      45.213 -52.836  37.211  0.00  0.00
E
ATOM  29719  CB  ILE E 198      44.169 -54.285  35.943  0.00  0.00
E
ATOM  29720  HB  ILE E 198      44.866 -54.234  35.079  0.00  0.00
E
ATOM  29721  CG2 ILE E 198      43.694 -55.679  35.986  0.00  0.00
E
ATOM  29722 HG21 ILE E 198      42.910 -55.813  36.761  0.00  0.00
E
ATOM  29723 HG22 ILE E 198      43.301 -56.077  35.026  0.00  0.00
E
ATOM  29724 HG23 ILE E 198      44.402 -56.480  36.288  0.00  0.00
E
ATOM  29725  CG1 ILE E 198      42.957 -53.457  35.562  0.00  0.00
E
ATOM  29726 HG11 ILE E 198      42.573 -53.793  34.575  0.00  0.00
E
```

```
ATOM  29727 HG12 ILE E 198      42.147 -53.697  36.284  0.00  0.00
E
ATOM  29728  CD  ILE E 198      43.219 -51.967  35.412  0.00  0.00
E
ATOM  29729  HD1 ILE E 198      43.532 -51.433  36.335  0.00  0.00
E
ATOM  29730  HD2 ILE E 198      44.081 -51.856  34.720  0.00  0.00
E
ATOM  29731  HD3 ILE E 198      42.292 -51.523  34.992  0.00  0.00
E
ATOM  29732  C   ILE E 198      44.186 -54.043  38.495  0.00  0.00
E
ATOM  29733  O   ILE E 198      44.577 -54.931  39.288  0.00  0.00
E
ATOM  29734  N   ILE E 199      43.208 -53.123  38.697  0.00  0.00
E
ATOM  29735  HN  ILE E 199      43.084 -52.466  37.957  0.00  0.00
E
ATOM  29736  CA  ILE E 199      42.322 -53.160  39.783  0.00  0.00
E
ATOM  29737  HA  ILE E 199      42.931 -53.431  40.633  0.00  0.00
E
ATOM  29738  CB  ILE E 199      41.544 -51.906  40.113  0.00  0.00
E
ATOM  29739  HB  ILE E 199      40.938 -52.090  41.025  0.00  0.00
E
ATOM  29740  CG2 ILE E 199      42.636 -50.967  40.495  0.00  0.00
E
ATOM  29741 HG21 ILE E 199      43.214 -51.306  41.382  0.00  0.00
E
ATOM  29742 HG22 ILE E 199      43.345 -50.564  39.740  0.00  0.00
E
ATOM  29743 HG23 ILE E 199      42.112 -50.033  40.789  0.00  0.00
E
ATOM  29744  CG1 ILE E 199      40.778 -51.335  38.961  0.00  0.00
E
ATOM  29745 HG11 ILE E 199      41.450 -50.911  38.184  0.00  0.00
E
ATOM  29746 HG12 ILE E 199      40.243 -52.243  38.610  0.00  0.00
E
ATOM  29747  CD  ILE E 199      39.659 -50.322  39.405  0.00  0.00
E
ATOM  29748  HD1 ILE E 199      38.878 -50.252  38.619  0.00  0.00
E
ATOM  29749  HD2 ILE E 199      39.168 -50.629  40.354  0.00  0.00
E
ATOM  29750  HD3 ILE E 199      40.114 -49.347  39.682  0.00  0.00
E
ATOM  29751  C   ILE E 199      41.345 -54.345  39.615  0.00  0.00
E
ATOM  29752  O   ILE E 199      40.853 -54.677  38.550  0.00  0.00
E
ATOM  29753  N   ARG E 200      41.136 -55.123  40.706  0.00  0.00
E
```

ATOM 29754 HN ARG E 200 41.717 -54.886 41.481 0.00 0.00
E
ATOM 29755 CA ARG E 200 40.381 -56.378 40.713 0.00 0.00
E
ATOM 29756 HA ARG E 200 40.147 -56.746 39.725 0.00 0.00
E
ATOM 29757 CB ARG E 200 41.252 -57.489 41.254 0.00 0.00
E
ATOM 29758 HB1 ARG E 200 42.041 -57.573 40.477 0.00 0.00
E
ATOM 29759 HB2 ARG E 200 41.727 -57.223 42.223 0.00 0.00
E
ATOM 29760 CG ARG E 200 40.592 -58.872 41.279 0.00 0.00
E
ATOM 29761 HG1 ARG E 200 41.086 -59.481 42.065 0.00 0.00
E
ATOM 29762 HG2 ARG E 200 39.534 -58.944 41.611 0.00 0.00
E
ATOM 29763 CD ARG E 200 40.652 -59.463 39.849 0.00 0.00
E
ATOM 29764 HD1 ARG E 200 40.240 -58.705 39.149 0.00 0.00
E
ATOM 29765 HD2 ARG E 200 41.717 -59.625 39.579 0.00 0.00
E
ATOM 29766 NE ARG E 200 39.936 -60.785 39.780 0.00 0.00
E
ATOM 29767 HE ARG E 200 39.036 -60.762 40.215 0.00 0.00
E
ATOM 29768 CZ ARG E 200 40.212 -61.743 38.891 0.00 0.00
E
ATOM 29769 NH1 ARG E 200 41.324 -61.687 38.139 0.00 0.00
E
ATOM 29770 HH11 ARG E 200 41.648 -62.507 37.667 0.00 0.00
E
ATOM 29771 HH12 ARG E 200 41.880 -60.870 38.293 0.00 0.00
E
ATOM 29772 NH2 ARG E 200 39.392 -62.809 38.971 0.00 0.00
E
ATOM 29773 HH21 ARG E 200 39.362 -63.614 38.378 0.00 0.00
E
ATOM 29774 HH22 ARG E 200 38.702 -62.806 39.695 0.00 0.00
E
ATOM 29775 C ARG E 200 39.055 -56.199 41.561 0.00 0.00
E
ATOM 29776 O ARG E 200 38.145 -57.019 41.697 0.00 0.00
E
ATOM 29777 N GLU E 201 38.999 -55.011 42.165 0.00 0.00
E
ATOM 29778 HN GLU E 201 39.750 -54.367 42.041 0.00 0.00
E
ATOM 29779 CA GLU E 201 37.837 -54.589 42.996 0.00 0.00
E
ATOM 29780 HA GLU E 201 36.959 -55.015 42.535 0.00 0.00
E

ATOM 29781 CB GLU E 201 38.022 -54.874 44.546 0.00 0.00
E
ATOM 29782 HB1 GLU E 201 38.967 -54.431 44.926 0.00 0.00
E
ATOM 29783 HB2 GLU E 201 37.190 -54.411 45.117 0.00 0.00
E
ATOM 29784 CG GLU E 201 38.045 -56.354 44.896 0.00 0.00
E
ATOM 29785 HG1 GLU E 201 37.077 -56.774 44.546 0.00 0.00
E
ATOM 29786 HG2 GLU E 201 38.865 -56.916 44.400 0.00 0.00
E
ATOM 29787 CD GLU E 201 38.204 -56.703 46.433 0.00 0.00
E
ATOM 29788 OE1 GLU E 201 37.311 -57.137 47.153 0.00 0.00
E
ATOM 29789 OE2 GLU E 201 39.379 -56.545 46.856 0.00 0.00
E
ATOM 29790 C GLU E 201 37.814 -53.132 42.787 0.00 0.00
E
ATOM 29791 O GLU E 201 38.706 -52.556 42.156 0.00 0.00
E
ATOM 29792 N GLN E 202 36.772 -52.394 43.218 0.00 0.00
E
ATOM 29793 HN GLN E 202 35.972 -52.863 43.585 0.00 0.00
E
ATOM 29794 CA GLN E 202 36.861 -50.979 42.943 0.00 0.00
E
ATOM 29795 HA GLN E 202 36.991 -50.751 41.895 0.00 0.00
E
ATOM 29796 CB GLN E 202 35.551 -50.250 43.348 0.00 0.00
E
ATOM 29797 HB1 GLN E 202 34.711 -50.714 42.788 0.00 0.00
E
ATOM 29798 HB2 GLN E 202 35.321 -50.276 44.434 0.00 0.00
E
ATOM 29799 CG GLN E 202 35.620 -48.709 42.988 0.00 0.00
E
ATOM 29800 HG1 GLN E 202 36.455 -48.164 43.477 0.00 0.00
E
ATOM 29801 HG2 GLN E 202 35.698 -48.696 41.880 0.00 0.00
E
ATOM 29802 CD GLN E 202 34.336 -47.954 43.374 0.00 0.00
E
ATOM 29803 OE1 GLN E 202 33.889 -47.827 44.496 0.00 0.00
E
ATOM 29804 NE2 GLN E 202 33.718 -47.446 42.298 0.00 0.00
E
ATOM 29805 HE21 GLN E 202 32.815 -47.026 42.386 0.00 0.00
E
ATOM 29806 HE22 GLN E 202 34.206 -47.598 41.439 0.00 0.00
E
ATOM 29807 C GLN E 202 38.048 -50.337 43.638 0.00 0.00
E

ATOM 29808 O GLN E 202 38.208 -50.568 44.821 0.00 0.00
E
ATOM 29809 N ASP E 203 38.746 -49.409 42.902 0.00 0.00
E
ATOM 29810 HN ASP E 203 38.507 -49.361 41.935 0.00 0.00
E
ATOM 29811 CA ASP E 203 39.859 -48.628 43.545 0.00 0.00
E
ATOM 29812 HA ASP E 203 39.468 -48.350 44.513 0.00 0.00
E
ATOM 29813 CB ASP E 203 41.157 -49.482 43.668 0.00 0.00
E
ATOM 29814 HB1 ASP E 203 40.898 -50.562 43.639 0.00 0.00
E
ATOM 29815 HB2 ASP E 203 41.883 -49.288 42.850 0.00 0.00
E
ATOM 29816 CG ASP E 203 41.948 -49.197 44.914 0.00 0.00
E
ATOM 29817 OD1 ASP E 203 41.536 -48.258 45.683 0.00 0.00
E
ATOM 29818 OD2 ASP E 203 42.832 -49.998 45.224 0.00 0.00
E
ATOM 29819 C ASP E 203 40.082 -47.283 42.723 0.00 0.00
E
ATOM 29820 O ASP E 203 39.506 -46.923 41.708 0.00 0.00
E
ATOM 29821 N CYS E 204 41.036 -46.491 43.258 0.00 0.00
E
ATOM 29822 HN CYS E 204 41.624 -46.763 44.016 0.00 0.00
E
ATOM 29823 CA CYS E 204 41.146 -45.049 42.877 0.00 0.00
E
ATOM 29824 HA CYS E 204 40.315 -44.800 42.234 0.00 0.00
E
ATOM 29825 CB CYS E 204 41.268 -43.985 44.041 0.00 0.00
E
ATOM 29826 HB1 CYS E 204 40.435 -44.205 44.741 0.00 0.00
E
ATOM 29827 HB2 CYS E 204 42.233 -44.135 44.570 0.00 0.00
E
ATOM 29828 SG CYS E 204 41.213 -42.237 43.426 0.00 0.00
E
ATOM 29829 HG1 CYS E 204 41.294 -41.668 44.620 0.00 0.00
E
ATOM 29830 C CYS E 204 42.398 -45.047 41.956 0.00 0.00
E
ATOM 29831 O CYS E 204 43.576 -45.311 42.304 0.00 0.00
E
ATOM 29832 N LEU E 205 42.256 -44.838 40.638 0.00 0.00
E
ATOM 29833 HN LEU E 205 41.390 -44.755 40.152 0.00 0.00
E
ATOM 29834 CA LEU E 205 43.223 -44.648 39.555 0.00 0.00
E

ATOM 29835 HA LEU E 205 44.149 -45.136 39.819 0.00 0.00
E
ATOM 29836 CB LEU E 205 42.518 -45.034 38.183 0.00 0.00
E
ATOM 29837 HB1 LEU E 205 41.765 -44.246 37.971 0.00 0.00
E
ATOM 29838 HB2 LEU E 205 43.309 -45.128 37.408 0.00 0.00
E
ATOM 29839 CG LEU E 205 41.674 -46.362 38.230 0.00 0.00
E
ATOM 29840 HG LEU E 205 40.853 -46.262 38.972 0.00 0.00
E
ATOM 29841 CD1 LEU E 205 41.001 -46.669 36.925 0.00 0.00
E
ATOM 29842 HD11 LEU E 205 40.466 -47.628 37.091 0.00 0.00
E
ATOM 29843 HD12 LEU E 205 40.184 -45.966 36.655 0.00 0.00
E
ATOM 29844 HD13 LEU E 205 41.604 -46.907 36.023 0.00 0.00
E
ATOM 29845 CD2 LEU E 205 42.695 -47.493 38.623 0.00 0.00
E
ATOM 29846 HD21 LEU E 205 42.854 -47.321 39.709 0.00 0.00
E
ATOM 29847 HD22 LEU E 205 42.211 -48.480 38.464 0.00 0.00
E
ATOM 29848 HD23 LEU E 205 43.649 -47.643 38.075 0.00 0.00
E
ATOM 29849 C LEU E 205 43.534 -43.166 39.340 0.00 0.00
E
ATOM 29850 O LEU E 205 42.643 -42.324 39.319 0.00 0.00
E
ATOM 29851 N VAL E 206 44.786 -42.866 39.027 0.00 0.00
E
ATOM 29852 HN VAL E 206 45.616 -43.403 39.160 0.00 0.00
E
ATOM 29853 CA VAL E 206 45.129 -41.531 38.558 0.00 0.00
E
ATOM 29854 HA VAL E 206 44.386 -40.747 38.543 0.00 0.00
E
ATOM 29855 CB VAL E 206 46.367 -40.952 39.291 0.00 0.00
E
ATOM 29856 HB VAL E 206 47.025 -41.808 39.555 0.00 0.00
E
ATOM 29857 CG1 VAL E 206 47.233 -39.801 38.694 0.00 0.00
E
ATOM 29858 HG11 VAL E 206 48.108 -39.612 39.352 0.00 0.00
E
ATOM 29859 HG12 VAL E 206 47.493 -40.026 37.637 0.00 0.00
E
ATOM 29860 HG13 VAL E 206 46.562 -38.925 38.560 0.00 0.00
E
ATOM 29861 CG2 VAL E 206 45.730 -40.356 40.534 0.00 0.00
E

ATOM 29862 HG21 VAL E 206 45.005 -39.544 40.310 0.00 0.00
E
ATOM 29863 HG22 VAL E 206 45.283 -41.119 41.206 0.00 0.00
E
ATOM 29864 HG23 VAL E 206 46.479 -39.887 41.206 0.00 0.00
E
ATOM 29865 C VAL E 206 45.641 -41.606 37.123 0.00 0.00
E
ATOM 29866 O VAL E 206 46.510 -42.374 36.689 0.00 0.00
E
ATOM 29867 N ILE E 207 45.062 -40.737 36.221 0.00 0.00
E
ATOM 29868 HN ILE E 207 44.509 -40.098 36.749 0.00 0.00
E
ATOM 29869 CA ILE E 207 45.111 -40.920 34.741 0.00 0.00
E
ATOM 29870 HA ILE E 207 45.791 -41.625 34.287 0.00 0.00
E
ATOM 29871 CB ILE E 207 43.757 -41.483 34.245 0.00 0.00
E
ATOM 29872 HB ILE E 207 43.010 -40.755 34.629 0.00 0.00
E
ATOM 29873 CG2 ILE E 207 43.646 -41.696 32.707 0.00 0.00
E
ATOM 29874 HG21 ILE E 207 43.795 -40.759 32.129 0.00 0.00
E
ATOM 29875 HG22 ILE E 207 44.391 -42.452 32.380 0.00 0.00
E
ATOM 29876 HG23 ILE E 207 42.678 -42.128 32.372 0.00 0.00
E
ATOM 29877 CG1 ILE E 207 43.330 -42.766 35.008 0.00 0.00
E
ATOM 29878 HG11 ILE E 207 44.068 -43.582 34.855 0.00 0.00
E
ATOM 29879 HG12 ILE E 207 43.211 -42.587 36.098 0.00 0.00
E
ATOM 29880 CD ILE E 207 41.935 -43.215 34.669 0.00 0.00
E
ATOM 29881 HD1 ILE E 207 41.326 -42.295 34.795 0.00 0.00
E
ATOM 29882 HD2 ILE E 207 41.783 -43.643 33.655 0.00 0.00
E
ATOM 29883 HD3 ILE E 207 41.472 -43.996 35.309 0.00 0.00
E
ATOM 29884 C ILE E 207 45.356 -39.599 34.022 0.00 0.00
E
ATOM 29885 O ILE E 207 44.651 -38.608 34.214 0.00 0.00
E
ATOM 29886 N MET E 208 46.221 -39.578 32.967 0.00 0.00
E
ATOM 29887 HN MET E 208 46.750 -40.401 32.773 0.00 0.00
E
ATOM 29888 CA MET E 208 46.477 -38.414 32.128 0.00 0.00
E

ATOM 29889 HA MET E 208 45.932 -37.557 32.495 0.00 0.00
E
ATOM 29890 CB MET E 208 47.954 -38.058 32.157 0.00 0.00
E
ATOM 29891 HB1 MET E 208 48.184 -37.811 33.215 0.00 0.00
E
ATOM 29892 HB2 MET E 208 48.586 -38.904 31.815 0.00 0.00
E
ATOM 29893 CG MET E 208 48.369 -36.751 31.372 0.00 0.00
E
ATOM 29894 HG1 MET E 208 48.347 -36.937 30.276 0.00 0.00
E
ATOM 29895 HG2 MET E 208 47.670 -35.912 31.576 0.00 0.00
E
ATOM 29896 SD MET E 208 50.122 -36.429 31.770 0.00 0.00
E
ATOM 29897 CE MET E 208 50.390 -35.048 30.540 0.00 0.00
E
ATOM 29898 HE1 MET E 208 50.176 -35.298 29.479 0.00 0.00
E
ATOM 29899 HE2 MET E 208 49.759 -34.194 30.868 0.00 0.00
E
ATOM 29900 HE3 MET E 208 51.445 -34.701 30.552 0.00 0.00
E
ATOM 29901 C MET E 208 45.910 -38.747 30.725 0.00 0.00
E
ATOM 29902 O MET E 208 46.038 -39.839 30.280 0.00 0.00
E
ATOM 29903 N GLY E 209 45.308 -37.806 30.048 0.00 0.00
E
ATOM 29904 HN GLY E 209 45.103 -36.897 30.403 0.00 0.00
E
ATOM 29905 CA GLY E 209 44.882 -38.064 28.658 0.00 0.00
E
ATOM 29906 HA1 GLY E 209 44.272 -38.954 28.612 0.00 0.00
E
ATOM 29907 HA2 GLY E 209 45.752 -37.926 28.033 0.00 0.00
E
ATOM 29908 C GLY E 209 43.931 -36.959 28.247 0.00 0.00
E
ATOM 29909 O GLY E 209 43.640 -36.059 28.966 0.00 0.00
E
ATOM 29910 N HSE E 210 43.448 -36.958 27.020 0.00 0.00
E
ATOM 29911 HN HSE E 210 43.731 -37.648 26.359 0.00 0.00
E
ATOM 29912 CA HSE E 210 42.336 -36.083 26.628 0.00 0.00
E
ATOM 29913 HA HSE E 210 42.660 -35.106 26.954 0.00 0.00
E
ATOM 29914 CB HSE E 210 42.331 -35.945 25.091 0.00 0.00
E
ATOM 29915 HB1 HSE E 210 43.318 -35.490 24.865 0.00 0.00
E

ATOM 29916 HB2 HSE E 210 42.246 -36.929 24.584 0.00 0.00
E
ATOM 29917 ND1 HSE E 210 40.171 -35.459 23.794 0.00 0.00
E
ATOM 29918 CG HSE E 210 41.275 -34.979 24.518 0.00 0.00
E
ATOM 29919 CE1 HSE E 210 39.558 -34.317 23.511 0.00 0.00
E
ATOM 29920 HE1 HSE E 210 38.563 -34.343 23.069 0.00 0.00
E
ATOM 29921 NE2 HSE E 210 40.256 -33.246 23.818 0.00 0.00
E
ATOM 29922 HE2 HSE E 210 39.935 -32.299 23.792 0.00 0.00
E
ATOM 29923 CD2 HSE E 210 41.347 -33.663 24.493 0.00 0.00
E
ATOM 29924 HD2 HSE E 210 42.171 -33.095 24.908 0.00 0.00
E
ATOM 29925 C HSE E 210 41.068 -36.384 27.261 0.00 0.00
E
ATOM 29926 O HSE E 210 40.845 -37.501 27.719 0.00 0.00
E
ATOM 29927 N LYS E 211 40.189 -35.372 27.384 0.00 0.00
E
ATOM 29928 HN LYS E 211 40.561 -34.507 27.055 0.00 0.00
E
ATOM 29929 CA LYS E 211 38.919 -35.432 28.070 0.00 0.00
E
ATOM 29930 HA LYS E 211 39.104 -35.772 29.078 0.00 0.00
E
ATOM 29931 CB LYS E 211 38.240 -34.092 28.184 0.00 0.00
E
ATOM 29932 HB1 LYS E 211 38.950 -33.398 28.682 0.00 0.00
E
ATOM 29933 HB2 LYS E 211 38.075 -33.773 27.133 0.00 0.00
E
ATOM 29934 CG LYS E 211 36.907 -33.958 28.864 0.00 0.00
E
ATOM 29935 HG1 LYS E 211 36.402 -33.061 28.448 0.00 0.00
E
ATOM 29936 HG2 LYS E 211 36.337 -34.839 28.499 0.00 0.00
E
ATOM 29937 CD LYS E 211 36.962 -33.805 30.400 0.00 0.00
E
ATOM 29938 HD1 LYS E 211 37.299 -34.726 30.922 0.00 0.00
E
ATOM 29939 HD2 LYS E 211 37.704 -33.007 30.622 0.00 0.00
E
ATOM 29940 CE LYS E 211 35.636 -33.339 31.037 0.00 0.00
E
ATOM 29941 HE1 LYS E 211 35.300 -32.315 30.767 0.00 0.00
E
ATOM 29942 HE2 LYS E 211 34.812 -34.020 30.734 0.00 0.00
E

ATOM 29943 NZ LYS E 211 35.674 -33.215 32.432 0.00 0.00 E
ATOM 29944 HZ1 LYS E 211 34.828 -32.709 32.763 0.00 0.00 E
ATOM 29945 HZ2 LYS E 211 35.706 -34.114 32.953 0.00 0.00 E
ATOM 29946 HZ3 LYS E 211 36.504 -32.608 32.588 0.00 0.00 E
ATOM 29947 C LYS E 211 37.979 -36.488 27.581 0.00 0.00 E
ATOM 29948 O LYS E 211 37.294 -37.214 28.309 0.00 0.00 E
ATOM 29949 N LYS E 212 38.005 -36.634 26.307 0.00 0.00 E
ATOM 29950 HN LYS E 212 38.736 -36.175 25.808 0.00 0.00 E
ATOM 29951 CA LYS E 212 37.112 -37.584 25.573 0.00 0.00 E
ATOM 29952 HA LYS E 212 36.103 -37.553 25.959 0.00 0.00 E
ATOM 29953 CB LYS E 212 37.052 -37.392 24.070 0.00 0.00 E
ATOM 29954 HB1 LYS E 212 38.038 -37.168 23.612 0.00 0.00 E
ATOM 29955 HB2 LYS E 212 36.630 -38.314 23.615 0.00 0.00 E
ATOM 29956 CG LYS E 212 36.239 -36.203 23.667 0.00 0.00 E
ATOM 29957 HG1 LYS E 212 35.206 -36.398 24.027 0.00 0.00 E
ATOM 29958 HG2 LYS E 212 36.634 -35.271 24.127 0.00 0.00 E
ATOM 29959 CD LYS E 212 36.169 -36.057 22.150 0.00 0.00 E
ATOM 29960 HD1 LYS E 212 37.208 -36.244 21.803 0.00 0.00 E
ATOM 29961 HD2 LYS E 212 35.677 -37.005 21.842 0.00 0.00 E
ATOM 29962 CE LYS E 212 35.518 -34.752 21.646 0.00 0.00 E
ATOM 29963 HE1 LYS E 212 34.487 -34.600 22.031 0.00 0.00 E
ATOM 29964 HE2 LYS E 212 36.076 -33.827 21.903 0.00 0.00 E
ATOM 29965 NZ LYS E 212 35.405 -34.820 20.225 0.00 0.00 E
ATOM 29966 HZ1 LYS E 212 35.469 -33.866 19.817 0.00 0.00 E
ATOM 29967 HZ2 LYS E 212 36.268 -35.298 19.898 0.00 0.00 E
ATOM 29968 HZ3 LYS E 212 34.557 -35.377 19.997 0.00 0.00 E
ATOM 29969 C LYS E 212 37.509 -39.015 25.806 0.00 0.00 E

ATOM 29970 O LYS E 212 36.633 -39.885 25.954 0.00 0.00
E
ATOM 29971 N ASP E 213 38.789 -39.412 25.807 0.00 0.00
E
ATOM 29972 HN ASP E 213 39.369 -38.617 25.649 0.00 0.00
E
ATOM 29973 CA ASP E 213 39.311 -40.757 26.172 0.00 0.00
E
ATOM 29974 HA ASP E 213 38.914 -41.537 25.539 0.00 0.00
E
ATOM 29975 CB ASP E 213 40.815 -40.678 25.938 0.00 0.00
E
ATOM 29976 HB1 ASP E 213 41.246 -39.830 26.512 0.00 0.00
E
ATOM 29977 HB2 ASP E 213 41.323 -41.588 26.325 0.00 0.00
E
ATOM 29978 CG ASP E 213 41.129 -40.529 24.453 0.00 0.00
E
ATOM 29979 OD1 ASP E 213 40.866 -41.476 23.680 0.00 0.00
E
ATOM 29980 OD2 ASP E 213 41.735 -39.526 24.001 0.00 0.00
E
ATOM 29981 C ASP E 213 39.008 -41.115 27.594 0.00 0.00
E
ATOM 29982 O ASP E 213 38.604 -42.243 27.893 0.00 0.00
E
ATOM 29983 N ILE E 214 39.013 -40.138 28.550 0.00 0.00
E
ATOM 29984 HN ILE E 214 39.266 -39.197 28.336 0.00 0.00
E
ATOM 29985 CA ILE E 214 38.702 -40.364 29.936 0.00 0.00
E
ATOM 29986 HA ILE E 214 39.159 -41.284 30.269 0.00 0.00
E
ATOM 29987 CB ILE E 214 39.136 -39.281 30.895 0.00 0.00
E
ATOM 29988 HB ILE E 214 38.711 -38.296 30.606 0.00 0.00
E
ATOM 29989 CG2 ILE E 214 38.757 -39.553 32.328 0.00 0.00
E
ATOM 29990 HG21 ILE E 214 39.003 -38.696 32.991 0.00 0.00
E
ATOM 29991 HG22 ILE E 214 37.657 -39.646 32.460 0.00 0.00
E
ATOM 29992 HG23 ILE E 214 39.182 -40.491 32.745 0.00 0.00
E
ATOM 29993 CG1 ILE E 214 40.618 -38.962 30.831 0.00 0.00
E
ATOM 29994 HG11 ILE E 214 41.068 -39.634 31.593 0.00 0.00
E
ATOM 29995 HG12 ILE E 214 40.969 -39.095 29.786 0.00 0.00
E
ATOM 29996 CD ILE E 214 41.005 -37.500 31.214 0.00 0.00
E

ATOM	29997	HD1	ILE	E	214	40.503	-36.755	30.560	0.00	0.00	E
ATOM	29998	HD2	ILE	E	214	40.680	-37.264	32.250	0.00	0.00	E
ATOM	29999	HD3	ILE	E	214	42.112	-37.519	31.296	0.00	0.00	E
ATOM	30000	C	ILE	E	214	37.232	-40.614	30.086	0.00	0.00	E
ATOM	30001	O	ILE	E	214	36.818	-41.530	30.798	0.00	0.00	E
ATOM	30002	N	LYS	E	215	36.307	-39.813	29.463	0.00	0.00	E
ATOM	30003	HN	LYS	E	215	36.559	-38.972	28.991	0.00	0.00	E
ATOM	30004	CA	LYS	E	215	34.851	-40.080	29.264	0.00	0.00	E
ATOM	30005	HA	LYS	E	215	34.425	-40.093	30.256	0.00	0.00	E
ATOM	30006	CB	LYS	E	215	34.202	-38.960	28.484	0.00	0.00	E
ATOM	30007	HB1	LYS	E	215	34.544	-37.998	28.922	0.00	0.00	E
ATOM	30008	HB2	LYS	E	215	34.429	-39.020	27.398	0.00	0.00	E
ATOM	30009	CG	LYS	E	215	32.651	-38.955	28.614	0.00	0.00	E
ATOM	30010	HG1	LYS	E	215	32.306	-38.005	28.153	0.00	0.00	E
ATOM	30011	HG2	LYS	E	215	32.101	-39.776	28.106	0.00	0.00	E
ATOM	30012	CD	LYS	E	215	32.223	-38.916	30.074	0.00	0.00	E
ATOM	30013	HD1	LYS	E	215	31.116	-38.970	29.999	0.00	0.00	E
ATOM	30014	HD2	LYS	E	215	32.523	-39.886	30.527	0.00	0.00	E
ATOM	30015	CE	LYS	E	215	32.697	-37.668	30.903	0.00	0.00	E
ATOM	30016	HE1	LYS	E	215	33.807	-37.721	30.892	0.00	0.00	E
ATOM	30017	HE2	LYS	E	215	32.380	-36.733	30.393	0.00	0.00	E
ATOM	30018	NZ	LYS	E	215	32.151	-37.809	32.200	0.00	0.00	E
ATOM	30019	HZ1	LYS	E	215	32.144	-36.936	32.765	0.00	0.00	E
ATOM	30020	HZ2	LYS	E	215	31.195	-38.201	32.082	0.00	0.00	E
ATOM	30021	HZ3	LYS	E	215	32.592	-38.574	32.749	0.00	0.00	E
ATOM	30022	C	LYS	E	215	34.488	-41.464	28.697	0.00	0.00	E
ATOM	30023	O	LYS	E	215	33.595	-42.091	29.174	0.00	0.00	E

ATOM 30024 N ARG E 216 35.287 -41.962 27.666 0.00 0.00
E
ATOM 30025 HN ARG E 216 35.972 -41.336 27.301 0.00 0.00
E
ATOM 30026 CA ARG E 216 35.154 -43.256 26.930 0.00 0.00
E
ATOM 30027 HA ARG E 216 34.140 -43.410 26.591 0.00 0.00
E
ATOM 30028 CB ARG E 216 35.962 -43.459 25.622 0.00 0.00
E
ATOM 30029 HB1 ARG E 216 35.696 -42.600 24.970 0.00 0.00
E
ATOM 30030 HB2 ARG E 216 37.040 -43.495 25.890 0.00 0.00
E
ATOM 30031 CG ARG E 216 35.685 -44.792 24.837 0.00 0.00
E
ATOM 30032 HG1 ARG E 216 36.146 -45.641 25.386 0.00 0.00
E
ATOM 30033 HG2 ARG E 216 34.585 -44.908 24.735 0.00 0.00
E
ATOM 30034 CD ARG E 216 36.259 -44.762 23.429 0.00 0.00
E
ATOM 30035 HD1 ARG E 216 35.933 -45.695 22.923 0.00 0.00
E
ATOM 30036 HD2 ARG E 216 36.004 -43.897 22.780 0.00 0.00
E
ATOM 30037 NE ARG E 216 37.724 -44.863 23.565 0.00 0.00
E
ATOM 30038 HE ARG E 216 38.075 -45.637 24.091 0.00 0.00
E
ATOM 30039 CZ ARG E 216 38.567 -43.905 23.305 0.00 0.00
E
ATOM 30040 NH1 ARG E 216 38.260 -42.890 22.503 0.00 0.00
E
ATOM 30041 HH11 ARG E 216 38.971 -42.201 22.369 0.00 0.00
E
ATOM 30042 HH12 ARG E 216 37.546 -42.966 21.807 0.00 0.00
E
ATOM 30043 NH2 ARG E 216 39.726 -43.856 23.950 0.00 0.00
E
ATOM 30044 HH21 ARG E 216 40.245 -43.001 23.954 0.00 0.00
E
ATOM 30045 HH22 ARG E 216 39.843 -44.619 24.585 0.00 0.00
E
ATOM 30046 C ARG E 216 35.453 -44.379 27.917 0.00 0.00
E
ATOM 30047 O ARG E 216 34.739 -45.387 28.089 0.00 0.00
E
ATOM 30048 N PHE E 217 36.512 -44.152 28.697 0.00 0.00
E
ATOM 30049 HN PHE E 217 37.042 -43.321 28.547 0.00 0.00
E
ATOM 30050 CA PHE E 217 37.051 -45.005 29.695 0.00 0.00
E

ATOM 30051 HA PHE E 217 37.060 −46.009 29.295 0.00 0.00
E
ATOM 30052 CB PHE E 217 38.504 −44.571 29.943 0.00 0.00
E
ATOM 30053 HB1 PHE E 217 38.997 −44.604 28.947 0.00 0.00
E
ATOM 30054 HB2 PHE E 217 38.423 −43.537 30.340 0.00 0.00
E
ATOM 30055 CG PHE E 217 39.308 −45.431 30.848 0.00 0.00
E
ATOM 30056 CD1 PHE E 217 40.073 −46.469 30.328 0.00 0.00
E
ATOM 30057 HD1 PHE E 217 39.879 −46.638 29.279 0.00 0.00
E
ATOM 30058 CE1 PHE E 217 40.919 −47.257 31.114 0.00 0.00
E
ATOM 30059 HE1 PHE E 217 41.520 −48.060 30.714 0.00 0.00
E
ATOM 30060 CZ PHE E 217 40.945 −46.917 32.491 0.00 0.00
E
ATOM 30061 HZ PHE E 217 41.523 −47.525 33.171 0.00 0.00
E
ATOM 30062 CD2 PHE E 217 39.379 −45.125 32.142 0.00 0.00
E
ATOM 30063 HD2 PHE E 217 38.846 −44.237 32.447 0.00 0.00
E
ATOM 30064 CE2 PHE E 217 40.270 −45.737 32.999 0.00 0.00
E
ATOM 30065 HE2 PHE E 217 40.317 −45.611 34.071 0.00 0.00
E
ATOM 30066 C PHE E 217 36.218 −45.103 30.898 0.00 0.00
E
ATOM 30067 O PHE E 217 35.968 −46.217 31.275 0.00 0.00
E
ATOM 30068 N GLU E 218 35.738 −44.015 31.480 0.00 0.00
E
ATOM 30069 HN GLU E 218 36.000 −43.210 30.954 0.00 0.00
E
ATOM 30070 CA GLU E 218 34.811 −43.935 32.605 0.00 0.00
E
ATOM 30071 HA GLU E 218 35.139 −44.551 33.430 0.00 0.00
E
ATOM 30072 CB GLU E 218 34.624 −42.446 33.050 0.00 0.00
E
ATOM 30073 HB1 GLU E 218 35.589 −41.941 33.265 0.00 0.00
E
ATOM 30074 HB2 GLU E 218 34.105 −41.959 32.197 0.00 0.00
E
ATOM 30075 CG GLU E 218 33.688 −42.321 34.238 0.00 0.00
E
ATOM 30076 HG1 GLU E 218 32.891 −43.077 34.075 0.00 0.00
E
ATOM 30077 HG2 GLU E 218 34.108 −42.598 35.229 0.00 0.00
E

```
ATOM  30078  CD  GLU E 218      32.977 -40.950  34.333  0.00  0.00
E
ATOM  30079  OE1 GLU E 218      33.562 -39.888  34.013  0.00  0.00
E
ATOM  30080  OE2 GLU E 218      31.836 -40.946  34.755  0.00  0.00
E
ATOM  30081  C   GLU E 218      33.493 -44.534  32.182  0.00  0.00
E
ATOM  30082  O   GLU E 218      32.923 -45.291  32.949  0.00  0.00
E
ATOM  30083  N   ASN E 219      32.907 -44.267  31.024  0.00  0.00
E
ATOM  30084  HN  ASN E 219      33.256 -43.650  30.322  0.00  0.00
E
ATOM  30085  CA  ASN E 219      31.629 -44.802  30.653  0.00  0.00
E
ATOM  30086  HA  ASN E 219      30.979 -44.774  31.515  0.00  0.00
E
ATOM  30087  CB  ASN E 219      31.015 -44.086  29.442  0.00  0.00
E
ATOM  30088  HB1 ASN E 219      31.791 -44.021  28.650  0.00  0.00
E
ATOM  30089  HB2 ASN E 219      30.168 -44.671  29.024  0.00  0.00
E
ATOM  30090  CG  ASN E 219      30.620 -42.613  29.664  0.00  0.00
E
ATOM  30091  OD1 ASN E 219      30.607 -42.103  30.783  0.00  0.00
E
ATOM  30092  ND2 ASN E 219      29.989 -41.996  28.578  0.00  0.00
E
ATOM  30093 HD21 ASN E 219      29.240 -41.378  28.821  0.00  0.00
E
ATOM  30094 HD22 ASN E 219      29.773 -42.513  27.750  0.00  0.00
E
ATOM  30095  C   ASN E 219      31.742 -46.289  30.331  0.00  0.00
E
ATOM  30096  O   ASN E 219      30.836 -47.067  30.630  0.00  0.00
E
ATOM  30097  N   GLU E 220      32.850 -46.805  29.711  0.00  0.00
E
ATOM  30098  HN  GLU E 220      33.578 -46.134  29.596  0.00  0.00
E
ATOM  30099  CA  GLU E 220      33.096 -48.237  29.552  0.00  0.00
E
ATOM  30100  HA  GLU E 220      32.221 -48.634  29.060  0.00  0.00
E
ATOM  30101  CB  GLU E 220      34.264 -48.345  28.570  0.00  0.00
E
ATOM  30102  HB1 GLU E 220      33.983 -47.831  27.626  0.00  0.00
E
ATOM  30103  HB2 GLU E 220      35.056 -47.662  28.946  0.00  0.00
E
ATOM  30104  CG  GLU E 220      34.743 -49.811  28.359  0.00  0.00
E
```

ATOM 30105 HG1 GLU E 220 35.700 -49.847 27.795 0.00 0.00
E
ATOM 30106 HG2 GLU E 220 34.912 -50.423 29.271 0.00 0.00
E
ATOM 30107 CD GLU E 220 33.665 -50.505 27.502 0.00 0.00
E
ATOM 30108 OE1 GLU E 220 32.982 -51.452 27.953 0.00 0.00
E
ATOM 30109 OE2 GLU E 220 33.464 -49.966 26.368 0.00 0.00
E
ATOM 30110 C GLU E 220 33.349 -48.891 30.867 0.00 0.00
E
ATOM 30111 O GLU E 220 32.843 -49.989 31.189 0.00 0.00
E
ATOM 30112 N GLY E 221 34.316 -48.429 31.713 0.00 0.00
E
ATOM 30113 HN GLY E 221 34.799 -47.561 31.637 0.00 0.00
E
ATOM 30114 CA GLY E 221 34.526 -49.050 33.030 0.00 0.00
E
ATOM 30115 HA1 GLY E 221 35.360 -48.518 33.464 0.00 0.00
E
ATOM 30116 HA2 GLY E 221 34.649 -50.113 32.885 0.00 0.00
E
ATOM 30117 C GLY E 221 33.402 -48.953 34.132 0.00 0.00
E
ATOM 30118 O GLY E 221 33.368 -49.922 34.962 0.00 0.00
E
ATOM 30119 N MET E 222 32.632 -47.880 34.353 0.00 0.00
E
ATOM 30120 HN MET E 222 32.822 -46.993 33.939 0.00 0.00
E
ATOM 30121 CA MET E 222 31.546 -47.969 35.286 0.00 0.00
E
ATOM 30122 HA MET E 222 31.860 -48.538 36.148 0.00 0.00
E
ATOM 30123 CB MET E 222 31.209 -46.586 35.911 0.00 0.00
E
ATOM 30124 HB1 MET E 222 32.056 -46.376 36.599 0.00 0.00
E
ATOM 30125 HB2 MET E 222 31.094 -45.798 35.136 0.00 0.00
E
ATOM 30126 CG MET E 222 29.887 -46.709 36.747 0.00 0.00
E
ATOM 30127 HG1 MET E 222 29.003 -46.747 36.075 0.00 0.00
E
ATOM 30128 HG2 MET E 222 29.915 -47.577 37.440 0.00 0.00
E
ATOM 30129 SD MET E 222 29.781 -45.230 37.847 0.00 0.00
E
ATOM 30130 CE MET E 222 29.064 -46.234 39.203 0.00 0.00
E
ATOM 30131 HE1 MET E 222 29.919 -46.818 39.608 0.00 0.00
E

ATOM 30132 HE2 MET E 222 28.745 -45.584 40.045 0.00 0.00
E
ATOM 30133 HE3 MET E 222 28.323 -46.997 38.882 0.00 0.00
E
ATOM 30134 C MET E 222 30.261 -48.562 34.650 0.00 0.00
E
ATOM 30135 O MET E 222 30.322 -49.155 33.575 0.00 0.00
E
ATOM 30136 N GLN F 8 17.376 -36.335 32.016 0.00 0.00
F
ATOM 30137 HT1 GLN F 8 16.623 -35.747 31.606 0.00 0.00
F
ATOM 30138 HT2 GLN F 8 16.849 -36.903 32.709 0.00 0.00
F
ATOM 30139 HT3 GLN F 8 17.742 -36.949 31.261 0.00 0.00
F
ATOM 30140 CA GLN F 8 18.555 -35.593 32.635 0.00 0.00
F
ATOM 30141 HA GLN F 8 19.267 -36.295 33.043 0.00 0.00
F
ATOM 30142 CB GLN F 8 19.357 -34.788 31.537 0.00 0.00
F
ATOM 30143 HB1 GLN F 8 19.297 -35.081 30.467 0.00 0.00
F
ATOM 30144 HB2 GLN F 8 18.937 -33.761 31.581 0.00 0.00
F
ATOM 30145 CG GLN F 8 20.891 -34.715 31.853 0.00 0.00
F
ATOM 30146 HG1 GLN F 8 21.424 -34.068 31.123 0.00 0.00
F
ATOM 30147 HG2 GLN F 8 20.942 -34.363 32.906 0.00 0.00
F
ATOM 30148 CD GLN F 8 21.657 -36.063 31.735 0.00 0.00
F
ATOM 30149 OE1 GLN F 8 21.599 -36.772 32.721 0.00 0.00
F
ATOM 30150 NE2 GLN F 8 22.278 -36.369 30.536 0.00 0.00
F
ATOM 30151 HE21 GLN F 8 23.069 -36.980 30.551 0.00 0.00
F
ATOM 30152 HE22 GLN F 8 21.980 -35.978 29.665 0.00 0.00
F
ATOM 30153 C GLN F 8 18.156 -34.670 33.858 0.00 0.00
F
ATOM 30154 O GLN F 8 17.557 -35.164 34.769 0.00 0.00
F
ATOM 30155 N PHE F 9 18.476 -33.338 33.859 0.00 0.00
F
ATOM 30156 HN PHE F 9 18.834 -32.823 33.084 0.00 0.00
F
ATOM 30157 CA PHE F 9 18.265 -32.454 35.005 0.00 0.00
F
ATOM 30158 HA PHE F 9 18.156 -33.019 35.919 0.00 0.00
F

ATOM 30159 CB PHE F 9 19.540 -31.422 35.090 0.00 0.00
F
ATOM 30160 HB1 PHE F 9 19.655 -30.607 34.343 0.00 0.00
F
ATOM 30161 HB2 PHE F 9 19.538 -31.024 36.126 0.00 0.00
F
ATOM 30162 CG PHE F 9 20.843 -32.275 35.064 0.00 0.00
F
ATOM 30163 CD1 PHE F 9 21.015 -33.282 36.076 0.00 0.00
F
ATOM 30164 HD1 PHE F 9 20.225 -33.366 36.808 0.00 0.00
F
ATOM 30165 CE1 PHE F 9 22.132 -34.165 35.979 0.00 0.00
F
ATOM 30166 HE1 PHE F 9 22.216 -35.000 36.658 0.00 0.00
F
ATOM 30167 CZ PHE F 9 23.104 -34.032 34.970 0.00 0.00
F
ATOM 30168 HZ PHE F 9 23.877 -34.785 35.015 0.00 0.00
F
ATOM 30169 CD2 PHE F 9 21.816 -32.116 34.085 0.00 0.00
F
ATOM 30170 HD2 PHE F 9 21.666 -31.381 33.308 0.00 0.00
F
ATOM 30171 CE2 PHE F 9 22.946 -32.980 34.042 0.00 0.00
F
ATOM 30172 HE2 PHE F 9 23.720 -32.989 33.289 0.00 0.00
F
ATOM 30173 C PHE F 9 17.126 -31.504 34.892 0.00 0.00
F
ATOM 30174 O PHE F 9 16.887 -30.830 33.869 0.00 0.00
F
ATOM 30175 N ALA F 10 16.437 -31.308 36.040 0.00 0.00
F
ATOM 30176 HN ALA F 10 16.378 -31.900 36.840 0.00 0.00
F
ATOM 30177 CA ALA F 10 15.709 -30.058 36.260 0.00 0.00
F
ATOM 30178 HA ALA F 10 15.733 -29.477 35.350 0.00 0.00
F
ATOM 30179 CB ALA F 10 14.297 -30.378 36.640 0.00 0.00
F
ATOM 30180 HB1 ALA F 10 13.821 -30.854 35.756 0.00 0.00
F
ATOM 30181 HB2 ALA F 10 14.098 -31.116 37.446 0.00 0.00
F
ATOM 30182 HB3 ALA F 10 13.731 -29.450 36.872 0.00 0.00
F
ATOM 30183 C ALA F 10 16.287 -29.328 37.422 0.00 0.00
F
ATOM 30184 O ALA F 10 16.927 -29.895 38.306 0.00 0.00
F
ATOM 30185 N VAL F 11 16.157 -27.995 37.341 0.00 0.00
F

ATOM 30186 HN VAL F 11 15.707 -27.614 36.537 0.00 0.00
F
ATOM 30187 CA VAL F 11 16.481 -27.081 38.384 0.00 0.00
F
ATOM 30188 HA VAL F 11 16.761 -27.499 39.340 0.00 0.00
F
ATOM 30189 CB VAL F 11 17.645 -26.174 37.976 0.00 0.00
F
ATOM 30190 HB VAL F 11 17.426 -25.444 37.167 0.00 0.00
F
ATOM 30191 CG1 VAL F 11 18.143 -25.329 39.262 0.00 0.00
F
ATOM 30192 HG11 VAL F 11 17.386 -24.628 39.675 0.00 0.00
F
ATOM 30193 HG12 VAL F 11 18.350 -26.062 40.071 0.00 0.00
F
ATOM 30194 HG13 VAL F 11 19.027 -24.711 38.998 0.00 0.00
F
ATOM 30195 CG2 VAL F 11 18.919 -26.944 37.566 0.00 0.00
F
ATOM 30196 HG21 VAL F 11 19.797 -26.264 37.526 0.00 0.00
F
ATOM 30197 HG22 VAL F 11 19.107 -27.649 38.404 0.00 0.00
F
ATOM 30198 HG23 VAL F 11 18.758 -27.335 36.539 0.00 0.00
F
ATOM 30199 C VAL F 11 15.325 -26.105 38.681 0.00 0.00
F
ATOM 30200 O VAL F 11 14.893 -25.331 37.851 0.00 0.00
F
ATOM 30201 N ILE F 12 14.742 -26.239 39.901 0.00 0.00
F
ATOM 30202 HN ILE F 12 15.098 -26.957 40.495 0.00 0.00
F
ATOM 30203 CA ILE F 12 13.666 -25.379 40.350 0.00 0.00
F
ATOM 30204 HA ILE F 12 13.272 -24.906 39.463 0.00 0.00
F
ATOM 30205 CB ILE F 12 12.520 -26.200 41.032 0.00 0.00
F
ATOM 30206 HB ILE F 12 13.001 -26.549 41.971 0.00 0.00
F
ATOM 30207 CG2 ILE F 12 11.363 -25.386 41.615 0.00 0.00
F
ATOM 30208 HG21 ILE F 12 10.795 -26.048 42.303 0.00 0.00
F
ATOM 30209 HG22 ILE F 12 11.822 -24.510 42.121 0.00 0.00
F
ATOM 30210 HG23 ILE F 12 10.670 -25.203 40.766 0.00 0.00
F
ATOM 30211 CG1 ILE F 12 12.054 -27.440 40.206 0.00 0.00
F
ATOM 30212 HG11 ILE F 12 11.380 -27.029 39.425 0.00 0.00
F

ATOM 30213 HG12 ILE F 12 12.885 -27.873 39.608 0.00 0.00
F
ATOM 30214 CD ILE F 12 11.376 -28.473 41.115 0.00 0.00
F
ATOM 30215 HD1 ILE F 12 11.250 -29.392 40.504 0.00 0.00
F
ATOM 30216 HD2 ILE F 12 11.967 -28.716 42.024 0.00 0.00
F
ATOM 30217 HD3 ILE F 12 10.395 -28.127 41.504 0.00 0.00
F
ATOM 30218 C ILE F 12 14.156 -24.303 41.221 0.00 0.00
F
ATOM 30219 O ILE F 12 14.622 -24.635 42.307 0.00 0.00
F
ATOM 30220 N GLY F 13 13.961 -23.018 40.834 0.00 0.00
F
ATOM 30221 HN GLY F 13 13.422 -22.856 40.011 0.00 0.00
F
ATOM 30222 CA GLY F 13 14.313 -21.858 41.638 0.00 0.00
F
ATOM 30223 HA1 GLY F 13 14.501 -22.226 42.636 0.00 0.00
F
ATOM 30224 HA2 GLY F 13 13.446 -21.221 41.535 0.00 0.00
F
ATOM 30225 C GLY F 13 15.532 -21.248 41.071 0.00 0.00
F
ATOM 30226 O GLY F 13 16.669 -21.704 41.320 0.00 0.00
F
ATOM 30227 N LEU F 14 15.440 -20.212 40.322 0.00 0.00
F
ATOM 30228 HN LEU F 14 14.590 -19.691 40.302 0.00 0.00
F
ATOM 30229 CA LEU F 14 16.541 -19.737 39.506 0.00 0.00
F
ATOM 30230 HA LEU F 14 17.386 -20.410 39.527 0.00 0.00
F
ATOM 30231 CB LEU F 14 16.101 -19.541 38.001 0.00 0.00
F
ATOM 30232 HB1 LEU F 14 15.429 -18.661 38.098 0.00 0.00
F
ATOM 30233 HB2 LEU F 14 16.935 -19.212 37.345 0.00 0.00
F
ATOM 30234 CG LEU F 14 15.466 -20.710 37.213 0.00 0.00
F
ATOM 30235 HG LEU F 14 14.641 -21.051 37.874 0.00 0.00
F
ATOM 30236 CD1 LEU F 14 14.924 -20.165 35.890 0.00 0.00
F
ATOM 30237 HD11 LEU F 14 14.254 -19.306 36.110 0.00 0.00
F
ATOM 30238 HD12 LEU F 14 15.791 -19.894 35.251 0.00 0.00
F
ATOM 30239 HD13 LEU F 14 14.465 -20.918 35.215 0.00 0.00
F

ATOM 30240 CD2 LEU F 14 16.597 -21.753 36.955 0.00 0.00
F
ATOM 30241 HD21 LEU F 14 16.613 -22.474 37.800 0.00 0.00
F
ATOM 30242 HD22 LEU F 14 16.237 -22.231 36.019 0.00 0.00
F
ATOM 30243 HD23 LEU F 14 17.577 -21.256 36.795 0.00 0.00
F
ATOM 30244 C LEU F 14 17.130 -18.397 40.028 0.00 0.00
F
ATOM 30245 O LEU F 14 17.047 -17.396 39.362 0.00 0.00
F
ATOM 30246 N GLY F 15 17.640 -18.420 41.333 0.00 0.00
F
ATOM 30247 HN GLY F 15 17.596 -19.297 41.805 0.00 0.00
F
ATOM 30248 CA GLY F 15 18.195 -17.294 41.960 0.00 0.00
F
ATOM 30249 HA1 GLY F 15 17.889 -17.293 42.996 0.00 0.00
F
ATOM 30250 HA2 GLY F 15 17.864 -16.407 41.440 0.00 0.00
F
ATOM 30251 C GLY F 15 19.710 -17.405 41.828 0.00 0.00
F
ATOM 30252 O GLY F 15 20.220 -18.043 40.882 0.00 0.00
F
ATOM 30253 N ARG F 16 20.364 -16.837 42.806 0.00 0.00
F
ATOM 30254 HN ARG F 16 19.867 -16.504 43.604 0.00 0.00
F
ATOM 30255 CA ARG F 16 21.858 -16.971 42.785 0.00 0.00
F
ATOM 30256 HA ARG F 16 22.248 -16.515 41.887 0.00 0.00
F
ATOM 30257 CB ARG F 16 22.572 -16.279 43.918 0.00 0.00
F
ATOM 30258 HB1 ARG F 16 22.171 -16.596 44.904 0.00 0.00
F
ATOM 30259 HB2 ARG F 16 23.649 -16.549 43.928 0.00 0.00
F
ATOM 30260 CG ARG F 16 22.360 -14.786 43.868 0.00 0.00
F
ATOM 30261 HG1 ARG F 16 23.099 -14.302 43.194 0.00 0.00
F
ATOM 30262 HG2 ARG F 16 21.372 -14.448 43.488 0.00 0.00
F
ATOM 30263 CD ARG F 16 22.681 -14.027 45.152 0.00 0.00
F
ATOM 30264 HD1 ARG F 16 23.626 -14.408 45.595 0.00 0.00
F
ATOM 30265 HD2 ARG F 16 22.705 -12.945 44.902 0.00 0.00
F
ATOM 30266 NE ARG F 16 21.612 -14.398 46.075 0.00 0.00
F

ATOM 30267 HE ARG F 16 20.835 −14.915 45.717 0.00 0.00
F
ATOM 30268 CZ ARG F 16 21.213 −13.757 47.197 0.00 0.00
F
ATOM 30269 NH1 ARG F 16 21.904 −12.800 47.804 0.00 0.00
F
ATOM 30270 HH11 ARG F 16 21.303 −12.270 48.403 0.00 0.00
F
ATOM 30271 HH12 ARG F 16 22.659 −12.371 47.308 0.00 0.00
F
ATOM 30272 NH2 ARG F 16 19.981 −14.004 47.640 0.00 0.00
F
ATOM 30273 HH21 ARG F 16 19.577 −13.361 48.290 0.00 0.00
F
ATOM 30274 HH22 ARG F 16 19.364 −14.582 47.106 0.00 0.00
F
ATOM 30275 C ARG F 16 22.416 −18.467 42.693 0.00 0.00
F
ATOM 30276 O ARG F 16 23.176 −18.849 41.807 0.00 0.00
F
ATOM 30277 N PHE F 17 21.768 −19.408 43.550 0.00 0.00
F
ATOM 30278 HN PHE F 17 21.021 −19.197 44.176 0.00 0.00
F
ATOM 30279 CA PHE F 17 22.215 −20.825 43.456 0.00 0.00
F
ATOM 30280 HA PHE F 17 23.288 −20.911 43.544 0.00 0.00
F
ATOM 30281 CB PHE F 17 21.584 −21.519 44.675 0.00 0.00
F
ATOM 30282 HB1 PHE F 17 21.566 −20.851 45.563 0.00 0.00
F
ATOM 30283 HB2 PHE F 17 20.506 −21.628 44.430 0.00 0.00
F
ATOM 30284 CG PHE F 17 22.110 −22.827 44.968 0.00 0.00
F
ATOM 30285 CD1 PHE F 17 23.467 −23.198 44.902 0.00 0.00
F
ATOM 30286 HD1 PHE F 17 24.290 −22.522 44.721 0.00 0.00
F
ATOM 30287 CE1 PHE F 17 23.822 −24.504 45.277 0.00 0.00
F
ATOM 30288 HE1 PHE F 17 24.835 −24.863 45.180 0.00 0.00
F
ATOM 30289 CZ PHE F 17 22.891 −25.502 45.498 0.00 0.00
F
ATOM 30290 HZ PHE F 17 23.225 −26.496 45.758 0.00 0.00
F
ATOM 30291 CD2 PHE F 17 21.144 −23.853 45.271 0.00 0.00
F
ATOM 30292 HD2 PHE F 17 20.120 −23.513 45.312 0.00 0.00
F
ATOM 30293 CE2 PHE F 17 21.503 −25.184 45.440 0.00 0.00
F

ATOM 30294 HE2 PHE F 17 20.715 -25.884 45.672 0.00 0.00
F
ATOM 30295 C PHE F 17 21.846 -21.481 42.147 0.00 0.00
F
ATOM 30296 O PHE F 17 22.692 -21.857 41.278 0.00 0.00
F
ATOM 30297 N GLY F 18 20.530 -21.538 41.939 0.00 0.00
F
ATOM 30298 HN GLY F 18 19.839 -21.180 42.563 0.00 0.00
F
ATOM 30299 CA GLY F 18 19.955 -22.360 40.820 0.00 0.00
F
ATOM 30300 HA1 GLY F 18 18.878 -22.320 40.889 0.00 0.00
F
ATOM 30301 HA2 GLY F 18 20.371 -23.342 40.990 0.00 0.00
F
ATOM 30302 C GLY F 18 20.296 -21.852 39.507 0.00 0.00
F
ATOM 30303 O GLY F 18 20.447 -22.531 38.535 0.00 0.00
F
ATOM 30304 N GLY F 19 20.480 -20.574 39.393 0.00 0.00
F
ATOM 30305 HN GLY F 19 20.541 -20.052 40.240 0.00 0.00
F
ATOM 30306 CA GLY F 19 20.865 -20.050 38.088 0.00 0.00
F
ATOM 30307 HA1 GLY F 19 20.732 -18.981 38.000 0.00 0.00
F
ATOM 30308 HA2 GLY F 19 20.366 -20.567 37.281 0.00 0.00
F
ATOM 30309 C GLY F 19 22.240 -20.249 37.642 0.00 0.00
F
ATOM 30310 O GLY F 19 22.417 -20.560 36.441 0.00 0.00
F
ATOM 30311 N SER F 20 23.272 -20.271 38.543 0.00 0.00
F
ATOM 30312 HN SER F 20 23.063 -20.052 39.493 0.00 0.00
F
ATOM 30313 CA SER F 20 24.651 -20.590 38.415 0.00 0.00
F
ATOM 30314 HA SER F 20 25.032 -19.909 37.669 0.00 0.00
F
ATOM 30315 CB SER F 20 25.412 -20.408 39.789 0.00 0.00
F
ATOM 30316 HB1 SER F 20 25.247 -19.377 40.169 0.00 0.00
F
ATOM 30317 HB2 SER F 20 24.982 -21.183 40.459 0.00 0.00
F
ATOM 30318 OG SER F 20 26.856 -20.541 39.873 0.00 0.00
F
ATOM 30319 HG1 SER F 20 27.088 -20.722 40.786 0.00 0.00
F
ATOM 30320 C SER F 20 24.864 -22.030 37.906 0.00 0.00
F

ATOM 30321 O SER F 20 25.606 -22.230 36.939 0.00 0.00
F
ATOM 30322 N ILE F 21 24.052 -22.990 38.447 0.00 0.00
F
ATOM 30323 HN ILE F 21 23.386 -22.631 39.096 0.00 0.00
F
ATOM 30324 CA ILE F 21 23.872 -24.258 37.945 0.00 0.00
F
ATOM 30325 HA ILE F 21 24.763 -24.867 37.977 0.00 0.00
F
ATOM 30326 CB ILE F 21 22.889 -25.038 38.762 0.00 0.00
F
ATOM 30327 HB ILE F 21 21.936 -24.472 38.839 0.00 0.00
F
ATOM 30328 CG2 ILE F 21 22.563 -26.481 38.211 0.00 0.00
F
ATOM 30329 HG21 ILE F 21 23.508 -27.039 38.035 0.00 0.00
F
ATOM 30330 HG22 ILE F 21 21.933 -27.048 38.929 0.00 0.00
F
ATOM 30331 HG23 ILE F 21 22.028 -26.412 37.239 0.00 0.00
F
ATOM 30332 CG1 ILE F 21 23.409 -25.083 40.257 0.00 0.00
F
ATOM 30333 HG11 ILE F 21 24.381 -25.613 40.355 0.00 0.00
F
ATOM 30334 HG12 ILE F 21 23.571 -24.028 40.566 0.00 0.00
F
ATOM 30335 CD ILE F 21 22.441 -25.671 41.272 0.00 0.00
F
ATOM 30336 HD1 ILE F 21 22.344 -26.771 41.152 0.00 0.00
F
ATOM 30337 HD2 ILE F 21 22.676 -25.517 42.347 0.00 0.00
F
ATOM 30338 HD3 ILE F 21 21.458 -25.160 41.190 0.00 0.00
F
ATOM 30339 C ILE F 21 23.525 -24.497 36.474 0.00 0.00
F
ATOM 30340 O ILE F 21 24.276 -24.989 35.606 0.00 0.00
F
ATOM 30341 N CYS F 22 22.330 -24.081 36.179 0.00 0.00
F
ATOM 30342 HN CYS F 22 21.742 -23.659 36.865 0.00 0.00
F
ATOM 30343 CA CYS F 22 21.780 -23.996 34.846 0.00 0.00
F
ATOM 30344 HA CYS F 22 21.541 -25.029 34.638 0.00 0.00
F
ATOM 30345 CB CYS F 22 20.585 -23.065 34.804 0.00 0.00
F
ATOM 30346 HB1 CYS F 22 20.723 -22.033 35.191 0.00 0.00
F
ATOM 30347 HB2 CYS F 22 20.229 -22.913 33.763 0.00 0.00
F

ATOM 30348 SG CYS F 22 19.185 -23.858 35.715 0.00 0.00
F
ATOM 30349 HG1 CYS F 22 19.283 -22.944 36.669 0.00 0.00
F
ATOM 30350 C CYS F 22 22.717 -23.400 33.784 0.00 0.00
F
ATOM 30351 O CYS F 22 22.948 -24.046 32.758 0.00 0.00
F
ATOM 30352 N LYS F 23 23.251 -22.173 33.959 0.00 0.00
F
ATOM 30353 HN LYS F 23 22.943 -21.581 34.700 0.00 0.00
F
ATOM 30354 CA LYS F 23 24.212 -21.465 33.125 0.00 0.00
F
ATOM 30355 HA LYS F 23 23.769 -21.254 32.163 0.00 0.00
F
ATOM 30356 CB LYS F 23 24.536 -20.093 33.687 0.00 0.00
F
ATOM 30357 HB1 LYS F 23 23.612 -19.479 33.731 0.00 0.00
F
ATOM 30358 HB2 LYS F 23 24.970 -20.218 34.702 0.00 0.00
F
ATOM 30359 CG LYS F 23 25.487 -19.258 32.867 0.00 0.00
F
ATOM 30360 HG1 LYS F 23 26.514 -19.676 32.935 0.00 0.00
F
ATOM 30361 HG2 LYS F 23 25.190 -19.197 31.798 0.00 0.00
F
ATOM 30362 CD LYS F 23 25.657 -17.788 33.311 0.00 0.00
F
ATOM 30363 HD1 LYS F 23 24.626 -17.431 33.517 0.00 0.00
F
ATOM 30364 HD2 LYS F 23 26.215 -17.845 34.270 0.00 0.00
F
ATOM 30365 CE LYS F 23 26.441 -16.804 32.399 0.00 0.00
F
ATOM 30366 HE1 LYS F 23 26.013 -16.815 31.374 0.00 0.00
F
ATOM 30367 HE2 LYS F 23 26.282 -15.785 32.813 0.00 0.00
F
ATOM 30368 NZ LYS F 23 27.892 -17.057 32.349 0.00 0.00
F
ATOM 30369 HZ1 LYS F 23 28.308 -16.859 33.281 0.00 0.00
F
ATOM 30370 HZ2 LYS F 23 28.037 -18.045 32.058 0.00 0.00
F
ATOM 30371 HZ3 LYS F 23 28.366 -16.363 31.736 0.00 0.00
F
ATOM 30372 C LYS F 23 25.484 -22.288 32.804 0.00 0.00
F
ATOM 30373 O LYS F 23 25.989 -22.247 31.648 0.00 0.00
F
ATOM 30374 N GLU F 24 26.044 -22.987 33.888 0.00 0.00
F

ATOM 30375 HN GLU F 24 25.587 -23.037 34.772 0.00 0.00
F
ATOM 30376 CA GLU F 24 27.269 -23.812 33.722 0.00 0.00
F
ATOM 30377 HA GLU F 24 27.984 -23.389 33.033 0.00 0.00
F
ATOM 30378 CB GLU F 24 27.820 -24.185 35.106 0.00 0.00
F
ATOM 30379 HB1 GLU F 24 27.631 -23.265 35.700 0.00 0.00
F
ATOM 30380 HB2 GLU F 24 27.153 -24.922 35.602 0.00 0.00
F
ATOM 30381 CG GLU F 24 29.249 -24.753 35.261 0.00 0.00
F
ATOM 30382 HG1 GLU F 24 29.507 -24.980 36.318 0.00 0.00
F
ATOM 30383 HG2 GLU F 24 29.300 -25.731 34.737 0.00 0.00
F
ATOM 30384 CD GLU F 24 30.430 -23.997 34.679 0.00 0.00
F
ATOM 30385 OE1 GLU F 24 31.587 -24.333 35.095 0.00 0.00
F
ATOM 30386 OE2 GLU F 24 30.182 -23.037 33.968 0.00 0.00
F
ATOM 30387 C GLU F 24 26.958 -25.200 32.975 0.00 0.00
F
ATOM 30388 O GLU F 24 27.716 -25.733 32.128 0.00 0.00
F
ATOM 30389 N LEU F 25 25.803 -25.882 33.272 0.00 0.00
F
ATOM 30390 HN LEU F 25 25.167 -25.404 33.872 0.00 0.00
F
ATOM 30391 CA LEU F 25 25.409 -27.114 32.623 0.00 0.00
F
ATOM 30392 HA LEU F 25 26.214 -27.834 32.648 0.00 0.00
F
ATOM 30393 CB LEU F 25 24.311 -27.736 33.410 0.00 0.00
F
ATOM 30394 HB1 LEU F 25 23.556 -26.942 33.592 0.00 0.00
F
ATOM 30395 HB2 LEU F 25 23.880 -28.547 32.784 0.00 0.00
F
ATOM 30396 CG LEU F 25 24.644 -28.438 34.786 0.00 0.00
F
ATOM 30397 HG LEU F 25 25.406 -27.868 35.359 0.00 0.00
F
ATOM 30398 CD1 LEU F 25 23.366 -28.575 35.497 0.00 0.00
F
ATOM 30399 HD11 LEU F 25 23.600 -28.937 36.521 0.00 0.00
F
ATOM 30400 HD12 LEU F 25 22.852 -27.601 35.643 0.00 0.00
F
ATOM 30401 HD13 LEU F 25 22.652 -29.271 35.007 0.00 0.00
F

ATOM 30402 CD2 LEU F 25 25.326 -29.826 34.703 0.00 0.00
F
ATOM 30403 HD21 LEU F 25 24.763 -30.444 33.971 0.00 0.00
F
ATOM 30404 HD22 LEU F 25 26.382 -29.714 34.377 0.00 0.00
F
ATOM 30405 HD23 LEU F 25 25.336 -30.404 35.651 0.00 0.00
F
ATOM 30406 C LEU F 25 24.982 -26.895 31.181 0.00 0.00
F
ATOM 30407 O LEU F 25 25.250 -27.748 30.313 0.00 0.00
F
ATOM 30408 N HSE F 26 24.415 -25.777 30.781 0.00 0.00
F
ATOM 30409 HN HSE F 26 24.169 -25.073 31.444 0.00 0.00
F
ATOM 30410 CA HSE F 26 24.086 -25.450 29.398 0.00 0.00
F
ATOM 30411 HA HSE F 26 23.607 -26.364 29.079 0.00 0.00
F
ATOM 30412 CB HSE F 26 23.188 -24.205 29.323 0.00 0.00
F
ATOM 30413 HB1 HSE F 26 22.370 -24.175 30.075 0.00 0.00
F
ATOM 30414 HB2 HSE F 26 23.735 -23.242 29.412 0.00 0.00
F
ATOM 30415 ND1 HSE F 26 21.782 -25.268 27.531 0.00 0.00
F
ATOM 30416 CG HSE F 26 22.405 -24.126 28.030 0.00 0.00
F
ATOM 30417 CE1 HSE F 26 21.460 -24.854 26.324 0.00 0.00
F
ATOM 30418 HE1 HSE F 26 20.837 -25.444 25.652 0.00 0.00
F
ATOM 30419 NE2 HSE F 26 21.718 -23.559 26.100 0.00 0.00
F
ATOM 30420 HE2 HSE F 26 21.432 -23.098 25.260 0.00 0.00
F
ATOM 30421 CD2 HSE F 26 22.340 -23.059 27.209 0.00 0.00
F
ATOM 30422 HD2 HSE F 26 22.845 -22.101 27.200 0.00 0.00
F
ATOM 30423 C HSE F 26 25.412 -25.321 28.521 0.00 0.00
F
ATOM 30424 O HSE F 26 25.482 -25.759 27.384 0.00 0.00
F
ATOM 30425 N ARG F 27 26.443 -24.698 29.146 0.00 0.00
F
ATOM 30426 HN ARG F 27 26.195 -24.333 30.040 0.00 0.00
F
ATOM 30427 CA ARG F 27 27.738 -24.486 28.652 0.00 0.00
F
ATOM 30428 HA ARG F 27 27.614 -23.887 27.762 0.00 0.00
F

ATOM 30429 CB ARG F 27   28.613 -23.722 29.699 0.00 0.00
F
ATOM 30430 HB1 ARG F 27   28.066 -22.930 30.254 0.00 0.00
F
ATOM 30431 HB2 ARG F 27   28.761 -24.386 30.577 0.00 0.00
F
ATOM 30432 CG ARG F 27   29.966 -23.137 29.266 0.00 0.00
F
ATOM 30433 HG1 ARG F 27   30.596 -24.009 28.990 0.00 0.00
F
ATOM 30434 HG2 ARG F 27   29.772 -22.478 28.393 0.00 0.00
F
ATOM 30435 CD ARG F 27   30.775 -22.378 30.405 0.00 0.00
F
ATOM 30436 HD1 ARG F 27   31.712 -21.902 30.045 0.00 0.00
F
ATOM 30437 HD2 ARG F 27   30.087 -21.606 30.812 0.00 0.00
F
ATOM 30438 NE ARG F 27   31.132 -23.410 31.409 0.00 0.00
F
ATOM 30439 HE ARG F 27   30.796 -23.264 32.339 0.00 0.00
F
ATOM 30440 CZ ARG F 27   32.138 -24.262 31.344 0.00 0.00
F
ATOM 30441 NH1 ARG F 27   32.813 -24.585 30.241 0.00 0.00
F
ATOM 30442 HH11 ARG F 27   33.368 -25.414 30.178 0.00 0.00
F
ATOM 30443 HH12 ARG F 27   32.688 -24.101 29.375 0.00 0.00
F
ATOM 30444 NH2 ARG F 27   32.487 -24.859 32.492 0.00 0.00
F
ATOM 30445 HH21 ARG F 27   33.295 -25.448 32.468 0.00 0.00
F
ATOM 30446 HH22 ARG F 27   32.136 -24.519 33.364 0.00 0.00
F
ATOM 30447 C   ARG F 27   28.504 -25.817 28.345 0.00 0.00
F
ATOM 30448 O   ARG F 27   29.123 -26.047 27.308 0.00 0.00
F
ATOM 30449 N   MET F 28   28.274 -26.756 29.277 0.00 0.00
F
ATOM 30450 HN MET F 28   27.685 -26.481 30.032 0.00 0.00
F
ATOM 30451 CA MET F 28   28.701 -28.126 29.202 0.00 0.00
F
ATOM 30452 HA MET F 28   29.678 -28.205 28.748 0.00 0.00
F
ATOM 30453 CB MET F 28   28.626 -28.869 30.581 0.00 0.00
F
ATOM 30454 HB1 MET F 28   27.691 -28.564 31.097 0.00 0.00
F
ATOM 30455 HB2 MET F 28   28.777 -29.965 30.476 0.00 0.00
F

ATOM 30456 CG MET F 28 29.732 −28.543 31.596 0.00 0.00
F
ATOM 30457 HG1 MET F 28 30.682 −28.993 31.238 0.00 0.00
F
ATOM 30458 HG2 MET F 28 29.902 −27.445 31.575 0.00 0.00
F
ATOM 30459 SD MET F 28 29.457 −28.862 33.342 0.00 0.00
F
ATOM 30460 CE MET F 28 30.980 −27.991 33.740 0.00 0.00
F
ATOM 30461 HE1 MET F 28 31.119 −26.946 33.391 0.00 0.00
F
ATOM 30462 HE2 MET F 28 31.316 −27.876 34.792 0.00 0.00
F
ATOM 30463 HE3 MET F 28 31.805 −28.482 33.181 0.00 0.00
F
ATOM 30464 C MET F 28 27.866 −28.912 28.166 0.00 0.00
F
ATOM 30465 O MET F 28 28.359 −29.907 27.658 0.00 0.00
F
ATOM 30466 N GLY F 29 26.659 −28.491 27.808 0.00 0.00
F
ATOM 30467 HN GLY F 29 26.223 −27.672 28.173 0.00 0.00
F
ATOM 30468 CA GLY F 29 25.732 −29.215 26.949 0.00 0.00
F
ATOM 30469 HA1 GLY F 29 26.335 −29.710 26.203 0.00 0.00
F
ATOM 30470 HA2 GLY F 29 25.036 −28.465 26.603 0.00 0.00
F
ATOM 30471 C GLY F 29 24.946 −30.307 27.537 0.00 0.00
F
ATOM 30472 O GLY F 29 24.547 −31.249 26.832 0.00 0.00
F
ATOM 30473 N HSE F 30 24.722 −30.319 28.862 0.00 0.00
F
ATOM 30474 HN HSE F 30 24.937 −29.474 29.345 0.00 0.00
F
ATOM 30475 CA HSE F 30 23.884 −31.315 29.419 0.00 0.00
F
ATOM 30476 HA HSE F 30 23.875 −32.227 28.841 0.00 0.00
F
ATOM 30477 CB HSE F 30 24.184 −31.631 30.871 0.00 0.00
F
ATOM 30478 HB1 HSE F 30 24.063 −30.746 31.531 0.00 0.00
F
ATOM 30479 HB2 HSE F 30 23.490 −32.405 31.264 0.00 0.00
F
ATOM 30480 ND1 HSE F 30 25.975 −33.306 30.588 0.00 0.00
F
ATOM 30481 CG HSE F 30 25.509 −32.108 31.107 0.00 0.00
F
ATOM 30482 CE1 HSE F 30 27.181 −33.428 30.979 0.00 0.00
F

ATOM 30483 HE1 HSE F 30 27.821 -34.283 30.761 0.00 0.00
F
ATOM 30484 NE2 HSE F 30 27.605 -32.396 31.725 0.00 0.00
F
ATOM 30485 HE2 HSE F 30 28.518 -32.346 32.130 0.00 0.00
F
ATOM 30486 CD2 HSE F 30 26.514 -31.553 31.868 0.00 0.00
F
ATOM 30487 HD2 HSE F 30 26.700 -30.554 32.244 0.00 0.00
F
ATOM 30488 C HSE F 30 22.527 -30.757 29.452 0.00 0.00
F
ATOM 30489 O HSE F 30 22.316 -29.554 29.613 0.00 0.00
F
ATOM 30490 N GLU F 31 21.544 -31.638 29.277 0.00 0.00
F
ATOM 30491 HN GLU F 31 21.817 -32.596 29.260 0.00 0.00
F
ATOM 30492 CA GLU F 31 20.154 -31.213 29.202 0.00 0.00
F
ATOM 30493 HA GLU F 31 20.172 -30.351 28.551 0.00 0.00
F
ATOM 30494 CB GLU F 31 19.260 -32.264 28.689 0.00 0.00
F
ATOM 30495 HB1 GLU F 31 19.666 -32.527 27.689 0.00 0.00
F
ATOM 30496 HB2 GLU F 31 19.247 -33.176 29.324 0.00 0.00
F
ATOM 30497 CG GLU F 31 17.815 -31.790 28.535 0.00 0.00
F
ATOM 30498 HG1 GLU F 31 17.287 -31.704 29.509 0.00 0.00
F
ATOM 30499 HG2 GLU F 31 17.729 -30.829 27.985 0.00 0.00
F
ATOM 30500 CD GLU F 31 16.944 -32.774 27.749 0.00 0.00
F
ATOM 30501 OE1 GLU F 31 16.769 -33.987 28.124 0.00 0.00
F
ATOM 30502 OE2 GLU F 31 16.327 -32.365 26.732 0.00 0.00
F
ATOM 30503 C GLU F 31 19.569 -30.759 30.572 0.00 0.00
F
ATOM 30504 O GLU F 31 19.385 -31.556 31.527 0.00 0.00
F
ATOM 30505 N VAL F 32 19.367 -29.444 30.762 0.00 0.00
F
ATOM 30506 HN VAL F 32 19.525 -28.818 30.002 0.00 0.00
F
ATOM 30507 CA VAL F 32 19.018 -28.851 32.046 0.00 0.00
F
ATOM 30508 HA VAL F 32 18.700 -29.615 32.739 0.00 0.00
F
ATOM 30509 CB VAL F 32 20.205 -28.100 32.536 0.00 0.00
F

ATOM 30510 HB VAL F 32   21.003 -28.854 32.709 0.00 0.00
F
ATOM 30511 CG1 VAL F 32   20.860 -27.100 31.568 0.00 0.00
F
ATOM 30512 HG11 VAL F 32   20.971 -27.590 30.577 0.00 0.00
F
ATOM 30513 HG12 VAL F 32   20.120 -26.276 31.476 0.00 0.00
F
ATOM 30514 HG13 VAL F 32   21.825 -26.680 31.926 0.00 0.00
F
ATOM 30515 CG2 VAL F 32   19.945 -27.484 33.956 0.00 0.00
F
ATOM 30516 HG21 VAL F 32   20.955 -27.490 34.419 0.00 0.00
F
ATOM 30517 HG22 VAL F 32   19.721 -26.398 34.013 0.00 0.00
F
ATOM 30518 HG23 VAL F 32   19.165 -28.083 34.473 0.00 0.00
F
ATOM 30519 C   VAL F 32   17.782 -27.903 31.875 0.00 0.00
F
ATOM 30520 O   VAL F 32   17.672 -26.894 31.210 0.00 0.00
F
ATOM 30521 N   LEU F 33   16.702 -28.378 32.440 0.00 0.00
F
ATOM 30522 HN LEU F 33   16.692 -29.313 32.785 0.00 0.00
F
ATOM 30523 CA LEU F 33   15.489 -27.591 32.471 0.00 0.00
F
ATOM 30524 HA LEU F 33   15.444 -27.090 31.515 0.00 0.00
F
ATOM 30525 CB LEU F 33   14.298 -28.524 32.514 0.00 0.00
F
ATOM 30526 HB1 LEU F 33   14.173 -29.068 31.553 0.00 0.00
F
ATOM 30527 HB2 LEU F 33   14.491 -29.409 33.157 0.00 0.00
F
ATOM 30528 CG LEU F 33   12.907 -27.982 32.921 0.00 0.00
F
ATOM 30529 HG LEU F 33   12.887 -27.624 33.973 0.00 0.00
F
ATOM 30530 CD1 LEU F 33   12.248 -26.850 32.085 0.00 0.00
F
ATOM 30531 HD11 LEU F 33   12.890 -26.013 31.737 0.00 0.00
F
ATOM 30532 HD12 LEU F 33   11.723 -27.213 31.175 0.00 0.00
F
ATOM 30533 HD13 LEU F 33   11.488 -26.335 32.711 0.00 0.00
F
ATOM 30534 CD2 LEU F 33   11.900 -29.222 32.961 0.00 0.00
F
ATOM 30535 HD21 LEU F 33   10.954 -28.893 33.442 0.00 0.00
F
ATOM 30536 HD22 LEU F 33   11.610 -29.635 31.971 0.00 0.00
F

```
ATOM  30537 HD23 LEU F  33      12.414 -29.989  33.579  0.00  0.00
F
ATOM  30538  C   LEU F  33      15.399 -26.578  33.707  0.00  0.00
F
ATOM  30539  O   LEU F  33      15.776 -26.888  34.811  0.00  0.00
F
ATOM  30540  N   ALA F  34      14.964 -25.314  33.383  0.00  0.00
F
ATOM  30541  HN  ALA F  34      14.665 -25.013  32.481  0.00  0.00
F
ATOM  30542  CA  ALA F  34      15.045 -24.216  34.302  0.00  0.00
F
ATOM  30543  HA  ALA F  34      15.575 -24.475  35.207  0.00  0.00
F
ATOM  30544  CB  ALA F  34      15.663 -23.059  33.548  0.00  0.00
F
ATOM  30545  HB1 ALA F  34      15.178 -22.904  32.561  0.00  0.00
F
ATOM  30546  HB2 ALA F  34      15.600 -22.031  33.964  0.00  0.00
F
ATOM  30547  HB3 ALA F  34      16.732 -23.334  33.419  0.00  0.00
F
ATOM  30548  C   ALA F  34      13.678 -23.860  34.575  0.00  0.00
F
ATOM  30549  O   ALA F  34      12.922 -23.291  33.767  0.00  0.00
F
ATOM  30550  N   VAL F  35      13.204 -23.950  35.773  0.00  0.00
F
ATOM  30551  HN  VAL F  35      13.780 -24.437  36.424  0.00  0.00
F
ATOM  30552  CA  VAL F  35      11.869 -23.650  36.288  0.00  0.00
F
ATOM  30553  HA  VAL F  35      11.382 -23.216  35.427  0.00  0.00
F
ATOM  30554  CB  VAL F  35      11.155 -24.940  36.743  0.00  0.00
F
ATOM  30555  HB  VAL F  35      11.901 -25.327  37.469  0.00  0.00
F
ATOM  30556  CG1 VAL F  35       9.708 -24.628  37.273  0.00  0.00
F
ATOM  30557 HG11 VAL F  35       9.269 -25.591  37.613  0.00  0.00
F
ATOM  30558 HG12 VAL F  35       9.641 -23.897  38.107  0.00  0.00
F
ATOM  30559 HG13 VAL F  35       9.102 -24.233  36.430  0.00  0.00
F
ATOM  30560  CG2 VAL F  35      11.137 -25.874  35.485  0.00  0.00
F
ATOM  30561 HG21 VAL F  35      12.142 -26.296  35.268  0.00  0.00
F
ATOM  30562 HG22 VAL F  35      10.305 -26.603  35.590  0.00  0.00
F
ATOM  30563 HG23 VAL F  35      10.890 -25.303  34.565  0.00  0.00
F
```

```
ATOM  30564  C   VAL F  35      11.867 -22.627  37.413  0.00  0.00
F
ATOM  30565  O   VAL F  35      12.327 -22.933  38.499  0.00  0.00
F
ATOM  30566  N   ASP F  36      11.218 -21.482  37.136  0.00  0.00
F
ATOM  30567  HN  ASP F  36      10.758 -21.318  36.267  0.00  0.00
F
ATOM  30568  CA  ASP F  36      10.940 -20.402  38.135  0.00  0.00
F
ATOM  30569  HA  ASP F  36      10.857 -20.895  39.092  0.00  0.00
F
ATOM  30570  CB  ASP F  36      11.953 -19.152  38.022  0.00  0.00
F
ATOM  30571  HB1 ASP F  36      12.887 -19.560  37.580  0.00  0.00
F
ATOM  30572  HB2 ASP F  36      11.589 -18.323  37.379  0.00  0.00
F
ATOM  30573  CG  ASP F  36      12.094 -18.517  39.414  0.00  0.00
F
ATOM  30574  OD1 ASP F  36      13.241 -18.562  39.941  0.00  0.00
F
ATOM  30575  OD2 ASP F  36      11.144 -17.797  39.791  0.00  0.00
F
ATOM  30576  C   ASP F  36       9.501 -19.929  37.973  0.00  0.00
F
ATOM  30577  O   ASP F  36       8.828 -20.349  37.010  0.00  0.00
F
ATOM  30578  N   ILE F  37       8.943 -19.115  38.904  0.00  0.00
F
ATOM  30579  HN  ILE F  37       9.510 -18.756  39.642  0.00  0.00
F
ATOM  30580  CA  ILE F  37       7.509 -18.708  38.809  0.00  0.00
F
ATOM  30581  HA  ILE F  37       6.908 -19.246  38.091  0.00  0.00
F
ATOM  30582  CB  ILE F  37       6.775 -19.001  40.195  0.00  0.00
F
ATOM  30583  HB  ILE F  37       6.803 -20.084  40.441  0.00  0.00
F
ATOM  30584  CG2 ILE F  37       7.413 -18.389  41.438  0.00  0.00
F
ATOM  30585 HG21 ILE F  37       6.887 -18.908  42.267  0.00  0.00
F
ATOM  30586 HG22 ILE F  37       8.489 -18.663  41.484  0.00  0.00
F
ATOM  30587 HG23 ILE F  37       7.279 -17.292  41.554  0.00  0.00
F
ATOM  30588  CG1 ILE F  37       5.262 -18.639  40.063  0.00  0.00
F
ATOM  30589 HG11 ILE F  37       4.708 -18.568  41.024  0.00  0.00
F
ATOM  30590 HG12 ILE F  37       5.079 -17.627  39.643  0.00  0.00
F
```

```
ATOM  30591  CD  ILE F  37       4.516 -19.583  39.041  0.00  0.00
F
ATOM  30592  HD1 ILE F  37       5.067 -20.416  38.554  0.00  0.00
F
ATOM  30593  HD2 ILE F  37       3.750 -20.052  39.694  0.00  0.00
F
ATOM  30594  HD3 ILE F  37       4.058 -18.933  38.265  0.00  0.00
F
ATOM  30595  C   ILE F  37       7.468 -17.259  38.516  0.00  0.00
F
ATOM  30596  O   ILE F  37       6.491 -16.671  38.107  0.00  0.00
F
ATOM  30597  N   ASN F  38       8.665 -16.659  38.447  0.00  0.00
F
ATOM  30598  HN  ASN F  38       9.499 -17.176  38.622  0.00  0.00
F
ATOM  30599  CA  ASN F  38       8.831 -15.340  37.881  0.00  0.00
F
ATOM  30600  HA  ASN F  38       7.874 -14.840  37.857  0.00  0.00
F
ATOM  30601  CB  ASN F  38       9.845 -14.489  38.633  0.00  0.00
F
ATOM  30602  HB1 ASN F  38      10.860 -14.880  38.861  0.00  0.00
F
ATOM  30603  HB2 ASN F  38      10.045 -13.536  38.099  0.00  0.00
F
ATOM  30604  CG  ASN F  38       9.297 -14.045  39.944  0.00  0.00
F
ATOM  30605  OD1 ASN F  38       8.487 -13.036  39.909  0.00  0.00
F
ATOM  30606  ND2 ASN F  38       9.517 -14.636  41.103  0.00  0.00
F
ATOM  30607 HD21 ASN F  38       9.240 -14.139  41.926  0.00  0.00
F
ATOM  30608 HD22 ASN F  38      10.097 -15.445  41.011  0.00  0.00
F
ATOM  30609  C   ASN F  38       9.236 -15.420  36.417  0.00  0.00
F
ATOM  30610  O   ASN F  38      10.280 -16.013  36.109  0.00  0.00
F
ATOM  30611  N   GLU F  39       8.472 -14.819  35.529  0.00  0.00
F
ATOM  30612  HN  GLU F  39       7.613 -14.433  35.856  0.00  0.00
F
ATOM  30613  CA  GLU F  39       8.803 -14.697  34.103  0.00  0.00
F
ATOM  30614  HA  GLU F  39       8.842 -15.628  33.556  0.00  0.00
F
ATOM  30615  CB  GLU F  39       7.620 -13.917  33.463  0.00  0.00
F
ATOM  30616  HB1 GLU F  39       6.726 -14.519  33.731  0.00  0.00
F
ATOM  30617  HB2 GLU F  39       7.521 -13.017  34.107  0.00  0.00
F
```

ATOM 30618 CG GLU F 39 7.753 -13.558 31.984 0.00 0.00
F
ATOM 30619 HG1 GLU F 39 8.722 -13.938 31.594 0.00 0.00
F
ATOM 30620 HG2 GLU F 39 6.987 -14.188 31.484 0.00 0.00
F
ATOM 30621 CD GLU F 39 7.660 -12.145 31.586 0.00 0.00
F
ATOM 30622 OE1 GLU F 39 7.890 -11.911 30.395 0.00 0.00
F
ATOM 30623 OE2 GLU F 39 7.256 -11.254 32.385 0.00 0.00
F
ATOM 30624 C GLU F 39 10.125 -14.034 33.738 0.00 0.00
F
ATOM 30625 O GLU F 39 10.772 -14.496 32.783 0.00 0.00
F
ATOM 30626 N GLU F 40 10.541 -12.973 34.433 0.00 0.00
F
ATOM 30627 HN GLU F 40 9.863 -12.618 35.072 0.00 0.00
F
ATOM 30628 CA GLU F 40 11.852 -12.354 34.537 0.00 0.00
F
ATOM 30629 HA GLU F 40 11.917 -11.670 33.703 0.00 0.00
F
ATOM 30630 CB GLU F 40 11.913 -11.631 35.933 0.00 0.00
F
ATOM 30631 HB1 GLU F 40 11.085 -10.890 35.940 0.00 0.00
F
ATOM 30632 HB2 GLU F 40 11.812 -12.278 36.831 0.00 0.00
F
ATOM 30633 CG GLU F 40 13.144 -10.715 36.074 0.00 0.00
F
ATOM 30634 HG1 GLU F 40 14.118 -11.248 36.116 0.00 0.00
F
ATOM 30635 HG2 GLU F 40 13.163 -10.026 35.202 0.00 0.00
F
ATOM 30636 CD GLU F 40 12.982 -9.816 37.254 0.00 0.00
F
ATOM 30637 OE1 GLU F 40 12.230 -8.801 37.124 0.00 0.00
F
ATOM 30638 OE2 GLU F 40 13.604 -10.122 38.276 0.00 0.00
F
ATOM 30639 C GLU F 40 12.976 -13.324 34.412 0.00 0.00
F
ATOM 30640 O GLU F 40 13.773 -13.301 33.477 0.00 0.00
F
ATOM 30641 N LYS F 41 12.995 -14.212 35.345 0.00 0.00
F
ATOM 30642 HN LYS F 41 12.230 -14.299 35.978 0.00 0.00
F
ATOM 30643 CA LYS F 41 14.034 -15.305 35.521 0.00 0.00
F
ATOM 30644 HA LYS F 41 15.034 -14.903 35.583 0.00 0.00
F

ATOM 30645 CB LYS F 41 13.828 −15.838 36.951 0.00 0.00
F
ATOM 30646 HB1 LYS F 41 12.813 −16.281 37.041 0.00 0.00
F
ATOM 30647 HB2 LYS F 41 14.496 −16.721 37.044 0.00 0.00
F
ATOM 30648 CG LYS F 41 14.010 −14.817 38.032 0.00 0.00
F
ATOM 30649 HG1 LYS F 41 13.105 −14.175 37.976 0.00 0.00
F
ATOM 30650 HG2 LYS F 41 13.917 −15.369 38.992 0.00 0.00
F
ATOM 30651 CD LYS F 41 15.376 −13.997 38.012 0.00 0.00
F
ATOM 30652 HD1 LYS F 41 16.099 −14.838 37.955 0.00 0.00
F
ATOM 30653 HD2 LYS F 41 15.554 −13.397 37.094 0.00 0.00
F
ATOM 30654 CE LYS F 41 15.672 −13.149 39.223 0.00 0.00
F
ATOM 30655 HE1 LYS F 41 15.058 −12.228 39.327 0.00 0.00
F
ATOM 30656 HE2 LYS F 41 15.614 −13.669 40.203 0.00 0.00
F
ATOM 30657 NZ LYS F 41 17.023 −12.597 39.100 0.00 0.00
F
ATOM 30658 HZ1 LYS F 41 16.996 −12.068 38.205 0.00 0.00
F
ATOM 30659 HZ2 LYS F 41 17.197 −11.928 39.878 0.00 0.00
F
ATOM 30660 HZ3 LYS F 41 17.798 −13.290 39.118 0.00 0.00
F
ATOM 30661 C LYS F 41 13.990 −16.359 34.472 0.00 0.00
F
ATOM 30662 O LYS F 41 14.959 −16.766 33.846 0.00 0.00
F
ATOM 30663 N VAL F 42 12.786 −16.798 34.169 0.00 0.00
F
ATOM 30664 HN VAL F 42 11.984 −16.370 34.578 0.00 0.00
F
ATOM 30665 CA VAL F 42 12.595 −17.773 33.112 0.00 0.00
F
ATOM 30666 HA VAL F 42 13.255 −18.574 33.412 0.00 0.00
F
ATOM 30667 CB VAL F 42 11.183 −18.344 33.023 0.00 0.00
F
ATOM 30668 HB VAL F 42 10.437 −17.554 32.790 0.00 0.00
F
ATOM 30669 CG1 VAL F 42 11.132 −19.556 32.039 0.00 0.00
F
ATOM 30670 HG11 VAL F 42 11.218 −19.145 31.010 0.00 0.00
F
ATOM 30671 HG12 VAL F 42 12.067 −20.142 32.169 0.00 0.00
F

```
ATOM  30672 HG13 VAL F  42      10.199 -20.156  32.106  0.00  0.00
F
ATOM  30673  CG2 VAL F  42      10.875 -18.807  34.455  0.00  0.00
F
ATOM  30674 HG21 VAL F  42      11.733 -19.411  34.819  0.00  0.00
F
ATOM  30675 HG22 VAL F  42      10.731 -17.986  35.190  0.00  0.00
F
ATOM  30676 HG23 VAL F  42       9.949 -19.408  34.575  0.00  0.00
F
ATOM  30677  C   VAL F  42      12.976 -17.231  31.689  0.00  0.00
F
ATOM  30678  O   VAL F  42      13.604 -17.838  30.782  0.00  0.00
F
ATOM  30679  N   ASN F  43      12.691 -15.938  31.453  0.00  0.00
F
ATOM  30680  HN  ASN F  43      12.091 -15.381  32.023  0.00  0.00
F
ATOM  30681  CA  ASN F  43      13.361 -15.219  30.411  0.00  0.00
F
ATOM  30682  HA  ASN F  43      13.397 -15.866  29.547  0.00  0.00
F
ATOM  30683  CB  ASN F  43      12.603 -13.965  30.075  0.00  0.00
F
ATOM  30684  HB1 ASN F  43      12.367 -13.456  31.034  0.00  0.00
F
ATOM  30685  HB2 ASN F  43      13.207 -13.244  29.483  0.00  0.00
F
ATOM  30686  CG  ASN F  43      11.285 -14.173  29.418  0.00  0.00
F
ATOM  30687  OD1 ASN F  43      10.724 -15.254  29.173  0.00  0.00
F
ATOM  30688  ND2 ASN F  43      10.661 -13.059  29.044  0.00  0.00
F
ATOM  30689 HD21 ASN F  43       9.699 -13.095  28.772  0.00  0.00
F
ATOM  30690 HD22 ASN F  43      11.138 -12.206  29.253  0.00  0.00
F
ATOM  30691  C   ASN F  43      14.827 -14.948  30.485  0.00  0.00
F
ATOM  30692  O   ASN F  43      15.527 -15.055  29.480  0.00  0.00
F
ATOM  30693  N   ALA F  44      15.399 -14.544  31.683  0.00  0.00
F
ATOM  30694  HN  ALA F  44      14.755 -14.354  32.419  0.00  0.00
F
ATOM  30695  CA  ALA F  44      16.833 -14.334  31.960  0.00  0.00
F
ATOM  30696  HA  ALA F  44      17.176 -13.622  31.224  0.00  0.00
F
ATOM  30697  CB  ALA F  44      17.060 -13.867  33.441  0.00  0.00
F
ATOM  30698  HB1 ALA F  44      16.658 -12.839  33.571  0.00  0.00
F
```

ATOM 30699 HB2 ALA F 44 16.704 -14.550 34.242 0.00 0.00
F
ATOM 30700 HB3 ALA F 44 18.137 -13.781 33.701 0.00 0.00
F
ATOM 30701 C ALA F 44 17.615 -15.637 31.656 0.00 0.00
F
ATOM 30702 O ALA F 44 18.672 -15.559 31.057 0.00 0.00
F
ATOM 30703 N TYR F 45 17.154 -16.820 32.034 0.00 0.00
F
ATOM 30704 HN TYR F 45 16.319 -16.765 32.576 0.00 0.00
F
ATOM 30705 CA TYR F 45 17.881 -18.058 31.801 0.00 0.00
F
ATOM 30706 HA TYR F 45 18.935 -17.846 31.699 0.00 0.00
F
ATOM 30707 CB TYR F 45 17.922 -18.970 33.027 0.00 0.00
F
ATOM 30708 HB1 TYR F 45 16.919 -19.337 33.335 0.00 0.00
F
ATOM 30709 HB2 TYR F 45 18.496 -19.896 32.806 0.00 0.00
F
ATOM 30710 CG TYR F 45 18.594 -18.263 34.087 0.00 0.00
F
ATOM 30711 CD1 TYR F 45 17.958 -17.549 35.118 0.00 0.00
F
ATOM 30712 HD1 TYR F 45 16.885 -17.454 35.037 0.00 0.00
F
ATOM 30713 CE1 TYR F 45 18.651 -17.042 36.232 0.00 0.00
F
ATOM 30714 HE1 TYR F 45 18.096 -16.539 37.010 0.00 0.00
F
ATOM 30715 CZ TYR F 45 20.037 -17.130 36.262 0.00 0.00
F
ATOM 30716 OH TYR F 45 20.661 -16.706 37.416 0.00 0.00
F
ATOM 30717 HH TYR F 45 19.995 -16.149 37.826 0.00 0.00
F
ATOM 30718 CD2 TYR F 45 20.040 -18.316 34.187 0.00 0.00
F
ATOM 30719 HD2 TYR F 45 20.527 -18.892 33.415 0.00 0.00
F
ATOM 30720 CE2 TYR F 45 20.756 -17.782 35.289 0.00 0.00
F
ATOM 30721 HE2 TYR F 45 21.808 -17.966 35.456 0.00 0.00
F
ATOM 30722 C TYR F 45 17.320 -18.764 30.518 0.00 0.00
F
ATOM 30723 O TYR F 45 17.515 -19.963 30.338 0.00 0.00
F
ATOM 30724 N ALA F 46 16.586 -18.104 29.560 0.00 0.00
F
ATOM 30725 HN ALA F 46 16.263 -17.162 29.610 0.00 0.00
F

ATOM 30726 CA ALA F 46   16.067 -18.774 28.384 0.00 0.00
F
ATOM 30727 HA ALA F 46   15.419 -19.565 28.732 0.00 0.00
F
ATOM 30728 CB ALA F 46   15.187 -17.713 27.619 0.00 0.00
F
ATOM 30729 HB1 ALA F 46   15.873 -16.889 27.330 0.00 0.00
F
ATOM 30730 HB2 ALA F 46   14.779 -18.230 26.724 0.00 0.00
F
ATOM 30731 HB3 ALA F 46   14.513 -17.141 28.293 0.00 0.00
F
ATOM 30732 C   ALA F 46   17.041 -19.325 27.408 0.00 0.00
F
ATOM 30733 O   ALA F 46   16.746 -20.315 26.763 0.00 0.00
F
ATOM 30734 N   SER F 47   18.216 -18.689 27.239 0.00 0.00
F
ATOM 30735 HN SER F 47   18.386 -17.876 27.792 0.00 0.00
F
ATOM 30736 CA SER F 47   19.190 -19.095 26.219 0.00 0.00
F
ATOM 30737 HA SER F 47   18.766 -19.863 25.589 0.00 0.00
F
ATOM 30738 CB SER F 47   19.720 -17.861 25.359 0.00 0.00
F
ATOM 30739 HB1 SER F 47   18.784 -17.438 24.936 0.00 0.00
F
ATOM 30740 HB2 SER F 47   20.232 -17.101 25.987 0.00 0.00
F
ATOM 30741 OG SER F 47   20.575 -18.332 24.346 0.00 0.00
F
ATOM 30742 HG1 SER F 47   20.609 -17.741 23.590 0.00 0.00
F
ATOM 30743 C   SER F 47   20.313 -19.750 27.042 0.00 0.00
F
ATOM 30744 O   SER F 47   21.171 -20.356 26.423 0.00 0.00
F
ATOM 30745 N   TYR F 48   20.368 -19.634 28.384 0.00 0.00
F
ATOM 30746 HN TYR F 48   19.614 -19.341 28.968 0.00 0.00
F
ATOM 30747 CA TYR F 48   21.411 -20.194 29.214 0.00 0.00
F
ATOM 30748 HA TYR F 48   22.170 -20.585 28.553 0.00 0.00
F
ATOM 30749 CB TYR F 48   21.846 -19.148 30.321 0.00 0.00
F
ATOM 30750 HB1 TYR F 48   20.985 -18.939 30.992 0.00 0.00
F
ATOM 30751 HB2 TYR F 48   22.726 -19.620 30.807 0.00 0.00
F
ATOM 30752 CG TYR F 48   22.346 -17.826 29.690 0.00 0.00
F

```
ATOM  30753  CD1 TYR F  48      21.578 -16.703  29.853  0.00  0.00
F
ATOM  30754  HD1 TYR F  48      20.649 -16.777  30.399  0.00  0.00
F
ATOM  30755  CE1 TYR F  48      22.058 -15.449  29.342  0.00  0.00
F
ATOM  30756  HE1 TYR F  48      21.502 -14.528  29.442  0.00  0.00
F
ATOM  30757  CZ  TYR F  48      23.147 -15.456  28.504  0.00  0.00
F
ATOM  30758  OH  TYR F  48      23.571 -14.292  27.919  0.00  0.00
F
ATOM  30759  HH  TYR F  48      24.424 -14.488  27.525  0.00  0.00
F
ATOM  30760  CD2 TYR F  48      23.664 -17.733  29.101  0.00  0.00
F
ATOM  30761  HD2 TYR F  48      24.288 -18.604  28.964  0.00  0.00
F
ATOM  30762  CE2 TYR F  48      24.016 -16.612  28.418  0.00  0.00
F
ATOM  30763  HE2 TYR F  48      24.834 -16.406  27.743  0.00  0.00
F
ATOM  30764  C   TYR F  48      20.928 -21.370  29.971  0.00  0.00
F
ATOM  30765  O   TYR F  48      21.536 -21.784  31.001  0.00  0.00
F
ATOM  30766  N   ALA F  49      19.754 -21.995  29.652  0.00  0.00
F
ATOM  30767  HN  ALA F  49      19.311 -21.662  28.823  0.00  0.00
F
ATOM  30768  CA  ALA F  49      19.344 -23.299  30.098  0.00  0.00
F
ATOM  30769  HA  ALA F  49      20.180 -23.865  30.482  0.00  0.00
F
ATOM  30770  CB  ALA F  49      18.294 -23.037  31.183  0.00  0.00
F
ATOM  30771  HB1 ALA F  49      17.289 -22.755  30.803  0.00  0.00
F
ATOM  30772  HB2 ALA F  49      18.180 -23.935  31.828  0.00  0.00
F
ATOM  30773  HB3 ALA F  49      18.451 -22.146  31.827  0.00  0.00
F
ATOM  30774  C   ALA F  49      18.736 -23.997  28.910  0.00  0.00
F
ATOM  30775  O   ALA F  49      18.510 -23.285  27.902  0.00  0.00
F
ATOM  30776  N   THR F  50      18.484 -25.254  28.877  0.00  0.00
F
ATOM  30777  HN  THR F  50      18.592 -25.747  29.737  0.00  0.00
F
ATOM  30778  CA  THR F  50      18.015 -26.092  27.766  0.00  0.00
F
ATOM  30779  HA  THR F  50      18.510 -25.785  26.857  0.00  0.00
F
```

ATOM 30780 CB THR F 50   18.279 -27.575 27.832 0.00 0.00
F
ATOM 30781 HB THR F 50   17.439 -28.024 28.404 0.00 0.00
F
ATOM 30782 OG1 THR F 50   19.518 -27.898 28.338 0.00 0.00
F
ATOM 30783 HG1 THR F 50   20.176 -27.457 27.795 0.00 0.00
F
ATOM 30784 CG2 THR F 50   18.301 -28.208 26.429 0.00 0.00
F
ATOM 30785 HG21 THR F 50   18.525 -29.286 26.572 0.00 0.00
F
ATOM 30786 HG22 THR F 50   17.480 -28.195 25.680 0.00 0.00
F
ATOM 30787 HG23 THR F 50   19.200 -27.870 25.869 0.00 0.00
F
ATOM 30788 C   THR F 50   16.546 -25.900 27.379 0.00 0.00
F
ATOM 30789 O   THR F 50   16.152 -25.960 26.227 0.00 0.00
F
ATOM 30790 N   HSE F 51   15.763 -25.684 28.450 0.00 0.00
F
ATOM 30791 HN HSE F 51   16.160 -25.680 29.364 0.00 0.00
F
ATOM 30792 CA HSE F 51   14.359 -25.573 28.432 0.00 0.00
F
ATOM 30793 HA HSE F 51   14.006 -25.087 27.535 0.00 0.00
F
ATOM 30794 CB HSE F 51   13.638 -26.906 28.912 0.00 0.00
F
ATOM 30795 HB1 HSE F 51   13.997 -27.198 29.923 0.00 0.00
F
ATOM 30796 HB2 HSE F 51   12.529 -26.904 28.844 0.00 0.00
F
ATOM 30797 ND1 HSE F 51   13.448 -28.052 26.695 0.00 0.00
F
ATOM 30798 CG HSE F 51   14.009 -28.082 27.980 0.00 0.00
F
ATOM 30799 CE1 HSE F 51   13.871 -29.214 26.188 0.00 0.00
F
ATOM 30800 HE1 HSE F 51   13.732 -29.620 25.186 0.00 0.00
F
ATOM 30801 NE2 HSE F 51   14.707 -29.895 27.001 0.00 0.00
F
ATOM 30802 HE2 HSE F 51   15.190 -30.696 26.649 0.00 0.00
F
ATOM 30803 CD2 HSE F 51   14.748 -29.196 28.200 0.00 0.00
F
ATOM 30804 HD2 HSE F 51   15.316 -29.418 29.095 0.00 0.00
F
ATOM 30805 C   HSE F 51   14.004 -24.635 29.579 0.00 0.00
F
ATOM 30806 O   HSE F 51   14.545 -24.599 30.646 0.00 0.00
F

ATOM 30807 N ALA F 52 13.028 -23.723 29.422 0.00 0.00
F
ATOM 30808 HN ALA F 52 12.407 -23.729 28.642 0.00 0.00
F
ATOM 30809 CA ALA F 52 12.810 -22.688 30.367 0.00 0.00
F
ATOM 30810 HA ALA F 52 13.237 -22.836 31.348 0.00 0.00
F
ATOM 30811 CB ALA F 52 13.450 -21.347 29.898 0.00 0.00
F
ATOM 30812 HB1 ALA F 52 13.509 -20.578 30.698 0.00 0.00
F
ATOM 30813 HB2 ALA F 52 14.502 -21.564 29.612 0.00 0.00
F
ATOM 30814 HB3 ALA F 52 12.970 -20.983 28.965 0.00 0.00
F
ATOM 30815 C ALA F 52 11.282 -22.618 30.426 0.00 0.00
F
ATOM 30816 O ALA F 52 10.576 -22.297 29.381 0.00 0.00
F
ATOM 30817 N VAL F 53 10.722 -22.892 31.575 0.00 0.00
F
ATOM 30818 HN VAL F 53 11.205 -23.019 32.437 0.00 0.00
F
ATOM 30819 CA VAL F 53 9.287 -23.099 31.743 0.00 0.00
F
ATOM 30820 HA VAL F 53 8.777 -22.619 30.921 0.00 0.00
F
ATOM 30821 CB VAL F 53 8.887 -24.621 31.812 0.00 0.00
F
ATOM 30822 HB VAL F 53 9.668 -25.021 32.493 0.00 0.00
F
ATOM 30823 CG1 VAL F 53 7.516 -24.884 32.398 0.00 0.00
F
ATOM 30824 HG11 VAL F 53 7.471 -24.490 33.436 0.00 0.00
F
ATOM 30825 HG12 VAL F 53 6.714 -24.365 31.832 0.00 0.00
F
ATOM 30826 HG13 VAL F 53 7.279 -25.969 32.349 0.00 0.00
F
ATOM 30827 CG2 VAL F 53 9.151 -25.256 30.419 0.00 0.00
F
ATOM 30828 HG21 VAL F 53 9.133 -26.356 30.571 0.00 0.00
F
ATOM 30829 HG22 VAL F 53 8.345 -25.029 29.688 0.00 0.00
F
ATOM 30830 HG23 VAL F 53 10.115 -24.892 30.003 0.00 0.00
F
ATOM 30831 C VAL F 53 8.961 -22.440 33.063 0.00 0.00
F
ATOM 30832 O VAL F 53 9.711 -22.503 34.005 0.00 0.00
F
ATOM 30833 N ILE F 54 7.796 -21.748 33.060 0.00 0.00
F

ATOM 30834 HN ILE F 54   7.216 -21.692 32.251 0.00 0.00
F
ATOM 30835 CA ILE F 54   7.235 -21.040 34.166 0.00 0.00
F
ATOM 30836 HA ILE F 54   7.935 -20.825 34.959 0.00 0.00
F
ATOM 30837 CB ILE F 54   6.758 -19.597 33.757 0.00 0.00
F
ATOM 30838 HB ILE F 54   7.666 -19.044 33.434 0.00 0.00
F
ATOM 30839 CG2 ILE F 54   5.802 -19.611 32.526 0.00 0.00
F
ATOM 30840 HG21 ILE F 54   5.503 -18.617 32.129 0.00 0.00
F
ATOM 30841 HG22 ILE F 54   6.058 -20.337 31.725 0.00 0.00
F
ATOM 30842 HG23 ILE F 54   4.886 -20.065 32.960 0.00 0.00
F
ATOM 30843 CG1 ILE F 54   6.206 -18.799 34.933 0.00 0.00
F
ATOM 30844 HG11 ILE F 54   5.227 -19.232 35.232 0.00 0.00
F
ATOM 30845 HG12 ILE F 54   6.822 -18.914 35.850 0.00 0.00
F
ATOM 30846 CD ILE F 54   5.957 -17.355 34.502 0.00 0.00
F
ATOM 30847 HD1 ILE F 54   6.894 -16.820 34.236 0.00 0.00
F
ATOM 30848 HD2 ILE F 54   5.274 -17.264 33.631 0.00 0.00
F
ATOM 30849 HD3 ILE F 54   5.539 -16.739 35.326 0.00 0.00
F
ATOM 30850 C   ILE F 54   6.019 -21.746 34.846 0.00 0.00
F
ATOM 30851 O   ILE F 54   4.851 -21.776 34.396 0.00 0.00
F
ATOM 30852 N   ALA F 55   6.439 -22.334 36.022 0.00 0.00
F
ATOM 30853 HN ALA F 55   7.351 -22.175 36.390 0.00 0.00
F
ATOM 30854 CA ALA F 55   5.534 -23.188 36.793 0.00 0.00
F
ATOM 30855 HA ALA F 55   4.494 -22.929 36.657 0.00 0.00
F
ATOM 30856 CB ALA F 55   5.657 -24.693 36.418 0.00 0.00
F
ATOM 30857 HB1 ALA F 55   5.298 -24.859 35.379 0.00 0.00
F
ATOM 30858 HB2 ALA F 55   6.723 -25.005 36.413 0.00 0.00
F
ATOM 30859 HB3 ALA F 55   5.018 -25.315 37.080 0.00 0.00
F
ATOM 30860 C   ALA F 55   5.771 -23.121 38.402 0.00 0.00
F

ATOM 30861 O ALA F 55 6.922 -22.891 38.840 0.00 0.00
F
ATOM 30862 N ASN F 56 4.675 -23.280 39.205 0.00 0.00
F
ATOM 30863 HN ASN F 56 3.747 -23.298 38.842 0.00 0.00
F
ATOM 30864 CA ASN F 56 4.802 -23.381 40.619 0.00 0.00
F
ATOM 30865 HA ASN F 56 5.743 -22.880 40.788 0.00 0.00
F
ATOM 30866 CB ASN F 56 3.602 -22.639 41.387 0.00 0.00
F
ATOM 30867 HB1 ASN F 56 3.479 -21.651 40.894 0.00 0.00
F
ATOM 30868 HB2 ASN F 56 2.700 -23.269 41.238 0.00 0.00
F
ATOM 30869 CG ASN F 56 3.944 -22.467 42.887 0.00 0.00
F
ATOM 30870 OD1 ASN F 56 4.400 -23.391 43.570 0.00 0.00
F
ATOM 30871 ND2 ASN F 56 3.770 -21.310 43.528 0.00 0.00
F
ATOM 30872 HD21 ASN F 56 4.184 -21.139 44.422 0.00 0.00
F
ATOM 30873 HD22 ASN F 56 3.526 -20.504 42.989 0.00 0.00
F
ATOM 30874 C ASN F 56 4.841 -24.841 40.992 0.00 0.00
F
ATOM 30875 O ASN F 56 4.030 -25.672 40.614 0.00 0.00
F
ATOM 30876 N ALA F 57 5.809 -25.360 41.702 0.00 0.00
F
ATOM 30877 HN ALA F 57 6.426 -24.788 42.236 0.00 0.00
F
ATOM 30878 CA ALA F 57 6.120 -26.735 41.941 0.00 0.00
F
ATOM 30879 HA ALA F 57 5.791 -27.201 41.024 0.00 0.00
F
ATOM 30880 CB ALA F 57 7.687 -26.830 42.048 0.00 0.00
F
ATOM 30881 HB1 ALA F 57 8.013 -26.385 43.013 0.00 0.00
F
ATOM 30882 HB2 ALA F 57 8.016 -27.887 41.964 0.00 0.00
F
ATOM 30883 HB3 ALA F 57 8.144 -26.201 41.254 0.00 0.00
F
ATOM 30884 C ALA F 57 5.424 -27.279 43.197 0.00 0.00
F
ATOM 30885 O ALA F 57 5.604 -28.427 43.625 0.00 0.00
F
ATOM 30886 N THR F 58 4.550 -26.445 43.838 0.00 0.00
F
ATOM 30887 HN THR F 58 4.571 -25.481 43.584 0.00 0.00
F

ATOM 30888 CA THR F 58 3.948 -26.818 45.099 0.00 0.00
F
ATOM 30889 HA THR F 58 4.492 -27.559 45.666 0.00 0.00
F
ATOM 30890 CB THR F 58 3.933 -25.560 45.985 0.00 0.00
F
ATOM 30891 HB THR F 58 3.547 -25.821 46.994 0.00 0.00
F
ATOM 30892 OG1 THR F 58 3.057 -24.613 45.406 0.00 0.00
F
ATOM 30893 HG1 THR F 58 3.500 -24.232 44.644 0.00 0.00
F
ATOM 30894 CG2 THR F 58 5.299 -24.972 46.174 0.00 0.00
F
ATOM 30895 HG21 THR F 58 5.101 -23.913 46.444 0.00 0.00
F
ATOM 30896 HG22 THR F 58 5.859 -25.468 46.995 0.00 0.00
F
ATOM 30897 HG23 THR F 58 5.933 -25.001 45.262 0.00 0.00
F
ATOM 30898 C THR F 58 2.522 -27.358 44.892 0.00 0.00
F
ATOM 30899 O THR F 58 1.779 -27.652 45.799 0.00 0.00
F
ATOM 30900 N GLU F 59 2.082 -27.504 43.650 0.00 0.00
F
ATOM 30901 HN GLU F 59 2.586 -27.105 42.887 0.00 0.00
F
ATOM 30902 CA GLU F 59 0.779 -27.987 43.333 0.00 0.00
F
ATOM 30903 HA GLU F 59 0.214 -28.392 44.159 0.00 0.00
F
ATOM 30904 CB GLU F 59 0.062 -26.792 42.660 0.00 0.00
F
ATOM 30905 HB1 GLU F 59 0.380 -25.938 43.296 0.00 0.00
F
ATOM 30906 HB2 GLU F 59 0.487 -26.537 41.665 0.00 0.00
F
ATOM 30907 CG GLU F 59 -1.463 -27.015 42.563 0.00 0.00
F
ATOM 30908 HG1 GLU F 59 -1.633 -27.813 41.809 0.00 0.00
F
ATOM 30909 HG2 GLU F 59 -1.976 -27.336 43.495 0.00 0.00
F
ATOM 30910 CD GLU F 59 -2.173 -25.897 41.909 0.00 0.00
F
ATOM 30911 OE1 GLU F 59 -2.954 -26.171 40.948 0.00 0.00
F
ATOM 30912 OE2 GLU F 59 -2.029 -24.654 42.252 0.00 0.00
F
ATOM 30913 C GLU F 59 0.847 -29.194 42.360 0.00 0.00
F
ATOM 30914 O GLU F 59 1.556 -29.166 41.333 0.00 0.00
F

ATOM 30915 N GLU F 60 0.051 -30.266 42.640 0.00 0.00
F
ATOM 30916 HN GLU F 60 -0.669 -30.138 43.318 0.00 0.00
F
ATOM 30917 CA GLU F 60 0.019 -31.491 41.810 0.00 0.00
F
ATOM 30918 HA GLU F 60 1.024 -31.879 41.733 0.00 0.00
F
ATOM 30919 CB GLU F 60 -0.698 -32.586 42.602 0.00 0.00
F
ATOM 30920 HB1 GLU F 60 -1.772 -32.318 42.507 0.00 0.00
F
ATOM 30921 HB2 GLU F 60 -0.466 -33.611 42.240 0.00 0.00
F
ATOM 30922 CG GLU F 60 -0.181 -32.665 44.060 0.00 0.00
F
ATOM 30923 HG1 GLU F 60 0.846 -33.074 43.947 0.00 0.00
F
ATOM 30924 HG2 GLU F 60 -0.167 -31.648 44.508 0.00 0.00
F
ATOM 30925 CD GLU F 60 -0.855 -33.682 44.976 0.00 0.00
F
ATOM 30926 OE1 GLU F 60 -1.166 -33.517 46.192 0.00 0.00
F
ATOM 30927 OE2 GLU F 60 -1.094 -34.804 44.469 0.00 0.00
F
ATOM 30928 C GLU F 60 -0.504 -31.381 40.338 0.00 0.00
F
ATOM 30929 O GLU F 60 0.091 -31.953 39.409 0.00 0.00
F
ATOM 30930 N ASN F 61 -1.497 -30.537 39.959 0.00 0.00
F
ATOM 30931 HN ASN F 61 -1.795 -29.834 40.599 0.00 0.00
F
ATOM 30932 CA ASN F 61 -1.886 -30.315 38.601 0.00 0.00
F
ATOM 30933 HA ASN F 61 -1.826 -31.302 38.166 0.00 0.00
F
ATOM 30934 CB ASN F 61 -3.382 -29.797 38.631 0.00 0.00
F
ATOM 30935 HB1 ASN F 61 -3.443 -28.937 39.332 0.00 0.00
F
ATOM 30936 HB2 ASN F 61 -3.763 -29.390 37.670 0.00 0.00
F
ATOM 30937 CG ASN F 61 -4.294 -30.896 39.120 0.00 0.00
F
ATOM 30938 OD1 ASN F 61 -4.366 -31.920 38.482 0.00 0.00
F
ATOM 30939 ND2 ASN F 61 -4.910 -30.694 40.343 0.00 0.00
F
ATOM 30940 HD21 ASN F 61 -5.404 -31.482 40.711 0.00 0.00
F
ATOM 30941 HD22 ASN F 61 -4.826 -29.824 40.830 0.00 0.00
F

ATOM 30942 C ASN F 61 -0.977 -29.417 37.768 0.00 0.00
F
ATOM 30943 O ASN F 61 -0.753 -29.667 36.559 0.00 0.00
F
ATOM 30944 N GLU F 62 -0.193 -28.482 38.353 0.00 0.00
F
ATOM 30945 HN GLU F 62 -0.231 -28.304 39.334 0.00 0.00
F
ATOM 30946 CA GLU F 62 0.908 -27.735 37.730 0.00 0.00
F
ATOM 30947 HA GLU F 62 0.481 -27.521 36.762 0.00 0.00
F
ATOM 30948 CB GLU F 62 1.242 -26.530 38.626 0.00 0.00
F
ATOM 30949 HB1 GLU F 62 1.377 -26.873 39.674 0.00 0.00
F
ATOM 30950 HB2 GLU F 62 2.195 -26.026 38.360 0.00 0.00
F
ATOM 30951 CG GLU F 62 0.135 -25.515 38.689 0.00 0.00
F
ATOM 30952 HG1 GLU F 62 -0.790 -25.806 39.231 0.00 0.00
F
ATOM 30953 HG2 GLU F 62 0.482 -24.656 39.302 0.00 0.00
F
ATOM 30954 CD GLU F 62 -0.245 -24.992 37.359 0.00 0.00
F
ATOM 30955 OE1 GLU F 62 0.444 -24.094 36.712 0.00 0.00
F
ATOM 30956 OE2 GLU F 62 -1.250 -25.429 36.813 0.00 0.00
F
ATOM 30957 C GLU F 62 2.043 -28.672 37.512 0.00 0.00
F
ATOM 30958 O GLU F 62 2.658 -28.643 36.456 0.00 0.00
F
ATOM 30959 N LEU F 63 2.404 -29.623 38.436 0.00 0.00
F
ATOM 30960 HN LEU F 63 2.138 -29.545 39.393 0.00 0.00
F
ATOM 30961 CA LEU F 63 3.463 -30.682 38.254 0.00 0.00
F
ATOM 30962 HA LEU F 63 4.317 -30.093 37.956 0.00 0.00
F
ATOM 30963 CB LEU F 63 3.688 -31.524 39.564 0.00 0.00
F
ATOM 30964 HB1 LEU F 63 2.750 -32.018 39.897 0.00 0.00
F
ATOM 30965 HB2 LEU F 63 4.431 -32.330 39.387 0.00 0.00
F
ATOM 30966 CG LEU F 63 4.347 -30.543 40.666 0.00 0.00
F
ATOM 30967 HG LEU F 63 3.583 -29.761 40.863 0.00 0.00
F
ATOM 30968 CD1 LEU F 63 4.535 -31.387 41.887 0.00 0.00
F

```
ATOM  30969 HD11 LEU F  63       3.737 -32.128  42.111  0.00  0.00
F
ATOM  30970 HD12 LEU F  63       5.499 -31.896  41.676  0.00  0.00
F
ATOM  30971 HD13 LEU F  63       4.749 -30.599  42.641  0.00  0.00
F
ATOM  30972  CD2 LEU F  63       5.689 -29.912  40.187  0.00  0.00
F
ATOM  30973 HD21 LEU F  63       6.210 -30.751  39.679  0.00  0.00
F
ATOM  30974 HD22 LEU F  63       5.554 -29.134  39.405  0.00  0.00
F
ATOM  30975 HD23 LEU F  63       6.313 -29.492  41.004  0.00  0.00
F
ATOM  30976  C   LEU F  63       3.193 -31.709  37.156  0.00  0.00
F
ATOM  30977  O   LEU F  63       4.121 -32.000  36.398  0.00  0.00
F
ATOM  30978  N   LEU F  64       1.962 -32.154  37.048  0.00  0.00
F
ATOM  30979  HN  LEU F  64       1.315 -32.045  37.798  0.00  0.00
F
ATOM  30980  CA  LEU F  64       1.477 -32.950  35.882  0.00  0.00
F
ATOM  30981  HA  LEU F  64       2.118 -33.813  35.779  0.00  0.00
F
ATOM  30982  CB  LEU F  64       0.103 -33.568  36.221  0.00  0.00
F
ATOM  30983  HB1 LEU F  64       0.341 -34.274  37.046  0.00  0.00
F
ATOM  30984  HB2 LEU F  64      -0.534 -32.732  36.580  0.00  0.00
F
ATOM  30985  CG  LEU F  64      -0.685 -34.318  35.059  0.00  0.00
F
ATOM  30986  HG  LEU F  64      -0.704 -33.579  34.230  0.00  0.00
F
ATOM  30987  CD1 LEU F  64       0.156 -35.516  34.600  0.00  0.00
F
ATOM  30988 HD11 LEU F  64       0.503 -36.235  35.373  0.00  0.00
F
ATOM  30989 HD12 LEU F  64      -0.349 -36.181  33.867  0.00  0.00
F
ATOM  30990 HD13 LEU F  64       1.148 -35.254  34.174  0.00  0.00
F
ATOM  30991  CD2 LEU F  64      -2.095 -34.697  35.588  0.00  0.00
F
ATOM  30992 HD21 LEU F  64      -1.968 -35.285  36.522  0.00  0.00
F
ATOM  30993 HD22 LEU F  64      -2.681 -33.777  35.797  0.00  0.00
F
ATOM  30994 HD23 LEU F  64      -2.692 -35.331  34.898  0.00  0.00
F
ATOM  30995  C   LEU F  64       1.548 -32.129  34.606  0.00  0.00
F
```

ATOM 30996 O   LEU F 64   2.257 −32.600 33.741 0.00 0.00
F
ATOM 30997 N   SER F 65   1.055 −30.879 34.476 0.00 0.00
F
ATOM 30998 HN SER F 65   0.615 −30.451 35.262 0.00 0.00
F
ATOM 30999 CA SER F 65   1.366 −30.044 33.343 0.00 0.00
F
ATOM 31000 HA SER F 65   1.005 −30.669 32.540 0.00 0.00
F
ATOM 31001 CB SER F 65   0.464 −28.710 33.382 0.00 0.00
F
ATOM 31002 HB1 SER F 65   0.472 −28.443 34.460 0.00 0.00
F
ATOM 31003 HB2 SER F 65   0.928 −27.907 32.769 0.00 0.00
F
ATOM 31004 OG SER F 65   −0.802 −28.874 32.885 0.00 0.00
F
ATOM 31005 HG1 SER F 65   −1.290 −28.216 33.387 0.00 0.00
F
ATOM 31006 C   SER F 65   2.781 −29.678 33.053 0.00 0.00
F
ATOM 31007 O   SER F 65   3.216 −29.741 31.945 0.00 0.00
F
ATOM 31008 N   LEU F 66   3.631 −29.305 34.058 0.00 0.00
F
ATOM 31009 HN LEU F 66   3.207 −29.283 34.960 0.00 0.00
F
ATOM 31010 CA LEU F 66   5.092 −29.250 33.910 0.00 0.00
F
ATOM 31011 HA LEU F 66   5.290 −28.433 33.232 0.00 0.00
F
ATOM 31012 CB LEU F 66   5.689 −28.977 35.280 0.00 0.00
F
ATOM 31013 HB1 LEU F 66   5.309 −28.031 35.721 0.00 0.00
F
ATOM 31014 HB2 LEU F 66   5.469 −29.856 35.924 0.00 0.00
F
ATOM 31015 CG LEU F 66   7.213 −28.814 35.411 0.00 0.00
F
ATOM 31016 HG LEU F 66   7.754 −29.773 35.266 0.00 0.00
F
ATOM 31017 CD1 LEU F 66   7.726 −27.776 34.482 0.00 0.00
F
ATOM 31018 HD11 LEU F 66   7.666 −28.264 33.485 0.00 0.00
F
ATOM 31019 HD12 LEU F 66   7.173 −26.817 34.569 0.00 0.00
F
ATOM 31020 HD13 LEU F 66   8.828 −27.724 34.612 0.00 0.00
F
ATOM 31021 CD2 LEU F 66   7.531 −28.502 36.881 0.00 0.00
F
ATOM 31022 HD21 LEU F 66   8.593 −28.497 37.207 0.00 0.00
F

```
ATOM  31023 HD22 LEU F  66       7.053 -27.533  37.142  0.00  0.00
F
ATOM  31024 HD23 LEU F  66       7.148 -29.367  37.464  0.00  0.00
F
ATOM  31025  C   LEU F  66       5.694 -30.514  33.303  0.00  0.00
F
ATOM  31026  O   LEU F  66       6.702 -30.465  32.614  0.00  0.00
F
ATOM  31027  N   GLY F  67       5.211 -31.723  33.649  0.00  0.00
F
ATOM  31028  HN  GLY F  67       4.453 -31.836  34.286  0.00  0.00
F
ATOM  31029  CA  GLY F  67       5.868 -32.945  33.151  0.00  0.00
F
ATOM  31030  HA1 GLY F  67       6.391 -32.803  32.217  0.00  0.00
F
ATOM  31031  HA2 GLY F  67       5.152 -33.747  33.050  0.00  0.00
F
ATOM  31032  C   GLY F  67       6.993 -33.496  34.035  0.00  0.00
F
ATOM  31033  O   GLY F  67       7.907 -34.175  33.587  0.00  0.00
F
ATOM  31034  N   ILE F  68       7.020 -33.176  35.369  0.00  0.00
F
ATOM  31035  HN  ILE F  68       6.278 -32.586  35.678  0.00  0.00
F
ATOM  31036  CA  ILE F  68       8.260 -33.287  36.172  0.00  0.00
F
ATOM  31037  HA  ILE F  68       9.038 -32.876  35.545  0.00  0.00
F
ATOM  31038  CB  ILE F  68       8.255 -32.391  37.398  0.00  0.00
F
ATOM  31039  HB  ILE F  68       8.011 -31.344  37.119  0.00  0.00
F
ATOM  31040  CG2 ILE F  68       7.267 -32.817  38.404  0.00  0.00
F
ATOM  31041 HG21 ILE F  68       7.454 -33.876  38.681  0.00  0.00
F
ATOM  31042 HG22 ILE F  68       7.343 -32.354  39.411  0.00  0.00
F
ATOM  31043 HG23 ILE F  68       6.226 -32.708  38.032  0.00  0.00
F
ATOM  31044  CG1 ILE F  68       9.761 -32.285  37.910  0.00  0.00
F
ATOM  31045 HG11 ILE F  68       9.674 -31.774  38.893  0.00  0.00
F
ATOM  31046 HG12 ILE F  68      10.198 -33.278  38.149  0.00  0.00
F
ATOM  31047  CD  ILE F  68      10.759 -31.393  37.108  0.00  0.00
F
ATOM  31048  HD1 ILE F  68      11.701 -31.339  37.694  0.00  0.00
F
ATOM  31049  HD2 ILE F  68      11.054 -31.834  36.132  0.00  0.00
F
```

```
ATOM  31050  HD3 ILE F  68      10.230 -30.438  36.902  0.00  0.00
F
ATOM  31051  C   ILE F  68       8.567 -34.716  36.513  0.00  0.00
F
ATOM  31052  O   ILE F  68       9.688 -35.093  36.747  0.00  0.00
F
ATOM  31053  N   ARG F  69       7.595 -35.590  36.442  0.00  0.00
F
ATOM  31054  HN  ARG F  69       6.641 -35.304  36.405  0.00  0.00
F
ATOM  31055  CA  ARG F  69       7.781 -36.983  36.564  0.00  0.00
F
ATOM  31056  HA  ARG F  69       8.311 -37.074  37.501  0.00  0.00
F
ATOM  31057  CB  ARG F  69       6.386 -37.705  36.845  0.00  0.00
F
ATOM  31058  HB1 ARG F  69       5.654 -37.055  37.370  0.00  0.00
F
ATOM  31059  HB2 ARG F  69       5.925 -38.186  35.955  0.00  0.00
F
ATOM  31060  CG  ARG F  69       6.591 -38.914  37.839  0.00  0.00
F
ATOM  31061  HG1 ARG F  69       7.370 -39.540  37.354  0.00  0.00
F
ATOM  31062  HG2 ARG F  69       7.011 -38.527  38.793  0.00  0.00
F
ATOM  31063  CD  ARG F  69       5.281 -39.715  38.064  0.00  0.00
F
ATOM  31064  HD1 ARG F  69       4.354 -39.154  38.308  0.00  0.00
F
ATOM  31065  HD2 ARG F  69       5.121 -40.275  37.119  0.00  0.00
F
ATOM  31066  NE  ARG F  69       5.557 -40.788  39.062  0.00  0.00
F
ATOM  31067  HE  ARG F  69       6.444 -40.714  39.517  0.00  0.00
F
ATOM  31068  CZ  ARG F  69       4.817 -41.861  39.343  0.00  0.00
F
ATOM  31069  NH1 ARG F  69       3.592 -42.042  39.033  0.00  0.00
F
ATOM  31070 HH11 ARG F  69       3.102 -41.428  38.415  0.00  0.00
F
ATOM  31071 HH12 ARG F  69       3.129 -42.815  39.467  0.00  0.00
F
ATOM  31072  NH2 ARG F  69       5.425 -42.780  40.072  0.00  0.00
F
ATOM  31073 HH21 ARG F  69       4.891 -43.578  40.351  0.00  0.00
F
ATOM  31074 HH22 ARG F  69       6.415 -42.751  40.207  0.00  0.00
F
ATOM  31075  C   ARG F  69       8.534 -37.728  35.470  0.00  0.00
F
ATOM  31076  O   ARG F  69       9.016 -38.838  35.640  0.00  0.00
F
```

ATOM 31077 N   ASN F 70   8.839 -37.038 34.349 0.00 0.00
F
ATOM 31078 HN ASN F 70   8.510 -36.105 34.223 0.00 0.00
F
ATOM 31079 CA ASN F 70   9.749 -37.536 33.273 0.00 0.00
F
ATOM 31080 HA ASN F 70   9.645 -38.611 33.246 0.00 0.00
F
ATOM 31081 CB ASN F 70   9.335 -37.032 31.899 0.00 0.00
F
ATOM 31082 HB1 ASN F 70   9.665 -35.971 31.863 0.00 0.00
F
ATOM 31083 HB2 ASN F 70   9.983 -37.568 31.173 0.00 0.00
F
ATOM 31084 CG ASN F 70   7.843 -37.136 31.527 0.00 0.00
F
ATOM 31085 OD1 ASN F 70   7.339 -38.259 31.529 0.00 0.00
F
ATOM 31086 ND2 ASN F 70   7.201 -35.982 31.290 0.00 0.00
F
ATOM 31087 HD21 ASN F 70   6.276 -36.050 30.917 0.00 0.00
F
ATOM 31088 HD22 ASN F 70   7.636 -35.114 31.528 0.00 0.00
F
ATOM 31089 C   ASN F 70   11.191 -37.073 33.559 0.00 0.00
F
ATOM 31090 O   ASN F 70   12.097 -37.287 32.799 0.00 0.00
F
ATOM 31091 N   PHE F 71   11.465 -36.413 34.664 0.00 0.00
F
ATOM 31092 HN PHE F 71   10.764 -36.156 35.325 0.00 0.00
F
ATOM 31093 CA PHE F 71   12.807 -36.011 35.025 0.00 0.00
F
ATOM 31094 HA PHE F 71   13.447 -36.043 34.156 0.00 0.00
F
ATOM 31095 CB PHE F 71   12.873 -34.617 35.692 0.00 0.00
F
ATOM 31096 HB1 PHE F 71   11.908 -34.243 36.095 0.00 0.00
F
ATOM 31097 HB2 PHE F 71   13.774 -34.701 36.336 0.00 0.00
F
ATOM 31098 CG PHE F 71   12.941 -33.618 34.495 0.00 0.00
F
ATOM 31099 CD1 PHE F 71   11.736 -33.282 33.931 0.00 0.00
F
ATOM 31100 HD1 PHE F 71   10.868 -33.592 34.493 0.00 0.00
F
ATOM 31101 CE1 PHE F 71   11.687 -32.496 32.780 0.00 0.00
F
ATOM 31102 HE1 PHE F 71   10.713 -32.128 32.493 0.00 0.00
F
ATOM 31103 CZ PHE F 71   12.875 -32.030 32.177 0.00 0.00
F

ATOM 31104 HZ PHE F 71 12.815 -31.553 31.210 0.00 0.00
F
ATOM 31105 CD2 PHE F 71 14.178 -33.341 33.883 0.00 0.00
F
ATOM 31106 HD2 PHE F 71 15.059 -33.869 34.217 0.00 0.00
F
ATOM 31107 CE2 PHE F 71 14.116 -32.578 32.655 0.00 0.00
F
ATOM 31108 HE2 PHE F 71 15.072 -32.473 32.163 0.00 0.00
F
ATOM 31109 C PHE F 71 13.228 -36.965 36.074 0.00 0.00
F
ATOM 31110 O PHE F 71 12.507 -37.188 37.039 0.00 0.00
F
ATOM 31111 N GLU F 72 14.384 -37.603 35.916 0.00 0.00
F
ATOM 31112 HN GLU F 72 14.871 -37.634 35.047 0.00 0.00
F
ATOM 31113 CA GLU F 72 15.013 -38.424 36.929 0.00 0.00
F
ATOM 31114 HA GLU F 72 14.213 -39.016 37.350 0.00 0.00
F
ATOM 31115 CB GLU F 72 16.012 -39.251 36.190 0.00 0.00
F
ATOM 31116 HB1 GLU F 72 17.030 -38.808 36.230 0.00 0.00
F
ATOM 31117 HB2 GLU F 72 16.227 -40.085 36.892 0.00 0.00
F
ATOM 31118 CG GLU F 72 15.824 -39.784 34.733 0.00 0.00
F
ATOM 31119 HG1 GLU F 72 16.219 -40.819 34.654 0.00 0.00
F
ATOM 31120 HG2 GLU F 72 14.740 -39.727 34.498 0.00 0.00
F
ATOM 31121 CD GLU F 72 16.494 -38.772 33.801 0.00 0.00
F
ATOM 31122 OE1 GLU F 72 17.565 -38.993 33.256 0.00 0.00
F
ATOM 31123 OE2 GLU F 72 15.911 -37.643 33.612 0.00 0.00
F
ATOM 31124 C GLU F 72 15.810 -37.625 37.992 0.00 0.00
F
ATOM 31125 O GLU F 72 16.198 -38.149 39.042 0.00 0.00
F
ATOM 31126 N TYR F 73 16.134 -36.354 37.711 0.00 0.00
F
ATOM 31127 HN TYR F 73 15.866 -35.981 36.827 0.00 0.00
F
ATOM 31128 CA TYR F 73 17.053 -35.672 38.589 0.00 0.00
F
ATOM 31129 HA TYR F 73 17.132 -36.119 39.569 0.00 0.00
F
ATOM 31130 CB TYR F 73 18.413 -35.435 37.988 0.00 0.00
F

ATOM 31131 HB1 TYR F 73   18.555 -36.167 37.164 0.00 0.00
F
ATOM 31132 HB2 TYR F 73   18.501 -34.481 37.426 0.00 0.00
F
ATOM 31133 CG TYR F 73   19.586 -35.681 38.963 0.00 0.00
F
ATOM 31134 CD1 TYR F 73   20.464 -36.762 38.697 0.00 0.00
F
ATOM 31135 HD1 TYR F 73   20.347 -37.480 37.899 0.00 0.00
F
ATOM 31136 CE1 TYR F 73   21.673 -36.797 39.434 0.00 0.00
F
ATOM 31137 HE1 TYR F 73   22.405 -37.564 39.229 0.00 0.00
F
ATOM 31138 CZ TYR F 73   21.898 -36.024 40.584 0.00 0.00
F
ATOM 31139 OH TYR F 73   23.033 -36.219 41.420 0.00 0.00
F
ATOM 31140 HH TYR F 73   22.885 -35.742 42.240 0.00 0.00
F
ATOM 31141 CD2 TYR F 73   19.866 -34.819 40.009 0.00 0.00
F
ATOM 31142 HD2 TYR F 73   19.057 -34.121 40.161 0.00 0.00
F
ATOM 31143 CE2 TYR F 73   20.982 -35.022 40.868 0.00 0.00
F
ATOM 31144 HE2 TYR F 73   21.156 -34.275 41.629 0.00 0.00
F
ATOM 31145 C   TYR F 73   16.464 -34.285 38.835 0.00 0.00
F
ATOM 31146 O   TYR F 73   16.131 -33.511 37.897 0.00 0.00
F
ATOM 31147 N   VAL F 74   16.176 -33.948 40.090 0.00 0.00
F
ATOM 31148 HN VAL F 74   16.224 -34.527 40.901 0.00 0.00
F
ATOM 31149 CA VAL F 74   15.529 -32.705 40.409 0.00 0.00
F
ATOM 31150 HA VAL F 74   15.581 -32.051 39.551 0.00 0.00
F
ATOM 31151 CB VAL F 74   14.027 -32.913 40.692 0.00 0.00
F
ATOM 31152 HB VAL F 74   13.627 -33.478 39.823 0.00 0.00
F
ATOM 31153 CG1 VAL F 74   13.894 -33.829 41.930 0.00 0.00
F
ATOM 31154 HG11 VAL F 74   12.810 -33.993 42.109 0.00 0.00
F
ATOM 31155 HG12 VAL F 74   14.438 -34.772 41.706 0.00 0.00
F
ATOM 31156 HG13 VAL F 74   14.384 -33.440 42.848 0.00 0.00
F
ATOM 31157 CG2 VAL F 74   13.210 -31.612 40.706 0.00 0.00
F

ATOM 31158 HG21 VAL F 74 12.148 -31.920 40.601 0.00 0.00
F
ATOM 31159 HG22 VAL F 74 13.379 -31.147 41.701 0.00 0.00
F
ATOM 31160 HG23 VAL F 74 13.419 -30.899 39.880 0.00 0.00
F
ATOM 31161 C VAL F 74 16.295 -31.952 41.550 0.00 0.00
F
ATOM 31162 O VAL F 74 16.493 -32.412 42.686 0.00 0.00
F
ATOM 31163 N ILE F 75 16.842 -30.771 41.255 0.00 0.00
F
ATOM 31164 HN ILE F 75 16.868 -30.410 40.326 0.00 0.00
F
ATOM 31165 CA ILE F 75 17.498 -29.870 42.307 0.00 0.00
F
ATOM 31166 HA ILE F 75 17.648 -30.378 43.248 0.00 0.00
F
ATOM 31167 CB ILE F 75 18.825 -29.192 41.913 0.00 0.00
F
ATOM 31168 HB ILE F 75 18.825 -28.442 41.094 0.00 0.00
F
ATOM 31169 CG2 ILE F 75 19.426 -28.575 43.174 0.00 0.00
F
ATOM 31170 HG21 ILE F 75 19.393 -29.252 44.055 0.00 0.00
F
ATOM 31171 HG22 ILE F 75 20.466 -28.218 43.012 0.00 0.00
F
ATOM 31172 HG23 ILE F 75 18.700 -27.814 43.531 0.00 0.00
F
ATOM 31173 CG1 ILE F 75 19.931 -30.201 41.453 0.00 0.00
F
ATOM 31174 HG11 ILE F 75 20.969 -29.805 41.475 0.00 0.00
F
ATOM 31175 HG12 ILE F 75 19.908 -31.044 42.177 0.00 0.00
F
ATOM 31176 CD ILE F 75 19.731 -30.867 40.052 0.00 0.00
F
ATOM 31177 HD1 ILE F 75 18.878 -31.578 40.071 0.00 0.00
F
ATOM 31178 HD2 ILE F 75 19.667 -30.034 39.319 0.00 0.00
F
ATOM 31179 HD3 ILE F 75 20.664 -31.434 39.844 0.00 0.00
F
ATOM 31180 C ILE F 75 16.452 -28.720 42.589 0.00 0.00
F
ATOM 31181 O ILE F 75 15.851 -28.121 41.730 0.00 0.00
F
ATOM 31182 N VAL F 76 16.212 -28.562 43.920 0.00 0.00
F
ATOM 31183 HN VAL F 76 16.641 -29.175 44.579 0.00 0.00
F
ATOM 31184 CA VAL F 76 15.490 -27.436 44.458 0.00 0.00
F

```
ATOM  31185  HA  VAL F  76      14.894 -26.970  43.687  0.00  0.00
F
ATOM  31186  CB  VAL F  76      14.505 -27.877  45.537  0.00  0.00
F
ATOM  31187  HB  VAL F  76      15.065 -28.251  46.420  0.00  0.00
F
ATOM  31188  CG1 VAL F  76      13.492 -26.704  45.947  0.00  0.00
F
ATOM  31189 HG11 VAL F  76      13.151 -26.243  44.996  0.00  0.00
F
ATOM  31190 HG12 VAL F  76      12.614 -27.153  46.458  0.00  0.00
F
ATOM  31191 HG13 VAL F  76      13.929 -25.847  46.503  0.00  0.00
F
ATOM  31192  CG2 VAL F  76      13.553 -29.023  45.081  0.00  0.00
F
ATOM  31193 HG21 VAL F  76      13.210 -28.831  44.042  0.00  0.00
F
ATOM  31194 HG22 VAL F  76      14.189 -29.931  45.004  0.00  0.00
F
ATOM  31195 HG23 VAL F  76      12.692 -29.161  45.769  0.00  0.00
F
ATOM  31196  C   VAL F  76      16.463 -26.437  45.034  0.00  0.00
F
ATOM  31197  O   VAL F  76      17.244 -26.703  45.979  0.00  0.00
F
ATOM  31198  N   ALA F  77      16.389 -25.121  44.483  0.00  0.00
F
ATOM  31199  HN  ALA F  77      15.685 -24.867  43.825  0.00  0.00
F
ATOM  31200  CA  ALA F  77      17.439 -24.142  44.712  0.00  0.00
F
ATOM  31201  HA  ALA F  77      18.147 -24.472  45.457  0.00  0.00
F
ATOM  31202  CB  ALA F  77      18.194 -23.866  43.410  0.00  0.00
F
ATOM  31203  HB1 ALA F  77      17.426 -23.679  42.630  0.00  0.00
F
ATOM  31204  HB2 ALA F  77      18.869 -22.997  43.565  0.00  0.00
F
ATOM  31205  HB3 ALA F  77      18.851 -24.723  43.152  0.00  0.00
F
ATOM  31206  C   ALA F  77      16.751 -22.923  45.215  0.00  0.00
F
ATOM  31207  O   ALA F  77      17.372 -21.889  45.302  0.00  0.00
F
ATOM  31208  N   ILE F  78      15.423 -23.065  45.649  0.00  0.00
F
ATOM  31209  HN  ILE F  78      15.016 -23.974  45.608  0.00  0.00
F
ATOM  31210  CA  ILE F  78      14.509 -22.033  46.198  0.00  0.00
F
ATOM  31211  HA  ILE F  78      14.432 -21.216  45.496  0.00  0.00
F
```

ATOM	31212	CB	ILE	F	78	13.096	-22.549	46.309	0.00	0.00	F
ATOM	31213	HB	ILE	F	78	13.118	-23.448	46.962	0.00	0.00	F
ATOM	31214	CG2	ILE	F	78	12.141	-21.481	46.976	0.00	0.00	F
ATOM	31215	HG21	ILE	F	78	11.099	-21.831	47.136	0.00	0.00	F
ATOM	31216	HG22	ILE	F	78	12.445	-21.303	48.030	0.00	0.00	F
ATOM	31217	HG23	ILE	F	78	12.109	-20.466	46.524	0.00	0.00	F
ATOM	31218	CG1	ILE	F	78	12.515	-22.817	44.939	0.00	0.00	F
ATOM	31219	HG11	ILE	F	78	12.225	-21.889	44.402	0.00	0.00	F
ATOM	31220	HG12	ILE	F	78	13.325	-23.100	44.233	0.00	0.00	F
ATOM	31221	CD	ILE	F	78	11.269	-23.595	44.880	0.00	0.00	F
ATOM	31222	HD1	ILE	F	78	11.279	-24.647	45.236	0.00	0.00	F
ATOM	31223	HD2	ILE	F	78	10.483	-23.099	45.488	0.00	0.00	F
ATOM	31224	HD3	ILE	F	78	10.829	-23.604	43.860	0.00	0.00	F
ATOM	31225	C	ILE	F	78	15.110	-21.492	47.464	0.00	0.00	F
ATOM	31226	O	ILE	F	78	15.496	-22.204	48.400	0.00	0.00	F
ATOM	31227	N	GLY	F	79	15.148	-20.097	47.602	0.00	0.00	F
ATOM	31228	HN	GLY	F	79	14.621	-19.552	46.954	0.00	0.00	F
ATOM	31229	CA	GLY	F	79	15.914	-19.398	48.536	0.00	0.00	F
ATOM	31230	HA1	GLY	F	79	16.226	-18.514	48.000	0.00	0.00	F
ATOM	31231	HA2	GLY	F	79	16.659	-20.039	48.983	0.00	0.00	F
ATOM	31232	C	GLY	F	79	15.109	-18.919	49.661	0.00	0.00	F
ATOM	31233	O	GLY	F	79	14.959	-19.634	50.624	0.00	0.00	F
ATOM	31234	N	ALA	F	80	14.518	-17.679	49.529	0.00	0.00	F
ATOM	31235	HN	ALA	F	80	14.687	-17.111	48.727	0.00	0.00	F
ATOM	31236	CA	ALA	F	80	13.762	-17.041	50.606	0.00	0.00	F
ATOM	31237	HA	ALA	F	80	14.459	-16.921	51.421	0.00	0.00	F
ATOM	31238	CB	ALA	F	80	13.379	-15.651	50.152	0.00	0.00	F

ATOM 31239 HB1 ALA F 80   12.835 -15.179 50.998 0.00 0.00
F
ATOM 31240 HB2 ALA F 80   14.211 -14.933 49.990 0.00 0.00
F
ATOM 31241 HB3 ALA F 80   12.708 -15.634 49.267 0.00 0.00
F
ATOM 31242 C   ALA F 80   12.473 -17.802 51.129 0.00 0.00
F
ATOM 31243 O   ALA F 80   12.234 -17.790 52.298 0.00 0.00
F
ATOM 31244 N   ASN F 81   11.650 -18.406 50.278 0.00 0.00
F
ATOM 31245 HN ASN F 81   11.780 -18.483 49.293 0.00 0.00
F
ATOM 31246 CA ASN F 81   10.473 -19.074 50.766 0.00 0.00
F
ATOM 31247 HA ASN F 81   10.183 -18.593 51.689 0.00 0.00
F
ATOM 31248 CB ASN F 81   9.282 -19.108 49.801 0.00 0.00
F
ATOM 31249 HB1 ASN F 81   9.355 -18.175 49.202 0.00 0.00
F
ATOM 31250 HB2 ASN F 81   9.375 -19.893 49.021 0.00 0.00
F
ATOM 31251 CG ASN F 81   7.930 -19.202 50.443 0.00 0.00
F
ATOM 31252 OD1 ASN F 81   7.531 -20.252 50.913 0.00 0.00
F
ATOM 31253 ND2 ASN F 81   7.299 -18.012 50.649 0.00 0.00
F
ATOM 31254 HD21 ASN F 81   6.390 -17.939 51.059 0.00 0.00
F
ATOM 31255 HD22 ASN F 81   7.871 -17.235 50.386 0.00 0.00
F
ATOM 31256 C   ASN F 81   10.878 -20.467 51.251 0.00 0.00
F
ATOM 31257 O   ASN F 81   11.219 -21.286 50.400 0.00 0.00
F
ATOM 31258 N   ILE F 82   10.897 -20.672 52.616 0.00 0.00
F
ATOM 31259 HN ILE F 82   10.598 -19.917 53.195 0.00 0.00
F
ATOM 31260 CA ILE F 82   11.099 -21.988 53.250 0.00 0.00
F
ATOM 31261 HA ILE F 82   11.961 -22.451 52.794 0.00 0.00
F
ATOM 31262 CB ILE F 82   11.447 -21.890 54.745 0.00 0.00
F
ATOM 31263 HB ILE F 82   12.363 -21.271 54.852 0.00 0.00
F
ATOM 31264 CG2 ILE F 82   10.307 -21.163 55.577 0.00 0.00
F
ATOM 31265 HG21 ILE F 82   9.481 -21.887 55.746 0.00 0.00
F

ATOM 31266 HG22 ILE F 82   10.636 -20.782 56.567 0.00 0.00
F
ATOM 31267 HG23 ILE F 82   9.837 -20.331 55.009 0.00 0.00
F
ATOM 31268 CG1 ILE F 82   11.711 -23.240 55.393 0.00 0.00
F
ATOM 31269 HG11 ILE F 82   10.777 -23.839 55.334 0.00 0.00
F
ATOM 31270 HG12 ILE F 82   12.551 -23.703 54.832 0.00 0.00
F
ATOM 31271 CD ILE F 82   12.132 -23.092 56.872 0.00 0.00
F
ATOM 31272 HD1 ILE F 82   12.268 -24.072 57.378 0.00 0.00
F
ATOM 31273 HD2 ILE F 82   13.015 -22.423 56.953 0.00 0.00
F
ATOM 31274 HD3 ILE F 82   11.328 -22.672 57.513 0.00 0.00
F
ATOM 31275 C   ILE F 82   10.005 -22.979 52.933 0.00 0.00
F
ATOM 31276 O   ILE F 82   10.228 -24.076 52.488 0.00 0.00
F
ATOM 31277 N   GLN F 83   8.714 -22.615 53.177 0.00 0.00
F
ATOM 31278 HN GLN F 83   8.534 -21.688 53.496 0.00 0.00
F
ATOM 31279 CA GLN F 83   7.517 -23.387 52.826 0.00 0.00
F
ATOM 31280 HA GLN F 83   7.782 -24.211 53.472 0.00 0.00
F
ATOM 31281 CB GLN F 83   6.146 -22.763 53.247 0.00 0.00
F
ATOM 31282 HB1 GLN F 83   6.185 -22.559 54.339 0.00 0.00
F
ATOM 31283 HB2 GLN F 83   5.889 -21.847 52.673 0.00 0.00
F
ATOM 31284 CG GLN F 83   4.898 -23.688 53.033 0.00 0.00
F
ATOM 31285 HG1 GLN F 83   4.101 -22.929 53.186 0.00 0.00
F
ATOM 31286 HG2 GLN F 83   4.792 -23.985 51.968 0.00 0.00
F
ATOM 31287 CD GLN F 83   4.827 -24.786 54.158 0.00 0.00
F
ATOM 31288 OE1 GLN F 83   4.461 -24.493 55.311 0.00 0.00
F
ATOM 31289 NE2 GLN F 83   5.230 -25.977 53.725 0.00 0.00
F
ATOM 31290 HE21 GLN F 83   5.702 -26.119 52.855 0.00 0.00
F
ATOM 31291 HE22 GLN F 83   5.106 -26.860 54.177 0.00 0.00
F
ATOM 31292 C   GLN F 83   7.397 -24.029 51.442 0.00 0.00
F

```
ATOM  31293  O   GLN F  83       6.925 -25.169  51.177  0.00  0.00
F
ATOM  31294  N   ALA F  84       7.752 -23.184  50.408  0.00  0.00
F
ATOM  31295  HN  ALA F  84       7.882 -22.205  50.541  0.00  0.00
F
ATOM  31296  CA  ALA F  84       7.738 -23.574  49.001  0.00  0.00
F
ATOM  31297  HA  ALA F  84       6.729 -23.846  48.726  0.00  0.00
F
ATOM  31298  CB  ALA F  84       8.139 -22.446  48.100  0.00  0.00
F
ATOM  31299  HB1 ALA F  84       9.213 -22.174  48.184  0.00  0.00
F
ATOM  31300  HB2 ALA F  84       7.864 -22.764  47.072  0.00  0.00
F
ATOM  31301  HB3 ALA F  84       7.556 -21.512  48.252  0.00  0.00
F
ATOM  31302  C   ALA F  84       8.674 -24.703  48.780  0.00  0.00
F
ATOM  31303  O   ALA F  84       8.179 -25.751  48.259  0.00  0.00
F
ATOM  31304  N   SER F  85       9.939 -24.585  49.169  0.00  0.00
F
ATOM  31305  HN  SER F  85      10.102 -23.812  49.778  0.00  0.00
F
ATOM  31306  CA  SER F  85      10.901 -25.653  49.025  0.00  0.00
F
ATOM  31307  HA  SER F  85      11.117 -25.845  47.984  0.00  0.00
F
ATOM  31308  CB  SER F  85      12.228 -25.176  49.684  0.00  0.00
F
ATOM  31309  HB1 SER F  85      12.490 -24.237  49.151  0.00  0.00
F
ATOM  31310  HB2 SER F  85      12.081 -24.993  50.770  0.00  0.00
F
ATOM  31311  OG  SER F  85      13.300 -26.071  49.586  0.00  0.00
F
ATOM  31312  HG1 SER F  85      13.830 -25.855  48.816  0.00  0.00
F
ATOM  31313  C   SER F  85      10.441 -26.927  49.791  0.00  0.00
F
ATOM  31314  O   SER F  85      10.590 -28.027  49.241  0.00  0.00
F
ATOM  31315  N   THR F  86       9.805 -26.811  50.985  0.00  0.00
F
ATOM  31316  HN  THR F  86       9.591 -25.938  51.416  0.00  0.00
F
ATOM  31317  CA  THR F  86       9.514 -27.972  51.816  0.00  0.00
F
ATOM  31318  HA  THR F  86      10.311 -28.697  51.742  0.00  0.00
F
ATOM  31319  CB  THR F  86       9.307 -27.692  53.337  0.00  0.00
F
```

ATOM 31320 HB THR F 86   9.142 -28.655 53.866 0.00 0.00
F
ATOM 31321 OG1 THR F 86   8.270 -26.768 53.514 0.00 0.00
F
ATOM 31322 HG1 THR F 86   8.207 -26.655 54.466 0.00 0.00
F
ATOM 31323 CG2 THR F 86   10.507 -27.116 53.967 0.00 0.00
F
ATOM 31324 HG21 THR F 86   11.316 -27.861 53.809 0.00 0.00
F
ATOM 31325 HG22 THR F 86   10.972 -26.180 53.589 0.00 0.00
F
ATOM 31326 HG23 THR F 86   10.405 -27.020 55.069 0.00 0.00
F
ATOM 31327 C   THR F 86   8.254 -28.685 51.345 0.00 0.00
F
ATOM 31328 O   THR F 86   8.053 -29.892 51.480 0.00 0.00
F
ATOM 31329 N   LEU F 87   7.268 -28.046 50.808 0.00 0.00
F
ATOM 31330 HN LEU F 87   7.294 -27.052 50.873 0.00 0.00
F
ATOM 31331 CA LEU F 87   6.122 -28.575 50.165 0.00 0.00
F
ATOM 31332 HA LEU F 87   5.645 -29.249 50.861 0.00 0.00
F
ATOM 31333 CB LEU F 87   4.984 -27.480 49.955 0.00 0.00
F
ATOM 31334 HB1 LEU F 87   4.680 -27.189 50.983 0.00 0.00
F
ATOM 31335 HB2 LEU F 87   5.436 -26.594 49.459 0.00 0.00
F
ATOM 31336 CG LEU F 87   3.686 -27.956 49.253 0.00 0.00
F
ATOM 31337 HG LEU F 87   4.018 -28.091 48.201 0.00 0.00
F
ATOM 31338 CD1 LEU F 87   3.031 -29.213 49.725 0.00 0.00
F
ATOM 31339 HD11 LEU F 87   3.752 -30.057 49.670 0.00 0.00
F
ATOM 31340 HD12 LEU F 87   2.630 -29.203 50.761 0.00 0.00
F
ATOM 31341 HD13 LEU F 87   2.230 -29.551 49.034 0.00 0.00
F
ATOM 31342 CD2 LEU F 87   2.690 -26.771 49.288 0.00 0.00
F
ATOM 31343 HD21 LEU F 87   2.380 -26.685 50.352 0.00 0.00
F
ATOM 31344 HD22 LEU F 87   3.118 -25.839 48.861 0.00 0.00
F
ATOM 31345 HD23 LEU F 87   1.746 -27.003 48.750 0.00 0.00
F
ATOM 31346 C   LEU F 87   6.447 -29.145 48.761 0.00 0.00
F

ATOM 31347 O LEU F 87 6.034 -30.167 48.305 0.00 0.00
F
ATOM 31348 N THR F 88 7.411 -28.483 47.997 0.00 0.00
F
ATOM 31349 HN THR F 88 7.707 -27.577 48.291 0.00 0.00
F
ATOM 31350 CA THR F 88 7.882 -29.079 46.741 0.00 0.00
F
ATOM 31351 HA THR F 88 7.131 -29.275 45.990 0.00 0.00
F
ATOM 31352 CB THR F 88 8.753 -28.021 46.142 0.00 0.00
F
ATOM 31353 HB THR F 88 9.516 -27.674 46.871 0.00 0.00
F
ATOM 31354 OG1 THR F 88 7.953 -26.942 45.706 0.00 0.00
F
ATOM 31355 HG1 THR F 88 7.814 -26.500 46.546 0.00 0.00
F
ATOM 31356 CG2 THR F 88 9.455 -28.374 44.876 0.00 0.00
F
ATOM 31357 HG21 THR F 88 9.943 -27.470 44.454 0.00 0.00
F
ATOM 31358 HG22 THR F 88 10.223 -29.176 44.917 0.00 0.00
F
ATOM 31359 HG23 THR F 88 8.762 -28.589 44.034 0.00 0.00
F
ATOM 31360 C THR F 88 8.730 -30.319 46.983 0.00 0.00
F
ATOM 31361 O THR F 88 8.570 -31.335 46.249 0.00 0.00
F
ATOM 31362 N THR F 89 9.572 -30.401 48.082 0.00 0.00
F
ATOM 31363 HN THR F 89 9.725 -29.491 48.461 0.00 0.00
F
ATOM 31364 CA THR F 89 10.244 -31.631 48.561 0.00 0.00
F
ATOM 31365 HA THR F 89 10.766 -31.973 47.680 0.00 0.00
F
ATOM 31366 CB THR F 89 11.314 -31.415 49.606 0.00 0.00
F
ATOM 31367 HB THR F 89 10.914 -31.154 50.609 0.00 0.00
F
ATOM 31368 OG1 THR F 89 12.112 -30.372 49.224 0.00 0.00
F
ATOM 31369 HG1 THR F 89 11.627 -29.554 49.358 0.00 0.00
F
ATOM 31370 CG2 THR F 89 12.182 -32.668 49.714 0.00 0.00
F
ATOM 31371 HG21 THR F 89 12.734 -32.708 48.750 0.00 0.00
F
ATOM 31372 HG22 THR F 89 12.923 -32.517 50.527 0.00 0.00
F
ATOM 31373 HG23 THR F 89 11.639 -33.590 50.012 0.00 0.00
F

ATOM 31374 C THR F 89 9.223 -32.649 49.053 0.00 0.00
F
ATOM 31375 O THR F 89 9.377 -33.853 48.813 0.00 0.00
F
ATOM 31376 N LEU F 90 8.115 -32.240 49.645 0.00 0.00
F
ATOM 31377 HN LEU F 90 7.905 -31.278 49.801 0.00 0.00
F
ATOM 31378 CA LEU F 90 6.954 -33.166 49.877 0.00 0.00
F
ATOM 31379 HA LEU F 90 7.422 -33.932 50.477 0.00 0.00
F
ATOM 31380 CB LEU F 90 5.882 -32.622 50.797 0.00 0.00
F
ATOM 31381 HB1 LEU F 90 6.288 -32.221 51.750 0.00 0.00
F
ATOM 31382 HB2 LEU F 90 5.368 -31.855 50.179 0.00 0.00
F
ATOM 31383 CG LEU F 90 4.875 -33.623 51.230 0.00 0.00
F
ATOM 31384 HG LEU F 90 4.345 -34.101 50.378 0.00 0.00
F
ATOM 31385 CD1 LEU F 90 5.563 -34.787 51.888 0.00 0.00
F
ATOM 31386 HD11 LEU F 90 4.826 -35.398 52.452 0.00 0.00
F
ATOM 31387 HD12 LEU F 90 6.193 -35.452 51.259 0.00 0.00
F
ATOM 31388 HD13 LEU F 90 6.292 -34.464 52.661 0.00 0.00
F
ATOM 31389 CD2 LEU F 90 3.920 -32.942 52.204 0.00 0.00
F
ATOM 31390 HD21 LEU F 90 3.228 -33.676 52.668 0.00 0.00
F
ATOM 31391 HD22 LEU F 90 4.425 -32.393 53.027 0.00 0.00
F
ATOM 31392 HD23 LEU F 90 3.291 -32.154 51.737 0.00 0.00
F
ATOM 31393 C LEU F 90 6.288 -33.710 48.550 0.00 0.00
F
ATOM 31394 O LEU F 90 6.133 -34.881 48.386 0.00 0.00
F
ATOM 31395 N LEU F 91 6.077 -32.880 47.545 0.00 0.00
F
ATOM 31396 HN LEU F 91 6.212 -31.898 47.650 0.00 0.00
F
ATOM 31397 CA LEU F 91 5.374 -33.275 46.297 0.00 0.00
F
ATOM 31398 HA LEU F 91 4.546 -33.908 46.579 0.00 0.00
F
ATOM 31399 CB LEU F 91 4.793 -32.184 45.417 0.00 0.00
F
ATOM 31400 HB1 LEU F 91 5.195 -31.250 45.864 0.00 0.00
F

```
ATOM  31401  HB2 LEU F  91       4.950 -32.304  44.324  0.00  0.00
F
ATOM  31402  CG  LEU F  91       3.335 -32.074  45.683  0.00  0.00
F
ATOM  31403  HG  LEU F  91       2.832 -32.922  45.170  0.00  0.00
F
ATOM  31404  CD1 LEU F  91       2.889 -31.764  47.126  0.00  0.00
F
ATOM  31405 HD11 LEU F  91       3.036 -32.663  47.760  0.00  0.00
F
ATOM  31406 HD12 LEU F  91       3.553 -30.992  47.569  0.00  0.00
F
ATOM  31407 HD13 LEU F  91       1.808 -31.529  47.237  0.00  0.00
F
ATOM  31408  CD2 LEU F  91       2.801 -30.800  44.964  0.00  0.00
F
ATOM  31409 HD21 LEU F  91       2.663 -31.118  43.909  0.00  0.00
F
ATOM  31410 HD22 LEU F  91       1.804 -30.545  45.382  0.00  0.00
F
ATOM  31411 HD23 LEU F  91       3.493 -29.953  45.157  0.00  0.00
F
ATOM  31412  C   LEU F  91       6.170 -34.247  45.417  0.00  0.00
F
ATOM  31413  O   LEU F  91       5.674 -35.233  44.968  0.00  0.00
F
ATOM  31414  N   LEU F  92       7.482 -33.940  45.222  0.00  0.00
F
ATOM  31415  HN  LEU F  92       7.870 -33.175  45.731  0.00  0.00
F
ATOM  31416  CA  LEU F  92       8.366 -34.660  44.310  0.00  0.00
F
ATOM  31417  HA  LEU F  92       7.885 -34.904  43.375  0.00  0.00
F
ATOM  31418  CB  LEU F  92       9.570 -33.813  43.855  0.00  0.00
F
ATOM  31419  HB1 LEU F  92      10.141 -33.509  44.758  0.00  0.00
F
ATOM  31420  HB2 LEU F  92      10.290 -34.426  43.271  0.00  0.00
F
ATOM  31421  CG  LEU F  92       9.153 -32.488  43.141  0.00  0.00
F
ATOM  31422  HG  LEU F  92       8.354 -32.000  43.739  0.00  0.00
F
ATOM  31423  CD1 LEU F  92      10.399 -31.622  43.216  0.00  0.00
F
ATOM  31424 HD11 LEU F  92      10.387 -30.702  42.594  0.00  0.00
F
ATOM  31425 HD12 LEU F  92      10.553 -31.434  44.300  0.00  0.00
F
ATOM  31426 HD13 LEU F  92      11.280 -32.192  42.850  0.00  0.00
F
ATOM  31427  CD2 LEU F  92       8.555 -32.745  41.719  0.00  0.00
F
```

ATOM 31428 HD21 LEU F 92 9.359 -32.944 40.978 0.00 0.00
F
ATOM 31429 HD22 LEU F 92 7.910 -33.649 41.692 0.00 0.00
F
ATOM 31430 HD23 LEU F 92 7.999 -31.834 41.411 0.00 0.00
F
ATOM 31431 C LEU F 92 8.870 -35.928 44.946 0.00 0.00
F
ATOM 31432 O LEU F 92 9.282 -36.809 44.205 0.00 0.00
F
ATOM 31433 N LYS F 93 8.940 -36.036 46.282 0.00 0.00
F
ATOM 31434 HN LYS F 93 8.860 -35.298 46.947 0.00 0.00
F
ATOM 31435 CA LYS F 93 9.195 -37.345 46.899 0.00 0.00
F
ATOM 31436 HA LYS F 93 10.125 -37.705 46.486 0.00 0.00
F
ATOM 31437 CB LYS F 93 9.359 -37.149 48.470 0.00 0.00
F
ATOM 31438 HB1 LYS F 93 10.032 -36.287 48.666 0.00 0.00
F
ATOM 31439 HB2 LYS F 93 8.438 -36.878 49.029 0.00 0.00
F
ATOM 31440 CG LYS F 93 9.900 -38.440 49.232 0.00 0.00
F
ATOM 31441 HG1 LYS F 93 10.026 -38.409 50.335 0.00 0.00
F
ATOM 31442 HG2 LYS F 93 9.170 -39.223 48.934 0.00 0.00
F
ATOM 31443 CD LYS F 93 11.345 -38.785 48.648 0.00 0.00
F
ATOM 31444 HD1 LYS F 93 11.289 -39.088 47.581 0.00 0.00
F
ATOM 31445 HD2 LYS F 93 11.975 -37.885 48.815 0.00 0.00
F
ATOM 31446 CE LYS F 93 12.017 -39.974 49.322 0.00 0.00
F
ATOM 31447 HE1 LYS F 93 11.234 -40.749 49.466 0.00 0.00
F
ATOM 31448 HE2 LYS F 93 12.853 -40.421 48.743 0.00 0.00
F
ATOM 31449 NZ LYS F 93 12.567 -39.766 50.697 0.00 0.00
F
ATOM 31450 HZ1 LYS F 93 12.765 -38.782 50.968 0.00 0.00
F
ATOM 31451 HZ2 LYS F 93 11.796 -39.965 51.367 0.00 0.00
F
ATOM 31452 HZ3 LYS F 93 13.392 -40.375 50.868 0.00 0.00
F
ATOM 31453 C LYS F 93 8.164 -38.457 46.640 0.00 0.00
F
ATOM 31454 O LYS F 93 8.449 -39.621 46.472 0.00 0.00
F

ATOM 31455 N   GLU F 94   6.911 -38.100 46.617 0.00 0.00
F
ATOM 31456 HN GLU F 94   6.669 -37.153 46.812 0.00 0.00
F
ATOM 31457 CA GLU F 94   5.761 -38.945 46.480 0.00 0.00
F
ATOM 31458 HA GLU F 94   6.003 -39.911 46.898 0.00 0.00
F
ATOM 31459 CB GLU F 94   4.462 -38.315 47.215 0.00 0.00
F
ATOM 31460 HB1 GLU F 94   4.200 -37.391 46.656 0.00 0.00
F
ATOM 31461 HB2 GLU F 94   3.627 -39.041 47.109 0.00 0.00
F
ATOM 31462 CG GLU F 94   4.668 -37.918 48.675 0.00 0.00
F
ATOM 31463 HG1 GLU F 94   5.466 -37.178 48.896 0.00 0.00
F
ATOM 31464 HG2 GLU F 94   3.714 -37.465 49.019 0.00 0.00
F
ATOM 31465 CD GLU F 94   5.004 -39.148 49.547 0.00 0.00
F
ATOM 31466 OE1 GLU F 94   5.964 -39.162 50.298 0.00 0.00
F
ATOM 31467 OE2 GLU F 94   4.248 -40.179 49.421 0.00 0.00
F
ATOM 31468 C   GLU F 94   5.418 -39.076 45.010 0.00 0.00
F
ATOM 31469 O   GLU F 94   4.459 -39.804 44.660 0.00 0.00
F
ATOM 31470 N   LEU F 95   6.190 -38.455 44.146 0.00 0.00
F
ATOM 31471 HN LEU F 95   6.880 -37.803 44.451 0.00 0.00
F
ATOM 31472 CA LEU F 95   6.163 -38.870 42.752 0.00 0.00
F
ATOM 31473 HA LEU F 95   5.182 -39.219 42.466 0.00 0.00
F
ATOM 31474 CB LEU F 95   6.330 -37.449 42.044 0.00 0.00
F
ATOM 31475 HB1 LEU F 95   7.048 -36.792 42.580 0.00 0.00
F
ATOM 31476 HB2 LEU F 95   6.870 -37.582 41.082 0.00 0.00
F
ATOM 31477 CG LEU F 95   5.033 -36.691 41.815 0.00 0.00
F
ATOM 31478 HG LEU F 95   4.334 -36.866 42.661 0.00 0.00
F
ATOM 31479 CD1 LEU F 95   5.137 -35.150 41.579 0.00 0.00
F
ATOM 31480 HD11 LEU F 95   4.176 -34.593 41.615 0.00 0.00
F
ATOM 31481 HD12 LEU F 95   5.815 -34.710 42.341 0.00 0.00
F

ATOM 31482 HD13 LEU F 95   5.673 -34.993 40.619 0.00 0.00
F
ATOM 31483 CD2 LEU F 95   4.413 -37.367 40.512 0.00 0.00
F
ATOM 31484 HD21 LEU F 95   3.359 -37.064 40.335 0.00 0.00
F
ATOM 31485 HD22 LEU F 95   4.989 -37.074 39.608 0.00 0.00
F
ATOM 31486 HD23 LEU F 95   4.483 -38.453 40.733 0.00 0.00
F
ATOM 31487 C   LEU F 95   7.267 -39.839 42.488 0.00 0.00
F
ATOM 31488 O   LEU F 95   7.412 -40.251 41.320 0.00 0.00
F
ATOM 31489 N   ASP F 96   8.062 -40.176 43.516 0.00 0.00
F
ATOM 31490 HN ASP F 96   7.784 -39.911 44.437 0.00 0.00
F
ATOM 31491 CA ASP F 96   9.262 -41.029 43.444 0.00 0.00
F
ATOM 31492 HA ASP F 96   9.690 -40.805 44.410 0.00 0.00
F
ATOM 31493 CB ASP F 96   9.050 -42.636 43.451 0.00 0.00
F
ATOM 31494 HB1 ASP F 96   8.431 -42.897 42.567 0.00 0.00
F
ATOM 31495 HB2 ASP F 96   9.983 -43.205 43.253 0.00 0.00
F
ATOM 31496 CG ASP F 96   8.243 -43.098 44.670 0.00 0.00
F
ATOM 31497 OD1 ASP F 96   7.014 -43.207 44.511 0.00 0.00
F
ATOM 31498 OD2 ASP F 96   8.809 -43.091 45.753 0.00 0.00
F
ATOM 31499 C   ASP F 96   10.324 -40.689 42.440 0.00 0.00
F
ATOM 31500 O   ASP F 96   10.938 -41.517 41.701 0.00 0.00
F
ATOM 31501 N   ILE F 97   10.706 -39.381 42.422 0.00 0.00
F
ATOM 31502 HN ILE F 97   10.255 -38.744 43.043 0.00 0.00
F
ATOM 31503 CA ILE F 97   11.687 -38.866 41.522 0.00 0.00
F
ATOM 31504 HA ILE F 97   11.411 -39.075 40.499 0.00 0.00
F
ATOM 31505 CB ILE F 97   11.530 -37.401 41.395 0.00 0.00
F
ATOM 31506 HB ILE F 97   11.588 -36.853 42.360 0.00 0.00
F
ATOM 31507 CG2 ILE F 97   12.630 -36.840 40.311 0.00 0.00
F
ATOM 31508 HG21 ILE F 97   12.526 -35.737 40.231 0.00 0.00
F

ATOM	31509	HG22	ILE	F	97	13.639	-37.047	40.725	0.00	0.00	F
ATOM	31510	HG23	ILE	F	97	12.496	-37.280	39.300	0.00	0.00	F
ATOM	31511	CG1	ILE	F	97	10.115	-37.140	40.789	0.00	0.00	F
ATOM	31512	HG11	ILE	F	97	10.041	-37.714	39.840	0.00	0.00	F
ATOM	31513	HG12	ILE	F	97	9.323	-37.558	41.446	0.00	0.00	F
ATOM	31514	CD	ILE	F	97	10.008	-35.636	40.559	0.00	0.00	F
ATOM	31515	HD1	ILE	F	97	10.647	-35.074	41.273	0.00	0.00	F
ATOM	31516	HD2	ILE	F	97	10.371	-35.255	39.581	0.00	0.00	F
ATOM	31517	HD3	ILE	F	97	8.936	-35.347	40.613	0.00	0.00	F
ATOM	31518	C	ILE	F	97	13.099	-39.420	41.930	0.00	0.00	F
ATOM	31519	O	ILE	F	97	13.516	-39.067	43.045	0.00	0.00	F
ATOM	31520	N	PRO	F	98	13.844	-40.212	41.203	0.00	0.00	F
ATOM	31521	CD	PRO	F	98	13.524	-40.576	39.815	0.00	0.00	F
ATOM	31522	HD1	PRO	F	98	12.432	-40.738	39.687	0.00	0.00	F
ATOM	31523	HD2	PRO	F	98	13.916	-39.799	39.125	0.00	0.00	F
ATOM	31524	CA	PRO	F	98	15.094	-40.951	41.639	0.00	0.00	F
ATOM	31525	HA	PRO	F	98	14.596	-41.673	42.269	0.00	0.00	F
ATOM	31526	CB	PRO	F	98	15.700	-41.397	40.225	0.00	0.00	F
ATOM	31527	HB1	PRO	F	98	16.484	-42.181	40.301	0.00	0.00	F
ATOM	31528	HB2	PRO	F	98	16.117	-40.515	39.694	0.00	0.00	F
ATOM	31529	CG	PRO	F	98	14.422	-41.860	39.518	0.00	0.00	F
ATOM	31530	HG1	PRO	F	98	14.565	-42.152	38.455	0.00	0.00	F
ATOM	31531	HG2	PRO	F	98	13.960	-42.694	40.089	0.00	0.00	F
ATOM	31532	C	PRO	F	98	16.186	-40.232	42.491	0.00	0.00	F
ATOM	31533	O	PRO	F	98	16.890	-40.915	43.214	0.00	0.00	F
ATOM	31534	N	ASN	F	99	16.393	-38.889	42.264	0.00	0.00	F
ATOM	31535	HN	ASN	F	99	15.716	-38.511	41.638	0.00	0.00	F

ATOM 31536 CA ASN F 99   17.352 -38.056 42.934 0.00 0.00
F
ATOM 31537 HA ASN F 99   17.603 -38.579 43.845 0.00 0.00
F
ATOM 31538 CB ASN F 99   18.593 -37.698 42.129 0.00 0.00
F
ATOM 31539 HB1 ASN F 99   18.482 -37.071 41.219 0.00 0.00
F
ATOM 31540 HB2 ASN F 99   19.323 -37.136 42.750 0.00 0.00
F
ATOM 31541 CG ASN F 99   19.319 -38.974 41.648 0.00 0.00
F
ATOM 31542 OD1 ASN F 99   20.226 -39.587 42.238 0.00 0.00
F
ATOM 31543 ND2 ASN F 99   18.933 -39.422 40.423 0.00 0.00
F
ATOM 31544 HD21 ASN F 99   19.285 -40.304 40.109 0.00 0.00
F
ATOM 31545 HD22 ASN F 99   18.195 -38.940 39.951 0.00 0.00
F
ATOM 31546 C   ASN F 99   16.664 -36.785 43.339 0.00 0.00
F
ATOM 31547 O   ASN F 99   16.333 -35.985 42.507 0.00 0.00
F
ATOM 31548 N   ILE F 100   16.554 -36.460 44.627 0.00 0.00
F
ATOM 31549 HN ILE F 100   16.789 -37.076 45.375 0.00 0.00
F
ATOM 31550 CA ILE F 100   16.092 -35.220 45.150 0.00 0.00
F
ATOM 31551 HA ILE F 100   15.822 -34.572 44.329 0.00 0.00
F
ATOM 31552 CB ILE F 100   14.937 -35.491 46.091 0.00 0.00
F
ATOM 31553 HB ILE F 100   15.190 -36.317 46.789 0.00 0.00
F
ATOM 31554 CG2 ILE F 100   14.423 -34.229 46.885 0.00 0.00
F
ATOM 31555 HG21 ILE F 100   13.641 -33.617 46.387 0.00 0.00
F
ATOM 31556 HG22 ILE F 100   13.900 -34.627 47.781 0.00 0.00
F
ATOM 31557 HG23 ILE F 100   15.312 -33.668 47.244 0.00 0.00
F
ATOM 31558 CG1 ILE F 100   13.799 -36.018 45.304 0.00 0.00
F
ATOM 31559 HG11 ILE F 100   13.463 -35.143 44.708 0.00 0.00
F
ATOM 31560 HG12 ILE F 100   14.105 -36.819 44.598 0.00 0.00
F
ATOM 31561 CD ILE F 100   12.628 -36.451 46.079 0.00 0.00
F
ATOM 31562 HD1 ILE F 100   13.009 -37.173 46.833 0.00 0.00
F

ATOM 31563 HD2 ILE F 100 12.139 -35.547 46.501 0.00 0.00
F
ATOM 31564 HD3 ILE F 100 11.841 -36.771 45.363 0.00 0.00
F
ATOM 31565 C ILE F 100 17.287 -34.568 45.825 0.00 0.00
F
ATOM 31566 O ILE F 100 17.787 -35.070 46.809 0.00 0.00
F
ATOM 31567 N TRP F 101 17.794 -33.334 45.361 0.00 0.00
F
ATOM 31568 HN TRP F 101 17.325 -32.881 44.606 0.00 0.00
F
ATOM 31569 CA TRP F 101 18.813 -32.552 45.979 0.00 0.00
F
ATOM 31570 HA TRP F 101 19.112 -32.982 46.924 0.00 0.00
F
ATOM 31571 CB TRP F 101 20.150 -32.267 45.086 0.00 0.00
F
ATOM 31572 HB1 TRP F 101 19.745 -31.950 44.102 0.00 0.00
F
ATOM 31573 HB2 TRP F 101 20.702 -31.387 45.481 0.00 0.00
F
ATOM 31574 CG TRP F 101 21.042 -33.478 44.856 0.00 0.00
F
ATOM 31575 CD1 TRP F 101 20.756 -34.792 45.026 0.00 0.00
F
ATOM 31576 HD1 TRP F 101 19.935 -35.262 45.546 0.00 0.00
F
ATOM 31577 NE1 TRP F 101 21.904 -35.539 44.665 0.00 0.00
F
ATOM 31578 HE1 TRP F 101 21.959 -36.510 44.741 0.00 0.00
F
ATOM 31579 CE2 TRP F 101 22.910 -34.643 44.270 0.00 0.00
F
ATOM 31580 CD2 TRP F 101 22.419 -33.367 44.382 0.00 0.00
F
ATOM 31581 CE3 TRP F 101 23.177 -32.300 44.043 0.00 0.00
F
ATOM 31582 HE3 TRP F 101 22.678 -31.342 44.023 0.00 0.00
F
ATOM 31583 CZ3 TRP F 101 24.509 -32.499 43.566 0.00 0.00
F
[truncated: 545,659 more chars]
